# PPAR**γ** agonist treatment reduces fibroadipose tissue in secondary lymphedema by exhausting fibroadipogenic PDGFR**α^+^** mesenchymal cells

**DOI:** 10.1172/jci.insight.165324

**Published:** 2023-12-22

**Authors:** Ziyu Chen, Soheila Ali Akbari Ghavimi, Mengfan Wu, John McNamara, Olga Barreiro, David Maridas, Radomir Kratchmarov, Ashley Siegel, Sarah Djeddi, Maria Gutierrez-Arcelus, Patrick J. Brennan, Timothy P. Padera, Ulrich von Andrian, Babak Mehrara, Arin K. Greene, C. Ronald Kahn, Dennis P. Orgill, Indranil Sinha, Vicki Rosen, Shailesh Agarwal

**Affiliations:** 1 Department of Surgery, Brigham and Women’s Hospital and Harvard Medical School, Boston, Massachusetts, USA.; 2 Department of Bone and Joint Surgery, Peking University Shenzhen Hospital, Shenzhen Peking University-The Hong Kong University of Science and Technology Medical Center, Shenzhen, Guangdong, China.; 3 Rutgers University, Rutgers, New Jersey, USA.; 4 Harvard Medical School, Boston, Massachusetts, USA.; 5 Harvard School of Dental Medicine, Boston, Massachusetts, USA.; 6 Division of Allergy and Clinical Immunology, Department of Medicine, Brigham and Women’s Hospital and Harvard Medical School, Boston, Massachusetts, USA.; 7 Boston Children’s Hospital and Harvard Medical School, Boston, Massachusetts, USA.; 8 Broad Institute of MIT and Harvard, Cambridge, Massachusetts, USA.; 9 Massachusetts General Hospital and Harvard Medical School, Boston, Massachusetts, USA.; 10 Memorial Sloan Kettering Cancer Center, New York, New York, USA.; 11 Joslin Diabetes Center and Harvard Medical School, Boston, Massachusetts, USA.

**Keywords:** Cell Biology, Adipose tissue, Fibrosis

## Abstract

Secondary lymphedema occurs in up to 20% of patients after lymphadenectomy performed for the surgical management of tumors involving the breast, prostate, uterus, and skin. Patients develop progressive edema of the affected extremity due to retention of protein-rich lymphatic fluid. Despite compression therapy, patients progress to chronic lymphedema in which noncompressible fibrosis and adipose tissue are deposited within the extremity. The presence of fibrosis led to our hypothesis that rosiglitazone, a PPARγ agonist that inhibits fibrosis, would reduce fibrosis in a mouse model of secondary lymphedema after hind limb lymphadenectomy. In vivo, rosiglitazone reduced fibrosis in the hind limb after lymphadenectomy. Our findings verified that rosiglitazone reestablished the adipogenic features of TGF-β1–treated mesenchymal cells in vitro. Despite this, rosiglitazone led to a reduction in adipose tissue deposition. Single-cell RNA-Seq data obtained from human tissues and flow cytometric and histological evaluation of mouse tissues demonstrated increased presence of PDGFRα^+^ cells in lymphedema; human tissue analysis verified these cells have the capacity for adipogenic and fibrogenic differentiation. Upon treatment with rosiglitazone, we noted a reduction in the overall quantity of PDGFRα^+^ cells and LipidTOX^+^ cells. Our findings provide a framework for treating secondary lymphedema as a condition of fibrosis and adipose tissue deposition, both of which, paradoxically, can be prevented with a pro-adipogenic agent.

## Introduction

Secondary lymphedema is a morbid condition, characterized by progressive limb swelling due to impaired drainage of lymphatic fluid. Patients develop retention of protein-rich lymphatic fluid, which progresses to chronic lymphedema, characterized by limb hypertrophy caused by irreversible fibroadipose tissue deposition (1). As a result, patients experience limb heaviness, pain, open wounds, and disability, all of which severely impact quality of life for patients with cancer (2). Patients report reduced physical, functional, social, and emotional well-being (3). In the United States, secondary lymphedema affects over 5 million individuals who have undergone removal of lymph nodes in the affected extremity during cancer surgery (1). In fact, the morbidity of secondary lymphedema has led to the development of myriad clinical trials studying the efficacy of surgical treatment without lymphadenectomy (e.g., Z0011 breast cancer and MSLT melanoma trials) (4–7). However, despite these studies, lymphadenectomy remains a component of surgical management of cancers of the breast, skin, head/neck, prostate, and uterus, among others.

Strategies to manage secondary lymphedema are inadequate. Nonsurgical approaches such as sleeve, pneumatic pump compression, or manual lymphatic drainage demand 40+ h/wk of patient commitment and cause pain (8); even short periods of nonadherence lead to relapse (9). Surgical procedures to address secondary lymphedema (10) have focused on reconstituting lymphatic drainage but are not curative and require continued long-term compression. Therefore, despite attempts to eliminate lymphatic fluid retention, almost all patients progress to some degree of chronic lymphedema.

To date, pharmacologic therapies targeting the fibroadipose tissue deposition have not been identified. Others have focused primarily on antiinflammatory strategies including nonsteroidal antiinflammatory drugs (11) and therapies targeting T cells (12); these strategies have limitations including the risk of immunosuppression in patients with a history of malignancy. However, a therapeutic strategy addressing fibroadipose tissue would halt the progression of secondary lymphedema to its chronic, irreversible phase. Here, we report on our findings with use of rosiglitazone, a pro-adipogenic agent, which functions as an agonist of peroxisome proliferator–activated receptor gamma (PPARγ), for the reduction of fibrosis in secondary lymphedema.

## Results

### A mouse model of secondary lymphedema that recapitulates fibrosis and adipose tissue deposition.

**Figure 1:**
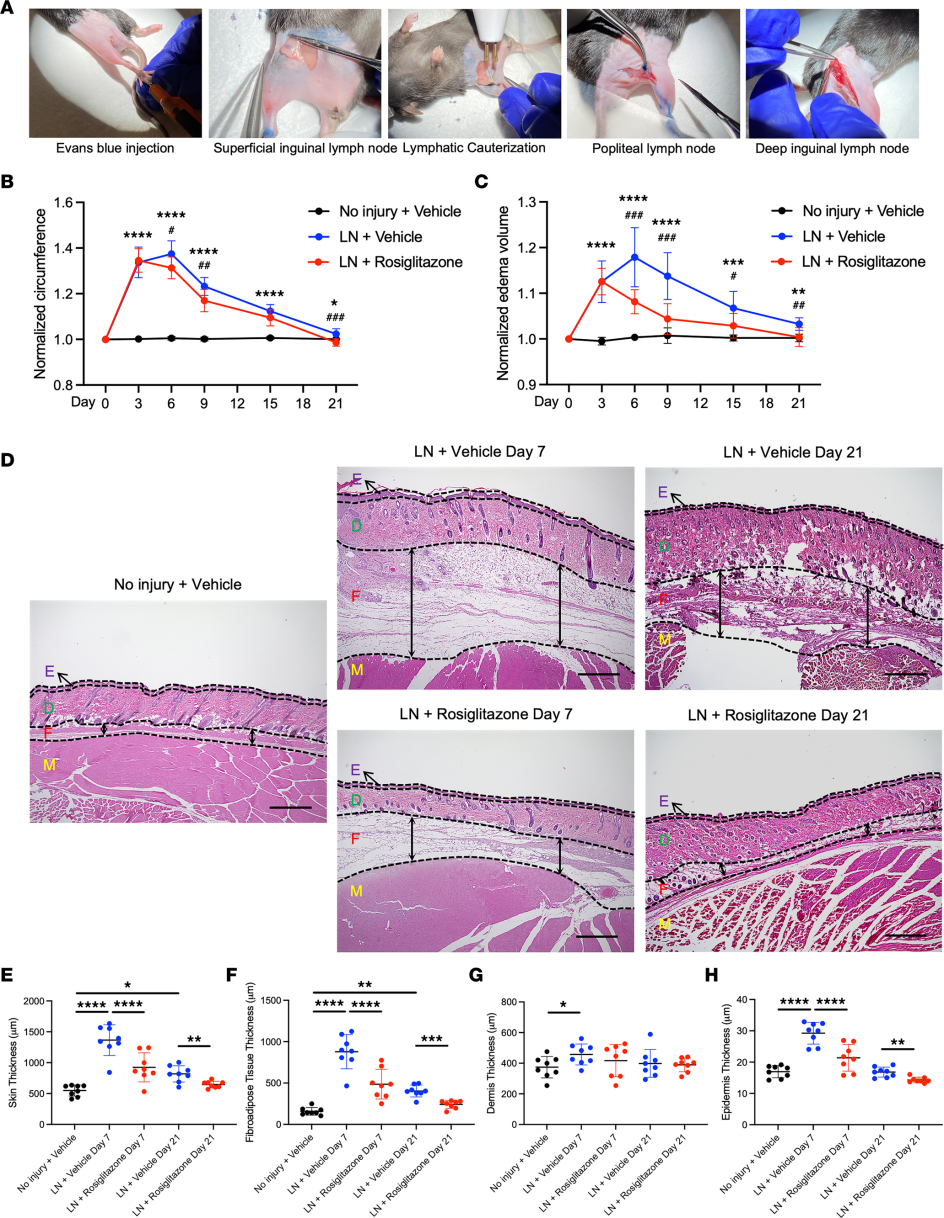
A mouse model of hind limb lymphedema recapitulates augmented swelling and adipose tissue deposition observed in patients with lymphedema. (**A**) Surgical procedure for hind limb lymphadenectomy in mice. (**B**) Normalized circumference of hind limbs with/without lymphadenectomy and rosiglitazone treatment (*n* = 8); comparison between No injury + Vehicle and LN + Vehicle is indicated by *; comparison between LN + Vehicle and LN + Rosiglitazone is indicated by ^#^. (**C**) Normalized edema volume of hind limbs with/without lymphadenectomy and rosiglitazone treatment (*n* = 8); comparison between No injury + Vehicle and LN + Vehicle is indicated by *; comparison between LN + Vehicle and LN + Rosiglitazone is indicated by ^#^. (**D**) H&E staining of No injury + Vehicle and LN + Vehicle day 7 and day 21, as well as LN + Rosiglitazone day 7 and day 21 hind limbs, original magnification 4×, scale bar: 500 μm. (**E**) Quantification of overall hind limb skin thickness (*n* = 8). (**F**) Quantification of hind limb fibroadipose tissue thickness (*n* = 8). (**G**) Quantification of hind limb dermis thickness (*n* = 8). (**H**) Quantification of hind limb epidermis thickness (*n* = 8). Statistical significance is established using 1-way ANOVA and Tukey’s post hoc tests, *P < 0.05, **P < 0.01, ***P < 0.001, ****P < 0.0001, ^#^*P* < 0.05, ^##^*P* < 0.01, ^###^*P* < 0.001. LN, lymphadenectomy; E, epidermis; D, dermis; F, fibroadipose tissue; M, muscle.

Hind limb lymphadenectomy consisting of surgical excision of the hind limb popliteal, superficial inguinal, and deep inguinal lymph nodes and femoral lymphatic cauterization was performed (Figure 1A) as previously described (13, 14). Limb circumference, normalized to the uninjured hind limb at day 0, increased by nearly 40% within the first week after surgery (1.37 vs. 1.00, *P* < 0.0001) (Figure 1B). Similar findings were noted based on edema normalized to baseline (normalized edema area: 1.16 vs. 1.00, *P* < 0.0001, normalized edema volume: 1.18 vs. 1.00, *P* < 0.0001) (Figure 1C and Supplemental Figures 1 and 2; supplemental material available online with this article; https://doi.org/10.1172/jci.insight.165324DS1). We noted a gradual improvement in both circumference and edema area and volume based on gross measurements, though a significant increase persisted even after 3 weeks (normalized circumference: 1.02 vs. 1.00, *P* = 0.0305; normalized edema area: 1.02 vs. 1.00, *P* = 0.0336; normalized edema volume: 1.03 vs. 1.00, *P* = 0.0017). Consistent with previous demonstrations of CD4^+^ T cells in lymphedema (15), we noted a marked increase in the presence of CD4^+^ T cells of the affected hind limb when compared with the control hind limb based on immunohistochemistry (Supplemental Figure 3, A and B).

**Figure 2:**
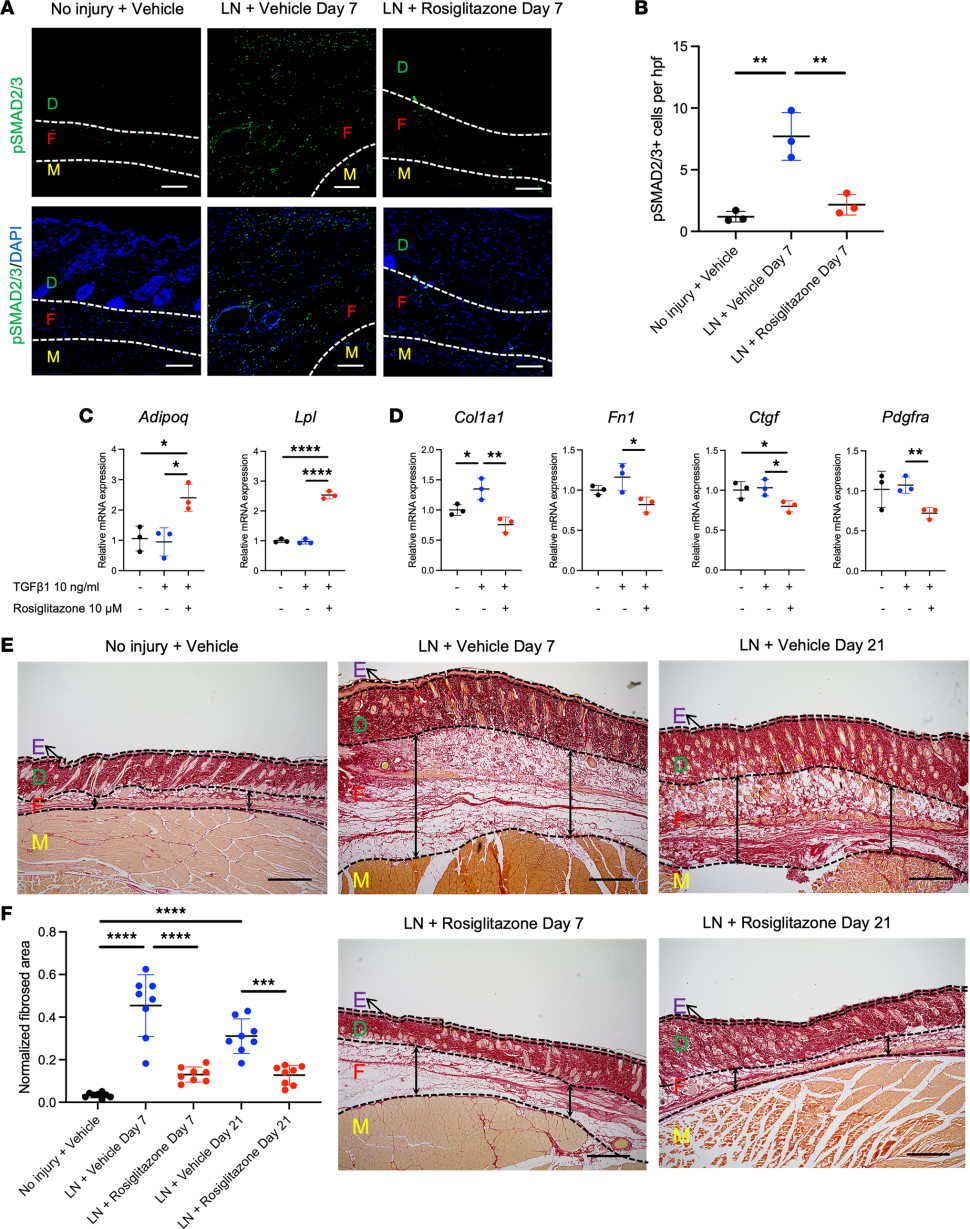
Treatment effect of rosiglitazone on fibrosis in hind limb lymphedema. (**A**) Immunostaining of No injury + Vehicle and LN + Vehicle day 7 and LN + Rosiglitazone day 7 sections for phosphorylated (p-) SMAD 2/3; original magnification 20×, scale bar: 100 μm. (**B**) Quantification of p-SMAD2/3^+^ cells in fibroadipose tissue layer (*n* = 3). (**C**) Expression levels of adipogenic genes among adipose-derived mesenchymal cells treated with TGF-β1 with or without rosiglitazone (*n* = 3). (**D**) Expression levels of fibrogenic genes among adipose-derived mesenchymal cells treated with TGF-β1 with or without rosiglitazone (*n* = 3). (**E**) Picrosirius red staining of No injury + Vehicle and LN + Vehicle day 7 and day 21, as well as LN + rosiglitazone day 7 and day 21 hind limbs; original magnification 4×, scale bar: 500 μm. (**F**) Normalized quantification of hind limb fibrosis (*n* = 8). Statistical significance is established using 1-way ANOVA and Tukey’s post hoc tests, *P < 0.05, **P < 0.01, ***P < 0.001, ****P < 0.0001. LN, lymphadenectomy; E, epidermis; D, dermis; F, fibroadipose tissue; M, muscle.

Histologic evaluation was performed to further characterize changes in skin thickness and fibrosis after lymphadenectomy. Based on H&E staining, we noted increased thickness of the skin (marked E, D, and F) and of the subdermal layer alone (marked F) at both 1 and 3 weeks after lymphadenectomy (1 week: skin: 1,365.04 μm vs. 548.88 μm, *P* < 0.0001, subdermal layer: 878.36 μm vs. 157.77 μm, *P* < 0.0001; 3 weeks: skin: 819.10 μm vs. 548.88 μm, *P* = 0.0251, subdermal layer: 403.73 μm vs. 157.77 μm, *P* = 0.0049) (Figure 1, D–F). We also noted an increase in the dermis thickness (marked D) and epidermis thickness (marked E) at 1 week after lymphadenectomy (1 week: dermis: 457.43 μm vs. 374.16 μm, *P* = 0.0306, epidermis: 29.24 μm vs. 16.95 μm, *P* < 0.0001), which later improved after 3 weeks (3 weeks: dermis: 398.55 μm vs. 374.16 μm, *P* = 0.5571, epidermis: 16.82 μm vs. 16.95 μm, *P* = 0.8854) (Figure 1, G and H). Based on Picrosirius red staining (Figure 2E), we noted increased normalized fibrosis in the affected hind limb at both 1 and 3 weeks after lymphadenectomy (1 week: 0.454 vs. 0.033, *P* < 0.0001; 3 weeks: 0.311 vs. 0.033, *P* < 0.0001) (Figure 2F).

### Augmented PPARγ signaling reduces fibrosis and adipose tissue deposition.

Next, we sought to reverse the fibrosis observed in secondary lymphedema through augmented PPARγ activity. Previous studies have established that PPARγ agonists reverse the effects of pro-fibrotic TGF-β ligands (16–18). First, we examined the presence of increased TGF-β signaling in the hind limb after lymphadenectomy; immunostaining for p-SMAD 2/3 verified increased signaling after lymphadenectomy (Figure 2, A and B). Based on this validation, we designed an in vitro experiment in which adipose-derived mesenchymal stem cells (AdMSCs) were treated with rosiglitazone and either TGF-β1 or tumor necrosis factor-α (TNF-α), with subsequent evaluation of adipogenic and fibrogenic gene expression; rosiglitazone augmented adipogenesis despite the presence of TGF-β1 ligand (*Adipoq*: 2.40 vs. 0.95, *P* = 0.0177; *Lpl*: 2.53 vs. 0.97, *P* < 0.0001) (Figure 2C). Furthermore, rosiglitazone reduced fibrogenic gene expression, which had been induced by TGF-β1 (*Col1a1*: 1.35 vs. 0.76, *P* = 0.0091; *Fn1*: 1.16 vs. 0.82, *P* = 0.038, *Ctgf*: 1.03 vs. 0.80, *P* = 0.0306, *Pdgfra*: 1.07 vs. 0.72, *P* = 0.0083) (Figure 2D). Similarly, rosiglitazone rescued adipogenic gene expression, which was reduced among TNF-α–treated AdMSCs (Supplemental Figure 4). These findings provided support for in vivo delivery of rosiglitazone to mice that had undergone lymphadenectomy. We noted modest initial reductions in limb circumference and edema after 1 week (circumference: 1.31 vs. 1.37, *P* = 0.0257, edema area: 1.08 vs. 1.16, *P* = 0.0015, edema volume: 1.08 vs. 1.18, *P* = 0.0003) (Figure 1, B and C, and Supplemental Figure 2). Furthermore, Picrosirius red staining and quantification verified the desired reduction in fibrosis (1 week: 0.130 vs. 0.454, *P* < 0.0001; 3 weeks: 0.128 vs. 0.311, *P* < 0.001) (Figure 2, E and F). Upon detailed histological evaluation, however, we noted an unexpected reduction in subdermal adipose tissue and overall skin thickness (1 week: skin: 924.66 μm vs. 1365.04 μm, *P* < 0.0001, subdermal layer: 486.12 μm vs. 878.36 μm, *P* < 0.0001; 3 weeks: skin: 646.12 μm vs. 819.10 μm, *P* = 0.0068, subdermal layer: 242.36 μm vs. 403.73 μm, *P* = 0.0003) (Figure 1, D–F). While we did not identify a reduction in the dermis thickness, we did note a decrease in epidermis thickness after 1 and 3 weeks (1 week: dermis: 417.15 μm vs. 457.43 μm, *P* = 0.3786, epidermis: 21.38 μm vs. 29.24 μm, *P* < 0.0001; 3 weeks: dermis: 389.46 μm vs. 398.55 μm, *P* = 0.8032, epidermis: 14.30 μm vs. 16.82 μm, *P* = 0.0015) (Figure 1, G and H).

### Increased presence of adipogenic PDGFRα^+^ cells after lymphadenectomy is reduced by rosiglitazone.

Given the unexpected finding of reduced subdermal adipose tissue in rosiglitazone-treated mice, we first verified that PPARγ levels were not reduced with rosiglitazone treatment (Supplemental Figure 5).

**Figure 3:**
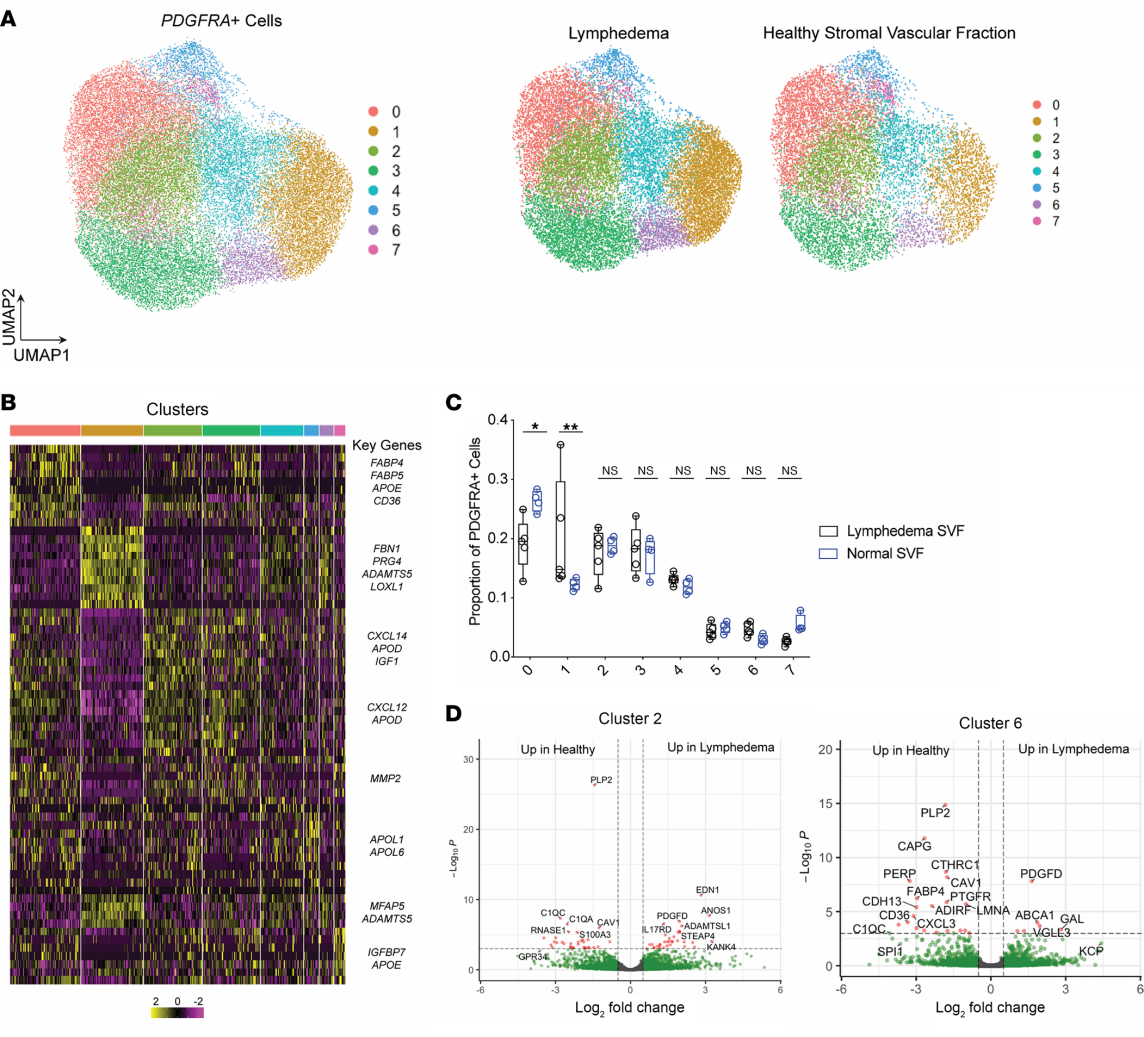
Lymphedema modifies the presence of fate of PDGFRα^+^ cells. (**A**) Left: Uniform manifold approximation and projection (UMAP) of *PDGFRA*^+^ cells subclustered from total lymphedema scRNA-Seq data set (Supplemental Figure 6). Right: *PDGFRA*^+^ UMAP split by lymphedema and healthy SVF (LSVF and NSVF, respectively). (**B**) Heatmap of top 10 differentially expressed transcripts identifying each cluster in the *PDGFRA*^+^ UMAP space. (**C**) Proportion of cells in each cluster for LSVF and NSVF samples, respectively. Box plots show the interquartile range (box), median (line), and minimum and maximum (whiskers). Statistical significance is established using Mann-Whitney nonparametric *t* test, *P < 0.05, **P < 0.01. (**D**) Volcano plots of top differentially expressed genes between lymphedema and healthy samples for cluster 2 and cluster 6; fold-change cutoff 0.5, *P*-adjusted cutoff 0.001. Labeled transcripts colored in red meet both fold-change and significance cutoff; transcripts to the right are increased in lymphedema; and transcripts to the left of plot are increased in healthy.

Next, we sought to examine how rosiglitazone modifies PDGFRα^+^ cells, which have been previously shown to contribute to both fibrosis and adipogenesis in muscle and normal development. We reanalyzed single-cell RNA-Seq (scRNA-Seq) data from the stromal vascular fraction (SVF) of adipose tissue from patients with lymphedema and healthy controls (Supplemental Figure 6A) (19). We found significant heterogeneity in the adipose-derived stromal cell compartment, with 6 distinct clusters expressing the lineage-defining transcript *PDGFRA* (Supplemental Figure 6B). The relative abundance of 2 of these clusters (cluster 1 and cluster 3) differed significantly between diseased and healthy SVF samples (Supplemental Figure 6C). These clusters exhibited varying degrees of adipogenic or fibrogenic gene expression, with adipogenic clusters expressing transcripts such as *APOD*, *APOE*, *CEPB*, and *CEPD* while fibrogenic clusters expressed *FBN1*, *COL1A1*, and *COL1A2*. Cluster 1 was noted to be adipogenic while subclusters 6, 10, and 11 exhibited more fibrogenic transcriptional features (Supplemental Figure 6D). Although we observed expansion of the *PDGFRA^+^* compartment in lymphedema, as previously suggested (19), there was significant patient-patient variability, with some clusters exhibiting patient-specific changes (Supplemental Figure 6C). We therefore reclustered *PDGFRA-*expressing cells using the Harmony algorithm to decrease donor and batch effects (Figure 3A) (20). Within the *PDGFRA*^+^ space, 8 distinct clusters were observed, all of which were present in both healthy and diseased samples. The transcription profiles of these clusters were heterogeneous and suggested differential cell states associated with adipogenesis and fibrosis (Figure 3B and Supplemental Data 1). Cluster 0, defined by expression of adipocyte markers such as *FABP4*, *FABP5*, *APOE*, and *CD36* was more abundant in healthy samples, while cluster 1, defined by *FBN1*, *PRG4*, and *ADAMTS5* expression, was more abundant in lymphedema; the relative proportions of the other clusters were unchanged between tissue types (Figure 3C). We also performed pseudobulk expression analysis to identify differentially expressed transcripts within each cluster between healthy and lymphedema. Lymphedema samples exhibited aberrant expression of metalloproteinases such as *ADAMTSL1* and a decrease in transcripts associated with adipocyte identity (*FABP4*, *CD36*) (Figure 3D and Supplemental Data 2–9). These alterations were independent of cluster frequency, and fibrogenic metalloproteinase expression was increased in lymphedema in clusters with a globally adipogenic profile, e.g., cluster 6. Taken together, these findings suggest that the adipocyte stromal cell compartment is heterogeneous and composed of distinct adipogenic and fibrogenic cells, with expansion of *PDGFRA-*expressing cells in lymphedema. Moreover, differentially expressed transcripts in healthy and lymphedema tissues suggest a role for fibrogenic metalloproteinases and loss of adipocyte identity in disease progression.

**Figure 4:**
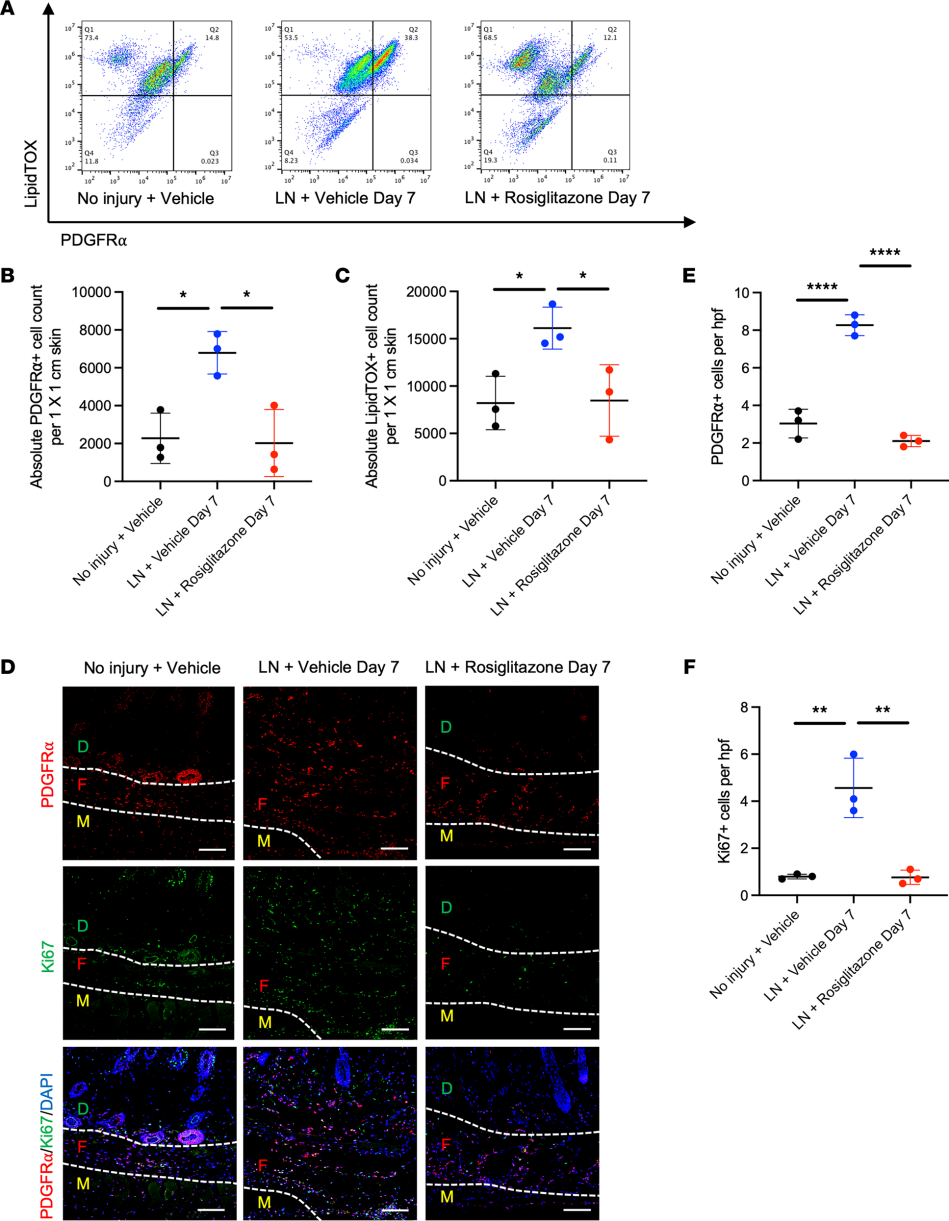
Rosiglitazone reduces presence of PDGFRα^+^ adipocytes after lymphadenectomy. (**A**) Representative flow cytometry plots showing detection of PDGFRα^+^ and LipidTOX^+^ cells in hind limb tissue from No injury + Vehicle, LN + Vehicle, and LN + Rosiglitazone mice. (**B**) Quantification of PDGFRα^+^ cells in 1 cm × 1 cm area of skin from No injury + Vehicle, LN + Vehicle, and LN + Rosiglitazone hind limbs (*n* = 3). (**C**) Quantification of LipidTOX^+^ cells in 1 cm × 1 cm area of skin from No injury + Vehicle, LN + Vehicle, and LN + Rosiglitazone hind limbs (*n* = 3). (**D**) Immunostaining for PDGFRα and Ki67 in No injury + Vehicle and LN + Vehicle day 7 and LN + Rosiglitazone day 7 sections; original magnification 20×, scale bar: 100 μm. (**E**) Quantification of PDGFRα^+^ cells in fibroadipose tissue layer (*n* = 3). (**F**) Quantification of Ki67^+^ cells in fibroadipose tissue layer (*n* = 3). Statistical significance is established using 1-way ANOVA and Tukey’s post hoc tests, *P < 0.05, **P < 0.01, ****P < 0.0001. LN, lymphadenectomy; D, dermis; F, fibroadipose tissue; M, muscle.

Next, the hind limbs of mice euthanized 1 week after lymphadenectomy were evaluated for the presence of PDGFRα^+^ cells. Flow cytometry performed over a standard 1 cm × 1 cm area of skin demonstrated a significant increase in the absolute count of PDGFRα^+^ cells after lymphadenectomy relative to the uninjured hind limb (6.79 × 10^3^ vs. 2.28 × 10^3^, *P* = 0.0195) (Figure 4, A and B). Upon staining with LipidTOX, we noted a significantly increased presence of LipidTOX^+^ cells indicative of their adipogenic features (1.61 × 10^4^ vs. 0.82 × 10^4^, *P* = 0.0414) (Figure 4, A and C). Immunostaining similarly verified a visible increase in the presence of PDGFRα^+^ cells in the hind limbs of mice with lymphadenectomy (Figure 4, D and E). Costaining with Ki67 verified that these cell populations exhibited proliferation within the site (Figure 4, D and F).

The hind limbs of mice treated with rosiglitazone after lymphadenectomy were compared with hind limbs of those that did not receive rosiglitazone. Flow cytometry quantified a significant and substantial reduction in PDGFRα^+^ cells within a standardized 1 cm × 1 cm area of skin (2.02 × 10^3^ vs. 6.79 × 10^3^, *P* = 0.0153) (Figure 4, A and B). Flow cytometry for LipidTOX^+^ cells showed a reduction in rosiglitazone-treated mice (0.85 × 10^4^ vs. 1.61 × 10^4^, *P* = 0.0472) (Figure 4, A and C). Immunostaining verified a reduction in the presence of PDGFRα^+^ cells (Figure 4, D and E) with rosiglitazone treatment; there was a corresponding reduction in PDGFRα^+^Ki67^+^ cells (Figure 4, D and F).

## Discussion

Our results show that rosiglitazone, a pro-adipogenic agent, which augments signaling through PPARγ (21), reduces fibrosis and adipose tissue deposition in the hind limb after lymphadenectomy. These findings are consistent with previous studies indicating that rosiglitazone reduces fibrosis in other disease contexts, including liver (16), lung (18), and dermal fibrosis (22). For example, rosiglitazone reduces expression of pro-fibrotic peptides including Ctgf, α-SMA, and Col1 by dermal fibroblasts in scleroderma (22). Our in vitro studies demonstrate that rosiglitazone indeed reduced the expression of pro-fibrotic genes by AdMSCs that were exposed to pro-fibrotic TGF-β1; rosiglitazone also reversed the antiadipogenic effect of TNF-α in culture.

Unexpectedly, our histological examination of the hind limb demonstrated reduced total amount of fibroadipose tissue in the subdermal layer with reduced number of adipocytes after rosiglitazone treatment. Analysis of previously collected scRNA-Seq data obtained from the SVF of lymphedema and normal tissues (23) verified an enrichment in PDGFRα^+^ cells as previously reported by Liu et al.; we noted that these cells exhibited adipogenic and fibrogenic properties, though there was a marked reduction in the presence of PDGFRα^+^ cells expressing adipogenic genes. Previous studies have demonstrated that these cells possess both fibrogenic and adipogenic potential in various contexts. For example, in muscle injury, Contreras et al. showed that TGF-β1 reduces pro-adipogenic *Pparg* and *Adipoq* gene expression in muscle-resident PDGFRα^+^ mesenchymal cells (24). Scherer et al. showed that adipocytes undergo reversible dedifferentiation into proliferative PDGFRα^+^ mesenchymal cells; these PDGFRα^+^ cells are then capable of undergoing redifferentiation into mature adipocytes (25). Shin et al. showed that embryonic PDGFRα^+^ cells contribute to mature adipocytes during development. Using scRNA-Seq, Leinroth et al. showed that a subset of intramuscular PDGFRα^+^ cells known as fibroadipogenic progenitors undergo robust adipogenesis; this cluster of cells simultaneously expressed TGF-β receptor (*Tgfbr2*) and adipogenic genes including *Pparg* and *Fabp4* (26). Our findings with the human lymphedema samples and the previous literature supported our decision to examine how rosiglitazone may impact PDGFRα^+^ cells. In our mouse experiments immunostaining and flow cytometry verified the increased presence of PDGFRα^+^ cells in the lymphedematous hind limb relative to control. Moreover, these PDGFRα^+^ cells were proliferative and exhibited lipid accumulation based on staining with LipidTOX. However, rosiglitazone reduced the total number of both PDGFRα^+^ and LipidTOX^+^PDGFRα^+^ cells and correspondingly reduced proliferation based on immunostaining.

We recognize several limitations of this study. First, while the model of secondary lymphedema presented here is representative of the human condition both based on the surgical lymphadenectomy and the development of fibroadipose tissue deposition, the timeline of this development is accelerated in the mouse model. Although other models such as the tail model have gained in popularity, the circumferential skin incision is not representative of the extensive lymphadenectomy performed in patients. In addition, the tail model also experiences gradual resolution of the underlying lymphedema over the course of several weeks. Importantly, our model does re-create the increased CD4^+^ T cell infiltration observed in the tail lymphedema model. Second, the question of when to administer rosiglitazone therapy is critical. Based on our clinical experience, patients are often intermittently adherent to therapy; because of this, all patients progress to some degree of fibroadipose tissue deposition. As a result, this study paves the way for inquiries into the use of rosiglitazone as an adjunct therapy initiated simultaneously with nonsurgical compression therapy. Third, rosiglitazone is associated with adverse effects including heart failure; however, these findings have been noted in patients with an underlying history of diabetes, for which rosiglitazone was originally indicated (27). Fourth, questions remain regarding the source of PDGFRα^+^ cells present in the lymphedema site; it remains unclear whether these cells are derived from local mesenchymal cells or from cells that traffic to the injury site. Improved understanding of the source of these cells may provide clues to additional therapeutic strategies.

The present study presents a therapeutic agent that simultaneously addresses the fibrosis and adipose tissue deposition present in secondary lymphedema by modifying the fate and function of mesenchymal cells in the injury site. Based on these preclinical findings, we anticipate a clinical trial examining rosiglitazone therapy in patients with early-stage lymphedema who would benefit from prevention of progression to chronic lymphedema.

## Methods

### Animals.

Male 6- to 8-week-old C57BL/6J mice (Jackson Laboratory; weight: 23 ± 2 g) were acclimatized for 1 week in the Brigham and Women’s Hospital (BWH) vivarium.

### Mouse hind limb lymphedema model.

Evans blue (MilliporeSigma) (4% in PBS) was filtered and injected into mouse foot pads to stain lymph nodes in hind limbs. Mouse hind limb secondary lymphedema was induced by surgical excision of the ipsilateral superficial inguinal, popliteal, and deep inguinal lymph nodes and the femoral lymphatic vessel (13). During surgical procedures, electrocauterization was used to prevent bleeding.

### Animal experimental setup and rosiglitazone treatment.

A total of 32 mice that underwent lymphedema surgery were randomly assigned into 2 groups, lymphedema (LN + vehicle) group and rosiglitazone (LN + rosiglitazone) group (*n* = 16). Rosiglitazone (Combi-Blocks) was dissolved in DMSO at the concentration of 25 mg/mL and was diluted in corn oil to the final concentration of 2.5 mg/mL before injection. Mice in the LN + rosiglitazone group were intraperitoneally injected with rosiglitazone (10 mg/kg) twice a week from the day of surgery (day 0, 3, 7, 10, 14, 17). Mice in the LN + vehicle group were injected with corn oil only at the same time points. On day 7, 8 mice in the LN + vehicle group and LN + rosiglitazone group were sacrificed (*n* = 8 per group); the rest of them were sacrificed on day 21 (*n* = 8 per group). In addition, 8 mice without surgery were assigned into the control group (no injury + vehicle), received corn oil injection at the same time points, and were sacrificed on day 21. For flow cytometry experiments, another 9 mice were randomly divided into 3 groups: no injury + vehicle, LN + vehicle, and LN + rosiglitazone; skin from these mice was isolated and used for flow cytometry analysis at day 7 (*n* = 3 per group).

### Circumference and edema evaluation.

Circumference of hind limbs of each mouse was measured by tape measure every 3 days. Edema area and volume of hind limbs in each group were measured by using Vernier caliper every 3 days as previously described (Supplemental Figure 1) (13). Area of the cross-sectioned thigh was calculated by using the following equation: S (mm^2^) = π × length × width × 1/4, where S = cross-sectional area. Edema volume of the hind limb was calculated by using the equation V (mm^3^) = S × height × 1/3, where V = hind limb volume. All measurements were blinded and normalized to the day 0 values of the same mice before lymphadenectomy.

### Tissue procurement.

Sacrifice was performed via CO_2_ asphyxiation. Skin and muscle of hind limb were harvested, then fixed in 10% formalin for 48 hours followed by 70% ethanol for histological assessment. Formalin-fixed tissues were then dehydrated, embedded in paraffin, and sectioned with 5 μm thickness.

### Histology.

Sections were stained either with H&E or with Picrosirius red according to standard protocol, then used to assess the overall skin thickness, epidermal thickness, dermal thickness, fibroadipose tissue thickness, and fibrosis. Quantitative histological analysis was performed using samples from days 7 and 21. Images were captured by using Olympus BX53 (UCMAD3, T7) and all-in-one Keyence microscopes and assessed by ImageJ (version 1.52a; Media Cybernetics; NIH) by 2 independent researchers under blinded conditions. Fibrosis was assessed with the image thresholding plugin in ImageJ software; normalization is based on the length of the section.

### scRNA-Seq analysis of SVF of healthy and lymphedema patients.

FASTQ files from the scRNA-Seq data set from Liu et al. 2022 (23) were downloaded from Genome Sequence Archive (accession code HRA000901) (https://ngdc.cncb.ac.cn/gsa-human/browse/HRA000901), processed with Cell Ranger count, and then aggregated together with Cell Ranger aggr with default parameters (v6.1.2). The human GRCh38 reference genome and gene feature files from GENCODE (version 32) were used. Merged data were then analyzed in the R statistical environment with Seurat (version 4). Data were filtered as follows: cells expressing >200 genes and genes expressed in >3 cells were included. Counts were normalized in Seurat with the LogNormalize method, and normalized counts were used to perform principal component analysis with the 2,000 most variable genes. The first 10 principal components were used to perform UMAP. Clusters were identified with the FindClusters function in Seurat, using a resolution parameter of 0.5. Six clusters that expressed *PDGFRA* were identified and further analyzed after removal of 1 cluster that also expressed T cell markers. Expression of key selected genes was visualized using the Dot Plot function. Frequencies of each cluster were quantified as a proportion of the total detected cells. The *PDGFRA*-expressing clusters were then reclustered with the algorithm Harmony to remove batch effect and decrease patient-patient variability. The first 16 principal components were used for UMAP generation. Cluster frequency was then quantified as a proportion of the total *PDGFRA*^+^ space. To identify differentially expressed transcripts between healthy and lymphedema patients for each cluster, pseudobulk analysis was performed. Counts were aggregated for each gene across biological replicates and transformed from a genes-by-cells matrix to a genes-by–data set matrix. Differential expression analysis was then performed with DESeq2. Tissue type, i.e., lymphedema or healthy SVF, was used to design contrasts with DESeq2. Results were visualized as volcano plots for each cluster with fold-change cutoff of 0.5 and *P*-adjusted value cutoff of 1 × 10^–3^.

### Immunofluorescence staining.

Tissue sections were deparaffinized, rehydrated, and blocked with 10% goat serum, 1% bovine serum albumin (BSA), and 0.3% Triton (MilliporeSigma) in PBS for 1 hour and incubated with primary antibodies at 4°C overnight. The sections were probed with antibodies for APC-PDGFRα (1:100), FITC-CD4 (1:50), Ki67 (1:200), p-SMAD2/3 (1:200), and PPARγ (1:200) (Supplemental Table 1). After thorough washing, the sections were incubated for 1 hour in the dark using the following secondary antibodies: Alexa Fluor Plus 488 goat anti-rabbit IgG (1:1,000) for Ki67 and p-SMAD2/3 or Alexa Fluor 594 goat anti-rabbit IgG (1:1,000) for PPARγ. ProLong Diamond Antifade Mountant with DAPI (P36971, Invitrogen) was used to stain the nuclei and mount the samples. Sections incubated with conjugated primary antibodies were mounted without secondary antibody incubation. Fluorescent images were taken by Olympus FluoView confocal microscope at original magnification, 20×. Quantification of immunofluorescence staining was performed by using ImageJ software. Each staining image was divided into 25 small squares (5 by 5). In total 10 squares, 2 squares from each row, were randomly selected, and positive stained cells in the fibroadipose layer were counted.

### Flow cytometry.

Flow cytometry was performed using single-cell suspensions obtained from skin and subcutaneous tissue. Tissue in 1 cm × 1 cm sections harvested from the mouse hind limb was minced with scissors and digested by using collagenase type I (1 mg/mL) and dispase (2 mg/mL) (Thermo Fisher Scientific) at 37°C on a shaker. All suspensions were filtered through 100 μm filters. Cells were then stained with following antibodies: PDGFRα (1:100) and HCS LipidTOX red neutral lipid stains (1:200) (Supplemental Table 1). After incubation on ice for 20 minutes, cells were washed and stained with Alexa Fluor Plus 488 goat anti-rabbit IgG (1:1,000). Flow cytometry was performed on the CytoFLEX FCM using CytExpert software (Beckman Coulter), and data were analyzed with FlowJo software (Tree Star). Gating strategy is based on unstained and single stained cells.

### Cell culture and rosiglitazone treatment.

Mouse AdMSCs were isolated from adult male C57BL/6J mice’s inguinal fat pads as previously described (28). Briefly, the inguinal fat pads from 8- to 10-week-old male C57BL/6 mice were harvested, washed with PBS, and minced, followed by digestion in 10 mL of type I collagenase (1 mg/mL in 1% BSA/PBS) for 30 minutes at 37°C. The digested fat pads were filtered through a 40 μm cell strainer and centrifuged at 500*g* for 5 minutes at room temperature. The supernatant containing adipocytes and debris was discarded. Pelleted cells were resuspended and washed with PBS 2 times and used as AdMSCs. AdMSCs were cultured in DMEM/F12 (MilliporeSigma) supplemented with 10% fetal bovine serum (MilliporeSigma) and antibiotics (penicillin and streptomycin, Hyclone). AdMSCs were maintained at 37°C under a 5% CO_2_ atmosphere. For the rosiglitazone treatment experiment, AdMSCs were treated with/without recombinant TGF-β1 (10 ng/mL) (Invitrogen) and rosiglitazone (10 μM) for 7 days after seeding (10 × 10^4^ cells/mL) in 6-well plates.

### Real-time PCR analysis.

Total RNA was isolated from AdMSCs using TRI reagent and RNA extraction kit (Zymo Research). First-strand cDNA was synthesized by using iScript RT-PCR mix (Bio-Rad) according to the user manual. SYBR green qPCR Master Mix (Bio-Rad), primers, and cDNA were mixed in a final reaction volume of 10 μL. Real-time PCRs were performed by using Applied Biosystems 7300 Fast Real-Time PCR System according to the manufacturer’s instructions. The oligonucleotide primers purchased from Integrated DNA Technology are listed in Supplemental Table 2.

### Statistics.

Data are expressed as means ± SD. Error bars in the charts represent SD. Statistically significant differences between 2 groups were established using 2-tailed Student’s *t* test. Statistical significance among 3 or more groups was established using 1-way ANOVA and Tukey’s post hoc tests. Mann-Whitney nonparametric *t* tests were used in the comparison of proportion of PDGFRA^+^ cells acquired from scRNA-Seq data. Significance was set at *P* < 0.05. Data were analyzed and graphically presented using Prism (V9, GraphPad Software).

### Study approval.

The experiments were carried out according to the protocol approved by the Institutional Animal Care and Use Committee at BWH (protocol number 2020N000036).

### Data availability.

All the numbers for all data points displayed for each figure subpanel are available in the Supporting Data Values file. Other data are available upon reasonable request made to the corresponding author.

## Author contributions

ZC, IS, VR, and SA designed the study. ZC, SAAG, MW, JM, OB, DM, RK, and SD conducted experiments. ZC, SAAG, MW, JM, and As acquired and analyzed data. MGA, PJB, TPP, UVA, BM, AKG, CRK, DPO, VR, and SA provided reagents. ZC, RK, and SA wrote the manuscript, which was reviewed and edited by UVA, BM, AKG, CRK, DPO, IS, VR, AS, and SA.

## Supplementary Material

Supplemental data

Supplemental data set 1

Supplemental data set 2

Supplemental data set 3

Supplemental data set 4

Supplemental data set 5

Supplemental data set 6

Supplemental data set 7

Supplemental data set 8

Supplemental data set 9

Supplemental table 10

Supplemental table 11

Supporting data values

## Version 1 12/22/2023

Electronic publication
