# Supplementary figures and images for: PPARγ agonist treatment reduces fibroadipose tissue in secondary lymphedema by exhausting fibroadipogenic PDGFRα+ mesenchymal cells
Source: JCI Insight. 2023 Dec 22;8(24):e165324. doi: 10.1172/jci.insight.165324 (PMC10807713; doi:10.1172/jci.insight.165324)

**Fig S1**

**A**

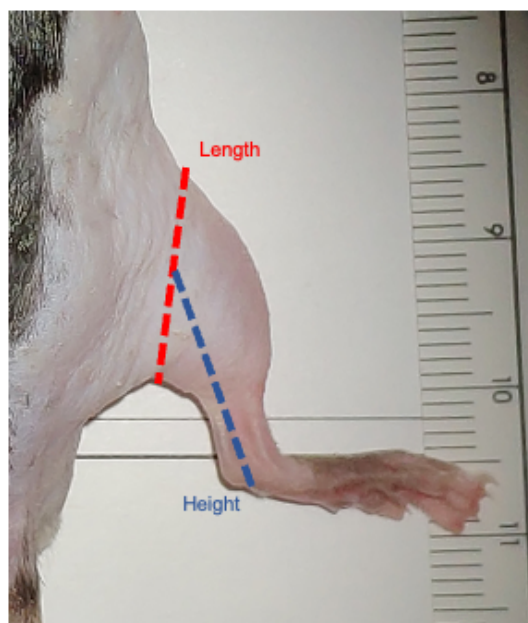

**B**

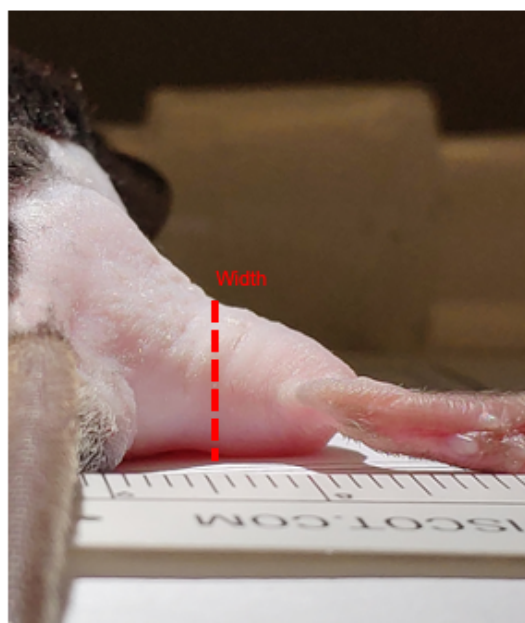

Fig S2

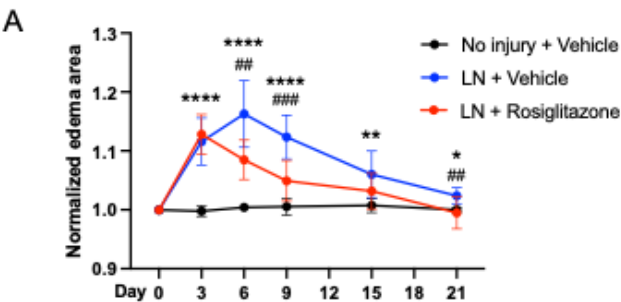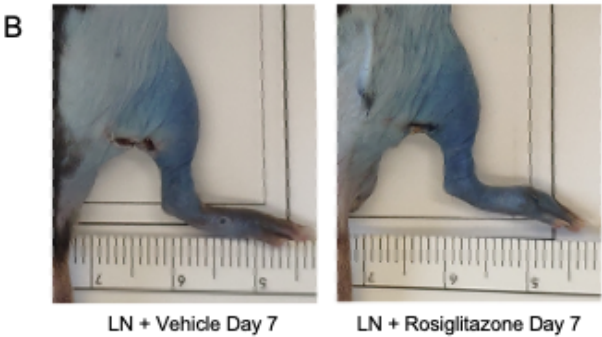

**Fig S3**

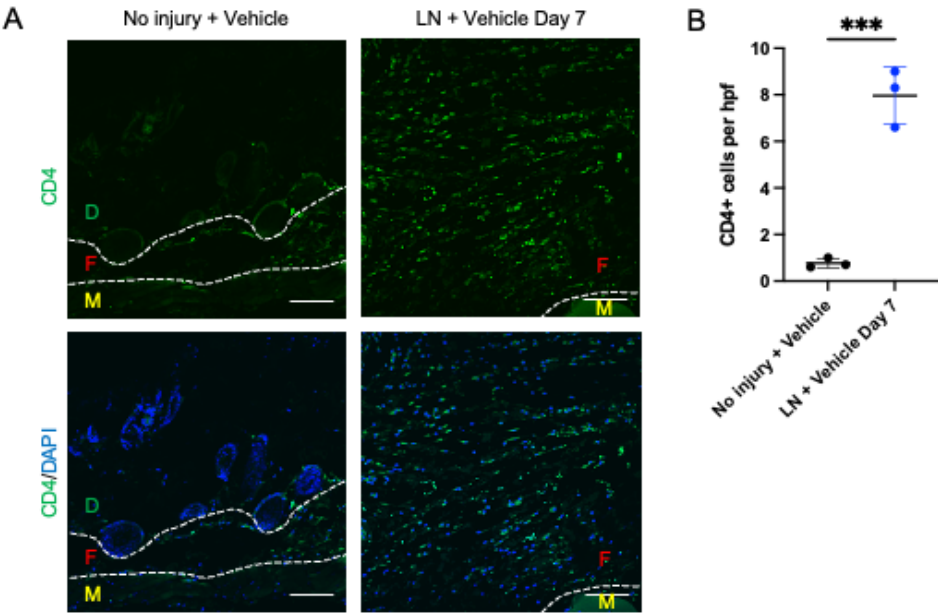

Fig S4

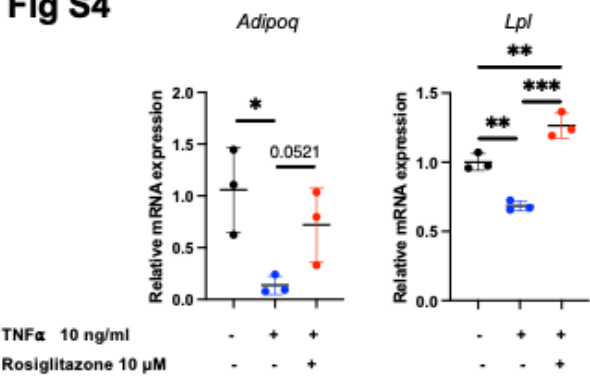

**Fig S5**

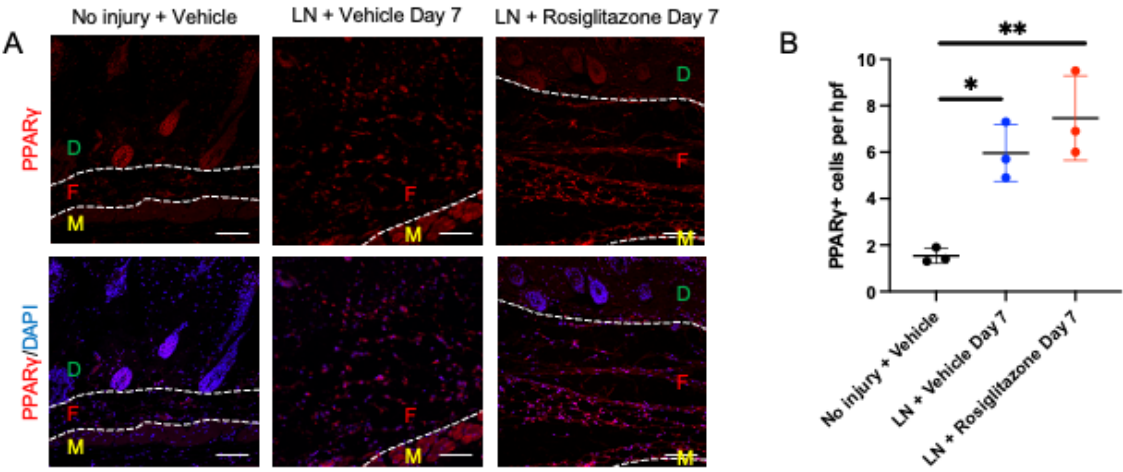

Fig S6

A

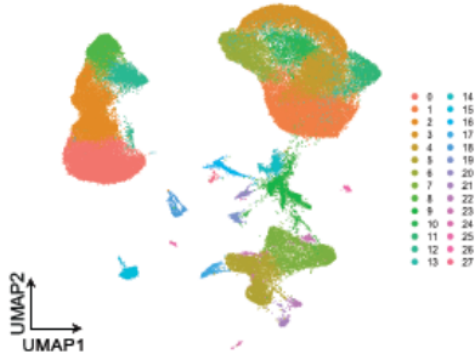

B

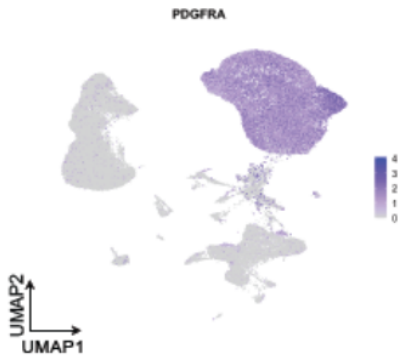

C

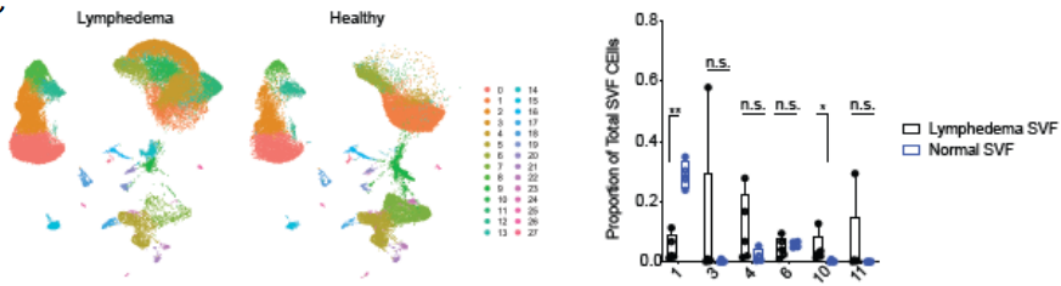

D

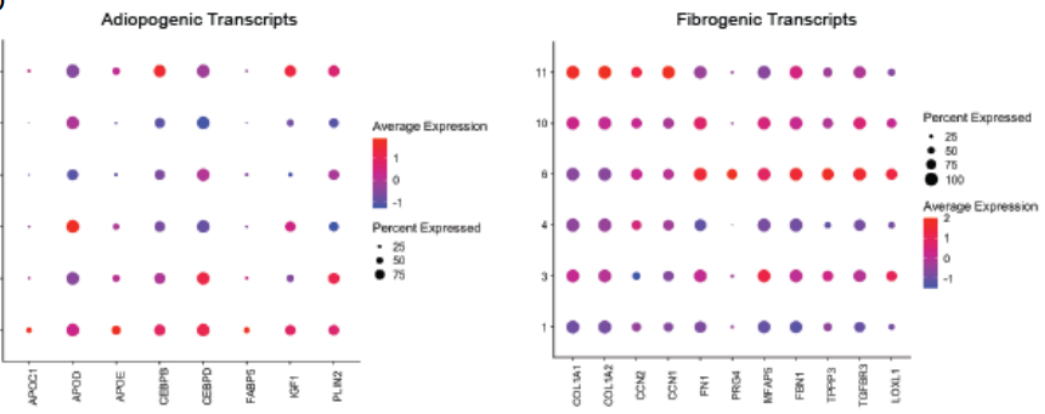

Supplement: Supplemental data [file jciinsight-8-165324-s182.pdf]
