# Supplementary material for: PPARγ agonist treatment reduces fibroadipose tissue in secondary lymphedema by exhausting fibroadipogenic PDGFRα+ mesenchymal cells
Source: JCI Insight. 2023 Dec 22;8(24):e165324. doi: 10.1172/jci.insight.165324 (PMC10807713; doi:10.1172/jci.insight.165324)
Supplement: Supplemental data set 9 [file jciinsight-8-165324-s191.pdf]

"","p_val","avg_log2FC","pct.1","pct.2","p_val_adj","cluster","gene"
"FABP4",0,1.55254092018778,0.603,0.311,0,"0","FABP4"
"APOE",0,1.33936935083613,0.743,0.45,0,"0","APOE"
"SLC5A3",0,1.03452044271795,0.67,0.363,0,"0","SLC5A3"
"MRPS6",0,0.900112930172584,0.82,0.638,0,"0","MRPS6"
"C7",0,0.891789590708529,0.317,0.132,0,"0","C7"
"CD36",0,0.862049937677205,0.347,0.127,0,"0","CD36"
"FOSB",0,0.784018177773044,0.997,0.943,0,"0","FOSB"
"IGF1",0,0.776514446520001,0.88,0.623,0,"0","IGF1"
"COL4A1",0,0.770276613058331,0.843,0.6,0,"0","COL4A1"
"RASD1",0,0.737448026893231,0.828,0.65,0,"0","RASD1"
"FABP5",0,0.717140432686359,0.462,0.214,0,"0","FABP5"
"IRF1",0,0.691505637194444,0.88,0.685,0,"0","IRF1"
"IGFBP3",0,0.688201301242345,0.764,0.528,0,"0","IGFBP3"
"COL15A1",0,0.661972440921955,0.578,0.255,0,"0","COL15A1"
"COL4A2",0,0.646002620915887,0.832,0.587,0,"0","COL4A2"
"RND3",0,0.643078476491037,0.822,0.643,0,"0","RND3"
"CXCL14",0,0.63298024467721,0.922,0.796,0,"0","CXCL14"
"JUN",0,0.632440515826816,1,0.988,0,"0","JUN"
"APOC1",0,0.575501717856178,0.399,0.179,0,"0","APOC1"
"CDKN1A",0,0.572106093284514,0.838,0.657,0,"0","CDKN1A"
"FOS",0,0.571446350989516,1,0.981,0,"0","FOS"
"ENPP2",0,0.569176822690537,0.563,0.313,0,"0","ENPP2"
"JUNB",0,0.568244970028835,0.999,0.956,0,"0","JUNB"
"ATF3",0,0.559786970491563,0.909,0.733,0,"0","ATF3"
"FST",0,0.553899514473906,0.749,0.509,0,"0","FST"
"ZFP36",0,0.553315285059833,1,0.972,0,"0","ZFP36"
"EGR1",0,0.550970924648254,0.991,0.918,0,"0","EGR1"
"SRPX",0,0.537924200085008,0.951,0.833,0,"0","SRPX"
"MGP",0,0.531869793269502,1,0.998,0,"0","MGP"
"APOD",0,0.525788938560806,0.996,0.965,0,"0","APOD"
"VIM",0,0.525143579417085,1,1,0,"0","VIM"
"SOCS3",0,0.522076548223873,0.984,0.871,0,"0","SOCS3"
"CYP26B1",0,0.521634119097152,0.503,0.261,0,"0","CYP26B1"
"BTG1",0,0.511964915402062,0.978,0.913,0,"0","BTG1"
"HSD3B7",0,-0.501980588291409,0.252,0.473,0,"0","HSD3B7"
"EMILIN2",0,-0.50582012358446,0.366,0.611,0,"0","EMILIN2"
"CST3",0,-0.506138743365382,0.999,1,0,"0","CST3"
"RAB32",0,-0.512610428117082,0.534,0.699,0,"0","RAB32"
"TRIOBP",0,-0.5198198496738,0.656,0.789,0,"0","TRIOBP"
"SMIM14",0,-0.532122964068382,0.703,0.827,0,"0","SMIM14"
"LINC02802",0,-0.539325767665827,0.538,0.745,0,"0","LINC02802"
"DPP4",0,-0.542489780756313,0.167,0.449,0,"0","DPP4"
"TMSB4X",0,-0.55301158497929,1,1,0,"0","TMSB4X"
"SMURF2",0,-0.560158973633768,0.326,0.54,0,"0","SMURF2"
"NTM",0,-0.573059337768875,0.253,0.513,0,"0","NTM"
"PRSS23",0,-0.592696989519188,0.508,0.706,0,"0","PRSS23"
"CPE",0,-0.597550569030084,0.909,0.956,0,"0","CPE"
"CTSH",0,-0.609991779204511,0.362,0.597,0,"0","CTSH"
"VSIR",0,-0.621227814330864,0.537,0.737,0,"0","VSIR"

"LRRC17",0,-0.647579702885451,0.209,0.488,0,"0","LRRC17"
"SMOC2",0,-0.647649011092731,0.599,0.795,0,"0","SMOC2"
"TRAC",0,-0.649001967282582,0.099,0.34,0,"0","TRAC"
"TRIO",0,-0.6557460993373,0.409,0.641,0,"0","TRIO"
"DBN1",0,-0.668220182027324,0.539,0.754,0,"0","DBN1"
"EMP3",0,-0.670655534016969,0.888,0.935,0,"0","EMP3"
"ACTB",0,-0.689244476018388,0.993,0.997,0,"0","ACTB"
"C1QTNF3",0,-0.693541531901558,0.053,0.293,0,"0","C1QTNF3"
"COL14A1",0,-0.697553198897193,0.69,0.858,0,"0","COL14A1"
"TPM1",0,-0.70262124266977,0.629,0.784,0,"0","TPM1"
"TNXB",0,-0.709089382995034,0.979,0.993,0,"0","TNXB"
"F10",0,-0.711267353644665,0.711,0.863,0,"0","F10"
"TIMP1",0,-0.712945212896418,0.973,0.985,0,"0","TIMP1"
"COL1A1",0,-0.725124912933382,0.994,0.997,0,"0","COL1A1"
"SH3BGRL3",0,-0.725810716015866,0.881,0.929,0,"0","SH3BGRL3"
"PTGIS",0,-0.729713587049931,0.488,0.744,0,"0","PTGIS"
"METRNL",0,-0.730344540378786,0.703,0.853,0,"0","METRNL"
"PPIC",0,-0.739717689806623,0.77,0.89,0,"0","PPIC"
"CLU",0,-0.740348425265318,0.614,0.815,0,"0","CLU"
"LINC01133",0,-0.765124333295063,0.323,0.599,0,"0","LINC01133"
"IGFBP6",0,-0.7947178427415,0.963,0.985,0,"0","IGFBP6"
"TPPP3",0,-0.804794270106173,0.523,0.725,0,"0","TPPP3"
"LTBP4",0,-0.813217429209035,0.798,0.923,0,"0","LTBP4"
"CREB5",0,-0.822395283780439,0.319,0.581,0,"0","CREB5"
"CD248",0,-0.924157863807644,0.849,0.932,0,"0","CD248"
"SCARA5",0,-0.941169060051136,0.85,0.958,0,"0","SCARA5"
"LOXL1",0,-0.944990486453664,0.371,0.649,0,"0","LOXL1"
"FSTL1",0,-1.01751910470333,0.988,0.995,0,"0","FSTL1"
"MARCKS",0,-1.02508356928237,0.902,0.972,0,"0","MARCKS"
"CLEC3B",0,-1.05094892753494,0.759,0.893,0,"0","CLEC3B"
"FN1",0,-1.05433358681314,0.842,0.946,0,"0","FN1"
"PROCR",0,-1.11412100377942,0.468,0.76,0,"0","PROCR"
"ADAMTS5",0,-1.12241575678114,0.65,0.786,0,"0","ADAMTS5"
"ACKR3",0,-1.15142099215685,0.664,0.837,0,"0","ACKR3"
"C17orf58",0,-1.23063307905173,0.519,0.727,0,"0","C17orf58"
"SFRP4",0,-1.38693102249602,0.224,0.542,0,"0","SFRP4"
"PI16",0,-1.40933734795426,0.271,0.6,0,"0","PI16"
"SEMA3C",0,-1.47827060472743,0.488,0.754,0,"0","SEMA3C"
"IGFBP5",0,-1.51235579472579,0.911,0.922,0,"0","IGFBP5"
"CD55",0,-1.55140055950407,0.425,0.698,0,"0","CD55"
"MFAP5",0,-1.60412417811409,0.96,0.99,0,"0","MFAP5"
"FBN1",0,-1.71707137764677,0.948,0.984,0,"0","FBN1"
"HLA-
B",6.42228261759175e-308,-0.562542847824177,0.959,0.979,1.697537741481
85e-303,"0","HLA-B"
"SPARC",5.13449343960043e-302,-0.567346513007204,0.968,0.985,1.3571493
0595519e-297,"0","SPARC"
"TGFBR3",3.06532604695511e-288,-0.532224585710978,0.828,0.885,8.102269
80731174e-284,"0","TGFBR3"
"FNDC1",2.04808348390046e-285,-0.619528044873964,0.257,0.465,5.4134942

646457e-281,"0","FNDC1"
"TNFAIP2",8.49282055286269e-280,-0.520803325842434,0.652,0.793,2.24482232853267e-275,"0","TNFAIP2"
"COL5A1",5.80051918575028e-278,-0.510514174000719,0.643,0.783,1.53319323117751e-273,"0","COL5A1"
"AADAC",5.91134668697669e-277,0.556144108526286,0.659,0.455,1.56248715630168e-272,"0","AADAC"
"FMO2",7.89408513464801e-277,0.52447119465658,0.296,0.126,2.08656458279016e-272,"0","FMO2"
"VASN",2.8655441112892e-275,-0.503296632286375,0.425,0.613,7.57420619495962e-271,"0","VASN"
"ABLIM1",3.06966477254607e-273,-0.50456874798832,0.629,0.773,8.11373792679378e-269,"0","ABLIM1"
"PRG4",5.48875571719598e-256,-1.54069688925229,0.097,0.281,1.45078791116924e-251,"0","PRG4"
"BTG2",2.65959356688003e-251,0.536004172893661,0.837,0.68,7.0298377159773e-247,"0","BTG2"
"PDGFD",3.9024745317695e-250,-0.500922894403794,0.471,0.647,1.03150206823731e-245,"0","PDGFD"
"PPP1R14B",9.46050242597591e-227,-0.53339347008201,0.711,0.788,2.50060000123395e-222,"0","PPP1R14B"
"IFI27",1.85615809677413e-221,-0.636300758147685,0.723,0.822,4.90619708139338e-217,"0","IFI27"
"YWHAH",1.63090636460458e-216,-0.519709836222234,0.5,0.644,4.31081170292283e-212,"0","YWHAH"
"PCOLCE2",3.57610823288233e-211,-0.6720248674365,0.875,0.895,9.45236928115458e-207,"0","PCOLCE2"
"COL3A1",9.44974622407287e-168,-0.537688872625463,0.993,0.996,2.49775692194694e-163,"0","COL3A1"
"HTRA3",6.25037900191707e-150,-0.617163933685175,0.679,0.756,1.65210017778672e-145,"0","HTRA3"
"CFB",2.0212308774023e-149,-0.566367224168704,0.353,0.502,5.34251745514977e-145,"0","CFB"
"DEPP1",1.01458346970211e-122,0.576043147454368,0.658,0.54,2.68174702711662e-118,"0","DEPP1"
"CCL2",7.0859310408753e-100,0.503787448384668,0.466,0.346,1.87295329272416e-95,"0","CCL2"
"PTX3",5.6432996567375e-62,-0.571346661943588,0.182,0.273,1.49163696526886e-57,"0","PTX3"
"PRG4.1",0,2.35145563394945,0.629,0.153,0,"1","PRG4"
"IGFBP5.1",0,2.28849415872127,0.999,0.901,0,"1","IGFBP5"
"CD55.1",0,2.07801194744984,0.988,0.559,0,"1","CD55"
"C17orf58.1",0,1.79812607580981,0.971,0.616,0,"1","C17orf58"
"FBN1.1",0,1.78536901686633,1,0.971,0,"1","FBN1"
"SEMA3C.1",0,1.64213407722386,0.985,0.631,0,"1","SEMA3C"
"ADAMTS5.1",0,1.60382098525806,0.987,0.704,0,"1","ADAMTS5"
"MFAP5.1",0,1.56327937836918,1,0.979,0,"1","MFAP5"
"LOXL1.1",0,1.50490307578838,0.931,0.511,0,"1","LOXL1"
"C1QTNF3.1",0,1.46703291190709,0.715,0.133,0,"1","C1QTNF3"
"PCOLCE2.1",0,1.44408645856992,0.998,0.866,0,"1","PCOLCE2"

"CREB5.1",0,1.4386502828827,0.931,0.432,0,"1","CREB5"
"ACKR3.1",0,1.43586066633172,0.985,0.757,0,"1","ACKR3"
"HTRA3.1",0,1.37287546620672,0.946,0.692,0,"1","HTRA3"
"PROCR.1",0,1.33455836880505,0.975,0.634,0,"1","PROCR"
"CLEC3B.1",0,1.32918288215727,0.988,0.836,0,"1","CLEC3B"
"TRAC.1",0,1.28980356761281,0.755,0.181,0,"1","TRAC"
"LINC01133.1",0,1.26827912474934,0.922,0.452,0,"1","LINC01133"
"CD248.1",0,1.21020562089941,0.996,0.896,0,"1","CD248"
"IGFBP6.1",0,1.15781125486837,0.999,0.975,0,"1","IGFBP6"
"FSTL1.1",0,1.1393966611391,1,0.992,0,"1","FSTL1"
"EMP3.1",0,1.13331491112674,0.982,0.912,0,"1","EMP3"
"TPPP3.1",0,1.13077471407358,0.923,0.627,0,"1","TPPP3"
"TPM1.1",0,1.10716579998922,0.96,0.703,0,"1","TPM1"
"PI16.1",0,1.08311239414349,0.761,0.477,0,"1","PI16"
"PPP1R14B.1",0,1.04505825519473,0.954,0.729,0,"1","PPP1R14B"
"TMSB4X.1",0,1.04214781139041,1,1,0,"1","TMSB4X"
"FNDC1.1",0,1.04105106133005,0.8,0.334,0,"1","FNDC1"
"SMURF2.1",0,1.03258996015678,0.838,0.415,0,"1","SMURF2"
"SH3BGRL3.1",0,1.02279890665551,0.984,0.904,0,"1","SH3BGRL3"
"UAP1",0,0.988088678404726,0.899,0.617,0,"1","UAP1"
"METRNL.1",0,0.980403184202494,0.978,0.785,0,"1","METRNL"
"HSD3B7.1",0,0.975711409149465,0.807,0.339,0,"1","HSD3B7"
"SCARA5.1",0,0.9728507115356,0.998,0.92,0,"1","SCARA5"
"DBN1.1",0,0.96244247407637,0.954,0.652,0,"1","DBN1"
"GFPT2",0,0.953795719924037,0.877,0.502,0,"1","GFPT2"
"ACTB.1",0,0.952461696962804,1,0.996,0,"1","ACTB"
"ADI1",0,0.94936405248724,0.917,0.632,0,"1","ADI1"
"FN1.1",0,0.948278364553513,0.998,0.907,0,"1","FN1"
"GAS7",0,0.937562945139762,0.922,0.65,0,"1","GAS7"
"PPIC.1",0,0.928333912707716,0.971,0.839,0,"1","PPIC"
"RAB32.1",0,0.925492597184427,0.915,0.606,0,"1","RAB32"
"YWHAH.1",0,0.92163242115368,0.827,0.564,0,"1","YWHAH"
"TRIO.1",0,0.920122905239173,0.875,0.526,0,"1","TRIO"
"LRRC17.1",0,0.916213760568327,0.784,0.347,0,"1","LRRC17"
"SDC2",0,0.915886826895824,0.979,0.859,0,"1","SDC2"
"HAS1",0,0.913557563763204,0.363,0.086,0,"1","HAS1"
"EFHD1",0,0.895419383985065,0.743,0.254,0,"1","EFHD1"
"IFI27.1",0,0.893890773761524,0.925,0.772,0,"1","IFI27"
"KCNAB1",0,0.889860715365419,0.539,0.071,0,"1","KCNAB1"
"RHOB",0,0.885111355250559,0.987,0.897,0,"1","RHOB"
"CADM3",0,0.876156006995543,0.577,0.178,0,"1","CADM3"
"KLF2",0,0.86790075334393,0.976,0.848,0,"1","KLF2"
"TRIOBP.1",0,0.862556513336076,0.952,0.717,0,"1","TRIOBP"
"NTM.1",0,0.84770328499328,0.829,0.373,0,"1","NTM"
"MARCKS.1",0,0.847487265397717,0.998,0.947,0,"1","MARCKS"
"RHOA",0,0.819456810790567,0.993,0.942,0,"1","RHOA"
"RAP1B",0,0.816189282695716,0.979,0.869,0,"1","RAP1B"
"OLFML2A",0,0.815853352445973,0.726,0.261,0,"1","OLFML2A"
"SMIM14.1",0,0.813299144887379,0.953,0.765,0,"1","SMIM14"
"DPP4.1",0,0.809476651345145,0.788,0.297,0,"1","DPP4"

"TGFBR3.1",0,0.801721021544739,0.98,0.849,0,"1","TGFBR3"
"VSIR.1",0,0.799371640063353,0.904,0.646,0,"1","VSIR"
"ALDH1A3",0,0.798660251089918,0.654,0.263,0,"1","ALDH1A3"
"CAMK2N1",0,0.79148161627783,0.828,0.522,0,"1","CAMK2N1"
"TNXB.1",0,0.783131897505428,0.998,0.988,0,"1","TNXB"
"MYL9",0,0.779111368733103,0.943,0.832,0,"1","MYL9"
"TAGLN2",0,0.765020379112166,0.972,0.836,0,"1","TAGLN2"
"GLIPR2",0,0.760569479839712,0.847,0.493,0,"1","GLIPR2"
"C12orf75",0,0.750946982519628,0.682,0.231,0,"1","C12orf75"
"PTGIS.1",0,0.744705679898611,0.929,0.635,0,"1","PTGIS"
"ABLIM1.1",0,0.740663617666155,0.933,0.698,0,"1","ABLIM1"
"DEFB1",0,0.739892645393303,0.419,0.075,0,"1","DEFB1"
"GUCY1A2",0,0.739417064620435,0.508,0.052,0,"1","GUCY1A2"
"PMEPA1",0,0.739067586440298,0.535,0.134,0,"1","PMEPA1"
"LTBP4.1",0,0.736695342349091,0.992,0.874,0,"1","LTBP4"
"DLGAP4",0,0.734857604493142,0.851,0.508,0,"1","DLGAP4"
"SSR3",0,0.727897535384812,0.946,0.762,0,"1","SSR3"
"TPM4",0,0.723516732531876,0.902,0.695,0,"1","TPM4"
"VASN.1",0,0.720665413404271,0.799,0.521,0,"1","VASN"
"CYP4B1",0,0.720581563815391,0.649,0.318,0,"1","CYP4B1"
"EMILIN2.1",0,0.718603642043855,0.845,0.493,0,"1","EMILIN2"
"FABP3",0,0.713016843254615,0.405,0.067,0,"1","FABP3"
"CRIP1",0,0.707671641725493,0.983,0.954,0,"1","CRIP1"
"PAMR1",0,0.705263792798376,0.734,0.296,0,"1","PAMR1"
"ZNF385A",0,0.702475017588362,0.665,0.286,0,"1","ZNF385A"
"F10.1",0,0.700169157597481,0.938,0.805,0,"1","F10"
"ARL4C",0,0.697193880083701,0.635,0.231,0,"1","ARL4C"
"JAG1",0,0.682888650168783,0.685,0.387,0,"1","JAG1"
"ARL4D",0,0.681824840233389,0.689,0.314,0,"1","ARL4D"
"UGDH",0,0.675896866430569,0.812,0.547,0,"1","UGDH"
"AXL",0,0.675091555752594,0.927,0.735,0,"1","AXL"
"ACTG1",0,0.672029511510353,1,0.996,0,"1","ACTG1"
"MYL6",0,0.658305354428506,0.996,0.991,0,"1","MYL6"
"UGP2",0,0.65775717772842,0.896,0.775,0,"1","UGP2"
"DACT2",0,0.657260682640392,0.464,0.027,0,"1","DACT2"
"S100A11",0,0.651013795248555,0.997,0.988,0,"1","S100A11"
"CMTM3",0,0.649913467264525,0.876,0.617,0,"1","CMTM3"
"S100A10",0,0.647741645476215,1,0.999,0,"1","S100A10"
"PLAC9",0,0.646582134955291,1,0.994,0,"1","PLAC9"
"CITED4",0,0.645339487195722,0.554,0.213,0,"1","CITED4"
"DST",0,0.642795274640282,0.939,0.795,0,"1","DST"
"VCAN",0,0.640864590792592,0.991,0.921,0,"1","VCAN"
"MYL12A",0,0.63987902222554,0.972,0.924,0,"1","MYL12A"
"TBC1D12",0,0.635562734032933,0.7,0.308,0,"1","TBC1D12"
"ADGRD1",0,0.63275075108443,0.742,0.369,0,"1","ADGRD1"
"MEDAG",0,0.62715530834115,0.961,0.789,0,"1","MEDAG"
"CTSH.1",0,0.625772826625999,0.751,0.499,0,"1","CTSH"
"AIF1L",0,0.619415393654371,0.543,0.121,0,"1","AIF1L"
"AHNAK",0,0.618671859665136,0.999,0.993,0,"1","AHNAK"
"GAPDH",0,0.616851164633312,0.994,0.969,0,"1","GAPDH"

"APBB1IP",0,0.611824644321848,0.775,0.43,0,"1","APBB1IP"
"EDIL3",0,0.607141154912884,0.448,0.095,0,"1","EDIL3"
"CD70",0,0.605043557917705,0.503,0.087,0,"1","CD70"
"IER2",0,0.599486591605062,0.927,0.809,0,"1","IER2"
"PRSS23.1",0,0.597643004027945,0.855,0.62,0,"1","PRSS23"
"HAS2",0,0.597427966466143,0.474,0.244,0,"1","HAS2"
"S100A16",0,0.593314478887758,0.871,0.672,0,"1","S100A16"
"ITGA11",0,0.588507441145,0.8,0.453,0,"1","ITGA11"
"S100A6",0,0.581211227113528,1,0.999,0,"1","S100A6"
"FAM107B",0,0.575856823462483,0.632,0.278,0,"1","FAM107B"
"CAPZB",0,0.57461770159234,0.924,0.749,0,"1","CAPZB"
"S100A4",0,0.572111146283291,1,0.998,0,"1","S100A4"
"LTBP1",0,0.57092132864483,0.568,0.199,0,"1","LTBP1"
"ANXA2",0,0.566043678631916,0.999,0.996,0,"1","ANXA2"
"GPX4",0,0.562948687509113,0.997,0.99,0,"1","GPX4"
"SERF2",0,0.560068799124211,0.998,0.994,0,"1","SERF2"
"DBNDD2",0,0.558719932666371,0.722,0.43,0,"1","DBNDD2"
"GYPC",0,0.555507172590129,0.972,0.892,0,"1","GYPC"
"ZYX",0,0.553794329792108,0.822,0.53,0,"1","ZYX"
"RRAS2",0,0.553593323464366,0.612,0.282,0,"1","RRAS2"
"ADAMTSL4",0,0.553287033902185,0.845,0.568,0,"1","ADAMTSL4"
"CYTOR",0,0.552265360494323,0.816,0.576,0,"1","CYTOR"
"ACE",0,0.548594286927403,0.697,0.351,0,"1","ACE"
"WNT2",0,0.547247052881528,0.415,0.066,0,"1","WNT2"
"SPARC.1",0,0.547176001704462,0.997,0.977,0,"1","SPARC"
"GUK1",0,0.543477452384494,0.98,0.924,0,"1","GUK1"
"VEGFA",0,0.541380186922486,0.611,0.305,0,"1","VEGFA"
"KPNA2",0,0.539270186544265,0.517,0.186,0,"1","KPNA2"
"CD151",0,0.538247303770964,0.937,0.808,0,"1","CD151"
"SDCBP",0,0.529438126163582,0.982,0.94,0,"1","SDCBP"
"OSR2",0,0.526558455297547,0.846,0.639,0,"1","OSR2"
"CST3.1",0,0.52519058540632,1,0.999,0,"1","CST3"
"HEG1",0,0.524911621024004,0.701,0.423,0,"1","HEG1"
"PLXDC1",0,0.524175367816794,0.66,0.318,0,"1","PLXDC1"
"LINC02802.1",0,0.523737801472553,0.886,0.658,0,"1","LINC02802"
"GADD45G",0,0.52352427017008,0.363,0.108,0,"1","GADD45G"
"SPRYD3",0,0.517195215469401,0.624,0.337,0,"1","SPRYD3"
"NEDD9",0,0.517053233197354,0.462,0.101,0,"1","NEDD9"
"PXN",0,0.513724698503413,0.786,0.508,0,"1","PXN"
"ACSL3",0,0.510495998902192,0.677,0.375,0,"1","ACSL3"
"STXBP6",0,0.508370874519903,0.573,0.238,0,"1","STXBP6"
"RRAS",0,0.504945637230661,0.933,0.799,0,"1","RRAS"
"CLTB",0,0.503297520068824,0.891,0.733,0,"1","CLTB"
"NHSL1",0,0.503121603033246,0.625,0.299,0,"1","NHSL1"
"BIN1",0,0.502402008893659,0.898,0.696,0,"1","BIN1"
"ARHGAP29",0,0.501026307585937,0.796,0.536,0,"1","ARHGAP29"
"COX6C",0,0.500493457260143,0.974,0.919,0,"1","COX6C"
"ANXA4",0,0.50042010139601,0.919,0.788,0,"1","ANXA4"
"ARRDC3",0,-0.508774112490521,0.297,0.542,0,"1","ARRDC3"
"EMP2",0,-0.521559308278156,0.558,0.767,0,"1","EMP2"

"HEBP2",0,-0.523350603164117,0.287,0.568,0,"1","HEBP2"
"MT-ATP6",0,-0.534178109634145,1,1,0,"1","MT-ATP6"
"VIM.1",0,-0.534640011368771,1,1,0,"1","VIM"
"MT-ND4",0,-0.542757713622909,1,1,0,"1","MT-ND4"
"WNT11",0,-0.544665945437482,0.597,0.777,0,"1","WNT11"
"TCF4",0,-0.547646105730578,0.921,0.954,0,"1","TCF4"
"CD302",0,-0.550471243298548,0.584,0.776,0,"1","CD302"
"MT-ND2",0,-0.554864200423092,1,1,0,"1","MT-ND2"
"PLEKHH2",0,-0.559898797967971,0.176,0.453,0,"1","PLEKHH2"
"COLEC12",0,-0.563674033278903,0.49,0.737,0,"1","COLEC12"
"PODN",0,-0.56617755799734,0.85,0.923,0,"1","PODN"
"FIBIN",0,-0.568159577834791,0.156,0.457,0,"1","FIBIN"
"EMILIN1",0,-0.573105793732895,0.41,0.679,0,"1","EMILIN1"
"FAM13A",0,-0.573384233510545,0.334,0.615,0,"1","FAM13A"
"NID2",0,-0.573527782343568,0.228,0.523,0,"1","NID2"
"HSPB6",0,-0.582944045347994,0.371,0.637,0,"1","HSPB6"
"CP",0,-0.593030547561046,0.203,0.458,0,"1","CP"
"SAMHD1",0,-0.60439212873971,0.498,0.747,0,"1","SAMHD1"
"IGFBP4",0,-0.604820774211381,0.978,0.985,0,"1","IGFBP4"
"TSC22D1",0,-0.609981944354579,0.418,0.632,0,"1","TSC22D1"
"CAV1",0,-0.616641501988528,0.695,0.845,0,"1","CAV1"
"SLC40A1",0,-0.617235003162843,0.097,0.444,0,"1","SLC40A1"
"ANGPT1",0,-0.623469160558977,0.16,0.458,0,"1","ANGPT1"
"FGF7",0,-0.624608150067638,0.808,0.912,0,"1","FGF7"
"BGN",0,-0.624686494045573,0.149,0.392,0,"1","BGN"
"IGSF10",0,-0.64101398293426,0.051,0.382,0,"1","IGSF10"
"MT-ND1",0,-0.678184340119613,1,1,0,"1","MT-ND1"
"LHFPL6",0,-0.680556587150449,0.951,0.976,0,"1","LHFPL6"
"PTGFR",0,-0.699515696407819,0.229,0.563,0,"1","PTGFR"
"HELLPAR",0,-0.705630467572851,0.099,0.467,0,"1","HELLPAR"
"DIO3OS",0,-0.709501886192806,0.119,0.529,0,"1","DIO3OS"
"ABCA10",0,-0.711358925729679,0.291,0.603,0,"1","ABCA10"
"AEBP1",0,-0.71419206379958,0.919,0.973,0,"1","AEBP1"
"CDH11",0,-0.714691356526339,0.221,0.594,0,"1","CDH11"
"PIK3R1",0,-0.718132545118596,0.827,0.928,0,"1","PIK3R1"
"PDK4",0,-0.722452637799137,0.872,0.942,0,"1","PDK4"
"BOC",0,-0.722720971253552,0.253,0.605,0,"1","BOC"
"GDF10",0,-0.725552052910042,0.154,0.529,0,"1","GDF10"
"ABCA8",0,-0.740812139853765,0.888,0.93,0,"1","ABCA8"
"CYP26B1.1",0,-0.751143424772635,0.066,0.369,0,"1","CYP26B1"
"ABCA6",0,-0.754092198440424,0.612,0.82,0,"1","ABCA6"
"GSN",0,-0.761681177901138,1,1,0,"1","GSN"
"CCN5",0,-0.765420354220562,0.976,0.99,0,"1","CCN5"
"COL6A3",0,-0.768740462108596,0.98,0.997,0,"1","COL6A3"
"EMP1",0,-0.786876909184073,0.802,0.915,0,"1","EMP1"
"TNNT3",0,-0.790300511826399,0.448,0.678,0,"1","TNNT3"
"FBLN1",0,-0.793207830297885,0.981,0.992,0,"1","FBLN1"
"SELENOP",0,-0.802844406746827,0.986,0.994,0,"1","SELENOP"
"LUM",0,-0.809196281880361,0.982,0.996,0,"1","LUM"
"COL15A1.1",0,-0.815397574153873,0.043,0.388,0,"1","COL15A1"

"MT-ND3",0,-0.817507291266574,1,1,0,"1","MT-ND3"
"ITIH5",0,-0.855187095593383,0.568,0.832,0,"1","ITIH5"
"EPHX1",0,-0.858286877813532,0.8,0.939,0,"1","EPHX1"
"RND3.1",0,-0.886075613042452,0.499,0.723,0,"1","RND3"
"F3",0,-0.908749753662171,0.343,0.672,0,"1","F3"
"OGN",0,-0.924525437737169,0.709,0.879,0,"1","OGN"
"FGL2",0,-0.935497669537719,0.449,0.8,0,"1","FGL2"
"C3",0,-0.941765589417885,0.975,0.997,0,"1","C3"
"CYGB",0,-0.942964272089304,0.361,0.766,0,"1","CYGB"
"COL4A2.1",0,-0.946006825228537,0.342,0.707,0,"1","COL4A2"
"NTRK2",0,-0.963535812504898,0.826,0.944,0,"1","NTRK2"
"ADH1B",0,-1.00401104290328,0.762,0.94,0,"1","ADH1B"
"COL4A1.1",0,-1.02423169865374,0.4,0.71,0,"1","COL4A1"
"SOD3",0,-1.03041342932603,0.89,0.967,0,"1","SOD3"
"CTHRC1",0,-1.03567489943554,0.359,0.607,0,"1","CTHRC1"
"FST.1",0,-1.08328230745813,0.217,0.639,0,"1","FST"
"DHRS3",0,-1.11281688408572,0.321,0.747,0,"1","DHRS3"
"SRPX.1",0,-1.15934758882573,0.61,0.915,0,"1","SRPX"
"RARRES2",0,-1.16072503692028,0.459,0.83,0,"1","RARRES2"
"GGT5",0,-1.17622304309805,0.125,0.656,0,"1","GGT5"
"SLC5A3.1",0,-1.20738835692706,0.233,0.473,0,"1","SLC5A3"
"DEPP1.1",0,-1.25739297907714,0.384,0.607,0,"1","DEPP1"
"SVEP1",0,-1.25774945808289,0.671,0.859,0,"1","SVEP1"
"DPT",0,-1.27685358462266,0.646,0.91,0,"1","DPT"
"SFRP1",0,-1.29769504051193,0.332,0.684,0,"1","SFRP1"
"IGFBP3.1",0,-1.3407007547968,0.311,0.64,0,"1","IGFBP3"
"CFH",0,-1.39157995290261,0.926,0.979,0,"1","CFH"
"SFRP2",0,-1.48759478788598,0.321,0.678,0,"1","SFRP2"
"SLIT3",0,-1.4915826932997,0.551,0.924,0,"1","SLIT3"
"GAS6",0,-1.5904069129214,0.343,0.848,0,"1","GAS6"
"GPC3",0,-1.66375584012417,0.706,0.969,0,"1","GPC3"
"IGF1.1",0,-1.71944820478499,0.307,0.763,0,"1","IGF1"
"IGFBP7",0,-1.73339888451005,0.703,0.914,0,"1","IGFBP7"
"CXCL12",0,-1.89660524547241,0.881,0.991,0,"1","CXCL12"
"MGP.1",0,-1.96621256058484,0.997,0.999,0,"1","MGP"
"MYOC",0,-2.1215720878963,0.268,0.705,0,"1","MYOC"
"APOE.1",0,-2.2215111800614,0.271,0.567,0,"1","APOE"
"APOD.1",0,-2.36331587978744,0.889,0.991,0,"1","APOD"
"CXCL14.1",0,-4.09397950749997,0.534,0.889,0,"1","CXCL14"
"LAMA2",6.98812714113415e-304,-0.535585435415827,0.641,0.791,1.8471017
6594458e-299,"1","LAMA2"
"SFRP4.1",4.32541359952441e-303,0.770667573814121,0.66,0.431,1.1432933
2262629e-298,"1","SFRP4"
"VCAM1",9.94519814976346e-301,-0.524379787603358,0.041,0.252,2.6287147
7494548e-296,"1","VCAM1"
"SPARCL1",1.39003036704988e-293,-0.757696636555106,0.081,0.302,3.67412
826618625e-289,"1","SPARCL1"
"NRP1",1.43593595063764e-293,-0.570513316053639,0.337,0.55,3.795465904
72541e-289,"1","NRP1"
"MEG3",3.69296414784168e-281,0.5436912982154,0.955,0.861,9.76124283557

512e-277,"1","MEG3"
"CD9",5.06033546538031e-263,-0.540996528925293,0.298,0.542,1.337547870
20932e-258,"1","CD9"
"FOSB.1",6.85241432980479e-237,-0.639966859398264,0.969,0.951,1.811230
155654e-232,"1","FOSB"
"APOC1.1",1.30612853543598e-236,-0.542354852476612,0.074,0.261,3.45235
894486437e-232,"1","APOC1"
"SOD2",5.58738498850198e-224,-0.798812543106519,0.805,0.862,1.47685760
016084e-219,"1","SOD2"
"H19",2.78025579773397e-202,-0.899802459781135,0.483,0.628,7.348772124
57043e-198,"1","H19"
"FABP4.1",1.02595923586257e-193,-1.09932279514371,0.237,0.405,2.711815
45223196e-189,"1","FABP4"
"CCDC71L",1.43974569121922e-193,-0.637545096251842,0.432,0.603,3.80553
581103064e-189,"1","CCDC71L"
"ADM",3.74845300454845e-174,-0.625875123436739,0.593,0.722,9.907910981
62247e-170,"1","ADM"
"ADIRF",5.18099991186738e-172,-0.681303876475953,0.454,0.611,1.3694418
9670479e-167,"1","ADIRF"
"NABP1",7.02142018349742e-169,-0.528405202859368,0.358,0.517,1.8559017
8290204e-164,"1","NABP1"
"MRPS6.1",8.04026653085806e-167,-0.819289102071804,0.634,0.687,2.12520
32494364e-162,"1","MRPS6"
"PTGDS",1.43593490095764e-161,-1.37008216005264,0.235,0.392,3.79546313
021123e-157,"1","PTGDS"
"AADAC.1",5.51810682068751e-156,-0.598241784825694,0.374,0.527,1.45854
599484412e-151,"1","AADAC"
"CCL2.1",1.78593657820592e-141,-0.864127789335875,0.248,0.4,4.72058756
351389e-137,"1","CCL2"
"SLPI",1.6800575770618e-131,0.644943636924035,0.394,0.257,4.4407281876
8975e-127,"1","SLPI"
"KCNQ1OT1",1.17804551011139e-83,-0.598104080458138,0.599,0.662,3.11380
989232642e-79,"1","KCNQ1OT1"
"MT1X",1.14923890387382e-76,-0.65447644763455,0.682,0.725,3.0376682707
1927e-72,"1","MT1X"
"MT2A",5.25803242807944e-42,-0.551144466815111,0.977,0.962,1.389803131
38996e-37,"1","MT2A"
"PTX3.1",4.6311537610457e-07,-0.54186971364783,0.237,0.258,0.012241065
621196,"1","PTX3"
"CXCL14.2",0,0.885271403350854,0.96,0.794,0,"2","CXCL14"
"MYOC.1",0,0.80907652764155,0.833,0.579,0,"2","MYOC"
"SFRP2.1",0,0.792349810020124,0.81,0.569,0,"2","SFRP2"
"H19.1",0,0.786145081225515,0.792,0.56,0,"2","H19"
"DHRS3.1",0,0.782864760289589,0.913,0.615,0,"2","DHRS3"
"SFRP1.1",0,0.750042782019293,0.81,0.577,0,"2","SFRP1"
"MGP.2",0,0.749694855529322,1,0.999,0,"2","MGP"
"TNNT3.1",0,0.65297724524981,0.841,0.592,0,"2","TNNT3"
"GAS6.1",0,0.637076653773125,0.948,0.713,0,"2","GAS6"
"SVEP1.1",0,0.631170555277135,0.955,0.796,0,"2","SVEP1"
"APOD.2",0,0.580966133094499,0.998,0.966,0,"2","APOD"

"RARRES2.1",0,0.563103186373334,0.933,0.724,0,"2","RARRES2"
"DPT.1",0,0.557525889712556,0.966,0.838,0,"2","DPT"
"GPC3.1",0,0.536015996532925,0.996,0.904,0,"2","GPC3"
"FBLN1.1",0,0.529153903150591,0.999,0.988,0,"2","FBLN1"
"OGN.1",0,0.508878843018311,0.96,0.824,0,"2","OGN"
"S100A10.1",0,-0.530327703759504,1,0.999,0,"2","S100A10"
"SDC2.1",0,-0.609575153732971,0.845,0.889,0,"2","SDC2"
"PPP1R14B.2",0,-0.632147677517457,0.69,0.789,0,"2","PPP1R14B"
"TRAC.2",0,-0.65145220842018,0.089,0.331,0,"2","TRAC"
"FNDC1.2",0,-0.675507805801519,0.223,0.463,0,"2","FNDC1"
"FN1.2",0,-0.763993772464274,0.912,0.927,0,"2","FN1"
"PCOLCE2.2",0,-0.823864015850217,0.851,0.9,0,"2","PCOLCE2"
"SH3BGRL3.2",0,-0.893351479802016,0.905,0.922,0,"2","SH3BGRL3"
"ADAMTS5.2",0,-1.04784238227777,0.664,0.776,0,"2","ADAMTS5"
"CD55.2",0,-1.35506480983005,0.505,0.668,0,"2","CD55"
"IGFBP5.2",0,-1.69509206449132,0.887,0.926,0,"2","IGFBP5"
"TMSB4X.2",5.3882180306939e-302,-0.560341290545429,0.999,1,1.424213789
87301e-297,"2","TMSB4X"
"IGF1.2",2.26650235537433e-296,0.531436538278748,0.86,0.64,5.990819025
72544e-292,"2","IGF1"
"DEPP1.2",1.98879209614639e-290,0.818095383773059,0.738,0.529,5.256775
26853413e-286,"2","DEPP1"
"CD248.2",8.46178211764582e-285,-0.692059537655497,0.892,0.919,2.23661
824933614e-280,"2","CD248"
"ACTB.2",1.42074131370567e-275,-0.547173322131734,0.998,0.996,3.755303
44038683e-271,"2","ACTB"
"EMP3.2",1.71338388527493e-252,-0.638199504521707,0.918,0.927,4.528816
2855587e-248,"2","EMP3"
"CREB5.2",2.28834641800953e-247,-0.722428074853013,0.388,0.554,6.04855
725208279e-243,"2","CREB5"
"C17orf58.2",1.45900102138161e-243,-1.00528914654163,0.602,0.7,3.85643
149971586e-239,"2","C17orf58"
"PCSK5",8.50782635307631e-243,0.588573167118525,0.565,0.357,2.24878866
164513e-238,"2","PCSK5"
"RAMP2",1.68522735031289e-233,-0.552623732441757,0.753,0.832,4.4543929
3234704e-229,"2","RAMP2"
"FBN1.2",2.85890524287373e-224,-0.94923018521239,0.977,0.977,7.5566583
3796384e-220,"2","FBN1"
"CLEC3B.2",2.21196489855414e-222,-0.720290453229258,0.855,0.866,5.8466
6561985831e-218,"2","CLEC3B"
"CTHRC1.1",4.43009697517172e-222,0.547054593611066,0.725,0.525,1.17096
323247739e-217,"2","CTHRC1"
"ALDH1A1",1.24980845526045e-221,0.502024530963463,0.707,0.531,3.303493
70894442e-217,"2","ALDH1A1"
"ADM.1",3.83729242047271e-210,0.540462067742513,0.834,0.669,1.01427313
257935e-205,"2","ADM"
"TPPP3.2",5.14382218288616e-204,-0.706400566187368,0.591,0.701,1.35961
507938047e-199,"2","TPPP3"
"HTRA3.2",7.10754379462499e-198,-0.711753669567553,0.669,0.754,1.87866
597579528e-193,"2","HTRA3"

"LINC01133.2",1.92563231931514e-196,-0.581022249427639,0.425,0.564,5.0
8983134641377e-192,"2","LINC01133"
"TPM1.2",4.59788299564657e-196,-0.56725327458038,0.692,0.763,1.2153124
334093e-191,"2","TPM1"
"SEMA3C.2",2.15485595869356e-195,-0.907348190096281,0.627,0.711,5.6957
1527001882e-191,"2","SEMA3C"
"GAS7.1",5.68914230081996e-188,-0.511148092762444,0.619,0.718,1.503754
09295273e-183,"2","GAS7"
"C1QTNF3.2",6.07152012504101e-169,-0.587584776186207,0.113,0.269,1.604
82419945084e-164,"2","C1QTNF3"
"PRG4.2",5.77757590392151e-160,-1.40335235638095,0.116,0.269,1.5271288
6292453e-155,"2","PRG4"
"IGFBP6.2",3.95278604807146e-156,-0.62366346965058,0.983,0.979,1.04480
040822625e-151,"2","IGFBP6"
"LOXL1.2",4.64284860266835e-147,-0.660521513308263,0.522,0.604,1.22719
77426573e-142,"2","LOXL1"
"PROCR.2",4.60005687772944e-146,-0.646939438756136,0.659,0.706,1.21588
703392145e-141,"2","PROCR"
"CRIP1.1",5.09787513966228e-143,-0.530022748010953,0.959,0.959,1.34747
035691553e-138,"2","CRIP1"
"MFAP5.2",4.92921134855578e-114,-0.716327609990555,0.992,0.981,1.30288
914365026e-109,"2","MFAP5"
"CCN2",2.97472887685491e-88,0.566034135480247,0.783,0.696,7.8628033673
0289e-84,"2","CCN2"
"ACKR3.2",6.44608270942959e-60,-0.550960384341747,0.822,0.795,1.703828
58175643e-55,"2","ACKR3"
"FABP4.2",8.94669218723174e-19,-0.726873199142279,0.348,0.379,2.364789
67892909e-14,"2","FABP4"
"APOE.2",1.10382341574262e-07,-0.731480281530124,0.515,0.512,0.0029176
260524909,"2","APOE"
"CXCL12.1",0,0.68659800953505,0.993,0.966,0,"3","CXCL12"
"APOD.3",0,0.638333565815023,0.995,0.967,0,"3","APOD"
"GPC3.2",0,0.623230401713948,0.983,0.906,0,"3","GPC3"
"C3.1",0,0.583372377664096,0.996,0.992,0,"3","C3"
"SLIT3.1",0,0.563454789509474,0.94,0.837,0,"3","SLIT3"
"TUBB4B",0,-0.509880461432491,0.582,0.828,0,"3","TUBB4B"
"MIDN",0,-0.512257127496334,0.282,0.566,0,"3","MIDN"
"PABPC1",0,-0.530505157895921,0.802,0.95,0,"3","PABPC1"
"CSRNP1",0,-0.534647037248455,0.105,0.451,0,"3","CSRNP1"
"ABL2",0,-0.577758272010527,0.257,0.533,0,"3","ABL2"
"INTS6",0,-0.59494135082854,0.328,0.638,0,"3","INTS6"
"TMSB4X.3",0,-0.597641883139898,0.999,1,0,"3","TMSB4X"
"DNAJA1",0,-0.599106234226586,0.704,0.896,0,"3","DNAJA1"
"HLA-E",0,-0.617337360617529,0.944,0.99,0,"3","HLA-E"
"NFIL3",0,-0.619993503695236,0.312,0.634,0,"3","NFIL3"
"ABHD5",0,-0.632777513628892,0.256,0.593,0,"3","ABHD5"
"UAP1.1",0,-0.634494260043966,0.466,0.712,0,"3","UAP1"
"ZFP36L1",0,-0.636935066664368,0.933,0.982,0,"3","ZFP36L1"
"PPP1R15A",0,-0.642652840998817,0.652,0.918,0,"3","PPP1R15A"
"DDX5",0,-0.662774012554512,0.957,0.993,0,"3","DDX5"

"CEBPD",0,-0.710295024991088,0.936,0.987,0,"3","CEBPD"
"AC020916.1",0,-0.730045348898392,0.285,0.65,0,"3","AC020916.1"
"PNRC1",0,-0.752537451132682,0.929,0.985,0,"3","PNRC1"
"NAMPT",0,-0.771896969419247,0.531,0.799,0,"3","NAMPT"
"MCL1",0,-0.812260570016656,0.776,0.955,0,"3","MCL1"
"DNAJB1",0,-0.855584777172097,0.526,0.843,0,"3","DNAJB1"
"SLC2A3",0,-0.858782166400877,0.285,0.666,0,"3","SLC2A3"
"JUND",0,-0.869380461214063,0.97,0.998,0,"3","JUND"
"RHOB.1",0,-0.898374629668561,0.764,0.946,0,"3","RHOB"
"CCNL1",0,-0.957893254290442,0.66,0.936,0,"3","CCNL1"
"MAFF",0,-0.979389206160515,0.239,0.695,0,"3","MAFF"
"JUN.1",0,-0.987311638902238,0.957,0.998,0,"3","JUN"
"DUSP1",0,-0.987922947091366,0.893,0.989,0,"3","DUSP1"
"SERTAD1",0,-0.997971103990692,0.258,0.744,0,"3","SERTAD1"
"BTG2.1",0,-1.05787326875794,0.409,0.777,0,"3","BTG2"
"CDKN1A.1",0,-1.07061505920482,0.377,0.763,0,"3","CDKN1A"
"KLF4",0,-1.07831345139891,0.753,0.954,0,"3","KLF4"
"KLF2.1",0,-1.1502938344534,0.657,0.917,0,"3","KLF2"
"GADD45B",0,-1.16477469122633,0.713,0.919,0,"3","GADD45B"
"MYC",0,-1.19289833702668,0.475,0.819,0,"3","MYC"
"FOSB.2",0,-1.21744456900723,0.83,0.981,0,"3","FOSB"
"IRF1.1",0,-1.26916584467673,0.403,0.795,0,"3","IRF1"
"C11orf96",0,-1.27783432327037,0.431,0.716,0,"3","C11orf96"
"NFKBIZ",0,-1.32557749090175,0.349,0.845,0,"3","NFKBIZ"
"FOS.1",0,-1.32989230720984,0.933,0.996,0,"3","FOS"
"IER2.1",0,-1.34704075622818,0.504,0.9,0,"3","IER2"
"ZFP36.1",0,-1.36553776209074,0.904,0.993,0,"3","ZFP36"
"IGFBP5.3",0,-1.40512945573481,0.852,0.934,0,"3","IGFBP5"
"SOCS3.1",0,-1.46557747800618,0.618,0.953,0,"3","SOCS3"
"ATF3.1",0,-1.48818941725003,0.393,0.851,0,"3","ATF3"
"JUNB.1",0,-1.58502413709948,0.847,0.99,0,"3","JUNB"
"EGR1.1",0,-1.77905349546936,0.742,0.974,0,"3","EGR1"
"ABCA8.1",7.82066948589615e-305,0.625557085084786,0.942,0.918,2.067159
35851207e-300,"3","ABCA8"
"CEBPB",2.59248969404488e-304,-0.592035857294057,0.706,0.876,6.8524687
5929943e-300,"3","CEBPB"
"SAT1",1.35096666093535e-302,-0.633850590930812,0.775,0.918,3.57087507
818431e-298,"3","SAT1"
"CD55.3",1.63458489367179e-302,-1.11370231240663,0.465,0.676,4.3205347
9095327e-298,"3","CD55"
"NNMT",2.96311037494854e-299,-0.605566629483345,0.909,0.97,7.832093343
06399e-295,"3","NNMT"
"GPRC5A",8.92087090293612e-294,-0.559801944762421,0.137,0.385,2.357964
59706408e-289,"3","GPRC5A"
"ERRFI1",9.39861338103987e-294,-0.530744574380497,0.619,0.829,2.484241
48887646e-289,"3","ERRFI1"
"ABCA6.1",8.5025096078418e-273,0.61674671711648,0.846,0.768,2.24738333
954475e-268,"3","ABCA6"
"LUC7L3",3.88049123377441e-266,0.526529404853448,0.772,0.688,1.0256914
4291125e-261,"3","LUC7L3"

"GAS6.2",2.98327584800839e-257,0.52945240747235,0.873,0.729,7.88539472145577e-253,"3","GAS6"
"CREB5.3",3.0830752266107e-257,-0.64040223026519,0.329,0.566,8.1491844389774e-253,"3","CREB5"
"SVEP1.2",5.76485988805565e-248,0.735216365213247,0.877,0.813,1.52376776561087e-243,"3","SVEP1"
"ADAMTS5.3",5.64929376185522e-245,-0.84054475544068,0.62,0.785,1.49322132713357e-240,"3","ADAMTS5"
"CCN1",4.63816058082358e-244,-0.6249640992228,0.597,0.808,1.22595860472329e-239,"3","CCN1"
"CXCL14.3",2.45071729543201e-235,0.704289779259685,0.907,0.805,6.47773595528588e-231,"3","CXCL14"
"KLF6",1.93474435162486e-231,-0.583253833190632,0.861,0.95,5.11391627021482e-227,"3","KLF6"
"SOD2.1",1.47735543478775e-229,-0.856657597263233,0.741,0.874,3.90494588523097e-225,"3","SOD2"
"MT1A",7.21848013457055e-222,-0.749777523669602,0.226,0.443,1.90798866916969e-217,"3","MT1A"
"ABCA10.1",1.28162524194819e-207,0.548488950970217,0.653,0.522,3.38759183951746e-203,"3","ABCA10"
"TRAC.3",2.942911998342e-207,-0.525368824329269,0.127,0.322,7.77870499401756e-203,"3","TRAC"
"OGN.2",2.26691768166007e-200,0.528179542829331,0.894,0.838,5.99191681616391e-196,"3","OGN"
"C1QTNF3.3",2.31764489249504e-188,-0.563729985727415,0.096,0.272,6.12599897984289e-184,"3","C1QTNF3"
"C17orf58.3",3.67645747919725e-180,-0.785719349742513,0.555,0.709,9.71761240901416e-176,"3","C17orf58"
"NABP1.1",2.05036063973263e-172,-0.505294691997663,0.322,0.523,5.41951324294129e-168,"3","NABP1"
"LOXL1.3",1.74552549755704e-170,-0.606163649244134,0.444,0.62,4.61377299514277e-166,"3","LOXL1"
"SEMA3C.3",2.22731961586488e-155,-0.690157117875945,0.575,0.722,5.88725120865405e-151,"3","SEMA3C"
"PRG4.3",1.17926341071375e-151,-1.22163511632183,0.113,0.269,3.11702904719858e-147,"3","PRG4"
"IER3",3.01451109056424e-148,-0.604789879536595,0.212,0.386,7.9679557145794e-144,"3","IER3"
"NR4A1",7.79200434141236e-146,-0.586353767910685,0.232,0.407,2.05958258752212e-141,"3","NR4A1"
"MT2A.1",5.19457981725485e-142,-0.845367556487957,0.939,0.97,1.3730313372968e-137,"3","MT2A"
"CCL2.2",8.63861322361475e-141,-0.908449285679528,0.236,0.4,2.28335824726585e-136,"3","CCL2"
"MYOC.2",2.70892550826922e-139,0.552076641977057,0.734,0.601,7.1602319034572e-135,"3","MYOC"
"SFRP2.2",1.18904789611552e-138,0.691987865946204,0.692,0.594,3.14289139901254e-134,"3","SFRP2"
"PCOLCE2.3",6.27636298605603e-136,-0.547667546342976,0.828,0.904,1.65896826447433e-131,"3","PCOLCE2"

"ACKR3.3",6.65666714226294e-132,-0.585090799130245,0.715,0.817,1.75949
025904294e-127,"3","ACKR3"
"SFRP1.2",3.60561279274449e-130,0.524181805422893,0.71,0.599,9.5303557
3378222e-126,"3","SFRP1"
"PROCR.3",1.75642619596255e-127,-0.50588674396766,0.591,0.72,4.6425857
211682e-123,"3","PROCR"
"TNFAIP6",2.3390221049198e-121,-0.592212925314337,0.222,0.379,6.182503
227724e-117,"3","TNFAIP6"
"HTRA3.3",6.9777703311169e-118,-0.524343183572836,0.628,0.763,1.844364
25392082e-113,"3","HTRA3"
"MFAP5.3",4.21133888530969e-110,-0.547395990365202,0.974,0.985,1.11314
109416506e-105,"3","MFAP5"
"MT1M",1.58024290262167e-99,-0.592198916174843,0.702,0.809,4.176898040
2096e-95,"3","MT1M"
"IGFBP3.2",5.67528969450181e-95,0.508349875359588,0.65,0.563,1.5000925
7205072e-90,"3","IGFBP3"
"MT1X.1",1.53481672363009e-85,-0.543609530474926,0.617,0.738,4.0568275
6389906e-81,"3","MT1X"
"FBN1.3",1.76714490296888e-66,-0.572598532330574,0.967,0.979,4.6709174
0752733e-62,"3","FBN1"
"PTGDS.1",1.0000171037241e-39,0.687516376443578,0.416,0.352,2.64324520
856354e-35,"3","PTGDS"
"IGFBP7.1",1.49892481670466e-270,-1.19951103416726,0.824,0.882,3.96195
807551375e-266,"4","IGFBP7"
"MRPS6.2",1.40283592981852e-245,-0.913764107502262,0.537,0.698,3.70797
592969631e-241,"4","MRPS6"
"MMP2",1.19240356148884e-242,0.521115711801501,0.996,0.986,3.151761093
7273e-238,"4","MMP2"
"MGP.3",3.22290415228352e-233,-1.00317581981003,0.998,0.999,8.51878025
531579e-229,"4","MGP"
"SLC5A3.2",1.47874932542106e-218,-1.11548831114765,0.242,0.456,3.90863
021695294e-214,"4","SLC5A3"
"CXCL14.4",3.41825825892395e-211,-1.68394933631025,0.788,0.828,9.03514
022998779e-207,"4","CXCL14"
"NRP1.1",3.0808118236691e-180,-0.531636154900309,0.345,0.534,8.1432018
1232216e-176,"4","NRP1"
"COL15A1.2",5.08457395382165e-163,-0.545944109860391,0.153,0.349,1.343
95458747414e-158,"4","COL15A1"
"RARRES2.2",4.27638337087155e-152,-0.572482560717191,0.697,0.77,1.1303
3365258877e-147,"4","RARRES2"
"GGT5.1",4.70185549758052e-147,-0.560056269824132,0.431,0.575,1.242794
44512048e-142,"4","GGT5"
"APOE.3",7.5649798039396e-142,-1.38769434379232,0.377,0.532,1.99957546
177731e-137,"4","APOE"
"IGF1.3",1.99327740750844e-139,-0.758506396257183,0.599,0.69,5.2686308
4352631e-135,"4","IGF1"
"APOD.4",2.41433306270132e-132,-0.815750572149882,0.983,0.97,6.3815651
5133212e-128,"4","APOD"
"CPB1",1.99016148913819e-128,0.512316172430553,0.616,0.448,5.260394848
09005e-124,"4","CPB1"

"POSTN",1.13194821052329e-124,0.79088340369739,0.291,0.152,2.991965510
05517e-120,"4","POSTN"
"CXCL12.2",2.05355389271455e-114,-0.568827051204139,0.989,0.968,5.4279
536492231e-110,"4","CXCL12"
"GAS6.3",1.76759100175794e-99,-0.534830232300036,0.732,0.757,4.6720965
3584659e-95,"4","GAS6"
"IGFBP3.3",4.76234273277972e-66,-0.589790740901192,0.504,0.59,1.258782
43112833e-61,"4","IGFBP3"
"FABP4.3",6.68372901010923e-58,-0.887516805694443,0.288,0.386,1.766643
25195207e-53,"4","FABP4"
"MYOC.3",1.46599928663126e-22,-0.58692837327797,0.602,0.627,3.87492931
442376e-18,"4","MYOC"
"TNFSF14",0,0.695304533298553,0.355,0.052,0,"5","TNFSF14"
"MT2A.2",1.71257861407412e-281,1.7816528359707,0.995,0.964,4.526687792
72071e-277,"5","MT2A"
"IL32",7.05699960072783e-276,1.30870008656335,0.748,0.403,1.8653061344
6438e-271,"5","IL32"
"SLC39A14",2.95304044480623e-275,1.09010853780937,0.54,0.198,7.8054765
0371182e-271,"5","SLC39A14"
"SOD2.2",2.23294117163147e-273,1.82271794662452,0.963,0.846,5.90211010
485631e-269,"5","SOD2"
"MT1X.2",1.72714874773898e-229,1.89015670459962,0.916,0.707,4.56519957
002366e-225,"5","MT1X"
"NAMPT.1",8.41024540111066e-211,1.13527349479462,0.926,0.744,2.2229960
6442157e-206,"5","NAMPT"
"NNMT.1",4.03817086338265e-198,1.09551746790657,0.986,0.958,1.06736932
26093e-193,"5","NNMT"
"PTMA",1.34051836435136e-184,0.741294964381163,0.997,0.982,3.543258140
65353e-180,"5","PTMA"
"TYMP",3.43940617738517e-161,0.863169507112717,0.879,0.675,9.091038408
06448e-157,"5","TYMP"
"APOL1",1.34934823885202e-158,0.622192664212095,0.652,0.36,3.566597264
93366e-154,"5","APOL1"
"IFI16",2.75336338252941e-158,0.829159768813045,0.947,0.855,7.27769009
270173e-154,"5","IFI16"
"DDX21",1.9552419908281e-152,0.839144458153121,0.792,0.554,5.168095630
15684e-148,"5","DDX21"
"EIF4A1",5.45376461596523e-152,0.70642553413091,0.983,0.956,1.44153906
329193e-147,"5","EIF4A1"
"MT1M.1",1.89572178809097e-151,1.48418198043221,0.913,0.785,5.01077183
028205e-147,"5","MT1M"
"WARS",6.48110792107888e-151,0.754773418995429,0.455,0.193,1.713086445
69957e-146,"5","WARS"
"IFITM3",6.68959570994371e-148,0.537742950011834,0.993,0.99,1.76819393
805232e-143,"5","IFITM3"
"NOVA1",4.20148454983782e-143,-0.670002883466388,0.963,0.984,1.1105363
9621313e-138,"5","NOVA1"
"MT1A.1",1.12834709580688e-142,1.38482648437805,0.664,0.393,2.98244704
363675e-138,"5","MT1A"
"STAT1",1.24361229002863e-142,0.63077662681559,0.679,0.388,3.287116005

00367e-138,"5","STAT1"
"PLA2G2A",4.88791945677384e-141,1.20812543573172,0.954,0.883,1.2919748
7081446e-136,"5","PLA2G2A"
"APOL6",1.8242032354561e-140,0.642449928991677,0.807,0.55,4.8217339919
5755e-136,"5","APOL6"
"PSME2",1.09410837492419e-136,0.754040343971467,0.764,0.534,2.89194725
659961e-132,"5","PSME2"
"LAP3",1.78033588575799e-134,0.704037427885851,0.65,0.396,4.7057838132
3551e-130,"5","LAP3"
"OSMR",5.91920360868464e-128,0.514544131730499,0.596,0.327,1.564563897
84752e-123,"5","OSMR"
"PTPRC",9.81769764916202e-128,0.516280113419254,0.297,0.105,2.59501384
26265e-123,"5","PTPRC"
"CCL2.3",1.21994880862692e-127,1.47153158420595,0.623,0.36,3.224568690
96267e-123,"5","CCL2"
"ICAM1",8.42124259466881e-125,0.603143405873681,0.425,0.184,2.22590284
262286e-120,"5","ICAM1"
"PPA1",1.77397476533011e-122,0.736659808255456,0.909,0.798,4.688970099
72054e-118,"5","PPA1"
"PLSCR1",6.01037293157614e-122,0.601948903322494,0.727,0.472,1.5886617
7327421e-117,"5","PLSCR1"
"PDPN",1.08732753314355e-119,0.706891579856796,0.564,0.316,2.874024135
60502e-115,"5","PDPN"
"STOM",1.05432839996258e-116,0.551194259070414,0.88,0.734,2.7868008267
811e-112,"5","STOM"
"CCL5",3.33620580501e-114,0.731150436297299,0.255,0.088,8.818259183802
44e-110,"5","CCL5"
"DNAJA1.1",1.46345256066968e-110,0.534766157256389,0.945,0.859,3.86819
780836211e-106,"5","DNAJA1"
"PTX3.2",6.00851918511228e-110,2.54524461053514,0.466,0.244,1.58817179
100888e-105,"5","PTX3"
"NABP1.2",1.04540989967602e-109,0.734678575446916,0.711,0.477,2.763227
44682366e-105,"5","NABP1"
"CTSC",9.87662396665449e-108,0.523113955005528,0.482,0.238,2.610589246
86611e-103,"5","CTSC"
"STEAP4",3.18309069396065e-102,0.762336168460133,0.545,0.316,8.4135453
2227678e-98,"5","STEAP4"
"GBP1",7.19335716005215e-102,0.928238545756078,0.518,0.289,1.901348164
54498e-97,"5","GBP1"
"HLA-
B.1",1.82310168378983e-99,0.518697592139252,0.995,0.974,4.818822370593
27e-95,"5","HLA-B"
"TNFAIP6.1",3.30294260834426e-98,1.57158750156083,0.566,0.342,8.730337
90237555e-94,"5","TNFAIP6"
"CHI3L2",1.37358623432652e-96,1.30817099313534,0.287,0.113,3.630663134
57185e-92,"5","CHI3L2"
"ACSL4",7.7382022228975e-95,0.502762496668237,0.559,0.327,2.0453616115
5627e-90,"5","ACSL4"
"PNRC1.1",1.00932107411859e-94,0.504212351630595,0.994,0.974,2.6678374
6311026e-90,"5","PNRC1"

"MARCH3",1.01038115912944e-94,0.589910547410366,0.488,0.276,2.67063947981094e-90,"5","MARCH3"
"CCDC71L.1",7.15643124852345e-91,0.902809033517359,0.764,0.562,1.89158790760972e-86,"5","CCDC71L"
"SLC25A5",5.37925330926145e-90,0.50739016374767,0.806,0.627,1.42184423470399e-85,"5","SLC25A5"
"TNFAIP2.1",4.53808229738953e-88,1.03709832966655,0.876,0.758,1.199505912846e-83,"5","TNFAIP2"
"RARRES1",1.77158395043645e-87,0.819065165313272,0.469,0.258,4.68265069779363e-83,"5","RARRES1"
"MT1E",5.37003139418013e-86,0.932047872973333,0.93,0.854,1.41940669810969e-81,"5","MT1E"
"CFB.1",1.6093864729056e-81,0.874893026647374,0.665,0.461,4.25393032518409e-77,"5","CFB"
"CHI3L1",1.10284710470445e-80,0.993682817886633,0.262,0.107,2.9150454671548e-76,"5","CHI3L1"
"CXCR4",5.12826381555797e-80,0.735005300040364,0.405,0.222,1.35550269172828e-75,"5","CXCR4"
"RGS16",5.23222681068504e-78,0.665342616011205,0.372,0.181,1.38298219060027e-73,"5","RGS16"
"PCOLCE2.4",1.91055442587058e-75,-0.675932159813021,0.828,0.894,5.04997745846113e-71,"5","PCOLCE2"
"SRGN",1.65421932397637e-73,0.775147163530266,0.502,0.317,4.37243251713433e-69,"5","SRGN"
"WTAP",6.51784167456448e-73,0.505783507064693,0.767,0.575,1.72279591142088e-68,"5","WTAP"
"TNFAIP3",1.1148773229173e-67,0.612006876809289,0.517,0.327,2.946843739935e-63,"5","TNFAIP3"
"CTSL",3.54230007989049e-67,0.806115341735449,0.947,0.897,9.36300757116654e-63,"5","CTSL"
"HSPA5",2.23905998913532e-64,0.694217778023969,0.891,0.8,5.91828336328248e-60,"5","HSPA5"
"CRISPLD2",3.60049597475472e-59,0.560995907501046,0.621,0.445,9.51683096047168e-55,"5","CRISPLD2"
"PLIN2",6.15716946818706e-59,0.928085522001934,0.891,0.803,1.6274630338312e-54,"5","PLIN2"
"CD74",1.2273952832221e-57,0.540680093437308,0.674,0.487,3.24425121261264e-53,"5","CD74"
"CYP1B1",1.21484350757405e-52,0.552614938332605,0.405,0.245,3.21107435921973e-48,"5","CYP1B1"
"IGFBP6.3",3.28334605561752e-51,-0.564729314056145,0.97,0.98,8.67854029420822e-47,"5","IGFBP6"
"IFI6",3.4425043649524e-51,0.695169175821642,0.805,0.687,9.09922753744219e-47,"5","IFI6"
"C11orf96.1",2.57972934214773e-48,0.722692222215085,0.789,0.66,6.81874059716487e-44,"5","C11orf96"
"IFIT3",4.34055517004823e-40,0.758643572145169,0.566,0.434,1.14729554254715e-35,"5","IFIT3"
"DEPP1.3",1.47076324614426e-35,0.674264763917126,0.697,0.559,3.8875214122085e-31,"5","DEPP1"

"TPPP3.3",1.21747406154084e-33,-0.519134445285004,0.613,0.685,3.21802743946474e-29,"5","TPPP3"
"GAL",3.77098164354764e-31,0.570484942083933,0.254,0.147,9.96745868022513e-27,"5","GAL"
"CCL4",3.74259096242035e-29,0.947373260443841,0.455,0.336,9.89241643186947e-25,"5","CCL4"
"CXCL2",3.05020458895486e-28,0.772395957736528,0.395,0.28,8.06230076952549e-24,"5","CXCL2"
"ISG15",1.32717334003826e-24,0.705356447519534,0.468,0.343,3.50798457238913e-20,"5","ISG15"
"IGFBP5.4",1.16142051733306e-22,-0.580239114617053,0.893,0.921,3.06986671141476e-18,"5","IGFBP5"
"CD55.4",1.09929610562753e-16,-0.597902753217039,0.617,0.64,2.90565946639469e-12,"5","CD55"
"G0S2",5.19542224620171e-10,0.86168957998886,0.424,0.355,1.37325400811604e-05,"5","G0S2"
"FOS.2",0,-1.34147500915989,0.956,0.986,0,"6","FOS"
"ZFP36.2",0,-1.35495514462492,0.929,0.98,0,"6","ZFP36"
"JUNB.2",0,-1.40853890944947,0.899,0.968,0,"6","JUNB"
"EGR1.2",7.5871820789305e-307,-1.52069769205783,0.803,0.939,2.00544396710291e-302,"6","EGR1"
"SOCS3.2",3.93375568320052e-298,-1.45089827764176,0.673,0.904,1.03977030218356e-293,"6","SOCS3"
"ATF3.2",4.38334023451111e-263,-1.46964128252775,0.398,0.787,1.15860449078598e-258,"6","ATF3"
"JUN.2",2.95841472011532e-252,-1.10293874533674,0.982,0.991,7.81968178820882e-248,"6","JUN"
"FOSB.3",3.02019527218811e-249,-1.35996575220146,0.843,0.96,7.9829801434476e-245,"6","FOSB"
"TNXB.2",1.3925424980196e-242,0.736369346212507,0.996,0.99,3.6807683307654e-238,"6","TNXB"
"FBN1.4",3.82447746914291e-239,0.90051884099201,0.999,0.976,1.01088588464385e-234,"6","FBN1"
"MFAP5.4",3.14040795500636e-238,0.86473510244024,0.996,0.983,8.30072630667282e-234,"6","MFAP5"
"CD55.5",1.46173112814644e-236,0.935641528899361,0.923,0.627,3.86364771791666e-232,"6","CD55"
"IRF1.2",8.93797921369048e-224,-1.35339587877961,0.346,0.743,2.36248666576267e-219,"6","IRF1"
"FN1.3",4.20358593679176e-221,0.823974770521523,0.991,0.921,1.1110918348128e-216,"6","FN1"
"MAFF.1",4.23853905885003e-204,-0.969482138182888,0.216,0.633,1.12033064403524e-199,"6","MAFF"
"CLEC3B.3",8.18912508113238e-204,0.808578842676365,0.959,0.86,2.16454954144491e-199,"6","CLEC3B"
"SERTAD1.1",1.65888446435918e-198,-0.940402182807109,0.279,0.676,4.38476341619418e-194,"6","SERTAD1"
"CCNL1.1",3.40104269284527e-197,-0.940249201465825,0.669,0.897,8.98963604572861e-193,"6","CCNL1"
"MCL1.1",2.17255769093805e-189,-0.844428003756531,0.758,0.931,5.742504

48868746e-185,"6","MCL1"
"SEMA3C.4",3.31352608595246e-185,0.932737808030363,0.922,0.687,8.75831
215038953e-181,"6","SEMA3C"
"CD248.3",1.877974011325e-184,0.740873507657771,0.975,0.912,4.96386090
673424e-180,"6","CD248"
"IGF1.4",4.86989753272196e-183,-1.31350069400403,0.336,0.693,1.2872113
1584907e-178,"6","IGF1"
"CDKN1A.2",2.5111157032459e-182,-1.04327447171518,0.357,0.71,6.6373810
2681956e-178,"6","CDKN1A"
"SCARA5.2",1.2347756079069e-180,0.661192200863347,0.988,0.933,3.263758
88681951e-176,"6","SCARA5"
"MGP.4",6.22373168245655e-180,-1.38641070623095,0.996,0.999,1.64505675
830692e-175,"6","MGP"
"CXCL14.5",8.14303158114232e-180,-2.66242851288313,0.642,0.831,2.15236
610752754e-175,"6","CXCL14"
"PNRC1.2",6.70479405929563e-175,-0.771162880405493,0.916,0.977,1.77221
116575302e-170,"6","PNRC1"
"IER2.2",4.45340748640209e-170,-1.04642940488902,0.594,0.841,1.1771246
668058e-165,"6","IER2"
"FSTL1.2",8.02086993949335e-169,0.661696714067587,0.998,0.993,2.120076
34240688e-164,"6","FSTL1"
"NFKBIZ.1",8.64567919566832e-169,-0.937186096866715,0.464,0.772,2.2852
2592499905e-164,"6","NFKBIZ"
"ACTB.3",5.2984209886225e-167,0.573620779202958,0.999,0.996,1.40047863
57127e-162,"6","ACTB"
"BTG2.2",5.89540861023252e-159,-0.980696676950473,0.398,0.727,1.558274
40385666e-154,"6","BTG2"
"MYC.1",1.23528425577093e-154,-1.10711957379482,0.5,0.77,3.26510334485
371e-150,"6","MYC"
"DUSP1.1",1.73373440583238e-153,-0.838069748005287,0.934,0.974,4.58260
678149614e-149,"6","DUSP1"
"JUND.1",2.00321146932047e-152,-0.624867048098102,0.988,0.994,5.294888
55570785e-148,"6","JUND"
"NFIL3.1",1.41737976121451e-150,-0.701843281867895,0.23,0.593,3.746418
18484219e-146,"6","NFIL3"
"C17orf58.4",4.93008425709564e-147,0.732083598439244,0.878,0.674,1.303
11987083552e-142,"6","C17orf58"
"IGFBP5.5",3.54944113466201e-145,0.705227011461702,0.991,0.917,9.38188
280713862e-141,"6","IGFBP5"
"BTG1.1",1.08735902735721e-144,-0.693131364486037,0.782,0.933,2.874107
38111057e-140,"6","BTG1"
"ADAMTS5.4",9.60041583147763e-142,0.705390757675905,0.925,0.749,2.5375
8191257617e-137,"6","ADAMTS5"
"MARCKS.2",6.79973709026555e-141,0.739098466411151,0.992,0.955,1.79730
650769899e-136,"6","MARCKS"
"GGT5.2",7.47426952553072e-141,-0.797994582451386,0.225,0.571,1.975598
92098828e-136,"6","GGT5"
"CEBPB.1",2.02452951209594e-138,-0.83517542731476,0.645,0.855,5.351236
406372e-134,"6","CEBPB"
"GADD45B.1",8.61528497489255e-137,-1.05212649508148,0.729,0.89,2.27719

21245636e-132,"6","GADD45B"
"CEBPD.1",4.91639796152196e-136,-0.745630401663436,0.925,0.98,1.299502
30918948e-131,"6","CEBPD"
"METRNL.2",1.17333044633082e-133,0.564601931988117,0.932,0.816,3.10134
703574162e-129,"6","METRNL"
"LTBP4.2",1.21273283166007e-132,0.622098564462282,0.973,0.893,3.205495
42064391e-128,"6","LTBP4"
"HLA-
E.1",3.53246347275292e-131,-0.583010877408818,0.944,0.983,9.3370074511
8052e-127,"6","HLA-E"
"RARRES2.3",1.87084929959777e-130,-0.817309760414129,0.514,0.771,4.945
02886869682e-126,"6","RARRES2"
"NAMPT.2",3.55742764214456e-129,-0.827531264968981,0.504,0.763,9.40299
274371651e-125,"6","NAMPT"
"GAS6.4",2.56941393915265e-128,-0.883526518946566,0.517,0.764,6.791474
92396827e-124,"6","GAS6"
"F10.2",2.20865968826014e-125,0.619451231630824,0.92,0.826,5.837929288
00921e-121,"6","F10"
"NTM.2",2.70669334165652e-125,0.600752478795141,0.712,0.447,7.15433184
066651e-121,"6","NTM"
"PCOLCE2.5",5.61658393670765e-125,0.54697228701706,0.98,0.887,1.484575
46615057e-120,"6","PCOLCE2"
"IGFBP7.2",2.36133109429062e-122,-1.25993074139886,0.72,0.881,6.241470
34842898e-118,"6","IGFBP7"
"SOD2.3",2.67461670771724e-122,-1.10825327594658,0.675,0.859,7.0695468
8183821e-118,"6","SOD2"
"DPP4.2",4.5436005569473e-121,0.547340722168521,0.646,0.378,1.20096449
921231e-116,"6","DPP4"
"FNDC1.3",2.90446128401518e-119,0.681221961016635,0.667,0.41,7.6770720
6590893e-115,"6","FNDC1"
"LINC02802.2",4.19516942921211e-119,0.564694393578262,0.846,0.694,1.10
886718352934e-114,"6","LINC02802"
"ITGA11.1",4.37378404302445e-119,0.643089175977185,0.731,0.508,1.15607
859825222e-114,"6","ITGA11"
"DBN1.2",8.00232462736759e-113,0.524499462011931,0.867,0.701,2.1151744
455058e-108,"6","DBN1"
"DDX5.1",1.17561565841896e-112,-0.523060363216656,0.961,0.988,3.107387
30833299e-108,"6","DDX5"
"DNAJB1.1",1.12552140805559e-110,-0.687480723821843,0.571,0.797,2.9749
7818577253e-106,"6","DNAJB1"
"ACKR3.4",3.66608118130043e-110,0.615815670484803,0.932,0.794,9.690185
77841331e-106,"6","ACKR3"
"SLC2A3.1",6.10832690289862e-110,-0.736704829216427,0.31,0.612,1.61455
296697416e-105,"6","SLC2A3"
"PPIC.2",9.81391015751812e-110,0.540460324369246,0.923,0.861,2.5940127
3283519e-105,"6","PPIC"
"IGFBP6.4",8.31773863522623e-108,0.610211498318233,0.996,0.979,2.19854
4676063e-103,"6","IGFBP6"
"TPPP3.4",2.39898041004012e-107,0.586100092936404,0.859,0.674,6.340985
01981803e-103,"6","TPPP3"

"NFKBIA",5.36565405605543e-107,-0.582146415298434,0.903,0.964,1.418249
68009657e-102,"6","NFKBIA"
"RND3.2",2.55371808588142e-106,-0.777836480465815,0.402,0.694,6.749987
64460177e-102,"6","RND3"
"EMP1.1",1.09773887686261e-105,-0.660615314895725,0.733,0.901,2.901543
39932326e-101,"6","EMP1"
"APOD.5",9.58423718675175e-105,-1.11723807407099,0.947,0.973,2.5333055
7320222e-100,"6","APOD"
"AC020916.1.1",4.85398880851234e-103,-0.645152885679652,0.294,0.599,1.
28300632186598e-98,"6","AC020916.1"
"APOE.4",1.74804227397514e-96,-1.85816703689179,0.263,0.523,4.62042533
85711e-92,"6","APOE"
"COL15A1.3",5.97029609024422e-96,-0.665851473371051,0.066,0.335,1.5780
6866257335e-91,"6","COL15A1"
"C11orf96.2",7.74028778939928e-96,-1.11688183849025,0.439,0.676,2.0459
1286849402e-91,"6","C11orf96"
"PROCR.4",7.98766745535009e-96,0.540458827090196,0.865,0.691,2.1113002
6179813e-91,"6","PROCR"
"PPP1R15A.1",1.06288541659074e-95,-0.534200095714526,0.714,0.878,2.809
41873313264e-91,"6","PPP1R15A"
"ABHD5.1",7.62565023541171e-95,-0.591556927767797,0.267,0.546,2.015611
87022402e-90,"6","ABHD5"
"FST.2",9.99248486358837e-94,-0.711106034493993,0.295,0.572,2.64121359
914368e-89,"6","FST"
"HELLPAR.1",2.97603173890918e-93,-0.526628610058556,0.131,0.41,7.86624
709228474e-89,"6","HELLPAR"
"NABP1.3",1.08957841594753e-91,-0.665422632479776,0.222,0.499,2.879973
66903252e-87,"6","NABP1"
"DPT.2",1.5600635361992e-91,-0.661775772035649,0.718,0.867,4.123559938
88173e-87,"6","DPT"
"TRIO.2",2.01433929233443e-91,0.588883604236628,0.764,0.584,5.32430161
749838e-87,"6","TRIO"
"DNAJA1.2",1.11609252171101e-90,-0.53382797743325,0.72,0.869,2.9500557
5338653e-86,"6","DNAJA1"
"TPM1.3",2.5517013297963e-89,0.545835887430118,0.867,0.746,6.744656954
91759e-85,"6","TPM1"
"PTGIS.2",2.44333498003904e-87,0.567873391470937,0.835,0.683,6.4582230
1923918e-83,"6","PTGIS"
"ARID5B",7.93952928435258e-87,-0.501214170382018,0.796,0.926,2.0985763
8044007e-82,"6","ARID5B"
"SLC5A3.3",1.06237637160996e-86,-1.07252880592507,0.182,0.439,2.808073
22543944e-82,"6","SLC5A3"
"INTS6.1",2.10557940288313e-86,-0.539241186271714,0.326,0.595,5.565467
47770068e-82,"6","INTS6"
"NOVA1.1",3.1650662241921e-86,0.611213715796009,0.985,0.983,8.36590304
378455e-82,"6","NOVA1"
"KLF9",5.28540075374386e-86,-0.515083479358313,0.693,0.865,1.397037127
22958e-81,"6","KLF9"
"COL14A1.1",1.5322706638569e-85,0.559859235522712,0.913,0.818,4.050097
81870657e-81,"6","COL14A1"

"CYGB.1",2.13424440132477e-85,-0.522149095736304,0.449,0.701,5.64123480158163e-81,"6","CYGB"
"CXCL12.3",9.30970859623292e-84,-0.749783406325407,0.951,0.971,2.46074217615629e-79,"6","CXCL12"
"MRPS6.3",1.46678351486615e-83,-0.868915367081479,0.465,0.686,3.87700218649421e-79,"6","MRPS6"
"COL4A2.2",1.48426033685582e-78,-0.606884062938722,0.415,0.649,3.92319692237729e-74,"6","COL4A2"
"NRP1.2",3.31627952436327e-78,-0.537091500619261,0.269,0.52,8.76559003879701e-74,"6","NRP1"
"MT1A.2",6.16510800803268e-78,-0.874820399311928,0.171,0.416,1.6295613486832e-73,"6","MT1A"
"SOD3.1",7.08210380399504e-78,-0.547518815616101,0.886,0.956,1.87194167747197e-73,"6","SOD3"
"ID4",3.49179540466318e-77,-0.513486642257829,0.159,0.419,9.22951361360571e-73,"6","ID4"
"CILP",8.32314536710621e-77,0.513361150369704,0.943,0.888,2.19997378343351e-72,"6","CILP"
"DEPP1.4",4.19669820936723e-71,-1.30348444516001,0.363,0.574,1.10927127069995e-66,"6","DEPP1"
"TNNT3.2",4.19848050294852e-71,-0.589962168560246,0.425,0.645,1.10974236653935e-66,"6","TNNT3"
"CCN1.1",2.09854438962104e-70,-0.745680677606581,0.599,0.779,5.54687253064632e-66,"6","CCN1"
"MYOC.4",2.47552312517591e-69,-1.11366172364506,0.426,0.632,6.54330272446497e-65,"6","MYOC"
"COL3A1.1",2.47855104665382e-69,0.649538529711799,0.999,0.995,6.55130612651537e-65,"6","COL3A1"
"SFRP4.2",4.27969508079359e-69,0.712598717958724,0.654,0.466,1.13120900375536e-64,"6","SFRP4"
"CTSH.2",7.80120501380146e-69,0.582291319194753,0.696,0.54,2.062014509248e-64,"6","CTSH"
"NNMT.2",2.62837497771985e-68,-0.605673301518432,0.92,0.961,6.94732074110911e-64,"6","NNMT"
"ZFP36L1.1",3.58304922491368e-67,-0.568875901001649,0.934,0.975,9.47071571129185e-63,"6","ZFP36L1"
"SLIT3.2",7.32839311973348e-67,-0.547826368303781,0.73,0.86,1.93704086940795e-62,"6","SLIT3"
"IGFBP3.4",1.38866116992519e-66,-0.840905417932927,0.371,0.588,3.67050920434626e-62,"6","IGFBP3"
"COL5A1.1",8.02369692545805e-66,0.513712610705519,0.832,0.75,2.12082357133707e-61,"6","COL5A1"
"DST.1",1.00699029139593e-65,0.538103903636656,0.881,0.82,2.66167673821771e-61,"6","DST"
"MT1X.3",3.70063069339454e-64,-0.965426449757023,0.558,0.724,9.78150704878045e-60,"6","MT1X"
"COL1A1.1",1.83865332058665e-60,0.661457078563151,0.996,0.996,4.85992845697464e-56,"6","COL1A1"
"CCL2.4",2.04728630050387e-60,-1.07926517149934,0.173,0.38,5.41138714949184e-56,"6","CCL2"

"RASD1.1",1.9502343348614e-59,-0.71947786723212,0.484,0.697,5.15485939
390566e-55,"6","RASD1"
"SAT1.1",2.32112998260206e-58,-0.66622447781524,0.808,0.897,6.13521077
001376e-54,"6","SAT1"
"CYP26B1.2",8.62209912481087e-58,-0.503380661966428,0.117,0.321,2.2789
9324067001e-53,"6","CYP26B1"
"CCDC71L.2",3.11514192465749e-50,-0.613356884561713,0.378,0.579,8.2339
4313525469e-46,"6","CCDC71L"
"COL4A1.2",9.01469791496007e-50,-0.586813635184818,0.48,0.659,2.382764
95288225e-45,"6","COL4A1"
"FABP4.4",4.68168945728907e-47,-0.947580073156833,0.2,0.381,1.23746415
735065e-42,"6","FABP4"
"MT1M.2",1.93484744923716e-45,-0.785289434108826,0.672,0.796,5.1141887
7782367e-41,"6","MT1M"
"MT2A.3",5.5065584203908e-45,-1.03384115363855,0.951,0.966,1.455493521
6777e-40,"6","MT2A"
"NR4A1.1",1.87471046590153e-38,-0.560523925310625,0.217,0.383,4.955234
70347091e-34,"6","NR4A1"
"IER3.1",1.74273430716457e-36,-0.557477567374688,0.201,0.363,4.6063953
206974e-32,"6","IER3"
"SFRP2.3",1.46837617013912e-34,-0.604534187210235,0.459,0.618,3.881211
89291172e-30,"6","SFRP2"
"MT1E.1",2.61032444843903e-26,-0.521658436433285,0.794,0.86,6.89960958
211403e-22,"6","MT1E"
"PTGDS.2",2.91612124713874e-10,-0.540071939863228,0.285,0.366,7.707891
68043711e-06,"6","PTGDS"
"IGFBP7.3",0,2.32464908550825,0.985,0.87,0,"7","IGFBP7"
"BGN.1",0,1.36296122382956,0.776,0.331,0,"7","BGN"
"NOTCH3",2.04465359220929e-300,0.506541249928428,0.451,0.102,5.4044283
7492759e-296,"7","NOTCH3"
"GPX3",1.0867284334355e-274,-1.109431997065,0.766,0.966,2.872440595256
71e-270,"7","GPX3"
"SPARCL1.1",9.95635135856933e-270,1.67848393046075,0.653,0.247,2.63166
279109704e-265,"7","SPARCL1"
"SERPINE2",5.66687041639927e-254,0.598212792039289,0.44,0.113,1.497867
18846265e-249,"7","SERPINE2"
"A2M",5.60563947672987e-233,0.940248175710749,0.524,0.169,1.4816826264
8924e-228,"7","A2M"
"PCOLCE2.6",4.1314712122556e-230,-1.40415582619734,0.636,0.9,1.0920304
708234e-225,"7","PCOLCE2"
"CCN5.1",2.13873136132442e-205,-0.978646949383395,0.914,0.99,5.6530947
3425272e-201,"7","CCN5"
"ACKR3.5",4.01673314117295e-189,-1.30817399905204,0.48,0.811,1.0617029
0387483e-184,"7","ACKR3"
"CRLF1",7.21090782380046e-189,0.78633783462526,0.386,0.11,1.9059871559
8694e-184,"7","CRLF1"
"MFAP4",6.01859338539062e-171,-0.742502980579518,0.92,0.982,1.59083460
362645e-166,"7","MFAP4"
"APOE.5",2.35511719170773e-168,1.78907263043026,0.795,0.502,6.22504576
112187e-164,"7","APOE"

"IGFBP5.6",2.51901964846589e-164,-2.00245192766778,0.781,0.925,6.65827
273482505e-160,"7","IGFBP5"
"NRP1.3",1.31303978398973e-145,0.81791237751249,0.787,0.5,3.4706267570
4166e-141,"7","NRP1"
"GGT5.3",1.80488242193845e-143,0.818546049944422,0.829,0.547,4.7706652
1766771e-139,"7","GGT5"
"TIMP2",4.21452091153721e-142,-0.535038877320105,0.99,0.997,1.11398216
733752e-137,"7","TIMP2"
"THY1",4.24890253011948e-142,0.893963415165744,0.944,0.872,1.123069916
76118e-137,"7","THY1"
"PRELP",1.23736566828623e-140,-0.69122860208347,0.626,0.858,3.27060493
441415e-136,"7","PRELP"
"CHL1",1.02710570621256e-138,-0.734561835820123,0.509,0.789,2.71484580
266103e-134,"7","CHL1"
"PHLDA3",3.45535063124058e-138,0.731832194983066,0.758,0.477,9.1331827
8849509e-134,"7","PHLDA3"
"CXXC5",1.19281388082301e-135,0.643108856320682,0.61,0.32,3.1528456497
9139e-131,"7","CXXC5"
"ACKR4",1.05534935093522e-134,-0.760611609972802,0.182,0.544,2.7894994
0439198e-130,"7","ACKR4"
"TIMP1.1",8.75654895560805e-132,1.40303380415408,0.987,0.982,2.3145310
1994632e-127,"7","TIMP1"
"COL4A2.3",6.14262846505673e-130,0.821024294626462,0.881,0.63,1.623619
5558838e-125,"7","COL4A2"
"CFD",8.06889775077564e-126,-0.547556917312786,1,1,2.13277105348502e-1
21,"7","CFD"
"COL4A1.3",4.66217230364933e-121,0.918072046585746,0.87,0.644,1.232305
38330059e-116,"7","COL4A1"
"VEGFD",1.64308750881443e-119,-0.73901895435586,0.189,0.543,4.34300890
32983e-115,"7","VEGFD"
"TGFBR3.2",2.18962611563781e-116,-0.711248262927858,0.754,0.877,5.7876
1974885386e-112,"7","TGFBR3"
"NOVA1.2",2.56088253381638e-116,-0.702379319484279,0.959,0.984,6.76892
471338345e-112,"7","NOVA1"
"FSTL1.3",3.34547288487213e-112,-0.820625851804721,0.988,0.994,8.84275
392929402e-108,"7","FSTL1"
"PLPP3",1.11186496075339e-111,-0.613157222802654,0.926,0.977,2.9388814
6426336e-107,"7","PLPP3"
"PTMS",8.99313540469005e-111,0.515147527396148,0.962,0.907,2.377065550
16767e-106,"7","PTMS"
"PDGFRL",1.23370840208074e-109,-0.549224642702523,0.767,0.915,3.260938
0483798e-105,"7","PDGFRL"
"ANGPTL2",4.05761464728535e-109,-0.602396305795635,0.811,0.93,1.072508
70357046e-104,"7","ANGPTL2"
"CYGB.2",2.32952212284177e-108,0.656264560361297,0.877,0.684,6.1573928
7509537e-104,"7","CYGB"
"ADM.2",5.87804877869269e-108,-1.1590307965702,0.453,0.707,1.553685853
18405e-103,"7","ADM"
"MFAP5.5",3.60355933376912e-107,-1.11579245363597,0.973,0.984,9.524928
03101854e-103,"7","MFAP5"

"C17orf58.5",4.14327200984883e-107,-1.17121721480905,0.464,0.69,1.0951
4965764324e-102,"7","C17orf58"
"ADH1B.1",2.07627646664332e-106,-0.681808922089446,0.751,0.912,5.48801
395663162e-102,"7","ADH1B"
"CAV1.1",4.66966772252747e-106,0.957939180452469,0.928,0.813,1.2342865
7241846e-101,"7","CAV1"
"LMO4",1.5893398445383e-104,0.701008535896742,0.834,0.65,4.20094307708
362e-100,"7","LMO4"
"THBS2",2.42579387576836e-103,-0.635778207699044,0.495,0.736,6.4118583
7243092e-99,"7","THBS2"
"TPM2",9.6671936747422e-103,0.749669422304679,0.748,0.512,2.5552326321
0786e-98,"7","TPM2"
"FBN1.5",2.01146938215506e-99,-1.20235255717626,0.964,0.977,5.31671587
091226e-95,"7","FBN1"
"APOC1.2",3.67995507911147e-98,0.841632414380712,0.463,0.218,9.7268572
6510744e-94,"7","APOC1"
"CDKN1C",3.22734584024543e-95,-0.605727726179412,0.811,0.91,8.53052052
493672e-91,"7","CDKN1C"
"CPE.1",3.13932788632066e-92,-0.588472746674066,0.862,0.949,8.29787146
912277e-88,"7","CPE"
"SEMA3C.5",5.82497553980143e-91,-1.10391795976647,0.493,0.704,1.539657
53468031e-86,"7","SEMA3C"
"GYPC.1",4.61493532315947e-90,-0.502759482210723,0.835,0.91,1.21981970
461751e-85,"7","GYPC"
"RGS16.1",1.75201813154652e-85,0.832820629720214,0.4,0.182,4.630934325
30377e-81,"7","RGS16"
"LIFR",5.62751647995831e-85,0.528213616406584,0.573,0.319,1.4874651559
8258e-80,"7","LIFR"
"TMEM37",1.22930276128225e-84,-0.566161621106793,0.184,0.465,3.2492930
5862124e-80,"7","TMEM37"
"PLP2",1.88760484365507e-84,0.508656743361798,0.765,0.535,4.9893171227
4908e-80,"7","PLP2"
"GPC3.3",4.01450213177891e-84,-0.727742247844659,0.902,0.92,1.06111320
34718e-79,"7","GPC3"
"C11orf96.3",1.57171219555699e-83,0.783645418757793,0.852,0.659,4.1543
4967529623e-79,"7","C11orf96"
"TMEM176B",3.63686310900564e-83,-0.573366012157333,0.804,0.902,9.61295
656972372e-79,"7","TMEM176B"
"MXRA5",9.22435588802545e-83,0.507421894178358,0.666,0.399,2.438181748
32289e-78,"7","MXRA5"
"IGFBP3.5",1.92092863010991e-82,0.830136633389318,0.77,0.572,5.0773985
5510651e-78,"7","IGFBP3"
"TNXB.3",4.24620620218988e-82,-0.531150567492948,0.988,0.99,1.12235722
336283e-77,"7","TNXB"
"CD9.1",8.0054398963393e-81,0.673690138524481,0.734,0.488,2.1159978734
004e-76,"7","CD9"
"SLIT2",1.85106069579743e-78,0.506775247497199,0.573,0.338,4.892723631
13178e-74,"7","SLIT2"
"NTM.3",1.69223167966127e-76,-0.579344764901606,0.209,0.467,4.47290677
568066e-72,"7","NTM"

"LAMB1",2.45774026459352e-76,0.520949067103349,0.643,0.402,6.49629906737359e-72,"7","LAMB1"
"GALNT15",5.33762215931052e-75,-0.637097129150597,0.286,0.545,1.41084028914896e-70,"7","GALNT15"
"CPB1.1",1.1613242387645e-73,-0.639304378110404,0.216,0.479,3.06961222790233e-69,"7","CPB1"
"MARCKS.3",2.48185696769486e-72,-0.79102179154698,0.956,0.957,6.56004433701106e-68,"7","MARCKS"
"LMNA",7.49477565635193e-71,0.601514020249379,0.99,0.976,1.98101910148694e-66,"7","LMNA"
"PDGFD.1",3.04777430344791e-70,-0.55069171565807,0.381,0.618,8.05587703887353e-66,"7","PDGFD"
"METRNL.3",4.30417870083895e-70,-0.585825270352094,0.73,0.824,1.13768051420575e-65,"7","METRNL"
"RAB13",4.79211444178029e-70,0.565766281532813,0.719,0.531,1.26665168925137e-65,"7","RAB13"
"NR2F2",6.37399359617762e-69,0.676987911366195,0.724,0.531,1.68477398734167e-64,"7","NR2F2"
"TSPAN8",1.2570463202818e-68,0.647459797988224,0.348,0.166,3.32262483376885e-64,"7","TSPAN8"
"PTGIS.3",1.41463908053723e-68,-0.539086985263889,0.51,0.696,3.73917401767602e-64,"7","PTGIS"
"RASD1.2",2.57119346914651e-68,-0.87597275172532,0.489,0.695,6.79617857764805e-64,"7","RASD1"
"CLEC3B.4",1.01411287931931e-67,-0.782087398641404,0.807,0.866,2.6805031626168e-63,"7","CLEC3B"
"ADAMTS5.5",2.13349566177688e-65,-0.927903796163253,0.641,0.761,5.63925573320866e-61,"7","ADAMTS5"
"PRSS23.2",1.03444675583852e-63,0.727409646949563,0.796,0.659,2.73424966503237e-59,"7","PRSS23"
"COL15A1.4",1.66909523161842e-63,0.792839108666134,0.533,0.316,4.41175251621381e-59,"7","COL15A1"
"VSIR.2",1.03738541677723e-62,-0.550974405744467,0.57,0.698,2.74201713362557e-58,"7","VSIR"
"RHOB.2",1.2385091937078e-61,-0.563300622473128,0.881,0.915,3.27362750080846e-57,"7","RHOB"
"OSR2.1",2.29948209553917e-60,-0.560111032835296,0.503,0.684,6.07799107492914e-56,"7","OSR2"
"SH3BGRL3.3",7.00668845394702e-60,0.794269013193706,0.946,0.918,1.85200789214728e-55,"7","SH3BGRL3"
"PLA2G2A.1",3.01462448374964e-56,-0.780658563323253,0.772,0.89,7.96825543544705e-52,"7","PLA2G2A"
"DEPP1.5",9.93359406575498e-56,-1.00954197713155,0.368,0.573,2.62564758346036e-51,"7","DEPP1"
"CCL2.5",1.58323233547182e-55,0.893993348235291,0.562,0.365,4.18479970911912e-51,"7","CCL2"
"DHRS3.2",4.79801290752446e-53,-0.539986042173981,0.52,0.673,1.26821077171687e-48,"7","DHRS3"
"F10.3",4.85147812321871e-51,-0.512121285078618,0.748,0.833,1.28234269752917e-46,"7","F10"

"CD55.6",3.05756179175379e-50,-1.06909904779796,0.533,0.643,8.08174732796362e-46,"7","CD55"
"TAGLN",7.35640629965238e-48,-0.506437854954371,0.623,0.767,1.94444531312412e-43,"7","TAGLN"
"COL6A3.1",5.43563171548182e-45,0.50735153065874,0.998,0.994,1.43674617503615e-40,"7","COL6A3"
"SVEP1.3",4.50562696438739e-44,-0.518577782462672,0.735,0.827,1.19092731922687e-39,"7","SVEP1"
"LINC01133.3",6.05066415717491e-44,-0.555233382057361,0.4,0.544,1.59931155002447e-39,"7","LINC01133"
"ASPN",4.58337397379033e-41,-0.521785603158481,0.619,0.733,1.21147740875226e-36,"7","ASPN"
"KLF2.2",8.63099220684843e-36,-0.514649312952405,0.831,0.874,2.28134386011418e-31,"7","KLF2"
"MEG3.1",9.76730102280338e-35,-0.508798070894801,0.805,0.881,2.58169300634739e-30,"7","MEG3"
"GADD45B.2",3.28212365521607e-34,-0.572140638773533,0.838,0.885,8.67530924546711e-30,"7","GADD45B"
"CREB5.4",3.81023156874317e-32,-0.517314072828392,0.393,0.529,1.0071204082502e-27,"7","CREB5"
"PTX3.3",6.80486279662097e-18,-0.55434162388007,0.152,0.258,1.79866133440285e-13,"7","PTX3"
"APOD.6",8.25055979639271e-15,0.621771162195317,0.983,0.971,2.18078796538252e-10,"7","APOD"
"MYOC.5",1.66631613676238e-13,0.600878507953643,0.673,0.622,4.40440681269032e-09,"7","MYOC"
"PTGDS.3",3.12885051278212e-05,0.819886620776356,0.41,0.361,0.827017767538571,"7","PTGDS"
