# Supplementary material for: PPARγ agonist treatment reduces fibroadipose tissue in secondary lymphedema by exhausting fibroadipogenic PDGFRα+ mesenchymal cells
Source: JCI Insight. 2023 Dec 22;8(24):e165324. doi: 10.1172/jci.insight.165324 (PMC10807713; doi:10.1172/jci.insight.165324)
Supplement: Supplemental table 10 [file jciinsight-8-165324-s192.pdf]

Supplementary Table 10. Antibody information.

Antibody	Cat No.	Company
APC-PDGFRα	135908	BioLegend, San Diego, CA, USA
Ki67	AB9260	EMD Millipore Corporation, Temecula, CA, USA
FITC-CD4	100406	BioLegend, San Diego, CA, USA
PDGFRα	3174	Cell Signaling Technology, Massachusetts, USA
pSMAD2/3	8828S	Cell Signaling Technology, Massachusetts, USA
PPAR-gamma	ABN1445	EMD Millipore Corporation, Temecula, CA, USA
Alexa Fluor Plus 488 goat anti-rabbit IgG	A32731	Invitrogen, Carlsbad, CA, USA
Alexa Fluor 594 goat anti-rabbit IgG	A32740	Invitrogen, Carlsbad, CA, USA
