# Supplementary material for: PPARγ agonist treatment reduces fibroadipose tissue in secondary lymphedema by exhausting fibroadipogenic PDGFRα+ mesenchymal cells
Source: JCI Insight. 2023 Dec 22;8(24):e165324. doi: 10.1172/jci.insight.165324 (PMC10807713; doi:10.1172/jci.insight.165324)
Supplement: Supplemental table 11 [file jciinsight-8-165324-s193.pdf]

Supplementary Table 11. Real-Time PCR primer sequences.

Gene name	Forward sequence	Reverse sequence
Adipoq	CCA CTT TCT CCT CAT TTC TG	CTA GCT CTT CAG TTG TAG TAA C
Col1a1	GAT CTG TAT CTG CCA CAA TG	TGG TGA TAC GTA TTC TTC CG
Ctgf	GAG GAA AAC ATT AAG AAG GGC	AGA AAG CTC AAA CTT GAC AG
Fn1	CCT ATA GGA TTG GAG ACA CG	GTT GGT AAA TAG CTG TTC GG
Lpl	GAG ACT CAG AAA AAG GTC ATC	GTC TTC AAA GAA CTC AGA TGC
Pdgfra	CTA GTT CCT GCA TCC ATT TTG	ATA TTT GAG ACA TTG CTG GC
Actb	GAT GTA TGA AGG CTT TGG TC	TGT GCA CTT TTA TTG GTC TC
