# Supplementary material for: PPARγ agonist treatment reduces fibroadipose tissue in secondary lymphedema by exhausting fibroadipogenic PDGFRα+ mesenchymal cells
Source: JCI Insight. 2023 Dec 22;8(24):e165324. doi: 10.1172/jci.insight.165324 (PMC10807713; doi:10.1172/jci.insight.165324)
Supplement: Supplemental data set 1 [file jciinsight-8-165324-s183.pdf]

"","gene","baseMean","log2FoldChange","lfcSE","stat","pvalue","padj"
"1","C1QA",94.2094543460429,-2.508374526504,0.277674656130756,-9.03350
187394423,1.6626413300135e-19,2.81119396078683e-15
"2","PLP2",822.927428725872,-1.25533179605696,0.164764013530593,-7.618
96829991871,2.55711246537447e-14,2.16178287822758e-10
"3","C1QB",76.9372135785642,-2.5252090509851,0.376227615014989,-6.7119
1839781484,1.92082111990229e-11,1.08257478317693e-07
"4","GSTM1",18.5955089030212,7.56366033129122,1.15116281761154,6.57045
22553851,5.01626671555836e-11,2.12037594066652e-07
"5","CCN3",98.9951405080189,-2.84438818516812,0.4352710513139,-6.53475
156820585,6.37152135553083e-11,2.15459366158631e-07
"6","AIF1",81.938293399756,-1.81814202372464,0.28467506117343,-6.38672
743664397,1.69473307727961e-10,4.77575781177394e-07
"7","FOLR2",25.065488474076,-2.73911176527422,0.430905883205108,-6.356
635803857,2.06220132080838e-10,4.98109999031829e-07
"8","MS4A4A",26.6331842408457,-3.12529061769987,0.496007631809285,-6.3
0089219857316,2.95937173576733e-10,6.25463216354425e-07
"9","ADD3",2929.48550322076,0.683432015729626,0.111031710568421,6.1552
8673953444,7.49417789686829e-10,1.26711559880249e-06
"10","ANOS1",48.6436331092208,3.45931015114133,0.560765597389119,6.168
90580885777,6.87641729617193e-10,1.26711559880249e-06
"11","RNASE1",83.893899344073,-2.59224910746032,0.42429958011455,-6.10
947837082581,9.99573008116199e-10,1.53643458374806e-06
"12","LUZP2",43.627417873464,2.73080952705641,0.460961048765611,5.9241
6546770955,3.13887282247489e-09,4.42267180686712e-06
"13","CEP152",90.8332597992814,1.46480380027865,0.24789610182963,5.908
94245398567,3.44310944718455e-09,4.4781611179228e-06
"14","LARP6",1076.35044836372,-0.929319510831961,0.163967680662647,-5.
66769931169532,1.44727657185003e-08,1.74789659120288e-05
"15","CAV1",3861.3521532817,-1.07591344179671,0.190322519708507,-5.653
10633468149,1.57573754181342e-08,1.77617135713209e-05
"16","VEGFC",300.235821420056,2.1233125454716,0.378910475756675,5.6037
3143875579,2.09785675072723e-08,2.216910121331e-05
"17","CD9",1547.00946746801,-1.98778912857994,0.357245647202834,-5.564
20811322392,2.63345515324507e-08,2.61920351359222e-05
"18","EYA2",33.8483422659416,2.94573989087591,0.539255000957947,5.4626
1024124582,4.69183980939287e-08,4.40720152762304e-05
"19","PLXNC1",82.4396324291277,2.17087033415776,0.398991014070604,5.44
090031504722,5.30119731851694e-08,4.71750759270971e-05
"20","VEGFD",700.823153416884,1.24047389474923,0.230789436003622,5.374
91627099327,7.66182775506676e-08,6.46569231838411e-05
"21","KLHL13",64.9625659275044,2.02007689588612,0.376427586762789,5.36
644222401026,8.03049081417473e-08,6.46569231838411e-05
"22","PRR16",83.4904942951988,-1.27485164516339,0.24237975194694,-5.25
972831857042,1.44268395612731e-07,0.00011087681968273
"23","FXYD5",687.770523798884,-1.35720350802595,0.260199798360303,-5.2
1600522590186,1.82823019532845e-07,0.00013439876583745
"24","PI16",513.628624448226,1.57237125975884,0.303812861256767,5.1754
5983160321,2.27350341829225e-07,0.000160168315818689
"25","TYROBP",108.197743143638,-1.54612372680108,0.301180148406114,-5.

13355124825915,2.84325563212943e-07,0.000192295064912178
"26","MXRA5",594.317804210379,1.41947290322525,0.277722768860919,5.111
11461637525,3.20263596288145e-07,0.000208269880232306
"27","F13A1",21.9122727285787,-2.47169864901264,0.4849742848823,-5.096
55609804653,3.45888098374404e-07,0.000216602813604238
"28","SCARA5",2744.19204128251,0.77096077875821,0.15221632913561,5.064
90192698945,4.08610657521054e-07,0.000245380049896423
"29","CD163",35.6767858885383,-2.29906562259164,0.454426458242477,-5.0
5926884513595,4.20867130766281e-07,0.000245380049896423
"30","PTGDR",47.8873862532031,-1.92043136494834,0.380448202580618,-5.0
4781295304291,4.46896351029201e-07,0.000251870783440058
"31","C1QC",36.1499334446798,-2.55132333090325,0.507957257199638,-5.02
271262934339,5.0946744870826e-07,0.000277873407185783
"32","KCP",66.1335343451424,2.93071363715048,0.585099849362349,5.00891
196664025,5.47386020761013e-07,0.0002892250887196
"33","MMP23B",359.017306763699,2.02307087987565,0.404973393316485,4.99
556492664355,5.86637824788007e-07,0.000300571889136837
"34","S100A3",35.6860699885806,-1.49002041380447,0.298872434114261,-4.
98547287648087,6.18104504470443e-07,0.00030493588634646
"35","ESR1",53.898001740371,1.4096090806392,0.282973892529352,4.981410
36277044,6.31225220139941e-07,0.00030493588634646
"36","SLCO2B1",18.2655591442961,-2.96410515702779,0.597398236407153,-4
.96169050456926,6.98822838135063e-07,0.00031934314992399
"37","CKB",906.42710463476,-0.785652805799048,0.15818059130095,-4.9668
0913465726,6.80635171204701e-07,0.00031934314992399
"38","MS4A7",38.6328178319978,-2.00176582308503,0.404361915855855,-4.9
5043114742439,7.40492516944099e-07,0.000329480196749759
"39","TMCC3",61.7482871494527,1.65255322734779,0.334447293859292,4.941
14695406402,7.76643426596837e-07,0.000336704796330752
"40","PHACTR2",778.591455310327,1.02846072340515,0.209706348300604,4.9
042898879289,9.37659281068298e-07,0.00039634857810757
"41","CDH12",25.6262149181097,-3.15297712654155,0.650181876851021,-4.8
4937713399848,1.23849754230564e-06,0.000510744303543995
"42","MARCKS",6095.65784711579,1.52462180233683,0.315730571226544,4.82
886974300275,1.37310186249149e-06,0.000552771578357289
"43","VGLL3",713.362122776783,1.60137084909588,0.333245600231813,4.805
3773192562,1.54459927286558e-06,0.000607350802456075
"44","FLRT2",406.587843275701,1.18053516057686,0.248741732649093,4.746
02772926035,2.07450285120248e-06,0.000797174868366625
"45","IL17RE",57.3011894254766,-1.16273684485419,0.245344586481013,-4.
73919910576131,2.14564593150066e-06,0.000806190697995849
"46","CX3CL1",16.6972934127281,-2.68156763374734,0.567932658215683,-4.
72162957166828,2.33962506843179e-06,0.000859964796892276
"47","IL17RD",139.035930729285,1.21462844111441,0.257730609127516,4.71
278303041396,2.44356336545211e-06,0.000860745195480506
"48","IGSF10",545.240722226288,1.76557685634222,0.374299562405389,4.71
701555031473,2.39329404649793e-06,0.000860745195480506
"49","EDN1",15.8364490582004,2.22982171071459,0.473684170969019,4.7074
0178240077,2.50894097563926e-06,0.000865738245226705
"50","NTM",301.401909271661,1.33777250084896,0.284901141332466,4.69556

736274302,2.65867730864889e-06,0.00089905831869271
"51","PCSK5",432.272144603389,1.71735844412103,0.369533012640184,4.647
37489040858,3.36185862848462e-06,0.00111455501353761
"52","CCL18",12.4388948174529,-3.79867948491883,0.822094841615426,-4.6
2073144438466,3.82389495540496e-06,0.00124335415203821
"53","STEAP4",370.883602662814,1.44578884397853,0.31738272767373,4.555
34822129579,5.22988672465969e-06,0.00165197429186207
"54","LRRC4C",119.102849731283,-1.54732951685329,0.339810829103589,-4.
55350266774928,5.27600022241256e-06,0.00165197429186207
"55","ADIRF",3090.86682314392,-1.61974956203048,0.356203549728805,-4.5
4725833940643,5.43492697862118e-06,0.00167079537008231
"56","STAC",85.7291365533254,1.18183859697401,0.261820114212207,4.5139
3354758115,6.36361637446561e-06,0.00192135760106187
"57","IL10",17.8017006068211,-2.80141206621756,0.622227443479385,-4.50
223161253153,6.72436428070407e-06,0.00199465879400254
"58","KCND2",230.935553966094,1.12402490025786,0.250077169375063,4.494
71218451,6.96641078805475e-06,0.00199640802719372
"59","TET1",273.742979100902,0.84233532855097,0.187355784395123,4.4959
1311669637,6.92720201502862e-06,0.00199640802719372
"60","ST8SIA4",25.2824470852703,-1.99018745206073,0.444039089641825,-4
.48200957637778,7.39434246918717e-06,0.00208372570781694
"61","RBMS3",1377.34031902294,0.848134964913384,0.189786960979229,4.46
887900273723,7.86305914728242e-06,0.0021443323235847
"62","C3AR1",13.4266754875872,-2.49598227251773,0.558361529864983,-4.4
7019026028044,7.81500457502566e-06,0.0021443323235847
"63","LRRC61",20.376121547252,-1.68728988791864,0.378643976656658,-4.4
5613819825428,8.34492483477504e-06,0.00223961887470439
"64","S100A6",39753.3550653754,-0.93514830036114,0.211340936745727,-4.
42483275961937,9.6517024955706e-06,0.00254985915304856
"65","ECHDC3",27.9835975676673,-2.49828180956309,0.565051796297168,-4.
42133239100298,9.80941396652287e-06,0.00255165494378413
"66","FHDC1",32.9541211781072,1.69406303639146,0.386296317421243,4.385
397840964,1.1577398390519e-05,0.0029659189694984
"67","GAB1",233.913659175779,0.676859716249143,0.154962367053023,4.367
89737483516,1.25448411345472e-05,0.00314973162723314
"68","SYNE2",565.112756860899,1.06322152243168,0.243713941164937,4.362
57982349943,1.28537663992836e-05,0.00314973162723314
"69","TOX2",32.6757612024217,-2.31060904171082,0.529402136370184,-4.36
456312313618,1.27377065382261e-05,0.00314973162723314
"70","TAGLN2",1860.16649731315,-0.904018504049353,0.207566165192383,-4
.35532690605649,1.32868475975306e-05,0.00320934313112925
"71","LYVE1",18.3311356380656,-2.59326263436183,0.596878306155911,-4.3
4470914358284,1.39460319811312e-05,0.00332111984136573
"72","SNTA1",383.654520883973,-0.589293519417925,0.13573107631242,-4.3
4162562788138,1.41432372616729e-05,0.00332130355028286
"73","NEGR1",2425.8620987721,0.795960112407001,0.183471909016785,4.338
32141755381,1.43575078701805e-05,0.00332543483656182
"74","IL7",46.4783298369151,-1.406432581804,0.324434278660889,-4.33503
077297838,1.45739733150946e-05,0.00332995595691377
"75","STAB1",23.1319687100723,-2.7107216891684,0.625739689716647,-4.33

202773248393,1.47742326633078e-05,0.00333070301161611
"76","CAPG",342.86020023659,-1.55795485235828,0.360064584307839,-4.326
87612238559,1.51238961190608e-05,0.00336466888922474
"77","UBXN11",86.495192336276,-0.988109849463266,0.229223472776161,-4.
31068353295635,1.62750684689816e-05,0.00357375139835768
"78","MUC12",30.3891830882277,-2.23651272459518,0.522311261430487,-4.2
8195386496148,1.85259397596467e-05,0.00392245415214535
"79","A2M",375.449740015994,-1.42171022996235,0.332054516913858,-4.281
55666477855,1.85590449592872e-05,0.00392245415214535
"80","CRIP1",7361.95023001666,-1.06146437385374,0.247678238000289,-4.2
8565861265735,1.82198598102635e-05,0.00392245415214535
"81","PERP",315.42295358118,-1.3977137483123,0.327810801592275,-4.2637
8185686129,2.00995679068339e-05,0.00419559869344133
"82","PDGFD",713.832385599678,1.31842245610611,0.309552411843597,4.259
12512926,2.05228567300774e-05,0.00423171294624571
"83","FBN1",6132.59483029909,1.25730365654419,0.296211923823656,4.2446
085232298,2.18975372573013e-05,0.00446076578248735
"84","IGF2-
AS",7.68045324617594,3.80681420000592,0.898584078362588,4.236458548144
71,2.27072817370417e-05,0.0045706514239274
"85","SMOC2",1472.2781338819,1.57034258326157,0.370992405825946,4.2328
1597844433,2.3078333503294e-05,0.00459068779851406
"86","ANKRD33B",98.5461999310754,1.02487834663958,0.242425674806777,4.
22759820079475,2.36199097880787e-05,0.00464378412438179
"87","MAF",1319.80160927337,0.795194689579076,0.18822839952591,4.22462
652597551,2.39337356527894e-05,0.00465139772893521
"88","AL161431.1",38.3643859353258,-1.46589778923952,0.34786080825953,
-4.21403548325527,2.5084785010049e-05,0.00481969937443078
"89","C16orf89",289.061710135931,1.25037669347058,0.297494260250697,4.
20302795898278,2.63368043685376e-05,0.00500340099172172
"90","SERPINA5",328.918324395104,-1.34384222777833,0.320140875732692,-
4.19765899841044,2.69688305709764e-05,0.00506654430326742
"91","CTSO",564.887577382676,0.606045944589627,0.144680547994207,4.188
8557445462,2.80364541165354e-05,0.00518208022590798
"92","GAB2",100.809095038064,1.06346755597479,0.253958686485136,4.1875
6125531084,2.81967932803131e-05,0.00518208022590798
"93","HRCT1",311.802063035641,-1.58082327757119,0.380919023477587,-4.1
5002449376016,3.32439698758847e-05,0.00604396820066084
"94","ADAMTSL3",110.485348460097,1.45926133138377,0.351873827555212,4.
14711529278149,3.36690540932984e-05,0.00605613155967543
"95","MAN1C1",585.403288764503,0.925635029593518,0.223418862237494,4.1
4304781755432,3.42720450987441e-05,0.00609970251083753
"96","TBC1D12",223.443896981966,0.735283200276381,0.177613805961886,4.
1397862981111,3.47629507309743e-05,0.00612262469749285
"97","ADAMTSL1",187.873389931761,1.69551345696445,0.412619273687987,4.
10914750009123,3.97122380725333e-05,0.00685157674826931
"98","ARHGAP12",431.926030972244,0.532671944668063,0.12962008586746,4.
10948612711716,3.96540534589618e-05,0.00685157674826931
"99","AQP7",133.860333366335,-1.75492204288176,0.427446110682535,-4.10
559834099215,4.03269674183001e-05,0.00688735722331938

"100","GPC1",167.213562802557,-1.0015003945451,0.24456884985311,-4.094
96301408216,4.22235788120789e-05,0.00691234551609653
"101","PLEKHA2",295.575669599437,1.01841089614856,0.248650041871791,4.
09576000262117,4.20785675077528e-05,0.00691234551609653
"102","GRK5",192.904046947439,0.713150957059711,0.174314164494849,4.09
11819135661,4.29180305038315e-05,0.00691234551609653
"103","SAA1",9.92991847372806,-3.2841343185124,0.802347047794919,-4.09
31593473649,4.25535068368991e-05,0.00691234551609653
"104","ACSM5",33.4586367261448,-1.75379808459203,0.4286822374352,-4.09
11377506215,4.29262052986832e-05,0.00691234551609653
"105","DBNDD2",558.285600066595,-1.61774836845724,0.39490890836082,-4.
09651019312824,4.19425028618934e-05,0.00691234551609653
"106","FRMD6",804.08127863377,0.937462711491959,0.229832731158172,4.07
889123001715,4.52510009098094e-05,0.0072179615413496
"107","PPP1R9A",33.9389771817812,2.20634072351488,0.542617327749257,4.
06610812202892,4.78047636175222e-05,0.00755404619855201
"108","LILRB5",21.3799220536672,-2.64892146633464,0.652962211620265,-4
.05677605716507,4.97547463817145e-05,0.00778938196131508
"109","IL33",608.0964166671,0.678674064173734,0.168137765903365,4.0364
1656904014,5.42738137575134e-05,0.00841891415607373
"110","VSIG4",23.5810343252459,-1.77063959164629,0.442173122212111,-4.
00440348519626,6.21741681485614e-05,0.00955673486414434
"111","CRIP2",420.659156467061,-1.28506145379689,0.321445508427351,-3.
99775831394862,6.39451919907102e-05,0.00974040816377412
"112","ACP5",25.2113936397373,-2.41964578572398,0.605956821903322,-3.9
9309934018703,6.52152300963642e-05,0.0098451706291904
"113","APBB2",282.702094152655,0.967149361947946,0.242448026175329,3.9
8909975554324,6.6324527220236e-05,0.00992402748884734
"114","KCNIP4",9.36170744638403,-2.81914474366552,0.707823080740126,-3
.98283811361127,6.80971433852809e-05,0.0100998815820906
"115","NAV2-
AS3",14.2186709402637,-2.40470534361971,0.604483752985017,-3.978114104
38241,6.9464036758141e-05,0.0102130255087535
"116","LTBP2",858.981981920757,1.04340407109697,0.262809999501698,3.97
01840610149,7.18171299815914e-05,0.0103784960147756
"117","TNFRSF12A",85.3479532378178,-1.88336608021252,0.474319027638012
,-3.97067368262918,7.16696866015784e-05,0.0103784960147756
"118","ADAMTS16",51.1799808277588,2.56455275407794,0.64697034890998,3.
96394171448308,7.37222595767238e-05,0.0105635251264682
"119","PTGER2",64.8585531302021,-1.51556234946937,0.383660506804746,-3
.95026937250197,7.80632863977034e-05,0.0110915466085073
"120","FBLL1",12.2600046123409,2.09243076970706,0.530316505372305,3.94
562633542414,7.95916530142332e-05,0.0112144639097055
"121","MUC20",56.4993842621389,2.19298149344946,0.55739751636919,3.934
32232661214,8.34317583997995e-05,0.0115628210739657
"122","GPD1",141.973114358262,-2.08612884006032,0.530181521950779,-3.9
347445236954,8.32852387388315e-05,0.0115628210739657
"123","GPX3",8244.17397379865,0.839691981497213,0.213779639985621,3.92
783887910791,8.57125995005719e-05,0.0117823466045176
"124","CTHRC1",1738.03696245748,-1.27279524085781,0.324675551829959,-3

.92020659912332,8.84730896727555e-05,0.0120637338724754
"125","PLEKHG3",42.8570003541456,1.15742066625636,0.295881672741935,3.91176869973238,9.16226416296761e-05,0.0123932449973965
"126","CDH13",58.7605934137461,-1.9147064629988,0.490428192677491,-3.90415251730425,9.45561397609599e-05,0.0126885334212564
"127","RGL1",306.335863796591,0.602815323797056,0.154693511398678,3.89683651464522,9.74573587353306e-05,0.0128735079804451
"128","CYBB",14.7224525685284,-2.08684385830934,0.535444005498319,-3.89740820119406,9.72276588448577e-05,0.0128735079804451
"129","SLC25A33",147.812802743394,-1.52897475300992,0.392671928431566,-3.89377147258028,9.86976350219288e-05,0.0129362760693858
"130","EBF1",3160.93028776645,0.465180496266227,0.119721465602553,3.88552290038378,0.000102109866544119,0.0132805663348304
"131","FCGR2B",25.5213163645491,-1.94051276317785,0.500689760110537,-3.87567894887534,0.000106327803473671,0.013723591611701
"132","UCP2",172.181266070409,-1.72409009271448,0.445569454886024,-3.86940817825024,0.000109099859483606,0.0139747001829455
"133","LMNA",6776.77079693155,-0.875473045981118,0.226533186434609,-3.86465691742623,0.000111245454064478,0.0141423920099413
"134","AC097518.2",8.33749005705661,3.94803342782219,1.02870712690752,3.83785950787636,0.000124111452636583,0.0155880247488754
"135","CXCL12",15588.2536438057,0.659341784950029,0.171830268542065,3.83716903048788,0.000124460807966535,0.0155880247488754
"136","ZNF790-AS1",24.7384866373772,1.36608915642756,0.356465660186862,3.83231629019033,0.000126942387568578,0.0157819256544817
"137","TRIL",182.775580630873,1.29462568167772,0.338640931785056,3.82300413258799,0.000131835569128305,0.0162706262979663
"138","AL513534.2",60.3782171839686,1.24146461581101,0.325839681953758,3.81004734710978,0.000138940180630794,0.0170231925659817
"139","NATD1",90.020064420443,0.851883901315143,0.224295578756774,3.79804143281364,0.000145843948666089,0.0177404998852248
"140","MRC1",25.0958936722171,-2.20327581981917,0.580645436933674,-3.79452877724215,0.000147924125375772,0.0178650079418111
"141","RFX2",138.846744026719,-1.01746989768972,0.268661284126282,-3.78718467381206,0.000152363858885339,0.0182706959293142
"142","TUBA1C",240.795953177114,-0.875299358309562,0.231373671776507,-3.7830551401503,0.00015491507850354,0.018445803854492
"143","FOLH1",43.3958453730123,1.54781955214101,0.409916771595632,3.77593613970955,0.000159407831532992,0.0188480252836352
"144","FAM43A",378.066638439096,1.19060777838617,0.315800603573305,3.77012508815489,0.000163165745799941,0.0191583779860098
"145","TRIO",471.408585330974,0.924243038340564,0.245610070087167,3.76305026097891,0.000167853450638998,0.0194388092013985
"146","SLC6A15",17.1751217699936,-2.83981809700913,0.754410943107581,-3.76428539770553,0.000167026037651122,0.0194388092013985
"147","NRG2",26.5858238718809,-1.25954781452618,0.336557426409618,-3.74244546603231,0.00018223808961048,0.0209610994498911
"148","NCF4",14.0104274772567,-2.00119869086971,0.537274047255677,-3.72472614504937,0.000195527364883165,0.0223376803070578

"149","IL1RN",15.6919412666152,-2.6859047710602,0.721691412342799,-3.72168038184222,0.000197901419114946,0.0224571623784933
"150","PRDM6",34.8669579942229,1.45940036632556,0.392977331299637,3.71370114784762,0.000204249979291568,0.0226355696681956
"151","CAV2",393.69245841618,-0.728229766819813,0.1961305162956,-3.71298551889933,0.000204828611262948,0.0226355696681956
"152","ABCA1",789.218363937738,1.51419879043559,0.407524934609532,3.71559789804375,0.000202723754386085,0.0226355696681956
"153","MAOA",271.965774659546,-1.36438724589474,0.367297494845374,-3.71466526464908,0.000203472856455757,0.0226355696681956
"154","SIGLEC1",7.90836545983337,-2.87445262708703,0.775807205016789,-3.70511205425687,0.000211297301335135,0.0231987972141199
"155","SEMA3E",109.828323361803,-1.98877766988217,0.537144328054637,-3.70250148053295,0.000213484114411041,0.0232513795656175
"156","DLGAP2",11.5887343587476,2.36787011782509,0.639746026271199,3.70126584705243,0.000214526568029118,0.0232513795656175
"157","TGFBR2",2237.92419105874,0.548844473129436,0.148530917342479,3.69515305600601,0.000219754368125484,0.0236662857086987
"158","SPON1",79.6178122116548,1.42766878524998,0.38752721293759,3.68404782319094,0.000229559142606572,0.0245657340708349
"159","ENHO",29.960667703455,-1.59615782476255,0.433697659165395,-3.68034687536517,0.000232916893557705,0.0247682945677589
"160","SNCG",329.582480892602,-1.53675294950458,0.41820096340093,-3.67467577551057,0.00023815164315991,0.0251666748909235
"161","HLA-
DMB",28.6220367257834,-1.40563718693656,0.383487325324539,-3.66540715719092,0.000246945388363151,0.0259338672449948
"162","ST3GAL5",676.232180587091,0.721751028115571,0.196999701915407,3.66371634625871,0.000248582064183741,0.0259446021062882
"163","MAN2A1",193.529164595759,0.823353313870097,0.225269762693074,3.65496595737929,0.000257216106232845,0.0266810424796622
"164","LY86",6.33447037584935,-2.46359894490292,0.675078851822456,-3.64934990668445,0.000262904796579448,0.0269039675230778
"165","DOCK4",115.566862818292,1.00282826649088,0.274868826813805,3.64838849903556,0.000263890385826063,0.0269039675230778
"166","AL592183.1",44.9236737763294,-1.48751416901546,0.407745159852543,-3.64814672368742,0.000264138786895607,0.0269039675230778
"167","SUSD1",61.4298706138549,1.17967776633692,0.324570038382416,3.63458615039197,0.000278427210233024,0.0281895046144908
"168","SGIP1",25.1214592455146,-2.47807625327158,0.683995120052042,-3.62294434656645,0.000291268508739199,0.0284668667385108
"169","AC091938.1",9.59829022842867,-3.13835003721769,0.865518979422925,-3.62597483340013,0.000287873391855334,0.0284668667385108
"170","WASF1",146.618910507334,0.735051621206994,0.202875103845593,3.62317311130712,0.000291010915739622,0.0284668667385108
"171","LHFPL6",5671.29010671532,0.530202060753471,0.146231473686889,3.62577253299615,0.000288098873262716,0.0284668667385108
"172","REM1",60.9816324725717,-1.24993431769278,0.344835596674366,-3.62472531765074,0.0002892687333816,0.0284668667385108
"173","PIM3",258.138873555895,-0.886420037561824,0.244571973313172,-3.

62437292202231,0.000289663399570789,0.0284668667385108
"174","FMNL2",263.070247288552,0.710515385489086,0.196469454458313,3.61641654397652,0.000298709562901628,0.0287775364445682
"175","EMILIN2",396.662915039335,0.871732459327502,0.24109738782941,3.61568603947008,0.000299553253740478,0.0287775364445682
"176","MYADM",3308.6316469542,-0.856435476217061,0.236782921938788,-3.61696472534646,0.000298077907052443,0.0287775364445682
"177","SERTAD4-AS1",84.849191837388,-1.77326114387908,0.491729242600955,-3.60617386612922,0.000310744972780058,0.0296840451964137
"178","TBC1D4",235.319665270537,0.699061110952433,0.194090773206049,3.60172253119061,0.000316115663640191,0.0300274361844289
"179","LIFR",435.223783794497,0.906782421887786,0.251996120521691,3.59839834046069,0.00032018295846972,0.0301462654337826
"180","MT-CYB",75496.583662307,-0.56965214951716,0.158333907305629,-3.59779000727603,0.000320932563170148,0.0301462654337826
"181","NOX4",190.769623634025,0.908662337544261,0.253058548419624,3.59071978883523,0.00032976601616604,0.0308048828803061
"182","LINC01151",11.3405133408113,-2.38361392372937,0.664664287140947,-3.58619226253676,0.000335541612230677,0.0311721845032763
"183","SSC5D",631.627064063278,-0.714792142885186,0.199759092493334,-3.57827087600049,0.000345874890760852,0.0319565718742322
"184","FBXL7",247.272691582411,0.845407158265623,0.236521243462804,3.57433922589102,0.000351113477858935,0.0321911329231049
"185","PLIN4",32.4133615016306,-2.47006497857411,0.691214437303181,-3.57351473764239,0.000352221409437805,0.0321911329231049
"186","H19",2841.06546693733,1.55915987451104,0.436715736016452,3.5701939406468,0.000356717024234564,0.032426728203
"187","FCGR1A",7.38329989648781,-2.72833276055904,0.766258225370021,-3.56059180864459,0.000370019908874998,0.0331797267269627
"188","VAMP8",59.0031702151948,-1.1056649133155,0.310582061357238,-3.55997673685261,0.000370887647941564,0.0331797267269627
"189","RGCC",223.995806260193,-1.00941630496662,0.283402829525535,-3.56177214834641,0.000368360007243567,0.0331797267269627
"190","WLS",229.060452521346,0.644265494535642,0.181069282145136,3.55811591509614,0.000373524480554647,0.0332397469327261
"191","HDAC9",12.8881447423984,-1.78281495659098,0.50137397039217,-3.55585862424506,0.000376746642473491,0.0333509540887004
"192","LMO7DN",8.60160457214184,2.69354171846747,0.760799486469329,3.54040948551042,0.00039950664672764,0.0351815540774528
"193","IGFBP6",10826.5670292897,-0.884236470274573,0.250030707400013,-3.53651149280606,0.000405448859786578,0.0355198410428573
"194","PODN",3033.43849786799,0.634596417731523,0.179641137181882,3.53257849336041,0.000411528038046532,0.0358057029133481
"195","SDS",4.31054882547641,-4.48958004224559,1.27123497202748,-3.53166813455833,0.000412947247936059,0.0358057029133481
"196","AQP3",182.36608019349,-1.45300098456992,0.411777928481821,-3.5286033662051,0.000417758759034823,0.0360380872334734
"197","ROR1",158.583762823561,1.09017391438171,0.309926453282047,3.517

52457022313,0.000435592022285889,0.0364603461030189
"198","PNMA2",11.4757884137249,2.07489969435063,0.589406038571317,3.52032310252548,0.000431021395902349,0.0364603461030189
"199","TMC3-AS1",12.1983803652219,1.80795493197789,0.51394166103888,3.51782131910322,0.000435105229396171,0.0364603461030189
"200","SHMT1",127.886396431232,-0.795571713468631,0.226117722085404,-3.51839610859049,0.000434163777281247,0.0364603461030189
"201","TNFSF14",60.2600020740121,1.28363986238951,0.364280755503488,3.52376523600687,0.000425461032486342,0.0364603461030189
"202","APCDD1L",5.11558084992365,4.22871390050413,1.20129956969225,3.52011605363967,0.000431358012953144,0.0364603461030189
"203","TRIM47",327.033460258347,-0.930291155289231,0.264709782589131,-3.5143814716254,0.000440779330470661,0.036712792707379
"204","PLAGL1",1836.72350201038,0.870725530732996,0.248628125836854,3.50211999467973,0.000461571689175207,0.0382561476498745
"205","CPB1",1110.59938652724,1.18307909856397,0.338594380227931,3.49408958816021,0.000475681363787249,0.03869584059987
"206","HLA-DPA1",432.200867939024,-0.990452066631552,0.283311371285171,-3.49598416095554,0.0004723167351328,0.03869584059987
"207","GALNT8",45.3091916497795,1.2748928699031,0.364891754091478,3.49389334126604,0.000476031159496863,0.03869584059987
"208","AC018926.2",97.639674052157,-1.16316910263014,0.33255450862966,-3.49767954559735,0.000469324686218362,0.03869584059987
"209","AIF1L",39.6902861673452,1.58953065128574,0.456027849589043,3.48559995341989,0.000491034737141917,0.0396145004379941
"210","SWAP70",499.219337432952,0.667715758683408,0.191593511042888,3.48506457786005,0.00049201828081256,0.0396145004379941
"211","FBLN5",3797.15988070156,0.704863788935487,0.202494101044006,3.48091023541622,0.000499712932124097,0.040043347186513
"212","AL138756.1",11.1757963101525,2.01137454324245,0.578609228483794,3.4762227151356,0.000508529789019989,0.040557649399764
"213","LGALS2",15.2648405307282,-2.02362916126489,0.58434189151489,-3.46309102710177,0.000534007706051682,0.0423896821310884
"214","ENPP5",13.9627807809875,2.11074356526806,0.610861829478904,3.45535350779509,0.000549571745151205,0.0430767143478597
"215","SCN2B",7.52557379342057,2.30140819977833,0.666110689399766,3.45499364655464,0.000550305790107506,0.0430767143478597
"216","EHD4",213.03604435606,0.667924890987174,0.193246934775404,3.45632851441318,0.000547587507486149,0.0430767143478597
"217","ATP11A",195.985396830118,0.727131630183467,0.21056200399843,3.45328984515596,0.00055379361924778,0.0431499655034169
"218","MYO10",20.4627216401047,2.15942272262751,0.625564815780465,3.45195680472115,0.000556536813967725,0.0431647910576436
"219","HS6ST3",3.53098639515349,-4.83786191641913,1.40212601744007,-3.45037596923838,0.000559806336111903,0.0432201165798176
"220","IFRD2",140.917556064774,-0.760435207508556,0.220600225494293,-3.44711890391167,0.000566599154158398,0.0433067014404606
"221","MAST4",333.501285316561,0.918744549136269,0.266599437677853,3.4

4616086642478,0.000568611764832165,0.0433067014404606
"222","ITGA7",67.0360902611124,-1.29197482675941,0.374734088533725,-3.44771096703452,0.000565358688061851,0.0433067014404606
"223","RMRP",14.3103203793219,1.71961874509477,0.500023113772635,3.43907851003203,0.000583697930203164,0.0442563435151349
"224","EMCN",307.523642195409,-1.33197053482257,0.387847618315322,-3.43426250909621,0.000594168476287279,0.0444522150312624
"225","CYP2U1",160.946810891962,0.56925457905648,0.165733749668576,3.43475351396344,0.000593093025236388,0.0444522150312624
"226","GLIS2",159.965960246631,-0.608855901657621,0.177248711430409,-3.43503711109725,0.000592472686457335,0.0444522150312624
"227","C1orf162",38.1200972022384,-1.17826940442693,0.343589910669912,-3.42928988260922,0.000605162801713676,0.0450752980236776
"228","S100A4",24901.8370691717,-0.732006396684698,0.213581229458425,-3.42729742000661,0.00060962097180175,0.0452082078562455
"229","ANKRD36B",254.766239401145,-1.53274049903482,0.448177504386662,-3.41994072445113,0.000626347821344822,0.0456427649725506
"230","IER3",651.202365289006,-1.02065099621961,0.298157361750597,-3.42319569178832,0.00061889504674515,0.0456427649725506
"231","PLAT",161.808260414999,0.818953829300209,0.239354015922787,3.42151697828372,0.000622728367522445,0.0456427649725506
"232","TEAD1",659.478403744821,0.828643025349768,0.242378292216184,3.41880049476822,0.000628978249266874,0.0456427649725506
"233","ARHGAP20",302.864760528034,1.12761479276522,0.32972356329346,3.4198793119363,0.00062648923443159,0.0456427649725506
"234","MERTK",10.9525098926112,-2.3746193692232,0.695391328921001,-3.41479577104846,0.0006382985128828,0.046121159212916
"235","MEG8",334.206103960543,1.26621785897421,0.370990791929372,3.41307085383206,0.000642352390969375,0.0462165711766391
"236","TGFBR3",1882.45935305644,0.55796277148138,0.16356158584265,3.41133138693184,0.000646464704250422,0.0463153610994328
"237","ACADVL",1185.75465130769,-0.581149715046124,0.170564507712492,-3.40721362750172,0.00065629738005492,0.0466246895040697
"238","ABCA8",3944.21181925035,0.920394225961726,0.270123244198438,3.40731220185401,0.000656060381331482,0.0466246895040697
"239","TP53INP1",133.33056498534,0.658738992663826,0.19344680496232,3.4052720218984,0.000660981770327756,0.0467610032330615
"240","SLC43A2",107.756681184812,1.16817633174951,0.343328903107201,3.40249923958415,0.00066772541064638,0.0470412551800375
"241","GPRC5B",348.071199414601,0.786143114339888,0.231356374610964,3.39797472908115,0.000678866907081352,0.0476277247507531
"242","A4GALT",455.119882638777,-0.736597149077479,0.216995157992378,-3.39453265175324,0.00068745841896437,0.0480311857349156
"243","CRIM1",326.469608536651,0.818794536747626,0.241291942875507,3.39337703111818,0.00069036546461407,0.0480357994884555
"244","DTNA",28.7686288790491,-1.72811756437264,0.510898092545166,-3.38250932933335,0.000718268381884175,0.0497724663971214
"245","PLGLB1",8.97244578632363,1.84587827771442,0.546611190105383,3.37694930350501,0.000732945577621555,0.0505822196996949
"246","DHRS4",178.657296851935,-1.2836001431634,0.380250076160301,-3.3

75673599135,0.000736352207838385,0.0506107444314285
"247","NRN1",485.338235703459,-0.795121330700751,0.235825869441005,-3.37164592071889,0.000747204442193916,0.0511487154194928
"248","PARP16",109.971646676282,-1.01464936073271,0.301044368444029,-3.37043129548315,0.000750506181843245,0.0511675746879258
"249","FAM184A",207.231527393102,-0.723983670072393,0.215143139381918,-3.36512552597456,0.000765088433658479,0.0519522700252914
"250","SH3BGRL3",3359.32628679752,-1.00706994336007,0.299464711157189,-3.36290022109304,0.00077128235684353,0.0519555461733482
"251","HDGF",538.325307918686,-0.508644573093395,0.151207293796532,-3.36388913737083,0.000768524078054396,0.0519555461733482
"252","IL6ST",2988.13611603467,0.770136232896874,0.229479661324783,3.35601084841632,0.000790754668775821,0.0528461657694134
"253","MBNL2",739.011300384664,0.608872862924624,0.181380087385265,3.356889235759,0.000788246837876439,0.0528461657694134
"254","AGTR1",1138.0946051758,0.715894357062388,0.213423469536482,3.35433754599334,0.00079555251612659,0.0529574879632614
"255","MBP",633.221431594638,0.588942911928945,0.176273897455756,3.34106705774045,0.000834570634508025,0.0553369423069086
"256","AMPH",143.240233869064,1.8272787788575,0.547392991542815,3.3381479249622,0.000843388369306649,0.0557031662040501
"257","SCRN1",286.186836302503,0.463522623108663,0.138968185457209,3.33545855537843,0.000851588465288375,0.0560259057241083
"258","PDE10A",41.653941742723,1.01720583304859,0.30576048874218,3.32680601484224,0.000878475044611569,0.0575707599003582
"259","NLGN1",72.670752389866,0.717677626413681,0.215894821499803,3.32420028154466,0.000886724897014091,0.057870493477254
"260","RPL39L",77.5400645136425,-0.742063128946605,0.223476616260726,-3.32054038298509,0.000898433602778211,0.057870493477254
"261","FZD8",209.875607622689,1.11154387084625,0.334886713093917,3.3191638467141,0.00090287436521066,0.057870493477254
"262","LMO2",81.2568907623204,-1.31695935021377,0.396800685910279,-3.31894423819506,0.000903584710077777,0.057870493477254
"263","ZDHHC7",267.084988482493,0.585590812293811,0.176420309837996,3.31929363932956,0.000902454781892133,0.057870493477254
"264","DDX39A",130.07992019308,-0.676113186733078,0.203679327199451,-3.31949833117329,0.000901793437489261,0.057870493477254
"265","SPRED2",206.691781938204,0.71277360215597,0.214997137696813,3.31527019285772,0.000915545846225802,0.0584152798791919
"266","NFATC2",97.9590932810085,-1.51876691796266,0.458928673225785,-3.30937465137519,0.000935046243482611,0.0594351950556541
"267","SEPTIN11",1212.76821600972,0.842080582117874,0.254790993951321,3.30498566318539,0.000949812584363958,0.0601476823087108
"268","TM4SF19",3.93496558589746,-3.78593137767524,1.14629902826822,-3.3027432496343,0.000957440072594783,0.0604044654754947
"269","GPR34",9.48618156406884,-2.40381739731742,0.728185956401505,-3.30110375816148,0.000963052603383656,0.0605326892862857
"270","RERG",156.313224992382,-1.36224022978196,0.412944314975533,-3.2988472788702,0.000970827130039876,0.0605476941674071
"271","CMKLR1",124.555687322477,-1.09380215261845,0.331562977915841,-3

.29892727919727,0.000970550504150869,0.0605476941674071
"272","TBC1D2B",632.834315429638,0.656603344615419,0.199096123728071,3
.29792128706744,0.000974034351403757,0.0605476941674071
"273","S100A10",28624.5616583229,-0.875168907858854,0.265595987714191,
-3.29511343673093,0.000983819570530929,0.0609031089304447
"274","IQSEC3",13.3739332639326,1.73842125307267,0.5277187994768,3.294
21891885642,0.000986955988108697,0.0609031089304447
"275","LAMC1",3412.91734650359,0.875317368718183,0.265800052164205,3.2
9314220065477,0.000990743526472853,0.0609145147112837
"276","RECK",644.630179891599,0.574230418740622,0.174522469307805,3.29
029506067698,0.00100082376020864,0.061089993276562
"277","MAN2B1",355.080635842029,-0.47582026154804,0.144593213924712,-3
.29075098777316,0.000999203201833405,0.061089993276562
"278","PTMA",9287.45694715785,-0.603004964630858,0.183809037233683,-3.
2806056421711,0.00103584464696495,0.0629507322420203
"279","NRXN3",10.4537476345934,-2.41251758835492,0.735565284386913,-3.
27981436802817,0.00103875409838678,0.0629507322420203
"280","HRH2",26.5566403586831,1.95399799069088,0.596107406961107,3.277
92939304706,0.00104571549079955,0.0631462768515669
"281","MS4A6A",54.6375163361624,-1.71382817075082,0.524042736617602,-3
.27039771949252,0.00107396371718196,0.0646212759078738
"282","KCNS2",3.80891077983753,4.5112156538928,1.38263380969799,3.2627
6966630679,0.00110329150689563,0.0661505418389766
"283","ZEB1",1011.61499568332,0.601429453802493,0.184712203537421,3.25
603529319951,0.00112979706829003,0.0675003845605929
"284","ECM2",2000.91739610694,0.549191392494321,0.168798017041679,3.25
354173063962,0.00113975983403262,0.0676177518379773
"285","KCNK15-
AS1",41.9468130240556,-1.24978905381299,0.384125327656908,-3.253597104
45667,0.00113953771491233,0.0676177518379773
"286","ANTXR2",1286.6940556772,0.575189662198108,0.176898258605753,3.2
5152811978785,0.00114786420367976,0.0678604473979627
"287","PALMD",208.805658140939,-0.892082231779942,0.274642327645977,-3
.24816003209042,0.00116153921698469,0.0684296344277948
"288","MMP9",14.4300512864493,-2.14054895039025,0.659787827900755,-3.2
4429893955581,0.00117740107082558,0.0691232545330517
"289","ANGPTL1",679.720587362717,-0.737940434033073,0.227652040641876,
-3.24152786837496,0.00118890809045459,0.069317441356573
"290","CXXC5",285.838302255649,-0.557287877467616,0.171907070335183,-3
.241797305841,0.00118778469378308,0.069317441356573
"291","AL356218.2",42.4078767553812,-1.64926578371885,0.50939681817023
2,-3.23768371707358,0.00120504323489264,0.070016738885102
"292","TNFAIP6",871.035850257942,-1.35675863826954,0.41949485817711,-3
.2342676240783,0.00121955118468117,0.0706170254472234
"293","TAF9B",68.5807001706493,0.862421296402771,0.267412630219464,3.2
2505820198166,0.00125947061130298,0.0726796214877503
"294","PLD1",143.204127941443,0.843014571927676,0.261534190082552,3.22
33436540805,0.00126703444902349,0.0727093970899403
"295","STK3",289.598465792748,0.515869505046605,0.160059152613795,3.22
299285371915,0.00126858718603811,0.0727093970899403

"296","CD86",7.76141964518099,-2.35163711067861,0.730332987891744,-3.2
1995192558273,0.00128212095731611,0.0731130573025192
"297","OGN",2932.51890925771,1.02383209985804,0.318012630879141,3.2194
6992176906,0.00128427833089947,0.0731130573025192
"298","NMUR1",16.5950887072059,1.86297266880333,0.57931414847048,3.215
82456379151,0.00130070320315484,0.073799630063564
"299","KCNJ12",65.9350437005062,1.0926382132366,0.340158401485032,3.21
214530779326,0.00131747720472619,0.0745013531020416
"300","CAB39L",815.721554998935,-0.902271758828697,0.281410198522882,-
3.20625110093632,0.00134476585321912,0.0757910034874294
"301","RBP4",11.3933771383821,-2.33200924815778,0.728480872504812,-3.2
0119489224115,0.00136858929888087,0.0768364233246736
"302","KCNB1",11.2172296458864,1.65422953522758,0.516883264493625,3.20
039291047308,0.00137240358670756,0.0768364233246736
"303","TMOD2",244.367230736498,0.640777510699265,0.200433826458066,3.1
9695293964428,0.00138887583805362,0.0775020220125761
"304","TMEM238",35.6594241320325,-0.873131605193851,0.27349885029766,-
3.19245073331601,0.00141071007765098,0.0784614670819828
"305","AKAP12",1045.33695827367,0.766404388474572,0.240184976411355,3.
19089228612694,0.00141834147736283,0.0786272711450843
"306","VIM",116485.818397383,-0.702608414135739,0.221198454416941,-3.1
7637126347809,0.00149129962712211,0.0824016146907866
"307","LYZ",213.537954671733,-1.4643671428272,0.461226778320723,-3.174
93955610904,0.00149867715611552,0.0825395223309486
"308","AC096667.1",2.23174975374964,-4.16156267085544,1.31266182620543
,-3.17032352718403,0.00152269294107637,0.0830506201539332
"309","P2RY14",10.7865185877902,-2.14656553724179,0.676826808620073,-3
.17151376083676,0.00151646683212037,0.0830506201539332
"310","ZNF667",61.9531540118108,0.781549361653152,0.246505601410437,3.
17051359961535,0.0015216970954785,0.0830506201539332
"311","MYO1B",309.433206634333,0.552913908349563,0.174470218086581,3.1
6910194996822,0.00152910748493407,0.0831323130394381
"312","PCED1B",21.1492910947931,1.26874295710766,0.401321636926384,3.1
6141179634525,0.00157006360999731,0.084813532005861
"313","ADGRD1",228.764542295155,0.764377866812183,0.241722977931459,3.
16220606478265,0.00156578720580867,0.084813532005861
"314","SNAI2",937.478758742878,0.792695083019179,0.251020865183467,3.1
5788523173088,0.00158918109603038,0.0855728470435721
"315","GRID1",33.587457915491,1.02080364132712,0.323399298883735,3.156
48068765327,0.00159685461489609,0.0857130724719463
"316","PRICKLE4",2.90265994789324,-3.84061888179965,1.21763684193113,-
3.15415791436515,0.00160961962508152,0.0859054519169326
"317","ABCA6",2037.05509155527,0.687675822001498,0.218034287285862,3.1
5398018615253,0.00161060020449891,0.0859054519169326
"318","MAZ",751.146238058352,-0.558725722465367,0.177310642581501,-3.1
5111216298564,0.00162650015981454,0.0864807066105166
"319","ARSI",11.151146857332,-2.2130133649936,0.703180721119936,-3.147
14738121517,0.00164871835198901,0.0871141559232193
"320","CABLES1",109.765354822328,0.760040166955905,0.241482643019452,3
.14739046025218,0.00164734816874562,0.0871141559232193

"321","CDA",39.0721804211088,-1.78182270804533,0.566442037354733,-3.14
563996056223,0.00165723879397884,0.0872915686249044
"322","PLAUR",254.396251528623,-0.740455968253806,0.235780599635288,-3
.14044484321087,0.00168691469652626,0.0885787381641801
"323","SLC4A4",18.4563591627611,2.55241218315311,0.813236981414329,3.1
3858351438239,0.00169766550783126,0.0888672706080834
"324","CSF1R",17.5965900633658,-1.57547633175045,0.503685211257412,-3.
12789872828981,0.00176060833284513,0.0915834782815924
"325","EYA4",7.6973941032901,2.26322480388686,0.724385295882477,3.1243
3841044454,0.00178205372336432,0.0915834782815924
"326","TPP1",460.743621959569,0.589649979382345,0.188629941230489,3.12
596174041027,0.00177224607069219,0.0915834782815924
"327","SPI1",20.2248110607109,-1.36045568330985,0.435415870485847,-3.1
2449723477425,0.00178109195915375,0.0915834782815924
"328","LMO7",43.5188552701835,1.41596330072879,0.452798120569094,3.127
14041071803,0.00176515603364703,0.0915834782815924
"329","AC024132.3",10.0280160549151,-2.27083364522225,0.72676526790600
6,-3.12457645611643,0.00178061241095227,0.0915834782815924
"330","OXT",62.5485983413461,-0.894464973665158,0.28732890875725,-3.11
303508419631,0.00185174003485621,0.0948764257859052
"331","STK17A",215.391453700741,0.786404420566581,0.252721700783126,3.
11174077307053,0.00185987737878395,0.0950054583700272
"332","FCER1G",80.7080275281327,-1.3847686654919,0.445345410700011,-3.
10942614927876,0.00187451138910757,0.0952556219041834
"333","CYP26B1",1046.43824439927,-1.07191358587072,0.344757096469455,-
3.10918497936035,0.00187604223409588,0.0952556219041834
"334","HS6ST1",115.896829935166,0.770623146252018,0.248013766097532,3.
10717892146748,0.00188882041895551,0.0953318675931335
"335","MSRA",87.0417730919234,-0.786890450283703,0.253201303688617,-3.
1077661876947,0.00188507140001935,0.0953318675931335
"336","ARHGEF4",49.144334803252,0.867956148030883,0.279814998099063,3.
10189287181669,0.00192287530864935,0.096545089578225
"337","ITGBL1",356.753014097838,-1.10485079919022,0.356210788802181,-3
.10167696746487,0.00192427816346474,0.096545089578225
"338","NLRP3",40.1285856447808,-1.38477525985625,0.447496111406304,-3.
09449674435035,0.00197147098382305,0.0983292961489087
"339","ZNF415",30.1174641864983,1.23124915337449,0.397854978701254,3.0
947184760481,0.00196999786969183,0.0983292961489087
"340","ACTR3C",4.08154107530467,-2.48126206455998,0.802504318860822,-3
.09189870539538,0.00198880701678779,0.0989022030583763
"341","CCDC71L",1827.84542436845,-1.20922562894997,0.391249865233699,-
3.09067359864183,0.0019970302824323,0.0990199062034173
"342","DYNLT3",231.424677890555,-0.812450498207842,0.263043225054661,-
3.08865775972376,0.00201062911317212,0.0994026814196323
"343","ATP23",66.8209041301388,-0.874197532428744,0.283423755574698,-3
.08441870250479,0.00203950341747086,0.100266973147117
"344","APPBP2",335.104223069294,0.395622261054013,0.128267610882011,3.
08435043214403,0.00203997153788789,0.100266973147117
"345","POLG2",237.4690530458,1.18681602590974,0.385320662008496,3.0800
7367090938,0.00206949398347139,0.101423200789954

"346","TWIST1",1332.66748269528,-1.03340085592069,0.335872765314384,-3
.07676287761352,0.00209261694407186,0.101965323603363
"347","MIR497HG",129.080704037599,0.943489972559215,0.306635195995563,
3.07691349486465,0.00209155989207201,0.101965323603363
"348","PTGER3",125.156125484197,-1.44728241544364,0.471118392156156,-3
.07201425276541,0.00212619572719787,0.1033037855042
"349","BCL7A",104.244593010287,0.970357001025483,0.31624995531437,3.06
832296643614,0.0021526383196444,0.104288850167758
"350","DHCR7",79.8194317992879,-0.709790394640074,0.231782801399147,-3
.06230829188126,0.00219637114058134,0.106103552128427
"351","FCGR2A",52.113933083914,-1.42169424498037,0.464734162187984,-3.
05915588018532,0.00221961624896356,0.10692100153127
"352","SEMA3G",11.300972345944,-2.00896384620457,0.658973138141929,-3.
0486278270297,0.00229889087284912,0.11011231410236
"353","ABRACL",127.383345279342,-1.03628063109142,0.339845359867514,-3
.04927109052012,0.00229397378324039,0.11011231410236
"354","GBE1",269.382286756456,-0.476155628897957,0.156329459579675,-3.
04584708588005,0.0023202580513684,0.110821816758579
"355","ZNF528-
AS1",59.5249130412194,1.03529250367292,0.34000028128432,3.044975432850
23,0.00232699317039876,0.110830424014373
"356","MPEG1",15.9500212120893,-1.94270724767309,0.638329566948856,-3.
04342356716926,0.00233902851050056,0.111090713639167
"357","LINC01871",16.5017672517888,2.03951681669239,0.670574086787364,
3.0414488971136,0.00235442527636279,0.111508746702359
"358","TUSC1",437.064716574862,-0.622050881357236,0.204851591561832,-3
.03659286517906,0.00239268392681116,0.113004189481908
"359","PAK3",105.092087491006,0.846964336830327,0.27944178244935,3.030
9151673975,0.00243813739202363,0.11483015884216
"360","AC013275.1",5.04687209538321,2.77380977438556,0.916922857767042
,3.02512883269214,0.00248527238889415,0.116401622026101
"361","CRISPLD1",110.170155471941,1.1059800857088,0.365518019307498,3.
02578813434194,0.00247986000064801,0.116401622026101
"362","OSMR",270.89326439527,0.536707627501969,0.177874291538033,3.017
34232002386,0.00255001666825976,0.118775982994314
"363","ZNF610",4.28500236362531,2.79325565475383,0.925729175625148,3.0
1735726635982,0.00254989092650697,0.118775982994314
"364","LRRFIP2",385.08939337604,-0.530416024836207,0.175955380574613,-
3.0144916461437,0.002574102998235,0.118915118836496
"365","DDIT4L",39.9483718627868,-1.35736275242455,0.450235347108464,-3
.01478495889296,0.00257161513035098,0.118915118836496
"366","MT-
CO2",54589.3154957295,-0.638036705217355,0.211544659457362,-3.01608514
653122,0.00256061344132325,0.118915118836496
"367","DENND2A",214.353205975246,0.61298565163832,0.203515758637867,3.
01198126248817,0.00259548617916941,0.119457451261808
"368","ACAD10",58.2965814290403,0.765681806260759,0.254256270738565,3.
01145692114732,0.0025999729160365,0.119457451261808
"369","SCHLAP1",4.95282248138317,-2.88132850829141,0.957556148591187,-
3.00904392137274,0.00262071226425517,0.119759467470342

"370","TMEM64",388.48251076473,1.16526849116406,0.38724133109007,3.009
15320139993,0.0026197697599389,0.119759467470342
"371","ZNF528",51.8595160291182,0.78775137504745,0.261902510330778,3.0
0780383529938,0.0026314293376861,0.119925086904573
"372","TYMP",1255.57346079615,-0.697321423688625,0.231992980016871,-3.
00578674250365,0.0026494706923846,0.120398916792161
"373","RGPD2",11.8846745617974,2.66607917800325,0.889669524093407,2.99
670732311533,0.00272912592217167,0.123710619549809
"374","APBB1IP",382.83087883165,0.965909984290805,0.322558023754274,2.
99453094686195,0.00274867170837405,0.12426347926521
"375","KANK1",221.181859970433,0.78334782829425,0.261704283239313,2.99
325566474556,0.00276018420748769,0.124451185547205
"376","TMEM245",315.889403312314,0.652173380790616,0.218069958429512,2
.99066127901071,0.00278374085811787,0.12517949582196
"377","LGALSL",120.136696742137,-0.655993870748692,0.21968432522223,-2
.98607499686242,0.0028258333135633,0.126050459281916
"378","FBLN2",7476.05519649294,0.488360517548969,0.163676036241897,2.9
8370200526619,0.00284783980634562,0.126050459281916
"379","HLA-
DQA1",103.979822259803,-1.32886554343303,0.445352144782581,-2.98385347
191215,0.00284643048355277,0.126050459281916
"380","C8orf82",289.050264048606,-0.544271877792726,0.182369575566578,
-2.98444450562438,0.00284093729309098,0.126050459281916
"381","SVEP1",3614.94175020557,1.09501090857225,0.36673881321915,2.985
80588992065,0.0028283211001404,0.126050459281916
"382","NET1",185.776833239014,0.991841913394819,0.332040619286049,2.98
71101780483,0.0028162820533198З,0.126050459281916
"383","GPR183",93.7021117442378,-1.71039059395192,0.574279755041451,-2
.97832298446332,0.00289830389852687,0.127949144428962
"384","SLC12A6",124.460374333083,0.656796669931559,0.221464414690691,2
.96569844346722,0.00301996443974212,0.132972809237395
"385","NIPAL2",163.345472883797,0.7205063442043,0.243333648327293,2.96
098114320462,0.00306660713988081,0.134675827327545
"386","ZNF727",83.3125399478392,0.80098089305898,0.270705054379874,2.9
5886936759955,0.00308769960181412,0.135250841625578
"387","FILIP1",422.650174473809,0.66109244644432,0.223580577741209,2.9
5684201697307,0.00310807320162319,0.13579147724301
"388","KANK4",12.5286321009828,3.02706646125329,1.02591423655417,2.950
60381598813,0.00317153440537823,0.137392201164698
"389","AC096537.1",2.1725599001829,-4.06946119916803,1.37945453266869,
-2.95005098232215,0.00317721496660733,0.137392201164698
"390","HLA-
DMA",82.8373958984758,-0.945774725430316,0.320551459337399,-2.95046145
597121,0.00317299631920186,0.137392201164698
"391","PLEKHG2",229.064999571529,1.2967469740098,0.439468514430611,2.9
5071644823043,0.00317037820503539,0.137392201164698
"392","SPP1",51.0056902379205,-2.88686931837188,0.980973691777255,-2.9
4286110073111,0.00325194287080895,0.140047244695807
"393","SREBF1",178.443637401273,1.03276829942031,0.350976939808953,2.9
4255314888346,0.00325517903746465,0.140047244695807

"394","MT-
CO3",83117.6357941143,-0.482368225311538,0.164074036408305,-2.93994245
44609,0.00328273208180658,0.140874198068999
"395","CIDEC",61.5527142385351,-1.65451292499204,0.563387180294886,-2.
9367244816008,0.00331698655122599,0.141983819261086
"396","CCRL2",4.94531803193644,-2.50367444722309,0.852891422305068,-2.
93551369112909,0.00332995916076169,0.142179165379188
"397","DUSP10",33.3421484074353,-1.00323691189402,0.341920728929613,-2
.93412135331678,0.00334493400602042,0.142458801445323
"398","XPO1",403.610508142329,0.438989445110119,0.149847061928846,2.92
958326616087,0.00339416862693217,0.143657772823838
"399","SCIN",263.860713072016,2.01256253363068,0.687073690586391,2.929
18003003875,0.00339857517917763,0.143657772823838
"400","TFCP2",136.671647350112,0.539553500058037,0.18416405650386,2.92
974378551837,0.00339241592420689,0.143657772823838
"401","TUBB6",643.222336557459,-0.686284944137882,0.234398517954706,-2
.92785530440298,0.00341308842862731,0.143911469205064
"402","LMCD1-
AS1",17.2553872769448,1.14525224846034,0.391271648152661,2.92700034328
452,0.00342248502972385,0.143948698712863
"403","PPP1R1A",211.296686572951,-1.40177445840259,0.479146460073545,-
2.92556571989999,0.00343830543768028,0.144255256427539
"404","LRATD1",12.1274466176627,-1.94813264845328,0.666354382637687,-2
.92356844828096,0.00346044134212628,0.144824609437305
"405","ANXA1",10600.4763363328,-0.536853785491184,0.183800990798232,-2
.92084271776595,0.00349086011652255,0.145561854186264
"406","MED16",123.276910808352,0.706435216166542,0.241892682082252,2.9
2044889529287,0.00349527518332288,0.145561854186264
"407","HPSE2",50.6042055946312,-1.57499665983585,0.539568328051718,-2.
91899390300182,0.00351163091943112,0.145883674657841
"408","EHBP1",422.331495663166,0.65650794373867,0.225143655167951,2.91
595134337201,0.0035460579736389,0.145906951728258
"409","SLC4A3",24.5707375219648,-1.12689544832819,0.38628223939601,-2.
917285169751,0.00353092788715423,0.145906951728258
"410","AGA",139.491890297041,-0.668943279302841,0.229260715176168,-2.9
1782776124036,0.00352478989553999,0.145906951728258
"411","SERPINB7",6.40624988043708,3.26610309175148,1.12010348278648,2.
91589405974027,0.0035467090821099,0.145906951728258
"412","ZDHHC14",217.342487301686,0.830688543562,0.285016304410299,2.91
452990831771,0.00356224674000081,0.146190456019257
"413","TLR5",133.67938074531,0.807860136065652,0.277377215958864,2.912
49637528073,0.00358552361772538,0.146789426945522
"414","KRT222",634.508449656053,1.28532584350776,0.441465999637621,2.9
1149453086494,0.00359704204583033,0.146905282393476
"415","PROSER2",23.7573877728203,1.29751034871532,0.446263324714768,2.
90749939969779,0.00364331043585205,0.148436368311775
"416","KCNAB1",17.7998022386484,2.14978103061379,0.739729560996564,2.9
0617158481216,0.00365880753464992,0.148709417778511
"417","CD52",82.9504973632347,-0.909569842664895,0.313524692069812,-2.
90111071207866,0.00371842477755014,0.149672745273372

"418","DPYD",422.022410971047,0.465026042610482,0.160304768326197,2.90088715055701,0.00372107859169157,0.149672745273372
"419","PRELP",1842.90400843033,0.624138980460247,0.215135389503123,2.90114509705614,0.00371801675920569,0.149672745273372
"420","AL356124.1",8.30287068923906,1.90692652164019,0.656839410067693,2.90318530284848,0.00369388003585651,0.149672745273372
"421","SESN3",470.27872387516,0.777221372126368,0.267969651348168,2.90040819255513,0.00372676991720427,0.149672745273372
"422","PRG4",181.227327639565,-2.30965390471818,0.797123704941176,-2.89748490780188,0.00376167836690764,0.150380864686851
"423","PRIMA1",27.5580005625782,1.42119626850748,0.49050028988067,2.89744226013247,0.00376218983691376,0.150380864686851
"424","VILL",96.4303547492542,-0.907799889731166,0.313680354526683,-2.89402851224445,0.00380333624249505,0.151667002802137
"425","MYH10",447.029884891967,0.685373872797044,0.236900925932928,2.89308228787205,0.00381481338233339,0.151766740396454
"426","SNHG9",635.708673208174,-1.09860043398644,0.380347205613469,-2.88841463213721,0.0038718911245204,0.153614602677751
"427","KIFC3",54.0525214530271,-0.837732863145571,0.290093529777203,-2.88780264692206,0.00387943194602554,0.153614602677751
"428","STARD10",132.649704097444,-0.820441768681746,0.284343419523166,-2.88539038482972,0.00390928570964252,0.154272792502158
"429","NRK",20.6639003708392,2.32357061182074,0.805400818846876,2.88498665192256,0.00391430257768072,0.154272792502158
"430","FBXO31",86.2573286857403,0.595199714225369,0.206505886778262,2.88224090611263,0.00394857718424772,0.155261727979675
"431","NQO2",524.091482539915,-0.643419870098443,0.223360789604401,-2.88063035252525,0.00396880800124171,0.155695140800452
"432","MGST2",217.673357242758,-0.806065355069595,0.280152499402009,-2.87723777867468,0.00401173176583387,0.156652103225679
"433","BDKRB2",187.401228033078,1.18544544636101,0.411923064015262,2.87783217284742,0.00400418100994545,0.156652103225679
"434","KCNB2",3.42288823202987,4.3745339372547,1.5231816499982,2.87197126965117,0.00407920051085106,0.158754957321881
"435","PXN",433.846207862918,0.589924577706334,0.205436139409387,2.87157157159555,0.004084362812575,0.158754957321881
"436","LASP1",574.108809889795,0.455204489372225,0.158747517204373,2.86747470063542,0.0041376188391369,0.160456099385612
"437","ROBO2",78.09287927144,0.956817716863144,0.333910280516252,2.86549343549359,0.00416359905322331,0.160903116885123
"438","DMD",205.305387204629,0.753130022470098,0.262859272214188,2.86514535373292,0.00416817868439105,0.160903116885123
"439","CCL3",112.319801325332,-1.52637165479011,0.53321717657415,-2.86257030314899,0.00420220023834118,0.161112928865925
"440","MSL1",399.790146122065,0.560754098292961,0.195850510969846,2.86317403777056,0.00419420117095977,0.161112928865925
"441","SLC7A4",20.841649045034,1.98938272766931,0.694700299669342,2.86365606667537,0.00418782453985977,0.161112928865925
"442","MID1IP1",554.791827375748,-0.898245755301688,0.314393241981268,-2.85707717392731,0.00427561867779982,0.16355692444398

"443","SRPRA",383.711853749311,-0.400775085087566,0.140549837086392,-2.85148025352189,0.00435161890165329,0.166088425257684
"444","VIT",1261.71778004428,0.548922476575825,0.192749318143836,2.84785690482288,0.00440147101896461,0.166487856797883
"445","C11orf96",4561.76496272269,-0.985897241406702,0.345931352929258,-2.84997943394947,0.00437220560944538,0.166487856797883
"446","SLC38A4",6.30300725115495,2.44038135637367,0.856770879690071,2.84834769040756,0.00439468833620305,0.166487856797883
"447","PGP",153.065644190436,-0.744427029127849,0.261378172956215,-2.84808414072338,0.00439832942815002,0.166487856797883
"448","PDE1B",62.7477643799181,-1.13336031029074,0.398149296617813,-2.84657117297047,0.00441928491119663,0.16678854749668
"449","SPINT1",3.72213616154716,-2.27235680486894,0.800026835548434,-2.84035072812426,0.00450639578194355,0.169697416216262
"450","NDRG1",1144.39506184618,0.51555432027529,0.181680324666575,2.83770034659201,0.00454398196819332,0.170732549151584
"451","AKR1C1",3037.39133178613,-0.85884151266619,0.303154770315084,-2.83301335411464,0.00461114595874805,0.172871964236169
"452","NT5DC1",142.748539997335,-0.647022203451573,0.228687660407251,-2.82928340908007,0.00466523658540507,0.173744097326055
"453","ST6GALNAC4",132.70167161234,-0.685011878392916,0.241997559301703,-2.83065614533284,0.00464526309290545,0.173744097326055
"454","KCNQ1OT1",2818.52157059444,1.16570720896136,0.411928999828118,2.8298741031774,0.00465663239739839,0.173744097326055
"455","LAIR1",9.87531172526406,-1.56033048777488,0.551944230157625,-2.82697128173489,0.00469905418529104,0.174618919043738
"456","DOCK7",245.162272638348,0.576894669811494,0.204338162669173,2.82323508382277,0.00475416976797203,0.176279610607173
"457","SLC6A2",5.62561505847352,-2.50812735863931,0.8903874535897,-2.81689431777986,0.00484904784839933,0.179404159782792
"458","EHD1",172.388613221074,0.566830707489358,0.201275847062274,2.81618840890522,0.00485971578135846,0.179406276050674
"459","ZNF423",123.671084605333,0.665343892866427,0.236385042772544,2.8146615583738,0.004882862726617,0.179868067498127
"460","TXNIP",16659.0345848082,0.727343730514433,0.258543100783904,2.81323975889948,0.00490450673482981,0.180272608418484
"461","EDEM3",245.208020446716,0.573365840757503,0.204021303907986,2.81032338179788,0.0049491744373341,0.181314523357971
"462","CCL13",18.2266485057762,-1.39013633194974,0.494788953550853,-2.80955409770859,0.00496101808053612,0.181314523357971
"463","PLP1",182.46274418485,0.760244137377479,0.270617479416531,2.8092942814212,0.00496502391262955,0.181314523357971
"464","PLEKHG1",21.9374920189901,1.01225042506557,0.360623361292422,2.80694634268233,0.00500135716900034,0.181955006668857
"465","SGMS1",192.670418144812,0.53189991308571,0.189506012008189,2.8067706530742,0.00500408552762114,0.181955006668857
"466","ROR2",14.8345706690352,1.57782607557948,0.562295273869591,2.80604541582083,0.00501536229491397,0.181973703181127
"467","DEPTOR",239.29529446985,1.02574471755609,0.366102610083511,2.80179569690068,0.00508190451875279,0.183993236837414

"468","TRABD2B",45.0263225551759,1.07940992390746,0.385763158524594,2.79811563145588,0.0051401707359879,0.185705142743768
"469","MID1",131.424721488195,0.945698095565485,0.338339219329099,2.79511815816308,0.00518807493438647,0.187036185481037
"470","MAP4K3",201.340883717231,0.731061832035466,0.26195523778753,2.7907891371442,0.00525797143752914,0.187876058282091
"471","LIMS2",168.1333944659,0.731603152335472,0.262200951826915,2.79023835435357,0.00526692512784044,0.187876058282091
"472","RANBP17",12.5411604931961,-1.30579772726812,0.467987927983909,-2.79023805783514,0.00526692995184003,0.187876058282091
"473","HMGA1",113.64009713547,-0.929836914093883,0.333126538153808,-2.79124238869426,0.00525061356023114,0.187876058282091
"474","SEPTIN1",308.181023450307,-0.675987397969342,0.242210256527774,-2.79091153141083,0.00525598362781747,0.187876058282091
"475","PLAAT3",1116.74989691321,-0.52492105156225,0.188324649805911,-2.78731993981265,0.00531459798812754,0.189177311122654
"476","INSYN1",32.0825225168802,-0.886795542291884,0.3182603375588,-2.78638409389623,0.00532996752854878,0.189325821371224
"477","PYCARD-AS1",16.8447813380355,1.64143876932128,0.589307361177161,2.78536953287407,0.00534667515744254,0.189521139543058
"478","SLITRK4",82.5695576251268,-0.896334571988783,0.322069930423751,-2.78304333102275,0.00538516135036462,0.190486000234236
"479","HERC4",242.26451574017,0.428454784119616,0.153998964955748,2.78219262215648,0.0053992983777513,0.190587342319455
"480","HPRT1",77.7514424160804,-0.805553458642454,0.289626031974083,-2.78135723212387,0.00541321343753586,0.190680443337201
"481","REEP5",1415.25587486286,-0.487779280507823,0.175449600740309,-2.7801675150564,0.00543308640033543,0.190982588060024
"482","ALCAM",62.8913129900285,-0.880531348996613,0.317081408135714,-2.77698826359361,0.00548651600534041,0.1924606070919
"483","AL583785.1",32.6350942301363,1.57799931622432,0.568972215149403,2.77342069473456,0.00554703588507596,0.194180709616696
"484","SCN9A",3.79697111097866,-2.90674907495186,1.05037610879415,-2.76734119389756,0.00565155725119968,0.195294324266896
"485","COL4A4",13.7180965204217,-1.84674422795401,0.666732982653329,-2.76984081484121,0.00560836951007948,0.195294324266896
"486","MPPED2",3.28504326927149,-3.04959360327341,1.10202084085098,-2.76727398450877,0.00565272260642368,0.195294324266896
"487","PRG2",49.1284359391057,-0.845017772797731,0.305132154079616,-2.76935013730886,0.00561682373510011,0.195294324266896
"488","NMB",191.409273965195,-0.715912220441169,0.258428604799585,-2.77025146266749,0.00560130299907123,0.195294324266896
"489","ZNF559",130.052431844291,0.608036121258492,0.219657087641281,2.76811519167306,0.00563815238033068,0.195294324266896
"490","IQCN",143.17934281608,1.38959838386546,0.502227238499712,2.76687180093331,0.00565970066777732,0.195294324266896
"491","FSTL1",7965.85020893084,0.776325502214929,0.280720069329246,2.76547916246204,0.005683923633858,0.195332887807462
"492","CEBPG",211.634088807479,-0.598765130197302,0.216486159778284,-2

.76583561189561,0.00567771481499047,0.195332887807462
"493","AFAP1",166.797809597789,0.530461401889777,0.192028131827289,2.76241505263862,0.00573754897888961,0.195980763909223
"494","DOCK9-DT",7.40683184816657,-1.73353118902987,0.627423170571548,-2.76293779117325,0.00572836831407344,0.195980763909223
"495","GALC",167.761040974848,0.600823207251692,0.217430680047189,2.76328624424712,0.00572225592174849,0.195980763909223
"496","PTGS1",72.3708535503762,1.06188978969538,0.384615294949372,2.76091409686439,0.0057639835066325,0.196486760343029
"497","MLIP",14.8068840534302,-2.21833910774463,0.804072207172441,-2.75888046864041,0.00579997454816199,0.197315834326606
"498","NOG",8.90113637996923,-1.75731435938439,0.637390152136881,-2.75704661186387,0.00583260365326611,0.19802743487836
"499","LINC00882",78.1970588506095,1.0162215214963,0.368964611024004,2.75425200990398,0.00588264523000341,0.199326183464725
"500","CLIP4",163.155148733876,0.601221460473582,0.218448401480133,2.75223556867392,0.00591899266181889,0.200156655852068
"501","SGCD",346.17084147369,0.588890456510187,0.214049442533837,2.75118892877796,0.00593793858949785,0.200396538265928
"502","LRRN2",4.04061925721781,3.8442831002881,1.39806124213619,2.74972439291297,0.00596454087060504,0.20089334071751
"503","AC083862.2",34.6400490904629,0.889606609524305,0.323744325828236,2.74786780354658,0.00599841887627697,0.201232671349387
"504","TNKS",195.206204320463,0.540936566309333,0.19684108814517,2.74808766506306,0.00599439793620508,0.201232671349387
"505","SYCE1L",139.9960951654,-1.02277184364384,0.372320647203292,-2.7470188702304,0.00601396742086403,0.201354774558355
"506","TKT",777.909541044527,-0.431167476277606,0.157091761849335,-2.74468547046493,0.00605689178829921,0.202391158807437
"507","MYLK",382.435585123336,-1.21812794348176,0.444217520412945,-2.74218797662322,0.00610314037289884,0.20353431444768
"508","SH3RF3-AS1",5.74227014610397,2.15432717889124,0.786369602636853,2.73958603138696,0.00615166125733267,0.203645227222775
"509","PPP1R16A",50.6268157693287,-0.794407166501987,0.289990296911816,-2.73942671517579,0.00615464342978697,0.203645227222775
"510","MAN1B1-DT",22.3520445734096,-1.00093572558018,0.365276029621188,-2.7402173819569,0.00613985606620355,0.203645227222775
"511","CCNQ",114.15914126721,-0.692062709917517,0.252589560250425,-2.73987059968505,0.00614633778206347,0.203645227222775
"512","ENPEP",100.241194009609,1.45410303303229,0.53099215988956,2.73846422390629,0.00617268760177741,0.203843363224321
"513","AGAP2-AS1",59.9086174655205,-0.89978161028383,0.328865824047442,-2.73601433925231,0.00621883160682776,0.204966870971235
"514","PEG10",362.717382222091,0.938404165929177,0.343183627288142,2.7344083205382,0.00624924954121406,0.205568698916045
"515","FGFRL1",75.5286384705764,-0.707939995261338,0.259075225960844,-

2.73256538766209,0.00628431960589502,0.20605604500339
"516","ALDH5A1",34.561770524749,0.798868185394786,0.292442352344899,2.73171166552724,0.00630062545935373,0.20605604500339
"517","LINC01615",5.08204019486265,-2.18858786148668,0.801174114477329,-2.73172562859757,0.00630035846258559,0.20605604500339
"518","AL441992.1",11.2845383178474,-1.23367402066529,0.451889476132891,-2.73003485547534,0.00633276295734969,0.206707251125229
"519","RANBP3L",33.6056515268047,-1.00711591076359,0.369098569399457,-2.72858253664386,0.00636071703240813,0.206821160738378
"520","ZFYVE1",102.851146479059,0.571780209326163,0.209519254477726,2.72901032772121,0.00635247144125582,0.206821160738378
"521","PILRA",31.0860894933943,1.12479693635041,0.412336308115771,2.72786294636611,0.00637460872649877,0.20687501794173
"522","UBE2E3",1502.36347111273,-0.739043619988431,0.271021850546405,-2.72687836238463,0.006393660358407,0.207095803333229
"523","TFEC",6.88466263004408,-1.90502521612626,0.69894684805044,-2.72556521492286,0.00641914940121637,0.207523858653473
"524","PIK3CD-AS2",20.567050606927,-1.28904906751574,0.473523380947359,-2.72225009235403,0.00648390531378466,0.208433278760189
"525","AC092683.1",337.091645309812,-1.16228583307698,0.427059094385605,-2.72160421908148,0.00649658965617574,0.208433278760189
"526","RHOBTB3",2212.30408648981,0.626465933124969,0.230133846601023,2.72218077600318,0.00648526555362155,0.208433278760189
"527","DOK2",15.0995702079056,-1.35460294919933,0.497573686118459,-2.72241677361699,0.00648063546945447,0.208433278760189
"528","RAB13",909.8530766503,-0.832342580263445,0.305947981734838,-2.72053626745225,0.00651761219300892,0.208711717726126
"529","STBD1",70.4008729212016,-0.609392022856733,0.22408860196417,-2.71942444870163,0.00653956324072279,0.209018781236561
"530","MCM6",126.716746373875,0.48020737776164,0.176645812292088,2.71847586722071,0.0065583439754713,0.20922354705145
"531","SEC11C",80.0786933627983,-0.877240092255137,0.322883256096873,-2.71689558281692,0.00658973938139553,0.209829215556753
"532","GNG4",18.4286534994369,1.7535015439389,0.645632189329362,2.71594504257961,0.00660868873316808,0.210037047181214
"533","FAM72B",5.20848817199255,2.55326479747162,0.94069413034238,2.71423485606562,0.00664290531664207,0.210210668324845
"534","FZD7",296.424221897318,0.455518893657672,0.167823775102181,2.71426913963963,0.00664221782592957,0.210210668324845
"535","GTF3A",788.477061116371,-0.603978391816548,0.222557439296865,-2.71380904509291,0.0066514494649747,0.210210668324845
"536","GNS",603.021687786895,0.513423481133221,0.18924886058791,2.71295414692721,0.00666863333845609,0.210360545683984
"537","PM20D2",98.240420476655,0.630778126358131,0.232746318037002,2.71015297547198,0.00672521824903696,0.211541282902876
"538","SLC27A6",3.35488282687919,-3.09877299290775,1.14351618694837,-2.70986368909849,0.00673108647987622,0.211541282902876
"539","ICAM4",48.8683885741107,1.19356369767449,0.44057747815994,2.70908922230746,0.00674681935145723,0.21164234062048

"540","IGSF3",36.0232619527431,0.937867084530353,0.346537274347341,2.70639597514209,0.00680178884892286,0.212185693464184
"541","NCOA2",293.541751105822,0.527495472575027,0.194880850827732,2.70675887515144,0.00679435862985222,0.212185693464184
"542","KCTD12",1495.26987483586,1.02347373552141,0.378161691201934,2.7064447809836,0.0068007891458533 2,0.212185693464184
"543","ANXA5",5118.19647370101,-0.503660550188879,0.186325322511532,-2.70312453186661,0.00686910054316404,0.213497705852606
"544","NUTM2A-AS1",355.017401649893,-0.49385008959814,0.182688731191053,-2.70323235800286,0.006868724594873,0.213497705852606
"545","RNASE6",11.5109993178093,-1.54694299008154,0.572818941062619,-2.70057932653527,0.00692188279687221,0.214743475833973
"546","HECW2",123.819663959095,0.621314671275881,0.230138397308261,2.69974362619574,0.00693929276519832,0.21488930782779
"547","SLCO4C1",4.252701479527,2.39060593886568,0.889386777757444,2.68792610667487,0.0071897304997481,0.222237592851446
"548","FAM43B",36.7734726997715,-1.1787842535483,0.438691538041773,-2.68704579716798,0.00720870682258182,0.222414330660433
"549","HLA-DQB1",113.09467194945,-1.21846889510596,0.453562397228153,-2.68644160660665,0.00722175701044345,0.222414330660433
"550","ASIC1",63.2321170810821,0.640896359195156,0.238889489625802,2.68281522221451,0.00730053145073774,0.224024293591785
"551","DPEP2",4.51766817700342,-2.11052679475877,0.786589727209502,-2.68313546662509,0.00729354400682404,0.224024293591785
"552","CDK6",159.766463897603,0.773647390694198,0.288704329300319,2.67972216616616,0.0073683289359404,0.225018738069305
"553","ZNF268",109.981438352383,0.527879670967296,0.1970055911704,2.67951618952128,0.00737286378580522,0.225018738069305
"554","MEG3",3960.04363832192,1.02075736615314,0.380858131341765,2.68015116956281,0.00735889188756297,0.225018738069305
"555","TRMT61B",100.889894352113,-0.518971715304633,0.193993236714009,-2.67520519836327,0.00746835234151268,0.225911847984846
"556","EFHD1",164.018704954181,0.711953773679319,0.265984417411949,2.67667474886947,0.00743567788306653,0.225911847984846
"557","AC091563.1",29.4265029124318,1.10207017208885,0.411939236982199,2.67532216683809,0.00746574691805522,0.225911847984846
"558","C20orf194",154.110136072317,0.740912055362995,0.2767214993186,2.67746473326944,0.00741816615543534,0.225911847984846
"559","CDH19",5.99393810724113,-2.79989641960773,1.04662016593854,-2.67517912489004,0.00746893322826645,0.225911847984846
"560","GALNT2",301.782357856737,0.487097442799057,0.182224560043992,2.6730614286102,0.00751624855549174,0.226937018886169
"561","LINC01354",53.1820666196148,0.787854618554127,0.294942791869152,2.67121163925121,0.00755779781695058,0.227784751317291
"562","CSF1",979.549286778905,0.402056424842941,0.150591253019586,2.66985244349252,0.00758845867555959,0.22799939686053
"563","EBF2",367.097546640702,0.672564473437634,0.251925048517422,2.66970068040345,0.00759188907218347,0.22799939686053

"564","FPR3",9.7952293876039,-2.05819729925278,0.772771264598719,-2.66339781710381,0.00773559019617355,0.231903118859756
"565","IL6R",170.697206648415,0.784702716890229,0.29476642602491,2.66211701065275,0.00776508799780124,0.23237541215367
"566","AC139099.2",6.57620768795136,-1.61725376706217,0.608407200311406,-2.65817657357507,0.00785647187186118,0.234694746306411
"567","HELZ",399.100904017372,0.504732273385502,0.19001550358164,2.65626890370367,0.00790105836811813,0.235195589591798
"568","ZNF532",229.574363420269,0.562901085413046,0.21188167217575,2.65667662347942,0.0078915100499420‌9,0.235195589591798
"569","BEND6",66.8347152539233,-0.849410392449135,0.319957012517631,-2.65476410648236,0.00793638858836577,0.235418347810682
"570","C14orf180",46.6763868514135,-1.44816122133719,0.54540369896808,-2.65520975394402,0.00792591078460264,0.235418347810682
"571","MIR3142HG",2.05157591998177,-4.0263991618978,1.51715518565885,-2.65391385136997,0.00795641370983111,0.235599024528589
"572","STXBP5",138.750086246472,0.72564495569884,0.273760142579116,2.65065962072667,0.00803347568806574,0.236261180799905
"573","NRBP2",222.606774188025,0.615504715384731,0.232365829807544,2.64886070337675,0.00807636125317654,0.236261180799905
"574","LDHA",3680.25018713507,-0.598512782464594,0.225898496422413,-2.64947660981957,0.00806165523910845,0.236261180799905
"575","VPS51",626.490116638646,-0.489036767642028,0.184622213081265,-2.64885118361554,0.00807658874511149,0.236261180799905
"576","TAFA2",21.018069361189,1.56658739342381,0.59097421331827,2.65085575329515,0.00802881230436926,0.236261180799905
"577","RAB20",34.2611300427514,-0.884113824212481,0.333551767696614,-2.65060452330337,0.00803478615879565,0.236261180799905
"578","AL442128.2",10.2948004387367,-1.56788799913506,0.591894421260509,-2.64893187503957,0.0080746606591484,0.236261180799905
"579","MT-ATP6",103325.254918917,-0.482170363451263,0.182151261733959,-2.6470876943773,0.00811882960509181,0.237086651058536
"580","LEPR",1103.58784350939,1.10099488706585,0.416521606443296,2.64330798218924,0.00821003185440755,0.239054030505122
"581","FOCAD",85.1673286788003,0.553119485229955,0.209267269086283,2.64312468760654,0.00821447786393871,0.239054030505122
"582","Z83843.1",145.136849839443,0.78097929755528,0.295555801132443,2.64240896156631,0.00823185921752912,0.239148239948423
"583","ELMO1",47.6121919989048,0.713377497864936,0.270054062302883,2.64160994943611,0.0082513020395141,0.239301912322649
"584","MLPH",14.6058133185992,1.69699461586122,0.643200273058292,2.63836115583774,0.0083307806885068,0.240780922873971
"585","KLF3-AS1",86.3678597621067,0.570329132429275,0.216139484524533,2.63870867316952,0.008322246417254,0.240780922873971
"586","MME",531.545269300963,0.66797533974901,0.253441075284229,2.63562383879523,0.0083982774304217,0.242274596284682
"587","PPIP5K1",11.3897851492254,-1.31658287700088,0.499631977620732,-2.63510530945297,0.00841111828833145,0.242274596284682

"588","TTPA",13.1778874458452,1.584077111559,0.601289958289818,2.63446460350746,0.00842700897945492,0.242319503103102
"589","MAP4K3-DT",236.795759209574,0.682568148067861,0.259460115297852,2.63072475430104,0.0085203010674283З,0.243684172235807
"590","EN1",130.642378132929,1.07152367700117,0.407394971931959,2.63018385307939,0.0085338702396998,0.243684172235807
"591","GRB10",83.9843931508694,0.588978555994839,0.223889389481932,2.6306675691854,0.00852173471385721,0.243684172235807
"592","DDX11",37.4535036220033,1.22417827076791,0.465523676843523,2.62967993178873,0.00854652910668522,0.243684172235807
"593","NID2",783.374822456233,0.885568883709999,0.336729745102223,2.62990988052202,0.00854075054723512,0.243684172235807
"594","ENPP2",938.972099345608,-1.00211737331496,0.381274785711807,-2.62833371329314,0.00858042946160244,0.24423889113935
"595","AC009236.1",8.98789110508124,2.17194236940598,0.826921759588782,2.62653914257385,0.00862580710133532,0.244813422794596
"596","DNASE1L3",213.309432299128,1.92823196050196,0.734556387561008,2.62502919197855,0.00866415386641531,0.244813422794596
"597","LINC02664",6.34889089667042,1.73814446348001,0.662034684274897,2.62545831021488,0.00865324049463359,0.244813422794596
"598","SGPL1",93.0735211442916,0.576733451171193,0.219650003391889,2.6256928853409２,0.00864727995538743,0.244813422794596
"599","HHEX",2.73481321303198,-2.70435776417423,1.03038957728413,-2.62459735986683,0.00867514867493697,0.244813422794596
"600","ATP8B4",348.798813174171,0.935273014441557,0.356414876949382,2.62411328743268,0.00868748838873655,0.244813422794596
"601","TMEM131",251.711853972064,0.447060935295656,0.170424069143397,2.62322650516866,0.00871013446164415,0.245043183822761
"602","AC074387.1",10.5224520218098,-2.26770329833302,0.866154939796048,-2.61812661238992,0.00884139930513014,0.247427771898277
"603","ASAP1-IT2",20.5074937805738,1.07297386569608,0.41011290640971,2.61628895098504,0.00888912978815097,0.247427771898277
"604","OGA",562.696371094265,0.639230286417338,0.244332804313476,2.61622784633215,0.00889072083573844,0.247427771898277
"605","FXYD6",24.9498167242352,-1.55208817618808,0.593345027085447,-2.61582739441172,0.00890115413080936,0.247427771898277
"606","ABCC9",310.758557659485,0.760276215496261,0.290290103181627,2.61902216838779,0.00881822167311887,0.247427771898277
"607","STXBP6",225.027561890497,-0.84386926445244,0.322368200618246,-2.6177186919617,0.00885197460740109,0.247427771898277
"608","DPY19L3",122.996235521636,0.58896196786606,0.22523560784008,2.6148705949027,0.00892612668921574,0.247427771898277
"609","LYPD3",7.07715544818101,-1.59584600534788,0.610300580583728,-2.61485251058015,0.00892659929370411,0.247427771898277
"610","SPECC1L",315.609722071204,0.565126394364022,0.216035148594216,2.61590022753896,0.00889925573768184,0.247427771898277
"611","RIPOR3",272.991542771023,0.667614846978937,0.255562317398938,2.61233680213024,0.00899256146422527,0.248848165690869

"612","SIPA1L2",157.972509612976,0.738997225345084,0.283040967094143,2
.61091965920002,0.00902991046766682,0.24920307679429
"613","ZDHHC23",2.65423746491843,-3.06143446581549,1.17263448103984,-2
.61073208686542,0.00903486432900993,0.24920307679429
"614","HIVEP3",63.6334034214419,-0.860767322515921,0.329989205611156,-
2.60847114959939,0.00909476779148358,0.249699696978499
"615","PBX1",866.532694484789,0.503569736383577,0.193058379761854,2.60
838062043592,0.00909717372479035,0.249699696978499
"616","AL133230.1",4.03011252356839,2.88690628176467,1.10656090766333,
2.60889957504536,0.009083389517269,0.249699696978499
"617","SMCR8",71.9396693953146,0.719629428683462,0.276012717000174,2.6
0723287138617,0.00912772599239687,0.250132238378357
"618","SCARB2",1105.88530401499,0.359312063709362,0.137847286102371,2.
60659512326215,0.00914474195476483,0.250193037170168
"619","LINC00342",1042.5898018194,-0.995436247165062,0.382610141244888
,-2.60169854339521,0.00927633528197871,0.253219886714918
"620","NECAB1",63.9270637760268,1.40405953412297,0.539739288878815,2.6
0136618373582,0.00928532823298138,0.253219886714918
"621","AUP1",573.572875928125,-0.424958208773224,0.163411557561006,-2.
60053949130602,0.00930773053439141,0.253422073873575
"622","PLD5",12.0566207632969,1.5511376533944,0.596661683474439,2.5996
9375670635,0.00933069874674119,0.253638994228135
"623","ENPP4",12.2191564918288,1.5303445628546,0.588817724463569,2.599
01239258514,0.00934923980066917,0.25373506669296
"624","KREMEN1",290.772410409867,0.663820481197005,0.255902860666975,2
.59403306186906,0.00948573646938182,0.257026974718442
"625","DUXAP8",283.844142850404,0.707482794846311,0.272820659210702,2.
59321561971564,0.0095083137774968,0.257226510959865
"626","LYL1",13.3838123765693,-1.24650417555115,0.480985145510726,-2.5
9156480649224,0.00955405450513278,0.258051044045982
"627","SERPINB8",53.5969938238329,-0.666045928948662,0.257174005432652
,-2.58986489644686,0.00960136055674736,0.258915158362814
"628","PREPL",233.888371814068,0.452580309439158,0.174838477388026,2.5
8856240457145,0.00963774822461839,0.259070026998168
"629","DLL1",45.6950292237033,0.872586535272068,0.33707434626974,2.588
70645283041,0.00963371791611521,0.259070026998168
"630","SNCA",2.79358341378446,-2.74425000336279,1.06048602671901,-2.58
772858314134,0.00966110715878762,0.259285714032986
"631","EFHD2",194.582500356174,-0.629212939830207,0.243539564439443,-2
.58361692186841,0.00977703214245117,0.260013422591916
"632","RGS2",279.531021242525,-0.857831155888917,0.331785976067205,-2.
58549552352134,0.00972391354901273,0.260013422591916
"633","GREM2",9.73733094725507,1.80219348782958,0.697579651814773,2.58
349492153495,0.00978049070076051,0.260013422591916
"634","ATF2",264.527938828976,0.395115845649353,0.152868194731609,2.58
468314055163,0.00974685251974482,0.260013422591916
"635","TBCK",110.270145509041,0.563413397178171,0.218070927576511,2.58
362452730199,0.0097768165739265,0.260013422591916
"636","MYO5B",3.00042493937188,2.85807859747007,1.10567800637776,2.584
9104178469,0.00974043011703365,0.260013422591916

"637","NNT-
AS1",278.375145641737,0.404715328547633,0.156857735576106,2.5801426181
5776,0.00987595192957022,0.261918988093674
"638","MEGF9",155.230596064913,0.527623603197418,0.204513913904688,2.5
7989098699325,0.00988315084006173,0.261918988093674
"639","MDGA2",4.37584946500598,2.95361477149523,1.14512878155115,2.579
286119675,0.00990047460589929,0.261967487694124
"640","HAS2",559.516172788832,-0.909426767716596,0.352763007183331,-2.
57801058840608,0.00993709529716329,0.262525636381933
"641","VCAM1",600.127529578202,1.16997047571262,0.45394326227602,2.577
34957854981,0.00995612041684767,0.262617915769205
"642","CCDC18-
AS1",323.649496221039,0.528123922573732,0.20566938143833,2.56782958591
282,0.0102337452380174,0.262876665763108
"643","NPR1",265.532688639841,-0.544345798802701,0.211649805603438,-2.
57191731053429,0.0101137044546774,0.262876665763108
"644","DYSF",11.0618808746055,-1.49541672871786,0.581031366930277,-2.5
737280529595,0.0100609317891121,0.262876665763108
"645","ZMAT3",257.039417023608,0.847175384863247,0.330058425521255,2.5
6674370158956,0.0102658459607507,0.262876665763108
"646","C5orf38",274.50093872937,-0.768644850997039,0.29936479708834,-2
.56758596359016,0.0102409393738112,0.262876665763108
"647","SNHG18",181.027420439106,-0.617167244330592,0.239758435518558,-
2.57412108564924,0.0100495096028465,0.262876665763108
"648","ULBP2",12.1143943151974,1.488991843973,0.578656399552872,2.5731
88242839,0.0100766384164252,0.262876665763108
"649","C9orf72",65.3112165700604,-0.679997521427433,0.264224493288022,
-2.57355975203325,0.0100658264294129,0.262876665763108
"650","LINGO2",2.70195719194022,4.01889267306563,1.56542475734865,2.56
728575052845,0.0102498108184995,0.262876665763108
"651","FBP1",14.8120906240463,-1.10229450002071,0.429059090233482,-2.5
6909718290987,0.010196385813768,0.262876665763108
"652","LINC00963",366.327628610453,-0.47230504446333,0.184036140317952
,-2.56637116844196,0.0102768793511601,0.262876665763108
"653","DHH",3.8178169733058,3.44312618513903,1.33887569926983,2.571654
84967482,0.0101213740879937,0.262876665763108
"654","WASHC4",394.587677909238,0.379184727437037,0.14773927478474,2.5
6658040314275,0.0102706811056469,0.262876665763108
"655","GALNT16",268.414007327491,0.797598882660023,0.309670955606782,2
.57563348521716,0.0100056643939025,0.262876665763108
"656","AC007262.2",31.9795735219926,1.53786214224925,0.598067313976942
,2.57138637459217,0.0101292248267974,0.262876665763108
"657","ARHGAP23",131.364321612048,0.591357023862148,0.230182177496172,
2.56908258621362,0.0101968153249293,0.262876665763108
"658","EPB41L3",546.173551342349,0.304096720093383,0.118134738979357,2
.5741515384947,0.0100486250745656,0.262876665763108
"659","MN1",109.446941005958,0.834710623643395,0.324652332613955,2.571
09079402781,0.0101378744536033,0.262876665763108
"660","ATP7A",112.072794009524,0.459306532695767,0.178652304331559,2.5
7095218790654,0.0101419327738234,0.262876665763108

"661","CMC4",254.125646794041,-0.598491408335394,0.23301029602767,-2.5
6851915360995,0.0102134067747237,0.262876665763108
"662","ALOX5",14.8697446694744,-1.43633813232966,0.560012663814435,-2.
56483152103432,0.0103225914396718,0.263249435990907
"663","ANKFN1",5.95755529746001,1.75362700532616,0.68365579333554,2.56
507298324828,0.0103154104900766,0.263249435990907
"664","CDO1",686.018230213121,-0.883661200325626,0.344700884229096,-2.
56355942428719,0.0103604963859539,0.263818182068837
"665","LAMC2",58.5005814242262,0.68562963947643,0.267612365341287,2.56
20252584444,0.0104063749956305,0.264587952520483
"666","AOX1",487.449378041988,0.362377151765607,0.141556929898705,2.55
993932635383,0.0104690439127062,0.265745766177108
"667","YPEL1",47.7656540563709,0.639871323944436,0.25000200038694,2.55
946481609778,0.0104833467021606,0.265745766177108
"668","SDK2",8.07303507578927,1.85511083220873,0.725409367441024,2.557
32957895606,0.010547922815738,0.266982453545656
"669","MEF2B",37.4687215639005,-0.657194912203075,0.257166556922328,-2
.55552246010576,0.0106028518169613,0.267971627086968
"670","NPR3",42.9709033044671,1.48541701545775,0.5816387802915,2.55384
796507775,0.0106539765272837,0.268461155176323
"671","SELENOM",4638.96522976139,-0.551729384079239,0.216017061908745,
-2.55410095482325,0.0106462383602104,0.268461155176323
"672","SLC37A3",191.479172868047,0.505245536714962,0.197925800958104,2
.55270174110302,0.0106890986437355,0.268945356946846
"673","LINC01260",7.82981379648209,-1.47298240927766,0.577277614054264
,-2.55160147114105,0.0107229094635391,0.269395175645646
"674","RFTN2",208.186831791439,0.462530223819621,0.181317608237008,2.5
5093936169193,0.0107433015978278,0.269507037709308
"675","ZNF804A",2.66374589162964,-3.75853263910898,1.47397510976648,-2
.54992951658759,0.0107744699492695,0.269888500595925
"676","SMAD5",649.463847756738,0.412187141010254,0.161804235033086,2.5
4744346417119,0.0108515434873415,0.270616367675471
"677","SCAI",99.5243051033435,0.617969320663911,0.242547307000269,2.54
783006377896,0.0108395258939363,0.270616367675471
"678","ITGAM",15.1787594808383,-1.32199617424802,0.51884208755446,-2.5
4797404828739,0.0108350531059627,0.270616367675471
"679","CDKN2C",501.115806172848,-0.531405510562134,0.208692304268038,-
2.54635892025809,0.0108853201765379,0.270660284624857
"680","ABCA9",2027.82296693492,0.737081027218193,0.289429936581597,2.5
4666478500365,0.0108757849751743,0.270660284624857
"681","JUNB",30247.6916900227,-0.495419285869762,0.19463282062784 2,-2.
54540464589503,0.010915117037498,0.271002641512505
"682","ANGPT1",846.383449287412,0.835198152818453,0.328368386454954,2.
54347917543221,0.0109754599069754,0.271702893275462
"683","AP000842.2",2.85396106815715,-3.1454768239566,1.23656914562723,
-2.54371284863421,0.0109681209855274,0.271702893275462
"684","IQCJ-
SCHIP1",2.75268726189292,-2.6520544134677,1.04312087734488,-2.54242290
71305,0.011008688356019,0.272127050765451
"685","ZNRF1",274.745814690889,-0.598075984066545,0.23531390910187,-2.

54160914817675,0.0110343487894964,0.272363166909204
"686","PYGO1",105.196655957974,0.555472856630091,0.218618045154694,2.5
408371767163,0.0110587406327423,0.27256732743208
"687","AC023509.2",7.39736234934433,1.87906880804696,0.740354607219415
,2.53806593451787,0.0111466981759942,0.274335331527963
"688","KIDINS220",847.683619355604,0.417630420720829,0.164896863518495
,2.53267655799886,0.0113195347556768,0.275418504820243
"689","LINC00968",11.5565999114873,1.60738204472504,0.634172469809434,
2.53461340762404,0.0112571482452761,0.275418504820243
"690","GTF2A1",218.505115179757,0.408172568069654,0.16114105396789,2.5
3301413897286,0.011308639131054,0.275418504820243
"691","CCL3L1",42.4183636746617,-2.42754993867839,0.957196450329206,-2
.53610420080798,0.0112093374903588,0.275418504820243
"692","AC015813.1",34.5924642997926,1.0244672461111,0.404507195675254,
2.53263046260754,0.0113210232345676,0.275418504820243
"693","MBD2",767.551875610976,-0.458284684914969,0.18073640886227,-2.5
3565226729831,0.0112238122229742,0.275418504820243
"694","ZNF507",148.002104450403,0.560663931745724,0.221162645484585,2.
53507517292202,0.0112423197949242,0.275418504820243
"695","COL4A5",8.54679457355174,-1.6927452674875,0.668289456215722,-2.
53295222862395,0.0113106366264562,0.275418504820243
"696","LILRB4",6.87543714211011,-2.10053377113075,0.830019331574174,-2
.53070463689922,0.0113833661030121,0.276537290330071
"697","KDM4A",153.858017496458,0.40780316396166,0.161195992519933,2.52
985919554564,0.0114108308793545,0.276806784086263
"698","PCDHB5",33.4183464040964,0.849951581263813,0.33641409806296,2.5
265040500911,0.0115204060079985,0.278267178261771
"699","DCTN4",209.469142800122,0.44444735937175,0.175910725783698,2.52
655065455332,0.0115188775877125,0.278267178261771
"700","KIAA0513",26.3723054347559,0.868161526390391,0.343617118128356,
2.52653747612799,0.0115193097635414,0.278267178261771
"701","UPP1",210.461607523312,-0.831440152516334,0.329218822499498,-2.
52549397450567,0.0115535762721043,0.278670281895491
"702","EDNRA",8.33650658138442,1.75973049909072,0.69698134719373,2.524
78851288626,0.0115767934394396,0.278832512071288
"703","FREM1",138.021543541945,0.746527134333303,0.295744517665368,2.5
2422983264898,0.0115952093080736,0.278878803671278
"704","GTDC1",98.6245579563861,0.490931252339363,0.194564548173818,2.5
2323075784998,0.0116282067888615,0.279275171002941
"705","RIN1",37.8379994256508,-0.813551520168353,0.32274471739508,-2.5
207276101516,0.0117112469900971,0.280581969618795
"706","TRIM29",9.88541556382516,-1.26117891034981,0.500449464699696,-2
.52009243552015,0.0117324019706936,0.280581969618795
"707","PAQR4",9.81967750991716,-1.45723759428947,0.578170822977554,-2.
52042741760083,0.0117212409137177,0.280581969618795
"708","MTX3",128.237820478311,0.588517727495747,0.23357858624432,2.519
57055207178,0.0117498090610239,0.280601372321741
"709","WFIKKN2",4.78728620910645,1.99704054375011,0.792930497869308,2.
51855685853474,0.0117836856372,0.281013479201379
"710","SLC6A6",99.5883898445008,-0.994292594133482,0.395110168695923,-

2.51649457014783,0.0118528726552619,0.282265311063617
"711","AC068888.1",319.533660130228,-0.498905180612956,0.198513842086967,-2.51320097061237,0.0119641157366432,0.284513739627514
"712","MPZL1",1209.50541905807,0.436766955386031,0.173854645428749,2.51225358004616,0.0119962853106388,0.284878078697023
"713","HSD11B1",171.153092259657,-0.77148663321605,0.307355584997875,-2.51007845919372,0.0120704339568214,0.285050003692367
"714","SGPP2",3.2924162245059,2.99959851481159,1.19487986752166,2.51037664651021,0.0120602449502564,0.285050003692367
"715","PRPH2",10.6188963871346,1.62880700944193,0.648695093070424,2.51089768805312,0.0120424593529458,0.285050003692367
"716","TSPAN19",2.56699138022542,-2.90147380954978,1.15639048167633,-2.50907790709564,0.0121046784156091,0.285050003692367
"717","CLEC10A",33.4526566282492,-1.12208607402652,0.447046666017204,-2.50999763407996,0.0120731970503122,0.285050003692367
"718","A1BG",84.3999097137606,-1.09388309790299,0.435944668578999,-2.50922462584209,0.0120996515026107,0.285050003692367
"719","TSPAN9",108.352119925893,0.493951083144091,0.196915679545118,2.50843957314692,0.0121265707184409,0.28516836955132
"720","CAMK2D",1034.15834253144,0.442850453613638,0.17675855409823,2.50539757961322,0.0122313817110617,0.287233613848098
"721","COA6-AS1",35.0884948273314,-1.03308068686336,0.413045013716887,-2.50113341780096,0.0123796531250194,0.289910214733834
"722","AMOT",110.977331167829,0.563110062175656,0.225123054885604,2.50134337623395,0.0123723154541604,0.289910214733834
"723","PARM1",6.97322288072022,-1.79226330604686,0.717188916736001,-2.49901143788394,0.012454029132902,0.291248581713841
"724","ASAP3",238.67554336398,0.406683539504145,0.163167458328114,2.4924304372404,0.0126872179897951,0.29146125240687
"725","HDAC1",122.732427030629,-0.469366834561916,0.188163679546617,-2.49446033205166,0.0126148825740501,0.29146125240687
"726","CACHD1",211.564251933872,0.60175289205077,0.240966060690179,2.49725164750264,0.0125160101833688,0.29146125240687
"727","SERTAD4",61.7686484402646,-1.43374178421106,0.574626006133403,-2.49508683719094,0.0125926308811526,0.29146125240687
"728","HEATR1",133.691483878903,0.458069861078553,0.183528114276196,2.49591111904083,0.0125634076569453,0.29146125240687
"729","ISOC1",112.411619410183,-0.67130924568641,0.268886673873997,-2.49662519906432,0.0125381399687938,0.29146125240687
"730","FAM171A1",114.534524475061,0.582263070520345,0.233183552785198,2.49701603550363,0.012524329302107,0.29146125240687
"731","ESAM",5.16405727075027,-2.42909219255311,0.974544646323019,-2.49254069756387,0.012683279447634,0.29146125240687
"732","APAF1",92.8788128557341,0.494291433293741,0.198254621411524,2.49321518850106,0.0126592099166526,0.29146125240687
"733","NECAB2",3.81049582361879,2.71250539180562,1.08766556277102,2.49387815947307,0.0126355908937706,0.29146125240687
"734","HCST",52.2703324369835,-0.842241205307796,0.33777418161167,-2.49350379975488,0.0126489230446228,0.29146125240687

"735","CLCN4",76.2831172384414,0.731059479759968,0.292857892721642,2.49629427080127,0.0125498442500136,0.29146125240687
"736","SLITRK2",95.7426325581242,-1.09224456238766,0.437899434502461,-2.49428173760641,0.0126212321167271,0.29146125240687
"737","DNASE1",93.1073045260535,0.849601133214194,0.341297738939018,2.48932540794241,0.0127985762702913,0.293222666094966
"738","SEC14L2",19.7385032904257,-0.913437470642463,0.366883529938078,-2.48972056825945,0.0127843564345154,0.293222666094966
"739","ACVR2A",322.877989644628,0.721191504527407,0.290123021477524,2.48581274541593,0.0129255958115018,0.295732035156796
"740","SHISA2",5.10030891535417,-2.02917907099431,0.816804100436944,-2.48429099450996,0.0129809683962564,0.296597586005275
"741","RFX7",76.2779271212378,0.610107758813432,0.245972587659836,2.48038923612566,0.0131239031553365,0.299118539095887
"742","UBN1",239.902060169719,0.557905148287877,0.22493329865378,2.48031372690004,0.0131266829908415,0.299118539095887
"743","BTN3A1",115.41651109564,0.584600292706543,0.235830036189417,2.47890515624141,0.0131786343735609,0.299898183025797
"744","PHLDB2",278.313177831437,0.54809886419807,0.221240358013526,2.47739096573221,0.0132346839442581,0.300768865765479
"745","TENM3-AS1",2.32771751290871,-3.50040952889464,1.41442855802839,-2.47478708558738,0.013331562640369,0.302563840434039
"746","RASA2",215.07805527036,0.468457896206002,0.189352172279706,2.47400328481053,0.013360846833566,0.302821981584361
"747","INO80C",62.4275707275346,-0.575260519080274,0.232677068917697,-2.47235587828277,0.0134225822720464,0.303813950543186
"748","BBS1",101.576491093945,0.465416003821963,0.188477207380953,2.46934900134241,0.013535913181173,0.305524293876939
"749","TMEM135",282.096196070019,0.447298412817909,0.181124079515869,2.46956900492471,0.0135275925459326,0.305524293876939
"750","NINL",60.2135482708296,0.721526132243028,0.292244262986749,2.46891461570195,0.0135523551222915,0.305524293876939
"751","CCDC170",54.3692405945855,1.09135050715225,0.442160008327369,2.46822527274839,0.0135784836796056,0.305705728435115
"752","LPAR4",53.6607553999184,0.767379512908684,0.311267099335193,2.46534090672499,0.013688294756857,0.3077682017938
"753","SNX7",465.599110963565,-0.541542077573196,0.21984047516945,-2.4633410983841,0.013764889452713,0.308646050898689
"754","PCNX4",318.944558408437,0.602093513937984,0.244465958245269,2.46289306805617,0.013782101279188,0.308646050898689
"755","PEG3",141.183065867275,0.68333454837081,0.277406799703798,2.46329415537198,0.0137666919555071,0.308646050898689
"756","ABCA10",940.196135017671,0.836678652796239,0.339795228547266,2.46230253548088,0.0138048165984455,0.308745818844598
"757","EXOC4",221.755888030045,0.374364102755216,0.152081810957892,2.46159682342861,0.013832005746813,0.308945248569504
"758","CLCF1",20.5373517810078,-0.990843573246357,0.402635476423126,-2.46089485717619,0.0138590974774124,0.309141979087189
"759","ENO1",1627.30644459268,-0.480629076049948,0.19544296971861,-2.4

5917812619167,0.0139255505928831,0.309805773028253
"760","FMO4",70.8073637315399,0.644361211764831,0.262270734632705,2.45685517550108,0.0140159177015463,0.309805773028253
"761","ANKRD50",123.241586022253,0.53168743676681,0.216222270125914,2.45898554509297,0.0139330227540857,0.309805773028253
"762","LIN7A",7.02945333158839,-1.57173362414534,0.639622367656719,-2.45728370929779,0.013999208105456,0.309805773028253
"763","MYO9A",484.721615945405,0.518008786185585,0.210844852950971,2.45682443244674,0.0140171171260122,0.309805773028253
"764","AC093525.6",15.6234669100323,1.29162536492275,0.525362669888916,2.45854043111942,0.0139503067562429,0.309805773028253
"765","SPINT2",9.15220199054864,-1.74575919150953,0.710484317897933,-2.45713965464375,0.0140048231908499,0.309805773028253
"766","PTENP1-AS",6.88200611155333,-1.65534245719018,0.673935555980229,-2.45623256185453,0.0140402263214432,0.309911418593945
"767","SOD3",7813.12847664783,-0.70916570806831,0.28885693605694,-2.45507592010364,0.0140854837170295,0.310100727457729
"768","PTMS",2982.45058847649,-0.467942795449626,0.190567647097443,-2.4555206645877,0.01406806642059,0.310100727457729
"769","PNLIPRP3",2.63098726126851,-3.06934187724005,1.25099325749885,-2.45352391696874,0.0141464131785853,0.311037131369987
"770","LINC00622",8.04288375630525,1.42116110380744,0.579623417299089,2.45186971642678,0.0142116107028486,0.311429252136662
"771","MTHFD2L",50.2418960033311,-0.656333908240567,0.267616817760014,-2.4525136863003,0.014186198268167,0.311429252136662
"772","AGPAT3",126.857713741788,0.451169570733559,0.184025415896939,2.4516698877411,0.0142195045333276,0.311429252136662
"773","DAAM2",48.9715268446525,0.773510003659689,0.315718413824617,2.44999965092115,0.0142856353201506,0.31192666320048
"774","NBR1",377.944630762529,0.374374447273527,0.152773309665159,2.45052259517099,0.0142649009279541,0.31192666320048
"775","CSF2RA",3.60992286137164,-1.95956938866999,0.799922460470329,-2.44969917148948,0.0142975611533222,0.31192666320048
"776","SDC4",178.186265980253,-0.78804675413916,0.321760594922594,-2.4491711122325,0.0143185407620893,0.311981813409027
"777","RCAN3",51.8310617562509,-0.737335354042999,0.301254449264232,-2.44755008878318,0.0143831131809321,0.312985428137965
"778","FGF12",32.9076624801059,1.64365252958631,0.672014992058168,2.44585693624532,0.01445083291844,0.314054862448565
"779","APBA1",28.7335132597573,0.787544668846606,0.322167206222827,2.44452152061033,0.0145044427081562,0.314815298215026
"780","SPOCD1",2.7807092663847,-2.64871943992377,1.08478580404416,-2.44169810302565,0.0146183654893662,0.315667080069225
"781","AL358472.4",21.3138294506102,-0.991183852444714,0.405741594102349,-2.44289436146565,0.0145700014040916,0.315667080069225
"782","CNTN1",107.795414103362,1.34560008140568,0.551030237341146,2.44197140232907,0.014607303689413,0.315667080069225
"783","EMP2",1782.82045587136,-0.547686622542247,0.22424291678405,-2.44238092510043,0.0145907420526206,0.315667080069225

"784","SNX18",361.474734579836,0.456061776917953,0.186916629457783,2.4
3992082588328,0.0146904811808605,0.316819714038251
"785","CREBL2",456.859985970369,0.351598869663529,0.144248241198158,2.
43745689197366,0.0147909776632061,0.318175382098586
"786","BCL3",176.523630765343,-0.683895501952043,0.280560688233854,-2.
437602738492,0.0147850121942157,0.318175382098586
"787","NCKAP5",15.5728651398336,1.12641573849101,0.463724416388165,2.4
2906281981954,0.0151379091688804,0.31866840113645З
"788","NOSTRIN",38.9753074453672,1.04656960760075,0.43088623010817,2.4
2887689248742,0.0151456740976495,0.318668401136453
"789","FLNB",210.410054830106,0.873124990451277,0.358523126562508,2.43
533799011163,0.0148778853574098,0.318668401136453
"790","GNB4",409.98758438511,0.540803760742636,0.222672291007672,2.428
6980580085,0.0151531461150762,0.318668401136453
"791","PCDHGB4",2.41047000941,3.0083696865196,1.23712290305818,2.43174
682085578,0.0150262065435712,0.318668401136453
"792","PDE7B",101.478815347488,0.632949967334854,0.260564426836553,2.4
2914957739757,0.0151342870906546,0.318668401136453
"793","CASD1",198.813815642104,0.398676876356565,0.164075296510809,2.4
2984096225783,0.0151054494430502,0.318668401136453
"794","AC079781.5",27.5969828461614,1.2465184427728,0.512632191526454,
2.43160391285821,0.0150321357308802,0.318668401136453
"795","CTNNAL1",180.824924959282,-0.599382537535357,0.246738727195454,
-2.42921954063804,0.0151313667221589,0.318668401136453
"796","EMX2",687.560630406334,-0.696274480303814,0.285893459781113,-2.
43543339829074,0.0148739624964701,0.318668401136453
"797","AL161772.1",97.0271758144257,-0.61796127542529,0.25364657424963
,-2.43630838403169,0.014838028533368,0.318668401136453
"798","FAN1",95.3343030849536,0.527692636445247,0.216749735011976,2.43
457107994199,0.0149094512329688,0.318668401136453
"799","PDPR",154.044570345276,0.625615589942609,0.257582275514151,2.42
879906505151,0.0151489254652262,0.318668401136453
"800","EFNB3",4.0744647312133,-2.69684977175132,1.10983050900638,-2.42
996543153762,0.0151002629756304,0.318668401136453
"801","JUND",19405.2736453256,-0.421893237732747,0.173499199256087,-2.
43167253532985,0.0150292883588396,0.318668401136453
"802","STX16",433.324427019458,0.504142838301006,0.207257034208416,2.4
3245224571749,0.0149969689614293,0.318668401136453
"803","MRPS6",3555.77216204483,-0.810955233769672,0.333559305351068,-2
.43121753990988,0.0150481764864729,0.318668401136453
"804","AC096576.6",2.0349528125258,-3.38335295526417,1.39165831750798,
-2.43116640967065,0.0150503003514404,0.318668401136453
"805","PIK3AP1",7.84972167698606,-1.54984757085294,0.638498935365582,-
2.4273299218041,0.0152104167331942,0.319475436179935
"806","KANSL3",119.29060325316,0.543630315008902,0.224061822552026,2.4
2625141943882,0.0152556974409199,0.319508896810011
"807","AJUBA",9.97960287984128,1.17268398689084,0.483393203674147,2.42
594223083315,0.0152687005336225,0.319508896810011
"808","UBE2O",77.1389973717102,0.531598200152827,0.219124424435952,2.4
2601070839644,0.0152658198320066,0.319508896810011

"809","LINC02518",7.18870660203,1.97250717463168,0.813840933456808,2.4
2370111104318,0.0153632440790766,0.321089902211405
"810","CLIC4",586.754886404205,0.373250301156118,0.154061418815325,2.4
2273700986447,0.0154040737797736,0.321149296508523
"811","EPB41L2",1556.65310326005,0.390251095645263,0.161063238751997,2
.42296813766527,0.0153942768194269,0.321149296508523
"812","PLXNA4",454.45473841695,0.799048513319084,0.330015302490136,2.4
212468551908,0.0154673697609333,0.321280451987544
"813","TRAPPC8",126.033415577699,0.47381498699786,0.195669762098891,2.
42150336319413,0.0154564580150048,0.321280451987544
"814","ARVCF",28.7195042631708,0.766669101513125,0.31657376583846,2.42
177079797679,0.0154450886629337,0.321280451987544
"815","FAR2",26.0064190317437,-1.03281292169488,0.426663282488465,-2.4
2067448520791,0.0154917425921185,0.321391881898822
"816","KLF4",6168.94911773193,0.820738382872144,0.339232888489805,2.41
939508437965,0.0155463446702574,0.322129406476363
"817","IRF4",101.394689544084,1.15624082522129,0.478109794048007,2.418
35837628791,0.0155907132795683,0.322653341653538
"818","C17orf49",578.669637567998,-0.655065368552704,0.270928005894821
,-2.41785771238065,0.0156121803568678,0.322702622828755
"819","AC093418.1",4.08378879507941,2.44678420147769,1.01295839457512,
2.41548341430546,0.0157143381494374,0.324417618352489
"820","AC067817.1",8.22747878405387,1.62522179427536,0.673503654500881
,2.41308533875704,0.0158181154351005,0.326161824117901
"821","NR4A2",266.858605326224,-0.995234733812072,0.41251149141681,-2.
41262305298174,0.0158381901140296,0.326177976185156
"822","SLC30A1",159.71371621803,0.51725620359798,0.214566572073161,2.4
1070264860088,0.0159218233994953,0.32678903653238
"823","TXN",2783.64791783395,-0.703699629805634,0.291895121555375,-2.4
1079613135,0.0159177432678402,0.32678903653238
"824","SERTAD3",282.120198570027,-0.653971656657709,0.271288804814792,
-2.41061055617158,0.01592584374868,0.32678903653238
"825","RGS4",3.52388763787495,-2.28369106356786,0.947825372131128,-2.4
0940064564121,0.0159787460656227,0.327267654818269
"826","RNF150",35.6794895658673,1.41932967019706,0.589323472216061,2.4
0840512403127,0.016022390209932,0.327267654818269
"827","TEX26-
AS1",65.7200054994977,-1.13294935015683,0.47043346971638,-2.4083094063
0113,0.0160265920386519,0.327267654818269
"828","SH3GL1",466.695639583622,-0.42019414659407,0.174435848323349,-2
.4088749567988,0.0160017794743049,0.327267654818269
"829","AREG",122.049971789846,-1.05990667720803,0.440404582999356,-2.4
0666586616693,0.0160988916418298,0.327951879373564
"830","CDK2",64.6128609864859,-0.585974704211664,0.243445675654618,-2.
40700395534239,0.0160839956641854,0.327951879373564
"831","AC002467.1",24.022853695766,-0.875850439389165,0.36405303561650
5,-2.40583199067674,0.0161356834763404,0.32825229077642
"832","MAP7D3",1390.82184829736,0.472521963189032,0.196437896773275,2.
40545216045766,0.0161524666386315,0.32825229077642
"833","MAP3K2-

DT",30.580847662858,-0.720049121025458,0.29958827202986,-2.40346231228
2,0.0162406409579642,0.329647967967897
"834","IGFBP2",28.1101376205388,1.64901749519371,0.686988236792925,2.4
0035768718813,0.0163790582508069,0.332058893171034
"835","KIAA1109",420.679136292367,0.552088652938817,0.230289476409059,
2.39736813660626,0.0165133234752844,0.333887068771554
"836","SLC2A8",74.0972752661913,-0.556640074939602,0.232160596209765,-
2.39765095381069,0.0165005804584576,0.333887068771554
"837","ADAMTS1",1338.11668873474,0.978696267275984,0.408295021458431,2
.39703208670064,0.0165284762574988,0.333887068771554
"838","TM6SF1",7.50321473427928,-1.72325803256224,0.719266378829802,-2
.39585511471545,0.0165816432765006,0.334561365774548
"839","MEGF6",226.104763769342,0.766052627103678,0.319945272662552,2.3
9432394399413,0.0166510351720838,0.334840157841127
"840","AC120053.1",22.6966562415613,1.05413887908683,0.440361475253254
,2.39380358711121,0.0166746754732807,0.334840157841127
"841","CYP11A1",30.3892301947138,1.1962620569957,0.499567793060154,2.3
9459403431088,0.0166387763218288,0.334840157841127
"842","PHKG2",76.1702561751095,-0.565022607830251,0.235998456614341,-2
.39417924988208,0.0166576058055038,0.334840157841127
"843","ALPK2",26.5162599135011,1.782929317073,0.745006732780291,2.3931
7208640422,0.0167034047234925,0.335019178012825
"844","AC092803.1",42.4259922708597,-0.963860912823024,0.4032355837793
1,-2.39031710393531,0.0168338312597691,0.337235093531013
"845","FCN1",17.4751044246265,-1.08738034708127,0.455030522578708,-2.3
8968661029367,0.0168627548449238,0.337414744281624
"846","STOX2",23.7886161455265,1.02562272207559,0.429814848975595,2.38
619657864315,0.0170236486559591,0.339733027706386
"847","KCTD16",12.6809135385476,1.90899083099502,0.800081596212729,2.3
8599517853108,0.0170329743689345,0.339733027706386
"848","SAMD9L",164.238569869335,0.769016486462505,0.322321552982483,2.
3858674027433,0.0170388932750778,0.339733027706386
"849","AC079140.6",5.46691121764044,1.53003320854957,0.641654556770089
,2.38451233986608,0.0171017744467316,0.339784726610269
"850","GASAL1",66.7101104972005,-1.33543317484519,0.559889787472144,-2
.38517151897798,0.0170711601072684,0.339784726610269
"851","PDE3A",60.8778996449328,0.785295195822144,0.329318890496433,2.3
8460415871785,0.0170975072066794,0.339784726610269
"852","SLC2A10",255.164944018922,0.372836251827337,0.156477424435186,2
.38268397612697,0.0171869416076463,0.341076066551741
"853","AC108463.3",30.9803506441821,1.43435559343039,0.60255540342818,
2.38045428730663,0.0172913062729077,0.342662685387231
"854","LINC01277",8.79795740539197,-1.26356989778271,0.530886965472125
,-2.38011098400014,0.017307424492589,0.342662685387231
"855","PPIP5K2",265.799489270584,0.384517902878886,0.161596347461872,2
.37949625049298,0.0173363193878416,0.342833319543422
"856","YDJC",50.4886223281165,-0.799410487770136,0.336044940251258,-2.
37887970332904,0.0173653420166333,0.343006078057518
"857","ACVR1B",127.711834466619,0.522444642945359,0.219792576869577,2.
37698947974651,0.0174545859351232,0.344366556582338

"858","PTP4A2",1427.71125541333,-0.334867605303362,0.141059709041605,-2.37394226585703,0.0175993021029022,0.345207656561334
"859","NOTCH2",834.063625021653,0.334073536214449,0.140720438537027,2.37402284762314,0.0175954616724559,0.345207656561334
"860","SPATA18",14.48371993421,1.44391093212713,0.608010305301904,2.37481325486772,0.0175578307341572,0.345207656561334
"861","IL15",14.5281499716007,-1.17935925046213,0.496607238551593,-2.37483298451681,0.0175568923175116,0.345207656561334
"862","CLU",3087.849628898,-1.09997418463241,0.463216540056035,-2.3746435835373,0.0175659027587985,0.345207656561334
"863","PIGX",157.209105930345,-0.368924143431335,0.155477139727822,-2.37285136629843,0.0176513654177838,0.345481014699077
"864","LRRC23",78.0438931531533,-0.52822085666202,0.222665443197276,-2.37226239095408,0.0176795304147798,0.345481014699077
"865","AC009271.1",18.5676837158477,1.38818622033676,0.58518355176606,2.37222358035744,0.0176813877328971,0.345481014699077
"866","EBP",169.613127513833,-0.552097451966232,0.232761993631865,-2.37193986591912,0.0176949703530519,0.345481014699077
"867","LAMB3",17.2393866181228,-1.1247583817307,0.474358206485097,-2.37111610245966,0.0177344592567392,0.345852637961876
"868","AKR1C2",2142.2317276393,-0.826931681807236,0.348951202430387,-2.36976309595093,0.0177994861867323,0.346321878533107
"869","ZNF558",40.589890289415,0.637476352836573,0.268999294757057,2.36980678113785,0.0177973833707455,0.346321878533107
"870","KLF7",380.995989300034,0.620133781300059,0.261982521232498,2.3670807440994,0.0179290207934544,0.346846548713646
"871","AC055822.1",3.43681813685966,2.17778386356142,0.919864329069074,2.36750550569277,0.0179084535771385,0.346846548713646
"872","ISLR",2910.37524798398,0.436550985073039,0.184389249276462,2.36755118200249,0.0179062431335927,0.346846548713646
"873","THRA",507.071789428244,0.378134719107768,0.159727417493681,2.36737515099891,0.0179147632317977,0.346846548713646
"874","TMEM221",124.05078120885,1.03505812843473,0.437241138236356,2.36724781343702,0.0179209287263419,0.346846548713646
"875","FNDC4",416.647973366561,-0.75826930143454,0.32040277999139,-2.36661274117196,0.0179517057384613,0.346888503572461
"876","C6orf120",278.977548598257,0.393404846778298,0.166339366814312,2.36507361013021,0.0180264876037398,0.347905898869244
"877","BCL2A1",51.4106190350986,-1.47925404073752,0.625584047798844,-2.36459680508537,0.018049709452853,0.347905898869244
"878","AC005332.7",12.9280640572616,1.08323428736278,0.458170372516029,2.36426087835892,0.0180660858296189,0.347905898869244
"879","DKK3",74.8227157413721,-2.14653957783688,0.908574601765781,-2.36253530933526,0.0181504123225379,0.349132163309978
"880","SEMA3C",755.873014054927,0.730769543189189,0.309384744688172,2.36200897340859,0.0181762022373828,0.349176401471623
"881","KHDRBS3",539.087808891865,-0.649024264496524,0.274818628560277,-2.36164581672153,0.0181940152410989,0.349176401471623
"882","AK1",387.599927073205,-0.687218907135708,0.291208308199528,-2.3598877085088,0.0182804675581004,0.350437806658006

"883","NID1",1779.11549493104,0.736778808895933,0.312553011406297,2.35
729230565042,0.0184087498889815,0.35249733083001
"884","MYLIP",540.043340100488,0.683333828109338,0.290232560757036,2.3
5443544420704,0.0185508656295335,0.354415859959494
"885","PELI3",84.6320147814882,-0.44553078313848,0.189197443778498,-2.
35484567994525,0.0185303994333867,0.354415859959494
"886","USB1",82.723875699,-0.480431785094532,0.204225851054491,-2.3524
5333836971,0.018650029550838,0.355506989453854
"887","AC008969.1",38.4130162828361,0.647916464264617,0.27541070875792
5,2.35254637405588,0.0186453646620045,0.355506989453854
"888","DDR2",2199.64441033291,0.429140758063883,0.182863013928247,2.34
678817134816,0.018936016879067,0.355866322015561
"889","PARP15",13.3763410308689,1.02351291460175,0.435980862722435,2.3
4760972812093,0.0188943069852225,0.355866322015561
"890","IL20RB",6.34542311437056,-1.41913285510517,0.604585765378291,-2
.34728128972276,0.0189109719359436,0.355866322015561
"891","DDX60",88.1098629344863,0.478778842303303,0.204037801721791,2.3
4652029311768,0.0189496342755894,0.355866322015561
"892","PPIL6",38.0391565286106,-0.679844091210389,0.289487334056544,-2
.34844157664459,0.018852156470052,0.355866322015561
"893","GEM",677.88827825306,-0.704128277791076,0.300108347681282,-2.34
624689126897,0.018963541290278,0.355866322015561
"894","THNSL1",31.6443274498372,0.720756172938234,0.307161981524057,2.
34650189897211,0.0189505696428114,0.355866322015561
"895","SBF2",457.69860356504,0.50011889194439,0.212695152921169,2.3513
4127447534,0.0187058684834407,0.355866322015561
"896","PKM",913.394604997455,-0.524059636493311,0.223360821161769,-2.3
4624691012289,0.0189635403309363,0.355866322015561
"897","HSD3B7",252.315831577006,-0.646024112848503,0.27521858207009,-2
.34731284490079,0.0189093702695497,0.355866322015561
"898","HSF4",38.2528381686117,0.904481021744129,0.385297615452486,2.34
748668423999,0.0189005487215885,0.355866322015561
"899","ZNF28",39.2392229883971,0.62727098892803,0.267176205592672,2.34
778013834191,0.0188856654392579,0.355866322015561
"900","ZNF347",74.3775904474424,0.587972991539615,0.250493113215612,2.
34726210230585,0.0189119459019612,0.355866322015561
"901","RGN",74.626976142132,-0.573277554942,0.244119131286188,-2.34835
160981272,0.018856711200888,0.355866322015561
"902","CPZ",8.03978690018862,1.81678537409511,0.774899814563229,2.3445
4227495091,0.0190504507295013,0.357100910126839
"903","SYNGR2",15.0039603458382,-1.17014539006344,0.49947783605477,-2.
34273736609831,0.0191428529329596,0.358435611728107
"904","PDS5B",363.269958305334,0.426592482440202,0.182315251910379,2.3
3986173932339,0.0192908798800169,0.360807740056776
"905","ZNF251",66.7129808518907,0.575865237550704,0.246202331605446,2.
33899181131055,0.0193358572931875,0.361249364765983
"906","AC108863.2",45.3919398886138,0.692440881588685,0.29614541419255
2,2.3381786392899,0.0193779831200464,0.36163679756484
"907","NAV2",190.116374950993,-1.0443119159826,0.447057512561881,-2.33
596771475359,0.0194929242504786,0.363380775333068

"908","SERP2",77.5752704522174,-0.665052556269576,0.284829115370029,-2
.33491774675349,0.0195477180721425,0.363780717585854
"909","PDGFB",5.21683538965556,-1.67458876758406,0.717250863140602,-2.
33473231423047,0.0195574090540301,0.363780717585854
"910","G0S2",682.49841967226,-0.934658827055287,0.400706434828548,-2.3
3252762076357,0.0196729516081563,0.364326687613041
"911","MMAA",47.5171764744585,0.579038378447392,0.248169915538756,2.33
323357180643,0.0196358897376469,0.364326687613041
"912","BX322234.1",114.603717843875,-0.932205145874603,0.3994726543402
75,-2.33358938527126,0.0196172329370099,0.364326687613041
"913","KLRD1",29.9522935839261,1.08325971393732,0.464404060783654,2.33
258019344057,0.0196701894803715,0.364326687613041
"914","AL512625.1",23.1743328672862,1.00437800716979,0.430810446577823
,2.33136873803351,0.0197339245016357,0.365056012553235
"915","COG5",141.770729027365,0.41385687645029,0.177755886396253,2.328
23162619618,0.0198998076935333,0.366920336403774
"916","HSPA12A",142.257652073162,0.656643206916908,0.281949224753965,2
.32894134569765,0.0198621731984393,0.366920336403774
"917","RASA3",255.609407389487,0.49731714115924,0.213587486532154,2.32
840017565529,0.0198908643419358,0.366920336403774
"918","ALDOA",106.208340634033,-0.721892807239383,0.310128427551683,-2
.32772214059312,0.0199268627009601,0.367018948309186
"919","PARD3B",251.378194084377,0.615702507394642,0.264912203009092,2.
32417570954068,0.020116078620963,0.367254523195385
"920","LGI2",4.50320593590438,-1.62350517001567,0.697942931530449,-2.3
261288232487,0.0200116793212007,0.367254523195385
"921","ID4",1025.09418075826,-0.594491864226434,0.255652935343216,-2.3
2538642057179,0.0200513069383476,0.367254523195385
"922","AC004918.1",20.0944129379838,1.10512250266783,0.475409843940957
,2.32456798434548,0.0200950723909506,0.367254523195385
"923","KCNMA1",22.7140161107295,-1.03664437181983,0.446172681452957,-2
.32341515944904,0.0201568605113152,0.367254523195385
"924","GUCY1A2",14.234135526237,1.45198156936851,0.623996925422016,2.3
2690500580034,0.0199703217286706,0.367254523195385
"925","NR4A1",1122.2410984656,-1.67477604408106,0.720702702662419,-2.3
2380985653878,0.0201356872369864,0.367254523195385
"926","RTN4RL1",9.51971080280449,1.98801657086313,0.854905798199424,2.
32542178921961,0.020049417494935,0.367254523195385
"927","YES1",332.74540231566,0.297793786406217,0.12812739976936,2.3242
0065452253,0.0201147422523186,0.367254523195385
"928","FTX",977.272878196532,0.74039203145995,0.318602113332907,2.3238
7671165983,0.0201321027593712,0.367254523195385
"929","CHIT1",3.58129660525788,-2.94389224392893,1.26847698008833,-2.3
2080856818065,0.0202971778771683,0.367468908318911
"930","RNF212",152.44326696993,-0.421552997599861,0.181586629140779,-2
.32149800673398,0.0202599815502422,0.367468908318911
"931","GRK6",81.1870943847023,-0.491264501444475,0.211681290712306,-2.
3207743102443,0.0202990277010801,0.367468908318911
"932","KCTD21",38.2820719062119,0.697996287849517,0.300554103557774,2.
32236485739861,0.0202132979600402,0.367468908318911

"933","ENOSF1",263.373427919514,0.451384408715634,0.194493212129382,2.
32082345586107,0.0202963740337482,0.367468908318911
"934","HBZ",3.09293062428293,-2.50899062503531,1.08054594660036,-2.321
96569977349,0.0202347826218274,0.367468908318911
"935","DAB2",2306.36607841304,0.453145457709976,0.195303103837412,2.32
021636526174,0.0203291757364115,0.367621073102936
"936","PTPRQ",9.99918349580827,1.87116148820953,0.806740949268728,2.31
940809488553,0.0203729191476757,0.368018501013783
"937","SLAMF8",3.43855500434738,-2.19475735775869,0.946972284635733,-2
.31765743661963,0.0204679459765868,0.368447718851649
"938","AC090136.3",13.2527967338863,1.19374117322767,0.515094813888202
,2.31751735999183,0.0204755661001927,0.368447718851649
"939","SLC39A4",112.083396413604,-0.512821339778309,0.221275955757501,
-2.31756468082016,0.02047299158562,0.368447718851649
"940","KIF22",211.181443158491,-0.473066720029981,0.204139906789192,-2
.31736522011103,0.020483845263813,0.368447718851649
"941","METAP1D",54.854295204417,0.678899509278521,0.293035570790856,2.
31678190960326,0.0205156149765017,0.368627011713805
"942","ZNF518B",78.5218076411579,0.610829988211784,0.264070196994776,2
.31313489808117,0.0207152235282693,0.371818470717597
"943","FBLN1",11676.3604459268,0.556529678577348,0.240737337368488,2.3
1177134656719,0.0207902872401709,0.372770070685906
"944","PLCE1",90.8655736775577,0.51325558832557,0.222101829911838,2.31
09021142658,0.0208382622599066,0.373234468528074
"945","FSTL3",380.219132040703,-0.470664369563768,0.20376214344292,-2.
30987150807832,0.0208952688952367,0.373859477757314
"946","DDX60L",115.94044019256,0.384245461518236,0.166535655182118,2.3
0728645525211,0.0210388561098568,0.376030633303868
"947","PKIB",4.88826502622414,-1.5982174996982,0.693025959700224,-2.30
61437704145,0.0211026002633357,0.376096993558655
"948","C14orf39",8.06412370451114,1.56016063892162,0.676502031548155,2
.30621722650446,0.0210984974939126,0.376096993558655
"949","HMG20B",489.437702194329,-0.443963624712253,0.192523432140975,-
2.30602384226748,0.0211093001470998,0.376096993558655
"950","PRXL2C",375.344212745348,-0.30856224553804,0.133881408365698,-2
.30474305061985,0.0211809682776232,0.376428496797935
"951","HS3ST3A1",13.9799714952152,-1.24859518521823,0.541659195370775,
-2.30513059851878,0.0211592602692263,0.376428496797935
"952","BCORL1",85.4675295193101,0.49669793840829,0.215534102357945,2.3
0449814193861,0.0211946965313245,0.376428496797935
"953","ANKRD37",68.482054521264,-0.557671691454375,0.242141271191958,-
2.30308401665356,0.0212741164167897,0.377046918632159
"954","AQP1",78.5617566895652,-1.20311148906051,0.522315021776458,-2.3
0342119008675,0.021255156638052６,0.377046918632159
"955","SUFU",60.3663988099464,0.517052397284962,0.22467906065802,2.301
29321250795,0.021375063157162,0.377678098242532
"956","CADM1",16.1229684161463,-1.18938267726914,0.516838977361057,-2.
3012635063672,0.0213767411886742,0.377678098242532
"957","CYGB",2153.4646885583,-0.411383606131796,0.178736525363319,-2.3
0162024966958,0.0213565971621625,0.377678098242532

"958","SUPT20H",185.169437907596,0.372837095537856,0.162046453152056,2
.30080380215421,0.0214027234490926,0.377742430143276
"959","IQSEC1",215.248777550335,0.570022218230371,0.247814238486428,2.
30019962416965,0.0214369131165558,0.377951331569057
"960","SH2D3C",14.0954001974476,-1.38516625593981,0.602408953671984,-2
.29937859903397,0.0214834501081185,0.378298833401636
"961","FERMT3",37.2913661585306,-0.661877663541537,0.287960694021545,-
2.29850002893803,0.0215333462406517,0.378298833401636
"962","THOC6",91.9847606490482,-0.51872097869313,0.225681313328285,-2.
29846667871247,0.0215352422670361,0.378298833401636
"963","RGS19",60.3039048256562,-0.72582593766159,0.315813297621616,-2.
29827541502454,0.0215461187938121,0.378298833401636
"964","C5AR1",51.5225904642537,-1.0246391733161,0.446182527901671,-2.2
9645741202557,0.0216497415481132,0.379330393881345
"965","MIR155HG",21.0491537096281,-1.07973878215978,0.470118531435377,
-2.29673733316382,0.0216337583695434,0.379330393881345
"966","ITIH4",19.8893408276085,1.33736390956908,0.582842880316439,2.29
455305148961,0.0217587514490647,0.380190625409727
"967","PDZRN3",170.583704767233,0.802873159087994,0.349923994318562,2.
29442156617896,0.0217662955640298,0.380190625409727
"968","BMPR1A",225.86558121233,0.398550506888391,0.173649634612353,2.2
9514163838062,0.0217250085126253,0.380190625409727
"969","COBLL1",317.757814272862,-0.509333663366502,0.222090591936428,-
2.29335992545013,0.0218272918098551,0.3804348408796
"970","OSBPL10",105.303808568667,0.569762884399174,0.248605754619927,2
.29183304815385,0.0219152788630666,0.3804348408796
"971","PHC3",293.178239005422,0.407030794898695,0.177584502070852,2.29
204007192193,0.0219033309614543,0.3804348408796
"972","H2AFY",521.448822676639,-0.283157606349682,0.123532985148412,-2
.29216193561176,0.02189630052866,0.3804348408796
"973","EIF5AL1",25.7229316046966,-0.776996404436904,0.338945425265355,
-2.29239383841397,0.0218829272574491,0.3804348408796
"974","HSD11B1L",74.3025742973815,-0.642516509573034,0.280309668530094
,-2.29216677734415,0.0218960212450476,0.3804348408796
"975","MTREX",331.139038136005,0.356462560909151,0.155595907405218,2.2
9095075091414,0.021966262226499,0.380928781257072
"976","KLHL8",171.171454374515,0.449450224180087,0.196359753785191,2.2
8891213966262,0.0220844577776707,0.382586077976288
"977","KIRREL1",410.077248764114,0.638199763277804,0.278915867567325,2
.28814433844914,0.0221291169120603,0.382967357982719
"978","AC093627.6",21.983755514775,-3.59044606299333,1.57030774889965,
-2.28646013210419,0.0222273539300579,0.383906422790708
"979","ADGRA2",310.086393620976,0.430256048644608,0.188201760474815,2.
28614252894933,0.0222459216473055,0.383906422790708
"980","FCGBP",4.90928284241007,-1.86692500196742,0.816660733161956,-2.
2860472239666,0.022251495998042,0.383906422790708
"981","SUSD5",7.13575162707367,1.7480338212487,0.765184539926967,2.284
46045370381,0.022344484186985,0.384334220380002
"982","CTNND2",15.2041536446162,1.52822414528572,0.668787361866145,2.2
8506732098144,0.0223088806204707,0.384334220380002

"983","AC026124.2",21.841120898321,-0.90322881963062,0.395312922346981
,-2.28484516587045,0.022321908243804,0.384334220380002
"984","NUDT2",282.442147514206,-0.551800634102891,0.241744535716009,-2
.28257748398963,0.0224552685098745,0.385847235736747
"985","DUSP14",220.23221189245,-0.592363128696824,0.259657007473136,-2
.28132925993961,0.0225289705429512,0.38672064359413
"986","POMT1",81.424638800416,0.488162830484763,0.214099661588146,2.28
007287290262,0.0226033668047951,0.387397693405559
"987","WDR81",123.008051839528,0.494007993974893,0.216680665045606,2.2
798895963833,0.0226142372481244,0.387397693405559
"988","LINC02631",2.17249872125721,2.86619271132421,1.2574270127099,2.
27941079868106,0.0226426569988224,0.387491947911021
"989","OLR1",12.6179329055756,-1.62354452045345,0.712580218519911,-2.2
7840245667454,0.0227026101713383,0.38812510897572
"990","BICRAL",205.370323723087,0.449224404594339,0.197577680931398,2.
27365966882826,0.0229864568592405,0.391789327193587
"991","ATP6V1C1",231.514332603532,0.364877216233563,0.160450457698824,
2.27408024549522,0.0229611622026071,0.391789327193587
"992","ACSS1",58.1261373632444,0.772188797600516,0.339579064961064,2.2
7395878391106,0.022968464757749,0.391789327193587
"993","CENPA",26.9783736117011,2.90233219522899,1.27776004208093,2.271
42194124518,0.0231214474150526,0.392504114019623
"994","SPEGNB",7.59070620063198,-1.27794784793885,0.562436261147624,-2
.27216475220012,0.023076561330966,0.392504114019623
"995","KLHL36",257.780074846439,0.338480079213194,0.149041783425478,2.
27104152562987,0.0231444642581952,0.392504114019623
"996","RAMP2",2816.96224672934,-0.78031725349378,0.343557062048511,-2.
27128864370017,0.0231295102484111,0.392504114019623
"997","DOCK11",363.571274127896,-0.503701242088662,0.221704637334241,-
2.27194725444233,0.0230896962985591,0.392504114019623
"998","FAM198B-
AS1",10.3350227592576,1.44969292089849,0.638748982690507,2.26958157301
82,0.0232329827084044,0.392823271633701
"999","NT5E",907.987520836617,0.510162848300082,0.224750999307507,2.26
990246927477,0.0232135012701755,0.392823271633701
"1000","AC006387.1",3.4211455894529,-2.36903182142645,1.04375180341537
,-2.26972716470954,0.0232241421570672,0.392823271633701
"1001","ALG10",25.2274485169512,-0.72497658628808,0.319671476326714,-2
.26788012061211,0.0233365143044334,0.394179604255105
"1002","RUNX1T1",1369.45184650138,0.50020286188659,0.220655007321748,2
.26690011687439,0.0233963280936591,0.394401909678553
"1003","IL4R",166.387406196299,0.464257086045349,0.204773697796765,2.2
6717147290136,0.0233797527756209,0.394401909678553
"1004","ANPEP",647.808307582059,0.483865052051676,0.2135267714206,2.26
606269945687,0.0234475446289265,0.394871598193116
"1005","PPP1R3C",7.80058035814408,-1.32437467283748,0.584684042911321,
-2.26511171100722,0.023505825156159,0.395043150591195
"1006","IFI30",108.055766397932,-0.658994028049575,0.290978286951265,-
2.26475327404738,0.0235278242633862,0.395043150591195
"1007","HSPA12B",110.821042213762,0.515632239471206,0.227619720390225,

2.26532322677147,0.0234928517303658,0.395043150591195
"1008","AC159540.2",61.2468643962249,-1.18673639414013,0.524259324401185,-2.26364384743301,0.0235960287190458,0.395100365031255
"1009","RASL12",143.29456665027,-0.721896299576648,0.318853211634598,-2.26403960579808,0.0235716789387069,0.395100365031255
"1010","ATP1B2",8.6267520237218,1.55401979282747,0.686538639610737,2.26355765453869,0.0236013347930901,0.395100365031255
"1011","AC083805.1",11.5062297747673,-0.970549371368874,0.428968132142463,-2.26252091623102,0.0236652380449255,0.395681315942573
"1012","RBL2",393.929953921965,0.375352366492869,0.165920977659375,2.26223574491854,0.02368284195256,0.395681315942573
"1013","ADAMTS18",2.32372538341034,-2.78237749926195,1.23043302828032,-2.26129942492738,0.0237407218298993,0.396256786475753
"1014","ALPL",535.023634105771,1.69761775836547,0.751207805911053,2.2598510625254,0.0238304960281642,0.396471687149969
"1015","ITPR3",111.682545890708,0.511081108836089,0.226184217799366,2.25957900073045,0.0238473921121078,0.396471687149969
"1016","PPM1N",12.0305628659501,-1.39831974939326,0.618797034839946,-2.25973892999493,0.023837458631288,0.396471687149969
"1017","HPS4",188.444993722837,0.421223398065274,0.186388272404226,2.2599243645102,0.0238259454691046,0.396471687149969
"1018","AC245297.3",650.856358747484,-0.412278100739558,0.182508201215236,-2.25895657287942,0.0238860863604373,0.396724899982588
"1019","AL683813.1",31.9556395223864,1.02712868818529,0.454888421696309,2.25797940592785,0.0239469434000204,0.397345357220359
"1020","NOTCH4",30.5273201142404,-0.731927276994243,0.324339909211382,-2.25666733019039,0.0240288697146909,0.397923730789415
"1021","DPY19L4",224.473733114401,0.341069748827131,0.151119291861591,2.2569570345758,0.0240107596274199,0.397923730789415
"1022","AC092155.1",6.84099095898065,2.05960171807076,0.914222658165285,2.25284475250711,0.0242689393410346,0.398927031903824
"1023","FAM117B",56.2331849214462,0.599958746399644,0.266052371333061,2.25504002611793,0.0241308165210675,0.398927031903824
"1024","TBC1D5",430.986981668765,0.412316301346571,0.18288024771351,2.25456989752378,0.0241603387126893,0.398927031903824
"1025","SEC24B",143.480724976439,0.455605102025845,0.20218069667771,2.25345500095942,0.024230474957881,0.398927031903824
"1026","BCL7B",243.521628340042,-0.520158070240941,0.230881616824978,-2.25292111773131,0.0242641230925578,0.398927031903824
"1027","SNX30",68.4548349789221,0.58137009542685,0.258077262808839,2.25269785140847,0.024278206519342,0.398927031903824
"1028","WDR34",133.196347061533,-0.410456297284066,0.182187061246968,-2.25293878980605,0.0242630086574415,0.398927031903824
"1029","ARFGAP3",378.507304262242,-0.335451316014958,0.14886292038824,-2.2534242586407,0.0242324114027115,0.398927031903824
"1030","LGALS12",4.2318167898235,-2.16207357822157,0.960492510008725,-2.25100514131227,0.0243852117462905,0.399133746569486
"1031","ULK2",157.628655648907,0.49139211737093,0.218258448622837,2.25142312002814,0.0243587511509475,0.399133746569486
"1032","CEP131",97.5180138762206,0.777408521893639,0.345330928840837,2

.25119865313873,0.024372958175261,0.399133746569486
"1033","MT-
ND4",61622.3390188658,-0.417493050999652,0.185383402163845,-2.25205194
276597,0.024318989728365,0.399133746569486
"1034","SH3BP5",2156.79470500869,0.336994714768743,0.149768938814539,2
.25009749976293,0.0244427567718685,0.399302542510872
"1035","PCDH18",1314.98942189204,0.353254291339929,0.156977320319832,2
.25035241154706,0.0244265833378051,0.399302542510872
"1036","SESN1",206.974339049235,0.491252719650971,0.218485940362508,2.
24844087832788,0.0245480910291348,0.40063621922839
"1037","TCEA3",289.259344725818,0.652055055379839,0.290171876144091,2.
2471338850773,0.0246314719426706,0.401222473609513
"1038","AFF3",249.439731391793,-0.931156553209487,0.414318318115377,-2
.24744239512525,0.0246117681510702,0.401222473609513
"1039","CFH",11539.6228842746,-0.65112512589389,0.289893609736305,-2.2
4608305952715,0.0246986882167575,0.401930144724673
"1040","CMTM8",214.069790683116,0.713802382095182,0.317961436278202,2.
2449338210645,0.0247723813473904,0.402354873988163
"1041","ANKFY1",266.402112828183,0.513802421985784,0.228865717695301,2
.24499513146758,0.0247684451094318,0.402354873988163
"1042","LRTOMT",52.1118837748462,-0.594396743987998,0.265041398898707,
-2.24265622826404,0.0249189913236462,0.404347701823618
"1043","POU3F3",5.28975111506973,-1.59319865603749,0.711300171188431,-
2.23984011331755,0.0251013046592067,0.406708220415071
"1044","EPHB3",57.0098702252419,0.792613164600634,0.353897830371959,2.
23966663985357,0.025112572871619,0.406708220415071
"1045","PFKP",202.621898415216,-0.750750567733014,0.335356105766088,-2
.23866676295635,0.0251776066774791,0.40737126670126
"1046","LINC01625",16.9450488911127,0.939875487592891,0.42031221041046
4,2.23613652973592,0.0253428292372105,0.409261276736156
"1047","BICC1",1017.95184293696,0.536762703004237,0.240030443278333,2.
23622760377033,0.0253368659237783,0.409261276736156
"1048","RDH5",97.5934573517627,-0.591149909451522,0.264507758986577,-2
.23490574233598,0.0254235374977362,0.410006538885295
"1049","AL161421.1",29.901098872203,-0.820983144142302,0.3673806452441
27,-2.23469351140355,0.0254374768920437,0.410006538885295
"1050","FGD4",213.403586737028,0.599247364874588,0.268214436779556,2.2
3420995554801,0.0254692616937955,0.410127882589233
"1051","CISD2",249.87817448233,-0.348143917567669,0.155859326416404,-2
.23370603205062,0.0255024218485187,0.410271121422221
"1052","CBWD2",79.9570979960512,-0.92462818782588,0.414184924054352,-2
.23240425743875,0.0255882566887803,0.410576143922433
"1053","HLA-
DPB1",536.29931952801,-0.530304035542543,0.237516660408309,-2.23270247
497969,0.0255685711436047,0.410576143922433
"1054","LINC00484",3.00750597149049,2.34849410141001,1.05239117080365,
2.23157906163029,0.0256427967815288,0.410576143922433
"1055","CYB5R2",42.7118565991282,-0.617773961855564,0.27682182385966,-
2.23166639552508,0.0256370198232708,0.410576143922433
"1056","GDPD5",103.99116059936,0.643851928315296,0.288517890588895,2.2

3158406919283,0.0256424655110949,0.410576143922433
"1057","SIDT1",2.94650096281758,-2.19346084029897,0.983654627815965,-2
.2299095416947,0.0257534488145541,0.41163109557318
"1058","AC009271.2",5.31004123175336,1.85782127067227,0.83315959587372
9,2.22985041506241,0.0257573751547448,0.41163109557318
"1059","ERMP1",31.9193555935801,0.690631830684231,0.309791209738355,2.
2293461175594,0.0257908843823752,0.411777406172993
"1060","TIAM2",64.7285159885515,0.773076693259707,0.346931875123563,2.
22832420049143,0.0258589036734768,0.412150859591557
"1061","CD36",1414.49067912365,-1.75121687766669,0.785911451157785,-2.
22826232533811,0.0258630270893448,0.412150859591557
"1062","GRIP2",8.17187487683634,-1.17120241847909,0.526283333502565,-2
.22542182874082,0.0260529334922091,0.414786251870313
"1063","RABEP1",341.372806724794,0.382169082328891,0.171770864195617,2
.22487721720761,0.0260894818499672,0.414977383931557
"1064","WFS1",124.43887275568,0.469902686833428,0.211320706585856,2.22
364714951638,0.0261721936474993,0.415121435452081
"1065","AL080250.1",15.017834422603,0.904976782400305,0.40694224244781
1,2.22384576483569,0.0261588230993854,0.415121435452081
"1066","NUDT11",32.0699409383935,-0.735504632760291,0.330741561982141,
-2.2238046780465,0.0261615885289448,0.415121435452081
"1067","FMO1",207.827406939666,-0.889556513768947,0.400379564452304,-2
.22178300979424,0.0262979734785508,0.415945870510138
"1068","CD24",18.0608708090015,1.36196258324701,0.612921165286508,2.22
208443823336,0.0262775997437041,0.415945870510138
"1069","ZNF260",91.3452872101436,0.43987723120923,0.197952390148078,2.
22213649898432,0.0262740823070367,0.415945870510138
"1070","PLEKHO1",228.698089671942,-0.698051565291811,0.314260080744313
,-2.22125433061209,0.026333740199515,0.416122317096635
"1071","ZNF8",34.858787875256,0.651629649759993,0.293582254926168,2.21
958118662134,0.0264472105256595,0.417525149923296
"1072","BACH2",60.9358454758937,0.803484464639087,0.362078089107585,2.
2190916512497,0.0264804899173236,0.41766056298704
"1073","PXDN",488.763043969665,0.530382685566655,0.239105153219231,2.2
1819847220255,0.0265413028748985,0.417840175985832
"1074","AGPAT2",405.004824387373,-0.53898832057021,0.242977277947912,-
2.21826635446033,0.0265366768135312,0.417840175985832
"1075","AC114964.2",2.55677287916924,2.50658474049525,1.13026758434295
,2.21769143450432,0.0265758786791244,0.417995308564312
"1076","LINC00294",70.7823958497103,0.718708420447041,0.32416783980706
5,2.21708736090167,0.0266171223177433,0.418254929505951
"1077","VEPH1",5.85823856310133,1.84835265162003,0.834049230877071,2.2
1611936465229,0.0266833283631329,0.418517361747542
"1078","FZD9",3.14556649009691,-2.35303127610852,1.06160835703968,-2.2
1647772505296,0.0266588017633476,0.418517361747542
"1079","FAM53C",548.728293068866,0.570789600492088,0.257708477082537,2
.2148654439073,0.0267693016064939,0.4194766928291
"1080","OLA1",323.682450364318,-0.491877696260024,0.222123104253831,-2
.21443734055658,0.0267987086118297,0.419548671489646
"1081","SERGEF",122.091907623807,-0.636688382590238,0.287565017041076,

-2.21406758423363,0.0268241301274731,0.419558179644141
"1082","NR2F2-
AS1",64.3409957767763,0.709849169583911,0.320682304348835,2.2135589022
4533,0.0268591370978511,0.419717458456993
"1083","C3orf70",29.1030661117005,-0.868797957096312,0.392870393652592
,-2.21141111962887,0.0270073805222312,0.420644006047515
"1084","USP30",55.6583186358051,0.50576093367791,0.228758030487874,2.2
108991435154,0.0270428220116897,0.420644006047515
"1085","PECAM1",13.6999950170952,-1.11691966619237,0.505157459255034,-
2.21103271015639,0.0270335720073362,0.420644006047515
"1086","PIEZO2",9.97725374260641,1.73725151244993,0.785631132226519,2.
21128140317768,0.0270163563344636,0.420644006047515
"1087","EMP3",2499.00381344862,-0.517686224269278,0.234111221502828,-2
.2112832565056,0.0270162280741304,0.420644006047515
"1088","LINC00654",18.5801794911039,0.794267405452891,0.35946877187941
,2.20955884790833,0.02713579400516,0.421702210514012
"1089","AC114296.1",10.2657498939695,-1.1903866742213,0.53926148966441
4,-2.20743868612254,0.0272834261468278,0.42360713433477
"1090","ZSWIM8",180.880443798712,0.428520941390594,0.194209967183539,2
.20648274444957,0.0273502171170428,0.424254560564183
"1091","RELL1",192.665415774309,0.471742903947831,0.21387055312529,2.2
0574032775552,0.027402186530829,0.424671099783004
"1092","PID1",800.07613996193,0.556167327430926,0.252367069369426,2.20
38030905561,0.0275381951444771,0.426388098445805
"1093","ZMYM2",288.692955315256,0.423709058303725,0.192317655946672,2.
20317295475573,0.0275825607416407,0.426684297364741
"1094","POU5F2",49.7564378356898,0.727441828719254,0.330326918333635,2
.20218755525254,0.0276520627602051,0.427368443463938
"1095","APP",2569.85477358796,0.285788547296054,0.12982433259196,2.201
3480954629,0.0277113904748019,0.427894237578037
"1096","CRAT",292.272903760187,-0.318806397743796,0.144848219682879,-2
.20096869980017,0.0277382397587014,0.42791802722639
"1097","ADIPOQ",2.07165975559909,-3.00104818138363,1.36376129741712,-2
.20056705456258,0.0277666880544108,0.42796641898266
"1098","AC254633.1",37.7038400438594,1.25429661983467,0.57057041715263
5,2.19832045638484,0.0279262776460726,0.4300341552275
"1099","IGDCC4",112.226421126833,0.678200539543647,0.308998414726982,2
.19483501280379,0.0281754349175509,0.4330820487145
"1100","KCNJ2",2.80030960179378,-2.76694418378419,1.26061307157319,-2.
19491947702173,0.0281693744098434,0.4330820487145
"1101","SYBU",36.6382069278391,-0.926266385789549,0.422109028365896,-2
.19437710056899,0.0282083107676059,0.433193568082362
"1102","ADAMTS19",7.13914979561386,-2.03086468039054,0.9260436938215,-
2.19305492164174,0.0283034223081147,0.433866060186404
"1103","STAMBPL1",83.223779452799,0.762134243822251,0.347508182881282,
2.19314042478999,0.0282972632544295,0.433866060186404
"1104","DNAH7",4.12767242954159,-1.67366532027544,0.763526819797162,-2
.19201903178734,0.0283781324401634,0.434278904518327
"1105","HFE",64.708650854247,0.572750731645694,0.261295052651324,2.191
96929231562,0.0283817240059588,0.434278904518327

"1106","TRIM23",227.711449090417,0.454700634968186,0.207547744603244,2
.19082426473682,0.0284645119904967,0.434758779345363
"1107","ZNF189",73.9039970561676,0.627473298952289,0.286364487504932,2
.19117008683377,0.0284394864023748,0.434758779345363
"1108","ADAM28",3.81246382971853,-2.27146628816599,1.0370511545926,-2.
19031267465136,0.0285015682493952,0.434931873610806
"1109","LINC02381",321.777156420705,0.350028491916007,0.15983359910873
3,2.18995564054017,0.0285274540914426,0.434934349664663
"1110","CCDC34",170.012452622718,-0.577868513727997,0.263917530515643,
-2.18957987595199,0.0285547198078626,0.434957840100307
"1111","PDCD4",984.943765344713,0.364496700613144,0.166562414800087,2.
18834904051207,0.0286441872356972,0.435144580216684
"1112","CTNND1",347.715109756553,0.344293292632396,0.157286911697925,2
.18895068200985,0.0286004247768374,0.435144580216684
"1113","APOL1",309.012829184215,0.701351603238821,0.320479891218593,2.
18844184130243,0.0286374332925632,0.435144580216684
"1114","PIK3CA",297.883828987467,0.426686315494735,0.195108221153767,2
.18692125309501,0.0287482731300355,0.436333754113681
"1115","MTA3",169.045983227642,0.37629054034634,0.172249725991849,2.18
456394156555,0.0289208342059916,0.438559161215162
"1116","SDF2L1",217.46085024713,-0.651064531993471,0.298130018080693,-
2.18382749977646,0.0289749260520687,0.438985707606073
"1117","HLA-
DOA",7.99849231355114,-1.34240642609932,0.615458629298332,-2.181148110
03586,0.029172463386707,0.441331150883757
"1118","CCND1",622.026602731327,-0.449811259537325,0.206272309828912,-
2.1806671962437,0.0292080410361322,0.441331150883757
"1119","PRTG",40.0803812902338,0.596206965387417,0.273394205296969,2.1
8075933518708,0.0292012217747316,0.441331150883757
"1120","NOC4L",127.960778614373,-0.502863826412528,0.230654921716224,-
2.18015649816137,0.0292458629760407,0.441508081427586
"1121","SLC35E2B",214.488758915875,0.541607815020413,0.248757455061607
,2.17725259685696,0.0294617259011858,0.443976135159052
"1122","ADGRL4",10.3558387303177,-1.09547348825329,0.503309090974578,-
2.17654222404781,0.0295147399777424,0.443976135159052
"1123","NMNAT3",101.558096775177,0.572649567562082,0.263142512377911,2
.17619556181661,0.0295406406466722,0.443976135159052
"1124","FZD10-
AS1",39.4703707348955,-1.2521965810621,0.575266776604601,-2.1767232734
2272,0.0295012207442503,0.443976135159052
"1125","BAHD1",44.718506500276,0.677102065532779,0.311127387994703,2.1
7628563623691,0.0295339089090754,0.443976135159052
"1126","UBTD1",67.0945262313727,-0.719816803958151,0.331067283674661,-
2.17423115920301,0.0296877794129982,0.445791273814363
"1127","ARHGEF6",146.682490865969,0.528924752671901,0.243429089602966,
2.17280832596704,0.0297947464075933,0.447000507772483
"1128","EML2-
AS1",5.11047415108624,-1.88705021649423,0.868805855219795,-2.172004487
71934,0.0298553244210946,0.447512256482152
"1129","KSR1",63.340489719722,0.633394973763175,0.291736853716335,2.17

111744949112,0.0299222953299352,0.44811883918383
"1130","NBL1",944.708304539511,-0.587655870881913,0.270738736496035,-2
.17056442859819,0.0299641134309837,0.448347991054046
"1131","LNCTAM34A",5.48348379212432,1.64687315199807,0.759297068385112
,2.1689444363333,0.030086902568815,0.448641833571854
"1132","AC118549.1",185.385603375226,0.38947391039987,0.179614194473661,2.16839159923401,0.0301289043316901,0.448641833571854
"1133","GPBAR1",7.06797701328005,-1.82335409576885,0.840534248543127,-
2.16927995370708,0.030061436204187,0.448641833571854
"1134","ATP1B3",560.568807968263,-0.438532641450999,0.202209570361154,
-2.16870369027421,0.0301051870454531,0.448641833571854
"1135","AL356599.1",86.9970791860112,0.678074804439346,0.3127698301444
82,2.16796742871943,0.0301611648133808,0.448641833571854
"1136","BOP1",77.269366409425,-0.518612198946701,0.239227936958264,-2.
16785800831104,0.0301694916472201,0.448641833571854
"1137","AP005329.3",35.7966267552899,-0.673010816476165,0.310207463850
485,-2.16955068753776,0.03004090053354,0.448641833571854
"1138","CAPN2",1161.17244089353,-0.37307914209681,0.172232905911059,-2
.16613161186206,0.0303011311820565,0.448985012426098
"1139","IL1B",148.038899796561,-1.87600766318598,0.866606752086144,-2.
164773882352,0.0304050058686943,0.448985012426098
"1140","LHFPL2",180.514546109422,0.504597818082009,0.23297651757037,2.
16587415480445,0.0303208047764774,0.448985012426098
"1141","RGS22",64.4551811364448,-0.780996150639909,0.360643129287227,-
2.16556503428601,0.0303444407347746,0.448985012426098
"1142","GSN",106749.999983845,-0.547745932490355,0.252937208235436,-2.
16554114877598,0.0303462677261408,0.448985012426098
"1143","ARHGAP21",988.686661530137,0.336446029173437,0.155417692348899
,2.16478590106811,0.0304040850224783,0.448985012426098
"1144","KL",12.9057648373687,1.29301811442696,0.597064984333709,2.1656
2375680076,0.0303399494791381,0.448985012426098
"1145","TUG1",474.534075152648,0.505280739454938,0.233343427389983,2.1
6539520785589,0.0303574327309705,0.448985012426098
"1146","LRP6",610.626723828665,0.603376567513173,0.278919008577915,2.1
6326800596891,0.0305205723539199,0.450298287399718
"1147","PHLDA2",35.4275625508764,-0.939076904504841,0.434198805271875,
-2.16278094988501,0.0305580314855743,0.450457886973051
"1148","IGF2R",420.683871598981,0.438663242353628,0.202938526544236,2.
16155724506066,0.0306523200157511,0.451454204552543
"1149","ADAM12",166.620935237468,1.03315400090011,0.478076997416222,2.
16106193454991,0.0306905554993209,0.451623944632304
"1150","BORCS7",818.315785113213,-0.415201156801717,0.192228657998665,
-2.15993370148067,0.0307778023498589,0.45214438985402
"1151","GLB1L2",6.97350975393589,-1.230289438363,0.569601387920063,-2.
15991299258502,0.0307794057678009,0.45214438985402
"1152","ECSIT",279.652073370288,-0.458573932822961,0.212352412172484,-
2.15949481398159,0.0308117992365715,0.452227345045096
"1153","BNC2",347.859080064498,0.644081690180451,0.298355557968123,2.1
5877221985342,0.0308678427070297,0.45226471792934
"1154","TNNI2",2.75778060393217,-2.31124771214696,1.07053673228575,-2.

15896161471463,0.0308531450290904,0.45226471792934
"1155","UBA6",182.69093138786,0.362133495400356,0.167843054954025,2.15757211699674,0.0309611145114333,0.453095854506481
"1156","CEP128",34.0761754733597,0.942336885119745,0.436802346743379,2.15735307318156,0.0309781646445169,0.453095854506481
"1157","RAB6B",36.8219123952418,0.800646332224337,0.371509051227061,2.15511931561256,0.0311524985745235,0.453756264354807
"1158","CXCL3",163.236753488163,-1.87344414551418,0.869758961181485,-2.15398084886562,0.0312416738207945,0.453756264354807
"1159","GFPT2",542.239237338534,0.574658545200714,0.266825661548437,2.15368545088905,0.031264847881083,0.453756264354807
"1160","ZFAND1",383.464308692347,-0.56105627158518,0.260354437074922,-2.15497103828396,0.0311641006401739,0.453756264354807
"1161","RASSF4",219.633003104287,0.572962830056759,0.265965495223277,2.15427504825677,0.0312186084408481,0.453756264354807
"1162","ZNF215",17.0846696148734,-1.02155694641321,0.473862530873854,-2.1558086572689,0.0310986092044909,0.453756264354807
"1163","ALYREF",192.683943648826,-0.594165651108599,0.275735381007743,-2.15484008231759,0.0311743504668434,0.453756264354807
"1164","ZNF358",618.021069624933,-0.296491023874611,0.137633301400378,-2.15420992490846,0.0312237128843759,0.453756264354807
"1165","LINC01278",102.600982171605,0.448182932986318,0.20788461331583,2.1559216232392,0.0310897857097264,0.453756264354807
"1166","HIST1H2BD",13.491939860659,1.67401154711864,0.777622328929864,2.15273081139832,0.0313398405176803,0.454065144364129
"1167","APPL2",324.376295208507,0.36018763485331,0.167311934353997,2.15279105010661,0.0313351038494859,0.454065144364129
"1168","EXOC3L4",30.1578292465896,0.737960703352135,0.342885454367309,2.15220766571687,0.0313810021575784,0.454272246986589
"1169","SNHG14",315.73691193096,0.587489442741827,0.273095019921296,2.15122722820481,0.0314582689075109,0.454612316827516
"1170","ZNF578",7.99635963474834,1.23815518360673,0.575506022679112,2.15142002831323,0.0314430617543367,0.454612316827516
"1171","VPS52",83.6291422622599,0.483640406431673,0.224911001878169,2.15036348774817,0.0315264741035633,0.45482049841557
"1172","CD209",4.9261780759621,-1.8102766491086,0.841723078138543,-2.15067959537476,0.0315014979888129,0.45482049841557
"1173","WDFY1",228.261526980711,0.389615458785059,0.181304167914936,2.1489602984078,0.03163754723677,0.455645356626326
"1174","AL121929.3",11.4921214353888,-0.986392734456651,0.458990362894878,-2.14904889992769,0.0316305238378537,0.455645356626326
"1175","HDAC4",202.282283372019,0.492906416649379,0.229450823457561,2.14820068728386,0.0316978161412514,0.456065236670474
"1176","CBWD1",177.473435778775,-0.531189753735601,0.247305032158179,-2.1479132434145,0.0317206481147669,0.456065236670474
"1177","CDK19",128.82705936336,0.526764542548355,0.24529372702608,2.14748476830126,0.031754708528511,0.456167044859868
"1178","HTR2B",19.6806312300122,1.28321649727373,0.597928490373569,2.14610361930072,0.0318647125935195,0.457358710128376
"1179","MDFI",26.2727470816128,1.39131078702251,0.648521201907067,2.14

535898430332,0.0319241558082638,0.457823262430979
"1180","PPARGC1A",17.930735859604,0.952717827864252,0.444350723080675,
2.14406723873223,0.0320274996265368,0.45828244899639
"1181","IRX1",261.549397641959,-0.602659883516874,0.281199231786695,-2
.14317756022187,0.0320988434264468,0.45828244899639
"1182","VEGFB",1742.34761612005,-0.47237370775591,0.220444811679184,-2
.14282070944523,0.0321274977322668,0.45828244899639
"1183","AL355001.2",55.3706062658805,-0.63113189207677,0.2944631028149
4,-2.14333098457301,0.0320865305345903,0.45828244899639
"1184","GALK2",182.250700790068,0.356863439880179,0.166556911767232,2.
14259159883383,0.0321459063466832,0.45828244899639
"1185","CES2",194.572550097742,0.434153122057555,0.202465790929439,2.1
4432828412411,0.0320065920638413,0.45828244899639
"1186","PTPRT",43.3184876425066,-2.3095924731426,1.07736155036468,-2.1
4374874651952,0.0320530240543465,0.45828244899639
"1187","STX19",3.90290544836968,2.33983768725622,1.09243702277301,2.14
185132733496,0.0322054475961164,0.458358340029576
"1188","PCDHB2",60.6987460595106,0.519505793640591,0.242538532111555,2
.14195158648707,0.0321973780593541,0.458358340029576
"1189","AC073896.2",42.4307602406818,-0.623088342221238,0.290973787278
512,-2.14138994460293,0.0322426051514737,0.458501234567802
"1190","AC087854.1",20.3237844769765,1.10851636829063,0.51791742180285
4,2.14033419542429,0.0323277683610575,0.459325972646017
"1191","LPP",1919.18818953534,0.428724471598424,0.200432916498683,2.13
899233263535,0.0324362893436987,0.45970895240843
"1192","BAALC",3.04587233191719,-2.26763242232297,1.05995415153563,-2.
13936840479156,0.0324058436861946,0.45970895240843
"1193","PTGDS",3807.49143191334,1.73558213929189,0.811283133037953,2.1
3930509413252,0.0324109674100408,0.45970895240843
"1194","UBAC2",307.060207416498,-0.422563876197257,0.197627567344921,-
2.13818285512645,0.0325019053251109,0.460253111588756
"1195","MIR4435-2HG",262.632652769901,0.616522554829624,0.288472424932
669,2.13719753274,0.0325819286371449,0.460649374148843
"1196","AC009509.1",11.7997415756051,6.90102788277118,3.22905348169176
,2.13716741512612,0.0325843773055368,0.460649374148843
"1197","ANO8",47.8778651270331,0.547456432637971,0.256413356238961,2.1
3505427590822,0.0327565769349494,0.462572046108498
"1198","MT-
ND1",64707.0526517696,-0.334327639641345,0.156606388412659,-2.13482759
566863,0.0327750952944157,0.462572046108498
"1199","SMYD2",94.7343126204255,-0.481361076407724,0.225659939771111,-
2.13312596332328,0.0329143944614202,0.462926031525992
"1200","ARHGEF3",119.907477293724,0.549951342976345,0.257848168276222,
2.13284952401603,0.0329370721508025,0.462926031525992
"1201","NPR2",154.724569179368,0.489203403791043,0.22935148049678,2.13
298559368973,0.0329259080085819,0.462926031525992
"1202","CKAP5",179.822404083567,0.357919712347226,0.167792782167902,2.
13310553483208,0.0329160698544823,0.462926031525992
"1203","LRRK2",237.00226916621,0.564957386190195,0.26479601716239,2.13
355696299512,0.0328790640854019,0.462926031525992

"1204","FGD2",5.5018843791654,-1.57329765555212,0.738286426806266,-2.1
3101256968519,0.0330881066641202,0.464284594385564
"1205","GPR107",169.771025732214,0.44635686017577,0.209458289592437,2.
13100594416334,0.0330886524860778,0.464284594385564
"1206","IFNAR2",124.693994729898,-0.523666833377381,0.245796929478765,
-2.13048565939316,0.033131538516771,0.46450087333463
"1207","C3orf14",177.057147455666,-0.470144918401267,0.220883186699115
,-2.12847761492003,0.0332975040023045,0.466440925990857
"1208","EXOC1",346.429080588523,0.36471824894918,0.171386408846586,2.1
2804650849328,0.0333332276890293,0.466554812720287
"1209","CDC42EP3",32.0279954016089,0.83373284100751,0.391856476704399,
2.12764849012932,0.0333662386390519,0.466630573125798
"1210","TBX19",35.7733500801153,0.692816575525,0.325943099640962,2.125
57521938081,0.0335386447836042,0.467011927095622
"1211","AC147067.1",61.6230159205835,-0.663714827275181,0.312166039781
319,-2.12615961601759,0.033489971393152,0.467011927095622
"1212","CKMT2",5.90714701502004,-1.49891084030388,0.705590429059998,-2
.12433556149671,0.0336420941094433,0.467011927095622
"1213","UBQLNL",4.83626494755582,1.77247688729192,0.833755178359392,2.
12589610631226,0.0335119111774639,0.467011927095622
"1214","CCDC85B",927.894969765862,-0.619660300029018,0.291682664782038
,-2.12443307349809,0.0336339468651547,0.467011927095622
"1215","RAB30",318.703943384481,0.368791710078325,0.173425589376919,2.
12651265250597,0.033460596879793,0.467011927095622
"1216","LINC02384",51.9080837481467,0.984921826359955,0.46352478881510
8,2.12485254322142,0.0335989189089401,0.467011927095622
"1217","SLC7A8",113.391749501599,0.592920835943887,0.278954327176162,2
.12551223688118,0.0335438940980279,0.467011927095622
"1218","AC139100.2",3.51139423243481,2.1043807056364,0.99016258676336,
2.12528804235595,0.0335625854296948,0.467011927095622
"1219","ABCB5",59.2359495642367,1.06875599304194,0.503599860108965,2.1
222325058047,0.0338182181297366,0.467226076995038
"1220","ZNF706",1117.16540323158,-0.459908857303527,0.216716138209422,
-2.12217170859282,0.0338233214006344,0.467226076995038
"1221","HSPB2",1019.94039711372,-0.444117663321677,0.209145414963429,-
2.12348744723538,0.033713026280433,0.467226076995038
"1222","KLHL33",29.614028461715,0.88548885145335,0.417214205682043,2.1
2238423187387,0.0338054852326905,0.467226076995038
"1223","LINC02345",2.67224663295196,-2.45674028207439,1.15695457672166
,-2.12345439614041,0.0337157931013185,0.467226076995038
"1224","SUGP1",82.6755966853738,0.416315772391705,0.196091422744244,2.
12306977309605,0.0337480055015279,0.467226076995038
"1225","ITGB1BP1",1583.92425443459,-0.535005075261362,0.25217134000747
3,-2.12159349768101,0.0338718888969381,0.467515018342391
"1226","AKTIP",157.601190594928,-0.395691705277997,0.186572056935085,-
2.12085192058355,0.0339342657940264,0.467834526126228
"1227","TCF4",4901.79542588929,0.453527913220997,0.213861623180018,2.1
2066057704631,0.033950376363667,0.467834526126228
"1228","PNMA6A",37.0829877759446,0.855184774130264,0.403370551230042,2
.12009719480625,0.0339978494864998,0.468107197978614

"1229","MTUS1",407.740553929938,0.637514423500525,0.300789846520682,2.
11946789718745,0.0340509440207861,0.46815596104
"1230","RRP12",44.2561859232937,-0.612174168758646,0.288843295469757,-
2.11939892100678,0.0340567679251951,0.46815596104
"1231","LINC02550",18.9673433054721,1.35216098819877,0.638249688534703
,2.11854547285886,0.0341288980629037,0.468766375668218
"1232","CADM3",177.340030792658,-0.987712805753107,0.466341494082191,-
2.11800326217385,0.0341747914110353,0.468955258889054
"1233","GFRA1",688.460722664291,0.617457399274945,0.291565979037095,2.
1177278683683,0.0341981212568136,0.468955258889054
"1234","MYZAP",17.7390438795532,-1.24042643881713,0.585904748332906,-2
.11711279409589,0.0342502761132433,0.469289844832024
"1235","USF2",480.611743122132,-0.284913786059985,0.134620273567808,-2
.11642554653162,0.0343086312529258,0.469708775080542
"1236","CLDN23",101.113405776762,-0.858566716304891,0.405941749679633,
-2.11499979241472,0.0344299650073478,0.470393320190358
"1237","ASS1",349.986546204428,-0.598939997766954,0.283121860258185,-2
.1154848206379,0.0343886473166225,0.470393320190358
"1238","SLC43A1",205.263938882263,0.40353633510882,0.190831657197513,2
.11461945588596,0.0344623940778389,0.470393320190358
"1239","ACTN1",492.769182598777,-0.795114588280875,0.376024034938402,-
2.11453129162641,0.034469915052984,0.470393320190358
"1240","ARHGEF9",96.6677674439815,0.393856250984491,0.186353327936699,
2.11349190993936,0.0345586867225819,0.471224415407593
"1241","BAZ2B",508.078427024496,0.520420450184314,0.246281202122751,2.
11311478788758,0.0345909442693915,0.471284194767826
"1242","HSDL2",331.298483325529,-0.303721011276466,0.143776046618065,-
2.11245905295537,0.034647094515509,0.471289681470817
"1243","WBP1L",346.23380794865,0.473392133981768,0.224087356128898,2.1
125338892814,0.0346406823862907,0.471289681470817
"1244","DUSP23",228.194600360884,-0.535821299805903,0.253871234859154,
-2.11060264508964,0.0348064801317246,0.472317789781059
"1245","GAL",132.005387221079,1.16668352268274,0.552698681330761,2.110
88530168672,0.0347821717474869,0.472317789781059
"1246","AC022467.1",6.8337392834101,1.74388246525096,0.826137650845585
,2.11088607747876,0.0347821050495546,0.472317789781059
"1247","CHPF",196.283361275776,-0.480562382993956,0.227903148166276,-2
.10862547033945,0.0349769225784699,0.472989968479777
"1248","AC010207.1",9.98755423947448,1.51005650152833,0.71614119483992
6,2.10860164505111,0.0349789807760355,0.472989968479777
"1249","MSANTD3",165.599933488681,-0.457468044553667,0.217006716863858
,-2.10808241866848,0.0350238609259925,0.472989968479777
"1250","UBAC1",331.380691366901,-0.384585727886176,0.182400035693216,-
2.10847397274101,0.0349900117784282,0.472989968479777
"1251","PRICKLE1",512.967693196101,0.357702197211282,0.169679131598158
,2.1081095467792,0.0350215148484619,0.472989968479777
"1252","MARCH2",346.786001548507,-0.661167527688212,0.313432934725477,
-2.10943858936618,0.0349067415381217,0.472989968479777
"1253","FADS2",16.420276233341,1.41594067272336,0.672059364094522,2.10
686845295443,0.0351289838354131,0.474031012521281

"1254","KDM5B",496.642022004498,0.299894740047388,0.142390056842351,2.10614945100711,0.0351913724503065,0.474116115848432
"1255","DANCR",313.562177055834,-0.609048195456339,0.289139369515965,-2.1064173878359,0.0351681122216979,0.474116115848432
"1256","WDYHV1",92.7024253070787,-0.459504834671801,0.218253811340012,-2.10536911979031,0.0352591896684036,0.474651575567968
"1257","VPS8",102.556573459094,0.480951273053973,0.228540294051241,2.10444847395768,0.0353393447808249,0.474974277865013
"1258","CRNDE",790.46645919234,-0.387709658379441,0.184216717609982,-2.10463883739525,0.035322758237433,0.474974277865013
"1259","FNDC3B",1050.33956423209,0.514033529602763,0.244387332688418,2.10335586524908,0.0354346734002763,0.47568070927568
"1260","AC005162.3",10.1365161760513,1.37290827378564,0.652770725888294,2.10320135284465,0.0354481720893871,0.47568070927568
"1261","SORT1",120.625426511388,-0.783712498975364,0.37271055684855,-2.10273759241498,0.0354887140085604,0.475847086801537
"1262","TBXAS1",7.61693152787379,-1.209936609175,0.575931334705865,-2.10083483266791,0.0356554675847597,0.477195354783692
"1263","LRFN4",81.9695923338768,-0.48050621402716,0.228744453013787,-2.10062455153043,0.0356739370976216,0.477195354783692
"1264","ZNF185",24.4730312395316,0.775885351477026,0.369332756940929,2.10077589083473,0.0356606437689564,0.477195354783692
"1265","FKBP1B",56.7688329311065,-0.743003571685109,0.354167226132802,-2.09788912372853,0.0359149410165566,0.477826612544054
"1266","AC116366.1",73.4325674629699,0.822932104523371,0.392206554354538,2.09821099465736,0.035886510808051,0.477826612544054
"1267","ERLIN2",216.237314610535,0.46659022041412,0.222258401065146,2.09931421344725,0.0357892112989046,0.477826612544054
"1268","NF1",457.401146787244,0.383850397417476,0.182941158398336,2.09821781373921,0.0358859086999972,0.477826612544054
"1269","ZNF91",335.680871138222,0.388675373411329,0.185242057076457,2.09820264115782,0.0358872484131305,0.477826612544054
"1270","THBD",25.1236840885773,-0.872673227144872,0.41598580951758,-2.09784374172983,0.0359189510612428,0.477826612544054
"1271","ELMO2",60.1810628846841,0.511843005465088,0.243853069513381,2.09898118767375,0.0358185591114512,0.477826612544054
"1272","S1PR1",26.9522128464937,0.870619064985301,0.415081694842577,2.09746436858771,0.0359524881738542,0.477829081186997
"1273","CRTC1",93.6843832600254,0.399725885020714,0.190599569906465,2.09720245023048,0.0359756576976016,0.477829081186997
"1274","RFTN1",810.705900316009,0.489316138949682,0.233453207607529,2.09599235737338,0.0360828689162551,0.478876881974914
"1275","POLR3F",90.8692803984498,-0.400533170107876,0.191137975029797,-2.09551853861294,0.0361249222269986,0.479058968638504
"1276","PAGR1",294.686158478182,-0.359342518914953,0.171509366683338,-2.09517722480089,0.03615524107651,0.479085279092187
"1277","TEDC1",43.5913522514445,-0.708167331648849,0.33809675752266,-2.09457001846989,0.0362092327373991,0.479424986001523
"1278","CDK14",373.745402610026,0.34463615815824,0.164662153992487,2.09298949274018,0.0363500925245268,0.480913430676604

"1279","WDR36",160.788766816212,0.378586571344339,0.18106869622843,2.0
9084496232705,0.0365419641126962,0.483051683325778
"1280","EGR2",98.7758989976962,-1.16181916095867,0.555749361229607,-2.
09054520258579,0.0365688522981426,0.483051683325778
"1281","TTC28",340.01675117984,0.375887372840758,0.179860776774444,2.0
8987962568483,0.036628614233528,0.483463395363382
"1282","WDR26",295.074885640666,0.392921488270665,0.188272570854072,2.
08698211581342,0.0368897512023425,0.483513111107912
"1283","TFPI",1877.48865853508,0.36756141545071,0.176006552832908,2.08
83393801801,0.0367672315231406,0.483513111107912
"1284","JAGN1",497.807962345666,-0.492627200348597,0.236038962976613,-
2.08705882340877,0.036882817581547,0.483513111107912
"1285","CCT6A",460.288980251015,-0.379937026635156,0.181994721962616,-
2.08762662201379,0.0368315286146586,0.483513111107912
"1286","TMEM120B",50.8818325565533,0.51744687041071,0.247900575898587,
2.08731612879508,0.0368595677815561,0.483513111107912
"1287","ZNF770",194.570646054623,0.468664224929819,0.224341577046895,2
.08906539349081,0.0367018370177308,0.483513111107912
"1288","MCRIP2",91.2871602282934,-0.571748438985863,0.273704047879497,
-2.08892942364369,0.0367140767415403,0.483513111107912
"1289","LDLR",232.007882088189,-0.883228138300212,0.423098101029012,-2
.0875256498484,0.0368406449370465,0.483513111107912
"1290","CD33",5.54602646123247,-1.78479368217374,0.854851256794807,-2.
08784120978622,0.0368121608309495,0.483513111107912
"1291","RGS3",35.0995823019067,-0.732299375533618,0.350958186201944,-2
.08657157554446,0.0369268789234593,0.483624840308172
"1292","COL24A1",28.2146753159873,1.20389436089898,0.578556859279989,2
.08085746731482,0.037446954226252,0.48415009433485
"1293","SH3RF3",53.6066296590655,0.745614275997306,0.358271227793619,2
.08114472543359,0.0374206611128428,0.48415009433485
"1294","DIRC3",16.001508265336,1.56134141088844,0.750194351611783,2.08
124922233008,0.0374111002727489,0.48415009433485
"1295","MAPKAPK3",69.4246488087556,-0.491872656916698,0.23634494158462
8,-2.08116430848414,0.0374188692226966,0.48415009433485
"1296","FAM20C",305.941868821701,-0.331725692217977,0.159320062617259,
-2.08213383028159,0.0373302472069483,0.48415009433485
"1297","PDE7A",115.763019819111,0.572849715898081,0.27526945703213,2.0
8105077139458,0.0374292591199553,0.48415009433485
"1298","TIGAR",16.802966603641,-0.828671660284432,0.397565755644208,-2
.08436377761377,0.0371270900612903,0.48415009433485
"1299","DENND5B",140.774303988677,0.426419854010434,0.20462704333436,2
.08388806807741,0.0371703499435126,0.48415009433485
"1300","HELLPAR",1078.60361170611,-0.412477474802194,0.197987740114043
,-2.08334856776789,0.0372194627290544,0.48415009433485
"1301","SDR39U1",174.513875632621,-0.408568087745802,0.195900201481998
,-2.08559299406003,0.0370155065117878,0.48415009433485
"1302","AKT1",341.949823842532,-0.31319384234888,0.150342713602363,-2.
08319934398175,0.037233056892775,0.48415009433485
"1303","BBS2",158.547519533876,0.352556435455735,0.169211684884064,2.0
8352298895488,0.0372035784589268,0.48415009433485

"1304","FAM20A",143.645474877553,0.495003808226589,0.237499092692412,2
.08423452323531,0.0371388399005931,0.48415009433485
"1305","RHBDF2",7.71374011560939,-1.37157646632128,0.657856471920705,-
2.08491749319843,0.0370767904891691,0.48415009433485
"1306","AXL",1095.02075421174,-0.401137532054592,0.192536861823381,-2.
08343237889982,0.0372118294573055,0.48415009433485
"1307","PLXNB2",204.492196942921,0.416581114195731,0.200119886624199,2
.08165775637291,0.0373737418048506,0.48415009433485
"1308","TAF1",200.048836619962,0.518512319637465,0.249190957735472,2.0
8078304425432,0.0374537688307301,0.48415009433485
"1309","AC022424.1",4.86067519782255,2.14830231490671,1.03282792940497
,2.08001957900615,0.0375237370778531,0.484683992751979
"1310","ZNF728",8.50237982269228,1.49236695156824,0.717650515308393,2.
07951770358157,0.0375697923301365,0.484908434135839
"1311","PEAR1",109.189135907354,0.934786820990713,0.449667749326386,2.
07883892583146,0.0376321577675009,0.485342885990012
"1312","AFF1",491.781421580864,0.495043489194997,0.238198316653967,2.0
7828290371233,0.0376833101478511,0.485632170716362
"1313","VASH2",86.8499378701475,0.723954121579883,0.348592986862682,2.
07678911757643,0.0378210270610609,0.487035739183868
"1314","AC053503.5",2.06711514983783,-2.25602480396892,1.0871424516543
,-2.07518784731102,0.037969128497517,0.487828286197582
"1315","NIM1K",5.60909711446953,-1.31048758785285,0.631498652001154,-2
.0752025102509,0.037967770088267,0.487828286197582
"1316","LMO3",935.096646905125,0.355177043029056,0.17111451619779,2.07
566868621777,0.0379246039914921,0.487828286197582
"1317","FOXRED2",102.063748790372,0.531499073568046,0.25636112716781,2
.07324362878205,0.0381496119493951,0.489774972543942
"1318","RBM3",2322.19507250468,-0.515727139329026,0.248854062490954,-2
.07240795736567,0.0382274119043563,0.490401426766963
"1319","HOXB6",12.4906808602976,-0.888573942826552,0.4288727024235,-2.
07188272372046,0.0382763794982951,0.490657334766622
"1320","CRTAC1",10.2408470717516,-1.86723011117243,0.901786690449676,-
2.07058956507923,0.0383971681522976,0.49183281751443
"1321","SHE",69.4591706914742,0.492282041151005,0.238401827499997,2.06
492561870572,0.0389300386701302,0.49453726058194
"1322","AGAP1-
IT1",2.93601699748767,2.77173062338618,1.3419657382668,2.0654257738099
8,0.0388827319853861,0.49453726058194
"1323","SUCLG2-
AS1",91.2745617398187,0.557096076703052,0.269610757905521,2.0662976545
5528,0.0388003827563635,0.49453726058194
"1324","ZFHX4",1694.94908468159,0.432533239214005,0.209461329864734,2.
06497896052377,0.0389249910580761,0.49453726058194
"1325","CDC14B",249.939323717941,0.330345146540956,0.15986650521136,2.
06638123542018,0.0387924963236706,0.49453726058194
"1326","FAM160B1",240.710168698406,0.416954461210111,0.201670130041866
,2.06750727598356,0.038686379311411,0.49453726058194
"1327","NCAM1",11.0029496524008,-1.5174878278252,0.734591030472569,-2.
06575872135137,0.0388512675478803,0.49453726058194

"1328","AC106739.1",50.8923764694142,-0.853867742399763,0.413210873565788,-2.0664212803292,0.0387887182921277,0.49453726058194
"1329","KRT24",18.8453617856797,1.360637081026,0.657958920621665,2.06796661369136,0.0386431626403075,0.49453726058194
"1330","ZNF490",36.2333287764491,0.605053005883802,0.292963918989745,2.06528164959789,0.0388963588227618,0.49453726058194
"1331","AC020910.2",2.12435324607513,2.36838970860456,1.14621557913659,2.06626899137821,0.0388030876390231,0.49453726058194
"1332","AP000240.1",4.53426405482027,-1.73762852183335,0.841634425361213,-2.06458822200339,0.0389619786173097,0.494571422268373
"1333","ZBTB37",147.089537861739,0.513500536591832,0.248933091293909,2.06280544672767,0.0391311163449671,0.496345772813731
"1334","BATF3",26.7187457509393,-1.36012092982293,0.659743610404357,-2.06159015164892,0.0392467724476101,0.496423573514256
"1335","POLR2J",775.384165928094,-0.419014671472447,0.203169161929433,-2.06239306936741,0.0391703286136288,0.496423573514256
"1336","ZBTB44",529.950136118896,0.432759952858126,0.209924070783165,2.06150705463945,0.039254691139612,0.496423573514256
"1337","CTXN1",19.7834775564586,-0.971639169975472,0.47131483763279,-2.06155014099619,0.0392505850753639,0.496423573514256
"1338","QPCT",28.4951071407289,-0.94913055384414,0.461936787043694,-2.05467626840978,0.0399102836053079,0.500392370737257
"1339","SP110",311.978011561604,0.301840617606586,0.146927480151701,2.05435101245144,0.0399417307424041,0.500392370737257
"1340","LINC02006",2.56203502736209,-2.33604999179569,1.13656597430265,-2.05535802110299,0.039844436995105,0.500392370737257
"1341","RSRC1",582.631120112099,-0.298006127977482,0.144952599446389,-2.05588674584412,0.0397934339697574,0.500392370737257
"1342","CEP162",166.466061908546,0.33327312657482,0.162326837685551,2.05309936007264,0.0400629420234758,0.500392370737257
"1343","C6orf58",2.11085730377335,2.90000763585822,1.41285521465762,2.05258656780411,0.0401126913732574,0.500392370737257
"1344","TMEM120A",215.514554860865,-0.369676661060629,0.179778031410325,-2.05629496641267,0.0397540931951452,0.500392370737257
"1345","RBM33",324.55568876848,0.457002511873051,0.222150201519512,2.05717802075867,0.0396691049464747,0.500392370737257
"1346","OSR2",1119.90948306167,0.814884457055822,0.396719536487206,2.05405678850934,0.0399701956783415,0.500392370737257
"1347","LINC00702",108.452107953414,-0.893324401480889,0.435323106676993,-2.0520950709463,0.0401604238875359,0.500392370737257
"1348","HBB",308.886008185514,3.46692365451612,1.68611671463224,2.05615876079628,0.0397672158461542,0.500392370737257
"1349","QSER1",269.057703469634,0.39929594680952,0.194145192165517,2.05668727798885,0.039716316697211,0.500392370737257
"1350","DZIP1",193.459545941586,0.448476972911186,0.21853024112779,2.0522421546633,0.0401461345651706,0.500392370737257
"1351","LGALS3",11051.9637266464,-0.569942936967226,0.277568101002808,-2.05334451224084,0.0400391766979352,0.500392370737257
"1352","DACT1",87.3895899074172,-0.496746182256892,0.242027422978911,-2.05243759629741,0.0401271538977243,0.500392370737257

"1353","AC009060.1",4.9274655616272,1.839622868781,0.895916915600526,2
.0533409256459,0.0400395243002435,0.500392370737257
"1354","MED1",253.547267872546,0.525493625298858,0.255548115974982,2.0
5633926626289,0.0397498259438197,0.500392370737257
"1355","RELCH",88.1521990551019,0.545988746453667,0.265858886359945,2.
05367875390275,0.0400067942055339,0.500392370737257
"1356","RBFADN",6.52224440249667,1.49525784985002,0.727316645298984,2.
05585539601579,0.0397964565610534,0.500392370737257
"1357","TPGS1",536.99481772569,-0.558356156215546,0.271952919143704,-2
.05313536612748,0.0400594508067458,0.500392370737257
"1358","AP003068.2",10.6275773252927,-0.972812321171514,0.474269088140
117,-2.05118222017478,0.0402492047053929,0.501129273312801
"1359","ARID1A",388.079133951133,0.326574397464122,0.159248723495747,2
.05071909083682,0.0402943107264903,0.5012069329219
"1360","CCDC6",287.243910569886,0.386415963099051,0.188448746630109,2.
05050959483172,0.0403147284583501,0.5012069329219
"1361","STMN1",107.07472808456,-0.730950383005899,0.356802424628754,-2
.04861383373849,0.0404998908412547,0.501371650906795
"1362","ZFAND5",2521.84890666685,0.68363174470824,0.333551662411011,2.
04955280320519,0.0404080900879336,0.501371650906795
"1363","CAND1",343.206806740826,0.308143893159513,0.150312974020089,2.
05001527757897,0.0403629399869205,0.501371650906795
"1364","PEAK1",319.07138375136,0.665313062827405,0.32477228115745,2.04
855248254656,0.0405058951465982,0.501371650906795
"1365","AC020765.2",14.55589230442,-0.858782921385319,0.41913608686403
1,-2.04893577122103,0.0404683958999749,0.501371650906795
"1366","CANT1",131.4871306724,0.354248669563375,0.172853907261444,2.04
941083008073,0.0404219591216812,0.501371650906795
"1367","PTGFR",970.439396986681,-0.610204197481832,0.298320143551874,-
2.04546763157388,0.0408087771959262,0.501714371965884
"1368","DHX57",77.80296594736,0.5384671066803,0.263449284375979,2.0439
1182141847,0.0409622590919005,0.501714371965884
"1369","ATOH8",254.54736004105,0.5165479814282,0.252542194416299,2.045
39278128195,0.0408161500603785,0.501714371965884
"1370","AC137936.1",17.9212529593674,-1.09093565628058,0.5330963135423
3,-2.04641380659999,0.0407156748276304,0.501714371965884
"1371","LSAMP",122.031546681276,1.71837308317714,0.840519146224664,2.0
4441872727767,0.0409121987982856,0.501714371965884
"1372","NHSL1",217.809780780764,0.445372041525307,0.217909839236285,2.
04383630902678,0.040969720878097,0.501714371965884
"1373","ARHGEF10",415.681467732153,0.504657950248129,0.246672405342622
,2.04586301231049,0.0407698503338467,0.501714371965884
"1374","KLHL9",174.890938845754,0.386428950484165,0.188814376284654,2.
04660766880161,0.0406966212829193,0.501714371965884
"1375","AC074032.1",80.042657088756,-0.468579521867404,0.2292342538919
4,-2.04410778019366,0.0409429007210896,0.501714371965884
"1376","CFL2",453.263990156544,0.405736304058016,0.198525831898579,2.0
4374564346515,0.040978681552217,0.501714371965884
"1377","PEX11A",90.4489939610999,-0.573061872671169,0.280011833967643,
-2.04656304896524,0.0407010060274036,0.501714371965884

"1378","KRT25",10.6399347646643,1.90040545317833,0.928034628649746,2.0
4777429043067,0.0405821206066563,0.501714371965884
"1379","AC087645.2",30.2023922496519,-1.12972883750987,0.5523429144770
34,-2.04533960316937,0.0408213888690071,0.501714371965884
"1380","ADAM33",402.269847351803,0.485359156342339,0.237146834180091,2
.04666091377697,0.040691389479047,0.501714371965884
"1381","AC010998.3",2.47529873360777,-2.96419434358871,1.4500241058835
1,-2.04423797615599,0.0409300432122049,0.501714371965884
"1382","GNA13",244.6336625078,0.334784689138302,0.163882862864062,2.04
282914813369,0.0410693540456118,0.502097352279974
"1383","F8A1",180.604776239106,-0.519810843178897,0.254422427176188,-2
.04310150228591,0.0410423912426851,0.502097352279974
"1384","MAP1A",869.008699471367,-0.378096081446795,0.185121396647516,-
2.04242236874821,0.0411096527638431,0.502226885065794
"1385","TSC22D1-
AS1",9.84682781200101,1.00630262296204,0.492844797967376,2.04182458070
431,0.0411689350651393,0.502563682493724
"1386","RNF24",545.918845253831,-0.558634759229851,0.273633307363343,-
2.04154517815359,0.0411966680823457,0.502563682493724
"1387","DYRK3",147.915581584358,-0.794253081013603,0.3893125851399,-2.
04014232092751,0.0413361526004107,0.502851519900686
"1388","SEL1L3",21.834855195766,0.824910433180225,0.404323854298939,2.
04022202600573,0.0413282169104783,0.502851519900686
"1389","AL596244.1",15.7912809537239,0.865705169706132,0.4244020410621
16,2.03982329476928,0.0413679287766052,0.502851519900686
"1390","ZEB1-
AS1",102.039357656905,0.480488909526097,0.235606221436671,2.0393727576
3004,0.0414128391649186,0.502851519900686
"1391","AC026369.3",2.30738031042435,-2.80436266995982,1.3750994987261
2,-2.03938891153531,0.0414112281999055,0.502851519900686
"1392","RANBP10",65.9765874915397,0.54801441608443,0.26864187731355,2.
03994411282648,0.0413558924217487,0.502851519900686
"1393","C19orf44",40.7077097054435,0.624054118143021,0.306026448591134
,2.03921628674908,0.0414284461332893,0.502851519900686
"1394","MAP1LC3C",11.5064936122746,1.2842526731125,0.630063622263629,2
.038290464221,0.0415208929624168,0.502890586109272
"1395","HARBI1",10.178550282993,-1.02098156250495,0.500830668405459,-2
.03857636305609,0.0414923262741815,0.502890586109272
"1396","NEO1",111.93746626075,0.464458097919558,0.227865932189109,2.03
82954725066,0.0415203923969085,0.502890586109272
"1397","CRABP2",1346.20870248019,-0.78932400105451,0.387450388501843,-
2.0372259893882,0.0416274005622473,0.503806812438085
"1398","ATRN",145.849682840835,0.394417873958076,0.193632619351148,2.0
3693920621302,0.0416561345983228,0.503806812438085
"1399","AC008494.3",4.03420699990872,-1.6001346186595,0.78580298333541
7,-2.03630509503485,0.0417197285334928,0.504215275228231
"1400","MALL",45.3420034073539,0.691748643419781,0.339811412091498,2.0
3568396706324,0.0417821000663357,0.504608391372574
"1401","AC093599.2",2.45055112346877,2.53171742212502,1.24403194138949
,2.0350903685779,0.0418417809267085,0.504968473882076

"1402","AC018638.6",6.46395461083214,2.06322257574699,1.0145549639745,
2.03362326242471,0.0419895945589593,0.505648185498574
"1403","EIF4G2",1493.34851349296,0.372192550319257,0.183044506926004,2
.03334454865513,0.0420177253741126,0.505648185498574
"1404","RASGRP1",13.9287722268192,1.17536000387847,0.577868838951957,2
.03395636630995,0.0419559950125764,0.505648185498574
"1405","ADAMTS5",1610.05151111964,0.550969632538,0.270920811498422,2.0
3369253727933,0.0419826050632203,0.505648185498574
"1406","PLIN1",77.4797082380876,-1.06648799894121,0.524684353767944,-2
.03262779094209,0.0420901415861121,0.506159398248922
"1407","DVL1",172.821669334145,-0.392426465838656,0.193248695971087,-2
.03068105513824,0.042287359365269,0.506629411722455
"1408","FILIP1L",2140.44110352294,-0.573581477350941,0.282446806126501
,-2.03075929665159,0.0422794179079503,0.506629411722455
"1409","LTC4S",68.1596918224392,-0.691638405951486,0.34050903689161,-2
.03118957507035,0.0422357675022539,0.506629411722455
"1410","ZNF438",146.545860607439,0.406050871756646,0.199978974495767,2
.03046781683162,0.0423090093063701,0.506629411722455
"1411","AC084018.2",6.88366558754327,-1.17988594663359,0.5810266448070
61,-2.0306916338155,0.0422862855633679,0.506629411722455
"1412","CIC",142.585239074876,-0.42399009576647,0.208710319841879,-2.0
3147643148498,0.0422066879991277,0.506629411722455
"1413","PINK1",615.537827387381,-0.434643640987089,0.214245836833277,-
2.02871452445222,0.0424873756500678,0.507010983084723
"1414","CYP1B1-
AS1",27.6088279016941,0.806541388354676,0.397452615113066,2.0292768437
9491,0.0424301004993805,0.507010983084723
"1415","KRIT1",336.925297700153,0.26188646121388,0.129097388258099,2.0
2859612225695,0.0424994438598207,0.507010983084723
"1416","AQP5",9.75845279779829,-1.27720712875104,0.629666478141572,-2.
02838673025862,0.0425207933530954,0.507010983084723
"1417","DIP2B",97.7305843146288,0.500201499046933,0.24646958172563,2.0
2946544374696,0.0424109052437036,0.507010983084723
"1418","GMFG",52.7613645570683,-0.672280077495926,0.331352802263262,-2
.02889510184916,0.0424689757792381,0.507010983084723
"1419","KRBOX1",34.4079816799283,-0.778945548649859,0.384267763231222,
-2.02709054254221,0.042653153896129,0.507158597802918
"1420","AC016405.2",4.02588888775008,-1.52725556553422,0.7533583022287
74,-2.02726320399724,0.0426355024381791,0.507158597802918
"1421","ADCY9",119.244661765789,0.399732109753865,0.197167834426474,2.
02736978329459,0.0426246097489604,0.507158597802918
"1422","PPL",1303.22140926796,-0.780896560815945,0.385106512044375,-2.
02774177115438,0.0425866100336006,0.507158597802918
"1423","PPM1G",496.063237083264,-0.375065398522957,0.185065394423604,-
2.0266641404846,0.0426967721395755,0.507227750949913
"1424","AGAP9",33.0715598854045,0.787067615201234,0.388397772087234,2.
02644729647023,0.0427189683790321,0.507227750949913
"1425","SMARCE1",1064.67870286334,0.404878664419321,0.199842729674604,
2.02598645984554,0.042766172200048,0.507431887409412
"1426","MBLAC1",35.9838930338988,-0.692730462343531,0.342080323303478,

-2.02505205693744,0.0428620191215892,0.507856355506538
"1427","SULT1A1",294.663829728576,1.01881766152709,0.503061128493545,2.02523630592969,0.0428431053136231,0.507856355506538
"1428","PRKD3",254.815570377664,0.344838387168725,0.170374009116798,2.02400817446471,0.042969310670095,0.508059513853123
"1429","PABPC4L",20.6953473403593,0.731193901931405,0.361161105877055,2.02456435655149,0.0429121173574498,0.508059513853123
"1430","CNTROB",47.2080333657855,0.50582678491495,0.249902473272178,2.02409675379257,0.0429601975698098,0.508059513853123
"1431","TRABD2A",69.9070507471575,-0.94753095533034,0.468279732333011,-2.02342935195947,0.0430289005494915,0.508315241130825
"1432","LINC00612",2.44143257874701,-2.05959117287571,1.01811611546573,-2.02294329849946,0.0430789937781471,0.508315241130825
"1433","STAT3",1668.05559619453,-0.290074914064672,0.143393976753316,-2.02292258456361,0.0430811296747381,0.508315241130825
"1434","DNAJC3",701.765144220761,-0.267815847640781,0.13249514077359,-2.02132580921159,0.0432460491475629,0.50990529915411
"1435","AC104825.1",29.814954512293,0.619613657596271,0.30663755023925,2.02067117061438,0.0433138160951727,0.509993037978538
"1436","HRAS",232.437402570051,-0.391483880385003,0.193736403214049,-2.02070377012457,0.0433104393341095,0.509993037978538
"1437","RTN1",9.55968932688799,-1.08374760737924,0.536792536860069,-2.01893195780728,0.043494292082771,0.511761649641957
"1438","HLA-DRA",1023.37924074499,-0.699720215511751,0.34667876920478,-2.01835323552344,0.0435544860682474,0.512113526037501
"1439","SNX33",233.583554561683,0.376257640273797,0.186503028330478,2.01743448158429,0.0436501919044761,0.512882171453011
"1440","ADCY3",540.399821100687,0.370142175354189,0.183507183653413,2.01704460820057,0.0436908583372805,0.513003494976901
"1441","PLPP7",46.9809607082811,0.474418491729535,0.235268428600178,2.01649874805674,0.0437478490021779,0.513316190790302
"1442","AC106738.1",3.72492677148133,-2.0149241956927,0.999441509379907,-2.01605014078597,0.0437947329421306,0.513509947701487
"1443","NCEH1",40.1627072798086,-0.672741106935633,0.333887005018165,-2.01487658047378,0.0439175824328182,0.514593543848988
"1444","AIDA",819.427098223302,0.287452914774959,0.142710583906862,2.01423683447726,0.0439846741470739,0.514724000675003
"1445","VPS13B",194.738340664326,0.419192513091995,0.208119659696749,2.0141898833719,0.043989601429819,0.514724000675003
"1446","SLC39A1",784.959564755173,-0.384389172381716,0.190885330219408,-2.01371772225707,0.0440391782796814,0.51494773606698
"1447","NR1H2",439.823399442765,-0.306224703094431,0.152164394919663,-2.01245963785487,0.0441715072946698,0.516138110116293
"1448","SERINC2",7.56061568454082,-1.44465564225287,0.718264745456805,-2.01131358790845,0.0442923441608064,0.517192648529637
"1449","ELL2",222.80192659723,1.00819173537311,0.501386769610405,2.01080642027413,0.0443459077268301,0.517460736953239
"1450","SNX32",4.48663916401339,1.55102129706992,0.77173672551587,2.00978033802024,0.044454442676474,0.518369459844015

"1451","IQCA1",10.1854065742221,1.34661980165421,0.670636580925258,2.00797248458519,0.044646215663985,0.519393658998516
"1452","WNT5A",92.8085314552424,0.983423656394138,0.489704829694228,2.00819676826179,0.0446223863051662,0.519393658998516
"1453","SASH1",1625.81137650321,0.530668664783284,0.264312580912375,2.00773138740305,0.0446718433705482,0.519393658998516
"1454","ORC5",57.5908477358166,0.432700801765106,0.21544807332253,2.00837628804108,0.0446033206862784,0.519393658998516
"1455","PRRC2B",587.858471780446,0.362320653091411,0.180483022814074,2.00750545642544,0.0446958702296452,0.519393658998516
"1456","FBXL15",265.169242682755,-0.494781283010034,0.24652619736929,-2.00701300019999,0.0447482787640019,0.519645533888561
"1457","LAPTM5",69.25062514742,-0.723807555603268,0.360798039515611,-2.00612940296188,0.0448424435319298,0.520024715526659
"1458","CALR",2196.46467294267,-0.477733298058355,0.238113927558097,-2.0063223640784,0.0448218654566125,0.520024715526659
"1459","LONRF2",16.7762352393799,0.978360503715127,0.488120756860944,2.00434111838813,0.0450335320804076,0.520660107581492
"1460","NCKAP1",529.252901489381,0.339490038163961,0.16943367183348,2.00367515199467,0.0451048696855078,0.520660107581492
"1461","OXSM",33.1834860273346,-0.682645317594441,0.340627065026754,-2.00408419554337,0.0450610421001719,0.520660107581492
"1462","LINC02066",12.5463965033954,0.985930713860076,0.491728048821973,2.00503248944627,0.0449595738275538,0.520660107581492
"1463","VLDLR",96.3135645944041,1.11054269082356,0.553979138693679,2.00466518187362,0.04499885317251,0.520660107581492
"1464","TRIM34",82.0165186946666,0.446859786125894,0.222940752749774,2.00438807447396,0.045028505787325,0.520660107581492
"1465","NCLN",125.976641240188,0.517508099999782,0.258288966497053,2.00360126496416,0.0451127902535419,0.520660107581492
"1466","AMOTL2",796.717868703238,0.4479768016952,0.223840634230347,2.00132028411875,0.0453578851695643,0.523131734274893
"1467","ITGB3BP",146.56916092299,-0.565080756114497,0.282458731494014,-2.0005781132189,0.0454378741922132,0.523353853071583
"1468","DBF4B",16.6251925369105,0.881505339666969,0.440627705083091,2.00056721240608,0.0454390499354793,0.523353853071583
"1469","ICAM3",111.680852055477,-0.428817755636806,0.214386238672507,-2.00021119961837,0.0454774629693676,0.523439716736602
"1470","PTPRD",352.699901155913,0.523295861408742,0.261763443046944,1.99911742952929,0.0455956497093761,0.524443024004171
"1471","NLRC5",33.1953884617988,0.708998747748187,0.35488378758485,1.9978335797565,0.0457347054910529,0.525684840545698
"1472","CCNG2",713.998091955368,0.435605979839169,0.21811777157913,1.99711365417622,0.0458128379933336,0.52586793264853
"1473","SEMA4G",5.63482042899509,1.25730219989856,0.629512126501381,1.99726446396866,0.0457964615164066,0.52586793264853
"1474","PDZRN4",16.0492849744551,2.53856647046104,1.2715074600821,1.99650143641086,0.0458793695620468,0.525917546139042
"1475","MRTFA",124.000353698474,0.382155959204598,0.19139531834657,1.99668394454983,0.0458595273372048,0.525917546139042

"1476","CEP83",121.842185620957,0.465130352345229,0.233066554622242,1.
99569755128152,0.0459668537893797,0.526563390156391
"1477","CGNL1",10.3434133986,-1.31975085147349,0.661730870501553,-1.99
439214687565,0.0461092160649803,0.527479448732535
"1478","LDHC",2.58248342894286,-2.82599144096581,1.41693656515187,-1.9
9443751433072,0.0461042622479896,0.527479448732535
"1479","TASOR2",261.279032582489,0.460031570772919,0.230808282789963,1
.99313285126579,0.0462469015784199,0.528696830214959
"1480","RAP2B",332.486834712566,-0.285885802309752,0.143464388904585,-
1.99273007394112,0.0462910123773029,0.52884353869962
"1481","PLEC",501.641222743976,-0.479760995592556,0.240970058348853,-1
.99095688020296,0.0464856279886004,0.529635443417289
"1482","METTL15",141.232457112643,0.333722732659268,0.167615062968877,
1.99100681494977,0.0464801380263565,0.529635443417289
"1483","SLC30A4",79.2969686179927,0.417133002572972,0.209458037790248,
1.99148720657209,0.0464273503374949,0.529635443417289
"1484","GNB5",138.974629293789,-0.490280919126119,0.246227627441003,-1
.99116940784231,0.0464622659019757,0.529635443417289
"1485","SETD9",77.4520344760487,-0.534544180972701,0.268661615505782,-
1.98965594681759,0.0466288486203933,0.529839094404308
"1486","RNF38",180.272195213751,0.413384750535323,0.207745258114929,1.
98986371234827,0.0466059506849656,0.529839094404308
"1487","ARAP1",277.456237275401,0.354969354477478,0.178374194682155,1.
9900263886825,0.0465880286574148,0.529839094404308
"1488","PRDM10",28.5010376323716,0.758914851865462,0.381395626560023,1
.9898362724042,0.0466089743113963,0.529839094404308
"1489","KANSL1L",150.267426158965,0.552493108468358,0.277794547646337,
1.98885512026586,0.0467171966875547,0.52990132223629
"1490","STARD5",33.6662664162871,0.633581935817031,0.318496669782549,1
.98928904421388,0.046669308250382,0.52990132223629
"1491","C16orf74",63.296433185289,0.706172422100244,0.355082815099871,
1.98875414993436,0.0467283458395025,0.52990132223629
"1492","SATB1",175.699356436334,0.538779188300776,0.271080911613942,1.
9875216779117,0.0468646160638134,0.530069617649353
"1493","FABP4",4024.18236909261,-1.4777212514185,0.743240711326499,-1.
98821354764211,0.0467880772815317,0.530069617649353
"1494","AL022345.4",17.9523884231157,-0.851190457750573,0.428244178430
99,-1.98762878895209,0.0468527599098863,0.530069617649353
"1495","TMEM72-
AS1",14.1684218177339,0.928451110052547,0.467281629803106,1.9869197735
0738,0.0469312880069246,0.530069617649353
"1496","CDKL1",45.594980011709,0.513984173178261,0.258650251074205,1.9
871783268859 2,0.0469026387112914,0.530069617649353
"1497","ADAP2",9.98817034315964,-1.22871387186429,0.618233364073868,-1
.98745966048748,0.0468714819454035,0.530069617649353
"1498","GIPC3",5.57398245124067,-1.32026867863905,0.664684068439645,-1
.98631010028328,0.0469989018238587,0.530125038050569
"1499","LINC00205",69.4314734194413,0.636002291724241,0.32018224578377
3,1.9863758846696,0.0469916022795246,0.530125038050569
"1500","RAD23A",955.10467267908,-0.294926713721033,0.148652434660657,-

1.98400190615304,0.0472556274360284,0.532665432458912
"1501","POU2F1",219.662127439076,0.478260777316862,0.241453485263037,1
.98075739845234,0.0476184864244045,0.536397980322339
"1502","TFR2",3.46478204392562,-1.97282540293753,0.996298205765915,-1.
98015553126577,0.0476860549034175,0.536801475570561
"1503","ITGB2",53.6491195253583,-0.784791366735202,0.396468388627178,-
1.97945508203729,0.0477647921302101,0.537330076738252
"1504","AC096642.1",21.8648176475482,0.973886841050723,0.4927525669165
59,1.97642164939881,0.0481070415660882,0.538322824704413
"1505","STON1",61.659011450035,0.73221963625165,0.370531340690976,1.97
613415072039,0.0481395854597275,0.538322824704413
"1506","GAP43",6.60333068760624,-1.40549638617773,0.711226238376284,-1
.97615935737473,0.0481367314107739,0.538322824704413
"1507","RADIL",62.0289067585632,0.43131257696496,0.218150787141432,1.9
7713050966591,0.0480268799100282,0.538322824704413
"1508","C8orf48",33.6708798273364,-0.557782521111393,0.282022919729175
,-1.97779145626543,0.0479522377287631,0.538322824704413
"1509","RNF170",233.975578929516,0.414503315559007,0.209663553504247,1
.97699270393513,0.0480424549136394,0.538322824704413
"1510","CYP7B1",622.558105101276,0.53734112623105,0.271643155400849,1.
9781139909015,0.0479158485770356,0.538322824704413
"1511","CRAMP1",72.3488259245304,0.594586860684836,0.300757793073484,1
.97696244080219,0.0480458758651216,0.538322824704413
"1512","FBF1",23.7548193378156,0.636327202264455,0.321988026604472,1.9
7624492120049,0.0481270444202902,0.538322824704413
"1513","GPR1",9.62718207648292,1.44234242398966,0.730223409956074,1.97
520704530196,0.0482446567289601,0.538785109625665
"1514","VPS35L",165.587967891749,0.310435336681337,0.157161513042737,1
.9752630950869,0.0482382989940412,0.538785109625665
"1515","SAP25",14.0010098092432,-1.11755944518661,0.56607276884544,-1.
97423283134778,0.0483552744485564,0.538826010357726
"1516","SLC25A3",3756.02119598819,-0.419789287217939,0.21265357469224,
-1.97405234229179,0.0483757915615702,0.538826010357726
"1517","VTN",4.15888890680042,-1.56422647658421,0.792304874959697,-1.9
7427344702855,0.0483506584642169,0.538826010357726
"1518","ISYNA1",99.9003901788022,-0.505020087003288,0.255783499055301,
-1.9744044821832,0.0483357688026266,0.538826010357726
"1519","NEK6",184.180574117979,0.535979158506713,0.271625757805847,1.9
7322655567084,0.0484697562262872,0.539517207553696
"1520","POLR3GL",523.711472252595,-0.432953786034645,0.219621543009421
,-1.97136300975753,0.0486823688979417,0.54033717971345
"1521","AC092490.1",6.22795862182573,1.27221126954301,0.64528602097807
6,1.97154630378432,0.0486614221482831,0.54033717971345
"1522","TNFRSF19",38.8553175556775,0.730984353570204,0.370826545497,1.
97122984437509,0.0486975917188965,0.54033717971345
"1523","PKN1",450.388169085876,-0.458086171796111,0.23241923682388,-1.
97094774966168,0.0487298526202237,0.54033717971345
"1524","OPA3",35.6649529397266,-0.522801438226348,0.265260081241691,-1
.97090129724419,0.048735166729537,0.54033717971345
"1525","DDX17",2116.57932380425,0.341219939818349,0.173117620385798,1.

97102951772286,0.0487204996190935,0.54033717971345
"1526","FDXR",47.8266724149211,0.651094970922312,0.330566473022558,1.96963401935162,0.048880329839277,0.541591492085515
"1527","LST1",41.1211364429867,-0.643993083399784,0.327263074891506,-1.96781468124163,0.04908936433682,0.543195662439105
"1528","RTL8B",112.864260154347,-0.444138136182801,0.225695916108987,-1.96786075636534,0.0490840612493497,0.543195662439105
"1529","WNT2",14.5331984444725,-1.31674810872685,0.669338036111921,-1.96723932853963,0.0491556259456401,0.543573135048321
"1530","TRIM17",2.89269864045294,1.83073905383076,0.930981289511288,1.9664617049305,0.0492453017377292,0.544208863909494
"1531","AC104695.3",10.1254239444002,1.10851436549564,0.564033210490598,1.96533527614704,0.0493754452653209,0.545290678344903
"1532","TPPP",41.7528321846896,-1.08029067935537,0.55023848804108,-1.96331355009596,0.0496097519417837,0.545419956749236
"1533","PTGER4",442.291161937937,0.406733952614653,0.207049213406841,1.96443128627319,0.049480097599076,0.545419956749236
"1534","PPIL1",45.8076082081522,-0.660665666977387,0.336272116460304,-1.96467573324765,0.0494517803303706,0.545419956749236
"1535","ADAMTS14",3.97766472469447,2.31072839112974,1.17696986535897,1.9632859422658,0.0496129579773081,0.545419956749236
"1536","JDP2",1204.66470030195,-0.39179286932417,0.199555522681215,-1.96332761960213,0.0496081181483667,0.545419956749236
"1537","PCSK6",26.3670666983818,0.909014298296794,0.462804154006583,1.96414463964354,0.0495133206937894,0.545419956749236
"1538","TVP23A",5.38309396283406,2.04554888951929,1.04166820920476,1.96372402598416,0.0495621047906278,0.545419956749236
"1539","PDLIM4",411.307112041842,-0.533739327581065,0.272200682550002,-1.96083023224242,0.0498988301059407,0.546598753232813
"1540","LAMB1",588.230635379108,0.725862364739188,0.370010571962796,1.96173412259197,0.0497934469394306,0.546598753232813
"1541","COL5A1",1076.9354006983,0.664764319515419,0.338991110816028,1.96100811586233,0.0498780761647969,0.546598753232813
"1542","FAM149B1",100.760987217772,0.43279182809846,0.220733442017603,1.96069895047414,0.0499141515845436,0.546598753232813
"1543","AC087392.1",21.1720275534991,-1.18020853724037,0.601881760140985,-1.9608644345094,0.0498948391154518,0.546598753232813
"1544","LINC01597",9.69213804075989,-1.18612034452584,0.604558420352921,-1.96196149882987,0.0497669668973965,0.546598753232813
"1545","TMEM74",13.4909061540495,-0.859042052880133,0.438313254793029,-1.95988153104284,0.0500096387796102,0.546768447191315
"1546","CD2BP2-
DT",3.18676225419709,-1.66226370771821,0.848207999479796,-1.95973594771291,0.0500266612139203,0.546768447191315
"1547","CEP192",91.7663679799547,0.448202091872199,0.228671856093708,1.96002297584242,0.0499931048970511,0.546768447191315
"1548","MEX3B",93.9150608191466,0.487443631484135,0.248771362366435,1.95940411648403,0.0500654789709736,0.546839223799239
"1549","CCL20",22.4527624251301,-1.15734953623955,0.591561637373984,-1.95643101769947,0.0504144012289382,0.549674611794246

"1550","MAP3K5",74.0667028511447,0.712979470861489,0.364398158619908,1
.95659460399517,0.0503951499381087,0.549674611794246
"1551","SNTB1",32.1721572004762,0.587293387564274,0.30019678282765,1.9
5636136414378,0.0504226001237802,0.549674611794246
"1552","CEP170",357.129516364303,0.294133055891566,0.150446033150379,1
.95507352192905,0.0505743929914987,0.549715126569659
"1553","CA3-
AS1",3.91285473189633,-1.57134962110121,0.803554088914074,-1.955499502
49738,0.0505241420276792,0.549715126569659
"1554","RCVRN",30.5053795019513,-0.759483813028046,0.388475730345533,-
1.95503542100948,0.0505788896207997,0.549715126569659
"1555","CAPS",66.7880821737041,-0.628760255950492,0.32162459269653,-1.
95495080360276,0.0505888772736214,0.549715126569659
"1556","GPR108",331.700791878399,-0.337846027420414,0.172792476040432,
-1.95521260625585,0.0505579812525907,0.549715126569659
"1557","CEP70",70.1784638356874,-0.529833039665745,0.271119177620816,-
1.95424404985015,0.0506723621473094,0.549829990768264
"1558","MBLAC2",52.3452059066043,0.446344788590707,0.22846303369322,1.
95368494138994,0.0507384882482047,0.549829990768264
"1559","CLTB",1076.90212133848,-0.452736237193572,0.231750059207568,-1
.95355392245262,0.0507539943882805,0.549829990768264
"1560","CYTH3",259.796678715138,0.378712371676809,0.193891662031179,1.
95321638748916,0.0507939600640228,0.549829990768264
"1561","PTPN3",6.91037701924506,-1.58946096365844,0.813868044494731,-1
.95297133781093,0.0508229915741002,0.549829990768264
"1562","ZNF516",108.381589940652,0.445219342498343,0.22797426661282,1.
95293683411418,0.0508270804099123,0.549829990768264
"1563","CECR2",2.96177282586312,3.58389704035271,1.83468577107943,1.95
341191219036,0.0507708058385853,0.549829990768264
"1564","CPT1A",199.491720236707,0.462332973051668,0.236787553200933,1.
95252227915604,0.0508762284938376,0.550009764305503
"1565","KLKB1",2.80462821281528,2.2864499801836,1.17123678314738,1.952
16715619141,0.0509183621907621,0.550113525828375
"1566","FAM210A",109.629559256212,0.330029786540889,0.169097269472484,
1.95171564609203,0.0509719739694508,0.55034108293453
"1567","FBXO4",123.247178484035,-0.391437275527666,0.200656208497843,-
1.95078576665059,0.0510825357023606,0.551182842154124
"1568","ANKRD31",4.94894017491407,1.42763226322708,0.732257919962207,1
.94963034787082,0.051220193545265,0.551707363817802
"1569","HIF3A",112.126740637587,-1.01540610401297,0.520839614530063,-1
.94955620825643,0.0512290372127957,0.551707363817802
"1570","DIP2A",178.207806406258,0.50892313201899,0.261020105574114,1.9
4974686298442,0.051206297742782,0.551707363817802
"1571","TP53TG5",2.42266027584706,-2.25755320969593,1.15824101585835,-
1.94912214192562,0.051280840023991,0.551913713001681
"1572","ACAP2",503.505163119811,0.262275597238034,0.134593810342203,1.
94864530970036,0.0513377971885014,0.552175238462583
"1573","NCOA1",478.068447828185,0.300874846061577,0.154447195811679,1.
94807580986087,0.0514058927987517,0.552556157305336
"1574","MPRIP-

AS1",5.60255837179691,-1.77143643323376,0.909533771712555,-1.947631290
14806,0.0514590969458595,0.552776627166832
"1575","PLSCR4",1079.20861098258,0.415402021561306,0.213378150078275,1
.94678799778198,0.0515601564754813,0.552808576846821
"1576","SPG11",202.293099187298,0.387503907978921,0.199013570718575,1.
94712303577976,0.0515199859021919,0.552808576846821
"1577","AXIN2",20.9202071046109,0.862977823368319,0.443278372540979,1.
94680786798039,0.0515577733363208,0.552808576846821
"1578","PPM1B",235.096924026651,0.366852818504564,0.188501668567044,1.
94615157145989,0.051636535265473,0.552917066683603
"1579","NFIB",4626.83404791738,0.37750242915514,0.19395124422245,1.946
37797075521,0.0516093537923118,0.552917066683603
"1580","AC022087.1",3.10533333236247,2.47016778053638,1.26943058175514
,1.94588645967633,0.051668379782357,0.552917066683603
"1581","WARS2-
AS1",60.571670632277,0.530858515871342,0.272948792037496,1.94490150298
381,0.0517868341214056,0.553386344458968
"1582","LSM12",291.793264513445,-0.349026523133284,0.179440714451436,-
1.94507987889084,0.0517653651775758,0.553386344458968
"1583","NEDD4L",25.0421844094417,0.732845436456912,0.376841324028765,1
.94470560877493,0.0518104201134698,0.553386344458968
"1584","CCL4L2",94.0062761385828,-1.51317521347642,0.778496734733385,-
1.94371427131887,0.0519299166768721,0.554312519679643
"1585","SPATA33",81.3607363806903,-0.479808037622109,0.246920050168575
,-1.94317163508811,0.0519954240429643,0.554661596036871
"1586","DOCK2",13.3979878862683,-1.01951543236938,0.524844679522747,-1
.94250884527677,0.0520755301834995,0.555142350094146
"1587","AC090360.1",4.05020025742941,1.75331147003434,0.90278958537337
2,1.9421042272095,0.0521244838810917,0.555142350094146
"1588","TAB3",59.9658919560493,0.644373151814512,0.331811705812942,1.9
4198438610172,0.0521389905340374,0.555142350094146
"1589","TTC32",161.859894947992,-0.842532144777061,0.434229920907953,-
1.94029039504088,0.0523444080131986,0.556032559528078
"1590","MKLN1",486.04250471249,0.345984311149772,0.178339490919435,1.9
4003195459424,0.0523758065486393,0.556032559528078
"1591","FAM89B",621.501524448368,-0.479144817053898,0.246959972178081,
-1.94017197535311,0.0523587931443384,0.556032559528078
"1592","SPATA13",107.507028787602,0.459309955267479,0.236765077470448,
1.93993962359085,0.0523870278760485,0.556032559528078
"1593","SOS2",241.58658057726,0.375963742921165,0.193761936092642,1.94
033849218666,0.052338566315749,0.556032559528078
"1594","MED19",238.085642457202,-0.364062679244525,0.187744610595962,-
1.93913784309905,0.0524845557614927,0.556718236396059
"1595","GATA6",70.8192834308018,0.69655280266927,0.359315877053502,1.9
3855280869082,0.0525558145921721,0.557124585031001
"1596","FUCA1",197.461791926221,-0.388917103217966,0.201004031115968,-
1.93487215683541,0.0530059856908446,0.558064351734159
"1597","ZMYM4",270.680306783266,0.290845699023217,0.150275490255802,1.
93541673714146,0.0529391770729063,0.558064351734159
"1598","TYW3",339.587367887086,-0.298780165763403,0.154609532948749,-1

.93248217018058,0.0533000206515076,0.558064351734159
"1599","SYT11",42.5984742511062,0.625714374178541,0.323704510487184,1.9329800911233,0.0532386504021979,0.558064351734159
"1600","RCOR3",243.579655447483,0.3254126166906,0.168261827415478,1.93396578231068,0.053117335086874,0.558064351734159
"1601","STAT4",10.0129521789539,1.2349518370207,0.638689952638481,1.93357016486483,0.0531659984804695,0.558064351734159
"1602","PLCXD3",31.3437903464302,-1.35311732220626,0.698742981849379,-1.93650220088785,0.0528062232542052,0.558064351734159
"1603","HINT3",96.1734382686519,0.448273625138104,0.232031343990588,1.93195288803864,0.0533653210428091,0.558064351734159
"1604","ATXN7L1",67.3383879751723,0.438570747252284,0.227014859758074,1.93190325831384,0.0533714475546197,0.558064351734159
"1605","ARFIP2",167.989707472274,-0.491206858580143,0.253895218871712,-1.9346833735705,0.0530291618878681,0.558064351734159
"1606","PARVA",1003.30317279533,0.287143420056878,0.14830249225124,1.9362009073349,0.0528430994054977,0.558064351734159
"1607","NXF1",197.390405300061,0.529952918600706,0.273762162122062,1.93581506842577,0.0528903547211568,0.558064351734159
"1608","AL354811.1",7.42240794288809,-1.18279668924929,0.611073305332644,-1.93560523578333,0.0529160686295506,0.558064351734159
"1609","LINC02328",25.55673288226,0.662000913298643,0.342050310842238,1.93539047419247,0.0529423973720312,0.558064351734159
"1610","HBA2",110.465419999193,2.89832270134745,1.4992478347594,1.93318451702989,0.0532134713937371,0.558064351734159
"1611","ZNF213-AS1",32.1193477260402,-0.675859266268189,0.349029640257101,-1.93639504590596,0.0528193357837719,0.558064351734159
"1612","CES1",246.587431783929,0.889690551536453,0.45954848949964,1.93601017491137,0.0528664547966109,0.558064351734159
"1613","MEAK7",79.8850492600302,0.438617712136885,0.226490634354876,1.93658211689953,0.0527964457221049,0.558064351734159
"1614","TUBB3",51.736293797695,-1.00981344293426,0.522599215980891,-1.93229039013939,0.0533236738743992,0.558064351734159
"1615","RUNDC1",81.6608219798884,-0.438356882483286,0.226683319010533,-1.93378535481438,0.0531395241675904,0.558064351734159
"1616","WDR7",69.589975237371,0.497201435217774,0.257411193671506,1.93154550944772,0.0534156270317646,0.558064351734159
"1617","CHERP",136.96556461598,0.383254183792002,0.198237320736169,1.9333099457194,0.0531980273469735,0.558064351734159
"1618","JAG1",371.570727567448,0.368325497726044,0.190502033492738,1.93344654108422,0.0531812126058838,0.558064351734159
"1619","TAFA5",21.3243131032058,1.00132928443248,0.518453912808375,1.93137569163757,0.0534366090287203,0.558064351734159
"1620","DUXA",2.40201050642883,-2.23198167215232,1.15584129224085,-1.93104510726134,0.0534774744304581,0.558146381277893
"1621","DHRS3",1535.74201744255,0.587716928378394,0.304925140697762,1.92741381387418,0.0539280794447171,0.558606126029577
"1622","GABPB2",223.089422285581,0.623973816764821,0.323617057417648,1.9281240047849,0.0538397038332864,0.558606126029577

"1623","TMEM63A",112.730065544548,0.582447017422963,0.302140315528719,
1.92773684108899,0.0538878671851434,0.558606126029577
"1624","POLN",12.23507933906,1.04559190109585,0.542445306814698,1.9275
5267297948,0.0539107904075508,0.558606126029577
"1625","PCDHGA8",24.2132450812045,0.935951122189137,0.485010765892608,
1.92975329210811,0.0536374133366011,0.558606126029577
"1626","NRM",58.1344721687868,-0.572135152885242,0.29686921610782,-1.9
2722964134297,0.0539510174950496,0.558606126029577
"1627","OLFML2A",131.047890157191,0.629034319510509,0.326043078525137,
1.92929818463241,0.0536938549935763,0.558606126029577
"1628","BDNF",23.9753166794271,-1.27726603470165,0.662021750834844,-1.
9293414953375,0.0536884815392777,0.558606126029577
"1629","TIAF1",3.60722558642688,1.72438700622543,0.894602647057897,1.9
2754516420946,0.0539117251893973,0.558606126029577
"1630","PLEKHM1",159.229719441866,0.342427280065062,0.177608001339169,
1.9279946707533,0.0538557890509743,0.558606126029577
"1631","ODF3L2",3.32704029806119,-1.87850215164744,0.973823336000832,-
1.92899685415409,0.0537312527673901,0.558606126029577
"1632","MED29",371.87497670958,0.273637874579138,0.141806169573789,1.9
296612792065,0.0536488206234188,0.558606126029577
"1633","CYTH4",9.05000028456995,-1.22032130925766,0.632613870994562,-1
.92901446713323,0.0537290662428628,0.558606126029577
"1634","EPB41L4A",72.4413005486082,0.486330112393947,0.252650645457665
,1.92491141874181,0.0542404411247821,0.559888509485846
"1635","AC105046.1",9.39839386554535,1.52809304354307,0.79380752126080
6,1.92501708867157,0.0542272204043926,0.559888509485846
"1636","FRY",11.964665293213,-1.07530187745429,0.558573097731941,-1.92
508712256372,0.054218459710251,0.559888509485846
"1637","PATL2",13.6100408549632,0.874594503441001,0.454198230979525,1.
92557884154425,0.0541569827481747,0.559888509485846
"1638","AL022323.4",31.8407187713538,0.712218681485697,0.3698120037713
13,1.92589389804157,0.054117623524239,0.559888509485846
"1639","AC245407.2",83.5687532328476,-0.72095089969013,0.3751756602371
17,-1.92163558593987,0.0546516275817266,0.560008029064414
"1640","NPL",30.178667336245,-0.663268003602744,0.34511715670662,-1.92
186331717661,0.0546229586412527,0.560008029064414
"1641","KCTD3",312.291563789808,0.27723631709927,0.144240502107333,1.9
220420969882,0.0546004609909165,0.560008029064414
"1642","AL390728.5",6.70593408514657,1.76680289309674,0.91904693985650
8,1.92242943910198,0.0545517442583767,0.560008029064414
"1643","SYNPO",29.5789379343172,-0.731231594214551,0.380519713735364,-
1.92166546914595,0.0546478648887231,0.560008029064414
"1644","CYREN",107.788936323716,-0.468765151733691,0.243971924439969,-
1.92138973699424,0.0546825914351401,0.560008029064414
"1645","EBF3",626.333307534205,0.451669303569676,0.234849486573593,1.9
23228831195,0.0544513179375268,0.560008029064414
"1646","AC009318.4",2.82944754559206,-1.7283498083969,0.89945436702418
5,-1.92155363491656,0.0546619474140974,0.560008029064414
"1647","SLC35E3",228.385016617436,0.337724923826656,0.175713774297414,
1.92201735565148,0.054603573948337,0.560008029064414

"1648","MTUS2",3.06087969488267,-1.84857910757258,0.961406341296172,-1
.92278647245067,0.0545068716667456,0.560008029064414
"1649","AP000525.1",29.928416575403,0.825938623092706,0.42934328957314
4,1.92372547365037,0.0543890032683315,0.560008029064414
"1650","GAS2L1",137.563587206163,-0.449740417436631,0.233727304649905,
-1.92421000237986,0.0543282658793388,0.560008029064414
"1651","SHOX",3.95768112388346,2.43114661363239,1.26396211213627,1.923
43313956097,0.0544256757726297,0.560008029064414
"1652","ERCC3",112.243818697812,0.348407418188788,0.181502616531941,1.
91957242736208,0.0549119296159078,0.561676289138396
"1653","ELP1",91.0568665953084,0.405762283549985,0.211353813795154,1.9
1982475387576,0.0548800390172601,0.561676289138396
"1654","NOTCH2NLC",44.7100015381797,0.605660037518923,0.31560184047886
7,1.91906370571207,0.0549762720122716,0.56192426929845
"1655","LCORL",95.4568469345388,0.383278702176384,0.199798048725677,1.
9183305573851,0.0550691101389782,0.56192426929845
"1656","PAOX",55.0793595028158,-0.495692316281447,0.258382065135878,-1
.91844707186148,0.0550543472526183,0.56192426929845
"1657","ARHGAP5-
AS1",22.0802237927257,-0.841947205053975,0.438834525255911,-1.91859837
045179,0.0550351819931447,0.56192426929845
"1658","CMPK1",1068.3705368597,-0.423087343913334,0.220669531047762,-1
.91728935981542,0.0552011809318727,0.562253956142231
"1659","ZDHHC11",15.5482936367986,-1.46282781125985,0.762883534991617,
-1.91749820800094,0.0551746684193062,0.562253956142231
"1660","EFHC1",314.72909454564,-0.339023929947141,0.176821960531574,-1
.91731801258138,0.0551975429397443,0.562253956142231
"1661","PROCR",803.546145255643,0.828177860005137,0.432096646144577,1.
91664959076779,0.0552824633680902,0.562742860100945
"1662","ANKRD36C",718.66867072079,-0.859735949882146,0.448625544935993
,-1.91637761065257,0.0553170485534835,0.562756111276955
"1663","NBDY",460.466253770663,-0.502861090883026,0.262463949041757,-1
.91592442588381,0.0553747159076773,0.563004026799163
"1664","ARHGEF26",177.682001954535,-0.662992637780407,0.34635925159945
8,-1.9141762049628,0.0555976450703859,0.563576128807005
"1665","ZKSCAN8",85.7691016662794,0.411546616712147,0.214915665140962,
1.91492144810484,0.055502522085249,0.563576128807005
"1666","FLNC",118.535541338732,0.629584223626993,0.328901775394389,1.9
1420135349544,0.0555944328935261,0.563576128807005
"1667","CPNE2",129.700601589056,-0.35167914435074,0.183681995398669,-1
.91460868871467,0.055542426230043,0.563576128807005
"1668","ZNF431",180.786590824344,0.33452889965416,0.174727490111174,1.
91457508741932,0.0555467147523234,0.563576128807005
"1669","NCAPH2",126.66960294353,-0.334181510932463,0.174636087260323,-
1.91358794264734,0.0556728268833391,0.56400009403445
"1670","ADGRL3",2.8485695286007,2.46975537501588,1.29083402037432,1.91
330204815929,0.0557093956900391,0.564032612171964
"1671","CNIH3",50.9359490279421,-0.544491225307336,0.284710369681101,-
1.91243903731786,0.0558199049288634,0.564460570294753
"1672","PRSS12",26.0457430237678,1.06332430224062,0.556076674614791,1.

91219008237886,0.0558518177255218,0.564460570294753
"1673","NUDT10",8.16631192085184,-1.37809998980398,0.720501046557242,-1.91269672180066,0.0557868891164952,0.564460570294753
"1674","MORC2-AS1",29.369602044327,-0.607235401551665,0.31771794464492,-1.91124049423872,0.0559736821837783,0.56535425230784
"1675","PRKG2",50.4523469977959,1.23561364392551,0.647418521539834,1.90852377992941,0.05632355266326,0.566898247474887
"1676","IQGAP2",22.4705235275404,-0.946124397512626,0.495812056387936,-1.90823193047237,0.0563612463925529,0.566898247474887
"1677","AC091982.3",3.45497517571278,-1.57809940096915,0.826491431600006,-1.90939596060192,0.0562110314874097,0.566898247474887
"1678","NAPEPLD",63.1521485679611,0.437524996979947,0.229198991250555,1.90893072693176,0.0562710285815739,0.566898247474887
"1679","TMEM140",371.279341992371,0.48241331983946,0.252616059225392,1.90967003965902,0.0561757108610051,0.566898247474887
"1680","HSPA5",1570.49576589904,-0.408853702741983,0.214240006528457,-1.90839101140373,0.0563406977395789,0.566898247474887
"1681","AC005899.5",8.65412506465399,1.25239279656465,0.656199882367442,1.90855382668807,0.056319673174989,0.566898247474887
"1682","CARD19",210.507347220703,-0.419931641575264,0.220124109151602,-1.90770399114279,0.0564294855925184,0.566910126202199
"1683","SHISA6",4.1104554890133,2.00090814919818,1.04878223970503,1.90783946700026,0.0564119680008358,0.566910126202199
"1684","LAMA4",2808.71938204795,0.386385474960777,0.202593145136974,1.90719915374995,0.0564948030476817,0.567170239310354
"1685","EMX2OS",806.845403132502,-0.390183760347329,0.204607618384965,-1.90698549461245,0.0565224658882155,0.567170239310354
"1686","LINC00645",28.2808031048263,1.14658641579575,0.601604161904994,1.90588178805988,0.0566655444059079,0.568268697992343
"1687","HIST1H2AC",144.941159453389,0.809194020375041,0.424871756516103,1.90456063027191,0.0568372083331287,0.5689778084645
"1688","KPNA5",159.316847759013,0.361222900348338,0.189641549592006,1.90476665649206,0.0568104099836826,0.5689778084645
"1689","TSPAN12",22.7699599624179,0.817816652871371,0.429295311256085,1.90502116242197,0.0567773202727112,0.5689778084645
"1690","SLC1A4",18.5836271594792,-1.03615091931515,0.544124139569272,-1.90425464331607,0.0568770282378981,0.569039522749338
"1691","LRRFIP1",481.783334234512,-0.588193749332451,0.308989280635982,-1.90360567888242,0.0569615586670357,0.569548216405819
"1692","ERCC6L2",194.244351528069,0.388561571089852,0.204149566639975,1.90331812839502,0.0569990467651981,0.569586219093363
"1693","SLC25A27",49.9885882902756,0.819095237340097,0.430568863998744,1.90235594309599,0.057124636611197,0.570109689940465
"1694","CFAP43",2.86135778070792,1.7714969945839,0.931133518024102,1.90251662118562,0.0571036480062991,0.570109689940465
"1695","HEXIM1",350.911400149182,0.503680135634736,0.264796277903195,1.90214205283834,0.05715258602135 6,0.570109689940465
"1696","ARRB2",62.0593205144719,-0.574760579421242,0.302212215567595,-1.90184429951571,0.0571915129009152,0.570161615641907

"1697","MXRA7",1687.64309481862,-0.420175428946215,0.221086545036341,-1.90050203587536,0.0573672680606066,0.571576763917935
"1698","MFSD13A",7.74697583486005,-1.09864719590245,0.578314754391474,-1.89973917760146,0.0574673562624947,0.572236784267527
"1699","NR2F1-AS1",40.6888756765181,0.776676537407496,0.408892249117016,1.89946505243054,0.057503357358697,0.572258249688552
"1700","AL136441.1",9.87327164641652,1.16177126155564,0.611823625242608,1.8988662967942,0.0575820576706057,0.572704371232119
"1701","ITGAX",17.8331785845936,-1.06590461944525,0.561679271555129,-1.89771044335332,0.0577342360960852,0.573852494196292
"1702","DANT2",15.0779903930008,1.04292226064264,0.549637108563664,1.89747425054333,0.057765374090495,0.573852494196292
"1703","RIPOR2",54.9616266514606,0.588807566162963,0.310492952385259,1.89636370693649,0.057911967507686,0.574296508281499
"1704","KMT2D",92.5278564471169,0.535115198786571,0.282147026048573,1.89658280748438,0.0578830214507513,0.574296508281499
"1705","AC009088.1",7.93310998531778,1.30044930160719,0.685691572477433,1.8965513852075,0.0578871720070286,0.574296508281499
"1706","HOTAIRM1",67.8701848633358,-0.720418906189166,0.380048794574625,-1.89559581946709,0.0580135104999656,0.574966257639753
"1707","ZNF518A",234.615647305534,0.411947152539759,0.217432185726064,1.89460061381509,0.0581453334230457,0.575935147930203
"1708","NGF-AS1",4.95931383104755,1.34557887102477,0.710583782037244,1.89362451696687,0.0582748668998821,0.576310398757758
"1709","AC009315.1",3.55335709072956,1.62411524909492,0.8575128147089,1.89398364810007,0.0582271803538127,0.576310398757758
"1710","PTDSS1",98.8642652937834,-0.385528154148603,0.203606212094806,-1.89349897619572,0.0582915442443647,0.576310398757758
"1711","TRMT1",108.298085475202,-0.382361819513089,0.201956483337934,-1.89328816383321,0.0583195583318266,0.576310398757758
"1712","PAM",1509.38581062637,0.429749094479722,0.227156712361641,1.89186174606871,0.0585094037982733,0.576884203473941
"1713","NMUR2",3.57130531539491,-1.71118253796529,0.904785026010409,-1.89125868440886,0.0585898210003211,0.576884203473941
"1714","AL355353.1",9.74245665004554,-0.938558788758271,0.496313836003867,-1.89105908534647,0.0586164573910711,0.576884203473941
"1715","RHOBTB1",30.1883180001007,0.724612972516825,0.383081274369713,1.89153848281683,0.0585524989718841,0.576884203473941
"1716","KCNE3",53.1256171517468,0.556726394401545,0.294379282604738,1.8911874146696,0.0585993307557546,0.576884203473941
"1717","GANC",83.030026852821,0.403209976460276,0.213084419103477,1.89225461982028,0.0584570641122883,0.576884203473941
"1718","RALGAPA2",81.1945416598941,0.483392167580328,0.25551560505708,1.89183031491303,0.0585135928214141,0.576884203473941
"1719","AC022400.4",3.34077993247496,1.76820813433465,0.935267892646384,1.89059000981144,0.0586790948664421,0.57710953919149
"1720","PPIF",149.063788401824,0.5191840791075,0.274682954285666,1.89012121432026,0.0587417504701061,0.57710953919149

"1721","AC011815.2",5.47301212553189,1.57487318548298,0.833202109002356,1.89014546226806,0.0587385083138615,0.57710953919149
"1722","AL359258.2",16.7954069992173,-0.758086489567394,0.401444901156306,-1.88839486411169,0.0589729603224734,0.578053658107509
"1723","CARS",137.152109396482,-0.343046550266296,0.181670326254893,-1.88829159576112,0.0589868149530857,0.578053658107509
"1724","CDKN1B",378.865252688171,0.32776871698549,0.173534314795238,1.88878330704933,0.0589208704482698,0.578053658107509
"1725","AL139384.1",20.7654083506457,0.767217389658058,0.406337627921941,1.88812784477209,0.0590087895607736,0.578053658107509
"1726","PSTPIP2",56.767101464242,-0.441280563419431,0.233683960834079,-1.88836478911255,0.0589769949488265,0.578053658107509
"1727","TRIM69",184.975331314101,0.475729448263828,0.252133902129413,1.88681269851466,0.0591855225361307,0.57877895606761
"1728","GCOM1",5.31692253803091,-1.30629336346181,0.692276511971289,-1.88695317676181,0.0591666237500493,0.57877895606761
"1729","ZFP36",28725.9731892634,-0.409072343695189,0.21678534838499,-1.88699257926194,0.0591613237618413,0.57877895606761
"1730","BOK",109.556369709171,-0.389475538079385,0.206549335771856,-1.88562958396332,0.0593448878901406,0.579019801797529
"1731","ABHD18",59.0518248885767,0.533861247980479,0.283049818827213,1.88610347885922,0.0592810116193922,0.579019801797529
"1732","PTAR1",195.383493880293,0.368090431335606,0.195283288771651,1.88490491762468,0.0594426760716367,0.579019801797529
"1733","KIF5B",901.649317990032,-0.266544373672407,0.141387078252728,-1.88521028205959,0.0594014531887614,0.579019801797529
"1734","CAPN1",490.200399003952,-0.37218134769901,0.197428542281089,-1.88514458648596,0.0594103198048173,0.579019801797529
"1735","HOXC10",355.530167882025,-0.288723922741739,0.153107465197429,-1.88575992927213,0.0593273129603632,0.579019801797529
"1736","CORO6",27.1119490892127,0.623496902764426,0.33079361999447,1.8848516569783,0.0594498684599309,0.579019801797529
"1737","CDK5",40.1720068629487,-0.54801394564288,0.290849308013169,-1.88418514517513,0.0595399361717698,0.579381686176273
"1738","C12orf4",81.9187516997976,0.376348259793967,0.19975284037203,1.88406962871234,0.0595555577581241,0.579381686176273
"1739","ZBTB20",2713.36488632858,0.40281106578977,0.213876764078096,1.88337927930631,0.0596489863707756,0.579811539160953
"1740","OTOA",4.74305808422198,-1.75269077174015,0.930680129808275,-1.88323648007959,0.0596683273089696,0.579811539160953
"1741","AC068491.3",26.9410943045828,1.00346460367347,0.533089847645366,1.882355494305,0.0597877644054959,0.579972186212349
"1742","CARF",74.4096595164967,0.4976065149183,0.264324485894223,1.88255928403633,0.0597601185873447,0.579972186212349
"1743","EFCC1",5.50258034300647,-1.46740139332996,0.779432292437405,-1.88265409012137,0.0597472609462218,0.579972186212349
"1744","IGSF6",16.5978277182424,-0.901337005767249,0.478928459460721,-1.88198673092462,0.0598378172734521,0.580124893612115
"1745","SCLY",31.8514939732304,-0.551301215128806,0.293109635189887,-1.88087032612099,0.0599895606724381,0.580265155520356

"1746","GPD1L",119.376986969493,0.493516167698001,0.262376748398845,1.88094475104858,0.0599794348078822,0.580265155520356
"1747","CXCL16",100.146278180438,-0.450258430851541,0.239339270496058,-1.88125596739026,0.0599371077020931,0.580265155520356
"1748","SNAPC2",205.51411178276,-0.501404970270319,0.26651544485894,-1.88133550960133,0.0599262935067168,0.580265155520356
"1749","CD84",14.271807285996,-1.16534552554415,0.619793646268586,-1.88021534676906,0.0600787348692388,0.580795454070377
"1750","YTHDF3",273.789363986103,0.341839303485807,0.181861227609296,1.87967115354682,0.0601529093182888,0.581180223287787
"1751","BLNK",2.1591748954968,-2.02467400459182,1.07732159529362,-1.8793589708373,0.060195494606521 7,0.581259521877252
"1752","OMA1",106.158093149613,0.363631542333953,0.193644395418622,1.87783148356993,0.0604042218271012,0.581283200143783
"1753","CLK1",867.89860765553,0.290861765390118,0.154850820891952,1.87833531469018,0.060335308390148,0.581283200143783
"1754","TNIK",16.778225208204,1.01216000461803,0.538958412023534,1.87799277650728,0.0603821532694591,0.581283200143783
"1755","TSPAN33",5.82923257218308,-1.57970884476178,0.840825993391459,-1.87875833665661,0.0602774982794547,0.581283200143783
"1756","USP44",47.1564227658065,0.802534863863884,0.427351574276161,1.87792654145056,0.060391214929111,0.581283200143783
"1757","ZNRF3",97.0241028145142,0.465309824077786,0.247707609817896,1.87846398590605,0.060317719341673,0.581283200143783
"1758","CCNI",6484.83244994656,-0.269565389414916,0.143607303982995,-1.87710082940374,0.0605042757600486,0.581338081180671
"1759","MPDZ",348.598106655147,0.429112424911691,0.22869913556425,1.8763185258789,0.060611554651857,0.581338081180671
"1760","AL590399.3",3.79208567026472,1.64190098093625,0.874929798228425,1.87660882537182,0.0605717269053608,0.581338081180671
"1761","FGF14-AS2",78.5416179495521,-0.484950129801087,0.258438413422117,-1.87646303573687,0.0605917258564456,0.581338081180671
"1762","CMIP",114.098366459968,0.423478886938214,0.225630630265057,1.8768678988342,0.0605362015429852,0.581338081180671
"1763","C2CD2",131.139618484074,0.532876359021301,0.284006156258394,1.87628453566507,0.0606162193708021,0.581338081180671
"1764","MAN2B2",302.586178467926,0.356528215186122,0.190099050813764,1.87548656166308,0.0607258165280112,0.581729238445107
"1765","EPS8L1",5.8737766781898,-1.54717088611207,0.824855369777504,-1.87568747540482,0.0606982067222747,0.581729238445107
"1766","AC090630.1",5.89139061828976,-1.66249621026614,0.887308234040464,-1.8736400119897,0.0609800592430228,0.583343002615343
"1767","AC130650.2",11.5275155522526,1.09854274745446,0.586205723697486,1.87398843621896,0.0609320189751243,0.583343002615343
"1768","PATZ1",100.818588601539,0.417280657460136,0.222726491944017,1.87351156038062,0.0609977778935372,0.583343002615343
"1769","NPLOC4",205.475570589243,0.312075225359388,0.166652086129158,1.87261517457108,0.0611215443598048,0.584196196741425
"1770","SLC17A5",175.706751643845,0.329820740098692,0.176173270593444,

1.87213837256743,0.0611874624291952,0.584495827543973
"1771","C3orf80",2.5222826519807,-2.27400224555915,1.21504392279919,-1
.87153912948295,0.0612703915156754,0.58495752667817
"1772","TNFSF12",863.628269058575,-0.440580307991683,0.235449734126186
,-1.87122873434871,0.06131338359339,0.585037635325642
"1773","AL592295.5",190.991492724335,-0.675650733766942,0.361122038522
204,-1.87097618448285,0.0613483820920426,0.585041423808379
"1774","TMEM205",529.815113515108,-0.380388595043288,0.203661458713445
,-1.86774953614812,0.0617969904207526,0.58898732470918
"1775","ZNF718",36.3980699666034,0.621124049412218,0.332619019432368,1
.86737382147359,0.0618494029337726,0.589154763269987
"1776","PLEKHA8",66.6107441273067,0.547964492373056,0.293734723505938,
1.86550805377281,0.062110224365608,0.591272190506154
"1777","AL118506.1",14.0285446889374,1.02641972276539,0.55027535488255
4,1.8652838322815,0.0621416301472342,0.591272190506154
"1778","KLRC2",3.52942904249716,2.24728161331512,1.20505814230387,1.86
487401265029,0.0621990658500487,0.591485829804625
"1779","AL391001.1",6.08535113414782,1.3176653697259,0.707018273308713
,1.86369351326589,0.062364756857307,0.592691858542902
"1780","STK38",261.909335298577,0.365641799643613,0.196231665612701,1.
86331700595802,0.0624176789134736,0.592691858542902
"1781","TCAF1",535.300343062609,0.393949246592033,0.211434420548637,1.
86322191802924,0.0624310503941867,0.592691858542902
"1782","USP40",126.636149032234,0.472668950493442,0.253734672756912,1.
86284730170196,0.0624837528501931,0.592859311555031
"1783","ADGRG4",16.7924036303769,0.948836365630664,0.509497018483015,1
.86230013368036,0.0625607966567016,0.593257403180881
"1784","LIMCH1",242.779893077777,0.477277413869901,0.256418063654596,1
.86132523999094,0.0626982609135555,0.594044600858911
"1785","TIFA",159.420778799366,-0.567710539719072,0.305021769564024,-1
.86121318662113,0.0627140769182137,0.594044600858911
"1786","ACP6",14.6596305389145,-0.72582256706163,0.39031405371052,-1.8
5958604401148,0.062944115331623,0.594193348791516
"1787","ZNF148",622.38729228902,0.307948217315881,0.165776126178058,1.
8576149920714,0.0632237079923382,0.594193348791516
"1788","TMCC1",127.747421292937,0.416252477726694,0.224106105208495,1.
85739017390641,0.0632556634392759,0.594193348791516
"1789","BZW2",291.214086767412,-0.373737608603383,0.201046961494654,-1
.85895676226533,0.0630332673243116,0.594193348791516
"1790","CACNA2D1",249.022004732541,0.404093250625426,0.217532908920792
,1.85761893513116,0.0632231476485254,0.594193348791516
"1791","MRRF",92.6286607256538,-0.351835564535898,0.189400813624969,-1
.85762435652765,0.0632223772266351,0.594193348791516
"1792","AC006064.2",4.09585663157341,1.4654973825572,0.78862208993916,
1.85830120821275,0.0631262523623406,0.594193348791516
"1793","GNAO1",14.0656543185169,1.08031933444974,0.581619511060692,1.8
5743310515766,0.0632495601998951,0.594193348791516
"1794","CAMTA2",118.424261073724,-0.405834710129871,0.218467348497867,
-1.85764469116461,0.0632194875883848,0.594193348791516
"1795","SNHG30",170.175679819977,-0.51250832271467,0.275876053292767,-

1.85774849465018,0.0632047383730667,0.594193348791516
"1796","CD300A",6.73352095384667,-1.26697556661092,0.681066823439641,-
1.86028084617634,0.0628458020002813,0.594193348791516
"1797","C3",15516.156208078,0.392757556293596,0.211153321531533,1.8600
5862207071,0.0628772325169706,0.594193348791516
"1798","ZNF480",135.215737580765,-0.378241128039923,0.203642150855365,
-1.85738132528646,0.0632569214469322,0.594193348791516
"1799","ARFGEF2",118.808068378893,0.424933937782529,0.228772884361764,
1.85744887978319,0.0632473177526325,0.594193348791516
"1800","DONSON",37.9814688356786,0.662112373662991,0.356473342099966,1
.85739660015675,0.0632547498326903,0.594193348791516
"1801","LINC00311",6.30970531208281,1.53705672216314,0.82786963205593,
1.85664102492323,0.0633622431443372,0.594852197159607
"1802","NINJ1",135.616217267488,-0.406406439145015,0.218939176156674,-
1.85625271036093,0.0634175461309911,0.595040993331186
"1803","GALNT13",31.1810779475179,-1.08040188231881,0.582192902748557,
-1.85574553935334,0.0634898364729672,0.595134701251755
"1804","HTATSF1",416.476500259838,-0.302800226228721,0.163174034573964
,-1.85568878663392,0.0634979300365605,0.595134701251755
"1805","ATP6AP1L",31.4012976677201,0.633248905450727,0.341298416889596
,1.8554112006197,0.0635375291453267,0.595175923982927
"1806","ESRRG",2.42239841274369,2.30267825647959,1.24205389344209,1.85
392781153659,0.0637494883981561,0.595840989406315
"1807","AC233280.1",4.61454725422861,-1.73453653703677,0.9354097702055
06,-1.85430662826592,0.0636953043877635,0.595840989406315
"1808","CCNC",300.901132563288,-0.352178161945245,0.189962696109889,-1
.85393326772704,0.0637487077023897,0.595840989406315
"1809","FOXO3",1115.34987498322,0.572589467904082,0.308799658536225,1.
85424255524853,0.0637044663909814,0.595840989406315
"1810","FAM228B",200.614950902495,-0.344973403879725,0.186198081116273
,-1.8527226586417,0.0639221205952257,0.5962852039224
"1811","KMT2C",649.983373622738,0.490077448885643,0.264533326227339,1.
85261137367801,0.0639380810688024,0.5962852039224
"1812","CNN2",415.772522212624,0.493779553022814,0.266513249369848,1.8
5273923225326,0.0639197438917468,0.5962852039224
"1813","MBTPS2",89.0146392197723,0.422910149353771,0.228222758374014,1
.85305861854803,0.0638739572323398,0.5962852039224
"1814","ZNF672",85.6268971540106,-0.36694794791095,0.198229217252759,-
1.85112948028776,0.064150927877155,0.596303043952038
"1815","GTF2E1",45.8845633828844,0.480755384979972,0.259609790145569,1
.85183842531671,0.0640490280468647,0.596303043952038
"1816","LOX",842.798979044193,0.587406016139552,0.317323830233436,1.85
112481375077,0.0641515990625005,0.596303043952038
"1817","CDKN2AIPNL",162.761651629639,-0.440436709247902,0.237781352055
943,-1.85227607396344,0.0639861896029251,0.596303043952038
"1818","RC3H2",196.479490302088,0.364909882264903,0.197121290226967,1.
85119467229919,0.0641415519537017,0.596303043952038
"1819","SMIM25",9.27471771967755,-1.06058556002974,0.572809535279493,-
1.85155011344607,0.0640904522613402,0.596303043952038
"1820","LSM10",336.894923844498,-0.427843218100139,0.23148755575518,-1

.84823420293324,0.0645684694961609,0.597129327760355
"1821","LRRC8D",43.5872737016042,0.602113842758569,0.325448048824886,1
.85010739788625,0.0642980718985555,0.597129327760355
"1822","RDH10",347.526689540951,0.722926560273,0.391181314883808,1.848
06004982045,0.0645936562854086,0.597129327760355
"1823","CISD1",269.34124892656,-0.475087365484212,0.257071408082937,-1
.84807547843259,0.0645914246043632,0.597129327760355
"1824","ANXA11",1304.56100332273,-0.280252415167647,0.151541444795286,
-1.84934501281966,0.0644080099406481,0.597129327760355
"1825","IL17D",194.448087903221,-0.484213016886681,0.261914992375012,-
1.84874112205604,0.0644952027510613,0.597129327760355
"1826","AF001548.2",9.12030152003966,1.10674757776129,0.59840148608237
7,1.84950673335884,0.0643846764335874,0.597129327760355
"1827","IRX3",302.581484232659,0.539336839890978,0.2917490050919,1.848
6330046647,0.0645108235659572,0.597129327760355
"1828","ZNF772",8.37912491422296,1.01682991773815,0.55014420251153,1.8
4829707028828,0.0645593793340014,0.597129327760355
"1829","DYRK1A",192.631491651997,0.392676381325179,0.212300452311631,1
.84962574054613,0.0643675101894566,0.597129327760355
"1830","CFAP36",542.993567223408,-0.399112263685528,0.216137396854755,
-1.84656736637637,0.0648098674732662,0.598306918495997
"1831","SOWAHC",178.020181302787,0.417717156293911,0.226227427196614,1
.84644789303497,0.0648271986447047,0.598306918495997
"1832","MAGT1",427.431883741759,0.371222881422353,0.201020542561319,1.
84669127190877,0.0647918973929984,0.598306918495997
"1833","CAMK2N1",821.055548527448,-0.558452539399846,0.302907437494108
,-1.84364089577929,0.065235491878558,0.599482138209869
"1834","AL357568.2",22.9318969715298,0.868354009253604,0.4711284441970
95,1.84313645238183,0.0653090902875904,0.599482138209869
"1835","AC012603.1",2.92368579903193,2.1215736927622,1.15095828090068,
1.84331068116732,0.065283662530237,0.599482138209869
"1836","TPBG",399.771360459136,-0.419712497787187,0.227669066439863,-1
.84352008970903,0.0652531112803061,0.599482138209869
"1837","LAMA2",1566.05073657068,0.469785239124532,0.254649519443188,1.
84483065254454,0.0650621769804993,0.599482138209869
"1838","CREB3L1",206.84065984809,0.800423886911644,0.434110100901485,1
.84382691222679,0.0652083693039187,0.599482138209869
"1839","ABHD15",63.2631251324818,-0.376608353216309,0.204222383127373,
-1.84410909053695,0.0651672433733385,0.599482138209869
"1840","LENG9",89.0740145417196,-0.40267397047174,0.218417000072825,-1
.84360178162634,0.0652411961952168,0.599482138209869
"1841","NCOA6",173.286230074737,0.397077271294925,0.215416664229579,1.
84329876574331,0.0652854012623758,0.599482138209869
"1842","ABCG1",24.6290497553802,2.16762284904731,1.17485758128225,1.84
500903222802,0.0650362247289715,0.599482138209869
"1843","HERPUD1",1429.14161512075,-0.360065494041111,0.195451422402687
,-1.84222498672468,0.0654422469741548,0.600378465457954
"1844","LSM14B",97.5909212713325,0.350872039588464,0.190563113309481,1
.84123796832934,0.0655866935907092,0.601377340147349
"1845","TMEM61",2.90592045780977,2.14874813184262,1.16795503348083,1.8

3975244786501,0.065804589622846,0.602203466168115
"1846","RBPJ",2858.46444024747,0.392593027929128,0.213374394218337,1.8
3992568258872,0.0657791488729085,0.602203466168115
"1847","AGBL3",16.5086346067897,-0.722511333555152,0.392793792604458,-
1.83941637357472,0.0658539676481869,0.602203466168115
"1848","LINC01465",5.6995507668248,-1.10074242198034,0.598582425150247
,-1.83891537026675,0.0659276347134648,0.602203466168115
"1849","AL049870.3",2.40514459446171,-1.97642091623925,1.0745025709995
2,-1.83938221236711,0.0658589885245246,0.602203466168115
"1850","AL627171.2",253.295014855007,0.622412765284199,0.3386298692995
64,1.83803267730524,0.0660575903032085,0.602203466168115
"1851","ACACA",62.5290144899315,0.45588054978484,0.248036415109689,1.8
379581465215,0.0660685728496483,0.602203466168115
"1852","DUS1L",256.081522815255,-0.324413371827846,0.176444106266984,-
1.8386183516777,0.0659713401105622,0.602203466168115
"1853","ZNF561",87.2798922762954,0.378784535114522,0.205954372935408,1
.83916723746049,0.0658905919085104,0.602203466168115
"1854","ICAM5",4.22806990950579,1.55120490004409,0.843014344753331,1.8
4006940059599,0.0657580490157718,0.602203466168115
"1855","LCA5L",7.81361824313332,-0.980144742856682,0.533159958156316,-
1.83836900701631,0.06600804886513,0.602203466168115
"1856","ECM1",1741.53507979176,-0.573835550862301,0.312592200504514,-1
.8357321453835,0.0663972812824846,0.60226342222767
"1857","TMEM37",808.885173291522,0.639362107086069,0.348420428190837,1
.83503048430868,0.0665011727630796,0.60226342222767
"1858","DNAJC13",209.7010854905,0.291682413946593,0.158983854100631,1.
83466689492864,0.0665550602855761,0.60226342222767
"1859","CPLANE1",191.920422779329,0.525240844354807,0.28618814763784,1
.83529908100695,0.0664613871742759,0.60226342222767
"1860","AFF4",810.198623835084,0.441121196161403,0.240215157169901,1.8
3635871007675,0.0663046219839009,0.60226342222767
"1861","PANK3",197.322342807699,0.402385800610242,0.219396189553688,1.
83406011484888,0.0666450711490401,0.60226342222767
"1862","HSP90AB1",4790.20306011779,-0.236304631847553,0.12874064392902
8,-1.83550916506074,0.0664302823723167,0.60226342222767
"1863","LARP4B",142.030259365024,0.379553844050685,0.206859314636321,1
.83484047947262,0.0665293288641198,0.60226342222767
"1864","COX15",133.274614853781,0.362537182489273,0.197442447903326,1.
83616636817014,0.0663330550607899,0.60226342222767
"1865","SCUBE2",14.9669045985277,0.996072542107985,0.542280580469447,1
.83682133932529,0.0662362746071137,0.60226342222767
"1866","LARP4",191.505618118434,0.527337609538659,0.287403934540704,1.
83483086402902,0.066530754001464,0.60226342222767
"1867","NEMP1",46.0712472334445,0.497268778494796,0.271117798618674,1.
83414287453035,0.0666327885259606,0.60226342222767
"1868","AC068338.3",13.3963708993475,-0.838319332379024,0.456815422946
882,-1.83513797973608,0.0664852477680382,0.60226342222767
"1869","LINC02130",3.24422742115181,-1.57570990059281,0.85900253575390
2,-1.83434836919298,0.066602298481724,0.60226342222767
"1870","OSCAR",15.6826150406419,-0.828266043342686,0.450756899379466,-

1.83750053406374,0.0661360377027728,0.60226342222767
"1871","GPC4",395.826668775531,0.443890031207062,0.242013321844746,1.8
3415535898401,0.0666309358309922,0.60226342222767
"1872","MAP3K7",168.721404897096,0.286313705330404,0.156156614298772,1
.83350354140366,0.0667277223967368,0.60236643368074
"1873","ARHGAP28",92.6273815630822,0.653188242602128,0.356223241724787
,1.83364858351037,0.0667061754924767,0.60236643368074
"1874","AC003991.1",4.87606762353555,1.82840686094641,0.99802814295385
5,1.83201934119302,0.066948539095717,0.602919688363496
"1875","SETX",430.784794201999,0.317742441675682,0.173439051236321,1.8
320121069086,0.0669496168713198,0.602919688363496
"1876","POGLUT3",613.316730389509,0.268609910383317,0.14658307848813,1
.83247557053502,0.0668805981471742,0.602919688363496
"1877","AP000919.4",3.86764002872782,1.47397033091325,0.80421386819153
,1.83280889476307,0.0668309959289049,0.602919688363496
"1878","GREB1L",9.39172811468771,1.03292323589523,0.563855536229214,1.
83189340092838,0.0669673039239795,0.602919688363496
"1879","PGM1",278.092706058454,-0.355608618463605,0.194198501700602,-1
.83116046390435,0.0670765959055455,0.603582269063844
"1880","AL139424.3",2.01518910440722,2.581666328344,1.41314568584253,1
.82689325963217,0.0677158199120425,0.603707036071873
"1881","TRMT1L",119.404815798503,0.349458578525578,0.191045037992367,1
.82919473961705,0.0673704402096189,0.603707036071873
"1882","ACTR3",677.642240796327,-0.271164148182461,0.148374013644,-1.8
2757169886282,0.0676138564701104,0.603707036071873
"1883","PTPN23",174.55094114779,0.329611229469582,0.18023877752284,1.8
2874758694929,0.0674374300508024,0.603707036071873
"1884","AC113410.4",3.28134590636123,-1.55473500922588,0.8505118391621
28,-1.82799925602154,0.0675496633030172,0.603707036071873
"1885","ARHGEF37",5.75988506344398,-1.12720483775028,0.616987856500729
,-1.82694817389642,0.0677075620796387,0.603707036071873
"1886","AL512308.1",10.7764106554064,1.25387646294685,0.68594656738549
2,1.82795063429801,0.0675569608106149,0.603707036071873
"1887","CCND3",115.702837841068,-0.456214516838657,0.249525774499063,-
1.8283262230306,0.0675006065215459,0.603707036071873
"1888","TCTE3",13.0637414091634,-0.925107598948742,0.505404024450351,-
1.83043180147771,0.0671853959802251,0.603707036071873
"1889","TTC39B",104.971891679986,0.322004217679917,0.176165405321434,1
.82785159828846,0.0675718268729538,0.603707036071873
"1890","PSD",6.51320650635731,-1.338149589958,0.731296044830448,-1.829
8329375872,0.0672749239226034,0.603707036071873
"1891","HPS5",83.9840821492929,0.395893302563472,0.216491113028234,1.8
2868154274693,0.0674473290574988,0.603707036071873
"1892","MGA",200.037656935148,0.38812947592206,0.212440487733819,1.827
00331778739,0.0676992705503334,0.603707036071873
"1893","ERI2",37.9089541583693,0.530133860952014,0.290201583409305,1.8
2677797524042,0.0677331587076143,0.603707036071873
"1894","SGCA",37.0046277324812,1.67993562571429,0.918607064181225,1.82
878587724737,0.0674316914735299,0.603707036071873
"1895","MED13",436.073778151951,0.356530788534321,0.194954771105518,1.

82878719260146,0.0674314943481981,0.603707036071873
"1896","B9D2",49.157925630638,-0.532948914876016,0.291474194718584,-1.82846003019435,0.0674805391011658,0.603707036071873
"1897","SYS1",361.813927355424,-0.295054592997691,0.161422397992822,-1.82784171630762,0.0675733103815085,0.603707036071873
"1898","BRWD1",347.652157490725,0.327050767156928,0.179063256518751,1.82645381031971,0.0677819327497813,0.603823455707746
"1899","LINC02591",53.6911391331629,0.468293722654367,0.256503135976263,1.82568420020293,0.0678978444555083,0.603875511765564
"1900","KLHL24",439.152490482935,0.490404373943213,0.268646031397215,1.82546666106566,0.0679306377678083,0.603875511765564
"1901","TSPAN11",33.8686383260231,0.91112228868216,0.499089173580189,1.8255701323799,0.0679150381820926,0.603875511765564
"1902","NEDD4",130.586767577079,0.317671215686303,0.173991589453601,1.82578489387855,0.0678826696226324,0.603875511765564
"1903","LINC01914",31.8732762957209,-0.639064535906645,0.350162260252138,-1.82505257832892,0.067993095380231,0.60411311439251
"1904","LINC01686",25.643036319142,-0.601384673944712,0.329717266735353,-1.82394049271133,0.0681610690818855,0.605287476909937
"1905","TMEM53",59.5224557172241,-0.558055889071082,0.306130852227952,-1.8229325303532,0.0683136102469986,0.605325835606866
"1906","AC011379.1",37.4504383992597,-0.696681673302664,0.382181503680035,-1.82290787647832,0.0683173447834347,0.605325835606866
"1907","PCDHGA5",58.9305769962815,0.539930662603346,0.296067477231466,1.82367434495761,0.0682013196260504,0.605325835606866
"1908","HIVEP1",211.206468388332,0.325223839455779,0.178426841800731,1.82272933922685,0.0683443943797911,0.605325835606866
"1909","CSNK1G1",176.051231475105,0.412225594412133,0.22611707556111,1.82306264747673,0.0682939030570827,0.605325835606866
"1910","ACKR1",5.38081306722085,-1.48789364968578,0.816631236051111,-1.82198963742878,0.0684565580086784,0.606001823461117
"1911","GRPEL2",46.1243966778644,0.47822120628966,0.262578416174514,1.82125101239024,0.0685687093025451,0.606535055599815
"1912","SPPL3",374.979406637335,0.253154759189186,0.139010437353786,1.82112051446108,0.0685885395260732,0.606535055599815
"1913","PRKCI",165.087453331738,0.36492738163803,0.200457773545684,1.82047009294386,0.0686874466789074,0.607069207641976
"1914","KCNIP2",2.25045290546608,-1.92576485095977,1.05796650256535,-1.82025125208614,0.0687207513263983,0.607069207641976
"1915","ABHD12",230.285126075261,-0.420890856103938,0.231269334340443,-1.81991640744016,0.0687717358825025,0.607202355248748
"1916","SNHG10",41.9256021201249,-0.490022900273323,0.269290857495628,-1.81967893314491,0.0688079133420141,0.607204696652805
"1917","NAA10",343.682418697613,-0.450662568077277,0.247782980529579,-1.81877934922766,0.068945100173934,0.608097941440207
"1918","VENTX",5.91281301031189,-4.21224321947754,2.31667901897598,-1.81822478857664,0.06902978275873,0.6085222755753
"1919","KCTD10",203.559837655412,0.403982856263504,0.222223737235944,1.81791045946896,0.0690778194088991,0.6085222755753
"1920","AC108134.2",62.4334410766546,0.705603665339606,0.3881725722192

15,1.81775765687311,0.0691011810447466,0.6085222755753
"1921","LMX1A",10.0840266973288,1.02865226352718,0.566196333890299,1.8
1677662315362,0.0692513236647111,0.609209875402152
"1922","MEX3D",190.712191528723,-0.408000760240135,0.224546070917144,-
1.81700244664127,0.0692167387177929,0.609209875402152
"1923","BGN",721.06043624065,-0.624400702569115,0.343745848083304,-1.8
1646034723246,0.0692997852921229,0.609319173020912
"1924","PELI1",103.863395706858,-0.588355545165071,0.324030216390926,-
1.81574283941239,0.0694098292655842,0.609969539096933
"1925","AC098818.2",117.703422685437,-0.726562435799592,0.400330550305
176,-1.81490629492485,0.0695383110031558,0.61007952413875
"1926","AASS",424.712543122291,0.533116247783373,0.293777816220447,1.8
1469198267622,0.0695712578233381,0.61007952413875
"1927","DDB1",430.780542700507,0.237275572439816,0.130727964792292,1.8
1503301773889,0.0695188355792772,0.61007952413875
"1928","IFNG",10.8517370694413,1.27306434252614,0.701701797761368,1.81
425264490926,0.0696388385135905,0.61007952413875
"1929","ZNF146",191.914788518341,0.268997026570313,0.148245800354839,1
.8145338749998,0.0695955723726,0.61007952413875
"1930","ID1",696.458794111433,-0.607394922661097,0.334753277387921,-1.
81445549211825,0.0696076290509398,0.61007952413875
"1931","LIMK1",66.9711536997007,-0.40559057271768,0.223619746326861,-1
.81375115292741,0.0697160458779426,0.610097990593317
"1932","AC015912.3",71.3480237381386,-0.930955426143186,0.513337102054
776,-1.81353621707213,0.0697491579052718,0.610097990593317
"1933","AL050403.2",34.9870681673265,0.609306465162725,0.3359770506709
48,1.81353596606059,0.0697491965824984,0.610097990593317
"1934","PIKFYVE",131.810572302128,0.429346962407463,0.236896962561695,
1.81237850314627,0.0699277320990564,0.611343378661244
"1935","PPP1CC",1157.96666375869,-0.286975233310425,0.15837019013956,-
1.81205334828187,0.0699779537863595,0.611466275255694
"1936","SEMA3B",61.2865057649958,0.764718210388234,0.422465536767148,1
.81013158195133,0.070275384423566,0.61374803710416
"1937","FAM71A",41.2878690363957,0.996222589880708,0.550635883510736,1
.80922206436858,0.0704165111073741,0.613924691974099
"1938","ZNF703",509.01627908338,0.578131846018016,0.319586120846525,1.
80900173163544,0.0704507343338182,0.613924691974099
"1939","STXBP1",286.242658328704,0.313037963894743,0.172979578591799,1
.80968161931679,0.0703451745301329,0.613924691974099
"1940","IQCH-
AS1",129.326956250881,0.655127230172701,0.362182532123136,1.8088316582
589,0.0704771603455008,0.613924691974099
"1941","ODF3B",235.821650404193,-0.537156462603972,0.296960657587309,-
1.80884722901734,0.0704747406220013,0.613924691974099
"1942","DCAF6",339.716228481475,0.276463351357795,0.152979175472551,1.
80719598274603,0.0707317270924118,0.614055564851636
"1943","LEFTY1",2.27901165846186,2.12631418046793,1.17644999758946,1.8
0739868657805,0.0707001386034319,0.614055564851636
"1944","ARFIP1",283.600042970049,0.261072525506151,0.144463119293884,1
.8071915294522,0.0707324212043898,0.614055564851636

"1945","GOLGA6L4",74.7291458180203,0.594905420371388,0.328959934917833
,1.80844339150293,0.0705375197297294,0.614055564851636
"1946","IKZF3",10.7750842626994,0.998233388305134,0.552394659010056,1.
8071018103145,0.0707464064543995,0.614055564851636
"1947","WNT3",15.552763371009,0.917572107636807,0.507618774187286,1.80
760081048198,0.0706686520091813,0.614055564851636
"1948","IER2",4192.21019890735,-0.466994491592564,0.258382199783745,-1
.80737872803706,0.0707032483425847,0.614055564851636
"1949","MLLT3",133.738005664902,0.323400250672901,0.17899140415488,1.8
0679207585334,0.070794704707019,0.614140500792882
"1950","MMP2",8728.72483177994,-0.425652541648681,0.235613215193171,-1
.80657329131434,0.070828837032536,0.614140500792882
"1951","PROSER1",54.9348255912516,0.441224281989326,0.244276115020965,
1.80625224840938,0.0708789469972111,0.614259987610889
"1952","ALPK3",9.7241479392204,-1.03748235795341,0.574475847114631,-1.
80596340675467,0.0709240556706253,0.614336031392896
"1953","MIB1",303.091732568088,0.394085501801342,0.218270912861646,1.8
054879444754,0.0709983603263655,0.614664760060516
"1954","PIK3R4",80.0862185762254,0.370218885051406,0.205086442327021,1
.80518458875538,0.0710458017255787,0.614760703979572
"1955","SMIM17",7.06455241320092,1.08079259690159,0.59896224370433,1.8
0444194648621,0.0711620522661306,0.61545165202851
"1956","USP49",41.1525133838465,0.741743808336894,0.411380294359101,1.
80306110552153,0.0713786186297407,0.615798652614592
"1957","RIC1",121.380135941197,0.450066969032901,0.249550411735525,1.8
0351122605994,0.0713079640041763,0.615798652614592
"1958","HOXB2",274.915963585405,-0.444060466253938,0.246286351270361,-
1.80302507208964,0.0713842772134256,0.615798652614592
"1959","ACSF2",69.0782789858366,0.487100853283654,0.270142056763833,1.
80312854325195,0.0713680293956348,0.615798652614592
"1960","AL359851.1",11.5169954000055,-0.824320695933932,0.457079477181
592,-1.80345155948982,0.0713173264632715,0.615798652614592
"1961","SLC25A30",24.3846877014956,0.689530987312879,0.38257083977447,
1.80236159065172,0.0714885341026843,0.616383546460064
"1962","LYST",277.481050508422,0.378623305643827,0.210126732258011,1.8
0188071063191,0.0715641756839059,0.616407072064942
"1963","WNK2",14.2388171543121,-1.16551188275833,0.646814884588947,-1.
80192495647192,0.0715572131526626,0.616407072064942
"1964","TRIM72",2.80724655728303,3.29635548830808,1.82998108132767,1.8
0130577411027,0.0716546982537695,0.616872524477971
"1965","PIWIL4",28.0053752434985,0.600827128279579,0.333630543731644,1
.80087566791503,0.071722478914606,0.617141818569038
"1966","CSRP1",705.913722075211,-0.386105079948789,0.214567741344556,-
1.79945539590118,0.0719466735440049,0.617665591191178
"1967","DRD4",6.00723849845166,1.178173493799,0.654867510111922,1.7991
0207119245,0.0720025360916615,0.617665591191178
"1968","SLC3A2",598.637736631634,-0.526011332965533,0.292237805400148,
-1.79994279742585,0.0718696709954359,0.617665591191178
"1969","AC023157.3",52.8793339977731,-0.847719233396115,0.471184990212
768,-1.7991218969291,0.0719994005949378,0.617665591191178

"1970","NFATC4",193.828769733112,0.42518453604508,0.236312105240206,1.79924991829297,0.0719791563442005,0.617665591191178
"1971","SLC7A5",7.98270528307064,1.01592686266667,0.564624258033941,1.79929722857496,0.0719716762633433,0.617665591191178
"1972","NPY5R",15.4278306943403,1.27048466003089,0.706398264735179,1.79853876128529,0.0720916719019946,0.618116627037995
"1973","SLC6A9",10.9634167139085,-0.974941550415384,0.542912089884875,-1.79576319735691,0.0725321861389382,0.618365009152653
"1974","AL160006.1",21.8314664470802,0.666303651265941,0.371201673599913,1.79499096758948,0.0726551390444962,0.618365009152653
"1975","SNED1",636.763786315263,0.497250747007188,0.276979594751794,1.79526129877106,0.0726120780519093,0.618365009152653
"1976","RUFY3",581.479367897804,0.406228535391755,0.226151737768767,1.79626537208886,0.0724523223020213,0.618365009152653
"1977","SORBS2",17.2922831796652,1.07701884524898,0.599701803964407,1.79592397109564,0.0725066094950392,0.618365009152653
"1978","FER",234.006337294879,0.370846441309577,0.206630777800081,1.79472992967378,0.0726967395494823,0.618365009152653
"1979","WDR86-AS1",78.5526901329141,0.867511389246717,0.482617180677132,1.79751451871142,0.0722539753164106,0.618365009152653
"1980","ADAM9",434.600732797916,0.290665515842026,0.161922228782251,1.79509334838088,0.0726388283730279,0.618365009152653
"1981","SLC45A4",21.3063931551166,0.842074187042839,0.468805166718728,1.79621353778309,0.0724605624867432,0.618365009152653
"1982","AL512625.3",7.82214840438489,-0.960353135074471,0.534155616469539,-1.79789017556691,0.0721944133094111,0.618365009152653
"1983","PAPPA",80.368076564829,0.731256251974353,0.407459303135935,1.79467310316975,0.0727057983318828,0.618365009152653
"1984","RAD9A",87.8229848550259,-0.397893606318966,0.221680262259478,-1.79489866289146,0.072669847025073,0.618365009152653
"1985","TCIRG1",201.515138627816,-0.336909624497711,0.187494543027257,-1.79690362747642,0.0723509206635039,0.618365009152653
"1986","ZNF429",116.628236920755,0.358662402508393,0.199747046368075,1.79558300875941,0.0725608601670752,0.618365009152653
"1987","ZFP30",37.7052330932878,0.622070548377601,0.34596975279446,1.79804894316057,0.0721692521166106,0.618365009152653
"1988","LTBP4",2342.63050399228,0.685507053707936,0.381392521315476,1.79737938054875,0.0722754118911456,0.618365009152653
"1989","RXYLT1",233.704867360103,-0.342323717320909,0.190775239030058,-1.79438232687511,0.0727521657965056,0.618448275157022
"1990","GFOD1",15.3027020605686,-0.753423468122171,0.419947306025278,-1.79409049019312,0.0727987266832256,0.618533100884411
"1991","SLC35E4",31.4470509729896,-0.644950826933518,0.359648928129952,-1.79327887973151,0.0729283427608091,0.6193231639376
"1992","ANP32B",935.2717548424,-0.430503818067953,0.240290831249024,-1.79159485957166,0.0731978871561751,0.621300138572595
"1993","RAI14",70.5232867898894,0.540824933180525,0.301970027014439,1.79098878960813,0.0732950939798588,0.621576835901179
"1994","POR",364.247453725278,-0.354183249041461,0.19776463093324,-1.7

9093322890999,0.073304010574104,0.621576835901179
"1995","ELOVL6",42.5253046005106,0.552648801162631,0.308945503435645,1
.78882293160726,0.0736433366101649,0.622465790892943
"1996","USP53",1995.64890856006,0.502047565673669,0.28049767550769,1.7
898457260474,0.0734787159363569,0.622465790892943
"1997","SLC25A46",191.063022353137,0.279782431773967,0.156339213656687
,1.78958576821523,0.0735205280818633,0.622465790892943
"1998","CENPP",32.1261440398992,0.512084321808449,0.286248932297401,1.
78894753492535,0.0736232653550871,0.622465790892943
"1999","ADM",3032.57751049317,0.674642242720838,0.377173489306425,1.78
86788489865,0.0736665511933274,0.622465790892943
"2000","NPIPA2",2.98804066215514,2.13460431398609,1.1927856178316,1.78
959595259594,0.0735188896392458,0.622465790892943
"2001","ZNF324",88.5368365967287,-0.432158350703182,0.241516902684042,
-1.78935033490613,0.073558412442677,0.622465790892943
"2002","HLA-F-
AS1",173.808955050015,-0.608622608160994,0.340444027968892,-1.78773178
014627,0.073819291844002,0.622822647953287
"2003","ZSCAN29",62.7422347423319,0.497076637794321,0.278046605935823,
1.7877457490312,0.0738170371008769,0.622822647953287
"2004","EVA1C",41.7623012447768,-0.652606160081159,0.365016421150565,-
1.78788164659575,0.0737951045661118,0.622822647953287
"2005","C8orf31",25.5020061125093,0.604708617140902,0.338499782972598,
1.7864372373611,0.0740284908812185,0.624161428660195
"2006","TNRC6B",1364.22145784825,0.363085903895176,0.203262211707274,1
.78629318674378,0.074051799496827,0.624161428660195
"2007","TRNP1",4.25614678160966,-1.75004644820899,0.979859568068521,-1
.78601761440024,0.074096406161541,0.624187429038742
"2008","LHX9",5.23294280247053,1.93037815568367,1.08094893457042,1.785
81808441379,0.0741287176194579,0.624187429038742
"2009","STEAP1",432.767807851972,-0.442775905888104,0.248053629074613,
-1.78500071754612,0.0742612004929236,0.624535984468342
"2010","TMEM9B-
AS1",31.6056047159429,0.794927259334806,0.445396760271408,1.7847621048
0383,0.0742999124971706,0.624535984468342
"2011","FAM177A1",455.598164664539,-0.29187020514634,0.163586354019098
,-1.78419652969504,0.0743917360255052,0.624535984468342
"2012","SEC14L5",12.627803504529,0.845700455010743,0.473944693850382,1
.78438637668917,0.0743609032239344,0.624535984468342
"2013","LONP2",412.115790864742,0.326328450265506,0.18286654042292,1.7
8451700081818,0.0743396947951043,0.624535984468342
"2014","VAT1",1585.03088823374,0.25034809674784,0.14027106788094,1.784
74506917087,0.0743026769512368,0.624535984468342
"2015","PUSL1",67.1953842960317,-0.505011277982617,0.283619030462799,-
1.78059729334297,0.0749782622759161,0.624652059130127
"2016","PDCL3",137.810108862763,-0.450189057342156,0.252707594015342,-
1.78146232247704,0.0748369547833664,0.624652059130127
"2017","GRPEL1",186.721500890496,-0.432665660606602,0.242615529775088,
-1.78333868820226,0.0745311872109593,0.624652059130127
"2018","AL445250.1",21.3866224628176,0.688298538838938,0.3867185473920

02,1.77984361877848,0.0751015569143552,0.624652059130127
"2019","LRRC17",237.1470766805,0.86754343606075,0.486563264926922,1.78300233206271,0.0745859237053941,0.624652059130127
"2020","WHRN",19.190621641072,-0.617745122102051,0.347090270247167,-1.77978230753097,0.0751115941813439,0.624652059130127
"2021","TRMT112",1609.84080154597,-0.374568536069654,0.209989498200668,-1.7837488982983,0.0744644766090649,0.624652059130127
"2022","ARID2",157.433273454721,0.333977082505973,0.187494223867611,1.78126598044852,0.0748690092876471,0.624652059130127
"2023","CACNB3",44.8622453363206,-0.594820164898886,0.333925225631088,-1.7812974859109,0.0748638649980637,0.624652059130127
"2024","AC068987.3",3.00401066508499,1.94494924817961,1.09200343025812,1.78108346025971,0.0748988173155116,0.624652059130127
"2025","TPT1-AS1",304.514539924122,0.32871248934417,0.184713347002255,1.7795816852377,0.0751444457221835,0.624652059130127
"2026","RAD51B",62.4686407673025,0.463986252276016,0.260675464820409,1.77993833288329,0.0750860534169677,0.624652059130127
"2027","CPSF2",134.026694971639,0.33508307375619,0.188287138324694,1.77963867706329,0.0751351122201538,0.624652059130127
"2028","IRX5",146.462639028147,-0.408015546415581,0.229090923716077,-1.78102012858988,0.0749091625028262,0.624652059130127
"2029","C19orf12",301.125372934473,0.268167510286753,0.150532520054403,1.78145898434329,0.0748374996683802,0.624652059130127
"2030","ZNF175",37.9469252950054,0.562847660416674,0.316126991205458,1.78044797209634,0.0750026768006039,0.624652059130127
"2031","RWDD2B",118.208618784316,0.377518218132047,0.211740053061297,1.78293248100188,0.0745972949473683,0.624652059130127
"2032","MT-ND3",76404.4227967716,-0.694981803287745,0.390434290510201,-1.78002245238136,0.0750722863135928,0.624652059130127
"2033","AC011043.1",26.4920052126714,0.679548349789649,0.381450365318085,1.78148564420166,0.0748331480585761,0.624652059130127
"2034","AL354822.1",16.0708369797104,1.09020455358915,0.611871051952768,1.78175540436142,0.0747891275303942,0.624652059130127
"2035","TBC1D9",162.297821797114,0.327677007279105,0.184229084377107,1.77863885274688,0.075298990006548,0.62470820560879
"2036","PDGFRL",2523.08204973745,-0.271791891290218,0.152763439894353,-1.77916844159952,0.0752121505978881,0.62470820560879
"2037","ADAMTS15",89.0755740590109,1.45257298521858,0.81667130410205,1.77865069817253,0.0752970467566035,0.62470820560879
"2038","METTL16",157.260804675797,0.360531186274069,0.202675849023328,1.77885617853054,0.0752633440819146,0.62470820560879
"2039","BAALC-AS1",10.8866482705021,-0.84001595369596,0.472519650699241,-1.77773760827279,0.0754469598638529,0.625624445850258
"2040","BPIFB4",11.7894775059242,2.75823448201909,1.55173566194083,1.77751568754257,0.0754834320756167,0.625624445850258
"2041","NYNRIN",123.485652158228,0.366420412866314,0.206177409963257,1.77720931178452,0.075533807943286,0.625735239933895

"2042","RNLS",56.0682563202704,-0.389022893097112,0.218968139627709,-1
.77661870698875,0.0756309956344927,0.626233532903037
"2043","C7",1773.3834245013,-0.673907652642516,0.379460493619708,-1.77
596262054595,0.0757390783672316,0.62682150613468
"2044","SERPINE1",102.9950669957,-0.995927433333905,0.560935442864729,
-1.77547603026767,0.0758193199250991,0.627178601415643
"2045","SAMD5",2.86827532334754,1.66351982000209,0.937209438830877,1.7
7497126157548,0.0759026324971492,0.627437980243305
"2046","GPATCH2L",292.355031410861,0.38671525976778,0.217887826919586,
1.77483646165557,0.0759248939896973,0.627437980243305
"2047","IRF5",4.06849075475049,-1.34324069385143,0.757070113271852,-1.
7742619478747,0.0760198316360045,0.627915639131198
"2048","MOV10",69.52962096392,0.443335393877413,0.250148757986992,1.77
228700811885,0.0763469267451341,0.62821337925264
"2049","DOK1",95.3875325272543,-0.423234114210705,0.238809331187833,-1
.77226790974015,0.0763500954672809,0.62821337925264
"2050","TWF2",508.043633682517,-0.426286319872993,0.240481760714656,-1
.77263472542021,0.0762892537124414,0.62821337925264
"2051","ANKIB1",411.28175371345,0.380337572591989,0.214516983816309,1.
77299515323072,0.0762295100006764,0.62821337925264
"2052","TSC1",195.41289645927,0.483411427860035,0.272801571237056,1.77
202581960192,0.0763902713356652,0.62821337925264
"2053","RIC3",5.27181650473345,-1.24439128401121,0.701728740178279,-1.
77332238621874,0.0761753016447822,0.62821337925264
"2054","CTTN",854.41619157454,-0.300291804997874,0.169452671012333,-1.
77212789390625,0.0763733295774076,0.62821337925264
"2055","RBMS2",187.278366991858,0.307766587636227,0.173622991430062,1.
77261424366253,0.0762926498694012,0.62821337925264
"2056","ADGRE5",236.013962429729,-0.400374479095076,0.225926944611786,
-1.77214134322512,0.0763710975584082,0.62821337925264
"2057","SPAST",113.840836612393,0.316570201536146,0.17881179701576,1.7
7041004463617,0.076658857661517,0.630115685630009
"2058","RASSF1",267.143643724317,-0.391979583625543,0.221749199968642,
-1.76767079060927,0.0771159558502918,0.630806280366102
"2059","POLH",39.1213697065117,0.591122866053004,0.334350186395889,1.7
6797528491006,0.0770650355393837,0.630806280366102
"2060","DDIT4",1360.02631877109,1.2233181369406,0.691671487808453,1.76
864040010765,0.0769539042123124,0.630806280366102
"2061","ADRB1",52.7704174080008,-0.874444092694047,0.494256346654785,-
1.76921166235384,0.076858558686813,0.630806280366102
"2062","RPS6KA4",81.4860055728516,-0.479096586367754,0.270929764695786
,-1.76834238536215,0.0770036820928253,0.630806280366102
"2063","HBA1",31.5932531063945,3.1572627143222,1.78554905418073,1.7682
3073380689,0.0770223381873723,0.630806280366102
"2064","AC135048.3",4.11258074548468,-1.4688508207211,0.83016847323621
8,-1.76934064358663,0.0768370446304119,0.630806280366102
"2065","GAREM1",149.784304845176,0.377311061011933,0.213228963988123,1
.76951129881656,0.0768085869001341,0.630806280366102
"2066","TTI1",68.7346764325389,0.374028730856775,0.211568395506435,1.7
6788565211479,0.0770800219053005,0.630806280366102

"2067","USP9X",362.788321116725,0.310988007329562,0.175929734523536,1.7676830364793,0.0771139074553084,0.630806280366102
"2068","CLSTN2",175.138188667056,0.639015977318812,0.361680868734022,1.76679507421981,0.0772625539575856,0.631159063109765
"2069","TOLLIP-AS1",26.022250548783,-0.647215341532917,0.366470780650998,-1.76607624865263,0.0773830575956082,0.631159063109765
"2070","AP003307.1",46.1138063115678,-0.559631463567948,0.316828610758903,-1.7663539357366,0.0773364881030459,0.631159063109765
"2071","MON2",267.992191047171,0.411272921598512,0.232850844957291,1.76625050114784,0.0773538319240549,0.631159063109765
"2072","ATG14",213.457829974559,0.494135484004034,0.279721749224891,1.76652507491207,0.0773077985943849,0.631159063109765
"2073","CPT1B",13.7315442401445,-0.877695068054569,0.496800718034023,-1.7666944434538,0.0772794144500779,0.631159063109765
"2074","MMACHC",27.294530818289,0.590764088398527,0.334750551724478,1.76478899095218,0.0775992355749806,0.631547806514426
"2075","NFKBIZ",3139.23685913102,0.500177406413749,0.283561095055407,1.7639140740235,0.0777464469520008,0.631547806514426
"2076","GM2A",145.704551395125,0.304986271310566,0.172859652433223,1.76435777243266,0.0776717629459699,0.631547806514426
"2077","PCOLCE-AS1",4.53288649050481,1.20140978993535,0.681319373629202,1.76335773858268,0.0778401728264704,0.631547806514426
"2078","ASPH",1664.60221568802,0.339166967130593,0.19224771295695,1.76421847580856,0.0776952032750145,0.631547806514426
"2079","PTK2",289.142685356923,0.294196800677842,0.166839773046766,1.7633493219592,0.0778415914819058,0.631547806514426
"2080","SURF1",451.451697360198,-0.339876275809165,0.192744331834162,-1.76335289642446,0.0778409889889668,0.631547806514426
"2081","AP000662.1",9.74702297096222,0.986394688275824,0.559120403131019,1.76419011495934,0.0776999764413908,0.631547806514426
"2082","LINC02762",344.282084207287,-0.51708629047771,0.292922646593159,-1.76526566481524,0.0775191271952004,0.631547806514426
"2083","SLC5A3",2975.21794745174,-0.712932792613774,0.404061280530508,-1.76441749547924,0.077661714733329,0.631547806514426
"2084","PLS3",1017.60515264717,0.280482962048593,0.159032316182976,1.76368532371672,0.077784973401614,0.631547806514426
"2085","LRRC10B",11.204737271254,1.14373588074145,0.64880210668492,1.76284242753991,0.0779270693828527,0.631938076319076
"2086","RHOB",5934.96568483267,0.525953499947697,0.298488354338306,1.76205701932204,0.0780596641738045,0.632011118662441
"2087","RSPH9",4.8345759415624,-1.45147063893853,0.823690977862171,-1.76215434908091,0.0780432227298754,0.632011118662441
"2088","LAT2",5.33193970198979,-1.10887230198835,0.62915795980891,-1.7624704332202,0.0779898476154238,0.632011118662441
"2089","NIN",215.254676523058,0.361262335735123,0.205055270713286,1.76178029698271,0.0781064249433348,0.632011118662441
"2090","JAM2",119.156662655586,-0.585859314474808,0.33255671707336,-1.7616823970077,0.0781229736222203,0.632011118662441

"2091","SLC30A9",173.190725864856,0.300178507018105,0.170427276779543,
1.76132901194215,0.0781827323818346,0.632192079919684
"2092","MTF1",118.955171408659,0.45925084815299,0.260875690569066,1.76
042024901283,0.0783365785574907,0.632238486244568
"2093","CMPK2",17.1727913469056,-0.774351792931643,0.439753024245001,-
1.76087883479847,0.0782589129415705,0.632238486244568
"2094","TMEM158",15.4105207006802,-0.874090253686978,0.496526068391988
,-1.76041160641926,0.0783380428603247,0.632238486244568
"2095","DDX25",2.51578950416877,-1.955315862796,1.11057673036118,-1.76
063103911793,0.0783008715750817,0.632238486244568
"2096","INTS6",1405.11851464118,0.406606349816667,0.231027973724129,1.
75998751693246,0.078409923136304,0.632516689116712
"2097","HSDL1",100.316509656998,0.391984950249173,0.222760042333761,1.
75967353095517,0.0784631761951504,0.632644436388938
"2098","CDC14A",73.928850968375,0.437383080968435,0.248757962726013,1.
75826765975801,0.0787019776564624,0.633663351531174
"2099","HUS1",79.6128058590383,-0.354303032613562,0.201484594411059,-1
.75846214768525,0.0786689067091001,0.633663351531174
"2100","AL049874.3",5.49582532667366,1.42364007933599,0.80951845003513
7,1.75862585871168,0.0786410778665753,0.633663351531174
"2101","HSPD1",1018.93951511329,-0.274217855637052,0.155996488734566,-
1.75784633270589,0.0787736593819015,0.633938616291857
"2102","ZNF75D",51.7549905752538,0.491128455547886,0.279461340062854,1
.75741108032126,0.078847766040379,0.634233124743448
"2103","RBM12",168.528683231771,0.283367267270802,0.161272241648642,1.
75707402820235,0.0789051919468585,0.634393240816682
"2104","RNF144A",117.605490913002,0.603320411086537,0.343665368524227,
1.75554613977348,0.0791659357280418,0.634681574512246
"2105","PDE11A",36.2863170502707,-1.12788956852828,0.6423224520566,-1.
75595538489583,0.0790960268543304,0.634681574512246
"2106","DTX3",87.9245535230528,0.407106919153563,0.231926869606388,1.7
553245117501,0.0792038160764632,0.634681574512246
"2107","FOS",63497.1483963549,-0.189100457742905,0.107726783051511,-1.
75537087794113,0.0791958900135317,0.634681574512246
"2108","LINC01578",1362.49434951761,-0.348691789963301,0.1986213549132
38,-1.75556042357891,0.0791634948657069,0.634681574512246
"2109","USP22",494.944911190325,0.262300508026155,0.149397575767556,1.
7557213139406,0.0791360056335703,0.634681574512246
"2110","ATG4A",51.4130140457816,-0.433486198407789,0.246938784257503,-
1.75543991484043,0.0791840897043972,0.634681574512246
"2111","CLPTM1L",335.677287884798,-0.28628953547574,0.163204089489889,
-1.7541811382948,0.0793994741015002,0.63594803794797
"2112","AL135791.1",12.1548064312928,-0.798546505470897,0.455304819916
86,-1.75387228629979,0.0794523932888793,0.636070580363812
"2113","TCTN2",42.5498533083226,0.566941764661251,0.323302447913331,1.
75359564494616,0.0794998177976579,0.636149038960152
"2114","MDM2",245.168421512982,0.353448883905796,0.201586468112578,1.7
5333635841276,0.079544288068743,0.636203795017174
"2115","MAP3K7CL",34.4921143017565,-0.705425873489514,0.40245447400234
1,-1.75280912266716,0.0796347767196214,0.636626385236576

"2116","DMTF1",176.304809474935,0.447828483571963,0.255645743216976,1.75175411855723,0.0798160966869655,0.637774368044996
"2117","OTUD7A",19.0194000264577,0.718032054530907,0.409958805296124,1.75147367309809,0.0798643523070103,0.637858511481781
"2118","ZNF385B",34.8301785325907,0.985352741181667,0.563167274255353,1.74966264239084,0.0801765435335817,0.640049574157601
"2119","SPAG17",58.641837405794,-1.77917787077105,1.01763403474222,-1.74834745107728,0.080403881304381,0.640690464657157
"2120","ARL3",463.99269571958,-0.365046607399736,0.208801900348064,-1.74829159500569,0.0804135479010697,0.640690464657157
"2121","SIGIRR",435.073536061778,-0.508113634730997,0.29066582179288,-1.748102448361,0.0804462891215486,0.640690464657157
"2122","GANAB",554.25489400501,0.225797903053563,0.129158766402327,1.7482197247859,0.0804259873366048,0.640690464657157
"2123","GLI1",36.8624671551559,-0.713669518201631,0.408247138048545,-1.74813109924794,0.0804413289668944,0.640690464657157
"2124","CA2",9.06399650416848,-1.06136331170894,0.607426447503387,-1.74731165570959,0.0805832923553136,0.641479428975349
"2125","ITGAV",576.623977996471,0.23235447844315,0.133017631262879,1.74679458833509,0.0806729756529134,0.641589215587704
"2126","AC004825.2",19.8830044339691,-0.720399498395854,0.412404370336255,-1.74682799265312,0.0806671793575732,0.641589215587704
"2127","LRAT",5.42605258140194,1.76741731845331,1.01204810625117,1.74637678538837,0.080745501120958,0.641864096357855
"2128","ZRANB2-AS2",14.6506304893791,1.03748389595826,0.595514855607186,1.7421629136362,0.081479941385828,0.64220332817928
"2129","DPYD-AS1",2.18859895720103,-1.82571070686365,1.04711675311566,-1.74355982886466,0.0812358732065323,0.64220332817928
"2130","PIP5K1A",120.004310446591,0.324300804586109,0.185813056540168,1.74530687253402,0.080931466287359,0.64220332817928
"2131","UAP1",846.495188417737,-0.399692991600338,0.229143537478122,-1.74429091912967,0.0811083743021524,0.64220332817928
"2132","SOS1",315.884749264341,0.377749700566748,0.216869891397865,1.74182639246006,0.0815388270135304,0.64220332817928
"2133","FBLN7",241.225575658846,0.309911218641781,0.17770718609883,1.74394308663143,0.0811690144776111,0.64220332817928
"2134","WWC2",206.629428658103,0.388396848725975,0.222943977934281,1.74212756193157,0.0814861257255498,0.64220332817928
"2135","SSBP2",608.637156418668,-0.307241008007209,0.176228843854501,-1.74342066421814,0.0812601613247504,0.64220332817928
"2136","ANKHD1",547.599069202953,0.356509913369833,0.204727100748288,1.741390915354,0.0816150795391117,0.64220332817928
"2137","ICA1",2.23575195782221,-2.04845200161533,1.17562941667139,-1.74243003157849,0.0814332247445046,0.64220332817928
"2138","AC007032.1",54.918333464913,-0.585920341025747,0.336124124550351,-1.74316658112404,0.0813045211259558,0.64220332817928
"2139","DGAT1",132.505456057627,-0.301003721505944,0.17285740316502,-1.74134122111385,0.0816237847325097,0.64220332817928

"2140","CDNF",21.9523510367225,-0.649819242187896,0.373077450671548,-1
.74178107258481,0.0815467598781569,0.64220332817928
"2141","ABCC2",184.958659225174,1.37825261263062,0.790043596595894,1.7
445272875689,0.0810671874590569,0.64220332817928
"2142","TMEM86A",25.8580891852697,0.603044141305678,0.345878102426548,
1.74351639226349,0.0812434534741624,0.64220332817928
"2143","NDUFA4L2",10.9395638389003,-1.00552305761186,0.576856984621408
,-1.74310632343611,0.0813150442641134,0.64220332817928
"2144","SH2B3",62.6101398659621,-0.615665130677508,0.353508195316759,-
1.74158658507434,0.0815808103986368,0.64220332817928
"2145","SPTSSA",276.376748747979,-0.327547555679012,0.187879291866908,
-1.7433936035433,0.0812648848528137,0.64220332817928
"2146","SERPINA3",615.091689692723,-0.692167493277197,0.39656593463160
2,-1.7454033058089,0.0809146906484073,0.64220332817928
"2147","HYKK",76.7358166350383,-0.47242341888715,0.270905370493256,-1.
74386878350539,0.0811819730524244,0.64220332817928
"2148","ENGASE",57.2645615091826,0.496441911187618,0.285076644395289,1
.74143312315424,0.0816076863751757,0.64220332817928
"2149","CHM",262.424895078126,0.237254497772032,0.136026917273693,1.74
417315724846,0.0811289004704993,0.64220332817928
"2150","LRCH3",300.535110543159,0.368996293780825,0.211941827510962,1.
74102629063034,0.0816789702275134,0.64233861795665
"2151","CAPN7",215.732306558962,0.381225165974908,0.2190253101465,1.74
055302430535,0.0817619579467221,0.642393673310027
"2152","AC108471.3",7.12206010918457,1.33572888296226,0.76732258647158
3,1.74076575681735,0.0817246466307274,0.642393673310027
"2153","TRPT1",147.242258797196,-0.367086580611259,0.211011154194455,-
1.73965486333,0.0819196394442368,0.643333610647076
"2154","KAZN",184.027149097971,0.428440961172073,0.246421767022707,1.7
3864900957631,0.0820965202283741,0.64422384965843
"2155","PLEKHM3",105.163141174967,0.426893499141712,0.245622736466839,
1.73800481699033,0.082209965006426,0.64422384965843
"2156","LMTK2",44.7281213040954,0.568612990732987,0.327206641103273,1.
73777949254251,0.0822496754984168,0.64422384965843
"2157","AL132800.1",5.22888431478847,1.2249120662663,0.704899437258053
,1.73771179479305,0.0822616093809173,0.64422384965843
"2158","APCDD1L-
DT",2.96325207167417,1.77812489432216,1.02317364135751,1.7378525232168
9,0.0822368030923075,0.64422384965843
"2159","APOOL",219.914878017614,0.324507437398196,0.186656241534801,1.
73852979536017,0.0821175047290836,0.64422384965843
"2160","PCBP4",116.66071162085,-0.396430584899034,0.228251019839448,-1
.73681846056102,0.0824192195095565,0.645159334938695
"2161","TSHR",4.26586613946094,1.87681678039541,1.08076614552741,1.736
56140892489,0.082464616221202,0.645215979207813
"2162","TRAM2-
AS1",89.0462241184551,-0.330391932407185,0.190395062220116,-1.73529674
853237,0.0826882573803073,0.646367570867423
"2163","PIAS1",371.595838966064,0.313849085942682,0.180858438518747,1.
73533006539891,0.0826823593638026,0.646367570867423

"2164","RAB34",1121.09821449793,-0.377853829854609,0.217792056787281,-1.73492934236652,0.0827533211573063,0.646577243127419
"2165","C1orf167",36.8401605364722,0.566076176381816,0.326331093389421,1.73466821841062,0.0827995886896697,0.646598340014948
"2166","SLF2",176.128752588753,0.359234172888799,0.207113170572973,1.73448251453535,0.0828325055874365,0.646598340014948
"2167","AC007364.1",20.1033976484845,0.702598655586944,0.405507307984322,1.73264116762628,0.0831594673404596,0.647655584427679
"2168","ASPN",2058.84999062741,0.613016427362164,0.353739172299901,1.73296167166481,0.0831024814812521,0.647655584427679
"2169","CDK1",3.0091585403183,-1.46076360808644,0.842710126145286,-1.7334117186514,0.083022516196805,0.647655584427679
"2170","CLEC7A",23.9107845757889,-0.893929757948226,0.515832792608302,-1.73298357676735,0.0830985878928862,0.647655584427679
"2171","GATD3B",350.108094795989,-0.428509171746333,0.24731186583915,-1.73266725513701,0.0831548277781905,0.647655584427679
"2172","CYP27C1",39.1178759050961,0.870459580887158,0.502486527871932,1.73230431584628,0.0832193939577204,0.647823901029989
"2173","GPR88",11.8941919757143,1.34988914595904,0.780533179340676,1.72944492519754,0.0837294954307013,0.648001729375355
"2174","AC023481.1",7.53630086563512,1.23825327952713,0.715579326724576,1.73042070009902,0.0835551380884542,0.648001729375355
"2175","AC124017.1",4.1469538312636,-1.19435376738397,0.690615029034733,-1.72940598911279,0.083736458872691,0.648001729375355
"2176","KIT",21.0192325626914,0.840654044579014,0.486147723531236,1.72921522386806,0.0837705826577577,0.648001729375355
"2177","SFRP2",2272.84998788821,0.708760515936038,0.409373467664029,1.73132987826586,0.0833929453841811,0.648001729375355
"2178","UBR2",270.122189024468,0.367333692410459,0.212481887798287,1.72877649110109,0.0838491052059889,0.648001729375355
"2179","GSAP",30.2509467061607,-0.557120124052483,0.322095725657901,-1.72967251556825,0.0836888018905414,0.648001729375355
"2180","ZNF783",69.3384181431718,0.541728288169854,0.313289952324393,1.72915947080527,0.0837805578032998,0.648001729375355
"2181","RBM12B",146.109139131729,0.357776880180319,0.206743606199241,1.73053419526564,0.0835348771908703,0.648001729375355
"2182","TMEM67",72.3650596651901,-0.448920504461449,0.259319892427749,-1.73114565280227,0.0834257896411227,0.648001729375355
"2183","NXNL2",4.2129287752782,-1.24149621777449,0.717553480366193,-1.7301793549115,0.0835982357087334,0.648001729375355
"2184","ZNF462",261.895313775098,0.400614187593545,0.231772592815107,1.72847955285692,0.0839022837719958,0.648001729375355
"2185","LINC02666",4.20634364800429,1.31877101810343,0.762044871217949,1.73056872096742,0.0835287145308935,0.648001729375355
"2186","TSPAN18",19.2789124176725,0.738709369693506,0.426583464873915,1.73168777161076,0.0833291690441716,0.648001729375355
"2187","ARG2",3.83254874318153,-1.6268193349605,0.941275776647324,-1.72831318442611,0.08393209056849,0.648001729375355
"2188","MRC2",1827.73089017312,0.447407661946223,0.258822876825636,1.72862487054277,0.0838762554680939,0.648001729375355

"2189","CHMP1B",381.964637494056,-0.329765148977376,0.190499292399392,
-1.73105708070561,0.083441584263857,0.648001729375355
"2190","LRRC25",4.20496812760586,-1.27288143502018,0.736022907615654,-
1.72940464467836,0.0837366993236078,0.648001729375355
"2191","TRIM2",230.821989721905,0.418621120247849,0.242281775607677,1.
7278275231304,0.084019151353381,0.648274740657786
"2192","LINC01068",3.09770504584095,1.85457695793153,1.07357702175892,
1.72747452706563,0.084082476121512,0.648274740657786
"2193","ZNF426",148.248986703076,0.376871295758614,0.218155884040015,1
.727532115015,0.0840721426517453,0.648274740657786
"2194","TLCD3A",63.487055844557,0.604447906515366,0.349970541209632,1.
72713938843585,0.0841426331484982,0.648442862932911
"2195","MMP14",1148.62023053815,0.436397193203048,0.252729242555143,1.
72673802521222,0.0842147232555935,0.648702752075433
"2196","AGPS",465.062949143677,-0.40012315528575,0.231757935107313,-1.
72647014265327,0.0842628662914555,0.648717170927508
"2197","NBEAL1",592.884347972213,0.341669056890842,0.197919781583971,1
.72630069696133,0.0842933300525038,0.648717170927508
"2198","AL096803.2",25.0438669537071,0.575913276879167,0.3337029442733
24,1.72582617792985,0.0843786887968354,0.649078648852089
"2199","ZDBF2",61.9299394076913,0.463517606167901,0.268673645459217,1.
72520682248405,0.0844902066188077,0.649107490252584
"2200","CHST2",10.2320249696325,-1.30348926696398,0.75546235341151,-1.
72541922317863,0.0844519494601505,0.649107490252584
"2201","CDIP1",109.93907237559,0.395429789833733,0.229212639119382,1.7
2516572974748,0.0844976097732397,0.649107490252584
"2202","STXBP3",312.504236751559,-0.300896553303868,0.174541635588539,
-1.72392422180113,0.0847215240068641,0.649211162782751
"2203","B3GNT5",23.0676150534792,-0.813439832259271,0.471870633193932,
-1.72386195503071,0.0847327668623712,0.649211162782751
"2204","BRPF3",114.636222544909,0.368688530396075,0.213938090733621,1.
72334215534875,0.0848266687189197,0.649211162782751
"2205","CDCA7L",51.6683509558082,0.476698050961029,0.276418261098973,1
.72455339624014,0.0846079882880736,0.649211162782751
"2206","PIGO",57.1495317899845,-0.472963308657663,0.274336027622677,-1
.72402915051383,0.0847025808635814,0.649211162782751
"2207","LPXN",30.727136541263,-0.612310047391903,0.355294864293212,-1.
7233855845622,0.0848188200067083,0.649211162782751
"2208","NLK",26.6499259676436,0.581136715162726,0.337247422749171,1.72
317614890996,0.0848566755234138,0.649211162782751
"2209","FBXL20",231.78918921952,0.390215946603688,0.226380757338621,1.
72371517434233,0.0847592742824896,0.649211162782751
"2210","CD300E",4.93369061777774,-1.34180747403417,0.778243878468948,-
1.7241478039942,0.0846811640686235,0.649211162782751
"2211","SCYL3",69.0043796369103,0.469787947414187,0.272809112297265,1.
72203906041923,0.0850624429620716,0.649692590313411
"2212","BAP1",135.725925941844,-0.308512479623483,0.179297772633365,-1
.72067101053364,0.0853105397105574,0.649692590313411
"2213","HCLS1",32.1483355349794,-0.698055850130033,0.405531060551825,-
1.72133756950739,0.0851895858180894,0.649692590313411

"2214","COL6A6",252.610360852086,1.31059321346966,0.761258494279075,1.72161391080544,0.0851394815607893,0.649692590313411
"2215","HEBP2",1203.03231608017,-0.496713038766977,0.288706529168672,-1.72047733107131,0.0853457108360817,0.649692590313411
"2216","TPST1",505.975578423495,0.526842448738313,0.306253057603199,1.72028469809083,0.0853807035531345,0.649692590313411
"2217","GTF2I",1011.63367392505,0.314618447921453,0.182877892219363,1.72037442089538,0.0853644035268642,0.649692590313411
"2218","PRUNE2",20.9862566050971,-1.11917341990205,0.650050682836444,-1.72167101651002,0.0851291305282387,0.649692590313411
"2219","AC080038.2",3.2767409157804,2.00049160685497,1.16165613864425,1.72210307362531,0.085050848429293,0.649692590313411
"2220","FPR1",8.26198580069301,1.34643273352565,0.782449376784134,1.72079213489757,0.0852885501483697,0.649692590313411
"2221","ZNF787",164.824030801779,-0.441155732750089,0.256346242304554,-1.72093699827272,0.0852622568976757,0.649692590313411
"2222","SPIN3",53.9405779659584,0.480802775390954,0.279476805887348,1.72036736238054,0.0853656857627136,0.649692590313411
"2223","LINC01750",11.1311086000239,1.01201763288432,0.588401654710515,1.71994355349359,0.0854427025856408,0.649871891731001
"2224","DCLK1",2176.94397833478,0.37958207815752,0.220774919850594,1.71931702393731,0.0855566617827797,0.650446059992464
"2225","GPS2",484.439944205328,-0.299840190521644,0.174426638741481,-1.71900457799935,0.0856135383357107,0.650585935361886
"2226","RAB3GAP2",267.899939084815,0.312894600661747,0.18215857495955,1.71770448210428,0.0858505314164647,0.651436938570069
"2227","EFEMP1",7740.0725744628,0.480631812758926,0.279965217835602,1.71675544724687,0.0860238644638684,0.651436938570069
"2228","LINC02656",2.70456157728502,2.09061588984626,1.21737005719482,1.71732159624795,0.0859204282297723,0.651436938570069
"2229","DNAJC4",360.811620665206,-0.449282444178586,0.261690901065767,-1.71684396495572,0.0860076855195311,0.651436938570069
"2230","AMIGO2",52.760063903481,0.686908560634619,0.399951059311264,1.71748153841,0.0858912247801403,0.651436938570069
"2231","LINC01232",17.2376818654694,0.660940200197363,0.38488838325212,1.71722563984067,0.0859379525265628,0.651436938570069
"2232","TTC9",3.82472407774153,1.45890622530485,0.849831113783507,1.71670135588435,0.0860337522963665,0.651436938570069
"2233","SPECC1",175.442430948663,-0.576425795074629,0.335757909534217,-1.71678992126881,0.0860175631354198,0.651436938570069
"2234","ACKR4",868.645177034663,0.553844695402498,0.322918139865679,1.71512413527736,0.0863224701831743,0.652798191409548
"2235","SMCHD1",409.603755325796,0.339347724803119,0.197827085010939,1.71537544914214,0.0862764136168907,0.652798191409548
"2236","ZBED1",267.549662559017,0.350434299580306,0.204324554677486,1.71508656966582,0.0863293562805624,0.652798191409548
"2237","S100PBP",153.058241306997,0.314017382942349,0.183395179679006,1.71224447388404,0.086851624637344,0.652951209145492
"2238","STX6",98.5191664884573,0.312046709437718,0.182185100394001,1.71280038138615,0.0867492699552518,0.652951209145492

"2239","GALNT3",5.16356139684053,-1.24449049384641,0.726311458294868,-1.71343915841291,0.0866317774601789,0.652951209145492
"2240","IFT57",662.30807952542,-0.358263433573294,0.209167604546338,-1.71280554821254,0.0867483190869407,0.652951209145492
"2241","ANKRD17",485.12604244033,0.366859346257913,0.214222682369909,1.71251401672041,0.0868019837597872,0.652951209145492
"2242","AL512329.2",7.32407751913587,1.0730260089812,0.626000077377092,1.71409884400833,0.086510574082891,0.652951209145492
"2243","C6orf47",132.727782269291,-0.317608262998427,0.185341505768309,-1.71363808490615,0.0865952144630214,0.652951209145492
"2244","FAM120B",90.3306232730175,0.370851876827863,0.21653199539888,1.71268858509665,0.0867698462605861,0.652951209145492
"2245","ZP3",3.74572771879399,-1.35212439709763,0.788933936156322,-1.71386263808751,0.086553956214053,0.652951209145492
"2246","EXOC6",27.1197480825876,0.603387497921519,0.352151908896333,1.71342958160464,0.0866335380068121,0.652951209145492
"2247","CIB2",107.133990810586,-0.467419188983406,0.272610548426441,-1.71460419151584,0.0864178197486216,0.652951209145492
"2248","SULF2",153.93569327361,0.44118563010643,0.257645840544479,1.71237241468397,0.0868280593159035,0.652951209145492
"2249","RHOXF1",2.6152913428128,1.86353925519626,1.08756215296524,1.71350138483149,0.0866203388086312,0.652951209145492
"2250","AC092645.2",7.09218657300791,1.2398904590655,0.724243175108657,1.7119808673095,0.0869001944159559,0.653025994304437
"2251","TJP2",80.0538946918666,-0.725558453242272,0.423870838115844,-1.71174421073048,0.0869438173068115,0.653063555319222
"2252","TNFAIP2",1094.46024567391,0.476462765209738,0.278446326708133,1.71114760551022,0.0870538679607123,0.653599822149078
"2253","INPP5B",65.2285689851905,0.443631457757313,0.259359455729372,1.71048885227543,0.0871755131090608,0.653642383879379
"2254","N4BP2L1",273.671669650802,0.327985112331537,0.191708747575698,1.71085105129086,0.0871086125955394,0.653642383879379
"2255","PIP4K2B",222.915612510568,0.318528195256932,0.186205215047092,1.71062982944046,0.0871494687826905,0.653642383879379
"2256","PUM2",318.397863098551,0.327418205357429,0.191499129825403,1.70976341070557,0.0873096315809716,0.654120516417428
"2257","HECW1",6.62271877479349,1.31124442542751,0.766932742981912,1.70972544519257,0.0873166551664381,0.654120516417428
"2258","SRGAP2",148.552379951263,0.410255884669847,0.2400456706881,1.70907429196217,0.0874371889068849,0.654733387970598
"2259","OTUD4",159.107928193128,0.433035156915042,0.253462501042699,1.70847819749909,0.0875476484908522,0.655270314600854
"2260","SET",1405.73778705133,-0.250856195475315,0.146910724385351,-1.70754175043963,0.0877214046047813,0.655990052657073
"2261","FTO",217.489276790947,0.281133243525775,0.164628902135903,1.70767854172833,0.087696005885076,0.655990052657073
"2262","JADE3",22.5558978878838,0.607609898463868,0.355920852806891,1.70714891715978,0.0877943769044691,0.656245501636058
"2263","ZNF676",36.1976706994094,0.65732187937058,0.385102601413362,1.70687467952215,0.0878453480161502,0.65633634302124

"2264","STAR",4.06800609615768,1.34427924185206,0.787683206110212,1.70662422586164,0.0878919193743215,0.656394245928016
"2265","FCGR3B",7.38814221370895,1.61616723203547,0.947181559356295,1.7062908542411,0.087953940051577,0.65656742533866
"2266","SENCR",9.69690429905525,-0.817346010604123,0.479082353705769,-1.70606578238968,0.0879958325223363,0.656590263145482
"2267","SLC25A36",830.039500002205,0.188377628442173,0.110464355164418,1.70532501784658,0.0881338241633857,0.656868022764521
"2268","CBLL1",105.950784442753,0.346879331986899,0.203444469855962,1.70503200324142,0.0881884558596795,0.656868022764521
"2269","IFIT5",154.315314429115,0.401025598131016,0.235176159739374,1.70521365165346,0.0881545848411397,0.656868022764521
"2270","RALGAPB",118.604319548543,0.418373160106569,0.245310364436779,1.70548505387097,0.0881039974497242,0.656868022764521
"2271","PTPN12",639.152947914842,0.258432716194422,0.151614731110744,1.70453566286811,0.0882810593317285,0.657268230374665
"2272","Z69720.1",2.30273804590155,2.09692765322288,1.23064865215926,1.70392065155532,0.0883959122724713,0.65754425195906
"2273","AC092140.2",3.63306003222493,1.5103685866618,0.88640666601559,1.70392286584545,0.0883954985393552,0.65754425195906
"2274","HOXC12",6.04584429596026,1.83589561941688,1.07804728925785,1.70298245513956,0.0885713518060193,0.658270073114802
"2275","LINC01415",8.58210653814266,0.854020872889271,0.501477235972377,1.70301024977396,0.0885661502732058,0.658270073114802
"2276","AC079610.1",7.35893825458288,-0.932268079923173,0.547582781591272,-1.70251532967126,0.0886588072681572,0.658630541867312
"2277","VAMP7",167.683555984948,0.262612365756728,0.154313830682298,1.70180705511352,0.0887915435261101,0.659326929266346
"2278","MAVS",302.219702243112,0.460060763655836,0.270408338764057,1.70135568214581,0.0888762179050093,0.659665975565363
"2279","MOB3A",182.042842216303,0.347148169786668,0.20421173962342,1.69994227769095,0.0891417835096277,0.661346764186391
"2280","RICTOR",335.565943558617,0.359755781852629,0.21169145891486,1.69943456243702,0.0892373344078952,0.661765285161707
"2281","SMIM4",218.28791457207,-0.389304413676187,0.229147279839096,-1.69892662024856,0.0893330105497053,0.662162255040801
"2282","LINC02447",24.2157936726943,0.628565381410105,0.370019754636802,1.69873465817273,0.0893691900877163,0.662162255040801
"2283","MEF2C",127.146226268343,-0.467439329010424,0.275261560363677,-1.69816420568437,0.0894767742365961,0.662668987644488
"2284","PHKG1",94.396033507672,-0.614354144171694,0.361884035779143,-1.69765472756757,0.0895729471055787,0.66309080107755
"2285","IDH3A",131.643652821035,-0.31580605770514,0.186129158848063,-1.69670383544221,0.089752667195525,0.66383993741992
"2286","PTPRM",168.717143624412,0.359427107054849,0.211826162150915,1.69680224295796,0.0897340545653697,0.66383993741992
"2287","DGAT2",5.2685632326673,-1.24199046143321,0.732289785417217,-1.69603685066507,0.0898789014993818,0.664482932466789
"2288","IGSF8",209.908395442098,-0.448774901799642,0.264668515464932,-1.69561121016339,0.0899595333860623,0.664788369970079

"2289","TFRC",112.194702924456,-0.73851732903611,0.43598620541221,-1.6
9390067820578,0.0902841580737353,0.665163330752447
"2290","TLR3",225.524853803298,0.305831013956146,0.180547299545844,1.6
9391076313767,0.0902822413970962,0.665163330752447
"2291","AC008592.5",2.22393861571942,2.38482694088478,1.40765585474224
,1.69418322869937,0.0902304707532246,0.665163330752447
"2292","RNF130",842.592428459272,-0.310956833533149,0.183563807256612,
-1.69399860560993,0.0902655480120822,0.665163330752447
"2293","ANGPTL8",3.93442823362208,-1.47191238229598,0.868952496691851,
-1.69389280530251,0.0902856543693438,0.665163330752447
"2294","MPV17L2",23.3375338194971,-0.612815501348666,0.361526336274115
,-1.69507844895709,0.090060539891955,0.665163330752447
"2295","MAOB",126.173450453282,-0.512972070690442,0.302675497410241,-1
.69479219520426,0.0901148485925386,0.665163330752447
"2296","GLCE",197.996264908431,0.355633228542843,0.210022706340087,1.6
9330847478449,0.0903967658345191,0.665402053430583
"2297","POLR2I",756.245906015054,-0.411450038549849,0.242966389789549,
-1.69344426159617,0.0903709359281748,0.665402053430583
"2298","POLR1A",101.784137430626,0.509156106720935,0.300875777962671,1
.69224691388785,0.0905989049643201,0.66598648403864
"2299","TDO2",2.54710222805915,-2.11899006855658,1.25276372379944,-1.6
9145228928725,0.0907504527453842,0.66598648403864
"2300","AC009549.1",5.98521217775567,-1.35203389759686,0.7989961454760
49,-1.6921657322781,0.0906143782691254,0.66598648403864
"2301","STARD13",59.6828003338461,0.646323947991616,0.382103718742263,
1.69148824334676,0.0907435913226523,0.66598648403864
"2302","INTS6-
AS1",34.5882100566551,-0.558171574240826,0.32999692221125,-1.691444788
33505,0.0907518842692824,0.66598648403864
"2303","SPHK1",37.9792393022146,-1.08326713274908,0.640068793795959,-1
.69242297585657,0.0905653546613081,0.66598648403864
"2304","AC017100.1",3.26480417315019,-1.47413241769715,0.8714551454157
98,-1.69157578040783,0.0907268876162826,0.66598648403864
"2305","GJA9",6.85526043780364,1.73140088908065,1.02389988245236,1.690
98651025697,0.0908393790930493,0.666186953587823
"2306","AC092164.1",26.6136808341903,0.590442384302936,0.3492880852352
45,1.69041661958001,0.0909482777099615,0.666186953587823
"2307","TTC30B",23.8528418160844,-0.659560449169998,0.390092731414708,
-1.69077861763289,0.0908790924843908,0.666186953587823
"2308","FICD",44.0838553506671,-0.482051993447064,0.285187278880889,-1
.69029977542721,0.0909706180552586,0.666186953587823
"2309","SIK1B",2.93342501756034,1.77940771697939,1.05273543860688,1.69
027055775204,0.090976205100206,0.666186953587823
"2310","EVA1B",1125.04574426926,-0.352538204076825,0.208742718448124,-
1.68886467847949,0.0912453655406783,0.666898846637475
"2311","DDX59-
AS1",2.02763462704827,2.85408536157451,1.68963891312904,1.689168815536
47,0.0911870832483366,0.666898846637475
"2312","CDS1",17.2396565057364,1.05310505618156,0.623516359673427,1.68
897742592212,0.0912237560676549,0.666898846637475

"2313","FZD6",55.5648255222375,-0.659000459058052,0.390052482778132,-1
.68951740638683,0.0911203190175817,0.666898846637475
"2314","ATF5",173.600472552361,-0.383859747727493,0.227306377890343,-1
.68873285162581,0.0912706370427678,0.666898846637475
"2315","GULP1",443.504886451059,0.516915163826383,0.306406069298096,1.
68702651683929,0.0915982532773528,0.668137733569232
"2316","C1QTNF3",41.0815261147554,0.547378175535539,0.324384556005874,
1.68743599348678,0.0915195477110655,0.668137733569232
"2317","SLC25A45",78.2712376542954,0.538737971894543,0.319305818637411
,1.68721626869665,0.0915617742844543,0.668137733569232
"2318","ZKSCAN2",22.1675231054414,0.579901157448619,0.343694800970391,
1.68725612319802,0.091554113908875 3,0.668137733569232
"2319","AREL1",46.5935363526096,0.480495138834872,0.284922247436658,1.
68640793464783,0.0917172542002284,0.668544989530718
"2320","SLC35B1",209.969184634659,-0.524203602558473,0.310855569654635
,-1.68632527041697,0.0917331662947282,0.668544989530718
"2321","DDI2",239.690805770826,0.523444205789419,0.310613215716327,1.6
8519618388505,0.0919507270914321,0.668938343465763
"2322","FBXO42",160.023894781359,0.396891698973567,0.2356560546683,1.6
8419903122038,0.0921432104288954,0.668938343465763
"2323","EFCAB14",565.869205309664,0.311541481679817,0.184913850982087,
1.68479256705328,0.0920285994944813,0.668938343465763
"2324","LINC01807",7.617148590224,1.01471343675959,0.602322303656331,1
.68466854141028,0.0920525392017156,0.668938343465763
"2325","AL023806.1",11.9109888952244,-0.805076503229062,0.477896657318
284,-1.68462467962581,0.0920610066989075,0.668938343465763
"2326","UBAC2-
AS1",13.3520236080054,-0.682094764657369,0.404958400764383,-1.68435761
147287,0.0921125775867106,0.668938343465763
"2327","PTPN21",154.126689274071,0.366833809335743,0.217690779448148,1
.68511413421219,0.0919665531763562,0.668938343465763
"2328","SLC46A1",38.1421536561269,0.521253299216609,0.309418354804966,
1.68462307139202,0.0920613171795876,0.668938343465763
"2329","PCDH19",7.57747111568186,1.03392442805733,0.613888409178361,1.
68422210388554,0.0921387529888516,0.668938343465763
"2330","AC002074.1",27.3280325358402,-0.782973970600403,0.465102955536
943,-1.68344225999702,0.0922895081455726,0.669712877135339
"2331","UBL7-
AS1",79.0063590668609,-0.411101222947663,0.244293537969539,-1.68281660
810415,0.0924105989199868,0.670303906709196
"2332","RBM26",416.835751656095,0.37234567316117,0.221295573676503,1.6
8257171607724,0.0924580308787642,0.670360371397146
"2333","TOMM22",538.014904385758,-0.339800864439918,0.202110417437073,
-1.68126348334179,0.0927117474595005,0.671911798562038
"2334","ACVR2B",71.3785810100542,0.46080081896376,0.2743462021176,1.67
963257886192,0.0930288249179359,0.673632279105979
"2335","FAM107A",48.5809449605999,-0.728468511660674,0.433680101141495
,-1.67973699909971,0.0930084976820064,0.673632279105979
"2336","GNG12",1072.25871603249,0.275760709572639,0.164233457345944,1.
67907754016145,0.0931369328181744,0.67399046886865

"2337","PTCD3",250.355596838404,0.289424330388823,0.172424133042116,1.
67856045022496,0.093237739926885,0.67399046886865
"2338","TRIP12",397.078388617806,0.311871623223999,0.185795753314207,1
.67857239824303,0.0932354096631787,0.67399046886865
"2339","SHKBP1",83.1339933738631,-0.418040647280908,0.249025189104048,
-1.67870828161982,0.0932089111414515,0.67399046886865
"2340","POFUT1",148.744269016114,0.369050197065431,0.219900629505372,1
.67825893857395,0.0932965602697409,0.67412745343623
"2341","TMEM225B",47.745999482095,-0.460669401223881,0.274607965827342
,-1.67755294292346,0.0934344057829748,0.674817678581813
"2342","CPED1",530.16767706363,0.281750685812223,0.168033940985975,1.6
7674866255585,0.0935916404231342,0.674817678581813
"2343","ZNF566",112.144616721397,0.366628893178241,0.218646698654074,1
.6768096451266,0.0935797110632319,0.674817678581813
"2344","ISM1",572.187249763612,0.457471714325795,0.272755836212251,1.6
7722062588535,0.0934993471547869,0.674817678581813
"2345","C21orf62-
AS1",46.5458454224916,0.486154391458936,0.289905192954296,1.6769426808
2869,0.0935536909624935,0.674817678581813
"2346","FAM228A",7.68420826506668,1.11331673996151,0.664139975300101,1
.67632845690163,0.0936738738715139,0.675122702224875
"2347","AL590617.2",9.02895794823247,-1.17398733468005,0.7005761567961
24,-1.67574548932543,0.0937880554842248,0.675657623403184
"2348","KIAA0319L",162.467409953003,0.393283819596145,0.23478672971235
7,1.67506834853046,0.0939208222210704,0.675766084299229
"2349","ATP6V1E2",77.7939729137922,-0.391856769824593,0.23389107465178
4,-1.67538145868964,0.0938594121103825,0.675766084299229
"2350","AC084036.1",10.9685444782974,-0.87513928244512,0.5226495900942
87,-1.67442833407225,0.0940464481209324,0.675766084299229
"2351","CD2AP",183.425761222922,0.287309122422854,0.171584989979592,1.
6744420503042,0.0940437544040334,0.675766084299229
"2352","AC012673.1",5.31705700516226,-1.16357929797342,0.6949883640662
22,-1.67424284798896,0.0940828816205574,0.675766084299229
"2353","AL359397.2",3.39999629472831,1.5010733147073,0.89644756975515,
1.67446860848459,0.0940385388457551,0.675766084299229
"2354","RAB8A",401.834123324348,-0.303410880084599,0.181162714925399,-
1.67479759954768,0.0939739500325567,0.675766084299229
"2355","TNKS2",275.908036720392,0.289992418023519,0.17322947898133,1.6
7403619596855,0.0941234858972745,0.675770657983489
"2356","ASCC2",220.335763210462,-0.287393035406004,0.171720086501246,-
1.67361338595598,0.0942066060247681,0.676080345783862
"2357","NBPF12",135.783740264842,0.672365968896073,0.402050790088839,1
.6723408720264,0.0944571243886249,0.676506356699381
"2358","ATP1B1",160.429811301929,0.598901685583686,0.358338503276336,1
.67132942764411,0.0946566269368849,0.676506356699381
"2359","LTBP1",111.897704884289,0.47365632514921,0.28340905410073,1.67
128155680186,0.0946660775934904,0.676506356699381
"2360","ATP6V1A",184.377198268054,0.2856181812598,0.170886414258239,1.
67139197401721,0.0946442801778445,0.676506356699381
"2361","DROSHA",112.502859309867,0.31052337130807,0.185669865748577,1.

6724489461772,0.0944358272259695,0.676506356699381
"2362","EMB",25.9413373321245,-0.724840013647333,0.433395349685418,-1.67246836906178,0.0944320001477101,0.676506356699381
"2363","LATS2",438.578195043011,0.34517615741755,0.206417893214548,1.67222013577466,0.0944809213056485,0.676506356699381
"2364","F10",1372.85518611257,0.492906160926983,0.29484616790215,1.67174009563713,0.0945755839662672,0.676506356699381
"2365","AC093249.6",9.75627403778631,-1.04056849417006,0.622362566490542,-1.67196510554571,0.0945312031401717,0.676506356699381
"2366","NFIC",3023.34706606326,-0.284030766610012,0.16981155529284,-1.67262331541691,0.0944014740245823,0.676506356699381
"2367","DUSP2",113.134450000863,-0.755170073274267,0.451971547527452,-1.67083542626851,0.0947541890008074,0.676849948299811
"2368","UBR3",191.700067338963,0.37890127808222,0.226972600296988,1.66937012479232,0.0950440505675889,0.677403920234094
"2369","LINC00578",6.99868808419995,0.982569451768629,0.588708229180753,1.669026188297,0.095112189888558,0.677403920234094
"2370","CASTOR3",42.8595124284575,0.607816193995692,0.364000230606715,1.66982365088776,0.0949542595818307,0.677403920234094
"2371","C9orf16",992.987387414698,-0.461933017368529,0.276688315368305,-1.66950677607632,0.0950169886201526,0.677403920234094
"2372","DENND4A",143.225960176434,0.410711016556888,0.245983781221606,1.66966705901183,0.0949852546090206,0.677403920234094
"2373","AC100793.4",102.808225927392,-0.736865818879276,0.441255251207272,-1.66993099087935,0.0949330179121564,0.677403920234094
"2374","CHMP4B",650.561283572175,-0.261772308981249,0.156837303317648,-1.66906917833873,0.0951036707343547,0.677403920234094
"2375","UTRN",663.941990146003,0.429614139846293,0.257626402656077,1.66758583521353,0.0953979720611799,0.678581788645532
"2376","DENND11",74.0443141992003,0.518247005910694,0.310754703080715,1.66770446520349,0.0953744085873045,0.678581788645532
"2377","SYT7",13.1335153859346,1.06286566495476,0.6372986002586,1.66776714168755,0.0953619610398122,0.678581788645532
"2378","CLIC1",3312.78778367586,-0.407687358756049,0.244540567637449,-1.66715634422048,0.0954833208498011,0.67864714988368
"2379","ZMYM3",68.7378129250343,0.36520149693492,0.219059257958667,1.66713564328711,0.0954874360996732,0.67864714988368
"2380","CHP1",404.524254023627,-0.338638269062435,0.20315259103832,-1.66691582584127,0.0955311435537465,0.678672510591069
"2381","DBT",179.712399850518,0.393027501877493,0.23610999031683,1.66459496843018,0.0959935898778632,0.678779865362097
"2382","ARNT",345.804937999272,0.2859574624293,0.171749582728914,1.66496743622748,0.095919252713821,0.678779865362097
"2383","HMCN1",438.24704308647,0.525162419659321,0.315268902275574,1.66576029500138,0.0957611673117851,0.678779865362097
"2384","RBSN",169.709294589522,0.360031674493075,0.216331740113664,1.66425728514877,0.0960610245904542,0.678779865362097
"2385","FOXP2",160.414192011107,0.456834161481385,0.274470565819273,1.66441949838144,0.0960286261842959,0.678779865362097
"2386","IMPDH1",120.486002369931,-0.291710514798743,0.175283453161941,

-1.66422163379699,0.0960681463101194,0.678779865362097
"2387","AC022034.1",43.8688674303307,0.542015853623956,0.325437673547381,1.66549818192774,0.0958134060463857,0.678779865362097
"2388","C15orf56",11.6549092151989,-0.797341353000874,0.478845118812157,-1.66513413560274,0.0958859977637062,0.678779865362097
"2389","CLN6",61.7008195988133,-0.399947554701372,0.240293318414798,-1.6644139643158,0.09602973134401,0.678779865362097
"2390","FLYWCH1",149.518639091424,0.288057174806047,0.172856473261346,1.66645292114995,0.0956232377057822,0.678779865362097
"2391","EEF2K",336.312314289666,0.439668540824773,0.264006158628796,1.66537228945089,0.09583850433273,0.678779865362097
"2392","MT1F",72.5152303497511,-0.862275384909728,0.517534576767427,-1.66612130593396,0.0956892557163296,0.678779865362097
"2393","TRPV1",42.2009044182236,0.510075919006265,0.306370867294972,1.66489693850416,0.0959333191207774,0.678779865362097
"2394","VLDLR-AS1",8.95109311355625,1.28699556730482,0.773553194750679,1.66374539726338,0.0961633199245717,0.679168510143968
"2395","ASXL2",193.122740913655,0.358418664826588,0.215516890029137,1.66306531603222,0.096299361731143,0.679301528875696
"2396","FAM92A",394.236390561656,-0.361257244027665,0.217199314664736,-1.6632522796073,0.0962619569836442,0.679301528875696
"2397","CCNT1",177.082134470698,0.362182874376686,0.21778247956815,1.6630487222612,0.0963026830325907,0.679301528875696
"2398","PHF21A",217.017682566612,0.425702490493124,0.256048845253409,1.66258312968297,0.0963959103904154,0.679392268812481
"2399","IL27RA",6.6565235337255,-1.31593391438551,0.791459789176646,-1.66266679922485,0.0963791516024295,0.679392268812481
"2400","ATF7IP",402.926137653404,0.360517692202591,0.216901414219766,1.66212697828384,0.0964873173180193,0.679753150505446
"2401","ASPSCR1",134.378097473857,-0.366112641276097,0.220323022381854,-1.66170851015999,0.0965712339714611,0.680060984585366
"2402","SRP19",435.770622180144,-0.385997418207144,0.232367008585864,-1.66115413954951,0.0966824934459504,0.680561032133276
"2403","HIST1H2BC",15.2992310060835,0.785410692035336,0.472918367703046,1.66077434431244,0.096758775660267,0.680814556331167
"2404","FARP2",98.8860605657125,0.399870312413152,0.240837435109849,1.66033288068678,0.0968475045147126,0.681155410289002
"2405","IKBIP",533.039581876812,-0.290440271667123,0.174951553595837,-1.6601182767321,0.0968906608153008,0.681175589632061
"2406","PRXL2B",16.3742788164577,0.739527237559757,0.446098887605617,1.65776525812266,0.0973648562068461,0.681741540510423
"2407","ACTR1B",174.555248253241,-0.307153819413751,0.185376434895217,-1.65691944387305,0.0975357627092855,0.681741540510423
"2408","ARL10",64.5405140389437,0.485851768707087,0.293197377295183,1.65708088247305,0.0975031236808519,0.681741540510423
"2409","SDHAF3",220.949968657661,-0.37107688264285,0.223938117086735,-1.65705100797613,0.0975091629321363,0.681741540510423
"2410","KLF10",564.341945735799,-0.452659318027317,0.27320211246155,-1.65686609795531,0.0975465498988283,0.681741540510423

"2411","SFMBT2",52.7832712329406,0.653474649309694,0.393802103861122,1.65939857330002,0.0970355035981212,0.681741540510423
"2412","ZSWIM8-AS1",12.9285479548119,1.17158320344787,0.706935735978172,1.65726974012252,0.0974649522559072,0.681741540510423
"2413","ACER3",162.592157034554,0.397612692074801,0.24005407751216,1.65634633743999,0.0976517016698449,0.681741540510423
"2414","AC020656.1",6.58886266071694,-1.37503923754212,0.830019710916094,-1.65663443826471,0.0975934053506613,0.681741540510423
"2415","BBS10",83.9725401734466,0.399507781615017,0.241201097474073,1.65632654991531,0.0976557066359315,0.681741540510423
"2416","GSTZ1",181.619827603009,-0.49878568596486,0.3008043163239,-1.65817330037172,0.0972824922178466,0.681741540510423
"2417","STARD9",202.176770208759,0.562064420014257,0.33875596280773,1.65920155428606,0.0970751845210325,0.681741540510423
"2418","ARRDC4",88.2806963485116,0.468748467638683,0.282799269165584,1.65753068960098,0.0974122294698202,0.681741540510423
"2419","PITPNM3",2.99510723496704,1.73662939357999,1.0467634452182,1.6590466561602,0.0971063911300283,0.681741540510423
"2420","DHRS11",40.2271074961425,-0.532436882311782,0.32104080431306,-1.65847105775558,0.0972224245673209,0.681741540510423
"2421","TM9SF4",203.117557107307,0.246416570798317,0.1486406225254,1.65780098745354,0.0973576419574637,0.681741540510423
"2422","MX2",591.149783921396,0.624777125899454,0.377207510748611,1.65632207232436,0.0976566129120088,0.681741540510423
"2423","KLHL6",3.09180510595819,-1.724620332992,1.04136676111644,-1.65611232986067,0.0976990728653205,0.681756468843103
"2424","HNRNPA0",1707.16736702257,-0.2984207895187,0.180416664263084,-1.65406444431066,0.0981144194300941,0.68257363367
"2425","RAB24",96.5593406901475,-0.414352844652297,0.250320821596455,-1.65528717111787,0.0978662599826497,0.68257363367
"2426","DENND1A",83.4416907915284,0.349414924990994,0.211268402641925,1.65389107231152,0.0981496469293696,0.68257363367
"2427","GABPB1-AS1",594.562851488839,0.642920412864278,0.38868198709736,1.65410395697919,0.098106392254624,0.68257363367
"2428","MINK1",124.8974370451,0.308351571789999,0.186317328043214,1.65498064527031,0.0979284240172287,0.68257363367
"2429","FAM174C",331.013881010148,-0.418829103400595,0.25317171183199,-1.65432820424479,0.0980608453558383,0.68257363367
"2430","AC008760.2",68.7965902700782,0.562021601430518,0.339728462381211,1.65432592103476,0.0980613090138668,0.68257363367
"2431","ZNF616",48.2836909001733,0.426196055750944,0.257715833743119,1.65374416294406,0.0981795053871208,0.68257363367
"2432","CDKL5",102.897520114702,0.294485086336791,0.178053127556136,1.65391695376958,0.0981443874280679,0.68257363367
"2433","TCP11L1",55.0042305315342,0.415245945683381,0.251158197754804,1.65332427687178,0.0982648847457972,0.682605863304001
"2434","LINC01257",3.10243022974322,1.65701436993291,1.00218300780698,1.65340497396664,0.0982484712496721,0.682605863304001

"2435","SMG7-
AS1",6.07361569469183,-0.971471256185765,0.588034198737051,-1.65206591
431628,0.0985211145764612,0.683543293089375
"2436","FZD1",199.953392763484,0.333876757642488,0.202070483638016,1.6
5227870806004,0.0984777477259944,0.683543293089375
"2437","PNKP",158.139914767103,-0.318408022767947,0.192718789772917,-1
.65218982094653,0.0984958608506145,0.683543293089375
"2438","TGFBI",146.377141226684,0.462504266492154,0.280025352075797,1.
65165140607328,0.0986056341379167,0.683674227236781
"2439","SUSD2",28.2007823442531,0.751360990975144,0.454935549622296,1.
65157678180778,0.0986208564129708,0.683674227236781
"2440","TRAFD1",113.971726419224,0.322992681491792,0.195634506003864,1
.65100057290206,0.0987384579142943,0.684169348696469
"2441","USPL1",80.3314605273901,0.316654375778961,0.191815205243375,1.
65083041971147,0.0987732067759688,0.684169348696469
"2442","LINC02649",5.00050788777592,1.19121887375791,0.72183076460632,
1.65027445790232,0.0988868139432135,0.684675778112962
"2443","ZNF844",95.2197456567093,0.699708765630188,0.424140736286067,1
.64970894273702,0.0990024802612815,0.684915685866509
"2444","SYNGR1",134.16470683204,0.477800113656118,0.289623183217501,1.
6497302058078,0.0989981293160664,0.684915685866509
"2445","FAM3C",979.274444353679,-0.305934197940609,0.185471682530099,-
1.64949276227632,0.0990467247444488,0.684941522281857
"2446","OSGIN2",223.84041562941,-0.43359084537587,0.262953421287132,-1
.64892642679256,0.0991627083856559,0.685463235234943
"2447","ZKSCAN1",595.77960941171,0.365424345929616,0.22166814274746,1.
64851990638065,0.0992460292308835,0.6854787018937
"2448","ANXA2",11769.7649485182,-0.376602354344574,0.228434072453839,-
1.64862601405784,0.0992242759052907,0.6854787018937
"2449","C11orf54",224.753378121255,0.321410174922354,0.195021112507997
,1.64807887099493,0.0993364874734258,0.68582332797088
"2450","LINC00623",262.239122210314,-0.344594259121299,0.2091348366866
81,-1.64771333451996,0.0994115104141497,0.686061150237732
"2451","SLC26A2",193.489853484895,0.349919106578172,0.212449961969651,
1.64706598831097,0.0995444830789527,0.686698539330449
"2452","LINC00857",7.78876117156852,-0.847935808012813,0.5149440992219
87,-1.64665603372081,0.0996287660181401,0.686719598791159
"2453","BSG",1673.62107279165,-0.365055111528926,0.221691309867529,-1.
64668209929863,0.0996234054780859,0.686719598791159
"2454","SMAD2",326.190168532577,0.291954257051402,0.177385721104867,1.
64587236916778,0.0997900387378854,0.687270865572369
"2455","NAT14",77.3082245000166,-0.510570652582647,0.31019635365909,-1
.64595955613254,0.099772085978975,0.687270865572369
"2456","NCSTN",334.590394985384,0.388954757465331,0.236765952751279,1.
64278162863191,0.10042812257616,0.688281037055264
"2457","PANTR1",4.49189735671016,-1.51558034623166,0.923533308568841,-
1.64106733581736,0.100783438305944,0.688281037055264
"2458","IL1A",2.81256675700994,-2.03016341263953,1.23672653469783,-1.6
4156210421696,0.100680786633803,0.688281037055264
"2459","INHBB",525.390151114965,0.714157729421657,0.434569620910104,1.

64336781739603,0.100306854541312,0.688281037055264
"2460","MFSD1",224.42937834992,-0.252357669728179,0.153563865291434,-1.64334017803767,0.100312569821089,0.688281037055264
"2461","ZNF721",255.13393996216,0.396499277161071,0.241499431759508,1.64182281619576,0.100626729157653,0.688281037055264
"2462","LDB2",454.294491129465,0.369429690720247,0.224934981925544,1.64238433505435,0.100510379266665,0.688281037055264
"2463","ZNF608",12.7547625388535,0.841749790373065,0.511808522600861,1.64465762722266,0.10004043563252,0.688281037055264
"2464","ZSCAN31",25.6419118822599,0.545972467542812,0.332194720067947,1.64353144273678,0.100273025331914,0.688281037055264
"2465","FBXO30",90.9604892699514,0.400810887428713,0.244119491708317,1.64186351783665,0.100618291940244,0.688281037055264
"2466","FAM225B",21.6318186229956,0.738607131145944,0.450186014444079,1.64067098365556,0.100865731299598,0.688281037055264
"2467","ZNF618",208.631894992386,0.390384125110384,0.237340167375628,1.64482956857674,0.10000496264996,0.688281037055264
"2468","USP6NL",38.1426770299548,-0.433605376633017,0.2638392306444,-1.64344542535991,0.100290808117081,0.688281037055264
"2469","AC007406.5",27.8989130762527,0.572185839889077,0.348530595575566,1.64170906988572,0.100650311106984,0.688281037055264
"2470","ATG101",269.380950424431,-0.422459736000758,0.257153464147275,-1.64283120743344,0.100417861419813,0.688281037055264
"2471","GLIPR1",225.629809553169,0.292227580304284,0.178048602142614,1.64127983476228,0.100739340046852,0.688281037055264
"2472","OIP5-AS1",569.367726268896,0.27951145642471,0.170228698772451,1.64197610884837,0.100594955404289,0.688281037055264
"2473","SGSM2",96.9743442217616,0.387917911321184,0.236081542396103,1.64315222352422,0.100351442030936,0.688281037055264
"2474","STAT5B",218.659730018786,0.324531259867462,0.197432667661912,1.64375664732037,0.10022647961323,0.688281037055264
"2475","AC087289.2",2.44416172191736,-1.59869836096789,0.972623329292476,-1.64369732127529,0.100238739563406,0.688281037055264
"2476","MFSD12",111.796983990092,-0.407365273173838,0.247730172056992,-1.64439103154589,0.10009545645526,0.688281037055264
"2477","RELB",79.0405281571687,-0.455864128300551,0.277868435668544,-1.64057542989276,0.100885578747804,0.688281037055264
"2478","AR",769.352208996195,0.344269703873877,0.209842510268695,1.64060991947272,0.100878414566916,0.688281037055264
"2479","G6PD",199.474735925734,-0.371697788753406,0.226584186979072,-1.64044011062316,0.100913691202981,0.688281037055264
"2480","ADD1",854.475481968625,0.219203551029259,0.133642615410856,1.64022194833111,0.100959027363173,0.688312594619569
"2481","JTB",1354.81978782354,-0.329543098344809,0.20099904769069,-1.63952567005158,0.101103829036177,0.688490353111623
"2482","ZBTB49",18.0058786381781,0.677027041096169,0.412994237137915,1.63931353083284,0.101147979484816,0.688490353111623
"2483","MSMO1",97.2465206638498,-0.454907013345999,0.27745483825743,-1.63957138467315,0.101094316912569,0.688490353111623

"2484","GRAMD1B",4.07581780672076,-1.32211157342718,0.806412241482169,-1.63949839228279,0.101109505229609,0.688490353111623
"2485","AC015908.2",20.2749785147232,-0.821371525300572,0.501157183363396,-1.63894991944071,0.101223690067153,0.688728431249665
"2486","KCNAB2",32.2849770912914,-0.469247646387772,0.286427873563223,-1.63827507620062,0.101364324471674,0.688853697012486
"2487","COL8A2",111.871757694575,-0.420709816127646,0.256745143070078,-1.63862813955088,0.101290728203247,0.688853697012486
"2488","DHRSX-IT1",2.68439249135147,1.6307898094677,0.995320933656571,1.63845625498558,0.101326552326588,0.688853697012486
"2489","SMIM10L2A",6.42020870003037,1.12590766825882,0.687369383696229,1.63799507944974,0.101422720237567,0.688973625462751
"2490","NPY1R",183.683512699501,1.16458209512137,0.711481213950685,1.6368416653684,0.101663557360618,0.689337578133187
"2491","MTRF1L",156.423732177708,-0.310979428481267,0.18999995609827,-1.63673421229858,0.101686017125079,0.689337578133187
"2492","PTBP3",286.467114847588,0.373969559948182,0.228399938865602,1.6373452716563,0.101558346562285,0.689337578133187
"2493","CCND2-AS1",25.8510217293774,-0.640596235378965,0.391426764027343,-1.63656728218568,0.101720916574539,0.689337578133187
"2494","SCNN1A",8.46969848838861,-0.877768376441659,0.536216484989368,-1.63696641377794,0.101637487491268,0.689337578133187
"2495","KLF16",29.2681614417313,-0.636273963370907,0.388774601673744,-1.63661401910422,0.101711144502085,0.689337578133187
"2496","UGGT1",189.23542788275,0.30894891164346,0.188986699768984,1.63476536719841,0.102098243493726,0.691251622881658
"2497","SEPTIN8",222.89702943549,0.293906990378653,0.179762788322106,1.63497124806509,0.102055075076591,0.691251622881658
"2498","LGALS1",14946.2305903424,-0.478486216465153,0.292717827708019,-1.6346329849866,0.102126008632504,0.691251622881658
"2499","CTSD",2087.44725351761,-0.361302205514722,0.221067652623462,-1.63435130027873,0.102185107677134,0.691374870190068
"2500","ELOVL4",43.9574406337253,-0.432681454043748,0.264870747916194,-1.63355696107541,0.102351911134766,0.692083480882596
"2501","ZNF439",8.02417402842775,1.14011012571699,0.697971295810472,1.63346276925775,0.102371704854943,0.692083480882596
"2502","CELF2",2012.64345521636,0.365390438860313,0.223719669917671,1.63325128717907,0.102416157360135,0.692107269642351
"2503","TCP11",2.98306240746447,-1.54432233671115,0.945948118320117,-1.63256557817745,0.102560395696798,0.692528422700262
"2504","CAPN6",40.892760024954,1.02191921714154,0.625930927493138,1.63263895783891,0.102544952626622,0.692528422700262
"2505","AMBRA1",61.7396977367634,0.423202556952738,0.259310247226776,1.63203175145883,0.102672797557415,0.693010643154
"2506","C5orf56",260.684453717551,0.28624335542049,0.175456150720653,1.63142388707833,0.102800907988338,0.693598464591707
"2507","KAT2B",125.509025459738,0.444405592206416,0.272615836577372,1.63015325076424,0.103069111528219,0.694023312512598

"2508","PMEL",4.27272275112645,-1.94570001423984,1.19324633707781,-1.6
3059374563407,0.102976069753017,0.694023312512598
"2509","ZNF43",163.585337341353,0.322062291229696,0.197479497607583,1.
6308644448229,0.10291892553795,0.694023312512598
"2510","AC010642.2",304.691065632737,-0.368283248079008,0.225874285629
008,-1.63047886151991,0.103000329251895,0.694023312512598
"2511","CYB5R3",1869.36417772505,-0.323696604818947,0.198556244345582,
-1.63025144782433,0.103048364457322,0.694023312512598
"2512","TSBP1-
AS1",15.6956338950963,-0.63533570503345,0.389877524195949,-1.629577663
76432,0.103190788328975,0.694289633532158
"2513","PPP1R15A",3428.80083303363,-0.276720470842105,0.16981068742074
7,-1.62958218381428,0.10318983236368,0.694289633532158
"2514","WDR19",114.475732954831,0.457560209887002,0.280837781041219,1.
62926871231704,0.103256146523529,0.69445303318211
"2515","STAT1",450.402340274263,0.565720464667949,0.34758386160206,1.6
2757977905093,0.103614019635182,0.694793497694344
"2516","ZBTB20-
AS5",2.13068347147795,2.26756530323672,1.39329969294065,1.627478506401
51,0.103635509887931,0.694793497694344
"2517","MCC",147.667505248069,0.431071346176359,0.264679856959995,1.62
865187826331,0.103386735248792,0.694793497694344
"2518","CHCHD7",179.584466835362,-0.325227870572639,0.199785542377717,
-1.62788491450377,0.103549290711599,0.694793497694344
"2519","CPXM2",133.96373853845,0.658286999916673,0.404243700772875,1.6
2844095939675,0.103431418577482,0.694793497694344
"2520","CD44-
AS1",4.99857633353308,1.84851045589193,1.13510002854952,1.628500052329
34,0.103418898145272,0.694793497694344
"2521","PSMC4",395.080410563734,-0.407258938329446,0.250184943842937,-
1.62783152364724,0.103560614288032,0.694793497694344
"2522","GSTT2B",21.5148460718257,-1.07308463280161,0.659079115249444,-
1.62815754280952,0.103491484777177,0.694793497694344
"2523","SPSB4",18.6426898622103,1.07077754774794,0.658110319696255,1.6
2704871159315,0.103726752601538,0.69512958104907
"2524","TYMS",22.756676276573,0.754842559318999,0.464154084874386,1.62
62758077919,0.103890995796748,0.695954420337326
"2525","AC011477.2",9.94203070414633,0.964154147653886,0.5930073216829
64,1.62587224879046,0.103976834757112,0.696253592900296
"2526","ART4",6.06994888721503,1.12260717162971,0.690638557088368,1.62
546263904301,0.104064018354568,0.696561529033667
"2527","TRIM7",40.0047754373068,-0.704202565744979,0.433467350549133,-
1.62458040923467,0.104251994261377,0.697543616530021
"2528","SGSH",100.758339905285,0.329727237130277,0.203002625616093,1.6
2425109591359,0.104322229841882,0.69773744547727
"2529","AC080038.1",53.556703564075,-0.461911112373246,0.2845608929870
74,-1.62324171647236,0.104537743336452,0.698902397917254
"2530","PDGFC",27.1330712456317,-0.833525182572189,0.51381200585335,-1
.62223765322076,0.104752472380148,0.699508216036154
"2531","KATNAL1",206.596666050462,0.258343638210822,0.159223555532083,

1.62252147521456,0.104691738713343,0.699508216036154
"2532","PUDP",49.6611382684854,0.418489669086943,0.257946579063082,1.6
2238890938964,0.104720102338982,0.699508216036154
"2533","AC004837.4",8.40657135902958,-1.00673656413571,0.6208012327938
44,-1.62167294611354,0.104873394460531,0.699864197921532
"2534","MDK",618.782010593045,0.561376803716626,0.346186523309472,1.62
16021304064,0.104888566213222,0.699864197921532
"2535","RND2",13.4542618697483,-0.701326681814816,0.432680195708694,-1
.62088925901982,0.10504139081974,0.700331165607322
"2536","UPF1",253.068511483671,0.405947541036794,0.250423684542184,1.6
2104292083608,0.105008434029857,0.700331165607322
"2537","SLC39A8",95.1450463646341,0.414550332283867,0.255801538249767,
1.6205935864197,0.105104828628419,0.700477904000517
"2538","GIPC2",10.5667928175082,-0.854758296399103,0.528284900689307,-
1.61798736871679,0.105665319846492,0.701067976733601
"2539","SRGAP2C",227.002944197535,0.285008622995014,0.176098767426002,
1.61845893166048,0.105563730550996,0.701067976733601
"2540","ANKRD35",664.271917181696,-0.446020875443455,0.275930817442201
,-1.61642283952891,0.106002923591942,0.701067976733601
"2541","LIX1L",284.215040779164,0.276076580695632,0.170725921791722,1.
61707476989015,0.105862142228158,0.701067976733601
"2542","NOTCH2NLA",57.3027003780697,0.549266572553349,0.33989112837303
8,1.61600738207711,0.106092717148851,0.701067976733601
"2543","NUP133",119.199463044607,0.29162075378439,0.180180050036745,1.
61849635253692,0.10555567225233,0.701067976733601
"2544","EMILIN1",966.411803662519,0.359331674875241,0.22216764514909,1
.61738976273572,0.105794174128084,0.701067976733601
"2545","C2orf68",185.124717864652,0.302603867766271,0.187255202815578,
1.61599711632203,0.106094936668104,0.701067976733601
"2546","PMS1",134.466368365728,0.297689350997376,0.184134277226955,1.6
1669709453639,0.10594368140255,0.701067976733601
"2547","TASOR",351.101881491852,0.247249775979316,0.152785878422846,1.
61827636514308,0.105603051849252,0.701067976733601
"2548","LATS1",228.036945161399,0.318723549124447,0.197336372874302,1.
61512824261478,0.10628292597743,0.701067976733601
"2549","AC100814.1",8.72746563669764,0.945951553405265,0.5847194542378
18,1.61778703709852,0.105708500960943,0.701067976733601
"2550","GNA14",14.1921020250939,0.636986029295929,0.394504237993224,1.
6146493952414,0.106386642105011,0.701067976733601
"2551","PTER",164.338099367054,0.295711462276963,0.183147632959884,1.6
1460706588402,0.106395814306744,0.701067976733601
"2552","AL132656.3",8.95481136180774,-1.01125093333797,0.6249694537689
33,-1.61808057536177,0.105645234094696,0.701067976733601
"2553","TIMM10",395.186478136305,-0.466001899733713,0.288576463558878,
-1.61482989287044,0.106347537734729,0.701067976733601
"2554","AP001160.3",24.8872645070715,-0.562455776134426,0.347846986855
364,-1.6169631975806,0.105886225231788,0.701067976733601
"2555","RSF1",970.718272119814,0.230412982052556,0.142396501401846,1.6
1810844918391,0.1056392279582,0.701067976733601
"2556","KDM5A",571.189995419119,0.337542267277055,0.208714917992436,1.

61724073450892,0.1058263266237,0.701067976733601
"2557","TPCN1",261.330267521862,0.37549061306342,0.232513376279652,1.61492047929236,0.106327916702812,0.701067976733601
"2558","RTRAF",1348.70729515993,-0.412806032416583,0.255042948440509,-1.61857457710843,0.105538828767345,0.701067976733601
"2559","MEG9",14.385810182243,1.1259097090149,0.696557404758565,1.61639184555816,0.106009620292777,0.701067976733601
"2560","SNX1",402.195922567266,0.227661363582483,0.140917596511274,1.61556377073369,0.106188662231548,0.701067976733601
"2561","EDC3",68.6329809997195,0.352693855302785,0.218346879682352,1.61529148397211,0.106247586982612,0.701067976733601
"2562","VPS53",188.693127612186,0.605597210545867,0.375049170205548,1.6147141725816,0.10637260694136,0.701067976733601
"2563","RNF213",634.37913955771,0.36278820018067,0.224292042482248,1.61748137011764,0.105774413892803,0.701067976733601
"2564","TRIB3",13.1141018668057,1.03726521361049,0.640547174767529,1.61934242233906,0.105373607834452,0.701067976733601
"2565","HSPA13",118.867260419521,-0.307095854285725,0.190105336970476,-1.61539838480925,0.106224449806748,0.701067976733601
"2566","Z95114.1",19.9213841966478,-0.985019302417224,0.608160886316182,-1.61966894711659,0.105303410161807,0.701067976733601
"2567","RTCA-AS1",97.9610901321297,0.431052540237266,0.267161393899985,1.61345370281544,0.106645973892336,0.701106957636822
"2568","ALKAL2",38.8626909845064,2.08842491237353,1.29367984142693,1.61432902136745,0.106456078393674,0.701106957636822
"2569","SLC1A2",41.0485573676798,0.982202585376835,0.608604391728101,1.61386049579419,0.106557689048785,0.701106957636822
"2570","METTL7A",2068.4428494106,0.334187546589712,0.20712827959218,1.61343273476564,0.106650526084806,0.701106957636822
"2571","ALDH2",1115.28250180898,-0.412595015349098,0.25571073741939,-1.61352244928379,0.106631050019603,0.701106957636822
"2572","GAS6-DT",28.7358698500758,0.528123101737192,0.32731430400013,1.61350449791825,0.106634946844019,0.701106957636822
"2573","AC022730.4",2.55327379589833,2.82053060167003,1.74842768868952,1.61318115694225,0.106705155986309,0.701193461879716
"2574","AC024060.2",10.4383533234423,-0.73695932778866,0.457110539027375,-1.61221250631573,0.106915704726442,0.701214404776833
"2575","RSPH3",34.2808365730817,-0.648774022597852,0.402261835256482,-1.61281525050517,0.106784651828278,0.701214404776833
"2576","GNG10",803.862936116465,-0.321055620530104,0.199132054703757,-1.61227493488042,0.106902125153442,0.701214404776833
"2577","AL356515.1",3.61137190096707,1.73331729950121,1.07508047344859,1.6122674928149,0.106903743893083,0.701214404776833
"2578","ELP5",290.783906363633,-0.496549115526355,0.307917893679148,-1.61260233886817,0.10683093003032,0.701214404776833
"2579","PEX6",69.0585958874165,-0.487812990943956,0.30265102807569,-1.61180021110637,0.107005422242947,0.701385657827689
"2580","RXRA",581.081830286686,0.325431689470508,0.201940735170055,1.6

1152077215358,0.107066263476063,0.701385657827689
"2581","ASIP",38.0504478902248,-0.726418195837022,0.450756525704331,-1
.61155336509428,0.10705916572127,0.701385657827689
"2582","AL445183.2",7.05686127762749,1.02967210707123,0.63922566008033
1,1.61081159811675,0.107220792418296,0.701468699295479
"2583","PER2",400.684018663101,-0.528369020092417,0.328022246042581,-1
.61077191095091,0.107229445464888,0.701468699295479
"2584","WDR86",222.553438467488,0.635872626722502,0.394766469042636,1.
61075642585497,0.107232821851384,0.701468699295479
"2585","ZNF469",41.9437818998639,0.501202345612343,0.31117030372002,1.
61070108432746,0.107244889264183,0.701468699295479
"2586","DCP1B",71.6524617244943,0.36664072254963,0.227665888999354,1.6
1043327202464,0.107303301869263,0.701579361177685
"2587","EVI5",323.400700470267,0.224062320061091,0.139256293019911,1.6
089924211113,0.107617998844009,0.701696360979751
"2588","AC000032.1",2.84408464383067,1.83376734887193,1.14220005954159
,1.60546949157743,0.108390521988594,0.701696360979751
"2589","CEPT1",180.602939837878,0.322620907441652,0.200646403101986,1.
60790775440748,0.107855383279135,0.701696360979751
"2590","RALGPS2",193.044604435169,0.459181725414126,0.286085959767289,
1.60504809738877,0.108483220227175,0.701696360979751
"2591","PGBD2",35.1098482878428,-0.463669751203817,0.288380355782225,-
1.60784097081136,0.107870012722226,0.701696360979751
"2592","NBAS",192.130451920949,0.283803876973046,0.176581482795026,1.6
0721199347093,0.108007871968398,0.701696360979751
"2593","FEZ2",682.556951844324,0.256505767295211,0.159644132234884,1.6
0673470239304,0.108112577664754,0.701696360979751
"2594","WDFY3-
AS2",229.358962225087,0.322970968771345,0.201148907436539,1.6056312355
2257,0.108354958227827,0.701696360979751
"2595","C4orf36",32.2560490256721,0.596108428454127,0.370378000582051,
1.60945959942907,0.1075158822226,0.701696360979751
"2596","SLC9A3",2.27110196405779,-1.67024235365627,1.03867203942863,-1
.60805556542666,0.107823009762528,0.701696360979751
"2597","TREM2",7.95436994232758,-1.51497113470553,0.941008766198444,-1
.60994369991453,0.107410147698588,0.701696360979751
"2598","KLHL7",164.362621891352,0.289941326751902,0.180101536421053,1.
60987703111015,0.107424704234528,0.701696360979751
"2599","AC007036.1",2.79263216578286,1.98745834418984,1.23812637335851
,1.60521444898933,0.108446618732739,0.701696360979751
"2600","ZNF862",38.4025347454281,0.527456910830756,0.32788550798082,1.
60866185907067,0.107690300037058,0.701696360979751
"2601","NEIL2",77.1660817307878,-0.406790811089018,0.252916260821266,-
1.60840117502961,0.107747344495388,0.701696360979751
"2602","SDC2",1894.55343744859,-0.322090938127948,0.200195165081934,-1
.60888470006818,0.107641555583412,0.701696360979751
"2603","ZNF79",10.2846542302292,0.804886865383439,0.500732488162219,1.
60741890013473,0.107962506782998,0.701696360979751
"2604","JMJD1C-
AS1",18.6237457039927,-0.606118437393394,0.377573900988547,-1.60529749

489168,0.108428350223418,0.701696360979751
"2605","YLPM1",250.593660487922,0.430720491802207,0.268000594628475,1.60716244827482,0.108018737206975,0.701696360979751
"2606","C15orf61",1001.58420761104,-0.397716103855979,0.247663120157307,-1.6058753665195,0.10830129694382,0.701696360979751
"2607","RPUSD1",67.1766839602955,-0.388250918833544,0.241885419618323,-1.60510261199776,0.10847122457913,0.701696360979751
"2608","CDH11",945.298136828981,0.440272449689065,0.274217438415189,1.60555963265347,0.108370700907006,0.701696360979751
"2609","SOCS7",72.7303542421846,0.507243323133254,0.315607597009523,1.60719617632636,0.108011340566868,0.701696360979751
"2610","CCDC40",14.7489956560107,-0.74445017569648,0.463089528650121,-1.6075728981964,0.107928751831021,0.701696360979751
"2611","AC006213.4",3.65570405670278,1.41153600131051,0.878577959089358,1.60661440081375,0.108138981488586,0.701696360979751
"2612","HCK",7.86584587678435,-1.27040348188785,0.791196480392546,-1.60567382865195,0.108345594517835,0.701696360979751
"2613","ZNF512B",80.3503539937944,0.396668778999276,0.24708881378695,1.60536923108668,0.108412571593677,0.701696360979751
"2614","Z95331.1",49.111125085064,0.733875590357089,0.456122202679172,1.60894511612557,0.10762834312799,0.701696360979751
"2615","AC108673.3",20.1709357304568,-0.789393302594796,0.492000462567211,-1.6044564236298,0.10861348240365,0.701891118329627
"2616","AC067930.3",4.72771707319737,1.59192535370331,0.992148557003429,1.60452317595601,0.108598780102314,0.701891118329627
"2617","ARHGAP42",146.974704724526,0.355491705548305,0.221580485848738,1.60434572650491,0.108637867084731,0.701891118329627
"2618","NR2C1",139.651108882831,0.267338557949886,0.166748788639421,1.60324137962994,0.108881372997013,0.702927168626765
"2619","BCL2L1",185.125966554896,0.368757342773566,0.230001473887774,1.60328252050027,0.10887229379553,0.702927168626765
"2620","BX284668.6",3.59594350183646,1.7672372835747,1.10315729062824,1.60198123929206,0.109159758156204,0.704455416375994
"2621","PIK3R1",3567.8514705555,0.405809762463651,0.253377739646774,1.6015998999335,0.109244112965721,0.704730813439301
"2622","ENSA",983.07676768147,-0.328115883366158,0.20519203762806,-1.59906732814319,0.109805643307522,0.706466444841543
"2623","RBM5",544.546890913938,0.299306250411129,0.187167701759134,1.59913407921366,0.109790813822182,0.706466444841543
"2624","CXCL8",513.394840694819,-1.58149727501707,0.988828241027642,-1.59936499525286,0.109739525489795,0.706466444841543
"2625","CD83",113.265189525235,-0.750099253582238,0.46894719184979,-1.59953885345475,0.109700922664983,0.706466444841543
"2626","DSE",1144.84201783287,0.342428620537638,0.214078946444284,1.59954365539051,0.109699856613464,0.706466444841543
"2627","SPA17",39.6162468888733,-0.535269772269581,0.334599641813336,-1.59973205401156,0.109658037727307,0.706466444841543
"2628","CERS1",2.80195256496707,1.61049021256799,1.00647486298059,1.60012959270405,0.109569837317723,0.706466444841543
"2629","KCNJ5",2.01575829140152,-1.81435249820031,1.13538625468455,-1.

59800463561575,0.110041945429586,0.707717464177805
"2630","LINC01554",14.5441170887307,0.984143687075761,0.616311533035129,1.59682828297757,0.110303989582076,0.708959172840744
"2631","MEIS2",447.703869573463,0.24309599198341,0.152243143241143,1.59676151456203,0.110318877675893,0.708959172840744
"2632","TMEM123",639.437813768525,0.280067811816707,0.175428268457514,1.59648051183117,0.110381553369468,0.709092440870426
"2633","SRM",645.549824914801,-0.424452465494797,0.265929298948498,-1.59611019610517,0.110464192655306,0.709353805323175
"2634","DUSP28",28.2022103998734,-0.535752404661202,0.335721379588073,-1.59582450578085,0.11052798040763,0.709493960794309
"2635","TRMT5",114.542489182502,0.29890953614701,0.18735220229683,1.59544180683521,0.110613473425427,0.70977328602547
"2636","DYNC2LI1",311.319070593601,-0.317728220977934,0.199295431589382,-1.59425742197927,0.110878389983486,0.709914669849026
"2637","PRDM1",51.7298885803381,0.789221325078677,0.495142508685336,1.59392762938928,0.110952245234174,0.709914669849026
"2638","EEPD1",75.0165380360417,-0.412309477209376,0.258549633986788,-1.59470145384327,0.110779012789888,0.709914669849026
"2639","ZDHHC12",128.560519908336,-0.32858097245334,0.2061556621634,-1.5938488858623,0.11096988515983,0.709914669849026
"2640","AC091271.1",88.4172314752794,-0.588309528426955,0.369157458041399,-1.59365472811599,0.111013389347103,0.709914669849026
"2641","AL121894.1",9.71511017822,-0.778636238632021,0.488247842839144,-1.59475612652844,0.110766781554981,0.709914669849026
"2642","HSD17B10",415.988660913824,-0.407122389046095,0.255293106466982,-1.59472535189175,0.110773666247706,0.709914669849026
"2643","STAG2",728.600578183693,0.317254294403557,0.199058331771464,1.59377551082761,0.110986324442194,0.709914669849026
"2644","HAUS7",66.1336577790504,-0.438692651484167,0.275168240761264,-1.59427065518356,0.110875427289804,0.709914669849026
"2645","AC103858.1",6.16029585969051,1.38686510508432,0.870595671011296,1.59300712289704,0.111158593141729,0.710574477444371
"2646","CDK5R1",7.0268633059297,0.968255386326802,0.60819525249571,1.59201404870162,0.111381547952186,0.711730617073151
"2647","LBX2-AS1",45.6356257885648,0.418694417733972,0.263647904001184,1.58808172331267,0.112267860564559,0.712012373002839
"2648","PPA2",150.580073404975,-0.323143496297567,0.203468609416023,-1.58817371006281,0.112247064196224,0.712012373002839
"2649","ARRDC3",870.383778059476,0.491487731282686,0.309216402608247,1.58946203091743,0.111956119667416,0.712012373002839
"2650","KDM3B",174.267938783214,0.331001703200073,0.208160467736616,1.59012759146414,0.111806047766653,0.712012373002839
"2651","ZBTB22",80.5565169804263,-0.434230151127254,0.27337741567692,-1.58839072368887,0.112198013783784,0.712012373002839
"2652","BEND3",6.22195644434497,0.951354206015431,0.5978994883464,1.59116076290109,0.111573400673937,0.712012373002839
"2653","AL591926.2",4.77849022976617,1.48394109244883,0.934368315993831,1.58817574081635,0.112246605117655,0.712012373002839

"2654","PRF1",18.0085873773181,0.99347215375691,0.62462236153962,1.590
51647031675,0.1117184360765,0.712012373002839
"2655","HTRA1",4228.73818182904,-0.351683590232893,0.221358904151906,-
1.58874833420549,0.112117221898316,0.712012373002839
"2656","FTH1",55116.5174888076,-0.411873985154451,0.259173087549007,-1
.58918500778585,0.112018630198374,0.712012373002839
"2657","SYTL2",61.386306293116,0.389107942943167,0.244984421655967,1.5
8829667744994,0.112219268469113,0.712012373002839
"2658","AC007529.1",10.5984867988804,0.875255441068597,0.5501666203141
48,1.59089157493565,0.111633978953682,0.712012373002839
"2659","FRS2",218.631037185019,0.258501351367713,0.162760735679884,1.5
8822918984669,0.112234522793053,0.712012373002839
"2660","AL162377.1",52.8650938553979,-0.444404408475565,0.279759192049
202,-1.58852477811491,0.112167722636005,0.712012373002839
"2661","NDRG2",452.53466624369,-0.247444012615265,0.15575793028175,-1.
58864471406152,0.112140627188567,0.712012373002839
"2662","AHSA1",455.100437676744,-0.334767670543015,0.21060517716497,-1
.58955100273146,0.111936048932342,0.712012373002839
"2663","AC005856.1",3.6891426244973,1.46003553270111,0.918290670154884
,1.58994921777312,0.111846252242249,0.712012373002839
"2664","AL118522.1",13.2271224523148,-0.968036745071898,0.609128833008
529,-1.58921510953717,0.112011836376932,0.712012373002839
"2665","OPRL1",8.24084545086754,0.941330213944682,0.591879385880796,1.
5904088508571,0.111742676566946,0.712012373002839
"2666","PCMTD2",394.792562130981,0.338885854779933,0.213378242881614,1
.58819310817904,0.112242679057171,0.712012373002839
"2667","AL139246.5",2.99038468345417,-1.43583751234718,0.9051055333293
41,-1.58637579759964,0.112654087242303,0.712273365514872
"2668","KCND3",44.6194940268359,0.604399006343932,0.380954840070878,1.
58653715026033,0.112617511728829,0.712273365514872
"2669","CCT3",712.488623283345,-0.338237592089259,0.2132271210582,-1.5
8627847344493,0.112676153264443,0.712273365514872
"2670","Z97200.1",163.805371211495,0.478094224240342,0.301177729169678
,1.58741559529786,0.112418549493607,0.712273365514872
"2671","SLC25A37",389.90940115637,0.253684142708497,0.159852471323599,
1.58698918201239,0.112515094759718,0.712273365514872
"2672","FBXW5",459.198777944899,-0.269509398304152,0.169849178197457,-
1.58675715222381,0.112567656642177,0.712273365514872
"2673","ZNF280D",522.891182283487,0.224340631007217,0.141386199614417,
1.58672226581541,0.112575561160256,0.712273365514872
"2674","NUP88",86.3929388928887,0.338612240940823,0.213286474112842,1.
58759359846549,0.112378266693857,0.712273365514872
"2675","TRABD",153.174686043135,-0.338047083868577,0.213114131799497,-
1.5862255638055,0.112688150742387,0.712273365514872
"2676","PLCL2",126.076714649243,0.355610247529635,0.224217985024757,1.
58600233380195,0.112738780149069,0.71232709071766
"2677","DBR1",108.395098123139,0.284152225071411,0.179243478519663,1.5
8528626769671,0.112901307653244,0.712910244346089
"2678","NCKAP1L",16.3041693128198,-0.804749147811364,0.507656359008489
,-1.58522420438726,0.112915403025717,0.712910244346089

"2679","CAMTA1",513.206185945209,-0.342104321178094,0.215960825321647,
-1.58410360151463,0.113170144952884,0.713452949613484
"2680","C5orf30",47.8574705796718,-0.3840321352338,0.242406348745937,-
1.58424949354894,0.113136954315191,0.713452949613484
"2681","RSU1",422.422830413012,0.312982186744421,0.197572308445906,1.5
8413994960287,0.113161874995492,0.713452949613484
"2682","AL590807.1",23.7981469073484,1.15181268065384,0.72697764847360
7,1.58438527384196,0.113106071003933,0.713452949613484
"2683","AC025280.2",13.8590248991714,2.27452276868025,1.43608802725728
,1.58383241522058,0.113231860590382,0.713575959322465
"2684","AC021148.2",3.06444989068303,1.54825607464433,0.97816398492927
7,1.58281852378389,0.113462833413491,0.713802913438731
"2685","TRAPPC11",134.55421447697,0.384300271075276,0.242805752700527,
1.58274780066379,0.113478958559458,0.713802913438731
"2686","PARD3-
AS1",3.86531045219617,-1.38265695734505,0.873495674892379,-1.582900748
20966,0.113444088194317,0.713802913438731
"2687","PEX16",184.45942958534,-0.332038112462779,0.209768345646753,-1
.5828799690394,0.113448825122006,0.713802913438731
"2688","ESR2",6.51688691500802,1.20614589146735,0.761722584927961,1.58
344509580404,0.11332005137909,0.713802913438731
"2689","CLEC4A",8.79549538031955,-0.91944798404233,0.581139217861526,-
1.58214754018101,0.113615892969736,0.714398482087131
"2690","LINC01137",12.3339184882915,-0.851140225490778,0.5382440112876
96,-1.58132781348465,0.113803103279833,0.714737847758384
"2691","RHBDD1",71.0402511482949,0.380318380025959,0.240570525108661,1
.58090181602329,0.11390048904161,0.714737847758384
"2692","SYNPO2",267.958549254529,0.661317195817506,0.418271055281271,1
.58107329557575,0.113861279824275,0.714737847758384
"2693","AL137026.3",44.9460381900308,0.543432937319904,0.3438438184874
79,1.58046446700828,0.114000538095044,0.714737847758384
"2694","FBXO3",226.139112381972,-0.230316214082888,0.145665217958948,-
1.58113389943093,0.113847425147312,0.714737847758384
"2695","AL356966.1",10.9028675201883,1.03523182949491,0.65490262083809
4,1.58074161952521,0.113937127965588,0.714737847758384
"2696","KLHL25",13.0578895932494,0.717878145659517,0.454229155389893,1
.58043167670142,0.114008042075015,0.714737847758384
"2697","MIER2",41.7140693450809,0.416107180500616,0.263283330093577,1.
58045395564057,0.114002943552987,0.714737847758384
"2698","CDKN2B-
AS1",4.68005977984773,1.15829517849616,0.733168011952946,1.57984958374
111,0.114141317336332,0.715308151787512
"2699","PQBP1",390.929349008511,-0.325555685327741,0.206103592312325,-
1.579573076215,0.114204669100156,0.715439994496269
"2700","WDR48",214.396567441268,0.266275912712893,0.168715572385892,1.
5782533227215,0.114507424603696,0.716540168467539
"2701","ARMC4",2.03625712174511,-1.82193859248351,1.15419647309025,-1.
57853418803598,0.114442940388044,0.716540168467539
"2702","ZNF587",71.4760167326115,0.493800839892289,0.312866354683392,1
.57831237683577,0.11449386392293,0.716540168467539

"2703","AC096921.2",20.5721469810724,0.616584882612675,0.390776380985483,1.57784582849592,0.114601032541769,0.716625420575076
"2704","C3orf18",45.4666932182716,0.380413166392472,0.241127901136708,1.57764059903128,0.114648199825856,0.716625420575076
"2705","TCEAL3",207.162296654056,-0.399758016025348,0.253383994249731,-1.57767666899809,0.114639908865235,0.716625420575076
"2706","EMC1-AS1",53.6759681479796,-0.51164209645116,0.324500422634906,-1.57670702643986,0.11486295288506,0.716798999809495
"2707","BTN3A2",239.074562244991,0.535931966426629,0.339830948372741,1.57705461787076,0.114782958229088,0.716798999809495
"2708","TRDV3",3.08724594956798,1.68075904167501,1.06595732939308,1.57676015289654,0.114850723529097,0.716798999809495
"2709","PTPRH",7.4443201961178,0.975450941076212,0.618608557282216,1.57684682759925,0.114830773789588,0.716798999809495
"2710","PSMG1",163.282058924137,-0.431556171862709,0.273726109774638,-1.57659849189401,0.114887939997855,0.716798999809495
"2711","AL591686.2",2.06280104822304,1.91239016521434,1.21318833497461,1.57633411901736,0.114948822521395,0.716914308812893
"2712","CRYAB",2091.33774260688,-0.345947780400739,0.219496773737781,-1.57609505829922,0.11500389774797,0.716993327110132
"2713","TNFAIP8L2",2.27243306076485,-1.75760729549217,1.11651346271384,-1.5741926579372,0.11544291622454,0.717082439731693
"2714","GBA",154.067603362649,0.322737609455962,0.204878642953937,1.57526233482776,0.115195904134147,0.717082439731693
"2715","AL121987.2",2.13539638148991,-1.62473100135034,1.03200230950209,-1.57434822227696,0.115406967113862,0.717082439731693
"2716","AL137796.1",10.4127134272306,-0.910451545916537,0.5781058038736,-1.5748873991855,0.115282437721208,0.717082439731693
"2717","CLASP2",157.507623225866,0.330328639989669,0.209827916874655,1.57428356011845,0.115421908717899,0.717082439731693
"2718","POPDC2",7.39604389308106,-0.853795534196177,0.542367416753414,-1.57420137682119,0.115440901158901,0.717082439731693
"2719","AC036214.2",5.30182273691599,-0.954885648246117,0.606657217699849,-1.57401184785468,0.115484710396818,0.717082439731693
"2720","PAAF1",148.272378099373,0.309855536204326,0.196660223504192,1.57558824394258,0.115120727175254,0.717082439731693
"2721","MEOX1",3.76335228691998,-1.43629077629717,0.911800027564793,-1.57522563377539,0.115204372328225,0.717082439731693
"2722","TMEM100",219.292273333375,-0.689793897899812,0.438217467811082,-1.57409037422759,0.115466557598491,0.717082439731693
"2723","PGK1",899.609463503408,-0.372129161617611,0.236286411951438,-1.57490715841117,0.115277876098428,0.717082439731693
"2724","THEMIS2",21.5387883461418,-0.846697215548135,0.538098311710133,-1.57349911182074,0.115603293815422,0.717152019133265
"2725","SRD5A3-AS1",5.12925616636078,1.00022808684315,0.636002097159893,1.57268048534703,0.115792820690431,0.717152019133265
"2726","TMEM184C",108.376420033765,0.315118015723599,0.200350190768393,1.57283611517934,0.11575677077092,0.717152019133265

"2727","SNX13",239.610111038202,0.218521784608729,0.138924853184697,1.
5729495450192,0.115730501569206,0.717152019133265
"2728","SRRT",148.468237847368,-0.380469617131721,0.24188030490764,-1.
57296650207631,0.115726574889261,0.717152019133265
"2729","PLCD3",152.43031815978,0.308054889090092,0.195764521585762,1.5
7359917208051,0.115580144787273,0.717152019133265
"2730","CCDC137",115.946703414935,0.377085460321391,0.239685954420974,
1.5732480496504,0.115661393314264,0.717152019133265
"2731","ALDH1A1",852.796024213106,0.644520657181321,0.409874024966938,
1.57248475853846,0.115838171156398,0.717170193303691
"2732","LAYN",53.0846438224723,-0.685067751822861,0.435757432081872,-1
.57213096412352,0.115920181767724,0.71741523913934
"2733","CPS1",14.6712623605256,-0.624082094294175,0.397069635237957,-1
.57171951443775,0.116015614432806,0.717616732695943
"2734","ZNF107",88.154241902296,0.370199650938965,0.235558774803085,1.
57158081352916,0.116047798979068,0.717616732695943
"2735","PRR5",131.466910484685,-0.452640632546455,0.288041616764645,-1
.57144178549831,0.116080066472877,0.717616732695943
"2736","SRSF8",257.320382522699,-0.277351395917314,0.176574962010873,-
1.57072890039889,0.116245633209766,0.718377619265614
"2737","PHLDA3",684.673343974962,-0.336794688358897,0.214507770238721,
-1.57008153123817,0.116396144651603,0.718463801922006
"2738","SULT1C2",3.22692136997567,1.68154134532283,1.0709716508979,1.5
7010817598489,0.116389946806097,0.718463801922006
"2739","ZNF292",798.304062528638,0.332604128282611,0.211858137260366,1
.56993794330332,0.116429549164082,0.718463801922006
"2740","CNKSR3",30.7716960262,0.562568316183319,0.358223623177883,1.57
043890961922,0.116313036300901,0.718463801922006
"2741","PAFAH1B3",54.310646703416,-0.626047861235995,0.398830501055132
,-1.56970908588923,0.116482806463889,0.718530204922087
"2742","MED12L",5.71346671401341,1.16610605774587,0.742981881395501,1.
56949460941852,0.116532734550821,0.718576030556268
"2743","CHMP1A",268.40805778922,-0.289681433943298,0.184628007093454,-
1.56900049187374,0.116647824404361,0.718761448625703
"2744","APOC1",1102.15119526987,-0.996415334467556,0.635024028948888,-
1.56909863098701,0.116624958740741,0.718761448625703
"2745","VPS13D",231.754982273176,0.351586211650297,0.22431838733824,1.
56735350954603,0.117032084347809,0.719156421635731
"2746","FOXJ3",202.615800388899,0.339681342922618,0.216564318766727,1.
56850096478038,0.116764264981605,0.719156421635731
"2747","RGS13",4.02712996914455,-1.52987703711179,0.975593140351737,-1
.56815067043237,0.116845973602492,0.719156421635731
"2748","TRANK1",135.19220160501,0.321456837009922,0.205061993868431,1.
56760807278686,0.116972626957213,0.719156421635731
"2749","SBDS",1302.50304150935,-0.321064639809898,0.204814703195046,-1
.56758589496451,0.1169778060054,0.719156421635731
"2750","GTF3C4",111.164374042853,0.339307133029274,0.216398900971465,1
.56797068518392,0.11688797388729,0.719156421635731
"2751","C11orf1",100.913583185164,-0.440297715481699,0.280908086706932
,-1.56740847386518,0.117019244527636,0.719156421635731

"2752","EGFL6",13.1058474479769,2.4422461310392,1.55828295047696,1.567
26744028847,0.117052192591763,0.719156421635731
"2753","USP48",334.973931265528,0.265567496638746,0.169467231611175,1.
56707284419482,0.117097665813441,0.719174476416147
"2754","ITGA4",55.61055512287,-0.541640016913785,0.345839657349018,-1.
56615936143832,0.117311313896658,0.719633459848939
"2755","AC004839.2",6.40894131349035,-0.975359910480872,0.622825046560
77,-1.56602550887572,0.117342645422494,0.719633459848939
"2756","DLEU2",137.933397631502,0.401706389015785,0.256473373578151,1.
56626936906329,0.117285568789122,0.719633459848939
"2757","BRIP1",4.71651819544339,1.16411296529647,0.74310489901261,1.56
655267223143,0.117219287708311,0.719633459848939
"2758","AC093677.2",2.46645609100534,-1.47439040221433,0.9420809466703
18,-1.56503579381942,0.11757451676883,0.720010840104085
"2759","PTCD2",53.3573196509067,0.387046161785403,0.247302221645602,1.
56507353314465,0.117565668575826,0.720010840104085
"2760","HAUS6",100.797682765805,0.286990881633647,0.183346569904896,1.
56529179565515,0.117514505976728,0.720010840104085
"2761","AC005736.1",22.8619241031496,0.795645936257097,0.5083138471388
81,1.56526512259209,0.117520757433388,0.720010840104085
"2762","FAM171A2",27.8507599328568,0.640926531861486,0.409624634549308
,1.56466793694346,0.117660790219754,0.720163228914589
"2763","LTN1",356.893497776031,0.213076744140396,0.136188997501415,1.5
6456650720395,0.117684587265851,0.720163228914589
"2764","FAM49A",183.27058821567,0.409739043643606,0.262007169617981,1.
56384668496295,0.117853577653416,0.720429936553295
"2765","TNPO1",452.262582138519,0.251275250082681,0.160697370471693,1.
56365502027268,0.117898606248105,0.720429936553295
"2766","RPL26L1",291.545375067251,-0.394425387258946,0.252216838427915
,-1.56383447559421,0.117856445649491,0.720429936553295
"2767","SLC26A11",88.0206245278648,0.363964458956281,0.232684350601973
,1.56419827124032,0.117771013084997,0.720429936553295
"2768","CD1E",2.90799819494675,-1.52687578313151,0.977516103248381,-1.
56199552933968,0.118289042051768,0.720472306560261
"2769","RNF144B",15.6906762014296,-0.784800005378329,0.502150926112652
,-1.56287674594922,0.118081588220415,0.720472306560261
"2770","STRBP",43.3281889531337,0.499690844920929,0.319720930326276,1.
56289688138651,0.118076851323022,0.720472306560261
"2771","OLFML1",833.981260863529,0.219276512544426,0.14036675746763,1.
56216839727877,0.118248323387687,0.720472306560261
"2772","FEN1",32.7165855514469,-0.525384275560649,0.336203769610415,-1
.56269596908283,0.11812412297676,0.720472306560261
"2773","SF3B3",253.765280487335,0.238232343952202,0.152495549901094,1.
56222489185235,0.118235018598494,0.720472306560261
"2774","ARID3A",79.0969271864341,0.342628468421916,0.2191560695172,1.5
6339940379805,0.117958680309346,0.720472306560261
"2775","KIAA0355",202.232069012194,0.313129214794418,0.200415916886047
,1.56239693762675,0.118194508085374,0.720472306560261
"2776","ZGPAT",144.642872151066,-0.341731342892381,0.2187174938408,-1.
56243260148692,0.118186111907837,0.720472306560261

"2777","IFIT2",286.174399774077,0.691194422853182,0.442691168623806,1.
56134676235331,0.118441955781754,0.720884301064756
"2778","SMURF2",328.413678839013,0.298766887956708,0.191336952350047,1
.56146987963998,0.118412925249077,0.720884301064756
"2779","CARNMT1",65.0473167460326,0.388862607808421,0.249228707397647,
1.5602641119026,0.118697480550173,0.721815197950285
"2780","STRAP",596.982086134955,-0.393894726461114,0.252459025690656,-
1.56023230060217,0.118704995114148,0.721815197950285
"2781","USP10",159.3184372489,0.272523600563882,0.174677126539765,1.56
015619195477,0.118722975248388,0.721815197950285
"2782","LINC01359",35.8431409380825,0.647419534220883,0.41557826028057
4,1.55787632823667,0.119262567656602,0.721980880922186
"2783","CLCA2",2.78186069929594,1.45865462882707,0.936642557216453,1.5
5732260678177,0.119393910678183,0.721980880922186
"2784","CDC42SE1",169.581654181434,-0.2592811039294,0.166426261834894,
-1.55793383250189,0.11924893410682,0.721980880922186
"2785","RXRG",17.3096093174473,-0.728779772745347,0.46802764864086,-1.
55712974406898,0.119439684418536,0.721980880922186
"2786","ZRANB3",34.4844888524551,0.48524039917402,0.311583709954241,1.
55733558485866,0.119390830974973,0.721980880922186
"2787","TNIP2",290.51100684567,-0.294909441005246,0.189398543946486,-1
.55708399262336,0.119450545013927,0.721980880922186
"2788","MTTP",2.64752850996389,-1.43992674412607,0.923726924850071,-1.
55882296530415,0.119038287119698,0.721980880922186
"2789","HIST1H4J",10.6008971831326,-0.87760613393882,0.563509441941061
,-1.55739384049329,0.11937700765467,0.721980880922186
"2790","MRPL2",328.351477792692,-0.361530737635017,0.231856351366569,-
1.55928761711354,0.11892832122299,0.721980880922186
"2791","JAZF1",277.407559079197,0.258648852515767,0.165927401700703,1.
55880734504789,0.119041985240702,0.721980880922186
"2792","COPG2",66.0024810867701,-0.333467363886696,0.214176275525729,-
1.55697620134699,0.119476135842221,0.721980880922186
"2793","LYPLA1",249.174221238458,-0.226119425622299,0.144972255488381,
-1.55974275809223,0.118820683403979,0.721980880922186
"2794","AL162426.1",4.91362497416053,1.29751328730287,0.83279128743274
8,1.55802937288492,0.119226285364408,0.721980880922186
"2795","DLG5",161.506596352893,0.392970067995126,0.252206326860817,1.5
5812930185527,0.119202599873225,0.721980880922186
"2796","AC106779.1",2.29081335574633,1.82113045992455,1.16817923066376
,1.55894781564451,0.119008731835184,0.721980880922186
"2797","ZNF614",35.9246209119549,0.432719977659088,0.277472920338507,1
.55950345399899,0.118877267708087,0.721980880922186
"2798","LRP5L",17.0237346842947,-0.739743658141193,0.474661874487454,-
1.55846445206912,0.119123188396054,0.721980880922186
"2799","PGBD1",40.1736608527683,0.410388987383114,0.263626779234504,1.
55670447658908,0.119540665334616,0.722112743650478
"2800","CPA1",7.41840509815222,-1.24624305060177,0.800689171251857,-1.
55646297632988,0.119598039994963,0.722201307226728
"2801","CPEB1",60.1439469622895,0.4552286338778,0.292524297951729,1.55
620793576922,0.119658654924168,0.722309367175232

"2802","ZNF436-
AS1",24.0208128908193,-0.585302959373029,0.376288117205658,-1.55546490
205306,0.119835387305926,0.722344644765987
"2803","P2RX7",47.6200542898443,0.446357835934809,0.286874649065037,1.
55593335761647,0.119723940208296,0.722344644765987
"2804","MAFG",327.711650580396,-0.30758673345781,0.197743242353721,-1.
55548543554071,0.119830500620511,0.722344644765987
"2805","ARSA",234.445112129355,0.463088662196472,0.297696576495969,1.5
5557268292181,0.119809738693748,0.722344644765987
"2806","SLC25A35",37.4925631964527,-0.486273895048568,0.31269737637263
7,-1.55509425979028,0.119923621865151,0.722618887560933
"2807","LINC00886",9.73576473722253,1.03587596241058,0.666554749407935
,1.55407483530901,0.120166567006952,0.723052069378483
"2808","LPCAT1",111.036756517052,0.312035649738768,0.200777147557716,1
.5541392709998,0.120151199551887,0.723052069378483
"2809","PAQR8",47.5540560284137,0.426082348673336,0.274159766458714,1.
55413886646092,0.120151296026741,0.723052069378483
"2810","SRRM3",117.448100207478,-0.404113340087326,0.260032098420227,-
1.55409021633266,0.120162898602117,0.723052069378483
"2811","C19orf18",11.4901254490426,0.7807994744249,0.502536542214146,1
.55371681228343,0.120251980991502,0.723308607116442
"2812","AHCY",200.73006556113,-0.296514706065707,0.190884979608603,-1.
55336845609168,0.120335134366344,0.723551369795924
"2813","SF1",1110.69717799634,-0.216609157631586,0.139503920465637,-1.
55271018125216,0.120492388887024,0.723981986958706
"2814","CLTC",718.920318480578,0.245989892635193,0.15842395071934,1.55
273171460661,0.120487242265015,0.723981986958706
"2815","PPM1M",177.503144742017,0.29489862858842,0.189962853210589,1.5
5240155432653,0.120566171785641,0.724027741392291
"2816","CCDC159",40.9477692445721,-0.408305893073227,0.263029441468854
,-1.55232011592731,0.120585647016838,0.724027741392291
"2817","TRIP10",815.201354988969,-0.228852506633768,0.147492555366418,
-1.55162073140049,0.120752999669578,0.724775192904943
"2818","NFATC3",148.950382667643,0.363357816932996,0.234297170941378,1
.55084167458389,0.120939630694489,0.725637784166935
"2819","ELOVL1",361.769541604915,-0.276576089585339,0.178412248166008,
-1.55020797298621,0.121091606788738,0.726087577047408
"2820","SSH3",105.348820279468,-0.295946224759381,0.190911988729371,-1
.55017098050821,0.121100483041974,0.726087577047408
"2821","AL135925.1",135.043330991681,-0.409503686887897,0.264201885016
195,-1.54996504609646,0.121149905797457,0.726126411635379
"2822","BOLA3",274.905509829291,-0.383398985911504,0.247453124136779,-
1.54938026039866,0.121290336121683,0.726678125375042
"2823","HMCES",153.168425725076,-0.307927822917111,0.198786079759559,-
1.54904117677437,0.121371821898304,0.726678125375042
"2824","CD4",12.0979433270276,-0.759886649960032,0.490608299692405,-1.
54886627567543,0.121413869421841,0.726678125375042
"2825","ZNF441",56.3691153498577,0.439606791512636,0.283774505996268,1
.54914124497997,0.121347769881121,0.726678125375042
"2826","NOL9",142.396294365741,0.275057787441174,0.177695107142409,1.5

4791987165261,0.121641589627577,0.72677941874452
"2827","HECTD3",124.576707758253,0.361205389810048,0.233351476774466,1
.54790273797646,0.121645715344629,0.72677941874452
"2828","KLHL29",80.8851238792306,0.577739924813576,0.373112864842263,1
.54843206775467,0.121518305483008,0.72677941874452
"2829","GDF10",811.293381724063,-0.458693135518247,0.29628903311386,-1
.54812728199081,0.121591654770679,0.72677941874452
"2830","SCO2",182.665220716545,-0.393733947076228,0.254268656129229,-1
.54849580388751,0.121502971215911,0.72677941874452
"2831","DSEL",367.077243399128,0.271916709254509,0.175715731761906,1.5
4748073224858,0.121747367088073,0.72692199948388
"2832","BPI",3.09890045877685,2.05819374013017,1.3302112618789,1.54726
831678075,0.1217985583474,0.72692199948388
"2833","OR7D2",2.44806039564139,-1.97397581071593,1.27577089341612,-1.
54728080167296,0.121795549073993,0.72692199948388
"2834","BTBD1",161.409311902126,-0.244260429077501,0.157900611375097,-
1.54692516355908,0.12188129242254,0.727159101016339
"2835","PCYOX1",1362.61104101928,0.332174026125133,0.214791491116964,1
.54649527501185,0.121985000332479,0.727264592955417
"2836","CIZ1",403.249630731317,-0.230613584183831,0.149113309759963,-1
.54656606144055,0.121967918808827,0.727264592955417
"2837","NAPB",46.0276414260551,0.475698820192274,0.307685532225004,1.5
460552101761,0.122091234646661,0.727641380121868
"2838","HOMER3",476.481217047438,-0.355909540772757,0.230275149975788,
-1.54558379751431,0.122205116728257,0.72806346498991
"2839","AOC3",95.7640838291626,-0.657365760238952,0.425429196209818,-1
.54518252648261,0.122302119658478,0.728384726729675
"2840","TNFRSF4",5.36303173066831,-1.1672391564486,0.756972493673198,-
1.54198358091532,0.123077583564997,0.729093649347359
"2841","CNNM4",29.0032534894893,0.486061572706391,0.315290234701401,1.
54163218269897,0.12316300038032,0.729093649347359
"2842","TTN-
AS1",107.021524461348,0.401885738308069,0.260769297174639,1.5411543562
1596,0.123279223258959,0.729093649347359
"2843","AC117453.1",8.24985840040214,1.19178218147507,0.77246905343623
1,1.54282191134206,0.122873991583794,0.729093649347359
"2844","PDE1C",30.0197074662189,1.50637761519045,0.975748134710001,1.5
4381808338086,0.122632409243215,0.729093649347359
"2845","ADCY1",50.8812949965311,0.57937525521993,0.375883179744407,1.5
4137052797599,0.123226632681426,0.729093649347359
"2846","PDCD1LG2",102.929270909381,0.35135048643725,0.227676048158922,
1.54320355293589,0.122781395537686,0.729093649347359
"2847","CRACR2B",2.60882052003335,-1.45151189899762,0.941177150424703,
-1.54223027869156,0.123017644661473,0.729093649347359
"2848","DENND5A",226.140471800406,0.283577629770633,0.183626060836013,
1.54432126071626,0.122510524514763,0.729093649347359
"2849","VWCE",37.9997098937073,0.820108638761091,0.532187778263765,1.5
4101366520789,0.123313460183888,0.729093649347359
"2850","ARNTL2",7.59269146675508,-0.891391403318281,0.577649073080479,
-1.54313655964976,0.122797645882757,0.729093649347359

"2851","IPO8",121.258950808743,0.365681465841514,0.23717876258165,1.54
179683653432,0.12312297106227,0.729093649347359
"2852","RAB21",501.449204699964,0.256226459692354,0.166277243658959,1.
54095926811179,0.123326699617545,0.729093649347359
"2853","PRKAB1",49.2034289693188,0.414902575848423,0.268784902282373,1
.5436230693216,0.122679672996925,0.729093649347359
"2854","AC007613.1",3.90381467990716,-1.43867740104756,0.9328122106617
52,-1.54230120982972,0.123000415104182,0.729093649347359
"2855","MMP28",272.349671608474,-0.493423144916783,0.319744434886566,-
1.54317977447155,0.122787163210669,0.729093649347359
"2856","AC005332.6",126.368998002686,0.31243870223937,0.20259622177006
7,1.5421743777333,0.123031224634818,0.729093649347359
"2857","MBD3",198.664643652781,-0.286709486694082,0.18567268776889,-1.
54416618911099,0.122548077440372,0.729093649347359
"2858","AP1M1",258.789453678592,0.306931607457536,0.199033245536196,1.
54211225682756,0.123046316988257,0.729093649347359
"2859","THAP8",38.7394976765819,0.508222340511794,0.329231844417772,1.
5436609463175,0.12267049198635,0.729093649347359
"2860","AL021707.2",14.3663762056213,0.639536846449099,0.4147713670097
05,1.54190211117957,0.123097382861132,0.729093649347359
"2861","FAM87B",17.3396915416655,-0.811872704792822,0.527219478043815,
-1.53991409385173,0.123581294868331,0.72921274765380 9
"2862","COL7A1",9.96140197557359,-0.796811408032571,0.517238918976952,
-1.54050938318522,0.123436237380739,0.72921274765380 9
"2863","MAP4K5",379.902500721525,0.241607103380459,0.156834086734942,1
.54052673376285,0.123432011462796,0.72921274765380 9
"2864","AC104041.1",9.85853239539335,1.10440206361777,0.71723066407384
2,1.53981434277337,0.12360561478447,0.72921274765380 9
"2865","PSMC5",1175.47910629781,-0.370279016232814,0.240453497895273,-
1.53991944169632,0.123579991136984,0.72921274765380 9
"2866","PRPF31",415.782640594077,-0.305898441133664,0.198626858328889,
-1.54006584863339,0.123544303298937,0.72921274765380 9
"2867","PSPN",9.13833564339957,-0.722811211045374,0.469509665613199,-1
.53950230204814,0.123681716323382,0.729407205997815
"2868","AC060766.4",4.80121544503986,1.1224248778875,0.729203995664482
,1.53924674653585,0.123744069295753,0.729520475471618
"2869","SDHB",415.153129709463,-0.321167630126787,0.209057926965538,-1
.53626143140571,0.124474274741103,0.7299264316527
"2870","FAM72A",13.5973250325687,0.691329004915663,0.449688402800908,1
.53735119831795,0.124207330180027,0.7299264316527
"2871","ALS2",69.1317409927762,0.442760079814302,0.288256264543331,1.5
3599464877457,0.124539692789179,0.7299264316527
"2872","KLHDC8B",257.767967825077,-0.365345954186392,0.237745318439264
,-1.53671145486604,0.124363984761062,0.7299264316527
"2873","SIAH2",108.269430460014,-0.30812368060459,0.200582940345235,-1
.53614101016896,0.1245038000391,0.7299264316527
"2874","SLC7A11",2.39979964118515,-1.5790278112796,1.0278875276325,-1.
53618734426764,0.124492439037023,0.7299264316527
"2875","OR2A1-
AS1",45.2632747448987,-0.759365462236817,0.493809005509179,-1.53777159

```
542446,0.124104470997889,0.7299264316527
"2876","LINC00863",70.5402780965839,-0.53161841685935,0.345716711299817,-1.5377284333771,0.124115028457371,0.7299264316527
"2877","MS4A2",5.95664966375194,-1.27917484409199,0.831551416583077,-1.53829915815457,0.123975485493242,0.7299264316527
"2878","CARD16",32.5103793724624,-0.574179948349689,0.373187753016894,-1.53858197035661,0.123906382914616,0.7299264316527
"2879","ZC3H12C",79.5960116125611,0.43106242984912,0.280172451329599,1.53856108194596,0.123911485778702,0.7299264316527
"2880","PDE8A",179.868659273276,0.366471582849299,0.238593619775999,1.53596556015771,0.124546827260352,0.7299264316527
"2881","MARF1",265.545965474329,0.406594125784112,0.264586437579081,1.53671567410778,0.12436295108671,0.7299264316527
"2882","ZNF629",65.9310483798655,0.450822558668227,0.293393728908102,1.53657871402369,0.124396508435362,0.7299264316527
"2883","IRGQ",95.3824924683118,0.376236187705603,0.244613093256415,1.5380868730163,0.124027375184122,0.7299264316527
"2884","ISOC2",314.121816010761,-0.461411458867101,0.300322120866546,-1.53638852021872,0.124443120587144,0.7299264316527
"2885","AL034405.1",2.23253366127752,1.75570513284829,1.14298182885854,1.53607440513875,0.124520132838288,0.7299264316527
"2886","INTS11",331.500344566708,-0.259808531412432,0.169274077984575,-1.53483944207989,0.124823271729786,0.730079420419606
"2887","INPP4B",6.6512277519535,0.904850408729129,0.589412635502334,1.53517307608779,0.124741319939308,0.730079420419606
"2888","AC008467.1",17.0205664946607,0.76543156138535,0.498442506382027,1.53564664246089,0.124625068082095,0.730079420419606
"2889","CCDC136",32.2389469421123,0.565708016885402,0.368579263492258,1.5348340856872,0.124824587783231,0.730079420419606
"2890","AP001830.1",6.63108541476935,0.973437882686297,0.634242520251728,1.53480388274811,0.124832008778867,0.730079420419606
"2891","ZNF544",39.3599696703953,0.573497076091151,0.373540890093483,1.53529932411851,0.124710320099935,0.730079420419606
"2892","HIST2H4B",5.51580390330859,1.17226714743113,0.763980643564276,1.5344199585503,0.124926370633926,0.730123003862185
"2893","MRPL41",772.336880991342,-0.330345102783542,0.215274627972351,-1.53452873613127,0.124899629361848,0.730123003862185
"2894","CENPV",110.360998033126,-0.498433491113205,0.324871832255413,-1.53424656010601,0.124969007166854,0.730123003862185
"2895","RARB",3.62060074759695,1.34826814562611,0.879834480876756,1.53241112383156,0.125421014160406,0.731752418020753
"2896","CD74",882.444561844461,-0.481222082031002,0.314020825781297,-1.53245276275452,0.125410745770915,0.731752418020753
"2897","WASHC5",190.035128920536,0.220006201916063,0.143551788673822,1.53259115716043,0.125376621650006,0.731752418020753
"2898","AL390755.3",3.02290009362354,2.19009934745893,1.42879574800797,1.53282885290803,0.125318029533601,0.731752418020753
"2899","ABCC3",16.9760015951355,-0.641193344185296,0.418603282334324,-1.53174466432683,0.125585455961557,0.731954115614618
"2900","FP236383.4",2.62478465426101,1.69784602026782,1.10819990075586
```

,1.53207559313964,0.125503781806075,0.731954115614618
"2901","MPP1",86.4269125406078,-0.351628225352768,0.229545679871164,-1
.53184423052582,0.125560878390713,0.731954115614618
"2902","G2E3",120.619443682003,0.331300539396619,0.216347170646096,1.5
313375183379,0.125685997545823,0.7322876797053
"2903","GBP4",135.920317764213,0.304522042704576,0.199020161161686,1.5
3010650241198,0.125990369004528,0.732515931646749
"2904","CENPF",42.3077478795888,-0.444578194802176,0.290590511816821,-
1.52991297624481,0.126038270967413,0.732515931646749
"2905","PHIP",889.890568182597,0.316511271091448,0.206900172832023,1.5
2977770273984,0.126071762543887,0.732515931646749
"2906","DPYS",4.91773408615628,1.54294084927978,1.00836170790514,1.530
1462135896,0.125980541372509,0.732515931646749
"2907","CALCOCO1",572.254135116743,0.312734639020742,0.20442517625306,
1.52982448029593,0.126060180378194,0.732515931646749
"2908","HOXC-
AS3",53.277006576701,-0.400948081969451,0.262092635061828,-1.529795302
62217,0.12606740469966,0.732515931646749
"2909","SLC39A9",149.670488173743,0.325397472452776,0.212644856449579,
1.53023909388531,0.125957557899559,0.732515931646749
"2910","SOX18",2.40930806789636,-2.03077372985592,1.32742182768996,-1.
52986314334605,0.126050607997224,0.732515931646749
"2911","SPTBN1",4489.15234480692,0.30809351240697,0.201505825942094,1.
52895585508037,0.12627538801114,0.73324109788234
"2912","FIGN",74.6425321191203,0.725670184272768,0.474682347003452,1.5
2874904418447,0.126326668921886,0.73324109788234
"2913","ICE2",228.978368375314,0.242754579177779,0.158788248460254,1.5
2879436313288,0.126315430230436,0.73324109788234
"2914","NDUFA8",422.645223538646,-0.393743097213367,0.257636392616865,
-1.52828990195849,0.126440575974958,0.733293624501163
"2915","SLC2A14",2.45530240420932,-4.90283821818797,3.20900230007749,-
1.52783879839213,0.126552566613106,0.733293624501163
"2916","AC023509.3",19.0954783820613,-0.526516045798948,0.344499280844
949,-1.52835165434182,0.126425251380504,0.733293624501163
"2917","PFKL",385.133174399833,-0.213597812754446,0.139788971323868,-1
.52800189264985,0.126512068043318,0.733293624501163
"2918","SMS",465.483614855774,-0.367102630355361,0.240244872685403,-1.
52803523443381,0.126503790057615,0.733293624501163
"2919","GASK1A",63.7350545181728,-0.40885532588028,0.267658397625803,-
1.52752661417287,0.126630114441543,0.733491598142379
"2920","FAM210B",603.976818517756,0.269584702176774,0.176511453537263,
1.52729297036729,0.126688176712449,0.733576606799345
"2921","ZNF449",41.585407570739,0.417146201743583,0.273163811536813,1.
5270917454136,0.126738199260032,0.733615019886551
"2922","PRAC2",16.3141147319426,-1.23072176934599,0.806045730553468,-1
.52686345537854,0.126794968535716,0.733692446270321
"2923","PCGF1",112.355385572905,-0.342644079090802,0.224571709192408,-
1.52576689344797,0.127067928396598,0.734606164271828
"2924","VHL",139.107949754872,0.290137966129459,0.19017263503239,1.525
65570793108,0.127095630581602,0.734606164271828

"2925","RASGRP2",11.8827217188895,-0.846285360143977,0.554707365055669
,-1.52564291274381,0.127098818840104,0.734606164271828
"2926","APOBEC3A",5.01479228626279,-1.1388900443216,0.74655311121665,-
1.52553117415259,0.127126664103346,0.734606164271828
"2927","FAM13A-
AS1",30.1763993744119,0.612706226763881,0.40172598911445,1.52518443756
778,0.127213101101725,0.734854497242217
"2928","AC092620.1",9.80548750925964,0.845127898818477,0.5543087327838
6,1.52465196529389,0.127345928659768,0.735234046098128
"2929","EXOSC6",317.971070101236,-0.255143109594013,0.167353877206287,
-1.52457244405227,0.127365774841579,0.735234046098128
"2930","ASNS",9.57564819256919,0.878748996864831,0.576514544363739,1.5
2424428048845,0.127447700348329,0.735455876276294
"2931","UXT",1231.62471077291,-0.348503611926234,0.228698265669834,-1.
52385769479057,0.127544263432872,0.735761994583074
"2932","TARS",305.083185924921,0.240327429016064,0.157752688265011,1.5
2344427001037,0.127647593467624,0.736106927131848
"2933","FABP3",32.5214810302818,-0.627143478963461,0.411853585254178,-
1.52273405262799,0.127825254864506,0.736612405703147
"2934","NFIA-
AS2",7.40716644543888,-0.941996072523415,0.619179917275728,-1.52136082
944682,0.128169312946798,0.736612405703147
"2935","ADGRL2",197.283106083751,0.332801561431595,0.218790401521531,1
.52109763096186,0.128235338900916,0.736612405703147
"2936","HSPA6",16.0650204249739,-1.06708202007556,0.70246390435418,-1.
51905601620427,0.128748396655951,0.736612405703147
"2937","TNFSF4",8.43808789535988,0.907532216259423,0.597401339594279,1
.51913321265025,0.128728968218449,0.736612405703147
"2938","AC079148.1",4.60491970280106,1.17478447632058,0.77216222174304
3,1.52142185053897,0.128154008974527,0.736612405703147
"2939","EXOC6B",122.168197549973,0.320293516799217,0.210417881715783,1
.52217822072672,0.127964430713319,0.736612405703147
"2940","CYBRD1",4632.85334752005,0.32368068202053,0.212978994826421,1.
51977748925114,0.128566908560466,0.736612405703147
"2941","AC078802.1",3.53399666022126,-1.22558905889337,0.8065479336715
82,-1.519548941517,0.128624378705535,0.736612405703147
"2942","SSR1",669.931505023498,0.230623801025416,0.1516966779117,1.520
29565973526,0.128436684387063,0.736612405703147
"2943","PPP1R11",167.292730399126,-0.359254277503857,0.23625131967418,
-1.52064453226891,0.128349065146059,0.736612405703147
"2944","HIP1",125.932928306877,0.427930628820945,0.281687841440722,1.5
1916613309346,0.128720683649498,0.736612405703147
"2945","AC006333.2",77.6891721239634,-0.429138968976463,0.282305444464
721,-1.52012289309599,0.128480091889999,0.736612405703147
"2946","SARAF",1465.90865053852,-0.310975465142523,0.204658587353324,-
1.51948407913934,0.128640692502568,0.736612405703147
"2947","MARCH8",103.15236183399,0.367701797142283,0.241740629089848,1.
52105915553657,0.128244993058319,0.736612405703147
"2948","ERCC6",107.148116963178,0.398606396787364,0.261935211106333,1.
52177477439468,0.128065524220225,0.736612405703147

"2949","UBXN1",1465.89331556213,-0.370571418783986,0.243607734696826,-1.52118084117965,0.128214461933573,0.736612405703147
"2950","ERG28",172.914567707077,-0.300560078358738,0.197619742406316,-1.52090107344018,0.128284664552134,0.736612405703147
"2951","PTPN9",171.242563084096,0.282612922287974,0.185986618177949,1.51953363664892,0.12862822794942,0.736612405703147
"2952","RNF112",17.285477065529,-0.644063284529319,0.423936444428036,-1.51924490803869,0.128700861282764,0.736612405703147
"2953","CACNB1",23.3319252944888,-0.51105582584212,0.335579583895944,-1.52290499889465,0.12778247496853,0.736612405703147
"2954","PHLPP1",84.1401191943228,0.410990594793285,0.270579527239859,1.51892716712805,0.12878082985915,0.736612405703147
"2955","C20orf27",191.859329954561,-0.457178769037115,0.300606807554385,-1.52085301313212,0.128296727418002,0.736612405703147
"2956","ANKRD54",99.5994468974686,-0.27545966225928,0.181228363258822,-1.51995889222859,0.128521307546981,0.736612405703147
"2957","SPATA6",465.810559170142,0.271889669117153,0.179073420653826,1.5183139302552,0.128935277594936,0.736748115436021
"2958","AFAP1L2",13.544096540761,-0.677688988595418,0.446250136022312,-1.51863032387184,0.128855573822853,0.736748115436021
"2959","CD44",4612.79481802776,-0.311662947732912,0.205263722270325,-1.51835377574642,0.128925237876618,0.736748115436021
"2960","TMEM39B",125.168947090089,-0.274043488124427,0.180647926357339,-1.51700323192386,0.129265868048138,0.736749884554642
"2961","PTPN13",337.015378963714,0.320555083650503,0.211364068172584,1.51660159847397,0.129367301580378,0.736749884554642
"2962","PRRT1",6.05899392483023,-0.972446631428649,0.641499522421339,-1.51589611128337,0.129545623774601,0.736749884554642
"2963","AC103770.1",2.59715938143839,4.71113536878288,3.10678071420654,1.51640421457492,0.129417174026848,0.736749884554642
"2964","ZHX2",186.895679083569,0.312969631596489,0.206316986862077,1.51693583914983,0.129282883947503,0.736749884554642
"2965","AP2A2",348.900071364545,0.281720741882878,0.185655420855246,1.51743881533378,0.129155930204844,0.736749884554642
"2966","BBS4",146.905637077246,0.287145404730562,0.189231880971383,1.51742615069173,0.129159125635908,0.736749884554642
"2967","LPCAT2",86.286439193602,-0.406512772369281,0.268077565325195,-1.5163998221043,0.129418284030114,0.736749884554642
"2968","AC016588.2",14.4781224850932,0.674773615313187,0.444797399725953,1.51703588134491,0.129257625070822,0.736749884554642
"2969","GPX4",9988.22242255345,-0.42372106356811,0.27950931684877,-1.51594611709264,0.129532977794079,0.736749884554642
"2970","ZNF85",56.723455734979,0.480792413402025,0.317098350483381,1.51622489574326,0.129462494965059,0.736749884554642
"2971","ZFP82",55.8152325043661,0.401718097333808,0.264670509862213,1.51780452436103,0.129063684207023,0.736749884554642
"2972","TGFB1",222.158449185969,-0.372899889266183,0.245639600157129,-1.51807725231456,0.128994924963944,0.736749884554642
"2973","RAI2",289.873970331429,-0.43747395324247,0.288411476366656,-1.51683961662577,0.129307182041877,0.736749884554642

"2974","MAATS1",15.8763433811588,-0.738937888078153,0.487646358437875,-1.51531509523677,0.129692627344086,0.737337909594419
"2975","HIST2H2BF",10.7326326673862,0.750591358571364,0.495666178196403,1.51430820093993,0.129947689549008,0.737503483779459
"2976","UHMK1",208.866997961542,0.320352703701249,0.211511454763143,1.51458796432557,0.129876782039224,0.737503483779459
"2977","GPR158",7.66875027735723,0.855898100265979,0.56545523921772,1.51364430091774,0.130116078787621,0.737503483779459
"2978","SIRT1",254.061800559106,0.425959685215919,0.281203514517703,1.51477368960516,0.129829725575529,0.737503483779459
"2979","CCSER2",579.10158277116,0.183555100132153,0.121161083404447,1.51496747119229,0.129780642028039,0.737503483779459
"2980","RTN4RL2",51.4150776108957,-0.420995945631431,0.278041427073472,-1.51414826942383,0.129988238534997,0.737503483779459
"2981","COX5A",619.194284725911,-0.423595647950984,0.279809171794191,-1.51387334887847,0.130057964753994,0.737503483779459
"2982","COASY",139.3982056403,-0.284385820203907,0.187845624423996,-1.51393369462791,0.130042657187868,0.737503483779459
"2983","AC012313.1",21.2741932551776,0.931287923307343,0.615392970340565,1.51332232929466,0.130197803480528,0.737503483779459
"2984","AP001627.1",5.44388606849174,1.1515882165108,0.760974340854988,1.51330755149633,0.130201555422385,0.737503483779459
"2985","GRIN2A",2.41807481177556,-4.85807810945996,3.20948775956528,-1.51366151653994,0.130111710140742,0.737503483779459
"2986","CTR9",324.51374321153,0.266985111800051,0.176473732069616,1.51288868132924,0.130307937456462,0.737858876930293
"2987","AC009309.1",6.20135750385629,-1.44451596670499,0.95515430856402,-1.51233780107915,0.130447949256981,0.737983539160403
"2988","COX17",502.192127900439,-0.330391955176595,0.218744459363445,-1.51040148005599,0.130941011206601,0.737983539160403
"2989","FAM184B",10.8503183539498,1.28570747141576,0.850969387510924,1.51087394010311,0.130820571568268,0.737983539160403
"2990","SEMA6A",206.907479392003,0.507004898915666,0.33556637799738,1.5108930219452,0.130815709025972,0.737983539160403
"2991","BTN3A3",121.60480619884,0.393260512274068,0.260181905305571,1.51148294425857,0.130665450853894,0.737983539160403
"2992","AC008080.4",3.9714260409276,-1.32405934247017,0.875940589154378,-1.5115857843149,0.130639270336823,0.737983539160403
"2993","PAPPA-AS1",2.45138950939206,1.59229462159947,1.05371822564079,1.51111993970795,0.130757895311,0.737983539160403
"2994","ST14",2.56632422186132,-1.72980453399571,1.1448722279808,-1.51091492283515,0.13081012829083,0.737983539160403
"2995","MARS",75.6079714342695,-0.376103829218359,0.248822453664531,-1.5115349265281,0.130652216954061,0.737983539160403
"2996","WDR66",46.7948210486402,0.647826489508475,0.428392645580877,1.51222598284818,0.130476383239653,0.737983539160403
"2997","AL139383.1",4.81261447428222,1.09492807269646,0.724779629223028,1.51070481088194,0.130863676155571,0.737983539160403
"2998","HSPB9",3.48516641737141,1.31817209274859,0.871865449826323,1.5

1189853091572,0.13055967784555,0.737983539160403
"2999","ARHGDIA",841.967759106428,-0.23735737126947,0.15714002623980 8,-1.51048321009724,0.130920170414079,0.737983539160403
"3000","CD99L2",194.670871110821,0.298108006045512,0.197248399423098,1.51133295335934,0.130703642097911,0.737983539160403
"3001","KLHL21",179.962029549968,0.472154370015519,0.312880865552927,1.5090547936868,0.131284780761094,0.738036163575539
"3002","CD58",142.119427132694,-0.297095447594202,0.197260994417913,-1.50610336559888,0.132040640779676,0.738036163575539
"3003","KCNK1",58.0053067342863,0.561659488509138,0.371955214631039,1.51001912707764,0.13103854367031,0.738036163575539
"3004","KIAA2012-AS1",2.28605993983727,1.68168660105228,1.1168360587271,1.50575958567184,0.132128901534372,0.738036163575539
"3005","SEPTIN2",1567.15772945135,0.309689883812143,0.205552465513182,1.50662208326702,0.131907553643099,0.738036163575539
"3006","GUCY1A1",10.8527115336232,1.2808889089537,0.850275377173308,1.50644008204958,0.13195423776356,0.738036163575539
"3007","APC",361.158999634627,0.353764066139411,0.234833064719472,1.50644913041531,0.131951916515191,0.738036163575539
"3008","AC114284.1",5.76106232094052,-1.09217615253537,0.724006191497097,-1.50851769689562,0.131422080857956,0.738036163575539
"3009","KDM7A-DT",121.225605920245,-0.456341943499563,0.302807285498739,-1.50703752965502,0.131801037750579,0.738036163575539
"3010","RHOBTB2",56.7514447877871,0.394125852923099,0.261609132567001,1.50654470299181,0.131927400463109,0.738036163575539
"3011","TMEM246",4.28061883437827,1.22141694742971,0.81058846213101,1.50682745251351,0.13185489088924,0.738036163575539
"3012","FAM225A",20.5122277156114,0.568419424700803,0.377425842251079,1.50604267400076,0.132056219189175,0.738036163575539
"3013","P4HA1",395.549785535607,0.328235887435434,0.217806671583201,1.50700566263439,0.131809205743717,0.738036163575539
"3014","MMRN2",14.593168751051,-0.77014544935462,0.510321123965947,-1.50913887978898,0.13126329558279,0.738036163575539
"3015","TNKS1BP1",436.46124953784,0.253965003802602,0.168646634359812,1.5059022010615,0.132092790060187,0.738036163575539
"3016","SLC46A3",47.8608914910109,0.40331787867068,0.267767374258874,1.50622487069974,0.132009456948874,0.738036163575539
"3017","HEATR5A",133.351514713084,0.385029459062478,0.255128163338631,1.50916094101077,0.131257659082716,0.738036163575539
"3018","AC022613.2",4.45331784408844,-1.38676386454897,0.92006794183204,-1.50724071723186,0.131748966964842,0.738036163575539
"3019","RPS17",7231.16825767106,0.330988308616579,0.219343142297606,1.50899775187634,0.131299357295706,0.738036163575539
"3020","IGF1R",292.595841204313,0.367519754338524,0.24394619104212,1.50656074099172,0.131923286779034,0.738036163575539
"3021","CDK12",361.612277901736,0.288607883106073,0.191272064749876,1.5088867445619,0.13132772783871,0.738036163575539
"3022","SLC16A3",13.6139732758137,-0.827545578973187,0.549271467544093

,-1.50662400629204,0.131907060446981,0.738036163575539
"3023","ZNF681",38.0190062367875,0.420789522569549,0.279089897857688,1
.5077203646551,0.131626111182279,0.738036163575539
"3024","CR381653.1",5.20329642120834,1.01604803882656,0.67279355702228
,1.51019287896199,0.130994215210325,0.738036163575539
"3025","PCBP3",20.4069284842768,0.933059717328125,0.619338190301152,1.
50654316484896,0.131927794995943,0.738036163575539
"3026","UFD1",464.641044352525,-0.368532041527871,0.244304606553748,-1
.50849403425715,0.131428132387411,0.738036163575539
"3027","CENPI",2.70541272583611,1.35866105575182,0.901242367382898,1.5
0754237142359,0.131671691591526,0.738036163575539
"3028","SELENOO",109.014879400505,-0.35303965483602,0.23449380370476,-
1.50553937570357,0.132185461417938,0.738108250216146
"3029","TUB",105.657027219492,0.387323776160694,0.25736149921534,1.504
97948349536,0.132329351540211,0.738423985426367
"3030","SPESP1",35.3115734691075,-0.486897264275332,0.323499291979216,
-1.50509530112546,0.132299576910718,0.738423985426367
"3031","TEX9",23.242326340069,-0.468875164693722,0.311607439593407,-1.
50469823604186,0.132401676808029,0.73858381770708
"3032","LINC00517",6.58280935074295,-1.15598494037275,0.76836390461691
8,-1.50447585242709,0.132458886392681,0.738659251691113
"3033","AL049775.1",17.6352663328262,0.603179361031436,0.4009685667329
99,1.50430585106959,0.132502633222715,0.738659585403782
"3034","AC107223.1",11.9409921935799,0.647574082360907,0.4308198324954
66,1.50312040792069,0.132807997092821,0.739143388691709
"3035","SCAF8",186.810916698394,0.251885643432156,0.167533064429807,1.
50349809626799,0.132710647487398,0.739143388691709
"3036","SIAE",59.899082810241,0.383707383983891,0.255195427333015,1.50
358252102682,0.132688894466766,0.739143388691709
"3037","TMED2",1246.06485138043,-0.244723290397496,0.162794794419729,-
1.50326238175978,0.132771396689043,0.739143388691709
"3038","ABCD4",245.579651235912,0.237510188861817,0.157985855750162,1.
50336362539577,0.132745301172905,0.739143388691709
"3039","HSPB7",11.4979389469918,-0.751385559732753,0.500498989353709,-
1.50127288109615,0.133284996199824,0.739266539060901
"3040","PRKACB",170.560978804465,0.267476888560343,0.178372740779151,1
.49953904050572,0.133733848554357,0.739266539060901
"3041","ISG20L2",169.638848017377,-0.233564056731448,0.155678783457139
,-1.50029471932347,0.133538076734736,0.739266539060901
"3042","PIK3C2B",55.6638545195397,-0.481238299235247,0.320940470831434
,-1.49946280688298,0.133753610531522,0.739266539060901
"3043","DUSP19",18.3964453583724,0.541153213417003,0.360275338805667,1
.50205455419445,0.133083020308752,0.739266539060901
"3044","POMGNT2",120.867860677282,0.329518203920992,0.219828161871809,
1.49898084538026,0.133878601297005,0.739266539060901
"3045","AC078850.1",5.23626274228724,-1.35664270955435,0.9043284051549
15,-1.50016598154069,0.133571412829608,0.739266539060901
"3046","TCERG1",269.326669632222,0.347993447709693,0.231674899572859,1
.50207660972894,0.133077324835411,0.739266539060901
"3047","GRIA1",60.7537703984938,0.684771572191326,0.456034888221355,1.

50157715972587,0.133206345704053,0.739266539060901
"3048","DYNLT1",860.155557865416,-0.359153296480702,0.239598860363691,
-1.49897748234502,0.133879473775992,0.739266539060901
"3049","TRIM4",188.399969359368,0.255857655043614,0.1704269993918,1.50
127418752128,0.133284658435862,0.739266539060901
"3050","RDH10-
AS1",4.17582523276669,1.43009766995905,0.953275034375171,1.50019419201
135,0.133564107298773,0.739266539060901
"3051","UBR5-
AS1",18.0613190499097,0.603167686655097,0.401708357599393,1.5015064417
8683,0.133224621801089,0.739266539060901
"3052","PYCR3",33.5776521496776,-0.526470508803517,0.351133595068669,-
1.49934530958383,0.133784073680732,0.739266539060901
"3053","IDNK",95.2070299641816,-0.401311519782358,0.267444214549933,-1
.50054290932299,0.133473827171793,0.739266539060901
"3054","FRRS1L",2.16524179764436,2.14362581558119,1.42947851037193,1.4
9958589795341,0.133721702859032,0.739266539060901
"3055","ZBTB34",53.6240075414441,0.393878178451604,0.262542564896053,1
.50024503115352,0.133550942509594,0.739266539060901
"3056","ARHGDIB",400.56826173513,-0.377924159807194,0.251852957405653,
-1.50057463569301,0.133465615812057,0.739266539060901
"3057","AC027807.2",3.83466692868615,1.14363344334123,0.76107517891667
9,1.50265502675976,0.132928025649163,0.739266539060901
"3058","AC099518.5",109.009373607076,-0.65101608875293,0.4343038924872
4,-1.49898746019656,0.133876885214757,0.739266539060901
"3059","EXOC7",468.554467507145,0.198722617095844,0.132489580401798,1.
49991128731168,0.13363738377489,0.739266539060901
"3060","LINC01894",34.7645255379675,0.505642317040858,0.33716211362981
8,1.49970087563284,0.133691903659794,0.739266539060901
"3061","PLCG1",64.7286140306086,0.355479182460833,0.236927013951856,1.
50037421453793,0.133517495006246,0.739266539060901
"3062","WDR45",568.34445909236,-0.34349910971787,0.228800977706965,-1.
50130088236687,0.133277756879031,0.739266539060901
"3063","RNF13",801.107110400766,-0.233449889728093,0.155768156643169,-
1.4987009845849,0.133951221192529,0.739320837681214
"3064","ALOX5AP",30.2720796536444,0.49711516870811,0.331719143299022,1
.49860259424339,0.133976759324299,0.739320837681214
"3065","CBX7",250.666413353451,0.354012834093825,0.236348097325442,1.4
9784507723946,0.134173506066814,0.740164972456016
"3066","TRIR",2193.22706796679,-0.320983496869489,0.214454936928706,-1
.49674100054035,0.134460663377522,0.741265372151009
"3067","PIK3IP1",389.445097009546,0.300883012136161,0.201003359415275,
1.49690539009616,0.134417877513798,0.741265372151009
"3068","CERS2",630.99861950358,-0.330888577129749,0.221293658586773,-1
.495246539114,0.134850111728789,0.741502721771969
"3069","PM20D1",3.50947833781577,1.28736840858077,0.860337519703161,1.
49635274424036,0.13456175710416,0.741502721771969
"3070","FGFBP2",335.977598494595,-1.27987143601688,0.855510919655675,-
1.49603167722512,0.134645400540492,0.741502721771969
"3071","RBM22",339.712551800972,-0.215572478436793,0.144153137067008,-

1.49544077099471,0.134799446744205,0.741502721771969
"3072","LINC00242",7.64402142624582,-0.910296024259272,0.608772313458031,-1.49529800244772,0.134836686191608,0.741502721771969
"3073","EPB41L4B",6.51556431066045,-1.12400541678185,0.75145899564896,-1.49576413788374,0.134715129798637,0.741502721771969
"3074","NUDT15",132.018152745284,-0.261270648162109,0.174744740001413,-1.4951560096172,0.134873731188857,0.741502721771969
"3075","CPPED1",144.647527127602,-0.269581124940926,0.18031143586951,-1.49508611941851,0.134891967971324,0.741502721771969
"3076","ZNF821",55.4753957842885,-0.346222779920286,0.231577629679216,-1.49506142022387,0.13489841330557,0.741502721771969
"3077","ETAA1",119.706235624631,0.268983856719916,0.179939510190133,1.49485711301367,0.1349517370564,0.741554751429839
"3078","ARPC4",624.736363626804,-0.278744379030974,0.186551898195937,-1.49419213487823,0.135125407743893,0.742026760030446
"3079","TBRG1",309.001226676128,-0.204424905755022,0.136811193968989,-1.49421183913765,0.135120259149804,0.742026760030446
"3080","FNDC10",21.2034394010843,1.10264405412527,0.738084528275806,1.49392652451487,0.135194824789147,0.742166914784055
"3081","MAFIP",7.52523191856596,0.88046137934412,0.589592741901438,1.49333822615358,0.135348674168112,0.74277032873562
"3082","AL353593.1",11.3846675246612,0.693567700969816,0.464507576481912,1.49312462505512,0.135404567710584,0.742835960691288
"3083","SLC37A4",50.0788593450747,-0.373507119810795,0.2502335659705,-1.49263396524041,0.135533027432961,0.743299522489948
"3084","RGS14",42.9775854774486,-0.46885441321568,0.314149113206088,-1.4924581783177,0.135579073131779,0.7433109495824
"3085","INKA1",19.8726702989517,-0.65063842925025,0.436043248571376,-1.49214196385785,0.135661932893022,0.743524136581917
"3086","PNISR",2507.60230325659,0.283047682219088,0.189727430863327,1.49186483436328,0.135734583116506,0.743590103307812
"3087","N4BP2L2-IT2",9.18954187111192,0.928906486285763,0.622691409147912,1.49176056171527,0.135761926242679,0.743590103307812
"3088","HIST1H2BN",8.28744447426435,0.827544821652396,0.554866855991707,1.49142954335114,0.135848756453076,0.743616485148235
"3089","GCNA",6.98323107376332,0.879640818898677,0.589806062647559,1.49140687864429,0.135854703254252,0.743616485148235
"3090","STK39",64.1273623449591,0.383642482757724,0.257310556512265,1.49097063081218,0.135969205817603,0.744002372803891
"3091","NKD2",5.4679266491882,1.30684828567571,0.876713312909805,1.49062215256923,0.13606072490084,0.744021583642756
"3092","XG",886.762830996768,-0.368136777623449,0.246964682928521,-1.49064543665946,0.136054608435339,0.744021583642756
"3093","HP1BP3",1546.27967373197,0.237556767585553,0.159563414451332,1.48879220466926,0.136542095694503,0.744254040144349
"3094","TERC",4.8291019476914,-0.956970265170526,0.64254458713733,-1.48934452850039,0.136396667728134,0.744254040144349
"3095","ZNF595",105.808867792753,0.364491376871662,0.244789774848267,1.48899755758831,0.136488011893111,0.744254040144349

"3096","AL357497.1",5.13693517812395,0.956872885623179,0.642675681459328,1.48888920683976,0.136516546170695,0.744254040144349
"3097","AL035530.2",22.1478551499605,-0.547214577102924,0.367431116546989,-1.48929840848943,0.136408806642584,0.744254040144349
"3098","BRF2",113.044887421505,0.386269807355449,0.259392873055108,1.489130378973,0.136453039529068,0.744254040144349
"3099","PIP5KL1",7.90932723906132,-0.876929220752218,0.588538438111621,-1.49001180545815,0.136221131870657,0.744254040144349
"3100","HDAC7",576.598428499752,0.276159309302837,0.18533618781213,1.49004526618822,0.136212334185442,0.744254040144349
"3101","SSH1",98.5962956538121,0.427199847254624,0.28694486797183,1.48878720248296,0.136543413326696,0.744254040144349
"3102","BCAP31",769.093022809702,-0.258413276643512,0.173466649455164,-1.48970005159582,0.136303121050226,0.744254040144349
"3103","ZBTB20-AS1",2.86229434526926,1.6966525409822,1.14048689542826,1.4876563227367,0.136841551739167,0.745638722786282
"3104","NME3",884.653681900908,-0.34492164454782,0.231931382420928,-1.48717108028886,0.136969632058,0.745898541447317
"3105","LINC00667",349.730423259351,-0.228149036246412,0.153414486047872,-1.48714141750095,0.136977464584452,0.745898541447317
"3106","DACT2",3.71523800908386,1.68370692122104,1.13245948283987,1.48677012002126,0.137075535760235,0.746192259701885
"3107","IP6K1",142.961524075406,0.2625901646337,0.176662935650981,1.48639081347815,0.137175778296437,0.746497605225672
"3108","BCL9",74.4318289007382,0.326497176962307,0.219832446027203,1.485209225766,0.137488408864679,0.746756831700608
"3109","AC004083.1",2.01321198310757,1.83160593346153,1.2329409009371,1.48555857954702,0.137395917887818,0.746756831700608
"3110","LRP4",40.2862281376375,0.529146342201923,0.356203995505447,1.4855148984252,0.137407479784931,0.746756831700608
"3111","AL110292.1",49.9361394273673,-0.507243727835266,0.341374884163318,-1.48588473073665,0.137309613082322,0.746756831700608
"3112","MT1G",111.934955751904,0.752507065032191,0.506451826743944,1.48584134816962,0.137321090389997,0.746756831700608
"3113","STRN4",124.031707971414,-0.329480259339111,0.221823766462583,-1.48532442935814,0.137457903553572,0.746756831700608
"3114","AL662884.5",33.4920544667794,-0.660845692015046,0.445143286135664,-1.48456848075126,0.137658170074171,0.747014328572474
"3115","GALNT17",23.4042205079799,0.923325964608135,0.621861226271234,1.48477815564177,0.13760260030078,0.747014328572474
"3116","HECTD4",163.919306975635,0.34735922265804,0.233985985010678,1.48453003560101,0.137668361002592,0.747014328572474
"3117","ZDHHC20",120.353934447651,0.365424498963368,0.246272785588345,1.48382005786945,0.137856664402912,0.747316724046508
"3118","TRDC",20.1123477773167,-0.668478401352244,0.450511796238499,-1.48381997304762,0.137856686911584,0.747316724046508
"3119","SECISBP2L",392.961148266569,0.34349474341348,0.231478561278386,1.48391601155832,0.137831203548717,0.747316724046508
"3120","AC011346.1",21.443654135525,0.552923362977312,0.37268823726510

9,1.48360830230334,0.137912865562218,0.747381644527559
"3121","AC127502.2",100.587679310745,-0.457156796076918,0.308258599489
13,-1.48303014687848,0.138066401311879,0.747973954944327
"3122","IFFO2",38.7969657188485,0.401571660297463,0.27198158101181,1.4
764663798319,0.139818734244549,0.748056121249249
"3123","AL513166.1",13.9639827882022,0.623247450215592,0.4226031597936
81,1.47478180361895,0.140271214832211,0.748056121249249
"3124","GSTM2",113.690757781058,0.351101857688774,0.237399555363856,1.
47894909554759,0.1391539203398,0.748056121249249
"3125","OAZ3",4.59861666033303,-1.08885241064594,0.735613667027131,-1.
48019600430532,0.138820946465041,0.748056121249249
"3126","KLHDC9",22.9791128942884,-0.676604435811091,0.459816265257388,
-1.47146694654734,0.141164880075548,0.748056121249249
"3127","NCF2",18.8401664063928,-0.774522570088552,0.523828478603258,-1
.47858049290055,0.139252469471449,0.748056121249249
"3128","MTR",367.724108650365,0.351472844460754,0.238557303975859,1.47
33266959469,0.140662965647918,0.748056121249249
"3129","LPIN1",188.329575808055,0.316898695024171,0.215054734523344,1.
47357227789734,0.140596789992201,0.748056121249249
"3130","LINC01121",10.4961187570098,0.891425013192798,0.60136106328717
2,1.48234574470132,0.138248322329102,0.748056121249249
"3131","TIA1",299.154367784862,0.272241139618733,0.184872855433646,1.4
7258578865001,0.14086275910939,0.748056121249249
"3132","FAM126B",169.845973880652,0.347681608960413,0.236346485040656,
1.47106739878364,0.141272890573449,0.748056121249249
"3133","EPHA4",71.3179889561026,0.464432274870742,0.313523738023219,1.
4813304976491,0.138518525416846,0.748056121249249
"3134","U73169.1",2.12014759898504,2.01703347213794,1.3682701848208,1.
47414852308728,0.140441606277591,0.748056121249249
"3135","AC046134.2",31.8919422275839,0.490415666384133,0.3315968780150
31,1.47895139821522,0.139153304870354,0.748056121249249
"3136","AC069243.1",3.42192665608251,-1.40575310078823,0.9553934150821
12,-1.47138663360727,0.1411865861251,0.748056121249249
"3137","ACTRT3",18.7749075174286,-0.5858061875141,0.397265440363724,-1
.47459639826146,0.140321083815217,0.748056121249249
"3138","RPL22L1",860.932763786585,-0.387962137159431,0.2624516539725,-
1.47822325097666,0.139348032481059,0.748056121249249
"3139","MAP3K13",188.2145497742,-0.251344009406593,0.169863174215767,-
1.47968510871772,0.138957301478093,0.748056121249249
"3140","SENP5",181.008698187736,-0.280110292275675,0.189924749643357,-
1.4748488167112,0.140253193493224,0.748056121249249
"3141","INTU",131.439228042554,0.304383281098295,0.206781904292602,1.4
7200153775344,0.141020462320607,0.748056121249249
"3142","GUCY1B1",27.95864464772,0.703112052417954,0.477259750661177,1.
47322721315571,0.140689779559234,0.748056121249249
"3143","PDZD2",14.8813106468425,0.835458555468724,0.5666622034504,1.47
435023966947,0.140387314831509,0.748056121249249
"3144","MAP3K1",73.2131061470583,0.423478944872326,0.287348697084669,1
.47374583274183,0.14055003753573,0.748056121249249
"3145","B3GALT4",149.025410691618,-0.238372393294551,0.160915967045715

,-1.48134705132668,0.138514116475284,0.748056121249249
"3146","TXLNB",7.52747563647071,-1.14943907868299,0.778383206390903,-1.4767007680093,0.139755866216058,0.748056121249249
"3147","AC004160.1",53.1125895977296,0.651022311078968,0.441457252872056,1.47471200630075,0.140289986806317,0.748056121249249
"3148","ITGB8",31.6918650807962,0.60593183833972,0.410138035649097,1.47738513786158,0.139572427760496,0.748056121249249
"3149","GLI3",475.836111270004,0.302944471165933,0.204735802668406,1.47968487786471,0.138957363114703,0.748056121249249
"3150","AC002429.2",2.64727550586522,1.71306272339708,1.1593227250613,1.47764094187536,0.139503909667674,0.748056121249249
"3151","ARHGEF35",61.8632205959664,-0.429172213153876,0.290372777438445,-1.47800429826743,0.139406627767152,0.748056121249249
"3152","ZNF777",59.4245298770885,0.335577369889044,0.22660351443707,1.48090099450879,0.138632957991609,0.748056121249249
"3153","MICU3",230.292457916332,0.351190487245266,0.236908408998146,1.4823892859287,0.138236743131623,0.748056121249249
"3154","ZNF395",275.541749950246,0.296828870004189,0.201337334147503,1.4742862830732,0.140404526820057,0.748056121249249
"3155","UBXN8",84.4865835234289,0.386281100305577,0.262587112724602,1.47105886613219,0.14127519791364,0.748056121249249
"3156","AP3M2",76.2101935077506,0.390554535297323,0.265517175268761,1.47092004463364,0.141312741125437,0.748056121249249
"3157","MSC-AS1",107.82433777318,0.277212181440734,0.188466014420699,1.47088684552926,0.141321720705108,0.748056121249249
"3158","TMEM70",301.360836486825,-0.415238066919224,0.281472229043597,-1.47523636107954,0.140149008960872,0.748056121249249
"3159","TRMT10B",136.605626388202,0.320322134734255,0.216660668493996,1.4784507818646,0.13928716162639 7,0.748056121249249
"3160","SHB",14.289009186126,-0.788740305883873,0.534056768080472,-1.47688476773508,0.139706528714298,0.748056121249249
"3161","ADIRF-AS1",26.4026988629448,-0.715338467962008,0.484130670286604,-1.47757312615317,0.139522071848088,0.748056121249249
"3162","NHLRC2",241.463703777218,0.319393583008748,0.216262743382718,1.47687751488254,0.139708473233091,0.748056121249249
"3163","SHTN1",87.7972325961304,-0.56847472858713,0.385962286069231,-1.47287636410455,0.140784376387216,0.748056121249249
"3164","MRPL23",185.354466050939,-0.549116895926298,0.372813403133883,-1.47290009240655,0.140777977159628,0.748056121249249
"3165","SOX6",3.79680979909396,-1.31913283883669,0.894586405670147,-1.47457286459491,0.140327414704699,0.748056121249249
"3166","GPR137",117.330546201385,-0.361402840969177,0.244834025665693,-1.47611362426664,0.13991339201167,0.748056121249249
"3167","NUMA1",860.370213063034,0.258007099355315,0.174528888075963,1.47830598246302,0.139325897133483,0.748056121249249
"3168","TMEM25",66.2976288276923,-0.356901375859703,0.241755985393463,-1.47628765128126,0.139866687768019,0.748056121249249
"3169","HMBS",42.8196553058108,-0.466332718948166,0.316171709297887,-1

.47493499650471,0.140230020411934,0.748056121249249
"3170","SCAF11",1295.52386731134,0.243602590846092,0.164830501570411,1
.47789752821951,0.139435208037562,0.748056121249249
"3171","UTP20",115.474611388561,0.322939960661059,0.21915174862258,1.4
7359061787466,0.140591848977398,0.748056121249249
"3172","KDM2B",61.3736771151641,0.374260736088265,0.254078682684577,1.
47301116384048,0.140748025550508,0.748056121249249
"3173","ITM2B",16883.6056435876,-0.324552536151506,0.219548884055829,-
1.47827003333333,0.139335515224492,0.748056121249249
"3174","ANKRD10-
IT1",15.6052333332461,0.801253579653682,0.544018384769799,1.4728428341
4931,0.140793419379944,0.748056121249249
"3175","GZMB",27.7025891245599,0.672468772309756,0.453541024445455,1.4
82707706832,0.138152086137157,0.748056121249249
"3176","DLST",254.326888197112,0.238824104497764,0.162249788482284,1.4
7195325634487,0.141033500689838,0.748056121249249
"3177","GLRX5",765.869261350306,-0.403344466228077,0.274152130102989,-
1.47124323300554,0.141225349155057,0.748056121249249
"3178","BTBD6",534.730519562198,-0.299554337322669,0.203672902814322,-
1.47076185974409,0.141355530363813,0.748056121249249
"3179","GOLGA8B",78.457014768369,-0.875430402451132,0.594392764523322,
-1.47281470216615,0.140801006889965,0.748056121249249
"3180","VPS18",66.3064123842411,0.302830691990437,0.204698332231832,1.
47939989881044,0.139033467165678,0.748056121249249
"3181","PLEKHO2",85.46867150988,0.313811430500578,0.212442889469155,1.
47715666683276,0.139633646503706,0.748056121249249
"3182","LCTL",4.85273044142934,1.40168356181528,0.948956089618773,1.47
707947411812,0.139654334938391,0.748056121249249
"3183","STX4",476.259932667917,-0.283976697182988,0.192912787822184,-1
.47204703425229,0.141008176862774,0.748056121249249
"3184","AC004687.1",11.9335595340302,-1.01138961876522,0.6860923828831
8,-1.47413037077461,0.140446492712917,0.748056121249249
"3185","TCF4-
AS2",10.1587204643091,1.00307129813408,0.677921729563838,1.47962700469
777,0.138972815627555,0.748056121249249
"3186","AC036176.1",116.270671429878,0.453168920096467,0.3064427869204
1,1.47880432967791,0.139192618351767,0.748056121249249
"3187","DOK6",52.3068935821299,0.603626327116807,0.410347508321549,1.4
7101253175834,0.141287727838037,0.748056121249249
"3188","AC011471.2",3.13329984611868,-1.36343254461315,0.9204923849409
8,-1.48119915701482,0.138553510791737,0.748056121249249
"3189","AC008764.7",12.1437260071105,1.11973473417939,0.75869931220838
3,1.47586101128802,0.139981207998295,0.748056121249249
"3190","PHLDB3",27.75042560065,-0.626024905354748,0.425406558694754,-1
.4715920395669,0.141131076494649,0.748056121249249
"3191","AC007785.3",2.34970822334226,1.80561722647209,1.22607464414314
,1.47268132091092,0.140836985583018,0.748056121249249
"3192","ZNF615",45.6461325900169,0.375103810940343,0.253952235389868,1
.47706441868678,0.139658370222427,0.748056121249249
"3193","RRBP1",1661.09130920787,0.308898151433432,0.209750128854889,1.

47269588400175,0.140833056943881,0.748056121249249
"3194","LRCH2",61.3209186549082,0.36852041800326,0.249374696033331,1.47777791357791,0.139467231913219,0.748056121249249
"3195","FMR1",256.712926434224,0.336604525288834,0.227243373129771,1.48125122705608,0.138539640013084,0.748056121249249
"3196","MMP11",56.9462454219387,0.456592787160383,0.310607991323794,1.46999690901194,0.141562591136497,0.748917487777188
"3197","CHSY1",101.96475416305,0.312833081423493,0.212863392521985,1.4696424674862,0.141658612152025,0.749191058575678
"3198","PLOD1",185.449490256792,0.332880086950208,0.226657054680816,1.46865089824351,0.141927501913978,0.749287064056556
"3199","WDR82",441.095545048762,0.205934336812099,0.140216466828777,1.46868867451618,0.141917250718047,0.749287064056556
"3200","PXYLP1",95.3731475260234,0.366421005815465,0.249442989968063,1.46895691822159,0.141844474853949,0.749287064056556
"3201","SLC33A1",159.590550369472,0.251299631041982,0.171057553619176,1.46909403136589,0.14180728644292,0.749287064056556
"3202","NPEPPS",654.766555845241,0.312385573412312,0.21273414075392,1.46843178206014,0.141986973813414,0.749287064056556
"3203","AC006942.1",20.1967768942884,-0.677411971973335,0.461083291261487,-1.46917484283586,0.141785371920066,0.749287064056556
"3204","EBF4",16.0612021028105,-0.543755120734342,0.370268678524106,-1.46854204061157,0.141957045354859,0.749287064056556
"3205","NUP153",192.671818678003,0.33147446724221,0.225768713969613,1.46820372678752,0.142048892261097,0.74937992834653
"3206","NDUFB2",1631.67517671245,-0.366923819181117,0.249954007826068,-1.46796533639278,0.142113638926392,0.749487650332949
"3207","SLC45A1",13.9735155322586,-0.737205529958495,0.502836215187944,-1.46609473958225,0.142622478637786,0.749783594240056
"3208","DESI2",506.705289870799,-0.241502053212195,0.164575479751335,-1.46742426986749,0.142260676312602,0.749783594240056
"3209","EDEM1",128.448156498548,-0.342664764486554,0.233702616000082,-1.4662427419573,0.142582168153888,0.749783594240056
"3210","ARIH2OS",50.8665767712526,-0.545972695034521,0.372405132804836,-1.46607188499909,0.14262870417776,0.749783594240056
"3211","CNOT6L",212.245989547916,0.326035132883655,0.222355897270106,1.46627607761446,0.14257308993579,0.749783594240056
"3212","MAT2B",248.09952525817,0.316591713637726,0.216006141204557,1.46566070701626,0.142740743827654,0.749783594240056
"3213","ANKRD6",64.6979676952565,0.323005961708199,0.220271783922347,1.4663973567403,0.14254006603129,0.749783594240056
"3214","BBS9",92.4932049760368,0.330165350396919,0.225242618180698,1.46582095814589,0.142697069839009,0.749783594240056
"3215","UBR5",372.115653901795,0.357448030694405,0.243808823989631,1.46609964662152,0.142621141997901,0.749783594240056
"3216","SMG1",376.485634391069,0.320935758652146,0.218998818793206,1.46546798937393,0.142793279654048,0.749783594240056
"3217","SNHG16",866.616159478626,-0.22705302389567,0.154907137783575,-1.4657363575647,0.142720125152134,0.749783594240056
"3218","GYS1",42.6649890661608,0.396442321565408,0.270234404520423,1.4

6703126964519,0.142367549200865,0.749783594240056
"3219","ZNF579",89.1328876831893,-0.359372269478213,0.245276727658256,-1.46517067847923,0.142874357248408,0.749783594240056
"3220","ZNF417",21.4383172570358,0.514241897203625,0.350981786320087,1.46515265819135,0.14287927257165,0.749783594240056
"3221","SH3KBP1",500.093309770452,0.279042188963326,0.190303593211409,1.46630015889052,0.142566532216743,0.749783594240056
"3222","AP000439.1",2.57127851273933,1.88244724198967,1.28450988104311,1.46549845179936,0.142784974451897,0.749783594240056
"3223","PHKA2",37.4756851364575,0.418514634865543,0.285684780059483,1.46495250736985,0.142933875652336,0.749837409100125
"3224","ISG15",347.289564220033,-0.513416653382187,0.350696058189956,-1.46399322545021,0.143195799369949,0.750978466422797
"3225","C1RL",427.507743042681,0.311022948248729,0.21252654274435,1.46345460775157,0.143343025668569,0.751517481551681
"3226","FHL2",221.933084026247,0.678475522183957,0.464434091186309,1.46086502920343,0.144052485384332,0.751848737318425
"3227","DNAJC10",349.23212177744,0.256903333082566,0.17577657227002,1.46153341008336,0.143869113677195,0.751848737318425
"3228","EREG",34.3402902736165,-0.9857788692098,0.673934592867041,-1.46272187188985,0.143543498825814,0.751848737318425
"3229","NDNF",105.228478896243,-0.485251475020821,0.332150983345079,-1.4609364396091,0.144032885243215,0.751848737318425
"3230","LRRC1",12.9769368072944,-0.735065790870586,0.502880076110972,-1.46171189870003,0.143820175241978,0.751848737318425
"3231","DST",1535.66079575631,0.403212359068955,0.27576757795246,1.46214563025413,0.143701306888928,0.751848737318425
"3232","GIMAP2",29.0176361489097,0.557306583956906,0.381348356228291,1.46141074126796,0.143902754708786,0.751848737318425
"3233","LPCAT3",149.83209058532,0.422373779551173,0.289093086318673,1.46103037236104,0.144007106467341,0.751848737318425
"3234","AC025263.1",3.58210173773398,-1.25836484154617,0.860605702055399,-1.4621851081637,0.14369049132579,0.751848737318425
"3235","DLEU1",29.8985937372898,-0.447925552311115,0.306579423832898,-1.46104244933038,0.144003792337064,0.751848737318425
"3236","LINC00641",37.7275288092105,0.799936237610593,0.547605407844618,1.46078951403923,0.14407321439033,0.751848737318425
"3237","TRMT61A",97.2960545106359,-0.272348214616726,0.186265676155061,-1.46214922812727,0.143700321171972,0.751848737318425
"3238","TEAD2",133.364747117154,-0.303897799055058,0.207907977204442,-1.4616937894414,0.143825139899811,0.751848737318425
"3239","LILRB1",2.28803462682542,-1.47165856187095,1.00664895142743,-1.46193820575101,0.143758144188765,0.751848737318425
"3240","PLCXD1",22.9353365142728,0.829060420314627,0.567035723565801,1.46209557151194,0.14371502212698,0.751848737318425
"3241","CCL5",75.783648632616,0.620748247101316,0.42500910123122,1.46055283358181,0.144138198284777,0.751955771860234
"3242","AL049597.2",36.2470084697456,-0.509750927236669,0.349105162374595,-1.46016439221171,0.144244898935857,0.752020235976281
"3243","CD8B",9.20098604742415,-0.817708166815891,0.560124756669679,-1

.45986792599156,0.144326375737911,0.752020235976281
"3244","PAXIP1-
AS1",112.532005690408,-0.338043326499686,0.231558676921974,-1.45986032
997413,0.144328463788918,0.752020235976281
"3245","C8orf88",71.6011396209751,-0.367149686585092,0.251454892387208
,-1.4601015836261,0.14426215746137,0.752020235976281
"3246","DEXI",438.112552950805,-0.325715934140557,0.223177321374273,-1
.45944907007072,0.144441548604406,0.752370386106063
"3247","CEP295NL",9.49114367347533,1.28747123663833,0.882257238477534,
1.45929234750179,0.144484660733758,0.752370386106063
"3248","KCNIP4-
IT1",8.1543571974901,-1.20999270879626,0.829286095972771,-1.4590775302
6645,0.144543769893823,0.752446447464518
"3249","LMBRD2",74.1065472952868,0.355347691904641,0.243610341550364,1
.45867244240605,0.144655284382421,0.752565802322284
"3250","BCL2L13",183.801734130975,0.253807365563178,0.173999063355649,
1.45867087252305,0.144655716675386,0.752565802322284
"3251","HIBCH",318.408245313612,-0.289449262924927,0.198483701292639,-
1.45830242503474,0.144757202101355,0.75286212646254
"3252","DDHD1",249.180133879581,0.314527866208418,0.215707671560879,1.
45812090934211,0.144807218960251,0.752890669797022
"3253","NRBP1",557.943546787584,-0.236931188213592,0.162514212327487,-
1.45791057176061,0.144865194284181,0.752960561007357
"3254","NR2F2",742.959870237939,0.305209774918486,0.209373767937167,1.
45772690593255,0.144915832617545,0.752973743266964
"3255","DLX4",3.04931500857338,-1.5878775184164,1.08945824446251,-1.45
749277357553,0.144980404690568,0.752973743266964
"3256","ZNF607",11.3888469874716,0.652477265869043,0.447694314512935,1
.45741691309817,0.145001331208732,0.752973743266964
"3257","CARHSP1",1393.09447509969,0.305265084749715,0.209496178140984,
1.45713915861645,0.145077971012886,0.753140415684949
"3258","ZNF704",545.904445663763,0.346918506724341,0.238112552191032,1
.45695177987096,0.145129691286531,0.753177661225496
"3259","MADD",90.5362419698847,0.375086581616384,0.257488371619106,1.4
5671270223898,0.145195701988499,0.753289024001701
"3260","BRINP3",5.4222192305916,1.66246301391811,1.14294283460774,1.45
454607490379,0.145794969096438,0.753623154228852
"3261","CLASP1",162.446818160543,0.340010147994497,0.233483704537878,1
.45624787249055,0.145324109957071,0.753623154228852
"3262","C1QTNF7",363.400715243152,0.650569063073642,0.446775797038247,
1.45614213524183,0.145353331725756,0.753623154228852
"3263","FGF1",12.264499873334,-1.35694682059833,0.93209143916124,-1.45
580869385455,0.14544551175903,0.753623154228852
"3264","LINC02541",2.55212414491876,1.41622921641584,0.972955479193385
,1.45559508806091,0.145504586703457,0.753623154228852
"3265","BICD2",208.689257174077,0.277465542857145,0.190666819119763,1.
45523769756111,0.145603467913983,0.753623154228852
"3266","ADK",350.19048464033,0.288782467812549,0.198505782962169,1.454
78113283775,0.145729863041092,0.753623154228852
"3267","AL161891.1",7.18966854747746,1.09613299686397,0.75353343585080

3,1.45465741095661,0.145764128599462,0.753623154228852
"3268","CIITA",18.0674722526944,-0.663267903194621,0.455749195372063,-1.45533532462552,0.145576451786892,0.753623154228852
"3269","TCF25",1373.99339224962,-0.17033667678826,0.117074811132349,-1.45493872798736,0.145686224997021,0.753623154228852
"3270","IL4I1",3.55422035967989,-1.32021396017956,0.907316489972494,-1.45507546128648,0.14564837169816,0.753623154228852
"3271","HMOX1",101.067176594645,0.390257599037965,0.268139502515449,1.45542747479171,0.145550954791281,0.753623154228852
"3272","DMRT3",16.2117381011042,0.876608070150377,0.602866616403436,1.45406636608944,0.145927907335129,0.754079785214657
"3273","TGIF2",41.6719730257152,0.416271611237416,0.286323697535042,1.45384966323464,0.145987991046403,0.754159777761253
"3274","AIMP2",113.600386076654,-0.385265755209076,0.265057277109458,-1.45351887490331,0.146079742961873,0.754249419389357
"3275","AVL9",120.315163940462,0.402174148092109,0.27670017474996,1.45346546475995,0.14609456165721 2,0.754249419389357
"3276","NOTCH1",28.8782346118413,0.50828816478377,0.349747967424813,1.45329840949838,0.146140918725864,0.754258441336055
"3277","AC093726.2",5.77886844911088,1.15717162568154,0.796328855406408,1.45313285814687,0.146186869570817,0.754265361825868
"3278","LINC01341",16.7722953406169,0.620808671222933,0.427378005304099,1.45259855097409,0.146335248626463,0.754267020288489
"3279","PSMD6-AS2",19.5894972851063,0.584738583432203,0.402598883428546,1.45240984886135,0.146387679419507,0.754267020288489
"3280","AC010623.1",5.53460409001603,1.11571441791864,0.768648985122224,1.4515265609063 8,0.146633291677801,0.754267020288489
"3281","CDKL3",45.0717369731689,0.401066914128,0.27606963428968,1.45277446090706,0.146286385076154,0.754267020288489
"3282","EYS",3.72074587415107,1.60655768880799,1.10668856610207,1.45168002816414,0.146590595054996,0.754267020288489
"3283","PHTF2",175.081747659525,0.272129395367405,0.187385546166056,1.45224325427027,0.146433979592857,0.754267020288489
"3284","ART5",12.4571706617437,-0.959300845189559,0.660429124280225,-1.45254170344935,0.146351042171892,0.754267020288489
"3285","PPP1R14B",1254.80002693368,-0.422997830472886,0.291366264493609,-1.45177353050138,0.146564586129691,0.754267020288489
"3286","ATP2B1-AS1",119.984667322258,-0.533863283689448,0.367679706211258,-1.45197919458385,0.146507390337452,0.754267020288489
"3287","ADAL",60.8517143643618,0.47797771938595,0.329238552302096,1.45176716409376,0.146566356919088,0.754267020288489
"3288","CEP104",107.571118587568,0.46933517514597,0.323573814883638,1.45047328787946,0.146926582906948,0.754428342368181
"3289","AGO3",386.781384568885,0.364039104064493,0.251527109542436,1.44731557853439,0.147808557912918,0.754428342368181
"3290","RPS6KC1",124.993393095942,0.307823090605018,0.212786156137648,1.44663119157946,0.148000245359158,0.754428342368181
"3291","ITPKB",66.8594264740843,0.330725330169926,0.228335620092662,1.

44841759702543,0.147500296352746,0.754428342368181
"3292","AC097448.1",83.901801299385,-0.456531609362282,0.315043302818533,-1.44910748864656,0.147307567030171,0.754428342368181
"3293","GMPPB",75.6913604545859,-0.519588029137937,0.358377438045928,-1.44983465468981,0.147104633127304,0.754428342368181
"3294","NCK1",373.591153748718,0.281146221969591,0.194202016418941,1.44769980844632,0.147701023545312,0.754428342368181
"3295","TSPAN5",26.909050522102,-0.913577579883997,0.630069719990153,-1.44996268015907,0.147068926554003,0.754428342368181
"3296","TRIM61",18.0185718198808,0.732815249061955,0.506527177091829,1.44674418709246,0.147968583773959,0.754428342368181
"3297","TMEM161B-AS1",224.0551385017,0.295788231372905,0.204118646189114,1.44909951587108,0.147309793218471,0.754428342368181
"3298","SPATA24",12.5560308791595,-0.63182372904913,0.436363153775372,-1.44793098038332,0.147636354303508,0.754428342368181
"3299","PURA",944.332195249526,-0.227276765483506,0.156881449259158,-1.44871663639504,0.147416732541939,0.754428342368181
"3300","TRERF1",132.616131125105,0.347722723533708,0.240395457168076,1.44646129186457,0.148047861366077,0.754428342368181
"3301","RBAK",62.3343122559188,0.495671063214298,0.3426214488631,1.44670178956704,0.147980463044892,0.754428342368181
"3302","NDUFB2-AS1",8.16948929200003,0.909160183842605,0.627450842447186,1.44897436155587,0.147344742647116,0.754428342368181
"3303","AP001330.4",9.31461297484603,-0.731098035431158,0.504005507358641,-1.45057548926925,0.146898104577628,0.754428342368181
"3304","CLIC3",21.3008435394038,-0.567392667485538,0.391798063630904,-1.44817629323471,0.147567752877422,0.754428342368181
"3305","SLC35C1",130.162287927976,0.310680032715142,0.214186519364175,1.45051160846823,0.146915904413145,0.754428342368181
"3306","MRE11",211.23428301643,0.312622805610438,0.215552454409685,1.45033285038016,0.146965722582078,0.754428342368181
"3307","AL132780.2",3.51266398680217,1.20588185670618,0.831135178967843,1.45088535201184,0.146811787392674,0.754428342368181
"3308","DNAJA2",567.696162947513,-0.247212465217184,0.170902391834256,-1.44651261204662,0.148033477164618,0.754428342368181
"3309","ZBTB4",442.815780045898,0.274766501801407,0.189552521666136,1.44955339758213,0.147183099604481,0.754428342368181
"3310","RETREG3",228.477243925766,0.258816967902528,0.178445184156842,1.45040040797652,0.146946893407967,0.754428342368181
"3311","IMPA2",271.789613706434,-0.416394115228739,0.287844042029134,-1.44659626196673,0.148010033758872,0.754428342368181
"3312","ABHD17A",591.901936028096,-0.301853499165135,0.208356519583891,-1.44873556041331,0.147411445616278,0.754428342368181
"3313","LILRA6",5.64761320293662,-1.16712906241046,0.805114080966652,-1.44964432991801,0.147157727358896,0.754428342368181
"3314","ZNF548",66.6359662898855,0.341124884082776,0.235792241313423,1.44671801829705,0.147975915866061,0.754428342368181
"3315","COL6A2",20496.7059680145,-0.272609506976899,0.188041624224038,

-1.44972959099792,0.147133940564168,0.754428342368181
"3316","HIC2",116.872526981425,0.547043631078084,0.377977156036077,1.44729283858062,0.147814924015015,0.754428342368181
"3317","SMIM10L2B",8.56727244196114,0.764254618995319,0.528036134618018,1.44735287774989,0.147798116365853,0.754428342368181
"3318","CCL17",4.11179352640287,-1.22801610063996,0.847940434340285,-1.44823392175581,0.147551640669606,0.754428342368181
"3319","APOBEC3F",48.3442629277624,0.340878116939951,0.235697185714609,1.44625450620653,0.148105830798917,0.754496350451367
"3320","BOC",1203.73642708667,0.385129712505367,0.26646207017847,1.44534534407623,0.148360907276574,0.755499832533977
"3321","MRPL3",443.974239407048,-0.292191135679212,0.2021756628321 5,-1.44523396924284,0.148392177894803,0.755499832533977
"3322","CD48",40.3104963037846,-0.566161653016177,0.391915005915789,-1.44460315239327,0.148569386820537,0.755946792766066
"3323","FGD1",55.63798921732,0.375707727598672,0.260050630651562,1.44474838094923,0.148528574934543,0.755946792766066
"3324","AC010245.1",3.4493182613614,1.32875635951339,0.920627987212836,1.44331519133602,0.148931702309408,0.756879237345199
"3325","ATP7B",14.8087885978395,0.638896438177928,0.442593133466294,1.44352993724559,0.148871245463899,0.756879237345199
"3326","GLG1",1361.50732666678,0.263909421741472,0.18284579708125,1.44334420563246,0.148923532896659,0.756879237345199
"3327","HOXB7",54.4413206143643,-0.640474104724918,0.443627736452391,-1.44371970482881,0.148817836296029,0.756879237345199
"3328","STK38L",97.6161659899137,-0.292489976650523,0.202791409560216,-1.44231936295937,0.149212300234914,0.758077395544451
"3329","MATN3",5.41474756101953,1.08729678587072,0.75403703762711,1.44196734591758,0.149311585732523,0.758143086662919
"3330","AC016396.2",7.0802416963596,-0.839321232080958,0.582071494660723,-1.44195556693629,0.149314908835316,0.758143086662919
"3331","DPM2",262.424185191544,-0.400065142608578,0.2775149638835,-1.44159845296315,0.149415685130708,0.758251971410093
"3332","CFAP300",51.7897282118472,-0.458457448078254,0.318028310259646,-1.44156175185775,0.14942604499281,0.758251971410093
"3333","PYCARD",506.553496293731,-0.394556386668863,0.273732196121598,-1.44139561315466,0.149472948912749,0.758262412306257
"3334","RPAP2",208.569792930909,0.231751155904349,0.160837339942581,1.44090393429215,0.149611824448807,0.758287060146616
"3335","NABP2",195.418906130805,-0.292311618110711,0.202865761984936,-1.44091154293654,0.149609674624518,0.758287060146616
"3336","TUFM",1010.31017286635,-0.364795642085183,0.25319959892947,-1.44074336463226,0.149657198941877,0.758287060146616
"3337","DNAJC28",8.73287795866801,-0.777893766211026,0.539895185419099,-1.44082367692755,0.149634502621874,0.758287060146616
"3338","PTPMT1",362.935400317964,-0.337930409397498,0.234695445463263,-1.43986777728243,0.149904811072128,0.759314123908791
"3339","YBX1",5699.36354882892,-0.289046842416579,0.200782729869293,-1.43960012200623,0.149980565098572,0.759470318863927
"3340","AC026356.1",4.11927866692192,1.20317713111911,0.83592138644697

6,1.43934244371128,0.150053522939394,0.759612265227326
"3341","CDKN1C",3519.43811651029,0.348677424791056,0.242275672345888,1.43917637877096,0.150100556139649,0.75962292822783
"3342","AC080013.3",2.63282103812361,1.42914207887556,0.993828444769759,1.43801687946923,0.150429264899761,0.759694746393414
"3343","ETV5",97.8761596056183,0.40296154064561,0.280068152380984,1.43879815402021,0.150207719572069,0.759694746393414
"3344","FAM131B",6.30002322325068,0.986663119600839,0.685847212788367,1.43860483968358,0.150262514366742,0.759694746393414
"3345","TMC5",20.3958826191259,0.977626216615274,0.679473734175688,1.43879912856572,0.150207443376526,0.759694746393414
"3346","SMG6",276.96174500838,0.279816343053088,0.194566098402572,1.4381556979887,0.150389882066156,0.759694746393414
"3347","COPS3",275.686923626706,-0.319457708191012,0.222089506989385,-1.43841873720887,0.150315279363329,0.759694746393414
"3348","CA11",58.3596045928519,-0.478811708900506,0.33290092599609,-1.43830092231744,0.15034869031779,0.759694746393414
"3349","NEK9",169.187562044061,0.308588205730827,0.214672861237238,1.43748121654652,0.150581306259183,0.76023551096753
"3350","HK1",147.397739946729,-0.26760837791771,0.186190192674718,-1.43728503673248,0.15063701879493,0.760289765308861
"3351","SHPRH",395.806969730042,0.292394694869378,0.203569583481554,1.43633783529293,0.150906232880374,0.760626694224889
"3352","FAM204A",743.847405161702,-0.270375338274714,0.188205332607319,-1.43659764858437,0.15083235214368,0.760626694224889
"3353","INTS4",67.0406628865033,0.305446736113596,0.212593641302605,1.43676327401921,0.150785269139203,0.760626694224889
"3354","QPRT",65.8723451035794,0.778511545678328,0.542041261867722,1.43625882464334,0.150928705886237,0.760626694224889
"3355","DLGAP1",3.48812000018638,1.28878657719892,0.89719248356261,1.43646608817023,0.15086975933297,0.760626694224889
"3356","FLVCR1-DT",27.1453777204755,-0.61329285031467,0.427247337295278,-1.43545154475898,0.151158466750944,0.760650998757429
"3357","PSMA1",910.268730644318,-0.349192977853691,0.24320842403559,-1.4357766563324,0.15106590433764,0.760650998757429
"3358","SOX9",326.719016123973,-0.464479019579766,0.323570861277682,-1.4354785154191,0.151150786299449,0.760650998757429
"3359","AL109809.5",8.82049744315934,0.755510818582262,0.52611864005547,1.43600846094829,0.150999933699015,0.760650998757429
"3360","STEAP2-AS1",2.75559629765989,-1.35755556101144,0.945630922278055,-1.43560825796712,0.151113843589696,0.760650998757429
"3361","FAM8A1",316.545805286027,0.264564292678583,0.184333471079276,1.43524825485873,0.151216367308959,0.760715959077622
"3362","KIF2C",2.99081120025562,1.23757950706886,0.862415376594931,1.43501558605691,0.151282656233074,0.760823067099587
"3363","FMO3",196.881914574118,0.662984634468425,0.462240363546597,1.43428546434498,0.151490816616146,0.760846508244506
"3364","CXCR6",2.66881428288074,1.43545059450087,1.00130522257463,1.43

357945423468,0.151692310107182,0.760846508244506
"3365","BMP5",57.9171415892428,1.48747151976047,1.03734533069576,1.433
92125625398,0.151594735206968,0.760846508244506
"3366","CCZ1",318.717220708369,-0.466550291953732,0.325306786762325,-1
.43418554711741,0.15151932034808,0.760846508244506
"3367","DIS3",370.555212294793,0.302756870790642,0.211099372356585,1.4
341912408874,0.151517695956937,0.760846508244506
"3368","AL121839.2",48.5485123770253,0.723790465238259,0.5048157957306
22,1.43377142981573,0.151637500571132,0.760846508244506
"3369","TMEM220",259.234901987233,-0.325068787363364,0.226752838428053
,-1.43358199887102,0.151691583508126,0.760846508244506
"3370","SYNRG",272.243036663895,0.302022256786533,0.210492140003491,1.
43483864424355,0.151333082977428,0.760846508244506
"3371","SAMD1",220.763844478526,-0.28341827528704,0.197676464072106,-1
.43374820375003,0.151644130872184,0.760846508244506
"3372","FH",245.488837904206,-0.357813118493176,0.249682840433,-1.4330
7052207775,0.151837684599498,0.760934579539111
"3373","WNK1",557.075280173767,0.298080058536416,0.20797141419242,1.43
327418190572,0.151779497233857,0.760934579539111
"3374","CISD3",355.104657539213,-0.302866069287326,0.211344374260902,-
1.43304533345866,0.151844882384963,0.760934579539111
"3375","PGM3",254.460112049586,0.26006765571577,0.181530141287828,1.43
264173029765,0.151960249630725,0.76128708170557
"3376","FAAP20",540.133138971564,-0.309058074146931,0.21584661368827,-
1.43184119901588,0.152189273556701,0.76198289526109
"3377","HIF1AN",94.0963539581711,0.347363202010404,0.242582749345788,1
.43193694913259,0.152161866587417,0.76198289526109
"3378","VCPIP1",184.214774298069,0.305624819467426,0.213479390964919,1
.43163617849017,0.15224797010439,0.76205111856869
"3379","PUM1",739.85761633793,0.216645751111589,0.151353435861026,1.43
138971295316,0.152318554999234,0.762178788969238
"3380","SH3TC1",4.87676392936103,-0.969165549453486,0.677577290953267,
-1.43033947919062,0.152619609104018,0.762631209583363
"3381","ACHE",3.01390280799085,-1.22478666167515,0.856093713801484,-1.
43066891151027,0.152525127187623,0.762631209583363
"3382","APOA1-
AS",2.34914433701619,1.40870563602561,0.985019017293194,1.430130394737
66,0.152679598086094,0.762631209583363
"3383","CHEK1",22.6153036338828,-0.493173921921927,0.344686234745867,-
1.43079088228034,0.152490156995132,0.762631209583363
"3384","C12orf66",36.9013467328679,-0.476948348037164,0.33348376778813
6,-1.43019959022465,0.152659743036978,0.762631209583363
"3385","TOMM34",50.5878529247455,-0.359716837856504,0.25146440709555,-
1.4304880838258,0.152576983454251,0.762631209583363
"3386","FEM1B",276.028109870031,0.25740874053585,0.180009589705408,1.4
2997237512239,0.152724947821372,0.762632432889475
"3387","ID2",1859.05214804022,0.235833225858509,0.164945482078478,1.42
9764688834,0.152784566883194,0.762704888355783
"3388","CCDC110",15.1017990619737,-0.721556514064366,0.504785038991577
,-1.429433240545,0.15287975012156,0.762870525317426

"3389","AL137060.3",2.57901198303863,-1.45291351376775,1.01671956014908,-1.42902091266417,0.152998222814726,0.762870525317426
"3390","LINC01303",13.9777641013426,-1.26474761741629,0.885038946729207,-1.4290304648065,0.152995477441876,0.762870525317426
"3391","AC004494.1",6.11709325966199,0.921043954710182,0.644397460196447,1.42931034276485,0.152915054597138,0.762870525317426
"3392","CLCN6",98.9072536721726,0.286823382344243,0.201171364387186,1.4257664514926,0.153935767804354,0.763518737314348
"3393","SH3D21",18.0729711536835,-0.527945189750401,0.370780828572718,-1.42387402224293,0.15448294300641,0.763518737314348
"3394","AL591845.1",2.68778192894056,1.37326633223077,0.96352601233521,1.42525091658139,0.154084682879624,0.763518737314348
"3395","DPH5",171.505568121456,0.342503282135175,0.239860220421083,1.42792865583922,0.153312394596113,0.763518737314348
"3396","STAG1",482.388663844381,0.281363973938458,0.197481670470711,1.42475994489923,0.154226604490801,0.763518737314348
"3397","DLG1",251.623986198111,0.301679845921524,0.211833238888976,1.4241383812275,0.154406417830117,0.763518737314348
"3398","UGDH-AS1",13.0799453752323,-0.691583124273981,0.484783097670467,-1.42658258424696,0.153700247099277,0.763518737314348
"3399","TMEM167A",630.700195409316,-0.263681717796129,0.184972332815043,-1.42551977251532,0.154007008720101,0.763518737314348
"3400","RREB1",262.32046904865,0.396782910935634,0.278272609616466,1.4258784272103,0.153903437471889,0.763518737314348
"3401","DDAH2",2888.81873014212,-0.300204458401866,0.21034453842823,-1.42720348550574,0.153521250131979,0.763518737314348
"3402","TFPI2",8.18523418845604,-1.53929903439286,1.07767644001249,-1.42834989913579,0.15319117202029,0.763518737314348
"3403","KIAA1549",15.9002748386324,0.636132174673137,0.446375347067808,1.42510597606212,0.15412656945139,0.763518737314348
"3404","SPAG1",43.5237209831451,-0.418932278410597,0.2933796826422,-1.42795259248241,0.153305504314842,0.763518737314348
"3405","SQLE",67.348805234056,-0.488578141671242,0.342445268061561,-1.42673351697011,0.153656720760363,0.763518737314348
"3406","LYNX1",18.9686646508064,0.634517790183057,0.444271831473809,1.42821971872071,0.153228626682622,0.763518737314348
"3407","HSD17B12",522.341737533658,-0.182787262088874,0.128161856211378,-1.42622202496352,0.153804263987915,0.763518737314348
"3408","CCDC88B",11.5292303819719,-0.767783825312381,0.538872267717887,-1.42479743588203,0.154215763743482,0.763518737314348
"3409","EMSY",121.949587255794,0.395451647283015,0.277479997410377,1.4251537082803,0.154112774292605,0.763518737314348
"3410","GLYR1",201.635006944713,0.269391263473535,0.188834021791785,1.42660343150756,0.15369423455863,0.763518737314348
"3411","DOK4",66.7668230733542,-0.294974730727877,0.206609081057816,-1.42769489713443,0.153379695577741,0.763518737314348
"3412","SPATA2L",58.3889981623825,-0.36961719201679,0.259448428205005,-1.42462683075009,0.15426509994062,0.763518737314348
"3413","ITGAE",412.941767412403,-0.314620202172478,0.22038951319384,-1

.42756430473059,0.153417303945068,0.763518737314348
"3414","TAOK1",517.211430030605,0.337358563676982,0.236485007745478,1.42655370373445,0.153708576798708,0.763518737314348
"3415","MYO1D",268.258601439413,-0.445025399820574,0.312538887123733,-1.42390409051527,0.154474237557286,0.763518737314348
"3416","COPE",1179.19305221672,-0.395799365479996,0.277650350692161,-1.42553166056984,0.154003574874028,0.763518737314348
"3417","TM6SF2",10.8802738646044,0.675656739600632,0.474346936918995,1.42439359678212,0.154332566771263,0.763518737314348
"3418","ZNF134",52.1435533681997,0.406781967780626,0.285535436852171,1.42462866348609,0.154264569879802,0.763518737314348
"3419","ETS2",90.921363871609,-0.471704454286471,0.331242156545462,-1.4240471659945,0.154432819056037,0.763518737314348
"3420","ZC3H7B",221.209023462725,0.278084493857076,0.19499272201205,1.42612755485249,0.153831526290251,0.763518737314348
"3421","SCML2",16.4286262780141,0.51340437613907,0.359686161009814,1.42736761041263,0.153473961761591,0.763518737314348
"3422","CNNM2",50.1290505607105,0.448377600980712,0.314961305233932,1.42359583075669,0.154563503450118,0.763693663452539
"3423","POMT2",41.9737506738402,0.38396987045931,0.2697748785598,1.42329735262654,0.154649974114454,0.76389768107718
"3424","LINC00377",30.860762198235,0.918529904409289,0.645472559403677,1.42303478440335,0.154726071869711,0.764050357235127
"3425","DDX54",233.432129832041,-0.220571434981814,0.155021135488285,-1.42284749938813,0.154780368355386,0.764095319168718
"3426","AC087500.1",18.1289424725244,0.515025187660055,0.362075218406353,1.42242595316769,0.154902633310845,0.76447569294214
"3427","ZBTB11",162.282687915595,0.250469983616084,0.176128805119407,1.42208415850137,0.15500182101894,0.76474198709899
"3428","AP2M1",1512.71085844061,-0.257360078314807,0.181054528451994,-1.42145065641397,0.155185788807254,0.765203067119583
"3429","DLGAP1-AS1",110.097566907232,0.406467003858429,0.285937282477542,1.42152502932301,0.155164182472363,0.765203067119583
"3430","HADH",313.8980130697,-0.306114964802191,0.215386115811697,-1.42123815014063,0.155247537339284,0.765284361904555
"3431","DISP1",46.8276421353273,0.430439336038408,0.303203153510846,1.41964003690024,0.155712503269282,0.765314776981401
"3432","ENAH",676.293121735902,-0.328562133466614,0.231757915763828,-1.41769541024624,0.15627971067849,0.765314776981401
"3433","LMAN2L",87.9038898075701,0.31077671399222,0.219014835759269,1.41897562744933,0.155906121803945,0.765314776981401
"3434","ADAM23",11.4311020874226,1.11219587548882,0.782799884536429,1.42079207912436,0.155377214093306,0.765314776981401
"3435","TADA3",478.587463288973,-0.309359451886317,0.218366848226517,-1.4166960525318,0.1565718116706,0.765314776981401
"3436","MANF",477.129210073581,-0.378181170963802,0.2666456877975,-1.41829096914181,0.156105832208957,0.765314776981401
"3437","KCTD6",94.2154340942712,-0.318864557252344,0.224883647753741,-1.41790904068542,0.156217322570731,0.765314776981401

"3438","CD200R1",3.12294265999859,-1.44860338023496,1.02111219995494,-1.41865250488525,0.156000350397945,0.765314776981401
"3439","PTX3",294.237642356488,1.25008491866878,0.882023725806092,1.41729171460361,0.156397656560082,0.765314776981401
"3440","AC139887.4",9.4422724118129,0.834181207663724,0.588973128856089,1.41633152141233,0.156678463001307,0.765314776981401
"3441","ADAMTS3",54.8917954499256,-0.614619395640013,0.433976433903389,-1.41625062474438,0.156702138508967,0.765314776981401
"3442","NDST1",164.114286862911,0.346913980038842,0.244945432990982,1.41629086855281,0.156690360273248,0.765314776981401
"3443","TREM1",10.7995117400597,-0.837097167277402,0.590757678928382,-1.41698905851867,0.156486126456551,0.765314776981401
"3444","GNAI1",338.160380847813,-0.354830124186658,0.250216929673819,-1.41808999354764,0.15616449232296,0.765314776981401
"3445","GRIN3A",6.18964514521077,1.04800496370033,0.739637677846144,1.41691668108656,0.156507288844858,0.765314776981401
"3446","NRARP",4.02378210589709,-1.10605854721816,0.780955373157149,-1.41628905470838,0.156690891120273,0.765314776981401
"3447","NSMCE4A",276.332651311905,-0.361526668414311,0.254973768294903,-1.41789749915045,0.156220692649341,0.765314776981401
"3448","CTBP2",387.495462447934,0.320509292060329,0.225850383411552,1.41912219593751,0.155863393927273,0.765314776981401
"3449","NTF3",10.8490918996351,1.29818582478445,0.913753191308698,1.42071823894279,0.155398688011379,0.765314776981401
"3450","BICD1",63.4070245875837,0.446481290019081,0.315244195847839,1.41630296734975,0.156686819425453,0.765314776981401
"3451","ACSS3",104.824910674231,0.261414043552485,0.184502574272076,1.41685851584375,0.156524297313711,0.765314776981401
"3452","AL138966.2",2.59843935041327,-1.41907143803093,1.00152906204697,-1.41690490252032,0.156510732977192,0.765314776981401
"3453","AL160153.1",2.26879570965279,-1.36942377978332,0.966106671984871,-1.41746643460171,0.15634660114517,0.765314776981401
"3454","AL121603.2",69.2454381731439,-0.448650367679806,0.31609718064943,-1.41934314870522,0.155798998223566,0.765314776981401
"3455","MIA2",123.811727069013,-0.323638144299362,0.228416505415541,-1.41687722483361,0.156518826345987,0.765314776981401
"3456","AL138995.1",13.0632460869737,-0.645780272860074,0.455860449526908,-1.41661833907781,0.156594543775493,0.765314776981401
"3457","FAM161B",46.9137193665007,0.36224066235232,0.255429593269396,1.41816246784793,0.15614333682834,0.765314776981401
"3458","UBFD1",153.751079820503,0.353174277296703,0.249363768843571,1.41630148972545,0.156687251865424,0.765314776981401
"3459","DYNLRB2",19.1995161348814,-0.619296747648702,0.437225873914217,-1.41642291684276,0.156651718151714,0.765314776981401
"3460","MAPK7",92.364976383021,0.265781310038646,0.187258105249911,1.41933140722502,0.155802419717854,0.765314776981401
"3461","COQ8B",106.256780156297,-0.301260253056383,0.212444117752087,-1.41806822539535,0.15617084694523,0.765314776981401
"3462","PRELID3B",148.155754166787,-0.238777055109786,0.168135586341095,-1.42014584958464,0.155565224514098,0.765314776981401

"3463","STARD4-
AS1",30.6631946035541,0.564446705597276,0.398658040755734,1.4158668530
2335,0.156814491472601,0.765438992394792
"3464","KIF7",84.7999849832849,-0.321157401885941,0.226829385157422,-1
.41585448315285,0.156818113890203,0.765438992394792
"3465","RN7SL832P",94.1614042026137,-0.577213956856977,0.4077585108249
27,-1.41557795002054,0.156899110968932,0.765613324173938
"3466","TMEM241",39.8391154820734,0.424210656470427,0.29972301399316,1
.41534228826388,0.156968161772529,0.765729278491031
"3467","LIN54",56.4990482055049,0.365779937875784,0.258492402101199,1.
41505102239943,0.157053536859611,0.765924776816357
"3468","CDK9",185.41320777199,-0.272058580584058,0.192360956831573,-1.
41431288898332,0.157270054222233,0.766241876797792
"3469","DHTKD1",101.519712583249,0.400278238904934,0.283070783117318,1
.41405705843912,0.15734515000248,0.766241876797792
"3470","UNC119",82.5326260899985,-0.291143657638101,0.205819300075738,
-1.41455955554686,0.15719767413554,0.766241876797792
"3471","PTPRS",160.251543722033,0.324740803878945,0.229591872855161,1.
41442638992631,0.157236746174066,0.766241876797792
"3472","C1QTNF6",98.7746794096286,0.575111223486811,0.406680985875823,
1.41415813244442,0.157315477775901,0.766241876797792
"3473","NABP1",1380.28631324001,0.666342191801646,0.471393442721394,1.
41355846605501,0.157491583496527,0.766292861513459
"3474","MCU",95.4646251369884,0.322622707550161,0.228197224478988,1.41
378892003074,0.157423887762475,0.766292861513459
"3475","EDC4",76.4449513218009,0.28292680572302,0.200138138021149,1.41
365762927765,0.157462451648853,0.766292861513459
"3476","AKR1A1",749.217835948037,-0.356186559224099,0.252127745242897,
-1.41272258188385,0.15773730914375,0.766390799900609
"3477","CAAP1",104.040241078254,-0.247380522732522,0.175095324934435,-
1.41283339703762,0.157704716019423,0.766390799900609
"3478","AC104564.5",10.3094081057165,0.734054609262535,0.5194399419355
5,1.41316550769524,0.157607065678831,0.766390799900609
"3479","CRKL",131.425574826889,0.35205950042992,0.249207016913142,1.41
27190509753,0.157738347743915,0.766390799900609
"3480","ZNF182",45.3586871696012,0.394427019214937,0.279094545018942,1
.41323800932106,0.157585754153339,0.766390799900609
"3481","FBXO2",9.12757200048125,-0.875626189148782,0.620061957873122,-
1.41215918511155,0.157903095203802,0.766750584062575
"3482","MARCH7",496.185122177684,0.21836105677077,0.154614603896592,1.
41229257306646,0.157863832324806,0.766750584062575
"3483","HMBOX1",183.106546940208,0.365187995735722,0.258720187043083,1
.41151720671456,0.158092165668048,0.767448273647819
"3484","AC123768.3",12.1389035722953,0.582891359612749,0.4130034254790
89,1.41134751833254,0.158142169602912,0.76747066694777
"3485","RCCD1",65.5195290066631,-0.307482910774795,0.217913409359056,-
1.41103253663548,0.15823502046172,0.767700925671956
"3486","AL031985.3",2.86763260382739,1.31597585013708,0.93296648032916
2,1.41052854296844,0.158383674467185,0.768100968286512
"3487","CCDC112",186.597353986946,-0.260251272866309,0.184537278942784

,-1.41029104990217,0.158453760195372,0.768100968286512
"3488","AP000915.2",13.4057283985116,0.856624766661377,0.6074008815021
,1.41031202414943,0.158447569615266,0.768100968286512
"3489","TPMT",78.6960809516954,-0.35570154251464,0.252265287523768,-1.
41002967949416,0.158530919417918,0.768143952714105
"3490","RNF5",325.199711727705,-0.274878123400005,0.194955488392992,-1
.40995324453705,0.158553489175078,0.768143952714105
"3491","SMARCA2",651.599771002774,0.223524416731618,0.158588896450755,
1.40945817603963,0.158699732185686,0.768192119036812
"3492","ZNF551",17.9431452713948,0.59466263293656,0.421904721152385,1.
40947138802407,0.158695828045831,0.768192119036812
"3493","SYTL4",110.594161206242,0.410734732441598,0.291349916485005,1.
40976437335872,0.15860926959469,0.768192119036812
"3494","AKR1B1",471.081204229979,-0.235286703748642,0.166964141181193,
-1.40920500703983,0.158774557640468,0.768334350482267
"3495","MAP3K21",8.39279574084431,0.759540826466715,0.539203451857679,
1.40863494818129,0.158943139344914,0.768406102085871
"3496","TSPYL1",488.442266758942,-0.261043415908177,0.185329048277325,
-1.40854020637689,0.158971170161839,0.768406102085871
"3497","NDUFAB1",833.821273902321,-0.365737563798308,0.259613060410645
,-1.40877952449618,0.158900371432638,0.768406102085871
"3498","E2F4",183.978639680616,-0.275748170699209,0.195716157713695,-1
.40891878279457,0.158859184907226,0.768406102085871
"3499","F11R",3.85716233900163,-1.0761597955746,0.764616349463931,-1.4
0745067291472,0.159293794299181,0.769255677529511
"3500","ATXN1",279.735884271462,0.360276540596021,0.25595460184774,1.4
0757985203305,0.15925516923954,0.769255677529511
"3501","SNAI1",24.8268802160237,0.777953983332743,0.552786336381436,1.
40733215011294,0.15932892019803,0.769255677529511
"3502","DMC1",15.4943757546819,-0.557362380758471,0.395961561685411,-1
.40761739191566,0.159244394696146,0.769255677529511
"3503","AL445426.1",111.254305776982,0.36354430977644,0.25844884662432
5,1.40663931963635,0.159534367504455,0.769550791960731
"3504","SEC16B",21.1983329131571,0.545501459975267,0.388220511469361,1
.40513302069131,0.159981726623017,0.769550791960731
"3505","INO80B",102.173198714009,-0.310514330424715,0.220895412473514,
-1.40570746557241,0.159811009184887,0.769550791960731
"3506","AC021016.2",4.99346087713711,0.947519236762612,0.6739956643948
81,1.40582393451047,0.159776412956929,0.769550791960731
"3507","PYURF",1025.40829109217,-0.305211110887601,0.217199706490597,-
1.40520959175796,0.159958962750667,0.769550791960731
"3508","SLC16A12",13.4727638864895,0.770623120739421,0.548300955755734
,1.40547469897669,0.159880167736682,0.769550791960731
"3509","AC135279.4",4.93846143948748,-0.979386178869084,0.696666634631
026,-1.40581754627562,0.159778310387546,0.769550791960731
"3510","FANCM",58.6768429282072,0.352291156274665,0.250452619063874,1.
40661797665138,0.159540699590201,0.769550791960731
"3511","CREBBP",565.444986942102,0.292978165668343,0.208282430787831,1
.40663888240669,0.159534497220847,0.769550791960731
"3512","TGFB1I1",607.293866773032,-0.295355573202351,0.210133651134853

,-1.40556056398985,0.159854653280767,0.769550791960731
"3513","STXBP2",13.1828741043892,-0.64667954350548,0.460042121213037,-1.40569637797582,0.159814302967913,0.769550791960731
"3514","ZNF829",44.8305806612764,0.426106090739071,0.303195251798412,1.40538510485112,0.159906793566705,0.769550791960731
"3515","URB1",77.9183152121026,0.363289261445155,0.258361978832967,1.40612509273288,0.159686982414026,0.769550791960731
"3516","COLEC12",1432.79620741591,0.329818255360037,0.234825023781172,1.40452772046725,0.160161763022576,0.770197693169998
"3517","BCLAF3",178.83811650837,0.273311386969457,0.194615557364862,1.40436556393618,0.160210019765752,0.770210694967111
"3518","KCNS3",66.3840614867315,-0.549354521351819,0.391322749294169,-1.40383998206772,0.160366505105471,0.770488792446576
"3519","AC018647.1",9.68137227258014,0.826295223591703,0.588686585926376,1.40362502449655,0.160430539275277,0.770488792446576
"3520","AP003120.1",7.33202711659046,0.873957433440922,0.622612245524447,1.4036945783242,0.160409817622534,0.770488792446576
"3521","AC244636.3",2.14132899686489,1.44448893562072,1.02916137031757,1.40355922528942,0.160450144204187,0.770488792446576
"3522","AC008280.3",17.3988460668129,0.567813563990116,0.404730704126724,1.40294165527983,0.160634237965764,0.770497502276637
"3523","CLHC1",25.5064115492957,0.570460448996774,0.406613060652855,1.40295653091134,0.160629801757691,0.770497502276637
"3524","VSIG10L",9.44398201186117,-0.684121730797343,0.487549973585617,-1.40318278712245,0.160562339084848,0.770497502276637
"3525","AL034397.3",2.69352304380868,-1.41517133826073,1.00851164607986,-1.40322756188447,0.160548991160418,0.770497502276637
"3526","BTNL9",7.23346574882904,-1.17393792693265,0.837149849003424,-1.40230321767382,0.160824719977816,0.770857714963908
"3527","HAUS4",257.171652112774,-0.305473267264933,0.21784794712479,-1.4022315624115,0.160846109438885,0.770857714963908
"3528","GABRB3",7.78559609933781,0.757469638898137,0.540120816129472,1.40240778780976,0.160793509115874,0.770857714963908
"3529","AMMECR1",77.5449251970586,0.285306682046699,0.20354401908101,1.4016952369067,0.161006273589996,0.771406651702934
"3530","OSBPL1A",1167.69910986106,0.232753579636842,0.166105513393661,1.40123933806597,0.161142514446242,0.771840689591237
"3531","TSSK3",8.59861996119026,0.911238405423628,0.651371443299169,1.39895356911602,0.161826906946756,0.771904492392169
"3532","FALEC",10.5091528606094,0.888123349501975,0.634515314413938,1.39968780788574,0.161606826367354,0.771904492392169
"3533","TARBP1",99.859824897684,0.412603031763718,0.294831236151212,1.39945494632768,0.161676599759467,0.771904492392169
"3534","ZNF512",102.550926454069,0.31087701971906,0.22213463614699,1.399498183225,0.161663642767598,0.771904492392169
"3535","RBM43",397.685375478715,0.265365936639405,0.189567621906757,1.39984842332374,0.161558713666968,0.771904492392169
"3536","PARP14",371.909411582424,0.28650844481087,0.204804152112975,1.39893865361101,0.161831380060767,0.771904492392169
"3537","CYFIP2",10.8060299132075,0.885106508357488,0.632501340358782,1

.39937491334867,0.161700585664369,0.771904492392169
"3538","AC002454.1",2.30249141725722,1.79494913121007,1.28226829767895
,1.39982337117679,0.161566217379542,0.771904492392169
"3539","XPO7",143.919320761845,0.264449946765751,0.188856550012412,1.4
0026886411072,0.161432820935692,0.771904492392169
"3540","PTGES2",113.849701193634,-0.501120830822365,0.358222915554081,
-1.39890780032109,0.161840633163605,0.771904492392169
"3541","STAM",233.790380918662,0.253641230370755,0.181099072056413,1.4
0056615139223,0.161343848821052,0.771904492392169
"3542","NBR2",28.2506871299476,0.465935089206222,0.33266927472058,1.40
059550013321,0.161335067340941,0.771904492392169
"3543","FBXO27",42.7139484158197,0.673344097657801,0.480860470842792,1
.40028997700237,0.161426501049176,0.771904492392169
"3544","RHBDD3",77.015940486838,0.412317043731983,0.294689304865538,1.
39915849311232,0.161765460321218,0.771904492392169
"3545","PRR34-
AS1",45.1608102729117,-0.440176534687131,0.314487855071425,-1.39966147
369081,0.161614715864342,0.771904492392169
"3546","ZMYM5",181.486512883633,0.254330241188955,0.1818394124666,1.39
865300783278,0.16191706236062,0.772051238125597
"3547","PAX8-
AS1",119.914320106468,-1.09489004657203,0.783412039289104,-1.397591550
37441,0.162235757172396,0.772319822715872
"3548","ZNF736",57.0737248333974,0.401514136486726,0.287296723773312,1
.39755905049421,0.162245522486111,0.772319822715872
"3549","CASTOR2",71.6373736159373,0.35923774373547,0.25697611657547,1.
39794214545213,0.162130441261907,0.772319822715872
"3550","PTPA",142.187438109294,-0.261913922499471,0.187392220171357,-1
.39767767445185,0.162209881417906,0.772319822715872
"3551","AL359546.1",2.67908678084842,-1.37284311952637,0.9818465529327
84,-1.3982257364205,0.162045290594476,0.772319822715872
"3552","PORCN",53.6020160646903,-0.352180139018247,0.251997768277641,-
1.39755261098276,0.162247457433569,0.772319822715872
"3553","PSMG4",279.105350746775,-0.350236869086981,0.250682721841378,-
1.39713206604082,0.162373860684398,0.772503026171741
"3554","NR6A1",2.97520624883341,1.4747560025676,1.05583899427447,1.396
7622057575,0.162485091039733,0.772503026171741
"3555","LINC02680",3.31566138512237,1.29341795947557,0.926315861393139
,1.39630337056986,0.162623159247018,0.772503026171741
"3556","VRK1",74.8984238389309,-0.345057316372771,0.247165524792451,-1
.39605762843552,0.162697141956327,0.772503026171741
"3557","PLCB2",12.615968111558,-0.818697079658233,0.586046332022777,-1
.39698354024749,0.162418520853427,0.772503026171741
"3558","NPIPB13",10.1992909106577,0.800725252831916,0.573348526741386,
1.39657680361162,0.162540869792578,0.772503026171741
"3559","RAB31",248.351772065277,-0.317450671408071,0.227384183058445,-
1.39609829997048,0.162684895700209,0.772503026171741
"3560","OCEL1",212.737796662095,-0.363263336219614,0.260127648527844,-
1.39648106718164,0.162569678019649,0.772503026171741
"3561","THAP7-

AS1",62.0073676866383,-0.501350842387411,0.359004722133551,-1.39650208
333724,0.162563353679255,0.772503026171741
"3562","KAT6B",630.182527813331,0.264266456421359,0.189325860659219,1.
39582862848848,0.162766107141377,0.772558950678516
"3563","DUSP16",102.801319636572,0.316160502460162,0.226522233304795,1
.39571510419799,0.162800304073075,0.772558950678516
"3564","KDM3A",147.451496026767,0.337705197698861,0.242019881321511,1.
39536138872094,0.162906888563339,0.772631043991284
"3565","FAHD2B",147.292349647593,-0.373389690856894,0.267585330589882,
-1.39540418764276,0.162893989238192,0.772631043991284
"3566","RAP2A",172.909493651546,0.247243488542263,0.177314849625943,1.
3943755363064,0.163204231557274,0.77382421401301
"3567","NUDC",1062.75937544858,-0.291689228802127,0.209486882942811,-1
.39239853447892,0.163801748018161,0.773885072583381
"3568","RNF11",418.256097391095,0.177890760542713,0.12773727715986,1.3
9262997065522,0.163731715140209,0.773885072583381
"3569","SRGAP2B",196.383501373074,0.349419913036024,0.250846235633322,
1.39296454720092,0.163630511751753,0.773885072583381
"3570","LINC01305",3.0351408483803,1.75390989041009,1.26000463776331,1
.39198685294012,0.163926379157824,0.773885072583381
"3571","FAM114A1",1023.8748183357,-0.239547866769783,0.172183425771545
,-1.39123650082104,0.164153722120974,0.773885072583381
"3572","CSNK1G3",390.983116465582,0.342958912140649,0.246470328396191,
1.39148154007957,0.164079453604669,0.773885072583381
"3573","NDFIP1",2444.10120625384,-0.273298490232559,0.196311031975341,
-1.39217081935002,0.163870676932854,0.773885072583381
"3574","EBAG9",313.027863125422,-0.199634363018222,0.143258079705759,-
1.39352951978872,0.163459724741767,0.773885072583381
"3575","IGF2",311.614976882194,1.2774635572382,0.917625522970857,1.392
14039415812,0.163879888232722,0.773885072583381
"3576","KCNA1",31.9543136103533,-0.835396665821677,0.600505550233139,-
1.39115561129676,0.164178244343304,0.773885072583381
"3577","PTPN6",15.8310435160144,-0.720355396474516,0.517686330838147,-
1.39149008494824,0.164076864212378,0.773885072583381
"3578","LINC00173",5.68974649520519,1.28980565387531,0.926717583414432
,1.39180013086954,0.163982930342979,0.773885072583381
"3579","PRKCH",18.2428924216882,0.555848102176371,0.399550138817781,1.
39118485560049,0.164169378410576,0.773885072583381
"3580","ATG2B",123.355627493414,0.315410277943289,0.22658109687458,1.3
9204144694329,0.163909847437232,0.773885072583381
"3581","SLTM",718.621573944645,-0.213770074766985,0.153475083879649,-1
.39286501341559,0.163660614003948,0.773885072583381
"3582","AP3S2",182.227320175846,0.251292230553514,0.18033009813272,1.3
9351241504104,0.163464893406441,0.773885072583381
"3583","LRRC59",293.813820450591,-0.277831542660741,0.199630869101769,
-1.39172635930922,0.164005277061282,0.773885072583381
"3584","UTP18",159.344617662216,-0.315182129454819,0.226325630778742,-
1.39260466598653,0.163739371265826,0.773885072583381
"3585","EPS15L1",137.717744845371,0.279947681867186,0.201017055720101,
1.39265636373159,0.163723729995781,0.773885072583381

"3586","ERVK9-11",3.45978418918145,1.18051826209073,0.847730445779075,1.39256324692434,0.163751903509583,0.773885072583381
"3587","MT-ND2",43820.1704738256,-0.288528588617294,0.207248592085869,-1.39218600094397,0.163866080814761,0.773885072583381
"3588","SMIM3",312.217795444615,-0.449348105963382,0.323041273129944,-1.39099286481152,0.164227590434908,0.77390192281868
"3589","AC026748.3",12.5700406102504,-0.702313846186949,0.5050406079371,-1.39060866621327,0.164344126974718,0.774235302002936
"3590","TSNAX",408.494734709305,-0.267603621424072,0.192508695618136,-1.39008588970389,0.164502797507213,0.774613232520319
"3591","UBXN7",351.122218745663,0.300365337233116,0.216145766217106,1.38964247364168,0.164637471360635,0.774613232520319
"3592","PRR7",5.83381881987934,-0.958883584055522,0.689838487732955,-1.39001172173901,0.164525317942302,0.774613232520319
"3593","AC068587.4",61.0965477070645,0.53100403425285,0.38210139867843,1.38969403433075,0.164621807138581,0.774613232520319
"3594","NMRK1",146.727479523585,-0.443165566421745,0.318918219346576,-1.3895899937286,0.164653415997044,0.774613232520319
"3595","AL157392.4",5.00414223682246,1.02232364148569,0.7358217793461,1.38936311778403,0.164722359644563,0.774722018600909
"3596","TRNAU1AP",229.380540461731,-0.201360589835347,0.145389906570729,-1.38496952494697,0.166061786088527,0.775000247745891
"3597","FAM102B",258.196263882088,0.322644499915834,0.232741380601574,1.38627904965539,0.165661711535357,0.775000247745891
"3598","SOX13",96.0859202230471,0.382088018381866,0.27527972426571,1.38799913215933,0.165137309376556,0.775000247745891
"3599","WDR6",163.928092351051,0.308271659340496,0.222437670607463,1.38587883292711,0.165783905231478,0.775000247745891
"3600","JCHAIN",94.1706844065795,-0.793157064398625,0.571903232526225,-1.38687284716869,0.165480538856174,0.775000247745891
"3601","ARSJ",39.1811323021429,-0.435400682983771,0.314207882865211,-1.38570897398697,0.165835786852561,0.775000247745891
"3602","CYSTM1",1381.8208159756,-0.315285379019736,0.227492231603143,-1.38591712252288,0.165772211765137,0.775000247745891
"3603","JAKMIP2",2.21867853625866,-1.39585111940688,1.00688839612957,-1.38630172397702,0.165654790667273,0.775000247745891
"3604","SYPL1",1025.40235948762,-0.312414389975603,0.225504913157346,-1.38539948243885,0.165930349180781,0.775000247745891
"3605","AC067817.2",4.23758686271996,-1.13455403840947,0.817555770863836,-1.38773901285131,0.165216531751555,0.775000247745891
"3606","CCDC171",81.5731290077155,0.338139353977308,0.24344300290218,1.38898777104358,0.164836468707681,0.775000247745891
"3607","TMEM250",149.398813417707,-0.221763063968072,0.1599862758017,-1.38613804750942,0.165704754409323,0.775000247745891
"3608","FGFBP3",36.6269592859795,-0.469158553329272,0.338508250923249,-1.38595898932947,0.16575942654525,0.775000247745891
"3609","ZBED5",234.682182407455,0.206867780345915,0.148986546348301,1.38849973649499,0.164984924949864,0.775000247745891
"3610","DYNC2H1",311.637412202079,0.332674526022829,0.240082029687221,

1.38567024969023,0.165847616489918,0.775000247745891
"3611","RIMKLB",172.567783222493,0.26401691586248,0.190372320129313,1.38684508169645,0.16548900701405,0.775000247745891
"3612","TMEM106C",230.525667173355,0.487461901731455,0.351129385020596,1.38826860561061,0.165055268246655,0.775000247745891
"3613","AC025259.3",13.4784458647963,-0.924539621412057,0.667337770930768,-1.38541479545292,0.165925669475017,0.775000247745891
"3614","LINC02397",6.86550068144162,-0.968212998794214,0.699046696638569,-1.38504767056329,0.166037891352094,0.775000247745891
"3615","RIMBP2",2.64966025729832,1.49785914709063,1.08011946507898,1.38675322084035,0.165517025869799,0.775000247745891
"3616","ACIN1",370.849554461818,0.27247086372618,0.196342072745901,1.38773549609412,0.165217603017009,0.775000247745891
"3617","EIF5A",951.966652843513,-0.274351958062546,0.197855822371224,-1.38662564879085,0.165555943063593,0.775000247745891
"3618","CCL8",179.923236604016,-1.34443825139424,0.969847928867103,-1.38623614215963,0.165674808757053,0.775000247745891
"3619","ZADH2",222.778644310959,0.219729049137932,0.15849981135236,1.38630479912342,0.165653852059139,0.775000247745891
"3620","ALDH16A1",251.999213310639,-0.327849828259944,0.236691878656977,-1.38513340685878,0.166011678574106,0.775000247745891
"3621","CTSZ",1625.65970617866,-0.240256935633701,0.173475817991983,-1.38495923186714,0.166064933616239,0.775000247745891
"3622","PAXBP1-AS1",16.4327805769323,0.574147954925218,0.413796714106045,1.38751211731005,0.165285658796025,0.775000247745891
"3623","KCNJ15",31.9440983069207,-0.681196691164489,0.491218131121583,-1.38674989379796,0.165518040731598,0.775000247745891
"3624","TOR1AIP1",508.188671217394,0.21055575671833,0.152099591976594,1.38432821536255,0.166257978276094,0.775054099871989
"3625","GPSM3",24.2616427001454,-0.610073940063307,0.440578210268408,-1.38471201217972,0.166140544570004,0.775054099871989
"3626","CLEC2D",71.5947867617268,0.43296411523404,0.312736673747976,1.38443665734883,0.166224790997616,0.775054099871989
"3627","KIAA0895L",25.3027674635435,0.44326852216426,0.320240831371121,1.38417240633055,0.166305670353417,0.775054099871989
"3628","CYTH2",353.910638579888,-0.269368720457744,0.19459670626524,-1.38424090328943,0.166284702636518,0.775054099871989
"3629","PRPS2",142.143148376936,-0.393442629816875,0.284311584720122,-1.38384311776877,0.166406497170485,0.77531029323741
"3630","KIF1B",415.259703324788,0.351820604033266,0.254765782385873,1.38095705293889,0.167292166061159,0.776056550089055
"3631","BCL11A",2.09146775118277,-1.41543181538754,1.02488246863536,-1.38106744793108,0.167258223286714,0.776056550089055
"3632","GCA",87.031322358266,-0.34362827464817,0.248746502112194,-1.38143962520197,0.167143829328826,0.776056550089055
"3633","CREB1",352.641159230059,0.241642493531699,0.174984297495028,1.38093815839998,0.167297976019708,0.776056550089055
"3634","IRS1",250.52049498798,0.382532222928001,0.276902903614557,1.38146699776207,0.167135418306084,0.776056550089055

"3635","MFSD10",713.016255561622,-0.2997038712621,0.216848676870116,-1
.38208761790883,0.166944799889166,0.776056550089055
"3636","HAVCR2",9.20457574774366,-0.904596527104229,0.655205755463348,
-1.38062970229026,0.167392845882114,0.776056550089055
"3637","EGFR",1565.96512355299,0.232895200141185,0.168669477906852,1.3
8077856783195,0.167347055219941,0.776056550089055
"3638","AC093673.1",75.7228177903628,-0.425400221393562,0.307616916878
485,-1.38288955532833,0.166698733370615,0.776056550089055
"3639","HSPA14.1",103.932412146265,-0.335310694802187,0.24277949454109
1,-1.38113268353244,0.167238167949673,0.776056550089055
"3640","TESPA1",5.38706573426824,-0.909562734584475,0.658673874198001,
-1.38089997222367,0.167309718505024,0.776056550089055
"3641","CNOT2",552.395620399664,0.221348779759199,0.160045239285262,1.
38303882544529,0.166652961432317,0.776056550089055
"3642","MBTD1",147.821716533531,0.313413452086737,0.226753613174827,1.
3821762206942,0.166917599599541,0.776056550089055
"3643","R3HDM4",120.876048663527,-0.300811261662332,0.217563448390556,
-1.38263694516524,0.166776214854464,0.776056550089055
"3644","CYP2S1",3.5452575068052,-1.34392466777987,0.97212123404823,-1.
38246611709461,0.166828627185887,0.776056550089055
"3645","ZNF432",47.5178780213319,0.386439991726049,0.279865859568471,1
.38080433362578,0.167339130682714,0.776056550089055
"3646","AL133227.1",5.01973497566333,1.042113301802,0.753741915380916,
1.38258637411103,0.166791729438825,0.776056550089055
"3647","EDA2R",23.7779176226154,0.827364958964737,0.598951318700932,1.
38135593516884,0.167169547521458,0.776056550089055
"3648","DGUOK",540.976995450218,-0.36591814361847,0.26508409965721,-1.
38038510831714,0.167468102804013,0.776103653824733
"3649","AL158151.4",4.26933199192643,1.0792343139648,0.781969306074183
,1.38014920225323,0.167540710696728,0.776103653824733
"3650","ENOX1",10.6305245365238,0.849690766101126,0.615646195835263,1.
38016083238901,0.167537130583767,0.776103653824733
"3651","RPS6KA1",9.6606533790971,-0.938430825902023,0.680401922469023,
-1.37923012106825,0.167823813857503,0.776208215477474
"3652","TUSC2",180.958457805333,-0.256234749071225,0.185984098895089,-
1.37772395916364,0.168288531112251,0.776208215477474
"3653","EIF2A",718.754824106883,-0.267813215783003,0.194408187699167,-
1.37758197816969,0.168332388272729,0.776208215477474
"3654","TBL1XR1",978.219995496458,0.231074945351469,0.167710990353508,
1.37781635457759,0.168259995276565,0.776208215477474
"3655","CD14",201.549622274944,-0.664767622158061,0.482326035649268,-1
.37825365629124,0.168124986229225,0.776208215477474
"3656","AL035587.1",2.02682927223852,1.5240848616375,1.10586582629768,
1.37818243894919,0.16814696775825,0.776208215477474
"3657","AL021368.2",100.267755323848,0.475275825902704,0.3449164203634
37,1.37794491025364,0.168220297577954,0.776208215477474
"3658","UBN2",221.780300941947,0.329687902695543,0.239329987186667,1.3
7754531544934,0.168343714582204,0.776208215477474
"3659","ZBTB10",245.240414549548,0.28438712080252,0.206380470221223,1.
37797496292978,0.168211018394368,0.776208215477474

"3660","AC104211.1",7.98941619648234,0.705447214930524,0.511574580169546,1.37897237719811,0.16790327076754,0.776208215477474
"3661","KMT5B",390.361435665463,0.220571855020638,0.160009586375005,1.37849150177601,0.168051589864658,0.776208215477474
"3662","MLF2",661.718086799661,-0.260573723905499,0.18910225964573,-1.37795140255683,0.16821829295611,0.776208215477474
"3663","PHC1",169.432241251544,0.272186403641946,0.197578147303517,1.37761390799872,0.168322524573701,0.776208215477474
"3664","TTC7B",71.850106492629,0.324114161981599,0.235208511270461,1.37798653726823,0.168207444758176,0.776208215477474
"3665","GCHFR",191.046588736472,-0.347280147820339,0.251810811299072,-1.3791312057999,0.167854303974707,0.776208215477474
"3666","PRR14L",188.399646208657,0.315864362498641,0.229208338650879,1.37806662863062,0.168182717702175,0.776208215477474
"3667","RTL5",75.1721499143357,0.444547275353222,0.322589504153171,1.37805870814118,0.168185162917753,0.776208215477474
"3668","NBPF19",214.359771579893,0.658975422419925,0.47893276227403,1.37592471079036,0.16884494389274,0.77688179079505
"3669","ILF2",563.317913459825,-0.36798476807017,0.267526976094196,-1.37550527966422,0.168974850022577,0.77688179079505
"3670","MPC2",790.006535222856,-0.334123643473108,0.242819802424442,-1.37601480660572,0.168817049213056,0.77688179079505
"3671","SFT2D2",825.03287087277,0.376694774327941,0.273823795798104,1.37568312216987,0.168919759521632,0.77688179079505
"3672","LINC01412",3.54706472972894,-1.18580734431763,0.861876441353384,-1.3758437838904,0.168870002721081,0.77688179079505
"3673","NSUN7",19.0513464066104,0.523101311439336,0.380194550990741,1.37587798161809,0.168859413132986,0.77688179079505
"3674","RRAS2",239.187573843922,0.376497463675869,0.273673462180585,1.37571783787876,0.168909007164139,0.77688179079505
"3675","SAAL1",44.5720848110442,-0.38337530979721,0.278729310312279,-1.37543952362846,0.168995222766986,0.77688179079505
"3676","ANKRD52",55.7857076121339,0.405561420103364,0.294784489568485,1.37578954950119,0.168886797836442,0.77688179079505
"3677","AL161945.1",2.52180037847745,-1.30551696858697,0.948232874800016,-1.37678939771235,0.168577369720757,0.77688179079505
"3678","FAM104B",172.277180193687,-0.390912728633965,0.284115903741682,-1.37589175222441,0.168855149100065,0.77688179079505
"3679","EP400",175.788269579271,0.337219920221752,0.245341777758412,1.37449040804543,0.169289486064958,0.777892081615197
"3680","CDR2L",49.9643341972656,0.32785500539958,0.238538205714893,1.37443393781305,0.169307006171276,0.777892081615197
"3681","RP9",174.942571505357,-0.289042590378454,0.210332335596324,-1.37421851737145,0.169373853678919,0.777984686975984
"3682","CBFA2T2",143.517719881946,0.335952411353796,0.244493955236745,1.3740724633805,0.169419187215849,0.777984686975984
"3683","SH3GLB2",296.824366002568,-0.269425322086634,0.196122882640726,-1.37375770975277,0.169516914180669,0.778222097465859
"3684","PTPRO",8.56697633067288,0.920126387561408,0.66989106675228,1.37354628719308,0.169582581903648,0.778312240723911

"3685","ANKRD28",773.365209503323,-0.370702252070444,0.27001013439767,
-1.37291977168707,0.169777289262892,0.77836073938638
"3686","STK32C",89.9737833040816,-0.331759877462737,0.24162008139274,-
1.3730641739313,0.169732397337754,0.77836073938638
"3687","TEX22",5.46311971418989,0.949917945409867,0.691775541355068,1.
37315919488732,0.169702861975801,0.77836073938638
"3688","SLC23A2",111.227574028506,0.33890600971405,0.246834963284057,1
.3730065028269,0.169750325125282,0.77836073938638
"3689","KCNA2",19.2173723298322,0.71723248600031,0.523265151222273,1.3
7068651394987,0.170472700630574,0.77841226805298
"3690","GCC2",1023.82748742806,0.224543219726982,0.163757186904614,1.3
7119612257247,0.170313826599272,0.77841226805298
"3691","FOXF2",63.5296968763023,0.423492816602922,0.308891339884078,1.
37100903107822,0.170372140778884,0.77841226805298
"3692","AL080317.1",6.24446847040766,0.920437331617061,0.6719369976896
87,1.36982683611974,0.170740962787421,0.77841226805298
"3693","TNPO3",220.687665151532,0.244909056496088,0.178800742541768,1.
36973176405504,0.170770649399191,0.77841226805298
"3694","COL27A1",45.2250836995385,0.646164554473691,0.47176995855912,1
.36966023959475,0.170792985730779,0.77841226805298
"3695","AC011092.2",2.62038025985851,1.45221782784753,1.05984358334117
,1.37021901219556,0.170618545186445,0.77841226805298
"3696","LBHD1",3.23382795034856,1.10469481692367,0.806314685368115,1.3
7005419468371,0.170669984892487,0.77841226805298
"3697","IGSF9B",7.49419486213286,0.937991589100203,0.683581507576031,1
.37217227017493,0.170009815648794,0.77841226805298
"3698","PYM1",163.681899200138,-0.307756303376609,0.224509850581228,-1
.37079198342463,0.170439810679386,0.77841226805298
"3699","AC068338.2",10.7745404330997,-0.691301411112634,0.504525540768
106,-1.37020102106263,0.170624159671774,0.77841226805298
"3700","HCFC1R1",800.762960218814,-0.361823212843652,0.264170583819051
,-1.36965746758348,0.170793851444594,0.77841226805298
"3701","IST1",355.553239038842,0.184334004386012,0.134574408429759,1.3
6975526429474,0.170763311000762,0.77841226805298
"3702","DLG4",72.6288353059632,-0.302628797502251,0.220736907426643,-1
.37099319289332,0.170377078038347,0.77841226805298
"3703","CDC37",763.945821065718,-0.238353487180579,0.173782163610748,-
1.37156473500044,0.170198978282464,0.77841226805298
"3704","KXD1",400.202364309019,-0.233214859875791,0.169978533054458,-1
.37202537099831,0.170055539841538,0.77841226805298
"3705","ZNF737",94.4179447604638,0.411462834717143,0.300375792573781,1
.36982687982779,0.170740949140305,0.77841226805298
"3706","TMEM86B",9.95523794134688,0.650122328831447,0.474297422452306,
1.37070601284328,0.170466619672632,0.77841226805298
"3707","CTNNBL1",369.759284463222,-0.239277330988076,0.174552025289062
,-1.37080810487204,0.170434783732649,0.77841226805298
"3708","DNAL4",128.385687675351,-0.306245280507817,0.223316454937045,-
1.37135116440099,0.170265513250842,0.77841226805298
"3709","ALG13",302.800171631217,-0.208820271935066,0.152460027714878,-
1.3696722679704,0.170789229243377,0.77841226805298

"3710","LINC00893",30.3749863307155,0.593016023427616,0.432974270428791,1.3696334030203,0.170801367073371,0.77841226805298
"3711","DLEU2L",21.9828535989075,0.550237409783182,0.401795921152454,1.36944498641241,0.170860220223258,0.778427950899948
"3712","IBA57",57.6147910604006,0.41084740428773,0.300164925968422,1.36873887900864,0.171080912575706,0.778427950899948
"3713","METTL8",151.869974874246,-0.271693098783305,0.198462695857038,-1.36898825046203,0.171002947694391,0.778427950899948
"3714","NUS1",168.476033279972,0.29417160189397,0.214921703227289,1.36873846371332,0.171081042438148,0.778427950899948
"3715","AC008264.2",8.36497471126733,0.919021267284493,0.671206694009958,1.36920754141177,0.170934409339742,0.778427950899948
"3716","KANK2",872.817359907598,0.29918600703367,0.218534037750856,1.36905907250368,0.170980810348767,0.778427950899948
"3717","SYNM",112.093634674539,-0.606137319028968,0.442906334676518,-1.36854515632897,0.171141497483308,0.778493526889365
"3718","YEATS2",85.642555846643,0.372205177755939,0.272065610159019,1.36807139108243,0.171289730700911,0.778748794485346
"3719","TRBC1",26.6843370544085,0.691766548192927,0.50564126794544,1.36809748738224,0.171281563104526,0.778748794485346
"3720","MINAR1",2.2181909299842,1.34989721171449,0.987064897039739,1.36758709155082,0.171441359257189,0.778935222565436
"3721","TPPP3",1246.25058387666,-0.496463763293061,0.362968758145326,-1.36778648892499,0.171378918091809,0.778935222565436
"3722","SLC47A1",8.23113891809518,1.07150589408285,0.783551488398031,1.36749902201518,0.171468943599985,0.778935222565436
"3723","YY1AP1",204.22214424943,0.228899424985169,0.167414447223733,1.36726207792131,0.171543173578199,0.779063115460703
"3724","PNO1",91.7564655342626,-0.306878859898144,0.224529463660584,-1.36676432079331,0.171699189548544,0.779376520732218
"3725","WDR76",20.6684234836756,-0.693343342498682,0.507294285075438,-1.366747788999,0.171704373061717,0.779376520732218
"3726","TMEM51",2.24002336784708,-1.50773900003657,1.10353030022046,-1.36628690642692,0.171848929081191,0.779404960543125
"3727","FAXDC2",452.835692047678,0.259242427276909,0.189736503768912,1.36632868281715,0.171835822145847,0.779404960543125
"3728","SNRPB",918.062830084707,-0.302526642340892,0.221398293382681,-1.36643619839464,0.171802093621312,0.779404960543125
"3729","KCNMB4",64.7441548722543,0.329753598817201,0.241392938798823,1.36604492433815,0.171924863321403,0.779540249138719
"3730","AC093627.4",4.70194271389777,-1.0748550180149,0.787159441153951,-1.36548577304642,0.172100421633153,0.779708984183643
"3731","MCOLN1",210.465347099119,-0.243166282363558,0.178075019733959,-1.36552719593598,0.172087411373066,0.779708984183643
"3732","FAM120C",67.7502142931287,0.296326705621155,0.216987492251862,1.365639570032,0.172052120191868,0.779708984183643
"3733","KIAA1191",155.82571749127,0.223701139455032,0.163894761285759,1.3649071983759,0.172282219518789,0.780183451261666
"3734","MTRNR2L1",2.86468124723394,1.31308111196057,0.962063703493459,1.36485880009036,0.172297433582391,0.780183451261666

"3735","TAX1BP3",989.477631588889,-0.337902343933891,0.247608623015139
,-1.36466307117758,0.172358971470185,0.780253143137319
"3736","FIBP",494.329684859833,-0.367415829984445,0.269280377277107,-1
.36443595964792,0.172430396772169,0.780367545134858
"3737","RBM17",602.794407198303,-0.180782153777364,0.132640059319778,-
1.36295290204539,0.172897354464536,0.78160456527777
"3738","PAMR1",198.158814575649,0.474863466879349,0.348376226076103,1.
36307655728383,0.172858384101719,0.78160456527777
"3739","STX3",62.1042825734273,-0.509618700572923,0.373860154491945,-1
.36312654464466,0.172842632281654,0.78160456527777
"3740","UNC93B1",121.542935766322,-0.31171718675742,0.228693141605738,
-1.36303688238633,0.172870887063013,0.78160456527777
"3741","CBARP",3.64609036659119,-1.09560055853521,0.803999993880177,-1
.36268727223211,0.172981090801361,0.78160456527777
"3742","ARHGAP4",20.8092730363878,-0.679114728708904,0.498334893891758
,-1.36276776327128,0.172955713849186,0.78160456527777
"3743","TMEM214",236.321919234988,-0.296447642098803,0.217611542265101
,-1.362279036365,0.173109840937051,0.781977341855105
"3744","ZNF343",35.6091740803389,0.533846044163163,0.391953533178512,1
.3620136035871,0.173193592008947,0.782146702373738
"3745","AC079848.1",13.6373650716662,-0.8360369093211,0.61389220941963
,-1.36186271220396,0.173241215737509,0.782152864002619
"3746","BCAS3",79.1888097224095,0.306421704501176,0.225089206115816,1.
36133451172026,0.17340800134211,0.782487986840778
"3747","AC018697.1",2.05780256207333,-1.972350761709,1.44881351729904,
-1.36135585301963,0.173401260246755,0.782487986840778
"3748","SOX4",2101.46747203775,0.480260049202324,0.352886708087807,1.3
6094683703083,0.173530490588752,0.782831786252566
"3749","DCAF10",277.246767899122,0.261263768499948,0.192035275517163,1
.36049883437482,0.17367212150573,0.783261731240035
"3750","C1orf54",180.314293907806,0.571922405612999,0.421129890839714,
1.35806652069416,0.174442577371313,0.78343823564962
"3751","GATB",62.0974336542981,0.364499554446411,0.268051411694625,1.3
5981210523026,0.173889390712587,0.78343823564962
"3752","AL390955.2",2.82387042530382,-1.22699778095988,0.9035231778257
18,-1.35801472621055,0.174459011416834,0.78343823564962
"3753","NOL8",254.967461338132,0.279340939242535,0.205761214122588,1.3
5759764265441,0.174591391395349,0.78343823564962
"3754","AL445222.1",9.0105305416071,0.709714846319291,0.52242785731527
7,1.3584934960522,0.174307144965568,0.78343823564962
"3755","PIP4K2A",213.097925776574,0.22071273905505,0.162347043196823,1
.35951191169812,0.173984430501608,0.78343823564962
"3756","AL117339.4",4.313803947877,1.03148603380059,0.759422044056943,
1.35825137270212,0.174383934481654,0.78343823564962
"3757","ELOVL3",8.31185725005922,-0.709455782915236,0.522277966260817,
-1.35838735069468,0.174340805898607,0.78343823564962
"3758","CD81",14098.9153643667,-0.288635409648542,0.21241608462017,-1.
3588208734977,0.17420335723604,0.78343823564962
"3759","HEBP1",1181.05649383766,-0.326626093201222,0.240513866710092,-
1.35803435231835,0.174452784047206,0.78343823564962

"3760","RAB3IP",16.4653761774769,0.627133379294903,0.461699710117565,1
.35831443154949,0.174363932911025,0.78343823564962
"3761","CTNS",61.437220964211,0.328569026469873,0.242013256909644,1.35
76488770305,0.174575125852992,0.78343823564962
"3762","NDEL1",196.393102567053,-0.225436727830205,0.165986240451326,-
1.35816515403464,0.174411284864277,0.78343823564962
"3763","AC025048.4",2.31820711678779,1.30334416962658,0.95964652014467
1,1.35815025873287,0.174416010293112,0.78343823564962
"3764","CBX4",133.732731797887,0.375314398062487,0.276185467280223,1.3
5892160350959,0.174171432315458,0.78343823564962
"3765","C19orf54",31.2390198028213,0.447781544627613,0.329197342156741
,1.36022223537397,0.173759608230472,0.78343823564962
"3766","ETHE1",204.438949452965,-0.382697768991615,0.281422307018515,-
1.35987005808476,0.173871047592257,0.78343823564962
"3767","UBQLN2",239.95659348491,0.246405125478782,0.181392346586167,1.
35840971306765,0.174333713914624,0.78343823564962
"3768","MBNL3",90.7212912105916,0.378077654200893,0.278490365038914,1.
35759689261804,0.17459162952021 3,0.78343823564962
"3769","ZNF354C",80.7121170074015,0.312584178592792,0.230347400352174,
1.35701196590406,0.174777408477749,0.784035628655146
"3770","N4BP2L2",1322.02193011887,0.202488142507437,0.149230055763229,
1.35688579268984,0.174817501776076,0.784035628655146
"3771","MBOAT1",233.806604690356,-0.398364880305471,0.293654733042498,
-1.35657571794635,0.174916061515499,0.784269628242923
"3772","ITSN2",229.653154227476,0.314746233302454,0.232106960725202,1.
35603961345688,0.175086564448768,0.784283331387063
"3773","UHRF1BP1",57.1470831223049,0.474242446265927,0.349671466492284
,1.35625148664051,0.175019165381592,0.784283331387063
"3774","ZNF891",76.1465332980694,0.323935091164507,0.23889203931988,1.
35598947577635,0.175102516601007,0.784283331387063
"3775","KCNE2",3.85704088429598,-1.11164025705853,0.819804133555557,-1
.35598274216703,0.175104659095467,0.784283331387063
"3776","PDCD4-
AS1",7.13246426941966,0.901597256189878,0.665043203706865,1.3556972707
4044,0.175195508162978,0.784482429030623
"3777","STK32B",16.8132408478652,-0.591910092727588,0.436764604827036,
-1.35521534067989,0.175348958466347,0.784961659981202
"3778","VDAC2",1461.3997302726,-0.327171660329556,0.24149696570517,-1.
35476509766579,0.175492409948666,0.785395888674441
"3779","OSTF1",270.2528248015,-0.398456064194711,0.294154487807827,-1.
35458094542153,0.175551107733018,0.785450682601181
"3780","ZNF433",22.631779600296,-0.448649554227023,0.331254362339361,-
1.35439591212807,0.175610101097443,0.785506769670783
"3781","LNPK",440.844333234502,0.197870292840094,0.146135601750074,1.3
5401839435744,0.175730509315576,0.785629680462124
"3782","CELF2-
AS2",6.00404071144964,1.12729983463676,0.832514588901812,1.35409018612
372,0.175707606795976,0.785629680462124
"3783","DNAJB11",328.954875604456,-0.308691228270558,0.22811609208287,
-1.35321986911128,0.175985399518765,0.785924510667402

"3784","AC007920.2",65.0628521713663,-0.504703324330562,0.372994330089
421,-1.353112590772,0.176019663886871,0.785924510667402
"3785","SPSB3",483.861497233417,-0.24948017143331,0.184335622256124,-1
.3534018459366,0.175927288060799,0.785924510667402
"3786","AATK",7.41920480553635,0.872412267652737,0.644682074608615,1.3
5324418347195,0.175977634278962,0.785924510667402
"3787","ZNF653",15.3081815094229,-0.550180221371532,0.406612090590677,
-1.3530837722319,0.176028869286577,0.785924510667402
"3788","LCOR",230.112544117253,0.297477645699515,0.219943731634011,1.3
5251704374336,0.176209970220059,0.786525389778445
"3789","NIBAN1",1017.8993044936,0.34243308072456,0.253312562845006,1.3
5182036326435,0.176432788253577,0.786540237729903
"3790","AHCTF1",356.572217116601,-0.357749046997736,0.264846902780069,
-1.35077678176518,0.176766947810422,0.786540237729903
"3791","DNMT3A",107.69267357345,0.330638355014258,0.244805352158441,1.
35061734598133,0.176818041377535,0.786540237729903
"3792","KCMF1",372.513764918827,-0.234389955259017,0.173490410936274,-
1.35102541975713,0.176687290027269,0.786540237729903
"3793","C6",192.505177442529,-0.524298838461139,0.388117951017167,-1.3
5087500355774,0.176735476653833,0.786540237729903
"3794","MCM9",40.9477350739662,0.424485109974344,0.314101940654812,1.3
514246651562,0.1765594371306,0.786540237729903
"3795","CHCHD3",347.417290955107,-0.32897738788851,0.243293631057289,-
1.35218249018218,0.176316943672427,0.786540237729903
"3796","FKTN",101.322192344689,0.334618437173546,0.247715031351963,1.3
5082007477417,0.176753075821555,0.786540237729903
"3797","TM9SF3",1032.69337399181,0.173500864318252,0.128459020979683,1
.35063199917811,0.176813345083634,0.786540237729903
"3798","SLC22A18",63.7356800970461,-0.434890380805071,0.32188779866451
9,-1.35106202412576,0.176675565105141,0.786540237729903
"3799","CPSF7",239.275611290988,0.189002801322615,0.139882613103759,1.
35115292121702,0.17664645192645,0.786540237729903
"3800","AC012645.1",17.5347157439431,-0.564170541633135,0.417365690891
475,-1.35174153972285,0.176458011460474,0.786540237729903
"3801","ZNF317",48.3048196155646,0.38363726545846,0.283735432646372,1.
35209501992865,0.176344920254818,0.786540237729903
"3802","AC110772.2",9.4846005579628,-0.883330116700851,0.6542087206757
45,-1.35022675299779,0.176943259206413,0.786683309666586
"3803","AC034111.1",7.3540010705106,-0.851047980012576,0.6302892966115
8,-1.35024977353699,0.176935877360066,0.786683309666586
"3804","GOLGA5",199.980798552863,-0.228961145393383,0.169631924317252,
-1.34975268549787,0.177095326364147,0.786988921566546
"3805","COL1A1",23684.8646861646,0.514059794373035,0.3808633889832,1.3
49722260639,0.177105089103425,0.786988921566546
"3806","GATM",7.24374396223611,-0.792677677008732,0.587588009466462,-1
.3490365089793,0.177325239702067,0.787553231647634
"3807","PRR34",6.43640163421656,0.80247189880212,0.594837248495078,1.3
4906127824435,0.177317284343298,0.787553231647634
"3808","ATP6V0D1",427.293356821091,-0.34142785491738,0.25311794210468,
-1.34888839597226,0.177372815983247,0.787557660883597

"3809","ZC3H7A",267.292462143862,0.257424362564595,0.190888411094629,1.34855940750107,0.177478526383029,0.787820142841757
"3810","AC092828.1",19.7901208201528,0.582549903159329,0.432149091479456,1.34802991524286,0.177648761062741,0.788302835087088
"3811","MIR762HG",38.5828864083443,-0.433906197990086,0.321905300835632,-1.34793119859695,0.177680512450727,0.788302835087088
"3812","GSPT2",143.075339206282,0.279414786044794,0.207323594149425,1.34772304710968,0.177747476492884,0.788393056805267
"3813","PEF1",263.485618723088,-0.337922998279883,0.250809471157677,-1.34732949565306,0.17787413657158,0.788541138215069
"3814","TTF2",83.9244867141569,0.293409179283195,0.21776540480252,1.34736359776371,0.177863158535383,0.788541138215069
"3815","CCL27",7.03358222342417,-0.843733584531725,0.626325669594834,-1.34711640523616,0.177942745342183,0.788638515922837
"3816","GLDN",42.3040311752781,0.626085011876714,0.464816271225377,1.34695158202227,0.177995827020459,0.788667044879959
"3817","ZNF511",414.709496570071,-0.275482009793828,0.204599192081445,-1.34644720241205,0.178158336804415,0.789180287840989
"3818","MANEA-DT",14.1711529493251,0.781576089983976,0.580625865790803,1.34609244271177,0.178272705579167,0.789479724787234
"3819","COX6C",2563.28056296975,-0.356604774304779,0.264946938178456,-1.345947897177,0.178319320378664,0.789479724787234
"3820","AP4B1",45.9191751898724,0.347254724202066,0.258076827348315,1.34554786561054,0.178448374702764,0.789844272113701
"3821","AP000926.2",4.08684156285463,-1.05300126808589,0.782736358073112,-1.34528217224775,0.178534128531544,0.790017023085934
"3822","PFN1",1923.74028378069,-0.363001145338569,0.269868170941021,-1.3451054419378,0.17859118607866,0.790062735274199
"3823","TMUB1",141.213274203764,-0.342953977290845,0.255192511725708,-1.34390298121078,0.178979761874923,0.790906031679815
"3824","WNT10B",11.9557004757378,0.751188325302365,0.558862371340951,1.34413831351705,0.178903664673935,0.790906031679815
"3825","BCL2L2",97.665657335345,-0.307363614746347,0.228728584482579,-1.34379188085151,0.179015695720289,0.790906031679815
"3826","SOCS6",118.238997267211,0.288796241011124,0.214881241116717,1.34398070073627,0.178954627778923,0.790906031679815
"3827","ANKRD27",81.4236827810486,0.297570755041997,0.221437808816042,1.3438118658824,0.179009231447973,0.790906031679815
"3828","TPRG1-AS1",5.11501415057465,-1.00135304719831,0.745560640815965,-1.34308732566998,0.179243699073449,0.791180689981937
"3829","SGCZ",2.95897126209516,2.26587915133369,1.68715040968078,1.34302142733227,0.179265035682564,0.791180689981937
"3830","SLC2A13",119.456946975399,0.301141415202933,0.224171517453161,1.34335270878404,0.179157792260556,0.791180689981937
"3831","PCIF1",122.681242616589,-0.289557687514317,0.215588046173328,-1.34310641361589,0.179237519117901,0.791180689981937
"3832","FRZB",35.6749944234233,0.786585900887842,0.586218465562575,1.34179652654404,0.179661978763444,0.791595553483927

"3833","AC006994.2",7.03161310478868,-0.819131032731818,0.610573799541143,-1.34157579861339,0.179733577586042,0.791595553483927
"3834","DAPP1",8.33640589971747,-0.870177416080687,0.648523471377829,-1.34178245581758,0.17966654233535,0.791595553483927
"3835","AC005906.2",10.5088849560006,-1.06526646728025,0.793870839171986,-1.3418637071886,0.179640191189715,0.791595553483927
"3836","LINC02352",19.6194306843945,-0.719857408553456,0.536442094540983,-1.34191074093359,0.17962493869147,0.791595553483927
"3837","SLA2",5.03978126900989,1.04229748187303,0.776863139429101,1.34167452279817,0.179701551218106,0.791595553483927
"3838","MATN4",12.4036575519495,0.992449121160204,0.739373772079657,1.34228337362944,0.179504132250755,0.791595553483927
"3839","UCKL1",168.067957028926,-0.237341815063999,0.17687886683551,-1.341832516853,0.179650306384828,0.791595553483927
"3840","STIM1",194.815550117117,0.229307292084341,0.170952417176959,1.34135156361654,0.179806335729704,0.791709772009855
"3841","GPN2",104.682550456644,0.241563885133891,0.18013101856678,1.34104546266325,0.179905692477377,0.791755407545348
"3842","AL022068.1",3.96845684089872,1.04468534547621,0.779100280111004,1.34088688214483,0.17995718187821,0.791755407545348
"3843","SCART1",4.79651598565635,1.00835782975047,0.75197761574942,1.34094128419706,0.179939516878998,0.791755407545348
"3844","FNDC5",57.3681497569094,0.65933471308558,0.492519876813309,1.33869665799397,0.180669444705149,0.792227577323169
"3845","MSH2",107.089986667746,0.316520088592043,0.236303443684707,1.33946456156757,0.180419483832266,0.792227577323169
"3846","KYNU",5.96325823175422,-1.15665277248244,0.862956924872298,-1.34033662532299,0.180135929314373,0.792227577323169
"3847","HACD2",137.031447865187,0.280620530980514,0.209417576747335,1.34000467075925,0.180243826422601,0.792227577323169
"3848","AC093849.4",5.53954057494076,0.995206746885006,0.743070950238926,1.339315911307,0.180467851029831,0.792227577323169
"3849","AL359715.1",8.14412835128658,0.808390045162354,0.603502433691992,1.33949757288788,0.180408744054194,0.792227577323169
"3850","CPSF4",130.204646990074,0.260606127776252,0.194619116726748,1.33905719108854,0.180552055295433,0.792227577323169
"3851","LIPC",25.1401946466499,-0.611166399459568,0.456473059775752,-1.33888821338068,0.180607067304041,0.792227577323169
"3852","TMEM92",2.33868378898118,1.32494720831967,0.989215076876715,1.33939245295672,0.180442945026592,0.792227577323169
"3853","AC023421.1",3.52313066299167,-1.0879278668423,0.812401777248524,-1.33915003303776,0.180521835173781,0.792227577323169
"3854","MRPL4",362.451118421197,-0.311845393413969,0.232854488905834,-1.33922860958923,0.180496261384881,0.792227577323169
"3855","ZNF133",56.7847289394077,0.314122161613956,0.234649998392826,1.33868384302345,0.180673618296554,0.792227577323169
"3856","AP000547.3",25.7594857440585,-0.63772419677048,0.475842420429918,-1.34020038859567,0.180180205316928,0.792227577323169
"3857","AL358115.1",2.19308522538885,-1.42133298026403,1.06209093528954,-1.33824038322722,0.180818088804033,0.792606971590544

"3858","AC026894.1",2.57214247073975,1.31748072764268,0.984568186774994,1.33813050770831,0.180853897350149,0.792606971590544
"3859","PANK4",56.6725345406475,-0.330176170323378,0.247466122774699,-1.33422775861721,0.182129224849429,0.792644231371872
"3860","AL034417.3",78.1994123716782,1.10855136007986,0.831858689532629,1.33261979952712,0.182656604269567,0.792644231371872
"3861","TMEM234",80.7895708276452,-0.274538622160295,0.206188768808232,-1.33149164111665,0.183027293598843,0.792644231371872
"3862","RABGGTB",257.810704368806,-0.291010164448056,0.218763199250092,-1.33025191369308,0.183435284824133,0.792644231371872
"3863","CHTOP",285.209830643269,0.207605588418002,0.156115504924618,1.32982043339158,0.183577441869548,0.792644231371872
"3864","PLA2R1",337.532747462663,0.384401718987186,0.288064882483458,1.33442756254491,0.182063772000486,0.792644231371872
"3865","AC064836.4",58.0663633168057,-0.470588311401449,0.352411828033576,-1.33533631384419,0.181766298472818,0.792644231371872
"3866","TMEM43",883.401354962291,-0.251214410465806,0.188838446236986,-1.3303139030838,0.183414868279322,0.792644231371872
"3867","TRAK1",139.377612181648,0.37340479285208,0.280015819166351,1.33351320637442,0.182363444544931,0.792644231371872
"3868","AC026304.1",22.3332433892389,-0.472730119519166,0.354545248756827,-1.33334213665742,0.182419551801,0.792644231371872
"3869","STIM2-AS1",4.787528263262,1.03728625136821,0.778258393612645,1.33283014983387,0.182587549152205,0.792644231371872
"3870","FRYL",172.11864473743,0.31169093081795,0.234390653673394,1.32979248930407,0.183586651241671,0.792644231371872
"3871","AC110792.4",4.7811442765516,-1.27898969702768,0.957688387431944,-1.33549671669018,0.181713829161524,0.792644231371872
"3872","SEMA5A",30.238395222409,0.856806582763193,0.642925053282591,1.33266945873175,0.182640300088902,0.792644231371872
"3873","NIPBL",646.291595980555,0.304259272159936,0.228823817088881,1.32966609870752,0.183628309337806,0.792644231371872
"3874","RASA1",165.220747610721,0.285138327258491,0.213868001786337,1.33324445394761,0.18245159540862,0.792644231371872
"3875","PRRC1",306.914929109494,0.259638254543072,0.194237694633907,1.3367037486335,0.181319357830899,0.792644231371872
"3876","UBE2D2",1084.4916288286,-0.271822314291748,0.203857787197253,-1.33339186120338,0.182403241880145,0.792644231371872
"3877","MYOZ3",5.14290490953276,-0.9501242257895,0.714015857531145,-1.33067664501843,0.183295430988339,0.792644231371872
"3878","PHF10",303.974926362975,0.254051535243947,0.190383840465712,1.33441753576613,0.182067056210925,0.792644231371872
"3879","HOXA11-AS",35.5477178851451,0.442202147614952,0.331204668167808,1.33513259357476,0.181832953531978,0.792644231371872
"3880","FAM200A",64.9674902327926,0.384095971073796,0.288165722107078,1.33289958384111,0.182564759170599,0.792644231371872
"3881","UBE3C",196.919576679654,0.272133884768775,0.204330438820912,1.33183233168354,0.18291529106833,0.792644231371872

"3882","SULF1",155.795003011712,0.33038207424309,0.247491435176539,1.3
3492326313202,0.181901463067015,0.792644231371872
"3883","CHRAC1",267.338626474592,-0.230672085932785,0.173061860198731,
-1.3328880532539,0.182568543652303,0.792644231371872
"3884","SYK",5.93826183897042,-0.90817194540713,0.682959692428866,-1.3
2975921635627,0.183597617261949,0.792644231371872
"3885","AL158152.1",126.53609834183,-0.380239829580571,0.2846585630662
91,-1.33577513173922,0.181622783607831,0.792644231371872
"3886","C9orf116",24.7130721486925,-0.532919288022713,0.40073436153194
,-1.3298567309912,0.183565479989475,0.792644231371872
"3887","AL133482.1",83.7398118577021,0.522360303108501,0.3926909034352
43,1.33020729163552,0.183449982384967,0.792644231371872
"3888","APBB1",110.100947310835,0.257537029992408,0.192674106708526,1.
33664577141134,0.181338290882419,0.792644231371872
"3889","C11orf91",3.78244780729935,-1.05521798356393,0.78996296430121,
-1.33578158882089,0.181620672454931,0.792644231371872
"3890","HNRNPUL2",399.017979954318,0.282072441137652,0.212014336713011
,1.33044041035524,0.183373207641473,0.792644231371872
"3891","ARL2",1255.25874486598,-0.36148907563052,0.271357468588407,-1.
33215082492837,0.182810631891706,0.792644231371872
"3892","SIPA1",96.4165884993239,0.346974673075305,0.259398559795974,1.
33761218006844,0.181022891879133,0.792644231371872
"3893","C1RL-
AS1",72.4683702805558,0.494681189189107,0.371694197087577,1.3308821958
0827,0.183227776420651,0.792644231371872
"3894","AC092111.1",6.52048007658242,0.886176478726706,0.6660346167235
42,1.33052615656243,0.183344974186598,0.792644231371872
"3895","KLRC3",5.75646587788567,1.05107497383928,0.790408943757792,1.3
2978628612453,0.183588695634272,0.792644231371872
"3896","PTHLH",20.1351534134092,-0.53257542412591,0.400252947431313,-1
.33059713249783,0.183321606541344,0.792644231371872
"3897","AC022506.2",4.8157130406494,-0.934926352092149,0.6988194396728
93,-1.33786540415901,0.180940316551275,0.792644231371872
"3898","EXOSC8",309.934634100187,-0.324933965750454,0.242961030687708,
-1.33739128793914,0.181094946732724,0.792644231371872
"3899","SAV1",1333.56896828564,0.212406344006236,0.159632620763512,1.3
3059485580272,0.183322356071054,0.792644231371872
"3900","DICER1",430.062236620798,0.307898645273183,0.231108181021896,1
.33227064447369,0.182771269818293,0.792644231371872
"3901","C15orf48",8.50472850037628,-1.01638933655993,0.761077597608741
,-1.33546085149972,0.18172556003419,0.792644231371872
"3902","AC022467.2",3.62430205232651,1.30815900720699,0.97910414889398
1,1.3360774833655,0.181523948808146,0.792644231371872
"3903","TCF12",361.320496406566,0.242314522254017,0.181674758169917,1.
33378199973925,0.182275311847057,0.792644231371872
"3904","IL16",161.059844671543,-0.419235784071688,0.314967766868937,-1
.33104345323736,0.183174713432982,0.792644231371872
"3905","TXNL4B",84.8409563374267,0.30954167181125,0.232668834386052,1.
33039593647359,0.183387852721973,0.792644231371872
"3906","AC005838.2",32.4473564801598,0.511841737266139,0.3846993334832

71,1.33049811298515,0.183354207675829,0.792644231371872
"3907","HOXB5",18.5364358582756,-0.64912566264053,0.486721369970589,-1
.33366994483878,0.182312048871877,0.792644231371872
"3908","CYBC1",237.902458585812,-0.262947352355616,0.197329411337772,-
1.33252995877804,0.182686103653859,0.792644231371872
"3909","SH2D3A",8.75940766600192,-0.732400473993392,0.547720234907683,
-1.33717987270789,0.181163930168006,0.792644231371872
"3910","EIF3G",2407.6453106949,-0.299319889462749,0.223761367323378,-1
.33767456394818,0.181002546157375,0.792644231371872
"3911","SIN3B",147.641110294124,0.29828010266973,0.224257042902982,1.3
3008131565693,0.18349148091295,0.792644231371872
"3912","ZNF793-
AS1",3.59128363941652,-1.09967718547614,0.825249738327491,-1.332538666
05862,0.182683244446828,0.792644231371872
"3913","SLC24A3",9.63004575657006,0.635948581043425,0.477465545424873,
1.33192559575692,0.182884639224175,0.792644231371872
"3914","SOGA1",150.542124591877,0.337344764044834,0.252431642413103,1.
33638065664039,0.181424885502874,0.792644231371872
"3915","AL391097.1",7.66422468367827,1.0334429959615,0.773895131612028
,1.33537859814266,0.181752465781838,0.792644231371872
"3916","PPP2R3B",74.499767094694,-0.271892666146323,0.204288030683219,
-1.33092802959139,0.183212693306049,0.792644231371872
"3917","GPR173",18.5040234671233,0.535172792089216,0.401327356799726,1
.33350688165592,0.182365518691248,0.792644231371872
"3918","MARCO",6.58558709944035,-1.07952778787453,0.812427593449178,-1
.32876799923963,0.183924522850119,0.792761825956267
"3919","PLEKHA3",418.808959862157,-0.181154669314621,0.13634264996423,
-1.32867205795214,0.18395618736363,0.792761825956267
"3920","HOXA13",17.4887005506994,0.746024940518424,0.56124470031894,1.
32923293546376,0.183771132224415,0.792761825956267
"3921","NCAPG2",48.2056309540723,-0.34067149798149,0.2562528313465,-1.
32943505908366,0.183704477648483,0.792761825956267
"3922","AJM1",4.50858501043702,-0.922049846300812,0.693963700807915,-1
.32867157925891,0.183956345361917,0.792761825956267
"3923","ACTA2",398.885417325452,0.337816177741862,0.254265855871111,1.
3285943430529,0.183981839389483,0.792761825956267
"3924","LINC01569",8.27530014931979,0.662619770948388,0.49873964572768
3,1.32858852634744,0.183983759466075,0.792761825956267
"3925","ARHGEF19",24.6820030219285,-0.470347137462581,0.35416271027052
5,-1.32805381205522,0.184160330395698,0.793033429689181
"3926","SYNJ2",74.0245285689419,-0.641464584758032,0.48302275285559,-1
.32802146682687,0.184171015313234,0.793033429689181
"3927","FBXO10",24.966366350753,0.426160519622083,0.320910877711648,1.
32797156226348,0.184187501679052,0.793033429689181
"3928","INTS3",155.598474339769,0.292673323689456,0.220509736576907,1.
32725805323966,0.184423334509565,0.793708951262903
"3929","SOAT1",182.653472156657,0.259072611998478,0.195242255134613,1.
32692900837401,0.184532167460047,0.793708951262903
"3930","AC099522.2",8.1441994362783,-0.724260829444201,0.5457213353663
85,-1.32716238583186,0.184454972000469,0.793708951262903

"3931","NDUFV3",407.581788946921,-0.31172430007871,0.23490761243044,-1
.32700808140484,0.18450600938986,0.793708951262903
"3932","AC003986.2",5.4751017947323,-0.897636289809126,0.6767892844105
98,-1.3263157536409,0.184735130844285,0.794290205944984
"3933","WDR25",44.3991774428535,-0.394625458829727,0.297552779831979,-
1.32623684125069,0.184761259757607,0.794290205944984
"3934","PAIP1",603.801260061122,-0.276400811059197,0.208490007845804,-
1.32572689653126,0.184930174993076,0.794752325283972
"3935","AC006042.4",2.21960508215244,1.51546027152229,1.1433236083574,
1.32548673048004,0.185009767702727,0.794752325283972
"3936","AGO2",383.615367088003,0.332546724193681,0.250882842677197,1.3
2550604355818,0.185003366276577,0.794752325283972
"3937","DFFA",312.680434212943,0.232140930336841,0.175168301263922,1.3
2524508522281,0.185089876207537,0.794894495025917
"3938","RRAGC",504.906568645986,0.286275143520353,0.216108110312466,1.
32468486771012,0.185275694205045,0.795323863092125
"3939","RRP36",235.710774525821,-0.331540890291664,0.250337324076532,-
1.32437658473295,0.185378007123614,0.795323863092125
"3940","AC026904.2",8.82770391100929,0.711138669227927,0.5368952072297
1,1.32453905278329,0.185324082039049,0.795323863092125
"3941","MYCBPAP",5.11888194119316,0.992108737149272,0.749089771931549,
1.32441901401362,0.185363923219577,0.795323863092125
"3942","ABO",5.23949788176343,-1.16193154655378,0.87747174765349,-1.32
418114846544,0.185442890128235,0.79540040240695
"3943","PHLPP2",65.1011221733197,0.341457787604513,0.257936129842672,1
.32380751705,0.185566978991283,0.795529026568106
"3944","GEMIN8",111.102058578758,-0.369965043970066,0.279443231701181,
-1.32393632051065,0.185524194405138,0.795529026568106
"3945","SPATS2L",646.17137602533,-0.193734466092715,0.146406897551914,
-1.3232605111656,0.185748758974692,0.796001460599238
"3946","CCDC180",36.0349734801689,0.448095023816297,0.338692186562751,
1.32301553325995,0.185830212251224,0.796001460599238
"3947","MTIF3",633.364119943497,-0.253435714378322,0.191574499026441,-
1.3229094460184,0.185865493638857,0.796001460599238
"3948","LINC02254",47.5612383584659,0.446904878666777,0.33775406563160
4,1.32316654081146,0.185780000276052,0.796001460599238
"3949","STK24-
AS1",3.8601512242831,-0.934848212650766,0.70678070839426,-1.3226849594
8999,0.185940167351719,0.79611961245451
"3950","VPS11",144.592733178085,0.207783108542775,0.157113042742075,1.
32250706189862,0.185999359363691,0.796171434967416
"3951","DCAF5",384.442830756487,0.265378344300339,0.200691041019412,1.
32232282493701,0.186060675360671,0.796232320677859
"3952","HNRNPAB",698.255422571885,-0.247397154168023,0.187134993614984
,-1.32202507606367,0.186159800897613,0.796454937645961
"3953","AC010136.1",2.88831149024237,1.52124832805316,1.15124914490488
,1.32138932288097,0.186371584309386,0.796740589935231
"3954","NUP155",71.706128368113,0.289925728407321,0.219520343470389,1.
32072373714388,0.18659349631762,0.796740589935231
"3955","AFDN",217.629555964236,0.315145415113053,0.238774295666224,1.3

1984648612925,0.186886277483033,0.796740589935231
"3956","MOSPD3",213.777781595384,-0.42623196360483,0.322678264026041,-1.32091935256734,0.186528256206672,0.796740589935231
"3957","CYHR1",442.173309429488,-0.229701741709335,0.173941255556999,-1.3205707925574,0.186644516930488,0.796740589935231
"3958","CDC26",747.500521349068,-0.337268705735178,0.255445534561955,-1.32031552758804,0.186729693439591,0.796740589935231
"3959","AL731577.1",8.6710391952128,1.30308787270522,0.986122909583581,1.3214254126349,0.186359557251539,0.796740589935231
"3960","DBX2",56.6657957488074,0.46491054788469,0.351901444588204,1.32113850350552,0.186455186735948,0.796740589935231
"3961","B3GNT9",86.7204207589791,0.378312163459084,0.286488494967171,1.32051433165732,0.186663354263192,0.796740589935231
"3962","PEMT",227.669727696769,-0.314344274581931,0.238113725313513,-1.32014344896771,0.186787128628619,0.796740589935231
"3963","RECQL5",33.092424111559,0.371940022043945,0.281682794877632,1.3204215124517,0.186694325051775,0.796740589935231
"3964","SMAD4",343.893851013548,0.20151980692274,0.152680426051522,1.31987977852994,0.186875160005009,0.796740589935231
"3965","FAM118A",96.8792813638427,0.377449145699407,0.285926047820781,1.32009359964292,0.18680376942244,0.796740589935231
"3966","RLIM",330.324809871204,0.309364079515085,0.234163598658188,1.32114505110023,0.186453003957436,0.796740589935231
"3967","IL7R",51.7772084340527,0.503477608419857,0.38152217049956,1.31965491746026,0.186950258345853,0.796812444696666
"3968","ERBIN",329.895350418892,0.305136300162813,0.231303092118366,1.3192054518954,0.187100436140645,0.797251556014625
"3969","DGKA",57.6482808584858,-0.314125324350769,0.238155956866726,-1.31898999497441,0.187172457320021,0.797357497698897
"3970","GPAT2",49.9569844221546,-0.799722229618847,0.606779357052258,-1.31797863642545,0.187510799597187,0.797446191513882
"3971","CD302",1549.94393430106,0.216709507511577,0.164394140303546,1.31823133787758,0.187426217973276,0.797446191513882
"3972","DNER",25.462600388875,0.537895212899032,0.408046830800674,1.31821931282635,0.187430242235999,0.797446191513882
"3973","AC097534.2",55.501774671485,-0.465427065939835,0.353147138066484,-1.31794092538338,0.187523424264206,0.797446191513882
"3974","ECI1",589.326261755765,-0.263492269187541,0.199858321094149,-1.3183952899485,0.187371356694997,0.797446191513882
"3975","MYLPF",7.35793828740421,-0.686239095323766,0.520504549198125,-1.3184113306617,0.187365989823938,0.797446191513882
"3976","XIST",4033.2584504822,0.396134013846647,0.300392492212817,1.31872141986159,0.18726226307732,0.797446191513882
"3977","MYSM1",235.733603660463,0.304858416589473,0.231375180260435,1.31759342659971,0.187639787268326,0.797648027954525
"3978","FGD6",39.7237901662902,0.451858285342823,0.342998561748883,1.31737661825427,0.187712414432875,0.797648027954525
"3979","SCPEP1",1654.97825299424,0.298206537791713,0.226352968049716,1.31744036917693,0.18769105678831,0.797648027954525
"3980","LERFS",36.2356228615107,1.77633693938984,1.34885927495686,1.31

691791157876,0.187866141870638,0.798100685112753
"3981","AC016394.1",22.2799359755142,-0.443860609041571,0.337180412052
27,-1.31638906999367,0.188043489067328,0.798653432090022
"3982","TOMM70",284.724059634074,0.193627057332099,0.147215401136904,1
.31526359223811,0.188421329554606,0.798695478213434
"3983","SGMS2",169.549926886221,0.251884039415177,0.191512727192755,1.
31523394349483,0.188431290666749,0.798695478213434
"3984","LNPEP",420.378054810222,0.255645593264691,0.194249336816544,1.
31606932334669,0.188150776117622,0.798695478213434
"3985","HCG11",319.938439141074,0.227940283526266,0.173307770852435,1.
31523406253,0.188431250673631,0.798695478213434
"3986","IER3-
AS1",10.9865153354191,-0.564344942327789,0.428762430196221,-1.31621826
583434,0.188100794682118,0.798695478213434
"3987","DNAJB9",276.419768693983,-0.346446048841365,0.263327254728978,
-1.31564827650649,0.188292122084233,0.798695478213434
"3988","GDAP1",25.5303115155249,-0.436035751325966,0.331398613099026,-
1.31574404385233,0.188259965982442,0.798695478213434
"3989","ARL2BP",579.120840707734,-0.212127597240617,0.161200982033756,
-1.3159200059724,0.188200893191136,0.798695478213434
"3990","GREB1",6.95378155294032,0.712473821531236,0.54190097486946,1.3
147675582937,0.188588033617787,0.798959276474452
"3991","SLC25A21",15.0118420929513,-0.599342989247363,0.45583040889273
5,-1.31483766232981,0.188564466863978,0.798959276474452
"3992","DHPS",360.089952127892,-0.277921061075101,0.211444413606803,-1
.31439301863948,0.188713978641953,0.799292572865266
"3993","CFLAR-
AS1",3.25678924096593,1.37751345952751,1.04815368624271,1.314228512104
42,0.188769316238926,0.799326721504572
"3994","VDAC1",821.833068118504,-0.263724096563117,0.200712481313336,-
1.31393969541641,0.188866498901143,0.79953799785191
"3995","RAET1G",21.258009863582,0.657347454086883,0.500474153756624,1.
31344935428283,0.189031576117471,0.799728013078883
"3996","PDK4",6319.65308638467,0.415649991355419,0.316473087334987,1.3
1338179450139,0.189054328986961,0.799728013078883
"3997","ZDHHC13",44.5479475769857,-0.443655540113359,0.337831642236716
,-1.31324448229895,0.189100579387827,0.799728013078883
"3998","RAD51-
AS1",100.330898835784,0.431472356634407,0.328476987294629,1.3135542924
5762,0.188996238908301,0.799728013078883
"3999","TRIM8",520.715534865511,-0.214280649343163,0.163222160597206,-
1.31281591028535,0.189244987483391,0.799738627435437
"4000","FECH",119.105642391413,-0.23894902577452,0.181995093027381,-1.
31294213376715,0.189202447816234,0.799738627435437
"4001","GNRH2",3.04523907215591,1.25588550861263,0.956458026180802,1.3
130586750654,0.189163177484411,0.799738627435437
"4002","TTC37",482.005966449514,0.209223775702697,0.159480490922074,1.
31190827475524,0.189551085216644,0.800329264923171
"4003","PRAG1",2.7285006049237,-1.38040199880674,1.05217152681666,-1.3
1195528830088,0.189535221065588,0.800329264923171

"4004","NPFF",21.1254223393144,0.613121205121855,0.467251305069227,1.3121872501373,0.18945696267895,0.800329264923171
"4005","SMAD6",126.976809272343,0.359351144336382,0.273929073053218,1.31184010638611,0.189574089544435,0.800329264923171
"4006","CACNB4",129.118630385902,0.450843644769711,0.343825480128886,1.31125722445209,0.189770874858457,0.800937934155788
"4007","FBXL4",155.398655086884,0.296591506991253,0.226210172756887,1.31113248965159,0.189813005805668,0.800937934155788
"4008","ZKSCAN3",31.0186751005003,0.410401444765278,0.313156700289144,1.31053062056902,0.190016392471708,0.801396149641217
"4009","CD40LG",5.73324314006795,0.866449626480633,0.661091504394558,1.31063494345484,0.189981127654251,0.801396149641217
"4010","UFC1",900.050587401819,-0.319836875503617,0.244280314155873,-1.30930270254824,0.190431834553415,0.801548782332374
"4011","GLYCTK",15.8065185650312,-0.645952090367342,0.493319700396911,-1.30939852969104,0.190399389269382,0.801548782332374
"4012","NR2F1",52.2541901812527,0.563181202955931,0.430060160177678,1.30954051340923,0.19035132371361,0.801548782332374
"4013","POLD2",356.777602088028,-0.321188598205686,0.245175863240503,-1.31003351618923,0.190184497529694,0.801548782332374
"4014","FAM107B",235.340840941243,0.260718348104405,0.199029914174187,1.30994553851955,0.190214260213732,0.801548782332374
"4015","PPP2R3C",236.932440905366,-0.281917759000931,0.215292641903773,-1.30946304763604,0.190377546989088,0.801548782332374
"4016","AC016355.1",9.28473194386296,-0.668491457064453,0.510558429241075,-1.30933389555068,0.190421272737425,0.801548782332374
"4017","ANGPTL4",1717.43490636699,-0.483507978670505,0.369235412285342,-1.30948430888003,0.190370349488144,0.801548782332374
"4018","USP33",737.239109864973,0.262129897382902,0.200227733510927,1.30915879027616,0.190480568213649,0.801554367186754
"4019","TLCD2",163.076279312953,0.38163047756361,0.291546684564938,1.30898582548829,0.190539152208276,0.801601389782914
"4020","SLPI",281.566758965174,0.587535084330165,0.448944025978509,1.30870453849917,0.190634453775551,0.801688318452671
"4021","AC115618.1",2.26668776694519,-1.39857696548233,1.06872147168187,-1.30864495805569,0.190654644458138,0.801688318452671
"4022","RPRD2",320.722906208528,0.229321293086947,0.175303191770272,1.30814100286014,0.190825488298603,0.801742367210026
"4023","SUCNR1",44.9350383806531,-0.724985939206368,0.554250432072063,-1.30804758508895,0.190857169861625,0.801742367210026
"4024","CARD11",3.46387470027648,1.38003847838143,1.05481632143088,1.30832112695164,0.19076441221255,0.801742367210026
"4025","CRHR1",3.1704476738379,1.57717800342615,1.20553256112902,1.30828320551465,0.190777269335081,0.801742367210026
"4026","FOXN2",214.720235838428,-0.30422497002747,0.232717276845484,-1.30727281683287,0.191120072830575,0.801974453258533
"4027","ZSCAN25",42.04876085427,0.381894324627289,0.292120861319859,1.30731616667778,0.191105355844692,0.801974453258533
"4028","H2AFY2",40.1194697640621,0.525103987288279,0.401706051527982,1.30718465726599,0.191150004993776,0.801974453258533

"4029","ADAM10",189.973355505704,0.328578238757118,0.251318798616354,1
.30741608095423,0.191071438784454,0.801974453258533
"4030","SSH2",245.534167397271,0.348988871626966,0.267005758180162,1.3
0704623752528,0.191197008580858,0.801974453258533
"4031","PNPO",66.4300034686728,0.289124427900666,0.221161411885886,1.3
0730051610381,0.191110669014932,0.801974453258533
"4032","ABI1",329.881702710975,0.206749497252103,0.158206065670606,1.3
0683672826153,0.191268168418811,0.802073956256265
"4033","AC016575.1",6.58324363188234,-0.742305882359013,0.568293537439
28,-1.3062015199114,0.191484036093754,0.802382176523716
"4034","TWNK",17.6516105089218,0.539252416866165,0.412766176863583,1.3
0643557319471,0.191404475145525,0.802382176523716
"4035","AC005840.4",2.0116333578898,-1.46123305076157,1.11862411352886
,-1.30627708904996,0.191458345470614,0.802382176523716
"4036","TMEM243",611.829128840133,-0.353953359253143,0.271135613591766
,-1.30544768562227,0.191740449827054,0.80325756334882
"4037","GPD2",97.034748523576,0.268363315657337,0.205661793631078,1.30
487686078794,0.191934781580875,0.80328624386284
"4038","SMARCAL1",127.226060492857,0.257742390212897,0.197517760230484
,1.30490741648821,0.191924375522662,0.80328624386284
"4039","LGALS9",79.7687695961726,-0.46949331529923,0.359795309882481,-
1.30489003720638,0.191930294165079,0.80328624386284
"4040","AL022328.2",3.04276505237951,1.33674633831954,1.02442924151744
,1.30486936934705,0.191937332931504,0.80328624386284
"4041","ADRA2B",4.793164260405,1.59366485367901,1.22241233081182,1.303
70482488558,0.192334243719658,0.803574448643094
"4042","SELENOK",1163.30004227826,-0.259618163435772,0.199127033232827
,-1.30378160725278,0.192308055478985,0.803574448643094
"4043","TCOF1",132.428764628784,-0.260038058631079,0.199452217942343,-
1.30376117805945,0.192315023028929,0.803574448643094
"4044","RWDD1",1826.6479385824,-0.29875938579216,0.229211678979849,-1.
30342130523997,0.192430966769148,0.803574448643094
"4045","LRRC18",2.20617293859828,1.46846373599225,1.12710673656421,1.3
0286128931196,0.192622121892457,0.803574448643094
"4046","AL589863.1",4.78490060825842,1.05515529323021,0.80956106787778
3,1.30336713942562,0.192449449565564,0.803574448643094
"4047","PWWP2B",14.4853210543067,-0.608010417093504,0.466664261275093,
-1.3028861808106,0.192613622497432,0.803574448643094
"4048","AL161717.1",3.08796505040217,1.35885768247611,1.04239180127385
,1.30359590397346,0.192371397984061,0.803574448643094
"4049","DEGS2",9.335054181246,-0.758752630668392,0.581945690520329,-1.
30382034445513,0.192294844336589,0.803574448643094
"4050","TMOD3",722.683964223087,-0.24820832521735,0.190375192213671,-1
.3037850275091,0.192306888989646,0.803574448643094
"4051","AC022898.2",2.06880743721228,-1.33581839583907,1.0253003610182
2,-1.30285567685987,0.192624038345781,0.803574448643094
"4052","HS3ST2",2.60987725825031,-1.65119435290048,1.2673207768021,-1.
30290166714305,0.192608334708346,0.803574448643094
"4053","ZNF564",41.3172881432449,0.479093368416387,0.367606410613732,1
.30327805659462,0.192479849801598,0.803574448643094

"4054","ANTXR1",714.202225654336,0.325843092402874,0.2501533127902,1.3
0257356486082,0.19272038764387,0.803579855556732
"4055","RAB33B",131.967656064753,-0.242121082319449,0.185870596229669,
-1.30263251547477,0.192700251400401,0.803579855556732
"4056","FBXO28",117.644065293345,0.28941307600233,0.222263072859629,1.
30211947616287,0.192875546267592,0.803863558376279
"4057","TSGA10",48.1868133344561,-0.320388288034352,0.246082065633276,
-1.30195708171521,0.19293105748113,0.803863558376279
"4058","ZNF582-
AS1",25.3125945741107,0.559022899193404,0.429333330378541,1.3020719791
322,0.192891780939598,0.803863558376279
"4059","AL139420.1",14.46447420651,-0.667386545458213,0.51281612143161
3,-1.30141490793053,0.193116473495592,0.804292483566244
"4060","CD1D",4.84487971882227,-0.985084265079297,0.756981662085702,-1
.30133174212583,0.193144926639021,0.804292483566244
"4061","CFLAR",1103.9177539172,0.308873971272611,0.237375161949426,1.3
0120594225616,0.193187971836004,0.804292483566244
"4062","ATP6V1E1",474.802422390568,0.194701808699573,0.149644016626649
,1.30109985743927,0.19322427657003,0.804292483566244
"4063","MGST3",3452.60140238973,-0.346183593307008,0.266179732458218,-
1.3005633077693,0.193407973336858,0.804660928439862
"4064","WDFY3",209.801859129359,0.280365688409613,0.215559823464371,1.
30063981266877,0.193381772765387,0.804660928439862
"4065","TAPT1",146.716947027767,0.235661398168875,0.181272467068275,1.
30003966945579,0.193587373324227,0.805209177900624
"4066","AL020997.2",16.2498273206136,-0.703850066126545,0.541629004091
302,-1.29950586251821,0.193770382861443,0.805574043132844
"4067","PARD6G",14.0923891914672,0.599844113854954,0.461572850140979,1
.29956541783544,0.193749958716239,0.805574043132844
"4068","PES1",112.410207286825,0.245147283959933,0.188672121023415,1.2
9932966582545,0.193830817735829,0.805627204099653
"4069","LYPLAL1-
DT",3.28486332630522,1.28949567007342,0.992943027019961,1.298660280583
75,0.194060540780896,0.80597293008941
"4070","GLS",631.899198766069,0.367967991293436,0.283432451321494,1.29
825639081833,0.194199246368228,0.80597293008941
"4071","PTGR2",67.8421624019096,-0.336163156471175,0.258934777556758,-
1.29825417675881,0.194200006930699,0.80597293008941
"4072","LRRC37B",24.4640190884032,0.451845492964064,0.348025301441141,
1.29831219481174,0.194180077586038,0.80597293008941
"4073","STXBP4",85.6283280638489,0.274320965279405,0.21123881888794,1.
29862951669375,0.194071103291117,0.80597293008941
"4074","MSL3",131.064143433115,-0.277140906001011,0.21346101304398,-1.
29832095355001,0.194177069068198,0.80597293008941
"4075","NTNG2",13.1494177974494,0.572705433948175,0.441264978455216,1.
29787194069447,0.194331343490394,0.806122265882135
"4076","LUC7L3",1017.40048339154,0.320581175124426,0.247003027720373,1
.29788358500346,0.1943273415359,0.806122265882135
"4077","NFKBIE",33.0701637283941,-0.49160950542738,0.378830029290662,-
1.29770468921878,0.194388831702226,0.806162954726817

"4078","KIAA0556",38.7827851218569,0.334762016357332,0.258051587245353
,1.29726780575484,0.194539057556707,0.806588127800099
"4079","ASAP1",409.252667728192,0.321491083725616,0.247906551039228,1.
2968236715727,0.194691863925688,0.806825989033219
"4080","ZFYVE26",55.219552150196,0.329793017253367,0.254288094533812,1
.29692669197895,0.194656411448934,0.806825989033219
"4081","VPS54",119.204673748658,0.262595488234032,0.202935649095183,1.
2939840259947,0.195670938522271,0.807124720306066
"4082","AC019080.6",3.76716172268331,1.13610141656795,0.87766079370615
9,1.29446527031298,0.195504757944785,0.807124720306066
"4083","NR2C2",156.297675574806,0.284313872573148,0.219376095148652,1.
296011182898,0.194971632171952,0.807124720306066
"4084","SLC12A7",89.3327266340078,0.260345787234317,0.200869289804353,
1.29609552305329,0.19494257720087,0.807124720306066
"4085","KIF2A",229.547150130642,-0.233323165028969,0.180123348787608,-
1.2953521384065,0.19519878072511,0.807124720306066
"4086","LY6G5C",7.02208274811806,-0.795747787065143,0.614757008052467,
-1.29441027372107,0.195523743819993,0.807124720306066
"4087","UST",310.578117955484,0.291592274538174,0.225154045236535,1.29
507899461385,0.195292980268764,0.807124720306066
"4088","HOXA-
AS3",6.53963898829559,0.958262627251562,0.740262144393931,1.2944909239
3629,0.195495902285722,0.807124720306066
"4089","AC009812.4",4.93277591156987,0.909253842386109,0.7017646302058
45,1.29566781118536,0.195089955650842,0.807124720306066
"4090","ECPAS",252.359221309927,0.225192304739048,0.174021188593217,1.
29405106676662,0.19564778217524,0.807124720306066
"4091","RNF214",162.651241559048,-0.206668555092792,0.15968945629721,-
1.29419036099757,0.195599675405885,0.807124720306066
"4092","EDNRB",145.172551244547,-0.684008618805074,0.527779121506729,-
1.29601303070181,0.194970995574165,0.807124720306066
"4093","LINC00519",18.1766499432372,0.59389440507553,0.458307357574542
,1.29584305217909,0.195029562257783,0.807124720306066
"4094","MAP2K3",207.114301427683,-0.224050949372289,0.172958130694161,
-1.29540570583799,0.195180310746551,0.807124720306066
"4095","TBCD",151.290883325987,0.294102422837572,0.226992274643889,1.2
9564948101854,0.195096273577832,0.807124720306066
"4096","ERCC1",492.433899484154,-0.274891710410805,0.212368358078081,-
1.29440992480497,0.195523864276819,0.807124720306066
"4097","YWHAB",2669.80204444429,-0.242686889394537,0.187540028304922,-
1.29405381660683,0.195646832404271,0.807124720306066
"4098","TP53RK",110.376720039729,-0.339284874475253,0.262010181628753,
-1.2949301144182,0.195344338857257,0.807124720306066
"4099","ADRM1",428.588537755136,-0.31674166675711,0.24473378486112,-1.
29422942948744,0.195586184237504,0.807124720306066
"4100","HMOX2",302.33888671267,-0.286706166867822,0.2215994676716,-1.2
9380349998271,0.195733303445615,0.807185047477675
"4101","POU2F2",8.62478484406017,-0.86028245274032,0.665098748638682,-
1.29346575151605,0.195850021917858,0.807469439304351
"4102","SORBS3",694.300684943299,-0.16850592599656,0.130291663110738,-

1.29329783635767,0.195908068699061,0.807511854111097
"4103","LINC01138",24.6788122516982,0.433396341306118,0.33516550435106
,1.29308158411245,0.195982843755822,0.80762318357871
"4104","COLEC11",11.1312813184612,1.02176325181853,0.790414073174935,1
.29269364817141,0.196117035497225,0.807674784147954
"4105","PRKCE",109.111512807929,0.36763138376507,0.284469817804482,1.2
9233880276798,0.196239839764591,0.807674784147954
"4106","MFSD6",16.2264730798162,-0.737104287285015,0.570632816808053,-
1.29173132980355,0.196450203692868,0.807674784147954
"4107","NTMT1",234.125306650572,-0.302354018429622,0.234080764514558,-
1.29166537479767,0.196473053418532,0.807674784147954
"4108","STIP1",217.940965798752,-0.29950486510467,0.231719498293484,-1
.29253199368373,0.196172973610591,0.807674784147954
"4109","CD27",7.46713821946924,-0.830320932870657,0.642482089794643,-1
.29236432588503,0.196231004889596,0.807674784147954
"4110","RIC8B",73.8263032096361,0.303631973674639,0.235008034813153,1.
29200677719826,0.196354797595574,0.807674784147954
"4111","RTTN",43.0304787591534,0.453263883529163,0.35087555217111,1.29
180810895631,0.196423606456927,0.807674784147954
"4112","FBXW9",54.6704214560132,-0.330508427840333,0.255833126639,-1.2
9189066397451,0.196395011333686,0.807674784147954
"4113","REPS2",60.3668424180359,0.41178392538529,0.318668078517744,1.2
9220324577431,0.196286767957229,0.807674784147954
"4114","CLSTN1",432.707920203544,0.18042194809387,0.139750750895597,1.
29102668098476,0.196694425862605,0.807772676787275
"4115","TSPAN13",2.23282206736635,-1.28913145539617,0.998624155751139,
-1.29090754311518,0.196735739473031,0.807772676787275
"4116","GNG11",1995.72095266122,-0.349811195485692,0.270898626806921,-
1.29129925688038,0.196599928225064,0.807772676787275
"4117","CHAMP1",103.744300466797,0.274198437405734,0.212380824466651,1
.29106965327178,0.196679525860076,0.807772676787275
"4118","LGMN",998.467307460768,-0.242070842062625,0.187518062121887,-1
.29092013496427,0.196731372679103,0.807772676787275
"4119","GTF2H1",191.007103872445,0.241327397837904,0.186965718787245,1
.29075746828497,0.196787790181529,0.807790229761905
"4120","VANGL2",39.5487331833996,0.483072554284291,0.374486076626282,1
.28996132148959,0.197064087787164,0.807878936974867
"4121","DERL1",456.739078483433,-0.215110928828022,0.166751005652181,-
1.29001278275175,0.197046219907235,0.807878936974867
"4122","EXOC5",258.999112998182,0.231675517196275,0.179609327255167,1.
28988578008055,0.197090318690546,0.807878936974867
"4123","AC055811.3",3.87573574549822,-1.04762662133006,0.8117681262671
8,-1.29054909577128,0.196860077095061,0.807878936974867
"4124","MFAP4",5941.19751141241,-0.291986789272765,0.226342812476289,-
1.2900201516377,0.197043661451268,0.807878936974867
"4125","AC006141.1",33.5349229027387,0.51169327850933,0.39670168815335
7,1.28986917320987,0.197096085582051,0.807878936974867
"4126","LRIG2",110.685722250029,0.289468005100206,0.22446581316274,1.2
8958615577838,0.197194385030515,0.80808595785166
"4127","PRRG4",3.15508595433599,-1.27652146492311,0.989994560202786,-1

.28942270618299,0.197251171748897,0.808122803956956
"4128","MTMR11",59.5017319886955,-0.375557339751988,0.291291925087038,-1.28928167040597,0.197300180932993,0.808127775972637
"4129","VPS45",122.361443318095,0.268593192289889,0.208470153272905,1.28840118392525,0.19760634683861,0.808794023807127
"4130","EFNA1",10.6905208575411,0.646809277617456,0.502007731228121,1.28844485329955,0.197591153778538,0.808794023807127
"4131","CFP",8.21690313437873,-0.847996517884833,0.658072833306777,-1.28860587303642,0.197535140611483,0.808794023807127
"4132","TSTD3",139.721702968762,0.226558339211308,0.175961449752476,1.28754530910041,0.197904287772485,0.809516526400726
"4133","TRIM35",54.3719275781796,-0.317409079884206,0.246588240710731,-1.28720282430886,0.19802360311308,0.809516526400726
"4134","ANAPC5",626.619701597435,-0.188095678609993,0.146132386272555,-1.28715942720029,0.198038725615158,0.809516526400726
"4135","AL442663.3",7.28875969151794,-0.762699806405129,0.592388728523225,-1.28749884945731,0.197920470365198,0.809516526400726
"4136","LINC00672",11.1023297486312,0.836249247131636,0.649613954839779,1.28730185197128,0.197989098308205,0.809516526400726
"4137","FAM227A",9.21878671828981,-0.62092542127443,0.482433559586826,-1.28706929469462,0.198070136605146,0.809516526400726
"4138","PRDM16-DT",5.40443248795593,-0.966754003081245,0.753737726296643,-1.2826132610228,0.199627601902233,0.809619451418317
"4139","PPCS",619.688415302089,-0.311648621475702,0.242524413855129,-1.28501958430405,0.198785440443302,0.809619451418317
"4140","PTRHD1",348.706054420782,-0.361580631200508,0.281039587652973,-1.28658255664318,0.198239826779191,0.809619451418317
"4141","ZNF638",653.104213975311,0.282528194219613,0.219791782854999,1.28543565437113,0.198640088599547,0.809619451418317
"4142","HTRA2",320.856521795486,-0.301370202372065,0.234751232975898,-1.28378538656283,0.199217058147309,0.809619451418317
"4143","GGCX",274.772373726112,0.332383766085761,0.258520801065557,1.28571381767254,0.198542957092323,0.809619451418317
"4144","ALPK1",129.984697832424,0.224341134140778,0.17472743127114,1.2839491344244,0.199159753662357,0.809619451418317
"4145","HAND2",112.705255733409,-0.567474560273734,0.442383639085258,-1.28276570410048,0.199574172897467,0.809619451418317
"4146","UBE2QL1",14.6009411238543,0.790357422579545,0.614552433528473,1.28606995832997,0.198418647466601,0.809619451418317
"4147","SRFBP1",263.058128563657,-0.262474322647967,0.204319627920227,-1.28462607983236,0.198922980641972,0.809619451418317
"4148","TCF7",154.68880738617,0.59653508511603,0.464872514242717,1.28322296294026,0.199413973216584,0.809619451418317
"4149","ADAMTS2",470.07404218265,0.322879087877064,0.251269462644697,1.28499135740034,0.198795304176058,0.809619451418317
"4150","CFB",688.018111465158,-0.496379503482406,0.38580396238289,-1.28661069320427,0.198230014711288,0.809619451418317
"4151","PIM1",502.661455015441,-0.948619210699181,0.73853527587313,-1.28446025760609,0.198980960713687,0.809619451418317

"4152","GNA12",277.762287489471,0.216219544547317,0.168378577185031,1.28412740006535,0.199097382294908,0.809619451418317
"4153","DUSP4",26.1547593319251,-0.811378467215851,0.632480192505609,-1.28285197991976,0.199543939150491,0.809619451418317
"4154","FSBP",19.5711222310944,0.569504318973781,0.443995831329241,1.28267942802253,0.199604410081308,0.809619451418317
"4155","SLC52A2",176.937253452908,-0.292980010988568,0.228018685998835,-1.28489474318813,0.19882906817724,0.809619451418317
"4156","DIPK1B",2.48773980558415,-1.2820192101385,0.997182212229517,-1.28564187609418,0.198568074955226,0.809619451418317
"4157","AP000821.2",26.0184213192942,-0.51221864617226,0.398822937836374,-1.28432594411711,0.199027932745965,0.809619451418317
"4158","ADAMTS8",2.07308732556579,-1.86440474303016,1.44997691548231,-1.2858168451668,0.198506989860706,0.809619451418317
"4159","CSRNP2",74.4059421837225,0.311806165400851,0.242772675874704,1.2843544450685,0.199017964733141,0.809619451418317
"4160","SPRYD3",340.491389528249,-0.232881055103886,0.181135122702469,-1.28567586246878,0.19855620857773,0.809619451418317
"4161","TDRD9",3.17310222448941,1.0645857006362,0.829134978732,1.28397152206058,0.199151919919305,0.809619451418317
"4162","HAPLN3",24.4542296140493,-0.648252132974087,0.504972367576173,-1.28373783319203,0.199233701974161,0.809619451418317
"4163","ADAMTSL5",11.1978200945468,0.603705179410656,0.470362584728799,1.28348894876224,0.199320828870243,0.809619451418317
"4164","YIPF2",301.53638604286,-0.262690490530511,0.204456144475302,-1.28482560993536,0.19885323091423,0.809619451418317
"4165","SELENOW",2138.95502077249,-0.32980933213937,0.25693820919588,-1.28361341495898,0.199277253547565,0.809619451418317
"4166","PLTP",4272.53943502998,-0.331861866596552,0.25813241755743,-1.28562646155328,0.198573457128882,0.809619451418317
"4167","RTL10",49.2806134946281,0.426571365596106,0.33240993028307,1.28326902037147,0.199397842301341,0.809619451418317
"4168","ZNF74",35.4754932069769,0.357282139281622,0.278501957507566,1.2828711958763,0.199537205733605,0.809619451418317
"4169","RAB9B",53.4966694721122,0.363304748888881,0.282996593608087,1.28377781603976,0.199219707785797,0.809619451418317
"4170","ADAM17",316.726062886108,-0.375247256148191,0.292921807047163,-1.28104923266356,0.200176373923611,0.809706797851338
"4171","FAM168B",219.93706365069,0.199606980372809,0.155667878569756,1.28226183980122,0.199750809556844,0.809706797851338
"4172","STAM2",237.491292475438,0.187610409671108,0.14634659498473,1.28195951324104,0.199856849146813,0.809706797851338
"4173","ZCWPW2",53.0955701652924,0.32726493523957,0.255412037597745,1.28132150041804,0.200080763996839,0.809706797851338
"4174","LMLN",58.3541480426216,0.365947065448706,0.285685112774366,1.28094552038395,0.200212802445322,0.809706797851338
"4175","PRELID1",998.179541568118,-0.323706604157679,0.252436206099823,-1.28233033271651,0.199726791706109,0.809706797851338
"4176","CSNK2B",1082.61329719981,-0.286922878601361,0.223866660963072,-1.28166863867546,0.199958910804226,0.809706797851338

"4177","SERINC1",1555.98577046067,0.217840181623844,0.170031967605066,
1.28117191544723,0.200133288336915,0.809706797851338
"4178","GNAQ",482.219610368753,0.261005911945162,0.203754673728082,1.2
8098122693119,0.200200260117707,0.809706797851338
"4179","BMS1",201.891586986071,-0.211030368465959,0.164759730536488,-1
.28083705756744,0.200250904751496,0.809706797851338
"4180","USP8",604.922171109899,0.226813225699214,0.177001468017746,1.2
8142002571682,0.200046173932362,0.809706797851338
"4181","LRRC28",105.909246183874,-0.285855881429332,0.223213023529789,
-1.28064159030212,0.200319584540581,0.809706797851338
"4182","AC010654.1",5.80860441605379,-0.820913408251391,0.640963487652
146,-1.28074909736029,0.200281808507024,0.809706797851338
"4183","RBM42",365.549251913815,-0.255535079607949,0.199367676737461,-
1.28172772933725,0.199938174076071,0.809706797851338
"4184","PUF60",514.339712935713,-0.28910728441858,0.225880372555065,-1
.27991326182226,0.200575642973664,0.809962354795062
"4185","B4GALT1",734.88984310153,-0.325286767014461,0.254114960205804,
-1.28007720108653,0.200517986025009,0.809962354795062
"4186","BMI1",393.517799600236,0.188079573278338,0.146943483218113,1.2
7994497720709,0.200564487823819,0.809962354795062
"4187","KLRG1",15.8193256886459,0.507577534715851,0.396612949070235,1.
27978054147184,0.200622329186286,0.809962354795062
"4188","LIMA1",3035.10458465519,-0.203727072180158,0.159134387021207,-
1.28022029677978,0.200467669586742,0.809962354795062
"4189","HMGXB4",316.434372037626,0.185132519745857,0.14468228763817,1.
27957971060596,0.200692989291186,0.81005420456807
"4190","GNRH1",13.8214114603981,0.588101469988831,0.459843487812873,1.
27891660005013,0.20092642634518,0.81060940506903
"4191","RPS6KL1",3.15638385850763,-1.09745550897592,0.858065063195768,
-1.27898868751114,0.200901039558402,0.81060940506903
"4192","AKR7A3",4.87129192774585,-0.873020726143449,0.682784090736877,
-1.27861902171924,0.201031248230559,0.810633718823073
"4193","MBOAT2",88.8690347819939,0.315837937871278,0.247039586196766,1
.27849120350985,0.201076284406433,0.810633718823073
"4194","TENM4",3.99875910117552,1.20720400341728,0.944237398375368,1.2
784962822849,0.201074494782287,0.810633718823073
"4195","PCDHA3",2.12268326797592,1.4410170730714,1.12760613461252,1.27
794362662507,0.201269303990545,0.810831877977634
"4196","AL355377.4",2.19150496866025,1.34886599415175,1.05533825173371
,1.27813617286763,0.201201416485229,0.810831877977634
"4197","AL022344.1",9.39642523865312,-0.621296438670556,0.486155321046
26,-1.27797930367903,0.201256723797076,0.810831877977634
"4198","ARHGAP10",584.864984998034,0.191365156644945,0.149814497792352
,1.2773473826958,0.20147963226031,0.810937352598881
"4199","TMEM168",178.353573975602,0.207854493082273,0.162716154271953,
1.27740539353505,0.201459161586713,0.810937352598881
"4200","SLC7A7",50.8621548918939,-0.358308464114936,0.280498895771011,
-1.27739705758929,0.201462103054427,0.810937352598881
"4201","BECN1",276.655401687488,-0.179391571106899,0.140458050830565,-
1.27718966656671,0.201535294276112,0.810937352598881

"4202","SGK2",2.85767001801202,-1.18564289693406,0.928226254772004,-1.27732100965543,0.201488939193373,0.810937352598881
"4203","AC008033.3",2.60583469571808,1.35799999723937,1.06367548367337,1.27670517755054,0.201706352853351,0.811239537118092
"4204","SPRY3",8.46674265162261,0.756612523728669,0.592584223155438,1.27680166660496,0.201672277015237,0.811239537118092
"4205","PFKM",124.800061451949,-0.26250959990904,0.205724157642192,-1.27602709821572,0.20194594011375,0.812009977513265
"4206","PCYOX1L",32.6274860510842,0.385862424521501,0.302440421585565,1.27582954189321,0.202015782133471,0.812097150585815
"4207","AC142472.1",33.3415671230826,-0.474699732905148,0.372110946363371,-1.27569408410146,0.202063680654987,0.812097150585815
"4208","AP1S2",190.711084852515,-0.328669591365286,0.257694121873716,-1.27542525601865,0.202158764063546,0.812286212639362
"4209","SIX2",36.6177960043173,0.342803571081004,0.268816305866682,1.27523354647622,0.20222659087285,0.812365692201983
"4210","ATXN7",263.619539403376,0.434625185686745,0.341000496604755,1.27455880567384,0.202465445986716,0.812938912548893
"4211","DCBLD2",226.318943317377,0.250411232314478,0.196463424878882,1.27459466039979,0.202452748410742,0.812938912548893
"4212","STK17B",324.498016260557,0.42412009574003,0.332888541827894,1.27406036089793,0.202642025098181,0.813112728627271
"4213","ATP9B",148.615300718215,0.290570567986457,0.228060189435679,1.27409596872412,0.202629406944909,0.813112728627271
"4214","KDM5C",212.837193897812,0.223409326785351,0.175356496179697,1.27402937246426,0.202653006768117,0.813112728627271
"4215","ROMO1",1080.20935846855,-0.306573300357292,0.240658767194991,-1.27389209182184,0.20270166144755,0.813114992112734
"4216","EMC1",179.364996190547,0.208473020219392,0.164310408239581,1.26877549908718,0.204521145996091,0.813725518844753
"4217","RUNX3",20.8759123174441,0.898972830589109,0.70660843570498,1.27223619923559,0.203289209496677,0.813725518844753
"4218","SLC2A1-AS1",2.40673429119851,1.40198800762394,1.10816320772507,1.26514578164173,0.205819074005429,0.813725518844753
"4219","ATP6V0B",620.68416615641,-0.312907091643528,0.247349438701464,-1.26504063759445,0.205856760791165,0.813725518844753
"4220","AL713852.1",3.7042505105345,1.14168526592328,0.897408960357433,1.27220176793037,0.203301439647262,0.813725518844753
"4221","RAB4A",692.49782991212,-0.268970441014111,0.212063565882915,-1.2683481950059,0.204673633090555,0.813725518844753
"4222","SLC30A6",91.8977063927196,0.254118178039274,0.200215717203167,1.26922192517689,0.204361923329592,0.813725518844753
"4223","TEX261",239.897080356683,0.191995484761753,0.15158904484898,1.26655250683206,0.205315343511374,0.813725518844753
"4224","TPRKB",392.761513013458,-0.348692525663557,0.274140947287676,-1.2719461616861 2,0.203392248857521,0.813725518844753
"4225","EAF1-AS1",9.28342641559571,0.710586068491642,0.558146943828027,1.27311647290966,0.202976714333399,0.813725518844753

"4226","EFHB",6.00049503625093,-1.57456629992814,1.24108112872275,-1.26870537589158,0.204546164385502,0.813725518844753
"4227","DZIP3",149.29000878433,0.241121786324951,0.190403327899502,1.266373802312,0.205379285606052,0.813725518844753
"4228","CEP63",286.118112085697,-0.210674925935066,0.165636440167335,-1.27191169842959,0.203404494874925,0.813725518844753
"4229","CHIC2",348.929781973769,-0.257404200771864,0.202694167359449,-1.26991419696549,0.204115195514622,0.813725518844753
"4230","BBS12",25.2487276951928,-0.509487961812467,0.401933300552393,-1.26759330742752,0.204943223170842,0.813725518844753
"4231","TMEM192",173.701913375979,0.237033565895949,0.187004419630579,1.26752921863665,0.204966122837383,0.813725518844753
"4232","HPF1",566.846782836935,-0.274316781767726,0.216761718928153,-1.26552226622013,0.205684171728623,0.813725518844753
"4233","ARHGEF28",23.8716897501641,0.797076447620535,0.626760993763317,1.27173907686019,0.203465841519602,0.813725518844753
"4234","TSLP",20.2320987217168,0.782776171086016,0.61592847757711,1.27088809753567,0.203768461364417,0.813725518844753
"4235","AC136424.2",2.31232756336413,1.71575385052478,1.35343746444309,1.2677008695269,0.204904794169163,0.813725518844753
"4236","MICAL1",67.0184810190267,-0.320477143124898,0.252535591860506,-1.26903752759698,0.204427679775567,0.813725518844753
"4237","GTF2IRD2B",169.172596444048,0.203133566843882,0.160136052174598,1.26850614889896,0.204617256270786,0.813725518844753
"4238","SEM1",1910.00683245312,-0.311141149723782,0.245892379587225,-1.26535499085449,0.205744102047618,0.813725518844753
"4239","GAL3ST4",65.2524413013413,0.361642463395975,0.285637470596473,1.26608901360451,0.205481215492408,0.813725518844753
"4240","PAXIP1-AS2",113.484819646834,0.372287293315738,0.293615376474218,1.26794208731922,0.204818632687769,0.813725518844753
"4241","AGPAT5",50.682435926246,-0.295723652540648,0.233601985369898,-1.26592953425624,0.205538311451377,0.813725518844753
"4242","TRMT9B",25.356455076378,0.543205249894471,0.427856366558974,1.26959721147353,0.204228143214864,0.813725518844753
"4243","KAT6A",363.596222711687,0.254824924819596,0.201450342945903,1.26495155626529,0.205888694138927,0.813725518844753
"4244","TMEM68",95.5213576420384,0.250815593877998,0.198224486828609,1.26531084978851,0.205759918734245,0.813725518844753
"4245","AC016877.3",4.82866588251118,0.883082959388821,0.697482477743049,1.26610056534517,0.205477080246603,0.813725518844753
"4246","ZNF367",2.05611633384177,-1.23501500224225,0.972988776948357,-1.26930035731317,0.20433395898,0.813725518844753
"4247","TRMO",70.4775675514587,-0.311027259543113,0.244300197609389,-1.27313552173385,0.202969955913483,0.813725518844753
"4248","NALT1",2.84031429081074,1.23867356690386,0.976442908676406,1.26855708193213,0.204599079687872,0.813725518844753
"4249","DIP2C",221.165656001443,0.347851964791505,0.27456082507711,1.26693953769192,0.205176909973465,0.813725518844753
"4250","ZNF32",391.180860008044,-0.299086302543248,0.23618343455244,-1

.2663305667902,0.205394757841264,0.813725518844753
"4251","ANAPC16",1064.87760387088,-0.266999210842384,0.211056993075159
,-1.26505739967262,0.205850752422823,0.813725518844753
"4252","ATP5MD",1744.99558914712,-0.338118576111753,0.267270554679101,
-1.26507978597835,0.205842728248339,0.813725518844753
"4253","CALHM2",765.932834564957,-0.183301606229597,0.144578623434965,
-1.26783338971304,0.204857455528829,0.813725518844753
"4254","TRUB1",89.1337879661782,0.236708405286832,0.186767061300584,1.
26739909938333,0.205012621674952,0.813725518844753
"4255","ESRRA",184.208675997725,-0.212572098278721,0.167608441699761,-
1.2682606179839,0.204702952495236,0.813725518844753
"4256","PCF11",679.260665100034,0.200345906095695,0.158072593334199,1.
26742974142344,0.205001670881484,0.813725518844753
"4257","BACE1-
AS",35.0730110563155,0.432900376636811,0.342261008176366,1.26482528332
219,0.205933965882227,0.813725518844753
"4258","VPS26B",228.960876921528,0.20354489602742,0.160012747962358,1.
27205424954831,0.203353844888228,0.813725518844753
"4259","SHMT2",227.661248666948,-0.308477159222748,0.243883218153194,-
1.26485603051613,0.205922941642723,0.813725518844753
"4260","TCP11L2",195.836070204603,-0.359355863076055,0.283516862813016
,-1.26749379035368,0.204978782571256,0.813725518844753
"4261","SETD1B",94.8934935038977,0.334325473033345,0.264140339319827,1
.26571153006863,0.205616378816591,0.813725518844753
"4262","NUP58",155.63725075387,0.268765528676458,0.212239265862434,1.2
663327286981,0.205393984162214,0.813725518844753
"4263","PCNX1",267.184124074476,0.266628364492696,0.20955719973284,1.2
7234170351873,0.203251737248503,0.813725518844753
"4264","MLH3",256.701127611133,0.243123596338379,0.191497351358456,1.2
6959247537208,0.204229831118715,0.813725518844753
"4265","ANP32A",576.059404299317,-0.226362269940122,0.178202806095688,
-1.27025087258488,0.203995281643944,0.813725518844753
"4266","CSPG4",16.054901435004,-0.850978266035469,0.670396785446958,-1
.26936507529361,0.204310886399,0.813725518844753
"4267","MNT",131.974129976002,0.375088161043252,0.29506706239224,1.271
19631043277,0.203658818842148,0.813725518844753
"4268","BCL6B",2.2799732797585,-1.25133501531503,0.986464237912402,-1.
26850519990786,0.204617594950457,0.813725518844753
"4269","FLCN",211.991063593666,0.300295584424264,0.236280594810806,1.2
7092783334457,0.203754323482047,0.813725518844753
"4270","NUFIP2",777.977884205231,0.344425051839292,0.271644745646585,1
.2679245866489,0.204824882930862,0.813725518844753
"4271","SMG8",42.6110177686554,0.325757484584354,0.256834750259267,1.2
6835439618475,0.204671419556069,0.813725518844753
"4272","AC007423.1",6.70799518127234,-1.30567200820232,1.0291010413582
1,-1.26875006022644,0.204530221755942,0.813725518844753
"4273","PKIG",677.499886544311,-0.303980806800366,0.240179811315362,-1
.26563846118287,0.205642549627101,0.813725518844753
"4274","FAM209B",2.39487534466945,-1.24285209273195,0.981201366030971,
-1.26666363884039,0.205275586770776,0.813725518844753

"4275","NPEPL1",261.573471633091,-0.191880202898521,0.151575312072199,-1.26590669862599,0.205546487887346,0.813725518844753
"4276","PLAC4",2.25124969735329,1.88277790842293,1.48010100152463,1.2720604245815,0.203351651038271,0.813725518844753
"4277","RFPL1S",2.04212185552029,1.47827272288186,1.16153114413354,1.27269314331179,0.203126951685904,0.813725518844753
"4278","PPARA",166.853544650242,0.309341192357333,0.243686332017555,1.26942364717873,0.204290006593857,0.813725518844753
"4279","KDM6A",140.578011660664,0.29672996738157,0.234508784357364,1.26532559620192,0.205754634681125,0.813725518844753
"4280","TTN",17.9468454298637,0.90560380104404,0.716485684749263,1.26395240033436,0.206247112217772,0.813821744078901
"4281","RAB11FIP1",36.8765330106858,-0.530386678333276,0.419595250488817,-1.26404356988167,0.206214389023725,0.813821744078901
"4282","SLC20A2",89.6591005859863,0.246266141927963,0.194796753238416,1.26422097819337,0.206150723233912,0.813821744078901
"4283","DYDC2",3.50027281688778,-1.08362188941684,0.857214008778885,-1.26412060269579,0.206186742821256,0.813821744078901
"4284","UBE2M",680.508734318713,-0.34711079757239,0.274513944083309,-1.26445597775189,0.206066411892625,0.813821744078901
"4285","PPM1F",113.012763388442,0.271148998610523,0.214514639507957,1.26401162751629,0.206225853565624,0.813821744078901
"4286","AL034417.4",10.7324342871199,0.916292909640928,0.725156579322686,1.26357939204905,0.206381034167115,0.814115627727621
"4287","S100A13",7776.34311567623,-0.303126626454995,0.239940138255634,-1.26334271814098,0.206466040436245,0.814115627727621
"4288","AC108488.3",6.51150956242818,0.742959022462541,0.58807674865749,1.26337085109831,0.206455934578681,0.814115627727621
"4289","PRDX6",2833.88640115307,-0.32664764016724,0.258645682679848,-1.2629154942113,0.206619550999967,0.814334431216049
"4290","ACAD11",93.8429535377667,0.401531191077508,0.318028561227189,1.26256330415138,0.206746162612054,0.814334431216049
"4291","C4orf46",70.7460301397641,0.371581669092459,0.294198160954317,1.26303192340539,0.206577707324349,0.814334431216049
"4292","TPK1",57.0960757769263,-0.309339997032613,0.245018227456229,-1.26251830422647,0.206762344050775,0.814334431216049
"4293","EIF6",579.687445110378,-0.33660828940891,0.266573746649137,-1.26272108052693,0.206689435362851,0.814334431216049
"4294","C1orf174",193.386429986107,-0.280666766830442,0.222397271277697,-1.2620063421551,0.206946504265417,0.81466732957679
"4295","RBMS3-AS3",20.1383969646172,0.555105848602057,0.439918881278642,1.26183683452872,0.207007504854409,0.81466732957679
"4296","CEP97",95.9662689384876,0.267739161932933,0.212230788803785,1.2615472215036,0.207111757881633,0.81466732957679
"4297","SEPSECS-AS1",34.3007515401461,0.362752657788218,0.287542332967449,1.26156261599673,0.207106215312713,0.81466732957679
"4298","FSIP1",3.86021621041613,-0.950601676564634,0.753399788593229,-1.26174932745817,0.207039001068603,0.81466732957679

"4299","ZFYVE19",137.362082095122,-0.213380528094531,0.169150940600066
,-1.26147999731813,0.207135962257548,0.81466732957679
"4300","RCC1",51.1168181461201,-0.353648077667911,0.281232580593637,-1
.25749327094825,0.208575074147621,0.81483071942883
"4301","AKIRIN1",434.49609668375,-0.341513268540467,0.270982610339375,
-1.26027743297904,0.207569297916131,0.81483071942883
"4302","SLC27A3",573.551371787538,-0.336842647284537,0.267575525950514
,-1.25886942046723,0.208077500680327,0.81483071942883
"4303","MAP4",830.713780186647,-0.217329070198769,0.172742629642951,-1
.25810907619026,0.208352311352037,0.81483071942883
"4304","ABHD6",49.0670255969964,0.319674376935674,0.253788222692949,1.
25961076342947,0.207809810782889,0.81483071942883
"4305","SEC61A1",391.602129724314,0.231631604997532,0.184120095624907,
1.2580462996794,0.208375012372677,0.81483071942883
"4306","N4BP2",131.613829019412,0.293274128037316,0.232947758610014,1.
25896952083706,0.208041341064481,0.81483071942883
"4307","LINC01088",50.7316490977712,-0.699334818399154,0.5553191171296
39,-1.25933863399825,0.207908044370935,0.81483071942883
"4308","BHMT2",422.393108749625,0.238556413222684,0.189570320818208,1.
2584059160371,0.208244993453578,0.81483071942883
"4309","GJA1",696.811447915525,0.365475239560399,0.290257953760625,1.2
5913944760255,0.20797996824216,0.81483071942883
"4310","ZDHHC4",275.521281591337,-0.273195418280978,0.217030259825801,
-1.25878952778409,0.208106363870834,0.81483071942883
"4311","R3HCC1",319.416770137578,-0.300630750261702,0.238978163265531,
-1.25798418631107,0.208397475354646,0.81483071942883
"4312","GSN-
AS1",7.87551492563577,0.857927064353033,0.680416960745605,1.2608843016
0958,0.20735053491912,0.81483071942883
"4313","LINC01001",9.78225868588815,0.767682167652893,0.61002043911313
,1.25845319014061,0.208227905925787,0.81483071942883
"4314","IRF7",182.26317328025,-0.45938130260199,0.364875455354556,-1.2
590085078636,0.208027258873948,0.81483071942883
"4315","BRMS1",195.763875262876,-0.304924039123896,0.242442073868573,-
1.2577191502214,0.208493344025554,0.81483071942883
"4316","AC010186.3",7.61242790495258,0.677612874203497,0.5387934368121
94,1.25764871638496,0.20851882667963,0.81483071942883
"4317","TMEM260",110.6478603555,0.319292966481426,0.253670131094429,1.
25869358408053,0.208141029722251,0.81483071942883
"4318","TRIP11",705.330900734788,0.199440704576072,0.158186209780934,1
.26079703693685,0.207381981641682,0.81483071942883
"4319","MPI",107.928554664114,0.278758216552666,0.22114360177732,1.260
53032650414,0.207478114938614,0.81483071942883
"4320","SNRNP25",219.851207709658,-0.423913978034401,0.336392917267778
,-1.26017509963491,0.207606203360751,0.81483071942883
"4321","MPG",790.7202299451,-0.330861941862944,0.263023828222521,-1.25
791622796635,0.208422054153236,0.81483071942883
"4322","TWSG1",305.734544207893,0.197905391602073,0.157156479922582,1.
25928877829005,0.207926044989814,0.81483071942883
"4323","FXYD7",7.6487921138153,-0.7195616913085,0.570694892902299,-1.2

6085181461697,0.207362241536841,0.81483071942883
"4324","ZNF337-
AS1",39.0715539060279,0.351836774921564,0.279790692069842,1.2574999272
4467,0.208572665361261,0.81483071942883
"4325","CFAP298",374.352538674994,-0.201398326714358,0.159985184210601
,-1.25885611038358,0.208082309072846,0.81483071942883
"4326","ADA2",25.3213492045637,-0.523363757602204,0.41552033888953,-1.
25953824306382,0.207835985975381,0.81483071942883
"4327","KIAA1671",75.9948962807756,0.393815522154111,0.312439295643384
,1.26045451915117,0.207505444893968,0.81483071942883
"4328","IGBP1",1063.76511994858,-0.315900899698415,0.251137638341034,-
1.25787955077221,0.208435320231175,0.81483071942883
"4329","MSN",659.22252090466,-0.208486973149941,0.165841077119471,-1.2
5714917420458,0.208699623640653,0.815128952764184
"4330","AC011389.1",7.95803214887843,1.25669455679751,1.00000430577962
,1.256689145771,0.208866220031315,0.8152146925876
"4331","ATP6V0E2",45.4366665042863,-0.36416137000206,0.289754033391061
,-1.25679482608125,0.208827940051701,0.8152146925876
"4332","POU2F3",2.81986435661053,1.67230767819415,1.33050656569706,1.2
5689547222791,0.208791488295078,0.8152146925876
"4333","EPAS1",224.704612341891,0.341557331534205,0.271908523297638,1.
25614794046131,0.209062337455704,0.815603599838727
"4334","LMBR1L",165.745882497936,0.251313222306507,0.200055078444017,1
.25622015827423,0.209036160062165,0.815603599838727
"4335","PELO",114.478870500228,-0.292808235526968,0.233140414079529,-1
.25593083757278,0.209141046853169,0.815722449871597
"4336","NBPF14",154.901033663924,0.59139273780324,0.471232766693605,1.
25499069589904,0.209482137538329,0.815816415286969
"4337","AL451085.1",4.20092719944341,-1.11204921275176,0.8864410199728
82,-1.2545101001596,0.209656656924394,0.815816415286969
"4338","CISH",59.4069245483232,-0.663233266595669,0.528547921353678,-1
.25482144532334,0.209543585725722,0.815816415286969
"4339","FASTKD3",48.1310293896135,-0.396860365277077,0.316265251594561
,-1.25483391955382,0.209539056380684,0.815816415286969
"4340","SFXN4",112.367788620831,-0.316243101894671,0.25200297075672,-1
.25491815015136,0.209508474430778,0.815816415286969
"4341","SCYL1",261.653965287376,-0.178238854140775,0.142090686099391,-
1.2544020937171,0.209695891935011,0.815816415286969
"4342","FRMD6-
AS1",48.067578928974,0.478129070958724,0.380845054495651,1.25544250953
161,0.209318165802598,0.815816415286969
"4343","AL133467.1",167.705227066122,-0.35559798619326,0.2832309991263
75,-1.25550517877669,0.209295429288586,0.815816415286969
"4344","COTL1",735.843799639797,-0.306092451855859,0.243817364110604,-
1.2554169510134,0.209327438992201,0.815816415286969
"4345","PLD6",24.2099720757679,0.507774506750282,0.404677972276159,1.2
547619132671,0.209565202562599,0.815816415286969
"4346","MOSPD2",171.860660655496,0.244873430314602,0.195178385472172,1
.25461346409956,0.209619113352591,0.815816415286969
"4347","CFI",57.2336483150767,0.700500846620037,0.558564459099198,1.25

410923521654,0.209802304064842,0.815914267353593
"4348","DUSP15",8.67349787037162,-0.645843700546814,0.514999247921767,
-1.2540672693272,0.209817555858376,0.815914267353593
"4349","GARS-
DT",269.007169360518,0.289623218812016,0.23097669002185,1.253906698483
81,0.209875920030299,0.815953565387973
"4350","CHL1",2083.23630058583,0.271773494735277,0.216876642102434,1.2
5312478144564,0.21016029863126б,0.816495939627169
"4351","FAM71F2",16.752103095559,0.751656030898467,0.599708216627727,1
.2533695721649,0.210071239722761,0.816495939627169
"4352","TPM2",1083.25378571643,-0.318254308139284,0.253953525048662,-1
.25319901772697,0.210133287358962,0.816495939627169
"4353","CASP9",38.9292944503656,-0.355354920657591,0.283653056275255,-
1.25278015799963,0.210285724646488,0.816690365959103
"4354","FDPS",543.841481162462,-0.313718788496336,0.250509297987085,-1
.25232392975892,0.210451852642761,0.816690365959103
"4355","SLC19A2",372.032366337471,0.604357387927634,0.482543738099051,
1.252440639492,0.210409345679983,0.816690365959103
"4356","DEGS1",563.187772895373,-0.253764572034854,0.202588539297202,-
1.25261070006816,0.210347418877947,0.816690365959103
"4357","CELF1",488.924682485857,0.295756060581195,0.236163513652385,1.
25233595997613,0.210447470818713,0.816690365959103
"4358","PTTG1",36.2368825619368,-0.53020075910096,0.423655756000851,-1
.25148956810089,0.21075591729812,0.817069129068991
"4359","MAPK13",87.1035039320203,0.32723478006667,0.261410069773926,1.
25180632999208,0.210640443030348,0.817069129068991
"4360","PCMT1",823.361983995075,-0.277856345476936,0.22202380132981,-1
.25147098560027,0.210762692895361,0.817069129068991
"4361","C9orf170",6.27230112955169,-0.730529657979552,0.58375535538528
9,-1.2514311881514,0.210777204469501,0.817069129068991
"4362","RASGRP4",2.4804665623859,-1.2223962490521,0.976828309780878,-1
.25139314331124,0.210791077655485,0.817069129068991
"4363","NOP14-
AS1",70.4020977551711,-0.278565378248235,0.222647660730917,-1.25114891
094633,0.210880153584805,0.817227054048104
"4364","PRR26",3.57780329983109,1.12656123810939,0.90091213701524,1.25
04673783636,0.211128864741841,0.817239015018792
"4365","LOH12CR2",38.3929885783038,-0.386421540430268,0.30908499620690
5,-1.25021125312596,0.211222387141954,0.817239015018792
"4366","TP53BP1",258.168499161853,0.240927306153983,0.192711703375114,
1.25019550932523,0.211228136861527,0.817239015018792
"4367","ARID3B",19.9100329797804,0.473080616931002,0.378211667006247,1
.25083559868921,0.21099446405378,0.817239015018792
"4368","STAP2",30.3052908331113,-0.481927592233013,0.385365026640054,-
1.25057428390655,0.211089837764196,0.817239015018792
"4369","IL10RB",480.924257228621,0.182911056163591,0.146319349342637,1
.25008112040785,0.2112699156995,0.817239015018792
"4370","FOXO4",29.6669123560609,0.484633581205683,0.38743732810916,1.2
5086961437319,0.210982051427071,0.817239015018792
"4371","RBM41",118.253592223524,0.345015523574794,0.275993820390987,1.

25008423408186,0.211268778397625,0.817239015018792
"4372","SMYD5",56.7579455583243,0.343874554288595,0.275219440759311,1.24945590086176,0.211498373349324,0.817856048232793
"4373","ALMS1",83.1232282681789,0.388021273486877,0.310621933556106,1.24917538515287,0.211600932897514,0.817856048232793
"4374","CARMN",18.9171191629465,0.873669407469968,0.699880304312324,1.24831260729419,0.211916599036433,0.817856048232793
"4375","STXBP5-AS1",4.3871880973583,-0.848364902264664,0.679405796874633,-1.24868658196216,0.211779730470286,0.817856048232793
"4376","AC104232.3",10.4247331525487,0.605163138638044,0.484484587763619,1.24908645996661,0.211633452401403,0.817856048232793
"4377","SURF2",216.024356543949,-0.335856453544614,0.268990277695734,-1.24858212877313,0.211817952184541,0.817856048232793
"4378","AC079949.2",4.97296863269577,-0.817814031982802,0.655102059893712,-1.24837652337025,0.211893202278056,0.817856048232793
"4379","CCNB1IP1",332.607850706484,-0.313943908613938,0.251585240868017,-1.24786298087587,0.212081239508218,0.817856048232793
"4380","TOM1L1",2.1813802791527,-1.4001973680373,1.12076427466221,-1.24932369784833,0.211546703709294,0.817856048232793
"4381","AC022966.1",12.2995524938056,0.72614436450749,0.581537462367769,1.24866308965023,0.211788326388296,0.817856048232793
"4382","AC008532.1",5.6605572785157,0.830282092913628,0.665348833609582,1.2478899052235,0.212071377973254,0.817856048232793
"4383","NORAD",1546.04402250307,0.166722652280295,0.133577321317798,1.24813591585386,0.211981287422487,0.817856048232793
"4384","PABPC1L",22.6748568558743,0.527778971826515,0.422969741495798,1.2477936836807,0.212106622397729,0.817856048232793
"4385","SBF1",102.156051690879,0.248554537373594,0.199192326904817,1.24781180698975,0.212099983793701,0.817856048232793
"4386","SAMMSON",4.14761458757648,-0.955859008502474,0.766516024154687,-1.24701764657379,0.212391027191021,0.818185480690385
"4387","AC073343.2",4.1575975671263,0.925002105231497,0.74184119850917,1.24690042436362,0.212434011132647,0.818185480690385
"4388","SRPK2",501.751651098004,0.211623971305709,0.16966206844649,1.24732636613147,0.212277853590535,0.818185480690385
"4389","LINC01229",3.56738180699877,1.17740267668693,0.944158442995408,1.24703929242166,0.212383090612246,0.818185480690385
"4390","TMEM160",518.62906704632,-0.288975284096444,0.231682626383509,-1.24728940019049,0.212291402650156,0.818185480690385
"4391","EBNA1BP2",359.450926849949,-0.233331960659156,0.187151802548849,-1.24675240890748,0.212488295554362,0.818208176095003
"4392","ANKMY2",166.511858721765,0.255745204076052,0.205187335445999,1.24639858264234,0.212618101347318,0.818521597809758
"4393","S100A14",4.28577890411057,0.891141293113715,0.715379376178159,1.24569050043705,0.212878042522281,0.818592257055389
"4394","KIAA0040",106.46676162956,0.507972302102875,0.407690437732174,1.24597551252006,0.212773385318322,0.818592257055389
"4395","PDCD6IP",470.781244257279,0.181566906170035,0.145739734315874,1.2458298145138,0.212826881375504,0.818592257055389

"4396","RALGPS1",27.7768957305467,0.440504303052315,0.353622966941309,
1.24568917811672,0.212878528168473,0.818592257055389
"4397","NDUFS8",576.696276684184,-0.333020498741427,0.267335088819511,
-1.24570440869535,0.212872934509915,0.818592257055389
"4398","MAPK1IP1L",330.332392413883,0.183665807174315,0.14746571907806
4,1.24548137914743,0.212954856060107,0.818699569409798
"4399","UTP25",108.565513160365,0.271611877911252,0.218282762917354,1.
24431207613992,0.213384728226757,0.818768955945889
"4400","RBMS1",1541.86884211306,0.215174078126701,0.172901719284448,1.
24448778772818,0.213320091226866,0.818768955945889
"4401","DYNC1I1",9.25622306528784,-0.642362403788677,0.5159048328536,-
1.24511801960763,0.213088371321868,0.818768955945889
"4402","RCE1",90.7999678331536,-0.303814966697479,0.244070850775894,-1
.24478185629976,0.21321194722322,0.818768955945889
"4403","AP002490.1",6.33282698102277,1.07323662046663,0.86234425076733
4,1.24455705422938,0.213294614839936,0.818768955945889
"4404","STAT2",404.659974773523,0.404468095952459,0.324861001806605,1.
24504970957778,0.213113478363208,0.818768955945889
"4405","MARK3",372.383931949083,0.248659561796285,0.199835430657747,1.
24432169499591,0.213381189483227,0.818768955945889
"4406","UBALD1",65.5018688581946,-0.317018144836477,0.254739430891993,
-1.24448007018941,0.213322929892299,0.818768955945889
"4407","AC002550.1",2.94514360909883,1.27905701162639,1.02797690052559
,1.24424684151213,0.213408728936216,0.818768955945889
"4408","SLMAP",190.108178178922,0.250077161164688,0.201048391183775,1.
24386551761112,0.213549062180377,0.818935709536362
"4409","OXA1L",449.224564003297,-0.20605909123494,0.165650003241196,-1
.24394257291324,0.213520699239791,0.818935709536362
"4410","PPARG",518.928955909327,-0.357223730263215,0.287253137872978,-
1.24358512811504,0.213652292437963,0.819145796041062
"4411","LIMK2",48.0107589115143,0.302503262245633,0.24328146601266,1.2
4342913253364,0.213709740503916,0.819180297538021
"4412","ATXN1L",154.101885316893,0.308159763156175,0.247867063764911,1
.24324611134478,0.213777155423974,0.819252979127053
"4413","AL355336.1",2.90140420253851,1.10029877248306,0.88562411293205
3,1.24239929380455,0.214089276064432,0.82012025232226
"4414","SLC13A3",12.643625226094,-0.507460000882796,0.408461599153354,
-1.24236893243978,0.214100472779185,0.82012025232226
"4415","SMO",97.8311983257365,0.265056437564031,0.213435109604238,1.24
185958934082,0.214288372155823,0.820654087522231
"4416","AL354743.1",7.13352553698509,-0.785486659867929,0.635388031138
613,-1.23623143870107,0.216372547723531,0.820848773793561
"4417","PPOX",74.3626241738116,-0.332896692773619,0.26903478137784,-1.
23737418287968,0.215948198862326,0.820848773793561
"4418","HIST3H2A",102.231923297703,0.500231883578579,0.403532501520791
,1.23963220234642,0.215111464168408,0.820848773793561
"4419","FMN2",2.76651373220928,1.12087621410688,0.905788663706389,1.23
745886763516,0.215916775726795,0.820848773793561
"4420","AC079779.2",2.84887898114203,1.4847865403361,1.19649585824103,
1.24094582535278,0.214625762531454,0.820848773793561

"4421","ODC1-
DT",6.57713483039325,0.761261147319863,0.61569785824058,1.236420002329
14,0.216302484816953,0.820848773793561
"4422","PPP4R3B",400.602910179576,0.216607053459358,0.175169239651783,
1.236558735369,0.216250947446826,0.820848773793561
"4423","CCDC93",191.925744625848,0.226165299283633,0.182653141862441,1
.23822287959307,0.215633430202487,0.820848773793561
"4424","CHMP2B",603.719651280006,-0.221344366150707,0.178406964512693,
-1.24067110695648,0.214727272030806,0.820848773793561
"4425","LRRC34",79.6149779718651,-0.27475714179075,0.222280039364298,-
1.23608553685941,0.216426770379211,0.820848773793561
"4426","SLIT2",354.978319256603,0.355440233496994,0.286302343589883,1.
2414855884175,0.21442641878321,0.820848773793561
"4427","SNHG8",1489.56534965357,-0.344935944106101,0.278129048770005,-
1.24020107080344,0.214901032444943,0.820848773793561
"4428","SLC10A7",65.5486251405524,0.284900065420194,0.229920247250603,
1.23912560475662,0.215298986061128,0.820848773793561
"4429","CCDC152",146.750734119212,0.466791667148452,0.376093548142419,
1.24115840182318,0.214547238457028,0.820848773793561
"4430","AC114980.1",26.2079770831763,0.498074707831198,0.4029344658392
38,1.2361184015217,0.216414555771435,0.820848773793561
"4431","AC008906.1",10.2296683102187,0.906832938044418,0.7318020042200
15,1.23917799188178,0.215279589012617,0.820848773793561
"4432","LARP1",527.07952059395,0.234914937848995,0.189689194594066,1.2
3842023975964,0.215560279712792,0.820848773793561
"4433","UBTD2",316.771634913034,0.264553094723263,0.213734861051694,1.
23776296211818,0.215803965532083,0.820848773793561
"4434","AHI1",619.149408713789,-0.272281155437469,0.219714768457439,-1
.23924830974761,0.215253554841875,0.820848773793561
"4435","PINX1",90.6912526598439,-0.249541726123909,0.201430799622115,-
1.23884592918288,0.215402561067543,0.820848773793561
"4436","AC090578.1",6.19913732555902,0.745468879648362,0.6014920272651
67,1.23936618584592,0.215209918013603,0.820848773793561
"4437","ABHD17B",66.0855634701479,0.346609948103044,0.280067102549091,
1.23759607946917,0.215865868876946,0.820848773793561
"4438","DNM1",542.65477281017,-0.268524479907403,0.217228278066889,-1.
23613961449678,0.216406671938399,0.820848773793561
"4439","HSPA14",73.3240475300345,0.30912101622047,0.249802417846852,1.
23746206656008,0.215915588797871,0.820848773793561
"4440","DRAP1",1016.2613600057,-0.308446369742726,0.248625490626657,-1
.24060637935914,0.214751194162808,0.820848773793561
"4441","AP002748.4",21.1346469042362,-0.596171688474394,0.480495688018
241,-1.24074305626601,0.214700683140971,0.820848773793561
"4442","ZPR1",175.151125942334,-0.233665192168069,0.188313434732068,-1
.24083123703058,0.214668099148924,0.820848773793561
"4443","RASSF9",93.0524351834164,-0.310560714427805,0.25061305162002,-
1.23920407345216,0.215269932424499,0.820848773793561
"4444","TMPO",213.709512028027,0.240138275564419,0.194235976200478,1.2
363223346254,0.216338772296045,0.820848773793561
"4445","CCDC62",2.50020466595512,1.57824958773663,1.27241304638857,1.2

403594824936,0.214842460352569,0.820848773793561
"4446","SBNO1",299.204677136226,0.220002979355504,0.17772092782095,1.23791261981905,0.21574846247279,0.820848773793561
"4447","FNDC3A",524.461028765846,0.165033205916321,0.133274990432474,1.23829088549017,0.21560822216183,0.820848773793561
"4448","ARHGAP5",392.639581824543,0.282979307951272,0.228612195587555,1.23781370116318,0.215785146957292,0.820848773793561
"4449","LINC02256",72.5547674118063,-0.46290544118766,0.374076780824629,-1.23746103718925,0.215915970735056,0.820848773793561
"4450","TRPM7",232.465999796138,0.261110026650639,0.211144588276114,1.23664086672771,0.216220440970045,0.820848773793561
"4451","ABCC1",97.2437872379615,0.257464967885772,0.207563453388433,1.24041570749911,0.214821674147467,0.820848773793561
"4452","AC008915.1",3.0315818407204,1.13024760177009,0.914046250631034,1.23653217874893,0.21626081217292,0.820848773793561
"4453","MTSS2",32.4214451109384,0.420764250855942,0.339544484831314,1.23920213595864,0.215270649762324,0.820848773793561
"4454","DEF8",196.430088548647,-0.277103152797756,0.223544296337359,-1.23958945648771,0.215127282404563,0.820848773793561
"4455","USP32",90.1146544467668,0.299922289824723,0.242369915448178,1.23745675807214,0.21591755846151,0.820848773793561
"4456","MALT1",195.222844089538,0.302851113584375,0.244422076498303,1.23904975329214,0.215327073333197,0.820848773793561
"4457","ZNF320",2.61961320690231,1.17275112928556,0.946698383208532,1.23878011211014,0.215426940968611,0.820848773793561
"4458","DSTN",5616.03418299141,-0.195505194701828,0.158022179835445,-1.23720097334067,0.216012480270163,0.820848773793561
"4459","ITPRID2",149.354652825279,0.249868564256302,0.202195024794021,1.23577998277083,0.216540357550183,0.820868953940449
"4460","MASP1",311.397528178904,-0.518387352870152,0.419693359245175,-1.23515738681803,0.216771935139822,0.820868953940449
"4461","SAP30L-AS1",8.48049142981958,0.688999850013055,0.557588900381918,1.23567712617867,0.216578603276598,0.820868953940449
"4462","HOXA9",66.4925160249213,-0.348769004159602,0.282283514423224,-1.23552735579414,0.216634301906587,0.820868953940449
"4463","TNC",208.468247318991,1.17768217089942,0.953459337939167,1.23516769309208,0.216768100221112,0.820868953940449
"4464","TMTC4",44.3254634632446,0.377902389399117,0.305794525792213,1.23580495242711,0.216531073680283,0.820868953940449
"4465","SNAI3-AS1",12.2106950719209,0.593334299264719,0.480352204143354,1.23520677983117,0.216753556663079,0.820868953940449
"4466","MARCH1",8.47843165629592,-0.819661420024453,0.663758287676127,-1.23487937588569,0.216875400190987,0.821074479588357
"4467","PCGF6",89.390806099844,-0.244742921252392,0.198212431166007,-1.23475061484623,0.216923332169458,0.821074479588357
"4468","AL034376.1",3.89833744738855,1.01028215761789,0.818338334454426,1.23455313662097,0.216996859291642,0.821095387924819
"4469","ZNF277",235.97423988566,-0.263644622316042,0.213608713596255,-

1.23424095336467,0.21711313114265,0.821095387924819
"4470","UNC119B",68.3921834442498,0.386504995630982,0.313191358946595,
1.23408575808405,0.217170949900453,0.821095387924819
"4471","SEC23B",71.3451532234438,-0.277161657085174,0.22458092120027 7,
-1.23412824029698,0.217155121844259,0.821095387924819
"4472","URB1-
AS1",44.3086883644956,-0.436997841878922,0.354107096837057,-1.23408382
88254,0.217171668724852,0.821095387924819
"4473","UQCRHL",5.91105832889919,0.744414694036617,0.603381145090442,1
.233738740585,0.217300273028199,0.821313435781309
"4474","CEP78",85.8616005624638,0.283434742169124,0.229768770216179,1.
23356512681185,0.217364994514575,0.821313435781309
"4475","FERMT2",1015.07301554644,0.263607272386982,0.213712765129048,1
.23346526459384,0.217402228435351,0.821313435781309
"4476","SLC2A4RG",951.815299227821,-0.317290975002557,0.25724741248037
8,-1.23340783855993,0.217423641977593,0.821313435781309
"4477","AL356441.1",2.28532124222057,-1.65874539929619,1.3466239945312
1,-1.23178066485711,0.218031027838412,0.821416527538398
"4478","LINC01873",6.92851712662736,0.848541007520715,0.68951800379818
6,1.23062922628061,0.218461569133374,0.821416527538398
"4479","BMPR2",551.677983682574,0.239529780390978,0.194546846907658,1.
23121903129415,0.218240955358827,0.821416527538398
"4480","DHFR2",81.7356196736044,0.249232956844462,0.202370708390931,1.
23156636069586,0.218111113365121,0.821416527538398
"4481","HSPA4L",13.220174529528,0.726620840736573,0.589352497897121,1.
23291382208312,0.217607917990989,0.821416527538398
"4482","ANKRA2",114.515495601074,0.270466649901457,0.219692406290845,1
.23111515080495,0.218279799744,0.821416527538398
"4483","TAB2",260.073609784902,0.286291831238293,0.232643414364091,1.2
3060363441125,0.218471145276802,0.821416527538398
"4484","RHBDD2",143.593028471432,-0.357009264188577,0.28969887431818,-
1.23234605253063,0.217819843744088,0.821416527538398
"4485","AP003352.1",3.98841370851788,0.999151068965473,0.8115685274830
73,1.23113580077354,0.218272077635282,0.821416527538398
"4486","ANKS6",64.0789011119317,0.381914935003023,0.310199742460872,1.
23119036777149,0.218251673111212,0.821416527538398
"4487","GPAM",65.2283681427321,0.289349106589353,0.234892288041128,1.2
3183740514584,0.218009827515439,0.821416527538398
"4488","AC104389.5",6.73027642642907,0.769554546245884,0.6250912937881
1,1.2311074460537,0.218282681005782,0.821416527538398
"4489","FOXO1",608.363631792398,0.299826969876727,0.24320424026336,1.2
3281966446001,0.217643053021877,0.821416527538398
"4490","ZNF205",126.73514540918,-0.30715690025025,0.2494116630403,-1.2
3152580960345,0.218126269698467,0.821416527538398
"4491","CKLF",537.027835621219,-0.306967900037723,0.249116481319929,-1
.23222638025101,0.217864531564559,0.821416527538398
"4492","TRPV2",162.105428596621,-0.260030652386929,0.210960467234611,-
1.23260369961992,0.217723655969504,0.821416527538398
"4493","EVI2B",26.7004498203354,-0.565018941947475,0.45828584407408,-1
.23289634461444,0.217614439421287,0.821416527538398

"4494","KCTD15",20.7162655941453,0.658610221473377,0.534262701090192,1
.23274602574622,0.217670534239226,0.821416527538398
"4495","CD3EAP",44.9692882211139,0.333064790852043,0.270536079629317,1
.23112891747526,0.218274651640514,0.821416527538398
"4496","PANK2",191.393770223961,0.23352741282425,0.189724367442066,1.2
3087727724568,0.218368767327943,0.821416527538398
"4497","CFAP410",226.624482473994,-0.263112224725575,0.21378789655445,
-1.2307161863046,0.21842903208106,0.821416527538398
"4498","HIST2H2AB",3.99455649605248,0.993532356217466,0.80745964433218
4,1.23044211954292,0.2185315889902,0.821461117529192
"4499","PIGC",314.30580262864,-0.256986449965925,0.208976545018194,-1.
22973824619194,0.218795139798366,0.821553904783398
"4500","XPR1",93.5344240635135,0.345876916432251,0.281394892907657,1.2
2915136397218,0.219015060171878,0.821553904783398
"4501","BX323046.2",5.11552417358104,1.01267295499775,0.82431498461079
6,1.22850242189384,0.219258420786743,0.821553904783398
"4502","AC112715.1",12.6674277415409,-0.648067165942267,0.527067570958
15,-1.22957131428927,0.218857677473011,0.821553904783398
"4503","BHLHE40",216.481168834765,-0.483114700494991,0.393277763995717
,-1.22843126340663,0.219285117831055,0.821553904783398
"4504","ADPRH",16.9909250277764,-0.534600554311787,0.435151478065748,-
1.22853898299528,0.21924470480145,0.821553904783398
"4505","TMEM128",234.814905347658,0.267508988620164,0.217739857748418,
1.22857152285482,0.2192324979129,0.821553904783398
"4506","AC124852.1",120.791270794497,0.351545671342295,0.2859651186685
74,1.22933060150503,0.21894787827117,0.821553904783398
"4507","LINC01003",236.112498511301,-0.368440496579761,0.2994907761733
3,-1.23022318512583,0.218613540001889,0.821553904783398
"4508","AC107959.1",46.0346213480003,0.349094463975083,0.2839299151333
67,1.22950927453737,0.218880922688349,0.821553904783398
"4509","TGFBR1",193.687370094484,0.243763949492267,0.198309484375893,1
.22920973880511,0.218993178462766,0.821553904783398
"4510","WDR11",288.664137384844,0.289054092430043,0.235181705509332,1.
22906708157439,0.219046656058612,0.821553904783398
"4511","RNF227",21.0026298095692,0.422149770376293,0.343534258888758,1
.22884329423748,0.219130565608664,0.821553904783398
"4512","PTRH2",131.832784677282,-0.241863916234294,0.196717141593944,-
1.22950096913029,0.218884034714419,0.821553904783398
"4513","CHKB",239.899084196249,-0.260718954736281,0.211972740209308,-1
.22996454392598,0.218710382406595,0.821553904783398
"4514","SNRPG",751.796199901034,-0.256215640691444,0.20877809970977,-1
.22721512001316,0.219741747980124,0.822010341198326
"4515","SEMA6A-
AS2",2.5174979180269,1.29937439490978,1.05889780299106,1.2271008507520
3,0.219784688156084,0.822010341198326
"4516","CNOT6",82.583121954071,0.293182634145272,0.23892899512082,1.22
707013435944,0.219796231808561,0.822010341198326
"4517","TRA2A",754.147774457971,0.223030468160144,0.181815505411188,1.
22668563198582,0.219940770024472,0.822010341198326
"4518","CSGALNACT1",95.4054683095844,-0.908080624822409,0.739741758837

803,-1.22756436820476,0.219610544594906,0.822010341198326
"4519","ZNF487",57.3591474219223,-0.402570254496043,0.328121193323947,
-1.22689500918215,0.219862054626099,0.822010341198326
"4520","AL158212.3",21.6053680633217,0.500240038260731,0.407412137866332,1.22784765540013,0.21950416233519,0.822010341198326
"4521","AC000403.1",6.50784417630174,0.71680509070959,0.584169959131032,1.22704887422807,0.219804221919833,0.822010341198326
"4522","SALL2",32.9677615943916,0.398979071002251,0.325003666893902,1.22761405991305,0.219591881293724,0.822010341198326
"4523","AP4E1",49.1309715130541,0.322054771478751,0.262314792290526,1.22774155687743,0.219544000962299,0.822010341198326
"4524","AC002553.2",7.2831919496922,0.665338220764139,0.542388032672001,1.22668307684895,0.219941730753562,0.822010341198326
"4525","OR2C3",3.78561008621953,1.24119819668511,1.01246763838858,1.22591394492427,0.220231060534836,0.822909783761992
"4526","CKAP4",441.428872584533,0.287872076869625,0.234849556509903,1.22577228225461,0.220284380542761,0.82292715559368
"4527","VPS13C",625.343910358563,0.223952687875315,0.182754765476515,1.22542734954889,0.22041424750221,0.82323041678095
"4528","LINC01886",44.5470011681594,-0.518166617324414,0.422949773729198,-1.22512565205007,0.220527881444951,0.82347292832845
"4529","MIER3",65.7871021867292,0.2980379979141,0.243370701829351,1.22462562532725,0.220716308325509,0.823530204634544
"4530","FKBP6",2.09782661777347,-1.29611892873071,1.05859693958005,-1.2243743395337,0.220811044835237,0.823530204634544
"4531","LAMTOR4",1767.23538418038,-0.311639971834583,0.254494877851829,-1.22454319892452,0.220747380445796,0.823530204634544
"4532","AL450326.1",52.1676250914497,-0.362867880027396,0.296313449333507,-1.22460820068609,0.22072287659707,0.823530204634544
"4533","CAMKK1",31.4108310750609,-0.624417240884816,0.510015803519502,
-1.22430959310644,0.220835459416432,0.823530204634544
"4534","APOL6",1167.51199344521,0.373902298827286,0.305308460732065,1.22467060994886,0.220699351883334,0.823530204634544
"4535","COL4A2",2373.41316679951,-0.354093073676925,0.289256864283005,
-1.22414752215002,0.220896581614611,0.823576494363804
"4536","SPSB1",354.726581488116,-0.308255427118293,0.253409231016229,-1.21643329993196,0.223819905199359,0.824329314790353
"4537","MRTO4",238.203744383991,-0.266415551725694,0.218175515282359,-1.22110655442204,0.22204567836231,0.824329314790353
"4538","SNRNP40",235.991596816084,-0.374228850202109,0.306227093919589,-1.22206316042165,0.221683743054837,0.824329314790353
"4539","CCDC24",8.78924250128108,-0.716195792877402,0.586950843899264,
-1.22019722830541,0.222390117338797,0.824329314790353
"4540","GPBP1L1",238.272510899424,0.197201952083712,0.161545456839666,
1.22072112668222,0.222191625878282,0.824329314790353
"4541","AL513218.2",3.38960468129927,-1.05903315808453,0.868813539435518,-1.21894182124809,0.222866274949295,0.824329314790353
"4542","BCL10",249.355371265711,-0.301703447659692,0.248212748423128,-1.21550343234336,0.224174140480742,0.824329314790353
"4543","MLLT11",16.4343000498235,-0.504737085213568,0.416206443793153,

-1.21270848335163,0.225241295257659,0.824329314790353
"4544","S100A1",4.00831172979216,-1.02060582936047,0.835067531792924,-1.22218358456494,0.221638210119517,0.824329314790353
"4545","GATAD2B",250.316013472561,0.29198409855837,0.239047176913875,1.22144968339688,0.22191580563836,0.824329314790353
"4546","TSTD1",13.0660342123384,-0.61822802959212,0.507748307569991,-1.2175875731637,0.223380739117094,0.824329314790353
"4547","DUSP12",146.598082276984,-0.316793596312136,0.260055764307768,-1.21817563688848,0.223157235819138,0.824329314790353
"4548","C1GALT1C1L",72.795659009564,-0.366195515409546,0.301089147889155,-1.21623618113383,0.223894964624184,0.824329314790353
"4549","PCBP1",1919.74573303171,-0.142515009254698,0.117438084882874,-1.21353315150562,0.224926047560594,0.824329314790353
"4550","TEKT4",5.05337709066345,-0.800273748290872,0.654180181837812,-1.22332313101053,0.221207673925529,0.824329314790353
"4551","RPL14",20479.4386642874,-0.287313088850207,0.2355661857666,-1.21967033560104,0.222589871290483,0.824329314790353
"4552","NDUFB4",1672.33145769031,-0.284790850495922,0.233700461345258,-1.2186148407948,0.22299041355542,0.824329314790353
"4553","NPHP3",328.375942343525,0.350020809452216,0.286321837818553,1.22247332623658,0.221528684886504,0.824329314790353
"4554","PSMD2",618.766140617692,-0.199050805108182,0.163745937612946,-1.21560759314035,0.224134440254945,0.824329314790353
"4555","WDR1",306.17157772697,-0.233302388915988,0.191559855112542,-1.21790856846766,0.223258719727761,0.824329314790353
"4556","CLOCK",229.388805190873,0.241908043223019,0.198990126708591,1.2156786229765,0.224107370566869,0.824329314790353
"4557","PAQR3",107.90620090199,-0.426704261531131,0.351866267465216,-1.21268874281423,0.225248845381315,0.824329314790353
"4558","LINC00989",3.68782918875458,-1.27735141912473,1.05107380120684,-1.21528233094391,0.224258428533942,0.824329314790353
"4559","NDUFS6",1003.31730444786,-0.291549611393634,0.240190471757528,-1.21382671535761,0.224813902375437,0.824329314790353
"4560","MTRR",92.7669511833648,0.220873905867297,0.182186482160996,1.21235068182564,0.225378170940798,0.824329314790353
"4561","NREP",187.823480110962,0.332274754318136,0.273381068920573,1.21542707997339,0.22420324489412,0.824329314790353
"4562","ADRA1B",56.4224126893885,-0.606310567835544,0.497029802586594,-1.21986763103589,0.22251505821045,0.824329314790353
"4563","MRNIP",217.024448797103,0.27801819067241,0.228956355231328,1.21428466308137,0.22463904020247,0.824329314790353
"4564","GNL1",361.470874348525,-0.196052528265808,0.161083578090942,-1.21708575504279,0.223571589945657,0.824329314790353
"4565","SNHG32",1479.92005147483,0.302726285484578,0.249415068507149,1.2137449725733,0.224845125161489,0.824329314790353
"4566","TFEB",83.295688482056,-0.292816858854831,0.240777995885402,-1.21612798452811,0.223936171668232,0.824329314790353
"4567","SENP6",565.270812506916,0.192192810064574,0.158403920796783,1.21330841495482,0.225011926831268,0.824329314790353
"4568","LCA5",255.633259608771,0.210786927302585,0.172811225489391,1.2

1975251726645,0.222558706376999,0.824329314790353
"4569","EZR",38.4429280021488,-0.47835794807996,0.394057665746805,-1.2
1392879687645,0.224774915278541,0.824329314790353
"4570","ZNF316",73.7658678758265,0.377029815287727,0.310751554539517,1
.21328376247843,0.225021348783452,0.824329314790353
"4571","GTF2IRD1",21.3772951009431,0.442183359220045,0.362907974841336
,1.2184448672239,0.22305496381283,0.824329314790353
"4572","ST7",79.5646028472315,-0.272486149060168,0.223280250166801,-1.
22037730097762,0.222321878175099,0.824329314790353
"4573","AC058791.1",215.704089246709,-0.593048704045931,0.487513840234
645,-1.21647562612887,0.223803790463508,0.824329314790353
"4574","AC144652.1",22.6299432648179,-0.441960058528622,0.364413502558
33,-1.2127982509591,0.225206964300973,0.824329314790353
"4575","SOX7",4.02973097232497,-1.06233653499481,0.875737021817379,-1.
21307710937033,0.22510034080422,0.824329314790353
"4576","REEP4",44.7832792585416,0.296840322677105,0.244852542395555,1.
2123228118153,0.225388835005666,0.824329314790353
"4577","CYP7A1",2.59117943892634,-1.31275745837471,1.07705250693553,-1
.218842581881,0.222903946090995,0.824329314790353
"4578","AC022868.2",6.42050469253072,-0.727641009336854,0.599246795030
923,-1.21425932582469,0.224648712407559,0.824329314790353
"4579","AC027373.1",6.79560991861714,0.757939530788094,0.6232603239405
,1.21608820852914,0.223951321855391,0.824329314790353
"4580","AP003696.1",5.03074070095534,1.00605631480811,0.82907184484158
7,1.21347301933807,0.224949023756291,0.824329314790353
"4581","ZNF572",6.55525465864456,0.745205617531268,0.610278201549344,1
.22109165236343,0.222051319955479,0.824329314790353
"4582","TRAPPC9",58.841061505641,0.300034830181314,0.245348852395387,1
.22289070135041,0.22137098112144,0.824329314790353
"4583","UHRF2",152.16811725443,0.31047294835656,0.255701430492007,1.21
420106160206,0.224670955230535,0.824329314790353
"4584","AL158151.1",21.2930322352521,0.778863663278432,0.6417539844025
97,1.21364834844535,0.224882036077624,0.824329314790353
"4585","ATP5F1C",1433.86037524584,-0.340604588564333,0.279968256476621
,-1.21658288282684,0.223762958632789,0.824329314790353
"4586","KCNQ1",2.2841188425228,-1.33637446365458,1.10214931207056,-1.2
1251671531146,0.22531464804523,0.824329314790353
"4587","TKFC",51.6919210599656,0.357125789485229,0.294326741603605,1.2
1336507698713,0.224990272186291,0.824329314790353
"4588","YIF1A",578.531736644415,-0.338886801133114,0.277337972609975,-
1.22192715964537,0.221735173646079,0.824329314790353
"4589","PAK1",133.971484969617,0.222360813519301,0.182825697532625,1.2
1624485244817,0.223891662359418,0.824329314790353
"4590","LINC02728",3.85469610421523,0.975177378171417,0.80193460983158
,1.2160310407057,0.223973097657427,0.824329314790353
"4591","ST3GAL4",344.371821549406,-0.373401467446181,0.307351219418381
,-1.21490153236676,0.224403649286408,0.824329314790353
"4592","TRPV4",7.95469560325301,0.675577072328178,0.55357795248199,1.2
2038290957796,0.222319753017336,0.824329314790353
"4593","OGFOD2",55.509421246147,0.341849820192509,0.281462036412796,1.

21455036902791,0.224537627948641,0.824329314790353
"4594","ZNF26",75.3307243646541,0.343609475302359,0.282043326730031,1.2182861381126,0.223115255874366,0.824329314790353
"4595","LINC00327",5.31659559940205,0.811285192040579,0.66485293280176 1,1.22024759463982,0.222371029331177,0.824329314790353
"4596","AL359232.1",11.2406850528006,-0.556701872170078,0.456328502120 682,-1.21995858155459,0.222480576458258,0.824329314790353
"4597","AL118558.4",2.70749411187149,1.40669767728889,1.15974847403137 ,1.21293341512156,0.225155278818381,0.824329314790353
"4598","MOK",121.622976145095,-0.244070852592857,0.199549486723807,-1.22310939807463,0.221288379440004,0.824329314790353
"4599","AL138976.2",5.3465638598321,0.819997077874635,0.67593726786864 2,1.21312600570204,0.225081648660185,0.824329314790353
"4600","AC011330.2",3.82881359946462,1.05784036498034,0.86667672497287 3,1.22057087089013,0.222248540907665,0.824329314790353
"4601","EME2",16.366065443333,-0.513327674700843,0.423125517393392,-1.21318061331571,0.225060774511491,0.824329314790353
"4602","IL32",378.72416351111,0.393323584015134,0.322996553446521,1.21 773306810302,0.223325426467666,0.824329314790353
"4603","BOLA2B",27.5650644789602,-0.579544650484255,0.477304585663305, -1.21420298042783,0.224670222678722,0.824329314790353
"4604","AC027279.1",53.3182140972347,0.351247060210194,0.2889978155465 06,1.21539693836776,0.224214735179575,0.824329314790353
"4605","C17orf100",12.6770490666399,-0.592886682969409,0.4853892216729 44,-1.22146651902563,0.221909434831671,0.824329314790353
"4606","TVP23B",70.8914976350347,-0.344488621392709,0.283232598528552, -1.21627462086778,0.223880326026609,0.824329314790353
"4607","MRPS23",279.526127043724,-0.330637518921086,0.271951450003616, -1.21579612433282,0.224062595571551,0.824329314790353
"4608","ARL16",384.095865272454,-0.279102176384503,0.230198988847808,-1.21243875909909,0.225344471787746,0.824329314790353
"4609","HEXD-IT1",6.99649372511874,0.845189480117669,0.695241366473124,1.2156777787 91,0.224107692274823,0.824329314790353
"4610","AC125437.1",3.66213335320557,-0.898652668794589,0.738197436207 643,-1.21736086406809,0.223466946393775,0.824329314790353
"4611","P2RY11",10.5820162690409,0.674783502174704,0.553867440533881,1 .21831227617256,0.223105326726535,0.824329314790353
"4612","ZNF69",26.5102504733844,-0.372818391555763,0.30490210129903,-1 .2227478589599,0.22142494457384,0.824329314790353
"4613","MAU2",171.685222846355,0.283122961854898,0.23168963649898,1.22 19923434346,0.221710522420051,0.824329314790353
"4614","ZNF738",59.4838849780993,0.277272824001745,0.226767898838387,1 .22271637838542,0.221436838672934,0.824329314790353
"4615","LILRB2",12.7563865620773,-0.783185391418811,0.64018398407761,-1.22337548407625,0.221187908635295,0.824329314790353
"4616","LENG8",403.53956467095,0.263292261414447,0.215722019605981,1.2 2051639371518,0.222269178763969,0.824329314790353
"4617","CENPB",185.434224320572,-0.314728818458393,0.259138977213837,-1.21451748340692,0.224550177662042,0.824329314790353

"4618","AP000331.1",10.3043674211394,-0.803426747558625,0.657792240512437,-1.22139894343043,0.221935007051237,0.824329314790353
"4619","HSCB",212.751309214634,-0.296534394538948,0.243155799615751,-1.21952425156031,0.222645276961092,0.824329314790353
"4620","LINC01315",86.0617665716258,-0.453251586415821,0.372008285990392,-1.21839110440547,0.223075383910059,0.824329314790353
"4621","GTPBP6",399.39381976044,-0.300807226147814,0.247166242790365,-1.21702390565909,0.223595120495065,0.824329314790353
"4622","PRKX",45.2957605882086,0.351039091695644,0.288573664601423,1.21646267402986,0.223808721592228,0.824329314790353
"4623","TMSB4X",29761.5228282786,-0.3352869097721,0.276120522920123,-1.2142773967913,0.224641813994342,0.824329314790353
"4624","ZSCAN16",12.1912229635257,-0.592397232355906,0.488816536193521,-1.21190096588995,0.225550292380815,0.824409619325982
"4625","AP5Z1",128.799231075212,0.210842165085112,0.173978938835503,1.21188326872405,0.225557067601253,0.824409619325982
"4626","SAMD4B",673.69939588105,0.20763177091576,0.171300257247272,1.2120925809004 7,0.225476943388668,0.824409619325982
"4627","DIPK1A",97.0752915254859,0.302588270183891,0.249849891536861,1.21108025431842,0.225864648237394,0.825355407909629
"4628","HNRNPR",962.1446892743,-0.186792410796468,0.154362400754177,-1.21009008595258,0.226244326962349,0.825376110222837
"4629","C1orf53",13.7601941948969,-0.625544626636276,0.516748224426451,-1.21054044710184,0.226071580161436,0.825376110222837
"4630","KIAA2026",391.592233260427,0.259715560601583,0.214655180383761,1.20991983579088,0.226309655014968,0.825376110222837
"4631","TSPAN15",422.943371718983,-0.428565640759733,0.354191467588583,-1.20998296112977,0.226285431115911,0.825376110222837
"4632","OLFM4",49.3936320255638,0.598771593210432,0.494524434237418,1.21080284765659,0.225970973557495,0.825376110222837
"4633","RALGAPA1",276.249823319486,0.274707240348993,0.226982755607203,1.21025599329836,0.226180678273237,0.825376110222837
"4634","RPAP1",61.2115582620232,0.294213502901849,0.243146303585137,1.21002663237622,0.226268673668971,0.825376110222837
"4635","TLCD1",9.15590464830477,-0.613348275851619,0.506887893379689,-1.21002747128582,0.226268351772858,0.825376110222837
"4636","AL121832.2",15.9265621690349,-0.673921177378687,0.556650558281228,-1.21067187906818,0.226021184032858,0.825376110222837
"4637","PRDM16",14.7206708497816,-0.780408630866305,0.645227069112606,-1.20951005967499,0.226466948670387,0.825385749911411
"4638","RCHY1",149.680247413517,0.246939613846733,0.204182850126052,1.20940428490583,0.226507563259342,0.825385749911411
"4639","MFAP3L",4.97293729412252,-0.865901356255107,0.71584349899319,-1.20962383184728,0.226423269099404,0.825385749911411
"4640","LRP11",376.976981881476,0.25505918634747,0.210833087418549,1.20976830283343,0.226367812259457,0.825385749911411
"4641","AL713852.2",2.10835250907862,1.36287912476252,1.12800786615048,1.20821774888288,0.226963516414002,0.825515043826717
"4642","ANAPC1",100.663956275414,0.253214309812306,0.209588103362802,1.20815211240299,0.226988757798043,0.825515043826717

"4643","BCL6",863.585174847833,0.371836940643448,0.30771915521984,1.20836462188323,0.226907041692919,0.825515043826717
"4644","MDN1",141.86057292213,0.312304904299614,0.258381194561797,1.20869827554312,0.226778784441111,0.825515043826717
"4645","TBC1D32",43.5282144771082,0.351646789176369,0.291270217469621,1.20728714467021,0.227321579698741,0.825515043826717
"4646","ARRDC1",79.0812079158611,-0.284243693180324,0.235162810401724,-1.20871022375841,0.226774192478187,0.825515043826717
"4647","ZNF488",7.58707284005189,0.660055197056694,0.546399471881908,1.20800848284742,0.227043999442598,0.825515043826717
"4648","FADS1",37.9878061141717,0.550849941315673,0.456168858817467,1.20755709353692,0.227217671571128,0.825515043826717
"4649","GXYLT1",127.087017352938,0.281726851002403,0.233028871330271,1.20897830982974,0.226671178518634,0.825515043826717
"4650","MED13L",859.708873729459,0.315450867649282,0.261192356818701,1.20773391492555,0.227149628225667,0.825515043826717
"4651","AC133919.1",3.52299194216963,1.12169422959261,0.928613887507776,1.20792316880272,0.227076816782159,0.825515043826717
"4652","ZNF830",183.789251373218,-0.258687551019366,0.214295498172133,-1.20715345504634,0.227373051756625,0.825515043826717
"4653","CST7",22.6458541441782,0.518832283382311,0.429607817625446,1.20768818000108,0.227167226271753,0.825515043826717
"4654","TIAM1",44.0124113280057,0.424805175540473,0.351837807677943,1.20738921818579,0.227282285802298,0.825515043826717
"4655","PICK1",37.0236591038015,-0.328832379860577,0.272377427914301,-1.20726736564984,0.227329194327705,0.825515043826717
"4656","SPIN4",20.6008688334187,0.493369149352606,0.408638796994694,1.20734779218482,0.227298232440246,0.825515043826717
"4657","MT-ND5",13388.902292114,-0.257362589135392,0.212860244365456,-1.20906837207953,0.226636578956613,0.825515043826717
"4658","DDX47",27.0759839251834,-0.399542964885722,0.331016016740904,-1.20702003733691,0.227424427406391,0.825524306266049
"4659","AKAP5",5.07305320978588,0.825146723651967,0.683757954512195,1.20678190024223,0.22751614830086,0.825679981856825
"4660","NAA35",153.004072726767,0.262070910434457,0.217208865832902,1.20653873602041,0.227609832647014,0.825842714677192
"4661","UBE2S",558.91885785421,0.372563864015406,0.308972318385064,1.20581632025394,0.227888321315132,0.826675764169974
"4662","B3GALNT2",117.315257423866,-0.254105656387376,0.210756448445224,-1.20568389846168,0.227939395756778,0.826682797871253
"4663","MTMR12",96.5437026905446,0.263155784813342,0.218331399507007,1.20530434654634,0.228085832272853,0.826682797871253
"4664","ZFAND2A",124.505333657747,-0.345899369781769,0.286959058351902,-1.20539623933944,0.228050372581792,0.826682797871253
"4665","SINHCAF",42.2284354311661,-0.439359702935574,0.364453248548306,-1.20553103775487,0.22799836352881,0.826682797871253
"4666","AC079834.2",4.79909359699188,-0.898133580765276,0.74571698061049,-1.20438933820443,0.228439130977669,0.826722779659765
"4667","AC102953.2",7.15672812144313,0.703069604575609,0.5837178195008

34,1.20446829116308,0.228408630678951,0.826722779659765
"4668","LGI1",4.56422520216233,1.10078500694865,0.913910050492156,1.20447850021548,0.228404687034103,0.826722779659765
"4669","EI24",635.848932716758,-0.185512436951834,0.15402027100377 8,-1.20446767002042,0.228408870621104,0.826722779659765
"4670","LINC02076",5.19289680836584,-0.904523785051412,0.75095372286928,-1.20450003443004,0.228396368762488,0.826722779659765
"4671","CU633967.1",3.01980731141285,1.28322768602662,1.06492364081542,1.20499502203185,0.228205223616901,0.826722779659765
"4672","AL022328.1",2.07661585138915,1.23048724936948,1.02162779649905,1.20443791132754,0.228420366362712,0.826722779659765
"4673","PIGW",17.6087783100097,-0.434524096230205,0.360882083064401,-1.20406115077944,0.228565944070506,0.827004704118149
"4674","WSB1",2769.06500877082,0.319752815232028,0.265611085415581,1.20383836665452,0.228652057415232,0.827139278300542
"4675","ZNF524",265.774904005475,-0.356136564029113,0.295892214030365,-1.20360234957911,0.228743310924911,0.827292385265966
"4676","LINC01915",44.556730671634,-0.433463450574201,0.360221453832699,-1.2033249157212,0.228850610945996,0.827503449502759
"4677","IMP4",277.036830506555,-0.302766010291956,0.25163503932066,-1.20319495690797,0.228900885998978,0.827508270359359
"4678","PLAC9",10517.0120468985,-0.294897960455667,0.245123888753044,-1.20305679693573,0.228954342322768,0.827524587429107
"4679","EIPR1",74.162895234339,-0.29364109574173,0.244369451504801,-1.20162767454573,0.229507814276955,0.827590949527423
"4680","CCT7",847.613358264318,-0.258201217314297,0.214708224957563,-1.20256789121763,0.229143579200121,0.827590949527423
"4681","ABHD5",1450.67117810437,-0.37601685664117,0.313432271927048,-1.1996749866545,0.230265591178258,0.827590949527423
"4682","CCDC36",79.2236002921667,-0.360676540641024,0.300394287774813,-1.20067709447059,0.22987648265751,0.827590949527423
"4683","SH3D19",1213.77572337153,0.190816318235461,0.158973174029829,1.20030514204653,0.230020853479343,0.827590949527423
"4684","AC025171.2",73.2984491552481,0.33853897648478,0.282240906729031,1.19946814375068,0.230345964496986,0.827590949527423
"4685","AC011374.2",13.6386025712927,0.531834649677717,0.442612378253379,1.20158105784665,0.229525884057273,0.827590949527423
"4686","HLA-
C",4251.58017898397,-0.3499897983844,0.291769039228765,-1.19954399311706,0.230316489256791,0.827590949527423
"4687","BMPER",411.004959545374,0.359922836430305,0.299247959970531,1.20275786162669,0.229070035633842,0.827590949527423
"4688","ARPC1A",759.40949163743,-0.325751581651278,0.271256210324344,-1.20089999510711,0.22978999617072,0.827590949527423
"4689","GFRA2",4.20519237358421,-1.1040529441136,0.918418626709047,-1.20212385943187,0.229315543517548,0.827590949527423
"4690","ZNF483",67.259358589756,0.351062748823531,0.292745386526729,1.19920847596851,0.230446892307202,0.827590949527423
"4691","PDLIM1",1609.02355944414,0.301006100157844,0.250523493660049,1.20150847236028,0.229554021993959,0.827590949527423

"4692","PPP1CA",422.269225056052,-0.259056083551984,0.216042558510383,-1.1990974618065,0.23049005094184,0.827590949527423
"4693","MRGPRF",392.258833301655,-0.218940833403817,0.182255351937946,-1.20128616842133,0.229640213913066,0.827590949527423
"4694","KCNRG",7.70207250368292,0.689616679142985,0.57452453662796,1.20032589589738,0.230012796314101,0.827590949527423
"4695","EMC9",40.7728238874767,-0.402039730131459,0.334620814555233,-1.20147854719031,0.229565623271809,0.827590949527423
"4696","ALDH6A1",303.434416333866,0.23527541555205,0.195981233704258,1.20049971675904,0.229945322577599,0.827590949527423
"4697","NUBP2",327.951590210541,-0.263965409162009,0.219879309426391,-1.20050135617866,0.229944686255141,0.827590949527423
"4698","MAPK3",658.886200497102,-0.205141545686963,0.170708589881184,-1.20170605257618,0.229477435304115,0.827590949527423
"4699","AC134312.5",9.33314692731534,-0.611620097035345,0.509372461753733,-1.20073255418948,0.229854961864567,0.827590949527423
"4700","KCTD11",54.5179473418483,-0.286927705432938,0.239059374688895,-1.20023615809393,0.230047636221672,0.827590949527423
"4701","ZNF846",76.2489318782509,-0.35087651470391,0.291879688349819,-1.20212720757528,0.229314246507397,0.827590949527423
"4702","CRLF1",108.360840099187,0.582686752054967,0.485759541967747,1.1995374289398,0.230319040005344,0.827590949527423
"4703","ZC3H4",134.41326927712,0.293614922997386,0.244560113998367,1.20058384908729,0.229912669168726,0.827590949527423
"4704","ZNF304",24.9042578446345,0.449652389612666,0.374942709697581,1.19925625430974,0.230428319436731,0.827590949527423
"4705","AL121894.2",2.57807179326663,1.24194627951396,1.03330679591551,1.20191436311381,0.229396708981879,0.827590949527423
"4706","CEBPB",3962.65083277529,-0.36297678807609,0.302702940165135,-1.19911880564514,0.230481752717151,0.827590949527423
"4707","TNFRSF6B",14.4486160566252,-0.514014790627311,0.428453966543544,-1.19969665533502,0.230257172494129,0.827590949527423
"4708","SCML1",224.468729300492,0.321399970630767,0.26737433110249,1.20205993337321,0.229340308316945,0.827590949527423
"4709","NKRF",52.193154050499,0.283882488666273,0.23667435020307,1.19946453184597,0.230347368158452,0.827590949527423
"4710","LIG4",49.3869676065542,0.422733439340206,0.352624855424026,1.1988191780522,0.230598263715384,0.827627986181216
"4711","RBBP6",1010.11848838731,0.303124187866373,0.252849993760262,1.19883011804136,0.230594008934837,0.827627986181216
"4712","REPS1",94.812761299099,0.267256952212912,0.223016523622228,1.19837287332854,0.230771888123171,0.827899657200632
"4713","HERC1",323.364922786739,0.251435379435801,0.209800077370993,1.19845227221334,0.230740993053211,0.827899657200632
"4714","PCNP",899.014064250217,-0.164667793525823,0.137441164912981,-1.19809660832023,0.230879409102145,0.827925885488224
"4715","LINC02268",3.31634570106157,-1.14797096901073,0.959192285858181,-1.19681005147331,0.231380599596289,0.827925885488224
"4716","SPCS3",350.110259898334,-0.193280918879071,0.161453013860891,-1.19713416465302,0.23125426545518,0.827925885488224

"4717","RNF44",114.629848303176,0.317045268215113,0.264809392700764,1.19725839397765,0.231205855841111,0.827925885488224
"4718","MYO6",172.316044001294,-0.266158962680619,0.222178585373664,-1.19795056860673,0.230936261419585,0.827925885488224
"4719","MAGI2-AS3",817.124787649846,0.187779526625503,0.156871794219573,1.1970254280554,0.231296643781096,0.827925885488224
"4720","NRF1",50.6747332765725,0.328233832165009,0.274274840556399,1.19673328949585,0.231410527367856,0.827925885488224
"4721","ZNF883",8.06486841257315,-0.711398381149309,0.594519288594398,-1.19659428179571,0.231464730346749,0.827925885488224
"4722","SRGAP1",560.56930696017,0.291407153889452,0.24323698185065,1.19803802724529,0.230902213141605,0.827925885488224
"4723","AC130895.1",15.2053268188911,0.697181903060406,0.582175376267423,1.19754618879682,0.231093735774777,0.827925885488224
"4724","TTC23",91.7017833103946,0.222007445795163,0.185522400997053,1.19666112880185,0.231438663709438,0.827925885488224
"4725","WDR59",116.701399101789,0.267209425583222,0.223211202087933,1.1971147643296,0.231261826012394,0.827925885488224
"4726","NOTCH3",148.565588540891,0.449761656852501,0.375604729337114,1.19743342328586,0.231137662735374,0.827925885488224
"4727","ZNF486",46.4264987961051,0.299952368486237,0.250484408066664,1.19748918027028,0.231115942278135,0.827925885488224
"4728","B4GALT1-AS1",39.3229831008519,-0.409356613080295,0.342181565023987,-1.19631404763608,0.23157402887203,0.828141641321549
"4729","TNF",11.3636069700701,-0.766602053948944,0.641040111414786,-1.19587220877184,0.231746431830791,0.828582928609645
"4730","PTCH2",100.773362076693,0.341322495610925,0.285736441191668,1.19453610532641,0.232268326075505,0.828871434631623
"4731","AL355816.2",5.7182131085762,0.884462876117189,0.740168914581663,1.19494734065815,0.232107605003262,0.828871434631623
"4732","RTN4",5600.57854879235,-0.20818916123998,0.174183884896068,-1.19522630560343,0.231998623462064,0.828871434631623
"4733","ACSL3",335.173115481308,0.321373747008282,0.268909195730622,1.19510136548181,0.232047428568438,0.828871434631623
"4734","PCBP2",3051.79535416667,-0.198475717275939,0.166049385909894,-1.19528124833681,0.231977163600562,0.828871434631623
"4735","POLR2A",569.816046797373,0.340488076975177,0.284963971586926,1.19484605397323,0.232147183048428,0.828871434631623
"4736","UQCRFS1",705.444417437097,-0.285729764764863,0.239175069318436,-1.19464694033158,0.232225001204861,0.828871434631623
"4737","PSMA7",2489.34532632914,-0.261398836965677,0.218683884376786,-1.19532739099921,0.2319591420122,0.828871434631623
"4738","LINC01695",2.17631540354992,1.37386696215746,1.15003347846183,1.19463214583544,0.232230783970527,0.828871434631623
"4739","NOCT",51.173766726875,-1.04951363523679,0.878891190114247,-1.19413375289422,0.232425651918914,0.828960981153247
"4740","ZDHHC17",262.704663463659,0.207001303751802,0.173354019437694,1.19409578401037,0.232440502226611,0.828960981153247

"4741","MATK",9.99454388946954,-0.749987706919289,0.6279916279195,-1.19426386208994,0.232374768998437,0.828960981153247
"4742","LRP5",258.78667431377,-0.3332215786204,0.27916765570483,-1.19362530655314,0.232624570215833,0.829442478534227
"4743","CDC42BPA",334.258210127692,0.266839054005515,0.223617439505077,1.19328373760159,0.232758269264391,0.829569312125277
"4744","C15orf39",39.3066697399661,0.419804808181594,0.351779972839204,1.19337324633169,0.232723227929337,0.829569312125277
"4745","COL9A2",4.50660699569632,-1.05738732531296,0.886222323003359,-1.19314002577766,0.232814538132739,0.829594986459085
"4746","AL590723.1",2.50804399918567,1.20450140343384,1.01022283388232,1.19231258989158,0.233138699766782,0.829760081336346
"4747","SLC35F5",292.386709664034,0.197705767093819,0.165844757294596,1.19211345790466,0.233216760765554,0.829760081336346
"4748","SCN7A",2.61379007283283,1.35172029431495,1.13321764479416,1.1928161377689,0.232941388489875,0.829760081336346
"4749","ACSL1",238.763823235487,0.359986992803859,0.301834189397643,1.19266473265427,0.233000702936612,0.829760081336346
"4750","TAP1",186.782035728335,0.314142414027429,0.263541010316759,1.19200580452299,0.233258969290024,0.829760081336346
"4751","CREB3L2",361.115471009316,-0.23684587178432,0.198686495962833,-1.19205822537947,0.233238415547923,0.829760081336346
"4752","ARHGEF17",86.2480173438968,0.303531092124514,0.254649456804191,1.19195656622943,0.233278276347844,0.829760081336346
"4753","RNF126",167.342617043415,-0.234574515466768,0.196758651166926,-1.19219416313115,0.233185121572736,0.829760081336346
"4754","INE1",17.053654434118,-0.49631789592072,0.41641086630015,-1.19189467923964,0.233302544752365,0.829760081336346
"4755","RC3H1",242.114860510409,0.308871715623772,0.259318710507944,1.19108920069348,0.233618568924662,0.82979325660823
"4756","MOCS2",371.250986385063,-0.25589841107069,0.214924043590429,-1.1906458058194,0.233792661409884,0.82979325660823
"4757","ITK",10.2686462221389,0.743443517220921,0.624416918828221,1.1906203928876,0.2338026422097,0.82979325660823
"4758","RGS17",12.9533425742407,-0.597997340269259,0.501926650912361,-1.19140384194039,0.233495085417379,0.82979325660823
"4759","AC092171.4",2.2118398292618,1.19069875775323,0.999843063316388,1.19088565139792,0.233698478152444,0.82979325660823
"4760","KLHDC10",228.746358395733,0.274593686485822,0.230554223750746,1.19101564056656,0.233647444868005,0.82979325660823
"4761","NUCB2",1178.73055334134,-0.265150285820739,0.222517015594047,-1.19159555107674,0.233419870303504,0.82979325660823
"4762","TTC9C",147.173564684768,0.272047822068241,0.228432245475631,1.19093441252918,0.233679333780101,0.82979325660823
"4763","PSMD7",755.213176860392,-0.219272952589869,0.184024620516209,-1.19154139253098,0.233441117112374,0.82979325660823
"4764","TOB2",511.233706628822,0.281761262760497,0.236648881957289,1.19063001874376,0.23379886166808,0.82979325660823
"4765","ECHDC2",631.914188526341,0.276349374995388,0.23227839084854,1.18973346588915,0.234151167493061,0.829998907422177

"4766","HERC5",46.6425098863284,0.373423052634662,0.313814111998406,1.18994984086809,0.234066107229849,0.829998907422177
"4767","TMEM200A",12.5161831837197,-0.619152736339459,0.520412373478662,-1.18973484854092,0.234150623882303,0.829998907422177
"4768","KBTBD11",28.8654876935731,0.44400059606462,0.373132999296168,1.18992583583368,0.234075542890457,0.829998907422177
"4769","LRRC14",45.6993119310543,0.288936214343818,0.242741220451723,1.19030551879952,0.233926332460494,0.829998907422177
"4770","ZHX3",298.106739109732,0.308931837360445,0.259666940034711,1.18972340999262,0.234155121149975,0.829998907422177
"4771","AMD1",791.884109919723,0.32998164551975,0.277480863767259,1.18920505378175,0.234358986299642,0.830547419902399
"4772","FAM120AOS",460.17130344339,-0.179083407915209,0.150608529707108,-1.18906550819849,0.234413889882015,0.830567906564357
"4773","LINC00648",31.3654182401113,0.435599154842296,0.366380671389911,1.18892504124138,0.234469165176059,0.830589701403061
"4774","HAAO",84.4680834898731,0.340894481407133,0.286793079526643,1.18864263381036,0.23458032354833,0.83080250394812
"4775","DBN1",732.523927198918,-0.282852825137785,0.238061851855476,-1.18814847037946,0.234774920976113,0.83080250394812
"4776","AC083864.5",7.54250013008754,-0.775790013623236,0.652883025115926,-1.18825269424869,0.234733868977989,0.83080250394812
"4777","ANK1",8.49415640293191,-0.664080066395628,0.558915105870061,-1.18815909504156,0.234770735870859,0.83080250394812
"4778","HHIPL1",35.5845372172145,0.338759666135227,0.285083502114298,1.18828225282362,0.23472222728617,0.83080250394812
"4779","ODF2L",752.490639828104,0.198459271710697,0.167100530342592,1.187663924847,0.234965841930187,0.831304133784391
"4780","STARD7",202.746076518881,0.223207034261601,0.187991364656578,1.18732599590069,0.235099057969246,0.831581954747337
"4781","LINC01023",31.3680539884237,-0.396099632868292,0.333698951483304,-1.18699693573388,0.235228829193679,0.831581954747337
"4782","HIST4H4",8.75299137158367,0.705789499388573,0.594616526025311,1.18696583175445,0.235241098270435,0.831581954747337
"4783","IL34",102.340297509928,0.475462100758208,0.400550326952061,1.18702212622364,0.235218893046214,0.831581954747337
"4784","TRIM32",69.6136170599594,0.280658965656151,0.236495611574997,1.18674069166458,0.23532991909611,0.831722046838843
"4785","ZNF99",9.24597140704934,0.854367530080673,0.720114140355423,1.18643348630675,0.235451154074317,0.831976617155393
"4786","LTB",33.9123601816657,-0.465078277681313,0.392130216090586,-1.18603019761648,0.23561037430097,0.832365275528793
"4787","LRIG3",319.538565551485,0.257115127423674,0.216848912370527,1.18568788108259,0.235745582210652,0.832617356976598
"4788","IRF9",524.091416393602,-0.368014516634757,0.310403550497882,-1.1856002163779,0.235780216773359,0.832617356976598
"4789","CACNA1C",84.5929388073599,0.473350863808169,0.399354407631267,1.18529019528244,0.235902728753955,0.832763193624431
"4790","IFT52",326.383313933678,-0.244024201461773,0.205884777622774,-1.1852464484231,0.23592001995866,0.832763193624431

"4791","NAALADL1",157.460686616874,-0.256525381200557,0.216487077347652,-1.18494546807803,0.236039008523846,0.833009299962679
"4792","C1orf112",20.1454738235014,-0.400803530064273,0.338361878469104,-1.18454103599874,0.236198962187817,0.833119319809797
"4793","PSMD14",373.181029592551,-0.256047481253626,0.216166328102878,-1.18449290183514,0.236218004445716,0.833119319809797
"4794","PAPSS2",291.786310860107,0.244318095704682,0.206245968222043,1.18459574172937,0.236177321483899,0.833119319809797
"4795","PXMP2",154.615055162576,-0.259524581139338,0.219172306365157,-1.18411210541788,0.23636868880817,0.833303125598109
"4796","TBL1X",147.835180828666,0.246831358407976,0.208435449685925,1.18421006973578,0.2363299170067,0.833303125598109
"4797","NDUFS5",2290.74383460073,-0.253664875168762,0.214415675575763,-1.18305191300778,0.236788573936316,0.833387241794858
"4798","MARK1",56.4000432731151,0.284887458182715,0.240831672719022,1.18293185844826,0.236836154295262,0.833387241794858
"4799","LINC02580",38.2374269241885,0.404702797986473,0.342083687534575,1.1830520212852,0.236788531026557,0.833387241794858
"4800","FAM172A",343.340683801759,0.197867248966887,0.167235667120414,1.18316416811024,0.236744090793176,0.833387241794858
"4801","SLC22A4",24.1538954035592,0.508830332367485,0.429873297474828,1.18367513254828,0.236541686406146,0.833387241794858
"4802","LACTB2",152.112519275681,-0.239273418456528,0.202224685388857,-1.18320578912723,0.236727599203138,0.833387241794858
"4803","TBCEL",59.7437384745617,0.298799996429168,0.252415406259143,1.18376291232558,0.23650692720102,0.833387241794858
"4804","NOMO1",63.8567412663978,-0.447329583427911,0.378009153122296,-1.18338294121462,0.236657414913968,0.833387241794858
"4805","TASP1",220.985332837768,0.266608202715977,0.225264458564572,1.18353425309459,0.236597479673179,0.833387241794858
"4806","DCAF12",116.226411694259,0.209444744520793,0.177130855210461,1.18242947719039,0.237035332749885,0.833914566403466
"4807","MAPK14",284.829725589366,0.19637118527333,0.166122432116282,1.18208710751281,0.237171139428848,0.834218769599117
"4808","ZNF655",338.228074075566,0.219667459586098,0.185902942617382,1.18162443527432,0.237354753481341,0.834690967525482
"4809","AC105446.1",5.18083916013784,-0.898761753960267,0.760882220760707,-1.18121008670923,0.237519275241513,0.834748681310228
"4810","CCDC186",561.830566614454,-0.230535529654781,0.195165713576852,-1.18122966083385,0.237511501302605,0.834748681310228
"4811","AACS",104.592188135479,0.31775535821397,0.269007007364559,1.18121591451091,0.237516960688863,0.834748681310228
"4812","RHOC",912.480083161889,-0.318062617694525,0.269417338351178,-1.18055734512506,0.237778617018414,0.834910451476502
"4813","AL392172.1",42.6171457161315,-0.356510209980713,0.302038058953706,-1.18034863293621,0.237861582964414,0.834910451476502
"4814","LSR",28.1224354288389,-0.41805395665282,0.354172414986784,-1.18036848428306,0.237853690903236,0.834910451476502
"4815","CLDN5",7.33942555506414,-0.900068077210723,0.762376283108662,-1.18060870616359,0.237758203437977,0.834910451476502

"4816","Z99716.2",2.71890634783066,-1.17150956786284,0.992304630608569,-1.18059468002721,0.237763778040074,0.834910451476502
"4817","ARMCX5-GPRASP2",49.8669722002336,-0.365741125851647,0.309698391461425,-1.18095907481393,0.2376189815136,0.834910451476502
"4818","FP236383.5",2.3611102942327,1.42777795772264,1.20985870440211,1.18011958960796,0.237952654341395,0.835056762059841
"4819","LINC01358",33.7565287655409,-0.485064247724357,0.411133313738773,-1.17982229003354,0.23807090223781,0.835298363776073
"4820","AC093627.5",9.80456542944788,-0.719885165036802,0.610382107553818,-1.17940083126262,0.238238604287442,0.835713344666405
"4821","AURKAIP1",1005.55457431002,-0.276724147188229,0.234719871106449,-1.17895492138682,0.238416126417579,0.83616259395736
"4822","FRAS1",6.2589127558185,1.41569555329925,1.20098280963911,1.17878086342023,0.238485446335379,0.836170292272281
"4823","SQOR",305.299788251655,0.252588544410824,0.214293980874365,1.17870106934506,0.238517229691815,0.836170292272281
"4824","TBX15",431.188484212317,-0.198377555306341,0.168323986165498,-1.17854596855432,0.238579017564882,0.836213521763478
"4825","SEC61B",2081.15229836697,-0.30477187032724,0.258689361006046,-1.17813840175714,0.238741434765308,0.836526693017084
"4826","TECTA",3.12708883982261,0.95937684011711,0.814360790198537,1.17807346776018,0.238767318458744,0.836526693017084
"4827","TRIT1",59.4201914996421,-0.282065222267543,0.23956137786232,-1.17742360970077,0.239026470990185,0.836766788504486
"4828","AC092903.2",26.654955050747,0.424080519165052,0.360220247788319,1.17728118219012,0.239083295201394,0.836766788504486
"4829","PCDHB16",76.029258512281,0.391023572469025,0.332072087425977,1.17752616758609,0.238985559438318,0.836766788504486
"4830","DENND4C",222.99793131023,0.235696322509549,0.200196597255134,1.17732431890026,0.239066083970289,0.836766788504486
"4831","ALG10B",65.7848944735164,0.282788827000181,0.240121934184022,1.17768844383646,0.238920835618178,0.836766788504486
"4832","LRBA",90.0358409752299,0.262459403279054,0.223029284901099,1.1767934573948,0.239277954633155,0.836928352697018
"4833","AC073957.3",3.23636211802908,1.35495307077453,1.15129476941532,1.1768950113989,0.239237413455549,0.836928352697018
"4834","PSKH1",94.8208372590039,0.252580535385905,0.214604745785951,1.17695689562164,0.239212711150487,0.836928352697018
"4835","MAD2L1",23.1954221331043,-0.389218358378301,0.330805699646865,-1.17657694167238,0.239364405633581,0.837057574033628
"4836","GALNT12",114.301500191761,0.371532394479986,0.315902098762417,1.17609979780289,0.239554998722231,0.83716237845084
"4837","CA12",282.257824765731,-0.532829034096665,0.453003849680651,-1.17621303764259,0.239509755868333,0.83716237845084
"4838","TTC39C",285.753421811952,-0.24082436637068,0.204717462608094,-1.17637432245684,0.239445327948845,0.83716237845084
"4839","OPHN1",210.920624348722,0.246341047803045,0.209472588269944,1.17600612966881,0.239592426621931,0.83716237845084
"4840","BANK1",23.1935746056157,-0.766424471284975,0.651960066772422,-

1.17556965579076,0.239766887163881,0.837278344512329
"4841","AC012531.1",3.21721952687933,1.06060301118459,0.90231250992965
2,1.17542758136787,0.239823694255572,0.837278344512329
"4842","FAM216A",59.6410918842578,0.313260513520391,0.26649635581244,1
.17547766296986,0.239803668525932,0.837278344512329
"4843","STX10",438.883041614755,-0.271334274545911,0.230828026005336,-
1.17548236772444,0.23980178733392,0.837278344512329
"4844","PHYHIP",21.4102411289261,-0.486650659863402,0.41420594538801,-
1.17490022845406,0.240034634214928,0.837495995729673
"4845","TEN1",178.687214284719,-0.319498856070374,0.271929739284938,-1
.17493164561744,0.240022063759341,0.837495995729673
"4846","PTPRA",1312.06949932727,0.20099282258928,0.171050409556013,1.1
7505022707041,0.239974621807906,0.837495995729673
"4847","BTBD8",104.658310638012,0.310129563171883,0.264289684059873,1.
17344558594888,0.240617165633381,0.837643997863448
"4848","PRKCD",57.8631518192365,-0.305998170093933,0.260688656633032,-
1.17380700045066,0.240472339373628,0.837643997863448
"4849","CARD6",323.518546751489,0.252001056154629,0.214584585675047,1.
17436700013599,0.240248057251766,0.837643997863448
"4850","DPYSL3",706.635925527554,-0.288132123132862,0.245378206921601,
-1.17423681078948,0.240300185451677,0.837643997863448
"4851","CORO1B",451.520408784803,-0.269153350281646,0.229302271810592,
-1.17379277647965,0.240478038051521,0.837643997863448
"4852","ABCD2",13.392502715418,1.02478745513336,0.872864463826318,1.17
40510670363,0.240374571597023,0.837643997863448
"4853","RHEBL1",9.45009346231641,0.66713800158003,0.568189720723796,1.
1741465521942,0.240336329938417,0.837643997863448
"4854","TINF2",270.255889908191,-0.227134567095832,0.193463203974004,-
1.17404530903123,0.240376877806242,0.837643997863448
"4855","AC087393.2",3.71881906489023,1.04156999247617,0.88781292395605
1,1.17318633731416,0.240721089757422,0.837643997863448
"4856","CUEDC1",256.104096471047,-0.212171459844152,0.180827822133817,
-1.17333415478034,0.240661830795704,0.837643997863448
"4857","ZSCAN30",102.891871872686,0.298838704535095,0.254701758429794,
1.17328873729573,0.24068003724571,0.837643997863448
"4858","AC027307.3",9.99265977985634,0.581889587584903,0.4953675125390
81,1.17466239278054,0.240129810715838,0.837643997863448
"4859","CXorf40B",60.1559038149533,-0.438854668309446,0.37401942142285
1,-1.17334727335802,0.24065657214854,0.837643997863448
"4860","FOXM1",2.29418466016238,-1.12330810454756,0.957585750485288,-1
.17306267765397,0.240770671923229,0.837644140098345
"4861","EARS2",27.6022102669722,0.408131122204892,0.347956825608758,1.
17293610059483,0.240821431289099,0.837648376925751
"4862","AC082651.1",10.3647164607303,0.592109396166682,0.5048967366058
39,1.17273365668221,0.240902630107485,0.837758467679424
"4863","AL451085.2",41.6560243439765,-0.411010230557833,0.350629040693
031,-1.17220818260078,0.241113484010386,0.837761343476003
"4864","ZC3H11B",5.40812047228053,0.797771563269723,0.680459806152307,
1.17240071501175,0.241036212579805,0.837761343476003
"4865","SLC5A6",33.9782869746583,-0.313650766869518,0.267706757447187,

-1.17162065634968,0.241349391061723,0.837761343476003
"4866","CDK15",18.8716252161786,-0.49577184919535,0.423026447556466,-1
.17196419292242,0.241211432483429,0.837761343476003
"4867","NPM1",4581.922733251,-0.302314023834925,0.257957470319615,-1.1
7195297139622,0.241215937984469,0.837761343476003
"4868","AC132192.2",13.8909759980368,-0.683142929781302,0.582797708798
235,-1.17217847542672,0.24112540831372,0.837761343476003
"4869","VASH1",35.961048654777,0.342044755839822,0.291715647120765,1.1
7252797104237,0.240985148905184,0.837761343476003
"4870","GID4",121.990816190987,0.19520942998134,0.166589310196554,1.17
180045797067,0.241277178792125,0.837761343476003
"4871","GPRASP2",50.4739056546573,0.287667463911799,0.2455284829532,1.
17162563158357,0.24134739269415,0.837761343476003
"4872","TMEM176B",3410.48663141988,0.394834750802012,0.33705798952984,
1.171414899118,0.241432046339824,0.837876239637469
"4873","UBE4B",163.539034895106,0.281383318338989,0.240393429206597,1.
17051168689459,0.2417951138115,0.838060567779905
"4874","GSTM3",1125.05419482924,0.355552666922013,0.303737562247805,1.
17059169202107,0.24176293836173,0.838060567779905
"4875","PRUNE1",67.9445110756914,0.268390416303174,0.229311418409821,1
.17041889219625,0.241832436583831,0.838060567779905
"4876","FYCO1",156.911433224238,0.278478183905104,0.238005356058543,1.
17005007163202,0.24198081925133,0.838060567779905
"4877","SMARCAD1",154.88250923011,0.204687648135785,0.174918298457717,
1.1701900255179,0.241924505927471,0.838060567779905
"4878","SPDYE16",5.70227233694289,0.838205235307671,0.715725062955662,
1.17112740448995,0.24154756993174,0.838060567779905
"4879","MDP1",273.75471471078,-0.357457952090032,0.305382373533538,-1.
17052581638532,0.241789431172554,0.838060567779905
"4880","PNN",974.328319516113,0.162030981584956,0.138474529044083,1.17
011397477564,0.241955105363386,0.838060567779905
"4881","CNTNAP4",28.9604474971462,-0.507438612209907,0.43354469312042,
-1.17044129535444,0.241823425482625,0.838060567779905
"4882","KIAA1328",157.33707112696,0.208654102237343,0.178259560857607,
1.17050721562146,0.241796912100474,0.838060567779905
"4883","PIGN",90.5362320174178,0.35911022306077,0.307038886369148,1.16
959199307744,0.242165201070672,0.838184077728336
"4884","SLC7A10",17.5357414434911,-0.497102731155012,0.425004991761568
,-1.16963974727594,0.242145974850234,0.838184077728336
"4885","LINC01426",34.8388062287176,0.703517777627556,0.60141560767422
,1.16976973768303,0.242093645117582,0.838184077728336
"4886","RHBDL2",5.75013102548085,1.28979800624504,1.10338390299777,1.1
6894763711959,0.242424728917512,0.838395851204192
"4887","CCT5",577.088499412916,-0.274863353515938,0.235112999276648,-1
.16906914701266,0.242375773304969,0.838395851204192
"4888","RPL41",49529.6386383657,-0.289741273074405,0.247820381901814,-
1.1691583672452,0.242339831431957,0.838395851204192
"4889","MGAT4C",7.39191207646185,1.06903428986544,0.914390948673868,1.
16912168850299,0.242354606804847,0.838395851204192
"4890","PIK3R3",7.64942538550305,0.732514590800311,0.626730744330626,1

.16878675161,0.242489559431032,0.838448562548033
"4891","THBS3",320.942174931837,-0.226396837148258,0.193804043470242,-1.16817396115381,0.242736601471297,0.838616766995645
"4892","SLC25A44",61.1481929054862,0.445177611194956,0.381084375737586,1.16818646876644,0.242731557349274,0.838616766995645
"4893","SRPRB",238.53723116901,-0.233553201667987,0.199877783071748,-1.16848004855122,0.242613182439599,0.838616766995645
"4894","FAM27C",8.26547868787203,-0.64266200364522,0.550027555233293,-1.16841783203505,0.242638265498706,0.838616766995645
"4895","DCLRE1B",21.7605034208411,0.467717500316291,0.400466109338788,1.16793278983967,0.242833876453142,0.838702006958653
"4896","INPP4A",62.202897052587,0.316294436753603,0.271047429023623,1.16693391224174,0.243237059469777,0.838702006958653
"4897","ZXDC",83.1425635600492,0.276294088060571,0.236748451894545,1.16703651428158,0.243195623919383,0.838702006958653
"4898","LINC02615",10.8473837518958,0.836381539271222,0.716706030429882,1.16697991053537,0.243218482572412,0.838702006958653
"4899","EPB41L4A-AS1",1350.40223773698,-0.295118958304623,0.252938663630718,-1.16676096120871,0.243306916497054,0.838702006958653
"4900","MTFR1",89.7041704918724,-0.246651592813175,0.211351376439446,-1.16702146429523,0.243201701504306,0.838702006958653
"4901","USP20",47.4616936071268,0.334770201520117,0.286797581009723,1.16726996211578,0.24310136514193,0.838702006958653
"4902","DDX23",225.063274887071,0.227866946983096,0.195264741369153,1.16696411950946,0.24322485983283,0.838702006958653
"4903","CCDC65",4.22886110246806,0.84281368712314,0.721915503920568,1.16746860615405,0.243021179249403,0.838702006958653
"4904","CNOT1",277.88522477746,0.190296574096957,0.163027977281572,1.16726329596968,0.243104056362551,0.838702006958653
"4905","PLEKHA4",262.199079557791,-0.308329234931159,0.264253143527118,-1.16679495583566,0.243293184540298,0.838702006958653
"4906","SSU72",824.590568879485,-0.259740018220459,0.223099001160297,-1.16423658048489,0.244328149603322,0.839152995709419
"4907","RHD",3.17952230064508,1.30512426865628,1.11959602359368,1.1657099892755,0.243731720009239,0.839152995709419
"4908","MRPS15",685.527258328437,-0.292912361801351,0.251389408985333,-1.16517383522088,0.243948634399623,0.839152995709419
"4909","NEK7",356.655972733524,0.24113879539461,0.207127944569042,1.16420213552706,0.244342105022653,0.839152995709419
"4910","MEMO1",44.3583333998404,0.393868135311937,0.338038504600091,1.1651576076456,0.243955201780089,0.839152995709419
"4911","ANKRD53",18.6866176609932,-0.391857889420002,0.336526791449394,-1.16441810689812,0.244254613187509,0.839152995709419
"4912","AMMECR1L",89.0230964352087,0.272159821439752,0.233352387376473,1.166303994142,0.243491558893815,0.839152995709419
"4913","KALRN",24.6763494802518,-0.547492305053109,0.46984797269788,-1.1652541606371,0.243916128132556,0.839152995709419
"4914","AL031118.1",3.15008672870963,0.992670741754841,0.852131140083474,1.1649271984798,0.244048462914736,0.839152995709419

"4915","AL360178.1",12.7140982950523,0.798613371885232,0.684902537212753,1.16602484075359,0.24360440221679,0.839152995709419
"4916","C8orf89",10.6870999436413,0.609988193637619,0.523518439009521,1.16517040888129,0.243950021045963,0.839152995709419
"4917","RFLNA",3.63823956165753,0.906595878735765,0.777451647650689,1.16611223537223,0.24356907038038,0.839152995709419
"4918","GAN",19.2246556287156,0.476944792077625,0.409438472359738,1.16487537023286,0.244069444527147,0.839152995709419
"4919","AC010538.1",6.2978649281788,-0.679406897277776,0.583283699039677,-1.1647966476628,0.244101316183009,0.839152995709419
"4920","TP53",144.966056644688,-0.250643795119648,0.215309963301016,-1.16410681269419,0.244380728109367,0.839152995709419
"4921","NSRP1",618.012179613299,-0.208584941389062,0.178966364242768,-1.16549801004013,0.243817465253975,0.839152995709419
"4922","SLC25A39",201.356292817426,-0.318567092082143,0.273338229144221,-1.16546848598356,0.243829409366181,0.839152995709419
"4923","QTRT1",235.728623711445,-0.242634511759496,0.208372433712082,-1.16442711464778,0.244250964549611,0.839152995709419
"4924","ZNF526",47.7566612300279,0.431419493145219,0.370462809048518,1.16454197994465,0.244204441093059,0.839152995709419
"4925","SP140L",96.1193731764483,0.237441363670813,0.204141907335495,1.16311915946094,0.244781159614586,0.839332757476109
"4926","ENDOG",260.22975938455,-0.303265654976632,0.260640446240613,-1.16354026917476,0.244610369732626,0.839332757476109
"4927","ANXA7",847.187541747479,-0.224367578806088,0.192961512802219,-1.16275818710055,0.244927626133389,0.839332757476109
"4928","PTDSS2",134.538423529978,-0.254454332549394,0.218819358032002,-1.16285110621785,0.244889917814412,0.839332757476109
"4929","TSPAN8",149.686275710811,0.614684521073901,0.528446051831619,1.16319256988178,0.244751380456268,0.839332757476109
"4930","CHRNA5",4.14407298138399,-1.08391900426775,0.931527837607128,-1.1635927135061,0.244589105692073,0.839332757476109
"4931","LRRC36",20.9603430974591,-0.579007980885256,0.497556372611462,-1.16370327616605,0.2445442812874,0.839332757476109
"4932","SLC39A3",230.622971477571,-0.310594634615244,0.267119909508272,-1.16275359327203,0.244929490500776,0.839332757476109
"4933","AL354993.1",2.749158128386,1.44396723309129,1.24109491153169,1.16346237477457,0.244641955134568,0.839332757476109
"4934","CCDC117",150.925893980665,0.216852590435263,0.186404134933999,1.16334645962679,0.24468896286979,0.839332757476109
"4935","RIOK2",157.93904011009,-0.260868333899655,0.22448701245291,-1.16206425952761,0.245209363784204,0.839951362006346
"4936","LUM",22294.2264140039,-0.325244429442877,0.279877864452306,-1.16209415160198,0.245197222786304,0.839951362006346
"4937","NRAS",130.203679984914,0.210843088859761,0.181844669591283,1.15946807422871,0.246265442657105,0.840137296358603
"4938","IER5",727.789379214726,0.2479705111218,0.213736684856085,1.16016822890635,0.245980319601611,0.840137296358603
"4939","AC104463.2",4.2439388396411,1.01188041714021,0.873155154924179,1.15887813458316,0.246505862741603,0.840137296358603

"4940","ZIC1",66.8112560974403,-0.548439373600354,0.473168540953112,-1.15907826943783,0.246424282685288,0.840137296358603
"4941","RTP4",64.766022916396,0.464765279756111,0.400732616741119,1.15978899730131,0.246134724841103,0.840137296358603
"4942","AC105285.1",9.87260279846472,0.628129413596145,0.541496412628577,1.15998813463422,0.246053637033902,0.840137296358603
"4943","MIR3945HG",3.40882823257516,1.06779795591914,0.919294351978359,1.16154086405643,0.245422015011349,0.840137296358603
"4944","CCL28",33.9141379016742,-0.450302717133984,0.388546134912919,-1.1589427269297 2,0.246479531190453,0.840137296358603
"4945","ZBED3-AS1",16.3182697503846,0.582364304261413,0.501732788062192,1.16070609319881,0.245761443082091,0.840137296358603
"4946","MGAT4B",249.060917128164,-0.228893280713495,0.197409409327794,-1.15948516077784,0.246258481768167,0.840137296358603
"4947","TMEM170B",32.557541649265,0.424384487609406,0.365866727726207,1.15994283013073,0.24607208317381,0.840137296358603
"4948","WDR46",293.345506793641,-0.26187806599701,0.225712766426256,-1.16022708924871,0.245956360532932,0.840137296358603
"4949","CGAS",64.6667869196563,0.349714417520218,0.30138737737211,1.16034858715546,0.245906910053759,0.840137296358603
"4950","PIP4P2",718.82210091169,-0.228917644141486,0.19713703571077,-1.16121074518612,0.245556206066854,0.840137296358603
"4951","GLIS3",149.421544635668,-0.31546186572551,0.272037691656953,-1.1596255791029,0.246201281972672,0.840137296358603
"4952","RABGAP1",233.650026124717,0.199335317310235,0.17159842739207,1.16163836895074,0.245382389782655,0.840137296358603
"4953","ATP5MC2",3094.51872069189,-0.308787595050712,0.266407602416105,-1.15907951668892,0.246423774333354,0.840137296358603
"4954","AC020661.4",2.163136093508,1.3102552546221,1.13011077182357,1.1594042701742,0.246291437008684,0.840137296358603
"4955","AC015712.2",38.6414853128121,0.40145855499826,0.345837593629645,1.16082971427386,0.245711156482845,0.840137296358603
"4956","AC007216.1",5.09121539625754,-0.867216876595665,0.747161936704513,-1.16068128473016,0.245771535544008,0.840137296358603
"4957","GGA2",166.662290065195,0.173598163596928,0.1496978606456,1.15965694398205,0.246188506674344,0.840137296358603
"4958","GLOD4",337.686870105238,-0.215780622395972,0.185821150417187,-1.16122745936899,0.245549410629714,0.840137296358603
"4959","DRC3",32.5393082122011,-0.385984185600403,0.333040646510605,-1.15897020272002,0.24646833107745,0.840137296358603
"4960","AC011451.1",3.19294575711801,-0.965166214151286,0.830747376991328,-1.16180470848644,0.245314801051098,0.840137296358603
"4961","RAB4B",73.9716044849783,-0.344266138293011,0.296581163798009,-1.16078220843276,0.245730480062257,0.840137296358603
"4962","DIPK2A",200.095970094076,0.318338398358493,0.274734720494279,1.15871193049705,0.246573626136554,0.840156265984065
"4963","AL133453.1",67.0537559606681,-0.47908877928793,0.413499223646217,-1.15862074676549,0.246610808379401,0.840156265984065
"4964","LYPLA2",105.268546422651,-0.276198034573656,0.238422730182495,

-1.1584383517555,0.24668519588627,0.840185267440166
"4965","POLR3C",139.184740774079,0.202852527338505,0.175152247067146,1
.15814972822324,0.24680293947572,0.840185267440166
"4966","CRMP1",2.07608261844619,-1.30392005113292,1.12584453656376,-1.
1581706077401,0.246794420383539,0.840185267440166
"4967","GPR137C",3.60230662937819,0.917486240083967,0.792225417167599,
1.1581126030571,0.246818087495582,0.840185267440166
"4968","AC140912.1",5.37532802989841,0.790248400001127,0.6825027955522
46,1.15786837087121,0.246917756727092,0.840355360455247
"4969","DIRC1",4.35838536487422,-1.04783863499846,0.90523746880788,-1.
15752901432414,0.24705629225799,0.840465621439385
"4970","ZNF451",322.871888392028,0.254824419703304,0.220140156253246,1
.15755536854511,0.247045531724485,0.840465621439385
"4971","ST5",361.278112287522,0.248106421222863,0.214360922665916,1.15
742374187081,0.247099278695007,0.840465621439385
"4972","PBRM1",446.866733838386,0.200227301775322,0.173035752977978,1.
15714410651771,0.247213489059663,0.840537441816912
"4973","SCAMP2",356.333840697829,-0.200754049978914,0.173493290261394,
-1.15712861100535,0.247219818911492,0.840537441816912
"4974","SMIM14",1077.43341104747,0.178013944760114,0.153858891362922,1
.15699484887237,0.247274464900606,0.840554212412433
"4975","MAPK1",380.186860749472,0.19742675674015,0.170671943483708,1.1
5676163703495,0.247369759413099,0.840709124051593
"4976","RAB5B",311.699309670006,0.194702445556676,0.168394034281857,1.
1562312547889,0.24758657878127,0.841276903945681
"4977","RAPGEF1",193.565877760894,0.249545649185791,0.215883970213601,
1.15592486528242,0.247711890902854,0.841533584767021
"4978","ANO7",9.02202921072545,-0.667618770608976,0.577662567209851,-1
.15572448087405,0.247793871358585,0.841642984518071
"4979","ECE1",461.561227487386,-0.295366374117439,0.255763749258433,-1
.15484064873865,0.248155687761354,0.842026162686929
"4980","HPGDS",6.28385033221311,-0.939826917380373,0.813609872085774,-
1.15513214579246,0.248036316108636,0.842026162686929
"4981","NCOA4",427.874147886392,0.141588021836049,0.122602550599927,1.
15485380314865,0.248150300001685,0.842026162686929
"4982","TUBGCP3",119.516193252435,0.215767742016979,0.186775145927761,
1.15522727044439,0.247997370083158,0.842026162686929
"4983","BLOC1S3",31.3548414263162,0.340139123139877,0.294507503008071,
1.15494213106875,0.248114124930373,0.842026162686929
"4984","MTHFD2",103.148647697813,-0.515950497674774,0.446962882691674,
-1.15434752561028,0.248357719295289,0.842453743317416
"4985","AC017104.1",3.30537607429893,1.23323227033363,1.06840678116672
,1.15427222297009,0.248388580756668,0.842453743317416
"4986","AC130324.1",2.96053751039705,1.006860221559,0.872368636615464,
1.1541682945702,0.248431178387783,0.842453743317416
"4987","COL18A1",700.794956030929,-0.416754864265859,0.36112904754321,
-1.15403307239081,0.248486610199778,0.842472750202096
"4988","LRRC4B",6.73567029968638,0.759138071038205,0.657910656508413,1
.15386194695039,0.248556772285629,0.842541681195955
"4989","STK32A",43.7627963831386,-0.642950190211895,0.557309233114309,

-1.15366864930447,0.24863604172497,0.842641449886909
"4990","ZC3HAV1",983.36474916631,0.516526103311035,0.447953800626475,1
.15307896168904,0.248877975980007,0.84311508874326
"4991","AC068790.9",5.80479836232727,0.808190679927945,0.7008609098465
24,1.15313990061869,0.24885296661946,0.84311508874326
"4992","XAF1",321.968973218336,0.319837316387427,0.277410184597291,1.1
5294006545479,0.248934985616601,0.84311508874326
"4993","CLIC6",76.2494792861949,0.344687642640331,0.298989500173452,1.
15284196415047,0.248975256570564,0.84311508874326
"4994","AL133245.1",9.71889206903324,0.880112528105076,0.7636964175767
98,1.15243768053498,0.249141264577174,0.843508310266491
"4995","ADSS",277.999662548217,-0.236638645708348,0.205383535731757,-1
.15217923805447,0.249247427445891,0.843613371843299
"4996","TBC1D31",22.9270267194175,0.38600887067025,0.335042470143892,1
.15211922388367,0.249272084559328,0.843613371843299
"4997","POLR1D",1106.03651443066,-0.239511913687581,0.207966432994573,
-1.15168544384194,0.249450355894738,0.843720166968934
"4998","LPAR6",185.610308183868,0.258676618846139,0.224589841910506,1.
15177345798754,0.249414177365408,0.843720166968934
"4999","GRB2",362.809282853137,-0.176844809213356,0.153570201343183,-1
.15155679726017,0.249503243130155,0.843720166968934
"5000","ATG4D",44.9018246555037,-0.293540075628241,0.254893165690652,-
1.15162003199604,0.249477246043569,0.843720166968934
"5001","AC011462.5",11.1889088457358,0.772999387005006,0.6713403626633
18,1.15142695120906,0.249556631418681,0.84373195841373
"5002","FO704657.1",2.31265569691298,-1.20904635548104,1.0512880642894
1,-1.15006190648446,0.250118374601074,0.844557626695398
"5003","FBXL2",32.6513030102832,0.393974280496198,0.342507630080003,1.
15026424492842,0.250035052581233,0.844557626695398
"5004","CTDSPL",577.196083636428,-0.166644111742668,0.144837527223344,
-1.15055893964344,0.24991373336908,0.844557626695398
"5005","SLC2A9",4.82325208736739,-0.772608710942627,0.671729956506837,
-1.15017754301205,0.250070753648436,0.844557626695398
"5006","AC004951.1",34.2398605203083,0.506072370505634,0.4400690862642
83,1.14998391457498,0.250150496480397,0.844557626695398
"5007","ITPK1",93.6695037494925,-0.259614726271767,0.22570044827415,-1
.15026234221929,0.250035836017594,0.844557626695398
"5008","EDEM2",107.751846368055,0.348106648940235,0.30255280558299,1.1
5056493450612,0.249911265845264,0.844557626695398
"5009","NUDT21",368.468170160029,0.164238140529038,0.142839500548209,1
.14980898070003,0.250222555519655,0.844632255685031
"5010","Z98885.3",7.77549735260566,-0.714184479201562,0.62139790431938
2,-1.14931909849907,0.250424425709439,0.845144948082874
"5011","RFC5",59.9859073013304,-0.252699925850934,0.219905941090549,-1
.14912732506341,0.250503482487602,0.84524304168836
"5012","STK24",567.995276521045,0.206434484299548,0.179668620008362,1.
14897350628029,0.250566905412994,0.84528835529188
"5013","ZNF669",58.9808539521733,-0.281183473839445,0.244858152252837,
-1.14835251043265,0.250823069881795,0.845347340314425
"5014","CGGBP1",527.910678072288,0.148750926214032,0.129502956133406,1

.14862958078577,0.250708754142406,0.845347340314425
"5015","NECTIN3",329.617596821401,-0.181378473832631,0.157918161919634
,-1.14855993527164,0.25073748558203,0.845347340314425
"5016","MFAP3",19.7673382352588,0.376533726145205,0.327967385100369,1.
14808283765769,0.250934368407742,0.845347340314425
"5017","SLC31A2",263.675809599642,-0.294271132205382,0.256303044525169
,-1.14813748213781,0.250911812920274,0.845347340314425
"5018","TOGARAM1",174.311165926953,0.22373507701989,0.194811147258853,
1.14847163608458,0.250773915679754,0.845347340314425
"5019","NUP50",149.599604757213,0.219296628083376,0.191007119992544,1.
1481070867512,0.250924358985618,0.845347340314425
"5020","FCRL6",2.60019485433956,1.18043821182248,1.03009026481512,1.14
595608961933,0.2518133212363,0.845488523928433
"5021","MPZ",4.739957672079,0.739902235057139,0.645853761357385,1.1456
18837153,0.251952899573598,0.845488523928433
"5022","DNM3",12.830634085922,-0.497277752069588,0.434677208107326,-1.
14401616370648,0.252616933700336,0.845488523928433
"5023","TMSB10",27973.9864898347,-0.292271597172505,0.255104423902524,
-1.14569395818938,0.25192180463724,0.845488523928433
"5024","LINC02035",23.0827949677147,0.37391334656139,0.325984955756447
,1.14702638866793,0.251370714806556,0.845488523928433
"5025","MXD4",877.776663395315,0.231480469453647,0.202481768339815,1.1
4321635647297,0.252948772836183,0.845488523928433
"5026","FAT4",239.768812181405,0.242885743274552,0.212013433046855,1.1
4561487818969,0.251954538386032,0.845488523928433
"5027","FRG1",616.598507481842,-0.263871540225193,0.230068200059148,-1
.14692747697141,0.251411595507942,0.845488523928433
"5028","AC008883.3",6.89360684667229,0.677323088438572,0.5923317054068
4,1.14348612822161,0.252836810912916,0.845488523928433
"5029","FAM174A",190.942864200286,-0.230668132391684,0.201339093765987
,-1.14566986508733,0.251931777230969,0.845488523928433
"5030","LSM2",632.115416935148,-0.280756160584823,0.244767946530905,-1
.14702993003773,0.251369251226613,0.845488523928433
"5031","STMP1",712.765418901691,-0.198192661275454,0.173274451855884,-
1.14380775211048,0.252703374200299,0.845488523928433
"5032","ADCK2",68.0262274443814,-0.302621104790513,0.264341476959849,-
1.14481128073775,0.252287341351373,0.845488523928433
"5033","SCARA3",390.90865235125,-0.372789399114214,0.325871513122663,-
1.14397664141293,0.2526333243277,0.845488523928433
"5034","PTPN20",18.5215940113086,-0.556956601799254,0.486313472361621,
-1.14526253836765,0.252100419185349,0.845488523928433
"5035","PTPRJ",15.8022608218232,0.562357190483975,0.491074670191367,1.
14515617403934,0.25214446924353,0.845488523928433
"5036","C11orf52",22.8734987134773,-0.429960069346364,0.37594133389164
3,-1.14368926900252,0.252752525275457,0.845488523928433
"5037","PIH1D2",21.1259941886952,-0.389029615436416,0.340132968238562,
-1.14375744712744,0.252724241711642,0.845488523928433
"5038","PTS",153.514733599015,-0.225763139966434,0.196814850519692,-1.
14708386775847,0.251346960540142,0.845488523928433
"5039","VDR",41.7241186616638,0.554014696854885,0.48361403742281,1.145

57199333428,0.251972291043664,0.845488523928433
"5040","BAZ2A",288.898568508776,0.293431201285114,0.25637693795317,1.1
4453040756229,0.252403734795551,0.845488523928433
"5041","EPSTI1",451.101339820378,0.342813417061081,0.29987522546553,1.
14318685889737,0.252961017151412,0.845488523928433
"5042","TSC22D1",1646.15664809775,-0.342506709839545,0.299527135266455
,-1.14349142202043,0.252834614201438,0.845488523928433
"5043","THSD1",60.9872114908688,0.414378263489832,0.361809251085549,1.
14529482661529,0.252087048290332,0.845488523928433
"5044","AL589743.4",2.09387535238325,1.75973019581821,1.53844113609835
,1.14383979635455,0.252690082234076,0.845488523928433
"5045","ADCY4",6.39622659652703,-0.831330573872336,0.726016410809488,-
1.14505755172314,0.252185317794567,0.845488523928433
"5046","KHNYN",135.440985425954,0.257926630322057,0.225077546804018,1.
1459456262274,0.251817650899022,0.845488523928433
"5047","SENP8",13.0258106453078,0.478144943715284,0.418257731706454,1.
14318255819085,0.252962802390459,0.845488523928433
"5048","STUB1",1244.57451805125,-0.248989246939374,0.217423578769154,-
1.1451805197436,0.252134386163307,0.845488523928433
"5049","HSD11B2",54.9564270358016,-0.592781068517707,0.51851655228939,
-1.14322496726559,0.252945198611506,0.845488523928433
"5050","GALNS",92.8087742173835,0.280640550958802,0.245263021753717,1.
14424322489433,0.252522781577665,0.845488523928433
"5051","AC025682.1",5.60370966402319,-0.767806926619551,0.671362628553
619,-1.14365455264275,0.252766928137882,0.845488523928433
"5052","ELOF1",349.627513414467,-0.30863603407293,0.269649487347734,-1
.14458231353846,0.252382222214105,0.845488523928433
"5053","CD37",31.3036944538313,-0.425243897869815,0.37149376830052,-1.
14468649047651,0.252339049638434,0.845488523928433
"5054","LINC02246",17.1748345757642,0.59149858788138,0.515706406049388
,1.14696769507403,0.251394972604448,0.845488523928433
"5055","RGL4",3.21569706193879,0.935980350416466,0.815737556813585,1.1
4740377293963,0.25121478259288,0.845488523928433
"5056","TOM1",212.44116113525,-0.183158028028118,0.159869778024961,-1.
14567012158809,0.251931671058974,0.845488523928433
"5057","PIR",130.196811230989,0.330297995954701,0.288491251247897,1.14
491512143251,0.252244319389933,0.845488523928433
"5058","RPL39",29574.9884452476,-0.266819183489845,0.233045027804158,-
1.14492545069045,0.25224004018229,0.845488523928433
"5059","ATP11C",181.356427056318,0.230217814006425,0.201389053186483,1
.14314959211436,0.25297648702117,0.845488523928433
"5060","GNB1",937.963747872545,0.205392460063028,0.1797860361195,1.142
42721234761,0.253276485373138,0.845949206113594
"5061","DTWD2",28.0257065051592,-0.403838533551762,0.353505357227644,-
1.14238306519272,0.253294827352373,0.845949206113594
"5062","BMT2",72.476264542889,0.268535030806067,0.235145620583774,1.14
199460801949,0.253456260951556,0.845949206113594
"5063","PRRT4",4.39867667842837,0.893917202746174,0.783008877358849,1.
14164376496142,0.253602124577606,0.845949206113594
"5064","TRIM22",489.774016493412,0.240488316953511,0.210700890275154,1

.14137304611982,0.25371471635924,0.845949206113594
"5065","AL139317.3",3.50274178346407,0.854833153755854,0.748799777139981,1.14160444467661,0.253618475697465,0.845949206113594
"5066","DCTN5",139.989140148832,0.238347548891936,0.208770841085064,1.14167068376575,0.253590930967084,0.845949206113594
"5067","AC015726.1",9.90102044962826,0.560163491477912,0.49044491253507,1.14215374073812,0.253390120502287,0.845949206113594
"5068","DHX58",63.3619004838653,0.287196297206444,0.251615669554261,1.14140863212221,0.253699914178862,0.845949206113594
"5069","KANSL1",462.699112856943,0.238528563420099,0.208817219854364,1.14228397249258,0.253336001125967,0.845949206113594
"5070","ARRDC2",79.1166389350369,0.312108352509481,0.273385737559765,1.14164094767837,0.253603296104548,0.845949206113594
"5071","TSIX",76.6963652295445,0.68575392522823,0.600614275293117,1.14175428962884,0.253556167445853,0.845949206113594
"5072","LINC02019",5.00198504034996,-0.862470913007593,0.75608749506562,-1.14070252270571,0.253993736414746,0.846398859196947
"5073","OXCT1-AS1",5.64192778643022,-0.730974176334652,0.640706043231714,-1.14088853079585,0.253916312818205,0.846398859196947
"5074","AVEN",166.259492875101,-0.234692976849052,0.205746849408539,-1.14068807140291,0.253999752280891,0.846398859196947
"5075","TMSB15A",14.7853304719403,-0.761299319122387,0.667735815101869,-1.14012054154418,0.254236085090721,0.847019453539685
"5076","ZRANB1",230.734727725283,0.24163915357313,0.211969145565497,1.13997323963579,0.254297450080314,0.847056990929462
"5077","CXCL1",25.5040869789186,-1.29771231162251,1.13894529112658,-1.13939828517916,0.25453707087814,0.847241877293875
"5078","RMDN1",198.245250176976,-0.225505275189603,0.197938895454223,-1.13926711913857,0.25459175826802,0.847241877293875
"5079","AC025159.1",55.5413485992421,0.38281355253915,0.33589946174157,1.13966706154973,0.254425034795134,0.847241877293875
"5080","MAFG-DT",14.9776760217495,-0.461888331857764,0.405346186246495,-1.13949100183931,0.254498419216067,0.847241877293875
"5081","CDR1",24.9551399948456,0.428715180916234,0.376317169779595,1.13923895943235,0.254603500031357,0.847241877293875
"5082","SRSF11",1685.17196873883,0.208176786431032,0.182826151486447,1.13865978547639,0.254845081966021,0.847554832850207
"5083","TCTEX1D2",134.65322621031,0.324085019498333,0.284765430452321,1.13807711485048,0.255088283230627,0.847554832850207
"5084","TBC1D30",2.56043439512633,1.43963281056676,1.26438234569948,1.13860559304972,0.254867694573782,0.847554832850207
"5085","KLF13",572.509402143668,-0.31684753656255,0.278368528154632,-1.13823045537153,0.255024264692191,0.847554832850207
"5086","CCDC113",36.2911694284623,-0.338644987030057,0.297443958724556,-1.13851694444282,0.254904687550785,0.847554832850207
"5087","HSBP1L1",89.0646579325021,-0.45699440261649,0.401643009985108,-1.13781241364921,0.255198820324131,0.847554832850207
"5088","MCM3AP",141.097529919358,0.275223355468061,0.241869208485153,1

.13790158404953,0.255161579762933,0.847554832850207
"5089","NIPSNAP1",89.4041848540632,-0.26459397502188,0.232432760643527
,-1.13836781996355,0.254966925473715,0.847554832850207
"5090","MORC2",73.5611052174852,0.234697189774101,0.206259998282192,1.
13787060859471,0.255174515727383,0.847554832850207
"5091","ASMTL",244.946678809814,-0.220140982143759,0.193300205098774,-
1.13885539868553,0.25476347101083,0.847554832850207
"5092","RBP7",15.2481009458466,-0.669014018988394,0.593158594110204,-1
.12788388405968,0.259368960858161,0.848075849193036
"5093","CENPS",57.0237834870951,-0.331614457106593,0.296836120019916,-
1.11716342702614,0.263924454973981,0.848075849193036
"5094","PIGV",121.104624633627,-0.262935087155778,0.235147025727923,-1
.11817313590012,0.263493055381658,0.848075849193036
"5095","FAM76A",268.351183591077,-0.205084350109808,0.184873246933318,
-1.10932410996049,0.267290387795747,0.848075849193036
"5096","KPNA6",330.158743793829,0.230004262000538,0.206762510834012,1.
11240795574003,0.265962785518156,0.848075849193036
"5097","SCMH1",111.832215350776,0.250920460100779,0.221176558310047,1.
13448035369569,0.256593110145088,0.848075849193036
"5098","PLPP3",7476.05936707661,0.284665923325773,0.254977260057283,1.
11643651383586,0.264235331132292,0.848075849193036
"5099","IFI44L",170.219397907054,-0.386320680132659,0.340581978591094,
-1.13429571855439,0.256670524498506,0.848075849193036
"5100","AL359504.2",14.1468686775562,-0.51520013062601,0.4612216187611
18,-1.11703378521129,0.263979879897576,0.848075849193036
"5101","ALX3",2.80113190268444,1.14092044992354,1.01080106201488,1.128
72897823167,0.259012180973699,0.848075849193036
"5102","ATP1A1-
AS1",67.2969227851523,0.550336680653647,0.496016618452102,1.1095125852
2559,0.267209118287706,0.848075849193036
"5103","AC245297.2",19.0818405739313,-0.437693191622471,0.386367509344
871,-1.13284161073644,0.257280773773801,0.848075849193036
"5104","SEMA6C",79.566809608205,0.254566185386198,0.224313964660777,1.
13486552551987,0.256431666386097,0.848075849193036
"5105","FLG-
AS1",13.5542524014223,0.461078775880247,0.410054672607767,1.1244324395
7687,0.260829616656568,0.848075849193036
"5106","UBAP2L",253.745186083684,0.202890021145048,0.180025955860454,1
.12700427099699,0.259740675211296,0.848075849193036
"5107","CD247",22.3916208106935,0.662851248043683,0.597021465381535,1.
11026367807409,0.266885419926674,0.848075849193036
"5108","C1orf21",1272.12653236528,0.172831102681688,0.154178809012106,
1.12097832243676,0.262297089818194,0.848075849193036
"5109","SWT1",69.5043624946386,0.267390035356571,0.23576912902455,1.13
411809452258,0.256745014516514,0.848075849193036
"5110","HSD11B1-
AS1",22.9754220926633,-0.566759215272265,0.508729867909749,-1.11406711
306522,0.265250395964413,0.848075849193036
"5111","AL136171.2",3.38950216741791,1.04943351403987,0.94631512312001
8,1.10896834299749,0.267443838886756,0.848075849193036

"5112","YWHAQ",1997.05982044963,-0.190132475308475,0.168428012544289,-
1.12886492238622,0.258954820150754,0.848075849193036
"5113","LDAH",44.8329475749984,0.265157924978467,0.234672329109659,1.1
2990707504574,0.258515382426352,0.848075849193036
"5114","ANXA4",1239.80016209339,-0.242580676929714,0.214485287013297,-
1.13098982362681,0.258059374641128,0.848075849193036
"5115","RPIA",235.550853593401,0.238884396654954,0.213253774840993,1.1
2018836165068,0.262633502439833,0.848075849193036
"5116","ECRG4",58.6658501535374,-1.04528772089777,0.927620873301968,-1
.12684799467368,0.259806754352764,0.848075849193036
"5117","CHCHD5",399.407375735557,-0.292526828025814,0.261888339029563,
-1.11699065758248,0.263998319752074,0.848075849193036
"5118","AC079922.2",23.0087192140337,-0.442728837522155,0.398202047885
728,-1.11181958976064,0.266215727286403,0.848075849193036
"5119","SMPD4",131.748692122664,0.211102376897527,0.188932583037168,1.
11734235304451,0.263847973078477,0.848075849193036
"5120","KLF7-
IT1",3.59022227817009,0.918244866489441,0.824165338794407,1.1141512791
9921,0.265214292759931,0.848075849193036
"5121","CXCR2",6.81730079091514,1.04458167947328,0.933874374752625,1.1
1854624959592,0.263333765191308,0.848075849193036
"5122","TUBA4A",30.776398592806,0.475383114435708,0.420664481789501,1.
1300766644558,0.258443921692958,0.848075849193036
"5123","SRGAP3",20.7700029783958,0.51619412660388,0.461802043849796,1.
11778224778012,0.263660005148556,0.848075849193036
"5124","TTLL3",323.082075685966,0.287709110933825,0.257366791501667,1.
11789523914534,0.263611738620423,0.848075849193036
"5125","RBMS3-
AS2",2.51747146219494,1.20610691697009,1.07598084182336,1.120937167362
77,0.262314608760949,0.848075849193036
"5126","KIF9",70.0024994233912,-0.28088108212541,0.247644351397896,-1.
13421154385271,0.256705822890786,0.848075849193036
"5127","DCAF1",70.3174412187379,0.300132941465586,0.268544560588382,1.
11762807933251,0.263725871127245,0.848075849193036
"5128","ESRG",61.9865520270381,0.348751786619276,0.306730740485525,1.1
3699652687969,0.255539737572186,0.848075849193036
"5129","AC126121.3",2.75203735001646,1.25825714990973,1.12222753565302
,1.121213933837,0.262196810023856,0.848075849193036
"5130","ARL6IP5",3272.45214905833,-0.198509396647223,0.175816746458735
,-1.12906990173325,0.258868346982958,0.848075849193036
"5131","ZNF654",118.164239278373,0.207187835696501,0.184596466579766,1
.12238245690891,0.261699860248601,0.848075849193036
"5132","AC091607.2",33.8403771164933,-0.3793976856566,0.339488936926559,-1.11755537335426,0.263756937520339,0.848075849193036
"5133","AC106707.1",55.9400929291889,-0.430605826391578,0.380658185957
649,-1.13121388762013,0.257965078087737,0.848075849193036
"5134","AC108734.4",94.1861789474974,0.371844442160805,0.3306528702821
,1.12457648362031,0.260768543488893,0.848075849193036
"5135","POLR2H",359.036397746328,-0.227959519260533,0.200680697924532,
-1.1359314653483,0.255985248290786,0.848075849193036

"5136","FYTTD1",331.421591537565,0.156340178269243,0.138260955762523,1
.13076159069645,0.258155450247441,0.848075849193036
"5137","NSD2",54.0127518512575,0.342982844369534,0.302368662955136,1.1
3432007476391,0.256660311428496,0.848075849193036
"5138","C4orf48",97.0386380842437,0.499825358877311,0.443738151095604,
1.1263970827012,0.259997481072123,0.848075849193036
"5139","RGS12",162.63211192317,0.246322391970261,0.218407811378899,1.1
2780944241475,0.259400404762022,0.848075849193036
"5140","HMX1",2.12918476887229,1.43430848071004,1.26830005715133,1.130
89049600106,0.258101183972876,0.848075849193036
"5141","PPAT",69.862807956612,0.250273137181656,0.221415893593043,1.13
033049760037,0.258336988345348,0.848075849193036
"5142","IGFBP7",8461.87751527556,-0.343114971700626,0.308899400206069,
-1.11076606646608,0.266669056284911,0.848075849193036
"5143","USO1",500.459810421024,0.174106008424566,0.15485772567234,1.12
429656104439,0.260887236800405,0.848075849193036
"5144","SLC9B2",131.151507092282,-0.290995503262422,0.257445799991289,
-1.1303175397395,0.258342446414407,0.848075849193036
"5145","SETD7",644.720001427508,0.19157054042055,0.16935727540218,1.13
11621538881,0.257986847927266,0.848075849193036
"5146","PRMT9",43.8558518757825,-0.27391671632271,0.246031986657054,-1
.11333782263249,0.265563368083668,0.848075849193036
"5147","TMEM154",18.6131081475072,0.476993467170024,0.426309194132282,
1.11889087482832,0.263186696379972,0.848075849193036
"5148","AC099343.3",106.427863142422,-0.347233655036516,0.306778491349
917,-1.13187092585463,0.257688703608296,0.848075849193036
"5149","LINC02427",6.04008450979277,0.8782236828213,0.784908995754721,
1.11888599515521,0.263188778384642,0.848075849193036
"5150","LRP2BP",11.660452632961,-0.509825795392603,0.459071066328422,-
1.11055963398022,0.266757945967399,0.848075849193036
"5151","ICE1",254.728987208956,0.223470348061959,0.199338655568149,1.1
21058770187,0.262262847054439,0.848075849193036
"5152","ZNF622",275.05431635397,-0.247469584407792,0.221441102980467,-
1.1175413284932,0.263762939012048,0.848075849193036
"5153","SREK1",674.445091244385,0.169011889439346,0.151656451202893,1.
11443916891629,0.26509082757598,0.848075849193036
"5154","DMGDH",47.8477724479261,0.300201583795054,0.266941951291126,1.
12459500031022,0.260760693323626,0.848075849193036
"5155","GLRX",523.483202107973,-0.343310262062218,0.3090464868241,-1.1
1086932451563,0.266624601088412,0.848075849193036
"5156","AC109454.2",12.9061593121566,0.512232865325268,0.4554655574437
45,1.12463578629331,0.260743402650565,0.848075849193036
"5157","AC011379.2",29.1693050432728,0.489214436776631,0.4316909123546
14,1.1332516455078,0.257108591562709,0.848075849193036
"5158","PCDHB13",25.3972595847109,0.45534173599488,0.404854386193029,1
.12470495942158,0.260714079439438,0.848075849193036
"5159","PCDHGA9",12.978566225637,0.517095631492671,0.461465090948407,1
.12055200195086,0.262478605627342,0.848075849193036
"5160","MRPL22",456.048847737995,-0.270929614492254,0.240895389748999,
-1.12467745760742,0.260725737472341,0.848075849193036

"5161","UBLCP1",212.975404267102,-0.235455769337174,0.210755029257232,
-1.11720118930018,0.26390831222361,0.848075849193036
"5162","NSD1",301.128848677385,0.199598141880122,0.176300733438009,1.1
3214583959915,0.257573125732606,0.848075849193036
"5163","TRIM52",116.84703962543,0.258420843781764,0.232473055022693,1.
11161632799353,0.266303149103866,0.848075849193036
"5164","C6orf62",520.828712234614,0.212064558785015,0.190053690986047,
1.11581394544231,0.264501783202594,0.848075849193036
"5165","CARMIL1",245.305227496769,0.257495608788896,0.231231884944246,
1.11358175733845,0.265458656171368,0.848075849193036
"5166","HIST1H2BK",5.87367164042899,0.850804410594993,0.76205132332442
9,1.11646602342134,0.264222705966947,0.848075849193036
"5167","NELFE",543.424475781751,-0.224858339848748,0.200488000213667,-
1.12155510359278,0.262051649934178,0.848075849193036
"5168","TBCC",125.673236376719,-0.275246106602337,0.244022532786064,-1
.12795365026248,0.259339494242005,0.848075849193036
"5169","AK9",47.3546021143881,-0.355753257498283,0.319249639405464,-1.
11434192427218,0.265132527754502,0.848075849193036
"5170","STX7",385.086190395604,0.175187202089634,0.154643405434412,1.1
3284625100897,0.2572788247784,0.848075849193036
"5171","IFNGR1",1214.50642717692,-0.268262545957931,0.240380487598205,
-1.11599135453261,0.264425835301299,0.848075849193036
"5172","WTAP",822.323125768304,-0.237479193688596,0.213817424059102,-1
.11066343041787,0.266713248747254,0.848075849193036
"5173","TWISTNB",282.694372369512,-0.231653207780426,0.207299560916024
,-1.11748045561113,0.263788951614749,0.848075849193036
"5174","NOD1",50.5695386382607,0.33595460785181,0.300725744183456,1.11
714615176698,0.263931840089862,0.848075849193036
"5175","DTX2",91.3993986009441,0.237632734936775,0.213031079083455,1.1
1548388131519,0.264643121933996,0.848075849193036
"5176","SEMA3D",322.359215342617,0.316218219899307,0.283984637612261,1
.1135046689781,0.265491744205306,0.848075849193036
"5177","AC002456.1",25.0192195990714,-0.437432885160165,0.390085983797
067,-1.12137555136492,0.262128038462795,0.848075849193036
"5178","AC007566.1",22.2087626123036,0.468049897842799,0.4219906160905
38,1.10914764451155,0.267366494140286,0.848075849193036
"5179","SMIM30",335.695298448519,0.292096945484932,0.258503102605823,1
.12995527922284,0.258495068999396,0.848075849193036
"5180","POT1-
AS1",3.98824591062498,1.03565220323641,0.914657054144124,1.13228471648
919,0.257514753472015,0.848075849193036
"5181","FAM66B",23.028787458251,0.358221255974848,0.320338407583949,1.
11825883969587,0.263456460723951,0.848075849193036
"5182","PPP2CB",498.861316938681,0.167320446205879,0.148623755573381,1
.1257988035652,0.260250690719217,0.848075849193036
"5183","MSC",60.4478310617713,-0.258843725016911,0.232143917415699,-1.
11501403051367,0.264844409333181,0.848075849193036
"5184","UBE2W",180.987402878464,0.204868527564926,0.182626974719375,1.
12178678905309,0.261953104621532,0.848075849193036
"5185","AC011029.1",5.25449134587408,-0.841185620466085,0.756317620412

452,-1.11221211533767,0.266046960007126,0.848075849193036
"5186","AC083837.1",4.59794314220667,-0.866190730107898,0.777908051546419,-1.11348729247111,0.265499202979763,0.848075849193036
"5187","RNF19A",771.143869222597,0.198558064631655,0.177418026921258,1.11915383164406,0.263074517700268,0.848075849193036
"5188","RNF139",370.61125423893,0.212452518337152,0.187533608041604,1.13287703764554,0.257265894113519,0.848075849193036
"5189","DENND3",18.337021208744,0.605775322023873,0.539809604288643,1.12220182303381,0.261776637701665,0.848075849193036
"5190","THEM6",42.9934558485129,-0.390996597717238,0.345486719675345,-1.13172685214835,0.257749288727353,0.848075849193036
"5191","ZNF696",42.624618120018,0.278546926870478,0.245463273733282,1.13478046077534,0.256467314979893,0.848075849193036
"5192","LURAP1L",156.379057654275,0.422666151517349,0.380854021355541,1.10978518754506,0.26709160371482,0.848075849193036
"5193","AL391834.1",4.06397375901999,0.827274511939605,0.735999609696366,1.12401487859606,0.261006714050052,0.848075849193036
"5194","LINC01410",4.90636663895544,-0.921901684303382,0.822765137574184,-1.12049191464467,0.26250419616155,0.848075849193036
"5195","TMEFF1",16.9481270512143,-0.465618362657083,0.410799982644175,-1.13344299495843,0.257028267284871,0.848075849193036
"5196","ZBTB43",207.413128533506,0.278084548667232,0.249295978639992,1.11547948019175,0.264645006916978,0.848075849193036
"5197","MIGA2",45.855725483327,0.307797812091161,0.276090131364852,1.11484539693456,0.264916678870596,0.848075849193036
"5198","FNBP1",382.409151494631,0.253003215301739,0.225069958972987,1.12410921677959,0.260966695746943,0.848075849193036
"5199","VAV2",44.7356088286154,0.342863175592146,0.303374712352988,1.13016399070603,0.258407129937723,0.848075849193036
"5200","SNHG7",1513.56591721927,-0.35112858091324,0.311665658070716,-1.12661941353054,0.259903427482119,0.848075849193036
"5201","NOXA1",7.71806706094269,-0.656913368145203,0.581961012765901,-1.12879274338855,0.258985274551062,0.848075849193036
"5202","ARRDC1-AS1",62.5572298632278,-0.319655235055285,0.284053525305465,-1.1253345112036,0.260447310721413,0.848075849193036
"5203","KLF6",5662.5091893969,-0.310589535825141,0.279996666928391,-1.10926154669039,0.267317368493819,0.848075849193036
"5204","SUV39H2",21.7172676285237,-0.452402998703698,0.402652508196839,-1.12355688712744,0.26120105456475,0.848075849193036
"5205","RET",41.4664914958994,-1.07358587414584,0.944020539236479,-1.13724842789348,0.255434447293808,0.848075849193036
"5206","TFAM",348.590564899765,-0.15338156151887,0.137545894380053,-1.11513006048048,0.264794691469115,0.848075849193036
"5207","NRBF2",343.071132027207,-0.245520569716656,0.216341083679586,-1.13487722970033,0.256426761714157,0.848075849193036
"5208","DNAJC12",2.19464785754755,-1.34406338663849,1.18975548819684,-1.12969715203878,0.258603857695646,0.848075849193036
"5209","ECD",112.390515164235,0.215524210218152,0.194076191226052,1.11051339608741,0.266777858765078,0.848075849193036

"5210","AL512662.2",2.60298426719569,-1.71689585675907,1.5246830651354
7,-1.12606737493115,0.260137002172748,0.848075849193036
"5211","BTAF1",132.697963090965,0.283318094861134,0.251430564405665,1.
12682439993262,0.25981673205264,0.848075849193036
"5212","ZDHHC16",94.9591522543374,-0.274887046117942,0.243360787911324
,-1.12954534901533,0.258667850480793,0.848075849193036
"5213","HOGA1",4.28981674368536,-0.777557515373215,0.699239350017214,-
1.11200480258165,0.266136085426767,0.848075849193036
"5214","TRIM6",23.4670713774014,0.425146741363747,0.382107988630002,1.
1126350508610 2,0.265865200345227,0.848075849193036
"5215","ABTB2",9.61224675071891,-0.773743401196632,0.695166103794687,-
1.11303384467829,0.265693894088523,0.848075849193036
"5216","LDLRAD3",48.7123056893891,-0.400182829763016,0.356899708520324
,-1.12127530566539,0.262170693588909,0.848075849193036
"5217","MAPK8IP1",71.1193161432976,-0.251070682844152,0.22249223370891
8,-1.12844695142314,0.259131208571821,0.848075849193036
"5218","CD6",11.0978652434942,0.693154304029833,0.612095192380067,1.13
24289304325,0.25745414767346,0.848075849193036
"5219","PYGM",35.4476287986627,0.436810944654983,0.385643265775583,1.1
3268137530289,0.257348081736157,0.848075849193036
"5220","FZD4",568.223652924601,0.265437217755708,0.233525582626047,1.1
3665155984543,0.255683976775512,0.848075849193036
"5221","RAB38",5.67032218212154,-0.778960698314138,0.695799618014484,-
1.11951872083081,0.262918908846861,0.848075849193036
"5222","PGR",76.5108565171988,0.348205337415596,0.31124638460605,1.118
74500279361,0.263248940347301,0.848075849193036
"5223","BIRC3",55.3731753154376,-0.476430059136569,0.426360399202817,-
1.11743506204462,0.263808350645706,0.848075849193036
"5224","BCO2",22.6335443274857,-0.35883029881198,0.315686581163301,-1.
13666630203189,0.255677811547653,0.848075849193036
"5225","IL10RA",21.6892827389267,-0.535034088627122,0.470820339863562,
-1.13638694705112,0.255794656232126,0.848075849193036
"5226","AP003392.5",4.57228020750135,0.992226712466103,0.8809239773671
4,1.12634771893895,0.260018366837657,0.848075849193036
"5227","TULP3",440.995915977786,0.180130793107287,0.158492919536312,1.
13652265119652,0.255737891171477,0.848075849193036
"5228","ANO2",3.30672522729732,0.865810274737554,0.779733971386715,1.1
103918855783,0.266830193320449,0.848075849193036
"5229","TPI1",1626.67755263812,-0.222250713675227,0.197994257173483,-1
.12251090939719,0.261645271681657,0.848075849193036
"5230","RASSF8-
AS1",284.319951672561,-0.203811805977897,0.181998782792096,-1.11985257
731487,0.262776589723407,0.848075849193036
"5231","PRR13",478.752160763499,-0.19961873377578,0.176969350657493,-1
.12798477834799,0.259326347659283,0.848075849193036
"5232","HMGA2-
AS1",35.8231687987619,0.51644571133628,0.462698670204523,1.11615992133
282,0.264353686667862,0.848075849193036
"5233","OSBPL8",1168.3366012304,0.216951113971266,0.194329597971469,1.
1164079802353,0.264247539134927,0.848075849193036

"5234","DCN",103027.12727739,0.240089486943743,0.21572201353936,1.1129
5775060034,0.265726575264933,0.848075849193036
"5235","AC138123.1",4.0303066231922,1.04180031206352,0.935338169760197
,1.11382208675458,0.265355519685991,0.848075849193036
"5236","ALDH1L2",258.247282374338,0.222440658740818,0.198688268637455,
1.11954601178142,0.262907273036352,0.848075849193036
"5237","COX6A1",1778.88864556323,-0.275248483605705,0.248097924499791,
-1.10943484981043,0.267242635316424,0.848075849193036
"5238","RILPL2",239.588660690498,-0.233760100191628,0.206499605058857,
-1.13201233544733,0.257629248394962,0.848075849193036
"5239","PAN3-
AS1",10.043485372327,-0.623942330031013,0.554994730672734,-1.124231088
23881,0.260915004097037,0.848075849193036
"5240","BRCA2",27.7353517542936,0.39715183003479,0.353059058268992,1.1
2488780767156,0.26063657914239,0.848075849193036
"5241","AL354809.1",2.08760154663013,1.57476954665226,1.41315409932065
,1.11436505573547,0.265122608174963,0.848075849193036
"5242","HTR2A",16.4746273843282,-0.538178643720063,0.476745461775856,-
1.12885950023598,0.258957107826084,0.848075849193036
"5243","KLF12",114.365056364446,0.255073713468991,0.226395377712306,1.
12667368056042,0.259880474237955,0.848075849193036
"5244","PNP",36.8941652951022,-0.590636873901602,0.522222166966262,-1.
13100689948261,0.258052187485266,0.848075849193036
"5245","CHD8",281.039902537415,0.250394884434483,0.225020393556613,1.1
1276529418871,0.265809244535743,0.848075849193036
"5246","COCH",127.024453597161,-0.388433211396968,0.34569598049953,-1.
12362663527555,0.261171451724996,0.848075849193036
"5247","EGLN3",18.1644071088557,-0.698415987635511,0.629547070065019,-
1.10939438978467,0.267260081531365,0.848075849193036
"5248","DAAM1",697.877180033884,-0.250808427785642,0.223197224840741,-
1.12370764450411,0.261137072316057,0.848075849193036
"5249","DHRS7",970.451891401042,-0.200968577378399,0.177518175407749,-
1.1321014139357,0.257591800513574,0.848075849193036
"5250","AC004816.2",65.0775238680923,-0.51492172033269,0.4589734629407
13,-1.12189867587008,0.261905523746899,0.848075849193036
"5251","ZC2HC1C",8.02746493999022,-0.637920963829588,0.564766571093935
,-1.12953031655885,0.258674188031806,0.848075849193036
"5252","STON2",28.6898487010659,0.409965471152744,0.364020370282839,1.
12621574126252,0.260074212182451,0.848075849193036
"5253","GON7",113.976446993013,-0.335431078349411,0.298513592127821,-1
.12367103942718,0.261152606713007,0.848075849193036
"5254","BAG5",268.894347822287,-0.190736458257786,0.171085309425992,-1
.11486169617792,0.264909693080357,0.848075849193036
"5255","AC022306.2",14.5454094656284,0.480179196625243,0.4241243405241
68,1.13216609080204,0.257564613244482,0.848075849193036
"5256","CCNB2",3.38107735166831,1.15354185905636,1.01753820646744,1.13
36595046009,0.256937402318866,0.848075849193036
"5257","TLE3",315.692514925486,-0.244170199538446,0.215692357610844,-1
.13202990705392,0.257621861133628,0.848075849193036
"5258","ETFA",485.500510988053,-0.205193071452969,0.180868597711955,-1

.13448699248364,0.256590326916953,0.848075849193036
"5259","AC048382.5",20.4849962209767,-0.365562873741323,0.329622662770566,-1.10903440518522,0.267415340053153,0.848075849193036
"5260","AC012409.3",3.98153069143439,0.827259769226034,0.741527306387713,1.11561605634722,0.264586516226148,0.848075849193036
"5261","AC087477.2",51.464833232804,0.404330480806604,0.359834094974475,1.12365805923779,0.261158115375986,0.848075849193036
"5262","ABAT",32.3838852674802,0.463179245886302,0.41422578229544,1.1181806292201,0.263489855669578,0.848075849193036
"5263","LINC02185",25.3907739082317,-0.818571886072434,0.724830159165277,-1.12932923074712,0.258758974383428,0.848075849193036
"5264","FUS",2277.94120419685,0.321236394539356,0.285282828364984,1.12602779627652,0.260153753994492,0.848075849193036
"5265","NKD1",6.11700412306142,0.9338182832492,0.836362097517038,1.11652391472723,0.264197939382199,0.848075849193036
"5266","NOD2",2.01712239703325,-1.26526522072821,1.1152805155257,-1.13448159733322,0.256592588764143,0.848075849193036
"5267","SALL1",3.87194046160626,-1.11812441272063,0.992856134603198,-1.12616961687757,0.260093731320836,0.848075849193036
"5268","AC012181.1",6.03323060102151,0.85931650177108,0.767256863817923,1.11998542117312,0.262719974732593,0.848075849193036
"5269","COQ9",184.597125422251,-0.222615973564813,0.197763823986963,-1.1256658021514,0.260307004078641,0.848075849193036
"5270","AC044802.2",2.42148744668917,-1.14710791821989,1.02325059850076,-1.12104299758105,0.262269560455855,0.848075849193036
"5271","ZDHHC1",151.955453537075,-0.26478256273748,0.233781415597891,-1.13260740619739,0.257379157073785,0.848075849193036
"5272","SLC7A6",122.160481210258,0.307407227968352,0.276215898918309,1.11292372804097,0.265741188299636,0.848075849193036
"5273","PRMT7",107.542960870374,0.242445792037709,0.217293814685266,1.1157510046426,0.264528731442325,0.848075849193036
"5274","TERF2",160.447598691385,0.191797218993901,0.17103153656832,1.12141434756557,0.262111531741462,0.848075849193036
"5275","COG4",174.637675709905,0.200195615229343,0.1802260971156,1.1108025887113,0.266653331953735,0.848075849193036
"5276","CDYL2",23.1768098255729,0.385033144838243,0.346663673581095,1.11068212270638,0.266705199949777,0.848075849193036
"5277","PAFAH1B1",670.69303173077,0.147685852313905,0.133022841223122,1.11022927307791,0.266900241545117,0.848075849193036
"5278","AC127024.5",11.0438515991247,0.692586435365866,0.613560978795969,1.12879804828034,0.258983036179773,0.848075849193036
"5279","AC116407.2",29.5813651467621,-0.523505492180788,0.466036804039827,-1.12331362596858,0.26130431877524,0.848075849193036
"5280","ZNF207",603.797574773235,0.199058533003992,0.179283834874735,1.1102982772712,0.266870515217728,0.848075849193036
"5281","IGFBP4",11749.4435803488,-0.331070313562854,0.294608161225216,-1.12376490924759,0.261112771688516,0.848075849193036
"5282","TUBG1",63.4815184990446,-0.33955108997219,0.305799568757648,-1.11037138264015,0.266839024628681,0.848075849193036
"5283","ARL17B",39.18074519635,0.495333002659373,0.444546516085074,1.1

1424335752657,0.265174799484527,0.848075849193036
"5284","NGFR",44.9820461304022,0.601643807178609,0.533229790012897,1.12830119105697,0.259192740646042,0.848075849193036
"5285","ACE",236.61119076587,0.355448363873436,0.315497639860529,1.12662764777121,0.259899944567392,0.848075849193036
"5286","MRPL38",344.973149405535,-0.262774270030369,0.233320597132994,-1.12623691718304,0.26006525119447,0.848075849193036
"5287","BIRC5",2.18721843221002,-1.30646753887112,1.17784238014786,-1.10920405046652,0.267342165656054,0.848075849193036
"5288","RAC3",47.4556763405012,-0.557725916937441,0.500549348735395,-1.11422763479065,0.265181542829505,0.848075849193036
"5289","ZNF397",217.724536545974,0.263808125026953,0.232711671931484,1.13362652950482,0.256951239897967,0.848075849193036
"5290","SMAD7",286.008079971002,-0.328260554807259,0.295781385803694,-1.10980802228414,0.267081761631427,0.848075849193036
"5291","POLI",136.531708253436,0.247541077867514,0.22009929293109,1.12467911446229,0.260725035120519,0.848075849193036
"5292","AC005306.1",5.01946915967663,0.927569846690445,0.818548880002833,1.13318809584986,0.25713527216045,0.848075849193036
"5293","ALKBH7",863.802668131713,-0.32655415466037,0.29215369243199,-1.11774782629655,0.263674710187203,0.848075849193036
"5294","CD70",17.4281320471008,0.939192763241827,0.830134820550357,1.13137377205689,0.257897805888313,0.848075849193036
"5295","RETN",3.77514129098443,-2.65790220673937,2.34833825167014,-1.13182255786579,0.257709041959614,0.848075849193036
"5296","COL5A3",325.883166443906,0.482312704418871,0.434763347067677,1.10936836711718,0.267271302824102,0.848075849193036
"5297","SLC44A2",262.866118884826,0.361888881491238,0.325002382540872,1.11349608781938,0.265495427604629,0.848075849193036
"5298","ZNF44",83.85531481714,0.242458603952076,0.215928808026513,1.12286362421037,0.261495418633502,0.848075849193036
"5299","SSBP4",576.612567466397,-0.266893986586799,0.239300341600106,-1.11530967654365,0.264717740184015,0.848075849193036
"5300","LRP3",123.897239221559,-0.295886885196716,0.26615888499016,-1.11169268389314,0.266270306516241,0.848075849193036
"5301","AC020910.5",65.5885156801723,-0.457113499200212,0.402709442905997,-1.13509505985663,0.256335491222525,0.848075849193036
"5302","PSMD8",1292.38830503595,-0.291486193572461,0.261947266961428,-1.11276669137908,0.265808644311579,0.848075849193036
"5303","PAF1",278.428596373362,0.170877670083729,0.152800893476119,1.11830282007111,0.263437682908247,0.848075849193036
"5304","ZNF546",53.8370343961402,0.287863948468988,0.254116861213279,1.13280144849336,0.257297643044515,0.848075849193036
"5305","RABAC1",2554.15893010363,-0.280145560952373,0.250454419713185,-1.11854908079957,0.263332556744208,0.848075849193036
"5306","DHX34",25.8802982991067,0.356647098079158,0.319624952006962,1.1158299620844,0.264494925944663,0.848075849193036
"5307","RASIP1",4.05236135734274,-0.913306058005311,0.804884082540632,-1.13470508091357,0.256498907747575,0.848075849193036
"5308","HSD17B14",76.7706483289361,-0.55149542045157,0.48807785275149,

-1.12993330335021,0.258504329580022,0.848075849193036
"5309","LENG1",167.403878177618,-0.321644714410079,0.288321549828156,-
1.11557639240557,0.264603501957908,0.848075849193036
"5310","PSMF1",536.797563421757,-0.190967099570006,0.171030283967421,-
1.11656892066192,0.264178686417503,0.848075849193036
"5311","TRMT6",117.780992228936,-0.221495084331726,0.196896154610839,-
1.12493352025847,0.260617206336371,0.848075849193036
"5312","PET117",104.24330880769,0.217961611284876,0.192460735001447,1.
13249911096535,0.257424657938625,0.848075849193036
"5313","AL121761.1",2.04083608557856,-1.18259964301912,1.0609567193036
1,-1.11465399247893,0.264998723549592,0.848075849193036
"5314","COMMD7",365.619596198797,-0.199844564965851,0.178776692142524,
-1.11784462823896,0.263633357330507,0.848075849193036
"5315","CNBD2",35.855436546764,-0.383850163840837,0.338793965624637,-1
.13298996672839,0.25721846674184,0.848075849193036
"5316","LPIN3",155.029587827685,-0.256334032656524,0.229637587674318,-
1.11625468309686,0.264313133444367,0.848075849193036
"5317","OSER1",440.818455035821,-0.276592798278186,0.248761182888758,-
1.1118808612591,0.266189378611942,0.848075849193036
"5318","RAB22A",439.148184818785,-0.152212291846386,0.135087802221484,
-1.12676562460336,0.259841588007202,0.848075849193036
"5319","FNDC11",6.91499180010691,-0.661168840808354,0.59037397844656,-
1.11991528242501,0.26274986520103,0.848075849193036
"5320","AF165147.1",11.573976327552,0.528718832714088,0.47123063640301
5,1.12199588029736,0.261864191538571,0.848075849193036
"5321","LRRC75B",75.1670274435268,0.257244903117591,0.228625674729182,
1.12517941575158,0.260513013931073,0.848075849193036
"5322","MYH9",625.088015041452,0.218368874460586,0.194254100126863,1.1
241403621235,0.260953484808755,0.848075849193036
"5323","H1F0",501.191063399892,-0.192200140328559,0.170865195660242,-1
.12486419241716,0.260646587580173,0.848075849193036
"5324","RTL6",62.0661547022126,0.26983508015184,0.240117168066418,1.12
376421196672,0.261113067574309,0.848075849193036
"5325","CERK",161.282287568512,0.2436155301861,0.216900412159593,1.123
16766833458,0.261366291246431,0.848075849193036
"5326","MED14OS",19.3085303539458,-0.49314883349935,0.444016333819443,
-1.11065471231039,0.266717002773926,0.848075849193036
"5327","NDUFB11",1383.14522160349,-0.276457863499276,0.245586760886985
,-1.12570344794155,0.260291063836844,0.848075849193036
"5328","ARMCX6",273.794081488837,0.328322718043491,0.292351395433742,1
.12304139187152,0.261419915443784,0.848075849193036
"5329","BEX5",25.47846230787,-0.6055146014069,0.534402120749303,-1.133
06923362858,0.257185180263736,0.848075849193036
"5330","SLC25A5-
AS1",9.36871272700677,-0.512035143135313,0.454581045866366,-1.12638911
760927,0.260000851017125,0.848075849193036
"5331","Z82195.3",5.17595463902948,0.921264644895262,0.810404752108255
,1.1367957091794,0.255623697512915,0.848075849193036
"5332","FAM3A",293.410546148414,-0.249100744360763,0.224387470387262,-
1.11013660402182,0.266940166045443,0.848075849193036

"5333","TRIB2",23.6786553283404,-0.530693362961091,0.47865649853552,-1
.10871442168817,0.267553398460699,0.848264177981155
"5334","MAB21L3",6.98211889152628,0.810671919703768,0.731818795389379,
1.107749520525,0.267970006272436,0.849137227232603
"5335","CD160",5.26679395567036,-0.793111738637714,0.715876308293384,-
1.10788935106465,0.267909605133896,0.849137227232603
"5336","THORLNC",12.8535104242801,0.54178494728207,0.489095783234039,1
.10772770049178,0.267979432488359,0.849137227232603
"5337","TMEM251",173.64962589265,-0.308078842336802,0.278166533504098,
-1.10753381600545,0.26806320025187,0.849243505688331
"5338","CUL7",82.5599438587515,0.292516946006485,0.26424196478635,1.10
700412874615,0.268292143263941,0.849809583796687
"5339","CYB561D1",51.1760252617282,0.295253577608197,0.266793233560637
,1.1066756591527,0.268434182828835,0.849812497723699
"5340","CFL1",3632.44672157172,-0.277599533449777,0.250820965172196,-1
.10676367607148,0.268396116752863,0.849812497723699
"5341","AEBP2",223.861520696473,0.183122958168971,0.165474548688954,1.
10665331689885,0.268443846128594,0.849812497723699
"5342","TMEM178A",4.1567432737986,0.926457747853752,0.838020212986065,
1.10553150568118,0.268929350486902,0.849936602973445
"5343","RCAN2",84.2400773172693,-0.269773869038548,0.243898941035821,-
1.1060887263095,0.268688117816157,0.849936602973445
"5344","ZDHHC21",147.06118275324,0.25078973898443,0.22669942486683,1.1
0626543993992,0.2686116457678,0.849936602973445
"5345","RAD23B",661.360804845021,0.197321652174159,0.178487604982977,1
.10552019672726,0.268934247912788,0.849936602973445
"5346","AC110048.2",12.224293670695,0.724705857851976,0.65514988754416
4,1.1061680260203,0.268653799365366,0.849936602973445
"5347","NME1",292.534322665882,-0.405071838398351,0.3663594229142,-1.1
0566785801826,0.268870306921462,0.849936602973445
"5348","RYR1",11.8933443763445,0.97034409566997,0.87722020264625,1.106
15794385811,0.268658162444752,0.849936602973445
"5349","SNRNP70",688.337500843958,0.204433370493214,0.184883655120345,
1.1057406365108,0.268838795874372,0.849936602973445
"5350","CHKB-
DT",23.6968288703639,-0.460161634905401,0.416240974300382,-1.105517388
52438,0.268935464035246,0.849936602973445
"5351","CFAP44",67.8856754297327,0.413358277696899,0.374034605903291,1
.10513377952995,0.269101625520049,0.849989280708899
"5352","AC025175.2",3.74645348645814,-0.960803024808956,0.869564856764
839,-1.10492393676484,0.269192549409038,0.849989280708899
"5353","RESF1",268.228877222457,0.203546065510996,0.184260160279578,1.
10466671255553,0.269304032218924,0.849989280708899
"5354","STAC3",4.41399270246225,-0.803101895355808,0.726965473717451,-
1.10473182618842,0.269275808510431,0.849989280708899
"5355","CBX1",500.963060097666,0.146508975519624,0.132614958851624,1.1
0476960358254,0.269259434711011,0.849989280708899
"5356","AC005253.1",13.0847894582944,-0.533735521381804,0.483054314345
15,-1.10491823700066,0.269195019384324,0.849989280708899
"5357","ZNF831",7.46988977120781,0.684495413899146,0.61932351796212,1.

1052307784976,0.269059603446194,0.849989280708899
"5358","ARHGAP29",559.826874379307,0.319184192928657,0.289139242816884
,1.10391169949491,0.269631443255861,0.850074948328723
"5359","LINC02478",19.42578949605,-0.788701072029135,0.714206602061351
,-1.10430381034392,0.269461370510495,0.850074948328723
"5360","SLC1A3",187.894118537252,0.405906500495287,0.367706099529743,1
.10388840711209,0.269641548326819,0.850074948328723
"5361","PLXDC2",1056.53328488514,0.361707246924672,0.327577405941995,1
.10418862950737,0.269511320993529,0.850074948328723
"5362","FUT11",165.624610877986,0.206409119949108,0.186999910581269,1.
10379261309542,0.269683109938211,0.850074948328723
"5363","NAT10",90.541847600093,0.275056859025491,0.249179831966174,1.1
0384880210863,0.269658730994099,0.850074948328723
"5364","ARPIN",228.618172817379,0.196627075697247,0.178068848483335,1.
10421939251014,0.269497979377731,0.850074948328723
"5365","DHX30",202.681907978543,-0.149071147451076,0.135172919198895,-
1.10281814090092,0.270106148468876,0.850331518435499
"5366","SCAANT1",4.34660581980454,-1.21352308461812,1.10086977053807,-
1.10233119038685,0.270317714193921,0.850331518435499
"5367","LINC00877",3.16992172342017,-1.00632252291188,0.91211273443173
7,-1.10328743906732,0.26990235965403,0.850331518435499
"5368","MLF1",353.744076502287,-0.264711986250651,0.240098276479511,-1
.10251514559806,0.270237777696468,0.850331518435499
"5369","AC016596.1",15.03686319116,0.585221665481061,0.530493672504364
,1.10316427096733,0.269955834164083,0.850331518435499
"5370","EPHB6",115.336155911376,-0.235002065403153,0.213175286512213,-
1.10238888028744,0.270292643691597,0.850331518435499
"5371","CACFD1",31.4637301948312,-0.377651662669334,0.342569925788422,
-1.10240752103434,0.270284543257689,0.850331518435499
"5372","NARS2",78.1306171583587,0.255549737005384,0.23160385856082,1.1
0339153498289,0.269857171165541,0.850331518435499
"5373","PRPF8",462.63991783093,0.191247773267865,0.173414111203037,1.1
0283858643976,0.270097267966257,0.850331518435499
"5374","AATF",268.718128274267,-0.222536681634825,0.201806908960236,-1
.10272082745528,0.270148419226881,0.850331518435499
"5375","TXNL4A",474.423510264144,-0.254653736251556,0.230964790480895,
-1.10256518199739,0.270216037520668,0.850331518435499
"5376","AC107021.2",2.4417588536161,1.38381828131262,1.25582853314325,
1.10191657920769,0.270497940133479,0.850423795421506
"5377","CASP6",145.340292409702,0.230088805000847,0.208791436067925,1.
10200307701314,0.270460333894792,0.850423795421506
"5378","KPNA3",220.559837815815,-0.185267214423746,0.168108826011199,-
1.10206714792835,0.270432480399216,0.850423795421506
"5379","NXN",109.189731707694,0.279458818719894,0.253646992868623,1.10
176279071694,0.270564810874905,0.850475891852184
"5380","MRTFA-
AS1",2.09932813430122,1.76432275190839,1.60218119702342,1.101200510395
58,0.270809399631307,0.851086492372889
"5381","LINC01719",23.4037220846016,0.493488718563122,0.448522964336759,1.10025295871496,0.271221922012958,0.851117345470507

"5382","DUSP22",251.068535680368,0.186562685869794,0.169562830572811,1
.10025696810766,0.271220175593026,0.851117345470507
"5383","C1GALT1",383.669650395078,0.186219015875611,0.169244155642161,
1.1002980585595,0.271202277769345,0.851117345470507
"5384","TMX2",324.581314850449,-0.227998824305748,0.207175141652795,-1
.10051245765697,0.271108904790156,0.851117345470507
"5385","DDX51",107.886725904986,-0.232588472825447,0.211350117910227,-
1.10048896648471,0.271119134359079,0.851117345470507
"5386","RNF111",107.817942126894,0.216997349701484,0.197206839846435,1
.10035407428292,0.271177880228361,0.851117345470507
"5387","ITGA3",2.81327163443309,-0.9718019446152,0.883165093127692,-1.
10036272060256,0.271174114474383,0.851117345470507
"5388","PHF5A",319.573186647586,-0.294447039971666,0.267432830853625,-
1.10101306197827,0.270890972300559,0.851117345470507
"5389","TRMT13",148.950140056285,0.297281490363211,0.270720654117772,1
.09811159895427,0.272155761113822,0.851303286077425
"5390","TAF1B",180.611951840211,-0.231879082447994,0.211037728718384,-
1.0987565297266,0.271874277866002,0.851303286077425
"5391","HEATR5B",95.146347388146,0.305121707596325,0.277766187991812,1
.09848397964593,0.271993209377701,0.851303286077425
"5392","DNAH5",4.20519967337092,-0.783531039302523,0.712627269004311,-
1.0994962912341,0.271551650798601,0.851303286077425
"5393","LPL",317.459180723958,0.465389700264486,0.423553367791977,1.09
877464247447,0.271866375345707,0.851303286077425
"5394","ADAM32",32.3869469794798,0.351202805442476,0.319584017029877,1
.09893732704925,0.27179540375884,0.851303286077425
"5395","TEX10",66.9353872237879,0.240484856464504,0.218630667089214,1.
09995939575289,0.271349813735116,0.851303286077425
"5396","PAXX",250.826762359244,-0.250505702036925,0.227958449190983,-1
.09890948515381,0.27180754896136,0.851303286077425
"5397","MICU1",407.247291042007,0.231991977952118,0.210967358461905,1.
09965816344053,0.27148108957288,0.851303286077425
"5398","ARHGAP19",56.8810098679291,0.291001836503044,0.265056179549949
,1.09788738748574,0.272253666039993,0.851303286077425
"5399","NDUFV1",679.871695643238,-0.247869677441857,0.225536482930883,
-1.09902253604716,0.271758236165307,0.851303286077425
"5400","CD3G",11.7008072870914,0.565427385204396,0.515091678256616,1.0
9772184073745,0.27232596970244,0.851303286077425
"5401","GSEC",32.4288382897106,0.462760417272205,0.421481324349509,1.0
9793813044126,0.272231506341963,0.851303286077425
"5402","HMGB1",6182.3269632872,-0.224660933736553,0.204608277456929,-1
.09800510775448,0.272202258910324,0.851303286077425
"5403","UPF3A",661.366827337654,-0.243427866405044,0.22135132155272,-1
.09973531984116,0.271447460978628,0.851303286077425
"5404","TBC1D24",68.9463148029226,0.240798822807627,0.219221062923788,
1.09842922753887,0.272017105615812,0.851303286077425
"5405","AC107982.3",2.42936903058352,-1.06872554707641,0.9736104831827
77,-1.09769313861812,0.272338506883889,0.851303286077425
"5406","MOCOS",10.1344339645141,-0.487811169335767,0.443959348770212,-
1.09877440510494,0.271866478908075,0.851303286077425

"5407","POLR2E",839.028343320034,-0.238582656009894,0.217212311516167,-1.09838459129945,0.272036587905861,0.851303286077425
"5408","HDGFL2",351.021637475055,-0.220418284799032,0.200683468051895,-1.09833802922937,0.272056911777265,0.851303286077425
"5409","AC092296.2",7.43532136918093,0.904868673351142,0.823954621157568,1.09820207341018,0.272116261061963,0.851303286077425
"5410","MAP6D1",4.04134511242975,-0.801990028314727,0.730773669835464,-1.09745337225313,0.272443253064095,0.851460154448022
"5411","AC005670.3",161.254798106806,0.268512078198032,0.244691875955792,1.09734774458366,0.272489407127883,0.851460154448022
"5412","SERP1",1250.94911281968,-0.215605934478741,0.196526991781009,-1.09708052072049,0.272606194618928,0.851667690062238
"5413","PTPRG",510.58822448364,0.245627826737042,0.22395995345897,1.09674887382061,0.272751185169251,0.851714703751157
"5414","AC127035.1",2.97788924591514,-1.12475711961347,1.02562905849592,-1.09665098731009,0.272793989617589,0.851714703751157
"5415","USP2",9.02384382247766,-0.748139759864377,0.682244957958415,-1.09658525304921,0.272822736900654,0.851714703751157
"5416","BRCA1",66.9124508798561,0.249476889825028,0.22750029052213,1.09660031313569,0.27281615055447,0.851714703751157
"5417","LVRN",132.562806296839,0.340564772500754,0.310652730882002,1.09628771501163,0.272952883856367,0.851963699509591
"5418","GLIPR2",464.296262292469,-0.262606643661741,0.239581096459107,-1.09610752911202,0.273031720123034,0.852052477637551
"5419","NBPF15",60.1349229396367,0.366574557628687,0.334536907964381,1.09576716021933,0.273180683327351,0.85228185069409
"5420","S1PR2",270.783173911164,-0.199839552013005,0.182386262850509,-1.09569409937854,0.273212665811112,0.85228185069409
"5421","PRX",17.7813324125292,0.448525614958891,0.409390313285409,1.09559410763634,0.27325644148407,0.85228185069409
"5422","VOPP1",176.08107838987,-0.201110560666286,0.183624072595308,-1.09522982375801,0.273415962936374,0.852413522762256
"5423","PABPC1",3794.70898680292,-0.168877527805532,0.154220806320703,-1.09503725103311,0.27350031706797,0.852413522762256
"5424","KDM2A",332.671481249539,0.268196495080455,0.244898348599747,1.09513394685558,0.273457958419536,0.852413522762256
"5425","MKKS",124.380795611354,-0.26101579251455,0.238347083667955,-1.09510797655983,0.273469334547266,0.852413522762256
"5426","ERI3",318.760733342065,-0.27917092186457,0.255207874639405,-1.0938961905428,0.274000509715992,0.852461549134257
"5427","AC080013.1",9.61410052133481,0.553027069364425,0.50511937093976,1.09484431043603,0.273584850182486,0.852461549134257
"5428","FBXO25",186.220392959997,0.226917606338393,0.20738359223852,1.09419266919345,0.273870485949606,0.852461549134257
"5429","AC246817.1",12.3724914458766,0.712781747005372,0.65129659019262,1.09440423570246,0.273777727063065,0.852461549134257
"5430","RIPK2",179.962018773678,-0.267941895903016,0.244950046876118,-1.09386341958337,0.274014884346371,0.852461549134257
"5431","AL358781.1",10.1627007043367,0.712675897434948,0.651718901278968,1.0935326504055,0.274160001552881,0.852461549134257

"5432","SLC22A18AS",2.2726336741868,-1.07780429718857,0.985399187286318,-1.09377429075898,0.274053982357196,0.852461549134257
"5433","TXNRD1",400.941962272899,0.192752537404717,0.176245580195404,1.09365884347858,0.2741046311128,0.852461549134257
"5434","GCN1",110.268653360441,0.22838857576518,0.208848375125559,1.09356165987824,0.274147272227965,0.852461549134257
"5435","RDH11",364.200088341828,-0.199861435753871,0.182805143779155,-1.09330312934368,0.274260729380642,0.852461549134257
"5436","CIAO2B",880.294603122574,-0.268416874936298,0.245494430933045,-1.09337256212344,0.274230255379097,0.852461549134257
"5437","CBFB",169.851596702389,0.189706415729334,0.173297762427332,1.0946847384073,0.273654777010183,0.852461549134257
"5438","C18orf54",25.5288222680262,0.427729128853201,0.390784227302224,1.09454041122904,0.27371803383959,0.852461549134257
"5439","AC022098.1",18.9283826025946,-0.417300535370632,0.381696821048001,-1.0932774714363,0.274271991204776,0.852461549134257
"5440","EMD",317.640544329073,-0.167464836281214,0.153124367418048,-1.09365242844736,0.274107445687557,0.852461549134257
"5441","SLC35A3",280.820545855139,0.234384212916976,0.214412538720482,1.09314601802524,0.274329693975304,0.852484187784312
"5442","DNAH14",36.3231010979126,-0.340270287546828,0.311571894628393,-1.0921084135418,0.274785451759632,0.852510896490693
"5443","SNHG31",3.29007595206967,-1.33532657329042,1.22283134334557,-1.09199570370602,0.274834989583156,0.852510896490693
"5444","TF",6.74694793044924,0.68813206346996,0.63023584202243,1.09186437455182,0.274892718594396,0.852510896490693
"5445","AL441883.1",4.99593389915439,0.693955172738908,0.635480090815525,1.09201717373764,0.274825552681602,0.852510896490693
"5446","CAVIN4",4.45911889406219,0.868306624187796,0.795212810885546,1.09191729849127,0.274869453558549,0.852510896490693
"5447","ZNF140",73.7201169399366,0.218033295138503,0.199674364908354,1.09194435268932,0.27485756122001,0.852510896490693
"5448","CLEC14A",112.758189798991,0.386158982150526,0.353474813245981,1.09246534032907,0.274628616803013,0.852510896490693
"5449","GMFB",242.161447058225,-0.176260702622684,0.161281005649709,-1.09287948641336,0.274446716105621,0.852510896490693
"5450","CCL2",2334.63466035683,-0.705549775751169,0.645782607789223,-1.09254998081561,0.274591434427608,0.852510896490693
"5451","VASP",133.282662757362,-0.256485397629053,0.234926783010235,-1.09176737680811,0.27493536169153,0.852510896490693
"5452","ZNF274",47.5901652727442,-0.295308776249777,0.27018647776195,-1.09298133161927,0.274401996398507,0.852510896490693
"5453","AC012313.5",2.49424645588149,1.12673796878553,1.03204831967242,1.09174924013554,0.274943335614132,0.852510896490693
"5454","AC244453.2",2.78652507735722,1.06451126981407,0.975193651240643,1.09158962269678,0.275013519414353,0.852561458744555
"5455","PSMD6",275.068472710253,-0.240361171490786,0.220215259615978,-1.09148281508711,0.275060489558289,0.852561458744555
"5456","IPO13",44.7264789158839,-0.247573581654823,0.227097984700681,-1.09016194917418,0.275641811500988,0.85259302527552

"5457","PRDX1",2859.86742483782,-0.307894698132961,0.282574710205176,-1.08960457894268,0.27588736518434,0.85259302527552
"5458","DHCR24",33.4963066001162,-0.481441885700739,0.442640313540583,-1.08765937257226,0.276745509461619,0.85259302527552
"5459","AC103591.3",351.417520895585,-0.448316584282022,0.410984397726092,-1.09083601898876,0.27534504420343,0.85259302527552
"5460","SLC30A7",231.157797693903,0.185820603565454,0.170789936438965,1.08800674934299,0.276592127966589,0.85259302527552
"5461","BPNT1",67.1562955464828,0.248110995113881,0.228459509721935,1.08601736656033,0.277471308956102,0.85259302527552
"5462","PSEN2",43.4177074582701,-0.315461709608134,0.290265081396756,-1.0868055781637,0.277122742015307,0.85259302527552
"5463","ACP1",514.32187654707,-0.213149681009289,0.195949781258845,-1.08777708063743,0.276693529893933,0.85259302527552
"5464","KRTCAP3",3.92252944899319,0.827160564306724,0.760559755206571,1.08756814785981,0.276785798628942,0.85259302527552
"5465","TTC27",90.4210743177315,0.244103585991289,0.22482521761288,1.08574824738568,0.277590388561525,0.85259302527552
"5466","COX5B",2206.47848230551,-0.273659317725257,0.250936673675337,-1.09055130809345,0.275470364969269,0.85259302527552
"5467","SAP130",67.6166289300268,0.246720441341286,0.227148589587363,1.08616321056396,0.277406790574308,0.85259302527552
"5468","AC112220.2",7.88032240920853,-0.653222180171208,0.599972200403865,-1.08875407849147,0.276262347096393,0.85259302527552
"5469","ATP11B",195.317000815071,0.241162476565715,0.222164470385403,1.08551325127441,0.277694397885846,0.85259302527552
"5470","QDPR",328.557412720338,-0.268974411485296,0.246958125793321,-1.08914987357168,0.276087799572352,0.85259302527552
"5471","PGM2",99.0722709648811,-0.316296375858933,0.29067054900109,-1.08816107082712,0.276524007116455,0.85259302527552
"5472","METTL14",159.35470104015,0.217092156244792,0.199330296292729,1.08910767847342,0.276106404230018,0.85259302527552
"5473","HSD17B4",397.356208913908,-0.145146603238311,0.133592626411818,-1.08648663580336,0.277263750373285,0.85259302527552
"5474","FKBPL",40.2145954806852,-0.329798812220592,0.303879568326611,-1.08529446068622,0.277791258496013,0.85259302527552
"5475","GUSB",687.378292128263,-0.261477643743122,0.240741431403044,-1.08613478876165,0.277419362992226,0.85259302527552
"5476","PIK3CG",4.85254141212514,-1.04943209901083,0.964332425627539,-1.08824723831921,0.276485975893047,0.85259302527552
"5477","BRAF",213.742039119253,0.268691863421078,0.246747464666504,1.0889346473498,0.276182706034249,0.85259302527552
"5478","RPL30",30735.2545482133,-0.287335616413804,0.264371232726152,-1.08686415481309,0.277096849866795,0.85259302527552
"5479","AL359091.5",8.91577508000408,-0.655945245645546,0.602034575765502,-1.08954746463107,0.275912535753465,0.85259302527552
"5480","MORN4",58.4470717008403,-0.458713930551803,0.421891978629406,-1.08727815125099,0.276913901461929,0.85259302527552
"5481","PYROXD2",84.5050924322859,0.322181021416868,0.296408115526586,1.08695074304728,0.27705857900995,0.85259302527552

"5482","FGFR2",105.860130587343,-0.810688371165245,0.743229632097334,-1.09076432929288,0.275376596083048,0.85259302527552
"5483","DOCK1",384.551793103477,0.217178260342933,0.200110981419125,1.08528906711052,0.277793646572205,0.85259302527552
"5484","PLAAT5",52.1257158085849,-0.253100385048182,0.233045448817478,-1.08605590168127,0.277454260819295,0.85259302527552
"5485","AC084824.4",6.19910719881409,0.724048629705239,0.665085640005582,1.08865473279375,0.276306170835956,0.85259302527552
"5486","SP1",348.21343127666,0.225660918329681,0.20739089163753,1.08809464363596,0.276553328117214,0.85259302527552
"5487","R3HDM2",455.739994358592,-0.206169159476459,0.189935833924306,-1.08546741926867,0.277714686232721,0.85259302527552
"5488","AL627232.1",2.36467783050721,-1.13923541612235,1.04862653937252,-1.08640719393202,0.277298880208451,0.85259302527552
"5489","EFS",239.080369435114,0.190035304827705,0.17452514063573,1.08887065860814,0.276210926891121,0.85259302527552
"5490","SOCS4",85.0021257674176,-0.276259269958724,0.253947554601546,-1.08785954010144,0.276657119978612,0.85259302527552
"5491","AC005225.2",9.89724662398704,-0.543814470264188,0.499542024374052,-1.08862606893907,0.276318816022047,0.85259302527552
"5492","GSC",479.102713596854,-0.299550583287842,0.275334916860615,-1.08794985649963,0.276617244587408,0.85259302527552
"5493","C14orf132",213.222061827318,0.288904391098748,0.266166106450344,1.08542892613807,0.277731726676749,0.85259302527552
"5494","TTBK2",173.9323691862,0.304181490784323,0.280214476264218,1.08553096485103,0.277686556928714,0.85259302527552
"5495","GABPB1",78.5594223081037,0.280124234213018,0.25755268003573,1.08763859174036,0.276754686912751,0.85259302527552
"5496","PARD6A",27.7128097751445,-0.351829127367026,0.323672904965939,-1.08698974170869,0.277041343292683,0.85259302527552
"5497","ASGR1",3.96940315273923,-0.822283275426934,0.757576386410507,-1.08541302260359,0.277738767187239,0.85259302527552
"5498","CNP",316.429405849412,0.197721634691103,0.181236491239126,1.09095929489291,0.275290794107263,0.85259302527552
"5499","SEPTIN4",126.181727977367,-0.301242280879083,0.276401551658564,-1.08987188773529,0.27576958165341,0.85259302527552
"5500","TRIM37",104.57634604218,0.201592184265146,0.185321978134445,1.08779426107193,0.276685943616445,0.85259302527552
"5501","AP000897.1",6.34847695850277,0.698008406716509,0.640772203856549,1.08932379169302,0.276011124639808,0.85259302527552
"5502","AP001094.2",8.79951242720754,0.669524245504763,0.615822565726809,1.08720316981982,0.27694703026273,0.85259302527552
"5503","AC011444.2",2.77575206274694,-1.0381300579516,0.954455171535943,-1.08766769662006,0.276741833365631,0.85259302527552
"5504","ZNF611",62.5643638420729,0.312472903893378,0.286436944272694,1.09089595508286,0.275318667181678,0.85259302527552
"5505","NRIP1",530.833743924095,0.346978089919427,0.318316617535637,1.09004076697498,0.275695186548069,0.85259302527552
"5506","STS",89.9064175972009,0.261050936931907,0.23935609160515,1.09063836721793,0.275432040155058,0.85259302527552

"5507","TCEAL1",225.071179867453,-0.229860525403069,0.211339245173623,
-1.08763767569166,0.276755091472658,0.85259302527552
"5508","AC005052.2",4.71144951183996,0.849446879635367,0.7811318675311
33,1.08745643974321,0.2768351396853,0.85259302527552
"5509","MTMR1",43.0482727851949,0.294834960930562,0.270334690636016,1.
09062939808762,0.275435988341046,0.85259302527552
"5510","ATP13A3",229.702314468821,0.288234326969649,0.265714726888995,
1.08475104238412,0.278031934305969,0.852806276003371
"5511","RAC1",3658.84733288851,-0.201991558395416,0.186214728729123,-1
.08472385494943,0.278043979140373,0.852806276003371
"5512","RNF31",77.3087883689255,0.223502264457411,0.206097528954894,1.
08444902561799,0.278165756574319,0.852806276003371
"5513","C14orf28",88.5911077716407,0.22805269563693,0.210289720095707,
1.08446906264908,0.278156876898456,0.852806276003371
"5514","GFER",255.826621196346,-0.256493331173509,0.236491238558501,-1
.08457857778126,0.278108347225604,0.852806276003371
"5515","STK26",30.3556430146006,-0.450229701897504,0.415141857216435,-
1.08452013226596,0.278134245593562,0.852806276003371
"5516","SMG7",135.738675087555,0.263993809313124,0.243626492587893,1.0
836005826332,0.278541932398557,0.852939063755892
"5517","ORC4",265.559742359326,0.220836394847004,0.203805166524729,1.0
8356622460898,0.278557173072089,0.852939063755892
"5518","FAM162A",560.367709570796,-0.313790909522647,0.289564218168104
,-1.08366603963642,0.278512898282375,0.852939063755892
"5519","OCIAD2",7.60925515999972,-0.739828814874442,0.68277925265809,-
1.08355491470231,0.278562190091083,0.852939063755892
"5520","BTBD3",48.8812586725076,0.290561712505567,0.268088378779619,1.
08382807874124,0.278441033056845,0.852939063755892
"5521","AP000253.1",2.42718572774108,1.10312191342033,1.01799855804653
,1.08361834572452,0.278534053196042,0.852939063755892
"5522","RTCB",409.778323516581,-0.237426282503692,0.219002181871205,-1
.08412747523823,0.278308282280362,0.852939063755892
"5523","AC079414.3",20.0074926125277,0.483583561963655,0.4463590720360
03,1.0833958403889,0.278632761188358,0.85300067466463
"5524","STRADB",98.5006238551934,0.286753787695153,0.264716408485217,1
.08324901103048,0.278697910777282,0.853045668975793
"5525","AP000254.2",15.3843840367975,0.428187650841898,0.3953289532423
65,1.08311735664701,0.278756335896704,0.853070068297098
"5526","IPO9",203.279926328124,0.214695628377138,0.198427387071254,1.0
8198586669915,0.279258808059856,0.853569661527173
"5527","AC079921.1",3.46476651537235,-0.995719841175681,0.920297760803
047,-1.08195399748319,0.279272969456371,0.853569661527173
"5528","PLPP1",1176.83363746706,0.360003883816471,0.332636879406629,1.
08227291110553,0.279131279087794,0.853569661527173
"5529","PITX1",6.7185610923048,-1.28748181627726,1.18971161718307,-1.0
8217974648822,0.279172666208611,0.853569661527173
"5530","MED17",156.490638558512,0.216719051079007,0.200300368014539,1.
08197030902747,0.27926572120179,0.853569661527173
"5531","CTDSPL2",225.270360126413,0.18366086130421,0.169646781341512,1
.08260740258011,0.278982720160879,0.853569661527173

"5532","FGF7",3029.79297780541,0.171734048432682,0.158694580127352,1.0
821670676772,0.279178298921461,0.853569661527173
"5533","AUTS2",287.761008077887,0.260455520498301,0.240898504670867,1.
08118363314108,0.279615436926318,0.853702631623438
"5534","COL1A2",33918.6682054328,0.317250099766057,0.293413092998141,1
.08124043315295,0.279590176589475,0.853702631623438
"5535","SPRYD7",138.904328741339,-0.203182864949213,0.187878079461278,
-1.0814612621750 2,0.279491983330597,0.853702631623438
"5536","AC144831.1",23.5147689562065,-0.342120458316956,0.316272655509
998,-1.08172632808005,0.279374150777832,0.853702631623438
"5537","AC245452.1",69.2050369058798,-0.34395158282935,0.3180813825444
66,-1.08133201659883,0.279549450486775,0.853702631623438
"5538","BAIAP2L2",2.05151468135209,1.34167656019016,1.24094338491551,1
.08117467444456,0.279619421216619,0.853702631623438
"5539","MFAP2",536.182349623356,-0.351530849624846,0.325356448335634,-
1.08044838644848,0.27994255889907,0.85371304554824
"5540","CEP350",446.075155339639,0.259826934448259,0.240582133227299,1
.07999264518441,0.280145455341392,0.85371304554824
"5541","UBE2E2-
AS1",2.15128836399173,-1.1365625313761,1.05197978340014,-1.08040339682
44,0.279962583878807,0.85371304554824
"5542","BRD2",1468.12449153775,0.309233947891932,0.28616032791232,1.08
063179179289,0.279860934852607,0.85371304554824
"5543","RPS6KA2",62.5632643924066,0.44817117887515,0.415000256326571,1
.07992988448295,0.280173404290091,0.85371304554824
"5544","AL162231.2",114.446881307119,-0.2017339797321,0.18671266356582
8,-1.08045151239019,0.279941167571767,0.85371304554824
"5545","SLC2A6",8.01897405479009,-0.593746641091149,0.549717489882342,
-1.08009414293555,0.280100259800785,0.85371304554824
"5546","RPS13",21777.2771452253,-0.27898077522429,0.258237378935538,-1
.08032685420777,0.279996655401064,0.85371304554824
"5547","FAM168A",171.122401980317,0.222529573927059,0.205921235638855,
1.08065383949682,0.279851123671484,0.85371304554824
"5548","MMAB",185.215778847755,-0.227866474734248,0.211003296957551,-1
.07991902505717,0.280178240462928,0.85371304554824
"5549","ATF7IP2",37.1485414858095,-0.539317499644973,0.499151136401465
,-1.08046934147657,0.279933232102667,0.85371304554824
"5550","CCNL2",356.09208527145,0.262372710437159,0.24357522007899,1.07
717324591587,0.281402875279122,0.853724410822663
"5551","UBR4",136.12289066616,0.292062624433925,0.271166525830687,1.07
706002257921,0.281453451464681,0.853724410822663
"5552","IQCC",10.1832169688463,0.517747746798913,0.481543093429552,1.0
7518465919947,0.282292062292459,0.853724410822663
"5553","ZBTB8A",209.953753383481,0.174678631921056,0.162098753408399,1
.07760626314604,0.281209505980144,0.853724410822663
"5554","ST3GAL3",84.3611331074079,0.224940796617076,0.209116397238643,
1.07567268558273,0.282073667433801,0.853724410822663
"5555","AL604028.1",12.3772079287822,-0.501124769720734,0.464881783339
039,-1.07796172635842,0.281050836826275,0.853724410822663
"5556","TUT4",474.47856933336,0.211979312552149,0.196322701065781,1.07

974936877586,0.280253803120736,0.853724410822663
"5557","RASSF5",70.5324551328613,0.367080100447406,0.34033294917028,1.07859113066288,0.28077003688992,0.853724410822663
"5558","TTLL4",30.5545686668515,-0.398225681217671,0.369009430930897,-1.07917480648956,0.280509808372765,0.853724410822663
"5559","DNPEP",456.937295436354,-0.171052173633171,0.158753486701909,-1.07747034214345,0.281270193501886,0.853724410822663
"5560","TOP2B",301.504141794392,0.171886290201241,0.159257238536267,1.0792997026763,0.280454145408697,0.853724410822663
"5561","AZI2",750.645585825243,0.172730443332404,0.160669961446518,1.0750637006277,0.282346209735438,0.853724410822663
"5562","CRYBG3",426.657902218241,0.235208175311792,0.218405617692257,1.0769328087669,0.281510284470204,0.853724410822663
"5563","AC021086.1",2.35281649210441,-1.05395592316466,0.978367541000396,-1.07725969944483,0.281364261159034,0.853724410822663
"5564","TBL2",165.046751711584,-0.231156454614282,0.214618948915496,-1.07705519844521,0.28145560651341,0.853724410822663
"5565","TRRAP",159.325315600127,0.262655920444716,0.244213532906433,1.07551746751622,0.282143116058903,0.853724410822663
"5566","SNAPC3",174.623218932898,0.189577200206353,0.176177711625872,1.0760566615199,0.28190191636786,0.853724410822663
"5567","MTAP",252.561497198053,0.153819750578858,0.142990480451114,1.07573420337899,0.282046145970052,0.853724410822663
"5568","GCNT1",6.77801361892922,-0.697209412346949,0.648489962078177,-1.07512753183202,0.282317634640788,0.853724410822663
"5569","TYSND1",129.2995211145,-0.254354173871546,0.235757326981626,-1.07888131040512,0.280640641434247,0.853724410822663
"5570","PDCD11",123.220873614686,0.258625450841252,0.23957985365711,1.07949582109437,0.280366755699315,0.853724410822663
"5571","LINC01089",32.5139755382941,0.373515076610273,0.347325938378122,1.07540219528217,0.28219469937591,0.853724410822663
"5572","TEP1",36.703381754517,0.39645521630475,0.368036111070536,1.07721825217517,0.281382772966141,0.853724410822663
"5573","STYX",88.1738876526564,0.199468322083045,0.185035663932695,1.07799933182394,0.281034054320244,0.853724410822663
"5574","GCH1",50.2130406528151,0.641539482301283,0.596570621833256,1.07537893892568,0.28220510716736,0.853724410822663
"5575","PPP2R5E",478.877778413086,0.173105783566994,0.160412847929973,1.07912667720083,0.28053126034079,0.853724410822663
"5576","CDCA4",74.8892464160946,-0.225407540868514,0.20941084135812,-1.07638907043517,0.281753288343611,0.853724410822663
"5577","RSL24D1",1510.52820103921,-0.268233179630955,0.249491423407939,-1.07511984166434,0.282321077167682,0.853724410822663
"5578","RAB8B",493.331900637939,0.205961957216736,0.19145040622731,1.07579796394998,0.28201762306919,0.853724410822663
"5579","ZFHX3",526.377547995433,0.230799711039922,0.214457377052853,1.07620317944596,0.281836398124545,0.853724410822663
"5580","NCBP3",324.49008239853,0.175833135722364,0.163002413275824,1.07871492322527,0.280714830994315,0.853724410822663
"5581","AC004771.4",2.72369565459781,-0.960630719577324,0.893569891101

637,-1.07504821854842,0.282353140839859,0.853724410822663
"5582","KIF1C",101.411215053743,-0.277581370742452,0.257951111321711,-1.07610069722188,0.281882223890582,0.853724410822663
"5583","DVL2",144.483816197965,0.193501274384439,0.179915294444388,1.07551320182093,0.282145024806311,0.853724410822663
"5584","RANGRF",276.659159800833,-0.275837389727447,0.256504476265812,-1.07537066698829,0.282208809125525,0.853724410822663
"5585","C17orf75",70.970017406933,-0.267179377131166,0.24781133916984,-1.0781563831026,0.280963973084358,0.853724410822663
"5586","GJC1",9.33265711526526,-0.83312145801144,0.773582164356624,-1.07696570112153,0.28149558900367,0.853724410822663
"5587","AC010327.8",2.42907285517707,1.31418620257121,1.21978393705378,1.07739261245352,0.281304903113745,0.853724410822663
"5588","AL353194.1",16.5578875789689,-0.424022629270951,0.39344786988439,-1.07770981043955,0.281163278997118,0.853724410822663
"5589","ACTR5",40.3413362673465,-0.313647457420574,0.291267864222423,-1.07683509218532,0.281553944821182,0.853724410822663
"5590","AP000704.1",3.8529528886199,-0.798839820538845,0.741687202388791,-1.07705757624775,0.281454544294348,0.853724410822663
"5591","L3MBTL2",85.3814611648863,-0.212217137228649,0.19737815751369,-1.07518045513182,0.282293944136987,0.853724410822663
"5592","YY2",16.6492567393673,0.520506778751727,0.482864749278684,1.07795563774178,0.281053554108208,0.853724410822663
"5593","C17orf58",817.031397406098,0.424275658572851,0.394704958488129,1.0749184915183,0.282411222300284,0.853747353236761
"5594","ZNF665",42.5601773071747,0.307411436505447,0.286029457591965,1.07475446442997,0.282484672197794,0.853816738920326
"5595","ZBTB7A",963.101313205067,-0.231137495262254,0.215096876024267,-1.07457392935906,0.282565529195753,0.85390848393955
"5596","LINC02777",4.1465822938145,-0.79077618173558,0.736096878472364,-1.07428275389062,0.282695972226705,0.854147257899578
"5597","AC104964.3",2.07202936725605,-1.15991710833121,1.07982432294327,-1.07417205158857,0.282745576204397,0.854147257899578
"5598","GATA2",7.10436701167785,-1.09359674759559,1.01855246869129,-1.07367738158911,0.282967302201653,0.854312199656013
"5599","CCDC96",17.3365107058259,-0.394860775832965,0.367791350783122,-1.0735999500592,0.283002020008322,0.854312199656013
"5600","TRAM2",525.606321124278,0.322924727000949,0.300756350872774,1.07370875482378,0.282953236274267,0.854312199656013
"5601","INTS9",45.6021841059192,0.292619211223082,0.272571034273178,1.07355211827025,0.283023467685643,0.854312199656013
"5602","AP000781.1",2.180985713713,1.03745576734949,0.966435622001879,1.0734866800548,0.283052811833037,0.854312199656013
"5603","UBE2J2",296.037604263707,-0.224309774473375,0.209317069548753,-1.07162676678469,0.283887705893582,0.854747677273205
"5604","COPA",718.953976631272,0.194639762987861,0.181754921959845,1.07089129080566,0.284218312304651,0.854747677273205
"5605","COG2",111.575310252401,-0.211293742717732,0.197293356657382,-1.07096227819097,0.284186391156294,0.854747677273205
"5606","SRSF7",2184.17353664174,0.197280013221273,0.184230017346813,1.

07083533976927,0.284243473714998,0.854747677273205
"5607","AC008074.2",3.3109476223429,-0.831672998823058,0.776177585308806,-1.07149834595156,0.283945414026839,0.854747677273205
"5608","LIPT1",95.7819173536109,0.217473133871416,0.203093794443913,1.07080147114724,0.284258705305609,0.854747677273205
"5609","PAX8",11.1768737108837,-0.717091975942845,0.668762395511818,-1.07226719198833,0.283600038242915,0.854747677273205
"5610","AC017071.1",7.87624752539458,0.664782889743948,0.62012646728244,1.07201179891128,0.283714732612623,0.854747677273205
"5611","HACL1",130.670713467268,-0.233111656519336,0.217340493874705,-1.07256430848879,0.283466645838081,0.854747677273205
"5612","NCK1-DT",27.4433603224013,-0.338366860202346,0.31558720087529,-1.07218182253233,0.283638373281712,0.854747677273205
"5613","LY86-AS1",4.10620929235741,-1.03047388219893,0.96220014245345,-1.07095586119058,0.284189276611762,0.854747677273205
"5614","GCC1",39.7454451364811,0.329647173412097,0.30764829660877,1.07150657762719,0.28394171474213,0.854747677273205
"5615","ZDHHC2",152.582574518229,-0.271190340251463,0.252937756048435,-1.07216235522993,0.283647115538865,0.854747677273205
"5616","ZNF169",16.8967219500742,0.503556730819495,0.469882241863352,1.07166580465481,0.283870165122097,0.854747677273205
"5617","BDNF-AS",56.0894609970058,0.257142559719709,0.23980536555887,1.07229694014742,0.283586680696717,0.854747677273205
"5618","ANO4",2.02840293502561,-1.32300960354573,1.23500343098719,-1.07125986078289,0.28405260254955,0.854747677273205
"5619","AC004812.2",32.0876279140953,-0.365770227362421,0.341076550617183,-1.07239922152536,0.28354075746775,0.854747677273205
"5620","SCAPER",224.821450758833,0.235134612255568,0.219527417788985,1.07109451121766,0.284126935934385,0.854747677273205
"5621","EPG5",121.286530909196,0.24070647350501,0.224533301468167,1.07203017071896,0.283706480976905,0.854747677273205
"5622","CARD8",101.854014767582,0.217727636174373,0.203055697744062,1.07225573373865,0.283605183350559,0.854747677273205
"5623","SLC37A1",29.1596624575772,0.334354612097089,0.312015825796721,1.07159504247365,0.283901961025527,0.854747677273205
"5624","AAMP",458.003846458889,-0.181919680375331,0.169936716862527,-1.07051426986493,0.284387889314313,0.854785221678267
"5625","GLRX3",365.471578646938,-0.265838314035676,0.248371253040586,-1.0703264197497,0.284472406354726,0.854785221678267
"5626","SMPD1",175.652984705131,0.299541870787689,0.279868644052087,1.07029450120157,0.284486768754905,0.854785221678267
"5627","SPTY2D1",311.659939804863,0.178579531827782,0.166824055468637,1.07046631450195,0.284409463652353,0.854785221678267
"5628","AC018362.1",16.6020712557496,0.648515268936783,0.605969066463126,1.07021183890126,0.284523966619665,0.854785221678267
"5629","CREM",212.513600448632,-0.267592920065847,0.250099669821182,-1.06994511531012,0.284644014126797,0.854993958226308

"5630","TMEM18",466.79675100522,-0.227334627909319,0.212500718671638,-1.06980639562261,0.284706462915603,0.855029640315635
"5631","TTC28-AS1",102.037072796254,-0.282717091830787,0.26432564684614,-1.06957873821208,0.28480896974938,0.855185590574056
"5632","TINAGL1",4.01394171001025,-0.905682385755219,0.847047296595919,-1.06922292225587,0.284969232266149,0.85521082652286
"5633","CHI3L1",489.336499676945,-1.13318609328579,1.05980706697246,-1.06923809870692,0.28496239541916,0.85521082652286
"5634","CYB5A",2573.44398944109,-0.311218386116707,0.29113075557893,-1.06899865490965,0.28507027550762,0.85521082652286
"5635","PNPLA6",66.7934803662481,-0.264327032555137,0.247212451610028,-1.06923025451852,0.284965929137644,0.85521082652286
"5636","EID2",140.38095780433,-0.234217350300152,0.219077911141778,-1.06910527437235,0.285022235283241,0.85521082652286
"5637","SRP14-AS1",52.1402309300806,-0.401096773564457,0.375360224462265,-1.06856493422834,0.285265756299182,0.85564545103895
"5638","SMIM12",198.201060327189,-0.25618046914875,0.239775442446109,-1.06841829394738,0.285331868545702,0.855691953418009
"5639","TMEM173",529.312917412097,-0.235148302772254,0.220165752581317,-1.06805122965436,0.285497403588778,0.855868183761879
"5640","AC040970.1",5.98376046724165,-0.68639677910363,0.642692315743448,-1.0680021563812,0.285519539089576,0.855868183761879
"5641","LMAN1",1217.69337497706,-0.12042462197822,0.112762280922243,-1.06795127761969,0.285542490217693,0.855868183761879
"5642","CAMK1",331.848022172128,-0.225077315299876,0.210808847456395,-1.06768438808733,0.285662903039666,0.855881029910204
"5643","DAG1",218.285805997404,0.188382386166265,0.176453229773303,1.0676052028534,0.285698635723515,0.855881029910204
"5644","GCK",6.53074997752057,-0.773911852240488,0.724826569925149,-1.0677200372503,0.285646817184778,0.855881029910204
"5645","AGO4",105.149258459846,0.253498084190174,0.237498413273321,1.06736748551848,0.285805924859935,0.856008850772011
"5646","THOC3",114.564978792777,-0.279744079718369,0.262107806742766,-1.06728633227209,0.285842558047006,0.856008850772011
"5647","AL355377.3",9.05224127795454,0.830445227983767,0.77820479509626,1.06712941531162,0.285913400545073,0.856069377796369
"5648","BX284613.2",10.0548296338196,0.601092559552767,0.563417086210868,1.06686959672323,0.28603072559947,0.856269034779717
"5649","RRNAD1",109.452242945772,-0.266581268368268,0.249989666539677,-1.0663691506063,0.286256801353773,0.856468141646398
"5650","TMEM87B",135.382789551818,0.236633005516306,0.221945082148483,1.06617818798074,0.286343100232895,0.856468141646398
"5651","NOL7",1128.68188311849,-0.23494629088963,0.220366450767877,-1.06616179582213,0.286350508914543,0.856468141646398
"5652","MFAP5",9226.32922564379,0.426303580741048,0.399731329967539,1.06647527672066,0.286208848964738,0.856468141646398
"5653","AC008915.2",41.5814726667315,-0.354305206190886,0.332244335838084,-1.06639953784962,0.286243070522596,0.856468141646398

"5654","RIF1",580.243389488178,0.243154406279071,0.228096120173839,1.0
6601728294965,0.286415829274648,0.856511998828397
"5655","AL645504.1",2.88202504918714,1.05578888556836,0.990695619084839,1.06570460717657,0.286557194339339,0.856758446802334
"5656","PCNX2",155.847162420186,0.306816989830417,0.287959411266608,1.
06548693262312,0.286655635847743,0.856758446802334
"5657","DTX3L",170.992032112637,0.215247536785558,0.20215354464575,1.
06477250815814,0.286978888836655,0.856758446802334
"5658","BLOC1S4",285.375776236172,-0.24052934994578,0.22577375686692,-
1.06535566083332,0.286715013461518,0.856758446802334
"5659","MAP9",156.606279211708,-0.227684896811536,0.213843681912725,-1
.06472585383402,0.287000006902052,0.856758446802334
"5660","KCTD20",368.571051672759,0.206986121686445,0.194312243576814,1
.0652242899178,0.286774444226435,0.856758446802334
"5661","LRRN4CL",788.894938609116,0.260522516292548,0.244687580454212,
1.06471491445926,0.287004958758482,0.856758446802334
"5662","VIPAS39",101.909791404922,0.238808761310896,0.224247737015453,
1.06493275914057,0.286906359269017,0.856758446802334
"5663","SMIM26",1151.7281263004,-0.270689422839709,0.254147183827109,-
1.06508920840081,0.286835562299854,0.856758446802334
"5664","PRMT2",695.427743852065,0.143300939042475,0.134540797261059,1.
0651114157174,0.286825513922936,0.856758446802334
"5665","TBC1D8-
AS1",5.88320413236293,-0.66890198678395,0.628605089034603,-1.064105268
08053,0.287281014638839,0.856842451424164
"5666","HEG1",384.75255376556,0.252927232323101,0.237617898329059,1.06
44283705129,0.287134687306038,0.856842451424164
"5667","TBRG4",122.422863057318,-0.21911289071846,0.205893872208377,-1
.06420306912633,0.287236716978111,0.856842451424164
"5668","RNASEH2B",335.094805350013,-0.191581824908493,0.18004233628296
,-1.06409319532156,0.287286483151383,0.856842451424164
"5669","FASN",72.9880843121615,0.210230725792385,0.19756323953404,1.06
411864012871,0.287274957678419,0.856842451424164
"5670","KLF15",75.361007105379,0.410746679213111,0.386092731822578,1.0
638549896398,0.287394395868168,0.85699687795251
"5671","JADE1",566.387565047967,0.351344413588197,0.330594453543235,1.
06276560245512,0.287888262182699,0.85699687795251
"5672","HULC",21.2717789692206,0.410409435339079,0.385853222995612,1.0
6364133012243,0.287491211869286,0.85699687795251
"5673","AC004889.1",206.607085299863,0.306619687023609,0.2885149663393
75,1.0627514091,0.28789470041962,0.85699687795251
"5674","AL157392.3",39.696468322074,0.395263114498128,0.37172095613776
2,1.0633328790633,0.28763101978172,0.85699687795251
"5675","ZNF37A",352.350421245928,0.279176515565336,0.262526045930569,1
.06342406741299,0.287589683157041,0.85699687795251
"5676","DDB2",130.825666999617,0.282739975866393,0.265984648626856,1.0
6299358750979,0.287784859365427,0.85699687795251
"5677","LINC02289",24.3592423791225,0.472898329927112,0.44497770788646
4,1.06274611412168,0.287897102295542,0.85699687795251
"5678","MNS1",190.220461523275,0.305563418666299,0.28735853749911,1.06

335249798258,0.287622125983711,0.85699687795251
"5679","RSL1D1",1151.53924537514,-0.198425789540299,0.186729564393059,-1.06263724325206,0.287946490634505,0.85699687795251
"5680","XYLT1",189.077651328266,-0.288169023755919,0.27112818243754,-1.06285160459963,0.287849252892984,0.85699687795251
"5681","REXO5",30.1644372066538,0.334475748025646,0.314664183822273,1.06296097624686,0.287799648696003,0.85699687795251
"5682","C1orf109",81.740365244045,0.234882111654448,0.221208058397698,1.06181534866224,0.288319520486899,0.857167780892721
"5683","PLGLB2",15.6829933326116,0.530602402506223,0.499858304086067,1.06150562703238,0.288460177033332,0.857167780892721
"5684","LETM1",95.9110935617105,-0.268086053536541,0.252441548104034,-1.06197278360082,0.288248040934512,0.857167780892721
"5685","JAKMIP3",6.182509225162,0.689031215685514,0.648576093956591,1.06237528966282,0.288065347054785,0.857167780892721
"5686","TCHP",99.333767116173,0.315219094974129,0.296787942901456,1.06210209179149,0.288189340594574,0.857167780892721
"5687","BBOF1",87.7163148670537,0.239489584451778,0.225498689166211,1.06204424219626,0.288215600818552,0.857167780892721
"5688","AC015712.1",12.8096858858633,-0.727351289617487,0.685181248867584,-1.06154581845256,0.288441921946681,0.857167780892721
"5689","MGAT5B",3.92098581181873,0.835639883792361,0.787006654229382,1.06179519487113,0.288328671694464,0.857167780892721
"5690","FCHO1",6.1152349361253,-0.821091732090138,0.773512894737502,-1.06151007653051,0.288458156017047,0.857167780892721
"5691","SLC25A26",203.126411381338,-0.227420343969904,0.214427646921442,-1.06059245267576,0.288875153973451,0.857496682475968
"5692","UBE3D",72.7996015291748,0.281789094858286,0.265554094254,1.06113632196067,0.288627953174484,0.857496682475968
"5693","TCF21",25.3738682786472,-0.711038540344766,0.670392841625928,-1.0606296729247,0.288858231988291,0.857496682475968
"5694","GALNT11",515.217380776636,0.170507480660393,0.160756215150218,1.06065871544104,0.288845028429073,0.857496682475968
"5695","UQCRC2",691.42641477342,-0.237460014125782,0.223855064441596,-1.06077570645151,0.28879184508612,0.857496682475968
"5696","RINL",61.3003410083052,-0.268393588141903,0.253007703642768,-1.06081192105067,0.288775383505495,0.857496682475968
"5697","YBEY",268.557043892226,0.352369756526314,0.332373227529574,1.06016287516708,0.289070507415446,0.857925950391499
"5698","GPR171",3.74963973096696,0.969170024521233,0.914296105037559,1.06001766734139,0.289136561835775,0.857971391281026
"5699","MINPP1",108.518754938397,0.220095294241949,0.207662119470414,1.05987213654201,0.289202773377477,0.858017282376976
"5700","ACAP3",147.11851370585,-0.196607136971871,0.185697871797461,-1.0587473893417,0.289714839440171,0.85809455398308
"5701","PTPRU",55.5815020603595,-0.321955124136442,0.304370327736566,-1.05777434525449,0.29015833155058,0.85809455398308
"5702","FAM72D",3.9682895749215,0.929928748874017,0.879446531131423,1.05740225921143,0.290328040900185,0.85809455398308
"5703","RSAD2",18.2452097449181,-0.507177462446547,0.479598470435336,-

1.05750433688034,0.290281476379514,0.85809455398308
"5704","TCF19",46.5299333490915,-0.278068322053255,0.262605186822792,-1.05888358648795,0.289652800207522,0.85809455398308
"5705","AL596202.1",2.93850864027587,0.89197756371888,0.84181882671116,1.05958376721471,0.289334001739462,0.85809455398308
"5706","AL353748.3",4.43014263535578,0.75136101921052,0.71058605078217,1.05738216839955,0.29033720626871,0.85809455398308
"5707","AC048341.2",13.9843822821541,-0.560893996333228,0.52985372502536,-1.05858271791216,0.289789860956143,0.85809455398308
"5708","OAS2",39.1341218603713,0.460921643861743,0.435913626787309,1.05736920237787,0.290343121432546,0.85809455398308
"5709","NOVA1",6680.43120725226,-0.205787062550608,0.194251332676494,-1.05938558935565,0.289424209878965,0.85809455398308
"5710","EVL",409.679251716856,-0.152507413769289,0.144035932907173,-1.05881505184942,0.289684017337952,0.85809455398308
"5711","HACD3",442.928828526466,0.208055819065417,0.196639668516934,1.05805619300818,0.290029824556174,0.85809455398308
"5712","LINC00924",2.55705070992654,1.1244225769002,1.06119143308989,1.05958504925562,0.289333418231768,0.85809455398308
"5713","SLC38A7",72.0978396547785,0.235256129001654,0.222450219031365,1.05756753140568,0.290252651606417,0.85809455398308
"5714","ENKD1",106.488074263745,0.212893760986104,0.201067813529167,1.05881571619727,0.289683714720487,0.85809455398308
"5715","MLLT6",378.606439557023,0.25613704111582,0.242266528870941,1.05725311007476,0.290396086934657,0.85809455398308
"5716","PCGF2",224.196816413027,-0.206548968719162,0.195138642586553,-1.05847291946569,0.289839890537427,0.85809455398308
"5717","SNHG25",193.468920997374,-0.484908688025085,0.458281518911599,-1.05810221013652,0.290008846928281,0.85809455398308
"5718","SOCS3",11037.2269360665,-0.292104613004101,0.276003309758861,-1.05833735566181,0.289901668106382,0.85809455398308
"5719","ZNF382",5.83758266312245,0.754735246518367,0.713312480601055,1.05807099559286,0.290023076454929,0.85809455398308
"5720","KLHL22",108.422574445284,0.223394939297345,0.210924030286748,1.05912512193914,0.289542800270347,0.85809455398308
"5721","Z73429.1",4.51783088000832,1.00597145404646,0.950443975061818,1.05842267449907,0.289862786558727,0.85809455398308
"5722","OGT",468.607925442563,0.261279760547382,0.247111880266524,1.05733386944237,0.290359240905773,0.85809455398308
"5723","PRKAG2",283.122371204805,0.304604732193281,0.288151094752569,1.05710072854257,0.290465618761237,0.858150040540799
"5724","LINC02614",36.3577189763598,0.305429761104492,0.288962070811096,1.05698910672661,0.29051655900133,0.858150590425323
"5725","TLK2",211.788329821118,0.229006319685298,0.21670503744563,1.05676509593255,0.290618807721936,0.858302672657204
"5726","LINC02696",3.7647880853977,-0.95718795592853,0.90591915095188,-1.05659313518517,0.290697314856652,0.858306204534811
"5727","ZNF276",103.861600588598,0.280305538105084,0.26530515781897,1.05654010049948,0.290721530244314,0.858306204534811
"5728","AC022150.4",11.1440619913715,0.596678596910733,0.5648464268474

55,1.05635544202862,0.290805855019612,0.858405271765294
"5729","PTPDC1",66.0569366859068,0.238347419642398,0.225658398275129,1.05623110623961,0.290862642551267,0.85842303373308
"5730","SHBG",6.39678875367534,-0.797907457733339,0.755515095795361,-1.05611054256083,0.290917714380002,0.858435726830206
"5731","H6PD",649.732916024815,0.264885458235368,0.250984003098644,1.05538781342674,0.291247994121461,0.858531061578424
"5732","NBPF10",55.4683622147335,0.498463510761413,0.472195531976801,1.05562945222003,0.291137539647904,0.858531061578424
"5733","COX18",54.4954767246732,0.264696880769816,0.250753865545227,1.05560438796934,0.291148995350642,0.858531061578424
"5734","PKN3",8.19078357351573,-0.580558154806763,0.550097504623989,-1.05537318371149,0.291254682352368,0.858531061578424
"5735","JMJD8",283.055032607607,-0.226138523846495,0.214225440451631,-1.05561003104836,0.291146416135359,0.858531061578424
"5736","CNOT3",146.156874317918,0.168900270024407,0.160007849427613,1.05557490228514,0.291162472272926,0.858531061578424
"5737","SYTL1",12.4569265940447,0.552360088988265,0.523607433183507,1.05491261961264,0.291465290106217,0.858634858233759
"5738","ELOVL2-
AS1",2.15054186374556,1.17774329524987,1.1169227881838,1.05445363610583,0.291675276906888,0.858634858233759
"5739","DDX43",8.07142083493222,0.818570047764267,0.776129743616514,1.05468197101943,0.291570800059382,0.858634858233759
"5740","RNF217",189.571735381452,0.291969169610296,0.276903406653356,1.05440800869525,0.291696157185635,0.858634858233759
"5741","AC147651.4",3.13300279117919,-0.90369851224151,0.856758510388121,-1.0547879026403,0.291522338564933,0.858634858233759
"5742","EEF1D",7323.29351035637,-0.239043369197293,0.226694202440821,-1.05447500034632,0.291665500425261,0.858634858233759
"5743","AC123768.4",9.75940691378246,0.509594448288309,0.483096901946062,1.05484934023693,0.291494234634036,0.858634858233759
"5744","COX10-
AS1",44.435253091286,0.289193436478083,0.274174531808041,1.05477862794549,0.291526581326974,0.858634858233759
"5745","CENPU",13.2093421790121,0.482571754397307,0.45783456115059,1.05403085600298,0.291868790446842,0.858993474129713
"5746","FBXL19",27.1458598687738,0.337531283123171,0.320282366381428,1.05385534313556,0.291949150964187,0.859080446310909
"5747","SIM1",104.212805822446,0.565105287266501,0.536378116603993,1.05355768584369,0.292085470646942,0.85933202326405
"5748","ATAD3C",4.99400924043365,-0.759537741197919,0.72144451312773,-1.05280132758241,0.292432056398686,0.859454408063442
"5749","CD8A",7.49777911150857,-0.74650112676412,0.708992590615227,-1.05290398890677,0.292384997746386,0.859454408063442
"5750","ANAPC15",84.4077820248715,-0.30831704327834,0.292783797722105,-1.05305363779378,0.292316409701023,0.859454408063442
"5751","AMN1",43.5556651108558,0.273978348352457,0.260206987392976,1.05292463933216,0.292375532467117,0.859454408063442
"5752","CIT",5.23946201311212,0.740961497670196,0.703751709220734,1.05

287346085548,0.292398990887503,0.859454408063442
"5753","PLEKHG4",72.110760381625,-0.226564597391272,0.215157023342328,
-1.05301976143625,0.292331935185834,0.859454408063442
"5754","TLR9",2.32154347168864,-1.09817867621019,1.04403648529253,-1.0
5185852379717,0.292864463583182,0.859672640370306
"5755","MCM4",78.6392235831103,0.219990354362809,0.209016081959491,1.0
5250444033031,0.292568174383475,0.859672640370306
"5756","BBIP1",272.634358832324,-0.223527861336759,0.212475638339626,-
1.05201642448753,0.292792014159354,0.859672640370306
"5757","TUBA1A",3422.14146221415,-0.280143551874902,0.266342786640083,
-1.05181580251868,0.292884067415232,0.859672640370306
"5758","ZNF10",41.2912727232567,0.330868681429209,0.314587932812555,1.
05175261641824,0.292913063707909,0.859672640370306
"5759","BATF",9.28813341868198,0.849665393724398,0.807486154787241,1.0
5223524723872,0.29269163181817,0.859672640370306
"5760","PSMD3",483.619736087194,-0.212915145646065,0.202429113421317,-
1.05180100849883,0.292890856262932,0.859672640370306
"5761","DDX3X",2621.31339918396,0.252608591335125,0.240172412021282,1.
05178021575909,0.292900398049853,0.859672640370306
"5762","UBXN10-
AS1",5.51945455687502,-0.960818907146992,0.91379697823671,-1.051457741
74152,0.293048408070258,0.859708363778802
"5763","RCN2",639.957916449838,-0.201474820325593,0.191626415719176,-1
.05139377350172,0.293077774356578,0.859708363778802
"5764","HOXB-
AS3",5.4969084890133,-0.750422567689487,0.713619677207194,-1.051572135
21119,0.29299589769319,0.859708363778802
"5765","PCDHGA6",36.495335879352,0.42172592026519,0.401202000421182,1.
05115607554913,0.293186913097467,0.859879328126967
"5766","ROR1-
AS1",2.77643141301881,-1.32839083150858,1.26446533198294,-1.0505553595
7273,0.293462852701876,0.860036866064884
"5767","AL162595.1",12.8058424119735,0.489597781920556,0.4660681027290
67,1.05048549568981,0.293494956067801,0.860036866064884
"5768","AC027031.2",24.2972146563069,-0.423536233602283,0.403157775391
778,-1.05054710451932,0.293466645883891,0.860036866064884
"5769","SMIM10L1",297.275931167164,-0.265877441902065,0.25302479863753
5,-1.05079598258249,0.293352301320955,0.860036866064884
"5770","COX7B",1091.37416178365,-0.245443520038088,0.233598039864952,-
1.05070881664925,0.293392345444126,0.860036866064884
"5771","SNRK",215.702789135717,0.262601921211798,0.250033399226236,1.0
5026737237688,0.293595201738432,0.860070046880371
"5772","AC067838.1",12.3836932448128,0.44126382764819,0.42019970522376
5,1.0501288367473,0.293658882223822,0.860070046880371
"5773","MMP15",4.49203983312799,-0.819503517706725,0.780337901903711,-
1.05019058501127,0.293630497338416,0.860070046880371
"5774","ALG14",152.205684856957,-0.223908867512853,0.213460121173441,-
1.04894940695233,0.294201404336435,0.860112889880595
"5775","OXNAD1",42.6465585127755,0.318175461934597,0.303347651288442,1
.04888058497627,0.294233082272851,0.860112889880595

"5776","LINC02482",57.5361048046299,0.395693725167827,0.376898368019648,1.04986850234172,0.29377857485196,0.860112889880595
"5777","ECT2L",2.63841729378351,1.16310705909606,1.10872941581388,1.04904500819279,0.294157404017951,0.860112889880595
"5778","AL137026.1",119.51667250017,0.263486233316872,0.251124143381039,1.04922700688749,0.294073651605021,0.860112889880595
"5779","TMEM117",27.732814813009,0.314344435779146,0.29966970053832,1.04896969968757,0.294192064268485,0.860112889880595
"5780","RCBTB2",115.36930797133,-0.215256907162879,0.205212970675221,-1.04894396516268,0.294203909044179,0.860112889880595
"5781","POLR3E",105.556714394733,0.241666949029308,0.230373557963123,1.04902208033785,0.294167956124395,0.860112889880595
"5782","SULT1A2",11.0212267589718,0.839911440451673,0.800374386096035,1.04939820044528,0.293994886110778,0.860112889880595
"5783","KIAA0753",41.090308897942,0.284851112500886,0.271556060678765,1.04895877407004,0.29419709294045,0.860112889880595
"5784","ZNF530",11.2723286712425,-0.507742055914444,0.483717182857371,-1.04966718964821,0.293871153799798,0.860112889880595
"5785","ILKAP",168.988253845187,-0.192853038129921,0.183910617148906,-1.04862373428814,0.294351327780632,0.860309809872934
"5786","MRPL20",1202.0339611434,-0.242390943193736,0.232611877508186,-1.04204026806501,0.297393009947427,0.860556075801595
"5787","AL645728.1",16.5791045361411,-0.40531393439963,0.392248863427588,-1.03330811683653,0.30145972997219,0.860556075801595
"5788","CORT",3.92711526616106,-0.768429150497997,0.754200880723385,-1.01886535820664,0.308266891788922,0.860556075801595
"5789","PRDM2",603.033447859747,0.303131877718203,0.290980444002387,1.0417603105854,0.297522819484188,0.860556075801595
"5790","SLC66A1",48.3699499865095,0.274485481443816,0.267480056017226,1.02619045894823,0.304801817766746,0.860556075801595
"5791","MICOS10",1467.02300018897,-0.264013689284612,0.254495732949721,-1.03739927669739,0.299549815287713,0.860556075801595
"5792","AL031432.3",2.06359564117103,-1.24582866193489,1.20297000053997,-1.03562737339724,0.300376016447924,0.860556075801595
"5793","KHDRBS1",1130.46284352595,0.118903912859535,0.116323698690835,1.02218132846307,0.306695086244742,0.860556075801595
"5794","TXLNA",353.87889153359,0.182947584345768,0.179332205264408,1.0201602332165,0.307652474124695,0.860556075801595
"5795","EIF3I",1292.50143342223,-0.223692510188767,0.213922111039438,-1.04567269414955,0.295712173948688,0.860556075801595
"5796","ZNF362",132.680446087382,0.181006516265018,0.174069913061329,1.03984952414634,0.298409816312459,0.860556075801595
"5797","TRAPPC3",376.846228461039,-0.189614892275507,0.184635246606253,-1.0269701791006,0.304434506308102,0.860556075801595
"5798","FHL3",162.416129789643,-0.324977002470946,0.311987499555914,-1.04163469027933,0.297581078899737,0.860556075801595
"5799","TOE1",59.0019114626715,-0.228458293813468,0.222736149363991,-1.02569023692749,0.305037617743731,0.860556075801595
"5800","GPX7",237.524458767035,0.242103779407105,0.233854991550701,1.03527308868502,0.300541393977601,0.860556075801595

"5801","MAGOH",592.130696939558,-0.267355638733054,0.259356774760436,-1.03084116071387,0.302615312633644,0.860556075801595
"5802","NEXN-AS1",18.1170462834162,0.436369530426544,0.417071625745112,1.04627000133839,0.295436391547342,0.860556075801595
"5803","MTF2",192.260017968956,0.200535215523249,0.196114234806589,1.0225428853801 5,0.306524025968857,0.860556075801595
"5804","AC104506.1",25.7817072543834,-0.367231851269855,0.353412870244737,-1.03910152172882,0.298757523314784,0.860556075801595
"5805","NTNG1",41.4120270637204,0.365081392288217,0.358942978457137,1.01710136205329,0.309105212424429,0.860556075801595
"5806","AHCYL1",1012.07005908594,0.152659983452666,0.14623865264364,1.04390994236437,0.296527057801485,0.860556075801595
"5807","MAN1A2",504.95481837569,0.187399387040484,0.180604553549934,1.03762271413977,0.299445738851817,0.860556075801595
"5808","CHD1L",13.3211437986751,-0.511379542031459,0.501962670148124,-1.01876010397458,0.308316870525088,0.860556075801595
"5809","HIST2H2BE",21.5702736343839,0.397503322506083,0.390390067396902,1.01822089162414,0.308572993234991,0.860556075801595
"5810","SETDB1",87.1283363043059,0.20455521838671,0.196980819574311,1.0384524687671,0.299059452995301,0.860556075801595
"5811","MINDY1",91.0542774916621,0.235332877145207,0.229346286155277,1.02610284687966,0.304843108534267,0.860556075801595
"5812","PSMD4",826.062715773149,-0.216001323970974,0.210734669814852,-1.02499187324396,0.305367022254785,0.860556075801595
"5813","SNAPIN",240.062842093092,-0.233922916651955,0.224249825499465,-1.04313533413435,0.296885617903009,0.860556075801595
"5814","ASH1L",887.271474079634,0.208492569990644,0.203095976962334,1.02657163922706,0.304622214192879,0.860556075801595
"5815","SMG5",68.7320060829958,0.229849043404289,0.222668116159681,1.03224946331992,0.301955268818117,0.860556075801595
"5816","AL592435.1",9.39374584016709,-0.508537385403042,0.487182887544841,-1.04383261071795,0.296562840993831,0.860556075801595
"5817","PRRX1",5862.61485748843,0.132739040171123,0.130549094955469,1.0167748785727,0.309260535363786,0.860556075801595
"5818","RABGAP1L",333.204218402347,0.195959421382539,0.192197064050664,1.01957551927476,0.307929820213559,0.860556075801595
"5819","TRAF3IP3",9.68778370992965,-0.542771264584552,0.531462789572867,-1.02127801839292,0.307122737807508,0.860556075801595
"5820","COQ8A",236.606402187613,0.200805007533562,0.196062330506836,1.02418963915437,0.305745711519738,0.860556075801595
"5821","ARF1",1530.73117533659,-0.169070131113051,0.163204854113963,-1.03593812837817,0.300231008224637,0.860556075801595
"5822","TBCE.1",33.5161356721964,-0.314668182640147,0.305555259055678,-1.02982414248943,0.303092565409634,0.860556075801595
"5823","LINC01376",9.78184939673962,0.479206393780884,0.457339147077276,1.04781407155577,0.294724278017303,0.860556075801595
"5824","TCF23",3.60143837734403,0.960890317262405,0.919263878016297,1.04528236150857,0.295892487325334,0.860556075801595
"5825","PLB1",2.08696867457762,-1.11831089901747,1.07895741373429,-1.0

3647362238976,0.299981239085662,0.860556075801595
"5826","CYP1B1",230.74750558399,0.486791268827698,0.470971417516505,1.03358983310413,0.301327954345721,0.860556075801595
"5827","SFXN5",55.4887307191846,0.307917623446206,0.300137573099525,1.02592161409962,0.304928533669532,0.860556075801595
"5828","MAT2A",736.527102350803,0.291467977478321,0.285415960331257,1.02120420014368,0.307157702820283,0.860556075801595
"5829","RFX8",22.7288583731673,0.496627636129844,0.481059739892203,1.03236166934512,0.301902721295735,0.860556075801595
"5830","MBD5",224.79108001097,0.19681766611346,0.192304727338517,1.02346764345007,0.306086790919544,0.860556075801595
"5831","ARL5A",799.853285951326,-0.156367670265818,0.153818817669926,-1.01657048620255,0.30935780029504,0.860556075801595
"5832","SP3",547.350156483832,0.160126616246399,0.155078339644912,1.03255307358233,0.301813098280607,0.860556075801595
"5833","COQ10B",588.48831200168,-0.228507694996699,0.223797292018967,-1.02104763169937,0.307231872307159,0.860556075801595
"5834","HSPE1",1091.17652706912,-0.29165729825898,0.285299264328697,-1.02228549009842,0.306645798673009,0.860556075801595
"5835","INO80D",267.764971747381,0.228724365497366,0.223273211122129,1.0244147264593,0.305639429139618,0.860556075801595
"5836","MDH1B",5.42876049815927,0.662000053625451,0.64136144202932,1.03217937693733,0.301988094259319,0.860556075801595
"5837","SPAG16",331.147909003876,-0.234089796924939,0.22973467631615,-1.0189571756368,0.308223297737184,0.860556075801595
"5838","MFF",505.693788997738,-0.166676264719815,0.161793107159864,-1.03018149317774,0.302924815646281,0.860556075801595
"5839","GIGYF2",384.900335936171,0.20871599960281,0.204229949026239,1.02196568425915,0.306797142212541,0.860556075801595
"5840","HDLBP",1477.24620291433,0.194153171555436,0.190576604669255,1.0187670826248,0.308313556629084,0.860556075801595
"5841","CNTN4",110.199218213464,0.774981933934645,0.752066753668069,1.03046960945263,0.302789611286818,0.860556075801595
"5842","TRNT1",94.5979400625099,-0.255075938947021,0.250032981252217,-1.02016916996129,0.307648236461247,0.860556075801595
"5843","PRRT3",47.8593818794076,-0.299033563198695,0.288377724710621,-1.03695097635841,0.299758704898369,0.860556075801595
"5844","ZNF619",23.0022913196478,0.413491340697837,0.401719462797508,1.02930372807519,0.303336971988226,0.860556075801595
"5845","NKTR",869.120775155427,0.295862861622615,0.286455646052266,1.03284004242888,0.301678761327598,0.860556075801595
"5846","AC137630.4",4.31124651147645,0.775463962551105,0.756996225566266,1.02439607538469,0.305648234927323,0.860556075801595
"5847","NDUFAF3",894.466775611615,-0.257100531125083,0.247578995746076,-1.03845857501083,0.299056611513942,0.860556075801595
"5848","TMEM115",307.828096060977,-0.22046316232918,0.211128331959594,-1.04421401089539,0.296386386067382,0.860556075801595
"5849","DCP1A",337.24558648019,0.216182664885077,0.210897696689046,1.02505939267712,0.305335164356717,0.860556075801595
"5850","FHIT",80.6603692554149,0.313846293370939,0.304944122002961,1.0

2919279541939,0.303389087152529,0.860556075801595
"5851","ST3GAL6-
AS1",4.94554462115602,-0.843020299205127,0.820695965108436,-1.02720171
055519,0.304325492820318,0.860556075801595
"5852","GRAMD1C",2.42868731005879,1.05784217761803,1.02582159332418,1.
03121457425173,0.302440207614721,0.860556075801595
"5853","RABL3",140.215055687421,0.165566265548593,0.159561979410648,1.
03762980479511,0.299442436442533,0.860556075801595
"5854","EAF2",20.3860006052028,-0.417960478866138,0.400686453669777,-1
.04311107859563,0.29689685028244,0.860556075801595
"5855","WDR5B",34.0884957439701,0.355120745003444,0.345325975168762,1.
02836383747239,0.303778712513015,0.860556075801595
"5856","SLC12A8",4.35518480707566,-0.892103338160682,0.863441475488221
,-1.03319491069879,0.301512694091317,0.860556075801595
"5857","H1FX-
AS1",35.3859759274391,-0.293802834740551,0.283484988421865,-1.03639644
686699,0.300017227325037,0.860556075801595
"5858","CP",983.597207436588,-0.317850922247899,0.311444289938027,-1.0
205707168725,0.307457869115667,0.860556075801595
"5859","AADAC",2234.56483170044,-0.730042329225231,0.703088707807074,-
1.03833601808544,0.299113645640135,0.860556075801595
"5860","SKIL",354.404882570238,-0.261450833121077,0.251061724550401,-1
.04138069468486,0.297698898710869,0.860556075801595
"5861","TM4SF19-
AS1",5.99452098913791,0.640983348699136,0.627623678385989,1.0212861158
2581,0.307118902509262,0.860556075801595
"5862","SH3BP2",88.7646324880119,0.238708371288883,0.23161708939359,1.
03061640189789,0.302720741411706,0.860556075801595
"5863","TADA2B",79.3043887508815,0.261790522372131,0.25380002229239,1.
0314834490855,0.302314165735694,0.860556075801595
"5864","FAM200B",686.271839803644,-0.24327906798537,0.235679573046376,
-1.03224503015159,0.301957345052762,0.860556075801595
"5865","AC110792.2",3.00797503840359,1.21399414161196,1.18421997889336
,1.02514242560443,0.305295989702793,0.860556075801595
"5866","CEP135",71.9436585615421,0.309098785325535,0.302830352899098,1
.02069948526106,0.307396838477948,0.860556075801595
"5867","AC020741.1",3.94034200133795,0.837033286619283,0.8027106666232
28,1.04275839530131,0.297060204782464,0.860556075801595
"5868","PPM1K",117.021398057017,0.297349975139543,0.289837862575138,1.
02591832722496,0.304930083103862,0.860556075801595
"5869","ANAPC10",200.518531922207,-0.266598301991301,0.26124367279717,
-1.02049668471124,0.307492960784268,0.860556075801595
"5870","NEK1",192.51109008202,0.1829846396011,0.178067730595738,1.0276
1257746652,0.304132105450804,0.860556075801595
"5871","C5orf34",12.1467912563015,-0.430529851050112,0.41611141615868,
-1.03465041892995,0.300832198710993,0.860556075801595
"5872","CWC27",285.068558870386,0.170378951551955,0.165967055794546,1.
02658296091527,0.304616880742562,0.860556075801595
"5873","CMYA5",232.682589819089,0.418880027478816,0.407439909604061,1.
02807804931498,0.303913115186693,0.860556075801595

"5874","XRCC4",93.8775671887378,-0.233433034262279,0.228020792444119,-
1.02373573813225,0.30596011035899,0.860556075801595
"5875","MALINC1",29.5132014158881,-0.322883315829559,0.312834246297775
,-1.03212266448034,0.30201465766837,0.860556075801595
"5876","RPS14",30712.5454031512,-0.261214331221948,0.254875315138558,-
1.02487104755493,0.305424037307664,0.860556075801595
"5877","ERGIC1",761.468264541525,0.161078046805936,0.157529322555741,1
.02252738850533,0.306531356573292,0.860556075801595
"5878","HIGD2A",1479.94496525463,-0.309229800657394,0.297847217014882,
-1.03821618263414,0.299169420296526,0.860556075801595
"5879","MAPK9",188.631316943658,-0.183960921193205,0.175798022717771,-
1.04643339185071,0.295360982607952,0.860556075801595
"5880","TUBB2B",25.1541778821252,-0.66533894944686,0.649049919650997,-
1.02509672877646,0.305317548898904,0.860556075801595
"5881","PAK1IP1",190.626850712411,-0.21213469372561,0.203889194749855,
-1.04044107872353,0.29813502512998,0.860556075801595
"5882","HIST1H1D",7.73546094876636,-1.24093403049594,1.2079934986863,-
1.02726879891776,0.304293910006443,0.860556075801595
"5883","ZNF311",2.63254321163387,0.953033660489067,0.93743038447158,1.
01664473039913,0.30932246710511,0.860556075801595
"5884","VARS",108.821212314552,-0.248596882082222,0.239964587449095,-1
.03597320223326,0.300214644572195,0.860556075801595
"5885","C6orf89",556.059824810811,0.164265208773194,0.156707714474635,
1.04822668956596,0.294534176631529,0.860556075801595
"5886","UNC5CL",8.68339538646125,0.548266914529269,0.527329282336224,1
.03970504368786,0.298476956615853,0.860556075801595
"5887","CUL9",48.8615468823103,0.263152692890959,0.25315217830575,1.03
950396418525,0.298570415374069,0.860556075801595
"5888","AL355347.1",3.68720844572602,0.778353465548292,0.7625885242050
81,1.02067293283707,0.307409422520176,0.860556075801595
"5889","SMPD2",39.6580705170642,-0.330862905659772,0.323531168706892,-
1.02266160933484,0.30646786892844,0.860556075801595
"5890","ADGRG6",2.06008554374281,-1.31036987441916,1.28035974205287,-1
.02343882846408,0.306100408695337,0.860556075801595
"5891","AL078581.2",4.25694606367448,-0.716157896745143,0.684363740302
826,-1.04645797924397,0.295349636007225,0.860556075801595
"5892","RAET1E",2.93502796844996,0.944429650549894,0.917160315040338,1
.02973235437946,0.303135663116688,0.860556075801595
"5893","AL354892.2",2.4242769157534,-1.09172382120482,1.07365175272701
,-1.0168323373309,0.309233195897753,0.860556075801595
"5894","AC013470.2",2.56020328490508,-1.09573467105907,1.0714088188996
7,-1.02270454725619,0.306447560749854,0.860556075801595
"5895","URGCP",116.227825200641,0.191735678919581,0.186041992124866,1.
03060430997155,0.302726414126368,0.860556075801595
"5896","PURB",249.163097385435,0.233849058102645,0.224560763264751,1.0
413620558768,0.2977075458397,0.860556075801595
"5897","SLC25A40",56.8271740751731,0.27844330887896,0.26741562842471,1
.04123798043971,0.29776511261179,0.860556075801595
"5898","C7orf61",7.39794309314885,0.621223664596174,0.608386882295422,
1.02109970263054,0.307207203988364,0.860556075801595

"5899","AHCYL2",47.1457982622265,0.280931917135687,0.272451221678843,1
.03112739008688,0.302481084901316,0.860556075801595
"5900","MRPS33",459.765403158085,-0.268112912094263,0.262749550884355,
-1.02041244672676,0.307532893296245,0.860556075801595
"5901","AC005229.4",24.1055734326461,-0.35981413526657,0.3480611802489
48,-1.0337669228416,0.301245138472518,0.860556075801595
"5902","ZNF596",23.0462698336393,0.429229095754279,0.420087380955636,1
.02176146014633,0.306893814236245,0.860556075801595
"5903","DEFB1",30.5950800279987,2.1002569308024,2.06727118078109,1.015
95617949303,0.309650254297385,0.860556075801595
"5904","TNFRSF10D",71.1199706903084,0.561518169074475,0.54205612285771
8,1.03590411655928,0.300246876953587,0.860556075801595
"5905","BHLHE22",121.050954085149,0.335772735349471,0.330203195009662,
1.01686700923547,0.309216699422935,0.860556075801595
"5906","PKIA",49.5703606567617,-0.340764777308014,0.325107696233137,-1
.04815967525927,0.29456504587412,0.860556075801595
"5907","RBIS",1481.083172752,-0.254660241595966,0.243080140904785,-1.0
4763902410159,0.294804950722449,0.860556075801595
"5908","CA13",13.5288257029023,-0.511158009148538,0.4951376722185,-1.0
3235531818506,0.301905695463036,0.860556075801595
"5909","CPNE3",1082.98233269026,0.122970686355538,0.119547152861322,1.
02863751592802,0.303650041903501,0.860556075801595
"5910","MTDH",2165.84446971996,-0.12944317900479,0.126979899865817,-1.
0193989689831,0.308013595437045,0.860556075801595
"5911","TOP1MT",68.9379014975786,-0.250659083812772,0.23952411608958,-
1.04648787731682,0.295335839025157,0.860556075801595
"5912","GSDMD",409.385645317334,-0.151185140451333,0.147809152397209,-
1.02284018275845,0.306383415621326,0.860556075801595
"5913","ZNF623",81.6533679592608,0.217919984780768,0.212387023855307,1
.02605131342313,0.304867397515449,0.860556075801595
"5914","TONSL",8.86775979668104,0.508391208505223,0.494895691237093,1.
02726941759059,0.304293618767416,0.860556075801595
"5915","ZNF658",33.2424184894052,0.298952511365416,0.294162577448325,1
.01628328782893,0.309494504569675,0.860556075801595
"5916","AL354733.3",18.8401973776687,0.808931299868894,0.7852921663411
44,1.03010234221219,0.302961965857153,0.860556075801595
"5917","AL158071.3",2.81942003726335,1.13736466403248,1.09624721314004
,1.03750746218516,0.29949941982559,0.860556075801595
"5918","FAM120A",636.342144562124,0.146502387124086,0.142667849676557,
1.0268773760607,0.304478208655708,0.860556075801595
"5919","ZBTB6",56.9260900013837,0.260036703536303,0.253279552521863,1.
02667862820808,0.304571816020462,0.860556075801595
"5920","DOLPP1",20.9234082260321,-0.359241110193452,0.348912427064041,
-1.02960250861892,0.303196637170499,0.860556075801595
"5921","WDR5",91.3857084371116,0.209184787115547,0.204948177184958,1.0
2067161557024,0.3074100468239,0.860556075801595
"5922","UAP1L1",69.8257804178928,0.287996233515774,0.278329124168095,1
.03473265464609,0.300793781518912,0.860556075801595
"5923","SSNA1",496.607578035361,-0.212780744965017,0.203926459036099,-
1.04341901473095,0.296754270734155,0.860556075801595

"5924","RPP38",119.745801116701,-0.276541487848944,0.26962702878706,-1
.02564453234896,0.305059168487613,0.860556075801595
"5925","VIM-
AS1",49.827172584584,-0.330958993502882,0.324907760781071,-1.018624463
47008,0.308381285729562,0.860556075801595
"5926","RTKN2",9.35544509575727,-0.467800574959439,0.458578337531509,-
1.02011049513933,0.307676059842398,0.860556075801595
"5927","AC010997.4",2.90405759635457,0.902804300948052,0.8880743736603
08,1.01658636677808,0.309350242424347,0.860556075801595
"5928","ALDH18A1",89.4330406733345,0.239780397208459,0.234201858064958
,1.02381936330306,0.305920602762415,0.860556075801595
"5929","FRAT2",83.7558536863503,-0.246759528644057,0.236722166444773,-
1.04240144617646,0.297225596294992,0.860556075801595
"5930","AL139243.1",10.924633056819,-0.477577407308662,0.4631720964408
56,-1.03110142208155,0.302493261006065,0.860556075801595
"5931","NPM3",144.475550062234,-0.221933663863002,0.213113234888559,-1
.04138846176802,0.297695295366439,0.860556075801595
"5932","CUEDC2",539.71573702702,-0.244566138644431,0.234905684428293,-
1.04112482096654,0.297817621233729,0.860556075801595
"5933","ABLIM1",1147.56450714532,0.30527242583817,0.299243125746066,1.
02014849990981,0.307658037932358,0.860556075801595
"5934","NANOS1",45.0285463368624,0.678073415125563,0.647162773872453,1
.0477633178252,0.294747666955803,0.860556075801595
"5935","C10orf143",27.4000665271375,-0.382689412978098,0.3695288714448
91,-1.03561437968771,0.300382080745417,0.860556075801595
"5936","IFITM10",10.0846818140577,-0.722500674944883,0.691367744427121
,-1.04503092712773,0.296008675896688,0.860556075801595
"5937","ZNF195",109.357632517583,-0.228831064380541,0.223666177565125,
-1.02309194385866,0.306264375631337,0.860556075801595
"5938","DGKZ",114.613857411164,-0.211662770975504,0.208339927878893,-1
.01594914201248,0.309653605700077,0.860556075801595
"5939","PSMC3",833.244444873295,-0.225573004390225,0.217339028242018,-
1.03788540058731,0.299323411212642,0.860556075801595
"5940","TMEM138",126.793309302851,-0.227410468887374,0.223394188992828
,-1.01797844390068,0.308688200325334,0.860556075801595
"5941","TMEM258",2049.01182057921,-0.267601559836681,0.257601217944103
,-1.03882101945165,0.298887983779681,0.860556075801595
"5942","AP001453.1",3.36831464055998,-0.783679041395294,0.759605407863
436,-1.0316922882363,0.302216291218215,0.860556075801595
"5943","SF3B2",865.88617437713,-0.160646134075304,0.153750845219634,-1
.04484716065021,0.296093614242715,0.860556075801595
"5944","C11orf80",16.0386413049901,-0.404717032560187,0.39521920857643
3,-1.02403178736672,0.305820261055265,0.860556075801595
"5945","RPS6KB2",125.253641038118,-0.224469007544371,0.214721606733877
,-1.04539552846479,0.295840202503815,0.860556075801595
"5946","MYO7A",5.96504771761004,0.807194034247367,0.771502048312069,1.
04626298272751,0.295439631106247,0.860556075801595
"5947","MTMR2",113.579425369977,-0.186422845794478,0.181141964688137,-
1.02915327276831,0.303407655976298,0.860556075801595
"5948","ATM",367.893459357552,0.215782346450356,0.206349762357681,1.04

571163050978,0.295694191411647,0.860556075801595
"5949","NNMT",7911.30515767927,-0.337288995906974,0.331236634773648,-1
.01827201612968,0.308548703358222,0.860556075801595
"5950","FEZ1",506.36628273309,0.284258711741416,0.272380314373485,1.04
360960297462,0.296666048157641,0.860556075801595
"5951","VSIG10L2",18.0661781199256,0.724878338174567,0.697495700411825
,1.03925850402601,0.298684528081604,0.860556075801595
"5952","CRACR2A",34.2835240834264,0.365855342306976,0.35954108563708,1
.01756198921942,0.308886158978789,0.860556075801595
"5953","CLEC12A",9.98735898276424,0.869075759585818,0.835177975888692,
1.04058749712725,0.298067036399864,0.860556075801595
"5954","KLHL42",167.293036868377,-0.21453837368175,0.206593505960758,-
1.03845652206755,0.299057566829198,0.860556075801595
"5955","YARS2",135.425067272406,-0.231950963154811,0.226239713698796,-
1.02524423923033,0.305247958929879,0.860556075801595
"5956","NCKAP5L",184.041312242741,0.339791137372572,0.32454880578336,1
.04696468240708,0.295115867416304,0.860556075801595
"5957","KRT7",3.62546212571344,-0.901117813828919,0.859799583176456,-1
.04805565327191,0.29461296650424,0.860556075801595
"5958","ZNF740",93.5014435103012,0.246786864679705,0.240660750719515,1
.02545539287929,0.305148362772094,0.860556075801595
"5959","HOXC9",512.627459639041,-0.235492424861877,0.229063437083629,-
1.02806640754238,0.303918591007097,0.860556075801595
"5960","COQ10A",87.2757228831285,-0.246967851554023,0.242333787398791,
-1.01912264981691,0.308144742452001,0.860556075801595
"5961","STAT6",513.782809965632,0.190485381522898,0.1869263577259,1.01
903971082675,0.308184114286511,0.860556075801595
"5962","PAWR",189.792802077765,0.421874485865949,0.40910599465848,1.03
12107164749,0.302442016299526,0.860556075801595
"5963","AC025164.1",27.1419915725133,-0.373014964585511,0.361465980370
905,-1.03195040430293,0.30209535172594,0.860556075801595
"5964","GLTP",349.36318709404,-0.182641720564563,0.176749682184628,-1.
03333549631948,0.301446921259671,0.860556075801595
"5965","GIT2",181.112951769763,0.185829711638088,0.180823101807124,1.0
2768788822296,0.304096666939483,0.860556075801595
"5966","SRSF9",1270.10637888498,-0.25448071340675,0.246573099607111,-1
.03207005878678,0.302039298910634,0.860556075801595
"5967","DYNLL1",3894.92793846798,-0.30424287956917,0.298285498249703,-
1.01997207827542,0.307741703200146,0.860556075801595
"5968","EIF2B1",337.115599055268,-0.157042281689588,0.150754807074791,
-1.04170662771422,0.297547715264288,0.860556075801595
"5969","KBTBD7",120.81044757396,0.197376696870323,0.190025622200481,1.
03868464991572,0.298951422429766,0.860556075801595
"5970","GTF2F2",259.734457214956,-0.173470644283108,0.16742214816466,-
1.03612721605089,0.300142796703219,0.860556075801595
"5971","RUBCNL",6.46647604876519,-0.653895325030748,0.632035790482774,
-1.03458591250232,0.300862335786099,0.860556075801595
"5972","VCPKMT",50.5647378978362,-0.27896016620119,0.270352687254508,-
1.03183796334371,0.302148031633446,0.860556075801595
"5973","AL355073.2",4.1630852576508,0.89439833230654,0.875534369029767

,1.02154565707989,0.306995989224335,0.860556075801595
"5974","ARID4A",500.153024429373,0.213638790857059,0.209161539870386,1
.02140570866636,0.307062261804666,0.860556075801595
"5975","L3HYPDH",142.005786041428,-0.218530371394111,0.211283037129509
,-1.03430154338495,0.300995215569833,0.860556075801595
"5976","AC004817.5",3.13718205689442,-0.831086024935888,0.817944630980
527,-1.01606636128855,0.309597786432501,0.860556075801595
"5977","RGS6",4.4534616714762,1.27546402551754,1.22501960752917,1.0411
7845761679,0.297792731811903,0.860556075801595
"5978","COQ6",94.133242020828,-0.254685240207701,0.244265299183715,-1.
0426582943169,0.297106580121041,0.860556075801595
"5979","TC2N",172.204518765803,0.443053516539183,0.426114825858186,1.0
3975146991631,0.298455381172388,0.860556075801595
"5980","SLC25A29",114.525692092296,0.274157410403866,0.265697645170165
,1.03183982013948,0.302147161653227,0.860556075801595
"5981","AMN",9.86197975712779,-0.590227225434869,0.57158139021189,-1.0
3262148758214,0.301781068443415,0.860556075801595
"5982","ZNF106",1750.99364047678,0.20067076416489,0.196487027681165,1.
02129268549226,0.307115790851303,0.860556075801595
"5983","POLR2M",241.252052064854,0.168169156499898,0.165058148176944,1
.01884795362917,0.308275155778328,0.860556075801595
"5984","UBAP1L",22.5066716269746,0.501863069647999,0.485660848478207,1
.03336118449853,0.301434904105086,0.860556075801595
"5985","SNAPC5",187.106025250126,-0.234733910963347,0.227294636224634,
-1.03272965373173,0.301730432194709,0.860556075801595
"5986","AC024337.2",9.0333728740782,0.52264365411714,0.503668364218164
,1.03767417460978,0.299421772150052,0.860556075801595
"5987","MRPL28",317.450303490584,-0.247649303543567,0.237845353920875,
-1.04121984920485,0.297773525509371,0.860556075801595
"5988","METTL22",47.6192441750309,0.323666391778653,0.313268468653375,
1.03319173222244,0.301514181248509,0.860556075801595
"5989","AC087190.1",34.4744292027286,-0.46004139646157,0.4412374195931
76,-1.04261646005847,0.297125962762219,0.860556075801595
"5990","ARL6IP1",1113.74901673447,0.172800808036165,0.167701542677517,
1.03040678861526,0.302819087807287,0.860556075801595
"5991","COQ7",143.239500077875,-0.202732067489319,0.196943338665352,-1
.02939286427861,0.303295100912767,0.860556075801595
"5992","USP31",51.826695663583,0.308491916658646,0.298975515696572,1.0
3183003444246,0.302151746647332,0.860556075801595
"5993","COG7",92.0019260792402,0.214636935761048,0.209814445943514,1.0
2298454615862,0.306315152646206,0.860556075801595
"5994","AC109460.2",4.91808640202339,0.754939104441838,0.7367609228825
5,1.02467310764551,0.305517456166425,0.860556075801595
"5995","ZNF668",42.7202159705509,-0.289439879479215,0.279479700292544,
-1.03563829207003,0.300370920654458,0.860556075801595
"5996","PRSS36",26.6740118550409,-0.446037766494178,0.43064369961447,-
1.03574664367199,0.3003203555999,0.860556075801595
"5997","AC007336.1",14.7028670944411,0.747702695783735,0.7312515660916
3,1.02249722319233,0.306545626233616,0.860556075801595
"5998","ZNF319",34.1606245811552,0.29708915837342,0.288167770067511,1.

03095900802445,0.302560043147762,0.860556075801595
"5999","CDH5",14.9706924635263,0.598360764861729,0.579513255374906,1.03252299979684,0.30182717884067,0.860556075801595
"6000","AC027682.1",2.87783175368717,-1.06157538319258,1.04470847970316,-1.0161450814434,0.309560303996544,0.860556075801595
"6001","SLC7A6OS",175.82312836816,0.184799212933568,0.176660728223381,1.04606844312278,0.295529433628617,0.860556075801595
"6002","AC137932.3",19.8483107973462,0.379551076960084,0.369408125868001,1.0274573036753,0.304205180323871,0.860556075801595
"6003","RFLNB",734.902225124184,0.668272043956649,0.655396488360953,1.01964544489382,0.307896643840358,0.860556075801595
"6004","YWHAE",1943.25993645736,-0.170353589129392,0.166955324546425,-1.02035433486293,0.307560442878023,0.860556075801595
"6005","VMO1",25.9279065197568,-0.420657315727954,0.412051743994586,-1.02088468707823,0.307309074903941,0.860556075801595
"6006","BORCS6",81.3417366540494,-0.294212663625223,0.283198419705096,-1.03889232126223,0.298854817994506,0.860556075801595
"6007","LRRC75A",245.931186149152,-0.483925339800292,0.469059040348362,-1.03169387683241,0.302215546787862,0.860556075801595
"6008","KRT10",1926.77313209649,-0.275455812341824,0.266020433831097,-1.03546862312358,0.300450112420579,0.860556075801595
"6009","RAB5C",916.462865801262,-0.243452389919076,0.234370050989981,-1.03875213104546,0.298920029311688,0.860556075801595
"6010","AC003102.1",8.13461975121485,-0.564373783385077,0.54008353690561,-1.04497498038662,0.296034533204128,0.860556075801595
"6011","NMT1",678.680910150818,0.182862793442345,0.177938079863023,1.02767655795271,0.304101998378567,0.860556075801595
"6012","TBKBP1",36.373574490202,0.30301769449948,0.293147349977245,1.03367024986922,0.301290345631242,0.860556075801595
"6013","SNF8",796.715102415505,-0.277932787202199,0.267170648202057,-1.04028189126525,0.298208954865959,0.860556075801595
"6014","NXPH3",244.82299780987,0.248047480069352,0.237500983513315,1.04440611739802,0.296297534534058,0.860556075801595
"6015","SPATA20",216.613054206362,-0.222046611449122,0.212080472684262,-1.04699225081273,0.295103152229546,0.860556075801595
"6016","AC007448.5",3.79436204547669,0.771865538062561,0.757853914483687,1.01848855473475,0.308445837243,0.860556075801595
"6017","AC005332.2",5.36018767919158,0.638784197467408,0.628231718890075,1.01679711205282,0.309249956256626,0.860556075801595
"6018","AC139530.1",51.9364470787525,-0.386695877477765,0.379374874846891,-1.01929754213119,0.308061730510154,0.860556075801595
"6019","SIRT7",101.196946567003,-0.213462045966707,0.208090463250051,-1.02581369003058,0.304979411859456,0.860556075801595
"6020","SPIRE1",81.8789533059955,-0.232528161003491,0.22852526711631З,-1.01751619826415,0.308907930490915,0.860556075801595
"6021","SLC39A6",408.937724746641,0.181231111826022,0.174995071848567,1.0356355176839,0.300372215467422,0.860556075801595
"6022","ZBTB7C",25.0288600633858,0.383747268239009,0.373648414725762,1.02702768997604,0.304407425661921,0.860556075801595
"6023","LINC01905",2.29902403047547,1.35374969977767,1.31208093037362,

1.03175777380758,0.302185605105115,0.860556075801595
"6024","NARS",542.978642398313,-0.182753174757515,0.17968525036635,-1.01707387993678,0.309118284920902,0.860556075801595
"6025","ZNF236-DT",8.65432014428756,0.655096744905403,0.629906228706551,1.03999089872564,0.298344129079522,0.860556075801595
"6026","NDUFS7",734.367463596168,-0.242359183398936,0.235042455533129,-1.03112938830226,0.302480147974176,0.860556075801595
"6027","AMH",3.14175063849807,-1.03715920607717,0.992518234607981,-1.04497748244074,0.296033376779503,0.860556075801595
"6028","AC092068.2",14.5846906818259,-0.478024164277921,0.46984162205963,-1.01741553288196,0.30895579585824,0.860556075801595
"6029","AC007292.2",2.95261058955042,-0.928882790423417,0.887622498041366,-1.04648405428332,0.295337603205921,0.860556075801595
"6030","GTF2F1",309.590496298935,-0.207014784271358,0.20369908848252,-1.01627742084434,0.309497297626332,0.860556075801595
"6031","LRRC8E",15.6890574067508,-0.398730990733842,0.387690821235449,-1.02847673685751,0.303725628196413,0.860556075801595
"6032","CDKN2D",95.1582806983265,-0.218440270222014,0.20999491150142,-1.04021696840277,0.29823910981459,0.860556075801595
"6033","C19orf53",1437.96782115889,-0.24865812298942,0.240557243012774,-1.03367547730091,0.301287901013088,0.860556075801595
"6034","GIPC1",308.495031290363,-0.230837112324899,0.223889069743672,-1.03103341574102,0.302525149956872,0.860556075801595
"6035","AC024075.1",20.4018310067813,0.532974605748697,0.517767865604904,1.02936980286721,0.30330593347263,0.860556075801595
"6036","FOSB",20952.0351295611,0.3219362389935,0.316441516549018,1.01736410097008,0.308980253102505,0.860556075801595
"6037","AC007193.1",6.43884006235893,0.89033702994681,0.87319680404728,1.01962928153205,0.307904312374894,0.860556075801595
"6038","DACT3",60.7164383749591,-0.345776536621511,0.333537712843909,-1.03669397284417,0.299878501819411,0.860556075801595
"6039","AC073548.2",9.89728787204303,1.66349762873754,1.63184973973852,1.01939387446549,0.308016013070374,0.860556075801595
"6040","GRIN2D",11.2677046968648,-0.552700803010533,0.533359929493925,-1.036262329521,0.300079775338677,0.860556075801595
"6041","LMTK3",8.70609211674653,-0.60921282558981,0.586816531103309,-1.03816575249575,0.299192893919995,0.860556075801595
"6042","RPL13A",34607.7055078167,0.198635169969712,0.19409930309421,1.02336879526713,0.306133507604896,0.860556075801595
"6043","CPT1C",12.903606455708,-0.694865223295539,0.681049800428134,-1.02028548111859,0.307593087068214,0.860556075801595
"6044","VRK3",140.467431867278,0.21032218580221,0.206486797199522,1.01857449800522,0.308405016398513,0.860556075801595
"6045","ZNF160",151.277891935287,0.236824014124169,0.23188208309411,1.02131225907632,0.307106520139311,0.860556075801595
"6046","PPP1R12C",279.103033171368,-0.182598882479284,0.177982849181468,-1.02593527027489,0.304922096197747,0.860556075801595
"6047","PPP6R1",110.593392697066,0.202641975741368,0.198130865826993,1.02276833493634,0.306417392989867,0.860556075801595

"6048","ZNF264",237.5843099927,0.197712678414761,0.191408187432054,1.0
3293741541199,0.301633187760679,0.860556075801595
"6049","ZNF419",19.7962838506411,0.43474280761244,0.4210561303055,1.03
250558849959,0.301835331017817,0.860556075801595
"6050","CDC25B",94.5835839318003,0.253864098979095,0.245409278785025,1
.03445191736811,0.300924944060115,0.860556075801595
"6051","AP5S1",59.8064627540659,0.265192521270126,0.25885895657649,1.0
2446724184243,0.305614635798321,0.860556075801595
"6052","GPCPD1",156.176464938268,0.28574772196488,0.273067010387927,1.
04643809429392,0.295358812499876,0.860556075801595
"6053","HM13",780.656856606113,-0.22766909283978,0.224075251935739,-1.
01603854452018,0.309611032045079,0.860556075801595
"6054","UQCC1",87.7306066070902,0.213020022805509,0.206098299283517,1.
03358457370126,0.30133041413304,0.860556075801595
"6055","NCOA3",280.3518682016,0.193030555043918,0.187273021476469,1.03
074406298385,0.302660855770534,0.860556075801595
"6056","ZFAS1",4372.68417241462,-0.238243554721833,0.230927552766987,-
1.03168094004888,0.302221609115853,0.860556075801595
"6057","SPATA2",49.622130554317,-0.269020815189979,0.258408725149644,-
1.0410670732352,0.297844419905524,0.860556075801595
"6058","GNAS",8751.45892921794,-0.199397397185514,0.191265311707498,-1
.04251730439471,0.297171906923407,0.860556075801595
"6059","USP25",164.514668181279,0.189223588655341,0.185715149117043,1.
01889150968555,0.30825447489747,0.860556075801595
"6060","CHODL",7.69283468729181,-0.700199167141976,0.668544356459401,-
1.04734885632753,0.294938710589748,0.860556075801595
"6061","NCAM2",74.8440797191222,0.454666928847173,0.441871101998383,1.
02895827944149,0.303499280236758,0.860556075801595
"6062","LINC00310",97.0698217804567,-0.485915149849407,0.4688175703452
48,-1.03646957918315,0.299983124429195,0.860556075801595
"6063","PEX26",148.820668005473,0.217938682298888,0.211398868906455,1.
03093589585537,0.302570882048707,0.860556075801595
"6064","GRK3",38.075845312012,-0.336042250594134,0.324891103102934,-1.
03432272347472,0.300985317206422,0.860556075801595
"6065","XBP1",817.775330500448,-0.273176083258439,0.261891150186091,-1
.04309016575906,0.296906534934338,0.860556075801595
"6066","RNF215",91.5621935325586,-0.212207995357476,0.203820182888349,
-1.04115300236837,0.297804543830697,0.860556075801595
"6067","IL2RB",8.1802407694874,0.771318499519809,0.739414969495024,1.0
4314698963503,0.296880220514998,0.860556075801595
"6068","POLR2F",795.369972478652,-0.236821361060285,0.232731383461208,
-1.01757381208434,0.30888053791016,0.860556075801595
"6069","SERHL2",28.0856725333724,0.31958089130283,0.313263225348961,1.
02016727608813,0.307649134502811,0.860556075801595
"6070","SLC25A6",4590.8547280076,-0.24051385257092,0.230113253361723,-
1.0451977409265,0.295931587337924,0.860556075801595
"6071","BX842570.1",2.59128133825985,0.973655097794544,0.9553630974783
42,1.01914664734747,0.308133351243622,0.860556075801595
"6072","ATP6AP2",1709.82192255797,-0.202909610124954,0.194991371073931
,-1.04060815105516,0.298057446674541,0.860556075801595

"6073","MED14",75.2850369972834,0.270175113730243,0.260493469065078,1.03716655430907,0.299658242273119,0.860556075801595
"6074","WDR13",555.608495527955,-0.212744324321445,0.20338516725364,-1.0460169106439,0.295553224888303,0.860556075801595
"6075","PIM2",16.3897209688553,0.471588220414341,0.451777322357415,1.04385102367147,0.296554320620228,0.860556075801595
"6076","SPIN2A",60.397212142098,0.300152066554663,0.291351777650319,1.03020502903849,0.302913769464622,0.860556075801595
"6077","MAGEE1",31.3528369980408,0.2907459925597,0.282376228855958,1.02964046845463,0.303178810831399,0.860556075801595
"6078","BTK",3.76003955327586,-0.793371535162528,0.766015047524898,-1.03571272878519,0.300336182241744,0.860556075801595
"6079","BEX2",2.26892127004591,-1.00240364453875,0.970431289731264,-1.0329465415489,0.301628916681349,0.860556075801595
"6080","WDR44",82.5144056691811,0.218934422360871,0.211031131275252,1.03745083030101,0.299525799645188,0.860556075801595
"6081","C1GALT1C1",338.483183435694,-0.28194774708084,0.276641847570437,-1.01917967059938,0.308117676138518,0.860556075801595
"6082","XIAP",431.172374613565,0.194518724562323,0.188215996574774,1.03348667542743,0.301376202937686,0.860556075801595
"6083","MOSPD1",58.2147512944425,-0.281604105672957,0.275608926684883,-1.02175248479861,0.306898063291516,0.860556075801595
"6084","AC007325.4",78.1333002508539,-0.298225623303681,0.287564792550905,-1.03707279551925,0.299701932544562,0.860556075801595
"6085","SESN2",133.844322812685,0.29059533246041,0.286256016117569,1.01515886513651,0.310030105321248,0.860705377218362
"6086","BX470102.2",10.198813759345,0.492551458165675,0.485154320731454,1.01524697836983,0.309988111903288,0.860705377218362
"6087","BCAP29",571.196698814005,-0.170768157112352,0.16820166284376,-1.01525843576812,0.30998265175866,0.860705377218362
"6088","APEX1",849.673309392882,-0.248766558326182,0.244952908096726,-1.01556891183325,0.309834715278062,0.860705377218362
"6089","STX1B",5.12700014021058,-0.710108482953613,0.699150521180936,-1.01567325123947,0.309785009824161,0.860705377218362
"6090","PSMG2",1253.47421982794,-0.257312157034285,0.253473103734095,-1.01514580144258,0.310036331597518,0.860705377218362
"6091","PTTG1IP",810.668345323039,0.209115382768876,0.206007054966529,1.01508845317386,0.310063665284897,0.860705377218362
"6092","NBPF9",70.4244984170436,0.522257273880131,0.5148816184232,1.0143249539176,0.310427720647634,0.860867951568017
"6093","NCBP2",337.163163791775,-0.147610979866454,0.145502395481695,-1.01449175030953,0.310348163917516,0.860867951568017
"6094","ECSCR",2.39719777180056,-0.97277647479804,0.958910023265847,-1.01446063884593,0.31036300210404,0.860867951568017
"6095","NDUFAF6",71.6766683786677,-0.252134943128254,0.248553978522527,-1.01440719085252,0.310388494485953,0.860867951568017
"6096","SYCP3",3.71872216745026,0.900441506688555,0.887426240345619,1.01466630774617,0.310264919823667,0.860867951568017
"6097","DGCR11",6.44104326232616,0.643851096865861,0.634669504849163,1.01446672944981,0.310360097237059,0.860867951568017

"6098","CRY1",76.4165759752981,-0.317044624027481,0.31263240841332,-1.01411311014285,0.310528782993926,0.860902307792529
"6099","AC091057.6",6.16058416577126,-0.57385917155674,0.565888335827499,-1.014085527523,0.310541943176404,0.860902307792529
"6100","CYP3A5",37.3859430383305,-0.341381087231849,0.33670826862537,-1.01387794432716,0.310640996801399,0.860909486160841
"6101","MGST1",7848.45579457168,-0.253121588743016,0.249704926060251,-1.01368280048244,0.310734133684985,0.860909486160841
"6102","ZNF609",183.532932941731,0.301137288438679,0.297081142147436,1.01365332805012,0.310748201682021,0.860909486160841
"6103","PAM16",254.196058920813,-0.225138828916842,0.222087330579783,-1.01374008291735,0.310706792416291,0.860909486160841
"6104","CXCL14",47861.7776319289,-0.409706921729892,0.404240927753878,-1.01352162435997,0.310811072586521,0.860942597525048
"6105","AC027796.1",10.2276864783952,0.630273672267918,0.621940394008491,1.01339883747656,0.310869694467647,0.860963930230789
"6106","PPP4R3A",279.130701942544,0.188553168332733,0.186081643118166,1.01328193997619,0.310925511371572,0.860977488743946
"6107","NBPF11",89.1597144147522,0.316386981709363,0.312366856688985,1.01286988338965,0.311122315297262,0.861381383174407
"6108","CHROMR",93.1082948124036,0.262870920886002,0.259674322898076,1.01231002723816,0.311389841981968,0.861398724728747
"6109","FAM193A",172.95678590458,0.279279815201497,0.275893311049792,1.01227468741022,0.311406734171163,0.861398724728747
"6110","TMED2-DT",6.26243133679032,0.583501146891473,0.576452155824893,1.01222823263883,0.31142894014444,0.861398724728747
"6111","AC009090.6",5.77906617487113,0.771973892239599,0.762656433365991,1.01221711175042,0.311434256225859,0.861398724728747
"6112","RPS15",22613.8822074226,-0.231725136863424,0.228831875124694,-1.01264361329537,0.311230419967309,0.861398724728747
"6113","RRP7A",236.093532311473,-0.330818605669184,0.326809016565933,-1.0122689059971,0.311409497703488,0.861398724728747
"6114","ZNF227",65.1652929581971,0.232391173262452,0.229623825635261,1.01205165718119,0.311513354993288,0.861476579363185
"6115","PPP1R12B",262.752846555138,0.337376723038932,0.333584019426996,1.01136956026386,0.311839584527143,0.861577373103828
"6116","NDUFC1",1099.12160872314,-0.234821214841333,0.232137593286888,-1.01156047806151,0.311748250733142,0.861577373103828
"6117","ZFYVE16",398.976469099951,0.173451785021308,0.17145991764831,1.01161710212112,0.31172116555399,0.861577373103828
"6118","HSPA8",1994.93160059856,-0.284486337490541,0.28132711602111,-1.01122970836979,0.311906499927167,0.861577373103828
"6119","IREB2",341.328788704186,0.194110857925466,0.191943302915548,1.01129268370916,0.311876366736165,0.861577373103828
"6120","CRYM",10.7696462496243,0.520896466157647,0.514979854389258,1.01148901596434,0.311782435655841,0.861577373103828
"6121","NUP62",206.765981396679,0.183985744809697,0.181921399092838,1.01134745954656,0.311850158501098,0.861577373103828
"6122","RCSD1",9.48140627694022,-0.534993257654538,0.52916631919426,-1

.0110115444784,0.312010904425553,0.861676004774189
"6123","EAF1",69.8319160849697,-0.223663326055349,0.221377275166975,-1
.01032649302712,0.312338892307311,0.861676004774189
"6124","ADCY6",102.935717810006,0.199406287580443,0.197359479666463,1.
01037096326682,0.312317594022039,0.861676004774189
"6125","AC087284.1",4.69899823676068,0.936252702930093,0.9266291212618
71,1.0103855808623,0.312310593375597,0.861676004774189
"6126","NOB1",363.390790823957,-0.244594803876169,0.242028006416125,-1
.01060537372535,0.312205342829214,0.861676004774189
"6127","C1QBP",743.771725012211,-0.234064629302786,0.231607714289792,-
1.01060808799279,0.312204043214609,0.861676004774189
"6128","PMAIP1",20.9021001724175,-0.608672374966337,0.602464881238655,
-1.01030349472806,0.312349907337415,0.861676004774189
"6129","SLC25A5",948.293653213887,-0.237251035415696,0.234730154720712
,-1.01073948380421,0.312141134032934,0.861676004774189
"6130","LAGE3",203.879464981397,-0.259471369030668,0.25686837012086,-1
.0101335906347,0.312431290794017,0.861759912682746
"6131","HOXA1",2.98463411059743,1.09665382176221,1.08579627381645,1.00
999961798321,0.312495473067627,0.861773998375155
"6132","MALAT1",329760.339128053,-0.149267645640536,0.147849606595305,
-1.00959109109511,0.312691239651737,0.861773998375155
"6133","RABGGTA",66.4124870618994,-0.261685614994036,0.259148608472117
,-1.00978977482023,0.312596020080431,0.861773998375155
"6134","SPG7",391.325803423827,0.218005198414913,0.215894471331358,1.0
0977666111845,0.312602304259365,0.861773998375155
"6135","AC008555.4",16.2792543491161,-0.832985179215634,0.825009033059
671,-1.00966795009066,0.312654402559476,0.861773998375155
"6136","ZSWIM5",12.3031874949582,0.537235481833508,0.532254387144841,1
.00935848498194,0.312802740867571,0.86187474845901
"6137","SRP9",1275.89708777632,-0.243169022280137,0.240927872971426,-1
.00930215869617,0.312829745167551,0.86187474845901
"6138","DICER1-
AS1",19.5739332210735,0.361689908568593,0.358396447663126,1.0091894351
2678,0.312883792416319,0.86188321312726
"6139","NDUFS1",425.018539030025,0.144362681681811,0.143089558745481,1
.00889738529835,0.313023849317714,0.862128562349552
"6140","AKNAD1",15.2763342449516,-0.445007078566849,0.442403166010821,
-1.00588583616954,0.314470490338002,0.86219151804509
"6141","KLHL12",76.2865949198565,0.209629751296362,0.20814507172505,1.
00713290763508,0.313870909178263,0.86219151804509
"6142","U91324.1",2.17828694174047,-1.07854455341947,1.0724469513903,-
1.00568569104632,0.314566788464694,0.86219151804509
"6143","DNAJC27",119.186971967876,-0.277678713326389,0.275359516518582
,-1.00842243201589,0.313251708470774,0.86219151804509
"6144","GPR155",53.5767308988107,0.265322147697226,0.263520760163308,1
.00683584675758,0.314013665191514,0.86219151804509
"6145","COL6A3",12462.7132233092,0.22394766645627,0.222666630917004,1.
00575315454314,0.314534326810155,0.86219151804509
"6146","ZIC4",33.5609465823794,-0.467574265188769,0.463855954552135,-1
.00801608904692,0.313446738425936,0.86219151804509

"6147","KCNMB2",9.8693627447927,0.889197540346206,0.882929869185558,1.
00709871913885,0.313887336677884,0.86219151804509
"6148","DHX16",118.726253204561,-0.225409154868375,0.224097614199957,-
1.00585254186261,0.314486508266947,0.86219151804509
"6149","GLCCI1",45.4528674473423,0.290290179010239,0.288377276488888,1
.00663333305814,0.3141100995268,0.86219151804509
"6150","TAX1BP1",998.544254763315,-0.179264686735747,0.177851136800019
,-1.00794793871527,0.313479455948801,0.86219151804509
"6151","CAPZA2",706.699053137912,-0.211533381423887,0.210288818266224,
-1.00591835156964,0.31445484765978,0.86219151804509
"6152","FRMPD1",18.9295222899352,0.470028551536909,0.466278324036073,1
.00804289478519,0.313433870177726,0.86219151804509
"6153","AL589863.2",2.2239345944847,0.967917866762081,0.96214541740413
1,1.00599956020528,0.314415781620511,0.86219151804509
"6154","PDZD7",2.30968366308135,-1.15142375081951,1.14259614483355,-1.
00772591963125,0.313586058170368,0.86219151804509
"6155","AP000873.2",89.6056294966927,-0.295517790760547,0.293184469915
794,-1.00795854175163,0.313474365509218,0.86219151804509
"6156","ATP5F1B",1352.51308372096,-0.20312838878647,0.201966052390071,
-1.00575510776511,0.314533387004088,0.86219151804509
"6157","TMPO-
AS1",6.85248238594478,0.713726753687155,0.708981651940319,1.0066928413
9279,0.314082403287393,0.86219151804509
"6158","ARPC3",1885.37361495738,-0.27017474716854,0.268652693361602,-1
.00566550734293,0.31457650075823,0.86219151804509
"6159","SPART",968.956285695874,0.184058187796246,0.182743933066599,1.
00719178310105,0.31384262096808,0.86219151804509
"6160","CBLN3",30.5572710380284,0.347952961404853,0.345711631824643,1.
00648323450496,0.314183172484232,0.86219151804509
"6161","PHF12",107.580164982433,0.232981634298946,0.231586161506973,1.
00602571752514,0.314403199119975,0.86219151804509
"6162","COX11",396.33789106248,-0.200055922415879,0.198841921133647,-1
.00610535884642,0.314364891157067,0.86219151804509
"6163","AC040904.1",3.17582604290407,-0.872463867083294,0.865664561390
431,-1.00785443461142,0.313524348934998,0.86219151804509
"6164","PSMD12",525.420457329053,-0.15528183511251,0.154197436797385,-
1.00703253139382,0.313919141355885,0.86219151804509
"6165","ZNF407",95.9216421426416,0.255216193305191,0.253455586596069,1
.00694641113564,0.313960527221824,0.86219151804509
"6166","REEP6",11.0364070667551,0.680139815912939,0.674463895367488,1.
00841545497755,0.31325505652311,0.86219151804509
"6167","HIRA",65.5810519594583,0.24233168350619,0.240724006900759,1.00
667850550566,0.314089294634678,0.86219151804509
"6168","ARSD",141.836348354504,0.285917034657466,0.28356098557653,1.00
830879140918,0.313306243811206,0.86219151804509
"6169","SRPX",4723.74798006284,-0.338224449781327,0.335573225569485,-1
.00790058327014,0.313502191612958,0.86219151804509
"6170","REP15",6.58524671393099,0.63054185095025,0.627250136800778,1.0
0524784923326,0.314777519924858,0.862481163317898
"6171","LINC00482",3.27037740207276,-0.881479122510869,0.8769819436422

51,-1.00512801763049,0.314835210551104,0.862481163317898
"6172","ZCCHC3",138.39877750369,0.183337729295307,0.182389347572382,1.00519976487414,0.314800668382324,0.862481163317898
"6173","TMEM182",18.6131951562981,-0.550550623749476,0.547949753107489,-1.00474654952801,0.315018907321623,0.862704840459021
"6174","AEN",27.497599338574,0.32055149055301,0.319024782803291,1.00478554592626,0.315000125292195,0.862704840459021
"6175","AC239799.1",3.87752459102008,0.772083748794267,0.768834830891295,1.00422576836068,0.315269804286134,0.862762399185907
"6176","LIMD1",60.871847493625,0.278960061343117,0.27780069847876,1.00417336194871,0.31529505941387,0.862762399185907
"6177","RBM47",66.1339357772176,-0.300820403919635,0.29955804490452,-1.00421407148493,0.315275441001983,0.862762399185907
"6178","OLFM2",193.94109583431,-0.415285572197514,0.413442808182402,-1.00445711953054,0.315158329932592,0.862762399185907
"6179","SDCBP2",29.7583779312281,-0.359780136577692,0.358234327371034,-1.00431507839576,0.315226768028457,0.862762399185907
"6180","POLK",382.769107839663,0.185495224947128,0.18476203101121,1.00396831498282,0.31539388618964,0.862893176002336
"6181","TWF1",255.158595020376,-0.162822006250447,0.162212169145697,-1.00375950280402,0.315494548600889,0.8630289318466
"6182","AL591623.1",4.61565201947448,-0.862996934218269,0.859987034790364,-1.00349993582012,0.315619707877204,0.863231643608502
"6183","NUTM2A",2.49531773827789,-0.879269879914796,0.876504754750064,-1.00315471781499,0.315786217300041,0.863547365697734
"6184","AL078459.1",16.5587472236099,0.380392313768521,0.379352573296162,1.00274082883721,0.315985924900211,0.863706538846809
"6185","TRIM33",250.010742603262,0.192342466691985,0.191841635368709,1.00261064978055,0.316048755372913,0.863706538846809
"6186","GYPE",6.7588968098858,-0.667862989180303,0.665959557129464,-1.00285817964539,0.315929292966061,0.863706538846809
"6187","GAS7",956.17390561439,-0.252704775761321,0.252043832616945,-1.0026223341294,0.316043115627228,0.863706538846809
"6188","AC097662.1",12.7772525846318,0.423512496373795,0.42289936596087,1.00144982580319,0.316609385683905,0.864121306399267
"6189","HNRNPDL",1522.9373509884,0.211171924250658,0.210725867983218,1.00211676085006,0.3162872037416,0.864121306399267
"6190","TRAF3IP2",172.364179230789,-0.191759340805167,0.191477229900985,-1.0014733391763,0.316598023219528,0.864121306399267
"6191","PPDPFL",139.264334015441,0.415757051390047,0.415145815919865,1.00147233922815,0.316598506423151,0.864121306399267
"6192","AC079630.1",26.7368785042505,-0.293400331979693,0.292939466704135,-1.00157324405872,0.316549748754181,0.864121306399267
"6193","ZNF385A",241.391371409867,-0.389928391010144,0.389286087355513,-1.00164995276095,0.316512686060214,0.864121306399267
"6194","DHRS1",164.240904440199,-0.286321637929935,0.285901435798961,-1.00146974473842,0.316599760157259,0.864121306399267
"6195","AP5M1",112.930546762856,0.178657574639874,0.178358953713209,1.00167426933411,0.316500937821933,0.864121306399267
"6196","CCNDBP1",595.914248134032,-0.200793016468739,0.200534201986981

,-1.00129062513623,0.316686323918051,0.864191795481989
"6197","LTO1",54.7413733582568,0.233601084344861,0.233353038762639,1.0
0106296272608,0.316796369541173,0.8643525925774
"6198","NKX3-2",3.03716545017413,1.1313336481995,1.13025200723586,1.00
095699096902,0.31684760187108,0.86435289648858
"6199","LRTM1",10.9232520262666,-0.556153450744451,0.555765423151643,-
1.00069818591917,0.316972744709396,0.864415349604269
"6200","SELPLG",8.50100076917849,-0.519440027777482,0.519060343406463,
-1.00073148406701,0.316956641874584,0.864415349604269
"6201","NFATC1",109.475293213362,-0.351818413789623,0.351653770839513,
-1.00046819617409,0.317083981369826,0.864579254475248
"6202","CNST",169.579973035777,-0.24224840322764,0.242403564848843,-0.
999359903715525,0.317620376127373,0.864609082731927
"6203","AC012065.4",2.52823562154297,1.02104013260386,1.02176110953034
,0.999294378187086,0.317652107983261,0.864609082731927
"6204","SETD2",370.830226594383,0.227015408840825,0.227160442940627,0.
999361534526325,0.3176195864059,0.864609082731927
"6205","SLC9B1",14.4008521676484,0.387687023782715,0.387859883366895,0
.999554324663125,0.31752623669304,0.864609082731927
"6206","PPP1R3G",57.6014284250573,-0.266679702145366,0.266812015571847
,-0.999504094947907,0.317550556378288,0.864609082731927
"6207","GMPR",28.1361055523711,0.346196918373702,0.346445176225176,0.9
99283413744771,0.317657417904585,0.864609082731927
"6208","EGR3",191.119875932844,-0.413776111358616,0.413679241157767,-1
.00023416742058,0.317197197810396,0.864609082731927
"6209","BAG1",831.305928207317,-0.215348670806681,0.215498090798487,-0
.999306629626031,0.317646174856848,0.864609082731927
"6210","CDK2AP1",1480.6501758312,-0.224969544459163,0.225010924311329,
-0.999816098474806,0.317399513616891,0.864609082731927
"6211","TNRC6C-
AS1",3.73696383048058,0.838851320465957,0.839428518778307,0.9993123913
47877,0.317643384595513,0.864609082731927
"6212","ZNF112",13.3797522120038,0.437906349268407,0.438067172140669,0
.99963288079434,0.317488204675241,0.864609082731927
"6213","ARNTL",119.61542039317,0.961852175477992,0.962667829842118,0.9
99152714634434,0.31772071807942,0.864642185946699
"6214","TNFAIP8",69.6748731280125,-0.229725410434989,0.229966978368985
,-0.998949553819818,0.317819129308933,0.86470952856284
"6215","ISCU",1766.26184532367,-0.231729829586991,0.232001158468495,-0
.998830484798889,0.317876815703219,0.86470952856284
"6216","TRIM16",33.3317485081914,-0.326112389200326,0.32650912198968,-
0.998784925863825,0.317898889847801,0.86470952856284
"6217","ADGRB2",8.75300882735025,-0.500364629687157,0.501604469140986,
-0.997528252776629,0.318508167122792,0.865153588827331
"6218","SNRNP27",396.24152008553,0.143530131834344,0.143834908728292,0
.997881064502053,0.318337034998762,0.865153588827331
"6219","ANKRD36",172.8230734392,-0.407189349727908,0.408189486329798,-
0.997549822728452,0.318497702840118,0.865153588827331
"6220","TMEM14C",1677.25593582174,-0.247968557661675,0.248410200122997
,-0.99822212428836,0.318171660441852,0.865153588827331

"6221","IQUB",8.25160363088618,-0.497359379399795,0.498691335819043,-0
.997329096530099,0.318604794909016,0.865153588827331
"6222","NR1H3",266.470259755698,0.311679821881834,0.312383327479354,0.
997747941277157,0.318401599643025,0.865153588827331
"6223","IQCD",16.039310605773,-0.389640591986799,0.390403729845583,-0.
998045259815816,0.318257412231914,0.865153588827331
"6224","PAK6",20.2746235741544,-0.54187064631575,0.543275779608084,-0.
997413591135339,0.318563796980657,0.865153588827331
"6225","TOB1",457.372991760421,0.273983389029502,0.274728598600189,0.9
9728746998134,0.318624993945339,0.865153588827331
"6226","SIX5",160.308224774957,0.217711262012135,0.2180917065271,0.998
255575505262,0.318155443501246,0.865153588827331
"6227","LINC00653",9.08343270897223,-0.504464349495441,0.5056955043889
86,-0.997565422506508,0.318490135022525,0.865153588827331
"6228","ERRFI1",2084.87954411308,0.317577247258442,0.318556898964485,0
.996924719856238,0.318801051789614,0.865214796734959
"6229","FAM180A",154.075680389965,0.307822103062703,0.308757974223386,
0.996968916631101,0.318779597835107,0.865214796734959
"6230","GP1BB",71.7634864452875,0.377629625847325,0.3787783575061,0.99
6967272189632,0.318780396061318,0.865214796734959
"6231","TARDBP",329.687611909908,-0.179217747168536,0.179881722402372,
-0.99630882323691,0.319100118086123,0.865350521106025
"6232","STK36",37.1665030631865,0.292888328703281,0.293875885507479,0.
996639544607437,0.318939504003103,0.865350521106025
"6233","ADAT2",69.1785081448411,0.264778603120472,0.265859648244347,0.
995933775091426,0.319282323414976,0.865350521106025
"6234","AZIN1-
AS1",72.0260800883051,-0.242894870922134,0.243784493816334,-0.99635078
1461638,0.319079738237831,0.865350521106025
"6235","BAD",857.269678669996,-0.244950987189318,0.245867597425225,-0.
996271935604748,0.319118035762101,0.865350521106025
"6236","ARRB1",176.298191501447,0.163882516354905,0.164524005402084,0.
996100939521795,0.319201103433003,0.865350521106025
"6237","TMEM219",1143.2756549781,-0.233551067430524,0.234518842450338,
-0.995873359216246,0.319311680930949,0.865350521106025
"6238","MAFTRR",9.6445972658487,0.710119191240702,0.712594274135287,0.
996526659019836,0.318994320682414,0.865350521106025
"6239","MIR22HG",663.901314269643,-0.337342920123878,0.338736221731144
,-0.995886765223558,0.319305166479483,0.865350521106025
"6240","AC095055.1",8.94239628107267,-0.671515562215445,0.674400690137
066,-0.995721938064693,0.319385267844617,0.865411235371281
"6241","SLC39A7",481.83413051075,-0.241567889412551,0.242665685946252,
-0.995476094902252,0.319504765138774,0.865454938697894
"6242","TMEM229B",5.66735871024996,0.613116420348451,0.615984107151731
,0.995344544169264,0.319568720181214,0.865454938697894
"6243","NLRP1",78.2237835050475,0.293089399473763,0.294447747976986,0.
995386792690535,0.319548179618623,0.865454938697894
"6244","TNFSF13",147.312722244304,-0.358631515406977,0.360336780501209
,-0.995267579701798,0.319606141307644,0.865454938697894
"6245","ADCY2",13.2391066518118,0.644692455771166,0.647893776737362,0.

995058879894915,0.319707628297258,0.865534775255291
"6246","AC040168.1",3.22706339902378,-0.91685025373619,0.921460857840747,-0.994996418930522,0.319738006047111,0.865534775255291
"6247","FAM41C",4.30975673592865,0.672474622566816,0.67634376992994,0.994279318395253,0.320086901546807,0.866271749550022
"6248","POLE",54.4533504850308,0.298117051087401,0.299848294092714,0.994226270285942,0.320112721267361,0.866271749550022
"6249","LRRC40",134.233084682216,0.176908164272397,0.178001958506428,0.993855156183626,0.320293389024077,0.866511829063876
"6250","ARHGAP24",512.489435210727,-0.213036384001536,0.214373128471299,-0.993764402846125,0.320337580192415,0.866511829063876
"6251","AC005261.1",422.544083087738,0.35922794764145,0.361495153449098,0.993728253930884,0.320355183550881,0.866511829063876
"6252","LINC01772",5.69979485154982,0.744075267684097,0.748939338261339,0.993505387781428,0.320463726161293,0.866515788549432
"6253","TACSTD2",4.05986292234585,-0.751668849965424,0.757532652825597,-0.992259339794395,0.321071032392737,0.866515788549432
"6254","AC024560.4",3.60677442012074,-0.784540970137735,0.790239315149568,-0.992789089453549,0.320812748060092,0.866515788549432
"6255","SPOCK1",785.619626914171,-0.242570405522917,0.24440208566389,-0.992505464362153,0.320951015199187,0.866515788549432
"6256","AGER",10.1307602343455,0.588360210481693,0.592564285348556,0.992905284758446,0.320756114127739,0.866515788549432
"6257","ARMT1",157.998438419757,0.199214041794942,0.200646009745723,0.992863212417748,0.32077661955542,0.866515788549432
"6258","LSM8",708.863049037743,-0.209520512700403,0.211178707808544,-0.992147905793399,0.321125380355497,0.866515788549432
"6259","ZYX",504.699319193028,0.243050946461912,0.24492651142892,0.992342335845696,0.321030557930914,0.866515788549432
"6260","ATG13",145.099410984053,0.174703456929492,0.175936838174687,0.992989636178582,0.320715005091268,0.866515788549432
"6261","RECQL",423.023491469392,-0.196204591729422,0.19763372603618,-0.992768773147067,0.320822650958499,0.866515788549432
"6262","PKP2",2.25843053627804,-1.02345604892965,1.03076757918838,-0.992906713010421,0.320755418034186,0.866515788549432
"6263","RNASE4",3305.32729490184,-0.227986154587225,0.229676846397529,-0.99263882347384,0.320885997827279,0.866515788549432
"6264","SIPA1L1",65.9929049934681,-0.288693046229236,0.290960474056844,-0.992207093300361,0.321096513010059,0.866515788549432
"6265","SIVA1",1058.80040884829,-0.249381273920845,0.251156602701682,-0.992931387183376,0.320743392627992,0.866515788549432
"6266","MICOS13",1019.33551256706,-0.278645089525103,0.280451449686093,-0.993559098507024,0.320437565201857,0.866515788549432
"6267","TCHH",12.8007147816543,-0.600254266485073,0.606148585730696,-0.990275784874567,0.322039339098948,0.86667952868102
"6268","QSOX1",231.976786107801,-0.191103620777415,0.192904165932337,-0.990666115756388,0.321848641849343,0.86667952868102
"6269","LINC02817",2.18180958476423,1.13476257727151,1.14424402295544,0.991713790508217,0.321337161917667,0.86667952868102
"6270","LINC01126",2.88337665146042,-1.06096499956763,1.0717270375198,

-0.989958228564364,0.322194536515449,0.86667952868102
"6271","SLC16A14",5.68335643687753,0.688115383906191,0.694348938576617,0.991022446605586,0.321674619784009,0.86667952868102
"6272","AC018809.2",15.3683005024375,0.481208789427539,0.486361013631527,0.989406584698231,0.322464254168422,0.86667952868102
"6273","WWTR1",347.027983015557,0.188079502150788,0.19023572832543,0.988665503616896,0.322826825974115,0.86667952868102
"6274","RGMB",71.2778693983583,-0.303486768472045,0.306777211940186,-0.989274159422303,0.322529023356168,0.86667952868102
"6275","SAR1B",339.907444619745,-0.149336157367925,0.150864782311393,-0.989867582612407,0.322238846327675,0.86667952868102
"6276","FAF2",319.529478003891,0.131762690939192,0.132900802149508,0.991436385695883,0.32147254060263,0.86667952868102
"6277","FAM50B",151.787573239425,-0.253633904719507,0.256583010836682,-0.988506229981639,0.32290478484091,0.86667952868102
"6278","AL121944.1",37.6962872650986,-0.316664760229745,0.319810060872905,-0.990165097887866,0.32209342895498,0.86667952868102
"6279","GINM1",1353.86768248863,-0.147740313314914,0.149432390430223,-0.988676637572097,0.322821376751983,0.86667952868102
"6280","VPS13B-DT",52.9790230206493,-0.280197241426372,0.283349754728302,-0.9888741272956,0.322724730558632,0.86667952868102
"6281","WDR31",28.7287586503813,0.292136122620632,0.295547609592227,0.988457064578186,0.322928852063545,0.86667952868102
"6282","MKI67",3.41954411915809,1.22628559626412,1.23924979674918,0.989538670477034,0.322399659481739,0.86667952868102
"6283","TNNT3",1863.00380877372,0.282766832837447,0.285083898429101,0.991872337917287,0.321259804569946,0.86667952868102
"6284","TSSC4",297.753866963591,-0.221205845577514,0.223759095305125,-0.988589291871558,0.322864127423026,0.86667952868102
"6285","ROBO3",522.385167112596,-0.220156991720461,0.222696642788317,-0.988595916687124,0.3228608848294,0.86667952868102
"6286","PCCA-DT",54.0707741073607,-0.247971492399556,0.250582486780687,-0.989580299825916,0.322379302985621,0.86667952868102
"6287","AL136295.7",10.0561975403535,0.480114019948402,0.485351202724204,0.989209498716791,0.322560651983304,0.86667952868102
"6288","ITGAL",14.5477442182547,0.562464656506464,0.568522171996863,0.989345155230933,0.322494298258883,0.86667952868102
"6289","TMEM208",509.538808135645,-0.238800702049234,0.241227543090513,-0.989939618792335,0.322203633072831,0.86667952868102
"6290","SENP3",33.945965415026,-0.352227278119122,0.356329204281179,-0.988488380652573,0.322913522227312,0.86667952868102
"6291","SLFN12",61.727435880083,0.289031568265494,0.291968239298782,0.989941813396072,0.322202560329919,0.86667952868102
"6292","PSMC3IP",38.146370203894,-0.294339314339804,0.296848049343931,-0.991548756983004,0.321417696846767,0.86667952868102
"6293","ITGA2B",4.15267575650212,0.745732619376472,0.753027255082821,0.99031291941014,0.322021193743859,0.86667952868102
"6294","ATP5PD",1610.62258021472,-0.24198438504223,0.244722503517104,-

0.988811333508271,0.322755458113825,0.86667952868102
"6295","AC015802.6",33.8809772337444,0.498952256785869,0.503718232195541,0.990538409958087,0.321911024761449,0.86667952868102
"6296","ATP5F1D",2033.93796656163,-0.257718433073066,0.260712889707256,-0.988514351409505,0.32290080938938,0.86667952868102
"6297","ZNF781",24.7957956691973,0.571054398880399,0.577706596538833,0.988485162367387,0.322915097619525,0.86667952868102
"6298","DEDD2",72.9569042204811,-0.226914247787573,0.229049856110405,-0.990676229362907,0.321843701798326,0.86667952868102
"6299","ZNF586",21.9619276986018,0.411902679890133,0.415418543543615,0.991536575080422,0.321423642031917,0.86667952868102
"6300","TFE3",178.512421879687,0.188238799400736,0.190326409667257,0.989031420966899,0.322647768740647,0.86667952868102
"6301","LINC01116",259.35068342423,0.40747721573521,0.412466171134814,0.98790457072909,0.323199386739825,0.86682714453278
"6302","USP46",160.395254834953,0.196796119474811,0.199249629797565,0.987686249027177,0.323306331071385,0.86682714453278
"6303","MOCS1",62.2602916861357,0.280059229205577,0.283527583487456,0.987767136307456,0.323266705952928,0.86682714453278
"6304","WRN",152.111914987831,0.237995927333433,0.240837322157039,0.988202016206802,0.323053720943446,0.86682714453278
"6305","MTA2",54.0761700375701,0.281048488825044,0.284573799712098,0.987611962553755,0.323342725370726,0.86682714453278
"6306","ETFB",1417.22501104158,-0.260155134689835,0.263382145757044,-0.987747798705439,0.323276178783217,0.86682714453278
"6307","MORF4L2",1158.66599535368,-0.180571103050404,0.182742685927885,-0.988116717960808,0.323095489051013,0.86682714453278
"6308","MTX1",338.632057897106,-0.267005386905378,0.270566262516396,-0.986839173598731,0.323721487157387,0.867422550963629
"6309","ATF6",274.304166227784,0.169778149717587,0.172059830999413,0.986739024044296,0.323770593926922,0.867422550963629
"6310","DNAJC3-DT",21.8877543371999,-0.337672961125822,0.342246902502258,-0.986635550700399,0.323821335562008,0.867422550963629
"6311","ZNF652",360.226867122284,0.201458738795267,0.20418179505376,0.986663569796827,0.323807594944021,0.867422550963629
"6312","ZNF329",41.5730135552073,0.262310598806699,0.265851300145822,0.986681647457878,0.323798729826386,0.867422550963629
"6313","Z99289.1",6.23926280983057,0.725200902850146,0.735245003542323,0.986339110577038,0.323966733629455,0.867674565532525
"6314","AL590560.3",10.5325286708747,0.661860954986982,0.671194856569672,0.986093603830051,0.324087182063147,0.867722260383799
"6315","EPS8L2",133.731096026288,-0.162285308269393,0.164568618383255,-0.98612548287581,0.324071540189086,0.867722260383799
"6316","RNF4",165.462084041538,0.162228538861399,0.164546443980271,0.985913368512848,0.324175626146414,0.867821641368519
"6317","LRRC58",528.250857986313,0.194089235156249,0.196916185976596,0.985643888000741,0.324307893459206,0.867943397318425
"6318","RPL36AL",3753.41848881408,-0.279078204493696,0.283152331541432,-0.98561536286642,0.324323774796416,0.867943397318425

"6319","PDSS2",104.891581755888,0.21518023179453,0.218390420391748,0.985300689510742,0.324476394141514,0.86821441242993
"6320","PITHD1",543.17132038849,-0.167517478136726,0.170457622280288,-0.982751465706073,0.325729774139625,0.869532332877775
"6321","CLSPN",2.25334721648107,-0.960478386348405,0.977507314859759,-0.982579231630817,0.325814570066359,0.869532332877775
"6322","AMY2B",25.6597296475562,-0.346576531610302,0.352462868399989,-0.983299413023538,0.325460098935813,0.869532332877775
"6323","BCAN",5.68270523798572,-0.69282661192963,0.705295345948059,-0.982321258618742,0.325941604652382,0.869532332877775
"6324","IARS2",407.17356041577,0.168277748647659,0.171247566047881,0.982657754099748,0.325775909353673,0.869532332877775
"6325","SLC23A3",6.42009833277597,0.731407487768516,0.743467542864468,0.983778639415129,0.325224364179614,0.869532332877775
"6326","KLHL5",73.1074904266467,-0.229490493564034,0.23368491642664,-0.982050947375021,0.326074749541403,0.869532332877775
"6327","EPDR1",228.856151455625,0.235472040777638,0.239556255424212,0.982950916312576,0.325631596642588,0.869532332877775
"6328","WASHC2C",222.925354461817,0.207321726578167,0.211119113195573,0.982013060968628,0.326093413747854,0.869532332877775
"6329","BHLHE41",149.000687543045,-0.400576753107182,0.407388125615715,-0.983280385263467,0.325469461115199,0.869532332877775
"6330","SUOX",58.7776445924258,0.264376567151274,0.268920580843935,0.983102767075689,0.325556862580113,0.869532332877775
"6331","UBR1",178.246809028481,0.201439028080308,0.205011242487337,0.982575519451084,0.325816397840173,0.869532332877775
"6332","PAQR5",5.36764549271998,-0.771850041557296,0.784398308239245,-0.984002685179018,0.325114192644239,0.869532332877775
"6333","ABR",193.891198596067,0.260054208160544,0.264249709882939,0.984122965643922,0.325055056338361,0.869532332877775
"6334","CHD3",450.339190937961,0.173968140824638,0.176966514239129,0.983056831811467,0.325579468658089,0.869532332877775
"6335","RNFT1",181.627586053626,-0.218877982328696,0.222846369233383,-0.982192274801971,0.326005132694372,0.869532332877775
"6336","WBP2",376.097137156964,-0.156926710656655,0.159803329615302,-0.98199900486697,0.326100338465695,0.869532332877775
"6337","ZNF571",47.1813666925056,0.250809562673217,0.255087328592282,0.983230190450179,0.325494159179377,0.869532332877775
"6338","EIF3K",2873.00657951635,-0.261226155694983,0.265668475189317,-0.983278710463603,0.325470285171015,0.869532332877775
"6339","PNMA8A",6.88256324545005,0.680673406545341,0.692616981021402,0.982755874020808,0.325727603984329,0.869532332877775
"6340","MIR646HG",2.2259057291685,-1.20782782427133,1.22921481270535,-0.98260109769834,0.325803803959577,0.869532332877775
"6341","AC245060.5",70.48700160752,0.396182260635742,0.403308648669723,0.982330187915664,0.325937207028389,0.869532332877775
"6342","AL136454.1",24.4716837006855,0.446479653495983,0.454774883507083,0.981759700652046,0.326218246001674,0.869538373735279
"6343","CCNA2",5.63914369240647,0.609099778805965,0.620454147161228,0.981699907386851,0.326247711056404,0.869538373735279

"6344","FIS1",1687.71764633486,-0.256683901422771,0.261494623351794,-0
.981602979566619,0.326295479032045,0.869538373735279
"6345","ZWILCH",42.619786406707,0.293021907391657,0.298553325639372,0.
981472595437117,0.326359742117582,0.869538373735279
"6346","ZNF200",49.3784194006793,0.284567205238023,0.289916283209385,0
.98154957730505,0.326321798680471,0.869538373735279
"6347","HOPX",14.5267980890931,-0.433561195915277,0.441819639612931,-0
.981308110918543,0.326440824166244,0.86961737119944
"6348","ELOA-
AS1",45.1180274629143,-0.323064791543272,0.329293087648586,-0.98108585
8346469,0.32655040355885,0.869772246908164
"6349","AC016877.1",2.11195766073224,1.0612402848239,1.08196305203788,
0.980847065733952,0.326668164481814,0.869811862213938
"6350","TMEM35A",40.8323247690411,0.364984505039227,0.372101345210933,
0.980873919798189,0.326654919984462,0.869811862213938
"6351","RNF7",1149.96620270505,-0.248157408999406,0.253081043544462,-0
.98054522584505,0.326817056731759,0.869827163780175
"6352","CPNE8-
AS1",3.58452629673044,-0.829155308482699,0.845494995476209,-0.98067441
3117836,0.326753325552258,0.869827163780175
"6353","NHLRC3",312.000655761228,0.182497364658686,0.186122557925363,0
.980522547577867,0.326828245297815,0.869827163780175
"6354","FAHD1",166.257896638091,-0.204883058251461,0.20899651766605,-0
.980318048068335,0.326929148558637,0.869958773029498
"6355","PDPN",190.161509833259,0.479143584003403,0.488925003872311,0.9
79994028140434,0.327089066487245,0.870124577757994
"6356","RNF187",608.9467573701,-0.212243578671537,0.216578779981104,-0
.979983259163502,0.327094382317826,0.870124577757994
"6357","ZNF90",28.290843803143,0.321925933363522,0.328552514341786,0.9
79830983818402,0.327169555160191,0.870187641756883
"6358","CXorf56",83.8815749466297,0.218645083490375,0.223191646168891,
0.979629333102032,0.327269120103661,0.870315552487056
"6359","SLC50A1",129.990104182447,-0.239117873371344,0.244188458316139
,-0.979234952463516,0.327463902207285,0.870432388399603
"6360","AC073648.7",3.19758812568854,3.13031147624266,3.19690191795934
,0.979170320696234,0.327495830603876,0.870432388399603
"6361","ELL",92.4075210706986,0.311011507472799,0.317666960032038,0.97
9048961973985,0.327555787841919,0.870432388399603
"6362","ZNF724",22.3132789509366,0.329684525386154,0.336722316175239,0
.979099125745434,0.327531003582829,0.870432388399603
"6363","CLCN5",49.0166723519479,0.276141429874476,0.28205923789721,0.9
79019272451943,0.327570457025471,0.870432388399603
"6364","NAT1",14.9373873689422,-0.388762476218793,0.397188934029662,-0
.978784761888056,0.327686340447479,0.870603495330921
"6365","BIRC6",320.900065038002,0.235471530081383,0.240764788039489,0.
978014816862516,0.328066995918037,0.870657160097657
"6366","RUNX2",30.4718461823411,0.384187376049045,0.392633237900333,0.
978489182687503,0.327832438838897,0.870657160097657
"6367","STMN2",9.19360413617718,-0.897071549477411,0.917005995521839,-
0.978261378724046,0.327945066236268,0.870657160097657

"6368","IGF1",5096.2319576673,-0.211528232349956,0.216160385488731,-0.978570758336213,0.327792113541841,0.870657160097657
"6369","FLYWCH2",365.450489743794,-0.269630045279517,0.275673260614376,-0.978078340563784,0.328035579388372,0.870657160097657
"6370","ERBB2",153.356937769238,0.204909433896443,0.209511235276046,0.978035538888693,0.328056747337374,0.870657160097657
"6371","CCR10",24.5304869251681,0.362028141298421,0.370144080017384,0.978073568761166,0.328037939281898,0.870657160097657
"6372","AL121658.1",11.509856469524,-0.413382238970482,0.422864256652846,-0.977576686766061,0.328283732405256,0.870685701569894
"6373","PHYHIPL",16.0075915548351,-0.430426742171884,0.440268546997061,-0.977645905226923,0.328249484879315,0.870685701569894
"6374","POC1B-AS1",40.8222011466085,0.314308886477086,0.321493057261941,0.97765372961382,0.328245613718376,0.870685701569894
"6375","SMARCA1",628.172706621501,0.190616535703108,0.194930182856884,0.977870809483915,0.328138223997343,0.870685701569894
"6376","AAGAB",60.3333873135045,-0.243723812799014,0.249371941371245,-0.97735058506914,0.328395617896863,0.870845844949837
"6377","HDAC4-AS1",30.5898621034267,-0.362774925046016,0.371727051063134,-0.975917474955038,0.329105361580329,0.871415649524054
"6378","RNPEPL1",221.239212416443,-0.160275414728133,0.16423152686174,-0.975911372139056,0.329108386106578,0.871415649524054
"6379","QRICH1",188.520560093218,0.173382601810749,0.177631165037138,0.976082106844815,0.329023777605,0.871415649524054
"6380","HAUS3",105.807943058242,0.207896596072802,0.213164216083995,0.975288441428101,0.329417202329379,0.871415649524054
"6381","ARMC10",328.94546819859,-0.2527871411274,0.259189764961794,-0.975297543730794,0.329412688536193,0.871415649524054
"6382","ST7-AS1",50.1708187885926,-0.36693881264845,0.3758451931856,-0.976303061210758,0.328914303410156,0.871415649524054
"6383","TMEM71",3.91303046722547,0.845284225925089,0.866410077824021,0.975616798050192,0.329254397027368,0.871415649524054
"6384","AL590399.1",13.9539713838737,0.384552270956212,0.394253471543022,0.975393493559255,0.329365109861908,0.871415649524054
"6385","COQ4",275.626289122255,-0.184349515989507,0.189010962748903,-0.975337691043939,0.32939278013252,0.871415649524054
"6386","RNF121",125.35557457079,-0.214536018266063,0.219980010662397,-0.975252331428016,0.329435109519621,0.871415649524054
"6387","TMEM126A",298.234621545661,-0.249073024516698,0.255318457462023,-0.975538654716127,0.329293137213159,0.871415649524054
"6388","PICALM",376.394530123666,0.163978032356081,0.168051517629585,0.975760496953779,0.329183164850256,0.871415649524054
"6389","SLC66A2",255.452511440039,-0.176341272232379,0.180601745726643,-0.976409566379758,0.32886154272359,0.871415649524054
"6390","C19orf25",181.643677216917,-0.27078570377445,0.277435601512477,-0.976030842106152,0.329049180640811,0.871415649524054
"6391","KMT5C",36.0430260501215,0.265103968637217,0.271633022176841,0.

975963697317429,0.329082454584722,0.871415649524054
"6392","NOP56",507.47938386087,0.207872260834292,0.212888296948417,0.976438178208825,0.328847369888732,0.871415649524054
"6393","COL11A1",4.57921933654564,1.03285052607671,1.06015852121837,0.974241592558935,0.329936597540834,0.872200684427495
"6394","IDH1",228.268088229611,0.133577762398246,0.137087678030752,0.974396563696126,0.329859675019241,0.872200684427495
"6395","GRAMD2B",169.745537641836,0.280018971824471,0.287391249193063,0.974347592735367,0.329883981321253,0.872200684427495
"6396","NOM1",116.634497460179,0.198669541517046,0.203922941986418,0.974238305811996,0.329938229098548,0.872200684427495
"6397","ST6GAL2",3.43216144845163,1.03204995490249,1.05965014565994,0.973953487506707,0.330079634180193,0.872431837081438
"6398","TMBIM4",2390.53266515631,-0.229957353079844,0.236153915360248,-0.973760493147229,0.330175473294355,0.872431837081438
"6399","L3MBTL4",41.0170354445151,0.343971430042937,0.35324392793241,0.973750439409541,0.330180466375924,0.872431837081438
"6400","C12orf10",489.437633703582,-0.261882903288609,0.269003541847988,-0.973529573215051,0.330290169547658,0.87258534167372
"6401","TMEM144",19.8468172510627,0.372799575350874,0.383010659880513,0.973339946901675,0.330384374843116,0.872697861247837
"6402","FGGY",53.2200555347602,0.266728787226671,0.274193855081878,0.97277448886308,0.330665394483784,0.872750542279076
"6403","AC096677.1",3.22702990033329,-0.940967272850384,0.96770679177343,-0.972368160324634,0.330867425897071,0.872750542279076
"6404","MAP4K4",668.191329700905,0.255790441296577,0.26307846798273,0.972297137268446,0.330902747608049,0.872750542279076
"6405","GALNT5",46.4986669061896,-0.883726825670377,0.908904759477613,-0.972298600546765,0.330902019855056,0.872750542279076
"6406","RIOX2",138.79163695308,0.204110629424719,0.209840182432441,0.972695634642963,0.330704595502337,0.872750542279076
"6407","LINC01235",42.4702042355033,1.03243506008403,1.06189035460175,0.972261453934416,0.330920494827961,0.872750542279076
"6408","DEPP1",6490.95447319438,0.473839203199794,0.487068291983562,0.972839355380962,0.330633149464811,0.872750542279076
"6409","CASP1",68.2203145360838,0.278826432006045,0.286559551351625,0.973013918715656,0.330546384478907,0.872750542279076
"6410","CCDC59",655.913018706494,-0.19329477459356,0.198701557455483,-0.972789428874331,0.330657967653105,0.872750542279076
"6411","RTF2",758.265112330212,-0.195500784532379,0.201031906284489,-0.972486348787429,0.330808652944586,0.872750542279076
"6412","C2orf27A",102.841510815398,0.210960992575814,0.217034075936407,0.972017834828981,0.331041676018186,0.872933976624374
"6413","MCOLN3",147.01940493943,0.441049353822978,0.453927064657012,0.971630440578014,0.331234433047534,0.872943053846518
"6414","CHI3L2",480.798135023386,-0.661327161151326,0.680464271380256,-0.971876392290058,0.331112045635712,0.872943053846518
"6415","FSIP2",24.709692596083,0.639598916366962,0.658446949329295,0.971375016648596,0.331361564867983,0.872943053846518
"6416","CHL1-

AS2",91.691314264576,0.398469360515527,0.41010369384789,0.971630752156
36,0.331234277985314,0.872943053846518
"6417","GPR150",10.1762707529441,-0.505722998093126,0.520727312491624,
-0.97118585094624,0.331455738400386,0.872943053846518
"6418","PCDHGA7",9.62051361485319,0.494621511437737,0.509229099917789,
0.971314309252141,0.331391785321488,0.872943053846518
"6419","ANKDD1A",36.167388070777,-0.337962498470724,0.347991256190676,
-0.971181006586967,0.331458150324973,0.872943053846518
"6420","LINC01270",6.36303031366181,0.605554473997311,0.62328627608843
5,0.97155111098803,0.331273914283822,0.872943053846518
"6421","SLC35B4",161.254828524585,0.173004589805495,0.178166747864292,
0.971026254221528,0.331535204882772,0.872950974524163
"6422","MORN3",3.88520714372806,-0.982230578823309,1.01159975492532,-0
.970967592707471,0.331564416749123,0.872950974524163
"6423","AP000692.2",71.1744049176484,-0.390141741506038,0.401963791520
54,-0.970589266337195,0.331752853140549,0.873311107099548
"6424","CDH24",3.21455035403049,0.792473675358097,0.816594987540185,0.
970461106729607,0.331816702432929,0.873343213688662
"6425","TTC21A",17.1352297322968,-0.391686185764057,0.403655576374915,
-0.970347515774834,0.331873300241312,0.873356227312079
"6426","KAZALD1",226.94750315964,0.347452430515043,0.358167577526409,0
.970083425514483,0.33200490991934,0.873566607052007
"6427","ARID5A",108.467779299755,-0.384484302323007,0.396505090188319,
-0.969683143639837,0.332204455131826,0.873863777630571
"6428","AC073111.4",58.9410132088541,0.233831776149549,0.2411508183063
58,0.969649523861408,0.332221218512498,0.873863777630571
"6429","PGBD4",11.9153048546651,0.409700927296339,0.422652300376144,0.
969356908578804,0.33236714443628,0.874111631377916
"6430","LYG2",5.1410113934598,-0.69797767739696,0.720355268532324,-0.9
68935340500867,0.332577451320928,0.874310539320885
"6431","CDH23",33.6824724418051,-0.412689948817657,0.425932409526315,-
0.968909478564017,0.332590355814602,0.874310539320885
"6432","LINC00630",38.7909385761194,0.347724259634797,0.35888769532304
5,0.9688943481938,0.332597905660748,0.874310539320885
"6433","TCP1",793.598537811804,-0.165282990544163,0.1706514505332,-0.9
68541374994099,0.332774065829668,0.874637634859012
"6434","TRIM62",11.192478812833,0.430295649576412,0.44464853802185,0.9
67720823935933,0.333183815277244,0.87484762882733
"6435","CAMSAP2",208.366701646788,0.183491155919979,0.189608531391594,
0.967736813176509,0.333175827798599,0.87484762882733
"6436","PLEK",32.5564850404903,-0.6705305093782,0.69296290332661,-0.96
7628290286937,0.333230043198623,0.87484762882733
"6437","PXK",580.098868476808,0.212074349401216,0.219170645669257,0.96
7622049721249,0.333233161006795,0.87484762882733
"6438","ACAD9",145.540475505083,-0.210753001642608,0.217890686451839,-
0.967241900397569,0.33342312042603,0.87484762882733
"6439","NAF1",94.9170558237612,-0.177574803136481,0.183585118772211,-0
.967261422516563,0.333413363582989,0.87484762882733
"6440","NUDCD2",472.008429185736,-0.253811885176543,0.262344173294503,
-0.967476738626163,0.333305764254839,0.87484762882733

"6441","DNMBP",204.021237837746,0.185203525600592,0.19147063860468,0.967268542844173,0.33340980500291,0.87484762882733
"6442","EIF3M",1028.69297440935,-0.247091796179658,0.25537761166773,-0.9675546519761,0.333266834352696,0.87484762882733
"6443","AC018653.3",2.74286221595867,-0.89689303777707,0.926949037846171,-0.967575347897292,0.333256493996986,0.87484762882733
"6444","WBP2NL",3.22779726445465,0.861178713124655,0.889675535441145,0.967969421231348,0.333059641643745,0.87484762882733
"6445","ATXN3",482.796826926836,0.186114388568481,0.192468595583112,0.966985746451881,0.333551159142106,0.875047788793597
"6446","DNAJC11",85.2521547485627,0.208470492453805,0.215760426982604,0.966212828595363,0.333937694880897,0.875518459458242
"6447","ZBTB14",89.6059364421622,-0.21534277607283,0.22286274499683,-0.966257397914994,0.333915397945966,0.875518459458242
"6448","CCDC124",476.989190804661,-0.246842695646425,0.255416944498451,-0.966430383587655,0.333828866562415,0.875518459458242
"6449","GPC3",11506.8951283811,0.178210491337659,0.184429866971818,0.966277828335095,0.333905177430006,0.875518459458242
"6450","AL645608.7",2.41487287759096,-1.07115990467829,1.11071437073353,-0.964388264798338,0.334851303512984,0.875715879391365
"6451","TCTEX1D4",2.22894674319931,-1.03097685707411,1.06887602092056,-0.964542974952502,0.334773773503968,0.875715879391365
"6452","PRKAA2",49.4972159179186,0.328536716349861,0.340210428440121,0.965686789368024,0.334200931920229,0.875715879391365
"6453","SIKE1",252.213788672668,0.159347584584056,0.165069871668224,0.965334151978566,0.334377471255834,0.875715879391365
"6454","AC108488.1",68.3260946939168,-0.218646488521608,0.226785111587797,-0.96411306276145,0.334989244295843,0.875715879391365
"6455","ACVR1C",2.60850175975721,-0.878377807119692,0.910872027350221,-0.964326250829048,0.334882383865194,0.875715879391365
"6456","PLCL1",49.9777231795806,0.362183387547268,0.375338484389936,0.96495137751715,0.33456916601499,0.875715879391365
"6457","MREG",27.0568502740296,-0.396494369313637,0.411101939371652,-0.96446728010979,0.33481170506866,0.875715879391365
"6458","ZFAND2B",170.856255804617,-0.210535105043865,0.2182934704435,-0.96445901297976,0.334815847991264,0.875715879391365
"6459","PHF3",942.831165952159,0.211513890301985,0.219385771169377,0.964118544126937,0.334986496488531,0.875715879391365
"6460","PPIL4",513.763928370596,-0.146705814294728,0.152024260949571,-0.965015803256513,0.334536896427746,0.875715879391365
"6461","SMC2",197.747261665995,-0.144392164890912,0.149769260252396,-0.964097469985348,0.334997061030479,0.875715879391365
"6462","FADS3",155.797126607151,-0.219571524768123,0.22743052749622,-0.965444380687955,0.334322281474625,0.875715879391365
"6463","COG6",137.886003046794,0.198141614009017,0.205473604293326,0.964316631766274,0.334887204943227,0.875715879391365
"6464","AC093525.7",7.32072327279655,0.714858708676171,0.741089406933973,0.964605217653396,0.334742585038576,0.875715879391365
"6465","AC092376.1",2.32481694089657,-0.979560764656333,1.0147356116205,-0.965335949028142,0.33437657145428,0.875715879391365

"6466","SHPK",72.611497803669,0.257802085780302,0.266933739846877,0.965790558841258,0.334148993702619,0.875715879391365
"6467","BCAM",27.3279593561709,-0.380996612380674,0.394449847037011,-0.965893675058073,0.334097387605814,0.875715879391365
"6468","MAGEH1",422.020258071007,-0.221870126176192,0.230031111898692,-0.964522252424295,0.3347841575282,0.875715879391365
"6469","TTC34",2.90333320953937,0.920386203922444,0.956865466801555,0.961876288627024,0.336111750613527,0.875814167393096
"6470","LYPLAL1-AS1",121.400612702432,0.719416608366467,0.747340200596864,0.96263603616118,0.335730206540097,0.875814167393096
"6471","RAB3GAP1",295.537909076181,0.160083670939989,0.166446910932737,0.961770152674571,0.336165074134936,0.875814167393096
"6472","FLACC1",2.48531591543753,1.00858807017823,1.04876158246846,0.961694332666467,0.336203170025268,0.875814167393096
"6473","VIL1",4.14636641276437,0.683500025224337,0.710928223419809,0.961419173846365,0.336341447369737,0.875814167393096
"6474","ZNF852",12.9232104895797,0.430241777489465,0.447614418651788,0.961188379019047,0.336457458402233,0.875814167393096
"6475","PRICKLE2-AS3",2.12080289534162,1.43276253260321,1.48687242998252,0.963608244871457,0.33524237164463,0.875814167393096
"6476","GXYLT2",289.340932180945,0.237138149915397,0.246141043894486,0.96342384091396,0.33533486676008,0.875814167393096
"6477","CCDC50",876.755110031832,0.168041142253622,0.174776088772512,0.961465286434823,0.336318271560076,0.875814167393096
"6478","SEC31A",1274.07824883104,0.17329185592394,0.180229222396104,0.961508092972201,0.336296758264272,0.875814167393096
"6479","AC104113.1",4.75594064404584,-0.763576914134359,0.794110038314124,-0.961550512263281,0.336275440460434,0.875814167393096
"6480","CLINT1",630.548115476125,0.152429878055369,0.158307804375041,0.962870268191287,0.335612631821212,0.875814167393096
"6481","AL031775.1",6.399746684458,0.627921503939878,0.652710640167503,0.962021246932235,0.336038931236905,0.875814167393096
"6482","C6orf226",175.42860209387,-0.301096768229275,0.312846554109529,-0.962442335624573,0.335827456166975,0.875814167393096
"6483","Z95118.2",7.66542071318708,0.60648954963039,0.630502190416878,0.961915055726275,0.336092275081015,0.875814167393096
"6484","CROT",98.2991754433917,-0.204464528106116,0.212429930372981,-0.962503389927779,0.335796801180923,0.875814167393096
"6485","AC083862.3",61.7421907609879,0.291265405016722,0.302766357676394,0.962013769469181,0.336042687270008,0.875814167393096
"6486","TCEA1",1156.87024208605,-0.141332812634328,0.146989179609462,-0.961518480542836,0.336291537912927,0.875814167393096
"6487","PITX3",4.39746335576493,0.950333056521757,0.98897094414951,0.960931220622482,0.336586751629165,0.875814167393096
"6488","ATP5MG",2893.45633090491,-0.225701065941481,0.234874256009882,-0.960944250663149,0.336580199660961,0.875814167393096
"6489","OBI1",118.675368015059,0.224633266570759,0.233724303686151,0.961103586695889,0.336500086467995,0.875814167393096

"6490","LINC01551",7.92301604697997,0.798870717374537,0.831353791359566,0.960927496424948,0.336588624303308,0.875814167393096
"6491","C17orf64",2.80501548348625,-0.862071524491961,0.895412661973514,-0.96276450077435,0.335665719374155,0.875814167393096
"6492","AP001020.3",3.5815325601134,-0.974579748503365,1.01351672856441,-0.961582301541091,0.336259465336998,0.875814167393096
"6493","PIAS2",179.938222635943,0.227914750209428,0.236855354041763,0.962252895365166,0.33592258440942,0.875814167393096
"6494","PLD3",1213.18828716718,0.205704784606841,0.213883047449277,0.961762921652893,0.336168707254083,0.875814167393096
"6495","PTOV1",476.977672928053,-0.158847303883278,0.165015923840813,-0.962618032163455,0.33573924489405,0.875814167393096
"6496","SNX21",627.332415142169,-0.207001242575215,0.214846567673015,-0.963484056632732,0.33530466137208,0.875814167393096
"6497","CCDC22",108.331849215451,-0.2830792865702,0.294445457767896,-0.961398041987608,0.336352068412367,0.875814167393096
"6498","NDUFA1",1481.55152342333,-0.237704378691875,0.246914549380477,-0.962698955117423,0.335698621241339,0.875814167393096
"6499","CDK5RAP2",177.838671503156,0.212633614146566,0.221307582346433,0.960805824599862,0.33664980940599,0.875838587080547
"6500","TBC1D20",207.391972104628,0.194032738369509,0.201993688755043,0.960588123150775,0.336759302768816,0.875988660186945
"6501","MAP2K6",89.0698031062091,0.26444846820006,0.275395253453231,0.960250639341432,0.336929086165908,0.876295491292597
"6502","C1orf43",671.029002760764,-0.215978585286786,0.224979311629557,-0.959993093242319,0.337058691038171,0.876497746550815
"6503","SLIT3",4965.95211140611,0.262832413431657,0.274000632678075,0.959240169859245,0.337437768314426,0.876825425065515
"6504","CPNE8",159.350941737458,0.213814994100113,0.22290320838914,0.95922797901966,0.337443908337244,0.876825425065515
"6505","FAM222B",90.8840198804683,0.218667873762767,0.227958549870031,0.959244011191682,0.337435833608674,0.876825425065515
"6506","PIGA",26.9217936259286,0.306077710324537,0.31902021875227,0.959430444633405,0.33734194406882,0.876825425065515
"6507","ARMCX2",196.035939109834,0.20775870859037,0.216589538385636,0.959227810072973,0.337443993429223,0.876825425065515
"6508","AC093702.1",7.92973305438951,0.566084701509894,0.590239120975601,0.959076891708258,0.337520010740231,0.876888190165307
"6509","LINC02062",5.43983716717136,0.673559714956049,0.702851135440961,0.958324858554102,0.337898972526575,0.87719097944963
"6510","GPER1",43.648837125805,-0.297092752232974,0.310107311256621,-0.958032079376299,0.338046582624414,0.87719097944963
"6511","KIAA0895",6.46017819695017,0.70149953181642,0.73231593516726,0.957919250598034,0.338103478416917,0.87719097944963
"6512","AL355512.1",3.13339189440522,-0.866360961970727,0.904210210935589,-0.958141095392299,0.337991615322374,0.87719097944963
"6513","C1R",11690.5562941541,0.21867948582907,0.228262462784414,0.958017727319473,0.338053819546968,0.87719097944963
"6514","LINC02453",3.92643435954206,-0.720323725862032,0.751499859327185,-0.958514784695949,0.337803239854666,0.87719097944963

"6515","LINC00640",16.8976372811512,0.461942518914365,0.481844757811997,0.958695744687551,0.337712042801347,0.87719097944963
"6516","PITPNA-AS1",248.225024523648,-0.269280859638376,0.280909774958388,-0.958602667629724,0.337758948174073,0.87719097944963
"6517","ARHGAP35",257.560240479957,0.181510226809381,0.189428755214941,0.95819785440402,0.337962998958529,0.87719097944963
"6518","SPEN",369.525220868498,0.24909243160332,0.260173746869614,0.95740801906563,0.338361352499036,0.877247440196222
"6519","GPATCH3",43.6354482760377,-0.254327696648581,0.266842896587125,-0.953098995331668,0.340539915127891,0.877247440196222
"6520","MKNK1",173.11801906315,0.199090826738402,0.209500454931884,0.950312145160417,0.341953669523776,0.877247440196222
"6521","HS2ST1",140.324692767611,0.189490007934262,0.199083282714758,0.951812755698623,0.341191951777134,0.877247440196222
"6522","DAP3",301.094793332579,-0.208827813594519,0.219204006420727,-0.952664219073204,0.340760227963947,0.877247440196222
"6523","ARPC5",1099.89753181348,-0.153317087123843,0.160383505272038,-0.955940493156016,0.339102299449381,0.877247440196222
"6524","MAPKAPK2",180.059515160402,-0.223471959335413,0.234515502070741,-0.952909114161686,0.340636121802548,0.877247440196222
"6525","AL160408.2",4.20914268110182,0.749994005002216,0.788273123626123,0.951439269617845,0.341381433647624,0.877247440196222
"6526","EFCAB2",133.285817974684,-0.193995341591985,0.203551203746952,-0.953054258687429,0.340562580177344,0.877247440196222
"6527","TCF7L1",837.548775307041,0.191404839102204,0.200119500959783,0.956452710426605,0.338843565174992,0.877247440196222
"6528","RNF103",240.725653588941,0.186441852500614,0.195353548831406,0.954381702384722,0.339890464820912,0.877247440196222
"6529","RGPD8",8.76071961932181,0.47819402862733,0.502271615888866,0.952062616122701,0.341065226898525,0.877247440196222
"6530","GRM7",5.78930750858032,-0.884549877488836,0.924505095268634,-0.956782046973804,0.338677275647589,0.877247440196222
"6531","AC124045.1",3.39982838023442,-0.857987313257175,0.897813862842216,-0.955640527248085,0.339253878871395,0.877247440196222
"6532","CCDC12",393.453519646999,-0.212294750887836,0.22271041303997,-0.953232262425627,0.340472403379773,0.877247440196222
"6533","TEX264",518.372314803567,-0.266499470241108,0.278519667423698,-0.956842555163961,0.338646729380499,0.877247440196222
"6534","NIT2",341.622565338839,-0.273161863302946,0.285747747387627,-0.955954564122574,0.339095190145203,0.877247440196222
"6535","CLDN11",63.5658237920589,0.881641185257411,0.925292100058356,0.952824719028518,0.34067888768745,0.877247440196222
"6536","ADGRA3",113.508013678173,0.277909397155591,0.290771135087516,0.9557667994519,0.339190065431443,0.877247440196222
"6537","KLF3",520.21666635414,0.158193203802706,0.166183653462837,0.951917956467855,0.341138592095691,0.877247440196222
"6538","MED10",645.174263393408,-0.19193070378359,0.201684835906969,-0.951636759999756,0.341281231839428,0.877247440196222
"6539","HK3",2.20812508916267,-0.896192658461108,0.942679573167306,-0.

950686409221738,0.341763589377939,0.877247440196222
"6540","RPS10",12631.9436930655,-0.326228290469727,0.342822454699088,-0.95159545705976,0.341302186387658,0.877247440196222
"6541","DLK2",3.3819196892618,-0.812460770843121,0.852283623973659,-0.953275116392746,0.340450695820109,0.877247440196222
"6542","FIGNL1",57.6008250735468,0.210685994983304,0.221546918376705,0.950976869942584,0.341616117632879,0.877247440196222
"6543","AC016831.7",41.1039731901533,0.656700106446893,0.687978230032669,0.954536172482826,0.339812308318827,0.877247440196222
"6544","EIF4EBP1",307.124146403072,0.28255210892876,0.296361659108327,0.953403047407968,0.340385897989807,0.877247440196222
"6545","SFRP1",2376.20624691185,0.619628702109636,0.651413072446415,0.95120704253384,0.341499284101044,0.877247440196222
"6546","AF235103.3",3.25948314197431,0.837376667116885,0.878650752708773,0.953025607199852,0.340577096467164,0.877247440196222
"6547","BX088651.4",3.9355022374913,0.85881865719829,0.896840499227557,0.957604677685704,0.338262139526612,0.877247440196222
"6548","AL499604.1",39.4827205143437,-0.360285395931876,0.378725225819124,-0.95131079571643,0.341446628284091,0.877247440196222
"6549","ASTN2",25.3272706501484,-0.320761417058461,0.337549554076204,-0.9502646742826,0.341977783727691,0.877247440196222
"6550","DNLZ",24.0983797066874,-0.500647586374875,0.526827478025364,-0.950306518276885,0.341956527805009,0.877247440196222
"6551","PARG",84.224086047306,0.208516879665776,0.218775340621035,0.953109610406096,0.340534537325314,0.877247440196222
"6552","TIMM10B",147.614616457833,-0.161325474910314,0.169417187021639,-0.952237950271886,0.340976318452533,0.877247440196222
"6553","C11orf65",8.49240778170635,0.546044245102673,0.571454000084587,0.95553490748485,0.339307261223699,0.877247440196222
"6554","USP28",96.4398649996885,0.184621274935242,0.194290014287826,0.950235531208204,0.341992588333239,0.877247440196222
"6555","MPZL3",3.04172231266824,0.918363861285037,0.965940726981042,0.950745564021612,0.341733552198218,0.877247440196222
"6556","KMT2A",923.393354964739,0.228473530512821,0.240399711600158,0.95039020218471З,0.341914020568028,0.877247440196222
"6557","COX14",730.80619507595,-0.221717495369114,0.232777686024145,-0.952486035736758,0.34085054462587,0.877247440196222
"6558","AC138331.1",13.1894281453433,-0.392620634847937,0.41214255220008,-0.952633094428294,0.340776003160403,0.877247440196222
"6559","ACACB",486.686126929823,-0.307061861093731,0.320882745967361,-0.956928550857527,0.338603319334596,0.877247440196222
"6560","UBE2L5",8.12913712557887,0.490066762811643,0.512936552204944,0.955413999460575,0.33936837718462,0.877247440196222
"6561","LRCH1",64.5200258983192,0.273060136229756,0.286345733177797,0.953602951227522,0.340284661323046,0.877247440196222
"6562","SPRY2",1057.57631244116,0.239439537912714,0.251972885757904,0.950259140750436,0.341980594716021,0.877247440196222
"6563","ANKRD10",410.727762900421,-0.200977921712295,0.210457074052994,-0.954959212545584,0.339598323739311,0.877247440196222
"6564","OSGEP",192.897575437188,-0.218638112204693,0.22939908126693,-0

.953090618311084,0.340544159127397,0.877247440196222
"6565","CMTM5",2.64810562527278,0.896449737979816,0.936489329570862,0.957245010352233,0.338443603476627,0.877247440196222
"6566","ERO1A",172.924790738791,0.140223277918159,0.146485003233373,0.957253471843538,0.338439333659377,0.877247440196222
"6567","ATP6V1D",343.704204530591,-0.180354825141179,0.188942180213161,-0.954550354704842,0.339805133184838,0.877247440196222
"6568","EMC7",883.686358010422,-0.219443769614209,0.229669802727041,-0.955475064673672,0.33933750937501,0.877247440196222
"6569","LPCAT4",67.8218148768558,0.247272663513403,0.259519067534896,0.952811158972565,0.340685759349761,0.877247440196222
"6570","GPR176",26.636078599893,0.406592129748346,0.424978662602279,0.956735397628327,0.338700826780306,0.877247440196222
"6571","AC104590.1",3.75449773202523,-0.706676967270115,0.742417216626874,-0.951859616727178,0.341168182378848,0.877247440196222
"6572","POLR3K",133.540778341575,-0.297149453341915,0.312265767772766,-0.951591509569979,0.341304189142086,0.877247440196222
"6573","HAGH",212.209922644365,-0.210029251779732,0.220962424129512,-0.950520219024334,0.341847985226843,0.877247440196222
"6574","TBC1D10B",147.782770618748,0.172432781010176,0.181075026666317,0.952272570021115,0.340958765223678,0.877247440196222
"6575","LINC01572",7.6068162949118,-0.691632870082336,0.72596991109012,-0.952701840002951,0.34074116081874,0.877247440196222
"6576","MBTPS1",599.697659533796,0.149434996240933,0.156734169262681,0.953429599581984,0.340372450143968,0.877247440196222
"6577","EPN2-AS1",2.3861558401609,1.03571811276008,1.08797998738929,0.951964305194058,0.341115084901771,0.877247440196222
"6578","SRP68",265.292781298708,0.162708225867547,0.170337969718065,0.955208202474493,0.339472418613641,0.877247440196222
"6579","PRCD",79.3662781160238,-0.24617183975128,0.257047517424628,-0.957690010849698,0.33821909532205,0.877247440196222
"6580","AC139530.3",2.05702257987304,1.00473916679648,1.05546783195114,0.951937270261584,0.34112879638017,0.877247440196222
"6581","AP000894.4",7.50676294638995,-0.552014323655663,0.580421420574844,-0.951057807461608,0.341575031562451,0.877247440196222
"6582","CTIF",97.8389242845339,0.214155154531813,0.223725683062409,0.957222039063231,0.338455195367243,0.877247440196222
"6583","CACTIN",63.5179703429011,0.223041363299984,0.233452245830738,0.955404658911257,0.339373098890824,0.877247440196222
"6584","NDUFA7",293.759910409332,-0.254213211043379,0.267150428302081,-0.95157328647786,0.341313434704569,0.877247440196222
"6585","GCDH",130.64693027124,-0.212950851034297,0.223197640219588,-0.954090960929471,0.340037601149304,0.877247440196222
"6586","ZNF765",78.9894253208712,0.2359804788062,0.24694148916084,0.955612925183662,0.33926782898979,0.877247440196222
"6587","ZNF471",56.8637453955384,0.295259088313418,0.310239971357082,0.951711950661503,0.341243086878914,0.877247440196222
"6588","AC005498.2",4.02152992819279,-0.785135585921677,0.82085339168662,-0.956486985219672,0.338826256608525,0.877247440196222

"6589","USP27X-
AS1",4.35674187813865,-0.662218904845371,0.696120530872134,-0.951299201038807,0.341452512445385,0.877247440196222
"6590","ARMCX5",56.9692286394314,0.206798146777249,0.217587794215625,0.950412441666265,0.341902724593017,0.877247440196222
"6591","VMA21",190.197789660749,-0.17613866579458,0.18537235070325,-0.950188445722138,0.342016508503282,0.877247440196222
"6592","AC005730.2",2.07759797812532,-1.30395955401772,1.3663526497882,-0.954336023148818,0.339913579134603,0.877247440196222
"6593","AZIN2",45.8508752884636,-0.309974032183136,0.326827083792935,-0.948434348175146,0.342908380263708,0.877324982545183
"6594","MRPL9",351.346884936537,-0.199857639385723,0.210979542168556,-0.947284449153141,0.343493852833356,0.877324982545183
"6595","RABGAP1L-
IT1",3.6497740461578,-0.763717985203426,0.805741935185973,-0.947844405078845,0.343208670839675,0.877324982545183
"6596","CR1",5.13214289341813,-0.644270181358082,0.678298354198417,-0.949833030509786,0.342197099567932,0.877324982545183
"6597","PUS10",27.4766478387748,0.289404842316338,0.305546256530744,0.947171945754859,0.343551168353597,0.877324982545183
"6598","LINC01117",21.3766385128763,0.514406532668128,0.541759689827422,0.949510534517606,0.342361016716496,0.877324982545183
"6599","SEC13",402.698927526414,-0.218403702324834,0.230683690923555,-0.94676698404834,0.343757529046427,0.877324982545183
"6600","NGLY1",184.055812119325,-0.175830621392037,0.185780013030495,-0.946445306595896,0.343921506082283,0.877324982545183
"6601","AC092053.4",15.4132404785409,-0.451472705682735,0.476455174597521,-0.947565961612464,0.343350461413291,0.877324982545183
"6602","DAP",295.350766224971,-0.209981930202649,0.221884512673579,-0.946356857774747,0.343966602158269,0.877324982545183
"6603","CDC23",93.9661199257331,0.216213373424437,0.228219580286912,0.947391863365179,0.343439135728828,0.877324982545183
"6604","TUBB2A",461.828198710405,-0.291008867084854,0.307296239339282,-0.946997814586187,0.343639892548132,0.877324982545183
"6605","TMEM30A",537.260736068488,0.156050328228468,0.164511176007206,0.948569769032789,0.342839472561296,0.877324982545183
"6606","CD164",1700.12588625529,0.174322960854193,0.183967898095694,0.947572715993723,0.343347021465854,0.877324982545183
"6607","PSMG3-
AS1",49.8046411133029,0.341440103502057,0.360069334135315,0.948262101581087,0.342996039203982,0.877324982545183
"6608","NDUFA5",814.911317384418,-0.215625982236859,0.227572546902764,-0.947504368042206,0.343381831496955,0.877324982545183
"6609","SOX17",2.47750530960866,-0.92513453978569,0.974592031320021,-0.949253133675489,0.342491883551619,0.877324982545183
"6610","RHPN1",2.13194649799842,-1.16818872422563,1.23368364443617,-0.946911089803362,0.343684086456892,0.877324982545183
"6611","HABP4",174.883851423245,-0.20693733529026,0.21862361252891,-0.946546134228271,0.343870103211551,0.877324982545183
"6612","DEAF1",216.396686985195,-0.224234231932805,0.236858883651525,-

0.946699690870393,0.343791828022613,0.877324982545183
"6613","PUS3",102.139941644669,-0.229441661453572,0.241921217407464,-0
.948414793511589,0.342918331212147,0.877324982545183
"6614","RB1-
DT",4.57763128153348,0.655389165564915,0.691454487109475,0.94784136596
5638,0.343210218232311,0.877324982545183
"6615","PCDH17",2.22926383080149,1.27912310075082,1.34890273621402,0.9
48269335075224,0.342992357679552,0.877324982545183
"6616","GAS6-
AS1",6.14286529124597,0.705113298962984,0.742768568421016,0.9493041694
82992,0.342465933563223,0.877324982545183
"6617","AC006449.2",14.7699190311463,-0.521882791111554,0.551001675401
57,-0.947152820780818,0.343560912291905,0.877324982545183
"6618","KRT27",55.3292084678785,1.03200340656121,1.08869594440783,0.94
7926197265799,0.343167027264455,0.877324982545183
"6619","MLX",231.653335257247,-0.166155849599193,0.17554211778203,-0.9
46529822578012,0.343878418710921,0.877324982545183
"6620","FAM215B",48.7478775998491,-0.326960450009837,0.345241693394059
,-0.947047984834913,0.343614328048468,0.877324982545183
"6621","ICAM2",81.4339105289244,-0.310046444330626,0.327019474163232,-
0.948097800976422,0.343079667651417,0.877324982545183
"6622","GAPLINC",9.44809377585119,0.645035940957805,0.679524425526559,
0.94924614440161,0.342495437459749,0.877324982545183
"6623","MPPE1",199.562578700633,-0.187999165573772,0.198566269939862,-
0.946782983991742,0.343749374281606,0.877324982545183
"6624","PRDX2",1797.19246936099,-0.23731791952419,0.250568861415606,-0
.947116565815266,0.343579384233983,0.877324982545183
"6625","SRRD",130.015927463326,-0.199957190538955,0.211081449345876,-0
.947298737802901,0.34348657383354,0.877324982545183
"6626","RNF185",124.55954768873,0.182673026424941,0.192690658115325,0.
948011845574846,0.343123423848429,0.877324982545183
"6627","ZXDB",50.9692049024817,0.248807326115388,0.262328458670454,0.9
48457240881932,0.342896730891234,0.877324982545183
"6628","SMIM10",308.747175534432,-0.272511399696871,0.286939443802856,
-0.949717459841811,0.342255835655987,0.877324982545183
"6629","FGF13",3.26910290619033,0.778306841370704,0.821322078516953,0.
947626834500881,0.343319460177307,0.877324982545183
"6630","AC124798.1",2.97437889515609,-0.832407207362204,0.880264063778
549,-0.945633522501283,0.344335539946872,0.877844609510013
"6631","PPFIA1",348.446980987849,0.187031188184443,0.197868931670127,0
.94522766462523,0.344542658672444,0.877844609510013
"6632","AASDHPPT",235.172405101281,0.165499710910433,0.175024227808912
,0.945581723069343,0.344361969978942,0.877844609510013
"6633","USP15",323.814926393679,-0.147289256786802,0.155798313457291,-
0.945384154156316,0.34446278900655,0.877844609510013
"6634","IGHA2",23.1241899211681,-1.06138780930395,1.12299131425765,-0.
945143382525256,0.344585679756207,0.877844609510013
"6635","SEZ6",2.4471039761196,0.98679998695976,1.04405307041917,0.9451
62669330186,0.344575834667663,0.877844609510013
"6636","SBNO2",93.2304032035718,-0.231845846291067,0.245207549949109,-

0.945508596041129,0.344399284362271,0.877844609510013
"6637","RNASEH2A",32.2066831693722,-0.33148144807582,0.35047869240725,
-0.945796293061503,0.344252496667756,0.877844609510013
"6638","USP47",404.109313763629,0.186360937342746,0.19723922140911 7,0.
944847257109135,0.344736861639553,0.877924409130362
"6639","PGPEP1",125.332448356575,0.245226803146089,0.259560534023253,0
.94477692484683,0.344772774818169,0.877924409130362
"6640","SLC25A1",284.727496075612,-0.180914073007713,0.191447608108986
,-0.944979541894949,0.344669320624222,0.877924409130362
"6641","RERE-
AS1",6.28587323639182,0.560438351452509,0.593332650267527,0.9445601067
12168,0.344883501878561,0.877941922577945
"6642","AC018521.5",115.394370356625,-0.263048917095227,0.278476478258
035,-0.944600128314923,0.344863061501235,0.877941922577945
"6643","LCK",13.8472777983842,0.548446444104846,0.580812821451193,0.94
4273996456418,0.345029650507375,0.87804956814851
"6644","CSRNP1",668.622667870592,-0.342728482713158,0.362940935765716,
-0.944309249630618,0.345011640620353,0.87804956814851
"6645","DHRS9",29.3541430224298,-0.989011784764752,1.04772691714006,-0
.94395950756369,0.34519034085148,0.878144897469428
"6646","CCDC85C",17.9103534080798,0.557904629872537,0.591047620868329,
0.943925007350337,0.345207971885799,0.878144897469428
"6647","SLX4",31.4988211026498,0.365743162947442,0.387482579481343,0.9
43895757680255,0.345222920125342,0.878144897469428
"6648","L3MBTL1",23.1824782053163,0.39447569021484,0.417972768861004,0
.943783230878425,0.345280431548034,0.878159075904656
"6649","KIF3C",47.5320634471261,0.316977871094586,0.335909340209416,0.
943641135125844,0.345353064118439,0.878211702228088
"6650","AL359921.2",5.20013488439922,0.638279564812631,0.6772474014068
17,0.942461445384302,0.345956441309251,0.878247030118705
"6651","EIF5B",925.95088792035,-0.233559620371696,0.247567653009166,-0
.943417354944384,0.345467469619761,0.878247030118705
"6652","CYB561D2.1",3.58169272426461,-0.749026763945247,0.794233745521
138,-0.943081011313327,0.345639467557169,0.878247030118705
"6653","AC109587.1",3.15446973824864,-0.738759545925494,0.784153355656
541,-0.942111055951497,0.346135784665316,0.878247030118705
"6654","TMEM165",884.294313730074,-0.174270067911142,0.184785962655405
,-0.943091484909631,0.345634110792084,0.878247030118705
"6655","MANBA",184.806419994396,0.22551805243088,0.239241536152915,0.9
42637537182241,0.345866332826018,0.878247030118705
"6656","ZNF318",89.6770537612495,0.28143764294206,0.298660304090006,0.
942333611423779,0.34602186499334,0.878247030118705
"6657","LCNL1",3.62289587745614,0.818608345264124,0.868631064749369,0.
94241200722003,0.34598174215535,0.878247030118705
"6658","BLOC1S2",298.989671198435,-0.259732206623976,0.275766006966588
,-0.94185722700567,0.346265741477876,0.878247030118705
"6659","POLR2L",2534.97576701471,-0.288343343618498,0.306090063368849,
-0.94202124840307,0.346181761301562,0.878247030118705
"6660","PRR5L",9.29705548755202,0.725536360223372,0.769119262720057,0.
943334012539811,0.345510083809281,0.878247030118705

"6661","MRPL49",119.863916785798,-0.196404177509313,0.208279138167832,-0.942985357232705,0.345688392689155,0.878247030118705
"6662","EML1",237.024776800744,-0.230839955228978,0.245130510924378,-0.941702256314358,0.346345099545808,0.878247030118705
"6663","C16orf91",120.796922852761,-0.282126868313818,0.299554656856147,-0.941821006138797,0.346284288580282,0.878247030118705
"6664","PHKB",312.450644396731,0.158706657839468,0.168491125638663,0.94192888342275,0.346229051262982,0.878247030118705
"6665","PROCA1",22.7336843969144,0.307347736364599,0.326270323363795,0.942003346169805,0.346190926747453,0.878247030118705
"6666","GSK3A",210.137245087624,-0.140818932862864,0.149332355253056,-0.942990101671102,0.345685965900634,0.878247030118705
"6667","PRKD2",81.0105255977412,0.223204770014536,0.236839384016729,0.942430968317203,0.345972038342555,0.878247030118705
"6668","AC003681.1",79.2433952749644,0.438286245497804,0.465427592726195,0.941685135018718,0.346353867804088,0.878247030118705
"6669","DCUN1D4",154.641912846462,0.169608495778307,0.180225044361576,0.941092823026475,0.346657293134145,0.878314701384491
"6670","AC004540.2",4.22489697782702,0.95192200945498,1.01158032979028,0.941024633854168,0.346692235453046,0.878314701384491
"6671","ERI1",69.6147406981536,-0.202845499465736,0.215486197891367,-0.941338709628155,0.346531311573955,0.878314701384491
"6672","GPR135",22.4789647369348,0.364925073488756,0.38773859528533,0.941162623288026,0.346621527563795,0.878314701384491
"6673","NPTN",613.690298380679,-0.185946220717187,0.197551765957165,-0.941253143530518,0.346575148599207,0.878314701384491
"6674","AC145207.5",19.3907420339324,-0.374863957978818,0.398331720717696,-0.941084875950639,0.346661365355255,0.878314701384491
"6675","ARL8B",330.570126356164,0.136514447709352,0.145099340563137,0.940834377189683,0.346789740925706,0.878430103306642
"6676","LRPAP1",1373.529717463,-0.189093775926051,0.201052502829396,-0.940519383071332,0.346951212015685,0.878707473451348
"6677","TMEM60",225.449827078847,0.221031037033131,0.235067028826934,0.940289406541411,0.347069131929092,0.878874476959277
"6678","AC239809.3",23.4563960090488,-0.369846084610748,0.39347808370138,-0.939940748749385,0.347247954013503,0.879195628400765
"6679","PPP1CB",868.348470659915,-0.212944359709768,0.226634948356344,-0.93959189107476,0.347426937278292,0.879385427470264
"6680","MSX1",1198.11441316476,-0.210690970643485,0.224215467082681,-0.939680805186337,0.347381313853816,0.879385427470264
"6681","TMEM51-AS1",2.41353956037743,-1.04273761428979,1.11006410279128,-0.939349008465189,0.347551584126249,0.879437621132388
"6682","SF3A1",260.180579965146,0.132944596078016,0.141517460032324,0.939421863900394,0.347514191882875,0.879437621132388
"6683","MAD2L2",196.606408125597,-0.235270592214665,0.25148637472459,-0.935520234336021,0.349520260489029,0.879462133548233
"6684","COA7",57.6179700470551,0.231186560265077,0.246933975064573,0.93622823754658,0.34915568742536,0.879462133548233
"6685","CCN1",3659.48087556332,0.409803100807004,0.438111332922299,0.9

35385757025565,0.349589534373175,0.879462133548233
"6686","LRIF1",81.4461515600086,-0.241893665759198,0.258781143845944,-0.934742238805471,0.349921153348704,0.879462133548233
"6687","ADAMTSL4-AS1",65.2194858596324,0.450127756393461,0.479713398920067,0.938326420330955,0.348076686160285,0.879462133548233
"6688","ERO1B",122.655007689886,0.320317471459241,0.342033361567004,0.936509438704245,0.349010955155166,0.879462133548233
"6689","WDCP",34.5364321734181,0.326905050779315,0.349112759760924,0.936388148640523,0.349073377629328,0.879462133548233
"6690","STRN",289.734094221679,0.164928584406703,0.176636328223633,0.933718369631715,0.35044918675361,0.879462133548233
"6691","LRPPRC",320.653916705311,0.136668703939355,0.145569083376587,0.938858037498205,0.347803636255543,0.879462133548233
"6692","RAPGEF4",10.9156765318181,0.490328119668333,0.52236082076427,0.938677060333373,0.347896574687681,0.879462133548233
"6693","HEMK1",90.4383866469854,0.19634223443529,0.20987455209208З,0.935521874748989,0.349519415509734,0.879462133548233
"6694","LAMTOR3",300.616362162543,0.152993742433105,0.163583808096532,0.935262140020747,0.349653221427806,0.879462133548233
"6695","ARRDC3-AS1",2.06406575706537,-1.0246354691404,1.09716379581176,-0.933894713853825,0.350358205888502,0.879462133548233
"6696","SRA1",238.060347699741,-0.296493909508748,0.315686298203701,-0.93920423913182,0.347625892974339,0.879462133548233
"6697","HSPA1B",724.721697072672,0.253933010955377,0.271053096665603,0.936838627114646,0.348841572601543,0.879462133548233
"6698","ZBTB24",90.9558408368877,-0.184998173608342,0.198018126318074,-0.934248682422045,0.350175628925919,0.879462133548233
"6699","CHCHD2",4579.80343484147,-0.256623191322253,0.273753497030212,-0.937424340168089,0.348540325477939,0.879462133548233
"6700","MAGI2",190.431550170111,-0.178011727623752,0.189726665867733,-0.938253601883524,0.348114097875512,0.879462133548233
"6701","PILRB",127.163322012842,0.203525105770296,0.217152687528393,0.937244240846364,0.348632937535756,0.879462133548233
"6702","FSCN3",5.53250827729008,-0.695257880593703,0.744338662864373,-0.934061221431389,0.35027231377243,0.879462133548233
"6703","AKR1C3",1318.88064712708,-0.296434807529653,0.316697703215038,-0.936018179229969,0.349263827838764,0.879462133548233
"6704","ZFAND4",25.51399673284,0.305478443094564,0.325881512812055,0.937391140904462,0.348557396283155,0.879462133548233
"6705","AL356419.1",2.34070408200714,1.15841527655335,1.23602771562486,0.937208172526887,0.348651486743322,0.879462133548233
"6706","MIR210HG",4.77377602554489,-0.614008729826997,0.65400083672353,-0.938850067689685,0.347807728713057,0.879462133548233
"6707","AC132192.1",7.71644165561805,-0.79541187234086,0.850277131134677,-0.935473674658754,0.349544243992611,0.879462133548233
"6708","SPCS2",1964.13541769459,-0.227019972037746,0.241783560667834,-0.938938823676396,0.347762154678386,0.879462133548233
"6709","AP001267.3",16.7939657307642,-0.351734225415089,0.376274651932

864,-0.934780548219991,0.349901406085855,0.879462133548233
"6710","FOXRED1",94.834135859557,-0.236415602294487,0.252083475777965,
-0.937846487418009,0.348323306946444,0.879462133548233
"6711","GPRC5D-
AS1",3.53745331958409,-1.0485855037123,1.12187583936881,-0.93467161598
0482,0.349957558981858,0.879462133548233
"6712","AC124947.1",5.47047090940273,0.689104760201377,0.7371609775740
48,0.934809059574992,0.349886709864446,0.879462133548233
"6713","CCDC92",209.893135229796,-0.140635962166249,0.150354111353715,
-0.935364925508401,0.349600266183095,0.879462133548233
"6714","NRDE2",163.785258401464,0.210320665227904,0.224064084549634,0.
93866299746631,0.347903797148423,0.879462133548233
"6715","CATSPER2",13.7526960734113,0.588275561473816,0.628511299103137
,0.935982475276521,0.349282210754938,0.879462133548233
"6716","AC032011.1",2.48627645732634,1.10774849038119,1.18617389533335
,0.933883720371272,0.350363877292549,0.879462133548233
"6717","NLRC3",2.71644707111365,0.978147383135808,1.04747820084248,0.9
33811684433235,0.350401041198331,0.879462133548233
"6718","DCUN1D3",356.094328876322,0.375836778676821,0.400695880255046,
0.937960176774462,0.348264875915371,0.879462133548233
"6719","TENT4B",224.775917934628,0.216462802395752,0.231461475912021,0
.935200130141872,0.349685171476931,0.879462133548233
"6720","MYO18A",55.0889327551151,0.334152974647838,0.356688012806134,0
.936821431196948,0.348850419397972,0.879462133548233
"6721","AP005329.1",2.32195148457696,-1.0150749690603,1.08593038880782
,-0.934751416409568,0.349916422526521,0.879462133548233
"6722","NDUFV2",1239.80038080158,-0.251649379802039,0.269002358541013,
-0.935491350956582,0.349535138575549,0.879462133548233
"6723","RNF152",32.2665451184954,0.450743697290383,0.482303219776652,0
.934564976570375,0.350012535497826,0.879462133548233
"6724","TMEM259",502.204305648263,-0.146277186301967,0.156162960469096
,-0.936695781525701,0.348915066802089,0.879462133548233
"6725","RPL36",14669.3714956221,-0.225223411195706,0.240634105344666,-
0.935957980158768,0.349294822926391,0.879462133548233
"6726","SLC27A1",351.097338375434,0.178353544160691,0.190255812039536,
0.937440713367686,0.348531906700644,0.879462133548233
"6727","PIK3R2",137.663032924671,0.172700065643202,0.184350236839986,0
.936804143045952,0.348859313789401,0.879462133548233
"6728","PBX4",47.3140344519464,-0.272969822905808,0.291377009658236,-0
.936826907606682,0.34884760192996,0.879462133548233
"6729","CEBPA",382.518211635189,-0.172627436719759,0.184182132264728,-
0.937264839955479,0.348622344111678,0.879462133548233
"6730","CD93",13.3276567882267,-0.60794302110299,0.650772382700683,-0.
934186878951513,0.350207502801814,0.879462133548233
"6731","NOL4L",33.5263384633164,0.26557914128087,0.283527194695144,0.9
36697241922163,0.348914315376869,0.879462133548233
"6732","CRYZL1",210.815648916377,-0.145376325199694,0.155657935168767,
-0.933947408733605,0.35033102204886,0.879462133548233
"6733","LRRC3",35.2802866666606,0.341517088477481,0.365396173507022,0.
934648781895135,0.349969330326932,0.879462133548233

"6734","MCM3AP-
AS1",10.336823109013,0.499216136627964,0.533059639759648,0.93651085055
5215,0.349010228581491,0.879462133548233
"6735","CABIN1",272.108067950226,-0.230222585708045,0.245885914248293,
-0.936298390299693,0.349119576725996,0.879462133548233
"6736","MFNG",4.96100346876761,-0.658057811982663,0.70144093919405,-0.
93815141833433,0.348166600721128,0.879462133548233
"6737","LMF2",501.235120076106,-0.163013334930267,0.174056776859196,-0
.936552646049154,0.34898872001445,0.879462133548233
"6738","INE2",4.46316018733877,0.767117873656801,0.821615540031692,0.9
33670112455773,0.350474086577241,0.879462133548233
"6739","RBM8A",1035.1711465599,-0.201534454936825,0.215931649858693,-0
.933325221516674,0.350652076680324,0.879647672479364
"6740","LPIN2",404.792802764306,0.134672158514431,0.144289067207271,0.
933349706398574,0.35063943871887,0.879647672479364
"6741","ZNF589",42.8598939719482,0.260781108969968,0.279489614628473,0
.933061893253621,0.350788012782935,0.879703147510257
"6742","FAM149A",120.471764075,0.203888238430211,0.218500150704305,0.9
33126305739405,0.35075475849095,0.879703147510257
"6743","NIBAN2",88.2216400830211,-0.244977181189721,0.262574944967695,
-0.932980034404506,0.350830277008615,0.879703147510257
"6744","AL139260.1",26.6584739870592,0.398291854801582,0.4270254792223
69,0.932712154616367,0.350968607551177,0.879887112743453
"6745","FUCA2",650.196911498981,-0.208751090541909,0.223829014369069,-
0.932636419502352,0.351007722702543,0.879887112743453
"6746","MRPL47",295.746219672762,-0.228519785434469,0.245176310456784,
-0.932063073339824,0.351303930219097,0.880499088666542
"6747","ZNF354B",62.137837401425,0.225726703022419,0.242206286009876,0
.93196054793233,0.351356914553129,0.880501365238523
"6748","UTS2",2.08766396986273,-1.01754456856085,1.09212376163486,-0.9
3171177508091,0.351485499476447,0.880561602061442
"6749","PGM5-
AS1",23.3905348798504,-0.526983763696962,0.565730016960392,-0.93151105
2795802,0.351589269902815,0.880561602061442
"6750","TOMM40",239.111589428388,-0.192986085727565,0.207151889148483,
-0.93161634451345,0.351534833236161,0.880561602061442
"6751","XRN2",581.929043581285,-0.133753348449932,0.143575751089521,-0
.931587314953592,0.351549841219454,0.880561602061442
"6752","PODXL",9.18096064521998,0.688645887289441,0.73943795907664,0.9
31309894002973,0.351693285464797,0.880691657381336
"6753","CEP85",11.0783757270853,-0.448559708819829,0.48396854532114,-0
.926836492074468,0.354011434902592,0.880807653986341
"6754","KIAA1324",2.20599228652289,-0.916799701022591,0.98632677546605
3,-0.929509087482077,0.352625317912925,0.880807653986341
"6755","LINC01136",2.05329447665687,-1.04857595537603,1.12871511641154
,-0.928999656449808,0.352889264373551,0.880807653986341
"6756","ACADL",99.9003379946811,0.276731586957042,0.297258752477323,0.
930945126596913,0.35188194978138,0.880807653986341
"6757","THRB-
AS1",7.25754272756315,-0.485532600634696,0.523358984654241,-0.92772382

7948544,0.353550845044236,0.880807653986341
"6758","ACVR2B-
AS1",3.47900349208124,0.807052792845953,0.869150489157231,0.9285535737
64319,0.353120491387922,0.880807653986341
"6759","LINC02517",4.80040540088443,0.752152619748341,0.81171840863810
3,0.926617669063708,0.354125077718874,0.880807653986341
"6760","NOA1",275.429223287853,-0.220438401512201,0.237727123533633,-0
.927274928647397,0.353783808054513,0.880807653986341
"6761","PDLIM3",405.696980668508,-0.607269289439723,0.653249533401964,
-0.929613047371366,0.352571469555353,0.880807653986341
"6762","ITGA1",44.2164794863577,0.465582153964693,0.501538161624305,0.
928308530814158,0.353247550196576,0.880807653986341
"6763","RUFY1",238.49398932156,-0.1253155453636,0.135203902100098,-0.9
26863377588192,0.3539974738588,0.880807653986341
"6764","RBM24",9.75305008176193,-0.502809604230314,0.541599475288268,-
0.928379046088794,0.353210983902091,0.880807653986341
"6765","HMGCLL1",82.212893298199,-0.337483963684313,0.363970048677596,
-0.927230042445761,0.353807107754762,0.880807653986341
"6766","OSBPL3",13.8041723509305,-0.567462141144487,0.610101933969541,
-0.930110379182664,0.352313937377521,0.880807653986341
"6767","POM121C",184.792665324722,0.184166760476638,0.197921141805685,
0.930505749898358,0.352109288453655,0.880807653986341
"6768","AC064807.1",35.8526746890284,0.265076874320361,0.2860757620069
43,0.926596760455113,0.354135937532478,0.880807653986341
"6769","KIF24",2.35027433185207,0.914632482374051,0.984218625681875,0.
929298083279399,0.352734628274056,0.880807653986341
"6770","AL353150.1",6.37701668885652,0.6183500060303,0.66725474912246,
0.926707538377993,0.35407840249862,0.880807653986341
"6771","PDSS1",22.202718542282,-0.382973179827087,0.412833389266202,-0
.927670071715394,0.353578737533703,0.880807653986341
"6772","TCF7L2",1766.15549431729,0.210005364117951,0.226491086933676,0
.927212487524717,0.35381622049374,0.880807653986341
"6773","IKZF5",99.9593322069817,0.255813167943852,0.27594526037386,0.9
27043166449997,0.353904122480444,0.880807653986341
"6774","MMP21",3.28851260912993,0.760541379294072,0.820127822892182,0.
927344930954814,0.353747472926072,0.880807653986341
"6775","AL354950.1",2.84456454465282,0.964905593790943,1.0409145938330
9,0.926978639273131,0.353937624995829,0.880807653986341
"6776","TPBGL",3.00561693039831,0.982063896449435,1.0556743483417,0.93
0271629685899,0.3522304629714,0.880807653986341
"6777","AC024257.3",2.0923626144032,1.09430974139367,1.17695664658883,
0.929779142303415,0.352485447756641,0.880807653986341
"6778","PCBP2-
OT1",23.0023134040454,0.391881629069867,0.421459002967192,0.9298214685
43578,0.352463528806368,0.880807653986341
"6779","VEZT",251.048776864085,0.153700455320819,0.165675343259237,0.9
27720759753125,0.353552437000951,0.880807653986341
"6780","DNAH10",3.0154101305619,0.791389266695661,0.853795357968585,0.
926907436670298,0.353974595718635,0.880807653986341
"6781","FAM124A",10.7238388595897,0.418239114696125,0.449297045798033,

0.930874392804556,0.351918541998982,0.880807653986341
"6782","GZMH",18.6277147832284,0.485537654292227,0.523241647969035,0.927941527928526,0.35343790130812,0.880807653986341
"6783","SERPINA1",22.8728118027628,-0.454754961591852,0.488872354468976,-0.930212063404193,0.35226129713773,0.880807653986341
"6784","AL049840.2",48.7887485982948,-0.299030907856844,0.321336824844632,-0.93058400014199,0.352068794053044,0.880807653986341
"6785","FAH",116.06933032147,-0.258557213763143,0.278063243868737,-0.929850382833042,0.352448555827901,0.880807653986341
"6786","RRN3",144.005410635038,0.206547613368616,0.222707509960997,0.927438923836872,0.353698689051926,0.880807653986341
"6787","ZNF624",68.2900217738318,0.222374885800975,0.238960178982864,0.930593903752146,0.352063669157714,0.880807653986341
"6788","CENPX",516.458861498641,-0.228435518351145,0.245389803459866,-0.93090876283499,0.351900761290812,0.880807653986341
"6789","UQCR11",1692.41075615236,-0.221515991284673,0.238751208268852,-0.92781097482543,0.353505630111298,0.880807653986341
"6790","SYMPK",148.397845899294,0.163062032883046,0.175383165493848,0.929747347323174,0.352501913585001,0.880807653986341
"6791","SIGLEC6",2.02386548345327,1.42531892023331,1.53614873407617,0.927852159504911,0.353484263290929,0.880807653986341
"6792","PRDM15",23.1775513750275,0.371227679058462,0.399996224813626,0.928077956814296,0.353367133012263,0.880807653986341
"6793","S100B",4.0907688870136,-0.866072845598631,0.931161531096809,-0.930099468970211,0.352319585709832,0.880807653986341
"6794","BCOR",48.7928793777256,0.30022781760703,0.323188877512131,0.928954671702033,0.352912577879698,0.880807653986341
"6795","RBM10",169.112637509791,0.162587328959442,0.175144998215496,0.928301296731277,0.353251301627067,0.880807653986341
"6796","TSR2",305.688524726317,-0.211132402207323,0.227689188757037,-0.927283387322439,0.353779417401896,0.880807653986341
"6797","GLUD2",9.44213043294111,-0.519077931288042,0.558814570201581,-0.92889119032955,0.352945478985151,0.880807653986341
"6798","FAM50A",561.331586168099,-0.16818130251867,0.181257596702938,-0.927857952316897,0.35348125801622,0.880807653986341
"6799","C4orf3",1737.04662056267,-0.221442014615123,0.239099475525463,-0.926150147876594,0.354367955835749,0.880913110998604
"6800","DMTN",2.78538135006551,0.824784070462017,0.890550919701179,0.926150377497527,0.354367836521432,0.880913110998604
"6801","LAMTOR1",1268.29988851233,-0.233701910020373,0.252369143067707,-0.926032030618236,0.354429334613761,0.880913110998604
"6802","CUL5",371.694121832964,0.137072304589863,0.147971316131846,0.926343754810749,0.354267363916305,0.880913110998604
"6803","ZNF720",193.537319005852,0.152600369233534,0.164792769770631,0.926013741051461,0.354438839254998,0.880913110998604
"6804","TIGD7",20.4576744744533,0.376618729503066,0.406795241023344,0.925818917044445,0.354540094524171,0.881035261348424
"6805","GPR89A",61.2361939149476,0.229481812334674,0.248183287938755,0.924646515245234,0.35514980888897,0.881078624494093
"6806","TFB2M",86.9945368282099,-0.195132587955282,0.211072155578242,-

0.924482850050527,0.355234976530413,0.881078624494093
"6807","FKBP14",280.772372470604,0.24966946083353,0.270053911193338,0.
924517107455575,0.355217148690258,0.881078624494093
"6808","MAMDC2",118.30873200254,-0.759453454974277,0.821093032361701,-
0.924929849654027,0.355002398580564,0.881078624494093
"6809","KIF1BP",262.198591111956,0.161702738026737,0.174868561912136,0
.924710172363546,0.355116686654883,0.881078624494093
"6810","CHFR",55.2673130394464,0.286870553609359,0.310135805708211,0.9
24983663057787,0.354974405461747,0.881078624494093
"6811","FARP1",359.808873410617,0.228401338184106,0.246968475835835,0.
924819807107403,0.355059645795949,0.881078624494093
"6812","AL049840.6",3.91062210227866,-0.680111061671665,0.735372088709
113,-0.924852971868358,0.355042391944881,0.881078624494093
"6813","RAD51",3.53832305219168,-0.708301920663678,0.766153084782402,-
0.924491377418189,0.355230538764168,0.881078624494093
"6814","CASC4",689.6650556103,0.142298464592462,0.153781831784535,0.92
5326892918259,0.354795893988987,0.881078624494093
"6815","CORO1A",53.2504900889514,-0.316607726188868,0.342178613745444,
-0.925270351420624,0.354825296930394,0.881078624494093
"6816","TTPAL",125.135658783561,-0.227227145445934,0.245748313136215,-
0.924633591767466,0.355156533503953,0.881078624494093
"6817","SNX12",106.916537746491,0.16646703666777,0.180021469489956,0.9
24706576051241,0.355118557845308,0.881078624494093
"6818","FAHD2A",259.910769203588,-0.179027492513094,0.193733589275123,
-0.92409113557926,0.355438868156628,0.881196537066316
"6819","AC010680.4",3.71011313805122,0.733951454245967,0.7942014815606
83,0.924137603978881,0.355414676992045,0.881196537066316
"6820","AL357075.4",4.61948795084763,-0.625673455709456,0.677018222730
595,-0.924160435720545,0.355402791307602,0.881196537066316
"6821","DDX19B",205.258149545254,-0.188685518653373,0.204210446378367,
-0.923975839628552,0.355498895009575,0.881216143794442
"6822","MYORG",30.446898266328,0.436598430549302,0.472986543986472,0.9
23067339018822,0.355972113940885,0.881872015020143
"6823","ALKBH3",144.069228473772,-0.215480752826891,0.233378940096948,
-0.923308473066927,0.355846473562398,0.881872015020143
"6824","AC024909.1",4.45128267216749,-0.613145130092714,0.664135471732
591,-0.923222981138391,0.355891015039034,0.881872015020143
"6825","AC005562.1",98.5924044262929,0.211282903033207,0.2288771431655
5,0.923128015803585,0.355940496302151,0.881872015020143
"6826","C1orf159",25.3435064201827,-0.292857844516861,0.31873202707582
4,-0.918821516631563,0.358188936177613,0.882045395544532
"6827","VWA1",184.010667867066,0.233351436228175,0.253959765704895,0.9
18851990513071,0.358172994321183,0.882045395544532
"6828","LINC00339",50.7680402597482,-0.27737166103895,0.30377043417936
8,-0.913096305070855,0.361191893468801,0.882045395544532
"6829","HMGCL",257.556847201436,0.235726311617354,0.258597902770677,0.
911555388082149,0.362002815873517,0.882045395544532
"6830","AL031432.4",4.08264671479051,0.721786156551804,0.7919915799201
07,0.911355846263688,0.362107910043662,0.882045395544532
"6831","AL357079.1",4.87698056200109,-0.688248947201625,0.749046837129

973,-0.91883299292565,0.358182932510853,0.882045395544532
"6832","UQCRH",2081.72464597752,-0.222445398500538,0.244446177619362,-0.909997450837287,0.362823854152603,0.882045395544532
"6833","ANKRD13C",150.36522581011,0.16107280284966,0.176608928261034,0.912030917324796,0.36175244241233,0.882045395544532
"6834","AL356488.2",3.58882139524695,0.822638728340243,0.896171742172245,0.91794763171871,0.358646283093053,0.882045395544532
"6835","FCGR3A",19.9289909007691,-0.438725858091374,0.481691972348721,-0.910801680900255,0.362399876675326,0.882045395544532
"6836","POGK",110.785299807861,0.209135748149083,0.227219540827633,0.920412687162907,0.357357141164807,0.882045395544532
"6837","IKBKE",32.5526235530306,-0.240888423821469,0.264547289023312,-0.910568483656779,0.362522782649005,0.882045395544532
"6838","NENF",2243.62613872176,-0.211151473040197,0.229393543697286,-0.920476965641363,0.357323564739998,0.882045395544532
"6839","RNASEH1",273.893298537565,-0.192625418121369,0.209632026999805,-0.918873994962366,0.358161483371586,0.882045395544532
"6840","AC010969.2",32.1924264005361,0.277341878033586,0.301230152316234,0.920697599164741,0.357208330003535,0.882045395544532
"6841","HS1BP3",157.615344893596,0.189530680697182,0.20725710861919,0.914471315169322,0.360469243792507,0.882045395544532
"6842","MXD1",127.310052001314,0.322158570326112,0.35203585119955,0.915130005163871,0.36012338467188,0.882045395544532
"6843","C2orf69",168.253734092747,-0.169903821433364,0.186405040081436,-0.911476542475126,0.362044339790273,0.882045395544532
"6844","TMEM237",261.063400885671,-0.179004173896,0.195254149948937,-0.916775259029387,0.359260419792831,0.882045395544532
"6845","BHLHE40-AS1",8.96434923093629,0.474417321629674,0.518533138403314,0.914921895041302,0.36023263477495,0.882045395544532
"6846","UBE2E2",318.487678248587,-0.194333689830221,0.212919565928632,-0.912709402645312,0.361395397409006,0.882045395544532
"6847","EIF4A2",3136.00078139304,0.190117560718503,0.206920831620485,0.918793720427336,0.358203477645182,0.882045395544532
"6848","PI4K2B",74.6032193499768,-0.21164138402526,0.229956237929888,-0.920355046379685,0.357387252020936,0.882045395544532
"6849","MRPS18C",562.40469333625,-0.21843119236172,0.238333039508186,-0.916495643291698,0.359406991483796,0.882045395544532
"6850","EIF4E",319.420301565477,-0.194870593543435,0.212450430526998,-0.917252052914388,0.359010575995024,0.882045395544532
"6851","ADH1B",8299.29797558549,0.238794357366146,0.261602526064473,0.912813652675869,0.361340556633388,0.882045395544532
"6852","H2AFZ",1394.33074309417,-0.229471743406916,0.249546427082277,-0.91955531517691,0.357805186867376,0.882045395544532
"6853","AC109347.1",7.26091772124851,-0.627644663208302,0.682689556087445,-0.919370536156126,0.357901795030319,0.882045395544532
"6854","ANK2",1708.68912992657,-0.241711891441038,0.264357946647612,-0.914335636610308,0.360540510575371,0.882045395544532
"6855","RNF175",21.8605224175175,-0.570791497636969,0.62721061361191,-0.910047574529964,0.362797420657376,0.882045395544532

"6856","NLN",50.9981832840019,0.250391524641435,0.273901901694371,0.914164973271454,0.360630166086715,0.882045395544532
"6857","SCAMP1",325.433536161092,0.148381514368649,0.16214841676176,0.915096905242442,0.36014075951284,0.882045395544532
"6858","MCTP1",5.20669994850007,-0.639271677114028,0.700025725794528,-0.913211691453847,0.361131216155088,0.882045395544532
"6859","PCDHGB7",52.9774803232091,0.241678473483914,0.26224599665323,0.921571641009599,0.356752056508157,0.882045395544532
"6860","C1QTNF2",125.940549591742,-0.265957870889027,0.29175716890002,-0.911572702366626,0.361993697732056,0.882045395544532
"6861","HCG17",3.54370657555378,-0.881576293547967,0.959164667682556,-0.919108390093172,0.358038881225822,0.882045395544532
"6862","GPANK1",210.339406092776,-0.200227024110426,0.218059101451562,-0.918223650274476,0.358501789362527,0.882045395544532
"6863","CUTA",1130.7455309083,-0.29264634987556,0.320187841426677,-0.913983331070915,0.360725604551337,0.882045395544532
"6864","ORC3",149.142771381896,0.172252077920243,0.186841535250673,0.921915342266608,0.35657273578437,0.882045395544532
"6865","CASP8AP2",256.228994566881,0.161497393051002,0.177461635749069,0.910041161118112,0.362800802800846,0.882045395544532
"6866","THEMIS",4.64576073457045,0.838897752684088,0.910781160981136,0.921074994327273,0.357011274452162,0.882045395544532
"6867","TMEM181",148.173235371307,0.171931633022075,0.188269517773147,0.91322076486775,0.361126445062676,0.882045395544532
"6868","PDGFA",23.2025582548172,0.524370338590641,0.570714932780691,0.918795546553784,0.358202522303398,0.882045395544532
"6869","HOXA11",51.3340651689032,-0.245023841194927,0.266202036943583,-0.92044314915162,0.357341228831221,0.882045395544532
"6870","CHN2",9.89250368968452,0.550285624810892,0.601757656079151,0.914463853100543,0.360473163103449,0.882045395544532
"6871","GPC2",7.50110977897135,0.556664689830697,0.60732527431508,0.916584099778301,0.359360619441842,0.882045395544532
"6872","PDIA4",405.204419081757,0.17325505903298,0.18878118182599,0.917755982652334,0.358746631539868,0.882045395544532
"6873","DNAJB6",851.983461664981,-0.1364676745526,0.149178375997803,-0.914795282089746,0.360299112061666,0.882045395544532
"6874","BIN3",132.503712544138,-0.179411307094462,0.196751506068991,-0.91186751592921,0.361838463518233,0.882045395544532
"6875","KIF13B",202.618896865207,-0.312136766018728,0.340286638492694,-0.917275998262359,0.358998031322607,0.882045395544532
"6876","BAG4",169.38109584302,0.149990759267292,0.164231650989225,0.91328777591801,0.361091209724615,0.882045395544532
"6877","AC044893.1",2.8288584689192,0.978218248737105,1.06918970080734,0.914915517796753,0.360235982920559,0.882045395544532
"6878","SNHG6",2392.94350151431,-0.230633449497602,0.252173751566663,-0.914581506063819,0.360411371216073,0.882045395544532
"6879","MTERF3",79.8911471660715,-0.225008683601007,0.245246962688462,-0.917477962354364,0.358892235788869,0.882045395544532
"6880","AC068228.3",4.4573132357537,-0.721186749053977,0.791162234519698,-0.911553557017035,0.362003780167274,0.882045395544532

"6881","LRRC6",43.1059457917089,0.275319385080117,0.302328981695082,0.910661569845111,0.362473718696316,0.882045395544532
"6882","C9orf40",59.4727449356765,0.288731143721345,0.313584528431549,0.920744225378362,0.35718398058292,0.882045395544532
"6883","SLC44A1",747.79222113041,0.172668760384794,0.18906877052651,0.913259021592801,0.36110632888282,0.882045395544532
"6884","ARID5B",4250.58076197283,-0.196362101441538,0.214726078727218,-0.914477191617658,0.360466157317633,0.882045395544532
"6885","SLC29A3",48.0563481684447,0.232989286495827,0.252898901631202,0.921274410418718,0.356907177699101,0.882045395544532
"6886","SEC24C",100.726746686834,0.208863257790664,0.227536771014209,0.917931887930419,0.358654525956912,0.882045395544532
"6887","MMS19",94.1275424425475,0.179837340538645,0.195914261586717,0.917938995773637,0.358650804538971,0.882045395544532
"6888","AL391121.1",73.2302301463162,-0.274859308884437,0.29861676775092,-0.920441644836572,0.357342014625087,0.882045395544532
"6889","GSTO1",1108.85152284176,-0.228762876997163,0.251314011486995,-0.910267102274161,0.362681663551786,0.882045395544532
"6890","MTG1",233.051281988069,-0.189912453213929,0.20761086845192,-0.914751981098287,0.360321848725785,0.882045395544532
"6891","AMPD3",20.6436584261369,0.383478242662809,0.417860560166738,0.917718203674905,0.358766414891483,0.882045395544532
"6892","APLNR",7.27611266252997,0.797544769749718,0.871617840556131,0.915016573365282,0.360182929583665,0.882045395544532
"6893","CSKMT",243.804682490858,-0.221725862469437,0.243550438592208,-0.910389912459515,0.362616915759342,0.882045395544532
"6894","SNX15",234.565458839942,-0.182219525529642,0.199725036534133,-0.91235194491253,0.361583477832582,0.882045395544532
"6895","AP002387.2",30.2555270734566,-0.278663175114788,0.304527759226321,-0.915066579883409,0.360156678389938,0.882045395544532
"6896","SLC37A2",2.44740737208973,-1.01415622906641,1.10239402431272,-0.919958024716868,0.357594694785227,0.882045395544532
"6897","GAPDH",4704.27051691776,-0.291115487556998,0.319358590938845,-0.911563038593017,0.361998786902658,0.882045395544532
"6898","MANSC1",110.915593037635,0.243731485343917,0.265177606129776,0.919125445399174,0.35802996134977,0.882045395544532
"6899","RERGL",4.20489218594371,0.646306981568866,0.707886730230637,0.91300903657043,0.361237788902815,0.882045395544532
"6900","ST8SIA1",147.992241711005,0.382425632895437,0.418382321258581,0.914057820954339,0.360686464152033,0.882045395544532
"6901","BCAT1",104.52923150036,-0.284664398861689,0.309611202364628,-0.919425384765119,0.357873116775359,0.882045395544532
"6902","AC048341.1",34.7192649928804,0.347381503913847,0.381264858375178,0.91112909118944,0.362227360022366,0.882045395544532
"6903","GLIPR1L2",25.3065460190642,0.428279710347295,0.468452524190728,0.914243574815114,0.360588872167334,0.882045395544532
"6904","DUSP6",150.209058986383,-0.251578713917141,0.273558750282783,-0.919651495911133,0.357754907112667,0.882045395544532
"6905","TEX26",6.06663971231845,0.640962766395816,0.699729382868238,0.91601522258286,0.359658910792678,0.882045395544532

"6906","KBTBD6",73.3850527821206,0.21218571963046,0.231074377163039,0.918257239229723,0.358484208284876,0.882045395544532
"6907","SMIM2-AS1",2.62645667497857,-0.868767483977664,0.943603643858355,-0.920691107577023,0.357211720162263,0.882045395544532
"6908","F7",6.58167782441624,0.584839577502246,0.639301971110886,0.914809595356004,0.360291596590529,0.882045395544532
"6909","CUL4A",199.100543486645,0.154934188258188,0.169867779271482,0.912086970952701,0.361722936467515,0.882045395544532
"6910","ACOT1",10.3373307985224,-0.78025462051863,0.854689771593782,-0.912909743922232,0.361290012411696,0.882045395544532
"6911","ACOT2",173.79079179188,-0.374156435033735,0.40947991868069,-0.91373573639273,0.360855721294293,0.882045395544532
"6912","ADCK1",17.8375760851762,-0.344135504971571,0.373158482156986,-0.922223455788405,0.356412030693629,0.882045395544532
"6913","AL357093.2",8.26186062442983,-0.588051561785685,0.638974780192852,-0.92030480703511,0.357413497756904,0.882045395544532
"6914","DYNC1H1",1145.65131055758,0.168134842757803,0.183918051040433,0.914183473599553,0.360620446531487,0.882045395544532
"6915","RGMA",273.302529835854,0.199205218032937,0.217898876383192,0.914209477990236,0.360606784829465,0.882045395544532
"6916","TRAF7",221.698130954634,-0.178384628825408,0.194989614696749,-0.914841690942536,0.360274744508729,0.882045395544532
"6917","SDR42E2",22.3753999934975,-0.314743007110631,0.344366745279559,-0.913976193767258,0.360729354957747,0.882045395544532
"6918","AC009093.2",11.6317108002972,0.478494493365205,0.524701639867463,0.911936340595524,0.361802229866727,0.882045395544532
"6919","C16orf54",5.60712557653105,-0.655992899341371,0.717349747772966,-0.914467317201854,0.360471343646218,0.882045395544532
"6920","NAE1",380.74313804598,-0.170926784524064,0.186130666196932,-0.918316084160025,0.358453409071968,0.882045395544532
"6921","TRADD",332.682338589623,-0.226630627114807,0.245950131204758,-0.921449506876385,0.356815791750951,0.882045395544532
"6922","AC092376.3",5.93673211826819,0.571593407763311,0.62015503509008,0.921694375472232,0.356688015212004,0.882045395544532
"6923","PITPNA",192.096201988654,-0.138229919923182,0.150514602271297,-0.918382122646329,0.358418846761091,0.882045395544532
"6924","PIGS",123.933624792755,0.16471319804518,0.180745444292583,0.911299306546004,0.362137691711489,0.882045395544532
"6925","DCAKD",106.167991819023,-0.209228611172284,0.229143249983847,-0.913090877374888,0.361194747845278,0.882045395544532
"6926","VMP1",2409.01256903472,-0.340701660121028,0.374105251971537,-0.910710711291884,0.362447818855242,0.882045395544532
"6927","DCAF7",313.94522576285,0.176328004983802,0.193033728463713,0.913456971417037,0.36100225398432,0.882045395544532
"6928","TEX2",64.2522859385404,0.189256859925608,0.205932155643984,0.919025294198327,0.358082342140351,0.882045395544532
"6929","ARSG",79.2080158756751,-0.271377480680913,0.294203981571666,-0.922412671749679,0.356313362528099,0.882045395544532
"6930","PRELID3A",15.7048955858251,-0.413800694038838,0.45299278873753

8,-0.913481857386901,0.360989171167877,0.882045395544532
"6931","SS18",331.194060802705,0.141321061757916,0.153544809426194,0.920389704386888,0.357369146900782,0.882045395544532
"6932","TPGS2",351.103699220396,0.138751845622655,0.151132697668602,0.918079593384255,0.358577197541186,0.882045395544532
"6933","C18orf25",101.167865377756,-0.236953805645191,0.25722337682102,-0.92119856512912,0.35694676727768,0.882045395544532
"6934","AC125437.3",2.78926984975374,1.17732522012699,1.2830032964394,0.91763226438647,0.358811420442057,0.882045395544532
"6935","FZR1",96.8572097481889,0.191603058328604,0.210307226328808,0.911062647124828,0.362262366085049,0.882045395544532
"6936","MAP2K2",1522.73480257022,-0.210663361492292,0.228975145477204,-0.92002719794435,0.357558546506,0.882045395544532
"6937","KDM4B",177.741947859799,0.265491892009326,0.288034505026707,0.921736414825402,0.356666081272805,0.882045395544532
"6938","SLC25A23",139.586112056618,0.161893288210697,0.177878795967107,0.910132583990696,0.362752592375857,0.882045395544532
"6939","TIMM29",127.694680272842,0.187451091319366,0.204951274692942,0.914612956665946,0.36039485433872,0.882045395544532
"6940","PTGER1",2.16441264981755,-0.967408877958042,1.06198172779823,-0.910946820115007,0.362323394624725,0.882045395544532
"6941","NR2C2AP",132.716582295138,-0.253606508768861,0.277772321787965,-0.913001364342015,0.361241823984353,0.882045395544532
"6942","HPN",9.89801599760729,0.58286317890143,0.637006897265554,0.915002932312752,0.360190090736925,0.882045395544532
"6943","IGFLR1",33.472483377941,-0.273506689235001,0.297626002287513,-0.918961001837424,0.358115970685505,0.882045395544532
"6944","NFKBID",19.841062275363,-0.407347216706082,0.446329663714064,-0.912659968231563,0.36142140422861,0.882045395544532
"6945","CLEC11A",524.793507001203,0.277163614829168,0.30094891561226,0.920965653806387,0.357068359172494,0.882045395544532
"6946","PDXK",259.132969105644,-0.131621922858221,0.144614749490716,-0.910155591471467,0.362740460371717,0.882045395544532
"6947","ESS2",69.3363627265969,0.228342652032521,0.24875753269249,0.917932613179575,0.358654146240724,0.882045395544532
"6948","CRELD2",330.135108436804,-0.242702991637362,0.266435507138557,-0.910925853103907,0.362334442702383,0.882045395544532
"6949","CXorf38",123.802491723308,0.159951796573431,0.175330839580796,0.912285579398726,0.36161840354965,0.882045395544532
"6950","CSTF2",24.3106638888226,-0.308785880617048,0.338649852840563,-0.911814601503533,0.361866322560412,0.882045395544532
"6951","CUL4B",200.343224914869,0.17350981688343,0.18952398965799,0.915503188786504,0.359927529200863,0.882045395544532
"6952","LINC00629",6.68424835789448,0.674464417494576,0.73474294010741,0.917959711727176,0.358639958529245,0.882045395544532
"6953","PRRG3",227.021234102128,0.246759456406802,0.270214144350175,0.913199629132005,0.361137558972493,0.882045395544532
"6954","GABRE",242.000847179538,0.32540102546668,0.356208272995704,0.913513385666381,0.360972596846387,0.882045395544532
"6955","DNASE1L1",23.1903190672443,-0.331617631775755,0.35942296068982

6,-0.922638974258334,0.35619537790089,0.882045395544532
"6956","FCER1A",18.4399967884794,-1.02819219365375,1.13004191143687,-0
.909870849255829,0.362890624799745,0.882080891908294
"6957","SSX2IP",18.0639960469384,0.378533852356832,0.416368243143319,0
.909132381228546,0.363280251793921,0.882183187819237
"6958","RPL5",20738.7992942512,-0.227212505897424,0.250116237995397,-0
.908427648354465,0.363652323619485,0.882183187819237
"6959","PARP1",296.600421660396,0.182219334180299,0.200576887333723,0.
908476228754709,0.363626667391529,0.882183187819237
"6960","YIPF4",612.85718663413,0.140343547970626,0.154478496587032,0.9
08498924260034,0.363614681854078,0.882183187819237
"6961","RETREG2",420.999227656029,-0.115877876733343,0.127537578538627
,-0.908578303438995,0.363572763509674,0.882183187819237
"6962","SH3RF1",189.104274500799,0.202270497594152,0.222562977452126,0
.9088236503201,0.363443220497717,0.882183187819237
"6963","HTRA4",7.78510712155395,0.716575701551787,0.788375652901332,0.
908926726636837,0.363388804877651,0.882183187819237
"6964","NPDC1",508.251021159284,0.266861151461039,0.293522069528484,0.
909168948998374,0.36326095192261,0.882183187819237
"6965","PHB2",1230.76093850086,-0.226454653278882,0.249042645283181,-0
.909300706396631,0.363191417841999,0.882183187819237
"6966","THAP2",105.625625507301,0.289245959809752,0.318223980995321,0.
908938285873574,0.363382702891408,0.882183187819237
"6967","LCP1",67.7195249215937,-0.376947494458513,0.414954360266346,-0
.908407117873305,0.363663166495155,0.882183187819237
"6968","AC016876.1",134.596033337627,-0.30036584852233,0.3306070537041
98,-0.908528251762816,0.363599194312759,0.882183187819237
"6969","GGA3",101.195884662328,0.199391266380729,0.21932776685446,0.90
9101794270488,0.363296395583323,0.882183187819237
"6970","RTL3",2.79342693588844,1.10951987787711,1.22137492437744,0.908
418746555369,0.363657024950655,0.882183187819237
"6971","PEPD",568.543669321126,-0.224070527211032,0.246741501208266,-0
.908118521261251,0.363815606007814,0.882426375897307
"6972","ZSCAN26",93.2196841300572,0.234172756587946,0.257954143754746,
0.907807694729609,0.363979832269991,0.882523417982379
"6973","CD72",13.8093615747102,-0.579536449221341,0.638436521871553,-0
.907743259302352,0.364013882748429,0.882523417982379
"6974","ZNF22",851.211249564966,0.163830494097864,0.180519065041688,0.
907552307896286,0.364114801461803,0.882523417982379
"6975","MRPL34",694.673228275875,-0.239604240607628,0.263946183127459,
-0.907776872423738,0.363996119866646,0.882523417982379
"6976","TAB1",93.3008742776172,0.215158684040828,0.237076679763455,0.9
07548917318668,0.364116593556013,0.882523417982379
"6977","AC022706.1",17.5744552860538,-0.433507206796926,0.477731231098
76,-0.907429070106803,0.364179942498121,0.882550446862295
"6978","AC022211.2",3.1038505618811,0.869427703156404,0.95824853717684
2,0.907309189031356,0.364243316232543,0.882577528068192
"6979","FBXO17",649.801619754324,0.154541880809568,0.170369525198295,0
.907098148155874,0.364354897282944,0.882721393216795
"6980","HAT1",328.565379583374,-0.210520682821293,0.232165370273998,-0

.906770387731986,0.364528232378565,0.883014807028191
"6981","MNDA",25.8355949543992,-0.459840091808794,0.507499042696309,-0
.906090560024889,0.364887921438593,0.883044842722411
"6982","GNLY",61.6918759925155,0.775732842500743,0.855982499209074,0.9
06248484306067,0.364804345745041,0.883044842722411
"6983","NCL",2222.83330540483,-0.1590459315495,0.175466169751726,-0.90
6419350092044,0.364713934700559,0.883044842722411
"6984","HIST1H4D",2.45138219374058,-1.07296748967801,1.18436586159827,
-0.905942601410402,0.36496623401652,0.883044842722411
"6985","COMMD5",227.636982069291,-0.235830400449878,0.260339070406831,
-0.905858656103161,0.365010669847819,0.883044842722411
"6986","AL162231.1",48.1748255673233,-0.302271389751902,0.333409134338
798,-0.906608003860939,0.36461412767789,0.883044842722411
"6987","C9orf153",3.46603783146606,-0.766532007912507,0.84596358118899
1,-0.906105209440761,0.364880168263712,0.883044842722411
"6988","ARL4D",230.522249995448,0.312703831308402,0.345150849307389,0.
905991777032858,0.364940204831857,0.883044842722411
"6989","ELK1",91.887462101767,-0.187944879370286,0.207461264511715,-0.
905927570684756,0.36497419017229,0.883044842722411
"6990","BTF3",7542.18662414191,-0.236170969140497,0.260755157982818,-0
.905719261576638,0.365084464780121,0.883097014378009
"6991","MTHFS",251.226392847868,-0.247822630798892,0.27365348717329,-0
.905607428426298,0.365143675560805,0.883113898781589
"6992","HAUS1",215.942900225536,-0.237170883600249,0.26192320033441,-0
.905497807362776,0.365201720958128,0.883127960234557
"6993","SNX25",17.9916094318379,0.340541241899827,0.376205809566107,0.
905199317077497,0.365359803612361,0.883383892389217
"6994","SDF4",1177.75365074419,0.168253279559777,0.186047781261473,0.9
04355206060278,0.365807082235483,0.8834484901068
"6995","TIGD6",34.4403363529111,-0.274259560855506,0.303314123311048,-
0.904209661790966,0.365884237945181,0.8834484901068
"6996","MRPL18",778.925535155719,-0.236339775075116,0.261176031103765,
-0.904906066901747,0.365515152666295,0.8834484901068
"6997","HGF",137.699503368454,-0.338195133775698,0.373950913770932,-0.
904383760866709,0.365791945995924,0.8834484901068
"6998","MTCH2",175.473972473247,-0.207897683181367,0.229718334942836,-
0.905011274929797,0.36545941405215,0.8834484901068
"6999","AC009318.2",7.06788906675276,0.542325050116976,0.5995846698837
65,0.904501194505373,0.365729701261373,0.8834484901068
"7000","PFDN5",7374.2242124464,-0.23873175620114,0.264036218993218,-0.
904162910344022,0.365909023906903,0.8834484901068
"7001","C12orf49",140.285780936625,0.144579693604558,0.159888387170541
,0.904253874612831,0.365860798836546,0.8834484901068
"7002","PRKCSH",637.229071090635,0.120061189958646,0.132717009810891,0
.904640559109357,0.365655840777486,0.8834484901068
"7003","GRWD1",127.105990347034,-0.225500246783123,0.249304660628505,-
0.90451677162645,0.365721445233067,0.8834484901068
"7004","AC007952.4",453.758806577385,-0.347122365911839,0.383983268005
695,-0.904003884634605,0.36599334155029,0.883497543361678
"7005","FGF16",36.3624501787374,0.308911002845237,0.341743119540988,0.

903927497531334,0.366033847365067,0.883497543361678
"7006","PRKCQ-
AS1",6.09708015679538,-0.582778854084804,0.644809481444559,-0.90380006
9408427,0.366101424943053,0.8835345265397
"7007","RAD17",152.587662019375,0.168622131570687,0.186651848571906,0.
903404562348746,0.366311219491393,0.883666036864034
"7008","AMOTL1",138.09471264223,0.226893433832013,0.251108403868493,0.
903567663752257,0.366224694163099,0.883666036864034
"7009","ZNF677",228.649977620841,-0.162748670922429,0.180150934425881,
-0.903401758314992,0.366312707143365,0.883666036864034
"7010","AC026979.2",45.0445667727911,-0.274290632476992,0.303661586099
058,-0.90327734897464,0.366378715063267,0.883699188914368
"7011","AC253572.1",8.48168783546921,-0.468670148332654,0.519262562328
943,-0.902568724058639,0.366754831960761,0.883849871549678
"7012","XCL1",8.90936745936012,-0.667609994632729,0.739289095032606,-0
.90304320612667,0.366502964458047,0.883849871549678
"7013","SRI",2115.35555393408,-0.22599027134536,0.250361606303345,-0.9
02655461762551,0.36670878125819,0.883849871549678
"7014","RELA-
DT",19.7231181941809,-0.356064951673281,0.394425529326207,-0.902743167
465714,0.366662220292283,0.883849871549678
"7015","ZNF14",36.8164291248934,-0.32104235875723,0.355567021997813,-0
.902902515968438,0.366577635224265,0.883849871549678
"7016","RAC2",24.5953123666342,-0.431207078905012,0.47773306720234,-0.
902610910796295,0.366732433766189,0.883849871549678
"7017","NUDT17",22.7710376082644,0.345991676055718,0.383700253002819,0
.901723867388685,0.367203571067122,0.883879828909721
"7018","LOXL3",42.4689484271282,-0.335058539429217,0.371596192169895,-
0.901673769778641,0.367230190767859,0.883879828909721
"7019","ATP2C1",217.30180114881,0.157258006426482,0.174328880099521,0.
902076617119935,0.367016169177754,0.883879828909721
"7020","G3BP2",385.350617724013,0.143538501013346,0.159123389338098,0.
902057840839233,0.367026142765895,0.883879828909721
"7021","USP38",95.0588166279397,0.208654741411048,0.231354966897565,0.
901881399863925,0.367119872966783,0.883879828909721
"7022","FAM214B",45.8234846357847,-0.252462011112057,0.279802842784401
,-0.90228536851067,0.36690529595613,0.883879828909721
"7023","CPM",98.4212085246276,-0.256629266880774,0.284618807747989,-0.
901659552688457,0.367237745333025,0.883879828909721
"7024","NPIPB5",92.5646090297767,0.397063761521333,0.440334319428221,0
.901732488253298,0.36719899043422,0.883879828909721
"7025","VMAC",63.4245373717403,-0.206354527153532,0.228783883913158,-0
.901962689084603,0.367076688089075,0.883879828909721
"7026","PML",333.355066545927,0.267844606298245,0.297191550359395,0.90
1252427851125,0.367454121208353,0.884148894462905
"7027","AC012146.1",59.8908421708745,-0.216345312911127,0.240045796660
116,-0.901266824586197,0.367446468377388,0.884148894462905
"7028","FAM122A",140.21939468884,0.165256842439737,0.183415251618754,0
.900998368354009,0.36758918722019,0.884348033226946
"7029","MMP27",35.4484676205165,-0.494010835460467,0.548352832049514,-

0.900899578860679,0.367641715168518,0.884348573064347
"7030","SULT1C4",7.01752585047086,0.662298023697586,0.735390388480636,
0.900607397202914,0.367797100174713,0.884596496408826
"7031","ZNF117",115.282736501515,0.307026389181747,0.340952016923381,0
.900497354297049,0.367855632648026,0.884611440309034
"7032","TEX41",14.5749325766898,-0.428821856513413,0.476267985806783,-
0.90037934375749,0.367918409598524,0.884636585536383
"7033","AL050343.2",8.45038844165451,0.533445088298627,0.5927662793573
88,0.89992482176437,0.368160259686208,0.884869805152611
"7034","UIMC1",131.649909766918,-0.164253949479236,0.182524264157921,-
0.899901995151301,0.36817240828298,0.884869805152611
"7035","PAPLN",126.317378400168,0.359694616312011,0.399661058656858,0.
899999158088701,0.368120698734045,0.884869805152611
"7036","DDX18",760.424341382007,-0.171358021327609,0.190513644559278,-
0.899452749035467,0.368411553229419,0.884910342636692
"7037","ASNSD1",238.794492256806,-0.206231654793315,0.229523435938413,
-0.89852112029489,0.368907790546609,0.884910342636692
"7038","BRK1",2104.79878133326,-0.181446309084239,0.201790030652543,-0
.89918371337515,0.368554813914512,0.884910342636692
"7039","AC006043.1",3.32552972965572,0.803037618198163,0.8929925725873
65,0.899265730588816,0.368511136320623,0.884910342636692
"7040","CCDC25",514.933116257894,-0.183272974755264,0.203800568490522,
-0.899276072253879,0.368505629180482,0.884910342636692
"7041","EXOSC3",179.802440693211,-0.230414536398041,0.256394116511146,
-0.89867325948575,0.368826724353202,0.884910342636692
"7042","STX17-
AS1",7.63622368039371,-0.563008048502164,0.626743626948301,-0.89830677
8552381,0.369022019694899,0.884910342636692
"7043","SLC25A25",197.528592938702,-0.464067957593646,0.51579338939498
,-0.89971676088752,0.368271001405062,0.884910342636692
"7044","SPTAN1",1019.89313392359,0.135072839480413,0.150254128300774,0
.898962584309359,0.36867259043359,0.884910342636692
"7045","MYCBP2",1647.0963024428,0.176591530030993,0.196456514364381,0.
89888355498081,0.368714688269498,0.884910342636692
"7046","SYNJ2BP",263.984370201303,0.156416689283773,0.174125599559738,
0.898298065759771,0.36902666346885,0.884910342636692
"7047","TMEM204",244.301086539033,0.244341195917581,0.271950473322167,
0.898476817976047,0.368931398778692,0.884910342636692
"7048","LINC02182",3.83546597715941,-0.779032393459318,0.8668417768362
75,-0.898701948010124,0.368811439136418,0.884910342636692
"7049","VPS25",340.675241693263,-0.224849822822961,0.249939631557995,-
0.899616525083932,0.368324359946085,0.884910342636692
"7050","ERN1",103.961563554407,-0.288026352868234,0.320552184511536,-0
.898531867150226,0.368902063803923,0.884910342636692
"7051","OCRL",94.1902250970007,0.217845430217955,0.24237147834346,0.89
8808026863829,0.368754923853033,0.884910342636692
"7052","SNHG15",184.3946591996,-0.280667041882585,0.312593645132111,-0
.897865475684788,0.369257272502806,0.88502386220787
"7053","SMARCD3",147.348393523924,0.18019642631972,0.200675987515694,0
.897947126362727,0.369213738560925,0.88502386220787

"7054","ZFYVE27",71.4045460620691,-0.208567491771308,0.232305251526399,-0.897816516849627,0.369283377565444,0.88502386220787
"7055","DIDO1",242.9659324671,0.181130179999475,0.20169746564318,0.898029032848178,0.369170071436089,0.88502386220787
"7056","APLP1",4.75430056318006,0.660569425897722,0.735867777009607,0.897674074793871,0.369359334811481,0.885080446852681
"7057","EID3",103.502417870137,-0.313157494507139,0.348933913179222,-0.897469356457458,0.369468517903936,0.885186073417812
"7058","NDUFA6",612.503524444795,-0.224578225167699,0.250255681783276,-0.897395110342335,0.369508120782051,0.885186073417812
"7059","C2orf73",8.51588013854806,0.511414853478119,0.570319565396287,0.896716305222252,0.369870317776854,0.885358337843774
"7060","AC009950.1",34.1642360273527,0.325096723341887,0.362683945264249,0.896363700645816,0.370058547642752,0.885358337843774
"7061","SUN1",595.842659283816,0.14834694101664,0.16549230290466,0.896397828859157,0.370040326477898,0.885358337843774
"7062","AC072061.1",10.40110534274,0.435987240690235,0.486225179873323,0.896677627439664,0.369890962118395,0.885358337843774
"7063","ELN",1588.56961420317,0.231955353844099,0.258744164957013,0.896466028065348,0.370003916368329,0.885358337843774
"7064","SELENOH",1192.36135857322,-0.224789718808312,0.250655944903679,-0.896805854314341,0.369822523522997,0.885358337843774
"7065","AP004609.3",16.0124319191457,-0.378662403239782,0.422356401105944,-0.896547092096274,0.369960640897159,0.885358337843774
"7066","NTN4",105.918983012169,0.231137450096536,0.257790228474327,0.896610594840892,0.369926742593847,0.885358337843774
"7067","KNTC1",23.7433202250858,-0.273407156304512,0.305001406182103,-0.89641277306529,0.370032347892792,0.885358337843774
"7068","BBC3",44.0202282868181,0.283961411262368,0.316822492226499,0.896279204379725,0.370103662874367,0.885358337843774
"7069","TTC19",273.725122133118,-0.14022000742558,0.156492023209744,-0.896020158405426,0.370241997015902,0.885563967399189
"7070","SENP2",175.035087789509,0.18255356240189,0.203836113328598,0.895589890431243,0.370471837043627,0.885616773975331
"7071","SARNP",459.886028914367,-0.199817106438869,0.223113101396122,-0.89558661140256,0.370473588971346,0.885616773975331
"7072","HNRNPC",1487.28143448272,0.13312321929322,0.148640150079721,0.895607406355697,0.370462478678458,0.885616773975331
"7073","CSNK1G2",240.810272941869,-0.163492455739787,0.182530095844995,-0.895701363563768,0.370412281957552,0.885616773975331
"7074","ZSCAN20",13.7629519217161,0.403150871368737,0.450318308735395,0.895257562369347,0.3706494202808,0.88564206744693
"7075","TCEANC2",44.9760490073778,0.28403797857144,0.317607421949173,0.894305230111705,0.371158602399862,0.88564206744693
"7076","THEM4",130.578856648587,-0.160315735089985,0.179245060360519,-0.894394159412479,0.371111036332345,0.88564206744693
"7077","AFTPH",181.974935937439,0.198406874156391,0.221986470526988,0.893779128454902,0.371440078458248,0.88564206744693
"7078","AC007405.1",6.77133940471816,0.648634934463114,0.724943003267357,0.894739216103448,0.370926509923221,0.88564206744693

"7079","RAB5A",438.345213042167,-0.122054891264549,0.136332205701997,-0.895275556029246,0.370639803813147,0.88564206744693
"7080","TNK2",39.0769837611261,-0.32892406405908,0.367900881369948,-0.894056200230535,0.371291822408938,0.88564206744693
"7081","SLC13A4",4.09828920089105,0.776727755090966,0.868879612070011,0.893941743253127,0.371353061796844,0.88564206744693
"7082","RFX3",62.6531558139094,0.269789800510378,0.301701252981091,0.894228306460787,0.371199749995834,0.88564206744693
"7083","CD82",55.5538734819852,-0.281081480986185,0.314445752848495,-0.893894983283855,0.3713780821872,0.88564206744693
"7084","PC",31.6691366619727,-0.304980662886204,0.341100593996374,-0.894107686278161,0.371264277210424,0.88564206744693
"7085","NKAPD1",293.533741293156,0.166978259547828,0.186688516619894,0.894421695405094,0.37109630878295,0.88564206744693
"7086","IL18",20.1505780593663,-0.420145352290815,0.469527271986744,-0.894826301596976,0.370879948104017,0.88564206744693
"7087","FBRSL1",140.816562581728,0.173417545532895,0.193992141960069,0.893941083286722,0.371353414925323,0.88564206744693
"7088","ALKBH1",33.9428849169518,0.240083920209509,0.268397988767744,0.894507150786674,0.371050605518292,0.88564206744693
"7089","MIS12",63.9538092409316,-0.22832650213591,0.25519460419656,-0.894715242333437,0.370939328565718,0.88564206744693
"7090","LSM4",367.740704728568,-0.252718467359601,0.28277594724623,-0.89370566987985,0.371479390958932,0.88564206744693
"7091","ZNF550",21.4839885785954,0.365768094478371,0.408931923528156,0.89444739682493,0.371082562776999,0.88564206744693
"7092","PARVB",194.014731729619,-0.224472505748104,0.251123791888861,-0.893871918943657,0.371390423872061,0.88564206744693
"7093","TRMT10A",81.7530467637859,0.244853502260286,0.274046109018768,0.893475565615558,0.371602551544806,0.885756023965768
"7094","OSTM1",180.188334109549,0.154269530369071,0.172672900726972,0.893420621994414,0.371631963213459,0.885756023965768
"7095","PCDH9",415.264515011283,-0.431232792233048,0.482838456583403,-0.893120227590155,0.371792791767003,0.885764763026135
"7096","SCG5",27.8805644570758,0.334017272597534,0.373987749818351,0.893123565570715,0.371791004404113,0.885764763026135
"7097","ZNF555",28.1970019833574,0.374508878043667,0.419252987170851,0.893276588369421,0.371709072179217,0.885764763026135
"7098","MIR34AHG",8.66281775309099,0.546792714663039,0.612701682189665,0.892428943085187,0.372163063400756,0.885833815335774
"7099","RAB11FIP5",94.2688239111108,0.196905711488381,0.220808735728072,0.891747832526297,0.372528109059573,0.885833815335774
"7100","AC012360.3",19.5689939862779,0.301080962646011,0.33754533835468,0.891971917353649,0.372407984848181,0.885833815335774
"7101","LINC01990",2.22394720174425,-0.89700940137897,1.00597656104614,-0.891680220109851,0.372564358477949,0.885833815335774
"7102","NDUFAF2",496.886645032757,-0.220989884071522,0.247527556562353,-0.892789017677929,0.371970168697242,0.885833815335774
"7103","MCCC2",100.641421550411,-0.226255369814789,0.253599547192265,-0.892175764191152,0.372298730384175,0.885833815335774

"7104","NSA2",1920.24708931093,-0.209958485868109,0.235218926710448,-0.892608808331844,0.372066700467665,0.885833815335774
"7105","MRPS10",380.868475328771,-0.167915259895288,0.188330369127448,-0.891599483786152,0.372607646952214,0.885833815335774
"7106","RB1CC1",1006.96997697429,0.245684113115032,0.275478761907273,0.891844116816997,0.372476491403094,0.885833815335774
"7107","CD151",1303.55029293974,-0.247150013273067,0.276943861001383,-0.892419179755106,0.372168294554389,0.885833815335774
"7108","AC025423.4",2.23798457929899,-0.876483260642564,0.982298553158866,-0.892277869924554,0.372244012906393,0.885833815335774
"7109","PPP1R13B",12.6733957697922,0.463410810298217,0.519643056587627,0.891786784069292,0.372507226745846,0.885833815335774
"7110","FAM174B",26.0692277115519,0.293436952296747,0.328893056744422,0.89219564317155,0.372288077039526,0.885833815335774
"7111","PSMB3",872.704764203152,-0.271947976850129,0.304693941296141,-0.892528337430298,0.372109810897796,0.885833815335774
"7112","ZFP64",71.957352997258,0.184488483510019,0.206607136672658,0.892943421418771,0.371887472429601,0.885833815335774
"7113","RPL35A",23282.9631506099,-0.220869024003108,0.247766766447649,-0.891439264312211,0.37269356121352,0.885856367359331
"7114","PLIN2",2560.02915262308,-0.320669973266791,0.359742955578628,-0.891386386568738,0.372721918464294,0.885856367359331
"7115","DDOST",989.188131644707,-0.23432812518125,0.266311239534491,-0.879903250012478,0.378911723956375,0.885934653684056
"7116","MTFR1L",264.926585567746,-0.16721406681324,0.189248433536107,-0.883569093222303,0.376928869275582,0.885934653684056
"7117","P3H1",224.233362705727,0.182316550054941,0.206432256063284,0.883178595883049,0.377139784258328,0.885934653684056
"7118","AC135803.1",4.51798863801818,0.615701865763242,0.695330535225317,0.885480839071346,0.375897349435034,0.885934653684056
"7119","SPATA1",10.1666962989544,-0.461243405861561,0.523372031567305,-0.881291658784878,0.378159979615961,0.885934653684056
"7120","SLC44A3",20.2742020569577,-0.323018747251232,0.362960488544734,-0.889955676846133,0.37348968607414,0.885934653684056
"7121","RBM15",48.8490637306805,0.27144348828419,0.306085860708569,0.886821389448751,0.375175069871795,0.885934653684056
"7122","C1orf56",191.682126333804,-0.273982469591867,0.310853161791062,-0.881388717467904,0.378107462236147,0.885934653684056
"7123","S100A12",3.19972342051517,0.95708716345223,1.07630198722502,0.889236640656812,0.373875914769612,0.885934653684056
"7124","S100A2",7.48805980621994,-0.45477396146549,0.514909139598783,-0.883212059160282,0.37712170726054,0.885934653684056
"7125","C1orf220",14.5424822834412,0.438825952492378,0.496018906080351,0.88469602088371,0.376320602372057,0.885934653684056
"7126","MR1",206.373936313313,0.178446018877485,0.200462506528869,0.890171543633708,0.373373781927777,0.885934653684056
"7127","ELK4",266.433505868996,0.156668567051079,0.176390857153907,0.888189839195462,0.374438643449238,0.885934653684056
"7128","AL591686.1",2.35504985466418,-0.98293503013847,1.1111507394751,-0.884609977043081,0.376367023751832,0.885934653684056

"7129","AL390728.6",90.9524089472428,0.286465552953846,0.322146731014122,0.889239360126515,0.37387445354708,0.885934653684056
"7130","FANCL",99.9007689203587,0.176559561940485,0.199957231545062,0.882986629571818,0.377243495559717,0.885934653684056
"7131","AC007389.1",4.38149322839052,0.653727695868817,0.733626502631276,0.891090621077771,0.372880556085841,0.885934653684056
"7132","PRADC1",152.187994834324,-0.257551883637594,0.291769441079032,-0.882723984681558,0.377385419998213,0.885934653684056
"7133","NPAS2",67.1349464347223,0.385571792545196,0.437870185794441,0.880561876679595,0.378555000510474,0.885934653684056
"7134","AC010680.3",9.16162481526589,0.47242613024698,0.530223175813023,0.890994871211694,0.372931921716724,0.885934653684056
"7135","SF3B1",1218.65221299573,0.126576558845966,0.143783347249704,0.880328363938726,0.37868145133356,0.885934653684056
"7136","ARPC2",2168.27904452812,-0.176652195913836,0.198784297561141,-0.888662726790592,0.374184368608276,0.885934653684056
"7137","GMPPA",141.261355682842,-0.249278639899964,0.283307982958657,-0.879885689406574,0.378921237910735,0.885934653684056
"7138","RAD18",65.4682197165174,0.212516140402441,0.240160794154628,0.884891062883526,0.376215388646783,0.885934653684056
"7139","IP6K2",603.577026436327,-0.18235333634905,0.205959885343484,-0.885382782404131,0.375950215384275,0.885934653684056
"7140","NSUN3",95.1211057491989,0.166134253160826,0.187593366961061,0.885608355200055,0.375828607674215,0.885934653684056
"7141","ABI3BP",2682.74334643691,0.184664682444694,0.208890114732336,0.884027866427838,0.376681170163627,0.885934653684056
"7142","SLC41A3",228.83217056674,0.156823723279446,0.177563563489934,0.883197657205933,0.377129487189077,0.885934653684056
"7143","TIPARP-AS1",18.0043349841386,-0.401344816248245,0.456146918680879,-0.879858659155003,0.378935882600452,0.885934653684056
"7144","PCGF3",177.861477795957,0.211378178062681,0.24023658385215,0.879875057633894,0.378926998045389,0.885934653684056
"7145","DHX15",306.72727216553,0.118537598370801,0.133797313458597,0.885949017260964,0.375645000662433,0.885934653684056
"7146","SMIM20",541.501926860195,-0.229699345934054,0.259140327921942,-0.886389809629491,0.37540750853486,0.885934653684056
"7147","NAP1L5",74.6836189312418,0.259365942721896,0.292552255380353,0.886562786483014,0.375314336620849,0.885934653684056
"7148","TET2",115.475137141938,0.22691616215582,0.255762471691867,0.887214455876857,0.374963451001675,0.885934653684056
"7149","FAM241A",208.651949682983,-0.299664919918112,0.340477628760143,-0.880131011865153,0.378788340856416,0.885934653684056
"7150","HHIP",9.75963344734287,0.74196365699255,0.837144097924021,0.886303396073027,0.375454059515232,0.885934653684056
"7151","SMAD1",85.2790624557751,0.173110330197245,0.196732453851247,0.879927672371415,0.378898492697846,0.885934653684056
"7152","WWC2-AS2",17.687020973534,0.305812327762878,0.344417245015497,0.887912356854019,0.374587897302142,0.885934653684056

"7153","AL133351.1",3.16520039890619,-0.736018685068765,0.830635016849
754,-0.886091568665347,0.375568186015705,0.885934653684056
"7154","PSORS1C1",48.1759609919712,-0.282970706873255,0.32136647579651
5,-0.880523415430638,0.378575826093527,0.885934653684056
"7155","AGPAT1",241.356890645579,0.174818048853695,0.197575301753764,0
.884817319279962,0.376255166863258,0.885934653684056
"7156","MED20",36.3493930868098,0.251535920186774,0.284151911489894,0.
885216357925927,0.376039951436183,0.885934653684056
"7157","TAF8",92.1595640096499,0.179001511857268,0.201249443843153,0.8
89450964131734,0.373760765545712,0.885934653684056
"7158","RAB23",81.1918446999138,0.22654445269592,0.257507542530871,0.8
79758512971562,0.378990143724673,0.885934653684056
"7159","SEC63",978.615244808796,0.0957617367011832,0.107797950911334,0
.88834468458449,0.374355370278957,0.885934653684056
"7160","AL080276.2",22.5637158590756,0.328798851105459,0.3718686517818
55,0.884180071457969,0.376599014381454,0.885934653684056
"7161","SOD2-
OT1",6.22748611669742,-0.564387345719166,0.637188449046002,-0.88574635
4260136,0.375754223490633,0.885934653684056
"7162","PMS2",84.282139697983,0.213054463069888,0.241011215237085,0.88
4002277073721,0.376694983627147,0.885934653684056
"7163","AC005082.1",6.6452137243659,0.555354900673496,0.62569660827622
7,0.887578569753606,0.374767485422447,0.885934653684056
"7164","CREB5",389.835782282697,0.309047213090722,0.349779862573051,0.
883547757201655,0.376940391383951,0.885934653684056
"7165","SEC61G",1381.1670106893,-0.227730548253285,0.257723018703429,-
0.883625177909865,0.376898582852632,0.885934653684056
"7166","POMZP3",30.7724875397561,0.312633390628861,0.354413336838706,0
.882115197519052,0.377714514581397,0.885934653684056
"7167","GIMAP4",8.03872928918675,-0.501050044609297,0.564514172731022,
-0.887577440589849,0.374768093038925,0.885934653684056
"7168","CRYGN",5.77605213920129,0.576809599279447,0.653446013335801,0.
882719593520618,0.377387793113151,0.885934653684056
"7169","AC019257.1",2.52140295839589,0.830519890271391,0.9400952526484
67,0.883442276654013,0.376997357320122,0.885934653684056
"7170","ATP6V1H",188.879762389778,0.144385721961849,0.163831925108395,
0.881303945286123,0.37815333127769,0.885934653684056
"7171","ARFGEF1",239.5962936341,0.163580877996763,0.184391730493877,0.
887137820978338,0.375004703862522,0.885934653684056
"7172","EIF3H",1874.84279381489,-0.202291319241634,0.228450708270119,-
0.885492195552514,0.375891227035685,0.885934653684056
"7173","PVT1",22.759904238056,0.457889465130005,0.514001365777166,0.89
083316819145,0.373018678290674,0.885934653684056
"7174","AL136366.1",4.399961905495,0.662259061533381,0.749240517850378
,0.883907164328816,0.376746329453238,0.885934653684056
"7175","BX005266.2",3.29992424563183,-0.763041807192096,0.859781601489
54,-0.887483293280705,0.374818756970332,0.885934653684056
"7176","TRPM3",10.017804635501,0.604995762182221,0.680042822262873,0.8
89643625925012,0.373657273191537,0.885934653684056
"7177","SUSD3",4.85753711162369,-0.654621497336608,0.735030530305259,-

0.890604499196439,0.373141384646221,0.885934653684056
"7178","NCBP1",64.95405389116,0.25584448571885,0.289702664506182,0.883
12783092608,0.377167208713539,0.885934653684056
"7179","PHF19",166.232959110375,-0.206099035874158,0.232900170916703,-
0.884924365074295,0.376197425881862,0.885934653684056
"7180","LRRC8A",153.097630416817,0.164105326726081,0.18644891752746,0.
880162399987717,0.378771339228582,0.885934653684056
"7181","SEC16A",162.699176244448,0.167862403523895,0.190493094624559,0
.88119941489078,0.378209895930364,0.885934653684056
"7182","WDR37",54.2701421689762,0.257201786381765,0.291982735859104,0.
880880116507566,0.378382710496608,0.885934653684056
"7183","AL353796.2",6.49762311574034,-0.602511015234358,0.683906270277
827,-0.880984780252996,0.378326057751428,0.885934653684056
"7184","CCNY",527.167875532736,-0.133771284602255,0.151757326836786,-0
.881481556051161,0.378057232509368,0.885934653684056
"7185","AC022387.2",11.5360536407052,-0.435767396388042,0.490765727923
001,-0.887933634307105,0.3745764511571,0.885934653684056
"7186","GLUD1",582.85220570578,-0.150926663275038,0.170003249661657,-0
.887786930987581,0.374655374182803,0.885934653684056
"7187","SH3PXD2A",576.007854304593,0.274434922911779,0.30842010918748,
0.889808785927631,0.373568568125388,0.885934653684056
"7188","INPP5A",83.2550318067536,-0.175035422109175,0.197751333278076,
-0.88512891017044,0.376087108550574,0.885934653684056
"7189","TUBGCP2",318.574617318295,-0.129359841840224,0.145947775221157
,-0.886343362508967,0.37543252915417,0.885934653684056
"7190","FAR1",219.163162247803,0.159693284379218,0.180852537207592,0.8
8300273164492,0.377234795613251,0.885934653684056
"7191","HTATIP2",233.700061277517,0.305914640575575,0.347721804606375,
0.879768356551219,0.378984810072427,0.885934653684056
"7192","TTC17",398.576479043711,0.136422332026176,0.153541659225519,0.
888503698047201,0.374269867511353,0.885934653684056
"7193","AC090559.2",7.47057921231597,0.538740269119627,0.6072726830445
98,0.887147214359488,0.374999647218471,0.885934653684056
"7194","ZBTB3",18.743587728681,0.324464918252045,0.364655343050815,0.8
8978517505729,0.373581248386329,0.885934653684056
"7195","AP001160.1",15.7549370352046,0.400630287529768,0.4524339906881
36,0.885499975190687,0.375887032985834,0.885934653684056
"7196","NAA40",34.3188836628098,-0.240559866558555,0.272027229133049,-
0.884322747120643,0.376522012309436,0.885934653684056
"7197","PRDX5",2934.43996331288,-0.222791049311303,0.252315840132976,-
0.882984790783992,0.377244489064233,0.885934653684056
"7198","NCAPD2",47.8122446970084,0.255611066555278,0.289852290039905,0
.881866644973161,0.377848926476535,0.885934653684056
"7199","AC006064.4",2.829089057669,-0.835250211220589,0.94634749446875
1,-0.882604134424714,0.377450193968538,0.885934653684056
"7200","AC004241.1",11.0350793245313,0.431549665790816,0.4898400803780
23,0.881001132977475,0.378317206765379,0.885934653684056
"7201","ZBTB39",25.9877598538688,0.348126243034637,0.392759853582814,0
.886359030483836,0.375424088850926,0.885934653684056
"7202","BTG1",5347.96393349483,-0.180863179509083,0.203955081325746,-0

.886779472879216,0.375197641243127,0.885934653684056
"7203","SOCS2-
AS1",4.80696769707656,0.591042224772292,0.667329834395284,0.8856823032
76427,0.375788747088899,0.885934653684056
"7204","PRDM4",103.774552757418,0.212802164404213,0.241252887324037,0.
8820709536976,0.377738438537009,0.885934653684056
"7205","NAA25",112.31359210099,0.186233181663085,0.209127426367626,0.8
90524905784977,0.373184101229904,0.885934653684056
"7206","MRPL57",644.338078259962,-0.221770820466845,0.250638755304116,
-0.884822541501038,0.376252349846894,0.885934653684056
"7207","NIPA2",188.751484362218,-0.164294056255896,0.184801272403251,-
0.889030979707726,0.373986430527421,0.885934653684056
"7208","NDUFB10",1719.399261049,-0.215744453895791,0.242480567834362,-
0.889739148265131,0.373605967924756,0.885934653684056
"7209","ABCC6",4.14705797348138,0.7682768714189,0.866715081183969,0.88
6423794967779,0.375389201610977,0.885934653684056
"7210","BRD7",527.589715287231,-0.128884097934822,0.144945164797503,-0
.889192116997354,0.37389983867687,0.885934653684056
"7211","AC027682.6",15.5522445897174,0.393560596773884,0.4428865847630
39,0.888626141124716,0.374204037154144,0.885934653684056
"7212","ZCCHC14",232.641528150205,0.189475157670959,0.21329277985198,0
.888333668877355,0.374361293957175,0.885934653684056
"7213","PER1",561.626216727868,0.224696529891487,0.253344845138064,0.8
86919683599779,0.375122143406804,0.885934653684056
"7214","CCL14",12.9011940247212,0.596208755413358,0.675731268930027,0.
882316362771568,0.377605750295304,0.885934653684056
"7215","FZD2",58.4332733905631,0.346180236327955,0.392849574617589,0.8
81203032140068,0.378207938437074,0.885934653684056
"7216","PRSS57",2.04727311402433,-0.867327911792471,0.98438936150385,-
0.881082166986705,0.3782733486231,0.885934653684056
"7217","SF3A2",207.05004769098,-0.277445799499921,0.315076810892853,-0
.880565595143944,0.378552987113818,0.885934653684056
"7218","BRD4",795.800257572822,0.191105748691041,0.215238610266165,0.8
87878566279159,0.374606075289665,0.885934653684056
"7219","GPR42",2.31459376539291,-0.939082312486843,1.05980050865988,-0
.886093472133086,0.375567160386771,0.885934653684056
"7220","SIPA1L3",35.0561761497608,-0.243144467732295,0.273595979921493
,-0.888698977967675,0.374164880515139,0.885934653684056
"7221","AP2A1",427.953384795966,-0.143039085877702,0.160562922842938,-
0.890860002702009,0.373004280228957,0.885934653684056
"7222","TRIM28",479.432699526571,-0.110109069901232,0.124027889229784,
-0.887776697523529,0.374660879935619,0.885934653684056
"7223","AL035461.2",3.69090115352428,0.767284786017501,0.8705835274256
64,0.881345398627493,0.378130901026461,0.885934653684056
"7224","COX4I2",2.47505546071672,-1.33890329981813,1.50939435949154,-0
.887046709429308,0.37505375320953,0.885934653684056
"7225","AP001062.1",8.21655874412433,-0.478985768215103,0.543652273039
018,-0.881051716269981,0.378289829136899,0.885934653684056
"7226","CASTOR1",59.293404247343,-0.258690233524169,0.293264514791989,
-0.882105472964079,0.377719772858641,0.885934653684056

"7227","SFI1",41.7020396990078,0.284531039777674,0.321096444990169,0.886123294782616,0.375551091534287,0.885934653684056
"7228","ATF4",1824.20598182108,-0.14255287671565,0.161032081734419,-0.885245195741519,0.376024401143657,0.885934653684056
"7229","MIRLET7BHG",69.3937571551157,-0.353403876443949,0.399054285756798,-0.885603510744725,0.375831219095255,0.885934653684056
"7230","ASMTL-AS1",2.02829077759424,0.985376695411724,1.10778699243477,0.889500149524233,0.37373434288656,0.885934653684056
"7231","HCCS",81.622074483552,-0.228434933916716,0.258621385261756,-0.883279368740185,0.377085347971183,0.885934653684056
"7232","LANCL3",16.3768151720089,-0.552908362657658,0.624457768803091,-0.885421545347136,0.375929316310009,0.885934653684056
"7233","ZNF674-AS1",15.7019452615805,-0.396583233924309,0.4505754054751,-0.880170619845838,0.378766886954241,0.885934653684056
"7234","LINC00595",2.89884964539109,-0.944656877931411,1.07411840476754,-0.879471828932909,0.379145501070565,0.886092498545256
"7235","GPR180",82.4323541213739,0.238553338624488,0.271255802520692,0.87944050010245,0.379162480895134,0.886092498545256
"7236","TBCB",735.111350388231,-0.239947653066187,0.272919047912028,-0.879189836333925,0.379298354290768,0.886287531004465
"7237","AL162586.1",3.52697120946715,0.771850383097964,0.878164169484065,0.878936319562477,0.379435804629587,0.886394673810453
"7238","ARHGAP22",17.8749806718557,0.597490127958738,0.679907664255426,0.878781280709721,0.379519877842395,0.886394673810453
"7239","B4GALNT1",4.71711108034449,0.628575077685704,0.715331533223306,0.878718536079802,0.379553905748029,0.886394673810453
"7240","MRPL54",612.935351619316,-0.231627667719568,0.263595475985026,-0.878723987405329,0.379550949290076,0.886394673810453
"7241","RPS7",15454.944366102,-0.240461695052878,0.27414434502681,-0.877135346451744,0.380413127989203,0.886871538775395
"7242","KLF11",241.439853570522,0.192434069216332,0.219468664279327,0.876817972389045,0.380585515636203,0.886871538775395
"7243","AC007405.3",78.3619351543447,-0.36175611336438,0.41239138875582,-0.877215488072616,0.380369605159487,0.886871538775395
"7244","GHRLOS",4.87220988623011,-0.577954691643142,0.658235254588688,-0.878036670953289,0.379923818403815,0.886871538775395
"7245","NAALADL2",191.051687267897,0.148450378588793,0.169314894082775,0.876770938510689,0.380611067047375,0.886871538775395
"7246","THG1L",131.927511932441,-0.200773574536092,0.229010454056109,-0.876700477991721,0.380649347080601,0.886871538775395
"7247","PXDC1",953.44038980125,-0.21769258925126,0.248015081890005,-0.877739319691077,0.380085201193928,0.886871538775395
"7248","DPY19L1",97.835443691285,0.168250174767845,0.191666693085161,0.877826877792939,0.380037676014807,0.886871538775395
"7249","CYC1",622.198910366828,-0.215682427641425,0.24558829830035,-0.878227623767518,0.379820203936653,0.886871538775395
"7250","FKBP15",167.104526174866,0.182975354338973,0.208640777552057,0.876987502087503,0.380493426446069,0.886871538775395

"7251","RGS10",424.515320599131,-0.297972838020526,0.339439104515399,-0.877838864340418,0.38003117018707,0.886871538775395
"7252","COLCA2",70.5638586300782,0.338367621477166,0.385609539808964,0.877487682604527,0.38022180623288,0.886871538775395
"7253","ZFP90",192.151943592745,0.154313704225556,0.175839423856285,0.877583085984619,0.380170011499055,0.886871538775395
"7254","ZFP1",52.7644594889933,0.227282045152816,0.259247331606046,0.876699651042873,0.380649796362257,0.886871538775395
"7255","TMEM107",179.542108120188,0.170188017734499,0.193839833233847,0.877982687537633,0.379953113936339,0.886871538775395
"7256","ZNF841",32.3399212896627,0.34439236189538,0.392526641451667,0.877373216303806,0.380283955996418,0.886871538775395
"7257","PDHA1",324.572825666992,-0.155730719756372,0.177491648580466,-0.877397449411666,0.380270798049193,0.886871538775395
"7258","HYAL3",7.66413948539496,-0.466673703707107,0.532372890109475,-0.876591788156497,0.380708401105636,0.886885870197588
"7259","AC114956.3",2.23735749389632,1.09676022427225,1.25130616665134,0.876492303404311,0.380762458700855,0.886889606242465
"7260","DDX55",129.218987462488,-0.158719418795087,0.181116953376023,-0.876336620269688,0.380847062590795,0.886964481306496
"7261","REEP2",14.0053707201402,-0.381876818004854,0.435837896994075,-0.876190025325049,0.380926738179294,0.88697240992987
"7262","EIF3F",2767.94332767994,-0.204700032096888,0.23363920994144,-0.876137323646123,0.380955384487268,0.88697240992987
"7263","RPP21",471.911290212716,-0.217338299836107,0.248133866673357,-0.875891319270058,0.381089119112244,0.887039485951243
"7264","TMEM106B",540.32395753773,0.136288569508493,0.155596140731077,0.875912274354192,0.381077726239468,0.887039485951243
"7265","AL121749.1",4.08296996819481,0.885204810411917,1.01114590970661,0.875447155464206,0.381330651545989,0.887479512228436
"7266","HDC",5.46980340927583,-0.813239917748702,0.929127983085381,-0.875272225735957,0.381425802602138,0.887578787558071
"7267","BMP8A",2.66796277932527,0.987229536738396,1.12848287524254,0.874828992443692,0.381666959587203,0.88763130302333
"7268","NFIA",5189.73980299662,0.174512448899674,0.199468712065973,0.874886327244921,0.381635759263325,0.88763130302333
"7269","PIFO",46.0686292216463,0.282544200099714,0.322992614922718,0.874769846262022,0.381699147263973,0.88763130302333
"7270","CD1C",14.4970815523407,-0.670108988646284,0.766346424040606,-0.874420454803058,0.381889321989958,0.88763130302333
"7271","XCL2",9.69352404611576,0.68242582877966,0.780742115783419,0.874073288713129,0.382078342997622,0.88763130302333
"7272","RC3H1-IT1",3.24673341660736,-0.747890781725718,0.855627926787938,-0.87408411800364,0.382072445922978,0.88763130302333
"7273","LINC02104",21.2464654828627,-0.444450997801635,0.508245085829168,-0.874481643194921,0.381856012783638,0.88763130302333
"7274","TMEM141",340.113496619048,-0.211124474164104,0.241512395427524,-0.874176556405612,0.382022110995362,0.88763130302333
"7275","ACADS",312.676616053978,-0.197202296667875,0.225544420108769,-

0.874339061781152,0.381933632779773,0.88763130302333
"7276","AC105020.1",5.37833563283119,0.672881968489696,0.769620484812386,0.874303610374569,0.381952933698131,0.88763130302333
"7277","ZNF177",17.7651653439477,0.324952422549766,0.371684332320133,0.874269895966137,0.381971289492239,0.88763130302333
"7278","SUPT5H",319.550479253877,0.179952852624796,0.205719166800868,0.874750055734899,0.381709917749338,0.88763130302333
"7279","PLA2G2A",21664.1200096233,-0.354559949303442,0.40620076768757,-0.872868732675937,0.382734630641219,0.887764097901293
"7280","CLK2",71.7254252848685,0.220601641721437,0.252595339381321,0.873340110952774,0.382477723709308,0.887764097901293
"7281","CENPO",9.2179131439043,-0.423419571486922,0.485016995255309,-0.872999452862548,0.382663375944154,0.887764097901293
"7282","MAGEF1",301.044747638092,-0.148894392946056,0.170518260828288,-0.87318737725101,0.382560953867244,0.887764097901293
"7283","PCDHB9",29.9763706059279,0.370339367343284,0.42413847252966,0.873156743208164,0.382577648809687,0.887764097901293
"7284","TEAD3",93.0875197046211,0.211535901572349,0.242139984567532,0.873609957273917,0.382330701752006,0.887764097901293
"7285","EXT1",307.501552786486,0.219645216880782,0.251578787388978,0.873067316844872,0.382626386948864,0.887764097901293
"7286","NDUFS3",559.909128658688,-0.258541534364675,0.296025490575257,-0.873375917263948,0.382458213152291,0.887764097901293
"7287","PDE2A",2.02853757393516,-1.21921328049292,1.3966693022873,-0.872943422251955,0.382693916858574,0.887764097901293
"7288","ARHGAP32",160.335958991724,-0.218183235652293,0.249787384639828,-0.873475800096529,0.382403791063968,0.887764097901293
"7289","SIRT4",14.5880351699172,0.346232768719713,0.396686546830037,0.872811975819433,0.382765570954603,0.887764097901293
"7290","AC090517.2",12.2057380319253,-0.42911487435203,0.491267478257648,-0.87348520580672,0.382398666520073,0.887764097901293
"7291","LINC01290",15.0001055974736,0.599388398477364,0.686938337602263,0.872550512422281,0.382908124355239,0.88785490284771
"7292","MIEF1",103.480232438828,0.18955494047504,0.217243108631386,0.872547542102581,0.382909744000799,0.88785490284771
"7293","PPP1R13L",26.4850583819809,0.292258576403321,0.334998174768958,0.872418414234307,0.382980158451936,0.887896410133736
"7294","IL1RL2",3.65982389971818,-0.661898541365065,0.759574346291547,-0.871407182979043,0.38353186517426,0.888009722993113
"7295","IGF2BP2",164.637452296434,-0.297670333906729,0.341350476632581,-0.872037258723771,0.383188051842415,0.888009722993113
"7296","WDR17",15.7130866303248,-0.577094515282188,0.662440451780215,-0.871164364632818,0.38366441423072,0.888009722993113
"7297","AL662844.4",94.2492597258572,-0.397021299151376,0.455740987185805,-0.871155569313565,0.383669215923317,0.888009722993113
"7298","ATG5",224.511536183863,-0.156613173296065,0.179681234792748,-0.871616746605229,0.383417491664656,0.888009722993113
"7299","WDFY2",287.247641893511,0.264745971506142,0.303688866061075,0.871767131077169,0.383335429227183,0.888009722993113
"7300","CCDC32",316.998766007778,-0.212050387124991,0.243343353695592,

-0.871404063043587,0.383533568098786,0.888009722993113
"7301","AC015871.4",4.52133654495681,-0.646578481374368,0.742266832162318,-0.871086317424156,0.383707024396164,0.888009722993113
"7302","PSMB6",1163.40436881505,-0.229166760011679,0.263072968729716,-0.871114813195145,0.383691466687656,0.888009722993113
"7303","ACTG1",13532.6095892682,-0.231739545005861,0.265948017320055,-0.871371583594004,0.38355129632103,0.888009722993113
"7304","AC008686.1",2.38082684878142,0.861624090083247,0.988839985494028,0.871348350312489,0.38356397802535,0.888009722993113
"7305","EXOSC5",115.677660169221,-0.244215980657501,0.280360769874665,-0.871077578958985,0.383711795374242,0.888009722993113
"7306","ZNF256",14.4805052023494,-0.359247796564913,0.412272506540907,-0.87138431708462,0.383544345954057,0.888009722993113
"7307","ZNF83",328.086659671982,0.182101212751915,0.209084314490531,0.870946312714249,0.383783467750537,0.888054040334758
"7308","CKS1B",988.236730389059,-0.228929043868327,0.263319304646293,-0.869397115322931,0.384629960368507,0.888083618198738
"7309","AL354953.1",12.3020795150746,-0.38771686148535,0.446015473943108,-0.869290157262135,0.384688445132205,0.888083618198738
"7310","TSEN15",82.1114466929044,-0.224149308738043,0.258019417698209,-0.868730387571908,0.384994616401532,0.888083618198738
"7311","NAV1",1061.76395688621,0.195087714591799,0.224596183491523,0.868615448219146,0.385057501985645,0.888083618198738
"7312","ZNF692",44.3969636971755,0.225108217010548,0.258642709147628,0.870344336217345,0.384112256486986,0.888083618198738
"7313","LAPTM4A",10560.2797270364,-0.223347779794157,0.256695757549453,-0.870087538361941,0.384252567289292,0.888083618198738
"7314","TCAIM",53.6856152780508,-0.241700712539462,0.278021134835972,-0.869360930715074,0.384649745535904,0.888083618198738
"7315","PDGFRB",2159.200882934,-0.169591955700367,0.195265307511342,-0.868520669963435,0.385109361759708,0.888083618198738
"7316","MPC1",623.349452853119,-0.202346485909535,0.232533990519823,-0.870180249593596,0.384201907535877,0.888083618198738
"7317","RASA4",21.8734696592277,-0.306099375055627,0.352219754298458,-0.869057942719047,0.384815438972193,0.888083618198738
"7318","ARF5",648.058888052035,-0.38368577486688,0.440817428763376,-0.87039610920837,0.384083972237639,0.888083618198738
"7319","EFR3A",309.038012987485,-0.159233968698052,0.183276253968809,-0.86881942013694,0.384945909246857,0.888083618198738
"7320","AP001107.4",3.06086141864065,-0.698945978633902,0.804617771144684,-0.868668333834525,0.38502856644381,0.888083618198738
"7321","MIR100HG",401.614958330536,0.151157607808987,0.17370795653657,0.870182407431431,0.384200728487571,0.888083618198738
"7322","AC009318.1",18.4641809472778,0.346128604525858,0.397995058585044,0.869680658238368,0.384474945165249,0.888083618198738
"7323","CSAD",408.339335446088,0.249861100436958,0.28695004427953,0.87074773263864,0.383891909569766,0.888083618198738
"7324","RIN3",61.6468284454107,0.232693572007298,0.267588353813009,0.86959528952409,0.384521612909969,0.888083618198738
"7325","TMEM87A",664.697588128127,-0.114970464634247,0.132309259038154

,-0.868952524336131,0.384873098795693,0.888083618198738
"7326","WFIKKN1",4.08674133889384,0.67772504802592,0.778831133611673,0.87018227543512,0.38420080061064,0.888083618198738
"7327","AC106820.4",3.74918767797544,-0.653054887275059,0.751160186982297,-0.869394968733147,0.384631134072254,0.888083618198738
"7328","USP7",361.457327330209,0.160351394551845,0.184252953681313,0.8702785564523,0.384148194655101,0.888083618198738
"7329","TAOK2",73.8217862199351,0.26658166386057,0.306702854556593,0.869185466975756,0.384745695141114,0.888083618198738
"7330","RGS9",240.539036756029,-0.237215269271983,0.272758210924524,-0.869690662905925,0.38446947623072,0.888083618198738
"7331","NEFH",2.30596396314719,0.842551653313904,0.969489175886454,0.869067622692657,0.384810144661646,0.888083618198738
"7332","UBE2A",506.107292705428,-0.183343003019432,0.210738300965164,-0.870003232349015,0.384298637767142,0.888083618198738
"7333","KCTD7",67.256316902689,0.265366978276965,0.305588611299212,0.868379803647643,0.385186447403896,0.888140249925689
"7334","TP53TG1",320.869850369322,-0.219690465414892,0.253225007334309,-0.867570180874179,0.385629676496059,0.888559189629478
"7335","STN1",119.573828937233,-0.244511037367463,0.281879929682803,-0.867429751535021,0.385706586425496,0.888559189629478
"7336","UNKL",151.731942997718,0.227261093043899,0.261894459251625,0.867758308798545,0.385526657828908,0.888559189629478
"7337","MYOM1",37.6353767601687,0.305389672872747,0.352084518706837,0.867376032307258,0.385736009692475,0.888559189629478
"7338","DNMT1",249.031667632494,-0.158573876768137,0.18281606943754,-0.867395723231618,0.385725224357334,0.888559189629478
"7339","FKBP1A",2331.5886388664,-0.224401916251365,0.258692254845473,-0.867447370565496,0.385696936372438,0.888559189629478
"7340","FP565260.1",36.8099928405741,-0.302273687648126,0.348395833573175,-0.867615678832847,0.385604760316801,0.888559189629478
"7341","CCDC47",524.285426993802,-0.113357350640461,0.130721541661168,-0.867166567957751,0.385850751270067,0.888702425074824
"7342","RERE",589.314897528133,0.196885802098291,0.227507877829006,0.865402130146323,0.38681811225182,0.888807120228646
"7343","YARS",133.628868451479,-0.194130595611312,0.224268866523671,-0.865615449083396,0.386701080612782,0.888807120228646
"7344","DENND2D",46.0912012322375,0.422270638361386,0.487988571710921,0.865328949981097,0.386858265538847,0.888807120228646
"7345","COL3A1",22973.960185174,0.327520365264585,0.378563506233889,0.865166239933947,0.386947552164837,0.888807120228646
"7346","SCOC",417.750972861587,-0.218233815113843,0.252009451812845,-0.865974722550942,0.386504023829494,0.888807120228646
"7347","AC106845.1",20.8919576705457,0.537160114914547,0.620838092104602,0.86521771416056,0.386919304485778,0.888807120228646
"7348","IPO7",308.77359781089,0.184358271633631,0.212869262856209,0.866063372231263,0.386455410071435,0.888807120228646
"7349","GSTP1",4677.45001771069,-0.222039171550284,0.256386981741147,-0.866031379761936,0.386472953688087,0.888807120228646
"7350","METTL21C",3.17456273060112,1.19412175454259,1.37987527213867,0

.865383834795311,0.386828150505117,0.888807120228646
"7351","AL928654.1",20.6166297492833,0.279996913591302,0.323519954381086,0.865470304998507,0.386780707622158,0.888807120228646
"7352","AC093525.4",15.0459902372649,0.448136618284251,0.5172176644992
5,0.866437186978366,0.386250458396661,0.888807120228646
"7353","PRR14",100.282899350522,-0.1944189107925,0.22464486159211,-0.865450068230398,0.386791810442193,0.888807120228646
"7354","TOM1L2",123.486529058126,0.16574278881551,0.191216659624559,0.866780065821328,0.386062526330426,0.888807120228646
"7355","MED24",125.261308837124,0.19804267368646,0.228713564445534,0.865898243362045,0.386545966525854,0.888807120228646
"7356","EIF1",25106.8472330128,-0.182251639089092,0.210282504549097,-0.8666990127395,0.386106946525235,0.888807120228646
"7357","TTYH2",22.315788539617,0.320389578968672,0.370289182775742,0.865241529787569,0.386906235532217,0.888807120228646
"7358","SNRPD1",621.924673395946,-0.237394165151479,0.273838624032876,-0.866912642399849,0.385989876011788,0.888807120228646
"7359","AC020916.1",1714.56070888227,-0.341826882177751,0.394565207333024,-0.866338125675737,0.386304764343476,0.888807120228646
"7360","TMEM59L",2.40825167330637,0.80536187920725,0.92924067422412,0.866688148234252,0.386112900927619,0.888807120228646
"7361","ZNF45",32.1937114149338,0.272804646153964,0.31487974065503,0.86637725750936,0.386283311501764,0.888807120228646
"7362","KLHL23",38.5544882691729,0.299757206423361,0.34659499599892,0.864863053084284,0.387113958526376,0.889006279386606
"7363","EXOC3L1",7.52576699608735,-0.555901026261026,0.642796375274528,-0.864816678568869,0.387139415372817,0.889006279386606
"7364","CNRIP1",430.158826245924,0.17810203603183,0.205966566134791,0.864713333693559,0.387196149225885,0.889015818999357
"7365","AC012306.3",10.7702345554124,0.424012412293866,0.490466611473417,0.864508209886267,0.387308772285654,0.889153662159651
"7366","SLC15A3",62.7124790127168,-0.202666590457817,0.234462052721257,-0.864389730046248,0.387373832645762,0.889182291932465
"7367","MTHFR",153.1195336676,0.166353289274947,0.192477282738303,0.864274925894111,0.38743688094658,0.889206296056031
"7368","AC073263.2",10.679949951371,-0.546981938823795,0.633115782448456,-0.863952461757382,0.387614005705058,0.889492074981151
"7369","AC244093.4",2.19957878481013,-0.853422759800499,0.987922810510352,-0.863855708888459,0.387667160243867,0.889493329543127
"7370","USP37",92.4121859644963,0.205835658121949,0.238357034940667,0.86356023925699,0.387829514217792,0.889503720346505
"7371","AC113139.1",5.71371388113638,-0.709046302230362,0.820996014546718,-0.863641588591432,0.387784810439204,0.889503720346505
"7372","AC006449.6",17.439286274032,-0.361828897573166,0.418940928902765,-0.863675216744329,0.387766331727901,0.889503720346505
"7373","ZNF554",25.9851359564117,0.265988273436971,0.30808097636368,0.863371301196411,0.387933353186188,0.889621203807414
"7374","MED21",345.979434157028,-0.208076455861557,0.241039619346206,-0.863245869811534,0.388002298708793,0.889654315986771
"7375","AC020659.1",4.65478266564869,0.693788255793568,0.8037830860141

87,0.863153589401758,0.388053026993283,0.889654315986771
"7376","PDE4C",10.0992433278701,1.31362662032108,1.52248942458232,0.862814939211456,0.38823922402317,0.889960520578059
"7377","SLC25A34",10.3322497623145,0.551290176561519,0.639041202498821,0.862683304935312,0.388311614015718,0.889972972321864
"7378","IL6",556.284562313861,0.746133658612682,0.864968537594889,0.862613639898815,0.388349928423865,0.889972972321864
"7379","DOHH",90.9783091410271,-0.205418335607956,0.238181000079971,-0.862446356086276,0.388441940653088,0.890019290026606
"7380","GTPBP3",93.3659620447325,-0.172960564380081,0.200560613068658,-0.862385498995613,0.388475417577262,0.890019290026606
"7381","IDH3B",410.927969603748,-0.179696417033492,0.20841371381226,-0.862210138414228,0.388571891640944,0.89007621643287
"7382","SAT1",5940.09797426843,-0.266707785230414,0.309352323154244,-0.862148965008526,0.388605549426747,0.89007621643287
"7383","TMEM79",26.9461249271635,0.294205532464655,0.341989614787433,0.860276218175575,0.389636799050036,0.890147142053509
"7384","POMC",17.6345919083948,-0.494296384035814,0.573556436411595,-0.861809497123484,0.388792357902165,0.890147142053509
"7385","AC010883.3",16.4097173237287,0.500005838821094,0.580483812427693,0.861360520511287,0.389039512756107,0.890147142053509
"7386","PPP1R21",100.468529787573,0.171616390427432,0.199181726856431,0.861607101896109,0.388903761587302,0.890147142053509
"7387","WDPCP",83.5137960987407,-0.197788596323124,0.229865960118601,-0.860451874740712,0.389540001057573,0.890147142053509
"7388","CD38",22.8680150347342,-0.285510796222114,0.331812690077983,-0.860457736426574,0.389536771146313,0.890147142053509
"7389","CERT1",529.179236735069,0.192956360344924,0.224027688112245,0.861305858980459,0.389069609632797,0.890147142053509
"7390","COX7C",4224.21825159595,-0.192965051475671,0.223984336615481,-0.861511364551079,0.388956464722417,0.890147142053509
"7391","GCLC",80.6259193017592,-0.238900110928309,0.277631979680352,-0.860492048514598,0.389517864796404,0.890147142053509
"7392","UBE2H",700.999263942386,0.169218341839756,0.196495493005083,0.861181797362538,0.38913792375704,0.890147142053509
"7393","LONRF1",157.081424759965,0.179249043852998,0.208162875283345,0.861099961311594,0.389182990506288,0.890147142053509
"7394","FAU",16229.4000138989,-0.212307110595044,0.246763591059824,-0.860366432840465,0.389587083177379,0.890147142053509
"7395","SCN4B",4.84911470475907,-0.647939806946839,0.752216680049487,-0.861373888843055,0.389032152310129,0.890147142053509
"7396","ARHGEF12",397.955295260776,0.20481580629176,0.23790863582609,0.860901100040265,0.389292515759174,0.890147142053509
"7397","UBE3B",104.615339071761,0.201550466801821,0.233998082403372,0.86133383971234,0.389054203143061,0.890147142053509
"7398","FOXN3-AS1",44.4714800600618,0.273258354052746,0.317419177458006,0.860875376973396,0.38930668441942,0.890147142053509
"7399","LCMT1-AS2",2.21276586863057,1.05997821911437,1.23019163585119,0.861636665559

799,0.388887487753312,0.890147142053509
"7400","C16orf87",127.840795510426,0.153971043353327,0.178908168829097
,0.860614942073484,0.389450153630809,0.890147142053509
"7401","MIR99AHG",1105.64513860426,0.141452627943848,0.164372333117847
,0.860562268970374,0.389479174268493,0.890147142053509
"7402","SNX3",2537.46040670315,-0.203592201463635,0.23669563976692,-0.
860143438485463,0.389709978852644,0.890194045182452
"7403","GBP1",340.127950662995,0.378249599458497,0.439951150738995,0.8
5975363133644,0.389924864288902,0.890295842809725
"7404","VANGL1",149.396153244649,0.155386884575019,0.180846483889575,0
.859219826855459,0.390219246675501,0.890295842809725
"7405","HPS3",100.149876080275,0.184742179105265,0.215010023252525,0.8
59225892405442,0.390215900888623,0.890295842809725
"7406","SMARCA5",564.153623589245,0.12240141762637,0.142386760016635,0
.859640444182239,0.389987273434543,0.890295842809725
"7407","PHACTR1",92.878082981339,0.270947420988674,0.315377105891102,0
.859122034946415,0.390273191575056,0.890295842809725
"7408","FAM49B",96.9864896743553,-0.214120813809156,0.249211232710504,
-0.859194071953769,0.390233453398771,0.890295842809725
"7409","ZNF714",57.9839039196008,0.345308515241686,0.401797319719033,0
.859409703088989,0.390114518292315,0.890295842809725
"7410","POP4",191.003703841691,-0.18338756800058,0.213356334113933,-0.
859536553072996,0.390044562270963,0.890295842809725
"7411","HUNK",38.3418755773987,0.330943672735087,0.385217907972006,0.8
59107704720564,0.390281096930783,0.890295842809725
"7412","AL355596.1",2.31471286781854,-1.31420203879217,1.5290309598633
7,-0.859499953427755,0.39006474568906,0.890295842809725
"7413","SUCO",225.204562271558,0.145884406156785,0.16995070286354,0.85
8392484989727,0.390675775857414,0.890486643093868
"7414","ST3GAL6",22.5470547926589,-0.460207931041887,0.536247360072405
,-0.858200832876359,0.390781576213871,0.890486643093868
"7415","AC012020.1",5.15555826039306,0.658567320280917,0.7676430619791
71,0.857908255671548,0.390943125201535,0.890486643093868
"7416","KHDC1",17.8278775274161,-0.320380390755306,0.373173939806226,-
0.858528306991817,0.390600806703953,0.890486643093868
"7417","SH2D4A",95.9304920090926,0.189359828261543,0.220811232514141,0
.857564291931641,0.39113309956481,0.890486643093868
"7418","GDF6",15.9256340833941,0.359394007514716,0.41871516326388,0.85
832575231631,0.390712613239544,0.890486643093868
"7419","LINC01230",19.9603568131502,0.415281218231089,0.48422700164298
,0.857616813647406,0.391104087708436,0.890486643093868
"7420","RHOD",192.31866491674,0.250074539568868,0.291584283701549,0.85
7640667028651,0.391090912049625,0.890486643093868
"7421","AC092868.2",10.2110801478351,0.630526255205948,0.7351508663653
28,0.857682802338714,0.391067638839251,0.890486643093868
"7422","AC103691.1",9.54613057207379,0.419099857775752,0.4886496484023
11,0.857669414366798,0.391075033521889,0.890486643093868
"7423","SELENOS",1036.61440848116,-0.200468103228177,0.233744509211317
,-0.857637699831243,0.391092550996747,0.890486643093868
"7424","TRPV3",10.2093415321047,0.388665969599009,0.45303214521204,0.8

57921394114529,0.390935869828987,0.890486643093868
"7425","C19orf48",147.589074474211,-0.27711522882396,0.323156980324668
,-0.857525121523135,0.391154737299394,0.890486643093868
"7426","IL3RA",4.8441507792861,0.740687739633137,0.86360940193449,0.85
7665210654252,0.391077355408645,0.890486643093868
"7427","PNPLA4",101.020337264888,-0.274767850288226,0.32006984022151,-
0.858462172187383,0.390637309788294,0.890486643093868
"7428","NDE1",108.395154480223,0.176499256760146,0.205973672753235,0.8
56902022481287,0.391499034958347,0.89115046891165
"7429","AC008063.1",5.28204952544793,0.700307894950328,0.8174909964035
2,0.856655177893422,0.39163548148633,0.891316934774922
"7430","SRSF3",3359.16051442339,0.166137981824544,0.193956950329065,0.
856571427539343,0.391681782130344,0.891316934774922
"7431","AC087286.2",6.91352026150658,-0.542477788887483,0.633377869942
907,-0.856483648436253,0.39173031359785,0.891316934774922
"7432","SLC35A1",190.611309298373,-0.1872973016425,0.218709246797938,-
0.856375779189348,0.391789957537013,0.89133269672172
"7433","PIAS3",91.9591742174035,0.202104849918132,0.236260252379801,0.
855433141556278,0.392311402823485,0.891375426810016
"7434","PLK2",427.268509531345,-0.237252566074593,0.277424034018925,-0
.855198313706333,0.392441369598637,0.891375426810016
"7435","GMNN",122.633905537878,-0.223490959553671,0.261332068855693,-0
.855199136226493,0.39244091432474,0.891375426810016
"7436","ASAH2B",9.46677139598902,0.412697021243859,0.482145839183545,0
.855958898126574,0.392020514589836,0.891375426810016
"7437","AP002813.1",9.25988228464589,0.440849763264565,0.5150040336097
97,0.856012253291559,0.391991001798302,0.891375426810016
"7438","ARHGAP11A",9.82603607331757,0.433131387113688,0.50623269709798
6,0.855597415174174,0.392220500197391,0.891375426810016
"7439","GOLGA6L10",23.7409290986091,0.413738196071042,0.4836950701645,
0.855369884027003,0.392346410484851,0.891375426810016
"7440","NPIPB2",30.3432636276715,-0.294868675797738,0.344693417544962,
-0.855452006881667,0.392300962837396,0.891375426810016
"7441","SPATA22",12.6174181898492,-0.527722467728533,0.616791216803245
,-0.855593356960652,0.392222745700892,0.891375426810016
"7442","ATAD5",32.8519480920311,0.27730894188316,0.324091805701872,0.8
55649346896023,0.392191765861601,0.891375426810016
"7443","KHSRP",486.122270562936,0.147607060936385,0.172461640254861,0.
855883434242266,0.392062258864755,0.891375426810016
"7444","AL031668.2",4.71917469122553,-0.670600564463613,0.783838324063
879,-0.855534290524129,0.392255429410355,0.891375426810016
"7445","DENND1B",135.721486424246,0.180110394905116,0.21068415111483,0
.85488345446046,0.392615671156443,0.891625959975544
"7446","IDE",75.9094166922272,0.208301453498263,0.2436819929323,0.854
808560089799,0.392657138513006,0.891625959975544
"7447","TFDP1",201.451694541459,0.169667534711532,0.198530326083275,0.
85461772042002,0.392762814218359,0.891746161246679
"7448","FBXO5",56.1876263953081,-0.261118933062817,0.305615225656992,-
0.85440420221695,0.392881068382872,0.891766673106706
"7449","INTS8",107.496067814668,0.174087862148117,0.203792548876511,0.

854240565260343,0.392971711098259,0.891766673106706
"7450","CLNS1A",486.718141052412,-0.220090062667711,0.257650171709698,
-0.854220516164425,0.392982817679091,0.891766673106706
"7451","CCND2",447.537456974566,-0.251538443466925,0.294422676061814,-
0.85434466811963,0.392914044382809,0.891766673106706
"7452","GNG5",1367.17700458995,-0.21990105606151,0.257576025547807,-0.
853732623577174,0.393253153759515,0.89222116669478
"7453","SQSTM1",3205.04287280509,-0.15047979396238,0.176333591908571,-
0.853381323057289,0.39344787537619,0.89222116669478
"7454","KCTD1",108.792373904161,0.252058409103687,0.295396706959027,0.
853287809801645,0.393499718479006,0.89222116669478
"7455","CFD",225197.812766725,0.226954339163758,0.26594214400707,0.853
397418491614,0.393438952595918,0.89222116669478
"7456","MHENCR",25.1940018510828,0.278488354822796,0.326347722818203,0
.853348546200618,0.393466046168168,0.89222116669478
"7457","NAP1L3",29.4534878573819,-0.306767474869239,0.359381913790642,
-0.853597421288003,0.39332808783677,0.89222116669478
"7458","DDX49",230.639626627403,-0.197788227236737,0.231867426583356,-
0.853022911200647,0.39364659889029,0.892434525883216
"7459","SREBF2",85.2801123445893,0.224768538051613,0.263532815126068,0
.852905312547468,0.393711815379697,0.89246271275505
"7460","DNAJC6",2.54844647078135,0.955811286698379,1.12118101569228,0.
852503987599371,0.393934426741511,0.892699760257316
"7461","PSME4",106.703746262562,0.253396071176614,0.297295953358402,0.
852336092416081,0.39402757929976,0.892699760257316
"7462","AC106791.1",36.9416327378647,0.339785381413708,0.3986232406879
09,0.852397318398535,0.393993608008393,0.892699760257316
"7463","PPA1",1361.93624880998,-0.213937777666196,0.250972844673893,-0
.852433967285109,0.393973274188972,0.892699760257316
"7464","PRKAG2-
AS1",155.237533455708,-0.273511786969346,0.320955094362665,-0.85218085
574392,0.394113720452638,0.892775292793837
"7465","GPR161",125.558223308002,0.19652201327261,0.230703915201062,0.
851836489646643,0.394304850597853,0.892877842262192
"7466","POGLUT1",110.77014651695,-0.163247737900302,0.191668919496094,
-0.851717317181559,0.394371006785838,0.892877842262192
"7467","SMIM15",427.67813551526,-0.184925351515251,0.217241498958901,-
0.851243212744707,0.394634262788346,0.892877842262192
"7468","PSMG3",236.227282201238,-0.206718204064543,0.242626338927263,-
0.852002321670919,0.394212803471925,0.892877842262192
"7469","AC027644.3",231.436347290516,-0.216045918487086,0.253718654971
554,-0.85151767224727,0.394481850688415,0.892877842262192
"7470","TNFAIP1",78.7385334551187,0.203926424778358,0.239540253205561,
0.851324243209174,0.394589261467432,0.892877842262192
"7471","POLDIP2",248.108429339462,-0.16479526140094,0.193559543107789,
-0.851393110125133,0.394551017771399,0.892877842262192
"7472","CD7",13.4721707776436,-0.395408212090863,0.464333124273718,-0.
851561500613027,0.394457515338032,0.892877842262192
"7473","LRFN3",69.8510822207853,-0.19214162579925,0.225625757049625,-
0.851594376066696,0.394439262100834,0.892877842262192

"7474","AC009107.2",138.884097872464,-0.291533833109221,0.342564763431
76,-0.851032751263382,0.394751159806963,0.893022827136222
"7475","C14orf93",55.1402078504643,-0.25345701601595,0.297866442210346
,-0.850908259873614,0.394820316151311,0.893059786687139
"7476","ZNRD2",268.622580676694,-0.229016314244979,0.269183647408898,-
0.850780931343487,0.394891056137033,0.89310031797284
"7477","ZNF879",14.1284852796294,0.427849270521254,0.503014751413867,0
.85057002666l565,0.395008245433807,0.893126426022306
"7478","ALG5",437.285475042987,-0.238801423555904,0.280745700891919,-0
.850596902453859,0.394993310716863,0.893126426022306
"7479","ATF3",5322.01630230436,-0.248949795534729,0.292754073985934,-0
.850371754507814,0.395118434632877,0.893136696894744
"7480","OTUD1",236.090724887281,-0.203799361132634,0.239646656501049,-
0.850416042135526,0.395093820295089,0.893136696894744
"7481","TTC14",377.361329403715,0.184753259982383,0.217338300170246,0.
850072259871644,0.395284913174784,0.893228291754492
"7482","NFE2L3",5.39137427447843,-0.683948926391538,0.8047821232l395,-
0.849856012780382,0.395405143685788,0.893228291754492
"7483","PDZD8",270.088021121085,0.167927700206707,0.197564375357398,0.
849989781320251,0.395330767547111,0.893228291754492
"7484","MRVI1",21.2347525542224,-0.514573267477711,0.605486997812707,-
0.849850235160428,0.395408356268852,0.893228291754492
"7485","WDR93",6.1017949054232,0.526469548549228,0.619567889362508,0.8
49736659352905,0.395471512070619,0.893228291754492
"7486","LENG8-
AS1",20.7726929866694,-0.298022031747548,0.350726090981838,-0.84972871
8252047,0.395475928085766,0.893228291754492
"7487","CBLB",573.68505440137,0.208213137614798,0.245078872567095,0.84
957603825191,0.395560838631052,0.893245981351603
"7488","BTG3",407.518024705703,-0.186451932654263,0.219477954631405,-0
.849524650288423,0.395589419704329,0.893245981351603
"7489","AC104083.1",2.48447573317416,1.04233193601793,1.22745884737652
,0.849178722566333,0.395781851033767,0.89356116134049
"7490","FPGS",115.685437379203,-0.177229097523845,0.208734227959629,-0
.849065815684634,0.39584467067333,0.893583670459901
"7491","WIPF1",514.790636156814,0.161704771444515,0.190552988968216,0.
848607898097505,0.396099510665688,0.893800951065722
"7492","ARPC1B",1478.30276742233,-0.24032071089697,0.283183721667647,-
0.848638860601663,0.396082276311015,0.893800951065722
"7493","OSGIN1",39.8111581629833,-0.2938813444488,0.346232550479313,-0
.848797561182391,0.395993947474105,0.893800951065722
"7494","FBXW2",240.817387563061,-0.143687655119662,0.169434339486231,-
0.848043292495254,0.396413860951517,0.894198510894023
"7495","ZNF121",72.5123274812065,0.212929867051864,0.251094617752053,0
.848006496348422,0.396434352830707,0.894198510894023
"7496","PLXNA3",92.4797886670451,0.252195321946217,0.297347470250758,0
.848150218777837,0.396354317028453,0.894198510894023
"7497","TMCO4",100.300758315998,-0.157885261679836,0.186650851317853,-
0.845885569581296,0.397616583380392,0.894240648017514
"7498","ZMYM6",82.6580979527977,0.190799089004511,0.225545666541313,0.

845944379825015,0.39758377320713,0.894240648017514
"7499","MACF1",1033.0088621047,0.226223585149481,0.266911611149158,0.847559925083439,0.396683100611468,0.894240648017514
"7500","ZNF514",88.3636824453042,0.205412324727836,0.242419533618761,0.847342298128811,0.396804356650279,0.894240648017514
"7501","LARS2",29.6875303241255,0.290086061814182,0.342839937031405,0.846126808696763,0.397482006715937,0.894240648017514
"7502","AC124016.2",19.7171524010899,-0.312873131900555,0.369539190302863,-0.846657513223789,0.397186046742872,0.894240648017514
"7503","HINT1",2380.09691043026,-0.207885966522858,0.245678345692289,-0.846171305562414,0.397457186877118,0.894240648017514
"7504","GTF3C6",546.530709326638,-0.230335184080225,0.271824170957836,-0.847368294249131,0.396789871116851,0.894240648017514
"7505","AC092171.5",2.75982271741089,0.773431877975111,0.912985270431597,0.847146063604603,0.396913712560236,0.894240648017514
"7506","ZNF713",28.532394385072,0.305794914439168,0.361265276275911,0.846455318350668,0.397298789835649,0.894240648017514
"7507","MAP11",15.7039388537471,-0.348162273725258,0.411145694125709,-0.84680997198722,0.397101049077713,0.894240648017514
"7508","FCHSD2",64.8683408378914,0.250794001419375,0.29591559072999,0.847518715728003,0.396706059668529,0.894240648017514
"7509","CCDC77",53.2822756145406,0.226429204938208,0.267501831191605,0.846458523029782,0.397297002768247,0.894240648017514
"7510","LINC00621",2.20044763136368,-0.917123735351504,1.08204359002201,-0.84758483281884,0.396669224099943,0.894240648017514
"7511","VWA8",62.0772469869251,0.193270140442969,0.228442849102821,0.846032787640374,0.397534453650917,0.894240648017514
"7512","DOCK9",140.33309811514,0.179556787073864,0.212218060679038,0.846095692795106,0.397499363362476,0.894240648017514
"7513","C15orf41",53.087225692043,0.251222774888799,0.296833417634644,0.846342628436855,0.397361633678048,0.894240648017514
"7514","ZNF598",90.716159542896,-0.185586182967351,0.218919001442507,-0.847739034731942,0.396583322183598,0.894240648017514
"7515","MT1M",4537.26312086498,-0.539160696218635,0.636461054831362,-0.847122839843659,0.396926655703767,0.894240648017514
"7516","ZNF529",55.9470522082369,0.243995276746833,0.288281904901462,0.846377357018761,0.397342265932871,0.894240648017514
"7517","ZNF418",2.98568184018997,0.704412380617285,0.831960808249801,0.846689379634552,0.397168279913759,0.894240648017514
"7518","MORC3",330.258369295289,0.135537933152176,0.160129064167506,0.846429309112766,0.397313293888697,0.894240648017514
"7519","CAPN5",110.819237439534,-0.167508481845556,0.198065116002275,-0.84572430131327,0.397706563152613,0.894248469549657
"7520","GUCD1",207.404950325168,0.119496802343523,0.14130099305136,0.845689756052088,0.397725839307631,0.894248469549657
"7521","ZNF644",500.422528778725,0.107869173372351,0.127789366479698,0.844116974235787,0.398604043346913,0.894537257068681
"7522","ATP5PB",877.221670129432,-0.180057170011021,0.213460029235632,-0.843517030592463,0.398939345072302,0.894537257068681
"7523","ASH1L-

AS1",23.129398858591,-0.356397497339081,0.422381976586243,-0.843780078
448284,0.398792309700598,0.894537257068681
"7524","RBM34",26.8400057515353,-0.260662603348678,0.309370485816958,-
0.842558082618462,0.399475642708571,0.894537257068681
"7525","C2orf76",180.18898121816,-0.218574995969976,0.258599539123263,
-0.845225775386207,0.397984793740405,0.894537257068681
"7526","COL5A2",2172.75135630458,0.239240326330442,0.283709302314484,0
.843258660814904,0.399083797310667,0.894537257068681
"7527","FZD5",28.1233145201444,-0.433129568504075,0.514789070579596,-0
.84137289087435,0.400139067528969,0.894537257068681
"7528","AC012076.1",8.0439511218727,-0.705595893951159,0.8373127168804
02,-0.842691003882057,0.399401279780683,0.894537257068681
"7529","RPL29",24153.3612475132,-0.241697297965284,0.286401523014157,-
0.843910658789818,0.398719331569275,0.894537257068681
"7530","TMEM156",3.75634513076837,-0.788397500748669,0.935576385957553
,-0.842686404426242,0.399403852811517,0.894537257068681
"7531","PIGY-
DT",2.07913035241368,-0.849755513530179,1.00822083777449,-0.8428267713
70741,0.399325333127927,0.894537257068681
"7532","PDLIM5",325.318694511801,0.120483876244802,0.142895880894546,0
.8431584975757,0.399139806150943,0.894537257068681
"7533","WDR70",221.216985559806,0.184801581937589,0.219485377968645,0.
841976735069749,0.399800976015904,0.894537257068681
"7534","MARCH3",261.960937237472,0.270735928358917,0.321070120915253,0
.843229907495437,0.399099874981527,0.894537257068681
"7535","DINOL",19.1635916901991,-0.378178257342531,0.44933316078124,-0
.841643329161387,0.399987628268272,0.894537257068681
"7536","AL133338.1",12.2541059964113,-0.373281822195619,0.442333482180
797,-0.843892305767273,0.398729588135099,0.894537257068681
"7537","SOBP",430.781450187635,-0.171474587354137,0.202907622745073,-0
.845086966346124,0.398062284847123,0.894537257068681
"7538","FGFR1OP",54.4278297772085,0.223956708054985,0.265032253775217,
0.845016804048803,0.398101456893685,0.894537257068681
"7539","AC004130.2",41.3411178302243,-0.373053273608342,0.441732342584
09,-0.844523340595841,0.398377026212041,0.894537257068681
"7540","CYCS",734.668197385701,-0.225912664127849,0.268312834881759,-0
.841974869474301,0.399802020295534,0.894537257068681
"7541","INMT",376.906697983423,0.362969550686632,0.429774608350214,0.8
44557923233231,0.398357710169808,0.894537257068681
"7542","MDH2",736.172573091616,-0.23424149845071,0.277618758292654,-0.
84375241749256,0.398807769758446,0.894537257068681
"7543","UFSP1",7.66007176499561,0.497232087466683,0.590714808776445,0.
841746440209626,0.399929897512904,0.894537257068681
"7544","DDHD2",143.261216059718,0.161298816364559,0.191548671851949,0.
842077445930974,0.399744604866749,0.894537257068681
"7545","NDUFB9",1048.86373307948,-0.204143482452201,0.242167778676186,
-0.842983668463886,0.399237577659826,0.894537257068681
"7546","BRD3",354.920400140563,0.157334027915863,0.187008916565652,0.8
41318322170099,0.400169628934896,0.894537257068681
"7547","WAC",924.18030085891,0.155408100252093,0.184012401645474,0.844

552317465582,0.398360841218446,0.894537257068681
"7548","AIFM2",171.305522406477,-0.180315993623834,0.213699459433204,-0.843783105966141,0.398790617604501,0.894537257068681
"7549","SFR1",101.719497483186,-0.237326795849546,0.281547505421088,-0.842936951242367,0.399263706259077,0.894537257068681
"7550","EXT2",363.85468609144,0.133957548821964,0.158848376305574,0.843304489082549,0.399058172838517,0.894537257068681
"7551","SMAGP",23.6054307875822,-0.316602953846819,0.375243560501485,-0.843726547695323,0.398822229041707,0.894537257068681
"7552","SNRPF",837.544037038911,-0.193857241622939,0.230370806205723,-0.841500903763924,0.400067378940398,0.894537257068681
"7553","RPL6",19939.3711329728,-0.206589659821036,0.245505878373399,-0.841485593704711,0.400075952330917,0.894537257068681
"7554","PTPN11",289.799108673176,-0.151323515494394,0.179697099057625,-0.842103274276387,0.399730148670796,0.894537257068681
"7555","EFNB2",9.45280168756342,-0.630321590613997,0.746117602216963,-0.844801930340609,0.398221436667761,0.894537257068681
"7556","AL049828.1",6.56903689142626,0.529681345983683,0.629202900034404,0.841829155515209,0.399883589734021,0.894537257068681
"7557","UBR7",117.168390850933,-0.183175440672309,0.217229685160244,-0.843233927891512,0.399097626918345,0.894537257068681
"7558","GOLGA8N",34.9635672196014,0.336654306697457,0.399326999655954,0.843054206170649,0.399198128387664,0.894537257068681
"7559","BPTF",1227.12936292712,0.19538693539245,0.232245183368709,0.841295963853239,0.400182151198693,0.894537257068681
"7560","SLC35E1",251.324971327035,0.171896209688308,0.204269284425058,0.841517657302868,0.400057997350674,0.894537257068681
"7561","ZNF575",22.9729290930457,-0.279520510911204,0.331448360151652,-0.843330498854517,0.399043630144154,0.894537257068681
"7562","NAA20",497.870248629166,-0.20429772780302,0.242120975608635,-0.843783679994945,0.398790296777186,0.894537257068681
"7563","BCAS4",9.77435810473445,0.517569108369151,0.613918377444458,0.843058503189989,0.399195725290851,0.894537257068681
"7564","IDH3G",691.48297788004,-0.222029251421945,0.263574428842796,-0.842377814861435,0.399576506984382,0.894537257068681
"7565","AL109936.2",3.18380743799864,0.717703192717206,0.857550183587004,0.83692267397712,0.402636034387777,0.894725283376139
"7566","NCDN",51.7223304495568,-0.20831478979418,0.247832715818652,-0.840545967089394,0.400602339731022,0.894725283376139
"7567","FAF1",196.705396333826,-0.143762317918848,0.171242201054844,-0.839526220950669,0.40117408142571,0.894725283376139
"7568","TTC39A",4.53887027514254,0.756420321558026,0.899550261123875,0.840887223592124,0.400411116623027,0.894725283376139
"7569","ADAR",510.812272312745,0.145068056373452,0.172664915422081,0.840171009952032,0.400812510244478,0.894725283376139
"7570","RGS1",134.241563757699,-0.418755988868889,0.499439681343399,-0.838451577861242,0.401777132155409,0.894725283376139
"7571","HHAT",20.5195743672633,0.301903904278036,0.360480231439337,0.837504744913708,0.402308910954991,0.894725283376139
"7572","LHCGR",7.98185356985414,-0.486049609040293,0.580837622973324,-

0.836808067893729,0.40270046170407,0.894725283376139
"7573","IL1R1",1199.63954986214,0.16083579314284,0.192119599803898,0.837164939480455,0.402499861988403,0.894725283376139
"7574","FAP",303.81143624238,-0.409917566128544,0.489442594636282,-0.837519191465476,0.402300794031085,0.894725283376139
"7575","LINC00607",4.44751538248179,0.596258660460576,0.712129719223586,0.837289393160924,0.402429919900399,0.894725283376139
"7576","SS18L2",364.915135785767,-0.247462766056793,0.295196120704129,-0.838299519202766,0.401862505866804,0.894725283376139
"7577","DHX29",484.576178634991,-0.120826242891656,0.144332656300539,-0.837137249383564,0.40251542461807,0.894725283376139
"7578","HARS",242.378897096413,-0.157195412664737,0.187382287981716,-0.838902194854597,0.401524196045962,0.894725283376139
"7579","PCDHA10",5.51357961670738,-0.586645068767769,0.699274057786333,-0.838934409528777,0.401506117268091,0.894725283376139
"7580","DIAPH1",238.746418158555,-0.271040602178885,0.3236828651727,-0.837364690386909,0.402387606930568,0.894725283376139
"7581","TBC1D7",69.8100803150644,0.224661751576206,0.267311791594586,0.840448340254798,0.400657055027576,0.894725283376139
"7582","FOXP4",56.3921387478047,-0.259609591549519,0.309024740150026,-0.840093228210411,0.400856116664103,0.894725283376139
"7583","TUBE1",152.499643137949,0.183331245350283,0.218779562006979,0.837972448927543,0.402046177165308,0.894725283376139
"7584","RAB32",604.449701234684,0.231283822720832,0.276382956922351,0.836823750987696,0.402691644881705,0.894725283376139
"7585","MTHFD1L",36.2042744522199,0.32215984719873,0.38430007075234,0.838302856848403,0.401860631820549,0.894725283376139
"7586","SP4",65.6448764838874,0.250074706125401,0.298063543182205,0.838997964848494,0.401470451647399,0.894725283376139
"7587","ENTPD1",20.9517007379588,0.276102184287638,0.329207428987296,0.838687587145225,0.401644645713169,0.894725283376139
"7588","AP003555.1",14.1741133297663,-0.455237402527556,0.542582001026495,-0.839020464494411,0.401457825877085,0.894725283376139
"7589","EED",111.792423800453,0.155694033701175,0.185287842303194,0.840281972987769,0.40075030648105,0.894725283376139
"7590","NUDT4",537.948008206132,0.319236867740541,0.380286176331622,0.839464823097214,0.401208521028844,0.894725283376139
"7591","LACC1",40.8291149115396,-0.221824623032964,0.264445550150403,-0.838829100761204,0.401565218042408,0.894725283376139
"7592","AL157813.1",2.05313155663073,-0.907838787979,1.08340582803726,-0.837948961031229,0.402059369089172,0.894725283376139
"7593","RIPK3",20.1878957087611,-0.388767856950073,0.463334270976812,-0.839065619148922,0.401432487873192,0.894725283376139
"7594","AL049838.1",16.2992077182731,0.364878352542972,0.435826288182372,0.837210518128012,0.402474246265157,0.894725283376139
"7595","AL049840.5",34.22301687741,0.253575370818925,0.302376753572062,0.838607359274041,0.401689679581245,0.894725283376139
"7596","VPS39",132.451265572487,0.179469410548225,0.213814356364616,0.839370253708208,0.401261570854869,0.894725283376139
"7597","ELOB",2669.27396456811,-0.200593883731723,0.238704308943892,-0

.840344628126812,0.400715185765901,0.894725283376139
"7598","ZNF688",240.432843138064,-0.190070338099137,0.22666015658103,-0.838569693792601,0.401710823183212,0.894725283376139
"7599","VPS4A",304.415931748375,-0.132969316979333,0.15833832709034,-0.839779726253307,0.401031902709797,0.894725283376139
"7600","CENPN",20.2212308993207,-0.384882191559766,0.459101612996083,-0.838337702732161,0.40184106660605 7,0.894725283376139
"7601","APRT",1733.43320196525,-0.24906625892973,0.296598272760988,-0.839742782758679,0.401052620623828,0.894725283376139
"7602","LINC00304",2.84148066970309,-0.895401266031043,1.06676454613559,-0.839361665397189,0.401266388778894,0.894725283376139
"7603","AC005224.2",2.12419826264955,1.09011318097277,1.2976248096924,0.840083491645926,0.400861575430089,0.894725283376139
"7604","MAPT",24.2462228745507,-0.388593940917968,0.464178940557708,-0.837164091182326,0.402500338750957,0.894725283376139
"7605","AC027097.2",29.9969990716296,0.302955557229493,0.360627318682421,0.84007933269272,0.400863907144201,0.894725283376139
"7606","ANKLE1",2.03014261875599,-0.98245106678313,1.17334081380936,-0.837310911902496,0.402417827258852,0.894725283376139
"7607","SLC17A7",100.1580220529,0.189661057552735,0.226617017986371,0.836923278039697,0.402635694822429,0.894725283376139
"7608","OSER1-DT",135.846888512919,0.196030452178203,0.233322369528274,0.840169987020675,0.400813083707283,0.894725283376139
"7609","ADA",385.244989543554,-0.216824760868597,0.258144906125709,-0.839934299393107,0.400945225054417,0.894725283376139
"7610","GMEB2",45.4156762367053,0.212992234673217,0.254417197197048,0.837177034492102,0.402493064357712,0.894725283376139
"7611","SMURF1",75.7940862623043,0.179534072664617,0.214593719903254,0.836623144170092,0.402804432035074,0.894838698837081
"7612","TNFRSF14-AS1",6.93348199505734,0.480247323594093,0.583433455739781,0.823139843746447,0.410428473128414,0.895096093460411
"7613","LZIC",512.256413027945,0.110966342301322,0.133858626383721,0.828981630091021,0.407114802069143,0.895096093460411
"7614","NMNAT1",68.1370937399335,0.207014869590795,0.250009669258439,0.828027452717439,0.407654952852083,0.895096093460411
"7615","MUL1",125.165276366986,0.168961785067133,0.2052533881588,0.823186338519344,0.410402036518055,0.895096093460411
"7616","ZNF593",264.166584245995,-0.216399454783362,0.262891411904119,-0.823151479981731,0.41042185674957,0.895096093460411
"7617","STX12",552.468675543763,-0.145890581575848,0.17632956835053,-0.827374461019656,0.408024851343322,0.895096093460411
"7618","LRP8",5.98269240967945,0.614570694079408,0.743440905605953,0.826657087934236,0.408431450221645,0.895096093460411
"7619","AC119674.1",38.2983011264295,0.354647828450166,0.431998914791035,0.820946109602416,0.411676965143363,0.895096093460411
"7620","PATJ",39.9971637264155,-0.309825464027457,0.371602826595022,-0.833754325461872,0.404419438205511,0.895096093460411
"7621","RAP1A",687.409764805153,-0.126213333867937,0.152671932902139,-

0.826696377446396,0.408409175130744,0.895096093460411
"7622","ATP8B2",158.013344695493,0.169479006697248,0.203158146392448,0.834222056593583,0.404155864217197,0.895096093460411
"7623","CFHR3",2.13356773327448,0.904787107631665,1.0838159349864,0.834816206723351,0.40382119936713,0.895096093460411
"7624","TRIM11",68.1011269136788,0.186644552802647,0.223607513824913,0.834697142372381,0.403888251029723,0.895096093460411
"7625","ARV1",110.79933605836,-0.241741104080311,0.291137397299137,-0.830333396955966,0.406350310778318,0.895096093460411
"7626","ZNF496",81.6552028884122,0.210240377851036,0.251646359046976,0.835459645222959,0.403458959207879,0.895096093460411
"7627","DTNB",19.6345557536563,0.342298882496671,0.414686541998331,0.825440056113633,0.409121801623141,0.895096093460411
"7628","SELENOI",44.7897799321248,-0.191382825260995,0.231797672474441,-0.825646018003473,0.409004922630965,0.895096093460411
"7629","OXER1",14.2864316157279,0.411465865358837,0.498185386365567,0.825929215548896,0.408844246497317,0.895096093460411
"7630","MEIS1-AS2",17.9574653851569,-0.300125913030686,0.365637812226458,-0.8208284345734,0.411743999650874,0.895096093460411
"7631","PAIP2B",69.7408407068211,0.231889005110085,0.281863571323578,0.822699450025339,0.410678928167475,0.895096093460411
"7632","UNC50",514.244724384898,-0.227111193819613,0.271707915834004,-0.835865208867758,0.40323073680279,0.895096093460411
"7633","PSD4",7.55176764004989,-0.551547856672914,0.663968318764917,-0.830683996638391,0.406152169289652,0.895096093460411
"7634","AC009404.1",27.9106179681776,0.255974362916969,0.308317965467928,0.830228502995219,0.406409602835941,0.895096093460411
"7635","CCDC115",418.897265178649,-0.192173357354984,0.231722887856519,-0.829324022036083,0.406921081344074,0.895096093460411
"7636","MZT2A",1218.39407458967,-0.197580566790661,0.237943199342391,-0.830368623002124,0.406330400163368,0.895096093460411
"7637","AC019080.1",30.6480042290296,0.256750724993901,0.309399145331686,0.829836568289987,0.406631192408357,0.895096093460411
"7638","CWC22",316.759756341629,0.156373105364332,0.188203483505453,0.830872534619166,0.406045640863721,0.895096093460411
"7639","ITM2C",505.562608969895,0.250444758352738,0.300529548399533,0.833344873029887,0.404650255603576,0.895096093460411
"7640","EGOT",2.94722203001915,-0.883866373117457,1.05899202219946,-0.834629869337189,0.40392613910984,0.895096093460411
"7641","ZMYND10",2.12584602236946,-0.832364074814912,1.00793601735414,-0.825810428919777,0.408911637196671,0.895096093460411
"7642","AC135507.1",16.701432154023,0.329817048740336,0.396687883660761,0.831427079891325,0.40573240637014,0.895096093460411
"7643","PRICKLE2",350.586290910466,0.227605901731356,0.272274215762121,0.835943650023069,0.403186604620674,0.895096093460411
"7644","PPM1L",64.3441999877974,-0.342390991433261,0.412271775540076,-0.830498258059818,0.406257132302555,0.895096093460411
"7645","DVL3",274.575052425106,0.139501421218563,0.168841098797605,0.826229053305248,0.408674170259512,0.895096093460411

"7646","MB21D2",45.9541107744528,0.325488574649223,0.394537443933724,0
.824987791789669,0.409378521734592,0.895096093460411
"7647","AC139887.2",60.1854112678459,0.351566277986323,0.4252274774419
27,0.826772249294112,0.408366161823543,0.895096093460411
"7648","CXCL2",577.908311681889,-0.644400554169018,0.782516965238608,-
0.823497231107985,0.410225291118383,0.895096093460411
"7649","MRPL1",286.919024466683,0.187286564950914,0.225330414551496,0.
83116416096644,0.405880897935313,0.895096093460411
"7650","AC092445.1",2.95959668664024,0.706485433992757,0.8494880307108
66,0.831660257062784,0.405600739533455,0.895096093460411
"7651","LEF1",2.55733411586229,1.02185829463162,1.24232615016116,0.822
536251449792,0.410771763406357,0.895096093460411
"7652","CLCN3",271.390867119245,0.148552080495202,0.180616875064977,0.
822470660295501,0.41080907833669,0.895096093460411
"7653","C5orf49",12.645742867722,-0.388794464018862,0.469616131025843,
-0.827898443713098,0.40772801639609,0.895096093460411
"7654","SHROOM1",47.1964300550943,0.251433728223647,0.30588379590676,0
.821991003081082,0.411082017407415,0.895096093460411
"7655","CAMLG",1630.36500926931,-0.177036544979301,0.211965210813439,-
0.835215101100334,0.403596608644174,0.895096093460411
"7656","EGR1",15376.2968636031,0.206662937349689,0.249544464148776,0.8
28160777096935,0.407579453519372,0.895096093460411
"7657","HBEGF",51.6025726191437,-0.415655625401402,0.498179018862137,-
0.834349921742547,0.404083828052805,0.895096093460411
"7658","SLC35A4",157.283388803611,0.145715218834506,0.174834475565312,
0.833446712173608,0.404592839264265,0.895096093460411
"7659","PCDHB15",25.377591025707,-0.394293174341676,0.475938030423779,
-0.82845485995434,0.407412948862097,0.895096093460411
"7660","RACK1",17660.8242261924,-0.214436106420697,0.256687254499487,-
0.835398340438932,0.403493463914173,0.895096093460411
"7661","TRIM39",44.5452103914647,0.20234861548314,0.245513117667622,0.
824186574654157,0.409833554131448,0.895096093460411
"7662","LEMD2",117.241376598311,0.1708710153609,0.206882658808639,0.82
5932034830193,0.408842647127093,0.895096093460411
"7663","NUDT3",312.114802761261,0.185407574575828,0.224442745650473,0.
826079604571248,0.408758936433967,0.895096093460411
"7664","BVES-
AS1",4.82338158784231,-0.583357144598264,0.709768054132361,-0.82189828
2406321,0.411134790614786,0.895096093460411
"7665","WAKMAR2",16.6567774116781,-0.41719885172036,0.505437456913716,
-0.825421317738983,0.409132436238487,0.895096093460411
"7666","GSDME",46.886772672152,0.337637524464092,0.411200527306185,0.8
21101876196485,0.411588241434745,0.895096093460411
"7667","POM121",205.279659268116,0.202351935946715,0.242612652101245,0
.834053517795399,0.404250826681655,0.895096093460411
"7668","LRCH4",66.5328582801803,-0.208078423771921,0.252630991172488,-
0.823645677065217,0.410140914135925,0.895096093460411
"7669","AC005072.1",4.47033587019097,0.628561159343525,0.7634704929825
73,0.823294633022408,0.410340464926098,0.895096093460411
"7670","TTC26",38.9910501676081,0.261089670734525,0.312995567236048,0.

834164116252874,0.40418850894829,0.895096093460411
"7671","GSTK1",967.490687560906,-0.215703896287879,0.259767191890819,-0.83037390025196,0.406327417384032,0.895096093460411
"7672","AGAP3",177.082305375831,0.156263354384685,0.189045005398099,0.826593403277798,0.4084675576155,0.895096093460411
"7673","FGFR1",2567.16824908067,0.143540608717564,0.173203317323717,0.828740528388878,0.40725124714341,0.895096093460411
"7674","PREX2",114.320754523073,0.317504483073131,0.380526055934991,0.83438302875999,0.404065177604062,0.895096093460411
"7675","C8orf34",4.54479105131842,0.713923398643356,0.869644955724817,0.820936629303308,0.411682365430623,0.895096093460411
"7676","AC021546.1",24.4804639290917,-0.446125630443807,0.54345173400109,-0.820911228232298,0.411696834915459,0.895096093460411
"7677","AL159169.2",7.2368978168028,0.544149985831102,0.654542231823773,0.831344349340024,0.405779127499028,0.895096093460411
"7678","UBE2R2",939.38099759165,-0.117783727753346,0.142765686406643,-0.825014264407065,0.409363492366014,0.895096093460411
"7679","TMEM38B",105.016312293341,-0.248566016551944,0.301085118966621,-0.825567259534671,0.409049614040314,0.895096093460411
"7680","AKNA",136.730808744522,-0.206633927959237,0.247074996538401,-0.836320675318197,0.402974524860771,0.895096093460411
"7681","ARPC5L",219.82338185889,-0.213227686766702,0.259103454356294,-0.822944206963341,0.41053972195258,0.895096093460411
"7682","AL731533.2",2.0180819576078,-0.872114980055791,1.05910234900631,-0.823447309765708,0.410253668828145,0.895096093460411
"7683","AL133415.1",4.26154689761809,0.800778920445828,0.961624307547608,0.832735730742937,0.404993788354224,0.895096093460411
"7684","BAMBI",38.2251206087917,0.545202270322679,0.663874730244583,0.82124269155691,0.411508043623853,0.895096093460411
"7685","PRKG1-AS1",2.01222719995786,0.893656240656775,1.0728999777708,0.832935277446417,0.404881232512431,0.895096093460411
"7686","COL13A1",2.15757418530932,-0.893004229438998,1.07169324559782,-0.833264773392183,0.404695418754717,0.895096093460411
"7687","CHST3",135.94217774066,0.229310984653577,0.2764275874389,0.829551734608482,0.40679227510713,0.895096093460411
"7688","AL139339.1",24.8459098197663,-0.320964382260255,0.38809861656251,-0.827017589248629,0.408227092038054,0.895096093460411
"7689","CACUL1",330.484655927353,0.131830117592091,0.160431068251156,0.821724364421174,0.41123378920004,0.895096093460411
"7690","BUB3",432.145507311917,-0.120012522789421,0.146134310768533,-0.821248084438654,0.411504972429451,0.895096093460411
"7691","SIRT3",83.5010143788934,-0.167813699803502,0.203585100452107,-0.824292639445784,0.409773299868064,0.895096093460411
"7692","LSP1",2084.59096266224,0.192108804323908,0.231568182991505,0.829599307824407,0.406765368260202,0.895096093460411
"7693","CYP2R1",32.5151967381964,0.230879131430279,0.281106838442522,0.821321646636096,0.411463080822134,0.895096093460411
"7694","PIK3C2A",269.363794080295,0.188019861159172,0.228954774508868,0.821209610336778,0.411526883358898,0.895096093460411

"7695","UEVLD",98.8300343677816,0.180458679437976,0.216907608511974,0.831961039430363,0.405430936160008,0.895096093460411
"7696","CSTF3",116.33144938079,0.169891095130948,0.20367164497723,0.834142107262603,0.404200909661142,0.895096093460411
"7697","TMEM132A",19.8040897165296,-0.445476730886354,0.536171013263145,-0.830848217950418,0.406059379420409,0.895096093460411
"7698","AP000759.1",6.13931471210014,0.77225994042968,0.930400192303187,0.830029858998594,0.406521901917045,0.895096093460411
"7699","SLC36A4",114.469771266897,-0.213679703552739,0.257848152727797,-0.828703643179924,0.407272123747887,0.895096093460411
"7700","NPAT",111.572336444314,0.203960865257538,0.245247499931559,0.831653188368717,0.40560473059778,0.895096093460411
"7701","FXYD2",6.20322532587544,-0.515080926313082,0.620406894903434,-0.830230821972496,0.406408291961717,0.895096093460411
"7702","SORL1",15.1220839502346,0.450811619551512,0.54370841297951,0.829142254910263,0.407023915873633,0.895096093460411
"7703","ETS1",241.132047148312,0.210994365350066,0.257059931438712,0.820798341340764,0.411761143537679,0.895096093460411
"7704","PLBD1-AS1",21.1903832182559,0.303315019800132,0.368801612798894,0.82243409267716,0.41082988259715,0.895096093460411
"7705","MRPS35",224.954756440433,-0.17076680175294,0.205045816651946,-0.832822656620241,0.404944754849956,0.895096093460411
"7706","AC022364.1",9.18743332130284,0.410383140692382,0.496391019700248,0.826733612022629,0.408388065748361,0.895096093460411
"7707","YAF2",223.317895063471,-0.143159524950542,0.173667794054516,-0.824329725208593,0.409752233089398,0.895096093460411
"7708","LRP1",6387.56799629854,0.140938803687025,0.171632581616952,0.821165785419291,0.411551842411292,0.895096093460411
"7709","AC025165.5",3.06549893530666,0.719101775472055,0.86892657540509,0.827574844441618,0.407911319411224,0.895096093460411
"7710","ZFC3H1",314.299787675448,0.157502048967965,0.191379800991126,0.822981569383427,0.410518474434796,0.895096093460411
"7711","CAPS2",43.3600673782208,0.198367728762492,0.240539489001413,0.824678432576727,0.40955417938239,0.895096093460411
"7712","AC008149.2",14.8714671303274,-0.395185979345125,0.480070880116248,-0.823182566810617,0.410404181048171,0.895096093460411
"7713","HCFC2",204.38199337144,0.159149667891869,0.190642127758047,0.834808495705907,0.40382554166191,0.895096093460411
"7714","RAN",1325.17192694121,-0.223553776647205,0.267449192311002,-0.835873814818802,0.403225894823058,0.895096093460411
"7715","ULK1",171.461471029294,0.253680299009135,0.304281121746943,0.833703706469536,0.404447968984234,0.895096093460411
"7716","AC127070.2",5.48538982143906,-0.501645059238792,0.600997371968562,-0.834687608692294,0.403893620255742,0.895096093460411
"7717","CENPJ",70.010260228341,0.211480184167736,0.256968913490309,0.822979640981792,0.410519571075498,0.895096093460411
"7718","CPB2-AS1",10.8671255632206,0.420689796692016,0.510103689727341,0.824714279006454,0.409533823079656,0.895096093460411

"7719","AL132780.1",2.37376147334214,0.923249266318412,1.1176882042262
8,0.826034723125252,0.408784394888976,0.895096093460411
"7720","BRMS1L",141.13869776694,-0.159133479349332,0.190802957746738,-
0.834019981810541,0.404269723981273,0.895096093460411
"7721","AL352979.4",8.52472018569421,-0.446547631498992,0.538144950040
35,-0.829790619544993,0.40665717534093,0.895096093460411
"7722","CGRRF1",190.201004829556,-0.192552384336052,0.234246077423158,
-0.822009002047074,0.411071773522267,0.895096093460411
"7723","TMED8",77.4945405398461,0.191024635665434,0.23212466079202,0.8
22939859184496,0.410542194519765,0.895096093460411
"7724","ALDH1A2",2.56186213701284,-0.864197775005728,1.0408997386915,-
0.830241129748095,0.406402465200652,0.895096093460411
"7725","MINDY2",479.683909186071,0.168661959042987,0.205216342569832,0
.821873915746229,0.411148659891365,0.895096093460411
"7726","SMAD3",485.230359226633,0.169145349743963,0.203652496533137,0.
830558685129799,0.406222982491293,0.895096093460411
"7727","NOX5",4.21916045039741,0.687707275862979,0.8269828340455,0.831
585913940677,0.405642715672161,0.895096093460411
"7728","CERS3-
AS1",8.47838994424426,0.597361009514322,0.72257557691857,0.82671076714
4626,0.408401017108242,0.895096093460411
"7729","AC008870.2",4.90070969470368,0.628049274344989,0.7623016444272
44,0.82388550377177,0.410004617946764,0.895096093460411
"7730","AC007906.2",17.6101806669425,0.491201917292683,0.5893026445447
2,0.83353082128483,0.404545422692439,0.895096093460411
"7731","AC026461.3",11.1789138891427,0.543555220705695,0.6542539597202
2,0.830801575795027,0.406085732322285,0.895096093460411
"7732","AC126773.6",2.19366649455771,0.920817965426618,1.1062959832889
3,0.832343223997888,0.405215239360992,0.895096093460411
"7733","AC135178.6",5.42677534429727,-0.524826560578822,0.630989490691
355,-0.831751666741369,0.405549130712368,0.895096093460411
"7734","TMEM97",79.1952364846368,-0.230951932247387,0.28045775398458,-
0.82348207159958,0.410233908394138,0.895096093460411
"7735","AC068025.2",2.01485819334464,0.919851198891512,1.1118017955276
,0.827351783916664,0.408037700774137,0.895096093460411
"7736","AC005899.8",11.0272091961609,-0.672180496012488,0.809616493942
225,-0.830245555818006,0.406399963255024,0.895096093460411
"7737","PSMD11",536.218497885579,-0.138085413723515,0.16673506065378,-
0.828172630171914,0.407572741727713,0.895096093460411
"7738","TAF15",317.352183100683,-0.15339938901769,0.185313983867925,-0
.827780968364586,0.407794554705916,0.895096093460411
"7739","AC015849.3",2.09575067562467,1.03183980887058,1.25348647712053
,0.823175860054655,0.410407994413202,0.895096093460411
"7740","ZNHIT3",372.479822772637,-0.236807205611266,0.285197092938787,
-0.830328258858141,0.406353215004393,0.895096093460411
"7741","AC107993.1",2.79451409340588,-0.739713592612475,0.901083368976
805,-0.820915819867403,0.411694219310709,0.895096093460411
"7742","G6PC3",176.556735069471,-0.22212514730732,0.267589516550982,-0
.830096597842616,0.406484170491418,0.895096093460411
"7743","AC099850.1",25.919718689414,-0.265232971149598,0.3200869568108

56,-0.828627863478762,0.407315016190348,0.895096093460411
"7744","RNFT1-
DT",29.5406671766196,-0.360428432624116,0.438611861308438,-0.821748029223171,0.411220317755106,0.895096093460411
"7745","FTSJ3",205.6986635461,-0.227142705031659,0.272756635099115,-0.832766927738055,0.404976190217315,0.895096093460411
"7746","THOC1",158.496416601706,0.136543086933357,0.164521311198657,0.829941640621155,0.406571780204011,0.895096093460411
"7747","AP001269.4",8.90931818579636,-0.493680610915721,0.600323037369702,-0.822358264108552,0.410873025417968,0.895096093460411
"7748","SERPINB2",3.33292396779822,0.741880553205589,0.903629525241779,0.821000789020355,0.41164581878562,0.895096093460411
"7749","LINC00909",105.936589835954,-0.17905862087149,0.214309825263766,-0.835512887246819,0.40342899396907,0.895096093460411
"7750","NDUFA11",1984.67805398715,-0.199596885133196,0.24033039817047,-0.830510358459187,0.406250293734219,0.895096093460411
"7751","KANK3",4.2243021815087,-0.650893686547165,0.784443667131476,-0.829751980696497,0.406679025466101,0.895096093460411
"7752","AC008555.1",2.18120062998442,-0.887267405077278,1.0622330959727,-0.835285031544601,0.403557243199003,0.895096093460411
"7753","ACTN4",573.139227050715,-0.127038501069292,0.153368884057943,-0.828319915409289,0.407489347107634,0.895096093460411
"7754","ZNF428",789.492958141044,-0.211982043192504,0.256142777209919,-0.827593288015208,0.407900870717701,0.895096093460411
"7755","ZNF230",34.3505521709022,0.228628030919212,0.277432187357034,0.824086177949443,0.409890593268605,0.895096093460411
"7756","SPHK2",57.1168376403992,0.230025187705095,0.27521214459023,0.835810454686066,0.403261544049667,0.895096093460411
"7757","DBP",208.556118535269,-0.410300153677932,0.499741873447233,-0.821024163630055,0.411632504623604,0.895096093460411
"7758","FLT3LG",164.074913027433,-0.169181110585786,0.205674224050782,-0.822568366875251,0.41075349361491,0.895096093460411
"7759","U2AF2",194.442189386362,0.149325546996941,0.17981451186594,0.830442134215898,0.406288851719698,0.895096093460411
"7760","A1BG-
AS1",10.7148733806649,-0.411191274339197,0.499519718492397,-0.823173258465581,0.410409473645018,0.895096093460411
"7761","NECAB3",167.174470144533,-0.26300304173084,0.317480663757934,-0.828406488186535,0.40744033333828,0.895096093460411
"7762","MAFB",1761.60921967853,0.180868493589142,0.218914824751538,0.826204866638988,0.408687888041069,0.895096093460411
"7763","AP001437.1",4.17004867367331,-0.570462341841637,0.689411248016115,-0.827463061392208,0.407974650400565,0.895096093460411
"7764","ZBTB21",151.744591079168,-0.268980455357737,0.324187157630883,-0.829707312662878,0.406704285946079,0.895096093460411
"7765","AC006946.2",8.78550666689218,0.543475798870458,0.654359737094156,0.830545903212039,0.40623020593341,0.895096093460411
"7766","SMARCB1",397.83536295784,-0.194948179153272,0.235958538158248,-0.82619675759530,0.408692487251758,0.895096093460411
"7767","MTFP1",10.7111297763246,-0.429011983786568,0.518093139666785,-

0.828059572575096,0.407636763159803,0.895096093460411
"7768","EIF3D",1193.88275013054,-0.190586719360922,0.228990427801606,-0.832291206189821,0.405244593066679,0.895096093460411
"7769","RBX1",1353.42968171281,-0.21457100375393,0.25873903454641,-0.82929506222395,0.4069374642844,0.895096093460411
"7770","DESI1",156.070237289051,-0.14852162645616,0.17772147292733,-0.835698826989183,0.403324355346079,0.895096093460411
"7771","SNU13",1167.22373534544,-0.204422551224645,0.247893176316495,-0.824639686586818,0.40957618295055,0.895096093460411
"7772","APOO",39.3996206862743,-0.25304602872695,0.304807603119679,-0.830182797728948,0.406435439678083,0.895096093460411
"7773","ZNF41",20.936908474265,0.312693819537576,0.377838619226926,0.827585650660488,0.407905197429202,0.895096093460411
"7774","BEX4",385.137764306013,-0.223423363686519,0.271912306163956,-0.821674336253839,0.411262269138612,0.895096093460411
"7775","RNF113A",96.9407700791074,-0.196177029982985,0.238853001856057,-0.821329556080732,0.411458576766042,0.895096093460411
"7776","THOC2",599.51518266111,0.157825712594586,0.191065295277704,0.82603024460928,0.408786935324546,0.895096093460411
"7777","LINC00894",16.2360699568531,0.415095382422642,0.504146629422332,0.823362407278755,0.410301934194578,0.895096093460411
"7778","IRAK1",163.348274372234,-0.212945888853961,0.255542548095507,-0.833308935991252,0.404670517868043,0.895096093460411
"7779","TTC30A",19.4336828769182,-0.33222030267674,0.405257332780889,-0.8197761664102,0.412343720304174,0.895435390263348
"7780","CCNYL1",63.0696768043384,-0.256462078654302,0.312695141336159,-0.820166496858343,0.412121198334638,0.895435390263348
"7781","NKIRAS1",122.996917713397,0.205799002867371,0.251043773829761,0.819773379470181,0.412345309356051,0.895435390263348
"7782","ZBTB2",54.8964859047491,-0.305519831403294,0.372777258008346,-0.819577441595039,0.412457037921043,0.895435390263348
"7783","FMC1",414.625221167868,-0.218705489230606,0.266737207041352,-0.819928691825512,0.412256759204871,0.895435390263348
"7784","EXOSC4",123.036100298763,-0.238491519287641,0.290852673704735,-0.819973618429756,0.412231146747725,0.895435390263348
"7785","RNF20",205.513249044257,-0.169830299405451,0.207226684849806,-0.819538755486731,0.412479099806641,0.895435390263348
"7786","IPMK",122.26843861404,-0.165086309603372,0.201409071910386,-0.8196567713535,0.412411799992174,0.895435390263348
"7787","PKNOX2",25.766803594733,0.344217785526814,0.420032626160446,0.819502495968798,0.412499778493093,0.895435390263348
"7788","RILPL1",185.701437556534,0.179359747954117,0.218806785335617,0.819717485812909,0.412377179456118,0.895435390263348
"7789","LINC02126",10.7508347759441,0.560383020734973,0.683394429475033,0.819999397954481,0.412216450381436,0.895435390263348
"7790","CFAP69",86.706613676577,-0.307503754290833,0.375356236100926,-0.819231771623346,0.412654191136892,0.895651322708197
"7791","AC132217.1",50.4175128665637,0.897729430291549,1.09593823225091,0.819142360284062,0.412705196074022,0.895651322708197
"7792","AC108519.1",34.8264063415116,-0.430026583952562,0.525140163760

5,-0.818879631817085,0.412855091845306,0.895861639235169
"7793","CTNNBIP1",164.112754492712,-0.164907135720941,0.208421078316118,-0.791221008226538,0.428815034276093,0.89592121772305
"7794","FHAD1",4.08810059771328,-0.640379911092715,0.795294327560765,-0.805211214138562,0.420697806287197,0.89592121772305
"7795","ALDH4A1",13.3302463682073,0.365679171967819,0.459694590474901,0.795482869594013,0.426332671787802,0.89592121772305
"7796","GALE",18.3541955374558,-0.351565469363609,0.44120548229343,-0.796829331168184,0.425550157856457,0.89592121772305
"7797","TMEM222",244.79333213445,-0.210876195246335,0.263851133961189,-0.799224138552892,0.424160457671632,0.89592121772305
"7798","MATN1-AS1",9.28964753488656,-0.38191328805025,0.475987094053139,-0.802360595112297,0.422344400119531,0.89592121772305
"7799","SVBP",691.557404998478,0.207290366637576,0.255987672511385,0.80976698840198,0.41807410895265,0.89592121772305
"7800","TIE1",11.1082037273224,-0.411674196311212,0.513499151654696,-0.801703751573174,0.422724345187053,0.89592121772305
"7801","IPP",101.413845002059,0.145565499940507,0.182457819667569,0.79780357019349,0.424984489167804,0.89592121772305
"7802","LRRC41",330.547330912767,0.130671170279933,0.165733619752357,0.788440935974156,0.430438834081166,0.89592121772305
"7803","BTF3L4",561.244354384534,-0.199048066655818,0.252677371594375,-0.787755806544288,0.430839555667472,0.89592121772305
"7804","SASS6",92.1570947147395,-0.172134728083015,0.212590782142455,-0.809699867267385,0.418112694334693,0.89592121772305
"7805","PSMA5",546.214343495269,-0.202774991761216,0.256248150967583,-0.791322750995648,0.428755675450748,0.89592121772305
"7806","GSTM5",610.871456738468,0.223291644717419,0.281315297673405,0.793741565297492,0.42734589722117,0.89592121772305
"7807","KCNC4",24.0571543790733,0.33180757736465,0.407374846054083,0.81450187849988,0.415357496516775,0.89592121772305
"7808","PTPN22",8.38818796896405,0.485728770945,0.604063100209218,0.804102701815039,0.421337664344049,0.89592121772305
"7809","ATP1A1",869.768559829418,0.168251171112056,0.209548869638603,0.802920919603283,0.422020443824725,0.89592121772305
"7810","AC244033.2",20.9229552000815,-0.299347288633481,0.36969993618022,-0.809703381954483,0.418110673823007,0.89592121772305
"7811","IL6R-AS1",5.10317502619523,0.571946943602955,0.720980580750395,0.793290358816147,0.427608672685034,0.89592121772305
"7812","PBXIP1",272.068471268253,0.151145602034893,0.186303674226304,0.811286211410384,0.417201326583266,0.89592121772305
"7813","EFNA4",74.5766707968139,0.266677912868783,0.326381214047037,0.817074946079311,0.413885599603571,0.89592121772305
"7814","DPM3",491.358589514418,-0.210524380508395,0.25871134605677,-0.813742356944018,0.415792563468497,0.89592121772305
"7815","KHDC4",102.77632312665,-0.173167696608596,0.215722852816606,-0.802732275916135,0.422129493482712,0.89592121772305
"7816","MEX3A",10.593001415857,0.368667254388481,0.46092988755845,0.79

9833693452414,0.423807158780443,0.89592121772305
"7817","GLMP",282.616692971106,0.177552003807794,0.220479032512248,0.805301083666223,0.420645956603184,0.89592121772305
"7818","FAM20B",154.831090165202,0.163442189343297,0.201870509394692,0.809638762161834,0.41814782315451,0.89592121772305
"7819","TOR3A",184.525330847233,-0.193130051756222,0.238945445729081,-0.80826002423664,0.418940910112287,0.89592121772305
"7820","EIF2D",306.601564897898,-0.150457467034335,0.188315761803523,-0.798963748936284,0.424311432347857,0.89592121772305
"7821","FCMR",5.75376018375232,-0.584049111230299,0.718702477966789,-0.81264379786553,0.416422312773224,0.89592121772305
"7822","GGPS1",259.459012689957,-0.176617601351405,0.218303610412485,-0.809045718564552,0.418488849293217,0.89592121772305
"7823","LGALS8",226.270098467572,0.213403246760939,0.262984561986609,0.81146682204032,0.417097638600989,0.89592121772305
"7824","AKT3-IT1",4.71662235492374,0.545734088693291,0.684968181989297,0.796729108070918,0.425608374935062,0.89592121772305
"7825","SILC1",4.8595641689049,-0.508807910323581,0.642992192675141,-0.791312734617676,0.428761518999719,0.89592121772305
"7826","GAREM2",45.4441365927763,-0.226113569525208,0.277851000979104,-0.813794331236591,0.415762783129483,0.89592121772305
"7827","SUPT7L",147.764520490706,0.173764959910955,0.21520309801234,0.807446368179098,0.419409361805756,0.89592121772305
"7828","AL133243.3",51.1057742206467,0.26325649676264,0.33093703727252,0.795488165762037,0.426329592206682,0.89592121772305
"7829","LGALSL-DT",2.53336622664255,-0.707802688709142,0.898055296289099,-0.788150453133443,0.430608706571954,0.89592121772305
"7830","BOLA3-AS1",65.5530252354151,-0.183626225044779,0.228498741450806,-0.803620290767826,0.421616300975572,0.89592121772305
"7831","PARTICL",17.0355615148599,-0.381845507918433,0.479935956030989,-0.795617630060994,0.426254316188547,0.89592121772305
"7832","RNF181",632.518119326563,-0.230213887251803,0.288688919352959,-0.797446219161383,0.425191925506472,0.89592121772305
"7833","CHMP3",1046.85171125907,-0.148584088270856,0.187953847401897,-0.790534965496835,0.429215410457607,0.89592121772305
"7834","RPL31",8102.16488436986,0.151219720557087,0.185382287189957,0.81571828058274,0.414661281131615,0.89592121772305
"7835","SFT2D3",100.190291317646,0.151192042324467,0.1860658570925,0.812572734659774,0.416463069147566,0.89592121772305
"7836","CCNT2",170.304169759286,0.172933265851316,0.219139003573216,0.789148727663802,0.430025084951628,0.89592121772305
"7837","LINC01124",7.65753244233409,-0.497542877811127,0.612051112727504,-0.812910666225096,0.416269278679193,0.89592121772305
"7838","RBM45",45.1604397313951,-0.214488231057191,0.265729117013234,-0.80716871928833,0.419569284932793,0.89592121772305
"7839","GTF3C3",110.192371995303,0.170584615774323,0.215000715503408,0.793414177133841,0.427536553503625,0.89592121772305

"7840","TYW5",70.543427638041,0.208543126422807,0.257524931006066,0.80
9797814945903,0.418056388650209,0.89592121772305
"7841","AC064836.3",22.0268173565442,-0.255582375891624,0.321747201129
836,-0.794357728658183,0.4269872054854,0.89592121772305
"7842","ICA1L",115.850427733495,0.21189127020695,0.266586111596779,0.7
94832367439469,0.426711020175531,0.89592121772305
"7843","PNKD",584.244563105243,-0.196022938736785,0.246315460214736,-0
.795820686878094,0.42613626598542,0.89592121772305
"7844","OBSL1",251.900316890559,0.146906166200274,0.182212967110261,0.
806233324280255,0.420108327355403,0.89592121772305
"7845","AP1S3",4.11596443767416,0.584911682955444,0.742598729732749,0.
787655108386659,0.430898470695756,0.89592121772305
"7846","DGKD",54.9323247755027,0.227932366052054,0.282988587399953,0.8
05447202469382,0.420561662247895,0.89592121772305
"7847","TIMP4",170.730576160787,-0.226173193934868,0.2857035274153,-0.
791635987070266,0.428572957083926,0.89592121772305
"7848","RPL32",34108.5714579405,-0.210139974907018,0.258756978762798,-
0.812113265163963,0.416726642214548,0.89592121772305
"7849","HDAC11",71.5556594825271,-0.173050610806868,0.213378504875243,
-0.811003015078986,0.41736393927712,0.89592121772305
"7850","CHCHD4",72.7661302270872,-0.185787305440555,0.228950389112046,
-0.811474075938926,0.417093474478504,0.89592121772305
"7851","AC093495.1",16.7796392242445,0.35019084015368,0.43795112977661
5,0.799611683459525,0.423935816153913,0.89592121772305
"7852","EIF1B-
AS1",37.3373831594665,0.26546473644434,0.334819045632689,0.79286032233
5326,0.42785920663516,0.89592121772305
"7853","CTNNB1",892.676037100348,0.182516654998824,0.227799623713027,0
.801215787909954,0.423006732818034,0.89592121772305
"7854","ULK4",32.4574476504994,-0.267370068475181,0.331688974368924,-0
.806086693065041,0.420192863768167,0.89592121772305
"7855","KIAA1143",308.417670536153,-0.132624976532468,0.16429598711766
,-0.807231989406342,0.419532838791507,0.89592121772305
"7856","ATRIP",40.4751557915409,0.213979117165392,0.2694476608781,0.79
4139820950972,0.427114037653341,0.89592121772305
"7857","MST1",8.52517279447984,-0.515253177526911,0.636531460863913,-0
.809470087821896,0.41824480165764,0.89592121772305
"7858","GNAI2",1934.51822648163,-0.12841698435225,0.160125836870858,-0
.801975414222622,0.422567180168796,0.89592121772305
"7859","NEK4",77.4908516447218,-0.179419878690207,0.225962541009975,-0
.794024876372259,0.427180949454188,0.89592121772305
"7860","COL8A1",216.541173835201,0.691130157298627,0.87542232744663,0.
789481985585708,0.429830354557059,0.89592121772305
"7861","PARP9",160.209961877966,0.144372673842274,0.179400232135824,0.
804751878654034,0.420962875731448,0.89592121772305
"7862","MRAS",130.367551181538,-0.175479839706057,0.217052187414493,-0
.808468423176736,0.418820976721535,0.89592121772305
"7863","XRN1",390.699534745323,0.154654924485995,0.190354862982707,0.8
12455863026966,0.416530102820969,0.89592121772305
"7864","HLTF",276.678130535233,0.119956149005457,0.147521966268809,0.8

13140931072444,0.416137261332457,0.89592121772305
"7865","ERICH6-
AS1",26.0457282469999,-0.275247496876719,0.338919301451571,-0.812132846072355,0.416715407688173,0.89592121772305
"7866","SLC66A1L",510.466420647701,0.354856766910167,0.440928900153467,0.804793622705741,0.420938782377703,0.89592121772305
"7867","ACTL6A",214.324719972006,-0.183630426305738,0.230201319642452,-0.797694933247785,0.425047544855168,0.89592121772305
"7868","DNAJC19",724.39060613103,-0.178856745892207,0.221807723887505,-0.806359412366176,0.420035642540621,0.89592121772305
"7869","ST6GAL1",11.2403129222984,-0.50217958709717,0.616397450569972,-0.814700947631781,0.415243510808264,0.89592121772305
"7870","AC072022.1",2.56681632425305,0.760170449218725,0.961636337742284,0.790496801528364,0.429237689419115,0.89592121772305
"7871","UTS2B",4.66421273453389,0.563108069996578,0.688140860934879,0.818303492734849,0.413183912231689,0.89592121772305
"7872","MUC4",8.91919166470411,-0.536428971141319,0.660466924840142,-0.812196570284204,0.416678847222897,0.89592121772305
"7873","NCAPG",4.99505393546057,0.504111044224074,0.634270737551108,0.794788430837004,0.426736581861891,0.89592121772305
"7874","PACRGL",94.7436259998129,0.151370853717206,0.187103973689699,0.809019983553345,0.418503651761443,0.89592121772305
"7875","TLR6",7.0052847611705,0.510871922089913,0.624254893820028,0.818370712264206,0.413145539977113,0.89592121772305
"7876","SRP72",840.956686241437,-0.112944340046396,0.141652054498379,-0.797336406071601,0.42525568205694,0.89592121772305
"7877","COQ2",76.1827064237821,-0.199908271252228,0.253422462965848,-0.788834063534327,0.430208997570793,0.89592121772305
"7878","ADH1C",562.851378574677,-0.279812444082272,0.345743928131806,-0.809305446357978,0.418339474248958,0.89592121772305
"7879","PDE5A",91.4308680778758,-0.332794828553627,0.413965142634527,-0.80391993015566,0.421443219029015,0.89592121772305
"7880","AC084871.1",2.12778938131173,0.960032534206069,1.19574529614184,0.802873770278407,0.422047697987298,0.89592121772305
"7881","AC106897.1",25.3135273399887,-0.478806298936877,0.601063999280238,-0.796597865635337,0.42568461739752,0.89592121772305
"7882","LINC02241",2.28900890558154,0.872618983287868,1.08913935238623,0.801200490438637,0.423015587345865,0.89592121772305
"7883","FGF10",204.122154159565,-0.222624228327312,0.281330617362479,-0.79132598653659,0.428753787847977,0.89592121772305
"7884","LINC01948",3.7600817632199,0.690196184291032,0.857057112223092,0.805309441398549,0.42064113485044,0.89592121772305
"7885","MRPS36",605.904941622307,-0.213020468095107,0.262713027579709,-0.810848514280378,0.417452670125699,0.89592121772305
"7886","AC008522.1",7.4257744620827,0.639002613471505,0.7886373653647,0.810261650709388,0.417789811127891,0.89592121772305
"7887","KIF3A",147.079115804307,-0.153987842594568,0.189466349210882,-0.812745077085823,0.416364230890694,0.89592121772305
"7888","SMAD5-
AS1",14.1066563912383,0.396024209768743,0.489375947443367,0.8092433063

73518,0.418375209434509,0.89592121772305
"7889","PCDHGB6",50.6282936237731,-0.237982179599038,0.295127086356941
,-0.806371866902148,0.420028463392237,0.89592121772305
"7890","ABLIM3",56.3161238314293,0.260091573477704,0.324436486555628,0
.801671773230441,0.422742847872803,0.89592121772305
"7891","ANXA6",1175.16565880605,0.129968113464202,0.164041869104189,0.
792286226522172,0.428193800933721,0.89592121772305
"7892","STK10",63.7885252479735,0.244657009174698,0.307465753125206,0.
795721171180547,0.426194118583165,0.89592121772305
"7893","TSPAN17",64.6414557243538,-0.188373325054935,0.236975043210965
,-0.794907862458903,0.426667100338625,0.89592121772305
"7894","NHP2",752.438775203344,-0.199195833524279,0.250163934100669,-0
.796261196644439,0.42588023443772,0.89592121772305
"7895","RIOK1",182.496872186015,-0.145114124151957,0.177748408832948,-
0.816401818192017,0.414270357072877,0.89592121772305
"7896","SMIM13",93.6926663942336,-0.166866657521164,0.211226145713429,
-0.789990542873191,0.429533291381449,0.89592121772305
"7897","AL137003.2",5.16773942766696,0.571597204130379,0.7161009833024
72,0.798207539800212,0.424750062726338,0.89592121772305
"7898","CDKAL1",93.1334181819289,0.15546227091617,0.19423593264664,0.8
00378533455967,0.423491514486776,0.89592121772305
"7899","HCG18",247.555168629537,0.19823312695691,0.246058326960702,0.8
05634702167871,0.420453510234246,0.89592121772305
"7900","PPP1R10",737.935584462509,-0.170811092432115,0.209404076890538
,-0.815700892592473,0.4146712283936,0.89592121772305
"7901","TUBB",3235.91605312892,0.210395066231918,0.261751961263358,0.8
03795567438873,0.421515050122419,0.89592121772305
"7902","ABHD16A",225.181542785627,-0.134260710655855,0.164326376166152
,-0.81703688591114,0.41390734901601,0.89592121772305
"7903","SMIM29",119.960934535711,0.224126469496804,0.282915914800419,0
.792201702950899,0.428243075785395,0.89592121772305
"7904","MEA1",371.461308624082,-0.14278886100632,0.180446110770025,-0.
791310272064006,0.428762955659163,0.89592121772305
"7905","MYMX",9.26814511687647,0.583598918500584,0.72113044283192,0.80
928343034411,0.418352134914617,0.89592121772305
"7906","FBXO9",363.241783088334,-0.118741659501277,0.150250687523124,-
0.790290290571901,0.429358255879191,0.89592121772305
"7907","AL034374.1",2.17296448401841,-0.808259150180581,0.995253629831
321,-0.812113742622136,0.416726368271279,0.89592121772305
"7908","COX7A2",1650.43293627391,-0.172851093711242,0.213335191754201,
-0.810232443554819,0.417806594224194,0.89592121772305
"7909","HMGN3",1202.71457178737,-0.175019037271717,0.221203096574272,-
0.791214227929906,0.428818990210527,0.89592121772305
"7910","TENT5A",1241.10562279635,-0.175411299634316,0.222494124969804,
-0.788386208661115,0.430470835286729,0.89592121772305
"7911","DOP1A",77.3713566884096,0.194999249989977,0.242522836424779,0.
804044900944651,0.421371043950976,0.89592121772305
"7912","TBX18",178.34682882801,0.164684525192441,0.203577999131285,0.8
08950504942523,0.41854361656005,0.89592121772305
"7913","AL359711.2",7.33598103061049,-0.538770250586079,0.664773685091

431,-0.810456645124239,0.417677773073059,0.89592121772305
"7914","Z97989.1",43.8369575730146,-0.313920357223789,0.389646047525534,-0.805655181715187,0.420441698381973,0.89592121772305
"7915","CCN6",11.6074050973197,-0.481542431037088,0.610943559941266,-0.788194626494437,0.430582871767673,0.89592121772305
"7916","SMPDL3A",190.310869672067,0.265479587288373,0.332629572580488,0.798123826540208,0.424798635919917,0.89592121772305
"7917","SAMD3",8.55330621592426,0.592615026284685,0.73826450413107,0.802713692678733,0.422140236827915,0.89592121772305
"7918","MOXD1",46.595857816637,-0.78797922340895,0.986722127890428,-0.798582702400339,0.424532420882649,0.89592121772305
"7919","AL355881.1",2.42002963035014,-0.897153555473308,1.133964521039,-0.791165454322407,0.428847447584044,0.89592121772305
"7920","MTFR2",5.90602481277495,0.925668059749282,1.17240602044713,0.78954563829027,0.429793166620118,0.89592121772305
"7921","BCLAF1",1822.35307335548,0.176230646206629,0.217383001356493,0.810691935923835,0.41754260547162,0.89592121772305
"7922","AL355304.1",11.8338542059308,-0.54528117268099,0.678388210833937,-0.80378928167204,0.421518680930956,0.89592121772305
"7923","IPCEF1",5.48950234083477,0.504057675359615,0.622825184519601,0.809308434995938,0.418337755601642,0.89592121772305
"7924","FAM221A",38.2082023872506,-0.279905368713129,0.353944237038352,-0.790817703532208,0.429050377547037,0.89592121772305
"7925","GGCT",289.914149864569,-0.182972540653703,0.230674246493073,-0.793207492537305,0.427656942910882,0.89592121772305
"7926","AC004938.2",4.23918760226658,-0.803974619949486,0.996513752894662,-0.806787279768202,0.419789048924545,0.89592121772305
"7927","AC006001.2",8.12375380682609,0.590138114950062,0.736375593998231,0.801409117520915,0.422894838550627,0.89592121772305
"7928","SAMD9",170.563789206347,0.123989437563339,0.157374281620732,0.787863406183165,0.4307766079846,0.89592121772305
"7929","AP4M1",114.124035748945,-0.163169926015411,0.200058381764896,-0.815611545869468,0.414722343786269,0.89592121772305
"7930","PCOLCE",4698.52478933891,0.297870857520188,0.368057087289038,0.80930613159547,0.41833908019565,0.89592121772305
"7931","UPK3BL1",257.473992520365,-0.287840946523372,0.360547254959887,-0.798344579146486,0.424670554984607,0.89592121772305
"7932","PUS7",47.5355099146817,-0.23729528259736,0.29652175512495,-0.800262640079703,0.423558643928705,0.89592121772305
"7933","ING3",172.824219938758,-0.137959054030336,0.170755662817531,-0.80793252624224,0.419129425716016,0.89592121772305
"7934","AC009275.1",13.7802345463198,0.387530140106394,0.486868075999735,0.795965394343508,0.426052149715654,0.89592121772305
"7935","WEE2-
AS1",9.31061953247432,0.431756669601611,0.527713648596375,0.818164682209769,0.413263158871377,0.89592121772305
"7936","EZH2",20.7229863466701,0.410717643465387,0.512403456058919,0.801551274896477,0.422812572532733,0.89592121772305
"7937","AC073140.1",6.13298576248181,0.535693155291182,0.656509761048359,0.815971348903861,0.414516522642449,0.89592121772305

"7938","ABCB8",84.145742038252,-0.227164702952416,0.286325715609271,-0
.793378626397679,0.427557259650072,0.89592121772305
"7939","LINC01006",23.8172243510493,-0.345895502500197,0.4279349182370
87,-0.808289970645868,0.418923674739372,0.89592121772305
"7940","DLC1",1450.2770647031,0.175944346718426,0.217107759896316,0.81
0401004563133,0.417709740697641,0.89592121772305
"7941","SLC7A2",82.7515658436528,0.317115307173087,0.399049682370853,0
.794676255069359,0.426801848166484,0.89592121772305
"7942","FAM160B2",100.064064813334,0.200830375818296,0.249202757864092
,0.805891465807227,0.420305432473959,0.89592121772305
"7943","PPP2R2A",395.421373451039,0.113397530406189,0.142130631904993,
0.797840190297539,0.424963235145728,0.89592121772305
"7944","TACC1",2536.39735685082,0.173146822665947,0.212166453838766,0.
816089535047458,0.414448928814545,0.89592121772305
"7945","RAB2A",1617.85937183504,-0.129960547257614,0.16409530348865,-0
.791982125598143,0.428371098591541,0.89592121772305
"7946","EYA1",19.6787451794932,0.570772921717575,0.713956418235253,0.7
99450648722233,0.424029151950437,0.89592121772305
"7947","NUDCD1",98.667097153631,0.172917326103008,0.214829779664353,0.
804903893553171,0.420875141405864,0.89592121772305
"7948","TRPS1",147.794963900669,0.18782088427174,0.233298678787632,0.8
05066214895757,0.420781470639856,0.89592121772305
"7949","LRATD2",5.95217657372455,-0.504208074548456,0.629296590333087,
-0.801224863274053,0.423001479840092,0.89592121772305
"7950","SCRIB",84.3492017387474,0.189480919906253,0.232625003995673,0.
814533763145159,0.415339238330147,0.89592121772305
"7951","SHARPIN",466.025633269108,-0.188864640411292,0.234152931981233
,-0.806586698758184,0.419904639555122,0.89592121772305
"7952","AL391834.2",17.4330172457533,0.345398212812207,0.4257131918249
84,0.811340168556968,0.417170348367169,0.89592121772305
"7953","ACO1",298.923956567366,0.199237862529871,0.245407333342099,0.8
11865969189002,0.416868543405318,0.89592121772305
"7954","PHF24",2.49588067306939,0.862148516360708,1.07962443165625,0.7
98563362481604,0.424543638892299,0.89592121772305
"7955","VCP",866.995775338879,0.125213234167695,0.154013772593024,0.81
3000240560087,0.416217920243473,0.89592121772305
"7956","TMEM8B",125.290713781639,0.143460264760913,0.181128835059285,0
.792034381019219,0.428340629470412,0.89592121772305
"7957","BX664615.2",18.2454994527862,0.54543710660501,0.69005436505320
9,0.790426282663907,0.429278857951089,0.89592121772305
"7958","S1PR3",161.147672944105,-0.347557722819289,0.434322570020398,-
0.800229476453333,0.423577854591033,0.89592121772305
"7959","AUH",170.077202578055,0.133043013086212,0.167859187729035,0.79
2587018239215,0.428018474635162,0.89592121772305
"7960","NFIL3",1434.05228723764,-0.310813361898199,0.381816962431164,-
0.814037595184721,0.415623413987728,0.89592121772305
"7961","FANCC",21.7800117138842,0.286626422170357,0.362148716223935,0.
791460550126929,0.428675288221037,0.89592121772305
"7962","CNTRL",150.239684536055,0.232063765834293,0.284731688619995,0.
81502612848971 2,0.415057354094417,0.89592121772305

"7963","MIR181A2HG",3.00838024991836,0.714801842539067,0.888237749484903,0.804741571672208,0.420968824721788,0.89592121772305
"7964","ZER1",142.873994129244,0.15165740417024,0.189872179478928,0.798734204170609,0.424444549138866,0.89592121772305
"7965","CAMK1D",294.821406878114,0.235496547690465,0.296884265175867,0.793226773237588,0.427645711475833,0.89592121772305
"7966","CCDC3",5.91277106594404,-0.615017591661649,0.757727165503446,-0.811661003671449,0.41698617650147,0.89592121772305
"7967","YME1L1",806.767704212473,0.110759461186479,0.135583890308557,0.816907236799416,0.413981441836136,0.89592121772305
"7968","EIF4EBP2",1045.81303660548,0.154545060238588,0.189749091712998,0.814470619297312,0.415375397014844,0.89592121772305
"7969","CCNJ",24.9889809033301,0.277410703728659,0.348772711614,0.795391079895263,0.426386047122067,0.89592121772305
"7970","AL353719.1",7.22047673372378,-0.515553002359226,0.641895819665258,-0.803172394280558,0.421875099006289,0.89592121772305
"7971","SCD",23.9139451753907,-0.476577451083829,0.587785976064432,-0.810800989630259,0.417479966087844,0.89592121772305
"7972","SEC31B",29.2173651175953,0.304118916276066,0.379089499005376,0.802235137280217,0.422416954576958,0.89592121772305
"7973","SHOC2",304.946725298746,0.133219075695677,0.169108719857016,0.7877717707775,0.430830215972909,0.89592121772305
"7974","RAB11FIP2",330.325226487339,0.159614487644333,0.201792385628351,0.790983699148596,0.428953503847064,0.89592121772305
"7975","EEF1AKMT2",231.724516807057,-0.194488683059204,0.245981965270633,-0.790662367646444,0.429141041833253,0.89592121772305
"7976","EDRF1",109.583230602455,0.193462925016786,0.237001866555432,0.816292832746688,0.414332672655291,0.89592121772305
"7977","FUOM",291.195896746103,0.243390569112655,0.302377352072963,0.804923276971899,0.420863955174234,0.89592121772305
"7978","SYCE1",8.85957433939662,0.856372767863847,1.06826588156698,0.801647588527009,0.42275684146982,0.89592121772305
"7979","RHOG",304.868110956383,-0.139356768248106,0.176551959676378,-0.789324392114075,0.429922433722651,0.89592121772305
"7980","RRM1",157.239937508111,0.137836139378914,0.173068552957973,0.796425098743303,0.425784994750839,0.89592121772305
"7981","TRIM3",68.5911772370529,-0.179299172354551,0.220164691507914,-0.814386589995543,0.415423518427125,0.89592121772305
"7982","DCHS1",164.697047880872,-0.18606204254851,0.228211541921185,-0.815305137427136,0.414897668801861,0.89592121772305
"7983","TMEM9B",381.81491687114,-0.175924746587671,0.219671587997251,-0.800853438496891,0.423216498140835,0.89592121772305
"7984","PDHX",111.189778730187,0.165800261001753,0.207604843096859,0.798633878326233,0.424502737414267,0.89592121772305
"7985","NUP160",174.160137635498,0.186946475474051,0.230898898821412,0.809646457511452,0.418143399066003,0.89592121772305
"7986","SDHAF2",246.304119010326,-0.205496865477156,0.253115269586931,-0.811870677784531,0.416865841294547,0.89592121772305
"7987","ATL3",688.1764519145,0.126304065512188,0.158707017273153,0.795831637959673,0.426129899947836,0.89592121772305

"7988","DDIAS",3.30032095510064,-0.755282543036297,0.928893260466205,-0.813099389543667,0.416161076438096,0.89592121772305
"7989","NAALAD2",104.41233888778,0.265459889322277,0.32453016613067,0.817982169384499,0.413367368760279,0.89592121772305
"7990","CEP295",99.481525563403,0.186400757904251,0.229877750069737,0.810869072138139,0.41744086296842,0.89592121772305
"7991","ANGPTL5",317.380022163936,-0.308880757613054,0.384167051402731,-0.804027197244583,0.421381268026907,0.89592121772305
"7992","YAP1",420.017670735938,0.154422017870318,0.193151436495131,0.799486768891884,0.424008215640596,0.89592121772305
"7993","FDX1",398.189067187916,-0.171043981190732,0.209971398594726,-0.814606095570525,0.415297820184948,0.89592121772305
"7994","JHY",68.3279313815154,0.209986153332999,0.264337445816108,0.794386707810895,0.426970339958061,0.89592121772305
"7995","RBP5",48.9411244516677,-0.437364116838605,0.550507507572117,-0.794474391035094,0.42691931171356,0.89592121772305
"7996","TMTC1",72.8537729532445,-0.239295971842682,0.302434519649003,-0.791232337235849,0.428808424462483,0.89592121772305
"7997","ZCRB1",856.365763699594,-0.155011358688181,0.194052316616991,-0.798812203794163,0.424399313134251,0.89592121772305
"7998","KANSL2",93.4938664389275,-0.151330720611194,0.191318120599603,-0.790990002080901,0.428949825757347,0.89592121772305
"7999","HOXC6",381.319875306885,0.12083289150116,0.151440228717451,0.797891633712491,0.424933378838786,0.89592121772305
"8000","HOXC5",56.3897113726097,0.183849937557988,0.227445082838642,0.808326718975139,0.418902525155721,0.89592121772305
"8001","RNF41",197.381398313085,-0.149201652720836,0.188073697692304,-0.793314825792043,0.427594421095716,0.89592121772305
"8002","CS",166.207298683503,0.166640878562215,0.211496639618005,0.787912653662933,0.430747799122041,0.89592121772305
"8003","PTGES3",1891.31610138703,-0.164475473099702,0.207559938097509,-0.792423984162267,0.428113499196845,0.89592121772305
"8004","AC025034.1",3.3162523930084,0.680826718658534,0.842137566500558,0.808450716060157,0.418831166364766,0.89592121772305
"8005","NEDD1",132.053849136032,0.150156756214922,0.183774638159471,0.817070068638217,0.413888386770445,0.89592121772305
"8006","GLT8D2",837.855281973123,0.206320137844154,0.256492925307708,0.804389195517329,0.421172239094645,0.89592121772305
"8007","C12orf76",319.149111511133,-0.208580452985146,0.259401575993881,-0.804083214167002,0.421348918154023,0.89592121772305
"8008","ATXN2",381.9587505407,0.144167334118236,0.181859312116452,0.792741006443043,0.427928733744114,0.89592121772305
"8009","PSPC1",162.263105187591,0.143015135363691,0.181399634412652,0.788398145491062,0.430463855236527,0.89592121772305
"8010","MIPEP",54.2824168732596,-0.198140815935755,0.251044824629322,-0.78926469099022,0.429957319058194,0.89592121772305
"8011","ATP8A2",3.06715791855479,0.716363474433958,0.893633796880605,0.801629791682631,0.422767139138338,0.89592121772305
"8012","AKAP11",253.689567933679,0.171529885781307,0.209654748634989,0.81815406947897,0.413269218027668,0.89592121772305

"8013","COMMD6",3201.96824294696,-0.214483496613204,0.271074619086896,
-0.791234152926906,0.42880736511857,0.89592121772305
"8014","GPC6",214.216802287815,-0.257207169452177,0.326514543581844,-0
.787735721143169,0.430851306571684,0.89592121772305
"8015","NGDN",184.953552947776,-0.176283234240053,0.215474894805537,-0
.818114956729163,0.413291549234338,0.89592121772305
"8016","PELI2",145.501531005939,-0.271891638258739,0.341991669876081,-
0.795024154703117,0.426599451543821,0.89592121772305
"8017","JKAMP",305.19609832709,-0.196518664615927,0.245102313920743,-0
.801782167913251,0.422678975459643,0.89592121772305
"8018","SGPP1",105.615651506323,0.14767183975003,0.187305992671795,0.7
88398906215387,0.430463410405912,0.89592121772305
"8019","FNTB",33.3258924932984,-0.228953286300703,0.288235832191611,-0
.794326245143947,0.427005528961827,0.89592121772305
"8020","AC005520.2",32.1431473239849,0.223615046278932,0.2811066431559
07,0.795481187383077,0.426333649951522,0.89592121772305
"8021","SNW1",604.625348482497,-0.146983436119425,0.185267359132701,-0
.793358510681561,0.42756897609273,0.89592121772305
"8022","PSMC1",889.054003754266,-0.176842282575525,0.217553646928785,-
0.812867470033327,0.41629404703014,0.89592121772305
"8023","CCNK",252.751523610831,0.143452058986978,0.175476792352368,0.8
1749875333325,0.413643461434543,0.89592121772305
"8024","MTA1",258.344147241427,-0.139680053602454,0.176759731652013,-0
.790225535516441,0.429396065615239,0.89592121772305
"8025","TUBGCP5",90.6677067443599,-0.178229067576442,0.218776158441736
,-0.814664033073366,0.415264646451697,0.89592121772305
"8026","LRRC57",130.557397905183,0.180586910324988,0.223818850798359,0
.80684406018902,0.419756330955238,0.89592121772305
"8027","DUOX1",10.0744881017865,0.461939155644764,0.572079104412189,0.
807474267250865,0.419393294187097,0.89592121772305
"8028","TLN2",547.527243940865,0.177367034918095,0.216982335432779,0.8
17426149296115,0.413684937094406,0.89592121772305
"8029","AC079322.1",4.58424081063391,0.591315856453669,0.7350337276211
96,0.804474453665351,0.421123017272898,0.89592121772305
"8030","ADPGK",244.089049271388,0.144605247821527,0.176621282483966,0.
818730595700745,0.412940136494908,0.89592121772305
"8031","UBE2Q2",316.3616290203,0.121448461801374,0.152881285852322,0.7
94397176373102,0.426964247472974,0.89592121772305
"8032","EFL1",46.0074419196505,0.19781721717317,0.249887437640889,0.79
1625297536772,0.428579191807197,0.89592121772305
"8033","SNHG21",37.7462593598328,-0.30297481439614,0.376293183710609,-
0.805156265145491,0.420729510627078,0.89592121772305
"8034","CIB1",811.199706230406,-0.200035652325278,0.247927098149328,-0
.806832548029081,0.419762964361821,0.89592121772305
"8035","MCTP2",8.3122994042073,-0.515450154916708,0.636339255906228,-0
.810024134347395,0.417926304957203,0.89592121772305
"8036","AXIN1",81.6680764261008,-0.153958315855427,0.189840955780382,-
0.810985781348124,0.41737383617389,0.89592121772305
"8037","DECR2",54.0746994396539,0.257820688755368,0.324164737568994,0.
795338477247218,0.42641663710326,0.89592121772305

"8038","AC009041.2",27.0880075119296,0.391631891787,0.4962751628067,0.
789142639281227,0.430028643012337,0.89592121772305
"8039","ERCC4",116.244872393803,0.135286980572106,0.170061408147918,0.
795518407412191,0.426312007738438,0.89592121772305
"8040","NPIPA5",9.36078970350965,0.799822947638668,0.979963449174527,0
.816176305669767,0.414399306516647,0.89592121772305
"8041","NFATC2IP",268.804418062986,0.173930507409033,0.216350448779248
,0.803929496751364,0.421437693723232,0.89592121772305
"8042","KCTD13",55.6933367528834,-0.206777798866219,0.25719346597976,-
0.80397765191473,0.421409881757781,0.89592121772305
"8043","TMEM265",90.5222048895917,-0.277070523966086,0.342666785312992
,-0.808571288031344,0.418761785543906,0.89592121772305
"8044","ARMC5",42.6971623047221,-0.266590269924897,0.326652537309905,-
0.816127963126689,0.414426952168975,0.89592121772305
"8045","AC007611.1",7.60934218491862,0.429511546146909,0.5302397567874
99,0.81003270812649,0.417921377395761,0.89592121772305
"8046","C16orf70",48.4273440004105,0.18551493140585,0.234482803059463,
0.791166469290309,0.428846855381255,0.89592121772305
"8047","NRN1L",5.63533981732072,-0.523122356657644,0.64496835087953,-0
.811082211932218,0.417318460301135,0.89592121772305
"8048","CHTF8",13.5874835849636,-0.355606555402293,0.436492477262706,-
0.814691143435831,0.415249124195239,0.89592121772305
"8049","BCO1",2.21162424862187,0.794900969505708,1.00889516903958,0.78
7892532246353,0.430759569642608,0.89592121772305
"8050","CBFA2T3",3.24512534554871,-0.722407681489287,0.893280050901641
,-0.808713550425891,0.418679932084565,0.89592121772305
"8051","SERPINF2",20.7511988571007,0.34995475875203,0.444293216643851,
0.787666220509858,0.43089196914531,0.89592121772305
"8052","SLC16A11",4.90756820974414,0.594670086048284,0.739335671698705
,0.80433030463952,0.421206240353406,0.89592121772305
"8053","TMEM88",15.0329537331863,-0.581744969368256,0.721458842618536,
-0.806345331158201,0.420043759435803,0.89592121772305
"8054","CTC1",33.6088213211445,0.239289017511061,0.302898203088829,0.7
89998141523759,0.429528853692244,0.89592121772305
"8055","MED9",57.7243739968384,0.217398661961993,0.266246073946899,0.8
16532836481759,0.414195450912991,0.89592121772305
"8056","ALDH3A2",682.576980627483,0.114408222639577,0.140251950458715,
0.815733558537958,0.414652541087708,0.89592121772305
"8057","EFCAB5",3.45741068728976,-0.676102033334843,0.84219610047378,-
0.802784568765516,0.422099262789385,0.89592121772305
"8058","SLFN12L",14.4862389254027,0.377910248304216,0.477160464427181,
0.791998240587447,0.428361702119784,0.89592121772305
"8059","RPL19",29878.5160516303,-0.212863359688544,0.264797564405305,-
0.803872045298462,0.42147087615978,0.89592121772305
"8060","MIEN1",305.787898440763,-0.217345971719764,0.273291717155418,-
0.795289275438091,0.42644525055535,0.89592121772305
"8061","GSDMB",7.35245320475144,0.440182650409775,0.544170677328977,0.
808905493714545,0.418569508671951,0.89592121772305
"8062","ZNF385C",3.48571544617076,0.610997800716509,0.759192204196353,
0.804799887748168,0.420935166462111,0.89592121772305

"8063","RAMP2-
AS1",254.495741416099,-0.285148479741012,0.355696934676639,-0.801661335654293,0.422748887162474,0.89592121772305
"8064","AC002558.3",82.4411221034765,0.283407236577743,0.353918756562637,0.800769191580229,0.423265277694227,0.89592121772305
"8065","DYNLL2",302.80463813385,-0.139141154949309,0.176207502576508,-0.789643760423281,0.429735844206461,0.89592121772305
"8066","AC005828.5",6.47426462107991,0.560883837595875,0.711756341424356,0.78802787548537,0.430680400859489,0.89592121772305
"8067","MILR1",41.0374000109425,0.326931419835442,0.407971153680926,0.801359157101424,0.422923752711228,0.89592121772305
"8068","NUP85",132.119323619282,0.151248301361326,0.184958040870443,0.817743855036132,0.413503463231627,0.89592121772305
"8069","RNF157",8.25028808119023,0.509335543026934,0.645802006726531,0.788686838569418,0.430295062234125,0.89592121772305
"8070","TK1",3.0629890060895,-0.676013820837089,0.84486051364878,-0.800148438607366,0.423624799452048,0.89592121772305
"8071","C1QTNF1",169.437431156577,-0.26293510718536,0.332463331014542,-0.790869496443382,0.429020150257679,0.89592121772305
"8072","SECTM1",52.4478388564976,0.273788408556778,0.34426687741726,0.795279553498664,0.426450904509362,0.89592121772305
"8073","FN3K",42.7052550682829,0.31039765871644,0.387667468896654,0.800680179845546,0.423316819685888,0.89592121772305
"8074","MTCL1",13.3462701982399,-0.445641289293507,0.563180753707214,-0.791293534731102,0.428772720331646,0.89592121772305
"8075","AP001033.2",14.1705403080643,-0.382627427820305,0.482638083067439,-0.792783332364679,0.427904069060144,0.89592121772305
"8076","CELF4",11.6476764714153,0.506666542361979,0.631900419915148,0.801813903573627,0.422660614813384,0.89592121772305
"8077","AC015961.2",11.0165847493746,0.560998457369719,0.68812435986856,0.81525737219487,0.414925003719013,0.89592121772305
"8078","IER3IP1",706.396961170269,-0.185770581893032,0.23435895265067,-0.792675422858444,0.427966953055455,0.89592121772305
"8079","VPS4B",634.002241600791,0.152691127530287,0.189940719497182,0.803888328603242,0.421461471198873,0.89592121772305
"8080","C18orf65",2.21594128203089,-0.835095100281505,1.0388371008278,-0.803874928625532,0.421469210789955,0.89592121772305
"8081","ADNP2",132.665005133465,0.185829177486379,0.232837741617508,0.798105909271566,0.424809032531592,0.89592121772305
"8082","LSM7",898.699101001023,-0.223430866082835,0.27374639195788,-0.816196569696569,0.414387718451148,0.89592121772305
"8083","TLE5",1958.6372196235,-0.148220205719185,0.185512198370358,-0.798978218258604,0.42430304216733,0.89592121772305
"8084","CD320",187.070435220707,-0.243120424341968,0.300812190592217,-0.808213336910749,0.418967781393812,0.89592121772305
"8085","AC011511.5",7.84415390135424,0.773482674905393,0.944949243539823,0.818544149533251,0.413046543096948,0.89592121772305
"8086","SMARCA4",187.454599513502,0.201290637973142,0.251759304938385,0.799536041070678,0.423979657015339,0.89592121772305
"8087","ZNF433-

AS1",45.8041307101244,-0.247718684732897,0.310440448623962,-0.79795878
9941577,0.424894405101831,0.89592121772305
"8088","RFX1",159.203106223281,-0.18313880147855,0.228015173162126,-0.
803186906111431,0.421866712499236,0.89592121772305
"8089","DNAJB1",2780.48584411519,0.193723959581396,0.239477339959434,0
.808944844694749,0.418546872490686,0.89592121772305
"8090","ILVBL",279.556982949503,-0.190649636890015,0.23365610168520З,-
0.815941186705541,0.414533774257427,0.89592121772305
"8091","FKBP8",1773.08641953626,-0.18439112450082,0.225399801820661,-0
.818062496113152,0.413321502452861,0.89592121772305
"8092","CEP89",184.988811934142,0.166298630090559,0.20385179704466,0.8
15782016648723,0.414624820427461,0.89592121772305
"8093","AC092295.2",2.16199891790767,0.846682339251291,1.0728135112145
6,0.78921670020052,0.429985362851102,0.89592121772305
"8094","YIF1B",138.902067114389,-0.219863683898638,0.276579516056689,-
0.794938421446852,0.426649323150072,0.89592121772305
"8095","KDELR1",2020.65297109711,-0.182415465783597,0.224693060928446,
-0.81184289817338,0.41688178326472,0.89592121772305
"8096","RUVBL2",210.64553487104,-0.196730457464679,0.241892655195546,-
0.813296531495105,0.416048065205165,0.89592121772305
"8097","TSEN34",342.427983845257,-0.187474949779959,0.235444199606964,
-0.796260643043734,0.425880556143242,0.89592121772305
"8098","CHMP2A",1257.60422181046,-0.197208872635461,0.245693913947875,
-0.802660796381388,0.42217081812403,0.89592121772305
"8099","AL035071.1",49.600113231829,0.312911428136878,0.39592395714876
8,0.790332139510571,0.429333821791954,0.89592121772305
"8100","PXMP4",124.549514591547,0.150580593947715,0.188357246400622,0.
799441470000261,0.424034472303268,0.89592121772305
"8101","ZNF341",16.2902460591679,0.367160233290531,0.465996613856591,0
.787903221553288,0.430753316644757,0.89592121772305
"8102","SCAND1",905.085367344443,-0.20485938103689,0.253808254306981,-
0.807142311412427,0.419584497485601,0.89592121772305
"8103","NNAT",40.8631307562294,-0.502173611063756,0.631133229154042,-0
.795669737967781,0.42622402063632б,0.89592121772305
"8104","CHD6",392.695231392195,0.154880885343151,0.19548214027779,0.79
2301972564132,0.42818462180117,0.89592121772305
"8105","CD40",329.087689161405,-0.172778996227843,0.218820576040025,-0
.789592091176288,0.429766028544496,0.89592121772305
"8106","UBE2V1",1078.64377315624,-0.138387681571343,0.173305431569599,
-0.798519009577418,0.424569366195887,0.89592121772305
"8107","PARD6B",6.11311419833471,0.467579216746763,0.586353130058584,0
.797436208279549,0.425197737508458,0.89592121772305
"8108","ATP5F1E",4791.66838364772,-0.206290733093793,0.257946308892125
,-0.799742915414484,0.423859762948888,0.89592121772305
"8109","LAMA5",12.0173379689011,-0.443913149937332,0.559273657306854,-
0.793731555451704,0.427351725775339,0.89592121772305
"8110","CU633906.5",2.20852700548818,-0.814076713383254,1.019441053547
66,-0.798552020786553,0.424550217658475,0.89592121772305
"8111","GABPA",284.314075719893,0.12638644785742,0.15629529539606,0.80
8638849538949,0.418722911531128,0.89592121772305

"8112","BCR",63.5012747588309,-0.180687005466355,0.226131131207404,-0.799036402027425,0.424269304709241,0.89592121772305
"8113","GGT5",1718.384688143,0.213459102344119,0.264360927022407,0.807453297839384,0.419405370845682,0.89592121772305
"8114","EMID1",19.3047692018715,0.459283528729385,0.571649731322262,0.803435221891968,0.421723223903692,0.89592121772305
"8115","IFT27",186.321109540197,-0.203392673128015,0.254153304299766,-0.800275541128199,0.423551170888346,0.89592121772305
"8116","PLA2G6",39.510416379385,0.248062285489308,0.303940810816296,0.816153266233268,0.414412481944209,0.89592121772305
"8117","APOBEC3C",391.383190391989,0.129090788819387,0.161434834741499,0.799646427154934,0.423915680270603,0.89592121772305
"8118","GPM6B",17.0200460064272,0.399864376088516,0.493407650271853,0.810413814759869,0.417702380623278,0.89592121772305
"8119","NHS",26.7748524460836,0.299514941356766,0.375958958536957,0.796669249543426,0.425643147466877,0.89592121772305
"8120","ACOT9",352.295996862977,-0.135054077773475,0.165536181341498,-0.815858362075301,0.414581148937493,0.89592121772305
"8121","CDK16",181.034679440748,0.12594839864111,0.157163034031429,0.801386912751527,0.422907689225909,0.89592121772305
"8122","PPP1R3F",41.1841329448172,-0.233021693502737,0.287875525791972,-0.809452949713849,0.418254655871797,0.89592121772305
"8123","USP27X",27.9297849921329,-0.266950684382925,0.327832613882408,-0.814289588889648,0.415479072551787,0.89592121772305
"8124","KANTR",84.968296398762,0.294479231526734,0.360780279901394,0.816228735138236,0.414369324907094,0.89592121772305
"8125","TRO",19.7857852528695,0.335175359777954,0.415574844464138,0.806534284360125,0.419934847955978,0.89592121772305
"8126","ATRX",1368.22723320034,0.156918405625151,0.196201567545761,0.799781610249122,0.423837339551768,0.89592121772305
"8127","ITM2A",7411.5060228449,0.235187906971692,0.293718321223782,0.800726035719454,0.423290266513819,0.89592121772305
"8128","SRPX2",793.905628320836,0.156563806437249,0.195885213077914,0.799263017239462,0.42413791838867,0.89592121772305
"8129","CHRDL1",2296.66957904933,0.172460580569125,0.216413625588669,0.796902598438582,0.425507601681885,0.89592121772305
"8130","RAB33A",10.5912009910894,-0.45885911487664,0.578220730036169,-0.793570847672544,0.427445309380689,0.89592121772305
"8131","F8A3",17.6923143463138,-0.45610487819112,0.56193282389424,-0.811671535807922,0.416980131457366,0.89592121772305
"8132","MT-CO1",60432.4277058345,-0.188439862128182,0.231585297436349,-0.813695274329646,0.415819542050798,0.89592121772305
"8133","SLC44A5",2.25065124907936,-0.951470326705237,1.20837713412934,-0.787395176416332,0.431050569549878,0.896034936466271
"8134","FARS2",123.043231648352,-0.198590181086827,0.252245287553681,-0.787289954998919,0.431112148577781,0.896034936466271
"8135","AL158071.1",69.3676258139789,0.348027788525431,0.442022844980964,0.787352492019771,0.431075549245087,0.896034936466271
"8136","FUBP1",254.425993686276,0.183841361223182,0.234062061415993,0.

785438529042286,0.432196498179508,0.896066888326212
"8137","ZCCHC4",44.3704868026696,-0.191592998024544,0.243623962971859,
-0.786429198866102,0.431616082859349,0.896066888326212
"8138","MFSD4B",69.0069063095199,0.31825964740622,0.404951236218311,0.
785920918227905,0.431913818733264,0.896066888326212
"8139","AC073073.2",4.29570095657568,0.528920099339028,0.6728521263159
7,0.786086687776399,0.431816702725573,0.896066888326212
"8140","AC011586.2",2.77776794779428,0.702137285148088,0.8937510671945
76,0.785607213149461,0.432097637326736,0.896066888326212
"8141","POMK",5.41718582001223,0.587378929614547,0.747382075025591,0.7
85915195510187,0.431917171610552,0.896066888326212
"8142","MROH1",69.9912983246527,0.223298047317437,0.283971164625893,0.
786340569513853,0.431667990767241,0.896066888326212
"8143","PTCH1",79.2640065886048,0.293260622773456,0.37291846051092,0.7
86393417938259,0.431637038383285,0.896066888326212
"8144","SRGN",341.729030383969,-0.245794670148768,0.312714403502751,-0
.786003674265056,0.431865334568264,0.896066888326212
"8145","KLLN",4.54818332254305,-0.52724737602414,0.671245884941947,-0.
785475766558687,0.432174673226681,0.896066888326212
"8146","DNAJC24",117.726372058724,-0.139959802702085,0.178079128098242
,-0.785941644013851,0.431901675849728,0.896066888326212
"8147","AC079174.2",6.69348629492654,-0.448715242524987,0.570857926404
331,-0.786036633232571,0.431846025821654,0.896066888326212
"8148","KIF23",6.31399206056519,0.574837748716117,0.731643856617778,0.
785679731356538,0.432055140539572,0.896066888326212
"8149","CCDC102A",180.889780817895,-0.191201390495983,0.24337769424243
5,-0.7856159172316,0.432092536472573,0.896066888326212
"8150","ADGRG1",5.06559716175745,0.742154974676736,0.944982719476992,0
.785363540920079,0.432240450744534,0.896066888326212
"8151","OMG",36.8474146897847,-0.376334872788963,0.478888452240391,-0.
785850798924781,0.431954901900347,0.896066888326212
"8152","TNRC6C",507.374129722847,0.197262653022235,0.250664098577788,0
.786960135661465,0.431305202737419,0.896066888326212
"8153","GMIP",21.9938409169118,-0.308655980314296,0.392923143633594,-0
.785537796170442,0.432138319014507,0.896066888326212
"8154","SNRPD2",1723.95685958852,-0.166069333876832,0.211031096099592,
-0.78694247883951,0.431315539272238,0.896066888326212
"8155","APMAP",274.865498797256,0.15052500888791,0.191363737630421,0.7
86591079123978,0.431521283138883,0.896066888326212
"8156","CITED1",4.6707065749255,-0.802061042848685,1.01919956707129,-0
.786951906929705,0.43131001992689,0.896066888326212
"8157","TDRKH",4.59126815850866,-0.562817939538926,0.716816446810553,-
0.785163261868729,0.432357852398168,0.89613049684696
"8158","PITRM1",116.76229609951,0.186870121747584,0.238038990736785,0.
785039968322745,0.432430135070638,0.89613049684696
"8159","BRAP",108.240413501084,0.168124487409962,0.214142170833871,0.7
85106860340885,0.432390917763262,0.89613049684696
"8160","HES4",64.6474751254184,-0.341590491334188,0.435415380779315,-0
.784516363943788,0.432737183859127,0.896131007126556
"8161","CYB5RL",36.7398091725771,0.238727734163903,0.304141913803697,0

.784922180498889,0.432499196466673,0.896131007126556
"8162","TRIM59",8.18714011850324,-0.513574936286921,0.655628777311532,-0.783331900702839,0.433432234767215,0.896131007126556
"8163","RWDD4",300.569662472934,-0.150051137960958,0.191292318050785,-0.784407546993712,0.43280101144522,0.896131007126556
"8164","ITGA2",10.9190485087053,0.527149518133335,0.67296667336872,0.783321877582055,0.433438119154628,0.896131007126556
"8165","KLC4",46.8659092140266,-0.19413201846472,0.24772622285264,-0.78365550578067,0.43324227708334,0.896131007126556
"8166","HIVEP2",262.531830533512,0.198706491927252,0.253215416433367,0.784732994246979,0.432610133576818,0.896131007126556
"8167","PPP1R35",195.174300154041,-0.176545151313734,0.225045638846329,-0.784485992347032,0.432754998051297,0.896131007126556
"8168","TRBC2",37.7020049966014,0.358455956013219,0.457418530795865,0.783649834626376,0.433245605662618,0.896131007126556
"8169","URM1",325.143012972487,-0.21029709709723,0.268529770728099,-0.783142578668372,0.433543390010364,0.896131007126556
"8170","TSPAN31",363.585090778272,0.159592950017106,0.203746887257684,0.783290248823601,0.433456688112176,0.896131007126556
"8171","IRAK3",239.602305105734,0.197049229802451,0.251329469971228,0.784027554846666,0.433023942075546,0.896131007126556
"8172","PSME2",662.660314907011,-0.235958915699901,0.300911213372765,-0.784147965292334,0.432953293487302,0.896131007126556
"8173","NR2E3",3.47463892591934,0.757642459779802,0.966082187427826,0.784242241125478,0.432897983552361,0.896131007126556
"8174","AP1G1",197.597940280102,0.144882220174462,0.184841876326134,0.783817082222387,0.433147448989709,0.896131007126556
"8175","ZNF557",59.9732189084666,-0.25712801910713,0.32830006401608,-0.7832103837011,0.433503578249483,0.896131007126556
"8176","EPOR",53.917168614771,-0.200912773723055,0.256183916443268,-0.784252097135642,0.432892201444637,0.896131007126556
"8177","LILRA2",2.58921905717062,-0.772098097943794,0.985676205762806,-0.783318186469028,0.433440286149936,0.896131007126556
"8178","CPNE1",613.086251605826,0.198701263673032,0.25359946977663,0.783523971276625,0.433319482638563,0.896131007126556
"8179","AP000550.1",4.60299310855304,0.551673811351605,0.703289029921794,0.784419758990058,0.43279384811467,0.896131007126556
"8180","TST",101.705692022278,0.264070939435469,0.337160518050379,0.783220232791346,0.433497795526778,0.896131007126556
"8181","EIF4G3",351.568813617744,0.16449802096409,0.210316601365078,0.782144727978683,0.43412952302006,0.89630225036177
"8182","KIAA1522",42.8397460118194,0.280827391101289,0.358797175006941,0.782691198992456,0.433808471703769,0.89630225036177
"8183","CCDC30",57.620799942617,-0.204780607991554,0.262488380953464,-0.780151133729074,0.43530192158175,0.89630225036177
"8184","DPH2",36.2427888629677,-0.223054257984148,0.285594462527033,-0.781017446943794,0.434792233343645,0.89630225036177
"8185","FOXD2-AS1",7.76575829135696,0.414671828358356,0.532080508364595,0.779340385222705,0.43577923093253,0.89630225036177

"8186","LRRC8B",25.4125500287528,-0.253602160934343,0.326094279363522,
-0.777695829038553,0.436748351676909,0.89630225036177
"8187","HFM1",8.25746584388439,0.470514739261015,0.603997602322893,0.7
79001005056111,0.435979122703277,0.89630225036177
"8188","DR1",262.842927605606,0.106206918820841,0.136555493169537,0.77
775647361899,0.436712592392561,0.89630225036177
"8189","RWDD3",224.794653136469,-0.171386423120002,0.219968516896605,-
0.77914069494118,0.435896840255692,0.89630225036177
"8190","VAV3",4.90877914271706,-0.725532483531253,0.927897708504251,-0
.781909985208169,0.434267476368651,0.89630225036177
"8191","RNF2",103.293637381139,-0.198963664777259,0.254607548461946,-0
.781452340981934,0.434536496982272,0.89630225036177
"8192","PLA2G4A",43.8621651784846,0.205303391358299,0.263134345859212,
0.78022270596384,0.435259799586848,0.89630225036177
"8193","ATP5MC3",2090.46586227948,-0.24457399682402,0.313343352043946,
-0.780530351860534,0.435078769305436,0.89630225036177
"8194","C2orf88",52.484250496983,0.388733563861701,0.499660697333768,0
.777995079332866,0.436571914090012,0.89630225036177
"8195","SLC39A10",266.492712474446,0.140004084524867,0.180078185073442
,0.777462769672898,0.436885791623879,0.89630225036177
"8196","ANKRD44-
IT1",2.40142737091975,-0.978632364625013,1.25764328546626,-0.778147807
040689,0.436481881860667,0.89630225036177
"8197","TATDN2",82.734999459578,0.173497697287409,0.222085603634065,0.
78121991902404,0.434673160260349,0.89630225036177
"8198","ZBTB47",162.208793221809,-0.154546967687298,0.197990184026845,
-0.780578938531331,0.435050183076386,0.89630225036177
"8199","AC099661.1",4.57522453439572,0.571130918009149,0.7304959992066
63,0.78183989868447,0.434308669641657,0.89630225036177
"8200","HS3ST1",3.37089345372143,0.648118122876503,0.830458859284832,0
.780433751329529,0.435135607967761,0.89630225036177
"8201","CAST",2967.08138292668,-0.156903356974034,0.200806022174052,-0
.781367786061889,0.434586212100084,0.89630225036177
"8202","GMDS-
DT",95.2393008995067,0.172030447792532,0.220789411407941,0.77916076996
4101,0.43588501606943,0.89630225036177
"8203","HSPA1A",1648.53851553769,-0.26369394330125,0.337435080607963,-
0.781465705421462,0.434528639494309,0.89630225036177
"8204","XPO5",47.6549338337176,0.201312244092232,0.258849489036682,0.7
77719302600992,0.436734510210436,0.89630225036177
"8205","ZNF3",106.203013411776,0.17021613066091,0.217695074749335,0.78
1901615628673,0.434272395460603,0.89630225036177
"8206","LY6E-
DT",19.3935715558876,0.346329937360903,0.443204361080188,0.78142267489
6113,0.434553939152663,0.89630225036177
"8207","FSD1L",33.006465521654,-0.249805543043846,0.320467774951342,-0
.779502847304303,0.435683560982296,0.89630225036177
"8208","NAIF1",32.6883475922536,0.230386120748345,0.296165968689523,0.
777895319194703,0.436630727975042,0.89630225036177
"8209","IER5L",694.018817606616,-0.188207827982817,0.242055796252514,-

0.777539025698347,0.436840819120862,0.89630225036177
"8210","GPSM1",60.2122417387292,0.201254099769949,0.258664280868485,0.
778051376456862,0.436538725968503,0.89630225036177
"8211","ENTR1",139.467212085646,-0.139521395387882,0.17901386768399,-0
.779388754586189,0.435750746129777,0.89630225036177
"8212","PI4K2A",102.484283721084,0.193923671294875,0.248877271874989,0
.779193977151451,0.435865457445781,0.89630225036177
"8213","LINC02749",3.28207296643496,0.629138416029307,0.80644793216194
1,0.780135196506364,0.435311301343778,0.89630225036177
"8214","CAPRIN1",556.636357856763,0.138641584885973,0.178284049457795,
0.77764435633819,0.436778704054025,0.89630225036177
"8215","SSRP1",310.497572964312,-0.110910556475671,0.142592304078437,-
0.777815865957684,0.436677573131569,0.89630225036177
"8216","ROM1",54.8021625925042,-0.196233809393784,0.251326269835227,-0
.78079306839845,0.434924211535525,0.89630225036177
"8217","BATF2",7.49532497380887,0.452501211725036,0.580585430986544,0.
779387817148866,0.435751298178052,0.89630225036177
"8218","P4HA3",16.6104317111887,-0.431546074082837,0.551535451499277,-
0.782444850842744,0.433953184066608,0.89630225036177
"8219","GALNT6",2.32158996830377,-0.764720795487354,0.983668956915077,
-0.777416823120682,0.436912890198824,0.89630225036177
"8220","PPP1R12A",846.519535826862,0.114458944300839,0.146875082076912
,0.77929450443406,0.435806251201551,0.89630225036177
"8221","MAPKAPK5",122.487667978752,-0.147230763417844,0.18816048391893
,-0.782474408820501,0.433935819342583,0.89630225036177
"8222","MPHOSPH9",46.7816106425255,0.183994346172652,0.236489278089223
,0.778024051066006,0.43655483457418,0.89630225036177
"8223","IPO5",692.117429080158,0.159395791416024,0.204308821804339,0.7
80170870784392,0.43529030561144,0.89630225036177
"8224","SIX4",13.6934654599356,0.346468619369173,0.443177499883733,0.7
81782963846469,0.434342134688329,0.89630225036177
"8225","ZFP36L1",10064.9120178238,0.194709000768264,0.25030956068499,0
.777872807716289,0.436644000315038,0.89630225036177
"8226","CIAO2A",222.148991218034,0.183035573973028,0.23466747279208,0.
779978459712653,0.435403554107796,0.89630225036177
"8227","COX10",43.7604296554963,0.19218663654464,0.247084056584103,0.7
77818849186745,0.436675814185127,0.89630225036177
"8228","SP2",108.864620570947,0.152751328166481,0.196223795102835,0.77
845466237379,0.436301024501681,0.89630225036177
"8229","RNF125",62.1007046715671,0.272822681943251,0.350848273613479,0
.777608734206891,0.43679971039274,0.89630225036177
"8230","MYDGF",1257.55420308877,-0.209197201236758,0.268805799325314,-
0.778246606888057,0.436423645538015,0.89630225036177
"8231","ARHGEF18",60.3830202315056,0.181046185704893,0.231968372382392
,0.780477889487644,0.435109637041926,0.89630225036177
"8232","PIN1",541.557356313333,-0.186690265499246,0.239574350131558,-0
.779258152622467,0.435827660319195,0.89630225036177
"8233","WDR83",90.8647312271123,-0.156715623821267,0.201219825705781,-
0.778827947353525,0.436081072433015,0.89630225036177
"8234","AC006504.7",6.13984881790001,-0.493439021838248,0.632715699831

379,-0.779874787317196,0.435464580215135,0.89630225036177
"8235","ZNF284",22.7677130119201,0.309028233246469,0.397240386405152,0
.777937601065783,0.436605800015358,0.89630225036177
"8236","APOE",9792.80495586755,-0.508600103875263,0.65341097462452,-0.
778377045423101,0.436346767048459,0.89630225036177
"8237","FPR2",2.28260840313045,0.860196322312528,1.10046580177022,0.78
1665655514975,0.434411090644148,0.89630225036177
"8238","PPDPF",2151.54885943013,-0.239401455216204,0.305909355287823,-
0.782589519012771,0.433868198221716,0.89630225036177
"8239","SEPTIN5",26.8942012654188,0.3267695826514,0.419734085992846,0.
778515716393282,0.436265045046362,0.89630225036177
"8240","NAGA",127.750756931134,0.186237818484355,0.238047258439238,0.7
82356493855159,0.434005094433966,0.89630225036177
"8241","RABL2B",106.476290558648,-0.154719039938297,0.197801727352271,
-0.782192562265915,0.434101414929897,0.89630225036177
"8242","TBC1D8B",131.478338432745,0.152029270560886,0.195032362723293,
0.77950791570208,0.435680576521554,0.89630225036177
"8243","JAZF1-
AS1",3.15256751659521,0.638584832463602,0.821573753635285,0.7772702446
22596,0.436999346428472,0.896370853986728
"8244","ZSCAN23",4.60203544889337,0.549845266515978,0.707738247495936,
0.776904835172321,0.43721491835707,0.896378057424681
"8245","THSD7A",26.5266610837901,0.385314093035666,0.495791955252117,0
.777168909164186,0.437059122766381,0.896378057424681
"8246","WASF3-
AS1",9.67111499013945,0.483568541543786,0.622370633523072,0.7769784040
20181,0.437171511771081,0.896378057424681
"8247","TMEM185A",175.526413678753,0.128879109212113,0.165856257450121
,0.77705304094946,0.437127477538437,0.896378057424681
"8248","ZNF493",168.353061815367,0.190939075095885,0.245817598490046,0
.776751039261399,0.437305667977875,0.896455411514295
"8249","ZNF346",102.328830589992,0.181496409381524,0.233713288937601,0
.776577190824529,0.437408262951365,0.896557026303998
"8250","BUD23",314.431172588023,-0.190532613403258,0.245383099041,-0.7
76469993849994,0.437471531113075,0.896578017946652
"8251","MAP3K10",36.695680507563,-0.20023902830407,0.257951017035289,-
0.776267644165466,0.437590973207375,0.896714116469554
"8252","ZNF875",65.3845970842422,0.210895426620188,0.271713760150333,0
.776167635027037,0.437650013096438,0.8967264204356
"8253","AC006460.2",10.5124373319622,-0.486935990546117,0.627808825465
518,-0.775611891382789,0.437978177035042,0.897181368707112
"8254","FAM86B1",26.5488814632688,0.313132875350707,0.403689453751775,
0.775677621598828,0.437939356281755,0.897181368707112
"8255","AC073225.1",3.09274837478273,1.02548080192009,1.32237326701013
,0.775485127764793,0.438053050157337,0.897226041436735
"8256","CDKN2A",29.8230595607194,0.772167620156495,0.996215532739639,0
.775100964379664,0.438280001792871,0.897364769958085
"8257","MRPL52",371.599983151857,-0.2107571673627,0.271879519419739,-0
.775185890472775,0.438229824310579,0.897364769958085
"8258","FARSA",113.51905754362,-0.177501237204488,0.228973652921934,-0

.775203762264319,0.438219265414695,0.897364769958085
"8259","RAB36",16.3053629626362,0.376205743980325,0.485473047215982,0.774926118221667,0.438383317781462,0.897467627684824
"8260","AC026401.1",4.63218875812209,-0.647662806048775,0.836116001085225,-0.774608792569632,0.438570860167881,0.897742869699581
"8261","ZFP69B",7.68267345626004,0.423284188886989,0.546856511041383,0.774031542718447,0.438912138364733,0.897888201561433
"8262","AL139220.2",5.37304822891555,-0.489768171131197,0.632998289181983,-0.773727479997014,0.439091965835839,0.897888201561433
"8263","AC093326.1",2.1268696281547,-0.980595989941953,1.27214450524782,-0.770821228167732,0.440812902794848,0.897888201561433
"8264","RPS27A",30060.7653035165,-0.194014297003578,0.251797328950681,-0.770517692987837,0.440992863847772,0.897888201561433
"8265","ZNF142",37.8028876980603,0.208759743108991,0.270038653568483,0.773073559471177,0.439478848046769,0.897888201561433
"8266","GNL3",396.203683321242,-0.140770351265088,0.182368986339881,-0.771898523374665,0.440174531973273,0.897888201561433
"8267","EBLN2",20.6038797513703,0.353297395646271,0.456385661013045,0.774120279901108,0.438859665777452,0.897888201561433
"8268","ZBTB20-AS4",6.14942055917956,0.436404217244464,0.566982936460854,0.76969550436302,0.441480537476511,0.897888201561433
"8269","LINC00887",14.8674485034769,-0.413123235785718,0.535107804844156,-0.77203739516757,0.440092279575929,0.897888201561433
"8270","PDS5A",307.976419517321,0.146074901843675,0.189745077746892,0.769848175131743,0.441389958868656,0.897888201561433
"8271","C4orf47",4.67833984279133,-0.561924220571275,0.729321194610101,-0.770475648759504,0.441017794503679,0.897888201561433
"8272","MEF2C-AS1",4.03792918558932,-0.664708398343779,0.862770684932265,-0.770434612525071,0.441042128235688,0.897888201561433
"8273","SLC25A48",16.9359141803473,-0.34100014994752,0.441068320586926,-0.773123196637098,0.439449474104549,0.897888201561433
"8274","ECI2",712.651196108238,-0.190963399806978,0.247679842502024,-0.771009048931458,0.440701568015212,0.897888201561433
"8275","TDP2",177.287880028559,0.133123320820048,0.172531458723326,0.771588681885121,0.440358079822879,0.897888201561433
"8276","ZFAND3",305.623091067869,0.106850121400226,0.138732935316544,0.770185689190739,0.441189751680073,0.897888201561433
"8277","CHST12",376.876118541163,-0.122365719972253,0.158936988699007,-0.769900832863948,0.441358719835998,0.897888201561433
"8278","AC004854.2",11.0388502076083,-0.424321545728292,0.550161636419754,-0.771267056150294,0.440548654995257,0.897888201561433
"8279","AC009812.1",21.2455537453376,-0.287546587941929,0.373076550554238,-0.770744201196117,0.440858566842821,0.897888201561433
"8280","RNF183",2.06655782910367,-0.871007201936353,1.12816366191041,-0.772057487174693,0.440080380007868,0.897888201561433
"8281","MRPS2",249.79125190435,-0.200966924483972,0.260471644702769,-0.77155010371014,0.440380936326174,0.897888201561433
"8282","OLMALINC",34.9184075667966,0.227497268890206,0.29406598662369

,0.773626598241455,0.439151638239346,0.897888201561433
"8283","TACC2",80.3871269739156,-0.191515141119253,0.247886293070484,-0.772592702674357,0.43976346453687,0.897888201561433
"8284","DUSP8",28.443889489277,0.351556768093779,0.456164381146518,0.770680006207808,0.440896625752644,0.897888201561433
"8285","BBOX1-AS1",7.21192070510965,-0.53983799289396,0.698285964532454,-0.773090138300883,0.439469037015058,0.897888201561433
"8286","KBTBD4",73.5924216625763,-0.178797985011344,0.231155524259746,-0.773496482871991,0.439228609447579,0.897888201561433
"8287","KCTD14",5.6330427445902,-0.501311935864067,0.649026687728015,-0.77240573514621,0.439874158143128,0.897888201561433
"8288","MCAM",7.7266895331005,0.473744028282798,0.61414300616878,0.77139041481261,0.440475554824403,0.897888201561433
"8289","AP000842.1",5.70238757727327,0.910087962495819,1.17988789866515,0.771334262793472,0.440508828659612,0.897888201561433
"8290","AC092794.1",3.55271925135397,-0.657228007829596,0.853791227111449,-0.769776014275918,0.441432770124391,0.897888201561433
"8291","IRAK4",89.8674517201909,-0.171559593087178,0.222362549591556,-0.771530967792484,0.440392274081354,0.897888201561433
"8292","HELB",66.9994093274099,0.341633153265948,0.441563017585682,0.773690593777268,0.439113783803678,0.897888201561433
"8293","UBE2N",462.715435902348,-0.172629080538012,0.224296524325412,-0.769646703430677,0.441509493008147,0.897888201561433
"8294","UBE3A",508.683815257606,0.113080161089867,0.146340893365258,0.772717444109256,0.43968962060849,0.897888201561433
"8295","AC078909.1",10.8900327270001,0.359425958130803,0.466828303523638,0.76993180451537,0.441340346593498,0.897888201561433
"8296","AC051619.6",5.26626290755694,-0.762409245910168,0.984535293230879,-0.774384881021607,0.438703221701591,0.897888201561433
"8297","AC068870.2",8.48577641814862,-0.519946558104343,0.675435206254078,-0.769794871943284,0.441421582105978,0.897888201561433
"8298","LMF1",124.872367350672,-0.187664246006231,0.243501653779175,-0.770689821172299,0.44089080668972,0.897888201561433
"8299","SOX8",4.5896000322313,0.534431051979033,0.69209555958889,0.772192574528999,0.440000378798351,0.897888201561433
"8300","TSC2",201.036916554254,0.163119841469396,0.211787914070936,0.7702037303921,0.441179051433253,0.897888201561433
"8301","APOBR",9.34295122275413,0.59048474825102,0.767191922152041,0.769670184475689,0.441495560635669,0.897888201561433
"8302","FAM192A",211.256905619001,-0.123596063976127,0.160249452080389,-0.771272927124427,0.440545175801741,0.897888201561433
"8303","KATNB1",53.7918414074448,-0.225399195187354,0.291321477996503,-0.773712933002696,0.439100570217462,0.897888201561433
"8304","CENPBD1",30.2703667118058,-0.248584049873538,0.322376065685867,-0.771099583167462,0.440647907666387,0.897888201561433
"8305","NLGN2",114.018368007054,0.137670851650476,0.178580703283847,0.770916729069283,0.440756290575929,0.897888201561433
"8306","UBL5",2008.65766980167,-0.191255171042523,0.247374461945472,-0.773140321512577,0.439439340324508,0.897888201561433

"8307","LINC00663",16.5511782710415,0.317626007308555,0.411515715195407,0.771844174062056,0.440206724937417,0.897888201561433
"8308","PRRG2",3.69838858230815,0.678691528140703,0.879434048046917,0.771736697763713,0.440270390815387,0.897888201561433
"8309","LILRB3",8.86624635143635,-0.538085581256748,0.69834945780951,-0.770510487606796,0.440997136312068,0.897888201561433
"8310","PIGU",87.5451274384344,0.159863631882264,0.207645821325818,0.769886101543173,0.441367459017748,0.897888201561433
"8311","ZNF335",112.486120055281,0.221400836468828,0.285936012906193,0.774302034285771,0.438752200969108,0.897888201561433
"8312","UBE2L3",960.630094402936,-0.166244517508726,0.215448809206685,-0.771619569961251,0.440339779981217,0.897888201561433
"8313","SLC38A5",6.26513081680338,-0.538048294487288,0.698950819536645,-0.769794210762893,0.441421974373295,0.897888201561433
"8314","PRPS1",138.847889627755,-0.151699462015902,0.196851997019144,-0.770626990393952,0.440928058349905,0.897888201561433
"8315","ZFR",545.409033410808,0.121302447111884,0.1576577232358,0.769403773074022,0.441653649420118,0.898073349897217
"8316","CDK2AP2",115.272490876376,-0.20060584627837,0.260807287183186,-0.769172703895612,0.441790792339446,0.89813619296325
"8317","AC004148.1",74.7963597403066,-0.283197559133185,0.368178604016422,-0.76918527052852,0.441783333233135,0.89813619296325
"8318","CD34",2520.14626682365,-0.166068028343294,0.21628637024523,-0.767815503838739,0.442596802297155,0.898154912774879
"8319","IAH1",818.911175217322,-0.197709060165989,0.257466339972013,-0.767902554514429,0.442545079670889,0.898154912774879
"8320","AC012511.1",20.2911467890107,0.338174111954652,0.440262289503794,0.768119641443281,0.442416108874989,0.898154912774879
"8321","CASP8",80.0648508334968,-0.176933069757179,0.230269455744719,-0.768374030263614,0.442265004483681,0.898154912774879
"8322","NISCH",233.193095717032,0.150677909720642,0.196107003543622,0.768345377767834,0.442282022301159,0.898154912774879
"8323","FAM193B",91.2075235307932,0.174916899152041,0.227490253700987,0.768898431059608,0.441953608897581,0.898154912774879
"8324","ELOVL2",32.1198598955125,0.337937332765011,0.440031675194049,0.767984106180503,0.442496627510526,0.898154912774879
"8325","GOPC",652.392634915543,0.10918080582418,0.142005657696143,0.768848281086096,0.441983383135335,0.898154912774879
"8326","GVQW2",2.90980031765195,-0.867354877996116,1.12925896501481,-0.768074378745128,0.442442997555302,0.898154912774879
"8327","AC136475.5",2.74011350718634,-0.66837565952724,0.87030735275378,-0.767976574496817,0.442501102170474,0.898154912774879
"8328","CIPC",35.3724209014861,0.213306690034061,0.277484537026366,0.768715591578327,0.442062166958433,0.898154912774879
"8329","AL049775.3",7.25629114966393,-0.47178199818392,0.613531999423038,-0.76896070396912,0.441916638822667,0.898154912774879
"8330","ZNF225",26.2933246222317,0.279536740376625,0.363737697400016,0.768511876483364,0.442183137557718,0.898154912774879
"8331","AL157700.1",11.0329223619518,0.363260604965305,0.47308637171785,0.767852609336957,0.44257475501719,0.898154912774879

"8332","GRIA3",91.550294808532,-0.260798821684613,0.339647991706252,-0
.767850327553733,0.442576110785025,0.898154912774879
"8333","ZBTB41",172.990049359913,0.132231106495145,0.172279806475603,0
.767536887812043,0.442762369945185,0.898186860698205
"8334","DSTYK",220.607833163558,0.195288553166681,0.254440719828552,0.
767520832743559,0.442771911752989,0.898186860698205
"8335","RING1",264.872857170226,-0.125585950999737,0.163603436846822,-
0.767624161326877,0.442710503826795,0.898186860698205
"8336","ERAP1",188.11954432487,0.178213186995609,0.232242010054386,0.7
67359819844285,0.442867611035503,0.898273220655984
"8337","DGCR2",157.715107248514,0.122609391493244,0.159813164568768,0.
767204578071442,0.442959891400348,0.898352626100166
"8338","JARID2",86.7419975286486,0.189734902546822,0.247340183591865,0
.767101001509332,0.4430214665279,0.898369747667755
"8339","CIART",6.22644947559263,0.756153147874863,0.986991971496423,0.
766118843629928,0.443605591710047,0.898658486195155
"8340","AC009403.1",21.5536185213562,-0.294234494411371,0.384024327415
19,-0.766187122549813,0.443564969517593,0.898658486195155
"8341","IMPAD1",501.721180626055,-0.170102872755351,0.22185578600998,-
0.766727232201637,0.443243708954195,0.898658486195155
"8342","PLAU",331.77712043216,0.279055085962687,0.36414504090585,0.766
32949680849,0.443480271519734,0.898658486195155
"8343","RELA",282.346380481731,-0.113448093224924,0.148088852266447,-0
.766081251145111,0.443627958072736,0.898658486195155
"8344","HOXB3",107.06160620355,0.239629959385541,0.312705356981716,0.7
66312293778685,0.443490505057162,0.898658486195155
"8345","ZNF791",342.756303923911,0.195356754039268,0.254958143022012,0
.766230690746761,0.443539049953359,0.898658486195155
"8346","U2AF1L4",92.3496140774385,-0.140121008852412,0.182911914580858
,-0.766057307822176,0.443642203943161,0.898658486195155
"8347","SNHG11",78.3468161211546,-0.236362971978566,0.30851339237213,-
0.766135207814459,0.443595855728949,0.898658486195155
"8348","TMEM164",30.4243830048612,0.246772748546127,0.322184968474767,
0.765935014641918,0.443714970383628,0.898698217446859
"8349","GNG12-
AS1",9.74578967449294,-0.443794402372121,0.580040861152839,-0.76510886
0589706,0.444206724081527,0.898709250678215
"8350","ZNF670",38.0536236764949,-0.244256894402357,0.319139679795412,
-0.765360467112523,0.444056926780953,0.898709250678215
"8351","TRAPPC13",65.0144718048254,-0.165672245288206,0.21665189592617
2,-0.764693263264411,0.444454218653547,0.898709250678215
"8352","CLEC2L",21.2956979740594,0.355930538315886,0.465249411548037,0
.765031678667983,0.444252681148939,0.898709250678215
"8353","OXR1",556.808957944267,0.102511731104015,0.133918625997478,0.7
65477769357827,0.443987099182085,0.898709250678215
"8354","GTPBP4",212.751492200854,-0.134323766801466,0.175524272096446,
-0.765271749582637,0.444109742654207,0.898709250678215
"8355","DDX21",988.506103042025,-0.166608103294185,0.217840072371517,-
0.764818435287894,0.444379668517086,0.898709250678215
"8356","ITPRIP",49.6498018328554,-0.267586479598935,0.34987097052815,-

0.764814752121327,0.444381862040901,0.898709250678215
"8357","SLC11A2",135.598908818819,0.145318885420186,0.190050992744245,
0.764631025188825,0.444491289153731,0.898709250678215
"8358","GDPD3",29.0567509799209,0.250211990730898,0.326887099394376,0.
765438560268869,0.44401043884367,0.898709250678215
"8359","TANC2",93.1812593712806,0.262870295243994,0.343807061732232,0.
764586666485419,0.444517711345039,0.898709250678215
"8360","PWWP3A",387.215520558731,0.176895061950337,0.230993755878208,0
.765800189177428,0.443795201637776,0.898709250678215
"8361","CYP4F12",61.4217208603179,-0.295475937448523,0.38619822559014,
-0.765088801216044,0.444218667938623,0.898709250678215
"8362","MEIS3",151.742685993607,-0.160934248661096,0.210291731436002,-
0.765290425648873,0.444098624003974,0.898709250678215
"8363","SMDT1",1231.39924326343,-0.208902104960171,0.272917681420832,-
0.765439981288898,0.444009592952905,0.898709250678215
"8364","MCM2",22.7579337474425,0.275349875483677,0.360368207055251,0.7
64079266963362,0.444820006811878,0.899105400499132
"8365","ELANE",204.974329986286,-0.281955526719079,0.368974611362712,-
0.764159695643419,0.444772081673031,0.899105400499132
"8366","INKA2",7.02684943058825,-0.488989837145564,0.640394850190391,-
0.763575530003381,0.445120236173908,0.899376035094481
"8367","VIPR1",3.69934193903936,-0.768985671781111,1.00740387164519,-0
.763334044493279,0.445264203520196,0.899376035094481
"8368","ETFDH",203.399247397326,0.122325303301647,0.160210210567483,0.
763530007659047,0.445147373375029,0.899376035094481
"8369","OARD1",255.804014160376,-0.175744632872768,0.230198304782395,-
0.763448857883196,0.445195751469711,0.899376035094481
"8370","NSMCE1-
DT",7.92479977216844,0.452619383662303,0.592962135568238,0.76331920119
7485,0.445273053570848,0.899376035094481
"8371","AL121672.3",3.14782265867083,-0.660052571998684,0.864457043679
62,-0.763545831252788,0.44513794036096,0.899376035094481
"8372","GSTA4",295.67101653117,0.213530826605275,0.279790623758326,0.7
6318078046002,0.445355589296838,0.899435296683103
"8373","ATG4C",122.350679640469,0.145253368924305,0.190621082947886,0.
762000544105691,0.446059679418472,0.899513047101593
"8374","MIGA1",347.237162189128,0.179531856365816,0.235974161476234,0.
760811502592829,0.446769663015208,0.899513047101593
"8375","ADAMTS4",88.0426913898906,0.788366537685706,1.03908726518241,0
.758710614692513,0.448025685791079,0.899513047101593
"8376","CCDC80",18583.9001162626,-0.179162202257778,0.235728990646108,
-0.760034655757504,0.447233869848766,0.899513047101593
"8377","AC007620.2",14.4365764841618,0.35218314621215,0.46380288939964
7,0.759337973655189,0.447650407227694,0.899513047101593
"8378","AC004067.1",3.67182806737304,0.591813245394554,0.7799107881801
61,0.758821719565499,0.447959211175386,0.899513047101593
"8379","CDK7",75.923069799701,-0.184812180865445,0.243633580657105,-0.
758566123631182,0.448112143917209,0.899513047101593
"8380","TMEM161B",84.3866155377882,0.183957350781023,0.241256970826629
,0.762495484174911,0.445764337329389,0.899513047101593

"8381","AC011405.1",3.23722107410556,-0.613655923261993,0.808999358543243,-0.758536971360517,0.448129588710544,0.899513047101593
"8382","TRIM52-AS1",180.020962926759,-0.189666209216832,0.249849349439523,-0.759122285658549,0.447779409027351,0.899513047101593
"8383","HLA-B",6111.83723133915,0.317004369803901,0.417943986635625,0.758485299323791,0.448160510337344,0.899513047101593
"8384","HLA-DRB1",600.969441642751,-0.240622910210466,0.316550301162614,-0.760141150795673,0.447170217245052,0.899513047101593
"8385","TAPBP",652.298087623195,0.102883503672236,0.135179166812942,0.761089937879288,0.446603350055236,0.899513047101593
"8386","AL451165.2",74.054607741815,-0.241425282829512,0.317050173299171,-0.761473429638222,0.446374343214515,0.899513047101593
"8387","AL590428.1",12.6187127228272,0.390228241226674,0.512164188689709,0.761920200287747,0.446107632928139,0.899513047101593
"8388","SCML4",3.83413787186679,0.742017176374245,0.975515438487338,0.760641141184642,0.446871439439561,0.899513047101593
"8389","NDUFA4",2916.55955954006,-0.215825039841972,0.28351785587904,-0.761239672798782,0.446513926042594,0.899513047101593
"8390","AC004160.2",4.15623634957625,0.560485357165488,0.736197144980428,0.761325089328333,0.446462918533863,0.899513047101593
"8391","TMEM176A",2327.79523545498,0.231738790986278,0.304723828860165,0.760487920662814,0.446962986910601,0.899513047101593
"8392","UBXN2B",43.9143814921289,-0.198684095270345,0.260467276467379,-0.762798682295235,0.445583467099306,0.899513047101593
"8393","MINCR",68.464282645484,0.205606051419154,0.270721416837108,0.759474643052959,0.447568676949858,0.899513047101593
"8394","RCL1",94.9516860998045,-0.155616092934237,0.204042941937334,-0.762663444550952,0.445664136845105,0.899513047101593
"8395","LINC00476",70.1962082435099,0.190460949382482,0.249612415481435,0.763026746947403,0.445447444656564,0.899513047101593
"8396","STX17",358.854503654271,0.114710539425176,0.15093637814285,0.759992659401238,0.447258972692262,0.899513047101593
"8397","COMTD1",157.988052658795,-0.21098326872234,0.276880779516954,-0.762000414367591,0.446059756850778,0.899513047101593
"8398","ACSL5",195.402387917974,-0.177877952136247,0.233855531048575,-0.760631794076745,0.446877023921725,0.899513047101593
"8399","TMEM223",266.774017804233,-0.184445478630247,0.242591275115529,-0.760313735695603,0.447067073349961,0.899513047101593
"8400","NUDT8",133.836615187748,-0.205152482409085,0.268981898828935,-0.762699956027731,0.445642356812755,0.899513047101593
"8401","KLHL35",18.3799820683824,0.32848450037178,0.431912190747947,0.760535375959034,0.44693463178533,0.899513047101593
"8402","KLRF1",6.66135291224265,0.505595944711188,0.664110311523426,0.761313197428576,0.446470019726195,0.899513047101593
"8403","AC008124.1",168.47992178004,-0.147669190969291,0.194435652466796,-0.759475893931072,0.447567928945818,0.899513047101593
"8404","ORAI1",73.9937125054501,0.21707616865267,0.28598473463984,0.75

9048097186196,0.447823785625524,0.899513047101593
"8405","DNAJC15",1083.33141769004,-0.188973966731085,0.248735894504667
,-0.759737419914437,0.447411556419325,0.899513047101593
"8406","SUCLA2",426.642583164097,-0.143102110895766,0.188646936239724,
-0.758571083889314,0.448109175724458,0.899513047101593
"8407","AL358334.2",5.44299000913946,0.606654339862952,0.7972668399280
85,0.760917561700764,0.446706308397903,0.899513047101593
"8408","EIF3C",50.337462282652,0.262641370471715,0.345866719018977,0.7
59371619266163,0.44763028587831,0.899513047101593
"8409","MT1A",1951.42699360082,-0.594238262398497,0.782620348120224,-0
.759293141081494,0.447677219603269,0.899513047101593
"8410","ATMIN",293.081979415729,0.108059988072823,0.141782296436246,0.
762154308323069,0.445967912853221,0.899513047101593
"8411","AC005726.1",8.01127673583951,0.423282338803175,0.5558372178513
5,0.761522124120115,0.446345269487032,0.899513047101593
"8412","FMNL1",147.503436723142,0.169090134525928,0.222191243904888,0.
761011692244315,0.446650083614503,0.899513047101593
"8413","KRI1",83.2144060317758,0.171145840218299,0.224889515055062,0.7
61021874125147,0.446644002152508,0.899513047101593
"8414","AKAP8L",219.786787792236,-0.115152326086223,0.151652061682651,
-0.759319225921188,0.447661619305558,0.899513047101593
"8415","COLGALT1",266.262094320023,0.144916872695118,0.190910902339436
,0.759081178284195,0.447803997517606,0.899513047101593
"8416","CLIP3",157.381946232846,0.191194753859159,0.251704018861286,0.
759601514207552,0.4474928137557,0.899513047101593
"8417","ZNF880",65.086564404276,0.275931704356166,0.362528183809847,0.
761131731763225,0.446578389075189,0.899513047101593
"8418","ZNF324B",16.2764110184334,0.289747317036503,0.381742700754539,
0.759012068767257,0.447845337296562,0.899513047101593
"8419","CTSA",731.00377398451,0.180279445151779,0.236529135534043,0.76
2187054650741,0.445948371216667,0.899513047101593
"8420","CSE1L",168.027594076114,0.143716049307547,0.18935384228857,0.7
58981426363281,0.447863667596344,0.899513047101593
"8421","BID",113.213617824075,-0.220798086760459,0.290566624998336,-0.
759887983562199,0.447321544969762,0.899513047101593
"8422","Z82215.1",5.8270084140064,-0.576741145607198,0.759019639245157
,-0.759850095816711,0.447344194427039,0.899513047101593
"8423","TSPO",3023.37845228983,-0.214074447654373,0.2815708480563,-0.7
60286262346197,0.447083491657496,0.899513047101593
"8424","SH3BGRL",1525.32525100301,-0.169618432015715,0.222513608518404
,-0.762283408844567,0.445890873899207,0.899513047101593
"8425","RBBP4",511.293275136731,0.106179356615252,0.140140091599493,0.
757665814281772,0.448651069312226,0.899673835219914
"8426","POLR3G",36.325061656173,0.244440074595695,0.322746923715999,0.
75737383266522,0.44882592855926,0.899673835219914
"8427","FOXK1",171.140594326774,0.151927983670703,0.200533457936664,0.
757619128667736,0.448679025362855,0.899673835219914
"8428","UBE4A",229.843194985318,0.121656463173644,0.160615344498723,0.
757439854537751,0.448786386607705,0.899673835219914
"8429","SDSL",16.473091325138,0.500240895773371,0.660377967671458,0.75

7506943390703,0.448746207643648,0.899673835219914
"8430","SETDB2",96.1932901901356,0.211846201296307,0.27965967803624,0.757514285877333,0.448741810412559,0.899673835219914
"8431","TSPAN10",35.2491734234102,0.237452287612933,0.313336789291341,0.757818091357762,0.448559890372787,0.899673835219914
"8432","AC018529.1",4.09355003155636,-0.68192650969438,0.899640187730394,-0.757999163437484,0.448451483543749,0.899673835219914
"8433","PPP6R2",183.365601370863,0.198329869277245,0.261748646601707,0.757711154774512,0.448623919687061,0.899673835219914
"8434","PHKA1",10.3282535866021,-0.358525853075154,0.472845160388521,-0.758230987878918,0.448312713281015,0.899673835219914
"8435","RTL8C",804.961457535849,-0.137151281765256,0.180985822614166,-0.757801245336448,0.448569976746804,0.899673835219914
"8436","AC233992.3",3.39147176757281,-0.562305751242145,0.742562696342258,-0.757250201244919,0.448899979454021,0.899715606046538
"8437","AL008721.2",8.46794103364361,0.491364061601569,0.649153398558381,0.756930584809038,0.449091450643921,0.899943470790891
"8438","SYAP1",554.905559308822,-0.115604349024468,0.152737463802483,-0.756882732935551,0.449120121039362,0.899943470790891
"8439","C1QTNF12",3.02197238224855,0.736534769075804,0.973930389146287,0.756249909935991,0.449499373877725,0.900033739296134
"8440","HMGN2",2071.060442053,0.172009027589469,0.227440322225658,0.756282025571562,0.449480122501675,0.900033739296134
"8441","SF3A3",335.297757185967,-0.123928950149431,0.164900237293133,-0.751538943689513,0.452328372710364,0.900033739296134
"8442","SZT2",108.709027572582,0.222960517856378,0.294846504678533,0.756191829709727,0.449534190638823,0.900033739296134
"8443","HAX1",544.27022689795,-0.173256668908678,0.229468065559584,-0.75503608088634,0.450227333511605,0.900033739296134
"8444","ARHGEF2",146.93521093038,-0.172911345156434,0.228614835187108,-0.75634350244557,0.449443272147844,0.900033739296134
"8445","TOR1AIP2",417.043694397205,0.149962480026084,0.199784670329875,0.75062055451238,0.45288104735277,0.900033739296134
"8446","AL356275.1",3.30041483658202,0.632430686025732,0.84039332780511,0.752541298343571,0.451725603961242,0.900033739296134
"8447","LINC01118",9.46731635118343,0.423540203424058,0.560927497467968,0.755071208553553,0.450206257306083,0.900033739296134
"8448","PPP3R1",157.339927853091,0.141663352043398,0.18735202796611,0.75613460703518,0.449568494827613,0.900033739296134
"8449","AC007878.1",14.6411023678232,0.394391665629511,0.5238370678915,0.752889953391384,0.451516045810467,0.900033739296134
"8450","EIF2AK3",96.5418390347825,0.140122800472577,0.185588388890059,0.75501921920118,0.450237450531755,0.900033739296134
"8451","MMADHC",432.14134401047,-0.139670919409928,0.185628418839875,-0.752422071376957,0.451797277617443,0.900033739296134
"8452","TTC21B",132.366370127047,0.131342408717969,0.174739094477171,0.751648674333311,0.452262363737164,0.900033739296134
"8453","CTLA4",7.02159574435698,0.485977665884963,0.64544340445227,0.752936140539493,0.451488289278489,0.900033739296134
"8454","MAP2",3.54414780126634,-0.628395512816381,0.832693031455492,-0

.754654463383687,0.450456335745229,0.900033739296134
"8455","COPS7B",85.5134668399035,0.200688147282643,0.265739300960287,0
.755206875902161,0.450124863670432,0.900033739296134
"8456","EIF1B",1443.88041810453,-0.175260518485877,0.233527915916117,-
0.750490654611208,0.452959250191842,0.900033739296134
"8457","ZNF621",149.040374844303,0.205185154129854,0.272937378615832,0
.751766413125328,0.452191543480189,0.900033739296134
"8458","ABHD14B",619.205075124828,-0.162827406962867,0.215892980233671
,-0.754204267256082,0.450726575659824,0.900033739296134
"8459","U2SURP",868.835061493442,0.100808345794927,0.134319362720992,0
.750512388927319,0.452946165083977,0.900033739296134
"8460","PLOD2",144.838112315337,0.183651811023764,0.243000903803767,0.
75576595868166,0.449789529913378,0.900033739296134
"8461","IFT80",113.752657874702,0.146335343316974,0.1943147751061,0.75
3083975405739,0.45139945323565,0.900033739296134
"8462","PDCD10",441.218148094397,-0.157291604882531,0.208186761145457,
-0.755531254807476,0.449930286328994,0.900033739296134
"8463","TPRG1",71.2796400933995,-0.206154803455893,0.274636497025946,-
0.7506460564723,0.452865695459961,0.900033739296134
"8464","EVC2",127.679412984114,0.211677345157837,0.280033219088849,0.7
55900838645419,0.449708651107594,0.900033739296134
"8465","HHIP-
AS1",37.6372415974215,0.512863155799113,0.683541307431727,0.7503030910
10104,0.453072181405778,0.900033739296134
"8466","PLRG1",311.454531512039,-0.175272874265018,0.232161379076886,-
0.754961376271686,0.450272157299273,0.900033739296134
"8467","AC010457.1",10.219995288276,-0.605095860671026,0.8030440884650
38,-0.753502664875628,0.451147910536192,0.900033739296134
"8468","CEP120",103.388150857054,0.206204953398009,0.274749796614854,0
.750519039280924,0.452942161292603,0.900033739296134
"8469","CTNNA1",800.952698353339,0.102507302498581,0.13609778446954,0.
753188620212427,0.45133657668266,0.900033739296134
"8470","IK",691.413000276135,-0.143656976973,0.190341536655793,-0.7547
32674207547,0.450409397373271,0.900033739296134
"8471","FAM153CP",45.9440026230298,0.298711785461479,0.396788851910769
,0.752823029233327,0.451556266125134,0.900033739296134
"8472","AL590062.1",23.3457631228849,-0.318644252616989,0.423281592213
948,-0.752794967884949,0.451573131135448,0.900033739296134
"8473","TBP",64.0079830348657,0.211582140016035,0.280445984645654,0.75
4448812249429,0.450579770877051,0.900033739296134
"8474","GARS",274.420592646651,-0.15269372745323,0.20345786613942,-0.7
50493113638556,0.452957769727922,0.900033739296134
"8475","KIAA1324L",65.2301552333852,-0.233939186689756,0.3102731944447
86,-0.753978077636951,0.450862385485761,0.900033739296134
"8476","AC083880.1",5.70295040011236,-0.544832901924467,0.722624303940
753,-0.753964264630016,0.450870679906293,0.900033739296134
"8477","ELP3",116.099764338721,0.16516639896101,0.219249078612995,0.75
3327676475904,0.451253031436738,0.900033739296134
"8478","DCTN6",501.015117853609,-0.182152357675624,0.241418258677537,-
0.754509450417856,0.450543372877916,0.900033739296134

"8479","AC024451.4",5.0394606872956,-0.483882843740916,0.645130363901453,-0.750054362368892,0.453221964332851,0.900033739296134
"8480","TOX",3.14146419111848,0.721952882627,0.958362036408099,0.753319575692761,0.451257898161346,0.900033739296134
"8481","AL162253.2",57.3901907634359,-0.191223892185837,0.253341206914753,-0.754807693997377,0.450364376709694,0.900033739296134
"8482","CLTA",2154.50314057637,-0.187239847081084,0.24746169654306８,-0.756641733636932,0.44926453127829,0.900033739296134
"8483","NR4A3",63.4221210149166,-0.676576610935283,0.900744470026444,-0.751130463132812,0.452574143915419,0.900033739296134
"8484","AL161908.1",4.56970161217437,0.588450221185133,0.784616397379021,0.749984607957248,0.453263975047704,0.900033739296134
"8485","GDI2",1270.01821958305,-0.133929627923075,0.178525111820111,-0.750200498728873,0.453133958488599,0.900033739296134
"8486","SEPHS1",215.939207071058,-0.172345240039927,0.228348309432023,-0.754747168781786,0.450400698731791,0.900033739296134
"8487","RUFY2",250.563176411778,0.136553867319086,0.181862299059827,0.75086407696938,0.452734461571013,0.900033739296134
"8488","UROS",327.630276444191,-0.178213656894374,0.236035193569506,-0.755030019885127,0.450230970100759,0.900033739296134
"8489","TALDO1",1046.74742541256,-0.190703060867186,0.25407375934037,-0.750581490045616,0.452904564291897,0.900033739296134
"8490","SIK2",369.184666625302,0.18983372841741,0.252736538651746,0.751113113402998,0.452584584427285,0.900033739296134
"8491","LDHB",1301.70206172494,-0.192921036597782,0.256350037202567,-0.752568787206129,0.451709079859514,0.900033739296134
"8492","IFT88",109.999002486627,0.136726400260075,0.182240819153365,0.750251238417734,0.453103404426496,0.900033739296134
"8493","PCOTH",7.60425498413402,-0.498422658327442,0.661935621638344,-0.752977543486488,0.451463408670458,0.900033739296134
"8494","TGDS",84.4459929001213,-0.176451266004885,0.233232412836645,-0.756546930415159,0.449321345947277,0.900033739296134
"8495","AL162311.3",3.02015255957663,-0.643052515894815,0.85246778672299,-0.754342305844544,0.450643705269559,0.900033739296134
"8496","NPC2",2271.03387023679,-0.152402146979667,0.202160190835917,-0.753868238595816,0.45092834391046,0.900033739296134
"8497","LINC00524",4.42150578608378,-0.687025347531548,0.914874242427973,-0.750950584976861,0.45268239543697,0.900033739296134
"8498","RPUSD2",51.5706507891739,-0.184095561855142,0.244926214806892,-0.75163682254384,0.452269492973359,0.900033739296134
"8499","RMDN3",138.907721162363,-0.120827833690308,0.160733918634527,-0.75172580073185,0.452215971255585,0.900033739296134
"8500","RPLP1",50111.9188616255,-0.187718788535974,0.249066599289921,-0.753689130020455,0.451035910455807,0.900033739296134
"8501","ADORA2B",3.41441924498115,-0.664682531237152,0.885129770242217,-0.750943594468934,0.452686602652775,0.900033739296134
"8502","MIDN",947.646677242029,-0.289216981877676,0.385490083822648,-0.750257902900391,0.453099391342522,0.900033739296134
"8503","AC119403.1",8.05943991892004,-0.506515372593522,0.671140517132832,-0.754708380232202,0.450423977151294,0.900033739296134

"8504","TLE2",243.353407016259,-0.149232712300975,0.198253924440021,-0
.752735224397154,0.451609038456234,0.900033739296134
"8505","ZNF709",26.2929873503425,0.25227915768434,0.33445693214978,0.7
54294898487445,0.450672164933949,0.900033739296134
"8506","ZNF333",91.333393565528,0.175911587028105,0.234021864316651,0.
751688683199624,0.452238297574059,0.900033739296134
"8507","RPL28",26034.0505064823,-0.179287287639924,0.23774232251783,-0
.754124405537757,0.45077452395959,0.900033739296134
"8508","SOX12",36.1398775034864,0.212487002271923,0.282467394483123,0.
752253203102417,0.451898804301095,0.900033739296134
"8509","GSS",142.047154362879,-0.210821107438955,0.279468449424557,-0.
754364608502496,0.450630316852425,0.900033739296134
"8510","ADNP",356.552603148602,0.114253797030791,0.151727922211591,0.7
53017607869558,0.451439333194923,0.900033739296134
"8511","DPM1",325.290175045423,0.151482944344041,0.201946465760299,0.7
50114362109431,0.453185830283725,0.900033739296134
"8512","RPS21",12747.9019867292,-0.214387775633444,0.284818343054663,-
0.75271758600287З,0.451619639878265,0.900033739296134
"8513","MRPL39",154.196052541339,-0.233991167475496,0.311924807006118,
-0.750152479763839,0.453162875275918,0.900033739296134
"8514","PARVG",17.2336594548475,-0.315362433336528,0.418012189708471,-
0.754433581366294,0.450588913460874,0.900033739296134
"8515","MORF4L2-
AS1",5.07801230330722,0.503372259566031,0.669552942229263,0.7518035211
52582,0.452169224183131,0.900033739296134
"8516","PEBP4",19.8810315136045,0.293861085081411,0.391973179755505,0.
749696918714458,0.453437263739109,0.900092824088428
"8517","SCX",8.58809651340206,0.457689429746277,0.610549593136417,0.74
9635140030327,0.453474480806522,0.900092824088428
"8518","PPFIA2",3.25492739720743,-0.645133004819112,0.860657345020004,
-0.74958171048214,0.453506669529768,0.900092824088428
"8519","LYRM9",163.243600505076,-0.176140425047895,0.234918129334205,-
0.749794941527521,0.453378215813936,0.900092824088428
"8520","SRPK1",163.099494597781,-0.150929221058371,0.201421579739233,-
0.749320014537515,0.453664347322066,0.900192316999826
"8521","HACE1",52.2182203044046,0.200944865078101,0.268179873131607,0.
749291371987075,0.453681606991376,0.900192316999826
"8522","AC037459.2",13.1268584211403,0.396417114138063,0.5290969351451
4,0.749233434945752,0.453716520314201,0.900192316999826
"8523","PAXIP1",59.0014215347407,0.171341925910956,0.228727282130148,0
.749110138131492,0.453790825001565,0.900234104086174
"8524","TNNI3K",15.2162372518315,0.33503559070043,0.447349710974845,0.
748934407424418,0.453896740777759,0.900338584358324
"8525","SAFB2",347.406264270337,0.175792804238251,0.234776384285349,0.
748766980006774,0.453997664993496,0.900433140141939
"8526","ATAD1",312.202409418941,-0.139828046844593,0.18678419056719,-0
.748607504842838,0.454093807405587,0.900507338617979
"8527","THAP7",234.771149561365,-0.173246814787432,0.231449936225977,-
0.748528245945517,0.454141594298291,0.900507338617979
"8528","EIF2S1",347.899831420788,-0.142629767774002,0.190599855604973,

-0.748320439809822,0.454266898544944,0.90065017830651
"8529","PFN2",225.839363368634,-0.132089809798903,0.176653839323821,-0
.747732459733135,0.454621548024575,0.90073001459399
"8530","RFC4",73.8214831747909,0.18523419570955,0.247859816897948,0.74
7334513628792,0.454861664007184,0.90073001459399
"8531","ATP10D",151.191491307878,0.132065813761737,0.176668525853165,0
.747534475221133,0.454741000613973,0.90073001459399
"8532","MRPL14",432.616319799372,-0.191075150511819,0.255611788517199,
-0.747520885559478,0.454749200491038,0.90073001459399
"8533","ATP6V1B2",349.719015125372,-0.11699182330583,0.15638693631679,
-0.748092046952323,0.45440463878053,0.90073001459399
"8534","NDUFB6",685.423786997227,-0.202653013803737,0.271083887431572,
-0.747565691652891,0.454722165221115,0.90073001459399
"8535","IGHMBP2",39.9785740013796,-0.218053038692067,0.291837608210026
,-0.747172511553551,0.454959434604032,0.90073001459399
"8536","WNT5B",104.112874118821,0.223342321273164,0.298689342336282,0.
747741179937089,0.454616287156227,0.90073001459399
"8537","LEMD3",153.690133969802,0.145468221235219,0.194730209163365,0.
747024418348886,0.455048821350875,0.90073001459399
"8538","AL132639.2",7.22291935024623,-0.435674652242387,0.583110012082
493,-0.747156871284781,0.454968874359057,0.90073001459399
"8539","AC116158.3",5.64971866559609,0.485233925064037,0.6497151400170
08,0.746841031057601,0.455159524762896,0.90073001459399
"8540","METRN",293.161770610274,-0.208569667021549,0.279211589255973,-
0.746995021149851,0.455066566217777,0.90073001459399
"8541","NAT9",131.190184189358,-0.158189073050121,0.211461776977174,-0
.74807407424367,0.454415478844919,0.90073001459399
"8542","ATP5F1A",1139.51267188329,-0.139307318674952,0.186528140532263
,-0.746843442911269,0.455158068727016,0.90073001459399
"8543","PRMT1",663.34062883793,-0.204762929779702,0.274112179693871,-0
.747004128048528,0.455061069029783,0.90073001459399
"8544","AL031846.2",24.6578724669277,0.36941883831294,0.49412567979222
,0.747621209381955,0.454688667980156,0.90073001459399
"8545","ADAMTS9-
AS1",20.6219018062157,0.389762352369,0.522152981911312,0.7464524112115
51,0.455394168790078,0.900946789800338
"8546","RABGEF1",175.141487048057,0.171732452222138,0.230082575468206,
0.746394862247485,0.455428921955494,0.900946789800338
"8547","POP5",225.304119392105,-0.197389184436805,0.264400859457535,-0
.746552733761091,0.455333588712121,0.900946789800338
"8548","SV2A",3.28185321695425,-0.763712836954505,1.02366381237227,-0.
746058254403516,0.455632225507475,0.901032709810572
"8549","ZBED3",161.675694267739,-0.13085806722851,0.175394553989174,-0
.746078280381428,0.455620128839463,0.901032709810572
"8550","RB1",133.270660131387,0.163973937043044,0.219739714821236,0.74
6218939878216,0.455535168731676,0.901032709810572
"8551","MARC2",146.97916281056,0.175141097856317,0.235024142912607,0.7
45204708273068,0.456147977012581,0.901336415574323
"8552","GARNL3",45.8940699631467,0.193596388333274,0.259746879669649,0
.745327099133947,0.456074002714123,0.901336415574323

"8553","FAM160A2",92.1468719759159,0.144654105264904,0.194117943216811
,0.745186678097752,0.45615887520669,0.901336415574323
"8554","CLEC2B",1748.95563266273,-0.200512755743462,0.269098983607458,
-0.745126395705588,0.456195313475825,0.901336415574323
"8555","AC127070.1",6.31476993180239,-0.612324914475323,0.821729184972
891,-0.745166322035311,0.456171179440501,0.901336415574323
"8556","TXNDC17",439.902171613267,-0.194941672294667,0.261419779129969
,-0.745703607215385,0.455846479656493,0.901336415574323
"8557","PNMA8B",42.0592138772167,-0.228448462271355,0.306601757380984,
-0.745098345889401,0.456212269013784,0.901336415574323
"8558","ZDHHC8",170.160846173638,-0.164719005836432,0.221045107514728,
-0.745182771464221,0.456161236559133,0.901336415574323
"8559","AL450998.2",36.4757829036295,-0.215900517415628,0.290082788385
459,-0.744272070112419,0.456711894701458,0.901440828696635
"8560","AC017083.2",3.30041837963912,-0.647022001712024,0.868627382419
895,-0.744878661215463,0.456345076144288,0.901440828696635
"8561","NUP210",4.45121261463097,-0.555462403903155,0.746872689930473,
-0.743717652810231,0.457047307537982,0.901440828696635
"8562","P2RY13",3.87860446424042,-0.635060560873898,0.853969288800301,
-0.743657376445075,0.457083782029734,0.901440828696635
"8563","ALG3",180.616703083399,-0.191447239635686,0.257459775386735,-0
.743600585171449,0.457118149115505,0.901440828696635
"8564","CRYGS",5.51751877823026,0.45777823847114,0.61542974243224,0.74
383509100804,0.456976247919579,0.901440828696635
"8565","AC073316.1",7.93040731564261,0.64070165750748,0.86135751753983
4,0.743827788648574,0.456980666257336,0.901440828696635
"8566","CTTNBP2",27.202372186331,-0.446672214191424,0.600533477627911,
-0.743792362676875,0.457002101301233,0.901440828696635
"8567","ACTR3B",26.7208026275503,0.232151593789041,0.311780852087541,0
.744598625074826,0.456514399374182,0.901440828696635
"8568","MOB2",262.001883021405,-0.148749050787884,0.199947452827621,-0
.74394071384407,0.456912342853498,0.901440828696635
"8569","SMAD9-
IT1",8.25288759799929,0.44620588522496,0.59980264360686,0.743921171373
538,0.456924166270975,0.901440828696635
"8570","AC092338.3",50.2160327730556,-0.21174221566054,0.2844213844195
85,-0.744466581135026,0.456594251699312,0.901440828696635
"8571","WDR83OS",1908.6723845198,-0.185563668140829,0.249196665768033,
-0.744647475795535,0.456484859351776,0.901440828696635
"8572","ZNF98",6.18460034290337,0.569073891693035,0.765112940071108,0.
743777633195102,0.457011013773142,0.901440828696635
"8573","ASXL1",470.978204652737,-0.184778218848178,0.248460000320156,-
0.743694029663044,0.457061602204581,0.901440828696635
"8574","NOL12",91.3838621556917,-0.193410337944741,0.260020989508865,-
0.743825866942742,0.456981829001324,0.901440828696635
"8575","AL360012.1",121.06028700716,0.285952089020363,0.38469439785179
9,0.743322727383528,0.457286314925561,0.901457037747627
"8576","LINC00824",19.1872598413136,-0.298507407162572,0.4015241425762
76,-0.743435762659939,0.457217899231287,0.901457037747627
"8577","NRSN2",86.2131860198028,0.234584358741618,0.315580277602548,0.

743342900018172,0.457274104821915,0.901457037747627
"8578","BCDIN3D-
AS1",6.35520075549442,0.45228038510263,0.608645432495397,0.74309336923
5217,0.457425153829022,0.901625612140487
"8579","C11orf71",130.477386117243,-0.195591067953654,0.26333067106061
6,-0.742758400173715,0.457627965411726,0.901817494980671
"8580","METTL1",50.5520206948569,-0.254318586647749,0.342398377559142,
-0.742756401069163,0.457629175948318,0.901817494980671
"8581","AC022893.1",5.20559717087812,-0.502030900412382,0.676247827482
712,-0.742377099060205,0.457858890785347,0.902165030345956
"8582","USP3-
AS1",7.71223571380295,0.420826481928115,0.566941852425126,0.7422745033
33994,0.457921036454181,0.902182344950744
"8583","FAM157C",11.5670228174256,0.407276498238204,0.548961176822309,
0.74190400967104,0.458145496282568,0.902519404770553
"8584","ZNF780B",44.6161393566973,0.212511165777587,0.286479539981343,
0.741802244556197,0.458207160448194,0.902535725635841
"8585","EPHA2",53.0302289083234,0.221463868946515,0.299082450993433,0.
740477644913303,0.459010220858609,0.902540390077609
"8586","KRTCAP2",2426.61502108576,-0.198785199892311,0.268390866503403
,-0.740655606064712,0.458902283131571,0.902540390077609
"8587","RNASEL",116.007115892285,0.136145053755228,0.18385749237412,0.
740492280174229,0.459001343683337,0.902540390077609
"8588","CALM2",4092.34673640601,-0.150931908238415,0.203669402308419,-
0.741063245277546,0.458655093742336,0.902540390077609
"8589","ALG1L",13.6742385959089,-0.384468690665524,0.518911901120159,-
0.740913225993821,0.458746055639673,0.902540390077609
"8590","KLHL2",50.9624416253184,-0.179658499191555,0.242382929350542,-
0.741217624825911,0.458561498622398,0.902540390077609
"8591","CPE",4383.28395520455,0.223397734945903,0.301616195993129,0.74
0668896145723,0.458894222948009,0.902540390077609
"8592","ACSL6",3.84201975076948,-0.584505300883826,0.789188441704734,-
0.740640980018955,0.458911153644266,0.902540390077609
"8593","TLE1",61.1492851415287,0.266093920582494,0.359276596908322,0.7
40638056784963,0.458912926560417,0.902540390077609
"8594","PBX3",222.127174802006,0.120803308538117,0.163059377905261,0.7
40854712498078,0.458781537143561,0.902540390077609
"8595","DNAL1",105.195449384289,-0.1524010451176,0.205756510587738,-0.
740686380626645,0.458883619060836,0.902540390077609
"8596","SLIRP",784.653296477275,-0.205054173884982,0.276632932678161,-
0.741250045321049,0.458541844528902,0.902540390077609
"8597","BLCAP",369.051805177646,0.179427161416865,0.242197315144834,0.
740830513788178,0.458796211244227,0.902540390077609
"8598","TCFL5",92.949341403246,-0.155402473276502,0.209809912183729,-0
.740682228303955,0.458886137325125,0.902540390077609
"8599","BX322562.1",6.89830576675896,-0.518851959516501,0.700686159561
068,-0.740491234822629,0.459001977749428,0.902540390077609
"8600","DHX9",345.959892143938,-0.108498204940475,0.146590623703246,-0
.740144234327811,0.459212480701466,0.902564454271727
"8601","AC100858.3",4.59599660521281,-0.617269573303387,0.833978964864

364,-0.74015005091139,0.45920895170753,0.902564454271727
"8602","TACR2",2.37586393129391,-0.709068414793572,0.95813030737999,-0
.740054259146151,0.459267071703577,0.902564454271727
"8603","SEC11A",3112.26570468478,-0.193898958602315,0.261958238440287,
-0.740190343914355,0.459184505856318,0.902564454271727
"8604","TRAF4",50.0251635847163,-0.219682823298401,0.296861651895559,-
0.740017519594243,0.459289363884193,0.902564454271727
"8605","AC124067.2",10.0001475333063,-0.436092131848946,0.589387490940
919,-0.7399073420319,0.459356219126211,0.902590930039044
"8606","PFDN2",645.688382802532,-0.182745024968123,0.247082168471096,-
0.739612356888881,0.459535241594781,0.9026280047496
"8607","HNRNPLL",175.777367481128,-0.146327462323724,0.197828180091758
,-0.739669455867476,0.459500585952783,0.9026280047496
"8608","FAAP100",85.5192607281393,-0.158365661038176,0.214088797265667
,-0.739719513869087,0.459470204963898,0.9026280047496
"8609","SRPK3",5.29744891074918,-0.472824350049104,0.639382014846698,-
0.739502111523219,0.459602158034618,0.902654581025592
"8610","MT1H",2.51502101878361,0.667353184314622,0.902681328500021,0.7
3930097282898,0.459724258722823,0.902789519917014
"8611","FOXP1",1337.58170402119,0.213409577005629,0.288753504981522,0.
739071814969956,0.459863390495744,0.902853019798193
"8612","AL158206.1",70.2961773416804,0.225702152547326,0.3053779136925
88,0.739091278141785,0.459851572635858,0.902853019798193
"8613","MATN2",288.633547226111,-0.285782749142522,0.386749818435461,-
0.738934410618766,0.459946825997054,0.902907769760066
"8614","EMC8",168.225951769859,-0.186709176821138,0.252732485214846,-0
.738762081425415,0.460051480747751,0.902907769760066
"8615","NLGN3",4.03624013207504,0.550341391502062,0.744937614656688,0.
738775141265611,0.460043549098387,0.902907769760066
"8616","SYDE2",218.416574168442,0.218223804148031,0.296109597929043,0.
736969708764131,0.461140770410273,0.902967848320923
"8617","SUCLG1",607.712519154699,-0.178464336117338,0.242049773042868,
-0.73730429024501,0.460937323801199,0.902967848320923
"8618","IL1RL1",6.2538223386177,-0.65152323916731,0.882453828328871,-0
.738308587091881,0.460326949499394,0.902967848320923
"8619","SETD5",491.372199511792,0.154254610387861,0.208970123234323,0.
738165858355223,0.460413667148716,0.902967848320923
"8620","MRFAP1L1",271.425687166257,0.116486567320006,0.157884675290167
,0.737795274341367,0.460638865407343,0.902967848320923
"8621","SLC36A1",39.7114581573649,0.223777831744079,0.303097927959439,
0.738302083589384,0.460330900631476,0.902967848320923
"8622","TDRD6",14.8306094273163,0.38492771246891,0.521150114410155,0.7
38611969613739,0.460142653840115,0.902967848320923
"8623","SFRP4",357.194051521301,-0.350556592957395,0.474875164725104,-
0.738207889141404,0.46038812956927,0.902967848320923
"8624","ABHD11",84.386870059624,-0.188508255426038,0.255554524849194,-
0.737643974557992,0.460730825684503,0.902967848320923
"8625","ZCWPW1",80.8329433369127,-0.144826974804059,0.196434427173307,
-0.737278983567801,0.460952710100392,0.902967848320923
"8626","SWI5",426.065877864305,-0.177209592789372,0.240346069452836,-0

.737310134477346,0.460933770585821,0.902967848320923
"8627","BEST1",43.5328906844979,0.227051330114971,0.307919398372058,0.
737372608920941,0.460895787913327,0.902967848320923
"8628","HDDC3",175.968236045679,0.167736799105934,0.227539710744273,0.
737175935388483,0.461015365704156,0.902967848320923
"8629","PMM2",117.933415167092,-0.186971644445262,0.253270339915461,-0
.738229531763062,0.460374979989381,0.902967848320923
"8630","GCSH",717.513682642586,-0.216124454407657,0.29303695352737,-0.
737533105658194,0.460798218515029,0.902967848320923
"8631","ANKRD11",1039.50875181525,0.16836879435641,0.228348962123998,0
.737331112829765,0.460921016153232,0.902967848320923
"8632","RILP",250.929805186671,-0.215483072348938,0.292232944155469,-0
.737367489389903,0.460898900375765,0.902967848320923
"8633","STX8",523.038266087771,-0.192002730673128,0.260478254848425,-0
.737116158831976,0.461051713372318,0.902967848320923
"8634","TMEM94",74.2469743421995,0.181550439298911,0.246352396733222,0
.73695422373144,0.461150187500069,0.902967848320923
"8635","IDS",638.933661722993,-0.188488709773605,0.255670861188869,-0.
737231880461985,0.460981349254349,0.902967848320923
"8636","CCDC121",73.2825841853367,0.206491662236182,0.280292903840804,
0.736699571793162,0.461305067306283,0.903166521307855
"8637","PTN",90.8284303349611,0.55672069126859,0.755892089581499,0.736
508158958006,0.461421504096849,0.903289891312901
"8638","VAV1",4.08640960587132,-0.756561327757862,1.02750119265518,-0.
736311873081941,0.461540922215711,0.903419068398152
"8639","RNF19B",89.1854530094016,-0.276793704063181,0.376925522613201,
-0.734345878581498,0.462737963026187,0.903552292493395
"8640","SERBP1",2189.42403745514,-0.0851951816307167,0.116162822102787
,-0.733411775717116,0.463307319158228,0.903552292493395
"8641","MRPL19",371.44650035524,-0.133014978081056,0.181114894941239,-
0.734423185482404,0.46269086026832,0.903552292493395
"8642","IL18R1",18.1920841599253,0.306843302676008,0.418072584941473,0
.733947438143938,0.462980773454085,0.903552292493395
"8643","NEMP2",41.271364334976,0.193997396000234,0.263544642786376,0.7
36108288710254,0.461664798892265,0.903552292493395
"8644","ZNF445",112.838418521737,0.215135857293749,0.29307808518985,0.
734056444904056,0.462914337451192,0.903552292493395
"8645","MTRNR2L12",84.598297832512,-0.2486335108334,0.339195605138106,
-0.733009234397854,0.463552797217099,0.903552292493395
"8646","AC092910.3",3.0240747177631,-0.626620533719886,0.852275043051036,-0.735232761804997,0.462197749608155,0.903552292493395
"8647","CCDC39",20.0212528158472,0.365597842035677,0.497216623025146,0
.73528885621587,0.462163593528495,0.903552292493395
"8648","PAPSS1",222.337563942731,0.169319639310293,0.230672545280437,0
.734025972204214,0.462932909015217,0.903552292493395
"8649","AL357054.4",16.1858281878163,-0.38563431294025,0.526141834958711,-0.732947443668896,0.463590484901256,0.903552292493395
"8650","PON2",205.415442774567,0.117916289143632,0.160240136426611,0.7
35872371137406,0.46180837284318,0.903552292493395
"8651","TRIM56",657.885112677716,0.230839599501812,0.314647445680842,0

.733645235868085,0.463164983506233,0.903552292493395
"8652","BMP1",247.067286548796,0.152598829552968,0.20818396225727,0.732999928997363,0.463558472700376,0.903552292493395
"8653","KCTD9",266.175823910877,0.125155227474909,0.170713789276647,0.733128987442787,0.463479761751758,0.903552292493395
"8654","RGP1",69.1573176676116,0.186804215474232,0.254242605328944,0.734747880798899,0.462493054031924,0.903552292493395
"8655","DPCD",185.305465982126,-0.193528065875174,0.26295022564140 7,-0.73598744934752,0.461738335805218,0.903552292493395
"8656","BCCIP",216.563288153141,-0.111831233330359,0.152587688885939,-0.732898139731014,0.463620557809009,0.903552292493395
"8657","SIDT2",299.717742578412,0.115292368646025,0.157182351372615,0.733494362689062,0.463256964864742,0.903552292493395
"8658","CBL",72.3711617801797,0.27751159009408 3,0.377414096187783,0.735297364081512,0.462158413182887,0.903552292493395
"8659","LETMD1",576.680093258144,-0.174810235332669,0.238399578695307,-0.733265705792586,0.463396387257258,0.903552292493395
"8660","MZT1",169.194645446943,-0.186692838419434,0.254117337483792,-0.734671786931267,0.46253940661665,0.903552292493395
"8661","MIPOL1",99.8075574017378,-0.177868426959452,0.242702260317884,-0.732866792120046,0.46363967883089,0.903552292493395
"8662","BMP4",282.987816840538,0.17718769546477,0.24105633042772,0.735046846313373,0.462310964261658,0.903552292493395
"8663","MPP5",105.554800875694,0.188482198402929,0.256334826859633,0.735296879913005,0.462158707986884,0.903552292493395
"8664","PPP2R5C",430.930767596951,0.116926603395587,0.159265768628247,0.734160293217268,0.462851050294922,0.903552292493395
"8665","ABHD17C",14.1208788369509,-0.291667337603556,0.396999924573409,-0.734678571833391,0.462535273487822,0.903552292493395
"8666","NSMCE1",410.08849218365,0.175604066884519,0.239056516858461,0.734571344016063,0.462600595393208,0.903552292493395
"8667","GPT2",5.12416795606036,0.468263428254862,0.637188073176444,0.734890447525993,0.462406216476404,0.903552292493395
"8668","CTU2",67.5974009317833,-0.175260292254831,0.238576485975153,-0.734608406769324,0.462578016624775,0.903552292493395
"8669","RAB11FIP4",7.78333276362929,0.535026469470202,0.728627968989985,0.734293071691783,0.462770139571179,0.903552292493395
"8670","DNAJC7",538.743567396264,-0.132759999000832,0.180677123973862,-0.734791411778491,0.462466538297997,0.903552292493395
"8671","AC002546.1",32.1098973233403,-0.33950830752416,0.462558100809069,-0.733979811250348,0.462961042565127,0.903552292493395
"8672","MRPS12",323.244711428125,-0.176807675528829,0.240957858448679,-0.733770115102872,0.4630888573192,0.903552292493395
"8673","ZNF549",15.955464513101,0.370750845067057,0.505555219643521,0.73335380718348,0.4633426651086,0.903552292493395
"8674","ANKEF1",2.77359048399484,-0.686005503403208,0.934376335687296,-0.734185442419842,0.462835724613636,0.903552292493395
"8675","LINC01638",12.7257722194888,0.355661285266599,0.48514834678048,0.733098005232467,0.463498656691113,0.903552292493395
"8676","ZRSR2",125.222157808406,-0.153071342185269,0.208148342063398,-

0.735395442826283,0.462098696456525,0.903552292493395
"8677","CHMP4A",570.245542613916,-0.155981726924485,0.212928176504731,
-0.732555594496526,0.463829523072482,0.903713940551916
"8678","HNRNPL",446.155450917495,-0.151402559336289,0.20665453717618,-
0.732636028248505,0.463780450796831,0.903713940551916
"8679","ATAD3A",70.5420487252155,-0.171312735762816,0.234439616852293,
-0.73073287724553,0.464942329705003,0.903857751569502
"8680","GORAB-
AS1",11.3672030637625,-0.328775820842132,0.449313260226823,-0.73172961
9277558,0.464333614173426,0.903857751569502
"8681","FAM98A",153.651597713857,0.139264743257963,0.191036915537657,0
.728993885113848,0.46600540297189,0.903857751569502
"8682","AC016727.1",17.730497720389,0.288468705382055,0.39652449236524
4,0.727492780234979,0.466924136506932,0.903857751569502
"8683","AC007389.5",6.07754315107891,0.485041206983317,0.6648197879261
39,0.729582987438402,0.465645124335011,0.903857751569502
"8684","METTL5",534.824645265675,-0.187161889122464,0.258396981148284,
-0.7243191785397,0.468869805411552,0.903857751569502
"8685","PPP1R1C",19.8855511345176,0.538224454097505,0.738920924412381,
0.728392492776575,0.466373357471247,0.903857751569502
"8686","SATB2",22.0650878853057,0.305417010233078,0.419672271852168,0.
727751225700855,0.466765886443097,0.903857751569502
"8687","AC007038.1",6.87982193274324,0.445969713541278,0.6114641690417
29,0.729347255523066,0.465789272857169,0.903857751569502
"8688","DPH3",206.970304112782,-0.219851435629844,0.302292549112261,-0
.727280365577914,0.467054223489987,0.903857751569502
"8689","TMA7",3516.94068664906,-0.171850497783878,0.23511651951734,-0.
730916305398965,0.464830275871569,0.903857751569502
"8690","AC083798.2",28.3378786993781,-0.371627295900963,0.512880630295
465,-0.724588284191728,0.468704648483264,0.903857751569502
"8691","FBXO45",83.5374816935391,0.155049027181996,0.213732942493447,0
.725433456224231,0.468186154487458,0.903857751569502
"8692","SCLT1",125.177572954015,-0.127009823028291,0.175274687627259,-
0.724633001762304,0.46867720730182,0.903857751569502
"8693","ZDHHC11B",16.5912999166606,-0.475432130006479,0.65378771697316
2,-0.727196485439624,0.467105598899208,0.903857751569502
"8694","OXCT1",129.441512120358,-0.134916831125316,0.184324498042369,-
0.731952792809473,0.46419738173815,0.903857751569502
"8695","AC113349.2",3.19451632675126,-0.594044372408704,0.818262366591
25,-0.725982761352447,0.467849338554531,0.903857751569502
"8696","RAD50",550.924185911278,0.126059019161578,0.172769104397418,0.
729638667753969,0.465611079805782,0.903857751569502
"8697","ZMAT2",501.194560548439,0.107150798921988,0.146744701947693,0.
73018512763876,0.46527703208699,0.903857751569502
"8698","DDX39B",1009.34482480774,-0.114332348383024,0.157675426711081,
-0.725112027713248,0.468383306196353,0.903857751569502
"8699","SNHG5",2709.70689537773,0.244811467452288,0.335710549705463,0.
729233763035073,0.465858681609462,0.903857751569502
"8700","FRK",22.065869187912,-0.29373173211938,0.402890098133249,-0.72
9061680791751,0.465963933135115,0.903857751569502

"8701","SLC18B1",86.2447931429944,0.209410968093422,0.286544609775364,
0.730814543179119,0.464892439210942,0.903857751569502
"8702","PAPOLB",6.03949506000942,0.507684228820749,0.693907847544367,0
.731630620142669,0.464394053612701,0.903857751569502
"8703","C7orf31",22.4253452214267,-0.267245148719154,0.36523691912897,
-0.731703545623181,0.464349531837849,0.903857751569502
"8704","HOXA10-
AS",38.231950433457,-0.232498157189265,0.318775601321588,-0.7293474037
07715,0.465789182235441,0.903857751569502
"8705","TRIM73",51.0204087759275,0.216123416384745,0.296631276493445,0
.728592813743699,0.466250775643396,0.903857751569502
"8706","GTPBP10",94.9416766900828,0.155190272451512,0.212504678199912,
0.730291087076763,0.465212275128558,0.903857751569502
"8707","AKR1B15",2.38217100653958,-0.662749823877409,0.913564184769504
,-0.725455129400266,0.468172862660859,0.903857751569502
"8708","AC004918.4",2.35734846794278,-0.641670885769235,0.878305492899
72,-0.730578245219397,0.465036804033532,0.903857751569502
"8709","ANGPT2",45.6142779669022,-0.235333172385365,0.323843584448786,
-0.72668777053566,0.467417246779943,0.903857751569502
"8710","IDO1",19.6765151114973,-0.354320985067799,0.484974978244735,-0
.730596424479856,0.465025696638234,0.903857751569502
"8711","CEBPD",13709.1018608563,-0.205446703905931,0.281750431729084,-
0.729179730604557,0.465891728313636,0.903857751569502
"8712","AL161729.1",16.0962148364865,-0.305366940571257,0.420567080700
284,-0.72608379158633,0.467787404740122,0.903857751569502
"8713","PSMB7",1064.41479538287,-0.174643903892471,0.240997618712522,-
0.724670661998523,0.468654097581913,0.903857751569502
"8714","HMCN2",98.3596110369122,-0.532221281729394,0.733976577226432,-
0.725120253483516,0.468378260256752,0.903857751569502
"8715","CCDC7",59.1963871474263,0.231360214613243,0.316975010668224,0.
729900486872786,0.465451014689825,0.903857751569502
"8716","SLC16A9",4.78008876673847,0.670732121529612,0.92523184923847,0
.724934103902358,0.468492457481134,0.903857751569502
"8717","UNC5B",12.8632592774009,0.35194380130957,0.484364274863497,0.7
26609743067353,0.467465058000988,0.903857751569502
"8718","ZMIZ1-
AS1",14.6070237492425,-0.293852770417932,0.404318599614571,-0.72678519
0436591,0.467357556678446,0.903857751569502
"8719","C11orf68",276.362593419419,-0.163035042458419,0.22274317266657
3,-0.731941816696976,0.464204081396545,0.903857751569502
"8720","KDM4D",9.93129571516593,0.41425187484059,0.565842999341037,0.7
32096845455389,0.464109459100656,0.903857751569502
"8721","CRTAM",5.42383923725512,-0.43547949366812,0.596424249292729,-0
.730150550022964,0.465298165231572,0.903857751569502
"8722","FOXJ2",137.662429428765,0.141604646076699,0.19510884070158,0.7
25772576821795,0.46797820097323,0.903857751569502
"8723","DERA",380.588624573828,-0.171446759600593,0.235983210849661,-0
.726521005385496,0.467519435184679,0.903857751569502
"8724","NACA",16594.7088684904,-0.199715442209258,0.275641067135191,-0
.724548937083115,0.468728794786785,0.903857751569502

"8725","LNX2",33.0251320364544,0.277230570151144,0.380896103677402,0.727837768553134,0.466712901598295,0.903857751569502
"8726","DAD1",2551.44451003865,-0.212352100147299,0.291587089296697,-0.728263040244644,0.466452582498093,0.903857751569502
"8727","PSME1",1684.01782517606,-0.159622839283828,0.219231010270314,-0.728103378655288,0.466550305773332,0.903857751569502
"8728","PYGL",219.41566912792,-0.136963665145957,0.188388711138034,-0.727026923845784,0.467209462569598,0.903857751569502
"8729","RIOX1",24.0000197521565,-0.239644522528608,0.32823737553236,-0.730095170118682,0.465332013428833,0.903857751569502
"8730","HSP90AA1",7469.46805595365,0.131561553418068,0.181109360801572,0.72642050546581,0.467581024333299,0.903857751569502
"8731","CLBA1",24.7844942895301,-0.232731653898474,0.321301018735131,-0.724341475214339,0.468856120156438,0.903857751569502
"8732","PACS2",239.218614555712,0.124754834377535,0.172037836318787,0.725159284998003,0.468354317539687,0.903857751569502
"8733","DPH6-DT",3.19376491390284,0.787654998563394,1.0782845154444,0.730470471644282,0.465102655930002,0.903857751569502
"8734","SPTBN5",2.85614699360237,0.821676979643972,1.13255818669726,0.725505311157676,0.468142087760488,0.903857751569502
"8735","DUOXA1",3.37605545290896,0.820949811302324,1.12718243501523,0.72832026635620,0.466417559162479,0.903857751569502
"8736","AC009269.4",7.23643884204836,-0.460673579323741,0.630123817030837,-0.731084220073522,0.4647277121873,0.903857751569502
"8737","MORF4L1",3221.97611553148,-0.106872583659773,0.14601636008697,-0.731921981866399,0.464216188423293,0.903857751569502
"8738","MESD",737.499110274751,0.119979165236847,0.164328455619636,0.730118011420725,0.46531805265929,0.903857751569502
"8739","AC022167.2",4.94593411905614,-0.495340110355918,0.68327515718406,-0.724949685566402,0.468482898003229,0.903857751569502
"8740","KAT8",249.39388365579,-0.144363810381758,0.19908453741079,-0.725138236546613,0.468367228999492,0.903857751569502
"8741","CHRNE",4.73556915418351,0.502351655475081,0.692536977170335,0.72537882024388,0.468219662824041,0.903857751569502
"8742","CLDN7",7.48494143816168,-0.499256480778634,0.689174766244436,-0.724426524637958,0.468803920542481,0.903857751569502
"8743","NTN1",19.7735277934368,-0.373591452709054,0.512846672536704,-0.728466172669408,0.466328268668556,0.903857751569502
"8744","TUBG2",94.9958252907618,0.14805358953278,0.203129742139876,0.728862194049506,0.466085962739316,0.903857751569502
"8745","GRN",1453.79077954901,0.205106900466703,0.281282478154912,0.729184774722241,0.465888643232313,0.903857751569502
"8746","AC005921.2",110.875248738249,-0.339672018716994,0.467739677293991,-0.726198856342688,0.467716872980142,0.903857751569502
"8747","SOX9-AS1",8.14329929410914,-0.385190352812885,0.529280156093171,-0.727762695008498,0.466758864302376,0.903857751569502
"8748","AC100786.1",7.68192863331442,-0.420870612795779,0.579495205236688,-0.726271087305854,0.467672600253466,0.903857751569502

"8749","RMC1",47.508909012208,-0.188322673733159,0.257628705210505,-0.730984823990334,0.464788422623816,0.903857751569502
"8750","AC012615.1",18.6213963717908,-0.301583645697294,0.415757308570374,-0.725383870542943,0.468216565411617,0.903857751569502
"8751","MKNK2",567.666542836234,0.12998814594915,0.178286279960371,0.729097864277856,0.465941800951535,0.903857751569502
"8752","DAPK3",196.659809730099,-0.146409839046721,0.202136726478063,-0.724310923589684,0.468874872191631,0.903857751569502
"8753","UBXN6",543.51694929906,-0.125535094004467,0.171623769459711,-0.731455173136355,0.464501175592248,0.903857751569502
"8754","ZNF506",234.485211460382,-0.1302960159499,0.179777076931835,-0.724764348011421,0.468596611100236,0.903857751569502
"8755","AC092279.1",42.1640497245556,0.259567297909557,0.357544336646958,0.725972337707186,0.467855728742949,0.903857751569502
"8756","PDCD5",1078.59080113032,-0.173976817385237,0.238521654680283,-0.729396320927075,0.465759267641315,0.903857751569502
"8757","GPATCH1",89.5924980259631,0.170634851048059,0.235508582842971,0.724537717429315,0.468735680124451,0.903857751569502
"8758","SERTAD1",2059.53331516379,-0.293586934193643,0.403660590446602,-0.72731136291711,0.467035238847187,0.903857751569502
"8759","BCKDHA",169.30371623185,-0.162214174499174,0.223143591449401,-0.726949734229571,0.467256748725941,0.903857751569502
"8760","CCDC9",70.6186743392631,-0.197892282182822,0.27228371724149,-0.726787059423427,0.46735641157372,0.903857751569502
"8761","RPRD1B",118.295541263906,0.160994106362681,0.220769118115143,0.729241968882235,0.465853662955898,0.903857751569502
"8762","AL162458.1",24.1605073698797,-0.244832929216622,0.337641301940778,-0.725127310578744,0.468373931242227,0.903857751569502
"8763","ZNF334",8.9140999666983,-0.375819344933426,0.516947614015135,-0.726996962060499,0.46722781676555,0.903857751569502
"8764","IFNAR1",531.370453709186,0.110157963386505,0.152022256658029,0.724617340961482,0.468686817536616,0.903857751569502
"8765","DSCR9",2.13412200216301,0.757183223432696,1.04058277534735,0.727653043439957,0.466826001419704,0.903857751569502
"8766","DGCR6L",533.739578815585,-0.190540911780458,0.261323957405381,-0.729136791254388,0.465917991321959,0.903857751569502
"8767","AL096701.3",2.76038358622759,0.740608994067567,1.01782529134314,0.727638623609213,0.466834830746861,0.903857751569502
"8768","LARGE1",102.845266679856,0.181093682606877,0.248884633237243,0.727620987488828,0.466845629548558,0.903857751569502
"8769","AL021707.1",4.16947568152713,0.557319015487984,0.768135319813315,0.725547961553745,0.468115932488975,0.903857751569502
"8770","MORC4",275.48541403325,-0.14528484654147,0.198943269123241,-0.730282794596433,0.465217342885283,0.903857751569502
"8771","MCTS1",502.531349903994,-0.198841768741613,0.272377965577029,-0.73002149171784,0.465377047788727,0.903857751569502
"8772","TAGLN",3204.78787309884,0.319956862201048,0.441803557554834,0.724206169755293,0.468939171353035,0.903878649023839
"8773","SDAD1",224.000000333044,0.132095748108065,0.182454350243225,0.723993414966384,0.46906977781159,0.90392773165941

"8774","RNASEH2B-
AS1",2.72452229945392,-0.703073803911795,0.971342787163564,-0.72381636
3494965,0.469178481963744,0.90392773165941
"8775","ABCA3",12.0339077713835,0.352957915995752,0.48757410284447,0.7
23906199973752,0.469123323390601,0.90392773165941
"8776","CDC42EP4",968.258317849237,-0.180219494909253,0.24895662261432
3,-0.723899179771748,0.469127633585349,0.90392773165941
"8777","RSRP1",1506.33645113976,0.130877025475954,0.180967099360642,0.
723208947584083,0.469551522505957,0.903930009203455
"8778","USP24",124.732358181607,0.182179322749857,0.253144478469609,0.
719665401557349,0.471731033476692,0.903930009203455
"8779","HIPK1-
AS1",7.80637661142483,0.41775087260706,0.579316954312718,0.72110935041
1927,0.470842238559673,0.903930009203455
"8780","TSPAN2",5.06988282842765,-0.594242999071918,0.825476978392255,
-0.719878342615077,0.471599903597579,0.903930009203455
"8781","TRIM45",16.5459578167036,0.298610999166872,0.414669934606873,0
.720117313182999,0.471452768586542,0.903930009203455
"8782","AL358472.3",2.51472635494537,0.805535570244247,1.1141872340246
2,0.722980434208107,0.469691904948005,0.903930009203455
"8783","SDCCAG8",362.737731534257,0.136411578699987,0.189508647740432,
0.719817171018116,0.471637571232314,0.903930009203455
"8784","ATL2",151.284042106866,0.145233926534149,0.201675920247545,0.7
20135186966708,0.471441764653245,0.903930009203455
"8785","PAPOLG",86.6308917560839,0.178047012451464,0.246918514715964,0
.721075990013447,0.470862762475873,0.903930009203455
"8786","STAMBP",233.110164507687,0.113095171497518,0.157158074921298,0
.719626856934679,0.471754771543932,0.903930009203455
"8787","TMEM185B",156.611333429496,0.152351297816871,0.211936010908301
,0.718855173143695,0.472230158770362,0.903930009203455
"8788","CXCR4",211.757651338399,0.257756249644665,0.3578242331545,0.72
0343190209177,0.47131371858972,0.903930009203455
"8789","HIGD1A",277.271324130918,-0.199047435600218,0.275121114835414,
-0.723490218914292,0.469378761162003,0.903930009203455
"8790","PSMD6-
AS1",5.66038066510845,0.594307683400963,0.823841085595672,0.7213863132
00504,0.47067186514445,0.903930009203455
"8791","B4GALT4",157.084052058298,0.131147346698816,0.181755593481668,
0.721558793248606,0.470565781392928,0.903930009203455
"8792","ZNF141",158.067392852391,0.138627823675126,0.192116311893731,0
.721582786535105,0.470551025382197,0.903930009203455
"8793","AC093827.4",62.9320813166343,-0.25289732443145,0.3509913238201
5,-0.720523008030353,0.471203038833169,0.903930009203455
"8794","HMGCR",69.1836432812135,-0.166116784421467,0.230279662384272,-
0.721369758412554,0.470682047851295,0.903930009203455
"8795","CHSY3",28.4115155044426,0.252394421214784,0.349618283790542,0.
721914250245549,0.470347199417371,0.903930009203455
"8796","LYRM7",226.162382188959,0.148315273483859,0.205705754887034,0.
721006923531662,0.470905254984846,0.903930009203455
"8797","PCDHGA10",66.0874825447178,0.21020922990293,0.290681653984448,

0.723159604404125,0.469581833499131,0.903930009203455
"8798","AL365272.1",8.43565964895829,-0.371429194345253,0.516657864622784,-0.718907462323129,0.47219793826932,0.903930009203455
"8799","HLA-DQA2",13.3598934705348,-1.56635355851211,2.17457326494736,-0.720303879276299,0.471337916791419,0.903930009203455
"8800","SOD2",5885.03270276313,-0.251875223027032,0.349023049848227,-0.721657847917381,0.470504863845363,0.903930009203455
"8801","QKI",1264.74089693495,0.122227830566188,0.169105379038516,0.722790908610593,0.469808353655181,0.903930009203455
"8802","CRPPA",25.975929612725,0.268220829889217,0.371732035033469,0.721543489963322,0.470575193135807,0.903930009203455
"8803","HOXA-AS2",5.0964157069454,-0.529167718822852,0.734028632452132,-0.720908824843914,0.470965612925089,0.903930009203455
"8804","SGCE",1622.93316959354,0.188944141256875,0.262523176001658,0.719723660724269,0.47169515527233,0.903930009203455
"8805","POP7",265.014848929359,-0.156080572896833,0.217071309987108,-0.719029027401653,0.472123034761441,0.903930009203455
"8806","AC021097.2",14.6906802261505,-0.359121048892182,0.497444065737626,-0.721932521920159,0.47033596509056,0.903930009203455
"8807","JRK",44.1275607181448,0.2919912576897,0.404034587850505,0.722688765937382,0.469871118982225,0.903930009203455
"8808","EDF1",3322.41741139868,-0.195887518691621,0.270826909747622,-0.723294147077793,0.469499187905011,0.903930009203455
"8809","ZNF25",132.881825337783,0.141935148738187,0.197317767644587,0.719322696746924,0.471942114544485,0.903930009203455
"8810","TMEM254-AS1",21.8272197934522,0.360921189157504,0.501318429529576,0.719943987489514,0.471559483297818,0.903930009203455
"8811","PPP2R2D",240.146041690952,-0.137220596365971,0.189872482672982,-0.722698699854818,0.46986501451743,0.903930009203455
"8812","POLR2G",517.572922633325,-0.192060868325047,0.265897296154202,-0.722312227701876,0.470102536787527,0.903930009203455
"8813","PCNX3",55.1135759974756,-0.180108110685588,0.250007535247691,-0.720410728849228,0.471272146166477,0.903930009203455
"8814","ANKRD13D",177.433310971902,-0.121037364364633,0.167351187659883,-0.723253692173513,0.469524037313403,0.903930009203455
"8815","LINC00937",7.62475773727527,-0.437331485837562,0.605086575442009,-0.722758533385238,0.469828247303075,0.903930009203455
"8816","PRH1",141.623476795711,0.137392166600441,0.191036635216533,0.719192768678701,0.472022154325993,0.903930009203455
"8817","ITPR2",147.095190662234,0.196140561992777,0.272554132161977,0.719638922502967,0.471747340779785,0.903930009203455
"8818","BLOC1S1",1565.63475593293,-0.187884196634783,0.260641657004132,-0.720852525242365,0.471000254745387,0.903930009203455
"8819","TMEM19",119.012495872271,-0.156571608283762,0.217726397510922,-0.719120924581082,0.47206641576394,0.903930009203455
"8820","PEBP1",4204.84378511565,-0.144964016340787,0.20078864176838,-0.721973190635007,0.470310960490662,0.903930009203455

"8821","AL122035.2",7.85730978811571,0.375250063498493,0.518520623703904,0.723693612836461,0.469253855266455,0.903930009203455
"8822","AC091057.3",6.93948268045171,0.40076356806853,0.555123661530388,0.721935661981492,0.47033403444089,0.903930009203455
"8823","MYO5A",111.773686632349,-0.195465792349357,0.271041027969342,-0.721166805681783,0.470806892203339,0.903930009203455
"8824","AC135050.6",34.0405101070098,-0.245364738802356,0.341082607998149,-0.719370419507544,0.471912717699419,0.903930009203455
"8825","C17orf97",62.3347499577474,-0.413652837604219,0.575095698531627,-0.719276528515837,0.47197055477197,0.903930009203455
"8826","PPM1D",79.1577817204114,0.189304954704731,0.262238505300186,0.721880848459049,0.470367736866328,0.903930009203455
"8827","ZNF20",16.6457639772847,-0.346461965446833,0.478788017399385,-0.723622882896481,0.46929728902476,0.903930009203455
"8828","HAUS5",46.226232434633,0.210665955618048,0.292688768722508,0.719760982075044,0.471672172159707,0.903930009203455
"8829","SNRPA",291.435318818877,0.164963360490034,0.22928737474265,0.719461159495533,0.471856825364471,0.903930009203455
"8830","FAM182A",42.4876590190695,-0.199702470720012,0.27787133955156,-0.71868682478121,0.472333902963835,0.903930009203455
"8831","TLDC2",2.49450359341467,0.658960927652762,0.915672438395105,0.719647004782321,0.471742363220931,0.903930009203455
"8832","DEPDC5",46.4893866176465,0.205702599985734,0.286217087888686,0.718694336187693,0.472329273813669,0.903930009203455
"8833","NFAM1",2.08936643328663,-0.877917114718323,1.22130433789634,-0.718835664033181,0.472242180560425,0.903930009203455
"8834","GK",35.2971257112468,-0.237538496878838,0.329611034616154,-0.720663060189905,0.471116845191879,0.903930009203455
"8835","PHF6",136.44369836112,0.138475764429158,0.191897938597745,0.72161152663255,0.470533350391413,0.903930009203455
"8836","CXCL9",38.5575162256883,-0.499415953654314,0.695265560818137,-0.718309638502212,0.472566388488648,0.904200849708652
"8837","THAP1",115.576435450832,-0.176509372225133,0.245737684835919,-0.718283694839032,0.472582381646283,0.904200849708652
"8838","KTI12",57.6791309233893,-0.186925010094019,0.260370365437541,-0.717919682525696,0.472806811077891,0.904323253586537
"8839","AL359541.1",24.5314266235666,0.254026846943032,0.353815786628845,0.71796357467087,0.472779746550045,0.904323253586537
"8840","CXorf40A",47.7603403425422,-0.223717407867694,0.311553153749388,-0.718071395443652,0.472713266332024,0.904323253586537
"8841","CETN3",275.125610235174,0.177973283973667,0.247986801504828,0.717672403908973,0.472959302564676,0.904410301714945
"8842","APCDD1",50.7111395140536,0.349262532953461,0.48661272486698,0.717742293009158,0.472916200692639,0.904410301714945
"8843","ZMYND12",2.67788035255585,0.632328651825824,0.8813450764408,0.717458653515605,0.473091139714907,0.90456010294014
"8844","NKAIN1",3.11878770249182,-0.554532390135122,0.773614628306553,-0.716807011973127,0.473493184512065,0.904595287663597
"8845","RLF",235.640669254283,0.172616880981974,0.240864343534282,0.716656016615452,0.473586371277289,0.904595287663597

"8846","MDH1",919.634842772759,-0.198921388051358,0.277438865051324,-0
.716991788495671,0.473379163427883,0.904595287663597
"8847","AL359715.2",2.02764739621394,0.803638567897482,1.1210125408423
1,0.716886331435366,0.473444236556817,0.904595287663597
"8848","FAM220A",2.04423831789822,-0.793248370483799,1.10702025091379,
-0.716561751990544,0.473644551791213,0.904595287663597
"8849","AP006621.3",80.5421335799759,-0.167082224690317,0.233156948644
728,-0.716608386160122,0.473615768498869,0.904595287663597
"8850","ROBO4",82.7636403405225,0.334028697245823,0.465987896853502,0.
716818397004065,0.47348615865059,0.904595287663597
"8851","AL606834.1",52.8842574073571,0.196355358380665,0.2737751610784
32,0.717213926958159,0.473242107197218,0.904595287663597
"8852","AC093462.1",5.25291285008687,-0.576738028130094,0.804441853602
73,-0.716941846756414,0.473409979767295,0.904595287663597
"8853","ZNF93",30.8167997490803,0.251277553411527,0.350391262546353,0.
71713418760916,0.473291302714682,0.904595287663597
"8854","PRPF3",121.71778610561,-0.155104566911024,0.216518836515488,-0
.716355996582909,0.473771558525253,0.904628031544144
"8855","AC072022.2",36.0530041351814,0.249182942705575,0.3480140727137
82,0.716013984039409,0.473982714185567,0.904628031544144
"8856","GHITM",976.727972410449,-0.120496293956756,0.168270777870576,-
0.716085677392153,0.473938447029625,0.904628031544144
"8857","RPS25",17582.9869678991,-0.158624209076621,0.221479921261477,-
0.716201306976945,0.473867056177211,0.904628031544144
"8858","TM9SF1",470.360901624237,-0.15609103392401,0.217953721322357,-
0.716165950170443,0.473888885192736,0.904628031544144
"8859","AC104024.4",3.20821770432474,0.579505772527139,0.8091493881031
74,0.716191325171276,0.473873218809885,0.904628031544144
"8860","COL16A1",641.681172867575,0.14530458291559,0.203157196304879,0
.71523227115977,0.474465531289963,0.904671270140335
"8861","GCLM",207.118981744343,0.281087118245975,0.392836566802635,0.7
15531959088716,0.474280400128158,0.904671270140335
"8862","OSGEPL1",59.9802678679829,-0.212274209634117,0.296840901278938
,-0.715111053495439,0.47454042434792,0.904671270140335
"8863","OSMR-
AS1",44.7421638651937,-0.262122520269828,0.366438449799725,-0.71532482
580114,0.474408351750434,0.904671270140335
"8864","LYRM2",314.312016720785,-0.131526967544336,0.183772491925431,-
0.715705414702137,0.474173266669927,0.904671270140335
"8865","HNRNPF",1324.27360667683,-0.152576793429285,0.213317958627534,
-0.715255266883988,0.474451324356494,0.904671270140335
"8866","CEP290",275.772678309318,0.126062167551514,0.176176750949193,0
.715543718863722,0.474273136377915,0.904671270140335
"8867","OAZ2",395.953337916051,0.131253404186911,0.183355323039367,0.7
15841798379263,0.474089039732826,0.904671270140335
"8868","SULT2B1",9.4238268122319,-0.553246410977355,0.773603421390618,
-0.715155072585961,0.474513226865425,0.904671270140335
"8869","MPST",637.857110985605,-0.122828434639643,0.171611322312543,-0
.715736193768988,0.474154257617224,0.904671270140335
"8870","COPS9",1354.44477444378,-0.171184908671569,0.239579402938609,-

0.714522644984778,0.47490405858855,0.905193610760219
"8871","GGH",91.3219909019643,0.223044745081568,0.312171418174716,0.714494447908535,0.474921488115324,0.905193610760219
"8872","LUCAT1",25.4813786568414,-0.414363417173936,0.580070050109594,-0.714333410414224,0.475021037339536,0.905281300646627
"8873","SP100",950.57439778337,0.0843708439235708,0.11812884401306,0.714227288250133,0.475086645583637,0.905304294323018
"8874","AC092329.4",31.6578893220593,-0.217565731820217,0.304703472939675,-0.714024457027736,0.47521205641287,0.905441227161236
"8875","UVSSA",83.467408614027,0.233110781563197,0.326582833035745,0.71378761521639,0.475358519024858,0.905516205461052
"8876","SNAP23",342.812457422003,-0.121613155097224,0.170376541200845,-0.713790491578669,0.4753567401383,0.905516205461052
"8877","NBPF26",126.022689837008,0.330072874080759,0.462638224683328,0.713457852097482,0.475562485270446,0.905596261704672
"8878","NME7",166.017671517803,-0.159564922815066,0.223748912444291,-0.71314278613441,0.475757405811974,0.905596261704672
"8879","AC007365.1",9.44639019238896,0.410894016842103,0.576530877162956,0.712700799069196,0.476030921920979,0.905596261704672
"8880","KCTD18",123.812596400862,-0.1406282175911,0.197391551751294,-0.712432808513942,0.476196805242572,0.905596261704672
"8881","CENPH",35.0053829962801,-0.222791259941009,0.31233825885352,-0.713301216312067,0.475659385001091,0.905596261704672
"8882","KDM7A",244.312905188719,0.241162941498137,0.338159822991353,0.71316260862930 4,0.475745141020681,0.905596261704672
"8883","PLGRKT",252.419511980554,-0.208833472717082,0.29300013819703,-0.712741891529929,0.47600548892318,0.905596261704672
"8884","SLC35G1",28.9332527038609,-0.235173440569417,0.33007170482108,-0.712491974120885,0.476160179632405,0.905596261704672
"8885","MEN1",72.2822633744316,0.148290974672156,0.208097481177512,0.712603409868575,0.476091201142788,0.905596261704672
"8886","SDHD",1036.25357245631,-0.184904486358909,0.259311395362701,-0.713059625090066,0.475808862014201,0.905596261704672
"8887","HINFP",40.9278213218214,0.196454086030335,0.27545366314101,0.713201936725621,0.475720808023139,0.905596261704672
"8888","ELK3",344.360339279404,-0.145424426801441,0.20395493821187,-0.713022337563473,0.475831934799325,0.905596261704672
"8889","AC073578.1",10.1597752280066,0.334356654083257,0.469324356925302,0.712421269319448,0.476203948593343,0.905596261704672
"8890","PLA2G4C",66.8126811592982,0.338824099534381,0.47547394802254 5,0.712602869081514,0.476091535875597,0.905596261704672
"8891","ALG12",104.921815255956,-0.1435742278148,0.20140537290975,-0.712861954676529,0.475931183556254,0.905596261704672
"8892","AC132938.3",20.2157932774768,-0.273840877974242,0.384556940752046,-0.712094488370731,0.476406267068794,0.905777258922655
"8893","AC011455.6",2.57188000294499,0.818433739798513,1.149316382186,0.712104823775199,0.476399867433424,0.905777258922655
"8894","CAMP",2.01502010468714,0.887928199663287,1.24784976451742,0.711566588311754,0.476733203014664,0.905788176922374
"8895","CFAP97",594.766611373123,-0.11123349675347,0.156341063380618,-

0.711479724828708,0.476787010563587,0.905788176922374
"8896","AL022069.3",18.2258648353925,-0.353425084959299,0.496733095285828,-0.711498968587813,0.476775089738142,0.905788176922374
"8897","DIXDC1",485.860050994767,-0.18003965610768,0.25293679972348,-0.711797003458988,0.476590488558087,0.905788176922374
"8898","XPO6",130.956746703507,0.147711488601452,0.207573976418603,0.711608897945714,0.476706995533712,0.905788176922374
"8899","TPM4",1035.20758389917,-0.172858543045733,0.242873415592877,-0.711722782107582,0.476636457198706,0.905788176922374
"8900","ARMCX3-AS1",2.07653644078767,0.755802977129543,1.06201083028085,0.711671628555493,0.476668140321204,0.905788176922374
"8901","FGR",13.8446746557184,-0.385670696477867,0.542203957469843,-0.711301884031929,0.476897184345898,0.905885725780567
"8902","DDX59",150.330479124358,-0.154285978732322,0.217062192670422,-0.710791579289829,0.477213399546221,0.905885725780567
"8903","SRF",83.1988298013397,0.20737905602904,0.291744317680421,0.710824662080325,0.477192896001638,0.905885725780567
"8904","POP1",26.8260527397248,0.263786572545087,0.371065371601857,0.710889758875485,0.477152552715956,0.905885725780567
"8905","AC109322.1",6.41816250878133,-0.44726045956339,0.629226311251199,-0.710810167289453,0.477201879301507,0.905885725780567
"8906","PCYT2",68.097262845659,-0.16393448041065,0.230615648206638,-0.710855840379747,0.477173573234649,0.905885725780567
"8907","AC104971.1",3.48598766730914,0.588585904837534,0.82773818824668,0.711077383156962,0.477036284318704,0.905885725780567
"8908","C2CD3",48.3736669208974,0.192533253082346,0.270956325035439,0.710569325359593,0.477351157178297,0.905943805766151
"8909","CSF2RB",4.22233877994441,-0.64964236576877,0.914215727351038,-0.710600732773569,0.47733168888642,0.905943805766151
"8910","PRKAB2",147.835376124628,0.150712798498444,0.212229208164672,0.710141642622081,0.477616305093269,0.90604022063469
"8911","POLR2B",265.89715074355,0.0951778583810293,0.133983720444368,0.71036882738712,0.477475448679961,0.90604022063469
"8912","OTULIN",159.13994411218,0.180469675051066,0.254116542873511,0.710184677511911,0.477589621364548,0.90604022063469
"8913","LYRM1",352.764034037606,0.18311036109867,0.257816689256488,0.710234708337689,0.477558600839791,0.90604022063469
"8914","SLC2A5",8.03011277365495,0.547067133111596,0.774993187401638,0.705899280154679,0.480250775259349,0.906043677312476
"8915","CAPZB",1027.18580709318,-0.149299355730472,0.210866310808853,-0.708028490458156,0.478927565068747,0.906043677312476
"8916","KDM1A",226.966150500672,0.116051440506388,0.163536255920711,0.709637381955561,0.477929032032801,0.906043677312476
"8917","SRRM1",1237.13353149876,-0.101240869453356,0.14340111709154,-0.705997773983369,0.480189521766185,0.906043677312476
"8918","NBPF20",31.8313899825946,0.342923528161846,0.484106096402316,0.708364407534459,0.47871898940468,0.906043677312476
"8919","ODR4",263.952881188477,0.117026697835694,0.165855930096181,0.705592484801895,0.480441599145771,0.906043677312476

"8920","EPHX1",4245.32892473213,-0.188984059514674,0.266482722275411,-0.709179409085136,0.478213149694659,0.906043677312476
"8921","AL591848.4",2.90974184181833,0.606151803161375,0.857726239360677,0.706696117415251,0.479755342876306,0.906043677312476
"8922","FOSL2",456.77788904858,0.187634004014715,0.265179237339305,0.707574265230393,0.479209678835913,0.906043677312476
"8923","TCTA",105.674731495072,0.20467761687558,0.290158230293432,0.705400004227328,0.480561341382878,0.906043677312476
"8924","ADAMTS9-AS2",51.39804345405,0.339365336788274,0.48019164471015,0.706728949840682,0.479734935346273,0.906043677312476
"8925","KBTBD8",4.19033489390955,-0.571415307464892,0.807082234057296,-0.708001345280915,0.478944422060004,0.906043677312476
"8926","TBC1D23",314.736016776527,0.101528376794519,0.143796101609758,0.706057922697045,0.480152117265256,0.906043677312476
"8927","QTRT2",74.7219517127058,-0.149171427992066,0.211325214761249,-0.705885609346698,0.480259277496564,0.906043677312476
"8928","CCNL1",5049.84320491612,0.218647197271833,0.308890307664823,0.707847387393869,0.479040035108081,0.906043677312476
"8929","CPEB2",78.578139712331,0.192332158780827,0.272377733138309,0.706122914545163,0.48011170276426,0.906043677312476
"8930","TIGD2",11.6874292515996,0.31806767490932,0.449813022661948,0.707110863591782,0.479497585412086,0.906043677312476
"8931","AC098487.1",109.819827264946,0.208946614750756,0.296083748009347,0.705701059769617,0.480374061791283,0.906043677312476
"8932","SAP30",225.834392919823,-0.197696536826084,0.279273064348982,-0.707896901145614,0.479009284259039,0.906043677312476
"8933","AFAP1L1",30.0447683141847,-0.330830664997188,0.467160767430334,-0.708173048899111,0.478837800606074,0.906043677312476
"8934","ZC2HC1B",16.6963673916105,0.516546010846889,0.730288596758682,0.707317645571259,0.479369102277868,0.906043677312476
"8935","SDK1",218.658040444402,0.204505477600435,0.288660820148169,0.708462885595152,0.478657852389911,0.906043677312476
"8936","WDR97",2.10841620691806,0.816619544151818,1.15233890232314,0.708662653413413,0.478533845908852,0.906043677312476
"8937","NOL6",52.9064100882801,0.200198109065301,0.282399353462224,0.708918439829505,0.478375091341098,0.906043677312476
"8938","LRSAM1",45.8542312286436,-0.16969783862158,0.239253215783446,-0.709281328010144,0.47814991314612,0.906043677312476
"8939","MED22",77.2747104164705,0.146681348474892,0.207115159498319,0.708211551632378,0.478813893644089,0.906043677312476
"8940","PHRF1",121.786485003875,0.144586912368736,0.204746569613019,0.706175017447238,0.480079304458975,0.906043677312476
"8941","TAF10",691.626772727051,-0.190135024043167,0.268168853927608,-0.709012330322647,0.478316825160921,0.906043677312476
"8942","CYB561A3",230.602071240082,-0.13084606484223,0.184374629443902,-0.709674998327475,0.477905699653345,0.906043677312476
"8943","TMEM179B",808.458095655565,-0.163024459828049,0.231163149513178,-0.70523550216102l,0.480663691065538,0.906043677312476
"8944","AP003168.2",4.00458753853039,0.513725759528603,0.7277519250747

2,0.705907798836612,0.480245477307141,0.906043677312476
"8945","MSANTD4",102.178750035631,0.185413862118568,0.26171565591991,0
.708455371027971,0.478662517422903,0.906043677312476
"8946","ESD",1823.10689642201,-0.176940344473722,0.24997468291 2405,-0.
707833058981115,0.479048934066413,0.906043677312476
"8947","PCK2",67.5646052495776,-0.188277315878668,0.265898454005729,-0
.708079768958005,0.47889572231386,0.906043677312476
"8948","GMPR2",288.546685540819,-0.131741185448446,0.185814354292471,-
0.708993586367855,0.478328456898255,0.906043677312476
"8949","AF111167.2",3.91659540801172,-0.520556253295351,0.735274557743
092,-0.707975337665955,0.478960572935843,0.906043677312476
"8950","CCDC9B",19.4250225979101,0.379751273849747,0.536163337999727,0
.708275346215373,0.478774284003553,0.906043677312476
"8951","SRRM2-
AS1",6.07472981946628,-0.39286057161618,0.556385375351193,-0.706094352
980081,0.480129463246348,0.906043677312476
"8952","CORO7",51.8657618728451,0.17252343152617,0.244177459448901,0.7
06549375669434,0.479846558391423,0.906043677312476
"8953","NPIPA1",114.227920616252,0.264560843331251,0.375145883583433,0
.705221235014332,0.480672568340011,0.906043677312476
"8954","AC092375.2",5.19976523495303,0.523308830327352,0.7394845151623
74,0.707667056709693,0.479152039804613,0.906043677312476
"8955","CCL22",5.95805857616738,-0.736608318463771,1.04311999611393,-0
.706158755663732,0.480089416132958,0.906043677312476
"8956","KPNB1",868.472754422782,0.12725816692612,0.180052292120048,0.7
06784487038198,0.479700416372423,0.906043677312476
"8957","MAP3K3",244.374715564296,0.155421258614031,0.22016933383913,0.
705916922688208,0.480239803022188,0.906043677312476
"8958","SUMO2",5101.91084999302,-0.174348953821858,0.245860981373027,-
0.709136329189753,0.478239880392303,0.906043677312476
"8959","PGLS",1387.89649185106,-0.184531082246234,0.26162100572155,-0.
705337408734816,0.480600285565734,0.906043677312476
"8960","ZNF527",32.4768518040847,0.218445834808671,0.308091726541109,0
.709028565165068,0.478306750601781,0.906043677312476
"8961","CD79A",6.76484622224836,0.508089041418219,0.717809436154578,0.
707832769850637,0.47904911363783,0.906043677312476
"8962","RPL18",20593.0884302194,-0.183690779386015,0.259938381326459,-
0.706670474935815,0.479771281707352,0.906043677312476
"8963","AL121890.5",3.03454742685634,-0.5886116467917,0.82967848914770
7,-0.709445471337162,0.478048078495885,0.906043677312476
"8964","PTGIS",775.927429875864,-0.25453175717003,0.360791911436686,-0
.705480774656159,0.480511092089012,0.906043677312476
"8965","AP001469.3",2.66835881515037,0.733271462584971,1.0372259761626
4,0.70695439512401,0.479594818939395,0.906043677312476
"8966","MIF",3428.77532051839,-0.202430831962559,0.286552471727908,-0.
706435476692639,0.479917365171506,0.906043677312476
"8967","BRWD3",88.7712897105074,0.256824487201746,0.363813616629497,0.
705923240534707,0.480235873862934,0.906043677312476
"8968","PABPC5",89.9909262193066,0.172899927397757,0.244188364117951,0
.70805964904307,0.478908216174918,0.906043677312476

"8969","SEPTIN6",234.785444859529,-0.132225389752873,0.187348862181972,-0.705770978339022,0.480330572781668,0.906043677312476
"8970","DCAF12L2",58.0622457250045,-0.209965353519353,0.296912819038284,-0.70716163148307,0.479466039250994,0.906043677312476
"8971","CD53",50.3893434206479,-0.292114617530253,0.415228560313117,-0.70350319185649,0.481742218311765,0.906379154805846
"8972","SLC38A9",65.4194398585132,0.167174392881115,0.237461603858114,0.704005995769334,0.481429039711188,0.906379154805846
"8973","RANBP9",231.311721048741,0.125074346399325,0.177653410840411,0.70403571655419,0.481410531164803,0.906379154805846
"8974","ARMC2",31.0650603593085,0.208771423276533,0.29661865988123,0.703837794156738,0.481533794153281,0.906379154805846
"8975","TOMM5",1058.54611351982,-0.182604694024823,0.259489777086258,-0.703706697332137,0.481615448669461,0.906379154805846
"8976","MAMDC2-AS1",4.61288417301323,0.522876791907165,0.742518202729055,0.704193903914249,0.481312026887907,0.906379154805846
"8977","TUT7",190.523521691026,0.124230999818427,0.176481797414166,0.703930952872621,0.481475774215725,0.906379154805846
"8978","NINJ2",224.278506869447,0.211165430692385,0.300199210180072,0.703417675768431,0.48179549425479,0.906379154805846
"8979","ANO6",631.011420700428,0.148088819562658,0.210666000966447,0.70295547873548,0.482083495337649,0.906379154805846
"8980","GPN3",241.348831222239,-0.154948624570735,0.220410887099021,-0.702998960759696,0.482056397124836,0.906379154805846
"8981","NEDD8",1930.53694336772,-0.176671268892574,0.251093317726593,-0.703608007143168,0.481676923470487,0.906379154805846
"8982","AL049840.4",17.6293512274298,0.476429565118833,0.677657118037726,0.703054026051431,0.482022081351047,0.906379154805846
"8983","ATP5MPL",1547.62021624049,-0.167094098564021,0.237563113594839,-0.703367185399826,0.48182695091679,0.906379154805846
"8984","ZC3H18",206.415067225643,0.145953202698686,0.207602405355407,0.703041963549604,0.482029598387894,0.906379154805846
"8985","AC026254.2",3.96400776186373,0.502275348360268,0.714001962988541,0.703464940429484,0.481766048295262,0.906379154805846
"8986","DHX40",308.45723858452,0.137053743106956,0.194638329513057,0.704145701670551,0.481342041569892,0.906379154805846
"8987","TYMSOS",5.90241545385712,0.519104884892201,0.737424272666154,0.703943312057494,0.481468077109792,0.906379154805846
"8988","DNASE2",458.814209167177,-0.138311574240872,0.196245564290317,-0.704788282685767,0.480942001518852,0.906379154805846
"8989","ZNF667-AS1",234.371514504283,-0.158227966698608,0.224672624611086,-0.704260107222697,0.481270804922806,0.906379154805846
"8990","RPS5",15068.733192418,-0.169723306390505,0.241222348844991,-0.70359693951728,0.481683817837389,0.906379154805846
"8991","ZSWIM3",13.0559599195315,0.330418031139434,0.469989048489161,0.70303346897466,0.482034892023924,0.906379154805846
"8992","ZNFX1",244.44313989829,0.171569702407147,0.244008513940165,0.703129983608764,0.481974748045034,0.906379154805846

"8993","PFDN4",421.945153759516,-0.173140813812712,0.245715890117186,-0.704638245943875,0.481035390976385,0.906379154805846
"8994","SDCBP2-AS1",31.0806690032217,0.207488720382618,0.295384632348174,0.702435731788676,0.48240746832411,0.906786600825353
"8995","MED15",168.877832757249,0.132483982516087,0.188604505621254,0.702443359344421,0.482402712997241,0.906786600825353
"8996","FGF14",82.9104224651213,0.220138391777424,0.313525738693764,0.702138180726667,0.482592993577246,0.906933681827729
"8997","MANBAL",351.849233535895,-0.165695777840707,0.235961641912116,-0.702214887546936,0.482545162607589,0.906933681827729
"8998","SLC11A1",21.8143070566081,-0.248317541850905,0.353730225211249,-0.701996957434466,0.482681060893681,0.906998374926691
"8999","CNIH4",641.960192912042,-0.169338445242759,0.241451941247142,-0.701333956430815,0.483094627233025,0.907372134776271
"9000","ST3GAL5-AS1",2.76750696380027,0.73962848360897,1.0543140831737,0.701525755382624,0.482974967228879,0.907372134776271
"9001","LINC01473",39.7320613935107,0.186796357374573,0.266340919036387,0.701343068314085,0.483088942124548,0.907372134776271
"9002","IGLC3",108.526213212533,-0.854512772896199,1.21811631297311,-0.701503430990552,0.482988894197942,0.907372134776271
"9003","GUK1",2756.74985392381,-0.187590922660249,0.267716016380181,-0.700708628481356,0.48348487016359,0.907734601811862
"9004","ANKRD39",132.863653472606,-0.172436185078369,0.246151563037865,-0.700528499393861,0.483597313476252,0.907734601811862
"9005","DCTN3",1090.44047741759,-0.191039208259295,0.272748485600555,-0.700422617704558,0.483663415408272,0.907734601811862
"9006","BTRC",85.0126575161273,0.189842373390965,0.271033401292334,0.700439032553787,0.483653167297815,0.907734601811862
"9007","PCLAF",5.02351411794285,-0.484372834423085,0.691402140556895,-0.700566003502598,0.483573900839357,0.907734601811862
"9008","NUPR1",6114.5463029648,0.146693463086007,0.20942874179551,0.700445706870747,0.483649000425285,0.907734601811862
"9009","LINC00266-1",5.73576232280139,0.466239630981903,0.665210559838364,0.700890303207442,0.483371476380179,0.907734601811862
"9010","BMP2K",106.465168284427,0.145261625419801,0.207442031962713,0.700251651246415,0.483770160106101,0.907738908900851
"9011","ZNF599",64.9229669838221,-0.17840860969335,0.254779553226635,-0.700246968149164,0.483773084226731,0.907738908900851
"9012","CFAP57",13.6107389315076,-0.502375640474786,0.717633479537941,-0.700044876387663,0.483899279229216,0.907874945983976
"9013","AC104248.1",21.7397275636549,-0.320300334281129,0.45788777437681,-0.699517113591997,0.484228921768507,0.908308386289088
"9014","ARL6IP4",2273.60829854388,-0.163743358837433,0.234085287954326,-0.699502989993042,0.484237745091663,0.908308386289088
"9015","AC022007.1",14.4024407768545,-0.293822190796184,0.420696908528816,-0.69841775596518,0.484915975290999,0.908505333067958
"9016","SUCLG2",557.708516579723,-0.0987273128891082,0.14138170515481,-0.698303311457475,0.484987528759841,0.908505333067958

"9017","MSL2",106.715252168841,0.160797821253819,0.230168163510886,0.6
98610176147206,0.484795682439961,0.908505333067958
"9018","ZNF165",5.67151872265549,-0.50079721228763,0.71714807469448,-0
.698317725388833,0.48497851650724,0.908505333067958
"9019","ZC2HC1A",517.356346266413,-0.139124615433378,0.199217016037644
,-0.698357089170982,0.484953904922933,0.908505333067958
"9020","SPACA9",60.5653939297257,0.19135439604348,0.273860714941187,0.
698728899778758,0.484721469579734,0.908505333067958
"9021","NMT2",255.235250895099,0.116320749170129,0.166574667984252,0.6
98309956596316,0.484983373902241,0.908505333067958
"9022","ANO9",2.44949285600752,0.719805900957729,1.02962382432158,0.69
9096003758468,0.484492035794986,0.908505333067958
"9023","BANF1",818.210357017221,-0.183198084605527,0.262044696670173,-
0.699110063792331,0.484483249681903,0.908505333067958
"9024","VASN",533.167295280325,-0.180818375742989,0.258702631946706,-0
.698942930662717,0.484587696738021,0.908505333067958
"9025","BAX",371.196869102332,-0.175464236519134,0.251213053686322,-0.
698467830171868,0.48488466948678,0.908505333067958
"9026","RBL1",63.0256060065967,0.200515252518086,0.28695845171387,0.69
876057429395,0.484701671222546,0.908505333067958
"9027","SNHG17",124.406133665696,0.178194728135513,0.255220023130523,0
.698200423108581,0.485051861920811,0.908525189028146
"9028","CPAMD8",8.61430176979201,0.359683993606004,0.515380927713499,0
.697899309549058,0.485240166227202,0.908676567789293
"9029","LINC01431",9.63870558545189,0.384339279566112,0.55067650104229
9,0.697940222324086,0.485214578700114,0.908676567789293
"9030","PSEN1",214.877695849169,0.138862025938107,0.199042918565273,0.
697648662605244,0.485396940924995,0.908768849203833
"9031","AC006115.2",85.6800026230733,-0.140617191343805,0.201538130795
421,-0.697720033369486,0.485352297133758,0.908768849203833
"9032","SPR",143.706880526751,-0.154742854065295,0.221897117108644,-0.
697363066639258,0.48557560896084,0.908906739733888
"9033","GPALPP1",197.815338672591,0.110504193822467,0.158460966734657,
0.697359079018534,0.485578103857122,0.908906739733888
"9034","AC253572.2",63.6156833915793,-0.260572506726398,0.373849558698
14,-0.696998299620287,0.485803857937862,0.909027478849297
"9035","PHAX",480.636955087742,-0.122503371072953,0.175758509381955,-0
.696998236408183,0.485803897497176,0.909027478849297
"9036","AC009754.1",2.98530266737057,-0.553205149097811,0.793661954586
313,-0.69702868570305,0.485784841962344,0.909027478849297
"9037","PCAT1",17.2058846391231,-0.485537405732625,0.696924861064226,-
0.696685443235867,0.485999670639388,0.909127958613497
"9038","ATXN7L3B",540.759081648995,-0.102937667152503,0.14775995118827
5,-0.696654718174211,0.48601890335388,0.909127958613497
"9039","AC234775.3",4.1900331192407,0.51790560750261,0.743255568176301
,0.696806898834778,0.485923648105981,0.909127958613497
"9040","ZNF280C",39.4159266556793,0.192985899165521,0.277150199529513,
0.696322425504767,0.486226932166994,0.90935436323122
"9041","MT-
ND4L",3898.47546710504,-0.24278263343488,0.348680554884736,-0.69628956

9445985,0.486247504020195,0.90935436323122
"9042","CDC42",2378.98345281493,-0.152756635290552,0.221176522815011,-0.69065483689838,0.489782477495937,0.909571469198784
"9043","LUZP1",327.38623727869,-0.137298955061106,0.199165649865421,-0.689370657811129,0.490590043480331,0.909571469198784
"9044","AL020997.5",17.9766913326937,0.276226998864599,0.397910972799037,0.694192967139175,0.487561202501513,0.909571469198784
"9045","CTPS1",29.7107553125426,-0.237527214696224,0.343360960084499,-0.69177117467801 2,0.489081041485197,0.909571469198784
"9046","DDAH1",26.7239468553475,0.294536534980405,0.423465921665143,0.695537751472976,0.486718361266315,0.909571469198784
"9047","HIPK1",281.099270657211,0.156847549504245,0.226022792704431,0.693945719489247,0.48771625022841,0.909571469198784
"9048","SNX27",479.695199249255,0.119153524714192,0.172592993607568,0.690372895351226,0.48995971731329,0.909571469198784
"9049","STUM",5.78773531163397,0.470036843504213,0.675822006380007,0.695503903493662,0.486739565764525,0.909571469198784
"9050","TOMM20",1978.89119329135,-0.141551104579908,0.204803661322954,-0.691155146668481,0.489468047571712,0.909571469198784
"9051","SRBD1",130.883622257251,0.129450931662482,0.186037832622534,0.695831217971317,0.486534536364903,0.909571469198784
"9052","AC016745.2",2.77543545332238,-0.634179416490611,0.919754151011712,-0.689509708429178,0.490502565877815,0.909571469198784
"9053","NIFK",475.651604410015,-0.143609815224293,0.20835724890674,-0.689247991024169,0.49066722087545,0.909571469198784
"9054","ZNF860",7.57352115956778,0.42178075497342,0.610247478854704,0.6911634534976,0.489462827892115,0.909571469198784
"9055","DLEC1",2.1787424571206,0.961702364882032,1.39081049586662,0.691469015901255,0.489270845511117,0.909571469198784
"9056","HESX1",4.90520276443763,-0.450435531082667,0.65196685932099,-0.690887158822438,0.489636456506599,0.909571469198784
"9057","USF3",196.054328140258,0.169639312231652,0.24595933691275,0.689704706318299,0.490379905628866,0.909571469198784
"9058","EEFSEC",91.6563588898794,-0.136303441096037,0.196382028013955,-0.694072886783464,0.487636500950478,0.909571469198784
"9059","AC105345.1",8.41457209274199,0.441514102208716,0.635097537205837,0.695191016093674,0.486935601699022,0.909571469198784
"9060","RHOH",12.5505983391511,0.490496054421831,0.708517025151313,0.692285487871063,0.488758061795561,0.909571469198784
"9061","GAR1",163.998620555188,-0.143866455457642,0.208605868417481,-0.689656798962736,0.490410039442883,0.909571469198784
"9062","LINC02511",544.532679556429,-0.183652926528105,0.264324824672511,-0.694800145070161,0.487180557271672,0.909571469198784
"9063","PDCD6",943.975561290883,-0.156216541670148,0.226160130202914,-0.69073422238477,0.489732578806379,0.909571469198784
"9064","MRPL36",472.74580299234,-0.203623825057022,0.295036229594125,-0.690165493699342,0.490090120374721,0.909571469198784
"9065","AC008875.3",288.991264642167,-0.121928014562312,0.175368356743657,-0.695268045081467,0.486887336131776,0.909571469198784
"9066","MAP1B",2319.92662943011,-0.207498436773489,0.299708849187467,-

0.692333367319759,0.488728000188584,0.909571469198784
"9067","CSNK1A1",1177.23938096321,0.0930565607859793,0.134832659161207
,0.690163357786485,0.490091463419559,0.909571469198784
"9068","TMED9",929.095822344005,-0.171659889752644,0.24754723177514,-0
.693442978625475,0.488031598475008,0.909571469198784
"9069","TRIM38",445.410047601987,0.13101708250236,0.189519429264274,0.
691312141509588,0.489369403353951,0.909571469198784
"9070","AARS2",53.2991315254554,0.196568250479402,0.28309092652395,0.6
94364361631251,0.487453737515228,0.909571469198784
"9071","AL137009.1",7.12285224763738,-0.424596419045692,0.615952275820
117,-0.689333306676006,0.490613542738943,0.909571469198784
"9072","AC092171.3",2.99852882486663,-0.553357551361545,0.800841070255
007,-0.690970495788063,0.489584082545577,0.909571469198784
"9073","ZNF12",210.907335730725,0.117712582066358,0.169714705149154,0.
693590941120313,0.487938776390745,0.909571469198784
"9074","HOXA3",61.1351991640902,0.32174023171016,0.46450204547428,0.69
2656221527823,0.488525318851411,0.909571469198784
"9075","KBTBD2",150.29009302589,0.108678816356076,0.157039514104775,0.
69204758417405,0.488907446867365,0.909571469198784
"9076","CDK13",349.578619583009,0.166648896475402,0.240997077713187,0.
691497581865836,0.489252899820115,0.909571469198784
"9077","GIMAP6",3.63252334729276,-0.641388549379317,0.927192130366854,
-0.691753659649316,0.489092042640793,0.909571469198784
"9078","SNX16",105.095411568997,-0.137179395869664,0.197752103256595,-
0.693693738830505,0.487874293374338,0.909571469198784
"9079","ANKRD26",241.009433313572,0.161853534169015,0.234240912745541,
0.690970387162205,0.489584150810636,0.909571469198784
"9080","JCAD",30.1079892541204,0.345897724492095,0.501454608513956,0.6
89788703941024,0.4903270733743,0.909571469198784
"9081","PGAM1",861.908705162737,-0.162439927305695,0.234722576840904,-
0.692050715751122,0.488905480320058,0.909571469198784
"9082","BNIP3",638.000051888454,-0.131463845649152,0.190685451809523,-
0.689427769143458,0.49055411337362,0.909571469198784
"9083","DTX4",89.7014295464184,0.227445956523414,0.328217808941722,0.6
92972624662788,0.488326731337948,0.909571469198784
"9084","LINC02739",5.91104957353988,-0.433868835468395,0.6287226981710
83,-0.690079802638737,0.490144003778468,0.909571469198784
"9085","C12orf60",7.71628476097737,0.407826765267263,0.589080323675273
,0.692310961470977,0.48874206780839,0.909571469198784
"9086","LINC02823",14.6350663431261,0.267010116363261,0.38533026954933
1,0.692938337482667,0.488348249267599,0.909571469198784
"9087","NT5DC3",18.5322480402821,0.330477948204474,0.477470764215063,0
.692142792758761,0.488847660307428,0.909571469198784
"9088","PPTC7",178.196870744109,-0.153517283461195,0.221484716737189,-
0.693128111604002,0.488229157345293,0.909571469198784
"9089","FZD10",5.40849051680445,-0.657268136025431,0.952397033572963,-
0.690119889978719,0.490118796057841,0.909571469198784
"9090","LINC00346",17.9372480803359,-0.377641489579216,0.5446577809990
62,-0.693355539484832,0.488086456604934,0.909571469198784
"9091","RAB2B",148.588098802755,-0.136398212664642,0.197686324206074,-

0.689972931675618,0.490211209783758,0.909571469198784
"9092","METTL3",108.97758728659,0.13133824213736,0.190460838679883,0.6
89581349361305,0.490457499398514,0.909571469198784
"9093","EID1",5823.28211299153,-0.131418643912095,0.190369459629788,-0
.690334700574687,0.489983730742651,0.909571469198784
"9094","ARPP19",464.01911037503,-0.0960078842500392,0.138653236864704,
-0.692431611558571,0.48866631964746,0.909571469198784
"9095","NTHL1",188.379411316323,0.177012414792764,0.254690630399017,0.
695009527894464,0.487049330546698,0.909571469198784
"9096","CAPNS2",2.08135046333025,0.704187160186807,1.01537427571509,0.
693524719927415,0.487980318099851,0.909571469198784
"9097","GABARAPL2",1888.17036916323,-0.171252484624645,0.2480608220001
06,-0.690364900204076,0.489964743888056,0.909571469198784
"9098","RPL13",57555.2123717373,-0.170625126708402,0.247433690129822,-
0.689579202488069,0.490458849879269,0.909571469198784
"9099","CPD",226.492736434225,0.134198612379394,0.192897287169563,0.69
5699842898407,0.486616823945684,0.909571469198784
"9100","CWC25",272.484824186131,-0.152085069199968,0.219411406555835,-
0.693150240396761,0.488215271537603,0.909571469198784
"9101","CAVIN1",4618.12819317917,-0.137104580382373,0.198003538583376,
-0.692435000724196,0.48866419190728,0.909571469198784
"9102","AC103810.2",7.17273518254464,-0.418667493277525,0.602332515566
355,-0.695077025492912,0.487007031773311,0.909571469198784
"9103","TMEM104",25.3896660751046,-0.2164166667703,0.313334788039623,-
0.690688283048012,0.489761454188142,0.909571469198784
"9104","GALK1",264.232956265832,-0.18455545023761,0.267589565690981,-0
.689695989307516,0.490385388570446,0.909571469198784
"9105","DOT1L",40.146476418678,0.214838109655216,0.311128685474601,0.6
9051206039552,0.489872228246281,0.909571469198784
"9106","GNG7",13.3490492195116,0.385058438449647,0.553239480994069,0.6
96006795751033,0.486424573892388,0.909571469198784
"9107","ZNF253",67.1970276902617,0.228293143466352,0.329601732384624,0
.692633323904828,0.48853969202268,0.909571469198784
"9108","GRAMD1A",210.124402038085,-0.11035320885301,0.159852116127246,
-0.690345623983895,0.489976863023859,0.909571469198784
"9109","COX6B1",2282.23964738859,-0.18230198930755,0.262120734980886,-
0.695488624052744,0.486749137928636,0.909571469198784
"9110","CAPN12",3.30003652229923,-0.552138832099025,0.796383528050095,
-0.69330769993562,0.488116471896379,0.909571469198784
"9111","AC074212.1",19.0786364296941,0.277015962880613,0.4016604776830
18,0.689676924348101,0.490397380417509,0.909571469198784
"9112","FCGRT",4380.87546024719,0.187136468956917,0.270411796699359,0.
692042548591076,0.488910609088793,0.909571469198784
"9113","ZSCAN22",16.2318700724236,0.332472449102994,0.480304896612398,
0.692211242167071,0.488804679699177,0.909571469198784
"9114","NRSN2-
AS1",25.7551781641463,0.201042401204791,0.290393590578319,0.6923100499
71885,0.488742640102094,0.909571469198784
"9115","DLGAP4",471.280226778658,0.105407300455503,0.152675834196435,0
.690399374663865,0.48994306988225,0.909571469198784

"9116","RNF114",420.878207048973,-0.153340484260032,0.220450736049363,
-0.695577102657967,0.486693709842288,0.909571469198784
"9117","TBC1D10A",108.648359078815,-0.137037958169679,0.19844593884501
,-0.69055561916391,0.489844845826896,0.909571469198784
"9118","AL078622.1",3.55907608051077,0.726761482102303,1.0515566073223
4,0.691129205067628,0.489484348430363,0.909571469198784
"9119","AL022328.4",8.20980386813713,0.371554431695153,0.5378025718925
57,0.690875148453887,0.489644004793113,0.909571469198784
"9120","AL050309.1",97.6396437496922,0.148644677936965,0.2145462208584
18,0.692832888606589,0.488414430019194,0.909571469198784
"9121","TMLHE",89.06349518208,0.171769072169175,0.249100675197578,0.68
9556830919603,0.49047292273069,0.909571469198784
"9122","DYNLRB1",1563.24154489398,-0.189579649162614,0.275111378961573
,-0.689101446396711,0.490759429877027,0.909642670506552
"9123","RABEPK",157.007973821596,-0.140577834327088,0.204030443144431,
-0.689004210158843,0.490820618128802,0.909655384079692
"9124","RAB5IF",477.087860835088,-0.158828913207838,0.230547835188491,
-0.688919560133728,0.490873889540047,0.909655384079692
"9125","RFNG",487.04936315764,-0.154541836264623,0.224390288971776,-0.
688718914587527,0.49100017091796,0.909789686562288
"9126","LINC02609",245.578340117873,-0.155723150545336,0.2266270542966
65,-0.6871339833129,0.491998300907784,0.909803790217168
"9127","AMPD2",82.4330935608707,-0.14669622834837,0.213524264092801,-0
.687023692467163,0.492067798416835,0.909803790217168
"9128","TGFB2",112.659141043947,0.281140528361094,0.408565636632084,0.
688115943079821,0.49137977138836,0.909803790217168
"9129","HK2",9.80505234710261,-0.336620895127575,0.490231845888126,-0.
686656523746916,0.49229920018316,0.909803790217168
"9130","TGFBRAP1",103.879882637665,0.129017695709344,0.187588318003517
,0.687770417062565,0.491597368160032,0.909803790217168
"9131","GAK",151.998195503672,0.140182630638459,0.203975289976733,0.68
7253003314515,0.491923308809017,0.909803790217168
"9132","MRPS30-
DT",53.2186597143327,0.255825147848159,0.372239490780947,0.68725955784
9291,0.491919179107254,0.909803790217168
"9133","LYRM4",365.051297965946,-0.141848891474011,0.206263857842319,-
0.687705994437711,0.491637944357137,0.909803790217168
"9134","STYXL1",71.5789061482317,0.176366303286799,0.256792653516812,0
.686804318080899,0.492206048330723,0.909803790217168
"9135","ZNF398",72.382310139848,0.186668687597862,0.271657299922185,0.
687147695465325,0.491989660844919,0.909803790217168
"9136","VPS28",1740.83833887077,-0.18290238854803,0.26611531287711,-0.
687305012893013,0.491890540550022,0.909803790217168
"9137","ST6GALNAC6",629.861586312063,-0.116816474100234,0.169953084546
072,-0.687345418956292,0.491865083810402,0.909803790217168
"9138","CHKA",44.1653520308838,0.21807287586466,0.316954924170883,0.68
8024886930257,0.4914371094436,0.909803790217168
"9139","AP003071.4",7.86641736828757,-0.358879718302565,0.521857574327
828,-0.68769667425986,0.491643814763132,0.909803790217168
"9140","CD3E",18.0048142651386,0.368243298598202,0.536167320102663,0.6

86806682898339,0.492204557909804,0.909803790217168
"9141","LRMP",8.78736192623101,0.546187353643192,0.794774859216652,0.687222736488579,0.491942378743072,0.909803790217168
"9142","AC023794.2",23.7547938170738,-0.282256858517213,0.410332485669053,-0.687873537619107,0.491532422048901,0.909803790217168
"9143","LINC01309",6.32544301677325,0.469201250612257,0.682213027163719,0.687763545886756,0.491601695842534,0.909803790217168
"9144","PCID2",350.655437250816,-0.12343084413741,0.179326520494898,-0.688302231018427,0.4912624770713,0.909803790217168
"9145","MPP3",29.534606725241,0.262642918140612,0.382063180942822,0.68743320801676,0.491809777140746,0.909803790217168
"9146","ZNF682",31.9940272444118,0.223085942979346,0.324728513804281,0.686992159591513,0.492087669178239,0.909803790217168
"9147","ZNF626",178.289154182685,0.132387761420058,0.192583270300968,0.68743126655375,0.491811000216657,0.909803790217168
"9148","CSNK2A1",453.700713544784,0.134739337849489,0.196196654469142,0.686756551553131,0.492236153596608,0.909803790217168
"9149","LINC01637",23.3740602739274,-0.388865590583132,0.566271333452583,-0.686712477942686,0.492263932245528,0.909803790217168
"9150","AL021368.5",3.74698230242181,0.583785433546306,0.850337668581485,0.686533661998256,0.492376644715225,0.909847465447544
"9151","AC239868.1",9.38143805301725,0.353267770713797,0.515430109883974,0.685384427373325,0.493101368190709,0.90992689807651
"9152","KIAA2012",3.13631998980959,0.56695478699982,0.827298661558914,0.685308478477993,0.493149282751973,0.90992689807651
"9153","EMC3",605.152078248589,-0.148439518507583,0.216536568116818,-0.68551709209459,0.493017678790631,0.90992689807651
"9154","PTH1R",257.747060965807,0.180398174141675,0.263144108200191,0.685548976853528,0.492997565938987,0.90992689807651
"9155","ARL13B",197.896117387535,-0.12105946291753,0.176632949664851,-0.685373047029064,0.493108547651778,0.90992689807651
"9156","TFG",742.910558053005,-0.128788317388283,0.188182321052836,-0.684380534089189,0.493734904695927,0.90992689807651
"9157","CCDC14",291.034304299797,0.129492408904477,0.188862868910254,0.68564249633426,0.492938576545589,0.90992689807651
"9158","AL022069.1",4.23395356152495,-0.476278099624639,0.695315140954333,-0.684981631452666,0.493355512127095,0.90992689807651
"9159","SND1-
IT1",34.9850290237364,0.267997941548268,0.391110916164531,0.685222351184715,0.493203621668179,0.90992689807651
"9160","PLPBP",374.151334981387,-0.120155383548552,0.175335964522397,-0.685286580399221,0.493163098252676,0.90992689807651
"9161","ELOC",820.514012697384,-0.208878103732528,0.30481290624937,-0.685266599445243,0.493175704419991,0.90992689807651
"9162","GPAA1",597.425199758297,-0.18687083744162,0.272966125621166,-0.684593507770878,0.493600464964738,0.90992689807651
"9163","GOLM1",381.826325642685,0.0880471820334306,0.128579766317609,0.684767009266002,0.493490956556347,0.90992689807651
"9164","ITIH5",3239.61573969471,-0.193979604056107,0.283282404737242,-0.68475698035687,0.493497286118022,0.90992689807651

"9165","AC069549.2",4.1442705626296,0.634610181738571,0.92534809796754
,0.685806976998652,0.492834836058819,0.90992689807651
"9166","REEP3",398.259742862107,-0.111989515000101,0.163268624303424,-
0.685921838797241,0.492762397893316,0.90992689807651
"9167","SVIP",20.0021634184834,-0.342351206953625,0.499242954660901,-0
.685740687489839,0.492876644464799,0.90992689807651
"9168","CASP4",699.354401399676,-0.121070615186642,0.1769177441235,-0.
684332799892177,0.493765039617458,0.90992689807651
"9169","SEPHS2",462.960197019036,-0.265715308049844,0.387468292464139,
-0.68577303799494,0.492856240952769,0.90992689807651
"9170","ACD",110.907209583005,-0.137532792367609,0.200855269476545,-0.
684735793718719,0.493510657818206,0.90992689807651
"9171","SLFN13",21.8690049888255,0.259382512032251,0.37867447828651,0.
684974897716764,0.493359761371318,0.90992689807651
"9172","AC090337.1",2.77235305848954,0.781239351119618,1.1410864618705
1,0.684645184413969,0.493567847015582,0.90992689807651
"9173","AC022137.4",22.9646652589637,0.35571786830407,0.51973886121082
7,0.684416530785018,0.493712180389407,0.90992689807651
"9174","TMX4",601.021373788137,0.0897919261450273,0.131138587602983,0.
684710181696242,0.493526822807222,0.90992689807651
"9175","AC093151.8",2.82299145955603,-2.15006912452012,3.1408441116742
6,-0.684551365197811,0.493627065927346,0.90992689807651
"9176","TMEM240",71.2054554162671,-0.1651148751035,0.25024135569191,-0
.65982249275689,0.509367747313404,0.909935336521942
"9177","AL691432.2",30.7882104536743,-0.193922631215679,0.291382297871
925,-0.665526466885495,0.50571382096483,0.909935336521942
"9178","RPL22",13300.8022700282,-0.161078491420922,0.243303105037693,-
0.662048646670446,0.507940045523613,0.909935336521942
"9179","RNF207",37.4316579886661,0.242649499088411,0.359684433809858,0
.674617737882658,0.499918660534372,0.909935336521942
"9180","PARK7",3148.86617323586,-0.183454215256155,0.271315424894559,-
0.676165814484934,0.49893537445466,0.909935336521942
"9181","EXOSC10",172.389505082305,0.128395069139192,0.194780864522569,
0.65917701645849,0.509782104000444,0.909935336521942
"9182","PLA2G5",164.084239435128,-0.327109494613284,0.485636580158092,
-0.673568483055371,0.500585696098777,0.909935336521942
"9183","EPHB2",56.864356243513,-0.197942074409811,0.298484501987179,-0
.663156958207207,0.50723003458253,0.909935336521942
"9184","RPA2",348.626329430301,-0.173893568887723,0.257733405398296,-0
.674703260211812,0.499864312809378,0.909935336521942
"9185","BSDC1",350.005910559661,0.116742716549366,0.174222490312281,0.
670078336844532,0.50280785377783,0.909935336521942
"9186","ZMYM1",128.305611630972,0.119767629834677,0.176697775374873,0.
677810626537792,0.497891772460583,0.909935336521942
"9187","OSCP1",65.404390468212,-0.191538254512058,0.281797098926895,-0
.679702719586009,0.496692713672781,0.909935336521942
"9188","ZC3H12A",79.7895149436266,-0.288924210921648,0.437266910767418
,-0.660750227851854,0.508772506636742,0.909935336521942
"9189","MEAF6",628.287321407846,-0.124845415880115,0.187324590511743,-
0.666465708207639,0.505113475251681,0.909935336521942

"9190","UTP11",256.279489774113,-0.131452064074896,0.19769711137744,-0.664916463164349,0.506103925170452,0.909935336521942
"9191","POU3F1",19.1005959200078,0.434594018086182,0.655769651287476,0.662723590872101,0.507507598004995,0.909935336521942
"9192","NASP",846.163575771675,0.180801783851161,0.269698552298646,0.670384702884699,0.502612583605875,0.909935336521942
"9193","ZYG11B",377.644460399625,0.154362961797483,0.229588207716061,0.672347083210773,0.50136276219911,0.909935336521942
"9194","NFIA-AS1",2.02640476340654,0.66425641968106,0.972674700689489,0.68291734041216,0.494659075992976,0.909935336521942
"9195","FPGT",70.1004392855556,0.196469534909245,0.296939453381988,0.661648469651162,0.508196536602557,0.909935336521942
"9196","LMO4",1257.12080812734,-0.113789369847042,0.168501910161289,-0.675300177535813,0.499485071160809,0.909935336521942
"9197","KYAT3",191.021076435381,-0.179653367342787,0.269134586163514,-0.667522409154941,0.504438500622148,0.909935336521942
"9198","AC099063.4",11.8656013215601,0.32952946621663,0.488407418168552,0.674702008934082,0.499865107948676,0.909935336521942
"9199","AC092802.1",24.0544292084355,0.222522963820684,0.328777196446859,0.676819944404663,0.498520202278932,0.909935336521942
"9200","SARS",717.300954515028,-0.114608522860559,0.171814628386258,-0.667047526377712,0.504741776238458,0.909935336521942
"9201","ST7L",95.3935677808341,-0.145349575380918,0.21973910911631,-0.661464297208846,0.508314603657767,0.909935336521942
"9202","ANP32E",297.513491438384,0.109399614747775,0.162586788592492,0.672869030102899,0.501030616513188,0.909935336521942
"9203","HORMAD1",2.32832803158866,-2.09999873364785,3.15606387968063,-0.665385370419168,0.505804039660247,0.909935336521942
"9204","TDRD10",23.9119825440021,0.238626016907611,0.362120282655753,0.658968934735035,0.509915717454029,0.909935336521942
"9205","RIT1",124.869140949375,-0.143079783355761,0.211132205970359,-0.67767862651825,0.49797548106963,0.909935336521942
"9206","NAXE",390.888716638992,-0.185712395966479,0.272782625031735,-0.680807276287752,0.495993445341409,0.909935336521942
"9207","NES",99.2771459694019,-0.172059585327183,0.256504781620475,-0.670785098976292,0.502357441447119,0.909935336521942
"9208","SH2D2A",14.6820468227354,0.350520685346213,0.513133929047056,0.683097853219659,0.494545011879536,0.909935336521942
"9209","PIGM",140.928823577523,0.133055684555338,0.201843219550929,0.659203142178203,0.509765329445933,0.909935336521942
"9210","PEA15",522.097906295623,0.105320944642556,0.159550459155223,0.660110570663363,0.509182875684564,0.909935336521942
"9211","DEDD",134.23975971644,0.129469934462129,0.1911095064478,0.677464647722757,0.498111192922376,0.909935336521942
"9212","DPT",6622.17218823667,-0.146809903989185,0.222669241457629,-0.659318292136548,0.509691398494788,0.909935336521942
"9213","PLEKHA6",112.66663428658,0.32476239722773,0.490102742768564,0.662641460427592,0.507560209958786,0.909935336521942
"9214","FLVCR1",42.3775497591469,0.223736961803931,0.333326882897508,0

.671223874471374,0.50207792167982,0.909935336521942
"9215","AC104794.5",9.15884699920636,-0.355941847518037,0.532959729140663,-0.667858804439039,0.50422372579133,0.909935336521942
"9216","OST4",3260.65391698269,-0.173461574618714,0.260070525213899,-0.666978983781601,0.504785557709795,0.909935336521942
"9217","SLC4A1AP",193.611112253908,0.115690277599578,0.172493337360115,0.670694180831177,0.502415370695614,0.909935336521942
"9218","DPY30",510.972761412357,-0.177622475848399,0.261243777410544,-0.679910838868577,0.496560918182243,0.909935336521942
"9219","LINC02613",5.85834515145317,0.448969900102826,0.676278402884976,0.663883244219449,0.506765041250299,0.909935336521942
"9220","THUMPD2",129.70842233039,0.114112827351382,0.17040674678582,0.669649702865387,0.503081121805496,0.909935336521942
"9221","AC007877.1",2.05716250264767,0.751958172270352,1.10030959752847,0.683405992240195,0.4943503346222,0.909935336521942
"9222","AC005041.4",15.0409974057313,-0.314864357150069,0.468196205662036,-0.672505144942059,0.50126216585980З,0.909935336521942
"9223","LBX2",19.5118168401,0.29862974191715,0.436868934105066,0.683568270948125,0.494247826032854,0.909935336521942
"9224","SLC20A1",343.132780151716,0.270278910143471,0.401836239333759,0.672609594872756,0.501195695923239,0.909935336521942
"9225","WDSUB1",85.7453328463547,-0.169321398385412,0.249717227694907,-0.678052531450817,0.497738386439018,0.909935336521942
"9226","DYNC1I2",1270.67020394851,0.11560511434748,0.174080784496961,0.664088886556561,0.506633422652306,0.909935336521942
"9227","PRKRA",328.479815207301,-0.136565101513403,0.204584119992107,-0.667525424351958,0.504436575326131,0.909935336521942
"9228","BZW1",1454.08247566113,-0.109296023074687,0.161741507132492,-0.675745051547939,0.499202527139877,0.909935336521942
"9229","FASTKD2",175.669856454057,0.108582205361538,0.163274913814062,0.665026872929121,0.506033305158032,0.909935336521942
"9230","AC007563.2",30.5682796124192,0.249682685018144,0.37158113622967,0.671946610507747,0.501617685714053,0.909935336521942
"9231","AC009974.1",3.47779077906308,-0.543003826911073,0.806039165684294,-0.673669283117384,0.500521594685086,0.909935336521942
"9232","MTERF4",205.119520142607,-0.127051833864255,0.186222338115156,-0.682258826466293,0.495075303120574,0.909935336521942
"9233","AC061958.1",4.49825251232929,0.532326287598568,0.807383593071961,0.659322646838976,0.509688602709062,0.909935336521942
"9234","CMC1",408.035183947016,-0.154966721942741,0.232311000379186,-0.667065794085511,0.50473010810776,0.909935336521942
"9235","CMTM6",504.875957248174,-0.10860396627345,0.163105890606082,-0.66584944216233,0.505507338767642,0.909935336521942
"9236","OXSR1",125.735520263432,0.138699082246273,0.209262879649339,0.662798306506584,0.507459738392928,0.909935336521942
"9237","RRP9",83.7548108546109,-0.179448129434864,0.264429396014648,-0.678623981067986,0.497376144051886,0.909935336521942
"9238","PPP4R2",406.901856925497,0.103732337216482,0.155955219939984,0.665141809657932,0.505959795128786,0.909935336521942
"9239","LNP1",19.7347016298001,0.271005678683154,0.40782705149999,0.66

4511286552456,0.506363127623051,0.909935336521942
"9240","RUVBL1",137.335395980588,0.145646751991448,0.214058921787008,0
.680404959417523,0.496248081656657,0.909935336521942
"9241","TOPBP1",163.056913553581,0.132986378808799,0.194570664071503,0
.683486277047023,0.494299618708773,0.909935336521942
"9242","RBP1",53.2375331242043,-0.231383029732572,0.348946464975355,-0
.663090338940429,0.507272697751186,0.909935336521942
"9243","TRPC1",117.360823740807,0.128460216709254,0.193249527931158,0.
664737544688935,0.506218375557879,0.909935336521942
"9244","MBNL1-
AS1",23.0252866074974,-0.233527829248156,0.343509326407751,-0.67982966
1949135,0.496612322790948,0.909935336521942
"9245","AC078795.2",2.99675944719792,0.591209821888643,0.8730683206320
19,0.67716329629354,0.498302352518242,0.909935336521942
"9246","TNFSF10",1470.19796883498,-0.187959231919987,0.284488034050692
,-0.660692927023059,0.508809260643002,0.909935336521942
"9247","USP13",101.617189776684,-0.178079208643096,0.264765976518552,-
0.672590983874464,0.501207539264429,0.909935336521942
"9248","PARL",349.027926526689,-0.151393161301764,0.226211876800487,-0
.669253813915744,0.503333583506262,0.909935336521942
"9249","LSG1",167.893538400178,-0.137463673689162,0.203006611938932,-0
.677138898956227,0.498317830459145,0.909935336521942
"9250","LNX1",52.4752838049863,-0.19160075568438,0.289728868426775,-0.
661310544319487,0.50841318072845,0.909935336521942
"9251","ABCG2",3.91793288021492,-0.50940974968301,0.765098178216445,-0
.665809649253796,0.505532776481598,0.909935336521942
"9252","ENPP6",2.85986183307932,0.67610317832111,1.00727435460246,0.67
1220482514864,0.502080082193346,0.909935336521942
"9253","UFSP2",349.028909849956,-0.163227033790936,0.24249422169836,-0
.673117209341075,0.500872726290875,0.909935336521942
"9254","RNF180",20.4362353320864,0.271343844914242,0.408002095775975,0
.66505502722523,0.506015298008549,0.909935336521942
"9255","CCNB1",30.8976617305074,-0.259270758336463,0.386383742462548,-
0.671018808100069,0.502208547958058,0.909935336521942
"9256","ENC1",18.5870185840784,-0.31287097684411,0.471700934084234,-0.
663282504308628,0.507149639623616,0.909935336521942
"9257","AC011416.3",6.4809253518126,0.376140147687848,0.57021449257901
1,0.659646769037053,0.509480533823777,0.909935336521942
"9258","PPP2CA",298.521170118642,-0.113435132417343,0.166379004565358,
-0.681787541124414,0.495373303441301,0.909935336521942
"9259","SEC24A",127.270012591769,0.135154957418644,0.199212047219015,0
.67844771089599,0.497487866883423,0.909935336521942
"9260","FAM13B",164.420023302057,0.149366508370571,0.221518350729542,0
.67428503272371,0.500130117815686,0.909935336521942
"9261","SNHG4",10.3198840503463,0.384120453452486,0.582889403104752,0.
658993715456953,0.509899804293663,0.909935336521942
"9262","PCDHGA4",4.34406743755576,-0.445779016070654,0.667384834383976
,-0.667948975019978,0.504166163708634,0.909935336521942
"9263","PCDHGA12",31.8420077268256,0.212719912215153,0.322813586449299
,0.658955883966681,0.509924098225142,0.909935336521942

"9264","FBXO38",76.4286737118462,0.145746368942325,0.217379173312593,0
.670470711252271,0.502557771198468,0.909935336521942
"9265","LINC02227",2.04633742190011,0.746044816638088,1.10222335840624
,0.676854478675569,0.498498288699009,0.909935336521942
"9266","ZNF454",11.2441006405205,-0.341244038896988,0.504757611127633,
-0.676055261721851,0.499005559749801,0.909935336521942
"9267","TRIM41",57.181399680007,0.174867209488065,0.264541362514585,0.
661020294996116,0.508599298392977,0.909935336521942
"9268","EXOC2",61.4925150512011,0.14220604201176,0.215201415302202,0.6
60804399506683,0.508737761030805,0.909935336521942
"9269","HMGN4",354.233259214,-0.14125162606268,0.212093362535166,-0.66
5987961029474,0.505418795500256,0.909935336521942
"9270","MICA",191.427734547299,-0.191874674115862,0.283477668820098,-0
.676859926619585,0.49849483177526,0.909935336521942
"9271","PPP2R5D",144.441575057397,0.13945276005739,0.210355981273241,0
.662936985263322,0.507370913046259,0.909935336521942
"9272","FAM135A",71.495877912153,0.177211165309228,0.26766804830973,0.
662055730701821,0.507935505667829,0.909935336521942
"9273","AL589935.1",4.81979801896559,-0.443300820906867,0.649245067444
745,-0.682794283908202,0.494736842143136,0.909935336521942
"9274","IRAK1BP1",89.995735710529,-0.137087719840054,0.201980548386468
,-0.678717435590639,0.497316916513378,0.909935336521942
"9275","AL451064.1",2.86518418996113,0.68462058521146,1.00552968011322
,0.680855671146748,0.495962819734428,0.909935336521942
"9276","SH3BGRL2",46.6326463444614,0.198327785327085,0.298793913114672
,0.6637611297355,0.506843207479044,0.909935336521942
"9277","COQ3",58.9523621329641,-0.205154873011459,0.304059296978583,-0
.674719947885392,0.499853708493136,0.909935336521942
"9278","DDO",9.52549435659248,0.432554582225535,0.642764195092364,0.67
2959983658359,0.500972749316258,0.909935336521942
"9279","ASF1A",194.87713787788,-0.160910760438909,0.243262054872584,-0
.661470859165399,0.508310396750413,0.909935336521942
"9280","AL121933.2",28.6304355954237,-0.328975887341364,0.482727144965
185,-0.681494485596186,0.495558654847649,0.909935336521942
"9281","CITED2",1861.09338036913,0.237950723090071,0.359221640416851,0
.662406426333186,0.507710786305769,0.909935336521942
"9282","ACAT2",175.864769897874,-0.157517870906637,0.23749306356904,-0
.663252511629022,0.507168845188842,0.909935336521942
"9283","MEOX2",194.158383605587,0.288209497355807,0.422109789245301,0.
682783258524051,0.494743810006698,0.909935336521942
"9284","AC018645.3",28.3676076188479,0.265183146893546,0.3901432638610
11,0.67970710110227,0.496689938802203,0.909935336521942
"9285","FKBP9",1201.8916938633,0.141256697724187,0.211943847735964,0.6
6648170840119,0.505103251476945,0.909935336521942
"9286","NSUN5",121.739706754336,-0.134282128123412,0.199437334602818,-
0.67330486737018,0.500753356828673,0.909935336521942
"9287","AC005081.1",2.52201567199786,-0.622359025119534,0.939807144931
21,-0.662219933606791,0.50783028094497,0.909935336521942
"9288","NCF1",14.4587745577784,-0.405819164499203,0.598535155697337,-0
.678020598516714,0.497758632894693,0.909935336521942

"9289","PDAP1",1070.74784832383,-0.130294890889084,0.194262419396911,-0.670715886755583,0.502401540265248,0.909935336521942
"9290","ZSCAN21",66.5692797905675,0.171868962078731,0.256388422989859,0.670346032299319,0.502637229073826,0.909935336521942
"9291","DNAJC2",263.428740224783,-0.14497933307435,0.219533694663015,-0.660396725417909,0.508999273023876,0.909935336521942
"9292","AC018638.7",10.899420080196,0.395814324837685,0.595221333756642,0.664986791282442,0.50605894147928,0.909935336521942
"9293","ZC3HAV1L",10.4125735853393,0.332467475115399,0.497754517885788,0.667934620719376,0.504175326813821,0.909935336521942
"9294","SSBP1",1160.97041175508,-0.170452421322586,0.255017182138354,-0.668395830795867,0.503880956102894,0.909935336521942
"9295","ZNF746",61.4785508600885,0.156644706815582,0.237741471372071,0.658886755901452,0.50996849118062,0.909935336521942
"9296","ZNF467",11.6515917447917,-0.286456654478757,0.431394679279579,-0.664024542345155,0.506674603363112,0.909935336521942
"9297","REPIN1",195.540223601632,-0.139862451021581,0.204490140318619,-0.683956941902723,0.494002355698167,0.909935336521942
"9298","WDR60",331.059742169495,-0.180052136363095,0.267891197647746,-0.672109191881131,0.501514185204855,0.909935336521942
"9299","FDFT1",270.281642246457,-0.12202852151539,0.183004310388344,-0.666806815951164,0.50489553845822,0.909935336521942
"9300","CHMP7",100.150715232087,0.119645017366475,0.176606379047897,0.677467133472152,0.498109616271635,0.909935336521942
"9301","PTK2B",39.4144302557317,0.217835489952766,0.323406988539095,0.673564572419352,0.5005881830629,0.909935336521942
"9302","AC044849.1",45.6703208625828,-0.25421812089799,0.374914504996473,-0.67806957989097,0.497727577386678,0.909935336521942
"9303","HOOK3",729.685821124114,0.135215377135636,0.20427198847069,0.661937929659102,0.508011002135415,0.909935336521942
"9304","PRKDC",641.088889633539,0.0938595540529393,0.137666127818384,0.681791196864078,0.495370991497325,0.909935336521942
"9305","FAM110B",190.205215394809,0.18009775647325,0.27233554185717,0.66130830829163,0.508414614408474,0.909935336521942
"9306","HEY1",2.28644033049316,-0.72435252486103,1.08564138586432,-0.66721159886913,0.504636983333263,0.909935336521942
"9307","CA3",72.7173331574994,-0.513662409813854,0.775602066226881,-0.66227571093602,0.507794540246278,0.909935336521942
"9308","AC016405.1",4.19697361073165,-0.465035119756943,0.699069781413558,-0.665219885226072,0.505909863579794,0.909935336521942
"9309","ZHX1",415.064066099113,0.0990129536427492,0.146574682635491,0.675511976982954,0.499350544582924,0.909935336521942
"9310","PEG13",2.39531858374876,0.604203620090784,0.908318378874961,0.665189248773264,0.505929456154591,0.909935336521942
"9311","HSF1",252.65395606569,-0.0969923421880266,0.141998274736867,-0.683052962212112,0.494573376713532,0.909935336521942
"9312","WASHC1",153.246978594844,0.180295048650536,0.271097808360881,0.665055353050032,0.506015089616895,0.909935336521942
"9313","HINT2",441.279257529361,-0.17840837395187,0.266506866712562,-0.669432559665676,0.503219587558655,0.909935336521942

"9314","ZBTB5",32.9196351175728,0.182750655886559,0.270954255595757,0.674470513425736,0.500012226069215,0.909935336521942
"9315","DAPK1",23.8868919748587,-0.294469829635276,0.439259993633829,-0.670377074860018,0.502617445034267,0.909935336521942
"9316","TSTD2",114.206578294767,0.156818700448591,0.234959739621061,0.667427963197039,0.504498809232288,0.909935336521942
"9317","TBC1D2",14.9705932901527,-0.269064826982384,0.407503357587771,-0.660276343672654,0.509076508152034,0.909935336521942
"9318","PRPF4",138.760047495677,-0.149012891694104,0.225129731742283,-0.661897877907507,0.508036671889101,0.909935336521942
"9319","FAM102A",188.06367204813,0.116406255435919,0.173073266591697,0.67258368509642,0.50121218397346,0.909935336521942
"9320","RALGDS",349.583058524494,-0.131254938374192,0.197055944766183,-0.666079567048499,0.505360244092165,0.909935336521942
"9321","GBGT1",49.82952976789,0.176114774645639,0.259986382685222,0.677399996210068,0.498152200746258,0.909935336521942
"9322","REXO4",225.524134158646,0.113843322127032,0.167682184377856,0.67892318166906,0.497186536558143,0.909935336521942
"9323","NSMF",85.6145724976465,0.17213048541579,0.25395847927898,0.677789872992189,0.497904932950627,0.909935336521942
"9324","PRPF18",282.905626692744,-0.0963944943054161,0.14438039250841,-0.667642556102632,0.504361786100635,0.909935336521942
"9325","MSRB2",739.437803940599,-0.16401108154862,0.24472455710832,-0.670186447517097,0.502738942118554,0.909935336521942
"9326","ACBD5",202.49224130999,0.13221085583944,0.199484292249251,0.66276323989583,0.507482200255805,0.909935336521942
"9327","AL353796.1",6.60925067875772,0.378287404910295,0.561945677281892,0.673174330195145,0.50083639006129,0.909935336521942
"9328","SYT15",3.56642412331023,0.555712810446967,0.824837771815685,0.673723766582242,0.500486949027277,0.909935336521942
"9329","AC016394.2",4.24691487988861,0.499432093401433,0.746473912447381,0.669054986481711,0.503460402664862,0.909935336521942
"9330","CHCHD1",421.441417814616,0.158959750999025,0.236844875476499,0.671155542965495,0.502121446518601,0.909935336521942
"9331","SAMD8",280.224683749346,0.169523211022478,0.248483262937743,0.682231909780385,0.495092320338735,0.909935336521942
"9332","WAPL",273.621764000239,0.137509566413207,0.206931646476988,0.664516852566093,0.506359566420489,0.909935336521942
"9333","IFIT3",725.751984448774,0.277681937426513,0.406788634615007,0.682619704184501,0.494847179850916,0.909935336521942
"9334","PCGF5",210.072327054515,-0.109354844106817,0.164047349851957,-0.666605368544528,0.50502423901976,0.909935336521942
"9335","CPEB3",30.8789293411312,0.237731606020926,0.352290982539427,0.674816040726562,0.499792647848557,0.909935336521942
"9336","SLC25A28",237.393035495989,-0.111780924283716,0.165766549797219,-0.67432738643868,0.500103196446814,0.909935336521942
"9337","ADD3-AS1",16.8255872977146,-0.258758452878961,0.383199949069705,-0.675257012708768,0.499512490101253,0.909935336521942
"9338","LRRC27",36.0207680204367,0.182728956154808,0.268142454450557,0

.681462234427714,0.495579055289174,0.909935336521942
"9339","TSPAN32",12.8585375222849,-0.304115760666498,0.455282008139015
,-0.667972279224441,0.504151287596097,0.909935336521942
"9340","CAVIN3",1812.56078986235,-0.175835730516523,0.258978849069001,
-0.678957880725133,0.497164549785942,0.909935336521942
"9341","ILK",789.967572509099,-0.134354613737618,0.201783585110579,-0.
66583519994449,0.505516443062732,0.909935336521942
"9342","TRIM66",44.8873865695614,0.222615736620784,0.333442152040899,0
.667629258203321,0.504370276583484,0.909935336521942
"9343","TRIM44",499.842007149204,0.125297651703163,0.183695665977868,0
.68209367399152,0.49517972044091,0.909935336521942
"9344","PPP1R32",8.36924386570694,0.344781731992332,0.514179054880736,
0.670547990470565,0.502508524500107,0.909935336521942
"9345","BSCL2",407.462120079789,-0.159062046912685,0.23400337043441,-0
.679742546517167,0.496667491069097,0.909935336521942
"9346","CDCA5",2.30505093768901,0.599987230026339,0.889093749622992,0.
674830106814669,0.499783710112152,0.909935336521942
"9347","RAB6A",405.976596145051,0.0988684399778367,0.149326736859973,0
.662094692865001,0.507910536847625,0.909935336521942
"9348","COA4",493.879412195564,-0.158284670326831,0.236584821650595,-0
.669039836209767,0.503470066735107,0.909935336521942
"9349","WNT11",1771.95267206326,-0.241025138773035,0.358384786133161,-
0.672531725951892,0.501245249771002,0.909935336521942
"9350","TMPRSS5",2.2983038011415,0.644433254073097,0.963061999649685,0
.669150329166253,0.503399587628272,0.909935336521942
"9351","C2CD2L",22.3068228840914,0.241098710374487,0.358546686456658,0
.672433240862293,0.50130792695338,0.909935336521942
"9352","THY1",4262.76582100656,-0.268526287193874,0.400835279778323,-0
.66991679809816,0.502910830741142,0.909935336521942
"9353","NRGN",3.2142190289756,-0.516240027736423,0.782798474116887,-0.
659480115005103,0.509587511125472,0.909935336521942
"9354","APLP2",2381.12135059788,0.103322576579119,0.152127093652297,0.
679185897124111,0.497020081951648,0.909935336521942
"9355","AP003486.2",5.12538666636331,-0.486833613134001,0.729644015304
022,-0.667220730826046,0.504631151100223,0.909935336521942
"9356","CHD4",535.358824171019,0.160835431808778,0.236260519265018,0.6
80754585273577,0.496026790821759,0.909935336521942
"9357","ATN1",604.712655003129,0.130732890014916,0.192518987859368,0.6
7906491441984,0.497096732026673,0.909935336521942
"9358","AC091814.1",2.4472169002733,0.710506985877449,1.05457960197325
,0.673734808209831,0.500479927887772,0.909935336521942
"9359","AC010168.2",12.6154983302741,0.429609849262613,0.6318276791252
51,0.67994781402644,0.496537504911652,0.909935336521942
"9360","MFSD5",122.242162642182,0.133741588035417,0.200938596961174,0.
665584362875087,0.505676804057619,0.909935336521942
"9361","AC009779.2",75.7328362763335,-0.242731685801254,0.355873280924
051,-0.682073363785511,0.495192562340144,0.909935336521942
"9362","KITLG",328.599785644287,0.159675287095375,0.242195129433053,0.
659283642363715,0.509713644466247,0.909935336521942
"9363","SOCS2",80.1131658865322,0.231931168103746,0.339253515451223,0.

683651480502028,0.494195268439325,0.909935336521942
"9364","TMEM116",66.0231689867107,-0.188148718527411,0.277801401463824
,-0.67727778742654,0.498229721491594,0.909935336521942
"9365","SLC8B1",144.386282130105,0.156373509132011,0.23076051201214,0.
677644141835602,0.497997350972988,0.909935336521942
"9366","AC069234.5",2.06145647094532,0.665309192684673,1.0040398537458
9,0.66263225528601,0.507566106860267,0.909935336521942
"9367","CAMKK2",376.202221109816,-0.172703680495153,0.255575046717848,
-0.67574547168455,0.499202260346966,0.909935336521942
"9368","PSMD9",679.265601266201,-0.161483912331668,0.236184043881555,-
0.68372066833038,0.494151569655162,0.909935336521942
"9369","FLT3",2.33860196051997,-0.837647030299532,1.2590174259748,-0.6
65318059161077,0.50584708223343,0.909935336521942
"9370","MAB21L1",32.6681299305576,0.340457471535101,0.501580436410821,
0.678769439197682,0.497283960445346,0.909935336521942
"9371","COG3",87.3292312035815,0.158986646633251,0.233766844208376,0.6
80107767941344,0.496436226275679,0.909935336521942
"9372","TPP2",238.079879397322,0.128081002863416,0.188957804131235,0.6
77828594866933,0.497880378315309,0.909935336521942
"9373","TNFSF13B",502.405680817456,0.20991729135117,0.309684705027872,
0.677841972635612,0.497871895243832,0.909935336521942
"9374","NRL",4.99486912714225,-0.415464045248027,0.622454329684413,-0.
667461089809863,0.504477655750373,0.909935336521942
"9375","LTB4R2",2.56081537372936,0.597827158195426,0.886477924212431,0
.674384710399359,0.500066760737987,0.909935336521942
"9376","AL139317.5",72.0995877700747,-0.259972820990411,0.388426019695
404,-0.669298161833434,0.503305299139826,0.909935336521942
"9377","SAMD4A",256.869532249404,0.196889724644177,0.297386043670358,0
.662067803230277,0.50792776894453,0.909935336521942
"9378","NAA30",114.511912774691,0.122088192839075,0.179760150661724,0.
679172733165,0.497028421833928,0.909935336521942
"9379","PLEKHH1",5.24834730559616,-0.581035244137374,0.857196967786406
,-0.677831660601668,0.497878434273787,0.909935336521942
"9380","SRSF5",3111.45583523279,0.116957698119197,0.176561646236988,0.
662418484489049,0.507703060587619,0.909935336521942
"9381","AC005480.1",6.83995391008776,0.476037175356143,0.7080080842866
89,0.672361214400179,0.501353768151645,0.909935336521942
"9382","EFCAB11",84.0462836122334,-0.1738773955879,0.254630163351003,-
0.682862522254339,0.494693717784625,0.909935336521942
"9383","CALM1",5005.32207426975,-0.12099200988148,0.17777153587383,-0.
68060395207111,0.496122125561295,0.909935336521942
"9384","OTUB2",5.51745230185255,0.457434981700333,0.678285696079609,0.
674398685309508,0.500057878352508,0.909935336521942
"9385","AC138649.1",4.14621683976418,-0.567210720410036,0.850727778028
921,-0.666735864349257,0.504940865990231,0.909935336521942
"9386","SNURF",4.27136628333269,-0.520950162286713,0.790333917650206,-
0.65915197444086,0.50979818301295,0.909935336521942
"9387","FMN1",20.2136690204387,0.36848602428237,0.54989126316476,0.670
107072008458,0.502789536987789,0.909935336521942
"9388","BCL2L10",4.78423238014828,-0.544434777397449,0.799586119549848

,-0.68089573353769,0.495937467907419,0.909935336521942
"9389","SPG21",365.698167928235,-0.120066891336473,0.176656319932288,-0.679663718696814,0.496717413801474,0.909935336521942
"9390","SKOR1",2.28365097017939,0.627838395302841,0.94296471778985З,0.665813241426876,0.50553048014851,0.909935336521942
"9391","EWSAT1",2.08244363163585,0.743951545451154,1.11165232302005,0.669230414982668,0.50334850729605,0.909935336521942
"9392","ZNF592",108.054738729126,0.138353579905178,0.202969442537803,0.681647336541359,0.495461975246977,0.909935336521942
"9393","PIGQ",77.5297950252821,-0.140757801156519,0.209299068854125,-0.672519958770686,0.501252738338665,0.909935336521942
"9394","TBL3",101.770240432167,-0.133852364041749,0.200800126602642,-0.666595018172602,0.505030852124138,0.909935336521942
"9395","AC108134.3",3.79723414809959,-0.599047475574876,0.880270608908766,-0.680526498910932,0.496171148949915,0.909935336521942
"9396","NAGPA",77.7657500187023,0.173418414603251,0.262721928282927,0.660083517720288,0.509200235193021,0.909935336521942
"9397","SYT17",3.50519850163531,0.52714248147187,0.798289413311272,0.660340062992073,0.509035626024426,0.909935336521942
"9398","THUMPD1",522.404657293668,-0.102265265771922,0.151271510503941,-0.67603784368411,0.499016618207057,0.909935336521942
"9399","EIF3CL",3.80383263947571,0.588498213936475,0.889850429903037,0.661345091444863,0.508391030324112,0.909935336521942
"9400","SLC9A5",2.69831613241909,0.588939913807871,0.873793559109378,0.674003496212713,0.500309090920416,0.909935336521942
"9401","NPIPB15",67.6612524397981,0.265147043512878,0.39375535495621,0.67338015896283,0.500705468009624,0.909935336521942
"9402","TERF2IP",943.411537243712,-0.110034524789364,0.165484636265126,-0.664922903254145,0.506099805834234,0.909935336521942
"9403","MON1B",177.604296835508,0.15291002653513,0.228220929101131,0.670008781128794,0.502852192463358,0.909935336521942
"9404","DNAAF1",3.99819520905154,0.48732082868732,0.718929229338191,0.67784255918475,0.497871523304771,0.909935336521942
"9405","BANP",97.2336351540365,0.147420874045402,0.217568694532995,0.677583116274315,0.498036054136687,0.909935336521942
"9406","GEMIN4",87.641857843406,0.149907538864773,0.223283643628222,0.671377161483339,0.501980290343241,0.909935336521942
"9407","SCARF1",19.4412897314854,0.308865328961418,0.461468941343204,0.669309028820876,0.503298368483516,0.909935336521942
"9408","DPH1",7.60818234807435,0.363087170485356,0.532619039348436,0.681701448242496,0.495427751522275,0.909935336521942
"9409","EMC6",283.436220928022,-0.192402946094026,0.288366255112004,-0.667217272073998,0.504633360069229,0.909935336521942
"9410","INCA1",9.03364375180657,-0.323426721251917,0.481070497478494,-0.672306289716666,0.501388726425206,0.909935336521942
"9411","ACAP1",37.9349124612465,0.211873863239646,0.320889876429765,0.660269702481624,0.509080769219612,0.909935336521942
"9412","MPRIP",384.396167097918,0.125410136240801,0.185387136624607,0.676477011966291,0.49873783644553,0.909935336521942
"9413","RHBDL3",5.33592853118863,0.472866762777725,0.696360725607391,0

.679054325422034,0.49710344111573,0.909935336521942
"9414","GGNBP2",683.818745692117,0.112337490786811,0.168312034673525,0
.667435878870527,0.504493754520971,0.909935336521942
"9415","NR1D1",101.207531207495,-0.654511959269505,0.965818810036375,-
0.67767572185186,0.497977323165558,0.909935336521942
"9416","ACLY",236.015883918619,0.134556241059845,0.197846683309887,0.6
80103597436052,0.496438866790694,0.909935336521942
"9417","TTC25",7.88230731656217,-0.359727115254016,0.531320432932526,-
0.677043631219992,0.498378271814405,0.909935336521942
"9418","AC008105.3",37.2656496717412,0.18230375073214,0.26985471065573
8,0.675562602887857,0.499318391873161,0.909935336521942
"9419","AC068234.2",7.12329823955594,-0.383096136183187,0.568286729253
788,-0.674124726942379,0.500232020232905,0.909935336521942
"9420","TBX2-
AS1",23.8684140369926,-0.25399060744562,0.384368366369244,-0.660799976
451817,0.508740597923935,0.909935336521942
"9421","USP36",161.940981740875,0.154057736304223,0.229291667923454,0.
671885453577202,0.50165662159686З,0.909935336521942
"9422","CBX2",2.72443688259106,0.701185589474377,1.04937979748795,0.66
8190478940901,0.504012012225659,0.909935336521942
"9423","AC027601.6",8.84138713389452,-0.357290799996322,0.530003242737
308,-0.674129460323717,0.500229011181757,0.909935336521942
"9424","AC105114.2",4.96932630880003,0.603217869385867,0.9154786354427
93,0.658909827091821,0.509953675000469,0.909935336521942
"9425","MEX3C",136.465522768924,0.143004714662943,0.210294588471474,0.
680020896887422,0.49649122938845,0.909935336521942
"9426","BCL2",173.988182005859,0.188275338006132,0.284681076579452,0.6
61355297191966,0.50838448685408,0.909935336521942
"9427","AC027575.2",10.059857981518,-0.373906057257558,0.5467621378006
05,-0.683855064949496,0.4940666911404,0.909935336521942
"9428","AC012615.2",5.76952484061311,0.577829044535544,0.8758887890633
82,0.659705948689486,0.509442548496079,0.909935336521942
"9429","ADAT3",6.27599434519376,-0.562835313560613,0.824292422905265,-
0.682810247820631,0.49472675330294,0.909935336521942
"9430","AP3D1",267.099296078558,0.159269146128128,0.232820910702767,0.
684084370460436,0.493921890691201,0.909935336521942
"9431","OAZ1",5961.70292937301,-0.154354972063926,0.227058637377635,-0
.679802247765666,0.496629683235303,0.909935336521942
"9432","ZNF57",12.8316285034713,-0.33080026197873,0.500514057413792,-0
.660921021255645,0.508662964263303,0.909935336521942
"9433","DUS3L",86.8851900160806,-0.164513961742906,0.245591198432912,-
0.669869127202644,0.502941221890041,0.909935336521942
"9434","MLLT1",211.864400908076,0.127515100313269,0.187387220427186,0.
680489843558026,0.496194350577579,0.909935336521942
"9435","MAP2K7",223.366445222026,0.151370488094712,0.226700779681739,0
.667710487397607,0.504318414298707,0.909935336521942
"9436","C19orf38",5.58613862369428,0.467436702610162,0.706059672533257
,0.66203569017482,0.50794834884819,0.909935336521942
"9437","PLPPR2",123.213376155384,-0.126316434502904,0.190175516648121,
-0.664209761220867,0.506556066844313,0.909935336521942

"9438","SWSAP1",18.6117538002174,-0.333874924674703,0.489370655044685,
-0.682253668529055,0.495078564038061,0.909935336521942
"9439","NDUFA13",2952.15049499777,-0.168350488767547,0.255107731325668
,-0.659919195285511,0.50930568534148,0.909935336521942
"9440","ZNF66",44.5926884046159,0.206940093850537,0.30730407985283,0.6
734049673198,0.500689689317245,0.909935336521942
"9441","ECH1",1098.46753307888,-0.1708432090479,0.254554290557221,-0.6
71146452389087,0.502127237056183,0.909935336521942
"9442","SIRT2",344.126091614317,-0.118562394543265,0.178085740080377,-
0.665760180965377,0.505564400145468,0.909935336521942
"9443","HIPK4",2.65125353835515,-0.733498336624212,1.09353847967176,-0
.670756768288926,0.502375492200113,0.909935336521942
"9444","DMWD",190.287397247958,-0.117291268259467,0.176687043517773,-0
.663836271886389,0.506795107778353,0.909935336521942
"9445","PPP5C",109.214270922691,-0.130646763887371,0.193727396753157,-
0.67438455312461,0.500066860701535,0.909935336521942
"9446","TULP2",2.18335953238196,0.939102437285374,1.38679580638957,0.6
77174269606614,0.498295391010309,0.909935336521942
"9447","SCAF1",201.131098544966,0.118508300462815,0.174392380250307,0.
679549761822841,0.496789588970303,0.909935336521942
"9448","BCL2L12",85.6938053554849,-0.189979672091362,0.282044686144415
,-0.673580043958307,0.500578343995209,0.909935336521942
"9449","TMC4",3.33896872788776,0.54867275743186,0.808748967610175,0.67
8421586185351,0.49750442627645,0.909935336521942
"9450","AC008735.2",6.15289457807602,0.69302931347098,1.01487202728225
,0.682873598681068,0.494686718041966,0.909935336521942
"9451","ZNF470",21.0838462656819,0.257759618253869,0.385885532050196,0
.667969117381523,0.504153305927799,0.909935336521942
"9452","AURKC",9.98909894843575,0.360674236111744,0.530480763688407,0.
679900687828891,0.496567346088448,0.909935336521942
"9453","ZNF460-
AS1",4.60194186915794,0.489626848366632,0.731336189361657,0.6694962665
44666,0.50317896149209,0.909935336521942
"9454","DTD1",402.655451746806,-0.162233717631184,0.24206148914935,-0.
670216969255637,0.50271948792505,0.909935336521942
"9455","GART",129.648566219135,0.131684004314563,0.196467330355401,0.6
70259040403064,0.502692672927871,0.909935336521942
"9456","GNB1L",26.9702444838548,0.27436219049457,0.405511223870387,0.6
76583468827151,0.498670270647767,0.909935336521942
"9457","DGCR8",106.551484253535,0.183328947335966,0.268934505957401,0.
681686222016469,0.495437381440581,0.909935336521942
"9458","RANBP1",794.313795766999,-0.155575476022187,0.234948002783761,
-0.662169817061069,0.507862395483083,0.909935336521942
"9459","ADORA2A",9.67337251907001,0.336107006448449,0.506245457561003,
0.663921031642932,0.506740854572967,0.909935336521942
"9460","AL022323.3",2.4164049572068,0.626007539367746,0.94896959652964
8,0.659670806796167,0.509465104656772,0.909935336521942
"9461","EIF4ENIF1",90.8308278921666,0.139513062819042,0.20636312090922
4,0.676056178082379,0.499004977969518,0.909935336521942
"9462","KDELR3",404.087120810087,-0.182639422344492,0.274774781702516,

-0.664687717019918,0.506250251693781,0.909935336521942
"9463","RPS19BP1",1066.9876925612,-0.147087176192395,0.221508926313712,-0.66402369710412,0.506675144334542,0.909935336521942
"9464","AL035681.1",28.4326880336301,0.247703401020267,0.36389214081839,0.680705553198221,0.496057821819027,0.909935336521942
"9465","PHETA2",131.329898795203,-0.135221336381536,0.20138225815825,-0.671465985227344,0.50192372145342,0.909935336521942
"9466","OGFRP1",3.94042716887053,-0.510949904107855,0.769482357930414,-0.664017698186215,0.506678983772564,0.909935336521942
"9467","TTLL12",52.3003453201313,-0.182126198027717,0.275598373074728,-0.660839162422536,0.508715464811718,0.909935336521942
"9468","AL033543.1",4.68539574503597,0.495606634022983,0.744343025693922,0.665830963568106,0.505519151170306,0.909935336521942
"9469","TBC1D25",66.2527047462762,0.134901195317946,0.203498128850292,0.662911232059482,0.507387407653618,0.909935336521942
"9470","CCDC120",27.5486365424407,-0.23370167930583,0.349644229888995,-0.668398501471125,0.503879251789023,0.909935336521942
"9471","IQSEC2",57.6575001639032,0.180657894632797,0.273509395840291,0.660518056711616,0.508921434892391,0.909935336521942
"9472","EFNB1",135.243504789033,0.179095937844816,0.264820364714108,0.67629216521230,0.498855166121498,0.909935336521942
"9473","TSC22D3",5908.76909841017,0.231426434396249,0.350425881203831,0.660414789002518,0.508987684227788,0.909935336521942
"9474","CCDC160",19.3546232729096,-0.401525787256578,0.591268410511102,-0.679092236484429,0.497079421247276,0.909935336521942
"9475","FAM122B",76.1672589672919,-0.148178260904247,0.223393382630431,-0.663306402183743,0.507134337089715,0.909935336521942
"9476","LINC00892",5.26629665504021,0.551820732711981,0.822964903664906,0.670527661938632,0.502521478745608,0.909935336521942
"9477","ADO",81.3499516212335,-0.147949938175687,0.224585788491348,-0.658768033229257,0.510044737730474,0.909963498963795
"9478","IL10RB-DT",6.61600013378822,-0.388526991236778,0.589843913701324,-0.658694583790372,0.510091911709182,0.909963498963795
"9479","SAC3D1",113.990338130943,-0.190456264104762,0.289179024766113,-0.658610230319444,0.510146091778052,0.909964143874175
"9480","MRPL20-AS1",55.5285767574585,-0.162387785748179,0.246727871164937,-0.658165552928649,0.510431756947711,0.910349179376782
"9481","FBLIM1",63.9878048795309,-0.175155840758847,0.266949244806794,-0.656139113207143,0.51173462024294,0.910349179376782
"9482","SYPL2",2.58885623036159,0.580647306694696,0.883596849949489,0.657140534993859,0.511090557112325,0.910349179376782
"9483","IVNS1ABP",213.834748504147,-0.201190281866797,0.306543260210511,-0.656319377984805,0.511618651929677,0.910349179376782
"9484","MYD88",119.044389882464,-0.119687434992561,0.182204755523381,-0.656884254468333,0.511255343275024,0.910349179376782
"9485","CYB561D2",154.332473995734,-0.14673951018563,0.223639678850312,-0.656142554577029,0.511732406205233,0.910349179376782
"9486","THOC7",894.950305432462,-0.145790753708974,0.221758647225543,-

0.657429847868323,0.510904564780325,0.910349179376782
"9487","NAA50",388.959734423816,-0.0900662972281492,0.136889920786411,
-0.657946886890812,0.510572260851937,0.910349179376782
"9488","WDR55",142.146086045579,-0.104948543156054,0.15954732026697,-0
.657789444413378,0.510673438063973,0.910349179376782
"9489","GALNT10",103.542114431487,0.218941781731928,0.333509212136126,
0.656478962993573,0.511515998817452,0.910349179376782
"9490","AL445309.1",3.36431357484772,0.595489667220332,0.9065571529949
01,0.656869415516797,0.511264885442355,0.910349179376782
"9491","AC015987.1",4.64563363915373,-0.497362092709981,0.757502019105
928,-0.65658187062922,0.511449809148169,0.910349179376782
"9492","BRD3OS",89.9163807953483,-0.14052092426222,0.214090559139418,-
0.656362077931291,0.511591184120636,0.910349179376782
"9493","ABCA2",78.9809777384467,0.136901585316622,0.208665511630462,0.
656081516523291,0.511771676353616,0.910349179376782
"9494","AC010997.5",2.76827190809258,0.54707228336906,0.83388089156071
1,0.656055665630072,0.511788308557409,0.910349179376782
"9495","VWF",6.27778069743827,-0.581649324239407,0.886176910275046,-0.
656358022303783,0.511593792971427,0.910349179376782
"9496","C12orf75",100.375044508263,0.277490830926586,0.422774106858277
,0.65635720453336,0.511594319016885,0.910349179376782
"9497","SNRPN",507.683196920882,-0.158693776805543,0.241807951060333,-
0.656280226145036,0.511643838006792,0.910349179376782
"9498","NPIPA9",5.51988441651494,-0.49142041778897,0.748103060549748,-
0.656888661072777,0.511252509632022,0.910349179376782
"9499","BCL7C",1129.37381415991,-0.172991776352134,0.263198054663207,-
0.65726844589903,0.511008321886848,0.910349179376782
"9500","VPS9D1",41.4176208306411,0.171294042288918,0.260388746246048,0
.657839652283044,0.510641171857674,0.910349179376782
"9501","P3H4",166.393507038785,0.14551207847034,0.221481423096679,0.65
6994507421157,0.511184448178627,0.910349179376782
"9502","AC008946.1",7.00824242895754,-0.378738323123656,0.577125375092
145,-0.656249646037806,0.511663510404491,0.910349179376782
"9503","ICAM1",335.976230345189,-0.236701798126499,0.360818682145101,-
0.656013144106852,0.51181566708988,0.910349179376782
"9504","NOSIP",374.149046903821,-0.150595822709858,0.228947935432897,-
0.657773228769718,0.510683859313427,0.910349179376782
"9505","FLRT3",2.5905922263937,0.664328697337233,1.01025139404217,0.65
7587508668663,0.510803223310104,0.910349179376782
"9506","SHANK3",15.1696229480844,-0.334219375900589,0.508415590926129,
-0.657374364330127,0.510940231079792,0.910349179376782
"9507","SGK3",123.87304125134,-0.11716183030504,0.178668940872187,-0.6
55748166038849,0.511986172323019,0.910462833265319
"9508","BRF1",89.5084152495484,0.177162059708989,0.27016852837658,0.65
5746473408805,0.511987261573613,0.910462833265319
"9509","KCNMB1",13.0318805143057,-0.363838054382727,0.555088211113386,
-0.655459883849716,0.512171706693485,0.910695048561726
"9510","AL031848.2",9.66009220555736,-0.318432910062664,0.486035762164
956,-0.655163539086637,0.512362466569285,0.910746907564494
"9511","HSPB11",357.37341302858,-0.153850531168285,0.234816476960388,-

0.655194785135281,0.512342351450616,0.910746907564494
"9512","RPL8",23298.6464748529,-0.161538494859735,0.246525950548048,-0
.655259596406062,0.512300629523049,0.910746907564494
"9513","ADPRHL2",184.860516766772,-0.168575825203822,0.258014781023275
,-0.653357239981594,0.51352599867753,0.910951867685214
"9514","DUBR",72.7483744906928,0.166827541365455,0.25497621045682,0.65
4286692341074,0.512927117867364,0.910951867685214
"9515","SLC49A3",121.668020867139,-0.172924191295487,0.264642605482254
,-0.653425365807481,0.513482090309136,0.910951867685214
"9516","AL139274.2",12.1219794324046,-0.275345142114523,0.420476664523
953,-0.654840483065232,0.512570462928688,0.910951867685214
"9517","SEPTIN7",2578.79692252306,-0.0898687283514192,0.13733824728758
,-0.654360530488192,0.512879556803062,0.910951867685214
"9518","FUT10",84.1548497214835,-0.138043142356458,0.21124753952503,-0
.653466273107062,0.513455725728973,0.910951867685214
"9519","MROH6",29.8991348811762,-0.281874441277995,0.431326825443688,-
0.653505473460971,0.513430461929277,0.910951867685214
"9520","CDKN2B",23.9889553876532,0.373449651164918,0.571421985014531,0
.653544422438387,0.513405360777397,0.910951867685214
"9521","MAP3K8",301.59299615687,-0.22139630992972,0.338506939363138,-0
.654037729171083,0.51308749843212,0.910951867685214
"9522","SSSCA1-
AS1",13.5396933354278,-0.26331251966831,0.402312759898324,-0.654497062
769912,0.512791618874815,0.910951867685214
"9523","RBM7",509.674824222261,-0.158925813745513,0.24307471169128,-0
.653814675495298,0.513231210439492,0.910951867685214
"9524","AC022509.1",6.22685717434868,0.490467124137439,0.7491756416094
12,0.654675748778746,0.51267654237877,0.910951867685214
"9525","MLXIP",352.206694741834,0.167301726398653,0.255756033706141,0.
654145765299441,0.513017899005214,0.910951867685214
"9526","VPS36",471.912977823563,0.150678873017314,0.230668055314347,0.
653228175925678,0.513609188232975,0.910951867685214
"9527","NME4",624.139068138183,-0.183635947818676,0.281035989737794,-0
.653425022147548,0.513482311799483,0.910951867685214
"9528","KDM6B",313.566236042855,-0.245045881106096,0.374523308825021,-
0.654287397691934,0.512926663521724,0.910951867685214
"9529","LINC00683",9.86310080278406,0.354275308709537,0.54134139540252
2,0.654439715341021,0.512828554340599,0.910951867685214
"9530","TNFAIP8L1",8.26484598632659,-0.350785804136223,0.5364045312910
36,-0.653957570589383,0.513139141662923,0.910951867685214
"9531","ZNF442",7.54493924338081,0.375071040351568,0.574124905943184,0
.653291707900032,0.513568237173445,0.910951867685214
"9532","ZNF208",103.23658408276,-0.347855650766923,0.532116715448935,-
0.653720585479907,0.513291838299553,0.910951867685214
"9533","ZCCHC12",3.6717195295413,-0.566023179070755,0.865040804899232,
-0.654331189771667,0.5128984556439,0.910951867685214
"9534","CCDC51",128.862021705297,0.179054999146412,0.274229596494527,0
.652938273021111,0.513796073680933,0.911092188127657
"9535","AC024560.1",5.56323224038698,-0.539782860205793,0.826608320697
414,-0.653009226607319,0.513750330293872,0.911092188127657

"9536","MXD3",15.3755450578681,0.257695937629878,0.394855732093945,0.6
52633143409874,0.513992813215807,0.911345478801684
"9537","HEXA",849.429791982455,0.213447508017595,0.327173381041607,0.6
52398759758671,0.514143964228383,0.911422326187199
"9538","TLNRD1",234.407547129466,0.146316703869295,0.224260234733701,0
.65244158886679,0.514116342559392,0.911422326187199
"9539","RHEX",2.97480999811831,-0.784977353359211,1.20345879728564,-0.
652267742883845,0.51422846540358,0.911476558658532
"9540","EVA1A",3.77248415142551,-0.521616848967898,0.799801430084192,-
0.652182941099504,0.5142831633564,0.911477958703356
"9541","LRRC47",195.666312576772,0.110169956644097,0.169787990988772,0
.648867779178698,0.51642384269897,0.911577838608056
"9542","PGD",303.739340386774,0.147346953048414,0.227345828918554,0.64
8118128004891,0.516908549936686,0.911577838608056
"9543","CSF3R",23.7345952689954,0.416296355205611,0.644747577702609,0.
645673391575934,0.518490896255836,0.911577838608056
"9544","AL358472.2",8.35700132575398,0.333030667317352,0.5115643976292
99,0.651004387445039,0.515043654686593,0.911577838608056
"9545","BTG2",3547.40044264679,0.168980250760402,0.261006718259421,0.6
47417246143251,0.517361937129992,0.911577838608056
"9546","MRPL33",787.073813269578,-0.162985791620133,0.251161581172793,
-0.648928036123499,0.516384892174706,0.911577838608056
"9547","CHAC2",5.6787324781633,-0.429050404127779,0.664470425746619,-0
.645702784507956,0.518471856935504,0.911577838608056
"9548","MRPL53",42.4944875740969,-0.254110285787319,0.39193642334173,-
0.648345677139989,0.516761396984064,0.911577838608056
"9549","FBXO36",37.3858745319656,0.195681883310955,0.300981933126781,0
.650144948163814,0.515598597521592,0.911577838608056
"9550","DTYMK",235.571127242953,-0.17081223042422,0.2620023468813,-0.6
51949238079179,0.514433919670432,0.911577838608056
"9551","SLC25A38",120.002178175182,-0.155180950816976,0.23813575940061
2,-0.65164908960992,0.51462757207442,0.911577838608056
"9552","TMEM129",175.014558818626,-0.106180420225021,0.16406857912429,
-0.647170962238809,0.51752130238529,0.911577838608056
"9553","FBXL5",384.013884257087,0.0847735835041595,0.1312734389973,0.6
45778644573356,0.51842272012246,0.911577838608056
"9554","AC010491.1",3.64751845909149,-0.520003573320442,0.798203733561
708,-0.651467227546162,0.514744925854975,0.911577838608056
"9555","AC008914.1",7.79203027153814,0.496275802041369,0.7676378179188
86,0.646497333061057,0.517957323734055,0.911577838608056
"9556","PJA2",964.025444086299,0.0877111224706266,0.13476838367096,0.6
50828629693856,0.515157116817089,0.911577838608056
"9557","ABCF1",394.15809770848,-0.13008564340469,0.200075210739022,-0.
650183713035659,0.515573560232164,0.911577838608056
"9558","MDGA1",72.658787693947,0.196412180349735,0.303496129418618,0.6
47165355044183,0.517524930981529,0.911577838608056
"9559","AC019205.1",50.6780209268176,-0.186854034632225,0.287430476027
357,-0.650084281996739,0.515637781592281,0.911577838608056
"9560","SF3B5",1448.18708575815,-0.173280922042849,0.268020877662044,-
0.6465202395962,0.517942493853687,0.911577838608056

"9561","PDCD2",425.550838046693,-0.144511987115874,0.223787111829201,-0.645756522503264,0.518437048994395,0.911577838608056
"9562","FSCN1",222.187294984573,0.204161975618141,0.315648038427693,0.646802611652882,0.517759701889763,0.911577838608056
"9563","AOAH",10.5262246989911,-0.331030535674636,0.510052879303124,-0.649012188945815,0.516330497731436,0.911577838608056
"9564","CCM2",290.727095442211,-0.143861130730519,0.222024016635142,-0.647953013871151,0.517015340605335,0.911577838608056
"9565","BAZ1B",451.987161944806,-0.131041295021762,0.201720369883599,-0.649618554127074,0.515938645100918,0.911577838608056
"9566","IFT22",320.725026866307,-0.171436115009559,0.264062888156408,-0.649224569974464,0.516193232743329,0.911577838608056
"9567","INSIG1",323.487283901324,0.217218831747184,0.335824688542635,0.646822104383809,0.517747084611602,0.911577838608056
"9568","SMIM19",1034.57434794787,-0.196417649744264,0.302139081291592,-0.650090180007873,0.515633972019158,0.911577838608056
"9569","FABP5",1074.59949675044,-0.462163651480127,0.714439749536174,-0.646889610747682,0.5177033902412,0.911577838608056
"9570","CCNE2",5.43133699560104,-0.384361058756775,0.593527233342541,-0.647587906947733,0.517251520932403,0.911577838608056
"9571","ENY2",1317.43007633588,-0.14769096714736,0.22678379062634,-0.651241284659107,0.514890743920463,0.911577838608056
"9572","PSIP1",873.525814565147,-0.0944367236615697,0.145427169394985,-0.649374694250401,0.516096216628399,0.911577838608056
"9573","SARDH",17.0370798719774,0.354787589375731,0.54933700734281,0.645846874747925,0.518378527471988,0.911577838608056
"9574","AKR1E2",10.0965489159637,0.320778261999676,0.492895276395537,0.650804090364743,0.515172959452991,0.911577838608056
"9575","PARD3",244.861297459024,0.165285356878186,0.255081384131366,0.647971067904604,0.517003663263558,0.911577838608056
"9576","R3HCC1L",97.8709342820351,0.141943703625062,0.218732304705368,0.648937996681649,0.516378453744673,0.911577838608056
"9577","FAM24B",38.3753534654603,-0.210450869446037,0.325767214322929,-0.646016112712373,0.518268920531565,0.911577838608056
"9578","WEE1",187.191697407496,0.18502303579729,0.286451209627229,0.645914660434035,0.518334624644846,0.911577838608056
"9579","MARK2",149.447105040076,0.121201427370637,0.187601908385735,0.646056473591039,0.518242782584545,0.911577838608056
"9580","RDX",956.557680563434,0.082777016308045,0.127707891776265,0.648174636326031,0.516872004742737,0.911577838608056
"9581","NFRKB",114.439061688702,0.135191347432331,0.209198844466244,0.646233719776334,0.518128004961097,0.911577838608056
"9582","AICDA",3.87649983700245,-0.460831777180616,0.710858148518887,-0.648275296753346,0.516806908711064,0.911577838608056
"9583","AC068792.1",3.77090706749469,0.507343525602379,0.780072814104417,0.650379703572734,0.515446984364321,0.911577838608056
"9584","DNM1L",272.302460304039,-0.14481107880814,0.222134261653633,-0.651907894487434,0.514460591839188,0.911577838608056
"9585","TNS2",828.905633381481,0.152384203632431,0.235967363470629,0.645785083967338,0.518418549246931,0.911577838608056

"9586","AC083805.3",2.38374437670128,-0.599713938793145,0.925252863627
708,-0.648162207725358,0.516880042481686,0.911577838608056
"9587","AC011611.4",12.2560099377003,-0.283457711708969,0.435124257377
518,-0.651440839950778,0.514761954662943,0.911577838608056
"9588","SCYL2",112.517098757222,0.118950764196226,0.18270135660158,0.6
5106667191106,0.515003449457265,0.911577838608056
"9589","SLC15A4",216.930890667002,0.105789544373813,0.1630071831756,0.
648987009731043,0.516346772683633,0.911577838608056
"9590","RAB15",6.02771675741877,-0.395089444448542,0.611633983840606,-
0.645957312521575,0.518307001107365,0.911577838608056
"9591","RPP25",111.681946051969,-0.201816923906692,0.311553514813631,-
0.647776110076682,0.51712976913717,0.911577838608056
"9592","NRG4",2.06708820666828,0.878711134087315,1.35026908667634,0.65
0767423143962,0.515196632349486,0.911577838608056
"9593","AC021739.2",8.47877174288732,0.383861854597202,0.5933988900796
,0.646886708105757,0.517705268974612,0.911577838608056
"9594","MESP2",17.4753598218867,0.3423332044237,0.529274608535226,0.64
6796953609981,0.51776336426432,0.911577838608056
"9595","TM2D3",455.286153069821,0.115512361753905,0.178306561049464,0.
647830125117274,0.517094828632624,0.911577838608056
"9596","METTL9",914.35068638435,-0.143449493725945,0.220104926049604,-
0.651732318310843,0.514573870060083,0.911577838608056
"9597","NOS2",2.98325243204009,0.544856999596003,0.836971993343847,0.6
50985939707738,0.5150555631936,0.911577838608056
"9598","CRLF3",59.2462110619261,0.149374010089005,0.230096181887164,0.
649180742000558,0.516221557858989,0.911577838608056
"9599","NFE2L1",863.400905463434,0.168732888144225,0.261297425775963,0
.645750288749103,0.51844108674866,0.911577838608056
"9600","AC027601.4",2.92350468841301,-0.567425514521854,0.877423265749
42,-0.646695314190475,0.517829156367154,0.911577838608056
"9601","HGS",202.863400355308,0.104307097367218,0.16045027921828,0.650
089846371136,0.515634187517437,0.911577838608056
"9602","AC024075.2",62.3100179583139,-0.173400502751268,0.267346499161
178,-0.64859836689587,0.516598011369717,0.911577838608056
"9603","BABAM1",382.733657430907,-0.142438203672959,0.220072555674714,
-0.647232923870318,0.517481205894599,0.911577838608056
"9604","DDA1",198.338116627138,-0.142825947794183,0.219908364901418,-0
.649479376822295,0.516028572297117,0.911577838608056
"9605","SUGP2",276.494311030469,0.196860437959871,0.302794331913889,0.
650145716782623,0.515598101083235,0.911577838608056
"9606","FAAP24",35.2996874278883,0.200272296198308,0.3100179797417,0.6
4600219756664,0.518277932221364,0.911577838608056
"9607","BLVRB",1646.39104994287,-0.198877225244244,0.306295928343097,-
0.649297645972859,0.516146007024834,0.911577838608056
"9608","TMEM91",231.418295747051,0.151669697214875,0.234012879585129,0
.648125425761879,0.516903830238908,0.911577838608056
"9609","CCDC8",184.674420256553,0.155425045336796,0.238882949598845,0.
650632644974454,0.515283651970128,0.911577838608056
"9610","LIG1",32.4764506041358,-0.188268425472701,0.290907305980015,-0
.647176683440308,0.517517600025104,0.911577838608056

"9611","MED25",135.087329863724,0.139388227979129,0.21452002432986,0.6
49767910546182,0.515842149876156,0.911577838608056
"9612","EPN1",452.246517699029,-0.118869194647279,0.182521060637862,-0
.651262896631561,0.514876795148878,0.911577838608056
"9613","ZNF416",14.3173299675472,-0.278542868379768,0.429015045191808,
-0.649261305638441,0.516169491862186,0.911577838608056
"9614","GTSE1",2.87235071620358,0.659716861317162,1.02151590032257,0.6
45821431765127,0.518395006651734,0.911577838608056
"9615","PRRG1",183.149378001922,0.157026159088442,0.242397103094301,0.
647805427886457,0.517110804284991,0.911577838608056
"9616","GAGE10",2.65497278005826,-0.693231150799462,1.06419479912952,-
0.651413774401549,0.514779421281566,0.911577838608056
"9617","ZDHHC15",32.9848068376445,0.191533379026298,0.294493199526286,
0.650383028655309,0.5154448370773,0.911577838608056
"9618","YPEL4",58.9492602241803,-0.208773382271562,0.323479762863925,-
0.645398588224464,0.518668918061176,0.911701223264202
"9619","KCNE5",12.2374776393407,0.368711316759685,0.571237828452135,0.
645460259098686,0.518628963979304,0.911701223264202
"9620","ACOX3",63.0503666986094,0.149938137653156,0.232376031590314,0.
645239255645358,0.518772150599935,0.911737007097391
"9621","MAP3K4",196.376419567622,0.127428018545704,0.197501359534989,0
.645200715811423,0.518797122384906,0.911737007097391
"9622","SPTY2D1OS",27.7840254937695,-0.24233888453339,0.37571076787869
3,-0.645014477231151,0.518917803937836,0.911759558243888
"9623","TXNRD2",82.4153373519973,-0.15112267464187,0.234283957866363,-
0.64504064221107,0.51890084830256,0.911759558243888
"9624","GBP5",13.7317529217951,0.301718886638841,0.468080175747029,0.6
44588047672208,0.519194182492669,0.911938613077295
"9625","COX7A2L",1168.92140617595,-0.156404138496198,0.242669131222407
,-0.644516002955701,0.519240883791634,0.911938613077295
"9626","LSM5",614.97198852937,-0.161066074727287,0.249889221265708,-0.
644549908601401,0.519218904981311,0.911938613077295
"9627","AP001922.5",2.56048789069505,0.636246334915848,0.9872837951364
22,0.644441180995919,0.519289387669044,0.911938613077295
"9628","TANGO6",43.4060404635475,-0.165605568495279,0.256902549532655,
-0.644624075535804,0.519170829087903,0.911938613077295
"9629","CEACAM19",4.74023450907772,-0.443205802882894,0.68800326447952
1,-0.644191424321485,0.519451311175043,0.912128234432197
"9630","Z99572.1",22.1556639223238,0.285649971608541,0.444200539693504
,0.643065341175943,0.520181702546931,0.912273854025881
"9631","HYAL1",11.6177088622094,-0.359506040518644,0.558600382498427,-
0.643583591745315,0.519845493104552,0.912273854025881
"9632","SENP7",219.925522448874,0.112889996739593,0.175427030368693,0.
643515406390532,0.519889721210621,0.912273854025881
"9633","DGKQ",36.0355248626213,0.166084923176487,0.25821535854298,0.64
3203115855103,0.520092311778095,0.912273854025881
"9634","ERV3-1",93.003499167557,0.178016679123691,0.27658667280862,0.6
43619872628018,0.519821960473634,0.912273854025881
"9635","VIPR2",35.5087271185849,0.429976118673388,0.667773048105302,0.
643895586821564,0.519643143741815,0.912273854025881

"9636","WASF3",330.755813651801,-0.141007217527993,0.219264228764326,-0.643092666426467,0.520163972787579,0.912273854025881
"9637","ARHGEF7",257.632699098211,0.170517073870588,0.264886064058396,0.643737429059299,0.519745714391771,0.912273854025881
"9638","NOP10",927.16351692691,-0.176231617530852,0.273942823569036,-0.643315328486567,0.520019511993216,0.912273854025881
"9639","NOL11",207.824632455544,0.106543891787293,0.165675794135754,0.643086652115224,0.520167875096186,0.912273854025881
"9640","BAHCC1",71.3000891410817,0.179720307414226,0.279192243485183,0.643715259316523,0.519760093053245,0.912273854025881
"9641","METRNL",1166.55999338838,0.173535390561974,0.269729517899048,0.643368185705664,0.519985221826169,0.912273854025881
"9642","AL050341.2",63.7650834828907,-0.173882531332678,0.270533610625713,-0.642739107094708,0.520393400852494,0.912451948871544
"9643","TP53BP2",174.001839828552,0.174164570179588,0.270999414694089,0.642675078749485,0.520434955041017,0.912451948871544
"9644","MAP2K4",120.373156824886,0.133702599364783,0.208045825686499,0.642659370470894,0.520445149924129,0.912451948871544
"9645","STK40",151.885482516191,-0.135140121767162,0.210383864351432,-0.642350221029401,0.520645812993177,0.912639828823393
"9646","CAPZA1",528.272229464187,0.116032088239012,0.180906111070632,0.64139396702696,0.521266751654549,0.912639828823393
"9647","USP19",116.527668114074,0.108570178937238,0.169280931813957,0.641360948181445,0.521288199079739,0.912639828823393
"9648","AC108676.1",2.87641746677772,-0.688889692142713,1.07431727673943,-0.641234863348289,0.521370101791784,0.912639828823393
"9649","ATP6V1G2",6.42061727446133,0.387347131074716,0.603505808622881,0.641828339579018,0.520984646811004,0.912639828823393
"9650","RPS10-NUDT3",14.7198018880468,0.512095796274365,0.798696678532569,0.641164299337302,0.521415941946651,0.912639828823393
"9651","IFRD1",1030.63210337926,0.187213524972405,0.291768720552928,0.641650429893989,0.521100181400545,0.912639828823393
"9652","PAG1",14.9793459221998,0.363065568033886,0.566154708696928,0.641283314360353,0.521338627997733,0.912639828823393
"9653","KIAA1958",11.8965807466238,0.29528493064993,0.459721639777851,0.642312445402001,0.520670335172668,0.912639828823393
"9654","EXD3",66.9626166836787,0.147077551674429,0.229214825147654,0.641658110812358,0.521095193137982,0.912639828823393
"9655","POLR3A",87.7266291503864,0.141961314672429,0.221387329107635,0.641235048295877,0.521369981647909,0.912639828823393
"9656","ACYP1",134.402152822512,-0.185878905588786,0.289867819986652,-0.641254022600181,0.521357655818296,0.912639828823393
"9657","ORAI3",339.746094229071,0.176314283380999,0.274825963515978,0.641548859231959,0.521166147326087,0.912639828823393
"9658","TSEN54",40.0351297233207,-0.157752837066056,0.245712167352023,-0.642022895187151,0.52085831745634,0.912639828823393
"9659","PRR22",2.72509444900838,-0.586898265454329,0.914747452105603,-0.641595955368208,0.52113555980754,0.912639828823393
"9660","HNRNPH2",389.221690132525,-0.12150176742547,0.189403513330619,

-0.641496904090575,0.521199891693908,0.912639828823393
"9661","RBMXL1",129.528540488469,-0.165800745003265,0.258768135756014,
-0.640730917347543,0.521697523258233,0.91278629543952
"9662","WDR12",91.1860478619476,-0.133031453123199,0.207568055386815,-
0.640905234070277,0.521584255038315,0.91278629543952
"9663","STEAP1B",9.79271668958547,0.380478391028747,0.5938025512399,0.
640749000209383,0.521685772715495,0.91278629543952
"9664","RAB11B",579.687139863589,-0.108902613567159,0.169973587261881,
-0.640703154657617,0.521715564178349,0.91278629543952
"9665","ENTPD3-
AS1",95.6209682720815,-0.172561278407743,0.269400378413797,-0.64053836
6812129,0.52182265422401,0.912879196856654
"9666","FBXL6",53.4714115781897,-0.173249378050949,0.27051139083208,-0
.640451322652411,0.521879225841233,0.912883710999749
"9667","UBE2T",12.5714598120416,0.309656170758604,0.483650574439804,0.
640247705933707,0.522011572448377,0.913020757934949
"9668","MINDY3",130.923630148943,0.114995145954131,0.17965266706979,0.
64009707080752,0.52210949323315,0.913097570499182
"9669","NUDT22",177.930513842717,-0.169432340301051,0.264810592215141,
-0.6398246342178,0.52228661537967,0.913216146142031
"9670","GRTP1",43.8875081672596,0.153411110415483,0.239796890442422,0.
63975437768372,0.522332297028366,0.913216146142031
"9671","ADSL",264.786963450471,0.143043187152094,0.223594569592935,0.6
39743565384936,0.522339327498201,0.913216146142031
"9672","RINT1",74.6196205668122,-0.159574336304483,0.249488299481861,-
0.639606493113656,0.522428460075125,0.913224111107964
"9673","ANAPC11",1247.72234843214,-0.145296698960313,0.227178571949916
,-0.639570438854353,0.522451906005875,0.913224111107964
"9674","AL137077.2",4.16162830938874,-0.456440032915784,0.713836396173
368,-0.639418269175686,0.522550867253724,0.913302673508989
"9675","PLK3",404.372333803009,-0.218036152601154,0.341202908465594,-0
.639021963739036,0.522808643690067,0.91346993360666
"9676","PLXNA1",257.235173766309,0.141571113801927,0.221522368811328,0
.639082701045437,0.522769132938842,0.91346993360666
"9677","DCUN1D2",32.77571842222,0.214962036373142,0.336322505041095,0.
639154481639211,0.522722440300355,0.91346993360666
"9678","SLC9A1",104.191720996475,0.169120786394801,0.264763488000943,0
.638761740418673,0.522977941174972,0.913482544358101
"9679","CACNA2D4",28.5929094096704,0.20120280373057,0.314964604336212,
0.638810840839099,0.522945995005505,0.913482544358101
"9680","SNRNP35",214.332399999797,0.16738282978959,0.262035533282631,0
.638779129275767,0.52296662736142,0.913482544358101
"9681","PEX10",132.089299427858,-0.153994151037494,0.241732665154756,-
0.637043201997164,0.524096703455582,0.913538662588792
"9682","SYF2",1086.61008179114,-0.143056364356913,0.22416324659047,-0.
638179391728145,0.523356910664797,0.913538662588792
"9683","HHLA3",90.5629955933221,0.16305608550982,0.256087301484154,0.6
3672069862437,0.524306788606673,0.913538662588792
"9684","PPM1J",2.61856751367731,-0.562397025579837,0.882053722455738,-
0.637599514929838,0.523734411516924,0.913538662588792

"9685","PLXNA2",21.7804007586705,-0.267625012323152,0.419603185222577,-0.637805006606876,0.523600620087341,0.913538662588792
"9686","AC093690.1",18.3208279085641,-0.332033796129201,0.521144248945436,-0.637124551985539,0.524043717260747,0.913538662588792
"9687","CALCRL",47.3202581065333,-0.280922428159572,0.440786949022593,-0.637320203745808,0.523916293425865,0.913538662588792
"9688","CAND2",11.5096190571096,-0.298777178978193,0.469172633581562,-0.636817149153378,0.524243954279195,0.913538662588792
"9689","POLQ",2.82898037064047,0.541578508722057,0.848781675172428,0.638065741242631,0.523430886335466,0.913538662588792
"9690","AC108471.2",18.7450360434918,0.297766041798602,0.466616716517396,0.638138393371299,0.523383596082165,0.913538662588792
"9691","AF213884.3",3.36730711285823,-0.471812908993756,0.739220014079857,-0.638257758187249,0.523305904668985,0.913538662588792
"9692","AC116351.2",8.56068338787475,0.327089145185084,0.513142720539322,0.637423336808341,0.52384913144785,0.913538662588792
"9693","PSMB1",2386.62053799274,-0.179098775903401,0.280657495154992,-0.638140006930847,0.523382545819315,0.913538662588792
"9694","ZBED5-AS1",62.2661117216515,-0.189249027911315,0.297214481520954,-0.636742284369386,0.524292725873021,0.913538662588792
"9695","NADSYN1",109.067975101266,-0.131636540841422,0.206609803645615,-0.637126305328715,0.524042575275043,0.913538662588792
"9696","PLK1",4.88705398853946,0.492177409655395,0.771886396183054,0.637629335209419,0.523714995055083,0.913538662588792
"9697","ATP2A1-AS1",2.50715037715271,0.678031741026115,1.06438411557963,0.63701790650726,0.524113179884333,0.913538662588792
"9698","FOXO3B",14.1554159489029,-0.295300867199389,0.463504612239761,-0.637104484834416,0.524056787468179,0.913538662588792
"9699","LIG3",58.7329142700316,0.158077288910497,0.247531009332856,0.638616104449076,0.523072702089117,0.913538662588792
"9700","SNHG22",5.38840163568366,0.501435897713423,0.787331198216037,0.636880513371748,0.524202676688545,0.913538662588792
"9701","REX1BD",1302.59181957925,-0.143650458288872,0.225352266034868,-0.637448474854234,0.523832761800455,0.913538662588792
"9702","CHEK2",163.640485918255,-0.222942715151663,0.349545703759376,-0.637807052851476,0.523599287907482,0.913538662588792
"9703","TMEM31",3.60261662722049,0.537389689495529,0.842883952386406,0.637560708059573,0.523759679844768,0.913538662588792
"9704","TMEM187",117.724763891342,0.165662841072259,0.259917866976798,0.637366114916015,0.523886394757503,0.913538662588792
"9705","TPRG1L",279.708655453264,-0.11280097285222,0.177396007229825,-0.635870979362464,0.524860519295284,0.913663434028285
"9706","EXTL2",256.536081030445,0.123599631632995,0.194408655230283,0.635772267888933,0.524924865326989,0.913663434028285
"9707","IPO9-AS1",13.8266787448042,-0.266373970570485,0.419122710815103,-0.63555126863072,0.525068940478559,0.913663434028285
"9708","AC009228.1",4.73044647982086,-0.423951247138892,0.666301861099

946,-0.636275045726308,0.524597166822378,0.913663434028285
"9709","CTBP1-
DT",77.1988966488342,0.129407482135069,0.203447253102938,0.63607387252
1603,0.524728274103391,0.913663434028285
"9710","JMY",184.77976630055,0.176549002221131,0.27781156753196,0.635
500043570873,0.52510233831364,0.913663434028285
"9711","OMD",814.967125012662,0.104180473718544,0.163709906440415,0.63
6372446749049,0.524533695295919,0.913663434028285
"9712","C11orf58",1954.09796142554,-0.105670125589028,0.16629178377583
,-0.635450069688814,0.525134921450608,0.913663434028285
"9713","AC008011.2",2.07191156036106,-0.73158539460396,1.1499638308992
8,-0.636181221484029,0.524658311254014,0.913663434028285
"9714","HOMEZ",37.9676997953052,0.171627244179154,0.270080501851558,0.
635466992258048,0.52512388776343,0.913663434028285
"9715","PPP1R9B",119.470586066497,-0.131721766987416,0.207105868357753
,-0.636011755880723,0.5247687597432,0.913663434028285
"9716","AL049539.1",2.12557697254397,-0.669899328714499,1.053955036962
33,-0.635605225290501,0.525033762864328,0.913663434028285
"9717","ZMYND8",254.360246159535,0.143158949745401,0.225116503718426,0
.635932716530029,0.524820277376492,0.913663434028285
"9718","AC002377.1",5.17458686220816,-0.475166598702462,0.747123562219
302,-0.635994663708635,0.524779900154914,0.913663434028285
"9719","TANK",627.986257100042,0.0857621094594941,0.135017003252881,0.
635194882076188,0.525301320752327,0.913691363880512
"9720","MRPS22",340.925858739438,-0.160240560504488,0.252295600744522,
-0.635130220390764,0.525343488757065,0.913691363880512
"9721","PLK4",6.60356665426391,-0.410962107741196,0.646922140400344,-0
.635257447653399,0.525260521337083,0.913691363880512
"9722","DLG3",43.3653725815984,-0.211689169166129,0.333319444914316,-0
.635093968851852,0.525367130331579,0.913691363880512
"9723","ADIPOR1",233.228793677666,-0.128348788017943,0.202261035349231
,-0.634570013924486,0.525708890242616,0.913909717892469
"9724","CEP19",40.4066120753334,-0.190786592488644,0.300543691304233,-
0.634804848708388,0.525555700577047,0.913909717892469
"9725","AC067852.5",3.38150892317884,0.515063932596388,0.8115749090636
64,0.63464743284219,0.525658385073144,0.913909717892469
"9726","IRF2BP1",162.582699897571,-0.129152368861299,0.203476561587522
,-0.634728481028251,0.525605514965129,0.913909717892469
"9727","COA8",484.865359634314,-0.133732094595731,0.210831632733339,-0
.634307541339755,0.525880135880022,0.914019463143444
"9728","ZNF468",36.9842295193474,0.197267250842395,0.310994692481602,0
.634310667067303,0.525878096386102,0.914019463143444
"9729","THAP5",191.944190665957,-0.174944062355363,0.275848223248168,-
0.63420405719987,0.525947660130573,0.914042865401144
"9730","AC234582.1",3.38460576277892,-0.568597924896857,0.897125988371
959,-0.6337994130888,0.526211736274594,0.914043966087976
"9731","COX20",472.747086799454,-0.129016328431558,0.203587005753215,-
0.633715928746206,0.526266227699869,0.914043966087976
"9732","PRKRIP1",184.899147077763,0.118852845921612,0.187594332204585,
0.633563096096075,0.526365991227677,0.914043966087976

"9733","PHF2",160.494331397998,0.141380468077455,0.223159337798374,0.633540453526499,0.526380772285227,0.914043966087976
"9734","C12orf43",161.19863110926,-0.117406879310948,0.185202060939103,-0.633939377972432,0.526120385660839,0.914043966087976
"9735","UBE2Q2L",12.7508037953924,0.322687898529801,0.509233214429047,0.633674099384109,0.526293531407328,0.914043966087976
"9736","LINC01684",23.559855139459,0.276256750386015,0.435761528008659,0.633963148716804,0.52610487205955,0.914043966087976
"9737","NKAP",381.909614903145,-0.125137123410237,0.197397181231754,-0.633935715947837,0.526122775644199,0.914043966087976
"9738","AP003059.1",4.25586446113523,0.475190385328823,0.750271921601126,0.633357548973361,0.526500180055578,0.914157429079863
"9739","TSC22D2",700.00572813607,-0.279164952859566,0.44087100242612,-0.633212325880625,0.526594997639025,0.914228177439227
"9740","TNFRSF18",5.37099043101486,-0.492489241754216,0.778287494115546,-0.6327857578052,0.526873558542014,0.914308536804506
"9741","XPA",444.113075311175,-0.112294800663019,0.17747737416763,-0.632727417732443,0.526911662090319,0.914308536804506
"9742","QSOX2",37.0270629843839,-0.175337461121774,0.277050710801288,-0.632871363566119,0.526817649549511,0.914308536804506
"9743","AGAP4",30.3518921740482,0.202760852685672,0.320401018631148,0.632834606930807,0.526841654881189,0.914308536804506
"9744","DRAM1",237.859445916688,0.111844116280653,0.17675017545654,0.632780793522629,0.526876800800337,0.914308536804506
"9745","B3GAT2",25.2595087877899,0.352254424509559,0.556910739024978,0.632515051022854,0.527050376661295,0.914455389285703
"9746","STIL",4.10014910533378,0.457418322713126,0.724052246726352,0.631747673985192,0.527551770480214,0.914599776141288
"9747","GLMN",55.0878541856796,0.159309868227156,0.252145008658556,0.631818448735928,0.527505517031036,0.914599776141288
"9748","WBP1",336.381959467122,0.103833818825747,0.164365436379616,0.631725386509688,0.527566336450555,0.914599776141288
"9749","MAML3",71.2786442518023,-0.161346605821755,0.255349495485185,-0.631865770931652,0.527474591692629,0.914599776141288
"9750","ICK",312.247143295417,0.132400346613965,0.209546251162651,0.631843069868114,0.527489426858256,0.914599776141288
"9751","PDP1",239.701416054144,0.142789713044652,0.226020791394132,0.63175476983291,0.527547133034932,0.914599776141288
"9752","TTC12",31.5254903430485,0.209071194904104,0.330941987647834,0.631745752148509,0.527553026487698,0.914599776141288
"9753","ATP5PF",2294.28644507405,-0.161341759609588,0.255184986240514,-0.63225412273088,0.527220836401152,0.914599776141288
"9754","C9orf24",5.65734939460602,-0.450608576897692,0.713527103035124,-0.631522719993316,0.527698798478885,0.914735624839142
"9755","AC021097.1",7.23313484526376,0.384241044084074,0.608764048412274,0.631182220905158,0.527921385497738,0.914933864903214
"9756","ZNF181",120.457272484745,0.142543054933404,0.225814959713049,0.631238316161775,0.527884712280893,0.914933864903214
"9757","CCDC191",68.5208321104489,0.166350018756576,0.263667302197891,0.630908790623286,0.528100163729456,0.915149899388915

"9758","AC011481.2",4.67851596896501,0.522477162226433,0.828299749490316,0.630782711872039,0.528182608794033,0.915198970023521
"9759","TIPRL",470.543850037023,-0.106237638162415,0.16908068374645,-0.62832510378138,0.529790989644586,0.915351025051686
"9760","H3F3A",8876.69900091376,-0.142962238884694,0.227516357874388,-0.628360264819391,0.529767960974209,0.915351025051686
"9761","SLC66A3",730.52133052037,-0.14437714479197,0.230040850586916,-0.627615244960241,0.530256019436183,0.915351025051686
"9762","HOXD8",144.971748432398,-0.240274832892596,0.382278104300431,-0.628534122644295,0.529654100524192,0.915351025051686
"9763","DCUN1D1",235.720426140558,0.116166445735229,0.184987382172703,0.62796956403641,0.530023878459581,0.915351025051686
"9764","TBCCD1",48.9736914495329,-0.177969260345264,0.283466970713788,-0.627830677758069,0.530114867114069,0.915351025051686
"9765","SEC24D",274.84466833792,0.140845099065349,0.2238394182204,0.629223843526381,0.52920252111106,0.915351025051686
"9766","CYP4V2",205.188336495736,0.125384851283909,0.198972529814795,0.630161617790247,0.528588848597141,0.915351025051686
"9767","SLCO6A1",3.50638009397563,-0.693253902430707,1.1015980521867,-0.629316565197789,0.529141828596166,0.915351025051686
"9768","AC011337.1",2.13834600120549,-0.612245767319081,0.975001097006656,-0.627943670216097,0.530040841692511,0.915351025051686
"9769","HOXA2",3.87610835852912,-0.636031964584711,1.01063569371038,-0.629338512921134,0.529127462866501,0.915351025051686
"9770","SND1",322.830897902545,-0.119788247354182,0.190744743586665,-0.62800287495082,0.530002056639186,0.915351025051686
"9771","PLPP5",201.527252182789,-0.15693140716183,0.24975677313636,-0.628336942342502,0.529783235939495,0.915351025051686
"9772","MYBL1",13.1412611205748,0.363625930073505,0.578470884530034,0.628598499592464,0.529611942861451,0.915351025051686
"9773","DNAJC25",108.898250776664,0.136323037751562,0.217217177454878,0.627588661950458,0.530273438040056,0.915351025051686
"9774","C5",28.903557620087,0.238487664429954,0.379017689169049,0.629225683246631,0.529201316857085,0.915351025051686
"9775","RAB18",480.769871045898,-0.118669770985525,0.188586759701622,-0.629258231984481,0.529180011159719,0.915351025051686
"9776","GRASP",211.803963595687,0.13376717288612,0.213129868525149,0.627632221667399,0.530244895545499,0.915351025051686
"9777","PIP4P1",150.182490712543,-0.108715017644709,0.17270383421174,-0.629488153178007,0.529029522165768,0.915351025051686
"9778","ZFHX2",11.4473247644167,0.289985168403087,0.461074883983249,0.628932909764874,0.529392979637296,0.915351025051686
"9779","CILP",3568.74586005855,0.220179528770628,0.350495960986598,0.628194197019712,0.529876731309535,0.915351025051686
"9780","AC009065.4",9.04494445203625,-0.333403215936112,0.531206411694518,-0.627634020592062,0.530243716817303,0.915351025051686
"9781","ZNF646",48.3199515093638,0.17503525553719,0.278079550703899,0.629443103939595,0.529059006268777,0.915351025051686
"9782","HYDIN",10.4370018773436,0.405245345658421,0.645519101713215,0.627782113004705,0.530146685243672,0.915351025051686

"9783","COPRS",1206.53764371542,-0.162644787108631,0.258753893828654,-0.628569428278185,0.529630980189231,0.915351025051686
"9784","TBX21",4.91548853075125,0.559040298151259,0.887927487839771,0.629601297186262,0.528955474665469,0.915351025051686
"9785","AC132938.6",4.75552436772179,-0.40586249133257,0.644212297522202,-0.630013573000105,0.52868570391746,0.915351025051686
"9786","AC090220.1",2.06568003345774,0.654227238539317,1.04092709119229,0.628504382367412,0.529673576713786,0.915351025051686
"9787","AZU1",2.29579601409137,-0.973366145449031,1.54977612472151,-0.628068873898767,0.529958822392905,0.915351025051686
"9788","PRTN3",5.6975874917146,-0.439399221347291,0.69747720184942,-0.629983632701092,0.528705292853,0.915351025051686
"9789","LGI4",227.047395514797,0.235447764825155,0.373568191306305,0.63026716488316,0.528519802055815,0.915351025051686
"9790","ZNF350",53.2472111156202,0.186097148860585,0.296401259659003,0.627855458761147,0.53009863173843,0.915351025051686
"9791","AC011468.1",12.259820330915,0.311055223221226,0.494167940824106,0.629452454367013,0.529052886474694,0.915351025051686
"9792","PPP2R1A",595.113418924547,0.10109436447769,0.160673623569042,0.629190792067061,0.52922415635055,0.915351025051686
"9793","PCNA",228.559858260793,-0.135306925153674,0.215172589748567,-0.62882974691053,0.529460523123753,0.915351025051686
"9794","SLC9A8",42.7608047083979,-0.174367963648616,0.277434107341554,-0.62850226066094,0.529674966182063,0.915351025051686
"9795","RPGR",112.09573597522,-0.210047699198731,0.333506165647641,-0.629816539645784,0.52881462303127,0.915351025051686
"9796","LANCL1",121.235744771363,0.135907304616032,0.216798052639227,0.62688434218639,0.530735051688947,0.915587006831622
"9797","RPP14",117.384893805158,0.14814533633992,0.236223151627415,0.627141477536393,0.530566500642349,0.915587006831622
"9798","RSPO3",268.661494192579,0.239111148085839,0.381285918917888,0.627117698876608,0.530582086304798,0.915587006831622
"9799","GATAD1",626.371741927918,0.130209096835801,0.207705675648187,0.626892339024715,0.530729809388568,0.915587006831622
"9800","AP002851.1",2.66871393981059,-0.576749141204664,0.919834514825893,-0.627014024706206,0.530650041993346,0.915587006831622
"9801","RAD52",25.9109976814603,0.191440943332793,0.30518700645047,0.627290609647443,0.530468757677192,0.915587006831622
"9802","MGLL",904.618956083329,-0.206888476526249,0.330182339120126,-0.626588560362036,0.530928968926624,0.915684883956078
"9803","TLN1",1396.35343803241,0.109721384446963,0.175119920438169,0.62654999027197,0.530954258475597,0.915684883956078
"9804","PBDC1",397.274882929681,-0.145227995719244,0.231755231792818,-0.626643871621734,0.530892703632283,0.915684883956078
"9805","ZNF606",27.5290000148027,0.190993786318896,0.304895764525259,0.626423219149288,0.531037383782494,0.915734837837268
"9806","WDR61",442.030562275281,-0.131781068315725,0.210426567200989,-0.626256798600219,0.531146517702944,0.915736241594919
"9807","SLFN5",803.233159194055,0.164608484148098,0.262844678848654,0.626257624347344,0.531145976173097,0.915736241594919

"9808","RAB42",122.455061704552,0.176398236110488,0.281750263651882,0.626080110180262,0.531262397453704,0.915749272723747
"9809","POGZ",375.69551678639,0.15145411008867,0.24188201922427,0.626148692550164,0.531217416707835,0.915749272723747
"9810","ZNF436",252.062268410182,0.134056783720676,0.214464965241746,0.625075445630789,0.531921542642326,0.916393070134647
"9811","SLC16A1",241.766083481023,0.213357696260703,0.341860724701414,0.62410707298141,0.532557269170987,0.916393070134647
"9812","UMPS",132.502265795036,0.145344416181921,0.23240218937874,0.625400374112041,0.531708316666408,0.916393070134647
"9813","EIF5A2",97.6712144020452,-0.144553438833379,0.231467149367814,-0.624509522099292,0.532293018836191,0.916393070134647
"9814","UBE2D3-AS1",48.2410912842902,-0.16949846350549,0.271556005893846,-0.624174976162187,0.532512678908002,0.916393070134647
"9815","GZMA",38.6220652488505,0.262641114761376,0.420472814891395,0.624632807305781,0.532212082368271,0.916393070134647
"9816","PEX3",125.730567887068,-0.143696071564916,0.22996252154231,-0.624867350562938,0.532058122441657,0.916393070134647
"9817","AC069185.1",6.48211301549749,-0.338191031696967,0.541644659386877,-0.624378041647799,0.532379342331387,0.916393070134647
"9818","LINC00861",3.29509727829818,0.60118599954693,0.96241800600127,0.624662044764504,0.532192888954949,0.916393070134647
"9819","ZFPL1",205.329879295771,-0.165504988999126,0.265060631460903,-0.624404265872802,0.532362124249693,0.916393070134647
"9820","DCUN1D5",256.467079575289,-0.159297728883882,0.255216858848338,-0.624166168342919,0.532518462668498,0.916393070134647
"9821","FBXL8",60.7670833727637,-0.208463696858063,0.333923195980847,-0.624286360957146,0.532439539485936,0.916393070134647
"9822","NUMBL",355.563307330456,-0.14398402206678,0.230292879806113,-0.625221336360906,0.531825800236817,0.916393070134647
"9823","SMIM11B",3.37348874171506,-0.509007354444702,0.81542602645053,-0.624222600130096,0.53248140660725,0.916393070134647
"9824","POLDIP3",232.073470246067,0.0863750877354521,0.138393123783966,0.624128463710995,0.532543222228097,0.916393070134647
"9825","ARAF",133.490466839962,-0.116743844623075,0.186706591230451,-0.625279717516662,0.531787489403942,0.916393070134647
"9826","GPRASP1",80.9912737022667,0.160552590915481,0.256882978573795,0.62500283906261,0.531969194759982,0.916393070134647
"9827","TMED5",592.880693313361,-0.194057295121441,0.31223213283706,-0.621516092396265,0.534260109690895,0.916451890638056
"9828","SELENBP1",939.35542301223,0.141544929796464,0.22766047048126,0.621736964248764,0.534114841510208,0.916451890638056
"9829","SPDYA",3.11225801288616,-0.511583794531298,0.82285465339641,-0.621718297903144,0.534127117658625,0.916451890638056
"9830","ARHGEF33",11.8791835565908,0.32179553536456,0.51743653016396,0.621903396079501,0.534005391898205,0.916451890638056
"9831","TNS1",527.767498497186,-0.146080428424168,0.234837389162055,-0.622049278206554,0.533909465586386,0.916451890638056
"9832","ACKR2",16.7222883898975,0.30702610987049,0.493046662517955,0.6

22712074152432,0.533473746842669,0.916451890638056
"9833","ZGRF1",11.7672435415057,0.2864981559258,0.460173438237519,0.62258733798956,0.533555734041917,0.916451890638056
"9834","AC010359.3",30.5409912279575,0.251857123197155,0.40485059482866,0.622098933320686,0.533876816334588,0.916451890638056
"9835","HIST1H3H",4.16933413305462,0.44332286425474,0.710496140486024,0.623962382049647,0.532652290267803,0.916451890638056
"9836","LINC02587",2.59206082087905,-0.709475360979029,1.13772993717054,-0.62358854926807,0.532897832588318,0.916451890638056
"9837","PEX1",129.523326284179,0.144660643377709,0.232531484441796,0.622112071081363,0.533868178157308,0.916451890638056
"9838","ADGRB1",4.00702474270103,0.46526093351518,0.748463865474551,0.621621102870729,0.534191041451801,0.916451890638056
"9839","DNAJA1",2722.36693400345,0.174337226738553,0.280511126627671,0.621498436922808,0.534271722617656,0.916451890638056
"9840","LMX1B",28.9055393157502,0.196497376108814,0.316105104430393,0.621620383077625,0.534191514863916,0.916451890638056
"9841","CAMSAP1",57.8466382459134,0.160535640284165,0.257473948821775,0.623502459253802,0.532954386681163,0.916451890638056
"9842","AP003486.1",21.1669387947599,0.209298067534929,0.336708569049089,0.621600062410101,0.534204879972654,0.916451890638056
"9843","TMEM119",495.506121051987,-0.150950775203096,0.242680683728233,-0.622013968660725,0.533932682947542,0.916451890638056
"9844","SBNO1-
AS1",20.9451636356878,0.297884843939676,0.477511143889735,0.623828046217205,0.532740518667629,0.916451890638056
"9845","AL138820.1",2.81389672251905,0.572192015588835,0.919953399708429,0.62197934783456,0.533955447944591,0.916451890638056
"9846","AC027020.2",12.8749726427113,0.357926833861731,0.574766828285774,0.62273397880187,0.533459349904153,0.916451890638056
"9847","CIAPIN1",143.346886577636,0.148203953950609,0.237926248106816,0.622898713907654,0.533351083238489,0.916451890638056
"9848","CTRL",4.07902975892188,0.477580628182184,0.76695643031564,0.622695904623469,0.533484374471047,0.916451890638056
"9849","AC040977.1",15.3172315848019,0.307922012688768,0.494046815265405,0.62326484692215,0.53311049428591,0.916451890638056
"9850","NEURL4",30.454719580804,0.193951384490088,0.311709329859261,0.622218733644123,0.533798049326868,0.916451890638056
"9851","TIMP2",7890.2143580278,0.0843349258205196,0.135560703326441,0.622119270194653,0.533863444715666,0.916451890638056
"9852","SLC38A10",401.198369229368,0.113091485562553,0.181331608237798,0.623672213915655,0.532842874671781,0.916451890638056
"9853","AC009831.1",5.68293198041943,-0.422546320830642,0.677972725980655,-0.623249733563321,0.53312042430962,0.916451890638056
"9854","MAPRE1",326.502635526752,-0.0936243705865151,0.150307669876781,-0.622884851207303,0.533360193608864,0.916451890638056
"9855","ITCH",215.182387017636,0.150894906169009,0.242029864768483,0.623455730611382,0.532985084845558,0.916451890638056
"9856","STMN3",293.32278032925,-0.127821576182413,0.205397971523008,-0.622311774720203,0.533736880193976,0.916451890638056

"9857","TIMM17B",152.94257358719,-0.162948515436094,0.261971798119803,
-0.622007852011517,0.533936704927437,0.916451890638056
"9858","ZNF687-
AS1",9.92916211635063,-0.283960930243734,0.45736765784171,-0.620859226
434437,0.534692250535549,0.91657919768826
"9859","SLC35G2",96.2934733362539,0.136306512415504,0.219481453599171,
0.62103886310337,0.53457405313728,0.91657919768826
"9860","AC008771.1",55.752116170228,-0.173260265762392,0.2790500398676
21,-0.620893176881771,0.534669910797931,0.91657919768826
"9861","ZDHHC6",175.576799991902,-0.120052652159135,0.193247638762227,
-0.621237356006449,0.534443464041838,0.91657919768826
"9862","ELP4",103.087826037052,0.117415712937757,0.189133443375159,0.6
20808836567604,0.534725408445528,0.91657919768826
"9863","DMKN",2.16117838693735,-0.690331502663066,1.11198037691112,-0.
620812666299637,0.534722888340728,0.91657919768826
"9864","HCFC1",159.892696015377,0.127995347013564,0.206066594526153,0.
621135838673353,0.534510250598055,0.91657919768826
"9865","AC092747.4",45.4890806380885,0.153399025644687,0.2471636348082
3,0.620637521226401,0.534838146381321,0.916679511304143
"9866","NT5DC2",105.211413621376,-0.184884918784809,0.29798892437465,-
0.620442250237328,0.534966663492048,0.91675165003971
"9867","AC080013.6",2.18922899279369,0.657762707323954,1.0604767431175
2,0.62025189292724,0.535091961665865,0.91675165003971
"9868","LRP12",105.313633850024,-0.136139185198723,0.219492927559337,-
0.620244063043531,0.535097115817477,0.91675165003971
"9869","N4BP1",299.29212968944,0.106717639469262,0.172052958066934,0.6
20260416724399,0.535086350762601,0.91675165003971
"9870","MRPS28",250.632178235404,-0.150825864558613,0.243289724868786,
-0.619943421942535,0.535295036769373,0.916997819827412
"9871","AC026150.1",2.14707415818048,-0.640348219878671,1.033371301902
07,-0.619669056707903,0.535475691735971,0.917168942299277
"9872","MSRB1",111.750371887179,-0.14464351974356,0.233436456174881,-0
.619626951649744,0.535503418404215,0.917168942299277
"9873","DCDC1",2.43565653192378,-0.756843434042316,1.22186165629492,-0
.619418270589904,0.535640847974575,0.917311400542298
"9874","AP001107.9",11.1635679560047,-0.319911487862924,0.516874414063
152,-0.618934656386061,0.535959406555051,0.917311921987169
"9875","PAPOLA",815.311608163387,-0.0765902847731833,0.123726885011331
,-0.619027018793604,0.535898559704557,0.917311921987169
"9876","ACSM3",7.89526174381132,-0.420818592088416,0.679763090815647,-
0.619066550941265,0.535872517632348,0.917311921987169
"9877","SOX15",29.7702553075703,-0.21298648395312,0.343916993655075,-0
.619296190309021,0.535721253735415,0.917311921987169
"9878","REXO1",108.432129583348,0.147419703909615,0.23807995118922,0.6
19202512320954,0.535782957010481,0.917311921987169
"9879","TRPM4",22.453773330337,-0.218656044761073,0.353284370482545,-0
.618923629319956,0.535966671239132,0.917311921987169
"9880","GASK1B",119.792477650145,0.470908700974802,0.760965734861067,0
.618830361738663,0.536028118352488,0.917324233310109
"9881","PUS1",29.9287642595868,-0.240918876966417,0.389520985491696,-0

.618500378515686,0.53624554836956,0.917603454289295
"9882","DUSP1",13557.6204521692,-0.182051523569533,0.294387488581424,-0.618407814974718,0.53630654760012,0.91761496729638
"9883","LLGL1",55.6198863653418,0.245110178686345,0.396553211574119,0.618101610407793,0.536508360816454,0.917867384871456
"9884","ZSWIM1",17.3558614716475,0.24352826228566,0.394047916550979,0.618016875757683,0.536564214453406,0.917870066570031
"9885","AC137630.3",6.55528321369985,-0.378580329986425,0.612778454994242,-0.617809465885974,0.536700942951667,0.918011081783185
"9886","TAF12",412.198631388476,-0.15924929436667,0.257831381913924,-0.617648996737856,0.536806739262082,0.918090360024319
"9887","AL592148.3",27.3465281687653,-0.219173450774008,0.354928484583768,-0.617514401615405,0.536895485074969,0.918090360024319
"9888","SERPINI1",74.7609475806533,-0.253319875567925,0.410239863519569,-0.617492101802635,0.536910189254818,0.918090360024319
"9889","RNF25",53.2623185863441,0.165774280578364,0.268615443575844,0.617143520757985,0.53714006496219,0.918303719736703
"9890","CD5",3.37194266263414,0.573376586268905,0.929181925397721,0.617076775383336,0.53718408659558,0.918303719736703
"9891","USP16",706.576308468663,-0.0983825318058972,0.159438622347677,-0.617055832252244,0.537197899924044,0.918303719736703
"9892","PIK3CD",19.4651183467448,-0.309546724766209,0.502056632901147,-0.616557385125031,0.537526710216142,0.91834933343812
"9893","GBP2",733.556762569759,0.113710804717464,0.184492343664146,0.616344301660918,0.537667305686772,0.91834933343812
"9894","MORN2",249.779858762399,-0.165761356665319,0.269144775269529,-0.615881755457897,0.537972563729373,0.91834933343812
"9895","KPNA4",558.930748829583,-0.107426304961162,0.174177199482368,-0.616764451836512,0.537390102378635,0.91834933343812
"9896","HELQ",129.587864663707,-0.12157610963986,0.197320445914402,-0.616135388689525,0.537805167335009,0.91834933343812
"9897","AL669970.3",8.85836406497418,-0.382971775606301,0.62103141931302,-0.616670531790391,0.537452061960182,0.91834933343812
"9898","SC5D",160.409547119907,0.106452538282299,0.172565131149519,0.616883246187569,0.537311738219229,0.91834933343812
"9899","AC073611.1",12.4658259462546,0.357080092291383,0.579580351040073,0.616101100823367,0.537827795589786,0.91834933343812
"9900","EPYC",3.69263509578345,-0.549493697033861,0.891509215284865,-0.61636345156374,0.537654669555927,0.91834933343812
"9901","SMOC1",503.57082890118,-0.205296007940226,0.333343971018837,-0.615868369578597,0.537981399055063,0.91834933343812
"9902","IGHG4",189.208179991475,0.524164691007935,0.85052295347616,0.616285179448279,0.537706318628089,0.91834933343812
"9903","SIN3A",208.309503643883,0.119448122258646,0.19397836303523,0.615780648880681,0.538039300747458,0.91834933343812
"9904","NQO1",166.625691795625,0.201677268198001,0.3273773802684,0.616039104573005,0.53786871118808,0.91834933343812
"9905","ACSF3",111.889197191941,0.122204555666509,0.198371989780778,0.616037353870161,0.53786986662023,0.91834933343812
"9906","POLRMT",62.3108878852205,-0.155811821687271,0.253005333891727,

-0.615844019138151,0.537997471706495,0.91834933343812
"9907","RUSC1",200.8849512933,-0.0945718369047768,0.153639123689326,-0
.615545276709666,0.538194678019378,0.9184353982724
"9908","ADAMTS9",16.8673760181347,0.336050082125398,0.546036854942704,
0.61543479910465,0.53826761585303,0.9184353982724
"9909","NEK5",18.2523370779911,0.245325474141937,0.398660031181138,0.6
15375144117392,0.53830700241776,0.9184353982724
"9910","ZNF576",135.873808139863,-0.127498976872825,0.207140364704042,
-0.615519708362938,0.538211557920866,0.9184353982724
"9911","LRWD1",55.393744618687,-0.218690468273231,0.355483774003273,-0
.615191140260646,0.538428498095977,0.918549999576913
"9912","LINC00910",44.4841378599283,0.298156855966142,0.48473414577464
6,0.615093569465097,0.538492928575872,0.918567235306784
"9913","ATP6V0E2-
AS1",3.29848227386186,0.544014666499626,0.884744343437905,0.6148834638
32857,0.538631684108042,0.918618571202216
"9914","AC009159.3",2.79665241392352,-0.546533028941359,0.888805252504
425,-0.614907514780509,0.538615799750618,0.918618571202216
"9915","USF1",43.1993733523374,-0.189908964029488,0.308909139650848,-0
.614772888377914,0.538704716271692,0.918650463209457
"9916","LINC01128",86.7045256119044,-0.162655575873602,0.2669693777445
92,-0.609266790250432,0.542347615820624,0.918705594323782
"9917","CDK11A",159.145224836873,-0.159659433671276,0.263361142698528,
-0.606237625016838,0.544356971502505,0.918705594323782
"9918","KIAA2013",273.532918238925,-0.101547217390963,0.16607337197886
9,-0.611459960022273,0.540895118044712,0.918705594323782
"9919","RCC2",121.618205565987,0.103923041796677,0.170429833948189,0.6
09770246142878,0.542014013930892,0.918705594323782
"9920","LURAP1",45.1488142328394,-0.219257687131406,0.36454447702476,-
0.601456614898909,0.547535900084113,0.918705594323782
"9921","ZFYVE9",150.861113874173,0.133472781777581,0.217593956055461,0
.613402983231579,0.539609913980283,0.918705594323782
"9922","ZNHIT6",221.553258201815,0.145171887106183,0.238132922705948,0
.609625437157401,0.542109957332418,0.918705594323782
"9923","PKN2",555.451899343666,0.0948938833345614,0.15735921006237,0.6
03039906573947,0.546482141775046,0.918705594323782
"9924","LRIG2-
DT",21.6099118173062,-0.214464979022206,0.355151028821384,-0.603869795
151478,0.545930211790614,0.918705594323782
"9925","TENT5C",52.7042532933408,0.178879362563278,0.297452511784543,0
.601371161702772,0.547592801949666,0.918705594323782
"9926","HIST2H2AA4",34.0760490121846,0.367054825047493,0.6038372469498
43,0.607870459965154,0.543273392982577,0.918705594323782
"9927","S100A16",821.826683742448,-0.159273364235348,0.26166827948231,
-0.608684264483482,0.542733739009826,0.918705594323782
"9928","TPM3",1130.24451463608,-0.092976513252553,0.153868825080233,-0
.604258290814086,0.545671931868904,0.918705594323782
"9929","AL021026.1",3.21991766875534,-0.538853918912848,0.893031819699
386,-0.603398341499452,0.54624372528251,0.918705594323782
"9930","GLUL",2832.63122836546,0.174761871819839,0.290713114662985,0.6

01148909372167,0.547740809781716,0.918705594323782
"9931","NUAK2",4.00729142384598,-0.451377965259707,0.746982093239129,-0.604268789499896,0.545664952967428,0.918705594323782
"9932","CDK18",2.49250000297557,0.732257761238254,1.19975337209511,0.610340240144125,0.541636445896631,0.918705594323782
"9933","TAF1A",46.1258264554199,0.158787299149886,0.264397601607821,0.600562554971335,0.548131384471916,0.918705594323782
"9934","NTPCR",424.45789334513,0.172111818767664,0.280443081200463,0.613713905976655,0.539404397518736,0.918705594323782
"9935","TRAPPC12",254.72332999137,0.0975018308595953,0.160112272791607,0.608959133235829,0.54255152708452,0.918705594323782
"9936","AC093110.1",7.90439711296027,0.445509955730786,0.738076584296616,0.60360938852349,0.546103369407728,0.918705594323782
"9937","NAGK",362.884845589742,0.106439069275732,0.174675966945257,0.609351538950345,0.542291452144028,0.918705594323782
"9938","MOB1A",645.576182983499,0.0765346213090123,0.127282247290766,0.601298475931014,0.547641204500655,0.918705594323782
"9939","DNAH6",2.36873536498137,-0.677715535451263,1.1270858421426,-0.601298951784294,0.547640887615904,0.918705594323782
"9940","LINC01127",2.62780289337395,0.66352078752019,1.08987077877661,0.608806842463468,0.542652477662465,0.918705594323782
"9941","SPOPL",170.596342098668,0.136022492508727,0.22291814753931,0.610190305321553,0.541735751051194,0.918705594323782
"9942","LRP1B",324.500208604159,0.255506592614722,0.416843328486048,0.612955936089244,0.539905475844756,0.918705594323782
"9943","CYTIP",29.2804823801987,-0.257070456472852,0.420872673927586,-0.610803391139342,0.541329748128142,0.918705594323782
"9944","HOXD9",15.936357511911,-0.281544743214219,0.461443470627052,-0.610139185264947,0.541769611074211,0.918705594323782
"9945","ING5",156.390718466375,0.127464687690384,0.20795626899857,0.612939866175711,0.539916101855952,0.918705594323782
"9946","RPUSD3",149.791408494739,-0.11770868814305,0.19485035478752,-0.604097889744205,0.545778562458756,0.918705594323782
"9947","IL17RC",228.232443191734,-0.143973955173623,0.236617198378498,-0.608467838180211,0.542877230585863,0.918705594323782
"9948","NR1D2",326.895627533995,-0.446978045363609,0.744371176972566,-0.600477368268764,0.54818813936552,0.918705594323782
"9949","KLHL18",57.9286250318982,-0.162679385191914,0.265857356764734,-0.611904771685045,0.540600764391445,0.918705594323782
"9950","UBA7",147.60760752771,0.125965745137286,0.205769498164452,0.612169180859904,0.540425829855535,0.918705594323782
"9951","DENND6A",194.382365263127,0.116914103747105,0.192030375533229,0.608831302977241,0.542636262634727,0.918705594323782
"9952","SLC35A5",135.583007772129,0.117172476719106,0.194505413085915,0.602412420611395,0.546899644271202,0.918705594323782
"9953","ZBTB38",794.217922044999,0.116460372342419,0.192079449098962,0.606313548319357,0.54430656363536,0.918705594323782
"9954","PLSCR1",546.036143399183,0.119832152449379,0.197806163667835,0.605805957849761,0.544643612995421,0.918705594323782
"9955","FAM53A",3.84131272317156,-0.451230853937062,0.750994418404714,

-0.60084448416485,0.547943572326152,0.918705594323782
"9956","STX18",199.349960934432,-0.109113430467323,0.179917358820747,-0.606464163227481,0.544206572530908,0.918705594323782
"9957","TAPT1-AS1",11.00879363327,0.32842427863076,0.535473512622256,0.613334312321894,0.539655309969955,0.918705594323782
"9958","PRDM8",186.624804987545,0.130525041029632,0.21505020690156З,0.606951478495338,0.543883113412867,0.918705594323782
"9959","LINC02510",2.30864416960042,0.781115166145837,1.28291905015353,0.608857718694221,0.542618751761745,0.918705594323782
"9960","LINC02362",3.77687885915118,0.491410669821461,0.810040109963517,0.606649798913777,0.544083344193266,0.918705594323782
"9961","GTF2H2C",74.0167113288748,-0.143311125550908,0.23474606203275,-0.61049426904088,0.541534438608759,0.918705594323782
"9962","DCP2",251.937169711469,0.106207511772783,0.173122745200235,0.613480982235712,0.539558353825854,0.918705594323782
"9963","MZB1",9.04010944456642,-0.436931233716728,0.721057334419891,-0.605959072683521,0.544541931023214,0.918705594323782
"9964","PCDHB6",11.4835448580849,-0.314099466726497,0.520786960892413,-0.603124675372556,0.546425752326613,0.918705594323782
"9965","NOP16",84.3246592598547,-0.18642299696418,0.308991205496945,-0.603327841206222,0.546290615168719,0.918705594323782
"9966","AC146507.3",6.14823340911686,0.378884277189558,0.630976979281602,0.600472425508987,0.548191432524996,0.918705594323782
"9967","TMEM14B",957.953457010982,-0.156624590210063,0.259685408161977,-0.603132040874511,0.546420852822782,0.918705594323782
"9968","BTN2A1",122.326681934027,0.121594480753944,0.199708929280237,0.608858508190784,0.542618228411969,0.918705594323782
"9969","PPP1R18",279.114038383038,-0.0998929563358305,0.165667441263803,-0.602972772282783,0.546526802535935,0.918705594323782
"9970","HSPA1L",8.13291228383336,0.305447917829591,0.505398394295786,0.604370574337098,0.545597294761014,0.918705594323782
"9971","CRIP3",3.19701686405336,0.607148427029713,0.998106853068361,0.608300028361922,0.542988502228831,0.918705594323782
"9972","CENPW",83.6222876868555,-0.214672056442527,0.352113037379958,-0.609668014680409,0.54208174665418,0.918705594323782
"9973","THBS2",1512.91125832041,0.144001951309704,0.238563825600099,0.60362023013117,0.546096159728046,0.918705594323782
"9974","HIBADH",506.255257976295,-0.129464834518586,0.212966613029108,-0.607911412390686,0.543246230030408,0.918705594323782
"9975","AC211476.2",4.40973062241499,-0.477989874423868,0.787398181935428,-0.607049756260507,0.543817892407822,0.918705594323782
"9976","HSPB1",2426.66045331255,-0.14064043361487,0.234078501023438,-0.600825932325958,0.547955929985852,0.918705594323782
"9977","BUD31",844.418112353679,-0.146295822931044,0.239526653459465,-0.610770537717224,0.541351500747987,0.918705594323782
"9978","CADPS2",91.1420073077477,0.166274387663717,0.273192853333149,0.608633738529579,0.542767236244895,0.918705594323782
"9979","ZNF282",77.143548503667,0.135984955359557,0.225132942824277,0.604020689525199,0.545829886902678,0.918705594323782

"9980","PTPRN2",117.320913874312,0.17178983476724,0.280738908586462,0.611920291463027,0.540590495640616,0.918705594323782
"9981","INTS10",334.109575910782,-0.0998948714001342,0.165975661668287,-0.601864576987079,0.547264285210938,0.918705594323782
"9982","TNFRSF10A",3.29828717106028,0.529256608047799,0.876718991932165,0.603678730491959,0.546057257745238,0.918705594323782
"9983","RAD21",628.438303866802,0.10235972859138,0.169442062664406,0.604098692979866,0.545778028462183,0.918705594323782
"9984","AC234917.3",5.28179324572542,0.374232490114477,0.611292804794968,0.612198421409518,0.540406485888775,0.918705594323782
"9985","RECQL4",6.57832296041179,-0.380119987694055,0.631513546477533,-0.601918976741346,0.547228071730821,0.918705594323782
"9986","AL589843.1",14.035638051408,0.252649961955614,0.417764893985275,0.604765899655798,0.545334554410474,0.918705594323782
"9987","DOLK",94.8231932445519,-0.156894705351957,0.257529667521024,-0.609229635025056,0.542372239815584,0.918705594323782
"9988","PMPCA",174.721179114601,-0.106067346994111,0.174090019980511,-0.60926724579608,0.542347313918936,0.918705594323782
"9989","PHYH",616.787747127468,-0.157003954510213,0.260122551355579,-0.60357686671923,0.546124996722318,0.918705594323782
"9990","BEND7",86.049214831962,-0.162337882665263,0.268094532790856,-0.605524778798474,0.544830365649469,0.918705594323782
"9991","WAC-AS1",257.349459918587,0.136809643371408,0.222845803671788,0.613920662257136,0.539267755633408,0.918705594323782
"9992","ZWINT",16.9613711602493,0.315722866770228,0.52475259072844,0.601660425024208,0.547400198108982,0.918705594323782
"9993","DNAJB12",358.532520136244,-0.105612833247424,0.175462090065371,-0.601912545371348,0.547232352980442,0.918705594323782
"9994","LRMDA",170.395484430449,0.151593033230223,0.246783517402767,0.614275356902436,0.539033384071681,0.918705594323782
"9995","TLL2",3.66191594313242,-0.469784652245565,0.7712826884377,-0.609095289299381,0.542461279816204,0.918705594323782
"9996","AS3MT",50.8307227278465,-0.180110000522785,0.295418415018445,-0.609677634725444,0.542075372782291,0.918705594323782
"9997","OAT",617.845402363042,0.101830909459406,0.16738211870291,0.608373882757136,0.542939529320985,0.918705594323782
"9998","PSMD13",683.659295407106,-0.125660185480374,0.206671559111764,-0.608018761848209,0.543175030420154,0.918705594323782
"9999","TRIM5",97.8776997271144,0.132563122179442,0.216427648979994,0.612505485339793,0.540203369906866,0.918705594323782
"10000","RPL27A",18088.0038473701,0.123504433604568,0.205011314460585,0.602427402260826,0.546889674278486,0.918705594323782
"10001","FNBP4",502.64130753242,0.161669798314014,0.265426588174759,0.609094211042525,0.542461994479441,0.918705594323782
"10002","STX5",323.614005346715,-0.124344722602146,0.202401386178477,-0.614347188771225,0.538985925948268,0.918705594323782
"10003","NRXN2",54.3993390017689,0.316920951224115,0.527141074552818,0.601207089568886,0.547702063058579,0.918705594323782
"10004","MUS81",115.509315015077,-0.131633318084858,0.217771655151475,

-0.604455699219892,0.545540713920855,0.918705594323782
"10005","RBM14",130.802585930227,0.141195140969157,0.232122122853287,0
.608279552304454,0.543002080308292,0.918705594323782
"10006","PITPNM1",24.3728309188709,-0.218622437967152,0.36285976349111
3,-0.602498430423263,0.54684240765949,0.918705594323782
"10007","ANO1",5.79776946385865,-0.501750741884422,0.832163092949493,-
0.6029476026202,0.546543546996394,0.918705594323782
"10008","UVRAG",243.300790708354,-0.111266363322535,0.184353754477029,
-0.60354812755603,0.546144108897334,0.918705594323782
"10009","ENDOD1",100.768269764964,-0.155507329130315,0.254863702898637
,-0.610158792176706,0.541756624060694,0.918705594323782
"10010","AP001527.2",15.1188278375747,-0.362664580370942,0.59619239457
4926,-0.608301252533614,0.542987690461682,0.918705594323782
"10011","TMEM218",218.413936105739,-0.172011378298505,0.28559037634975
8,-0.602301031628058,0.546973774267893,0.918705594323782
"10012","CD27-
AS1",219.69149578004,0.149440141633561,0.245509682188629,0.60869347514
6712,0.542727632719525,0.918705594323782
"10013","TAS2R14",24.3540420079455,0.205152231747558,0.337773564159342
,0.607366157437882,0.54360794252472,0.918705594323782
"10014","AC016957.2",7.23485968892128,0.385193976342496,0.641109946067
942,0.600823585260171,0.547957493411588,0.918705594323782
"10015","RAPGEF3",6.27181542998948,-0.509178450614635,0.84292685674221
2,-0.604060063506025,0.545803709894577,0.918705594323782
"10016","ACVRL1",719.443253731328,-0.148743925006802,0.245906124083546
,-0.60488092991237,0.545258115018897,0.918705594323782
"10017","MAP3K12",232.275936745233,0.141063243634558,0.229698938992,0.
614122312682826,0.539134504830965,0.918705594323782
"10018","AC023794.3",9.42649869397128,-0.356064208971754,0.58849813670
2317,-0.605038804314624,0.545153213686097,0.918705594323782
"10019","HMGA2",6.92492900435761,-0.490390731606369,0.802621510331723,
-0.610986280947904,0.541208662710362,0.918705594323782
"10020","LRRIQ1",4.02007649972653,-0.614229361739346,1.02267028571005,
-0.600613286923539,0.548097586133061,0.918705594323782
"10021","EEA1",1720.76856660072,-0.0945206412241001,0.156705524941199,
-0.603173635770451,0.54639318446505,0.918705594323782
"10022","RSRC2",1200.78572654984,0.0927409814277248,0.152925190558288,
0.606446727901091,0.544218147129772,0.918705594323782
"10023","LINC00545",12.698964751748,-0.346377352259301,0.5688689889762
19,-0.608887738603344,0.542598852026119,0.918705594323782
"10024","LINC01048",8.11264745502277,0.468188115126862,0.7687930813651
29,0.608991062062513,0.542530363276116,0.918705594323782
"10025","AL138963.4",5.26777440414477,0.564534626420597,0.918934030347
074,0.614336402589615,0.538993052065802,0.918705594323782
"10026","GAS6",3523.98312610143,0.153225754618716,0.250141002862966,0.
612557529013577,0.540168947955906,0.918705594323782
"10027","LTB4R",29.6301165341099,-0.199310608816991,0.330221701697196,
-0.603566052117779,0.54613218863101,0.918705594323782
"10028","MGAT2",185.12382686903,-0.114051107562103,0.189887770061821,-
0.600623765948548,0.548090604987797,0.918705594323782

"10029","AL365295.1",68.7500992482045,0.181148486558732,0.300665477683517,0.602491805691807,0.546846816088092,0.918705594323782
"10030","NUMB",361.10197625131,0.118083764004472,0.195168301794278,0.605035566323374,0.545155365103329,0.918705594323782
"10031","MKRN3",2.68602692719207,0.778917658645869,1.2895734001765,0.604011883727799,0.545835741347142,0.918705594323782
"10032","HAUS2",101.556470556084,0.119544244848795,0.19491479237413,0.61331540511987,0.539667809210238,0.918705594323782
"10033","LINC00926",21.9893569876166,-0.213180629024747,0.353632873416532,-0.602830350485116,0.546621553930406,0.918705594323782
"10034","CEMIP",3.18288652773367,0.495370290289466,0.814587294162872,0.608124253642508,0.543105067433612,0.918705594323782
"10035","AC105339.6",4.22035765612542,-0.422588565576805,0.699048368498669,-0.604519779488778,0.545498122943461,0.918705594323782
"10036","IQGAP1",1265.32127718712,0.109514953251557,0.178419557680803,0.613805765887405,0.539343686647104,0.918705594323782
"10037","SLCO3A1",459.49752687547,-0.132350102962164,0.220298098233874,-0.600777328643383,0.54798830628505,0.918705594323782
"10038","AL008727.1",4.91979008589544,0.454016084145389,0.748395724176609,0.606652429294543,0.544081598198475,0.918705594323782
"10039","JPT2",65.6867008452881,0.168261462475746,0.280196658249593,0.600512024400597,0.548165049671261,0.918705594323782
"10040","CCNF",8.41236448415945,-0.295052249847138,0.48116748984088,-0.613200717165473,0.53974363063661,0.918705594323782
"10041","ZNF213",52.1830026164016,-0.156623532158188,0.257323589991984,-0.608663714675623,0.542747362809312,0.918705594323782
"10042","TMEM159",1064.18809920108,0.0988508098624707,0.160873525509286,0.614462880387146,0.538909494820755,0.918705594323782
"10043","SLX1A",3.99964545160493,0.509927940467859,0.836963062282274,0.609259791080099,0.542352254359349,0.918705594323782
"10044","RNF40",173.887439373268,0.107503077914981,0.175818133784623,0.61144476739056,0.540905173167205,0.918705594323782
"10045","BCKDK",214.146643398757,-0.128950840065178,0.213504881086297,-0.603971391235113,0.545862662768141,0.918705594323782
"10046","HIC1",977.411485007239,0.0983270828304024,0.160396535509567,0.613024979112081,0.539859823278272,0.918705594323782
"10047","MED31",221.481663409046,-0.161865500315394,0.267983161388696,-0.604013698012229,0.545834535136122,0.918705594323782
"10048","TMEM102",3.84237662800039,-0.485592072647635,0.803361845613026,-0.604450006307048,0.545544497797541,0.918705594323782
"10049","EIF4A1",4601.42895028353,-0.142067144629388,0.233900726749908,-0.607382228364298,0.543597279642395,0.918705594323782
"10050","DHRS7B",282.69545664724,-0.133297909283345,0.221830098033359,-0.600900916805706,0.547905982539727,0.918705594323782
"10051","LINC02693",60.6046197037289,-0.165353249583076,0.271722818223598,-0.608536488264335,0.542831713236247,0.918705594323782
"10052","ERAL1",122.670612647853,-0.135767064346187,0.222187494967334,-0.611047279533657,0.541168280531357,0.918705594323782
"10053","STARD3",150.80659960732,-0.122063980670917,0.203061228887956,-0.601119087771644,0.54776067083288,0.918705594323782

"10054","HEXIM2",78.1776405046473,-0.1812938623421,0.301614645497865,-0.601077782688054,0.547788180419949,0.918705594323782
"10055","VEZF1",420.721681755088,0.136474893016096,0.222053259516829,0.614604322012902,0.538816059450836,0.918705594323782
"10056","AC005332.3",18.4838945411906,-0.263193176232671,0.435630220741618,-0.604166478130488,0.54573296536306,0.918705594323782
"10057","SLC16A6",3.03849505035922,-0.569497690010973,0.944181542549198,-0.603165455314223,0.546398625938178,0.918705594323782
"10058","RAB40B",223.939027736573,0.113115865178353,0.188230492630149,0.600943362564598,0.547877710256378,0.918705594323782
"10059","ABHD3",76.3350176053258,0.155118679942072,0.257249324392408,0.602989649471163,0.546515574898272,0.918705594323782
"10060","ME2",144.381976506184,-0.123355337070489,0.203011706620388,-0.607626718301285,0.543435076104435,0.918705594323782
"10061","CCBE1",377.566078750111,0.440114858907697,0.718050527605741,0.612930207537359,0.539922488549217,0.918705594323782
"10062","TNFRSF11A",18.9405459076317,-0.253057909052791,0.418586117966717,-0.604553993051705,0.545475383560211,0.918705594323782
"10063","RBFA",252.488377133726,-0.11935007752932,0.195058172100751,-0.611869147772359,0.540624335528057,0.918705594323782
"10064","ZNF562",54.594953461848,0.230759738348396,0.377342635490669,0.611539001015173,0.540842806899225,0.918705594323782
"10065","ZNF443",13.7314395115672,-0.255431683527361,0.415981241745339,-0.614046158561484,0.539184825611426,0.918705594323782
"10066","ZSWIM4",33.7042547117027,-0.206967412167283,0.342601768770159,-0.604104914315639,0.545773892484296,0.918705594323782
"10067","JAK3",12.5208957434132,-0.285744188582881,0.466818152971177,-0.612110276269663,0.540464798999166,0.918705594323782
"10068","UBA52",13743.4715822092,-0.13780135151659,0.227420294172396,-0.605932518107331,0.544559564965573,0.918705594323782
"10069","ZNF565",52.6796230921756,0.15078112316422,0.250292737183764,0.602419090784554,0.546895205391184,0.918705594323782
"10070","ZNF585A",159.337053046253,-0.11065243968685,0.181462637316036,-0.609780841519111,0.542006994281715,0.918705594323782
"10071","LRFN1",2.82424604581887,0.497243256571343,0.822681117981813,0.604417976422228,0.545565787165473,0.918705594323782
"10072","CTU1",132.060315515834,0.189533859242659,0.313458621912664,0.604653520410954,0.545409237298976,0.918705594323782
"10073","ZNF649",38.2413467571293,0.175586698445925,0.291625246923458,0.602097041659809,0.547109543636042,0.918705594323782
"10074","CCDC106",87.2170464755558,-0.193700420688428,0.315973496595368,-0.613027430387549,0.539858202483593,0.918705594323782
"10075","AC012313.6",6.21536278260342,0.391831270293313,0.641310093823438,0.610985658992591,0.541209074463902,0.918705594323782
"10076","RASSF2",101.768605671752,-0.258201406162291,0.425432459883897,-0.606915152249445,0.543907221928648,0.918705594323782
"10077","PLCB1",137.127864726387,0.195097401907039,0.324209044680992,0.60176421696997,0.547331097180854,0.918705594323782
"10078","EPB41L1",169.635386966701,0.107871042464277,0.177442687666877,0.607920472140217,0.543240220965032,0.918705594323782

"10079","MYL9",1809.04077122295,0.161708384618484,0.265526784754696,0.609009689052184,0.542518016691134,0.918705594323782
"10080","FITM2",57.5976446457856,-0.141723421773227,0.231164379937917,-0.613085034170443,0.539820115296984,0.918705594323782
"10081","CSTB",2333.36372683419,-0.149924202424098,0.248407589407539,-0.603541150983643,0.546148748521274,0.918705594323782
"10082","TSPAN7",9.46013591556063,-0.376679331783808,0.619413083613462,-0.608122982463301,0.543105910462931,0.918705594323782
"10083","SPIN2B",112.302228802079,-0.174489762980379,0.290450601193736,-0.60075538581513,0.548002923338541,0.918705594323782
"10084","P2RY10",4.84326868422072,0.450605650968874,0.743014495572711,0.606456070041474,0.5442119452503,0.918705594323782
"10085","HMGN5",75.1360095240115,-0.182421593631911,0.301130367204861,-0.605789430422366,0.544654589252487,0.918705594323782
"10086","BEX3",1400.88301894716,0.179767479304475,0.295107151833925,0.609160022680987,0.542418375715473,0.918705594323782
"10087","TCEAL4",1128.17343760025,-0.1187603371199,0.1959661900493,-0.606024626442056,0.5444984003364,0.918705594323782
"10088","ZFP92",6.52670701594154,-0.447705025820707,0.744733815099993,-0.601161135352227,0.54773266743716,0.918705594323782
"10089","FLNA",1232.00090313451,0.136550766490714,0.223864545880275,0.609970488867596,0.541881356782531,0.918705594323782
"10090","CD180",2.0134691800472,-0.628105637401038,1.04642283494691,-0.60024075968574,0.548345792989057,0.918817564713453
"10091","AC011603.2",20.2947666412837,0.270618540787228,0.450935083576543,0.600127492056831,0.54842127177006,0.918817564713453
"10092","C16orf86",84.4389094239395,0.197083642853172,0.328369402476612,0.600188815908964,0.548380406416021,0.918817564713453
"10093","BPGM",112.985674132291,0.121384742883104,0.202365827087881,0.599828264632797,0.548620694295548,0.918969556087688
"10094","AC005332.4",30.2149485910681,0.220311329280269,0.367263758458382,0.599872228626758,0.548591391894257,0.918969556087688
"10095","S100A8",55.214839591069,0.31125146439167,0.519951463392362,0.598616383077271,0.549428728465216,0.91926695607692
"10096","MAL",2.28192205492407,-0.608590989996277,1.01599971315727,-0.599007049032575,0.549168183940135,0.91926695607692
"10097","CCNT2-AS1",5.65601794837656,-0.394638747503427,0.659287720994656,-0.598583493877365,0.549450665845361,0.91926695607692
"10098","SEC22C",284.870916113096,-0.0766648675615216,0.128004729594264,-0.598922147677868,0.549224801512442,0.91926695607692
"10099","GRM3",2.1350273151792,-0.71700184438883,1.19696362491666,-0.599017237836905,0.549161389596985,0.91926695607692
"10100","LINC-PINT",765.848340238323,-0.201349691301454,0.33587788984468,-0.599472895922276,0.548857579133314,0.91926695607692
"10101","AC016065.1",29.3974767380691,-0.208226112443732,0.347407949345249,-0.599370604029559,0.548925775129054,0.91926695607692
"10102","MYOF",839.420992043744,0.14280810117087,0.238532989393154,0.598693294098165,0.54937742983655,0.91926695607692

"10103","PRC1",28.2934509138673,-0.205309807962874,0.342922246530911,-0.598706587396533,0.549368563622631,0.91926695607692
"10104","TMEM50B",399.694377828988,-0.1019694424912,0.170197370074227,-0.599124665949472,0.549089754327167,0.91926695607692
"10105","CHIC1",67.1270674639348,0.149176822972417,0.249130386384728,0.598790156179685,0.549312827476259,0.91926695607692
"10106","KLHL4",6.85144353578239,-0.411948125643417,0.687548053266403,-0.599155395301221,0.549069264212878,0.91926695607692
"10107","CYLD",203.394315308191,0.117550020359976,0.19641977857852,0.598463256657143,0.549530868784209,0.91931017407771
"10108","MGAT1",599.663863198081,-0.103966471654472,0.173747330765114,-0.59837737475819,0.549588158910595,0.919315056476093
"10109","AC124283.1",18.3981183296202,0.270046187033729,0.451447912183889,0.598177950867854,0.549721202022698,0.919446640003935
"10110","MAPKAP1",236.139921542051,-0.105954195405106,0.177200403108168,-0.59793427975685,0.549883785649776,0.919594431024963
"10111","AC106886.3",4.57150569758571,0.391562681902604,0.654915780832016,0.597882496288541,0.549918339962941,0.919594431024963
"10112","LINC00115",6.00784889689439,0.352631914525425,0.5903119707555,0.597365345774906,0.550263485241519,0.91971745046499
"10113","AL603840.1",19.5673077507651,-0.308077362782959,0.515712549047435,-0.597381939516509,0.550252408953318,0.91971745046499
"10114","CNBP",2314.09622885662,-0.106445284961589,0.178199449636789,-0.597337899631837,0.550281805726238,0.91971745046499
"10115","N4BP3",3.39711238060788,0.55577106273197,0.931135907612657,0.596874267427742,0.550591328718698,0.91971745046499
"10116","E2F3",58.8615296163371,0.163744742198091,0.274282173795807,0.5969937452807,0.550511556559105,0.91971745046499
"10117","AL391840.3",37.5874507178774,0.184315977936966,0.308717076428437,0.597038492555472,0.550481681467752,0.91971745046499
"10118","MARVELD1",411.917775253571,0.11177503237426,0.187292358126575,0.596794410045929,0.55064465052384,0.91971745046499
"10119","PLEKHA7",3.29515296877106,0.508830518869644,0.851494397010356,0.597573537366983,0.550124525737752,0.91971745046499
"10120","EEF1G",9358.52214335246,0.141061267799426,0.236057080496759,0.597572703612938,0.550125082200528,0.91971745046499
"10121","TMEM233",26.8807751997423,0.424505499308363,0.710785718109553,0.597234143135856,0.550351066597634,0.91971745046499
"10122","KATNBL1",354.491847313208,-0.0981547618919233,0.164445575746458,-0.596882959279233,0.55058552521057,0.91971745046499
"10123","ZNF843",13.8645995554971,-0.241845595515954,0.405112471425181,-0.596983831835993,0.550518175283935,0.91971745046499
"10124","MKS1",45.0626834145062,0.161389109073442,0.270535219260136,0.596554894090356,0.550804593661203,0.91989372477515
"10125","AC007066.2",2.89205408267103,0.573575325344357,0.961623552290566,0.596465554507388,0.550864258391963,0.919902506754698
"10126","LSS",187.851089184069,0.160241888763977,0.268753340256818,0.596241477820707,0.551013920214497,0.920061560634674
"10127","AC009951.6",110.23835643743,-0.182542471960706,0.306350556433254,-0.595861401676539,0.551267820495663,0.920151258970194

"10128","ZNF639",165.188554667668,-0.0974958835455315,0.16364109471761
2,-0.595790951617474,0.551314889199004,0.920151258970194
"10129","BAG2",100.381760193444,0.149142754446142,0.250445670240847,0.
595509414487842,0.551503007949133,0.920151258970194
"10130","CBWD6",6.70632733517305,0.350375271632017,0.58822132099737,0.
595652111076033,0.55140765635606,0.920151258970194
"10131","RGS11",55.0343685562231,0.262466076151294,0.440683416221878,0
.595588729890271,0.551450007433727,0.920151258970194
"10132","HOXB4",80.5943804853943,-0.151374718141749,0.254115776632502,
-0.595691932817947,0.551381048430871,0.920151258970194
"10133","TBC1D16",298.786372958236,0.104288751406029,0.175024103794214
,0.595853651841277,0.551272998175965,0.920151258970194
"10134","CABLES2",15.0034982001068,0.224441249546626,0.376872247612986
,0.595536686418753,0.551484783887348,0.920151258970194
"10135","MYH14",13.5034035224886,-0.334504319813981,0.561858621052138,
-0.595353185446524,0.551607411383826,0.920234643480783
"10136","LAMA1",8.35583151431767,0.456904042126859,0.767836672468683,0
.595053685906743,0.551807585578522,0.920477768050676
"10137","SMCO4",106.590756798876,-0.181266318958793,0.30468455966101,-
0.594931095820769,0.551889530458822,0.920523644174585
"10138","LINC00852",6.7514360230906,-0.480243754690499,0.8074374827548
33,-0.594775155906798,0.551993776542384,0.92060670485092
"10139","NOC2L",173.574460618679,-0.099441162017723,0.167309764597214,
-0.594353606659601,0.552275631287331,0.920717031681486
"10140","AL357873.1",9.10256642663482,-0.331213398610332,0.55840853822
0715,-0.593138134430563,0.553088711261762,0.920717031681486
"10141","MUTYH",67.6445884820111,-0.149283188724056,0.251198276249889,
-0.59428428790471,0.552321985707135,0.920717031681486
"10142","LINC01779",2.41010299078456,-0.591153543852649,0.996787607494
978,-0.593058681114901,0.553141881319388,0.920717031681486
"10143","AMT",41.3522117422216,0.195787486171183,0.329550288397612,0.5
94105036664265,0.552441862369164,0.920717031681486
"10144","RAB43",56.7796340574173,-0.143438696744095,0.241796219886746,
-0.593221419306224,0.553032979818908,0.920717031681486
"10145","SLC9A9",227.931571377432,0.125681074270455,0.211632569693641,
0.593864519305277,0.55260273162399,0.920717031681486
"10146","AC124016.1",20.2682651340267,-0.256604142086677,0.43231112297
4217,-0.593563589854665,0.552804039671499,0.920717031681486
"10147","SERPINB1",867.86586982042,-0.107036947359549,0.18014051773776
3,-0.594185853930796,0.55238781316178,0.920717031681486
"10148","SLC26A4",3.6524756984294,-0.486352414755618,0.819560822656661
,-0.593430531707304,0.552893060956387,0.920717031681486
"10149","ASB6",53.0161300761524,0.143825159552144,0.242470343550067,0.
593165982471772,0.55307007598107,0.920717031681486
"10150","ASCL2",7.02855084708006,0.449195507715941,0.756973393099004,0
.593409902925334,0.552906863075701,0.920717031681486
"10151","ELL3",7.02692784016973,0.323602867729177,0.544983058461713,0.
59378518782325,0.552655797268232,0.920717031681486
"10152","MT2A",19727.0206022155,-0.27629278013949,0.465276065617715,-0
.59382547385642,0.552628849213901,0.920717031681486

"10153","CTCF",346.198716421092,0.108541418929852,0.182716521257747,0.
594042718100677,0.552483541732142,0.920717031681486
"10154","PLD2",93.0175969667331,-0.127637683832202,0.214790710694233,-
0.594242103951607,0.552350195640553,0.920717031681486
"10155","ARHGAP44",37.6565156031104,0.195434137128571,0.32954184467177
9,0.593048015869493,0.553149018678763,0.920717031681486
"10156","AP005131.6",2.61573200118893,0.628027462629684,1.057286850142
43,0.593999123837664,0.552512698987508,0.920717031681486
"10157","TMSB15B",13.3319543162143,-0.299208458259587,0.50417658504658
3,-0.593459647143156,0.552873580950792,0.920717031681486
"10158","SLC25A14",29.7030832680853,-0.182351005286864,0.3071348459707
6,-0.593716433283589,0.552701789909006,0.920717031681486
"10159","WDR47",80.880692012562,-0.150688956715814,0.254182245239759,-
0.592838247115474,0.553289408588385,0.920860057132829
"10160","SHISA4",162.302620914864,-0.167527153034684,0.282684942438874
,-0.592628498671834,0.553429802362366,0.920924111269058
"10161","RRAGD",81.4615927946251,-0.19041807312822,0.321375914231787,-
0.592508849281357,0.55350989673124,0.920924111269058
"10162","STEAP2",220.023011492017,-0.177830815866056,0.300173835991004
,-0.59242610295784,0.553565291179483,0.920924111269058
"10163","ALKBH4",133.209236721027,-0.150997900230911,0.254881453970498
,-0.592424038229117,0.553566673444906,0.920924111269058
"10164","RPS4X",36896.6999483944,-0.157662194710235,0.266153168295128,
-0.592373916569006,0.553600228704679,0.920924111269058
"10165","LRRC4",9.84026534997552,0.431398118590267,0.728598460955882,0
.592093096140082,0.553788249732047,0.920965056208267
"10166","BLID",2.26680150113312,-0.610588772010288,1.03100843337725,-0
.592224808491817,0.553700058907658,0.920965056208267
"10167","BRICD5",3.8315599759,-0.45898336610895,0.775183320815529,-0.5
92096545145061,0.55378594028924,0.920965056208267
"10168","PDCD2L",53.6835783848559,-0.161450313220098,0.272737063759061
,-0.591963230060748,0.553875211079211,0.921019086243834
"10169","ZNF155",18.2281615353667,0.238979994945845,0.403837126747502,
0.591773215282574,0.55400246144104,0.921140094212322
"10170","AC008549.2",11.1977312360612,0.348130623730443,0.588482274615
803,0.591573678166812,0.554136104188433,0.921271705960474
"10171","PACC1",31.5656158569714,-0.283932839416592,0.48036167245433,-
0.591081378257934,0.554465896342426,0.921276471634998
"10172","OSTN-
AS1",3.55110722833143,-0.510398753576646,0.863209175416022,-0.59128050
0848082,0.554332492376771,0.921276471634998
"10173","ADAM19",46.8342139239149,0.263955969529699,0.446408629044375,
0.591287785128053,0.554327612505816,0.921276471634998
"10174","INVS",62.83737376408,-0.13664889401469,0.231162756061597,-0.5
91137155235673,0.55442852647247,0.921276471634998
"10175","POLE2",11.3032744603586,0.31283605766615,0.529139356086283,0.
591216763727432,0.554375191923686,0.921276471634998
"10176","SLC6A16",9.26793146849288,0.302887510939023,0.512428676309761
,0.591082281187419,0.554465291381275,0.921276471634998
"10177","B3GALNT1",87.313091844861,0.132146951920093,0.223698596331909

,0.590736616532102,0.554696909680906,0.921388677560149
"10178","ZCCHC9",296.982957274345,0.121063524058489,0.204911105848537,
0.590809968826065,0.554647754812984,0.921388677560149
"10179","FKRP",59.2737802375486,0.167661978902932,0.283765508727417,0.
590846927291599,0.55462298899863,0.921388677560149
"10180","RPARP-
AS1",33.3399145212858,-0.185249706575924,0.313839533612784,-0.59026886
9072707,0.555010406881461,0.92171811667205
"10181","TPSAB1",15.559654238335,0.794076595785296,1.34544396566722,0.
590196705361495,0.555058780692856,0.92171811667205
"10182","FUT2",2.05268491061418,0.688714146714874,1.16682097760286,0.5
9024834137777,0.555024167089944,0.92171811667205
"10183","RBM15B",216.037453784937,0.106157119049144,0.179896505199801,
0.590101063560084,0.555122895848401,0.921734059020404
"10184","ZNF664",172.396020108216,0.104066425179397,0.176470935040209,
0.589708583771517,0.555386039433587,0.922064505342229
"10185","KMT2B",193.224873506447,-0.127062703746815,0.215491429218252,
-0.589641565828239,0.555430978643873,0.922064505342229
"10186","PPIH",192.57078294093,-0.164323762265967,0.278950783866242,-0
.589077972782328,0.55580896897306,0.922079030100464
"10187","MAEA",248.115533512208,0.113664421875359,0.193088466821742,0.
58866499768882,0.55608602260776,0.922079030100464
"10188","AGGF1",273.2788570353,0.0926907211350838,0.157427516364089,0.
588783481286171,0.556006528328196,0.922079030100464
"10189","HDAC3",253.355002190831,-0.153969761324958,0.261304878694187,
-0.589234162386815,0.555704203224337,0.922079030100464
"10190","ZNF138",132.501045590054,-0.113637755833193,0.19304401864592,
-0.588662402649348,0.556087763761389,0.922079030100464
"10191","CEP41",57.9941991278896,0.152731494757101,0.25937365862823,0.
588847362391635,0.555963670836066,0.922079030100464
"10192","PLAG1",19.7925001820235,-0.390591406636119,0.663534343961958,
-0.588652886154922,0.556094148919708,0.922079030100464
"10193","FAM180B",127.124914253896,0.197636402198835,0.33562096344567,
0.588867870975013,0.555949912078973,0.922079030100464
"10194","VAMP1",34.0794005080535,0.217186979645722,0.368882569645895,0
.588769970492799,0.556015592853762,0.922079030100464
"10195","DNAH9",4.27462238985154,0.463693186608039,0.787587603413802,0
.588751250779163,0.556028152214872,0.922079030100464
"10196","AKT2",391.144723428238,0.104580156528439,0.177468102544145,0.
589289878176418,0.555666833630842,0.922079030100464
"10197","AC010487.2",3.43192185934553,0.522107075495158,0.886182690756
053,0.589164154232937,0.555751160705792,0.922079030100464
"10198","AC004263.2",3.80974249200911,-0.522668716222208,0.88813598557
5645,-0.588500775456633,0.556196213508654,0.92215783271272
"10199","CDON",685.074226405955,0.128396365519064,0.218237867405844,0.
588332203962991,0.556309333781971,0.922254948091535
"10200","DLG2",44.3718554584177,0.1566887313759,0.266368491533588,0.58
8240487731043,0.556370884874935,0.92226656092798
"10201","HEYL",4.97506992979714,0.504987764192284,0.863051061829511,0.
585119220086239,0.558467556131892,0.922339355816314

"10202","C1orf50",173.56448111336,-0.160465725084255,0.275727910772732
,-0.581971279710305,0.560586026355973,0.922339355816314
"10203","TESK2",31.2712579369946,0.158977350230381,0.27307723586121,0.
582169911486801,0.560452238109685,0.922339355816314
"10204","BEND5",162.636093144645,0.153458734385935,0.26353379282439,0.
582311409634647,0.560356941600431,0.922339355816314
"10205","CZIB",876.07012957373,-0.154434878114236,0.265944970990036,-0
.580702381922546,0.561441056108387,0.922339355816314
"10206","LINC01140",526.136795686201,-0.169967798615439,0.292084840076
441,-0.581912428494945,0.56062566850699,0.922339355816314
"10207","ABCD3",146.709157677835,-0.0974218085606797,0.167640694458292
,-0.581134603835214,0.561149738346226,0.922339355816314
"10208","CD2",22.484064770764,0.2400351309166,0.411619424470164,0.5831
48210815301,0.559793531312085,0.922339355816314
"10209","AC243772.2",2.03376608306744,-0.782033923613517,1.33062698100
846,-0.587718372447872,0.556721341570121,0.922339355816314
"10210","AL139011.1",5.97774695710818,0.37216762228112,0.6398894751991
77,0.581612351360016,0.560827821431647,0.922339355816314
"10211","RGS5",14.5950011403182,-0.258809502699318,0.443324196324188,-
0.583792865007664,0.559359679192054,0.922339355816314
"10212","FAM78B",16.2684623253863,-0.233248542978758,0.398648236982032
,-0.585098644219694,0.558481390435523,0.922339355816314
"10213","COLGALT2",10.6327451252647,0.461543080620415,0.78973142591962
5,0.58443043479365,0.558930755441422,0.922339355816314
"10214","AL513217.1",4.65633062354102,0.417224525303101,0.715931085110
213,0.582771909168984,0.560046857642287,0.922339355816314
"10215","CD55",509.409316429156,0.198376520749203,0.340390883603275,0.
582790345761465,0.560034444830627,0.922339355816314
"10216","LINC00954",16.5376802176969,0.245700320555049,0.4213842347619
82,0.583079053002143,0.559840084195013,0.922339355816314
"10217","GPATCH11",193.731791811274,-0.144093465570767,0.2479098217086
45,-0.581233387921643,0.561083168098174,0.922339355816314
"10218","KRCC1",482.440535787731,-0.0721681745743765,0.123338128473011
,-0.58512461205513,0.558463930837766,0.922339355816314
"10219","STEAP3",11.1924177307024,0.296857564970734,0.50645202127149,0
.586151407245741,0.557773772915369,0.922339355816314
"10220","MZT2B",3035.66066028999,-0.14814014572401,0.252346438850806,-
0.587050668908365,0.557169677421367,0.922339355816314
"10221","SLC26A6",20.2530005868016,-0.214405230134601,0.36896539754927
4,-0.581098475788555,0.561174085864134,0.922339355816314
"10222","CCDC66",365.519930614227,-0.131308967948027,0.223986456631578
,-0.586236194467816,0.557716801941404,0.922339355816314
"10223","PTPRG-
AS1",12.9608254626737,0.320257164280536,0.550011165046514,0.5822739330
27254,0.560382180670797,0.922339355816314
"10224","AC092040.1",199.760608728002,-0.196486177954229,0.33443081641
0158,-0.587524140458548,0.556851742338468,0.922339355816314
"10225","MBD4",314.740958529874,0.0904732203640847,0.15509895689345,0.
583325782301929,0.559674009459818,0.922339355816314
"10226","TMEM108",23.2798424360494,0.414924899364385,0.709234305835074

,0.585032190279967,0.558526072266271,0.922339355816314
"10227","TMEM44",108.853258542034,0.11895119149396,0.204439661606113,0.581840091885592,0.560674396272951,0.922339355816314
"10228","AC096586.1",6.44933031008444,0.416476382084629,0.712827770718802,0.584259479207246,0.559045749783325,0.922339355816314
"10229","CXCL10",26.92022837527,0.688465220068985,1.17826467980348,0.58430438582256,0.559015541955894,0.922339355816314
"10230","SDHA",500.42745900278,0.0966748111005407,0.164883828905051,0.586320755301061,0.557659985904968,0.922339355816314
"10231","AC094105.2",2.34088340395915,-0.714422929087731,1.22245543120451,-0.584416340139122,0.558940235864213,0.922339355816314
"10232","FCHO2",305.376734280294,0.110014444443871,0.189130264386907,0.581686092389806,0.560778141051158,0.922339355816314
"10233","SCAMP1-AS1",65.3716242441775,-0.141419313619929,0.24303368132055,-0.581891830183831,0.560639543840428,0.922339355816314
"10234","C5orf58",31.8752738610973,0.256047369139586,0.435811695135636,0.587518352530436,0.5568556283854,0.922339355816314
"10235","SH3PXD2B",310.205186653428,-0.196048354284133,0.33544550143012,-0.584441745226306,0.558923147813489,0.922339355816314
"10236","MAML1",155.521051203017,0.104100264086863,0.178741532593891,0.582406688452114,0.56029277741922,0.922339355816314
"10237","ZKSCAN4",42.6248440352193,0.157157697178893,0.2676692446924,0.587133935986913,0.557113757358794,0.922339355816314
"10238","MRPS18B",483.329573733606,-0.132450555160571,0.227876427782591,-0.581238509175411,0.561079717007498,0.922339355816314
"10239","BCKDHB",108.546673429998,0.118849669541474,0.204058917093372,0.582428208648642,0.560278285438063,0.922339355816314
"10240","AFG1L",21.0363002606783,0.18775342760112,0.32000076462619,0.586728059292124,0.557386359684533,0.922339355816314
"10241","RPS12",38984.5159321746,-0.148010427949713,0.252490153911969,-0.586202771302192,0.557739259589922,0.922339355816314
"10242","ZNRF2",110.864080423584,-0.158453755081053,0.272640808891294,-0.581181356251884,0.561118231580861,0.922339355816314
"10243","TRGC2",2.64405093799059,0.563346627299313,0.961023162489566,0.58619464055366,0.557744722858087,0.922339355816314
"10244","CCL26",47.0577506976138,-0.372794763277306,0.639639507344102,-0.582820102568737,0.560014410734981,0.922339355816314
"10245","DLD",217.407412945154,0.12700007940977,0.216258378525532,0.587260851004562,0.557028529704033,0.922339355816314
"10246","ATP6V1F",1471.9783761735,-0.155966699061381,0.265752548230444,-0.586886937114657,0.557279643522155,0.922339355816314
"10247","AC026979.3",2.75057628144286,-0.479799129823536,0.822757181703692,-0.583160062887584,0.559785553397859,0.922339355816314
"10248","STAU2",143.417489410794,0.0937472020338632,0.160102504332976,0.585544882164309,0.558181397124888,0.922339355816314
"10249","AC062004.1",148.735764795851,0.293860633083678,0.5056268098427,0.581180877602391,0.561118554141609,0.922339355816314
"10250","CDK20",21.4560220537497,0.223608678659164,0.382778977536842,0.584171785237713,0.559104742136554,0.922339355816314

"10251","GADD45G",113.465671250843,0.165110273919339,0.28392932675681,
0.581518914602148,0.560890774164675,0.922339355816314
"10252","TOR2A",69.6817656176411,-0.139944317862468,0.238079397587,-0.
587805241784219,0.556663025261828,0.922339355816314
"10253","NCS1",62.8985403312593,-0.217287579558859,0.371336767863422,-
0.585149649492231,0.558447097048957,0.922339355816314
"10254","DNA2",6.59919368846505,0.424468781178627,0.724915296665885,0.
585542591156364,0.558182937105435,0.922339355816314
"10255","CCAR1",436.0085415161,0.0949512800234391,0.163013260457853,0.
582475804463704,0.560246234440252,0.922339355816314
"10256","ENTPD1-
AS1",95.2915801748146,0.115680142839978,0.198372812406979,0.5831451469
40045,0.559795593689003,0.922339355816314
"10257","VTI1A",126.508185954335,0.107333153611392,0.184500779649399,0
.581749051767443,0.560735726109429,0.922339355816314
"10258","AC010998.2",3.45914175439785,0.485637593001718,0.831058944370
293,0.584359985884868,0.558978141947925,0.922339355816314
"10259","RPLP2",18351.5167966685,-0.0989317487662298,0.17004962221804З
,-0.581781643944945,0.560713769779158,0.922339355816314
"10260","AC087521.2",3.11124411985604,-0.514281053685436,0.88560108642
9033,-0.580714117864452,0.561433145112292,0.922339355816314
"10261","KAT5",220.725148968149,-0.119827774322214,0.204672243573475,-
0.585461771611437,0.558237264090929,0.922339355816314
"10262","RAB30-
DT",155.882144188159,-0.184368891196502,0.316075613681905,-0.583306282
470904,0.559687134024629,0.922339355816314
"10263","CREBZF",351.779216723159,-0.114339082685942,0.195454346460875
,-0.584991251186269,0.558553599469753,0.922339355816314
"10264","MNAT1",319.603013832973,-0.0915739524777389,0.155809371162918
,-0.58773071089535,0.556713058457839,0.922339355816314
"10265","DPH6",118.915160651408,-0.111155634412293,0.19127979013582,-0
.581115413883325,0.561162670830571,0.922339355816314
"10266","MAP2K1",199.245999735701,0.107482824122275,0.183908254765828,
0.584437192659643,0.558926209956731,0.922339355816314
"10267","FANCI",22.0998785943271,0.180880728895932,0.308168451120905,0
.586954077349618,0.557234549292289,0.922339355816314
"10268","RHOT2",182.441174650297,-0.115349735893179,0.197183900405019,
-0.584985567565351,0.558557421154823,0.922339355816314
"10269","ZNF267",111.853677696317,-0.153236272311139,0.26307810942402,
-0.582474431820392,0.560247158765122,0.922339355816314
"10270","SIAH1",299.328752784313,-0.117319369247378,0.201847867581684,
-0.581226696387572,0.561087677378746,0.922339355816314
"10271","SLC25A11",363.032236267728,-0.125044197430727,0.2132505164201
06,-0.586372307696494,0.557625349468768,0.922339355816314
"10272","KRBA2",42.4032071378624,0.158729314763854,0.27297944996188,0.
581469831469071,0.560923845147572,0.922339355816314
"10273","ZSWIM7",374.544005680083,-0.164794252847439,0.282643374770493
,-0.58304657939091,0.559861944125896,0.922339355816314
"10274","TP53I13",512.460450938654,-0.146082712209022,0.24990481511709
4,-0.584553411428163,0.558848041545428,0.922339355816314

"10275","PLEKHH3",267.245019858603,-0.100370405359257,0.171615659361526,-0.584855751116603,0.558644713613757,0.922339355816314
"10276","SRSF1",444.964488345304,0.0928491862282781,0.159906059727238,0.58064832806622,0.561477493576983,0.922339355816314
"10277","KATNAL2",24.3721635611424,0.204045060739033,0.349709645557553,0.583469925211006,0.559576997200961,0.922339355816314
"10278","FEM1A",54.7577836213632,0.158594830184047,0.273046699467306,0.580834086233067,0.5613522795960 7,0.922339355816314
"10279","AC011503.2",2.99383887920955,0.564913329399429,0.97128131378052,0.581616593858494,0.560824963142318,0.922339355816314
"10280","FAM98C",180.04747181697,-0.111187242372118,0.191326740336306,-0.581138016445991,0.561147438536104,0.922339355816314
"10281","AC011445.2",3.66500795533581,0.657525568062729,1.12543705178952,0.584240199855888,0.559058718880085,0.922339355816314
"10282","RDH13",81.733758525105,0.116886966527152,0.199088300405079,0.587111177750403,0.557129040942977,0.922339355816314
"10283","ZSCAN5A",29.1696284705618,-0.195258880068428,0.334642799543279,-0.583484480571278,0.559567201484899,0.922339355816314
"10284","FAM110A",18.4782768607791,-0.250794950053105,0.42958257932497,-0.583810801748978,0.559347610103082,0.922339355816314
"10285","LZTS3",35.2619073234201,0.195922071622379,0.335172397633278,0.584541188372986,0.558856262487513,0.922339355816314
"10286","EIF2S2",1133.41391265579,-0.0993598848011334,0.169731302457582,-0.585395170852263,0.558282035125307,0.922339355816314
"10287","Z93930.2",33.7537654749064,-0.192062238969054,0.327068310341313,-0.587223625451903,0.557053527242259,0.922339355816314
"10288","Z93241.1",69.6239293036727,0.229353009709648,0.394771793327855,0.5809761831671,0.561256505455436,0.922339355816314
"10289","TTC38",73.1251613727734,-0.134369031559662,0.229850316055727,-0.584593634089607,0.558820989135312,0.922339355816314
"10290","AL118516.1",166.305400736997,-0.204991802901903,0.35023474434666,-0.585298306952104,0.558347153069184,0.922339355816314
"10291","SLC35A2",83.8866810084666,-0.169398215157249,0.288823729105206,-0.586510726393759,0.557532355471062,0.922339355816314
"10292","HDAC8",181.43579903144,0.112682904466041,0.192001162846599,0.586886572953047,0.557279888113385,0.922339355816314
"10293","NXT2",61.4697585679668,-0.151756639584858,0.26136424260668,-0.580632752481112,0.561487993223168,0.922339355816314
"10294","AC002553.1",5.60160266161897,0.408726995527292,0.70413327885708,0.580468226399867,0.561598907577119,0.922431934069742
"10295","RER1",1099.30328403271,-0.121688243798772,0.209757269478648,-0.580138386150947,0.561821299473322,0.922528360832759
"10296","TLR2",14.1724457508525,-0.280497884622828,0.483386186799831,-0.580276996493863,0.561727837479312,0.922528360832759
"10297","HMGB2",521.020186468355,0.14411627477229,0.248416244924216,0.58014029966621 4,0.561820009179678,0.922528360832759
"10298","CALHM6",32.3980394108107,-0.222161580240754,0.383021495932811,-0.580023791353282,0.561898573973499,0.92256565243192
"10299","WARS2-
IT1",21.1565594430281,0.271562729310066,0.468529423751333,0.5796065637

36742,0.562179965698689,0.922754477041102
"10300","PPP1R7",508.558166416886,-0.134456800907425,0.232107443546115
,-0.57928689771085,0.562395604797052,0.922754477041102
"10301","PAK2",570.228556172079,-0.0804598996295196,0.138866027234376,
-0.579406649934044,0.562314818116587,0.922754477041102
"10302","MAF1",827.212383748926,-0.111401377460131,0.192236853627929,-
0.57950062830172,0.562251422798479,0.922754477041102
"10303","AP001107.2",4.10664880260218,0.459373031525764,0.792930594945
265,0.579335738151803,0.562362655612141,0.922754477041102
"10304","ALG11",49.5684994454616,0.179443411753386,0.309574893257974,0
.579644588955258,0.562154317449086,0.922754477041102
"10305","MPHOSPH6",136.125074692455,-0.124470249663237,0.2147806928656
63,-0.579522525989282,0.562236651694273,0.922754477041102
"10306","ERF",154.404678689038,0.148849673344457,0.256992374904294,0.5
7919879295987,0.562455045187479,0.922757018907593
"10307","ZNF577",36.4861106742842,-0.161018008518834,0.278037755409301
,-0.579122818344573,0.562506304345905,0.922757018907593
"10308","REV3L",1400.88359874588,-0.163040396950988,0.281579436774357,
-0.579020964097033,0.562575027719278,0.922780225909736
"10309","CUZD1",2.52973086359887,0.537637984368792,0.929059543989335,0
.578690556323442,0.562797989243166,0.922880374874221
"10310","NAV3",257.931269499194,0.0915732351670886,0.158242137770665,0
.578690584298113,0.562797970363854,0.922880374874221
"10311","SUMO3",1102.51016847786,-0.10928856017219,0.188855813214961,-
0.578687827034452,0.562799831164425,0.922880374874221
"10312","AC087071.1",7.13754280695897,0.432123356379028,0.747111466759
719,0.578392081509846,0.562999438831627,0.923028654297018
"10313","TMOD1",6.5328182220676,-0.377174988717323,0.652070726917373,-
0.578426500604307,0.56297620657987,0.923028654297018
"10314","AC008273.1",3.77386262145239,-0.515115642877487,0.89179595945
7781,-0.577616031351702,0.563523381067257,0.923039739130604
"10315","IRX2",184.981649946529,-0.228424162958799,0.395522892165021,-
0.577524506125161,0.563585188867477,0.923039739130604
"10316","CENPK",13.1734443805664,-0.236616957715254,0.409551284951097,
-0.577746832715974,0.563435055413265,0.923039739130604
"10317","AC073349.1",26.4593425974054,-0.279463936406414,0.48344204608
4031,-0.578071226262015,0.563216032434926,0.923039739130604
"10318","C8orf58",58.897955560606,-0.155990046183847,0.269932193117769
,-0.577886040127827,0.563341060776752,0.923039739130604
"10319","LINC02605",2.07958883524636,0.612357671376373,1.0601355282843
6,0.577622063442556,0.563519307657374,0.923039739130604
"10320","DNAH10OS",5.43411059209549,-0.469598395351385,0.8131254112821
47,-0.577522715236407,0.563586398303185,0.923039739130604
"10321","AL079343.1",39.5034732653072,0.183535984586521,0.317483952919
542,0.578095311270844,0.563199772434649,0.923039739130604
"10322","AL136038.5",46.2814104138591,-0.172266466198225,0.29830071420
2711,-0.577492637450279,0.563606710834182,0.923039739130604
"10323","CIAO3",78.1053446782237,-0.12910264362284,0.223269694252121,-
0.578236307687392,0.563104589061887,0.923039739130604
"10324","MISP3",18.3353439978057,-0.244495316445836,0.42333128435128,-

0.577550787016614,0.563567440813139,0.923039739130604
"10325","THNSL2",166.327846700092,-0.232564907900089,0.402847273118345
,-0.577302922022679,0.563734840113421,0.923160162386221
"10326","FYN",1997.89798319686,-0.0848298495724758,0.146987494930882,-
0.577122901593536,0.563856434598236,0.923269862113789
"10327","PROX1",18.8285644398504,0.32625787263874,0.566010387013055,0.
576416758640182,0.564333519571256,0.92343802676735
"10328","AUXG01000058.1",233.378179360017,0.383635376854554,0.66535758
3825207,0.576585262091695,0.564219657459258,0.92343802676735
"10329","NAXD",105.096521529384,0.108637412577261,0.188471367878545,0.
576413350208557,0.564335822850213,0.92343802676735
"10330","DIS3L",186.50451005147,0.0933398937243003,0.161835087433488,0
.576759312239145,0.564102058916421,0.92343802676735
"10331","DENND1C",3.25304907966267,0.517394130227161,0.897622507052892
,0.576405032362533,0.564341443730011,0.92343802676735
"10332","TMEM147",649.317589964576,-0.138795205661754,0.24068506026071
3,-0.576667307523821,0.564164221256752,0.92343802676735
"10333","CSTF1",97.0967416855972,0.127938287462449,0.22180884104883,0.
57679525693155,0.564077774033267,0.92343802676735
"10334","NUP205",68.1956025705464,0.148047356181288,0.257097856396511,
0.575840492240282,0.564723001159978,0.923865249728567
"10335","SPATS2",240.531243335851,0.0997062836035595,0.17317095332321,
0.575767943123034,0.564772044147526,0.923865249728567
"10336","PIGH",193.490336792823,0.0995762379053226,0.172966885986255,0
.575695384336374,0.564821095720618,0.923865249728567
"10337","GRAP2",4.24245888996858,0.487547378306664,0.84672387476662,0.
575804453891235,0.564747362720701,0.923865249728567
"10338","MBD6",151.816032377834,-0.102996537552089,0.178955048393511,-
0.57554418540686,0.564923316600613,0.923943068009592
"10339","MARC1",20.5733070702584,0.210730535708804,0.366221067876582,0
.575418931878111,0.565008003341553,0.923944322522058
"10340","EIF2S3",1132.48712899183,-0.100838427260141,0.175254927670912
,-0.57538140924341,0.565033374430925,0.923944322522058
"10341","TMEM175",124.147712926455,-0.137056154260988,0.23829662338679
8,-0.575149376072027,0.565190276814516,0.924012247995372
"10342","AL355574.1",5.50653998920521,0.399886560736057,0.695361126297
843,0.575077532540659,0.565238862137221,0.924012247995372
"10343","ASCC1",107.264627651733,0.135578298398107,0.235733712720714,0
.575133258766149,0.565201176222494,0.924012247995372
"10344","RPL7",16625.2474181045,0.100847234000383,0.175420028572136,0.
574890078523234,0.565365640328355,0.924130147590083
"10345","MIR1915HG",6.01265656459416,0.480376055516988,0.8357936894446
42,0.57475434617864,0.565457446842111,0.924190866235515
"10346","RPS6KA5",268.01418502884,-0.208502601557765,0.362949210506634
,-0.574467709315913,0.565651345541851,0.92441841778674
"10347","SMYD3",105.484162831205,-0.140934100166242,0.245445790873662,
-0.574196443396273,0.56583487580421,0.924442853138312
"10348","FRS3",41.2587916586373,-0.157186018632036,0.273689297290091,-
0.574322855107592,0.565749345931376,0.924442853138312
"10349","NSMCE2",232.643070009294,-0.117302467198904,0.204341349009122

,-0.574051545454303,0.565932921054759,0.924442853138312
"10350","PRDX3",792.840811246831,-0.147662494489866,0.257218859954225,-0.574073357280815,0.565918161552127,0.924442853138312
"10351","PEX5",88.7531861055048,-0.11853509281878,0.20649217620849l,-0.574041569008877,0.565939671920669,0.924442853138312
"10352","SOCS1",890.967003447914,-0.222135992810785,0.387148731418099,-0.573774301150661,0.566120541254168,0.924648967496665
"10353","AL590822.3",5.76534414686681,-0.420131016616462,0.739723204648833,-0.567957060122117,0.570064125368329,0.924673328713419
"10354","UBIAD1",76.0236370901803,0.125050904152101,0.218652306340602,0.5719166938825,0.567378413768722,0.924673328713419
"10355","ZBTB40",88.563785563869,0.153080082317864,0.271438483538465,0.563958655833604,0.572782269654503,0.924673328713419
"10356","MED8",283.225454933556,-0.136270306905576,0.240503533208751,-0.566604178689121,0.570983135306782,0.924673328713419
"10357","OSBPL9",459.29800578812,0.0836791645383329,0.148663655126832,0.562875737630306,0.573519502560663,0.924673328713419
"10358","PARS2",12.9777915983456,0.252840488215985,0.446256660423925,0.566580872934866,0.570998973041976,0.924673328713419
"10359","STRIP1",74.794153238621,0.120053833013113,0.212019821262402,0.566238723805595,0.571231509101265,0.924673328713419
"10360","ZNF697",31.6492191247438,0.176999157342934,0.313510714667426,0.564571317859729,0.572365378741155,0.924673328713419
"10361","PEX19",261.34823638346,-0.111116061737614,0.196280987744736,-0.566107104994399,0.571320973674152,0.924673328713419
"10362","ARHGAP30",12.8538692200495,-0.319642636141687,0.559922300462488,-0.570869629371194,0.568088019401104,0.924673328713419
"10363","TOMM40L",28.0524964112042,0.226460823842382,0.396415993121614,0.571270654493768,0.567816190680444,0.924673328713419
"10364","ELF3-AS1",4.42199854652731,0.598488367159012,1.05141558484506,0.569221510300521,0.569205824775225,0.924673328713419
"10365","ZC3H11A",13.4673697213542,0.285432869813183,0.504246552527163,0.566058148305947,0.571354252451446,0.924673328713419
"10366","GJC2",23.9187913768005,0.223172217058007,0.391543262601611,0.569980991564351,0.568690590607861,0.924673328713419
"10367","SLC35F6",418.188975920199,-0.121933411484031,0.216798999310864,-0.562426080708947,0.573825753709701,0.924673328713419
"10368","ATRAID",1812.35835080883,-0.146136903887259,0.25843632276557,-0.565465807296065,0.571756974987588,0.924673328713419
"10369","SNX17",640.910871211002,-0.132556404133462,0.231536675291468,-0.572507158818769,0.566978437327912,0.924673328713419
"10370","CCDC74A",95.7651528881932,-0.176956626378094,0.312520282726852,-0.566224453766917,0.571241208478523,0.924673328713419
"10371","AC114763.1",4.21334954738681,0.438892436680404,0.778389243490462,0.563846996025173,0.572858265013507,0.924673328713419
"10372","PPIG",1358.46262773952,0.0927240241901217,0.162836995218331,0.56942848930489,0.569065387559126,0.924673328713419
"10373","SGO2",56.835146980166,-0.147204660615441,0.261269213523342,-0.563421379160273,0.573147982555992,0.924673328713419

"10374","AC108066.2",2.03978697698337,0.593692535268452,1.04774458043291,0.566638612459485,0.570959735763043,0.924673328713419
"10375","XRCC5",1251.13357456191,-0.0828960376337561,0.145766679361989,-0.568689895362825,0.569566606368133,0.924673328713419
"10376","ABCB6",181.664790189116,0.140418327934591,0.249339267091895,0.563161709634927,0.573324773818648,0.924673328713419
"10377","THAP4",177.532414985046,-0.0983306671516318,0.174541885639456,-0.563364299585657,0.573186841926377,0.924673328713419
"10378","SUMF1",251.08704217635,0.0942204281571551,0.165121083292851,0.570614159489556,0.568261218191648,0.924673328713419
"10379","TMPPE",4.15664399101349,-0.43568067821411,0.767822994743698,-0.567423326986374,0.570426605088955,0.924673328713419
"10380","ACAA1",421.985661701389,-0.12868697021516,0.225423062400828,-0.570868698369203,0.568088650538929,0.924673328713419
"10381","RAD54L2",123.23375909973,0.128879695133972,0.22934361924797,0.561950210590446,0.574149942438355,0.924673328713419
"10382","CACNA2D3-AS1",5.79607913159353,0.55335927412638,0.974049049263063,0.568102062770899,0.569965667190727,0.924673328713419
"10383","FAIM",248.962546467421,-0.140301736252175,0.247080997780216,-0.567837015038189,0.57014564325756,0.924673328713419
"10384","AC092535.2",3.92296163222693,-0.412298138670238,0.729047545699752,-0.565529835608332,0.571713436726406,0.924673328713419
"10385","CC2D2A",204.626049560657,0.129612180460673,0.226489538882413,0.572265637963806,0.567142025377751,0.924673328713419
"10386","YTHDC1",624.768286411113,0.123450082772031,0.2184149766452,0.565208872890467,0.571931702259596,0.924673328713419
"10387","HSD17B11",784.094793486528,-0.132925462263503,0.234532301010009,-0.566768251925479,0.570871643076535,0.924673328713419
"10388","AC010245.2",8.60798565953878,0.322010171018925,0.567809392592171,0.567109623792731,0.570639704827721,0.924673328713419
"10389","ATG10",137.789297561307,-0.0959610573990481,0.170119963220327,-0.564078757028452,0.572700534452825,0.924673328713419
"10390","LYSMD3",149.96871828385,0.109082675464088,0.191783066148817,0.568781580431319,0.569504376317427,0.924673328713419
"10391","CDC42SE2",143.392811795285,-0.101341337980864,0.180479172148986,-0.561512648657356,0.574448110054752,0.924673328713419
"10392","ETF1",349.235447888723,-0.110951774760337,0.195743312061357,-0.566822812958013,0.570834569666664,0.924673328713419
"10393","AC011365.1",31.3244356944705,0.268203979499741,0.472320426219829,0.567843278865338,0.570141389601787,0.924673328713419
"10394","CPEB4",107.549216697433,0.135151962608366,0.237409196262276,0.569278548329936,0.56916712227987,0.924673328713419
"10395","AC008443.6",2.1883643629559,-0.625750028978422,1.11226773450629,-0.562589392432729,0.573714516832087,0.924673328713419
"10396","GMDS",63.2710600204665,-0.187757602952601,0.330029515245976,-0.568911549661435,0.569416166949418,0.924673328713419
"10397","SNRNP48",150.982113830992,0.119086922904021,0.208258150159019,0.571823589199703,0.567441494502802,0.924673328713419
"10398","EEF1E1",153.07015567872,-0.157630569359366,0.277502918266349,

-0.568032114199501,0.570013161923525,0.924673328713419
"10399","NHLRC1",12.3845842123512,-0.270937499160334,0.473133655892387
,-0.572644739570076,0.566885260593013,0.924673328713419
"10400","ABT1",177.053890398157,-0.124518577766024,0.219844559803016,-
0.566393718714687,0.571126163763215,0.924673328713419
"10401","HLA-
A",5931.3324640316,0.146599544504601,0.258144897428198,0.5678963480011
32,0.570105351884327,0.924673328713419
"10402","PSMB9",568.870173895983,0.180416957140288,0.318584159343087,0
.566308624736094,0.571183998414458,0.924673328713419
"10403","AL353135.2",15.9113907225474,-0.24320571701997,0.424781401034
738,-0.572543233831655,0.5669540047706,0.924673328713419
"10404","AC092428.1",9.533696910187,0.281274587462206,0.49706237167432
3,0.565873828901493,0.571479553601808,0.924673328713419
"10405","HOXA5",57.9857523001116,0.2580869577258,0.452677179997739,0.5
70134676828837,0.568586357224729,0.924673328713419
"10406","MTURN",1384.51828466503,0.121180306801297,0.214975512522588,0
.563693535972216,0.572962717310817,0.924673328713419
"10407","CALN1",4.46161492994828,-0.628142564464444,1.09745024386598,-
0.572365415175172,0.567074441062696,0.924673328713419
"10408","MTERF1",48.1452433621657,0.151934847870051,0.266907229686935,
0.569242159713174,0.569191813209912,0.924673328713419
"10409","COPS6",903.901742590684,-0.134593107254369,0.236270238986157,
-0.569657472866293,0.568910039326517,0.924673328713419
"10410","ABCF2",105.72170437166,-0.134780145377585,0.235595693639448,-
0.57208238102964,0.567266164920523,0.924673328713419
"10411","NUDT18",80.2010014016354,-0.169525054573671,0.301173670963735
,-0.562881390100279,0.573515653284497,0.924673328713419
"10412","DPYSL2",2802.81038768697,0.134412597830212,0.234867339153029,
0.572291568146197,0.567124461140137,0.924673328713419
"10413","AC009630.1",14.0606687040032,-0.242536289357527,0.42595321837
2993,-0.569396541441662,0.569087063407031,0.924673328713419
"10414","PCMTD1",1061.2108700609,0.103146338725136,0.183460029904151,0
.562227853004414,0.573960786714726,0.924673328713419
"10415","TERF1",470.679712895651,0.0878885653649566,0.153675213004689,
0.571911134180598,0.567382180510497,0.924673328713419
"10416","AC067930.8",12.2046856006438,0.349609045502996,0.618827449768
245,0.56495400395365,0.572105049988227,0.924673328713419
"10417","ZNF517",15.8495896247333,-0.206078653379977,0.360100502259851
,-0.572280938478861,0.56713166128947,0.924673328713419
"10418","KDM4C",62.3342782919212,0.145981927540765,0.254479628987097,0
.573648775431715,0.566205498390577,0.924673328713419
"10419","PTPRD-
AS1",78.5145740453131,0.220928260240055,0.387492083488572,0.5701490937
59667,0.568576579752076,0.924673328713419
"10420","PGM5",113.54829423909,0.231546766953154,0.405755227350153,0.5
70656275866872,0.568232663163256,0.924673328713419
"10421","AL158071.5",8.69771186170172,0.32249017703024,0.5719770103677
07,0.563816676518031,0.572878901212843,0.924673328713419
"10422","AL691447.2",19.2797144248047,0.314650884335329,0.559476970068

718,0.562401852388459,0.573842257269937,0.924673328713419
"10423","ZFP37",47.063567004479,0.164218944873651,0.290286428764306,0.565713476763967,0.571588572328748,0.924673328713419
"10424","DAB2IP",69.4337748871808,0.196632971693457,0.346304297229149,0.567804018797219,0.570168050679097,0.924673328713419
"10425","AL356113.1",2.89981623493657,0.698696994873294,1.2425246495945,0.562320429700381,0.573897721459431,0.924673328713419
"10426","RASGEF1A",3.62493714517951,-0.405294643116746,0.719978658099566,-0.562925912535449,0.573485334374499,0.924673328713419
"10427","AC073389.1",7.79739123806659,0.353743004261923,0.623735369041548,0.56713635592847,0.570621544111137,0.924673328713419
"10428","ZCCHC24",1029.22466739155,0.105264414026193,0.184736512514102,0.56980838597436,0.568807667139024,0.924673328713419
"10429","FAS",176.756117420797,-0.0939771895659319,0.165540070189688,-0.567700554060692,0.570238315282374,0.924673328713419
"10430","ERLIN1",200.703101762694,0.100771046808652,0.177161937323508,0.568807545972125,0.569486753133124,0.924673328713419
"10431","CASP7",228.311437602884,0.134895082850143,0.23820653491004,0.566294635456105,0.571193506582285,0.924673328713419
"10432","RNH1",2488.69366325496,-0.136583032200736,0.24126901150659,-0.566102672481025,0.571323986679549,0.924673328713419
"10433","TSG101",403.340919003989,-0.122952597886273,0.216842455712955,-0.567013491347981,0.570705015542974,0.924673328713419
"10434","KIF18A",4.84614730235155,-0.377116518236532,0.659712105554763,-0.571638014614585,0.567567235909319,0.924673328713419
"10435","PPP6R3",257.701696758988,0.0975110870237125,0.173654723144565,0.561522803745084,0.574441189247603,0.924673328713419
"10436","NDUFC2",1829.5958126239,-0.153498652164592,0.269860181580776,-0.568808081523675,0.569486389649358,0.924673328713419
"10437","ANKRD49",147.905237097146,0.159186768149978,0.280019894699319,0.568483779771828,0.569706516497445,0.924673328713419
"10438","ZBTB16",2306.99143955101,-0.244272757433141,0.434362136443147,-0.562371203515603,0.573863134628114,0.924673328713419
"10439","DDX6",667.886874677382,0.0998092859756479,0.177705899493517,0.561654319075036,0.574351563627916,0.924673328713419
"10440","MSANTD2",88.1990743506507,0.114408197417292,0.199513311519532,0.573436411565411,0.566349242434714,0.924673328713419
"10441","FLI1",45.6184765400206,-0.209147996829401,0.366739063851092,-0.570291025540497,0.568480326793221,0.924673328713419
"10442","CD69",85.0428624575739,0.306621454050494,0.545630412406255,0.561958144338545,0.574144536824748,0.924673328713419
"10443","TAS2R31",4.41385281417337,-0.408219524289426,0.726961682165838,-0.561542010127985,0.57442809998832,0.924673328713419
"10444","AC004241.3",3.13201610904468,-0.46128595306169,0.819293071507955,-0.563029237160113,0.573414975248357,0.924673328713419
"10445","AC004466.1",2.17600347298548,0.632037834806937,1.10649610919681,0.571206558752134,0.567859632814587,0.924673328713419
"10446","MCRS1",167.092324632557,-0.132517155425773,0.234631859757873,-0.56478755938142,0.572218269837921,0.924673328713419
"10447","ATF1",267.197912535597,0.0918170551910683,0.161374519532122,0

.568968728503584,0.569377362064301,0.924673328713419
"10448","CSRP2",151.325541852734,-0.220203647315524,0.385391226155538,-0.571376908374797,0.5677441785524,0.924673328713419
"10449","AC027288.3",10.6904963005918,0.454609457554564,0.809543292885001,0.561562873227514,0.574413881827183,0.924673328713419
"10450","MTERF2",230.775020843317,0.118059888069575,0.206507844098493,0.571696869845131,0.567527355407668,0.924673328713419
"10451","HVCN1",79.1882443279455,0.160458397865772,0.284328945709848,0.564340705675169,0.572522283783444,0.924673328713419
"10452","AC127164.1",2.55885753082842,0.743575161821378,1.3039748703447,0.570237340252436,0.568516733282176,0.924673328713419
"10453","VPS33A",81.5821744133675,0.118595281699721,0.20848162858237,0.568852433215068,0.569456288132578,0.924673328713419
"10454","SCARB1",44.6633177389695,0.183568099467139,0.325801845042915,0.563434806340521,0.573138841608277,0.924673328713419
"10455","TMEM132C",232.038659513808,-0.139878916646441,0.247059098730549,-0.566175936709771,0.571274186217144,0.924673328713419
"10456","LINC00598",7.71109709257892,0.363488937548817,0.641726541129816,0.56642341285879,0.571105982609717,0.924673328713419
"10457","LINC00562",9.50337808026637,0.333156905490564,0.591302631709533,0.563428754794079,0.573142961367874,0.924673328713419
"10458","MED4",607.646128161623,-0.115705885672528,0.205671368201416,-0.562576535005183,0.57372327406971,0.924673328713419
"10459","CDC16",261.684542343388,0.0789840885176766,0.139305224892186,0.566985829704562,0.570723809043236,0.924673328713419
"10460","ZFHX2-AS1",35.1404943198712,0.252948664007078,0.444635432143073,0.568890029271633,0.569430772263516,0.924673328713419
"10461","ZNF839",40.8059126092687,0.154821788475545,0.27283350212564,0.567458861427689,0.570402468802898,0.924673328713419
"10462","EIF3J",465.113056582754,-0.0859637263531846,0.150053707156358,-0.572886388362331,0.566721621418183,0.924673328713419
"10463","AC011939.2",78.556684391752,0.26038367251413,0.461584860471042,0.564107913436353,0.572680692813701,0.924673328713419
"10464","ZSCAN32",38.3146130955277,0.204517402054785,0.358361736404442,0.57070100202877,0.568202339444589,0.924673328713419
"10465","VPS35",613.617531061041,0.0758806573976985,0.134010357889663,0.566229794417641,0.571237578417084,0.924673328713419
"10466","DYNC1LI2",866.249642999385,-0.136599085372125,0.239161849642293,-0.571157505164106,0.567892880911316,0.924673328713419
"10467","THAP11",195.342364010307,-0.129947539835416,0.229294869255952,-0.566726766530309,0.570899832552608,0.924673328713419
"10468","CDT1",10.9194579188994,-0.286769478138317,0.509430062699916,-0.562922173494188,0.573487880559445,0.924673328713419
"10469","MYBBP1A",56.5018722750895,-0.144288525007871,0.25604730111867,-0.563522928683389,0.57307885144887,0.924673328713419
"10470","AC091153.3",2.27759000666757,-0.667413286941515,1.17422364067948,-0.56838685904442,0.569772311421417,0.924673328713419
"10471","CCDC144NL-AS1",10.7275717863106,0.334922091857356,0.586148445770829,0.5713946599

60086,0.567732148082208,0.924673328713419
"10472","GOSR1",431.218662237363,0.0823511877547227,0.146317885359131,
0.562823796643828,0.573554874432229,0.924673328713419
"10473","AC005899.6",19.6783912538167,0.311650883953964,0.546295553462
353,0.570480359905475,0.568351939258365,0.924673328713419
"10474","WIPF2",340.750610312907,0.107419551895427,0.189887123194426,0
.565702139715073,0.57159628043096,0.924673328713419
"10475","KAT7",143.358832068575,-0.118728250146695,0.209153507494151,-
0.567660813194899,0.570265305054696,0.924673328713419
"10476","C17orf67",29.8009156290394,0.226798078008008,0.39839084574349
1,0.569285364940424,0.569162497031706,0.924673328713419
"10477","KCTD2",85.9636784432263,0.140496769297347,0.245192738320118,0
.573005425282692,0.566641020596743,0.924673328713419
"10478","MCRIP1",952.799196446995,-0.149368480165785,0.261186817225947
,-0.571883687516165,0.567400776017068,0.924673328713419
"10479","AP001033.3",4.89824096080315,-0.399376036892274,0.71081629482
002,-0.561855488967647,0.574214482332365,0.924673328713419
"10480","B4GALT6",35.5189071449531,-0.161263658833087,0.28511483136827
2,-0.565609505682952,0.571659264510193,0.924673328713419
"10481","PIK3C3",162.907808034167,0.0930508321539856,0.163052454028036
,0.570680353808021,0.568216338556264,0.924673328713419
"10482","AC027307.2",22.8524327126646,0.19450780347368,0.3451055002567
88,0.563618381419449,0.57301387441255,0.924673328713419
"10483","ATP8B3",9.28128867185521,-0.324315115410724,0.57470224139787,
-0.564318514961556,0.572537383086514,0.924673328713419
"10484","LMNB2",64.4206083721799,-0.161642855693226,0.28776609588306,-
0.561716122940741,0.57430944755624,0.924673328713419
"10485","CCDC130",151.998546619285,-0.0977286085497964,0.1739188185609
98,-0.56192083961012,0.574169954396523,0.924673328713419
"10486","RPL18A",26006.7683035337,-0.131747717434279,0.229714193976487
,-0.573528849713851,0.566286671101419,0.924673328713419
"10487","ARMC6",75.8934367274532,-0.150225731989106,0.263567326405233,
-0.569970997687834,0.568697369033628,0.924673328713419
"10488","DMPK",55.3172550854516,-0.143537549609151,0.254470459710106,-
0.564063702217814,0.572710779742491,0.924673328713419
"10489","KCNJ14",11.7122176742001,-0.345377319008764,0.613288157419457
,-0.563156674118759,0.573328202413615,0.924673328713419
"10490","RPS11",21291.9375529393,0.0928275078107161,0.163782882132201,
0.566771732199634,0.570869278247792,0.924673328713419
"10491","ZIK1",7.02483441439756,0.392239494254603,0.690425244741862,0.
568112909025009,0.569958302807029,0.924673328713419
"10492","ZNF584",45.9700161703475,-0.155252684775127,0.272076892637147
,-0.570620618569688,0.568256838871661,0.924673328713419
"10493","ZNF446",54.8322775933358,-0.1220987887544,0.216175166249966,-
0.56481413139387,0.572200194162262,0.924673328713419
"10494","KIAA1755",70.295849902523,-0.153730641205172,0.27054630528004
1,-0.56822302949599,0.569883535838995,0.924673328713419
"10495","EWSR1",494.819366089227,0.114808840194319,0.202714982063191,0
.566355969479011,0.57115181992l805,0.924673328713419
"10496","MTMR3",226.952798716546,0.120092637592429,0.213105493432072,0

.563536095003146,0.573069888601823,0.924673328713419
"10497","TRIOBP",923.737474528337,0.10543731524885,0.187153138070999,0
.563374551640439,0.57317986230798,0.924673328713419
"10498","AC234772.2",14.1832630812249,-0.439765391881207,0.78223958635
8999,-0.562187595143494,0.573988212267779,0.924673328713419
"10499","CXCR3",3.42373020412354,-0.562853066236253,0.997272447700526,
-0.564392476232607,0.57248705810136,0.924673328713419
"10500","RPL36A",10211.3551789596,-0.149443865020587,0.265002897207316
,-0.563932947886508,0.572799765988678,0.924673328713419
"10501","LINC01285",3.80445187304835,0.564445916004441,0.9916541133059
37,0.56919636436813,0.569222887656009,0.924673328713419
"10502","AIFM1",113.070550748955,-0.109550740205776,0.193940348336349,
-0.564868224407758,0.572163398097143,0.924673328713419
"10503","MT-
ATP8",802.470441158828,-0.213525124796958,0.376578939222378,-0.5670129
2227834,0.570705402169617,0.924673328713419
"10504","AC145212.1",7.14120133259568,0.422185693942809,0.744004869984
368,0.567450175361997,0.570408368649961,0.924673328713419
"10505","UBE2L6",496.44296318244,0.173935797264157,0.309898842944524,0
.561266365538813,0.57461596686883,0.924726754382684
"10506","ZNF597",18.2058408255723,0.219814166225634,0.391669764084701,
0.56122322012607,0.574645375461253,0.924726754382684
"10507","CASKIN2",45.9494729245238,0.146392922514645,0.260802788066986
,0.561316554932857,0.574581757883929,0.924726754382684
"10508","LUC7L2",538.664080549368,-0.0730901379130693,0.13029182259540
8,-0.560972564947798,0.574816240060559,0.924913683569083
"10509","CLDN12",86.3843662750991,0.105182969524378,0.187584854405266,
0.560722078857901,0.574987013399836,0.924924405153118
"10510","IMP3",605.092208311112,-0.130097605217764,0.231984108408581,-
0.560803953814934,0.574931191054902,0.924924405153118
"10511","LPAR2",27.9061877711963,-0.259964116743046,0.463580611732694,
-0.560774351134738,0.574951373866172,0.924924405153118
"10512","PLEKHF2",101.078782743144,-0.137372456079023,0.24503654958030
3,-0.560620267932738,0.57505643166245,0.924948073301817
"10513","PNPLA8",399.079933823682,-0.0926638317877502,0.16531934181765
5,-0.560514158651548,0.575128784911857,0.924976457270967
"10514","CYB5B",247.904782423867,0.109033870259062,0.194670977219259,0
.560093095624912,0.575415939611718,0.925350266973077
"10515","UBQLN1",291.82496233434,0.0870209419543682,0.155411848847409,
0.559937627663189,0.575521982086621,0.925432779184079
"10516","RSBN1",320.285103740297,0.102932594200041,0.184051810069998,0
.559258798709416,0.575985109747197,0.925932956072946
"10517","GPATCH4",162.002902351459,-0.13187217802554,0.236166529359423
,-0.558386399559789,0.576580557909794,0.925932956072946
"10518","TIMM17A",209.541968265515,-0.1041131616378,0.186205402123028,
-0.559130725804676,0.576072506569672,0.925932956072946
"10519","AC092687.3",2.34670007252857,-0.508906896697394,0.91712636596
6807,-0.554892886718963,0.57896792763106,0.925932956072946
"10520","PREB",199.591106052425,0.140067054539098,0.251109843327821,0.
557791971365466,0.576986445720571,0.925932956072946

"10521","CNOT11",90.2699966889534,-0.131347040745806,0.236714093488823
,-0.554876301659695,0.578979272464501,0.925932956072946
"10522","SLC25A12",171.157736565224,-0.103499537917452,0.1855655928105
76,-0.557751770410927,0.577013900620544,0.925932956072946
"10523","STK25",406.650147567031,-0.0929204887873877,0.167310787224239
,-0.555376555982911,0.578637124742428,0.925932956072946
"10524","OSBPL10-
AS1",4.48043300011919,0.510584794665,0.920722626062864,0.5545478955462
74,0.579203936673107,0.925932956072946
"10525","EXOG",116.211568974845,-0.110193540937626,0.198750262426112,-
0.554432178315192,0.579283109140029,0.925932956072946
"10526","NBEAL2",24.8257416001831,-0.242791117029974,0.435547893160481
,-0.557438391604194,0.577227941112376,0.925932956072946
"10527","APEH",135.835993405229,-0.115183715948185,0.206656624428195,-
0.557367644356385,0.577276267281634,0.925932956072946
"10528","ARF4-
AS1",20.2164847411433,-0.22800687584912,0.408723636573229,-0.557850966
880084,0.576946156350302,0.925932956072946
"10529","ITGB5",953.90193603217,-0.100247606050709,0.180635334325144,-
0.554972295012132,0.578913610679169,0.925932956072946
"10530","NDUFB5",946.117270459119,-0.154626070637364,0.278460177010136
,-0.555289708918543,0.578696516763626,0.925932956072946
"10531","GOLPH3",476.579039844185,0.095983509121337,0.172010406416226,
0.558009896733101,0.576837626159322,0.925932956072946
"10532","GCNT4",3.77380142998567,-0.43216421032101,0.776890434208425,-
0.556274335854506,0.578023328778031,0.925932956072946
"10533","EDIL3",57.5964573827862,-0.288414307148179,0.518401878412006,
-0.556352743226286,0.577969737613118,0.925932956072946
"10534","ZSCAN12",15.2668928683798,0.231238576918851,0.413568278452571
,0.559130351544527,0.576072761973596,0.925932956072946
"10535","PRRC2A",363.705794116604,0.102715907080868,0.184274335318642,
0.557407557071117,0.577249003391616,0.925932956072946
"10536","SNRPC",455.085494285004,-0.158184804667209,0.284826352536632,
-0.555372785061608,0.578639703499388,0.925932956072946
"10537","YIPF3",835.663646503675,-0.117581283900814,0.211284257810395,
-0.556507546370682,0.577863937068478,0.925932956072946
"10538","LMBRD1",622.061427113876,0.0920212895120605,0.164706758981055
,0.558697712718912,0.576368040613706,0.925932956072946
"10539","ZNF853",75.6498528536553,-0.12054445794438,0.216322425789241,
-0.557244388807954,0.577360465476171,0.925932956072946
"10540","AC007285.1",8.99576152540918,-0.282353814117308,0.50783659868
8355,-0.555993433412586,0.578215343802909,0.925932956072946
"10541","TSC22D4",277.182119976376,0.110706319020064,0.198153690110264
,0.558689161723212,0.57637387743997,0.925932956072946
"10542","RHEB",998.051322489582,-0.129863171117945,0.233998088074559,-
0.554975351236914,0.578911520204618,0.925932956072946
"10543","TGS1",339.079555335984,0.0968773409054894,0.174399293727493,0
.555491589644078,0.578558461189537,0.925932956072946
"10544","DECR1",849.949500608336,-0.157587950991571,0.283429773531335,
-0.556003517302138,0.57820845029179,0.925932956072946

"10545","MED27",99.6843396351951,-0.142084170422093,0.254475731048268,
-0.558340749574833,0.576611723885704,0.925932956072946
"10546","PRXL2A",367.060195704273,0.129299161194591,0.233190742203538,
0.554478106518196,0.579251684956653,0.925932956072946
"10547","ADRA2A",74.5469261829566,-0.466948160119499,0.84046042708893,
-0.55558613477716,0.578493812085101,0.925932956072946
"10548","AHNAK",7709.06503545225,0.095611739617166,0.172435472776697,
0.554475088113292,0.579253750131745,0.925932956072946
"10549","INTS5",78.0007556288718,-0.131850493791635,0.236864737666559,
-0.556648892066175,0.577767342006606,0.925932956072946
"10550","MAP6",12.8107045288505,0.306947749774982,0.550283174199051,0.
55779962784025,0.57698121686603,0.925932956072946
"10551","SLC48A1",132.688387481376,0.109533968124068,0.196063340501044
,0.55866623431056,0.576389527605844,0.925932956072946
"10552","ZC3H10",41.6480188189472,0.15810984623379,0.284365383490869,0
.556009470255604,0.578204380773997,0.925932956072946
"10553","NUP37",150.528460296556,-0.156309477702005,0.281040218262564,
-0.556181882679764,0.578086523203312,0.925932956072946
"10554","CHST11",45.7386259569112,0.214746052805653,0.386870599288218,
0.555084964328518,0.578836546591592,0.925932956072946
"10555","COQ5",185.495966045074,-0.157106176560023,0.28201963562697,-0
.557075312187938,0.577475974318281,0.925932956072946
"10556","LINC00539",9.77178104305039,-0.273238250620465,0.492426420615
136,-0.554881377565278,0.578975800334173,0.925932956072946
"10557","RFXAP",108.961763512155,0.137290579212783,0.247347534964111,0
.555051333876049,0.578859548801683,0.925932956072946
"10558","AL355075.4",104.339472651194,-0.518316596725421,0.93243119725
4735,-0.555876506761516,0.578295279587981,0.925932956072946
"10559","TRAC",95.0958705464139,0.314979309503527,0.566578855874649,0.
555931987644124,0.578257349966163,0.925932956072946
"10560","SLC22A17",161.929691715569,0.140781552042594,0.25237280566272
7,0.557831703272875,0.576959311757248,0.925932956072946
"10561","LRR1",44.5796073154145,-0.235932553685175,0.424218803516861,-
0.556157699114809,0.578103053907268,0.925932956072946
"10562","NSMCE3",287.777707978713,-0.144224791028585,0.258646897680614
,-0.557612684791135,0.577108892710692,0.925932956072946
"10563","AC092756.1",2.20847337754079,0.557094334009333,0.998637763949
72,0.557854263197463,0.576943905259775,0.925932956072946
"10564","ALDH1A3",238.082374109371,-0.250757535578366,0.45099899525594
6,-0.556004643504935,0.578207680403617,0.925932956072946
"10565","GNPTG",528.669792916345,0.115403492616305,0.208146025152964,0
.554435245791971,0.579281010340341,0.925932956072946
"10566","SLC12A4",194.182257132952,-0.114885392540178,0.20719886458286
7,-0.554469218600524,0.579257766028162,0.925932956072946
"10567","SAT2",1404.53786252052,-0.145288528408252,0.260671505056967,-
0.55736252559136,0.57727976389165,0.925932956072946
"10568","SCO1",175.941167471572,0.109248562998191,0.195464010107064,0.
558919071282485,0.576216953122221,0.925932956072946
"10569","AARSD1",210.478967139604,-0.121392313206232,0.217646387213192
,-0.557750187175513,0.577014981890304,0.925932956072946

"10570","VAPA",1216.03318239313,-0.106927537115354,0.1923166860818,-0.555997190331541,0.57821277550741,0.925932956072946
"10571","AC004076.2",2.35491411696835,-0.558324458793472,0.99964982157136,-0.558520040463606,0.576489323691445,0.925932956072946
"10572","MZF1",96.3451940621439,0.114169229038868,0.205820894610141,0.554701840428412,0.579098617139662,0.925932956072946
"10573","KIZ",291.776347359229,-0.106200637624532,0.189859335234847,-0.559364844995197,0.575912748604871,0.925932956072946
"10574","BMP7",4.33831714981484,0.565807297587468,1.01854977602047,0.555502844247937,0.578550765215459,0.925932956072946
"10575","UCKL1-
AS1",3.57784133214017,0.553302273211932,0.994213985486438,0.556522319429271,0.577853840865924,0.925932956072946
"10576","LINC00323",8.39962851278747,-0.412612216224718,0.744072169662628,-0.554532521236216,0.579214455315662,0.925932956072946
"10577","LINC01679",2.02170545207171,-0.631892107145549,1.13807780147662,-0.555227512851659,0.578739052490245,0.925932956072946
"10578","AL021308.1",2.26715547916729,0.583660788669759,1.05077466992618,0.555457611773951,0.578581695778347,0.925932956072946
"10579","AC093772.2",11.4216223390933,0.283782398022636,0.512113806353788,0.554139323138241,0.579483500150116,0.926078168292832
"10580","EIF3E",3966.71379144379,-0.134617532240093,0.242922654640821,-0.554157999134064,0.579470719819332,0.926078168292832
"10581","NOP58",369.519816279764,0.0826905354038073,0.149247547874703,0.554049541056636,0.579544941542187,0.926088826348672
"10582","CENPC",448.037064222623,0.106249826567477,0.191940991122761,0.553554641694655,0.579883675391869,0.926095915504359
"10583","UTP3",175.582733410381,-0.10251255044745,0.185213581814406,-0.55348290035324,0.579932786453758,0.926095915504359
"10584","AC016885.2",20.7604612554511,0.360571451300401,0.651181444947201,0.553718866067563,0.579771261734241,0.926095915504359
"10585","RHOJ",143.863555308549,-0.118659712427045,0.21435482553119,-0.553566788771821,0.57987536021409,0.926095915504359
"10586","GREM1",28.7638648729555,0.588798820350298,1.06330142388878,0.553745915430925,0.579752747083761,0.926095915504359
"10587","JPT1",265.471774869071,0.127285528991177,0.229845876584507,0.553786436731562,0.579725011729238,0.926095915504359
"10588","AC008738.2",2.92561975456404,-0.46255071737544,0.835194512402918,-0.553823942215145,0.579699341146275,0.926095915504359
"10589","MARS2",6.62452093689948,-0.315362101043951,0.570059849883492,-0.553208757130334,0.580120471171782,0.926149851955738
"10590","OASL",64.313777790503,-0.533162355283563,0.963548646058761,-0.553332057975875,0.580036053071011,0.926149851955738
"10591","KCTD5",60.7782113448165,0.132027414586797,0.238664056817339,0.553193540524804,0.580130889641781,0.926149851955738
"10592","AC011447.3",12.0265820647938,0.250636440735749,0.453258939523192,0.552965245427719,0.580287208723815,0.926311945345758
"10593","ZBTB8OS",257.846754516764,-0.118345159021253,0.214129279116875,-0.552680882826204,0.580481946158919,0.926447871026524
"10594","AC007036.2",6.15328710752746,0.432612919119645,0.782668077285

234,0.552741234343181,0.580440613623446,0.926447871026524
"10595","TSPYL4",123.49298485146,-0.107056285654681,0.193812276865139,
-0.552371023065657,0.580694179426814,0.92652422249208
"10596","LYN",43.5665082249041,-0.231199082082905,0.418465364115895,-0
.552492755455082,0.580610796526436,0.92652422249208
"10597","AL731537.1",4.19730548468834,0.410536418316859,0.743145932638
437,0.55243041815395,0.580653494938249,0.92652422249208
"10598","FMO2",829.779115232693,0.2028290633322,0.367378390781177,0.55
2098513200281,0.580880860532191,0.926734628220257
"10599","LINC00900",18.618267416958,0.218104508685459,0.39515652881718
7,0.551944591016392,0.580986316334002,0.926815419999558
"10600","CAMKMT",44.7267531093855,-0.15017375209873,0.272484619205077,
-0.551127445419981,0.581546312493611,0.927283902021591
"10601","AC007098.1",21.7521418445616,0.24273950514291,0.4405554581702
31,0.550985127164433,0.581643870088213,0.927283902021591
"10602","ARSK",122.364491098265,0.112212911750435,0.20349222610658,0.5
51435865130606,0.581334920443874,0.927283902021591
"10603","PSMA2",1223.60327006633,-0.149589318035236,0.271414156439275,
-0.551147810407985,0.581532353144696,0.927283902021591
"10604","SLC25A13",118.534681580851,0.123446056166691,0.22407996385622
9,0.550901803277222,0.581700991244761,0.927283902021591
"10605","DPAGT1",101.755442683817,-0.116023424744623,0.210563808662976
,-0.551013136974208,0.581624669067813,0.927283902021591
"10606","ITFG2-
AS1",7.47395487486637,-0.30712111959577,0.557514145895479,-0.550875922
802769,0.581718733661167,0.927283902021591
"10607","AQR",185.546645047937,0.097934572847876,0.177655664806113,0.5
51260625180503,0.581455026167,0.927283902021591
"10608","GABARAP",4872.36955698528,-0.133654344723297,0.24268748314296
3,-0.550726156093364,0.581821411526569,0.927360145747666
"10609","AC138356.1",2.70197176083954,-0.475503005133627,0.86357526522
2134,-0.550621380999507,0.581893248833552,0.927387223232886
"10610","ASIC3",2.60028145597729,0.599996991716109,1.09000887124614,0.
55045147571154,0.582009750388397,0.927485472155233
"10611","RHOQ",797.773482831636,-0.115907108559947,0.210691509953312,-
0.550127096177875,0.582232202917306,0.927490304025421
"10612","LINC01184",156.566010085767,-0.090627638788075,0.164736741787
175,-0.550136161519801,0.582225985560873,0.927490304025421
"10613","CYP19A1",42.5395075476388,-0.23419954323697,0.425710382582593
,-0.55013819915828,0.582224588075139,0.927490304025421
"10614","LDOC1",112.465246599889,-0.136641739887586,0.248371252864869,
-0.550151188237266,0.582215679734559,0.927490304025421
"10615","CCDC28B",132.121820736959,0.174241005079712,0.31753801766242,
0.548724862498043,0.583194281242562,0.927649222772217
"10616","PSMB2",781.250624940136,-0.139443421764457,0.253810655373387,
-0.549399399955523,0.582731385787336,0.927649222772217
"10617","GDAP2",74.9281525834094,0.117527385319213,0.214222753474793,0
.548622326120191,0.583264660946974,0.927649222772217
"10618","SLAMF1",2.04754595063279,0.769438126724961,1.40233449158055,0
.548683735117818,0.583222510093243,0.927649222772217

"10619","LINC01142",14.5196174515939,-0.305444872857528,0.556438054909
786,-0.548928798385382,0.583054313936398,0.927649222772217
"10620","HADHA",1294.99229620663,-0.0864338738043839,0.157493622381619
,-0.548808723155454,0.583136723265773,0.927649222772217
"10621","HADHB",831.349143269724,-0.133995898049452,0.243912713318618,
-0.549360040427315,0.582758391210246,0.927649222772217
"10622","PFKFB4",2.51441079638665,0.549337916397559,1.00015419584934,0
.549253223830208,0.582831683328585,0.927649222772217
"10623","RORB",10.2045246885107,-0.332887558270167,0.60594144761985,-0
.549372484053956,0.582749853305961,0.927649222772217
"10624","TOR1A",215.525030073343,-0.104071713113182,0.18933703920634,-
0.549663782371522,0.582550002434871,0.927649222772217
"10625","AP003396.5",15.4886724639853,0.355350586099077,0.646916278536
18,0.549299187374218,0.582800144954383,0.927649222772217
"10626","PARPBP",21.5300600548804,-0.175121159279427,0.318781474099376
,-0.549345471766137,0.582768387231598,0.927649222772217
"10627","DIO3OS",897.943971612494,0.134436645782863,0.244579740515422,
0.549663866269355,0.582549944879741,0.927649222772217
"10628","ZNF768",79.9631405699306,-0.11017691480443,0.200724039046204,
-0.548897458062156,0.583075822719898,0.927649222772217
"10629","EZH1",423.418773559131,0.104158816091614,0.189485593746065,0.
549692533518935,0.582530278886215,0.927649222772217
"10630","ABHD8",100.837074718757,-0.145502292310816,0.265181786012933,
-0.54868886169927,0.583218991295526,0.927649222772217
"10631","GAB3",20.5833756835436,0.264929127221549,0.482408369100274,0.
549180205384208,0.582881787348443,0.927649222772217
"10632","AC100810.1",486.50672663883,-0.109455862633052,0.199710158873
499,-0.548073584490932,0.583641377708204,0.928161062292166
"10633","AL513548.4",2.18993708174301,0.707304026712112,1.290834326048
48,0.547943304914523,0.58373083262256,0.928216017867229
"10634","RNASEK",2612.00901957189,-0.1362465206601,0.248708571767927,-
0.547815942537087,0.583818290650256,0.928267788067946
"10635","TIMELESS",7.04027054351402,0.310972754423984,0.56789212814255
7,0.547591239626975,0.583972605989191,0.928425841284932
"10636","AGMO",14.918386672988,-0.262234642154815,0.479065672186819,-0
.547387670165923,0.584112424268304,0.92842648219393
"10637","ANKRD20A1",3.9236760841014,0.432545483977299,0.79006212154249
3,0.547482877843594,0.584047030527742,0.92842648219393
"10638","CHADL",52.8124863989494,0.189871916954374,0.346892545692548,0
.54735081313237,0.584137740571269,0.92842648219393
"10639","AL049795.2",2.81820778251806,0.567671005018259,1.041105109811
68,0.545258110510037,0.585576009975443,0.92849162003965
"10640","SFPQ",1786.63286388258,0.155841829269168,0.286122971896428,0.
544667309430788,0.585982352246624,0.92849162003965
"10641","CACYBP",681.840169582544,-0.118203391848993,0.217011916075617
,-0.544686181231656,0.585969370541615,0.92849162003965
"10642","GPATCH2",241.908802880602,-0.106922461985526,0.19622445018941
,-0.544898772208648,0.585823140757476,0.92849162003965
"10643","MFSD9",50.9971550116442,0.173941654015893,0.318827253854027,0
.545567080333511,0.5853635582457,0.92849162003965

"10644","C2orf49",269.300505784363,0.113562176442829,0.208274073590072
,0.545253542533306,0.5855791512451,0.92849162003965
"10645","PLEKHB2",149.3660076454,0.120597852125165,0.221408119234454,0
.544685770974197,0.585969652751807,0.92849162003965
"10646","MYOSLID",2.19483024071784,-0.601009128728466,1.10124704812626
,-0.545753225628225,0.585235579568175,0.92849162003965
"10647","LINC01963",10.8419520555601,0.258989674656187,0.4758271664251
12,0.544293585845416,0.586239460087697,0.92849162003965
"10648","AC012485.3",25.3112804906075,0.23106432756505,0.4226388991535
44,0.546718080204692,0.584572430822216,0.92849162003965
"10649","APPL1",803.206948408799,0.0755330574974919,0.138307846637273,
0.546122720683991,0.584981582717628,0.92849162003965
"10650","PROS1",767.455833483369,0.103016676800826,0.188908084262975,0
.545326989063205,0.585528645072055,0.92849162003965
"10651","CLDND1",430.983533154534,-0.105465169306028,0.193272046674239
,-0.545682477734559,0.585284218653134,0.92849162003965
"10652","CSTA",65.0010607397805,-0.256428421754361,0.470911425269891,-
0.544536420214046,0.586072393176465,0.92849162003965
"10653","SSR3",1160.36128453034,-0.124897030473172,0.229577386620801,-
0.544030195271226,0.586420694369206,0.92849162003965
"10654","ELF2",612.580742059895,-0.0905484011870836,0.166234038441684,
-0.544704333937292,0.585956883620248,0.92849162003965
"10655","EGFLAM",64.1303006748101,-0.199452575295923,0.365988262478179
,-0.544969868556411,0.585774241221445,0.92849162003965
"10656","RMND5B",184.409033435728,-0.141243163999079,0.258720008299083
,-0.545930579268536,0.585113657425971,0.92849162003965
"10657","SERPINB6",751.785220905724,-0.0937569703600165,0.171733253127
368,-0.545945346359221,0.585103506289973,0.92849162003965
"10658","ZBTB9",10.871955189623,0.286635807669055,0.526878220255065,0.
544026677607384,0.586423114985719,0.92849162003965
"10659","AL356776.2",5.70606727093134,0.385992525842152,0.705716521953
011,0.546951238684267,0.584412232435269,0.92849162003965
"10660","QRSL1",86.6309489561957,0.112582873630796,0.206494857804747,0
.54520909056849,0.585609720024799,0.92849162003965
"10661","CUL1",220.624289382038,-0.0789737888850809,0.144623334178867,
-0.546065331251509,0.585021029776371,0.92849162003965
"10662","AL358976.2",2.77873577055129,0.51616349458087,0.9451799269323
95,0.546100779198826,0.584996664216772,0.92849162003965
"10663","UBAP1",204.977123321506,0.117247919396294,0.215529355557489,0
.543999767887861,0.586441632573957,0.92849162003965
"10664","PTGES",888.084381261553,-0.143003612281668,0.261666703519601,
-0.546510543214589,0.584715042391049,0.92849162003965
"10665","LAMC3",5.86483854420241,0.405572325163689,0.742948337649032,0
.545895730041031,0.58513761367074,0.92849162003965
"10666","ST8SIA6",2.27854108454672,-0.683346795471647,1.24925487143462
,-0.547003506727898,0.584376322942321,0.92849162003965
"10667","SPOCK2",14.5861784768576,0.255256875655663,0.46874614855657,0
.544552475666596,0.586061348010819,0.92849162003965
"10668","POLA2",24.0582835722761,0.17391184028123,0.317964612452708,0.
546953445352655,0.584410716376463,0.92849162003965

"10669","FUT4",86.4102781281648,0.132973057943103,0.244464274607862,0.
543936565604122,0.586485125504108,0.92849162003965
"10670","GOLT1B",201.884605824574,-0.0940152086797266,0.17225629772870
4,-0.545786771917019,0.58521251719456,0.92849162003965
"10671","AC053513.2",2.59653175012494,0.47472956845086,0.8689071233471
41,0.54635248773441,0.584823662989029,0.92849162003965
"10672","CNIH1",604.097067217167,-0.113746395952156,0.208390844802918,
-0.545832020882347,0.585181410143897,0.92849162003965
"10673","PRKCB",9.00084500147917,0.369676203143128,0.677582167429739,0
.545581364021746,0.585353737458965,0.92849162003965
"10674","PLCG2",8.53597165003364,0.393184086530189,0.722763469923788,0
.544001049986171,0.586440750307827,0.92849162003965
"10675","SERPINF1",13150.4574549426,-0.169571964127758,0.3104923126761
97,-0.546139009581858,0.58497038664622,0.92849162003965
"10676","DDX42",487.136317351056,0.0942495455265122,0.173223278083601,
0.544092841153978,0.586377586518116,0.92849162003965
"10677","PGS1",69.8242902714565,0.1101213538903,0.201404518498842,0.54
6767047289126,0.584538784853182,0.92849162003965
"10678","AC007996.1",2.43813168874151,-0.493520847791744,0.90726985436
7222,-0.543962576752813,0.586467225636417,0.92849162003965
"10679","KDSR",1225.66376669668,0.0782614122318718,0.143844180446154,0
.544070757601263,0.586392782472835,0.92849162003965
"10680","ZNF461",12.0786882838546,0.260183221816162,0.475824922876004,
0.546804526848973,0.584513032731672,0.92849162003965
"10681","HERC2",321.538117243892,0.134241568316833,0.246834075063368,0
.543853470321591,0.586542310164277,0.928495213955396
"10682","ESYT1",181.406173651879,0.122243397133468,0.224900878923768,0
.543543438862697,0.586755690974832,0.928746042220789
"10683","IWS1",304.337295585783,0.108199179996769,0.1990957055214,0.54
3453108209505,0.586817868296059,0.928757513540182
"10684","ACBD6",459.906068256167,-0.0899172287387248,0.165872369215642
,-0.542086841611504,0.58775868295432,0.928821336991692
"10685","HPCAL1",99.7438064134061,-0.140134983082707,0.258518371263545
,-0.542069727570143,0.587770472149777,0.928821336991692
"10686","ASB3",151.408443434029,-0.0973917650798708,0.179324474173942,
-0.543103586548942,0.587058483380823,0.928821336991692
"10687","MRPL35",169.696227236194,-0.127260409193732,0.234574374062709
,-0.5425162475749,0.587462917743645,0.928821336991692
"10688","CCDC74B",44.1749746777427,-0.205841097312628,0.37957653772138
2,-0.542291413869527,0.587617769574506,0.928821336991692
"10689","C3orf67",16.2702618474402,-0.266162863905136,0.49094047474498
5,-0.542148952056544,0.587715898410481,0.928821336991692
"10690","AC007370.2",4.43050757189507,0.397818956251645,0.733484304558
734,0.542368737516441,0.587564511602021,0.928821336991692
"10691","TBC1D19",56.819201311127,0.136591380092482,0.252032851942402,
0.541958633724852,0.587847003025083,0.928821336991692
"10692","HAND2-
AS1",146.509582934859,-0.197954869206026,0.364864799902029,-0.54254307
145874,0.587444444339013,0.928821336991692
"10693","RNF145",318.714234360678,0.136008721505891,0.25070030157467,0

.542515189058844,0.587463646741111,0.928821336991692
"10694","DEF6",9.01047047542152,-0.282223684977509,0.519996285443535,-0.542741732735233,0.587307636199307,0.928821336991692
"10695","TMEM216",159.675247029697,-0.137046950247298,0.252268706833903,-0.543257829983373,0.586952294638026,0.928821336991692
"10696","FRMD8",72.3690939359433,-0.113941624557857,0.21007458021273,-0.542386539306543,0.587552250633623,0.928821336991692
"10697","ELF1",614.138117933755,0.103807631962735,0.191194241265293,0.542943298269618,0.587168843116793,0.928821336991692
"10698","KLHL28",160.859610651767,0.0959945415079572,0.177121433609759,0.541970215301308,0.587839024436491,0.928821336991692
"10699","RSPRY1",217.767520537219,-0.0814912892432404,0.150042396955064,-0.543121750232009,0.587045978148697,0.928821336991692
"10700","DDRGK1",464.69828183356,-0.118141678191324,0.217931992328378,-0.542103419186429,0.587747263413944,0.928821336991692
"10701","SPTLC3",65.518292263315,-0.167553094956766,0.308579156609999,-0.542982542299609,0.587141822406728,0.928821336991692
"10702","RMDN2",83.3073177937774,0.150853045468507,0.278574116555248,0.541518527758085,0.588150230670431,0.92887513702248
"10703","AAK1",370.136401159812,0.133143947948093,0.245791534503878,0.541694603993744,0.588028907614835,0.92887513702248
"10704","SEC22A",93.12571289918,0.122517694636068,0.226318629653823,0.541350461618962,0.588266045263603,0.92887513702248
"10705","RPL7L1",757.636052215204,0.0765495457599553,0.141425446793007,0.541271372979961,0.588320548993006,0.92887513702248
"10706","STOML2",705.106674025442,-0.125447482962825,0.231742016090612,-0.541323861244799,0.588284376581693,0.92887513702248
"10707","PLXDC1",260.901596337918,-0.171493479285554,0.316708023410009,-0.541487637222058,0.58817151658812,0.92887513702248
"10708","TIMM13",934.135920559922,-0.148232184488409,0.273721373053554,-0.541544062981909,0.588132635237059,0.92887513702248
"10709","HMGB3",17.9845289405809,-0.229108052197878,0.423059621241764,-0.541550270208723,0.588128358090015,0.92887513702248
"10710","AGRN",17.1657251894227,0.204879698585669,0.38017175291577,0.538913522675793,0.589946526004647,0.928890294575536
"10711","RPL11",35715.1414286447,-0.144839072905095,0.26830372517367,-0.539832508144796,0.589312546068165,0.928890294575536
"10712","ALG6",82.7044238533732,0.136051272073088,0.252585878958426,0.538633721861707,0.59013961440342,0.928890294575536
"10713","CREG1",1040.57719692457,0.0824849654676084,0.152868943396544,0.539579613981115,0.589486978665464,0.928890294575536
"10714","OPN3",56.9189812666484,0.134189651374526,0.248682492789189,0.539602325316404,0.589471312652415,0.928890294575536
"10715","ROCK2",824.839557129261,0.102256852883807,0.189504097623722,0.539602331379914,0.589471308469903,0.928890294575536
"10716","TXNDC9",226.338766521609,-0.163232633430058,0.302024169203831,-0.540462155265113,0.588878353300047,0.928890294575536
"10717","PTPN4",125.22270836672,0.131578908166623,0.244514047867371,0.538124125440815,0.590491357752532,0.928890294575536
"10718","THUMPD3",210.792369945386,-0.0849892369908214,0.1578323621164

58,-0.538477887875182,0.590247166862589,0.928890294575536
"10719","CD47",1125.80339268545,-0.105220855778794,0.195092772014145,-0.539337540250674,0.589653970216478,0.928890294575536
"10720","PLA2G12A",356.118655769063,0.0749706194135044,0.139263147693003,0.538337820560917,0.590343845287243,0.928890294575536
"10721","PSORS1C3",2.09563669905433,-0.736890627705514,1.36911691240997,-0.538223303668357,0.590422893504442,0.928890294575536
"10722","AL109615.2",4.02713741716922,-0.405646052809673,0.753037272942792,-0.538679913179398,0.590107736122933,0.928890294575536
"10723","AL390208.1",5.79468206882745,0.385406145900104,0.71371052797972,0.540003447883923,0.589194654648722,0.928890294575536
"10724","TPD52L1",17.0748300285407,-0.211759556135862,0.393552797229315,-0.538071531003435,0.590527665980153,0.928890294575536
"10725","AC090197.1",2.40979987013178,0.523040833328017,0.96893793531387,0.539808396663289,0.589329175825899,0.928890294575536
"10726","GSR",78.1309052500127,0.123021248803819,0.227992007462529,0.539585795892593,0.589482714436323,0.928890294575536
"10727","ADCK5",40.778788100179,-0.150780097067164,0.278748087961106,-0.540918856771504,0.588563512993112,0.928890294575536
"10728","UBAP2",165.080238068533,0.111623639613703,0.20666612188402,0.540115808997208,0.589117168874876,0.928890294575536
"10729","RUSC2",111.890675569981,0.104069486607922,0.192992154850765,0.53924205721417,0.589719843997815,0.928890294575536
"10730","CEL",4.86165165028522,-0.336373641854881,0.625073738966634,-0.538134336615981,0.590484308653317,0.928890294575536
"10731","DBH-AS1",2.10734678111052,-0.556793699699622,1.03464520922284,-0.538149401105188,0.590473909227675,0.928890294575536
"10732","MEIG1",3.51341139323471,0.427982101035693,0.793979172640588,0.539034417757238,0.589863106241407,0.928890294575536
"10733","FANK1",10.706971508709,0.258722499266041,0.478153302007722,0.541086923753718,0.588447670762262,0.928890294575536
"10734","TAF6L",50.2602840788158,-0.146064764129002,0.271114776507849,-0.538756190313268,0.590055096281023,0.928890294575536
"10735","AP002884.3",4.62282506420269,0.386477337977333,0.71447656678348,0.540923741862137,0.588560145735797,0.928890294575536
"10736","TSFM",175.660922585574,-0.141138322131337,0.26115546571905,-0.540437940836256,0.588895048382454,0.928890294575536
"10737","AC073655.2",2.11727289699255,-0.60124747200318,1.11193149886713,-0.540723482171111,0.588698190572486,0.928890294575536
"10738","ELMSAN1",519.242639484198,0.134375389574919,0.249244544176236,0.539130716056536,0.589796662586206,0.928890294575536
"10739","SLC24A4",4.04827790568705,-0.432007991851495,0.800398441244969,-0.539741170884259,0.589375542780913,0.928890294575536
"10740","PRPSAP2",89.3113710381082,0.122470870119285,0.226888081164232,0.539785384454088,0.58934504761425,0.928890294575536
"10741","AC015802.3",2.33235828257814,-0.550313197916387,1.02038474408598,-0.539319311765425,0.589666545793055,0.928890294575536
"10742","S1PR5",3.57204411489779,0.535470140087919,0.993792250041696,0.538814968687321,0.590014534036506,0.928890294575536

"10743","MAST3",15.027519396858,0.208829355822711,0.387352855450733,0.539119185218636,0.589804618422578,0.928890294575536
"10744","PLEKHF1",400.566016479522,0.16262093104823,0.301117546473359,0.540057970559405,0.589157054472286,0.928890294575536
"10745","SS18L1",53.9756752414488,0.127031463940022,0.235457979386506,0.539508001686784,0.58953637724427,0.928890294575536
"10746","C21orf58",31.4719922475786,0.154074730470373,0.285382589997607,0.539888331911434,0.589274045087422,0.928890294575536
"10747","EFHC2",8.93715576558486,0.321842473277894,0.597876832915335,0.538308988673375,0.590363746776865,0.928890294575536
"10748","ZNF275",110.798358216134,0.111406684148515,0.207024782262254,0.538132116025545,0.590485841594275,0.928890294575536
"10749","F8",44.2515264297611,-0.144427710060945,0.267535906583733,-0.53984420971823,0.589304475537525,0.928890294575536
"10750","SCAMP3",142.095838611156,0.0972197920719258,0.180772213248309,0.537802742606157,0.590713238343794,0.929057055253325
"10751","RPL15",23587.7662469288,-0.124668262352733,0.231881362572008,-0.537638130852448,0.59082690068035,0.929057055253325
"10752","ARID1B",867.371408737467,0.109057414742466,0.202859906902903,0.537599648976793,0.590853472624734,0.929057055253325
"10753","GADD45GIP1",1131.67660504305,-0.13238720466215,0.246231484274595,-0.537653440428896,0.590816328631514,0.929057055253325
"10754","AP000866.6",12.0627232919704,0.331598379248185,0.617238359855721,0.537229052526313,0.591109405521248,0.929200244380184
"10755","E2F1",12.8441582931825,-0.255383871970618,0.47523760180894,-0.537381450875367,0.591004153467004,0.929200244380184
"10756","AC008079.1",9.48066355171245,0.337815067157252,0.628792738921993,0.537243906054648,0.591099146734672,0.929200244380184
"10757","TMBIM1",485.236148090006,0.0717297806446495,0.133958857075838,0.535461276771279,0.592330927503376,0.929260097483811
"10758","P2RY1",42.8083289183242,0.186947813911556,0.349602170075499,0.534744432138917,0.592826592468775,0.929260097483811
"10759","FAM131A",59.089738964076,-0.125539011694926,0.234472231839535,-0.535410998180974,0.592365686626206,0.929260097483811
"10760","ARL9",7.42287579524555,-0.363571407081691,0.680246086148811,-0.534470413699897,0.593016113783435,0.929260097483811
"10761","LINC00271",19.5219648610063,-0.185238507526569,0.346582286384411,-0.534471941595746,0.593015056957036,0.929260097483811
"10762","SEMA4D",18.2067691440191,0.220717095644454,0.411742970385411,0.536055528617411,0.591920173987418,0.929260097483811
"10763","GOT1",80.6798940087526,-0.118928450470879,0.222264058910207,-0.535077290741485,0.592596412468644,0.929260097483811
"10764","NEURL1",4.55024444286124,-0.422081857751213,0.78866574999029,-0.53518471894641,0.592522131942915,0.929260097483811
"10765","HIKESHI",528.24718990296,-0.156028535156255,0.291877302718595,-0.534568922293645,0.592947978390385,0.929260097483811
"10766","POU2AF1",3.10288031615819,0.526566361987957,0.984590972599646,0.534807221111978,0.592783169196508,0.929260097483811
"10767","ARCN1",375.482300257097,0.0964898057605415,0.179882997976355,0.536403144521891,0.591679958630727,0.929260097483811

"10768","AL162171.3",7.42076937411657,0.324939111800958,0.607704489904687,0.534699211868456,0.592857866571986,0.929260097483811
"10769","RCOR1",177.995508016314,0.108987168301978,0.203876739154955,0.534573825114709,0.592944587352155,0.929260097483811
"10770","KLC1",405.291154237818,0.0988342591317964,0.184575946277602,0.535466625662859,0.592327229706572,0.929260097483811
"10771","PSMA4",1127.79283877197,-0.120877525066134,0.225254070099746,-0.536627484744705,0.591524955075151,0.929260097483811
"10772","LINC01197",3.6458624820947,0.471607679864886,0.878603990982185,0.536769334882806,0.591426956083432,0.929260097483811
"10773","E4F1",87.5896144738232,0.102695586967247,0.191658165231213,0.535826829205824,0.592078238095855,0.929260097483811
"10774","RPS15A",22405.120171677,-0.124953718291889,0.232715578811126,-0.53693748794232,0.591310795045429,0.929260097483811
"10775","YPEL3",1366.15151667201,-0.120093471718679,0.223929192497595,-0.536301097589003,0.591750472166064,0.929260097483811
"10776","POLR2C",382.135521824276,-0.120865712166651,0.225761498317654,-0.535369020259554,0.592394707960241,0.929260097483811
"10777","CFDP1",1067.38619027943,0.0987387433954325,0.184398110041707,0.535465050987235,0.592328318310541,0.929260097483811
"10778","KARS",296.878493300065,0.0705245307833066,0.131770162674792,0.535208649300681,0.592505586038426,0.929260097483811
"10779","AC130343.2",2.85914278573112,-0.44986819851916,0.839945531178295,-0.535592109036017,0.592240483554594,0.929260097483811
"10780","RPA1",218.406752521971,0.106358480926105,0.198849895272501,0.534868176723719,0.592741015225639,0.929260097483811
"10781","OR3A3",2.33522758146954,-0.561553072429542,1.04927670593415,-0.535181110238793,0.592524627090746,0.929260097483811
"10782","RPL23A",20866.0369967717,-0.128449093052902,0.239217108378314,-0.536956131288752,0.591297916767225,0.929260097483811
"10783","EPOP",11.4661637215836,-0.306299280251023,0.571246024812059,-0.536195031469665,0.591823767014117,0.929260097483811
"10784","ELAC1",86.14274604156,-0.13952308601866,0.260242229383787,-0.536127769689913,0.591870249067541,0.929260097483811
"10785","TMEM161A",152.089165159757,-0.159614484583273,0.297603219504631,-0.536333191720695,0.591728294986611,0.929260097483811
"10786","LIPE",20.986331355654,0.190427348286235,0.35546675749963,0.535710707875218,0.592158502012111,0.929260097483811
"10787","PYGB",129.596352012915,-0.133142240672438,0.248096443903723,-0.536655175614309,0.591505823895809,0.929260097483811
"10788","RALY-AS1",17.6325065328858,0.230698267989177,0.430197352147768,0.536261478220199,0.591777849842352,0.929260097483811
"10789","AL031667.3",2.58435602868556,0.586980460545952,1.09659101919815,0.535277464678824,0.592458006951154,0.929260097483811
"10790","AP000320.1",4.94385930340343,0.411667082644858,0.767071911797546,0.536673389174377,0.591493240593183,0.929260097483811
"10791","ZBTB48",62.2620391909912,-0.119629811413414,0.226865346757832,-0.527316371244277,0.597973907339274,0.929392804026028
"10792","CASZ1",3.42723985658876,0.477766071336291,0.903297707483554,0

.52891318928205,0.596865672091926,0.929392804026028
"10793","MIIP",90.5522752475135,-0.139628851898852,0.26787335222602,-0
.521249503687246,0.60219297282519,0.929392804026028
"10794","AKR7A2",867.525267489965,-0.13150736446764,0.25068422909680l,
-0.52459368880704,0.599865674998898,0.929392804026028
"10795","UBXN10",7.94285488814051,-0.363639686423563,0.706523738721559
,-0.514688561040514,0.60677066813731,0.929392804026028
"10796","MED18",41.746519912685,0.171796303013337,0.332443175185526,0.
516768927253395,0.605317473416842,0.929392804026028
"10797","LINC01715",9.0073543221028,-0.257738358931396,0.4875252833296
77,-0.52866665123726,0.597036715192886,0.929392804026028
"10798","AGO1",142.881253297549,0.127317441760622,0.239651854441147,0.
531259989861204,0.595238627018921,0.929392804026028
"10799","CYP4X1",84.550077688787,0.144354422474189,0.276218415564652,0
.522609697036659,0.601245891018353,0.929392804026028
"10800","EFCAB7",86.1575498284446,-0.113996695880176,0.219494977171358
,-0.519359018366875,0.603510404125514,0.929392804026028
"10801","GADD45A",750.283958481238,0.222192314967138,0.41963899479149,
0.529484432392994,0.596469440911691,0.929392804026028
"10802","CDC7",10.0523895980815,-0.241325316107109,0.457772071860588,-
0.527173523553449,0.59807309305339,0.929392804026028
"10803","AGL",412.075944277498,-0.107061500515185,0.201586845031057,-0
.531093685695069,0.595353859615868,0.929392804026028
"10804","GSTM4",142.177844392653,0.106602744110081,0.201794366026029,0
.528274134751268,0.5973090811802,0.929392804026028
"10805","AL139420.2",4.4972154160356,-0.482380944406399,0.935053765859
662,-0.515885783276763,0.605934184032734,0.929392804026028
"10806","AC245014.3",10.0611646061282,-0.272956057540142,0.51246107915
5563,-0.532637635603314,0.594284444322979,0.929392804026028
"10807","TARS2",75.6254423389428,-0.109199602711818,0.210976050267827,
-0.517592411902647,0.604742677176858,0.929392804026028
"10808","CREB3L4",47.6376113884756,-0.138334761013344,0.26860556398991
6,-0.515010779964845,0.606545487084829,0.929392804026028
"10809","AL353807.5",60.3371586786204,-0.168626182813185,0.32494978336
3157,-0.518929974557736,0.603809574184413,0.929392804026028
"10810","AL596325.2",21.7341190848435,0.268644821580815,0.514205836226
267,0.522446076365813,0.60135978200307,0.929392804026028
"10811","COP1",165.925170812782,0.0850163139955691,0.16442965665183,0.
517037593623309,0.605129916097073,0.929392804026028
"10812","TPR",1235.87952198112,0.093744505327697,0.177816451629001,0.5
27198155563732,0.598055989384068,0.929392804026028
"10813","KCNT2",77.1298757231885,0.238707449748097,0.456600024354023,0
.522793335558424,0.601118077862387,0.929392804026028
"10814","RNPEP",188.716957219216,-0.0977971637693982,0.185038925357744
,-0.528522112740995,0.597137003440785,0.929392804026028
"10815","ZBED6",9.72007405113091,-0.33493120337683,0.646887026943078,-
0.517758417508506,0.60462683412346,0.929392804026028
"10816","C1orf74",7.25708327419564,0.279842902874103,0.536124002488858
,0.52197421039719,0.601688286952366,0.929392804026028
"10817","TRAF5",99.6973387266712,0.12931090950055 8,0.246333243355485,0

.524942990800266,0.599622822417926,0.929392804026028
"10818","AL591895.1",46.4257649533516,0.184107606763689,0.356967825802014,0.515754063689205,0.606026189580441,0.929392804026028
"10819","WNT9A",21.7889035379586,0.199777503014548,0.377070702026298,0.529814440477571,0.596240592109235,0.929392804026028
"10820","C1orf198",127.654471866264,0.162797677432272,0.310826182420075,0.523757928514062,0.600446918820962,0.929392804026028
"10821","IRF2BP2",1300.32386465146,0.0972395835376699,0.18900442405807,0.514483108119172,0.606914267363788,0.929392804026028
"10822","E2F6",72.8260797507459,-0.151406296712227,0.284629775112888,-0.531941173941402,0.594766739041035,0.929392804026028
"10823","ATAD2B",148.502780791003,0.140611381981096,0.263827447620717,0.532967222512957,0.594056270091274,0.929392804026028
"10824","MPV17",308.68077389909,0.120890027249851,0.235392963072869,0.51356686993412,0.607554847440152,0.929392804026028
"10825","AC007743.1",3.95820558937108,-0.43586714250417,0.831512177524357,-0.524186120523055,0.600149093538322,0.929392804026028
"10826","AC016747.1",88.5768827232727,0.146436967294944,0.278716718415538,0.525397142042343,0.599307139868983,0.929392804026028
"10827","C2orf74",251.447968651815,-0.198831847120319,0.37587592131681,-0.528982666470759,0.596817474253666,0.929392804026028
"10828","LINC02245",5.66738190771956,-0.318751891772975,0.602393774963356,-0.529142074538145,0.59670689610191,0.929392804026028
"10829","MPHOSPH10",396.528757483952,-0.12161837863436,0.232092284568982,-0.524008709984553,0.600272481810698,0.929392804026028
"10830","TTC31",82.7023949649459,-0.12603360543754,0.24539265127526,-0.513599754444834,0.607531851297618,0.929392804026028
"10831","RMND5A",196.192475810417,0.100280403098049,0.189101655826555,0.530298915996943,0.595904698153675,0.929392804026028
"10832","TTL",106.518206020774,0.137048856194941,0.26483460969412,0.517488467059612,0.604815217674698,0.929392804026028
"10833","BIN1",978.129884933585,-0.134562417524967,0.259218101461564,-0.519108876911976,0.603684818385952,0.929392804026028
"10834","DAPL1",4.0582057935546,0.364645272822101,0.69837476584036,0.522134089972911,0.601576972482365,0.929392804026028
"10835","ITGA6",3.98558319722851,0.449374427580166,0.858417020390667,0.523491982225206,0.600631929114647,0.929392804026028
"10836","ANKRD44",65.8559116058727,0.169595846877443,0.326382388002001,0.519623157106144,0.603326254677988,0.929392804026028
"10837","MOB4",407.450540875686,-0.121921006222456,0.228945718162802,-0.532532371432072,0.594357327537656,0.929392804026028
"10838","CYP20A1",303.519006570178,-0.0828710356740152,0.158888759428727,-0.521566383751576,0.601972274024585,0.929392804026028
"10839","AC007563.4",3.96669016947194,0.422353354414109,0.815784629803999,0.517726540785138,0.604649077762423,0.929392804026028
"10840","CYP27A1",404.382418404026,-0.0848585436549586,0.163563117233942,-0.518812218121195,0.60389169679929,0.929392804026028
"10841","STK16",193.393308583392,-0.135996191264682,0.256022855843261,-0.531187697351286,0.595288717439697,0.929392804026028
"10842","PDE6D",92.3536727584156,-0.116827418236062,0.221069556249133,

-0.52846452590878,0.597176962283295,0.929392804026028
"10843","ARL4C",144.239466449671,0.254806996810803,0.496298097919536,0
.51341521935898,0.607660901731643,0.929392804026028
"10844","VGLL4",766.863578710168,-0.0893877507364826,0.168179665990296
,-0.531501535635351,0.595071277444566,0.929392804026028
"10845","CMTM7",93.7611364474103,-0.141824945796868,0.270290139698724,
-0.524713724129752,0.599782215293402,0.929392804026028
"10846","RPSA",10018.6337742759,-0.12791370120676,0.244671319047657,-0
.522798101978781,0.601114760577845,0.929392804026028
"10847","QARS",405.483109831699,-0.0868224657330976,0.163986635192929,
-0.529448425055809,0.596494413132168,0.929392804026028
"10848","USP4",160.93036817658,0.108499180265823,0.205157564891345,0.5
28857808988358,0.596904091875825,0.929392804026028
"10849","UBA3",183.555001659484,-0.100753406083076,0.192385086789539,-
0.523706945088194,0.600482384357085,0.929392804026028
"10850","TMEM45A",324.425018617015,0.23169002592335,0.450380511933767
,0.514431730024339,0.606950179932597,0.929392804026028
"10851","ARHGAP31",30.6475642694263,-0.15587677909107,0.30009083615943
2,-0.519431986281166,0.603459530602201,0.929392804026028
"10852","AC108673.2",2.67227717341854,-0.442233206861776,0.84419245424
5045,-0.523853541497551,0.600380410235471,0.929392804026028
"10853","SELENOT",412.794507443667,-0.112944308677214,0.21238406836550
4,-0.531792754260838,0.594869541713893,0.929392804026028
"10854","MBNL1",1408.99480401278,0.110235240331212,0.214256409643585,0
.514501482194104,0.606901424373155,0.929392804026028
"10855","C3orf33",66.0948357573086,0.156684389485426,0.294756970088609
,0.531571448296282,0.595022844033439,0.929392804026028
"10856","ABCF3",116.065245644451,-0.0981632683313483,0.190551551054567
,-0.515153341907136,0.6064458703202,0.929392804026028
"10857","OPA1",187.084896894132,0.0930141048400486,0.178616341202211,0
.520748013390039,0.602542322392414,0.929392804026028
"10858","NCBP2AS2",311.039673427201,-0.146383210070985,0.2781116274371
36,-0.526346961541812,0.598647159639848,0.929392804026028
"10859","HTRA3",1350.17880918642,-0.171617001292599,0.330981099073607,
-0.518509974656992,0.604102502496638,0.929392804026028
"10860","GNPDA2",225.601920690977,-0.117937888000246,0.222758292802219
,-0.529443310579507,0.596497960220906,0.929392804026028
"10861","PDGFRA",3357.95964266976,0.091460708906025,0.176025706092019,
0.519587229255102,0.60335130099168,0.929392804026028
"10862","REST",415.790358411079,0.0824256622494764,0.157109112321061,0
.524639602577825,0.599833750860156,0.929392804026028
"10863","AC009570.1",9.73335134079812,-0.353709142020269,0.67425864041
6183,-0.524589706113286,0.599868444227604,0.929392804026028
"10864","ABRAXAS1",345.687823217619,-0.116128434175981,0.2182985451659
72,-0.531970719675155,0.594746275200707,0.929392804026028
"10865","HERC3",124.640763195721,-0.106713715761598,0.205733285809744,
-0.518699321510297,0.603970434912638,0.929392804026028
"10866","C4orf33",69.0037185105639,0.128092861551358,0.243649656193906
,0.525725599421394,0.599078874613888,0.929392804026028
"10867","MGARP",2.24063175627671,-0.537628994633792,1.02648271893148,-

0.52375844689664,0.600446558224007,0.929392804026028
"10868","ELMOD2",122.005879811792,-0.100666184155118,0.195777260722149
,-0.51418731564533,0.607121035157919,0.929392804026028
"10869","AC093849.2",57.9342612122264,-0.222361408631236,0.42442491204
8438,-0.523912245296899,0.600339577403874,0.929392804026028
"10870","SELENOP",11688.3095655967,0.121269606570638,0.235525905844951
,0.51488861123613,0.606630859681215,0.929392804026028
"10871","TMED7",469.946137617384,-0.0848271426152405,0.16283805964613,
-0.520929460837238,0.602415911428727,0.929392804026028
"10872","SKP1",3402.92519305614,-0.1010278292231,0.19041038745457,-0
.530579400492226,0.595710273589523,0.929392804026028
"10873","PCDHB7",28.0024491469355,-0.212332786656127,0.405287634759303
,-0.523906402380694,0.600343641527704,0.929392804026028
"10874","AC034213.1",2.05119266482588,0.557358633753244,1.074696476627
65,0.518619578527151,0.604026053285108,0.929392804026028
"10875","HIST1H3D",5.44140819187553,-0.422170286893203,0.8189867814254
66,-0.515478755540358,0.60621851128524,0.929392804026028
"10876","APOM",42.7888605949886,-0.198227913143551,0.374785925055394,-
0.528909705225063,0.596868089104741,0.929392804026028
"10877","LY6G5B",31.2553343343387,-0.189131077635882,0.36419276105852,
-0.519315861979727,0.603540493839564,0.929392804026028
"10878","RNGTT",79.2654892966464,0.131906630687131,0.252855301076763,0
.521668440904415,0.601901201611285,0.929392804026028
"10879","NKAIN2",2.06715547334761,0.568235929135113,1.07232159545156,0
.529911858108041,0.596173044114803,0.929392804026028
"10880","ARHGAP18",326.402058859184,-0.113659539788801,0.2203111101190
08,-0.515904711874969,0.605920963004565,0.929392804026028
"10881","TBPL1",160.92234601862,-0.147789025067466,0.283840122347281,-
0.520677005933096,0.60259179518072,0.929392804026028
"10882","AIG1",434.722549742264,-0.107117534698339,0.202259465612232,-
0.529604557067812,0.596386134116461,0.929392804026028
"10883","RAET1E-
AS1",7.75214844133093,-0.28467663070886,0.549618899456634,-0.517952768
709916,0.604491223423927,0.929392804026028
"10884","SYTL3",9.743517199941,0.329593856134508,0.618972635227848,0.5
32485343254605,0.59438989040981,0.929392804026028
"10885","AL139393.3",52.3911012604453,0.137760984659595,0.264142083091
75,0.521541221478684,0.601989797566481,0.929392804026028
"10886","ADAP1",32.1666286866704,-0.350688952684767,0.656440488484085,
-0.534228096585893,0.593183732413398,0.929392804026028
"10887","TTYH3",79.7747848812633,-0.161977211171942,0.31451983644441,-
0.514998395659444,0.606554141103178,0.929392804026028
"10888","AC004982.2",17.9213273851891,-0.209318435090598,0.40287523107
7137,-0.5195614397315,0.603369279880933,0.929392804026028
"10889","ETV1",111.541883148572,0.172627674995489,0.328209309217985,0.
525968246929997,0.598910269232924,0.929392804026028
"10890","LINC01176",11.3607400536015,-0.316170000986202,0.594644850948
398,-0.531695516209285,0.594936897906557,0.929392804026028
"10891","NT5C3A",301.491905923994,-0.105377741828369,0.201089960188451
,-0.524032834506579,0.600255702634642,0.929392804026028

"10892","INHBA",14.1385580636162,0.505131766274669,0.949047637393087,0
.532251223618449,0.594552009777492,0.929392804026028
"10893","TMED4",624.844884086018,0.0747863196440772,0.140427882853233,
0.532560330075187,0.594337969025525,0.929392804026028
"10894","PPIA",4728.12982214366,-0.134153559348217,0.25629901300806,-0
.523425969432031,0.600677856086051,0.929392804026028
"10895","H2AFV",837.037121882842,0.110344214157388,0.207638945764511,0
.531423494523675,0.59512534413028,0.929392804026028
"10896","ADAM22",112.906590782609,0.188173751504574,0.363439005899781,
0.517758821837808,0.604626551984053,0.929392804026028
"10897","DLX5",5.25694565380084,0.345962226611101,0.650788837656634,0.
531604426186602,0.594999998557083,0.929392804026028
"10898","AC093799.1",4.62930633926948,0.423678371083564,0.811483198916
775,0.522103688220681,0.601598138666624,0.929392804026028
"10899","PMPCB",402.418786685242,-0.11223202781658,0.218410457931082,-
0.51385830550291,0.607351060042628,0.929392804026028
"10900","GIMAP1",8.69199356857346,-0.256023242353422,0.480120269347016
,-0.533248143640394,0.593861818996871,0.929392804026028
"10901","LMBR1",91.9225812703266,0.103480262458945,0.198586139545975,0
.521085019808182,0.602307545983363,0.929392804026028
"10902","MSR1",64.7280209341865,-0.175022829534011,0.328099233965904,-
0.533444797838813,0.593725714048733,0.929392804026028
"10903","ASAH1",976.504016683757,0.112506533138223,0.216978833913795,0
.518513861969234,0.604099791003867,0.929392804026028
"10904","TCIM",32.2376406480299,0.369651527777001,0.703039150382079,0.
525790814887204,0.599033557060587,0.929392804026028
"10905","UBE2V2",493.015775968127,-0.101795302651304,0.195677231183626
,-0.520220477546405,0.602909914355512,0.929392804026028
"10906","AF117829.1",169.615996733305,0.172441644523315,0.323743082414
927,0.532649665398269,0.594276115347112,0.929392804026028
"10907","NBN",446.562910682977,-0.0852238706026498,0.161560774724749,-
0.527503478166935,0.597844001709201,0.929392804026028
"10908","TSPYL5",109.188638828574,-0.101521950075664,0.196720022443465
,-0.516073294495687,0.605803219062862,0.929392804026028
"10909","AC103853.2",2.05114061053365,0.556137044355157,1.051107263975
49,0.5290963761888,0.596738595168896,0.929392804026028
"10910","C8orf76",150.511820875904,-0.133066379697786,0.25380773748726
2,-0.524280232805999,0.600083643504824,0.929392804026028
"10911","TRMT12",48.6985664408983,-0.151171226397972,0.287698246619671
,-0.525450635080916,0.599269961577032,0.929392804026028
"10912","PRNCR1",6.35879707600311,0.455480854271953,0.882488096682182,
0.516132575594376,0.605761817524354,0.929392804026028
"10913","SLA",26.6804467466659,0.253970323948615,0.492620485904875,0.5
15549659860586,0.606168977123692,0.929392804026028
"10914","MFSD3",38.1107828708887,-0.158040680916928,0.302484976187673,
-0.522474480910659,0.601340009832186,0.929392804026028
"10915","NFX1",206.565702091786,0.110882379538313,0.214441686857209,0.
517074740286607,0.605103985877757,0.929392804026028
"10916","FXN",101.530914988143,-0.140690676900509,0.270076888146643,-0
.520928235903393,0.602416764776639,0.929392804026028

"10917","MFSD14C",146.163553679169,0.103709076519835,0.196460334016365
,0.527888120719555,0.597576990221438,0.929392804026028
"10918","STOM",1331.9669838254,0.120263643565277,0.227682726272906,0.5
28207148315354,0.597355568505879,0.929392804026028
"10919","SEC61A2",24.8606124803109,-0.188738971383071,0.35774668817421
2,-0.527577131031778,0.597792869107035,0.929392804026028
"10920","CDC123",363.057861040155,-0.132731838050122,0.25246877410222,
-0.525735661854092,0.599071882232007,0.929392804026028
"10921","TMEM236",2.27094588067334,0.544898460254901,1.06128716411723,
0.513431688121984,0.607649384178161,0.929392804026028
"10922","ITGB1-
DT",9.16767097390102,-0.284182981670483,0.541585379153873,-0.524724249
599327,0.599774897259982,0.929392804026028
"10923","NRP1",1187.51165632983,0.131944647287556,0.249334232737807,0.
52918785294238,0.596675142272655,0.929392804026028
"10924","ZNF248",88.7608048185,0.10476567580536,0.198196766118968,0.52
8594274552766,0.597086932903934,0.929392804026028
"10925","CSTF2T",184.787856107366,-0.10652617103417,0.207373139346766,
-0.513693197536249,0.607466508651765,0.929392804026028
"10926","AL157893.2",12.348111583371,-0.256302740175486,0.488431371833
635,-0.524746678767362,0.599759303088057,0.929392804026028
"10927","AL355334.2",2.63470081918948,0.494546241509277,0.929228491488
255,0.53221166380425,0.594579405511347,0.929392804026028
"10928","LIPA",380.501288183666,-0.095259102738345,0.185299906539956,-
0.51408068421127,0.607195581435562,0.929392804026028
"10929","HELLS",36.711740428552,-0.211344345428056,0.403097878489812,-
0.524300316885438,0.60006967652535,0.929392804026028
"10930","AL138762.1",21.1628412623354,0.216174059475595,0.406987559420
452,0.531156430883112,0.595310382113213,0.929392804026028
"10931","ZNF143",125.124937559377,0.0956016950310312,0.18178971854033,
0.525891649971514,0.598963490861256,0.929392804026028
"10932","CHST1",4.10370389704676,0.434078960982397,0.836430033186318,0
.518966253912244,0.603784274196757,0.929392804026028
"10933","ZDHHC5",150.429249588134,0.0865820817903503,0.164906502231525
,0.525037403733124,0.599557189369383,0.929392804026028
"10934","UQCC3",374.887554150108,-0.135758094909222,0.263268334287318,
-0.515664351646112,0.606088856623062,0.929392804026028
"10935","COX8A",1416.75615382499,-0.121532267413964,0.231838655093968,
-0.524210543598542,0.600132108292001,0.929392804026028
"10936","MACROD1",194.20432796998,-0.125573012730999,0.243310879764883
,-0.516101100174161,0.605783799597908,0.929392804026028
"10937","EFEMP2",1553.38826178847,0.152630336831767,0.29728963852478,0
.513406177185167,0.607667225485435,0.929392804026028
"10938","KLC2",45.9578903647448,-0.14422788047969,0.276647822538047,-0
.521341101319727,0.602129173687403,0.929392804026028
"10939","RAB1B",462.583135245197,-0.103963541145359,0.200613582424748,
-0.518227828289525,0.604299320892622,0.929392804026028
"10940","INPPL1",119.744201123765,0.109511113711031,0.206051348202806,
0.53147487102702,0.595089750365886,0.929392804026028
"10941","AAMDC",818.398342331927,-0.121716148432359,0.235421899122058,

-0.517012856009854,0.605147184458416,0.929392804026028
"10942","TAF1D",804.898643459121,0.0966817438311891,0.182354916144857,0.530184465958616,0.595984040255239,0.929392804026028
"10943","NLRX1",51.2472302905971,0.124697054864448,0.233520711695942,0.533987130986527,0.593350437675599,0.929392804026028
"10944","RNF26",100.997631369294,0.109496948704583,0.211358883371454,0.518061729689154,0.604415200667558,0.929392804026028
"10945","USP2-AS1",3.46983072335888,-0.418838511010959,0.807074268988531,-0.518959068706118,0.603789284877251,0.929392804026028
"10946","ADIPOR2",150.281380775829,0.103591919370066,0.197075598751016,0.52564558994918,0.599134474491791,0.929392804026028
"10947","EMG1",255.166913507997,-0.118595100808611,0.226764858459246,-0.522987122495104,0.600983214674515,0.929392804026028
"10948","FGFR1OP2",636.751847877535,-0.0885729997221068,0.167194776384345,-0.529759371898657,0.596278777418297,0.929392804026028
"10949","CCDC91",232.547250724089,0.0963515180122804,0.180691567844899,0.533237489504691,0.593869193163101,0.929392804026028
"10950","PRKAG1",286.897555951747,-0.138197071754734,0.261305831897643,-0.528870981375064,0.596894953500682,0.929392804026028
"10951","AC121761.2",13.5334153783734,0.280349475068619,0.53782849534898,0.521261847397485,0.602184375066528,0.929392804026028
"10952","ACTR6",180.785091303424,-0.120388430397919,0.23005039687207,-0.52331329149963,0.600756252983676,0.929392804026028
"10953","AC025265.1",2.0584979566096,-0.659934931640507,1.2524265435673,-0.526925060020534,0.598245630492881,0.929392804026028
"10954","HSP90B1",3105.87752625973,0.10828288702381,0.207353461469177,0.522214031328849,0.601521317640064,0.929392804026028
"10955","ERP29",1427.06146710886,-0.136017327582568,0.260363450138694,-0.522413293840253,0.601382602015189,0.929392804026028
"10956","OAS1",141.356045559807,-0.192669339137798,0.363781087704167,-0.529629894598808,0.596368563145212,0.929392804026028
"10957","SACS",185.93793720307,0.12164288246112,0.233571046394278,0.520796067573295,0.602508842799706,0.929392804026028
"10958","MEDAG",1717.33509809821,0.132071355798532,0.255086928595024,0.517750386215236,0.604632438340778,0.929392804026028
"10959","WBP4",456.530243123554,0.0780058497325672,0.149897706481069,0.520393884361525,0.602789071712095,0.929392804026028
"10960","UCHL3",223.884847910267,-0.149186259510802,0.279658484264975,-0.533458728788107,0.593716072939301,0.929392804026028
"10961","FBXL3",514.690320296729,0.0900116676058277,0.174695661426695,0.515248443325526,0.606379421199581,0.929392804026028
"10962","GGACT",36.7010117792084,0.211922365428732,0.39811091075103,0.532319913134115,0.594504442681514,0.929392804026028
"10963","BIVM",132.58796894402,0.114360211576674,0.222052426322566,0.515014465145035,0.606542911931521,0.929392804026028
"10964","TXNDC16",61.2475781014927,0.164346495144878,0.309943892342794,0.530245954848862,0.595941412691735,0.929392804026028
"10965","GNPNAT1",160.661139339454,-0.0866965875492732,0.166670284758459,-0.520168233197149,0.602946324203981,0.929392804026028

"10966","KTN1",1355.80145910511,-0.10764351330945,0.205282000275285,-0
.524368981036327,0.600021926840015,0.929392804026028
"10967","ERH",1466.76189860521,-0.137904720571731,0.259384567664119,-0
.53166123880703,0.594960642479802,0.929392804026028
"10968","NDUFB1",1260.80859884114,-0.140471839353806,0.273346879794809
,-0.513895894693414,0.607324777883922,0.929392804026028
"10969","WARS",116.383728843122,0.133991033352978,0.256188926702263,0.
52301649051639,0.600962777621584,0.929392804026028
"10970","CDC42BPB",355.846467314481,0.122029102720064,0.23756337617040
9,0.513669677065585,0.60748295569094 4,0.929392804026028
"10971","CEP170B",58.6211839031121,-0.127123725174538,0.24732347708146
1,-0.513997808354713,0.6072535229574,0.929392804026028
"10972","SRP14",6509.46467461461,-0.115472586197699,0.218629933032739,
-0.5281645774479,0.597385112741452,0.929392804026028
"10973","AC090510.1",8.65743077872784,0.386639650247343,0.742056468974
485,0.521038042807814,0.602340270162102,0.929392804026028
"10974","HYPK",31.2094364509411,-0.195928480975559,0.370070781484746,-
0.5294351534306,0.596503617541611,0.929392804026028
"10975","AC090527.3",3.6439368969096,-0.587760385836824,1.107697930047
82,-0.530614321732505,0.595686069118216,0.929392804026028
"10976","BLOC1S6",741.018881822816,0.0812286293837111,0.15325898446108
5,0.530008923583443,0.596105743767707,0.929392804026028
"10977","DUT",1374.85695767964,-0.117331509276164,0.224333018844387,-0
.523023805771334,0.600957687022633,0.929392804026028
"10978","BNIP2",453.521321343388,0.0757531116751801,0.143490077219233,
0.527932754258955,0.597546009983566,0.929392804026028
"10979","DPP8",183.734136806867,0.0941501006804645,0.17878241002352,0.
526618366247992,0.598458635184498,0.929392804026028
"10980","AC116913.1",10.9737560330626,-0.250835425526234,0.48810010942
4476,-0.51390159658435,0.607320791196963,0.929392804026028
"10981","UACA",831.551970416582,0.129113292433615,0.245792455586721,0.
525293960408239,0.599378855271685,0.929392804026028
"10982","ARIH1",355.383342115696,0.149865719891834,0.287487187294862,0
.521295301199367,0.602161073783839,0.929392804026028
"10983","LOXL1-
AS1",17.3699324406377,-0.23040408600756,0.441904708762376,-0.521388619
399063,0.602096077825891,0.929392804026028
"10984","CPEB1-
AS1",3.72824000809712,-0.447244349841375,0.854664903549109,-0.52329790
0714226,0.600766961648965,0.929392804026028
"10985","AC090825.1",22.6600068201293,-0.221869231207488,0.42417334795
335,-0.52306264002209,0.600930663055348,0.929392804026028
"10986","AL031714.1",2.21067100309089,0.562830568528222,1.090970409201
33,0.515899023274382,0.605924936298885,0.929392804026028
"10987","TELO2",111.777941067386,-0.126307559618393,0.243544616548068,
-0.518621850109602,0.604024468891696,0.929392804026028
"10988","MRPS34",583.668825193048,-0.122044670677627,0.23653780658915,
-0.515962638013342,0.605880504228069,0.929392804026028
"10989","PKD1",364.00511702151,0.114239331635895,0.22079052748227,0.51
7410474709198,0.604869649141471,0.929392804026028

"10990","MLST8",123.415598406899,-0.137448842618288,0.267680389606834,-0.513481181121153,0.607614771462445,0.929392804026028
"10991","SRRM2",2853.31384924818,0.107116485005233,0.20377344964796,0.525664580887686,0.59912127716943,0.929392804026028
"10992","MOSMO",333.434105319821,0.0810372464149099,0.156144881576477,0.518987530021722,0.603769437183379,0.929392804026028
"10993","LINC02175",22.6999403062938,0.238660503908311,0.455834729609476,0.523568057468497,0.600579003379877,0.929392804026028
"10994","AC145285.6",3.13777821114574,-0.433922800631591,0.833131843239485,-0.520833292056584,0.602482908889682,0.929392804026028
"10995","RIPOR1",158.193171014512,0.102239206444133,0.197875059151813,0.516685664592489,0.605375604778059,0.929392804026028
"10996","LDHD",30.5258198056532,0.183751400975441,0.357283942534129,0.514300753826598,0.607041734754694,0.929392804026028
"10997","AC092139.1",3.63068393717267,0.449554530412694,0.872276688844547,0.515380654053925,0.606287048527178,0.929392804026028
"10998","MLYCD",135.513189405961,-0.105567883396218,0.204820721897806,-0.515416030263239,0.606262333030007,0.929392804026028
"10999","AC105429.1",20.2014493223134,-0.302997651570648,0.588346528529387,-0.514998622203162,0.606553982796388,0.929392804026028
"11000","SPIRE2",18.979151366968,-0.216784569437393,0.412251194799753,-0.525855527338603,0.598988590582769,0.929392804026028
"11001","PLSCR3",205.711638885402,-0.118937279163841,0.223873601812554,-0.531269779915478,0.595231843781669,0.929392804026028
"11002","CCDC144A",123.645432453986,-0.379157514730813,0.738304015570482,-0.513552014799541,0.607565235765006,0.929392804026028
"11003","AKAP10",107.040383134946,0.105791211696994,0.205813055828563,0.514016038832425,0.607240777156431,0.929392804026028
"11004","SUZ12",292.689893124199,0.0766960598475077,0.145624542046364,0.526669878371801,0.598422856624716,0.929392804026028
"11005","AP2B1",722.678037512964,0.0960432188081295,0.186350243182765,0.515390896023323,0.606279892955392,0.929392804026028
"11006","TBC1D3L",3.1457993059533,-0.528005048294921,0.994424711225876,-0.530965333357437,0.595442802249588,0.929392804026028
"11007","GPATCH8",429.992264655814,0.120679713977214,0.233557716735095,0.516701891353438,0.605364275569636,0.929392804026028
"11008","MRPL27",598.379371860149,-0.125294042002236,0.239539137904491,-0.523062924490415,0.600930465101637,0.929392804026028
"11009","CD79B",4.16317065199836,-0.413111816392694,0.802886397726981,-0.514533335677673,0.606879159921385,0.929392804026028
"11010","LINC01152",2.81843004174463,-0.444659902922538,0.833943879439124,-0.533201230784975,0.593894289630636,0.929392804026028
"11011","NT5C",694.681675967797,-0.133649266369858,0.255207650894256,-0.52368832165315 9,0.600495339589804,0.929392804026028
"11012","PRPSAP1",237.458679485899,-0.114621699294283,0.221482863920813,-0.517519492321825,0.604793565514113,0.929392804026028
"11013","MYL12B",4242.3126993471,-0.143459429261574,0.279062554189749,-0.514076242432829,0.607198686781617,0.929392804026028
"11014","ZNF519",95.2417390237349,0.127930987808095,0.249117143869549,0.513537470046968,0.607575407113807,0.929392804026028

"11015","LAMA3",7.2823049804271,0.35916914705323,0.697831524262996,0.5
14693209700667,0.606767419179301,0.929392804026028
"11016","CIRBP",5172.96675067837,-0.110041143579425,0.210529429473228,
-0.522687701452295,0.601191598129851,0.929392804026028
"11017","IZUMO4",20.093839842479,0.199628529692528,0.374643771670713,0
.532848921529619,0.594138165687847,0.929392804026028
"11018","YJU2",118.242057715747,-0.124292720553951,0.241094571029456,-
0.515535128075388,0.606179128964002,0.929392804026028
"11019","ADAMTS10",87.1156356548048,-0.141198437482636,0.2658787371574
74,-0.531063292206803,0.59537492044446,0.929392804026028
"11020","ZNF627",18.4200786006767,0.188704693867873,0.364655963132114,
0.5174869272589,0.60481629229231,0.929392804026028
"11021","PRKACA",347.511411818753,0.0919192652030437,0.17569374043087,
0.523178941820133,0.600849734277197,0.929392804026028
"11022","NDUFB7",1780.84538616301,-0.133799924542483,0.257080366134254
,-0.520459522267095,0.60274333322882,0.929392804026028
"11023","EPHX3",3.45100942052068,0.463872403558932,0.881083779508393,0
.526479336411973,0.598555205399279,0.929392804026028
"11024","HAUS8",39.691101865794,0.153018331798943,0.292674386432252,0.
52282788960203,0.601094029479403,0.929392804026028
"11025","SCGB1B2P",5.24302133934159,0.375503869325898,0.70802972736881
2,0.530350428535466,0.595868988832117,0.929392804026028
"11026","ETV2",21.4483093430195,-0.232287167991633,0.446223460008032,-
0.520562428491438,0.602671628195881,0.929392804026028
"11027","ARHGEF1",169.069542519826,-0.081288662428575,0.15454309392588
,-0.525993497111956,0.598892725196795,0.929392804026028
"11028","MYPOP",56.8039764144357,-0.132036345466942,0.249187122424962,
-0.529868253953218,0.596203278182301,0.929392804026028
"11029","AC008622.2",7.17617149025119,-0.28824023699361,0.541436575856
502,-0.532361960470883,0.594475325999927,0.929392804026028
"11030","BCAT2",101.58376908043,-0.109483369702327,0.205770110368843,-
0.532066438153129,0.594679981284922,0.929392804026028
"11031","C19orf73",2.61842928887555,-0.574822011099765,1.0902466102719
5,-0.527240356157934,0.598026687175909,0.929392804026028
"11032","NKG7",74.8554968680799,0.18345491772915,0.347901260929682,0.5
27318921578241,0.597972136593119,0.929392804026028
"11033","ZNF600",33.7763154284125,-0.187119591593003,0.352090364143657
,-0.531453316105681,0.595104683548608,0.929392804026028
"11034","ZNF813",33.5376262268485,-0.171616277054455,0.326821390348924
,-0.525107236314101,0.599508645939749,0.929392804026028
"11035","SLC27A5",73.7152641533392,0.152124242398594,0.293230045135242
,0.518788046867544,0.603908554303646,0.929392804026028
"11036","ESF1",469.161165696549,-0.0999037112987313,0.187517842915915,
-0.532769094104444,0.594193430314825,0.929392804026028
"11037","RBBP9",85.3304054650648,0.0999630908247624,0.192176916743313,
0.520161799443797,0.602950808048895,0.929392804026028
"11038","STAU1",749.084731089027,-0.0685569902350409,0.130205726341782
,-0.526528226992744,0.59852124516409,0.929392804026028
"11039","NKILA",6.6620950010813,0.294201762878267,0.557565104048289,0.
527654547858482,0.597739125562055,0.929392804026028

"11040","MRGBP",115.352084748525,0.114602152507519,0.218799208649082,0
.523777728516936,0.600433145625285,0.929392804026028
"11041","AP001442.1",2.86468316170836,0.502816158149262,0.961353946070
521,0.523029171726494,0.600953952930611,0.929392804026028
"11042","HLCS",53.2080007287109,0.192538281477657,0.360590580993262,0.
533952608931998,0.593374322543539,0.929392804026028
"11043","TUBA8",8.43010847181659,-0.328359517516348,0.623790156332965,
-0.526394195520259,0.598614347808234,0.929392804026028
"11044","C22orf39",282.922673059972,-0.0978091012352523,0.184832348052
482,-0.529177399226026,0.596682393342831,0.929392804026028
"11045","TBX1",36.9775844097661,-0.578135872103091,1.12473479009613,-0
.514019729089804,0.607238197135115,0.929392804026028
"11046","ASPHD2",7.00280126673059,0.349199640240205,0.668533657621777,
0.522336663620554,0.601435946060179,0.929392804026028
"11047","EP300",429.200633575279,0.118223862611205,0.222982587738268,0
.530193248766012,0.595977951433182,0.929392804026028
"11048","RPS6KA3",354.882402721203,0.112033444021228,0.211839128417118
,0.52886095622821,0.596901908464776,0.929392804026028
"11049","LINC01560",25.6149088137435,-0.189108160568785,0.366804841550
339,-0.515555246679677,0.60616507421704,0.929392804026028
"11050","OTUD5",208.061822969581,0.0881227756354715,0.165784956096993,
0.53154868638331,0.595038612611825,0.929392804026028
"11051","EDA",60.2319911597298,-0.179135144126259,0.346694613353904,-0
.516694339128364,0.605369548374261,0.929392804026028
"11052","IL2RG",24.8185067750897,0.238631757879581,0.456361457332605,0
.522900770968618,0.601043308070021,0.929392804026028
"11053","RBMX",1480.39954859428,0.0806506396428996,0.156511113349559,0
.51530295783387,0.606341332372073,0.929392804026028
"11054","CETN2",368.922832747378,0.117511545649883,0.228252785267132,0
.514830719425198,0.606671316870068,0.929392804026028
"11055","AC244090.1",72.5808841537032,-0.154484078341671,0.29283571541
3318,-0.527545207809187,0.597815031170616,0.929392804026028
"11056","ALDOC",22.1721926764211,-0.170608763584884,0.332405005957712,
-0.51325569870205,0.607772468744685,0.929469690804553
"11057","LTBR",481.437397365275,-0.0778474643077679,0.151712078620908,
-0.513126344424363,0.607862944427213,0.929523981584093
"11058","PPIE",334.476170374652,-0.114720687052049,0.224588394846734,-
0.510804163012688,0.609488193823449,0.92957218713389
"11059","CYP4Z1",7.31140027193962,0.344430212915902,0.674785255119142,
0.510429370385529,0.609750684819473,0.92957218713389
"11060","GTF2B",484.793281566867,0.154106512610724,0.300473383914198,0
.512879079681581,0.608035908231693,0.92957218713389
"11061","F3",1842.24696744004,0.186550783603889,0.365660172326965,0.51
0175287663211,0.609928663594948,0.92957218713389
"11062","AC009961.4",5.99958662848702,-0.294849764845934,0.57674290643
4661,-0.511232581374345,0.609188206879958,0.92957218713389
"11063","KCNE4",423.145090151595,-0.192794299178685,0.377743100954155,
-0.510384699791205,0.609781973813795,0.92957218713389
"11064","RUBCN",55.5479254015109,0.131512264767691,0.25762743218764,0.
510474616972876,0.609718993104864,0.92957218713389

"11065","MRPS17",221.27499093322,-0.13960489324325,0.272838807083284,-0.511675354161169,0.608878237721818,0.92957218713389
"11066","TBC1D13",115.12485660891,0.0987910591702116,0.193089412031114,0.511633745895362,0.608907363170083,0.92957218713389
"11067","PBLD",39.8591387963979,-0.143153351745828,0.28039967324286,-0.510533233117715,0.609677938143653,0.92957218713389
"11068","C10orf55",7.08555255223884,-0.378866380675629,0.741162687902651,-0.51117843202246,0.609226119692499,0.92957218713389
"11069","PANK1",7.04568021574716,-0.318442761204172,0.621814922998428,-0.512118235549289,0.608568262779085,0.92957218713389
"11070","AC012470.1",7.96477898737447,0.308279450433614,0.603217629618134,0.511058422859374,0.609310148166902,0.92957218713389
"11071","AKIP1",146.170272028299,-0.121378148318258,0.236934681679731,-0.512285273974059,0.608451369987662,0.92957218713389
"11072","PPP2R5B",40.7045962843131,-0.14796305511211,0.290021596829335,-0.510179437427136,0.609925756600755,0.92957218713389
"11073","LIPT2",12.9542239541422,-0.222103104769581,0.435235807756414,-0.510305220322967,0.609837646039692,0.92957218713389
"11074","CCDC89",40.0524495598631,-0.165036927755251,0.32289473750199,-0.511116808629418,0.609269266751515,0.92957218713389
"11075","CCDC153",5.66977138450983,-0.347714713557701,0.679344410873545,-0.511838631469106,0.608763950923066,0.92957218713389
"11076","AC008014.1",21.7708746558767,0.225140290543233,0.440400125510122,0.51121758941928,0.609198703432056,0.92957218713389
"11077","DCTN2",875.994889710583,-0.0972909344455569,0.189917472747154,-0.512280060587604,0.608455018142506,0.92957218713389
"11078","PGAM5",46.112775333499,-0.161866075865715,0.316451729374727,-0.511503211518371,0.608998740197886,0.92957218713389
"11079","DNAAF2",92.7200508589162,-0.119018570371675,0.232054341557261,-0.512890944306276,0.608027608324047,0.92957218713389
"11080","MAX",403.900436362982,0.0739612634706127,0.14435191629117,0.512367728610035,0.60839367226522,0.92957218713389
"11081","AL118558.3",24.5633218256925,-0.182962163201198,0.35809693983951,-0.510929144725998,0.609400672390537,0.92957218713389
"11082","CAPN3",27.7281560108925,0.219095327273742,0.427380224290906,0.512647321567715,0.608198044932389,0.92957218713389
"11083","FLII",337.677905813786,-0.0919064197959698,0.180082654877188,-0.510356868398279,0.609801468347515,0.92957218713389
"11084","H3F3B",15329.1904202631,-0.12240237950943,0.239189890908134,-0.511737260486653,0.60883490494418,0.92957218713389
"11085","IMPACT",170.019886872045,0.110068741323923,0.215543781991218,0.510656073244586,0.609591904456955,0.92957218713389
"11086","ACAA2",838.133131912005,-0.112547148739643,0.219461523326301,-0.512833170178563,0.608068024736868,0.92957218713389
"11087","DMAC2",139.229414444183,-0.108422806457091,0.211952794789018,-0.511542235453972,0.608971421917475,0.92957218713389
"11088","TRAPPC2B",134.008649724948,-0.17597233699542,0.343513660137007,-0.512271730111796,0.608460847553268,0.92957218713389
"11089","NXT1",265.121868965656,-0.146942969919493,0.286974970452592,-0.5120410664656,0.608622268758401,0.92957218713389

"11090","RBM39",2318.06825633417,0.0844474508834587,0.165182152291052,
0.511238349374826,0.609184168460953,0.92957218713389
"11091","CU638689.4",27.9028096539483,-0.178083904844049,0.34773278453
2308,-0.512128602091882,0.608561008024845,0.92957218713389
"11092","CHAF1B",9.29922006596935,-0.27464990487162,0.538070500968748,
-0.510434793167694,0.609746886543811,0.92957218713389
"11093","ICOSLG",38.1205480890455,0.219437646958056,0.430096586282939,
0.510205507219949,0.609907494320425,0.92957218713389
"11094","AP001505.1",17.1407809003522,0.220898380356044,0.431373130784
464,0.512081918394718,0.60859367869555,0.92957218713389
"11095","TTLL7",50.0311503974468,-0.157143751629278,0.308394759161662,
-0.509553897921146,0.610364029210242,0.929732883413222
"11096","AC079807.1",10.5774224582042,-0.267136226916736,0.52418196839
0575,-0.509624983356332,0.610314217484433,0.929732883413222
"11097","BRD8",198.435035450144,0.119242521543009,0.234000456987838,0.
509582430213829,0.610344035548451,0.929732883413222
"11098","PHF11",348.244376099362,-0.0898893131457139,0.176318303815101
,-0.509812714849946,0.610182676994799,0.929732883413222
"11099","AC010809.2",7.7534771692702,0.273061714163651,0.5355028038446
98,0.509916497548053,0.610109963502165,0.929732883413222
"11100","RPS2",31436.1941119004,-0.127507035922936,0.250138994739484,-
0.509744736344419,0.610230307005176,0.929732883413222
"11101","CTSS",314.340057667917,-0.107696619795018,0.211606918026469,-
0.5089465921031З,0.610789660397188,0.930213617185701
"11102","CARD8-
AS1",19.5023483878367,-0.209112930190522,0.410820759168374,-0.50901256
9408202,0.610743413731841,0.930213617185701
"11103","PIGG",112.768116134871,0.10399818380798,0.204415441159473,0.5
08758943150711,0.610921201059068,0.930282441432461
"11104","RUNDC3B",3.7355542482351,-0.406559230105772,0.799384785886603
,-0.508590152431853,0.611039532954124,0.930282441432461
"11105","ELAVL1",357.856927526077,-0.0765053250781905,0.15040220517862
3,-0.508671564937029,0.610982456888696,0.930282441432461
"11106","ZNF675",69.6228270695702,0.125940981493783,0.24770121391065,0
.508439096867778,0.611145440110604,0.930282441432461
"11107","AL021707.6",10.1894380589733,-0.322366295343521,0.63404485734
8339,-0.508428215460496,0.611153069530667,0.930282441432461
"11108","NEXMIF",10.7213625753181,0.237928283276396,0.467983917690518,
0.50841123868224,0.611164972760337,0.930282441432461
"11109","TTC7A",145.374883668398,0.122308930874329,0.24062539654861,0.
508296017912723,0.611245762256901,0.930321662457438
"11110","ANKS1B",30.7884020476087,-0.186024953933196,0.366057585993057
,-0.508184944258264,0.611323648394718,0.930356457880999
"11111","TBCE",34.9050928765743,-0.16232274207301,0.319594687087291,-0
.507901878946677,0.611522156959131,0.930440146085922
"11112","ZFP3",16.6855170614256,-0.224679967122823,0.442399097371457,-
0.50786714633409,0.611546516275958,0.930440146085922
"11113","ZNF337",68.4743387657006,0.134677854596753,0.265203505347158,
0.507828335151364,0.611573736559214,0.930440146085922
"11114","LINC01422",7.76480722788061,0.37120618917296,0.73101920830552

5,0.507792661198879,0.611598757014368,0.930440146085922
"11115","TK2",458.065977368349,0.0932981789185602,0.183779427667238,0.507663888732374,0.611689077262554,0.930461438933259
"11116","C17orf80",75.8241231168506,0.118987353615363,0.23440435849602,0.507615789991309,0.611722814950444,0.930461438933259
"11117","PRPF4B",793.842485410132,0.0898301380603961,0.177035933497788,0.507411892521348,0.611865843000578,0.930539616982325
"11118","ZFHX4-AS1",40.6377065310181,-0.193829449985267,0.382016059742045,-0.507385606029627,0.611884283274751,0.930539616982325
"11119","ZNF396",26.9860336110529,0.156962115746888,0.309437538062689,0.507249756217646,0.611979587389245,0.930600851117668
"11120","IGIP",75.4492601183203,0.130514527240092,0.257385045612951,0.507078905572302,0.612099455321598,0.930615735147701
"11121","BYSL",55.1734021705529,-0.149862507309803,0.295528851618793,-0.507099413437687,0.612085066560402,0.930615735147701
"11122","NHLRC4",27.9302070491713,0.160819032547525,0.317228781277967,0.506949690692189,0.612190118713065,0.93066989095491
"11123","CCDC17",17.1915726377039,0.202618726302146,0.400353635521117,0.506099378961325,0.612786886515573,0.93069299070208
"11124","CLCC1",193.411234145511,-0.0845186719052885,0.167011392672432,-0.506065308197623,0.612810803494574,0.93069299070208
"11125","LGALS8-AS1",5.08152554641955,-0.383761467427342,0.758055416773872,-0.506244608158796,0.612684943216237,0.93069299070208
"11126","MAN1A1",1326.30069035793,-0.0904134141808058,0.178570661022157,-0.506317295703951,0.612633923176194,0.93069299070208
"11127","PRKAR1B",14.9640983974301,-0.228197366512903,0.450665663888327,-0.506356229902284,0.612606595685311,0.93069299070208
"11128","POLB",48.9782333975254,0.126215291316559,0.249266847656866,0.506346080527741,0.612613719369141,0.93069299070208
"11129","TPRN",47.3488033535839,-0.126028653333484,0.248793465538435,-0.506559338528989,0.612464044683186,0.93069299070208
"11130","PGF",19.5034515109637,-0.18490313610216,0.365124389636892,-0.506411352816069,0.61256790643224,0.93069299070208
"11131","PPP4C",435.657802777469,-0.139015230614963,0.274296423419717,-0.50680657400424,0.612290543215271,0.93069299070208
"11132","TCAP",2.39818534217125,-0.540939115910553,1.06765155855278,-0.506662601273965,0.612391575749303,0.93069299070208
"11133","RTEL1",19.8411791527774,-0.184129180862834,0.363773196469236,-0.506164782479804,0.612740975739513,0.93069299070208
"11134","MAP3K6",99.9346124777164,-0.12841958567344,0.25449440834216,-0.504606708296647,0.613835097238115,0.930721996626084
"11135","MOB3C",88.9743015091626,-0.0989815783363944,0.196158293975986,-0.504600526085896,0.613839440267747,0.930721996626084
"11136","AL592295.4",14.1779626785081,-0.286796733815022,0.569300282841001,-0.503770580235458,0.614422603741601,0.930721996626084
"11137","DNM3OS",159.27260591261,-0.130300498650219,0.258195258412598,-0.504658758845208,0.613798532041221,0.930721996626084
"11138","APLF",32.2479384679415,-0.133080777262659,0.263174929281514,-

0.505674220663719,0.6130853684707,0.930721996626084
"11139","AC091488.1",7.19089261006653,0.319408851229807,0.633417530664653,0.504262726821986,0.614076766294526,0.930721996626084
"11140","AC099778.1",24.7416491759231,-0.166186862967947,0.329063903775918,-0.505029148019576,0.613538363616179,0.930721996626084
"11141","SHLD3",12.3112168642033,0.217065981515916,0.430512533293595,0.504203628766098,0.614118290691235,0.930721996626084
"11142","LEPROTL1",217.055280411446,-0.104826592604145,0.207410913859208,-0.505405384189676,0.613274137970079,0.930721996626084
"11143","DCLRE1A",15.0382897586277,0.202459835985696,0.401182930764728,0.504657153782117,0.613799659574024,0.930721996626084
"11144","ECHS1",902.931180916526,-0.111834507063011,0.221867454165366,-0.504059991510324,0.61421922049571,0.930721996626084
"11145","AL133330.1",155.266637660608,0.500935182946711,0.99243905604328,0.504751581365481,0.613733327163459,0.930721996626084
"11146","PATL1",114.615644363011,0.0985405701584697,0.195229943354489,0.5047410682261 2,0.613740712157165,0.930721996626084
"11147","MARCH9",112.017042679101,-0.113798991752108,0.22571783875139,-0.504164812057452,0.614145565365047,0.930721996626084
"11148","VPS29",931.579887621028,-0.12731833220528,0.252331036969779,-0.504568656056879,0.613861829315092,0.930721996626084
"11149","AL354696.1",2.2405861517656,-0.491213859044003,0.971168813984776,-0.505796574159457,0.612999463743243,0.930721996626084
"11150","NUFIP1",99.232500560329,0.106292615927523,0.210925160700305,0.503935213677754,0.614306904252745,0.930721996626084
"11151","HECTD1",521.697345836239,0.0867042939043964,0.171693851952089,0.50499358549304,0.613563341302149,0.930721996626084
"11152","NFKBIA",5034.40302480028,0.149389988916618,0.29625398101364,0.504263228482117,0.614076413815566,0.930721996626084
"11153","LINC01220",22.8294553626983,0.37464832778013,0.74072303854358,0.50578732979167,0.613005954053768,0.930721996626084
"11154","TNRC6A",1044.7827301445,0.1041102532595,0.205784006478784,0.50592004228586,0.612912781819518,0.930721996626084
"11155","AC004943.2",12.1089442998132,0.215641249320358,0.427673668280967,0.50421914023168,0.614107391663871,0.930721996626084
"11156","DDX52",235.833879214553,0.0926539007081722,0.183874835997655,0.503896578373311,0.614334055134242,0.930721996626084
"11157","C17orf113",12.2221003535346,-0.267454076396214,0.529909898456483,-0.504716136036055,0.613758226023141,0.930721996626084
"11158","AC120049.1",23.4066485995772,0.201677877884605,0.398908973004939,0.505573681046547,0.613155961591999,0.930721996626084
"11159","MED26",70.0453391796177,0.149418347215561,0.296257506546222,0.504352949423911,0.614013375071064,0.930721996626084
"11160","ZNF761",59.8404565784955,0.16562941034835,0.328567398975326,0.504095691979434,0.614194134111402,0.930721996626084
"11161","PISD",68.8425645020936,-0.102235801610953,0.202943277502947,-0.503765401194275,0.614426243573477,0.930721996626084
"11162","Z94277.1",3.58054492683753,-0.413935855461953,0.820238540772911,-0.504653018464483,0.613802564576988,0.930721996626084
"11163","KLHL17",13.6093502936373,0.234502765188117,0.465891391176142,

0.503342130010418,0.614723750799718,0.930792605039051
"11164","GFPT1",301.420267308253,0.109615191551399,0.217993200463878,0
.50283766336814,0.615078411172304,0.930792605039051
"11165","C2orf92",6.46898247056277,-0.270884337176137,0.53863314371067
,-0.502910636560539,0.615027102510288,0.930792605039051
"11166","ORC2",76.9909548762017,0.129386790310386,0.257187969055453,0.
503082592803897,0.614906204669907,0.930792605039051
"11167","AL355312.2",21.9236330192398,-0.223140694927066,0.44315191149
5373,-0.50353093180643,0.614591038683519,0.930792605039051
"11168","NUB1",553.095124846924,-0.0761561640125887,0.151442476364428,
-0.502871887998769,0.615054347032359,0.930792605039051
"11169","FJX1",28.1473685302789,-0.205304674402683,0.408139396360019,-
0.503025868695077,0.614946084719463,0.930792605039051
"11170","AC084757.3",3.61958779049108,0.439948973015056,0.874187504568
216,0.503266142236108,0.614777167507853,0.930792605039051
"11171","FBXL19-
AS1",14.983952507046,-0.234401857537407,0.465545081063134,-0.503499805
007324,0.614612917391814,0.930792605039051
"11172","ELMO3",2.81652871621621,0.477427123075479,0.94934507602157,0.
50290156354551,0.615033481795155,0.930792605039051
"11173","AC006305.1",7.7961328825798,0.280283395848665,0.5568619059351
21,0.50332657497552,0.614734685270826,0.930792605039051
"11174","ALX1",11.135001400713,0.392237290508764,0.780237429598414,0.5
02715296176764,0.615164453799835,0.930839501060283
"11175","AC020978.5",3.30364541880019,0.432887376493453,0.861350766521
544,0.502568051621541,0.615267995990192,0.930912865879389
"11176","SERPING1",9325.43006771219,-0.0692655307248226,0.137864693626
401,-0.502416745744383,0.615374402079511,0.930990550318574
"11177","DMXL1",209.905270037754,0.0811757646885948,0.161644052765028,
0.502188378106276,0.615535017287534,0.931148650250022
"11178","PCDHB3",19.2701140508842,-0.19134104179816,0.38111061609281,-
0.502061694737897,0.615624124001228,0.931148650250022
"11179","SPARC",6706.94427976821,0.169782941090723,0.338345847318183,0
.501802940501463,0.615806144504609,0.931148650250022
"11180","AL662884.3",17.2282002202772,-0.209298051463956,0.41710966445
7431,-0.501781831730528,0.61582099449898,0.931148650250022
"11181","PMCH",2.32852222134197,-0.512400992873349,1.02123404063548,-0
.501746879250617,0.615845583871426,0.931148650250022
"11182","AC026471.4",9.01074646864759,-0.346581354205007,0.69037239561
7139,-0.502020875117972,0.615652836966367,0.931148650250022
"11183","MAP1S",141.496703645057,0.102117283528894,0.20353435726418,0.
50172012677127,0.61586440476378,0.931148650250022
"11184","PSMD5",175.732780146686,0.0889938837949521,0.177443881444634,
0.501532558183585,0.615996370027199,0.931264898463866
"11185","THUMPD3-
AS1",292.232807343156,-0.159523901171421,0.318320244888968,-0.50114280
7385888,0.616270621771982,0.931515000844644
"11186","PCDH10",8.27789877522053,0.349607790748924,0.697623794091334,
0.501140863757799,0.61627198955809,0.931515000844644
"11187","AL391684.1",3.75713009414286,-0.366179007752854,0.73114592448

7776,-0.500828898156535,0.616491545848552,0.931734922327487
"11188","PRIM1",63.4559542582242,0.144884315488454,0.289363958410612,0.500699244937964,0.616582803756935,0.931734922327487
"11189","NUTF2",321.942146754696,-0.127265525381029,0.254164173488089,-0.500721732864496,0.616566974946622,0.931734922327487
"11190","AC064807.4",4.72431577461982,0.338752994191627,0.676672908067605,0.500615570910049,0.616641701828937,0.931740651878791
"11191","GAPVD1",204.939993392686,0.109942873408976,0.219665624063256,0.500501040514726,0.616722323669485,0.931779201912577
"11192","AC012306.2",67.4627053662808,-0.166354879699689,0.332438744124748,-0.500407616860879,0.616788091179386,0.931791456725578
"11193","RPS23",35585.9438044079,-0.126096310995086,0.252168262714724,-0.500048299645614,0.617041068601611,0.931791456725578
"11194","PCDHGA2",16.7295938401603,0.208556287027359,0.417025469593279,0.500104435421561,0.617001543190384,0.931791456725578
"11195","MMS22L",52.9175386348067,0.12193612188405,0.243862556411936,0.500019862327999,0.617061091847427,0.931791456725578
"11196","AC016831.6",10.9682936935014,-0.240711893262553,0.481281008271586,-0.500148331485209,0.616970636579753,0.931791456725578
"11197","ALDH1B1",57.0850384950652,0.121380923503675,0.242677329195107,0.500174136192536,0.616952468159297,0.931791456725578
"11198","NTN5",5.8891807104629,0.409613194698105,0.819340632114279,0.499930283746716,0.617124167658512,0.931803485155395
"11199","PRKCZ",3.34860841340069,-0.452608539360238,0.907416442895167,-0.498788117522053,0.617928659428408,0.931856634942677
"11200","NBPF3",12.2303833719884,0.289825984546651,0.581318376072424,0.498566700238876,0.618084669122528,0.931856634942677
"11201","SHISAL2A",6.12379580523519,-0.326878740035295,0.656168774197144,-0.498162596102272,0.618369443608453,0.931856634942677
"11202","GPR35",2.82828385103332,-0.48857182907871,0.98008348230514,-0.498500217481064,0.618131515958703,0.931856634942677
"11203","DALRD3",320.269498703599,-0.114914462774222,0.230398292053656,-0.498764386445453,0.617945379421772,0.931856634942677
"11204","TRA2B",1420.87126697003,0.0907454888549392,0.181654163572435,0.499550833684877,0.617391384729663,0.931856634942677
"11205","AC005674.2",4.55277334791483,0.443465653154939,0.890045743591851,0.498250406058118,0.618307558555801,0.931856634942677
"11206","GPAT3",20.3618657603423,-0.318386232847974,0.638967508940009,-0.498282351439353,0.618285045361216,0.931856634942677
"11207","HEXB",959.428221659792,-0.085465952399168,0.171323319668737,-0.498857671941107,0.617879655230737,0.931856634942677
"11208","YTHDC2",152.658368993036,0.123554250337488,0.247977151856483,0.498248525771417,0.618308883680607,0.931856634942677
"11209","PREP",80.3674585528054,0.103877968586269,0.208154403988609,0.499042857589279,0.617749191953267,0.931856634942677
"11210","LINC00265",7.15813493042201,-0.260341498077303,0.521164686321849,-0.499537871444588,0.617400513919051,0.931856634942677
"11211","MEST",1563.35736934417,0.320360585853015,0.643088813060701,0.498159164561267,0.618371862080485,0.931856634942677
"11212","LY6E",1848.71088122906,-0.145330205758806,0.291348555291834,-

0.498819036920342,0.617906875120143,0.931856634942677
"11213","MRPL21",534.816527679414,-0.134794835432154,0.269915697042089
,-0.499396059248584,0.6175003948355,0.931856634942677
"11214","XPO4",64.8236547277352,0.131119714833644,0.262864329476045,0.
498811364383288,0.617912280786247,0.931856634942677
"11215","RMI2",11.1685337671849,0.225813408697369,0.453155071589512,0.
498313762450663,0.61826290910802,0.931856634942677
"11216","SP2-
AS1",17.998285120741,0.250166827975622,0.501800448111087,0.49853847065
5652,0.61810456080714,0.931856634942677
"11217","RPS16",21338.9421096434,-0.102626714078928,0.205404128540207,
-0.499633161262575,0.617333403536382,0.931856634942677
"11218","PPP5D1",36.6874806410893,-0.129257326294881,0.259294476554143
,-0.498496258048486,0.618134306007616,0.931856634942677
"11219","FIZ1",34.6020355735084,0.139563086072799,0.279900988975601,0.
498615909088356,0.618049995283728,0.931856634942677
"11220","LAMP2",1302.95805294582,0.107551152499387,0.215550921368368,0
.498959372646735,0.617808005597777,0.931856634942677
"11221","DNTTIP2",305.118376821341,-0.086676128250113,0.17408742614173
6,-0.497888504477885,0.618562630170878,0.93197798529043
"11222","CDHR4",4.88899760566061,-0.40008585211698,0.80352119272483,-0
.497915743529109,0.618543430233159,0.93197798529043
"11223","SORBS1",15.2647452170944,-0.235848456736024,0.473901056653025
,-0.497674469016229,0.618713505996497,0.932122245334472
"11224","AK3",925.388619942139,0.102042667922913,0.205329842335553,0.4
96969494361922,0.619210563592078,0.932129633556044
"11225","PANX1",93.9948096094639,-0.178574478716421,0.359500601290562,
-0.496729290786612,0.619379964099962,0.932129633556044
"11226","BORCS5",104.020652270853,-0.0966219504106426,0.19429011167234
2,-0.497307606542475,0.618972148554535,0.932129633556044
"11227","AC103858.3",6.00771352496262,-0.302569575450265,0.60885237418
128,-0.496950637430178,0.619223861471384,0.932129633556044
"11228","NAA60",155.284747204116,0.0908208619169388,0.182657648240495,
0.497219047720139,0.619034590685874,0.932129633556044
"11229","AC009152.1",3.691221388896,-0.536386142392142,1.0797866908068
8,-0.496751948286493,0.61936398432363,0.932129633556044
"11230","AC002550.2",2.16271183730979,-0.510168183549876,1.02576502572
516,-0.497353848840004,0.618939544565918,0.932129633556044
"11231","ZNF19",16.0454515061666,0.188552029227614,0.379559706129711,0
.496765136505765,0.619354683080377,0.932129633556044
"11232","ELP2",296.462386992228,0.0894840703214392,0.180101182942946,0
.496854428489717,0.619291709808177,0.932129633556044
"11233","AC092069.1",3.53939030969926,-0.501411468227796,1.00863583297
427,-0.497118436442251,0.619105534245467,0.932129633556044
"11234","PKNOX1",118.413754318066,-0.112043040213869,0.225364393071148
,-0.497163898373677,0.61907347744702,0.932129633556044
"11235","CBX6",461.666898200222,-0.0957317408733583,0.192434548171259,
-0.497476891665839,0.618852794594106,0.932129633556044
"11236","SNHG20",41.7054363712189,0.131082008368879,0.263934457389746,
0.4966460600304,0.619438666245039,0.932135009689491

"11237","PPP1R26-
AS1",5.85722278180063,-0.309121174792209,0.622846044994214,-0.496304307102216,0.619679727936889,0.932138107309875
"11238","MARCH5",272.514023391593,-0.098106444964665,0.197710813481692,-0.496211832003563,0.6197449639729,0.932138107309875
"11239","H2AFX",328.2247500661,0.186786354377802,0.376302727227227,0.496372576819018,0.619631569371798,0.932138107309875
"11240","MMP19",357.455985829862,0.245625123664564,0.49503807029804,0.496174210433318,0.619771504753822,0.932138107309875
"11241","RPS27L",2266.97603325356,-0.115989198675266,0.23367110824867,-0.496378005584804,0.619627739902438,0.932138107309875
"11242","RRAD",47.5403370561574,-0.449383543842592,0.905036427081536,-0.49653641598904,0.619516001222974,0.932138107309875
"11243","LDLRAD2",29.1175917267889,0.215330632983009,0.434498403239235,0.49558440578307,0.62018765729273,0.932158929583916
"11244","IRAK2",15.8310690832774,-0.216974605823085,0.437611935446212,-0.495815100659552,0.620024869823647,0.932158929583916
"11245","SACM1L",138.08185454546,0.09908502172164,0.200091517620708,0.495198511660373,0.620460001118208,0.932158929583916
"11246","COPS4",349.420183971156,-0.11800440084229,0.238212626574076,-0.495374248373834,0.620335968909934,0.932158929583916
"11247","ANKS1A",101.000965987386,0.120348607498386,0.243009200380566,0.495243008535944,0.620428594902635,0.932158929583916
"11248","FAM53B",74.7581107533238,0.120964108088386,0.243936896524114,0.495882786950305,0.619977111220605,0.932158929583916
"11249","POLD4",655.944911721532,-0.117048508802324,0.236023206978731,-0.49591949156454,0.619951213572625,0.932158929583916
"11250","TIRAP",22.2559815330345,-0.164046500220829,0.331151421822045,-0.495382140647988,0.620330398920286,0.932158929583916
"11251","GNB3",6.86561110743879,0.274651661984814,0.55433526861433,0.495461280447408,0.62027454703881,0.932158929583916
"11252","BRI3BP",66.4367592130274,0.111165025763376,0.224145294651627,0.495950744521103,0.619929162818359,0.932158929583916
"11253","JUP",209.180270315654,0.144189374512111,0.291209932775468,0.495138929973535,0.620502055386029,0.932158929583916
"11254","YPEL2",428.824478404664,-0.154157952532679,0.311232780079965,-0.495313997751366,0.620378491635442,0.932158929583916
"11255","MROH8",12.988872503744,-0.214220520805184,0.432149317596154,-0.495709496885922,0.620099385731017,0.932158929583916
"11256","OR2L2",2.37494771828483,0.463487071839289,0.936295777734986,0.495022067663832,0.620584543375321,0.932200023044592
"11257","IFI6",1154.53665732033,0.198017609884433,0.400689623353799,0.494192008834699,0.621170582844748,0.932452473630616
"11258","PDE8B",7.69318522415507,0.279091500835647,0.564241028314587,0.494631703173564,0.620860119171332,0.932452473630616
"11259","MKLN1-
AS",46.9350474492588,0.148633644504852,0.300736146426727,0.494232722839874,0.621141832267168,0.932452473630616
"11260","ERBB3",2.34227841950246,-0.555341374231697,1.12344444969329,-0.494320279372345,0.62108000535835,0.932452473630616

"11261","XRCC3",9.19039219999988,-0.280723672822461,0.56791003486207,-0.494310111795508,0.621087184923427,0.932452473630616
"11262","GOLGA8Q",2.89200534878265,0.498239751317049,1.00835094137564,0.494113438955416,0.621226067339099,0.932452473630616
"11263","FAM214A",276.441510007451,0.127105071508681,0.257251656222277,0.494088447768267,0.621243716072607,0.932452473630616
"11264","NUBP1",186.643275302429,-0.0914439772054985,0.18507890089507,-0.494081047397956,0.621248942243251,0.932452473630616
"11265","TIMM44",214.849363594612,-0.125277495507364,0.253489063861829,-0.49421262439807,0.621156024898529,0.932452473630616
"11266","S100A9",106.666116365236,0.22056463390123,0.44661548354858,0.493858009912095,0.621406461159392,0.932458850888259
"11267","TAF9",440.074762888992,-0.126460186817667,0.256074821487755,-0.493840769205475,0.621418638029862,0.932458850888259
"11268","ZC3HC1",53.1602620319121,0.150388253522659,0.304472295486059,0.493930829675584,0.621355030720522,0.932458850888259
"11269","AC002310.1",6.37460056595163,0.301248466186383,0.610231518495885,0.493662580603684,0.621544496225042,0.932482195401332
"11270","TOP1",857.380903298352,-0.0837230135237949,0.169592641245859,-0.493671263733794,0.621538362898874,0.932482195401332
"11271","SLC22A23",30.9483447644451,0.188388283497662,0.381702951802245,0.493546834280871,0.621626256113928,0.932522113244104
"11272","ACBD3",563.828216583849,-0.0930046990161433,0.188515501011828,-0.49335305859176,0.621763144168208,0.932561983641982
"11273","DUSP3",162.958137464259,0.103744790112592,0.210281594796466,0.493361248344193,0.621757358454783,0.932561983641982
"11274","PEX11B",70.300107558477,0.141081690798751,0.286247147673095,0.492866713068078,0.622106768597458,0.932581012806616
"11275","TRG-AS1",4.2427679790935,-0.505740103165086,1.02591950885835,-0.492962750779423,0.622038907215674,0.932581012806616
"11276","UBC",14043.6073927509,-0.0927945415155892,0.188195974165191,-0.493073998671927,0.621960302156366,0.932581012806616
"11277","PDIA3",1971.92388595793,-0.109645695721186,0.222438260125971,-0.492926422186056,0.62206457705131,0.932581012806616
"11278","AC136469.1",12.4049033861704,-0.281255440387814,0.570606506970306,-0.49290612173557,0.622078921578644,0.932581012806616
"11279","C20orf96",59.6154080012753,-0.144935764937475,0.294062810072784,-0.492873494957088,0.622101976329122,0.932581012806616
"11280","AC092958.1",3.31130962283315,0.693201295501905,1.40767910143613,0.4924426986198,0.622406420609903,0.932851456039066
"11281","RELL2",3.2205331495918,-0.418516521039475,0.850098873767491,-0.492315110576117,0.622496599631092,0.932851456039066
"11282","AC005534.1",2.434743504581,0.498718444951891,1.0130393722179,0.492299172795249,0.622507864826637,0.932851456039066
"11283","KCNK6",37.1811321913954,-0.191262025705623,0.388447148436006,-0.492375929327055,0.622453612330512,0.932851456039066
"11284","ACADSB",123.908671281587,0.101270451132703,0.205784229833255,0.492119591548687,0.622634803151943,0.932958990756209
"11285","ZBTB45",42.7153522530512,0.139819135529135,0.284362472329161,

0.491693346115267,0.62293614278261,0.933302567966544
"11286","NONO",704.269258123014,0.0717375634672073,0.145915089432653,0
.491639101522243,0.622974496218974,0.933302567966544
"11287","SRSF4",720.575628723465,0.0974807488824781,0.198398122229028,
0.491339070084281,0.623186650796055,0.933454986858584
"11288","HIST1H4H",30.6877598539299,0.176710439984669,0.35963874768271
2,0.491355397946637,0.623175104431957,0.933454986858584
"11289","AL627309.5",6.90167250016336,0.289903088892667,0.590422716078
395,0.491009375144323,0.623419816735836,0.933494551508418
"11290","C7orf50",274.066071215845,0.140199187004929,0.285553673643377
,0.4909731512683,0.623445437186769,0.933494551508418
"11291","NDOR1",10.4230407393474,0.23644316245057,0.481707415085604,0.
49084393357024,0.623536834091443,0.933494551508418
"11292","NOP2",41.447438999677,0.138167433446936,0.281475086008469,0.4
90869140165317,0.623519004772263,0.933494551508418
"11293","IFT43",275.046809278716,-0.117261356362104,0.238902589510041,
-0.49083334175068,0.623544326043061,0.933494551508418
"11294","AC025040.1",4.83152954045662,0.392396394532761,0.799190927251
013,0.490992053529051,0.623432067924376,0.933494551508418
"11295","NOS1AP",2.71549722701403,-0.425640488060461,0.867356907785045
,-0.490732804731344,0.623615441210949,0.933518360335966
"11296","HAGHL",36.511035433597,-0.208039690704722,0.424029969018996,-
0.490624969706804,0.623691722545079,0.93354989773302
"11297","ZDHHC18",77.2796954139844,0.112219678900182,0.228773152157887
,0.490528184105858,0.623760191072831,0.933569736271525
"11298","CBR4",163.872781951898,0.111784931248969,0.228051249281353,0.
490174605932798,0.624010348642798,0.93384804299462
"11299","FCF1",176.595735755393,0.112939238210664,0.230436869716656,0.
490109236206572,0.624056602661238,0.93384804299462
"11300","PNRC2",1183.91750951275,0.0640134685652212,0.130888572831359,
0.489068428056729,0.624793252570735,0.934090224356151
"11301","AC009506.1",24.2404941434541,-0.156774805153896,0.32031573987
2643,-0.489438343605062,0.62453139547022,0.934090224356151
"11302","STX18-
AS1",34.8250935606127,-0.155961875740119,0.319027925250323,-0.48886590
6073096,0.624936634605914,0.934090224356151
"11303","VIRMA",255.607339427261,0.0825542084815761,0.168816475036076,
0.489017487564138,0.624829316215205,0.934090224356151
"11304","CPSF1",112.802458605323,0.114982309240474,0.234907165415524,0
.489479786779101,0.624502061485039,0.934090224356151
"11305","FAM219A",68.5768371118837,-0.102758927726683,0.21018995941196
5,-0.488885996334767,0.624922410416386,0.934090224356151
"11306","GATA3",6.82581902639699,0.327684671142294,0.669852524526887,0
.489189275465872,0.624707701475855,0.934090224356151
"11307","MRPS31",294.200606330134,0.09140999983196,0.186959590220376,0
.488929183703343,0.624891833621325,0.934090224356151
"11308","AMDHD2",149.977902086338,0.106128527884152,0.216969566105353,
0.489140158176006,0.624742472301643,0.934090224356151
"11309","SLC25A10",19.5309729039774,-0.186686044943409,0.3813191955887
91,-0.489579457585786,0.624431515706219,0.934090224356151

"11310","YJEFN3",21.6599515057506,0.221009776391967,0.451734396607848,
0.48924717278907З,0.624666716212819,0.934090224356151
"11311","SREBF2-
AS1",11.9388636964843,-0.239685100942386,0.489930991686036,-0.48922216
5998399,0.624684418268649,0.934090224356151
"11312","TRAPPC2",122.419717566494,-0.11914643731508,0.243514723240238
,-0.489278166550681,0.624644776340986,0.934090224356151
"11313","INPP1",206.36266428681,-0.0886208721002573,0.181363011149232,
-0.488638072001004,0.625097954127171,0.934235500129497
"11314","AC098829.1",3.36035537373659,-0.442864808271567,0.90668557207
3494,-0.488443647844513,0.625235631725287,0.934235500129497
"11315","LINC00240",9.12191089273769,0.238017200787121,0.4872294534335
61,0.488511519797883,0.625187568066181,0.934235500129497
"11316","DNAJC14",81.3851523801668,0.111026151255868,0.227318584480562
,0.488416516887829,0.625254845012147,0.934235500129497
"11317","AGFG1",315.668309578931,-0.0864362324466079,0.177188624462347
,-0.487820438297808,0.625677033274054,0.934404084981126
"11318","HDDC2",582.598602126628,-0.126234830325273,0.258894362948278,
-0.487592039037528,0.625838835570422,0.934404084981126
"11319","NOLC1",308.011868173061,-0.102634974325817,0.21038674634912,-
0.487839543635046,0.625663499513712,0.934404084981126
"11320","CTSC",249.830746335658,0.16435706642587,0.337030929438107,0.4
87661671585702,0.625789504664658,0.934404084981126
"11321","AC025031.4",3.75406320256146,0.38433500934591,0.7873405927984
75,0.488143267172156,0.625448366025947,0.934404084981126
"11322","NPIPB11",15.2434015287353,0.202397063975593,0.414789774765806
,0.487950948380704,0.625584585589764,0.934404084981126
"11323","RPGRIP1L",50.7804995603095,0.132287221078841,0.27120143854255
3,0.487782151118952,0.625704155368264,0.934404084981126
"11324","AC005790.1",3.37555844703902,-0.35367322587474,0.725517718662
226,-0.487477034367781,0.625920313845294,0.934404084981126
"11325","AKAP8",145.499618508591,-0.118031096502054,0.242111385250746,
-0.487507418867616,0.625898786652516,0.934404084981126
"11326","MEGF8",116.35169328935,0.101993467432807,0.209113961307733,0.
487741070921197,0.625733256560732,0.934404084981126
"11327","AL109811.2",23.9869027344236,0.17815455422367,0.3701641214105
22,0.481285310809719,0.630313735548959,0.934456788133943
"11328","SRSF10",932.268813483078,-0.0584848975096998,0.12194263675645
3,-0.479609913852424,0.631504795623983,0.934456788133943
"11329","PDIK1L",48.2040927119019,-0.136711882602134,0.28358239869927,
-0.482088744679504,0.629742905053814,0.934456788133943
"11330","AL445248.1",2.1033593418867,0.490923915363313,1.0234101523943
2,0.479694201014881,0.631444851961146,0.934456788133943
"11331","EIF2B3",214.966769155363,0.0895393826853631,0.185378520580114
,0.483008400353844,0.629089771657372,0.934456788133943
"11332","SCP2",1673.86358707715,0.111366581915632,0.230656153679713,0.
482825106284718,0.62921992276596,0.934456788133943
"11333","AC119800.1",2.46815730723361,0.446391173232044,0.927307867078
461,0.481384003177311,0.630243603860729,0.934456788133943
"11334","MFSD14A",260.996198972625,0.0855320316816998,0.17646988306963

6,0.484683449628332,0.627900908285844,0.934456788133943
"11335","PRPF38B",466.686209033961,-0.0866588571193016,0.179001461746552,-0.484123739961418,0.628298054064284,0.934456788133943
"11336","RPS27",42592.9729874961,-0.134634176916715,0.278502850455516,-0.483421181135162,0.628796711755574,0.934456788133943
"11337","PRCC",194.730727289724,0.0790353257996321,0.162278608690818,0.487034775792381,0.626233686855178,0.934456788133943
"11338","RGS16",453.769199479117,-0.334415155494561,0.69393808656194,-0.481909210591674,0.62987044278266,0.934456788133943
"11339","KIF21B",4.4244330628548,-0.443877739011878,0.921114256385988,-0.48189216042909,0.629882555479783,0.934456788133943
"11340","CCDC85A",55.6465351060953,0.258015813896556,0.535959778946513,0.481408911698028,0.630225904167369,0.934456788133943
"11341","DCTN1",312.390153336083,0.113129241325997,0.234055998574902,0.483342627468672,0.628852477604479,0.934456788133943
"11342","AC007681.1",2.82116002286636,0.427143478750351,0.890178508407552,0.479840250821683,0.631340989445758,0.934456788133943
"11343","TMEM127",173.420987872273,0.103933695491253,0.214020971636664,0.485623883942073,0.627233859367925,0.934456788133943
"11344","MRPL30",146.427074563936,0.113831119183683,0.237151555894066,0.479993136686528,0.631232273302869,0.934456788133943
"11345","TANC1",55.361986749071,0.212151732374829,0.435554967395462,0.487083716765894,0.626199005222042,0.934456788133943
"11346","CIR1",928.126939343793,0.0924292784796383,0.189934024016218,0.486638868198496,0.626514274022041,0.934456788133943
"11347","UQCRC1",437.806112160356,-0.0898317578508513,0.185357470010306,-0.484640612789204,0.627931299649102,0.934456788133943
"11348","TRMT10C",265.853408026006,-0.0912780785131361,0.188612893717705,-0.483944001462335,0.628425611566925,0.934456788133943
"11349","LEKR1",5.37286349061483,-0.315158537884529,0.65168801340553,-0.483603398254332,0.62866736258224,0.934456788133943
"11350","LPP-AS2",42.9675769979634,0.129836048617808,0.269668408266125,0.481465550423977,0.630185658164199,0.934456788133943
"11351","PCDH7",253.746147201518,0.194317144679017,0.401872816460593,0.483528959212576,0.628720202719725,0.934456788133943
"11352","ATP8A1",27.2549696129539,0.24828263718007,0.513628619092955,0.483389413967092,0.628819263256244,0.934456788133943
"11353","PPP3CA",633.399307862582,-0.108716611569593,0.224982907146488,-0.483221649806519,0.628938364713864,0.934456788133943
"11354","SPRY1",2884.92024757562,-0.113996050577551,0.23784370657366,-0.47928974964173,0.631732513299428,0.934456788133943
"11355","AC008875.2",7.7526502144593,0.287265482133722,0.596896908992409,0.481264817770023,0.630328298505846,0.934456788133943
"11356","TMEM267",90.9650967628195,-0.14423210705097,0.296769188695939,-0.486007687269536,0.626961715621225,0.934456788133943
"11357","MRPS27",166.287854649487,-0.0843331237720445,0.174352634663962,-0.483692855772346,0.628603864179921,0.934456788133943
"11358","MATR3.1",65.9022331181902,0.14424696456634,0.296259863930389,0.486893373448094,0.626333895150631,0.934456788133943

"11359","PCDHB12",36.4901642927923,0.178792947740965,0.371111528315344
,0.481776862477415,0.629964467525685,0.934456788133943
"11360","AL391422.4",91.0010189049085,-0.133056895494466,0.27796792229
7059,-0.478677159561853,0.632168317064189,0.934456788133943
"11361","AL590004.3",5.79884803214868,-0.347131457868522,0.72031278848
4078,-0.481917666072639,0.629864435916155,0.934456788133943
"11362","HIST1H1E",59.4222486570563,0.179843983116805,0.37608119449540
4,0.478205200762845,0.632504161260276,0.934456788133943
"11363","ZNF391",24.8611257350948,0.178775528033193,0.370102940591063,
0.483042711705247,0.629065409571951,0.934456788133943
"11364","ZSCAN9",36.2322780895497,0.141474436266836,0.292391863956905,
0.483852164531116,0.628490791033357,0.934456788133943
"11365","EHMT2",176.844002897945,-0.0882418641475136,0.183905699042302
,-0.479821259520707,0.631354494593156,0.934456788133943
"11366","BAK1",54.2701792428633,-0.141071864488574,0.289854287970146,-
0.486699249738559,0.626471476992758,0.934456788133943
"11367","CNPY3",260.986778892901,-0.100539267037381,0.209200163001325,
-0.480588856122185,0.630808737214066,0.934456788133943
"11368","RMND1",127.426634517464,-0.10346067652381,0.215604318733927,-
0.479863655474773,0.631324346032277,0.934456788133943
"11369","GTF2H5",855.900275333454,0.129256623418731,0.26848434901432,0
.481430757112166,0.630210381255166,0.934456788133943
"11370","AC073332.1",44.6313557488323,-0.172888114232673,0.35858059820
4306,-0.482145757741661,0.629702406327381,0.934456788133943
"11371","NUP42",179.469960266781,-0.110789343569262,0.230381871167719,
-0.480894364681183,0.630591578130062,0.934456788133943
"11372","SPDYE1",2.19047027522055,-0.536153608034917,1.1206925656032,-
0.478412746270282,0.632356463276672,0.934456788133943
"11373","SEMA3A",281.879579286518,0.210728951684868,0.435460131397022,
0.483922491385876,0.62844087766338,0.934456788133943
"11374","AC004522.5",7.10541451765918,-0.303134276484178,0.62823000303
7459,-0.4825211706199,0.629435762900379,0.934456788133943
"11375","AC083964.1",2.073064491972,0.513327432841356,1.05981974798079
,0.484353526926975,0.628134994140862,0.934456788133943
"11376","AP006545.1",3.19201659865121,0.421051904419827,0.870175591824
106,0.483870046891566,0.628478099150553,0.934456788133943
"11377","SPIDR",142.098339826767,0.0963317595650944,0.200867748428791,
0.479578032404961,0.631527469823982,0.934456788133943
"11378","COPS5",509.240660469998,-0.127180410571771,0.26555979958362,-
0.478914394314129,0.631999530309032,0.934456788133943
"11379","C9orf64",130.061074098669,0.0816212630320396,0.17058167383255
2,0.478487877379849,0.632303000480592,0.934456788133943
"11380","ANGPTL2",3493.61814726195,-0.115853776724691,0.24169750748943
1,-0.47933376693906,0.631701203803235,0.934456788133943
"11381","FBH1",322.480928697293,-0.0813366361338005,0.169937446307283,
-0.478626917734933,0.632204065361686,0.934456788133943
"11382","MSS51",10.3410382926379,0.25184418073891,0.520018055043778,0.
484298916732244,0.62817374462636,0.934456788133943
"11383","AGAP5",22.9980629949932,0.227239219130293,0.469805688287191,0
.483687670872521,0.628607544431204,0.934456788133943

"11384","VCL",545.45388361557,0.0919070670452974,0.192188368314665,0.4
78213472809242,0.632498274248743,0.934456788133943
"11385","CWF19L1",51.6688785735321,0.116805108072537,0.242194754211635
,0.482277613537702,0.629608747988955,0.934456788133943
"11386","NDUFB8",1494.22558158029,-0.126847160307507,0.265050785711898
,-0.478576812993816,0.632239716975397,0.934456788133943
"11387","NAP1L4",507.435512579515,0.0797465988880903,0.1658978236312 5,
0.480697076926984,0.630731808942836,0.934456788133943
"11388","PDE3B",9.71014478704562,-0.339465532063998,0.704753191209123,
-0.48168002117392,0.630033270783343,0.934456788133943
"11389","SLC5A12",2.89483505375759,-0.554884363577827,1.15407878035286
,-0.480802847278909,0.6306566264319,0.934456788133943
"11390","SLC39A13",265.578837751822,-0.0862654849053358,0.178377747443
861,-0.483611247151135,0.628661791197592,0.934456788133943
"11391","RBM4B",86.5922115974923,0.10727160548583,0.220239830402321,0.
487067236157385,0.626210683982368,0.934456788133943
"11392","AP002807.1",11.1537151371602,-0.237757679162683,0.49238646146
3199,-0.482868026988701,0.629189445148401,0.934456788133943
"11393","AP003119.2",8.46481702694772,0.26933016687127,0.5552379269612
18,0.485071631084888,0.627625534766064,0.934456788133943
"11394","BUD13",87.8974383981321,-0.113981015675316,0.236885217254537,
-0.481165591489153,0.630398813651434,0.934456788133943
"11395","EMP1",5293.83863557388,-0.135785169642095,0.281935916412011,-
0.481617139703701,0.630077948170743,0.934456788133943
"11396","C2CD5",121.688448748327,0.116809017310648,0.240824043484883,0
.48503885085702,0.627648786852767,0.934456788133943
"11397","CDK4",675.238657051041,-0.107617742019728,0.222697858310015,-
0.483245518553279,0.628921418893197,0.934456788133943
"11398","WASHC3",631.359252335764,-0.134864319072663,0.278040936553278
,-0.48505202415336,0.627639442558809,0.934456788133943
"11399","P2RX4",118.204648206234,0.0971400058078555,0.202139579317944,
0.480559057932265,0.63082991982559,0.934456788133943
"11400","CCDC122",91.8029238592954,-0.162903190330883,0.33526623177974
1,-0.485892031136334,0.627043718670579,0.934456788133943
"11401","FAM71D",3.74797272557412,-0.368536902387248,0.769351408223181
,-0.479022847619639,0.631922374806913,0.934456788133943
"11402","TMED10",1195.44350351631,-0.0772424180705033,0.16108409606930
5,-0.47951610342259,0.631571514929531,0.934456788133943
"11403","LINC00638",3.34632406097462,0.382706556169049,0.7979751771614
28,0.479597069084805,0.631513930827169,0.934456788133943
"11404","MYEF2",3.20785713569417,0.546400786265867,1.13299231665923,0.
482263452480418,0.62961880642778,0.934456788133943
"11405","DTWD1",766.301931530057,-0.0845314529658793,0.174845332150827
,-0.483464167593332,0.628766196236467,0.934456788133943
"11406","PARP6",107.240210068788,-0.12053849816378,0.247788758690743,-
0.486456685124365,0.626643408762654,0.934456788133943
"11407","CELF6",23.2700281407146,0.215943654679189,0.451172933379955,0
.478627237368718,0.632203837931658,0.934456788133943
"11408","CD276",678.536612259538,0.0820494264150806,0.16889194429297,0
.485810183301301,0.627101753587832,0.934456788133943

"11409","ISG20",67.2572939663058,-0.130147988115374,0.268913713658259,-0.483976760964927,0.628402361851375,0.934456788133943
"11410","SNRPA1",257.689219485581,0.123005419916596,0.253504155341751,0.485220527256334,0.627519922500607,0.934456788133943
"11411","MMP25",3.30325316447737,0.455339921899342,0.935609152119426,0.486677498684002,0.626486893488717,0.934456788133943
"11412","DNAJA3",96.6587603876171,0.10485196026222,0.216401619749345,0.484524840357799,0.628013439632262,0.934456788133943
"11413","C16orf72",220.052832731097,0.123186316352987,0.25445779812849,0.484112954128383,0.628305708282352,0.934456788133943
"11414","PDXDC1",263.494044656213,0.120445872570294,0.251372998927276,0.479151989610226,0.631830506255098,0.934456788133943
"11415","ITFG1",404.235420411511,-0.063231044345623,0.130628930012347,-0.484050847998573,0.628349782972429,0.934456788133943
"11416","FCSK",28.9043915705809,0.166163569375037,0.343954302951803,0.483097806740684,0.62902629129415,0.934456788133943
"11417","AC004771.3",3.32246768346613,-0.398475622879507,0.833342457677798,-0.478165511919199,0.632532407155961,0.934456788133943
"11418","SNHG29",5522.58144398105,-0.138927414924482,0.286387159614865,-0.48510350502904,0.627602925890546,0.934456788133943
"11419","TMEM11",292.85115350791,-0.113545747427353,0.237224664648287,-0.478642250778171,0.632193155432566,0.934456788133943
"11420","IFT20",482.504515239349,-0.10830473623958,0.225022024722865,-0.481307269246054,0.630298131397517,0.934456788133943
"11421","AC067852.3",16.3448739493886,0.254903685874817,0.524584814463018,0.485915106284091,0.627027357453991,0.934456788133943
"11422","GOSR2",164.915340367785,0.0870976123748328,0.180067778418338,0.483693491083594,0.628603413235482,0.934456788133943
"11423","SAP30BP",317.141358457843,-0.0908269213214381,0.188543033617444,-0.481730454733888,0.629997438634422,0.934456788133943
"11424","UNK",147.774584791339,0.114039876957301,0.235103906317166,0.485061599969574,0.62763265012466,0.934456788133943
"11425","AC116025.1",4.06925595980055,0.357077977154739,0.740718796490443,0.482069550342977,0.629756539832135,0.934456788133943
"11426","NARF",195.609377631864,0.086844238741646,0.180539154737628,0.481027170354564,0.63049718823966,0.934456788133943
"11427","ANKRD29",28.1686267281538,0.161378994549278,0.335806815076385,0.480570933358125,0.63082147791631,0.934456788133943
"11428","AC090229.1",2.51335151731122,0.42909816374662,0.884376392130611,0.485198573327869,0.627535493973669,0.934456788133943
"11429","RNF165",4.67745983132108,0.316811143458153,0.652411008294125,0.485600548474078,0.62725040750397,0.934456788133943
"11430","DYM",240.56012597044,0.0786580426540355,0.16418339457897,0.479086468249395,0.63187711588819,0.934456788133943
"11431","PSENEN",488.497055285561,-0.155967779798752,0.325906141666772,-0.478566556006252,0.632247015355397,0.934456788133943
"11432","ZNF569",45.4143416534326,0.124285606041892,0.257464352374122,0.48272937552649,0.629287902617766,0.934456788133943
"11433","NUCB1",1642.49145747264,0.105175889098351,0.219853686522758,0.478390382084697,0.632372377853017,0.934456788133943

"11434","AC010332.3",8.93941596789399,-0.354269810265704,0.737366723110246,-0.480452669156778,0.630905550790065,0.934456788133943
"11435","ZNF460",84.8363086729001,-0.15513433594696,0.324381159753684,-0.478247059924073,0.63247437136621,0.934456788133943
"11436","AL117332.1",15.5212382537704,-0.213101106493237,0.438501245613439,-0.485976057365859,0.626984141544737,0.934456788133943
"11437","WFDC3",11.6184180264442,0.308300264540753,0.643429343937532,0.47915170087531,0.631830711647935,0.934456788133943
"11438","ZBP1",3.78591882326715,0.362042105214579,0.747480565068097,0.484349857553281,0.628137597835259,0.934456788133943
"11439","AJ009632.2",7.65384750396492,0.362974023084527,0.756582831824846,0.479754506468313,0.631401965188116,0.934456788133943
"11440","PMM1",435.980493962343,-0.117450038551233,0.244750095511749,-0.479877396189188,0.631314574883245,0.934456788133943
"11441","ATP5MGL",4.47875682187858,0.342242281431929,0.712086339605368,0.480619080014366,0.630787252293851,0.934456788133943
"11442","SYP",19.2022307714531,0.208484129775131,0.430297417244022,0.484511692192888,0.628022768483396,0.934456788133943
"11443","GLA",229.167976278699,-0.118227968632214,0.242960304571116,-0.486614341552276,0.626531658296805,0.934456788133943
"11444","ZMAT1",80.5908900414798,0.21412038179595,0.445861989253133,0.480239147891087,0.631057353084969,0.934456788133943
"11445","TMLHE-AS1",2.18434152280828,0.47616118875048,0.977993420708956,0.486875656489903,0.626346451201026,0.934456788133943
"11446","PYGO2",64.1264911214515,0.112906049932569,0.236353856430025,0.477699207611603,0.632864308381661,0.934553465418543
"11447","GORASP2",328.169965570403,-0.0920166447288872,0.192588577298326,-0.477788693492191,0.632800609318209,0.934553465418543
"11448","GSTCD",38.9376433639313,-0.146098562305018,0.305757775913457,-0.477824519322677,0.632775108048946,0.934553465418543
"11449","RAB35",148.538775502985,0.0767795135167855,0.160758550497356,0.477607649977213,0.632929485007555,0.934553465418543
"11450","KLHDC1",102.106603523178,-0.0946057370449377,0.197927036194615,-0.477982891391932,0.632662382081782,0.934553465418543
"11451","IL21R",2.01991605762002,0.545225598021151,1.14140253970912,0.477680379228961,0.632877711405275,0.934553465418543
"11452","MIS18A",129.783722894939,-0.110100410539868,0.230691540091524,-0.477262453994573,0.633175243385666,0.934834702686417
"11453","AC079145.1",2.48519280645019,-0.451212909610656,0.945906471317677,-0.477016410493632,0.633350435992627,0.934903680660282
"11454","CHID1",623.950790297573,0.135811624046752,0.284672140968096,0.477080839680664,0.633304557900974,0.934903680660282
"11455","AC103703.1",12.0378668762299,-0.226364559748759,0.474594765174727,-0.476963878152809,0.63338784374045,0.934903680660282
"11456","AC009948.3",8.26060469444177,-0.251606743598856,0.527684089093527,-0.476813208507148,0.633495139268135,0.934977213381349
"11457","TAMM41",88.3491479694642,-0.116703772274216,0.244796135618961,-0.476738621625435,0.633548257257518,0.934977213381349
"11458","MYO7B",2.87424214081666,-0.438627886992493,0.920459654826073,

-0.476531355494745,0.633695874365449,0.935046947689514
"11459","WNT5A-
AS1",2.74971599547071,0.477264987915924,1.00163724074432,0.47648486747
6838,0.633728985620525,0.935046947689514
"11460","PSTK",37.4935405458355,-0.160383908308919,0.336630282222653,-
0.476439336502823,0.633761415928663,0.935046947689514
"11461","MRPS5",352.767273639568,0.0805190245557378,0.169049257754715,
0.476305105536566,0.633857028598914,0.935106416503834
"11462","SURF6",149.044032170112,0.116346944603894,0.244464424727338,0
.475925872378614,0.634127189273139,0.935423356851355
"11463","WDR92",116.030151420441,0.102969999704957,0.216505235994212,0
.475600505604909,0.634359015117388,0.935438882574987
"11464","AC114760.2",14.7259582536015,-0.254733400578622,0.53559802392
241,-0.475605564623076,0.634355410260938,0.935438882574987
"11465","GPBP1",754.914891268666,0.064047768451474,0.134619992719212,0
.475767136498543,0.634240285089071,0.935438882574987
"11466","UBE2D1",261.363706965347,0.115379927328484,0.24255949848425,0
.475676805276605,0.634304647907248,0.935438882574987
"11467","AL117336.2",14.9734346907649,0.258505712712124,0.543974895732
609,0.475216254904514,0.634632842326494,0.935679464427656
"11468","AC104938.1",3.10019724326094,0.587083657299558,1.235247501114
99,0.475276134353342,0.634590167358044,0.935679464427656
"11469","RRM2",2.44785661802345,0.683122815770763,1.43971978850964,0.4
74483174589073,0.635155393422764,0.935714528088149
"11470","TMA16",363.501316090064,0.105168078711468,0.221638377801757,0
.474503015924141,0.635141247814725,0.935714528088149
"11471","CDKN2AIP",278.470016395683,0.0950030344224813,0.2000792906822
41,0.474826925358117,0.634910339857223,0.935714528088149
"11472","AC005618.4",2.94776770750363,0.382429096668511,0.805941881876
548,0.474512002997122,0.635134840647975,0.935714528088149
"11473","FAM114A2",136.040649231136,-0.0861004012180562,0.181559395554
904,-0.474227185846845,0.635337909031115,0.935714528088149
"11474","CNOT8",212.316263747187,-0.0772991683383033,0.16297534496373,
-0.474299768198104,0.635286156801216,0.935714528088149
"11475","CSPP1",162.830125692617,0.0756513213531263,0.159373444246765,
0.474679591136852,0.635015366802469,0.935714528088149
"11476","ENO4",12.3497307395676,0.216734802719739,0.456467377659817,0.
474808964072917,0.634923143135153,0.935714528088149
"11477","AL391988.1",13.1665224812915,-0.201179632217707,0.42385749922
0144,-0.4746397848047,0.635043743938073,0.935714528088149
"11478","AC008115.3",3.79131272218166,-0.357378420002848,0.75341313422
9207,-0.474345885101234,0.635253275730948,0.935714528088149
"11479","TRIM25",301.598446883947,0.185535524836234,0.39128179137161,0
.474173674644692,0.635376064406201,0.935714528088149
"11480","AC010327.7",3.26267736514049,0.43296143061516,0.9119185661213
81,0.474780804668397,0.634943216121352,0.935714528088149
"11481","GPKOW",98.0812976079418,-0.0968635266544565,0.204123355973328
,-0.474534264795808,0.635118969629777,0.935714528088149
"11482","FSIP2-
AS1",2.12602106133133,-0.489113726820002,1.03189525635044,-0.473995518

25626,0.635503103173638,0.935749065144492
"11483","AC009996.1",16.0140095175845,0.340051582526213,0.717430255072473,0.473985561832574,0.635510203161474,0.935749065144492
"11484","Z97832.2",5.77958959992953,-0.325516718106776,0.687014883123878,-0.473813196923248,0.635633122964011,0.935767073842011
"11485","EXOSC2",89.2424192075825,0.124937108000425,0.26367825344884,0.473824088131203,0.635625355742966,0.935767073842011
"11486","ACADM",306.448982502679,-0.0699596377156316,0.148185614646549,-0.47210815896333,0.636849589076452,0.935873727549303
"11487","LIX1L-AS1",6.91038889958686,0.284602257971779,0.602335303342322,0.472498052816327,0.636571331265687,0.935873727549303
"11488","RUSC1-AS1",10.407778950068,-0.261399474977576,0.558923485193207,-0.46768382775545,0.640010707881347,0.935873727549303
"11489","RNASEH1-AS1",72.4827361588882,-0.199224932416619,0.426144882393667,-0.467505162323121,0.640138499544234,0.935873727549303
"11490","RTKN",59.3002699848294,0.10423112101026,0.222199239345346,0.469088559066856,0.639006336537628,0.935873727549303
"11491","USP39",100.41810036423,0.09551073438002,0.201885332210818,0.473093975347765,0.636146134823855,0.935873727549303
"11492","EPB41L5",59.3385828346777,-0.117383415260615,0.250841146925705,-0.467959171369049,0.639813787402052,0.935873727549303
"11493","RND3",3631.30983696383,-0.177276027466056,0.37790118540994,-0.469106830860429,0.638993276710858,0.935873727549303
"11494","CERS6",29.8864019263055,-0.172587925732988,0.366538460796082,-0.470858979868429,0.637741442779554,0.935873727549303
"11495","DCAF17",41.148541275212,0.139900097404154,0.29595098522529,0.472713741086744,0.636417421783484,0.935873727549303
"11496","PPIL3",268.693841811793,-0.120491381905614,0.254596404918666,-0.473264270735112,0.636024649446201,0.935873727549303
"11497","AC114730.2",3.8028731147035,0.382133421467077,0.812291308713554,0.470438889802072,0.63804148480921,0.935873727549303
"11498","SCAP",143.662105825159,0.0906917304811466,0.193935584795276,0.467638420132558,0.640043184979864,0.935873727549303
"11499","MON1A",40.838760329177,0.113442816279504,0.240583564282437,0.471531863025878,0.637260971348392,0.935873727549303
"11500","SNX4",252.24434438552,0.066300560274649,0.141202728621279,0.469541636496799,0.638682530933623,0.935873727549303
"11501","MRFAP1",2134.70637968661,0.0980173451804298,0.209801302774356,0.467191308558502,0.640363011422851,0.935873727549303
"11502","TMEM150C",303.267489840571,-0.13817226211191,0.295599872873893,-0.467430045786442,0.640192230344387,0.935873727549303
"11503","PGRMC2",815.598777307475,0.066638958272125,0.141772152659282,0.470042649576446,0.638324546852886,0.935873727549303
"11504","TRIP13",6.88424827143352,-0.306068687667343,0.650655458775315,-0.470400553072182,0.638068869105795,0.935873727549303
"11505","NIPBL-DT",130.385574782941,-0.0922998116755802,0.196766002487558,-0.46908414

3138073,0.639009492854581,0.935873727549303
"11506","LINC00603",12.9517570544994,0.250878827404827,0.536327052050332,0.467772092505383,0.639947579860297,0.935873727549303
"11507","AC025171.3",7.12578703370341,0.30579598212626,0.653986984323256,0.467587260077808,0.64007977724233,0.935873727549303
"11508","CCNJL",4.21091089132507,0.382205560885248,0.815955909062625,0.468414477596379,0.639488216778038,0.935873727549303
"11509","ATP6V0E1",2758.76294954253,-0.11772162777164,0.250426125552403,-0.470085249739671,0.638294112053397,0.935873727549303
"11510","STK19",262.145419393028,-0.096635279456264,0.204919289266067,-0.471577272214685,0.637228552458529,0.935873727549303
"11511","ETV7",28.9552402281362,0.190874493923963,0.405274204737968,0.470976172903415,0.637657750275854,0.935873727549303
"11512","GUCA1B",10.2957541076186,-0.281702263903193,0.599100735496482,-0.470208509541793,0.638206055087674,0.935873727549303
"11513","C6orf163",6.73458640118052,0.30226641180483,0.646914505890498,0.467243212283131,0.640325880385129,0.935873727549303
"11514","FIG4",73.2396997241339,0.124941131713128,0.265631291910314,0.47035547210798,0.638101071505911,0.935873727549303
"11515","HSF2",115.478406315484,-0.0994896661237907,0.210233969174283,-0.473233067494027,0.636046908491722,0.935873727549303
"11516","MAP7",8.95390298345155,-0.252779583327834,0.536533864895809,-0.471134442514494,0.637544730583392,0.935873727549303
"11517","USP42",126.142604347139,0.117177564207341,0.250725800563112,0.467353435283361,0.640247031720812,0.935873727549303
"11518","HOXA10",448.576748873702,-0.0955249776012526,0.202524934095562,-0.471670207067832,0.637162205832057,0.935873727549303
"11519","POLR2J3",394.921484283042,-0.119717088180245,0.254671879523254,-0.47008365589619,0.638295250730706,0.935873727549303
"11520","NAMPT",2621.34687560114,-0.186828708243619,0.399833505297874,-0.467266264002644,0.64030938986764,0.935873727549303
"11521","HILPDA",362.648092159287,0.154238075526923,0.329788030063055,0.467688519493665,0.640007352227616,0.935873727549303
"11522","AC004908.2",4.72201221636552,0.306715192240914,0.655261769894277,0.46808040134922,0.639727094085751,0.935873727549303
"11523","CTSB",1136.41541405189,-0.0698407736060617,0.147670349817931,-0.472950552986237,0.636248457106882,0.935873727549303
"11524","AC087623.1",2.26801542773514,-0.461099625320124,0.979305429557199,-0.47084352991754,0.63775247658762,0.935873727549303
"11525","PLAA",118.511449843654,-0.0807000561413249,0.171575045521879,-0.470348446627888,0.638106090034184,0.935873727549303
"11526","AL359644.1",12.3889241479908,0.212971091280784,0.45150320342726,0.471693422470024,0.637145632701776,0.935873727549303
"11527","AL158151.3",4.91114734534435,0.321849962248346,0.679753740660421,0.473480237027677,0.635870597425359,0.935873727549303
"11528","MALRD1",8.77408476659783,-0.309674640112524,0.655627983534298,-0.472332859319334,0.636689219612228,0.935873727549303
"11529","ATRNL1",2.72089577704603,0.450736279856911,0.963000004054059,0.468054286562192,0.639745768732867,0.935873727549303
"11530","MICAL2",8.4665880141382,-0.283220429279657,0.604920282193469,

-0.468194632609584,0.639645410156937,0.935873727549303
"11531","TUT1",42.1886458133852,0.127571645782126,0.272791349372194,0.467652827245882,0.640032880440654,0.935873727549303
"11532","CTSW",13.8411397105943,0.225364530432562,0.480477694098261,0.469042649015198,0.639039151397586,0.935873727549303
"11533","AP000766.1",29.2136666052627,0.201327479240754,0.426567138000071,0.471971376380899,0.636947219635682,0.935873727549303
"11534","AC006517.2",2.56844859654102,-0.494040292339783,1.04884097696954,-0.471034506839382,0.637616093266418,0.935873727549303
"11535","ETV6",126.094847303008,0.0848451571217557,0.180660790936908,0.469637914689447,0.638613731658472,0.935873727549303
"11536","HOXC-AS2",42.2335022997662,0.139635968037597,0.298184427942948,0.468287257657577,0.639579179499601,0.935873727549303
"11537","C12orf73",66.4566752510053,0.10859778100914,0.230871187206043,0.470382564075529,0.638081718990154,0.935873727549303
"11538","ZNF84",156.496450427872,0.0850441927173116,0.1810831929461,0.469641557196451,0.638611128826458,0.935873727549303
"11539","ABHD13",147.114889563964,0.0897683412311655,0.190106527080799,0.472200205903567,0.636783892781663,0.935873727549303
"11540","PRKD1",38.4562955021161,-0.135587911912461,0.287465679024762,-0.471666434659081,0.637164898915688,0.935873727549303
"11541","KLHDC2",539.934359305415,-0.0846231695877768,0.180689233465154,-0.468335428541716,0.639544736503683,0.935873727549303
"11542","TMEM30B",43.5068670662239,-0.144170920916149,0.307192741481516,-0.469317472219062,0.638842728165977,0.935873727549303
"11543","COX16",826.84762422762,-0.120905464859117,0.258198093378002,-0.46826296150032,0.639594167574215,0.935873727549303
"11544","IGHG3",117.613894493856,0.591987806722033,1.25018309956924,0.473520884201688,0.635841604942913,0.935873727549303
"11545","GOLGA8R",13.1091435555757,0.256659866981402,0.545290201647983,0.47068490540582,0.637865765241421,0.935873727549303
"11546","TYRO3",38.8965177520924,0.13981222918416,0.297771847977206,0.469528030046898,0.63869225419510,0.935873727549303
"11547","AP3B2",4.87473714955494,0.358065959183707,0.766491427964585,0.467149332817133,0.640393040809525,0.935873727549303
"11548","Z92544.2",2.95036074928925,0.401478680102566,0.848471559795211,0.473178712318263,0.636085683913222,0.935873727549303
"11549","NIP7",133.226225207193,-0.0943142318053642,0.200610317402104,-0.470136496600623,0.63825750060837,0.935873727549303
"11550","ZMYND15",10.5129663982605,-0.225122870414439,0.476715861361143,-0.472237004599883,0.636757629394135,0.935873727549303
"11551","MPP2",19.6348915250853,-0.168000350504506,0.359647472392927,-0.467125069409524,0.640410399085961,0.935873727549303
"11552","HOXB13",5.86011730261082,-0.469904998936751,0.999840136668445,-0.469980131526342,0.638369212675318,0.935873727549303
"11553","AC005332.1",3.42586607561837,0.37596572660405,0.803573303302537,0.467867368239961,0.639879440423024,0.935873727549303
"11554","SSTR2",8.79622487102682,-0.445390578330359,0.945895859275593,-0.470866400315419,0.637736143387499,0.935873727549303

"11555","TMC8",12.6310636408215,0.315926778360222,0.668387928770336,0.
47266978465851,0.636448786662738,0.935873727549303
"11556","USP14",389.014417382721,-0.0647188507614987,0.13702572160287,
-0.472311694508478,0.636704324289032,0.935873727549303
"11557","NACC1",38.3760009501361,0.134253645830305,0.286668494704302,0
.468323685059243,0.639553133220413,0.935873727549303
"11558","TSHZ3",96.3663304039109,-0.0984735470052097,0.20995029317202,
-0.46903267205503,0.639046282664706,0.935873727549303
"11559","AC010331.1",5.99546130161368,0.336893781865542,0.711817549197
811,0.473286704219652,0.636008646569625,0.935873727549303
"11560","TMEM143",29.5662650591359,-0.163120328917695,0.34747550587073
5,-0.469444108035569,0.63875222680724,0.935873727549303
"11561","HAS1",57.6123610559655,-0.408128963736327,0.86387013316899,-0
.472442498086098,0.636610976202058,0.935873727549303
"11562","ZNF581",278.326871224382,-0.104149977329295,0.221251998279501
,-0.470730109283467,0.63783348004725,0.935873727549303
"11563","MZF1-
AS1",19.8674037294778,0.157451988457106,0.33321679643541,0.47252116382
3224,0.636554839108012,0.935873727549303
"11564","NSFL1C",438.659972520162,-0.0710282975139914,0.15016252455143
5,-0.473009479070539,0.636206416438293,0.935873727549303
"11565","TRPC4AP",218.766986986745,0.0686937940090604,0.14568370065916
,0.471526970404025,0.637264464369393,0.935873727549303
"11566","SYNJ1",65.5012499385592,0.109409759493679,0.232550313913765,0
.470477797481067,0.638013693183125,0.935873727549303
"11567","AC016026.2",2.09408522269067,-0.488408467159168,1.04266255468
406,-0.468424290260582,0.639481200912396,0.935873727549303
"11568","CLTRN",4.45760283828923,0.32897504785318,0.700892009855254,0.
469366240772411,0.638807874669388,0.935873727549303
"11569","SHROOM4",13.3049251693787,-0.212131539547722,0.45398489542636
,-0.467265633030584,0.640309841244001,0.935873727549303
"11570","SH2D1A",6.17182434955659,0.308423412861249,0.654673473126879,
0.471110294706373,0.637561973891272,0.935873727549303
"11571","POLE4",796.396487038064,-0.113477091327362,0.243476898177623,
-0.466069233577049,0.64116594469606,0.935913219781791
"11572","TLK1",414.23305625433,0.0690143436584041,0.147974870504073,0.
466392323394562,0.640934705320478,0.935913219781791
"11573","AC025181.2",79.0916438944776,-0.133819451454329,0.28681532886
2887,-0.466570081818403,0.640807496276452,0.935913219781791
"11574","CCDC125",45.3368655091455,0.130751883878726,0.280222005124985
,0.466601057330981,0.640785330387446,0.935913219781791
"11575","AC027702.1",4.21644782299056,-0.327981639623872,0.70366949853
2615,-0.466101828071023,0.641142614834223,0.935913219781791
"11576","UGCG",458.871745644401,0.166505580251974,0.357057141560588,0.
466327545009263,0.640981065219512,0.935913219781791
"11577","IFITM1",672.887561554233,-0.343751560076572,0.737779797028179
,-0.465927044168496,0.641267722449257,0.935913219781791
"11578","AP001372.2",43.2242193871747,-0.120078541428495,0.25729018488
0445,-0.46670471119717,0.64071115863904,0.935913219781791
"11579","RHNO1",43.7771577662653,0.148311716535373,0.318296290333662,0

.465954901264799,0.64124778208782,0.935913219781791
"11580","CIDEB",43.5306801852902,0.118535664335556,0.254281847128834,0
.466158578262566,0.64110199611619,0.935913219781791
"11581","AC109630.1",8.60500519411479,-0.265176644240553,0.56877967602
6106,-0.466220322943435,0.641057803833356,0.935913219781791
"11582","ZFPM1",241.792220864852,-0.132093832217836,0.283299368030278,
-0.466269420705937,0.641022664188696,0.935913219781791
"11583","NAA38",1090.41656850629,-0.138404711716892,0.296924404591725,
-0.466127773859477,0.64112404410868,0.935913219781791
"11584","GATAD2A",145.551115426394,0.102822647086221,0.220418400776418
,0.466488490634314,0.640865883966321,0.935913219781791
"11585","POFUT2",143.600516983188,0.0866481184573865,0.185629654011828
,0.466779507394144,0.640657638843799,0.935913219781791
"11586","PCDHB8",2.42651217776399,0.457423363608632,0.982407727558116,
0.465614582191458,0.64149140328766,0.93600918003823
"11587","AC135050.1",2.56591391785939,0.498410464133685,1.070259346426
65,0.465691297906215,0.641436482117336,0.93600918003823
"11588","AC009283.1",14.6428135021597,0.198776365893204,0.426922246719
725,0.465603203910104,0.641499549224214,0.93600918003823
"11589","SPATA17",9.77993664211008,-0.221122084093263,0.47532686897184
,-0.465200051853924,0.641788201598176,0.936029224546454
"11590","AC246817.2",13.1254304398612,0.309540309105201,0.665016481401
188,0.465462612976148,0.641600204613802,0.936029224546454
"11591","FANCA",7.63480511099412,-0.262311675537156,0.563923843016612,
-0.465154433148217,0.641820867492909,0.936029224546454
"11592","NLE1",104.743252590999,0.122315082591005,0.262982487148261,0.
465107330595926,0.641854596643527,0.936029224546454
"11593","SNX11",61.0666568201534,-0.128796880479718,0.276957047511623,
-0.465042798646648,0.641900807819738,0.936029224546454
"11594","PTBP1",504.892114967327,-0.0990490023857384,0.212885307580841
,-0.465269320420929,0.641738602224044,0.936029224546454
"11595","MAGED2",1104.67578867137,-0.132892230044181,0.28556501985078,
-0.465365926518671,0.641669430631599,0.936029224546454
"11596","DCBLD1",14.8206009163802,-0.183116568218762,0.393888694830866
,-0.464894196309421,0.642007226875383,0.93603026825191
"11597","DISP2",5.54027159940543,0.43494281185151,0.935587859798784,0.
464887190760526,0.642012243962467,0.93603026825191
"11598","PRKAR2A-
AS1",4.29679137140956,0.37936678485535,0.816344062358492,0.46471433106
1985,0.642136044171297,0.93612574352075
"11599","LCP2",36.3915084322337,0.140290382821168,0.302011724782246,0.
464519657050797,0.642275479457117,0.93612574352075
"11600","CCDC167",146.384031527491,-0.139643549183503,0.30068760072209
5,-0.464414059136963,0.642351119251965,0.93612574352075
"11601","NDFIP2",205.596137422166,-0.0936015781962651,0.20154976808397
8,-0.464409257753474,0.642354558571548,0.93612574352075
"11602","IL13RA1",1074.06308712102,0.132143702389464,0.284491839999332
,0.464490308016477,0.642296501800683,0.93612574352075
"11603","CPT2",96.2970737053223,0.106678449451574,0.229760596517951,0.
46430263094847,0.64243093929547,0.93615636659552

"11604","GBA2",66.3573777527425,0.128484026539312,0.276918595865767,0.463977603734468,0.642663791638983,0.936343350103556
"11605","TGFB3",148.934744097821,-0.0840122565670388,0.181073026362837,-0.463968920465787,0.642670012890452,0.936343350103556
"11606","AL022322.2",5.40171840188098,0.317977691589216,0.685668242115645,0.463748606189035,0.642827868575092,0.936492641897954
"11607","AC011246.1",2.15086761804107,-0.463179363343023,1.00213788386182,-0.462191252124031,0.643944175958689,0.936517506184655
"11608","AC095057.3",4.28541459335605,-0.367514033974672,0.794579187794283,-0.462526629969852,0.6437037099983,0.936517506184655
"11609","AC113383.1",2.81340539303712,-0.452320548705062,0.977282009317696,-0.462835235267306,0.643482472909296,0.936517506184655
"11610","NACAD",48.1769542077554,0.138240952615088,0.29829078735733,0.463443587513435,0.643046441777308,0.936517506184655
"11611","IGFBP3",3429.97288067665,-0.157894219473548,0.341567607208903,-0.462263446946186,0.643892409112268,0.936517506184655
"11612","CALU",1329.85864316324,-0.0950501007484076,0.205429797118895,-0.462688967625257,0.643587327272853,0.936517506184655
"11613","TCAF2",21.1541305299579,-0.203391285653083,0.439837892386907,-0.462423290884107,0.643777800161878,0.936517506184655
"11614","CSGALNACT2",242.289153244338,0.0851340863445725,0.183706474187792,0.463424529380195,0.643060099662659,0.936517506184655
"11615","TRAF6",74.1054086097265,0.108440986068044,0.233997438903513,0.463428089538871,0.643057548289289,0.936517506184655
"11616","OVCH1-AS1",2.90482632715131,0.564315572272125,1.22047563645873,0.462373484086512,0.643813510991213,0.936517506184655
"11617","RARG",340.805572070191,-0.100595549954415,0.217691177454148,-0.462102098628244,0.644008105299798,0.936517506184655
"11618","LINC01550",49.4889468415608,-0.172920552519103,0.37328528912124,-0.463239665635308,0.64319258730418,0.936517506184655
"11619","PIF1",7.70292124467111,0.307969966915857,0.666416643788403,0.462128264331949,0.643989342372679,0.936517506184655
"11620","VPS33B",32.4166631441252,-0.145314492063265,0.313681805153568,-0.463254449814593,0.643181991401669,0.936517506184655
"11621","CHD2",559.557892001364,0.101912356983042,0.220288160299058,0.462632021823998,0.643628151725887,0.936517506184655
"11622","AC092338.2",2.16903392765501,0.519730776514326,1.12434487381022,0.462252097750971,0.643900546867391,0.936517506184655
"11623","ADAM11",3.43093375968026,-0.38299149726187,0.827512692919511,-0.462822504765039,0.643491598697125,0.936517506184655
"11624","LINC00526",35.6706771785494,-0.154313043423455,0.333842027685747,-0.462233723216879,0.643913722117561,0.936517506184655
"11625","PLIN5",3.36391306295043,-0.414241878804819,0.896033707605845,-0.462306133450774,0.643861801833401,0.936517506184655
"11626","HORMAD2-AS1",3.47321259130323,0.442056138379121,0.956326205948129,0.46224409163906,0.643906287544897,0.936517506184655
"11627","WNK3",12.7778972065304,-0.207302420899902,0.448605014472445,-0.462104555705174,0.644006343367138,0.936517506184655

"11628","TCEAL7",160.294084394215,-0.147756370969369,0.319817239630469
,-0.462002521002598,0.644079512590569,0.936540797977412
"11629","KPNA1",124.885615432792,-0.0809764391161759,0.175313858996581
,-0.461894111393413,0.644157257047439,0.936573299695426
"11630","GALT",171.586075052653,0.0873530476058546,0.189185248910937,0
.461732868226835,0.644272897562806,0.936580358695893
"11631","DPF2",240.882184056291,0.0783966940853045,0.16976486612703,0.
461795752406406,0.644227797207575,0.936580358695893
"11632","C5orf24",454.187905687916,0.0920928993892386,0.19952893983582
3,0.461551589784493,0.644402917275773,0.936688834705877
"11633","RAB29",179.322815745711,-0.10999936097376,0.23898011304835,-0
.460286672270114,0.645310466281234,0.936841679154529
"11634","LYPLAL1",305.214227256669,0.113604792734379,0.246309192450082
,0.461228391861187,0.644634753961587,0.936841679154529
"11635","MSTN",2.14768608566666,-0.488643018979867,1.06169815713695,-0
.46024664891345,0.645339190744783,0.936841679154529
"11636","AC008429.1",5.46775823587647,0.344059463477254,0.747176154280
094,0.460479716203947,0.645171927526673,0.936841679154529
"11637","SUMO4",20.2235923714112,-0.186825708591387,0.405777843068651,
-0.460413775130101,0.645219249018603,0.936841679154529
"11638","AC005076.1",16.5186880878644,-0.185591618218433,0.40321737538
5925,-0.460276837129849,0.645317524838759,0.936841679154529
"11639","AKR1B10",11.6961182824829,0.293844953785301,0.638233345128653
,0.46040363767905,0.645226524115628,0.936841679154529
"11640","DLG5-
AS1",7.82918165599377,-0.28595270129339,0.621100342394971,-0.460396946
797281,0.645231325816015,0.936841679154529
"11641","FIGNL2",13.4234869451831,-0.213997008308835,0.464415912833735
,-0.460787415752154,0.644951131169219,0.936841679154529
"11642","JMJD7",38.8981594457753,0.154205434424313,0.334953664671398,0
.460378406594221,0.645244631239773,0.936841679154529
"11643","INTS14",82.2485598243697,0.0988383269590633,0.21448583681009,
0.460815168166917,0.644931218372289,0.936841679154529
"11644","AC104564.3",2.79569496977504,0.538916000532152,1.169830263765
41,0.460678798646831,0.645029068110267,0.936841679154529
"11645","SDHAF1",270.395636329835,-0.122852691159214,0.266777054541011
,-0.460506962904218,0.645152374814396,0.936841679154529
"11646","POLD1",29.2341686370648,0.147598989546327,0.320084304046958,0
.461125358788832,0.644708669023305,0.936841679154529
"11647","FANCB",11.9742606127078,0.225989427909932,0.490925588542849,0
.460333364534342,0.645276956261404,0.936841679154529
"11648","IFT140",60.0352215013308,0.108834899412376,0.236521885260609,
0.460147268369931,0.645410517705165,0.936864786517765
"11649","HIST2H3PS2",14.8427742280846,0.207028487600346,0.450003657850
982,0.460059566157799,0.645473465655057,0.936875728156555
"11650","VAMP4",186.642625034295,-0.0830824870828371,0.180738031122014
,-0.459684586398693,0.645742634680128,0.937105524604893
"11651","GDE1",242.850842174433,-0.0767964681289224,0.167053578115985,
-0.459711602678769,0.645723240226527,0.937105524604893
"11652","TM2D1",670.775886282511,0.0878216791955808,0.191275006692092,

0.459138288448492,0.646134862846045,0.9371556902909
"11653","LINC00467",121.391189879258,-0.120425473334187,0.262819612677626,-0.458205809327864,0.646804586623587,0.9371556902909
"11654","UBXN2A",329.958322603851,0.0652931396894416,0.142437874221522,0.458397319156114,0.646667017347816,0.9371556902909
"11655","AC108047.1",12.6341419037842,-0.231683832521802,0.506234630727962,-0.457660970741259,0.64719603228979,0.9371556902909
"11656","SLC49A4",74.5220414950073,0.104193659290232,0.227646891994493,0.457698580364335,0.64716900807767,0.9371556902909
"11657","EIF4G1",440.015943694163,0.0991955272751553,0.216282983258556,0.458637687443824,0.646494368150555,0.9371556902909
"11658","AC027607.1",2.30957139459668,-0.509701310742141,1.11193509018208,-0.458391245354688,0.646671380220278,0.9371556902909
"11659","SCD5",131.559100680843,0.149426162347422,0.326537274936734,0.457608284923592,0.647233890210577,0.9371556902909
"11660","GALNT7",72.0841681848975,-0.108056280753016,0.235809496548133,-0.458235492356262,0.64678326330991,0.9371556902909
"11661","DNAJC21",494.272409580998,-0.0727799190444336,0.158613894011554,-0.458849582490749,0.646342186195907,0.9371556902909
"11662","KIAA0825",35.8721437516942,0.138835638961898,0.303327042505748,0.457709401097224,0.647161232978744,0.9371556902909
"11663","SLC4A2",151.541444754448,-0.0915536162975245,0.199766642412204,-0.45830282369471,0.646734895760705,0.9371556902909
"11664","RIDA",139.006125403541,-0.132522889862133,0.289488907525513,-0.457782272194501,0.647108873388709,0.9371556902909
"11665","C8orf33",298.254177450628,-0.103546518519625,0.225449396570058,-0.459289401945452,0.646026357313946,0.9371556902909
"11666","NANS",359.68606026946,-0.105349526637813,0.230245592312262,-0.457552848590197,0.647273725527391,0.9371556902909
"11667","TIMM8B",721.14274365486,-0.119194034486389,0.260319788623967,-0.457875427436541,0.647041941709226,0.9371556902909
"11668","FAM98B",130.094993392098,0.095355222753936,0.207524889386161,0.459488127115681,0.645883676160639,0.9371556902909
"11669","ORC6",9.627521961874,0.302625953853335,0.659814514340113,0.458653071850039,0.646483318649559,0.9371556902909
"11670","MYH3",4.73357436844882,0.339235614974097,0.739179290339932,0.458935496986245,0.646280487053658,0.9371556902909
"11671","BLMH",161.149852798828,0.0840759665345497,0.183211038202779,0.458902298460281,0.64630432815042,0.9371556902909
"11672","AC008026.3",2.55339936474829,-0.443701346685877,0.969726542136307,-0.457553059967197,0.647273573634659,0.9371556902909
"11673","LRRC45",64.3020590566891,-0.123300670151124,0.268643088958816,-0.458975775736509,0.646251561872087,0.9371556902909
"11674","CDC34",199.567915692311,-0.0902254342192716,0.196710114863452,-0.458672063111256,0.646469678714255,0.9371556902909
"11675","CARM1",219.461307822652,0.0841902396697619,0.183842474294447,0.457947707638664,0.646990010631688,0.9371556902909
"11676","MRPL40",660.730901465248,-0.126085521814088,0.275273624139615,-0.458037061153884,0.646925815285552,0.9371556902909
"11677","SAMM50",304.826810688737,-0.0948497907085875,0.20723884288359

3,-0.457683460247195,0.64717987250854,0.9371556902909
"11678","RBBP7",738.495879673779,-0.0858310640469288,0.187063587288318
,-0.458833625993919,0.646353645556437,0.9371556902909
"11679","CDC42-
AS1",5.59243041955617,0.286879887228865,0.628237519422382,0.4566423977
55278,0.647928099755014,0.937208779921142
"11680","YTHDF2",584.344563818689,-0.0900890252599508,0.19707699953791
6,-0.457126024199585,0.647580465878155,0.937208779921142
"11681","WARS2",181.277845841272,-0.0856311138232402,0.187833476433636
,-0.455888457420393,0.648470190162939,0.937208779921142
"11682","MEIS1",136.085820948473,0.0942531179789018,0.206609096844457,
0.456190552199446,0.648252958699812,0.937208779921142
"11683","UBXN4",1551.9015863483,0.0564811510879915,0.123522770750205,0
.457252948140314,0.647489244840288,0.937208779921142
"11684","AC012459.1",11.0673187842473,0.366580855073671,0.803770964402
666,0.456076259666957,0.648335141089441,0.937208779921142
"11685","ZDHHC3",353.248885597403,-0.0818091270291933,0.1790324191374,
-0.456951469590588,0.647705928022765,0.937208779921142
"11686","ATOX1",796.06170401192,-0.112033129784305,0.245181914479219,-
0.45693880000191,0.647715034753204,0.937208779921142
"11687","SYCP2L",4.69955326927015,-0.296887300616957,0.650867906985589
,-0.456140635343279,0.648288851038562,0.937208779921142
"11688","CYP39A1",16.5587400894955,-0.30611559069769,0.671241676024079
,-0.456043779210618,0.648358497041125,0.937208779921142
"11689","FBXL18",57.0939525265056,0.136408820669964,0.29923310604759,0
.45586139338563,0.648489652932542,0.937208779921142
"11690","CLN8",136.713974448751,0.0858765209725761,0.188386262246354,0
.455853414938902,0.648495390580757,0.937208779921142
"11691","ZP1",19.7582304505404,-0.191575783529627,0.419064540583421,-0
.457151023235981,0.647562498494189,0.937208779921142
"11692","TBC1D15",350.865950935354,0.0867194244001565,0.18996067810558
5,0.456512501771318,0.648021482928142,0.937208779921142
"11693","ENTPD5",88.4334222991291,0.126080380042752,0.276610047514708,
0.455805496494295,0.64852985125842,0.937208779921142
"11694","BEGAIN",5.81957893613035,0.356656457064715,0.781922701843399,
0.45612751263506,0.64829828695855,0.937208779921142
"11695","SNTB2",1604.81822896216,-0.0922976525258849,0.202277107064504
,-0.456293121180798,0.648179209812983,0.937208779921142
"11696","RSKR",10.8766102312635,0.228662163727017,0.501323971690101,0.
456116556637286,0.648306164942603,0.937208779921142
"11697","MGME1",74.9870404557061,0.0953791065379665,0.208555713147858,
0.457331545122173,0.647432759347086,0.937208779921142
"11698","TCEA2",401.795506488351,-0.102895054049756,0.225384805628034,
-0.456530571184865,0.648008492362941,0.937208779921142
"11699","THOC5",134.212322031749,-0.0877513306072082,0.192170593923489
,-0.456632457732558,0.647935245514368,0.937208779921142
"11700","FUNDC2",847.419529842908,-0.0965140671996367,0.21151447959094
8,-0.456300048045349,0.648174229400946,0.937208779921142
"11701","TDRP",18.8913198527193,-0.197342407679612,0.433050443463617,-
0.455703049513657,0.648603528803374,0.937235147851247

"11702","TTF1",198.510257315257,-0.114044132449699,0.250357039388517,-0.455525966948025,0.648730890679461,0.93729568694134
"11703","NFAT5",555.279808190537,0.11150245377221,0.244837790019167,0.455413577142161,0.648811729356603,0.93729568694134
"11704","ZNF345",64.4949764073789,0.137602823207662,0.302141017180224,0.455425828945241,0.64880291679454,0.93729568694134
"11705","FNBP1L",121.763923784913,-0.113389860416869,0.249476393466176,-0.454511382185113,0.649460801471096,0.93735238849964
"11706","ECT2",22.1957093286837,-0.162188150042815,0.356834309978008,-0.454519494083432,0.64945496428751,0.93735238849964
"11707","EPHA1-AS1",16.3134556314899,-0.228281078992924,0.501801147616216,-0.454923389628269,0.649164355165976,0.93735238849964
"11708","DOCK5",17.1094077603398,-0.219762112379954,0.483043236533682,-0.454953295603447,0.649142839474636,0.93735238849964
"11709","MLLT10",266.445998735905,0.0771964486594933,0.169655880052657,0.45501781980992,0.649096418882078,0.93735238849964
"11710","C12orf57",3469.92362853451,-0.116627676866205,0.256597327890037,-0.454516334309548,0.649457238004311,0.93735238849964
"11711","LBHD2",44.5923071001259,0.430105863134381,0.94621247627397,0.45455526524874,0.649429224224166,0.93735238849964
"11712","LOXL1",412.664257726744,-0.154710645244093,0.340005475957311,-0.455023981035876,0.64909198638804,0.93735238849964
"11713","AC005332.5",28.0974044142291,0.154108503177671,0.338993709154774,0.454605790655867,0.649392868079178,0.93735238849964
"11714","FAM104A",175.023712499764,0.0752757270090588,0.165355891807947,0.455234622643432,0.648940454595887,0.93735238849964
"11715","NHSL2",127.102585239961,0.167350180421317,0.368174606299212,0.454540257687715,0.649440023250287,0.93735238849964
"11716","DNM2",154.67006860994,-0.0905817355301421,0.199360902160809,-0.454360581981499,0.649569318642418,0.937428989382554
"11717","CLPB",65.1427198049983,-0.112726669559263,0.248220291405222,-0.454139622998167,0.649728336126101,0.937578450731425
"11718","AL139289.2",3.13785254121372,-0.396097233143112,0.874716249887228,-0.452829398326804,0.650671592764089,0.937651104001829
"11719","FAAH",16.9272913850654,-0.176746230088776,0.390030891339493,-0.453159567648019,0.650433844533187,0.937651104001829
"11720","AL365436.2",7.09448590746841,0.300455266292886,0.663899596662976,0.452561302647409,0.650864669177366,0.937651104001829
"11721","AC099332.1",2.23258699972245,0.490950444063335,1.0822474600512,0.453639728607086,0.650088153849187,0.937651104001829
"11722","ARMC1",268.195460833697,-0.0728818964355726,0.160750050282908,-0.453386461200516,0.65027048370753,0.937651104001829
"11723","POLE3",263.987832611366,-0.0800890230571648,0.176931578667465,-0.452655335245093,0.650796946370271,0.937651104001829
"11724","TPCN2",35.447994254748,-0.124185428704953,0.273987331587002,-0.453252447788882,0.650366969837257,0.937651104001829
"11725","ATP6V0A2",62.2144122613164,-0.116288304891603,0.25644564261449,-0.453461808537795,0.650216238181988,0.937651104001829
"11726","BORA",12.396314755943,0.181851033217626,0.401154581458579,0.4

53319098479254,0.650318982361464,0.937651104001829
"11727","DCAF4",41.5024820798831,0.126536836458679,0.279577690572114,0.452599905950077,0.650836866512357,0.937651104001829
"11728","CMTR2",106.936110236066,0.102795104597394,0.226856993479356,0.453127333748024,0.650457053944291,0.937651104001829
"11729","ZNF287",45.5381983888865,0.113166289965004,0.249954326408052,0.452747874346689,0.650730302001684,0.937651104001829
"11730","MIEF2",59.6775397766943,0.122823985572432,0.271194655694009,0.452899727165035,0.650620947413963,0.937651104001829
"11731","RARA-AS1",83.9153978184947,0.151374655798757,0.333518422821267,0.45387194661771,0.649920996079204,0.937651104001829
"11732","AC060780.1",94.6656836988995,0.0967583204204646,0.213838488902538,0.452483184468089,0.650920932522302,0.937651104001829
"11733","ZNF568",63.3167146234919,0.110355989436799,0.243753331082261,0.452736333681351,0.650738613150592,0.937651104001829
"11734","DM1-AS",3.29010548642794,-0.360000258697291,0.794780274117451,-0.452955704137281,0.65058063831252,0.937651104001829
"11735","GPR82",18.6466396784639,-0.183915430986591,0.405191499119629,-0.453897555566168,0.649902563037421,0.937651104001829
"11736","SUV39H1",12.1767899376741,0.190135082603761,0.420232434697542,0.452452183374681,0.650943261105599,0.937651104001829
"11737","PRICKLE3",25.9000450111478,-0.140196343138588,0.309707502044782,-0.452673384444901,0.650783947564522,0.937651104001829
"11738","MT-ND6",456.765036010677,-0.153757313838514,0.338882643842213,-0.453718467535638,0.650031473137943,0.937651104001829
"11739","CES4A",24.2431968126654,-0.18662000558397,0.412601618962308,-0.452300710921395,0.651052363867032,0.937728372796983
"11740","TMEM248",588.638193793291,0.0572318674976196,0.126571365199444,0.452170737097107,0.651145987528997,0.937783335361183
"11741","ABCA7",4.60584819861384,-0.324633395705183,0.718271321857375,-0.451964857605222,0.651294299357413,0.937917043994135
"11742","PRRT3-AS1",19.5310819889471,-0.170656018547738,0.377892991610021,-0.451598792083057,0.651558040360838,0.93821694314606
"11743","NDST1-AS1",4.00813571328189,0.645081729654332,1.4303969086895,0.450980931051748,0.652003292590068,0.938335142867719
"11744","AL122017.1",9.28718110777486,-0.274244237106844,0.608041804010379,-0.451028589314169,0.651968943964213,0.938335142867719
"11745","SPDYE3",2.21415747989373,0.453940711908682,1.00625598700194,0.451118520309292,0.651904130223654,0.938335142867719
"11746","PDCL",282.557992290328,0.0788748733807012,0.174851396177224,0.451096617500017,0.651919915451148,0.938335142867719
"11747","NT5C3B",372.162846198014,0.0898275131083407,0.19903469883931,0.451315844082355,0.651761927158689,0.938335142867719
"11748","FAM117A",153.476976791195,0.105928841070733,0.234903700881204,0.450945815980583,0.652028601463972,0.938335142867719

"11749","PCTP",70.9020329264233,0.13172052147575,0.292055194432642,0.4
51012425002867,0.651980593947023,0.938335142867719
"11750","AC002451.1",98.2160865063138,0.10888455345305,0.2415789387165
9,0.45072038991275,0.652191084832443,0.938489094667826
"11751","ATG7",114.686656080052,0.0876432578469731,0.194506055023497,0
.450593981952826,0.652282204817124,0.938492169582614
"11752","PRPF40B",48.7211744055232,-0.11607851499924,0.257629689688045
,-0.450563423570458,0.652304233317653,0.938492169582614
"11753","YRDC",77.1417500827022,-0.114964237073447,0.255328264011676,-
0.450260520582983,0.652522602199843,0.938566750999145
"11754","CLCN7",127.038076868233,0.0850038295717203,0.188781819618218,
0.450275507162858,0.6525117973712,0.938566750999145
"11755","ACTL10",9.10398620490627,-0.273921888587061,0.608240580481794
,-0.450351221830816,0.652457210713412,0.938566750999145
"11756","ABCB4",41.4898630875743,-0.170490095018379,0.378756342416611,
-0.450131326991349,0.652615749531564,0.9385957669644
"11757","ALAD",109.681119495212,0.102813104381484,0.228515633235302,0.
449917158515007,0.652770174867073,0.9385957669644
"11758","RBM25",1483.34756938207,0.105663181127243,0.234769497109684,0
.45007201714061З,0.652658513175413,0.9385957669644
"11759","METTL2A",116.775274617998,-0.0984456528332676,0.2188171187585
48,-0.4498992281399,0.652783104168502,0.9385957669644
"11760","ZDHHC9",385.508281799743,-0.13273059642448,0.295056808450818,
-0.44984759755715,0.652820334723287,0.9385957669644
"11761","SLC12A9",78.729708065806,-0.0912642732299259,0.20297167524562
7,-0.449640439334612,0.652969724175055,0.938730728369342
"11762","AL445228.2",3.57195189035173,-0.326594530248483,0.72657943496
273,-0.449495973231386,0.653073912258547,0.938800689378295
"11763","Z94721.3",5.77063170109089,0.29050495262042,0.646864516436817
,0.449097060108714,0.653361641129849,0.93894856489362
"11764","CKS2",59.6512525665711,-0.189627105167949,0.422116446431385,-
0.449229369694252,0.653266202886529,0.93894856489362
"11765","ST20-
AS1",14.9651261421043,-0.193226506562937,0.430305065514818,-0.44904539
1394035,0.653398912617597,0.93894856489362
"11766","RNF138",110.516301541465,0.102233271170297,0.227594985992283,
0.449189470165937,0.653294982825991,0.93894856489362
"11767","ERMAP",46.6034413044847,0.117347449765356,0.262232231012568,0
.44749438050478,0.65451814438378,0.938997714695765
"11768","HYI",715.288430313463,-0.11027867082601,0.245751688570995,-0.
448740236403915,0.6536190553406,0.938997714695765
"11769","AMIGO1",15.3562485014548,0.210810870272653,0.47106954600623,
0.447515387191628,0.654502980460395,0.938997714695765
"11770","LINC01348",2.0993925456022,0.624865229510762,1.39468623844155
,0.448032835119246,0.654129499619084,0.938997714695765
"11771","TMEM150A",111.394499884415,-0.103026406774882,0.2302020061506
9,-0.447547823312369,0.654479566346043,0.938997714695765
"11772","RPL24",18930.8273776247,-0.112419697824372,0.250979068877723,
-0.447924595174678,0.654207617318334,0.938997714695765
"11773","AC026329.1",11.0336896194779,0.248602145307716,0.554362268639

881,0.448447088431284,0.653830564438808,0.938997714695765
"11774","AC107027.3",5.69624642846869,0.272460347948601,0.608062245707812,0.4480796988661,0.654095678824881,0.938997714695765
"11775","AC068620.1",3.38798070305841,0.36191120764771,0.806534571525631,0.448723737859274,0.653630958461457,0.938997714695765
"11776","C6orf132",127.288003651266,0.109493103008941,0.244704975982551,0.447449434035001,0.654550589999155,0.938997714695765
"11777","C9orf92",2.43695212924975,-0.492492224598214,1.09907411336838,-0.448097374515401,0.654082922784361,0.938997714695765
"11778","FANCG",78.5002247290422,0.111824043487576,0.249952113356213,0.44738186841498,0.654599364982197,0.938997714695765
"11779","DPYSL4",7.18518830318892,-0.312810625063974,0.69724414766107,-0.44863858106706,0.653692397499216,0.938997714695765
"11780","SLC43A3",649.899040443115,0.115322001447128,0.257447447146048,0.44794385310687,0.654193718425975,0.938997714695765
"11781","ENDOV",158.395765731375,-0.089431333669495,0.199379434367216,-0.4485484370709,0.653757437269154,0.938997714695765
"11782","NDUFV2-
AS1",27.6987586766558,-0.152822921088411,0.341235563523177,-0.447851682018574,0.654260241505462,0.938997714695765
"11783","PODNL1",10.29990762106,-0.253958415349228,0.566616570670278,-0.44820153256162,0.654007756776817,0.938997714695765
"11784","ZNF101",23.6839985137277,-0.164991181589567,0.368614182631755,-0.447598571524288,0.654442934273783,0.938997714695765
"11785","ZNF701",64.2179073477431,-0.122810733053178,0.273770255549758,-0.448590489885622,0.653727095427058,0.938997714695765
"11786","LAIR2",2.20642022100017,0.527156299053868,1.17563021660996,0.448403155691228,0.653862264732055,0.938997714695765
"11787","MOCS3",27.342091497751,0.145501651793926,0.325183392044616,0.447444904486274,0.654553859791495,0.938997714695765
"11788","CITED4",200.38339431089,0.193479999905248,0.432639537830649,0.44720831774969,0.654724656325522,0.939058006086876
"11789","PPWD1",201.936889820963,0.0803688598657479,0.179729582197191,0.447165452026539,0.654755603852888,0.939058006086876
"11790","CLK4",212.17641123779,0.0960344309106184,0.214902057991874,0.446875343158648,0.654965067726955,0.939058006086876
"11791","OTUD6B",64.6229367323716,0.107640588674451,0.240880998958277,0.44686209846338,0.654974631285931,0.939058006086876
"11792","PCBD1",895.552848886637,-0.145425590598773,0.325281742657902,-0.447075785472893,0.654820341833369,0.939058006086876
"11793","PPM1A",351.486406387184,0.0827406090449515,0.185104149074872,0.44699489157039,0.654878748310918,0.939058006086876
"11794","AL359513.1",2.54339209802136,-0.446423026900856,0.999830509620618,-0.446498704135613,0.6552370484586,0.939354588378668
"11795","VPS41",182.748480018448,-0.0818508779923142,0.183468895247093,-0.446129453617075,0.655503738166533,0.939453731766719
"11796","GALNT4",81.0334207658908,0.112648459094646,0.252429887377464,0.446256424962074,0.655412028663254,0.939453731766719
"11797","RTN2",225.610654231333,0.128509118253486,0.288007995899919,0.446199828070545,0.655452907109623,0.939453731766719

"11798","NANP",38.5754758455953,-0.131932893552291,0.295750511532441,-0.446095233677453,0.655528455605852,0.939453731766719
"11799","FGL2",2021.91282840833,-0.10293005220268,0.230815004580382,-0.445941772242254,0.655639307130825,0.939532960841426
"11800","TMEM35B",1324.20766333815,0.111468317226196,0.250092724308699,0.445707957055986,0.655808216068873,0.939615737419922
"11801","FAM91A1",157.467508324621,0.103441072577191,0.232044995442139,0.445780234907003,0.655756000407221,0.939615737419922
"11802","EIF4EBP3",360.757971452126,0.118641068791753,0.266292333167823,0.445529420169161,0.655937203616183,0.939720914992579
"11803","RHOT1",158.921799832341,-0.0715025550165042,0.160519247296204,-0.445445366963139,0.655997933089757,0.939728293881353
"11804","TSPAN3",934.481435721751,0.0858996881401134,0.192908057518159,0.445288233395992,0.656111470120186,0.939811312842436
"11805","ZBTB17",78.9511094162701,-0.090430832886296,0.204833339530287,-0.441484931572503,0.658861967945666,0.939849671308979
"11806","PTAFR",5.52624735618833,-0.336672036597734,0.758359805318083,-0.44394762781042,0.657080448941628,0.939849671308979
"11807","ZNF691",35.0660769841909,0.16482424458745,0.372632253279672,0.442324149712677,0.658254657875214,0.939849671308979
"11808","SHC1",385.512990428462,0.0920485607971113,0.20765606296075,0.443272970886701,0.657568303772456,0.939849671308979
"11809","DCAF8",377.113946663129,0.0887714770362821,0.200838393020981,0.44200451766714,0.658485936805253,0.939849671308979
"11810","CDC73",325.463414742406,-0.0624586956996949,0.140884571889974,-0.443332402276617,0.657525322153944,0.939849671308979
"11811","ANGEL2",181.744751167225,0.0717424243086007,0.161466623128633,0.444317363666217,0.656813149200859,0.939849671308979
"11812","AL356512.1",16.4598594975663,-0.236939519148286,0.536243718953127,-0.441850432506413,0.658597441215204,0.939849671308979
"11813","FKBP7",408.267762102345,0.113904659223829,0.256870491826067,0.443432246398146,0.657453116027986,0.939849671308979
"11814","PGAP1",79.6731853763278,0.104555513140166,0.23550205597339,0.443968578991856,0.657065301157086,0.939849671308979
"11815","RAPH1",128.395969547569,0.128994353496805,0.291808108106145,0.442051985237791,0.658451588217154,0.939849671308979
"11816","RFT1",141.926345640063,-0.0751944309358707,0.170310054237715,-0.441514925659739,0.658840258491881,0.939849671308979
"11817","COMMD2",368.581897772407,-0.0877690319474763,0.198590387367868,-0.44196012259593,0.658518062716746,0.939849671308979
"11818","MYOZ2",5.35044721447165,0.285770332969309,0.646659020897208,0.441918110989647,0.65854846444557,0.939849671308979
"11819","MARCH6",607.054337626124,0.0880525996121781,0.198699362478022,0.443144852172929,0.657660964882773,0.939849671308979
"11820","FAM151B",7.71326846765621,0.235221900154887,0.532561432422126,0.441680312983015,0.658720557780622,0.939849671308979
"11821","CDX1",3.56222393180373,0.350913546586616,0.79436124006715,0.441755625635703,0.658666052400424,0.939849671308979
"11822","AL021807.1",2.15966535783724,0.49480502782703,1.11704923735973,0.442957222724182,0.657796676277839,0.939849671308979

"11823","BLVRA",302.26242001695,-0.129364047743118,0.29261430283699,-0
.442097486311817,0.658418663306239,0.939849671308979
"11824","ZNF7",121.626319126418,0.0781850952736026,0.176992938308876,0
.44174132607078,0.658676401162954,0.939849671308979
"11825","IPPK",27.7375784546903,0.142660740891953,0.322645912900253,0.
44215883477209,0.658374272158891,0.939849671308979
"11826","JMJD1C",1064.93274672064,-0.104155048616439,0.235849292417955
,-0.44161696458204,0.658766405779486,0.939849671308979
"11827","ATE1",180.549362537487,-0.0735841397582059,0.166105456182417,
-0.44299652431282,0.657768248711121,0.939849671308979
"11828","AP001160.4",6.76295592245355,0.270090371835237,0.609841153753
601,0.442886430626759,0.657847882761143,0.939849671308979
"11829","AQP11",7.18656647893101,-0.256892058011659,0.580212404802947,
-0.442755197726089,0.65794281250906,0.939849671308979
"11830","GDF11",132.971847062596,0.170436978559713,0.384651369812148,0
.443094687646501,0.657697247516391,0.939849671308979
"11831","DENR",481.734410142485,0.0599614037028189,0.135278809173858,0
.44324313666716,0.657589880727254,0.939849671308979
"11832","MTHFD1",108.320715643234,-0.0834508070033848,0.18900406428938
6,-0.441529166672375,0.658829951074149,0.939849671308979
"11833","RTF1",991.043676235357,-0.0845189049815886,0.191407584362146,
-0.441565078328754,0.658803959078014,0.939849671308979
"11834","AC124068.2",6.76938211830686,0.24251742147988,0.5479034141411
34,0.442628053084936,0.658034790199675,0.939849671308979
"11835","WDR90",64.6373768860019,-0.11683891818083,0.263965294154369,-
0.442629848575856,0.658033491287753,0.939849671308979
"11836","MAPK8IP3",55.2817120608992,0.140086921072963,0.31712523987620
4,0.441740055530266,0.658677320670606,0.939849671308979
"11837","TFAP4",77.7805845074057,-0.109581231282561,0.24708940456313,-
0.443488183867326,0.657412664087649,0.939849671308979
"11838","NMRAL1",194.32359747265,-0.104981506745113,0.236416601487533,
-0.444053023707174,0.657004248732476,0.939849671308979
"11839","TAF1C",73.1886429640063,0.112048479455797,0.253247051157543,0
.442447321473814,0.658165542142675,0.939849671308979
"11840","TEKT3",62.6519119748774,-0.188774560024123,0.424332999131227,
-0.444873626162984,0.656411083158626,0.939849671308979
"11841","ANKRD40",216.08270993136,-0.0997888757172837,0.22507515601852
8,-0.443358020860682,0.657506794782296,0.939849671308979
"11842","GDPD1",11.8846045985549,-0.272998321738056,0.617502988163319,
-0.44210040594306,0.658416550662268,0.939849671308979
"11843","ADCYAP1",4.36134132614961,-0.425887129305086,0.96321114070121
7,-0.442153450379571,0.658378168204587,0.939849671308979
"11844","CILP2",13.9171284691976,-0.207959459125458,0.46892253987023,-
0.44348360644598,0.65741597427435,0.939849671308979
"11845","PVR",45.5139047503353,0.188239919349043,0.423706212184293,0.4
44269906685171,0.656847455667514,0.939849671308979
"11846","KPTN",51.3232495428511,-0.125720439576582,0.283690481435621,-
0.443160584522862,0.657649586269336,0.939849671308979
"11847","FAM71E1",3.71869819447413,-0.369054059543065,0.83234073078308
7,-0.443393007087194,0.657481493067298,0.939849671308979

"11848","DHX35",41.5229254141436,-0.1236797433448,0.277925468475418,-0
.445010469976915,0.656312187814113,0.939849671308979
"11849","CBR1",684.944943641547,-0.0992008711173161,0.223387018198968,
-0.444076257954073,0.656987451077458,0.939849671308979
"11850","IGLC2",393.239817063801,-0.427389395915096,0.961917164914563,
-0.44430997959482,0.656818487069777,0.939849671308979
"11851","APOBEC3G",134.509736799232,-0.134675274249336,0.3027257144663
27,-0.444875568257403,0.656409679588708,0.939849671308979
"11852","CTPS2",63.4876989438117,0.0975922039450987,0.220272563889085,
0.443052018018186,0.657728109929393,0.939849671308979
"11853","MED12",97.1570627720152,0.0865666386667543,0.194540528659865,
0.444979970307922,0.656334229029012,0.939849671308979
"11854","DEK",2124.88084195845,-0.0817684358460437,0.185273152496152,-
0.441339906750613,0.658966939677993,0.939850523840021
"11855","AC105180.2",2.37365276806588,0.44794491794723,1.0151640523592
8,0.441253723382136,0.659029324026927,0.939850523840021
"11856","RTL8A",527.948929417955,-0.0907184242300128,0.205587936221683
,-0.441263363489346,0.659022345859569,0.939850523840021
"11857","IPO11",48.8987709939132,0.121932928446912,0.276390515091431,0
.441161768545407,0.65909588870389,0.939866179152009
"11858","DELE1",143.972008823614,0.0828196848212932,0.187786865480097,
0.441030231851178,0.659191110766242,0.939922693610695
"11859","PRRC2C",1706.61049910046,0.0628421304324939,0.142797703512724
,0.440078018669917,0.659880601727405,0.939995435508306
"11860","AC017083.1",7.79247737412239,-0.234470558127625,0.53265922823
5405,-0.440188671666085,0.659800463810619,0.939995435508306
"11861","NME6",147.57388339556,0.0804811698382583,0.183105545014808,0.
439534312473988,0.660274425261409,0.939995435508306
"11862","COMMD8",258.353155112318,-0.107547709802282,0.244406826805085
,-0.440035620969177,0.659911308332401,0.939995435508306
"11863","CEP44",164.329596238161,0.0750286169605587,0.170326605459125,
0.440498516120338,0.659576086778753,0.939995435508306
"11864","RPL37",31043.0867620723,-0.114483338528265,0.260222491025234,
-0.439944057399571,0.659977625353891,0.939995435508306
"11865","PCDHB11",71.7711226441943,-0.18967792752578,0.430555418489307
,-0.440542423531225,0.659544293254669,0.939995435508306
"11866","ASL",147.52197589459,-0.0812664028907597,0.18483127728935,-0.
43967884701429,0.660169725149536,0.939995435508306
"11867","CYP51A1",236.379524790607,-0.0794083307724219,0.1801540073190
51,-0.440780263254375,0.659372083291172,0.939995435508306
"11868","CDHR3",32.7266655710484,-0.129913991952939,0.295472609185752,
-0.439682014217662,0.660167430917441,0.939995435508306
"11869","LINC02669",2.08709211065584,-0.458509432399838,1.040719354184
74,-0.440569718009151,0.659524529528823,0.939995435508306
"11870","AL353751.1",5.1626875559662,0.285029383529793,0.6474913116803
28,0.440205726915629,0.659788112281043,0.939995435508306
"11871","ACOT4",29.3088601293595,0.166643419801891,0.379102173523544,0
.439573897065883,0.660245749715783,0.939995435508306
"11872","AC005520.3",4.88937290743667,-0.357652967321954,0.81275439381
9067,-0.440050487628091,0.659900541064832,0.939995435508306

"11873","ERVK13-1",49.5266904508595,0.162629518474163,0.370017790823856,0.439518105635038,0.660286165830805,0.939995435508306
"11874","ITPRIPL2",725.318845058835,-0.122176310833376,0.277988583305373,-0.439501181597678,0.660298426042829,0.939995435508306
"11875","NDUFA3",704.159604372571,-0.150586067072545,0.342226508566407,-0.440018710716926,0.659923555770424,0.939995435508306
"11876","NDUFAF5",66.7181586830078,0.109478845768612,0.248939383319486,0.43978113992557,0.660095628687116,0.939995435508306
"11877","ZNF70",29.5925926462769,0.136601086029825,0.309916168365937,0.440767859095792,0.659381064186205,0.939995435508306
"11878","SNRK-AS1",5.3518653342206,0.309625956665915,0.704673658524383,0.439389145486558,0.660379590210188,0.940031832907381
"11879","PAICS",150.176339727194,0.100283087175402,0.228539650025282,0.438799513188666,0.660806813088083,0.940085956726404
"11880","HIST1H3A",9.91281771020447,0.264023442585785,0.601691885533672,0.438801733800363,0.660805203918353,0.940085956726404
"11881","RBPMS-AS1",33.1702133760492,0.140788931078847,0.320629094066568,0.439102170340216,0.660587506570404,0.940085956726404
"11882","AL512598.1",8.33693439286167,-0.224270630081074,0.510703050081818,-0.43914096468612,0.660559398147366,0.940085956726404
"11883","CYSLTR2",2.87144938696036,0.388395045229554,0.884912701677571,0.438907752700639,0.660728378981439,0.940085956726404
"11884","ASB7",45.2324757343019,0.124785272374454,0.2842600107562,0.43898285953939,0.660673956149376,0.940085956726404
"11885","AC007114.2",6.50036891862664,0.289814296118653,0.660347388063498,0.438881566516903,0.660747354057268,0.940085956726404
"11886","LBR",224.0476998639,-0.0914282853650009,0.208579320951516,-0.438338206049933,0.661141134012625,0.94031430018951
"11887","SEMA4C",98.3214219969859,-0.117686146404301,0.268593505841184,-0.438157080662583,0.661272418639044,0.94031430018951
"11888","SLC4A7",214.796286006424,0.134389995717219,0.306905307941038,0.437887492460828,0.661467842823162,0.94031430018951
"11889","YWHAG",576.262966123949,-0.0863271768916181,0.197111581110931,-0.437960957976562,0.661414585466087,0.94031430018951
"11890","DNAJC17",162.636627708498,-0.103152622324989,0.235557359129344,-0.437908722980493,0.661452452006295,0.94031430018951
"11891","LINS1",79.1010733574439,0.115501043345456,0.2634696259244,0.438384663659853,0.661107461947832,0.94031430018951
"11892","ZNF174",53.6802951500618,-0.106157603260406,0.242143913967416,-0.438407067603156,0.661091224012961,0.94031430018951
"11893","DLGAP1-AS2",15.6981650597524,0.215159171116127,0.491180292968823,0.438045202945029,0.661353515869354,0.94031430018951
"11894","LONRF3",61.013902985217,-0.163927050665749,0.374281684108387,-0.43797775212072,0.66140241112761,0.94031430018951
"11895","MIR181A1HG",2.58766646041823,-0.415022952211375,0.948323194186247,-0.437638723544566,0.661648195598225,0.940491609178209
"11896","CENPQ",105.946960763245,-0.101156878463707,0.231224656036719,

-0.437483096299396,0.661761032402571,0.940572926686505
"11897","NDC1",41.8758528169076,0.108665253592705,0.248969924159038,0.436459359337281,0.662503479474636,0.9405983260288
"11898","PHTF1",59.3424917501102,0.10934768439362,0.250454326896036,0.436597305979106,0.662403416762474,0.9405983260288
"11899","ZNF678",68.5281412743259,0.112415500452058,0.257359545288636,0.436803306929926,0.662254000539919,0.9405983260288
"11900","AC116036.2",5.11308359080139,0.272408449257467,0.623364740955713,0.436996883782395,0.662113607993299,0.9405983260288
"11901","THAP6",111.620952865793,0.10447491149067,0.239325016761255,0.436539869105667,0.662445079163704,0.9405983260288
"11902","NSUN2",82.2313760702999,-0.0929517795378414,0.212773621683691,-0.436857627380256,0.662214603172698,0.9405983260288
"11903","MTMR7",3.86842911802834,0.39630704044621,0.908160055082386,0.436384575855693,0.662557727880472,0.9405983260288
"11904","MAMDC4",5.5957720344473,0.294019306196214,0.672483350236559,0.437214253844034,0.661955973461965,0.9405983260288
"11905","MTMR6",146.089318017624,0.0724255883681225,0.16582833897014,0.436750369797551,0.662292395517999,0.9405983260288
"11906","AL359317.2",2.4425330627041,0.448717442174411,1.02642855295599,0.437163834620695,0.66199253562961,0.9405983260288
"11907","LIN52",59.5142088957123,0.126594329853356,0.289686907823624,0.437003973719217,0.662108466207815,0.9405983260288
"11908","RABEP2",77.6590827327059,-0.0954130892908704,0.218634362248672,-0.436404819029996,0.662543043180308,0.9405983260288
"11909","AC116667.1",8.31341329553485,0.248937011847388,0.57023663986362,0.436550362507266,0.662437467593623,0.9405983260288
"11910","AP005205.2",6.07144131555157,-0.362103027631629,0.82860200413817,-0.437004769265859,0.662107889260061,0.9405983260288
"11911","LINC02021",3.23931637295557,-0.429390929028607,0.984398208127552,-0.436196374072401,0.662694258466025,0.940640524718125
"11912","MIR222HG",247.519546949245,-0.335202971280765,0.768478867478192,-0.436190226519505,0.662698718384333,0.940640524718125
"11913","AL137127.1",3.33576382220824,0.349519955936704,0.802220829061862,0.435690452397554,0.663061333828185,0.940782562195469
"11914","SUMO1",1202.80519791771,-0.11349692620282,0.260402562619659,-0.435851802152163,0.662944256480649,0.940782562195469
"11915","MICB",10.2330817993125,0.19579002163855,0.449411687658629,0.435658499801348,0.663084519997685,0.940782562195469
"11916","EPM2A",25.5052875979849,-0.150307553543925,0.345064845027201,-0.435592195814895,0.663132634033925,0.940782562195469
"11917","AL355474.1",3.40218522596012,-0.430781437723422,0.988448618852105,-0.435815711112726,0.662970443863784,0.940782562195469
"11918","LINC01436",94.4805403087458,-0.437208426210221,1.00303070376397,-0.435887380684913,0.662918441371658,0.940782562195469
"11919","DDIT3",479.470779465673,-0.139803281263191,0.32102626332665,-0.435488610229186,0.663207804552339,0.940810265909132
"11920","SOS1-IT1",2.50291157107928,0.43067647644489,0.989766617645042,0.435129321162196,0.663468561557828,0.941070845664473

"11921","TM7SF3",338.785987219775,-0.0611698969336257,0.140593907225406,-0.435082132226082,0.663502812347183,0.941070845664473
"11922","PCDHGA3",11.3642734141841,0.208170614735439,0.479115381733326,0.4344895252211,0.663932999663377,0.941365128579319
"11923","ANK3",6.74494515463193,0.325232249800664,0.748257081990898,0.434653085989262,0.663814255994281,0.941365128579319
"11924","ZNF408",29.1949484262179,0.127519186881934,0.293474942326924,0.434514735298529,0.663914696820262,0.941365128579319
"11925","LYSMD2",158.661960643481,0.130809655521678,0.301049604933745,0.434511965396754,0.663916707795064,0.941365128579319
"11926","ZNF226",126.659333399309,-0.107976086187185,0.248561112243575,-0.434404582489053,0.663994670664019,0.941373628340368
"11927","MAP10",27.2238079182752,0.136045666265124,0.313260640780298,0.434289050569038,0.664078553995622,0.941413615406891
"11928","LAP3",408.309332553502,-0.096285497356324,0.221766344466746,-0.434175427240098,0.664161055677032,0.941451637272574
"11929","RNF168",229.259164222774,-0.10473298569382,0.24138677485308,-0.433880380387723,0.664375307635635,0.94159746031042
"11930","LINC00957",5.48306109742834,0.34326727863537,0.791049717517114,0.43393894344946,0.664332779154241,0.94159746031042
"11931","CNN3",1272.96756382009,0.103744476103748,0.239184486342338,0.433742495971338,0.664475443532947,0.941660447511112
"11932","TRIM26",90.9741223635888,0.077627775183426,0.17902370753681,0.433617291539248,0.664566376024471,0.941706419248471
"11933","SASH3",7.49846185536478,0.294867348046727,0.680131742688096,0.433544458432888,0.664619274952212,0.941706419248471
"11934","CELSR3",2.31973658336588,-0.385409619778541,0.889544876613553,-0.433266077868689,0.664821479074796,0.941913990966705
"11935","MTOR",64.1546426554029,-0.139222711086115,0.323176421177052,-0.43079476707814,0.666617604838418,0.942356562235512
"11936","AL031293.1",14.3848010539639,-0.294947617617543,0.683875736776937,-0.431288320020845,0.666258741958253,0.942356562235512
"11937","AC004865.2",2.77392665968804,-0.39675598188042,0.92153991517173,-0.430535862146008,0.66680588541434,0.942356562235512
"11938","DNAJC27-
AS1",21.4478308846451,-0.155741021919217,0.360582232476195,-0.431915407616482,0.66580289607073,0.942356562235512
"11939","MTIF2",145.34126472621,-0.0922017413834469,0.214065308085166,-0.430717813214108,0.666673564954842,0.942356562235512
"11940","ACVR1",284.678253506536,0.0766565937370205,0.177344670705111,0.432246390219896,0.665562346181328,0.942356562235512
"11941","LY75",12.2561551250528,-0.237325235643818,0.549948632404812,-0.431540732460855,0.666075242114721,0.942356562235512
"11942","AP001816.1",109.404522543739,-0.121347610690323,0.281352430077301,-0.431301093283549,0.666249455518729,0.942356562235512
"11943","SMARCA5-
AS1",2.03001716217912,0.452015750541252,1.04505623552979,0.432527681452572,0.665357937788532,0.942356562235512
"11944","AC104118.1",4.13059396398486,0.32441624906344,0.752752996889748,0.430973042158417,0.666487972026682,0.942356562235512

"11945","TOMM6",1297.0303375357,-0.106962846957249,0.247884736508934,-0.431502352519368,0.666103142435675,0.942356562235512
"11946","PIWIL2",15.3405218535733,0.274250759277387,0.6357126926337,0.431406769843136,0.666172628326381,0.942356562235512
"11947","TPD52",5.46171323491445,0.316176548763391,0.730649836565645,0.432733346317507,0.665208501215835,0.942356562235512
"11948","ZNF239",17.2519235890082,0.164552812034969,0.381102919450291,0.431780507670535,0.665900947848134,0.942356562235512
"11949","RCN1",1069.64569295542,-0.0842621995655298,0.195564830137091,-0.430865813175417,0.666565942440623,0.942356562235512
"11950","ZFP91",542.880327969933,-0.0739035476621816,0.171207742109278,-0.431660080038966,0.665988485275692,0.942356562235512
"11951","PPP1R14B-AS1",5.68507541432194,-0.376262275735257,0.873052876299872,-0.430973066980676,0.666487953977857,0.942356562235512
"11952","ITFG2",132.362143784934,0.100103744559047,0.232367368049103,0.430799493919877,0.666614167584634,0.942356562235512
"11953","LINC02422",5.18808352246674,-0.264838974884623,0.615000187358737,-0.430632348295757,0.666735716402744,0.942356562235512
"11954","NFYB",484.269399598863,0.0759900173865259,0.17562695707865,0.432678551462324,0.665248313986595,0.942356562235512
"11955","RHOF",13.59243098767,-0.209886300062061,0.485996401422438,-0.431868012700826,0.665837344327035,0.942356562235512
"11956","MICU2",253.526017916183,-0.0731976210916741,0.169629944377026,-0.431513559474984,0.666094995484959,0.942356562235512
"11957","ZC3H14",319.25273897074,0.0711716687897341,0.164799096465932,0.431869290038534,0.66583641590477,0.942356562235512
"11958","AL161669.3",24.0843554203356,0.172431436187223,0.400471314803889,0.430571253952765,0.66678014658271,0.942356562235512
"11959","SGF29",185.684955065537,-0.114887729614935,0.265798333857376,-0.432236455163719,0.665569566231155,0.942356562235512
"11960","PTPN2",291.503680075093,-0.09296984526726,0.215800100250088,-0.430814652817671,0.666603144419797,0.942356562235512
"11961","DENND6B",34.9250104396395,-0.146749778486736,0.340210933005798,-0.431349390186221,0.666214343083529,0.942356562235512
"11962","P2RY8",3.0625836084963,-0.427333114844987,0.990855329636744,-0.431277000853042,0.666266971281943,0.942356562235512
"11963","TIMP1",5557.4934218133,-0.140184147181839,0.324872079367177,-0.431505678958025,0.666100724260855,0.942356562235512
"11964","GNL3L",172.408798903715,-0.111737046355519,0.258839556657312,-0.431684584066305,0.66597067321791,0.942356562235512
"11965","SLC16A4",110.919449182202,0.127612682890542,0.296659606247085,0.430165348444016,0.66707536651149,0.942422317594943
"11966","GAS5",3562.88064780617,-0.0985942238982691,0.229174503998511,-0.430214627622406,0.667039522425746,0.942422317594943
"11967","TSN",295.415917778027,0.0846865182597881,0.196766050455467,0.430391920068318,0.666910571901415,0.942422317594943
"11968","GEMIN7-AS1",6.83150680026893,-0.246440246666724,0.572811219562171,-0.430229433800355,0.667028753037669,0.942422317594943

"11969","LINC00649",24.2354929840043,-0.194564426471476,0.452518683667583,-0.429958880138531,0.667225553161074,0.94255574006579
"11970","CHD7",18.878206063109,-0.245329200320307,0.570727477207605,-0.429853494211682,0.667302216842988,0.942585286748641
"11971","AF129075.2",5.60306223648746,-0.272062578369439,0.633035225622325,-0.429774785600563,0.667359476202623,0.942587421571628
"11972","ZNF684",60.7116648270599,0.107190847406677,0.250057496373064,0.428664802940989,0.668167178431322,0.942956582015286
"11973","AL078645.1",2.65414527101163,0.408199627485161,0.951298616014673,0.429097257804552,0.667852447801012,0.942956582015286
"11974","NEB",7.38617200942417,0.353376176664578,0.823906000171961,0.428903511554501,0.667993444615032,0.942956582015286
"11975","CCDC174",289.032369711757,0.0817618753622094,0.190982045046195,0.428112890625047,0.668568932174075,0.942956582015286
"11976","NEK10",2.62434290312171,-0.374390849372771,0.873382401341064,-0.428667727673354,0.668165049682837,0.942956582015286
"11977","IRF1",4375.41113094019,-0.180694355650877,0.422039466416094,-0.428145635727652,0.668545093368804,0.942956582015286
"11978","SLC22A16",2.45702676217858,0.554128832217377,1.29423090106791,0.428152991680346,0.668539738197967,0.942956582015286
"11979","FBXL13",3.80819214563012,-0.355123634352181,0.829492679865718,-0.428121480722012,0.668562678453934,0.942956582015286
"11980","KMT2E-AS1",213.767988352586,-0.131046960502305,0.305488096232618,-0.428975669161646,0.667940931299013,0.942956582015286
"11981","CDC37L1",219.25092070638,-0.0818111067078149,0.190758192832765,-0.428873357903625,0.66801538967397,0.942956582015286
"11982","COLCA1",116.384672942218,0.145071435487259,0.338474001163141,0.428604368396781,0.668211165940383,0.942956582015286
"11983","NELL2",10.2649180072417,0.245876171950901,0.574162241894876,0.428234659143467,0.668480285004092,0.942956582015286
"11984","GSPT1",512.187255477795,-0.0513640120438032,0.119915063124634,-0.428336613477975,0.668406066064661,0.942956582015286
"11985","WRAP53",35.7704456739135,-0.151365931535786,0.353374886191268,-0.42834377158839,0.668400855349962,0.942956582015286
"11986","SPAG5-AS1",3.09853681452567,-0.346051747646719,0.807677276668053,-0.428452994337419,0.668321349116593,0.942956582015286
"11987","AC005899.7",9.49422148887866,0.234701246633791,0.547033104970587,0.42904395456361,0.667891237510408,0.942956582015286
"11988","B3GNT8",61.5058969622234,-0.106551375627587,0.248715912749015,-0.428405944959021,0.668355597192886,0.942956582015286
"11989","PHLDB1",513.788721593342,-0.0929024369451679,0.217191564063578,-0.427744223610695,0.668837348989629,0.943256476496509
"11990","PHF13",111.670448007669,0.146257355398172,0.342446292613876,0.427095747720897,0.669309590030768,0.943297962403235
"11991","FAM218A",3.60174749057143,-0.323076406990157,0.756523190267894,-0.42705420157147,0.66933984973621,0.943297962403235
"11992","RARS",262.034299374755,-0.0783249532865777,0.183185172651686,-0.42757256033766,0.668962346977397,0.943297962403235

"11993","AC005070.3",12.9874349794485,0.201847513773214,0.472588285457162,0.427110700761351,0.669298699270812,0.943297962403235
"11994","TTLL5",60.1038057611336,-0.140726753154179,0.329317020653962,-0.427329121570218,0.669139624597171,0.943297962403235
"11995","DNM1P35",2.44493201275473,0.454814356242655,1.06473217333275,0.427163156739245,0.669260494516326,0.943297962403235
"11996","RPS6KB1",242.282982686899,0.0741727350187554,0.173700801977378,0.427014349815238,0.669368875852496,0.943297962403235
"11997","AL022322.1",11.4398835274812,-0.18666457921211,0.436988948067102,-0.427160869943664,0.669262160018165,0.943297962403235
"11998","KRBOX4",135.159504698771,0.0914148654615597,0.214040618653719,0.427091203700235,0.669312899594763,0.943297962403235
"11999","SIL1",448.283052606559,0.08601379928677,0.201511464046928,0.426843205638857,0.669493534713402,0.943316390411183
"12000","RBM28",230.306415699554,0.105891594443638,0.24806653285637,0.42686771659339,0.669475680741545,0.943316390411183
"12001","GEMIN5",111.786654027564,-0.075163868882621,0.176153935284358,-0.426694236272877,0.669602049187223,0.943390671415513
"12002","AP002026.1",19.6408877961837,0.162526602973766,0.380997688258254,0.426581598740828,0.669684102867671,0.943397964914718
"12003","AC010864.1",4.61569174074338,-0.326344909461559,0.765108879380878,-0.426533946025611,0.669718817889245,0.943397964914718
"12004","AC233296.1",3.61054164922739,0.39192698189109,0.919720155727015,0.426137210814176,0.670007867030825,0.943726509143384
"12005","SNHG3",187.011582767515,-0.10983445879288,0.259378211198301,-0.423452911813431,0.67196484727483,0.943827131310708
"12006","LRRC42",128.120655862521,-0.102094917127475,0.24208020432501,-0.421740048560129,0.67321476914036,0.943827131310708
"12007","SSR2",2977.38295276602,-0.107344127944846,0.252460601627762,-0.425191603175843,0.670697004828429,0.943827131310708
"12008","UCK2",29.5714343075833,-0.129749378997754,0.3062118740432,-0.42372419228736,0.671766969753738,0.943827131310708
"12009","PCAT6",19.755836516188,-0.152518738022053,0.36087300815941,-0.422638253827731,0.672559212042453,0.943827131310708
"12010","YPEL5",578.238116014002,0.0853409372896181,0.202358954299059,0.421730471899435,0.673221760026307,0.943827131310708
"12011","LCLAT1",31.0284603005075,0.152346328269491,0.360114999220257,0.423049105422881,0.672259434006752,0.943827131310708
"12012","AC132153.1",2.37988482321786,-0.397013551732036,0.937746755885835,-0.423369688287539,0.672025556772424,0.943827131310708
"12013","CCT4",848.776562999424,-0.0769956880316049,0.182112754869803,-0.42279129809799,0.672447537141168,0.943827131310708
"12014","MOGS",110.105021649289,0.110030685687766,0.25980501554633,0.42351255404515,0.671921341062341,0.943827131310708
"12015","PECR",48.2594451650739,-0.117792425690044,0.277278254326054,-0.424816673692452,0.670970321843188,0.943827131310708
"12016","AC092053.3",12.5544655527442,0.275082406697597,0.64636754891963,0.42558201932845,0.670412444608433,0.943827131310708
"12017","ACTR8",88.5852378235415,0.103184494529524,0.243677097972004,0.423447650141414,0.671968685470687,0.943827131310708

"12018","AC107068.1",8.58628162921919,0.215430628051651,0.510014707284023,0.422400815064494,0.672732483072829,0.943827131310708
"12019","CENPE",22.6554551128776,0.167012247733722,0.395742027298707,0.42202302564054,0.673008210873751,0.943827131310708
"12020","AC096564.1",2.68714994512423,0.37221456097161,0.876231361115813,0.424790275136493,0.670989567568254,0.943827131310708
"12021","COL25A1",7.00747982196049,0.246123357550278,0.580468044411601,0.424008453040278,0.6715596485549,0.943827131310708
"12022","MIR4458HG",190.188976440077,0.109191722520432,0.25894635592732,0.421676999969366,0.673260794627834,0.943827131310708
"12023","SMN2",26.0935046981537,-0.231427323732947,0.548187472888491,-0.42216820919587 6,0.672902244148487,0.943827131310708
"12024","LINC01366",8.11540733321762,0.245692889551212,0.579333935578174,0.42409545594116,0.671496199323627,0.943827131310708
"12025","HLA-DRB5",78.2279307561634,0.587094314641576,1.38642642547015,0.423458687641853,0.671960634030667,0.943827131310708
"12026","LHFPL5",3.76196967507581,0.402088185257818,0.952247252189225,0.422251872434826,0.672841182885666,0.943827131310708
"12027","AL353135.1",3.23247833690167,0.41989622419345,0.986154596575233,0.425791478994964,0.670259796513387,0.943827131310708
"12028","STX11",40.6566848517586,-0.180083254479741,0.427489436174652,-0.421257788475894,0.673566850235365,0.943827131310708
"12029","MRPS24",1175.55206120643,-0.104032277288528,0.245462178541662,-0.42382202385151,0.671695614991228,0.943827131310708
"12030","POLR2K",729.142168816178,-0.115033644586544,0.272273409373347,-0.422493128694794,0.672665115099654,0.943827131310708
"12031","AL592211.1",2.06913011664458,-0.530819889819623,1.25412154136453,-0.423260323909334,0.672105338625709,0.943827131310708
"12032","AKR1C8P",19.3059263137297,0.212620064241309,0.501788274948108,0.423724656107792,0.67176663145311,0.943827131310708
"12033","PRKCQ",2.17294650962857,-0.437396813610258,1.03538123391139,-0.422450010956731,0.672696580923579,0.943827131310708
"12034","IGSF22",4.73383532395692,-0.293956209392527,0.693130002348344,-0.424099675957749,0.671493121821304,0.943827131310708
"12035","AP000442.1",3.89757042115592,0.312457989736182,0.741610023534962,0.4213238492204,0.673518617383626,0.943827131310708
"12036","KLRB1",33.6896112181178,0.162020793976342,0.383376702348901,0.422615127585118,0.67257608766291 2,0.943827131310708
"12037","SSPN",3099.26999090772,0.0996643091182279,0.23497835994084,0.424142500370333,0.671461891869563,0.943827131310708
"12038","ZNF641",96.7983465905553,0.119356768546671,0.283024095679247,0.421719459116085,0.673229799305245,0.943827131310708
"12039","ATF7",450.900308638416,0.0526784955910459,0.124221533290302,0.424068953230016,0.671515526898465,0.943827131310708
"12040","ARL1",860.115449208107,-0.079260137861493,0.187863572084857,-0.421902644466334,0.673096079735008,0.943827131310708
"12041","LINC00426",3.44071625375123,0.418662317721746,0.988703866290136,0.423445616019154,0.671970169290215,0.943827131310708
"12042","LMO7-

AS1",20.7664906514564,0.354298713914522,0.838413805015813,0.4225821566
8078,0.672600147389025,0.943827131310708
"12043","AHNAK2",732.990194475516,-0.117577537922008,0.27807348846119,
-0.422829010319041,0.672420020013316,0.943827131310708
"12044","NDUFAF1",90.5392252832272,0.108204747515898,0.255941163647022
,0.422771960453878,0.672461647231427,0.943827131310708
"12045","ZNF774",4.59366625885123,0.273855841204159,0.645060056714226,
0.42454316982377,0.67116972882952,0.943827131310708
"12046","METTL26",754.180328532641,-0.100123702780681,0.23742283409734
5,-0.421710502957057,0.673236337285838,0.943827131310708
"12047","ZNF48",20.9460897327684,-0.149153353461789,0.352751446320605,
-0.422828467515984,0.672420416072294,0.943827131310708
"12048","AC018553.1",5.69515054559636,-0.247205768755174,0.58062542809
5675,-0.425757737765558,0.670284385217445,0.943827131310708
"12049","RNF166",102.724772124647,-0.0829914335843001,0.19529911093003
4,-0.424945270816066,0.670876571889475,0.943827131310708
"12050","INPP5K",84.0038534010028,-0.0873272267484512,0.20532817055613
2,-0.425305629090859,0.670613890575594,0.943827131310708
"12051","CD68",562.751311504244,-0.0975989999950127,0.231078421955771,
-0.422363105862361,0.672760002977254,0.943827131310708
"12052","SLFN11",208.335519457861,0.0852703846345591,0.20098441932009,
0.424263656471582,0.671373541151334,0.943827131310708
"12053","C17orf53",4.54668112839792,0.275241665813633,0.65100166525477
,0.422797176265158,0.672443248044383,0.943827131310708
"12054","PRKAR1A",999.861407394022,0.0598148018802,0.141287860358307,0
.423354148958795,0.672036892564257,0.943827131310708
"12055","OXLD1",170.715344596598,-0.110914199846463,0.262894886498494,
-0.421895615102042,0.673101210760208,0.943827131310708
"12056","AP001020.2",12.0315436412232,0.30713634692373,0.7292628786963
24,0.421159990308003,0.673638257958198,0.943827131310708
"12057","PLIN3",570.839711818419,-0.102358948868468,0.241386663634852,
-0.424045584487252,0.671532569151172,0.943827131310708
"12058","NFIX",1952.56199410987,0.0900950763326634,0.212091487155574,0
.424793458431345,0.670987246792841,0.943827131310708
"12059","ZNF257",13.2074973282112,0.222130547991357,0.527091660166007,
0.421426793057961,0.673443457799641,0.943827131310708
"12060","SMG9",91.749347499516,0.0871624987061848,0.2069322137258,0.42
1212807502661,0.673599692907267,0.943827131310708
"12061","TFPT",79.250639073879,-0.11627669692419,0.27553438769403,-0.4
22004301885217,0.673021877460589,0.943827131310708
"12062","ZIM2",3.64723090139414,0.310124927407963,0.736391544059087,0.
421141347846708,0.673651870159547,0.943827131310708
"12063","ZNF749",66.3385794195657,0.218384385579771,0.513650900136007,
0.42516110751863\9,0.670719234000143,0.943827131310708
"12064","AVP",12.3596891434614,-0.280887396455902,0.66335588095251,-0.
4234339432592,0.671978684176354,0.943827131310708
"12065","SLC17A9",4.19982977005407,-0.354937142284172,0.84196016399537
7,-0.421560493551,0.673345847562875,0.943827131310708
"12066","AC004000.1",3.29515689730093,-0.355633082519823,0.84126096455
6528,-0.422738124676088,0.672486336446004,0.943827131310708

"12067","ENOX2",111.548511938226,-0.131635515140475,0.312369215455855,
-0.421410012982147,0.673455708757558,0.943827131310708
"12068","MECP2",400.009426116338,0.0907353422053868,0.214144973635029,
0.423709885248247,0.6717774050324,0.943827131310708
"12069","SLC35E2A",87.1073903967764,0.194591358245839,0.46382412007573
6,0.419536953391006,0.67482375386348,0.944041849721321
"12070","DFFB",28.6726516432256,-0.128300314520836,0.307000921091964,-
0.417915080073662,0.676009206493977,0.944041849721321
"12071","FAM229A",42.0759381790412,-0.110346131070565,0.26680005009413
3,-0.413591118261157,0.679173578923637,0.944041849721321
"12072","NSUN4",72.2342982904584,0.085656508690753,0.207833112947995,0
.412140815656197,0.680236213898648,0.944041849721321
"12073","NRDC",402.20219533936,0.0690141887935817,0.166136500070128,0.
415406540792964,0.677844319272051,0.944041849721321
"12074","PDE4B",40.4429804287329,-0.201890511992613,0.485305116885521,
-0.416007383742951,0.677404600186293,0.944041849721321
"12075","TLCD4",156.833695482849,-0.163658045189473,0.390845905364027,
-0.418727797690612,0.675415077815625,0.944041849721321
"12076","TMEM167B",251.735762003903,0.0622605759901678,0.1510387076753
06,0.412216026927427,0.680181091054489,0.944041849721321
"12077","SLC16A1-
AS1",166.121866965677,0.110976340141244,0.269146533085555,0.4123268424
41501,0.680099876736443,0.944041849721321
"12078","WDR3",104.093406622523,-0.071413277332785,0.171495505971063,-
0.416414861301582,0.677106455571688,0.944041849721321
"12079","APH1A",711.263613220936,-0.0730187924623298,0.176892035535298
,-0.412787338001768,0.679762429156956,0.944041849721321
"12080","FLAD1",68.7712330682901,-0.100396539025412,0.240800835584148,
-0.416927702023397,0.676731290367678,0.944041849721321
"12081","AL590560.2",2.59897050825975,0.41819800038461,0.9972083212710
41,0.419368743184548,0.674946663906053,0.944041849721321
"12082","HSD17B7",56.147343397805,-0.104350731812499,0.249919588150103
,-0.417537227013297,0.676285500755753,0.944041849721321
"12083","AL645568.1",29.8458001321496,0.129653271020589,0.310244735301
968,0.417906434075002,0.676015528146051,0.944041849721321
"12084","GLRX2",189.264315788909,-0.100013517380467,0.238938385935271,
-0.418574508189575,0.67552712303674,0.944041849721321
"12085","UGP2",1377.20379938934,0.0541158812160895,0.129990372546243,0
.416306839930304,0.677185488103101,0.944041849721321
"12086","AC233263.6",11.854947874097,-0.234320726143625,0.566885538560
297,-0.413347510572809,0.67935202550726,0.944041849721321
"12087","ACOXL-
AS1",5.1154604445796,-0.26266327599192,0.635455480771927,-0.4133464639
77062,0.679352792194396,0.944041849721321
"12088","PRPF40A",773.596221955053,0.06602042311934,0.159426299866846,
0.414112496962426,0.67879172099297,0.944041849721321
"12089","SSB",1157.39105325246,-0.076850339449143,0.184548697211003,-0
.416423093798795,0.677100432510328,0.944041849721321
"12090","HOXD-
AS2",222.079330878948,-0.157479119701142,0.380325307064896,-0.41406426

7551544,0.678827040770335,0.944041849721321
"12091","CNOT9",120.094561349444,-0.101403257272836,0.242478951384819,
-0.418194060530667,0.675805237994908,0.944041849721321
"12092","RBM6",433.124098668511,0.0961676376900884,0.229968963479522,0
.418176593202117,0.675818008029253,0.944041849721321
"12093","PHF7",52.7047950673898,-0.120274858046411,0.287626069069202,-
0.418163966971551,0.675827238886282,0.944041849721321
"12094","NEPRO",153.927553318747,0.0751387090490515,0.179215344431179,
0.419264931178401,0.67502252295295,0.944041849721321
"12095","AC073352.2",2.71718486848192,-0.392394868764355,0.94765439494
9427,-0.414069592095646,0.678823141419724,0.944041849721321
"12096","GK5",174.789986770988,0.135497336490702,0.32252168299425,0.42
0118533528542,0.67439886410525,0.944041849721321
"12097","LINC02043",2.21134869317912,-0.590299783259665,1.404955182094
13,-0.420155596977694,0.674371789875156,0.944041849721321
"12098","AC055764.2",2.12470480901181,0.459134147488855,1.097243807090
52,0.418443143193771,0.675623148510175,0.944041849721321
"12099","IQCG",49.0811486504139,-0.0946764417837992,0.225177359804981,
-0.420452757176811,0.67415473456034,0.944041849721321
"12100","OCIAD1",1278.21642590174,-0.0897782585238203,0.21466866662671
,-0.41821780483659,0.67578787913042,0.944041849721321
"12101","AC053527.2",2.45998219350668,-0.505622717467196,1.22414170844
249,-0.413042635489083,0.679575376767699,0.944041849721321
"12102","AC019131.2",9.7480909390185,-0.22600836617436,0.5409036171073
7,-0.417834821262616,0.676067889809938,0.944041849721321
"12103","RPL34",32284.3918403677,-0.101488812434437,0.246044571690825,
-0.412481412359572,0.679986601950312,0.944041849721321
"12104","AC123595.1",3.04507857848924,-0.37390773052693,0.904693374263
612,-0.413297743924871,0.679388482594969,0.944041849721321
"12105","NUDT12",85.321833226515,-0.0946397383423196,0.225294410546252
,-0.420071399520543,0.67443329531921,0.944041849721321
"12106","COMMD10",207.760922491448,-0.1166104502285,0.279235642480084,
-0.417605894408042,0.676235286441718,0.944041849721321
"12107","FOXC1",90.1170173751365,0.149106496453699,0.361361165427859,0
.41262457264093l,0.679881695027033,0.944041849721321
"12108","CAP2",38.0412605654675,-0.194945003852664,0.471330603078943,-
0.41360565721639,0.679162929471492,0.944041849721321
"12109","HIST1H4C",1491.98859841901,-0.155865593453753,0.3755206632777
67,-0.415065291196671,0.678094107226025,0.944041849721321
"12110","HIST1H4E",8.23296453073693,0.325621319210219,0.78459027995177
2,0.415020842764244,0.678126645204284,0.944041849721321
"12111","GTF2H4",94.3768608790797,-0.117657635306636,0.284470948493369
,-0.413601585433526,0.679165911952846,0.944041849721321
"12112","FKBP5",1149.18459067039,0.296103617148802,0.709979862873074,0
.417059176792085,0.676635123792818,0.944041849721321
"12113","AL590999.1",3.63976080857577,-0.330821648385766,0.78983573477
914,-0.41884867171561,0.67532673138994,0.944041849721321
"12114","AL080317.2",15.5468393078256,-0.217579804992651,0.52456726467
0719,-0.414779609111198,0.678303248071755,0.944041849721321
"12115","CEP85L",159.915491508988,0.0785985988985311,0.189938631803899

,0.41381049316855,0.6790128986296,0.944041849721321
"12116","HECA",218.152808904668,-0.106187538593207,0.254430743167191,-0.417353411271647,0.676419926532469,0.944041849721321
"12117","AL358852.1",13.7757342211067,-0.18751493174143,0.454479561344123,-0.412592661344009,0.679905078881273,0.944041849721321
"12118","ZMIZ2",101.373495768069,-0.106050355692016,0.256058160226024,-0.4141651084207,0.678753192921402,0.944041849721321
"12119","AP1S1",326.657839385826,-0.126772513392038,0.301413932559494,-0.42059274538351,0.674052492091718,0.944041849721321
"12120","ZNF786",23.1065294691922,-0.163118413887543,0.392292732392342,-0.415807891450826,0.677550583841396,0.944041849721321
"12121","CCAR2",72.4839005970775,-0.0851559880399144,0.205553120465051,-0.414277281936923,0.678671049564801,0.944041849721321
"12122","ZFPM2",233.035009845228,0.0771044618239424,0.183294138728037,0.420659724086137,0.674003575329169,0.944041849721321
"12123","TAF2",163.686257557942,0.0763165980114301,0.181874344417602,0.419611673410074,0.674769159240699,0.944041849721321
"12124","MTSS1",236.633923564223,-0.151088757059288,0.364818967363013,-0.414147208823568,0.678766300940549,0.944041849721321
"12125","ZC3H3",61.7130406992077,0.09641753091026,0.230961819499606,0.417460908123926,0.676341312057336,0.944041849721321
"12126","TRIM14",8.77411052962426,0.26732111262565,0.648833963098433,0.412002342400648,0.680337706343405,0.944041849721321
"12127","LPAR1",733.713360239347,0.0845623054850811,0.201712769047818,0.41922138040272,0.675054348004043,0.944041849721321
"12128","SLK",587.76786718691,0.0905520144964302,0.219624740107192,0.412303342748338,0.680117098848515,0.944041849721321
"12129","CASC2",16.0955272276563,0.150642806224922,0.359089993827981,0.419512681539898,0.674841488602975,0.944041849721321
"12130","INPP5F",54.8278251750482,0.110922383792277,0.268010529852669,0.41387323047812,0.678966949614079,0.944041849721321
"12131","SLC25A22",20.0922488358958,-0.174013268658349,0.42134142678596,-0.412998242270506,0.679607901553179,0.944041849721321
"12132","DNHD1",21.9139483062298,0.196066850732885,0.471360359440375,0.415959566404069,0.677439590659605,0.944041849721321
"12133","AC090559.1",2.69516313015882,-0.417001306991736,1.01028497755736,-0.412756119565345,0.679785303759332,0.944041849721321
"12134","MS4A4E",4.43040559640858,-0.341740547348638,0.818149463962838,-0.417699408728282,0.67616690466543,0.944041849721321
"12135","AP5B1",84.465416890215,0.109739032579548,0.262569968325583,0.417942056661535,0.675989482302478,0.944041849721321
"12136","AIP",766.997537144525,-0.0997974227792343,0.239583884432884,-0.416544806490067,0.677011387454732,0.944041849721321
"12137","CEP57",463.828979923964,0.0641970805834688,0.152731472544748,0.420326469154287,0.674246976059136,0.944041849721321
"12138","PGR-AS1",8.66067222305673,0.260713492303782,0.625908764886935,0.416535934515756,0.677017878042305,0.944041849721321
"12139","AP000880.1",6.92837410750914,0.280494066387126,0.670629917278906,0.418254627716634,0.675760959190491,0.944041849721321

"12140","FAM118B",31.9944990519261,-0.156942543224024,0.373521100429089,-0.420170488477716,0.674360912002026,0.944041849721321
"12141","AC004803.1",7.8000065536998,-0.228315858354264,0.552626981912822,-0.413146418519031,0.679499342238227,0.944041849721321
"12142","PLEKHG6",5.40231039237742,-0.302507235824609,0.724721418975751,-0.417411750093081,0.67637726181455,0.944041849721321
"12143","ZNF384",115.789183803173,0.0928562350612348,0.220913323982486,0.420328812166152,0.674245264665196,0.944041849721321
"12144","MYL6B",647.03083316557,-0.113182025587841,0.274685627468083,-0.412042037405077,0.680308611735793,0.944041849721321
"12145","LINC02356",12.9350889647221,0.226645381871265,0.545143271645569,0.415753791085257,0.677590175273601,0.944041849721321
"12146","RPLP0",18145.3948593544,-0.0784615969653777,0.189096659733133,-0.414928518970713,0.678194231723765,0.944041849721321
"12147","ABCB9",6.84423356398117,-0.262695696796528,0.636432640189708,-0.412762765778675,0.679780433872315,0.944041849721321
"12148","HNRNPA1L2",33.3650553765277,-0.116172369565948,0.280313188891336,-0.414437758085589,0.678553541391126,0.944041849721321
"12149","PRMT5",130.226572142299,-0.0860678530787377,0.207761577953626,-0.414262607776058,0.678681795064525,0.944041849721321
"12150","PSMB5",1089.7865652319,-0.118526232509328,0.287570463573614,-0.412164138960629,0.680219119909909,0.944041849721321
"12151","WDR89",46.4993278541717,0.105524576938945,0.252346802428097,0.418172831688695,0.675820758012953,0.944041849721321
"12152","SAMD15",3.76563505205372,-0.323563953999692,0.773915722049557,-0.418086808138229,0.675883649667409,0.944041849721321
"12153","FOXN3",1565.91300728469,0.0914612196458567,0.220395954879997,0.414985927013299,0.678152205310535,0.944041849721321
"12154","SETD3",181.035644943979,0.0725743344642268,0.175749297270157,0.412942387773349,0.679648824315779,0.944041849721321
"12155","SEMA6D",16.1231578939803,0.245855930547293,0.591160200018319,0.415887149607964,0.677492583180594,0.944041849721321
"12156","PIGB",39.5467007180807,-0.116349172218295,0.280560262670257,-0.414702962960369,0.678359363055486,0.944041849721321
"12157","AC009560.3",6.79995448871904,0.334970419765482,0.806523140046326,0.415326483684326,0.677902916313925,0.944041849721321
"12158","CLPX",230.734694646768,0.0656048602775427,0.15681220690503,0.418365773776585,0.675679706646884,0.944041849721321
"12159","EEF2KMT",57.4229709404218,0.115044477546957,0.278327203054788,0.413342556114829,0.679355654914581,0.944041849721321
"12160","AC007216.3",9.05807867360357,-0.237413169284467,0.575474029890788,-0.412552360233393,0.679934611035285,0.944041849721321
"12161","PALB2",51.2828173967203,0.114836105245311,0.278101536963851,0.412928696831467,0.679658855364992,0.944041849721321
"12162","CLN3",320.054280241987,-0.0792207172046441,0.190842903908748,-0.415109577469665,0.678061688551774,0.944041849721321
"12163","AC026471.1",21.8097972464692,-0.142882525967685,0.340590992582037,-0.419513519381371,0.674840876413438,0.944041849721321
"12164","ATPAF2",87.0579367867464,0.0986262242171174,0.234490062413405,0.420598737541551,0.674048115764317,0.944041849721321

"12165","TOP3A",84.4205052879892,0.110794707979748,0.264268584280142,0
.419250393615834,0.675033146319593,0.944041849721321
"12166","MAP3K14",30.3760641828717,0.155844523865022,0.373987534396162
,0.416710477039423,0.676890190066474,0.944041849721321
"12167","RNF213-
AS1",21.4874142524595,0.206846990774261,0.497406505400013,0.4158509961
74477,0.677519039816914,0.944041849721321
"12168","TAF4B",67.2184015751491,0.125037369294307,0.302257644315112,0
.413678104246564,0.679109864633672,0.944041849721321
"12169","SLC14A2",6.82417686593682,0.336108013952949,0.802414775363802
,0.418870669225355,0.675310653963735,0.944041849721321
"12170","ILF3",472.097757072608,0.0815404882762787,0.196103001256456,0
.415804387254856,0.677553148235858,0.944041849721321
"12171","AC010618.3",23.4887521637097,0.131640733602893,0.317886372499
949,0.414112541433068,0.678791688426172,0.944041849721321
"12172","COX7A1",1135.41830248993,-0.114663993909399,0.275314065390468
,-0.416484329439455,0.677055631925927,0.944041849721321
"12173","ZNF234",67.3436207487061,0.131095749325736,0.31236726778942,0
.419684655993192,0.674715835738204,0.944041849721321
"12174","SAE1",182.073893076815,-0.0866044020775258,0.208502968573396,
-0.415362921065748,0.677876246076347,0.944041849721321
"12175","ZNF845",36.7555137320107,0.146382961185881,0.351624153702523,
0.416305193043484,0.677186693055457,0.944041849721321
"12176","SPAG4",3.59114191690824,0.389750375596943,0.945142351512481,0
.41237214158612,0.680066679031503,0.944041849721321
"12177","DSN1",77.4723115356681,-0.0944996332731986,0.226472424136948,
-0.41726772534591,0.676482592791976,0.944041849721321
"12178","PTPN1",210.004789772016,0.100177362859607,0.243095838064395,0
.412089995687506,0.680273461161263,0.944041849721321
"12179","AURKA",6.99990468598705,0.2297067580954,0.555471224260225,0.4
13534937658244,0.679214730531996,0.944041849721321
"12180","TAF4",48.5306433409297,0.109003452008613,0.262961379223914,0.
41452266614329,0.678491370884273,0.944041849721321
"12181","DOP1B",42.2216054329264,0.112127053732969,0.267841189162022,0
.418632601220796,0.67548465974872,0.944041849721321
"12182","MIATNB",38.051743545587,-0.120078400468547,0.28835664094379,-
0.416423218399033,0.677100341350434,0.944041849721321
"12183","ASB9",5.83559609018331,0.261674357410577,0.631107967864179,0.
414626927142349,0.678415032958377,0.944041849721321
"12184","HUWE1",395.315395537817,0.0775898137380904,0.187275833740546,
0.414307666869523,0.678648799690315,0.944041849721321
"12185","PIN4",478.809383728717,-0.104449526226003,0.24938047843763,-0
.418836016677728,0.675335980705731,0.944041849721321
"12186","ACTB",11259.6800008969,-0.133606471986282,0.324531980130838,-
0.411689695210985,0.68056687894481,0.944091969159366
"12187","AC069287.2",3.79365255347908,-0.380371376030839,0.92361081940
2967,-0.411830792840556,0.680463449730764,0.944091969159366
"12188","MIS18BP1",289.942696594521,0.0859639399759722,0.2088285694203
06,0.4116483688731,0.680597173650551,0.944091969159366
"12189","DOK5",89.7252494403029,0.108135213252756,0.262649609184024,0.

411709020198814,0.68055271273651,0.944091969159366
"12190","USP35",16.0331933416186,0.166199776025625,0.403875986912769,0
.411511903186068,0.680697214915313,0.944153282181142
"12191","AC118553.1",12.0771555380263,-0.17505963766116,0.425606118617
83,-0.411318423310435,0.680839062187804,0.944195421349881
"12192","CHST7",375.732300455907,-0.149336161175782,0.363067301336108,
-0.411318123736885,0.680839281825038,0.944195421349881
"12193","CINP",282.234899425246,0.0831321361745012,0.202260647355929,0
.411014882337487,0.681061622078896,0.944414236977822
"12194","AC090206.1",3.39572950254664,-0.391295444169109,0.95217154779
7572,-0.410950574058003,0.681108777247904,0.944414236977822
"12195","GTF2H2",73.3448626927396,0.10280609971788,0.250227823544533,0
.410849993664208,0.681182532076927,0.944426112420014
"12196","PEX11G",80.8687939348204,0.131751486651822,0.32072979549674,0
.410786551488826,0.681229055303672,0.944426112420014
"12197","ZMIZ1",684.830893752118,-0.0963531249604688,0.234663165646649
,-0.41060182877425,0.681364522517657,0.944536471815082
"12198","NUP35",89.5057070610805,-0.114602164849687,0.2792283865243,-0
.410424478242344,0.681494592977455,0.944639332518676
"12199","TUBA1B",2869.05438438033,-0.129125012051872,0.314677550913723
,-0.410340717591496,0.681556027076847,0.944647045316447
"12200","TMEM39A",151.400564075017,-0.0722452994089307,0.1761612384122
5,-0.410108943715888,0.681726032215152,0.944710714349659
"12201","AC124242.3",3.03651002694475,0.393240744624557,0.959720867977
898,0.409744914115604,0.681993078977521,0.944710714349659
"12202","AL133477.2",3.94188054881543,-0.293090812620489,0.71460285668
2264,-0.410145033538268,0.681699559423618,0.944710714349659
"12203","SUPT16H",604.984075184223,-0.068008742261516,0.16595960575902
5,-0.409790936478034,0.681959315448763,0.944710714349659
"12204","TMX1",342.435207075355,-0.0806186502175609,0.1966295980831,-0
.410002619155483,0.681804026229272,0.944710714349659
"12205","SEL1L",468.551674281575,0.0615988662664418,0.150293510223833,
0.409857126729572,0.681910757205322,0.944710714349659
"12206","ZNF500",74.578944458507,0.12700499512122,0.309803074319316,0.
409953953492129,0.681839725892897,0.944710714349659
"12207","B4GALT7",183.859699003703,-0.128139868108367,0.31282990654913
1,-0.409615146844159,0.682088283972581,0.944716022068409
"12208","GET4",5.63482454794822,-0.279975207676154,0.683697781809337,-
0.409501412941882,0.682171730095071,0.944716022068409
"12209","RNF208",34.6698041784728,0.14588974159544,0.356301469237463,0
.409455907963739,0.682205118009476,0.944716022068409
"12210","SKIDA1",15.449493874035,-0.184136763615706,0.449733734098515,
-0.409435071586982,0.682220406284319,0.944716022068409
"12211","SMAP1",487.388550487317,-0.0779043010668552,0.190343648922625
,-0.409282376941945,0.682332446906891,0.944717294752193
"12212","KMT2E",999.83584720571,0.0884968523506415,0.216265124797016,0
.409205378970389,0.682388947291728,0.944717294752193
"12213","FP565260.3",2.23452540701163,0.605038326095771,1.478527562497
13,0.409216805585892,0.682380562436615,0.944717294752193
"12214","AC018647.2",57.5287156781242,-0.1053430748942,0.2575657975599

42,-0.4089948117808,0.682543468598453,0.944853853533867
"12215","XRCC6",1035.21042624082,-0.0688159633793029,0.168380609306656
,-0.408692922912369,0.6827650280493,0.945083184138974
"12216","MED30",335.943750919533,-0.110860240199636,0.271384554859023,
-0.408498708621148,0.682907578434717,0.945203121821725
"12217","CROCC",284.801992980647,0.0708185145251383,0.173462665722187,
0.40826372770357,0.683080066033852,0.94526182451621
"12218","DHFR",89.7596214746063,0.110736105424268,0.27127077037992,0.4
08212448651139,0.683117709590937,0.94526182451621
"12219","ZNF77",31.1835763764445,-0.128850492351851,0.315597581428334,
-0.408274650802768,0.683072047572747,0.94526182451621
"12220","ZNF285",4.04989722331002,0.325565078241464,0.797791144855745,
0.408083093351872,0.683212671819789,0.945315863758511
"12221","ZC3H15",824.523269260568,-0.0699375423336363,0.17157353023741
5,-0.40762431265977,0.683549512014905,0.945646633381628
"12222","CCDC82",476.530587545375,0.060752791249813,0.149048147213799,
0.407605142267669,0.683563588430935,0.945646633381628
"12223","FBXO44",83.7347397713751,0.106488135095132,0.261899600422453,
0.406599074314598,0.684302477237609,0.945670244856471
"12224","CSDE1",1799.58624055406,-0.0558055358296091,0.137017135774449
,-0.407288734465107,0.683795935917056,0.945670244856471
"12225","CRTC2",114.985253059344,-0.0728717501268329,0.179824083840654
,-0.405239101295276,0.685301765781699,0.945670244856471
"12226","BARD1",58.5044905666205,0.111073583201371,0.274399876991991,0
.404787292250181,0.68563387052779,0.945670244856471
"12227","NHEJ1",80.6307682539305,-0.104605617016423,0.256833407243242,
-0.407289760857913,0.683795182158434,0.945670244856471
"12228","PASK",9.37653529516618,0.21804352199743,0.537780867487668,0.4
0545050071427,0.685146396341356,0.945670244856471
"12229","TRAT1",4.36694760379973,-0.292081755210666,0.721573975984334,
-0.404784214691533,0.685636132912707,0.945670244856471
"12230","AC110609.1",6.67315211317412,0.276404557352892,0.682589667673
607,0.40493516155164,0.685525171687472,0.945670244856471
"12231","HPGD",119.964086407801,0.139441030452647,0.343033087387728,0.
406494404124455,0.684379367779902,0.945670244856471
"12232","LIFR-
AS1",46.8241886879997,-0.143077168145181,0.352390657507342,-0.40601861
9100878,0.684728919867065,0.945670244856471
"12233","ECHDC1",433.164249556445,0.0899499008562763,0.221672158169881
,0.405778973773252,0.684905009255975,0.945670244856471
"12234","AC104051.2",13.7676525666161,0.26171317322586,0.6465801525611
62,0.40476524401374,0.685650078761266,0.945670244856471
"12235","DCAF13",238.407133873727,0.0787751463668075,0.193702333385182
,0.406681452877293,0.684241964387758,0.945670244856471
"12236","TLE4",240.076662379969,0.145618382766288,0.35771985555441,0.4
07073805116583,0.683953781891951,0.945670244856471
"12237","CTSL",2535.30108579047,-0.141557199606255,0.348614334249935,-
0.406056738633034,0.684700911531776,0.945670244856471
"12238","PTGR1",488.340804643171,-0.128202080622782,0.316067232437596,
-0.405616487460762,0.685024412585915,0.945670244856471

"12239","TUBB4B",1711.91425081821,-0.0957021582194168,0.235378414499491,-0.406588507374042,0.684310239545542,0.945670244856471
"12240","ZNF214",30.9380654582296,0.125601267853163,0.308602987809273,0.40699951981926,0.684008340869554,0.945670244856471
"12241","AC068205.2",2.71559610237725,-0.440523290018272,1.08679334460457,-0.405342278000935,0.685225933697109,0.945670244856471
"12242","LTBP3",1300.68557611131,0.0762598340187367,0.188137695740355,0.40534053379702,0.68522721561340,0.945670244856471
"12243","MRPL48",123.430707471463,-0.0948404613951333,0.233442936401961,-0.406268284904664,0.684545485738235,0.945670244856471
"12244","EIF2B2",126.058385712115,0.0972857066555788,0.240176726999357,0.405058840925245,0.685434259880584,0.945670244856471
"12245","CCDC88C",7.23530507362041,0.278965666501431,0.688501205091409,0.405178181880443,0.68534654139065,0.945670244856471
"12246","UBL7",137.641111665565,0.0875003532926314,0.215591584611424,0.405861636252358,0.684844267620361,0.945670244856471
"12247","ALG1",78.2272569882592,-0.116640125094386,0.286525186612833,-0.407085068064176,0.683945509942743,0.945670244856471
"12248","IRX6",31.1260057652822,0.229252463496973,0.563895401165647,0.406551397693753,0.684337499995818,0.945670244856471
"12249","HS3ST3B1",4.62721596903405,-0.334387135730376,0.821186801870613,-0.407199841703085,0.683861217856127,0.945670244856471
"12250","RASL10B",5.31179455107485,0.28528202709491,0.70400715692868,0.405226032558375,0.685311371175669,0.945670244856471
"12251","SPAG9",854.210587547602,0.068725231918563,0.169427261702827,0.405632666359833,0.685012523142893,0.945670244856471
"12252","ARMC7",38.1877166302102,-0.117001225885378,0.288700794496632,-0.405268111885101,0.685280443463345,0.945670244856471
"12253","AC020911.2",18.1488642021932,0.19902425198855,0.490876893792969,0.405446364465649,0.685149436177696,0.945670244856471
"12254","AC093227.1",16.5941741709424,0.175813866459818,0.434176049298414,0.404936814787263,0.685523956429445,0.945670244856471
"12255","DERL3",3.83173208720855,-0.329266348997656,0.810445266104085,-0.406278329664977,0.684538106054495,0.945670244856471
"12256","GCAT",40.0336107160841,0.121289823567094,0.299035940237142,0.405602829783298,0.685034449310905,0.945670244856471
"12257","KLHL15",105.21955115394,0.130710738850929,0.321746853480321,0.406253355509268,0.684556454120947,0.945670244856471
"12258","HDAC6",86.2639557498875,-0.0861551491473273,0.211450981366637,-0.407447383741115,0.683679431378099,0.945670244856471
"12259","GDI1",299.700451401724,-0.0638009184961136,0.157500655749545,-0.405083510239912,0.685416126989633,0.945670244856471
"12260","PEX14",63.4331749752519,0.0981811306777036,0.242853279964127,0.404281674483484,0.686005599695887,0.946006253948133
"12261","SLC45A3",2.30511910172617,-0.37001065403793,0.915180553416607,-0.40430344881847,0.685989589682044,0.946006253948133
"12262","ANKRD42",97.2376356327695,-0.0998940630365943,0.247281836901164,-0.40396846079933,0.686235911892103,0.946216254903256
"12263","HSPA2",74.836521129237,-0.135356737721921,0.335105825551465,-0.403922365417468,0.686269809195566,0.946216254903256

"12264","EHBP1L1",140.137398891386,-0.078465134761826,0.194351570995392,-0.403727813261085,0.686412884568945,0.946336354557381
"12265","NAV2-AS1",3.16779738278725,-0.488621728369502,1.21054330418414,-0.403638371862139,0.686478664334581,0.946349878236372
"12266","AL121601.1",9.26021394160029,-0.198812197280073,0.492650852752037,-0.403555979187841,0.686539262210153,0.946356256762536
"12267","ABCC5",137.388536984651,0.09255767367308,0.229494073565359,0.403311825160139,0.686718843590173,0.946372337388755
"12268","TNFAIP3",363.587738762761,0.157396809406292,0.390251888342191,0.403321070590898,0.686712043022933,0.946372337388755
"12269","AL354892.3",7.19953288314626,0.310494364789667,0.769822175747329,0.403332580655064,0.686703576717824,0.946372337388755
"12270","AL031280.1",9.71130659457011,0.238866955331304,0.592876560179366,0.402894921767592,0.68702552731334,0.946557169846427
"12271","KIAA1614",60.8833454027469,0.117022735437777,0.291218033386523,0.401838904263382,0.687802588490345,0.946557169846427
"12272","NRP2",83.6876084944928,0.306583578501705,0.761007292312532,0.40286549366704,0.687047177247977,0.946557169846427
"12273","AC137695.3",2.01710920894106,-0.453954964009601,1.12807828213076,-0.402414416801069,0.687379061720152,0.946557169846427
"12274","SMN1",36.4892265205866,-0.132401613114628,0.329543620913963,-0.401772647722395,0.687851353785659,0.946557169846427
"12275","PKD2L2",3.87290861750001,-0.312518552916211,0.776054304322332,-0.402701912966141,0.687167526475141,0.946557169846427
"12276","PSMB8-AS1",57.5860708894171,0.105033286735625,0.26119752780359,0.402122055361127,0.687594201858556,0.946557169846427
"12277","TNRC18",379.472384537479,0.0843129735620307,0.209248215872465,0.402932819333661,0.686997646862848,0.946557169846427
"12278","RAMP3",2.23024180597689,-0.419509547018752,1.04437716770313,-0.4016839509632,0.687916637276608,0.946557169846427
"12279","TNS3",70.0339082458029,0.179875419478844,0.446748332570274,0.402632548047729,0.687218561871551,0.946557169846427
"12280","HSD17B6",2.3546819795082,0.477669257719676,1.18886533241871,0.401785841250727,0.687841643149479,0.946557169846427
"12281","DEPDC4",4.31346598440766,0.286936603059212,0.713341534340692,0.402242949899748,0.687505236074408,0.946557169846427
"12282","TSR3",473.625723519357,-0.08236307980444,0.2049053203586,-0.401956765496855,0.687715844975694,0.946557169846427
"12283","PEX12",28.1415240968181,0.131288791858773,0.326821250902168,0.401714366787223,0.687894250050342,0.946557169846427
"12284","MRO",17.0126939866728,0.303741293584834,0.754501138234537,0.40257234640568,0.687262856521915,0.946557169846427
"12285","GZMM",15.963445522972,0.180829589494474,0.449852065546336,0.401975678993183,0.687701925402626,0.946557169846427
"12286","ZNF136",116.45614889399,0.0930826481092451,0.231122986555124,0.40274076368014,0.687138942596811,0.946557169846427
"12287","AL031673.1",2.72664174584093,0.376505379875098,0.937208817299742,0.401730513974328,0.687882365205459,0.946557169846427

"12288","SLC35C2",263.440974531669,-0.0700985544700036,0.174209195739291,-0.402381482633718,0.68740329573534,0.946557169846427
"12289","TPTE2",2.24177499471949,0.492022426718334,1.22517376728707,0.401593994138341,0.68798285058967,0.946571245648152
"12290","RAB3D",61.0515431407458,0.100140285361845,0.249448332572352,0.401447002387956,0.688091049982702,0.946643081619815
"12291","CCDC18",83.934937404419,-0.0873493523066366,0.217748399482813,-0.401148079683274,0.688311104168083,0.946656499238022
"12292","DISC1",18.9722729478472,0.162435675908166,0.404833831510642,0.401240368923801,0.688243161936519,0.946656499238022
"12293","GSKIP",68.4857304881635,-0.102017932999866,0.254274308575609,-0.401212114473336,0.688263962256756,0.946656499238022
"12294","DNAJA4",74.8457966308585,0.114074011182776,0.284381980193629,0.401129533963811,0.688324757607774,0.946656499238022
"12295","CMTM1",7.17694860861812,-0.223362849495136,0.557030146502389,-0.400988799076027,0.688428370556652,0.946721991815525
"12296","GEN1",23.0146908882499,-0.167451567770456,0.417699622295774,-0.400889919052604,0.688501172284768,0.946745105806024
"12297","CCDC150",12.5186486282631,-0.178508199299657,0.445582514655242,-0.400617603762511,0.688701682943456,0.946943811922253
"12298","POGLUT2",70.6048544482658,-0.0964381221825702,0.240784479582989,-0.400516355329837,0.688776239569705,0.946969316851892
"12299","NPRL3",206.430293892774,-0.0866967264068522,0.216559332965941,-0.400337058761108,0.688908276164276,0.947073837985656
"12300","AC093635.1",4.52696981397036,-0.320457191101561,0.801097984877806,-0.400022465604431,0.689139970031108,0.947315334413494
"12301","AC099343.2",2.33808993434212,0.466401385861663,1.16637797455326,0.39987156482469,0.68925111688913,0.947324685747034
"12302","SMCR5",17.2576472444529,-0.188578384164146,0.471609733502269,-0.399861094392019,0.68925882919683,0.947324685747034
"12303","ARHGAP26",212.614222624985,0.135156725769224,0.338134974304964,0.399712351693398,0.689368393529854,0.947398260408256
"12304","CPLANE2",5.96772101299405,-0.318999929169796,0.801131922934468,-0.398186515900316,0.69049270470727,0.947692705971483
"12305","AL353622.1",4.75652652685001,0.250656983613781,0.629543800517853,0.398156543528179,0.690514796681651,0.947692705971483
"12306","BCS1L",108.62997962038,-0.0996793082861074,0.250713288577361,-0.397582867871601,0.690937691149464,0.947692705971483
"12307","LIMD1-AS1",9.86062207474348,0.223804833607619,0.562659369016565,0.397762564584667,0.690805214373542,0.947692705971483
"12308","DKK2",113.665076615961,-0.160790086421378,0.402723282747171,-0.399256991859399,0.689703853484463,0.947692705971483
"12309","AL646090.2",4.22560602272651,-0.44437476969486,1.11645563334191,-0.398022775311458,0.690613397497365,0.947692705971483
"12310","KIAA0408",250.14519160417,0.137223202135779,0.34503614043039,0.397706750268566,0.690846361027718,0.947692705971483
"12311","AC004540.1",34.6138171143641,0.151878911589402,0.380778108756503,0.39886460932691,0.689992967489905,0.947692705971483
"12312","HBP1",761.159330967108,0.0658199931893148,0.165608145760539,0

.39744417695786,0.691039943927278,0.947692705971483
"12313","PCM1",875.928100101395,0.0801434486314461,0.200934341260479,0.398853914809679,0.690000848022531,0.947692705971483
"12314","NKX3-1",3.9917944286026,-0.334152447790583,0.840103239792739,-0.397751647610622,0.690813262361519,0.947692705971483
"12315","RRAGA",968.010733566227,-0.0947837634475566,0.238172703699783,-0.397962327232224,0.690657955617787,0.947692705971483
"12316","ISCA1",517.573369454788,-0.0894212156439197,0.224763922932186,-0.397845056614797,0.690744402418693,0.947692705971483
"12317","AL356481.1",17.2032392034188,-0.172661787157047,0.433720443317768,-0.398094647871013,0.690560419439634,0.947692705971483
"12318","UCK1",181.510862572221,-0.0694576226643029,0.174023057804557,-0.399128848444381,0.689798266715211,0.947692705971483
"12319","AL731566.2",2.01953842252679,0.483375800918536,1.21463122745747,0.397960953079037,0.690658968560419,0.947692705971483
"12320","DHX32",125.583235305554,0.0766656984135583,0.192524657166867,0.398212361687832,0.690473654559096,0.947692705971483
"12321","OSBPL5",115.415195086858,0.0735079740499079,0.184884050632523,0.397589590872893,0.690932734619369,0.947692705971483
"12322","SLC26A10",4.28594464653465,0.352038310170722,0.884429213284818,0.398040119981149,0.690600612410955,0.947692705971483
"12323","USP30-
AS1",2.80112776251123,0.321124616884308,0.806430850511968,0.39820477686393,0.690479245083036,0.947692705971483
"12324","AC073592.1",3.23175748153032,0.372420497242278,0.933009048983028,0.399160648707763,0.689774836534757,0.947692705971483
"12325","ANKRD12",1784.82266802634,-0.0871638494423782,0.218463510711236,-0.398985849667091,0.689903630749005,0.947692705971483
"12326","AC005523.2",26.0691938913577,-0.154126699189886,0.38729450363097,-0.397957362536557,0.690661615293821,0.947692705971483
"12327","AP2S1",1227.39081490792,-0.105843101407925,0.265619167460652,-0.398476896150969,0.690278685483084,0.947692705971483
"12328","ZNF543",26.7713984434658,-0.158034617951354,0.397351972463326,-0.39771947518378,0.690836980062243,0.947692705971483
"12329","ZNF132",9.47630103227049,0.240204909042392,0.604296880127997,0.397494868733253,0.691002569701637,0.947692705971483
"12330","TXNDC12",416.24657193224,0.0885513528050652,0.222966672224262,0.397150623102988,0.691256391237415,0.947744054176256
"12331","PTPN7",7.68203591920247,-0.297098887610099,0.750770151752108,-0.395725491905539,0.692307547632894,0.947744054176256
"12332","AC021851.1",4.174565260433,0.288670551279681,0.729479216491607,0.395721419820605,0.692310551994969,0.947744054176256
"12333","ZFP2",11.1427384602927,-0.194548501788698,0.491501337418121,-0.395824969288323,0.692234155265771,0.947744054176256
"12334","IQCE",71.0061368774934,-0.101993713869921,0.256941706583244,-0.396952737748229,0.691402313126773,0.947744054176256
"12335","MIOS",77.0876451693951,-0.0934998471167345,0.235573615767772,-0.396902882404736,0.691439078574592,0.947744054176256
"12336","RFC2",135.098598012215,-0.0978334441566867,0.246239569066926,-0.397310003942122,0.691138871154381,0.947744054176256

"12337","MYOM2",21.8511835425411,0.190442118178769,0.480306020524178,0
.396501626131879,0.691735008476986,0.947744054176256
"12338","AL354920.1",11.9650564572647,0.247416181805889,0.624575190253
949,0.396135142200079,0.692005334626158,0.947744054176256
"12339","ANAPC2",75.7900448516748,-0.0892680605749296,0.22509220363960
6,-0.39658441799191,0.691673944921763,0.947744054176256
"12340","TIMM23B",52.8214944183325,-0.110299669149859,0.27828377349791
8,-0.396356811478568,0.691841822076596,0.947744054176256
"12341","COPB1",640.464474534197,0.0686657668786134,0.173391625047336,
0.396015475717859,0.692093611606925,0.947744054176256
"12342","ACAT1",538.1708149919,0.0735438243013976,0.185557936698724,0.
396338877279094,0.691855050559698,0.947744054176256
"12343","AC068987.1",2.06576657405445,-0.448891945536887,1.13332905610
263,-0.396082623241451,0.692044076915254,0.947744054176256
"12344","COMMD4",440.0423034743,-0.111844906283911,0.281763451163184,-
0.396946111435638,0.691407199609196,0.947744054176256
"12345","HOMER2",10.1002166891098,-0.190378426406257,0.480469550094173
,-0.396234113834982,0.69193232722584,0.947744054176256
"12346","MT1E",5237.29788198892,-0.250415570944634,0.631948102067649,-
0.396259708867402,0.691913447264246,0.947744054176256
"12347","CYB5D2",323.3626377795,-0.0990980245608863,0.250286523073279,
-0.39593831639058,0.692150533627182,0.947744054176256
"12348","NSF",122.56807224917,-0.0935533571396215,0.236029109220883,-0
.396363641113739,0.691836784479562,0.947744054176256
"12349","TOB1-
AS1",6.79132815517751,0.232186300742473,0.585524411356752,0.3965441854
15704,0.691703618411491,0.947744054176256
"12350","NAPA-
AS1",33.4662746735978,0.143896998571423,0.363102354962315,0.3962987201
95186,0.69188467125294,0.947744054176256
"12351","ZNF135",45.0917344563019,0.12715782781486,0.321133217306104,0
.395965975994482,0.692130128369397,0.947744054176256
"12352","MXRA8",4287.04395978335,-0.076002335259208,0.193521993439971,
-0.392732287985569,0.694517220503194,0.947749635107611
"12353","MCOLN2",3.58307441927372,0.406136985547458,1.03122403409767,0
.393839720679932,0.693699376681748,0.947749635107611
"12354","MIA3",510.027581357653,-0.0720276143942601,0.182620733240823,
-0.394410936349033,0.693277670807266,0.947749635107611
"12355","AC017002.3",7.88857805948182,0.22243030676044,0.5623368836760
52,0.395546358806114,0.692439715634825,0.947749635107611
"12356","RALB",287.329180862996,-0.0860926342348267,0.219187291844982,
-0.392781139408917,0.694481136009179,0.947749635107611
"12357","MAP3K20",1362.75987728557,0.0815097401717005,0.20619508606347
1,0.395303989672238,0.692618555361255,0.947749635107611
"12358","PAX3",12.3161455760013,-0.285187218708018,0.726209068219885,-
0.392706771628563,0.694536068639334,0.947749635107611
"12359","SH3BP5-
AS1",31.140053332103,0.16827396268784,0.428528305518178,0.392678757787
919,0.694556761806152,0.947749635107611
"12360","METTL6",91.1471013316494,0.0963894797302566,0.243797113138217

,0.395367600910065,0.692571616139918,0.947749635107611
"12361","LRRC2",113.352828537393,-0.159385659682869,0.404036566024774,-0.394483254946523,0.693224287627307,0.947749635107611
"12362","KIF9-AS1",14.0545691891977,0.163539027111877,0.413984893114384,0.395036219514153,0.692816157959159,0.947749635107611
"12363","RASSF1-AS1",3.95378354831728,-0.28240366619464,0.718813534917969,-0.392874719904749,0.694412013962139,0.947749635107611
"12364","SPCS1",1418.49044553169,-0.104732840155172,0.266500842830917,-0.392992528813953,0.694324999518078,0.947749635107611
"12365","LMOD3",3.05891301270984,0.309712090013548,0.783333270522668,0.395377167890363,0.692564556693739,0.947749635107611
"12366","HES1",570.025963669984,-0.112171076248477,0.283973595528466,-0.395005303361851,0.692838974065885,0.947749635107611
"12367","EXOSC9",149.747487912262,-0.0854716559018993,0.216546566317158,-0.39470335344277,0.693061827619745,0.947749635107611
"12368","TRIM27",128.026054601718,0.0730965132361562,0.185472269134783,0.394110200824883,0.693499680083846,0.947749635107611
"12369","HCG25",22.6554788331366,-0.141578905576282,0.359281381937648,-0.394061347717851,0.693535746957925,0.947749635107611
"12370","RNF8",222.327421776108,-0.0808915335825882,0.205640721406031,-0.393363401127497,0.694051097067979,0.947749635107611
"12371","HYMAI",713.096964264729,-0.093776550633747,0.238503661849678,-0.393187047555067,0.694181335499515,0.947749635107611
"12372","TRIM24",92.5751158656328,0.0793283731824782,0.200908803838152,0.39484767052012,0.692955311365116,0.947749635107611
"12373","MOB3B",138.836843949169,-0.18310249988233,0.46547015137198,-0.393371088012052,0.694045420451727,0.947749635107611
"12374","AL359091.4",6.56959026041761,-0.306575781948896,0.780526854269491,-0.392780569011716,0.694481557333632,0.947749635107611
"12375","FAM111A",209.155748789372,0.0594100730165775,0.150620761800633,0.394434819651324,0.693260040780584,0.947749635107611
"12376","AP002784.1",13.3440192413853,-0.252353668718115,0.641143050195154,-0.393599632159005,0.693876652842348,0.947749635107611
"12377","AC068790.7",6.36353775470344,0.235095089235261,0.595535316257381,0.39476263257183,0.69301807467722,0.947749635107611
"12378","SYNE3",870.99021178348,-0.122153191103781,0.310162617292617,-0.393835956667006,0.69370215581800 8,0.947749635107611
"12379","LINC02207",2.6602353787642,-0.341152318113617,0.864230680477267,-0.394746826073354,0.69302974109163,0.947749635107611
"12380","RAB11FIP3",79.3155112766106,-0.103947213705463,0.263694852080936,-0.394195081493507,0.693437016726379,0.947749635107611
"12381","MVP",558.927776755166,-0.069836239743983,0.177279872315838,-0.393932141487355,0.693631139628126,0.947749635107611
"12382","DLX3",7.13894445421486,-0.271103473535681,0.686055680614203,-0.395162493652076,0.692722970594762,0.947749635107611
"12383","TBX2",24.3456237261295,0.151713378822285,0.384755766490985,0.394310864281327,0.693351543185852,0.947749635107611
"12384","TRIM65",99.887775160243,0.100181995638812,0.254468298565112,0

.393691458636361,0.693808848182646,0.947749635107611
"12385","ESCO1",176.538738829038,0.0923631022938523,0.234653086908363,
0.393615543314468,0.693864903871546,0.947749635107611
"12386","ZNF266",50.0654942322893,0.10565082430445,0.267485148381782,0
.394978281761105,0.692858916223479,0.947749635107611
"12387","HOOK2",127.305632294266,-0.0859438306830163,0.218436170618884
,-0.393450546397677,0.693986742969534,0.947749635107611
"12388","SIRPB1",2.36222647842031,0.387136530454649,0.984280236170722,
0.393319418828095,0.694083577479381,0.947749635107611
"12389","SLC2A11",58.30132596776,0.104978931952217,0.26728201074618,0.
392764674506689,0.694493297863358,0.947749635107611
"12390","NUP50-
DT",14.4169470945089,-0.157007144373695,0.396897841809482,-0.395585785
142821,0.692410625287097,0.947749635107611
"12391","AL022328.3",3.19007959683618,-0.31239611112766,0.792351446395
736,-0.39426458113845,0.693385710007748,0.947749635107611
"12392","VAX2",5.4025210292548,-0.26947811711513,0.686596540591215,-0
.392483942437414,0.694700673629945,0.947762951959836
"12393","RXRB",155.096647165509,0.0665641240433403,0.169778295587069,0
.392064979879618,0.69501020225310,0.947762951959836
"12394","UQCC2",276.253981957428,-0.121555212948612,0.309842983124856,
-0.392312298709147,0.694827477490192,0.947762951959836
"12395","SGK1",391.700323441216,-0.260611339053613,0.663969292722093,-
0.392505108158206,0.694685037791738,0.947762951959836
"12396","AC027117.1",3.83297294720668,0.312575545963058,0.797267517703
659,0.392058548758336,0.695014953947836,0.947762951959836
"12397","ZNF16",29.0089641527916,-0.124826006103358,0.31828956379253,-
0.39217750219647,0.694927066014837,0.947762951959836
"12398","NIPSNAP3A",122.535283187217,-0.0936508883713159,0.23884056092
2317,-0.392106298903626,0.694979673587546,0.947762951959836
"12399","ADSSL1",19.3456680214042,0.249201664574248,0.635397050703158,
0.392198333779596,0.694911675164168,0.947762951959836
"12400","PNPT1",174.205934384899,-0.0629331335913211,0.16056787066118,
-0.391941011188462,0.695101799802282,0.947804937988466
"12401","RBM38",55.0618538927599,0.130869498495658,0.333978810892089,0
.391849704914194,0.695169266706376,0.947820495240013
"12402","PRRT2",80.0515052594192,-0.16447220506962,0.419934335375222,-
0.391661722356329,0.695308176055768,0.947933449504187
"12403","PHF1",246.250468561113,-0.0749928747524622,0.191513224666092,
-0.391580659159253,0.695368080705672,0.947938684880392
"12404","ANKRD13B",88.7978520715532,-0.0953262030453755,0.243488019555
425,-0.391502642386381,0.695425735883375,0.947940853137383
"12405","POLR3B",37.9009152894588,0.139921345967835,0.357547380550261,
0.391336515324202,0.695548511315725,0.948021669508713
"12406","POMP",1389.28439752462,-0.108699251392834,0.277847484295936,-
0.391219131129718,0.695635268371327,0.948021669508713
"12407","NPAS3",15.9897241785731,0.172893645351405,0.441963016459845,0
.391194826065527,0.695653232410374,0.948021669508713
"12408","AC090198.1",6.46997086076251,0.227167144275708,0.580823994360
977,0.39111184538035,0.69571456528887,0.948028841868489

"12409","AC024941.2",18.3812669465907,-0.187723309660849,0.480123314203775,-0.390989781390152,0.695804789128947,0.948075378724493
"12410","AC012368.1",6.44407720897488,-0.248099527257758,0.634850949060506,-0.390799647736074,0.695945335373103,0.948190469821791
"12411","CDK11B",142.619677236135,-0.110531734583672,0.283459672012301,-0.389938130524879,0.696582297195467,0.948279702211265
"12412","YIPF1",86.3693392108512,-0.0846762518141819,0.218274027139105,-0.387935536463155,0.698063739208147,0.948279702211265
"12413","LRRC8C",32.0095657898294,0.160495098071326,0.41225993904722,0.389305588222441,0.697050102941071,0.948279702211265
"12414","RASAL2-AS1",9.16553022987622,-0.282752058048768,0.724600829166211,-0.390217684920575,0.696375585478884,0.948279702211265
"12415","AL355999.1",3.55428460046236,0.289573406230788,0.743903797215095,0.389261900953921,0.697082416743277,0.948279702211265
"12416","C1orf35",282.850717287787,-0.0802049243631835,0.205661792941709,-0.389984562596495,0.696547962233291,0.948279702211265
"12417","RHOU",280.727839330916,-0.128220634892759,0.329616948844838,-0.388998913260119,0.697276950294393,0.948279702211265
"12418","STARD7-AS1",14.542432383675,-0.153844004441755,0.395389108381647,-0.389095200602386,0.697205723668314,0.948279702211265
"12419","MRPS9",196.52693561635,-0.0938138418439519,0.241941683520771,-0.38775394334188,0.698198131820916,0.948279702211265
"12420","ARHGAP15",32.1775713001514,0.131865703182147,0.338316006576482,0.389770807821168,0.696706031888133,0.948279702211265
"12421","CCDC173",4.92454951748506,-0.248581579051038,0.638697861591696,-0.389200581369647,0.697127773422258,0.948279702211265
"12422","AC093388.1",6.46806607127444,0.216015653143367,0.55644692662674,0.388205312684328,0.697864101927657,0.948279702211265
"12423","ARHGEF26-AS1",10.3005642995188,-0.20760508543923,0.535110713526802,-0.387966602408217,0.698040749012788,0.948279702211265
"12424","LXN",235.713871715041,0.109010599984048,0.279207639556328,0.390428428667892,0.696219769524812,0.948279702211265
"12425","AMACR",70.3092738790306,0.10860793941258,0.279521695860027,0.388549228990676,0.697609630509659,0.948279702211265
"12426","AC026741.1",2.34785792668367,-0.448788021409276,1.15308157507284,-0.38920752105585,0.697122640242472,0.948279702211265
"12427","SNX2",893.205356167439,-0.0756353509038253,0.195008110581783,-0.38785746232901,0.698121518798953,0.948279702211265
"12428","AC008608.2",37.0781720703261,-0.131265776936738,0.337064342502321,-0.38943833679421,0.696951917270347,0.948279702211265
"12429","TTC1",466.154661572533,-0.0788808521223638,0.203407530740437,-0.387797107782708,0.698166186019999,0.948279702211265
"12430","XRCC2",5.28922233298184,-0.261005252574363,0.67129935854816,-0.388806050908267,0.697419624365718,0.948279702211265
"12431","ZNF510",65.5071945852884,0.0997605745447865,0.256738028834293,0.388569527458572,0.697594612278672,0.948279702211265
"12432","IL15RA",401.37809654802,-0.101363254275294,0.260320574756494,

-0.389378574360133,0.696996119111598,0.948279702211265
"12433","LOXL4",67.640421040709,0.138046885796349,0.353742828064136,0.
390246458286697,0.696354310785033,0.948279702211265
"12434","RBM4",945.042581102769,-0.0934701956857931,0.239298274904692,
-0.390601209820758,0.696092031240668,0.948279702211265
"12435","CHORDC1",201.635392943881,-0.0669486477360322,0.1723296621913
8,-0.388491725015296,0.697652176629027,0.948279702211265
"12436","DPY19L2",2.73925878856254,-0.347447800605859,0.89212191527888
4,-0.389462241264687,0.696934237194023,0.948279702211265
"12437","ATP2B1",596.024754548581,0.093982858354748,0.242142785249317,
0.38812991375308,0.697919895836318,0.948279702211265
"12438","TCTN1",286.285901767195,-0.0727187491711497,0.18654148312938,
-0.389826155293909,0.696665101686464,0.948279702211265
"12439","SUGT1",627.381537430797,-0.0479559172605869,0.123608539204926
,-0.387966054522192,0.698041154470666,0.948279702211265
"12440","MYH11",13.9696272839562,0.208262125884669,0.535391249710192,0
.388990529817962,0.697283151903486,0.948279702211265
"12441","SULT1A3",12.8390346286406,-0.202148366777242,0.52117964079832
,-0.387866967457901,0.698114484333986,0.948279702211265
"12442","MSI2",104.929740888988,0.113589701250986,0.292197318535955,0.
388743133647234,0.697466171076983,0.948279702211265
"12443","BAIAP2",49.0563682666168,0.110957332758635,0.285870378636228,
0.388138614738498,0.697913457173836,0.948279702211265
"12444","XAB2",225.651986765515,-0.0610192155755881,0.15678740138857,-
0.389184430861014,0.697139719760252,0.948279702211265
"12445","NUDT19",60.1966297055486,-0.110353316152331,0.284146700612513
,-0.388367402874821,0.69774416351174,0.948279702211265
"12446","HELZ2",246.23052891315,0.161109640458184,0.414652244758701,0.
388541585134643,0.697615286001458,0.948279702211265
"12447","MX1",231.248208206085,-0.179837901867472,0.463397574627335,-0
.388085548380563,0.697952726209834,0.948279702211265
"12448","TTLL1",59.4832017113143,0.11830517616534,0.303906221955511,0.
389281849526129,0.697067661478942,0.948279702211265
"12449","CA5B",106.506873362651,-0.111597481403953,0.287773990904325,-
0.387795578930743,0.698167317507058,0.948279702211265
"12450","NADK",179.416569951268,0.0752315845979061,0.194408884166837,0
.386976062952679,0.698773929054715,0.948367425151352
"12451","AGBL5",36.1379020257319,-0.109003411447353,0.281715596826337,
-0.386927144522097,0.698810144913266,0.948367425151352
"12452","WDR53",30.3766432698505,0.124352491689209,0.32146283428667,0.
386833183889356,0.698879708858874,0.948367425151352
"12453","LINC00513",83.0190457600061,-0.187818722888595,0.484867531490
786,-0.387360898988479,0.698489046704631,0.948367425151352
"12454","AL731563.3",5.60289397461134,0.258967433381139,0.669078687682
508,0.387050788118998,0.698718608978456,0.948367425151352
"12455","ARF6",668.318771719175,-0.0709003927029487,0.183264150887836,
-0.386875405579687,0.698848449631689,0.948367425151352
"12456","LEO1",223.845433332028,-0.0916107226657461,0.236479198386004,
-0.387394423234683,0.698464231740453,0.948367425151352
"12457","TOP2A",8.13147119410402,-0.296149370036244,0.765120617222114,

-0.387062331572582,0.698710063342449,0.948367425151352
"12458","TMEM200C",3.00788012008978,0.324728374541229,0.83936669264987
7,0.386873076314314,0.698850174112,0.948367425151352
"12459","ZNF816",19.5248705527139,-0.151242669031259,0.390697580524563
,-0.387109305433107,0.698675288916858,0.948367425151352
"12460","EFCAB6",8.17802209493354,0.217719673454563,0.562530188545862,
0.387036425578097,0.69872924164234,0.948367425151352
"12461","RELN",24.6876878648513,0.294421278328693,0.761792499117916,0.
386484874384566,0.699137602564605,0.948641247424953
"12462","GLIS2-
AS1",8.06844209986865,0.217466770339885,0.563095699901271,0.3861985988
84725,0.699349591336186,0.948852743565418
"12463","DHX36",852.957447296743,0.0573355744775209,0.148543702454756,
0.385984552222835,0.699508109523128,0.948921133527094
"12464","MAPK10",239.948663839886,0.139977630547562,0.363182791351437,
0.385419226573739,0.699926840057705,0.948921133527094
"12465","CBX3",907.143596924382,-0.0610401464714098,0.158317921561891,
-0.385554243443926,0.69982682623832 4,0.948921133527094
"12466","NACC2",68.2030433625143,0.103114399626437,0.267227413169949,0
.385867596453735,0.699594729892571,0.948921133527094
"12467","BAG3",539.413496930846,0.122981819440286,0.318913358523599,0.
385627682733728,0.699772428230757,0.948921133527094
"12468","MDM1",76.8117433870147,-0.0876354501301095,0.227444102571654,
-0.38530544049829,0.700011131195897,0.948921133527094
"12469","PIK3R5",12.7154896934005,-0.233162033898706,0.605257353376441
,-0.385227924283789,0.700068556243223,0.948921133527094
"12470","MYO15A",7.78226382947236,-0.206780959154149,0.536481604352979
,-0.385439048564464,0.69991215658139,0.948921133527094
"12471","AC005726.3",3.51991011888055,0.350301114817187,0.909350344243
847,0.385221292359518,0.700073469340961,0.948921133527094
"12472","BST2",1000.58525908991,0.179218659426292,0.464871427303449,0.
385523069176084,0.69984991813183,0.948921133527094
"12473","SOD1",2853.07602016227,-0.0903120257842314,0.234350725243264,
-0.385371223794952,0.699962399396205,0.948921133527094
"12474","COL6A1",13163.9235834223,-0.0968117844918709,0.25101548444025
6,-0.385680527668456,0.699733285842904,0.948921133527094
"12475","ARHGEF11",123.267183790862,0.0813091835953107,0.2112130506665
42,0.384962876766927,0.700264919938899,0.949028286183644
"12476","UBA5",222.144126702989,-0.0602821957410328,0.15657374572055,-
0.385008326035856,0.700231246868297,0.949028286183644
"12477","MCUR1",309.537170628955,-0.0767845736931628,0.199635953584897
,-0.384622971535583,0.700516772210908,0.949028286183644
"12478","TNFRSF21",23.9951373244541,-0.171309877422516,0.4453643127382
85,-0.384651110389227,0.700495921518975,0.949028286183644
"12479","CREB3",255.155951907826,-0.0947465385617446,0.246421950364706
,-0.384489037691323,0.700616019337195,0.949028286183644
"12480","POLL",123.820909747349,-0.0767919150684286,0.19965066807178,-
0.384631395477323,0.700510530105872,0.949028286183644
"12481","PGGHG",47.5805652831594,0.159572786568513,0.415004381728997,0
.384508678929361,0.700601464545015,0.949028286183644

"12482","GIT1",133.019316764761,0.0641611474080722,0.166898206531799,0
.384432815315169,0.700657682542609,0.949028286183644
"12483","SNRPB2",823.439491142676,-0.080728097660771,0.209848199080846
,-0.384697595759065,0.700461476682408,0.949028286183644
"12484","UGGT2",220.333387759804,0.0639286427124724,0.166497157905822,
0.383962366184251,0.701006340919921,0.949424480316728
"12485","YOD1",29.355085274212,0.12371475431829,0.32296975253764,0.383
053686440411,0.701679958209469,0.949758281227565
"12486","KIAA1841",67.4166979593466,0.107950323185729,0.28250726236512
9,0.38211521460361,0.70237590696602,0.949758281227565
"12487","THRB",310.26861078256,0.0870161123453474,0.227534486852042,0.
382430433070707,0.702142120459717,0.949758281227565
"12488","DYNC1LI1",364.27083140759,0.0619438210036554,0.16166413824463
5,0.383163648266385,0.701598429453631,0.949758281227565
"12489","NICN1",51.4892835786084,0.0888905549061421,0.232386790445549,
0.382511220778576,0.702082207579899,0.949758281227565
"12490","MITF",644.856963087348,0.101637504745963,0.265587147925262,0.
382689846025848,0.70194974408693,0.949758281227565
"12491","AL583856.2",3.46830536797414,-0.372087019441124,0.97213941929
831,-0.382750675525221,0.701904636696235,0.949758281227565
"12492","METTL24",2.01269509096771,0.458619246558655,1.19788315856045,
0.382858080340487,0.701824994499111,0.949758281227565
"12493","AC091729.3",38.5955916339156,-0.129047544807006,0.33761121752
6673,-0.382237135816764,0.702285478927975,0.949758281227565
"12494","AGK",64.582023834034,0.0940413169519729,0.245954028658404,0.3
82353228629499,0.702199377686986,0.949758281227565
"12495","GRINA",1169.92238103022,-0.0514632223821391,0.13453524316626,
-0.38252595506547,0.702071280701829,0.949758281227565
"12496","AL359532.1",5.4411894809378,0.229495312154771,0.5986949663859
42,0.383325942324408,0.701478106401984,0.949758281227565
"12497","ARMH3",73.3614142964071,0.0914703835289175,0.239449074193337,
0.382003496305282,0.702458771278506,0.949758281227565
"12498","BET1L",164.843727225682,0.078706668658544,0.20535309527726,0.
383274810405352,0.701516014243819,0.949758281227565
"12499","CD248",2517.10235196785,-0.114796438398645,0.299448632957865,
-0.383359367063124,0.701453326593245,0.949758281227565
"12500","CCDC90B",519.066519382436,0.093809021443243,0.245598273231927
,0.381961241863683,0.702490113397792,0.949758281227565
"12501","NDUFA9",328.336634019211,-0.102083714224649,0.267087463306295
,-0.382210804509308,0.702305008302039,0.949758281227565
"12502","RORA",516.681985401907,0.100777831870743,0.262954789784143,0.
38325155420622,0.701533256015742,0.949758281227565
"12503","BNC1",3.18417382263927,0.362538621276261,0.949172674975728,0.
381952231489946,0.7024967968839,0.949758281227565
"12504","HIRIP3",139.842926050132,-0.0869424095380606,0.22766493752377
6,-0.381887569002498,0.702544761255805,0.949758281227565
"12505","AKAP1",94.712327077381,-0.109744532781832,0.286804214989405,-
0.382646164338575,0.70198213636959,0.949758281227565
"12506","HSH2D",4.64071547956462,0.336019102490424,0.879630262581916,0
.382000389009049,0.702461076089961,0.949758281227565

"12507","ALKBH6",51.4382992645544,-0.10638035633729,0.278283414032721,-0.382273434106936,0.702258557575788,0.949758281227565
"12508","SOCS5",252.523719724912,0.0836099903398241,0.219087320568164,0.38162861329901,0.702736857408693,0.949790246408247
"12509","HNMT",1053.52746951796,-0.0879393547274518,0.230513065224047,-0.381494014848917,0.702836711499719,0.949790246408247
"12510","AL359095.1",2.75756789559624,0.322803236488147,0.846193007535573,0.381477078649313,0.702849276263307,0.949790246408247
"12511","CAPRIN2",109.986630311055,0.0796063926236185,0.208557681048125,0.381699644067528,0.702684164123265,0.949790246408247
"12512","SLC38A1",33.5341043434504,0.21144745150804,0.554080592935439,0.381618584379254,0.702744297352207,0.949790246408247
"12513","LINC01091",126.6183353106,-0.110967914000755,0.29104058447741,-0.381279862394477,0.702995594615745,0.949836144619068
"12514","SPAAR",15.163362560408,0.202333464297094,0.530648589841077,0.381294642387894,0.702984628686409,0.949836144619068
"12515","GTF2H3",159.503936574739,-0.0756099804299988,0.198351262627489,-0.38119233237248,0.703060538268235,0.949847988896469
"12516","HERPUD2",225.003092061638,-0.071171445920816,0.18681895158113,-0.380964807469804,0.703229362462357,0.949848383169318
"12517","TMEM134",237.767061410522,-0.0796936751159141,0.209125828821005,-0.381080020412617,0.703143872266615,0.949848383169318
"12518","TRMU",88.1701528910369,-0.0813004975557386,0.213395410771556,-0.380985220168452,0.703214215578016,0.949848383169318
"12519","AC099791.2",13.1465843153848,-0.175303242883321,0.460595263006595,-0.380601488905915,0.703498976319216,0.949958701098169
"12520","BOD1",631.556964706844,-0.0877609875278993,0.230615024975138,-0.380551906959924,0.703535773311525,0.949958701098169
"12521","AL109628.2",12.5797795945657,-0.199621389825383,0.524510037060047,-0.380586405827978,0.703510170076303,0.949958701098169
"12522","MGRN1",135.444097907516,0.0765301716472718,0.201024094258003,0.38070148720108,0.70342476519902,0.949958701098169
"12523","LINC02847",2.24723878606953,0.363717682656126,0.956224653627458,0.380368442997528,0.703671936207265,0.950035211371479
"12524","INO80",207.37205868001,-0.0767569650459365,0.201819857502898,-0.38032414647223,0.703704813533026,0.950035211371479
"12525","DNAH1",10.2482982630031,-0.225799444634277,0.594458550270267,-0.379840519631888,0.704063802253029,0.950135189171995
"12526","ZNF184",28.1848842568811,-0.137553648161069,0.361862593504764,-0.380126740453646,0.703851336995326,0.950135189171995
"12527","SMIM8",199.7569662524,-0.0871786824448704,0.229535570389642,-0.37980467383283,0.704090412661201,0.950135189171995
"12528","ANKRD46",114.780538541876,0.0815003760421326,0.214546732560937,0.379872371251257,0.704040157259271,0.950135189171995
"12529","AF186192.1",2.04621732801509,-0.382684458983873,1.00767385080643,-0.379770159439598,0.704116035032239,0.950135189171995
"12530","AC138969.1",15.4207467417884,-0.162681180485658,0.428057348947186,-0.380045292729525,0.703911794296475,0.950135189171995
"12531","MAPK11",15.0636332052871,-0.234193013077608,0.616869016354263,-0.379647878023935,0.704206815509245,0.950181855927725

"12532","PMVK",507.536603607435,-0.0970548976316014,0.25610662014039б,
-0.378962861555069,0.704715442656986,0.950431645555802
"12533","AC108075.1",32.5138948062523,-0.257502791580501,0.67998741598
4463,-0.378687583810205,0.704919874562423,0.950431645555802
"12534","AC091978.1",6.27097122686656,-0.221376321235567,0.58454256662
6648,-0.378717195076336,0.704897883067177,0.950431645555802
"12535","GHR",636.679965938381,0.0871263031886682,0.229751401788486,0.
379219898161399,0.70452457657901,0.950431645555802
"12536","DDX56",190.80987367911,-0.0707056176109253,0.186847008550916,
-0.378414501571525,0.705122697057723,0.950431645555802
"12537","FNTA",647.394981781776,-0.0765700031169052,0.202140975731216,
-0.37879506042713,0.704840055731002,0.950431645555802
"12538","UCP3",3.24929736994289,-0.298744057957508,0.789132250532907,-
0.378572866279085,0.705005074532184,0.950431645555802
"12539","SLC35F2",37.5716874702199,0.111494864735261,0.294135474833023
,0.37905956361964,0.704643633022658,0.950431645555802
"12540","TRAPPC4",336.101391309462,-0.0997128355248975,0.2634695803007
95,-0.378460524403077,0.705088513704316,0.950431645555802
"12541","IFI27L2",1587.92280153516,0.106671284238941,0.281416019085517
,0.379051926701178,0.704649303998435,0.950431645555802
"12542","AC087741.1",19.0181486290234,0.185263654291183,0.488656024983
954,0.379128967656269,0.704592096154064,0.950431645555802
"12543","LINC01970",4.93125259715011,-0.307335827380435,0.811984140745
509,-0.378499790769633,0.705059349170606,0.950431645555802
"12544","ITPA",421.950243863723,-0.104924448491154,0.276897404751711,-
0.378928970407787,0.704740610383551,0.950431645555802
"12545","AC105942.1",28.8677139487851,-0.11025002525337,0.291485162971
151,-0.378235461899932,0.70525568402866,0.950535122005319
"12546","VCAN",3638.97230877718,0.0947171199288708,0.250535886044605,0
.378058095485801,0.705387437018745,0.950561152874229
"12547","PABPN1",586.6814579632,-0.0627294160679638,0.165898889931304,
-0.378118359284616,0.705342670305044,0.950561152874229
"12548","ATG16L1",49.5096112551688,-0.0937160803292181,0.2481138760478
41,-0.377713982877555,0.705643079182082,0.950624239184746
"12549","AL121612.2",7.14426161080874,0.203071595759864,0.537419389102
661,0.377864289747596,0.705531411723449,0.950624239184746
"12550","ZNF263",87.9769905530822,0.0778416338876188,0.20605343279403,
0.377774021194924,0.705598474164127,0.950624239184746
"12551","PTOV1-
AS1",6.58238257841537,-0.229039750024454,0.606418808594696,-0.37769235
8446511,0.705659145138854,0.950624239184746
"12552","EYA3",87.3307729674706,0.0825891945732713,0.218854946341236,0
.377369558942933,0.705898985905246,0.950655661948299
"12553","AL445524.1",28.1299659949212,-0.126459828934246,0.33537197804
0327,-0.377073331150641,0.706119109539378,0.950655661948299
"12554","RANBP2",296.266583845157,0.0780335944147087,0.206939800056515
,0.377083549870049,0.706111515711116,0.950655661948299
"12555","CRELD1",169.601700148802,-0.153383916496779,0.406925152908412
,-0.376933977662722,0.706222670098354,0.950655661948299
"12556","TBC1D22B",30.0404888603095,0.118080969682846,0.31313613203826

1,0.377091487061028,0.706105617373325,0.950655661948299
"12557","TIGD5",97.7162613556875,0.0741538879707352,0.196718186859111,
0.376954917868595,0.706207108034849,0.950655661948299
"12558","ATP2A3",12.3551131712149,0.19310256776635,0.512129071642532,0
.377058398866167,0.706130206207856,0.950655661948299
"12559","ZNF571-
AS1",2.22522835116596,0.398522096932574,1.05735618415305,0.37690430424
9936,0.706244722600697,0.950655661948299
"12560","AL031663.3",6.33520278135236,-0.261329575324695,0.69335598324
4898,-0.376905343921136,0.706243949940152,0.950655661948299
"12561","ATP5PO",1769.55017208604,-0.104127969505003,0.275890969060375
,-0.37742434940745,0.705858274405956,0.950655661948299
"12562","FBXW11",283.358833516318,0.0808888920494573,0.214752111285679
,0.376661684791787,0.706425040271426,0.950822685950428
"12563","AC026726.1",2.31842442595276,0.496595102565957,1.320823032610
88,0.375973987661568,0.706936234491704,0.951207850770788
"12564","PPP1R26",18.4309425362445,0.128084640537637,0.340623637385117
,0.376029806741867,0.70689473688791,0.951207850770788
"12565","GAS2",9.71809773738035,0.174787145221174,0.464862321817186,0.
375997659990846,0.706918635655018,0.951207850770788
"12566","NUDT16L1",168.556528251277,-0.0980423186957359,0.260646662970
953,-0.376150293190831,0.706805166562646,0.951207850770788
"12567","IL18RAP",4.44061587780384,0.307828400849469,0.819177645709176
,0.375777345075105,0.707082431515014,0.951228997541725
"12568","ZNF35",26.3703841147596,0.125376334716978,0.333674805508539,0
.375744085700139,0.707107159788658,0.951228997541725
"12569","C3orf62",57.7836069022825,-0.131515322377891,0.35010277817208
,-0.375647754252467,0.707178783756174,0.951228997541725
"12570","GFOD2",97.0756714511904,-0.0685554847075662,0.182488060321116
,-0.375671069038337,0.707161448603323,0.951228997541725
"12571","KIAA0930",510.35555482881,-0.0984957747875756,0.2622536222602
5,-0.375574506611895,0.707233246279691,0.951228997541725
"12572","HDAC2",375.212035330745,-0.0563124016738158,0.15003796116455,
-0.375321027003671,0.707421729975944,0.951406825519668
"12573","ALMS1-
IT1",8.42539829594327,-0.218400153275772,0.582287640540954,-0.37507262
4026288,0.707606456161839,0.951428227497763
"12574","TIGIT",5.85504759237813,0.266114547268766,0.709393796455691,0
.375129510010294,0.707564151079614,0.951428227497763
"12575","HSPA9",615.684208042097,-0.0728837385295604,0.194261891604998
,-0.375182893193269,0.707524451788548,0.951428227497763
"12576","FHL1",5025.02817076854,-0.0958924278465105,0.255731094934776,
-0.374973672524914,0.707680046762644,0.951451513252448
"12577","TNFRSF25",5.15825255001107,-0.284737204007518,0.7609786117185
09,-0.37417241381397,0.708276046378121,0.951534178008164
"12578","NECAP2",258.219853917463,0.0796194084609742,0.213133545369089
,0.373565823827006,0.708727364591436,0.951534178008164
"12579","MCL1",5418.50550385424,-0.111162794979809,0.297558682501346,-
0.373582763726972,0.708714759490668,0.951534178008164
"12580","LEFTY2",6.75046841199552,0.276937860096687,0.744040375121755,

0.372208107727176,0.709737909992665,0.951534178008164
"12581","CATSPERE",4.78489809602207,-0.291347019468892,0.780762349183795,-0.373157106990962,0.709031517886168,0.951534178008164
"12582","AC012065.3",2.18023014980976,-0.392853753479856,1.0540826778633,-0.372697286209274,0.709373756467927,0.951534178008164
"12583","AC074117.1",18.8733799875671,-0.152751887283101,0.410513105003467,-0.372099904780899,0.709818467228884,0.951534178008164
"12584","RBKS",28.2275860965376,0.135656637342355,0.364577471004464,0.37209275978738,0.709823786800152,0.951534178008164
"12585","IL1R2",9.86444671305249,-0.297643145506373,0.79877874872704,-0.372622764414686,0.709429227588059,0.951534178008164
"12586","PTPN18",239.496974541034,-0.0663565513555483,0.177929496800599,-0.372937329384528,0.709195088156726,0.951534178008164
"12587","AC093772.1",56.6646820200423,-0.182494301078211,0.489153443910297,-0.373081909879546,0.709087482096296,0.951534178008164
"12588","MFSD8",96.4721835854396,-0.0962757117750333,0.258079213094853,-0.373047137816748,0.709113361161512,0.951534178008164
"12589","PRIM2",64.6067630991936,-0.0973813962750578,0.26157586455583,-0.372287391424345,0.709678885235028,0.951534178008164
"12590","IBTK",290.797553989607,0.0725316810828725,0.194582275145301,0.372755848541243,0.709330166030948,0.951534178008164
"12591","TTLL11",22.2502859901295,-0.120972726985978,0.324702566289301,-0.372564739381192,0.709472420228676,0.951534178008164
"12592","CERCAM",644.547473327518,0.118148910225128,0.31671661951789,0.373042975783766,0.709116458771965,0.951534178008164
"12593","AL365203.2",10.2916988409662,-0.198428113699381,0.530475967061556,-0.374056745300877,0.708362098763408,0.951534178008164
"12594","ATG2A",97.3406559513627,0.105918063731143,0.283437997134116,0.373690418370494,0.708634654764627,0.951534178008164
"12595","FADD",108.601624115904,-0.105025511094272,0.281243790284404,-0.373432284453522,0.708826734987383,0.951534178008164
"12596","H2AFJ",2377.13486591976,-0.0944185678256038,0.252061413868134,-0.374585567765636,0.707968708072154,0.951534178008164
"12597","FKBP11",404.164963635062,0.111677781469944,0.300073431305903,0.372168175582652,0.709767639149255,0.951534178008164
"12598","EEF1AKMT1",219.505985966746,-0.115281932105663,0.3079926270468,-0.37430094743192,0.70818042727834,0.951534178008164
"12599","SAP18",2361.24345611516,-0.0978126657396474,0.261838218513693,-0.373561454454107,0.708730615886013,0.951534178008164
"12600","AL512506.1",4.19954320582141,-0.273115050298296,0.731174257789572,-0.373529356905911,0.708754500159023,0.951534178008164
"12601","PIBF1",207.046307577183,0.0707146037981604,0.188989419170921,0.37417229021803,0.708276138326176,0.951534178008164
"12602","MRM3",88.8940810245485,-0.10643146038794,0.285965697125426,-0.372182613012002,0.709756890549615,0.951534178008164
"12603","AC138150.2",6.86295700805002,-0.211597381139842,0.567941590872389,-0.37256891296659,0.709469313466442,0.951534178008164
"12604","RSAD1",143.857574641608,-0.0612960310849744,0.164633611753044,-0.37231784222119,0.709656215830644,0.951534178008164
"12605","CCDC57",56.1602150196657,0.121625928028707,0.325311900848669,

0.373874818939642,0.708497451663124,0.951534178008164
"12606","B3GNTL1",26.0740118084603,0.150327822146486,0.403779984699635
,0.372301322113111,0.709668514360672,0.951534178008164
"12607","PDE4A",270.651687647273,-0.0859251110301356,0.229754030525199
,-0.373987393534372,0.70841369527223,0.951534178008164
"12608","COMP",98.8509972705257,-0.446006057133755,1.19299348047938,-0
.373854563693454,0.708512522101095,0.951534178008164
"12609","RRAS",1458.72145780375,0.099290616653083,0.266179767658497,0.
373020900598541,0.709132888401292,0.951534178008164
"12610","ZNF808",42.6870603584485,0.105599484002895,0.28279456522113б,
0.373414121025697,0.70884025128672,0.951534178008164
"12611","AL035252.3",2.39463320881974,0.356042801097463,0.956731105679
785,0.37214510846753,0.709784812630049,0.951534178008164
"12612","DDX27",428.525087153798,-0.0840552503842073,0.225054645324455
,-0.373488182228042,0.708785139281339,0.951534178008164
"12613","UBE2G2",394.49632128303,-0.0877761581048219,0.235408238040291
,-0.372867826697716,0.709246818570971,0.951534178008164
"12614","THAP3",158.455124499072,0.0921659789492851,0.254530512737191,
0.362101886953132,0.717275893612377,0.951586393767242
"12615","MECR",146.836192688156,-0.0952786870639198,0.256700399884792,
-0.371166882118925,0.710513238313352,0.951586393767242
"12616","SDC3",168.935578998758,0.0884332192998556,0.238604482076859,0
.370626815263971,0.710915506637135,0.951586393767242
"12617","CSMD2",11.7544173503683,0.175044654174068,0.47504994071251,0.
368476320429649,0.712518098561852,0.951586393767242
"12618","CAP1",854.210525330405,-0.0632531498501104,0.175338836160144,
-0.360748087733049,0.718287773066433,0.951586393767242
"12619","PPT1",376.413788228921,-0.0541595262247008,0.148154949125878,
-0.365560020399217,0.714693412235613,0.951586393767242
"12620","POMGNT1",104.820446190703,-0.0761701434935036,0.2051602476866
81,-0.371271454155339,0.710435357237926,0.951586393767242
"12621","EPS15",667.776404707054,0.0676587993980268,0.186821876382626,
0.362156727617146,0.71723491411849,0.951586393767242
"12622","SLC1A7",30.5802330260155,0.15785738587395,0.436264322753664,0
.361838861535978,0.717472449742424,0.951586393767242
"12623","AC093425.1",19.088560785667,-0.218149472054828,0.587046915874
403,-0.371604834564024,0.710187089015883,0.951586393767242
"12624","BCAR3",22.4623945566792,0.131969272743812,0.355894186045633,0
.370810420395265,0.710778739476113,0.951586393767242
"12625","LAMTOR5",1063.75916738245,-0.0810604613533815,0.2243137604068
84,-0.3613708816006,0.717822212475718,0.951586393767242
"12626","PDE4DIP",821.409820026876,0.096681340083579,0.268091793403706
,0.360627749384299,0.718377742331044,0.951586393767242
"12627","SCNM1",165.256625840627,-0.0932204297782252,0.257160510445835
,-0.362499007396627,0.716979165149728,0.951586393767242
"12628","HCN3",15.3466420389356,0.171406224787708,0.462199717429158,0.
37084883076325,0.710750128833599,0.951586393767242
"12629","SEMA4A",251.611591967585,0.272636678796683,0.736117034123599,
0.370371375960993,0.711105798435471,0.951586393767242
"12630","AL596325.1",2.49657452046395,-0.403755834172662,1.09122994337

964,-0.370000691991822,0.711381974798192,0.951586393767242
"12631","ALDH9A1",692.048763366159,0.0881038860432898,0.23989273417602
9,0.367263670347935,0.713422350325751,0.951586393767242
"12632","METTL18",54.2335871689652,0.119007396648542,0.326583867346042
,0.364400720757109,0.715558797334016,0.951586393767242
"12633","KIFAP3",438.993981350168,0.0653451285645765,0.177705641471355
,0.367715554911687,0.713085340835956,0.951586393767242
"12634","PTPN14",134.528293783144,0.133520149949279,0.368684526883839,
0.362152843998651,0.717237816112017,0.951586393767242
"12635","AC010896.1",9.75446859216416,0.173082842285611,0.471927478511
246,0.366757288284257,0.713800069782846,0.951586393767242
"12636","ACYP2",146.650057109668,0.0900783867195599,0.244422621515297,
0.368535392350836,0.712474059981812,0.951586393767242
"12637","IMMT",239.562805784775,0.0674301371398596,0.186082325576087,0
.362367231445032,0.717077623429986,0.951586393767242
"12638","ZEB2-
AS1",11.5280435368319,-0.154920019462155,0.427323775248242,-0.36253545
5398329,0.716951933336066,0.951586393767242
"12639","BBS5",83.8968044309682,0.0771680741415725,0.212527280650848,0
.363097264055943,0.716532228326835,0.951586393767242
"12640","HOXD4",12.747320306794,0.16060230950516,0.44137945245303,0.36
38644903214,0.71595920225831,0.951586393767242
"12641","AC009948.2",6.52316430872382,0.232213986469027,0.637310360161
43,0.364365623069752,0.715585002494079,0.951586393767242
"12642","TRAF3IP1",131.998034567034,-0.0803429811661752,0.222529702853
774,-0.361043852285055,0.718066665480113,0.951586393767242
"12643","LSM3",982.042869704088,-0.0986345813616665,0.266385481186408,
-0.370270109776159,0.711181242571642,0.951586393767242
"12644","FGD5",158.946989304278,0.084550718558398,0.233973395806433,0.
361368942255072,0.717823662043021,0.951586393767242
"12645","BTD",391.655440348017,-0.0941647752183855,0.258344683485585,-
0.364492792914935,0.715490054635906,0.951586393767242
"12646","GOLGA4",1367.14560562274,-0.0730300553641752,0.20017415918140
2,-0.364832582101637,0.715236382002331,0.951586393767242
"12647","ZNF502",41.9861827204097,0.106332457361073,0.288461989542878,
0.368618609091537,0.712412022876282,0.951586393767242
"12648","NAA80",59.7010104197588,-0.100306097146242,0.277050628386821,
-0.362049700916735,0.717314890200727,0.951586393767242
"12649","FRMD4B",76.3480032538904,-0.0946948750537838,0.26107394061344
3,-0.362712857634432,0.71681939390668,0.951586393767242
"12650","IFT122",117.597087080691,-0.0808180622182046,0.21957859290196
5,-0.368059842037003,0.712828613662383,0.951586393767242
"12651","TMCC1-
AS1",22.4541906294665,-0.14113284377286,0.380754292063908,-0.370666455
282326,0.710885978064583,0.951586393767242
"12652","P2RY12",11.1921790832982,0.193982866573094,0.529833506382125,
0.366120421295496,0.714275219876371,0.951586393767242
"12653","UBE2K",554.139195963093,0.046757128364374,0.129809939450226,0
.360196827472541,0.71869994702087,0.951586393767242
"12654","AC021205.3",3.11085920223757,0.34740648725545,0.9624754807503

78,0.360951000003242,0.718136077422898,0.951586393767242
"12655","SCOC-
AS1",17.6605391366089,-0.181057491400613,0.500175124099383,-0.36198819
7087222,0.717360850558109,0.951586393767242
"12656","DCTD",427.458909543381,-0.0619925731051068,0.170595200957102,
-0.363389900520681,0.71631364510976,0.951586393767242
"12657","ING2",219.446449352569,0.0894165445764474,0.243291021604297,0
.367529159057417,0.713224345600298,0.951586393767242
"12658","LINC01060",10.882159571271,-0.733300175346674,1.9860381745932
2,-0.369227633550834,0.711958060224785,0.951586393767242
"12659","SRD5A1",41.7679903930585,0.100545153136743,0.276601514240261,
0.363501817453564,0.716230055508448,0.951586393767242
"12660","BRIX1",272.520841963996,-0.0758625372934611,0.208229168071517
,-0.364322337720745,0.71561732131,0.951586393767242
"12661","ARSB",45.0333898583654,-0.105567534566043,0.286173933790463,-
0.368892907777336,0.712207549894817,0.951586393767242
"12662","VCAN-
AS1",2.20452206289032,-0.369017835529306,1.00268220223681,-0.368030702
755163,0.712850340910389,0.951586393767242
"12663","LEAP2",32.0620909915466,0.190746247422281,0.523215926109199,0
.364565063683614,0.715436097620486,0.951586393767242
"12664","SMIM33",6.39077967828952,0.259107524261947,0.718366817366114,
0.36068971728394,0.718331412419531,0.951586393767242
"12665","TAF7",899.212631243908,0.0426260674223942,0.116795715109891,0
.36496259629292,0.715139327070121,0.951586393767242
"12666","YIPF5",510.004928910775,-0.0684681606672947,0.189386640389029
,-0.36152582107508,0.717706405969956,0.951586393767242
"12667","ADGRF5",2.57781768110758,0.401280568204226,1.10267607558019,0
.363915185149079,0.715921344928039,0.951586393767242
"12668","LINC00472",84.8208005014161,-0.165519951855917,0.445715392838
003,-0.37135794391574,0.710370945403907,0.951586393767242
"12669","CDC40",449.721545795153,0.0540977059337286,0.146779044744279,
0.368565594823011,0.712451544172574,0.951586393767242
"12670","ZUP1",44.4160657426291,0.0988541240781447,0.268641467736074,0
.367977903453325,0.712889710474432,0.951586393767242
"12671","AL606970.4",9.46405600211306,-0.245013484410508,0.67303753011
5949,-0.364041340114121,0.715827139331458,0.951586393767242
"12672","SNX8",74.143741184124,0.104169699593953,0.283338307216629,0.3
67651309197345,0.71313325102098,0.951586393767242
"12673","UMAD1",92.8126263695351,0.0811827965411908,0.220715181458593,
0.367817002911606,0.713009689962955,0.951586393767242
"12674","ARL4A",366.51586796269,-0.118582038143726,0.324741696222847,-
0.365158030283711,0.714993445254887,0.951586393767242
"12675","MALSU1",393.432062281823,-0.0912650416524834,0.24821637792922
2,-0.367683399515673,0.713109320052308,0.951586393767242
"12676","HNRNPA2B1",3762.31698814296,-0.0591875254146974,0.16015030582
4822,-0.369574851011767,0.711699292388075,0.951586393767242
"12677","HOXA6",37.8370793313913,0.156566248448028,0.432869761876195,0
.36169365993462,0.717580965388262,0.951586393767242
"12678","RBM48",106.41987608744,0.104193498483235,0.285705511285562,0.

364688444455988,0.715343985402377,0.951586393767242
"12679","ATP5MF",1244.37746148989,-0.0989886403463285,0.267024098302635,-0.370710512555082,0.710853159507757,0.951586393767242
"12680","GIGYF1",239.808019734937,0.086782370932323,0.234089194360085,0.370723523439664,0.710843467715422,0.951586393767242
"12681","PLOD3",232.877516364169,0.0634183806192891,0.175920814203388,0.360493901227451,0.71847781659920 3,0.951586393767242
"12682","TES",55.7158278892545,-0.157865197563603,0.429248992793396,-0.367770688374302,0.713044226865221,0.951586393767242
"12683","AC016831.1",117.325764267137,0.278468116685223,0.770727603730045,0.361305492806457,0.7178710880078,0.951586393767242
"12684","VDAC3",740.62594093589,-0.0910913327649608,0.246856155539186,-0.369005717382247,0.712123463201105,0.951586393767242
"12685","AC023632.2",15.2796130945173,-0.16618211759157,0.452243003258212,-0.367461998072499,0.713274433258998,0.951586393767242
"12686","UTP23",284.266129426589,0.0591152525694327,0.161150860349059,0.366831752814641,0.713744520953233,0.951586393767242
"12687","INSL6",3.47614410306402,-0.280577257676261,0.777405097690513,-0.360915124572491,0.718162896809069,0.951586393767242
"12688","RPP25L",180.779889664111,0.110408065325357,0.300340273060537,0.367609925236711,0.713164113028379,0.951586393767242
"12689","SIT1",9.0522466384847,0.181195944822506,0.500477196732699,0.362046354969658,0.717317390521532,0.951586393767242
"12690","AL161457.2",17.8305441389407,0.16026532034973,0.433591286876223,0.369623018728882,0.711663397460649,0.951586393767242
"12691","SMC5",391.836428103634,-0.0744073103396712,0.204716751116084,-0.363464689303705,0.716257785763237,0.951586393767242
"12692","SLC25A25-AS1",6.16557155010183,0.274426112603286,0.745312199447161,0.368202899143263,0.712721948757675,0.951586393767242
"12693","RPL7A",28429.0375195334,-0.093574772983176,0.255319822926113,-0.366500226699028,0.713991843478712,0.951586393767242
"12694","TRAF2",44.4717445950059,0.0976541573809945,0.269419997780961,0.362460686605705,0.71700779658989,0.951586393767242
"12695","PRTFDC1",192.234756386503,0.0744575867090576,0.203608714475468,0.365689587014355,0.714596717104602,0.951586393767242
"12696","VSTM4",263.562236799098,0.0975441960148446,0.270104308746122,0.361135283134372,0.717998318406297,0.951586393767242
"12697","PRKG1",148.432743942524,0.112439763331688,0.302996204456956,0.371092976340109,0.710568282208342,0.951586393767242
"12698","MYOZ1",2.94680137670761,-0.31941001204176,0.881953527047356,-0.362161953261977,0.717231009316184,0.951586393767242
"12699","AL157786.1",5.7808846617698,-0.255181399069509,0.70653804613326,-0.36117149029138,0.71797125318465,0.951586393767242
"12700","C11orf74",276.669582774218,-0.100140530707357,0.274345585648513,-0.365016008807428,0.715099456234319,0.951586393767242
"12701","PRDM11",51.3870445868091,0.0978709618148151,0.266263686421963,0.367571572113342,0.713192715214032,0.951586393767242
"12702","RAB3IL1",375.058655622796,0.0750203833514356,0.205019300508622,0.365918638710216,0.714425787800424,0.951586393767242

"12703","CATSPERZ",13.910720481558,-0.236590473655457,0.640686729275179,-0.369276376183281,0.711921732210666,0.951586393767242
"12704","MRGPRF-AS1",4.09722672534782,0.31960895408532,0.883032363453442,0.361944779504303,0.717393296107256,0.951586393767242
"12705","LRRC32",212.476389361179,0.108539658598553,0.297733453860263,0.36455311686101,0.715445016963889,0.951586393767242
"12706","AP002433.1",10.365661308211,0.196046737095625,0.531743439293142,0.368686706048004,0.712361258808547,0.951586393767242
"12707","FDXACB1",13.9203470641581,-0.1576092592594 8,0.434353507798155,-0.362859413887182,0.716709906329873,0.951586393767242
"12708","DLAT",88.9147104593606,-0.0726074070403369,0.201082787621156,-0.361082158743147,0.71803803007478,0.951586393767242
"12709","OAF",1898.86467061276,0.127338072655077,0.351008882300504,0.362777351446226,0.716771211886806,0.951586393767242
"12710","CCDC15-DT",4.69749786110287,-0.243079015603839,0.665341149465157,-0.365344929889338,0.71485394367471,0.951586393767242
"12711","FBXL14",39.1207260870899,-0.1150104227443,0.315995325061745,-0.363962418500422,0.715886073149801,0.951586393767242
"12712","AC008035.1",8.17940332758817,-0.193214044869548,0.52350843991128,-0.369075319783368,0.712071584295701,0.951586393767242
"12713","AAAS",115.292674330245,0.0728176036184155,0.197785827886328,0.368163909399341,0.712751019369847,0.951586393767242
"12714","LLPH",354.832482883345,-0.0904982232524247,0.245216448352855,-0.369054457236906,0.712087134281376,0.951586393767242
"12715","CPSF6",314.973984019024,-0.0529973293212075,0.144723874342685,-0.366196175730602,0.71421869562978,0.951586393767242
"12716","NDUFA12",948.783799572596,-0.100523220319839,0.271050654489462,-0.370865071361588,0.71073803185903,0.951586393767242
"12717","ALKBH2",166.567633764472,0.0983981289174199,0.26763430860722,0.367658875386672,0.713127608600914,0.951586393767242
"12718","DTX1",218.29134590037,-0.129042064913847,0.356632000943007,-0.361835350088142,0.717475073937058,0.951586393767242
"12719","TBX5-AS1",14.6496919283678,-0.231479588665173,0.625027922273722,-0.370350796206189,0.7111211302921,0.951586393767242
"12720","RNF10",412.904579231716,0.0459404737201269,0.125520241078017,0.366000521713247,0.71436468627230 1,0.951586393767242
"12721","SFSWAP",373.364367414471,0.0765825832449561,0.212656632663692,0.36012318207854,0.718755017435619,0.951586393767242
"12722","LINC02334",3.63529196692192,-0.288465079988117,0.781734582715758,-0.369006420294193,0.712122939271393,0.951586393767242
"12723","CLN5",372.317666950707,-0.0731812481687284,0.198065037569646,-0.369480899136454,0.711769307836412,0.951586393767242
"12724","FARP1-AS1",4.72258492947626,-0.390582205883333,1.05168295903554,-0.371387786145665,0.710348721371443,0.951586393767242
"12725","COL4A2-AS2",2.29857511371056,-0.467818552697747,1.29695741743559,-0.360704635

640807,0.718320258958234,0.951586393767242
"12726","LINC02588",4.70442332048377,0.241334169911614,0.664978757958401,0.362920118911093,0.716664557216899,0.951586393767242
"12727","GPHN",71.876499399614,0.0833104707603678,0.229144829297844,0.363571244507901,0.716178202867592,0.951586393767242
"12728","IRF2BPL",389.954090272484,-0.0648570437708952,0.175927512275166,-0.368657766668429,0.712382832019711,0.951586393767242
"12729","PIGBOS1",275.253401270241,-0.0763726497970434,0.209367375476956,-0.364778178181105,0.715276995618518,0.951586393767242
"12730","AC100830.1",3.15269594255471,0.306152590797548,0.840513834463434,0.36424455879776,0.71567539588227,0.951586393767242
"12731","ULK3",70.0475707430553,0.0890001070910024,0.244796413587399,0.363567855373122,0.716180734063546,0.951586393767242
"12732","CHTF18",24.0787205445363,-0.13626347052844,0.375669532149883,-0.362721644602455,0.716812829274606,0.951586393767242
"12733","NTAN1",1205.51654826263,-0.0836235789541728,0.228569763065121,-0.365855823765927,0.714472661883627,0.951586393767242
"12734","AC005632.3",15.947363477474,0.135214777100704,0.365323606258929,0.370123295577204,0.711290625374426,0.951586393767242
"12735","SNX20",2.95754318352819,-0.306490401141161,0.827829183826754,-0.370233868446589,0.711208243346067,0.951586393767242
"12736","CMTM4",34.0666492140145,0.135224108501942,0.37127089071508,0.364219527799488,0.715694085929422,0.951586393767242
"12737","RFWD3",44.507384949531,-0.12246263043864,0.334685311118779,-0.365903809848345,0.71443685337106,0.951586393767242
"12738","RPH3AL",20.4789729864953,0.161154221984794,0.447373704002302,0.360222830584527,0.71868050281145,0.951586393767242
"12739","SRR",62.5303902318834,0.0978769569699507,0.264389585593599,0.370199744253165,0.711233667127752,0.951586393767242
"12740","DERL2",418.859546833074,-0.0857179335662453,0.236092026561568,-0.363070006279487,0.716552589557583,0.951586393767242
"12741","RAI1",66.7899381513448,0.112203074779156,0.310581944007283,0.361267217699315,0.717899697669225,0.951586393767242
"12742","ALDH3A1",6.69213251868491,-0.204952658015092,0.564183004374022,-0.363273364185248,0.716400688508105,0.951586393767242
"12743","MRM1",13.8982078233871,-0.162333616931296,0.447586665713468,-0.362686445702154,0.716839126051387,0.951586393767242
"12744","CASC3",284.173499430262,0.0881119194238242,0.244048896725849,0.361042072330302,0.718067996067623,0.951586393767242
"12745","RPL27",10759.0885600958,-0.0828452729489512,0.2231668924309,-0.371225642148523,0.710469475818452,0.951586393767242
"12746","TACO1",118.639327147223,-0.108807373037722,0.298420516691042,-0.364610899559467,0.71540187750822,0.951586393767242
"12747","SLC25A19",50.4777376808964,-0.0996194184578605,0.27156068615325,-0.366840354798788,0.71373810416836,0.951586393767242
"12748","BAIAP2-DT",42.1219679348696,0.11362101953997,0.315396354168851,0.36024836063622,0.718661412517014,0.951586393767242
"12749","PYCR1",78.2821064691917,0.159382587744987,0.437386427936252,0.364397652887886,0.715561087899034,0.951586393767242

"12750","SCAMP4",182.693981580216,0.064697991551629,0.178704162684653,
0.362039644626505,0.717322404958627,0.951586393767242
"12751","CACNA1A",48.7971612103711,-0.130290973437496,0.35938084272504
4,-0.362542901423321,0.716946370146252,0.951586393767242
"12752","WIZ",148.126021388494,-0.0655703002476474,0.178099632812759,-
0.368166397718424,0.712749164075664,0.951586393767242
"12753","AC007773.1",5.39147923492872,0.22711676309442,0.6306059219541
96,0.36015640701661,0.718730172372834,0.951586393767242
"12754","GPI",366.321216621012,-0.0589673354666074,0.160980378867023,-
0.366301383321485,0.714140197482963,0.951586393767242
"12755","AC008982.2",15.0483579655425,-0.194596812349833,0.53002455188
2198,-0.36714678906626,0.713509527936424,0.951586393767242
"12756","ZNF235",16.4574761903996,-0.132479344645047,0.363028627243337
,-0.364928093002006,0.715165083152377,0.951586393767242
"12757","GEMIN7",62.0593200677098,0.123751455898653,0.33301174382784,0
.371612888110725,0.710181091924077,0.951586393767242
"12758","MAMSTR",15.3037907027085,-0.16911081852209,0.467917064308416,
-0.361411949726683,0.717791516269555,0.951586393767242
"12759","SPACA6",42.1954460718688,-0.116274867284951,0.316960043223261
,-0.366843928031172,0.713735438665621,0.951586393767242
"12760","RBCK1",609.883762947559,-0.0716841375064976,0.196484289919705
,-0.364833939323046,0.715235368819675,0.951586393767242
"12761","AL117335.1",16.8521326769837,-0.1728793980142,0.4719758906799
59,-0.366288620728357,0.714149719827692,0.951586393767242
"12762","RIN2",374.200903636167,0.108302673467391,0.300487689193013,0.
360422996889648,0.718530831610957,0.951586393767242
"12763","LINC00237",7.44233210442838,0.251222293419329,0.6941077831813
67,0.361935566069983,0.71740018128518,0.951586393767242
"12764","CBR3-
AS1",44.1669643215334,0.111927966920307,0.309718133810244,0.3613865469
97867,0.717810503383601,0.951586393767242
"12765","TRMT2A",104.156765326926,0.0804471053116149,0.222974738448593
,0.360790221669716,0.718256273147623,0.951586393767242
"12766","AC004832.6",5.61299731581162,-0.435006013231967,1.17295076742
216,-0.370864681889415,0.710738321960479,0.951586393767242
"12767","SLC25A17",97.5888939537474,-0.0689362017376414,0.188658630233
443,-0.365401792922704,0.714811503083107,0.951586393767242
"12768","CD99",6357.73210343933,-0.0889342629600029,0.242536651034802,
-0.36668380873801,0.713854885324027,0.951586393767242
"12769","AL022157.1",2.22335488976557,-0.413483216310472,1.13557962656
067,-0.364116444711841,0.715771057398758,0.951586393767242
"12770","CYSLTR1",16.7756215074783,0.207373541280869,0.567682168242541
,0.365298670421279,0.714888470784147,0.951586393767242
"12771","NSDHL",74.4118623890481,0.0990650549269116,0.27283879782248,0
.363090058003288,0.716537611141791,0.951586393767242
"12772","ZRANB2",882.542652630139,-0.0738844241630074,0.20568904469252
7,-0.359204469413785,0.719442133344659,0.951700080377261
"12773","PPP1R15B",778.509936097698,-0.229569603682271,0.6380935937118
84,-0.359774186646869,0.719016008441787,0.951700080377261
"12774","AL451123.1",4.11050650709172,-0.287785808932565,0.80016672763

8891,-0.359657305149085,0.719103423846289,0.951700080377261
"12775","TMEM268",36.7202569296173,0.0951856878142479,0.26489223120354
,0.359337408204729,0.71934269282761,0.951700080377261
"12776","TRAP1",158.677697140221,-0.0653097003862979,0.181805305358324
,-0.359228792897862,0.719423938602503,0.951700080377261
"12777","MIR193BHG",190.698476313085,-0.0661239646254828,0.18384932575
5214,-0.359663895169914,0.719098495087039,0.951700080377261
"12778","TMEM231",29.2199775117516,0.112556562133132,0.313145885997061
,0.359438099513106,0.719267377147213,0.951700080377261
"12779","UTP6",199.248800073245,-0.0791773610119073,0.220176475649433,
-0.359608631114498,0.719139828129145,0.951700080377261
"12780","AFMID",110.355381044127,-0.078238991156564,0.217826373008529,
-0.359180525645077,0.719460044202872,0.951700080377261
"12781","AC010336.8",11.5978709767035,-0.259015656474994,0.72105056766
5181,-0.359219821868675,0.71943064920006,0.951700080377261
"12782","CALM3",1258.88669915135,0.0711567203284056,0.197968270287582,
0.359434975236378,0.719269714021226,0.951700080377261
"12783","RO60",481.738002980821,-0.0444574534506141,0.124100172642512,
-0.358238449665014,0.720164875371092,0.951897834480044
"12784","CHST10",26.8320527324945,0.105657980812238,0.295303521453287,
0.357794516950763,0.720497094156977,0.951897834480044
"12785","TRMT44",48.5466646093587,0.0963881832585771,0.269008744280582
,0.358308736455206,0.720112280819191,0.951897834480044
"12786","GPX8",761.519879543878,0.0756654904249328,0.211560125955977,0
.357654780564263,0.720601677351921,0.951897834480044
"12787","LINC01011",23.4067385607054,-0.120094179284034,0.335512838518
893,-0.357942127681864,0.720386623228906,0.951897834480044
"12788","HIST1H2AG",4.36890884457648,0.269418822268293,0.752584346154,
0.357991530976068,0.720349651422688,0.951897834480044
"12789","AFDN-
DT",21.189330228556,0.121343247028661,0.339214978231847,0.357717833278
357,0.720554486030462,0.951897834480044
"12790","MPP6",110.637835213392,0.076149403225304,0.212475906541063,0.
358390767522563,0.720050899875696,0.951897834480044
"12791","APIP",241.722546774469,-0.0796846708046019,0.222414405200783,
-0.358271177321752,0.720140385591185,0.951897834480044
"12792","MCF2L",56.25605755564,-0.185141519452257,0.517695332772891,-0
.357626402503181,0.720622917041907,0.951897834480044
"12793","TIMM22",179.141858299767,-0.0949048339852076,0.26504440983405
7,-0.358071441856205,0.720289850123282,0.951897834480044
"12794","RPL26",29040.706132091,-0.0975311205286545,0.27199740695042,-
0.358573714441449,0.719914013673791,0.951897834480044
"12795","NPC1",76.4081882941194,0.0881089372806188,0.245939974496911,0
.358253827832797,0.720153368005386,0.951897834480044
"12796","AC011484.1",20.3445759022242,0.140334990715575,0.392150060620
024,0.357860433563857,0.720447761863761,0.951897834480044
"12797","UPB1",4.04372959525808,0.256348428724894,0.715793826977777,0.
358131656160334,0.720244789885061,0.951897834480044
"12798","ARHGAP6",377.048036009009,0.0843299638516465,0.23553800464599
5,0.358031239919823,0.720319935024616,0.951897834480044

"12799","SMC1A",339.561607708401,-0.0868542044688186,0.242549742106986
,-0.358088216109123,0.720277297330016,0.951897834480044
"12800","AL022162.1",2.05627927653125,-0.355390416377991,0.99292795541
6531,-0.357921654274408,0.720401945047659,0.951897834480044
"12801","AL445423.1",12.6893138702124,0.174879330791249,0.489364590815
924,0.357360001261371,0.720822316781223,0.952086847288253
"12802","AC012184.3",5.05968682744925,0.239569458059811,0.670582704727
912,0.357255647022713,0.720900430481309,0.952115644319479
"12803","ABHD14A",667.558141274642,-0.0932834170403258,0.2611813148310
11,-0.357159611899043,0.720972319534154,0.952136216408926
"12804","KDM8",8.05660152163103,0.197486093979025,0.553112151311266,0.
357045299964652,0.721057893281232,0.952174856263595
"12805","EFNA5",288.403149537398,0.0702003070344588,0.196861705103892,
0.356597068980029,0.721393472030649,0.952320489154764
"12806","HAS2-
AS1",102.506605795059,-0.090818902622355,0.254610284198718,-0.35669769
9419999,0.721318127995143,0.952320489154764
"12807","AP001992.2",2.21700223956032,0.462431446733816,1.296362285445
77,0.356714671450662,0.721305420960742,0.952320489154764
"12808","PLEKHA5",886.492534417701,-0.0525990979609997,0.1474834646450
5,-0.356644035231953,0.721358307116169,0.952320489154764
"12809","ARFGAP1",81.1491855116442,0.0875966960082287,0.24577397147397
5,0.356411606497168,0.721532338606691,0.952429446573654
"12810","LINC01355",20.2899834898435,0.218332684921558,0.6132361811226
26,0.35603359952093,0.721815402890132,0.952431962205325
"12811","NFYC-
AS1",4.09175607283489,-0.360721689955127,1.01316942306022,-0.356032941
524813,0.721815895652888,0.952431962205325
"12812","DDX46",687.026599027326,-0.0666752482748194,0.187195939026655
,-0.356178924721895,0.721706574002764,0.952431962205325
"12813","DNAJC9",157.09047723872,-0.0756060538268817,0.212224794986267
,-0.356254573513778,0.721649925535478,0.952431962205325
"12814","FRAT1",29.8723183709206,-0.131713569941232,0.369937415605017,
-0.356042845046695,0.721808479076139,0.952431962205325
"12815","RAB40C",56.7081250938469,-0.0901454405693571,0.25327893666499
8,-0.355913688506159,0.721905204235169,0.952475473523858
"12816","SMARCD1",175.284730773086,-0.0721700660151832,0.2028349350958
16,-0.355806882976501,0.721985194111154,0.952506683991213
"12817","STT3B",342.638146104363,0.0581209847293856,0.163520231697552,
0.355436046818272,0.722262948067376,0.952650122936515
"12818","C5orf51",112.63431401583,0.0674344399021261,0.189702136274212
,0.355475384866782,0.7222334823793,0.952650122936515
"12819","AL024507.2",6.79576512281184,0.255832192153622,0.719496288121
912,0.355571246686228,0.722161679971753,0.952650122936515
"12820","CPTP",83.3864580574175,-0.0920024430089434,0.259106466178125,
-0.355075827963689,0.722532784785463,0.952693559229267
"12821","TMEM69",104.729569561316,0.0817755996235846,0.230711934954072
,0.354448934945059,0.723002467016222,0.952693559229267
"12822","C1orf229",9.05073058890286,-0.18711094190731,0.52791322988638
1,-0.354435030824253,0.723012885477389,0.952693559229267

"12823","MELTF",102.236822950413,-0.164367068448717,0.463552790951192,
-0.354581121411095,0.722903421278426,0.952693559229267
"12824","UCHL1",99.0450104373049,-0.0914458281253605,0.258022558067993
,-0.354410206650471,0.723031486543162,0.952693559229267
"12825","MRPL32",483.982880338579,-0.0896268506262884,0.25248641956243
2,-0.354976916309459,0.722606884661156,0.952693559229267
"12826","PTP4A3",26.6843353127055,0.129478175142904,0.365290482825517,
0.354452637641672,0.722999692566758,0.952693559229267
"12827","AP000487.1",13.8810610218319,0.171991395286167,0.485213895334
816,0.354465106914315,0.722990349303927,0.952693559229267
"12828","TREH",3.80494883871922,0.39676037026188,1.11794450190848,0.35
4901669612898,0.722663257626147,0.952693559229267
"12829","HIF1A-
AS3",6.29892473465027,0.273196241961638,0.769004103999846,0.3552597971
07263,0.722394970833114,0.952693559229267
"12830","CDH22",6.30423020795396,-0.356961369593819,1.0062221336364,-0
.354754042533125,0.722773860558898,0.952693559229267
"12831","JOSD1",254.93311671658,0.0552815771085582,0.156013172119082,0
.354339164813358,0.723084719989897,0.952693559229267
"12832","ZNF674",20.040006705794,0.127856737541045,0.359991934058099,0
.355165561905091,0.722465562664218,0.952693559229267
"12833","WAS",18.3096727257204,-0.186095053274824,0.524704060217796,-0
.354666691920736,0.722839306794304,0.952693559229267
"12834","EIF2B4",148.236221867004,0.0712610950027311,0.201318614948949
,0.353971713051978,0.723360082281177,0.952868008628253
"12835","AGAP2",3.77962792731799,-0.277494105123505,0.784262481410533,
-0.353828101816651,0.723467711909755,0.952868008628253
"12836","AC023449.2",3.04223039489643,0.291662869228152,0.824078568268
101,0.353926045960783,0.72339430694936,0.952868008628253
"12837","RFXANK",260.621283926651,-0.0780327387173036,0.22048218226198
,-0.353918570275143,0.72339990956735,0.952868008628253
"12838","NR0B1",41.6499470676634,-0.315833396047376,0.892723189839952,
-0.353786481231655,0.723498905534038,0.952868008628253
"12839","PRMT6",61.8167360528887,0.0755739314145317,0.213669945943403,
0.353694718650558,0.723567680997268,0.952877172116528
"12840","BET1",243.167252983695,0.0711136568987634,0.201098033438397,0
.353626814160508,0.723618576412125,0.952877172116528
"12841","ZNF197",89.3200461124127,0.0808213235563172,0.228604420761092
,0.353542259975722,0.723681952726643,0.952886415131383
"12842","ELMOD3",110.695162447368,-0.0649461478793138,0.18438164220999
1,-0.352237604030813,0.724660077825171,0.952986901755307
"12843","LSM6",616.926545294063,-0.0865613966821581,0.245019320804032,
-0.353283963069143,0.723875567011043,0.952986901755307
"12844","AC093788.1",3.21904052915121,-0.337483548391363,0.95511312895
7322,-0.353344057535663,0.72383051980251,0.952986901755307
"12845","MDFIC",515.562037432279,0.083605094188037,0.237101663316951,0
.352612853990299,0.724378699748454,0.952986901755307
"12846","SLC39A14",165.560322133673,0.109685772462072,0.31125720787515
2,0.352395927505935,0.724541355739676,0.952986901755307
"12847","FIBIN",774.298141953856,0.097294559678961,0.276119744683891,0

.352363644948123,0.72456556293627З,0.952986901755307
"12848","MAP4K2",18.6082923148894,-0.165189696405308,0.46777148345113,
-0.353141870014328,0.723982084706192,0.952986901755307
"12849","U47924.2",11.5450062498547,-0.192128484953366,0.5446168010711
83,-0.35277737406462,0.724255347469323,0.952986901755307
"12850","HOXC11",34.0432272869065,-0.119206364586133,0.337884444552461
,-0.3528021680430б,0.72423675829929,0.952986901755307
"12851","DIABLO",7.25253752256451,-0.192880664359106,0.54727670477031,
-0.352437190689593,0.724510414788191,0.952986901755307
"12852","ANG",817.504891649566,-0.0867551564867361,0.24576603151786,-0
.352998972034228,0.72408921119078,0.952986901755307
"12853","GPR132",3.89526941506388,-0.2831981892202,0.803972529652378,-
0.352248589068894,0.72465184025663,0.952986901755307
"12854","SCRN2",350.543431141533,-0.0918583438412617,0.260121237014992
,-0.353136656181469,0.723985993270948,0.952986901755307
"12855","CFAP53",6.17646094407235,-0.202921499126009,0.575399682346024
,-0.352661819865204,0.724341985837564,0.952986901755307
"12856","CLTCL1",7.90988911302003,-0.175764581266109,0.498702699979687
,-0.352443612744163,0.724505599289443,0.952986901755307
"12857","UBA1",433.274452004939,0.0591346117735472,0.16785538877329,0.
352294985616554,0.724617048308175,0.952986901755307
"12858","AL391244.2",2.26918759609925,-0.342534693949267,0.97460350904
7227,-0.351460558852419,0.725242856935815,0.953083075759876
"12859","NCMAP-
DT",3.15974701353406,0.283562940100059,0.806472316678912,0.35160901897
7594,0.7251315004435,0.953083075759876
"12860","HIST1H1C",156.826848749987,0.0982839308105392,0.2800104284467
54,0.351000965770204,0.725587623861001,0.953083075759876
"12861","MMUT",230.703318654374,0.0505185212042272,0.143523114071097,0
.351988747813831,0.72484670111434,0.953083075759876
"12862","LZTS2",373.62011614704,0.053539732505431,0.152494533852303,0.
351092797577167,0.725518731148089,0.953083075759876
"12863","AL133215.2",4.59420783035071,-0.24631296966109,0.700689116063
282,-0.351529607088752,0.725191064734294,0.953083075759876
"12864","IMMP1L",107.630456129372,-0.0859073286358588,0.24457741443958
2,-0.351248003961055,0.725402299538149,0.953083075759876
"12865","AC092821.3",51.7609704789807,-0.0981091457813914,0.2792566155
19753,-0.351322548254732,0.725346380691692,0.953083075759876
"12866","AC009318.3",5.50879087644394,-0.218668688413812,0.62291090569
5939,-0.351043281493855,0.725555878102809,0.953083075759876
"12867","NIPA1",32.4692875988487,0.110006082636577,0.312725266030336,0
.351765893536423,0.725013838796106,0.953083075759876
"12868","MPV17L",50.0481295284815,-0.0999381377290207,0.28477460858119
4,-0.350937670415677,0.725635109667428,0.953083075759876
"12869","AC046158.1",2.78041043718188,-0.35025046271867,0.997717274412
54,-0.351051817685425,0.725549474207722,0.953083075759876
"12870","KANSL1-
AS1",61.3865192514619,-0.092349227107497,0.262915506955057,-0.35125059
0644253,0.725400359132498,0.953083075759876
"12871","NME2",6321.51973478101,-0.0972558679827478,0.277034852849933,

-0.351060045269577,0.725543301849678,0.953083075759876
"12872","CRLS1",206.45224362041,-0.0571710832461639,0.162641123462597,
-0.351516775271855,0.725200689621412,0.953083075759876
"12873","XPNPEP3",112.778200471116,0.0940881308471456,0.26772644915318
1,0.351433827867012,0.725262907844569,0.953083075759876
"12874","AL121929.2",2.53227833831373,-0.311840333930221,0.88955837162
3144,-0.350556347821467,0.72592121001951,0.953384792528342
"12875","RAD9B",5.68263256433034,0.216387672218743,0.617699064712373,0
.350312449185111,0.726104223392307,0.953551084203272
"12876","PPP2R3A",122.170703086309,-0.0777482031208563,0.2220188173089
09,-0.350187448357948,0.726198025890426,0.953600203615667
"12877","MIB2",161.938173519012,-0.0592097857891817,0.173835905634791,
-0.340607342153897,0.733399201394032,0.95363108755933
"12878","GPR157",32.5908743945295,-0.117712203655365,0.341889569285354
,-0.34429890300958,0.730621509190636,0.95363108755933
"12879","OTUD3",64.3452019258854,-0.0997575263558487,0.291753901596424
,-0.341923538331429,0.732408435073267,0.95363108755933
"12880","CCDC163",3.92454424891414,-0.329003852738806,0.94186488339778
,-0.349311093913942,0.726855770660391,0.95363108755933
"12881","SLC35D1",80.5403803493126,-0.0707130552281996,0.2064865878771
39,-0.342458345383064,0.732005986489227,0.95363108755933
"12882","NDUFS2",468.167343704008,-0.069604042279176,0.200932997720653
,-0.346404239566181,0.729038939409566,0.95363108755933
"12883","KLHL20",226.480963015539,0.0524579779194558,0.151792018463017
,0.34559114801044,0.729649999400354,0.95363108755933
"12884","TMEM183A",301.387665392138,-0.0553077153531404,0.162047904508
168,-0.341304724186376,0.732874191823768,0.95363108755933
"12885","RBBP5",138.436852803771,-0.0758547368080781,0.220033660105292
,-0.344741512602115,0.730288706546546,0.95363108755933
"12886","GPR137B",184.81811597828,0.0946516373266015,0.277384623984944
,0.341228853881025,0.73293130315318,0.95363108755933
"12887","AC104335.1",3.34256444807365,0.280810349059555,0.826217140449
379,0.339874756056044,0.733950847934283,0.95363108755933
"12888","GTF3C2",102.647324604323,0.0722844080182539,0.211200649783195
,0.342254666794144,0.732159248334261,0.95363108755933
"12889","NCAPH",2.89582549530568,0.304666606998861,0.896846993761458,0
.339708566921835,0.734076009566806,0.95363108755933
"12890","ANKRD23",11.136925534661,-0.157854462515282,0.463773290783043
,-0.340369886865968,0.733577993147721,0.95363108755933
"12891","PGM5P4-
AS1",8.78009325407926,0.195317981364493,0.573968978629689,0.3402936197
54157,0.733635421470136,0.95363108755933
"12892","DPP4",198.405559662805,0.240376724830412,0.702786060448375,0.
342033996344567,0.732325308087684,0.95363108755933
"12893","PHOSPHO2",57.7466878696197,-0.100228874029902,0.2943575416261
17,-0.340500445397825,0.73347968742506,0.95363108755933
"12894","SP5",110.376365452457,-0.144313004185619,0.421822609769459,-0
.342117754817578,0.732262276371536,0.95363108755933
"12895","TRAK2",187.122187412372,0.082797179483693,0.243965901884064,0
.339380129945534,0.734323385314804,0.95363108755933

"12896","ATG9A",134.485577518176,0.0673513052107244,0.19600943530744,0
.343612566941403,0.731137672527295,0.95363108755933
"12897","LINC02610",2.33227820518229,-0.330519619639777,0.952636210678
869,-0.346952609962455,0.728626921575298,0.95363108755933
"12898","CNOT10",93.6539235472476,0.0676602480823639,0.195219718975756
,0.346585111572496,0.728903032658078,0.95363108755933
"12899","SHISA5",555.424727847328,-0.0525486123720817,0.15149992824279
,-0.346855691494906,0.728699735520107,0.95363108755933
"12900","IMPDH2",1108.11636437351,-0.080666314375005,0.23556816589021,
-0.342433002651983,0.732025055529161,0.95363108755933
"12901","ASTE1",66.0810112298849,-0.0918631702672732,0.262742275222371
,-0.349632240146833,0.726614712075658,0.95363108755933
"12902","CDV3",849.47953587537,-0.0604752490349253,0.173546223309623,-
0.348467675536975,0.727488985554792,0.95363108755933
"12903","ARMC8",176.697574252211,0.0579209020911273,0.168152650372669,
0.344454291756685,0.7305046650344,0.95363108755933
"12904","PIK3CB",46.0862344735347,-0.101247328238932,0.295484385176993
,-0.342648658670359,0.731862791281281,0.95363108755933
"12905","TM4SF1",12.3355972567225,0.246382202429649,0.717970107120344,
0.343164986934966,0.731474343469599,0.95363108755933
"12906","PPP1R2",447.988540935208,-0.0576626259461433,0.16634224291537
1,-0.3466505256604,0.728853882781387,0.95363108755933
"12907","AASDH",183.999316086198,-0.0582957642898039,0.17176110077128,
-0.339400271819588,0.734308213844242,0.95363108755933
"12908","GPRIN3",15.546777948124,-0.235556561762556,0.685869498967888,
-0.343442246837083,0.731265781690722,0.95363108755933
"12909","UNC5C",8.16979966972716,0.271783386097907,0.785486399625785,0
.346006482387713,0.729337843059463,0.95363108755933
"12910","TMEM131L",55.2632677878283,0.0893739008254628,0.2568122647396
48,0.348012587779126,0.727830730033616,0.95363108755933
"12911","ADAMTS12",116.840789969865,-0.0800096935342778,0.232691606683
494,-0.343844346921832,0.730963347316764,0.95363108755933
"12912","ERCC8",40.2499220954084,0.105473729156131,0.306045309229597,0
.344634359603937,0.730369271317009,0.95363108755933
"12913","SREK1IP1",509.650878380657,0.0680766054002584,0.1990800858488
78,0.341955877254018,0.732384097562724,0.95363108755933
"12914","SERF1A",6.00573468151779,-0.193212652170263,0.561587976247523
,-0.344046988792908,0.730810948698947,0.95363108755933
"12915","PCBD2",66.4487020081606,0.0900877975337868,0.265035892209603,
0.339907915047752,0.733925875826577,0.95363108755933
"12916","FCHSD1",51.2259037507947,-0.0831182378554102,0.23863982526610
1,-0.348299944331283,0.72761493564749,0.95363108755933
"12917","RNF14",131.399444818097,-0.060035414738485,0.171899302670998,
-0.349247575793767,0.726903451796135,0.95363108755933
"12918","LINC02569",5.01254997637223,-0.218915289378122,0.637482058115
394,-0.343406197227428,0.731292897978841,0.95363108755933
"12919","HCG27",8.90032260690882,-0.209534500495706,0.607463572587722,
-0.344933441198975,0.730144409266721,0.95363108755933
"12920","TAF11",310.462210512489,-0.071365512494438,0.206842255603504,
-0.345023855431351,0.73007643663116,0.95363108755933

"12921","PLA2G7",19.860020296196,-0.225695212370995,0.661063869904662,
-0.341412112574756,0.732793357803275,0.95363108755933
"12922","OGFRL1",635.476377680963,0.0765934912078001,0.22152498215023,
0.345755546233865,0.729526435888357,0.95363108755933
"12923","AL603910.1",5.22202255039822,-0.257086106543254,0.74078899580
9391,-0.347043635903852,0.72855853686031,0.95363108755933
"12924","ENPP3",3.71918317823126,-0.287118288740089,0.840880256200273,
-0.34144967327156,0.732765085593769,0.95363108755933
"12925","LINC01013",3.40604372692069,0.300639131854316,0.8627457961068
29,0.348467802695719,0.72748889007353,0.95363108755933
"12926","YKT6",116.212765994173,-0.081607593427272,0.238578291390352,-
0.342057917137771,0.732307306516929,0.95363108755933
"12927","AC005089.1",12.4867427006023,0.199286066912606,0.586556231508
114,0.339756115795096,0.734040198473717,0.95363108755933
"12928","PON3",64.0848837947686,0.0817290470825804,0.238072730234197,0
.343294450406739,0.731376955362747,0.95363108755933
"12929","TAF6",143.241358501626,-0.0633195414638274,0.183248521985995,
-0.345539166033037,0.729689071087019,0.95363108755933
"12930","NYAP1",3.42079068553743,-0.334797958855087,0.967505025011245,
-0.346042604637837,0.729310696476591,0.95363108755933
"12931","PRKAR2B",97.6690493350563,-0.103836630631593,0.30522972503667
,-0.340191737941376,0.733712139714756,0.95363108755933
"12932","PSD3",151.549926372148,0.0980176837102289,0.28146857360443,0.
348236687510204,0.727662437380852,0.95363108755933
"12933","MRPL15",291.559376252976,-0.104221122814312,0.298019169649141
,-0.349712815242765,0.726554235093823,0.95363108755933
"12934","AC021321.1",10.017924177737,-0.246513064724251,0.719967089895
579,-0.342394906911654,0.732053720832904,0.95363108755933
"12935","LY96",405.802213692497,-0.123193273198547,0.355398748282111,-
0.346633953535362,0.728866334395916,0.95363108755933
"12936","BNC2-
AS1",22.7920273259661,-0.143550630124089,0.421627469810978,-0.34046792
5840897,0.733504173034299,0.95363108755933
"12937","DNAJB5",9.71832032809075,-0.164179222430154,0.47363143368695,
-0.346639202453505,0.728862390566533,0.95363108755933
"12938","PRRX2",672.311582040437,-0.0941403410281043,0.271757881513173
,-0.346412551142665,0.729032693926085,0.95363108755933
"12939","AL157938.3",26.496236488595,-0.121155963963175,0.355449060108
51,-0.340853240478928,0.733214067703238,0.95363108755933
"12940","CUBN",36.5847575434988,0.148311101274357,0.436945966044401,0.
339426640362405,0.734288352414642,0.95363108755933
"12941","ASAH2",2.57063116223403,-0.335510969611528,0.979107987860712,
-0.342670036166897,0.731846707044053,0.95363108755933
"12942","HNRNPH3",978.90325881001,0.052079907434621,0.149997589719209,
0.34720496197381,0.72843734334077,0.95363108755933
"12943","TAF5",37.4165224317163,-0.0996491459108993,0.29061264203861,-
0.34289336214651,0.731678685620075,0.95363108755933
"12944","CHST15",5.73800973220783,0.285219662076541,0.829626433318857,
0.343792881496726,0.731002054037701,0.95363108755933
"12945","SBF2-

AS1",47.7508640637467,0.0876232052783301,0.252862550653744,0.346525039
203281,0.728948169934172,0.95363108755933
"12946","LGR4",169.296053283329,0.111477905951546,0.323019124767397,0.
345112401724883,0.730009870350936,0.95363108755933
"12947","PACSIN3",59.6096044021287,0.112006635725186,0.322467230891006
,0.347342690963363,0.728333882105732,0.95363108755933
"12948","INCENP",19.0623046265772,0.12823997250855,0.366541490039717,0
.349864820199903,0.726440149878605,0.95363108755933
"12949","RCOR2",3.35729104270464,-0.34314015349705,0.992210015491006,-
0.345834196530704,0.729467323944231,0.95363108755933
"12950","AP001462.1",7.55523546819334,-0.18206170005761,0.526158325321
881,-0.346020753251853,0.729327118183263,0.95363108755933
"12951","THAP12",398.012098001111,-0.0623065980248682,0.18028417445940
2,-0.345602148450911,0.72964173112917,0.95363108755933
"12952","JAM3",266.982151193779,0.0775663358548858,0.221633239937639,0
.34997609508714,0.7263566378994,0.95363108755933
"12953","KLRK1",13.9214254575711,-0.22125210317358,0.648461273665926,-
0.341195553471964,0.732956370484174,0.95363108755933
"12954","PLBD1",929.740840251043,0.0743179966829909,0.215938142239931,
0.344163360451695,0.73072343516232,0.95363108755933
"12955","EPS8",1098.51769742824,0.0528732846823453,0.155338602916559,0
.340374405908276,0.733574590403803,0.95363108755933
"12956","MRPL42",345.596958006631,0.0756783602004913,0.216910408236214
,0.348892249181875,0.727170204263759,0.95363108755933
"12957","DGKH",80.3089859152443,0.110951252638599,0.325847323950581,0.
340500732961141,0.733479470905393,0.95363108755933
"12958","LINC00345",9.98194742049059,-0.184145484785657,0.536703342044
654,-0.343104785008653,0.731519631475299,0.95363108755933
"12959","DCT",2.59990233557358,0.328275740979892,0.945157902173993,0.3
47323701388745,0.728348146672259,0.95363108755933
"12960","LAMP1",2017.75189387187,0.0620394717461452,0.180098860536781,
0.344474537824603,0.730489441517775,0.95363108755933
"12961","ZNF219",87.1175634673099,-0.0887983253642585,0.25998353526791
5,-0.34155364982152,0.732686823580089,0.95363108755933
"12962","SRP54",293.745854335556,-0.0614550051652938,0.181039966339834
,-0.339455460624287,0.734266644493765,0.95363108755933
"12963","PSMA6",850.661873081471,-0.0884417773589522,0.256231228273441
,-0.345163928514483,0.729971135089648,0.95363108755933
"12964","AL606834.2",4.79468283566971,0.228462470464274,0.658562032213
453,0.346911087018489,0.728658117077826,0.95363108755933
"12965","GPR65",15.4134654183804,-0.192811225297597,0.552330556817531,
-0.349086652761989,0.727024256593349,0.95363108755933
"12966","DDX24",1390.86282981326,-0.0606341251432814,0.17526644238184,
-0.345953990503112,0.729377292350023,0.95363108755933
"12967","CYFIP1",415.26326639848,0.0396073647982901,0.115193367082217,
0.34383372759667,0.730971333967384,0.95363108755933
"12968","NDN",1585.74175762649,-0.0844244314117138,0.245946525264599,-
0.343263363126951,0.731400340184501,0.95363108755933
"12969","SPRED1",308.558915668547,0.0723882076582617,0.208080153258432
,0.347886170423744,0.727925671735521,0.95363108755933

"12970","PLA2G4B",21.7995924570299,-0.117044494528354,0.340839859961615,-0.343400254129711,0.731297368371547,0.95363108755933
"12971","FBXL22",7.51360933209037,0.194452511661529,0.569550326549931,0.341414099153329,0.732791862482062,0.95363108755933
"12972","CALML4",32.0246251204792,-0.108164320511833,0.31777623513442,-0.340378884739696,0.733571217943003,0.95363108755933
"12973","PPCDC",70.361458910295,-0.12074071164242,0.353413423579206,-0.341641555149816,0.732620660367553,0.95363108755933
"12974","ODF3L1",13.0504057316839,-0.167350655468686,0.478794062765397,-0.349525335594409,0.726694953699873,0.95363108755933
"12975","AC024270.5",2.96836647374753,-0.307059915405215,0.894111230892432,-0.343424738215996,0.731278951522711,0.95363108755933
"12976","MFGE8",1186.35160532608,0.0510324108661556,0.148349276231651,0.344001751558715,0.730844968841482,0.95363108755933
"12977","MMP25-AS1",21.6498127768413,0.141489552587068,0.407768521170326,0.34698498104018,0.728602602030588,0.95363108755933
"12978","NOMO2",7.22815569067887,0.236367944219288,0.695738376528389,0.339736820899146,0.734054730216562,0.95363108755933
"12979","AC145285.2",4.91643265536984,-0.242588977033214,0.700265948192924,-0.346424065969834,0.729024041486198,0.95363108755933
"12980","AC009093.1",36.9285747487258,0.164631219585047,0.485093838784145,0.33938014961741,0.734323370497299,0.95363108755933
"12981","AC009118.3",14.1967333552226,-0.191389137167405,0.556249660367806,-0.344070569033434,0.730793215659746,0.95363108755933
"12982","WWP2",82.3549733609689,0.0852616444735602,0.250068750684618,0.340952814936444,0.733139103786253,0.95363108755933
"12983","ZNF23",64.3176901334413,0.088150637082905,0.259817702818137,0.339278794811789,0.734399715584932,0.95363108755933
"12984","ADAT1",42.651020437542,0.120236845682375,0.346230102189882,0.347274384641559,0.728385192808359,0.95363108755933
"12985","NUDT7",89.4275432165028,0.119060740471584,0.344357709905485,0.345747276877472,0.7295326510598,0.95363108755933
"12986","AC009113.1",43.2250627706751,0.0886604147265786,0.255285210207445,0.347299456378742,0.728366359147354,0.95363108755933
"12987","TSR1",122.425089218933,0.0681009541080401,0.197980287936483,0.343978457743676,0.730862486894104,0.95363108755933
"12988","ENO3",5.08027010519482,-0.232233133555957,0.674397284682199,-0.3443565667163,0.730578148389701,0.95363108755933
"12989","SCIMP",3.85782451407808,0.323327048785878,0.92574087174065,0.349263015878227,0.72689186129342,0.95363108755933
"12990","PHF23",90.4217130810565,-0.0942285048115954,0.272496898984965,-0.34579661332878,0.729495570504602,0.95363108755933
"12991","GAA",364.96571348002,0.0872119723993066,0.253715136949793,0.343739728925061,0.731042030366527,0.95363108755933
"12992","TEPSIN",47.5220568907205,0.0936297718989159,0.275933945308401,0.33931951284308,0.734369044580936,0.95363108755933
"12993","AP000915.1",9.80026958687934,-0.291093724161206,0.833295023107032,-0.34932852841942,0.726842683288362,0.95363108755933
"12994","GNAL",85.3677188489455,0.101511768246403,0.291177046366867,0.

348625585405876,0.727370417074088,0.95363108755933
"12995","AC023043.1",14.9395542063018,-0.20965207066682,0.606955472044182,-0.345415900050669,0.729781725424072,0.95363108755933
"12996","THOP1",64.946160894566,-0.0942568334523326,0.271906760340149,-0.346651305522597,0.728853296826705,0.95363108755933
"12997","TRAPPC5",1300.53257860268,-0.0857761409219595,0.251473601117504,-0.341094017585884,0.733032804746703,0.95363108755933
"12998","AC011446.2",6.59098301919285,-0.191989213517947,0.565604689798553,-0.339440632266198,0.734277813443619,0.95363108755933
"12999","MAP4K1",4.78723984242114,-0.304004084436597,0.88896346330439,-0.341975904506328,0.732369025659851,0.95363108755933
"13000","ZNF283",39.673602476834,-0.0873306294493621,0.25535025998468,-0.342003291692758,0.732348415060807,0.95363108755933
"13001","NAPSA",3.18308079164656,-0.305851541012828,0.885728686368344,-0.345310641644539,0.729860847275723,0.95363108755933
"13002","PCED1A",151.079358894766,-0.0670547655127201,0.196201059096899,-0.341765563455004,0.732527327107789,0.95363108755933
"13003","TMEM230",1474.82943719674,-0.0983378705029309,0.282551069572088,-0.348035739704894,0.72781334297448,0.95363108755933
"13004","KAT14",53.3133033364976,0.0954718021509566,0.27713520522703 7,0.344495395569622,0.73047375817712,0.95363108755933
"13005","ATP9A",156.666124141261,0.0747628815986345,0.218466014957275,0.342217445643688,0.732187257254756,0.95363108755933
"13006","AL157838.1",2.3417681975396,-0.318892773548417,0.939669466552483,-0.339366963490248,0.734333302744396,0.95363108755933
"13007","FAM217B",204.680463462384,0.0590508919209222,0.173472212466312,0.340405480978054,0.733551191670854,0.95363108755933
"13008","BIRC7",5.47713269773996,-0.339555181112056,0.985241919887567,-0.344641426900314,0.730363957560596,0.95363108755933
"13009","ARFRP1",237.247718606121,0.0752093208405619,0.220503261179071,0.341080310732839,0.733043123204913,0.95363108755933
"13010","VPS26C",126.236724271989,-0.0698928673738317,0.203695370416794,-0.343124476667386,0.731504817960089,0.95363108755933
"13011","SNAP29",309.979551052076,0.0666905191328468,0.196524239726133,0.339350093534434,0.734346009843468,0.95363108755933
"13012","LINC00685",56.7560194161872,0.148531624600757,0.437699196432854,0.339346349756305,0.7343488298109,0.95363108755933
"13013","TCEANC",19.9624044363018,0.134882624813828,0.386589271688756,0.348904210984991,0.727161223704494,0.95363108755933
"13014","MAP7D2",18.5412254444439,0.16821223155876,0.488408821672496,0.344408667686915,0.730538971284226,0.95363108755933
"13015","GRIPAP1",127.666702656397,-0.0746147511311629,0.218749773522725,-0.34109635831651,0.733031042659887,0.95363108755933
"13016","PRAF2",186.506824479737,-0.0922744369266261,0.267901822520546,-0.344433778234373,0.730520089764844,0.95363108755933
"13017","PHF8",70.3682618650393,0.100340338991081,0.29024684576592,0.345706905879709,0.7295629937818,0.95363108755933
"13018","RAP2C-AS1",56.6143192476868,-0.0800040478510594,0.230965142067993,-0.346390139805198,0.729049534290847,0.95363108755933

"13019","MIR503HG",18.0395875665644,0.202845435050083,0.583141874249477,0.347849201039029,0.727953437195847,0.95363108755933
"13020","SSR4",2256.48226876389,0.0948886443130257,0.275474497226741,0.344455277233607,0.730503924027596,0.95363108755933
"13021","CLIC2",230.598786010922,0.0817098226484274,0.238038543297852,0.343262992271742,0.731400619154865,0.95363108755933
"13022","LAMB2",2215.12083784555,0.0586907441589473,0.17303284744754,0.339188454820645,0.73446776602209,0.953646213170135
"13023","SIGLEC7",2.29937263793004,0.3976752769095,1.17350423563449,0.338878433356897,0.734701311853665,0.953876202167072
"13024","AC012358.3",20.2181628099914,-0.124758627268911,0.369909038773335,-0.337268393555958,0.735914583602234,0.953894378547333
"13025","LINC01106",4.83474451050093,0.254171215307398,0.751048980160957,0.338421623650866,0.735045481132079,0.953894378547333
"13026","ANO10",130.595502491992,0.0745913117698114,0.220591984153424,0.338141533365657,0.735256532895921,0.953894378547333
"13027","FXR1",864.537506486334,0.0502542741390269,0.148805294004119,0.337718321618556,0.735575466505523,0.953894378547333
"13028","AC107294.2",10.3529811193737,0.15287006994596,0.451885114058995,0.338294104386016,0.735141566118468,0.953894378547333
"13029","GUF1",78.5717739605278,-0.0787052872558791,0.233389021341466,-0.337227890170237,0.735945114098491,0.953894378547333
"13030","BDP1",501.394302324075,0.0765929848990719,0.226859820143076,0.337622523242619,0.735647666770946,0.953894378547333
"13031","F2R",82.1854639171942,-0.152463626446643,0.45216730787798,-0.33718409931526,0.735978123080647,0.953894378547333
"13032","CMTR1",99.8469993175879,0.0795531259770588,0.235438145406641,0.337893954438254,0.735443103562626,0.953894378547333
"13033","AL035587.2",14.97946728357,-0.145475640664153,0.430812562587433,-0.337677341139812,0.735606351935772,0.953894378547333
"13034","GNMT",2.58932504254256,-0.312929177466366,0.927318403604525,-0.337456019690753,0.735773160891358,0.953894378547333
"13035","TRMT11",201.29520163247,-0.0596718225399555,0.1769848721895,-0.33715775705431,0.735997979772267,0.953894378547333
"13036","RNF139-AS1",10.8639023406993,-0.161210812830832,0.477589238358998,-0.337551183910161,0.735701434530784,0.953894378547333
"13037","CCDC107",766.006594372072,-0.084049775368623,0.249301196534839,-0.337141484023633,0.736010246406248,0.953894378547333
"13038","C9orf43",3.25461806572054,0.276979058357666,0.821725079691577,0.337070226044002,0.736063961566533,0.953894378547333
"13039","SVIL",482.796438932701,0.0771664109654506,0.228613070023404,0.337541554197014,0.735708692450952,0.953894378547333
"13040","LINC01588",11.4975727379669,0.181824674148993,0.538318422598306,0.337764168039018,0.735540914306419,0.953894378547333
"13041","CCP110",143.678723852482,0.0922699437700617,0.273239959993171,0.337688322646394,0.735598075549455,0.953894378547333
"13042","AC133552.5",26.9883575343279,-0.113880810320617,0.33755252338074,-0.337372119692811,0.735836399190002,0.953894378547333
"13043","CDK10",120.525773328442,0.0587699899849737,0.174300560608761,

0.337176138617765,0.735984123804187,0.953894378547333
"13044","LRRC37A3",21.0417191149671,-0.110674963701848,0.3272982697488
16,-0.338147109017059,0.735252331373207,0.953894378547333
"13045","WTIP",294.957337204938,0.0615044944321231,0.18247173805348,0.
337063125984457,0.736069313751305,0.953894378547333
"13046","CATSPERG",8.12920943740558,0.199384258238894,0.59152424346909
3,0.337068616274409,0.73606517504598,0.953894378547333
"13047","CHCHD10",522.55387730716,-0.0982039107276541,0.28996036614398
6,-0.338680461863153,0.734850460873339,0.953894378547333
"13048","TMEM184B",122.509201049752,0.0687644152597024,0.2041188232197
19,0.33688424308464,0.736204163935422,0.953996015007673
"13049","WRAP73",117.30880010847,-0.0636138190438784,0.189689169950966
,-0.335358202370343,0.737354893834637,0.954170866750042
"13050","ADAMTSL4",594.008019061913,0.0686780507000357,0.2046591580096
1,0.335572819550107,0.737193023441455,0.954170866750042
"13051","EEF1B2",6079.32974490375,0.0925324571772842,0.275798957242172
,0.3355069145386,0.737242729639366,0.954170866750042
"13052","DOCK10",27.8313018745527,-0.125832002215191,0.374715034356771
,-0.335807188604513,0.737016268937868,0.954170866750042
"13053","AC012063.1",28.1882515268419,0.108320638813282,0.321878217521
331,0.336526776019266,0.736473663446352,0.954170866750042
"13054","SFXN1",167.502876664021,0.0844488030229904,0.251340363690348,
0.335993796551642,0.73687554445561,0.954170866750042
"13055","RSPH4A",2.08929934658681,-0.350189760880447,1.04321823780139,
-0.335682169071816,0.737110553398425,0.954170866750042
"13056","COL14A1",1886.0504892067,0.124571059253451,0.370886400804482,
0.335873892877297,0.736965965006956,0.954170866750042
"13057","AVPI1",70.1671290954794,-0.104329287157868,0.310691192205527,
-0.335797376221893,0.737023668880011,0.954170866750042
"13058","PNPLA2",263.624568480155,-0.064572700733336,0.192067846875933
,-0.336197347883256,0.736722052716935,0.954170866750042
"13059","AVPR1A",11.9954606931601,-0.238482895793669,0.70882015970627,
-0.336450498096012,0.73653117465724,0.954170866750042
"13060","LINC00944",6.16694999415372,0.218352378715064,0.6499806064788
26,0.335936759556498,0.736918556165779,0.954170866750042
"13061","AC037198.1",31.680267515601,0.355070987196081,1.0567750726884
7,0.335994854886923,0.736874746370683,0.954170866750042
"13062","CHST14",205.858904587672,-0.0731899824915914,0.21769780734336
6,-0.336199906580371,0.736720123349726,0.954170866750042
"13063","GTF3C1",173.468795339079,0.0652979160343173,0.194297781774974
,0.336071340793495,0.736817069512497,0.954170866750042
"13064","SUPT6H",271.724109847773,0.0675906781724544,0.201463907949099
,0.335497702097249,0.737249677839407,0.954170866750042
"13065","GNA11",497.332518050504,0.108000806698981,0.321444669965743,0
.335985682109741,0.736881663521912,0.954170866750042
"13066","DIAPH2",497.578819710737,0.0740552270717888,0.220810245123474
,0.335379488530426,0.737338838685507,0.954170866750042
"13067","TEK",6.27043138317241,0.244156974449266,0.728606634298547,0.3
35101223288097,0.737548730108635,0.954275629681421
"13068","CHAF1A",46.2682708303206,0.107713759265591,0.321432700772125,

0.335105168226032,0.737545754362548,0.954275629681421
"13069","ZNF75A",81.2453871024197,0.0910957065592882,0.272135843028295
,0.334743507307182,0.737818578818837,0.954551727803879
"13070","FOPNL",206.939270771975,0.0594761510434216,0.177720800382648,
0.334660607623668,0.737881120120051,0.954559600534798
"13071","DNAJC8",1278.48771441185,-0.0618334977971365,0.18486857551324
1,-0.334472733537711,0.738022862785784,0.954577151409372
"13072","ZNF687",53.5303190101411,0.0823780848140045,0.246387720129018
,0.334343305627683,0.738120515585884,0.954577151409372
"13073","ZNF354A",91.1004482936134,-0.077565662659135,0.23195687284476
6,-0.334396914856861,0.738080067140839,0.954577151409372
"13074","AC008736.1",13.9232254969271,-0.163272859403087,0.48813739801
7622,-0.334481357228836,0.738016356398209,0.954577151409372
"13075","TSTA3",178.579912023686,-0.0994562532872362,0.297756866614072
,-0.334018336564991,0.738365721964814,0.954599870967902
"13076","DMRT2",89.8594144260079,0.102980521106308,0.308582252380996,0
.333721464250516,0.738589751123852,0.954599870967902
"13077","CEMIP2",185.244482421686,-0.257228798236271,0.770660061127653
,-0.333777253047064,0.738547649454138,0.954599870967902
"13078","CCDC15",39.5203829747063,-0.0916568158668861,0.27448463228893
8,-0.333923306024663,0.738437432574456,0.954599870967902
"13079","TSNAXIP1",6.4047130258467,-0.204188770879063,0.61101866536432
8,-0.334177632294281,0.738245521568148,0.954599870967902
"13080","EVI2A",14.4921544864772,-0.180656023522036,0.540904295014843,
-0.33398888710447,0.738387944459109,0.954599870967902
"13081","PRNP",1254.80048622423,0.0711633384003899,0.213135986885119,0
.333887014766526,0.738464818773688,0.954599870967902
"13082","USP18",108.178985205921,-0.131131319069323,0.392804541355653,
-0.333833510724597,0.73850519473177,0.954599870967902
"13083","USP21",42.499251101076,0.0995025518250386,0.299437156024964,0
.332298613658831,0.739663786621792,0.954601580352741
"13084","TAF1A-
AS1",7.49248429126638,0.244442406717523,0.734748007854928,0.3326887641
80748,0.739369231771852,0.954601580352741
"13085","EXOC8",53.065901136253,0.0767144369122855,0.230544357757233,0
.332753478153072,0.739320377876876,0.954601580352741
"13086","THADA",136.034167446767,0.0547645263364512,0.164439909603087,
0.333036709085026,0.739106573461225,0.954601580352741
"13087","CHDH",17.536227046865,0.171620715921534,0.51569209368284,0.33
2796872443587,0.739287619232878,0.954601580352741
"13088","IDUA",132.042720503615,-0.101879714555795,0.306330199007585,-
0.332581361177755,0.73945031480636,0.954601580352741
"13089","HLA-
DOB",3.4602599940652,0.276636518393437,0.829944136985693,0.33331944412
3268,0.738893163497643,0.954601580352741
"13090","AL136084.3",8.82348468352919,-0.18651022481456,0.559746457923
282,-0.333204832606771,0.738979670478364,0.954601580352741
"13091","TNFSF8",2.71499550084671,0.337970193465783,1.01487605482775,0
.333016225831781,0.739122035114414,0.954601580352741
"13092","LINC02716",11.7322377523989,0.163234760908129,0.4905993850923

88,0.332725164091654,0.739341752608744,0.954601580352741
"13093","USP5",121.681696372469,-0.0760493094486554,0.228313875466411,-0.333091054117048,0.73906555197126,0.954601580352741
"13094","ITGA5",271.687121725687,-0.0966654985192155,0.290727734689594,-0.332494932492161,0.739515565551599,0.954601580352741
"13095","AL138479.2",2.15533892827841,0.336206871492446,1.00772991761761,0.333627954886244,0.738660320820404,0.954601580352741
"13096","CHURC1",1347.01199297329,-0.0631392165020895,0.189990124158015,-0.332328939632549,0.739640889825796,0.954601580352741
"13097","TUBGCP4",158.683892066235,-0.0814076571071848,0.244978532696686,-0.332305268592566,0.739658761976555,0.954601580352741
"13098","PLA2G15",74.2577444950898,0.0892519562288927,0.268024137043304,0.332999696271655,0.739134512424442,0.954601580352741
"13099","ASPA",266.60253344547,0.0957869980720493,0.288220029838019,0.332339838164205,0.739632661244284,0.954601580352741
"13100","AC025048.2",6.75001909587869,0.201261777409437,0.604550003664863,0.332911713157492,0.739200927482449,0.954601580352741
"13101","PITPNC1",128.409706064578,-0.102316069215726,0.307545989288371,-0.332685428454047,0.739371750008345,0.954601580352741
"13102","ZSCAN16-AS1",358.832514591877,-0.0905148833536195,0.272996048297468,-0.331561148661722,0.740220660358525,0.954709297046668
"13103","TMEM14A",320.539574490154,0.0775112569557853,0.233444707845259,0.332032615651173,0.739864631019138,0.954709297046668
"13104","SLC26A7",11.8677424730599,0.283216459898086,0.853946170086093,0.33165610411899,0.740148950056671,0.954709297046668
"13105","FZD4-DT",13.4522981577724,0.145774604853788,0.43918117166021,0.331923621185136,0.739946933481124,0.954709297046668
"13106","TMTC3",187.468323803422,0.0581188517223063,0.175312832175095,0.331515103607817,0.740255434367271,0.954709297046668
"13107","THBS1",271.558099819842,-0.317443162701436,0.95692653249828,-0.331732010682865,0.740091627091342,0.954709297046668
"13108","SEPTIN9",194.473039256059,-0.0602407349085672,0.181501645534378,-0.331901866405708,0.7399633610194,0.954709297046668
"13109","NDRG3",158.966165261106,0.0601468495958983,0.181333725184046,0.331691468505662,0.740122243471127,0.954709297046668
"13110","MCM5",95.1479834754442,-0.0661696810125759,0.199459112874655,-0.33174558965455,0.740081372703172,0.954709297046668
"13111","ESPNL",11.6346479969577,0.230635547690773,0.695992781275456,0.331376350295066,0.740360226441318,0.954725378879585
"13112","RHOA",2875.12269992166,0.0752507932889691,0.227104288869315,0.331349062862795,0.740380835573049,0.954725378879585
"13113","GNAI3",856.3593550267,0.0634068899343938,0.191496902470468,0.33111183061654,0.740560015672849,0.954737990468665
"13114","INIP",149.57023596505,0.0665409234227832,0.200942758837141,0.331143673988834,0.740535963745749,0.954737990468665
"13115","RAPGEFL1",10.647640618744,-0.169735044621205,0.512611367205125,-0.331118378327502,0.740555070036916,0.954737990468665
"13116","AC146944.4",18.2663113626203,0.131992506382831,0.399219517596

314,0.330626386148535,0.740926712842025,0.955034530143876
"13117","IRF8",63.5953393353563,-0.131581858835724,0.397940393590951,-0.330657206342764,0.740903429998022,0.955034530143876
"13118","GYG2",4.64772870744027,-0.236635216298691,0.71581179989207,-0.330583005664856,0.740959484647939,0.955034530143876
"13119","SSBP3-AS1",2.06752815396584,0.336351566109027,1.01977415733638,0.329829466347302,0.741528821202599,0.955273533468729
"13120","LINC01133",366.181740816852,0.114038147343891,0.345510237581491,0.330057216660605,0.741356729540695,0.955273533468729
"13121","FGD5-AS1",738.876327574216,0.0440087731014299,0.133385556057849,0.329936571860476,0.741447889008291,0.955273533468729
"13122","SRD5A3",162.860045356462,0.0790128653801927,0.239584748222612,0.329790881791763,0.741557977570817,0.955273533468729
"13123","AC087672.2",14.0725276404806,-0.200963219724259,0.609112629335074,-0.329927849211791,0.741454480000868,0.955273533468729
"13124","STRADA",113.081090876217,-0.0581020898410889,0.176051546916559,-0.330028851542139,0.741378161958198,0.955273533468729
"13125","C18orf32",911.599209616302,-0.0831152170583396,0.252027830866186,-0.32978586838082,0.741561765975383,0.955273533468729
"13126","CCDC151",2.24798116069067,-0.350552918724974,1.06312120329822,-0.329739372742657,0.741596900893692,0.955273533468729
"13127","RAB1A",840.469891156952,0.0540397234533441,0.164077355446039,0.329355158769099,0.741887256837238,0.955429182618937
"13128","AC100793.2",16.3804866502301,-0.158288881276342,0.480589125270931,-0.329364259308005,0.741880379006032,0.955429182618937
"13129","ZNF726",7.62653288621348,0.200453529008975,0.608403328599473,0.329474740827624,0.741796883041052,0.955429182618937
"13130","AC010913.1",9.3780986673176,0.160691977080013,0.488504021004947,0.328947091877439,0.742195679005501,0.955680796635824
"13131","AL158042.1",2.7196560614064,-0.316332520558897,0.961537894604169,-0.328986015355245,0.742166258356884,0.955680796635824
"13132","ZNF625",25.511296772307,-0.107166704494826,0.326088480598782,-0.328643024427114,0.74242552399009,0.955831170305676
"13133","TNNC2",88.9837536167463,-0.123190694786414,0.374774473835227,-0.328706204362723,0.742377764302762,0.955831170305676
"13134","PPIC",1456.65976466386,0.118583048663771,0.360912285161233,0.328564733147822,0.742484708190054,0.955834585509169
"13135","COPB2",554.343079529743,-0.0620265612103025,0.188854189723538,-0.328436246509028,0.742581840812026,0.955854847122142
"13136","MELTF-AS1",37.0037156084072,0.112157947403164,0.341612124515951,0.328319574611371,0.742670045342061,0.955854847122142
"13137","AC090510.2",2.03206532322743,0.325601170254504,0.991560215212818,0.328372564024889,0.74262998466524,0.955854847122142
"13138","KIF15",5.39612399973825,-0.207856038070763,0.633584752283965,-0.328063510558734,0.742863642872624,0.956031243240244
"13139","DENND4B",66.546722141324,-0.0821523392338694,0.250540860082834,-0.327899964926711,0.742987300253338,0.956059648788049

"13140","LINC01703",11.9384026882339,0.153388473123751,0.468346215841609,0.327510862553068,0.743281528466933,0.956059648788049
"13141","CCDC58",104.624684953381,-0.0947515093394736,0.289056291945677,-0.327796045198284,0.743065877741544,0.956059648788049
"13142","AC026801.2",5.21424152419443,-0.245753074458048,0.750338981893504,-0.327522733575541,0.743272551380626,0.956059648788049
"13143","AC008443.8",3.44440552115106,0.251011042207675,0.766219501250235,0.327596781076574,0.743216556081504,0.956059648788049
"13144","LINC00535",16.3560358592397,-0.272384847870829,0.830926513733441,-0.327808588808865,0.743056392919069,0.956059648788049
"13145","OSBPL2",116.945721269977,0.0655733040201885,0.200206751294497,0.327527935977206,0.743268617239461,0.956059648788049
"13146","XPOT",164.424897595367,-0.061506617202228,0.187994174594259,-0.327172995306772,0.743537044543535,0.956315559800859
"13147","FOSL1",46.8734435045994,-0.387257077005995,1.18456545720891,-0.326919103245215,0.7437290719892,0.956372892739877
"13148","FBXW8",59.7215171767485,0.101882752422293,0.311673179457351,0.32688970093506,0.743751311014706,0.956372892739877
"13149","PPP1R3D",59.0648477851442,0.0784506491481981,0.239960731773772,0.326931196484927,0.743719925087448,0.956372892739877
"13150","NPHP4",42.3596845616483,-0.08611322306181,0.263804796407399,-0.326427814181299,0.744100696343384,0.956467503120683
"13151","TXNRD3",52.8148393702344,-0.0826833190942961,0.253202296430808,-0.326550431255235,0.744007939883041,0.956467503120683
"13152","XXYLT1",87.8782339588615,-0.0818542377279325,0.250693314546671,-0.326511450358976,0.744037427461523,0.956467503120683
"13153","CRYL1",310.603724977072,-0.0857163670255555,0.262611759416555,-0.326399576378421,0.74412205799438,0.956467503120683
"13154","ZNF410",152.467353020505,0.0634789945970465,0.194415627119945,0.326511790936862,0.744037169825566,0.956467503120683
"13155","RAD51D",89.572844946963,-0.0644261792836728,0.197418157986826,-0.326343736263471,0.744164301132753,0.956467503120683
"13156","CTBP1-AS",7.5764108647321,0.254200423954692,0.779113532678144,0.326268782780473,0.744221004775466,0.956467676249893
"13157","CCDC88A",452.403523545154,0.0794732597404468,0.243673456176215,0.326146561006526,0.744313470694888,0.956513807289592
"13158","MGAT5",45.004480461764,0.108960923833229,0.334254457729042,0.325981961687274,0.744438002836425,0.95652015397079
"13159","APH1B",247.738393184786,0.0685713544496956,0.210373239195921,0.325950937066834,0.744461476114742,0.95652015397079
"13160","DHODH",50.0689053554477,0.0851475935191564,0.261256483240637,0.325915714944111,0.744488125517837,0.95652015397079
"13161","PHF14",607.878499136785,0.0373776772461974,0.114828356876583,0.325509118678507,0.74479578237712,0.956625593000109
"13162","AC019270.1",28.5299486353194,0.186452190560278,0.572935023444705,0.325433396337434,0.744853083265107,0.956625593000109
"13163","BICDL1",2.11234979056521,-0.322435319557646,0.990179243429886,-0.325633284778583,0.744701826006791,0.956625593000109
"13164","WDR18",162.899637161374,-0.0829399065519786,0.254803309527142

,-0.325505609428294,0.744798437878618,0.956625593000109
"13165","LINC00665",31.4970967460526,0.156299577849915,0.479972819286307,0.325642560514831,0.744694807218436,0.956625593000109
"13166","GMEB1",127.042814059374,-0.0683243483916557,0.210413831733734,-0.324714149391643,0.745397424818435,0.956778881242793
"13167","CALHM5",74.6169565107598,0.0811247020933522,0.249613332511976,0.325001478394429,0.74517995278615 8,0.956778881242793
"13168","DAGLB",39.214596542794,0.0929679446814614,0.286203490603593,0.324831624119591,0.745308508724345,0.956778881242793
"13169","DMRTA1",3.03090398092446,0.376280196155402,1.1587457779213,0.324730586574754,0.745384983384229,0.956778881242793
"13170","GNE",109.620587004266,0.072958609813038,0.224368962871409,0.325172469843132,0.745050543334244,0.956778881242793
"13171","ZNF771",83.5842811545762,-0.0902762593925289,0.277687279100707,-0.325100449991406,0.745105048396773,0.956778881242793
"13172","ZZEF1",139.110049143185,0.0819048238640228,0.25216043069762,0.32481235710705,0.745323091607049,0.956778881242793
"13173","CCDC43",182.0776966407,0.061647621531671,0.18988874440229,0.324651267381425,0.745445021323397,0.956778881242793
"13174","TECR",496.610495321814,-0.0683391186552122,0.210531526622426,-0.32460277922068,0.745481723532798,0.956778881242793
"13175","RETSAT",120.726662312208,-0.0698967505502002,0.215389104813781,-0.324513863459485,0.745549028161617,0.956792635154203
"13176","LRRN3",71.701626482279,0.302404918945302,0.933072608637848,0.324095805777398,0.745865502211202,0.9569121029063 7
"13177","HECTD2",90.4112577764627,0.074423130779585,0.229635925144635,0.324091845527697,0.74586850036687,0.95691210290637
"13178","CEP164",210.993762964055,-0.069434886574078,0.214148637933335,-0.32423688165457,0.745758701496441,0.95691210290637
"13179","CDRT4",19.1168836250328,0.121216455777881,0.373869553028669,0.32422125523708,0.745770531149068,0.95691210290637
"13180","ERICH1",402.5378540472,0.0583365072403911,0.180054639196456,0.323993358353519,0.745943062529215,0.956935151839452
"13181","FGF2",286.908979200193,-0.0600672923404702,0.185541381213921,-0.323740676863967,0.746134372210581,0.956991724611172
"13182","EXOC3",243.434264542286,0.0495844058699534,0.153281178658569,0.323486590486115,0.746326761339184,0.956991724611172
"13183","DNAJC30",163.001250490025,-0.0838617748276842,0.259193307727021,-0.323549151647103,0.746279389810155,0.956991724611172
"13184","EBPL",624.251381924945,-0.107371589222089,0.331794488034644,-0.323608718933504,0.746234286144525,0.956991724611172
"13185","TICAM1",67.2393537018206,0.0820347502541157,0.253478736605341,0.323635628584663,0.746213910750592,0.956991724611172
"13186","ZNF497",5.63797411454346,0.223280486072285,0.690024371102831,0.323583478240671,0.746253398001318,0.956991724611172
"13187","PSPH",35.9892658909408,-0.110034130881043,0.340311979721847,-0.323333110315362,0.746442981119769,0.956995596358285
"13188","TRAPPC1",762.246544981781,-0.0967338818241868,0.299161617271946,-0.32334990934433,0.746430260110923,0.956995596358285
"13189","C6orf52",6.45481252969963,0.203521998934388,0.630043019559437

,0.32302873393741,0.746673481084786,0.957218531972216
"13190","SNRNP200",461.170201401391,0.054436108510249,0.168755449795833,0.322573929174482,0.747017940585594,0.95738426003874
"13191","D2HGDH",154.883274689577,0.0753510369166762,0.233705587707807,0.322418636437931,0.74713556759476,0.95738426003874
"13192","KDM1B",72.3564879108214,0.0761993350554831,0.236816255185106,0.321765644828403,0.747630242721744,0.95738426003874
"13193","ZNF782",28.9733550093883,-0.101537085372531,0.316072255837092,-0.321246434944497,0.748023645423033,0.95738426003874
"13194","MPP7",8.23862066416483,0.184454317100838,0.5721103540214,0.322410380802056,0.747141821019006,0.95738426003874
"13195","AP006333.1",2.24079989132184,0.3071744033486,0.953032634845481,0.322312575789604,0.747215906976989,0.95738426003874
"13196","SIK3",187.42288561687,0.0555502501339526,0.172847327012357,0.321383333454623,0.747919911740645,0.95738426003874
"13197","C1S",14095.3100596621,0.0656450901085411,0.204134350340587,0.321577872607015,0.747772509202754,0.95738426003874
"13198","YEATS4",160.144044079508,-0.0899124304715368,0.278990128593162,-0.322278178532445,0.747241962983548,0.95738426003874
"13199","TMEM263",522.395621604676,0.0659266023497567,0.204916794844846,0.321723763050625,0.747661973897327,0.95738426003874
"13200","ING1",248.906289039202,-0.0781591076840933,0.242474164501365,-0.322339940194549,0.747195178579526,0.95738426003874
"13201","VAMP2",1212.52952112162,0.0816845403481016,0.253598779593025,0.32210147256697,0.74737582273803,0.95738426003874
"13202","CNTNAP1",45.3401217580665,-0.0764582742962331,0.237923993099047,-0.32135588050762,0.747940713612097,0.95738426003874
"13203","PRKCA",85.6645949861417,0.106431161311386,0.331340686569194,0.321213680135114,0.748048465777845,0.95738426003874
"13204","MRPL12",208.967162658824,-0.0920015582924905,0.286206509496246,-0.321451662488121,0.747868137703652,0.95738426003874
"13205","DCXR",248.809270626748,-0.0656283642472587,0.204035881708061,-0.32165109243462,0.74771703284394,0.95738426003874
"13206","MYL12A",2434.1793110569,-0.087478932956599,0.272251431843142,-0.321316704798821,0.747970398468253,0.95738426003874
"13207","ZNF823",35.2006635888128,-0.103435042318787,0.321196526081491,-0.322030389246939,0.747429672507677,0.95738426003874
"13208","ZBED4",40.213066899607,0.0991544933600593,0.307972788891002,0.32195861756849,0.747484044999705,0.95738426003874
"13209","AL732509.1",18.7579167290275,0.116500163984081,0.361860604247017,0.321947630155823,0.74749236890936,0.95738426003874
"13210","RP2",91.0710844927516,-0.0765295095344205,0.237541786717986,-0.32217282942844,0.747321766984825,0.95738426003874
"13211","KCND1",14.4929352630956,0.13167771263938,0.408989705613189,0.321958501234056,0.747484133132885,0.95738426003874
"13212","ITPR1",40.0336563339619,-0.134475153793045,0.419397759530866,-0.320638703324184,0.748484203948045,0.957435933914198
"13213","NEU1",812.692025308545,0.0834451869001413,0.260153540548824,0.32075360851943,0.748397118218211,0.957435933914198
"13214","DMAC1",662.506310324969,0.0891493815657519,0.277969469775049,

0.320716450039989,0.748425279981938,0.957435933914198
"13215","ACER2",11.1880161409394,-0.15882191238899,0.495562847441964,-0.320487932476798,0.748598476836155,0.957435933914198
"13216","PCED1B-AS1",23.4788879207264,-0.160623336646486,0.500352904137364,-0.321020094653813,0.748195162860285,0.957435933914198
"13217","ADAMTS17",5.80267683846082,0.206027497136431,0.642652946357121,0.320589049352839,0.748521837289282,0.957435933914198
"13218","AC010615.2",2.11939840909286,0.372389582637151,1.16180350842373,0.320527163102123,0.748568742460579,0.957435933914198
"13219","PIGT",1152.91263971674,0.0622344282230421,0.194148624764266,0.320550445817511,0.748551095786271,0.957435933914198
"13220","C22orf31",5.95815511596448,0.233909657393599,0.729146683964009,0.320799178735818,0.748362581803935,0.957435933914198
"13221","RPL12",30112.6351105794,0.0857890880131068,0.268026657861201,0.320076699451042,0.748910188400367,0.957689719064696
"13222","PNPLA7",74.8171802533317,-0.146524773898096,0.457778158638707,-0.32007812328534,0.748909109073781,0.957689719064696
"13223","BEST4",3.73751485655373,-0.248002152975782,0.775799254954857,-0.31967310021484,0.74921615350598,0.957755035261913
"13224","AC006160.1",7.14301227878766,-0.177570620229391,0.556037180872787,-0.319350263503362,0.749460921657998,0.957755035261913
"13225","RPA3",343.894676055706,-0.090570391432963,0.283680745499332,-0.319268730324091,0.749522742429394,0.957755035261913
"13226","AC130352.1",2.07029756644786,0.336027592822492,1.05230397884402,0.319325593723997,0.749479626815313,0.957755035261913
"13227","PTPRE",86.7073409332851,0.150281225917857,0.469771117151107,0.319903077118122,0.749041805154487,0.957755035261913
"13228","BAZ1A",239.081821612153,-0.0659609153383744,0.206420993522824,-0.319545576313105,0.749312836487442,0.957755035261913
"13229","THAP10",17.719259594205,0.110462714910436,0.346008017947662,0.319249003435363,0.749537700158308,0.957755035261913
"13230","MVD",95.658828494315,0.0741092296374568,0.232180889455303,0.319187465477099,0.749584361344979,0.957755035261913
"13231","LINC02193",11.3758424520823,0.171986438015528,0.538727742298042,0.319245556729431,0.749540313600519,0.957755035261913
"13232","AC007448.4",7.51917559802721,-0.204606805192933,0.640972228594515,-0.319213214028918,0.74956483738156,0.957755035261913
"13233","AC240274.1",107.487223989304,0.119904007580256,0.374865929068375,0.319858376775515,0.749075692036347,0.957755035261913
"13234","WDR4",24.3408350210631,-0.130494657028946,0.409058615239347,-0.319012122388846,0.74971732035241,0.957852535327078
"13235","ZNF662",78.4348108006401,-0.0787781104544653,0.247144370717962,-0.318753408081327,0.749913511598523,0.957886050774936
"13236","PRR4",18.8420435109695,0.182696655001411,0.573002411367532,0.318840988060391,0.749847095116917,0.957886050774936
"13237","AKT1S1",210.225560939166,-0.0608730614113859,0.19093389034571,-0.318817478139515,0.749864923738221,0.957886050774936
"13238","NKIRAS2",166.577331601899,0.0597831853641909,0.187635460141633,0.318613471670358,0.750019636550409,0.957949238162435

"13239","MAIP1",150.842397782743,-0.0786463565506784,0.247047102411916
,-0.318345593948909,0.750222802808944,0.958076559301184
"13240","PPAN",90.1115897319534,-0.0884531281547271,0.277863860357721,
-0.318332610944268,0.750232649937762,0.958076559301184
"13241","COA6",311.455694037704,0.0864416578844857,0.27192068400758 8,0
.317892911309658,0.750566169866834,0.958115698616186
"13242","AC137630.2",3.75226990600958,-0.254636304031722,0.80113618452
326,-0.31784396829267,0.750603296893187,0.958115698616186
"13243","SERPINH1",1297.56015166984,0.0962529475044749,0.3026751781430
31,0.318007403497719,0.750479321030876,0.958115698616186
"13244","AC012645.4",3.41644873845847,0.363276051836718,1.142763434076
12,0.317892611020069,0.750566397657722,0.958115698616186
"13245","DDX5",9820.21879210106,0.0704999067219823,0.221774557536164,0
.317889966753676,0.750568403521705,0.958115698616186
"13246","AL031595.3",18.1385032770264,0.202967858530185,0.638287939540
874,0.317987926696816,0.750494095068856,0.958115698616186
"13247","VPS33B-
DT",3.04115064762945,-0.246663162519446,0.776455660567807,-0.317678362
134763,0.75072892612872 4,0.95820372031286
"13248","HIF1A",345.601739219898,0.0835021760955048,0.262977406833698,
0.31752604568159 8,0.750844479597027,0.958278869340771
"13249","WDR91",53.615285743233,-0.0753017212286513,0.237326918822939,
-0.317291108830479,0.751022723223442,0.958303528088082
"13250","EMC4",697.801426987526,-0.0679219928295752,0.214075866029229,
-0.317280009603238,0.751031144395958,0.958303528088082
"13251","SYCP2",5.5901858460026,-0.277144785744818,0.87351195442955,-0
.317276465810715,0.751033833137874,0.958303528088082
"13252","ITGA9-
AS1",92.5705129959345,0.0793687361438139,0.250685217644425,0.316607165
311165,0.751541698161029,0.958329559013091
"13253","FAM13A",1228.99682921781,-0.0668478015330308,0.21084867851855
4,-0.317041595909972,0.751212040079222,0.958329559013091
"13254","RAD1",181.60548654022,0.0684883403775898,0.21634354790249,0.3
16572142047234,0.751568276768647,0.958329559013091
"13255","FRMD3",3.79394703103806,-0.246785087894959,0.779429887947374,
-0.316622561837943,0.751530014070747,0.958329559013091
"13256","GAS1",3784.2062885668,-0.0800132792258333,0.252505049580356,-
0.316877937129611,0.751336223609264,0.958329559013091
"13257","RNF169",235.937432152042,0.0646082773774807,0.203737047066188
,0.317115999803862,0.751155584876989,0.958329559013091
"13258","REXO2",1161.24960112598,-0.0839675042329791,0.265025147435597
,-0.316828440793091,0.751373782470402,0.958329559013091
"13259","LACTB",262.028836621743,-0.148770202088989,0.469837406856787,
-0.316641884868771,0.751515350322291,0.958329559013091
"13260","RPS9",25974.224884483,-0.090652752414556,0.286420212744641,-0
.316502636269522,0.751621024489745,0.958329559013091
"13261","ZNF776",16.0002211838403,-0.11847881691351,0.374004190997394,
-0.31678473066719,0.751406951122971,0.958329559013091
"13262","AL024508.2",4.4134516624085,0.246188085615158,0.7783680485791
5,0.316287501863103,0.751784296682892,0.958393190704542

"13263","UBE2G1",181.789133924432,0.0638996448278652,0.201991646115731
,0.316347958228202,0.751738413344149,0.958393190704542
"13264","RAF1",264.595290930348,0.0599760684561516,0.189698044410473,0
.316165981797757,0.751876526951232,0.958438504047906
"13265","RFX5",45.0248051681337,0.0937015670066493,0.296591272421257,0
.31592826802254,0.752056955180597,0.958557789190714
"13266","TFDP2",656.220647027041,0.0597508369745537,0.18914878799729,0
.315893311330179,0.752083488964041,0.958557789190714
"13267","COA5",392.954414545733,0.0759425487736818,0.240813308221387,0
.315358604283885,0.752489393412755,0.958564658298497
"13268","ABTB1",311.639388265102,0.0656995249821604,0.208088705030009,
0.315728453270376,0.752208627980336,0.958564658298497
"13269","LINC01301",4.84569373392397,-0.220388486956805,0.698961012560
117,-0.315308698191302,0.752527281399242,0.958564658298497
"13270","SLC31A1",107.479060914398,-0.0759504749845528,0.2407399351725
53,-0.315487644084121,0.752391431019881,0.958564658298497
"13271","NT5C2",170.77127114974,0.0691843653656568,0.219317626608104,0
.315452827187856,0.752417862367257,0.958564658298497
"13272","SNAPC1",175.008742404735,-0.0556500020118799,0.17650487414133
,-0.315288754957102,0.752542422182058,0.958564658298497
"13273","CLMN",41.1536402777192,0.139192963649347,0.440931757098002,0.
315679153085837,0.752246051599211,0.958564658298497
"13274","ATP5MC1",880.407706370651,-0.089269443651966,0.28281260386873
6,-0.315648745603287,0.752269134116358,0.958564658298497
"13275","POT1",61.7111431448324,0.0686943792635125,0.218115786597831,0
.314944554610224,0.75280375200041,0.958620347007448
"13276","CBWD3",26.0508979114476,-0.11866452754986,0.376989879704865,-
0.314768469760405,0.752937453181061,0.958620347007448
"13277","ERGIC2",630.920782852791,-0.0529991744395284,0.16836462286692
7,-0.314788068520892,0.752922571480739,0.958620347007448
"13278","AC002070.1",4.04847320739066,0.270856471123099,0.860658356265
662,0.314708466084414,0.752983015649747,0.958620347007448
"13279","AC130371.2",8.75576604371663,-0.176842381771075,0.56161474844
3547,-0.31488201166578,0.752851239992983,0.958620347007448
"13280","TCN2",106.076619271921,0.113152368913392,0.359267307073029,0.
314953146823325,0.752797228124932,0.958620347007448
"13281","APOBEC3D",34.4180839573466,0.103235633193384,0.32795250153704
6,0.314788369381359,0.752922343032554,0.958620347007448
"13282","RAB28",209.085010384334,-0.0815743827734912,0.259277476712016
,-0.314621940200757,0.753048718687046,0.958631812645729
"13283","TYK2",107.545808357863,0.0681940846865307,0.216881373148258,0
.314430343632664,0.753194213036799,0.958744843335557
"13284","SLF1",68.6673421762273,-0.0824815663995066,0.262455320361754,
-0.314268982186448,0.75331675427937,0.958756468299253
"13285","RPL10",70926.9320328896,-0.0831046283356849,0.264384341272849
,-0.314332641394671,0.75326840940858,0.958756468299253
"13286","SLC16A5",39.3293385626923,-0.0922094707538062,0.2935509643565
07,-0.314117417246231,0.753431861511191,0.958830792897126
"13287","NCOR1",758.962104988057,-0.0426628758281274,0.135879639410274
,-0.313975486049911,0.753539657286424,0.95889580231797

"13288","APOD",50825.9688780169,0.109025515907468,0.347340253574868,0.313886786185488,0.753607026667552,0.95890936234911
"13289","HIST1H2AB",2.48572323093404,0.303760574344107,0.968284998510485,0.313709883775316,0.753741393306374,0.958914060647458
"13290","ORAI2",141.364532655671,0.0737255723832808,0.23504283245618,0.313668668867092,0.753772699257374,0.958914060647458
"13291","NR2F6",248.673148371124,-0.0720608961307725,0.229743584754431,-0.313657925237814,0.75378085995182,0.958914060647458
"13292","LRRCC1",140.346820516971,0.0698524718750747,0.222761811258143,0.313574716781807,0.753844064737814,0.958922317678827
"13293","LINC02298",7.85007228136276,0.193222899676963,0.616485960533665,0.313426277395998,0.753956822753029,0.958993602580923
"13294","PRORP",86.0312450831866,-0.0885378767053598,0.282680084905639,-0.313208752342474,0.754122069326903,0.959008148998552
"13295","SUN2",357.210027950948,-0.0761659213797304,0.243163886226634,-0.313228755148049,0.754106873388285,0.959008148998552
"13296","GRAMD4",62.2491597038483,0.0800518456034512,0.255603795109855,0.313187234051224,0.754138416671679,0.959008148998552
"13297","LINC00997",18.5512144496736,-0.133698239561811,0.427300224764114,-0.312890637105602,0.754363751199453,0.959222554356649
"13298","AL139089.1",6.74171857131502,0.19560777441964,0.626386345785836,0.3122797547163,0.754827924666033,0.959476402112176
"13299","LRFN5",49.5730938024865,0.128564988708512,0.411643577334917,0.312321133590555,0.754796480504268,0.959476402112176
"13300","AKAP13",1495.76337774397,0.0630612450224934,0.201938116142262,0.31228005008261,0.754827700213199,0.959476402112176
"13301","FKBP10",726.258445995517,0.0705852730406675,0.225982392316929,0.312348552101684,0.754775645166126,0.959476402112176
"13302","ADGRE2",4.84351613480362,-0.210530740732168,0.674228054175006,-0.312254495238671,0.75484711975965,0.959476402112176
"13303","CDK17",100.809525874429,-0.0784999429656503,0.251468889250473,-0.31216562493924,0.754914654969097,0.959490113975606
"13304","BCAR1",79.6901752516734,-0.0766806827875321,0.245702428102441,-0.312087606865494,0.754973944790739,0.95949334474758
"13305","PSMD1",392.629097095148,0.0552772032582948,0.177256862933525,0.311848028581127,0.755156021288261,0.959565163485134
"13306","IKBKB",97.5996282120046,0.0905459863125919,0.290613789209763,0.31156810060116,0.755368780282216,0.959565163485134
"13307","AC090739.1",4.4666232970938,0.28014463245359,0.899180746408611,0.311555417053252,0.755378420840266,0.959565163485134
"13308","POLR1E",208.696166774853,0.0888285261656383,0.284949529178446,0.311734244382662,0.75524250062026,0.959565163485134
"13309","AL450384.2",7.78790481843746,0.172916566203981,0.555205420021857,0.311446106194665,0.755461507821151,0.959565163485134
"13310","ABCC8",8.02959521865794,0.221077800487923,0.709497313564314,0.311597797851117,0.755346208035558,0.959565163485134
"13311","B3GAT3",247.725615061457,-0.0835518957201237,0.2682968249619,-0.311415894437024,0.755484472221085,0.959565163485134
"13312","FGF14-IT1",3.72480550366476,0.247890318141202,0.795396427960637,0.3116563130

37238,0.755301732501179,0.959565163485134
"13313","RNF149",378.284513369016,-0.0522595524187045,0.16787817593062
3,-0.311294497506937,0.755576749985646,0.959603598279523
"13314","GPR27",3.63078301488669,-0.300678171693862,0.966106424650493,
-0.311226759311364,0.755628241512513,0.959603598279523
"13315","PYCR2",187.966502555864,0.0632167544765066,0.203272212978957,
0.31099555394249,0.755804001600336,0.959628458459563
"13316","AC097500.1",3.26735695788806,0.259394689922407,0.835654996047
404,0.310408830377761,0.756250079896382,0.959628458459563
"13317","SEC62",2819.20465524965,-0.050721375417175,0.163152146144255,
-0.310883899573889,0.75588888469859,0.959628458459563
"13318","AC107214.1",6.26492723404061,0.19797010788764,0.6368959488501
68,0.310835872398073,0.755925397346784,0.959628458459563
"13319","ULBP3",5.42850236686752,0.188062826697242,0.605349468419752,0
.31066819499846,0.75605287832029,0.959628458459563
"13320","AC105219.1",4.79922059142031,0.234408375543552,0.754433779857
61,0.31070768807276,0.756022852116168,0.959628458459563
"13321","RPS24",28113.715521888,-0.0800661184873789,0.257458109438894,
-0.310986974393138,0.755810523934114,0.959628458459563
"13322","IFITM3",9218.36846576502,0.0829506581346588,0.26690458652246,
0.310787683401906,0.755962033566703,0.959628458459563
"13323","MRPL17",275.223023060696,-0.0826769823144693,0.26637356822732
5,-0.310379828091323,0.756272132066103,0.959628458459563
"13324","SLC2A3",1599.81647303974,0.0904574121141989,0.291263668586432
,0.310568814000074,0.756128438366302,0.959628458459563
"13325","QPCTL",8.51032631814293,-0.155736484001444,0.501532137383372,
-0.310521444974519,0.756164454177945,0.959628458459563
"13326","ERCC5",272.351750830813,0.0621325744456332,0.200243704027209,
0.310284783970988,0.756344401175742,0.959648141608844
"13327","GORAB",65.9475920893863,-0.0696720991473228,0.22546086517185,
-0.309020809860805,0.757305697058336,0.959774444351668
"13328","MTX2",298.952345072057,0.0893663754780596,0.289126063198059,0
.309091385569212,0.757252011887068,0.959774444351668
"13329","NR3C2",70.4780070111229,0.0844525211744073,0.273444057745357,
0.308847527610394,0.757437513486142,0.959774444351668
"13330","PCDHB10",33.6767020966868,-0.109661988802957,0.35405800299689
3,-0.309728880225083,0.75676713878527,0.959774444351668
"13331","PCDHGB5",4.84446729050544,0.215374536965052,0.696009601610127
,0.309441904920293,0.756985397964603,0.959774444351668
"13332","AL359643.3",23.6532077629429,-0.121682348218789,0.39408539774
0454,-0.308771522407256,0.757495333142083,0.959774444351668
"13333","ACOT13",378.632715961677,-0.0912943746846868,0.29494702374633
,-0.309528041765238,0.756919884517254,0.959774444351668
"13334","AL135905.2",4.89085703177036,-0.206857330753515,0.66875358477
6606,-0.309317715018477,0.757079856652494,0.959774444351668
"13335","RPF2",320.258335402756,-0.0712474170973473,0.229928811157492,
-0.309867287786504,0.756661879794023,0.959774444351668
"13336","UNC13B",50.4971561061462,0.0993991198837632,0.321955226390182
,0.308735848143369,0.757522472194997,0.959774444351668
"13337","TDRD7",41.0305833949396,-0.0965909314433615,0.312600815754866

,-0.308991296808085,0.757328147244519,0.959774444351668
"13338","AL157394.3",23.0459849724555,-0.109339841624898,0.353409521932676,-0.309385669709622,0.757028169947647,0.959774444351668
"13339","BCDIN3D",66.6465744918537,-0.0904813004154086,0.292167100121408,-0.309690243623631,0.756796522737778,0.959774444351668
"13340","RASSF3",35.8552908490284,-0.108542856138088,0.351090061162615,-0.309159580817112,0.75720013858491,0.959774444351668
"13341","C14orf119",376.498710913434,0.0932096739197278,0.30092336541579,0.309745551964497,0.756754459683463,0.959774444351668
"13342","LINC00923",2.52281813636342,0.315475141968754,1.02000929272599,0.309286537111484,0.757103571100419,0.959774444351668
"13343","FO393401.1",3.02737155534192,-0.304457737982355,0.985804341031548,-0.308841953022615,0.757441754212155,0.959774444351668
"13344","GID8",312.068384494276,0.0506767030128188,0.163599526578468,0.309760694744508,0.7567429434344,0.959774444351668
"13345","CCDC134",9.18544932787511,0.166990658815952,0.540262959246147,0.30909144511584,0.757251966591935,0.959774444351668
"13346","DCLRE1C",126.817974591005,0.0712626288499109,0.231029214428477,0.308457218392061,0.757734448940688,0.959971082173621
"13347","CD46",754.897965241361,0.0495462407572064,0.1607815030236,0.308158835596491,0.757961473666382,0.960088170666921
"13348","IKZF1",23.242147628674,0.155583847038289,0.504958974400198,0.308111856459419,0.757997219673097,0.960088170666921
"13349","AC068733.3",3.42646451690262,-0.237997771074328,0.772269498294751,-0.308179685459352,0.757945609357342,0.960088170666921
"13350","BOLA1",186.862837600394,0.08135866893789,0.264434251569718,0.307670691126182,0.758332923677443,0.960148346613407
"13351","CTSK",4854.24245984647,0.0610375571839586,0.198514029793229,0.307472259001215,0.75848393512441,0.960148346613407
"13352","LMOD1",536.548755830978,0.0630452758474527,0.205264486698091,0.307141663234623,0.758735546626393,0.960148346613407
"13353","SMARCC1",221.821272648755,0.0723583820414876,0.235371898283012,0.307421500057257,0.758522565337523,0.960148346613407
"13354","AC092902.4",10.5761690794456,0.158794963710733,0.516896837775186,0.307208232099492,0.758684880002056,0.960148346613407
"13355","ZFP62",83.8470724548795,0.0659648085928373,0.214568834759631,0.307429588582768,0.758516409505872,0.960148346613407
"13356","AL021408.1",2.49434724230417,-0.310048637636979,1.00768008872959,-0.307685585043033,0.758321589433353,0.960148346613407
"13357","ST3GAL1",145.095011271584,0.0705148166329697,0.229549682384528,0.307187602703137,0.758700581253724,0.960148346613407
"13358","MAPKAPK5-
AS1",204.065605656546,-0.0799171212266523,0.259598701882184,-0.307848693569053,0.758197467536312,0.960148346613407
"13359","PSMC6",669.563417560654,-0.061411553464504,0.199985926224354,-0.307079376153748,0.758782955254811,0.960148346613407
"13360","NPIPB4",20.5364367055501,0.151817882298337,0.493666784308261,0.307531085995725,0.758439165456803,0.960148346613407
"13361","AC009063.3",9.06249299712086,0.285323031324692,0.927953547813356,0.307475554134182,0.758481427376591,0.960148346613407

"13362","RIMS4",4.040054958426,-0.339840039801546,1.10667307374625,-0.30708259545083,0.758780504925766,0.960148346613407
"13363","AC018816.1",4.6599170016951,-0.20743887122374,0.676191227111527,-0.306775454792356,0.759014292229627,0.960227997651605
"13364","ADTRP",4.78954180567371,0.209602164163618,0.683248744870161,0.306772849181668,0.759016275645475,0.960227997651605
"13365","ITGA11",473.988346601612,-0.115872221715032,0.377558083736494,-0.306899061909376,0.758920203128311,0.960227997651605
"13366","FAM161A",82.9896172174835,-0.0780962688624889,0.254706512810603,-0.306612767772297,0.75913813419006,0.960310307712519
"13367","ARMH1",8.75704703998659,-0.178653690250042,0.584262850462207,-0.305776227444052,0.759775029832614,0.960353065237329
"13368","ZNF326",294.262365145516,0.0511007592772762,0.167110494475581,0.305790246373445,0.759764355249891,0.960353065237329
"13369","NUP43",66.3312140494191,0.0849011345475286,0.277194075014189,0.30628769587944,0.759385606609138,0.960353065237329
"13370","GBF1",161.13129313418,0.0558609782184151,0.182573050316865,0.305965081491849,0.759631232528128,0.960353065237329
"13371","CAT",1079.96002889155,-0.0749231953864343,0.244827791194106,-0.30602406295874,0.759586324544763,0.960353065237329
"13372","PCF11-AS1",24.9779532757329,0.165208708947207,0.540343187502369,0.305747741006695,0.759796720704977,0.960353065237329
"13373","ME3",82.4506479737778,-0.0815041994069423,0.26644679031007,-0.30589296764316,0.759686140495606,0.960353065237329
"13374","PPP2R1B",160.325256348388,-0.066616201206869,0.217758132789506,-0.305918315672108,0.759666840198498,0.960353065237329
"13375","PHLDA1",453.80661881891,-0.224360520008181,0.733314351588934,-0.30595408302325,0.759639606757509,0.960353065237329
"13376","AL390198.1",23.392250601011,-0.16567054325596,0.541614194680999,-0.305882941922408,0.759693774242457,0.960353065237329
"13377","IKBKG",119.373127894938,0.075840662942698,0.247823232352876,0.306027252661721,0.759583895955353,0.960353065237329
"13378","ABHD10",119.520038095602,0.0659210061053994,0.215695849715848,0.305620187835056,0.759893847822823,0.960404034907183
"13379","TUBD1",83.6840718302205,0.070482539525292,0.230697017453607,0.305519942577784,0.759970183610754,0.960428721465777
"13380","KRT8",6.27452805517477,-0.179448171037807,0.588159475337937,-0.305101215847457,0.760289065212047,0.960759903931636
"13381","EPB41",21.6838735944689,-0.163133464790404,0.535299660233807,-0.304751668848717,0.760555294148496,0.960809093952688
"13382","PFKFB2",18.3210730672562,0.115027219850576,0.377245825660979,0.304913168088831,0.760432286359054,0.960809093952688
"13383","DCLK2",21.4643947547543,-0.146264838472999,0.479773582396483,-0.304862218012092,0.760471092428383,0.960809093952688
"13384","ERC1",288.658205708544,0.0713583405511167,0.234097219004854,0.304823529533844,0.760500559867484,0.960809093952688
"13385","MCFD2",619.151693089596,0.0502613038006426,0.165214837990502,0.30421785604712,0.760961921988864,0.961077707404055
"13386","STX1A",6.55807493288542,-0.17496361753539,0.575208877584423,-

0.304174056336101,0.760995289018541,0.961077707404055
"13387","CUL2",198.68883826984,0.0563638279345286,0.185265506175377,0.304232714972711,0.760950602421314,0.961077707404055
"13388","ATP8B1",52.0642691412224,-0.0975087182167309,0.320359951741876,-0.304372371410821,0.760844214290221,0.961077707404055
"13389","PCOLCE2",4102.44191019257,-0.0907267613506831,0.298625510544951,-0.303814503942142,0.76126921619426,0.961144203137933
"13390","AC080013.5",3.7738737868638,-0.219799758321193,0.723491073568237,-0.303804381769559,0.76127692826617,0.961144203137933
"13391","ATPSCKMT",55.1691218617023,-0.0824924262763199,0.271352100796471,-0.304005113777224,0.761123995204353,0.961144203137933
"13392","TRIB1",152.746387751293,-0.291165307908381,0.958626105152892,-0.303731878720268,0.761332168951167,0.961144203137933
"13393","VASH1-AS1",12.1782497183536,0.139364422564062,0.45852658814593,0.303939675837748,0.761173849828003,0.961144203137933
"13394","SYNGAP1",89.3081099934498,-0.0674261672710561,0.222123138315649,-0.303553100241362,0.761468386980222,0.961244399511841
"13395","DDN-AS1",4.35891475118861,0.206741663183711,0.681359099244514,0.303425408735195,0.761565684454407,0.961295452986571
"13396","ZNF250",37.2377793705718,0.0990774980083508,0.326674870270542,0.303290846725669,0.761668221145869,0.961353111610507
"13397","AP003119.3",10.6736636446829,0.189242647198975,0.624134194811667,0.303208266382647,0.761731149709637,0.961360773254501
"13398","ZNF525",30.1413180717387,-0.1061186580392,0.350142146056778,-0.30307307827488,0.76183417028329,0.961419029045371
"13399","FOXD2",7.16871146608656,-0.18814657065632,0.62118329777353,-0.302884142781498,0.761978156312966,0.961441485782111
"13400","CCDC86",61.258477400821,0.0818858511028603,0.270472356383873,0.302751276313954,0.762079417580545,0.961441485782111
"13401","LIN37",115.094226843776,-0.0845342942911093,0.279118201235098,-0.30286199150412,0.761995038140748,0.961441485782111
"13402","TMEM191B",16.4849469479777,0.229618642089208,0.758367507803321,0.302780168884502,0.762057397394206,0.961441485782111
"13403","AC092053.2",3.87482397558091,-0.219074260550712,0.723913685137677,-0.302624836425142,0.762175784754548,0.961491320497643
"13404","KRR1",622.925425500798,-0.047592131488426,0.157502334396045,-0.302167784819963,0.762524161041951,0.961715538930128
"13405","RASD1",5676.29772983791,-0.0930177619661508,0.307762548144475,-0.30223873088835,0.762470080998734,0.961715538930128
"13406","TTC3",1735.04344312352,0.060572065922912,0.200390528155572,0.302270104682229,0.762446166073869,0.961715538930128
"13407","MANEAL",9.03607348357362,0.171174380549801,0.567053272411561,0.301866489230952,0.76275384247487,0.961756228856541
"13408","HLA-E",8561.67504344329,-0.0622408302224136,0.206142624997589,-0.301930909355314,0.76270473244397,0.961756228856541
"13409","FAM229B",283.113294130853,0.0935713441225461,0.31001778845439,0.301825726159298,0.762784918284029,0.961756228856541

"13410","AC079193.2",3.19282291585671,0.249323935858581,0.827431915843
132,0.301322599581533,0.763168509304442,0.961756228856541
"13411","PGM5P3-
AS1",3.25307060742321,0.288484980970727,0.957659990894438,0.3012394625
58404,0.76323189978143,0.961756228856541
"13412","EGFL7",56.5259244520689,-0.0821311968310071,0.272484830326318
,-0.301415666819506,0.763097549102016,0.961756228856541
"13413","AL731569.1",4.02486794930289,0.223107442060088,0.740511779867
712,0.301288174105676,0.763194757914744,0.961756228856541
"13414","RBM26-
AS1",31.9238856382499,-0.138372097958497,0.458439726959977,-0.30183269
4291299,0.762779606087575,0.961756228856541
"13415","ZNF211",17.4322851667803,0.113789764283665,0.377463066937795,
0.301459332715105,0.763064256216017,0.961756228856541
"13416","KCNK15",53.3630886364109,-0.133971456670459,0.44459254142086,
-0.301335367080842,0.763158774457711,0.961756228856541
"13417","HAR1B",5.01365711608068,0.202676472620773,0.672829315499578,0
.301230145524032,0.763239003950619,0.961756228856541
"13418","AP000962.1",2.31057247209311,-0.356916056280049,1.18338737405
109,-0.301605428709467,0.762952869100684,0.961756228856541
"13419","OTUD7B",104.549768383625,0.0863205420630301,0.286787844600584
,0.300990936987761,0.763421405500425,0.961914384395349
"13420","MORN1",23.3075699259781,-0.103891353102898,0.34538707082741,-
0.300796879437367,0.763569388121325,0.961981054910467
"13421","GKAP1",67.487702395183,0.0828456883262094,0.275443183159507,0
.300772331251465,0.763588108466607,0.961981054910467
"13422","TMEM8A",73.934578727966,0.0722846633832104,0.240450597361044,
0.300621683524756,0.763702994827878,0.96205410792354
"13423","SHOX2",405.668187735273,0.0595175791528758,0.198071155783782,
0.300485847711447,0.763806589848479,0.962112927151761
"13424","SGCG",97.2195706993359,0.102353874014408,0.340765752089136,0.
300364321786757,0.76389927503346,0.962157996295123
"13425","IKZF2",64.3227139352496,0.116802639355958,0.388993674860513,0
.300268736754761,0.76397217803976,0.962178144230634
"13426","LMNB1",12.7537830174079,-0.126388390636697,0.421389122445772,
-0.299932731778005,0.764228466700269,0.962429235436328
"13427","PCSK4",2.13354718832661,-0.333809662110253,1.11331145311196,-
0.299834930447521,0.764303069771224,0.962451500982487
"13428","DTNBP1",242.600032567286,0.0550261566717588,0.183640560969432
,0.299640539003354,0.764451358489218,0.962566545229051
"13429","AC079305.3",13.3467065745627,-0.134173668156512,0.44835969513
9986,-0.299254526245095,0.764745848372615,0.962865649287674
"13430","GPR89B",40.5349921949639,-0.0854504677618767,0.28585005084032
9,-0.298934590043532,0.764989954107694,0.962967049373218
"13431","PFN4",17.7156660454751,0.162759444108959,0.544540024810325,0.
298893445281,0.765021348537858,0.962967049373218
"13432","ARL6",47.261307885547,-0.0769235440167085,0.257393895571055,-
0.298855354926137,0.765050412726651,0.962967049373218
"13433","ATG3",354.620317298572,-0.0453233902557362,0.15165912804699,-
0.298850394561765,0.765054197671542,0.962967049373218

"13434","ARL6IP6",169.306099009438,-0.0688658265681597,0.230804305640898,-0.29837323171651,0.765418317116222,0.962997008780408
"13435","CNTFR",480.3761957435,-0.123768285422422,0.41491664228021,-0.298296748817408,0.765476685474845,0.962997008780408
"13436","STT3A",271.067550597551,0.0544006313504528,0.182348822743751,0.298332780721597,0.765449187356698,0.962997008780408
"13437","MT1X",4269.69576111204,-0.13312617819037,0.445914450279872,-0.29854645460988,0.7652861260069,0.962997008780408
"13438","RNMT",493.149844376488,-0.0404994514298379,0.135663096307656,-0.298529611457441,0.765298979175435,0.962997008780408
"13439","ASXL3",16.4277825516644,-0.187105026935619,0.6268777491779,-0.298471316266995,0.765343465284165,0.962997008780408
"13440","DOCK6",55.7766151179632,0.0979400614349766,0.327884092723368,0.298703302808922,0.765166436770164,0.962997008780408
"13441","JADE2",156.090625818669,0.0583121299963197,0.195877938834488,0.297696260963783,0.765934997435669,0.963225459607173
"13442","TMEM217",8.5492304039335,-0.274098643083315,0.920765489719922,-0.297685617177822,0.765943121860566,0.963225459607173
"13443","HNRNPA1",4218.45450529721,0.0699595767687892,0.235007283004771,0.297691100779072,0.76593893621263,0.963225459607173
"13444","GOLGA6L9",25.6638519791637,0.130321614534677,0.437641182381462,0.297781881096108,0.765869644339574,0.963225459607173
"13445","AC020915.2",80.0169755559698,0.066283748881122,0.222474176178116,0.297939068793559,0.765749668686446,0.963225459607173
"13446","HMGN1",804.982371544997,-0.0699040424701642,0.234889366174948,-0.297604117242578,0.766005331790939,0.963232050418057
"13447","TNFRSF1B",171.808895494587,0.0628669276122354,0.213496661215419,0.294463282256215,0.768403916791644,0.963254714597757
"13448","HPCA",2.4022978707724,-0.300895055232764,1.02246443838733,-0.294284127580366,0.768540800298702,0.963254714597757
"13449","LINC00853",6.31343880682393,0.220797591590312,0.755609855699202,0.292211105936406,0.770125220719924,0.963254714597757
"13450","AK5",5.09685056800275,0.233811472789747,0.798420315890997,0.292842589468462,0.769642472845716,0.963254714597757
"13451","MYOC",5332.31579275357,0.200395013734594,0.674628162298667,0.297045135281318,0.766432050430275,0.963254714597757
"13452","PTGS2",183.885012899276,-0.121347147619266,0.408961824778245,-0.296719987703168,0.766680295931687,0.963254714597757
"13453","RABL2A",67.2591819606653,-0.0688676194789037,0.234413609038497,-0.293786780389502,0.768920837280769,0.963254714597757
"13454","CHN1",6.18170209114176,-0.177853616358783,0.599204889486919,-0.296816029840934,0.766606966646679,0.963254714597757
"13455","AC017048.3",3.28927539223864,0.22999310875754,0.784823506588825,0.293050739212931,0.769483368934624,0.963254714597757
"13456","NDUFA10",798.172047515408,-0.0694268687037594,0.235201942454571,-0.295179827084842,0.76785651144456,0.963254714597757
"13457","CRTAP",1693.06203406308,-0.0432718059373106,0.147307340131677,-0.293751865308479,0.768947518963679,0.963254714597757
"13458","ALS2CL",6.44157961501892,0.182297461223934,0.614462009600047,0.296678164598967,0.766712229009523,0.963254714597757

"13459","RYK",399.075631333057,0.0596932299873731,0.202303055830281,0.
295068355455054,0.767941662742452,0.963254714597757
"13460","ATP5ME",2061.47965903,-0.0794064307286441,0.267192760798923,-
0.29718780737627,0.766323129903654,0.963254714597757
"13461","NFKB1",395.233711679945,0.0475969661570996,0.161034982975691,
0.295569107268354,0.767559168860983,0.963254714597757
"13462","ABCE1",336.496499592338,-0.0448871852854981,0.15275896722541,
-0.293843209998029,0.768877715034608,0.963254714597757
"13463","SUB1",2140.69808393656,0.0772180656687367,0.263639204901915,0
.292892954587179,0.769603974251458,0.963254714597757
"13464","MSH3",169.632543677323,0.0520983133330218,0.176932237865412,0
.294453480957223,0.768411405308079,0.963254714597757
"13465","FGF18",134.152777663596,-0.0808745507518076,0.274001439688016
,-0.295161043109457,0.767870860010284,0.963254714597757
"13466","AL138724.1",30.7684776915255,-0.135076097399018,0.45632096031
6491,-0.296011161322357,0.767221557671331,0.963254714597757
"13467","ATAT1",52.849621983194,-0.070244457311576,0.2379432349857,-0.
295215189941406,0.76782949894022,0.963254714597757
"13468","KIFC1",4.82737616538808,-0.224518958522389,0.768045648349957,
-0.292325018707856,0.77003813165502,0.963254714597757
"13469","CRYBG1",76.3601189204914,-0.0999171581184452,0.33835845578285
9,-0.295299722559813,0.767764928440819,0.963254714597757
"13470","AGPAT4",128.672668525748,0.0630761025256823,0.215824070915689
,0.292257032582445,0.770090108341007,0.963254714597757
"13471","COL28A1",2.86738960104644,-0.239090240329261,0.81729071183388
7,-0.292540018952101,0.769873766678668,0.963254714597757
"13472","ZNF800",247.053207661204,-0.0507935080343057,0.17246156337274
3,-0.294520744454375,0.768360014207504,0.963254714597757
"13473","RARRES2",2960.08050908618,0.0784739556418587,0.26829061457640
4,0.292496089607007,0.769907349269113,0.963254714597757
"13474","MCPH1",149.775927973601,0.0603829646647597,0.206263886093793,
0.292746179703518,0.76971616909187,0.963254714597757
"13475","LINC01504",63.6613009348551,0.0826461575656892,0.280263699051
488,0.294887128962449,0.768080104581293,0.963254714597757
"13476","ABL1",399.899029587388,0.0471541002755627,0.159327536160078,0
.295957004118776,0.767262916931952,0.963254714597757
"13477","NUP214",223.799687507266,0.0475665279070027,0.162712875930538
,0.292334135420905,0.770031161833035,0.963254714597757
"13478","RABL6",258.422439324504,-0.0540317496464401,0.184538922819,-0
.292793242862025,0.769680193450063,0.963254714597757
"13479","PPRC1",104.04719846366,-0.0930443661148207,0.316761596650345,
-0.29373625811568,0.768959445880283,0.963254714597757
"13480","AC136475.1",38.873702792087,-0.106289643369591,0.357472549966
957,-0.297336518228926,0.76620960410816,0.963254714597757
"13481","MRPL16",358.975841241011,-0.0795432700618171,0.26757414309790
7,-0.297275622901693,0.766256090965773,0.963254714597757
"13482","DPP3",43.9041703290504,-0.0821208709083939,0.279613048054285,
-0.293694702303201,0.768991202832167,0.963254714597757
"13483","AP000787.1",22.586524430518,0.106920003558076,0.3594158346725
26,0.297482729595078,0.766097991313318,0.963254714597757

"13484","AP002840.2",6.75013709168796,-0.203629336661431,0.688188126587349,-0.295891964412707,0.767312587916117,0.963254714597757
"13485","PAFAH1B2",430.574181681778,-0.0466846410293424,0.157750135826545,-0.295940417323474,0.767275584217675,0.963254714597757
"13486","TAS2R30",3.9315322891662,0.254174978787666,0.863729628197197,0.294276091139987,0.768546940727043,0.963254714597757
"13487","CASC1",5.69687611587945,-0.192895231236272,0.653304319767236,-0.295260915012774,0.767794571504347,0.963254714597757
"13488","AC010198.2",11.945770490913,0.154328275092035,0.526697148757161,0.29301141169304,0.769513429060361,0.963254714597757
"13489","NAA16",108.300215631094,0.06642565321743,0.226698831890703,0.293012772335129,0.769512389043094,0.963254714597757
"13490","SLAIN1",2.37281768731904,-0.275134023388671,0.938121066192354,-0.293282000909952,0.769306610267105,0.963254714597757
"13491","SCFD1",485.459490280617,0.0398639443480151,0.135060203033952,0.295156851926203,0.767874061551548,0.963254714597757
"13492","KIAA0586",154.429385529312,0.0556985536307441,0.188567329559789,0.295377538414382,0.767705489986831,0.963254714597757
"13493","AL136040.1",8.32168089917996,-0.183595270350591,0.625495006849821,-0.293519961534516,0.769124743968459,0.963254714597757
"13494","CORO2B",142.269723932783,-0.0540767558648939,0.184116392198225,-0.293709621502214,0.768979801535687,0.963254714597757
"13495","FAM169B",2.88748039676723,-0.354596351533376,1.21198213996361,-0.292575558534238,0.7698465981116,0.963254714597757
"13496","AC007014.1",2.60253762787495,0.282357740641483,0.958120712717931,0.294699547659824,0.768223408754314,0.963254714597757
"13497","ST3GAL2",119.454904349242,0.0699653610549373,0.23731510015965,0.294820519250015,0.768130990673959,0.963254714597757
"13498","CHRNB1",100.067891187059,-0.0888593894589316,0.302845877155325,-0.293414558895768,0.769205298535149,0.963254714597757
"13499","AC073508.3",25.8529989019579,0.106739871867734,0.360405707010438,0.296165875821279,0.767103407563411,0.963254714597757
"13500","AC103702.1",15.3549364081797,0.126323241425731,0.43083691153837,0.293204314771162,0.769365986244275,0.963254714597757
"13501","CSNK1D",331.773205785628,0.0615900955283118,0.207328587698679,0.297065138059127,0.766416779379581,0.963254714597757
"13502","METTL4",72.9538825097526,0.0668407528002211,0.226379288984647,0.295260017380627,0.767795257162802,0.963254714597757
"13503","FHOD3",9.69928325046661,-0.160789051999535,0.54425732576906,-0.295428365199003,0.767666667459974,0.963254714597757
"13504","AC018413.1",2.63217623540438,0.264498123220719,0.889946680337734,0.297206708069681,0.766308700844757,0.963254714597757
"13505","AC104532.2",30.3489137892634,-0.126183136717679,0.4289494966841,-0.294167816242029,0.768629672085428,0.963254714597757
"13506","CERS4",151.881669788549,0.0726018158678606,0.247474961440039,0.293370349248247,0.769239086751607,0.963254714597757
"13507","HNRNPM",990.337287907498,-0.0495221346994205,0.16701038174559,-0.296521295154324,0.766832006627879,0.963254714597757
"13508","AC011472.3",4.73159302318802,-0.216629993446169,0.738105083861753,-0.293494785746177,0.769143984481508,0.963254714597757

"13509","ITPKC",218.479038876755,0.121609802654497,0.410473830366229,0
.296266883922893,0.767026274093039,0.963254714597757
"13510","ZNF865",69.090362039512,0.0634627272735379,0.214044872310816,
0.296492630673176,0.766853893986177,0.963254714597757
"13511","KIF16B",130.497297423296,0.0694819802125695,0.234416537574997
,0.29640391813372,0.766921633461775,0.963254714597757
"13512","NFS1",73.7797723349848,0.0652153020511277,0.220828198174106,0
.295321442598153,0.767748337783289,0.963254714597757
"13513","AAR2",97.6589797661343,0.0600112289755665,0.205245680539326,0
.29238729320819,0.769990522521485,0.963254714597757
"13514","AL031651.2",7.58116751996557,-0.182266447455722,0.61651884996
998,-0.295638077350915,0.767506491239279,0.963254714597757
"13515","GET1",262.825125625459,-0.0734843192892411,0.250152798955091,
-0.293757733657953,0.768943034422807,0.963254714597757
"13516","FBXO7",407.166968730156,-0.0458427299614855,0.155169728418763
,-0.295436039159442,0.767660805984315,0.963254714597757
"13517","MCAT",98.8115117568543,-0.0759596716059837,0.258122361557915,
-0.294277764807062,0.768545661921766,0.963254714597757
"13518","USP11",274.210690641942,-0.0512131054715574,0.173790463160968
,-0.294683059933633,0.768236005057866,0.963254714597757
"13519","FAM85B",2.50838588908653,0.300517972356297,1.02966790705569,0
.291859123021151,0.770394338472786,0.963268857313989
"13520","NUTM2B-
AS1",260.717606124019,-0.0686616978114327,0.235200705405752,-0.2919281
1175027,0.770341589154415,0.963268857313989
"13521","CMTM2",4.70394893584755,-0.255726185540297,0.876465392307726,
-0.291769860834975,0.770462590610087,0.963268857313989
"13522","AC116407.1",2.4884465083571,-0.303600722790965,1.039974799627
85,-0.291930845727806,0.77033949875583,0.963268857313989
"13523","PAK4",59.9922595783456,0.067564796887335,0.231396752330668,0.
291986798461132,0.770296717675482,0.963268857313989
"13524","PIGP",488.481829969104,-0.0972996665940268,0.333504434325286,
-0.291749244026917,0.770478354998486,0.963268857313989
"13525","PPT2",34.9733050262037,0.0838734550522798,0.287617298935041,0
.291614778953969,0.770581174386375,0.963326173495366
"13526","PINK1-
AS",6.41023149143757,0.162965077638128,0.559275932492523,0.29138582257
9281,0.770756256295146,0.963339424964048
"13527","AKAP9",1573.72973050596,0.0684199282040887,0.234815497787025,
0.291377395652755,0.770762700550842,0.963339424964048
"13528","MTPAP",110.142750188514,0.0630160890283092,0.2162604659245,0.
291389777409936,0.770753231954787,0.963339424964048
"13529","EGLN1",211.721081829311,0.0445337353861197,0.153149935590728,
0.290785204801717,0.771215601496583,0.963351367445457
"13530","AL391832.2",12.010684108289,0.127206693040229,0.4371276887420
79,0.291005800630684,0.771046883148083,0.963351367445457
"13531","ADI1",758.97209020314,0.0668923103735271,0.229970925807077,0.
290872901166833,0.771148527373017,0.963351367445457
"13532","EPB41L4A-
DT",10.5770947540256,-0.148306839644366,0.509934031567362,-0.290835344

306247,0.771177252339411,0.963351367445457
"13533","TOMM7",5774.10827895341,-0.0651303846258353,0.224045308460326,-0.290701845414311,0.771279360090252,0.963351367445457
"13534","NECTIN1",33.4343933311581,0.0886015925594962,0.304949175169512,0.290545440925509,0.771398992420395,0.963351367445457
"13535","GATC",194.119308181708,0.0679638752485186,0.233875944661659,0.290597972129369,0.771358811187581,0.963351367445457
"13536","COPZ2",1638.80957833108,0.0807440976989721,0.277766306787754,0.290690755955041,0.771287842134769,0.963351367445457
"13537","TGIF1",289.495429281264,0.0664603236684921,0.228199479071106,0.291237841291405,0.770869423134842,0.963351367445457
"13538","MAPRE2",187.359619924446,0.0478552742821989,0.164624399531148,0.290693690719547,0.7712855974056,0.963351367445457
"13539","PRPF6",478.311376315519,-0.053813114957869,0.184825912292539,-0.291155684234874,0.77093225382235,0.963351367445457
"13540","ZBTB20-AS2",25.5339371376346,-0.107009144405882,0.368548220197083,-0.290353171014252,0.771546065325791,0.963403402203211
"13541","SARM1",32.1144720687588,-0.0998330261884742,0.343846323271367,-0.290341991267084,0.771554617295581,0.963403402203211
"13542","UFM1",614.84731540683,-0.0399717974865758,0.137713802187352,-0.290252660602577,0.771622951961724,0.963417580251723
"13543","SGCB",589.576066248809,0.0447848719546614,0.154581936450668,0.289716075389918,0.772033457142582,0.963858945090953
"13544","MFN2",91.8642115556456,0.0543976539907977,0.188417968953209,0.288707357865144,0.772805331606674,0.963885285538524
"13545","AK2",293.273547318398,-0.0474520142234744,0.16422454666873,-0.288945929131979,0.772622755646127,0.963885285538524
"13546","AL356488.3",28.8589900374713,-0.135626602418185,0.468702623089099,-0.289365998261978,0.772301311966631,0.963885285538524
"13547","SLAMF7",5.24811631996971,-0.263050868949395,0.909705637380127,-0.289160425241465,0.772458614882154,0.963885285538524
"13548","R3HDM1",105.858493413181,0.0566184941234949,0.196109273812912,0.288708907144844,0.772804145919419,0.963885285538524
"13549","MRPL44",160.047071246154,-0.0699057808408957,0.242360685005726,-0.288436966743365,0.77301227415452,0.963885285538524
"13550","DCK",73.121121381584,-0.0843606980577902,0.292351135142188,-0.288559502314779,0.772918490142134,0.963885285538524
"13551","FAT1",227.159906009709,-0.109579636997046,0.379708474296905,-0.288588863337726,0.772896018837122,0.963885285538524
"13552","SLC35B2",236.056291878712,0.0565452921694885,0.195526475575056,0.28919506682246,0.772432106751945,0.963885285538524
"13553","COL12A1",2168.83419802642,0.0920767950450775,0.319419088899617,0.288263282455277,0.773145211105611,0.963885285538524
"13554","MINDY4",102.315411231655,-0.0749786878170233,0.259898081542015,-0.28849265593714,0.772969651376997,0.963885285538524
"13555","NUDT5",323.556563565266,-0.0648983576569111,0.224738290595489,-0.28877303233441,0.772755070404478,0.963885285538524
"13556","ATG16L2",43.4491009291035,-0.0857181659252116,0.296362293528669,-0.28923438573983,0.772402019792723,0.963885285538524

"13557","AC016876.3",2.56075207121579,0.266377030756781,0.924818278576
331,0.288031753834757,0.773322432124137,0.963885285538524
"13558","AC005726.5",5.43500465798173,0.201877135487597,0.698178178497
386,0.289148446206204,0.772467781437426,0.963885285538524
"13559","MRPL45",247.71035098443,-0.0638622197388412,0.220807488209395
,-0.289221259010381,0.772412064369333,0.963885285538524
"13560","ORMDL3",106.309835752437,0.0511641941784026,0.177613035213811
,0.288065535937782,0.773296573250858,0.963885285538524
"13561","ZNF699",49.3531816748897,0.0889135373055979,0.308746576528466
,0.28798226139165,0.773360317100159,0.963885285538524
"13562","AC008569.2",3.54854350392317,-0.242515800607227,0.84083803314
2395,-0.288421540235153,0.773024081245392,0.963885285538524
"13563","AC010624.5",5.77113984272572,-0.218014483730406,0.75641111862
4925,-0.288222209275206,0.773176649253691,0.963885285538524
"13564","ERVK3-1",285.604396317677,-0.0550483097131079,0.1905825829890
94,-0.288842290044196,0.772702067951975,0.963885285538524
"13565","CSNK1E",677.321404951273,0.0406992608943439,0.141017670494701
,0.288611070879044,0.772879022538248,0.963885285538524
"13566","U62317.4",3.28438232887232,-0.306386445766559,1.0639334845502
3,-0.28797518850164,0.773365731228745,0.963885285538524
"13567","KTN1-
AS1",25.9582698560915,0.0999659657961731,0.347279020048051,0.287854894
840297,0.77345781484553,0.963928999293007
"13568","PER3",342.478685120124,-0.226305734995117,0.786742408092811,-
0.287649086495437,0.773615366506816,0.963983242456868
"13569","DAB1",5.09849347620672,-0.297292875739563,1.03334991033694,-0
.287698167644517,0.773577792758579,0.963983242456868
"13570","KHK",38.7381302780099,-0.118409767207606,0.411811830023745,-0
.287533670902019,0.773703724239318,0.964022296937243
"13571","ZNF17",34.7310716268638,-0.0902677816258182,0.314021374131951
,-0.28745744417986,0.773762082089456,0.964023969049335
"13572","SENP1",59.1027914306584,0.0660493866970026,0.229985460064177,
0.287189401793363,0.773967300821621,0.96420860022782
"13573","RFFL",64.5588261359135,0.0739384256393976,0.257703764193358,0
.286912478251271,0.774179335739927,0.964401695180925
"13574","C1orf122",909.842131046109,0.0889960440864832,0.3103082985613
77,0.286798788492214,0.774266390634911,0.964434556022173
"13575","MPLKIP",429.150053949747,-0.0632052961318967,0.22047762692961
6,-0.286674421400925,0.774361624662662,0.964434556022173
"13576","ZNF570",75.7047823586913,-0.0716083997901032,0.24980729521181
7,-0.286654557983925,0.77437683537716,0.964434556022173
"13577","RBM23",261.119966033845,0.0394285321882572,0.13758759769972,0
.286570394769946,0.774441285602675,0.964443784117996
"13578","BTN2A2",31.3043040357693,0.0881677197966284,0.308010085261603
,0.286249457454436,0.774687066172627,0.964590272025764
"13579","TRPA1",4.20983267965334,0.275711381750806,0.963374634407497,0
.286193316601465,0.774730062343854,0.964590272025764
"13580","ZNF711",36.9149854667172,-0.0942564224107687,0.32917770297241
2,-0.286339024665556,0.774618471439411,0.964590272025764
"13581","WDR77",65.7004651794564,-0.0672818338758302,0.236411586779825

,-0.284596177337497,0.775953540240234,0.964703524590007
"13582","MRPS25",344.349090612235,0.0558356258527247,0.19597079081713,0.284918102437152,0.775706886809292,0.964703524590007
"13583","AC106881.1",2.9724352397206,0.276007377089043,0.970008999057256,0.28454104792563,0.775995781707028,0.964703524590007
"13584","UTP15",50.9531045969378,-0.0842910462573295,0.295368630748882,-0.285375755860115,0.775356279635773,0.964703524590007
"13585","KLHDC3",508.85613396183,0.0601652969919262,0.210872428541011,0.285316090909557,0.775401986215058,0.964703524590007
"13586","L3MBTL3",47.0237752043395,-0.0858782067201545,0.300816288965563,-0.28548389788156,0.775273438981833,0.964703524590007
"13587","YWHAZ",1638.81930294849,-0.0451458488114885,0.158694285969654,-0.284483140244391,0.776040152663463,0.964703524590007
"13588","HACD4",167.896338292027,0.0840554030902444,0.294470846394708,0.285445585256942,0.77530278752928,0.964703524590007
"13589","RFK",260.135245493828,0.0714744704539059,0.250911531671329,0.284859248906626,0.775751977675023,0.964703524590007
"13590","PRMT3",64.0439744468437,0.0589799537678469,0.207181090797944,0.28467826644164,0.77589064284408,0.964703524590007
"13591","CACNA1C-AS2",2.75574007587118,-0.275224462851301,0.964313057352068,-0.285409868458119,0.775330147890278,0.964703524590007
"13592","SMAD9",170.23507895596,0.0909457409489478,0.319228491692207,0.284892305404355,0.775726651215174,0.964703524590007
"13593","C15orf65",22.9894520288909,-0.0997876353921966,0.349509367376793,-0.285507756605045,0.775255162691151,0.964703524590007
"13594","AC007950.2",14.7841120118476,-0.13536557038837,0.475899122788548,-0.284441731254285,0.776071882175225,0.964703524590007
"13595","PDCD7",582.280814037936,0.0342154147646509,0.119776318029718,0.285660933041551,0.775137829241975,0.964703524590007
"13596","TMED3",497.407736901984,-0.0858306987564304,0.30041151581496,-0.28571041467431,0.77509992731174,0.964703524590007
"13597","AC022558.1",4.51840390191375,0.227949177938274,0.801159734919266,0.284524006890142,0.776008839087867,0.964703524590007
"13598","SDF2",606.810912327278,-0.0721807117591848,0.253148547484242,-0.285131842455773,0.775543135406956,0.964703524590007
"13599","NOP53",4250.05594827394,-0.0688532736921836,0.242069457365576,-0.284436022790767,0.776076256297213,0.964703524590007
"13600","SCARF2",48.5427127359422,-0.138805277098044,0.487049156768417,-0.284992336336276,0.77565001329151,0.964703524590007
"13601","Z95115.1",10.5559694178401,-0.140360936357296,0.492079993313766,-0.285240079386438,0.775460216280108,0.964703524590007
"13602","TIMP3",11318.779905311,-0.0623506599480364,0.218863298897243,-0.28488403611841,0.775732986762295,0.964703524590007
"13603","SLC16A7",171.22941267332,-0.0721244436755885,0.254006194112693,-0.283947578237362,0.776450554236634,0.9650585993793
"13604","HSPB8",304.56838925101,-0.0773420192910931,0.272413257925539,-0.283914299473022,0.776476057839839,0.9650585993793
"13605","COL21A1",9.86457311002791,0.220000675334422,0.775289976312478,0.283765664533435,0.776589969052489,0.965118580298301

"13606","TAOK3",402.089326028935,0.0602304164663212,0.212357169787757,
0.283627892227604,0.776695559623787,0.965118580298301
"13607","NACA2",7.17607723608463,0.196696851704503,0.693442581417622,0
.283652687295883,0.776676556041287,0.965118580298301
"13608","AC023355.1",5.84251288249269,0.180421276959759,0.636480572189
811,0.283467060650445,0.776818828354351,0.96520081935739
"13609","MRPS11",320.667974195939,-0.0678745041813545,0.23954720216308
,-0.283345009119107,0.776912378021999,0.965246122977145
"13610","NXPE3",50.2521823721385,0.0867204426196116,0.306298517399875,
0.283123938554353,0.77708183171807,0.965314790293816
"13611","CHD9",1479.7272631197,0.0439259829166417,0.15513085047854,0.2
83154400179854,0.777058481823618,0.965314790293816
"13612","CTSG",32.0813322303942,-0.268541365082208,0.949201478389151,-
0.282912923332081,0.777243587746684,0.965444797356812
"13613","NOL10",145.623505915338,-0.0464769678190167,0.164878860480596
,-0.281885547265087,0.77803127196067,0.965720344144849
"13614","AC093323.1",91.1215532800556,0.0670031773655514,0.23751717833
7635,0.282098237418032,0.777868184706772,0.965720344144849
"13615","LINC00504",5.30618914420704,0.219680626019812,0.7804004708780
89,0.281497300703358,0.7783289981447,0.965720344144849
"13616","CASC15",14.7194270658965,-0.114441030316665,0.405369950734196
,-0.282312564385673,0.77770385226885,0.965720344144849
"13617","RTN4IP1",15.5976669488958,-0.119835984869071,0.42538671155990
8,-0.281710691971615,0.778165355400771,0.965720344144849
"13618","STKLD1",8.48887137238915,-0.162560329266527,0.575611971606191
,-0.282413044351592,0.777626813974006,0.965720344144849
"13619","ARHGAP1",460.368081480425,-0.0475646373573203,0.1688815380366
22,-0.28164497973133,0.778215746908298,0.965720344144849
"13620","OSBP",225.28759526551,-0.0515337901444251,0.183027490391394,-
0.281563114012125,0.77827852703451,0.965720344144849
"13621","UBASH3B",4.81959296094289,-0.201140491321528,0.71310530960068
,-0.282062815426464,0.777895345018867,0.965720344144849
"13622","SOX5",167.197717080397,0.0693416533047973,0.24636773796819,0.
281455899529144,0.778360748482104,0.965720344144849
"13623","AC087482.1",4.97304468273514,-0.197293545927874,0.70019841029
9813,-0.281768057490157,0.778121365349092,0.965720344144849
"13624","BOLA2",151.394908560653,0.0752100206956797,0.266811031638326,
0.28188497392278,0.778031711603184,0.965720344144849
"13625","RAP1GAP2",6.21169118512407,-0.210026422257748,0.7462783862354
,-0.281431736643515,0.778379279039863,0.965720344144849
"13626","TSPOAP1",4.83142464292783,0.216374282359552,0.7664175748696,0
.282319050938213,0.777698878943337,0.965720344144849
"13627","CCDC61",79.6668840678189,-0.0744926964165271,0.26426195483013
1,-0.281889598767297,0.778028165245123,0.965720344144849
"13628","GINS1",3.4278031154747,-0.23561069167257,0.837084825438988,-0
.281465730249034,0.778353209322498,0.965720344144849
"13629","ISCA2",244.530134501799,-0.0764982096917254,0.272009695511682
,-0.281233393345863,0.778531393611193,0.965756016200329
"13630","SCAMP5",6.00483680715363,-0.167602319637173,0.596245103870566
,-0.28109634536061,0.778636504189903,0.965756016200329

"13631","SCAF4",144.897355745754,0.0633961989252938,0.225493772993193,
0.281143900710764,0.778600030590876,0.965756016200329
"13632","SNRPD3",849.553648766214,-0.0619974129795848,0.22052562843550
5,-0.281134729869806,0.778607064326724,0.965756016200329
"13633","AL078604.2",2.3484577086586,0.327087134839804,1.1640738868397
3,0.280984857179291,0.778722014360673,0.965791228549127
"13634","JMJD4",95.8006732819521,0.0715359989390824,0.254705881346728,
0.280857271771048,0.778819874208749,0.965804852106178
"13635","FN1",3184.52119661101,0.0741178886116384,0.264169967435342,0.
280568943287542,0.779041039228306,0.965804852106178
"13636","TFB1M",61.8661830793813,0.0661538734123073,0.235726638218585,
0.280638089577997,0.77898799829764,0.965804852106178
"13637","ABHD4",88.5658975075924,0.0853043879457011,0.304089745085819,
0.280523724736679,0.779075726157805,0.965804852106178
"13638","IGHG1",164.736190185685,-0.350937089420197,1.24981880217685,-
0.280790374419843,0.778871186843351,0.965804852106178
"13639","CEP95",254.809443931507,0.0542795833192722,0.19347015651267,0
.280557912898145,0.779049500545557,0.965804852106178
"13640","AC110285.5",2.70305116490431,-0.260634727509717,0.92953904973
9592,-0.280391369875997,0.77917725745348,0.965859902421073
"13641","AL050343.3",2.31547397613633,0.28142782246049,1.0061574577262
3,0.279705547376727,0.779703421956132,0.966042140034872
"13642","ODC1",118.000882306222,0.0876319599729899,0.312989673885495,0
.279983549888772,0.779490125649818,0.966042140034872
"13643","OTULINL",22.8415470801345,-0.115050717791482,0.41118198976042
9,-0.279804856867673,0.779627225244646,0.966042140034872
"13644","LINCADL",3.58239925031499,0.23643396665352,0.845267246241725,
0.279715045986658,0.779696133912206,0.966042140034872
"13645","PURG",11.158735056052,0.166563836378701,0.595371454432267,0.2
79764565698792,0.779658138999181,0.966042140034872
"13646","AP002495.1",34.6449330346904,0.0896627960383709,0.32046128849
0229,0.279792908718536,0.779636392531059,0.966042140034872
"13647","CCL4",341.787371987114,-0.138057261263522,0.493697064767659,-
0.279639623396372,0.779754004297728,0.966042140034872
"13648","AC007114.1",21.7983713234922,-0.109927218113158,0.39319571549
6055,-0.27957379437483,0.77980451470953,0.966042140034872
"13649","ZGLP1",5.0822788917344,-0.191933778831847,0.686631525120299,-
0.279529517375742,0.77983848884173,0.966042140034872
"13650","TMEM44-
AS1",100.958090504057,0.0873881209977244,0.313631951625822,0.278632711
19131,0.780526706567761,0.96612197339137
"13651","MOB1B",78.2602045793595,-0.0713780270096361,0.255995179820646
,-0.278825667966266,0.780378615135994,0.96612197339137
"13652","THAP9-
AS1",90.7413958687313,0.0683258137775192,0.245223673138474,0.278626500
056284,0.780531473653071,0.96612197339137
"13653","CASP2",98.9958574304141,-0.0506078662683336,0.18123062371341,
-0.279245666275269,0.780056300282431,0.96612197339137
"13654","AL162390.1",58.2874566114316,0.0951954624701269,0.34094877406
2382,0.279207522396632,0.78008557107539,0.96612197339137

"13655","PANO1",2.65280987777275,-0.247485590685197,0.887720857306261,-0.278787626367344,0.780407810862425,0.96612197339137
"13656","ARF3",460.156622000902,0.0358484644599471,0.12858659487802,0.278788504306795,0.780407137068117,0.96612197339137
"13657","NPTN-IT1",13.1856450169543,-0.147992276963461,0.530535660393009,-0.278948783299188,0.780284130127038,0.96612197339137
"13658","CYBA",2222.3188438079,0.0873676371427331,0.313156527182893,0.278990311741795,0.780252259806069,0.96612197339137
"13659","RANBP3",181.518556102447,-0.047510794035349,0.170130454810715,-0.279260959410289,0.780044564746741,0.96612197339137
"13660","MMP24OS",564.236227524852,-0.0693331523731603,0.248827891756443,-0.27863898971995,0.780521887765812,0.96612197339137
"13661","CACNA2D2",5.99439011754918,0.169982378946728,0.610539525391002,0.278413389924048,0.780695042020208,0.966253698153698
"13662","ANKRD65",304.264141062605,-0.0743256614248453,0.268254318222931,-0.277071630821158,0.781725104688598,0.966270329778437
"13663","AL136115.2",2.09582167955056,0.298263619750936,1.07772413610112,0.276753215187297,0.781969607290977,0.966270329778437
"13664","KIF5C",6.03460930405164,-0.207400110585228,0.748074815545706,-0.277245144837463,0.781591877128817,0.966270329778437
"13665","PKD2",504.2948516777,0.0788355329828848,0.283502549521568,0.278076980668871,0.780953265882388,0.966270329778437
"13666","SPATA9",8.56432005697533,0.15700311950561,0.56585149059915,0.277463472508251,0.781424249847599,0.966270329778437
"13667","CYP21A2",6.30824917862437,-0.187104080115986,0.673282789137918,-0.277898207312795,0.781090500079089,0.966270329778437
"13668","MANEA",105.548826087029,-0.0555963361236542,0.200481286730216,-0.277314342053626,0.78153874789858,0.966270329778437
"13669","LINC00574",3.91581725147534,0.201953989917977,0.729796462062404,0.276726457877385,0.781990154474619,0.966270329778437
"13670","GPR146",124.178019273593,-0.0905340006358059,0.325678435715464,-0.277985861842271,0.781023211801054,0.966270329778437
"13671","MLXIPL",313.350803756602,-0.10566769215538,0.382010493665757,-0.276609396619964,0.782080048676243,0.966270329778437
"13672","EXTL3",40.012460402866,0.0785987981941411,0.28349326225455,0.277251027305076,0.781587360563891,0.966270329778437
"13673","SGMS1-AS1",40.6045156009585,-0.0948207161465539,0.342458655550206,-0.276882229751827,0.781870537959567,0.966270329778437
"13674","SMNDC1",240.506628253322,-0.0417613931369594,0.150057098469369,-0.278303349611176,0.780779505034274,0.966270329778437
"13675","EDRF1-DT",53.1519970506337,0.0963982924145138,0.347294840725496,0.277569031008749,0.781343207946515,0.966270329778437
"13676","CD63",17630.1745362598,-0.0824667056861075,0.296564780730365,-0.278073159877625,0.780956198816451,0.966270329778437
"13677","DYRK2",219.513136537916,-0.0635430287397996,0.229561208134741,-0.276802118511691,0.781932054372916,0.966270329778437
"13678","TOX4",489.174836551454,-0.0373555827931473,0.13453968750613,-

0.277654746235718,0.781277402345657,0.966270329778437
"13679","SNHG19",355.678343273157,-0.0794461279548457,0.28611143531267
6,-0.27767547238377,0.781261490628199,0.966270329778437
"13680","HEATR3",30.6112764854745,-0.0892515612632708,0.32185780602044
7,-0.27730121685351,0.78154882527447,0.966270329778437
"13681","AC004477.1",3.09390677603678,-0.234598209103181,0.84698310968
2431,-0.276980976859316,0.781794713175739,0.966270329778437
"13682","MMD",133.718814595038,-0.105477675568395,0.381311007934996,-0
.276618490873404,0.78207306487238,0.966270329778437
"13683","BFSP1",6.05475911294835,-0.169872667055151,0.612571539929994,
-0.277310740023222,0.781541513491949,0.966270329778437
"13684","HM13-
IT1",2.41216983204747,-0.378823253713215,1.36472936742315,-0.277581228
011896,0.781333843924048,0.966270329778437
"13685","CNKSR2",83.983303805603,-0.158942619458874,0.573434813376265,
-0.277176438805751,0.781644630238863,0.966270329778437
"13686","AL356056.2",4.39994312732724,-0.205097531966876,0.74212111047
2526,-0.276366659124257,0.78226646200859,0.966430026278039
"13687","C5orf22",44.932274940317,-0.0720201230235163,0.26095399840217
5,-0.275987811892121,0.782557427522577,0.966648230899455
"13688","DAGLA",20.0923306999089,0.08871406443731,0.321405176921857,0.
276019401077908,0.782533164960865,0.966648230899455
"13689","PWWP2A",184.419340988864,0.0449615696304083,0.163039301883206
,0.27577135764858,0.782723684234598,0.966782968298531
"13690","TSEN2",51.1811891227745,-0.0745797818545614,0.270644947363372
,-0.275563178182778,0.782883594521071,0.966828335212943
"13691","ROBO1",322.688728276597,-0.053814052957682,0.195324292439163,
-0.275511316517085,0.782923432797065,0.966828335212943
"13692","TM2D2",359.417785589548,0.0587619511661502,0.213291850803485,
0.275500216931823,0.782931959175279,0.966828335212943
"13693","AL136419.3",11.9368295077664,0.162885848360342,0.591567711076
385,0.275346076722078,0.783050367891045,0.966903937800467
"13694","ZNF322",291.632027451394,0.0476246509920379,0.173026568305153
,0.275244729514869,0.783128224375115,0.966929459451909
"13695","GHRL",3.4850552124955,-0.217290762029356,0.789769933183025,-0
.275131722416432,0.783215040728048,0.9669660393304
"13696","BCL2L11",155.703392095541,0.0755823399802167,0.27480276677495
7,0.275042136100882,0.783283866262598,0.966980403823599
"13697","BCAS2",452.540831231416,-0.0608155605511118,0.22117896602977,
-0.274960868308455,0.783346302480054,0.966986879048898
"13698","CNNM1",9.82028287398221,-0.2003119934854,0.729367825003257,-0
.27463782555051,0.783594502758542,0.967152044137632
"13699","PSMB10",468.64266059868,-0.0749199564042888,0.272745911387399
,-0.274687734174226,0.783556155515581,0.967152044137632
"13700","SPPL2A",864.75826639352,0.0411321775152674,0.149810541228457,
0.274561303750595,0.783653299230543,0.967154013386133
"13701","RASGRF2",2.3202452558459,0.278881681541961,1.01653757596373,0
.274344685465823,0.783819747267549,0.967218237250016
"13702","AL355312.3",16.8480448843871,0.169167803311894,0.616527904859
082,0.274387910066391,0.78378653298418,0.967218237250016

"13703","SART1",204.154802508288,-0.0564121881551243,0.205904878244148
,-0.273972081847593,0.784106076525789,0.967500951754947
"13704","CTH",19.6396671193077,-0.12003976496715,0.438600362318389,-0.
273688248529102,0.784324209385138,0.967677894393119
"13705","RBM44",2.18821098227396,-0.272202668503229,0.995702964126146,
-0.273377380916125,0.784563138205082,0.967677894393119
"13706","GLB1",229.623240937073,0.0559075051092852,0.204636400685481,0
.273204107001535,0.784696323105909,0.967677894393119
"13707","AL355297.4",4.57637993683275,0.232629634467498,0.851151378170
179,0.27331170510187,0.784613618383938,0.967677894393119
"13708","KNSTRN",26.956335685265,-0.086928566006001,0.318064428299631,
-0.273304897598012,0.784618850864424,0.967677894393119
"13709","MEF2A",454.632802730961,0.0574844893083752,0.210226521645247,
0.273440709851914,0.784514462757802,0.967677894393119
"13710","AC005632.6",2.97361230989195,0.268412892226658,0.982090093027
102,0.273307809672866,0.784616612542819,0.967677894393119
"13711","ZNF574",36.7300088922306,0.0866082034672716,0.317025779461251
,0.273189781646314,0.784707334399341,0.967677894393119
"13712","SDHAF4",89.9526288332223,-0.0779873525371791,0.28596543051555
7,-0.272716014647569,0.785071523279068,0.967917680874012
"13713","CRTC3",223.883063287783,0.0371056490980397,0.136118617614514,
0.272597898423583,0.785162327595984,0.967917680874012
"13714","AC090772.3",2.61947951333619,0.244300722076971,0.896794279632
566,0.272415566897979,0.785302504508499,0.967917680874012
"13715","ZNF221",17.1985478670655,0.11351973558999,0.416157663519198,0
.272780596252924,0.78502187605636,0.967917680874012
"13716","C5AR2",16.842248968465,0.217400880215714,0.797999194727603,0.
272432455636655,0.785289520113189,0.967917680874012
"13717","APOL2",205.22518143787,0.0600413613531824,0.220304818984334,0
.272537666810874,0.78520863303334,0.967917680874012
"13718","ZNF630",9.14358708886794,0.137962947525472,0.50632925106704,0
.272476747560462,0.785255467892603,0.967917680874012
"13719","RRP1",142.330817376952,-0.0554897158772295,0.203808270240654,
-0.272264299244128,0.785418804726812,0.967990462156202
"13720","CASP3",155.85102356621,0.0541980711371626,0.199152140720639,0
.272144054997576,0.785511256408868,0.968033842810579
"13721","ARL8A",290.523345803441,-0.0475836517301365,0.175192624750293
,-0.271607619315931,0.785923740265934,0.968117011720892
"13722","CCSAP",31.5605203946429,0.0900553163069156,0.33154763866474,0
.27162104568019,0.785913415538594,0.968117011720892
"13723","ACKR3",1699.79818499509,0.104508100247704,0.384878865343363,0
.271535045590174,0.785979549319416,0.968117011720892
"13724","SOX2-
OT",4.90342262741551,-0.183155502609704,0.67367571949877,-0.2718748758
61603,0.785718229990648,0.968117011720892
"13725","LINC02361",5.2757153681088,-0.165342491655784,0.6079468542471
68,-0.271968660583877,0.785646116589913,0.968117011720892
"13726","CCNE1",3.33225400222827,0.267106678626248,0.982649399693325,0
.271822970338769,0.785758142225958,0.968117011720892
"13727","DHRSX",207.692739143566,-0.0679833976327006,0.250182699004396

,-0.27173500766936,0.785825781531083,0.968117011720892
"13728","AC018690.1",2.21466082211217,0.268745371352443,0.990781817716151,0.271245764251031,0.786202017031001,0.968141378765173
"13729","PDIA5",92.0436333037561,0.0948250792719148,0.349620002733438,0.271223266776909,0.786219319126267,0.968141378765173
"13730","AC013643.2",4.57146181521978,0.195524109165021,0.720901184809817,0.271221789178503,0.786220455504127,0.968141378765173
"13731","MAN1B1",324.626876471209,0.0565296250364856,0.208481929652815,0.27114879994935,0.786276589959377,0.968141378765173
"13732","SKAP1",9.42970300515224,-0.166907736551783,0.615584400225066,-0.271137047155124,0.786285628885933,0.968141378765173
"13733","PLBD2",382.722373768794,-0.0457116637344809,0.168680729509427,-0.270995174537268,0.786394743608134,0.968205222815578
"13734","PSME3",128.208787436358,-0.0496232447675374,0.183226058762535,-0.270830716453112,0.786521234140761,0.968290449020823
"13735","SEPSECS",51.6823620702758,0.0738945280592579,0.272972548661987,0.270703147336471,0.786619355943306,0.968340740465192
"13736","AC009053.2",9.45362044526104,0.180606076117286,0.667664974004241,0.270504044916604,0.786772505487602,0.968458759666888
"13737","ARIH2",300.236889949009,0.046368202171294,0.171505564254217,0.270359754057681,0.786883499144705,0.968524874684332
"13738","CD109",473.488811661577,-0.0593829908879512,0.219811884037979,-0.270153686857491,0.787042020866245,0.968597018969007
"13739","DOCK8",40.4091073218829,0.160469546778078,0.594199260490361,0.270060159020817,0.787113972121727,0.968597018969007
"13740","THSD4",2.38715504359324,0.318403227740612,1.17874109269233,0.270121428458353,0.787066837147625,0.968597018969007
"13741","ITGB7",9.3945230946011,0.159685778563133,0.591671942888823,0.26988904997501,0.78724561154602,0.968688508843614
"13742","TMEM81",4.4993861422758,0.196480261580348,0.728404775456745,0.269740490728038,0.787359907650598,0.968712475557508
"13743","GSE1",250.892357951422,-0.0719662689401557,0.266823583845222,-0.269714797706569,0.787379675395483,0.968712475557508
"13744","AKAP7",91.744173928377,-0.0608753315103073,0.225787084612154,-0.26961387811387,0.787457322426024,0.96873751510326
"13745","TTC13",30.6627694997065,0.0812259762353593,0.30157646235523,0.269337917160399,0.787669656190518,0.968813250236759
"13746","TESK1",184.611154775587,-0.0602799097509224,0.223799874263281,-0.269347379882834,0.787662374987912,0.968813250236759
"13747","HCAR2",3.70844560897482,-0.251664375180125,0.934476789225868,-0.269310461299533,0.78769078252926,0.968813250236759
"13748","AL136418.1",2.72231102366726,-0.236771738059306,0.879492987286094,-0.269213901056707,0.787765083549088,0.968834160070409
"13749","PPP1R8",164.58344584006,0.0436771172740826,0.16330791440024,0.267452544688296,0.789120747512561,0.968913657634332
"13750","CTBS",314.111068377204,-0.0478853998217128,0.178153486266002,-0.268787329540186,0.788093344216351,0.968913657634332
"13751","OBSCN",22.5775505441819,0.113631787900011,0.423686976106336,0.268197500296757,0.788547299011335,0.968913657634332
"13752","AGT",23.8331034980213,0.295597397028538,1.10241131758082,0.26

8137121158379,0.788593773124345,0.968913657634332
"13753","AC097468.3",10.8842152879002,0.12277876317002,0.4598651249405
5,0.266988637561703,0.789477909559168,0.968913657634332
"13754","INHA",2.07560781282689,-0.252136535758429,0.939252950238104,-
0.268443698467502,0.788357806888263,0.968913657634332
"13755","OGG1",131.13808067794,0.0545099330342555,0.203746464106507,0.
267538056541491,0.789054916780669,0.968913657634332
"13756","ISY1",252.214295742729,-0.0416506360663373,0.155976236584156,
-0.267031933700137,0.789444573993993,0.968913657634332
"13757","ART3",4.58420264320157,0.23733141484845,0.888020333822611,0.2
67258986995065,0.789269762176591,0.968913657634332
"13758","ARAP3",5.82606346918416,0.204661255324137,0.766134855230435,0
.267134766062274,0.789365400466088,0.968913657634332
"13759","FNDC1",312.98441536639,-0.093301876473557,0.349165649811918,-
0.267213789568977,0.78930455952344,0.968913657634332
"13760","POLM",75.485346223077,-0.0674491312297035,0.252572749167575,-
0.267048331429266,0.789431948775408,0.968913657634332
"13761","AL360020.1",10.8280282267508,0.159710167555501,0.597455626911
136,0.267317203758224,0.789224941905162,0.968913657634332
"13762","ABITRAM",76.6797981675501,-0.0691719827369316,0.2574514919321
79,-0.26867967327668,0.788176195165962,0.968913657634332
"13763","SNAPC4",57.0204779655821,0.0590137986900618,0.219497789010561
,0.26885828306554,0.788038740557807,0.968913657634332
"13764","LINC00865",8.29191878491146,-0.175029683760222,0.653625730813
211,-0.267782731782083,0.788866563493891,0.968913657634332
"13765","EIF3A",1060.35687647617,-0.049877187398313,0.18590155094009,-
0.268298931052957,0.788469228964885,0.968913657634332
"13766","NEU3",22.6646525021,0.0850690827079199,0.318645530338238,0.26
697089589683,0.789491569743802,0.968913657634332
"13767","TBK1",185.252699288303,0.0490263527906092,0.182642090502999,0
.268428556942104,0.788369460552599,0.968913657634332
"13768","METAP2",816.147300663939,0.0503283886468504,0.187938894934134
,0.26779123429713,0.788860018400156,0.968913657634332
"13769","GEMIN2",139.297109567234,0.0641865759162557,0.239843280479281
,0.267618820873327,0.788992742279474,0.968913657634332
"13770","RPS29",22387.6905573587,0.0779621211556027,0.290864508635571,
0.268035868388752,0.788671709556542,0.968913657634332
"13771","SLC38A6",102.884199792578,-0.0511445535408408,0.1912580722349
47,-0.267411215344748,0.789152565172743,0.968913657634332
"13772","LTK",2.6004817501972,0.253413299079879,0.942680215033776,0.26
8822125508171,0.788066566175669,0.968913657634332
"13773","LINC00324",34.600904364112,-0.0857644196569356,0.319307809799
446,-0.268594807345311,0.788241508642701,0.968913657634332
"13774","CEP112",148.109880055035,-0.0608291794868871,0.22731232337565
8,-0.267601767398947,0.789005870379479,0.968913657634332
"13775","ZNF561-
AS1",41.0493436973271,-0.0774876680755057,0.289726145002073,-0.2674514
17182081,0.789121615525785,0.968913657634332
"13776","AC006504.1",4.90408195573894,0.22885244115643,0.8521710398341
89,0.268552239467043,0.788274269771537,0.968913657634332

"13777","MAGIX",4.32444967452795,-0.196304171310208,0.73530059906137,-0.266971319703527,0.789491243433325,0.968913657634332
"13778","NDST2",88.990634456702,0.0725450037795591,0.271866447327808,0.266840592109134,0.789591899052371,0.968914011736845
"13779","BACE2",339.503542202929,0.0488748882870585,0.183174358426446,0.266821670385073,0.789606468400874,0.968914011736845
"13780","ABL2",677.00315304321,0.18430820960644,0.692354309686668,0.266205044191686,0.790081298447169,0.968959804582616
"13781","TBC1D8",34.5087583697631,0.11295821724498,0.426143326718208,0.265070951867034,0.790954804204414,0.968959804582616
"13782","P4HTM",298.443902458841,-0.0513053193251256,0.193384254234468,-0.265302464920028,0.790776465797736,0.968959804582616
"13783","NUP54",316.981528989594,-0.0502041990307589,0.188356089909281,-0.266538762059348,0.789824310913636,0.968959804582616
"13784","AP3S1",1407.30482938489,0.059700458916664,0.225232237228806,0.265061785342994,0.790961865557681,0.968959804582616
"13785","LSM11",32.5846552968651,0.100548084022193,0.378623808053289,0.2655619691196,0.790576578359432,0.968959804582616
"13786","AC139795.3",9.95268877566345,0.180585966812869,0.67910647199391,0.265917016344514,0.790303119630158,0.968959804582616
"13787","C6orf136",61.9847289064502,-0.0744701970217476,0.279341862289722,-0.266591610764412,0.789783615583066,0.968959804582616
"13788","CNOT4",488.775330635447,0.0322686930535632,0.121237388243684,0.26616123558109,0.790115036025575,0.968959804582616
"13789","PPP1R3B",189.458134805694,0.0725091301034552,0.273150129508711,0.265455228719351,0.790658795266214,0.968959804582616
"13790","FBXO32",116.636469529424,0.0800558066820033,0.301888463128909,0.265183392078878,0.790868188246657,0.968959804582616
"13791","TNFSF15",4.21994891854159,0.356976956542651,1.34053322100284,0.266294748201465,0.790012217455151,0.968959804582616
"13792","B3GNT10",3.81268435935626,-0.220656399836341,0.830209667302046,-0.265783944137164,0.790405609329678,0.968959804582616
"13793","AC087521.1",2.71496225767763,0.348071249364453,1.30725089829033,0.266262008172779,0.790037430335984,0.968959804582616
"13794","ING4",257.269400826204,0.0657964574566071,0.247259989999819,0.266102321918945,0.790160406820028,0.968959804582616
"13795","PA2G4",1182.583156393,-0.0466241641809177,0.175650462931797,-0.265437183612896,0.79067269475888,0.968959804582616
"13796","CNPY2",930.929461653408,0.0665744186611794,0.249985374023163,0.266313255010714,0.789997965580271,0.968959804582616
"13797","TM9SF2",620.707197790417,0.0377106451990164,0.142118974655613,0.265345604205194,0.790743236089693,0.968959804582616
"13798","NUSAP1",17.0247047251179,-0.106166687464732,0.400469052158789,-0.265105847486651,0.790927922819768,0.968959804582616
"13799","GINS2",13.0763229701846,0.164396803529454,0.616588828498934,0.266623065373522,0.789759394718425,0.968959804582616
"13800","NAPG",168.984306035541,-0.0469850017710145,0.176894358002174,-0.26561051636501,0.790539185563002,0.968959804582616
"13801","AC011815.1",3.48741876162234,-0.206444612724969,0.778091927901551,-0.265321622448562,0.790761708921193,0.968959804582616

"13802","MYH7B",6.4475767617957,-0.165315957436162,0.622608148683253,-0.265521673280035,0.790607615998918,0.968959804582616
"13803","PCDHA4",3.09307909196529,0.226433752723734,0.854750438354377,0.264912122373028,0.791077159570949,0.969030834892821
"13804","ZNF281",137.909834538054,-0.0674053871845599,0.254752822067819,-0.264591326751292,0.791324302328011,0.96905274887825
"13805","DHX37",40.9350287005935,0.0975246435741303,0.368556239183654,0.264612651220193,0.791307873185682,0.96905274887825
"13806","MC1R",8.50072115580318,0.14217651883023,0.537189409637889,0.264667389712818,0.791265701095032,0.96905274887825
"13807","CTDNEP1",844.298924617504,-0.0424229809957124,0.160217283407677,-0.264784048845505,0.791175825621899,0.96905274887825
"13808","RNF167",306.68171645382,0.047282160015195,0.178768269570843,0.264488547820607,0.791403488234556,0.969079532087911
"13809","ABHD1",10.6013255992367,-0.137557684195844,0.521091900760305,-0.263979701076026,0.791795560306964,0.969338169610787
"13810","CSRNP3",3.02997027554963,0.246899458585439,0.935520036249615,0.263916804577729,0.791844026417328,0.969338169610787
"13811","AP003778.1",6.14586415141546,0.152403963724045,0.57739539295202,0.263950778936523,0.79181784671969,0.969338169610787
"13812","C18orf21",143.100038384193,-0.0683543757981615,0.25899943724774,-0.263917082309251,0.791843812404176,0.969338169610787
"13813","AC009093.6",3.83776247407218,-0.191752553851026,0.727262975702499,-0.2636632968505,0.792039379842365,0.96936750388468
"13814","AFG3L2",242.516893089869,0.053851058728842,0.20418104780967,0.263741710146576,0.791978953046439,0.96936750388468
"13815","LINC01480",4.42981530198408,-0.199830133094601,0.75790119684196,-0.263662511587602,0.792039984987394,0.96936750388468
"13816","DENND2C",67.1667585136811,0.0572927241510508,0.21811801025816,0.262668470536845,0.792806120509226,0.969523236365947
"13817","TENM3",30.2279988905882,0.100676919112551,0.382735877910498,0.263045418323951,0.792515572613144,0.969523236365947
"13818","NADK2",197.533564616599,-0.0558634058128983,0.212673386344148,-0.262672291880002,0.792803174908568,0.969523236365947
"13819","HOMER1",21.433616418173,0.100615665339478,0.382223033641944,0.263238100490123,0.792367066091039,0.969523236365947
"13820","DAXX",228.111777412669,-0.0487520454658016,0.185283319899592,-0.263121610149371,0.792456848151777,0.969523236365947
"13821","MTRES1",168.66258606169,-0.0537008504613722,0.204522702976849,-0.262566696409498,0.792884572006869,0.969523236365947
"13822","KATNA1",281.952153899139,-0.0489463879466758,0.185877083005973,-0.263326641214307,0.792298827350267,0.969523236365947
"13823","SLC29A4",5.74696936689473,0.195627378003729,0.744960132541974,0.262601137239658,0.792858023426546,0.969523236365947
"13824","KDELR2",1578.20490482691,0.0662862621573987,0.2524040189177,0.262619677933941,0.792843731503703,0.969523236365947
"13825","KCTD21-AS1",23.4833081959995,0.115592556575008,0.439302321939364,0.263127579350612,0.792452247470049,0.969523236365947
"13826","GABARAPL1",839.421746966065,0.0585643009041416,0.223076387970

684,0.262530254487704,0.792912663382323,0.969523236365947
"13827","CDK8",51.0103321354435,0.0714068161445723,0.271130842883172,0
.263366629134631,0.792268008998819,0.969523236365947
"13828","LINC01579",6.6659340879015,-0.318817560389212,1.2127411715390
6,-0.262890027873472,0.792635342658965,0.969523236365947
"13829","DHDDS",111.05336167916,0.0668439805798865,0.256358261815834,0
.260744397728468,0.794289625564715,0.969562372657199
"13830","THRAP3",1152.79636092082,-0.0342014574420148,0.13138828422775
1,-0.26030827362605,0.794625991108186,0.969562372657199
"13831","SMAP2",142.229279666038,0.0632026633861,0.241799728282391,0.2
61384344122536,0.793796129076806,0.969562372657199
"13832","AC093151.2",2.43821948383316,-0.232830770659859,0.90029881386
8141,-0.258614992126336,0.795932314641394,0.969562372657199
"13833","LYSMD1",36.712532971173,-0.100009735614314,0.386931734140298,
-0.25846868269029З,0.79604521554893,0.969562372657199
"13834","SHCBP1L",8.57534564844436,-0.12890582250006,0.500604045970609
,-0.257500560647942,0.796792382558998,0.969562372657199
"13835","RGS18",2.51750295950957,0.228653792967981,0.874085277641028,0
.261592088114182,0.793635944442501,0.969562372657199
"13836","ZNF124",31.7962365751423,-0.0983332941185714,0.37757059539727
,-0.260436843645379,0.794526826084778,0.969562372657199
"13837","ID2-
AS1",5.98112375939516,0.160455749956364,0.622817688529709,0.2576287618
53494,0.796693430070827,0.969562372657199
"13838","RDH14",474.662304471594,-0.0598796210030118,0.230235904417884
,-0.260079422253484,0.794802510544455,0.969562372657199
"13839","KCNK12",2.32247980179823,-0.270754991902392,1.05173855648165,
-0.257435643329593,0.796842490433439,0.969562372657199
"13840","NCK2",224.558705492015,-0.0476003173833889,0.183136456930069,
-0.259917212450851,0.794927633845458,0.969562372657199
"13841","AC010894.2",3.67152961993415,0.218512424477462,0.846157735036
019,0.258240769338545,0.796221095309615,0.969562372657199
"13842","AC008269.1",8.51326585066393,0.146891714927665,0.570290570666
254,0.25757345901065,0.796736115336567,0.969562372657199
"13843","GORASP1",66.0287329007589,-0.0667953828936724,0.2574786289763
55,-0.259421075680057,0.795310370146064,0.969562372657199
"13844","PLXNB1",39.519297103934,0.0839239269748955,0.324876712990787,
0.258325461995411,0.796155737138708,0.969562372657199
"13845","EOGT",45.477515721322,0.114039675516997,0.439376560576375,0.2
59548837487824,0.79521180575225,0.969562372657199
"13846","C3orf52",8.09353803810142,-0.130912459559838,0.50720719461753
7,-0.258104500387763,0.796326258422844,0.969562372657199
"13847","TIMMDC1",450.074602087453,-0.0593260032555916,0.2303405247443
05,-0.257557819326182,0.796748186871284,0.969562372657199
"13848","SLBP",340.344973752675,-0.0524476877400259,0.201554244127036,
-0.260216240879398,0.79469697726484,0.969562372657199
"13849","RASGEF1B",35.6747344677861,-0.128611740316494,0.4994281472499
84,-0.257518005392113,0.796778917588415,0.969562372657199
"13850","HPSE",8.6317737749704,-0.159459166403678,0.615678240756927,-0
.258997566988294,0.795637117724617,0.969562372657199

"13851","PRIMPOL",89.5409734515438,0.0522774906802191,0.20085738895432
,0.260271683070162,0.794654213716842,0.969562372657199
"13852","AC012640.2",8.38389811398604,0.167877707034771,0.645335436120
139,0.260140227296487,0.794755608896315,0.969562372657199
"13853","AK6",436.339455455425,-0.0707609912035012,0.26998467353311,-0
.262092622805211,0.793250034189531,0.969562372657199
"13854","SLU7",515.426850169208,-0.044504875370555,0.171567507853237,-
0.259401537782,0.795325443335081,0.969562372657199
"13855","LANCL2",59.0435419390283,0.0548523719089846,0.212529716061744
,0.258092717222889,0.796335352042297,0.969562372657199
"13856","MFHAS1",98.6269162176294,0.0580218735151216,0.225246121057035
,0.257593219553955,0.796720863174832,0.969562372657199
"13857","AC087623.3",9.86803351666519,-0.136818193257904,0.52157096668
1307,-0.262319419595883,0.793075191427409,0.969562372657199
"13858","RRS1",67.5898408831872,-0.058618401044718,0.226605260420643,-
0.258680672001638,0.795881633602049,0.969562372657199
"13859","TOPORS",522.149250954079,-0.0693780208819429,0.26533791341017
7,-0.261470439675516,0.793729742540657,0.969562372657199
"13860","ACTR1A",223.991893144487,-0.056707876988077,0.217060863275157
,-0.261253346791454,0.793897141329852,0.969562372657199
"13861","TP53I11",263.902271075098,0.0504214686649451,0.19307742998080
6,0.261146363248969,0.793979639076437,0.969562372657199
"13862","BACE1",417.859455627913,0.048988928155105,0.190270880853082,0
.257469392770257,0.796816440073325,0.969562372657199
"13863","HYLS1",30.2687137694364,0.0834845231159063,0.31837615077906,0
.262219776549284,0.793152007219249,0.969562372657199
"13864","FAM66C",41.5736428306026,0.0638059753459052,0.247036620619552
,0.258285493000527,0.796186581433921,0.969562372657199
"13865","AC022075.1",10.3663808137848,0.127999323576802,0.494599548443
998,0.258793854502062,0.795794299787508,0.969562372657199
"13866","MYL6",8475.03021660146,-0.0702522845440992,0.270140908687157,
-0.260057926381881,0.794819091448723,0.969562372657199
"13867","VSIG10",219.1319159555,0.0714038958293698,0.276980383846281,0
.257794053274898,0.796565854210854,0.969562372657199
"13868","AC079949.1",2.44197651828726,0.246942959145342,0.948033910035
236,0.260479036173046,0.79449428405057,0.969562372657199
"13869","IPO4",35.7442736778945,-0.0850647158668215,0.330272492432908,
-0.257559190716138,0.79674712835812,0.969562372657199
"13870","SNX6",1056.99142554575,0.0458992299869117,0.177262687793338,0
.258933397424412,0.795686629280879,0.969562372657199
"13871","BCL11B",17.3543066869872,-0.15348580140702,0.595144328723249,
-0.257896772260756,0.796486575987063,0.969562372657199
"13872","OIP5",4.69555590646216,-0.176092047059293,0.675397102788405,-
0.260723722876941,0.794305570409752,0.969562372657199
"13873","EIF3J-
DT",252.710569453403,-0.0545282111706297,0.20947248173066,-0.260312050
156268,0.794623078253437,0.969562372657199
"13874","AC013553.3",2.50167369263504,-0.241854453897521,0.92416321295
369,-0.261701018291496,0.793551955404582,0.969562372657199
"13875","LRRC49",62.7413646914422,0.0681026435458439,0.264226035223959

,0.257743880114311,0.796604578477987,0.969562372657199
"13876","CTSH",457.579845418777,0.120349436520607,0.464528355702709,0.259078773218376,0.795574462302129,0.969562372657199
"13877","CLUAP1",256.076670883115,-0.0397559964595318,0.152597893509577,-0.260527819520894,0.794456659128519,0.969562372657199
"13878","AC009152.6",7.65125435595966,-0.195910445990204,0.757590657835852,-0.258596702538341,0.7959464277223,0.969562372657199
"13879","DUS2",30.4257405414814,0.0838891216157536,0.320330282332714,0.261883206935214,0.793411486837041,0.969562372657199
"13880","C16orf46",22.7356856014643,-0.10116733063841,0.391646652511801,-0.258312767362059,0.796165533623222,0.969562372657199
"13881","AC090617.10",11.5283399903477,0.179229748203356,0.689297214664397,0.260018094358061,0.794849816243351,0.969562372657199
"13882","AC233723.1",34.2734067949463,-0.16625545024902,0.635597863748309,-0.261573330766977,0.793650407265372,0.969562372657199
"13883","AC127024.6",5.7482074425223,0.16544813974686,0.631913862314925,0.261820715786744,0.79345966714286,0.969562372657199
"13884","C17orf82",9.04382877705925,-0.158799308642569,0.616855268611053,-0.25743365862811,0.796844022382566,0.969562372657199
"13885","GADD45B",6560.43058516734,0.0669257260868664,0.258349200651571,0.259051415363686,0.795595570362276,0.969562372657199
"13886","RASAL3",9.44200255060917,-0.183638725580684,0.712485171896165,-0.257743926223697,0.796604542889961,0.969562372657199
"13887","LINC00662",164.854330410619,-0.0556680956627568,0.216129561931167,-0.257568169598594,0.796740197978789,0.969562372657199
"13888","MARK4",108.163229462313,0.0515361919308858,0.198507923734257,0.259617807498089,0.795158598821091,0.969562372657199
"13889","TPX2",3.4970291494462,0.235462394715758,0.90890160089303,0.259062581124741,0.795586955357447,0.969562372657199
"13890","CDH26",2.59485873036813,-0.241132263761865,0.925137094029202,-0.260644898273048,0.794366362256017,0.969562372657199
"13891","IL17RA",170.353229857469,-0.0703747944125994,0.27294233692028,-0.257837590190906,0.796532252285404,0.969562372657199
"13892","SH3BP1",27.4524814073148,-0.0786831237409083,0.299904674216868,-0.262360444852589,0.793043565242257,0.969562372657199
"13893","SYN1",3.66950724942876,0.232274538414046,0.892111969782844,0.260364781867668,0.794582406346343,0.969562372657199
"13894","ARMCX4",62.8540745353913,-0.0614524521559792,0.23715817303871,-0.25912011114181,0.795542568148907,0.969562372657199
"13895","ABCD1",66.1816122699693,0.0744464976258368,0.288623771364018,0.257936126584471,0.79645620298733,0.969562372657199
"13896","DKC1",207.195982400007,-0.0405329453145141,0.155098802967192,-0.261336287186485,0.793833185456028,0.969562372657199
"13897","B4GALT2",235.599060332157,-0.0658841612009278,0.256528067974932,-0.256830224158418,0.797309836966508,0.969649804844038
"13898","KIAA0232",204.382435690429,-0.0531717707360389,0.207089375637955,-0.256757598366595,0.797365904516224,0.969649804844038
"13899","ANKDD1B",3.32098089763412,0.200836799389595,0.782232967384544,0.256748063254235,0.797373265758178,0.969649804844038
"13900","TICAM2",99.356278141208,0.050065060684766,0.194970799878229,0

.256782352619134,0.797346793966366,0.969649804844038
"13901","PCDHGC3",97.2233331157688,-0.0541968880446479,0.211091257208322,-0.256746246914253,0.797374668000444,0.969649804844038
"13902","BDKRB1",16.7715370860584,0.125419507745831,0.488134528909991,0.256936357331438,0.797227903397146,0.969649804844038
"13903","ARL17A",54.7325588107314,0.096326108366775,0.374709893359738,0.257068495051177,0.797125897744159,0.969649804844038
"13904","AC132872.1",36.9353807450329,-0.0966201819540693,0.376276595758483,-0.2567796749604,0.797348861141919,0.969649804844038
"13905","TMEM52",4.55694699551249,-0.179370901148778,0.699304061417339,-0.25649915543924,0.797565432494501,0.969742293442904
"13906","XPNPEP1",185.677442073905,0.0465469717376279,0.181441446491846,0.256539906606839,0.797533970131553,0.969742293442904
"13907","UQCRQ",1344.91018900266,-0.0633745772126332,0.247307006806666,-0.256258720814072,0.797751069241103,0.969828521622705
"13908","TEX30",118.769946916683,0.0802023725748549,0.312894363223016,0.256324120858932,0.797700573502664,0.969828521622705
"13909","HOXC4",89.7221459606728,-0.0637332744383136,0.249047988045488,-0.255907606154493,0.798022181285892,0.969936157423231
"13910","ASB16-AS1",47.3137084396002,0.0745799177262832,0.291541481426765,0.255812371403545,0.798095720690982,0.969936157423231
"13911","AL672277.1",5.02605581428978,0.218476771248878,0.853994581546512,0.255829224177553,0.798082707002289,0.969936157423231
"13912","RIBC1",8.33115212808432,0.138062610531718,0.539786561177749,0.255772596914014,0.798126434719034,0.969936157423231
"13913","ZC3H12B",12.0913700922537,-0.136287295654912,0.532418608464857,-0.255977709058432,0.797968049608745,0.969936157423231
"13914","ASAP2",320.958916410401,0.0573551420008897,0.225035820840071,0.254871165784984,0.798822607325865,0.970084941798874
"13915","WDR75",141.472968667655,0.0447515429299315,0.175131444251671,0.255531170436884,0.798312871962535,0.970084941798874
"13916","ZNF501",33.2417157654834,-0.0737247185456845,0.288703588561787,-0.255364745942208,0.798441396975363,0.970084941798874
"13917","TMEM42",244.78251616863,0.067703118170233,0.26519836577234,0.255292365671487,0.798497295951825,0.970084941798874
"13918","LINC00689",2.21648276897359,-0.294085366150095,1.1534244143908,-0.254967176419118,0.798748450975085,0.970084941798874
"13919","GLIDR",39.3709232214301,0.0991171409467693,0.388802360012412,0.254929370654039,0.798777651040296,0.970084941798874
"13920","AC133550.3",2.30265264843216,-0.290249438318097,1.13800452294846,-0.255051216814227,0.798683541644676,0.970084941798874
"13921","AC010776.2",6.31085336519674,0.215276156230813,0.844568257772454,0.254894917313851,0.79880426203585,0.970084941798874
"13922","UBA2",598.893197931029,0.0464812579115536,0.182283195929636,0.254994749650406,0.798727154396653,0.970084941798874
"13923","FXYD1",3035.19499645305,0.0720858665917879,0.282550099999639,0.255125963826875,0.79862581130808,0.970084941798874
"13924","CLEC3B",1741.70938137224,0.101266337016185,0.397672775081421,0.254647396959603,0.798995448134415,0.970085813374745

"13925","MIR4453HG",9.45188960394053,-0.121415350136855,0.476590656908
037,-0.254758141765845,0.798909906739648,0.970085813374745
"13926","WDR24",38.3312672772191,0.0643613967827883,0.252675432500405,
0.254719646250868,0.798939641134025,0.970085813374745
"13927","MARCKSL1",550.273403829571,-0.0839253122033165,0.330731296698
022,-0.253756790002083,0.799683457512863,0.9702237855369
"13928","AL606760.1",6.29937628330229,0.16524522682463,0.6509965547089
98,0.253834257077591,0.799623606666268,0.9702237855369
"13929","AC013400.1",3.98056982846307,-0.237169963821245,0.93278093337
551,-0.254261161796032,0.799293802392924,0.9702237855369
"13930","KLHL41",3.49904136322383,-0.209526892189305,0.825507159748574
,-0.253815959940458,0.799637742877268,0.9702237855369
"13931","ATR",129.483952039173,-0.0589925941932134,0.232723710762858,-
0.253487682883014,0.799891378063286,0.9702237855369
"13932","GPRIN1",2.01268202838626,-0.279832602040896,1.10437210330164,
-0.253386155992447,0.79996982459013,0.9702237855369
"13933","HIST1H2BJ",6.87208501040043,0.153889800728817,0.6067296512767
26,0.253638173781338,0.799775102354221,0.9702237855369
"13934","POLR1C",82.8194900093318,-0.0592547305699819,0.23378980281279
3,-0.253453015730674,0.79991816401763,0.9702237855369
"13935","MRPL43",756.496505027157,-0.0612746490748429,0.24138881337187
5,-0.253842123911705,0.79961752883798,0.9702237855369
"13936","TMEM109",832.21716289577,-0.0419389234758282,0.16494776558001
,-0.25425578411661,0.799297956684476,0.9702237855369
"13937","FOLR1",2.50287105578437,0.236168443153834,0.929895648270089,0
.2539730598731,0.799516370957095,0.9702237855369
"13938","TARBP2",49.3953701856735,-0.07312114963484,0.288176761751847,
-0.253737147958535,0.799698633087947,0.9702237855369
"13939","RHPN2",4.09712404851789,-0.231849977032308,0.911341728773081,
-0.25440509274654,0.799182616929247,0.9702237855369
"13940","TXLNG",376.021265000146,0.0528902154772251,0.208657284195166,
0.253478883717066,0.799898176812786,0.9702237855369
"13941","ZNF81",12.8875361751643,-0.106013080084959,0.417425947475659,
-0.253968591856981,0.799519822780263,0.9702237855369
"13942","VTI1B",725.036973033494,-0.0510375335558305,0.201515568733907
,-0.253268439140915,0.800060783099775,0.970264504421962
"13943","PLXND1",362.902148536361,0.0540158927806997,0.213429250181608
,0.253085707487316,0.800201983199837,0.97029655277846
"13944","SH2B1",118.788146471038,0.0473567128988498,0.187090518717183,
0.253121928484452,0.800173994051994,0.97029655277846
"13945","PTBP2",171.823075539617,-0.0629084525235156,0.249934681777993
,-0.25169957236825,0.801273287560344,0.97034298424798
"13946","NAB1",169.570305041226,-0.0529934633573015,0.21037563597786,-
0.251899242566656,0.80111894505825,0.97034298424798
"13947","IQCB1",109.744836609613,0.050242235041341,0.199031514750933,0
.252433566132549,0.800705957993368,0.97034298424798
"13948","AC096751.2",3.54974425823207,0.237047037005829,0.937425305101
625,0.252870320137273,0.800368425340094,0.97034298424798
"13949","CHD1",437.300382162399,-0.0860178658350335,0.34117181349876,-
0.252124772421583,0.800944622709568,0.97034298424798

"13950","PCDHGA11",7.0310426282026,-0.159170305972105,0.630065839636794,-0.252624878161718,0.800558103461511,0.97034298424798
"13951","PFDN6",281.888716840352,-0.0588531179572187,0.23365683362428,-0.251878436612963,0.801135027431364,0.97034298424798
"13952","AEBP1",5393.7191312132,0.05037301910128,0.200072300569364,0.251774078460281,0.801215694399415,0.97034298424798
"13953","BRI3",3608.62035228891,-0.0575450382828592,0.22843331204720З,-0.251911762637177,0.801109267462916,0.97034298424798
"13954","POLR2J2",16.8267893883929,-0.138365049156086,0.549575619792817,-0.251767080221367,0.801221103987599,0.97034298424798
"13955","FASTK",293.616265755925,-0.0651978583057303,0.258874771310261,-0.251850954713508,0.801156270237881,0.97034298424798
"13956","HNRNPK",2508.65761530615,0.0272204810982601,0.108106228718869,0.251793827431045,0.801200428638917,0.97034298424798
"13957","RPL35",15014.8840641379,-0.0589386228585901,0.233064697143436,-0.252885244230348,0.800356892346302,0.97034298424798
"13958","MVK",55.8154732778613,0.0612578613836417,0.242310680046634,0.252807104382903,0.800417277494895,0.97034298424798
"13959","MMP17",69.3157737625608,-0.0615718498945256,0.244514318435729,-0.251812860238325,0.801185716537593,0.97034298424798
"13960","AC103740.1",15.8659019940887,0.137288920564103,0.543530040098988,0.252587548866848,0.80058695265685,0.97034298424798
"13961","SMOX",53.0664102535711,-0.0842584123367958,0.333535867806169,-0.252621743175644,0.80056052626203,0.97034298424798
"13962","DDT",881.627296019221,-0.0610550570648629,0.241973138450991,-0.252321631465837,0.800792469444555,0.97034298424798
"13963","PRKN",39.7763668854471,-0.06706200866914,0.266556531910936,-0.251586439050563,0.801360741606755,0.970379389750556
"13964","AL513477.2",7.4781666482292,-0.155172318271686,0.626568185608359,-0.247654320528615,0.804401877884058,0.970514832747317
"13965","DNAJC16",109.834962599418,-0.0649497158492165,0.260972499250431,-0.24887570926349,0.803456925416036,0.970514832747317
"13966","AL109627.1",3.22153492128298,-0.185314867163745,0.74873970647677,-0.247502390431186,0.804519441741686,0.970514832747317
"13967","ARHGEF10L",158.828808513562,-0.0427160496864621,0.170920093166193,-0.249918244807692,0.802650573532698,0.970514832747317
"13968","DIRAS3",6.33208389568096,-0.194552323775951,0.779321231806861,-0.249643299624829,0.802863210252774,0.970514832747317
"13969","AC092807.3",17.1178723670561,-0.109059539615289,0.441244190428717,-0.247163683921425,0.804781549525223,0.970514832747317
"13970","MRPL55",440.412783992176,-0.0592986694898232,0.239692432204144,-0.247394834056834,0.804602671783466,0.970514832747317
"13971","SH3YL1",252.856507386873,0.0436959355705612,0.175977244100021,0.248304465694016,0.803898844061018,0.970514832747317
"13972","GRHL1",45.8145170979721,-0.164742061194194,0.659512361766744,-0.249793742687206,0.802746858998938,0.970514832747317
"13973","CYS1",282.917366218764,-0.067175312024977,0.268856216460942,-0.249855900336736,0.802698788131907,0.970514832747317
"13974","AC009237.14",6.47304165228605,0.178809544926567,0.718020571023962,0.249031228550414,0.803336625312075,0.970514832747317

"13975","POLR1B",32.7797867556556,0.0769200299646598,0.308174125345284
,0.249599248082483,0.802897280129805,0.970514832747317
"13976","WDR33",522.553854092532,-0.0408916661401526,0.164191354605295
,-0.249048838402322,0.803323003713123,0.970514832747317
"13977","EIF4E2",512.590630013436,-0.0579405505817983,0.23319460977347
5,-0.248464364755608,0.803775138530366,0.970514832747317
"13978","EPHA3",4.87111369229831,0.265068615415577,1.05965651226343,0.
250145790025288,0.802474605980105,0.970514832747317
"13979","BBX",929.639196704993,0.037180908414259,0.150056160016194,0.2
47779953920229,0.804304665818774,0.970514832747317
"13980","SLC15A2",3.16327410647206,0.192978084193644,0.771451681044785
,0.250149282107068,0.802471905526369,0.970514832747317
"13981","TBC1D1",84.5875216963057,-0.0586186034393403,0.23382627703513
9,-0.250692968226711,0.80205149765589,0.970514832747317
"13982","ENOPH1",152.675237055802,0.0530927921112253,0.214127807516812
,0.247949076427436,0.804173807711665,0.970514832747317
"13983","PDE4D",44.4389707099278,0.0820659443480136,0.331589844379399,
0.247492333492926,0.804527223979594,0.970514832747317
"13984","CDYL",263.214833680977,-0.0526960812435383,0.210876174346861,
-0.249891109826664,0.802671558496709,0.970514832747317
"13985","SH2B2",12.1833697714774,-0.121054833191294,0.488770885329155,
-0.247671939603709,0.804388244489548,0.970514832747317
"13986","FAM185A",32.3844406096154,0.0741709724627963,0.29806692877909
2,0.248839992972743,0.803484553987643,0.970514832747317
"13987","RNF122",45.0425538865656,-0.142487440632593,0.573031031223992
,-0.248655714731959,0.803627107533884,0.970514832747317
"13988","ASH2L",184.049753908271,-0.0454156385894344,0.183118804627133
,-0.248011877763781,0.804125216735341,0.970514832747317
"13989","ARHGAP39",8.36933891317136,0.142795557436915,0.57475702679422
7,0.248445083365703,0.803790055271519,0.970514832747317
"13990","RPS6",25983.5784996972,-0.0612214701182487,0.244033294398535,
-0.250873432123843,0.801911965751389,0.970514832747317
"13991","DDX58",105.632248707128,0.0575364398468797,0.232484783370731,
0.24748475583079,0.804533087722285,0.970514832747317
"13992","MFSD14B",212.802879244148,-0.0501824109317468,0.2001699740947
7,-0.250698992986771,0.802046839302368,0.970514832747317
"13993","ERP44",681.706690323292,-0.0547432404595367,0.219844849416153
,-0.249008519439594,0.803354191385583,0.970514832747317
"13994","GLE1",83.5916663996906,0.0541161610859977,0.215586191345684,0
.251018679574076,0.801799667248949,0.970514832747317
"13995","FAM78A",10.3854438512467,0.130826139155694,0.527349981277838,
0.248082191713908,0.804070813958488,0.970514832747317
"13996","ZNF503-
AS2",21.3514954688592,-0.0952287284363218,0.385227878910799,-0.2472010
29960691,0.804752648243318,0.970514832747317
"13997","ABRAXAS2",120.395732132113,0.0495708332719576,0.1992840993329
57,0.248744548300045,0.803558387046711,0.970514832747317
"13998","PIDD1",37.6016836601794,0.0856492500802426,0.344938478187012,
0.248302974288091,0.803899997906807,0.970514832747317
"13999","PLAAT4",927.107864052611,0.0736552389843694,0.29685922536302,

0.248115041377942,0.804045398090494,0.970514832747317
"14000","AP002381.2",5.82358870882199,-0.160583497411521,0.646231642097601,-0.248492161247759,0.803753634341334,0.970514832747317
"14001","C11orf53",24.9539062367201,0.162019316380886,0.648869772706777,0.249694658613882,0.802823489200809,0.970514832747317
"14002","BIN2",12.4702428772154,0.128443716446889,0.517415382658269,0.248241008581921,0.803947938867201,0.970514832747317
"14003","HOTAIR",22.0567368186425,0.163151394136464,0.65155206399244,0.250403024231982,0.802275691101933,0.970514832747317
"14004","KMT5A",162.114367541047,-0.047811502389567,0.192515885762007,-0.248350946210604,0.803862884012846,0.970514832747317
"14005","AL133445.2",3.92146951936141,0.200360083756198,0.806616909116349,0.248395590883029,0.803828344672005,0.970514832747317
"14006","INAFM2",16.2834506707949,0.0966447572226211,0.390337147054173,0.247593030671017,0.804449303584204,0.970514832747317
"14007","RHBDF1",193.333038055738,0.0551070810376608,0.219596687298141,0.250946777547893,0.801855258004871,0.970514832747317
"14008","CCDC189",11.0495426026496,-0.10871671696576,0.43629946182326,-0.249179122319888,0.803222228158819,0.970514832747317
"14009","CMC2",211.054588889317,-0.0812765750696715,0.326977266896126,-0.248569497938496,0.803693805169983,0.970514832747317
"14010","EPN2",549.115910158454,0.0690115835867955,0.279243624189891,0.247137544454251,0.804801778445122,0.970514832747317
"14011","EFCAB13",50.9434801661115,0.0954384689091803,0.383235765475726,0.249033304056861,0.803335019860122,0.970514832747317
"14012","AMZ2",170.246133411969,-0.0436686563763661,0.175267059889212,-0.249154954752875,0.803240921699929,0.970514832747317
"14013","SPPL2B",173.26189634711,0.0435696752558235,0.175669135727314,0.248021230795234,0.804117980123985,0.970514832747317
"14014","TBXA2R",37.0799301667939,-0.0820429091426466,0.330580233803497,-0.248178507827587,0.803996294524931,0.970514832747317
"14015","U2AF1",70.7764176312793,0.0723302489601301,0.290785509935314,0.24874089832131,0.803561210592643,0.970514832747317
"14016","LINC01521",3.91036036634122,0.244465140214055,0.976526630509223,0.250341498712201,0.802323266584531,0.970514832747317
"14017","MICALL1",14.6765700450694,-0.124994331950925,0.500227895767453,-0.249874773095487,0.802684192652509,0.970514832747317
"14018","AMER1",22.3832138126656,-0.0857454795919343,0.345981437788002,-0.247832600905238,0.804263929760869,0.970514832747317
"14019","PJA1",166.785950980438,-0.0482272541019091,0.192236311225871,-0.250874841461371,0.801910876098182,0.970514832747317
"14020","LINC00891",73.9910684568969,0.0733136673393334,0.296386914981607,0.247357975786087,0.804631194223341,0.970514832747317
"14021","SLC6A8",79.3047119730135,0.0660548034913032,0.26353366321193,0.250650344575459,0.802084454527987,0.970514832747317
"14022","MTCH1",1804.75436187043,-0.0419902540099799,0.170142047358467,-0.246795278779689,0.805066664486308,0.970765023757987
"14023","SLC25A32",105.952989450559,-0.0547415048959024,0.222005362021329,-0.246577399741557,0.805235296955518,0.97080225620076
"14024","FLOT2",142.008552019859,0.067982428203589,0.275699061517018,0

.246582008039925,0.805231730163693,0.97080225620076
"14025","SAMHD1",1496.07040171968,0.0584174319479115,0.236955992371914,0.24653283237599,0.805269792004711,0.97080225620076
"14026","LUC7L",235.270070036949,0.0364950349099378,0.148152442118731,0.246334345813148,0.805423424797592,0.970918242298423
"14027","AOPEP",37.8720685078957,-0.0763842547534165,0.310303016011122,-0.246160207320314,0.805558217856317,0.971011502638811
"14028","CNPPD1",242.72933612191,0.0424380758604638,0.17261842516401,0.245849050123948,0.805799085537968,0.971042942168552
"14029","COLQ",15.2944010902492,-0.0931627716908787,0.379075920384771,-0.245762831878945,0.805865830590889,0.971042942168552
"14030","MICALL2",48.9566920540812,-0.0906691310888948,0.369051698772092,-0.245681381200978,0.80592888617526,0.971042942168552
"14031","KIAA1217",182.52100465275,0.100293398169594,0.408196829860754,0.245698620941781,0.805915539809908,0.971042942168552
"14032","AC022613.3",15.9498774187933,0.193941392895812,0.78907401808466,0.245783524043246,0.805849811815241,0.971042942168552
"14033","TMUB2",214.042946487675,0.0446539469742523,0.181657915172202,0.245813384635196,0.805826695473009,0.971042942168552
"14034","LINC01816",4.7884084583126,0.17810077827004,0.725281541140183,0.245560886590407,0.806022170193835,0.971086137497319
"14035","P4HA2",498.543580406458,-0.0513357882331556,0.20922029714874,-0.245367150954095,0.806172161432569,0.971197642002272
"14036","TADA1",35.9138726456852,0.0784718649293232,0.321243841790934,0.244275079303755,0.807017782779923,0.971312490085769
"14037","TMEM17",27.7615236609095,0.0961096829241481,0.393323023948377,0.244353056069157,0.80695739571844,0.971312490085769
"14038","CAPN10-DT",9.63913977324427,-0.136496462678137,0.558339335931727,-0.244468648174965,0.806867880559948,0.971312490085769
"14039","AC079848.2",5.06683050654146,0.185838860887185,0.758512776265434,0.245004259258717,0.806453133442929,0.971312490085769
"14040","AC010226.1",223.389564899352,-0.0674843731701517,0.275986141870646,-0.2445208759858,0.806827435887386,0.971312490085769
"14041","SKIV2L",61.9646394496527,-0.062303121584708,0.254800603307947,-0.244517166662317,0.806830308331808,0.971312490085769
"14042","DYRK4",32.6043178488266,-0.15361265665234,0.628945715975602,-0.24423833846147,0.807046236160607,0.971312490085769
"14043","KRAS",361.541630758778,0.0419861167421622,0.171748787915892,0.244462375843511,0.806872737822811,0.971312490085769
"14044","LRRK1",158.132787433514,0.0523842614857806,0.214243154528604,0.244508449294638,0.806837058940729,0.971312490085769
"14045","SETD1A",68.2011352276788,0.0583586158569832,0.238973494535146,0.244205391775783,0.80707175143216,0.971312490085769
"14046","MYO1C",751.838071293006,-0.0495750581981209,0.20297158116915,-0.244246302425987,0.807040068568999,0.971312490085769
"14047","AC005696.1",2.13396692028014,0.247832144895284,1.01407530388691,0.244392249712967,0.806927043663098,0.971312490085769
"14048","AC145207.2",3.38502819758079,0.18022763114363,0.735808787596979,0.244938133631458,0.80650433446938,0.971312490085769

"14049","GTPBP1",101.548347401078,-0.0451604675891465,0.184332704608672,-0.244994330686026,0.806460821076828,0.971312490085769
"14050","TMEM201",33.2023347020222,-0.0658008265350152,0.270503981853695,-0.243252709568631,0.807809636493492,0.971370836628402
"14051","AGMAT",2.92603322740866,0.224137777285593,0.920321860775951,0.243542815658661,0.807584921232875,0.971370836628402
"14052","PI4KB",221.457183135546,0.0430978163666446,0.177162973295082,0.243266499568514,0.807798954444022,0.971370836628402
"14053","MCUB",567.835429919477,-0.0519419773240374,0.213115697847344,-0.243726660441708,0.8074425238622,0.971370836628402
"14054","PTRH1",133.6543102487,0.0536954651423223,0.220475559183489,0.243543843776509,0.80758412488426,0.971370836628402
"14055","KCNQ1-
AS1",5.72014711208842,-0.153210724942162,0.628122259201017,-0.243918636376697,0.807293835300347,0.971370836628402
"14056","TIMM9",440.603299537019,-0.0587736550708271,0.240944288747549,-0.243930476112707,0.807284665455326,0.971370836628402
"14057","C16orf58",199.154906225104,-0.0458666953050911,0.188502826565373,-0.243320994919853,0.807756741448474,0.971370836628402
"14058","AP000919.2",2.18836981927597,0.287287750885452,1.17761353968501,0.243957581332069,0.807263672632513,0.971370836628402
"14059","PINLYP",58.793702320327,0.0749929051820313,0.308065331173866,0.243431823036611,0.807670893893037,0.971370836628402
"14060","AL110114.1",4.49914348713264,0.176457250817214,0.724889607099436,0.243426377049724,0.807675112305106,0.971370836628402
"14061","RRAGB",151.231739355609,-0.0433915418237833,0.178112944847387,-0.243618125908606,0.807526588746185,0.971370836628402
"14062","SMIM1",45.9800390952635,0.0919143834919598,0.378229964932684,0.243011902846773,0.807996176665176,0.971485248546108
"14063","IFI44",172.783474417066,0.0666111694303575,0.274182612784778,0.242944542521537,0.80804835908416,0.971485248546108
"14064","AL031055.1",3.99280212297639,-0.180648851826872,0.74369439963122,-0.242907371517725,0.808077154929765,0.971485248546108
"14065","MLEC",690.732787641711,-0.0426902206354714,0.175882940596729,-0.242719507023441,0.808222694846482,0.97152206202647
"14066","STARD8",31.9997343577104,0.0864764021365302,0.356194044093171,0.242778911019383,0.808176673444267,0.97152206202647
"14067","AC145124.1",119.468549952323,-0.0548782848882397,0.226243186067386,-0.242563260543433,0.808343745125245,0.971565935704653
"14068","TRAPPC6B",194.611612749677,-0.0421489058615955,0.173792679587488,-0.24252405775456,0.808374117784071,0.971565935704653
"14069","FAM234A",237.563960155852,0.0539966397873774,0.222766070754301,0.242391669451911,0.808476688762343,0.971620147387426
"14070","LINC01409",17.3838725080747,0.0893964092921169,0.371573913100862,0.240588497039755,0.809874066386369,0.971641369338097
"14071","IL22RA1",3.29468612061281,0.183133964814809,0.762650269329392,0.240128368374984,0.810230742707621,0.971641369338097
"14072","RHCE",3.61002343968741,-0.197448272610254,0.832988101030305,-0.237036126165589,0.812628764127924,0.971641369338097
"14073","LINC02574",2.22932943219084,0.304103384963378,1.2628359731660

4,0.240809884597257,0.809702468234892,0.971641369338097
"14074","ATXN7L2",8.64223666882179,0.119220776953071,0.50212802131156,
0.237431037291379,0.812322413742692,0.971641369338097
"14075","SF3B4",223.970939377441,-0.0523049473123222,0.217762420013014
,-0.240192716949033,0.8101808594774,0.971641369338097
"14076","UBQLN4",58.8619539794036,0.0614631294152391,0.257573934732675
,0.238623249976862,0.81139773498329,0.971641369338097
"14077","FCRLB",10.3457134630713,-0.112877822538861,0.478220223100683,
-0.236037325663445,0.81340370664719,0.971641369338097
"14078","ANKRD45",3.83789563815682,0.173814951198497,0.725356127664281
,0.23962705293219,0.8106193904974,0.971641369338097
"14079","UCHL5",188.743127781035,-0.0420737881324868,0.177603985122777
,-0.236896644539824,0.812736973176411,0.971641369338097
"14080","SNAP47",131.687673130093,-0.0512528563873062,0.21409227646294
8,-0.239396101690648,0.810798452541361,0.971641369338097
"14081","OSR1",268.916698446549,-0.0846731680790417,0.350728170990462,
-0.241421063611523,0.809228789082536,0.971641369338097
"14082","EIF2AK2",769.458504371666,0.0449650609267095,0.18604827322029
,0.241684914073182,0.809024319907466,0.971641369338097
"14083","AC019171.1",13.8272219629727,-0.0997243171400179,0.4165550622
64443,-0.239402485227054,0.810793503099198,0.971641369338097
"14084","REL",177.428504182569,0.0587026292111619,0.246944547470466,0.
237715834637664,0.812101501410235,0.971641369338097
"14085","VAMP5",1397.94341052312,-0.0607397278249513,0.254540841083088
,-0.238624684221596,0.81139662274291,0.971641369338097
"14086","FAM178B",4.47723584745339,-0.153902268897613,0.64599716361869
5,-0.238239852378756,0.811695069078167,0.971641369338097
"14087","TMEM177",30.4332733225326,-0.0995505048297336,0.4172954772314
29,-0.23856118808238,0.8114458636369,0.971641369338097
"14088","SH3BP4",97.3319508793598,0.0589256555006418,0.245960970629774
,0.239573194681111,0.810661147207228,0.971641369338097
"14089","XPC",360.822225306061,0.0513081761701656,0.21738570042212,0.2
3602369461531,0.813414283877306,0.971641369338097
"14090","ITGA9",66.0670628641801,-0.0744911883026027,0.30994274613882,
-0.240338543910425,0.810067816781994,0.971641369338097
"14091","CIP2A",29.1843092121332,-0.0779629077899035,0.325094993116031
,-0.239815775206595,0.810473076942587,0.971641369338097
"14092","MYLK-
AS1",13.0143772535526,0.117631396030926,0.498442430880534,0.2359979583
26144,0.813434254455855,0.971641369338097
"14093","AC096992.2",5.93137578027115,0.165116514699252,0.699003866069
659,0.236216883359551,0.813264379154512,0.971641369338097
"14094","GYG1",198.386834116652,0.0529959261958467,0.222968480232157,0
.237683488449429,0.812126591031567,0.971641369338097
"14095","SPON2",2478.82678560524,-0.0616691584218453,0.258645155260328
,-0.238431523527958,0.81154642007629,0.971641369338097
"14096","TBC1D14",103.156213815633,0.0539279785646369,0.22829524136360
8,0.23622033574824,0.813261700337197,0.971641369338097
"14097","PPID",217.090323461953,-0.055367823777125,0.234732327747264,-
0.235876431288747,0.813528557383466,0.971641369338097

"14098","SLC30A5",183.235087266841,-0.0525831335833918,0.220033784913879,-0.238977544307447,0.811122995356471,0.971641369338097
"14099","PGGT1B",200.888737803785,0.0522355118977945,0.219182437385694,0.238319787483137,0.811633075232229,0.971641369338097
"14100","DND1",2.64338052207842,0.238882916407073,1.01175189888423,0.236108196753094,0.813348713638584,0.971641369338097
"14101","PPARGC1B",10.6635973206474,-0.129553041229157,0.549443345082977,-0.235789626698622,0.813595917933815,0.971641369338097
"14102","MDC1",73.1211572111971,0.0615663197366524,0.254435825386409,0.241971898584456,0.808801937956871,0.971641369338097
"14103","AL645940.1",4.1907369901308,-0.198149793521491,0.831978814237737,-0.238166874120511,0.811751668557751,0.971641369338097
"14104","NFYA",65.1055780503598,0.0485491993715678,0.205830572322323,0.23586971956499,0.813533765646478,0.971641369338097
"14105","AL512274.1",4.7467815022783,0.185084214180336,0.768732374506317,0.240765473548839,0.809736890636804,0.971641369338097
"14106","SUPT3H",67.1342532191768,0.0663298379842324,0.279360291123944,0.237434739623763,0.812319541812185,0.971641369338097
"14107","KLHL7-DT",2.50272548802746,-0.223269475496172,0.938788969816211,-0.237827118420322,0.812015184584363,0.971641369338097
"14108","ZNF92",97.1048277845702,-0.0509692084680193,0.211681531296152,-0.240782500749728,0.809723693040583,0.971641369338097
"14109","TYW1B",45.4687999949671,-0.107929556696232,0.456184521370702,-0.236591886923159,0.812973414832744,0.971641369338097
"14110","SSC4D",3.31425061278852,-0.187005674253741,0.773722545137401,-0.241696038753184,0.809015699196431,0.971641369338097
"14111","AC007384.1",6.07657480966264,0.241269485280766,1.00005522033385,0.2412561630349,0.80935658431154,0.971641369338097
"14112","KRBA1",19.4602862442268,0.0878368283389024,0.368114249814928,0.238612953405262,0.811405719867923,0.971641369338097
"14113","DOCK8-AS1",5.85147571776731,-0.187354773354494,0.775845736312678,-0.241484569142476,0.809179574699463,0.971641369338097
"14114","UPF2",373.745924732556,0.0624566205468752,0.261168577540409,0.239142936470646,0.810994748990571,0.971641369338097
"14115","VSIR",718.093978572229,0.0741019390702633,0.310023524451642,0.239020374990356,0.811089783626295,0.971641369338097
"14116","SEC23IP",149.560210097328,0.0429059036847504,0.177427679825052,0.241821928388268,0.808918146710076,0.971641369338097
"14117","TTC36",2.78614294201948,-0.206830235278789,0.862994977513258,-0.239665630354855,0.810589481460773,0.971641369338097
"14118","RPUSD4",123.303124050489,-0.0550640385039713,0.232570384509134,-0.236762899197979,0.812840735406659,0.971641369338097
"14119","AC026356.2",14.851266585259,-0.124426892603458,0.515965609023084,-0.241153461446868,0.809436178919062,0.971641369338097
"14120","ARHGAP9",8.47511076817388,-0.146453173809008,0.621357042639393,-0.2356989037847,0.81366632058541,0.971641369338097
"14121","PWP1",373.831257395725,-0.0374530138414345,0.154656230070272,-0.242169447841945,0.808648867625388,0.971641369338097

"14122","SART3",155.598206307242,0.04917481446757,0.204293973245709,0.
240706143633646,0.809782877024512,0.971641369338097
"14123","UNG",120.894463074994,-0.0478221660749134,0.201738554820585,-
0.237050206478597,0.812617840900294,0.971641369338097
"14124","TRIAP1",200.616760912648,-0.0636081152986433,0.26904180790858
3,-0.236424650105891,0.813103170271854,0.971641369338097
"14125","PRPF39",102.448522495187,-0.0499441406073902,0.21083528522584
9,-0.23688701136477,0.812744446670611,0.971641369338097
"14126","NEMF",485.574711923084,0.0380029662925846,0.160441252401344,0
.236865305672884,0.812761286182365,0.971641369338097
"14127","EXD2",84.3956658957202,0.0532651851702651,0.224646282487212,0
.237106906824943,0.812573854264961,0.971641369338097
"14128","CYP46A1",68.2704160403509,0.0740214007092855,0.31352837573751
4,0.236091551634409,0.813361629473526,0.971641369338097
"14129","ANKRD9",162.534851823115,0.055516690268824,0.233269652519175,
0.237993625271339,0.811886038470424,0.971641369338097
"14130","CDAN1",33.7508411971111,0.0674609968018159,0.282612793070821,
0.238704681655761,0.81133458625089,0.971641369338097
"14131","LCMT2",19.0586591933889,-0.0928100097827147,0.391221355049873
,-0.237231451158599,0.812477238127363,0.971641369338097
"14132","B2M",29314.1732022782,0.0647271672355928,0.271203016614507,0.
23866684096511,0.811363930840733,0.971641369338097
"14133","SHF",70.2686479260039,-0.0548837492597464,0.228010210993628,-
0.240707418411538,0.809781888942196,0.971641369338097
"14134","SAXO2",12.9531842264802,0.0951934307000697,0.399167340029534,
0.238480008642557,0.811508818916086,0.971641369338097
"14135","ABHD2",278.625993195884,-0.0615041966476327,0.259851703190973
,-0.236689603694579,0.812897600873653,0.971641369338097
"14136","AC022819.1",6.46284533030965,0.154926114129017,0.651021853802
226,0.237973753452648,0.811901451189938,0.971641369338097
"14137","ZKSCAN2-
DT",18.7928128131636,-0.129755811715402,0.545865486260403,-0.237706568
708581,0.812108688594474,0.971641369338097
"14138","PIEZO1",114.126910853519,-0.0532168587514688,0.22205523993008
1,-0.239655946728505,0.810596989140458,0.971641369338097
"14139","RPAIN",342.659464291152,-0.0482443508743588,0.203322826700138
,-0.237279560083581,0.812439918056669,0.971641369338097
"14140","ZNF18",29.2434205500204,0.0717709213784902,0.303641234054387,
0.236367506547662,0.81314750790226,0.971641369338097
"14141","PIPOX",2.76690445118889,-0.198411937760836,0.839085317586838,
-0.236462173276321,0.813074056406769,0.971641369338097
"14142","KLHL11",17.1106017659748,-0.0831652510279014,0.35138082888789
9,-0.236681242090284,0.812904088190151,0.971641369338097
"14143","SETBP1",578.699682353614,0.0666986162143564,0.27656335472951,
0.241169392378792,0.809423832181615,0.971641369338097
"14144","ZNF236",110.977390648417,0.0530293007091246,0.221791625312508
,0.239095144527687,0.811031806712808,0.971641369338097
"14145","EEF2",6936.68160943613,-0.0280773262136968,0.117745098928489,
-0.238458555550997,0.811525456157009,0.971641369338097
"14146","INSR",491.47929842196,0.0680021627565051,0.281408082087844,0.

241649643649103,0.809051651735316,0.971641369338097
"14147","SPC24",7.09518117280188,0.161171230044929,0.676530703256774,0
.238231952029762,0.811701196275312,0.971641369338097
"14148","AC022098.4",2.91138394636792,-0.186120139710427,0.78952501690
6665,-0.235736849023024,0.813636874198639,0.971641369338097
"14149","AC008556.1",2.31845291750049,-0.281003065319658,1.16856335097
358,-0.240468833020942,0.809966822127644,0.971641369338097
"14150","AC011468.5",5.78941868923451,0.164266276398824,0.691453243369
974,0.23756671615022,0.812217168154975,0.971641369338097
"14151","ZNF837",15.5416053257806,-0.0976070462630164,0.41272296106981
9,-0.236495313975285,0.81304834307374,0.971641369338097
"14152","SLX4IP",48.4584182405117,-0.0610384610578086,0.25612575793829
,-0.238314418468271,0.811637239146519,0.971641369338097
"14153","WFDC2",8.86615001530143,-0.174919479540942,0.738142543509523,
-0.236972494105652,0.812678129072882,0.971641369338097
"14154","ZNRF3-
AS1",5.67492783171543,0.154158266976866,0.651095515493515,0.2367675146
09616,0.812837154626045,0.971641369338097
"14155","APOL4",98.5416062881703,-0.0872563785773051,0.364061793004378
,-0.239674638355296,0.810582497606999,0.971641369338097
"14156","RPL3",28014.6733821899,-0.0642060099332215,0.266425918521351,
-0.240990104452154,0.809562786045814,0.971641369338097
"14157","CDPF1",44.128327873422,0.070609186959638,0.295630393838951,0.
238842786232945,0.811227491560241,0.971641369338097
"14158","JPX",819.431022478516,0.0424478919439418,0.178484574669416,0.
237823868099316,0.812017705650613,0.971641369338097
"14159","SLC16A2",104.17659963619,0.063440502764905,0.26320465564777,0
.241031081341523,0.809531027119544,0.971641369338097
"14160","SFTA1P",25.5442971723639,-0.0739469313015346,0.31423966515758
8,-0.235320169605103,0.813960241536306,0.971666808456528
"14161","KLRC1",3.50727366298182,-0.244724666714511,1.04070351131689,-
0.235153109462311,0.814089898781356,0.971666808456528
"14162","SLC24A1",17.3314441250067,-0.0925856222408518,0.3934537180488
32,-0.235315153965227,0.813964134156303,0.971666808456528
"14163","AC135050.3",4.0095487794485,-0.196082553532604,0.832631623931
323,-0.235497365097409,0.813822723703526,0.971666808456528
"14164","MTHFSD",47.7701468029784,0.0636117279824873,0.270447876512347
,0.235208827678051,0.814046654684736,0.971666808456528
"14165","LINC01311",2.09721086489686,0.279551621280615,1.1875873843804
6,0.235394569660616,0.813902500461456,0.971666808456528
"14166","FAM122C",25.6356870002109,-0.0751680060350969,0.3196179075270
36,-0.235180833942286,0.814068381149797,0.971666808456528
"14167","CDC27",358.147827988182,-0.0333692319425881,0.141985435266951
,-0.235018696670187,0.814194221750409,0.971722728972677
"14168","ZNF835",24.3893372388269,-0.0785822387925778,0.33453548645640
8,-0.234899560656377,0.814286690575747,0.971764494936104
"14169","CLDN15",34.6234650977169,0.0661633289579501,0.281790545144822
,0.234796128180761,0.814366973012947,0.971791712873379
"14170","INTS12",80.8068476362257,0.053002605425262,0.226020736208252,
0.234503286355223,0.814594282170872,0.97185719185331

"14171","HCP5",7.11065947004876,-0.124430203695001,0.530413691939656,-0.234590859146143,0.814526304938834,0.97185719185331
"14172","REC8",27.404526934865,-0.13364353153894,0.569837332130686,-0.234529266517571,0.814574115264439,0.97185719185331
"14173","ELOA",246.079967707159,0.0362993308783764,0.155288319086771,0.233754419468558,0.815175637244095,0.971858302644142
"14174","LDLRAP1",61.6742923836343,-0.051515803936835,0.221143665519783,-0.232951750237795,0.815798872772829,0.971858302644142
"14175","TMEM200B",103.365517886457,0.0473047620222242,0.205477267383521,0.230218956211494,0.817921632507237,0.971858302644142
"14176","AL356055.1",6.55488121748119,0.15247291041529,0.652375815253498,0.233719440313774,0.815202794505823,0.971858302644142
"14177","ATPAF1",174.230483300056,0.0502325510087137,0.214752135020194,0.233909437053979,0.815055286719169,0.971858302644142
"14178","TTC4",157.109599896032,0.0493826641500491,0.213457535411407,0.231346548880889,0.817045586581753,0.971858302644142
"14179","AL445928.2",2.01475184664814,-0.271364755895797,1.17516543161382,-0.230916217066681,0.817379891783698,0.971858302644142
"14180","OLFML3",2748.4394162892,0.0556234463648012,0.241321052464388,0.230495623140918,0.817706664166321,0.971858302644142
"14181","C1orf61",4.32599010055085,-0.167845120263128,0.718197769750746,-0.233703204510618,0.815215399801674,0.971858302644142
"14182","MDM4",532.274462158861,0.0516381429776387,0.223189522523682,0.231364547913128,0.817031604677441,0.971858302644142
"14183","AC104794.3",77.5683404098948,-0.0482013181562659,0.207019239983572,-0.232834968189869,0.815889558351826,0.971858302644142
"14184","CGREF1",90.9348284376694,0.108503308517298,0.464978265996542,0.233351355218191,0.815488583398408,0.971858302644142
"14185","MSH6",199.798959676481,-0.0504102774829948,0.218640656857997,-0.230562230316273,0.817654912886434,0.971858302644142
"14186","ACTR2",975.698480451652,0.0368564234041864,0.158905658643555,0.231939024190825,0.816585373941367,0.971858302644142
"14187","LINC01960",8.73642056527602,0.125251010958511,0.541040220375899,0.231500369550142,0.816926098375246,0.971858302644142
"14188","AC026202.2",3.39727200334641,0.225464133114308,0.979248902895794,0.230241905247558,0.81790380073977,0.971858302644142
"14189","EOMES",2.45583950183601,0.270288785120459,1.17474898811745,0.230082160405687,0.818027926986895,0.971858302644142
"14190","ANAPC13",367.396904133007,-0.0551413323253037,0.236812986965454,-0.232847585902658,0.815879760112382,0.971858302644142
"14191","AC253576.2",2.01298026229721,-0.224434842078506,0.973157732230404,-0.230625349463255,0.81760587240732,0.971858302644142
"14192","BST1",345.549372201014,0.0726548876906623,0.314373904557255,0.231109791994297,0.817229508117669,0.971858302644142
"14193","UBA6-AS1",44.1600550652073,0.0668174787205859,0.286127309919468,0.233523597378356,0.815354847959337,0.971858302644142
"14194","AC112236.2",3.46677214654758,0.182497264994503,0.794020071324662,0.229839611850168,0.818216402776163,0.971858302644142
"14195","SLC22A5",51.6821905444022,0.0580078641486565,0.24983845323890

2,0.232181489264937,0.816397054375279,0.971858302644142
"14196","AC034243.1",6.19888839403723,0.23359834709587,1.0067589761793
7,0.232030061437715,0.816514665217203,0.971858302644142
"14197","HDAC2-
AS2",3.74589175008171,-0.221522273975868,0.961052902050964,-0.23049956
3034586,0.817703602994434,0.971858302644142
"14198","INTS1",208.339637523485,0.0440028304195105,0.188900375964947,
0.23294199492581,0.815806448038935,0.971858302644142
"14199","MRM2",128.859335106196,-0.0552486207032911,0.235957104467625,
-0.234146883722552,0.814870949443413,0.971858302644142
"14200","SEPTIN7-
AS1",5.70062550947782,-0.147965731787886,0.643120262275483,-0.23007474
7239893,0.818033687336572,0.971858302644142
"14201","NUDCD3",212.740586263032,0.0435848765884737,0.189210807248464
,0.230350883346953,0.817819124302601,0.971858302644142
"14202","GPR85",4.05323301436905,-0.195561101178119,0.839474162882809,
-0.232956664808539,0.815795056480985,0.971858302644142
"14203","HIPK2",117.927728211766,0.053050347749444,0.230094844108108,0
.230558611406863,0.817657724623053,0.971858302644142
"14204","TTI2",71.4634704540112,-0.0612495395228125,0.263820448253553,
-0.232163730780818,0.816410846806331,0.971858302644142
"14205","YTHDF3-
AS1",27.1630266101219,-0.107526766982729,0.466510012684155,-0.23049187
3827215,0.817709577266227,0.971858302644142
"14206","MRPL13",337.930894835091,-0.0640262318673549,0.27751007470453
5,-0.230716783653796,0.817534833795263,0.971858302644142
"14207","CCIN",2.7609250053692,-0.237934992747608,1.02995281460267,-0.
231015430390757,0.817302814518594,0.971858302644142
"14208","COL15A1",1340.08487524046,0.0803490015880376,0.34791170854583
2,0.230946529290068,0.817356342508542,0.971858302644142
"14209","PPP3CB-
AS1",5.39179415255294,-0.15334470482757,0.658743257090679,-0.232783718
356091,0.815929356521644,0.971858302644142
"14210","SFXN3",291.700024752266,-0.0608670143612837,0.260933631871575
,-0.233266267459309,0.815554650791444,0.971858302644142
"14211","RIC8A",310.884309115319,-0.0447151135051332,0.193775021814445
,-0.230757881415446,0.817502903908895,0.971858302644142
"14212","BTBD10",129.709144784523,0.0411543060051102,0.177466333597376
,0.231899229396819,0.816616283068181,0.971858302644142
"14213","CLP1",46.5923393828163,0.0620679971814646,0.266393922587982,0
.23299329270909,0.815766614102387,0.971858302644142
"14214","CDC42BPG",2.66397143437999,0.272922761842183,1.16910159394766
,0.233446574066002,0.815414651157372,0.971858302644142
"14215","SYVN1",197.756529711151,-0.0415950335221785,0.180049193066692
,-0.231020382894864,0.817298967042629,0.971858302644142
"14216","SNX19",109.404017088339,-0.0494947621033993,0.211986354662869
,-0.233480886928373,0.815388009494318,0.971858302644142
"14217","RBM19",97.3505240933788,-0.0524022187551248,0.22790841434684,
-0.229926652358596,0.818148765507396,0.971858302644142
"14218","MYO16",3.84505863555118,-0.20895591832817,0.901094793539203,-

0.231891161536357,0.816622549513639,0.971858302644142
"14219","AL442125.1",9.68917815399308,0.141136319045018,0.607250163309153,0.232418742015495,0.816212793417029,0.971858302644142
"14220","TTC5",176.669751198074,-0.0474480711834514,0.204338103634325,-0.232203736550098,0.816379775720213,0.971858302644142
"14221","DIO2",9.02152009152735,-0.123534806671985,0.536139448079563,-0.230415439704136,0.817768964746646,0.971858302644142
"14222","RAB11A",603.949450685151,0.0466037935565284,0.20130294798448,0.231510735551282,0.816918046197626,0.971858302644142
"14223","TMEM202-AS1",17.0860295359617,0.123069237649422,0.526215553584103,0.233876092812509,0.815081173716846,0.971858302644142
"14224","LAT",18.9484453785783,0.0961213594260755,0.418156851374187,0.229869148646476,0.818193450228312,0.971858302644142
"14225","PFAS",37.5923709572436,0.0748709712460404,0.325474522154552,0.230036350465822,0.81806352344395,0.971858302644142
"14226","FLJ45513",2.65195931300892,-0.208770637188533,0.89245328418188,-0.233928924783906,0.815040157399081,0.971858302644142
"14227","METTL23",285.265994757668,-0.0611620825169182,0.264640904045544,-0.231113488436362,0.817226636506931,0.971858302644142
"14228","NPB",47.1421084733432,-0.093587773535253,0.404383050948842,-0.231433472089543,0.816978063935051,0.971858302644142
"14229","AC100847.1",2.09029025915071,-0.252481835538984,1.09106078732208,-0.231409503918366,0.816996682467334,0.971858302644142
"14230","SHC2",262.313475559831,0.0673010740588596,0.29177146129289,0.230663663130852,0.81757610491632,0.971858302644142
"14231","SYDE1",205.879640508454,0.04653195628916,0.199453814349151,0.233296898537644,0.815530866778635,0.971858302644142
"14232","SMIM7",771.070896116943,0.0526625795254317,0.225089602668262,0.233962737066296,0.815013907360936,0.971858302644142
"14233","SRC",103.312138987845,0.0470533330721712,0.204172131035839,0.230459136775683,0.817735013042022,0.971858302644142
"14234","RUNX1",382.998894003548,-0.0578510929353931,0.24913545093826,-0.232207390467805,0.816376937864116,0.971858302644142
"14235","CTAGE9",2.38124199168541,-0.336927652990308,1.4486598548442,-0.232578856840444,0.816088446918179,0.971858302644142
"14236","FIP1L1",469.164666430288,0.0358871212158867,0.156317036752666,0.229579078271995,0.818418865786507,0.971893958612042
"14237","MRPL51",1308.94971901332,-0.059457167573456,0.258933462187552,-0.229623344434292,0.8183844652953,0.971893958612042
"14238","FUZ",135.262760714828,0.0490525513585635,0.213591414260093,0.22965600714096,0.818359082403011,0.971893958612042
"14239","CSNK2A2",471.828178600786,-0.0416618798608788,0.181594274486306,-0.229422871281223,0.818540261466045,0.971969853280981
"14240","ANKRD13A",129.259761147496,-0.0371655769223027,0.162073208423719,-0.229313513835909,0.818625250785474,0.972002509851179
"14241","METTL27",70.2239462946552,0.108716678008555,0.474250789405438,0.229238791873918,0.81868332367407,0.972003204598074
"14242","LIN9",26.5035687851736,-0.0750929889181284,0.328203055186111,-0.228800395765805,0.819024059172103,0.972179128728481

"14243","STK11IP",30.6319063690877,-0.0709231997961858,0.3098287847148
,-0.228910944673754,0.818938133794534,0.972179128728481
"14244","SHQ1",44.7082810537419,0.0612414777736563,0.267719687823539,0
.228752238102198,0.819061490935487,0.972179128728481
"14245","SLC9A3R2",302.604501947753,-0.061171137692808,0.267317304016
821,-0.228833348421887,0.818998446123465,0.972179128728481
"14246","SAMD11",27.4810056561123,-0.133290000905743,0.585182632700558
,-0.227775045699192,0.819821127435677,0.972193549072972
"14247","GPSM2",110.583895493364,-0.0586342873598275,0.256786328194842
,-0.228338820730898,0.819382847011055,0.972193549072972
"14248","GCFC2",143.420449792166,-0.0391113537180192,0.171582707814475
,-0.227944611763026,0.819689300337265,0.972193549072972
"14249","NDUFB3",624.299729229805,-0.0637512874615768,0.27919825892749
5,-0.228336980704928,0.819384277361147,0.972193549072972
"14250","CPOX",78.9437947852583,0.0549792568082745,0.240839543210203,0
.228281685289068,0.819427261710492,0.972193549072972
"14251","SLAIN2",329.631398295875,0.0415702424679646,0.182393918750919
,0.22791462978946,0.819712609091569,0.972193549072972
"14252","FRG1-
DT",3.9659510306765,-0.175504726656288,0.768812751611779,-0.2282801973
41096,0.819428418386665,0.972193549072972
"14253","VPS26A",497.376067408517,-0.0371831727659208,0.16312353281698
5,-0.227944871741088,0.819689098224356,0.972193549072972
"14254","DHRS13",11.5748585587778,-0.101010027736888,0.443294415053571
,-0.22786217084346,0.819753392403176,0.972193549072972
"14255","PPP1R37",105.12696178657,0.0469257047013983,0.205993100205958
,0.227802313060392,0.819799928409776,0.972193549072972
"14256","AP000317.1",4.0301209238158,-0.160590728914412,0.704814671307
17,-0.227848164137355,0.819764281760077,0.972193549072972
"14257","SLC19A1",16.5153265199963,0.0832249009209738,0.36496475410509
,0.228035447217485,0.819618683500492,0.972193549072972
"14258","ARHGAP8",4.85769367864508,0.195927068089807,0.860045627656942
,0.227810085638804,0.819793885638263,0.972193549072972
"14259","CYTOR",660.556985140529,0.0747002041457005,0.328676860342264,
0.227275519389811,0.820209507741235,0.972333465425061
"14260","CNNM3",141.373832631925,0.0599819237634229,0.263804964422907,
0.227372232719872,0.82013430995311,0.972333465425061
"14261","MGAT4A",16.3175366832427,-0.100916026552675,0.444108559821145
,-0.227232788742728,0.820242732748228,0.972333465425061
"14262","ZBTB11-
AS1",17.2049837635513,-0.0922967722311805,0.406506259534343,-0.2270488
33975908,0.820385769566394,0.972333465425061
"14263","HSPA4",231.112522621833,-0.0383241046884403,0.168835494196583
,-0.226990804693104,0.820430892354893,0.972333465425061
"14264","PCDHA5",2.3396272469737,-0.218210315768712,0.961458336035734,
-0.226957640898338,0.820456680341812,0.972333465425061
"14265","TVP23C",30.6610558379631,-0.0753305461864948,0.33148371820223
2,-0.227252628258915,0.820227306588702,0.972333465425061
"14266","SIRPA",300.871039545071,0.0404561450200104,0.177845089025128,
0.227479686067094,0.820050763388416,0.972333465425061

"14267","TMEM191C",11.0379266441448,-0.108831593581515,0.479466857286129,-0.226984601600039,0.820435715831654,0.972333465425061
"14268","AL109659.2",2.8639212642391,-0.205689071359584,0.910919982345305,-0.225803665904886,0.821354125732047,0.972365299118869
"14269","LINC01139",18.413624874376,0.162865725062606,0.719347131790689,0.226407693677989,0.820884344593986,0.972365299118869
"14270","AL451007.2",2.31435391638923,-0.221032996030154,0.98661913088116,-0.224030721797121,0.822733398186813,0.972365299118869
"14271","SEMA4F",53.1974655018774,-0.0712164494494198,0.314628925637713,-0.22635061066076,0.82092873802684,0.972365299118869
"14272","AC092944.1",125.903655150764,-0.0510057024209907,0.227717834563039,-0.22398641950405,0.822767870454494,0.972365299118869
"14273","SLC26A1",4.79361329199005,-0.178976633823507,0.792692981622554,-0.225783043338118,0.821370166015569,0.972365299118869
"14274","FBXO8",209.730442848245,-0.0498948821826005,0.221051720759862,-0.225715873240378,0.821422411601767,0.972365299118869
"14275","SLC35B3",174.657017042855,-0.0435368108613592,0.194491285560546,-0.223849673962929,0.822874276334171,0.972365299118869
"14276","HLA-F",193.647491026568,-0.0850853248256057,0.376417072392445,-0.226040025987178,0.821170289610028,0.972365299118869
"14277","AL645933.2",17.3694420912639,-0.100801198634698,0.448269329295222,-0.224867489357747,0.822082361336877,0.972365299118869
"14278","FAM126A",441.655180657853,0.0653875072466186,0.2889118677881,0.226323368946601,0.820949924095204,0.972365299118869
"14279","ADCYAP1R1",50.6758287353629,-0.11562216124954,0.513547094723939,-0.225144222287332,0.821867079569787,0.972365299118869
"14280","SMU1",345.370787299814,-0.0298865784451735,0.13313303350097,-0.224486573010864,0.8223787135175,0.972365299118869
"14281","RMI1",24.7406635072187,-0.0748850412326217,0.334734985780597,-0.223714414129706,0.822979529343478,0.972365299118869
"14282","PFKFB3",196.557171753485,0.108092440990289,0.483267093356312,0.22367018668617,0.823013945806136,0.972365299118869
"14283","TNKS2-AS1",4.52659779970753,0.167517944557008,0.746309778784637,0.224461677066339,0.822398083396888,0.972365299118869
"14284","TMEM41B",182.673900747972,0.0330492066663758,0.14722390803958,0.22448260684325,0.822381799321607,0.972365299118869
"14285","PLCB3",69.5054592447244,-0.0566005992052499,0.25021376461866,-0.226208974919955,0.82103889054406,0.972365299118869
"14286","CTSF",1767.28091982479,-0.0472175584118953,0.208276512444216,-0.226706112262859,0.82065227396124,0.972365299118869
"14287","BCL9L",382.838961502106,0.0596593038998467,0.26587926135867,0.224384946742298,0.822457782841954,0.972365299118869
"14288","AC021054.1",34.9697132381691,0.0630998389934163,0.280282994173411,0.22512903139024,0.821878896836822,0.972365299118869
"14289","TRHDE-AS1",37.2907458978133,0.145240009296463,0.64045702282142,0.226775574505583,0.820598257638334,0.972365299118869
"14290","GAS2L3",11.105605767158,0.121429573620602,0.538908085266105,0

.225325202832395,0.821726294718473,0.972365299118869
"14291","RASL11A",241.247059524965,-0.0733321265685807,0.325130275575698,-0.225546902510797,0.821553842258434,0.972365299118869
"14292","SEC23A",432.477983029354,-0.0374675091317096,0.166366178208286,-0.225211094798375,0.82181505874346,0.972365299118869
"14293","WDHD1",16.9292953474624,-0.0870090628042672,0.388222676685873,-0.224121536503314,0.822662734994022,0.972365299118869
"14294","ZNF710",24.6053513376666,-0.075607026893573 6,0.33730187028119,-0.224152409325641,0.822638713086761,0.972365299118869
"14295","BEAN1",7.74907678613912,-0.163734604114768,0.725466499236113,-0.225695610048395,0.82143817267442,0.972365299118869
"14296","AC104984.3",12.2308977533117,-0.132775282234952,0.593539320016965,-0.223700903642167,0.822990042761283,0.972365299118869
"14297","SPOP",998.769160844379,0.0427218682129985,0.188488463183202,0.226655082711745,0.820691956906269,0.972365299118869
"14298","MYO15B",115.906022193704,0.0733696572678013,0.326506804763251,0.224710959151376,0.822204138464589,0.972365299118869
"14299","JMJD6",106.690908339067,-0.0447591645641972,0.199133317593037,-0.224769843164418,0.822158327468498,0.972365299118869
"14300","PET100",828.850363926442,-0.0666250601794007,0.296204172765046,-0.224929512496263,0.822034109857082,0.972365299118869
"14301","GET3",646.807232974716,-0.0578251268723987,0.257325207584283,-0.224716138054446,0.822200109321062,0.972365299118869
"14302","CEBPA-DT",24.7402475584802,0.0912751899201842,0.404099514799,0.225873050022306,0.821300159142073,0.972365299118869
"14303","CLPTM1",378.704087385638,0.0326184359189853,0.145794497069132,0.223728855167411,0.822968291837221,0.972365299118869
"14304","STK4",268.715016752327,0.0406745293333607,0.179558938294786,0.22652467050448,0.820793373570552,0.972365299118869
"14305","AP001432.1",3.69055162096484,0.174993445776959,0.778700588662378,0.224724943482521,0.822193258781998,0.972365299118869
"14306","HDHD5",114.161102945232,-0.0598947065872199,0.267755421410924,-0.223691853825434,0.822997085049298,0.972365299118869
"14307","CRYBB2",3.31627149308003,-0.352082450302499,1.57115426953459,-0.224091584849139,0.822686040306229,0.972365299118869
"14308","TPST2",82.3631367215982,0.0614163530096255,0.272409925625999,0.225455635907872,0.821624834327081,0.972365299118869
"14309","TCEAL8",865.58001737778,0.0507556889598534,0.225556303661804,0.225024475644696,0.821960233693361,0.972365299118869
"14310","PGRMC1",1324.25612714405,-0.0487467586899725,0.217636862308628,-0.223982087284669,0.822771241436655,0.972365299118869
"14311","UPF3B",216.95593195786,-0.0524180192147361,0.232718124018863,-0.225242530790113,0.821790604627127,0.972365299118869
"14312","PIGZ",83.1073410667296,0.0570675051374312,0.255461575558636,0.22338977990188,0.823232157827986,0.972419262578984
"14313","PPP3CC",98.3706194276121,-0.0402738890844274,0.180204190352631,-0.223490302892612,0.823153929456714,0.972419262578984
"14314","TEFM",100.95010696122,-0.0530271169810419,0.23737485159546,-0.22338978465761,0.82323215412697,0.972419262578984

"14315","ZNF585B",40.9888442771979,-0.0595872002091004,0.266895216238959,-0.223260652808967,0.823332648881754,0.972470026356457
"14316","AL512343.2",6.24480924999075,0.14046908407386,0.629495807673272,0.223145384546814,0.823422356973971,0.972492353620955
"14317","NSD3",1002.45136959879,-0.0264261569469285,0.118455906038055,-0.223088555318118,0.823466585450154,0.972492353620955
"14318","ZNF717",49.8965690657732,-0.0604746824167518,0.271331453576087,-0.222881209014691,0.823627961584058,0.972615000311723
"14319","FBXO48",50.1010372384008,0.0560053800117247,0.251473787039032,0.222708619737898,0.823762292262343,0.972626774228057
"14320","MTPN",1153.76154104538,0.0245818484449448,0.11040083040543,0.22265999589561,0.823800138383279,0.972626774228057
"14321","CPQ",1775.99082208316,0.0418729054855495,0.188185737316283,0.222508390288759,0.823918142468214,0.972626774228057
"14322","MBIP",318.217626741771,0.0488100037092864,0.219371920293448,0.222498866965265,0.823925555196857,0.972626774228057
"14323","EID2B",69.017186834537,-0.0541956044790078,0.243567435161657,-0.222507596071035,0.823918760667813,0.972626774228057
"14324","AC025171.1",44.6921607098758,0.0548458430031593,0.246595936112859,0.222411787751677,0.823993336315233,0.972638880928369
"14325","CKMT2-AS1",65.9594834521149,0.0470290816458075,0.211561987043777,0.222294573344483,0.824084576276803,0.972678674742631
"14326","CYP4B1",356.707917926364,-0.0691002552950772,0.311168789755764,-0.222066793232425,0.824261887632702,0.97275214602455
"14327","CEACAM1",2.39521467602454,-0.239850972377871,1.07995511194892,-0.22209346455616,0.824241125353792,0.97275214602455
"14328","HNRNPA3",1393.92961621882,-0.0424263186164345,0.191265087895985,-0.221819460535877,0.824454429505103,0.97277567997713
"14329","SAPCD1",3.91117342518439,-0.175595427206072,0.791401490521273,-0.221879070622437,0.824408023684461,0.97277567997713
"14330","TMEM179",3.09915262693432,-0.197650241261258,0.891013730280181,-0.221826257603355,0.824449138029108,0.97277567997713
"14331","SCNN1D",14.0484127892959,0.0901660429836101,0.409641227475925,0.220109786163819,0.825785653446583,0.973128061592402
"14332","FAM189B",53.7044454389504,0.0521187167647402,0.236509796154823,0.220365995878761,0.825586125843407,0.973128061592402
"14333","ZBTB18",18.4740509579823,0.0827295254183961,0.375179887506222,0.220506290910981,0.825476873511533,0.973128061592402
"14334","HES6",16.9117487417464,-0.0882113562078449,0.400724074400526,-0.22012991443004,0.825769977814971,0.973128061592402
"14335","AC034206.1",2.15601863315859,-0.255601530888944,1.16044173379573,-0.220262270345008,0.825666902482239,0.973128061592402
"14336","BVES",23.2943376527504,0.0773992127113006,0.351406392079431,0.22025556294891,0.825672125955022,0.973128061592402
"14337","HGH1",57.2647299595046,-0.0566955543538738,0.256443924210824,-0.221083632721453,0.825027314148323,0.973128061592402
"14338","SPTLC1",483.481039845725,0.0360137193800293,0.162702030713707,0.221347694445189,0.824821716039022,0.973128061592402
"14339","CAMK2G",193.028147909078,0.0433419155633709,0.196207444248745

,0.220898425792772,0.825171523180867,0.973128061592402
"14340","CEP126",852.735929507515,0.0468436069440743,0.212887987430885
,0.220038751408095,0.825840974944279,0.973128061592402
"14341","SMUG1",141.419592604771,-0.0552127198847032,0.250422722633409
,-0.2204780752485,0.825498845699643,0.973128061592402
"14342","ZNF605",96.4571694895025,0.0544603672438304,0.246333862840248
,0.22108355958187,0.82502737109145,0.973128061592402
"14343","NOP9",42.7150425370287,0.0643272652479762,0.291742059951918,0
.220493628030796,0.825486734371917,0.973128061592402
"14344","FBXO22",161.601153088822,0.0439359652219794,0.199524728858615
,0.220203107019946,0.825712976964631,0.973128061592402
"14345","PIGL",76.8096605923036,-0.0510561208040336,0.231965286077881,
-0.220102419923694,0.825791390195824,0.973128061592402
"14346","OGFR",269.415107333308,-0.0309893968433343,0.140739493413005,
-0.220189771128385,0.825723362608191,0.973128061592402
"14347","C21orf91",121.521716826327,-0.0441280722811848,0.200553421787
711,-0.220031510247156,0.825846614371266,0.973128061592402
"14348","LINC01697",312.029747334462,-0.0870879303044359,0.39491314788
1338,-0.220524261528522,0.825462879447821,0.973128061592402
"14349","TMEM47",398.770700698665,0.0448107191247813,0.203478825343739
,0.220223008704134,0.825697478110213,0.973128061592402
"14350","BNIP1",78.1539679069132,0.0663272772886391,0.301979090603064,
0.219641953210009,0.826150015212153,0.973218508045612
"14351","RRM2B",108.709101114984,0.0512512105920331,0.233344709260388,
0.219637337201599,0.826153610478984,0.973218508045612
"14352","ZBTB26",22.5408232279541,0.0733846981201608,0.33410348361888,
0.219646611658418,0.826146386893807,0.973218508045612
"14353","NRIP2",2.29094450547798,0.198688474981253,0.903893213125965,0
.219814102037698,0.826015936393885,0.973218508045612
"14354","GBP3",209.058857545365,0.0600662185843072,0.273697350985856,0
.219462184664737,0.826290034089883,0.973243601281207
"14355","CEBPB-
AS1",16.2063939712305,0.0998325863707305,0.454761691224197,0.219527256
357029,0.826239350139426,0.973243601281207
"14356","GTPBP2",101.351298874741,0.0507003100467164,0.231408341073115
,0.219094565959043,0.826576383681949,0.973385061703758
"14357","AC011825.4",3.86536252383825,-0.176262177800551,0.80453318765
1851,-0.219086273264871,0.826582843384348,0.973385061703758
"14358","ZNF814",20.0374038664194,0.0826836384859775,0.377301676698131
,0.21914463569196,0.826537381459139,0.973385061703758
"14359","OGFOD3",255.110990469937,0.0527112737590899,0.240707200770604
,0.218985030735845,0.826661708520347,0.973410137729788
"14360","NSL1",331.040465365296,0.0355181269646105,0.162267938588438,0
.218885673125456,0.826739107056732,0.973433483434208
"14361","SLC29A1",488.534670658459,-0.048833609251091,0.22338034368918
,-0.218611935341276,0.826952354624516,0.973608075044885
"14362","AC107952.2",2.27482715035826,0.200675967603244,0.918225869231
945,0.218547499398052,0.827002553453669,0.973608075044885
"14363","FAM171B",162.420582068283,0.0629533595212317,0.28819160413553
2,0.218442725665338,0.827084178951637,0.9736234616592

"14364","HSBP1",1365.32457197804,-0.0583355636837338,0.267125148278699
,-0.218382896779418,0.827130790352067,0.9736234616592
"14365","AC079866.2",27.9419186477754,-0.108115728584097,0.49534658841
8913,-0.218262790360967,0.827224364522172,0.973665823553142
"14366","PSMB8",818.417716423726,0.0676194590517522,0.310217528973766,
0.21797433328621,0.827449109668614,0.97386256064854
"14367","CACNA1D",2.50925755504761,0.217009501428769,0.998300234649128
,0.217378994711988,0.827912999617178,0.973949788555413
"14368","SCFD2",63.1587447183699,-0.0598964326964205,0.275881736240354
,-0.217109089977009,0.828123330141939,0.973949788555413
"14369","SPARCL1",1021.82763732138,-0.178575039580822,0.82295619901585
9,-0.216992155590264,0.82821445823573,0.973949788555413
"14370","NAA15",231.062836793496,0.030959138321515,0.142661519904829,0
.217011134762676,0.828199667426656,0.973949788555413
"14371","ROPN1L",4.21399222989021,0.153641806532449,0.707694341704173,
0.217101928725995,0.828128910907334,0.973949788555413
"14372","GAS1RR",107.748349595923,0.0632309861292806,0.291352970930289
,0.217025369356572,0.828188574192596,0.973949788555413
"14373","ANTKMT",290.309174846046,-0.0612309554291314,0.28202689707563
6,-0.217110339701784,0.828122356231878,0.973949788555413
"14374","DHX38",229.992975123737,0.0442271338041295,0.203707663488592,
0.217110800088315,0.828121997452889,0.973949788555413
"14375","NAPA",347.691796405019,-0.0475994848460658,0.219267239688128,
-0.217084343806983,0.828142614877621,0.973949788555413
"14376","RCAN1",135.407688590409,-0.0635579754928513,0.292691825834248
,-0.217149813841553,0.828091594185173,0.973949788555413
"14377","MEI1",3.46758812203578,0.183888587412799,0.847135126707374,0.
21707113967465,0.82815290492455,0.973949788555413
"14378","MAMLD1",8.90750879651951,0.116455777579768,0.535949388771697,
0.217288759012608,0.827983316840695,0.973949788555413
"14379","MRPS21",1199.51013940253,-0.0547712920605261,0.25279395947111
,-0.216663769083397,0.828470385364238,0.97397978554711
"14380","NIT1",218.225790677386,-0.0460769896540758,0.212597064309219,
-0.21673389425104,0.828415731976095,0.97397978554711
"14381","SYNE1",1037.72752569979,0.052342909645272,0.24143728864196,0.
216797123342841,0.828366453888437,0.97397978554711
"14382","DHX33",97.1604523870693,0.050472048939143,0.232813385367298,0
.216791868987755,0.828370548885473,0.97397978554711
"14383","RRP15",243.518259965956,-0.0512341118352045,0.237044526150505
,-0.216137080519104,0.828880896673578,0.97439464652415
"14384","ZFP69",14.1962599683864,0.0905489458104444,0.419557070540404,
0.215820330935704,0.829127799969794,0.974508000783943
"14385","TIPARP",891.500359124762,0.0916184497814415,0.424569206929844
,0.215791555972595,0.829150230617329,0.974508000783943
"14386","UTP4",94.48358698681,-0.0410063088161639,0.190026639558996,-0
.2157924221116,0.829149555442971,0.974508000783943
"14387","PLEKHM2",318.75273680995,0.0393299489773645,0.18517486184292,
0.212393564579664,0.831800005976795,0.974645683953525
"14388","CDCA8",5.12788520697172,0.145504112886893,0.676232168493085,0
.215168871973562,0.829635659083374,0.974645683953525

"14389","TUFT1",39.4963720162408,0.0804413571801504,0.37864301586315,0.212446430569378,0.831758766014902,0.974645683953525
"14390","MSTO1",35.5306819471713,0.0735471901124286,0.349341660514369,0.210530831061311,0.833253391125863,0.974645683953525
"14391","ETV3",89.4097084926969,0.0563548984716851,0.267781035371055,0.210451417493386,0.833315365725928,0.974645683953525
"14392","B4GALT3",109.525769428548,0.0719430752702094,0.334956086341718,0.214783603593977,0.829936037008217,0.974645683953525
"14393","RASAL2",87.1787698867039,-0.0737591628511257,0.347039815071123,-0.212538042172508,0.831687302280694,0.974645683953525
"14394","AL512306.3",3.02541165048369,-0.220426418988106,1.04417100309477,-0.211101839004142,0.832807805250202,0.974645683953525
"14395","RAB7B",119.95833886177,-0.0449398230380724,0.215336762616468,-0.20869554502458,0.834685918124382,0.974645683953525
"14396","HLX-AS1",10.7939367358464,-0.163358611193101,0.768378940359988,-0.21260162481362,0.831637703999751,0.974645683953525
"14397","SPRTN",93.7367913440815,-0.0507899837853921,0.240183076656889,-0.211463623883658,0.832525514314628,0.974645683953525
"14398","ARID4B",925.571065829349,0.0530867570403397,0.248816623618535,0.213356954484392,0.831048552809922,0.974645683953525
"14399","PEX13",130.812197040157,-0.0387659066356263,0.184319129441742,-0.210319497238506,0.833418319000117,0.974645683953525
"14400","FAM136A",254.454269096705,-0.0347608854098145,0.16441103557624,-0.211426716509521,0.83255431114975,0.974645683953525
"14401","UXS1",276.537683186828,-0.0438185650761873,0.206843962371396,-0.211843577998711,0.832229069667391,0.974645683953525
"14402","ERICH2",21.938902867224,-0.0921132759377904,0.435912457476411,-0.21131140979786,0.832644280210554,0.974645683953525
"14403","ANKZF1",90.9786968256971,0.0552533155411901,0.264527695941139,0.208875351764621,0.834545546190287,0.974645683953525
"14404","LINC01985",28.2779161169041,0.0832213697765724,0.392218229240697,0.212181289833678,0.831965602968697,0.974645683953525
"14405","MYL5",132.132500073481,-0.0731315771655848,0.349152100521089,-0.20945478218931,0.834093231020112,0.974645683953525
"14406","UBE2D3",1831.18801617367,-0.032830652563346,0.154706539357623,-0.21221244234191,0.831941300215227,0.974645683953525
"14407","TBCA",1800.81314163619,-0.0468459944425549,0.222497347148127,-0.210546305576251,0.833241314885977,0.974645683953525
"14408","TXNDC15",546.066889679491,-0.0431570509881471,0.202573461266056,-0.213043953133947,0.831292679895383,0.974645683953525
"14409","LARS",388.443592175705,0.0368934989195039,0.173435924870854,0.2127212049463,0.83154442612665,0.974645683953525
"14410","AC091979.2",2.77425043533675,0.198348432263217,0.935749288551742,0.211967494594841,0.832132393646639,0.974645683953525
"14411","VARS2",42.526478121198,0.0577103579238769,0.271910379937581,0.212240363671018,0.831919518311841,0.974645683953525
"14412","NFKBIL1",313.341837100693,-0.0571130141313005,0.273564382261208,-0.208773575197254,0.834625000704786,0.974645683953525
"14413","GLO1",672.089446903886,0.0444607817844517,0.207910711114132,0

.213845556807533,0.830667497397813,0.974645683953525
"14414","KIAA1586",146.207018735742,0.0404502482172238,0.189350470828658,0.213626340828203,0.830838456535722,0.974645683953525
"14415","FAXC",7.80698539003236,-0.196248390224457,0.9121239060202,-0.215155406989312,0.829646156758654,0.974645683953525
"14416","Z99289.3",6.72703768500536,0.153658026041194,0.716107895933589,0.214573846921308,0.830099586120374,0.974645683953525
"14417","TMEM184A",6.54655681657835,0.149469758628368,0.704427342831474,0.21218619656012,0.831961775113347,0.974645683953525
"14418","SKAP2",499.969860402792,-0.0733338520126483,0.345970912520497,-0.211965368644405,0.832134052228156,0.974645683953525
"14419","SUMF2",613.407281168145,-0.0421142388422679,0.198600394733106,-0.212055161818102,0.832063999835652,0.974645683953525
"14420","AC008267.5",42.6274501333373,0.088608248838363,0.418050434587487,0.211955882609709,0.832141452862207,0.974645683953525
"14421","EPHB4",53.1737349499674,0.0514508779754784,0.245213796518369,0.20982048606561,0.833807783384891,0.974645683953525
"14422","DUS4L",38.6883037662128,-0.0615526858084998,0.287375008473584,-0.214189417985377,0.830399347827412,0.974645683953525
"14423","AC007938.3",18.160998295793,0.108325923428056,0.518253183978781,0.209021240538083,0.834431657285134,0.974645683953525
"14424","AC016831.5",197.237377500743,0.102744912711333,0.488718025168301,0.210233524077509,0.833485415756832,0.974645683953525
"14425","MCPH1-
AS1",5.13363854051114,0.135318934677261,0.640105735250292,0.211400909608081,0.832574447017721,0.974645683953525
"14426","MAK16",84.0756069655529,-0.0487129042172404,0.226290582678235,-0.215267041344384,0.829559124437683,0.974645683953525
"14427","LETM2",11.9906462747532,0.106422992687312,0.495876994862954,0.214615708713657,0.830066945526592,0.974645683953525
"14428","LY6K",27.3403208164854,0.137675692786163,0.642958027448615,0.214128585239828,0.830446784937247,0.974645683953525
"14429","IFT74",203.940441018608,-0.0359434734247092,0.170681618211946,-0.210587840689886,0.833208901269949,0.974645683953525
"14430","CHMP5",960.736926342125,-0.0474090034289365,0.226152023416764,-0.209633337401403,0.833953858248393,0.974645683953525
"14431","GRHPR",809.816665731419,-0.0513038608002459,0.24172623038537,-0.212239526999015,0.831920171012015,0.974645683953525
"14432","AK8",8.22246641081996,0.128792830625439,0.608465808912562,0.211668147558883,0.832365939587086,0.974645683953525
"14433","DPH7",109.67714417302,-0.0503849776906953,0.236306308695912,-0.213218927453743,0.831156205726216,0.974645683953525
"14434","DNAJC1",621.990037188043,-0.0353116543993418,0.168868387463009,-0.209107547776382,0.834364282680919,0.974645683953525
"14435","FRA10AC1",427.548997571954,0.0358801432241955,0.171346455419051,0.209401140726522,0.834135102333184,0.974645683953525
"14436","EXOSC1",195.012785388228,-0.0530424777969802,0.250798882259671,-0.211494075727424,0.832501754551186,0.974645683953525
"14437","ANKRD2",6.4963820790609,0.165558667109347,0.768442877824566,0.215446940673115,0.8294188758129,0.974645683953525

"14438","C11orf21",3.28149173623317,0.204706635951682,0.980100456307696,0.208862912606803,0.834555257050567,0.974645683953525
"14439","CRY2",232.511100316132,0.0442964618665038,0.206207843360735,0.214814631415415,0.829911844969882,0.974645683953525
"14440","CCDC84",121.483852185702,0.0507319284909145,0.242153891417985,0.209502842154808,0.834055716897192,0.974645683953525
"14441","PHETA1",49.1089069755269,0.0519549384629399,0.246330428507868,0.210915633840504,0.832953104498728,0.974645683953525
"14442","TBX3",319.359135917961,-0.0596481643442208,0.280057480411554,-0.212985435191968,0.831338323085729,0.974645683953525
"14443","ZCCHC8",165.569806544268,0.0413557764792636,0.197450928639123,0.209448376689322,0.834098230983848,0.974645683953525
"14444","NEK3",73.1849438479158,0.0611845236287754,0.290556809698269,0.210576801460316,0.83321751615428,0.974645683953525
"14445","CCDC168",5.35532831429486,-0.136294599340883,0.642368414234717,-0.212175126174685,0.83197041139301,0.974645683953525
"14446","AP4S1",70.5505266888886,-0.044578171392997,0.208424342511617,-0.21388178969792,0.830639241361982,0.974645683953525
"14447","TRIM9",16.0856774267982,0.127284295835719,0.604771280443518,0.210466832588963,0.833303335655464,0.974645683953525
"14448","AL163051.1",8.52388861067941,-0.112887988985716,0.53226622845672,-0.21208933227462,0.832037342009958,0.974645683953525
"14449","SORD",65.6309226669813,0.0554397612441709,0.259719532624656,0.21346011477809,0.830968095955274,0.974645683953525
"14450","USP3",225.009272951146,0.0412683416584843,0.197591251885704,0.208857129375119,0.834559771846709,0.974645683953525
"14451","UNC45A",212.718931644363,-0.0324612754025683,0.155370183578899,-0.208928602997267,0.834503974908727,0.974645683953525
"14452","AC015722.2",2.91509752241516,-0.238949309201289,1.13169842444746,-0.211142212482935,0.832776301849579,0.974645683953525
"14453","BMERB1",429.395315031188,0.038599113998346,0.182903341190582,0.21103558714177,0.832859502119375,0.974645683953525
"14454","NOMO3",6.35484439743156,0.141969209130444,0.670777658070029,0.211648684809985,0.832381124636163,0.974645683953525
"14455","ARHGAP17",136.338175904671,0.0381927631483057,0.180845549072491,0.211189953770974,0.832739049698481,0.974645683953525
"14456","LCMT1",250.906571925998,-0.0446556604387053,0.211232707161528,-0.211405047252259,0.832571218608146,0.974645683953525
"14457","AC010542.2",13.9719466308036,0.156352537610014,0.731348297812315,0.213786697908113,0.830713398678732,0.974645683953525
"14458","AARS",174.38368586839,-0.0376834582067157,0.175992714862537,-0.214119421000746,0.830453931224501,0.974645683953525
"14459","AC092718.4",4.68830559602527,-0.148999169095951,0.693569814620232,-0.21482937399393,0.829900350406139,0.974645683953525
"14460","FGF11",10.557240682634,-0.118877874910684,0.562482805959811,-0.211344904503939,0.832618145449976,0.974645683953525
"14461","TMEM99",182.473486290259,0.0581065684142912,0.273302878848144,0.212608695009675,0.831632188863376,0.974645683953525
"14462","GHDC",147.633898264397,0.0414107447946139,0.195294545197464,0.21204250611681 5,0.832073873135233,0.974645683953525

"14463","AC005837.1",2.3781433150564,0.214760166150525,1.0287058172515
1,0.208767329346228,0.83462987674522,0.974645683953525
"14464","PARD6G-
AS1",18.3866151515893,0.078599351803179,0.368530566442916,0.2132777005
76714,0.831110365780711,0.974645683953525
"14465","PIAS4",100.651681576148,0.0516986683775874,0.245162125139416,
0.210875429262118,0.832984477605823,0.974645683953525
"14466","CLPP",350.942408093307,-0.0508499233983351,0.243308268871286,
-0.208993815270765,0.834453066711883,0.974645683953525
"14467","SHFL",315.947855447567,-0.0298677265410018,0.142216676967022,
-0.210015640767135,0.833655465690497,0.974645683953525
"14468","ZNF799",34.9001686519545,-0.058072787239623,0.278245522990239
,-0.20871058989747,0.834674172656857,0.974645683953525
"14469","CC2D1A",93.2139041395788,-0.0612696980639151,0.29270071040105
7,-0.209325416327018,0.834194211887971,0.974645683953525
"14470","SLC25A42",55.0791563375402,0.0466614024093311,0.2227425170429
86,0.209485836062121,0.834068991284091,0.974645683953525
"14471","TIMM50",182.50911155187,-0.0356971172530973,0.170810200348075
,-0.208987034617101,0.834458360021339,0.974645683953525
"14472","LIN7B",29.585931555904,-0.0787790253153045,0.375876009366411,
-0.209587798508601,0.833989403518552,0.974645683953525
"14473","IRF3",297.895598776868,-0.0330109850843836,0.155810945311672,
-0.211865636386142,0.832211860187784,0.974645683953525
"14474","ZNF805",73.2603071902848,-0.0402672794068926,0.19037059606302
2,-0.211520477634908,0.832481154836119,0.974645683953525
"14475","LIME1",28.916007248098,-0.0629271069960835,0.297518881331582,
-0.211506263785497,0.832492244976885,0.974645683953525
"14476","CCT8",747.380272851803,0.0559271372528948,0.267677374290947,0
.208934869452604,0.834499082947608,0.974645683953525
"14477","LZTR1",148.283143293186,0.0417953340464658,0.19661321191224,0
.212576426782151,0.831657359898332,0.974645683953525
"14478","AP000350.5",4.7342860244556,-0.153755056941858,0.716509977159
974,-0.214588856879978,0.83008788247971,0.974645683953525
"14479","ZXDA",24.1706612823264,-0.0636207572888918,0.30366372067932,-
0.209510563680663,0.834049689748096,0.974645683953525
"14480","BRCC3",115.92772064262,0.0372543718808939,0.174498517648026,0
.213493916068893,0.830941734012185,0.974645683953525
"14481","ATG4B",160.261545487705,0.0433647454719942,0.208827699052513,
0.207658015046603,0.835496002139533,0.974793318654292
"14482","GOLGB1",1019.9137500023,-0.0527244706519448,0.253926878803493
,-0.207636430221107,0.835512857024221,0.974793318654292
"14483","TRAPPC3L",3.83297857934089,0.175930487464351,0.84462231390189
3,0.208294860991307,0.834998743986882,0.974793318654292
"14484","MTMR9",151.937716804424,-0.0439291641464008,0.211706944155655
,-0.207499873570998,0.83561949139906,0.974793318654292
"14485","AP003117.1",15.0125049278575,-0.108421749437521,0.52242902586
183,-0.207533931061089,0.835592896298838,0.974793318654292
"14486","PGAP2",75.8434654042414,-0.0420628577261227,0.202512498925372
,-0.207704995737687,0.835459316714697,0.974793318654292
"14487","ARHGEF40",232.138896029824,-0.0491466621703197,0.236368483487

698,-0.207923922196157,0.835288370138445,0.974793318654292
"14488","SPTLC2",130.143684859294,0.0345503043182702,0.16629475678794,
0.207765446040665,0.83541211391175,0.974793318654292
"14489","DCTPP1",252.905370229148,-0.049987782670703,0.239809792932825
,-0.20844762867839,0.834879470657963,0.974793318654292
"14490","FBRS",162.845033403521,0.0337856709963581,0.162577807272092,0
.207812318072503,0.835375514151164,0.974793318654292
"14491","COG8",91.4713985671442,0.0440016642571677,0.21165029135362,0.
207897962132501,0.835308640392852,0.974793318654292
"14492","TMEM170A",191.143099816574,-0.0432733614108469,0.208221161937
633,-0.207824031948339,0.83536636749324,0.974793318654292
"14493","STAT5A",163.324298944798,-0.0427517706619625,0.20589543543676
2,-0.20763826342859,0.835511425529484,0.974793318654292
"14494","ERGIC3",940.441697329076,-0.0327795675411389,0.15752738950617
2,-0.208088051505827,0.835160216481129,0.974793318654292
"14495","NTRK1",2.38533842146614,0.215942385221239,1.04233314652189,0.
207172136798879,0.835875426857113,0.974855483803379
"14496","NUDT6",56.2167526621546,-0.0528513048853124,0.254881863494916
,-0.207356083169749,0.835731777732313,0.974855483803379
"14497","AC007686.3",16.8898436093054,-0.0723187190284682,0.3491358882
27511,-0.207136308431697,0.835903406918701,0.974855483803379
"14498","AL022311.1",47.0444072992958,-0.0727258881233592,0.3511003719
01566,-0.207137029589216,0.835902843730737,0.974855483803379
"14499","AL353708.1",4.1082029367832,-0.170792458098707,0.829596120799
683,-0.205874224597474,0.836889159944029,0.974905494679695
"14500","TATDN3",111.245760943683,-0.0468989908003775,0.22851264621584
5,-0.205235865835094,0.83738784893735,0.974905494679695
"14501","AL356108.1",2.40597527109778,0.198645028884156,0.965782825219
043,0.205682917211851,0.837038603343012,0.974905494679695
"14502","POLR2D",105.35911330122,0.037815078416396,0.184186614108684,0
.205308505177701,0.837331099428806,0.974905494679695
"14503","AGAP1",191.789436864857,0.0677511627686747,0.330829423398367,
0.204791829193174,0.837734771081567,0.974905494679695
"14504","LINC02158",2.18133658974342,-0.228889061318052,1.115392815225
04,-0.205209373947663,0.837408545943035,0.974905494679695
"14505","STIMATE",92.9038330586172,-0.0526487807374979,0.2566625322499
92,-0.205128423989122,0.837471789463336,0.974905494679695
"14506","UGDH",548.589031288212,0.033931438567553,0.16557537115537,0.2
0493046961503,0.837626449079755,0.974905494679695
"14507","FST",2033.23447343256,-0.063709512097757,0.310367797319939,-0
.205271012804472,0.837360390251152,0.974905494679695
"14508","RFESD",23.9013223562818,0.0714844644700863,0.34809062557949,0
.205361647849842,0.837289582236063,0.974905494679695
"14509","PCDHB14",96.5807168994434,0.0457290415743152,0.22299540623532
8,0.205067191052613,0.837519629425314,0.974905494679695
"14510","C2",642.336665510708,0.0638565268170387,0.308983171053309,0.2
06666682199406,0.836270179377337,0.974905494679695
"14511","ZNF394",179.932489456566,-0.043873498840838,0.212623503773332
,-0.206343598248712,0.836522524744904,0.974905494679695
"14512","SLC1A1",7.40087922387888,-0.108473615454014,0.527052629921659

,-0.205811733583679,0.836937975339707,0.974905494679695
"14513","MUSK",6.9986228515052,0.151326947685245,0.732014072471574,0.206726828589926,0.836223203777922,0.974905494679695
"14514","TLR4",158.917364307472,0.0658125007799428,0.32066959522832,0.205234614566697,0.83738882649858,0.974905494679695
"14515","DENND10",401.26505835935,0.0397707511918272,0.194023239456358,0.204979317442913,0.837588284215611,0.974905494679695
"14516","FMNL3",49.3758923161176,-0.0656985123163392,0.318649139535421,-0.206178219756455,0.836651700459271,0.974905494679695
"14517","AP1G2",31.1307792227334,-0.0659616397289262,0.32059469621608 4,-0.205747757238215,0.836987951665457,0.974905494679695
"14518","IFI27",3353.08502699501,0.135456641568588,0.656589942344373,0.206303253877048,0.836554036979375,0.974905494679695
"14519","AC021752.1",2.47103938947214,0.188324310733145,0.910287644453508,0.20688439734476,0.836100142027269,0.974905494679695
"14520","PDP2",35.5418245242479,-0.0728139847546531,0.354121782485032,-0.20561848594482,0.837088936375438,0.974905494679695
"14521","CD300LG",2.31086335199717,-0.213816663291604,1.0394523336014,-0.205701268234966,0.837024267837579,0.974905494679695
"14522","GATA6-AS1",9.71423308296233,0.118799642033381,0.574736749662222,0.206702707114519,0.836242043088768,0.974905494679695
"14523","RPL17",24199.711661578,-0.0510909297639156,0.249565692712161,-0.204719363501785,0.837791390921219,0.974905494679695
"14524","AC008440.2",10.7355156298456,-0.112818555050876,0.549019698871301,-0.205490905486293,0.837188602863538,0.974905494679695
"14525","TTYH1",2.22257032324731,0.20136591612372 4,0.977752310527657,0.205947778343836,0.836831703603808,0.974905494679695
"14526","ZFP28",33.0360016724343,-0.0715659477908538,0.347169207084099,-0.206141404048883,0.83668045749055,0.974905494679695
"14527","STK35",74.1490625382191,-0.0577363032498466,0.281019753141176,-0.205452828865171,0.837218349015511,0.974905494679695
"14528","KIF3B",189.799807025521,-0.0502188897621442,0.244106082476834,-0.205725679805255,0.83700519802021,0.974905494679695
"14529","BTG3-AS1",7.97426636565452,0.106513357130009,0.520063923842361,0.204808201928451,0.837721978638741,0.974905494679695
"14530","TSPAN6",234.839996134712,0.0490086673471163,0.237697107902489,0.206181168040216,0.836649397541675,0.974905494679695
"14531","RNF220",161.342119534556,-0.0429681584128059,0.210714209197594,-0.203916758041282,0.838418549200602,0.974926346266497
"14532","SEC22B",493.406691380248,-0.0373557339675879,0.184193142961767,-0.202807408391646,0.839285567212145,0.974926346266497
"14533","BABAM2",169.218397730207,-0.048086398985436,0.236200095475996,-0.203583317307857,0.838679130979564,0.974926346266497
"14534","PKDCC",752.894655011132,0.0573981893825255,0.283424243197734,0.202516865653166,0.839512674686098,0.974926346266497
"14535","AC015982.2",12.9112156095083,-0.0859443481965791,0.423809462611925,-0.202790064353228,0.839299124087487,0.974926346266497
"14536","EIF2AK3-

DT",12.8853957937163,-0.0927033251560264,0.458288301384538,-0.20228167482338,0.839696525225029,0.974926346266497
"14537","INPP5D",9.97810856950283,-0.135244881487654,0.667914764121349,-0.202488234656065,0.839535055295932,0.974926346266497
"14538","AC018809.1",2.32268173351818,0.211639021917883,1.04305484278655,0.202903062462643,0.839210800573349,0.974926346266497
"14539","THRB-IT1",20.4499097641188,0.0938411500855232,0.460397194009896,0.203826503085738,0.838489081117613,0.974926346266497
"14540","AC006059.5",4.21890704438273,0.145907323536708,0.718612298444439,0.203040393063895,0.839103460607117,0.974926346266497
"14541","CHRD",115.412640174553,-0.0461593788433462,0.226107071989809,-0.204148319807647,0.838237595598933,0.974926346266497
"14542","FANCE",18.1712674739638,-0.0728295437561093,0.359784698281847,-0.202425350783141,0.839584211546375,0.974926346266497
"14543","CYB5R4",84.7283532956011,0.0458497431358247,0.225427816898026,0.203389908870764,0.838830286531548,0.974926346266497
"14544","TMEM242",321.425965072895,-0.0463337215060505,0.22913098743853,-0.202214995117064,0.839748650872741,0.974926346266497
"14545","AC007036.3",28.3390441483961,0.0750995211127016,0.369709058101766,0.203131406891388,0.839032324278945,0.974926346266497
"14546","TYW1",98.5813440863986,-0.0446961946826567,0.219623273710972,-0.203513015389606,0.838734073733421,0.974926346266497
"14547","CALD1",7221.07013065625,0.0364328499869789,0.179593244616217,0.202863142568833,0.839242003211957,0.974926346266497
"14548","AC124242.1",10.084411004019,-0.111720881366713,0.54942270143136,-0.203342310166029,0.838867487519152,0.974926346266497
"14549","APTX",147.068129203175,-0.0421595283719279,0.20806731188339,-0.202624467968116,0.839428564021181,0.974926346266497
"14550","RBM18",140.051267722752,0.0372938943713191,0.183179046691839,0.203592578107792,0.838671893485287,0.974926346266497
"14551","KIF11",6.04766133735319,0.12100879710358,0.597870114009167,0.202399809370175,0.839604177417931,0.974926346266497
"14552","AL356215.1",7.39437471723585,-0.12734726532585,0.626424345858506,-0.203292330778308,0.83890654952807,0.974926346266497
"14553","C11orf49",119.987954617096,-0.0489021794621755,0.240603368600605,-0.203248108065152,0.838941112667701,0.974926346266497
"14554","RITA1",53.2270624658027,0.059758160932814,0.295665299961679,0.202114218139765,0.8398274327757,0.974926346266497
"14555","VPS37B",77.1513644708972,-0.0435304027083549,0.215328495316456,-0.202158114950744,0.839793116463791,0.974926346266497
"14556","ABCC4",12.5164149863441,0.0903524921893855,0.445381131745028,0.202865558842557,0.839240114569594,0.974926346266497
"14557","SUSD6",85.1062997241782,-0.0657817486026838,0.323621894642715,-0.20326729956052,0.838926113135237,0.974926346266497
"14558","MFAP1",220.790383369962,0.0366448199464095,0.179833441502415,0.203770887329192,0.838532544041289,0.974926346266497
"14559","DAPK2",88.4607187146024,-0.0497179674307268,0.245426728351569,-0.202577640034002,0.839465168293418,0.974926346266497
"14560","PHB",937.81003364297,-0.0469354257763098,0.230109018614134,-0

.203970387857832,0.838376639498701,0.974926346266497
"14561","INTS2",53.0268492029257,0.0703124824107271,0.344594062083839,
0.204044381918631,0.838318816651891,0.974926346266497
"14562","AC061992.2",3.43416528173599,0.159211584834271,0.778698663991
396,0.204458530875341,0.837995195023514,0.974926346266497
"14563","SEH1L",94.213306955853,-0.045772726671005,0.225394453323545,-
0.203078318902994,0.839073817644568,0.974926346266497
"14564","TBC1D22A",138.243861266086,-0.0363197664650886,0.178702546631
252,-0.203241459899469,0.838946308700472,0.974926346266497
"14565","TUBGCP6",73.9787780708753,-0.0441734565042875,0.2172627543368
95,-0.2033181280386,0.838886387310509,0.974926346266497
"14566","GJD3",9.41609044369992,-0.138268085179131,0.685031688646481,-
0.201841881873126,0.840040338328906,0.975071806966157
"14567","CAPNS1",1110.56769144578,-0.0407041601909672,0.20169905123280
1,-0.201806403858521,0.840068074998581,0.975071806966157
"14568","VSIG8",2.11267792308952,0.199434420571692,0.990889198824811,0
.20126813452828,0.840488917664663,0.975174333366624
"14569","CPSF3",83.8988186325135,-0.0418132090887453,0.20748973014887,
-0.201519415244046,0.840292449654038,0.975174333366624
"14570","MCCC1",87.6912723286021,0.0389108337601412,0.1937350417136,0.
200845615826503,0.840819293339998,0.975174333366624
"14571","AC105916.1",2.11694151721335,-0.226337871842684,1.12537888317
62,-0.201121484707338,0.840603582860913,0.975174333366624
"14572","DDR1",17.9691659944371,-0.075822536569437,0.377394740320167,-
0.200910422082492,0.840768618233208,0.975174333366624
"14573","RPS3",20903.218085158,0.0524460120157081,0.261174585248055,0.
2008082523263,0.840848509945115,0.975174333366624
"14574","PPP1R12A-
AS1",6.89697517258406,0.114266340598148,0.567557367690997,0.2013300277
69738,0.840440524480727,0.975174333366624
"14575","SLC41A2",162.217760298155,0.0402900389240727,0.20050959207877
5,0.200938212014534,0.840746888160178,0.975174333366624
"14576","RAMAC",142.813858735526,0.0610437390381649,0.303836138353474,
0.200910067409916,0.84076889556684,0.975174333366624
"14577","OSBPL7",15.4651141742109,0.0749758980560495,0.37244049005794,
0.201309739562381,0.840456387389435,0.975174333366624
"14578","AC016590.3",4.67863835013503,-0.13672886210554,0.678311399392
515,-0.201572407935342,0.840251017706042,0.975174333366624
"14579","TMEM189",159.525949957496,-0.0407052490200625,0.2026039162409
59,-0.200910474858004,0.840768576965783,0.975174333366624
"14580","AC087627.1",2.29890763904184,-0.203607601474546,1.01513065529
041,-0.200572803523797,0.841032625539706,0.975320962457157
"14581","TRIM13",197.023571844537,0.0463052767872214,0.230959186262651
,0.200491167017544,0.841096465456577,0.975328100812002
"14582","ZNF563",36.0587664185008,0.0621161194339078,0.31035738024665,
0.200143845087693,0.841368083599198,0.975576159477112
"14583","OSBPL6",20.9839373517911,-0.0718646281899733,0.35990786994784
3,-0.199675067400965,0.841734714412626,0.975580318228843
"14584","ACOX2",153.081332309298,0.0575853737553749,0.288186816272981,
0.199819597926465,0.841621673492017,0.975580318228843

"14585","NCR3",3.3307203119411,0.177823136475937,0.890795556050404,0.199622837437994,0.841775565568997,0.975580318228843
"14586","C9orf85",177.59226449044,0.0478541703180085,0.239558881347863,0.199759533225235,0.841668651193237,0.975580318228843
"14587","AP001372.3",2.44589581526328,-0.246077700041319,1.23228921375878,-0.199691515022454,0.841721850154019,0.975580318228843
"14588","TP53INP2",94.9526217684596,-0.0881797007217978,0.440847929261464,-0.200022944123889,0.841462636898934,0.975580318228843
"14589","ACSS2",90.1661892874689,-0.0495841972319844,0.247947258091353,-0.199978808451738,0.841497154757158,0.975580318228843
"14590","DYRK1B",75.3952070649578,-0.0485391053871184,0.243291708989671,-0.199509903517424,0.841863897190037,0.975615817250798
"14591","JUN",51393.1221594654,0.0269610394543714,0.135555582668064,0.198892874227032,0.842346543703971,0.975640180911546
"14592","RNPC3",207.270081421472,0.0458373817624545,0.230169784130693,0.199145956258218,0.842148573219534,0.975640180911546
"14593","ORMDL1",516.394872665538,-0.0349055854028646,0.175494177746865,-0.198898823032254,0.842341890205648,0.975640180911546
"14594","PRKAR2A",360.569890810292,-0.0429251474007234,0.215634014464978,-0.199064825218913,0.84221203594833,0.975640180911546
"14595","AHR",478.946266635343,0.0544381385990896,0.273338740424915,0.199159981912785,0.842137602105559,0.975640180911546
"14596","AC084824.5",6.71190300863711,-0.113302500862286,0.569608582583688,-0.198912910245062,0.842330870397784,0.975640180911546
"14597","SIRT6",83.9385753487538,-0.0535197086265041,0.268459815663747,-0.19935836018579,0.84198243041034,0.975640180911546
"14598","ZNF773",26.0976157635933,0.0755359577766333,0.379621549248196,0.198977002033275,0.842280734596663,0.975640180911546
"14599","EPRS",534.450221030819,0.0307623466166772,0.156167765321246,0.196982690719799,0.843841083204428,0.975782994700526
"14600","MCEE",221.844020857033,-0.0462725233449169,0.235699131825633,-0.196320296076222,0.844359476515278,0.975782994700526
"14601","CASP10",20.3993571899349,0.078426281267263,0.398716904741117,0.196696654530323,0.844064928250789,0.975782994700526
"14602","GRK4",87.52454369172,-0.0453447689165567,0.231169395960563,-0.196153858204883,0.844489742219825,0.975782994700526
"14603","TXK",2.12189048606538,0.254943556144918,1.2843057348914,0.198506904718038,0.842648483060935,0.975782994700526
"14604","EXOC3-AS1",37.297218965703,-0.0538871610372931,0.271946709036559,-0.1981533853754,0.842925057338677,0.975782994700526
"14605","AC113370.1",10.1853157991234,0.106593686733361,0.54209558151126,0.196632642598188,0.844115024175454,0.975782994700526
"14606","TNXB",7068.253397449,-0.033015901246454,0.168188726013575,-0.196302701310605,0.844373247185646,0.975782994700526
"14607","ATF6B",238.487653388669,-0.0346978621450627,0.175322897210084,-0.197908331981791,0.843116785138018,0.975782994700526
"14608","AL109615.4",5.77151961155305,-0.128359691484737,0.650130443851611,-0.19743682625339,0.843485713566918,0.975782994700526
"14609","SLC2A12",22.5095859317988,0.0838586308485283,0.42349429339947

5,0.198015964218497,0.843032573398027,0.975782994700526
"14610","YAE1",123.313952007965,0.0466122556904383,0.237253035334993,0
.196466425074914,0.844245109437628,0.975782994700526
"14611","ENTPD4",128.869948023565,0.0593690718123901,0.299260446509576
,0.19838596281213,0.842743099238069,0.975782994700526
"14612","MYC",4158.81451986021,-0.0752897061317515,0.381231716545586,-
0.197490667392435,0.843443583969351,0.975782994700526
"14613","ITGB1",3892.76968512278,0.0298667968434855,0.15124564062807,
0.19747211701771,0.843458099210149,0.975782994700526
"14614","AC125494.2",2.52005909564608,0.199599407729068,1.004764947099
02,0.198652837467465,0.842534318884206,0.975782994700526
"14615","M6PR",367.381017104227,-0.0277863838230406,0.140138997775486,
-0.198277312269327,0.842828101479866,0.975782994700526
"14616","CCT2",543.392707393676,-0.0377497308591672,0.191989201955729,
-0.196624239668812,0.8441216003793,0.975782994700526
"14617","TESC",2.35165045461692,0.215078845472817,1.09569865478741,0.1
9629379349247,0.844380218971972,0.975782994700526
"14618","DHRS4-
AS1",227.507575166263,0.0320812095161775,0.162373643946045,0.197576458
448132,0.843376455136734,0.975782994700526
"14619","PPP1R36",3.86510840149117,0.175075485100144,0.88687646892824,
0.197406844395947,0.843509173955387,0.975782994700526
"14620","DMXL2",96.4805024288491,-0.0538596412773294,0.27273287051987,
-0.197481297999265,0.843450915296978,0.975782994700526
"14621","PPIB",4755.39146082771,0.0528300743312359,0.268584466617087,0
.196698174680943,0.844063738584149,0.975782994700526
"14622","BFAR",472.618253508799,-0.0372681160518336,0.188338925694416,
-0.19787792626736,0.843140574972515,0.975782994700526
"14623","GOT2",82.4249038399232,0.0517312333645944,0.263719094463346,0
.196160363245083,0.84448465084889,0.975782994700526
"14624","MPDU1",145.295467316746,-0.0523017948972587,0.26546583622295,
-0.197018929597153,0.843812724429875,0.975782994700526
"14625","MRPL10",291.193586443806,0.0513985089095575,0.261946643328968
,0.19621747488861,0.844439950933366,0.975782994700526
"14626","HEATR6",72.1232115911868,-0.0437837965399603,0.22137668791869
4,-0.197779616957866,0.843217494460415,0.975782994700526
"14627","LIMD2",63.194510188817,0.0657651753093859,0.335261584361936,0
.19616078422629,0.84448432135515,0.975782994700526
"14628","SAFB",339.476440289704,0.0300413468490271,0.152591158288687,0
.196874754644643,0.843925549901666,0.975782994700526
"14629","IL12RB1",2.54088406408598,-0.185089242993491,0.94029337805155
5,-0.19684201475185,0.843951171260497,0.975782994700526
"14630","ZNF583",54.7572836957553,-0.047437138049479,0.240807476375874
,-0.196991965379991,0.843833825289305,0.975782994700526
"14631","DNTTIP1",173.642135708545,0.0486599821901751,0.24636936857028
1,0.197508247362716,0.84342982809675,0.975782994700526
"14632","TRMT2B",30.8951858503916,-0.0590069522828742,0.29929465728726
6,-0.197153376601169,0.843707514543802,0.975782994700526
"14633","RENBP",53.4384084689302,0.0802958177206027,0.405465188532791,
0.198033813978358,0.843018607873288,0.975782994700526

"14634","SPRY4",97.9737330840024,0.0910120548653334,0.464790002610278,
0.195813279877378,0.844756315452171,0.975853533047471
"14635","DUOX2",2.49545439542871,0.193085268894877,0.98631770480493,0.
195763766537137,0.844795071398877,0.975853533047471
"14636","AC015917.2",6.38605148792053,-0.123244702904936,0.62936962221
795,-0.19582245242567,0.844749135796171,0.975853533047471
"14637","TMEM98",973.823576620954,0.0424012699743734,0.21665694579618
,0.195707176538649,0.84483936697119,0.975853533047471
"14638","AC008764.8",7.80016830142638,0.108308864139128,0.553336777070
354,0.195737692897571,0.844815480364567,0.975853533047471
"14639","OVGP1",3.49128101663569,0.190387285478388,0.974341232121219,0
.195401035286067,0.845079006191739,0.975934850308613
"14640","DUSP11",235.643492503308,-0.0509225839322646,0.26053484473133
6,-0.195454024527031,0.845037526598389,0.975934850308613
"14641","LLGL2",3.47629094006881,-0.187145546684311,0.957775605557319,
-0.195396025539211,0.845082927807452,0.975934850308613
"14642","MRPS7",403.004506936709,-0.0431309181176113,0.220835032353776
,-0.195308315251884,0.845151587794929,0.975947483023949
"14643","LHX4",5.13854082790791,-0.122706484434195,0.629561330797621,-
0.194907911956302,0.845465040137458,0.975976165675164
"14644","MCMBP",260.654795961505,0.0283001394362351,0.145120180523097,
0.195011743606059,0.84538375405496,0.975976165675164
"14645","MAGOHB",180.252443712366,-0.0564765300254175,0.28972597532170
7,-0.19493084789069,0.845447084273707,0.975976165675164
"14646","CALCOCO2",784.497830856047,0.0276578029212624,0.1417556658455
34,0.195108976817901,0.84530763514566,0.975976165675164
"14647","GGA1",168.285193224485,0.0442667384346199,0.227019403543428,0
.194990990830226,0.845400000528845,0.975976165675164
"14648","REV1",305.496769266488,0.0377995478018742,0.194480182903064,0
.194361951113111,0.845892480388312,0.97602254849371
"14649","ATG12",541.9038315993,-0.0246049994487656,0.126751952741851,-
0.19411929296961,0.846082475353224,0.97602254849371
"14650","DNPH1",516.9583578668,0.063052781481532,0.324263248403073,0.1
94449361104144,0.845824042851528,0.97602254849371
"14651","NSMAF",138.848570438895,-0.0316761499704184,0.162737130327595
,-0.194646113684402,0.845669999935923,0.97602254849371
"14652","UQCRB",3979.97810758176,-0.0468140034884135,0.24093331946411,
-0.194302737340515,0.845938842393832,0.97602254849371
"14653","SHC3",71.5375859342128,0.0670417368686116,0.344711596385798,0
.194486456421905,0.845794999473128,0.97602254849371
"14654","FAM86C1",11.8078126730241,0.0888537716232675,0.45690108834336
8,0.194470474879877,0.845807512023165,0.97602254849371
"14655","EAPP",697.373483507716,-0.0428155712459967,0.220524807132289,
-0.194153083286963,0.846056017883963,0.97602254849371
"14656","HNRNPA1P48",155.652418442706,-0.0628393546784415,0.3229199004
29368,-0.194597343164319,0.845708183145241,0.97602254849371
"14657","HSD17B1",16.6447587143616,0.0792689431843216,0.40818831107962
9,0.194196994457438,0.846021636150655,0.97602254849371
"14658","ALKBH8",47.2581169322764,0.0489082852736056,0.252308545930823
,0.193843157762143,0.846298692868097,0.976205369014449

"14659","CDC42-
IT1",9.17753077281621,-0.115044919381119,0.60006644614472,-0.191720300
510476,0.847961303456222,0.976211637268267
"14660","FRRS1",44.1758753507263,-0.0478455582856033,0.249736775583576
,-0.191583951437667,0.848068114538427,0.976211637268267
"14661","UBE2Q1",393.258119480586,0.0639887574255476,0.334164414974884
,0.191488843688988,0.84814262026556,0.976211637268267
"14662","EEF1AKNMT",119.851713353329,0.0351928963981407,0.182510809324
52,0.192826367536208,0.847094952694705,0.976211637268267
"14663","C1orf131",215.451010165375,-0.0424285790614622,0.220192151147
205,-0.192688880327516,0.847202632470192,0.976211637268267
"14664","LBH",79.8379948718783,0.0745764655975054,0.388066394446792,0.
19217450071609,0.847605519095456,0.976211637268267
"14665","ANKMY1",26.4033571541845,0.0589800436966432,0.307930863523961
,0.191536642419261,0.848105175416538,0.976211637268267
"14666","ARF4",1293.22524577339,-0.0489478430396536,0.255456595103614,
-0.191609236080987,0.848048307224732,0.976211637268267
"14667","NEK11",112.238084668024,0.0357999086824463,0.184807972048033,
0.193714092989136,0.8463997562601,0.976211637268267
"14668","NUDT9",249.079155823825,-0.0359198735054946,0.186524593718903
,-0.192574463181121,0.847292245982457,0.976211637268267
"14669","AC093297.2",35.0142662282574,0.0599774178788817,0.31050234858
0589,0.193162525671895,0.846831686173994,0.976211637268267
"14670","NDUFS4",1509.43222427354,-0.0458569969761802,0.23778006170312
1,-0.192854676913301,0.847072781187404,0.976211637268267
"14671","FEM1C",308.891606478784,0.0754347967910083,0.390405649232739,
0.193221581038235,0.846785437977018,0.976211637268267
"14672","HARS2",48.9286590268183,0.0530534142173099,0.275795908291128,
0.192364761848849,0.847456492862307,0.976211637268267
"14673","STC2",46.0392340707099,-0.178651601325472,0.927823978856994,-
0.192549023733528,0.84731217087156,0.976211637268267
"14674","SERAC1",21.0013481366054,-0.0636730740202356,0.33223811170616
6,-0.191648916174158,0.848017223093899,0.976211637268267
"14675","AL356417.2",7.68049306305443,0.098577750772682,0.511491648152
363,0.192726022269904,0.847173542675348,0.976211637268267
"14676","WDR27",123.132263574933,0.0409058731440786,0.211628076258098,
0.193291333869097,0.846730812924017,0.976211637268267
"14677","DNAAF5",97.0580409623427,-0.043548642394887,0.226489139558159
,-0.192276956324894,0.847525267800249,0.976211637268267
"14678","PENK",8.86374408169297,0.167966674434704,0.868183189464429,0.
193469162353075,0.846591554663503,0.976211637268267
"14679","TMEM273",27.3159015399583,-0.0858472195588344,0.4451733094603
11,-0.192839996770938,0.847084278454009,0.976211637268267
"14680","TMTC2",25.0163747868351,0.0677585271581219,0.349943913402688,
0.193626820078881,0.846468096218126,0.976211637268267
"14681","AL354696.2",5.04940739263096,-0.133404871047801,0.69584397184
3757,-0.191716644026278,0.847964167781293,0.976211637268267
"14682","FKBP3",623.712289112807,-0.04011265498716,0.209015226973018,-
0.191912596838403,0.847810670002382,0.976211637268267
"14683","AC012378.2",3.23455894840966,0.163470135130964,0.848099909509

875,0.192748676539106,0.847155799816361,0.976211637268267
"14684","ADAMTS7",72.897257729196,-0.0584850582339163,0.305000119837675,-0.191754213949302,0.847934737291032,0.976211637268267
"14685","COA3",561.223863417999,-0.0528896825381209,0.273181586647446,-0.193606323131791,0.84648414673273,0.976211637268267
"14686","NEURL2",27.5825358118965,-0.063398695587658,0.32980181124883,-0.192232708933866,0.8475599256449,0.976211637268267
"14687","DGCR6",36.8655924795651,0.0914604829059759,0.477652990426722,0.191478928718247,0.84815038755628,0.976211637268267
"14688","POLA1",38.7437503736799,0.0558800382545228,0.291307152379869,0.191825150182561,0.847879169798896,0.976211637268267
"14689","SLC25A43",171.338194720209,0.029764453227498,0.155446418800182,0.191477252787396,0.84815170046551,0.976211637268267
"14690","TMEM255A",24.6798729226089,0.0928523677053562,0.484178175287578,0.19177313733773,0.84791991368913,0.976211637268267
"14691","ZBTB25",121.369510647701,0.0410464076791576,0.214592044008619,0.191276465391741,0.848308998562156,0.976326223380909
"14692","AC010883.1",5.15233314164732,-0.137179784201283,0.717988104240478,-0.191061360753877,0.8484775195782,0.97635622095781
"14693","AP000941.1",4.15252859791233,0.1399284421866,0.732524983827556,0.191022074708568,0.848508298483207,0.97635622095781
"14694","AC004846.2",3.13454832631939,0.16223421483826,0.849179225248371,0.191048261679752,0.848487782106446,0.97635622095781
"14695","EHD2",820.019269422356,-0.0429271211263044,0.224896681332295,-0.190874853608345,0.848623641855826,0.976422493126799
"14696","PTPRF",143.506303437614,-0.0780106826953115,0.409880176111498,-0.190325581089071,0.849054009196349,0.976524477537057
"14697","AC007383.2",6.79005394051658,-0.122054661255711,0.640331659785128,-0.190611629755536,0.84882987802697,0.976524477537057
"14698","ZMYND19",65.4439037643546,-0.0514695201329839,0.270244266276232,-0.190455549130408,0.848952172252201,0.976524477537057
"14699","PPHLN1",388.203052161808,-0.030011933115562,0.157692407534621,-0.19031945535471,0.84905880910056,0.976524477537057
"14700","FXR2",98.3394914516472,0.0390431947195858,0.204875123428758,0.190570695290697,0.848861951154181,0.976524477537057
"14701","CYB561",221.194425187759,-0.0387305490874058,0.20349345127708,-0.190328233387076,0.849051930953157,0.976524477537057
"14702","ABCB10",63.3232684539405,0.0479935437120238,0.252312157058494,0.190214947513993,0.849140698532167,0.976552233082702
"14703","PABPC4",632.345078821149,0.0379665131867748,0.199894547741658,0.189932710099939,0.849361860036732,0.976562986864577
"14704","EXO5",28.6305149487333,-0.0549865738364711,0.295831423844156,-0.18587130847005,0.852545682619244,0.976562986864577
"14705","TMEM59",4506.56396608836,-0.0460565260540606,0.249202900624545,-0.184815369077307,0.853373852741765,0.976562986864577
"14706","C1orf52",289.881838976522,0.0396701897734304,0.211683319509746,0.187403475461863,0.85134429785369,0.976562986864577
"14707","LRRC8C-
DT",19.7640521822894,-0.0835584317476834,0.447667059969983,-0.186653071488633,0.851932652866899,0.976562986864577

"14708","MAGI3",215.011339530312,-0.0503616612112096,0.269812246865058
,-0.186654467305916,0.851931558398307,0.976562986864577
"14709","GOLPH3L",168.703454163918,-0.0363134047660092,0.1948954967555
26,-0.186322441362307,0.852191909934579,0.976562986864577
"14710","S100A11",8575.28371898284,-0.0614900322456217,0.3289620444111
41,-0.186921358528435,0.851722292653324,0.976562986864577
"14711","NMNAT2",7.96771615156827,-0.114003383622387,0.604634359513472
,-0.188549297320982,0.85044607475851,0.976562986864577
"14712","GEMIN6",152.047970924191,-0.050215356814563,0.270204568309971
,-0.185841997893083,0.852568668628533,0.976562986864577
"14713","FBXO11",346.810596020004,0.0321076189709827,0.173035256949428
,0.185555357544079,0.852793464990656,0.976562986864577
"14714","PCBP1-
AS1",146.472478194333,-0.0488098465855091,0.264896632870242,-0.1842599
73623064,0.853809512666148,0.976562986864577
"14715","ATIC",201.166637359381,-0.0393562908443339,0.208957013407325,
-0.188346350297493,0.850605153185401,0.976562986864577
"14716","ARMC9",42.6307250819627,0.0614418228644114,0.324682466305696,
0.189236651931067,0.849907342515439,0.976562986864577
"14717","CRBN",627.791240749598,0.0362501710035593,0.196114914946324,0
.184841479361633,0.853353372570835,0.976562986864577
"14718","AC097359.2",5.31418393186044,-0.145017904501397,0.77406304095
9088,-0.187346374685084,0.851389064872739,0.976562986864577
"14719","CMSS1",274.460140164761,-0.0354857772424461,0.192412579261377
,-0.184425453775772,0.853679703062105,0.976562986864577
"14720","CHCHD6",118.171551898245,-0.0532900944437284,0.28607696640581
7,-0.186278871428374,0.8522260756273,0.976562986864577
"14721","CPA3",11.5514981935453,-0.15505782716808,0.836883033473689,-0
.18528016576519,0.853009294114983,0.976562986864577
"14722","RGMB-
AS1",4.34167816485398,0.151246235000286,0.818510605766479,0.1847822544
20093,0.853399827087473,0.976562986864577
"14723","GNPDA1",232.707567401584,0.04313012852388,0.229214759143718,0
.188164709310177,0.850747536200455,0.976562986864577
"14724","RBM27",457.174521803101,0.0325073854380818,0.175103965868224,
0.185646197542696,0.8527222228487,0.976562986864577
"14725","AC008393.1",22.7084676774756,0.0684022453379459,0.36438449313
7448,0.187719967853144,0.851096176453144,0.976562986864577
"14726","LINC00847",70.4998694356952,-0.0489024731077387,0.26061404648
383,-0.187643274671969,0.851156300462598,0.976562986864577
"14727","AC008443.1",64.3266250184811,-0.0509541616854328,0.2721798903
29059,-0.187207664841919,0.851497815408622,0.976562986864577
"14728","AL031963.3",29.1758413433102,-0.0845192656779669,0.4464871121
66448,-0.189298332191184,0.849859002412639,0.976562986864577
"14729","TFAP2A-
AS2",2.99964606395293,-0.163177800264986,0.884011052616559,-0.18458796
3897058,0.853552227131379,0.976562986864577
"14730","SIRT5",65.9683888770537,0.0438283258752171,0.232105542949833,
0.188829294286566,0.850226611334605,0.976562986864577
"14731","AL662797.1",6.19682493083779,0.119416055754405,0.633400066751

222,0.188531801657211,0.850459788357173,0.976562986864577
"14732","FAM162B",57.0892675759434,0.104266270314726,0.557049294363217
,0.187176020811437,0.851522625184764,0.976562986864577
"14733","MED23",116.972215740935,-0.0369095793406092,0.199596420511988
,-0.184921048413252,0.853290961459566,0.976562986864577
"14734","COA1",230.171271985971,0.0357425127795867,0.190151896209638,0
.187968216420947,0.85090156666308,0.976562986864577
"14735","LINC02848",3.2073742719391,-0.166942809291298,0.8944418600012
72,-0.186644673909896,0.851939237464107,0.976562986864577
"14736","FAM66D",21.2977196464168,0.0880753700792316,0.468900606236906
,0.187833773101868,0.851006959844905,0.976562986864577
"14737","TUSC3",379.668127294235,0.0474553310157552,0.253126096060077,
0.18747703912951,0.851286624631448,0.976562986864577
"14738","RBPMS",817.178142619246,0.0579066560755542,0.313526958423083,
0.184694344520809,0.853468782282455,0.976562986864577
"14739","E2F5",13.8881762350091,0.0822309122106542,0.442516139670498,0
.185825792188922,0.852581377557542,0.976562986864577
"14740","CDC37L1-
DT",9.88261908795572,0.0957263529789932,0.5165605503101,0.185314873390
016,0.852982072795785,0.976562986864577
"14741","SLC25A51",41.2949734542641,-0.052241975457414,0.2767454099333
45,-0.188772689924637,0.850270977262631,0.976562986864577
"14742","NIPSNAP3B",30.8934015496312,-0.0710239885447476,0.37519770510
313,-0.189297502566615,0.849859652602926,0.976562986864577
"14743","ENG",480.285255224885,0.0336522372579719,0.18102896247194,0.1
85894217137703,0.85252771721745,0.976562986864577
"14744","AC069544.1",6.85917242535349,0.113621547821409,0.606367278156
503,0.187380737573513,0.85136212430477,0.976562986864577
"14745","MKX",13.7050903881262,0.240245206078234,1.30273458929437,0.18
4416079877301,0.853687056237204,0.976562986864577
"14746","SMC3",767.028380591423,0.0303709893002535,0.161312646860083,0
.188274074546654,0.850661807432503,0.976562986864577
"14747","AL158163.1",7.60135875429518,-0.116939712108077,0.62828747998
4873,-0.186124530304014,0.852347105616032,0.976562986864577
"14748","FANCF",48.6985535138937,-0.0497879399770206,0.266880711729497
,-0.186555032974749,0.852009526159138,0.976562986864577
"14749","LIN7C",230.910132624631,0.0360494162206925,0.194760927614383,
0.185095730762119,0.85315395010955,0.976562986864577
"14750","TSKU",372.067993524627,0.0714978015661859,0.386376799175093,0
.185046829206185,0.85319230538859,0.976562986864577
"14751","BIRC2",492.33500199779,0.0243109436778334,0.131048195102652,0
.18551147277374,0.852827882473793,0.976562986864577
"14752","PARP11",70.1662700852595,-0.0401616704356168,0.21296600364886
1,-0.18858254250681,0.850420016361358,0.976562986864577
"14753","EEF1AKMT3",92.3642184405068,0.0341442413190772,0.183693403077
635,0.18587625220622,0.852541805643232,0.976562986864577
"14754","CFAP54",3.40669283552502,-0.17777542125726,0.936468226193744,
-0.189836041720096,0.849437612197241,0.976562986864577
"14755","WSB2",174.856675189069,0.0367256499260827,0.199344442665745,0
.184232123228352,0.853831360078423,0.976562986864577

"14756","CLIP1",672.35088488785,0.0489894072167688,0.260041977292665,0
.188390381148477,0.8505706394327,0.976562986864577
"14757","MYCBP2-
AS1",8.81052051349968,-0.110552482778926,0.598773003701033,-0.18463170
8670227,0.853517913579086,0.976562986864577
"14758","MIR17HG",38.0102488035823,-0.119128669421077,0.64463287436241
9,-0.184800797723669,0.853385282143733,0.976562986864577
"14759","CMA1",7.02307412803397,0.212601898249622,1.1414174616357 6,0.1
8626129825011,0.852239855846222,0.976562986864577
"14760","DMAC2L",148.117024105805,-0.0410257351578105,0.22276949552470
4,-0.184162266297635,0.853886160267679,0.976562986864577
"14761","PSMA3",793.331943878414,-0.0464277663909131,0.248290593840079
,-0.186989630468307,0.851668763238402,0.976562986864577
"14762","MOAP1",138.991147242441,-0.0370069569436839,0.199835105471478
,-0.185187466718483,0.853081999180025,0.976562986864577
"14763","IGHG2",82.8856539537743,-0.211988274518288,1.14177390788562,-
0.185665719854166,0.85270691244513,0.976562986864577
"14764","AC048382.6",19.7146082699912,-0.0733067589802658,0.3856320831
77136,-0.190095072941826,0.849234630910788,0.976562986864577
"14765","GDPGP1",15.7516657128208,0.0735632160728809,0.399097260417398
,0.18432403168076,0.85375926235697,0.976562986864577
"14766","IQCK",134.626889403874,0.0422858637678014,0.226396538838894,0
.18677787206761 3,0.851834797121529,0.976562986864577
"14767","SRCAP",139.211989076849,0.0467685585380643,0.251321447782717,
0.186090598119181,0.852373714751718,0.976562986864577
"14768","AC009120.2",56.2267216340073,0.05604739446565,0.2961636837797
14,0.189244656030609,0.849901069504281,0.976562986864577
"14769","AC099811.4",2.38074023923348,-0.18446259501981,0.985433345856
077,-0.187189317060873,0.851512200547526,0.976562986864577
"14770","AP001029.2",2.84688035808936,0.21165156491247,1.1307119502356
4,0.187184335381228,0.851516106322833,0.976562986864577
"14771","GALNT1",582.00502011723,-0.0243496953781576,0.130878575472715
,-0.186047986006954,0.852407130800733,0.976562986864577
"14772","EGLN2",333.407333176168,-0.0375361839903374,0.199927624386245
,-0.187748862147336,0.851073524848722,0.976562986864577
"14773","HNRNPUL1",784.415622180954,0.0351681114013279,0.1882806476419
27,0.186785587588432,0.851828747480532,0.976562986864577
"14774","ZNF180",49.0744780904933,-0.0491749655172727,0.26591426165194
9,-0.184927898232239,0.853285588750066,0.976562986864577
"14775","SLC1A5",248.193373542634,-0.0526570589296256,0.28083704121401
9,-0.187500404868234,0.851268306278892,0.976562986864577
"14776","HSPBP1",88.3448008810766,-0.0385990068277343,0.20731122239453
7,-0.186188699202574,0.852296785739849,0.976562986864577
"14777","CU638689.5",8.45801351477663,-0.0934666995412441,0.4980559092
13811,-0.187663067162044,0.851140783956488,0.976562986864577
"14778","TCP10L",8.33473904118285,0.0966185858313287,0.508619256501201
,0.189962500625653,0.849338515592654,0.976562986864577
"14779","IGLC1",87.6235033400033,0.270807212041824,1.44792185154472,0.
187031649362092,0.851635818171775,0.976562986864577
"14780","C22orf23",5.37744071650405,0.111725309889584,0.5989433489885,

0.186537357962597,0.852023385518979,0.976562986864577
"14781","ATXN10",641.820116327083,-0.0405085511584422,0.21667933511732
6,-0.186951612789968,0.851698571360548,0.976562986864577
"14782","Z83745.1",8.41109042012127,-0.0938556946945367,0.498440669986
009,-0.188298628796023,0.850642560191964,0.976562986864577
"14783","PSMD10",165.998334083497,0.0320510023341292,0.17198461929464З
,0.186359701615059,0.85216269224601В,0.976562986864577
"14784","PDZD4",4.63048831291903,-0.129633987500095,0.697473874257929,
-0.185862140912472,0.852552872009252,0.976562986864577
"14785","AC083799.1",26.8522096856413,-0.0579210007947122,0.3149046665
82266,-0.183931859198348,0.854066911146985,0.976657317830632
"14786","PCDHB4",105.510538826112,-0.0479173832489574,0.26054819738488
1,-0.183909862858018,0.85408416734349,0.976657317830632
"14787","SLC25A24",273.894326928024,-0.0323548714403526,0.176760593201
723,-0.183043464916575,0.854763914631441,0.976681707219973
"14788","AL133243.2",19.4164714006892,-0.0735046172879492,0.4002010148
39627,-0.183669242611503,0.854272939222232,0.976681707219973
"14789","MLH1",124.598775752067,0.0435288257693401,0.237857293597888,0
.183003956325712,0.854794914344442,0.976681707219973
"14790","PHYKPL",490.689219101832,-0.0301259994419738,0.1639076924596,
-0.183798569730944,0.854171478213244,0.976681707219973
"14791","SERPINB9",136.157549210766,0.0754710584895764,0.4123539879300
79,0.183024926879994,0.854778460144157,0.976681707219973
"14792","AP003469.4",14.6377263843743,0.0839073691336141,0.45829473181
4881,0.183086043344497,0.854730506476389,0.976681707219973
"14793","STX2",304.768967085512,0.0440866903595215,0.240521699965145,0
.183296103286773,0.854565691730608,0.976681707219973
"14794","AQP9",8.25630161389867,-0.154660765155867,0.843618329822441,-
0.18333025692841З,0.854538895104094,0.976681707219973
"14795","SEMA7A",2.31998326467797,0.195338263435584,1.06684346065804,0
.183099274297559,0.854720125175463,0.976681707219973
"14796","TMEM101",243.657605273189,0.0475095263666289,0.25944101094631
7,0.183122653559423,0.854701781346548,0.976681707219973
"14797","ZNF708",168.283932301029,0.0421063425811731,0.230090346672499
,0.18299917050021,0.854798669472508,0.976681707219973
"14798","MMGT1",190.632792633122,-0.0301897260605964,0.16454952502169,
-0.183468934696815,0.854430091553319,0.976681707219973
"14799","ICMT",176.779948178943,0.0446714770665615,0.244944723153232,0
.182373706571405,0.855289458877142,0.976701411066147
"14800","SLC4A5",12.0512415022118,0.0869767102909649,0.477990943428927
,0.181963092578756,0.855611690160886,0.976701411066147
"14801","FARSB",107.265838485584,-0.0378518361707815,0.207354454681221
,-0.182546529945419,0.855153842124521,0.976701411066147
"14802","OSBPL11",169.640824574312,-0.0409198979014218,0.2246716749319
53,-0.182131983988704,0.855479148905704,0.976701411066147
"14803","AC006230.1",2.30916570973593,-0.182015425903957,1.00484959766
785,-0.181136984406817,0.85626005537222,0.976701411066147
"14804","RAPGEF6",107.946211861133,-0.0391214753514595,0.2150114419196
14,-0.181950667379301,0.855621441270829,0.976701411066147
"14805","AL359643.2",2.37397331870195,0.208040365744758,1.141302496129

58,0.18228328287222,0.855360417235231,0.976701411066147
"14806","HIST1H2AH",2.0670666902459,-0.18231653286846,1.00557645879716
,-0.181305490272259,0.856127796833095,0.976701411066147
"14807","CCHCR1",99.0371214977279,0.040771729348479,0.223401274023277,
0.182504462101818,0.855186852915514,0.976701411066147
"14808","SNX14",244.52817728877,-0.0302023039748023,0.165423320337177,
-0.182575853956032,0.855130831615898,0.976701411066147
"14809","AL513550.1",20.9656913007007,-0.0574633471605426,0.3170281639
45093,-0.181256284758646,0.856166417322517,0.976701411066147
"14810","ERICD",3.26138214979222,0.13917809106881,0.765032300843566,0.
181924463732243,0.855642005571806,0.976701411066147
"14811","GTF3C5",142.310748078121,0.0338263059372127,0.186567498453669
,0.181308674970592,0.856125297234672,0.976701411066147
"14812","LHPP",65.4020017543992,0.0551866120454204,0.303109890225844,0
.1820680018204,0.855529359842517,0.976701411066147
"14813","ALDH3B1",19.7779172756078,0.0613089269809378,0.33616135295836
3,0.182379462842451,0.85528494178744,0.976701411066147
"14814","LINC02156",3.67153742666805,0.151721514778605,0.8372720089892
1,0.181209347917614,0.856203257494162,0.976701411066147
"14815","RNF6",160.068563331989,-0.0320766768744538,0.176602055102212,
-0.181632523222274,0.855871123532265,0.976701411066147
"14816","AC099508.1",2.39742305044249,-0.179071253298782,0.98584924804
774,-0.181641618790493,0.855863985051893,0.976701411066147
"14817","ZNF594",27.1554253440306,0.0736706556115978,0.405314335569904
,0.181761781280228,0.85576967895886,0.976701411066147
"14818","RPL38",11580.2077071331,0.0421684056696156,0.230622066550663,
0.182846361149713,0.854918570825523,0.976701411066147
"14819","AC068473.5",16.9857525206579,0.0866726283303721,0.47716766657
2049,0.181639776544426,0.855865430902049,0.976701411066147
"14820","ZNF700",34.8857259391806,-0.0500527274344727,0.27437860511346
3,-0.182422122212388,0.855251466061872,0.976701411066147
"14821","C19orf47",24.229163993138,-0.0581669106305391,0.3207114672612
92,-0.181368353078404,0.856078457490888,0.976701411066147
"14822","LIF",8.46356776468595,-0.138200938382717,0.761793373036797,-0
.181415254154543,0.856041646451828,0.976701411066147
"14823","BX890604.2",7.97556163781366,0.105070333135712,0.579798323918
528,0.181218759698375,0.856195870273589,0.976701411066147
"14824","CCN5",15311.0271269256,-0.0498921822996926,0.275783792940939,
-0.180910494295715,0.856437831514746,0.976838292987812
"14825","UBL3",1115.17709602626,0.0340592876557895,0.188427613075102,0
.180755288993733,0.856559659141288,0.976911346830414
"14826","MRPS14",293.234519168309,0.0498164422132546,0.277067630554071
,0.179798853130671,0.857310484013786,0.97707781671896
"14827","C2orf81",43.8093607055088,0.0629335868961799,0.35019113921373
5,0.179712105330481,0.857378589494572,0.97707781671896
"14828","SCRG1",12.6679441701362,0.0818224181447802,0.455549921430348,
0.179612407544429,0.857456863288143,0.97707781671896
"14829","BHMT",3.01949384057099,-0.174114074690442,0.964894684231446,-
0.180448786314049,0.856800256925424,0.97707781671896
"14830","F12",2.1895231934212,-0.184754417939938,1.02758164472238,-0.1

79795366031332,0.85731322170626,0.97707781671896
"14831","PRR3",49.4612798968236,0.0437464310588537,0.242869311724319,0.180123337725394,0.857055741392716,0.97707781671896
"14832","RPS18",49830.7355275301,-0.049412555799734,0.274093928497877,-0.180275995424381,0.856935899796414,0.97707781671896
"14833","PTCD1",44.7352044463121,-0.045468056023741,0.2529686117736,-0.179737935489141,0.857358310190637,0.97707781671896
"14834","TIMM23",115.513267053385,0.0379025230015625,0.210783830467234,0.179817033012191,0.857296211168931,0.97707781671896
"14835","KIAA1549L",24.7726609932574,-0.10672761961528,0.59337029297359,-0.179866806409248,0.857257134806808,0.97707781671896
"14836","VPS37C",59.1531961636286,0.0439069173071562,0.243998813267365,0.179947257608359,0.857193974493156,0.97707781671896
"14837","ALG9",101.9754295926,0.0340941046253819,0.189341273981348,0.180066944245555,0.857100013066894,0.97707781671896
"14838","NCOA5",67.5804003679655,0.0409700709747941,0.228084566094374,0.179626669512754,0.857445665978995,0.97707781671896
"14839","NDUFAF8",695.188068027998,-0.0459287966634578,0.255819024302401,-0.179536282685395,0.857516630669427,0.977080072198846
"14840","SSBP3",391.382360135616,0.0325396842540032,0.181409438742159,0.179371506133441,0.85764600333522,0.977123015788492
"14841","AC005014.2",3.15758727586563,0.142224273652081,0.79483239549372,0.178936181336365,0.857987812643104,0.977123015788492
"14842","VSTM5",3.80110300649407,-0.146527693134561,0.817480872318798,-0.17924296224685,0.857746930940506,0.977123015788492
"14843","ADPRHL1",9.8542236768886,-0.0998645734395906,0.557795721221918,-0.179034312455509,0.857910759502424,0.977123015788492
"14844","SNX29",458.178255049773,0.047906245639658,0.267783059023844,0.17889946367142,0.858016643920732,0.977123015788492
"14845","ZNF764",34.6377716829555,0.0589553238984379,0.329120775608198,0.17912975499493,0.857835818749185,0.977123015788492
"14846","CDC42EP1",317.480193073414,-0.0374072367042301,0.208671076386719,-0.179264119167648,0.857730319198287,0.977123015788492
"14847","RANGAP1",78.8862769106928,-0.0371072804274484,0.207175291703261,-0.179110549923093,0.857850898322013,0.977123015788492
"14848","SNHG12",171.952243864818,0.0435587125857262,0.245520135286129,0.177414013457442,0.859183200771272,0.977135236338244
"14849","IGKC",1342.25466782967,0.144801355119189,0.81599786142626,0.177453106146743,0.859152496468865,0.977135236338244
"14850","NFE2L2",1491.66240116732,-0.0289952119319819,0.163259454296164,-0.177602038773096,0.859035523291113,0.977135236338244
"14851","SATB2-
AS1",12.1273714255507,0.0859825647285038,0.4816356030494,0.178522028239023,0.858313023024344,0.977135236338244
"14852","EPM2AIP1",274.563541091572,0.0332061309510407,0.18708576888897,0.177491485045811,0.859122353001017,0.977135236338244
"14853","MYL3",54.9402835707796,0.0573253382573984,0.323002822624256,0.177476276497076,0.859134298040305,0.977135236338244
"14854","AKIRIN2",367.995739945857,-0.039257890985102,0.220959571170125,-0.177670017991101,0.85898213276008,0.977135236338244

"14855","AC005532.1",2.19687276239489,-0.209090828255344,1.17340395738
513,-0.178191684917522,0.858572439696197,0.977135236338244
"14856","KLF14",18.5877903759434,0.107554400299403,0.606156416567149,0
.17743670999726,0.859165374357988,0.977135236338244
"14857","EFCAB1",6.40002023567611,0.12074607610221,0.675844476601646,0
.178659558940777,0.858205025480089,0.977135236338244
"14858","AL162274.1",3.69241370599756,-0.158682554622078,0.89076562166
6001,-0.178141758912175,0.85861164762427,0.977135236338244
"14859","EIF1AD",81.7648247448559,-0.0375447746823288,0.21150355823739
6,-0.177513678706945,0.859104921799733,0.977135236338244
"14860","EXPH5",11.9909411606749,0.0926695239297869,0.5218865209108,0.
177566425298855,0.859063494189427,0.977135236338244
"14861","SUDS3",267.656324555556,-0.0321135878542374,0.180453466970903
,-0.177960492493146,0.858754002835514,0.977135236338244
"14862","POSTN",844.058605442988,0.196105882261076,1.10071897059064,0.
178161626628318,0.858596045052706,0.977135236338244
"14863","AC005520.5",2.26914987746495,-0.16898883206385,0.949800985433
583,-0.177920253458894,0.85878560465776,0.977135236338244
"14864","CAPN15",71.3657981937282,0.0379962830470562,0.21250616230863,
0.17880085280479,0.858094075633485,0.977135236338244
"14865","COX4I1",4130.27057503253,-0.0446762859353541,0.25146265528439
,-0.177665689105318,0.858985532625669,0.977135236338244
"14866","ZNF100",78.5491485442192,0.0561959208379209,0.316339446124764
,0.177644367549905,0.859002278410003,0.977135236338244
"14867","AJ011932.1",10.5960210510738,-0.0802673562288453,0.4506312149
11231,-0.178122050965016,0.858627124780157,0.977135236338244
"14868","DARS2",34.2608258559086,0.0548930810660683,0.311223929930057,
0.176378085960179,0.859996919638891,0.977203611787315
"14869","CCDC138",24.1124319533051,0.0585794482856519,0.33173887774105
7,0.176583006141887,0.859835943443411,0.977203611787315
"14870","SLC40A1",752.148153397158,0.0378805190707264,0.21614999757422
,0.175251073309493,0.860882354552352,0.977203611787315
"14871","AC013468.1",2.06129940637328,-0.204472892041254,1.16173407871
327,-0.176006623019725,0.860288739258861,0.977203611787315
"14872","ACY1",81.573893247928,0.0652919349607599,0.369382467599623,0.
176759702172791,0.859697143564019,0.977203611787315
"14873","LINC00960",9.60285676632664,-0.107454559058426,0.606278574523
777,-0.177236279779192,0.85932279959755,0.977203611787315
"14874","ZNF330",274.091309087188,0.0314701230579208,0.179135736336504
,0.175677526447345,0.860547291991044,0.977203611787315
"14875","MAST4-
AS1",3.77370836876835,0.146687001451078,0.828540651243913,0.1770426124
90832,0.859474918296837,0.977203611787315
"14876","NCOA7",1043.67696122699,-0.0286005096367545,0.161868868514375
,-0.176689377637891,0.859752385015629,0.977203611787315
"14877","PTPRK",105.423068404502,0.0504688910080365,0.285958136764619,
0.176490487660363,0.859908621133444,0.977203611787315
"14878","LSMEM1",46.8756057951838,-0.086463796784622,0.489017145542192
,-0.176811380894951,0.859656549241534,0.977203611787315
"14879","CASC19",3.16797957967829,0.151677906698675,0.861095123971175,

0.176145355462205,0.860179749439934,0.977203611787315
"14880","KIFC2",19.35876845381,-0.0723934851819425,0.411915002001641,-0.175748600634006,0.860491451719526,0.977203611787315
"14881","ZNF34",69.6175086625848,-0.0444766460471425,0.253060572838924,-0.175754940993722,0.860486470375152,0.977203611787315
"14882","AL137186.1",4.0365775018776,0.162462730691119,0.926531021371055,0.175345160543801,0.860808428425517,0.977203611787315
"14883","AP002761.3",4.41147874671825,0.143445684240575,0.817922449185123,0.175378098967068,0.860782548368998,0.977203611787315
"14884","AC156455.1",13.1639433624203,0.111106418465079,0.631278754341915,0.176002150715342,0.860292252797444,0.977203611787315
"14885","ACTR10",642.49500662423,-0.0424250004505239,0.241518899186622,-0.175659133067438,0.860561743080095,0.977203611787315
"14886","GABPB1-IT1",350.743464126909,0.0332673695142406,0.189877974337248,0.175203941533278,0.860919387342314,0.977203611787315
"14887","ATP6V0C",1571.03084714178,-0.0443440801016107,0.251375684220641,-0.176405606767791,0.859975300176089,0.977203611787315
"14888","NPIPA3",3.95714373764854,0.164389516589077,0.936401910436746,0.175554443831072,0.860643994946636,0.977203611787315
"14889","ATXN7L3",81.6634914861637,0.0345324616048874,0.196300370328839,0.175916436362495,0.860359592372274,0.977203611787315
"14890","EFTUD2",104.932398007764,0.0409390916509907,0.232292853754766,0.176239135166037,0.860106076523415,0.977203611787315
"14891","SRSF2",1384.37493910928,-0.0415301501675075,0.236525803600833,-0.175584014662497,0.860620761687842,0.977203611787315
"14892","TSHZ1",244.183493244764,-0.0333049847699682,0.189318982240423,-0.17591994408502,0.860356836588162,0.977203611787315
"14893","FP565260.6",15.7139664573037,-0.0715755348512116,0.407843032749073,-0.175497750614386,0.860688538102336,0.977203611787315
"14894","Z93943.1",6.83172405361435,-0.113475702487233,0.645953337536408,-0.175671671455428,0.860551892065465,0.977203611787315
"14895","TMSB15B.1",34.7600270344952,0.0598092623719022,0.339928634469492,0.175946526144357,0.860335952888735,0.977203611787315
"14896","SLC9A6",49.0927176092189,0.0535840946614859,0.304008043989357,0.176258805386616,0.860090623840435,0.977203611787315
"14897","COMMD1",584.532450751213,-0.0517262334996073,0.295690900432051,-0.1749334640465,0.861131915181071,0.97737923218645
"14898","TMEM33",193.10291436679,0.0317982373524946,0.181870404839287,0.174840086712259,0.861205288810325,0.977396900470196
"14899","AC009126.1",5.33998810545922,-0.111752567454357,0.639978567117707,-0.174619234449773,0.861378833891705,0.977425011717425
"14900","PTK7",158.645389154106,-0.0470564529272165,0.269643280305071,-0.174513723738924,0.861461746272033,0.977425011717425
"14901","TSNARE1",13.963472969577,0.0724228393428139,0.416048818962816,0.174072935775565,0.861808142576495,0.977425011717425
"14902","TRIP4",122.625020612516,0.0475658549340508,0.272731790367473,0.174405245790971,0.861546991956996,0.977425011717425
"14903","AC040169.1",13.0418374721828,-0.0852281673261813,0.488037074464926,-0.174634616477905,0.861366746520696,0.977425011717425

"14904","ACBD4",74.7758890777208,0.0502462699221621,0.287988396779305,
0.174473244353201,0.861493556170355,0.977425011717425
"14905","HLF",163.082812931542,0.0813318439934962,0.466982423454586,0.
174164679243877,0.861736043137611,0.977425011717425
"14906","AC005776.2",2.11310271665804,0.172684680716475,0.991667688570
044,0.174135633042034,0.861758869868077,0.977425011717425
"14907","ZNF567",87.2967243446725,0.0383627492081794,0.220180544621456
,0.174233147048184,0.861682236349022,0.977425011717425
"14908","NECTIN2",252.177791872059,-0.0527733847302358,0.3029485752796
82,-0.17419915139563,0.861708952429904,0.977425011717425
"14909","COMT",1464.47124404921,0.0482283970250155,0.277222407563748,0
.173970053318743,0.861888997303228,0.97745114805842
"14910","BASP1",2348.99875135806,-0.0423466992491784,0.243831911885467
,-0.17367168604686,0.862123490594781,0.977461052006053
"14911","VNN2",4.11151956648348,0.171472928122279,0.986233739797519,0.
173866418479542,0.861970444786014,0.977461052006053
"14912","NFKB2",142.031182956292,0.0430694772252259,0.248148844798932,
0.173563077676722,0.862208851279359,0.977461052006053
"14913","RPAP3",164.205958592332,-0.031178711628747,0.179484986521857,
-0.173712087194268,0.862091737747568,0.977461052006053
"14914","ANAPC7",132.431746961572,-0.032255569278228,0.185892203426987
,-0.173517601510904,0.862244593723106,0.977461052006053
"14915","FBXL12",193.008504206908,0.04070191435389,0.234466592099961,0
.173593662062258,0.862184813336746,0.977461052006053
"14916","AL512353.1",10.6642206776857,0.0932953218482923,0.53820761952
5528,0.173344483548077,0.862380660083537,0.977484226097235
"14917","B3GNT7",2.25446570661299,0.178158372448646,1.02764454241448,0
.173365755468381,0.86236394066959,0.977484226097235
"14918","PNMA1",301.701161456839,-0.0314846821780207,0.181745891298391
,-0.17323462969696,0.862467004562774,0.977516564763868
"14919","SDE2",122.485883156036,-0.034446111416178,0.199391600230985,-
0.172756080879405,0.862843160268774,0.977549728881294
"14920","PARP10",167.844684774748,-0.0312227457400309,0.18062927814397
4,-0.172855397867139,0.862765091166079,0.977549728881294
"14921","VPS13A",135.036901605251,0.0454306074045744,0.262924072419092
,0.172789836193316,0.862816626419371,0.977549728881294
"14922","NUP98",185.515231366075,0.0395392992657444,0.228487663310763,
0.173047851655639,0.862613814773395,0.977549728881294
"14923","SYN3",4.17952760463813,-0.144979000323452,0.838824878398663,-
0.172835837439897,0.862780466727845,0.977549728881294
"14924","ABCB7",202.183197976836,-0.0290909656165543,0.1682443883677,-
0.172908980197163,0.862722972800964,0.977549728881294
"14925","AL357078.2",3.36785240520487,0.147121136485186,0.853535072338
165,0.172366832076582,0.863149146054258,0.977699856735137
"14926","GNAS-
AS1",3.06744705650093,0.148653436755365,0.862413098636743,0.1723691778
22494,0.863147302018125,0.977699856735137
"14927","ZCCHC18",8.15001807956427,-0.0961638220864671,0.5576696378591
55,-0.172438690504349,0.863092657095806,0.977699856735137
"14928","ARHGAP25",10.5086188634116,0.101034055365478,0.58668094685858

4,0.172212947951472,0.863270118963279,0.977771380722878
"14929","FBXW7",202.451997107387,-0.0418550248177003,0.243177604111792
,-0.172117103343361,0.863345466894075,0.977775541651855
"14930","TXNL1",879.63435353627,-0.0439409424682766,0.255379794644372,
-0.172061155149202,0.863389450961805,0.977775541651855
"14931","SAMD13",5.6995732610198,0.107797913212923,0.627381740312604,0
.171821884964665,0.86357755968199,0.977923071401988
"14932","CENPL",67.2675012280775,-0.0422753579156975,0.246394535538338
,-0.171575874535251,0.863770975491202,0.978011093122965
"14933","MAP1LC3B2",10.9308766940206,-0.0917718111868689,0.53464962999
3025,-0.171648507805132,0.863713869653109,0.978011093122965
"14934","IKZF4",108.757421352784,-0.0436862284588493,0.254774251464296
,-0.171470343677848,0.863853947387846,0.978039543486922
"14935","ADH5",2018.16780762854,0.0344526742671147,0.201069537752991,0
.171347060584776,0.863950878588541,0.978083793449953
"14936","AL627309.1",2.07370970474977,0.184575991919494,1.081275340139
19,0.17070211912512,0.864457996466675,0.978107155382004
"14937","SELENON",343.580359317517,-0.0369888771208411,0.2165535272270
29,-0.170807086795049,0.864375456511851,0.978107155382004
"14938","Z99127.4",47.6013023879638,0.0777636050100916,0.4570540328737
34,0.170140944870679,0.864899293571899,0.978107155382004
"14939","CYB5R1",300.750651051727,0.0402620138463904,0.23566246854137,
0.170846100762636,0.86434477876572,0.978107155382004
"14940","KCNIP3",26.7947593752945,0.0566371615158286,0.332975148249254
,0.17009426022819,0.864936007415872,0.978107155382004
"14941","SAYSD1",204.565088687293,0.0458283587758131,0.26885811456742,
0.170455553664611,0.864651885808485,0.978107155382004
"14942","EIF3B",493.938283661383,-0.0305254835796167,0.178922254121569
,-0.170607528557493,0.864532377789696,0.978107155382004
"14943","ATP6V1FNB",3.18146598263586,-0.16072165973452,0.9423382739126
04,-0.170556226128013,0.864572719973587,0.978107155382004
"14944","KIN",245.348716756686,0.0402328685146321,0.236257179758327,0.
170292680864925,0.864779967016774,0.978107155382004
"14945","MASTL",35.44619939604,-0.0532866546502549,0.311936876949124,-
0.170825120682816,0.864361275948933,0.978107155382004
"14946","NUDT14",93.4691959039886,0.0699473738134709,0.409858909858526
,0.170662079391211,0.864489481577648,0.978107155382004
"14947","SETD6",54.2960443843324,-0.0450916676302317,0.264738170106807
,-0.170325524317252,0.864754139035317,0.978107155382004
"14948","CRISPLD2",775.048257212714,0.0474246832906603,0.2772771148366
45,0.171037134884356,0.864194566381515,0.978107155382004
"14949","AC123912.4",2.67861259093043,-0.193956539927422,1.13579148464
259,-0.170767735583488,0.864406399649202,0.978107155382004
"14950","ZNF492",8.13154066220486,0.0927079609340022,0.542206514355232
,0.170982750076779,0.864237329297985,0.978107155382004
"14951","BRD1",258.3468893184,-0.0365541586979081,0.214660537754276,-0
.170288209842053,0.864783483025432,0.978107155382004
"14952","VBP1",413.001941399534,-0.0398855172402029,0.234523870735897,
-0.170070181406476,0.864954943652219,0.978107155382004
"14953","LINC00571",5.34103600027815,0.112745076236973,0.6635433865089

96,0.169913646235165,0.865078049032955,0.978115531165521
"14954","MAPKBP1",53.9283726630356,0.0494892722819829,0.291154074553314,0.1699762311687,0.865028829400533,0.978115531165521
"14955","HNRNPU",2247.44146899659,0.035539532154455,0.20941459673702 6,0.169708953951687,0.865239031989927,0.978204226024237
"14956","BAG6",336.980356203662,-0.0233075196905631,0.137372324792622,-0.169666777684284,0.865272202769014,0.978204226024237
"14957","TNFRSF14",379.752627037416,-0.0267134740477639,0.167602495725194,-0.159385896565432,0.873364849780612,0.97821882027368
"14958","AL139424.2",4.98153610828158,-0.122693726855153,0.74021733895219,-0.165753651527309,0.868350822177263,0.97821882027368
"14959","AGTRAP",443.438738379333,0.0488482028583195,0.288411219796325,0.169369981142953,0.865505633939679,0.97821882027368
"14960","NIPAL3",46.160775327686,-0.0522445659432251,0.322636599223163,-0.161930066424635,0.871360925691318,0.97821882027368
"14961","XKR8",58.048899296472,0.0473824975691626,0.294981307673264,0.16062881388283,0.872385759379539,0.97821882027368
"14962","MAP7D1",248.864078213628,-0.0333373906666965,0.197359718925706,-0.168916893721591,0.865862010875015,0.97821882027368
"14963","KDM4A-AS1",21.8303507410048,0.0912304474361085,0.548181703323361,0.166423736660714,0.867823495271321,0.97821882027368
"14964","UROD",471.603076634125,0.0450753004573348,0.270499427844434,0.166637322734959,0.867655424970973,0.97821882027368
"14965","RAVER2",44.3628976912644,-0.0456100248677243,0.289068695218773,-0.157782650359997,0.874628070402953,0.97821882027368
"14966","MIER1",688.484973323406,-0.0208316378811479,0.131419396895808,-0.158512657744607,0.874052847320259,0.97821882027368
"14967","PIGK",261.039570274878,-0.0354718838284411,0.21074711627139,-0.16831491911264,0.866335537545516,0.97821882027368
"14968","GON4L",326.432952966,-0.0334524305471365,0.202664637627894,-0.165062987498379,0.868894405046115,0.97821882027368
"14969","OLFML2B",638.522248850603,0.0374634584842459,0.224408013009387,0.166943497167718,0.86741450757863,0.97821882027368
"14970","BLZF1",186.510997227538,0.0337765287263342,0.202069757730319,0.167152814482076,0.867249810573455,0.97821882027368
"14971","NUCKS1",4187.48894984246,0.024284340799033,0.151936491026438,0.159832181426431,0.873013272844561,0.97821882027368
"14972","GNPAT",172.016241257654,-0.0254976312226841,0.156096150870156,-0.163345675601531,0.870246272876388,0.97821882027368
"14973","RNF144A-AS1",3.17942657435013,0.13885204405174,0.877143375523238,0.15830028240128,0.874220185624506,0.97821882027368
"14974","SDC1",6.41670200192122,0.109111801709077,0.67247415611536 5,0.162254267642604,0.871105626521124,0.97821882027368
"14975","AC009242.1",2.07778944429337,0.172527459293273,1.08506177951475,0.159002429677717,0.873666959579973,0.97821882027368
"14976","ZNF513",36.4650084882284,0.0638728706326793,0.383421030241621,0.166586769099307,0.867695204949161,0.97821882027368
"14977","PLEKHH2",759.91747254409,0.0646830024052396,0.40920176388658,

0.158071172007871,0.874400716395157,0.97821882027368
"14978","TET3",16.7099773565296,0.0725366656874805,0.440315856992035,0
.164737800230512,0.869150363156128,0.97821882027368
"14979","AC233266.2",6.20491895651591,0.117332010537221,0.728907450357
605,0.160969695781896,0.872117268613428,0.97821882027368
"14980","CIAO1",466.017907200112,0.0210434135981847,0.127214145634246,
0.165417245804462,0.868615580471445,0.97821882027368
"14981","NIFK-
AS1",103.048785254043,0.0418278054176437,0.259054199995448,0.161463529
324669,0.871728333805061,0.97821882027368
"14982","EPC2",263.765997666882,0.035052892654451,0.214639607686832,0.
163310458084673,0.870274000085664,0.97821882027368
"14983","AC009961.1",5.48789099209572,-0.106377806553855,0.66737392488
3969,-0.159397606929804,0.873355624200473,0.97821882027368
"14984","IFIH1",91.3650775240149,0.0420100752719136,0.251781398068055,
0.166851386139966,0.867486985062174,0.97821882027368
"14985","PJVK",33.830317648981,0.0480450642362427,0.3046097043465,0.15
7726636908424,0.874672209999125,0.97821882027368
"14986","FTCDNL1",2.99141792041927,-0.154325428675784,0.91675367134901
2,-0.168339035336169,0.866316566261825,0.97821882027368
"14987","IDH1-
AS1",7.36097626237414,0.0829650196505533,0.509792297258918,0.162742787
791508,0.870720956272953,0.97821882027368
"14988","GLB1L",76.0722889895642,-0.0540632898143804,0.333394480587489
,-0.162160122504467,0.871179761737501,0.97821882027368
"14989","TMEM198",15.9644250461519,-0.0660370116940464,0.4136809622178
56,-0.159632706663618,0.873170413161558,0.97821882027368
"14990","TIGD1",14.1217359287809,-0.0611223538651822,0.377102419551664
,-0.162084226184097,0.87123952762474,0.97821882027368
"14991","ASB1",119.60475371308,0.0297062820942762,0.188294549175886,0.
157764960400035,0.874642010363922,0.97821882027368
"14992","FANCD2",7.37261081492272,0.0834714898195164,0.524362693049191
,0.159186553364668,0.873521897634576,0.97821882027368
"14993","HRH1",39.7905302526168,0.0574855792809354,0.34403286644338,0.
167093277672051,0.867296655296541,0.97821882027368
"14994","XYLB",30.6200558543609,0.0662452786646099,0.413253459161649,0
.160301812836604,0.872643330906201,0.97821882027368
"14995","EXOSC7",318.378995807532,0.0405211814071014,0.251928917400544
,0.160843708714377,0.872216498815691,0.97821882027368
"14996","MUSTN1",158.775887377901,-0.11200322625374,0.693686670419008,
-0.16146083099173,0.871730458881518,0.97821882027368
"14997","PDE12",122.819196296343,0.0488972999431437,0.289049477746252,
0.169165847744825,0.865666192144672,0.97821882027368
"14998","PDHB",338.582969856931,-0.0336751031690426,0.209196038310981,
-0.160973904864215,0.872113953481784,0.97821882027368
"14999","AC107204.1",12.1133485675896,0.0842638940836606,0.52378858192
7712,0.160873865889826,0.872192746173864,0.97821882027368
"15000","CD200",84.1538339918134,-0.0520536659595696,0.315636028506156
,-0.164916743522372,0.869009513425524,0.97821882027368
"15001","COPG1",244.288248068831,-0.0373214915499538,0.224252306780802

,-0.166426343994909,0.867821443531105,0.97821882027368
"15002","DZIP1L",46.4619724048066,-0.0581038109625637,0.36634270177934
4,-0.158605073010464,0.873980031709186,0.97821882027368
"15003","MYNN",108.502283985469,0.0346394695347559,0.215040673267366,0
.16108333836775,0.872027762924318,0.97821882027368
"15004","IL1RAP",35.211647940575,0.0548105313367583,0.33821678537476,0
.162057395454296,0.871260656128933,0.97821882027368
"15005","DCAF16",180.823872678148,-0.0356090069187151,0.21264590616027
5,-0.167456818528526,0.867010621532696,0.97821882027368
"15006","AC024230.1",3.4293505245536,-0.15028918121089,0.9107178316903
26,-0.165022772126849,0.868926058218286,0.97821882027368
"15007","LARP7",727.534126557314,-0.0298548266183313,0.179106946784079
,-0.166687150634769,0.867616216392518,0.97821882027368
"15008","AC108865.2",3.64543275807179,0.128515833948065,0.810194663393
505,0.158623402195437,0.873965589949241,0.97821882027368
"15009","CEP72",8.87299525714747,0.082641459167305,0.499128031887693,0
.165571664758552,0.868494047714768,0.97821882027368
"15010","ANXA2R",84.2999266431494,-0.0568283094668917,0.35317783510270
9,-0.16090565097429,0.872167711469724,0.97821882027368
"15011","GAPT",3.27352560098308,-0.143774171514076,0.865745873604622,-
0.166069716180636,0.868102086177602,0.97821882027368
"15012","AC114971.1",3.33463346743377,0.18222728241526,1.1015695731909
5,0.165425123251541,0.868609380588328,0.97821882027368
"15013","NDUFA2",1129.95671637796,-0.0428975258567069,0.26071829547102
2,-0.164535924796558,0.869309268204065,0.97821882027368
"15014","MED7",124.397521067612,-0.046877249212939,0.287020304738521,-
0.163323808242921,0.870263489313592,0.97821882027368
"15015","ZBED8",31.9822592607489,0.0597478733374983,0.368243741873832,
0.162250885876478,0.871108289495791,0.97821882027368
"15016","HUS1B",5.38280031539333,-0.103127229118299,0.653892159840237,
-0.157712900462816,0.874683034622148,0.97821882027368
"15017","BPHL",155.188117387536,0.0344062664336302,0.209996995469958,0
.163841708099831,0.869855756996212,0.97821882027368
"15018","MRS2",91.1461586240935,0.0360850483850742,0.225180602237267,0
.160249364405963,0.872684644637779,0.97821882027368
"15019","ZNRD1",323.775169412152,0.036300339483649,0.22733011222748,0.
15968117522119,0.87313223060533,0.97821882027368
"15020","ILRUN",164.468319430162,0.0349979343400521,0.213698613701567,
0.163772397648434,0.869910321741336,0.97821882027368
"15021","Z84485.1",12.2347086985059,-0.083125785622605,0.5141486784008
74,-0.1616765521622,0.871560570248795,0.97821882027368
"15022","AL021368.3",7.57215382633963,0.118059757524792,0.745523705291
363,0.15835815372048,0.874174586136532,0.97821882027368
"15023","ZC3H12D",5.92481140088361,-0.114249397182963,0.70829986143324
3,-0.161300888795573,0.871856423269418,0.97821882027368
"15024","CAHM",22.2747300744093,-0.0636936537718437,0.380663093449642,
-0.167322902765907,0.867115984363923,0.97821882027368
"15025","OGDH",215.249374832489,-0.0305709389597591,0.181626117185808,
-0.168317967886107,0.866333139191195,0.97821882027368
"15026","RSBN1L",622.037753769801,-0.0341255859648593,0.20584721483885

4,-0.165781140111972,0.868329188741974,0.97821882027368
"15027","STAG3",7.1902095769999,0.0927912345153542,0.556361481046953,0
.166782276768588,0.867541364443466,0.97821882027368
"15028","GIMAP7",11.1504398349014,0.0830660003598184,0.511137295727575
,0.16251210986586,0.870902592744045,0.97821882027368
"15029","BNIP3L",1306.49575088865,-0.028580807199899,0.173866915498855
,-0.164383241733458,0.86942945527568,0.97821882027368
"15030","GTF2E2",187.269393831526,-0.0295802853700298,0.18196931768678
5,-0.162556444932904,0.870867682654134,0.97821882027368
"15031","AC087623.2",8.88560261004795,-0.0831473698991787,0.5217565687
61578,-0.15936046592865,0.873384884432356,0.97821882027368
"15032","SPAG8",15.98909484996,0.0765998176620415,0.47446165880407,0.1
61445748546088,0.871742337102958,0.97821882027368
"15033","ZCCHC7",338.986516660509,-0.0215580486103325,0.13186621376931
7,-0.163484246601981,0.87013717567347,0.97821882027368
"15034","RAB14",889.758595365251,0.0256935097637163,0.160276321880399,
0.160307582943469,0.872638785802925,0.97821882027368
"15035","KYAT1",12.2544124433316,-0.0648234301802351,0.39978485815752,
-0.16214578630863,0.871191050969757,0.97821882027368
"15036","OLFM1",196.112062762514,-0.0497948046750574,0.302866558145856
,-0.164411696622765,0.869407056296505,0.97821882027368
"15037","HACD1",15.2030152354943,-0.0834391658891382,0.499295383083268
,-0.167113834247538,0.867280480929415,0.97821882027368
"15038","DDX50",363.017733699349,-0.033244911164972,0.196775415066985,
-0.168948499758748,0.865837150189247,0.97821882027368
"15039","ZNF503",1473.09559888839,0.0228762469452767,0.135286925398374
,0.169094292577896,0.865722474150653,0.97821882027368
"15040","CH25H",220.500048296008,-0.165434179818321,1.0213677182465,-0
.161973182491357,0.871326972250842,0.97821882027368
"15041","NRIP3",18.3244116084029,-0.0995008846693215,0.619608542441053
,-0.160586689585203,0.872418939003033,0.97821882027368
"15042","HIPK3",542.596033141518,-0.0308327251853514,0.18842814279108,
-0.163631210967977,0.870021473059065,0.97821882027368
"15043","AC090469.1",2.65541325991732,-0.184144676702044,1.09329038264
534,-0.168431625874624,0.866243729645725,0.97821882027368
"15044","OTUB1",292.202431100215,-0.025390322711055,0.151717655602964,
-0.167352458816658,0.867092729921548,0.97821882027368
"15045","CDC42EP2",176.403263676204,-0.051223302936195,0.3042540905431
63,-0.168356990187871,0.866302441936816,0.97821882027368
"15046","NEAT1",17696.6660658259,0.0589722820460162,0.372206455548124,
0.158439707766948,0.874110326642251,0.97821882027368
"15047","MRPL11",654.314671316264,-0.0397704689326868,0.25030894640839
,-0.158885527278755,0.873759063402124,0.97821882027368
"15048","CWF19L2",477.513287931894,0.0427801454451027,0.27128111091592
4,0.157696734950194,0.874695773433751,0.97821882027368
"15049","CCDC84-
DT",6.43380703457245,0.115004495679197,0.69333778640188,0.165870803430
489,0.868258624659315,0.97821882027368
"15050","FAM234B",30.7135360580069,0.0512386416749028,0.31819154425499
1,0.161030808643492,0.872069135496041,0.97821882027368

"15051","CMAS",271.302793411754,0.03263252818184,0.201445343319926,0.161991970844491,0.871312176698274,0.97821882027368
"15052","HOXC8",300.56597643839,0.0287867497992773,0.171579896869354,0.167774607191869,0.866760599833511,0.97821882027368
"15053","AC026124.1",19.950001348657,0.0762895072470899,0.453971322343902,0.168049177320715,0.866544591249144,0.97821882027368
"15054","LINC01481",2.0877278437934,-0.179194586541946,1.0862886675605,-0.164960375536612,0.868975170452236,0.97821882027368
"15055","CHPT1",799.787676572091,-0.0466641350803325,0.282742738623227,-0.165040967303198,0.868911736925848,0.97821882027368
"15056","TBX5",57.6243703162829,-0.147295615372479,0.89321117107432,-0.164905702192817,0.869018204149576,0.97821882027368
"15057","PXN-AS1",56.0232450167276,-0.0505526272538196,0.315253922199193,-0.160355268226855,0.872601224347981,0.97821882027368
"15058","GOLGA3",356.960731717988,0.0383310953159899,0.240138323264999,0.159620900133006,0.873179714154006,0.97821882027368
"15059","PCCA",135.48213217475,-0.0293990445068802,0.182879476950216,-0.16075638993042,0.872285274202043,0.97821882027368
"15060","THTPA",2.73288781121699,-0.135121945925522,0.848805390892657,-0.1591907254305,0.873518610719518,0.97821882027368
"15061","DHRS4L2",481.131358568079,0.0451614293842183,0.271196267659297,0.166526736426013,0.867742444290113,0.97821882027368
"15062","STRN3",301.057477705767,-0.0327618304192575,0.197949076811059,-0.165506356215686,0.86854544729048,0.97821882027368
"15063","FBXO34",108.115605366311,-0.0333180461464565,0.207750066030991,-0.160375622414898,0.872585191545145,0.97821882027368
"15064","EIF2AK4",431.994471730571,0.0290175221439649,0.176292265360521,0.164598952112978,0.869259656059558,0.97821882027368
"15065","AC090515.2",2.41333802427538,0.176693278503913,1.06389030283089,0.166082234262077,0.868092234988716,0.97821882027368
"15066","MYO1E",184.412742737181,-0.0668595070953542,0.410688869601259,-0.162798439510349,0.870677136967906,0.97821882027368
"15067","CSK",114.195393306218,0.0345896626753101,0.209151081082895,0.165381228230902,0.868643927923948,0.97821882027368
"15068","ARNT2",5.50997042388149,-0.101403436884924,0.628360457301135,-0.161377813811616,0.87179583964795,0.97821882027368
"15069","TARSL2",117.255121840961,-0.0530734490321172,0.317753141710453,-0.167027299073818,0.867348569088581,0.97821882027368
"15070","Z69706.1",7.35216342185712,-0.0973684884174769,0.588405721366767,-0.165478486836101,0.868567381409197,0.97821882027368
"15071","RHBDL1",11.1762349517666,-0.0970838083427018,0.590873523326349,-0.164305565421453,0.869490600664507,0.97821882027368
"15072","CLEC16A",42.4166510220129,0.0426526334917336,0.25820483693767,0.165189134322955,0.868795117331682,0.97821882027368
"15073","AC026401.3",17.8295857464597,0.0691162326420548,0.429754272078748,0.160827331180992,0.872229398271114,0.97821882027368
"15074","CNEP1R1",88.2553584967395,-0.0372140791550736,0.221682411395908,-0.167871140162816,0.866684654711068,0.97821882027368
"15075","ADCY7",126.97870819525,-0.0406300265248083,0.253903166442544,

-0.160021740154243,0.872863948729057,0.97821882027368
"15076","PLLP",6.683880240399,-0.0960440513486743,0.608010494322864,-0
.157964463188481,0.874484801348945,0.97821882027368
"15077","CFAP20",333.024275111997,-0.0350425778630159,0.21851909285872
8,-0.160363917882776,0.872594411088821,0.97821882027368
"15078","GINS3",18.2651130355245,-0.0684252808879338,0.407547218127444
,-0.167895345237117,0.866665612111561,0.97821882027368
"15079","AC018557.1",8.36176838263841,-0.125578341950553,0.74205075443
201,-0.16923147264593,0.865614575212176,0.97821882027368
"15080","NOL3",65.4433519911749,-0.0474459428274479,0.300392017179546,
-0.157946749960034,0.874498759245241,0.97821882027368
"15081","CENPT",166.479260463131,0.034952058815465,0.208712264330498,0
.16746528493466,0.867003960375924,0.97821882027368
"15082","AC092123.1",2.96842210662872,-0.171929080687061,1.05843918746
885,-0.16243642782937,0.870962186484147,0.97821882027368
"15083","KCNAB3",2.9593275123249,-0.130231219232151,0.825820854436455,
-0.15769911662139,0.874693896617695,0.97821882027368
"15084","AC006441.4",6.12150859743077,-0.121938035295812,0.73779655394
0944,-0.165273251338028,0.868728911613303,0.97821882027368
"15085","RARA",271.573934894135,-0.0385519276340876,0.241003351791073,
-0.159964279947062,0.872909212294987,0.97821882027368
"15086","NAGLU",484.642419363887,0.0443900987268896,0.278046170446771,
0.159650099318263,0.873156711543548,0.97821882027368
"15087","CNTD1",12.0993926034577,0.0695896154610507,0.420942614413785,
0.165318532926306,0.868693272355006,0.97821882027368
"15088","IFI35",334.760625857451,-0.046838789217752,0.283692472983197,
-0.165104095731599,0.868862049327373,0.97821882027368
"15089","AC068234.3",2.61175481792265,-0.161940345434242,1.00821405345
466,-0.160620995987262,0.872391917206053,0.97821882027368
"15090","XYLT2",74.4838926459197,-0.0398808528583614,0.246582093418245
,-0.161734586260961,0.871514867198162,0.97821882027368
"15091","AP005482.3",6.27275099362935,-0.103647649160065,0.63787775283
6186,-0.162488264717209,0.870921368872082,0.97821882027368
"15092","RBBP8",70.3874017911347,-0.0455041576990945,0.271139644847946
,-0.167825541427603,0.866720528327038,0.97821882027368
"15093","AC027097.1",34.1188091287362,-0.0464478369636647,0.2843547674
98067,-0.163344674584999,0.870247060987527,0.97821882027368
"15094","AC110597.1",4.6813462393773,0.124268606502264,0.7505233489953
65,0.165575936669535,0.868490685625557,0.97821882027368
"15095","MADCAM1",4.32728060759425,-0.126098734805663,0.77468781705591
,-0.162773612840438,0.870696685055828,0.97821882027368
"15096","AC005944.1",4.56285643771667,0.117409279672411,0.743419265270
187,0.157931446166841,0.874510818555374,0.97821882027368
"15097","GNA15",5.84653731253587,-0.110702440764517,0.658079302354571,
-0.16822051744893,0.866409800358744,0.97821882027368
"15098","PRAM1",2.75442817812806,-0.137840040493045,0.863855518923066,
-0.159563766710531,0.873224723180955,0.97821882027368
"15099","TMED1",451.207283219846,-0.0406677681009434,0.257559483654156
,-0.15789660518015,0.874538273181963,0.97821882027368
"15100","ZNF573",42.9836056640604,0.0461072015011339,0.276708056270565

,0.166627607893173,0.867663069423732,0.97821882027368
"15101","SARS2",35.7877111147576,-0.0452961296897567,0.283900902223434
,-0.159549086794054,0.873236287909858,0.97821882027368
"15102","EMC10",318.873766251705,-0.0284853061580499,0.169046998225085
,-0.168505246807884,0.866185816335295,0.97821882027368
"15103","ZNF836",60.169795265819,-0.045604536990104 7,0.286436552137353
,-0.159213398743315,0.873500747843656,0.97821882027368
"15104","ZNF552",24.7454884190012,-0.0592737646650427,0.37593165278894
9,-0.157671651815708,0.87471553960952,0.97821882027368
"15105","ZNF587B",17.6838004686701,-0.0669289864841782,0.4032464568493
86,-0.165975386385544,0.868176320306576,0.97821882027368
"15106","CST3",21416.6785499916,-0.050287868387113,0.309153965918817,-
0.162662860357154,0.870783890596237,0.97821882027368
"15107","RPN2",1057.6122219981,-0.0346868074646549,0.212278675129636,-
0.163402223249566,0.870201752510968,0.97821882027368
"15108","ACOT8",89.3884958063208,0.0432941717477089,0.271235559830059,
0.159618347147529,0.873181725356673,0.97821882027368
"15109","ADARB1",151.861084675973,0.0489985993243345,0.305592069890545
,0.160339891483063,0.87261333649940 7,0.97821882027368
"15110","SPATC1L",206.439915980088,-0.0420830645600732,0.2548178694463
57,-0.165149581744432,0.8688262481751,0.97821882027368
"15111","AC002470.1",4.77292532473536,-0.114798283198355,0.69892832577
004,-0.16424900660862,0.869535123230683,0.97821882027368
"15112","TFIP11",73.3179434763794,-0.0403092229017177,0.24647327250969
1,-0.163543991976382,0.870090138792967,0.97821882027368
"15113","AP1B1",104.650535580219,-0.0334593411405317,0.211195734169988
,-0.158428110643564,0.874119464400423,0.97821882027368
"15114","POLR3H",114.483816835352,0.0315839698355201,0.186790884337529
,0.16908731894244,0.865727959328251,0.97821882027368
"15115","MAPK8IP2",9.90684002649748,-0.0922489650276886,0.544480589109
487,-0.169425626684992,0.865461867696933,0.97821882027368
"15116","ADGRG2",6.18539712934691,0.116167756403996,0.707679540427421,
0.164153052006893,0.869610658737821,0.97821882027368
"15117","ZC4H2",39.3726621017904,0.0415856904093296,0.251726143079816,
0.165202111709724,0.868784903200068,0.97821882027368
"15118","ELF4",46.3290442001618,0.0452960297974105,0.273907891453347,0
.165369568423426,0.868653104753731,0.97821882027368
"15119","PWP2",7.52924291030417,0.496074844252886,3.08305084200928,0.1
60903880498313,0.872169105936216,0.97821882027368
"15120","AL138955.1",2.64956712014294,-0.146028944822681,0.92689244217
3417,-0.157546807135752,0.87481392169373,0.97826413941783
"15121","PRR12",149.213652500759,0.0391663596007719,0.248748583844494,
0.157453598309757,0.874887374852455,0.978281577541519
"15122","AC097382.3",65.6741015802085,0.0526294895483885,0.33488902884
172,0.157155012603482,0.87512268239172,0.978317957154845
"15123","MAPK6",235.159334935604,-0.0389582945518059,0.247783806836251
,-0.157226959458056,0.875065981958025,0.978317957154845
"15124","AC009061.2",22.1103485286716,0.058214363985402,0.370502623777
027,0.15712267673558,0.875148166101466,0.978317957154845
"15125","WDR45B",377.087145426254,-0.0254295011470728,0.16184904985347

6,-0.157118631033635,0.875151354504792,0.978317957154845
"15126","ANKLE2",286.065322521674,0.0319124929798481,0.203282043794156
,0.156986285577504,0.875255656605747,0.978369869224512
"15127","AL603839.3",4.11898745517913,0.171837699672257,1.100205529672
32,0.15618690784388,0.875885696116107,0.978708214843516
"15128","C1D",519.402124329429,-0.0400009157138725,0.256734607758367,-
0.155806480720045,0.876185562140258,0.978708214843516
"15129","RNF123",49.3460617191139,0.0389004278603139,0.248868685932098
,0.15630904994985,0.875789423193555,0.978708214843516
"15130","SERPINB9P1",11.802414058348,-0.126217018190113,0.808689269620
623,-0.156076039254638,0.875973084795477,0.978708214843516
"15131","AC026979.4",17.9593767363386,0.0631172109853791,0.40524271293
3948,0.155751624818647,0.876228802956219,0.978708214843516
"15132","YY1",1234.60735424487,-0.0201031666386263,0.128557080897816,-
0.156375413149007,0.875737116210897,0.978708214843516
"15133","MLKL",40.8430404549826,-0.0494527490612865,0.316593720703254,
-0.156202558128558,0.875873360393167,0.978708214843516
"15134","SHLD1",35.5582889732924,-0.0602616781013161,0.386412606438187
,-0.155951635886797,0.876071143638804,0.978708214843516
"15135","CRNKL1",220.704288238886,-0.0379676495339117,0.24364933068496
8,-0.155829073805267,0.876167752979873,0.978708214843516
"15136","AP000692.1",4.45682774833116,0.123070049003149,0.790324106148
627,0.155720985916636,0.876252954595526,0.978708214843516
"15137","PDE9A",34.0242160661472,0.0734878619771466,0.471787595818095,
0.155764718336259,0.876218481801839,0.978708214843516
"15138","AL121672.2",11.8389353381525,-0.0736308969775116,0.4713195401
01768,-0.156222882169522,0.875857340810194,0.978708214843516
"15139","DNALI1",70.3082699460065,0.0466436193424609,0.300427339591589
,0.155257572116671,0.876618262500062,0.978792946070861
"15140","TAF5L",99.3398861369258,0.0402117860197701,0.258786554790447,
0.155385916599615,0.876517086239847,0.978792946070861
"15141","SERINC5",106.361629020357,0.0421490627552388,0.27112928404968
7,0.155457433906382,0.876460708738216,0.978792946070861
"15142","FAM133B",1161.43772266178,-0.040619515260805,0.26161471369342
1,-0.155264643518506,0.87661268793454,0.978792946070861
"15143","TAF3",253.216765136188,0.0345527980017991,0.222191413221126,0
.155509150875295,0.876419940346569,0.978792946070861
"15144","PODXL2",81.1361596691625,0.046080571014384,0.297432129182666,
0.154928020523579,0.876878063168424,0.978804966667389
"15145","AC011978.2",5.39698959992086,0.108672605676572,0.701047445727
05,0.155014623245462,0.876809788976114,0.978804966667389
"15146","STOML1",107.982409002935,0.042082917799281,0.271485752070411,
0.155009673540314,0.876813691105003,0.978804966667389
"15147","TMX3",333.658616404841,-0.0206310309795,0.133207468222207,-0.
154878936255172,0.876916759688979,0.978804966667389
"15148","OSM",8.57188133931127,-0.116166991079175,0.750060848855352,-0
.154876756007802,0.876918478535463,0.978804966667389
"15149","SMARCC2",533.519028628308,0.0314318245271124,0.20310084120099
9,0.154759696420981,0.877010765915033,0.978843357983456
"15150","ZNF485",23.8058747510412,0.0579483438327702,0.37477927738641,

0.154619925191391,0.877120960878209,0.978901729803879
"15151","HDHD5-
AS1",5.13099663409649,-0.11306253165178,0.731874291745025,-0.154483540
311551,0.877228488355241,0.978957117095269
"15152","DIS3L2",99.8989658311474,-0.0322258161072362,0.20872681528692
5,-0.154392314485023,0.877300413156978,0.978972768324854
"15153","DCPS",174.710360214691,-0.0399187478378244,0.258758916547284,
-0.154270037803818,0.877396820844691,0.978980493523697
"15154","TNFAIP8L3",15.2022782443724,0.0817489944788126,0.530023109091
813,0.154236660772939,0.877423136909043,0.978980493523697
"15155","C10orf25",13.0348960889264,0.0691051697418846,0.4489310109933
31,0.153932715828605,0.877662788024197,0.979010008050683
"15156","PDPK1",239.114095636116,0.0259341811822331,0.168501260011722,
0.153910903576798,0.877679986736364,0.979010008050683
"15157","NEK8",23.2245252680978,0.0594879926227557,0.386513139587131,0
.153909366926827,0.877681198369545,0.979010008050683
"15158","TAZ",121.993318599683,-0.0267461318112049,0.17363803050824,-0
.154033835404137,0.877583057144884,0.979010008050683
"15159","TMEM50A",2069.95828538934,0.0320731402482304,0.20888451764492
6,0.153544841953055,0.877968630767454,0.979029774645169
"15160","AC105206.1",4.33455089070837,-0.124486783405637,0.81096406402
6126,-0.153504685260167,0.878000295795141,0.979029774645169
"15161","KIF20B",99.2217746787537,0.0398348913189312,0.259106326223514
,0.153739555106687,0.877815095061541,0.979029774645169
"15162","PRCP",578.825877493947,-0.0267002371711542,0.17371185923874,-
0.153704170159499,0.87784299654759,0.979029774645169
"15163","DDX11-
AS1",2.31843061534159,-0.138018409107393,0.900131784084443,-0.15333133
6086278,0.878136990226911,0.979029774645169
"15164","CKAP2",53.9136084343876,0.0566347740832873,0.369438895319673,
0.15329943544326,0.878162145864008,0.979029774645169
"15165","AC006504.5",27.2865626264111,-0.0555784731265281,0.3617443397
50259,-0.153640201156702,0.87789343731431,0.979029774645169
"15166","TXN2",798.561043464823,-0.0381192001356934,0.248433983953561,
-0.153437945683063,0.878052922835435,0.979029774645169
"15167","SPICE1",76.8225949839796,-0.0368243081929926,0.24063266087899
2,-0.153031213877947,0.878373660079992,0.979201018305037
"15168","TP53I3",556.852725127213,0.0508661622898637,0.332648720808069
,0.152912544399088,0.878467243252744,0.97924077986006
"15169","NUF2",4.71741625797141,0.103089432231201,0.676334918059034,0.
152423643195964,0.878852810374775,0.97959105637147
"15170","IL1RAPL1",2.70399912045737,0.172630453975264,1.13298979832104
,0.1523671742067 6,0.878897345940099,0.97959105637147
"15171","PROSER3",37.6505398429198,-0.0524723774825786,0.3448671590852
14,-0.152152433481244,0.879066709643585,0.97971524135876
"15172","GPLD1",10.863955618317,0.0794951347123038,0.522919985648069,0
.152021603484486,0.879169896569877,0.979765661165534
"15173","HMGN3-
AS1",62.3235564048244,0.0387279696503403,0.255038155915027,0.151851669
062584,0.879303928611046,0.97977491486159

"15174","ACCS",125.16513398581,0.0428105675937501,0.282040650619021,0.151788642877507,0.87935364000908,0.97977491486159
"15175","ATXN2L",238.437194679277,0.0253054682766002,0.166793666878225,0.151717200959888,0.879409989823722,0.97977491486159
"15176","CDIPT",783.795475924156,-0.0223382091536466,0.147112500173916,-0.151844398859637,0.879309662901045,0.97977491486159
"15177","TGM2",3.49329209582471,-0.137507857546736,0.907057700627741,-0.151597695991746,0.879504250736102,0.979815370062991
"15178","VAMP3",479.953091665165,0.029765243419874,0.197906306822708,0.150400681502984,0.880448503625166,0.979815489804873
"15179","TENT5B",2.31390596665933,0.146681915363985,0.980739643354873,0.149562543288473,0.881109761834113,0.979815489804873
"15180","AC093424.1",2.67533817663113,-0.138988563579565,0.924983174381909,-0.150260639792113,0.88055898525992,0.979815489804873
"15181","SELL",12.0772376934168,0.0817025450903818,0.547552055978433,0.14921420566011,0.881384610985041,0.979815489804873
"15182","WDR43",271.824941906122,-0.0314003976583331,0.210313185740842,-0.149303038455355,0.881314517806666,0.979815489804873
"15183","CCDC13",6.78151702208035,-0.0953663681583578,0.631596029722799,-0.150992665676212,0.879981501508804,0.979815489804873
"15184","ZNF660",51.6496870993419,0.0424530665006601,0.282904584933611,0.150061429759516,0.880716149941973,0.979815489804873
"15185","EIF2B5",140.438363044806,0.0264831793482544,0.176504143986298,0.150042819109733,0.880730832860501,0.979815489804873
"15186","DNAJB14",462.618997016868,0.0205936198881363,0.137593471993607,0.14967003586546,0.88102494975935,0.979815489804873
"15187","GIN1",61.3467040789545,-0.045206704980782,0.298792652528758,-0.1512979137813,0.879740714900269,0.979815489804873
"15188","MIR3936HG",31.846024032053,-0.0510560165987069,0.341815406207339,-0.149367218889301,0.8812638770584,0.979815489804873
"15189","LRRTM2",8.73814036322629,-0.0903153944507113,0.597458294617417,-0.151166023242752,0.879844751776729,0.979815489804873
"15190","NR3C1",1909.6532998032,0.0208829567204041,0.139412110939317,0.149792988426192,0.880927941368367,0.979815489804873
"15191","ZNF76",84.9439021412121,0.0380190353642831,0.254691794731183,0.149274676887062,0.88133689629299,0.979815489804873
"15192","RPL10A",19162.2236409136,-0.0396401075962818,0.263614124543127,-0.150371713446624,0.880471356898523,0.979815489804873
"15193","CPVL",369.140136433861,-0.0528274137898163,0.34960508083464,-0.151105966949042,0.879892125606115,0.979815489804873
"15194","VPS37D",50.3849153927083,-0.0469001208326112,0.31397105949589,-0.149377209822822,0.881255993884105,0.979815489804873
"15195","CHPF2",112.801774936316,0.0341459110850942,0.227683038781958,0.149971255073569,0.880787293867095,0.979815489804873
"15196","VPS37A",221.734878808514,-0.0231513084819579,0.154664325003981,-0.14968745042764,0.88101120972829,0.979815489804873
"15197","AC090204.1",2.51675019904285,0.181260498439628,1.21135419977506,0.14963459776941,0.881052910408675,0.979815489804873
"15198","SIGMAR1",259.705713662044,0.0296262349125031,0.197269730491907,0.1501813523982,0.880621537660967,0.979815489804873

"15199","AL731571.1",3.46464192930253,-0.133729773219931,0.89691273769335,-0.149100093687879,0.881474651964864,0.979815489804873
"15200","LINC02709",21.0642520003965,-0.10180967463563,0.672781398957102,-0.151326530123228,0.87971814225236,0.979815489804873
"15201","POLD3",191.891267942784,0.0280673698410075,0.188033588213068,0.149267852130776,0.881342281330755,0.979815489804873
"15202","CXCR5",3.5461299033256,0.125229631916713,0.828978842395078,0.151064931349635,0.879924495707791,0.979815489804873
"15203","SPSB2",69.1217133572842,0.0467258693133501,0.312124551876466,0.149702639643175,0.8809992255175,0.979815489804873
"15204","SLCO1C1",9.05368335380026,-0.0794378163898739,0.531650721887337,-0.149417301847862,0.881224360079494,0.979815489804873
"15205","MAP2K5",114.984284098054,0.0375666543579724,0.251178127987182,0.149561805635757,0.881110343849778,0.979815489804873
"15206","AC087286.1",2.31338816272446,0.173843759139657,1.15625482194807,0.15035073224323,0.880487909302705,0.979815489804873
"15207","SKA2",226.628670679574,0.0418241166468539,0.277784838500351,0.150562992827994,0.880320455969444,0.979815489804873
"15208","SCN1B",330.645770780789,0.0323281036653097,0.216744505366785,0.149153048242688,0.881432867552106,0.979815489804873
"15209","INAFM1",331.847885426107,-0.0257307395922501,0.172364139164882,-0.14928128157584,0.881331684903549,0.979815489804873
"15210","BICRA",47.3337933697442,-0.0409378147491206,0.274361371721985,-0.149211291998509,0.881386910014357,0.979815489804873
"15211","SON",2525.69275182017,0.0275335513494016,0.183729652216956,0.149859051150267,0.880875819250568,0.979815489804873
"15212","BTBD19",49.4634637930505,0.0560041155385529,0.377342773519891,0.148417087774441,0.882013615322652,0.980081273579153
"15213","AL365361.1",3.60264598203821,0.114591065634591,0.772769519384035,0.148286213107797,0.882116895417265,0.980081273579153
"15214","SP140",4.84986280691672,-0.117629432744541,0.793139483225952,-0.148308633263477,0.882099202348886,0.980081273579153
"15215","IFT46",80.3770904025653,-0.0330143371518143,0.222187197644555,-0.14858793621687,0.881878792834577,0.980081273579153
"15216","FAIM2",14.3331229961722,-0.0844946436831934,0.568423384440725,-0.148647374467763,0.881831888831887,0.980081273579153
"15217","COPS2",675.61505213791,0.0296139690505718,0.199478389472721,0.148457028998731,0.881982095997713,0.980081273579153
"15218","AC110285.2",7.55171140166165,-0.0956056505363521,0.64475173880705,-0.148282889028335,0.882119518649606,0.980081273579153
"15219","CORO1C",154.535700283022,-0.0309367835083256,0.208738708880417,-0.14820817698,0.882178478791051,0.980082378566206
"15220","FBXO6",105.113424905067,0.0409081459881,0.27656006048073,0.147917764831956,0.882407668122402,0.980208189515379
"15221","UBTF",514.039373574991,-0.0257933380150222,0.17435584908657,-0.147935031432272,0.882394041279973,0.980208189515379
"15222","AC011825.2",2.04930828037606,-0.147133280513587,0.99651228302223,-0.147648235772227,0.882620385612898,0.980380073573964
"15223","SECISBP2",308.553645741602,-0.0247006753191932,0.167377563980337,-0.147574589639116,0.882678510023316,0.980380230406242

"15224","ZNF222",30.2879220585523,-0.0425771419645651,0.288687473731574,-0.147485242134731,0.882749027418488,0.98038069103163
"15225","UTP14A",114.108736607947,-0.0298411106644901,0.202412610570664,-0.14742713203667\9,0.88279489123235,0.98038069103163
"15226","RARRES1",290.040920728349,0.0537941813007877,0.365766314412691,0.147072541076301,0.883074763212793,0.980433915319582
"15227","AC087239.1",32.4742736201923,0.057904451246915,0.393609872065038,0.147111277832349,0.883044188308531,0.980433915319582
"15228","TDG",264.712818312521,0.0271047826074806,0.184262354085147,0.147098862065746,0.883053988048599,0.980433915319582
"15229","NCOR2",520.04756988802,0.0357210925666838,0.242736786562006,0.147159781888103,0.883005904324644,0.980433915319582
"15230","AC083843.3",28.970561211025,-0.0824103815079484,0.562250116308342,-0.146572457910803,0.883469494255945,0.980807761581059
"15231","PHGDH",401.552590570038,0.0420435386537823,0.288217386370898,0.145874401205199,0.88402054027311,0.980949517217287
"15232","GYPC",2210.6257842608,0.0337756922323448,0.231621557379907,0.145822749032577,0.884061316735097,0.980949517217287
"15233","NIF3L1",103.042742035051,-0.0334568992124483,0.228865766345016,-0.14618568668768,0.883774804541254,0.980949517217287
"15234","MACORIS",6.25765193284253,-0.107152109832564,0.732938159674204,-0.146195294129853,0.883767220388987,0.980949517217287
"15235","CEP83-DT",3.57200395406315,-0.10986688536659,0.753161555094698,-0.145874261137474,0.884020650848224,0.980949517217287
"15236","AL356019.2",9.26066864081839,-0.0702233636310006,0.481044324032473,-0.145981066863726,0.883936334755335,0.980949517217287
"15237","L2HGDH",26.3935925094823,-0.0538307485936638,0.369046859278957,-0.145864264226061,0.884028542819219,0.980949517217287
"15238","ZNF747",62.9094589786736,-0.032807458201604,0.224348776014885,-0.146234175128405,0.883736527686106,0.980949517217287
"15239","NELFA",138.848521815336,0.023854566484684,0.164077212046959,0.145386225101489,0.884405939886406,0.980956519242677
"15240","CAMK4",5.6921874063011,-0.0891181273242453,0.611911144682386,-0.145639000202394,0.8842063785083,0.980956519242677
"15241","C7orf26",107.387837759021,-0.0267600521116469,0.183959877330759,-0.145466786018413,0.884342337700265,0.980956519242677
"15242","AL121821.2",18.3529886612756,-0.0785959700059753,0.540920767044527,-0.145300337488255,0.884473748276237,0.980956519242677
"15243","IFI27L1",261.650727562516,0.0395720765489395,0.272248901246989,0.145352566595077,0.884432513223061,0.980956519242677
"15244","MRI1",141.851945360632,-0.0358908146422465,0.246865144397329,-0.145386319036114,0.884405865725353,0.980956519242677
"15245","RAE1",118.240714697596,0.0408428897771826,0.280471701817484,0.145622141244612,0.884219688122555,0.980956519242677
"15246","TMEM199",90.3558233711149,-0.0346115108385809,0.238403781079213,-0.145180209315056,0.884568591055072,0.980997359147262
"15247","MAPRE3",87.2057780815876,-0.03073976168136,0.211992134111512,-0.145004255984283,0.884707511518238,0.981087073178354
"15248","RAP1B",1704.49279997485,0.0326095260250774,0.225240562605523,

0.144776436570124,0.884887387084084,0.981093504315914
"15249","RIOK3",330.73224517684,-0.0327566903063291,0.226233506625858,
-0.144791506770488,0.884875488174596,0.981093504315914
"15250","NFKBIB",98.4733026307993,0.0397302397612589,0.274250195577081
,0.144868592263565,0.884814624538915,0.981093504315914
"15251","MSRB3",267.280552443935,-0.0275922866672285,0.190692777301425
,-0.144694975120185,0.884951706679603,0.981100482364351
"15252","AC007319.1",3.20271873706302,-0.153707045357565,1.06301240236
453,-0.144595721569818,0.885030075425565,0.981123034047696
"15253","OS9",1121.31936725831,0.0206821543176798,0.143208984087976,0.
1444193913489,0.885169305241714,0.981213047467835
"15254","MAN2C1",188.489295072687,0.0325276143932501,0.225354504271874
,0.144339757034578,0.885232185421089,0.981218420814198
"15255","GNB2",1118.32163912306,0.0241542854123511,0.16743844355556,0.
144257703902606,0.885296976283005,0.981225911176207
"15256","PREX1",17.0785443921545,-0.0803616933700435,0.558321997673043
,-0.143934313362132,0.885552339652618,0.98144460925842
"15257","AC073475.1",5.90008496017812,-0.0987776271745887,0.6873517457
07376,-0.143707538085807,0.88573141845951,0.981453840926401
"15258","AL122023.1",2.33066628529919,0.146122894767806,1.016989852224
09,0.143681762849694,0.885751772893231,0.981453840926401
"15259","ARHGAP33",44.7320624216889,-0.0389708186949857,0.271328341177
197,-0.143629738514986,0.885792856194516,0.981453840926401
"15260","UXT-
AS1",2.8069026214078,-0.128961312095449,0.896507712397623,-0.143848525
017765,0.885620083891929,0.981453840926401
"15261","AC018797.3",3.15252132993487,0.119151655721347,0.830980485082
218,0.143386827802049,0.885984685371083,0.981537744742122
"15262","MRPL58",171.024048493978,-0.0427724344339132,0.29821023984015
4,-0.143430468574251,0.885950221295891,0.981537744742122
"15263","AC121247.1",28.658422984367,0.0700229862396245,0.489218503959
822,0.143132333860731,0.886185669113332,0.981696081593934
"15264","AC022382.1",5.62196120101954,-0.0946421601333981,0.6622731929
45403,-0.142905014337792,0.886365198353488,0.981766313380988
"15265","CCNH",318.198721795472,-0.0314024328532323,0.219632978465586,
-0.142976856538658,0.886308459192017,0.981766313380988
"15266","AC016405.3",6.97794484547586,-0.0816233758441504,0.5716761690
92918,-0.142779042151893,0.886464689420359,0.981812194990137
"15267","CLUH",53.6077562023836,0.0381280465733615,0.267321139812767,0
.142630121209518,0.886582307401505,0.981878145905852
"15268","RPP40",48.3480894198202,-0.0499437379346643,0.350808862765398
,-0.142367377896219,0.886789828550688,0.981884958446244
"15269","RARS2",320.960345312519,-0.032116581623833,0.225768144405078,
-0.142254708734323,0.886878819812577,0.981884958446244
"15270","GIMAP5",4.16725720246073,0.113947525848947,0.800999557100971,
0.142256665236312,0.886877274465568,0.981884958446244
"15271","DET1",63.6255619902291,-0.0320753764876129,0.225020147792523,
-0.142544464583623,0.886649960263587,0.981884958446244
"15272","AC138150.1",43.6348924713514,-0.0404936889886405,0.2843531746
24109,-0.142406319332182,0.88675907115377,0.981884958446244

"15273","BX255925.3",23.043119858796,0.0451348234892072,0.317915254503
507,0.14197124186348,0.887102721229922,0.982068539943398
"15274","RAB12",156.82091833353,0.0395299801117993,0.278773047010522,0
.141799863852359,0.887238091612883,0.982089797249796
"15275","MAGED1",168.59581305083,0.0504723357027052,0.355879861490258,
0.141824084935154,0.887218959336477,0.982089797249796
"15276","ACOT11",9.06006730002177,-0.0808288189111856,0.5729217947706,
-0.141081766567372,0.887805347741948,0.982158266347944
"15277","RGPD5",68.5983475583073,0.0657035867727234,0.464690091978764,
0.14139269615523,0.887560060874284,0.982158266347944
"15278","TMEM41A",148.429182368778,0.0251219968769014,0.17737097729407
9,0.141635329861488,0.887368059043834,0.982158266347944
"15279","CMBL",1168.05565566363,-0.0290189825489789,0.205268196871688,
-0.141371059868171,0.887576815512302,0.982158266347944
"15280","TBC1D9B",388.453469617518,0.0210172525241972,0.14892411146824
7,0.141127264866565,0.887769404957619,0.982158266347944
"15281","KIF13A",248.017954401679,-0.0314812304372048,0.22240826257528
8,-0.141547036394604,0.887437804392495,0.982158266347944
"15282","ST7-
OT4",7.89287263322703,-0.0826572073136863,0.584794139988307,-0.1413441
10109139,0.887598104544503,0.982158266347944
"15283","PPP3CB",379.97269856336,0.0201875144758157,0.143113219579157,
0.141059746508252,0.887822743249467,0.982158266347944
"15284","AC090061.1",3.53197045465417,0.125226974813292,0.887303620770
655,0.141132045313337,0.887765628509689,0.982158266347944
"15285","SAXO1",5.65556202831708,-0.0949769448050616,0.674926528356251
,-0.14072190203632,0.888089641947392,0.982389248678214
"15286","CT70",6.35326664419342,-0.109662105646829,0.780552994949427,-
0.140492838226742,0.888270610638342,0.982525152732768
"15287","ARC",56.4120102161741,-0.0694029721323209,0.49516279520141,-0
.140161928167666,0.888532051829878,0.982621488150931
"15288","CBWD5",63.8397969061301,-0.0449990078468926,0.321015634763694
,-0.140176997547229,0.888520145743263,0.982621488150931
"15289","TEF",112.507480172579,-0.102869564703801,0.733666047265532,-0
.140213064359744,0.888491650007733,0.982621488150931
"15290","UBE2F",199.071635283322,-0.0304208351045927,0.217637189785198
,-0.13977774264875,0.888835599425904,0.982753990343152
"15291","MTBP",17.8008112962898,0.0508585819310647,0.36401393674833,0.
139716029516274,0.888884360913048,0.982753990343152
"15292","LINC00710",30.0841861823099,0.0821516775874652,0.587656295500
818,0.139795452233611,0.888821606603518,0.982753990343152
"15293","PDK3",51.0219768490881,0.0383827617091375,0.274372380421873,0
.139892950048837,0.88874457156096,0.982753990343152
"15294","SAP30L",310.882777994745,0.023000560823415,0.164874861597961,
0.139503139535612,0.889052575208884,0.982838809871315
"15295","NAP1L1",1922.48373231855,0.023687326173707,0.169835949947601,
0.139471803119512,0.889077335993717,0.982838809871315
"15296","NMD3",282.079126901104,-0.0249946147164471,0.179351760847425,
-0.13936085488288,0.889165003730172,0.982871462020774
"15297","CCDC148",4.59011343695889,-0.101390497715822,0.72845283345440

9,-0.139186084615823,0.889303104336133,0.982895599955245
"15298","NUDT1",445.573371232672,-0.0402850311328379,0.289405381273718
,-0.139199316044288,0.889292648960222,0.982895599955245
"15299","ADGRL1",57.5461864978697,0.0308394161057356,0.221920415044455
,0.1389661068341,0.889476931955396,0.983023463331057
"15300","MRTFB",150.885102042502,0.0335820483893098,0.241935585980175,
0.138805741425991,0.88960365690774 6,0.983099256927855
"15301","LINC01266",8.09002136589447,0.0696396901505358,0.502915755662
667,0.138471879964816,0.889867492038387,0.983133792171004
"15302","ARHGEF5",59.4975692612985,-0.0351980337331447,0.2541633439261
32,-0.138485877583411,0.889856430128535,0.983133792171004
"15303","NELFB",276.404252847073,0.0211977371994621,0.15293831850083,0
.138603179420644,0.889763730749692,0.983133792171004
"15304","AC027277.1",13.5924087810337,-0.0833349307151245,0.6016609651
37666,-0.13850812258704,0.889838850594874,0.983133792171004
"15305","FURIN",148.446511489441,0.0279174067728683,0.201853059093034,
0.138305591692822,0.889998906451076,0.983185182722892
"15306","HAR1A",9.34597886136327,0.0900523483724525,0.651298505311982,
0.138265860642987,0.890030305580588,0.983185182722892
"15307","AC007390.2",16.6816191669235,0.0706122492027823,0.51206576622
5437,0.137896836422561,0.890321950692307,0.983381520941051
"15308","GOLGA8O",10.0407686207322,-0.0787386369642633,0.5710093253475
97,-0.137893784687898,0.890324362583724,0.983381520941051
"15309","CEBPZ",465.440131896797,-0.022970427418898,0.167348197753691,
-0.13726127754723,0.890824276718186,0.983656395170287
"15310","NMI",267.441191570396,-0.0250135962317003,0.182638110566299,-
0.136957156171522,0.891064660336964,0.983656395170287
"15311","MKRN2",133.396616604464,-0.0268223731795772,0.196148798483338
,-0.136745029217477,0.891232335653538,0.983656395170287
"15312","AC011365.2",2.27073290486343,-0.220930890217225,1.61422390965
509,-0.136865083521425,0.891137438376869,0.983656395170287
"15313","SFT2D1",671.895460044395,0.0335601054533772,0.245334082561081
,0.136793490341978,0.891194029227956,0.983656395170287
"15314","PUM3",293.722216142274,-0.0254866429647764,0.186288738255873,
-0.136812580316958,0.891178939497062,0.983656395170287
"15315","CRADD",57.3373565461542,-0.0369136399593938,0.269275587334102
,-0.137084985404167,0.890963620333433,0.983656395170287
"15316","AC020663.3",7.76988641231097,-0.10345074639249,0.755844419563
535,-0.136867778229055,0.891135308355206,0.983656395170287
"15317","HAS3",3.47373451814982,-0.107344380414049,0.781871134175251,-
0.137291652961815,0.890800267933695,0.983656395170287
"15318","HDHD2",151.067634966962,0.0254410126358705,0.186114248852064,
0.136695673720784,0.891271349302626,0.983656395170287
"15319","AC005498.3",7.35088208332875,0.0789111310403266,0.57556594083
4277,0.137101807876167,0.890950323486169,0.983656395170287
"15320","RAB9A",463.191307059028,-0.0342394277772112,0.250337022914992
,-0.136773328125892,0.891209966557998,0.983656395170287
"15321","RAB7A",1116.60647250358,-0.0249001094000772,0.182473599515654
,-0.136458695757471,0.89145867506319,0.983787499718985
"15322","SERINC3",1348.86833496675,0.0238911877430039,0.17515766859969

9,0.136398183042754,0.891506509977187,0.983787499718985
"15323","FAM89A",120.722316876774,-0.0324314350154673,0.239579338330034,-0.13536824686773,0.892320728343433,0.983943761071939
"15324","POC1A",7.10895590149758,0.075277705214639,0.556391526714195,0.13529628256418,0.892377624139084,0.983943761071939
"15325","GRSF1",291.945725860834,-0.0190208991488975,0.140462577591462,-0.135416133428935,0.892282868991418,0.983943761071939
"15326","ZNF131",321.856194143493,0.0235034276159975,0.173850319828552,0.13519346780136,0.892458911619209,0.983943761071939
"15327","SMIM27",101.658521502139,0.038008435815957,0.280641395795962,0.135434174663209,0.89226860556608,0.983943761071939
"15328","WASHC2A",244.202882432808,0.0303448564793462,0.22397219483512 4,0.135484927053933,0.892228480841114,0.983943761071939
"15329","PYROXD1",124.074041092546,-0.0310994098524112,0.229699543831872,-0.135391691831893,0.892302192608197,0.983943761071939
"15330","RASSF8",434.898627476955,-0.0253802754555974,0.187126971881158,-0.135631305313464,0.892112756066991,0.983943761071939
"15331","HOXC-AS1",84.4064256937659,0.0471345758703306,0.348658144109274,0.135188512491933,0.892462829417983,0.983943761071939
"15332","TRAPPC2L",805.954361900593,0.043889106268314 3,0.323550875957879,0.135648238127558,0.892099369349609,0.983943761071939
"15333","PPFIA3",7.8875259304049,-0.0716016329495902,0.525940630573181,-0.136140143558708,0.89171049296848,0.983943761071939
"15334","ZNF547",3.86515878683694,-0.0966223033472293,0.712093579631321,-0.135687648521216,0.892068212462001,0.983943761071939
"15335","PDRG1",112.385839278338,0.0417664743702417,0.306988630390528,0.136052186418466,0.891780025673457,0.983943761071939
"15336","NELFCD",246.047854402257,0.0236636323424237,0.174789475552236,0.135383622312842,0.892308572413766,0.983943761071939
"15337","ALAS1",92.387412392925,0.0257540252344489,0.191061463030287,0.134794452141123,0.892774392355077,0.984169744276134
"15338","SNX24",74.2070564316768,-0.0349047189060301,0.25985302239388,-0.134324852504976,0.893145701868214,0.984169744276134
"15339","VPS50",98.4335719692426,0.0243626600490406,0.181265306419633,0.134403325877708,0.89308365182803,0.984169744276134
"15340","STRIP2",2.07033908336974,0.141828280395546,1.05486401181996,0.134451719658962,0.893045386485689,0.984169744276134
"15341","GVQW3",55.9838243499587,0.040751797185288,0.303513760270173,0.134266720391895,0.893191668199507,0.984169744276134
"15342","FLVCR2",32.1830518601532,0.0477512527466929,0.355147825047689,0.134454583074754,0.893043122368169,0.984169744276134
"15343","AC015871.1",41.9666662189614,-0.0542992247776103,0.403542009679134,-0.134556560346183,0.892962488986222,0.984169744276134
"15344","WHAMM",133.007604753723,0.0258889845178204,0.192625215302057,0.134400807688773,0.893085642986582,0.984169744276134
"15345","PPP4R1",152.58536708931,-0.0222423691395027,0.165237549160342,-0.134608442527306,0.892921466193895,0.984169744276134
"15346","AF230666.2",2.06068141317381,0.141709780065819,1.05606078835955,0.134187143039319,0.893254592331681,0.984174941166693

"15347","AC025171.4",5.93279915274821,0.0959469704167299,0.715581121863718,0.134082590338378,0.893337266221059,0.984201895957885
"15348","SELENOF",1475.13408347416,0.0289989419750905,0.2165860122406,0.133891111780923,0.89348867877227,0.984240444373024
"15349","GGT7",138.433114640495,-0.0259441872028852,0.193706563110734,-0.133935509392389,0.89345357081063,0.984240444373024
"15350","ZMYND11",423.369282173634,0.0211589805181111,0.158470969589583,0.13351960029594,0.893782464257643,0.984499928708028
"15351","AC006059.1",13.9253517560618,-0.0544757731090562,0.408565858735054,-0.133334129478456,0.893929137142388,0.984533210708931
"15352","AC244517.1",6.21483869698008,0.0862069315987062,0.646447322701852,0.133354920921323,0.893912694801529,0.984533210708931
"15353","LINC00884",12.4452624762753,0.069172575715101,0.519699858454063,0.133101009341944,0.894113496911001,0.984609557811432
"15354","MXI1",533.173049268799,0.0368507220256343,0.276866585299973,0.133099203667746,0.894114924925286,0.984609557811432
"15355","MED28",418.683798312788,-0.0219583284825874,0.165172936562386,-0.132941442705983,0.894239691230642,0.984682819884578
"15356","FAM47E",2.19486683279636,-0.152480664613888,1.14853892670104,-0.132760554360887,0.894382751200946,0.984752348110061
"15357","KCTD17",97.3867679365683,-0.0296669973178208,0.223540287087612,-0.132714320556426,0.894419316887048,0.984752348110061
"15358","WASF2",2474.70951391908,0.0174726577669347,0.134286152235436,0.130115112214258,0.896475353945072,0.985031388631088
"15359","PHACTR4",292.439389527728,0.027970448160294,0.21278154000334,0.131451479107891,0.895418167483856,0.985031388631088
"15360","TMEM54",381.246290834703,0.0315109347968368,0.239071942485261,0.131805240168572,0.895138341661915,0.985031388631088
"15361","MYCBP",49.2371582544949,-0.0445157974988223,0.336469631679356,-0.132302571488069,0.894744973503574,0.985031388631088
"15362","JAK1",1459.4690593164,0.0186576899219403,0.142956089008137,0.130513432840754,0.896160227172264,0.985031388631088
"15363","LPGAT1",203.849535249762,-0.0252824379488825,0.193621758316565,-0.130576429884221,0.896110389286464,0.985031388631088
"15364","COPS8",476.14553505038,-0.0330028730147416,0.250930328763516,-0.131522057048132,0.895362339128738,0.985031388631088
"15365","GLT8D1",686.471785680248,-0.0266757023413874,0.204820344000787,-0.130239515373946,0.896376932062853,0.985031388631088
"15366","AC012557.2",6.36185216554614,0.099529059052717,0.75983419488738,0.130987865145328,0.895784906902927,0.985031388631088
"15367","CTBP1",303.203334871738,-0.0264239396291747,0.201178351729106,-0.131345840156577,0.895501730664773,0.985031388631088
"15368","CRIPAK",36.7478663589833,-0.0453710311061233,0.348514269399337,-0.130184141912812,0.896420740723309,0.985031388631088
"15369","CCDC149",144.108665826494,0.0274105543021183,0.210391741619074,0.130283413651029,0.896342202207716,0.985031388631088
"15370","PLAC8",8.69074393440543,0.0992429986776654,0.752873779213287,0.131818906990451,0.895127531435559,0.985031388631088
"15371","AL135910.1",20.7077266170373,0.0503062385217117,0.383289849176835,0.131248554141861,0.895578687479375,0.985031388631088

"15372","ZNF789",28.15354952065,0.0386321953571843,0.296493141381769,0.130297096172761,0.896331377404399,0.985031388631088
"15373","AC005046.1",4.38600988931345,0.113980902363308,0.876221625333945,0.130082274926584,0.896501333516148,0.985031388631088
"15374","AC022915.1",4.44170584267478,-0.102535006731511,0.778510718031912,-0.13170660898635,0.895216357846235,0.985031388631088
"15375","OTUD6B-AS1",546.801114013649,-0.0276464377666163,0.211442930192529,-0.130751298903411,0.895972049987553,0.985031388631088
"15376","C12orf81",67.5827114158492,-0.0546737262924211,0.417912016080581,-0.13082592552658,0.895913013650353,0.985031388631088
"15377","C12orf29",179.054778039287,-0.030999505162536,0.234930284602308,-0.131951932953269,0.895022311152868,0.985031388631088
"15378","PPP1R3E",38.6061027984479,0.0483632904551253,0.366214557264619,0.132062719779266,0.894934682892922,0.985031388631088
"15379","ATL1",233.484830957921,0.0269710513599161,0.205311856149192,0.131366263331219,0.895485575307585,0.985031388631088
"15380","ZNF710-AS1",7.38816432330846,0.0922049076579763,0.707087837410486,0.130400924439107,0.896249235239548,0.985031388631088
"15381","UBE2I",1264.73358224079,-0.0246881711468883,0.18883399445852,-0.130740077906425,0.895980926850154,0.985031388631088
"15382","CCDC144NL",5.90338256129045,0.11100718779694,0.845928507251382,0.131225259398845,0.895597114623431,0.985031388631088
"15383","MTMR4",90.83580777872,-0.0378880677055091,0.291025946353725,-0.130187937468154,0.896417737863272,0.985031388631088
"15384","SLC9A3R1",54.4688366933679,-0.0389506294206649,0.297291070826633,-0.131018497502669,0.895760674647604,0.985031388631088
"15385","MIF4GD",142.7076850947,-0.0353115996410679,0.270032464796853,-0.130767978834075,0.895958854619726,0.985031388631088
"15386","ZNF473",22.9617274963461,0.0436558228438586,0.331048047762856,0.131871561058506,0.895085883134954,0.985031388631088
"15387","BACH1",241.42620779645,-0.0416325637037408,0.318056118413882,-0.130896911876303,0.895856857630096,0.985031388631088
"15388","SH3BGR",25.1986878238392,-0.0581382099240127,0.447089198015624,-0.130037160776989,0.896537026238692,0.985031388631088
"15389","MTM1",35.1004942154863,0.0365849029542215,0.279244045827809,0.131014084278026,0.895764165799182,0.985031388631088
"15390","CRIM1-DT",73.5222819608424,0.0475800986603101,0.367280411489968,0.129547063147989,0.896924787659421,0.985038378366764
"15391","FOXP1-IT1",5.79737929754657,-0.0836422573794594,0.646045932019662,-0.129467973148562,0.896987365359492,0.985038378366764
"15392","AC025449.2",17.028739427591,0.0476561110808256,0.36872106972875,0.129247051479548,0.897162166705279,0.985038378366764
"15393","NNT",206.322578631032,-0.024760758881802,0.190874438460375,-0.12972275953515,0.896785775212765,0.985038378366764
"15394","LINC01600",3.11168194821791,0.106879439546349,0.823398871298445,0.129802752070582,0.896722485501042,0.985038378366764

"15395","C9orf106",2.18748909421878,0.147888295773071,1.14045808409908
,0.129674468386883,0.896823983256415,0.985038378366764
"15396","AP006623.1",31.4413706669422,-0.0533626100576835,0.4126057360
62285,-0.129330751838186,0.897095939316887,0.985038378366764
"15397","LINC01852",22.3705973740237,0.0427823978240383,0.330642573154
71,0.129391679407298,0.897047731207463,0.985038378366764
"15398","CCR7",8.04085602804331,0.0936326772430853,0.725009810618655,0
.129146772735652,0.897241512581262,0.985038378366764
"15399","TMEM106A",47.2777815326714,-0.0522640413649423,0.403154251311
377,-0.129637827692349,0.896852973599694,0.985038378366764
"15400","ZNF254",164.167923472628,-0.0226555303535434,0.17551300678149
,-0.129081774445065,0.897292943234965,0.985038378366764
"15401","ERCC2",41.6488694062956,0.0325693077233967,0.251120767487432,
0.129695795569861,0.896807109121252,0.985038378366764
"15402","RAP2C",148.924512777752,0.0211554417448152,0.163904309664388,
0.129071906578499,0.897300751336935,0.985038378366764
"15403","EIF4E3",150.224085173023,-0.0338964697823835,0.26390190861728
1,-0.128443443095901,0.897798053236579,0.985071758527601
"15404","ARAP2",17.4298354450487,0.0709369601216643,0.550443923326135,
0.128872274023878,0.897458715816609,0.985071758527601
"15405","AC097376.3",91.1626554781784,-0.0310916527791882,0.2418902007
21937,-0.128536222990402,0.897724634160742,0.985071758527601
"15406","CCDC183",23.9187236079493,-0.0480411366906139,0.3748821345041
71,-0.128149976402995,0.898030286606602,0.985071758527601
"15407","PHPT1",1522.76547226596,-0.0338266141660719,0.263774277771975
,-0.128240761198535,0.897958443587943,0.985071758527601
"15408","FAM13C",202.342486835436,-0.0342320400644456,0.26708044292610
1,-0.128171271881249,0.898013434244414,0.985071758527601
"15409","NUAK1",479.56681324206,0.0545237252524851,0.425010679246085,0
.128287894669384,0.897921144601218,0.985071758527601
"15410","GOLGA8A",196.071733177248,-0.0570957005345695,0.4442283024895
69,-0.12852783178062,0.897731274301155,0.985071758527601
"15411","COG1",92.0657427305712,0.0282253045511533,0.219813504557404,0
.128405689213613,0.897827929088474,0.985071758527601
"15412","ZNF420",78.8594687172012,-0.0295825956259236,0.23002901249549
4,-0.128603758738924,0.897671191966339,0.985071758527601
"15413","CPXM1",1412.90494352969,-0.054522021811724,0.424730461717712,
-0.128368522453567,0.897857340473275,0.985071758527601
"15414","ATP6AP1",440.893428663677,-0.0218599630257964,0.1704243737588
16,-0.128267820756276,0.897937030028404,0.985071758527601
"15415","IVD",171.082345137793,-0.0256553621462131,0.200405744179531,-
0.128017099765514,0.898135440729423,0.985123193762769
"15416","AC091132.4",32.5338921489324,0.0732170530539055,0.57263659027
9569,0.127859543551277,0.898260127760917,0.985196045678619
"15417","TRAM1",1298.90835822443,-0.0181595746325853,0.142278205838051
,-0.127634267846023,0.898438411089434,0.98532766781476
"15418","PAQR7",56.3168342431198,-0.0449811892866987,0.354143019827996
,-0.12701419135282,0.898929166446137,0.985338580148876
"15419","PKP4",52.0348871650557,-0.0417728195590715,0.330477483797491,
-0.126401408891956,0.899414186964219,0.985338580148876

"15420","AC010680.2",4.60808478187341,-0.0924171197049013,0.732692539710618,-0.126133561754842,0.89962620118241,0.985338580148876
"15421","LINC02018",15.0834726018654,0.0532730093629259,0.41952757861152,0.126983330962984,0.898953591704701,0.985338580148876
"15422","METAP1",116.395403577499,0.0236380132275852,0.186224514125185,0.126932876365005,0.898993525515327,0.985338580148876
"15423","AC109347.2",2.37650404360109,0.128789323837738,1.02021049781295,0.126237991192823,0.899543539279437,0.985338580148876
"15424","RGL2",179.473796083923,-0.0250614428553644,0.196951905527161,-0.127246511214426,0.898745293921253,0.985338580148876
"15425","TJAP1",97.0640937914142,0.0233051943669321,0.184571111685719,0.126266749731753,0.899520775434107,0.985338580148876
"15426","EPHX2",134.247049093837,-0.0266210565454484,0.211014130933593,-0.126157695826665,0.899607097580515,0.985338580148876
"15427","MVB12B",37.7470975292014,-0.0393292741940845,0.30858824668013,-0.127449034813214,0.898585008463748,0.985338580148876
"15428","ZNF33B",80.1382514391246,-0.0414551080653903,0.328536935670139,-0.126180966474383,0.899588677486974,0.985338580148876
"15429","RRP8",126.508779526816,-0.0233245731518361,0.184619640085384,-0.12633852574433,0.899463961432498,0.985338580148876
"15430","ARL14EP",281.045272424548,0.0279992353902812,0.22033391748526,0.127076374395033,0.898879950350131,0.985338580148876
"15431","TNFRSF1A",1250.65131625522,-0.0361479252981185,0.286716511404376,-0.126075492203296,0.899672167041539,0.985338580148876
"15432","CLEC4E",10.2438343995576,0.0651710104427577,0.512992600216926,0.127040839215223,0.898908075379053,0.985338580148876
"15433","AC090517.5",48.0585668214307,-0.0350885041840304,0.277550998849476,-0.126421826365179,0.899398025821497,0.985338580148876
"15434","TMEM186",80.0863079136111,0.0273806609376841,0.216392504226808,0.126532390923234,0.899310510839327,0.985338580148876
"15435","LCAT",70.2805353010196,-0.0275263855949892,0.217379189874013,-0.126628430306244,0.899234493938579,0.985338580148876
"15436","AC015922.3",119.882453026991,0.0234605108659307,0.184923125770963,0.126866289806218,0.89904622784349,0.985338580148876
"15437","CCDC97",146.502468966147,0.0255966911571316,0.202590389033623,0.126347016160196,0.899457240913512,0.985338580148876
"15438","DDTL",10.4064732172351,-0.0858305461837842,0.67965072002701,-0.12628625800672,0.899505333689939,0.985338580148876
"15439","BOD1L1",1205.3523718018,0.0223423602691552,0.177591620331878,0.125807514044877,0.89988429368944,0.985507068961788
"15440","AC104596.1",7.02100334903297,0.0695654491436571,0.553930802336016,0.125585089058576,0.900060366704931,0.985606660891206
"15441","AC026471.2",8.68311559353429,-0.0707739342646874,0.5640628227299,-0.125471723029293,0.900150109858174,0.985606660891206
"15442","AC092376.2",40.767963305572,0.0348196830523254,0.277355599444401,0.125541662479778,0.900094744031475,0.985606660891206
"15443","CNTLN",367.848925413922,0.02465633534206,0.19701257007687,0.125151077073101,0.900403947471228,0.985756924620793
"15444","KIF21A",65.1536788828341,-0.0419050360291482,0.334708064131519,-0.125198764295927,0.900366195497307,0.985756924620793

"15445","GZMK",28.4512364474709,0.0647783461889094,0.518446521138025,0
.124947016804581,0.900565495969698,0.985809203359874
"15446","SURF4",605.594216347206,0.0249969010505908,0.200065694800207,
0.124943464573242,0.900568308203017,0.985809203359874
"15447","LYSMD4",44.4371201261124,0.0396502305248622,0.317770272418172
,0.124776399702626,0.900700571661189,0.985890157677697
"15448","C8orf37",34.2501401731418,0.0356079455810315,0.28582102497611
2,0.124581267539739,0.900855059182892,0.985928174289454
"15449","ATAD2",80.8855122074585,0.0338933215087481,0.272210209424209,
0.12451157353885,0.900910237329788,0.985928174289454
"15450","NUBPL",111.790579071318,0.0282253366700618,0.226637413024991,
0.124539617238525,0.900888034509261,0.985928174289454
"15451","NDUFAF7",104.925900822243,-0.0223009520221539,0.1795320082548
62,-0.12421713675979,0.90114335415072,0.985995769716441
"15452","LINC01119",9.65125533669463,0.0683077778630749,0.549755765398
739,0.124251135071828,0.901116435955384,0.985995769716441
"15453","AC008764.6",2.28025334913251,0.151384732950423,1.218755092322
98,0.124212595216221,0.901146949930693,0.985995769716441
"15454","SKI",545.31377666307,0.0267664352809741,0.216708367528471,0.1
235136214916,0.901700388491136,0.986022426819299
"15455","ATP5IF1",1434.2440955939,-0.0341639257416073,0.27660571648723
6,-0.123511278709179,0.901702243557138,0.986022426819299
"15456","BDH2",1197.75544040504,-0.0311926391416266,0.252076809472965,
-0.123742597372774,0.901519083007028,0.986022426819299
"15457","RNASET2",442.361388306012,0.0277093371900919,0.2246005089017,
0.123371658085687,0.901812799168064,0.986022426819299
"15458","LAPTM4B",345.671520202309,0.0323125491496083,0.26163016430523
8,0.123504677816546,0.901707470290175,0.986022426819299
"15459","ETNK1",317.819002742915,0.0168871473999311,0.136802587591314,
0.12344172502336,0.901757317920663,0.986022426819299
"15460","AC005476.2",2.4501943527112,-0.116896652797513,0.942977380306
089,-0.12396548977619,0.901342599417938,0.986022426819299
"15461","IGHA1",225.16870596114,-0.0942510344845881,0.7639414949836,-0
.123374676075962,0.901810409416731,0.986022426819299
"15462","AL021578.1",5.68228001493832,0.0897132822266804,0.72626954426
1248,0.123526152150488,0.901690466452394,0.986022426819299
"15463","HDAC10",71.9823178000488,-0.0315308696794881,0.25425718886592
7,-0.124011713572884,0.901306000568418,0.986022426819299
"15464","MAPK12",7.20281747826093,0.093218863473705,0.752201219995419,
0.123928093967028,0.901372208640544,0.986022426819299
"15465","NBPF1",113.34234404438,-0.0359452408196867,0.294662324693755,
-0.121987908895529,0.902908594054857,0.986036776029188
"15466","PTGFRN",251.763342871844,0.0319902014279919,0.26316279926835,
0.121560499876623,0.903247097952211,0.986036776029188
"15467","TMCO1",1065.54432998899,-0.0224886188447701,0.184625022779819
,-0.121806992931774,0.903051875635071,0.986036776029188
"15468","AC104695.4",102.110011126852,0.0375083260672932,0.30708810175
7298,0.122141906028444,0.902786634105074,0.986036776029188
"15469","RYBP",300.034636746876,-0.025659788951013,0.211018174900463,-
0.12159989928604,0.90321589274713,0.986036776029188

"15470","AC016924.1",2.0595838714965,0.145886086083333,1.1989007446004
5,0.121683205836987,0.903149914190545,0.986036776029188
"15471","P3H2",2.41421669729264,-0.119241783234284,0.971567768421563,-
0.122731308211272,0.902319870913102,0.986036776029188
"15472","TMEM232",4.43371401002556,0.0963731536891626,0.78429552341891
7,0.122878622676629,0.902203213955859,0.986036776029188
"15473","CDC5L",496.464700065468,-0.0193596153069912,0.158399438282319
,-0.122220227021804,0.902724607697555,0.986036776029188
"15474","AL021368.1",2.35862614011779,-0.123612942415859,1.00763181371
016,-0.122676696719914,0.902363117782036,0.986036776029188
"15475","PEX7",61.1736862239225,0.0362492384543081,0.297215991886796,0
.121962611177782,0.902928629117071,0.986036776029188
"15476","UBE2D4",177.147759535976,-0.0318116840782511,0.25922290496995
4,-0.122719418185435,0.9023292866061 8,0.986036776029188
"15477","LINC01474",2.85310830596477,0.123435227962863,1.0148632122682
,0.121627453306724,0.903194070305998,0.986036776029188
"15478","PHYHD1",417.202772388899,-0.0335975731301924,0.27611490622293
3,-0.121679678905368,0.903152707518566,0.986036776029188
"15479","FUBP3",211.349976829087,0.0175801554767618,0.144292859707829,
0.121836628037998,0.903028405047367,0.986036776029188
"15480","DDX31",26.2978846753648,0.0367637187130374,0.301123819059149,
0.122088378222302,0.90282902585869,0.986036776029188
"15481","RAD51AP1",14.0083101792996,-0.0520715520520398,0.428126970809
732,-0.121626423006135,0.903194886308943,0.986036776029188
"15482","RND1",31.320341956162,0.0359872304632932,0.293227113582019,0.
12272818165987,0.902322346822993,0.986036776029188
"15483","RFC3",55.6830637997794,-0.0337188731899597,0.277489444558116,
-0.121514075044025,0.903283867040223,0.986036776029188
"15484","SIX1",405.462939123147,0.0285363065048189,0.234468379751566,0
.121706417449786,0.903131530635107,0.986036776029188
"15485","AL049840.3",3.78843747520067,-0.104313268798917,0.85649912864
6439,-0.121790280118286,0.903065111985874,0.986036776029188
"15486","WDR73",180.667013198088,0.0226729333182246,0.185418657086399,
0.122279676028825,0.902677527379306,0.986036776029188
"15487","INO80E",179.297121535413,-0.0200439836877024,0.16364908022263
7,-0.122481493085286,0.902517702011508,0.986036776029188
"15488","PI4KA",128.058265295534,0.0250080999233132,0.205595881560262,
0.121637163806626,0.903186379549121,0.986036776029188
"15489","SMTN",101.368725711385,0.0333229292940961,0.27140136172889,0.
122780995208798,0.902280524005274,0.986036776029188
"15490","ZNF484",42.5908425031967,-0.0372057382043101,0.30697421332619
9,-0.121201510059004,0.903531428082253,0.986116020269476
"15491","B3GLCT",80.4255937752657,0.031130846928619,0.256620290902279,
0.121310933048835,0.903444760654519,0.986116020269476
"15492","POLG",104.978087293188,0.0249003403995007,0.205331921654401,0
.12126872528574,0.903478190771498,0.986116020269476
"15493","ALG2",172.506767621003,0.0238893205725611,0.197348228704672,0
.12105160876975,0.903650157823765,0.986118295371383
"15494","ATP6V0A1",173.34829195175,0.0285825065959812,0.23608021805718
,0.121071163146157,0.903634669602196,0.986118295371383

"15495","AL021155.5",111.900603742845,0.0518784041982898,0.440192882030454,0.117853800722522,0.90618349973054,0.986147517022834
"15496","PMF1",444.02346820782,0.0345178771293089,0.288556154424657,0.119622737550454,0.904782007098428,0.986147517022834
"15497","AKT3",398.270883388778,0.032393269305632,0.27428456634365,0.118100955290876,0.905987666538138,0.986147517022834
"15498","CAD",68.3494425853066,-0.0279287240066323,0.231346670155779,-0.12072394611629,0.903910920389863,0.986147517022834
"15499","SERTAD2",262.551655222748,-0.0241488130906439,0.205387202019327,-0.117577009926702,0.906402821990961,0.986147517022834
"15500","WDR54",75.3728929998337,0.037389727899408,0.316299064869122,0.118210048818447,0.905901227980004,0.986147517022834
"15501","CCR1",11.5608490452614,0.0861271483591088,0.731248846612132,0.117780901478525,0.906241262616128,0.986147517022834
"15502","SEMA3B-AS1",2.68263219093951,-0.106838064382078,0.906867947737052,-0.117809946474209,0.906218248288341,0.986147517022834
"15503","GSK3B",223.015745703748,0.0195486024254365,0.164818038693753,0.118607177833002,0.905586578410246,0.986147517022834
"15504","ZFYVE28",47.8935463616082,0.0287279709420456,0.242968653543015,0.118237354996741,0.905879592529136,0.986147517022834
"15505","LYAR",222.689124456643,0.021608254652635,0.181340833963477,0.119158240206323,0.905149990353503,0.986147517022834
"15506","ZNF827",175.73720797968,0.0326353278002131,0.273057926736261,0.119517965254803,0.90486500783606,0.986147517022834
"15507","AADAT",43.4994834692882,0.0314333304795148,0.264277915407302,0.118940435984105,0.905322545844041,0.986147517022834
"15508","AC112206.2",9.57858374231855,-0.0610805334187236,0.507807741119035,-0.120282793019506,0.90425913432469,0.986147517022834
"15509","APBB3",41.232310593676,0.0342256466450813,0.290547359161451,0.117797135530193,0.906228399261891,0.986147517022834
"15510","NKAPL",17.7554327655787,0.0518835273230564,0.431429229881187,0.120259648001469,0.904277468294592,0.986147517022834
"15511","ABCC10",31.6013399965754,0.0374022225213074,0.313946844818544,0.119135526088581,0.905167985414219,0.986147517022834
"15512","SNX9",1679.84159475894,-0.0213150291596412,0.180728775726984,-0.11793932136098,0.906115736713158,0.986147517022834
"15513","MAD1L1",113.818360153485,-0.0312984322101907,0.260728173119238,-0.120042386811329,0.904449570889819,0.986147517022834
"15514","WIPI2",517.912148893714,0.0161737846266485,0.137086174206327,0.117982609991767,0.906081436852742,0.986147517022834
"15515","RALA",522.469027878834,-0.0210679780328534,0.17540656608626,-0.120109403558432,0.904396483358701,0.986147517022834
"15516","AC092849.3",2.26239473848509,-0.120355751667398,0.995274278276268,-0.120927220058218,0.903748681891458,0.986147517022834
"15517","ZNHIT1",1193.6566979045,-0.0285291428683625,0.242331782385424,-0.117727615369029,0.906283485033694,0.986147517022834
"15518","MMP16",39.7870637644524,0.0466715884567008,0.392273540891092,0.118977151379319,0.905293457742605,0.986147517022834
"15519","FRMD4A",120.165291194212,-0.0317756798722776,0.26622733657355

4,-0.119355436151834,0.904993765641756,0.986147517022834
"15520","TRDMT1",95.1162674297763,0.0310780405249489,0.263022227670941
,0.118157468287546,0.905942889205962,0.986147517022834
"15521","CFAP70",20.2717239660667,0.0631533973175309,0.52351370919448,
0.12063370301172,0.903981172632676,0.986147517022834
"15522","ARFGAP2",341.21659157567,-0.0210208647715358,0.17873024654287
9,-0.117612240670707,0.906374905613846,0.986147517022834
"15523","GRK2",159.76843387537,-0.0246949541700358,0.209891484298397,-
0.117655817493423,0.90634037607401,0.986147517022834
"15524","UHRF1BP1L",172.824427707077,0.0224473889358807,0.188987089461
884,0.118777367278351,0.905451740024405,0.986147517022834
"15525","AL627171.1",9.03984903063297,0.0582342159485162,0.49298991908
6003,0.118124557306327,0.905968965754204,0.986147517022834
"15526","AC004846.1",55.6429502304961,0.0307544346347818,0.25793182306
2279,0.119234743001665,0.905089382042684,0.986147517022834
"15527","SPATA7",150.582998726559,-0.0257741065266729,0.21602005340600
1,-0.119313490207465,0.905026996205287,0.986147517022834
"15528","SNUPN",275.434349907061,-0.0241005807418631,0.203324594098252
,-0.118532540781648,0.90564571301561,0.986147517022834
"15529","AC012291.3",5.91736319292853,0.0839521395055173,0.70657917271
2449,0.118814908148563,0.905421997357888,0.986147517022834
"15530","GAS8",49.8521029714337,0.0279108509847195,0.233122054936784,0
.119726342461626,0.904699932187101,0.986147517022834
"15531","RNF135",166.638146526691,0.0231117380277244,0.194689082420817
,0.118711012144835,0.905504311815447,0.986147517022834
"15532","MYO19",80.3570378480343,0.0358987265204028,0.305341889819093,
0.117568953744512,0.906409205619772,0.986147517022834
"15533","LRRC37A2",20.2268966189487,0.0586790688628393,0.4994577183485
11,0.117485558250787,0.906475287632979,0.986147517022834
"15534","EIF4A3",702.530098700465,-0.0328835638334989,0.27357513691634
1,-0.120199387284067,0.904325203134013,0.986147517022834
"15535","MYO9B",157.435103298597,0.0264413519357133,0.223500964463588,
0.118305314695951,0.90582574645681,0.986147517022834
"15536","GDF15",97.0713172681948,-0.0488848222744853,0.414708953667023
,-0.117877421845432,0.90616478326126,0.986147517022834
"15537","XRCC1",124.047633198463,0.0231182277709702,0.194675185173046,
0.118752822813141,0.905471186045001,0.986147517022834
"15538","RALY",682.517289824762,-0.0275362401492657,0.22922368906456,-
0.120128247920791,0.904381555813594,0.986147517022834
"15539","LINC01547",8.81117479252244,-0.0762745817752966,0.63651603087
2228,-0.11983136021064,0.904616739076895,0.986147517022834
"15540","DRG1",256.440744307504,0.0229633849018065,0.195459637684971,0
.117484024700881,0.906476502813395,0.986147517022834
"15541","USP51",88.1569949668666,0.0294154107838945,0.248453028583839,
0.118394253237965,0.905755279006646,0.986147517022834
"15542","XPNPEP2",105.839811128976,0.0322507869869213,0.27234419987012
4,0.118419217307735,0.905735499702386,0.986147517022834
"15543","RBFOX2",819.697813370802,-0.0140087018055345,0.11946830883618
5,-0.11725872695447,0.906655030459633,0.986278276716945
"15544","PDE1A",136.797973467169,0.036916815264723,0.315664613660805,0

.116949489005415,0.906900080638088,0.986309201353757
"15545","AC083855.2",7.28707010010419,-0.100339600463635,0.86229292012
0906,-0.116363706719946,0.907364297791774,0.986309201353757
"15546","ARL15",45.1333433712253,0.02965808595194,0.254585013130908,0.
116495804632026,0.907259610879444,0.986309201353757
"15547","USP45",52.9474431129521,0.0278227043710734,0.239021142946424,
0.116402691528045,0.907333402365464,0.986309201353757
"15548","TRAF1",14.2345046671576,0.0528263323603398,0.453013917470626,
0.11661083759919,0.907168449159934,0.986309201353757
"15549","NCAPD3",47.4589961852404,0.0364586257264729,0.313579659094954
,0.116265914159416,0.907441798927079,0.986309201353757
"15550","DNAAF4",48.7942254861145,-0.0359505299572155,0.30698012493451
8,-0.117110285119873,0.906772659475375,0.986309201353757
"15551","LINGO1",10.3241648639832,-0.078121687154343,0.671652050501597
,-0.116312735286077,0.907404692819932,0.986309201353757
"15552","ZNF778",23.2010639668013,-0.0381748690360714,0.32653575299532
6,-0.116908695865282,0.906932407107279,0.986309201353757
"15553","PGAP3",42.8535150072749,-0.0282046496561576,0.241284506324359
,-0.116893745420363,0.906944254605913,0.986309201353757
"15554","ITGB3",54.9568161025604,-0.0393826861299242,0.337443771042698
,-0.116708884589074,0.907090749511109,0.986309201353757
"15555","TMEM147-
AS1",10.3698868138802,0.0566112874052702,0.486576102306504,0.116346214
162424,0.907378160673861,0.986309201353757
"15556","ZNF331",232.378183222458,0.0278085026704775,0.238098732630838
,0.116793997024728,0.907023300875627,0.986309201353757
"15557","FGF10-
AS1",37.9759236690398,0.036132550529244,0.311130484629039,0.1161331091
43338,0.907547049034309,0.986360191879674
"15558","AL020996.1",3.1956206276169,0.0945707768613169,0.818678903678
96,0.115516323232878,0.908035883017083,0.986393724692667
"15559","AHDC1",235.324952138416,-0.0292832987238663,0.253797083560585
,-0.115380753446979,0.908143333594503,0.986393724692667
"15560","SYNC",217.326058193868,0.0299113722550891,0.259831669836535,0
.115118269739431,0.908351379016986,0.986393724692667
"15561","NEXN",728.368940981655,0.0343872287637649,0.298108604689752,0
.115351345861192,0.9081666418275,0.986393724692667
"15562","AL157904.1",2.04654514713527,-0.135653903733545,1.17834198779
179,-0.115122693699272,0.908347872520204,0.986393724692667
"15563","AC007038.2",9.56330119701477,-0.0599777530641397,0.5180037418
9551,-0.115786331667539,0.907821883399595,0.986393724692667
"15564","SLC12A2",71.1600131110011,0.0298204878188576,0.25748626355028
1,0.115813897827736,0.907800035762913,0.986393724692667
"15565","CEP57L1",177.803244613811,0.0202474265759935,0.17524096700253
,0.115540486464567,0.908016731778964,0.986393724692667
"15566","PHF20L1",456.161875133858,0.0204319557206021,0.17670497396438
6,0.115627507603267,0.907947761209813,0.986393724692667
"15567","NAPRT",252.055213460519,0.036104711929257,0.311973338248847,0
.115730120182443,0.907866434183995,0.986393724692667
"15568","AL158211.1",3.45489579409116,-0.0886076180570657,0.7700745971

7359,-0.115063681339811,0.908394646746482,0.986393724692667
"15569","USP54",34.7155643101578,-0.0356670154365899,0.309478940815635
,-0.115248602514242,0.908248076057965,0.986393724692667
"15570","C19orf57",4.99537361560984,0.081318501399231,0.70557560454472
5,0.115251293944186,0.908245942822121,0.986393724692667
"15571","DNAJC5",65.763454178382,0.0243736869121788,0.210848378607421,
0.115598170937611,0.907971012569333,0.986393724692667
"15572","ZEB2",1189.61656376786,0.0201987365255527,0.175753279421465,0
.114926655093104,0.908503257366344,0.986410376231957
"15573","HPS1",239.653578248258,-0.0181709656001751,0.158235213516905,
-0.114835157082364,0.908575782057186,0.986410376231957
"15574","TMBIM6",3416.14997591704,-0.0184941550176937,0.16106590368821
3,-0.114823526234914,0.908585001149544,0.986410376231957
"15575","AMFR",266.903269102897,-0.0237415318127612,0.20721856900067,-
0.114572414659829,0.908784045605226,0.986563123152049
"15576","AC104162.2",16.6327365073878,-0.0436017365162656,0.3812473514
50688,-0.114366005036773,0.908947661193333,0.986637540551667
"15577","PGM2L1",65.747169521269,-0.035111861113717,0.307583930255433,
-0.114153756617123,0.909115909072766,0.986637540551667
"15578","THYN1",435.789623996032,-0.0327407022753156,0.286902519661432
,-0.114117862450118,0.909144362538146,0.986637540551667
"15579","SHCBP1",3.14655998686269,0.0910745131560435,0.796834169926556
,0.114295441377015,0.909003596073933,0.986637540551667
"15580","AL359555.4",13.2181274378239,-0.0567365393002118,0.4968936984
52542,-0.114182448835444,0.909093164707893,0.986637540551667
"15581","CDK5RAP3",320.729360339142,-0.022237929124064,0.1950395827842
13,-0.114017517914133,0.90922390671509,0.986660536213256
"15582","AC242426.2",26.2877799851639,0.0403059877759218,0.35591123165
6237,0.113247304920267,0.90983449295977,0.986993870905393
"15583","SNX10",64.2002846121744,0.0608912667910024,0.53682734966954,0
.113428026400827,0.909691221235425,0.986993870905393
"15584","MKRN1",410.007403986299,0.0166447828331184,0.147140119560084,
0.113121987958706,0.909933843011032,0.986993870905393
"15585","AP001160.2",8.55272814700653,-0.0556452372121748,0.4910900836
34548,-0.11330963313359,0.909785080291846,0.986993870905393
"15586","PACS1",278.183899154084,-0.0193360954135291,0.170821004573205
,-0.113195069083221,0.909875904844957,0.986993870905393
"15587","A2M-
AS1",25.7593149247158,-0.0347415043030132,0.307535830626263,-0.1129673
38577316,0.910056449456771,0.986993870905393
"15588","AC139768.1",20.29245792045,-0.0488012184620439,0.430670609643
48,-0.113314485291771,0.909781233604195,0.986993870905393
"15589","OAS3",60.5631716157075,0.0512801567946637,0.453797114336031,0
.113002386252927,0.910028663377417,0.986993870905393
"15590","TIPIN",56.4732616222547,-0.0329583059468024,0.291603121011645
,-0.113024530850224,0.910011107019148,0.986993870905393
"15591","RAB27A",76.4706149618384,-0.0267941155743235,0.23749523964627
4,-0.112819590044123,0.910173586892283,0.987057597791978
"15592","PARP2",105.918968325987,-0.0249179241489421,0.221184897419673
,-0.11265653505114,0.91030286180396,0.987134478410811

"15593","NME5",20.1556734432232,-0.0606897318431172,0.541519148168503,
-0.1120731040599,0.910765442900201,0.987230957079352
"15594","RWDD2A",48.8954346249976,-0.0353969388744889,0.31636138482586
6,-0.111887672049395,0.91091247150186,0.987230957079352
"15595","DUSP5",149.634020575957,-0.0905250354513139,0.808725182747068
,-0.111935472497369,0.910874570347922,0.987230957079352
"15596","PPFIBP1",539.139158046639,-0.0228779071407337,0.2042865524925
74,-0.111989295729906,0.910831893948316,0.987230957079352
"15597","IRS2",470.042124761378,0.034238539881118,0.304716323733462,0.
112362014156704,0.910536372859902,0.987230957079352
"15598","DDX28",28.4959567149135,0.0382126203252402,0.340708851188272,
0.112156230141859,0.910699533418407,0.987230957079352
"15599","DDX19A",142.883457445922,-0.0240740528302701,0.21517446909869
4,-0.111881548638691,0.910917326791753,0.987230957079352
"15600","ARHGAP27",23.8259926195469,0.0474048513823462,0.4223817549237
78,0.112232242112117,0.910639265151892,0.987230957079352
"15601","SLC25A53",33.9321007072999,-0.0342162846307037,0.304475568249
221,-0.112377767541259,0.910523882584349,0.987230957079352
"15602","LINC01002",8.72146644328673,-0.0579687770963833,0.51848075974
0401,-0.111805068958408,0.910977968279347,0.987233398773696
"15603","FOXO6",10.9998923374476,0.0509033146466504,0.458726300471843,
0.110966636520059,0.911642803497653,0.987312877741616
"15604","WDR78",22.3700289551885,-0.0425354427797798,0.391847475126723
,-0.108551019158727,0.913558612133404,0.987312877741616
"15605","TAF13",118.32102663646,0.0225343691946317,0.202966721122775,0
.111024945715119,0.911596565199686,0.987312877741616
"15606","LAMTOR5-
AS1",4.79543397885249,-0.0794200058014615,0.729837914459069,-0.1088186
90051646,0.91334629933318,0.987312877741616
"15607","EML4",163.887965369554,0.0253909065774542,0.229903343394855,0
.110441658666293,0.912059116343231,0.987312877741616
"15608","CRIPT",380.711588366841,-0.0209094945827682,0.191728549141385
,-0.109057804257149,0.913156642526309,0.987312877741616
"15609","AC092835.1",9.75168145316016,-0.0599885986397869,0.5463522887
68238,-0.109798384436226,0.912569272332058,0.987312877741616
"15610","LINC00691",3.37620661233401,-0.113822542000849,1.043731254269
94,-0.109053495845024,0.913160059760472,0.987312877741616
"15611","AC078785.1",10.882036839717,0.0535687495227169,0.488591480262
376,0.109639139622226,0.912695568826316,0.987312877741616
"15612","AC010210.1",6.25226939547684,0.0648958794747466,0.59552605749
4532,0.108972359241128,0.913224413879384,0.987312877741616
"15613","EEF1AKMT4",11.8100652859216,-0.0496558311928037,0.45427009031
5513,-0.109309048188304,0.912957370184891,0.987312877741616
"15614","CCDC127",242.684777767757,-0.0208745176716316,0.1913655704161
52,-0.109081887751475,0.913137540636849,0.987312877741616
"15615","OCLN",33.8275662359398,-0.076115598182069,0.691508228445182,-
0.110071861839173,0.912352383533032,0.987312877741616
"15616","HMGXB3",147.279978264789,0.0214956973373275,0.193511041730391
,0.111082536402633,0.911550896961013,0.987312877741616
"15617","ENPP1",199.556733927157,-0.0471747392202133,0.430306508784755

,-0.109630549985036,0.912702381299656,0.987312877741616
"15618","FZD3",26.5899272825401,0.0371152950158304,0.334990558995562,0
.110795047857818,0.91177887241187,0.987312877741616
"15619","NCALD",429.461343891654,-0.0325078583283134,0.295558528771447
,-0.109987887892931,0.91241898069392,0.987312877741616
"15620","OPTN",1006.3557413296,-0.017678460512399,0.1590741967068,-0.1
11133426277697,0.91151054257286,0.987312877741616
"15621","NOC3L",230.792450751605,-0.0192829045986581,0.172953010249705
,-0.111492159464689,0.911226082678839,0.987312877741616
"15622","HPS6",55.7927199177262,0.0258684002898701,0.233189448921936,0
.110932979212666,0.911669493362743,0.987312877741616
"15623","WDR74",477.94688051705,0.0338851674068051,0.307481467636095,0
.110202308019774,0.912248931889588,0.987312877741616
"15624","RTN3",1083.73358028411,-0.0203568881920491,0.187688459145606,
-0.108461054476752,0.91362997224398,0.987312877741616
"15625","PCSK7",364.307029835247,-0.0295676678169717,0.272602412353857
,-0.108464439333687,0.913627287358153,0.987312877741616
"15626","GPR162",5.08156226810902,-0.0739196533325378,0.67506935020833
9,-0.109499347451821,0.912806439315891,0.987312877741616
"15627","GPRC5A",553.300786364737,-0.0725683605294205,0.66423683201018
3,-0.109250732618675,0.913003622277949,0.987312877741616
"15628","CCPG1",922.091482336238,-0.0139199615676453,0.126509523898756
,-0.110030937898282,0.912384838978355,0.987312877741616
"15629","AC100839.1",2.27862761727503,0.118223242612763,1.066834869970
47,0.110816815179687,0.911761610895809,0.987312877741616
"15630","C15orf40",344.929149499429,0.0257864890259081,0.2378112323397
31,0.108432594929201,0.913652546541591,0.987312877741616
"15631","TPSB2",42.6743657761057,0.104294899229978,0.953363873893753,0
.1093967393625,0.912887819839993,0.987312877741616
"15632","VKORC1",1739.74643106005,-0.0279604541146696,0.25572032380750
4,-0.109339976183188,0.912932840243233,0.987312877741616
"15633","AC129507.1",6.13150527708735,0.0942803742807282,0.86031155155
5263,0.109588641591862,0.912735619094815,0.987312877741616
"15634","DRG2",211.695743775356,0.0242014694461942,0.223085154772445,0
.108485342607762,0.91361070679612,0.987312877741616
"15635","KAT2A",49.2382043364217,0.037904431292309,0.345621930002244,0
.109670214769251,0.912670923064732,0.987312877741616
"15636","AC067852.2",29.5420774176309,0.0320270248585349,0.29514912299
8768,0.108511333298688,0.913590090926786,0.987312877741616
"15637","ABI3",5.14621651089397,-0.0816604634189528,0.750702630097099,
-0.108778709631523,0.913378010849511,0.987312877741616
"15638","AC025887.2",2.26533490656119,-0.107450990113003,0.97535195201
1008,-0.110166376241374,0.912277427796186,0.987312877741616
"15639","CXXC1",156.267069511886,0.0180005297271546,0.16339183670088,0
.110167864506647,0.912276247516485,0.987312877741616
"15640","MPND",78.6558695508112,0.0274554762831002,0.251644913122855,0
.109104038473833,0.913119971775069,0.987312877741616
"15641","ZNF730",4.36912639972468,0.0805012680388646,0.722940256564118
,0.111352587309854,0.911336756007856,0.987312877741616
"15642","ZNF444",92.6493438532059,0.0213484883460522,0.192467919347401

,0.110919723237194,0.911680005229112,0.987312877741616
"15643","PAXBP1",215.466401201768,-0.0306031073527839,0.27875324674084
1,-0.109785653478813,0.912579369127824,0.987312877741616
"15644","SETD4",81.2895075492385,0.0212049872038226,0.192149304429407,
0.110356825213557,0.912126392413514,0.987312877741616
"15645","DUSP18",69.915983784605,0.0273389653680948,0.25012686238301,0
.109300397037051,0.912964231693224,0.987312877741616
"15646","PDXP",16.6874075997512,0.0505294882649646,0.454365987340607,0
.111208782507495,0.91145078740134,0.987312877741616
"15647","SLC10A3",143.183433175731,0.0265042510092532,0.24450587464651
6,0.108399240090123,0.913679003904842,0.987312877741616
"15648","CWC15",837.596740714153,0.0238612751584918,0.220789041982505,
0.108072732886728,0.913937997450078,0.987316487106894
"15649","GTF2A2",816.928620239978,-0.0300471500955967,0.27814493569725
9,-0.108026953718477,0.913974311343572,0.987316487106894
"15650","IQCH",5.36804302391691,-0.0745336216485828,0.689861360274732,
-0.108041449979022,0.913962812302168,0.987316487106894
"15651","SPN",8.7954334467451,-0.0831093699375022,0.767650853249721,-0
.108264544467934,0.913785846676453,0.987316487106894
"15652","RPS28",23156.1701983189,-0.030300481860097,0.280039105627814,
-0.108200894986316,0.913836335029003,0.987316487106894
"15653","USP34",513.82475322545,-0.0220935519433473,0.205749878149193,
-0.107380631969691,0.914487019106315,0.987365530550815
"15654","ZNF300",24.3046984468495,0.0377280159564894,0.350510979162405
,0.10763718741891,0.914283497050915,0.987365530550815
"15655","POU5F1",2.61843992822044,0.0930884436142891,0.867450627609941
,0.107312670775019,0.914540932765937,0.987365530550815
"15656","CUX1",310.44722286514,-0.0192344404619301,0.179092675491674,-
0.107399369678992,0.914472154538608,0.987365530550815
"15657","GOLGA2",732.467011530409,0.0229271721400005,0.212604841743558
,0.107839369752712,0.914123112430171,0.987365530550815
"15658","GATD1",116.68071925904,-0.0216442947722143,0.201918084030071,
-0.107193443698638,0.914635516632455,0.987365530550815
"15659","SMPD3",3.53022382049158,0.0884892552858677,0.825377871028665,
0.107210598190116,0.914621907752143,0.987365530550815
"15660","ABCA5",160.896405587033,0.0267397118920318,0.249306873931616,
0.107256215885031,0.91458571876561,0.987365530550815
"15661","FDX2",213.645302558315,0.0312213604865817,0.291032614859828,0
.107277875029983,0.914568536405155,0.987365530550815
"15662","ZNF529-
AS1",22.7673198769585,0.0433946405949915,0.403887543083822,0.107442384
243045,0.914438031329147,0.987365530550815
"15663","MTG2",107.455046209101,0.0253538890228704,0.236761142649017,0
.107086360283604,0.91472046785829,0.987365530550815
"15664","ACO2",224.605941757296,0.017155610209661,0.160119002383472,0.
107142874701247,0.914675633818767,0.987365530550815
"15665","ZBED6CL",4.15464746840093,0.0723254084267065,0.67652398362049
7,0.106907382706004,0.914862456208573,0.987455755478745
"15666","USP46-
AS1",31.4181177510148,-0.0366361242481596,0.343568672624083,-0.1066340

65231684,0.915079292506118,0.987563650138396
"15667","RPS3A",26522.645043027,-0.0275815219376227,0.259371926106465,-0.106339658079654,0.915312867359759,0.987563650138396
"15668","RNF34",126.464070463382,0.0186059962070917,0.174868555340758,0.106399896601394,0.915265075112929,0.987563650138396
"15669","ATP10A",51.4220075437862,0.0348585618697463,0.327623179648785,0.106398338197911,0.915266311520618,0.987563650138396
"15670","UNC13D",13.7818730678956,0.0531780966092705,0.49856994720737,0.10661255671618,0.91505772069926,0.987563650138396
"15671","BISPR",18.7949930106707,0.0607677562999851,0.57056967957614,0.106503654987639,0.915182755643708,0.987563650138396
"15672","LMCD1",340.16694086626,0.0713892311955905,0.6732157845021,0.106042123252337,0.915548931057862,0.987755316891675
"15673","HOXA4",30.8242976702238,-0.0380562842800974,0.359471357208327,-0.105867361938499,0.915687589902947,0.987841879032669
"15674","FNIP1",486.916706815704,-0.0259298738523302,0.245683451727808,-0.105541800515966,0.915945903169469,0.987978358332726
"15675","MAFK",116.606519308562,-0.0287422873748117,0.272297553773235,-0.105554702848149,0.915935665781407,0.987978358332726
"15676","LINC00511",2.30542799063301,-0.107709592569521,1.021069986611 4,-0.105486983244875,0.915989398227101,0.987978358332726
"15677","ZNF383",84.3938725096745,-0.0238686188563784,0.226478851478818,-0.105390056071574,0.91606630622785,0.987998284474102
"15678","AP001267.1",4.91250954535677,0.0908575763574324,0.862791478069996,0.105306529638742,0.916132581884381,0.98800674158063
"15679","AC011472.4",5.27116351671365,0.0684061150538241,0.650173620815744,0.105212073919576,0.91620753029595,0.988024550178195
"15680","MRPL37",291.298049290438,-0.0245388946970948,0.233776391319917,-0.104967377409441,0.916401694735816,0.988035548862003
"15681","AC034236.2",21.786070392757,-0.044293261641946,0.422224028327064,-0.104904644620641,0.91645147343286,0.988035548862003
"15682","MAK",6.34575973187735,-0.0737190095946072,0.702114277545231,-0.104995742078266,0.916379187375101,0.988035548862003
"15683","RPL21",27266.6673359003,-0.0235140398575231,0.223688145048101,-0.105119740934267,0.916280795087597,0.988035548862003
"15684","CNPY4",316.555378423197,0.0297559909339905,0.283925401510855,0.104802144421209,0.916532808420316,0.988060234938199
"15685","IFT172",60.8720516219139,-0.0363740161653329,0.34819700060568,-0.104463898603552,0.916801216232285,0.988223572871062
"15686","TRAF3",92.6955202936074,0.0233727005026448,0.22372850326209,0.104469033502023,0.916797141471274,0.988223572871062
"15687","ZNF2",24.2183462422251,-0.0358989702739825,0.3441622659414,-0.104308269170029,0.916924715864052,0.988230691983006
"15688","ZNF790",47.6168024161581,0.0294533323649564,0.282268257734123,0.104345180720601,0.916895424546576,0.988230691983006
"15689","MPP4",2.07117191037763,0.128819169442862,1.23974046293322,0.103908175375736,0.91724221921903,0.988446873330488
"15690","FUNDC1",156.416989928188,-0.0227305613153268,0.218697268766474,-0.103936191995148,0.917219985575374,0.988446873330488
"15691","AC093157.1",44.6792316304699,-0.0388643881404395,0.3751446759

93793,-0.103598399837301,0.917488057576551,0.988585781130787
"15692","AC016949.1",21.1120056846317,0.0562751117643062,0.54303847330
1673,0.103630064039761,0.917462928452967,0.988585781130787
"15693","ADCY5",4.34598780180141,-0.0717212925583741,0.694561340879647
,-0.103261279223431,0.917755605993472,0.988653289613354
"15694","SPEF2",87.7063105462049,-0.0231705889712455,0.224441622921468
,-0.103236595198533,0.917775196297373,0.988653289613354
"15695","HGSNAT",492.421147009033,0.0219596081113846,0.212735890742292
,0.103224745174694,0.917784601003738,0.988653289613354
"15696","ZNF414",139.020492962972,-0.0273361275672434,0.26468019282137
8,-0.103279838494343,0.917740876590665,0.988653289613354
"15697","COX19",182.436013875966,0.0197576753504526,0.191726004853038,
0.103051619761217,0.917922002348786,0.988738307683843
"15698","UQCR10",1630.18597943704,-0.0294988774113565,0.28663176553298
,-0.102915590519092,0.918029963960126,0.988791605977692
"15699","AC096586.2",2.0913163384573,-0.101307427644404,0.985100300123
371,-0.102839708435493,0.918090189550157,0.988793485248363
"15700","KIAA0100",178.956850876045,0.0163220120813928,0.1589159395922
72,0.102708464130596,0.918194355770887,0.98884268582001
"15701","SLC22A15",20.6288869511956,0.0570895362927242,0.5563113073184
68,0.102621563757004,0.918263327791629,0.988853980402577
"15702","SUPV3L1",86.1179450767532,-0.022687963715385,0.22141397382860
7,-0.102468526819122,0.918384793285278,0.988921798806997
"15703","C1orf216",111.070585768201,-0.0227768777375072,0.229019284537
795,-0.09945397298518,0.920777830888626,0.988959076174744
"15704","CTTNBP2NL",165.625714220574,-0.0168977593545267,0.17027509871
755,-0.0992379947613865,0.920949308278424,0.988959076174744
"15705","BROX",260.753354348059,-0.0183307221467673,0.181037625043988,
-0.101253660073774,0.919349100971697,0.988959076174744
"15706","CHML",72.6357263965267,-0.0248779013050876,0.253377003579795,
-0.0981853165583471,0.921785141812575,0.988959076174744
"15707","SH3BP5L",48.7676697172067,0.0279109990318282,0.27304386147400
3,0.102221668273929,0.918580729048411,0.988959076174744
"15708","ERLEC1",755.899028251652,0.0137246631958814,0.138895922690441
,0.0988125708086459,0.921287087281353,0.988959076174744
"15709","ABI2",328.270058101975,-0.0182793831251951,0.185098604929029,
-0.0987548400605384,0.921332925557449,0.988959076174744
"15710","SLC25A20",67.5201356450623,-0.0296924419306186,0.292048635461
657,-0.101669510914448,0.91901900347563,0.988959076174744
"15711","TPRA1",193.371527427553,-0.0209191422363512,0.21128377591755,
-0.0990096951150406,0.921130572341724,0.988959076174744
"15712","TACC3",8.40681951989055,0.0568159075735328,0.566384891627571,
0.100313247075263,0.920095640895984,0.988959076174744
"15713","SEC24B-
AS1",14.4105912503276,-0.043705370387477,0.444912924318889,-0.09823353
73924795,0.921746852252636,0.988959076174744
"15714","AC008966.1",21.2395700919946,0.0382628330652711,0.38697164981
8597,0.0988776130840793,0.921235443957961,0.988959076174744
"15715","AC008972.2",2.22369929360049,0.0914943300703547,0.91359754130
0376,0.100147303308332,0.920227381460691,0.988959076174744

"15716","FOXD1",10.0581432167961,0.0773964408854439,0.788256035825185,
0.098186930854797,0.92178385998402,0.988959076174744
"15717","TENT2",168.907834037993,0.0184330078201738,0.186827598915873,
0.0986631949837026,0.921405692383684,0.988959076174744
"15718","FBXL17",147.729881253416,-0.0203228354967927,0.19876777752246
1,-0.102244114967257,0.918562912527318,0.988959076174744
"15719","C5orf15",593.905805043771,-0.0207261082603204,0.2086884369681
34,-0.0993160357202021,0.920887346700248,0.988959076174744
"15720","AC005618.1",2.60339186730207,-0.0905596257733961,0.8874823369
55813,-0.102041045779038,0.918724095281752,0.988959076174744
"15721","SPDL1",56.752304365481,-0.0232940598651652,0.233383821864571,
-0.0998100882874493,0.920495098752897,0.988959076174744
"15722","GABRR2",2.05917311091942,0.126056726912655,1.24466305993921,0
.101277792335872,0.919329944686026,0.988959076174744
"15723","AL078600.1",2.1198777489974,-0.114443120436198,1.132641100480
08,-0.101040939082725,0.919517961764328,0.988959076174744
"15724","RNF146",578.568068219824,-0.018342427480534,0.186900380036523
,-0.0981401293937962,0.921821022663888,0.988959076174744
"15725","WIPF3",2.47259736354284,-0.101899828146642,1.03499543004889,-
0.0984543749549007,0.921571499390639,0.988959076174744
"15726","SUGCT",16.7026343228635,-0.03591812340826,0.361675023274543,-
0.0993104889662092,0.920891750597887,0.988959076174744
"15727","PSMC2",445.520264329707,-0.0207471569531996,0.208215415896762
,-0.0996427515409643,0.920627951877927,0.988959076174744
"15728","WASL",562.689877901818,0.0131150382804455,0.128920987146136,0
.101729272872998,0.918971566282892,0.988959076174744
"15729","SAMD12",2.25085189698705,0.0990212515426471,0.973164404206225
,0.101751822317643,0.918953667306691,0.988959076174744
"15730","IL11RA",895.754222121014,0.0300145795687103,0.301488485884779
,0.0995546462765453,0.920697901897633,0.988959076174744
"15731","AL450306.1",15.6120958332997,0.0773044878016374,0.76170243806
7945,0.101489090671312,0.919162217227521,0.988959076174744
"15732","FBXW4",140.234741226648,-0.0211217345769627,0.20788039857119,
-0.101605224552854,0.919070032321229,0.988959076174744
"15733","GSTO2",4.50124520489931,-0.0661872797171928,0.673866955200834
,-0.0982200999861566,0.921757522153007,0.988959076174744
"15734","TSPAN4",2263.46405752038,0.0216085922834934,0.216223815794793
,0.0999362267475709,0.920394955517375,0.988959076174744
"15735","ALG8",120.655604869808,0.0232918896728776,0.230908892748928,0
.10087047491152,0.919653281143202,0.988959076174744
"15736","AP001528.1",391.009180619824,-0.0254605167675974,0.2556209861
62638,-0.0996026075550711,0.920659823593151,0.988959076174744
"15737","AC079601.1",4.92976401505624,0.0651819494664864,0.66323596154
8121,0.0982786719138979,0.92171101353126,0.988959076174744
"15738","PUS7L",254.723774797906,0.0178273811528212,0.181767381650236,
0.0980779994241509,0.921870357203108,0.988959076174744
"15739","SLC38A2",1701.00610158829,0.0314465916574585,0.31836100517206
1,0.0987765183127967,0.921315712971188,0.988959076174744
"15740","HIP1R",9.97722639584649,0.0626299174840728,0.626458504995384,
0.0999745665270108,0.920364517235406,0.988959076174744

"15741","DHRS12",279.582499791781,-0.0230073648006417,0.2335540277258,
-0.0985098181550229,0.921527476120495,0.988959076174744
"15742","KLF5",91.5678394431196,0.0424635824374846,0.422618638620744,0
.100477306386837,0.91996539853125,0.988959076174744
"15743","LINC02323",3.0693069451446,-0.102476828735085,1.0161012170029
,-0.100852973129342,0.919667174694792,0.988959076174744
"15744","CERNA1",3.85865468749042,-0.0768722941390939,0.77608249929053
5,-0.0990517041801195,0.921097217913705,0.988959076174744
"15745","TPM1",983.451643038079,-0.0226943627040058,0.223783800451958,
-0.10141199969869,0.919223411201952,0.988959076174744
"15746","ROGDI",60.7681503719138,0.0327572711982727,0.328925090442618,
0.0995888491029761,0.920670746939066,0.988959076174744
"15747","CDR2",119.918576215836,-0.0229476270469582,0.231392535740374,
-0.099171855192018,0.921001820977906,0.988959076174744
"15748","AC007608.3",2.93153072302622,-0.0978479270114917,0.9763221702
80908,-0.100220941396157,0.920168920884987,0.988959076174744
"15749","CMTM3",784.631372767506,-0.0267067402130073,0.269205862014284
,-0.0992056414120367,0.920974995758409,0.988959076174744
"15750","C16orf95",50.2848440162838,-0.038223027763442,0.3771955669427
28,-0.101334774619039,0.919284712108743,0.988959076174744
"15751","SMYD4",368.388376382059,0.0239742299674198,0.236321909566846,
0.101447343631245,0.919195355513828,0.988959076174744
"15752","CHMP6",160.862854633042,-0.0245070251034253,0.244475985375582
,-0.100243077314018,0.920151347475213,0.988959076174744
"15753","CNDP2",193.916595210642,-0.0184212232741896,0.183491610593616
,-0.100392727572639,0.920032543155425,0.988959076174744
"15754","TCF3",180.451469521318,0.016479090571715,0.163379981879065,0.
100863584278722,0.919658751175343,0.988959076174744
"15755","RPS19",18602.9948450923,0.0231963719796215,0.230293055454849,
0.1007254514636,0.91976840677367,0.988959076174744
"15756","TPD52L2",276.560072821969,-0.0193209446882861,0.1965075414598
9,-0.098321644781403,0.921676891402366,0.988959076174744
"15757","MRAP",3.87991143943937,-0.106852135520916,1.05277618901756,-0
.101495585325338,0.919157061863754,0.988959076174744
"15758","RRP1B",203.364225849014,-0.016584149889402,0.164469127936796,
-0.100834424657344,0.919681899173619,0.988959076174744
"15759","TANGO2",64.3204975611843,0.0229793477153145,0.228638767496729
,0.100505036686936,0.919943384388672,0.988959076174744
"15760","HEPH",68.6145576685876,-0.0304346364819141,0.300411231703029,
-0.101309915442843,0.919304445308544,0.988959076174744
"15761","UBL4A",165.949381384938,-0.0220413793794716,0.218341660458828
,-0.100949032507829,0.919590919566695,0.988959076174744
"15762","EFNA3",5.34100166108503,-0.0622381473992653,0.635677260482782
,-0.0979084061493672,0.922005024919122,0.989040791862232
"15763","TM7SF2",8.23525796044661,-0.0506125908059011,0.51756376462753
2,-0.0977900584719737,0.92209900173587,0.989078850558275
"15764","TFAP2E",3.07030620104387,0.0815264365942184,0.835320271387576
,0.0975990160741487,0.922250705836337,0.989151435590095
"15765","RTCA",237.303803458266,0.0192682127893261,0.197792403588312,0
.0974163438017126,0.922395765982637,0.989151435590095

"15766","PCCB",67.635693364863,-0.0209493548648346,0.215063352619642,-
0.0974101566336378,0.922400679261239,0.989151435590095
"15767","CCNG1",653.06042785867,0.0171503407537611,0.175903921237201,0
.0974983424652279,0.922330650481814,0.989151435590095
"15768","MACO1",269.929684923398,-0.0141856226600491,0.151056075828306
,-0.093909646349961,0.925180931025511,0.989162445968089
"15769","GFI1",2.0765921338118,0.0999158429256378,1.04351175536503,0.0
957496093474157,0.923719439306092,0.989162445968089
"15770","RNF115",815.663348403296,0.0131463402851063,0.135747010479119
,0.0968444184421173,0.922849948878406,0.989162445968089
"15771","AL391069.2",4.5121938839158,0.0816720227944879,0.856489128126
114,0.0953567536498397,0.924031465081672,0.989162445968089
"15772","AC092803.4",2.64832996671217,0.0862824751557171,0.89999006793
2354,0.0958704748308426,0.923623444212221,0.989162445968089
"15773","PROM2",2.91043841853274,0.0764922447971889,0.810502589308188,
0.0943763114470486,0.924810232529989,0.989162445968089
"15774","ZC3H8",143.58273285697,-0.0192661134725973,0.205675452060302,
-0.0936724012496578,0.925369394468597,0.989162445968089
"15775","CD28",5.10458276530157,0.0666681265257135,0.700110647005058,0
.0952251287862958,0.924136010792364,0.989162445968089
"15776","AC097634.1",5.67786296119411,-0.0671674336663739,0.6994387758
45703,-0.0960304689787331,0.923496373637491,0.989162445968089
"15777","SLC7A14-
AS1",2.05019959107838,-0.103684692433004,1.10397829095509,-0.093919140
6955143,0.925173388967375,0.989162445968089
"15778","RPL9",27253.0837101775,0.0196566936991695,0.210246839824669,0
.0934934085837475,0.925511585948377,0.989162445968089
"15779","PRDM5",104.622057851702,-0.0213354141825512,0.220964680972276
,-0.096555766689375,0.92307918536051,0.989162445968089
"15780","ANKH",227.626733470493,-0.0182375118432131,0.191083305508843,
-0.095442727425338,0.923963179379538,0.989162445968089
"15781","C5orf17",2.35845574837908,-0.114768816080277,1.20066064578972
,-0.0955880551950535,0.923847752330117,0.989162445968089
"15782","AP3B1",261.590321423343,0.0192815984149391,0.199288280354913,
0.096752294618632,0.922923109496036,0.989162445968089
"15783","PFDN1",541.352650461986,-0.0232019296075807,0.247012962720351
,-0.0939300081747053,0.925164756136283,0.989162445968089
"15784","ADRB2",6.51857110169943,0.0710921477373356,0.754929393324417,
0.0941705918010071,0.924973645279226,0.989162445968089
"15785","AL133346.1",4.29759546132718,-0.0761014941509132,0.7977054283
51324,-0.0954004967826254,0.923996721503929,0.989162445968089
"15786","CCDC126",152.440592952275,0.023581386577491,0.251963276809622
,0.0935905695309263,0.925434401155988,0.989162445968089
"15787","MESTIT1",2.9928056191385,-0.082886388186116,0.88041994750425,
-0.094144150664778,0.924994648941783,0.989162445968089
"15788","TATDN1",417.397069909632,0.0223097261518975,0.238088354645211
,0.0937035588537817,0.925344643162552,0.989162445968089
"15789","AC139019.1",7.49102090569833,-0.0496299893327674,0.5261629615
24621,-0.0943243689919916,0.92485149255551,0.989162445968089
"15790","FGD3",6.26723239811253,-0.0691294640505253,0.7387391926398,-0

.0935776316449369,0.92544467899023,0.989162445968089
"15791","ADAMTS13",8.37636641876477,0.0535882436530237,0.572749678846574,0.0935631142752308,0.925456211578417,0.989162445968089
"15792","ZNF33A",209.669577494463,0.0293768539842665,0.312347386832987,0.094051864118762,0.925067957678774,0.989162445968089
"15793","CD59",3045.36867562545,0.021653902018275,0.228902714736134,0.0945987121351372,0.924633572817,0.989162445968089
"15794","CD3D",25.7081623825815,-0.0403903398871983,0.416582596671097,-0.0969563784228068,0.922761036151204,0.989162445968089
"15795","TAPBPL",193.395884544351,-0.0298317471212907,0.313820739592232,-0.095059833075574,0.924267302110455,0.989162445968089
"15796","NUP107",170.14456095831,-0.0212405436656285,0.223162958828517,-0.0951795216245999,0.924172235508963,0.989162445968089
"15797","IFT81",96.1277710689194,-0.021264847290363,0.218816003744716,-0.097181407787576,0.922582332587556,0.989162445968089
"15798","HCAR1",10.1649143747117,-0.0708076517142129,0.751320643851727,-0.0942442514972165,0.924915133568663,0.989162445968089
"15799","AL590064.1",5.13461207575949,-0.0654025671084066,0.690286132811985,-0.0947470389445307,0.924515754331615,0.989162445968089
"15800","FBXO33",159.547980731282,-0.0184687647381295,0.19346353397686,-0.0954638032216371,0.923946439761095,0.989162445968089
"15801","MED6",267.251768753812,-0.0183983145251112,0.192890015191911,-0.0953824100579092,0.924011087113281,0.989162445968089
"15802","TECPR2",102.000602286374,-0.0202122287313059,0.208898382498512,-0.0967562720666342,0.922919950771369,0.989162445968089
"15803","INF2",208.583249394202,0.0194709407725216,0.204636104304446,0.0951490981452318,0.924196400264343,0.989162445968089
"15804","CTF1",65.5505290340476,-0.0257646769402343,0.267703707877054,-0.0962432576842269,0.923327375587285,0.989162445968089
"15805","MED11",235.014129003355,-0.0245819884724297,0.26046985340546,-0.0943755607454664,0.92481082884165,0.989162445968089
"15806","DHX8",149.583348270379,-0.0211718679183707,0.224287478475381,-0.0943961208280051,0.924794497178322,0.989162445968089
"15807","AC011921.1",5.56814775448599,-0.0618565695722254,0.644509474692993,-0.0959746473885281,0.923540708047156,0.989162445968089
"15808","CD226",5.89827593322629,-0.0605028424197867,0.628546838653691,-0.0962582876868494,0.923315438800957,0.989162445968089
"15809","GAMT",298.685955948839,0.0280130434257458,0.298956815753397,0.09370264181852,0.92534537164566,0.989162445968089
"15810","TNFSF9",194.265537476719,0.0329153580192759,0.351728549926873,0.0935817067625567,0.925441441722502,0.989162445968089
"15811","AC007786.1",3.86833755008862,0.0712872716464379,0.740510142648689,0.0962677855990662,0.923307895594366,0.989162445968089
"15812","AC006213.1",2.47431908763651,-0.0904312024392664,0.932121186182516,-0.0970165722867277,0.922713233749748,0.989162445968089
"15813","PIH1D1",894.663637300471,-0.0241267324674616,0.249491107806977,-0.0967037770585701,0.922961640233034,0.989162445968089
"15814","SRXN1",37.6133359123367,-0.025526671839428,0.271016722879543,-0.0941885488401161,0.92495938103588,0.989162445968089
"15815","YWHAH",641.207006920065,-0.023857203498558,0.24764784363686,-

0.0963351957691227,0.923254358886005,0.989162445968089
"15816","NPTXR",2.50681661144826,0.0866327700454078,0.918158321345657,
0.0943549364323556,0.924827211557626,0.989162445968089
"15817","AKAP17A",170.409990364237,0.0204750546254116,0.21851728317043
6,0.0936999322357568,0.925347524109897,0.989162445968089
"15818","WWC3",161.910035058984,-0.0212551610635563,0.223903847477281,
-0.0949298607551309,0.924370538163609,0.989162445968089
"15819","KLF8",96.909081624963,-0.0259932741594122,0.26773219380083,-0
.0970868455914907,0.922657427206577,0.989162445968089
"15820","AL136169.1",25.1769611502759,-0.0425452837491389,0.4494613139
88436,-0.0946583886644213,0.924586170544779,0.989162445968089
"15821","PCDHGB2",7.20483549841863,-0.0530347095857991,0.5693147890636
56,-0.0931553344556964,0.925780157968318,0.989184826879558
"15822","TECPR1",43.0177158813019,0.029725858578337,0.319095636598137,
0.0931565811906514,0.925779167524634,0.989184826879558
"15823","ZNF707",13.2227842020792,-0.0373016679737433,0.39950177328746
2,-0.0933704690890142,0.925609250259781,0.989184826879558
"15824","PLPP6",26.8605285987125,0.0302378606983663,0.324478627771617,
0.0931890673540726,0.925753359581511,0.989184826879558
"15825","FTL",56726.4767713332,-0.026553184977031,0.285215021566126,-0
.0930988305988461,0.92582504644955,0.989184826879558
"15826","SPATA21",2.28940122625887,0.0917231060687857,0.99628035511450
1,0.0920655572479336,0.926645953530005,0.989686676917777
"15827","SPATA5",52.6579302938202,-0.0268448501099143,0.29139299260071
,-0.092125928871252,0.926597987789519,0.989686676917777
"15828","STARD4",9.26542243015196,0.0710221008027063,0.770833042447056
,0.0921368141890264,0.926589339345345,0.989686676917777
"15829","DDX41",145.27719028775,0.0186757565451057,0.202165526220789,0
.092378541951359,0.926397287560336,0.989686676917777
"15830","VTA1",192.553801435165,-0.0167664913634845,0.182062908792244,
-0.0920917471587642,0.926625145402371,0.989686676917777
"15831","ZNF793",12.4404167878415,-0.0678887210495767,0.73681967406306
4,-0.0921374977343047,0.926588796265193,0.989686676917777
"15832","CSNK2A3",2.88155803312404,-0.0790296755050532,0.8593203417930
35,-0.0919676535762577,0.926723739351557,0.989707237554075
"15833","GLI2",95.6224399649118,0.0227621505774076,0.247814994171362,0
.0918513855609066,0.92681611680792,0.98974337794406
"15834","DENND6A-
AS1",5.17310662214517,0.0724571624934764,0.790135618643478,0.091702184
769081,0.926934661513556,0.989773406853023
"15835","AC007541.1",65.3530775226467,0.0267869435167123,0.29242837599
2491,0.0916017244420909,0.927014481306038,0.989773406853023
"15836","AL136295.2",8.82600172945821,-0.0464828209962232,0.5074822782
02985,-0.091594963987355,0.927019852787111,0.989773406853023
"15837","CUTC",697.884870466371,-0.0281016033300633,0.307334669213273,
-0.0914364897458489,0.92714576855167,0.989845340321503
"15838","AC010198.1",2.27143061007762,-0.0987083423476673,1.0812742657
2869,-0.0912889037279971,0.927263034715184,0.98990803074658
"15839","ELP6",201.447924864497,-0.0264277669161009,0.289982464096435,
-0.0911357415988859,0.927384733112433,0.989975444628134

"15840","HIST2H2AC",156.259926714355,0.0290029219243552,0.319790808420
151,0.090693419450162,0.927736199601111,0.989977767837323
"15841","CTDSP1",689.060794177938,-0.0138333483125341,0.15360358993735
9,-0.0900587565575482,0.928240523486907,0.989977767837323
"15842","AC084198.2",15.6374452234067,0.0383915452065438,0.42776925292
5525,0.0897482578375679,0.928487266435799,0.989977767837323
"15843","RIPK1",138.133973070171,0.0190574126972396,0.2126055563247,0.
0896374160049436,0.928575350399995,0.989977767837323
"15844","NDUFAF4",133.92482386491,-0.0216889410363023,0.24076581282633
8,-0.0900831425429418,0.928221145051758,0.989977767837323
"15845","LINC01004",23.8487085554204,0.0465888188817083,0.519084203411
618,0.0897519488659237,0.928484333258572,0.989977767837323
"15846","JAK2",90.9428561571796,-0.0257114275878072,0.285731753376472,
-0.0899844951916511,0.928299535884032,0.989977767837323
"15847","KLF9",2989.89986075055,0.0255423987390723,0.282721132095249,0
.090344851655684,0.928013179387135,0.989977767837323
"15848","FKBP4",170.947082289748,0.0239297126253623,0.263762095143663,
0.0907246077656781,0.927711417092491,0.989977767837323
"15849","POU6F1",46.1295960959419,-0.0278241810511318,0.30958498635973
8,-0.0898757442287595,0.928385956430835,0.989977767837323
"15850","DAZAP2",725.780123968343,0.0109679910507183,0.122243752191606
,0.0897223036276488,0.928507891690173,0.989977767837323
"15851","NAB2",98.8775185442087,-0.0242536454730574,0.270626589295158,
-0.0896203345584984,0.928588924788932,0.989977767837323
"15852","CDADC1",234.164303323652,-0.0175998652115421,0.19657228299806
9,-0.0895338088519578,0.928657685888817,0.989977767837323
"15853","SHC4",5.75609763068922,-0.061641291849914,0.688168527946442,-
0.0895729597426626,0.928626573007732,0.989977767837323
"15854","LITAF",644.070785569953,0.0186652325329553,0.205278406462012,
0.0909264294021563,0.927551049548965,0.989977767837323
"15855","CD2BP2",367.922178805064,0.0203136797016795,0.226938095568953
,0.0895119863007197,0.928675028132706,0.989977767837323
"15856","FAM106A",60.4856166422073,-0.0478290595545512,0.5307843670441
72,-0.0901101511728791,0.928199682569777,0.989977767837323
"15857","JSRP1",2.68551430766378,0.0905286146033379,1.00495841102143,0
.0900819512633615,0.928222091706552,0.989977767837323
"15858","ZFP14",108.975616906019,-0.0241008866173286,0.267361350355494
,-0.0901434952557021,0.928173185674807,0.989977767837323
"15859","ZNF784",19.7195676882196,0.0357987259493226,0.395458911849521
,0.0905245143721649,0.927870414272344,0.989977767837323
"15860","IFNGR2",837.547734717685,-0.0189936678209549,0.20883272613387
3,-0.0909515868158469,0.927531059665321,0.989977767837323
"15861","PRDX4",1597.09919612294,0.02739872908485,0.305894582192704,0.
0895691871639284,0.928629571039335,0.989977767837323
"15862","AC111182.1",2.09332095065571,-0.0849649771482438,0.9520139299
14159,-0.0892476196812634,0.92888512130706,0.990076885271371
"15863","UPRT",51.9097138237225,0.0215451970674044,0.241336044746665,0
.0892746754427871,0.928863619760855,0.990076885271371
"15864","INTS7",40.1468016271221,0.029690482548574,0.336588798808172,0
.0882099542637932,0.929709805801284,0.990133216720046

"15865","B3GNT2",63.2448052227622,-0.028538175280364,0.322420227454947
,-0.0885123601134851,0.929469460946997,0.990133216720046
"15866","FBXO41",5.10979697857515,0.0752551482590762,0.849319027088843
,0.0886064551232574,0.929394677820591,0.990133216720046
"15867","MAGI1",41.5080123583716,-0.0300032965674952,0.342189483816479
,-0.0876803583583721,0.930130731248767,0.990133216720046
"15868","GMPS",247.753130233823,-0.014053304546781,0.158861032917368,-
0.0884628803470693,0.929508785828848,0.990133216720046
"15869","CLCN2",20.7978887952362,0.0303926426712156,0.34589856190835,0
.0878657676503106,0.929983364795976,0.990133216720046
"15870","NAAA",261.394965480626,-0.0251520921839679,0.286803814016054,
-0.0876978999399221,0.930116788799275,0.990133216720046
"15871","G3BP1",666.23772362729,0.0107698264179217,0.121744302591903,0
.0884626729024275,0.929508950699351,0.990133216720046
"15872","CLIP2",51.0644984367776,0.0262306460254904,0.299321085943904,
0.0876338061609408,0.930167732102035,0.990133216720046
"15873","ARL5B",287.214829766586,-0.0251193085824981,0.284658444184038
,-0.0882436797352055,0.92968300129437,0.990133216720046
"15874","SAR1A",842.578750587851,-0.0148408851425523,0.168597974268112
,-0.0880252874150884,0.929856577700004,0.990133216720046
"15875","RPP30",276.427836524045,-0.0194465150255817,0.221329123084978
,-0.087862431995067,0.929986016010199,0.990133216720046
"15876","C11orf95",159.455041329194,-0.0183123964606744,0.206257543847
545,-0.0887841293902441,0.929253470787714,0.990133216720046
"15877","NECAP1",175.385512332381,-0.0164935053467604,0.18785063131659
9,-0.0878011707022831,0.930034707274634,0.990133216720046
"15878","PSMA3-
AS1",367.697042188685,0.0189376766596283,0.214251793143365,0.088389816
4014725,0.929566854951011,0.990133216720046
"15879","TMEM62",43.1436209940684,-0.0262892091024826,0.29823452752584
7,-0.0881494484242914,0.929757895149338,0.990133216720046
"15880","KNOP1",282.056169659039,0.0229817775290324,0.259250869936212,
0.0886468675483598,0.929362559757196,0.990133216720046
"15881","PELP1",128.910460211803,-0.0217336094170156,0.247180917970768
,-0.0879259191827497,0.929935555852376,0.990133216720046
"15882","TSPOAP1-
AS1",3.053100784933,0.0702151139084288,0.795280532367792,0.08828974311
66547,0.929646390927549,0.990133216720046
"15883","VPS16",158.28314514803,-0.021171147053236,0.240191274877123,-
0.0881428647400569,0.929763127801618,0.990133216720046
"15884","SNX5",598.427253182136,-0.0128054405858939,0.145850389291691,
-0.0877984669638686,0.930036856246781,0.990133216720046
"15885","FBL",704.321841156047,-0.0137050586706697,0.156572553200308,-
0.0875316803011856,0.93024890482367,0.990157285663117
"15886","PACSIN2",121.538087750286,-0.0152957280172441,0.1749417117892
24,-0.0874332819817892,0.930327115469898,0.990178198940264
"15887","SDCBP",3138.43255731512,0.0244064334892264,0.279616529594807,
0.0872853744540562,0.930444679143077,0.990240991688245
"15888","LIMS1",1311.14218488705,0.0128422549574646,0.149020170539697,
0.0861779644390059,0.931324947360447,0.990243764303583

"15889","RAPGEF2",107.63864184521,-0.0234071890900975,0.27018182528013
6,-0.0866349506145646,0.930961683760717,0.990243764303583
"15890","AC008875.1",2.1925765437034,0.089972130472716,1.0465370974522
8,0.0859712767867919,0.931489250470277,0.990243764303583
"15891","ADAMTS6",7.98406184303864,-0.0509334205174449,0.5874145571250
29,-0.0867077941798504,0.930903780894618,0.990243764303583
"15892","LMAN2",977.883215124446,0.0195196028571311,0.22503912883171,0
.0867387061017659,0.930879209323765,0.990243764303583
"15893","HNRNPH1",1287.09147046274,-0.0205006739462898,0.2381855841881
27,-0.0860701709390511,0.931410635749154,0.990243764303583
"15894","HBS1L",234.967186573567,0.0145506955500494,0.167495968943269,
0.0868719148397996,0.930773323813086,0.990243764303583
"15895","AC074024.1",2.27971679389744,0.0879674332538686,1.02042132973
636,0.0862069722480188,0.931301888291321,0.990243764303583
"15896","NSUN6",110.440556508975,0.0172352933649748,0.198126944582834,
0.0869911631719983,0.930678536246694,0.990243764303583
"15897","TIMM23B-
AGAP6",2.20745769653371,0.0928109538980123,1.0684618316339,0.086864079
8858347,0.930779551659686,0.990243764303583
"15898","SLC18A2",2.71523452238457,-0.097386683370793,1.13053714824738
,-0.0861419578487684,0.931353570024738,0.990243764303583
"15899","WSCD2",25.4125479035602,-0.0382871318987305,0.44477601805482,
-0.0860818262328422,0.931401370557104,0.990243764303583
"15900","DIO3",120.239044176931,-0.0329870156120731,0.383766789749286,
-0.0859558890794679,0.931501482803908,0.990243764303583
"15901","ZBTB42",10.0414222610461,-0.0448870676427597,0.51601043847986
2,-0.0869886814208535,0.930680508919627,0.990243764303583
"15902","AC027348.1",2.36507172833065,-0.0855918591076072,0.9946569138
85603,-0.0860516404327244,0.931425366302154,0.990243764303583
"15903","PLEKHJ1",551.000463647297,0.0215186733882411,0.24963588301144
7,0.0862002414422703,0.931307238780424,0.990243764303583
"15904","FAM3B",15.0158228211827,-0.0385877903021514,0.447345990685476
,-0.0862593855888205,0.931260223696586,0.990243764303583
"15905","PDZD11",277.122243000351,0.0253239835557792,0.294566261817959
,0.0859704142609152,0.931489936127827,0.990243764303583
"15906","AL139353.2",2.27380960304926,0.0864836153362934,1.00724002124
242,0.0858619728290946,0.931576141119072,0.990260869737285
"15907","C9orf78",483.530725510909,-0.0133530893080525,0.1559618462400
94,-0.0856176663072857,0.931770354345418,0.990264377385027
"15908","AL158212.2",40.5195745215252,-0.0441357872317011,0.5167170139
88533,-0.0854157808565609,0.931930847702304,0.990264377385027
"15909","FBXO21",286.120898868745,-0.0182556733291202,0.21299420906358
7,-0.0857097167541779,0.931697177703136,0.990264377385027
"15910","SLC7A1",59.5631524768514,-0.0289300330349259,0.33817981133555
9,-0.0855463042594818,0.931827084885624,0.990264377385027
"15911","AL096870.8",84.5632907234914,0.0297328440930124,0.34726547775
0955,0.0856199248067373,0.931768558916416,0.990264377385027
"15912","LDLRAD4",78.4508650090997,0.0227758855090063,0.26664647263773
6,0.0854160390111347,0.93193064247478,0.990264377385027
"15913","TMC6",15.1765699226987,0.0458357221110683,0.537208270415975,0

.0853220708526629,0.932005345507834,0.990281303452928
"15914","FLOT1",713.191190101951,0.0155891080200666,0.183016638372687,
0.0851786381756267,0.932119373117665,0.99034022625823
"15915","STIM2",73.4246599085853,0.0252624916148311,0.297227208630779,
0.0849938729741684,0.932266261742903,0.99034756529099
"15916","TLR1",20.8720774798689,-0.036422996398822,0.430364430162497,-
0.0846329153760904,0.932553230250651,0.99034756529099
"15917","FYB1",53.0188431631826,-0.0351905153576913,0.416059192558475,
-0.0845805500445601,0.932594862465292,0.99034756529099
"15918","AL033523.1",3.10319290410147,-0.0703708103588639,0.8270099650
22512,-0.0850906438073553,0.932189328490583,0.99034756529099
"15919","AL023581.2",5.44539528444618,0.064146620550922,0.756210090905
075,0.0848264540799075,0.932399362018885,0.99034756529099
"15920","SLC26A4-
AS1",2.8785557883413,-0.0737076809629302,0.871378821560116,-0.08458741
38081117,0.932589405529967,0.99034756529099
"15921","LSM1",335.191488469136,-0.0189194746035782,0.223349434356226,
-0.0847079584423888,0.932493568789402,0.99034756529099
"15922","DDX10",135.532649278227,0.0167277085663345,0.197338208764544,
0.0847666991154932,0.932446868478386,0.99034756529099
"15923","CEP76",35.2944709600326,-0.0324592375085838,0.384491884488627
,-0.0844211251734337,0.932721611779815,0.990419959302463
"15924","WDR63",3.54803135325549,-0.0687431255048079,0.815816636272805
,-0.0842629611218428,0.932847360376804,0.990444500823461
"15925","ALKBH5",334.103447449072,-0.0131591030758621,0.15620096419001
3,-0.0842446981303812,0.932861880507075,0.990444500823461
"15926","CASS4",6.3621125044609,0.0538652765484481,0.640040264600477,0
.0841591998623268,0.932929856852414,0.99045447819042
"15927","TRIM68",29.1260696385505,-0.0286659430899765,0.34157371914418
8,-0.0839231518215127,0.9331175320005,0.99059152577789
"15928","CAVIN2",65.0946735452271,-0.0264904490348563,0.31667588922793
1,-0.0836516133244029,0.933333429208394,0.990633315539634
"15929","CCL19",16.1067825565151,0.0727955419396039,0.870673217327157,
0.0836083394905335,0.933367836200909,0.990633315539634
"15930","API5",216.642488249317,0.0138889046091076,0.165992644352305,0
.083671807647269,0.933317372760206,0.990633315539634
"15931","KCNJ8",116.104974126184,-0.0271849110132334,0.32526051349215,
-0.0835788848801947,0.933391255610475,0.990633315539634
"15932","IRF2",353.080763079655,-0.0125192595462529,0.150236087940542,
-0.0833305746832787,0.933588689749894,0.99067956080384
"15933","LINC00921",40.493822642362,-0.0208215967909659,0.249621472851
084,-0.083412683024218,0.933523404061908,0.99067956080384
"15934","SPTBN4",2.94146051825369,0.0678709776456187,0.814748187047617
,0.0833030115618435,0.933610605739791,0.99067956080384
"15935","WWOX",116.54231970865,0.0189004194103326,0.227268122495636,0.
0831635303833496,0.933721510789678,0.990735067739684
"15936","URB2",15.5172557861418,-0.0297467897471549,0.358849380384078,
-0.082894917403276,0.933935095457831,0.990775165892898
"15937","SLC9A3-
AS1",12.8888538402877,0.0441236648252784,0.532158332703935,0.082914542

7472362,0.93391949041937,0.990775165892898
"15938","TMEM220-
AS1",30.2896295907899,0.0249882170810388,0.301429212080554,0.082899122
1805037,0.933931752038606,0.990775165892898
"15939","SCCPDH",357.106083822853,-0.020279361084909,0.245340248316222
,-0.082658109397406,0.934123394694238,0.990850587044553
"15940","EIF2AK1",322.442362758931,0.0143788716712763,0.17392152534704
2,0.082674480013815,0.934110377386523,0.990850587044553
"15941","ZNF888",12.184242662052,0.0361608986627827,0.438081860453918,
0.0825437022781877,0.934214367488706,0.99088492098984
"15942","DSCC1",57.9205875420142,-0.024318816407805,0.295089157838674,
-0.0824117584865662,0.934319285937421,0.990934041314133
"15943","LZTS1",5.22571793214967,-0.0602603708305119,0.732065337016343
,-0.0823155636300351,0.934395778433876,0.990953008954399
"15944","PDIA6",1504.39327608477,-0.0193023245204883,0.235937853967932
,-0.0818110540376103,0.934796965692265,0.991254129565683
"15945","VEGFA",302.857476969557,-0.0263941689331121,0.322426594487309
,-0.0818610170016573,0.934757234280992,0.991254129565683
"15946","TWIST2",887.494510397414,0.0150474380164384,0.184854879178996
,0.0814013570172949,0.935122769950346,0.991288764534198
"15947","U91328.1",12.8408362490413,0.0332934755654814,0.4084009706201
11,0.081521538783145,0.935027196413053,0.991288764534198
"15948","ZNF680",171.663568177766,-0.0181773441255265,0.22258876805654
9,-0.0816633484440173,0.934914424678761,0.991288764534198
"15949","TMEM121",97.9843917433838,0.0260672088278733,0.31947591320180
7,0.0815936593360861,0.93496984360009,0.991288764534198
"15950","FN3KRP",206.860180298435,0.0228586698506879,0.280619820914688
,0.0814577878931697,0.935077893654962,0.991288764534198
"15951","DNAJB4",325.228188520114,-0.0153879509800187,0.19168734848542
2,-0.0802762994094467,0.936017507699105,0.991375672373841
"15952","VRK2",106.985327230978,0.0152850148591563,0.19240987415363,0.
0794398672437778,0.936682759346194,0.991375672373841
"15953","CEP68",251.093224545365,0.0169860167853697,0.212521621980823,
0.0799260641202073,0.936296059969225,0.991375672373841
"15954","GMCL1",136.665923784535,0.0163656248137447,0.206377244560995,
0.0792995606107524,0.936794355784457,0.991375672373841
"15955","ZAP70",8.7318052627157,-0.0520867518237623,0.650345487909192,
-0.0800908944432244,0.936164964665599,0.991375672373841
"15956","SORCS2",28.4167269987452,0.0439286141136131,0.542405942823626
,0.0809884454527398,0.935451141511268,0.991375672373841
"15957","RFC1",743.423268186875,0.0122637237624649,0.152752566799695,0
.0802848948426927,0.936010671600398,0.991375672373841
"15958","THAP9",20.4105567705149,0.0291123581143918,0.361413542885604,
0.0805513758060986,0.935798736962495,0.991375672373841
"15959","CREBRF",1086.64526512877,0.0219190478777619,0.275238844824246
,0.0796364622579286,0.936526394609661,0.991375672373841
"15960","BLOC1S5",195.197459623329,0.0150916224829766,0.19113709968540
1,0.0789570549506944,0.937066781556611,0.991375672373841
"15961","GABBR1",160.172069724879,0.0259079886342445,0.322099336674432
,0.0804347779841331,0.935891467669886,0.991375672373841

"15962","EIF4H",1016.56086923659,-0.0102280317187983,0.127404244068398,-0.0802801491707553,0.936014445915881,0.991375672373841
"15963","AC069113.2",3.24641635173423,-0.0826991796124018,1.04562769317873,-0.0790904641794585,0.936960668236412,0.991375672373841
"15964","NTRK2",5198.10384846535,-0.0203127063925391,0.250727562400969,-0.0810150515484795,0.935429982447548,0.991375672373841
"15965","MRPL50",229.05239751206,-0.0173252003288232,0.217069357366624,-0.0798141227255831,0.936385091848793,0.991375672373841
"15966","CHUK",50.0617409264094,-0.0213955934776231,0.270400832313455,-0.079125471969039,0.936932823325763,0.991375672373841
"15967","TLCD5",92.5358734250664,0.0201435964718201,0.254727779694102,0.0790789151305372,0.936969854272756,0.991375672373841
"15968","ATP2A2",355.930759486258,-0.0171679073093625,0.215141665635107,-0.0797981518767282,0.936397794227192,0.991375672373841
"15969","CARS2",269.248703399891,-0.0162706426665173,0.202249450500069,-0.0804483899772659,0.935880641953232,0.991375672373841
"15970","PSTPIP1",10.6040464397346,-0.0398631655819159,0.499659013804407,-0.0797807394254683,0.93641164319924,0.991375672373841
"15971","SNN",69.7029072190041,0.0214016987860875,0.269105951199943,0.0795288944397455,0.936611949953338,0.991375672373841
"15972","TXNDC11",115.948383297286,-0.018870663387506,0.232549742411122,-0.0811467825844536,0.935325221210636,0.991375672373841
"15973","TADA2A",59.1959246727718,-0.0209941341104844,0.261822073303744,-0.0801847370833731,0.936090329114441,0.991375672373841
"15974","SLC39A11",56.5037063891348,-0.0190588234739431,0.235361636450276,-0.0809767630841977,0.935460432177816,0.991375672373841
"15975","ZNF780A",70.4113811017906,0.0193003326733296,0.244189040733571,0.0790384884405511,0.937002009457925,0.991375672373841
"15976","CNFN",16.8871240917239,-0.051985051230338,0.654583959271573,-0.0794169342129731,0.936700999628766,0.991375672373841
"15977","ZNF224",163.94075322509,-0.0156518530193458,0.197996433273742,-0.079051186733784,0.936991909288509,0.991375672373841
"15978","CDK5RAP1",97.9008655303442,-0.0174914993946503,0.218400641957726,-0.0800890475314446,0.936166433569359,0.991375672373841
"15979","AL117379.1",2.6724118662491,-0.0698378154308942,0.867004898476544,-0.0805506584260477,0.935799307495016,0.991375672373841
"15980","KRTAP13-4",84.0145371271589,-0.0318014365642995,0.402860155398904,-0.0789391458502774,0.937081026489161,0.991375672373841
"15981","BRWD1-
AS2",3.93595879042741,-0.0624133967602508,0.772502331320814,-0.0807937972867179,0.935605940992682,0.991375672373841
"15982","MID2",11.8390972770823,-0.0348702245261463,0.431673613299234,-0.0807791429724811,0.935617595350827,0.991375672373841
"15983","VPS72",215.970263981264,-0.0155408402639136,0.201637031248881,-0.0770733439570013,0.938565198348672,0.991407619290458
"15984","TMEM9",520.453432828865,-0.0140356358082226,0.178419712585693,-0.0786663962452103,0.937297974530201,0.991407619290458
"15985","SLC41A1",114.922134528619,0.0306383470491169,0.394448731849744,0.0776738383856381,0.938087505160949,0.991407619290458
"15986","PPP2R5A",205.499709975323,0.01421839501211,0.183799586874693,

0.0773581445632058,0.938338636761393,0.991407619290458
"15987","ICOS",2.34633482635448,-0.0753288859509215,0.967330525189747,
-0.0778729544755608,0.93792911326499,0.991407619290458
"15988","H1FX",1760.82224487672,0.0166582249476526,0.213577166532855,0
.0779962821778987,0.937831010377178,0.991407619290458
"15989","GFM1",165.913669190283,0.0128073553561551,0.165539659721255,0
.0773672929962574,0.938331359178655,0.991407619290458
"15990","AP1AR",126.833327498982,-0.0188597811347632,0.243518207470068
,-0.0774471089069649,0.93826786580322,0.991407619290458
"15991","AC096733.2",17.4667756031068,0.0355940350429465,0.46013663156
9826,0.0773553605621705,0.938340851436531,0.991407619290458
"15992","UBE2B",1503.54393776872,0.0150669418737863,0.193017904731339,
0.0780598146827774,0.937780472852575,0.991407619290458
"15993","DNAJC18",137.181244360145,-0.0174843129677794,0.2250625068624
07,-0.0776864756885893,0.938077452427969,0.991407619290458
"15994","BTBD9",35.1731092226897,-0.0242186045234202,0.312885155737415
,-0.0774041340067451,0.938302052189858,0.991407619290458
"15995","CCZ1B",79.0079449348327,-0.0349602695203527,0.450321488111535
,-0.0776340246763748,0.938119176270274,0.991407619290458
"15996","AC090114.2",84.7389640098534,0.0191331495878344,0.24853195989
555,0.0769846646518841,0.938635744594372,0.991407619290458
"15997","EPC1",765.5881714895,-0.0136450950981914,0.176236134359834,-0
.0774250703339374,0.938285397398732,0.991407619290458
"15998","LDB1",104.848723143141,-0.0196292541056416,0.254677448063014,
-0.0770749599343591,0.938563912809289,0.991407619290458
"15999","ZNHIT2",37.8892353111331,-0.0277417469202442,0.35904081215707
4,-0.0772662772055775,0.938411717572462,0.991407619290458
"16000","RELT",11.8628120388676,0.0579891461153733,0.749166754924453,0
.0774048577759181,0.938301476433006,0.991407619290458
"16001","YBX3",5383.63368217315,0.0143468922654298,0.182140961090352,0
.0787680716053372,0.937217100283437,0.991407619290458
"16002","AC023794.1",3.57285986384168,-0.058071709006624,0.75281284737
0625,-0.0771396359791853,0.938512461970638,0.991407619290458
"16003","ZNF785",68.797834552947,0.0199864631053354,0.259512157862559,
0.0770155173844319,0.93861120054438,0.991407619290458
"16004","AC009163.7",9.13141310403577,0.048040791224279,0.623479925271
162,0.0770526672585091,0.938581647052565,0.991407619290458
"16005","PTGIR",116.614479101215,0.0419950346131791,0.533731218602741,
0.0786819903904409,0.937285570649653,0.991407619290458
"16006","ZSCAN18",287.200423989102,0.0137797174966438,0.17856787656433
1,0.077167952947458,0.938489935445635,0.991407619290458
"16007","CBR3",579.983569488989,-0.0186209914281153,0.241388830636889,
-0.0771410648081149,0.938511325317036,0.991407619290458
"16008","TCEAL9",1237.43522374101,-0.0171511980258585,0.21942221837928
2,-0.0781652749322396,0.937696584065215,0.991407619290458
"16009","ID3",2400.00312196986,-0.026089969126828,0.340941165104303,-0
.0765233764565988,0.939002716777114,0.991609389498935
"16010","AC063919.1",2.29662624456902,0.0901500725505691,1.17801009524
56,0.0765274193442066,0.938999500450935,0.991609389498935
"16011","BBS7",140.169399034841,0.0167869070474971,0.219265047854104,0

.0765598859087972,0.938973671655804,0.991609389498935
"16012","CDC42EP5",419.65264448877,-0.0175867974242067,0.230196745109486,-0.0763989839032783,0.939101677972592,0.991651959228115
"16013","UBP1",97.6557194173772,-0.0151160577536877,0.199763588028665,-0.0756697349244577,0.939681854968213,0.991964791922153
"16014","AC006480.2",11.8734582885753,0.0353401760077888,0.465559716453562,0.0759090074996081,0.939491490590223,0.991964791922153
"16015","CACNB2",4.41331009476772,-0.0494176399669299,0.651540919316655,-0.0758473313061592,0.939540559611857,0.991964791922153
"16016","AL135999.1",18.576854558213,0.0317814065516448,0.419733825210727,0.0757180018448333,0.93964345370658,0.991964791922153
"16017","AC145343.1",4.13248279594572,-0.0661656706128734,0.875899415109448,-0.0755402612120773,0.939784865213916,0.991964791922153
"16018","ZNF766",154.363032441139,0.0199319981373247,0.263963563177908,0.075510414760884,0.939808611414773,0.991964791922153
"16019","DNMT3B",4.36744064600765,-0.0580959109817407,0.768412502355438,-0.0756051089794317,0.939733271723276,0.991964791922153
"16020","GPAT4",179.506092633022,0.0142477609742347,0.189029340985624,0.0753732775025561,0.93991772017886,0.99201802826368
"16021","KCNMB3",13.8221784800819,-0.0400317042192565,0.537211213263654,-0.074517625899982,0.940598516717919,0.992141811761472
"16022","CD81-AS1",2.42155607946431,0.080028352396114,1.07543845714056,0.0744146276941759,0.940680469857355,0.992141811761472
"16023","NCR3LG1",2.53808955502397,-0.0841397825224906,1.12488313113639,-0.0747986881423763,0.940374885606156,0.992141811761472
"16024","C11orf98",11.8817776615467,-0.0490944116132162,0.658356837410819,-0.0745711274242922,0.9405559471189,0.992141811761472
"16025","AC107032.2",2.0868584641371,-0.093488213757369,1.25356007290402,-0.0745781680336964,0.940550345124441,0.992141811761472
"16026","AC009054.2",13.5582630895261,0.0365357940302238,0.489916219382046,0.074575595958648,0.940552391644414,0.992141811761472
"16027","ITGB4",8.39106100802814,-0.0357360519301872,0.475935490675966,-0.0750859152769457,0.940146354168856,0.992141811761472
"16028","RPTOR",48.7394183785313,-0.0241399867797484,0.322278394509199,-0.0749041424775354,0.940290980598574,0.992141811761472
"16029","GPS1",254.192580862591,0.0102930590829124,0.137173818779055,0.0750366154017432,0.940185579121846,0.992141811761472
"16030","DAZAP1",280.267140049582,-0.0111939979370292,0.150323511250192,-0.0744660488830546,0.940639555203339,0.992141811761472
"16031","Z82217.1",21.2520488108239,-0.0368550518833455,0.492905097444672,-0.0747710909755452,0.940396843470809,0.992141811761472
"16032","ZFP41",26.4513132502021,-0.0270330369274323,0.365781120689003,-0.0739049540788535,0.941086013847017,0.992507629873089
"16033","ANGEL1",27.8366599125174,-0.0210833171937063,0.285932851948312,-0.0737352040874181,0.94122108621043,0.992588169752757
"16034","AC068193.1",5.16675693499951,-0.048737601588345,0.665158502527775,-0.0732721620532994,0.941589543602327,0.992680476165901
"16035","PCDHGA1",5.6322801668083,0.0494701341038537,0.673824679797008,0.0734169222159639,0.94147435195291,0.992680476165901

"16036","CCN2",2482.04867363238,-0.0338315907043502,0.462117107512859,
-0.0732099940779811,0.941639013607031,0.992680476165901
"16037","TNFRSF10B",132.794735569673,0.0234195367395418,0.319489741531
91,0.0733029380763471,0.941565053745393,0.992680476165901
"16038","MGMT",930.815169874836,0.021131046271775,0.28849930284482,0.
0731825152400247,0.941660879892648,0.992680476165901
"16039","KLF2",5389.31916074346,-0.0180784264642763,0.246872719504543,
-0.0732297456784959,0.941623296297869,0.992680476165901
"16040","U73166.1",6.6375365302338,0.0416717600033291,0.57059483982926
3,0.0730321361051887,0.941780544869252,0.992708014513609
"16041","IARS",195.234516448232,-0.0142683413528609,0.195451044789156,
-0.0730021237197937,0.941804427538018,0.992708014513609
"16042","HLX",67.6502836203,0.0213211985529049,0.292601944327241,0.072
8675901382925,0.941911484681634,0.992758969143315
"16043","PLEKHA1",297.301216270772,0.0148251802707927,0.20478825668325
6,0.0723927265698769,0.942289372965439,0.993095351125079
"16044","MEPCE",171.943718157875,-0.0132825251673895,0.18459859392762,
-0.0719535554674783,0.942638869361616,0.993180324904027
"16045","IDI1",273.02274542427,-0.0141117483017438,0.196074297591414,-
0.0719714336610827,0.942624641514868,0.993180324904027
"16046","LTA4H",224.050829365561,0.0122383301601162,0.170191103472301,
0.0719093413840403,0.942674055948262,0.993180324904027
"16047","HDGFL3",340.554011777136,0.0158841735185573,0.220943373264106
,0.0718925093063101,0.94268745133307,0.993180324904027
"16048","AC007728.2",2.14195109493754,-0.0773004789645346,1.0758809580
1453,-0.0718485427116282,0.942722441115728,0.993180324904027
"16049","ZNF613",11.379475156172,-0.0303122714278226,0.42138855295937,
-0.0719342545376292,0.942654229472674,0.993180324904027
"16050","TXNDC5",638.961847993453,-0.0168447576729181,0.23479384324027
5,-0.0717427571372904,0.942806628535477,0.993207132416066
"16051","COMMD9",272.715192082898,0.0198548204378688,0.277404164248833
,0.0715736207191862,0.942941233835855,0.993287046395654
"16052","PPARD",71.702851188228,-0.0160026576584205,0.224414400192745,
-0.0713085151606855,0.943152218365588,0.993323639474608
"16053","NUP93",71.7303292020951,-0.0184471464353719,0.258430834552819
,-0.0713813677353646,0.943094238171499,0.993323639474608
"16054","ZNF217",307.257588523799,0.013453188498444,0.188318221636288,
0.0714385914520104,0.943048696516858,0.993323639474608
"16055","EGFL8",67.8180925776765,-0.0193787108934973,0.272185010900405
,-0.0711968334677626,0.943241101549976,0.993355374961506
"16056","ZNF775",73.2364811092213,-0.0194376977036416,0.27331705469264
7,-0.0711177636737666,0.943304030599723,0.993359775123325
"16057","GNPTAB",220.775158948056,0.0122421164151406,0.172429306271438
,0.0709978870753507,0.943399437118453,0.99339837346944
"16058","ZFP36L2",11430.8870544013,0.0124793657531798,0.17668868791717
3,0.0706291155381139,0.943692937423247,0.993451932128512
"16059","PALLD",1009.70585230183,0.0141883407412533,0.201327246067852,
0.0704740218642411,0.943816376677464,0.993451932128512
"16060","PDLIM2",1907.45136566283,0.0193296555352746,0.274789948837284
,0.0703433863467858,0.943920350700529,0.993451932128512

"16061","LATS2-
AS1",11.0718569101507,-0.0588312163116433,0.835854905402518,-0.0703844
841148743,0.943887640501663,0.993451932128512
"16062","DGKE",37.0756419650189,0.0232428359768745,0.329367682261991,0
.0705680527526265,0.943741537211418,0.993451932128512
"16063","RAB11B-
AS1",127.44940183786,0.0175025422059869,0.248040691293975,0.0705631891
06916,0.943745408189451,0.993451932128512
"16064","MIAT",13.6479977131606,0.0330523356960683,0.468355939413876,0
.0705709758638518,0.943739210706383,0.993451932128512
"16065","ARMCX1",721.663661647143,0.0152439368588154,0.215550611812833
,0.0707209166822132,0.943619873411617,0.993451932128512
"16066","AL034417.2",33.6472928547047,-0.0323995983997664,0.4670630099
63088,-0.0693687954486633,0.944696066691775,0.993503648532028
"16067","ATP13A2",36.2251907995563,0.0241886622181785,0.36209424729509
6,0.0668021168490574,0.946739238174106,0.993503648532028
"16068","RPS8",34036.459972312,-0.0160414383066253,0.231516645303675,-
0.0692884880289453,0.944759988937453,0.993503648532028
"16069","LEPROT",2878.7108140559,0.00926062818402029,0.137641322372466
,0.0672808719387368,0.946358104361183,0.993503648532028
"16070","RPF1",201.208122282984,-0.0126633776575934,0.190052511427714,
-0.0666309409039824,0.946875513193412,0.993503648532028
"16071","LAMTOR2",657.410945381197,0.0195826462518897,0.28874836128194
3,0.0678190732059899,0.945929660494944,0.993503648532028
"16072","RPL37A",26950.9847742597,-0.0174960227808124,0.26227407878456
7,-0.0667089285448744,0.946813426196902,0.993503648532028
"16073","SERPINE2",91.6236604745873,-0.0242515577996323,0.349212819962
608,-0.0694463559563165,0.944634331241975,0.993503648532028
"16074","CUL3",375.670307294729,0.0114491074335399,0.16813874511451,0.
0680932132908602,0.94571143282837,0.993503648532028
"16075","RPN1",503.237596154091,0.0115326096996068,0.170441137120677,0
.0676632994500696,0.946053665122536,0.993503648532028
"16076","FNIP2",217.270502209066,0.0192590969034267,0.282291189906322,
0.0682242223351633,0.945607145255962,0.993503648532028
"16077","PACRG",7.92918451194974,-0.0393250906396471,0.571815819752689
,-0.0687723026911974,0.945170865124576,0.993503648532028
"16078","SNHG26",98.3067297733859,0.0238997520030511,0.353176321636989
,0.0676708786485872,0.946047631626072,0.993503648532028
"16079","CNOT7",344.145274866551,0.00964250965240824,0.138888099227039
,0.0694264642260366,0.944650164331461,0.993503648532028
"16080","GOLGA7",467.611810970879,-0.0136734625274485,0.19920782865084
9,-0.0686391826066933,0.945276829188702,0.993503648532028
"16081","GINS4",8.30861610191012,-0.0378988662526426,0.546240386890903
,-0.0693812965173735,0.944686116256104,0.993503648532028
"16082","ZFAT",26.3855558105776,0.0242879390236389,0.362560244424042,0
.0669900779171814,0.946589602132117,0.993503648532028
"16083","GLI4",127.52261076038,0.0182691953557147,0.261335320241974,0.
0699071037883399,0.944267598959458,0.993503648532028
"16084","LINC01239",100.092365205026,-0.0319726640461081,0.47929990335
3424,-0.06670701125206,0.9468149525753,0.993503648532028

"16085","DPP7",1157.70162365129,-0.013868107867214,0.204764652434868,-0.0677270598333623,0.946002908130909,0.993503648532028
"16086","ANKRD16",120.338560836644,0.0126784156816151,0.186303446395785,0.0680525021243081,0.945743840465457,0.993503648532028
"16087","AL353801.3",6.70946529011375,0.0449540037878029,0.666595765490049,0.0674381778509423,0.946232876822949,0.993503648532028
"16088","ALG1L9P",6.84276929530612,0.0425159379082226,0.632233378649427,0.0672472212698496,0.946384893039704,0.993503648532028
"16089","XRRA1",271.926195876718,-0.0257128566281625,0.373830237463729,-0.0687821745041619,0.945163007164708,0.993503648532028
"16090","VWA5A",101.605542551389,-0.0149207239041486,0.212840340732526,-0.0701028942765099,0.944111763072719,0.993503648532028
"16091","TEAD4",3.80046549960994,0.0589085558334628,0.887562152774297,0.0663711894984812,0.947082307061268,0.993503648532028
"16092","LINC01252",18.2138090368377,0.0367003712935321,0.535421882659322,0.0685447727897289,0.945351980328142,0.993503648532028
[truncated: 503,654 more chars]
