# Supplementary material for: PPARγ agonist treatment reduces fibroadipose tissue in secondary lymphedema by exhausting fibroadipogenic PDGFRα+ mesenchymal cells
Source: JCI Insight. 2023 Dec 22;8(24):e165324. doi: 10.1172/jci.insight.165324 (PMC10807713; doi:10.1172/jci.insight.165324)
Supplement: Supplemental data set 2 [file jciinsight-8-165324-s184.pdf]

"","gene","baseMean","log2FoldChange","lfcSE","stat","pvalue","padj"
"1","PLP2",918.45084024194,-1.58450012229075,0.148780844482806,-10.649
8933232891,1.74564986321944e-26,2.7301963860752e-22
"2","TNFAIP6",950.97578538217,-2.80943092956846,0.351206422797531,-7.9
9937229846186,1.25055070291799e-15,9.7793064968187e-12
"3","PERP",91.9239616519527,-2.37104192137829,0.308384782796364,-7.688
58275002487,1.48773782516245e-14,7.75607319518026e-11
"4","C1QA",63.5951625799103,-2.6139379385686,0.348272389929846,-7.5054
4118382492,6.12220549336158e-14,2.39378234790438e-10
"5","CAV1",1777.1235749919,-1.71184506958293,0.237277514788287,-7.2145
2713760232,5.41217136866305e-13,1.6929272041178e-09
"6","PDGFD",1121.48557625189,1.75660101391291,0.245239805432342,7.1627
8913537765,7.90519005443755e-13,2.06061954085672e-09
"7","MATN2",245.701126161278,-2.13548005647349,0.302516007307219,-7.05
906466068359,1.67627063419991e-12,3.74526753126952e-09
"8","SFRP4",4094.51177236178,-2.51294756319657,0.362756506426037,-6.92
736730749429,4.2874436576409e-12,8.38195235068797e-09
"9","RGCC",442.863601971699,-2.14961946963603,0.319630031399912,-6.725
33635284879,1.75187284455495e-11,3.04436569875993e-08
"10","PTGFR",236.381756702683,-1.56352952835948,0.247679083894011,-6.3
1272331832655,2.74167412479913e-10,4.28797833118585e-07
"11","DLGAP2",41.6141082867175,2.58558318994753,0.413293063197566,6.25
605271461221,3.9484333341991e-10,5.61395430426127e-07
"12","C1QC",24.3839456760643,-2.7433254461876,0.445524003569901,-6.157
52557484186,7.38903139048004e-10,9.63037091225898e-07
"13","FABP4",261.068661962409,-2.916845749718,0.477902152044481,-6.103
43715180951,1.03811399569333e-09,1.23866010404055e-06
"14","LUZP2",126.002138255658,2.04143264285396,0.335050603681592,6.092
90841569111,1.10877502919231e-09,1.23866010404055e-06
"15","LMNA",5345.00892527003,-1.08542316140984,0.179668777660835,-6.04
124531563752,1.52929255062113e-09,1.59454236611429e-06
"16","GSTM1",13.1970322367721,6.53836464399277,1.11927978057981,5.8415
8202215158,5.17074089772438e-09,5.05439922752558e-06
"17","CTHRC1",579.224131417019,-1.53332864568604,0.265948960861066,-5.
76549966851369,8.14162621876132e-09,7.49029612126041e-06
"18","CD36",62.9273267327351,-2.49802228258045,0.434065691700381,-5.75
494062383704,8.66722529807129e-09,7.53085575899083e-06
"19","ACTN1",24.4028863478053,-2.59492987670631,0.457217007167081,-5.6
7548852301998,1.3829341597676e-08,1.13837317151396e-05
"20","RNASE1",48.9492350878641,-2.48707985292878,0.441066844121183,-5.
63878216211023,1.71257018804982e-08,1.33922988705496e-05
"21","RGS2",179.099482017568,-1.34085235889508,0.238323290733488,-5.62
619102299375,1.84232324150244e-08,1.37209216652848e-05
"22","TUBA1C",339.320708694234,-1.59121289092901,0.285177102855815,-5.
57973580274963,2.40884183737585e-08,1.71246756075265e-05
"23","VGLL3",667.631884372543,1.74536889442969,0.314201369204593,5.554
93726474881,2.77711483198178e-08,1.87269962601096e-05
"24","CTXN1",86.4931970035452,-1.40203073920408,0.252665410969588,-5.5
489619011319,2.87370786600147e-08,1.87269962601096e-05
"25","S100A3",27.5644542159004,-2.04869013025899,0.370269477029384,-5.

53297059940052,3.14852263040442e-08,1.96971575758101e-05
"26","C1QB",46.3428278295335,-2.43270383723333,0.444093775004173,-5.47790573558585,4.30389294290116e-08,2.588957139499e-05
"27","TNFRSF12A",215.500353805308,-1.87718162226742,0.345351300129346,-5.4355713198831,5.46211315838811e-08,3.16397962211815e-05
"28","CAPG",179.593535773592,-2.57442160522664,0.478132232923154,-5.38432974804358,7.27150925218631e-08,4.03094883643386e-05
"29","PHLDA2",62.3568907936442,-1.72149427820899,0.320017208673813,-5.37938033189857,7.47426574530576e-08,4.03094883643386e-05
"30","VEGFD",994.014254543323,1.45049533404334,0.2705909967759,5.36047152834366,8.30050088331832e-08,4.32732779383662e-05
"31","CDA",304.568037730766,-0.913681701883895,0.171870755691721,-5.316097542055,1.06016333171484e-07,5.34869500258714e-05
"32","DUSP10",46.6167611713813,-1.34854099234147,0.255464616538948,-5.27877797955582,1.30048262377051e-07,6.19106505715522e-05
"33","CLCF1",23.807299518116,-2.14297140188026,0.406022659511772,-5.27796011300725,1.30629889313377e-07,6.19106505715522e-05
"34","ARNTL2",33.0456688087147,-2.18446336828515,0.42046479158957,-5.195353836945,2.04330822975854e-07,9.3992178568893e-05
"35","ITIH5",978.246655756226,-1.12700088007725,0.218205046168702,-5.1648708399068,2.40605006704901e-07,0.000107516065853276
"36","GALNT13",29.2699373244844,-3.03901117172785,0.590355439686563,-5.14776517235337,2.63608205752879e-07,0.000114523120499306
"37","NFATC2",46.3210849365162,-1.93222175515169,0.379034701532916,-5.09774368240501,3.43725796223501e-07,0.000145293823052312
"38","SAA1",7.35559128435627,-3.88619492108883,0.763580899248268,-5.08943443309638,3.59133004803481e-07,0.000147811584082275
"39","AQP1",143.544942863982,-3.66762993557503,0.72161667412707,-5.08251827746597,3.72463457488496e-07,0.000148674554683954
"40","CCN3",571.945140427554,-1.35184477470293,0.266184941185333,-5.07859223246479,3.80241827836199e-07,0.000148674554683954
"41","AIF1",52.7334041384775,-1.67019599953859,0.331131373409862,-5.04390744476901,4.56119994336848e-07,0.000173993090522642
"42","CLIC3",59.7435242803087,-1.66521051601907,0.330459757421017,-5.03907201595361,4.67794453248444e-07,0.000174197744019183
"43","TUSC1",419.73167568883,-1.09542682373162,0.217604024919654,-5.03403750981207,4.80255636032888e-07,0.000174679026687311
"44","GPX3",4967.78193856446,1.10163107280663,0.220097482276475,5.00519615859493,5.58051731836824e-07,0.000196699347976258
"45","TUBB6",1075.4962931582,-0.79508861167523,0.15893863176099,-5.00248808528108,5.65950809394603e-07,0.000196699347976258
"46","SPP1",35.6545215933106,-2.94148113470333,0.588973741242361,-4.99424834882905,5.90653520379074e-07,0.000200822196928885
"47","BEND6",146.327896594585,-1.18333919832769,0.239741567646572,-4.93589497200656,7.97840369638975e-07,0.000254837575079705
"48","CCDC34",121.124062495093,-1.17279494925275,0.237499496131633,-4.93809447327306,7.88896251645031e-07,0.000254837575079705
"49","SCN2B",26.6355724684873,2.18683268223229,0.443059216278041,4.93575712204561,7.98404167449202e-07,0.000254837575079705
"50","CRIP2",455.157113500987,-1.96026769451029,0.398452312549228,-4.9

1970464914317,8.66749019328402e-07,0.000271119093245924
"51","NAV2",17.2280134206455,-2.1271866005499,0.433219123246407,-4.910
18629235348,9.09899102999383e-07,0.000279035724919811
"52","NTM",1292.80506482612,0.731766735543568,0.150070897996175,4.8761
4017983832,1.08181744574086e-06,0.000325377400988214
"53","EYA2",25.6713030942135,3.23938183882406,0.664957170778586,4.8715
6463781137,1.10717928290487e-06,0.000326722339332682
"54","RFX2",116.557971559829,-1.21882686129958,0.250628535868957,-4.86
308096192549,1.15572546071728e-06,0.000334732337141079
"55","STAB1",16.3025352636131,-3.0815157386979,0.635406094080788,-4.84
967923254777,1.23661286710611e-06,0.000351647731664355
"56","IL7",24.0760906225872,-2.76340073935987,0.578155414596357,-4.779
68495943113,1.75570089407504e-06,0.000490342178273815
"57","KLHL13",111.800321444202,1.57289772881471,0.330429423599944,4.76
016243250493,1.93437215756159e-06,0.000530764570951987
"58","EMP1",2158.49164671336,-1.06567910221101,0.22599801715547,-4.715
43562914492,2.41194140426483e-06,0.000650392475218998
"59","SLIT3",1088.15452202496,-1.06030325021953,0.225378218643191,-4.7
0455067309836,2.54425670257367e-06,0.000674443641156816
"60","PXDNL",280.831928449149,1.18425221791083,0.252072879716773,4.698
05486112369,2.62650890031021e-06,0.000684643320014194
"61","LAMC1",1855.39643292448,0.879472905627045,0.187422485167424,4.69
246208554653,2.69936572846904e-06,0.000692099672020586
"62","C16orf89",34.9541073420624,2.37347514273509,0.509654515746959,4.
65702759300872,3.20807461823885e-06,0.000809262694020251
"63","CPB1",479.200956220665,1.89971408321171,0.409262895652896,4.6417
9407268548,3.45397002812124e-06,0.000837470818048228
"64","RSPO3",120.731155097374,1.77792994510369,0.383395848383459,4.637
32185051067,3.52952592111347e-06,0.000837470818048228
"65","MS4A7",25.9705174723794,-2.29053902785663,0.493964171050409,-4.6
3705499730036,3.53408401478153e-06,0.000837470818048228
"66","CD9",1069.10682858892,-3.10425096256172,0.669140151746008,-4.639
16408911003,3.49821225420085e-06,0.000837470818048228
"67","LYVE1",13.2103362002522,-3.225355026812,0.696212369296698,-4.632
71721252267,3.60897357207047e-06,0.000842452935331076
"68","ARSI",42.0917277519662,-1.45300325007486,0.31478558456241,-4.615
85066576258,3.91488389982189e-06,0.000900423296959034
"69","CXXC5",368.737787930676,-0.942406356890317,0.2050417032259,-4.59
616917955487,4.30329338502415e-06,0.000975413167272141
"70","KLK1",6.46153319799502,-4.81067977203189,1.04895989933288,-4.586
14268771513,4.51510085026981e-06,0.00100880253283171
"71","DPT",2024.0136978363,-1.64273180962316,0.360044696116005,-4.5625
7744481224,5.05294457119894e-06,0.00109761184852155
"72","SERTM1",6.01116804435214,-5.46525774816276,1.19737921189249,-4.5
6434995186258,5.01044405190205e-06,0.00109761184852155
"73","TPPP3",4545.41280959219,-1.02834117378882,0.226108976364984,-4.5
4798916133641,5.41609242184229e-06,0.00115305821943564
"74","FPR3",7.60750394684019,-3.23250675338454,0.710994654871451,-4.54
645717972237,5.45564630679268e-06,0.00115305821943564
"75","MRC1",20.361815417441,-2.89049739084001,0.637828872915075,-4.531

77570596569,5.848993485281e-06,0.0012197101081306
"76","ADORA2B",14.8236629942889,-2.09664647405332,0.463108800555878,-4
.52733023327709,5.9733554555813e-06,0.00122925367533278
"77","MDGA2",15.7408596178307,2.84466827533449,0.629543136482011,4.518
62328486489,6.22430268089703e-06,0.00126426096011986
"78","GLIS3",59.0440344918355,-1.39392220113164,0.310724015389903,-4.4
8604591885991,7.25571105558223e-06,0.00145486308858085
"79","FMOD",164.717843905373,-1.41333728400519,0.315988383881566,-4.47
275075951815,7.72197640156307e-06,0.00152875583443603
"80","DBNDD2",1374.16237298794,-1.34682080042941,0.30163093626673,-4.
46512820302495,8.00208206507314e-06,0.0015644070437218
"81","LVRN",201.467992901195,1.16259893477719,0.260986503358404,4.4546
3240365585,8.40370547938978e-06,0.00162264140367477
"82","PDLIM4",354.896043622845,-1.09448020203718,0.246272051989373,-4.
44419167013076,8.82229286936506e-06,0.00166769922852071
"83","CSPG4",6.52501758198835,-3.58440291038723,0.806660334144174,-4.4
4350956489028,8.85032199278892e-06,0.00166769922852071
"84","NEGR1",1689.99804879965,0.785388740526508,0.17716482432504,4.433
09637519001,9.28893184625099e-06,0.00172951064375435
"85","CAV2",232.685631900531,-1.02199463184314,0.231221484525126,-4.41
998127441348,9.87094542434486e-06,0.0017745009935259
"86","COX4I2",7.79746587122026,-2.87713558213074,0.65051570287414,-4.4
2285339065428,9.74058422023411e-06,0.0017745009935259
"87","RIPOR3",74.0746363213219,1.08050966826181,0.244357987969292,4.42
183076248605,9.78681016621676e-06,0.0017745009935259
"88","CRIP1",12346.8134106296,-1.25912176814943,0.285525493203272,-4.4
0984009526963,1.03446976917062e-05,0.00183853490793506
"89","LILRB5",16.3256191862445,-3.52250442156178,0.79953179781671,-4.4
0570898015654,1.05438443432176e-05,0.00185287332053847
"90","BGN",262.551793088385,-1.99542550354774,0.454171023880057,-4.393
55528783077,1.11511812927493e-05,0.00193782750465111
"91","ENOSF1",133.400770517145,0.761065361811485,0.173530447474509,4.3
8577421361911,1.15573958080401e-05,0.00198634802678843
"92","RASSF2",99.6655344355018,-1.20901526994958,0.276980670424939,-4.
36498066126681,1.27134005416408e-05,0.00216127809207893
"93","KCNAB1",610.440197317482,1.56397537056612,0.358583823366919,4.36
153353456156,1.29153995786008e-05,0.00217200913343351
"94","LRRC8E",6.35392247333676,-3.07392672256381,0.706662444223023,-4.
34992229697957,1.36185780362082e-05,0.00226589957964145
"95","VSIG4",16.4003554006779,-2.2640173927665,0.520892881113833,-4.34
641646076121,1.38379719863015e-05,0.00227816717753426
"96","FOLR2",13.8489583974724,-2.75184001835671,0.633899455854953,-4.3
4113011604538,1.4175174556247e-05,0.00230937218812191
"97","PFKP",264.678195467385,-1.1967492963447,0.275982946560569,-4.336
31610669848,1.44890535618573e-05,0.00233617317224173
"98","IFIT2",128.899584896068,0.79821759383851,0.184564518128734,4.324
87025096423,1.52621665416603e-05,0.00243571719093436
"99","NLGN1",29.0922953561964,1.51717576390819,0.351768660776395,4.312
99297828183,1.61059235391579e-05,0.0025189664415243
"100","FABP5",114.021067986234,-2.11369467067376,0.489933436969197,-4.

31424865334646,1.60146618195005e-05,0.0025189664415243
"101","HES4",39.0033226926538,-2.1871156534246,0.508876639544743,-4.29792897426234,1.72401384276153e-05,0.00266966103968221
"102","EGFR",1535.28173503443,0.637788661703299,0.148488882525652,4.29519470316655,1.74540082916486e-05,0.00267628127138611
"103","THBS2",857.961267894819,0.696721730002417,0.162436460353828,4.28919547055372,1.79321501982704e-05,0.00271230932744892
"104","TYMP",1451.7912558147,-0.971227033336345,0.226503337187775,-4.287914895184,1.80358164996603e-05,0.00271230932744892
"105","ADAMTS19",14.514684083987,-2.13522309755733,0.500296294324619,-4.26791707589959,1.97306641005621e-05,0.0029389293955504
"106","RAB13",937.01178721519,-1.00958902937108,0.236978435608873,-4.26025695872758,2.04192013704194e-05,0.00298296947586094
"107","IL10",12.1851428438121,-2.6861199693984,0.631513863221086,-4.25346160367348,2.1049104455706e-05,0.00298296947586094
"108","LRRFIP1",340.666512698689,-1.10786679429125,0.26049691164622,-4.25289799902059,2.1102171068889e-05,0.00298296947586094
"109","STAC",57.8327419366454,1.35106424766765,0.317145175104591,4.26008135618673,2.04352506903045e-05,0.00298296947586094
"110","CPXM2",155.624777050891,1.0643973247324,0.250147605670595,4.25507700495058,2.08977081708242e-05,0.00298296947586094
"111","BBOX1-
AS1",22.5448839315212,-1.98350575250644,0.466468814477069,-4.25217225878225,2.1170691292875e-05,0.00298296947586094
"112","MUC12",50.6140389440041,-1.47901732917595,0.349442266200507,-4.23250840620208,2.3109927254982e-05,0.00322713627024928
"113","FAM214B",65.2170795844804,-0.944813723629884,0.223423936475343,-4.22879364912699,2.34947712899675e-05,0.0032518426811955
"114","LGALSL",167.853521840441,-1.0051609975431,0.238248330985517,-4.21896343779301,2.45428065095024e-05,0.00336710082288261
"115","SWT1",39.5765659471938,1.15263104669417,0.273402288453201,4.2158792935322,2.48806823872306e-05,0.00338377280466337
"116","PTGS1",170.446605985806,1.21091837835218,0.28824599004953,4.20098950255684,2.6575091473499e-05,0.00358305543659935
"117","BMPR1B",3.91341105703523,-5.33821641910935,1.27137928475827,-4.19875994764563,2.68380643176039e-05,0.00358758398228484
"118","TAGLN2",3355.93515274867,-0.769652501177465,0.184173499977381,-4.17895354799681,2.92853488153453e-05,0.00384141844004452
"119","PLA2G7",79.3601312525994,1.84228719663049,0.44087949859163,4.17866379025469,2.93226784773719e-05,0.00384141844004452
"120","ADGRG6",5.49656560205336,-3.42821851058271,0.820639892336538,-4.17749434629828,2.94737987727201e-05,0.00384141844004452
"121","ELN",1242.52735729856,-0.686110273784587,0.16437096582721,-4.17415734178893,2.99091005986658e-05,0.00386593663936473
"122","MAN1C1",396.845907414963,0.895099283591696,0.214832248641653,4.16650334971241,3.09307349938241e-05,0.00396521881396237
"123","CRIM1",462.450998104028,0.972370465590858,0.233517900182473,4.16400826159811,3.12708791538785e-05,0.00397623211355008
"124","FJX1",47.8375707693127,-1.30891942539923,0.315011813186679,-4.15514393621662,3.2508282953736e-05,0.0040351551221939

"125","TEDC1",61.584326220316,-1.50693626585165,0.362594481068808,-4.1559823564046,3.23892820290205e-05,0.0040351551221939
"126","ADAMTSL3",350.642399183768,1.2722078386543,0.306132202236189,4.15574653486719,3.24227113918479e-05,0.0040351551221939
"127","F13A1",13.8654110282667,-2.56236885111751,0.617620886321888,-4.14877298981445,3.34262074684153e-05,0.00411642428981114
"128","MLXIPL",112.184727638716,1.48268491705873,0.357791033255241,4.14399685640253,3.41304423506783e-05,0.00413798541367913
"129","A2M",32.1012673548834,-1.76230630025478,0.425103318216256,-4.14559525822913,3.38932057351182e-05,0.00413798541367913
"130","AC022893.2",26.3383240892531,1.75959247585178,0.426445489466132,4.12618381321048,3.68832508766551e-05,0.00443733879777605
"131","RGL1",223.436660119585,0.866583016194258,0.21031453180675,4.12041435629627,3.781916308829e-05,0.00451520389847982
"132","CACHD1",128.251637959814,1.12378146372739,0.273177289156905,4.11374410806867,3.89292961330014e-05,0.00454368799641897
"133","KCND2",69.7087856506064,1.16879114561377,0.284064182839055,4.11453191293736,3.87965884305059e-05,0.00454368799641897
"134","CLU",4009.23217422089,-1.37863412853418,0.334940989995853,-4.11605079614545,3.85419398181643e-05,0.00454368799641897
"135","FBLL1",8.79244428640427,3.83678406313264,0.933919456448684,4.10826012526002,3.98650959238848e-05,0.00461399260163004
"136","FZD10-AS1",14.0987235858735,-3.32691274132537,0.810437063483159,-4.10508464041206,4.04166871114652e-05,0.00461399260163004
"137","ACP5",14.7096498359826,-2.39072402232675,0.5822343730882,-4.1061196879295,4.0236105523937e-05,0.00461399260163004
"138","SEMA3E",65.1856192302742,-2.16688713790128,0.528116653032574,-4.10304641116407,4.07745415985184e-05,0.00462111471449876
"139","TUB",33.9730294224464,1.23997322100607,0.302433889202415,4.09998107115624,4.131839319615e-05,0.00464906237113515
"140","GPC1",50.5742175516299,-1.32474818815574,0.324108682181062,-4.08735791723,4.3631372241594e-05,0.00487424758470379
"141","TET1",155.904630419086,0.820128112142787,0.202322134915062,4.05357581110485,5.04406434486213e-05,0.00559331940230145
"142","LDHA",4117.06855275303,-0.632791424603956,0.156211048655158,-4.0508749544398,5.10264722633824e-05,0.00559331940230145
"143","ADAMTS5",7032.4543765921,0.959886186744311,0.236988422512017,4.05035054695821,5.11409638445721e-05,0.00559331940230145
"144","TSPAN5",25.6946985863105,-1.89312968292139,0.467972290823932,-4.04538841303674,5.22364430756921e-05,0.00567345812294323
"145","PTGER2",15.2334247469862,-1.76825022886081,0.438603630024299,-4.03154490254185,5.54114069732831e-05,0.0059767890004286
"146","ABCA8",1936.78556640072,0.919561956808115,0.228549082010496,4.02347692110128,5.73452059594993e-05,0.00612444812645634
"147","SSC5D",664.388758847489,-0.906450492421595,0.225340450565837,-4.02258223122155,5.75635469686113e-05,0.00612444812645634
"148","PPP1R14B-AS1",66.2198017016261,-1.45348861457632,0.361976775039787,-4.01541953739037,5.93401416052599e-05,0.00627080955882612

"149","PPP1R3C",22.3854515502207,-1.94509294867246,0.484710991615936,-4.0128921817677,5.9979313320301e-05,0.0062958151699967
"150","KCNB1",21.8253398541298,2.09829254713086,0.525361146025543,3.99400024726769,6.49677954288435e-05,0.00677397547004742
"151","ABCA9",1098.25337467437,1.01525268515901,0.254491561479943,3.98933732519465,6.62581407994345e-05,0.00686276372253745
"152","MMP9",10.4888196799135,-2.90814507976249,0.73030169119565,-3.98211467236407,6.83048080264462e-05,0.0070282052469317
"153","SVEP1",855.814978912463,1.35305127692232,0.340318973230855,3.97583262571869,7.01334406582335e-05,0.00712264293438164
"154","TSPAN9",50.1192634663174,1.00089682494332,0.25172067639527,3.97622014717471,7.00193101818294e-05,0.00712264293438164
"155","IGFBP6",26416.2373033518,-0.778162709631731,0.196110940762699,-3.96797193774791,7.24868668754399e-05,0.00731415869633471
"156","GSTM5",225.56745827188,1.60601940113546,0.405370591427643,3.96185474501084,7.43697821690532e-05,0.00743177725159117
"157","ABCA1",752.17573104988,1.6123360733239,0.407041717853155,3.96110767669658,7.46028790600903e-05,0.00743177725159117
"158","KRT25",22.5929429237671,3.3304157091136,0.841238988200392,3.95894122339496,7.52827592975428e-05,0.00745204022413651
"159","MYADM",3425.54568162332,-0.826517794457888,0.208855076281427,-3.95737469815757,7.57780146166479e-05,0.00745388772707153
"160","EHBP1",437.740180055022,0.616276488839561,0.155973969039685,3.95114962217036,7.77766778862166e-05,0.00755544870894676
"161","CD163",25.6745218751906,-2.23547866164525,0.565580487583252,-3.95253851701556,7.73264774215943e-05,0.00755544870894676
"162","SH3BGRL3",7950.321895498,-1.07700412147185,0.272716199001986,-3.9491754630388,7.84208539449155e-05,0.00757100096110172
"163","ESR1",49.93025042826,1.55043771829651,0.393684523599939,3.93827449481366,8.20696754476148e-05,0.00787466088343985
"164","HIST1H2AC",60.9353997918532,1.17088050527736,0.297671288308953,3.93346806112553,8.37289689134959e-05,0.00798488459638461
"165","MAF",1273.60903631669,0.798203619738078,0.203034064161615,3.93137783570499,8.44604114287756e-05,0.00800582324088516
"166","NQO1",224.121912149578,1.19195556379248,0.303675139007221,3.92510090779654,8.66933891827579e-05,0.00816797955914659
"167","F2R",105.765206434997,-1.20887289257554,0.308236613943223,-3.92189907977064,8.78537904657468e-05,0.00817876954097786
"168","TPP1",379.627568964246,0.596934720497418,0.152185958879597,3.92240338656794,8.7670051931312e-05,0.00817876954097786
"169","THOC6",94.2267755395872,-0.923210372165592,0.235676149820259,-3.91728383576228,8.95522858914163e-05,0.00828756065882693
"170","FXYD5",314.922630151539,-1.62891483682521,0.417051489087362,-3.90578832457781,9.39186997146822e-05,0.00864052037375077
"171","WASF1",169.348056244724,0.728820080565147,0.186740209978641,3.90285563376258,9.50644094954032e-05,0.00869477990940412
"172","IFI30",64.9203551112395,-1.4190406787609,0.364348324354633,-3.89473639346202,9.83055793936505e-05,0.00890704635864423
"173","FOXRED2",75.3321407926673,0.868585098491315,0.223045963182484,3.89419779716293,9.85242340182514e-05,0.00890704635864423

"174","DPYD",224.373971776252,0.600782802244349,0.15440139372877,3.891
04520196052,9.98133345399196e-05,0.00897172731151921
"175","FCER1G",53.4475589259741,-1.61021810478264,0.414659999858363,-3
.88322506471,0.000103080073451323,0.0092071247836638
"176","CTNNAL1",148.323512804102,-0.814074611698314,0.209706035584472,
-3.88197988402864,0.00010360944988443,0.0092071247836638
"177","WNT2",441.681662809865,-1.00396668907617,0.25961768636757,-3.86
709666480405,0.000110138778866627,0.00973203673149175
"178","ACADVL",769.350535239952,-0.643069259424453,0.166453486370931,-
3.86335710620932,0.000111839326254831,0.00982678125070536
"179","PRG4",8524.8883025659,-1.49761325585482,0.387953566899158,-3.86
028995125621,0.000113252558287743,0.0098953631934095
"180","MUC20",38.4930352324156,2.36642630664574,0.614031721567668,3.85
391539805807,0.000116243777006603,0.0101002926243515
"181","GRID1",22.6297616532308,1.95747716250656,0.508891546835297,3.84
655075266971,0.000119792325699631,0.0103511158781338
"182","HDGF",505.336909289382,-0.556514705017762,0.144888307018641,-3.
84099115014273,0.000122538532792225,0.0104157752873392
"183","SLCO2B1",14.1912660805921,-2.56475949166124,0.667585863851174,-
3.84184212179935,0.000122114376382573,0.0104157752873392
"184","SPRY2",577.829249658668,0.968207523924634,0.252020708049456,3.8
417776515993,0.000122146462242364,0.0104157752873392
"185","PTHLH",240.514544522258,-1.45952140086241,0.38136886552697,-3.8
2705966006341,0.000129683091588042,0.0109634786618215
"186","FAM133A",7.19856086854847,-2.3996351636048,0.627815888463418,-3
.82219565910942,0.000132268672827949,0.0111219464678985
"187","GEM",185.21843779376,-1.27355691147838,0.333367456600562,-3.820
27965316464,0.000133300447370635,0.0111487646891804
"188","EFHD2",276.796695056186,-0.734242303432744,0.192838949239849,-3
.80754150718541,0.000140355186874452,0.0116763570357257
"189","ANGPTL1",269.332218639423,-1.12052143182664,0.294572645066119,-
3.80388827881532,0.000142442441784555,0.0117873004736002
"190","RBP7",12.7286914537378,-2.52626388016758,0.665784860999347,-3.7
9441472486419,0.000147992132590433,0.0120551924672623
"191","HRH2",11.5274275110576,2.93851657310275,0.774010508446762,3.796
48149609698,0.000146764311986329,0.0120551924672623
"192","PLXNA4",17.0601656735055,1.57068786575202,0.413867158292317,3.7
9514980660204,0.000147554333675396,0.0120551924672623
"193","MAZ",588.103888954086,-0.759524478784917,0.200387044264259,-3.7
902873490329,0.000150473108622781,0.0121937793723331
"194","ADAMTSL1",254.138389681105,0.911577896515691,0.240985518440089,
3.78270819929919,0.000155131239057521,0.0124449836189292
"195","C11orf96",4339.24673795324,-1.21337090990674,0.320772294657001,
-3.78265495529839,0.000155164437704041,0.0124449836189292
"196","NGEF",2.47210198549434,-5.40009839184613,1.43211544677119,-3.77
071443787653,0.00016278085822239,0.0129892480744805
"197","TNIK",32.6318722430214,1.5694582188073,0.416653471510163,3.7668
1901417691,0.000165340771573428,0.0131265465350681
"198","INKA1",25.0522004743274,-1.48774568305028,0.395882995113052,-3.
7580439205918,0.000171246827758054,0.0135267696269493

"199","NBL1",1401.94133707928,-0.674592577301963,0.179860727184933,-3.7506385516186,0.000176384808053121,0.0138074801008006
"200","ID1",195.091210312701,-1.19131351276084,0.317651348954173,-3.75038077654349,0.000176566241698217,0.0138074801008006
"201","SLCO2A1",3.82907061420273,-3.990181278461,1.06528492525816,-3.74564699438886,0.000179929453997822,0.014000480898139
"202","PLEKHG4",181.697513238293,-0.957861272600232,0.256037190575743,-3.74110210491812,0.000183215063004809,0.0141855623039367
"203","KANK4",17.0401492401661,2.63267694196162,0.704426085358642,3.7373359628232,0.000185980346797838,0.014328732137528
"204","FLRT2",623.020004161452,0.908269316930282,0.243333279534129,3.73261445647385,0.000189502540256517,0.01452852808633
"205","PARP16",96.6019854212258,-1.21453556178376,0.325642681537942,-3.72965716916395,0.000191740475796094,0.0146283953241508
"206","LGI2",19.7238162481297,-2.12333252899228,0.570331726658938,-3.72297810158831,0.000196886604429988,0.0149480897732282
"207","TMOD3",932.22815303261,-0.700664735926941,0.188282706479153,-3.72134408427212,0.000198165203944116,0.0149724820757777
"208","MLIP",23.6182701153184,-2.29755025934547,0.617663704508644,-3.71974302937744,0.000199425574588689,0.014995269166188
"209","DKK3",155.177086682977,-2.76457893696763,0.744551264390082,-3.71308070940193,0.000204751554937639,0.0153220780824147
"210","DTX3",49.7137692318707,1.23695616503262,0.333317104659554,3.711049171317,0.000206402002080189,0.0153720348215912
"211","S100A6",51093.3023122755,-0.759205931273168,0.205252247569241,-3.69889216933936,0.000216542567529208,0.0160277440189165
"212","MS4A4A",18.9480337125576,-2.48501508781164,0.67197852703259,-3.69805728582621,0.000217255865218049,0.0160277440189165
"213","ALDH5A1",15.1972279436362,1.76058198860018,0.478904546377286,3.67626910606352,0.000236669864844609,0.0173780126111252
"214","HRCT1",306.811543408091,-1.38244908580137,0.376245674228054,-3.67432552849346,0.000238478533622562,0.0174289918965274
"215","AC073316.1",20.055878761188,2.12295238943267,0.578608848605505,3.66906312364417,0.000243440970043549,0.0176538584823844
"216","MEGF9",151.637127131106,0.848819827624003,0.231369728076306,3.66867279778305,0.000243812879296357,0.0176538584823844
"217","SYNJ2",83.4320116056674,-1.60737963397426,0.438443060985804,-3.66610804686976,0.00024626990719386,0.0177495914677971
"218","TRIM47",314.345642500061,-1.04333031122622,0.284932657260323,-3.66167332750841,0.000250573245014577,0.0179769062019632
"219","TXN",2755.81712430557,-1.01180028666268,0.276465255241158,-3.65977375992545,0.000252438026035932,0.0180279941881368
"220","VEGFC",92.1368979232117,1.57848434470132,0.431583674827602,3.65742366258838,0.000254763098623043,0.0180296879632654
"221","NATD1",96.7803817258991,0.82494496933415,0.225553834051695,3.65741940411919,0.000254767329915706,0.0180296879632654
"222","PDE1C",115.176722354965,1.58256757629611,0.433131418652052,3.653781527143,0.00025840616509528,0.0182048307301359
"223","LINC00702",18.6396134200157,-1.69082610477604,0.463278742023329,-3.64969499224486,0.000262551873785011,0.0184139520448322

"224","ULBP2",11.3724075466292,2.06583024846045,0.566884960403874,3.64
41789653207,0.000268246726289713,0.0187293696391568
"225","AL121839.2",54.3425267205299,1.77077505157591,0.487060818814409
,3.63563436674353,0.000277297409588406,0.019275251048723
"226","GAL",245.299426070458,1.59265787775776,0.438367081185943,3.6331
6030357261,0.000279970958720704,0.0193749813911142
"227","PLAUR",190.617911380518,-1.01588061764666,0.280268857765227,-3.
62466463718787,0.000289336656664187,0.0199349132609158
"228","SERPINE1",172.683550764638,-1.47809233600833,0.407929487729311,
-3.62340154480115,0.00029075390868478,0.0199446979466226
"229","ADIRF",1254.04815986381,-1.35591546042435,0.374351298721583,-3.
62204022012165,0.000292288662245125,0.0199624221725491
"230","MYEOV",2.92250625864496,-4.09972521635912,1.135211058647,-3.611
42114070433,0.00030452368562771,0.0206179672866554
"231","CCL3",83.993864244669,-1.92022388410262,0.531641290051849,-3.61
187876117626,0.00030398668647105,0.0206179672866554
"232","APBB2",187.533179943262,0.894442489504869,0.248345433919607,3.6
016063407649,0.000316257008260559,0.0213200845223929
"233","LMO7DN",25.0740417789208,2.10656451197316,0.585461796270952,3.5
9812463493047,0.000320520022838695,0.0215147345802455
"234","LSAMP",33.1490573884844,2.28474117165581,0.636324685615673,3.59
052732559828,0.000330009629029098,0.0220570538376713
"235","SYNE2",703.366357604717,0.881767165532119,0.245814525745806,3.5
8712392140708,0.00033434545074151,0.0222517568067967
"236","VIM",54309.3993470756,-0.800262452836625,0.223301923538255,-3.5
837687385596,0.000338671968750233,0.0224189312989771
"237","SNHG14",219.252462057477,1.285084689653,0.358665774169184,3.582
95879396293,0.000339724214696776,0.0224189312989771
"238","SCIN",73.7474210041646,4.08365745433007,1.14171835611239,3.5767
6429783886,0.000347873576753559,0.0226697614184402
"239","KIF21A",192.148155668127,-0.805232902022829,0.225011905421239,-
3.57862354223157,0.000345408583925724,0.0226697614184402
"240","EML1",287.827309677639,-0.61517782146508,0.171975261524614,-3.5
7712973372645,0.000347387785083892,0.0226697614184402
"241","LINC01088",81.1278759618329,-1.90515841771944,0.533365075900557
,-3.57195943979401,0.00035432029607039,0.0229069827299414
"242","TYROBP",72.3061566816702,-1.51917011609889,0.425315215952818,-3
.57186872022801,0.000354443083161498,0.0229069827299414
"243","KIAA0040",59.9031328127331,1.99876878732343,0.560794799447274,3
.56417140332514,0.000365007390839071,0.0233008799702983
"244","KCNE4",25.359668238736,-1.40808764104705,0.39495810983988,-3.56
515692668749,0.000363638546755765,0.0233008799702983
"245","LAMA4",1687.10029159984,0.59504472195969,0.166925549580232,3.56
473124369547,0.000364229209834755,0.0233008799702983
"246","REEP5",1359.14132215822,-0.714575113496528,0.200840384844886,-3
.55792543441108,0.000373795383109945,0.0237648772025998
"247","NIBAN2",51.9486370115213,-1.14354397372032,0.322336564216544,-3
.54767066683784,0.000388653860692151,0.0245102676662308
"248","PGPEP1",88.7172875121875,0.770448411521329,0.217166225493555,3.
54773588650963,0.000388557641792852,0.0245102676662308

"249","SEMA3B",378.31423011043,1.04298739424346,0.294153074256456,3.54
573004847857,0.000391527073054814,0.0245923029019168
"250","PXN-
AS1",49.1945702448331,1.4655624837287,0.413496597374828,3.544315704247
97,0.000393633593010711,0.0246257175787501
"251","USP2",11.5132490902521,-2.10966163572518,0.595983724897741,-3.5
3979739310358,0.000400434321479607,0.0249513656890082
"252","EMP3",6411.97881788172,-0.779119992563517,0.22049234335512,-3.5
3354670147744,0.00041002364353653,0.0254474991464735
"253","CADM1",8.42005162805074,-2.89012579072341,0.818707814780303,-3.
53010651486082,0.000415392396765219,0.0256788027091226
"254","ABRACL",148.862769535094,-0.99076667266368,0.28081258953409,-3.
5282131556406,0.000418375110939041,0.0257613650987661
"255","LPIN1",181.149722459561,0.915941980691837,0.260191684777136,3.5
2025846435629,0.000431126457352545,0.0263391320038821
"256","ZNF528-
AS1",52.7944082300803,1.06432532946527,0.302309121326925,3.52065238651
625,0.000430486555937836,0.0263391320038821
"257","SLC12A7",78.3445675752365,0.945921881741378,0.269226837853407,3
.51347543685979,0.000442285304858778,0.026869183038944
"258","MAP2K3",132.052945957367,-0.721088312672411,0.205268466528413,-
3.51290349106104,0.000443238441435266,0.026869183038944
"259","PPL",556.716451636338,-1.20784114144574,0.344219129355225,-3.50
893090604294,0.000449911768911376,0.0271684172423703
"260","PLEKHA2",367.220312243486,1.10145313072667,0.314008998444177,3.
50771199610217,0.000451978073713982,0.0271882195111026
"261","GAB1",123.069367978482,0.733688927692725,0.209921880851567,3.49
505694554779,0.000473960621195849,0.0284013184502034
"262","GRIA1",11.7178891544426,2.50796011332218,0.718717967626141,3.48
949132523532,0.000483940765040526,0.0288886777299001
"263","DIO3OS",74.0291227540809,1.4557822766366,0.41731790024309,3.488
42519285321,0.000485874758649929,0.0288938449630604
"264","MICAL2",7.16193005398354,-2.24059813798615,0.64381752233199,-3.
48017576450919,0.000501084932077691,0.0296854861276329
"265","MT1G",177.16175209907,-2.50919784438194,0.721266142399719,-3.47
887928862654,0.000503515338569863,0.0297169052650289
"266","GPC6",314.389506917315,1.32095982609429,0.380520021052391,3.471
45945814087,0.000517637488189257,0.0303215367613483
"267","MT-
CYB",44226.0275081405,-0.570204771172849,0.164209246266414,-3.47242791
826561,0.00051577349716276,0.0303215367613483
"268","LINC01137",9.76742829836479,-2.18558123780895,0.63048309830222,
-3.4665183629733,0.000527245719126255,0.0306547325172291
"269","PTMS",2583.17326524591,-0.553863204068967,0.159758236864145,-3.
46688355442956,0.000526529935464587,0.0306547325172291
"270","ANXA5",5185.15892858963,-0.659730710666395,0.190808358591804,-3
.45755665807993,0.000545097607838307,0.0314939361964563
"271","EDN1",27.7923891284483,1.28120277457072,0.370583756712294,3.457
25561729192,0.000545706950718648,0.0314939361964563
"272","FOLH1",11.6036639730338,2.1153968021887,0.613149253976372,3.450

05198729348,0.000560478608444153,0.0322275199855388
"273","TGFB1",258.477680843523,-0.792686561739026,0.230069466663675,-3
.44542269443258,0.000570167028739236,0.0326645140274053
"274","ADAP2",6.29540646823857,-2.1944580232192,0.637396905706641,-3.4
428438600378,0.000575631555003168,0.0328572172271881
"275","ODF3B",134.984543030446,-1.2627595886485,0.366897135811237,-3.4
4172648242797,0.000578014376791663,0.0328732540109877
"276","IMPG2",13.1253806470147,-1.68495132250807,0.49127972817604,-3.4
2971880554434,0.000604207056609398,0.0342383998745326
"277","ZNF727",69.2805925723307,1.01535950372939,0.296272561727355,3.4
2711285111773,0.000610035490961063,0.0344438811502925
"278","PRXL2C",186.331333418253,-0.648593188461804,0.189393635569403,-
3.42457752876352,0.000615756126797934,0.0346418195076248
"279","GLUD1",721.890053730662,-0.395197584253419,0.11553573382659,-3.
42056583850134,0.000624910075243077,0.0348285615675511
"280","NOX4",242.168322439207,0.894944133584574,0.261569414394256,3.42
144029208113,0.000622904005987711,0.0348285615675511
"281","LDLR",75.3074319617539,-1.49989480214239,0.43854036492447,-3.42
019782466483,0.000625756125350502,0.0348285615675511
"282","RBPJ",1957.75457908868,0.572618341242072,0.16750582569061,3.418
49806644767,0.000629677652052655,0.0349225477946934
"283","FAM53C",386.073466422554,0.796285355761625,0.233033420427019,3.
41704359101146,0.000633051424232785,0.034985598144879
"284","SLC30A1",155.367641077368,0.655810445756816,0.192257533529255,3
.41110402135177,0.000647004030159717,0.0355057650235016
"285","PKM",1336.99791975572,-0.672368917287399,0.19708271957909,-3.41
160766770105,0.000645809911076563,0.0355057650235016
"286","EBF2",686.332837707826,0.878260609947999,0.257625882607002,3.40
905424975389,0.000651885148213222,0.0356470604023282
"287","MIR497HG",85.2687907435472,1.013580909916,0.297402349918847,3.4
0811331918722,0.000654137233725588,0.0356470604023282
"288","HPRT1",66.1768163309287,-0.860587891677444,0.252752447837167,-3
.40486471660947,0.00066196838711098,0.0359485610222768
"289","FRMD6",665.464154952998,0.779110525757733,0.229041261419211,3.4
0161646390751,0.00066988578452543,0.0362526424566704
"290","AC025280.2",12.0900613299145,3.15869498232364,0.930121504248347
,3.3960025307406,0.000683777273608085,0.0368166385520673
"291","ABCA6",726.605125483783,0.997749543688976,0.293844028121191,3.3
9550730388664,0.000685015461550613,0.0368166385520673
"292","TMCC3",81.6047036318646,0.851080478711483,0.251005630882435,3.3
9068281344695,0.000697187388182592,0.0373425025725197
"293","SLC35E4",46.9418373532274,-1.11694083287539,0.329702737976269,-
3.38772082916638,0.000704759580835554,0.0376192486152494
"294","ZBTB37",103.627202522997,0.930968137418731,0.275264399650759,3.
38208696293416,0.000719373676071198,0.0381389976059442
"295","SASH1",1093.70191907494,0.778490609945531,0.230162619343005,3.3
8235032329628,0.000718684300806433,0.0381389976059442
"296","SCN9A",2.83708962684246,-3.7323348883708,1.10394173328788,-3.38
091656092642,0.00072244477275895,0.038172419749831
"297","S100B",3.5890970602229,-3.08531317150634,0.913220070952892,-3.3

7849908214018,0.000728826757634821,0.0383799679778067
"298","SQLE",99.5913301591447,-1.2909540342576,0.382913134156153,-3.37
140181180401,0.000747866923181762,0.0390907837447613
"299","TMEM135",100.130799338499,0.681706891366447,0.202245974107476,3
.37068213285758,0.00074982321761051,0.0390907837447613
"300","ST8SIA2",2.71799022902155,4.21507820684813,1.25038803730373,3.3
7101610147942,0.000748914804601327,0.0390907837447613
"301","SNHG18",58.6890028529092,-1.25370461640327,0.372205107959122,-3
.36831652654523,0.000756287149808479,0.0391666590165715
"302","HMGA1",54.1417016935358,-1.18621627503808,0.352148078601218,-3.
36851553968404,0.000755741366784909,0.0391666590165715
"303","S100A4",29912.2779686338,-0.643130258282502,0.191021406429921,-
3.36679679153364,0.000760467027479029,0.0392531495372013
"304","FABP3",564.50072024851,-1.06026104068803,0.315096474227197,-3.3
6487751342956,0.000765776458998423,0.0392680125204437
"305","CSF1R",10.0948309881241,-2.01689568391968,0.59930458679833,-3.3
6539337149839,0.000764346033207262,0.0392680125204437
"306","COL8A1",38.7397309140861,-1.89225872989771,0.56407781543597,-3.
35460583294737,0.000794781444761455,0.0405891805944304
"307","PRTG",20.6370265260817,1.38242584268654,0.412181151913908,3.353
9278452385,0.000796731358215481,0.0405891805944304
"308","CXCL3",104.758775040001,-2.64199988706004,0.788391585045347,-3.
35112644170103,0.000804835446863586,0.0408689168472289
"309","SCARB2",900.849030231317,0.459960303585663,0.137731439541999,3.
33954473368738,0.000839158346018499,0.0424739046334282
"310","ENO1",1502.26787507162,-0.709626817795176,0.212679311053929,-3.
33660483607283,0.000848084366850147,0.0427872241856009
"311","TUG1",391.621907192923,0.890246112762605,0.267064811777155,3.33
344594085067,0.000857773387157951,0.0431368995985542
"312","DHTKD1",63.064696402772,0.919671151708071,0.276869544817979,3.3
2167682910985,0.000894782646095337,0.0448538480286252
"313","PRSS23",2124.63681315134,-0.848376099937732,0.255599852135561,-
3.31915724070054,0.000902895725444252,0.0451159397634125
"314","FIGN",62.722653079968,1.43774798237123,0.433401228449165,3.3173
6019188479,0.000908723822792934,0.0452625496448455
"315","ZNRF3",58.2126220490154,0.866139398260103,0.261880872024329,3.3
0737938805869,0.000941732554827531,0.0467577687539765
"316","NCKAP5",52.8461220649271,1.17003449536205,0.353862002345488,3.3
0647113170322,0.000944790852118457,0.0467611674909262
"317","IL1RN",10.3456003045944,-2.65651297923368,0.804227947688081,-3.
30318411200503,0.000955936029420504,0.0470152185538889
"318","NID2",137.963975407221,1.03801056384216,0.314174068494991,3.303
9345634559,0.000953380826373473,0.0470152185538889
"319","PCOLCE2",11770.6543404591,-0.639957950356703,0.19401603976926 3,
-3.29847960569541,0.000972099412985686,0.0476205025216376
"320","ASPH",1435.40548320082,0.669426277517861,0.20298960032978,3.297
83533949671,0.000974332532412023,0.0476205025216376
"321","GAS2L3",5.46406960118051,-2.53349768475204,0.768678302779295,-3
.29591413676141,0.000981019933618658,0.0477979805663421
"322","MIR99AHG",742.768111383364,0.652053740737282,0.197983528056824,

3.29347470033029,0.00098957246883801,0.0480649484864176
"323","IFRD2",125.497548725767,-0.875300391394852,0.266203886201085,-3
.28808269438136,0.00100872205476685,0.0488433837045003
"324","FHIT",44.7409590874625,1.10218842978511,0.33546796309545,3.2855
2515004691,0.00101792454514151,0.0491368515000408
"325","STARD10",70.4780763487437,-0.947059647791186,0.28875246750138,-
3.27983222441782,0.00103868835856171,0.0499848797781699
"326","P2RY14",7.02198619303274,-3.09588011852143,0.944409618301076,-3
.27811159324138,0.00104504072827436,0.0501363097859235
"327","DYNLT3",71.0800287085486,-1.11989815396392,0.341733210885503,-3
.27711243242069,0.00104874599240551,0.0501602058752972
"328","AC018816.1",18.541960008356,-1.86882740513341,0.571620218950849
,-3.26935147354208,0.00107794312453596,0.0512432537013447
"329","SLC7A8",67.3561661987791,1.03534454498965,0.316656869510882,3.2
6961024590711,0.00107695761363976,0.0512432537013447
"330","ZEB1",1291.78144950309,0.709708370796235,0.217147364060967,3.26
832597699402,0.00108185684316187,0.0512734576577323
"331","ENPP6",41.6987611748901,5.52569744542146,1.69305316703502,3.263
7471480582,0.00109949241927327,0.0519518472430031
"332","OGA",474.491306088023,0.669190334342323,0.205132325576212,3.262
23735075679,0.00110536551007384,0.0520369887786277
"333","C20orf194",132.63847627777,0.797881047145064,0.244630551104063,
3.26157564353299,0.00110794867412295,0.0520369887786277
"334","AGTR1",837.426538945895,0.840063717855807,0.257827071719609,3.2
5824480824648,0.00112103652185336,0.0524940455143309
"335","APBB1IP",876.69564797076,0.766666910555158,0.23559399056049,3.2
5418703902939,0.001137173812977,0.0530907415968966
"336","AC074387.1",12.4701747216215,-2.44510351383532,0.75269578753943
6,-3.24846180131865,0.00116030786916458,0.0536899854252484
"337","PNP",44.3049893016508,-1.37701259936081,0.423888717887068,-3.24
852382536795,0.00116005493395926,0.0536899854252484
"338","CYSLTR1",10.2780732875315,-2.23638576256221,0.688441048230917,-
3.24847823689339,0.00116024083962772,0.0536899854252484
"339","NCS1",68.1188177599926,-0.892530333531389,0.274945053020829,-3.
24621346601852,0.00116951110514351,0.0539562055588335
"340","TPMT",69.7934756515006,-0.739543124817755,0.228083818681925,-3.
24241819999115,0.00118519966129413,0.0545191844195301
"341","ACOT7",55.9071496206219,-0.9505354054075,0.29328501912168,-3.24
099542572659,0.00119113095214725,0.054631343377076
"342","SBF2",294.048508291429,0.617057952320246,0.190465349074439,3.23
973864704956,0.00119639303605268,0.0547122429352745
"343","RPL39L",16.485221526994,-1.44467516462731,0.446152445333897,-3.
23807519097227,0.00120339088926366,0.0548718178078241
"344","KCNK15-
AS1",60.847920907035,-1.16437661267618,0.360200432743012,-3.2325797162
6844,0.0012267790266807,0.055775651096762
"345","HLA-
DPA1",236.815632582946,-1.13287379031596,0.351384129739543,-3.22403231
800959,0.00126399133876698,0.0573009406907697
"346","AC105446.1",22.7022246883565,-1.23036793214702,0.38207438748159

9,-3.22023138022114,0.00128087169461869,0.0574800978841984
"347","CCL18",7.2453170101049,-2.52851705877382,0.784695778412441,-3.2
2228961635221,0.00127170519629205,0.0574800978841984
"348","ANGPTL4",749.240056785129,-1.12996096702303,0.350937541152512,-
3.21983496924306,0.0012826441279786,0.0574800978841984
"349","MT-
CO2",31367.36114325,-0.697629861875528,0.216611025194311,-3.2206572183
9283,0.00127897020541399,0.0574800978841984
"350","TWIST1",620.072760627152,-0.988641755268219,0.307169318878824,-
3.21855632872705,0.00128837662758785,0.0575720298727829
"351","CYP1B1-
AS1",63.1046457869474,1.14938162826853,0.357363457565708,3.21628192232
607,0.00129863190172349,0.0578649656494457
"352","LINC02518",3.88584779964947,3.92332142215108,1.22090313979476,3
.21345837705898,0.00131146804993808,0.0582709099461125
"353","CMKLR1",39.55708448719,-2.18497363498328,0.681687236522071,-3.2
0524357494338,0.00134948229254738,0.0594785070110709
"354","CEP152",281.62662687306,1.14736725935289,0.357979353806677,3.20
512132097014,0.00135005562589068,0.0594785070110709
"355","LILRB4",4.60589140156738,-3.87480221505285,1.20848704741808,-3.
20632498571773,0.00134442058233097,0.0594785070110709
"356","AL360178.1",9.35723932340587,2.12239467703445,0.663006729037945
,3.20116611804853,0.00136872598189906,0.0601316695418575
"357","TBCK",82.293789089122,0.754103091877983,0.235794190565977,3.198
14109952373,0.00138316585081787,0.0602582560077756
"358","NRN1",268.494590703144,-0.949641443601193,0.296864023633052,-3.
19891050447739,0.00137947984790078,0.0602582560077756
"359","SLC7A4",25.2963943084846,1.98830821563356,0.621400235143964,3.1
9972234187024,0.00137560038708804,0.0602582560077756
"360","PAQR4",13.3325319522594,-1.81749487576093,0.568953566494794,-3.
19445203052007,0.00140096565418329,0.0608641745317409
"361","ZNF135",26.442605196168,1.28564174689028,0.402835669165437,3.19
147941778287,0.00141546195541748,0.0613236149106076
"362","GTF3A",745.923251850382,-0.731139455115345,0.229177487829745,-3
.19027606960465,0.00142136945058936,0.0614094425613744
"363","NINJ1",95.8903382940156,-0.790594195148541,0.2478957251845,-3.1
8922076836997,0.00142656884999767,0.0614642887437015
"364","MARCKS",11606.4424833466,0.906212259814372,0.284335439576275,3.
18712384627409,0.00143695231747597,0.0617415775970443
"365","CTSO",490.890103765531,0.559376715114711,0.175645590731414,3.18
468976525619,0.0014490926645869,0.0619402133865004
"366","LARP6",545.147512446534,-0.469173973016121,0.147325446114749,-3
.18460921306621,0.00144949604216491,0.0619402133865004
"367","KRT7",19.7255816275316,-2.36326357722558,0.743073586386947,-3.1
8038969561076,0.00147077119980675,0.062678096907296
"368","ABCB5",37.5654873124756,1.9473843385583,0.612754438110873,3.178
08279702078,0.0014825240809193,0.0630072734390703
"369","LGI4",94.8231834126478,1.20968053714066,0.380743164956671,3.177
15627876951,0.00148726869337099,0.0630376215835291
"370","CABLES1",66.4804728505408,0.802940207385019,0.252882117386031,3

.17515613869726,0.00149755896065163,0.0633022220124092
"371","SDC2",4235.71735859254,-0.486677700125559,0.153474508496664,-3.
1710653768677,0.00151880956910543,0.0638553270451854
"372","SIGLEC1",4.0967044060473,-2.59479328108861,0.818151345991769,-3
.17153213008919,0.00151637092662985,0.0638553270451854
"373","HLA-
DRA",530.898294147798,-1.31650776472612,0.415718976081075,-3.166821435
80901,0.00154114919144443,0.0646208400916647
"374","GSTM2",51.27998005687,0.898161966407105,0.28385310510225,3.1641
787609953,0.0015552124090152,0.0650361552860901
"375","TBC1D2B",401.530296601819,0.619909894639797,0.196650886206825,3
.15233715238799,0.00161969138279014,0.0675519286049009
"376","CCDC71L",589.339303214004,-1.20055015504229,0.381194515990797,-
3.14944235732729,0.00163582375965044,0.0680433074492895
"377","KCNB2",69.331534456348,1.13038177864607,0.359533043912946,3.144
02750396364,0.0016663977677744,0.0691311965198718
"378","MAP2K6",53.0452740995033,0.972311410679105,0.309351606260846,3.
14306242799738,0.0016719017855776,0.0691760421334225
"379","ETHE1",200.104657439997,-0.727275893295557,0.231534516921153,-3
.14111218908766,0.00168307546418488,0.0694546180998719
"380","GJB3",2.87843058249615,-4.77136678394211,1.51939922346295,-3.14
029829044364,0.00168775889083766,0.0694646027702656
"381","NID1",1831.81068393998,0.934704537576081,0.2979086677634,3.1375
5401812755,0.00170363878750601,0.0695167997973914
"382","WFS1",69.8950678369831,0.87980383244913,0.280380666030783,3.137
89051472092,0.00170168426444754,0.0695167997973914
"383","GNS",440.894180154987,0.525979445252444,0.167669066962813,3.137
00943638638,0.00170680633773646,0.0695167997973914
"384","ANOS1",469.818444326138,0.755781467274657,0.240794190300258,3.1
3870308221406,0.00169697300876205,0.0695167997973914
"385","PPP1R9A",27.5229580963096,1.56070416016592,0.498200060706748,3.
13268560817093,0.00173214870218394,0.0702556537681576
"386","CALR",2166.19176841546,-0.556809382322291,0.177758996451336,-3.
13238369611703,0.0017339310968356,0.0702556537681576
"387","PBX1",739.716080971476,0.670123755084026,0.214235397345603,3.12
79786785327,0.0017601294920659,0.071030411786764
"388","SLC19A3",15.2604650180394,1.9071638710084,0.609776544249636,3.1
2764387051863,0.00176213553537496,0.071030411786764
"389","CYP2U1",142.712941660654,0.544199892358491,0.174056496871808,3.
12657040753435,0.0017685815024448,0.0711069786587061
"390","PXN",927.038562548926,0.644433609613004,0.206290470752,3.123913
61202397,0.00178462844595894,0.0713851378383576
"391","AL049874.3",2.79772755697479,4.24518030376721,1.35888815537988,
3.12401008645222,0.00178404341012903,0.0713851378383576
"392","ODF2L",768.03650698471,0.647607450632933,0.207440060254202,3.12
190157407078,0.00179687001674228,0.0715090256026699
"393","IGSF10",36.3113601385592,1.85873631699175,0.59526306189651,3.12
25460405183,0.00179294060076459,0.0715090256026699
"394","TFEC",5.1548694048716,-2.32190840085114,0.744536914607077,-3.11
859406202379,0.00181716129080781,0.0721330014929802

"395","FAM114A1",1372.43611707048,-0.389936374207715,0.125260291564652
,-3.11300867447249,0.0018519057455708,0.0731848075569584
"396","PRDM6",126.29024159697,1.10450627687397,0.35490852515977,3.1120
8719592394,0.00185769620205323,0.0731848075569584
"397","MMP14",879.346830027444,0.809493682339475,0.260067555933823,3.1
1262848390616,0.0018542928033756,0.0731848075569584
"398","C9orf72",30.8053475184937,-0.991625952247531,0.318901799502004,
-3.10950252960645,0.0018740267982892,0.0736352793307
"399","ADD3",3210.486497644,0.447508350571164,0.144017786092142,3.1073
1308065553,0.00188796336391373,0.0736352793307
"400","TMTC1",290.95800679704,0.924312933092755,0.29743039768894,3.107
6612890772,0.00188574055515596,0.0736352793307
"401","SPIN4",30.0562356042534,1.32622591349197,0.4267690674845,3.1075
961557128,0.00188615615493089,0.0736352793307
"402","SLC2A8",49.5491934104991,-0.9268491433782,0.298354953637456,-3.
10653177391,0.00189295964181544,0.0736464895472476
"403","CRISPLD1",55.9356773395767,1.43121862019048,0.461464582194286,3
.10147013533512,0.00192562295218068,0.0747313721392206
"404","KCNQ1OT1",1278.91796454939,1.03264839081615,0.333053349045267,3
.10054948787139,0.00193161932774008,0.0747785304105319
"405","PDGFA",51.4572673936139,-1.22127451916601,0.394364720427828,-3.
0968148414521,0.00195612014547005,0.0753539878698314
"406","FAS",195.687725295217,-0.568313581556481,0.183515135267737,-3.0
9682130973746,0.00195607746532223,0.0753539878698314
"407","FSTL3",269.574795006444,-0.684347550239716,0.22116010801818,-3.
09435348161189,0.00197242331306755,0.0757953332097701
"408","FCGR2B",16.5566521898864,-2.13429713159241,0.691441354299202,-3
.08673630572124,0.00202367027889726,0.0773843598091764
"409","SMS",415.318355221887,-0.635214254464847,0.205764678302318,-3.0
8709084428749,0.00202125815387041,0.0773843598091764
"410","MAB21L1",48.1092827677628,0.970611280151476,0.314608151248822,3
.08514345956607,0.00203453993212043,0.0776102549716182
"411","CENPF",26.8996148257192,-1.09404609880138,0.354954215969967,-3.
08221750743748,0.0020546464541449,0.0781763384682248
"412","KCNJ12",95.6511519212354,0.710999811158558,0.230729256568675,3.
08153296956051,0.00205937669110669,0.0781763384682248
"413","FZD7",167.458410650852,0.500067999358139,0.162609014968599,3.07
527844907434,0.00210306110457494,0.0796413454613851
"414","RBMS3",1352.74786013728,0.667867151888966,0.217366781917689,3.0
7253549045903,0.0021224859253572,0.0801314630061869
"415","MEG8",502.153321059996,1.10648307379971,0.360182522020442,3.072
00656931631,0.0021262504570056,0.0801314630061869
"416","KPNA5",127.116441813537,0.714371313437037,0.232676082507728,3.0
7023956110019,0.00213887136023149,0.0802204989784665
"417","NR2F2-
AS1",23.0655994718908,1.26722698569688,0.412722413665198,3.07040990200
463,0.00213765171084625,0.0802204989784665
"418","BZW2",277.216789995858,-0.676526617145862,0.220543789377884,-3.
06753873711079,0.00215829484886206,0.0805626048596721
"419","DTNA",4.34302070319973,-2.40138289674398,0.782704792728735,-3.0

6805697250437,0.00215455536921743,0.0805626048596721
"420","POLG2",77.392376337228,0.901012106280529,0.293938339487697,3.06530991449055,0.00217444549513472,0.0809722084378737
"421","PCDHGA5",40.4444753133633,1.27351528885376,0.415685684269065,3.06364962048931,0.00218654835120118,0.0812294921918919
"422","PCDH17",5.06143160873247,4.36266372852281,1.42485526779609,3.06182938514927,0.00219988804721482,0.0815313958730799
"423","SNAI2",404.443862734948,0.958088970760237,0.313195472721608,3.05907669237562,0.00222020305249891,0.0818961692006673
"424","NPAS3",15.9218837741195,1.40001311581338,0.457615209119766,3.0593675383,0.00221804850147815,0.0818961692006673
"425","MPEG1",12.606031269472,-2.03033216348909,0.663880774005255,-3.05827829783336,0.00222612732413515,0.0819214855281735
"426","ZC3H7B",171.665719497022,0.632284218707917,0.206897278344833,3.05602965764536,0.00224289069391275,0.0823446254760456
"427","LINC01151",3.54290066095824,-2.94678858233521,0.964965481811307,-3.0537761587118,0.00225980625360397,0.082771357860342
"428","ENTPD1",46.9782806660128,1.20438122475124,0.394634463085002,3.05189064162352,0.00227404933645425,0.0828762499747769
"429","ATP11A",330.596359843908,0.708443720933437,0.232146147330193,3.05171431480094,0.00227538549493937,0.0828762499747769
"430","IL27RA",14.9603213946232,-1.87268629005086,0.613734949152676,-3.05129485071087,0.00227856697500985,0.0828762499747769
"431","APP",2970.18182555784,0.556492253877761,0.18243400150084,3.05037574848787,0.00228555227735133,0.0829374422686192
"432","QPCT",46.9785659052041,-1.50207308786175,0.492892215903248,-3.0474766006262,0.00230778357359855,0.0835503127108365
"433","OLA1",267.960327667129,-0.567307851587883,0.18632942423972,-3.04464983940496,0.00232951356680198,0.0841422452304455
"434","TBC1D12",772.16758253561,0.570487295441343,0.187513864783225,3.04237393912633,0.00234720108316655,0.0843913217028158
"435","NBR2",25.2294662154813,1.10674057539128,0.363746978834926,3.04261104500756,0.00234535265584302,0.0843913217028158
"436","MYO5B",108.501685116117,1.06407422181132,0.350257700257141,3.03797524231481,0.00238173521343927,0.0854365567389682
"437","TBC1D4",106.105212127045,0.843986710878332,0.278083072946026,3.03501648603453,0.00240522539111794,0.0858852171622935
"438","MYH10",136.275579755636,0.786792059911615,0.259184326529647,3.03564675552098,0.00240020384818594,0.0858852171622935
"439","TC2N",220.743871503136,1.06354139569596,0.350662993510688,3.03294449479323,0.00242180140466212,0.0862801229360263
"440","RELB",59.9845891861039,-0.748787792639149,0.247243150351035,-3.02854817848754,0.00245731891510099,0.0873465178004079
"441","CLTB",1548.77316406586,-0.685576528890902,0.226541789758464,-3.02626958859049,0.00247591442603759,0.0876092796905609
"442","PLEKHG3",37.5305764108179,0.918873684191574,0.303580521988522,3.02678735174688,0.00247167770614854,0.0876092796905609
"443","CRLF1",81.6988645548326,-2.59254646019627,0.857334550191962,-3.02396125248398,0.00249488389987631,0.0880812284290417
"444","DANT2",14.8218782797733,1.95742785188385,0.647788295272765,3.02

170920062647,0.00251351888956968,0.0885392689929499
"445","ZNF610",4.98343897060493,2.9322290707076,0.97132815580206,3.018
78315087691,0.0025379211714523,0.0891979485876717
"446","SUCNR1",16.3815366317741,-2.02221387550627,0.670839099930031,-3
.01445439855427,0.00257441908835257,0.0902778352955922
"447","FREM1",126.544246094744,1.37073229847821,0.455133734739384,3.01
17132478943,0.0025977866824764,0.0908931954617294
"448","EMCN",19.3403811100283,-1.50550122251545,0.50021691166518,-3.00
969676835547,0.0026150862759784,0.0912945298131746
"449","GULP1",271.581915121734,0.735492319552454,0.2445913504801,3.007
0250567274,0.00263818016312442,0.0914973857114475
"450","AC091182.1",8.05301608738555,2.13742316512808,0.710626775485781
,3.00779992938902,0.00263146315654925,0.0914973857114475
"451","HSD3B7",1290.00665963359,-0.725027135681811,0.241113576568638,-
3.00699423898022,0.00263844763144903,0.0914973857114475
"452","RHOBTB3",1020.83209153379,0.895301915266255,0.297871198202573,3
.00566795537375,0.0026499820090489,0.0916940677467363
"453","ADCY2",11.2883127055576,-1.49526049008705,0.498175116164808,-3.
001475669034‬4,0.00268674509474876,0.0925565931318737
"454","DOCK4",473.780718937277,0.956610466402944,0.318704984106876,3.0
015547735587,0.0026860471170256‬2,0.0925565931318737
"455","NMNAT3",39.4119902223542,0.88691983156522,0.295692551229217,2.9
9946626277268,0.00270453073759837,0.0927606595088563
"456","MAP3K1",112.08850895539,0.75227450780775,0.250748265866361,3.00
011848619796,0.00269874602415988,0.0927606595088563
"457","GPR34",5.28410216302693,-2.58481430278917,0.862809818368486,-2.
99581002413356,0.00273716902566134,0.0936746686243839
"458","CCL4L2",60.7777277801246,-1.62428038387771,0.544208056928666,-2
.9846680202506,0.0028388624252219,0.0969428129486254
"459","LMO2",39.1061891920207,-1.23301323751621,0.41324269658626,-2.98
375082657711,0.00284738547844688,0.0969980969863401
"460","CCL2",482.833124032381,-1.30578618861311,0.437719080361797,-2.9
8316031262292,0.00285288520548059,0.0969980969863401
"461","CHL1",1068.90265317427,0.596018297289703,0.19986061375112,2.982
16985379574,0.00286213158219634,0.0971013838298281
"462","KANK1",404.385193605165,0.658108554782965,0.220773465822117,2.9
8092233290943,0.00287381667810253,0.097286781050917
"463","IRX3",230.105236025376,0.945887307686335,0.317459979239056,2.97
954819361359,0.0028867381575837,0.0975131420833888
"464","FHAD1",4.15159132996907,-2.59969397064648,0.874004752057511,-2.
97446205472738,0.0029350275356477,0.0981123721993121
"465","TXNIP",9673.66937801338,0.995801322046626,0.334739000841212,2.9
7485897832083,0.0029312326665878,0.0981123721993121
"466","COBLL1",262.113612579823,-0.607686798379608,0.204209193478041,-
2.9758052907885,0.00292220331007808,0.0981123721993121
"467","CHL1-
AS2",26.0548073116998,1.8064455396392,0.607476045040096,2.973690163403
84,0.0029424202003959‬6,0.0981123721993121
"468","BAALC",2.65631685656179,-3.95759200907356,1.32991798659907,-2.9
7581659091182,0.00292209564210‬3,0.0981123721993121

"469","NR2F2",377.238322005058,0.466407925157845,0.156822316472998,2.9
7411704945802,0.00293832967057464,0.0981123721993121
"470","MAP7D3",486.772114323006,0.559882941985802,0.188318232867755,2.
97306815946481,0.00294838970164173,0.0981123721993121
"471","AC072022.2",29.6325606842644,1.24742213464029,0.420004550173284
,2.97002052507676,0.00297779852509449,0.0984262967365306
"472","SDK1",450.04201821977,0.543673100532982,0.183019739979366,2.970
57082801164,0.00297246853457056,0.0984262967365306
"473","AC008969.1",32.386146968972,1.10888449812526,0.373305130733218,
2.97045073006972,0.00297363100695217,0.0984262967365306
"474","PLCB1",355.566221227096,0.673614979090498,0.226845746767162,2.9
6948472118327,0.0029829964611966 4,0.0984262967365306
"475","PDZRN3",92.2449566384299,0.834835471142558,0.281542249309526,2.
96522270881179,0.00302463879274827,0.0995902120391219
"476","TNFRSF19",35.4459444748458,1.23962397534963,0.418407244542417,2
.96272110848683,0.00304932715270483,0.100192177874587
"477","KCNA1",16.8641932912496,-1.94010427857257,0.655784007586969,-2.
95845012401477,0.00309190272160684,0.101378110201113
"478","PTMA",6915.29549978196,-0.716309545561116,0.242229469633429,-2.
95715276363823,0.0031049424600710 3,0.101555101170067
"479","LASP1",704.14669917687,0.517173970823905,0.174958593169175,2.95
597924889479,0.00311678059856983,0.101555101170067
"480","AC011043.1",19.4813535793966,1.76076547564379,0.595645227645196
,2.9560641031319,0.00311592323095585,0.101555101170067
"481","S100A10",36993.7991657153,-0.690828371441528,0.233866063414986,
-2.9539487745842,0.00313736077936787,0.101861347470447
"482","SERTAD4",12.0304057132687,-2.991107688745,1.01264158288054,-2.9
5376739343111,0.00313920520976698,0.101861347470447
"483","SHE",128.577957197108,0.680852197878167,0.230650146273336,2.951
88279252731,0.00315842793394601,0.102272904527776
"484","PPP1R14B",3160.61450163846,-0.726178882681292,0.24615643888468,
-2.95007063789014,0.00317701283981389,0.102662150443573
"485","ANK1",25.9933974596593,-1.38937640923033,0.471100649100496,-2.9
4921353193454,0.00318583773049155,0.102735055886367
"486","PODN",1323.73955470698,0.604657683937684,0.205209789142404,2.94
653430747441,0.00321356768655574,0.103416046538543
"487","GNG4",6.92684068591693,2.99781852195465,1.01765232093171,2.9458
1799726059,0.00322101866369787,0.10344298131465
"488","PTPRS",69.9401336232984,0.773768981919978,0.262725924035945,2.9
4515657242151,0.00322791270507409,0.103451956367538
"489","CHST12",471.206945245371,-0.410794567510053,0.139513289388722,-
2.94448342025302,0.00323494278303572,0.10346524565783
"490","SERPINE2",34.5731754502551,-1.2956403535967,0.440144305868095,-
2.94367173748008,0.0032434381593525,0.103525250637292
"491","NR4A2",153.629033105287,-1.28335944699635,0.436499551497301,-2.
94011630159552,0.0032808907324265,0.104294981819411
"492","C3orf14",163.33837999711,-0.666039706290934,0.226499286193822,-
2.94058192183875,0.00327596362227399,0.104294981819411
"493","SAMD12",12.7892128483684,2.14862643175149,0.731142702935547,2.9
3872375820032,0.00329566668958404,0.104340540536628

"494","ZNF559",90.1901498938569,0.684934124820997,0.23304455636666,2.9
3906940157554,0.00329199350262012,0.104340540536628
"495","VPS13B",213.703800826327,0.689623365124488,0.234746316121406,2.
9377388174553,0.00330615424008924,0.10446111578787
"496","AC106897.1",9.64201158638313,-1.47958857418095,0.50385039521484
2,-2.93656328988301,0.00331871092585373,0.104646449355549
"497","RABGAP1",184.974617742656,0.547519560092608,0.186747292052762,2
.93187416039167,0.00336923238048436,0.10602574332148
"498","YDJC",28.5835087522819,-0.975047979650943,0.332953380052597,-2.
92848199798096,0.00340621556633288,0.106974320195675
"499","PTPRG",492.544881388474,0.618160923246993,0.211195484523489,2.9
2696088953664,0.00342291922101866,0.107283480193852
"500","HERC5",37.9396168714728,1.13582673867567,0.388275654426894,2.92
53102164033,0.00344112999347207,0.107638546195806
"501","SPI1",11.7941811097289,-1.53770623180491,0.526096359418844,-2.9
2286043093616,0.00346831943667964,0.108272487005329
"502","ACSM5",5.62545649531131,-2.13041480692354,0.729386690618854,-2.
92083038301121,0.00349099832202371,0.108763374016834
"503","CRABP2",1177.43272791144,-1.07961685459894,0.370888744537549,-2
.91089139398146,0.00360399266471312,0.111176420662945
"504","SYNPO2",121.3405352649,1.40282915024079,0.481877096306501,2.911
17623351103,0.00360070862032567,0.111176420662945
"505","IGF2R",419.008391797512,0.74463092583523,0.255745868819465,2.91
160490401069,0.00359577141355408,0.111176420662945
"506","KL",17.2561692284261,1.20424930468862,0.413581364995897,2.91175
910379948,0.00359399692596294,0.111176420662945
"507","TK1",6.59846460595762,-2.835323058657,0.973274206358502,-2.9131
8010909314,0.00357768185097882,0.111176420662945
"508","GAP43",98.2184035102038,-1.24701628313292,0.428733239791601,-2.
90860648859199,0.00363043511590402,0.111771663804604
"509","AC108734.4",55.2262720137899,1.013402382028,0.34854953901438,2.
90748449960277,0.00364348400512672,0.111953025226291
"510","AC012063.1",59.8813329552594,-0.825773711312552,0.2841800033505
77,-2.90581216685342,0.00366301264374828,0.112332387741614
"511","ATP1B3",425.260833247612,-0.508287180520453,0.175177063409023,-
2.90156239994529,0.003713068211082,0.113644592605328
"512","AL596087.2",3.58713757585208,-2.37510858196477,0.81984812883805
,-2.89701043207959,0.00376737227665437,0.114591150915663
"513","HLX",61.0183946831738,1.31544830670356,0.454148311978858,2.8965
1700117912,0.00377330196429452,0.114591150915663
"514","CPED1",366.972838192056,0.445526988717864,0.153767680995392,2.8
974033154029,0.00376265695307772,0.114591150915663
"515","AL391121.1",53.2012049244412,0.820170731613751,0.28302063631615
9,2.89791847792169,0.00375648218196864,0.114591150915663
"516","AOPEP",84.6009214847316,-0.92509669471432,0.319452704942561,-2.
89587998599247,0.00378096968507817,0.114601484253145
"517","PCSK5",290.86934647966,0.505844129779419,0.174722239239986,2.89
513305220768,0.0037899785254397,0.1146303232123
"518","MS4A6A",36.4919982868596,-1.55101808892829,0.535834030112208,-2
.89458675964177,0.00379657975856594,0.1146303232123

"519","KCNMA1",50.7463668769626,-1.09521212457009,0.378483432165611,-2
.89368577721749,0.00380748978559431,0.114693303786278
"520","PDCD4",662.314801101364,0.465053193729563,0.16073984218136,2.89
320424493668,0.00381333235095043,0.114693303786278
"521","SMAD1",83.64447121148,0.636902683455436,0.220300497029482,2.891
06330690757,0.0038394077031577,0.115255924140857
"522","AL357568.2",22.980465038351,1.62761003546707,0.563286717283707,
2.88948768988511,0.00385870114842057,0.115613191496739
"523","CCRL2",3.48722437555013,-2.38775915723677,0.827507112865178,-2.
88548475307884,0.00390811391279188,0.116869792726702
"524","SERINC2",17.1348740261812,-1.13971423574826,0.395168709175966,-
2.88412065349222,0.00392508338102531,0.117057281754605
"525","SLC25A45",44.6050482778953,0.950406979797113,0.329570054820823,
2.88377832237768,0.00392935248856571,0.117057281754605
"526","TMEM221",12.3468930447314,1.97154036947719,0.684052812360729,2.
88214642766137,0.00394976137574722,0.117441573986096
"527","AC113383.1",18.576141531756,-1.27574658989764,0.442797680040861
,-2.88110495470508,0.00396283657121599,0.117606762758668
"528","CHEK1",15.797031479699,-1.07041020492648,0.371780707234375,-2.8
7914403329079,0.00398756172301001,0.118116411643706
"529","NBEA",34.717407065227,1.5305029301579,0.532425566782563,2.87458
571797499,0.00404557939052825,0.11948625856514
"530","KSR1",62.0155122461696,0.767037750353347,0.26685959457025,2.874
31205757689,0.00404908676723301,0.11948625856514
"531","CLN8",183.924990276791,0.536935011570117,0.187042898308389,2.87
065168699877,0.00409626611805152,0.120650851386677
"532","ZNF880",55.9448251363448,0.890700778801966,0.310360069832663,2.
86989489106059,0.0041060826519235,0.120712655406172
"533","KCP",32.1107322311734,2.85496932690687,0.995152998908083,2.8688
747660304,0.00411934865749703,0.120875446535185
"534","GAS2L1",185.59148803654,-0.584074713379527,0.203823081291865,-2
.86559652458183,0.00416224361043366,0.121905412110829
"535","FN1",9456.47203746196,-0.566178756089634,0.197809243274317,-2.8
6224620608083,0.00420650000705952,0.122741903191065
"536","FBP1",63.7133264533974,1.08058260153223,0.377455166169921,2.862
81046964337,0.00419901654181956,0.122741903191065
"537","SMOC2",1768.70429232199,0.935562571410338,0.327197645768931,2.8
5931938541831,0.00424551102525544,0.122962578583324
"538","CCDC136",46.087933201771,1.19731706876704,0.418528409004088,2.8
6077848721457,0.00422602211415036,0.122962578583324
"539","ANXA2",14928.9376481959,-0.565602335867212,0.19779990318364,-2.
8594672027827,0.00424353295621639,0.122962578583324
"540","PNMA6A",27.379485872338,1.31237598147335,0.45894193741693,2.859
56866103764,0.00424217574138545,0.122962578583324
"541","TRIL",340.899471773196,1.02448805025064,0.358487724381281,2.857
80510900009,0.00426582305409156,0.123322500121982
"542","DISP1",94.4241581333156,1.16331242282159,0.407669085807057,2.85
357036705052,0.00432309525966246,0.124529772302133
"543","TMEM192",119.329294635575,0.693288517869294,0.242957348088647,2
.85354002800662,0.0043235080792876,0.124529772302133

"544","SCRN1",366.850990435939,0.472697653131974,0.165714415011474,2.8
5248361223883,0.004337904906686,0.124714766067223
"545","C6orf120",230.715057679682,0.431452150804088,0.151330613027685,
2.85105665120882,0.00435742052566962,0.125045976186189
"546","ZNF512B",59.3143493713346,0.740006624898259,0.260268270203919,2
.84324564157769,0.00446566381752299,0.127917549644798
"547","CAPS",199.060286751462,-1.24872789950422,0.439308315326924,-2.8
4248637218474,0.00447631447789271,0.127988223828596
"548","VEPH1",5.89508732866484,3.09774559305461,1.09033913948928,2.841
08446708207,0.00449604021863207,0.128000370686146
"549","ANK2",802.561483901085,-0.713484395314755,0.251266330055252,-2.
83955432929619,0.00451766014186398,0.128000370686146
"550","AC022424.1",37.3205386711892,1.35416469980628,0.476815835162781
,2.84001620739795,0.00451112417993807,0.128000370686146
"551","ACACB",93.7222272133829,0.665769102628148,0.234399196853375,2.8
4032160333984,0.00450680727768431,0.128000370686146
"552","CDH4",3.1716443189954,4.41810254841009,1.55574220427596,2.83986
802972043,0.00451322008425046,0.128000370686146
"553","C21orf62-
AS1",37.8009789714804,1.02180558940701,0.360084926548614,2.83767943079
689,0.00454427970997161,0.128521762502633
"554","CD58",228.319900445493,-0.524631561039711,0.185029857450981,-2.
83538866790025,0.00457699647586942,0.129213402315158
"555","ABCA10",236.669860704683,0.90472836884441,0.319177037322465,2.8
3456597139337,0.00458879822807685,0.129313160877697
"556","GDF10",172.281912096903,-1.39369658347116,0.491843306795053,-2.
83361909009753,0.00460241554670812,0.129463631565674
"557","PLEC",521.387485253827,-0.583672562892691,0.206068270855324,-2.
83242325696262,0.00461966538875315,0.129715559569298
"558","BMP2K",152.724110296083,0.580511618048918,0.205059109331493,2.8
309477201058,0.00464103062452235,0.129918755848271
"559","CTSH",1051.54758257093,0.906353665427334,0.320178461942898,2.83
077649860464,0.00464351563421891,0.129918755848271
"560","HLA-
DMA",41.3422162511135,-1.23331826358629,0.435967403777291,-2.828923109
61927,0.00467049185644447,0.130285371632481
"561","ZEB1-
AS1",118.90049401024,0.555812042484509,0.196527649052125,2.82816206862
116,0.00468160990137177,0.130285371632481
"562","SNHG9",383.555943039462,-1.01411902417498,0.358578620152487,-2.
8281636639232,0.00468158657057087,0.130285371632481
"563","FCGR2A",30.7712678352114,-1.67660429192452,0.592946489663341,-2
.8275811074899,0.00469011328145554,0.130290180678445
"564","NRP2",17.1442432724855,-1.21166716829146,0.428627010895327,-2.8
2685677171977,0.00470073478295322,0.130353709229412
"565","STK3",327.617182076796,0.56772504780296,0.201029672744173,2.824
08581804456,0.0047415687266704,0.131021439726369
"566","SSBP4",612.480357615669,-0.676518492300249,0.239534254999196,-2
.82430791496824,0.00473828401600345,0.131021439726369
"567","EN1",151.387882662754,0.67377211770476,0.2387294672691,2.822324

89106706,0.00476768506092558,0.131048496226496
"568","CCDC80",16234.472029688,-0.556762683869476,0.197233718476666,-2
.82285751224299,0.0047597720479531,0.131048496226496
"569","TEAD1",465.706509202634,0.568740955786052,0.201464117102559,2.8
2303848430003,0.00475708610033859,0.131048496226496
"570","SLC9A9",100.650181605115,0.575626951365803,0.204087926129105,2.
82048508348143,0.00479511027604413,0.131248671423509
"571","EGR2",102.004937211784,-1.39207314361218,0.49358275183647,-2.82
034398169852,0.00479721950209557,0.131248671423509
"572","RHOJ",69.9891738329512,1.34565809180784,0.47715859608069,2.8201
4848492907,0.00480014322597488,0.131248671423509
"573","EPB41L2",1634.0120402214,0.488269490602766,0.173215920197153,2.
8188488104732,0.00481962134857501,0.131551270317126
"574","FKBP1B",45.9636996369706,-0.917093741173367,0.325623870633856,-
2.81642048965318,0.00485620615826816,0.131894513107188
"575","AC092140.2",3.17131361480932,3.76085659362345,1.33537252028371,
2.81633516977302,0.00485749613489388,0.131894513107188
"576","INO80C",103.858076868564,-0.655605953171549,0.232726805869557,-
2.81706248114373,0.00484650963381346,0.131894513107188
"577","CDKN2C",297.045592407427,-0.765469713167831,0.272279090245623,-
2.81134226090332,0.00493352747564517,0.13372681060501
"578","RETREG1",141.626536412246,-1.0709320194291,0.381217369427126,-2
.80924245670768,0.00496582329282587,0.134369336158818
"579","RBP4",4.00543256263551,-2.39525002098455,0.853303934487686,-2.8
0703032551072,0.00500005343917933,0.135061892553998
"580","SLAMF9",5.76068357139259,-1.81828451108708,0.647951878050747,-2
.8062030108734,0.00501290988571299,0.135175707952675
"581","PNMA2",22.540473691612,2.01511237962758,0.718372699605579,2.805
10712716947,0.00502998590605884,0.135402718710431
"582","CLEC7A",17.2781115212871,-1.30309714658099,0.465022288247547,-2
.80222513955569,0.00507514422918308,0.136383600935435
"583","SIN3B",122.620814564087,0.856744002887046,0.305910721770388,2.8
0063411288377,0.00510023098842615,0.136822663222959
"584","SPHK1",10.6048399196025,-1.9447997793978,0.694645693117819,-2.7
9970033452427,0.00511500662671778,0.136984081578538
"585","KCNN4",7.26206843798103,-2.11180777730914,0.754501703732128,-2.
79894368278179,0.00512700786763718,0.13707077444418
"586","SHISA6",152.044862085091,1.24786940744082,0.446039666741609,2.7
9766464843072,0.00514735249103118,0.137379851467112
"587","GNAI1",122.631495358831,-1.19962479089391,0.42896046037469,-2.7
965859367226,0.00516456741658457,0.137604487896734
"588","SPATA6",200.783728520306,0.58015118004924,0.207566799592179,2.7
950095159202,0.0051898187542534,0.137696242548072
"589","WLS",300.071714612324,0.616956255618441,0.220712946055822,2.795
28802747439,0.00518534941809504,0.137696242548072
"590","MAN2B1",225.994990424663,-0.49923639947738,0.178635391124491,-2
.79472279448513,0.00519442347208202,0.137696242548072
"591","CPM",17.1679486839059,-1.15177392338714,0.412437894038212,-2.79
259966175763,0.00522863581131301,0.138368636360297
"592","DDB1",380.873319949549,0.341902183901681,0.12246573096352,2.791

81923964938,0.00524126267962417,0.138468493765747
"593","SPON1",39.894948428594,1.05350974530355,0.377722139314139,2.789
11304276868,0.00528526135986104,0.139128701728766
"594","LRRC36",20.9159835689818,-1.2803273608868,0.459122026317405,-2.
78864286071447,0.00529293973968132,0.139128701728766
"595","OXT",23.6783204860989,-1.16991675671173,0.419504942220419,-2.78
88032749255,0.00529031893927679,0.139128701728766
"596","AC005725.1",5.04862638033479,3.5793784504333,1.28449279990708,2
.78660841905243,0.00532627974889406,0.139770159853529
"597","NET1",143.818331279596,1.08285008367597,0.388804469291397,2.785
07622520257,0.005351514106499,0.139962676631512
"598","AL592183.1",25.190316633571,-1.38499539945011,0.497287106849862
,-2.7851021680887,0.00535108594500606,0.139962676631512
"599","WDFY1",205.882382905012,0.476325935364774,0.17112261590435,2.78
35358456127,0.00537699205765265,0.140394250052901
"600","VAMP8",38.8234878807278,-0.801064898892513,0.288222107214707,-2
.77933190702742,0.00544708369752836,0.141987315048906
"601","BCAP29",470.120012191727,-0.40465470500137,0.145649885442039,-2
.77826998471895,0.00546491896218819,0.14221519562167
"602","NMB",169.869783135122,-0.59861130737787,0.215533146444158,-2.77
735149907888,0.00548038766632632,0.142380835716518
"603","ANGPTL7",9.25842445798212,3.48512214471715,1.25534226316228,2.7
7623262355393,0.00549928463243556,0.142634845192856
"604","IQCN",56.6314938614789,0.883025307931488,0.318291485394875,2.77
426619451035,0.00553263869545577,0.14326236622008
"605","CD8B",5.66050423782092,-1.83849526659837,0.662880551588518,-2.7
7349405136811,0.00554578542857405,0.143276607027026
"606","C17orf49",612.093928107488,-0.699848586964236,0.25236517580687,
-2.77315831998871,0.00555151047687836,0.143276607027026
"607","GFRA1",382.001293881547,1.17619409420049,0.424392398573696,2.77
147776009529,0.0055802484129224,0.143781029947457
"608","HAS2",1171.79461733437,-0.932011002124409,0.337281444529213,-2.
76330351770562,0.00572195307231591,0.147189713899705
"609","AC104051.2",32.6771777415519,1.32223851693899,0.479048769294245
,2.76013341791269,0.00577777603606174,0.148381637445001
"610","UAP1",2660.51921792695,-0.612789452579886,0.222065944217941,-2.
75949315298197,0.00578911002543396,0.148428984914405
"611","IRF7",111.279525442279,-0.731543282220194,0.265452888276256,-2.
75583093847854,0.00585432477462915,0.149366459176509
"612","RAD23A",752.05513541538,-0.44208650452177,0.160400158843895,-2.
7561475481581,0.0058486607197769,0.149366459176509
"613","SCO2",209.147328912189,-0.663345972989007,0.240621027953955,-2.
75680799234198,0.0058368614631371,0.149366459176509
"614","DOCK7",248.487868105766,0.516365801367626,0.187619967869333,2.7
5219001064559,0.00591981620268103,0.150301826964174
"615","C5AR1",38.5399856263475,-1.25644752509022,0.456518732107846,-2.
75223651675569,0.00591897552468329,0.150301826964174
"616","LINC01260",9.32784122993981,-1.71169987121349,0.621918025132097
,-2.75229178451601,0.0059179766046864,0.150301826964174
"617","LMNB1",40.7773423394535,1.13423384469756,0.412272046758291,2.75

117814466463,0.00593813408405999,0.150522556036788
"618","ADD1",1001.66886389691,0.370053751565086,0.134615637029074,2.74896557140061,0.00597836653325342,0.151052750533253
"619","VWCE",14.8295057367458,1.81920778765349,0.661764378449072,2.74902646152854,0.0059772560556926,0.151052750533253
"620","SHC2",161.347726687802,0.662276372507862,0.241000793840277,2.74802568885639,0.00599553114295624,0.151242108186832
"621","SNX18",403.760589493167,0.459528911231852,0.167285655798274,2.74697139476196,0.00601483802373068,0.151484809486551
"622","LIFR",175.924823947132,0.718367818537636,0.261635482816112,2.74568193429074,0.00603852755417086,0.151654333066751
"623","ADGRE5",257.959672765114,-0.626632606520268,0.228235756840514,-2.74554966844282,0.00604096224428299,0.151654333066751
"624","DSTN",4702.64976421149,-0.426932234186732,0.155569009529703,-2.74432700624231,0.00606351036464813,0.15197644567804
"625","ZDHHC7",295.129616886073,0.570339408305078,0.207901498211654,2.74331552783927,0.00608222114145854,0.152201501843859
"626","LRRFIP2",283.147128888801,-0.435341038643059,0.158773698527537,-2.74189643927427,0.00610855974011959,0.15261641267647
"627","TJP2",16.5559374490782,-1.52857168511019,0.557714777523307,-2.74077673160868,0.00612941421958627,0.152649742666129
"628","RASA3",221.972511772681,0.449078838351075,0.163822402637106,2.74125413326931,0.00612051481742888,0.152649742666129
"629","DNAJB11",296.069387352174,-0.577403461236676,0.210809187020982,-2.7389862339311,0.00616289538783997,0.15323956099494
"630","COL8A2",310.333419471131,-0.722734394433187,0.264170492110836,-2.73586345188756,0.00622168372364957,0.15407438162401
"631","KCNA2",6.49793975548466,2.18083890946109,0.797243153435897,2.73547524373496,0.00622902716050398,0.15407438162401
"632","SLC4A4",192.766598516389,0.847306210305642,0.309788305273759,2.73511361107347,0.0062358749084398,0.15407438162401
"633","DHRS4",131.767426259452,-0.97672051377694,0.356911638592715,-2.73658913906002,0.00620797735417803,0.15407438162401
"634","CD55",7318.03268191151,-0.61013628713022,0.223749959186117,-2.72686658513624,0.00639388855710914,0.154321631224054
"635","CENPA",15.1546316301858,2.40900229737337,0.883145928006475,2.72775112354445,0.0063767699158808,0.154321631224054
"636","SUCLG2-AS1",76.4540368622241,0.906550558338172,0.331815421045547,2.73209290720016,0.00629333915025356,0.154321631224054
"637","IGF2BP2",57.8351234075614,-0.879282368163102,0.322197855962689,-2.72901371592282,0.00635240617273169,0.154321631224054
"638","ARHGEF10",772.301339351481,0.774541529689467,0.283580150644759,2.73129670016903,0.00630856492668131,0.154321631224054
"639","ECM2",936.000727396041,0.490665147486942,0.179647847553814,2.73126093169562,0.00630924970065322,0.154321631224054
"640","CIZ1",420.517448219412,-0.465214295141312,0.170450513445477,-2.72932175877619,0.0063464747191215,0.154321631224054
"641","ARL3",499.904627035357,-0.503922563508795,0.18471711348917,-2.72807729608843,0.00637046785171417,0.154321631224054

"642","HSPA12A",164.395980883073,0.637139340168178,0.233447545304493,2
.72926125368821,0.00634763936846095,0.154321631224054
"643","AL627171.2",143.475978530266,0.90742073989306,0.332264605632758
,2.73101836461029,0.00631389532179966,0.154321631224054
"644","FBLN5",2202.87840121308,0.516573024359926,0.189425043633182,2.7
2705770288885,0.0063901863204716,0.154321631224054
"645","PCLAF",9.222541760785,-1.70196268116169,0.622645029565092,-2.73
34397615773,0.00626765869315143,0.154321631224054
"646","CES1",181.062035801098,1.08173430176936,0.396351573691056,2.729
2292337725,0.00634825579071229,0.154321631224054
"647","TTC28",347.796852426181,0.573615576577077,0.210124826422809,2.7
2988007339437,0.00633573691311306,0.154321631224054
"648","GPRASP1",43.4576875718312,1.11802147402576,0.409703516864108,2.
72885495976005,0.00635546501592044,0.154321631224054
"649","AQP7",5.79747421833531,-2.68785853352913,0.987411348478723,-2.7
2212643461131,0.00648633210959471,0.156100352944666
"650","SLITRK2",8.04425736257458,-2.33689814709466,0.85850210101349,-2
.72206456377436,0.00648754663772588,0.156100352944666
"651","AUP1",548.260024659217,-0.463096546520692,0.170172458508474,-2.
72133664036847,0.00650185118583286,0.156204228181914
"652","RUNX1T1",876.915351079877,0.70866176978425,0.260807144761531,2.
71718694835686,0.0065839406992153,0.15793379223286
"653","EPDR1",295.996287323668,-0.492342935115417,0.181332571185092,-2
.71513789220398,0.0066248180103615,0.158670985730557
"654","STOX2",26.4341373996343,1.5019056062931З,0.553521040348394,2.71
336678610773,0.00666033411135471,0.159277714834232
"655","PCDHGA8",18.8917326251219,1.63043332109038,0.601034343552449,2.
71271240750338,0.00667349965285114,0.159348907741362
"656","FERMT3",34.9642048424068,-0.891088373522146,0.32876266867921,-2
.71043052759626,0.00671959237269944,0.159883525715959
"657","TRIM16",65.7045928551422,-0.627495292625365,0.23147000936743,-2
.71091401577339,0.00670980235280301,0.159883525715959
"658","TRIP10",666.514430504966,-0.325831217642934,0.120229064884581,-
2.71008693243791,0.00672655753971233,0.159883525715959
"659","DEPTOR",67.3812743856324,1.02224815705746,0.377348822874631,2.7
0902701980096,0.00674808439430465,0.159909151404431
"660","AL513534.2",33.6808333723726,0.832654375556206,0.30730596797547
8,2.70952881599309,0.00673788519374052,0.159909151404431
"661","MAPK8IP1",98.7696916095902,-0.533656946328009,0.197183273321443
,-2.70640068672587,0.00680169233453685,0.160935655237755
"662","CAB39L",587.743598942601,-0.684059071480142,0.252911090228461,-
2.70474130202125,0.00683576019128689,0.161497415999587
"663","TMEM255A",127.031802933641,1.09307242575946,0.404244778909983,2
.70398650220505,0.00685130721640657,0.161620580489591
"664","C1QTNF2",394.83503044168,-0.543409225236932,0.201150476089044,-
2.7015060356923,0.00690262274961698,0.162496709076023
"665","RAB37",13.660716438562,1.62659081847395,0.602176085774726,2.701
18800281029,0.00690922708027846,0.162496709076023
"666","TMEM167A",738.356222407907,-0.416646459586662,0.154316672976607
,-2.69994454617241,0.00693510345405355,0.162675688365898

"667","GNA14",9.33642610162403,1.46698038958194,0.543361711366484,2.69
98228967821,0.00693763965089858,0.162675688365898
"668","FTX",760.301292836798,0.852398897000501,0.315807129919249,2.699
11226265999,0.00695247190104247,0.162779431934587
"669","FHOD1",56.9712021491004,-0.764420757953253,0.28333994217955,-2.
6978926870425,0.00697799310044669,0.163132753499232
"670","NDFIP2",317.05823973267,-0.510302827415612,0.189219554682876,-2
.6968820863724,0.00699920494462592,0.163384425871566
"671","ANXA3",67.2132053957033,1.11205070919529,0.412537727882912,2.69
563396032209,0.00702548220487269,0.16350973464912
"672","CCL26",102.969105441526,-1.36483279955173,0.506223552249131,-2.
69610687509072,0.00701551534845285,0.16350973464912
"673","FAT1",247.809916574177,-0.716013162103479,0.265715363883087,-2.
69466225678437,0.00704600113715354,0.163743622266094
"674","BOLA3",228.292700476901,-0.53245494881191,0.197728367903803,-2.
69286068790572,0.0070841863072172,0.16423445215594
"675","MSL1",277.739711075441,0.508436203537242,0.188821942783805,2.69
267541706944,0.00708812373435162,0.16423445215594
"676","UBXN11",70.5441092425952,-0.691755301531698,0.256986520589565,-
2.69179605196689,0.0071068390535371,0.164424501179468
"677","ZNF496",53.5917551496085,0.704728182170269,0.261915413984643,2.
69067089809228,0.00713085013018344,0.16457887226764
"678","AC124242.1",4.07396090247063,2.84846371484961,1.05890620655022,
2.69000568438402,0.00714508019627413,0.16457887226764
"679","MARCH2",551.427380796118,-0.735925631728331,0.273557482959919,-
2.69020471955489,0.00714081982118296,0.16457887226764
"680","RXRA",275.517742160685,0.420777422480771,0.156473282201063,2.68
913271685632,0.00716379314934683,0.164767242434977
"681","ANK3",12.6931922766752,1.49924145291543,0.558043580855295,2.686
60281087293,0.00721827301357371,0.16577649035579
"682","TRIM29",27.6496218067672,-1.10437668753614,0.411256102120481,-2
.68537459223549,0.00724485573732919,0.166077026474942
"683","HAUS7",69.8300465135387,-0.735920847939012,0.274084169905535,-2
.68501770165221,0.00725259648864356,0.166077026474942
"684","PTGDS",500.614569095544,3.19535433436588,1.19046185734871,2.684
1299573279,0.00727188335388218,0.166275227565376
"685","RBL2",356.694968087662,0.503008994327451,0.187501881184937,2.68
268772104384,0.00730331508251155,0.166750142905811
"686","PLEKHG1",59.2014443653214,0.756683461067237,0.282380476318527,2
.67965927011786,0.00736971341067189,0.168020871345347
"687","PRKCE",88.1300192095891,0.751415176312765,0.280598707158824,2.6
7789963796038,0.00740854134044525,0.168281207014966
"688","USP54",119.537161685723,0.951378956045538,0.355299783703952,2.6
7767952495648,0.00741341122975138,0.168281207014966
"689","AC005520.2",20.8803808224944,1.06374461136045,0.397242940833493
,2.67781879050766,0.0074103297160825,0.168281207014966
"690","SLC25A27",26.9327877157387,1.05143378213715,0.392841593947292,2
.67648283261529,0.00743993771942494,0.168502898928787
"691","HGF",35.7374035388879,-1.06063663171185,0.39631192588496,-2.676
26725928941,0.00744472526597133,0.168502898928787

"692","THBS3",399.794608040146,-0.452692604573821,0.169290137990122,-2
.67406365160051,0.00749382265912637,0.169241010429602
"693","CCT6A",434.6918225744,-0.450865107109272,0.168621266145872,-2.6
7383300703741,0.00749897827542931,0.169241010429602
"694","CYP4X1",66.7363850677512,1.11995218636255,0.419472514597955,2.6
6990600668073,0.00758724828491447,0.169622354591137
"695","PLPPR5",4.39763906196402,4.16602761024111,1.56016022988601,2.67
025625345257,0.00757933787164741,0.169622354591137
"696","ECM1",1913.66897016375,-0.836955580015071,0.313190466465662,-2.
67235331094228,0.00753212976646988,0.169622354591137
"697","AADAC",719.551887694587,-2.39523128807471,0.89816519809734,-2.6
6680482961122,0.00765761270924916,0.169622354591137
"698","FGF12",13.0491913790806,1.99259826228032,0.746529341614244,2.66
914929019623,0.00760436419625836,0.169622354591137
"699","SPATA18",9.05394965467127,1.65209085519287,0.619220868547845,2.
66801546767512,0.00763007452952546,0.169622354591137
"700","FAM20C",298.356844060279,-0.448868512854595,0.168312836505198,-
2.66687034794718,0.00765612009766345,0.169622354591137
"701","PLEKHA8",60.9214173797215,0.802178356326622,0.300624567018799,2
.66837259603092,0.00762196796441167,0.169622354591137
"702","GTF2I",883.655155541711,0.396200721632337,0.148298640736114,2.6
7164095143222,0.00754813646281853,0.169622354591137
"703","LPL",9.96080219024586,-1.68401015638377,0.631576001368386,-2.66
636185151931,0.00766771129769396,0.169622354591137
"704","ATP23",40.4070260872439,-1.02574904816958,0.384248214927148,-2.
66949593601642,0.00759651924134345,0.169622354591137
"705","PLXNC1",185.9873870867,0.762425175071606,0.285451187976474,2.67
094763373149,0.00756374457059035,0.169622354591137
"706","MEIS2",250.323820296543,0.419957702357764,0.157372891605523,2.6
6855173132643,0.00761790462619258,0.169622354591137
"707","AR",352.784980141305,0.558998067806282,0.209609680481198,2.6668
5234442893,0.00765653022037192,0.169622354591137
"708","EXOC4",197.384536183195,0.441610945982239,0.165713548993859,2.6
6490548698951,0.00770099628018092,0.170118053420946
"709","PRRT4",7.37867424440267,-2.18153985611309,0.819605253172487,-2.
66169579522446,0.0077748108620639,0.171264847722084
"710","SDC4",115.340583476971,-0.835461150491877,0.313882300181746,-2.
6617020138062,0.00777466724000452,0.171264847722084
"711","ACVR2B",41.5830439537102,0.986769168136407,0.370948459914856,2.
66012472019132,0.00781117208749316,0.171795821196929
"712","DLG1",219.068810624096,0.683433178884817,0.256958147309971,2.65
970620523033,0.0078208839317272,0.171795821196929
"713","AVEN",139.980144193386,-0.726522336871648,0.273332289525992,-2.
65801869999176,0.00786015315635112,0.172416262784476
"714","PLAGL1",1093.47519528577,0.52480717231049,0.19754229002783,2.65
66826386216,0.00789136925977825,0.172858564738
"715","CD209",4.04200933710124,-2.73896244237939,1.03321071653651,-2.6
5092337752828,0.00802720498580634,0.175342857511189
"716","Z83843.1",110.748468381389,0.837674337333732,0.315962872401975,
2.65117964957612,0.00802111642844506,0.175342857511189

"717","ZC2HC1B",4.25885985290725,3.42283367878267,1.29204548984392,2.6
4915879950648,0.00806924060786115,0.176015234458784
"718","MAST4",294.419669457843,0.741924789329327,0.280117487819681,2.6
486200312025,0.00808211431169635,0.176050512304918
"719","PLPP3",3496.72792427336,0.704873021956432,0.266178588083501,2.6
4812067353559,0.00809406272575163,0.176065564715932
"720","HIST1H2BD",5.63155070389636,2.43169438836139,0.918455112832343,
2.64759197742663,0.00810673036892516,0.17609619856943
"721","EYA4",16.3210868003648,1.78485562107801,0.675106298553086,2.643
81420363487,0.0081977640868568,0.177334758393417
"722","HELZ",259.790358169941,0.528167524365134,0.199751839911777,2.64
411844515878,0.00819039899456691,0.177334758393417
"723","SNHG16",454.49240781302,-0.570675285791119,0.215812650612768,-2
.6443087750916,0.00818579449080532,0.177334758393417
"724","TTN-
AS1",68.5552252851865,0.708339716960676,0.268176386806887,2.6413202347
7052,0.0082583619775786,0.177912113906672
"725","COL6A6",216.382234077629,1.29955378856819,0.492010842225674,2.6
4131128226665,0.00825858022354502,0.177912113906672
"726","TBC1D16",237.531977448495,0.417688087241687,0.158085587516954,2
.64216424661034,0.00823780964762181,0.177912113906672
"727","NME1",290.672851421654,-0.910225828869315,0.344872550813766,-2.
6393107445679,0.00830747936269806,0.178719363456118
"728","AFF3",126.366160563145,-1.15491348835297,0.438014426821591,-2.6
3670193864044,0.0083716354837698,0.179605458115445
"729","PUS7",37.0545352115714,-0.943068703870309,0.357622065966477,-2.
63705401209418,0.00836295145314286,0.179605458115445
"730","METTL7A",940.212516123568,0.525332763498399,0.199284200252755,2
.63609841037127,0.00838654051286361,0.179678758385187
"731","NPL",16.0992559361777,-1.13339098859364,0.430529340425574,-2.63
255226106843,0.0084745983961316,0.180822263186219
"732","SEC63",1054.53422635642,0.309561502254287,0.117556320957534,2.6
3330376225464,0.00845586839424274,0.180822263186219
"733","STXBP6",599.469148240165,-0.513628795449276,0.195074207529196,-
2.63299183400451,0.00846363822359568,0.180822263186219
"734","ATL2",100.332943681591,0.539952538992179,0.205274781787748,2.63
03890535881,0.00852872025967055,0.181729134688348
"735","SDF2L1",194.088227844479,-0.827627118987143,0.314756258801813,-
2.62942227785297,0.00855300804403394,0.181998701780532
"736","MID1IP1",186.499290133248,-0.920758232465123,0.350292720360017,
-2.62853944415061,0.00857524099588313,0.182223871162517
"737","HMCN1",353.258778571782,0.742804776106132,0.282677161667698,2.6
2774952077429,0.0085951779109014,0.182261158668659
"738","VWC2",7.55553555349583,-2.24468002212627,0.854287382883515,-2.6
2754673321957,0.00860030275559274,0.182261158668659
"739","ADRA2B",15.6288538828288,1.70057279371061,0.647401657443564,2.6
2676620326517,0.00862005379978718,0.182432532379799
"740","DNASE1",82.0593749205907,0.940087616504714,0.357969652529728,2.
62616568153538,0.0086352773850868,0.182507754463186
"741","KIF22",123.962253783436,-0.521567283427616,0.198646368801653,-2

.62560693444338,0.00864946353618916,0.182560876796219
"742","FGFRL1",32.8681815140717,-0.803550774899619,0.306331166459747,-2.62314404435635,0.00871224297121698,0.183638113301662
"743","RFLNB",36.5633263302262,-1.39171316314023,0.530794271852104,-2.6219445780455,0.00874296474550822,0.183790280402888
"744","ALG13",226.073177049226,-0.411583012289195,0.156951389009256,-2.62235979488479,0.00873231891266614,0.183790280402888
"745","CATSPERB",3.86466212647218,3.12571780496537,1.19407405483971,2.61769175227993,0.008852673413964,0.185349146177238
"746","ELP5",195.705840242685,-0.643602907942554,0.245846954636557,-2.61790067277429,0.00884725537506784,0.185349146177238
"747","LGALS2",9.41181934006548,-1.82854072531679,0.698421967375447,-2.6181031106283,0.00884200827975098,0.185349146177238
"748","PTGER3",6.1096881835015,-2.10941983672799,0.806541830465987,-2.61538801466658,0.00891261422493415,0.185999953929138
"749","C1orf162",26.3101995824862,-1.03035005736049,0.3939962225506,-2.61512674078534,0.00891943513087301,0.185999953929138
"750","RGPD2",8.80932171491647,2.8001846695149,1.07075423159898,2.61515162572212,0.00891878527531184,0.185999953929138
"751","SYDE2",88.1591047636432,0.785138846491014,0.300599488403399,2.61191012220677,0.00900379213322062,0.187380585933761
"752","PNISR",1768.83404170118,0.427893769512676,0.16383791672392,2.61168951649764,0.00900960362034452,0.187380585933761
"753","AL161421.1",21.0302504728972,-0.985209723633293,0.377416115760483,-2.61040714079769,0.00904345202982384,0.187834780539767
"754","GALC",166.551942993635,0.475720520076912,0.182346024775552,2.60888890044338,0.00908367286266314,0.188419951687071
"755","DPF3",8.16202128289584,2.20677485106887,0.846063134394905,2.60828626299517,0.00909968200441569,0.188502021919287
"756","AC007262.2",5.72879320233669,2.6115265022551,1.00170080124417,2.60709235633178,0.00913147269008811,0.188910360943093
"757","KDM4A",115.492101418154,0.534522321980165,0.205178617873234,2.60515607094308,0.00918324184527423,0.189552665979763
"758","LHFPL6",2308.947187573,0.327451957512956,0.125700128112977,2.60502485103793,0.00918675964275322,0.189552665979763
"759","PFN1",2235.26697494803,-0.625041944779159,0.240005852375627,-2.60427793152694,0.00920680628711833,0.18971600834062
"760","VASH2",238.673558239146,0.817401786008323,0.313997647939069,2.60320990100836,0.0092355390870755,0.189807925521499
"761","SERPINB8",85.3728202101995,-0.785365844295064,0.301643427568346,-2.60362326017237,0.00922440917123258,0.189807925521499
"762","AC009061.2",29.2003250578765,0.888580321291767,0.341450933928711,2.60236605906395,0.00925829719311987,0.19002594238897
"763","AL512625.1",17.6200948257121,1.02837772193846,0.395269731066405,2.60171129007017,0.00927599053865904,0.190139570150232
"764","ZMYM2",242.550040511581,0.549014702393689,0.211098130226989,2.60075587502052,0.00930186215941414,0.190420319598478
"765","CLCN5",73.7637671826643,0.838152201150312,0.322352968613536,2.60010697204144,0.00931947045562838,0.190531395981736
"766","FZD6",17.4891986849612,-1.37931870794961,0.530677913652239,-2.5

9916358390882,0.00934512279736012,0.19059107399488
"767","PECAM1",10.0621192444887,-1.34391110467672,0.517067197329322,-2
.59910338853072,0.00934676174898163,0.19059107399488
"768","ACSL3",694.405429088663,0.557300506567594,0.214482369052022,2.5
9835113268645,0.00936726520749809,0.19061472238427
"769","BMPR1A",226.643588418622,0.497858922561623,0.191652227840007,2.
59772050746648,0.00938448441405932,0.19061472238427
"770","FOSL1",27.3739347552233,-1.85218003175906,0.712948735766909,-2.
59791474315007,0.00937917780659848,0.19061472238427
"771","SLC35E2B",183.865235202539,0.587528677796965,0.226354194338761,
2.59561648288996,0.00944213920368341,0.191385688154426
"772","ZBTB4",339.023206260594,0.44783512559419,0.172576301475798,2.59
499781699166,0.00945915197847641,0.191385688154426
"773","ISOC2",357.280993535879,-0.738422690205073,0.284523540217304,-2
.59529559361276,0.00945095997027594,0.191385688154426
"774","REEP3",393.891061268168,-0.350966948714229,0.13537494249391,-2.
59255473907233,0.00952660196259727,0.192252973800027
"775","PDGFB",4.22895686507088,-2.34864990026236,0.905904840893823,-2.
5926011146436,0.00952531761442091,0.192252973800027
"776","ALOX5",10.1069594596452,-1.90627705743261,0.735524667948878,-2.
59172416711537,0.00954963040107815,0.192469354990802
"777","IKBIP",424.203194581092,-0.489817487077879,0.189192867095056,-2
.58898495804168,0.00962592991373143,0.193757456693384
"778","CCDC186",850.456079272902,-0.40464816260544,0.156624383760449,-
2.5835578911157,0.00977870545503563,0.196579631512542
"779","TMEM246",9.46284916449194,2.14083417483736,0.828951873545975,2.
58257957205597,0.00980647452170056,0.196884802977403
"780","VANGL2",15.3528931963179,1.27914852151718,0.496609151003915,2.5
7576510406893,0.0100018567694842,0.198677516773879
"781","ZNF512",81.8919080933156,0.508029257444119,0.19708779542226,2.5
7767994388322,0.00994660782276159,0.198677516773879
"782","LRRC15",3.91633356157715,-3.83877653917259,1.48963610685071,-2.
57698945502085,0.00996649910437526,0.198677516773879
"783","CCNG2",387.729150124164,0.68967178030538,0.267610722023988,2.57
714554592308,0.00996199941329186,0.198677516773879
"784","PHIP",606.144214227053,0.519832047882119,0.201838779738939,2.57
548152319627,0.0100100621351272,0.198677516773879
"785","HILPDA",353.369250570158,0.969737841374506,0.376526974860562,2.
57548039349272,0.0100100948348987,0.198677516773879
"786","ZNF623",71.2633803955886,0.663522141964182,0.257522248659851,2.
57656239574312,0.00997881935662494,0.198677516773879
"787","PTGIR",22.1988888742138,-1.64478344928451,0.638144570697377,-2.
57744643582418,0.00995333065313185,0.198677516773879
"788","AL162457.2",13.4731864168768,2.46036247253678,0.954733869864072
,2.57701392000167,0.00996579372356113,0.198677516773879
"789","TRPC1",65.9525132074975,0.735306391140058,0.285620470117138,2.5
7441769085561,0.0100408974079555,0.19903629335922
"790","KLHL8",84.4546122134659,0.522587337522136,0.203138498198121,2.5
7256670772694,0.0100947500032845,0.199850493735911
"791","CD302",510.595883156188,0.362794958010457,0.14110149196591,2.57

116316033077,0.0101357561778272,0.200408630368164
"792","PHKG2",64.3060524678315,-0.682169255959758,0.265518100319114,-2.56920057480032,0.0101933439445553,0.201038965060334
"793","XG",396.780775268941,-0.640142415553339,0.249120392030674,-2.56961066227978,0.0101812868099062,0.201038965060334
"794","SOX13",109.208899860286,0.653100733916502,0.254305021155232,2.56817868144978,0.0102234443336909,0.201125370287957
"795","ACTR3",770.615522637406,-0.337371280512869,0.131365105851657,-2.56819555182214,0.0102229467666205,0.201125370287957
"796","SERPINA5",31.06305675132,-1.08454772824144,0.4224291533668,-2.5674073855876,0.0102462156182425,0.201320115916222
"797","CHMP1B",363.403156249675,-0.406992858383376,0.158565051487483,-2.56672485245277,0.0102664039661404,0.201463686361901
"798","ZNF546",37.294617147941,0.848093490940576,0.330576175664968,2.56550094462978,0.0103026940830996,0.201922475513381
"799","FBXO10",14.9207680056648,1.13055417605781,0.440980368889121,2.5637290360698,0.0103554352802473,0.202702137400586
"800","C14orf39",5.40576778040567,2.67143170320751,1.04241790683145,2.56272622112529,0.0103853906084413,0.203034386395028
"801","CARF",69.0061257474667,0.721873649944666,0.281795781525774,2.56169076072078,0.0104164019862289,0.203386425798526
"802","TMEM44-AS1",60.2411468425462,0.814150609494402,0.317962480565986,2.56052414751948,0.010451439993598,0.203816111595852
"803","DPY19L4",198.500886424666,0.42101359388558,0.164503162922613,2.55930394532071,0.0104881996477811,0.204278259640469
"804","HLA-DPB1",324.946808921086,-0.513408661646437,0.20066530725312,-2.5585322578896,0.0105115067340421,0.204477568806491
"805","LIN7A",10.4267498919339,-1.40410054544147,0.549067585499965,-2.55724537838622,0.0105504765347011,0.204980686959908
"806","PRSS12",54.5647136002437,1.19274714108578,0.467132209836679,2.55333953850624,0.0106695428376201,0.206546935292713
"807","CCDC85B",710.130373338681,-0.80840090097816,0.316663482903124,-2.55287061699335,0.0106839175608571,0.206546935292713
"808","CBX7",179.109928491861,0.563816234680646,0.220790392903845,2.55362666493461,0.0106607495089645,0.206546935292713
"809","MID1",93.3859614019706,0.874269905722238,0.342442534341482,2.55304122019614,0.0106786857518878,0.206546935292713
"810","POU2F1",184.635942592886,0.586362492337739,0.229738329584206,2.55230589252987,0.0107012519659173,0.206626642897466
"811","WNT5A",90.7635978520378,1.27979158952883,0.501622646657668,2.55130345102265,0.0107320838436865,0.206711565659184
"812","SMAD5",547.132835154324,0.409485300235354,0.160496350257408,2.55136829951966,0.0107300869256544,0.206711565659184
"813","LRRC32",202.414430300855,-0.703620505055146,0.275940748473556,-2.54989706648047,0.0107754728369252,0.207291998978487
"814","RTCA-AS1",53.070988952783,0.725706427879448,0.284843770623525,2.54773494358283,0.0108424816430345,0.207955877274817

"815","RNPEPL1",273.065706724243,-0.517600004415936,0.203150548818205,
-2.54786416983359,0.0108384662625423,0.207955877274817
"816","ZNF518B",73.8653761627722,0.648722902232008,0.254670270561375,2
.54730519114781,0.0108558446304649,0.207955877274817
"817","NAALAD2",36.0032234654203,0.845380131089733,0.331903000919827,2
.54706986302284,0.010863168269407,0.207955877274817
"818","MAP2",6.47575304227519,2.49037571739597,0.978055058786541,2.546
25309181034,0.0108886210624062,0.208188304909575
"819","CHIT1",2.61299195867005,-3.10487965632444,1.22023252172062,-2.5
4449836490698,0.0109434824108087,0.208472673453164
"820","AC069549.2",3.14686491781542,3.62062310383041,1.42261359563643,
2.5450502616704‌4,0.01092620099713,0.208472673453164
"821","APPL2",353.312370504627,0.358409963433689,0.140841444139931,2.5
4477625973215,0.0109347777217327,0.208472673453164
"822","LRRC59",318.184149202355,-0.47394771666632,0.18632542139905,-2.
54365568105318,0.0109699160318013,0.208722003330137
"823","CDH13",75.274532351929,-2.56292892712193,1.00781148468519,-2.54
306382301498,0.0109885155582163,0.208821850948364
"824","MBD2",499.935746754063,-0.438302769876331,0.172411138201141,-2.
54219521110633,0.0110158629953665,0.209087496659625
"825","SLC66A1L",84.0625610487355,1.24508204522954,0.490186838455122,2
.54001525041667,0.0110847635311603,0.20914483375202
"826","PPP1R16A",37.2169551134072,-0.831015327433709,0.327073197401532
,-2.54076253889282,0.0110611014883192,0.20914483375202
"827","MRPL49",116.34563415616,-0.579099788529013,0.227854036402597,-2
.54153842377318,0.011036581465076,0.20914483375202
"828","ASB16-
AS1",34.3674807353969,0.732215060773838,0.288227806962048,2.5404039550
9185,0.0110724500322343,0.20914483375202
"829","RBM12",139.100735445091,0.523075852659234,0.20593665174691,2.53
998425351733,0.0110857459834031,0.20914483375202
"830","LINC01914",33.8259115434331,-0.978573930848149,0.38537083305027
7,-2.53930460461308,0.0111073070278101,0.209299134837289
"831","MMD",51.611972407949,0.904874950454714,0.356425217426178,2.5387
5120562178,0.0111248904512814,0.209378202957931
"832","IL6R",167.670093987577,0.676206393062362,0.266622943081933,2.53
618981639762,0.0112065972237642,0.210662476658259
"833","CSF1",1328.75260441517,0.454627963992733,0.179304645737556,2.53
550577076604,0.0112285078418926,0.210727167198089
"834","ECRG4",31.8222043714566,3.11198787800484,1.22769501192854,2.534
82163547797,0.0112504593740668,0.210727167198089
"835","AC007563.2",137.143328542193,0.777758646976457,0.30678913498580
6,2.53515707788166,0.0112396914316835,0.210727167198089
"836","PHC3",232.861040115032,0.581995953810041,0.229661674801351,2.53
414486467299,0.0112722120927993,0.210882053984906
"837","ZNF790-
AS1",23.1693666590668,0.961273682460262,0.379819775789828,2.5308679108
6986,0.0113780687955647,0.212608119429668
"838","AKT1",329.08132534525,-0.372112663070905,0.147074103706063,-2.5
3010321799816,0.0114028976003245,0.212817802469064

"839","FARP2",94.9392458988465,0.637724297412412,0.252167094804607,2.5
2897507466847,0.0114396150928301,0.212840365829912
"840","AC013562.1",6.29283725488282,2.06461526261028,0.81625817456118,
2.52936549605793,0.0114268962553956,0.212840365829912
"841","NIPAL2",162.221486288761,0.557563693441201,0.220520817938631,2.
52839481847181,0.0114585414340656,0.212840365829912
"842","PRIMA1",22.610903375481,1.2303765913199,0.486561362955946,2.528
71823575375,0.0114479890374714,0.212840365829912
"843","SALL2",20.6420772970515,0.984545096800951,0.389475656589583,2.5
2787325765634,0.0114755770078861,0.212903943538955
"844","TLR4",194.709085400462,0.758548254135411,0.300410201870625,2.52
504159117102,0.0115684597229115,0.214372879225516
"845","RNF24",115.765804644176,-0.722365086347076,0.28665721647361,-2.
51996128070118,0.0117367744028768,0.217234499007092
"846","ZNF595",80.6511416994563,0.645687772944214,0.256458992911401,2.
5177037685993,0.0118122621070727,0.217636120693164
"847","ANKRD46",75.6472313544687,0.605462857905529,0.240413801497849,2
.51841971689361,0.0117882754135274,0.217636120693164
"848","AC110048.2",15.6347957982833,1.52013744372402,0.603823599189347
,2.51751909955963,0.0118184561637794,0.217636120693164
"849","NOVA2",163.987692339409,0.832229000338417,0.330464135348902,2.5
1836405623792,0.0117901386841396,0.217636120693164
"850","MBNL3",140.343086287719,0.820816288833364,0.326078758387525,2.5
1723323804451,0.0118280500376719,0.217636120693164
"851","RHOC",1341.62612792204,-0.634628193918802,0.252206886125351,-2.
51630002522366,0.0118594179346444,0.217956870150221
"852","PLEKHO1",167.150398514797,-0.819475656783845,0.325747335526513,
-2.51567877127623,0.0118803408992022,0.218077246858832
"853","SPATA22",11.5880396539044,-1.62625176332332,0.646549506480024,-
2.51527802128724,0.011893854959756,0.218077246858832
"854","GYG2",3.2794207389658,2.9382088345435,1.16837587633929,2.514780
46923511,0.0119106523381884,0.218129511205231
"855","LINC01871",15.0340438913243,1.71549822669235,0.682639946904777,
2.51303521639886,0.0119697385572601,0.218955217585435
"856","AZIN2",24.5478984365972,-1.08197392330943,0.430685185272267,-2.
51221532643486,0.0119975858585457,0.219208227602401
"857","PKN1",380.73020233857,-0.566968869597662,0.2257920568435,-2.511
02221009766,0.0120382122738177,0.219693862266638
"858","TRIO",1794.6707395826,0.767558899984814,0.30593872791689,2.5088
6478221,0.0121119838489508,0.220307353491467
"859","AC093627.6",7.09255723167269,-4.48135648404762,1.78654488831842
,-2.50839288357635,0.0121281733603678,0.220307353491467
"860","ARHGAP12",514.817865612989,0.363673035236151,0.14497571446845,2
.50851003955765,0.0121241522804483,0.220307353491467
"861","PDPR",126.007320073006,0.721132646874542,0.287406591458684,2.50
910267302693,0.0121038297494818,0.220307353491467
"862","DENND11",51.287251132111,0.7013495242832,0.279691002908679,2.50
758700490697,0.0121558651289853,0.22055421185305
"863","BCL7A",46.8573261032992,0.920120740867852,0.367048735399535,2.5
068080942066,0.0121826834536593,0.220784668847314

"864","SMO",55.1908302355036,0.758516673615092,0.302967482983837,2.50362403959885,0.012292858210387,0.222009587079044
"865","SHTN1",45.9127035690781,-0.856956443393142,0.342271113985054,-2.50373580585174,0.0122889759726061,0.222009587079044
"866","OGT",330.171494094078,0.497448715172557,0.198634700028724,2.50433944875,0.0122680270063253,0.222009587079044
"867","PUM2",244.860874409204,0.439155164104935,0.175442508297644,2.50312862239689,0.0123100797760102,0.222064184194694
"868","GASAL1",37.5285923022327,-1.26144587744239,0.504134129528257,-2.50220289315223,0.0123423170282992,0.222389214657373
"869","EPB41L5",71.6385711462216,0.601902630115297,0.240596823117614,2.50170647440786,0.0123596349152431,0.222444982824398
"870","YWHAQ",1980.25725140341,-0.436701012324745,0.174649817909719,-2.50043783355381,0.0124039900931435,0.222986672479039
"871","ARHGAP20",606.09813671938,0.73715255919905,0.294951711130566,2.49923133645675,0.012446303287829,0.223490451689605
"872","DGCR2",120.359137533191,0.442982887239942,0.177301112479957,2.49847776499439,0.0124727966864342,0.223709335064027
"873","FYCO1",147.180036742348,0.566094393217986,0.226713635322513,2.49695785792807,0.0125263842217067,0.224283886364157
"874","TMEM176B",1752.47283481487,0.925179318537233,0.370552534717815,2.49675614617447,0.0125335112968205,0.224283886364157
"875","ARHGAP4",14.3909660802152,-1.30248970654035,0.521761190840387,-2.49633305313963,0.0125484720948109,0.224294975500392
"876","LINC00882",305.408927212711,0.734840524949859,0.294645710958485,2.49398005000451,0.0126319643992833,0.225015857864226
"877","LOXL2",358.966659491636,-0.623134754205228,0.249780022606349,-2.49473415729201,0.0126051527963306,0.225015857864226
"878","NPEPPS",538.585551080105,0.501491661267993,0.201070137972663,2.49411308076078,0.0126272309467076,0.225015857864226
"879","AC008280.3",14.8027573400559,1.16518819935564,0.467324273203798,2.4933183790509,0.0126555310810137,0.225179187835101
"880","VWA2",8.02264293722084,-1.53159031129857,0.614397159541723,-2.49283429702211,0.0126727972365493,0.225230169067762
"881","TP53INP1",116.962233238622,0.567774544847238,0.227928780559566,2.49101734082615,0.0127377902761229,0.226128308647629
"882","PCDHB2",44.5795803070275,0.858667538890369,0.344821770684169,2.49017785967129,0.0127679182656761,0.226406169699743
"883","MGST2",141.359320896934,-0.787637465208752,0.316380510836333,-2.48952586594756,0.0127913610449125,0.226468198010603
"884","N4BP2L1",195.342343566116,0.518277747228492,0.208237885081892,2.48887346807558,0.0128148564731064,0.226468198010603
"885","AC245140.2",8.99213086771525,1.96778926245018,0.79050943429727,2.48926727130014,0.0128006694967921,0.226468198010603
"886","SPRED2",151.482580217835,0.651997924654771,0.262086272648477,2.48772252764747,0.0128563995182297,0.226690065913317
"887","FLYWCH1",105.389767235678,0.49642457289943,0.199529596344238,2.48797462629541,0.0128472898721477,0.226690065913317
"888","PCDHA10",3.99824033197577,2.68738368357413,1.080988513332,2.48604277513611,0.0129172438304462,0.22674039675441

"889","NF1",386.330556558016,0.562891649918739,0.226410536067736,2.486
15483932398,0.0129131767035677,0.22674039675441
"890","SLC4A11",4.60856419358451,2.55899755394304,1.02926801026363,2.4
8623053318016,0.0129104302001749,0.22674039675441
"891","TOMM34",42.4755644739757,-0.601965016512231,0.242087522584181,-
2.48655944794887,0.0128985017382635,0.22674039675441
"892","PLGLB1",7.4414910562927,1.53659830765265,0.618716761732566,2.48
352461528564,0.0130089343239305,0.228093870881472
"893","SIRT7",113.076093186439,-0.602956552571689,0.242862337092155,-2
.48270917504551,0.0130387490421717,0.22836062152247
"894","FHDC1",86.35322406235,0.589191384471382,0.237465577294363,2.481
16544378564,0.0130953576138451,0.228700490033946
"895","KCNK6",28.1410697671191,-1.45252970085401,0.585465146846829,-2.
48098406656139,0.0131020229584665,0.228700490033946
"896","ADAM33",425.003309661938,0.517068838613855,0.208377273529579,2.
48140706448229,0.0130864830689931,0.228700490033946
"897","SERTAD4-
AS1",17.4107716338139,-2.89102072700589,1.16564645123243,-2.4801866157
1803,0.0131313637033655,0.228957110725347
"898","PRKCD",41.7312290650571,-0.71346914014424,0.288059212907516,-2.
47681416936075,0.0132560901196182,0.230874442617849
"899","TESK2",11.9162486259259,-1.10438005724548,0.446027097770313,-2.
4760380316942,0.0132849425886297,0.231114195383728
"900","TBL2",149.704384695261,-0.536083840795994,0.216542713447363,-2.
47564941004724,0.0132994102202913,0.231114195383728
"901","ABCG1",132.07139059355,1.67685125803853,0.677447821876647,2.475
2478403331,0.013314374513138,0.231117444379
"902","LY6G5C",5.41370710979975,-1.80622815860542,0.729952104825039,-2
.47444749685098,0.0133442433078692,0.231210148199301
"903","PI16",4561.782748077,-0.631083250913973,0.255094729527239,-2.47
391724667752,0.0133640648319801,0.231210148199301
"904","ZNF532",188.632134264044,0.443827666136949,0.179383449122162,2.
47418403597925,0.0133540886087047,0.231210148199301
"905","AOX1",716.799475082421,0.404617256692238,0.163580464298484,2.47
350598023696,0.0133794564855803,0.231220662358537
"906","P4HA2",569.173075870213,-0.52570766830419,0.212594447137082,-2.
47281937691068,0.0134051875412987,0.231409639233897
"907","SRPRB",227.607529576218,-0.631791335710226,0.255694514839324,-2
.47088341377694,0.013477975097566,0.232153667980102
"908","SLC52A2",183.894223111682,-0.56681851152907,0.229371442441518,-
2.47118170202723,0.0134667374670166,0.232153667980102
"909","ZMYM4",297.275107486285,0.480151935898061,0.19438625844404,2.47
009196916194,0.0135078319377606,0.232411981855419
"910","LINC01239",87.0200858760041,1.00647512496966,0.407694680888687,
2.46869820027025,0.0135605532514155,0.233062695441911
"911","CD109",406.122434297029,-0.41208701222438,0.166981445458268,-2.
46786109135322,0.0135923054122556,0.233251816571168
"912","MSI2",72.7029156373592,0.883635891399175,0.35809207229304,2.467
62204407603,0.0136013847003136,0.233251816571168
"913","PTPRM",102.372699877734,0.586549606287752,0.23776485032966,2.46

693153119355,0.0136276412640388,0.233446121982001
"914","ICOSLG",6.44973810012523,-1.81790369485569,0.737157751789223,-2
.46609859347377,0.0136593730658955,0.233733692287315
"915","SPSB1",526.383369958914,-0.53341559435573,0.216403196291545,-2.
46491550724184,0.0137045563948236,0.23425055957928
"916","AC002451.1",57.1551679594444,0.552224121395025,0.22409696322255
5,2.46421956573593,0.0137311967663662,0.234449691513065
"917","CD74",331.346551380906,-0.807896578523154,0.328108061682457,-2.
46228810831548,0.0138053719646455,0.235399746840415
"918","SLC25A3",2372.96607740383,-0.534012340776857,0.216902930615662,
-2.46198767006561,0.0138169416623722,0.235399746840415
"919","SYBU",2.65711760021575,-2.39868300554933,0.974606466644735,-2.4
6118108964251,0.0138480449512778,0.235528862093136
"920","ABTB2",8.69365895702366,-1.6398514461107,0.66633262271269,-2.46
101029758192,0.0138546389466551,0.235528862093136
"921","LMO3",326.646923534035,0.710795227816433,0.289127539149871,2.45
841413068572,0.0139552145124036,0.236981058603684
"922","GABPB1-
AS1",293.183628162225,0.751396621875957,0.305885192835783,2.4564661496
3592,0.0140311020274675,0.237934842294263
"923","TM6SF1",5.62183911474112,-1.86104774217023,0.757696327072269,-2
.45619211242755,0.014041806869412,0.237934842294263
"924","FAM110B",164.614376145866,0.566838125685452,0.230915173178081,2
.45474612120144,0.0140984117453436,0.23863545421772
"925","GMDS-
DT",114.592751398336,0.494910701530612,0.20182659908011,2.452157960280
39,0.01420023101989,0.239745538074227
"926","AC069113.2",7.80847109335929,-1.47935939163305,0.60341208138283
1,-2.4516568979574,0.0142200178025001,0.239745538074227
"927","LINC00294",56.1411079964808,0.681502168923442,0.277991345511218
,2.45152296978951,0.0142253106990334,0.239745538074227
"928","WDR81",93.7997074347245,0.480128976907552,0.19579000211166,2.45
226503768937,0.014196005720782,0.239745538074227
"929","PER2",215.44363423182,-0.921785261775648,0.376151340457781,-2.4
5057018979069,0.0142630151516655,0.239928179386911
"930","CDC14B",190.241662245659,0.438887562097807,0.179103134098219,2.
45047393674934,0.014266829081191,0.239928179386911
"931","ZNF503",952.821179305194,0.370368453088293,0.151206321674086,2.
44942439567172,0.0143084745136233,0.240370076684284
"932","RALGPS2",275.806275166301,0.645127473088171,0.263525553465722,2
.44806419948221,0.0143626061580944,0.241020558275318
"933","IL17RD",229.971295737811,0.554704392081479,0.226711733120407,2.
4467387922392,0.0144155269877164,0.241649348432887
"934","AL161431.1",75.4367246601917,-0.839266823056773,0.3430777055494
81,-2.44628785106448,0.0144335713242677,0.241692778920286
"935","CYP26B1",42.7718432390429,-1.45730693887798,0.595872828619544,-
2.44566771445866,0.0144584185189484,0.2418499097715
"936","NAALADL1",75.9803019312751,-0.661082832517801,0.270494097456557
,-2.44398247035307,0.0145261323238619,0.242671955250681
"937","KXD1",366.123320654747,-0.399951365134631,0.163668115482181,-2.

44367306335958,0.0145385947615018,0.242671955250681
"938","CYP11A1",15.3783676239431,1.42794455847089,0.584520385850005,2.44293371632262,0.0145684127081071,0.242910420847329
"939","AC092807.3",15.9990445164446,-1.22162127012499,0.500334452546857,-2.44160933532872,0.014621959953845,0.243163771277742
"940","FAM117B",111.930545799147,0.713270583019311,0.292127819767977,2.44163867578866,0.0146207717863454,0.243163771277742
"941","HSDL1",76.4920915959203,0.574203026243807,0.235193710567422,2.44140468237225,0.0146302499215061,0.243163771277742
"942","LINC01894",56.3621393208703,0.776642220362899,0.318164330985612,2.44100970701842,0.0146462610780537,0.243171468429681
"943","ZNF668",137.362340618674,-0.533379655785732,0.218550950036367,-2.44052773825498,0.0146658196181073,0.243237983910072
"944","SAMHD1",543.062532332781,-0.595364474643162,0.244005478828281,-2.4399635512371,0.0146887438634312,0.243360120788203
"945","TLR3",117.871094360158,0.562662823939691,0.230712234471747,2.43880791683198,0.0147357986353788,0.243881365774947
"946","NLRP3",29.8956797041143,-1.30741615998204,0.536794854802106,-2.43559741358554,0.014867220873511,0.245496737680911
"947","TIAM2",26.2757190543224,0.8555320621608,0.351473273068296,2.43413120631382,0.0149275829625669,0.245496737680911
"948","CHST3",74.8113153617891,0.527901797504757,0.216751058292277,2.43552119959161,0.0148703532098925,0.245496737680911
"949","OLFM4",34.4039251944887,1.15684203141066,0.475211005321238,2.43437550573696,0.0149175104662148,0.245496737680911
"950","RBM3",1895.20658083357,-0.688260261198207,0.282691469214533,-2.4346693697923,0.0149054023477066,0.245496737680911
"951","MSN",797.972497193355,-0.402151978088318,0.165196099860392,-2.43439148035686,0.0149168520392278,0.245496737680911
"952","ZNF697",15.4039316568861,1.16622054783102,0.479303870181097,2.43315487394329,0.0149678971193217,0.245901166960285
"953","MT-ND4",34735.6220561068,-0.476213995936869,0.195883229671569,-2.43111162060847,0.0150525764908611,0.247032839787059
"954","MT-ATP6",56992.887388666,-0.477445213198479,0.196469137805332,-2.43012830682622,0.0150934785504744,0.247444449192265
"955","SLC1A7",34.9714489365877,1.67571468634645,0.689747026385305,2.42946271929321,0.0151212199476132,0.247639664901226
"956","RNF38",119.338131661506,0.47486240090858,0.195515555763936,2.4287704323738,0.0151501217965127,0.247793293457471
"957","ADM",1027.78164885592,1.57264001821925,0.647683214291968,2.42810062622732,0.0151781314023182,0.247793293457471
"958","GPRASP2",28.5322965010834,0.885378409267056,0.364596396631147,2.42837948330787,0.0151664647659489,0.247793293457471
"959","DHCR24",55.5705843330694,-1.06374607671472,0.438567906262024,-2.42549913371724,0.0152873522326462,0.248233690958829
"960","CNST",112.749911959092,-0.473559459042917,0.195251706688042,-2.42537935814069,0.0152923975001642,0.248233690958829
"961","RFTN2",120.091336599209,0.58596285490469,0.241564795844434,2.42

569639692882,0.015279046172952,0.248233690958829
"962","ATG101",263.592835455129,-0.491993099075119,0.202899270156417,-2.4248145333191,0.0153162091927922,0.248233690958829
"963","ANKFY1",208.520117825467,0.505314696750146,0.208290440482282,2.42601002513666,0.0152658485728907,0.248233690958829
"964","SPINT2",5.2701588338974,-2.07318089213019,0.854611478770841,-2.42587531718153,0.0152715159072069,0.248233690958829
"965","SOGA1",194.743008833675,0.650870727198607,0.268409889045362,2.42491336482989,0.0153120403330613,0.248233690958829
"966","PCYOX1",948.440434223428,0.512781589332589,0.211550717169175,2.42391799089257,0.0153540723502612,0.248459008519327
"967","CXCL14",1653.6416899552,-1.0498470896817,0.433152897583581,-2.4237332718734,0.0153618837108817,0.248459008519327
"968","CFB",1340.03925859896,-0.905703833647174,0.373968782355507,-2.42187015702873,0.0154408665254474,0.249336974492842
"969","LRR1",45.7274707002455,-0.666630988152033,0.275273852986979,-2.42170108391503,0.0154480516805348,0.249336974492842
"970","POLR3GL",448.970068853182,-0.515623907207465,0.213130985224475,-2.41928177014899,0.0155511888319197,0.249508605250955
"971","LIMD1",136.64308823734,0.715212852634656,0.295574231523823,2.41974020856758,0.01553159885707,0.249508605250955
"972","ADAMTS16",325.915059157386,1.07930387287782,0.445885812087119,2.42058357458332,0.0154956168846569,0.249508605250955
"973","LINC01554",5.30487573186232,2.60274362855591,1.07586663761742,2.41920656106585,0.0155544047391101,0.249508605250955
"974","STAC3",3.39168596872214,-2.18783323631874,0.904037760077516,-2.42006842295077,0.0155175869535105,0.249508605250955
"975","YIF1B",141.337295823366,-0.569011305334376,0.235063261909589,-2.42067305929428,0.015491803352984,0.249508605250955
"976","CD44",3592.07817896011,-0.495376971909547,0.2048429244053,-2.41832600929578,0.015592100300606,0.24985701711217
"977","WDR34",126.024936071685,-0.566498131074335,0.23442602518664,-2.41653259540323,0.0156691232456894,0.250577799143744
"978","SYNGR1",56.0124370147712,0.782175078375698,0.323667797308218,2.41659839156275,0.0156662915499686,0.250577799143744
"979","CAPN1",517.110270059714,-0.485221696921116,0.201053653359266,-2.41339408070374,0.0158047208366863,0.252136524475782
"980","C15orf65",34.9453812397423,-0.882175106606076,0.365568713150788,-2.4131581146612,0.0158149571937814,0.252136524475782
"981","REM1",10.4206612656015,-1.58902217627719,0.658364570261939,-2.41359005033484,0.0157962239792331,0.252136524475782
"982","TCIRG1",216.780787495204,-0.451586136278397,0.187263895909427,-2.41149600186046,0.015887226039089,0.253030769095064
"983","STAT5B",160.472109384611,0.525515315518165,0.218079979645433,2.40973663136147,0.0159640399002646,0.253995507670537
"984","SEPTIN11",774.012311403076,0.420081963569221,0.174468676172382,2.40777870724578,0.0160499063110848,0.254263755394683
"985","RICTOR",258.358868939221,0.540561753709014,0.224511616865603,2.40772286644126,0.0160523612001872,0.254263755394683
"986","RAB30",362.042855731641,0.560204029232709,0.23269123802226,2.40

749945719537,0.0160621860824774,0.254263755394683
"987","ERN1",81.038625316556,-0.506953287734046,0.210472927459169,-2.4
0863893448906,0.0160121304416667,0.254263755394683
"988","LANCL3",7.64232067395578,-1.72711044942604,0.717312542001785,-2
.40775164003998,0.0160510962059551,0.254263755394683
"989","CCDC18-
AS1",220.485158661257,0.457832314332654,0.190460653031187,2.4038157333
0889,0.0162249493122091,0.256059253929182
"990","PPIL6",31.236720526349,-0.734872431715483,0.305757064372162,-2.
40345201254618,0.0162410984589353,0.256059253929182
"991","LIPE-
AS1",37.64053971616,0.684806274106748,0.284879139304956,2.403848438244
82,0.0162234979097362,0.256059253929182
"992","PATZ1",78.6790483616531,0.556904789469869,0.231645636162105,2.4
0412381038833,0.016211281776782,0.256059253929182
"993","GSTT2B",4.09602886615117,-1.97737138481136,0.822924030724581,-2
.40286018026511,0.0162674059147733,0.256215738677799
"994","IL15",11.4828320949281,-1.48158298996422,0.616697859447881,-2.4
0244548812065,0.0162858616428116,0.256248366291322
"995","IDH3A",102.028492930791,-0.515487267763912,0.21462270290509,-2.
40183009898943,0.0163132832293865,0.256421859002617
"996","SPAG17",110.315392935087,-1.26287771031224,0.525967122199838,-2
.40105827343409,0.01634773292755,0.25670536444466
"997","TCF7L2",1265.64352828489,0.36050020112124,0.150167738539005,2.4
0065013050458,0.0163659758220425,0.256734064048892
"998","RCOR3",173.016885663691,0.398222178906248,0.165973647792317,2.3
9930967477766,0.0164260164921079,0.257076679015449
"999","ENPEP",10.4727531430653,1.4509831830242,0.605089384534994,2.397
96502815739,0.0164864391977299,0.257076679015449
"1000","EBF1",2807.61660369113,0.455645852087634,0.189942377556111,2.3
9886358141975,0.0164460404332552,0.257076679015449
"1001","LMBR1",78.1468344231124,0.531099509463063,0.221471810550155,2.
39804563905342,0.0164828113930809,0.257076679015449
"1002","ATM",343.334296579641,0.481937352896175,0.200964327844758,2.39
812387633523,0.0164792910808785,0.257076679015449
"1003","DZIP1",142.855127821325,0.63663112071356,0.26539437539625,2.39
881165440312,0.0164483726912524,0.257076679015449
"1004","SYS1",277.19924182292,-0.462128853818273,0.19279757318906,-2.3
9696406014977,0.0165315451196225,0.257523272580573
"1005","NOP16",53.6968505428917,-0.609963164109396,0.254528827609766,-
2.39644039473819,0.0165551858448702,0.257634931954
"1006","PLGLB2",11.8557201202744,1.33385063226577,0.556879846259867,2.
39522159263658,0.0166103233212485,0.257730496063014
"1007","HLA-
DQB1",66.1670627412033,-1.29785544684195,0.541854129281817,-2.39521187
844807,0.0166107634291252,0.257730496063014
"1008","AP003555.1",11.8609489777227,-1.27127387001927,0.5306784277976
42,-2.39556349651363,0.0165948396564316,0.257730496063014
"1009","SNED1",220.613901394208,0.669336281868336,0.279551283091679,2.
39432376938448,0.0166510430998254,0.258099419307501

"1010","CCDC39",12.11619038152,1.26103795802294,0.526997400686512,2.39
287320275242,0.0167170171671532,0.25886549355869
"1011","MICALL2",52.8970432562755,-0.671960327680527,0.280939955590708
,-2.39182898092105,0.0167646519971689,0.259346347414166
"1012","FUCA1",147.148692617671,-0.469824287981633,0.196537420957021,-
2.39050805538134,0.0168250800522722,0.259857945520173
"1013","PCDHGA10",53.6666590575733,0.842732888369448,0.35260531339641,
2.39001755320125,0.0168475675676122,0.259857945520173
"1014","TMEM250",137.767746846835,-0.435772235981794,0.182306444135076
,-2.39032820835953,0.0168333222402655,0.259857945520173
"1015","GTF2IRD2B",100.266093051107,0.463298846772492,0.19390294054789
,2.3893337845388,0.016878959580237,0.260006254228608
"1016","NRBP2",134.90453560707,0.480651935428875,0.201217130672279,2.3
8872273858089,0.0169070563011825,0.260006254228608
"1017","SLC25A23",103.126965060767,0.648776201725115,0.271582010303606
,2.3888776763963,0.0168999281686592,0.260006254228608
"1018","SREBF1",357.403658934632,0.721482298113999,0.302224257421151,2
.38724152809683,0.0169753347834603,0.260799838912887
"1019","TREM2",4.35670695155016,-2.17932284227861,0.913075409058039,-2
.38679392814541,0.0169960150872035,0.260861311053839
"1020","MATR3.1",49.0037594589162,0.747931725155462,0.313525727386905,
2.38555135933862,0.0170535409791158,0.261487628346442
"1021","STX4",712.633978034081,-0.374882671598892,0.157288748525741,-2
.38340424927179,0.0171533461914532,0.262760366732936
"1022","LUC7L3",847.034355137324,0.521272120245499,0.218914207320094,2
.3811708094546,0.0172577076960693,0.26410034086744
"1023","DDX11",29.000183523904,1.00936264686028,0.424047115233532,2.38
03077785458,0.0172981833034265,0.264460984228339
"1024","ROR1",207.187775164979,0.778029505287195,0.327004984295365,2.3
7925885736483,0.0173474891176272,0.26495579082001
"1025","LINC01235",5.5080059419407,2.77649491105457,1.16728009003629,2
.37860213221682,0.0173784219813162,0.26516928759784
"1026","BBS1",81.3149833775766,0.520875198976015,0.219107646186579,2.3
7725706081689,0.0174419281338383,0.265878904496327
"1027","ISYNA1",23.6640913400871,-0.75032649334047,0.315948634518138,-
2.37483695564886,0.0175567034407844,0.267367908290037
"1028","ZNF706",1155.56503051225,-0.533011762813192,0.224536026832539,
-2.37383626285824,0.0176043551978373,0.26783279697877
"1029","CBR3-
AS1",27.6774971033803,0.83186890074672,0.350501589064413,2.37336698805
621,0.0176267405064995,0.267912751721722
"1030","RGS16",512.96012910767,-1.45792877802336,0.61449526432195,-2.3
7256308172217,0.0176651463647318,0.268186394430964
"1031","LINC00324",28.5847225217228,-0.798443826718124,0.3365894021185
16,-2.37215973436082,0.0176844435160144,0.268186394430964
"1032","DDX17",1718.43551663246,0.511480229451391,0.215640250126008,2.
37191446936512,0.0176961866402017,0.268186394430964
"1033","RAB34",955.80878402594,-0.492984107990096,0.207976608840018,-2
.37038247108508,0.017769692475367,0.269039680846796
"1034","RFX7",64.2412293989762,0.672236507836879,0.283654745157217,2.3

6991102498317,0.0177923664022773,0.269122447322647
"1035","NCOA1",405.503832678749,0.44644230535996,0.188458996942685,2.3689094848347,0.0178406190453407,0.26933135315553
"1036","SERTAD3",144.714097093153,-0.615649212619023,0.259884144081262,-2.36893718466533,0.0178392829705387,0.26933135315553
"1037","IGSF3",93.1957239279179,0.829887786686785,0.350885338864618,2.36512528386654,0.0180239725086756,0.271591937440322
"1038","UST",166.704952789145,0.502720214944889,0.212557493369868,2.36510229291288,0.0180250915001953,0.271591937440322
"1039","SELENOM",2575.63035788158,-0.52023659274985,0.220066001032038,-2.36400257336485,0.018078687007419,0.272137309717068
"1040","PCNX1",148.222466786777,0.576152801290594,0.243839809094086,2.36283322001898,0.0181358292138531,0.272577472799255
"1041","TUBB3",67.5267127140153,-1.2770733939088,0.540516443018043,-2.36269110848524,0.0181427844746818,0.272577472799255
"1042","NMI",199.812164396035,-0.517593174152486,0.219215008375894,-2.36112106551096,0.018219781532105,0.273002804837595
"1043","RGS17",6.71295681241156,-1.46944539346691,0.62223177211428,-2.3615724225619,0.0181976171148436,0.273002804837595
"1044","AC083805.3",2.73533101837824,-2.15548557027415,0.912936639472971,-2.36104618554743,0.0182234608855786,0.273002804837595
"1045","CCNO",4.80633050881386,-1.8961014284708,0.803565452854235,-2.35961043588412,0.0182941348024541,0.273799299818547
"1046","HPS3",115.933862007339,0.540099902719845,0.22911133356114,2.35736877056637,0.0184049592407718,0.274931769365493
"1047","RECK",798.37048650192,0.479742823566146,0.203495011369176,2.35751638498798,0.0183976433813696,0.274931769365493
"1048","SRM",568.69505458276,-0.650346333683024,0.2760858693271,-2.35559442164897,0.0184930964958109,0.275131240661081
"1049","MYCBP",56.3815961463733,-0.686231866913774,0.291396443459035,-2.35497681017595,0.0185238616634347,0.275131240661081
"1050","PPM1G",463.796264949472,-0.39975402604476,0.169728727686504,-2.35525259332128,0.0185101185084051,0.275131240661081
"1051","AC068587.4",25.8621195647646,0.923270840660943,0.391899762657032,2.35588517431417,0.0184786286794178,0.275131240661081
"1052","SH2D3C",9.74028561485011,-1.12956059963004,0.479490097867654,-2.35575375727951,0.0184851667474265,0.275131240661081
"1053","GDPD1",13.1373792527127,1.14242610115518,0.484760275586727,2.35668258867222,0.0184390002671013,0.275131240661081
"1054","NPAS2",67.5670617553493,1.1550822124422,0.490948562545916,2.35275607377743,0.0186348538822375,0.275220734521953
"1055","NNT-AS1",191.629068257761,0.375107960476718,0.159434259391773,2.3527437698003,0.0186354704513266,0.275220734521953
"1056","HLA-DQA1",67.8113692852424,-1.15541935164877,0.49109204763072,-2.35275516519378,0.0186348994119936,0.275220734521953
"1057","SPART",624.094846931165,0.377773004585006,0.160536885347715,2.35318508744435,0.018613366579688,0.275220734521953
"1058","CPNE2",40.0021218294041,-0.872090180379256,0.370530743241411,-

2.35362435178844,0.0185913883329598,0.275220734521953
"1059","TAFA5",131.597259497766,-0.61827796145601,0.262624281891072,-2
.35422999352532,0.0185611227628133,0.275220734521953
"1060","ANXA1",9039.10864092325,-0.515130051949213,0.219049619582207,-
2.3516591945319,0.0186898901771294,0.275764039971985
"1061","SLC39A4",44.8477366963297,-0.732268033780567,0.311441950749155
,-2.35121836354781,0.0187120490514814,0.275830770183948
"1062","SLC12A8",5.36765807748708,-2.07573560124856,0.882971146373513,
-2.35085326374922,0.0187304186071486,0.27584156969473
"1063","MEOX1",7.76524325954122,-2.43620467457141,1.03670532025759,-2.
34994904238177,0.0187759813444147,0.276252444239553
"1064","ZDBF2",45.8414900881274,0.714375648029391,0.304147504303722,2.
34878023959064,0.0188350196833252,0.276860627675945
"1065","BMPER",58.7629831512294,1.1828265301187,0.50387094177792,2.347
47915000828,0.0189009309746169,0.277244311025348
"1066","GSN",50090.3385859225,-0.739241149135356,0.315028081775103,-2.
34658810405066,0.0189461863465231,0.277244311025348
"1067","DENND5B",84.8352085132906,0.545083249391131,0.232212814561794,
2.34734353665947,0.018907812542O916,0.277244311025348
"1068","USP10",156.445697370882,0.399218757547213,0.170132413809625,2.
34651791864854,0.0189497550182927,0.277244311025348
"1069","CFAP298",323.027125407055,-0.387843832599269,0.165267095505234
,-2.3467698238031,0.0189369492902269,0.277244311025348
"1070","IFT57",546.807950983361,-0.447305464918583,0.190778419234632,-
2.34463345861177,0.0190457929527595,0.277756261869724
"1071","SF3B3",228.036192142578,0.414604453760721,0.176845996601099,2.
34443788227742,0.0190557844620342,0.277756261869724
"1072","NECAB2",23.8173959561864,2.70336563902288,1.15275453902335,2.3
4513554057507,0.0190201638017167,0.277756261869724
"1073","AC022150.4",10.6733092380867,1.51789496890677,0.64742109464681
7,2.34452504167295,0.0190513311390263,0.277756261869724
"1074","CFL2",526.544057205463,0.332450552858324,0.141853189102033,2.3
4362410152934,0.0190974077203158,0.277884022059481
"1075","LINC02345",3.24912714647402,-2.93666932393578,1.25307417568847
,-2.3435718179431,0.0191000846364413,0.277884022059481
"1076","ALPL",298.952934658479,2.07316244594173,0.885338088822245,2.34
166187145481,0.0191980988978834,0.279050433794514
"1077","CCDC9B",8.22318275287699,1.64767047385183,0.703826822664152,2.
34101688198668,0.0192312974968335,0.279273438115576
"1078","ECHDC2",298.83205499046,0.43742793193834,0.187006284599676,2.3
3910819026613,0.0193298349108288,0.280120941766261
"1079","DNASE1L3",106.844065278185,1.89626884487103,0.810771041185869,
2.33884629389015,0.019343389840637,0.280120941766261
"1080","UBAC1",236.085294762833,-0.437477129865697,0.187045227568365,-
2.33888421294149,0.0193414267561176,0.280120941766261
"1081","ANKRD50",88.3220214694708,0.522894483266362,0.223679983199387,
2.33769010434992,0.0194033298853955,0.280327938015906
"1082","CARMIL1",310.934689754102,0.509802209220528,0.218126258898458,
2.33718861633184,0.0194293788241203,0.280327938015906
"1083","CRTAC1",100.915313525706,-7.10837032728685,3.0411595055752,-2.

33738819494848,0.019419008395685,0.280327938015906
"1084","FLRT3",5.65553410801957,2.29684899082122,0.982433080718663,2.3
3791902563078,0.0193914491078967,0.280327938015906
"1085","TAB3",50.9492198742185,0.776977017307764,0.332498864542509,2.3
3678096428034,0.0194505761220792,0.280375124930248
"1086","ZDHHC12",101.843960862694,-0.576495360427797,0.246744243189962
,-2.33640855395344,0.0194699585603952,0.280396088291511
"1087","CLIC1",3766.36691580781,-0.578213443396435,0.247524185592609,-
2.33598766121423,0.0194918846208907,0.280453611288621
"1088","MNDA",15.576263142819,-1.0592502189493,0.453673697124983,-2.33
482837039478,0.0195523884909696,0.280687833281706
"1089","MPDZ",215.872382770564,0.546580391843126,0.234072921396871,2.3
3508595774902,0.0195389307418481,0.280687833281706
"1090","ZBTB33",62.0427943589577,0.611072863113296,0.261764849138676,2
.33443437926827,0.0195729883706592,0.280687833281706
"1091","RENBP",31.7120574859521,1.24730094861104,0.534335883097769,2.3
3430130385388,0.019579950518564,0.280687833281706
"1092","HSPA5",1227.63496135773,-0.518096327100169,0.222011564408898,-
2.33364567507819,0.0196142828439108,0.280922512526342
"1093","RIPK2",138.558826030362,-0.553233371547865,0.237235281955246,-
2.33200292548489,0.0197005372583969,0.281642050019495
"1094","MIR155HG",13.3432148159595,-1.6011781697013,0.686585557279896,
-2.33208833585843,0.0196960445406818,0.281642050019495
"1095","IGF1R",224.864794701197,0.59597205654231,0.255668803070412,2.3
3103159003008,0.019751694002964,0.282115519823157
"1096","CITED4",526.062751125503,0.863120330582172,0.370540437110558,2
.32935529874339,0.0198402510980798,0.282785082922189
"1097","LRP1B",39.4872793373263,1.00084357374756,0.42981163294238,2.32
856325199027,0.0198822147381883,0.282785082922189
"1098","NT5DC1",95.1132251488233,-0.546874532935448,0.234812348493239,
-2.32898540662223,0.0198598388174158,0.282785082922189
"1099","ZNF629",52.3276267924337,0.62969656555038,0.270437593831681,2.
32843576452725,0.0198889764203586,0.282785082922189
"1100","HM13",817.617043149591,-0.460620040151058,0.197736627133684,-2
.32946241082409,0.0198345821044451,0.282785082922189
"1101","MYLIP",420.163704212598,0.792269077040666,0.340367516710473,2.
32768709745764,0.0199287247620661,0.282859739754329
"1102","SOX9",45.5817086867217,-0.988329949118551,0.424603171135395,-2
.32765560011184,0.0199303985427922,0.282859739754329
"1103","ANO1",4.85792685832778,-2.50634869800078,1.07725122320197,-2.3
2661485456571,0.0199857732128523,0.283131787182074
"1104","TOB2",357.017464368272,0.457564610473286,0.196640475549192,2.3
2690960086099,0.019970077110849,0.283131787182074
"1105","ST6GALNAC4",122.894592844093,-0.581828868267186,0.250223085618
168,-2.32524056215596,0.0200591005441717,0.283656720172554
"1106","PYCARD-
AS1",6.21306893655453,1.79752441890888,0.773033196319811,2.32528748760
904,0.0200565929031155,0.283656720172554
"1107","HCG11",233.74429908557,0.423590600106221,0.182225588053686,2.3
2453962492593,0.0200965903894439,0.283673893222836

"1108","PLSCR3",161.531215309803,-0.417615416807207,0.179632148537996,-2.32483672998473,0.0200806921732805,0.283673893222836
"1109","SCARA5",7277.35429450427,0.415969477972804,0.179028508512861,2.32348178191364,0.0201532852292331,0.28421765643391
"1110","ZNF740",71.1944270228252,0.552239567292777,0.237756130729883,2.32271431065717,0.0201945049794975,0.284542394485893
"1111","TDRP",5.55799676977573,1.99727424160752,0.860336413899269,2.32150378542662,0.0202596700319089,0.285203635732722
"1112","PPP2R2C",4.10541503231678,-2.33612689176024,1.00647434150138,-2.32109930221907,0.020281485046385,0.285253980328652
"1113","C11orf54",183.644034614276,0.464072325295959,0.200016126619345,2.3201745436214,0.0203314371024927,0.285314246913795
"1114","SECTM1",41.9523831274651,0.697774039066383,0.300739037166579,2.32019775563718,0.0203301819628578,0.285314246913795
"1115","RTL5",51.7699049471971,0.745496819527684,0.321333864194951,2.32000701636413,0.0203404977818978,0.285314246913795
"1116","AP000525.1",59.7753517493121,1.11485364306029,0.480609032948147,2.31966851771713,0.0203588161656252,0.285315308987794
"1117","DACT2",405.242729597777,0.775420834429675,0.33454310889267,2.31785026747763,0.0204574600858307,0.286410281540192
"1118","CREBL2",320.283500597277,0.427170230576404,0.184319430988468,2.31755397836015,0.0204735738338833,0.286410281540192
"1119","SGPL1",77.1206392181937,0.494765237091381,0.213593025523361,2.31639228799289,0.0205368594506219,0.286736146053721
"1120","TMOD2",319.650025889526,0.572909886488602,0.247348068258313,2.31620926139716,0.020546845763948,0.286736146053721
"1121","TLCD2",127.459068831637,0.639810066094086,0.276242521395269,2.31611723952735,0.02055186826894,0.286736146053721
"1122","APOB",9.88388639882803,2.02881916008049,0.876625021336151,2.31435232933251,0.0206484035208799,0.287178305393721
"1123","IL6ST",3312.54731871768,0.542951496081727,0.234591354592091,2.31445654519458,0.0206426922644223,0.287178305393721
"1124","POLR3G",111.426915340532,0.649061543769983,0.280379405375238,2.31494015368686,0.0206162074866437,0.287178305393721
"1125","ZNF91",256.11043461148,0.500340395902616,0.216204906521075,2.31419538045426,0.020657007261377,0.287178305393721
"1126","NCF4",8.88153325559413,-1.70234128126881,0.735774281621992,-2.31367326065822,0.0206856517121118,0.287321130352956
"1127","NAA10",336.083192766311,-0.527388743940142,0.228016155722175,-2.31294463442641,0.0207256833659524,0.287621728343829
"1128","FCGR1A",5.60506312361563,-1.51010737321917,0.653448636158055,-2.31098098558723,0.0208339051851418,0.288558864094405
"1129","KIAA1109",349.089108634607,0.523864811629304,0.226720786757127,2.31061659198673,0.0208540419603097,0.288558864094405
"1130","SHPRH",276.227589855943,0.468601038809553,0.202824048388468,2.31038204065448,0.0208670124866223,0.288558864094405
"1131","ZNF728",10.5922943063815,1.32906203597397,0.575107899018028,2.31097858026866,0.0208340380499998,0.288558864094405
"1132","KIAA1755",8.61354049412681,-1.59818894285556,0.692039315828262,-2.30939038621351,0.0209219280264975,0.289062680507439

"1133","MFAP4",6641.2112950978,-0.499520279307181,0.216389697533789,-2
.30842912116545,0.0209752808480615,0.289544035713753
"1134","INHBB",33.8854665873534,1.37469407596331,0.595806650118232,2.3
0728219581052,0.021039093408991,0.290168801513773
"1135","PIP4K2A",218.314051130655,0.475417415468467,0.206137308924782,
2.30631426182993,0.0210930788103545,0.290321771693246
"1136","SEPTIN1",149.570978258121,-0.537800368907389,0.233209238229316
,-2.30608518337754,0.0211058730444514,0.290321771693246
"1137","AL355596.1",2.75497296339025,-2.5637830832981,1.11166686785375
,-2.30625123176325,0.0210965984212953,0.290321771693246
"1138","LONRF2",23.3902817593775,1.02977180154118,0.446726203827914,2.
30515199851108,0.0211580621404258,0.290783912017802
"1139","XKR6",5.69515959914726,2.43861123226001,1.0584858479051,2.3038
6758319574,0.0212300779117982,0.291332733977281
"1140","SULT1A1",120.873414507367,1.61265387308232,0.700004791938768,2
.30377547647329,0.0212352504305691,0.291332733977281
"1141","ASAP3",216.71641166172,0.449075912424256,0.194973954764346,2.3
0326103282374,0.0212641606904321,0.291425156970704
"1142","DENND4C",258.022679447842,0.473122020986826,0.205437915999656,
2.30299270066397,0.0212792537890373,0.291425156970704
"1143","RPUSD1",51.5104935399704,-0.565752918732221,0.24580919948237,-
2.30159375614743,0.0213580925887453,0.292248965956234
"1144","LRRN3",43.7096191187142,-1.00466372998665,0.436952328419906,-2
.29925249195877,0.021490605842242,0.292552806793926
"1145","STMP1",716.165276839954,-0.390205908857018,0.169761033452457,-
2.29855992816101,0.0215299412161003,0.292552806793926
"1146","ZBTB34",42.9425318241649,0.660048636233552,0.287112059302937,2
.2989234162997,0.0215092884616337,0.292552806793926
"1147","ANXA7",1145.51196306546,-0.35965483593071,0.156454055896153,-2
.29878882890344,0.0215169334681271,0.292552806793926
"1148","VDAC2",1161.79479579676,-0.495663251264205,0.215628747746293,-
2.29868816864529,0.0215226528479228,0.292552806793926
"1149","PDS5B",347.830718021957,0.408213898599459,0.177444247159551,2.
30051920608285,0.0214188224604715,0.292552806793926
"1150","TLCD1",10.8552582475361,-1.35513044941373,0.589276964311227,-2
.29964945430654,0.0214680878933238,0.292552806793926
"1151","EBP",236.081198713444,-0.522635459228375,0.227173160932277,-2.
30060389653238,0.0214140305951199,0.292552806793926
"1152","PALMD",46.850500701011,-1.42279555847797,0.619260449214232,-2.
29757214477903,0.0215861525547631,0.293062001698347
"1153","MAP3K2-
DT",14.9045521338614,-1.06478743874654,0.463595936568617,-2.2968006290
7313,0.0216301456693281,0.29340457785628
"1154","FAM174C",317.040796056453,-0.601798444306483,0.262171952232849
,-2.2954341194057,0.0217082578933015,0.294208971794832
"1155","MARF1",193.393562747737,0.510584221558734,0.222496473417957,2.
2947969184195,0.0217447653386788,0.294448597313365
"1156","USP40",110.161685470154,0.51217012710208,0.223398344825672,2.2
9263170012182,0.0218692177344048,0.295621923393337
"1157","KLHL4",117.593008245564,-0.686138988950262,0.299260459405739,-

2.29278198099667,0.0218605599195446,0.295621923393337
"1158","SMIM1",7.85915366550745,1.89519027025838,0.827252612597459,2.2
9094504072673,0.0219665925242886,0.296258383781783
"1159","AKR1C2",879.885661043193,-0.813217080762487,0.354987645061733,
-2.29083206718664,0.021973128208879,0.296258383781783
"1160","ZNRF1",181.130731562009,-0.580142685407303,0.253237889969445,-
2.29090001293765,0.0219691972450343,0.296258383781783
"1161","FAM89B",578.490309698082,-0.539512451284374,0.235735555024312,
-2.28863419109066,0.022100615586689,0.297720609626025
"1162","NTRK3",17.7818267031956,1.97013431715661,0.861087115526673,2.2
8796167267187,0.0221397532208386,0.297991170717656
"1163","ZNF608",10.4451855181155,1.6803959414986,0.734872025539376,2.2
8665112169052,0.0222161947861746,0.298762929024738
"1164","DYNLT1",949.210366267691,-0.548085186785407,0.239749382611463,
-2.28607548772558,0.0222498427352771,0.298958368023827
"1165","ZNF146",153.159271051499,0.41207896617073,0.180395043275446,2.
2843142399513,0.0223530696075823,0.300087561083765
"1166","BCL2A1",30.9477104966848,-1.46135302742969,0.639842241923921,-
2.28392708651994,0.0223758164456451,0.300135308070232
"1167","CD52",58.0269906930787,-0.826636596505612,0.361990412926658,-2
.28358698735233,0.0223958152564159,0.300146144481871
"1168","SAMD9L",176.216096731648,0.696662278584065,0.30549009677823,2.
28047418208063,0.0225795803297638,0.301833022527783
"1169","VAMP1",28.8738717991745,0.858884545235466,0.376552947362742,2.
28091308606352,0.0225535904395054,0.301833022527783
"1170","IGSF6",11.5406376947723,-1.4420859891385,0.632314245437817,-2.
28064763611327,0.0225693060645553,0.301833022527783
"1171","KRT222",687.19918467881,0.60507891628623,0.265407272505148,2.2
7981287240158,0.0226187892274346,0.302098944079485
"1172","TRMT1L",90.3100841737038,0.485391889674583,0.213034926005918,2
.27846155921447,0.0226990922990587,0.302480886031632
"1173","PRELP",2212.90901391927,0.327446507595515,0.143720552189555,2.
27835547948383,0.0227054066624767,0.302480886031632
"1174","EYA1",4.61856356389256,2.92447853126154,1.28336839260181,2.278
75218691701,0.0226818005907782,0.302480886031632
"1175","PAXX",263.528404921264,-0.516309636089305,0.226744986224171,-2
.27704984655694,0.0227832491533968,0.303259588731171
"1176","LY86",3.43364873067604,-2.08946828419207,0.918020675292289,-2.
27605798042272,0.0228425395953117,0.303574481435829
"1177","THNSL1",16.7015012249431,1.0127484225344,0.444967626872805,2.2
7600472792124,0.0228457266400237,0.303574481435829
"1178","EGR3",144.0517361968,-1.01976261200811,0.448563492048027,-2.27
339636436333,0.0230023050423305,0.305395628915152
"1179","KIAA0355",127.894200280207,0.496498849774018,0.218576833277383
,2.2715071964829,0.0231162918199189,0.306648688773139
"1180","POLN",11.5079249478512,1.28709886427146,0.566987313182796,2.27
006642714156,0.0232035529526953,0.307234531281317
"1181","PRRC2B",408.739669816611,0.462477336221248,0.20375196810766,2.
26980549202293,0.0232193872106468,0.307234531281317
"1182","NDUFA4L2",30.4500122266256,-1.23727692185571,0.54508582441225,

-2.26987543326746,0.0232151420642291,0.307234531281317
"1183","MERTK",6.66267686061138,-1.45068959300388,0.639433193501116,-2
.26871173994089,0.0232858612085575,0.307853651142722
"1184","FMO4",57.4732183084966,0.723962025307252,0.319181631976808,2.2
6818197783967,0.0233181174371801,0.308019726957346
"1185","SEMA6D",14.4680028445662,1.20317845435938,0.530614644373696,2.
26751837160382,0.0233585779081143,0.308293804626926
"1186","PCDHB16",43.3718284391355,1.142219552561,0.50385743642687,2.26
694987507003,0.0233932879360304,0.308414278998042
"1187","TXNL4A",619.922087834541,-0.481734947565945,0.212524830727158,
-2.26672312085924,0.0234071450876391,0.308414278998042
"1188","SIPA1L2",231.382712320226,0.739236378185667,0.326269230655009,
2.26572507833974,0.0234682210799431,0.308698887880832
"1189","C1QTNF3",1813.16223290007,-0.672313634362639,0.296715539218152
,-2.2658524596797,0.0234604181871057,0.308698887880832
"1190","IFIT5",162.68669446847,0.430965658117318,0.190270383988708,2.2
6501701989992,0.0235116350973628,0.30880151459469
"1191","APOC1",55.8405919754019,-2.04905745409947,0.904679561158607,-2
.26495384893551,0.0235155117571787,0.30880151459469
"1192","ZNF704",399.060847343857,0.463668456378557,0.204770029415052,2
.26433749950165,0.0235533648528071,0.309039116021731
"1193","SLC25A32",94.8647553571003,-0.445146115201326,0.19670591473282
9,-2.26300320356882,0.0236354918063259,0.309856740864155
"1194","FMO3",6.80286951248451,2.2657995595172,1.00203705687258,2.2611
9338000224,0.0237472848693685,0.310590101855285
"1195","ZNF292",585.003934400855,0.471822220748054,0.208695859642178,2
.26081256023489,0.0237708664910982,0.310590101855285
"1196","TNKS",169.919842907082,0.455471931196716,0.201443908539826,2.2
6103600996537,0.0237570272818454,0.310590101855285
"1197","PALM2-
AKAP2",123.463751294959,-0.637430148831437,0.281864250159342,-2.261479
23502569,0.0237295971162693,0.310590101855285
"1198","MPP1",57.3172290608654,-0.581141114379623,0.257107486938084,-2
.26030412922037,0.0238023818880423,0.310742281075944
"1199","SNX30",47.5383298734518,0.584388902542151,0.258711300313872,2.
25884567791651,0.0238929860400357,0.311405251388465
"1200","KCTD10",210.397390568314,0.447956530506832,0.198290236520146,2
.25909524527356,0.0238774608521218,0.311405251388465
"1201","NEDD4",133.567521222587,0.395112459051881,0.175016159937313,2.
25757700999383,0.0239720433027608,0.312175484808642
"1202","DDX3X",1455.61635818473,0.438324936720371,0.194283473278805,2.
25611025643625,0.024063726964736,0.313108726895567
"1203","MLF1",172.676898654304,-0.542480344600406,0.240488952917254,-2
.25573914319074,0.0240869726097949,0.313150666348456
"1204","FGFBP2",137.799171482995,-2.53991783657863,1.12628767154122,-2
.25512353615928,0.0241255756977432,0.313392029827827
"1205","NUP133",106.252573805639,0.47274267447414,0.209833470041957,2.
25294217542899,0.024262795158594,0.313611666347447
"1206","KDM3B",153.395119065696,0.460487147016983,0.204361815068697,2.
25329348764195,0.0242406501082524,0.313611666347447

"1207","NDFIP1",1530.75517344052,-0.492088472544658,0.218284894959567,-2.25434046930186,0.0241747572514332,0.313611666347447
"1208","MSANTD3",107.193706861687,-0.554961955035344,0.24622752091909,-2.25385835411023,0.0242050803478533,0.313611666347447
"1209","CHST15",103.038821236003,1.1957345286789,0.530720919419157,2.25303824463442,0.0242567376715956,0.313611666347447
"1210","KIAA0930",280.226891221472,-0.432330057019831,0.191842677637094,-2.25356559001779,0.0242235101120097,0.313611666347447
"1211","VPS51",365.975681073592,-0.492177642713785,0.218548189326789,-2.25203258022808,0.0243202132112752,0.313943515406608
"1212","RNF126",152.196441340887,-0.468457549075271,0.208027720917636,-2.25189963630254,0.0243286151325325,0.313943515406608
"1213","MT1F",90.8448619981952,-1.69877731957882,0.75467171321708,-2.25101496429106,0.02438458960625,0.314147431171128
"1214","MAVS",333.408006284619,0.583986828677424,0.259422259937621,2.25110531693713,0.0243788677511335,0.314147431171128
"1215","DNAJC27",58.9244610263579,0.611269499552856,0.271709566826054,2.24971651419321,0.0244669465318311,0.314569125848519
"1216","NXF1",134.304329399814,0.434500089127954,0.193134590628508,2.24972692728932,0.0244662851017559,0.314569125848519
"1217","PPM1N",6.10012621827844,-1.47696683019258,0.65656163943564,-2.24954785884557,0.0244776615190312,0.314569125848519
"1218","ADAMTS9-
AS2",9.53964701455744,1.58650905835131,0.705761466747395,2.24793947119693,0.0245800498289214,0.314592454438896
"1219","AC092828.1",17.4910798334814,1.08081599877281,0.480568241895022,2.24903750300028,0.024510110145916 5,0.314592454438896
"1220","DBF4B",12.2108073440598,1.23485742008428,0.549297461906089,2.24806685943763,0.0245719269148046,0.314592454438896
"1221","PNMA8B",35.8587557747569,0.821170469792948,0.365240043373786,2.24830350529929,0.0245568433591829,0.314592454438896
"1222","CHRDL1",1938.59591415055,0.46535053714236,0.206999566461304,2.24807493608617,0.0245714119855371,0.314592454438896
"1223","PCDHB12",25.2480852324838,0.891682569635487,0.396732625999366,2.24756551692553,0.0246039084722321,0.314640334019387
"1224","PLD6",12.4522974899711,1.07548847664118,0.478609166341932,2.2471121580501,0.0246328601103888,0.314753212521635
"1225","CLEC10A",19.1420508037566,-1.27440113412789,0.567308231639465,-2.24639986351863,0.0246784070730743,0.315077784998272
"1226","CYP27C1",92.6205385085975,0.709841869392465,0.316077093973127,2.24578712892373,0.02471764612976 5,0.315321358458014
"1227","SCAI",61.5616817689057,0.547301571937965,0.243892999643597,2.24402329192614,0.0248309027587136,0.316508002564206
"1228","CASP9",44.2213440171033,-0.59456506293789,0.265169719855108,-2.2422057211614,0.0249480795699727,0.316867078532121
"1229","PNRC2",1053.94906810523,0.283986529272512,0.126660306830117,2.24211149001406,0.0249541675833671,0.316867078532121
"1230","RCE1",67.4451606697797,-0.641005616788079,0.2858476807751,-2.24247268702667,0.0249308386278544,0.316867078532121
"1231","GOLGA8B",20.0570866759978,-1.2411496279773,0.553580106379375,-

2.24204160097965,0.0249586837511193,0.316867078532121
"1232","AC018926.2",39.165603453498,-0.846248710171893,0.3774499895642
5,-2.24201545520998,0.0249603734495891,0.316867078532121
"1233","SNHG32",786.255100528053,0.517518683674775,0.23086538257269,2.
2416469628651 7,0.0249841982011716,0.316912295106507
"1234","LINC01473",31.6831647807185,0.739843387010039,0.33011837630332
,2.24114572261877,0.0250166373317091,0.317066619017772
"1235","SNAPC2",216.374898927441,-0.615435807914134,0.274741913333558,
-2.24005067318195,0.0250876333286986,0.317708975919713
"1236","BID",94.6387956056591,-0.653995550257323,0.292082208953207,-2.
2390804034288,0.0251506850923038,0.318249769290964
"1237","S100A16",1573.39704935288,-0.55425449794951,0.247687641792784,
-2.23771559185501,0.0252396078247249,0.319116787694985
"1238","CYP4B1",957.81396871492,0.749861390859724,0.335383331534241,2.
23583380673516,0.0253626595561249,0.319497427206165
"1239","GALNT2",211.396080962699,0.387819034635901,0.173466994942441,2
.23569350910002,0.0253718545134307,0.319497427206165
"1240","ZBTB20",2319.3543971056,0.551525083050289,0.246649475533957,2.
2360683389102,0.0253472950071365,0.319497427206165
"1241","PXYLP1",145.867208675089,0.591398894568832,0.264392996132614,2
.23681755273199,0.0252982668565399,0.319497427206165
"1242","ZNF681",26.7032457083613,0.74874394773372,0.334831081880242,2.
23618411865814,0.025339713075082,0.319497427206165
"1243","KIRREL1",262.526170860098,0.419099621763367,0.187660076253364,
2.23329133255564,0.0255297387235289,0.320767711646154
"1244","RASA1",163.954005750148,0.418682972530087,0.187481090056003,2.
23320107859955,0.025535687239503,0.320767711646154
"1245","GALNT16",716.010938621857,0.427996623576341,0.191676465939188,
2.23291170086645,0.0255547678204033,0.320767711646154
"1246","BBS2",173.109698050749,0.442832064149038,0.198266474886158,2.2
3351963262224,0.0255146971268288,0.320767711646154
"1247","ANTXR1",1057.13311676435,0.405427856461097,0.181730325825453,2
.23093121425699,0.0256856857267339,0.322152465730648
"1248","GCAT",23.0643581740594,-0.771221960557579,0.34577146877321,-2.
23043839705416,0.025718352865975,0.322303717006289
"1249","PCDHB5",16.5788895536916,1.2038628594628,0.539985428474545,2.2
2943582545127,0.0257849207763094,0.322763365438644
"1250","CCDC144NL-
AS1",41.5035093428084,-0.827353205241437,0.371132797140517,-2.22926459
643551,0.0257963047825003,0.322763365438644
"1251","TMEM131L",70.0933912258386,0.808498649579832,0.36274177465544,
2.22885453528976,0.0258235849967219,0.322846418344309
"1252","PCBD2",62.5718560179543,0.600314689319274,0.269499610802266,2.
22751597871408,0.0259128091075319,0.322929350152828
"1253","AL450326.1",64.7038886672458,-0.530500705179069,0.238137170354
289,-2.22771062740779,0.0258998178776846,0.322929350152828
"1254","RIN1",26.86438372322,-0.810400071623494,0.363762770910334,-2.2
2782576016624,0.0258921363459584,0.322929350152828
"1255","PGP",224.266729179656,-0.503768169880805,0.226155846997387,-2.
22752662188135,0.0259120986163973,0.322929350152828

"1256","DDR2",2235.98600781772,0.587187782800119,0.263752859213877,2.2
2628025550225,0.0259954151607667,0.323376328538521
"1257","LRBA",99.1162406426938,0.548627828880863,0.246439106634071,2.2
2622065294005,0.0259994052423142,0.323376328538521
"1258","TMEM173",294.430527075653,-0.584741262831009,0.262790096957958
,-2.2251267060667,0.0260727333943142,0.323376328538521
"1259","ZNF425",14.4122987438124,0.977765706326036,0.43924758821712,2.
22600130895363,0.0260140937414709,0.323376328538521
"1260","KCTD12",583.13361047636,0.755664056346068,0.339597336595971,2.
22517662806379,0.0260693831931059,0.323376328538521
"1261","RELCH",77.3678422402017,0.593891996160284,0.266868654370438,2.
22540934064106,0.0260537710605283,0.323376328538521
"1262","MDGA1",40.9976949869017,0.848776259311844,0.381706537714134,2.
22363563483816,0.0261729689831204,0.324362309743267
"1263","MIR193BHG",38.8134250703588,-0.655507515536277,0.2951312650435
84,-2.22107107303414,0.0263461479271126,0.326250002834553
"1264","SPRY3",4.17360749519377,2.11356036650555,0.951808670255778,2.2
2057271860907,0.0263799153076074,0.326409711559319
"1265","COLCA2",18.8709479435627,0.9659715739142,0.435285248757996,2.2
1916910042418,0.0264752223916323,0.32667409615246
"1266","MON2",210.071905343011,0.521378812080413,0.234876528321999,2.2
1979955087568,0.0264323774348792,0.32667409615246
"1267","ADCY4",3.60561527493132,-1.80351705597426,0.812741338780574,-2
.21905416879647,0.0264830395244189,0.32667409615246
"1268","SEC14L2",22.2259109242144,-0.928868375279133,0.41859248452123,
-2.21902783644464,0.0264848308133836,0.32667409615246
"1269","LINC01772",5.00965651718388,2.01267082258715,0.908080955076928
,2.21640021336715,0.0266641051017306,0.327884723367431
"1270","AC079922.2",24.1677759256659,-0.757080449503326,0.341636397145
112,-2.21604154542629,0.0266886569755949,0.327884723367431
"1271","EDNRA",4.75032620155467,2.08656171215626,0.941476519872406,2.2
162652685582,0.026673340180225,0.327884723367431
"1272","EXOC2",72.1001809245695,0.615264179506879,0.277677872667879,2.
21574795858068,0.0267087683868355,0.327884723367431
"1273","LRRC6",40.701986502381,0.615798267030565,0.277823380595823,2.2
1650987656229,0.0266566022290415,0.327884723367431
"1274","SPIN3",39.4606296198805,0.613278556537096,0.276715002045715,2.
2162822832272,0.0266721756127212,0.327884723367431
"1275","SLC7A7",11.3553497975535,-0.982859746241429,0.443813776250537,
-2.21457692130448,0.0267891175557502,0.328355641514054
"1276","ZNF772",11.2544052372938,1.08707177594003,0.490837488306685,2.
21472850350177,0.0267787051977803,0.328355641514054
"1277","SLC27A4",45.8918992160093,-0.687444650103102,0.310485085946299
,-2.21409878032596,0.0268219845272883,0.328464640754151
"1278","NFKB2",92.5015391787802,-0.638097342616031,0.288231439355957,-
2.21383671414137,0.0268400134836192,0.328464640754151
"1279","TAS2R4",3.94496410026372,2.40069640080251,1.08526559944154,2.2
1208191067503,0.0269610057746214,0.329687357556747
"1280","AL512308.1",26.3640684750706,1.08013751858957,0.48848552828645
2,2.21119655761055,0.0270222286702917,0.329920106481937

"1281","SHPK",66.0900271790627,0.639262401275799,0.289082028322081,2.2
1135296782809,0.0270114040546279,0.329920106481937
"1282","CRNDE",662.445706367238,-0.525958647127628,0.238034897966947,-
2.20958629016095,0.0271338876615427,0.331024963359226
"1283","SIAH2",103.345274893648,-0.383840236484366,0.173797670936374,-
2.20854649211546,0.0272062006274691,0.331648462832125
"1284","LINC01341",16.5606925574432,1.11558558481965,0.505573480095376
,2.20657457074132,0.0273437951597242,0.332907793158578
"1285","KIDINS220",765.955114109904,0.384053887642664,0.17408266587460
7,2.20615812443555,0.0273729301533265,0.332907793158578
"1286","BCL7B",242.841968014302,-0.515153487155828,0.233537156229284,-
2.20587376961144,0.027392839281328,0.332907793158578
"1287","KCNJ8",79.1588792289778,-0.828901483284268,0.375774539277912,-
2.20584791315847,0.0273946502426528,0.332907793158578
"1288","AL020996.1",2.68544280241855,2.57834733527478,1.16929070355135
,2.2050524539739,0.0274504139377252,0.333326454958091
"1289","FBXL7",201.547860022231,0.621057988386382,0.281866589579568,2.
2033756796531,0.0275682808885357,0.334288657471432
"1290","MYOM1",32.97729495512,0.801833341768212,0.363920980097949,2.20
331716394146,0.0275724020548687,0.334288657471432
"1291","AL359541.1",15.8016867534388,1.09631946713393,0.49766457989284
6,2.20292846111327,0.0275997912534128,0.334361529979378
"1292","CD47",1063.57021057447,-0.434953678245801,0.19765089919422,-2.
20061573217736,0.0277632389083688,0.336081313101306
"1293","PTPRH",23.9019983506393,0.790103857761671,0.359232400255982,2.
19942259439477,0.0278478875129335,0.336845290566342
"1294","GBE1",159.563246563441,-0.379438618839515,0.172718804243062,-2
.19685760622533,0.0280306173739228,0.338531934925215
"1295","AL035681.1",12.2541504431789,1.10740299031763,0.50407807348551
8,2.19688784052901,0.0280284574760718,0.338531934925215
"1296","FAM161A",27.222016979374,0.746262999897812,0.339761219073973,2
.19643372463687,0.0280609140113387,0.338636338840538
"1297","STXBP2",8.37813743343662,-1.1399606611125,0.519287579400717,-2
.19523960582317,0.0281464146069556,0.339406264034531
"1298","MSC",51.8239384558609,-0.690012798917256,0.314419793292964,-2.
1945590374278,0.0281952446387676,0.339471613664607
"1299","RRP7A",191.703383213452,-0.663715638189675,0.302399243026486,-
2.19483234001198,0.028175626715034,0.339471613664607
"1300","WNT9A",34.4531972773142,-0.696946571246168,0.317840674494665,-
2.1927545061822,0.0283250713132513,0.340561341929904
"1301","ZNF219",88.9579606472587,-0.461800655306259,0.210608628419234,
-2.1926958015557,0.0283293034431461,0.340561341929904
"1302","CLPTM1L",307.748759657062,-0.358430461006092,0.163616333280593
,-2.1906765285554,0.0284752087743917,0.341527810760342
"1303","MRNIP",150.599519890946,0.426935311518083,0.194885018166284,2.
19070360325905,0.0284732481813254,0.341527810760342
"1304","FAM3C",783.019122033438,-0.343176327170049,0.156616369626517,-
2.19119066537184,0.0284379978239032,0.341527810760342
"1305","CHCHD10",137.376611545514,0.65555143039936,0.299294479747134,2
.19032249092338,0.0285008568316603,0.341573487239209

"1306","RNASE6",8.20284510195737,-1.59302342293905,0.727731618832694,-2.18902598391191,0.0285949514913069,0.341915169207982
"1307","TTC7B",49.5144910447026,0.578672142084487,0.264316342100818,2.18931654957514,0.0285738403098055,0.341915169207982
"1308","MBP",744.566163921214,0.345790060632519,0.157960257491534,2.18909532134089,0.0285899125286504,0.341915169207982
"1309","TTC14",363.410045782823,0.49550451298495,0.226496091850361,2.18769564161979,0.0286917797571276,0.342810875020225
"1310","DYRK3",51.7659693711365,-1.06551379387586,0.487284328555344,-2.18663669532487,0.0287690563059789,0.342948201696272
"1311","KLHL33",7.73277769085908,1.6575531110912,0.757962701818013,2.18685313553751,0.0287532470380261,0.342948201696272
"1312","AP4E1",70.243238174401,0.705169789344418,0.322404866047463,2.18721819552378,0.0287265992045129,0.342948201696272
"1313","USP49",30.0082061678635,0.916441698500348,0.419456257262437,2.18483258416852,0.0289011239858222,0.343985118888296
"1314","CDK19",102.6695156278,0.524202913195398,0.239959466635177,2.18454775110986,0.0289220224640735,0.343985118888296
"1315","AP003068.2",12.6774778658905,-0.932634885842228,0.426833427707157,-2.18500901124851,0.0288881858805717,0.343985118888296
"1316","SDCBP2-AS1",25.5333844813581,0.763930300945694,0.349748918539241,2.18422491236376,0.0289457251838744,0.344005426957292
"1317","AKR1C1",1175.03983673623,-0.628378079442007,0.287766004531542,-2.18364250657388,0.0289885275299188,0.34425252131202
"1318","POU5F2",45.4062223768004,0.926939842600927,0.424561618266002,2.18328695463981,0.0290146846355188,0.344301720561087
"1319","PRKG1",70.8914157332669,0.665688664292608,0.30510549396869,2.18183112874697,0.0291219982621268,0.345313156042202
"1320","SLC39A7",494.057363139223,-0.362643289662589,0.166236103911787,-2.18149536189217,0.0291467971409055,0.34534538430588
"1321","TIMM17A",199.886927978955,-0.358960521489723,0.164632393400247,-2.18037601273908,0.029229600720264,0.345927759857524
"1322","UBAC2",274.225488907549,-0.460499677619312,0.211215797176144,-2.18023312543842,0.0292401853281104,0.345927759857524
"1323","MLH3",235.512136850705,0.436759390455815,0.200424106316441,2.17917594087327,0.0293186005040267,0.346593281846543
"1324","COL6A3",5436.65345718967,0.41221668948104,0.189283956661166,2.17776877001226,0.0294232560602451,0.347409198583359
"1325","TCF4",2214.02216558044,0.453975438122249,0.208470265522981,2.17765078863107,0.0294320452764035,0.347409198583359
"1326","CWC22",339.464046084751,0.349783604564896,0.160727513577202,2.17625219715033,0.0295364078347794,0.34837814369227
"1327","SRSF5",2092.0404325584,0.335364479756893,0.154151030975787,2.17555781258168,0.0295883407798114,0.34872769389318
"1328","AL590004.3",5.17136622587249,-1.95340909028501,0.898087034508255,-2.17507770987317,0.0296242935112143,0.348888516954361
"1329","ING5",131.483332879635,0.460190367541599,0.211695030438689,2.17383642208303,0.0297174221528682,0.34972195821735
"1330","KLHL24",301.7595424134,0.62417942659384,0.287244587036101,2.17

298934345243,0.0297811193463851,0.350208050058242
"1331","SPTSSA",248.435923603397,-0.405531086323103,0.186684554412245,-2.17227979893608,0.0298345647945549,0.350556615794357
"1332","RAMP2-AS1",233.854531661351,0.826331342012931,0.380447039260814,2.17200097973806,0.0298555890177802,0.350556615794357
"1333","EIF5A",965.335852289119,-0.48825544616429,0.224853167660543,-2.17144126206575,0.0298978327030851,0.350789274925919
"1334","PAXIP1-AS2",107.749982779065,0.558886679507779,0.257690867627933,2.16882610025097,0.0300958889095419,0.352584046850364
"1335","ENHO",5.25021332759673,-1.99307716122089,0.918920205137752,-2.1689338748647,0.0300877045039584,0.352584046850364
"1336","FEN1",32.2338632148793,-0.649808268390536,0.299674943963797,-2.16837704145542,0.0301300110385679,0.352719590301798
"1337","CDH11",184.338473104838,-0.528255854570396,0.243669033164448,-2.16792362866186,0.0301644977369645,0.352859195666511
"1338","LRP11",243.648375676357,0.327860368956619,0.151271252561754,2.16736731800892,0.0302068572113563,0.353090617926466
"1339","SOX4",1243.51203170329,0.603565860647092,0.278567432956463,2.16667775641032,0.030259433825009,0.353236140456918
"1340","DEPP1",799.392772224081,0.880085597238326,0.406259140990578,2.16631580299319,0.0302870629381539,0.353236140456918
"1341","ITGA5",457.497587328012,-0.436558767214119,0.20150595949848,-2.16648067531428,0.0302744749964385,0.353236140456918
"1342","GMPPA",142.311594460894,-0.570746933187997,0.263896323565462,-2.16276955084756,0.0305589086499919,0.35465327420353
"1343","JMY",133.880267063215,0.529939724055523,0.244903951957457,2.16386758898681,0.0304745130243463,0.35465327420353
"1344","ENPP4",32.2756660887762,1.2631436271111,0.583993500121673,2.16294124309249,0.030545699093844,0.35465327420353
"1345","ENPP5",4.04171399641652,2.09324651669589,0.968017198328678,2.16240632946395,0.0305868701311092,0.35465327420353
"1346","CTSC",75.3617672394391,-0.724545434734329,0.335012080623077,-2.16274420130394,0.030560859393764,0.35465327420353
"1347","MTCL1",11.016998104699,-0.974392855499953,0.450417712454013,-2.16330936496961,0.030517393280592,0.35465327420353
"1348","ZNF829",37.4281955833645,0.775442240714194,0.35848170256676,2.16312920621042,0.0305312433226763,0.35465327420353
"1349","MN1",156.201092272012,0.478162257823804,0.221129200709175,2.16236596654946,0.0305899787020819,0.35465327420353
"1350","PTPRT",3.12763590148537,-2.93111396743433,1.35658410656778,-2.16065775298679,0.0307217866254799,0.355917587275931
"1351","FAM177A1",401.065920693572,-0.398084088987296,0.184298510263534,-2.15999623880879,0.0307729607368082,0.356246562489771
"1352","AC005089.1",7.32106530273127,1.83274200159797,0.849003640056908,2.15869746032552,0.0308736459521291,0.357147797848594
"1353","PODXL",19.0404243109313,-1.27645206449562,0.591413528085042,-2.15830718081253,0.0309039568016679,0.357234208705163
"1354","SMG6",254.102426892904,0.389049411128204,0.180290639822597,2.1

5790132816115,0.0309355042229698,0.357334775514954
"1355","CLK1",610.535944362952,0.470249083138523,0.217959110984802,2.1
5751055789227,0.0309659053913737,0.357421963336594
"1356","MICOS10",1511.13201076353,-0.432543001071498,0.200648511645276
,-2.15572494171393,0.0311051494074844,0.357717222870199
"1357","MYLK",7.53427617276952,-1.53391812629405,0.7112780471109,-2.15
656610312182,0.0310394880515082,0.357717222870199
"1358","PTX3",166.157608573281,1.43999175595298,0.667960873365773,2.15
580255277084,0.0310990860730564,0.357717222870199
"1359","GSDME",120.55552707477,0.499220407527511,0.231579855555762,2.1
5571603294011,0.0311058454669738,0.357717222870199
"1360","ATRX",1148.05933893616,0.414559449301134,0.192272077195001,2.1
5610844460108,0.031075198266354,0.357717222870199
"1361","PPM1E",6.38953682735025,-2.03956944348317,0.946305738824348,-2
.15529649647592,0.0311386397922517,0.357822393505225
"1362","EDA2R",19.3103172747721,1.09484615205285,0.508045986253598,2.1
5501387999618,0.0311607480789077,0.357822393505225
"1363","ARPC2",2542.89510843539,-0.393750409534635,0.182818505398715,-
2.15377764234475,0.0312576138508262,0.358671372433545
"1364","ASS1",42.4218928438044,-0.860916779099015,0.399798279933448,-2
.15337789657906,0.0312889912802493,0.358768199137169
"1365","LAIR1",5.02843938696383,-1.96773886966461,0.914059459623003,-2
.15274712049498,0.0313385580460863,0.359073295121458
"1366","BMP8B",220.966325420205,1.6503092161771,0.767128118241284,2.15
128239590617,0.0314539168980814,0.359079752033572
"1367","ERLIN2",179.634296207265,0.376190603426918,0.174848446083718,2
.15152385882112,0.0314348747118093,0.359079752033572
"1368","AL441992.1",17.0163870661752,-0.848971398517994,0.394567282386
221,-2.1516517877095,0.0314247900249654,0.359079752033572
"1369","UBQLN2",215.828199318638,0.463326164495262,0.215346270787034,2
.15154022775471,0.0314335841871795,0.359079752033572
"1370","ARHGEF6",223.374105085383,0.397659472594128,0.184813454367974,
2.15168031978,0.0314225412086873,0.359079752033572
"1371","LINC02763",12.0815436256507,-1.24065802328917,0.57685306134700
8,-2.15073492093829,0.0314971283827281,0.35920478714837
"1372","AC130371.2",20.9314753548896,0.898249395048816,0.4176812761807
68,2.15056179501823,0.0315108035784887,0.35920478714837
"1373","PCNX4",247.325518878397,0.387660154066845,0.180371092723493,2.
14923660001952,0.031615649360639,0.359951028044299
"1374","LDLRAD4",61.7111745763258,-0.763724722492935,0.355360843968694
,-2.1491527146424,0.0316222961977537,0.359951028044299
"1375","PTP4A2",1394.53882380388,-0.311015610044184,0.144762393453847,
-2.14845584287291,0.0316775606687882,0.359986371788433
"1376","PLEKHO2",139.581453282298,0.388185073711684,0.180694334926861,
2.14829686757368,0.0316901795854283,0.359986371788433
"1377","GNAL",88.1469556407098,0.558208397947188,0.259844153835091,2.1
4824305149252,0.0316944522987642,0.359986371788433
"1378","CRAT",201.177663905085,-0.403549285318065,0.187888375314216,-2
.14781401267209,0.0317285333924478,0.360053288464897
"1379","BX255925.3",16.8417363766032,0.830684430201404,0.3867985294286

2,2.14758942188455,0.0317463864957221,0.360053288464897
"1380","VWA1",35.9975723566664,1.09370690906158,0.510056996906518,2.14
428370886956,0.032010161342141 8,0.361289687536302
"1381","MAP7D1",335.044783265662,-0.370703155215442,0.172944949675543,
-2.14347487978636,0.0320749860715084,0.361289687536302
"1382","LINC01121",20.7061131596726,1.04333737050949,0.486461001999732
,2.14475028053753,0.0319728184067601,0.361289687536302
"1383","CDH18",4.91466085206182,2.67286035379928,1.24558592665327,2.14
586589058606,0.0318836798384567,0.361289687536302
"1384","CMYA5",118.099605998124,0.971110978824741,0.45298749215159,2.1
4379203763923,0.0320495536176785,0.361289687536302
"1385","AMPH",58.0512495701659,1.11263217510139,0.519197315651595,2.14
298522268943,0.0321142849704183,0.361289687536302
"1386","TMEM140",200.831998585395,0.680201906033717,0.317413723805155,
2.14295052488424,0.0321170713118697,0.361289687536302
"1387","APBA1",55.4201406905679,0.735186177180938,0.343102897256516,2.
14275712347392,0.0321326058416238,0.361289687536302
"1388","LINC01550",47.8860052726307,0.91597000255591,0.427411413119272
,2.1430639763948,0.0321079615721768,0.361289687536302
"1389","ELL3",10.3000119300361,1.22298254742959,0.570400952541927,2.14
407521933389,0.0320268602733542,0.361289687536302
"1390","ZNF280D",294.801834521282,0.343035472472134,0.159937030786942,
2.14481581147461,0.0319675765077259,0.361289687536302
"1391","DCUN1D3",287.237002948786,0.731088572776223,0.341066749139128,
2.14353517199062,0.0320701499910851,0.361289687536302
"1392","WNT4",167.474696462426,-0.924806774121961,0.431742609317486,-2
.14203266984449,0.0321908531879867,0.361684586106402
"1393","AL499604.1",19.8790570299811,0.809589665051596,0.3781367303338
72,2.14099715818873,0.0322742672313663,0.361806050432963
"1394","CYB5R2",17.0433158919563,-0.822584716288295,0.384292895319437,
-2.14051502462604,0.0323131679038234,0.361806050432963
"1395","C2CD5",76.1911814930127,0.468141572666895,0.218634383759505,2.
14120745610555,0.0322573120362053,0.361806050432963
"1396","CCDC85C",14.1492146685299,1.1765302861514,0.549661467236225,2.
14046346029522,0.0323173307196195,0.361806050432963
"1397","EIF6",509.025166348969,-0.574968585047447,0.268486801849115,-2
.14151526662592,0.0322325086838019,0.361806050432963
"1398","HSD11B1",219.727058823627,-0.712035109281467,0.332741305579633
,-2.13990597903409,0.0323623658232807,0.362051074017246
"1399","DCT",2.76718186231903,2.60020031902624,1.21540445151897,2.1393
7040939466,0.032405681465247,0.362276524743719
"1400","DNAJB5",10.9811535991809,-1.03472639719824,0.483726360663837,-
2.13907382632247,0.032429689783902,0.362285963014448
"1401","SOD3",2397.19586254154,-0.751998641232715,0.351629153833461,-2
.13861289098024,0.0324670326167021,0.362444247055832
"1402","LAP3",276.216777507568,-0.465541775973155,0.217800555489872,-2
.1374682673608,0.0325599240315321,0.363221977070729
"1403","ZNF483",57.863187564798,0.759731977443701,0.35572455837918,2.1
3573102994445,0.0327013436523725,0.364539568583824
"1404","HPS6",41.8102411061254,-0.566784314110289,0.265538429479691,-2

.13447189252747,0.0328041720887639,0.364738320441279
"1405","SIGIRR",302.282477993429,-0.605036482560617,0.283473021432936,-2.13437059901574,0.0328124563210281,0.364738320441279
"1406","DUSP16",92.8300467274044,0.561017364379478,0.262830120039002,2.13452462866976,0.0327998598022895,0.364738320441279
"1407","MXRA7",1636.61548809767,-0.414582113784665,0.194179447464972,-2.13504631513308,0.0327572271287146,0.364738320441279
"1408","FOXP1-IT1",10.5813662132916,1.29392597957009,0.606335058634802,2.13401148613019,0.032841840598365,0.364805672555702
"1409","SMAD6",63.7541527070642,0.771178240267932,0.361504423489773,2.13324703698888,0.0329044663974205,0.365241912317712
"1410","LINC01278",108.132878593796,0.504115688104844,0.236346826919787,2.13294883064343,0.0329289239959069,0.365254164039705
"1411","GPD2",84.1603641579481,0.552327323656577,0.259001326433367,2.13252700772818,0.0329635466687364,0.365379071508886
"1412","AL356417.2",42.2858197921517,-0.677045453584056,0.317537550558582,-2.13217445430646,0.0329925076950944,0.365428934460222
"1413","RERGL",2.95879652232793,2.86478366833025,1.34381175269925,2.13183406275165,0.033020490331014,0.365428934460222
"1414","MIB1",219.041078095567,0.487027397793839,0.228477643502336,2.13161948945285,0.0330381402382835,0.365428934460222
"1415","ACVR2A",92.8785530858424,0.452471228898772,0.212448313091493,2.12979440652894,0.0331885906880111,0.36578216803594
"1416","CNOT6",85.8218574514912,0.534290411176237,0.250872215136633,2.12973130916569,0.0331938025788483,0.36578216803594
"1417","RBM12B",114.701983279819,0.575132235977835,0.269912444601805,2.1308103701047,0.0331047676871132,0.36578216803594
"1418","HDAC7",1016.26249989021,0.559385977788751,0.262550051708106,2.13058795513267,0.0331231027287834,0.36578216803594
"1419","AC022467.2",15.8599267962891,1.6517646560151,0.775647366268655,2.12953041271977,0.0332104014457184,0.36578216803594
"1420","CPEB1",52.0505791916601,0.719782986045465,0.337938883627419,2.12992058895191,0.0331781700232534,0.36578216803594
"1421","NXPH3",67.3113839168605,0.770603871647214,0.362170495468207,2.12773785079039,0.0333588247874599,0.367158353044245
"1422","AC234582.1",4.8268544963824,-1.45042821890541,0.681868755254254,-2.12713694201251,0.0334087066221261,0.367281643922256
"1423","CDIP1",88.9523558156966,0.534475893698904,0.251288489029395,2.12694141209303,0.0334249514444394,0.367281643922256
"1424","AXL",2172.25294609409,-0.333388433730349,0.156759238405921,-2.12675461504256,0.0334404770425379,0.367281643922256
"1425","RPL22L1",628.606594148765,-0.521671713546129,0.2453380479188,-2.12633840519831,0.0334750924265991,0.367403821440007
"1426","AL590617.2",3.53633027535904,-2.16423139200513,1.01802532223464,-2.12591115833393,0.0335106576170508,0.36753624483217
"1427","SORD",69.9839760855143,-0.515013291009901,0.242456201884759,-2.12414979285492,0.0336576199724548,0.368889401800416
"1428","AC008011.2",9.2577779169346,-1.73666452640172,0.81769747425559,-2.1238472431173,0.0336829190894656,0.368908161456052

"1429","HK1",150.911904166035,-0.460302425746356,0.216834557202601,-2.12282779869018,0.0337682844807219,0.369584303203983
"1430","MRTO4",160.857096594049,-0.555555823442487,0.261825575502345,-2.12185468274665,0.0338499429753063,0.36987985100914
"1431","MLLT11",18.0518550138175,-0.887329425591657,0.418223990847482,-2.12166074880972,0.0338662369977678,0.36987985100914
"1432","RBM5",448.087829107313,0.433512240766702,0.204317254045436,2.12176031237332,0.0338578709872408,0.36987985100914
"1433","WDR26",217.407096641913,0.403026694030429,0.190046402632456,2.12067520588574,0.0339491444270968,0.370010187344804
"1434","MAP4K3-DT",97.6354781498639,0.509738926172284,0.24034822511402,2.12083499235523,0.0339356908380504,0.370010187344804
"1435","ATP6V1A",161.72426207273,0.336774507000022,0.158796771278766,2.12078938562812,0.033939530336351,0.370010187344804
"1436","TMUB1",111.21662304384,-0.580654738820109,0.273858624987096,-2.12027186964613,0.0339831245323729,0.37012260980941
"1437","MINDY4",76.3810281814854,-0.568913107445523,0.268405529005797,-2.11960278744197,0.0340395572168589,0.370479244865465
"1438","CCT3",573.640848946912,-0.392707336184848,0.185329684416931,-2.11896619486701,0.034093323963305,0.370548705202286
"1439","LRRTM4",3.89165338064419,3.04369463850961,1.43632694200871,2.11908204844573,0.0340835335450668,0.370548705202286
"1440","GATD3B",222.632949534727,-0.557377356500822,0.263085581331106,-2.11861613122513,0.0341229213372928,0.370612840080042
"1441","THRA",283.876819951129,0.357984793813499,0.169016196159998,2.11805023392324,0.0341708135814544,0.370875450668943
"1442","TPCN1",198.609237476664,0.475812355481758,0.22480195611568,2.1165845871773,0.0342951193417641,0.371708708596806
"1443","MIF4GD",132.663931883549,-0.522412750601254,0.246814292169064,-2.11662276933059,0.034291876106735,0.371708708596806
"1444","TNC",20.0018346571536,-1.25182461585876,0.591519084681414,-2.11628778897807,0.0343203386533804,0.371724443586474
"1445","SNTB1",98.3134751528059,0.733143159556387,0.346510005321066,2.11579218001823,0.0343624864877208,0.371923383161213
"1446","JTB",1417.49530039576,-0.466969852688204,0.220899034648141,-2.11395153189337,0.0345194072037541,0.372539522198406
"1447","FAHD2A",184.941011816151,0.422864762770635,0.199978503795711,2.11455108796401,0.0344682261767183,0.372539522198406
"1448","DMTF1",153.585720167456,0.508694072839049,0.240616816504069,2.11412518970987,0.0345045762517675,0.372539522198406
"1449","THY1",2635.91423891371,-0.800931715152094,0.378919017865378,-2.1137279402446,0.0345385106897499,0.372539522198406
"1450","CD68",361.620451577665,-0.553822863624712,0.261907457644212,-2.11457462344219,0.0344662183967771,0.372539522198406
"1451","MT-CO3",45018.449389366,-0.420531194505725,0.199064753336142,-2.11253467757606,0.0346406148489901,0.373383333038046
"1452","EPHA2",27.7020456553454,0.775355438074792,0.367111296388804,2.11204461889841,0.0346826224272687,0.37357866030474

"1453","COL13A1",2.94558963383036,-2.42857549848609,1.15020219414114,-2.11143354695086,0.0347350641458143,0.373886031135951
"1454","PYGO1",65.9247737309392,0.550745300059281,0.260892502230067,2.1110047063507,0.0347719073381596,0.374025193100974
"1455","DLC1",876.984832362856,0.404521296007595,0.191680275257246,2.11039605126143,0.0348242563157853,0.374330837648716
"1456","RPRM",2.76625237611516,-2.55365364817512,1.21096788095654,-2.10877075134148,0.0349643744114451,0.375578857002062
"1457","EZH1",269.483046990914,0.379988470583376,0.180228426423924,2.10837146016903,0.0349988711064502,0.375691382364366
"1458","AC007389.5",4.20666817564237,2.31670463859591,1.09921143446539,2.10760602187754,0.0350650822688536,0.376143955202243
"1459","ATP1A1-AS1",47.7571580981122,0.946029227326027,0.449200764243087,2.1060276442764,0.0352019511210328,0.376321610070371
"1460","ITPR3",173.655844876451,0.5200309021842,0.2468237059433,2.10689204343954,0.0351269384629327,0.376321610070371
"1461","MSS51",9.7066448765051,1.10197488051557,0.523208999300519,2.10618487447427,0.035188296502439,0.376321610070371
"1462","NR1H3",94.3612031104091,0.704878598357921,0.334692758163849,2.10604675830138,0.0352002909267086,0.376321610070371
"1463","DNAAF2",79.7787147629145,-0.442348964182942,0.210003536721358,-2.10638816416635,0.0351706485596818,0.376321610070371
"1464","ADGRA3",76.5734885611669,0.575815941578745,0.273494064430338,2.10540562471846,0.0352560146039798,0.376616508701243
"1465","PPP1CA",506.719013706597,-0.375569421698313,0.178404523327987,-2.10515638669009,0.0352776972664527,0.376616508701243
"1466","KANSL1L",105.595967065534,0.475750640149603,0.226071372689115,2.10442673254273,0.0353412395533635,0.37678049530648
"1467","PRR5L",67.9398499267364,0.722695085954475,0.343392688576058,2.10457330629625,0.0353284672730342,0.37678049530648
"1468","HLA-DMB",17.7174456681456,-1.26177196145023,0.599793015033464,-2.10367898562445,0.0354064587652565,0.377218675128482
"1469","MIR29B2CHG",32.2411081686774,0.982868674698512,0.467419455041162,2.10275516797211,0.0354871768331005,0.377564248754893
"1470","CDS1",126.943484597634,0.512725671362523,0.243818090931171,2.10290249343009,0.0354742938378497,0.377564248754893
"1471","TTN",9.47381289577392,1.85873565225944,0.885195540907076,2.09980232204374,0.0357462339100736,0.378006151692732
"1472","PRELID1",968.659817932245,-0.511392045949549,0.243354334994545,-2.10142977712319,0.03560325632034,0.378006151692732
"1473","PM20D2",52.127293281531,0.820270473811939,0.390587928611431,2.10009171744775,0.0357207737762497,0.378006151692732
"1474","ENOX1",13.5970129359867,-1.17621991734736,0.560137586128018,-2.09987679183975,0.0357396808037426,0.378006151692732
"1475","KCTD11",57.6536799026374,-0.511667635501,0.243557627925595,-2.10080727037345,0.0356578879847174,0.378006151692732
"1476","NFATC1",71.7600618634006,-0.719473963245699,0.342325119339122,-2.10172705010571,0.0355771926189398,0.378006151692732

"1477","MLLT1",173.076181201589,0.445326940614252,0.212074013024499,2.
09986567549324,0.0357406589419841,0.378006151692732
"1478","APOL1",305.212326387316,0.542787340418123,0.258332339759916,2.
1011203665889,0.035630401491757,0.378006151692732
"1479","GEMIN8",90.9927354312993,-0.612384963411837,0.291551607408715,
-2.10043418677969,0.035690664342329,0.378006151692732
"1480","SLC38A7",56.0000448403132,0.547821687039166,0.260960631407207,
2.09925031252832,0.0357948409607654,0.378264400423223
"1481","FBXO44",38.7626714319169,0.691733545292399,0.32966333787084,2.
09830292249063,0.0358783945325671,0.378635688589305
"1482","APCDD1L-
DT",23.6943137515276,-1.30138657995952,0.62015226837364,-2.09849523468
232,0.0358614204206321,0.378635688589305
"1483","TNFRSF11A",5.26884181924018,-1.6096446757806,0.767342941547716
,-2.09768616954237,0.035932877421042,0.378954958101885
"1484","AC106739.1",46.1209886913345,-0.822508101744689,0.392159279988
41,-2.09738273124379,0.0359597085184362,0.378982372795379
"1485","PRRX2",855.769904087858,-0.526585999267651,0.251103708787688,-
2.09708570936675,0.0359859887963481,0.379003949343356
"1486","AC003681.1",71.6482742278377,0.836059434541927,0.3987641990217
19,2.09662611787371,0.0360266853882677,0.379177227101283
"1487","CREG1",465.589609627177,0.371939186406922,0.177431295880471,2.
09624341952326,0.0360606030671871,0.379197292869335
"1488","VIT",1460.22598336365,0.385351573398411,0.183869861879002,2.09
578431973804,0.0361013279464476,0.379197292869335
"1489","MAPK11",4.31536121131573,-1.86161515821292,0.888183978928566,-
2.09597921419234,0.0360840348631152,0.379197292869335
"1490","BAZ2B",420.809923915613,0.531604254372611,0.253821991238209,2.
09439793525891,0.0362245465503975,0.380236179898132
"1491","GBA",148.776195723073,0.39529554431065,0.188946900803533,2.092
0985876433,0.036429697483358,0.380749528235049
"1492","SH3BP5",1537.53592470905,0.29840303949122,0.142536248850584,2.
09352387127871,0.0363024154158535,0.380749528235049
"1493","BHMT2",209.954781735925,0.395042684737698,0.188860225995617,2.
09171985607422,0.0364635832229344,0.380749528235049
"1494","TIGAR",32.6669851780337,-0.845930546746467,0.404250360447029,-
2.09259070495575,0.0363857070252149,0.380749528235049
"1495","RAB8B",524.261605275333,0.451980318790464,0.216024011782607,2.
09226888742955,0.0364144692282918,0.380749528235049
"1496","HCFC1R1",1119.81341972339,-0.562919768212462,0.268988829918636
,-2.09272544284733,0.0363736706769923,0.380749528235049
"1497","ZNF793",6.0092281570043,-1.4769439438756,0.705621298066675,-2.
0931113444595,0.0363392162527824,0.380749528235049
"1498","CYB5R3",1570.28336293184,-0.413451574617234,0.197665948904147,
-2.09166817506705,0.0364682092900322,0.380749528235049
"1499","COA6-
AS1",28.601555186526,-0.752480898059188,0.360057348015497,-2.089891796
97786,0.036627520630634,0.382157720255581
"1500","WARS2-
AS1",36.2213518056766,0.687054600306814,0.329000950116409,2.0883058242

346,0.0367702564202058,0.382205241393276
"1501","IL1B",102.244276591408,-1.92985473171004,0.924149207625154,-2.0882501611069,0.0367752746342782,0.382205241393276
"1502","AP2M1",1450.04483560811,-0.366549354516293,0.17552994532283,-2.08824399644257,0.0367758304349257,0.382205241393276
"1503","DDX60L",260.549998333123,0.42680772274821,0.204329154992171,2.08882439103986,0.0367235339390847,0.382205241393276
"1504","CEBPG",161.590918627206,-0.422086982867092,0.202128400483934,-2.0882121555 2359,0.0367787012977545,0.382205241393276
"1505","TMEM47",343.279384311386,0.461125891048124,0.22075260119594,2.08888089449432,0.0367184460768793,0.382205241393276
"1506","CXCL8",392.612188378483,-1.85840810576091,0.89019708092214,-2.08763671055382,0.0368306178713688,0.382490613219261
"1507","PREPL",246.568563765343,0.431464491035343,0.206786686404674,2.08651968140242,0.0369315742996328,0.382523060957786
"1508","AL731577.1",7.43337729425221,2.14422598390173,1.02740927901633,2.08702220983898,0.0368861269537999,0.382523060957786
"1509","ASIC1",39.4848908682932,0.7993416085272,0.383089620397239,2.08656556055561,0.0369274231329297,0.382523060957786
"1510","CELF4",4.1582132476518,2.0770290280062,0.995383435538376,2.08666223874098,0.036918676947523,0.382523060957786
"1511","KIFC3",17.3129028373352,-1.1106453133897,0.532366309991529,-2.0862426726578,0.0369566466215401,0.382529419696153
"1512","C8orf82",165.211676565915,-0.397042717622678,0.190379565289001,-2.08553222096057,0.0370210165425209,0.382942261061526
"1513","PLEKHM1",187.72963662494,0.512950882353383,0.2460180015156,2.08501361361094,0.0370680648155415,0.383175501464024
"1514","GJA9",3.05068168802125,2.78572940748546,1.33744091668593,2.08288035211923,0.0372621309639008,0.383407715970663
"1515","CFAP36",418.843103444078,-0.404193307014857,0.194042047052133,-2.08301918659033,0.0372494747141152,0.383407715970663
"1516","MSX1",715.026874949243,-0.60795676831374,0.291665223987144,-2.08443351594271,0.03712075182927,0.383407715970663
"1517","ZNF76",70.5663083093159,0.529748492503634,0.254283687631279,2.08329719235388,0.0372241425098392,0.383407715970663
"1518","MEG3",5412.16513938761,0.754421486715474,0.362196670594983,2.08290563653217,0.0372598257461263,0.383407715970663
"1519","GOLGA6L4",52.5419981861007,0.726209132440651,0.348585737565698,2.08330133502316,0.0372237651359857,0.383407715970663
"1520","SPECC1",9.21633975720584,-1.21026656994386,0.581048400219099,-2.08290147513958,0.037260205138171,0.383407715970663
"1521","PINK1",600.945095583605,-0.30741071103984,0.147670453329812,-2.08173472829571,0.0373667066497174,0.383764701660352
"1522","LINC02482",41.6373759979577,0.857326039938424,0.411781139368311,2.08199443338662,0.0373429781863914,0.383764701660352
"1523","ZNF594",14.5169798283533,1.00305826295968,0.481847143221387,2.08169390868178,0.0373704373803527,0.383764701660352
"1524","MAP3K13",163.34012177712,-0.323739889580175,0.155595628233365,-2.08064900830392,0.0374660445971525,0.384494053477339
"1525","LRRC73",4.68843737527939,-1.40331718744499,0.674686765574951,-

2.07995363040673,0.0375297861938922,0.384643418133993
"1526","ZNF134",49.6891445410848,0.581073814404901,0.279341519063021,2
.0801555613858,0.0375112667529265,0.384643418133993
"1527","CXCL12",1967.31165287208,0.604305268872913,0.290750930938486,2
.07842935161836,0.0376698316410409,0.385573407634738
"1528","ARHGAP33",18.2234178838971,0.835054323580613,0.40174846940955,
2.07855010576118,0.0376587209758588,0.385573407634738
"1529","LINC02762",271.88656505132,-0.680088953626714,0.32726291071949
8,-2.07811191354228,0.0376990526230593,0.385620132782634
"1530","LUM",9517.19352669255,-0.668262702525814,0.321658982784599,-2.
07755025754502,0.0377508017333161,0.385897084385009
"1531","KAT6B",435.325518080479,0.387923382070371,0.18683420965311,2.0
7629739109673,0.0378664544572572,0.386184885831721
"1532","FGFR2",6.1768879505551,-1.49912246666453,0.721967060211889,-2.
07644164018308,0.0378531234187449,0.386184885831721
"1533","GLDN",2.52548470392611,-2.11270367616579,1.01772486745205,-2.0
7590847362814,0.0379024168639189,0.386184885831721
"1534","TLE3",201.32094650823,0.388112206484355,0.186876935907747,2.07
683310195084,0.037816965891917,0.386184885831721
"1535","PSMD7",657.958867440698,-0.414541580663969,0.19966753155942,-2
.07615919036192,0.0378792302721315,0.386184885831721
"1536","LRIG2",75.6818438270171,0.547878742275032,0.264232520208392,2.
07347203835068,0.038128370749806,0.38622261562627З
"1537","NR2F1-
AS1",18.421823544174,0.858573568872301,0.413991128373118,2.07389364174
609,0.0380891896899898,0.386222615626273
"1538","NR2F1",12.2994417902471,0.975336268625972,0.469976952082461,2.
07528531836353,0.0379600993257066,0.386222615626273
"1539","MAN2A1",268.572224141496,0.511403266432008,0.246459734411203,2
.07499723090168,0.0379867914119204,0.386222615626273
"1540","ELP1",79.7480024192636,0.420543853769658,0.202713878707518,2.0
7456863067789,0.0380265319259907,0.386222615626273
"1541","TIMM10",304.838442002005,-0.511974326875268,0.24685095405606,-
2.07402207065807,0.0380772611530443,0.386222615626273
"1542","SP1",242.871772477618,0.399188584891774,0.192507507944598,2.07
36260582972,0.0381140531728589,0.386222615626273
"1543","LINC02289",5.87190961843896,1.85078603005733,0.89234950775535,
2.07405956295405,0.0380737794503148,0.386222615626273
"1544","KATNB1",54.0782508039175,-0.530308933910472,0.255638669083108,
-2.07444724936379,0.0380377930225063,0.386222615626273
"1545","YARS",108.78894382336,-0.454156338877452,0.21917655956736,-2.0
7210269097173,0.0382558654395866,0.386762595652964
"1546","RUNX2",13.1190148384675,-1.08009968409875,0.52115423771063,-2.
0725144418733,0.038217490837681,0.386762595652964
"1547","EIF4EBP2",556.262010631894,0.344810302479465,0.166391990658551
,2.07227704359305,0.0382396120246546,0.386762595652964
"1548","AC010680.3",5.30386660457265,1.61947403189636,0.78183912441478
8,2.07136478761989,0.038324718951718,0.387208400778339
"1549","RNF19B",100.53634610079,-0.64831293179505,0.313121400984768,-2
.07048425868083,0.0384070186430644,0.387678651557093

"1550","HSF4",32.5430246711202,0.779213999023053,0.37637068980953,2.07
03365594632,0.038420838229763,0.387678651557093
"1551","CHI3L1",78.2127768690132,-2.24304352057393,1.08400475832601,-2
.06921925696874,0.0385255164520993,0.387797545086132
"1552","EHD4",221.777947764054,0.412458689123166,0.199337042275927,2.0
6915224794111,0.0385318021140569,0.387797545086132
"1553","ZNF667",38.9753920496317,0.625481470766876,0.302209370330541,2
.0696958207575,0.0384808383717273,0.387797545086132
"1554","PCMTD2",292.72066624096,0.443331016834881,0.214235251595237,2.
06936539870891,0.0385118109103938,0.387797545086132
"1555","GSTM3",760.224814751674,0.691430140733927,0.334458653410302,2.
06731126159772,0.0387048337730399,0.388787265105113
"1556","C5orf38",169.751273021531,-0.912433546625326,0.441344586538332
,-2.0673948983536,0.0386969585826829,0.388787265105113
"1557","GRK6",64.0560327077134,-0.550473805004217,0.266309078049804,-2
.067048592693,0.0387295753857907,0.388787265105113
"1558","LINC00632",380.090105974733,0.441845173922654,0.21373282497666
8,2.06727803261332,0.0387079629736874,0.388787265105113
"1559","ARHGAP32",170.900250968229,-0.54272810891219,0.262640469454159
,-2.06642986147615,0.038787908745649,0.389123087095542
"1560","TWF2",441.411669442385,-0.535989668656842,0.259498683503854,-2
.06548126341027,0.0388774865662515,0.389566174990454
"1561","USP3",210.463238782384,0.437550055481028,0.211871077477698,2.0
6517123851926,0.0389068008526272,0.389566174990454
"1562","SGSM2",120.589795680385,0.52161214809599,0.252551931660722,2.0
6536590184043,0.0388883923431875,0.389566174990454
"1563","STMN1",66.4259835100337,-0.854329434150981,0.413822703292584,-
2.06448178737779,0.0389720589594915,0.389725522565457
"1564","BTN3A1",84.2933820028068,0.534528890676202,0.258917391459231,2
.06447657943583,0.0389725522565457,0.389725522565457
"1565","FAM149B1",93.3302763045874,0.431787743352606,0.209272408500249
,2.06328080441666,0.039085956750509,0.390609816982723
"1566","AC004889.1",101.632218369369,0.667138683730998,0.3233864875530
08,2.06297637473688,0.0391148728638607,0.390649177261036
"1567","PAX8",15.7792366246233,-1.18533058315457,0.575107897298043,-2.
06105774016226,0.0392975318638454,0.392222972782732
"1568","GLTP",426.392950898289,-0.310321978465244,0.150585049648174,-2
.06077548329185,0.0393244644669001,0.39224146955505
"1569","FILIP1",642.091390730848,0.33907705316378,0.164579346278434,2.
06026491677839,0.0393732219095319,0.392477495643773
"1570","TPPP",34.6966752085449,-1.38262219104218,0.671204202378583,-2.
05991289408277,0.0394068688175284,0.392562693188627
"1571","AC002429.2",2.94166747674218,2.67715665374066,1.30031427759934
,2.05885354014821,0.0395082709059709,0.393137195939417
"1572","CYP3A5",86.2225687997079,-0.718125567410804,0.348810328007601,
-2.05878527597714,0.0395148127887957,0.393137195939417
"1573","BASP1",2900.07135664621,-0.480158555040769,0.233263209292393,-
2.05844100532328,0.0395478189010589,0.393215440313134
"1574","MCFD2",456.659650504022,0.327026893058287,0.158982844409744,2.
05699485546658,0.0396867207306028,0.393572739844029

"1575","DANCR",170.305571283436,-0.516521764418099,0.251165959235203,-2.05649589614334,0.0397347414458901,0.393572739844029
"1576","AC083862.2",17.2096082808695,0.862276937358531,0.419182542983455,2.05704400574851,0.0396819930876418,0.393572739844029
"1577","LINC00968",121.790304560864,0.763867886001303,0.371416275604049,2.05663546854266,0.0397213037900225,0.393572739844029
"1578","HMCN2",392.754032016052,-0.948508662931545,0.461080866959249,-2.05714166624784,0.0396726007862556,0.393572739844029
"1579","IL17D",216.487623827192,-0.356232140686359,0.173191558217232,-2.05686780783819,0.0396989433396929,0.393572739844029
"1580","IRX2",90.2048109144026,-0.710118265172127,0.34542164030272,-2.05580132313018,0.0398016704519789,0.39398615561326
"1581","N4BP2L2",1031.29497735757,0.293861165805742,0.142973033501853,2.05536078103801,0.0398441706161527,0.394157386740435
"1582","CASD1",133.95068462204,0.408642120796987,0.198854539272979,2.05498009897586,0.0398809269456583,0.394271616580338
"1583","AP000695.2",8.09563448054552,-1.20848953549144,0.588289568484482,-2.05424267271078,0.0399522101581371,0.394726826830868
"1584","DUSP23",142.27721782981,-0.572542114653289,0.278793536675371,-2.05364199429042,0.040010354516649,0.394802488732108
"1585","C11orf80",17.3877294551672,-0.868733956228663,0.423004378131274,-2.05372332094176,0.0400024780752982,0.394802488732108
"1586","RNF217",101.127237850699,0.57860952618235,0.281789130023761,2.05334224969416,0.0400393959770541,0.394829681329597
"1587","SLC25A37",433.644962129541,0.386106053252781,0.188060712942143,2.05309257426651,0.040063600017268,0.394829681329597
"1588","SEMA6C",49.4120412742287,0.636584813370229,0.310173650083458,2.0523497505315,0.0401356842560622,0.39529099607356
"1589","ANKRD33B",45.6170419844474,0.600116216693846,0.292472805820909,2.05187013886453,0.0401822845950203,0.395358426670429
"1590","TNKS1BP1",528.544968968225,0.380525534167953,0.185485853192329,2.05150704282223,0.0402175944819137,0.395358426670429
"1591","SLC25A18",3.39867301801358,1.98085484239767,0.965564566217511,2.05149910394646,0.0402183668051568,0.395358426670429
"1592","TRIM4",144.088916071188,0.345892574182223,0.168713526057861,2.05017690202028,0.0403471711279727,0.395450776633329
"1593","EIF5AL1",29.604212699399,-0.811210153131913,0.39550703763382,-2.05106376358079,0.0402607375899726,0.395450776633329
"1594","CBX1",470.7345500564,0.329709168749436,0.160845829984777,2.04984592252494,0.0403794686881986,0.395450776633329
"1595","AC009271.1",4.19018975885676,1.98538294507475,0.968238291272007,2.05051066764411,0.0403146238784235,0.395450776633329
"1596","ETS2",61.9214186352677,-0.83341539807794,0.406543988630714,-2.05000054455356,0.0403643776717034,0.395450776633329
"1597","LCA5L",5.82625027372494,-1.42685114623576,0.695807097271736,-2.05064184000199,0.0403018386646314,0.395450776633329
"1598","ZNF703",259.930048461709,0.575481116953352,0.280806327144272,2.04938799921585,0.04042418979396,0.395641006494077
"1599","ZDHHC17",203.228471299367,0.40256339969011,0.196491134360657,2.04876113622109,0.0404854777541282,0.395993040697038

"1600","LINC01220",5.11931591881121,1.72733711709003,0.843413366244032,2.04803147095281,0.0405569158228299,0.396443852168162
"1601","ERCC6",138.831793675317,0.514917966895886,0.251582707614448,2.04671446530817,0.0406861281283051,0.396797072056894
"1602","LINC00173",32.7531897643238,1.11436476431765,0.544405740560945,2.04693793854825,0.040664178469851,0.396797072056894
"1603","AL161772.1",117.571252937219,-0.462007129968542,0.225797488750191,-2.04611279127058,0.040745274790497,0.396797072056894
"1604","ALYREF",181.078031177318,-0.529973430199199,0.258975983706918,-2.04641921854409,0.0407151428177636,0.396797072056894
"1605","SLC17A9",3.02962856017629,-1.74652093312873,0.853490258978699,-2.04632790445511,0.0407241200476853,0.396797072056894
"1606","XIST",2684.53658089759,0.562441072875943,0.274792757627526,2.04678273813285,0.0406794212706449,0.396797072056894
"1607","WDR86",145.144617828374,0.913855911911748,0.4467099726459,2.04574772866364,0.0407811972321347,0.396899766465829
"1608","ESRRG",11.1367619469895,1.12613916342845,0.55065063669578,2.04510644023936,0.0408443654971121,0.397020432799772
"1609","MTUS1",598.00278711825,0.684205867047007,0.334516685519103,2.04535646999269,0.0408197271827118,0.397020432799772
"1610","ARL10",60.6790474186963,0.651530169200614,0.318629484997963,2.04478932389066,0.0408756327550465,0.397077575334737
"1611","AGAP1-IT1",2.63511260004091,2.81848713341994,1.37891769948945,2.04398502859416,0.0409550261958894,0.397229834596749
"1612","GNL3",323.960232594147,-0.416616261856362,0.203916114235818,-2.04307670052289,0.0410448459728667,0.397229834596749
"1613","GTF2E1",40.7308566407392,0.625496087518865,0.306061673437743,2.04369296061533,0.0409838890753017,0.397229834596749
"1614","HTRA3",4367.72788169966,-0.5492023886988,0.268827209142297,-2.04295685117236,0.0410567096977798,0.397229834596749
"1615","POLD4",865.471815674214,-0.444630002060146,0.217681076785069,-2.04257535210173,0.0410944931187685,0.397229834596749
"1616","NUMB",279.024493741069,0.407373678861357,0.199395730344846,2.04304113311164,0.0410483664396263,0.397229834596749
"1617","ZSCAN30",80.9421075597844,0.53295653843696,0.260718799964708,2.04418146489284,0.0409356235645746,0.397229834596749
"1618","CYTH4",8.31365797384437,-1.38393472059448,0.677514391954005,-2.04266468289051,0.0410856432149751,0.397229834596749
"1619","CLCA2",14.7127970339765,1.82983652924776,0.896030508921661,2.04215873346756,0.0411357884049965,0.397383403739435
"1620","ACTR1B",157.437183812447,-0.375510729229574,0.183969966776445,-2.0411523457298,0.0412356867192815,0.398102555734298
"1621","LIPH",4.5806867228182,1.8741711544658,0.918659723971177,2.04011464262757,0.0413389086414578,0.398116090611084
"1622","NRM",82.5951648046471,-0.429841915273611,0.210671354913034,-2.04034343183986,0.0413161318408359,0.398116090611084
"1623","FRY",18.3485970688358,-0.952489798300218,0.4668016322828,-2.0404594423594,0.0413045866322534,0.398116090611084
"1624","LTBP2",683.064025276303,0.615382971210114,0.301635756480152,2.

04015259460994,0.0413351296476133,0.398116090611084
"1625","AL033527.4",4.48097163664336,1.72698215330341,0.84766466677700
8,2.0373412045942,0.041615861382749,0.400044297496124
"1626","PDK1",60.2637423053108,0.738181296322574,0.362309371044347,2.0
3743362804773,0.0416066068294508,0.400044297496124
"1627","PYROXD2",77.7288223346108,0.54079706986872,0.265389200085274,2
.03775085683574,0.0415748553021021,0.400044297496124
"1628","RRS1",54.6829685525985,-0.599375200979987,0.294260379387095,-2
.0368872025123,0.0416613468702462,0.400235543642906
"1629","ARL6IP5",2967.746104139,-0.348745900276661,0.171286705928398,-
2.03603600399932,0.0417467400455576,0.400809707988042
"1630","HLA-
DRB1",219.028964911435,-0.718238028886661,0.352885261872224,-2.0353301
9507833,0.0418176598995501,0.40117164001296
"1631","CHMP4B",484.862765924673,-0.276414425215763,0.135820143797224,
-2.03515043857149,0.0418357381624768,0.40117164001296
"1632","APPBP2",283.447625484281,0.319185685109585,0.156865540219015,2
.03477248517385,0.0418737708311283,0.401290303798313
"1633","TFPI",1564.22967722954,0.412097375792906,0.202556105896987,2.0
3448508238248,0.0419027111633377,0.401321740719291
"1634","ITGB1BP1",1229.36611090381,-0.528371024729888,0.25979169285590
2,-2.03382571213683,0.0419691711339236,0.401325871185484
"1635","RANBP10",55.2031859951264,0.644550050102553,0.316897776027385,
2.03393680505619,0.041957967489454,0.401325871185484
"1636","APCDD1",229.944611615624,1.55609422464792,0.765147814682705,2.
03371713907752,0.0419801230984304,0.401325871185484
"1637","ARHGAP29-
AS1",2.50188830402356,2.5185241538582,1.23870338566536,2.0331938888706
5,0.0420329382323091,0.401585310906118
"1638","RGS3",2.71444036130062,-2.46402642531914,1.21279951076623,-2.0
3168487738126,0.04218556783579,0.402797485318532
"1639","RPRD2",234.088577298345,0.388048052366381,0.191024145016502,2.
03140839778583,0.0422135832484065,0.402819061626039
"1640","AL133445.2",2.67830720811834,2.68447288710469,1.32166316896709
,2.0311324020648,0.0422415653282744,0.402840293740373
"1641","URGCP",179.230516752942,0.397362350593708,0.195724392384278,2.
03021373960145,0.0423348178373262,0.403095418277448
"1642","AL158206.1",41.1264937129191,0.762868596805358,0.3757774829926
66,2.03010726116404,0.042345637610604,0.403095418277448
"1643","SYK",6.02683187228997,-1.60494800802896,0.790379537871823,-2.0
3060419852271,0.042295161485015,0.403095418277448
"1644","BATF2",7.63261149574509,1.39561489105722,0.687842437667292,2.0
2897468174575,0.0424608691532041,0.403946468099825
"1645","DZIP3",110.892363756706,0.399349015025548,0.196937043592323,2.
0278003962131,0.042580623915778,0.404839488171896
"1646","KCTD7",75.2413534562013,0.604877032777932,0.298449697335813,2.
02673026033372,0.0426900060259222,0.405078460317252
"1647","AL133215.2",6.58931423794644,-1.38380267981514,0.6826894845639
27,-2.02698695542243,0.0426637467392884,0.405078460317252
"1648","RIPK3",6.3181167064914,-1.40784983450269,0.694475765457599,-2.

02721232982844,0.0426407027441865,0.405078460317252
"1649","MYO9A",522.494108136155,0.446570562294561,0.220360963491735,2.02654115873527,0.0427093594030146,0.405078460317252
"1650","KIRREL3",8.14177083737965,-1.49520960573481,0.738444376182756,-2.02481006553804,0.0428868711143116,0.406515554077475
"1651","CYP51A1",221.510774122359,-0.281210358405303,0.138935455681884,-2.02403595990056,0.0429664519100617,0.406773966545264
"1652","MEX3B",112.204171531402,0.591677672751329,0.292361754848428,2.02378615854895,0.0429921589961203,0.406773966545264
"1653","GDE1",310.832688283369,-0.319981371112225,0.158076626134116,-2.02421685569595,0.0429478440152363,0.406773966545264
"1654","PNPLA4",65.0113192004652,-0.551582599197885,0.272614590150077,-2.02330549841163,0.0430416603619789,0.406996111282557
"1655","AHCY",120.692768274644,-0.392635084544341,0.194097938240601,-2.02287096969385,0.043086452279322,0.407173482567127
"1656","TEX261",173.677147279305,0.331949087224415,0.164118719214142,2.02261563345061,0.0431127911205168,0.407176360582659
"1657","ESRG",23.7520324065523,0.91043795398069,0.450190589334281,2.02233892833478,0.043141349598859,0.407200185712827
"1658","LINC01232",13.6909804282133,1.04697124424764,0.51800308404438,2.02116797466391,0.0432623796793138,0.407849748175647
"1659","SLC35B1",191.568596461996,-0.633636653530672,0.313539241103616,-2.02091658862334,0.0432884003818142,0.407849748175647
"1660","FBXO27",11.6478749787259,1.15486167864214,0.571324794411082,2.02137503910112,0.0432409565866575,0.407849748175647
"1661","ILF2",489.634885269961,-0.503587951760918,0.249275125951931,-2.02020939649642,0.0433616720259012,0.408048465995845
"1662","UBTD1",47.8005523621582,-0.711778868071701,0.35229735502018,-2.02039231328015,0.0433427101165625,0.408048465995845
"1663","PLB1",2.54631824894867,-2.06660310656859,1.02328955614607,-2.01956825822777,0.0434281904127909,0.408232885412162
"1664","BYSL",38.7117045686345,-0.724752071810429,0.358873897633466,-2.01951737529446,0.0434334732305522,0.408232885412162
"1665","FMNL2",238.718332904905,0.568257605790257,0.281566456578566,2.01820065037362,0.0435703684517758,0.409273611162627
"1666","HERPUD1",1125.42584251459,-0.428569348633628,0.212683444256384,-2.01505740200915,0.0438986349097151,0.412109633846305
"1667","INTS6",746.779343703116,0.582326343658347,0.289046482947108,2.01464601029207,0.0439417529213666,0.412227914641932
"1668","TAOK1",588.027824973613,0.480632839234375,0.238594439479413,2.01443436939714,0.0439639489528608,0.412227914641932
"1669","SLC40A1",66.0130762706237,-0.658993194614846,0.327333700498088,-2.01321524063085,0.044091990579678,0.4131807864986
"1670","HLTF",192.561982342741,0.347108253080282,0.172461507823146,2.01267087051233,0.0441492658067535,0.413469770788997
"1671","MRGPRF",296.75619535061,-0.476499273019719,0.236874319362544,-2.01161220980828,0.0442608313112193,0.414266547999683
"1672","ERO1B",69.66397929351,0.7539946135809,0.374958091477133,2.01087703057845,0.0443384470770443,0.414372089764177
"1673","SH3BP4",68.4071523410406,-0.677942176361019,0.337200449114209,

-2.01050199708187,0.0443780850610611,0.414372089764177
"1674","CALCOCO1",374.704761701638,0.400652546583284,0.199270054872947
,2.01060087447026,0.0443676316254386,0.414372089764177
"1675","SPPL3",301.528375410528,0.287000655096105,0.142705096471234,2.
01114509707758,0.0443101329466325,0.414372089764177
"1676","FAM182A",39.2838865769161,-0.618644588576811,0.307746551635041
,-2.01024052191645,0.0444057385434338,0.414382906216769
"1677","LRRK2",180.043748084327,0.475438001192705,0.236651210834021,2.
00902416479145,0.0445345710889948,0.415337323692235
"1678","VSIG10L",6.79088490147775,-1.27617168103638,0.635310581899508,
-2.00873669886116,0.044565064563589,0.41537402251164
"1679","MBD5",158.165673512968,0.476399595209342,0.237223254568983,2.0
0823311388643,0.0446185257196816,0.415624623142239
"1680","CPZ",4.49147317027312,1.87480035779726,0.933694559838986,2.007
93754021718,0.0446499293476781,0.415669580355765
"1681","CD70",446.988312034349,-0.790958744797053,0.393990761045406,-2
.0075565800029,0.0446904324868778,0.415799145802955
"1682","AL117332.1",13.2762006949683,-1.00752216993209,0.5019403219955
97,-2.00725489820466,0.0447225288450724,0.415850387120649
"1683","TEX26-
AS1",50.5911167878621,-0.810735538016801,0.403988242146772,-2.00682953
966332,0.0447678163821909,0.416024152238542
"1684","TRPS1",75.7113139116651,0.774877078002546,0.386273942486467,2.
00602989944032,0.0448530580628207,0.416568781533561
"1685","LAMA2",788.118737266872,0.496174801725147,0.247449774875618,2.
00515357904267,0.0449466310184944,0.417190094438725
"1686","FHL3",158.296909777564,-0.569129904333584,0.284048439338932,-2
.0036367939853,0.0451089814546075,0.417705429218509
"1687","AC011379.1",19.5990386687536,-0.83693054789766,0.4176741545697
09,-2.00378821323017,0.0450927519769476,0.417705429218509
"1688","PCBP2-
OT1",11.3644696954234,1.22052646744422,0.608960657160476,2.00427803191
,0.0450402857544902,0.417705429218509
"1689","AC116407.2",19.3879255490314,-0.874865190911907,0.436617882299
643,-2.00373192757026,0.0450987842339249,0.417705429218509
"1690","SMYD2",66.7588355430553,-0.635805690953976,0.317597745688398,-
2.0019212969407,0.0452931967585416,0.419163075327569
"1691","LPP",1852.32957444131,0.464096193561853,0.232006948241653,2.00
035471816327,0.0454619743278231,0.419588910476721
"1692","CAMK2D",344.196348579841,0.338247170495445,0.169076337348189,2
.00055889428733,0.0454399471309717,0.419588910476721
"1693","SERGEF",110.351497578725,-0.517279390734518,0.258517546444864,
-2.00094499521657,0.0453983178702062,0.419588910476721
"1694","CD27-
AS1",220.86848497598,0.408636913881027,0.20429299170666,2.000249301100
75,0.0454733505919465,0.419588910476721
"1695","RERG",419.154544166499,-0.638203856729871,0.319012372845317,-2
.00056145483524,0.0454396709476972,0.419588910476721
"1696","AL354822.1",18.7080468430472,1.2562130939964,0.628222988431822
,1.99962929903628,0.0455403077445371,0.419958970002689

"1697","FAM229A",22.879607974219,0.69795444021236,0.349182204535274,1.99882591709181,0.0456271925756439,0.420101558695535
"1698","PHLDA3",236.479530967328,-0.580918734584458,0.290640693658672,-1.99875223001872,0.0456351687361221,0.420101558695535
"1699","ZNF549",17.3707862149813,0.914925185621529,0.457750678721615,1.99874129772279,0.045636352188217,0.420101558695535
"1700","BTBD8",79.4941468258707,0.67352572760635,0.337277482546318,1.9969483954917,0.0458307891441432,0.42114779213537
"1701","AREG",82.6749317174099,-0.888497042993908,0.44489436865457,-1.99709662696083,0.0458146872932529,0.42114779213537
"1702","JDP2",1639.58322927135,-0.348450508244658,0.174476990342031,-1.99711439062299,0.0458127580103904,0.42114779213537
"1703","CDK5",38.0090623627389,-0.738930801660354,0.370150959819412,-1.99629578705094,0.0459017363577926,0.421353121983309
"1704","SPG11",214.356983328233,0.392502614757751,0.196620239471349,1.99624726230153,0.045907015336289,0.421353121983309
"1705","EIF1B-AS1",25.8362241785423,0.664623746718031,0.333014533486167,1.99578000323412,0.0459578743473494,0.421564674731671
"1706","FBXL20",254.01960005441,0.491660693381796,0.246379724615582,1.99554039663335,0.045983972831984,0.421564674731671
"1707","ZMAT3",249.981961496139,0.465421401545439,0.233265431121854,1.99524378433215,0.0460162977944592,0.421613882545602
"1708","PELI1",62.1275446971254,-0.771057306824279,0.386620272751106,-1.99435301552503,0.0461134893019092,0.421763141919217
"1709","AK1",394.529727551928,-0.520719170486887,0.261039555349856,-1.99479029064771,0.0460657567688705,0.421763141919217
"1710","AC129507.1",15.7115026866232,1.84240740498832,0.923709850136019,1.99457373407572,0.046089390673166,0.421763141919217
"1711","SLC19A2",190.84253969694,0.885408438475517,0.444239499173802,1.9930880530034,0.0462518059813714,0.422262887610395
"1712","ACADL",23.341753224864,1.04926896345701,0.526642773750106,1.99237322860314,0.0463301224513708,0.422262887610395
"1713","TBC1D5",317.084118719164,0.30453529560838,0.152784612495147,1.99323276496877,0.0462359648507242,0.422262887610395
"1714","PARP8",49.5841530546158,0.573847852736322,0.288021563271126,1.99237809217825,0.0463295892189837,0.422262887610395
"1715","TRIM7",55.7287910153958,-0.632695865285614,0.317497044270481,-1.99276143417137,0.0462875766406255,0.422262887610395
"1716","LY6E-DT",9.42108113317107,1.45192902922246,0.728577922981488,1.99282600175541,0.0462805034778139,0.422262887610395
"1717","FAM174B",20.3015164277123,0.791840853809349,0.397506922623542,1.99201777061694,0.0463691081262794,0.422372074021555
"1718","LAPTM5",48.439341443291,-0.872285392143305,0.438098136333148,-1.99107304916719,0.0464728568979481,0.422820079297248
"1719","TAF1",158.739633229605,0.491937123180224,0.247101313174684,1.99083168300468,0.0464993949099276,0.422820079297248
"1720","HS6ST2",9.42627758954514,2.07466653419364,1.04196859212959,1.99110275478976,0.0464695916703438,0.422820079297248

"1721","SMURF1",64.3553595858556,0.534854133291336,0.26878512876592,1.
98989481206429,0.0466025239853174,0.423511606699805
"1722","PPM1J",3.5598665366007,-1.65846201179684,0.833795486811113,-1.
98905131777542,0.0466955389441826,0.423864323323863
"1723","SLC43A1",113.430683329702,0.465585022963476,0.234072721423256,
1.98906143412411,0.0466944224553264,0.423864323323863
"1724","OSMR",273.994801428193,0.351766302949555,0.17689733908467,1.98
853360242567,0.0467527064998609,0.42396994374398
"1725","GARS",277.141441730574,-0.365952668880866,0.184038697580878,-1
.98845500262271,0.0467613908541154,0.42396994374398
"1726","RTTN",28.7889839634415,0.63693458641818,0.32041547151125,1.987
83967395225,0.046829424360389,0.424340786208855
"1727","CFLAR",796.626925945786,0.50300962205417,0.253160127659882,1.9
8692276980504,0.0469309559145164,0.424768605615183
"1728","EXOSC5",57.2117656205931,-0.569700301290662,0.286698594225046,
-1.9871053181498,0.0469107270345082,0.424768605615183
"1729","AC092687.3",2.73059984844727,-1.88792872634503,0.9505785805043
,-1.98608380734126,0.0470240189479323,0.424999507562278
"1730","FAM184B",10.1587015984566,1.83181545915633,0.922467829871361,1
.98577706434682,0.0470580835593205,0.424999507562278
"1731","KIF1C",77.2477467435362,-0.45896311460103,0.231132613771789,-1
.9857133405422,0.0470651628579197,0.424999507562278
"1732","NANP",36.7672288485544,-0.555883438442257,0.279855229331018,-1
.98632500014766,0.0469972484267038,0.424999507562278
"1733","NQO2",460.57758766119,-0.418859493649443,0.21097096790464,-1.9
8538925905089,0.0471011800606791,0.425079316877681
"1734","HIST2H4B",4.58993434283375,1.94356466527339,0.979892072217865,
1.98344768814627,0.0473174448295148,0.42613785553244
"1735","IL20RA",4.46051645596479,1.56928643909456,0.791186920635598,1.
98345852056537,0.0473162359318239,0.42613785553244
"1736","SORBS3",503.69290391998,-0.294046248438934,0.148284293464104,-
1.98298984720263,0.0473685636211467,0.42613785553244
"1737","NCALD",294.477539930945,-0.372635922350653,0.187820748421404,-
1.98399764393755,0.0472561025836159,0.42613785553244
"1738","MED13",378.604710573511,0.422540434191446,0.213084922903576,1.
98296729976833,0.047371082283422,0.42613785553244
"1739","MED14",85.1116478716411,0.485034945827558,0.24461257926641,1.9
8287000317879,0.0473819520953269,0.42613785553244
"1740","C1orf54",85.7698980505491,0.795549713375906,0.401624294895466,
1.98083065065317,0.0476102682798761,0.427699365822666
"1741","TMEM176A",1046.43522461144,0.71163050714693,0.359216388863304,
1.98106358509643,0.0475841433515538,0.427699365822666
"1742","SOAT1",143.710472566084,0.328873092156674,0.166146381034544,1.
97941772856489,0.0477689940868464,0.42794377266815
"1743","DESI2",419.215572842378,-0.283902211980521,0.143534502721343,-
1.97793705762639,0.0479358077502628,0.42794377266815
"1744","TFDP2",395.991730217904,0.312545132519734,0.157963215845849,1.
97859438886542,0.0478616919214021,0.42794377266815
"1745","AFF1",444.259425567039,0.555609584634249,0.280955645858649,1.9
7757045577856,0.0479771849533226,0.42794377266815

"1746","MTX3",115.603285988093,0.51474334835702,0.260094961092432,1.97905928740423,0.0478093315501374,0.42794377266815
"1747","CD83",63.8396829621845,-1.09683450666045,0.554196081077178,-1.97914518725676,0.0477996621340132,0.42794377266815
"1748","BICRAL",152.013812402819,0.444591822285517,0.224731689811849,1.97832278419541,0.0478923043796067,0.42794377266815
"1749","ELMO1",111.984795179162,0.718199987512429,0.363197323393446,1.97743744585478,0.0479922047851854,0.42794377266815
"1750","ZNF462",130.744855016454,0.55489613225176,0.280294718059566,1.97968815143294,0.0477385807675596,0.42794377266815
"1751","KLF6",4902.35708714655,-0.498613846155039,0.252069406057557,-1.97808156869774,0.0479195054804354,0.42794377266815
"1752","GNPNAT1",260.773310652986,-0.557823164379879,0.281800063196987,-1.97949978453321,0.047759763877599,0.42794377266815
"1753","ZNF469",33.0304524007514,0.846150203761539,0.427315977101587,1.98015110387595,0.0476865522417524,0.42794377266815
"1754","ZNF260",82.6954860523412,0.440672492181771,0.222878558004657,1.97718657248565,0.0480205448230565,0.42794377266815
"1755","ZNF449",41.9089902942592,0.605118695898369,0.306034065828151,1.97729195362899,0.0480086386761741,0.42794377266815
"1756","PKNOX2",20.9986019532938,0.845103660810692,0.427660339548836,1.97610950246694,0.0481423764206547,0.428541131029619
"1757","NPTN",682.539262416871,-0.340754721590753,0.172429958368384,-1.97619210034695,0.0481330242668736,0.428541131029619
"1758","PTPRU",31.2319821274262,0.911367121885924,0.462214933035108,1.97173880969506,0.0486394308174109,0.428752675116649
"1759","LINC02798",4.9705722672294,1.79110505452431,0.907480348689934,1.9737122209974,0.0484144747590694,0.428752675116649
"1760","LMX1A",59.6022945076116,0.557793607064829,0.282696260401439,1.97311986466585,0.0484819075637498,0.428752675116649
"1761","TRAF5",161.449942584785,0.517853053169879,0.26285824629151,1.97008486694223,0.0488286449787912,0.428752675116649
"1762","CAPN2",1155.80243102702,-0.327701308943563,0.166332375847834,-1.97015949103833,0.0488200945502204,0.428752675116649
"1763","DEGS1",463.40127697716,-0.417174923234865,0.211661028805995,-1.97095764670614,0.0487287204712744,0.428752675116649
"1764","LIPT1",53.5610722226733,0.550655532629526,0.278991485606339,1.97373597775851,0.0484117719749274,0.428752675116649
"1765","SH3RF3",60.2673639017952,0.695621793513177,0.352156438138312,1.9753203922399,0.0482318004975043,0.428752675116649
"1766","DPP4",1022.00382713797,0.412963344350471,0.209565029903141,1.97057373809618,0.0487726529766419,0.428752675116649
"1767","AFAP1",319.648425378697,0.405465867357603,0.205590970509471,1.97219686425346,0.0485871375099877,0.428752675116649
"1768","PDLIM3",218.437721478296,-1.31138435444433,0.665581010246205,-1.97028511068733,0.048805703890368,0.428752675116649
"1769","PDLIM7",578.261381132317,-0.42700190649433,0.216553841177634,-1.97180481386184,0.0486318926094554,0.428752675116649
"1770","ZNF398",60.8305878141905,0.476403933728899,0.241819756635921,1.9700786253216,0.048829360200623,0.428752675116649

"1771","SFRP1",423.546118252012,1.66766185487997,0.845230584318349,1.9
7302592431021,0.0484926088119757,0.428752675116649
"1772","PLAT",518.694384417576,0.640629333197396,0.324922829643812,1.9
7163533845766,0.0486512500414792,0.428752675116649
"1773","PIK3AP1",4.99775733582873,-1.56329822212811,0.792932887604612,
-1.97153913851488,0.0486622408495316,0.428752675116649
"1774","HSPA8",2160.41843963587,-0.417837202323387,0.21151380321168,-1
.97546068378914,0.0482158920936691,0.428752675116649
"1775","AC008014.1",11.9983268282937,1.04122051860407,0.52790313443463
2,1.97237040412572,0.0485673378519164,0.428752675116649
"1776","FMNL3",26.8257537548599,-1.07393162050867,0.544829532726121,-1
.97113327380606,0.0487086336981545,0.428752675116649
"1777","KPNA3",224.084727502087,-0.310696960190609,0.15731323001051,-1
.97502117380624,0.0482657451965291,0.428752675116649
"1778","RBM26",322.090419461313,0.448745337165917,0.227802744558166,1.
96988556058128,0.0488514876635466,0.428752675116649
"1779","SLC9A5",3.28385547362064,-1.62769173551719,0.824591336101831,-
1.9739374696949,0.0483888535238748,0.428752675116649
"1780","PLIN3",475.685355092304,-0.400190162789314,0.203122621959722,-
1.97019002082727,0.0488165968094824,0.428752675116649
"1781","DPY19L3",143.956036115582,0.415538433335261,0.210728573818922,
1.97191309087647,0.0486195286260968,0.428752675116649
"1782","CDR1",12.2399447373062,1.02013397768286,0.516709194493948,1.97
429035239435,0.0483487372733977,0.428752675116649
"1783","AL160006.1",14.8344598755671,0.828139477968071,0.4205752802547
92,1.96906360608348,0.0489457874287031,0.428793522549583
"1784","RHOU",329.376190714725,0.560213003519421,0.28453250833848,1.96
888927311248,0.0489658076261864,0.428793522549583
"1785","LINC01985",13.8880496278605,-1.01197443740467,0.51379812735474
6,-1.96959541798011,0.0488847572077562,0.428793522549583
"1786","PIM3",107.640493068245,-0.520707476414568,0.264454990802034,-1
.96898336021331,0.0489550019183636,0.428793522549583
"1787","SIX2",84.86112918602,0.701427649422542,0.356377549112767,1.968
21503253728,0.0490433013458394,0.428991741078819
"1788","KLF16",40.0801846353243,-0.772164677363986,0.392276203739222,-
1.96842089834567,0.0490196292873295,0.428991741078819
"1789","RPS6KA4",99.4499506871193,-0.425013306408016,0.215978095018339
,-1.96785376022474,0.0490848664499876,0.429115322122865
"1790","FRMD4A",237.953840186075,0.65446321741702,0.332658053118726,1.
96737524097588,0.0491399665593452,0.429117295917453
"1791","ANKRD27",79.274425602915,0.40251100556557,0.204586139679982,1.
96744024886137,0.0491324780436355,0.429117295917453
"1792","PEG3",74.7724532154199,0.552587467916684,0.280926301467105,1.9
670193393458,0.0491809812555274,0.429235796225697
"1793","GPR137",120.952734629542,-0.543469410981272,0.276485720220353,
-1.96563283828235,0.0493410379935859,0.430392545577068
"1794","SBSN",4.94673831592054,-6.28991701305293,3.20128490446481,-1.9
6481013116965,0.0494362171759987,0.430982406149732
"1795","CLASP1",148.914779497965,0.412558561790327,0.210035410105259,1
.96423337180894,0.0495030344044325,0.431084330782475

"1796","AHSA1",368.98196715723,-0.363563129856002,0.185090251303358,-1
.96424785906272,0.0495013551376443,0.431084330782475
"1797","STRBP",25.6643592727689,0.621703312587326,0.316552539852939,1.
96398143851934,0.0495322444896575,0.431098666565522
"1798","AL157392.3",30.7560738942862,0.83910716331419,0.42745944711782
9,1.96300998602773,0.0496450137084857,0.431839830033769
"1799","DDX21",635.930249107325,-0.327132314564881,0.166738930275526,-
1.96194322480248,0.0497690946384867,0.432678510364609
"1800","STRAP",541.125132202503,-0.429521027292039,0.218955454643923,-
1.96168224258468,0.0497994904958554,0.432702239641766
"1801","SENP7",191.293329659995,0.362339500578164,0.184804166180869,1.
96066738140276,0.0499178364989454,0.433249146971979
"1802","AL590399.1",11.0708799515074,1.17242247831213,0.59793529395344
7,1.96078487115264,0.0499041236088602,0.433249146971979
"1803","NT5E",1380.60351311669,0.306308998554276,0.156318891392397,1.9
5951363156338,0.0500526650602003,0.433736695989624
"1804","LAMA3",59.496116953441,0.866155752201131,0.442034647216969,1.9
5947480057143,0.0500572082008485,0.433736695989624
"1805","LINC00630",21.9228599750611,0.861208853981051,0.43947580666994
4,1.95962744913473,0.0500393506461322,0.433736695989624
"1806","CYBB",11.3129472717002,-1.32643684759649,0.677256927955574,-1.
95854304746736,0.0501663249756914,0.434441485393031
"1807","ARHGEF4",39.1161113367032,0.714261518144062,0.365020921539676,
1.95676871104065,0.050374667304001,0.434801212270737
"1808","FAM126B",136.959129832943,0.453524741305312,0.231648830716893,
1.95781148517681,0.05025213724359,0.434801212270737
"1809","KIF9",58.1490097242213,-0.624181412227173,0.318956078168872,-1
.95695098776798,0.0503532310322625,0.434801212270737
"1810","AC090921.1",5.43041633461592,1.85509986038896,0.94764423429828
8,1.95759103812057,0.0502780197961781,0.434801212270737
"1811","C3AR1",7.08411146903527,-1.16792320114646,0.596688007577804,-1
.9573431782005,0.0503071342184754,0.434801212270737
"1812","WFDC10B",4.35935068893507,-1.62291009050243,0.829310649031997,
-1.95693868443237,0.0503546776995876,0.434801212270737
"1813","ZNF433-
AS1",35.0517161457489,-0.674733318026604,0.344902162578923,-1.95630352
961967,0.0504294086537037,0.435033619053462
"1814","FAXDC2",287.634167752097,0.491136555073077,0.251161939677201,1
.95545772462299,0.0505290685199213,0.435267960083765
"1815","AP003071.4",7.81525634822004,-1.15369917269738,0.5900174528869
13,-1.95536448464772,0.0505400649304423,0.435267960083765
"1816","FBXL19-
AS1",7.84298268722091,1.40414470206702,0.718093769823469,1.95537792009
003,0.0505384802757219,0.435267960083765
"1817","TSPYL2",120.362434596214,0.655558562670765,0.335340947864311,1
.95490162130759,0.050594683170499,0.435498538682776
"1818","B3GNT2",34.9267422381841,-0.648876915477503,0.332273233329384,
-1.95284136785784,0.0508383950059426,0.437115172013712
"1819","GAREM1",57.4107875882377,0.626574647707228,0.320831036065991,1
.95297392481178,0.0508226850145247,0.437115172013712

"1820","TRIM33",202.720768498007,0.416861206690021,0.213569880200985,1.9518726437348,0.0509533268953486,0.437461575402643
"1821","LINC00377",6.54904578359279,1.62331690580073,0.831704782454003,1.95179460314153,0.05096259529307,0.437461575402643
"1822","SNN",48.4924590611438,0.481571480815785,0.246701910987597,1.95203790229252,0.0509337048201017,0.437461575402643
"1823","CORO6",25.1306821886652,0.754878398235944,0.386902920080287,1.95107960952918,0.0510475763970848,0.437950682858149
"1824","HS2ST1",114.591737354528,0.384459194015772,0.197118996333023,1.95039139386772,0.0511294868369939,0.438412924413698
"1825","VCAM1",40.9184313060326,-0.74745892513316,0.383581755391293,-1.94863002378899,0.051339623952048,0.439251487204612
"1826","ARHGAP23",149.82355478519,0.388458089001902,0.199303558620789,1.94907753624717,0.0512861658867901,0.439251487204612
"1827","AC005332.6",226.724012665579,0.42770009869482,0.219477804197995,1.94871686573365,0.0513292465113197,0.439251487204612
"1828","KDELR3",420.792959669644,-0.585145218905596,0.300195329484342,-1.94921493252651,0.0512697624244897,0.439251487204612
"1829","SEC61G",1323.6437125483,-0.469173307635516,0.240829908741684,-1.94815216302206,0.051396758795866,0.43949989478805
"1830","TRIM2",168.45499065779,0.466009493555555,0.239348856652449,1.94698859260578,0.0515361022973894,0.439764978629621
"1831","MSMO1",138.14774636141,-0.542446918551666,0.278630423362703,-1.94683305579141,0.0515547525599342,0.439764978629621
"1832","CUX1",358.016267180554,0.447445790832118,0.229836762281097,1.94679818141921,0.0515589350858902,0.439764978629621
"1833","MVK",22.5493387657974,-0.738956271802853,0.379590483348522,-1.94671969983077,0.051568348517054,0.439764978629621
"1834","COX5A",640.293741350411,-0.491496300936926,0.252445431461301,-1.94694076296751,0.05154183689976,0.439764978629621
"1835","HNRNPAB",654.050315531103,-0.33740476810364,0.173432462085529,-1.94545337156805,0.05172043651569,0.440484074016468
"1836","POU5F1",3.04682065768011,2.19556069232492,1.12842554405224,1.94568503336121,0.051692585556342,0.440484074016468
"1837","A2M-AS1",14.6523237861835,0.877280999208309,0.45097134492802,1.94531428454358,0.051737163936589,0.440484074016468
"1838","CISD1",171.295555931606,-0.569456084826515,0.292885130253244,-1.9442983818746,0.0518594796791729,0.441285235137249
"1839","CDC42SE1",164.985036705202,-0.377021745336806,0.194040732513964,-1.94300310276181,0.052015783427617,0.441589882640783
"1840","ODC1-DT",5.07021955945146,1.44261383550384,0.742787495895823,1.94216225161949,0.0521174612940695,0.441589882640783
"1841","PIK3R4",79.7588385190298,0.417694320095676,0.215137841820954,1.94151952329845,0.0521952936940025,0.441589882640783
"1842","ZNF395",133.091769929466,0.387801290992984,0.199631812865048,1.94258262461975,0.0520666079574831,0.441589882640783
"1843","AL117339.4",5.19020670609616,1.62619718340672,0.837278703073251,1.94224118855254,0.0521079089736087,0.441589882640783

"1844","AL590807.1",6.40260398302634,1.59200538765445,0.819714824657072,1.94214541419386,0.0521194990150505,0.441589882640783
"1845","AC005670.3",121.311607238957,0.538698996373338,0.277412816826544,1.94186772815968,0.0521531151109544,0.441589882640783
"1846","SLC26A11",62.9101196613193,0.495723349265838,0.255223579237494,1.94231015310914,0.0520995646294993,0.441589882640783
"1847","L3MBTL4",47.8876505137661,0.804887188986646,0.414451044551284,1.94205612355997,0.0521303063768367,0.441589882640783
"1848","SIPA1L3",46.5837505481679,0.487361153579877,0.250882986198188,1.94258351658362,0.0520665000990262,0.441589882640783
"1849","ZBED1",222.862100319157,0.41512885710577,0.213826078544921,1.94143230765259,0.0522058627239647,0.441589882640783
"1850","PCDHB13",18.3067661096617,0.960861256400047,0.494996823475323,1.94114630807919,0.0522405334911471,0.441632479625488
"1851","TLN2",388.504929804712,0.448769610844208,0.231214296022205,1.94092501443384,0.0522673733879014,0.441632479625488
"1852","CLCN4",28.9599082983156,0.933722275144102,0.481203915745333,1.94038794072959,0.0523325610510533,0.441773913499707
"1853","USP9X",383.821759072768,0.335623519046821,0.172973130129416,1.94032170658941,0.052340604968987,0.441773913499707
"1854","BLOC1S2",306.035992668998,-0.40280082474744,0.207638838216369,-1.93991079996173,0.052390531329702,0.441956801508381
"1855","SNX7",675.731918168922,-0.346430889919634,0.178623736983452,-1.93944486757506,0.052447191650351,0.442196268146356
"1856","PCBP1-AS1",105.762811946678,0.503419334204377,0.259659722481019,1.93876558672351,0.0525298882783031,0.442196484029317
"1857","SCRN3",66.5748467329732,0.430452641645163,0.222029834865413,1.93871531682258,0.05253601253801,0.442196484029317
"1858","DMRTA1",5.84190975332626,1.57980055093918,0.814953613863287,1.93851591558707,0.0525603109853261,0.442196484029317
"1859","TPM4",2137.72845333213,-0.482356042987366,0.248773441382166,-1.93893705175052,0.0525090036008611,0.442196484029317
"1860","MICU3",131.036794169641,0.429346295999082,0.221531241390398,1.9380846390079,0.05261289721465,0.442400920665122
"1861","NEK6",106.431502469477,0.678459867418036,0.350163967246857,1.93754906523474,0.0526782617768688,0.442695931263139
"1862","TPI1",1253.70832738876,-0.380919807589793,0.196620670474244,-1.93733347908449,0.0527045923281308,0.442695931263139
"1863","TRIM23",162.902568175684,0.357372965532008,0.184582645030229,1.93611357922345,0.052853791756579,0.443710844376219
"1864","CDKN1C",1768.18715853035,0.655823185924488,0.338796821636706,1.93574184892363,0.0528993262072454,0.443854861524313
"1865","HAS1",1146.20907252105,-1.38135342453844,0.713869666954799,-1.93502187931723,0.052987610854698,0.444357229902133
"1866","NBEAL1",376.466098860116,0.384824903843936,0.198943794721095,1.93433981885906,0.053071360506145,0.44451737607979
"1867","ZP3",2.66600230306977,-1.8547884958901,0.959130022488841,-1.93382383243214,0.053134791517778,0.44451737607979
"1868","IDNK",68.5963545433925,-0.56374639766591,0.29153619329026,-1.9

3370981250562,0.0531488167051923,0.44451737607979
"1869","TNFSF12",627.782084914663,-0.411003113388563,0.212542618665714
,-1.9337444695503,0.0531445533377206,0.44451737607979
"1870","C19orf54",19.7528599299926,0.748876298565964,0.387199262599261
,1.93408503295893,0.0531026738204943,0.44451737607979
"1871","GLIPR2",1349.4936872914,-0.343181568058253,0.177563267357438,-
1.93272839121293,0.0532696657893728,0.445289990884976
"1872","ZKSCAN1",507.085682502617,0.437437450576661,0.226407967047723,
1.93207622629489,0.0533500981689448,0.445403144512323
"1873","TMEM245",261.523137520262,0.448116912653993,0.231929901317507,
1.93212220635808,0.053344424063895,0.445403144512323
"1874","GREB1L",19.8448355711669,1.02767015340046,0.531940738221255,1.
93192602024967,0.0533686376480878,0.445403144512323
"1875","LSM10",337.863590334036,-0.43667827346703,0.226273752606333,-1
.92986711201433,0.0536233053315546,0.447289864205608
"1876","LYPLA1",175.559962687864,-0.328146631480845,0.170075570078396,
-1.92941661950383,0.0536791621306935,0.447517108594907
"1877","AC005332.7",13.2458313395352,0.880755447857295,0.4567635600251
09,1.92825243723225,0.0538237347141088,0.448483330276325
"1878","LMCD1",490.89037018349,-0.590784258764126,0.306579362829009,-1
.92701900516907,0.0539772614827958,0.448959360354859
"1879","PDHX",66.9098307452324,0.41864679721207,0.217267664137674,1.92
687116545235,0.0539956877766937,0.448959360354859
"1880","RAD9A",63.3362091697411,-0.559920825739941,0.290562872835559,-
1.92702123390975,0.0539769837394803,0.448959360354859
"1881","HOXB7",42.4953956864929,-0.9254549027495,0.480226752778439,-1.
92712067246382,0.0539645930254254,0.448959360354859
"1882","ANKRD28",405.597680600572,-0.491474656509188,0.255253293182355
,-1.92543904284939,0.054174455101125,0.449254091685521
"1883","PARP15",16.867299785398,1.00856221406657,0.52381212469785,1.9
2542739374021,0.054175911245088,0.449254091685521
"1884","YEATS2",65.9420429215523,0.554146252908105,0.287754976158839,1
.92575732418341,0.0541346824242272,0.449254091685521
"1885","TGFBI",53.3518388323273,-0.850957887444869,0.441933110215592,-
1.92553548891084,0.0541624005539672,0.449254091685521
"1886","SOCS7",44.8099502877794,0.614333998562509,0.319100252389173,1.
92520687139185,0.0542034828011878,0.449254091685521
"1887","ZNF516",60.1872169666861,0.556245823865255,0.288882036195426,1
.92551198818385,0.0541653376435989,0.449254091685521
"1888","FAM136A",230.798986377966,-0.333939455643974,0.17352115173315,
-1.92448846903416,0.0542933847148308,0.449369854513233
"1889","BATF",4.5518624918594,1.79795922989032,0.934119441257481,1.924
76374056616,0.0542589221439237,0.449369854513233
"1890","AC139099.2",14.1342838596915,-0.789700874497814,0.410360732311
654,-1.92440653385438,0.0543036461016631,0.449369854513233
"1891","GAB2",62.2334786378386,0.524154543259892,0.272716390552989,1.9
2197668133206,0.0546086919767982,0.450939779576095
"1892","FLVCR2",33.1845743252133,0.675836126306159,0.351631511977724,1
.92200102460946,0.0546056288267894,0.450939779576095
"1893","DLGAP1-

AS1",90.2548110192074,0.513095965845054,0.266953062146344,1.9220456274
9602,0.0546000167525395,0.450939779576095
"1894","ADAMTS10",79.8473200318394,-0.461633953563985,0.24011720731228
7,-1.92253590957187,0.0545383596355108,0.450939779576095
"1895","GALT",107.591631337908,0.454185326359596,0.236383991258825,1.9
2138783993326,0.0546828304205073,0.451313703312261
"1896","AL096803.2",19.7950349076398,0.937152700270225,0.4880446965036
89,1.92021900244774,0.0548302423398984,0.451725452363236
"1897","IQSEC1",471.130326428624,0.476361583981781,0.24815728479665,1.
91959540648638,0.0549090247314817,0.451725452363236
"1898","RBMS3-
AS3",18.7207695417678,0.835072236349986,0.435029748092692,1.9195750175
964,0.0549116021674606,0.451725452363236
"1899","SELENOS",1443.44041585523,-0.278201919560244,0.144850154386713
,-1.92061873001195,0.0547797920960655,0.451725452363236
"1900","VASN",1351.23579870742,-0.534993451559944,0.278695874108977,-1
.91963176085896,0.0549044292893101,0.451725452363236
"1901","TUFM",742.123238806607,-0.464015814993607,0.241751613382678,-1
.91939076848723,0.0549348983628437,0.451725452363236
"1902","ZNF529",49.4846743158666,0.584853395873155,0.304567196480977,1
.92027704437856,0.0548229143730275,0.451725452363236
"1903","AL008633.1",2.62998041081139,2.38733852356566,1.24446401637862
,1.91836685685199,0.055064510486183,0.452553307411404
"1904","POLR2A",432.811901406783,0.34683970307933,0.180821931689329,1.
91812851372054,0.0550947177653333,0.452563753072381
"1905","USP51",63.775699103863,0.461226672398675,0.240494380717255,1.9
1782723165133,0.0551329216051897,0.452639839320297
"1906","MMACHC",13.7340565018755,0.94714530430625,0.494075119068393,1.
91700668127581,0.0552370828505644,0.45325707019036
"1907","SUV39H2",13.4807570543693,-0.806966714676289,0.421092427940347
,-1.91636481953222,0.055318675525352,0.453688560679866
"1908","CELF1",410.659006689465,0.47157760292346,0.246188753812473,1.9
1551236854089,0.0554271933128367,0.454102306659385
"1909","PRPS2",106.643322244886,-0.39800639414721,0.207769670419187,-1
.9156135414,0.0554143046450187,0.454102306659385
"1910","ZNF783",55.9911575157617,0.577722295143982,0.301670074091346,1
.91507989940377,0.0554823148212496,0.45431591822217
"1911","PABPC4L",8.28343676857303,1.46147887113813,0.763504625128483,1
.91417160163527,0.0555982330618777,0.454491560739237
"1912","AC108863.2",62.754622806929,0.802378337441955,0.41926367030399
,1.9137797865009,0.0556482993213808,0.454491560739237
"1913","PRXL2A",92.4181226657677,0.711807042258142,0.371973127909336,1
.91359802321967,0.0556715378409169,0.454491560739237
"1914","RHNO1",39.1681453242978,0.557247477824773,0.29111051205799,1.9
1421283238913,0.0555929667664713,0.454491560739237
"1915","NEO1",117.072314736256,0.549025280903351,0.286915040193646,1.9
1354653465639,0.0556781221468273,0.454491560739237
"1916","TRMT1",63.6420080986948,-0.428895253597364,0.224115016503098,-
1.91372831811757,0.0556548787576263,0.454491560739237
"1917","SH3GL1",464.493601315153,-0.348003135524326,0.181912208717955,

-1.91302792691549,0.0557444773689225,0.454795840401642
"1918","PAM",1762.97107332548,0.326993123887931,0.170988299108368,1.91
237134700481,0.055828580445872,0.455244524595119
"1919","STRADB",60.6467577546281,0.510282014427697,0.267136405281055,1
.91019271181263,0.0561084052128854,0.455429865437052
"1920","AC010457.1",3.97147237531438,-1.49004138469452,0.7793533433934
09,-1.9118945178405,0.0558897249985914,0.455429865437052
"1921","LINC01625",11.6172144531177,1.02377156166744,0.53619092176253
,1.90934146796474,0.0562180561683676,0.455429865437052
"1922","PHACTR2",729.605288991486,0.414563165121288,0.217105902661195,
1.90949743899057,0.0561979517907456,0.455429865437052
"1923","MTRF1L",144.328501663352,-0.374151462345876,0.195967780470074,
-1.90924988510044,0.0562298638209045,0.455429865437052
"1924","SH3GL2",4.32163586481184,-1.46856234476403,0.768581457059664,-
1.91074391825983,0.0560374979046838,0.455429865437052
"1925","LRRC8A",201.495705476785,0.336571934448799,0.176151612374577,1
.91069459945161,0.0560438392447836,0.455429865437052
"1926","FCN1",13.0799198156835,-0.9593792152895,0.502233945259146,-1.9
1022375995409,0.0561044091889708,0.455429865437052
"1927","IL10RA",14.5367653319777,-1.17696994423153,0.616060982616462,-
1.91047636101355,0.056071907196907,0.455429865437052
"1928","SOAT2",2.68517060445843,-1.70684418673383,0.893686468030957,-1
.90989149751197,0.056147185040722,0.455429865437052
"1929","FTO",158.304992814343,0.359548202107863,0.188216723957569,1.91
028828123116,0.0560961057923531,0.455429865437052
"1930","AC006946.2",5.27040345683467,1.55642826760875,0.81511862470437
4,1.9094500118596,0.0562040644269765,0.455429865437052
"1931","SPECC1L",260.369896991913,0.417931803131266,0.218863291838258,
1.90955641588413,0.0561903513378505,0.455429865437052
"1932","ARFGAP3",356.711421566117,-0.272239604157598,0.142635112726961
,-1.90864366391137,0.0563080751657543,0.455827275151344
"1933","THPO",10.3630340605435,-0.916505241944616,0.480583426928504,-1
.90706793158093,0.0565117912871369,0.457146415534727
"1934","ICAM1",74.3862939827654,-0.839793940524687,0.44039024769768,-1
.90693128404876,0.0565294864222609,0.457146415534727
"1935","RPIA",133.517822834752,0.388763644295197,0.203932964050799,1.9
0633057340527,0.0566073299920909,0.457539349393437
"1936","HTRA4",7.99561446368847,-1.57243108071519,0.825123650360431,-1
.9056914439773,0.0566902500255517,0.457972887603114
"1937","A4GALT",463.510768169703,-0.496113894165393,0.260372825180858,
-1.90539812985778,0.0567283381410515,0.458043989946332
"1938","PHF21A",158.724847315052,0.434067752546816,0.227896445215282,1
.90467101027739,0.0568228496199956,0.45857036535435
"1939","ICAM2",77.1412349198286,-0.703776300030428,0.369609604571788,-
1.9041071750443,0.0568962274497408,0.458689173873168
"1940","AC011815.2",3.54830028489117,1.97059111653477,1.03480737051506
,1.90430719057783,0.0568701882994623,0.458689173873168
"1941","POP7",250.686914139637,-0.447728563964306,0.235305372651523,-1
.902755380887,0.0570724718247953,0.459222470485229
"1942","GPR107",151.990803140033,0.360922868606531,0.189689940191964,1

.90269904793728,0.0570798262546858,0.459222470485229
"1943","SYNRG",249.14620822754,0.366577217675434,0.192634087216046,1.90297170647844,0.0570442372167812,0.459222470485229
"1944","ATP7A",100.552458616026,0.403494859600053,0.212023334792513,1.90306816933577,0.0570316507239362,0.459222470485229
"1945","IL17RE",31.1538685729439,-0.777019238868578,0.408471791152238,-1.90225923968145,0.057137271605307,0.459448291983034
"1946","AGO1",104.048873035614,0.484154785894019,0.25465438786592,1.90122302604475,0.0572728063073001,0.459592965954938
"1947","PBXIP1",253.95352378712,0.344717246878159,0.181252726367627,1.90185965081145,0.0571895054051376,0.459592965954938
"1948","SHMT2",167.778848824355,-0.436105644106404,0.229357262640637,-1.90142504791621,0.0572463612817396,0.459592965954938
"1949","GPR183",69.89136336329,-1.4362190373271,0.755395620475986,-1.90128059839971,0.0572652689477773,0.459592965954938
"1950","AC007878.1",15.3511081200543,0.963132613061129,0.50706667080622,1.89942007335994,0.0575092662895285,0.461090080475463
"1951","CROT",82.0240210394365,-0.390179181887216,0.205451893317286,-1.89912672785473,0.0575478156705949,0.461090080475463
"1952","TRUB2",121.247201960974,-0.389342181001387,0.205001919837633,-1.89921236498544,0.0575365596277724,0.461090080475463
"1953","FGF10",26.7367455991589,-1.06602689695578,0.56153649459602,-1.89841071277602,0.0576419995092587,0.461471468569894
"1954","RABEP2",113.106520594699,-0.434523068893796,0.228899192604499,-1.89831630225355,0.0576544277228627,0.461471468569894
"1955","RGS10",544.090513135759,-0.464407959674441,0.244817259870184,-1.89695759163671,0.0578335354243871,0.462195449175991
"1956","AP3S2",142.315858682367,0.357589682018279,0.188490138311629,1.89712674212738,0.057811212526672,0.462195449175991
"1957","TMEM102",3.57953332656027,-1.52356041956882,0.803159230026429,-1.89695936074679,0.0578333019167375,0.462195449175991
"1958","SYNGR2",8.8898326886214,-1.33760521011907,0.705276879147428,-1.89656750372426,0.0578850428875494,0.462370822656421
"1959","AC005632.6",3.01482497871414,2.35606678009193,1.24263081477333,1.89603118809001,0.0579559205928562,0.462700662619842
"1960","KLF7",334.713855506501,0.400250106131765,0.211138954788915,1.89567153314703,0.0580034917935479,0.462844189617903
"1961","GOLT1B",214.442356300146,-0.338709831468177,0.178798101514936,-1.89437040213698,0.058175862214132,0.463622187403617
"1962","HOXB2",213.876849314667,-0.573312826779145,0.302657231369219,-1.89426442641229,0.0581899203243798,0.463622187403617
"1963","CDKN2D",100.026107823333,-0.435883599893101,0.230102195822646,-1.89430439085885,0.0581846185467837,0.463622187403617
"1964","PCDH7",65.4572786620907,1.60448059775404,0.847488923966598,1.8932171883078,0.0583289925273046,0.463657074640977
"1965","KMT2C",603.102487743764,0.478478336940185,0.252776292326674,1.89289245655137,0.0583721726322304,0.463657074640977
"1966","RASL12",22.152777387152,-0.822117278007926,0.434204848739592,-1.89338576110876,0.0583065875950844,0.463657074640977
"1967","CCDC113",12.8249554234483,-0.942156237302352,0.497681461483339

,-1.8930908828596,0.0583457844119556,0.463657074640977
"1968","VAT1",1475.16810631621,0.312131666782551,0.164851155663909,1.8
9341509633643,0.058302689394106,0.463657074640977
"1969","MAFB",813.816124308281,0.692248533405911,0.365593118058404,1.8
9349443195843,0.0582921479936018,0.463657074640977
"1970","POLR2J",633.819136447995,-0.391931390248469,0.207118238026049,
-1.89230747607642,0.0584500254302215,0.463816111482265
"1971","FRS2",180.240191102947,0.3157760075678,0.166894137129649,1.892
07370012342,0.0584811618825464,0.463816111482265
"1972","MT-
ND5",8302.76323268788,-0.375206088336742,0.198286785347258,-1.89223950
390666,0.058459077174894,0.463816111482265
"1973","CLSTN1",441.372444957497,0.33130898593941,0.175125991573392,1.
89183217729599,0.0585133446032564,0.463836142724243
"1974","OLFM1",168.308577876841,0.658179112222436,0.348133012243991,1.
89059666585468,0.0586782056705679,0.464436810064616
"1975","MGA",150.239567737807,0.46524288839849,0.246082503909709,1.890
59718186708,0.0586781367357719,0.464436810064616
"1976","NUP50-
DT",18.6985742594809,-0.731736145842468,0.386951411804602,-1.891028494
84361,0.0586205405663326,0.464436810064616
"1977","PDGFRA",2467.22313477784,0.386697412226722,0.204568016904545,1
.89031217136529,0.0587162219162205,0.46450263569534
"1978","FP565260.1",25.5315087465149,-0.790708367633152,0.418386889034
83,-1.88989757651638,0.0587716597468593,0.464706146835632
"1979","ATP9B",95.6071633992688,0.519758809934191,0.275064665389826,1.
88958770548584,0.0588131227364991,0.464799009398103
"1980","ASL",79.0349341356225,-0.474561865440713,0.251377933808113,-1.
88784217553067,0.0590471412893242,0.466412772608601
"1981","AP1G1",160.015937074168,0.372024627525396,0.197092149863226,1.
88756694664686,0.0590841109449049,0.466469205037008
"1982","RSRP1",1297.53075179793,0.302931555642814,0.160604585757958,1.
88619493156536,0.059268691283744,0.466513503612358
"1983","NFKBIZ",2722.5660569319,0.503240833386785,0.266784303335994,1.
88632099825225,0.0592517113017242,0.466513503612358
"1984","ARPC1B",1981.31776562143,-0.467859854833343,0.24799880570561,-
1.88654075773543,0.0592221214453126,0.466513503612358
"1985","CREM",152.129404861561,-0.465352723367632,0.246683035116086,-1
.88643991326214,0.05923569828283,0.466513503612358
"1986","GUCY1A2",590.251474747544,0.622089540860678,0.329726775787797,
1.88668190314346,0.0592031231716529,0.466513503612358
"1987","RAD51D",58.3139561133787,0.487579374339375,0.258488894248453,1
.88626817317237,0.0592588258453813,0.466513503612358
"1988","ADHFE1",10.8312676370058,0.907595040336769,0.481273664415565,1
.885819041104,0.0593193441217546,0.466677335042376
"1989","ADGRA2",209.37225073636,0.327263001906291,0.173580432893532,1.
88536804783188,0.0593801647847566,0.466920953863043
"1990","BATF3",17.2633143876666,-1.05550112978077,0.560623178111557,-1
.88272831197632,0.0597371965485187,0.467163213697922
"1991","CEP97",72.0628961786508,0.493759134077241,0.262158525323489,1.

88343725792617,0.0596411351454333,0.467163213697922
"1992","RARRES1",240.601761614923,-0.860833509169107,0.4569575682002,-1.8838368572374,0.0595870463689795,0.467163213697922
"1993","KLHL5",79.4930469200414,-0.547292042611285,0.290579606982727,-1.8834495933633,0.0596394648420675,0.467163213697922
"1994","NR3C2",41.8402703226165,0.695947536745289,0.369638872554995,1.88277691665599,0.059730606572167,0.467163213697922
"1995","AL691447.2",9.96020331981488,1.24401549841732,0.660599454078623,1.88316155990838,0.0596784766881212,0.467163213697922
"1996","TPT1-AS1",251.492141342904,0.398953380992149,0.211891364997849,1.88282038296463,0.0597247137832032,0.467163213697922
"1997","ATP10A",44.294612252788,-0.686760722586158,0.364772243761982,-1.88271101853429,0.0597395413935962,0.467163213697922
"1998","LINC01569",6.31212326486853,1.23597932006827,0.65592783531057,1.88432210607902,0.0595214190038295,0.467163213697922
"1999","CYB5D2",373.400901861042,0.407629625960045,0.216400940298924,1.88367770212537,0.0596085843337176,0.467163213697922
"2000","KANSL1",363.569679755517,0.371717605860804,0.197361338002488,1.88343679477953,0.0596411978594433,0.467163213697922
"2001","NORAD",1387.42745070083,0.270270594353603,0.143611278231784,1.88195939539926,0.0598415289530418,0.467726892966303
"2002","INTS3",130.374380005935,0.365254130892159,0.194138755673914,1.88140760264097,0.0599164934645939,0.468078899993131
"2003","ZNF283",30.5227283302754,0.626831322411475,0.3333865590634,1.88019374318048,0.0600816780283161,0.46891756567115
"2004","COMMD7",349.487100149537,-0.384134089084733,0.204307289548481,-1.88017808827903,0.0600838108443084,0.46891756567115
"2005","BANP",65.337910757195,0.43476322608585,0.231318799908536,1.87949801856899,0.0601765238211526,0.469406899033829
"2006","NDUFS5",1832.00998400107,-0.375861153901927,0.200074213231622,-1.87860868140363,0.0602979448899256,0.469594508602943
"2007","VDAC1",806.129113156492,-0.311461643852819,0.165798612204438,-1.87855398613814,0.0603054190510254,0.469594508602943
"2008","FBXW5",427.420269788979,-0.380188848710068,0.202371019556146,-1.87867239856737,0.0602892388460636,0.469594508602943
"2009","TBCD",150.320171462707,0.426700622166608,0.227183184215704,1.87822273747809,0.0603507009138053,0.469594508602943
"2010","DIDO1",199.860853809075,0.385376478803117,0.205168937314713,1.87833735382652,0.0603350296119187,0.469594508602943
"2011","KBTBD11",4.42257016257683,1.82990223760335,0.974662306976503,1.87747307401256,0.0604532843748305,0.470158810354226
"2012","TMED9",1137.44980019644,-0.446497267429571,0.237866290926478,-1.87709349521736,0.0605052807808634,0.470329319787626
"2013","HIPK2",164.13125954768,0.65434088598368,0.348825621289123,1.8758395199455,0.060677319495586,0.471432328321394
"2014","SENP8",10.9857075278368,0.880912180080394,0.469671906397948,1.87559053049685,0.0607115277080792,0.471463899381509
"2015","BEX4",357.945346011874,0.518789236138919,0.276723573843741,1.87475620140649,0.0608262711918987,0.472120536695432

"2016","LYZ",142.06955255369,-0.8999917393709,0.480136185663571,-1.874
45097087833,0.060868293754366,0.4722123582928
"2017","HAGHL",34.6286678105882,-0.71416443940693,0.381114011873109,-1
.87388659865046,0.0609460569593858,0.472581225009813
"2018","NBPF11",53.209910168256,0.646504199359294,0.345143738028856,1.
87314480352891,0.0610483919089231,0.472982867184302
"2019","FAM216A",39.0909015850205,0.537062918723704,0.28680826238711З,
1.87255037303917,0.0611304997424924,0.472982867184302
"2020","ODF3L1",4.73906171725078,-1.41594302693894,0.756162475319344,-
1.87253807634524,0.061132199232398,0.472982867184302
"2021","MIS12",67.4782560146931,-0.434772110520903,0.232163414567861,-
1.87269863914678,0.0611100114000527,0.472982867184302
"2022","MT-
ND3",40529.344846787,-0.676061067046515,0.361063482962517,-1.872416067
94309,0.0611490637753619,0.472982867184302
"2023","MAD2L2",145.950677326385,-0.469610739497316,0.251110529269115,
-1.87013559671978,0.0614649903875144,0.474203478305402
"2024","CAMK2N1",1632.11407203511,-0.305578699466173,0.163433274592574
,-1.86974592675791,0.061519108534633,0.474203478305402
"2025","Z99572.1",10.8794626804312,1.02858448713633,0.549835295957005,
1.87071382048337,0.0613847581566798,0.474203478305402
"2026","CPLANE1",163.303542353189,0.517844227251394,0.276932756574563,
1.86992768084467,0.0614938612549113,0.474203478305402
"2027","PRPH2",8.75200635996712,1.29923936843407,0.694830013431668,1.8
6986650449555,0.0615023582428599,0.474203478305402
"2028","IQCH-
AS1",42.0870058488326,0.609999687157912,0.326025251205219,1.8710197596
7482,0.0613423422374439,0.474203478305402
"2029","TMEM88",9.61214135318401,-1.15452214782431,0.617266419391109,-
1.87037899933577,0.0614312061482,0.474203478305402
"2030","ACKR1",17.5486614300352,-0.972298702280289,0.52032334250766,-1
.86864325093387,0.0616724641703268,0.475019141070243
"2031","DCUN1D4",108.907416627898,0.389587795970761,0.208497566897673,
1.86854840450951,0.0616856697898761,0.475019141070243
"2032","WSB1",1621.59713494224,0.485921907336478,0.260104674003797,1.8
6817829859292,0.061737222618509,0.475182166217264
"2033","RBBP4",382.898175808052,0.272290009409834,0.14599097429603,1.8
6511536567804,0.0621652352146356,0.475214132065195
"2034","ABHD10",99.0004057479766,0.359664369556886,0.192642397254563,1
.86700526302949,0.0619008528877173,0.475214132065195
"2035","PLD1",90.3510471256191,0.482007185433449,0.25841558099117,1.86
524041462469,0.0621477129979296,0.475214132065195
"2036","RBM47",25.1059701180803,-0.688568639823677,0.368876366326008,-
1.86666510159431,0.06194837010786,0.475214132065195
"2037","AGA",65.1305984911499,-0.730332709468751,0.391415539201475,-1.
86587561382642,0.0620587701513621,0.475214132065195
"2038","AC011379.2",30.5201143099384,0.808190539601875,0.4333218559968
56,1.86510449084696,0.0621667592202934,0.475214132065195
"2039","MMS22L",41.2843531412997,0.496263236214379,0.265771780928243,1
.86725330462517,0.0618662229268255,0.475214132065195

"2040","KLF4",5578.53443178429,0.488849950960666,0.262009349368138,1.8
6577292810191,0.0620731414296128,0.475214132065195
"2041","PAAF1",106.822958007007,0.373007741023315,0.199935729449104,1.
86563823310165,0.062091996709176,0.475214132065195
"2042","CACNA2D4",22.9073528453047,0.666209028123046,0.356960993331829
,1.86633565170451,0.0619944198007414,0.475214132065195
"2043","DIS3L",128.693692577691,0.365403467497041,0.195910648482785,1.
86515368269607,0.0621598656899951,0.475214132065195
"2044","ACTN4",571.673598650179,-0.292915262354867,0.156971826255568,-
1.86603716948523,0.0620361653633644,0.475214132065195
"2045","CABLES2",8.63945519286219,1.00544833767237,0.538988541515467,1
.86543545962103,0.0621203908917726,0.475214132065195
"2046","AC009509.1",9.33617484817136,6.02922292847409,3.22931251694664
,1.86702986992873,0.0618974167152016,0.475214132065195
"2047","ROR1-
AS1",4.32287546699696,-1.64624904544908,0.882893676132782,-1.864606226
04063,0.0622366195245323,0.47531798285871
"2048","CALU",1967.22250675532,-0.294213740536303,0.157791391497069,-1
.86457409206488,0.0622411271671763,0.47531798285871
"2049","ZNF426-
DT",3.53132098603894,1.82815303439724,0.980852002842508,1.863841873288
99,0.0623439134948395,0.475870574455485
"2050","NSD1",272.887874466928,0.375961059554011,0.201783823107411,1.8
6318731484181,0.0624359169610209,0.476235020565151
"2051","IQSEC3",5.65941462120407,2.31675585612929,1.24351586454295,1.8
630689983041,0.0624525592825527,0.476235020565151
"2052","DIP2A",177.732512170904,0.530807887565436,0.284954991509616,1.
86277799435397,0.0624935073051483,0.476315036185438
"2053","SNX15",170.896525310673,-0.362357607241247,0.194611603137939,-
1.86195273765055,0.0626097523981411,0.47696859596051
"2054","NOCT",10.0726484842368,-1.16187581698971,0.624122767638339,-1.
86161421636037,0.0626574879664809,0.477099859686349
"2055","SYPL2",3.01689859750894,1.99573243092934,1.07224248778333,1.86
12696788906,0.0627061027876892,0.47723768739633
"2056","ZNF638",548.13895258521,0.415865009361199,0.223497131370054,1.
8607174365591,0.0627840901758996,0.477366636048162
"2057","DIP2B",68.4918019955987,0.453912335470385,0.243921869140406,1.
86089233027771,0.0627593830991516,0.477366636048162
"2058","KDM5B",418.235535529452,0.31218260556248,0.16781295607828,1.86
030097352469,0.0628429559077336,0.477404882955087
"2059","ZNF528",53.9758909125876,0.568875881097164,0.305806151558183,1
.86024995965108,0.0628501696933839,0.477404882955087
"2060","PTS",194.23367861606,-0.421200943827947,0.226509897452982,-1.8
5952555965188,0.0629526797786706,0.477951413465247
"2061","TRPA1",3.47342390026161,2.22671359215579,1.19772767922368,1.85
911508165117,0.0630108279815303,0.478160771291186
"2062","ACADSB",81.2792824864992,0.452034275744424,0.243182403784933,1
.85882805955071,0.0630515138257267,0.478237476350323
"2063","AL359220.1",5.24244171756259,1.43397003582099,0.77154668965502
,1.85856546991622,0.0630887553473416,0.478287994974514

"2064","GPAT2",40.5099414302185,-1.0118718096373,0.544569183610647,-1.
85811434082316,0.0631527787448197,0.478541404829932
"2065","NFAT5",356.047509152035,0.418348265921307,0.225207828545072,1.
85760978481075,0.0632244479966138,0.478852477804862
"2066","IMP4",231.30532866092,-0.419379567168142,0.22598314648144,-1.8
5580019438567,0.0634820428683684,0.480570740784744
"2067","MGST3",2821.59767421919,-0.494692034153495,0.266746651387674,-
1.85453887267188,0.0636621041487455,0.480615368747819
"2068","MRPS10",383.960950726594,-0.326934624773605,0.176295696358233,
-1.85446741768031,0.0636723173942123,0.480615368747819
"2069","SND1",254.678494674619,-0.33480092777486,0.1805181971341,-1.85
466580704963,0.0636439644294212,0.480615368747819
"2070","MKLN1",390.612082769107,0.439279460419282,0.236818779092474,1.
85491818724287,0.0636079103999726,0.480615368747819
"2071","LRP6",249.567377911163,0.428372318243354,0.230922688138714,1.8
5504647332891,0.0635895904295766,0.480615368747819
"2072","ASPHD2",20.5884708934599,-0.717884606609158,0.386953625142784,
-1.85522129775697,0.0635646315417102,0.480615368747819
"2073","TSPAN17",67.0729875461975,-0.430590675206259,0.232231490440323
,-1.8541442178657,0.0637185300815506,0.480732180644212
"2074","TFRC",81.0203355635203,-0.605982589642499,0.326918021258473,-1
.85362246874542,0.063793190778585,0.481063405871296
"2075","FSIP2",11.955787936936,1.2730430465061,0.687087023062294,1.852
81194925243,0.0639093169278165,0.481474815390679
"2076","CD320",109.496418396684,-0.634619697137603,0.34249166030633,-1
.85294934355479,0.0638896196545023,0.481474815390679
"2077","HECTD4",151.070585830536,0.508214759182215,0.274457775156828,1
.85170472540563,0.0640682350985051,0.482036054477778
"2078","ZNF664",145.993640228736,0.349285602478839,0.188656762132821,1
.85143431133908,0.064107096759193,0.482036054477778
"2079","HCST",37.6339926730264,-0.721700568368595,0.389722396213115,-1
.85183242066987,0.0640498905599784,0.482036054477778
"2080","SLC25A5",826.98902978343,-0.376596514137286,0.203405120834877,
-1.85146033979648,0.064103355319422,0.482036054477778
"2081","KMT2A",792.278557499173,0.406509547823139,0.219671371876041,1.
85053493475941,0.0642364877201066,0.482776870707577
"2082","GPATCH4",126.877777146975,-0.515713293531491,0.279006717511793
,-1.84839023995791,0.0645459096201064,0.482874124986918
"2083","UFC1",700.830325075566,-0.425577280607529,0.230074254025187,-1
.8497388263223,0.0643512015827739,0.482874124986918
"2084","BIRC6",322.024342616792,0.446045559634554,0.24159209191999,1.8
4627549722229,0.0648522136527178,0.482874124986918
"2085","FAP",398.755323276802,-0.687571948284077,0.372320763924292,-1.
84671932082705,0.0647878300153123,0.482874124986918
"2086","AC093772.1",4.17453976107662,-1.96240086348959,1.0623378221287
5,-1.84724747873258,0.0647112810857604,0.482874124986918
"2087","AC116351.1",3.50405982481043,1.87779751492353,1.01636593068242
,1.84756046836666,0.0646659529468118,0.482874124986918
"2088","AC106786.1",4.61348908915932,1.53832301058673,0.83175546138467
9,1.84948952186713,0.0643871594255484,0.482874124986918

"2089","UPP1",61.7401973925104,-0.750815799842413,0.405880420474209,-1
.84984483598692,0.064335916551449,0.482874124986918
"2090","IFRD1",596.338358135909,0.522384923321157,0.282704040199189,1.
84781555634328,0.0646290296888189,0.482874124986918
"2091","ADAM28",2.92746514533358,-1.87527876897207,1.01556481369105,-1
.84653775287507,0.0648141629427202,0.482874124986918
"2092","POLB",22.1918354141003,0.711768187148017,0.385536848368184,1.8
4617421177932,0.0648669141047004,0.482874124986918
"2093","AL589863.1",3.90998089464937,1.90137685038396,1.02955529488661
,1.8467943002453,0.0647769582733563,0.482874124986918
"2094","CPSF7",192.824635523817,0.338385380939918,0.183001306780661,1.
84908723818839,0.0644452168394449,0.482874124986918
"2095","ITGBL1",779.770871035718,-0.486544378703077,0.263530210888972,
-1.84625655275654,0.0648549630215615,0.482874124986918
"2096","SGSH",85.5340960820499,0.477505079732199,0.258506116685579,1.8
471713004493,0.0647223174295689,0.482874124986918
"2097","HIF3A",41.0482655931702,-0.971876279798014,0.525944803899548,-
1.84786744272814,0.0646215214121666,0.482874124986918
"2098","CCDC8",70.0705395524344,0.584400112778925,0.316438434807437,1.
84680509222766,0.0647753935992349,0.482874124986918
"2099","SNRPB",695.241362425676,-0.45811637627183,0.247821962771202,-1
.84857052679701,0.064519851799982,0.482874124986918
"2100","PDXK",173.222869166519,-0.411293416287629,0.222468080797224,-1
.84877495600151,0.06449031505956,0.482874124986918
"2101","ATP6AP2",1608.34237533023,-0.315766620124646,0.170930393576165
,-1.84734039112794,0.064697822486244,0.482874124986918
"2102","CARD19",160.067749728431,-0.421705284623194,0.228593726164984,
-1.84478065823571,0.0650694521264159,0.483921175110387
"2103","ZNF189",48.5835400640714,0.559438121713766,0.303246147678033,1
.8448317513591,0.0650620170891101,0.483921175110387
"2104","DUSP2",72.4062038454204,-0.865190174083624,0.469254660117502,-
1.84375403723637,0.0652189939085584,0.484342385911611
"2105","DLL1",8.07264451784313,1.17142619039006,0.635280762322802,1.84
395036000606,0.0651903748386696,0.484342385911611
"2106","ACSS1",15.4746554163129,0.876351763971761,0.475279284674668,1.
84386694777078,0.0652025330417266,0.484342385911611
"2107","CCBE1",13.7339737315322,-1.12788798111317,0.611818805931736,-1
.8435000202315,0.0652560387665461,0.484387492315511
"2108","NT5DC2",110.040966787655,-0.517004421663007,0.280496164411981,
-1.84317822222928,0.0653029934580477,0.484434260675885
"2109","LSM14B",91.0199480481904,0.396623865213012,0.21520179248927,1.
84303234942984,0.0653242874530333,0.484434260675885
"2110","HSPA6",5.63282483416637,-1.40419089237562,0.762486553359346,-1
.84159430246877,0.0655345150740089,0.484516483189122
"2111","GALNT10",396.59380824253,0.602084496674303,0.326967761285113,1
.84141853713,0.0655602483645732,0.484516483189122
"2112","EEF1E1",145.360797153705,-0.433177758383621,0.235135162398195,
-1.84225002320174,0.0654385863891251,0.484516483189122
"2113","NUP153",140.90947790321,0.397814757760128,0.215978614844698,1.
84191734929952,0.0654872404483466,0.484516483189122

"2114","MOXD1",57.0465991069629,-1.10125642804017,0.597679843401256,-1
.84255239690397,0.0653943896405792,0.484516483189122
"2115","TNRC6A",569.407678898398,0.397934279349786,0.216015388797674,1
.84215708688469,0.065452175464312,0.484516483189122
"2116","AC116667.1",13.8691705491432,1.21462978808906,0.65967252332502
9,1.84126175510057,0.0655832093933102,0.484516483189122
"2117","BCL6B",4.22867958718146,-1.34278451476537,0.729203924043452,-1
.8414389589672,0.0655572580352891,0.484516483189122
"2118","PRKRA",300.603026590676,-0.313312920229404,0.170221474522928,-
1.84061923507308,0.0656773769508714,0.48475421213385
"2119","PLCB2",9.01960996175827,-1.09551347995003,0.595135585331258,-1
.84077965921036,0.0656538547995056,0.48475421213385
"2120","AP001627.1",3.85614065798709,1.89315622790433,1.02882567749102
,1.8401137037337,0.0657515458071414,0.485072724728156
"2121","LDB2",103.749333351172,0.492766879502456,0.267955322325433,1.8
3898895989828,0.0659168099085181,0.485833603661274
"2122","SEPHS2",277.277101781817,-0.460845150209491,0.250584226699748,
-1.83908283565541,0.0659030032040256,0.485833603661274
"2123","PRDX1",2868.75409920361,-0.449835304538069,0.244742424346016,-
1.83799480511027,0.066063170804402,0.485981587101155
"2124","IRX1",84.1189381554896,-0.708198252563703,0.385329826741822,-1
.83790146366792,0.0660769264065328,0.485981587101155
"2125","PTTG1",22.5803588677561,-0.782864745335291,0.425893743711196,-
1.83816916048939,0.0660374826083781,0.485981587101155
"2126","AC011611.4",4.9435914171893,1.42274133016853,0.773806729070452
,1.83862620563875,0.0659701841164213,0.485981587101155
"2127","EPS15L1",117.458266872574,0.379987152300091,0.206762255453132,
1.83779748130202,0.066092252926096,0.485981587101155
"2128","RGS22",22.0483970265891,-0.61119031036659,0.332661121941351,-1
.83727604476228,0.0661691544409736,0.486318409519185
"2129","PGK1",803.246533389217,-0.371339467360895,0.202141202137433,-1
.83703007320807,0.0662054559339139,0.486356660782721
"2130","GZMB",19.678306213705,0.938231459588812,0.510995264307625,1.83
608640847205,0.0663448781144438,0.487118113844655
"2131","GPC3",1959.83377032286,0.671405125602093,0.365707574542078,1.8
359070807948,0.0663714002943068,0.487118113844655
"2132","ANKS6",59.7981176445697,0.447551383881935,0.243816892776989,1.
83560449312794,0.0664161721645109,0.487218073476994
"2133","UGDH",1600.27538934711,-0.424061688729975,0.231097319806542,-1
.83499180814805,0.0665069032419504,0.487654930475435
"2134","JUNB",17646.5860895058,-0.452047995508898,0.246383892598524,-1
.83473030944234,0.0665456590424753,0.487710453338479
"2135","AC024060.2",9.3912219731419,-0.910826324967791,0.4965281907505
65,-1.834389953954,0.0665961297866797,0.487851742324904
"2136","UBE4A",221.522655285493,0.312802820999793,0.170646582409018,1.
83304474419561,0.0667959173066006,0.489086210990278
"2137","POGZ",283.94195262719,0.388523710699095,0.212166917686214,1.83
121720830061,0.0670681292186206,0.49046414789192
"2138","CISD2",201.780562079407,-0.312030923418307,0.170421954594755,-
1.83093149095891,0.0671107693143451,0.49046414789192

"2139","DPYSL3",1295.31482993519,-0.388532500056503,0.212202163753859,
-1.83095446899955,0.0671073392756089,0.49046414789192
"2140","POM121",129.273735455606,0.376828043962446,0.20583489343666,1.
83072965749806,0.0671409041327749,0.49046414789192
"2141","TNNC2",115.928036730284,0.756966513520885,0.413324281904472,1.
83141070259172,0.0670392650421416,0.49046414789192
"2142","LINC01359",15.7570881032868,0.98819098091049,0.540093641759359
,1.82966601438123,0.0672998958162881,0.490535316617065
"2143","BNIP3L",1149.1539393893,0.2894545876726,0.15821394637231,1.829
51373320436,0.0673226839000257,0.490535316617065
"2144","LINGO2",13.3312742499531,3.50336421595888,1.91502842899517,1.8
2940585263119,0.0673388315074705,0.490535316617065
"2145","MRPL48",114.691367135619,-0.392868620200573,0.21465443310511,-
1.83023762667038,0.0672144136483556,0.490535316617065
"2146","RASD1",1577.8775914104,0.70272304810625,0.383908515965593,1.83
044402216186,0.0671835700544895,0.490535316617065
"2147","LINC01436",105.871023404832,-1.65367680002373,0.90392164063343
7,-1.82944707338226,0.0673326611927055,0.490535316617065
"2148","EDEM3",247.321923637768,0.377137161936726,0.206201397171058,1.
8289748135114,0.0674033814275633,0.490776948569409
"2149","AC068193.1",6.61831389742564,-1.22335903729726,0.6691084361194
62,-1.82834197158268,0.0674982444138981,0.490830256981452
"2150","IMPDH1",72.9611437801695,-0.525888991737496,0.287636260931299,
-1.82831257100475,0.0675026542286116,0.490830256981452
"2151","SLC39A6",318.67491346617,0.278032908745028,0.152071993142215,1
.82829791995306,0.0675048518393288,0.490830256981452
"2152","ZNF281",100.831879408648,0.387914220194126,0.212347795358607,1
.82678713258608,0.0677317813074829,0.491008360138374
"2153","LNPEP",435.46760107876,0.383744772846306,0.210174614269691,1.8
2583788332255,0.0678746850779517,0.491008360138374
"2154","LAMB1",255.561123202083,0.705693037419362,0.386296111267361,1.
82681890093101,0.0677270030574968,0.491008360138374
"2155","ANGPT2",45.0080873577202,-0.748471855960137,0.409513833701977,
-1.82770835650167,0.0675933333219963,0.491008360138374
"2156","OSGIN2",104.368993893646,-0.493759302956705,0.270368131401282,
-1.82624816171054,0.0678128897335653,0.491008360138374
"2157","YIF1A",663.948811954179,-0.452578473598102,0.247868844923002,-
1.82587881804464,0.0678685174893021,0.491008360138374
"2158","PRKCB",13.088334337577,1.47158720455463,0.805738006778625,1.82
63842491905,0.0677924027216155,0.491008360138374
"2159","BCL3",106.382711242858,-0.464667376437325,0.254291829678869,-1
.82729966992698,0.0676547248438887,0.491008360138374
"2160","BCL2L1",147.679459809893,0.344904678383655,0.188883675552559,1
.82601634246407,0.0678478002149198,0.491008360138374
"2161","MCM3AP-
AS1",4.38674557375446,1.67487599480686,0.917104169950841,1.82626581547
071,0.0678102317968401,0.491008360138374
"2162","EMD",267.765325492744,-0.257761827978013,0.141112853327209,-1.
82663607106237,0.0677545061385695,0.491008360138374
"2163","GZMH",16.9433454577604,0.879708841966643,0.481939970969807,1.8

2534941062557,0.0679483182894381,0.491313776258351
"2164","PDE7B",35.8562307984661,1.03670459593028,0.568239362807484,1.8
2441531471573,0.0680893084399529,0.49165133148701
"2165","ALDH6A1",198.572224661616,0.365091602795001,0.20011228991104,1
.82443368649323,0.0680865331314205,0.49165133148701
"2166","AC009120.2",45.6219297276914,0.644956962802381,0.3534609604481
16,1.82469080032122,0.0680477023269597,0.49165133148701
"2167","ZNF593",204.94980965067,-0.459970136879249,0.252266696397736,-
1.82334863637346,0.0682506043733256,0.491989417726617
"2168","EREG",24.3624615570995,-1.26697930962142,0.694739885132065,-1.
82367435170442,0.0682013186054559,0.491989417726617
"2169","GIMAP2",22.4244107030552,0.682526679119476,0.374345192883145,1
.82325482494584,0.0682648049237459,0.491989417726617
"2170","ARHGAP42",213.186433505068,0.428014268570207,0.234720238094563
,1.82350815611294,0.0682264629116251,0.491989417726617
"2171","CBFA2T2",108.479796553191,0.467510003619297,0.256441642014607,
1.82306586382202,0.0682934159772688,0.491989417726617
"2172","DSTYK",211.865241595868,0.413512814343774,0.22689059315186,1.8
2252075151924,0.0683760079743718,0.492357626482125
"2173","LINC01091",33.4335345905465,0.802385926469169,0.44047092483558
1,1.82165469098484,0.068507396883249,0.493076708354356
"2174","ZNF718",35.1115077372861,0.711472780743901,0.390661961043053,1
.82119799645785,0.0685767649370046,0.493102689976319
"2175","SHROOM1",25.9827572725499,0.624044403624102,0.342769734687233,
1.82059365361859,0.0686686482588505,0.493102689976319
"2176","WDR86-
AS1",135.053076921001,1.27934397738436,0.70257449743411,1.820937113511
35,0.0686164167614146,0.493102689976319
"2177","PTDSS1",108.29337119361,-0.432168793255782,0.237354532820115,-
1.82077328846849,0.0686413263014744,0.493102689976319
"2178","NDRG1",1325.26079699153,0.337486826099193,0.185309276744151,1.
8212084792989,0.0685751720345978,0.493102689976319
"2179","HSD17B7",101.932036715949,-0.389967833805538,0.214299931599466
,-1.81972915667748,0.0688002608531623,0.493351793115199
"2180","SPIDR",94.2364064817338,0.351816842999013,0.193377118095806,1.
81933026235663,0.0688610591029718,0.493351793115199
"2181","AL162274.2",6.29758637086781,1.31339332324452,0.72159686798509
5,1.82012059851629,0.0687406413755288,0.493351793115199
"2182","FAM214A",129.61440881999,0.49504348228058,0.272023966968202,1.
81985244828978,0.0687814780495918,0.493351793115199
"2183","BX470209.1",3.41349239325697,-1.77457596832269,0.9753113429554
14,-1.81949690336352,0.0688356548246808,0.493351793115199
"2184","CPD",174.799202520526,0.304693749612416,0.167519169543626,1.81
885900248011,0.0689329439667273,0.493640679322168
"2185","ZNF660",43.1452870205136,0.528607902272957,0.290754032325686,1
.81805871459365,0.0690551592271719,0.494289560783967
"2186","PCF11",420.115026023684,0.325928938108477,0.179320803808053,1.
81757459919349,0.069129176855034,0.494593012814607
"2187","UGGT1",187.790248819064,0.406021507048854,0.223415274858466,1.
81733996167481,0.0691650746193823,0.494623578896726

"2188","CPNE3",748.44602863334,0.228770152188709,0.125915983343964,1.8
1684760038587,0.0692404519473873,0.494936320135804
"2189","FBXL4",119.837172485539,0.409418122411525,0.225433889023292,1.
81613387492607,0.0693498384793804,0.495039467739621
"2190","MRPL52",362.953012521151,-0.407921637259012,0.224590029442842,
-1.81629450902596,0.0693252071111652,0.495039467739621
"2191","SNX33",249.840956440476,0.397041883452225,0.218590552325856,1.
8163725706697,0.0693132398617547,0.495039467739621
"2192","CDHR4",12.0859402100365,-1.0768139207608,0.593407422543687,-1.
81462833097866,0.069581045644754,0.495998310160554
"2193","ADAMTS2",784.248395520383,-0.284424332512628,0.156721513991779
,-1.8148391070772,0.069548638599611,0.495998310160554
"2194","NRF1",38.3515267159803,0.58287251253554,0.321242151160254,1.81
44334746556,0.0696110160359601,0.495998310160554
"2195","RPH3AL",28.0991430611028,0.739864288935539,0.40769191972264,1.
81476319039848,0.0695603094399179,0.495998310160554
"2196","CMPK1",762.068383334329,-0.327131922458226,0.180585642040518,-
1.81150571419641,0.0700626053413133,0.496126936205069
"2197","ZNF678",63.1320815270758,0.469439223537977,0.258846484156161,1
.81358161022873,0.0697421637695838,0.496126936205069
"2198","AL390728.5",5.92940715800958,1.45450241451389,0.80238745399026
,1.8127182912453,0.0698752818545027,0.496126936205069
"2199","WDR35",60.2118464313255,0.438913819579246,0.242187718528107,1.
81228768430844,0.0699417565142913,0.496126936205069
"2200","SUPT7L",111.339970256526,0.369809681702046,0.204125729062294,1
.81167598715196,0.0700362760853418,0.496126936205069
"2201","TOP2B",318.863416119387,0.254996394525899,0.140584996397723,1.
81382367293662,0.0697048766915589,0.496126936205069
"2202","SOX2-
OT",3.97025668455973,1.5086366418991,0.832551723038461,1.8120635633221
9,0.0699763755754039,0.496126936205069
"2203","TMEM70",253.399925859822,-0.416029132950832,0.22941810507379,-
1.81341020499241,0.0697685767481763,0.496126936205069
"2204","FAM166B",4.76880426801059,1.46695638885635,0.809970711103915,1
.81112275881806,0.0701218512962385,0.496126936205069
"2205","TNKS2",261.026950449998,0.345032101087592,0.19054876557178,1.8
1072860825024,0.0701828721538997,0.496126936205069
"2206","FXYD6",13.4596311745477,-1.19639380869686,0.660866254645189,-1
.8103418056036,0.0702427977902729,0.496126936205069
"2207","ARMH4",84.7476655676459,0.392245086553062,0.216641784759234,1.
81056986300674,0.0702074607803757,0.496126936205069
"2208","GATM",64.4441615311676,0.794401688109005,0.438173519925207,1.8
1298424479098,0.0698342513645661,0.496126936205069
"2209","FHOD3",116.851518854622,0.909421443034865,0.501701108178919,1.
81267577091048,0.0698818435945058,0.496126936205069
"2210","LINC00683",5.81637698872603,1.32014051331494,0.727878574447315
,1.8136823361195,0.0697266460734956,0.496126936205069
"2211","POLR2I",475.392667693455,-0.395482855962918,0.218253283937139,
-1.81203622153436,0.0699805999129841,0.496126936205069
"2212","PLEKHG2",65.2953083950613,0.962684562981801,0.531441461213072,

1.81145927302015,0.0700697879361834,0.496126936205069
"2213","CR381653.1",4.98477397306783,1.37808322251016,0.761284363954562,1.81020823198257,0.0702635015149762,0.496126936205069
"2214","TCF20",67.7514196378464,0.544169250615672,0.300525555059743,1.81072538243044,0.0701833717424807,0.496126936205069
"2215","TSIX",45.6884360863973,1.07812034245149,0.595271053210305,1.81114189349066,0.0701188900491173,0.496126936205069
"2216","CFL1",3588.29346759302,-0.425178412442005,0.234905971724294,-1.80999405558336,0.0702967090081072,0.496137422782851
"2217","MTMR11",65.759120426159,-0.460040928770731,0.254263093308986,-1.80931067416725,0.070402751618355,0.496422374371913
"2218","ZNF589",60.1551143822536,0.566161817614687,0.313047912478496,1.80854685511943,0.070521431283926,0.496422374371913
"2219","HDAC9",10.2123315896112,-0.97634487787968,0.539640153568992,-1.80925172343547,0.0704119053484782,0.496422374371913
"2220","PEG10",120.336434588386,0.900148356048683,0.497742123086059,1.80846328710871,0.0705344257574287,0.496422374371913
"2221","AFAP1L2",144.321871364932,-0.47154017606886,0.260675868594047,-1.80891380016151,0.0704643961165687,0.496422374371913
"2222","RSPRY1",206.230550032041,-0.267488393352671,0.147880441975729,-1.80881521436468,0.0704797158298429,0.496422374371913
"2223","CMC4",159.829806132481,-0.445772443540783,0.246514164918671,-1.80830356619812,0.0705592671501767,0.496422374371913
"2224","ZNF626",152.898466322122,0.346274486457379,0.191540630811329,1.80783828992641,0.0706316724710363,0.496708344175813
"2225","JADE1",144.796871429563,0.460662398219934,0.254846012378533,1.80761077609366,0.0706670998769324,0.49673413126976
"2226","PSMC5",1036.28834850618,-0.364729737926918,0.201834217753723,-1.80707583672437,0.070750455591905,0.497096642164149
"2227","C1orf21",1067.771764884,0.306376256858569,0.169775904156183,1.80459210852867,0.0711385338732147,0.497462938760859
"2228","XPO1",350.997592885255,0.293816159566073,0.16274749151683,1.80534985103404,0.0710199533550883,0.497462938760859
"2229","SPATS2L",502.597290835727,-0.271405008773441,0.150391645978897,-1.8046548197998,0.071128713914499,0.497462938760859
"2230","ARHGEF37",11.9087804502135,-0.962851688797094,0.533638284548135,-1.80431523876215,0.0711819021993909,0.497462938760859
"2231","SRPRA",374.6495594215,-0.265856708106602,0.147260709337245,-1.8053471920861,0.0710203691752303,0.497462938760859
"2232","AC021054.1",29.3408437143232,0.670765554403898,0.371406289214861,1.80601560577197,0.0709159019587497,0.497462938760859
"2233","TFCP2",109.70813403201,0.363594580893723,0.201515580296149,1.80430009609868,0.0711842747408441,0.497462938760859
"2234","SLC7A6OS",134.793829742222,0.311961643622124,0.17283085697947,1.80501126404286,0.0710729194126487,0.497462938760859
"2235","PIGW",14.7112111218291,-0.792369866318096,0.438810994872292,-1.80572017469321,0.0709620597056677,0.497462938760859
"2236","MED1",214.240533117338,0.413224714093053,0.228948154788726,1.80488335655895,0.0710929367441445,0.497462938760859
"2237","C3",7150.68048025107,0.3743881661335,0.207454365065293,1.80467

721667687,0.0711252070565641,0.497462938760859
"2238","LILRA6",2.84164013588745,-1.99272289082489,1.10328327549403,-1
.80617520004786,0.0708909774111164,0.497462938760859
"2239","SCYL3",53.3061304190932,0.493713811367322,0.273692908138298,1.
80389698339515,0.0712474579808542,0.497682109343707
"2240","IL1R2",7.16525352038079,-1.35275659421649,0.75016087184991,-1.
80328866111154,0.0713428924629897,0.498126267018375
"2241","AL512625.3",2.57367420088721,-1.91492257304222,1.0620922328720
7,-1.80297201483524,0.0713926098371216,0.498250967359474
"2242","CDC37",626.975882490882,-0.288136059779276,0.15986668376508,-1
.80235214112958,0.0714900198645548,0.498578126512601
"2243","MPV17L2",25.6366924445394,-0.763966429269046,0.42389168077522,
-1.80226804138239,0.0715032441027983,0.498578126512601
"2244","CAMK2A",2.82417128132739,-1.83327264765647,1.01775615310996,-1
.80128868988365,0.0716573895646902,0.499430290905416
"2245","WDR4",17.5790648501709,-0.663930738711314,0.368816148993068,-1
.80016721210272,0.0718342393550037,0.500439867934191
"2246","ABHD15",64.5580779656455,-0.405617409792855,0.225359805440793,
-1.7998658145781,0.071881828705394,0.500548442098113
"2247","CSRP1",319.271451684406,-0.515659048932964,0.28657163632912,-1
.79940714139882,0.0719543007480727,0.500830112906034
"2248","PCNX2",142.808477555386,0.480568856466066,0.267152189818505,1.
79885800970806,0.0720411442742713,0.501211519772955
"2249","ARHGDIA",838.005369039539,-0.324712414156847,0.180530813403308
,-1.79865369260501,0.0720734783147117,0.501213517493148
"2250","PRKACB",139.756506021764,0.350161245332991,0.194827416572612,1
.79728937278438,0.0722896924509011,0.501460128840538
"2251","STXBP3",312.095046645292,-0.314618185046055,0.17538287910947,-
1.79389337570219,0.0728301889785597,0.501460128840538
"2252","LINC02591",72.406363656276,0.492806073368192,0.274308607393927
,1.79653886201422,0.0724088578609811,0.501460128840538
"2253","TPM3",1031.08741784298,-0.29984686142876,0.166795897009269,-1.
79768727411861,0.0722265792094505,0.501460128840538
"2254","AL445228.2",2.57976368456062,1.95616718236002,1.09045912247074
,1.79389317953321,0.0728302202954796,0.501460128840538
"2255","MFSD6",25.8738319870475,-0.855095377044382,0.476333985711643,-
1.79515928464955,0.0726283254032729,0.501460128840538
"2256","TYW5",57.3238664182696,0.490507000163253,0.273284399904336,1.7
9485912966476,0.072676147059021З,0.501460128840538
"2257","COX17",541.074154495685,-0.357153119801606,0.198986455851429,-
1.79486145563731,0.0726757763785445,0.501460128840538
"2258","TRIM61",11.8621932645963,1.48125389598081,0.825617101734922,1.
79411726436887,0.072794453999582,0.501460128840538
"2259","CITED2",962.151727610815,0.493243707350505,0.274556724878058,1
.79650929173043,0.0724135562946878,0.501460128840538
"2260","AIG1",239.383472108864,-0.45692592304143,0.254422702442607,-1.
79593219730265,0.072505301029849,0.501460128840538
"2261","LATS1",168.025693779657,0.395347609962443,0.22022648448171,1.7
9518649127452,0.0726239920299064,0.501460128840538
"2262","MTHFD1L",85.6878735729519,-0.61184982838802,0.340483197988249,

-1.7970044689522,0.0723349102963474,0.501460128840538
"2263","MFHAS1",82.4402381727804,-0.403487245069649,0.224924800198815,
-1.79387619645765,0.0728329315590898,0.501460128840538
"2264","RAD54B",3.49823290811239,1.63297769081056,0.908810543626218,1.
79682960575574,0.0723626747668633,0.501460128840538
"2265","UBR5",282.221299851561,0.360826788341651,0.200761669304146,1.7
9728924147873,0.0722897132854386,0.501460128840538
"2266","ZBTB44",355.083592049243,0.340059695631652,0.189495415120059,1
.79455368572479,0.0727248378234651,0.501460128840538
"2267","FBXO34",90.5240290008779,0.375485254959481,0.209251201547474,1
.79442341158693,0.0727456129214305,0.501460128840538
"2268","PAQR5",5.9423537154117,-1.11123524168269,0.619489478727029,-1.
79379195263515,0.0728463818878326,0.501460128840538
"2269","SLC14A2-
AS1",8.299744312433,-0.93743232663014,0.52195625664125,-1.795997872814
95,0.0724948553385209,0.501460128840538
"2270","DOK6",16.06647946883,1.1219131126658,0.624080739729679,1.79770
507442957,0.0722237568624548,0.501460128840538
"2271","AL683813.1",24.7303735432623,0.834293245594958,0.4647182716366
7,1.79526671644887,0.0726112152848177,0.501460128840538
"2272","BCAP31",587.444160174782,-0.303184833587735,0.168971125821283,
-1.79429965986264,0.0727653523744296,0.501460128840538
"2273","AC010623.1",3.17271032884392,2.03715291223766,1.13590540742729
,1.79341774316543,0.0729061525657695,0.501502198542272
"2274","VASP",120.570141469622,-0.372695111382193,0.207820365305674,-1
.79335220989536,0.0729166240080004,0.501502198542272
"2275","VAMP4",159.550258719854,0.312574075323306,0.174351126353121,1.
79278494989609,0.0730073168978502,0.501625120977658
"2276","ALKAL2",4.34865096679312,2.37975607778868,1.32824948298751,1.7
9164841264317,0.0731893029179504,0.501625120977658
"2277","KIF5C",5.23030614399934,1.47030641249634,0.820924485021837,1.7
9103734792029,0.0732873018819661,0.501625120977658
"2278","POC1A",6.23357819735941,-1.23932522904853,0.691804464909366,-1
.7914386100571,0.0732229377297838,0.501625120977658
"2279","CRHBP",9.88964900370077,-1.7838856322322,0.995892791973866,-1.
79124263837328,0.0732543666411486,0.501625120977658
"2280","IER3",1319.76400295631,-1.12476140486935,0.627542679624078,-1.
79232654828055,0.0730806730444734,0.501625120977658
"2281","CRYGN",3.70621834439786,1.83898981340821,1.02630740484655,1.79
185086721961,0.0731568580975493,0.501625120977658
"2282","NCOA2",272.807367071114,0.367768162191456,0.205315370431855,1.
79123541222414,0.0732555257439946,0.501625120977658
"2283","LCOR",215.270750139966,0.408281582345229,0.227904309524624,1.7
9146056165785,0.0732194179342464,0.501625120977658
"2284","LGALS3",6874.57098899177,-0.554315301053303,0.309364701774493,
-1.79178586914988,0.0731672732289226,0.501625120977658
"2285","ZNF616",46.8978673800702,0.504761633615828,0.281671885391391,1
.79201993452221,0.0731297728479248,0.501625120977658
"2286","AC012459.1",5.00401261218129,1.40895560440833,0.78705978758972
3,1.79015066787123,0.0734296932636102,0.501721451569622

"2287","BOLA2B",29.0540581827796,-0.811075906582137,0.452972820508907,
-1.79056197162317,0.0733636141454289,0.501721451569622
"2288","USP32",113.086263974232,0.424177478534253,0.236936151869936,1.
7902606891628,0.0734120127276804,0.501721451569622
"2289","PPARA",136.443910098849,0.482340438973784,0.269432222549462,1.
79021066749073,0.0734200508306673,0.501721451569622
"2290","ZNF285",3.03268830250575,1.94998971302254,1.08943927434135,1.7
8990216246927,0.0734696411575367,0.501775191137063
"2291","ENAH",511.270058448331,-0.350902824777753,0.196073210719063,-1
.7896520564481,0.073509864300073,0.501776704173326
"2292","LRRC23",70.4135530345211,-0.486778648146208,0.272019079473338,
-1.78950185806331,0.0735340285144031,0.501776704173326
"2293","ST5",251.663713574486,0.398488625316997,0.222736324321892,1.78
905989640519,0.0736051698662118,0.502043112388815
"2294","ARRDC3-
AS1",2.88147818808411,-1.6544098997364,0.925464776409189,-1.7876530170
6622,0.0738320061898689,0.503151449590218
"2295","PLXDC2",1428.90079056686,0.526882949379308,0.294703307328955,1
.78784199659894,0.0738015031363723,0.503151449590218
"2296","RELL1",182.296162749767,0.338695185033639,0.18949503755867,1.7
8735648910476,0.0738798893343783,0.503258479612228
"2297","AC007529.1",5.95601413012906,1.36899403573859,0.766262950824614,1.78658518497514,0.0740045579413443,0.503671382606685
"2298","FANCM",44.0926866774545,0.492762429888087,0.275812784781155,1.
78658299062921,0.0740049128663786,0.503671382606685
"2299","PIK3R2",116.477156253767,0.403333170464502,0.225815804178415,1
.78611577667005,0.0740805141994661,0.503966612474837
"2300","LINC01886",27.7910995397491,-0.858832818952624,0.480906279885179,-1.78586318140341,0.0741214137026037,0.504025613177705
"2301","TMEM37",322.862878943226,1.17176567338034,0.656358205513354,1.
78525333200317,0.0742202348455263,0.504478258576285
"2302","CBWD1",114.31306716821,-0.473472188426121,0.265278803272876,-1
.78480972691621,0.0742921850739118,0.504747947244127
"2303","GLI1",21.9842476254852,-0.812227121666936,0.455132966614106,-1
.7845930337883,0.0743273521863004,0.504767602342049
"2304","YBX1",5437.06775549191,-0.354123474999225,0.198456251750683,-1
.78439062450955,0.0743602134604489,0.504771587899922
"2305","DHRS9",2.59248633502146,-1.84894145628089,1.03639184093328,-1.
78401776553537,0.0744207784271137,0.504963546464234
"2306","DBT",127.482657936001,0.395363742144749,0.221663879439368,1.78
361825636501,0.0744857170157648,0.505041997235854
"2307","ZMAT1",71.9659828104628,0.778509983121393,0.436494783539329,1.
78354934006044,0.0744969237610688,0.505041997235854
"2308","LRCH3",268.655534974635,0.414231264068885,0.232306890880708,1.
78312086438106,0.0745666307510544,0.505295539404892
"2309","LPP-
AS2",29.038505442941,0.563428784085024,0.316021415815165,1.78288165259
839,0.0746055703300466,0.505340459056704
"2310","HS6ST1",156.475806572254,0.330384014070814,0.185516178848409,1
.78089057311159,0.0749303289418251,0.506639301719528

"2311","BHLHE40",377.699725559728,-0.836322592891465,0.46961946109611,-1.78085165154667,0.0749366888004807,0.506639301719528
"2312","AC008914.1",7.83007723663867,1.52373094337897,0.855607576206979,1.78087593629532,0.0749327205740703,0.506639301719528
"2313","PDLIM1",1429.21870527827,-0.410743042090589,0.230626781846647,-1.78098588031163,0.0749147574230867,0.506639301719528
"2314","TAFA2",8.68373668797203,1.00798098647268,0.566054605812598,1.78071333776301,0.0749592931060733,0.506639301719528
"2315","AC093702.1",9.31706088823897,1.0985160580118,0.617416165696336,1.77921492673077,0.0752045320969242,0.50691161691385
"2316","ZNF148",505.724324262712,0.305458057462502,0.171657942767878,1.77945775498168,0.0751647449406788,0.50691161691385
"2317","AC139019.1",3.56318513014217,1.56282222748032,0.87800884626987,1.77996182398368,0.0750822086263376,0.50691161691385
"2318","PGAM1",882.91287538059,-0.393532050807164,0.221061071280805,-1.7801960721853,0.0750438779944186,0.50691161691385
"2319","ZNF823",42.0204378935816,-0.481681778970247,0.270747505955415,-1.77908113048165,0.0752264618194352,0.50691161691385
"2320","FOSB",7827.13851610661,0.619416900527324,0.348071959570724,1.77956564295282,0.0751470731240534,0.50691161691385
"2321","ADRM1",398.133155536996,-0.433242042536997,0.243440560414989,-1.7796625254167,0.0751312068768558,0.50691161691385
"2322","AC080013.1",9.12661483698333,1.01949800349091,0.573126729275984,1.77883520592176,0.0752667834229422,0.506964897818612
"2323","AZI2",346.819558888403,0.32088200050856,0.180440436757112,1.7783264454214,0.0753502554165778,0.506981318904751
"2324","ENDOG",205.083217366948,-0.35733169902294,0.20093839636309,-1.77831467499746,0.0753521874761679,0.506981318904751
"2325","TBXA2R",24.8681848921197,-0.902155556978142,0.5073341095736,-1.778227680643,0.0753664684433214,0.506981318904751
"2326","GGCT",161.840423035975,-0.443144001874486,0.249255031455072,-1.777873847872,0.07542457629536,0.507154072768457
"2327","DVL1",155.766576125588,-0.315580039899076,0.177660694276243,-1.77630759119056,0.075682232698694,0.508015018946288
"2328","NRBP1",520.42617891262,-0.268826693451679,0.151308198248783,-1.77668293300057,0.0756204219018099,0.508015018946288
"2329","LINC00504",4.04791229527689,-1.35999916539726,0.765420129794319,-1.77680088680542,0.0756010059341098,0.508015018946288
"2330","PPWD1",142.617498831403,0.388622014502481,0.218781043538569,1.77630569914514,0.0756825443826631,0.508015018946288
"2331","CDC26",540.546556036822,-0.376447360823891,0.212034265718243,-1.77540813768339,0.0758305213183087,0.508789941406413
"2332","MBLAC1",20.6665123104548,-0.680736440181978,0.383501644844591,-1.77505481223643,0.0758888372217204,0.508962870560766
"2333","AP2A2",333.980021456236,0.317555151637485,0.178976360588578,1.77428544525757,0.0760159468258351,0.509596831699983
"2334","TMEM170B",31.4116787558633,0.535316315322732,0.301774089972426,1.77389753829312,0.0760801000197959,0.509698919723919
"2335","LRRC25",4.01134312777105,-1.63712999593454,0.922951089498958,-1.77379929940089,0.0760963540636414,0.509698919723919

"2336","TTC21B",105.456355982754,0.36492569526173,0.205764307905991,1.77351309843521,0.0761437233826086,0.509797874017123
"2337","COQ4",220.65904882582,-0.359704215554244,0.202857431984816,-1.77318726770221,0.0761976811277861,0.509940835617704
"2338","MTR",263.051620347824,0.434058719220109,0.245002269667783,1.77165182922053,0.0764523704873793,0.510860752910658
"2339","TUBB4B",1729.04065122888,-0.40657865386011,0.229483610685465,-1.77171107185243,0.0764425308143417,0.510860752910658
"2340","ZNF215",24.833304684759,-0.764466426585931,0.431518928167618,-1.77157101736447,0.0764657942815762,0.510860752910658
"2341","AC005736.1",22.3213288038875,0.865404843850372,0.488489427962788,1.77159380390172,0.0764620089767502,0.510860752910658
"2342","POLR1C",65.3637305867759,-0.496951313917411,0.280615665710479,-1.77093218462769,0.0765719795335667,0.510865618885246
"2343","HEY2",9.23113660345043,-1.21473872768487,0.685990499459826,-1.77078068667336,0.0765971787906586,0.510865618885246
"2344","SCAF8",158.95219434557,0.344160838987779,0.194340988648261,1.77091225778767,0.0765752936579743,0.510865618885246
"2345","ANO8",31.9393065509821,0.520748604985581,0.294068375676336,1.77084191317035,0.0765869939299762,0.510865618885246
"2346","TAS2R30",3.78279069472579,1.70972600175514,0.965986414760732,1.76992758451849,0.0767392049312325,0.51159469954155
"2347","TTC32",102.061157301022,-0.779952110843782,0.44106937553766,-1.76832070894296,0.0770073037634352,0.512125902779517
"2348","AC007098.1",15.3549337905755,0.784773904736363,0.443822402915313,1.76821606926883,0.0770247887897203,0.512125902779517
"2349","SELENOK",904.810015085699,-0.332441880144993,0.188033789268459,-1.7679901119812,0.0770625567212742,0.512125902779517
"2350","SEL1L3",15.2964734370785,1.11010246347624,0.62788571657851,1.76800082270614,0.0770607661222207,0.512125902779517
"2351","MAMDC2",219.360572737538,-0.857514027415151,0.485143031304974,-1.76754889193924,0.0771363485402104,0.512125902779517
"2352","ZNF618",151.969984829443,0.535953194222592,0.303052896960943,1.76851368060562,0.0769750671678591,0.512125902779517
"2353","SETX",510.623696731485,0.366844813371652,0.207507308202451,1.76786454679344,0.0770835510045546,0.512125902779517
"2354","MMAB",69.5602726408319,-0.633369854027865,0.358161994648288,-1.76838934195078,0.0769958371173316,0.512125902779517
"2355","GRK3",20.4150661292534,-0.662374952991362,0.374754732836432,-1.76748922682844,0.0771463316463262,0.512125902779517
"2356","STARD8",5.56886964091865,-1.28207732686307,0.725267656376807,-1.76772990714613,0.0771060677001713,0.512125902779517
"2357","MFSD4B",53.233521195379,0.65796947444345,0.37231278971723,1.76724918567309,0.0771865057293214,0.512175201360452
"2358","CEP72",5.76222250754293,1.40439821976609,0.794921829992981,1.76671235683449,0.0772764128780719,0.512554324602648
"2359","HSP90AB1",3215.81076651798,-0.247398488944724,0.140057302220204,-1.76640906988022,0.0773272445528552,0.512633601070736
"2360","CES2",143.485202600757,0.358977587633113,0.203242797864873,1.76624997984815,0.0773539193431545,0.512633601070736

"2361","FBXO31",66.9800954189884,0.434603360894399,0.246124355850626,1
.76578770269352,0.0774314723766391,0.512713051638711
"2362","MRPL4",265.901667094352,-0.42680017699512,0.241699448472639,-1
.76583016507559,0.0774243461162428,0.512713051638711
"2363","ZIC1",42.823875272126,-0.926241795747012,0.524722285577101,-1.
76520384440755,0.0775295127440868,0.5129213100697l
"2364","AP3M1",91.641868556063,0.380873419259998,0.215833076281475,1.7
646665924517,0.077619816376544,0.51292131006971
"2365","CREB3L1",260.865447741042,0.71704758113764,0.406217665319918,1
.7651806958541,0.0775334018880197,0.51292131006971
"2366","ZNF253",51.9353816774017,0.501095419415494,0.283967172858912,1
.76462446123821,0.0776269015943096,0.51292131006971
"2367","PFKL",241.468649408354,-0.355469205541912,0.201440340322494,-1
.76463763401525,0.0776246862681685,0.51292131006971
"2368","SPAG8",11.8705304106761,0.822469603891179,0.466257398995896,1.
76398188138655,0.0777350297267462,0.513293019843718
"2369","PICK1",17.2297086009808,-0.655361207597934,0.371540994831748,-
1.76390012600013,0.0777487956527984,0.513293019843718
"2370","PPP2R2B",12.0374876866099,1.45336573187287,0.824419992914315,1
.762894816191,0.0779182315142062,0.513718810053898
"2371","PAPSS2",576.01568335808,0.346297287150626,0.196427057902677,1.
76298159147811,0.0779035944791694,0.513718810053898
"2372","C17orf82",3.59035113695427,1.68338387197541,0.954982420672106,
1.7627380730115,0.0779446762313235,0.513718810053898
"2373","ARHGAP35",216.323108381705,0.42182143817733,0.239279274224779,
1.76288330672999,0.0779201730690531,0.513718810053898
"2374","ITGA6-
AS1",2.50054416931287,-1.82203998363266,1.03385559510585,-1.7623737708
2252,0.0780061672227244,0.513907521214579
"2375","GHR",105.845892794392,0.441627882166443,0.250783250437377,1.76
099433034792,0.0782393626183197,0.515226792147587
"2376","ZNF333",70.2836087741153,0.433505417378103,0.246225438417269,1
.76060369783344,0.0783055023290578,0.515445309943798
"2377","MPP4",2.83291129982566,-1.58087728113153,0.898081023712244,-1.
76028358176072,0.0783597365129706,0.515585308819041
"2378","ST3GAL5-
AS1",4.05516396962028,1.66168365849519,0.944173785197396,1.75993411864
086,0.0784189775864021,0.515758120038406
"2379","FDPS",509.344555173607,-0.456110696360097,0.259218101354379,-1
.75956344860556,0.0784818534927106,0.515911067192986
"2380","GTDC1",104.710706696978,0.358620667888016,0.203942092947605,1.
75844359889034,0.0786720602793263,0.515911067192986
"2381","EGFLAM",14.4348503612836,-1.04429592786489,0.593573937175016,-
1.75933588464983,0.0785204749654407,0.515911067192986
"2382","ZNF367",3.45897037409825,-2.07860708821844,1.18230145386742,-1
.75810245468202,0.078730078199151,0.515911067192986
"2383","ANP32B",921.01648016292,-0.318126628032678,0.180927844970294,-
1.75830662264789,0.0786953514566208,0.515911067192986
"2384","WASHC4",298.48079107885,0.266488132318062,0.151530680760839,1.
75864142482577,0.0786384322374746,0.515911067192986

"2385","NDUFAB1",765.635800796399,-0.414216240422207,0.235580409469184
,-1.75827965218131,0.0786999381240302,0.515911067192986
"2386","USB1",80.7492679756963,-0.441793970686129,0.251297820558676,-1
.75804935237381,0.0787391123650676,0.515911067192986
"2387","ZNF182",32.4677831464027,0.600865601896443,0.341619557405679,1
.75887354476871,0.0785989895392673,0.515911067192986
"2388","PPP1R11",158.454725087559,-0.35594698656289,0.20252169092048,-
1.75757463284588,0.0788199127175892,0.516224218971146
"2389","MTX1",268.61718422231,-0.469720226377113,0.26746316426098,-1.7
5620529905486,0.0790533602265414,0.517257878687995
"2390","FAM43A",269.065369253274,0.665374994377115,0.378872602843873,1
.75619717388566,0.0790547471026422,0.517257878687995
"2391","GLYATL2",3.99554879956747,-1.98365532043018,1.12965608992849,-
1.75598161078895,0.0790915485558341,0.517257878687995
"2392","CHADL",39.446691163931,0.763434541651432,0.434789059055476,1.7
5587339596331,0.0791100285052227,0.517257878687995
"2393","GLRX3",348.405259578944,-0.395763138295764,0.225477260130963,-
1.75522417677904,0.0792209700344821,0.517525008658414
"2394","MTERF2",97.5380963770526,0.418091615777392,0.238210934326604,1
.75513192523798,0.0792367446595508,0.517525008658414
"2395","LAGE3",125.802325989981,-0.478649467247983,0.272726421770678,-
1.75505352264863,0.0792501531801088,0.517525008658414
"2396","ADCY1",36.9555558988615,0.796860840204171,0.454164529267246,1.
75456423576239,0.0793338734033164,0.517855500846356
"2397","GPR146",22.5499992324639,1.05481372672837,0.601565916937506,1.
75344662493229,0.0795253737710643,0.518789606150057
"2398","NOC3L",197.75434242462,-0.29751384381447,0.169758695549735,-1.
7525690972767,0.0796759996146059,0.518789606150057
"2399","AL162274.1",3.25966606045999,1.89540964391036,1.08144180171365
,1.75266911349911,0.0796588203316102,0.518789606150057
"2400","EMILIN2",1229.69856816908,0.361206642341813,0.206095931902508,
1.75261412977661,0.0796682642361166,0.518789606150057
"2401","AC006942.1",14.2574810718031,-0.985644589206909,0.562376746284
223,-1.75264108219149,0.0796636348244785,0.518789606150057
"2402","PLCXD1",9.61546106349891,1.28146265297399,0.731099899258944,1.
75278734721876,0.0796385158046469,0.518789606150057
"2403","ALG1L",3.50054599748101,1.8402150464251,1.05083196464849,1.751
19820136101,0.0799117751914831,0.519655927930256
"2404","FAM66B",11.098358872509,0.931943545267021,0.532228053739743,1.
75102296603617,0.0799419541304473,0.519655927930256
"2405","AP001527.2",3.96115838794305,1.58550445738926,0.90530505581391
8,1.75134828553874,0.0798859351319123,0.519655927930256
"2406","GALNT8",24.3179037475184,0.733504277274348,0.418790066771412,1
.75148442017541,0.079862502644746,0.519655927930256
"2407","FAM171B",108.004956880729,0.512386590090925,0.292751887643242,
1.75024179763902,0.0800765992503303,0.520098842306963
"2408","CMTM8",14.0670415189044,0.972689366130208,0.555713012396022,1.
75034477227075,0.0800588396182605,0.520098842306963
"2409","INPP5B",54.3968095144232,0.556000693295205,0.317947455667917,1
.74871880049238,0.080339638607361,0.521374252207106

"2410","ZNF133",33.7462403341229,0.592517892265848,0.338825576400395,1
.74874015875844,0.0803359449390562,0.521374252207106
"2411","SPAST",106.474843637962,0.375012665090587,0.21451887019563,1.7
4815700245202,0.0804368447157773,0.521670076816099
"2412","METAP1D",41.0726532078352,0.490811367238965,0.280773314957178,
1.74806985241393,0.0804519325626874,0.521670076816099
"2413","PRRC2A",304.718682415621,0.275172447951164,0.157449155402926,1
.74769084817872,0.0805175743949666,0.521879346679352
"2414","PYGL",76.1370504294939,-0.424239786746473,0.24282662223088,-1.
74708927237437,0.0806218539393558,0.52233877200146
"2415","LHFPL5",4.55998981276006,2.36979158181016,1.35666453465677,1.7
4677786679941,0.0806758772898414,0.522472348162782
"2416","USP46",146.746672428351,0.371700283705684,0.212914080913866,1.
74577595859456,0.0808498900917486,0.523382566653538
"2417","CDC14A",75.6924674562156,0.532537108988069,0.305187612917384,1
.74494994700925,0.0809935820150519,0.523509910249525
"2418","SLC39A1",628.713476116316,-0.382689061963403,0.219309194014759
,-1.74497500518674,0.0809892198790475,0.523509910249525
"2419","ZNF354C",60.0447154436559,0.476942900797832,0.273408776151193,
1.74443157060213,0.0810838639497201,0.523509910249525
"2420","ZNF37A",265.03849640443,0.431699336809507,0.247543566421711,1.
74393276726922,0.0811708140892006,0.523509910249525
"2421","FRY-
AS1",3.35743216587165,-1.88023269281844,1.07803231197963,-1.7441338927
6402,0.0811357452982521,0.523509910249525
"2422","LINC01578",963.267470047846,-0.313427775594505,0.1797245689421
3,-1.74393393980222,0.0811706096074928,0.523509910249525
"2423","UPF1",188.800714531888,0.359547153681225,0.206140832480169,1.7
4418211741632,0.0811273385443142,0.523509910249525
"2424","ZNF714",43.2364943011326,0.675311987765606,0.387198085442233,1
.74409950140716,0.0811417409973773,0.523509910249525
"2425","TXLNG",174.458013731543,0.378332298025298,0.216916648864375,1.
74413674563932,0.081135247951595,0.523509910249525
"2426","PIK3CA",251.998337967911,0.332420630641809,0.190712549851764,1
.74304538899087,0.0813256867122495,0.524176023113263
"2427","DCTN5",144.363834120446,0.392706603533615,0.22531055157614,1.7
4295700217531,0.0813411258373331,0.524176023113263
"2428","ZNF587",55.7110942559515,0.673775644503043,0.386868962226144,1
.74161204513788,0.0815763522407593,0.52547534968924
"2429","MAP3K12",152.396623960458,0.381341388501067,0.219055011557458,
1.74084758796327,0.0817102978834492,0.525950152947862
"2430","AC009163.7",3.72938035021655,1.60270421309058,0.92076851233871
4,1.74061579171487,0.0817509476864611,0.525950152947862
"2431","LINC00205",34.342984089658,0.743103739132347,0.426900565957629
,1.74069513697038,0.0817370311670925,0.525950152947862
"2432","ARID1A",278.379934193474,0.371471322645233,0.213600785848203,1
.73909155422875,0.082018660013334,0.526520533596943
"2433","CPSF3",78.6155474127309,-0.442154660334615,0.254141826720772,-
1.73979492490394,0.0818950339580161,0.526520533596943
"2434","AC008883.3",4.19526639992533,1.43686917303366,0.82628253754780

3,1.73895623801748,0.0820424608548648,0.526520533596943
"2435","NUMA1",651.027375770223,0.30836076405774,0.177275789496388,1.7394409294904,0.0819572341041131,0.526520533596943
"2436","STARD13",108.749401239215,0.574274967022195,0.330174987026344,1.73930488252393,0.0819811489579088,0.526520533596943
"2437","AC092068.2",22.0098932216646,-0.828148375334496,0.476043958669809,-1.73964685456477,0.0819210465749654,0.526520533596943
"2438","LINC00893",17.4895955489919,0.902937236948794,0.519296589982251,1.73876981741716,0.0820752596489352,0.526520533596943
"2439","SPA17",47.8635418167948,-0.537320535711932,0.309088115434252,-1.7384056807135,0.0821393564421343,0.526715676406306
"2440","STARD7",173.728908714865,0.355475426563197,0.204575232697623,1.73762689586483,0.0822765775029265,0.526947449691143
"2441","PDCL3",120.601615182312,-0.494635043224223,0.284639009528335,-1.73776266311446,0.0822526420895047,0.526947449691143
"2442","AC021097.1",5.77092271834844,1.30641019930076,0.751792155632628,1.73772789395685,0.0822587712637838,0.526947449691143
"2443","UBE2E3",1241.39984163131,-0.448989351798921,0.258550172458084,-1.73656566356269,0.0824638646641651,0.52776273240786
"2444","ATXN7",218.768678094702,0.512868266369602,0.295342015396311,1.73652321591088,0.0824713630437858,0.52776273240786
"2445","MBNL1-AS1",31.2810604480846,-0.488332982664777,0.281313337732933,-1.73590412242871,0.0825807887688935,0.528246845131082
"2446","SPART-AS1",2.8890376399343,1.83666707789725,1.05882605128374,1.73462588653768,0.0828070912830805,0.529477885391406
"2447","PHLDB2",427.404997337177,0.394709226480784,0.227616206312421,1.73409983794834,0.0829003702815002,0.529711898312691
"2448","FOCAD",71.4819032031722,0.354040906025461,0.204171422804414,1.73403751202055,0.0829114275619865,0.529711898312691
"2449","TIA1",245.564160102235,0.339647556343253,0.195961775496019,1.7332337160323,0.0830541366161268,0.530269935734762
"2450","PPP1R1A",3.80763793771839,-1.38366964517699,0.798349092980383,-1.73316367156065,0.0830665820044864,0.530269935734762
"2451","NBPF15",40.7424821335485,0.66208107139555,0.382079969011402,1.73283376542514,0.0831252195120952,0.530427757310963
"2452","KLHDC10",182.994220955302,0.406292609047752,0.234565021353979,1.73211080962758,0.0832538349255195,0.531031801890344
"2453","TLR5",82.2236550006011,0.508516638135856,0.293636485027,1.7317896925823,0.0833110140287502,0.531179885613393
"2454","CEP192",86.9656222158385,0.440866825868714,0.254642875481041,1.73131419850597,0.0833957404100136,0.531503414838065
"2455","DIPK2A",142.262009961789,0.513190892879494,0.296509029458225,1.73077661013287,0.0834916151681557,0.531836941577763
"2456","NR6A1",9.91534713411766,1.65472947035928,0.956137585259427,1.7306394977772,0.0835160823858686,0.531836941577763
"2457","TUBB2A",546.153168442693,-0.585582167780775,0.33843371537449,-1.73027136830267,0.0835818025220287,0.532038824356748
"2458","MTCH2",174.272205408046,-0.385471585918546,0.222880741870097,-

1.72949705158112,0.0837201737315829,0.532702814142375
"2459","BRD7",508.509491636392,-0.265072013711467,0.153326896820011,-1
.7288030946236,0.083844342122713,0.533275929564551
"2460","NBPF12",91.6805645619354,0.709514166471077,0.410735968907951,1
.72742155589029,0.0840919820740351,0.53428314130413
"2461","TMEM131",223.530468767295,0.370467181082917,0.214512582575877,
1.72701841838054,0.084164355699061 7,0.53428314130413
"2462","NICN1",25.4531574717157,0.595047790653619,0.344597641938465,1.
72679008279423,0.0842053702097332,0.53428314130413
"2463","GPRC5D-
AS1",3.33126748823762,-1.55546728056353,0.90056824441951,-1.7272064501
521,0.0841305928547813,0.53428314130413
"2464","RABEP1",327.950139851317,0.331963625131906,0.192232197969677,1
.7268887763759,0.0841876405069569,0.53428314130413
"2465","PSMD8",1210.11829664495,-0.416987457055794,0.241483056355953,-
1.72677728760043,0.08420766900989,0.53428314130413
"2466","UBR3",178.649816877582,0.368740098488739,0.213566175129215,1.7
2658473780147,0.0842422688758048,0.534285922634869
"2467","MARCH7",373.750607205984,0.237446614134389,0.137563233001195,1
.72609067811255,0.0843311006198581,0.534458495312369
"2468","DLEU1",19.561345311874,-0.612773475426843,0.355045579365874,-1
.72590087312531,0.0843652476324697,0.534458495312369
"2469","AL049838.1",14.9611120746696,0.812591142172007,0.4708639180615
85,1.72574519091889,0.0843932641114342,0.534458495312369
"2470","TOB1",327.392730095514,0.331106404112038,0.191870829036015,1.7
2567349490051,0.0844061690167233,0.534458495312369
"2471","NAA30",93.2973673497084,0.336806951050319,0.1952137958199,1.72
532350818612,0.0844691877044463,0.53464107474607
"2472","KMT2D",73.6642358004461,0.521121922380064,0.302178491952713,1.
72455001351192,0.0846085983784988,0.535090367424069
"2473","DEDD2",62.437721523239,-0.385729447493224,0.223647630586548,-1
.72471957999999,0.0845780206728909,0.535090367424069
"2474","XRN1",304.041998035736,0.293384208083361,0.170188788979026,1.7
2387505571544,0.0847304013093425,0.535355239338144
"2475","CCNY",651.86222617891,-0.248944757337483,0.144422518964382,-1.
72372535199223,0.0847574360637904,0.535355239338144
"2476","TXNDC17",460.615780214071,-0.391274467734595,0.227013895611119
,-1.72357056241552,0.0847853966010952,0.535355239338144
"2477","NEDD4L",10.5604407184651,1.03348765463527,0.599624015872235,1.
72355947606921,0.0847873994782981,0.535355239338144
"2478","TET2",92.8347682991845,0.512008840261466,0.297222854821693,1.7
2264289894068,0.0849531222072557,0.536070148387484
"2479","RUFY2",242.001245797917,0.332223607386436,0.19286685385006,1.7
2255419090683,0.0849691750545124,0.536070148387484
"2480","PRRX1",3328.78297619913,0.21085672639853,0.122553810508935,1.7
2052362568651,0.0853373029225328,0.536613965067329
"2481","COX20",439.986685424275,-0.268791348197724,0.156146264235799,-
1.72140748620036,0.0851769067634514,0.536613965067329
"2482","PCDHGB5",3.46393965505348,1.78878302324663,1.03962472602216,1.
72060453976593,0.0853226091155653,0.536613965067329

"2483","STK32A",4.07158560540526,-1.46510348650558,0.851416871811919,-1.72078277399843,0.08529024941245,0.536613965067329
"2484","RASA4",8.37404953079528,-0.944174257367369,0.548818632811874,-1.72037573237973,0.0853641652869254,0.536613965067329
"2485","HMBOX1",138.28006108604,0.459597471311066,0.267079580093304,1.72082594689758,0.085282412556025,0.536613965067329
"2486","MAN1B1-DT",26.2550104310428,-0.538659176934961,0.31300820655509,-1.72091071625036,0.0852670266974645,0.536613965067329
"2487","SLC23A2",76.9430045411542,0.37540939390989,0.218172068239265,1.72070328222853,0.0853046804872904,0.536613965067329
"2488","ZHX3",266.232061598468,0.498514190940293,0.289691029757528,1.72084786801148,0.0852784336015338,0.536613965067329
"2489","KCNC4",11.9712585473497,0.922758465509513,0.536620797323172,1.71957268542798,0.0855101447930701,0.537315654706154
"2490","RGPD8",8.93635662030119,1.0322593985844,0.601829483581936,1.7152024398018,0.0863081177213801,0.537392162537619
"2491","TTC30B",23.2630707017691,-0.556985312222021,0.324715825872919,-1.7153007886964,0.086290094051213,0.537392162537619
"2492","CHPF",173.154859063741,-0.422666172381824,0.246195151982762,-1.71679323893193,0.0860169567365833,0.537392162537619
"2493","DOCK10",18.503680145265,-0.788027037220169,0.45944330690217,-1.7151779672959,0.0863126030878836,0.537392162537619
"2494","CHIC2",334.111644700095,-0.339858319589928,0.197992921113243,-1.71651752839964,0.0860673625906111,0.537392162537619
"2495","KDR",7.06184002442237,2.05400943651393,1.19614630043643,1.71718913962656,0.0859446192344263,0.537392162537619
"2496","PCDH18",777.442584969042,0.266437736778017,0.155320799890632,1.71540281125018,0.0862714003496241,0.537392162537619
"2497","TMEM200A",6.77630450847624,-1.09824388004501,0.639277068015419,-1.71794662282259,0.0858063517178791,0.537392162537619
"2498","SEC24C",106.789520929924,0.361043647944753,0.210296121696794,1.71683455230481,0.0860094058127522,0.537392162537619
"2499","CD151",1997.59819867661,-0.438582625046597,0.255567904488158,-1.71610995490601,0.086141919558275,0.537392162537619
"2500","STIP1",203.63849501236,-0.400426433245034,0.23292874793808,-1.71909408688137,0.0855972413330495,0.537392162537619
"2501","LRTOMT",48.8809448010464,-0.52515504051008,0.305686229597342,-1.71795452219692,0.0858049107498636,0.537392162537619
"2502","AASDHPPT",192.739901519342,0.254867972352001,0.148521622579014,1.7160327764155,0.086156043607732,0.537392162537619
"2503","TMPO",188.349102026843,0.393346177815021,0.22905974227964,1.71722090446962,0.0859388174111992,0.537392162537619
"2504","ZNF605",87.2731394277648,0.498134910792087,0.290120002280899,1.7169960942913,0.0859798856172345,0.537392162537619
"2505","PNN",668.035558702779,0.272543549790125,0.158868746818472,1.71552652896256,0.0862487358096196,0.537392162537619
"2506","FAM161B",36.58126997834,0.530446376931209,0.309224452452662,1.71540889707748,0.0862702853403751,0.537392162537619
"2507","SMAD3",602.215649417583,0.373540160021612,0.217707214468667,1.

71579137114619,0.0862002340561306,0.537392162537619
"2508","FAM222B",75.3828133955521,0.410743682949996,0.239000664473135,
1.71858803763354,0.0856894113942353,0.537392162537619
"2509","MSL3",138.533366677322,-0.330566636765994,0.192346317267838,-1
.71860133046211,0.0856869892589794,0.537392162537619
"2510","BCLAF3",134.456112055592,0.372587125821818,0.216727518141378,1
.71915006002499,0.0855870515017314,0.537392162537619
"2511","TMSB15A",4.67091316169087,1.69834591951487,0.989987345943446,1
.71552285640214,0.0862494085372398,0.537392162537619
"2512","GABRE",146.734525817866,0.591715038946445,0.344560672107207,1.
7173028927757,0.085923843775091,0.537392162537619
"2513","MTIF3",446.124531822373,-0.326217495038522,0.190231328601557,-
1.71484632650488,0.0863734053961201,0.537556729166462
"2514","SETD7",945.749919271535,0.289283531591659,0.16871356669016,1.7
1464297309845,0.0864107048776838,0.537574949994819
"2515","CFAP410",199.218227025742,-0.471667036970643,0.275115729350516
,-1.71443137069675,0.0864495312162026,0.537602651380282
"2516","ATP2B1",1039.87415756086,0.395491284277519,0.230732293917764,1
.71406991870187,0.0865158856285739,0.537801451204649
"2517","CEP170",401.084919422545,0.292515864981056,0.170687929594989,1
.7137466350148,0.0865752680407356,0.537956770821257
"2518","AC011468.5",4.54941475117139,1.26410898370823,0.73777524526725
1,1.7134066124031,0.0866377606535267,0.538131285393629
"2519","ATP8A1",31.509251230804,0.812144933905008,0.474114764077535,1.
71297119482276,0.0867178390474042,0.538414848233982
"2520","EPHB4",42.3687831632132,0.513460679569584,0.299913527782951,1.
71202907506452,0.0868913104503225,0.539277815652002
"2521","KDM5A",462.132311049825,0.335474861939455,0.196012935976268,1.
71149348010415,0.0869900537489189,0.539472029947764
"2522","ALDH16A1",176.312818707486,-0.395581245331811,0.23113331974415
3,-1.71148515397819,0.0869915894839041,0.539472029947764
"2523","POLD2",335.694727095908,-0.402475959819004,0.235324117002251,-
1.71030477005956,0.0872095301807611,0.540128505495174
"2524","LINC02381",441.805468607749,0.382735435029047,0.22376468829884
8,1.71043714689195,0.0871850668109949,0.540128505495174
"2525","FBXW9",49.91548316265,-0.668293590683336,0.39077753401259,-1.7
1016379529588,0.0872355885473664,0.540128505495174
"2526","LINC00894",17.4596104242304,0.979320643563896,0.57248887494298
1,1.71063698602254,0.0871481468328175,0.540128505495174
"2527","GIPC2",9.22705133342996,-1.00787851184688,0.58946330432339,-1.
70982401186747,0.0872984213656336,0.540303644700637
"2528","KCNS3",47.0172085583886,-0.632901782968281,0.37032311308596,-1
.70905288004903,0.0874411547042386,0.54037271382315
"2529","PCDH9",173.116954686923,0.836378675650091,0.489352823665604,1.
70915265060701,0.0874226769848937,0.54037271382315
"2530","AC060766.4",2.94697844718843,1.80594529492662,1.05651750504581
,1.70933778787538,0.0873883975166483,0.54037271382315
"2531","ARHGEF1",148.103025167237,-0.297324435347157,0.173973939189302
,-1.70901709033349,0.0874477838034778,0.54037271382315
"2532","RUFY3",519.750103443724,0.366094692101493,0.214268868384637,1.

70857621483449,0.0875294776093408,0.540450465775796
"2533","CAMKK1",24.3495881401188,-0.852825091752902,0.499129234057768,-1.70862580983224,0.0875202846314972,0.540450465775796
"2534","SHARPIN",347.964380721589,-0.380344507550753,0.222662352194726,-1.70816711402621,0.0876053386602556,0.540455815790173
"2535","RBM17",482.265772141891,-0.218031204391892,0.127636251185598,-1.70822319181758,0.0875949368155523,0.540455815790173
"2536","MBTD1",85.790827810404,0.379469850643229,0.222170410012177,1.70801255946924,0.0876340120744167,0.540455815790173
"2537","TDRD10",26.309121541615,0.629458592227752,0.368808703311231,1.70673464746455,0.08787138424404,0.54167949657747
"2538","CDK9",186.886018733023,-0.324803803393368,0.190325324737282,-1.70657164957798,0.0879016983576482,0.54167949657747
"2539","LRRC37A3",25.6835621617211,0.59382039841011,0.348046301323807,1.70615345185825,0.0879795127509871,0.54194548224712
"2540","AKAP9",1042.41020898837,0.347244317981391,0.203551625486558,1.70592751176198,0.0880215767183462,0.541947185872848
"2541","CKB",896.673962876273,-0.583289928542637,0.341949142928654,-1.70577976463692,0.0880490920270401,0.541947185872848
"2542","DYNC2H1",214.473653236095,0.374123364900185,0.219370941818808,1.70543720056231,0.0881129152568762,0.54212666979447
"2543","CCND3",88.8676543621392,-0.422129979354901,0.247692877215739,-1.70424755083784,0.0883348490682336,0.543278426829404
"2544","ACKR4",549.359728178822,0.597529503096817,0.350774890854765,1.70345574519534,0.088482813133002,0.543815672281818
"2545","KIF5B",977.172028112084,-0.261820118443886,0.153717381036256,-1.70325643514661,0.0885200894917359,0.543815672281818
"2546","PPAN",64.3648594203329,-0.562452616735715,0.330228583913928,-1.70322208353204,0.0885265154494572,0.543815672281818
"2547","PIKFYVE",99.6862647526972,0.40797388107545,0.239624060072166,1.70255808599764,0.0886507995151632,0.544030666133535
"2548","BAP1",106.263844999523,-0.346973554058725,0.203820779539437,-1.70234632034458,0.0886904664177513,0.544030666133535
"2549","ZNF454",8.00087273190541,-0.963205036083112,0.565736041786026,-1.70256968787472,0.0886486267216897,0.544030666133535
"2550","LPCAT3",111.759988714789,0.58016790617639,0.340852915226398,1.70210633460781,0.0887354366564353,0.544030666133535
"2551","UTP6",157.737078385745,-0.319864693063882,0.187906186963814,-1.70225737764281,0.0887071309928955,0.544030666133535
"2552","WDR6",131.576460572723,0.390998084260962,0.229895948650998,1.70076109020317,0.0889878584419514,0.544821771465922
"2553","TMEM161B-AS1",145.288705719536,0.314784123846663,0.185113689487476,1.70049078875909,0.089038647561822,0.544821771465922
"2554","AATF",213.859859499012,-0.281561893908954,0.165564150843677,-1.70062113370666,0.0890141530829688,0.544821771465922
"2555","PSMG1",92.6574553114188,-0.522502413896746,0.307131856759276,-1.70123157984967,0.0888995100186624,0.544821771465922
"2556","TMSB4X",51031.4020762079,-0.475291586110462,0.279418257227103,-1.70100404614635,0.0889422274095687,0.544821771465922

"2557","ERBIN",294.59423052591,0.323431691171328,0.190234075134448,1.700177483675,0.0890975462013887,0.544968956820383
"2558","ZDHHC1",155.87603951149,-0.366235005336563,0.215473337396567,-1.69967667351124,0.0891917593492239,0.545331945356474
"2559","JAGN1",356.084935101032,-0.392607628387451,0.231096032481937,-1.69889385019251,0.0893391859631018,0.545593466795358
"2560","WDR48",174.145443471769,0.31843672641247,0.187421138108578,1.69904381984914,0.0893109274691733,0.545593466795358
"2561","SCAPER",132.166975352399,0.482077868689314,0.28369884195719,1.69925920516115,0.0892703554237599,0.545593466795358
"2562","TNRC18",296.692009932502,0.391984502190315,0.230794650071862,1.69841242883344,0.0894299479284829,0.54589998477292
"2563","ETNK1",212.34043437399,0.29516797075696,0.173806376762493,1.69825743022253,0.0894591854842068,0.54589998477292
"2564","AL080250.1",11.7926363190605,0.90208808394825,0.531581678725725,1.6969886661833,0.0896988033131652,0.546332400090604
"2565","SCARA3",324.825214213021,-0.427462213849658,0.251808151232845,-1.69757099504848,0.0895887610380923,0.546332400090604
"2566","TMEM68",78.5625138966711,0.366699063629717,0.21607445354412,1.69709587419986,0.0896785361207392,0.546332400090604
"2567","BCAR1",96.6909334841061,-0.348101090221335,0.20507534270509,-1.69743024992489,0.0896153475784926,0.546332400090604
"2568","SMAD7",147.463105073106,-0.655355528934205,0.386194439113612,-1.69695744568039,0.0897047061018332,0.546332400090604
"2569","HOXB5",8.1829923586658,-1.01831621518826,0.600227529157367,-1.69655033420049,0.0897817063645035,0.546588512082847
"2570","TTC27",208.366376292536,0.532816401024477,0.314137091494638,1.69612699503067,0.0898618323253077,0.546863446524441
"2571","SLC22A23",38.1695531013356,0.5199888664321,0.3066333319777,1.69580020240564,0.0899237241555076,0.547027244571038
"2572","OSTF1",273.715076535039,-0.391463878843103,0.230903466399124,-1.69535730644444,0.0900076597605617,0.54718698947634
"2573","TNFSF8",3.18954753253643,1.8387788724588,1.08463815484611,1.69529244775618,0.0900199567725462,0.54718698947634
"2574","PARD6G",5.05093789458371,1.43044992383933,0.843872202940958,1.6951025509006,0.090055968420078,0.547193219149192
"2575","KAT6A",301.134378097469,0.380633390565402,0.224603481537264,1.69469051842035,0.0901341452812471,0.547455546484934
"2576","QSOX1",191.927577306945,-0.366256174837889,0.21620149978058,-1.69405011163011,0.0902557610795442,0.547931214675636
"2577","HES1",236.187200367222,-0.432598106542353,0.255456674519727,-1.69343043142506,0.090373566486571,0.547931214675636
"2578","TSPAN15",23.9055398400355,-1.00689804509853,0.594616695571586,-1.69335649771931,0.0903876300424003,0.547931214675636
"2579","RRM1",139.826071043674,-0.296748870166703,0.175216406923639,-1.6936134884676,0.0903387532335678,0.547931214675636
"2580","GDF11",70.7099219101943,0.560783952473203,0.331076054397821,1.69382214456188,0.0902990847882066,0.547931214675636
"2581","PAPOLG",64.8809333910947,0.392153234602519,0.231697959506004,1.69251915484546,0.0905470310508428,0.548049378850163

"2582","ARPC4",611.65931196477,-0.332139313662421,0.196439190778574,-1
.69079964311607,0.0908750753858248,0.548049378850163
"2583","AC079848.1",6.05827138119801,-1.05981368052975,0.6269919766028
69,-1.69031458149109,0.0909677869242342,0.548049378850163
"2584","TBC1D9",121.812056874865,0.40004283496243,0.236545597203176,1.
6911869833655,0.0908010962599586,0.548049378850163
"2585","HHIP-
AS1",19.995393431628,-1.05844043478766,0.625254059250147,-1.6928165745
2496,0.0904903867937091,0.548049378850163
"2586","FOXD1",15.397949346933,0.920961570534447,0.544795242329917,1.6
9047285838214,0.0909375265474646,0.548049378850163
"2587","ARID1B",613.171263274265,0.380446943124544,0.225048894210147,1
.69050794255087,0.0909308200304286,0.548049378850163
"2588","LINC00648",27.3662288950118,0.69263279323036,0.40973163272836,
1.69045477064632,0.0909409842654281,0.548049378850163
"2589","IL16",62.5911334535032,-0.81528805243937,0.48191002672051,-1.6
9178478810155,0.0906870150461552,0.548049378850163
"2590","JMJD8",252.325722475811,-0.415626997636344,0.245739172391925,-
1.69133391958148,0.0907730452039274,0.548049378850163
"2591","NUTF2",288.857728574404,-0.388783364304724,0.229814284394797,-
1.69172845512438,0.0906977603468895,0.548049378850163
"2592","NME2",4445.8324740486,-0.446565788839945,0.264163360578508,-1.
69049101988248,0.0909340548347791,0.548049378850163
"2593","CCDC47",444.019425980591,-0.261537625194164,0.154578455031301,
-1.69194099618349,0.0906572243062612,0.548049378850163
"2594","CCDC124",425.547739212326,-0.312904735170891,0.184909070781842
,-1.69220868315357,0.0906061915198415,0.548049378850163
"2595","GDF15",47.4807158316623,0.91174110097811,0.539289348650895,1.6
9063435660087,0.0909066586411815,0.548049378850163
"2596","AL022322.1",7.52411696045479,1.05134843367823,0.62156126720536
8,1.6914638815981,0.0907482404393164,0.548049378850163
"2597","GNG10",620.174605324605,-0.305766653243439,0.180946850802076,-
1.68981472674479,0.0910634054846627,0.548414193985416
"2598","AC019171.1",11.8546769557074,0.981210723034811,0.5807892627989
24,1.68944363452276,0.0911344449720318,0.548472176635568
"2599","DNAJC4",273.237873682825,-0.490043102242333,0.290097565256901,
-1.68923548809645,0.0911743106749675,0.548472176635568
"2600","U2AF2",155.985190678507,0.38224002021554,0.226282636901707,1.6
8921498109278,0.0911782390826391,0.548472176635568
"2601","GTPBP6",283.338856534031,-0.350010525979918,0.207245768205487,
-1.68886693808328,0.091244932418631,0.548662338726409
"2602","NKD2",2.5946515734453,2.40949358941016,1.42744751346414,1.6879
73509837,0.0914163141314729,0.548788898013371
"2603","NOTCH4",16.4048855638292,-0.955770159945376,0.566154647123246,
-1.6881786006736,0.0913769497551613,0.548788898013371
"2604","PHKG1",64.4373529120445,-0.754169018148595,0.446824225638966,-
1.68784272399313,0.0914414237994146,0.548788898013371
"2605","LCNL1",2.51776433382109,-1.75403787565498,1.03919194951431,-1.
68788632020752,0.0914330531150467,0.548788898013371
"2606","AC008149.2",4.92756856708419,-1.19477248398102,0.7076912525335

85,-1.68826798367742,0.0913597981758739,0.548788898013371
"2607","GDI2",1046.25630981952,-0.304403433341624,0.18042131731085,-1.68718108191819,0.091568537911982,0.549340979264825
"2608","AL162231.1",22.8304231920523,-0.614637412623662,0.36452923790979,-1.6861127961861,0.0917740757841454,0.549827143218016
"2609","TMX1",272.561008221259,-0.234863811434873,0.139270023441614,-1.6863916988808,0.0917203792591123,0.549827143218016
"2610","ZNF90",23.0693157332716,0.629028680450464,0.373123322360031,1.68584658946488,0.091825351539991,0.549827143218016
"2611","FCGBP",4.56142022281249,-1.59377090049043,0.945299151417282,-1.68599633047474,0.0917965061477638,0.549827143218016
"2612","CARD8-AS1",26.2390064692731,-0.641677211595255,0.380570526840626,-1.68609276425648,0.0917779334597846,0.549827143218016
"2613","ECT2",39.1362775034829,-0.456527513115525,0.270921875497309,-1.68508915080229,0.0919713725158065,0.550490725659094
"2614","FAM204A",563.693574049971,-0.287480739922825,0.170644166310466,-1.6846795653113,0.0920504111411139,0.550584109061812
"2615","EPS8L2",131.810023466897,-0.303880848557346,0.180402410462734,-1.68446113207628,0.0920925849939707,0.550584109061812
"2616","PRPF40B",33.1123913363719,0.498880020475709,0.296136358196226,1.68462941705098,0.092060092110322,0.550584109061812
"2617","INPP5F",48.0970822748554,-0.404580339486632,0.240260724370609,-1.68392208317226,0.0921967279238926,0.550996111856966
"2618","ELOVL1",322.552314850647,-0.421115982676749,0.250141855391259,-1.68350867158182,0.0922766621088575,0.551039701581152
"2619","TUT4",350.534762135293,0.368464821917192,0.218866741606409,1.68351216458373,0.0922759864949476,0.551039701581152
"2620","EFS",250.427750690998,0.299678202606009,0.178026184273574,1.68333778443227,0.0923097198300907,0.551039701581152
"2621","CALM1",5317.30119751325,-0.278504427356611,0.165477898901006,-1.68303096187619,0.0923690978085593,0.551183780895028
"2622","PCDHB10",17.5910318747456,0.741288218404773,0.440570317599205,1.68256504987505,0.092459322966822,0.551301487121658
"2623","FICD",56.9616030068406,-0.46326572682372,0.275307415245271,-1.68272157294055,0.0924290035029624,0.551301487121658
"2624","PHLPP1",177.636595188891,0.433570733011412,0.25772301435474,1.68231282757941,0.0925081950028784,0.551382686678741
"2625","TRAK1",154.495972337101,0.492849937207814,0.293047057357705,1.68181158907261,0.0926053807512907,0.551751678076261
"2626","CLIC5",5.48448893683537,-3.22698787951918,1.91905385085401,-1.68155149897597,0.0926558422347393,0.551842106835995
"2627","SUN1",601.845458906208,0.263540053164958,0.15676401248799,1.68112597389116,0.0927384482571008,0.551881700476806
"2628","DNAJB9",207.170229291032,-0.399623678983532,0.237733688967979,-1.68097201838886,0.0927683497796371,0.551881700476806
"2629","SLC1A1",8.88778006006414,-1.10966579882653,0.660033095477654,-1.68122751181665,0.0927187316080847,0.551881700476806
"2630","MAP4",949.815913519624,-0.333427077166576,0.198485945067591,-1.6798523293578,0.092986050761952,0.552756303275154

"2631","COQ2",66.9469294058824,-0.464130418523648,0.276272273221082,-1
.67997466091082,0.0929622459205746,0.552756303275154
"2632","AL121929.3",7.41964990684501,-0.91481676845361,0.5447986441343
47,-1.67918326945765,0.0931163315794095,0.553320450570655
"2633","TBL1XR1",622.206440212787,0.314691599332628,0.187432632661153,
1.67895843356977,0.0931601449895388,0.553370553602881
"2634","MARCH6",388.693244878789,0.340105507658012,0.202592082415278,1
.67876998747096,0.0931968799223273,0.553378588452999
"2635","USP33",406.204073648184,0.303756415586896,0.181065747180804,1.
67760286148202,0.0934246538288571,0.553710672175019
"2636","XPO7",115.374615127756,0.375776784661242,0.224023677723014,1.6
773976236827,0.0934647537521797,0.553710672175019
"2637","QSER1",211.883664808467,0.383642502139633,0.228651691417339,1.
67784677104969,0.0933770160602569,0.553710672175019
"2638","COPS3",219.720579327828,-0.419879823902836,0.250241513082159,-
1.67789835799539,0.0933669431554534,0.553710672175019
"2639","MED29",326.567764728611,0.263206616124387,0.156841430172544,1.
67817021200858,0.0933138751438809,0.553710672175019
"2640","ZNF347",69.6389686640621,0.451681690559099,0.269284625304242,1
.67733932098345,0.0934761476113758,0.553710672175019
"2641","RFPL1S",2.85340413271064,1.8097339924638,1.07901194217061,1.67
721405272237,0.0935006320469453,0.553710672175019
"2642","PTPRD",203.308301099128,0.391675834213063,0.233657603012838,1.
67628114455811,0.0936831364362441,0.55442088662313
"2643","NLGN3",6.59901240191399,1.19134088582959,0.7107227193422,1.676
23864188867,0.0936914580143819,0.55442088662313
"2644","CEP295NL",5.81463454237175,1.5137121536742,0.903304766405355,1
.6757491048098,0.0937873470017955,0.554568660532356
"2645","RUVBL2",161.344295590319,-0.413911934440226,0.246975033653425,
-1.67592621941317,0.0937526452657927,0.554568660532356
"2646","TRIM17",3.21561777944519,1.7291926684518,1.03237016542237,1.67
497349920447,0.0939394313207409,0.554903463336825
"2647","ITSN2",251.724359005921,0.419416557666579,0.250478658865431,1.
67446024969301,0.0940401803400042,0.554903463336825
"2648","TKT",487.19258791739,-0.285189668445896,0.170334998514855,-1.6
7428696939827,0.0940742141908227,0.554903463336825
"2649","ZBTB38",703.438104995753,0.325888406330296,0.194590847250206,1
.67473656102266,0.0939859306662059,0.554903463336825
"2650","ACSL1",89.7946646244815,0.340345889822951,0.203355078966063,1.
67365325495386,0.0941987656751452,0.554903463336825
"2651","ARC",87.3525666410843,-0.846174596367969,0.505551785783455,-1.
67376442960566,0.0941769056330516,0.554903463336825
"2652","MLLT3",140.571760697307,0.418376815942058,0.249770227350936,1.
67504678351525,0.0939250529405066,0.554903463336825
"2653","RASSF4",262.49426774035,0.552498063998054,0.330108415448271,1.
67368669849809,0.0941921893123724,0.554903463336825
"2654","CCND1",254.268872546331,-0.698224229323828,0.416898112546172,-
1.67480784467811,0.0939719392397385,0.554903463336825
"2655","TMEM160",544.625000303485,-0.377649876328369,0.225591876048657
,-1.67404023116026,0.094122692903184 8,0.554903463336825

"2656","CDKN2A",58.9012260324699,-0.84643997194064,0.505983068746873,-
1.67286224425839,0.0943544179380344,0.555193038581963
"2657","TKFC",35.3188145217337,0.57081898982478,0.341211931142981,1.67
291626618292,0.0943437811411752,0.555193038581963
"2658","GALNT1",694.130705426491,-0.248538972361515,0.148546813392482,
-1.67313567141181,0.0943005906186557,0.555193038581963
"2659","EXOC6B",113.944214424838,0.421363022348442,0.252036289651934,1
.67183473034915,0.0945569162353412,0.55554852363664
"2660","GRAMD1B",4.35749617895825,1.61042052269262,0.963191793782435,1
.67196246177361,0.0945317244993487,0.55554852363664
"2661","NEK9",123.335504848434,0.39103060890077,0.233885401135539,1.67
188976739153,0.0945460609356201,0.55554852363664
"2662","AC009159.3",3.66108611147107,1.5759095301392,0.942470574142349
,1.67210475677003,0.094503666807997,0.55554852363664
"2663","AURKAIP1",800.063815685685,-0.416250265689238,0.24988218246416
4,-1.66578609801014,0.0957560260446157,0.55589969368977
"2664","CAP1",1077.85900076538,-0.251507371971304,0.15055944243094,-1.
67048554318782,0.0948233373698734,0.55589969368977
"2665","GNG5",1437.1548064141,-0.354160372984799,0.212142102404883,-1.
66944877499548,0.0950284741994194,0.55589969368977
"2666","MCOLN3",137.896467290336,0.611794766653771,0.366195689633055,1
.67067713786259,0.0947854669732878,0.55589969368977
"2667","PIP5K1A",101.851382043085,0.370694208055062,0.22263105630126,1
.66506063535649,0.0959006592306054,0.55589969368977
"2668","SPATA17",8.67617804603771,-0.855042090238338,0.511695413548991
,-1.67099815163083,0.094722042902715,0.55589969368977
"2669","RNF144A",30.6769742573347,0.68876484118828,0.412593868362901,1
.66935307090524,0.0950474282936978,0.55589969368977
"2670","PPP1R1C",16.5539126972616,0.834450257298978,0.499658680522871,
1.67004054933212,0.0949113411539935,0.55589969368977
"2671","NABP1",348.455981014243,0.710476107574634,0.425586233288771,1.
66940575611279,0.095036993669159,0.55589969368977
"2672","UBE2E2",301.860321835874,-0.30982124812254,0.18599812174152,-1
.66572245580575,0.095768707197084,0.55589969368977
"2673","CMTM7",64.8215226382645,-0.529250087656877,0.317883743044177,-
1.66491712532568,0.0959292910766656,0.55589969368977
"2674","TUSC2",189.971560303101,-0.2883659281413,0.172816453891048,-1.
66862542106725,0.095191637645514,0.55589969368977
"2675","PPM1K",124.323154628902,0.486054592508812,0.290976117307635,1.
67042778976575,0.0948347552266578,0.55589969368977
"2676","HAND2",88.8329857818815,-0.735120002476446,0.440756669524374,-
1.66785905535978,0.0953437093407787,0.55589969368977
"2677","AC008906.1",5.06587974595414,1.42046734276307,0.85021431622356
3,1.67071680123246,0.0947776286687442,0.55589969368977
"2678","TPBG",335.745730259627,-0.324859898842759,0.19497192589723,-1.
66618807988794,0.0956759594135348,0.55589969368977
"2679","UBE3D",42.3506784320604,0.410380996754819,0.245812700464887,1.
66948654800462,0.0950209941281885,0.55589969368977
"2680","KDM7A-
DT",54.9900976540473,-0.690703594503444,0.413563773009687,-1.670125962

62213,0.0948944444036452,0.55589969368977
"2681","NDUFB2",1265.51331288372,-0.373378517768344,0.224238897846296,
-1.66509254796763,0.095894293239799,0.55589969368977
"2682","AC067817.1",4.5472357456195,1.53419396290892,0.920758837128297
,1.6662277906491,0.095668052745814,0.55589969368977
"2683","NUDT2",213.820869172093,-0.429409112411166,0.257694343582783,-
1.66635055485112,0.095643612912756,0.55589969368977
"2684","ERCC6L2",185.674158041882,0.34433318696036,0.206582989296224,1
.66680319678506,0.0955535443849071,0.55589969368977
"2685","HERC4",289.592433089723,0.35312938588783,0.21212375952404,1.66
473282710139,0.0959660706558935,0.55589969368977
"2686","CDK2AP2",119.288145998536,-0.355198022083912,0.212972440607708
,-1.66781214071816,0.095353025036331,0.55589969368977
"2687","CASC1",5.23443956898515,-1.20970712461693,0.72605081751141,-1.
66614663249508,0.0956842124137712,0.55589969368977
"2688","AGAP2-
AS1",32.2994597825186,-0.645601953888765,0.387732149325893,-1.66507202
204203,0.0958983877532319,0.55589969368977
"2689","NOC4L",90.524304636507,-0.48321117231855,0.290325106034094,-1.
66437956027752,0.0960366021011209,0.55589969368977
"2690","F10",2822.10276698921,0.437514263982168,0.262011565560711,1.66
982805910066,0.0949533871590895,0.55589969368977
"2691","PELI2",111.658986141481,-0.55567645717224,0.333528832269786,-1
.66605223719538,0.0957030105224987,0.55589969368977
"2692","C15orf61",475.715889856847,-0.42912539244331,0.257670087093908
,-1.66540632357886,0.0958317186720557,0.55589969368977
"2693","SMG1",356.023814686819,0.393380931050963,0.235829261691114,1.6
6807514992015,0.0953008095214513,0.55589969368977
"2694","LSM12",256.068855540606,-0.286334930921236,0.17199521499359,-1
.66478428444598,0.0959558004062969,0.55589969368977
"2695","METTL2A",117.758496653809,-0.305549916713028,0.183250051174874
,-1.66739334998299,0.0954362153200375,0.55589969368977
"2696","PITPNC1",70.3642853996021,-0.505382860576778,0.303594122065124
,-1.66466615736509,0.0959793784191616,0.55589969368977
"2697","RNF165",21.2477456666892,0.81984403788105,0.492585077562795,1.
6643704310684,0.0960384253420562,0.55589969368977
"2698","MFSD12",104.217004737892,-0.444051589363734,0.266248808970042,
-1.66780685735837,0.0953540741824398,0.55589969368977
"2699","MAU2",140.201349849439,0.414512795077026,0.248890541521073,1.6
6544213590346,0.0958245788931393,0.55589969368977
"2700","EML2-
AS1",4.47376649296664,-1.32905464665732,0.798169192414645,-1.665128971
76177,0.0958870277626519,0.55589969368977
"2701","FAM217B",124.695813227523,0.365361327990662,0.219062330789258,
1.66784187255885,0.0953471211919182,0.55589969368977
"2702","NRK",3.66826547929012,1.73212553505467,1.03779181996318,1.6690
4913079401,0.0951076433951867,0.55589969368977
"2703","LIMD1-
AS1",20.9956203984569,1.26804564464024,0.762489993320299,1.66303250632
638,0.0963059287973468,0.556418443439418

"2704","H2AFY",428.433411185194,-0.269983080846325,0.162342135595193,-1.66304995222891,0.0963024368470965,0.556418443439418
"2705","NUDT22",184.326791563708,-0.408708957044282,0.245758624084711,-1.66305031437433,0.096302364361589,0.556418443439418
"2706","OLR1",8.54006710145354,-1.39235137567155,0.836994690565205,-1.66351279329063,0.0962098321452198,0.556418443439418
"2707","AMH",4.75061155957136,-1.47459018651903,0.886355495385055,-1.66365549059796,0.0961812958097187,0.556418443439418
"2708","ARHGEF3",179.373507758548,0.437246481938977,0.262990667595373,1.66259314802648,0.0963939036198409,0.556721068173675
"2709","NUDT17",11.1918455044814,0.840758585278181,0.505745386985098,1.66241473855095,0.0964296457568678,0.556721912010857
"2710","TREM1",8.93050664350472,-1.37224252358109,0.825606094646892,-1.66210318998189,0.0964920860923415,0.556876836341041
"2711","PGM2",70.5616961460574,-0.572948992022361,0.34486060949949,-1.66139296933305,0.0966345489102955,0.557342410833552
"2712","RNF208",24.2695386715165,0.640853468011798,0.385743589640642,1.66134573644844,0.0966440292954343,0.557342410833552
"2713","METTL1",39.2081637207624,-0.66354170646789,0.399443105073698,-1.6611670048616,0.0966799102790475,0.557343824830189
"2714","YWHAB",2557.0560916313,-0.250359359987402,0.150780954922496,-1.66041765762852,0.0968304603274424,0.558006042564922
"2715","CPNE8-AS1",3.58668274819962,-1.29066073166651,0.777445076132027,-1.6601310771532,0.0968880862524715,0.558132474765619
"2716","ERICH6-AS1",17.145765984925,-0.663163446799809,0.3995413903497,-1.65981163107875,0.0969523531499559,0.558158476630656
"2717","PDZD2",22.9336326025575,0.881265098156126,0.531062669306175,1.65943710430162,0.0970277447179265,0.558158476630656
"2718","MTREX",256.846310819363,0.307496460051438,0.185305884352479,1.65939932844515,0.0970353515318896,0.558158476630656
"2719","HERPUD2",203.39219985931,-0.308845848109581,0.186080466967748,-1.65974351388053,0.0969660615334224,0.558158476630656
"2720","USP22",434.510927913766,0.264202565994017,0.15926944005464,1.65884030171374,0.0971479768659277,0.558600866979084
"2721","PDAP1",914.342565703709,-0.296415177291665,0.178707670386327,-1.65865951165319,0.0971844224206257,0.558605059411461
"2722","PUS10",19.4869883730915,0.626127283974315,0.37759232512622,1.6582097736362,0.0972751327430361,0.558859091131809
"2723","ADAMTS1",1121.59620763758,0.686795674437919,0.414209891088652,1.65808612786344,0.097300083449611,0.558859091131809
"2724","PFDN6",214.844269340706,-0.372282781990018,0.224587594368349,-1.65762843240323,0.0973924871763846,0.559184471159565
"2725","RGS12",95.0664653709898,0.450297769214349,0.271823345756883,1.65658239530718,0.0976039340248286,0.559603469194046
"2726","PRPF4",94.2047668403007,-0.317251478259329,0.191512285386553,-1.65655940880754,0.0976085846522606,0.559603469194046
"2727","ITGB2",43.1760388589661,-0.653141860422419,0.39420387177271,-1.65686312893194,0.0975471502992605,0.559603469194046

"2728","MMP11",38.4349222761362,0.725072900256661,0.437650923690442,1.65673796399781,0.0975724640299567,0.559603469194046
"2729","BCHE",17.5044762163959,1.07244429174023,0.647480939217033,1.65633337876646,0.0976543244716014,0.559660547722919
"2730","FAN1",72.9307326524836,0.442508732571714,0.267210442995849,1.6560308332657,0.0977155748943699,0.559806443717196
"2731","HEATR5B",72.4868187527052,0.528006805838408,0.319102197902512,1.65466364477915,0.0979927455086469,0.560983360086104
"2732","UEVLD",97.5522996309383,0.394303243532783,0.238284742778552,1.65475656953507,0.0979738869666394,0.560983360086104
"2733","DDIT4",904.835994544779,1.13320136905898,0.685326941408934,1.65351936512123,0.0982252082562856,0.562108399973767
"2734","NAALADL2",145.864180396965,0.317781612963413,0.192208997723341,1.65331288715639,0.0982672015489175,0.562143025685834
"2735","FIBP",461.051325843264,-0.470296636506855,0.284518388232961,-1.65295691230256,0.0983396330462784,0.562258483674549
"2736","TGFB1I1",464.472025810857,-0.455964153328451,0.27586368585783,-1.6528603680132,0.0983592846121206,0.562258483674549
"2737","RARB",6.35766253863319,1.32423107641023,0.801419505917064,1.65235693245938,0.0984618096148407,0.562297323696968
"2738","GTF2A1",211.749998254473,0.270700649042052,0.163818334635777,1.6524441518949,0.0984440412065998,0.562297323696968
"2739","TSPAN6",142.879147277159,0.404232396094972,0.244648689276691,1.65229741181158,0.0984739366755752,0.562297323696968
"2740","CTPS1",27.3794053716177,-0.742500956716798,0.449648455265303,-1.65129213282565,0.0986789379334555,0.563230468466173
"2741","ABHD16A",189.83997626854,-0.306994048138756,0.185928202376585,-1.65114299076027,0.0987093806947429,0.563230468466173
"2742","PIM1",248.831077460459,-1.0789711734558,0.653611802584263,-1.65078287936317,0.0987829172600862,0.563239090757473
"2743","TNRC6B",1213.23339001134,0.365798748335057,0.221575525381066,1.65089870691249,0.0987592599200599,0.563239090757473
"2744","AASS",276.004221764703,0.506642436732079,0.306953617838711,1.65055046524422,0.0988304006135237,0.56330446996921
"2745","MEGF8",83.9817243706889,0.392991166014291,0.238155759557343,1.6501434470648,0.0989136003327146,0.563449493637422
"2746","FITM2",51.4805937162592,0.407262277642111,0.246814623219116,1.65007353425957,0.0989278970286676,0.563449493637422
"2747","P2RX6",4.24276005025932,1.51883757755486,0.920668374630704,1.64971190431526,0.0990018742406677,0.563665567209335
"2748","LRRC8D",32.1890286439886,0.657758985916979,0.398812001957012,1.64929586544358,0.099087036283174,0.563739995441557
"2749","NR1H2",361.315894463169,-0.258826815000809,0.156927946851204,-1.6493353809454,0.0990789450538065,0.563739995441557
"2750","LINC01277",3.10011741523385,-1.3065762260525,0.792530350303237,-1.64861348912704,0.0992268434657921,0.564330120656359
"2751","WARS2",127.775942589683,-0.373520395572292,0.226664049496185,-1.64790312536342,0.0993725519790745,0.564953367122037
"2752","IRF4",74.8824638943038,0.558974775951707,0.339253547623857,1.64766081258924,0.0994222937623589,0.565030768329685

"2753","ADGRL2",95.2260087823118,0.408520707260057,0.247997383751677,1
.64727829414972,0.0995008572889272,0.56527185179761
"2754","STN1",92.052229155833,-0.482291461637011,0.292876887239932,-1.
64673787058487,0.0996119365564145,0.56549208266509
"2755","STX16",339.689061212142,0.32345347210126,0.196413726797003,1.6
4679667442773,0.099599845154309,0.56549208266509
"2756","COG4",157.045313918307,0.304003159335383,0.184631678213968,1.6
46538461201,0.09965294836102,0.565519634385469
"2757","SLC41A1",60.6475438880118,0.571414130350389,0.347308418328157,
1.64526426713471,0.0999153253255341,0.566483339771359
"2758","CCT5",588.128699148982,-0.314312078578647,0.191049552579225,-1
.64518615372474,0.0999314280325562,0.566483339771359
"2759","MECP2",264.809606266237,0.348104991077614,0.211588863127436,1.
64519524294602,0.0999295542265366,0.566483339771359
"2760","CENPW",124.292404088484,0.566975550194394,0.344741707803066,1.
64463868850554,0.100044343466368,0.566845673593035
"2761","ZNF518A",173.514119555389,0.339812498733256,0.206632630655957,
1.64452486354415,0.100067832787108,0.566845673593035
"2762","RC3H1",189.447089415124,0.455910965959943,0.277633888829483,1.
64213010119941,0.100563044672219,0.566857998175124
"2763","FH",200.141375531374,-0.442294538417879,0.269382032894399,-1.6
4188581423047,0.100613670264332,0.566857998175124
"2764","RPUSD3",111.562792164654,-0.303494131908866,0.184730022068552,
-1.64290638040546,0.100402304719058,0.566857998175124
"2765","TNIP2",249.995987772459,-0.353940011080257,0.215550300326068,-
1.64202977469687,0.100583833698804,0.566857998175124
"2766","ISOC1",79.8031708336296,-0.440705058454641,0.268164433599202,-
1.6434135300482,0.100297402626878,0.566857998175124
"2767","PCDHGA3",6.1816421297328,1.33260573551435,0.810555528499831,1.
64406470458689,0.100162837640912,0.566857998175124
"2768","PTK2",294.341429736055,0.302941715369933,0.184503644728165,1.6
41928081238,0.100604909472847,0.566857998175124
"2769","AL162426.1",4.3402365605205,1.51157894204223,0.920214441081642
,1.64263770982064,0.100457913698768,0.566857998175124
"2770","ZFAND4",23.3442578649466,0.55640301187168,0.338497925073458,1.
64374127773733,0.100229655680585,0.566857998175124
"2771","AGAP9",25.4640482907391,0.59860431044385,0.364335741207587,1.6
4300188737943,0.100382542761074,0.566857998175124
"2772","ZNF785",63.08451320439,0.50380217563993,0.306764581276004,1.64
23088139587,0.100526021422117,0.566857998175124
"2773","AC134312.3",5.18794074111562,1.20325034878718,0.73275231126838
8,1.64209696821612,0.100569909901773,0.566857998175124
"2774","TBC1D3L",2.74324203398695,1.82440811515813,1.11022924879652,1.
64327152895294,0.100326766234889,0.566857998175124
"2775","AP5S1",59.9633784976212,0.467660266564816,0.284644566210628,1.
64296221350933,0.100390751558415,0.566857998175124
"2776","DYRK1A",166.640528612843,0.362206575527612,0.220415014163371,1
.64329357009748,0.100322208021292,0.566857998175124
"2777","UBR2",239.577862350807,0.372461492902219,0.226931835837195,1.6
4129237983793,0.100736737145274,0.566993194017923

"2778","STEAP4",292.987872807465,0.732403239363218,0.446172729320339,1
.64152399112088,0.100688691154305,0.566993194017923
"2779","C9orf16",730.214405249574,-0.497438693290372,0.303150585860658
,-1.64089636138463,0.100818930470834,0.566993194017923
"2780","SLC27A1",96.9488933369549,0.455653694067326,0.277680970146484,
1.64092517332735,0.100812948772361,0.566993194017923
"2781","NEXMIF",4.25139458911296,1.457126114495,0.887888815327227,1.64
111326704569,0.100773905251253,0.566993194017923
"2782","UBN2",178.696079430006,0.392672657612359,0.239435646011417,1.6
3999247461105,0.101006731653219,0.567382549941396
"2783","ECPAS",234.244758595849,0.315088058327021,0.192121552219034,1.
64004534987201,0.100995738054111,0.567382549941396
"2784","SNAPC4",111.426449838993,0.337487489296352,0.205816196668261,1
.63975185024102,0.101056773289466,0.567382549941396
"2785","IREB2",212.695965390213,0.297220337489567,0.181218724521227,1.
64011935452483,0.100980352922645,0.567382549941396
"2786","CNOT1",240.154139604445,0.309025115441327,0.188465524519436,1.
63969042205095,0.101069551415392,0.567382549941396
"2787","AC005828.5",4.33962428314906,1.39041617647564,0.84817755273055
5,1.63929848414338,0.101151111587465,0.567636664954414
"2788","RBAK",51.4167872575125,0.491657450487765,0.300157661557638,1.6
3799733758705,0.101422249176184,0.568615493633178
"2789","RDH10",162.772935310999,0.641562896461977,0.391659410458759,1.
63806327469701,0.101408495056204,0.568615493633178
"2790","DNAJC3-
DT",13.1786233597689,0.775253381132254,0.473310546439887,1.63793810842
268,0.101434605322031,0.568615493633178
"2791","SMIM12",172.315857555514,-0.346689997804492,0.211740611278118,
-1.63733350778477,0.101560803219597,0.569118940291829
"2792","AC010654.1",5.76415177599182,1.23866701481826,0.75673703779084
1,1.63685263567161,0.101661264577846,0.569477857448968
"2793","PIAS2",136.558865330516,0.381188855674032,0.232971378775028,1.
63620466032496,0.101796761417869,0.570032706256884
"2794","NIN",282.456318620887,0.392130969854695,0.23971093969982,1.635
84928725299,0.101871133800471,0.570245000944655
"2795","PPP1CB",899.144905158618,-0.402266045130723,0.245960709005936,
-1.63548904520768,0.101946569301439,0.5702590643328
"2796","APOL6",467.363897510545,0.352125541904991,0.215283811582083,1.
63563409304807,0.101916190609266,0.5702590643328
"2797","OSBPL3",6.67864482219369,-1.04396929314287,0.638438541314497,-
1.63519152680447,0.102008903849189,0.570275463257391
"2798","TTPA",4.34389372327102,1.43718613224281,0.879225171202625,1.63
460530853205,0.102131814103833,0.570275463257391
"2799","PYCR3",26.5937439918831,-0.593134516444405,0.362841689157224,-
1.63469230292165,0.102113566862331,0.570275463257391
"2800","HNRNPC",1088.06430943148,0.222281813539586,0.135978080930157,1
.6346885617084,0.102114351535652,0.570275463257391
"2801","GATA6",37.7288014626091,0.520236901114447,0.318228091653012,1.
6347925112837,0.102092551180393,0.570275463257391
"2802","ARHGAP26",74.0210749438076,0.45712630578843,0.279865693899116,

1.63337742264763,0.10238964244966,0.570978451395266
"2803","NAT10",63.5215897483412,0.436339610784815,0.267079778958579,1.63374259364086,0.102312910726377,0.570978451395266
"2804","PTPN6",8.3854545195346,-1.0197089976053,0.624200816845815,-1.63362329892173,0.102337972550235,0.570978451395266
"2805","CD99",6897.97972457558,-0.403142550187016,0.246825444776088,-1.63331033618812,0.102403744000238,0.570978451395266
"2806","RBFADN",4.81992874422143,1.23834974905103,0.758266478957144,1.63313265641672,0.102441099692924,0.5709831786163
"2807","CNTN1",34.2404058898678,1.02986142984338,0.630682615097903,1.63293137497299,0.102483430537827,0.571015622946781
"2808","MCRIP2",68.4899163361106,-0.566312973877616,0.346871094065134,-1.63263236276262,0.102546340513558,0.571162665823379
"2809","AC079949.1",4.93520826659761,-1.83905268920188,1.1270674886118,-1.63171478885175,0.102739583143331,0.572035272467677
"2810","AC005229.4",16.3117602408168,-0.652579318136856,0.400109942818259,-1.63100000349973,0.102890318771626,0.572670671027838
"2811","P2RY13",2.51815208741475,-1.71071673459489,1.04920918299272,-1.63048204526319,0.102999656895663,0.572783294488758
"2812","STARD4",9.75473563628626,-0.997811130886152,0.612009992732631,-1.63038372368875,0.103020422467831,0.572783294488758
"2813","AL359711.2",6.57118217814277,-1.09000973633384,0.668465202062458,-1.63061552489309,0.102971471249148,0.572783294488758
"2814","ARF1",1445.86352518098,-0.248782276527245,0.152704676776162,-1.62917260806567,0.103276483937088,0.573188150736712
"2815","RFX8",8.79128880608724,-0.868754392825966,0.533118062065132,-1.62957223670247,0.103191936131514,0.573188150736712
"2816","C12orf60",6.60480606139376,1.15131069752496,0.706414477751522,1.62979487791575,0.103144856711225,0.573188150736712
"2817","SLC25A35",17.0125211479982,0.675606582243895,0.414655150681333,1.6293215727184,0.103244961655243,0.573188150736712
"2818","TAF9B",58.0484830144681,0.421947459548108,0.258967999767509,1.62934208059264,0.103240622600739,0.573188150736712
"2819","NBPF9",48.6127158359699,0.774142347005333,0.475423079221227,1.62832302603699,0.103456409543702,0.573372872169913
"2820","POMGNT2",139.240824691007,0.368835222074826,0.226497493638524,1.62842959606195,0.103433826344687,0.573372872169913
"2821","DUOX2",10.5937223998649,-1.33532139416739,0.819966516710817,-1.62850722188492,0.103417379163431,0.573372872169913
"2822","AC005899.7",4.24889354529709,1.5287641688257,0.93863575375151,1.62870864732734,0.10337471129898,0.573372872169913
"2823","NFATC3",119.62392798103,0.356569526947027,0.219038320670061,1.62788650796922,0.103548952771333,0.5736824730229
"2824","RAPH1",96.7883654941366,-0.343584958955032,0.211085102211242,-1.6277082340524,0.103586766330067,0.5736887483719
"2825","C1orf220",14.206041996259,0.748367724610151,0.46065441859827,1.62457515741923,0.104253114067958,0.57395212426944
"2826","TP53BP2",183.972052629306,0.473031346552028,0.291168021929883,1.62459923798205,0.104247979622429,0.57395212426944
"2827","POU3F3",3.70886518819518,-1.4459545438095,0.889152922326566,-1

.62621581451479,0.103903753093933,0.57395212426944
"2828","CCNT2",152.183212942488,0.327418694849385,0.20140497804873,1.6
2567329775814,0.104019173341069,0.57395212426944
"2829","SATB1",109.260642341046,0.473430259662659,0.291419472801828,1.
62456631710607,0.104254999045504,0.57395212426944
"2830","PAICS",107.175346620065,-0.353447423379087,0.217519040187963,-
1.62490337891187,0.104183148133509,0.57395212426944
"2831","AC004160.1",13.7071260389826,0.802428708166285,0.4935535740750
02,1.62581885800374,0.103988195456343,0.57395212426944
"2832","WASHC2C",178.147903325596,0.336245302228416,0.20704310033052,1
.62403529357724,0.104368276305773,0.57395212426944
"2833","PBLD",44.7743420501877,0.429456886372703,0.263997118832282,1.6
2674838374103,0.103790548182859,0.57395212426944
"2834","AMBRA1",45.4663306854646,0.483825905937969,0.297859935275712,1
.62434033127053,0.104303194112547,0.57395212426944
"2835","CD3G",9.59856309157302,1.13192398345145,0.696453092179291,1.62
526952089409,0.104105142826774,0.57395212426944
"2836","TMEM106C",529.08929625738,0.492321864786846,0.302945717029335,
1.62511577854449,0.104137891459795,0.57395212426944
"2837","ITGA7",9.56873707794441,-0.860531122824336,0.529455782127821,-
1.62531254142879,0.104095980494766,0.57395212426944
"2838","LRIG3",239.040007558108,0.310681017736688,0.191255922517473,1.
62442560547794,0.104285005955555,0.57395212426944
"2839","NRDE2",123.481402898167,0.49801020100864,0.306069390373926,1.6
2711534270127,0.103712603059715,0.57395212426944
"2840","ITGAM",11.3726420616648,-1.25387763494469,0.770556340329779,-1
.62723680192945,0.103686814362612,0.57395212426944
"2841","C16orf46",12.6459676238242,-0.765638991231521,0.47094825000367
5,-1.62573911512687,0.104005165292269,0.57395212426944
"2842","ZNF611",57.7947989169601,0.47156332108191,0.290344986138758,1.
62414831870576,0.104344157744505,0.57395212426944
"2843","APOBEC3F",30.4821310732484,0.486288054032724,0.299380477792032
,1.62431450981426,0.104308702074358,0.57395212426944
"2844","BEX3",1022.1760019712,0.483190041665928,0.297233976628098,1.62
562183215851,0.104030127916987,0.57395212426944
"2845","HEPH",38.5361941681767,-0.741286519121781,0.456512918940259,-1
.62380184298528,0.104418106611615,0.574024318947505
"2846","PMS1",106.307365211833,0.410676726612422,0.253023582150468,1.6
2307688130113,0.104572970997767,0.574548074672485
"2847","AC090559.1",2.51503059238537,-1.65880272469212,1.0220520764628
8,-1.62301194126322,0.104586852211801,0.574548074672485
"2848","GNAI2",2111.18195867643,-0.255635064832331,0.157601906530129,-
1.6220302816163,0.1047968644888,0.575114515696487
"2849","LINC01615",12.1184487883112,-0.913068397905673,0.5629225215555
1,-1.62201433224347,0.10480027939482,0.575114515696487
"2850","ASXL3",8.56650310911774,1.21740876704488,0.750471870726976,1.6
2219107008712,0.104762443146696,0.575114515696487
"2851","WDR5B",28.5443427988572,0.507606264874405,0.313033405238798,1.
62157219127197,0.10489498098009,0.57525207677574
"2852","BCL6",442.811564273374,0.502802358167873,0.310074410470208,1.6

2155386316918,0.104898908117929,0.57525207677574
"2853","MB21D2",56.87746163927,0.703379685409338,0.433927435149593,1.6
2096154433484,0.105025886323444,0.575746534209133
"2854","PPP1R14C",2.92231127034705,-4.75108507417966,2.93419669574083,
-1.61921151403249,0.105401761479334,0.57760460740602
"2855","ZC3H18",179.322860188953,0.368072736962428,0.227418082213876,1
.61848492160035,0.105558133765054,0.578258918418722
"2856","MARK1",40.5559143750212,0.523482458621439,0.32367859597854,1.6
1729093342998,0.105815495456906,0.578453110432018
"2857","CNNM3",110.396458186539,0.353778252043654,0.218673591826203,1.
61783711096138,0.105697706319301,0.578453110432018
"2858","INTS10",260.483144293722,-0.243364292083153,0.150463837696106,
-1.61742712275277,0.105786115005566,0.578453110432018
"2859","NHLRC2",158.843273742323,0.369182931005554,0.228252936301929,1
.6174290547448,0.105785698258817,0.578453110432018
"2860","NPIPB5",58.4783908862453,0.728085826111611,0.450054283290308,1
.61777335122474,0.105711451436783,0.578453110432018
"2861","AL121672.2",8.3684531365434,0.972501557285833,0.60111402145646
4,1.61783209602983,0.105698787370628,0.578453110432018
"2862","MIGA1",248.023332767202,0.346767740989759,0.214863184984299,1.
61390021755053,0.10654907148173,0.579022751207175
"2863","SRSF7",1295.64194679893,0.301087836984976,0.18653921028451,1.6
1407264738473,0.106511669527965,0.579022751207175
"2864","AC097448.1",72.9986157031684,-0.595947635596858,0.368992860785
656,-1.6150654902319,0.106296513287242,0.579022751207175
"2865","VHL",107.12185482258,0.377478337314982,0.233828564944874,1.614
33799760083,0.10645413243907,0.579022751207175
"2866","POPDC2",12.5737553134223,-0.793935814446095,0.491920407939145,
-1.61395177275164,0.106537887492878,0.579022751207175
"2867","SCD5",37.167929894782,0.514464155114354,0.318682894838438,1.61
434505411773,0.106452602678948,0.579022751207175
"2868","ZFAND3",283.364234478666,0.272392377943407,0.168600768652772,1
.61560578946345,0.106179571394193,0.579022751207175
"2869","AHI1",477.983255819484,-0.328119045341218,0.20323516429088 7,-1
.61447969147498,0.106423418407495,0.579022751207175
"2870","ANKIB1",307.358695074328,0.314517285721337,0.19466294140698,1.
61570190734855,0.10615877841529,0.579022751207175
"2871","NRBF2",502.396045432766,-0.356000840741903,0.220442567932845,-
1.61493691567934,0.106324356886683,0.579022751207175
"2872","CUZD1",2.70651739846374,1.62523483342756,1.00665081551559,1.61
44971109919,0.106419642980499,0.579022751207175
"2873","PANX1",63.7193969228788,-0.551726222248648,0.341448657580969,-
1.61583948274219,0.106129022639321,0.579022751207175
"2874","PCED1B",17.231777311976,0.741562857020082,0.459333299922657,1.
61443304272725,0.106433529368162,0.579022751207175
"2875","SLC22A17",106.208275873032,0.483075981349835,0.299136994299882
,1.61489882747687,0.106332606228103,0.579022751207175
"2876","RETREG3",175.118644800221,0.293121984162579,0.181342211078286,
1.61640239423373,0.106007341059428,0.579022751207175
"2877","NAT14",58.9062784742875,-0.564336425957049,0.349521289690809,-

1.61459814495497,0.10639774743192,0.579022751207175
"2878","EBF4",3.79209952858263,1.37989265644271,0.854613529322311,1.61463937686188,0.106388812896317,0.579022751207175
"2879","WDR13",454.370022033761,-0.352621805434178,0.218649682249341,-1.61272498458086,0.106804269974198,0.580207982770566
"2880","SLC25A28",141.887541487803,-0.347539770449258,0.215537881493465,-1.61243011224361,0.106868376616711,0.580354656349083
"2881","NOTCH3",22.3604946589987,-0.708387774514576,0.439397002843553,-1.61218162602442,0.106922422367877,0.580446610841235
"2882","G6PD",175.3503969018,-0.372369000886031,0.231008356673428,-1.61192870356825,0.106977455239294,0.580543858411714
"2883","MAP3K14",56.2454559585815,-0.461751396594389,0.286495085662101,-1.61172536529645,0.107021715463992,0.580582597938548
"2884","WRAP53",34.6554447125591,-0.476412769113599,0.295689543045852,-1.6111924831908,0.107137775654886,0.581010683509854
"2885","ARHGAP10",445.270494097288,0.233189799369008,0.144840149761976,1.60998038011022,0.107402139572841,0.582041906589044
"2886","CLDN15",29.2631464548725,0.575328243728291,0.357351316197778,1.60997936106627,0.107402362047058,0.582041906589044
"2887","UQCRC1",360.625094518148,-0.276442803228482,0.171730151274379,-1.60975111928249,0.107452200215613,0.58211029143477
"2888","DGUOK",509.027637472624,-0.376408898734514,0.233964068881192,-1.60883207637176,0.107653064965212,0.582449925890203
"2889","DAB2",1431.94370282407,0.280284719578251,0.17423947470983,1.6086177948196,0.107699940763102,0.582449925890203
"2890","AC005162.3",20.5370800275399,0.817531531166927,0.508275318484744,1.60844231745136,0.107738339872146,0.582449925890203
"2891","CNTFR",15.049180594524,1.22673347208641,0.762498320040153,1.60883432768981,0.107652572557215,0.582449925890203
"2892","PIAS1",304.132378526797,0.336434682976068,0.209089661109021,1.6090450440811,0.107606492589944,0.582449925890203
"2893","FOXO4",18.4282775331657,0.679199911848559,0.42223784624529,1.60857184614851,0.107709994505335,0.582449925890203
"2894","MRPL39",101.422509652843,-0.48492446523998,0.301526581230876,-1.60823123208722,0.107784545318647,0.582498372074512
"2895","FANCL",57.8862859123125,0.421186517466898,0.261995094620674,1.60761222677359,0.107920132709795,0.582640019720971
"2896","PCDHGA6",32.1857065269718,0.709609514627872,0.441343072409134,1.60784106285926,0.107869992557369,0.582640019720971
"2897","SCUBE1",11.7408515744553,1.37336139728021,0.854292261298905,1.60760135552684,0.10792251516187,0.582640019720971
"2898","SRSF8",275.385787970905,-0.288957497317547,0.17978986342011,-1.60719571070783,0.108011442675364,0.582918896978156
"2899","CCNB1",20.2983399200169,-0.612066563803594,0.380898611921975,-1.60690153402022,0.10807596985862,0.583065942941983
"2900","LSM2",508.463291596138,-0.420176508227905,0.261534614159288,-1.60658087105822,0.10814634151804,0.583244407359361
"2901","RALA",584.812875147905,-0.213881329902161,0.133171447638818,-1.60605996025695,0.10826073622919,0.583315125934388
"2902","ARHGAP11A",7.24732536013105,-0.892630237855255,0.5558055699436

38,-1.60601168128951,0.108271343387949,0.583315125934388
"2903","IRAK1",188.364814394495,-0.421681973850547,0.262562756979994,-1.60602356061746,0.10826873335697,0.583315125934388
"2904","LINC02604",10.0176287601793,0.805500107488445,0.501648042062608,1.60570766742455,0.108338155829593,0.583474089936236
"2905","CCN2",2887.20806741574,-0.460071829424809,0.2866742379316,-1.60485934398675,0.108524762512784,0.584076836097706
"2906","TRRAP",129.909196650586,0.386128321024294,0.240584020920263,1.60496245572464,0.108502067336941,0.584076836097706
"2907","SMURF2",1433.34741866568,0.48481467752258,0.302167571621627,1.60445634493718,0.108613499736748,0.584353331917009
"2908","LINC00339",41.9654454990054,-0.432710251473198,0.269800460121685,-1.60381583959508,0.108754652091167,0.584380731056204
"2909","SRSF11",1192.89092857594,0.263422812402989,0.164548638413671,1.60088114336596,0.109403246837121,0.584380731056204
"2910","MARC1",5.71123019592818,1.11301258330693,0.695048609559461,1.6013449534305,0.109300537576005,0.584380731056204
"2911","COQ8A",86.8382754450086,0.433332094755699,0.270678513081731,1.60091057772604,0.109396726425073,0.584380731056204
"2912","DMGDH",23.2417849995932,0.533518013447759,0.332607269770642,1.60404796267881,0.108703480851782,0.584380731056204
"2913","SRP19",405.913637406681,-0.304114419896345,0.189667985624759,-1.60340406892921,0.108845473294377,0.584380731056204
"2914","DOP1A",56.6713734821297,0.492226773470433,0.307447414685514,1.60101126228018,0.109374424720419,0.584380731056204
"2915","HECW1",4.74152906484297,1.4332826111165,0.894568847204668,1.60220492318192,0.109110301760853,0.584380731056204
"2916","INVS",49.3691877356874,0.45002190206222,0.280743039145719,1.60296726654953,0.108941881121219,0.584380731056204
"2917","BMI1",328.470534603346,0.226011336303767,0.141059552455933,1.60224055988249,0.109102424140975,0.584380731056204
"2918","C1RL",190.701684257604,0.320026833950213,0.19989823578167,1.60094876624998,0.109388267215059,0.584380731056204
"2919","AVIL",21.1628296628508,0.687123563607958,0.429032028450863,1.60156705803295,0.109251380223208,0.584380731056204
"2920","SYNM",18.3023284401067,1.01652609807349,0.634193281574555,1.60286481677902,0.10896450286062,0.584380731056204
"2921","MBTPS1",557.802826158738,0.211531357977411,0.132114447346705,1.60112207427469,0.109349883935301,0.584380731056204
"2922","CXCL16",60.5072946790697,-0.484394538614674,0.302154776383994,-1.60313381245074,0.108905114364478,0.584380731056204
"2923","BLMH",111.278626755344,-0.295083940766685,0.18421098411988,-1.60188026884788,0.109182088476728,0.584380731056204
"2924","AC138150.1",31.1961665276209,0.567161701346284,0.353881257800316,1.60268928869445,0.109003269519312,0.584380731056204
"2925","DDA1",188.617892547743,-0.332391433011532,0.207427512640576,-1.60244621738048,0.109056971604071,0.584380731056204
"2926","ZNF98",6.01263855225487,1.29331637453643,0.807339453170485,1.60194868398599,0.109166957595493,0.584380731056204
"2927","CRKL",123.509629864077,0.340329204807444,0.212481460923732,1.6

0168893478006,0.10922441325295,0.584380731056204
"2928","UXT",854.331675224443,-0.366881426734991,0.228722537043152,-1.60404580798163,0.108703955764442,0.584380731056204
"2929","MOB1B",58.6263991818981,0.462677319113801,0.289066849410587,1.60058934484258,0.109467903784944,0.584526464730805
"2930","B4GALT2",240.628324405754,-0.458403132213207,0.286615886782594,-1.59936400371595,0.109739745677728,0.584780109846563
"2931","SMG7",100.483104760471,0.372039005304357,0.232595800910978,1.59950869210553,0.10970761880856,0.584780109846563
"2932","AL133245.1",7.37480756506922,1.18892862456246,0.743131029335296,1.59989097161764,0.1096227725472,0.584780109846563
"2933","KLF3",820.528443113166,-0.324247171030742,0.202637314283427,-1.60013555340168,0.10956851526708,0.584780109846563
"2934","TMEM120A",206.140776097019,-0.333795455157829,0.20869675005514,-1.59942814188355,0.109725503406724,0.584780109846563
"2935","MET",6.99225281381823,-0.999402623348917,0.624789393892895,-1.59958320854633,0.109691075944949,0.584780109846563
"2936","PIGZ",58.2940227677396,-0.40580288563079,0.25389256021615,-1.59832523365518,0.10997061441645,0.585411984163814
"2937","BDNF-AS",41.4064235301419,0.406085899291311,0.254034699136496,1.59854500456694,0.10992173790566,0.585411984163814
"2938","ZNF160",129.024693402192,0.374012893052884,0.233996861131231,1.59836713725459,0.109961293834096,0.585411984163814
"2939","NTN5",2.93367601635282,1.98061798056743,1.23962531629812,1.5977553495617,0.110097435243153,0.585887678531101
"2940","SNRPG",668.679486989323,-0.320088752388633,0.200365112216637,-1.59752737813232,0.110148199880569,0.585958451065341
"2941","FER",291.26920775402,0.361034372131228,0.226075521555876,1.59696357060929,0.110273827865257,0.58642729269385
"2942","HSPA12B",228.361389387502,0.315253415136184,0.197511524860234,1.59612668354046,0.110460512296668,0.587220398477187
"2943","CIB1",843.257194256098,-0.375431467528334,0.235311747707778,-1.59546419244042,0.110608471156283,0.58780716577787
"2944","FBXO30",85.0052697383104,0.424724038736576,0.266307474433225,1.59486337978498,0.110742790225461,0.588321073072763
"2945","WDR59",87.5679977924418,0.360008366051563,0.225911396186121,1.59358213941082,0.111029657479477,0.589644768413927
"2946","CTSS",248.497244378263,-0.39506274669896,0.248083040323117,-1.59246172646227,0.111280996294306,0.58977796748321
"2947","IQGAP2",16.2800253121918,-0.870059702012967,0.546172505608201,-1.59301263443149,0.111157356732397,0.58977796748321
"2948","STXBP5",125.292257005753,0.473276302274494,0.297047413337308,1.59326855250906,0.11109995826744,0.58977796748321
"2949","AL139393.3",66.6171635320199,0.431913452292726,0.271200211874321,1.59259998105341,0.111249957798406,0.58977796748321
"2950","KBTBD4",62.7300291633625,-0.478319716157915,0.300349118893001,-1.5925457611491,0.111262129485833,0.58977796748321
"2951","TMEM109",1192.14883248842,-0.306491453137939,0.192405148828682,-1.59294829168444,0.111171791496724,0.58977796748321

"2952","DCTN4",153.424725695467,0.368027534186098,0.231145069052479,1.
59219288429874,0.111341371588394,0.589898052724418
"2953","TMEM185A",152.971617916924,0.270984559242347,0.170235917230794
,1.59181777647407,0.111425654715114,0.590144679899895
"2954","ARL2",1167.13267196465,-0.337404168788508,0.212084998861669,-1
.59089124926076,0.111634052259538,0.590848249522564
"2955","CEP57",300.702013978022,0.245366820004465,0.154230961627531,1.
59090507778216,0.111630939643971,0.590848249522564
"2956","NGF-
AS1",20.1214330644499,0.657732187941216,0.413860102879551,1.5892621283
493,0.112001225105983,0.590991619655054
"2957","AC108488.3",8.62702093304365,0.99756090889866,0.62756304442560
9,1.58957879652028,0.111929779643449,0.590991619655054
"2958","PLEKHM3",81.2946926131765,0.433794007571924,0.272864029489816,
1.58978084573113,0.111884212862233,0.590991619655054
"2959","HARS",216.592172049726,-0.312044712707269,0.196313393752139,-1
.58952329610913,0.111942298835262,0.590991619655054
"2960","GPNMB",4011.87722511113,0.273109591734326,0.171737220225526,1.
5902760704737,0.111772590088023,0.590991619655054
"2961","CCDC6",364.880664301868,0.348299179930234,0.219124816458498,1.
58950129683829,0.111947261502205,0.590991619655054
"2962","GRASP",108.597531710959,0.490486333533654,0.308524723098203,1.
5897796734349,0.1118844772,0.590991619655054
"2963","AC093525.4",11.706637125793,0.9141010647375,0.575152931631634,
1.58931827426197,0.111988555056992,0.590991619655054
"2964","NIP7",115.522497858292,-0.374127779259652,0.235223813765535,-1
.59051829519511,0.111718025071867,0.590991619655054
"2965","NOTCH2NLC",31.0498778614208,0.584889516585725,0.36813422589905
,1.58879418276668,0.112106867044175,0.591349544880572
"2966","FAM8A1",261.957459523387,0.344115435827425,0.21665910996444,1.
58828048303118,0.112222928768714,0.591592458777134
"2967","HSD17B12",452.918192736692,-0.232877767713346,0.14662655073301
5,-1.58823737276192,0.11223267310937,0.591592458777134
"2968","PIP4K2B",179.953330567188,0.299823231620224,0.18879507485627,1
.58808820541786,0.112266394990443,0.591592458777134
"2969","UBE2M",604.060737831328,-0.419323808283353,0.264112932691201,-
1.58766859316815,0.112361298512942,0.591893131944228
"2970","CRELD2",293.26646265684,-0.38947973971687,0.245393691708755,-1
.58716280359449,0.112475776789919,0.592296683163077
"2971","AL672277.1",3.51789822875996,1.80956366854315,1.1402959465321,
1.58692458220731,0.112529726658131,0.592381327813251
"2972","COPE",1105.29953982886,-0.42365608881561,0.267053893190434,-1.
5864067127211,0.112647078659784,0.592799566029279
"2973","VASH1",108.628279828342,0.589314390766146,0.371520741521116,1.
58622204605137,0.112688948443277,0.592820435133821
"2974","CMSS1",140.217357874652,-0.408573406425716,0.257644467872471,-
1.58580314104766,0.112783972953223,0.593046425649723
"2975","LCORL",80.2232888236711,0.356003053548559,0.224534771462509,1.
58551413319964,0.112849568370793,0.593046425649723
"2976","IRGQ",79.5145826465135,0.366676845518591,0.231254630915603,1.5

855978497244,0.112830564339437,0.593046425649723
"2977","MID2",7.55152941763358,1.04247565684763,0.65756220100713,1.58536432790865,0.112883581148288,0.593046425649723
"2978","LHPP",46.9505112815876,0.623872318299052,0.393611506099968,1.5849951249662,0.112967441903371,0.593216614985858
"2979","RTRAF",1163.35489765859,-0.359499654398483,0.226829722753323,-1.58488777411873,0.11299183478535,0.593216614985858
"2980","AL031280.1",9.31514647112081,1.13129595745292,0.713914303295935,1.58463831335225,0.113048534736723,0.593227304253193
"2981","TMEM108",92.5196981018987,0.635718635027511,0.401283935107733,1.58421152557936,0.113145591341897,0.593227304253193
"2982","FAM149A",95.8049469995154,0.37502128641624,0.236707112936164,1.58432622393302,0.113119501125719,0.593227304253193
"2983","GTPBP4",184.663578140697,-0.262527880289817,0.165681561552841,-1.58453287034049,0.113072507624918,0.593227304253193
"2984","SLC1A4",27.2311153488079,-0.532836541210074,0.336507985713403,-1.583429112627,0.11332369183473,0.593761397694964
"2985","AC108463.3",67.073403417833,0.649179849682594,0.41012335464373,1.5828892510804,0.113446709120426,0.593761397694964
"2986","MARCO",15.0057846289914,-0.679744668382375,0.429373042835679,-1.58310979164687,0.113396442232884,0.593761397694964
"2987","HIGD1A",436.179102471595,-0.337975614331494,0.213509649564906,-1.5829524099741,0.113432311785092,0.593761397694964
"2988","NUFIP2",554.940254966808,0.370413509591029,0.234029513087766,1.58276409117732,0.113475244099121,0.593761397694964
"2989","SLC25A19",39.319401205783,-0.523997044795105,0.330975536721371,-1.5831896519779,0.113378244329775,0.593761397694964
"2990","AMOTL2",638.664670684148,0.274336510764652,0.173350253609799,1.58255615467496,0.11352266366253,0.593810856080924
"2991","SLC27A3",401.205723006751,-0.43797373626809,0.276831153172165,-1.58209699757205,0.113627428939194,0.593853829115477
"2992","AL353746.1",6.12475970110095,3.64975578786231,2.30726371058813,1.58185463200995,0.113682759870316,0.593853829115477
"2993","SSNA1",496.633199193379,-0.36450114747189,0.230402624628203,-1.58201820860365,0.113645413767131,0.593853829115477
"2994","HPS4",142.869443939543,0.299427391425226,0.189267561534007,1.58203227747205,0.113642202161035,0.593853829115477
"2995","TMEM39B",82.0147926339973,-0.399446073035849,0.25255475085589,-1.58162169463118,0.113735958386192,0.593933351973302
"2996","TMEM72-AS1",14.64421920018,0.714589179228376,0.4519427196165,1.58114988517737,0.113843770859274,0.594297922643206
"2997","AL139423.1",2.60427038208064,1.60695592671287,1.01691428301933,1.58022751135095,0.114054773555791,0.594433694994917
"2998","PRKAA2",19.1442306781093,0.677392163443352,0.428525436725414,1.58075135193761,0.113934901777556,0.594433694994917
"2999","C4orf46",53.4477106061219,0.542755219709572,0.343425762371912,1.58041498098735,0.114011862996966,0.594433694994917
"3000","C7",15.0536702795508,-0.833516613913023,0.52732858685025,-1.58063991730781,0.113960393338208,0.594433694994917

"3001","PEX11A",26.2124374640825,-0.579388733024188,0.366654044703158,
-1.58020548632774,0.114059815772362,0.594433694994917
"3002","ZNF19",15.8714600061941,0.658514065076333,0.416872085590984,1.
57965497772004,0.114185901428855,0.594892571068383
"3003","LRRN4CL",1307.54180376779,0.339017670702287,0.214684434570081,
1.57914415817426,0.114302995021769,0.594908100545914
"3004","ATG2B",123.463360741239,0.374643230887611,0.237214821073447,1.
57934158241998,0.114257728869037,0.594908100545914
"3005","ZNF256",11.6084576651789,-0.721996819848757,0.457188966962016,
-1.57920875616568,0.114288182204703,0.594908100545914
"3006","IL1A",2.84530929586606,-1.71138805218881,1.08389676598916,-1.5
7892163339651,0.114354033236649,0.594972918258817
"3007","LIN54",44.7492472166129,0.503466516989694,0.318982660222508,1.
57835073743036,0.114485055801854,0.594972918258817
"3008","ZNF862",22.3273889157966,0.563557432595609,0.357038710975541,1
.57842109348814,0.114468902467538,0.594972918258817
"3009","TIMP2",9340.95516099614,0.236460549026185,0.149823474309189,1.
57826101761734,0.114505657542138,0.594972918258817
"3010","MT-
ND1",31920.6586831039,-0.306204141189122,0.193979493975494,-1.57853871
516855,0.114441901232159,0.594972918258817
"3011","ANTXR2",1112.77364111207,0.315246092054335,0.199793767842201,1
.57785748504087,0.11459835401068,0.595014079102756
"3012","PDCD1LG2",31.0485205069054,0.534832584588305,0.338988720962027
,1.57772973410586,0.114627712297737,0.595014079102756
"3013","ROBO3",183.758681654428,-0.377017051200231,0.238952683420196,-
1.57778956822698,0.114613961154369,0.595014079102756
"3014","SSH1",83.369578019521,0.482004318152469,0.305602110094693,1.57
722837058656,0.114742987232025,0.595220336996676
"3015","ZNF418",2.50613938903242,1.9100085946717,1.21099243362966,1.57
722586998079,0.114743562406968,0.595220336996676
"3016","XYLT1",88.0979741544165,-0.50107652151504,0.317791592835055,-1
.57674568117073,0.11485405472233,0.595595960164868
"3017","STARD4-
AS1",11.1845011980281,0.75227724914539,0.477237451824724,1.57631645686
869,0.114952890848147,0.595625620593801
"3018","LONP2",298.947231436984,0.336277912368502,0.213303096436427,1.
57652616387933,0.114904593920672,0.595625620593801
"3019","CYB561",315.130837056066,-0.317562093225074,0.201485827075957,
-1.57610139548603,0.115002437507998,0.595625620593801
"3020","PI3",9.17012186885862,-1.66685773945543,1.05761097005067,-1.57
605942700799,0.115012108324378,0.595625620593801
"3021","ABHD2",298.24639974107,-0.484960504190959,0.307897177241181,-1
.57507291406923,0.115239615270377,0.596606283624194
"3022","GALNT11",508.573056745374,0.231070778302936,0.14672368020502,1
.57487038206823,0.11528636641351,0.596650817573559
"3023","ABCB8",89.1370271545863,-0.367979495246296,0.23372426984446,-1
.57441713473394,0.115391045059768,0.596995019760098
"3024","PLA2G2A",18032.9719308334,-0.606358378632318,0.385470922137395
,-1.57303273427247,0.115711238759916,0.597146158973996

"3025","ADCY3",306.4335640194,0.268651000360898,0.170692376568331,1.57
388985824655,0.115512914962436,0.597146158973996
"3026","AC010680.2",3.32966908614437,1.4148812154488,0.899982339898595
,1.57212108807404,0.115922471720298,0.597146158973996
"3027","LINC00886",10.3195360262826,0.784199180316639,0.49875477799139
7,1.57231412092891,0.11587771977301,0.597146158973996
"3028","RNF130",573.354797331442,-0.252373476756708,0.160544789870403,
-1.57198173145595,0.115954788030948,0.597146158973996
"3029","BRD2",917.489047498973,0.35183406738993,0.223687504284328,1.57
288208170411,0.115746124824681,0.597146158973996
"3030","AL359715.1",5.49055448890165,1.14294924260549,0.72700502222998
1,1.57213390232115,0.11591950049679,0.597146158973996
"3031","CNOT2",424.220217936572,0.211825361733276,0.134708856282906,1.
57246796965165,0.115842061839932,0.597146158973996
"3032","AC087286.2",2.85753132326727,1.7091870968785,1.08624527075122,
1.57348173833381,0.115607313558112,0.597146158973996
"3033","MPG",850.050876470948,-0.359725253107189,0.228727980141214,-1.
5727208052338,0.115783480190254,0.597146158973996
"3034","FAHD1",148.379690391354,-0.39569477359026,0.251391582945721,-1
.57401759022177,0.115483382868255,0.597146158973996
"3035","AC005632.3",15.0946854035154,0.753629717914775,0.4790788130493
14,1.57308087393378,0.115700092977252,0.597146158973996
"3036","MKKS",112.649764052539,-0.297548282887901,0.189123687148994,-1
.57329992542652,0.115649386611381,0.597146158973996
"3037","C21orf58",16.9245990129975,0.70611023123952,0.449018196970918,
1.57256484481686,0.115819613247471,0.597146158973996
"3038","RASGEF1B",41.067490122038,-0.640566184262819,0.407717282796945
,-1.5711038292724,0.116158533297146,0.597605245682244
"3039","PMS2",64.0416317810833,0.384338972216824,0.244597895869783,1.5
7130939679643,0.116110799575371,0.597605245682244
"3040","IL33",658.309864089972,0.306539272510015,0.195129788539751,1.5
7095067239089,0.116194107050577,0.597605245682244
"3041","SLC25A39",243.451310089042,-0.417038754153126,0.26547097228990
4,-1.57093918990775,0.116196774432206,0.597605245682244
"3042","MRTFA",155.619147531992,0.307639664238247,0.195922412912911,1.
57021169586655,0.116365869464311,0.598278171736302
"3043","EIF2A",472.695872730528,-0.30038968213334,0.19134272686293,-1.
56990384248327,0.116437483536613,0.598449636054101
"3044","FAM13A-
AS1",18.7228211010593,0.785861434928155,0.50079427923679,1.56923005615
361,0.116594343195506,0.598981678714433
"3045","GPR150",7.4017145441197,-1.06763306084348,0.680397993074493,-1
.56913023217368,0.11661759665508,0.598981678714433
"3046","JCAD",10.8049024687288,-1.07327688970906,0.684148871830807,-1.
56877681729846,0.11669995203005,0.599207895518705
"3047","PIFO",18.4109394170533,0.752235107244883,0.479587064538602,1.5
6850583109156,0.116763130196402,0.599335528805949
"3048","WDR47",71.6156324273827,-0.39560729753197,0.252290179432027,-1
.56806459301186,0.116866058637388,0.599470369658495
"3049","EMSY",90.5437721002744,0.41207432529635,0.262783205692645,1.56

811514727588,0.116854262135686,0.599470369658495
"3050","AC026904.2",5.68957699736107,1.03342496682436,0.659324757538485,1.5673990017947,0.117021457151162,0.599770147622161
"3051","NIPA2",210.428594128267,-0.24732552220486,0.157785297180552,-1.56748142332836,0.117002205035267,0.599770147622161
"3052","ZNF846",59.6422026013799,-0.454532508851823,0.290005903584755,-1.56732157253821,0.117039545431128,0.599770147622161
"3053","SLC12A6",162.637051817299,0.425956111932686,0.271805489318796,1.56713579626455,0.117082953654198,0.599796067851835
"3054","RGMB",67.8885546608967,0.470269845701444,0.300202335282951,1.5665096184485,0.11722935859578,0.600349433018336
"3055","RC3H2",191.156917158916,0.329482257002542,0.210371519090592,1.56619231741469,0.117303600737215,0.600366592007791
"3056","DHX35",31.7443454455256,0.519032853376139,0.331425658472846,1.56606116668141,0.117334298183241,0.600366592007791
"3057","ZNF217",252.431265801277,0.318710304873529,0.203518297899271,1.56600319560097,0.117347869038863,0.600366592007791
"3058","AP001372.2",30.2224902373176,-0.447280288947434,0.285654885520523,-1.56580654355831,0.117393913869712,0.600405759621416
"3059","PEX11G",53.8082522708161,0.57366805240448,0.366414408309501,1.56562634927805,0.117436117669426,0.600425263272256
"3060","RAN",1175.28007838297,-0.331679626306884,0.211876797020311,-1.56543628642399,0.117480645719274,0.600456633676287
"3061","AC112253.1",2.68387767277611,-1.60265633041242,1.02405575564513,-1.56500885970099,0.117580831941079,0.600659783566732
"3062","ZNF117",58.8315604584272,0.534793602219658,0.34173446240543,1.56493904201323,0.11759720315098,0.600659783566732
"3063","NAA25",77.9419054298976,0.360388034088188,0.230316544527849,1.56475095971498,0.117641314554613,0.600688919240664
"3064","RARG",282.129836680089,-0.412538658250051,0.263743576202692,-1.56416571045889,0.117778657570493,0.601193930940768
"3065","KRT27",43.6821107260536,1.00536449578574,0.64286401650266,1.56388360520654,0.117844905374919,0.601335830363371
"3066","FBXO9",310.226641140661,-0.223544494831332,0.143002589729612,-1.56321990569547,0.118000879656401,0.601935341756722
"3067","ESPNL",4.44543972259011,1.26825939652585,0.812025140163833,1.56184745249386,0.118323929972006,0.602012447873184
"3068","KLF15",12.733482994082,0.989428064335027,0.633038765163324,1.56298179319201,0.11805687730965,0.602012447873184
"3069","LMX1B",28.2441845420734,0.533564266874034,0.341513753140041,1.56235074566745,0.118205383539978,0.602012447873184
"3070","UCP2",77.8068956233982,-0.472892923004166,0.302771592075741,-1.56188009503173,0.118316238472921,0.602012447873184
"3071","LINC00327",3.34264316655983,1.42893409599632,0.914885325593238,1.56187235276701,0.11831806273255,0.602012447873184
"3072","CD300E",4.55888047838551,-1.2924113200242,0.827385908103373,-1.56204173574434,0.118278157157719,0.602012447873184
"3073","ENGASE",50.6764266407246,0.498922491627246,0.319418091723883,1.5619731773319,0.118294307820999,0.602012447873184
"3074","SLC2A10",191.182123216865,0.310863810281828,0.199030599015268,

1.56188953768853,0.118314013590791,0.602012447873184
"3075","SMAGP",10.7665212786929,-0.766504845599606,0.490870081838735,-1.56152284272119,0.118400438496426,0.602118560567973
"3076","NEK8",18.6589608919135,-0.53826072788725,0.344722422103171,-1.56143231009834,0.118421783395594,0.602118560567973
"3077","ZBTB22",75.8732199961082,-0.36151607949217,0.231593771006812,-1.56099224051037,0.118525581700847,0.602450470523641
"3078","TLCD4",50.8176566055306,-0.481767517439155,0.308734958037845,-1.56045664702505,0.118652007266382,0.602648370386998
"3079","EFNA3",5.87573916520404,-1.13846512288887,0.729604288470218,-1.56038710418757,0.118668430439986,0.602648370386998
"3080","DERL1",318.229742864882,-0.285120109798845,0.182755142760542,-1.56012085620173,0.11873132379801,0.602648370386998
"3081","AC067930.8",6.77137443021191,1.26084195648252,0.808479519041023,1.55952244526623,0.11887277638388,0.602648370386998
"3082","LINC02254",11.5234965511021,0.771016932515979,0.494355239625362,1.55964146976632,0.118844630811315,0.602648370386998
"3083","TRADD",283.06236919366,-0.353081260418818,0.226356372464168,-1.55984678750191,0.118796091865343,0.602648370386998
"3084","CYBC1",242.983663894757,-0.312572688637953,0.200378774602737,-1.55990917330265,0.118781346383975,0.602648370386998
"3085","AC233296.1",3.00722668914312,1.65590130372588,1.06126521926305,1.56030865204058,0.118686959767423,0.602648370386998
"3086","AC104211.1",6.46567742037323,1.0509477198289,0.673987297369717,1.55929900152465,0.118925627953692,0.602702670492011
"3087","RPL41",29838.8324077492,-0.337634771626463,0.216572967164142,-1.55898852958212,0.118999095043435,0.602702670492011
"3088","ZNF415",20.3461639431406,0.764069121621954,0.490100896470784,1.55900372173161,0.118995499291965,0.602702670492011
"3089","AC105942.1",19.9991627501355,-0.556572079227961,0.357091339490717,-1.5586266528383,0.119084770960278,0.60282202580358
"3090","ZNF550",21.2344840265616,0.606661207152808,0.389243839433828,1.55856341370803,0.119099748064774,0.60282202580358
"3091","CCDC24",4.08644601656295,-1.1614646373453,0.745310450224937,-1.55836354769313,0.119147092612088,0.602866557247836
"3092","BRMS1",161.675754853069,-0.414320010457476,0.265905965870909,-1.55814484680881,0.119198915688974,0.602933713252118
"3093","CLIC4",592.444852813353,0.242075514184186,0.155427651546867,1.55748035677674,0.119356480756458,0.603123471688573
"3094","ZNF691",24.6806040872598,0.586436107420171,0.376460151300963,1.55776409639527,0.119289179955816,0.603123471688573
"3095","ANGPT1",126.379869577674,0.464736267166629,0.298417428131496,1.55733621215262,0.119390682119426,0.603123471688573
"3096","AP000487.1",10.4331259877049,0.803848383583967,0.516141623391844,1.55741824947472,0.119371216087004,0.603123471688573
"3097","C2orf49",211.416254310159,0.250194930379202,0.160678073571427,1.55711930581481,0.119442162209933,0.603188704218066
"3098","PPA2",120.215944834432,-0.360262419663661,0.231449389541521,-1.55654944857408,0.119577493788178,0.603639213937907
"3099","TCF7",98.6919607097098,0.624945404534184,0.401527819742573,1.5

5641869331706,0.11960856291519,0.603639213937907
"3100","ZNF318",76.0009065323062,0.45268715218405,0.290930876961612,1.55599555781692,0.119709148686378,0.60375720266203
"3101","LINC01303",3.73226962528119,-1.30691994041252,0.839895090419767,-1.55605141084863,0.119695867773098,0.60375720266203
"3102","CAP2",2.83000526217469,-1.50336859496767,0.966417579085539,-1.55560973589721,0.119800922190818,0.604025281452093
"3103","GABPB2",200.818403088075,0.438470481841076,0.282466460651618,1.55229219366283,0.120592324931943,0.604129051522988
"3104","AL365436.2",5.6702775478806,1.23527413071571,0.794375781227777,1.55502491378385,0.119940135923638,0.604129051522988
"3105","TMEM63A",75.8626543097523,0.517124505884697,0.33315213808009,1.55221728086397,0.120610242577601,0.604129051522988
"3106","CD200R1",3.76062495584431,-1.32598940835455,0.853517274948402,-1.55355895805941,0.120289655559731,0.604129051522988
"3107","SESN1",299.761175897902,0.438771410095683,0.282622956179338,1.55249741927284,0.12054324973551,0.604129051522988
"3108","AC090739.1",4.2376140706514,1.37980032054893,0.887513811967131,1.55468039138531,0.120022206894777,0.604129051522988
"3109","ZBTB26",14.9739441150822,0.644461953332144,0.414851107210198,1.55347772280527,0.120309047321949,0.604129051522988
"3110","AL137026.1",35.1415215307,0.656134804305901,0.422192908829982,1.55411138032668,0.120157851090642,0.604129051522988
"3111","DTX4",30.53100115326,0.577586015707316,0.371660161697389,1.55407029117529,0.120167650808692,0.604129051522988
"3112","EP400",166.25034057228,0.384468548714943,0.247203512381086,1.55527138353217,0.119881449698734,0.604129051522988
"3113","G2E3",99.3357644799545,0.36042251216473,0.231988241976902,1.55362405048362,0.120274119037311,0.604129051522988
"3114","AC092140.1",6.06847771814023,-1.00954248371772,0.649299968705335,-1.55481677556629,0.119989712655097,0.604129051522988
"3115","MT2A",11221.6267537743,-0.662261220713075,0.4265532862583,-1.55258731335161,0.120521758465149,0.604129051522988
"3116","ZNF491",4.13705275039096,1.20452609284966,0.775125857648563,1.55397485577856,0.120190414462051,0.604129051522988
"3117","DDX39A",119.657659230147,-0.355959804732286,0.229337293838199,-1.55212350671331,0.120632674418561,0.604129051522988
"3118","MRPS12",279.15452240946,-0.384175669583037,0.247157560392498,-1.55437555287788,0.120094861167254,0.604129051522988
"3119","TMEM238",29.3672993747781,-0.612178404084019,0.394320539017978,-1.55248926573441,0.120545199176878,0.604129051522988
"3120","DTD1",424.091611008264,-0.317904119624811,0.20474316129963,-1.55269713335906,0.120495507508272,0.604129051522988
"3121","CASS4",4.27657188914715,-1.18359025000137,0.761729929372205,-1.55381875434099,0.120227655713066,0.604129051522988
"3122","PI4KA",128.013850881339,0.342430983020241,0.220459104737536,1.55326305723647,0.120360302168936,0.604129051522988
"3123","AL121601.1",6.39118713828134,0.959960312110263,0.618476498419269,1.55213708938622,0.120629425086856,0.604129051522988
"3124","BAG4",143.923407026296,0.300703968614541,0.193757893006579,1.5

5195725938417,0.120672450691919,0.604134804360313
"3125","TEX41",5.09796892239745,-1.04396939927212,0.672787841600731,-1
.55170669670286,0.120732419617496,0.604241613701646
"3126","IGDCC4",105.808562239474,0.540249428883151,0.34826753976824,1.
55124829963386,0.12084219136831,0.604404180684478
"3127","COQ8B",81.9880406867199,-0.30807228685628,0.198577095049267,-1
.55139890015939,0.120806118644818,0.604404180684478
"3128","RPS6KA2",65.0106622557067,0.720380604987107,0.464529097011002,
1.5507760646693,0.12095535851882,0.604429198499596
"3129","AC004951.1",25.3385372246233,0.799583936342925,0.5156132302962
49,1.55074363759735,0.1209631324363,0.604429198499596
"3130","NPIPA2",3.15447605465937,2.28069241330919,1.47051007350915,1.5
5095327423814,0.120912882014878,0.604429198499596
"3131","RNASEL",92.113474676947,0.346542290901901,0.223555853788793,1.
55013740427169,0.12110854001723,0.604962493091496
"3132","NSUN4",57.7191437832509,0.421790297334116,0.272290349016983,1.
54904607841172,0.121370643672361,0.605232359194131
"3133","ATP10D",109.040984250575,0.370798711808971,0.239373714356097,1
.54903688070514,0.121372854568803,0.605232359194131
"3134","ANKHD1",426.126157911127,0.34976865642333,0.225834244700835,1.
54878484831506,0.121433449050587,0.605232359194131
"3135","PTPN12",552.142389515742,0.32427188115165,0.209359928342716,1.
54887271751845,0.121412320552868,0.605232359194131
"3136","FAM160A2",68.0180783014756,-0.329174282425082,0.21253392252322
7,-1.54880820208411,0.12142783326383,0.605232359194131
"3137","RAB22A",434.00127485433,-0.226073831222481,0.145935509845537,-
1.54913517252768,0.121349229326633,0.605232359194131
"3138","PEX26",128.377356677135,0.36996850671408,0.238788224533911,1.5
4935825431183,0.121295623143949,0.605232359194131
"3139","DNAJC7",456.423742194782,-0.202361243731508,0.13068449492134,-
1.54847171313866,0.121508767027831,0.605414818832519
"3140","HAX1",447.80538234853,-0.323343769943718,0.208910787725709,-1.
54776004371902,0.121680079771366,0.60543511596797
"3141","TRANK1",92.8572494869973,0.302251444200807,0.19527187944654,1.
54784931172619,0.12165858086324,0.60543511596797
"3142","WDR1",545.417296389576,-0.259181696083916,0.16742811437987,-1.
54801776896245,0.121618018466322,0.60543511596797
"3143","KLF3-
AS1",101.892922768438,0.403903679102877,0.260972831992116,1.5476847763
0299,0.121698209147935,0.60543511596797
"3144","KATNBL1",320.775471923269,-0.20011218083175,0.129309050421795,
-1.54754968951516,0.121730752286294,0.60543511596797
"3145","ATP11C",143.55959091404,0.281685843534823,0.182027555280735,1.
54749012093465,0.121745104841385,0.60543511596797
"3146","NIFK",379.492408931165,-0.255122864530298,0.164924124500163,-1
.54691052812013,0.121884821996418,0.605744714338728
"3147","MGAT4C",24.5104496848219,1.77815070340354,1.14946309232842,1.5
4694023259296,0.121877658363694,0.605744714338728
"3148","SLC25A6",2540.45677025135,-0.389750022948982,0.251985113880005
,-1.54671844279889,0.121931153899631,0.60578247998419

"3149","SESN3",512.486095517825,0.4034634099851,0.260880009111576,1.54654782234595,0.121972319918931,0.605794564475098
"3150","AC103858.3",2.51745832906631,1.80576668004439,1.16791286303549,1.54614846466463,0.122068716412375,0.605888519419088
"3151","DCAF7",348.516572914598,0.270643001268702,0.175041655250403,1.54616340254288,0.122065109652911,0.605888519419088
"3152","CCM2",289.241606097152,-0.343965800087515,0.222537392749272,-1.5456539498288,0.122188164342175,0.606288988043026
"3153","QKI",1192.115686594,0.235793516486132,0.152576585328621,1.54541088973958,0.122246907936479,0.606388087575812
"3154","WDR82",422.527807002971,0.282707428291546,0.182968078018608,1.54511886091297,0.122317515650588,0.606545955857702
"3155","MYDGF",1335.82958177315,-0.402061172973648,0.260324354103384,-1.54446238562058,0.122476356808345,0.607141115842319
"3156","CACNB2",2.65867181700178,1.72512237652897,1.1172982638885,1.5440124023151,0.122585328111931,0.60729633565746
"3157","ZNF136",97.251551509071,0.302324111122692,0.195788265801158,1.54413805079479,0.122554892521546,0.60729633565746
"3158","AGAP4",29.2488228093631,0.619757126936575,0.401500992230516,1.5436004864983,0.1226851471062,0.607598385288461
"3159","TRMT61B",78.3705065521268,-0.387237604262212,0.250988786714562,-1.54284822573608,0.122867605286075,0.607662487490331
"3160","TNFAIP8",38.16597726209,-0.452938956060502,0.293639695553309,-1.5424990657582,0.122952364789283,0.607662487490331
"3161","CD82",16.8731831739609,-0.621168174033528,0.402805034173101,-1.54210628302771,0.12304776840677,0.607662487490331
"3162","BBOF1",68.3380905985912,-0.404722525678419,0.262440381088972,-1.54215035048745,0.12303706191344,0.607662487490331
"3163","STARD9",219.001541054384,0.491708751273465,0.318640506501972,1.54314577475234,0.122795410505431,0.607662487490331
"3164","CCNDBP1",643.257852115262,-0.292893992492641,0.189877424777318,-1.54254247357809,0.122941824941601,0.607662487490331
"3165","HBA1",14.3044212393058,1.39078299507099,0.901834735871742,1.54217057710315,0.123032147961247,0.607662487490331
"3166","LIPE",15.898880359365,0.742104101433512,0.481185190816861,1.54224218782318,0.123014751737294,0.607662487490331
"3167","ZNF221",11.7584627336484,0.817557710114939,0.530085820146475,1.54231197863212,0.122997799468604,0.607662487490331
"3168","ERAP1",88.1799121996106,0.367893221357993,0.238722502649911,1.54109150697667,0.1232945166004,0.608631339154589
"3169","SEC23B",76.4902473044143,-0.325820730995756,0.211437426115637,-1.54097946130673,0.123321784768599,0.608631339154589
"3170","CERS6",18.0070884752292,-0.601605307422014,0.390455076118722,-1.54077983414176,0.123370379011105,0.608679093922295
"3171","LAMB3",35.8689081598544,-0.564763312236761,0.36661399079417,-1.54048488715162,0.123442203842883,0.608841396437305
"3172","ATXN7L1",50.133082580811,0.380166904220086,0.246839037980563,1.54014092475122,0.123526006027807,0.609062652671783
"3173","BTN3A3",114.020998687079,0.371734697935972,0.241519250139313,1.5391514246651,0.123767333154122,0.609292127960486

"3174","BACE1-
AS",24.287390159906,0.61697784456424,0.40083237275389,1.53924155458138
,0.123745336334624,0.609292127960486
"3175","AC099518.5",64.2526079915592,-0.714324302825658,0.463948946896
876,-1.53966143818931,0.123642901098986,0.609292127960486
"3176","FUS",1545.57914332559,0.352865175103634,0.229192961427773,1.53
959865479915,0.123658213589334,0.609292127960486
"3177","PNPLA6",66.2281302143945,-0.32533082582296,0.211368841182557,-
1.53916170426452,0.123764824193747,0.609292127960486
"3178","FLCN",160.765783726636,0.398432656716701,0.258905940670467,1.5
389089013752,0.123826537767999,0.609391771772029
"3179","SETD2",308.482463007148,0.332012567087654,0.215791306093551,1.
53858175798666,0.123906434793994,0.609593155136225
"3180","DPAGT1",70.8921816935895,-0.412190456747208,0.268101392610176,
-1.53744243076924,0.124185002552271,0.609621293131675
"3181","KCNJ5",13.1977385804113,1.06812663161194,0.694314850321082,1.5
3838943689305,0.123953423441045,0.609621293131675
"3182","DCLK1",1661.4226297085,0.282119497442082,0.183487603922601,1.5
3753981964409,0.12416117169772,0.609621293131675
"3183","ALDOA",93.4543484597095,-0.513549780176905,0.333972298015151,-
1.53770172924225,0.124121560653683,0.609621293131675
"3184","TRPV1",38.9226758443129,0.518768587885216,0.337411676425862,1.
53749447375513,0.124172267298244,0.609621293131675
"3185","HMG20B",319.038961519906,-0.34973936562083,0.227387541273442,-
1.53807620093071,0.124029984251287,0.609621293131675
"3186","KANTR",49.272617433308,0.573955048680334,0.373142751005264,1.5
3816480993955,0.124008322787151,0.609621293131675
"3187","SMIM13",93.6243226806073,-0.332757584457836,0.216491376261202,
-1.53704775776544,0.124281614749625,0.609904127607195
"3188","NAP1L5",51.700829799882,0.450535698976857,0.293216239061774,1.
53653051556241,0.124408319487351,0.610103561259124
"3189","SRRM2",1966.40542122139,0.283731995338502,0.184650178620128,1.
53659204371641,0.124393242143298,0.610103561259124
"3190","LSM4",394.790876492534,-0.395130823251763,0.257179400513677,-1
.53640152540425,0.124439932879732,0.610103561259124
"3191","ZNF524",234.584276688691,-0.444506976120794,0.289346400644848,
-1.53624505136456,0.124478290535669,0.610103561259124
"3192","LSM11",22.9165742709329,0.584151103347857,0.380292336032059,1.
53605804798183,0.124524144174697,0.610137097397324
"3193","ROBO4",102.360792073999,0.581254346208125,0.37851197658118,1.5
3562999897168,0.124629152320292,0.610269236784399
"3194","BBS10",83.9677443305556,0.354772652194677,0.231007009770572,1.
53576574384918,0.124595844177493,0.610269236784399
"3195","RGMA",178.784307800792,-0.400772650354664,0.261118518344331,-1
.5348304398165,0.12482548357152,0.611039299861838
"3196","EIF3I",1001.84575861015,-0.30059014845868,0.195903488329789,-1
.53437874445941,0.124936503641505,0.611342035893053
"3197","AL139384.1",21.1953648497291,0.672210905306507,0.4381334018636
77,1.53426080378063,0.124965504395786,0.611342035893053
"3198","CMTM6",491.195915459227,-0.233661878463079,0.152341858126785,-

1.53379958296567,0.125078965633631,0.61137210770957
"3199","RNF7",1004.11811133715,-0.386517827268456,0.252006864089821,-1
.53375912463516,0.125088922293518,0.61137210770957
"3200","STX17",238.811101518258,0.333068259214116,0.217137863281323,1.
53390225997846,0.12505369993763,0.61137210770957
"3201","PSMC4",385.671053044748,-0.361887001184648,0.236001647166712,-
1.53340879408782,0.125175163310868,0.611602484905334
"3202","Z97200.1",103.077326111683,0.48632098010655,0.317298250710321,
1.53269354311864,0.125351380844841,0.612272203751814
"3203","AC008966.1",8.90953239084106,0.773489241141344,0.5048013297403
37,1.53226466645644,0.125457136567483,0.612296882683773
"3204","NECAB1",4.41693341180996,1.19167093229857,0.777753186724741,1.
53219678509696,0.125473881649712,0.612296882683773
"3205","TNFAIP8L3",9.42502065252192,-0.953661475968337,0.6223083737368
51,-1.53245804847807,0.125409442329017,0.612296882683773
"3206","TRBC2",28.5031276236566,0.834139834051972,0.544596909179126,1.
5316646495649,0.125605210044701,0.612364552711698
"3207","TTC12",25.5902226818556,0.522393065948934,0.341053401951892,1.
53170460391016,0.12559534579398,0.612364552711698
"3208","ZNF724",12.891028685012,0.671151265627803,0.438100927003421,1.
53195582172909,0.125533336943919,0.612364552711698
"3209","LYPLA2",88.4444236936346,-0.418508077787981,0.27327194498949,-
1.53147106924597,0.125653011262049,0.612406698703161
"3210","RPL6",10604.9657708475,-0.396550364277244,0.259066040502935,-1
.53069218762678,0.125845485491945,0.612534990242508
"3211","BIVM",108.895346690434,0.34420889190047,0.224912294765605,1.53
041385425013,0.125914321843329,0.612534990242508
"3212","RAB31",196.521928778636,-0.34939970560416,0.228294113066273,-1
.53048057574191,0.125897817865617,0.612534990242508
"3213","OLFM2",6.81580477455445,-1.024584074565,0.669229625665803,-1.5
309903137442,0.125771786597764,0.612534990242508
"3214","RNASEH2A",20.2200937845848,-0.844811262154991,0.55185288657950
7,-1.53086317513223,0.125803212052472,0.612534990242508
"3215","CHKB-
DT",21.4133181628394,-0.520413057005365,0.339899543117353,-1.531079013
02971,0.125749865996063,0.612534990242508
"3216","SLAMF8",2.87600496422806,-1.55545023717418,1.01706840974392,-1
.52934672070467,0.126178513091873,0.612867063589096
"3217","AC103706.1",13.9009052216168,-0.826765724994446,0.540546645798
471,-1.52949931596224,0.126140708510146,0.612867063589096
"3218","ENTR1",117.924065077683,-0.293986931079817,0.192222140039758,-
1.52941243406723,0.126162231909359,0.612867063589096
"3219","MT1X",1916.58700210668,-0.554388279011624,0.362392524203619,-1
.5298005394287,0.126066108055283,0.612867063589096
"3220","MZF1-
AS1",15.6842794308753,-0.664917882391479,0.434679114614298,-1.52967524
786986,0.126097133359253,0.612867063589096
"3221","ILK",806.088506315926,-0.282171513522389,0.184549903588263,-1.
52897134073786,0.126271548833921,0.612938244494884
"3222","AC048341.2",11.5931752497704,-0.682478012244559,0.446363771000

27,-1.52897268233749,0.126271216231158,0.612938244494884
"3223","ADTRP",7.25308252626105,-1.20831046502713,0.790672134876586,-1
.52820671391908,0.12646122237109,0.613287912522122
"3224","PPDPFL",31.0741007850765,0.904631224884477,0.591935395502651,1
.52826006310418,0.126447981362759,0.613287912522122
"3225","RFWD3",41.6184124240551,0.507541939116429,0.332107805777544,1.
52824453471713,0.126451835321537,0.613287912522122
"3226","KIAA1614",42.6007433877751,0.399363005554527,0.261427208733968
,1.52762601677365,0.126605418418353,0.613796882846572
"3227","DHFR2",66.3794840808924,0.36255881615155,0.237408597768523,1.5
2715116284479,0.126723427070648,0.614178617720771
"3228","SRSF12",4.01241340711149,1.31429707293618,0.861021990189874,1.
52643845094635,0.126900707841822,0.614466585339349
"3229","CNNM2",29.6492165693217,0.526506604125577,0.344906882381326,1.
52651811552857,0.126880882405589,0.614466585339349
"3230","SOS2",200.625177592217,0.334853301890938,0.219352542144229,1.5
2655309401778,0.126872178372867,0.614466585339349
"3231","AC100793.2",13.7119881776584,-0.688330058468835,0.451116415996
682,-1.52583686618466,0.12705049690121,0.61500147679818
"3232","OBSL1",125.37238728025,0.335932018719864,0.220285971143873,1.5
2498144559764,0.127263725623682,0.615271922335206
"3233","BRF2",88.5433914242366,-0.367553605846762,0.241018216989135,-1
.52500342272191,0.127258243953723,0.615271922335206
"3234","KCNE3",12.3745458474011,0.927952834251387,0.608442707624892,1.
52512771148779,0.12722724654003,0.615271922335206
"3235","SIAE",42.388682123341,0.404820882608095,0.265393795675079,1.52
535925558605,0.127169515490272,0.615271922335206
"3236","UBA5",155.008717079878,-0.311512660325624,0.204346542555783,-1
.52443323204544,0.127400523890308,0.615669255057051
"3237","KCNMB4",45.7427163783265,0.733434228270701,0.481149775218867,1
.52433663288541,0.127424640576706,0.615669255057051
"3238","CCL5",52.4223802068117,0.74759352423299,0.490622869911039,1.52
376411716915,0.127567646218079,0.616169853876082
"3239","ZNF517",11.2758813431904,0.807416828302996,0.530050955930581,1
.52328152467051,0.127688287360837,0.616562153233557
"3240","MANF",459.956569354133,-0.367289089666487,0.241182088327143,-1
.5228705092241,0.127791105231418,0.61686817463561
"3241","SHLD1",31.521597440848,-0.514732381766484,0.338074012943803,-1
.52254347290529,0.127872961230467,0.61707285209642
"3242","MRPL2",239.32818789543,-0.38734618343063,0.254585509780792,-1.
52147772967971,0.128139995839963,0.617826435685696
"3243","BICC1",485.652395222639,0.321576132590074,0.21132185329469,1.5
2173628792491,0.12807517120535,0.617826435685696
"3244","NUCB2",992.032941555754,-0.373065612516148,0.245204429516124,-
1.52144728075402,0.128147631545038,0.617826435685696
"3245","TSSC4",269.165308302141,-0.30786901677721,0.202411414148375,-1
.52100620447981,0.1282582803288,0.617978898441908
"3246","LINC00987",2.89258899564517,1.70947284093831,1.1237935080754,1
.52116276580556,0.128218996716807,0.617978898441908
"3247","ME3",71.3904540688129,-0.41552176120157,0.273294725154482,-1.5

2041632331796,0.128406374503705,0.618501908604233
"3248","MTMR3",185.196518239983,0.28634879771373,0.188375231046117,1.5
2009792435837,0.128486366211597,0.618696664885894
"3249","IGFBP5",50647.0435581674,0.479226188958179,0.315402820803498,1
.51940996512757,0.128659335183548,0.619045560900195
"3250","PRDM5",106.786601827791,0.367069657989422,0.241634301420969,1.
51911237697136,0.128734211819382,0.619045560900195
"3251","PSMG3",271.470604898943,-0.320312723940611,0.210867604308423,-
1.51902291957616,0.128756726957055,0.619045560900195
"3252","PENK",93.9103670545435,-0.454426883247649,0.299144599745978,-1
.51908770418564,0.128740421298561,0.619045560900195
"3253","BPTF",1003.37163140104,0.33007740433949,0.21726225485244,1.519
25793352219,0.128697583871324,0.619045560900195
"3254","PDSS1",17.5221625688101,-0.705142985582019,0.464259919021178,-
1.51885389345844,0.128799276748482,0.619059830469039
"3255","AC119674.1",17.4116327819711,0.729043991902789,0.4801216768677
59,1.51845673092488,0.128899299460328,0.619350243797091
"3256","LINC02613",18.0877392580505,0.788401020840403,0.51941199671983
5,1.51787218204291,0.129046623927684,0.61947104621307
"3257","ANAPC1",89.7735458578032,0.315878624461318,0.208161029025184,1
.51747243920043,0.129147446824559,0.61947104621307
"3258","AUH",112.983217746687,0.302905752474018,0.199585813357421,1.51
767175922254,0.129097166803847,0.61947104621307
"3259","NFRKB",83.1693122218867,0.358437677497088,0.236211686974159,1.
51744260450712,0.129154974165898,0.61947104621307
"3260","AL049775.1",12.7633604903024,0.712743873151683,0.4697094422445
16,1.51741440356366,0.129162089622815,0.61947104621307
"3261","CYP46A1",27.7955467214905,0.622524162071139,0.410195454398259,
1.51762813409124,0.129108170281325,0.61947104621307
"3262","AC009065.4",9.41514999602904,-0.785232256911088,0.517589437172
757,-1.51709482558277,0.129242744492712,0.619667849131215
"3263","SZT2",74.4463878231727,0.488613843151294,0.32253113831424,1.51
49354127639,0.129788761213502,0.619755815322966
"3264","AC009242.1",2.96469538911717,1.63924742135467,1.08192042266967
,1.51512753341857,0.129740110228677,0.619755815322966
"3265","AC137936.1",3.22368371507125,-1.37975195810326,0.9110177858882
27,-1.51451703740123,0.129894756000614,0.619755815322966
"3266","SH3TC1",4.51769731054622,-1.28945596205033,0.850768101127186,-
1.51563741087839,0.129611061888407,0.619755815322966
"3267","PTCD2",44.6386173194483,0.478015540479268,0.315404541908233,1.
51556327498399,0.129629819249514,0.619755815322966
"3268","PKIB",3.52394680522763,-1.31027982426942,0.864119852105482,-1.
51631723432443,0.129439155826258,0.619755815322966
"3269","RHBDD2",125.887282659452,-0.480470476600082,0.316986732441684,
-1.51574317606013,0.129584305547586,0.619755815322966
"3270","ZBTB10",207.193493474415,0.38944095207268,0.256789778342714,1.
51657497656674,0.12937402719347,0.619755815322966
"3271","NYNRIN",110.782610421871,0.389085663886786,0.256783099100859,1
.5152308124997,0.129713962573076,0.619755815322966
"3272","TRIM69",209.203484900577,0.428913907155851,0.282807485554578,1

.51662855144998,0.129360492604506,0.619755815322966
"3273","AC103740.1",14.0530830011209,0.91206626120363,0.601885923763265,1.5153473859315,0.129684454039027,0.619755815322966
"3274","RFFL",62.1162637115238,0.450462658862538,0.297230185980974,1.51553469367803,0.129637051260571,0.619755815322966
"3275","CD300A",5.61112086009145,-1.15570249562947,0.762418979069526,-1.51583647227659,0.129560707150092,0.619755815322966
"3276","RHBDF2",4.88227978756727,-1.32359370978621,0.873938626744697,-1.51451562990918,0.129895112700044,0.619755815322966
"3277","QTRT1",135.933522546555,-0.371228946798358,0.244792588839063,-1.51650402718042,0.129391952784735,0.619755815322966
"3278","RABAC1",2302.38109430224,-0.365050079013122,0.241026022215406,-1.51456708142026,0.129882073884496,0.619755815322966
"3279","EIF2B5",133.822595879353,-0.265407854772553,0.175289112852394,-1.51411488399765,0.129996704304324,0.619890972146158
"3280","HDHD2",125.664447071817,0.319946399144501,0.211328723740022,1.51397497454298,0.130032186749974,0.619890972146158
"3281","PBDC1",417.570974505007,-0.349040340507225,0.230551746229819,-1.51393492443685,0.130042345243705,0.619890972146158
"3282","LCLAT1",38.1771710223022,-0.502652569872006,0.33227516155164,-1.51275998941584,0.130340635367495,0.62036747935107
"3283","ABHD18",48.6057607979807,0.50066580816676,0.330913653816928,1.51298020614086,0.130284686798455,0.62036747935107
"3284","RNF170",232.932002863593,0.280323257473352,0.185300454768445,1.51280393684759,0.130329468532769,0.62036747935107
"3285","RACGAP1",20.6488929763107,-0.520688668031996,0.344181772318065,-1.51283045736315,0.130322730154272,0.62036747935107
"3286","SLC39A9",138.867807937742,0.325557826908592,0.215177879448201,1.51297070007125,0.130287101538856,0.62036747935107
"3287","ZNF891",51.8132611130152,0.646588035551205,0.427614183651377,1.51208276121718,0.130512809739357,0.620708636447432
"3288","CHD8",234.892880961997,0.336514476003647,0.222561044808871,1.51200977822797,0.130531375017622,0.620708636447432
"3289","MAP1A",572.90301434594,-0.369923793862372,0.244617466100991,-1.5122542137267,0.130469204025326,0.620708636447432
"3290","ZDHHC16",82.1208455781485,-0.363946370254247,0.240851031962521,-1.5110849527557,0.130766807933631,0.621639172061395
"3291","ARHGAP44",22.1402245874698,0.543871809411305,0.359989066642619,1.51080090982662,0.130839182848983,0.621794232682495
"3292","ENTPD1-AS1",67.1068062736089,0.364729614862799,0.241455751317089,1.5105443248847,0.130904588099597,0.621916086840126
"3293","RERE",460.439490624975,0.399273441155396,0.265219614188686,1.50544461945918,0.132209804880027,0.621967472092388
"3294","NOTCH2NLA",57.8704752227603,0.509344407612012,0.338087120081753,1.50654780190635,0.131926605595939,0.621967472092388
"3295","AL445423.1",9.15252533325563,0.893495609802325,0.593323002917244,1.50591769644729,0.13208830309852,0.621967472092388
"3296","AC092164.1",23.2154726407552,0.533319155157102,0.353241159778475,1.50978769147841,0.131097606757278,0.621967472092388

"3297","LBX2-
AS1",42.5071037154062,0.453496729168158,0.300442332242643,1.5094301984
1127,0.131188880672264,0.621967472092388
"3298","CYTOR",1249.36049798476,-0.449060348656717,0.297587372337312,-
1.50900337312604,0.131297920778892,0.621967472092388
"3299","SH3BP5-
AS1",18.6698675544423,0.773841282204719,0.512615333875903,1.5095944874
5647,0.131146928834548,0.621967472092388
"3300","ZNF197",81.233095287712,0.327433297151905,0.217114177460851,1.
50811568816571,0.131524920699134,0.621967472092388
"3301","AP002026.1",4.27890824631471,1.3003604975112,0.861229836469346
,1.50988788642309,0.131072034161302,0.621967472092388
"3302","CCDC152",85.4710623835558,0.684250286116738,0.454630022576431,
1.505070611569,0.132305923716333,0.621967472092388
"3303","STX11",30.5484252730623,-0.518990552492183,0.344395216451531,-
1.50696214029797,0.13182036180201,0.621967472092388
"3304","EEPD1",14.5453298514214,-0.768259077509899,0.509908340271538,-
1.50666113266707,0.131897538966346,0.621967472092388
"3305","ZNF655",305.360262250065,0.315031154226277,0.208748460389319,1
.50914240823017,0.131262394076185,0.621967472092388
"3306","LY6K",18.3818198710133,1.01195818936706,0.672238004743982,1.50
535700484899,0.132232316702447,0.621967472092388
"3307","DRAP1",968.211155691705,-0.366549557873501,0.242931372083078,-
1.50886052604251,0.131334429294816,0.621967472092388
"3308","GVQW3",46.6211933351224,0.413368615406449,0.27395752824304,1.5
0887846761317,0.131329843398975,0.621967472092388
"3309","TAS2R14",14.748506562203,0.681896517909083,0.453067712608249,1
.50506535542667,0.132307274913771,0.621967472092388
"3310","NEMP1",31.4196488557482,0.443140853010202,0.294316842599305,1.
50565917022123,0.132154690439571,0.621967472092388
"3311","PPP1CC",889.414678707477,-0.269796755942109,0.179014970112808,
-1.50711840340556,0.131780310361855,0.621967472092388
"3312","ACAD10",45.7544455423531,0.458222498324681,0.304327433914327,1
.50568909424603,0.132147004881337,0.621967472092388
"3313","COX6A1",1657.41700562354,-0.357103466754209,0.236778418647434,
-1.50817573997713,0.131509554586394,0.621967472092388
"3314","AC007686.3",14.7772157049798,0.69798293834732,0.46253775238788
7,1.50902912193422,0.131291340797144,0.621967472092388
"3315","BEGAIN",5.81295509699051,1.50099465446753,0.99500105930466,1.5
0853573514431,0.131417467866043,0.621967472092388
"3316","INF2",87.2176319612731,-0.411299656899501,0.273069173278595,-1
.50621050322615,0.132013144009861,0.621967472092388
"3317","ZSCAN2",10.8012588995778,0.736936602180266,0.489288811205409,1
.50613826701811,0.13203168287645,0.621967472092388
"3318","CRAMP1",54.8445750178944,0.492528057664796,0.326680071742924,1
.50767708307712,0.131637193579572,0.621967472092388
"3319","CREBBP",405.422779151522,0.303434559912774,0.201291254163859,1
.50744035637915,0.13169782106264,0.621967472092388
"3320","MED31",202.344611432646,-0.38337855374066,0.254119716354816,-1
.50865331993904,0.131387400522388,0.621967472092388

"3321","FAM117A",56.1660008649264,0.41916447757666,0.278359214036597,1
.50584013907135,0.132108216460992,0.621967472092388
"3322","TRIB3",13.1847451948816,1.03274414508614,0.686047740739764,1.5
053531755276,0.13223330068145,0.621967472092388
"3323","AL391097.1",5.69898493978402,1.49431597078621,0.99271929877390
7,1.50527543146569,0.1322532789527,0.621967472092388
"3324","MBTPS2",57.7582236692653,0.37767130270539,0.250750060765937,1.
50616634569006,0.132024476461857,0.621967472092388
"3325","SLC9A7",13.6973610501385,0.973794790271398,0.646904179421372,1
.50531534846848,0.13224302099351 6,0.621967472092388
"3326","SPIN2A",23.605885838038,0.609104579836712,0.40368382786388,1.5
0886544814002,0.131333171186,0.621967472092388
"3327","MORF4L2-
AS1",2.61972436461997,1.6824559161531,1.11770024019112,1.5052836669923
3,0.132251162518484,0.621967472092388
"3328","GSK3A",166.580127086331,-0.239147593756158,0.158935544438187,-
1.50468288639591,0.132405624986057,0.622242780883992
"3329","SEC61B",1887.83773532587,-0.359613108950764,0.239112917006227,
-1.50394681079232,0.132595062632856,0.622758792666028
"3330","TRPV3",22.5424382833312,-0.635788988007218,0.422739802324512,-
1.50397238327505,0.132588477738497,0.622758792666028
"3331","SLC49A3",100.754275287182,-0.460999159719259,0.306621888761358
,-1.50347765967241,0.132715913628958,0.622772281325642
"3332","KRIT1",284.369706508151,0.218802880699794,0.145534001309932,1.
50344853251047,0.132723419452153,0.622772281325642
"3333","SYVN1",135.894303245241,-0.267490815332833,0.177951975452426,-
1.50316294411885,0.132797030576791,0.622772281325642
"3334","SERP2",40.738456221995,-0.531756669345677,0.353625833823843,-1
.5037268731067,0.132651706871723,0.622772281325642
"3335","OSCAR",8.03055174762644,-0.867099495735182,0.57679761656805,-1
.50329937369442,0.132761861568751,0.622772281325642
"3336","AKR1A1",606.964698605958,-0.39567869557929,0.263293240528084,-
1.50280612895979,0.132889044994725,0.622919285813251
"3337","PLEK",24.4706126478996,-0.919399666963774,0.612867253690329,-1
.50016118731697,0.133572654397562,0.622919285813251
"3338","MALL",36.9394172355049,0.542344275101993,0.361320387994669,1.5
0100656680905,0.13335386309302,0.622919285813251
"3339","HDAC4",222.918140906579,0.333551129582856,0.222187737653266,1.
50121304220388,0.133300467694143,0.622919285813251
"3340","GMPPB",90.3836193851492,-0.564296067919155,0.375569944261845,-
1.50250592876445,0.132966497944433,0.622919285813251
"3341","C3orf52",4.61225148777057,-1.05592395492208,0.702901317264439,
-1.50223641496581,0.133036063438677,0.622919285813251
"3342","RUBCN",58.9205112602052,0.444058627120093,0.295860530417904,1.
50090526266839,0.133380066818763,0.622919285813251
"3343","TMEM161B",69.9853479181938,0.37795576913924,0.251805007139905,
1.50098591538032,0.133359204549124,0.622919285813251
"3344","AC005062.1",12.0861511387644,-1.97664405768406,1.3170618652663
8,-1.50079818557672,0.133407768130217,0.622919285813251
"3345","PTRH1",116.549088785981,-0.398483359555841,0.265635552566072,-

1.50011305228703,0.133585120499849,0.622919285813251
"3346","TRMT112",1219.3706277442,-0.312775880337974,0.208408355898612,
-1.50078378090625,0.133411495021135,0.622919285813251
"3347","DYNC1H1",1384.04495642862,0.249355396486838,0.166170220855037,
1.50060218493884,0.133458485884888,0.622919285813251
"3348","COTL1",765.71230044992,-0.448163642404069,0.298707944020843,-1
.50034055462816,0.133526209434001,0.622919285813251
"3349","EMC6",269.164363342131,-0.396869642555509,0.264215411880392,-1
.50206848166438,0.13307942375035,0.622919285813251
"3350","MRC2",1864.35192237906,0.331113563920452,0.220462350354835,1.5
0190526131796,0.133121577661614,0.622919285813251
"3351","RGL3",4.79041450381656,1.55062281742801,1.03355064761642,1.500
28720992442,0.133540021084483,0.622919285813251
"3352","POFUT1",124.432852763422,0.351951736285768,0.23434341837886,1.
50186311491271,0.133132464232342,0.622919285813251
"3353","TIMP1",7546.23796051131,-0.466089390518434,0.310590222441184,-
1.50065699703955,0.13344430102702,0.622919285813251
"3354","PIM2",10.1205352428959,0.83420233766081,0.555753797035655,1.50
102858875707,0.133348167336906,0.622919285813251
"3355","KDELR2",1638.64876035338,-0.322096677713489,0.214821541554675,
-1.4993686172367,0.133778030353349,0.623337173827203
"3356","ZNF394",125.005340861053,-0.249776312152667,0.16659374911474,-
1.49931383068062,0.13379223602691,0.623337173827203
"3357","TMEM123",645.887718880445,0.218432894704421,0.145689348908739,
1.49930586100189,0.133794302591939,0.623337173827203
"3358","MAP3K7",150.242395151494,0.261067539659119,0.174257762052656,1
.49816878504517,0.134089403461556,0.623884922307856
"3359","LEMD3",112.255657701917,0.375926651101502,0.250937836358225,1.
49808676346778,0.134110709650373,0.623884922307856
"3360","UBAP1L",19.0637377169401,0.872748109433516,0.582382729964269,1
.49858171358732,0.133982179577926,0.623884922307856
"3361","TUBD1",62.3868663913716,-0.358526356615187,0.239347733463545,-
1.49793086162562,0.134151214432309,0.623884922307856
"3362","ZNF674-
AS1",17.9662579918388,-0.623218867641347,0.41601552930619,-1.498066355
07266,0.134116011407508,0.623884922307856
"3363","PAK3",202.653669069887,0.434402611752657,0.289968313314061,1.4
9810372998294,0.134106302159042,0.623884922307856
"3364","FOXJ2",96.5527711312933,0.313939981039728,0.209625839272952,1.
49762062791767,0.134231844245481,0.624074329369595
"3365","EIF2S2",1006.09656731792,-0.246305730918885,0.164552197559379,
-1.49682431819244,0.13443897687812,0.624851589412716
"3366","BCL10",228.385587959725,-0.302314405808626,0.202021238075392,-
1.496448634256,0.134536783910383,0.62512041008865
"3367","FAM193A",139.190605508741,0.450220704885899,0.300948605234677,
1.49600528812825,0.134652277137609,0.62520033684569
"3368","HIST1H3A",3.97350739842026,1.25414107523115,0.838409372100457,
1.49585765255601,0.134690753729515,0.62520033684569
"3369","ZNF248",67.825413657042,0.368501896425666,0.246406775179692,1.
49550228948427,0.134783402841693,0.62520033684569

"3370","ACER3",83.0828062970507,0.348312314400688,0.232912802097064,1.49546229861393,0.134793832215068,0.62520033684569
"3371","RPL4",4875.02888195365,-0.235499200340335,0.157447056724925,-1.49573580630202,0.13472251554372,0.62520033684569
"3372","PRC1",31.6079621045904,-0.549409371403794,0.36733070220897,-1.49568050832637,0.134736932045434,0.62520033684569
"3373","ERCC1",395.557705814068,-0.34213785278002,0.228843441425464,-1.49507388391315,0.134895160836035,0.625484825222528
"3374","KMT5B",310.737177982496,0.252706157316639,0.169062784001939,1.49474740291596,0.134980377881878,0.625694460602424
"3375","WWC2",230.456481722247,0.400565038184647,0.268053173926228,1.49434917079135,0.135084379496849,0.625727517720712
"3376","SELENOP",3970.49465231592,0.402841210574161,0.269592349457869,1.49426054331381,0.135107533717573,0.625727517720712
"3377","AC010642.2",238.225967176747,-0.333286023210887,0.223005230619795,-1.49452110286647,0.135039470419317,0.625727517720712
"3378","ZNHIT6",163.618260176506,0.336888480968077,0.22556885762627,1.49350617152234,0.135304739994592,0.625857282206535
"3379","TADA1",28.8054401435886,0.453686781487701,0.303950873026759,1.49263194071418,0.135533557669428,0.625857282206535
"3380","BRK1",1749.39156475435,-0.302159472011167,0.202366105974529,-1.49313280776969,0.135402426190458,0.625857282206535
"3381","PDE12",100.022184779307,0.312045837344445,0.208959087643026,1.49333460853125,0.135349620652139,0.625857282206535
"3382","DNAJC13",213.210686019236,0.271974618917297,0.18210317972903,1.49351932965693,0.1353012983183,0.625857282206535
"3383","SRGAP1",595.158451848002,0.286463326014289,0.191908764179738,1.49270580339933,0.135514213593284,0.625857282206535
"3384","DYNLL1",4008.13849512595,-0.369096105037424,0.247280078733848,-1.49262369588085,0.135535717060968,0.625857282206535
"3385","ADGRD1",936.243357936025,0.333737747448302,0.223563137123139,1.49281206080267,0.135486389327301,0.625857282206535
"3386","ARL6IP1",658.865434529196,0.254322035435924,0.170298540740374,1.49338939917076,0.135335286241606,0.625857282206535
"3387","CYGB",368.915091339555,-0.693044744606358,0.464074790783347,-1.49339019996435,0.135335076745462,0.625857282206535
"3388","GLRX2",195.365468316125,-0.313239097298784,0.2099855213195,-1.4917175971489,0.135773193958672,0.626306581982042
"3389","KCTD2",97.3148015504426,0.308016109911363,0.206487750152302,1.4916919269263,0.135779926473617,0.626306581982042
"3390","DOHH",81.7817998543002,-0.369415650675709,0.247617778322728,-1.49187854433553,0.135730988305034,0.626306581982042
"3391","MFNG",4.15583276462341,-1.23720733260386,0.829426827103661,-1.49164132648586,0.13579319817782,0.626306581982042
"3392","ADH1B",2174.54336853163,0.991564833238144,0.664934464157426,1.49122189732579,0.135903246457309,0.626576761031062
"3393","AKAP11",241.603711581231,0.282228614251297,0.189273828641967,1.49111272422752,0.135931902185319,0.626576761031062
"3394","KCNN3",3.77636127774615,1.39500323259935,0.936057989867541,1.4902957377638,0.136146492705668,0.626827065086531

"3395","RASSF1",198.207105683619,-0.348536347658885,0.233862129520197,
-1.49034967044026,0.136132318634763,0.626827065086531
"3396","SNHG8",719.682387203588,-0.429526013807487,0.288315326464094,-
1.48977863603439,0.136282450278913,0.626827065086531
"3397","LRRC10B",16.1119754437475,0.718968535889278,0.482627629101168,
1.48969618094236,0.136304139245533,0.626827065086531
"3398","SLC46A3",34.6170471600302,0.500756750019451,0.335987257930797,
1.49040399062571,0.136118043874,0.626827065086531
"3399","AL110292.1",15.7032985130074,-0.798477931164718,0.536004205134
462,-1.48968594558771,0.136306831736528,0.626827065086531
"3400","MGAT2",223.343139874143,-0.294203385356369,0.197468815283307,-
1.4898726410764,0.136257726466354,0.626827065086531
"3401","AC245060.5",73.9524998355738,0.633484870840795,0.4251241364864
89,1.49011739506568,0.136193371143094,0.626827065086531
"3402","TSPAN10",20.2738902141805,0.612743139371882,0.411480890039616,
1.48911688052607,0.136456593404546,0.627331311242532
"3403","AAK1",420.314815054609,0.346515059406593,0.232725615211928,1.4
8894250033905,0.136502510699287,0.627357998041979
"3404","B9D2",42.8900122341151,-0.438910385356803,0.294813019179338,-1
.48877545021107,0.136546509046047,0.627375852373731
"3405","S100A1",3.41120453595578,-1.16776443020768,0.785043807972634,-
1.48751498750549,0.136878847799597,0.628490833834203
"3406","SLC39A10",188.16110141528,0.263895320212299,0.177528947780157,
1.48649177225505,0.137149091549487,0.628490833834203
"3407","EPHB3",9.76547579291216,0.882351979497364,0.593462257810939,1.
48678701616516,0.137071071788843,0.628490833834203
"3408","AC016885.2",3.59246320616483,1.7154407402742,1.15358237606794,
1.48705526008588,0.13700021660471,0.628490833834203
"3409","CSAD",298.210907231326,0.409715266224897,0.275463277955541,1.4
8736800515031,0.136917642362008,0.628490833834203
"3410","QPRT",13.1010950198289,1.96230860476004,1.31978122720045,1.486
84385284259,0.137056056327503,0.628490833834203
"3411","ZNF417",16.6814365799701,0.704016329309587,0.47361141472465,1.
48648513828344,0.137150845004868,0.628490833834203
"3412","EPB41L1",103.493231552769,0.346620454987398,0.233131196958798,
1.4868042523226,0.137066518101215,0.628490833834203
"3413","ZDHHC9",187.819505430471,-0.256193494569989,0.17231012764526,-
1.48681623112381,0.137063353443756,0.628490833834203
"3414","TRAPPC3",348.713792327852,-0.304039134484877,0.204600616163614
,-1.48601279989179,0.137275735375772,0.62882270513083
"3415","COLGALT2",13.3553805229788,-0.92025137920891,0.619444911013034
,-1.48560648872543,0.137383237734797,0.62882270513083
"3416","LBH",93.2481624238446,-0.559934394154813,0.376948238253458,-1.
48544106944019,0.13742702318491,0.62882270513083
"3417","CCT7",668.190019156592,-0.293026806631119,0.197259499403273,-1
.48548894992409,0.13741434841385,0.62882270513083
"3418","AL137003.2",4.33018577818394,-1.10925539912391,0.7468592119254
07,-1.48522690945224,0.137483725977554,0.62882270513083
"3419","EBF3",331.711608828039,0.336360971339673,0.226415217383255,1.4
855934827486,0.137386679945793,0.62882270513083

"3420","ZNF337-
AS1",27.5697631205673,0.519366571438966,0.349707022396533,1.4851476755
5928,0.137504709178225,0.62882270513083
"3421","ADCY9",119.068272421883,0.310127335280276,0.208889772356949,1.
48464585786581,0.137637660937393,0.629246716474956
"3422","SCML1",140.637593379194,0.333313776039619,0.224566506248511,1.
48425418201397,0.137741500528306,0.629537424974488
"3423","RARRES2",485.608404250596,0.685886197313182,0.462237534568003,
1.48383925150993,0.137851571171802,0.629689706839204
"3424","ACOT1",2.58489753995037,-1.54483189593206,1.04111458887471,-1.
48382503947216,0.137855342469145,0.629689706839204
"3425","SLC66A2",221.215333086197,-0.263933099154707,0.177912967797442
,-1.48349556764857,0.137942793198553,0.62990519288332
"3426","CHD1L",15.1083617355104,-0.59554015390281,0.401746197718852,-1
.48237906739214,0.138239460545383,0.630343525501239
"3427","ABCB6",127.667475959929,-0.388224732693962,0.261911703019284,-
1.48227333188459,0.138267581188326,0.630343525501239
"3428","FBLN2",7755.92376485835,0.278246051729815,0.187716499700378,1.
48226742014652,0.138269153561087,0.630343525501239
"3429","PDE6B",2.62811121504779,1.53895178656756,1.03818278485752,1.48
235147896308,0.138246797337412,0.630343525501239
"3430","PCOTH",5.09727765782525,-1.06188372132766,0.716354786183831,-1
.4823433050326,0.138248971147941,0.630343525501239
"3431","CDYL2",6.61062953139903,0.86334914981806,0.582468596062152,1.4
8222437339083,0.138280603324473,0.630343525501239
"3432","EFHB",6.11737563456913,-1.47682807169836,0.996638136368701,-1.
48180971388397,0.138390933681988,0.63041088641088
"3433","ZIC4",20.2527071720408,-0.732988271203469,0.494689307321808,-1
.48171440205931,0.13841630332065,0.63041088641088
"3434","FGFR1",2733.43097823333,0.244226578793983,0.16480820845769,1.4
8188358504414,0.138371273479812,0.63041088641088
"3435","PXK",316.653653774572,0.259626523541301,0.175282553961103,1.48
11886161752,0.138556318863894,0.63057001847849
"3436","AC107959.1",32.1382810353895,0.472686648357962,0.3191164817677
07,1.48123545903857,0.138543840293996,0.63057001847849
"3437","ARVCF",18.1022027311788,0.693569714237633,0.468270964690553,1.
48112901831521,0.138572196516021,0.63057001847849
"3438","MMADHC",332.288468253996,-0.294354090593469,0.198986467187129,
-1.47926688057965,0.139069000853587,0.630808081098892
"3439","CTDSPL",313.100375327926,-0.267824733804164,0.180918137864541,
-1.48036419656658,0.138776079499128,0.630808081098892
"3440","NME6",148.912223462472,0.295805618552362,0.200027272381474,1.4
7882643716817,0.139186708163685,0.630808081098892
"3441","JAZF1",255.630938149718,0.246609811934922,0.16677853682426,1.4
7866635977735,0.139229507413899,0.630808081098892
"3442","MDH2",574.768557368535,-0.415130743706094,0.28044339254385,-1.
48026573184884,0.138802344590669,0.630808081098892
"3443","SEMA3C",6071.6287741537,0.453131789816183,0.306200467315802,1.
4798533581232,0.138912385320305,0.630808081098892
"3444","ACTA2",436.001535701078,-0.459404747338249,0.310413579885805,-

1.47997631903622,0.138879566530611,0.630808081098892
"3445","PKP3",6.19663121516881,-1.22269714021996,0.826607010523501,-1.47917586550059,0.139093318101987,0.630808081098892
"3446","SLC5A12",2.77566753429001,-1.6062581175157,1.08519213422615,-1.4801601180616,0.13883052092507,0.630808081098892
"3447","PPP1R32",9.77867936353872,0.736166849383927,0.497848308389631,1.47869709905247,0.139221287991195,0.630808081098892
"3448","AC090510.2",2.67830243535465,1.8393555222211,1.24343850471053,1.47924928756271,0.139073701069592,0.630808081098892
"3449","SNAI3-AS1",10.4311197723356,0.754943790527964,0.510170672473191,1.47978672875916,0.138930171475364,0.630808081098892
"3450","TMEM11",247.604595384597,-0.365513936752665,0.246877342718953,-1.48054873212391,0.138726865646311,0.630808081098892
"3451","SPATA2",34.9251883070306,-0.4353284683422,0.294342846044046,-1.4789843687149,0.139144492566946,0.630808081098892
"3452","KREMEN1",430.751745762162,0.357333781555351,0.241657987252601,1.47867565073211,0.139227023051624,0.630808081098892
"3453","MAP9",74.4364387308268,0.338127142328352,0.228790486463048,1.47788987014092,0.13943725812997,0.63101814732429
"3454","ZFYVE26",44.6585036019342,0.418025932271252,0.282783677002104,1.47825340098446,0.139339965336746,0.63101814732429
"3455","CENPB",190.160325371918,-0.324372992660586,0.219471413314095,-1.47797377235805,0.139414798500485,0.63101814732429
"3456","SULF2",132.254937055186,0.61783740346868,0.417973882852595,1.4781722706023,0.139361673960087,0.63101814732429
"3457","RADIL",121.983485822133,0.267400961146994,0.18099354031356,1.47740610346501,0.139566811068374,0.631056642124708
"3458","P2RY11",5.95459904136093,-0.867211700744839,0.586943541302903,-1.47750446119535,0.139540463322275,0.631056642124708
"3459","PUDP",41.6344305076914,0.408166435305513,0.276241957043204,1.47756857674475,0.13952329032061,0.631056642124708
"3460","TRDV3",3.14408104187635,1.61846729808476,1.09577167752917,1.47701143520537,0.139672572016816,0.631352319752312
"3461","OSM",5.56793862114262,-1.2197820610208,0.826046464423019,-1.47665066501168,0.139769303105937,0.631424581333582
"3462","HSD17B10",313.790607088385,-0.364109218028766,0.246568181166242,-1.47670805010836,0.139753913346529,0.631424581333582
"3463","PIGX",137.595341458094,-0.291095109438148,0.197154531008324,-1.47648196543785,0.139814553169303,0.631446610328589
"3464","SPAG1",19.8543910604298,-0.555345094036376,0.376245525238002,-1.47601780429171,0.139939112678388,0.631653523333568
"3465","FRMD8",72.1710829503571,-0.309623363426656,0.20977511193362,-1.47597758653387,0.13994990929366,0.631653523333568
"3466","DUS3L",60.8826418327303,-0.406702134880416,0.275569624173709,-1.47585981618949,0.139981528892209,0.631653523333568
"3467","NKTR",690.054768833118,0.396082729391866,0.26848680583855,1.47524094584388,0.140147776783517,0.631856797029174
"3468","E2F4",156.408180590802,-0.271542410996502,0.184059374364784,-1.4752979136957,0.140132467095866,0.631856797029174

"3469","LINC00654",15.937541618152,0.688228584678656,0.466500096151356
,1.47530212824514,0.14013133451808,0.631856797029174
"3470","RBPMS-
AS1",13.0385116908013,0.750224869444443,0.508709182069418,1.4747618008
2409,0.14027659438217,0.632255313007821
"3471","GYS1",30.6363885232007,0.50130138888349,0.339961786972995,1.47
458157973299,0.140325070183555,0.632291586767732
"3472","NLRC5",43.5580370920506,0.557757135000502,0.378340451224126,1.
47422019822589,0.140422313268084,0.632302349322657
"3473","SLC44A2",135.154352009368,0.464911660560214,0.315322687996405,
1.47439964917943,0.140374018863605,0.632302349322657
"3474","FERMT1",4.31416183156661,2.13041384979422,1.44520863294238,1.4
7412200649313,0.140448744344432,0.632302349322657
"3475","YTHDF3",227.062436893373,0.293792943275623,0.199388386998304,1
.47347068552253,0.140624162642498,0.632666120595921
"3476","HELLPAR",81.7809026352929,-0.689741187248765,0.468158532899617
,-1.4733068795665,0.14066830650615,0.632666120595921
"3477","TCF12",231.392318128459,0.285893977227048,0.194080200256115,1.
47307132231815,0.1407318052144,0.632666120595921
"3478","ZNF397",198.968768512043,0.357962334029584,0.242923303860223,1
.473561113081,0.140599797996032,0.632666120595921
"3479","LPAR4",29.3447586837425,-0.62492281049717,0.424203282537098,-1
.47316825734963,0.14070567195876,0.632666120595921
"3480","TANC1",10.7876080455685,-0.79697890118566,0.541113573990889,-1
.47284958184967,0.14079159949655,0.632688646435855
"3481","MAP1S",112.992039102289,0.285710401541919,0.193997536439853,1.
47275273070542,0.140817722394067,0.632688646435855
"3482","ZNF566",98.6319869528319,0.310603754656398,0.210927041139118,1
.4725648877402,0.140868398422524,0.632734563850739
"3483","C2CD2L",21.5165431203448,-0.498070434404903,0.338276446776847,
-1.47237692470344,0.140919120876651,0.632780663368022
"3484","SEC23A",485.529217901542,-0.19232082083339,0.130644662854266,-
1.47209091157381,0.140996329412125,0.632850825320028
"3485","FAM171A2",14.2971449080074,1.0471498525534,0.711369661680782,1
.47201927346642,0.141015673033267,0.632850825320028
"3486","PSMA5",490.544603742452,-0.36092496261644,0.245259221833537,-1
.47160608240618,0.141127282120981,0.632973391476425
"3487","MYCBP2",868.473709696737,0.230422458067886,0.156575263415911,1
.47164023895534,0.141118053352373,0.632973391476425
"3488","IRS2",157.199484106327,0.513332885928642,0.348857488938705,1.4
7146872922323,0.141164398303694,0.632973391476425
"3489","SLC10A3",133.686101713534,-0.266534259985305,0.181178773090454
,-1.47111195996585,0.141260841119408,0.633224292091586
"3490","RBMX",933.331091672411,0.242030474017789,0.164589330175799,1.4
705113251222,0.1414233207087,0.633770984494001
"3491","FLVCR1-
DT",25.9240789050077,-0.611641925956056,0.416081646432051,-1.470004580
11296,0.141560513527509,0.63420407664573
"3492","PUSL1",71.5749238260058,-0.479695306891433,0.327016661481568,-
1.46688338361154,0.142407781441642,0.634619039909079

"3493","KLHL21",87.8322762645047,0.382437163085131,0.26051810706216,1.46798687967544,0.142107786854436,0.634619039909079
"3494","ALS2",54.4123959984919,0.42317938798478,0.288369662132774,1.46748928044288,0.142243003209077,0.634619039909079
"3495","DST",2968.11301170503,0.389197253706088,0.264935378070235,1.46902711348316,0.14182543526032,0.634619039909079
"3496","RBM33",270.940872054118,0.332049917760196,0.2263734753349,1.46682343092077,0.14242409399494,0.634619039909079
"3497","FDFT1",167.750827432391,-0.249932312535943,0.170337072447597,-1.46728078007113,0.142299689934293,0.634619039909079
"3498","TRAPPC9",52.2233769681338,0.393173715670364,0.2678453312397,1.46791326864124,0.142127783506709,0.634619039909079
"3499","TSC1",135.492415947801,0.346258295785548,0.235818128988753,1.46832772047845,0.14201522461852,0.634619039909079
"3500","CCDC84-DT",4.41290200620787,1.08210849146181,0.737703897490458,1.46686020657198,0.142414087522164,0.634619039909079
"3501","ETFBKMT",59.328107056964,0.320604998513912,0.218350029371654,1.46830755844878,0.142020698737897,0.634619039909079
"3502","GPD1",12.6033671279823,-1.93587455723601,1.31764191572321,-1.46919624682209,0.141779568004764,0.634619039909079
"3503","RBMS2",151.317913034297,0.272351334664697,0.18540311932744,1.46896845993027,0.141841344173826,0.634619039909079
"3504","WDFY2",262.649723023987,0.40507735793918,0.27593549393994,1.4680146876188,0.142100233307776,0.634619039909079
"3505","DCTPP1",213.872157141076,-0.40330062620562,0.274516103991024,-1.46913284992128,0.141796759269481,0.634619039909079
"3506","PSKH1",70.3016663331061,0.355346855579451,0.241962237179332,1.46860460426345,0.141940065301653,0.634619039909079
"3507","FANCA",5.65897438888341,-0.955742239027369,0.651237691742896,-1.4675781993968,0.142218833350739,0.634619039909079
"3508","TEN1",128.973431138897,-0.504051001565605,0.343572354369801,-1.46708835898675,0.142352020440149,0.634619039909079
"3509","LPAR2",10.1159739278266,-0.837218532200375,0.570647971404819,-1.46713661338235,0.142338895870122,0.634619039909079
"3510","PSMA7",2279.25368054376,-0.283738459268267,0.193170912005611,-1.46884671362957,0.141874370442854,0.634619039909079
"3511","SERP1",1091.59136875864,-0.256288043537646,0.174797553386283,-1.46619926064571,0.142594009980147,0.634652907253701
"3512","ARL9",3.88645530602595,-1.13029352026183,0.770735180776799,-1.46651346461523,0.142508455743597,0.634652907253701
"3513","CYC1",497.827485269766,-0.340307948460821,0.232099009588304,-1.46621887385241,0.142588668369197,0.634652907253701
"3514","KLHL36",229.675754130972,0.23619319254013,0.161080448611291,1.46630577811523,0.142565002044736,0.634652907253701
"3515","KBTBD7",107.184755475407,0.306368043465679,0.209011652828319,1.46579408047324,0.142704394220655,0.634963506574976
"3516","YEATS4",116.269464374205,-0.365538201142401,0.249460309950087,-1.46531607058268,0.142834703962022,0.635181907866371
"3517","GATA6-

AS1",3.70813194937847,1.27313531042416,0.868773779777264,1.46543938141
246,0.142801079597217,0.635181907866371
"3518","ZFP82",61.0460292078087,0.363843300905265,0.248408310973335,1.
46469858226411,0.143003171922027,0.635750315196276
"3519","PIK3R1",1505.18744869879,0.391823910191092,0.267660129207817,1
.46388597865045,0.14322510506264,0.636556022500623
"3520","PREX1",30.2042088355829,0.753388582592058,0.514753666962594,1.
46359051123924,0.143305866724731,0.636734021470111
"3521","VPS11",114.893968727071,0.264390744158855,0.180714888888809,1.
46302690267835,0.143460017694611,0.636762918359429
"3522","VPS53",144.969317051871,0.386452698891455,0.264110298608091,1.
4632246486719,0.143405918273914,0.636762918359429
"3523","SLC43A2",18.4382308735988,0.698715648062524,0.47760037116695,1
.46297132549397,0.143475225338787,0.636762918359429
"3524","AC021092.1",5.53642467100703,1.22015600409345,0.83397287623408
2,1.46306437399167,0.143449765077102,0.636762918359429
"3525","ELK4",202.025951438481,0.307116078035394,0.209973381387132,1.4
6264291219447,0.14356511466399,0.63698110449498
"3526","ZNF84",125.190685249652,0.253074977236414,0.173044190474854,1.
46248756772445,0.143607648723118,0.636989116854669
"3527","BOP1",53.9644330750735,-0.398363240275783,0.272479381401797,-1
.46199407172156,0.143742834412575,0.637046735679421
"3528","ZCCHC8",133.20003564509,0.335709756109889,0.229618138966919,1.
46203500132999,0.143731618661699,0.637046735679421
"3529","SCRN2",149.813065725711,0.385654005708329,0.26376083350676,1.4
6213522523785,0.14370415760255,0.637046735679421
"3530","SUCO",183.414436948645,0.305649257115056,0.209120929130945,1.4
6159094828652,0.143853336320751,0.637355858372957
"3531","TMEM43",680.859140519394,-0.317128057403397,0.216999437704835,
-1.46142340624291,0.143899281152202,0.637378860725133
"3532","WDR46",221.856953019593,-0.34091170820527,0.23332866921666,-1.
46107938364279,0.143993657282729,0.637616308012991
"3533","ETNK2",33.8527800771561,-0.477890154808352,0.327192070851806,-
1.46057987763646,0.144130771828493,0.637808689118974
"3534","AC107204.1",4.73744610037899,1.13893814072236,0.77969331882463
2,1.46075144319472,0.144083665751286,0.637808689118974
"3535","ACVR1B",74.3487267071634,0.367127267725332,0.25140072606423,1.
46032699854469,0.14420022536602
9,0.637808689118974
"3536","MROH8",11.7476027050709,-0.675927756317081,0.462840714637108,-
1.46038957883609,0.144183035227526,0.637808689118974
"3537","AL445183.2",4.10968701591815,1.12199316162815,0.76854789625919
6,1.45988710279385,0.14432110437575,0.638162870352484
"3538","ZNF362",94.0155346902428,0.295796111789392,0.202658155367797,1
.45958158581165,0.144405103119141,0.638353819328254
"3539","MAST2",101.439052715091,-0.321738846425937,0.220588797671352,-
1.45854571865107,0.144690183026712,0.638703812413637
"3540","VPS45",104.963348114734,0.311748640013187,0.213756611155899,1.
45842806137032,0.144722590616301,0.638703812413637
"3541","CKAP5",180.663199579088,0.285145093060779,0.19549310413264,1.4
5859412446237,0.144676851716576,0.638703812413637

"3542","SCG5",2.52861130975606,-1.36926884989267,0.938881914485179,-1.45840369142001,0.144729303784778,0.638703812413637
"3543","WNT3",11.3286363474635,0.720139506603327,0.493780363002177,1.45842070799432,0.144724616219094,0.638703812413637
"3544","AC022966.1",15.4801454738241,0.792302768426169,0.543037645175169,1.45901996936252,0.144559611513071,0.638703812413637
"3545","TEKT4",3.29756735471305,-1.40680394119428,0.965219737190229,-1.45749603638392,0.144979504679332,0.639104647263679
"3546","TMEM165",729.642617824738,-0.206565764764684,0.14169040067531,-1.45786703813506,0.144877195673317,0.639104647263679
"3547","RSF1",775.580694830426,0.215922753674522,0.148147878540048,1.45748123970708,0.144983586220686,0.639104647263679
"3548","RBP5",3.69987236511531,-1.089560354114,0.747471900840444,-1.45766061960177,0.144934111689533,0.639104647263679
"3549","SNRNP40",195.941811298052,-0.378173894970313,0.25979015768593,-1.45568984729399,0.145478377767049,0.639623970625431
"3550","GK5",187.102244282738,0.460914172275108,0.31650415610143,1.45626578163289,0.145319160994356,0.639623970625431
"3551","CHRAC1",238.912785126019,-0.288668636154177,0.198209717382166,-1.45637983831841,0.145287645914694,0.639623970625431
"3552","CTTN",767.079026545408,-0.248987931071877,0.171016628539958,-1.45592819363586,0.145412470841235,0.639623970625431
"3553","NRAV",13.9976499740584,-0.699867617515496,0.480811569963175,-1.45559645656842,0.145504208169744,0.639623970625431
"3554","RNASEH2B",239.706175433309,-0.231259534928361,0.158829072755439,-1.45602773419479,0.145384952907701,0.639623970625431
"3555","GAS6",323.612641038173,0.479555149662068,0.32920688165278,1.45669843611551,0.145199641673727,0.639623970625431
"3556","PRPF8",386.924892756754,0.259436964645468,0.178118088446378,1.45654473898966,0.145242091353725,0.639623970625431
"3557","GPS2",437.754769106188,-0.23057412330031,0.158407673625481,-1.45557420308721,0.145510363649954,0.639623970625431
"3558","BRWD3",72.4510894987281,0.471123927154948,0.323534121246749,1.45618003238563,0.145342857873977,0.639623970625431
"3559","YME1L1",600.905405912345,0.195664905179423,0.134447412747754,1.45532666773234,0.145578847235194,0.639745201112234
"3560","PSIP1",834.293599083219,0.1685546346421,0.115854273153755,1.45488491752397,0.145701123971394,0.639996861553925
"3561","TICAM1",62.4388010350083,-0.350409504858015,0.24086038790973,-1.45482413234899,0.145717955498307,0.639996861553925
"3562","PTPRF",18.3868508008278,0.874936162826929,0.602109363722249,1.4531183461723,0.146190898077593,0.641173764984173
"3563","AL390728.6",78.4427200283373,0.524414203678614,0.360844342694917,1.45329756249495,0.146141153794109,0.641173764984173
"3564","AC091563.1",8.66154518254569,0.85280200643596,0.586830741031707,1.45323335470914,0.146158974176467,0.641173764984173
"3565","ZNF385A",803.903860041697,-0.440293165819222,0.302954374552324,-1.45333159974945,0.146131707687506,0.641173764984173
"3566","SNHG25",152.905037995046,-0.665300480229126,0.457718981020009,-1.45351298027128,0.146081378376691,0.641173764984173

"3567","ARHGEF19",21.3938524852672,-0.493836912534331,0.341175301679085,-1.44745797865181,0.147768697438037,0.641604858236797
"3568","LRRC41",251.527535609518,0.248684887572981,0.17164126289996,1.44886423795381,0.147375499981101,0.641604858236797
"3569","GSTM4",67.2700164666889,0.414305513378136,0.286138477694721,1.4479196112176,0.147639534267094,0.641604858236797
"3570","SELENBP1",665.314427592359,0.392752532838658,0.271066205703961,1.44891736621567,0.147360660735597,0.641604858236797
"3571","MPC2",730.692379163897,-0.334087547830439,0.230843832139653,-1.44724485265141,0.147828358473276,0.641604858236797
"3572","IER5",393.709199431539,0.343574288025693,0.237114500086386,1.44898050477943,0.147343026999789,0.641604858236797
"3573","GLUL",2592.68311975546,0.369549784404288,0.254801544902714,1.45034357835383,0.146962732434118,0.641604858236797
"3574","SPTBN1",3811.5745731717,0.278532093600183,0.192157266248725,1.44950070865213,0.147197802576994,0.641604858236797
"3575","AC110769.2",8.00559674754376,0.946350097432322,0.652113713529247,1.45120410412881,0.146723034414158,0.641604858236797
"3576","AC061958.1",3.652150018865,1.36255728395943,0.938208303551823,1.45229719114735,0.146418988163412,0.641604858236797
"3577","SNRK",120.985615401027,0.305065801828101,0.210797851177293,1.44719597531155,0.147842043455941,0.641604858236797
"3578","PTH1R",44.6073068532913,0.67735374197716,0.468347720446122,1.44626249345668,0.148103591364455,0.641604858236797
"3579","PTPN23",155.269375363845,0.266611391026741,0.183769194041577,1.45079479951586,0.146837008214936,0.641604858236797
"3580","MANBA",123.348887888108,0.326977508730887,0.225668069549122,1.44893120849653,0.147356794638135,0.641604858236797
"3581","CENPU",7.50788685870686,-0.975181351709361,0.673179415329083,-1.4486202778982,0.147443655087934,0.641604858236797
"3582","DHFR",68.1551506086735,0.430083601328502,0.296920807986294,1.44847915592482,0.147483091323692,0.641604858236797
"3583","ELL2",122.000034738609,0.746870065646907,0.514426748184243,1.4518492055149,0.146543538697699,0.641604858236797
"3584","PCDHB3",13.493019746997,0.66359130729203,0.458132170260261,1.44847131541767,0.147485282573157,0.641604858236797
"3585","PRRT1",2.81091834554777,-1.25672008675246,0.868711415032453,-1.44664852447635,0.147995388308185,0.641604858236797
"3586","COL21A1",9.57341163544841,1.23181818039905,0.852178702690619,1.44549280157998,0.148319513500864,0.641604858236797
"3587","FRK",30.8473290901448,-0.579448623776746,0.399507765029808,-1.45040641133349,0.146945220284148,0.641604858236797
"3588","IFNGR1",634.482648644435,-0.294517916074931,0.203142340781599,-1.449810585729,0.147111346751404,0.641604858236797
"3589","ACAT2",107.556201124692,-0.349837907114503,0.241045955009785,-1.45133282614222,0.146687204837586,0.641604858236797
"3590","FGL2",535.863913078484,-0.504010541589887,0.348121314469702,-1.44780144346419,0.147672588969859,0.641604858236797
"3591","CNOT4",323.308291684369,0.216424241227851,0.149552073758632,1.44714971707545,0.147854996017209,0.641604858236797

"3592","ZNF487",58.0865599293323,-0.38014475682124,0.262310416482278,-1.44921716003193,0.147276946784433,0.641604858236797
"3593","ADIRF-AS1",8.20736196538739,-0.793396743562968,0.548582909894015,-1.44626587750656,0.148102642565373,0.641604858236797
"3594","EIF3M",705.41932217586,-0.315337403115115,0.217472411937014,-1.45001106258225,0.147055434319819,0.641604858236797
"3595","ANAPC15",100.294507229539,-0.386206398226156,0.26620563984012,-1.45078217898804,0.146840523567093,0.641604858236797
"3596","ESAM",2.66463194984898,-1.64962947431173,1.14132305217844,-1.44536594714624,0.148355123121646,0.641604858236797
"3597","PHB2",806.987734841476,-0.389726842916189,0.269527647377127,-1.4459623964694,0.148187749177508,0.641604858236797
"3598","SDS",3.55215795793757,-1.46364942737696,1.01086512180443,-1.4479176260076,0.147640089537083,0.641604858236797
"3599","EXOSC8",249.149832025884,-0.338054555058914,0.233749050198438,-1.44622857193186,0.148113102329425,0.641604858236797
"3600","AL445223.1",3.3100893854221,1.41474958572463,0.974586845896505,1.45164034552839,0.146601634377855,0.641604858236797
"3601","PPP2R3C",213.700558683537,-0.293771764616428,0.202348639219553,-1.45180993432666,0.146554460873764,0.641604858236797
"3602","SLC16A11",4.63005915087452,1.12870376203015,0.780610542333894,1.44592431285326,0.148198431782553,0.641604858236797
"3603","IFT20",402.94733120059,-0.332121323990344,0.229459561301699,-1.44740677662877,0.147783028902717,0.641604858236797
"3604","ACACA",63.9614701028043,0.379753269494637,0.262368542161673,1.44740396987315,0.147783814545334,0.641604858236797
"3605","AC100793.4",97.9045557488584,0.589115029484979,0.407144015043533,1.44694508016283,0.147912305884515,0.641604858236797
"3606","CEP131",101.61388205286,0.626375250759285,0.433056332106452,1.44640593918232,0.148063377007871,0.641604858236797
"3607","NDUFV2",999.664907013849,-0.37704996277843,0.260885045254884,-1.44527242797705,0.148381379299392,0.641604858236797
"3608","SETBP1",198.627625595323,-0.437900920416518,0.301459644360038,-1.45260212638454,0.146334255339077,0.641604858236797
"3609","LMNB2",66.3877731280491,-0.430788004603831,0.297009250302582,-1.45041948749058,0.146941576035224,0.641604858236797
"3610","NAPA-AS1",25.3194062340953,0.685055403642586,0.473710665215308,1.44614730878229,0.148135888903751,0.641604858236797
"3611","PLEKHA4",177.792272857174,-0.439986181106228,0.304391344098767,-1.44546219738582,0.148328103882046,0.641604858236797
"3612","BCL2L12",63.4977855895618,-0.441759381749561,0.305314468600421,-1.44689959756775,0.147925045879378,0.641604858236797
"3613","RALGAPA2",49.7887706952369,0.417151547627622,0.288584355307171,1.44550991748532,0.148314709352798,0.641604858236797
"3614","ACSS2",58.0067258433948,-0.348597871186312,0.240630619047819,-1.4486845961903,0.147425684152095,0.641604858236797
"3615","BRWD1",291.157104272319,0.276170478074839,0.190766463229748,1.44768883062132,0.147704095077604,0.641604858236797

"3616","CERK",114.062837650581,0.312668302953243,0.215550391732162,1.45055780432891,0.146903032168974,0.641604858236797
"3617","PHF8",50.5123222458405,0.380717253632235,0.262805316883727,1.44866648113012,0.147430745444596,0.641604858236797
"3618","FADS3",79.067001159846,-0.3713007893052,0.256984365701052,-1.44483804799678,0.148503381126037,0.64195491454152
"3619","CORT",5.02811925386644,-1.14710362784492,0.794444085221404,-1.44390731731011,0.148765048053455,0.642026164114891
"3620","ALDH4A1",10.9992306934263,0.739631921937959,0.512358692236724,1.44358226598843,0.148856516331409,0.642026164114891
"3621","METAP1",78.5778263915307,0.298169857073453,0.206542768664076,1.44362283415693,0.14884509824965,0.642026164114891
"3622","SPEF2",46.929433651321,0.427556923339409,0.296172633076234,1.44360712500185,0.148849519578126,0.642026164114891
"3623","HINT1",2255.80404920188,-0.328138421176248,0.227204873634655,-1.44424023977538,0.148671409355357,0.642026164114891
"3624","PANK3",181.483006945256,0.337515614436564,0.233908828710932,1.44293661892374,0.149038326438007,0.642026164114891
"3625","CYCS",582.31426843483,-0.294503484356031,0.204135624635726,-1.44268539546473,0.149109115158078,0.642026164114891
"3626","TRIM73",41.8654573931633,0.580439552200206,0.401908957712548,1.44420655738493,0.148680880911308,0.642026164114891
"3627","ARPC5L",207.926993409783,-0.338680626715953,0.234695300125461,-1.44306522770121,0.149002097511327,0.642026164114891
"3628","SEC23IP",134.234481634112,0.274124708712717,0.190022424481143,1.44259136499924,0.149135617278094,0.642026164114891
"3629","TLNRD1",402.027630288693,0.367338016157856,0.254465763345979,1.4435655756897,0.148861214080379,0.642026164114891
"3630","LIG3",49.3622495016251,0.433571715307124,0.300419871202131,1.44321916380626,0.148958742748606,0.642026164114891
"3631","NDC80",11.1292477572678,-0.789141865804596,0.546694108978566,-1.44347973179922,0.148885377991435,0.642026164114891
"3632","AGPAT3",100.513620421225,0.282915430122046,0.19604333198843,1.44312702325801,0.148984692174049,0.642026164114891
"3633","NBDY",464.646835577704,-0.315296298454895,0.218522717054705,-1.44285364333981,0.149061704121325,0.642026164114891
"3634","HRAS",242.418508245649,-0.283020071060693,0.196214340143155,-1.44240258308443,0.149188835579461,0.642078532873628
"3635","LINC01376",6.37317304030936,1.03626470781668,0.718617398630725,1.44202563115117,0.14929514305095,0.642359294997758
"3636","FAM49A",92.4640547494479,-0.428802930160947,0.297602280696896,-1.44085901881134,0.149624515808873,0.642432376828034
"3637","PARD3B",214.560591046852,0.329038630188111,0.22832087017149,1.44112375684699,0.149549723029123,0.642432376828034
"3638","HMGCR",64.7273669881628,-0.330346904453704,0.2291437565177,-1.44165788967585,0.149398908701972,0.642432376828034
"3639","DKK1",306.447878024127,-1.06900927223528,0.741870605966009,-1.44096458821588,0.14959468732312,0.642432376828034
"3640","UQCRFS1",624.002309144432,-0.350713120623736,0.243415275303633,-1.440801610278,0.149640738413333,0.642432376828034

"3641","ZC3H4",106.740782301654,0.370478816885975,0.25697475510476,1.4
4169343301813,0.149388877031334,0.642432376828034
"3642","ZNF480",117.350129677627,-0.316032889958687,0.219334696681411,
-1.44087048123413,0.149621276897961,0.642432376828034
"3643","LENG8",337.823965253179,0.338160151210854,0.234674935040502,1.
44097259961952,0.149592423897596,0.642432376828034
"3644","HNRNPR",832.849421680735,-0.212177337357041,0.147292458883014,
-1.44051731477686,0.149721094914674,0.642600967196899
"3645","OMA1",89.0317162979419,0.379037401065085,0.263264910359794,1.4
3975663352578,0.149936264360071,0.642615503306069
"3646","PTCD3",192.857445379249,0.284393924621881,0.197605759553068,1.
43919856012854,0.150094273246616,0.642615503306069
"3647","EPHA3",7.48254773423043,1.70272162661405,1.18296954361028,1.43
936218460667,0.150047932636349,0.642615503306069
"3648","DAP",304.925107929048,-0.312271165851909,0.216954707926522,-1.
43933804818685,0.150054767702608,0.642615503306069
"3649","OSMR-
AS1",34.7072199686606,-0.461009516785054,0.320243260787742,-1.43956040
058752,0.149991809873159,0.642615503306069
"3650","TEX10",43.4460520333947,0.372510460803207,0.25864376014995,1.4
4024530337496,0.149798010111657,0.642615503306069
"3651","TIMM23",112.915917958378,-0.308801724639023,0.214427866152992,
-1.4401193752434,0.149833628335858,0.642615503306069
"3652","GIT2",180.625235401829,0.282001610626534,0.195923822571756,1.4
3934314329362,0.150053324826247,0.642615503306069
"3653","UBFD1",131.364858902625,0.284353057974435,0.197544167499836,1.
43944041260884,0.150025781290266,0.642615503306069
"3654","FBXL6",47.1475738192196,-0.410239266525598,0.285200732516325,-
1.43842290623189,0.150314097179845,0.642852742655942
"3655","SPATA33",66.816176822998,-0.357607866342172,0.248591423414035,
-1.43853662138041,0.150281854428397,0.642852742655942
"3656","P3H4",188.247445646961,-0.398138905705817,0.276787637836954,-1
.43842734024251,0.150312839863285,0.642852742655942
"3657","PRKAR1A",762.438056573058,0.214965250060778,0.149421368030898,
1.43865133142354,0.150249334928255,0.642852742655942
"3658","BTBD6",361.267687638367,-0.316412967478712,0.220002659228194,-
1.43822337688435,0.150370684404693,0.642918945896501
"3659","ATAT1",45.0559802398728,0.40053503324064,0.278554986265964,1.4
3790293833839,0.150461595889889,0.642956109212531
"3660","NABP2",174.088051102193,-0.302132826620007,0.210105773945761,-
1.43800344438894,0.150433076851222,0.642956109212531
"3661","GPN2",79.1249660963834,0.305329893694756,0.212614430610479,1.4
3607323744707,0.150981502499551,0.643853543421737
"3662","ZKSCAN8",77.4099065418102,0.379737211374396,0.26430831058342,1
.43672066359239,0.150797381105199,0.643853543421737
"3663","VLDLR",3.19220156756178,1.5127581849827,1.05323236875665,1.436
30050676141,0.150916849919987,0.643853543421737
"3664","AKAP5",3.85240260373006,1.40211302712989,0.976131018846697,1.4
363983933084,0.15088901008521,0.643853543421737
"3665","LACTB",139.08561047374,-0.535572909148998,0.372791407537737,-1

.43665572306621,0.150815841803097,0.643853543421737
"3666","FDXR",32.5375693828104,0.485316134862737,0.337943250635986,1.4
3608766841594,0.150977396613252,0.643853543421737
"3667","CSNK1G2",203.471417726589,-0.269234265917103,0.1874884029706,-
1.43600490297697,0.151000946117067,0.643853543421737
"3668","UBE2A",437.819517631436,-0.276035383773473,0.192182249934664,-
1.43632090823849,0.150911047229266,0.643853543421737
"3669","AADACL2",44.3526465942932,-0.507968470909123,0.353922535539787
,-1.43525325431565,0.151214943172592,0.644455186948061
"3670","MTHFD2L",60.4715597760463,-0.352812030676082,0.245945507769803
,-1.4345130101189,0.15142591909124,0.644455186948061
"3671","LPCAT1",146.896119807906,0.300421612260678,0.20946760502043,1.
43421514859721,0.151510875406085,0.644455186948061
"3672","THSD7A",6.37891138601732,1.47911531247247,1.03091228395802,1.4
3476349587537,0.151354503425734,0.644455186948061
"3673","SEC31B",25.691210596766,0.629070714567021,0.438618867374366,1.
43420805934028,0.151512897852175,0.644455186948061
"3674","AP003486.1",26.8937347891541,0.457234619031012,0.3186969953993
04,1.43470012466898,0.151372568682249,0.644455186948061
"3675","MYLPF",4.93791695021258,-1.12682207481866,0.785558963584535,-1
.43442074631409,0.151452230771388,0.644455186948061
"3676","XAF1",291.536109785545,0.45723929936333,0.318655186890113,1.43
490304936102,0.151314726645707,0.644455186948061
"3677","F8A1",153.207123991872,-0.404900101161789,0.282276064153721,-1
.43441174290035,0.151454798540512,0.644455186948061
"3678","BBS9",82.2353953153077,0.293896411948079,0.204955962356668,1.4
339490716383,0.151586796797942,0.644594209331106
"3679","LNPK",329.354884294515,0.221526931287251,0.154511517482633,1.4
3372439088335,0.151650928915435,0.64469163583512
"3680","NR4A1",1073.79131838627,-0.919493447899165,0.641571106337941,-
1.43319024004618,0.151803478088128,0.645164781874545
"3681","RPA1",162.273553362788,0.257037757480914,0.179409149881435,1.4
3269034857353,0.151946348889498,0.645596548935545
"3682","OTULINL",28.1843536743197,0.585308456539337,0.408710018709894,
1.432087371841,0.152118818034285,0.645809951960334
"3683","FNDC1",1685.57492617186,-0.443388243785727,0.30961100556932,-1
.43208166315152,0.152120451599864,0.645809951960334
"3684","MCAT",91.6097370326211,-0.381066886166829,0.266050327585164,-1
.43231128345387,0.152054755315718,0.645809951960334
"3685","ZNF445",87.3992461544954,0.373123872985695,0.260587249577051,1
.43185775048971,0.152184535688259,0.64587835967188
"3686","DSN1",67.2443407944348,-0.333847825916066,0.2331768130023,-1.4
3173680786508,0.152219158168194,0.64587835967188
"3687","RUSC2",104.590959825725,0.328847255516757,0.229740800199397,1.
43138378220736,0.152320253803464,0.646132023185836
"3688","JAML",8.91791281900545,-0.827028949546328,0.577945527245503,-1
.43098079413809,0.152435719587061,0.646304992062319
"3689","KRT24",20.1003615422004,0.591411864614192,0.413299285657044,1.
43095302880573,0.152443677475569,0.646304992062319
"3690","ANKH",212.231795345789,-0.346325313806063,0.242115737950718,-1

.43041223481539,0.152598738799317,0.64670472180538
"3691","WDR70",159.959365207407,0.346408973195327,0.242187163085526,1.
43033581459062,0.152620660369799,0.64670472180538
"3692","AL390195.2",3.4193645994152,1.41874788640778,0.992409096621501
,1.42959984066821,0.15283190130679,0.646723738213798
"3693","HSPD1",736.865244672838,-0.197839151050651,0.138373461105741,-
1.42974779607102,0.152789416950078,0.646723738213798
"3694","ZFAND2A",122.550095077557,-0.35034577295634,0.245021601301418,
-1.42985667833162,0.152758157912103,0.646723738213798
"3695","PARVA",654.148553563098,0.174487554612386,0.122049199921834,1.
42964931129525,0.152817695162981,0.646723738213798
"3696","C17orf113",5.51456264386072,0.99815125689895,0.698061906190078
,1.42988930931172,0.152748790822828,0.646723738213798
"3697","ARPC3",1791.53708349742,-0.378403527836353,0.264751656131666,-
1.42927728334272,0.152924552536428,0.64694076323228
"3698","FZD10",2.92757716537762,-1.35973882874628,0.951491201761711,-1
.42906085335176,0.152986743745977,0.647028845913217
"3699","MARC2",64.6243281129196,0.407900345936078,0.285557288131586,1.
42843612434124,0.15316636769881,0.647106742703004
"3700","GLYR1",195.449655311991,0.321469544750881,0.225018638678387,1.
4286351861294,0.153109115453576,0.647106742703004
"3701","AC015908.2",15.6784689507316,-0.760595018766805,0.532472510211
079,-1.42842119392285,0.153170662499138,0.647106742703004
"3702","ZNF358",396.641764431085,-0.262680765658916,0.183876884993982,
-1.4285687168755,0.153128230893339,0.647106742703004
"3703","NBPF19",110.224939021551,0.645673254955683,0.452383827269723,1
.42726865116404,0.153502472997714,0.648158390303524
"3704","MSRA",60.7120633846926,-0.366088356694425,0.256483350464014,-1
.42733770450253,0.153482577555034,0.648158390303524
"3705","CCDC93",169.076180794247,0.288270097622856,0.202033638670438,1
.42684208194205,0.153625418329777,0.648327453501811
"3706","EPS8L1",2.65446751353551,-1.27721457325226,0.895120440995154,-
1.42686337475693,0.153619279562337,0.648327453501811
"3707","RFC3",43.923333085653,0.415726904486483,0.291401751062212,1.42
664518305426,0.153682193568325,0.648392098033072
"3708","AC245297.3",571.191900739359,-0.270079001769514,0.189460736123
287,-1.42551436934018,0.154008569438079,0.64941157143445
"3709","TRAPPC11",134.095782984385,0.301744066086502,0.211664018509369
,1.42558035235046,0.153989510936151,0.64941157143445
"3710","DECR2",46.3164417994056,0.535842563429921,0.375930544010862,1.
42537650096992,0.154048397060218,0.64941157143445
"3711","FAM172A",317.154094936041,0.284015080700013,0.199306504045982,
1.42501661980127,0.154152396958899,0.649674882359791
"3712","POLR1B",30.5890945215377,0.500665902138394,0.351547394381684,1
.4241775366277,0.154395085790811,0.65003614852304
"3713","SLC10A7",54.9710234469542,0.395935280988612,0.278043789422073,
1.4240033262803,0.154445509189768,0.65003614852304
"3714","FAM92A",307.924509804896,-0.297887812585632,0.2092143428782,-1
.42384029931952,0.154492707003615,0.65003614852304
"3715","PTPMT1",297.868198375178,-0.330458818277824,0.232087091746397,

-1.42385694866183,0.154487886362599,0.65003614852304
"3716","TDRD9",3.7158261758134,1.3158517026177,0.92423828276125,1.4237
1477914382,0.154529053721781,0.65003614852304
"3717","HOXB6",8.94690142263189,-0.757740179018389,0.532167675745902,-
1.42387486792827,0.154482698156165,0.65003614852304
"3718","ZNF780A",62.9018675320519,0.360482120949331,0.253107233530169,
1.42422686195716,0.154380811352221,0.65003614852304
"3719","PPP2R5D",123.087764723463,0.254355217907889,0.178685499403286,
1.42347990607688,0.154597083068421,0.65014745339879
"3720","LAMC2",47.3062324115311,0.452520302936855,0.31809431404151,1.4
2259789930669,0.154852753324806,0.650984025475899
"3721","RXYLT1",126.200114324012,-0.286719371567033,0.201559264906175,
-1.42250653523915,0.154879255677482,0.650984025475899
"3722","CCDC117",117.344111973408,0.33569402875039,0.236025637081521,1
.42227782075404,0.154945614938635,0.65108796819996
"3723","ZC3H3",48.4187415719507,0.43267497731411,0.304484343830886,1.4
2100894867167,0.155314157948152,0.65220546495297
"3724","C11orf95",104.908224872487,0.295776289348831,0.208171753196661
,1.42082816139522,0.155366721605216,0.65220546495297
"3725","TMED2",1063.42242697598,-0.221731963715477,0.156062651496923,-
1.42078813597402,0.155378360768208,0.65220546495297
"3726","TMEM94",55.5117802592534,0.392738192452829,0.276386207678477,1
.42097608904459,0.155323710836386,0.65220546495297
"3727","MRPS15",518.958743366949,-0.381091081004121,0.268295379182402,
-1.42041611810628,0.155486573100445,0.652309550238992
"3728","IWS1",276.885366982275,-0.262026355525163,0.184469248878233,-1
.42043379651925,0.155481429520078,0.652309550238992
"3729","FANCE",13.6066886342027,-0.572773769618193,0.403291569275452,-
1.42024731795716,0.155535692448907,0.652340635532557
"3730","THBD",392.80671396798,1.23554753125143,0.870205782606593,1.419
83374041769,0.155656089629919,0.652577985455607
"3731","ARSA",217.035896870509,0.4011482133449,0.282545245076661,1.419
7662864086,0.155675732975375,0.652577985455607
"3732","RAD51",3.47227060541034,-1.15811665800472,0.816035777360056,-1
.41919838582485,0.155841186441614,0.653096504808907
"3733","MAN2B2",299.6427120241,0.277172473534099,0.195350793891401,1.4
1884487906501,0.155944245279187,0.653135882304647
"3734","HPGDS",2.80552397282894,-1.41994143663903,1.00075428199699,-1.
41887120763107,0.155936567855103,0.653135882304647
"3735","MTFR1",97.4386016775156,-0.270335407541093,0.19054659966287,-1
.41873645617078,0.155975864476206,0.653135882304647
"3736","RNF13",838.163885307004,-0.185413575841004,0.130746359560317,-
1.41811654614726,0.156156741276493,0.653368494800523
"3737","BTC",4.09230570522651,-1.77497512969555,1.25156292343226,-1.41
820686476385,0.15613037829159,0.653368494800523
"3738","CCDC57",38.327270415101,0.569624433407297,0.401618450792424,1.
41832237110468,0.156096668214638,0.653368494800523
"3739","DCAF16",109.721331261502,0.355629161090669,0.250830659884338,1
.41780578679917,0.156247474222154,0.653402690677747
"3740","PCYOX1L",35.293397327708,0.532463324996397,0.375555389800773,1

.41780237870867,0.156248469509896,0.653402690677747
"3741","EMC8",157.834228893357,-0.311024391605893,0.219438973428998,-1
.41736167803632,0.156377210833711,0.653766259673681
"3742","NHLRC3",275.645199121833,0.286886114379816,0.202503631813481,1
.41669614421561,0.156571784853478,0.654229953275017
"3743","RNF152",6.09703248711489,-0.947995693919709,0.669096158524903,
-1.41683027445513,0.156532556063437,0.654229953275017
"3744","POLR3B",35.1220150737618,0.50125674963531,0.353882557356188,1.
41644943842425,0.156643957846058,0.654356704250093
"3745","SARM1",21.7700445061865,0.550389885006882,0.388637148759372,1.
41620503022901,0.156715483557906,0.654480684337958
"3746","FIBIN",151.491988398645,-0.497551881069623,0.35137101757072,-1
.4160299404019,0.156766738570632,0.654519965628586
"3747","NBPF14",143.271865020217,0.641958223713008,0.453771762747751,1
.41471611152206,0.157151748559992,0.654723146651847
"3748","NCEH1",75.7625036874026,-0.44178564666614,0.312204415107629,-1
.41505252740849,0.157053095624844,0.654723146651847
"3749","NDST2",80.4008368276071,0.319250090768535,0.225608083539678,1.
41506494696316,0.157049454526961,0.654723146651847
"3750","COX15",97.4714099013201,0.311838751385695,0.220328888491879,1.
41533302110399,0.156970877596145,0.654723146651847
"3751","DIO3",4.07534593754896,1.40150455538308,0.990757819594164,1.41
457834363313,0.157192162127729,0.654723146651847
"3752","VPS18",61.600974409893,0.316732646404955,0.223890623528622,1.4
1467579755283,0.157163573659687,0.654723146651847
"3753","DPP8",187.883349759522,0.266785910106275,0.188576879334377,1.4
1473287206764,0.157146832469493,0.654723146651847
"3754","DUXAP8",559.293209623471,0.455846293318961,0.322046313587079,1
.41546813016291,0.156931286210929,0.654723146651847
"3755","PHETA2",149.362496213161,-0.283669508254179,0.200440834978033,
-1.41522813096078,0.157001619068705,0.654723146651847
"3756","TCEANC2",37.5465909854562,0.482336092228826,0.341228772835768,
1.41352702534546,0.157500820898146,0.654913132814117
"3757","LBR",108.666582408637,0.294984628212994,0.208626215340087,1.41
393845319071,0.157379974272563,0.654913132814117
"3758","ITPRID2",153.788766979963,0.317543027808817,0.224574693483943,
1.4139751139481,0.157369209503732,0.654913132814117
"3759","AC024560.1",3.49469171700037,-1.27817193119821,0.9043478732417
74,-1.41336311945579,0.157548983729112,0.654913132814117
"3760","LETM1",82.9921292938744,-0.324380208237467,0.229450355538101,-
1.41372719809799,0.157442016385368,0.654913132814117
"3761","STARD3NL",125.772488432728,-0.269883565590348,0.19107776856218
1,-1.41242786966356,0.157824015190307,0.654913132814117
"3762","CREB3",233.690699965796,-0.347565441385146,0.246009455545068,-
1.41281334335328,0.157710613861912,0.654913132814117
"3763","C9orf40",47.0742018190037,0.433437956716596,0.306825026576483,
1.41265515904242,0.157757142150936,0.654913132814117
"3764","AC009090.6",2.5988285646046,1.69965630098649,1.20269071400905,
1.41321146092568,0.157593557680875,0.654913132814117
"3765","POLDIP2",186.367424241007,-0.315420534698021,0.223256544014249

,-1.41281652500135,0.157709678119506,0.654913132814117
"3766","CACNB1",26.9046313952685,-0.526084509315707,0.372465729018649,
-1.41243735551672,0.157821223834996,0.654913132814117
"3767","ZNF844",72.3046854521179,0.584737547264162,0.413805426515646,1
.41307365683387,0.157634067960276,0.654913132814117
"3768","PRELID3B",154.39655065685,-0.314375288725924,0.222408300896978
,-1.41350519498616,0.157507234983317,0.654913132814117
"3769","KRBOX4",91.2784761531662,0.320298867818097,0.226637179517361,1
.41326709280532,0.157577205808121,0.654913132814117
"3770","ZNF302",263.617827963825,0.270473145541974,0.191579371417723,1
.41180725012522,0.158006723125679,0.655497387184514
"3771","FAM124A",10.9533974328938,0.731576511808126,0.518331318897395,
1.4114071157505,0.158124606014261,0.655812473631143
"3772","BRD3",286.916462930285,0.245795072499854,0.174168252902213,1.4
1125072109356,0.15817069927056,0.655829728682811
"3773","MAP4K3",96.1505973681869,0.27917447874744,0.19786882230158,1.4
1090685990914,0.158272079187933,0.655862629957015
"3774","MRPL3",367.50513203434,-0.264609571267775,0.187560326327963,-1
.41079713630422,0.158304439136044,0.655862629957015
"3775","CXCL10",9.94861270088048,-1.19258593930407,0.845227844877296,-
1.41096385611527,0.158255271707321,0.655862629957015
"3776","TBC1D24",88.1781270926846,0.328912956946014,0.233163492911742,
1.41065375560536,0.158346732865229,0.65586411599899
"3777","BRAF",161.84155903987,0.355883420772937,0.252329438621062,1.41
039199673958,0.158423967267146,0.655980790701873
"3778","AC133552.5",29.1687033500533,-0.523112238297981,0.370929501007
901,-1.41027401939335,0.158458786909954,0.655980790701873
"3779","ATP6V1FNB",3.19255001890952,-1.35310262984882,0.96015144746027
6,-1.40925958444155,0.158758424739503,0.656863476219641
"3780","ATP6V1C1",175.075520993668,0.262685511909318,0.186435401128119
,1.40898944256193,0.158838289922784,0.656863476219641
"3781","TK2",450.224801906066,0.276449824655762,0.196205133861937,1.40
898364489428,0.158840004287895,0.656863476219641
"3782","PIAS4",75.0104863372233,0.334585688434653,0.237419195958003,1.
40926131555865,0.158757913047267,0.656863476219641
"3783","AC040970.1",5.60898354760662,-1.02680789380755,0.7288726925448
86,-1.40876164563445,0.158905659804769,0.656961279235155
"3784","GHDC",117.500400814577,0.327302957807495,0.232410545797781,1.4
0829649826768,0.159043292135022,0.657182850460171
"3785","IER2",4711.04972888245,-0.253370795023139,0.179902674355995,-1
.40837703458351,0.159019455811042,0.657182850460171
"3786","KLRD1",27.517921731759,0.623564244414374,0.442868637433222,1.4
080117481979,0.159127591259626,0.657357508531577
"3787","PDIA6",1261.86854817331,-0.299845398102226,0.213094313550041,-
1.40710182785714,0.159397196194553,0.65829737218981
"3788","TMEM8B",82.701471249572,0.322735455864483,0.229423692642962,1.
40672243632107,0.15950971006738,0.658451724585837
"3789","ZBTB39",22.986527255296,0.588930859011053,0.418780072701245,1.
40630105728835,0.159634746324416,0.658451724585837
"3790","SQOR",285.286860929539,-0.304005741625697,0.216179359958305,-1

.40626626743798,0.159645072866336,0.658451724585837
"3791","ERI2",33.8154495848188,0.54287557435196,0.385988758491993,1.40645436533671,0.159589246484844,0.658451724585837
"3792","ZNF737",92.8323375322146,0.43149242073592,0.306758789700439,1.40661795268291,0.159540706701326,0.658451724585837
"3793","FNDC10",30.0119502581899,0.819685993591311,0.582985757852825,1.40601375342387,0.15972004080473,0.658587249719477
"3794","B3GLCT",80.3609023326724,-0.371435371189461,0.264339253537864,-1.40514647831619,0.159977725617771,0.659475916885067
"3795","SLC2A6",7.01848073181262,-0.919474702479625,0.654569489284367,-1.40470143740564,0.160110078168816,0.659847594877543
"3796","H6PD",361.835886619765,0.326693502665294,0.232626146278459,1.40437138254543,0.16020828799487,0.65992298471456
"3797","ZBTB11",150.592149657753,0.301030209350011,0.214549326527219,1.40308158605112,0.160592511497674,0.65992298471456
"3798","EIF4A2",1928.57626785126,0.304278019235484,0.216778637425076,1.40363470704373,0.160427654505053,0.65992298471456
"3799","FCHSD1",32.060977136366,-0.46012886324483,0.327873345240164,-1.40337380249007,0.160505400823529,0.65992298471456
"3800","CSNK2B",765.378498134397,-0.331297148046115,0.235961094249855,-1.4040329364438,0.160309041969675,0.65992298471456
"3801","VIM-
AS1",24.203436773501,-0.468878525996272,0.334065409610544,-1.40355305430423,0.160451982947265,0.65992298471456
"3802","SFXN3",297.038030984428,-0.247104498604125,0.17602108978981,-1.40383461379086,0.160368104037688,0.65992298471456
"3803","AP003396.5",7.42280898530787,-0.891070609183286,0.634961810594322,-1.40334520016133,0.160513925694108,0.65992298471456
"3804","AC074032.1",56.3658126683198,-0.327071074022825,0.23307644517527,-1.40327811236726,0.160533922428224,0.65992298471456
"3805","FBXO21",212.2652934622,0.284912740081697,0.203055133398838,1.40312995447436,0.160578090245924,0.65992298471456
"3806","SMARCE1",1222.80746481212,0.283655705052239,0.202034978466217,1.40399304717262,0.160320919991675,0.65992298471456
"3807","LRP8",7.48792295564373,0.870243382671893,0.620357894722143,1.40280858851917,0.160673925228818,0.660084105746971
"3808","LARP1",417.655385729369,0.250426178776815,0.178600489655352,1.40215841098794,0.160867947728761,0.660439589671379
"3809","GLI3",471.2791398898,0.286914749464163,0.204633014337037,1.40209413614758,0.160887137893092,0.660439589671379
"3810","NUP58",119.20008735215,0.377933921963272,0.269523321053475,1.40223087369975,0.160846315033282,0.660439589671379
"3811","SIPA1L1",48.1348253122531,0.705588272208065,0.503409030140653,1.4016202133102,0.161028687665705,0.66084719892197
"3812","COL6A2",13245.0228068356,-0.318908952756555,0.227565221982158,-1.40139582832019,0.161095739070805,0.660948939944227
"3813","HP1BP3",1280.85393667928,0.221286659341256,0.15808524820408,1.39979322457455,0.161575247351246,0.661070543280957
"3814","NKIRAS1",67.5958730554391,0.397574390997675,0.283823689128527,1.40077944944771,0.16128003581595,0.661070543280957

"3815","SLBP",299.226949103821,-0.262770899390701,0.187695312078617,-1
.39998648064602,0.16151736694307,0.661070543280957
"3816","MCUB",586.255041173353,-0.256883564171247,0.183482541811236,-1
.40004363159261,0.161500253171025,0.661070543280957
"3817","LINC02615",14.5624062697389,0.651388707406417,0.46518841902502
1,1.40026853800799,0.161432918552031,0.661070543280957
"3818","KDM4C",62.2283341033328,0.410043290252502,0.292813035143385,1.
40035873079117,0.161405921736247,0.661070543280957
"3819","RASSF8-
AS1",198.572584497882,-0.230538436554988,0.164700559110443,-1.39974288
976394,0.161590325253395,0.661070543280957
"3820","UBE2N",381.858539037252,-0.283540285231631,0.202521603394517,-
1.40004957732478,0.161498472808375,0.661070543280957
"3821","HERC1",225.439158668018,0.275828464620159,0.196920344982169,1.
40071085415342,0.161300555591732,0.661070543280957
"3822","SLC16A5",25.1765002203229,0.565169278324904,0.403661722758354,
1.40010619402532,0.161481520506833,0.661070543280957
"3823","AC092069.1",4.80982579829935,-1.16707093398124,0.8331387350279
57,-1.40081223560212,0.161270228776334,0.661070543280957
"3824","TFB1M",53.1081626494455,-0.357452755645156,0.255422561654652,-
1.39945646668619,0.161676144133621,0.661248664814287
"3825","AFDN",138.408754330432,-0.31410506747462,0.224484368373832,-1.
39922913007263,0.161744283854366,0.661286337438603
"3826","CDC37L1",166.404493497127,-0.270051640381605,0.193012093958212
,-1.39914362278289,0.161769918608702,0.661286337438603
"3827","LTBP1",665.449433628316,0.432199887109864,0.309190342437855,1.
39784407139667,0.162159896711473,0.661348365870859
"3828","MAPKAPK3",45.2783786235258,-0.421056698456667,0.30106167501330
3,-1.39857289519851,0.161941099091805,0.661348365870859
"3829","AC108471.2",26.2948889458499,0.57609069255253,0.41188714876718
6,1.3986614884121,0.161914518033143,0.661348365870859
"3830","MARCH3",191.62796428804,0.331784535131471,0.237262567037792,1.
39838550713574,0.16199733290753,0.661348365870859
"3831","NUDCD2",446.977943248268,-0.335395730589246,0.239965332262626,
-1.397684104728,0.162207949583159,0.661348365870859
"3832","PPP1R10",448.314530536394,0.269407567430666,0.192703375412387,
1.39804280466873,0.162100213587184,0.661348365870859
"3833","NUDT8",88.4128904691157,-0.45105246557268,0.322699065600544,-1
.39774952472599,0.162188296573464,0.661348365870859
"3834","NAV3",223.25165422578,0.348070842364602,0.249011459517987,1.39
781053867306,0.162169968817729,0.661348365870859
"3835","FAM219B",142.204582684666,0.270187869136365,0.193245574991977,
1.39815811641526,0.162065591026967,0.661348365870859
"3836","PIR",74.350171842645,0.464842050297035,0.332311919861256,1.398
81244853062,0.161869232311787,0.661348365870859
"3837","CCPG1",895.04961603578,-0.176436134286898,0.126269240450444,-1
.39730098682381,0.16232307921548,0.661645285100367
"3838","ODC1",128.120935395949,-0.402961906321121,0.288542583644163,-1
.39654224077394,0.162551269716045,0.661885409622219
"3839","DCAF13",182.525857629008,-0.257856380190902,0.184615489306521,

-1.3967212673189,0.162497406240685,0.661885409622219
"3840","VENTX",3.80038224297608,-1.59386637434812,1.14128430618399,-1.
39655506144423,0.162547411930542,0.661885409622219
"3841","LINC02617",7.05410765857269,1.24259012494798,0.889504220028683
,1.39694685755163,0.162429552387499,0.661885409622219
"3842","ISG20",68.4809137758613,-0.322127365202459,0.230794577715575,-
1.39573194652536,0.162795230315043,0.662706247300173
"3843","ZNF319",24.352729272998,0.503149269190587,0.360532744441226,1.
39557162823143,0.162843531102732,0.66273037378265
"3844","ABI2",277.974973627986,0.317217886910004,0.227413725664791,1.3
9489332045677,0.163048011674346,0.663389933035061
"3845","MTHFR",123.301792714655,0.339754842651353,0.244595517514793,1.
3890477066114,0.164818243719603,0.663443717980617
"3846","CROCC",156.355850312876,0.298260813257424,0.214086605530768,1.
39317830051987,0.163565879904028,0.663443717980617
"3847","LUZP1",295.105348710298,-0.232569369024801,0.167529801304807,-
1.38822685404885,0.165067977490948,0.663443717980617
"3848","KIAA0319L",137.300997550073,0.347065313386301,0.24987760788003
6,1.38894123539443,0.16485062014391,0.663443717980617
"3849","KCNT2",37.6343880706189,0.617000003242487,0.443794306927105,1.
39028372742022,0.164442737220999,0.663443717980617
"3850","ALMS1",77.8165767732949,0.435934353244511,0.313043480716885,1.
39256806193887,0.163750446584424,0.663443717980617
"3851","FAM168B",189.544115260081,0.2579851280014,0.185782635582578,1.
38863961743469,0.164942363989731,0.663443717980617
"3852","SEC22A",75.4418440944806,-0.324372284958201,0.233412525180502,
-1.38969528180785,0.164621428166893,0.663443717980617
"3853","AC010207.1",3.50578816974628,1.39189549100024,1.0021720278079,
1.38887880760831,0.164869605796938,0.663443717980617
"3854","SLC25A36",619.500576604003,0.191255102022126,0.137482801092469
,1.3911202019625,0.164188979797869,0.663443717980617
"3855","SMC4",134.997240128228,-0.340104223078544,0.244350691531843,-1
.39186928813837,0.163961983459242,0.663443717980617
"3856","SMARCAD1",124.307561667084,0.25463021939303,0.183025063329124,
1.39123142350805,0.164155261262575,0.663443717980617
"3857","DKK2",89.5318193049041,0.786079332217144,0.565426932562791,1.3
9024034220345,0.164455906847299,0.663443717980617
"3858","ADRB2",14.3808196850017,-0.766447573508299,0.552109785773656,-
1.3882158825247,0.165071317363228,0.663443717980617
"3859","CNOT8",172.411243205174,0.259397440067752,0.186697645382972,1.
38939856223495,0.164711587260841,0.663443717980617
"3860","RGS14",10.7297258786015,-0.642952356073423,0.461725793100208,-
1.39249824393909,0.163771573042867,0.663443717980617
"3861","TRIM52",88.4100234510485,0.360473660568909,0.259682872585425,1
.38813028745409,0.165097375344026,0.663443717980617
"3862","RREB1",161.867179575292,0.324791488883711,0.233889703562536,1.
38865236022188,0.16493848720859,0.663443717980617
"3863","MCM9",31.7690468623682,0.460485711562374,0.331643322923202,1.3
8849685711601,0.164985801132719,0.663443717980617
"3864","SP4",47.8435742674065,0.485537784985335,0.349737491517043,1.38

829206694209,0.165048126901913,0.663443717980617
"3865","PON2",141.586122482908,0.321004383309493,0.230561556210551,1.3
9227193199697,0.16384006763911,0.663443717980617
"3866","LINC01230",12.1717823135522,0.73428158072877,0.52660276171064
8
,1.39437472440039,0.163204476604813,0.663443717980617
"3867","RANBP6",82.3606770288782,0.291497790697192,0.2095676277676,1.3
9094856301208,0.16424102504446l,0.663443717980617
"3868","SET",1182.60554853047,-0.196097065700063,0.141076712622526,-1.
39000308452575,0.164527940705319,0.663443717980617
"3869","AGPAT2",159.726343693185,-0.387654476128371,0.278519331340782,
-1.39184046673606,0.16397071286147,0.663443717980617
"3870","CCDC7",43.3036144315013,0.403078295428162,0.289561374071212,1.
39203060740081,0.163913129681071,0.663443717980617
"3871","ADAMTS14",16.9263094108108,1.03488895752463,0.745415464583056,
1.3883384591484,0.165034006361694,0.663443717980617
"3872","ZRANB1",182.242340625281,0.282566740385487,0.203276554804348,1
.39006065238294,0.16451046033200 2,0.663443717980617
"3873","PSMC3",715.061014185452,-0.31290104801009,0.224495977185968,-1
.39379356339596,0.163379952414175,0.663443717980617
"3874","C11orf52",16.4549504694593,-0.67655997403426,0.487369275638221
,-1.38818757737301,0.165079934048336,0.663443717980617
"3875","ADAMTS15",72.6600147943809,0.74922241104578,0.539589787444692,
1.38850369017145,0.164983721868539,0.663443717980617
"3876","MCRS1",169.764311792628,-0.342978058455062,0.246532048182786,-
1.39121084249772,0.164161500321447,0.663443717980617
"3877","IQCD",13.0080888887579,-0.566812076762114,0.407587382565123,-1
.39065167619989,0.164331077931081,0.663443717980617
"3878","RILPL2",172.826303437955,-0.283074623286508,0.203669056785787,
-1.38987545655616,0.164566699690494,0.663443717980617
"3879","POLE",47.930014115824,0.404856981460538,0.290643300505338,1.39
296856578706,0.163629296488322,0.663443717980617
"3880","MTUS2",3.62006332927295,-1.54031194445939,1.10816404902874,-1.
38996743831331,0.164538765313009,0.663443717980617
"3881","RTN1",6.87913422922482,-0.949538020079572,0.681217746258774,-1
.39388327050257,0.163352856962641,0.663443717980617
"3882","LPCAT4",79.2667905539799,-0.346531582052346,0.24887280808242,-
1.39240435595352,0.1637999861559,0.663443717980617
"3883","ICE2",188.662867103112,0.228566992106546,0.164148688439188,1.3
9243873514849,0.163789581625247,0.663443717980617
"3884","ADAMTS17",15.4554954229192,-0.730766166699547,0.52515095287607
7,-1.3915354484218,0.1640631180183,0.663443717980617
"3885","ATF7IP2",14.456420592197,-0.819613565244612,0.588027646970714,
-1.39383508490959,0.163367410688293,0.663443717980617
"3886","IQCK",124.504178564193,-0.355713571017815,0.255504941383343,-1
.39219840169011,0.163862326650372,0.663443717980617
"3887","CDK12",294.1324626355,0.273684204951263,0.196743163497579,1.39
107351984117,0.16420313376093,0.663443717980617
"3888","AC110597.1",5.33046333588877,1.19033515172999,0.85737531056696
,1.38834782977697,0.165031154304715,0.663443717980617
"3889","RALY-

AS1",17.6875434749607,-0.569913105174023,0.409950678079749,-1.39019920
114184,0.164468395991828,0.663443717980617
"3890","SCAF4",112.310851712944,0.337377084711944,0.242442686641319,1.
39157460010776,0.16405125483884,0.663443717980617
"3891","NUDT11",27.0112211249968,-0.620622692180836,0.446392683980368,
-1.39030659429027,0.164435796279205,0.663443717980617
"3892","ARMCX2",127.628092150511,0.419528818996634,0.302173816850591,1
.38836919548218,0.165024651548996,0.663443717980617
"3893","LONRF1",112.50340502139,0.302537800921871,0.218058902548126,1.
38741320526962,0.165315800607824,0.664150814668987
"3894","AL049840.4",17.9560663189523,0.809460464856779,0.5836312360377
37,1.38693821521993,0.165460603620615,0.6644720402677
"3895","TBCB",734.56232261022,-0.312832395054006,0.225566847098356,-1.
38687222470064,0.165480728698382,0.6644720402677
"3896","DNM2",155.582773196789,-0.271822054514667,0.196045031783135,-1
.38652865641276,0.165585536214512,0.664722224434025
"3897","SEC13",391.089600885788,-0.309466632894661,0.223239021749347,-
1.38625689393196,0.165668474322197,0.664800701033196
"3898","CCNL1",2805.00748698746,0.366993378657953,0.264750447379419,1.
38618605668306,0.165690097994079,0.664800701033196
"3899","RIPOR2",90.4391004292508,0.561762652818456,0.405310065301016,1
.38600716071842,0.165744716959695,0.664849287830118
"3900","STK16",180.97494197736,-0.345661519247423,0.249492708044096,-1
.38545740257198,0.165912649126443,0.665352264701941
"3901","ARMCX4",54.1038233856908,0.429792233773271,0.310301684902395,1
.38507863374464,0.16602842439552,0.665645874787474
"3902","HTATIP2",169.081586297389,0.551497234489469,0.398303358903091,
1.38461607757531,0.166169892679869,0.666042317148425
"3903","LRP1",3780.71393525857,0.183695929547861,0.13270298603417,1.38
426372335405,0.166277717590435,0.666303741510224
"3904","GK",38.9801078940231,0.505513897961142,0.365308913814887,1.383
79842058084,0.166420186809587,0.666639822524177
"3905","AC234775.3",3.46514181786981,1.23580959309846,0.89320204410169
6,1.38357228497089,0.166489459512752,0.666639822524177
"3906","CXorf56",64.1972839910618,0.33140604614806,0.239521739560362,1
.38361572839422,0.166476149695022,0.666639822524177
"3907","SRGN",221.36245792099,-0.490304925823927,0.354417772133934,-1.
38340953635542,0.16653932812902,0.666668823122057
"3908","AL591848.4",3.68001590158932,1.31152942440985,0.94846238480000
1,1.38279540172424,0.166727609333709,0.666750991027165
"3909","TP53I11",244.849077358676,0.409959482662436,0.296476977543279,
1.38277004190854,0.166735387576746,0.666750991027165
"3910","RECQL",495.096126313454,-0.239577391443914,0.173231402789466,-
1.38299054089564,0.166667766291751,0.666750991027165
"3911","TMEM265",146.367747506958,-0.380342476512094,0.275066909735899
,-1.38272712220192,0.166748552328142,0.666750991027165
"3912","FMR1",200.890092606117,0.277925928055412,0.201009982491665,1.3
826473919868,0.166773010031859,0.666750991027165
"3913","TSLP",13.6785611720231,0.998108271575588,0.722199676422466,1.3
8203921181449,0.16695966154895,0.66698572327601

"3914","TAB2",211.866882504021,0.280749489496455,0.203117629795622,1.38220148481915,0.166909844341747,0.66698572327601
"3915","PHC1",100.624575679209,0.315290176141446,0.228124805146956,1.38209510332881,0.166942501801083,0.66698572327601
"3916","AC025171.2",56.0622213364675,0.403218094304551,0.291820579591337,1.38173289515501,0.16705373007529,0.667191097644928
"3917","ZFHX2-AS1",23.3851944687412,0.698517563924811,0.50565326417209,1.38141610747534,0.1671505609483,0.667242077822716
"3918","PRKX",26.0851839684512,0.49948499430599,0.361575263346773,1.38141362238863,0.16715181975124,0.667242077822716
"3919","PRPF39",73.8121425804255,0.317792645256779,0.230103589461175,1.38108512779371,0.167252787799943,0.667474764274333
"3920","EFCAB14",436.11143975866,0.248631191579681,0.180112724134768,1.38041991632776,0.167457391489116,0.667780112924472
"3921","GTF2F2",217.862072183271,-0.246111598188114,0.178280276187541,-1.3804757511662,0.167440210756554,0.667780112924472
"3922","BRCA1",72.1341337253439,0.317804247666749,0.230202918931975,1.38053960888594,0.167420562956027,0.667780112924472
"3923","ACYP2",120.068611099573,0.365247842553017,0.264774647425626,1.3794668262399,0.167750867604569,0.667928607264627
"3924","SBDS",1251.86992025386,-0.234678183378113,0.170071540969662,-1.37987920871474,0.167623839152694,0.667928607264627
"3925","LYNX1",8.81756152969863,0.904061916838115,0.655367621996114,1.37947296524129,0.167748976044086,0.667928607264627
"3926","PROSER2",98.2955015879039,0.324100493485369,0.234931642278867,1.3795523256959,0.167724524795795,0.667928607264627
"3927","RNF213",629.96390816518,0.313390630191038,0.227116448291758,1.37986760777648,0.167627411665857,0.667928607264627
"3928","ZNF440",16.323594810506,0.590490534859327,0.42786572544329,1.38008374998383,0.167560859987075,0.667928607264627
"3929","CD1D",2.65211708572477,-1.26831441348296,0.919718650544607,-1.37902434916584,0.167887246650606,0.6683014857764
"3930","UTP11",214.744970856405,-0.279974704751075,0.203165730917115,-1.37806067729648,0.168184554997118,0.668315546219381
"3931","ZNF385B",152.563941466303,0.676010334634613,0.490678054144651,1.37770647968561,0.168293929965358,0.668315546219381
"3932","AC021074.3",6.03142560753025,-1.07429987187787,0.779493875832816,-1.3782018116949,0.168140988066033,0.668315546219381
"3933","TP63",5.93169366508672,-0.823522181470284,0.598112947676884,-1.37686733696187,0.168553267351577,0.668315546219381
"3934","AC092535.2",4.30606550654008,1.39013781907184,1.00927523315848,1.37736246110139,0.168400212792671,0.668315546219381
"3935","CCDC167",156.777652092513,-0.443379888835323,0.321915250188631,-1.37731868426711,0.168413741040087,0.668315546219381
"3936","EPHA7",3.16107461874828,4.45581949984543,3.23596997653247,1.37696564929817,0.168522868383927,0.668315546219381
"3937","HINT3",87.8390719017915,0.297205574451728,0.21568303872363,1.37797379066306,0.168211380340892,0.668315546219381
"3938","MTDH",1838.36425734289,-0.177034546081967,0.128591084116707,-1

.37672488958327,0.168597320531463,0.668315546219381
"3939","RMRP",12.0672028004423,0.743103419146855,0.539841607681383,1.37652120283666,0.168660327567359,0.668315546219381
"3940","SWI5",340.706667062666,-0.30067176014876,0.218365974118548,-1.37691671682114,0.168537998186284,0.668315546219381
"3941","PRF1",14.5119790978037,0.921721098737814,0.669799591007431,1.37611475299869,0.168786108729292,0.668315546219381
"3942","CDCA3",2.54614178137199,-1.27547302567938,0.926847621706298,-1.37614101369897,0.168777979889492,0.668315546219381
"3943","TMEM120B",58.8546197316345,0.37341460638643,0.270846783899999,1.37869315267299,0.167989381578051,0.668315546219381
"3944","C16orf91",96.9730990793215,-0.346930503533604,0.252119379352628,-1.37605647143994,0.168804150480225,0.668315546219381
"3945","SLX4",26.6882574496189,0.600304907225544,0.435723281699428,1.37772052226405,0.168289592650616,0.668315546219381
"3946","PITPNA",207.037142798408,-0.261120347443369,0.189740784661461,-1.37619514913077,0.168761223523312,0.668315546219381
"3947","KRT10",1894.01300960715,-0.339201192242792,0.246267794634787,-1.37736723856168,0.168398736475235,0.668315546219381
"3948","AC008105.3",81.1288639915549,0.3025792132245,0.21984955876226,1.376301207644,0.168728399191073,0.668315546219381
"3949","SLC16A3",6.43707971077936,-1.06817201590997,0.776304624074892,-1.37597018333221,0.168830864649154,0.668315546219381
"3950","CHERP",101.104734234507,0.267407611741717,0.194182154994785,1.37709673553112,0.168482341864157,0.668315546219381
"3951","IFNAR2",93.9965517000858,-0.333836120280027,0.242463920062708,-1.37684864698091,0.168559046910015,0.668315546219381
"3952","HNRNPU",1658.71647319226,0.260368780857119,0.189345328530366,1.37510010348823,0.169100412301477,0.668454213942364
"3953","SOS1",261.172128497218,0.249533947755823,0.181752150782416,1.37293532253465,0.169772454371012,0.668454213942364
"3954","AC007370.2",2.71688724333567,1.48686207792848,1.08165462742425,1.37461814541409,0.169249860072947,0.668454213942364
"3955","TRMT10A",77.6416030850866,0.367015047721628,0.267015288761967,1.37450948754027,0.169283566885798,0.668454213942364
"3956","ANKRD31",2.92121730599215,1.28280739899189,0.933857806952224,1.37366458730855,0.169545835607105,0.668454213942364
"3957","DNAJC18",112.855298217807,0.377875236159981,0.275046311696571,1.37386040128707,0.169485025131346,0.668454213942364
"3958","PGBD1",30.7205927472077,0.441146351904797,0.321112699201545,1.37380537425558,0.169502112248417,0.668454213942364
"3959","AL355347.1",2.76046099011377,1.38898064528668,1.01160402340214,1.3730477668677,0.169737497535171,0.668454213942364
"3960","IMPG1",8.76906632066399,1.40524570769266,1.02367143571573,1.37275072710233,0.169829853334262,0.668454213942364
"3961","RCC1L",184.21323834649,-0.257737167944647,0.187611686947629,-1.37377991818065,0.169510017362838,0.668454213942364
"3962","POM121C",132.282147198746,0.30798982589329,0.22433339950522,1.37291115176153,0.169779969316136,0.668454213942364
"3963","GIMAP7",7.56081116285383,-0.780836812952128,0.568660972382041,

-1.37311482741872,0.16971665223563,0.668454213942364
"3964","AGAP3",217.756914094596,0.267861264868648,0.194937367528256,1.
37408885871931,0.169414097831392,0.668454213942364
"3965","MVB12B",24.7607332853298,0.491159256852136,0.357673396362834,1
.37320600818153,0.169688312417357,0.668454213942364
"3966","DYRK2",262.801669694441,-0.394758120902416,0.287046292984178,-
1.37524200991572,0.169056428174885,0.668454213942364
"3967","HVCN1",13.9643371330119,-0.603334557337678,0.438588632188394,-
1.37562744006214,0.168937006790266,0.668454213942364
"3968","AL138820.1",2.50548599943627,1.47970097652985,1.07680523615718
,1.37415841495208,0.169392507700807,0.668454213942364
"3969","CCNB1IP1",172.38999211066,-0.408450137424346,0.297106340396938
,-1.37476075696887,0.169205628111452,0.668454213942364
"3970","GLG1",1123.78760352158,0.219829168306178,0.160082868457931,1.3
7322107245941,0.169683630643012,0.668454213942364
"3971","CLDN7",5.37150375294596,-0.918676110024462,0.667909601177946,-
1.37544977404765,0.168992046828237,0.668454213942364
"3972","CISD3",272.123099340859,-0.3141389758255,0.228462970625629,-1.
37501046653317,0.169128199843984,0.668454213942364
"3973","KAT7",99.0478206236778,0.342496802921978,0.249452772663192,1.3
7299256795359,0.169754657168715,0.668454213942364
"3974","AC008735.2",6.8005816546866,1.4808679242076,1.07880769670185,1
.37268943180044,0.169848915997887,0.668454213942364
"3975","SLC25A42",38.9252567095863,0.418801826308968,0.305223802299042
,1.37211391495165,0.17002797833326,0.668990586448348
"3976","LSM1",306.974028942731,-0.320448417831519,0.233575341010033,-1
.37192743226159,0.170086029610154,0.669050679854831
"3977","ARPIN",155.837378357272,0.255223008785233,0.18605197818167,1.3
7178336548521,0.170130887161682,0.669058857231254
"3978","TCEA3",250.221679090325,0.346295607812924,0.252615056130818,1.
37084310459149,0.170423870595856,0.669580821374345
"3979","AL589743.4",3.15929807748144,2.14639583150086,1.56578898208437
,1.37080785218171,0.170434862525017,0.669580821374345
"3980","POLI",79.9174955685927,0.291317266623557,0.212484590682741,1.3
7100420170477,0.170373646234842,0.669580821374345
"3981","TNPO2",151.538027561497,0.264376268060256,0.192842663109429,1.
37094283908657,0.170392775612991,0.669580821374345
"3982","ZFYVE9",125.655267988279,0.297102284868977,0.216997144657506,1
.36915296898447,0.170951463771932,0.670144483604108
"3983","DDX59",111.958769304835,-0.34612645896055,0.253436944625894,-1
.36573008119033,0.172023699022558,0.670144483604108
"3984","CLIP4",151.296576863493,0.372906055393275,0.27377948146311,1.3
620672133661,0.173176674263361,0.670144483604108
"3985","CYP1B1",615.9182852736,0.463861853872558,0.340591780170585,1.3
6192909188893,0.173220264043895,0.670144483604108
"3986","MAT2A",499.252825318327,0.32458531788393,0.237218928088449,1.3
6829434522571,0.171219960110261,0.670144483604108
"3987","ARID5A",70.5382441228515,-0.463583966723103,0.340300776039121,
-1.36227713647591,0.17311044029479,0.670144483604108
"3988","CHROMR",65.4094706040859,0.412979861883734,0.301973280516491,1

.36760398528432,0.171436068334652,0.670144483604108
"3989","ITGA4",29.1201990249854,-0.667230781994895,0.487159988499045,-1.36963379125337,0.17080124582193,0.670144483604108
"3990","ITGA9-AS1",52.9076888599953,0.351165223421269,0.257411288464364,1.36421842847768,0.172498829860457,0.670144483604108
"3991","LRRC2",85.5815264378095,-0.711914140308462,0.522162096835347,-1.36339681609052,0.172757483997639,0.670144483604108
"3992","NAA50",301.970623806202,-0.192870436917777,0.140909456285704,-1.36875439024276,0.17107606228076,0.670144483604108
"3993","HCLS1",24.8287311808242,-0.646928899599664,0.474786172417329,-1.36256895668609,0.173018397993725,0.670144483604108
"3994","AASDH",117.256736276411,0.253073717097923,0.185516229161548,1.36415944977809,0.172517387457911,0.670144483604108
"3995","PPID",182.901356796853,-0.281109754911951,0.205876378031041,-1.36542986427305,0.172117982842157,0.670144483604108
"3996","C6",3.93736760987147,-1.03972885373649,0.76000557204269,-1.36805425115763,0.171295095297779,0.670144483604108
"3997","CETN3",220.276403403806,0.302872652967905,0.222204775032846,1.36303395335737,0.172871810130275,0.670144483604108
"3998","DCP2",200.301013703257,0.274429146459751,0.201307190799647,1.36323568656263,0.17280824364067,0.670144483604108
"3999","FAM193B",62.8013234273645,0.405891061437463,0.29802946804995,1.36191586722369,0.173224438048416,0.670144483604108
"4000","ID4",285.750146400016,-0.566275445802356,0.413965970806175,-1.36792752481458,0.171334763070808,0.670144483604108
"4001","HCG18",166.596042864291,0.34710848724012,0.254177578652001,1.36561410758953,0.17206011621922 5,0.670144483604108
"4002","OGFRL1",349.728982181143,0.281292992134819,0.205822108872764,1.36668015732319,0.171725580066802,0.670144483604108
"4003","NOD1",49.0138614099001,0.409630977474951,0.299317092083424,1.36855190802395,0.171139385685373,0.670144483604108
"4004","AC002074.1",16.4989665606905,-0.778826603688702,0.568529314530278,-1.36989700228945,0.17071905561501,0.670144483604108
"4005","AC079781.5",20.7284937556476,0.756016928833514,0.552975866957351,1.3671788843035,0.171569242226076,0.670144483604108
"4006","SLC26A4",3.4731524325537,-1.14258466401299,0.837332049196238,-1.36455384110732,0.172393320951637,0.670144483604108
"4007","PLPBP",353.768298150176,-0.20474905978878,0.149933877275047,-1.36559571132264,0.172065893412779,0.670144483604108
"4008","RAB2A",1614.20861628391,-0.194457669433231,0.142800153591523,-1.36174692073142,0.17327776801119,0.670144483604108
"4009","PDE7A",297.754899690417,0.341751808319269,0.250764195046753,1.36284132691093,0.172932523379366,0.670144483604108
"4010","ELOC",596.506185886569,-0.321174716061834,0.235030046648296,-1.36652619799904,0.17177386381193,0.670144483604108
"4011","AC016877.1",2.95603299997199,1.40449922644918,1.02965084100442,1.36405388168196,0.17255060809745,0.670144483604108
"4012","MFSD14C",133.615775895263,0.314204179671094,0.230363447339073,1.36394980757783,0.172583363283978,0.670144483604108

"4013","CCDC183",17.613153479704,0.590928434854671,0.433166927444352,1
.36420487672293,0.172503093776392,0.670144483604108
"4014","MRPL41",554.437770558276,-0.363508657818162,0.265900616265976,
-1.36708467593227,0.17159876591749,0.670144483604108
"4015","LARP4B",148.861361617186,0.31476048546471,0.230520972864149,1.
36543101286583,0.172117622050269,0.670144483604108
"4016","SPOCK2",12.6421115480872,0.802056462753882,0.588845797312074,1
.362082342126,0.173171900273717,0.670144483604108
"4017","COMTD1",138.816286984646,-0.359284854786978,0.262387021173236,
-1.36929354653471,0.170907534460715,0.670144483604108
"4018","SFXN4",78.438749623117,-0.363960243862689,0.266452626923517,-1
.36594729076235,0.17195550795049,0.670144483604108
"4019","NDUFS3",493.909594100463,-0.385994676158151,0.282777109560439,
-1.36501386819519,0.172248691271654,0.670144483604108
"4020","COX8A",1300.2630892709,-0.306024822056626,0.223795385799364,-1
.36743133002296,0.171490147731242,0.670144483604108
"4021","MRPL57",550.917892832765,-0.282233007216611,0.20612851111284,-
1.36920897401772,0.170933961653144,0.670144483604108
"4022","RNF6",154.661960353665,-0.233655835115819,0.171138246411214,-1
.36530460031936,0.172157333706844,0.670144483604108
"4023","COL4A1",384.862524835582,0.414884037752964,0.303771853648901,1
.365775113031,0.172009560002161,0.670144483604108
"4024","CNIH1",448.605124295402,-0.266499430592665,0.19564760252208,-1
.36214002705496,0.173153698279675,0.670144483604108
"4025","RAD51B",35.5634504707269,0.41595186969721,0.305010273286725,1.
36373068754374,0.172652342015562,0.670144483604108
"4026","AL049840.2",45.4620622027914,-0.397871764657515,0.290949424196
993,-1.36749459379623,0.171470330654366,0.670144483604108
"4027","THBS1",872.167041336905,-0.867998730955205,0.635354664084777,-
1.36616409703319,0.171887463664121,0.670144483604108
"4028","TTBK2",138.121489237887,0.353624181184738,0.259661458795233,1.
36186626550382,0.173240094146846,0.670144483604108
"4029","HAGH",236.88095476621,-0.300005232608919,0.219392506620167,-1.
36743609538277,0.171488654945183,0.670144483604108
"4030","MPHOSPH6",107.053674046209,-0.311601180263526,0.22804550816899
4,-1.36639911377957,0.171813726811519,0.670144483604108
"4031","ZNF778",19.3061620154838,0.615344732673293,0.451069447188093,1
.36419067287592,0.172507562950852,0.670144483604108
"4032","SMCR8",57.379734022969,0.405125383873184,0.29633382167027,1.36
712502673409,0.171586120029827,0.670144483604108
"4033","SLC39A11",73.1510432685385,-0.294643736861798,0.21609527801655
1,-1.36348993631981,0.172728153989108,0.670144483604108
"4034","AC027097.1",37.7359804455504,0.424295069405409,0.3106052157051
98,1.36602686610426,0.171930531021255,0.670144483604108
"4035","NDUFS7",579.837082009846,-0.340977251371663,0.249335909134136,
-1.36754169327542,0.17145557807921,0.670144483604108
"4036","RFX1",108.254542493584,-0.379366252738927,0.278487275966978,-1
.3622390876627,0.173122443875033,0.670144483604108
"4037","AC007785.3",3.84866307908087,1.36244546448239,0.99800431772566
7,1.36516990987298,0.172199653352833,0.670144483604108

"4038","ZNF264",205.259761002925,0.296948047571945,0.217089930051682,1
.36785730918635,0.17135674486834,0.670144483604108
"4039","ISM1",171.99447369684,0.580726732688326,0.424002030615112,1.36
963196106832,0.17080181741876,0.670144483604108
"4040","CD93",8.26835072060438,-1.08601528342906,0.793035544570079,-1.
36944086663582,0.170861507231868,0.670144483604108
"4041","AL118506.1",9.8723339357792,0.699764702433267,0.51146968924891
5,1.36814500867268,0.171266690682859,0.670144483604108
"4042","PAXBP1-
AS1",11.5795461208643,0.682737405623275,0.500199182776623,1.3649310697
2581,0.172274715899188,0.670144483604108
"4043","EIF4ENIF1",82.9707216111379,0.326222493103523,0.23839343121008
8,1.36842064585259,0.171180445419416,0.670144483604108
"4044","TRIOBP",2134.26680961339,0.25794585453236,0.189373049452516,1.
36210435052977,0.173164955538324,0.670144483604108
"4045","HUS1",66.9258743928144,-0.332403474842835,0.244174207940067,-1
.3613373732103,0.17340709746932,0.670478863886321
"4046","UBE3B",103.514886029543,0.338443463683573,0.248692110917763,1.
36089344545187,0.173547365194413,0.670855361255716
"4047","AP1G2",24.9024380987693,-0.551747297101713,0.405584301166242,-
1.36037636445786,0.173710853997987,0.671321412534845
"4048","LINC02453",5.70787895931821,-1.10038286332589,0.80920783583684
5,-1.35982724659101,0.173884598060193,0.671826856141656
"4049","SNX27",319.51862056309,0.187584727165962,0.13798375647693,1.35
946963581416,0.173997817953869,0.672098264459993
"4050","NPIPB13",7.83436721831796,0.909816560933123,0.66941931630703,1
.35911309812852,0.174110752914798,0.672233354216705
"4051","SNHG30",115.98149198762,-0.383588765055541,0.282239861640181,-
1.3590878440288,0.17411875434347,0.672233354216705
"4052","ZNF624",71.5696515821015,0.343237525083182,0.252575930923693,1
.35894787689362,0.174163106064866,0.67223864236291
"4053","ARRDC1",111.334228826132,-0.286964892656695,0.211205207500772,
-1.35870178606106,0.174241105914117,0.672243107384701
"4054","ABI1",446.91901324704,0.263472676632378,0.194033492410314,1.35
787215577826,0.174504254038191,0.672243107384701
"4055","RAB18",426.87961521476,-0.247393456082359,0.182114306167523,-1
.35845152030388,0.174320455781067,0.672243107384701
"4056","METRN",157.881934042968,-0.477261231882431,0.351466445123166,-
1.3579140726085,0.174490951442041,0.672243107384701
"4057","MAFTRR",7.15042755491821,0.925396186312999,0.68151076424663,1.
35785997061392,0.174508121226463,0.672243107384701
"4058","P4HB",1964.31666698159,-0.300572348025819,0.221328262724775,-1
.35803870832161,0.174451401908631,0.672243107384701
"4059","THOP1",53.5877371895975,-0.33644578811022,0.247772458857652,-1
.35788210546642,0.174501096367267,0.672243107384701
"4060","COX7B",1011.85456099979,-0.324562538376617,0.239015201682584,-
1.35791588188454,0.174490377272816,0.672243107384701
"4061","RABGGTB",183.006991383468,-0.313562388662765,0.230986274684351
,-1.35749359606434,0.17462442688322,0.672525495309913
"4062","CP",202.803383325956,0.647275534644497,0.476982852850099,1.357

02055278687,0.174774680125467,0.672938453264969
"4063","MICOS13",794.561078715775,-0.338691808087147,0.249698960569178
,-1.35640055254982,0.174971757567712,0.673531451725084
"4064","DHCR7",26.6708048331819,-0.607777626362679,0.44819806981095,-1
.35604695178415,0.175084229726645,0.673798561251162
"4065","ARL13B",185.437878678051,-0.245480819253087,0.181102667575425,
-1.35547875986338,0.175265071315497,0.673837946408067
"4066","AC026124.2",19.589148987724,-0.529483065575637,0.3906027929499
85,-1.35555371116723,0.175241208172471,0.673837946408067
"4067","EPN2",326.546483279655,0.315346196183556,0.232646552112807,1.3
5547332775707,0.175266800894375,0.673837946408067
"4068","TMC4",3.09920165408469,-1.11936793227339,0.82557724268O672,-1.
35586093511828,0.17514341894313,0.673837946408067
"4069","AL021368.2",54.0034795944058,0.4489242722277,0.331420833714774
,1.35454451428377,0.175562721746117,0.674763746072102
"4070","RGN",25.7219234740975,0.680195500143187,0.502194345359492,1.35
444675239478,0.175593890442037,0.674763746072102
"4071","UBA7",104.683512506597,0.272263563418908,0.2010580615244,1.354
1539262571,0.175687274719162,0.674791006043145
"4072","ZNF853",39.4294261363364,0.467787712162517,0.345430863477714,1
.35421516031585,0.175667743692554,0.674791006043145
"4073","SIKE1",215.289997038902,0.208761463434934,0.154192297744397,1.
35390331740821,0.175767225023655,0.674932334733602
"4074","RBM8A",698.516084822317,-0.262069690575066,0.193618724970968,-
1.3535348433596,0.175884826461564,0.675218135949647
"4075","PCBP4",91.9261682580594,-0.388384085926021,0.286998234599662,-
1.35326297901373,0.175971631750911,0.675385600143373
"4076","ATP6V0B",580.599143347694,-0.344322226090218,0.254543850858861
,-1.35270298193586,0.176150537432971,0.67554721453399
"4077","LINC00607",8.5296238426684,-0.751067920082282,0.55533659290573
,-1.35245530310259,0.176229708139302,0.67554721453399
"4078","ABL1",559.130550785813,0.270729699726953,0.20014302264667,1.35
268117842357,0.176157505875781,0.67554721453399
"4079","RHOG",312.957483927418,-0.290803527232042,0.21496803490404,-1.
35277566900518,0.176127307995953,0.67554721453399
"4080","LIME1",23.662071806842,0.463175449894493,0.342450022436677,1.3
5253444166481,0.176204408561046,0.67554721453399
"4081","CD86",6.10689132828286,-1.11626045528869,0.825601371666406,-1.
3520574136591,0.176356949302672,0.675810312811052
"4082","CCDC84",92.9840143479566,0.366737445970853,0.271330594570948,1
.35162585166915,0.176495036025715,0.675810312811052
"4083","ATXN2",342.776662178037,0.243737452979208,0.180337154359154,1.
35156537123675,0.176514394362733,0.675810312811052
"4084","NAE1",275.716455118729,-0.234450440100757,0.173429477566474,-1
.35184885170916,0.176423672730398,0.675810312811052
"4085","AC005899.5",5.29741232181841,1.11136659024669,0.82217388929128
,1.35174152903918,0.176458014879386,0.675810312811052
"4086","MICB",6.29067730910546,0.868758658879799,0.642847699104776,1.3
5142221103012,0.176560222820353,0.675820334045598
"4087","GBP4",58.1637533037017,0.397788414246687,0.294386778208034,1.3

5124415800217,0.176617233543548,0.675873142310029
"4088","SLC25A33",48.7005553444318,-0.471244592313641,0.34960773684931
6,-1.34792380901099,0.177682889419561,0.67634912937058
"4089","PGM1",248.113528589914,-0.259652235933061,0.192503597458726,-1
.34881757723376,0.17739556746082,0.67634912937058
"4090","TRMT13",112.361016345956,0.335977838062525,0.249343417785348,1
.34745019959483,0.177835282233522,0.67634912937058
"4091","PRDX6",1556.4538831282,-0.338846598859799,0.251326457290845,-1
.3482329019888,0.177583485339965,0.67634912937058
"4092","RBBP5",104.750692995337,0.298469552168512,0.221206172191681,1.
34928220678164,0.177246338576148,0.67634912937058
"4093","CACNB4",119.067144997456,-0.41691111758843,0.30893032960156,-1
.34953119729661,0.17716640673955,0.67634912937058
"4094","SESTD1",1137.09980912187,0.33577361326745,0.249222324348488,1.
34728545745339,0.17788831394625,0.67634912937058
"4095","NR2C2",141.567912196359,0.256904356190594,0.190585929098153,1.
34797126632726,0.177667624489149,0.67634912937058
"4096","MAATS1",8.66631075078427,-0.825997284406562,0.613112170195228,
-1.34722049986962,0.177909227508348,0.67634912937058
"4097","ZNF827",84.5462972802572,0.484608647723515,0.359262452340572,1
.34889867996591,0.177369512291906,0.67634912937058
"4098","DNAJC21",397.060500331599,-0.201046449846252,0.149057090721216
,-1.34878823190151,0.177404995682575,0.67634912937058
"4099","AC011346.1",20.4808429473444,0.70294499006689,0.52172687619860
6,1.34734287638902,0.177869829030155,0.67634912937058
"4100","ZNF3",80.9451607714497,0.344330272819065,0.255505682842371,1.3
4764232634111,0.177773450082376,0.67634912937058
"4101","GRINA",578.739571276394,0.224447747806752,0.166566229014246,1.
34749852437108,0.177819728363802,0.67634912937058
"4102","ZNF438",134.928301775587,0.30220642797101,0.22402377079748,1.3
4899268454957,0.177339315823896,0.67634912937058
"4103","RAB3IL1",262.124219931703,0.317574353505414,0.235428977380857,
1.34891786490526,0.177363349330278,0.67634912937058
"4104","AC004263.2",6.41189012048485,1.15420505351054,0.85559836560479
1,1.34900334071428,0.177335893056519,0.67634912937058
"4105","TP53BP1",192.959533894813,0.226368160115528,0.167607218162039,
1.35058717994281,0.176827709770909,0.67634912937058
"4106","AC090527.3",4.38238898886911,-1.03753961315212,0.7684355539623
33,-1.35019730386262,0.176952702803267,0.67634912937058
"4107","ETFA",348.031550585244,-0.316411104951478,0.234653212159808,-1
.34842008783579,0.17752330664186,0.67634912937058
"4108","ZCCHC14",134.212472391456,0.248476858329208,0.18403025013327,1
.35019573221939,0.17695320680027,0.67634912937058
"4109","APRT",1469.65035412906,-0.385600469054996,0.28555116774631,-1
.35037282785109,0.176896422226019,0.67634912937058
"4110","ARHGEF18",59.0206888578882,0.369188014558481,0.273718559024288
,1.34878692871433,0.177405414386021,0.67634912937058
"4111","ZNF234",61.2604280332878,0.38803135755134,0.288009905749165,1.
34728476280009,0.177888537585202,0.67634912937058
"4112","SLC13A3",5.64219909887839,-0.809054273510519,0.600510934119437

,-1.34727650662495,0.177891195621233,0.67634912937058
"4113","SLC35A2",85.7082802053631,-0.389662245132709,0.288899603896551
,-1.34878082170109,0.17740737652952,0.67634912937058
"4114","PLXNA3",69.1756770952362,0.345838744740445,0.256209448501725,1
.34982822359932,0.177071089405236,0.67634912937058
"4115","EBNA1BP2",269.568644391879,-0.304234455907898,0.22610658110460
8,-1.34553560724155,0.17845233047664,0.676721440907913
"4116","PKP4",21.3914424818448,-0.512902358079394,0.381383866785877,-1
.34484545033825,0.178675149269236,0.676721440907913
"4117","THUMPD3-
AS1",226.419314902778,-0.281150062249386,0.209023261447675,-1.34506590
463744,0.178603952590149,0.676721440907913
"4118","PDIA5",96.3573829130775,-0.464864889599807,0.345608698576207,-
1.34506131215706,0.178605435536457,0.676721440907913
"4119","AFF4",625.794897286817,0.365129920570324,0.271344693485845,1.3
4563132921327,0.178421442744889,0.676721440907913
"4120","TECPR1",48.6596689453677,0.414055623790272,0.307767330589039,1
.3453527474726,0.178511347043603,0.676721440907913
"4121","HACD1",7.48638454915814,0.807231846740653,0.599611166136298,1.
3462588629598,0.178219047587996,0.676721440907913
"4122","SGMS1-
AS1",34.9549986671977,-0.353882762035571,0.263004144216343,-1.34554063
050989,0.178450709460793,0.676721440907913
"4123","SORBS1",16.7642617180923,-0.746220659221713,0.55505025485958,-
1.34441999204288,0.178812612573853,0.676721440907913
"4124","DAZAP2",666.840795639167,0.19943104117872,0.148247293150913,1.
34525924177046,0.178541530891938,0.676721440907913
"4125","UHRF1BP1L",173.327975167047,-0.273798753012377,0.2034788647527
05,-1.34558816880139,0.178435369328902,0.676721440907913
"4126","PGF",12.3379569476927,-0.753572514746161,0.560364900907785,-1.
34478892865235,0.178693406594732,0.676721440907913
"4127","SPG21",285.905769582985,-0.222232482233999,0.165257715796351,-
1.34476312445138,0.17870174218785,0.676721440907913
"4128","TBX2",5.04637941875029,0.94701468805576,0.704378636916419,1.34
446821414337,0.1787970283189,0.676721440907913
"4129","MEX3D",163.460665966877,-0.310856358328803,0.2312285405202,-1.
3443684660616,0.178829265682379,0.676721440907913
"4130","TRIR",1574.03601714626,-0.273570600423566,0.203330997939019,-1
.34544463557697,0.178481689058298,0.676721440907913
"4131","CDC42EP1",117.337133995355,-0.456418431253164,0.33937337691180
3,-1.34488578746641,0.178662120639543,0.676721440907913
"4132","GPKOW",78.3424180961322,-0.282311438019459,0.209623265559702,-
1.34675622605953,0.178058757100764,0.676721440907913
"4133","JPX",638.080653573393,0.222496154645691,0.16527302165403,1.346
23396135062,0.178227075715985,0.676721440907913
"4134","DAD1",2441.17246390381,-0.379399875365527,0.282321832839609,-1
.34385595173247,0.178994972242008,0.677184655506775
"4135","NAT1",19.1891152905714,-0.499672876372116,0.371879866124341,-1
.34364057290762,0.179064642771317,0.677284404581233
"4136","CCDC17",7.95127004261269,0.811944792557255,0.604703652642137,1

.34271521101223,0.179364207390325,0.677319178259436
"4137","SEC16A",147.140340848746,0.278062294235506,0.207045164891328,1
.34300308042187,0.179270976393903,0.677319178259436
"4138","NRXN2",18.776034211472,0.913398706263025,0.680018445986162,1.3
431969553979,0.179208207268589,0.677319178259436
"4139","AAGAB",78.0639507329123,-0.316325483881629,0.235593351286084,-
1.3426757680335,0.179376984421393,0.677319178259436
"4140","NUBP2",273.563960838721,-0.303429945595332,0.225971929449302,-
1.3427771596888,0.17934414130218,0.677319178259436
"4141","ZNF224",132.192733503319,0.254273398026825,0.189276483603532,1
.34339667129192,0.179143564172463,0.677319178259436
"4142","CDS2",162.543752693902,0.204679741126773,0.152419009049343,1.3
4287542218905,0.1793123160555,0.677319178259436
"4143","POLE4",387.295363936222,-0.279747510387526,0.208390906490239,-
1.34241707135445,0.179460802561834,0.677447046506537
"4144","ACSF2",35.0721094700503,0.61874740526712,0.460959253481517,1.3
4230390342284,0.179497478307103,0.677447046506537
"4145","LINC01315",61.9332626736882,0.689518779318318,0.51378117708111
2,1.34204756825776,0.179580572649973,0.677597142640671
"4146","CLCN6",64.9761523596245,0.291219655326328,0.217217528971754,1.
3406821111762,0.180023685023485,0.677777613674638
"4147","ROBO2",29.7961887547932,0.522691769217259,0.389651461240874,1.
34143413078116,0.179779542389923,0.677777613674638
"4148","TBC1D1",64.1754841395298,-0.348280011328832,0.259835417698541,
-1.34038698193525,0.180119565806899,0.677777613674638
"4149","AC025171.1",52.2196157031967,0.345885003356404,0.2581414894159
08,1.33990473262951,0.180276319291227,0.677777613674638
"4150","PCDHB15",8.74578301667261,1.07073092242773,0.799316534170048,1
.33955808075395,0.180389059886893,0.677777613674638
"4151","HFE",54.7540953456327,0.392040263107046,0.292582082964548,1.33
993257254426,0.180267267266829,0.677777613674638
"4152","HEBP2",196.360672286329,-0.463054288745696,0.345594449726129,-
1.33987767775972,0.180285116385269,0.677777613674638
"4153","ST7-
AS1",37.6355996790361,-0.507858917962023,0.379017096286441,-1.33993670
190067,0.180265924653724,0.677777613674638
"4154","CALHM3",3.59412403424242,-1.24707213176773,0.930999297230455,-
1.33949846737536,0.180408453051632,0.677777613674638
"4155","CA12",242.558032525347,-0.344171491562164,0.256698583295438,-1
.34076116487971,0.179998008712761,0.677777613674638
"4156","ABAT",17.8479146941741,0.718980186203263,0.536484403446067,1.3
4016978235518,0.180190153247448,0.677777613674638
"4157","AC009088.1",3.16304486367021,1.41629995124627,1.05683616234351
,1.34013199179868,0.180202436855449,0.677777613674638
"4158","AC005306.1",4.97030615652455,1.02900472688378,0.76737087392457
2,1.34094837561547,0.179937214305276,0.677777613674638
"4159","SMG9",144.403510527905,-0.243243347536967,0.181426696273452,-1
.34072522144339,0.180009682652468,0.677777613674638
"4160","PRICKLE3",32.410617076475,-0.404601074438242,0.302020128022422
,-1.33964937068103,0.180359364846836,0.677777613674638

"4161","HTATSF1",438.405423610827,0.214557431658334,0.159950755941808,
1.34139679675157,0.179791657048308,0.677777613674638
"4162","ZNF185",20.8183077712056,0.496682523143182,0.370431815704559,1
.34082036716661,0.179978781840844,0.677777613674638
"4163","GRIN2A",4.58520496034646,-3.51116457494909,2.62089325238482,-1
.33968240475044,0.180348620328125,0.677777613674638
"4164","SGCA",90.2975142928312,1.6592396643203,1.23901729768644,1.3391
5778853009,0.180519310925448,0.678031225474064
"4165","ALG10B",48.5881962096979,0.330895961847989,0.247179229378541,1
.3386883787927,0.18067214107486,0.678442325668864
"4166","SYT11",98.5859943928561,0.4113488821748,0.307309898980886,1.33
854745173824,0.180718042772352,0.678451797637924
"4167","DIRC3",43.9174617391481,0.712987240833782,0.532888601559186,1.
33796676969191,0.180907269509955,0.678462834217935
"4168","EOGT",24.0250699358662,-0.48990092096091,0.366219137836224,-1.
33772615995835,0.180985720047059,0.678462834217935
"4169","ZNF141",117.012256171816,0.347180739999694,0.259562679843649,1
.33756031571574,0.181039808081295,0.678462834217935
"4170","APC",337.254353632604,0.293291882510862,0.21928794279948,1.337
47381988554,0.181068022380157,0.678462834217935
"4171","TRA2A",523.850059342053,0.271602477433577,0.203050026478661,1.
33761360263659,0.181022427907415,0.678462834217935
"4172","MTERF1",42.149307666991,0.358556677656254,0.268037544007151,1.
33771065163426,0.180990777385264,0.678462834217935
"4173","PHF19",131.410469957936,-0.272974954088891,0.204009721295083,-
1.33804875746119,0.180880543256949,0.678462834217935
"4174","ACTG1",17771.28514242,-0.333716146781233,0.249483534481194,-1.
33762794196099,0.181017751174818,0.678462834217935
"4175","SLC25A25",53.8116263758465,-0.617658641350804,0.46195544661385
9,-1.33705240598039,0.181205531169138,0.678815450894685
"4176","AC092645.2",3.44192941471581,1.33112469019346,0.99570601746180
3,1.33686516587164,0.181266653119094,0.678816378660982
"4177","PCDHGB7",40.5523341630807,0.500322237641887,0.374306420500534,
1.33666485595636,0.181332058468156,0.678816378660982
"4178","SOCS2",28.5968273568743,0.56581608577569,0.423308187546378,1.3
3665282747147,0.181335986575804,0.678816378660982
"4179","CAPZB",1633.66720521565,-0.280311003070988,0.20974844189147,-1
.33641518641664,0.181413605280312,0.678944433257735
"4180","TRPM7",225.924794460696,0.292905163420294,0.219200562256437,1.
33624275597264,0.181469940224486,0.678992790696402
"4181","NCKAP1",548.414724636595,0.22160956246018,0.16594076812905,1.3
3547388600634,0.181721296611453,0.679770647931864
"4182","PSMD6-
AS1",4.0843269034387,1.20097346341247,0.899423873342725,1.335269719880
83,0.181788085299518,0.679857879981938
"4183","PLSCR4",804.778391619628,0.278005592712885,0.208245927267098,1
.33498693761398,0.18188062169662,0.679945518494462
"4184","PCDH10",3.52935436077615,1.20579820596415,0.903265360609262,1.
33493241139107,0.181898468630488,0.679945518494462
"4185","MED13L",715.793052878764,0.329579550007115,0.246916393096783,1

.33478197163657,0.181947715681551,0.679967090384579
"4186","MOB3C",96.1959185529479,0.287332310299696,0.215396389969371,1.33396994415995,0.182213706887551,0.680310903729125
"4187","PTPRJ",38.6599343339514,0.397927111217038,0.298272072270423,1.3341078438489,0.182168515555854,0.680310903729125
"4188","PRH1",95.3070253610269,0.300441444503788,0.225175651198188,1.33425369441634,0.182120727674818,0.680310903729125
"4189","CHAMP1",134.099496526981,0.263711365912956,0.19767711718254,1.33405105088333,0.182187126264226,0.680310903729125
"4190","MOSPD2",117.918528175056,0.265401144399992,0.198977998396482,1.33382156087004,0.182262343103205,0.680330082609577
"4191","POLR1A",51.0726379906087,0.333160118133722,0.249916493513307,1.33308575776726,0.182503662630245,0.68073465726194
"4192","TNS2",473.430578532438,0.290094109430926,0.217573653943219,1.33331450832108,0.182428614512562,0.68073465726194
"4193","EIF2S1",295.579483831051,-0.243487477941733,0.182641079574713,-1.33314738671444,0.182483441250823,0.68073465726194
"4194","EIF4A1",3036.04279770334,-0.227644181578097,0.170780915786817,-1.33296030489883,0.182544830726124,0.68073465726194
"4195","CSNK1G1",145.862454194696,0.265948670505855,0.199554117106321,1.33271452557483,0.182625504612821,0.680873156649468
"4196","WDR76",16.6044719160994,-0.665039200636845,0.499161004661807,-1.33231401176345,0.182757024722561,0.681201112168936
"4197","ZBTB40",74.6626092224562,0.418634423484204,0.314256438472074,1.33214270969155,0.182813298065812,0.681248506492567
"4198","PTTG1IP",810.440031912026,0.208018519194754,0.156192614818777,1.33180764939564,0.182923403699905,0.681413948154507
"4199","MORF4L2",1063.72463254441,-0.239863896965819,0.180112825303648,-1.33174245954689,0.182944831732786,0.681413948154507
"4200","LMF2",370.747936108922,-0.279882845894691,0.210187926681829,-1.33158383696492,0.182996979061666,0.681445893458205
"4201","RFT1",108.445071121878,-0.26793295126763,0.201256896393421,-1.33129823657754,0.183090898241142,0.681471120535807
"4202","PRR5",82.4037446786431,-0.411604294094198,0.30915310251442,-1.3313930565358,0.183059712906225,0.681471120535807
"4203","TGFBR3",3788.52250485439,0.232384115864513,0.17463036313483,1.33071999446678,0.183281161499417,0.682016979740873
"4204","FAM122C",15.4191221157171,0.688647862433483,0.517553440535266,1.33058310214548,0.183326225626304,0.682022399808609
"4205","MME",218.19112788022,0.408700867785751,0.307285198593277,1.33003759913183,0.183505883469752,0.682350152187857
"4206","FAT3",4.16011730557423,1.96228701592212,1.47570410270312,1.32972932197431,0.18360747019621,0.682350152187857
"4207","MED17",110.204115726081,0.267404093800204,0.201071937604281,1.32989265924551,0.183553640395821,0.682350152187857
"4208","PCSK6",365.027865169627,0.899439781323284,0.676446752470374,1.32965348423737,0.183632467427026,0.682350152187857
"4209","LRRC75B",165.693027631418,-0.264156964986681,0.198626372335245,-1.32991889184197,0.183544996211701,0.682350152187857
"4210","VPS54",100.677585881576,0.322692317415655,0.242752610062169,1.

32930524344522,0.183747285067385,0.682614617209952
"4211","CDK15",13.7131976597483,0.637116685137279,0.479841124105009,1.3277659065292,0.184255453201025,0.6829897647878
"4212","PSMD6",247.126139712676,-0.263002043510196,0.198089121633862,-1.32769554098138,0.184278707219996,0.6829897647878
"4213","PCDHGA1",4.37467831607459,1.08316877042677,0.815584052061813,1.32808968945444,0.184148479198299,0.6829897647878
"4214","NUP42",155.536206716873,-0.283884445071051,0.213722243471088,-1.32828684773494,0.184083362991497,0.6829897647878
"4215","LINC01004",13.8434600044759,-0.667675229630117,0.502543951140214,-1.32859071950869,0.183983035508546,0.6829897647878
"4216","LINC01048",82.237898625909,-0.527909787933118,0.397423094358196,-1.32833193497637,0.184068474254229,0.6829897647878
"4217","KDELR1",1545.88298019053,-0.302612409924361,0.227844547788632,-1.3281529571868,0.184127581668417,0.6829897647878
"4218","ZNF71",38.2479974320783,0.373585737849323,0.281237154456667,1.32836551618177,0.184057385626807,0.6829897647878
"4219","ADARB1",47.6932643539097,0.386907778285821,0.291336151562183,1.3280458886107,0.184162947782643,0.6829897647878
"4220","RTCB",350.167537658785,-0.282573829271238,0.212833325567705,-1.32767661510485,0.184284962110263,0.6829897647878
"4221","SATB2",17.3202989851679,-0.492223128079481,0.370883972230349,-1.32716203700971,0.184455087364323,0.683134635656644
"4222","POLR1D",655.089924334702,-0.257813618104482,0.194248719087525,-1.32723458520371,0.184431095085692,0.683134635656644
"4223","HOMEZ",29.1328333098776,0.416386221451972,0.313681021882964,1.32741923292805,0.184370041001116,0.683134635656644
"4224","PITHD1",412.190322299339,-0.233321123166067,0.175981741601197,-1.32582574216597,0.184897424218564,0.683189091905716
"4225","ZNF684",62.1396420702719,-0.303226956092676,0.228759540313866,-1.32552703890136,0.184996407440314,0.683189091905716
"4226","RAB10",547.523024217094,-0.16379817278571,0.123470435870278,-1.32661856768531,0.18463489057586,0.683189091905716
"4227","SCAANT1",2.55225896478367,-1.5300302611981,1.15353300065614,-1.32638620683396,0.184711805191163,0.683189091905716
"4228","FBXO4",100.485888679074,-0.268227410714915,0.202404231057903,-1.32520653996695,0.185102656827371,0.683189091905716
"4229","DOCK2",10.5475002133044,-0.984377158833061,0.742922023639917,-1.32500737292743,0.18516870591997,0.683189091905716
"4230","ZSCAN23",4.28347839540911,1.09612304646986,0.826472459362159,1.32626687562682,0.184751314664764,0.683189091905716
"4231","TIMM23B",35.3566906249173,-0.407922865127386,0.307669586007823,-1.32584721948114,0.184890308652908,0.683189091905716
"4232","OLMALINC",28.9627976717841,0.428721797800341,0.323405181811849,1.32564912967215,0.184955944700172,0.683189091905716
"4233","SUFU",50.8566572403544,0.34454420650183,0.259666604661889,1.32687145869396,0.184551207137871,0.683189091905716
"4234","CDKN1B",259.079222354075,0.285446996425998,0.215419152223897,1.32507715065797,0.185145563783121,0.683189091905716
"4235","AC087284.1",3.32510573243169,1.34315543823044,1.01319391309821

,1.32566473294659,0.184950774009408,0.683189091905716
"4236","TRAF7",230.744783193546,-0.223732913599814,0.168713923522778,-1.32610817725194,0.184803867936654,0.683189091905716
"4237","LAMA1",2.59200376405674,1.487027846609,1.12151760798172,1.32590682128036,0.184870563274234,0.683189091905716
"4238","ARMC6",63.4088068878106,-0.443416192743542,0.334551174517007,-1.32540617555357,0.185036469844163,0.683189091905716
"4239","ZGPAT",123.028947538983,-0.292224428250435,0.220504525743794,-1.32525365302467,0.185087035432517,0.683189091905716
"4240","MORC4",370.50261174012,-0.32097150298102,0.242275040186769,-1.32482282423144,0.185229922740855,0.683253771619568
"4241","SMARCA5",467.288233820362,0.230318558084455,0.173931462532084,1.32419146445095,0.185439464898166,0.683629247273277
"4242","SMARCA2",559.286431945933,0.235121312791364,0.1775678382784,1.32412105182431,0.18546284502433,0.683629247273277
"4243","RIMKLB",133.950315048186,0.319079651970967,0.240932150533884,1.32435480804,0.18538523594977,0.683629247273277
"4244","GBP1",230.261132181179,0.36858972804778,0.278597034377009,1.3230210036945,0.185828393085446,0.684206203697837
"4245","FCGR3B",3.15096679747289,1.742758487206,1.3180053782944,1.32226963251187,0.186078381138032,0.684206203697837
"4246","BX284613.2",2.78636743215602,2.0871881661336,1.57729516482243,1.32327050299972,0.185745437326785,0.684206203697837
"4247","AC104825.1",20.9551940459248,0.547466353380537,0.413845613374781,1.32287581573264,0.185876679082003,0.684206203697837
"4248","HPF1",350.605304021432,-0.278764314155052,0.210828259810188,-1.32223409900565,0.186090209612767,0.684206203697837
"4249","EGR1",9487.19813437445,0.366365933025173,0.276812659592221,1.32351581594886,0.185663900183614,0.684206203697837
"4250","PCDHGA12",31.2481851596,0.47115637821224,0.356448030302697,1.32180945932604,0.186231607647865,0.684206203697837
"4251","C8orf48",29.8701938712424,-0.450254663210439,0.340363398642009,-1.322864517768,0.185880436900106,0.684206203697837
"4252","PTBP3",221.279796176295,0.286552820483543,0.216788405228347,1.32180879407139,0.186231829229008,0.684206203697837
"4253","MARK3",366.929061084473,0.277747572281181,0.210059146885708,1.32223507711522,0.186089884009979,0.684206203697837
"4254","HEXD-IT1",5.88970651458282,1.11625861940163,0.844232148209002,1.3222176172392,0.186095696289513,0.684206203697837
"4255","SUPT5H",258.922025471775,0.294859028413488,0.222898677096956,1.3228388443294,0.18588897635608,0.684206203697837
"4256","NPTXR",4.42638596444488,1.01267876198761,0.766007020498059,1.32202282079499,0.186160551862862,0.684206203697837
"4257","BCORL1",95.2837553764958,0.347665043815984,0.262966782871027,1.32208729946891,0.186139082468806,0.684206203697837
"4258","U2AF1",55.07140878556,0.40912442710486,0.309718321731126,1.32095648981345,0.18651587238947,0.685088831416465
"4259","ANKRD36B",78.6006447208379,-0.385339681562855,0.291832249881884,-1.32041500457478,0.186696496663748,0.685258495380005

"4260","TNPO1",385.366360359134,0.236181825323234,0.178867837528249,1.
32042645892632,0.186692674476266,0.685258495380005
"4261","C20orf27",114.548625721663,-0.417150017309892,0.31595268709831
4,-1.32029267147865,0.186737321439231,0.685258495380005
"4262","NINL",35.2507257359656,0.421267050166047,0.318992658448557,1.3
2061675718466,0.18662918255892,0.685258495380005
"4263","AC231981.1",8.44326097124775,0.823700820658961,0.6240705690394
01,1.31988409888779,0.18687371732469,0.685303631950197
"4264","HELLS",18.2964821403761,-0.658151434527943,0.498643577782162,-
1.31988350768545,0.186873914742106,0.685303631950197
"4265","DOC2B",4.60304347432926,1.18021064985006,0.8941924137253,1.319
86206965588,0.186881073546521,0.685303631950197
"4266","AC046134.2",24.0552446556328,0.507738928827178,0.3848552693616
95,1.31929836810938,0.187069383184535,0.685351406185553
"4267","CYP19A1",4.16765244031225,-1.27254007821956,0.964512200323034,
-1.31936130802012,0.18704835058674,0.685351406185553
"4268","CNBD2",33.3356218695912,-0.43913068430469,0.332788119665742,-1
.31955036359399,0.186985184483848,0.685351406185553
"4269","WAS",12.0807012750198,-0.712696541551661,0.540076206947234,-1.
31962217995153,0.186961193767081,0.685351406185553
"4270","BDKRB2",525.95476014645,0.469298717050857,0.3557870197335,1.31
904395332461,0.187154418642303,0.685502367111385
"4271","CEP19",44.1711197969411,-0.389590065083382,0.295419202564884,-
1.31877028202936,0.187245922249185,0.685568983808754
"4272","TMEM86A",8.60495216027747,0.769104891944347,0.583333447671195,
1.31846527061802,0.187347943529323,0.685568983808754
"4273","ZFHX2",14.8430094283034,0.662401253001508,0.502397004606005,1.
31848169262272,0.187342449594791,0.685568983808754
"4274","ZNF507",112.966908079083,0.313533259630823,0.237775472209236,1
.31861060654281,0.187299325944852,0.685568983808754
"4275","NDUFV1",447.71736883453,-0.330712374737015,0.250869299960143,-
1.31826562592377,0.187414743602695,0.685611674830918
"4276","C16orf74",26.1925831928667,0.674811840274888,0.511931437936316
,1.31816839183616,0.187447283988299,0.685611674830918
"4277","NME7",119.240624568824,-0.261789130704615,0.198679256442872,-1
.31764702260142,0.187621836703348,0.686065571018108
"4278","ZNF709",21.1893036755713,0.476457382548698,0.361627674369939,1
.31753573168543,0.1876591120726,0.686065571018108
"4279","CPT1A",262.782838291297,0.360961870790784,0.27399474161749,1.3
1740437301788,0.187703115894023,0.686066074452564
"4280","SERHL2",8.31707089339934,0.794485160542437,0.603146267805165,1
.31723464597327,0.187759984009869,0.686113586428585
"4281","WTAP",512.866452031774,-0.217996362075523,0.165524086639053,-1
.31700688704534,0.187836316054189,0.686168373009905
"4282","FIS1",1284.44699557922,-0.344467667079617,0.261595068603336,-1
.31679725049383,0.187906594731549,0.686168373009905
"4283","CLTRN",4.22116097541177,0.988245922228074,0.750488979072482,1.
31680271101307,0.187904763897849,0.686168373009905
"4284","LINC02693",53.8362679396065,0.370761653894662,0.28163034362209
2,1.3164833345947,0.18801186835869,0.686392535277756

"4285","AC012511.1",10.728190068031,0.700988444768158,0.53266101588449
1,1.31601229274149,0.188169916780151,0.686548106604832
"4286","BICD2",227.412961637362,0.261293591176238,0.198565631605915,1.
31590542161303,0.188205788832008,0.686548106604832
"4287","FGF13",3.19314080324118,1.58379727696024,1.20364602831251,1.31
583309353888,0.188230069125417,0.686548106604832
"4288","SLITRK4",154.559134196259,-0.499005091240422,0.379197922306898
,-1.31594890658858,0.188191192182952,0.686548106604832
"4289","ZNF548",52.0983008556671,-0.30417120275602,0.231200568859422,-
1.31561615205613,0.188302909519108,0.686653650006725
"4290","SENP2",129.742672996109,0.270239418335118,0.20545560790696l,1.
31531780070707,0.188403118096006,0.68673383377011
"4291","PURB",272.591091905792,0.301438781022143,0.229180604562409,1.3
1528923050754,0.188412716157771,0.68673383377011
"4292","HMGB3",12.1428570306097,-0.66921841034537,0.508885471654976,-1
.3150668423859,0.188487439024996,0.686846119839454
"4293","UNKL",105.877770831339,0.325781991922057,0.247842263299145,1.3
1447311522022,0.188687039652222,0.687105351486967
"4294","CTF1",51.9785627621984,0.488161898376308,0.371377380925007,1.3
1446319417844,0.188690376255532,0.687105351486967
"4295","SNRPD1",518.00899501857,-0.287776744374736,0.218914540827493,-
1.31456203542691,0.188657136322588,0.687105351486967
"4296","USP13",68.2937053480081,0.31537062887602,0.239952284794038,1.3
1430558849113,0.188743387377913,0.687138402837654
"4297","TMEM234",61.7406764754043,-0.377441957849292,0.287223777025409
,-1.31410415167649,0.188811157212988,0.687218089012649
"4298","TAF13",122.547146199107,-0.244919435491036,0.186477686436442,-
1.3133980808718,0.189048843857642,0.687218089012649
"4299","TRIM22",305.490319587177,0.327392821772765,0.249285115916166,1
.31332679277517,0.189072854029503,0.687218089012649
"4300","BHLHE41",55.5091975204788,-0.631027220178196,0.480243110558164
,-1.31397453978004,0.188854772317325,0.687218089012649
"4301","PITPNM2",48.7910673586545,0.351470612974788,0.267528876729312,
1.31376701188937,0.188924622044816,0.687218089012649
"4302","TPGS1",442.575246794887,-0.351968470444349,0.2679203947508,-1.
31370540406872,0.188945361668466,0.687218089012649
"4303","PARVG",10.7074107267685,-0.793736110552075,0.604279651671717,-
1.31352447224763,0.189006280163679,0.687218089012649
"4304","PDHA1",218.392002114248,-0.273168498002909,0.208033877030077,-
1.31309622212836,0.189150526720358,0.687340668658551
"4305","BAK1",46.376365275046,-0.346833127857721,0.264207786454873,-1.
31272863874116,0.189274403743331,0.687462153138468
"4306","ZNF688",206.785049182035,-0.332624685088903,0.253370939825409,
-1.31279729758316,0.189251260907522,0.687462153138468
"4307","ZNF780B",44.8656084566745,0.412336305732328,0.314135679753443,
1.31260576976153,0.189315824396891,0.687462153138468
"4308","CLHC1",18.2946105360331,0.674758659827807,0.514450351141829,1.
3116108451091,0.189651471923026,0.687722935514984
"4309","MAPK14",253.504699333027,0.242882004972261,0.185167655787249,1
.31168698949953,0.189625768385785,0.687722935514984

"4310","TRAF3IP2",104.53212774523,-0.290286257351426,0.221252144852914
,-1.31201556280689,0.18951488359559,0.687722935514984
"4311","NNMT",4447.59665366079,-0.417332674743225,0.318030752602643,-1
.31223999983628,0.1894391695136,0.687722935514984
"4312","NOP10",912.892501634933,-0.326579702478087,0.248932812234058,-
1.31191906582014,0.189547443816086,0.687722935514984
"4313","MCRIP1",870.880354858552,-0.334040014383489,0.254629003546331,
-1.31186946393053,0.189564182196258,0.687722935514984
"4314","LINC01094",6.41998316080028,0.89007357949781,0.678979889629726
,1.31089829476864,0.189892127062438,0.687808944727769
"4315","AJM1",8.00456480302365,-0.766315322572881,0.584576569557392,-1
.31088956088865,0.189895078218319,0.687808944727769
"4316","LINC02649",3.01428207548814,1.23250946971062,0.939997593130641
,1.31118364421101,0.189795726794531,0.687808944727769
"4317","AC110285.2",5.51443724751125,0.967295562256025,0.7378441147301
38,1.31097550681123,0.189866038778938,0.687808944727769
"4318","CENPX",367.153143510168,-0.376892956379824,0.28749828279289,-1
.31093985229586,0.189878085342357,0.687808944727769
"4319","TMEM54",395.151110473053,-0.326342337313106,0.249252525883137,
-1.30928397277752,0.190438176580205,0.689092267597878
"4320","PSRC1",5.60530483395508,1.02855906606764,0.786541390920137,1.3
076985876921,0.190975562566842,0.689092267597878
"4321","ADAMTSL4-
AS1",30.4836367741757,0.560961492101513,0.429352080096137,1.3065302769
1378,0.191372289740091,0.689092267597878
"4322","DNMT3A",74.5846573860626,0.435558392863528,0.333382522654177,1
.30648238364715,0.191388565944273,0.689092267597878
"4323","CBWD2",62.9794381725243,-0.602828253108286,0.461792719086806,-
1.3054087433435,0.191753702745046,0.689092267597878
"4324","HPSE",71.5093138885239,0.443397096467432,0.33952372864512,1.30
593846337875,0.191573485055247,0.689092267597878
"4325","ALPK1",94.965491318624,0.295709668025322,0.226582066134297,1.3
0508858476934,0.191862685322563,0.689092267597878
"4326","DDX60",80.3635779445285,0.341040845146789,0.261056861120907,1.
30638529737335,0.191421563185593,0.689092267597878
"4327","DCBLD1",33.3703387841778,0.416242169100338,0.31843389004252,1.
30715411303978,0.191160376252742,0.689092267597878
"4328","GARS-
DT",648.487552659138,0.436083853522552,0.33376538908325,1.306558042822
65,0.191362854149287,0.689092267597878
"4329","ZNF680",122.189153165782,0.266065062405249,0.20371211640928,1.
30608363947628,0.191524116006412,0.689092267597878
"4330","STEAP2",62.7972463983456,-0.476768184959716,0.364576044041122,
-1.30773316775015,0.190963829332019,0.689092267597878
"4331","AC005046.1",2.89945894859772,1.40396894896246,1.07429085943116
,1.30687973060282,0.19125356108097,0.689092267597878
"4332","TMEM168",111.700732372891,0.342508993515242,0.261932938242633,
1.3076209346301,0.191001912680915,0.689092267597878
"4333","ZFHX4",974.652563463441,0.271624371796491,0.207700164434324,1.
30777157801616,0.190950797105136,0.689092267597878

"4334","PAG1",9.72300052982644,0.818260337438937,0.626425796516004,1.30623665562603,0.191472090976239,0.689092267597878
"4335","HAS2-AS1",80.989813575322,0.311887596939187,0.238319102937681,1.30869742750225,0.190636863474841,0.689092267597878
"4336","ALAD",78.025134090503,0.305816993157635,0.233719422338179,1.30847915889136,0.190710838937505,0.689092267597878
"4337","ST6GALNAC6",441.872765580767,-0.265367573232031,0.203260326408721,-1.30555518590688,0.19170386860l587,0.689092267597878
"4338","PLPP7",34.1070757291218,0.370535168427213,0.28298543091143,1.30937895719156,0.190406015821988,0.689092267597878
"4339","H2AFY2",30.0278714710269,0.579509607295356,0.442640930933154,1.30920926375621,0.190463475106527,0.689092267597878
"4340","AC005383.1",5.46425048742273,-0.866641139598093,0.664044673982408,-1.30509463226423,0.19186062632l976,0.689092267597878
"4341","ZDHHC5",138.354352889492,0.222172059178012,0.169987053921388,1.30699399779443,0.191214749986087,0.689092267597878
"4342","AL354811.1",11.1104027532364,0.815713344575002,0.62471570666666,1.30573529026101,0.191642592473927,0.689092267597878
"4343","DNAJC3",619.680413849585,-0.208764045142238,0.159516643446636,-1.30872892402652,0.190626190436737,0.689092267597878
"4344","LIG4",48.5555794346128,-0.353292084733828,0.270663112475476,-1.30528346290941,0.191796343015414,0.689092267597878
"4345","PSMC6",565.188666138757,-0.195795815062343,0.149972543128098,-1.30554440818614,0.191707535915975,0.689092267597878
"4346","PDPK1",224.998776594658,0.206439704296261,0.15816903340575,1.30518407965915,0.191830173917232,0.689092267597878
"4347","MVP",514.966012983089,-0.240265289224343,0.18357640940124,-1.30880263977273,0.190601212544058,0.689092267597878
"4348","SNF8",689.100528244727,-0.340883191455318,0.260670231910775,-1.3077181424076,0.190968927461641,0.689092267597878
"4349","MYO15B",45.5575605726841,0.406563990836953,0.310938130310952,1.30753983253958,0.191029436014961,0.689092267597878
"4350","ZNF329",36.8463614101552,0.363400813163151,0.277697381052158,1.30862167942051,0.190662533573922,0.689092267597878
"4351","YTHDF1",125.182689127459,0.252103896251912,0.193106724806267,1.30551588249883,0.19171724254598,0.689092267597878
"4352","AF165147.1",16.5518624711065,0.668509918478545,0.511299904223944,1.30747123743983,0.191052717173465,0.689092267597878
"4353","SREBF2-AS1",7.85625117856834,0.757373413981662,0.580345457378511,1.30503892871464,0.191879592416161,0.689092267597878
"4354","DMD",90.7968197485384,0.28563026104498,0.218713922254875,1.30595372301963,0.191568295372641,0.689092267597878
"4355","ATG4A",45.6362757396741,-0.432962008219315,0.331348562061344,-1.3066663260158,0.19132605992436,0.689092267597878
"4356","SNHG10",29.6569502126194,-0.446948314907477,0.342548903346059,-1.30477228372834,0.191970399514963,0.689260112124432
"4357","SMC6",208.255986241411,-0.205328272596119,0.157432410624152,-1.30423126840325,0.192154741716228,0.689605360358376

"4358","AL512274.1",19.9046011357994,-0.772588596260093,0.592314380014
556,-1.30435562992934,0.192112356026294,0.689605360358376
"4359","TOR1AIP1",398.019671790758,0.223072628452736,0.171079807233584
,1.30390974867165,0.192264355992075,0.689840451414557
"4360","AL359258.2",11.2351350112725,-0.626698118114667,0.480975831356
373,-1.30297216046667,0.192584266198232,0.690088475843103
"4361","SREK1",525.848692279598,0.277432854510995,0.212929766817209,1.
30293128414103,0.192598222318104,0.690088475843103
"4362","ACTR1A",259.629516997594,-0.268618683810959,0.206112671461075,
-1.30326137595906,0.192485542595629,0.690088475843103
"4363","MED19",202.346525814762,-0.277854313952138,0.213194701283168,-
1.30328902303762,0.192476107223784,0.690088475843103
"4364","SPCS2",1590.47661172794,-0.270991783448641,0.207958669736478,-
1.30310404366424,0.192539243302065,0.690088475843103
"4365","GPATCH1",60.0918910880192,0.362627732089005,0.278288295507552,
1.30306497953006,0.192552578390465,0.690088475843103
"4366","LTBP4",5423.37550290483,0.266663005296773,0.204691829541437,1.
3027535387913,0.192658917321614,0.690147839420531
"4367","C1orf167",35.4727609099242,0.54879756495825,0.421306986597112,
1.3026073205927,0.192708857233178,0.69016865745979
"4368","SLC8B1",114.458955258201,0.273617556458352,0.210111359561473,1
.30225018309064,0.192830875308446,0.690286215275421
"4369","ASGR1",4.2196391925463,-1.02168518770688,0.784629876405099,-1.
30212373812208,0.19287408956225,0.690286215275421
"4370","RBM10",135.915000307326,0.240598649220566,0.184760657646419,1.
30221797370415,0.192841882620301,0.690286215275421
"4371","AC009236.1",57.4587944848269,0.543011825494546,0.4170976793272
27,1.30188167522393,0.192956837635256,0.690424374425854
"4372","HEG1",918.499150860487,0.316588517311244,0.243207672203824,1.3
0172093027527,0.193011801956566,0.690463079277378
"4373","ZSCAN20",13.6414485713322,0.577855678759411,0.444201840640359,
1.30088537662603,0.193297692286937,0.691071207669045
"4374","HMGN4",337.819823878068,-0.237178846825798,0.182292309696428,-
1.30109079873294,0.193227376905872,0.691071207669045
"4375","SWAP70",614.400273693673,0.289614132608552,0.222665207832103,1
.3006707937368,0.193371163445518,0.691071207669045
"4376","CARS2",187.749784228271,-0.285270253780241,0.219320188738131,-
1.30070220813486,0.193360406170351,0.691071207669045
"4377","APEX1",559.463802092275,-0.300061377495274,0.230727995517971,-
1.30049835010985,0.193430221380836,0.691071207669045
"4378","AP001469.3",3.11113012206791,1.1773745426524,0.905359600260568
,1.30044961395841,0.193446914781015,0.691071207669045
"4379","ATP8B4",21.5780015125483,0.76520719785888,0.588492369463085,1.
3002839757413,0.193503658089799,0.691116056753702
"4380","TP53",108.052999342407,-0.335443219895654,0.25811724621076,-1.
29957693575328,0.193746008896582,0.691823648206058
"4381","ADRA1B",181.284958189995,-0.427138553684514,0.328738177250178,
-1.29932749903718,0.193831561023063,0.691971151426776
"4382","MACF1",824.518723610174,0.309508850550122,0.238264039855592,1.
29901621217248,0.193938365492735,0.692194440051661

"4383","DPY19L1",116.58285356961,0.260834235354835,0.200839308851942,1.29872103646364,0.194039682025148,0.692240106494824
"4384","DIS3",254.212363198847,0.284858108844244,0.219320810716118,1.29881933189165,0.194005938647929,0.692240106494824
"4385","C6orf132",415.107268058662,-0.267954316435309,0.206393140925099,-1.29827142139647,0.194194083198234,0.692614444279446
"4386","NUTM2A-AS1",285.550637171143,-0.277426404571969,0.21370779818521,-1.29815761019416,0.194233181113149,0.692614444279446
"4387","CSF2",4.57888285707686,-1.64299638154829,1.26632438728687,-1.29745300496696,0.194475364936367,0.693007375422432
"4388","POP4",190.806229778751,-0.293513962844432,0.226223669373227,-1.29745027855679,0.194476302476282,0.693007375422432
"4389","VRK3",124.381131251691,0.256264914860289,0.197511965066252,1.29746526887285,0.194471147746453,0.693007375422432
"4390","BCL2",91.0535682367979,0.320186763313917,0.246825477804532,1.29721925857055,0.194555756149525,0.693132579995119
"4391","IFIT3",397.367241724064,0.292093285231456,0.225254310823133,1.29672672706718,0.194725229824859,0.693578363575675
"4392","AL138724.1",16.8736998148149,-0.644721526433798,0.497361638866334,-1.29628317918396,0.194877941530765,0.693963243596893
"4393","MAP3K3",215.263746916046,0.275062360321708,0.212214066143965,1.29615517632609,0.194922028716186,0.693963243596893
"4394","AC009309.1",3.03694832264601,-1.28058269264165,0.988364326375605,-1.2956585526894,0.195093146792434,0.693978766512258
"4395","RPP14",78.6047968745768,0.308993557591591,0.238442620668793,1.29588224087168,0.195016058514885,0.693978766512258
"4396","ZSCAN9",29.0505447712568,0.373051181876049,0.287886148029506,1.29582886995249,0.195034449375774,0.693978766512258
"4397","AC121761.2",10.3398715564279,0.673124548655236,0.519587204474239,1.29549870139,0.195148249048652,0.693978766512258
"4398","UBR1",145.633372407581,0.328441190024353,0.253518645012839,1.29553070941871,0.195137214664385,0.693978766512258
"4399","GALNT4",94.7523049975143,0.408706096079623,0.315515961436863,1.29535790905275,0.195196790952424,0.693993591838126
"4400","CAPZA2",682.71022475116,-0.23414140956802,0.180817981778077,-1.29490113353542,0.195354337424515,0.69439587211805
"4401","PABPC5",71.2616938777149,0.322404839207069,0.249069679382626,1.29443632001382,0.195514751973183,0.694808161976955
"4402","AP006621.3",45.9382120392163,0.333750930667447,0.25786227969493,1.29429915481356,0.195562108316098,0.6948185765706
"4403","MOCS2",251.27126822302,-0.249521107700412,0.1928266589913,-1.29401768928471,0.195659310737604,0.695006045863305
"4404","MEGF6",65.8960590978356,0.460159049917417,0.355872962717596,1.29304301850708,0.195996181053094,0.695043603431381
"4405","ATP2C1",211.920606104143,0.216070115079539,0.1671745844129,1.29248184368667,0.196190329641272,0.695043603431381
"4406","DAPP1",4.58896193827467,-1.05408532033658,0.815400497275626,-1.29272096823394,0.196107582953538,0.695043603431381
"4407","HNRNPA0",1216.33209053356,-0.190153667458766,0.147119119394268

,-1.2925149922164,0.196178857392938,0.695043603431381
"4408","ZNF346",74.8776436563828,0.291388737069279,0.225469097283452,1
.29236662842072,0.196230207876863,0.695043603431381
"4409","RSPH3",22.8773725449929,-0.554477788154835,0.428826988688113,-
1.29301047457652,0.196007436367964,0.695043603431381
"4410","NACAD",35.5410199326351,0.582919050045372,0.450885536137915,1.
29283155773502,0.196069323198083,0.695043603431381
"4411","OTUD1",275.949764580735,-0.421916281971788,0.326092950565612,-
1.29385281478784,0.195716265595614,0.695043603431381
"4412","ZNF25",118.251271132875,0.264040181599923,0.204315435476219,1.
29231636848433,0.196247605674743,0.695043603431381
"4413","LINC02550",14.3122185002855,0.763584705044261,0.59074604876092
5,1.29257691464185,0.196157428213339,0.695043603431381
"4414","PIP4P1",132.407543216729,-0.297333231764714,0.22996109524828,-
1.29297188919584,0.19602078173138,0.695043603431381
"4415","EIF4A3",557.272518418611,-0.28349212617854,0.219327624165071,-
1.29255093724618,0.19616641787002,0.695043603431381
"4416","LINC01431",5.15566213484551,0.855653398596088,0.66209357136321
1,1.29234512401978,0.196237651624236,0.695043603431381
"4417","ATOH8",105.69132095834,0.567742389966495,0.439500990243915,1.2
9178864796506,0.196430347732324,0.695061230437456
"4418","SENP6",417.152928971593,0.224618295592862,0.173857459591174,1.
29196812216773,0.196368184404117,0.695061230437456
"4419","DRD4",5.68652078135263,0.837691549878451,0.648345816831934,1.2
9204435060865,0.196341786008835,0.695061230437456
"4420","AP002807.1",12.9273928524222,-0.63743937965263,0.4934168818938
14,-1.29188806269869,0.196395912308124,0.695061230437456
"4421","MIIP",77.054165871235,-0.324740658046885,0.251505869928795,-1.
29118520430089,0.19663946435613,0.695158463185119
"4422","PTPN18",204.985325720797,0.2438921905556,0.188867938547869,1.2
9133717681673,0.196586784633877,0.695158463185119
"4423","EFNA5",313.465925722148,0.266533617477043,0.206458343741929,1.
29098012047509,0.196710570961133,0.695158463185119
"4424","DPP3",59.9442277929703,-0.334929671664965,0.259432086511053,-1
.29101097774463,0.196699870934209,0.695158463185119
"4425","NSRP1",436.627548923957,-0.211600505132513,0.163911969733199,-
1.29093992023241,0.19672451138474,0.695158463185119
"4426","PCIF1",106.557514135314,-0.265312898746444,0.205501361652507,-
1.29105177996375,0.196685723063868,0.695158463185119
"4427","RPL31",5083.82538607645,0.181781506688483,0.140839330948247,1.
29070129391115,0.196807275828678,0.695240388743412
"4428","TMEM258",1684.13040785533,-0.303944811557831,0.235664671552087
,-1.28973430576612,0.197142924141436,0.695240388743412
"4429","WNT1",14.1427445627383,-0.681960003357621,0.52867722641 5327,-1
.28993640974782,0.197072737826419,0.695240388743412
"4430","TMTC4",42.3523926207626,0.473330661319943,0.366881341501595,1.
29014645275408,0.196999813848299,0.695240388743412
"4431","SYNJ2BP",221.804092532858,0.224271094380108,0.173844392663643,
1.29006803696011,0.197027036397831,0.695240388743412
"4432","SLC25A29",53.3880620923601,0.424019759082219,0.328623392388583

,1.29029085848166,0.196949689672199,0.695240388743412
"4433","USP8",505.74213339156,0.22407250639902 5,0.173737280150891,1.28972035365362,0.197147770081652,0.695240388743412
"4434","AC068338.2",5.97324062683608,-0.738481863488333,0.572211754078355,-1.29057443896409,0.196851284200354,0.695240388743412
"4435","SETD1A",119.759941249405,-0.246092166564849,0.190730661689342,-1.29026012065998,0.196960358189548,0.695240388743412
"4436","CEP85",13.1771023791838,-0.535031799436688,0.414986506543109,-1.28927517160395,0.197302439447006,0.695472200349598
"4437","SERBP1",1722.94946875729,-0.151092000364071,0.117189881369294,-1.2892922033767,0.19729652047362,0.695472200349598
"4438","MRPL15",243.052104946019,-0.345884215756292,0.268390058678556,-1.28873706224175,0.197489513040133,0.695504612462887
"4439","TRAP1",146.062317038866,-0.281958970047753,0.218734674149306,-1.28904560351182,0.197382232869773,0.695504612462887
"4440","MMP28",113.086486123733,-0.368770409695312,0.286130529929198,-1.28881881212244,0.197461084353951,0.695504612462887
"4441","LSM7",717.471818391447,-0.328263528981426,0.254692900864109,-1.28886014438451,0.197446712116282,0.695504612462887
"4442","RPS6KA1",4.75187750101152,-0.975352522482381,0.757213802493457,-1.28808074981017,0.197717855575087,0.695634153297131
"4443","OXCT2",4.67026310983026,1.30478976241015,1.01340625279258,1.28752882549779,0.197910029148489,0.695634153297131
"4444","GATAD2B",199.317098604674,0.309092813602881,0.240014870715306,1.28780692913612,0.197813179649934,0.695634153297131
"4445","AL356512.1",8.5739292030518,-0.688939195015448,0.535212335967002,-1.28722592645534,0.198015553099669,0.695634153297131
"4446","TTLL3",221.732857274407,0.255430250596505,0.198301142264377,1.28809268408532,0.197713701709046,0.695634153297131
"4447","NHP2",530.945902911881,-0.299575247685712,0.232587747197676,-1.28800958474869,0.197742626744653,0.695634153297131
"4448","AC018638.7",8.31499624553309,0.912902685373637,0.70916965498522,1.28728390866169,0.197995350077359,0.695634153297131
"4449","AQP3",98.5921100350471,-0.925960852277607,0.719254694633079,-1.28738937567863,0.197958605557283,0.695634153297131
"4450","VEGFB",1474.73511793041,-0.34341441374417,0.266636454998645,-1.28794996822889,0.197763379839994,0.695634153297131
"4451","PATL2",8.26416659466144,0.828346801096305,0.643500998769957,1.2872502182276,0.198007088815715,0.695634153297131
"4452","SECISBP2L",272.217598821826,0.287741950089264,0.223499964659695,1.28743622186869,0.197942286028536,0.695634153297131
"4453","THAP6",82.0438754122819,0.289779973512731,0.225234291357067,1.28657129323767,0.198243754768136,0.695906253364279
"4454","MDM1",98.6501839207104,0.27841056602284 3,0.216380211863038,1.28667295232647,0.198208304339187,0.695906253364279
"4455","RB1",138.175232783409,0.290692626525281,0.225973504923296,1.28640137092157,0.198303020216344,0.695906253364279
"4456","RTF2",662.438815402879,-0.260495411137601,0.202504951192642,-1.28636563996795,0.198315484094923,0.695906253364279
"4457","AP001605.1",3.16853224307186,1.28752165392625,1.00053033789744

,1.28683919433358,0.19815034262729,0.695906253364279
"4458","ZRANB2-
AS2",9.40513266913652,0.948492362766407,0.738513339505866,1.2843266492
668,0.19902768612028,0.695988635348783
"4459","PEAR1",21.1130392452121,0.913796417454183,0.711594378593787,1.
28415350787337,0.199088248925067,0.695988635348783
"4460","ZBTB41",184.968894181117,0.1967656220472,0.153375441457732,1.2
8290174865724,0.199526500149253,0.695988635348783
"4461","RAP2B",207.538477713235,-0.230698353534197,0.17952640021308,-1
.28503859744517,0.198778796609722,0.695988635348783
"4462","ZDHHC11",12.6447721211316,-0.940007465895928,0.732815083133679
,-1.28273487750313,0.199584976313254,0.695988635348783
"4463","FAM174A",133.190857940549,-0.293442115339383,0.228291543797209
,-1.28538320105298,0.198658408602948,0.695988635348783
"4464","LYRM4",246.89624507129,-0.246432505561499,0.191987479649515,-1
.28358633600158,0.199286733234328,0.695988635348783
"4465","C8orf58",46.2220202657359,-0.375760663382725,0.292330856732825
,-1.28539514296347,0.198654237626834,0.695988635348783
"4466","SARAF",1197.76151280769,-0.245631324731128,0.191019183051877,-
1.28589872915758,0.19847840729269,0.695988635348783
"4467","DDHD2",117.590832752674,0.259775113180126,0.202383111404158,1.
28358098350093,0.199288607054099,0.695988635348783
"4468","GSN-
AS1",4.43732599946877,1.14328581866193,0.889360684739494,1.28551423317
843,0.198612646240567,0.695988635348783
"4469","PPP1R26-
AS1",4.61183282636395,1.03558153503041,0.80645292362526,1.284119016365
07,0.19910031525393,0.695988635348783
"4470","NACC2",64.4788959087367,0.388548736741786,0.302829399325638,1.
28306147820203,0.199470538323361,0.695988635348783
"4471","REXO2",1299.02795655471,-0.291797451194471,0.227277813976757,-
1.28388005009724,0.199183928641981,0.695988635348783
"4472","POU2F3",2.52162608252581,2.22304028719687,1.73137887849111,1.2
8397100993529,0.199152099116501,0.695988635348783
"4473","CD4",10.6238062723331,-0.677254274647015,0.52782071433357,-1.2
8311423984585,0.199452055609591,0.695988635348783
"4474","CSRNP2",72.5673834083078,0.34121583273763,0.265460632284369,1.
28537263624125,0.198662098650265,0.695988635348783
"4475","ANAPC5",576.346477726171,-0.197458334058453,0.153788002335875,
-1.28396449046266,0.199154380348261,0.695988635348783
"4476","DLEU2",78.6934204608998,0.360541370665276,0.280446075635682,1.
28559962854906,0.198582826451743,0.695988635348783
"4477","IRF9",347.601695783471,-0.348465565101071,0.271658220081592,-1
.28273521410988,0.199584858344548,0.695988635348783
"4478","KHNYN",120.821318480192,0.30321078097165,0.235930837252182,1.2
851680793535,0.198733555575129,0.695988635348783
"4479","OIP5-
AS1",462.647709357537,0.206430397527854,0.160719665924639,1.2844128087
2652,0.198997553581535,0.695988635348783
"4480","NPIPB4",15.2719294818492,0.685396242471425,0.533969507940058,1

.28358685707643,0.199286550815544,0.695988635348783
"4481","MIEN1",392.707161441106,-0.296994988685475,0.231327932031268,-1.2838699852525,0.199187450855915,0.695988635348783
"4482","ATP5F1D",1575.7017734087,-0.317405871407005,0.247311841019485,-1.28342367311882,0.199343684502336,0.695988635348783
"4483","UQCR11",1638.29669315568,-0.270599620709721,0.21094182682749,-1.28281633272769,0.199556430636475,0.695988635348783
"4484","AC008764.7",7.93479635194045,0.821944235279736,0.640170364818762,1.28394608755817,0.199160819820333,0.695988635348783
"4485","RPL13A",21749.4474427521,0.217168287353804,0.168916130962534,1.28565748052785,0.198562626586851,0.695988635348783
"4486","FKBP1A",1885.01788115735,-0.335125313574857,0.261296824537073,-1.28254644566991,0.199651022971075,0.696063753737766
"4487","HBA2",54.784091862612,2.08908523657888,1.62909362824836,1.28236044899709,0.199716231743839,0.696135918090849
"4488","WDTC1",139.509034887346,0.263210649002185,0.205325221098057,1.28192068950206,0.199870469365058,0.696518302332779
"4489","RPS7",8905.23428581843,-0.350525473533956,0.273586771793923,-1.2812223019247,0.20011559481699,0.696567553108305
"4490","HAVCR2",3.82662053255841,-1.30007048674838,1.01445620072012,-1.28154422618297,0.200002576103562,0.696567553108305
"4491","ERGIC1",792.729612458019,0.213889591655997,0.166890524560041,1.28161615058647,0.19997733180391,0.696567553108305
"4492","MAMDC4",6.41992122061093,-0.812645961881458,0.634325057408487,-1.2811191240048,0.200151827600302,0.696567553108305
"4493","AC079414.3",12.0028135097134,0.79642740666377,0.621638907987762,1.28117367885127,0.200132669087505,0.696567553108305
"4494","ABHD17A",583.883989230095,-0.283924847554442,0.221563224183954,-1.2814619781788,0.200031446671654,0.696567553108305
"4495","S100A8",32.1498478073542,0.936684478349273,0.731320691462467,1.28081221997989,0.200259630784896,0.696735895427354
"4496","CCDC102A",120.78756064611,-0.281481968168801,0.219782822530042,-1.28072778813424,0.200289295769909,0.696735895427354
"4497","ZNF791",283.023444608429,0.287629035313797,0.224643717849603,1.28037871731789,0.200411975255711,0.697007625750349
"4498","DCLRE1B",18.235620257396,0.475347658840885,0.37144706362831,1.27971844547018,0.200644175033962,0.69759179344719
"4499","MELTF",142.869130493997,0.574454981335701,0.448921659513167,1.27963302541176,0.200674229299623,0.69759179344719
"4500","MRPS17",195.464131419006,-0.301442114624061,0.235589867499734,-1.27952071039049,0.200713751311532,0.69759179344719
"4501","LZIC",407.425836233418,0.181474605937676,0.141914747565337,1.27875790959728,0.200982320049682,0.69786032544841
"4502","ZCCHC4",28.5976499261759,0.457102342223725,0.357511253884695,1.27856770173492,0.201049329713801,0.69786032544841
"4503","VTA1",186.809233984337,-0.200602555205615,0.156824649415252,-1.27915194424854,0.200843554661582,0.69786032544841
"4504","AC004839.2",5.77565555837148,-0.819982172645653,0.64134209895972,-1.27854100639221,0.201058735707835,0.69786032544841
"4505","CAPRIN2",72.9766761962481,0.317634972344998,0.248395917721904,

1.27874473645985,0.200986960381551,0.69786032544841
"4506","OAS1",130.346885631287,-0.447917113024882,0.350229265009769,-1
.27892542906826,0.200923316935239,0.69786032544841
"4507","CNOT7",256.252328395144,0.199749901494102,0.156340070208279,1.
27766286165787,0.201368325390481,0.69852247393343
"4508","GPAA1",426.8569301861,-0.31588254071192,0.247242978700443,-1.2
776198635539,0.201383493284261,0.69852247393343
"4509","LGALS1",15520.1409057387,-0.362485305266174,0.283715728151684,
-1.27763556721951,0.201377953605458,0.69852247393343
"4510","ATP6V1D",334.916956833266,-0.238518599065162,0.18671231861721,
-1.27746578710836,0.201437851713403,0.698556097737833
"4511","TBC1D7",53.897106509404,-0.399386024439706,0.312717112665515,-
1.27714796620962,0.201550013262431,0.698564334894329
"4512","AC092171.5",3.50724253416884,-1.08100952060402,0.8464701057778
25,-1.27707938322368,0.201574222722385,0.698564334894329
"4513","PRAF2",212.045885511529,-0.353280770618524,0.276602110181419,-
1.27721646948685,0.201525834055995,0.698564334894329
"4514","TTLL4",17.9040767286115,0.598331013336211,0.468765478815779,1.
27639734659589,0.201815093740623,0.698756535741942
"4515","PPIL1",32.4122348500144,-0.604544083716192,0.473560194064397,-
1.27659396058526,0.201745635172462,0.698756535741942
"4516","ARHGEF35",34.0567215365361,-0.45701464099684,0.358080599756318
,-1.2762898668843,0.201853070874814,0.698756535741942
"4517","AL139317.3",5.22690143091867,-0.88607141647862,0.6939877542831
67,-1.2767824950944,0.201679047244769,0.698756535741942
"4518","PAF1",282.149550172128,0.226488618518824,0.17745589316204,1.27
630936613647,0.201846180576323,0.698756535741942
"4519","SH3YL1",196.23619303484,-0.253719832513097,0.198913856002205,-
1.27552618813184,0.202123060959503,0.69878465224281
"4520","U73169.1",2.65408278624045,1.72242789826787,1.35005128595092,1
.27582404919874,0.20201772421543,0.69878465224281
"4521","TRA2B",932.252113315701,0.225172226728981,0.176541264900208,1.
27546512627664,0.202144660054851,0.69878465224281
"4522","HMGCLL1",19.045650255987,-0.748988286824308,0.587017982388445,
-1.27592051571715,0.201983618001454,0.69878465224281
"4523","FOXP2",65.2777423838788,0.418738657857272,0.328356534562815,1.
27525605182426,0.202218627624741,0.69878465224281
"4524","WAC-
AS1",372.422063271374,0.292779362632602,0.229568378035438,1.2753470889
0702,0.202186417574402,0.69878465224281
"4525","BRF1",60.3057190325284,0.319889271453641,0.250777726152836,1.2
7558885057713,0.202100897444593,0.69878465224281
"4526","DYNLRB1",1659.43478118816,-0.316708330663942,0.248222600208895
,-1.27590449216716,0.201989282915964,0.69878465224281
"4527","PLD2",60.4002742719936,-0.293722696809926,0.230393682608619,-1
.27487304983483,0.202354179448493,0.699043160206773
"4528","AC104564.5",11.6945674186025,0.747070036062336,0.5860328284557
35,1.27479212731299,0.202382827967792,0.699043160206773
"4529","NES",43.9415640404806,-0.465804722047587,0.365466151463437,-1.
27454955864548,0.202468720819166,0.699185425836112

"4530","MRPL33",936.746846794985,-0.281417789662929,0.220905395124114,
-1.27392900252534,0.202688578807256,0.69931170926384
"4531","TFG",767.570680341696,-0.227697934054639,0.178701101089543,-1.
27418316208664,0.202598511120522,0.69931170926384
"4532","NDUFS6",933.501938096364,-0.289650418538833,0.227475307016198,
-1.27332686056429,0.202902079021459,0.69931170926384
"4533","SETD9",60.5042545338076,-0.298091223701266,0.233980471268014,-
1.27400044151469,0.202663259698925,0.69931170926384
"4534","AIRN",3.26515541934078,1.46893870931206,1.15350231243544,1.273
45970049304,0.202854964169265,0.69931170926384
"4535","LINC02680",2.60078328491651,1.30797716581437,1.02652048860188,
1.27418515298787,0.202597805710865,0.69931170926384
"4536","SDR39U1",113.336441956104,-0.315404628241534,0.247671435859091
,-1.27348003271956,0.20284775356298,0.69931170926384
"4537","ARID3A",37.6439315059135,0.373334715475532,0.293258000966996,1
.27305892505742,0.202997132996025,0.69931170926384
"4538","JUND",11991.9252046601,-0.272695433690004,0.214187186434322,-1
.27316408712257,0.202959821375067,0.69931170926384
"4539","LRRC4B",3.84953348407813,1.50772824718579,1.18414583143446,1.2
7326230195765,0.202924979150452,0.69931170926384
"4540","LIF",24.2434739196989,-0.428159167001424,0.336199023250786,-1.
27352888435384,0.202830429617479,0.69931170926384
"4541","ATF6",235.100856235057,0.24497351217283,0.192548648044222,1.27
226814969154,0.203277861033849,0.699356506663497
"4542","CACNA2D1",180.12512139751,0.302539351441754,0.237752035330825,
1.27249952254154,0.203195693618699,0.699356506663497
"4543","TMEM259",378.870417567947,-0.185002520240934,0.145354420668062
,-1.27276844688071,0.20310022094257,0.699356506663497
"4544","LMTK3",6.0688676580138,-0.82431083699847,0.647907344891134,-1.
27226654165647,0.203278432179812,0.699356506663497
"4545","CSNK2A1",387.577261946993,0.21722086465088,0.17070338224557,1.
27250474942782,0.20319383767454,0.699356506663497
"4546","RBM38",32.6301097302213,0.395302987686787,0.310605881630736,1.
27268352296285,0.203130366835806,0.699356506663497
"4547","ABT1",139.852628709504,-0.297319477940834,0.233776424700328,-1
.27181121159655,0.203440204475331,0.699434248152445
"4548","RAD21",524.460971712399,0.212800664077583,0.167352208838902,1.
27157368016834,0.203524633205996,0.699434248152445
"4549","FAM86C1",11.1163188963112,-0.558892442798677,0.439498257396782
,-1.27166020204286,0.203493876714063,0.699434248152445
"4550","HSD11B2",49.8163194961813,-0.680070733380194,0.534635169719503
,-1.27202767774704,0.203363285446255,0.699434248152445
"4551","ITCH",186.217312179866,0.292557883854466,0.230028235379539,1.2
7183466573899,0.203431869263064,0.699434248152445
"4552","AGO2",326.398601670042,0.300691370396527,0.236504568270377,1.2
713977264607,0.203587191050258,0.69948905931372
"4553","VTI1A",97.8025004219742,0.270500017189622,0.212798077392247,1.
27115818199341,0.203672380184828,0.69948905931372
"4554","ATL1",178.438149307964,-0.2138390637695,0.168224686926251,-1.2
7115150384109,0.203674755506054,0.69948905931372

"4555","SLC50A1",122.017967823685,-0.266141988282749,0.209425070948466
,-1.27082200367615,0.203791978944107,0.699698336935962
"4556","LINC01266",7.32785041329216,0.804935629685254,0.63347333057564
8,1.27067011479993,0.20384603167404,0.699698336935962
"4557","C12orf73",55.9262407713427,0.383717727830749,0.301996545113235
,1.27060303847805,0.203869905461457,0.699698336935962
"4558","HMOX2",281.4516137752,-0.272367809807237,0.214396064550914,-1.
27039556615815,0.203943761837467,0.69979825255331
"4559","AP003100.2",3.22936535923938,-1.09111870238664,0.8590859094454
82,-1.27009265358679,0.204051628172373,0.700014798117111
"4560","CAPNS1",1285.77896747516,-0.201459831136168,0.158641137541364,
-1.26990914373417,0.204116995716345,0.700085485307815
"4561","NPEPL1",210.927010370181,-0.22660608289697,0.178482997443486,-
1.26962279960992,0.204219024009346,0.70028185387112
"4562","AC007193.1",4.13416724573424,1.39828628789166,1.10146023832732
,1.26948412592278,0.204268448657447,0.700297794169764
"4563","DCBLD2",380.645789698033,0.314940510872068,0.248189300954153,1
.26895281005786,0.204457895332803,0.700314767219429
"4564","SEPSECS-
AS1",31.5559243802186,0.464993276330326,0.366506733183773,1.2687168726
5066,0.204542062446186,0.700314767219429
"4565","NKAPL",14.3236874185571,0.570207801777906,0.4493696159357,1.26
890599977613,0.204474592191568,0.700314767219429
"4566","ROR2",9.85441519301164,1.0013285723295,0.788932638393642,1.269
21935232433,0.204362840706711,0.700314767219429
"4567","BAHD1",43.7189335316822,0.366932954147964,0.289199590985092,1.
26878794295003,0.204516706536403,0.700314767219429
"4568","DDX42",385.729699280045,0.221179248257995,0.174282910594769,1.
26908167589803,0.20441193502589,0.700314767219429
"4569","PPM1M",103.937703073236,0.269426962246922,0.212393849720866,1.
26852525438477,0.204610437916648,0.700395545856069
"4570","ATAD3A",64.3946254751639,-0.406801573632024,0.320779619752598,
-1.26816527167708,0.204738936103434,0.700409119199088
"4571","TMEM237",307.395484727017,-0.263732592203365,0.20795806677435,
-1.26820082670577,0.204726241891649,0.700409119199088
"4572","TWIST2",646.704153286821,-0.304923592118682,0.240449892379939,
-1.26813777748283,0.204748752747969,0.700409119199088
"4573","IFITM1",417.242181261721,-0.969836374906519,0.765050294539443,
-1.26767662443729,0.204913455820363,0.700626031291588
"4574","ARID2",137.168844726847,0.288776901150912,0.227785089946551,1.
26776033154177,0.204883552263498,0.700626031291588
"4575","CARD10",9.42442432970838,-0.888253221235241,0.700745063196166,
-1.26758398722616,0.204946553271037,0.700626031291588
"4576","XRN2",465.073299943054,-0.179393528189401,0.141598133517821,-1
.26692014741016,0.205183843891556,0.701283941972013
"4577","ARNT",310.252419585646,0.22918777282493,0.180967892137899,1.26
645544752373,0.20535007041486,0.701420164944214
"4578","ZFP62",63.046358244399,0.278819443370787,0.220182314323838,1.2
6631171185124,0.20540150552251,0.701420164944214
"4579","PTPRD-

AS1",35.1142598470455,0.645962804082402,0.510115368172826,1.2663072794
6144,0.20540309178033 9,0.701420164944214
"4580","LINC02175",12.2477065379703,0.577081165657214,0.45564094737657
,1.26652612979553,0.205324780543414,0.701420164944214
"4581","SYNPO",5.43446703533239,-0.920265267840277,0.726968512548273,-
1.26589426083178,0.20555094141446,0.701618665151058
"4582","TBC1D2",15.3862530209162,-0.478139429808302,0.377680144632246,
-1.26599037996523,0.205516526430015,0.701618665151058
"4583","PDGFC",129.611247390403,-0.45090890614093,0.35633096513077,-1.
26542161716272,0.205720230091509,0.70203133601868 2
"4584","RASSF9",19.4677301671737,-0.49510639111218,0.391332500903608,-
1.26518086274192,0.205806501000362,0.702031336018682
"4585","DUSP18",90.3704731589835,-0.317404266882664,0.250852728527341,
-1.26530123370003,0.205763364501297,0.702031336018682
"4586","MCUR1",215.031346872312,-0.262197713027317,0.207314798289212,-
1.26473225833856,0.205967322096042,0.702273581334664
"4587","PDCD5",1008.98528221462,-0.305923020469036,0.241887488822808,-
1.26473271502329,0.205967158331806,0.702273581334664
"4588","CAMSAP2",204.945117513203,0.264259092997156,0.209049324082059,
1.26409924623064,0.206194407144434,0.702408352259319
"4589","S1PR3",307.043436615844,-0.438909452590087,0.347173932631604,-
1.26423504571072,0.206145675492896,0.702408352259319
"4590","SSH2",162.076071008797,0.341673604176854,0.270294073247349,1.2
6408100655683,0.206200953089767,0.702408352259319
"4591","AC027097.2",27.7745922563062,0.477986414735146,0.3781549566599
16,1.26399616431581,0.206231403681253,0.702408352259319
"4592","ZNF570",56.7432398483598,0.316277662053569,0.250141201403486,1
.26439650996719,0.206087744921363,0.702408352259319
"4593","CGNL1",15.4174027951022,0.913233743313843,0.722703767081483,1.
26363495654905,0.206361080726107,0.702696995984391
"4594","AC087071.1",4.47219721213364,1.15262397253655,0.91269584212734
9,1.2628785180504,0.206632841209362,0.702752718034921
"4595","HIKESHI",508.970258770549,-0.347635335716033,0.275159345350927
,-1.26339643406504,0.206446745034817,0.702752718034921
"4596","HOXC9",370.478410991548,-0.279048146149371,0.220956021692149,-
1.2629126104477,0.2066205874785,0.702752718034921
"4597","SNHG21",29.5552141449362,-0.479298993848015,0.379435602828412,
-1.26318930083312,0.206521156976292,0.702752718034921
"4598","FANCB",5.74146794883435,-0.79647397500842,0.630701118644513,-1
.2628390079919,0.206647042854386,0.702752718034921
"4599","TRO",14.0778874885466,0.719507717225855,0.569659203778007,1.26
304940296592,0.206571425848141,0.702752718034921
"4600","C1orf174",172.436751861637,-0.256109012358819,0.20288556168768
2,-1.26233237214321,0.206829212767431,0.702901854569202
"4601","TMEM50A",1710.2173669672,-0.278116596858459,0.220380351161713,
-1.26198454350579,0.206954348198744,0.702901854569202
"4602","EVA1B",511.546782660174,-0.317625038970239,0.251604671845682,-
1.26239722275526,0.206805888075119,0.702901854569202
"4603","CNTN4",212.99078148874,0.62227250701005,0.493292388395548,1.26
146788729908,0.207140322743571,0.702901854569202

"4604","PBRM1",409.000517634995,0.205702249078016,0.163025672876583,1.26177825521837,0.207028588783077,0.702901854569202
"4605","SLC26A2",204.729038202985,0.298296955961516,0.236421855927827,1.26171480547288,0.207051427442231,0.702901854569202
"4606","DBN1",2449.77975460658,-0.203096997984341,0.160987852648681,-1.26156722164344,0.207104557135852,0.702901854569202
"4607","TM9SF1",392.621153683328,-0.260037442844726,0.206008676178632,-1.26226452044789,0.206853618897569,0.702901854569202
"4608","YLPM1",180.21595569595,0.304013070375432,0.24088030305307,1.2620918627309,0.206915732988767,0.702901854569202
"4609","AC007906.2",9.10455580458694,-0.695878138499479,0.551615478463385,-1.26152757793882,0.207118830424847,0.702901854569202
"4610","ABR",122.310707014154,0.320808183406638,0.254341665903508,1.2613276800992,0.207190812364606,0.70292067361875
"4611","CEP63",255.896748586871,-0.196278343904165,0.155663446050765,-1.26091480616558,0.207339543104675,0.702967798429897
"4612","CNP",245.612262267812,0.218961004711617,0.173648151194055,1.26094636312554,0.207328172518698,0.702967798429897
"4613","EMID1",13.7729324736998,0.851795714701696,0.67541287169999,1.26114818119731,0.207255464244292,0.702967798429897
"4614","SMYD3",69.0758407161112,-0.313623489076035,0.248830643407473,-1.26038933461447,0.207528947215483,0.70307638676439
"4615","TADA3",403.465557384785,-0.302887366159665,0.240389417233859,-1.25998627412539,0.207674313789357,0.70307638676439
"4616","TGFBR2",2086.54753571707,0.194751317573445,0.154678894261361,1.25906846246504,0.208005604502964,0.70307638676439
"4617","CXCL2",315.36576274989,-0.908921176280001,0.721625450987516,-1.25954700604888,0.207832822973804,0.70307638676439
"4618","PRKG2",9.67831693593475,0.747812259293616,0.593758248381019,1.2594557824378,0.207865751868267,0.70307638676439
"4619","FRG1",508.295996642566,-0.268820486936388,0.213328334135353,-1.26012556196978,0.207624070308497,0.70307638676439
"4620","CFAP69",22.0626118799249,-0.451876454793856,0.358715554925075,-1.25970688638869,0.207775120255387,0.70307638676439
"4621","SUSD1",19.4170250959404,0.625602203989422,0.496487505703654,1.26005628903547,0.207649057125073,0.70307638676439
"4622","SLC25A25-AS1",5.72826630763062,-0.792058861388177,0.629079599747558,-1.25907573811966,0.208002976797123,0.70307638676439
"4623","WASF3",110.630159503453,0.307871633155994,0.244392224197912,1.25974397985214,0.207761734443511,0.70307638676439
"4624","GPATCH2L",234.626111789402,0.31964633058117,0.253657938392171,1.26014715962477,0.207616280458785,0.70307638676439
"4625","AC130650.2",10.0533772885126,0.798759349686474,0.634461203301954,1.25895696305693,0.208045877106496,0.70307638676439
"4626","C1QBP",617.276459445822,-0.270054806081691,0.214455632317311,-1.25925723266673,0.207937435257212,0.70307638676439
"4627","SERTAD1",1165.09047409057,-0.443763052948281,0.352393126010761,-1.25928407847187,0.207927741937771,0.70307638676439
"4628","RAB4B",82.9963799969187,-0.359283743244854,0.285351759565983,-

1.25909068789806,0.207997577548201,0.70307638676439
"4629","ZNF687",51.9785054266154,0.331293118242675,0.263222876849691,1
.2586030599151,0.208173741254529,0.703356516141894
"4630","FASTKD3",36.141387136367,-0.404982096606324,0.321834573076784,
-1.25835485210504,0.208263451981273,0.703507643409743
"4631","PLGRKT",192.371179731488,-0.350296356578674,0.278437227243597,
-1.25808017859699,0.208362760975186,0.703539201565612
"4632","KIAA2026",333.802660492774,0.288846078029458,0.229591683350012
,1.25808598035806,0.208360662977021,0.703539201565612
"4633","ARPC5",1306.66918425503,-0.211869856309537,0.168559370085733,-
1.2569449933384,0.208773554562073,0.703724043521811
"4634","HHAT",9.99329656594437,0.642151369317692,0.510846546732836,1.2
5703378720014,0.208741401264719,0.703724043521811
"4635","RAB3GAP1",302.637222223511,0.214845080400706,0.170843668091765
,1.25755366177989,0.208553220599092,0.703724043521811
"4636","ITM2C",598.287529047468,0.329091378168317,0.261797279510701,1.
25704659262842,0.208736764565244,0.703724043521811
"4637","HOTAIRM1",75.1322658547298,-0.538167534611806,0.42808603817693
2,-1.25714806514988,0.208700025161973,0.703724043521811
"4638","PYGM",10.3136156947398,0.93869300522331,0.746811575470745,1.25
693419338287,0.208777465597264,0.703724043521811
"4639","MPP5",89.1473895733736,0.307039220707203,0.244243503671897,1.2
5710291611138,0.208716371360783,0.703724043521811
"4640","LINC02328",24.9278828142323,0.486554529636548,0.38706570121877
4,1.25703343929598,0.208741527237887,0.703724043521811
"4641","HIGD2A",1294.29300333277,-0.352376344259906,0.280444880195936,
-1.25649055890667,0.208938166778855,0.703906818265318
"4642","NHSL1",647.002544782349,0.228653023693064,0.181988919807185,1.
25641178559288,0.20896671081879,0.703906818265318
"4643","EDA",45.1576285771698,0.397250164190436,0.316141751641346,1.25
655710493154,0.208914055580636,0.703906818265318
"4644","HAAO",35.487240784971,0.425959272677454,0.339166489380437,1.25
590023193495,0.209152144476454,0.703925013903968
"4645","LINC01106",4.21434864155819,1.09911265806045,0.875009056707963
,1.25611575061363,0.209074006343699,0.703925013903968
"4646","HEIH",167.416254047349,0.273958337755455,0.218131527732825,1.2
5593187102695,0.209140672129489,0.703925013903968
"4647","NAP1L3",54.563528034262,-0.400191827948629,0.318582200726847,-
1.25616505578651,0.209056133306181,0.703925013903968
"4648","AL359636.2",3.53180216478816,-1.34424421745373,1.0706552980404
1,-1.25553408264458,0.209284943516498,0.704220420954827
"4649","DUS1L",175.00966171922,-0.23275401250324,0.185402321105785,-1.
2553996687584,0.209333709541059,0.704233000047787
"4650","HYAL1",27.4614328085337,-0.646366021105783,0.515083077701723,-
1.25487722095208,0.209523334359965,0.704259784441898
"4651","RNF5",240.007108463924,-0.290187739461687,0.231268110342806,-1
.25476763325279,0.209563125493872,0.704259784441898
"4652","LATS2-
AS1",6.51934329043779,1.44945939757618,1.15472540681071,1.255241626301
02,0.209391058655286,0.704259784441898

"4653","SHMT1",44.2362380369775,-0.436719890993332,0.347957978145264,-1.25509377115363,0.20944472138598,0.704259784441898
"4654","ARMCX6",235.119740325024,0.352514617573246,0.2809424352394,1.25475746400809,0.209566818209245,0.704259784441898
"4655","SFTA1P",9.39607600523724,-0.79934387481437,0.637139804001694,-1.25458160013535,0.209630686376749,0.704323079469895
"4656","UBXN7",267.725123820359,0.281832337978356,0.224743361797785,1.25401852016407,0.209835273925975,0.704556394204004
"4657","AC011939.2",30.6708307142529,0.635455026086815,0.506688893836535,1.25413253342755,0.20979383707015,0.704556394204004
"4658","LINC01697",41.6922614818881,0.801799178322448,0.639375358022986,1.25403515831716,0.20982922660459,0.704556394204004
"4659","LPAR6",83.7328650933538,0.331779017615722,0.2646549199028,1.25362875452145,0.209976974675518,0.704880850810282
"4660","PTPRA",1495.32434260629,0.240072230255714,0.191547167617858,1.25333218570303,0.21008483976762,0.705091608147121
"4661","TTF2",75.5182874841339,0.369066563120266,0.294592749397846,1.25280260248986,0.210277554305158,0.70510414300845
"4662","MZB1",5.96492505029826,0.969256252079461,0.77368046912987,1.25278624800953,0.210283507711784,0.70510414300845
"4663","GDPD5",119.030278826868,0.253337694017128,0.202170678509277,1.25308821182743,0.21017360560839,0.70510414300845
"4664","NCBP3",258.451359347283,0.238165528567869,0.190124046382828,1.25268493438387,0.21032039091784,0.70510414300845
"4665","AC092279.1",31.7139963753615,0.533254806653904,0.425581449320151,1.25300293869894,0.210204637220238,0.70510414300845
"4666","NAA20",383.683501275799,-0.29213541910384,0.233227199122722,-1.25257868808913,0.210359074889861,0.70510414300845
"4667","SOCS5",157.891184700623,-0.28677782875252,0.228995043358587,-1.25233203542948,0.210448900268018,0.705254081892393
"4668","EHMT1",196.271914446122,0.257236475854782,0.205549759507643,1.25145598063916,0.210768164156303,0.706172683677073
"4669","RTN4",4931.58565268944,-0.201406423184147,0.161079397999009,-1.25035495343349,0.211169912257603,0.706459342825434
"4670","TCTA",143.945009325617,0.264524975771552,0.211472164858698,1.25087373058437,0.210980549420691,0.706459342825434
"4671","LINC01366",2.58331636516731,1.39824437980453,1.1181750377769,1.25047003605486,0.21112789446083,0.706459342825434
"4672","DNAJC15",634.272551843546,-0.315920472814787,0.252647767560641,-1.25043841022248,0.211139440760749,0.706459342825434
"4673","AL049840.3",3.4454392475001,1.39666501947809,1.11676176012529,1.25063829130521,0.211066473696235,0.706459342825434
"4674","ZNF701",55.9741159344102,0.341790350310851,0.273341567947997,1.25041483034105,0.211148049855276,0.706459342825434
"4675","PCNA",191.409861026329,-0.281394574868107,0.225017819271104,-1.25054351597409,0.211101069382457,0.706459342825434
"4676","HMCES",98.8133847199597,-0.307821658314546,0.246251064584272,-1.25003178700657,0.211287935829046,0.706551917119152
"4677","IPO4",26.3184676526347,-0.401864869187201,0.321465598003851,-1.2501022556771,0.211262195909153,0.706551917119152

"4678","DAPK3",195.673682353246,-0.235154477496421,0.188154002614293,-1.24979790081042,0.211373383123432,0.70668655665893
"4679","FOXJ3",151.170272089677,0.317039189166652,0.253739620524589,1.24946663241316,0.211494450492968,0.706692388501362
"4680","ADAM15",249.650148579571,-0.220673686347067,0.176637545045134,-1.2493022720095,0.211554537251495,0.706692388501362
"4681","MINAR1",4.69580854676762,1.30828303032922,1.04721400810277,1.24929863447818,0.211555867197147,0.706692388501362
"4682","FBF1",22.3987959827949,0.423349085708607,0.338827224946693,1.2494541599342,0.211499009740251,0.706692388501362
"4683","OSR1",182.229783630483,0.410815030877361,0.328971021555269,1.24878789911391,0.211742661063559,0.706712594757591
"4684","TTC1",429.968750817239,-0.204532157756607,0.163772577154723,-1.24887915492333,0.211709276864238,0.706712594757591
"4685","BBIP1",267.26141720135,-0.197093548977401,0.157822494158529,-1.24883052969258,0.211727065005007,0.706712594757591
"4686","NDUFA6",583.417861576833,-0.307101285018924,0.245917118985893,-1.24879994644269,0.211738253559862,0.706712594757591
"4687","MRAP2",3.79290108025435,1.52896367348015,1.22478224912354,1.24835551345905,0.211900892841709,0.706788220099026
"4688","FUT4",69.7781245459432,0.331355206472373,0.265395196042868,1.24853505795505,0.211835178059341,0.706788220099026
"4689","CRISPLD2",314.910547537806,0.533240441816627,0.427133587890513,1.24841608558612,0.211878721291763,0.706788220099026
"4690","USP44",54.3431499104622,0.585743624288891,0.469482117205126,1.24763777537658,0.212163738119564,0.70706176522267
"4691","AC009996.1",7.25497411942059,0.991255729004877,0.794371707421038,1.24784873346387,0.212086458017606,0.70706176522267
"4692","PLCD3",176.713375532483,0.234833560202585,0.188211954425372,1.24770799452964,0.212138012536598,0.70706176522267
"4693","LPIN2",304.301564004011,0.179997663872837,0.144238042130035,1.24792087589877,0.212060034806516,0.70706176522267
"4694","SLC39A13",297.855093535239,-0.254529201879277,0.20408376402128,-1.24718006402869,0.212331481092751,0.707209958536555
"4695","FAIM2",17.7661603699487,0.860977622616479,0.690324370349453,1.24720734135554,0.212321481759458,0.707209958536555
"4696","TCEANC",21.1269451156854,0.453648256611284,0.363749030472728,1.24714629760449,0.212343859673124,0.707209958536555
"4697","GPALPP1",154.97497837963,0.216726714950924,0.173897138885024,1.24629258618348,0.21265699867987,0.708102077784366
"4698","HIST2H3PS2",12.0687285002673,0.677407411621801,0.544031299255953,1.24516257161722,0.213071997583518,0.708115114250478
"4699","CEP350",401.843573804887,0.306157337566059,0.245937920337843,1.24485616998588,0.213184624557735,0.708115114250478
"4700","CNIH4",555.353759368365,-0.291920658580881,0.234435811086569,-1.24520506158116,0.213056382533651,0.708115114250478
"4701","CNOT6L",171.33077906861,0.281002348278468,0.22561344660578,1.24550354824139,0.21294671205519,0.708115114250478
"4702","HIST1H4E",4.90980572022261,1.32665045742381,1.0646143981517,1.24613236466371,0.212715804641455,0.708115114250478

"4703","HIST1H2BN",6.95829412119581,0.909916300262168,0.731044524783122,1.24467972799894,0.213249500519166,0.708115114250478
"4704","KMT2E-AS1",125.820044520718,0.409438444549809,0.328851288532875,1.24505653110396,0.213110971046253,0.708115114250478
"4705","SEMA4D",9.68242895035931,0.756428806732513,0.60771136411098,1.24471723157439,0.213235709635522,0.708115114250478
"4706","AL355574.1",4.56201492157502,0.953618074099599,0.765554148522331,1.24565724833478,0.212890255204502,0.708115114250478
"4707","CCDC86",36.3184604982956,-0.397061967396074,0.318688769674973,-1.24592393952581,0.212792320306859,0.708115114250478
"4708","TPBGL",4.0517181739777,1.14479501522641,0.919666787663676,1.24479325619081,0.213207755699119,0.708115114250478
"4709","BRMS1L",101.663188013172,-0.223332888596622,0.179361288665923,-1.24515657897953,0.213074199942669,0.708115114250478
"4710","PLCG1",47.4780511816166,0.327306697048092,0.262913316680347,1.24492247551703,0.213160248357541,0.708115114250478
"4711","RNFT1",138.525324331865,-0.288108989226705,0.231505367491516,-1.24450241628745,0.2133147106244,0.7081812936034
"4712","FBN1",33821.3874238236,0.282840721991548,0.227337134690771,1.24414659477553,0.213445614875306,0.70839714644246
"4713","RWDD2B",119.253998163037,0.294030738291153,0.236344016341361,1.24407946874556,0.213470316571823,0.70839714644246
"4714","HOXB4",62.3883022701219,-0.378779548233177,0.304505214927463,-1.24391809947625,0.213529707262854,0.708443916332423
"4715","CASP1",86.6166272489596,0.423073960088215,0.34020455437159,1.24358699685752,0.213651604308833,0.708547729302405
"4716","PHLDB3",11.3344654422384,-0.607518990315816,0.488497985637487,-1.24364686892825,0.213629558395662,0.708547729302405
"4717","RBM15B",177.094772324453,-0.224404596674797,0.180537242686593,-1.24298229736651,0.213874356950217,0.708707314180832
"4718","DXO",43.6011609806304,-0.362899240573383,0.292033024094918,-1.24266507768461,0.213991277872363,0.708707314180832
"4719","AC055822.1",6.63593306235869,0.836846980622166,0.673472975232793,1.24258435215296,0.2140210390702,0.708707314180832
"4720","PLCE1",120.805495696678,0.345385694671562,0.277971749077932,1.24252085263071,0.214044451628194,0.708707314180832
"4721","AC073578.1",4.93427884412596,0.914598529217403,0.735831654644893,1.24294534414775,0.213887974806212,0.708707314180832
"4722","ACOT2",97.0339571995734,-0.497327288176592,0.40027223004755,-1.24247262448737,0.214062234794773,0.708707314180832
"4723","ADNP2",99.8789109123785,0.31190618058961,0.250935209746387,1.24297495319547,0.213877063345672,0.708707314180832
"4724","SMARCA1",701.4802001692,0.220944984192871,0.177785809544086,1.24275938984929,0.213956511452283,0.708707314180832
"4725","KIAA1522",36.3327265689112,-0.44015770750984,0.354421176621041,-1.24190577918111,0.214271327576694,0.708853146625214
"4726","S100PBP",132.875165427306,0.252788848089756,0.203637055552793,1.24136958965319,0.214469247848031,0.708853146625214
"4727","SLC2A1",7.67675091242784,-0.820834432860882,0.661321322967632,

-1.24120363937072,0.214530530736422,0.708853146625214
"4728","MAP10",14.6005617927343,-0.481599548969189,0.387994149886203,-1.24125466610886,0.214511686002352,0.708853146625214
"4729","GNLY",43.9998453939155,0.692435611473546,0.557738608992769,1.24150560909532,0.214419027372455,0.708853146625214
"4730","SP110",243.299180244845,0.20706313610725,0.166835015221275,1.24112516687591,0.214559513818655,0.708853146625214
"4731","FAM225B",12.0095038440615,0.824585532657749,0.664203032529085,1.24146607629594,0.214433622617445,0.708853146625214
"4732","TINF2",245.262059960958,-0.261922009903493,0.210970478199796,-1.24151024417475,0.214417316179064,0.708853146625214
"4733","AC137932.3",11.5961660684352,0.625750542780024,0.503931119414234,1.24173824293168,0.214333155026459,0.708853146625214
"4734","RILP",115.869403296249,-0.429158155348697,0.345582695608934,-1.24183924948122,0.214295878106614,0.708853146625214
"4735","STK32B",189.487219593561,0.348583139505078,0.280918910819293,1.2408674748469,0.214654709816413,0.709017879942703
"4736","SLC6A9",2.50157752594233,-1.39744910943919,1.12991110127865,-1.23677792691636,0.216169538892231,0.70941016750319
"4737","EIF2B3",164.686956947184,-0.323807845432199,0.261919026894329,-1.23628989184829,0.216350827061775,0.70941016750319
"4738","RNF11",341.940608630708,0.185828370603217,0.150063058296581,1.23833522195683,0.215591788914622,0.70941016750319
"4739","MOV10",43.7770408298548,0.411414322803282,0.33254465136233,1.23717016983388,0.216023913489771,0.70941016750319
"4740","PAX8-AS1",266.123730007146,-0.597897059726128,0.4830032148098,-1.23787387204363,0.215762831746388,0.70941016750319
"4741","PSMD14",306.788337845525,-0.273542437331141,0.221418627768734,-1.23540842108754,0.216678540291735,0.70941016750319
"4742","CYB561D2",156.426841113121,-0.30316187925862,0.244827156963996,-1.23826900176438,0.215616333665689,0.70941016750319
"4743","RRP9",54.7370863675664,-0.404181881069595,0.326961748365841,-1.23617482194691,0.216393587494641,0.70941016750319
"4744","ACAP2",447.864865468097,0.168587261412858,0.13611870262983,1.23853120956732,0.215519157199147,0.70941016750319
"4745","CBR4",150.312874685911,0.289815511687298,0.233874992461377,1.23918982802387,0.21527520669375 1,0.70941016750319
"4746","DROSHA",80.7221951122484,0.267617699428794,0.216127367666646,1.23824068334357,0.215626830603996,0.70941016750319
"4747","PAIP1",446.709185768273,-0.231317757407827,0.18684820417327,-1.23799829081214,0.215716694580897,0.70941016750319
"4748","SRFBP1",185.986561577029,-0.26431337992419,0.213849875439719,-1.23597630992634,0.216467369649717,0.70941016750319
"4749","NDST1",205.490079616663,0.282955236216097,0.228494784952417,1.23834439492796,0.215588389078786,0.70941016750319
"4750","AC139795.2",3.24348257505784,-1.03781189765096,0.838070121785623,-1.23833539780629,0.21559172373807,0.70941016750319
"4751","BLOC1S5",155.089556184217,0.225445130630914,0.182466094811891,1.23554532617871,0.216627618293492,0.70941016750319

"4752","PTCD1",35.912732233562,0.366402373539011,0.295449070689645,1.2
4015409046217,0.214918405451857,0.70941016750319
"4753","KIAA1549",3.34736061526976,1.10618967252753,0.895336624338369,
1.23550142198748,0.216643947567218,0.70941016750319
"4754","ASAH1",862.99538012991,0.2628755800587,0.212104520810602,1.239
36811461663,0.215209204050473,0.70941016750319
"4755","LRRCC1",116.690271577179,0.264867582290139,0.213974789033773,1
.23784481099936,0.21577360923278,0.70941016750319
"4756","SARDH",2.63726200677709,1.63924281690966,1.32670019182917,1.23
55789401444,0.2166151168592,0.70941016750319
"4757","POLR3A",70.6854304452518,0.337874556716979,0.273029542403136,1
.23750182395316,0.21590083760904,0.70941016750319
"4758","PWWP2B",5.3619344830335,0.905028490417898,0.730812018915711,1.
23838752920439,0.215572402469102,0.70941016750319
"4759","RIC8A",288.584927078088,-0.229723795641666,0.185678395025898,-
1.23721338505551,0.216007873584668,0.70941016750319
"4760","FAM111A",175.064605007556,0.233148468230513,0.188509904979015,
1.23679691131597,0.216162489054102,0.70941016750319
"4761","DLG2",23.6890159636037,0.575112519472668,0.464684271783235,1.2
3764145764104,0.215849035060736,0.70941016750319
"4762","ATF7IP",247.795030072926,0.289368117057628,0.234147455240487,1
.23583712135767,0.216519113498747,0.70941016750319
"4763","SINHCAF",23.3207088359959,-0.460830886377821,0.372186320849821
,-1.23817255111794,0.215652087043314,0.70941016750319
"4764","ZFC3H1",246.286436589202,0.247433987973954,0.199934850839624,1
.23757307410318,0.215874403475736,0.70941016750319
"4765","LATS2",413.461886356672,0.267872341121529,0.215966190738279,1.
24034387144492,0.214848231964574,0.70941016750319
"4766","ITM2B",14127.5331315098,-0.269633850821028,0.21784593076495,-1
.23772727759582,0.21581720125167,0.70941016750319
"4767","KCNRG",4.56491392911343,-0.832439281682931,0.673140806185156,-
1.23664955984552,0.216217212221509,0.70941016750319
"4768","HAUS4",169.692965231207,-0.306837838239834,0.247527814360099,-
1.23960953250066,0.215119853109994,0.70941016750319
"4769","RASGRP1",9.74480428232352,0.772015289833983,0.62470331787803,1
.23581109262608,0.216528790760891,0.70941016750319
"4770","CHP1",396.851089439843,-0.262452802301541,0.211718229399035,-1
.23963252029132,0.215111346515065,0.70941016750319
"4771","ARHGAP27",44.0549893384698,0.413918408448125,0.33485519473876,
1.23611165348964,0.216417063730703,0.70941016750319
"4772","ZNF726",7.16636832615431,0.741876003630598,0.599746572064556,1
.2369824825789,0.216093586064887,0.70941016750319
"4773","RBM42",299.063884256913,-0.264891734139611,0.213694034980681,-
1.23958412860499,0.215129254062658,0.70941016750319
"4774","SAMD4B",797.942702902808,0.216980047787381,0.17497077256244,1.
24009310017734,0.21494096074952,0.70941016750319
"4775","AC006213.4",4.0924248946036,0.987638457917894,0.79921825718638
,1.23575562624763,0.216549413729488,0.70941016750319
"4776","GABPA",242.637320338994,0.220930013311509,0.178748668974611,1.
23598130592452,0.216465512534974,0.70941016750319

"4777","SYAP1",486.315403535054,-0.215722132696478,0.174518945178475,-1.23609578590947,0.216422961124296,0.70941016750319
"4778","LYRM7",157.810409063949,0.244078514223866,0.197588789307246,1.23528523596716,0.216724366493661,0.709411697773305
"4779","PSMF1",379.345507552103,-0.208280919274618,0.168746425485672,-1.23428344437615,0.217097302840411,0.710483744805195
"4780","PRPF38B",413.043402744042,-0.214429058876273,0.173929496006302,-1.23285045837483,0.217631561786509,0.710516004981204
"4781","RUSC1",197.307116464022,-0.259836140799612,0.210565031115816,-1.23399473988013,0.217204864327793,0.710516004981204
"4782","ARHGEF26",28.7120994778735,-0.641270847692706,0.519807024020353,-1.23367099338695,0.217325526860024,0.710516004981204
"4783","TENT4A",86.6555696832562,0.403537143832753,0.327240413974109,1.23315191706327,0.217519090757163,0.710516004981204
"4784","MPC1",604.593730024542,-0.256005242746819,0.207623329110971,-1.23302734737477,0.21756556131436,0.710516004981204
"4785","HOXA-AS2",6.33895953327862,0.819011481932783,0.664353425885019,1.23279484988241,0.217652313290598,0.710516004981204
"4786","TMEM243",445.945746061414,-0.299585720621249,0.242966173431357,-1.23303469116818,0.217562821523955,0.710516004981204
"4787","PIK3CG",3.27057034911208,-1.11047655795858,0.900354841268534,-1.23337656117226,0.217435305612637,0.710516004981204
"4788","FNBP1",320.539461612857,0.257973194319104,0.209065608773683,1.23393415029999,0.21722744281264,0.710516004981204
"4789","KLF12",226.230237559355,0.399133452342365,0.323635916214922,1.2332792262689,0.21747160560187 5,0.710516004981204
"4790","GNG2",110.287961548165,-0.319579888605885,0.259225084392407,-1.23282779270742,0.217640019795681,0.710516004981204
"4791","UFD1",425.529732653016,-0.29370486461713,0.238115049692215,-1.23345779696315,0.217405012947662,0.710516004981204
"4792","PSMB6",983.853805052106,-0.309342849002818,0.251028751932937,-1.23230047004918,0.217836864300279,0.710907530944942
"4793","HEATR6",61.3106397728633,-0.267008553587671,0.216687258969601,-1.23223005753711,0.217863158300454,0.710907530944942
"4794","SP2",73.7351271949969,0.253314358107058,0.205598936969863,1.23208009652399,0.217919165538247,0.710941958493572
"4795","HEBP1",720.370561134785,-0.289352571294311,0.23487813418201,-1.2319263872817,0.217976583399959,0.710980972758157
"4796","CD84",10.0354928178918,-0.907023992863034,0.736579466321827,-1.23140005163643,0.218173277676654,0.711029394220227
"4797","SDHAF3",170.923667261698,-0.295950925319595,0.240302326500612,-1.23157744508496,0.218106970607315,0.711029394220227
"4798","SLC25A21-AS1",46.9221112875941,0.377713647950966,0.306719246365348,1.23146379768113,0.21814944867268,0.711029394220227
"4799","PKD1",289.387926670467,0.230300364210541,0.187009171774224,1.23149234888106,0.2181387765126,0.711029394220227
"4800","PLAU",517.299218337576,0.447429235045922,0.363425031152613,1.23114589445556,0.218268303148432,0.711190887758641

"4801","RNASEH1-
AS1",68.4560952511154,-0.424633002325599,0.344977772537844,-1.23089960
028951,0.218360417132261,0.711342829399827
"4802","YIPF4",554.752631449416,0.18224745229094,0.148137811014459,1.2
3025614488898,0.218601201170046,0.711526947339633
"4803","SLC4A5",7.63712120167709,0.963847822753137,0.783547387708273,1
.23010788865267,0.218656706378025,0.711526947339633
"4804","WDR36",114.328329241171,0.274217415022171,0.2228392108296,1.23
05617759159,0.218486808829384,0.711526947339633
"4805","TBXAS1",6.95637196398665,-0.743725812086166,0.604431854307099,
-1.23045436269859,0.218527006812938,0.711526947339633
"4806","AP3M2",61.3066403764673,0.306726833485729,0.249441405703996,1.
22965484667654,0.218826382142176,0.711526947339633
"4807","UROS",299.006926805687,-0.314516205859312,0.255759662376763,-1
.22973342604744,0.218796945388425,0.711526947339633
"4808","AEN",22.4661296972627,-0.50603010743275,0.411479303324275,-1.2
2978264847008,0.218778507540643,0.711526947339633
"4809","RPL28",16186.7193485604,-0.264139047850546,0.21469791468063,-1
.23028231663759,0.218591403858764,0.711526947339633
"4810","TENM1",87.7710286415269,-0.567561533519131,0.461428847847121,-
1.23000877853041,0.218693817565507,0.711526947339633
"4811","LUCAT1",13.0633755593458,-0.820081195922968,0.667142272701529,
-1.22924483948848,0.218980021785816,0.711730577874097
"4812","ORMDL2",146.019303858578,-0.293951700440747,0.239121480794257,
-1.22929859527621,0.218959873776107,0.711730577874097
"4813","AC233266.2",7.0049437430457,-0.709607175716362,0.5774465042594
47,-1.22887084861031,0.219120232790674,0.711742563000237
"4814","ANXA2R",57.1474336511861,-0.432672291624535,0.352029663270573,
-1.22907907136217,0.219042161112844,0.711742563000237
"4815","OGN",1469.56313778163,0.416236390409686,0.338686405260388,1.22
897283134136,0.219081992576126,0.711742563000237
"4816","CAAP1",111.775419541107,-0.218114312019262,0.177516227650814,-
1.22870069348424,0.219184046110269,0.711802010208599
"4817","HSF1",212.797346737853,-0.195388692591465,0.159061200529958,-1
.22838688467378,0.219301768902837,0.712036467851435
"4818","B4GALT1",642.571274850726,-0.296829158463485,0.241698152661253
,-1.22809858162011,0.21940996342519,0.712239897876705
"4819","FEM1B",208.805446126702,0.238885831217363,0.194542383013747,1.
22793721099058,0.219470539404722,0.712288698130286
"4820","GUSB",622.202910497759,-0.366219591538985,0.298308223743143,-1
.22765503057105,0.219576494316588,0.712336936550807
"4821","FASTK",245.787775699686,-0.259913663452629,0.211712512087005,-
1.22767266275607,0.219569872595541,0.712336936550807
"4822","MRPL37",222.026754175145,-0.31600288086478,0.257694801391922,-
1.22626796954347,0.220097850700231,0.712373537259211
"4823","RAB3GAP2",221.749051619086,0.252799614326408,0.206256141745423
,1.2256586019069,0.220327175061071,0.712373537259211
"4824","KDM3A",105.925919105481,0.329551356699502,0.268972637503544,1.
22522260910335,0.220491358125223,0.712373537259211
"4825","RNF212",131.152903743963,-0.296019521169787,0.24148111045145,-

1.22584959385178,0.220255280303619,0.712373537259211
"4826","FLOT1",576.83768772167,-0.188475928969477,0.153727587393988,-1
.22603842397156,0.220184215864025,0.712373537259211
"4827","ZHX2",133.27671975034,0.252451381391367,0.205868499566526,1.22
627493726784,0.220095229515267,0.712373537259211
"4828","ALDH1A1",510.397272540371,0.556430891008805,0.453354815154622,
1.22736292283347,0.219686215472788,0.712373537259211
"4829","TMEM80",156.869036786503,0.343423708141998,0.280083925451022,1
.22614572610327,0.220143841045952,0.712373537259211
"4830","VEZT",186.094166155613,0.221411384179975,0.180408184139343,1.2
2728015492336,0.219717311808666,0.712373537259211
"4831","ABHD4",86.8474076920579,0.276622108090733,0.225490348636626,1.
22675808416309,0.219913529326357,0.712373537259211
"4832","FRMD6-
AS1",21.2496953326483,0.459270730184904,0.374777414128331,1.2254493277
112,0.220405971099452,0.712373537259211
"4833","VPS13C",617.32539767652,0.21766207436495,0.177653654914152,1.2
252045952566,0.220498143538406,0.712373537259211
"4834","AC010531.6",3.03642715579523,-1.15840861596503,0.9451850682694
89,-1.22558920454163,0.220353302356605,0.712373537259211
"4835","COX10-
AS1",28.8131804429896,0.4009490679767,0.32672403167824,1.2271796045035
2,0.219755093376126,0.712373537259211
"4836","AC002553.2",6.34035202507926,0.870489591749425,0.7104860038718
21,1.22520301174922,0.220498740017381,0.712373537259211
"4837","GALR1",6.19273086054725,-1.11977321837141,0.912703730568567,-1
.22687481256799,0.219869646666098,0.712373537259211
"4838","MAP2K7",158.656446976596,0.312687462316762,0.255211611009349,1
.22520860661511,0.220496632536411,0.712373537259211
"4839","RPS5",8876.10609126663,-0.274773187221314,0.224083425487148,-1
.22620933085063,0.220119910849929,0.712373537259211
"4840","PPP6R2",156.164559398053,0.342029816044105,0.279134118770483,1
.22532429052622,0.22045305981581,0.712373537259211
"4841","PRRG3",370.105516806856,0.295283684209666,0.240983961266668,1.
22532504925882,0.2204527740571,0.712373537259211
"4842","CEP104",86.3400328306942,0.351686615944678,0.287217335049932,1
.22446166379038,0.220778119672027,0.712687263502682
"4843","MED10",541.649566895966,-0.249958101369078,0.204084995015286,-
1.22477451784417,0.220660188553207,0.712687263502682
"4844","PPP1R3B",106.355591952299,-0.260458474736848,0.212698905645341
,-1.22454073727649,0.220748308456684,0.712687263502682
"4845","EXOC3L4",11.8708886246241,0.763853562318712,0.623814870951394,
1.22448758099297,0.220768348401143,0.712687263502682
"4846","HLA-F-
AS1",100.777439802967,-0.429928428917894,0.351168860187778,-1.22427833
916709,0.220847245343719,0.712725201786684
"4847","CASTOR3",35.2453781275942,0.374475929851515,0.305957591014963,
1.22394717715372,0.220972154952918,0.712725201786684
"4848","NTNG2",37.700508326584,0.402968488717215,0.329235928825611,1.2
2395052737594,0.220970891043119,0.712725201786684

"4849","MFSD11",171.821498350406,-0.217722431646218,0.177874873587892,
-1.22402016234539,0.220944621624801,0.712725201786684
"4850","ZBTB3",17.9491196394334,0.486246252743923,0.397424053017908,1.
22349477604973,0.221142876068453,0.712981773182974
"4851","RALGAPA1",255.675465882382,0.314962608818964,0.257417357658624
,1.22354844942762,0.221122616580974,0.712981773182974
"4852","WDR5",56.4497140238203,0.309112167437537,0.25270833469133,1.22
319735838987,0.221255163087375,0.713038735514779
"4853","PRMT7",84.9449341012749,0.240452390421469,0.196594877681885,1.
2230857347695,0.221297315996722,0.713038735514779
"4854","BTBD3",63.2566617063354,0.336774942758894,0.275306189845007,1.
22327414050695,0.221226170866144,0.713038735514779
"4855","ITGB3BP",102.742561366559,-0.342239750086643,0.279894980125134
,-1.22274343731937,0.221426615145661,0.713116110763739
"4856","OLFML2B",246.974022789542,0.464407366686262,0.379780780595296,
1.22283009150257,0.221393877351218,0.713116110763739
"4857","APOL4",27.3581000509048,0.638353189796758,0.522101939044074,1.
22266006321587,0.221458117006361,0.713116110763739
"4858","DUSP12",112.261341912599,-0.296586565847415,0.242609320204167,
-1.22248628205142,0.221523788365656,0.713180743112157
"4859","CLSPN",4.0214274745204,-0.987329638512825,0.808106347918364,-1
.22178181257471,0.221790148117875,0.713450826113445
"4860","SSX2IP",20.0999985991439,-0.541898117496331,0.443467813998311,
-1.22195591290058,0.221724299488893,0.713450826113445
"4861","ASPRV1",7.34544893654198,0.754696423493381,0.617681158641228,1
.221821991711,0.221774950228422,0.713450826113445
"4862","EIF1AD",74.0355594045925,-0.29282998918414,0.239654202199691,-
1.22188547705974,0.221750938208762,0.713450826113445
"4863","HLA-
C",3988.65049992166,-0.38946099006067,0.318867077076926,-1.22138978294
932,0.221938473758585,0.713634401641502
"4864","WSCD2",168.52372818192,0.333642779390329,0.273140843689759,1.2
2150453547434,0.221895049427004,0.713634401641502
"4865","SHISA4",163.675273446924,-0.417215602096314,0.341656466175563,
-1.22115529311225,0.222027227678074,0.713773040264149
"4866","C19orf48",98.0473244437511,-0.420388837396271,0.34430249345172
5,-1.22098690945209,0.222090976225473,0.713831251164488
"4867","FAM102B",139.062472773295,-0.348666085210111,0.28564720220918,
-1.22061788987796,0.222230729576391,0.713840338996665
"4868","PRR16",3.46808164206577,-1.07490536489975,0.880581362299012,-1
.2206769424388,0.22220836123789,0.713840338996665
"4869","ZFP2",6.72518844913915,0.735801062858255,0.602694569197185,1.2
2085231967226,0.222141940137735,0.713840338996665
"4870","PUF60",421.732484477619,-0.303241419988644,0.248488021077663,-
1.22034623107191,0.222333651153224,0.713966227906769
"4871","SPRYD7",101.358048752436,-0.289976903992911,0.237656177776299,
-1.22015302402894,0.222406870994999,0.713966227906769
"4872","DEXI",351.423416300404,-0.282365417598346,0.231411926474444,-1
.22018524239514,0.222394659973332,0.713966227906769
"4873","AC242426.2",23.4565330908394,0.451337935318087,0.3702601107354

41,1.21897531554669,0.222853561583243,0.714142176439892
"4874","ADAM17",249.300937180422,-0.331917792472254,0.27230065778467,-1.21893863633164,0.222867483868888,0.714142176439892
"4875","DOK1",90.4503882635762,-0.3109924780389,0.255400221244269,-1.21766722254114,0.223350457687874,0.714142176439892
"4876","SLC23A3",7.1486699336086,0.84691923489177,0.695992511832806,1.21685107309778,0.223660883897761,0.714142176439892
"4877","EIF4E2",492.194236413674,-0.251949912523783,0.207013935181208,-1.21706740323177,0.223578571692926,0.714142176439892
"4878","SRA1",266.193295505382,-0.364482828462693,0.29936238981796,-1.21753046093844,0.223402454086646,0.714142176439892
"4879","FUCA2",527.656428356164,-0.304548898022071,0.250120210951854,-1.21761011180617,0.223372169984989,0.714142176439892
"4880","AP003117.1",21.2173351429498,-0.611175567354839,0.502020832278368,-1.21743068824671,0.223440392862121,0.714142176439892
"4881","DPM2",230.565830658153,-0.326813288154588,0.268003487271614,-1.21943670017761,0.222678487530601,0.714142176439892
"4882","AL135925.1",70.4349143096953,0.335522405518223,0.275777276725997,1.21664268173765,0.223740195943444,0.714142176439892
"4883","ENO4",6.60831197185139,0.801105520898582,0.657299857826619,1.2187824344698,0.222926780192186,0.714142176439892
"4884","TBRG1",291.728590817466,-0.198554940419948,0.163016650381116,-1.21800405023503,0.223222433612316,0.714142176439892
"4885","CHST11",19.166281524217,-0.571545444297876,0.468602739320081,-1.21968011780546,0.222586161521237,0.714142176439892
"4886","SUPT20H",168.588342436103,0.23160672722611,0.190049023732643,1.21866833450263,0.222970101206462,0.714142176439892
"4887","PSME2",489.595676607151,-0.329024629932702,0.270365044839088,-1.21696438283479,0.22361776757935,0.714142176439892
"4888","KTN1-AS1",9.44283519487114,0.65815537509903,0.54085462974635,1.21688035731097,0.223649740177491,0.714142176439892
"4889","AC091057.3",3.0555796760585,1.21992566055714,1.00207868435749,1.21739508044654,0.223453933918746,0.714142176439892
"4890","RPLP1",33010.0921342198,-0.283394787542642,0.232734988040684,-1.21767160979295,0.2233487898097,0.714142176439892
"4891","CERS3-AS1",5.68112540109506,0.875817990767945,0.719035027506199,1.21804634998869,0.223206359706253,0.714142176439892
"4892","ZNF75A",73.0758644567764,0.31365926229782,0.257398932948262,1.21857250418698,0.223006490327831,0.714142176439892
"4893","PIEZO1",150.487854943886,-0.232501551900232,0.190879336492275,-1.21805511362746,0.223203029627152,0.714142176439892
"4894","IKZF3",6.13816417554375,0.872592049858033,0.715675113676773,1.21925722046572,0.22274658004722,0.714142176439892
"4895","NBR1",330.652143145032,0.185195924851207,0.152217937699117,1.21664980915243,0.223737482975578,0.714142176439892
"4896","MYPOP",51.323825446125,-0.290113047770223,0.238113110331275,-1.21838334464912,0.223078331317261,0.714142176439892
"4897","PIK3IP1",374.989516702009,0.365682211331159,0.300553341244076,

1.21669654317432,0.223719694794398,0.714142176439892
"4898","AP1S2",280.693801140505,-0.283115882586147,0.232540688885625,-1.21748965285554,0.223417970888853,0.714142176439892
"4899","SYTL4",104.306342402757,0.374216806826877,0.307391138643852,1.21739620887527,0.223453504786984,0.714142176439892
"4900","ARMCX3",664.750388428854,0.152300389653339,0.125134099158241,1.21709742330701,0.223567150962304,0.714142176439892
"4901","ZNF107",66.738601515764,0.327191196150755,0.269001885265396,1.21631562480668,0.223864711684351,0.714254018900366
"4902","LINC02384",90.7924400669127,0.463154069625786,0.380785974786678,1.21631073698356,0.22386657293156,0.714254018900366
"4903","THEMIS2",10.5238521383522,-0.872743479709192,0.717757315540244,-1.21593115223394,0.224011149819905,0.714427385914672
"4904","RAPGEF1",227.423587927277,0.282623911610461,0.232434701946933,1.21592821228126,0.224012269854575,0.714427385914672
"4905","TMEM184C",94.1359541021996,0.259791082742083,0.213679438224513,1.21579822981901,0.224061793314595,0.714439642699342
"4906","DAP3",234.910804568407,-0.232573356134213,0.191368167282519,-1.2153189291449,0.224244475040225,0.714621038156241
"4907","SERPINA1",13.4498588291023,-0.847188495878428,0.69697124599136,-1.21552861864969,0.224164540419381,0.714621038156241
"4908","CFP",5.2656832152782,-1.18362940029207,0.973948644124774,-1.2152893352562,0.224255758009644,0.714621038156241
"4909","NUDC",788.055991456886,-0.225138955734652,0.185376961943491,-1.21449263907606,0.224559659014472,0.71483601548126
"4910","TBX15",225.185373865076,-0.284447024433643,0.234313318859015,-1.21396011894993,0.224762953683,0.71483601548126
"4911","GPR161",52.9771692383765,0.473393426918152,0.390064712288403,1.21362792378957,0.224889838955874,0.71483601548126
"4912","PTPN4",97.5657851534724,0.279691679324145,0.230280607633909,1.21456896521998,0.224530531546697,0.71483601548126
"4913","AC116366.1",19.3128044669405,1.61730164514659,1.33211706186216,1.21408372540907,0.224715754016073,0.71483601548126
"4914","ARL4A",281.41730824583,-0.399502073256684,0.329005887547006,-1.21427028627141,0.224644528347095,0.71483601548126
"4915","FAM171A1",190.775576200252,0.241152712749477,0.198715800587801,1.21355580198529,0.224917393362102,0.71483601548126
"4916","TMEM9B",410.54579823484,-0.249746508528349,0.205784408741806,-1.21363182981322,0.224888346713715,0.71483601548126
"4917","TOGARAM1",154.804409437164,0.233356272251375,0.19227474442073,1.2136605509705,0.224877374411154,0.71483601548126
"4918","EXD2",47.255594545986,0.349215114314693,0.287577510989163,1.21433387858987,0.224620253600921,0.71483601548126
"4919","DICER1",353.044977707473,0.247059853489647,0.203442991296775,1.21439353557894,0.224597482770474,0.71483601548126
"4920","TFAP4",43.5562052044242,0.423527475642213,0.34883915993025,1.21410530780689,0.224707513409399,0.71483601548126
"4921","RAB36",8.5091164079718,0.7093770925448,0.58452698313112,1.21359169553628,0.224903679794664,0.71483601548126
"4922","BORCS6",60.8807620302601,-0.348096658038082,0.286913487148268,

-1.2132460606782,0.225035758613549,0.715066896529035
"4923","DDOST",842.811233597295,-0.314183130942495,0.259101145102314,-1.21258873949951,0.225287096225182,0.715284243800619
"4924","TMEM14C",1441.85702560892,-0.284425452528489,0.234533212196818,-1.21272995779294,0.225233082176511,0.715284243800619
"4925","NLRP1",49.8679748909377,0.39362341704648,0.324599502950364,1.21264331420332,0.225266221057397,0.715284243800619
"4926","SH3KBP1",336.051944084444,0.173764062329587,0.143295215422916,1.2126298970747,0.225271353065495,0.715284243800619
"4927","TMSB15B.1",16.7381888367701,0.492047273864075,0.40587052641815,1.21232570939911,0.225387726269462,0.715458501898597
"4928","MACO1",217.628822519648,-0.187098325425127,0.154532671455761,-1.21073636831994,0.225996459277564,0.715486574941998
"4929","MAGOH",422.205884008647,-0.296545507188702,0.245087186573989,-1.20995924484685,0.226294531853534,0.715486574941998
"4930","MSTO1",34.3569980515737,-0.450806354004643,0.372474188604436,-1.21030226468496,0.226162928998902,0.715486574941998
"4931","LINC01354",35.8025585220916,0.558961084771748,0.461827935162934,1.21032324425015,0.226154881759969,0.715486574941998
"4932","RALB",238.721383171703,-0.1939967036892,0.160052539976584,-1.2120813810114,0.225481230165356,0.715486574941998
"4933","ZNF621",111.896881966101,0.377743521770444,0.311797906245858,1.21150114931364,0.225703394448293,0.715486574941998
"4934","CD96",5.38755458574132,1.07334351427763,0.887011731306506,1.21006687554929,0.226253232419735,0.715486574941998
"4935","RAB6B",22.8845318502441,0.453519899721183,0.374432803526158,1.21121839606529,0.225811714144311,0.715486574941998
"4936","AC079140.6",2.97735166551421,1.36264338987521,1.1262306861695,1.20991499042686,0.226311514465349,0.715486574941998
"4937","OTUD4",126.162968297885,0.237669979934194,0.196371016526027,1.21031089077595,0.226159620220404,0.715486574941998
"4938","DNAH5",11.4140300950975,-0.694285659868179,0.573624014485642,-1.21034971049936,0.226144730257105,0.715486574941998
"4939","GRPEL2",39.1553190807338,0.322094734295879,0.26585219385768,1.21155567543786,0.225682510349821,0.715486574941998
"4940","BTN2A2",30.0745869389214,0.415355981963684,0.343176965861867,1.21032593466914,0.226153849797001,0.715486574941998
"4941","EI24",515.245111268815,-0.192344494351521,0.158847856533846,-1.21087245713345,0.225944290055889,0.715486574941998
"4942","C12orf10",341.814514395175,-0.330767651367664,0.272944283727235,-1.21185044380052,0.225569634749152,0.715486574941998
"4943","AL096870.8",82.0832361844006,-0.403381829174092,0.333297824096576,-1.21027441528454,0.226173611651272,0.715486574941998
"4944","AL121612.2",3.1013005204543,1.1580440620435,0.956195990230191,1.21109487372428,0.225859045855093,0.715486574941998
"4945","SIN3A",135.556697927526,0.280382109180431,0.231694937837945,1.210134808282,0.226227168431141,0.715486574941998
"4946","AP2S1",1049.78643333021,-0.297663393368762,0.245781972111788,-1.21108717132995,0.225861997519295,0.715486574941998
"4947","ZNF8",40.8522280337613,0.37482906208713,0.309598541509377,1.21

069388847808,0.226012745552759,0.715486574941998
"4948","SKP2",38.9976138325293,0.338103201522943,0.279587792397083,1.2
0929171701014,0.226550791928402,0.716098299466494
"4949","CZIB",741.837286421008,-0.338142099182205,0.279983144406631,-1
.2077230573963,0.227153805936209,0.716343595223735
"4950","PODXL2",48.3838807568254,0.451013518973849,0.373560890342125,1
.2073360210722,0.227302763790004,0.716343595223735
"4951","YTHDC1",456.279568994792,0.267569785017997,0.221386435118656,1
.2086096642488,0.22681284178752,0.716343595223735
"4952","RAP1GDS1",81.316605749729,-0.243879959068966,0.201816611705906
,-1.20842361294003,0.226884361628285,0.716343595223735
"4953","SPRY1",1362.23548461725,0.375381964775141,0.310816785813226,1.
20772745201964,0.227152114986503,0.716343595223735
"4954","GZMA",30.2576060953793,0.711715364622437,0.588932404980279,1.2
0848395945587,0.226861162108393,0.716343595223735
"4955","CUTA",890.584132487991,-0.379833068735933,0.314587541227852,-1
.20740022714639,0.227278048117081,0.716343595223735
"4956","TEAD3",57.6787885291686,0.433512572865232,0.359068120856985,1.
20732682096804,0.227306305461793,0.716343595223735
"4957","AL356599.1",72.5168996200625,0.355960150822906,0.2948390857413
23,1.20730312918962,0.227315426029117,0.716343595223735
"4958","AC073316.3",2.51789383444161,1.24940986196309,1.0342871138022,
1.20799132589989,0.227050598850666,0.716343595223735
"4959","ADCK2",41.5105887519988,-0.386560813395991,0.319845635414174,-
1.20858555063735,0.226822110375353,0.716343595223735
"4960","BANF1",677.0188553916,-0.300113946905557,0.248246209369212,-1.
20893667487669,0.226687174852623,0.716343595223735
"4961","NUAK1",222.96310663412,0.45485092383979,0.376555786980968,1.20
792440208276,0.227076342358218,0.716343595223735
"4962","LINC00598",8.78650244839226,0.718121035144018,0.59469204492481
1,1.2075511035881,0.227219976841723,0.716343595223735
"4963","DNAJA2",422.610909759871,-0.187630172976316,0.155314026952013,
-1.20806971951276,0.227020445916686,0.716343595223735
"4964","AL731569.1",5.25333178500534,0.858932453550653,0.7115848239190
64,1.20706966292517,0.227405316931558,0.716399835912637
"4965","AC010331.1",5.23979904655782,-0.731634596539561,0.606150099032
643,-1.2070188517781,0.227424883970987,0.716399835912637
"4966","UBR4",131.308681473507,0.330151133256393,0.273585984118373,1.2
0675455769527,0.227526681244698,0.716409464391278
"4967","BTG2",1970.25639785964,0.415532424606274,0.344641972286908,1.2
0569303224725,0.227935872638232,0.716409464391278
"4968","AL591846.2",5.92003933660764,-0.93230240727095,0.7731372113427
37,-1.20586927338782,0.227867899831252,0.716409464391278
"4969","MIR4435-2HG",522.891821988826,0.236048680204946,0.195823384521
405,1.20541620083757,0.228042670336411,0.716409464391278
"4970","HAT1",251.269868049015,-0.259903442955334,0.215711950063864,-1
.20486344348742,0.228256023059855,0.716409464391278
"4971","LMLN",54.9454377051361,0.359448007669889,0.29797334234087,1.20
630927869613,0.227698261319548,0.716409464391278
"4972","AC147067.1",53.732965977901,-0.351246142163331,0.2915316027338

02,-1.20483041587795,0.228268775523089,0.716409464391278
"4973","MAD2L1",20.2492680722856,-0.40460135267821,0.33576378002661,-1.20501786299327,0.228196406068592,0.716409464391278
"4974","BTNL9",3.53811038385847,-1.19512294700542,0.991725849538163,-1.20509407671684,0.228166986200015,0.716409464391278
"4975","SRSF3",2286.58737950824,0.238914101819749,0.198267431623909,1.2050093142526,0.228199706204843,0.716409464391278
"4976","AL158071.1",17.6919393898882,0.809285027038633,0.671369625221025,1.20542395222632,0.228039679473669,0.716409464391278
"4977","SACS",172.384084960354,0.318399409429432,0.264260886081175,1.20486771292982,0.228254374600135,0.716409464391278
"4978","ELMSAN1",392.526711182854,0.401816011537178,0.33306312346548,1.20642599924103,0.227653276288994,0.716409464391278
"4979","AC127502.2",45.6641486226649,-0.413330497426388,0.342580052361063,-1.20652237215131,0.227616138173442,0.716409464391278
"4980","MPI",81.0124013553577,0.351182151950573,0.291484418990305,1.20480591438493,0.228278236250724,0.716409464391278
"4981","DEF8",207.766325769314,-0.268447466917926,0.222548765942807,-1.20624109408414,0.227724543144756,0.716409464391278
"4982","ATP5PD",1548.59279110097,-0.262021277848756,0.217141357112402,-1.20668527328546,0.227553372662534,0.716409464391278
"4983","TPGS2",289.836164032982,0.205626676287963,0.170544305009894,1.20570825438019,0.227930001185366,0.716409464391278
"4984","LINC00685",33.3928473904575,0.41311123868630,0.342934651921221,1.2046354498501,0.228344065216785,0.716409464391278
"4985","FAM120C",49.4958562562109,0.30996834590371,0.257298181584259,1.2047047670339,0.228317295093837,0.716409464391278
"4986","SRGAP2",228.893496559517,0.272359272939459,0.226165099320873,1.20424978812954,0.228493047627865,0.7165074394883
"4987","PILRB",69.5573681394544,0.286182249926341,0.23765364842291,1.20419884914653,0.228512730701256,0.7165074394883
"4988","RALY",599.589327353635,-0.236229068447759,0.196169962819225,-1.20420611317263,0.228509923772055,0.7165074394883
"4989","ARHGAP18",156.887888722341,-0.322287842798168,0.267850118026907,-1.20323950264526,0.228883652428355,0.71677340494226
"4990","EBAG9",212.751468752874,-0.201122256103408,0.167147939824893,-1.20325895918375,0.228876125496713,0.71677340494226
"4991","ADD3-AS1",24.89343430365,-0.403406850104625,0.335196184872079,-1.20349475414995,0.228784920164833,0.71677340494226
"4992","TSG101",355.179658502353,-0.277242363977577,0.230453150269995,-1.20303134781523,0.228964189967489,0.71677340494226
"4993","RPS6KB2",90.6660415152333,-0.293871770547329,0.244119657885878,-1.20380215625532,0.228666056100068,0.71677340494226
"4994","SPATA2L",45.4104835736255,-0.354003139901695,0.294236161669751,-1.20312587648226,0.228927613220243,0.71677340494226
"4995","UHRF1",2.78748208715866,-1.39529164391772,1.1597156790878,-1.20313251694175,0.228925043929521,0.71677340494226
"4996","TCEAL5",4.01791427272293,1.22031834548711,1.01378332511416,1.20372698510275,0.228695118685547,0.71677340494226

"4997","WAC",778.240426248795,0.239531590981304,0.199150621185785,1.20
276597459291,0.229066895221593,0.716951419104605
"4998","ATF6B",152.654597352688,0.249891533532932,0.207806907849023,1.
20251793417033,0.229162921946811,0.71710846323492
"4999","CRYBG3",256.851522227953,-0.243475545745979,0.202558016073044,
-1.20200400095832,0.229361977959214,0.717407776503709
"5000","GTF3C6",478.797201549316,-0.285412859223146,0.237478796686254,
-1.20184565193086,0.229423334307043,0.717407776503709
"5001","SMPD3",3.44568890235711,1.25413754797217,1.04355160012018,1.20
179735034448,0.229442052306365,0.717407776503709
"5002","TUBG1",84.7274229491467,-0.372470913019336,0.309871391208251,-
1.20201775183891,0.229356650382746,0.717407776503709
"5003","CD2",12.7178605152008,0.746148601452427,0.621142944837592,1.20
12510286944,0.229653840432279,0.717556511124108
"5004","AC136424.2",3.39585411273882,1.56114976605381,1.29949044474968
,1.2013553253595,0.229613397844879,0.717556511124108
"5005","MSRB3",198.77110730992,-0.243146666301124,0.202360367471421,-1
.2015528007749,0.229536837686726,0.717556511124108
"5006","CDK16",180.701296794019,-0.175731321914687,0.146296317790977,-
1.20120126444854,0.229673139046502,0.717556511124108
"5007","GTF2H4",63.4063117742481,-0.393607917617745,0.327764695714652,
-1.2008856437681,0.229795563859986,0.717769293629412
"5008","NKG7",53.5496832372829,0.404068610490487,0.336502571566367,1.2
0078907156522,0.229833032128906,0.717769293629412
"5009","AC004540.1",137.405155282916,0.440246551716254,0.3667131895149
38,1.20052009118783,0.229937414559006,0.717951919285855
"5010","SASS6",62.3219599634557,0.267983189016649,0.223489543096315,1.
1990860301735,0.230494495508955,0.717971302481587
"5011","AL356488.3",14.1589521612319,-0.5554786029585,0.46293772447255
7,-1.1998991950621,0.230178492637772,0.717971302481587
"5012","SGCB",404.024197157029,0.199187270983519,0.165951183751598,1.2
0027628896983,0.23003205530061,0.717971302481587
"5013","MRPL36",395.599064854968,-0.322228151386766,0.268686831622226,
-1.19927035292827,0.230422839085306,0.717971302481587
"5014","CENPK",6.35201484536034,-0.769897163148856,0.642024865029676,-
1.19917032047235,0.230461725254203,0.717971302481587
"5015","SURF1",324.534008100331,-0.25570893184133,0.213059394766764,-1
.20017674940481,0.230070703183708,0.717971302481587
"5016","MRPL17",291.48642232105,-0.299584568231758,0.249701501521228,-
1.19977079195213,0.230228370699121,0.717971302481587
"5017","CLMP",609.902295001703,-0.166719981780179,0.139009585031426,-1
.19934162628058,0.230395135447419,0.717971302481587
"5018","CTNS",50.4507468209517,0.342100215065463,0.285109492050165,1.1
9989065465863,0.230181809910516,0.717971302481587
"5019","KPNB1",685.407755375923,0.197626049303726,0.164738210342572,1.
19963698095763,0.230280357624687,0.717971302481587
"5020","TNRC6C",257.367806825323,0.311606723738898,0.259793665034416,1
.19943926922782,0.23035718590849,0.717971302481587
"5021","FXYD1",1605.23112996944,0.343799762810928,0.28658366697316,1.1
9964883708159,0.230275751063571,0.717971302481587

"5022","ILKAP",144.379591509474,-0.226509560769526,0.189042539400855,-1.19819359963857,0.230841656504872,0.718051612517143
"5023","ACTR8",74.4730827382948,-0.294298504224251,0.245594687453761,-1.19830973249232,0.230796459087531,0.718051612517143
"5024","AL121933.2",13.2415041076309,-0.683777136995155,0.570624007663663,-1.19829717609461,0.230801345573242,0.718051612517143
"5025","TMEM141",276.040795794754,-0.319648122342978,0.266672125091023,-1.19865592338859,0.230661763348304,0.718051612517143
"5026","TUBA1B",3597.87422272414,-0.350498338955753,0.292429802778869,-1.19857256553565,0.230694191061903,0.718051612517143
"5027","NUMBL",289.049630958439,-0.206887053544567,0.172600589326428,-1.19864627549617,0.230665516387287,0.718051612517143
"5028","RAE1",90.1116522927533,-0.271149216077683,0.226233576070167,-1.19853657793742,0.230708191890343,0.718051612517143
"5029","PLEKHH2",162.22876207659,-0.438487959716875,0.366249226633569,-1.19723927814756,0.231213304418444,0.718591143930898
"5030","C3orf35",6.71467808000923,0.893806034659368,0.746654025224739,1.1970819207602,0.23127462597687,0.718591143930898
"5031","ERICH1",432.452008780042,0.25161425856504,0.210148881947727,1.19731428610516,0.231184078176271,0.718591143930898
"5032","VCPIP1",150.677934278014,0.245136915409151,0.204785821052543,1.19704046964391,0.231290781237093,0.718591143930898
"5033","PPP6R3",216.625588064829,0.218042571571687,0.182102341585557,1.1973628107865,0.231165172326048,0.718591143930898
"5034","NPC2",1349.49968589619,-0.298699925414351,0.249508238836961,-1.19715455812877,0.23124631804295,0.718591143930898
"5035","BDNF",3.55831255346305,-0.98533312803568,0.823327631169782,-1.19676917272377,0.231396536960719,0.71864106659936
"5036","KBTBD6",70.8671830539933,0.292075743587831,0.244054688242466,1.19676350284923,0.23139874753161,0.71864106659936
"5037","PELO",79.9157045900865,-0.304102684179784,0.254157657830354,-1.19651198699182,0.231496823634862,0.718660246456777
"5038","PCMT1",655.005324502527,-0.282635166281755,0.236193332123594,-1.19662635579339,0.231452223000385,0.718660246456777
"5039","TOMM70",192.073820927485,0.193735686297524,0.161965614800535,1.19615318681137,0.231636785284445,0.718809389255698
"5040","DTX2",75.4562704759597,0.250496696045484,0.209403844784994,1.19623732937035,0.231603957357292,0.718809389255698
"5041","OXSM",27.2217320025144,-0.467294959940994,0.390739074104658,-1.19592585157181,0.231725495862332,0.71894202644056
"5042","COX7A2",1586.00066902446,-0.243206132277314,0.203429755974038,-1.19552880114722,0.231880490421999,0.718985543145158
"5043","AP000919.4",13.659148830488,0.754079506999688,0.63073716353884,1.19555268119737,0.231871166408171,0.718985543145158
"5044","LILRB2",10.512688670297,-0.876823069178822,0.73348603618034,-1.1954188981496,0.231923405701235,0.718985543145158
"5045","ZNF674",12.2364012471533,0.563150536642673,0.471067966203531,1.19547618824787,0.231901034174178,0.718985543145158
"5046","SESN2",103.485813230715,0.278177039551667,0.232845830045777,1.19468336408249,0.232210764588057,0.719070506968001

"5047","GINM1",1065.85016186261,-0.188899315346223,0.158122022807065,-1.19464266895137,0.232226670760574,0.719070506968001
"5048","PTAR1",173.993542176067,0.302769968556928,0.2533874779794,1.19488923040445,0.232130311155805,0.719070506968001
"5049","AGTPBP1",25.0124903846472,-0.435057884640266,0.364154600041654,-1.19470654658901,0.232201703777384,0.719070506968001
"5050","NFATC2IP",218.088129437312,0.32109399553784,0.26870862665779,1.19495231519591,0.232105661312626,0.719070506968001
"5051","CA5B",172.736142633544,-0.488790277927573,0.409138109050777,-1.19468283964452,0.232210969565908,0.719070506968001
"5052","MAD2L1BP",75.6943408391817,-0.319667959644008,0.267622163442439,-1.19447491019466,0.232292249416265,0.719131191779568
"5053","PGD",436.52499531469,0.263891328486971,0.221074149734179,1.1936779076354,0.232603985615024,0.719224465797767
"5054","MIR3945HG",3.25025626202866,-1.18532147407023,0.992498916417319,-1.19427986717502,0.23236851029845,0.719224465797767
"5055","FSD1L",28.1341132025533,-0.404379754905413,0.338714278623893,-1.19386686781644,0.232530049700232,0.719224465797767
"5056","SMCO4",125.000440349896,-0.294101064436245,0.246347178955056,-1.19384790880801,0.232537467185694,0.719224465797767
"5057","PAN2",48.5644838186484,0.367727323340364,0.30807819386098,1.19361685009845,0.232627879636092,0.719224465797767
"5058","FNDC3A",427.537093027106,0.154910596343197,0.129786219422971,1.19358277814031,0.232641213981838,0.719224465797767
"5059","CCDC189",10.4998164666457,-0.643899339103994,0.539471229060788,-1.1935749386024,0.232644282127296,0.719224465797767
"5060","HES6",34.755365393775,-0.378442219345742,0.317116546798725,-1.19338528110909,0.232718516778631,0.719311779133951
"5061","ZNF652",291.870675879333,0.20594707095781,0.172599289661249,1.19320926153294,0.23278742838912,0.719382608181356
"5062","GPR157",18.4618850640429,-0.54589279362761,0.457816281024138,-1.19238396766154,0.233110723741199,0.719509530060062
"5063","AC093425.1",323.396376331074,0.377932864459509,0.317027354854451,1.19211436701738,0.233216404345505,0.719509530060062
"5064","CHMP2B",467.583617964601,-0.199508070586561,0.167270774589053,-1.19272521500966,0.232977007092634,0.719509530060062
"5065","HIST1H4H",13.5028431716099,0.493785783421741,0.413924368465394,1.19293721520294,0.232893962918838,0.719509530060062
"5066","GCNT1",4.98384982321314,-1.0662579770248,0.893955595170742,-1.19274154419398,0.232970609919816,0.719509530060062
"5067","NTRK2",1606.78069278775,0.353796082991123,0.29689943674189,1.19163608686364,0.233403968703577,0.719509530060062
"5068","TSFM",130.366578017717,-0.32028031116478,0.268786579887241,-1.19157850551594,0.233426557258616,0.719509530060062
"5069","TMEM220",116.630218617701,-0.385381515677263,0.323372273142293,-1.19175806859509,0.233356121676954,0.719509530060062
"5070","MLX",224.755122678809,-0.23251465541529,0.195114920772032,-1.19168054649678,0.233386528719028,0.719509530060062
"5071","SBNO2",85.1289021696444,-0.320647653823106,0.268923905760827,-1.19233599897318,0.233129524476005,0.719509530060062

"5072","ZNF561",78.21388703989,0.241030317474762,0.202236713954074,1.1
9182275444555,0.233330751637267,0.719509530060062
"5073","AC006504.7",5.31320095619045,0.845964354632996,0.7098114091185
97,1.19181566225241,0.23333353312727,0.719509530060062
"5074","CHM",220.844238392204,0.184907087921518,0.155141520341272,1.19
186074440143,0.233315852741875,0.719509530060062
"5075","RASSF5",161.034313468461,0.244847758627714,0.205513786028679,1
.19139335301597,0.233499201135046,0.719591626749185
"5076","SLC30A7",234.474917912172,0.199212507173294,0.167230850876433,
1.19124256158029,0.233558375419981,0.719632189040287
"5077","FYTTD1",291.740247659733,0.175480067345633,0.147331688079072,1
.19105448144634,0.233632197610006,0.719717859094051
"5078","EEF2K",373.227240410355,0.328837534106917,0.276160919450637,1.
19074608659715,0.233753279591065,0.719949053328921
"5079","AP4M1",99.1663945955806,-0.26761419648857,0.224767775012898,-1
.19062528635706,0.233800720300962,0.719953389546574
"5080","RARS",232.071560771754,-0.2019731174683,0.169799181000442,-1.1
8948228300214,0.234249938736916,0.719980465534183
"5081","ZFAND5",1138.66659524411,0.372868317208386,0.313516340036187,1
.18931063422515,0.234317452202954,0.719980465534183
"5082","MED22",61.9683665655774,0.297627963209534,0.250230590299203,1.
18941478279557,0.234276486497563,0.719980465534183
"5083","FAM111A-
DT",28.6710860847662,0.368666949336613,0.310071233985122,1.18897501260
727,0.234449499835509,0.719980465534183
"5084","IL18BP",10.3828514868329,0.694058962544705,0.583168343822049,1
.19015198595295,0.233986660737622,0.719980465534183
"5085","RNF41",165.255500222371,-0.186548142942146,0.156899795162861,-
1.1889635849971,0.234453996864808,0.719980465534183
"5086","ARL1",854.638250043194,-0.211760115570176,0.17795368988059,-1.
18997316499743,0.23405693947081,0.719980465534183
"5087","AC130895.1",8.66626281335341,0.864395406284959,0.7269961650333
01,1.18899582674602,0.234441309146792,0.719980465534183
"5088","MAP4K5",342.917950813885,0.190945363997771,0.16058678610522,1.
18904779545596,0.234420859532542,0.719980465534183
"5089","AC026471.2",6.27501908796686,-0.678119051581475,0.569853626778
188,-1.18998812978588,0.234051057559971,0.719980465534183
"5090","HDGFL2",240.498519805549,-0.230752620885141,0.193954931402766,
-1.18972288673579,0.234155326879119,0.719980465534183
"5091","FBXO17",321.631386408811,0.216864226029094,0.182217968395336,1
.19013634022409,0.233992809095575,0.719980465534183
"5092","THAP7-
AS1",50.8587833390271,-0.390700691248962,0.328490421025759,-1.18938229
622935,0.234289264189864,0.719980465534183
"5093","YWHAH",1870.7061007897,-0.210002551559301,0.176404084467984,-1
.19046309042473,0.233864428672725,0.719980465534183
"5094","ORAI2",79.3318905181578,-0.307012265140314,0.25828922375898,-1
.18863753071944,0.234582332518915,0.720233152845666
"5095","C11orf53",3.05246874586118,1.29345998507623,1.0883419974858,1.
18846831976004,0.234648953918213,0.720296298190551

"5096","RIC1",97.6804453963578,0.23440648010358,0.197349065828063,1.18
777598019037,0.234921680040572,0.720709116483828
"5097","ANKRD26",189.595220050836,0.246360953625883,0.207395391249094,
1.18788056061472,0.234880469377554,0.720709116483828
"5098","WDR11",245.936689712198,0.273011375493764,0.229848299416099,1.
18778940800221,0.23491638842897,0.720709116483828
"5099","ISG15",491.140359261093,-0.445042486736054,0.375098919285264,-
1.18646699271772,0.235437929016432,0.720906391187631
"5100","ERRFI1",1709.98219229131,0.482923220562794,0.406861092978586,1
.18694863897495,0.235247880218642,0.720906391187631
"5101","ERMAP",52.9231079537664,0.311867476426047,0.26288228656836,1.1
863388762215,0.235488499731891,0.720906391187631
"5102","GGPS1",250.22692845498,-0.221488603015651,0.186672054104135,-1
.18651184334262,0.235420227193948,0.720906391187631
"5103","B3GALNT2",100.603663336067,-0.227666559351499,0.19182189351771
3,-1.18686431030604,0.235281146923642,0.720906391187631
"5104","RPSA",6550.66887830807,-0.25686206873324,0.216460751895412,-1.
18664499907747,0.235367678310235,0.720906391187631
"5105","MBLAC2",63.3149343163624,0.278274237761454,0.234358130380296,1
.18738887919056,0.235074264500246,0.720906391187631
"5106","CAVIN3",2170.27792772721,-0.286767270652097,0.241681371577071,
-1.18655098976318,0.235404777499358,0.720906391187631
"5107","TSKU",283.215761277303,0.384241444984023,0.323918346514448,1.1
8622933562945,0.235531744179312,0.720906391187631
"5108","PSENEN",471.837765394125,-0.3345335991536,0.281770580815376,-1
.18725524213898,0.235126956801816,0.720906391187631
"5109","CBR3",705.145403046427,-0.292587911628865,0.246473996109633,-1
.18709444504126,0.235190369231928,0.720906391187631
"5110","RPGR",67.8016376549968,-0.364702874676316,0.307482284120107,-1
.18609394268015,0.235585202388746,0.720906391187631
"5111","LAMP2",1011.43069702129,0.258777685742491,0.218166337625625,1.
18614855325002,0.23556363905649,0.720906391187631
"5112","ANGPTL5",737.659070855854,-0.524436164900272,0.442203037474714
,-1.18596237577916,0.235637158155925,0.720924325813511
"5113","UHMK1",204.324576797856,0.251631871265174,0.212214328904533,1.
18574401909672,0.235723405100404,0.721047145662101
"5114","SEC11C",62.3979944324386,-0.36686018205269,0.309504562856594,-
1.18531429283862,0.235893204435412,0.72142544336524
"5115","GEMIN7-
AS1",4.79780764101224,-0.824106934397664,0.695522124994104,-1.18487522
507591,0.236066784266461,0.721815152673989
"5116","SLC12A9",64.7481669933402,-0.27404080162451,0.231389086349354,
-1.1843289843444,0.23628285965286,0.722197605210219
"5117","KDM6A",114.955432695118,0.323489142184896,0.273142077124101,1.
18432555536992,0.236284216487256,0.722197605210219
"5118","TMEM214",218.400745207751,-0.277865356837676,0.234671394167805
,-1.18406147380275,0.236388729285019,0.722203261242189
"5119","LINC00852",5.0558544303563,-0.775494127621488,0.65494093780629
1,-1.18406726905633,0.23638643540741,0.722203261242189
"5120","RASSF3",26.6067650114097,-0.435463698943704,0.367835575134662,

-1.18385422286652,0.236470773709799,0.722203261242189
"5121","AC026471.4",6.41109892871165,-0.853074356970009,0.720531440500469,-1.18395160713248,0.236432219694661,0.722203261242189
"5122","AC005906.2",5.31521116827545,-1.13912745102861,0.962508356553437,-1.18349876473552,0.236611535790719,0.72249207726803
"5123","CYB5RL",26.1065241028844,0.357700894437085,0.302316541678908,1.18319987537103,0.236729942374682,0.722571655402641
"5124","EAF2",17.6365285115303,-0.491815252819998,0.41574210225593,-1.18298158919021,0.236816444048625,0.722571655402641
"5125","PURG",7.39427212674818,0.805241629728874,0.680787984083973,1.1828082289266,0.236885158555715,0.722571655402641
"5126","GABRB3",4.7487395950201,0.905540899911693,0.765528539021094,1.18289633077512,0.236850236017868,0.722571655402641
"5127","ETV2",9.37493560346584,-0.61796250711477,0.522467854905996,-1.1827761293103,0.236897883371103,0.722571655402641
"5128","RTEL1",20.0752180690027,-0.452140945927639,0.382284733477335,-1.182733461038,0.236914798523321,0.722571655402641
"5129","TASOR",345.042287678415,0.199477574257975,0.168674857097475,1.18261593749387,0.23696139326124,0.72257285837508
"5130","CMTR1",69.1189664931674,0.330350669058313,0.279423676053301,1.1822572579544,0.237103639529332,0.72286567684966
"5131","WDR61",314.324614916287,-0.269981787224008,0.228413952766893,-1.1819846552874,0.237211789509964,0.723054450971708
"5132","LINC01184",147.507496639072,-0.198339088801888,0.167835451469689,-1.1817472832175,0.237305990801205,0.723200642270236
"5133","AGO3",279.224628254613,0.29416000496151,0.249062951105875,1.18106688953695,0.237576152016838,0.723319083897141
"5134","PPCS",526.440671042743,-0.251399273518891,0.213040309935571,-1.18005495577302,0.237978358266196,0.723319083897141
"5135","SERTAD2",189.836940963967,-0.249662618185993,0.211625187355408,-1.17973962034445,0.238103790641188,0.723319083897141
"5136","IMMT",247.235556403486,-0.199014662411026,0.168575980750182,-1.18056357451037,0.237776141066806,0.723319083897141
"5137","CPS1",6.55350708006745,-0.695896088083355,0.589695901733482,-1.18009313959565,0.237963172884979,0.723319083897141
"5138","CAPN7",165.333534757223,0.222415499819536,0.188603807897989,1.17927364403923,0.238289229548818,0.723319083897141
"5139","DPH3",191.092942161661,-0.309439114432052,0.262423330755626,-1.17916007521529,0.238334440596647,0.723319083897141
"5140","COL25A1",6.19290403920313,0.787519206507144,0.667880116754743,1.17913258195757,0.238345386403161,0.723319083897141
"5141","DTWD2",21.9257320737968,-0.423690014132206,0.359136136141123,-1.17974765414783,0.238100594420157,0.723319083897141
"5142","CDC23",73.1739562765412,0.291362188653797,0.24718120406414,1.17873925631576,0.238502018789744,0.723319083897141
"5143","TNFRSF21",99.4477210574352,0.561979991074814,0.475752180190031,1.18124522487809,0.237505320110832,0.723319083897141
"5144","AC026979.4",17.0604279657098,0.548703021430831,0.465431506999353,1.17891249986132,0.238433019804024,0.723319083897141
"5145","FPGS",90.604022049177,-0.281122115045078,0.238520931893512,-1.

17860563772485,0.238555245677832,0.723319083897141
"5146","REXO4",182.528801282415,0.193254911143254,0.163808195732355,1.17976338289576,0.238094336879623,0.723319083897141
"5147","RBM7",381.591269270941,-0.286994493865794,0.24333921255037,-1.17942657721361,0.238228357362283,0.723319083897141
"5148","RESF1",217.437844203051,0.230814119632585,0.195781848721513,1.17893523398537,0.238423966362511,0.723319083897141
"5149","PA2G4",783.263274926498,-0.17534695519874,0.148563981458469,-1.18027905201072,0.237889246876051,0.723319083897141
"5150","ZNF268",88.73946905514,0.241604348294022,0.204892856661043,1.17917409240729,0.238328860111494,0.723319083897141
"5151","CLEC14A",33.3740269102948,0.692962113781614,0.586771094281178,1.180975205724,0.237612573147973,0.723319083897141
"5152","AC011330.2",3.40626005329363,1.07454258897967,0.911773732554531,1.17851891386376,0.238589796563659,0.723319083897141
"5153","HAPLN3",11.9338840134025,-0.698438035097817,0.591546461388613,-1.18069852612808,0.237722507230887,0.723319083897141
"5154","AC004494.1",3.41520884007721,1.19100825995041,1.00815935249478,1.18136905341715,0.237456146199354,0.723319083897141
"5155","SCARF1",9.1162705583926,-0.592688950505117,0.502914025316937,-1.17850948804143,0.238593552034869,0.723319083897141
"5156","WDR7",73.8477822589613,0.301549972456084,0.25546847103122,1.18038038603688,0.237848959354804,0.723319083897141
"5157","SMARCA4",154.174081234797,0.291731506470295,0.246940614520569,1.1813832529601,0.237450507837215,0.723319083897141
"5158","AL031846.2",20.4146105077268,0.668321359905725,0.566543644639801,1.17964673371393,0.238140747467484,0.723319083897141
"5159","NUP50",149.0378900569,0.255195296034367,0.216504542741672,1.17870642713885,0.238515095496514,0.723319083897141
"5160","DNAJB14",398.70232856018,0.169120689562104,0.143583190514266,1.17785855681554,0.238852999400394,0.723812782411753
"5161","AC092428.1",6.28878208613342,0.884628801252892,0.7511899840316,1.17763657670877,0.238941521457282,0.723812782411753
"5162","ALKBH8",40.1858495146581,0.307528112452166,0.26109852993316,1.17782399054829,0.238866782344397,0.723812782411753
"5163","RAB5IF",452.352198726391,-0.249221134769497,0.211609007673825,-1.17774350680595,0.23889887658436,0.723812782411753
"5164","TMEM185B",115.298240451407,-0.230163917399024,0.19556984062043,-1.1768886075115,0.239239969795923,0.72397473856859
"5165","WIPF1",333.807355523571,0.209103637311491,0.177674506333548,1.17689161842353,0.23923876788045,0.72397473856859
"5166","MRPS18C",395.443001537457,-0.286116107453679,0.2430966566773,-1.17696438677676,0.239209721030612,0.72397473856859
"5167","C5orf63",14.9874533538215,-0.534606706028653,0.454128116793584,-1.17721560559451,0.239109461456697,0.72397473856859
"5168","HOXA-AS3",4.37519731860492,0.882305318092054,0.749819287365863,1.17669061460344,0.239319015242942,0.72397473856859
"5169","LGALS9",26.0543452205869,-0.408752904141926,0.347324175820182,-1.17686280598431,0.239250269592203,0.72397473856859

"5170","RAP2C",164.381820265978,0.209887400694244,0.178354791649076,1.
17679709501279,0.239276502383041,0.72397473856859
"5171","SDCCAG8",287.744106093861,0.209374471356893,0.178061260030044,
1.17585639527411,0.239652266033682,0.724002596246242
"5172","NIPBL",562.602656180446,0.265804303403686,0.22600752746512,1.1
7608606396842,0.239560486227914,0.724002596246242
"5173","UBE2J1",368.193901224477,-0.171812823531486,0.146114218418311,
-1.17588024896798,0.239642732508273,0.724002596246242
"5174","PFKM",85.7642749144846,0.248470797037896,0.211227495870647,1.1
7631843342051,0.23946765237624,0.724002596246242
"5175","CD2BP2-
DT",5.65732779276883,-0.764281717759949,0.649863813834737,-1.176064433
02336,0.23956912929134,0.724002596246242
"5176","FKRP",53.3911773557929,0.373981174941637,0.318047455166612,1.1
7586595605905,0.239648444874887,0.724002596246242
"5177","CA11",36.4129448011194,-0.428882637976668,0.364729848195874,-1
.17589125238344,0.239638334900441,0.724002596246242
"5178","OXTR",7.41512713370938,-1.12849394388265,0.95996210472524,-1.1
7556092925735,0.239770376110876,0.724079683794959
"5179","ORMDL3",77.0022717551435,0.25504916943773,0.216942719435064,1.
17565212652399,0.239733916386163,0.724079683794959
"5180","OTUD7B",80.3709972274548,0.318999208605528,0.271526579931284,1
.17483602778873,0.24006032326059,0.724577589256255
"5181","TMSB10",24177.0851668524,-0.308707825572578,0.262742671276955,
-1.17494362096658,0.240017272373328,0.724577589256255
"5182","MSTN",72.2373835887383,2.41847597473982,2.06084291646611,1.173
53727225701,0.240580419219591,0.724577589256255
"5183","HTR2B",3.9434400560922,1.1246020342539,0.957835149231501,1.174
10812826841,0.240351718145999,0.724577589256255
"5184","AC092902.4",24.5582638414736,0.537543192436737,0.4580101850555
57,1.17364899291821,0.240535648699618,0.724577589256255
"5185","TBCCD1",41.2563959356962,-0.314260669458747,0.267915469935852,
-1.17298441009768,0.240802057539145,0.724577589256255
"5186","LRP2BP",6.88498327642404,0.756409285663237,0.644191461485645,1
.17419949019317,0.240315130198712,0.724577589256255
"5187","GM2A",98.6634657460083,0.23400265602213,0.199452271857448,1.17
322632549092,0.240705057770185,0.724577589256255
"5188","PXDC1",651.62598686728,-0.298920521686927,0.254773553295335,-1
.17327924276511,0.240683843428883,0.724577589256255
"5189","TFEB",69.8906729649678,-0.293386664075903,0.249787897509216,-1
.17454315041456,0.24017753884447,0.724577589256255
"5190","PHF10",288.08500549148,0.202231075525055,0.172388149946385,1.1
7311471576179,0.240749806020151,0.724577589256255
"5191","ZNF273",60.5437234233344,0.296829449797913,0.252811580479151,1
.17411334257448,0.240349629853696,0.724577589256255
"5192","SFR1",84.3723384448296,-0.239587307212635,0.204209579350432,-1
.17324225423085,0.240698671858158,0.724577589256255
"5193","CD6",8.09254487714847,1.0718461920181,0.913427499006511,1.1734
3324257688,0.240622112962492,0.724577589256255
"5194","IFT81",75.7050173188058,-0.272650031275249,0.232318280015331,-

1.17360558651371,0.240553042522547,0.724577589256255
"5195","GALNS",85.3123038246418,0.31753988785536,0.270668181014333,1.17317036182596,0.240727494831413,0.724577589256255
"5196","DUSP3",156.773047591205,0.220210676593184,0.187740744383224,1.17295090800152,0.240815492899873,0.724577589256255
"5197","BCAS3",70.30548520654,0.270065019661146,0.229928163614351,1.1745625912714,0.240169756961792,0.724577589256255
"5198","SYNJ1",64.2284135449001,0.322454703251896,0.274648670712161,1.17406249378806,0.240369994974721,0.724577589256255
"5199","SDHB",330.626521684612,-0.239926108197621,0.204695331956456,-1.17211323728994,0.241151596015713,0.724844135828008
"5200","LAPTM4A",10287.0206821747,-0.288110568603393,0.245847547285566,-1.17190743525595,0.241234221589331,0.724844135828008
"5201","ARFGEF1",216.879514825807,0.237732622121578,0.202745064015296,1.17256922271431,0.240968597596302,0.724844135828008
"5202","ZDHHC13",20.4251726643927,-0.545161454233234,0.465308413281151,-1.17161314662031,0.24135240746452,0.724844135828008
"5203","APLP2",2003.03314076809,0.175121707604954,0.149390771649637,1.17223912609316,0.241101063896895,0.724844135828008
"5204","CARHSP1",855.600374486801,0.332642348178908,0.283927371986327,1.17157548372943,0.241367535766769,0.724844135828008
"5205","PPP4C",440.638433223113,-0.295792413856662,0.252411717144815,-1.17186482942454,0.241251329504388,0.724844135828008
"5206","CDK5R1",4.72470672843034,0.921670460257572,0.786274831256806,1.17219885925173,0.241117226308923,0.724844135828008
"5207","CCDC9",51.0411120259594,-0.31626878281925,0.269835394920104,-1.17208041929745,0.241164770475312,0.724844135828008
"5208","HCK",5.34996620485536,-0.834000435852636,0.711735396383742,-1.17178440202653,0.241283626592404,0.724844135828008
"5209","AF131215.6",4.52515190361562,1.36637538495982,1.16660998619339,1.171235803851,0.241504007389108,0.724975561528915
"5210","MYO1E",184.841889311908,0.290550908714388,0.248048789313315,1.17134580466502,0.241459806922782,0.724975561528915
"5211","TAX1BP3",1287.85225727761,-0.308188005132969,0.263235441679584,-1.17076941906668,0.241691473128134,0.725399086494727
"5212","ZNF717",40.5621561452305,0.314663171596416,0.268807361152428,1.1705898612575,0.241763674601378,0.725476567683336
"5213","RASAL3",6.3942709398096,-0.788093218338144,0.67340227984403,-1.17031563736416,0.241873971270894,0.725577399708066
"5214","WFDC3",64.4678789205845,-1.04372301962925,0.891860843278347,-1.17027564052782,0.241890061513929,0.725577399708066
"5215","AC007365.1",6.12911322850811,0.806320674140273,0.689377027941614,1.16963670307935,0.242147200435964,0.72563381172593
"5216","PRRC1",274.894404827642,0.190994774181227,0.163258782791078,1.16988973527778,0.242045345215169,0.72563381172593
"5217","CCDC171",45.3992637571386,0.351150023890607,0.300143862982962,1.16993904323321,0.242025500370796,0.72563381172593
"5218","KLC2",45.8944144657138,-0.362858889134497,0.310289084032869,-1.16942202548143,0.242233640090862,0.72563381172593
"5219","MIPEP",40.4894073304395,-0.31583550950712,0.27005297703975,-1.

16953167104183,0.242189488737363,0.72563381172593
"5220","ITGAL",10.9878421379309,0.852183213161555,0.728627082829662,1.1695738921096,0.242172488945072,0.72563381172593
"5221","SELENOW",2136.65811852902,-0.255886788425916,0.218760141595348,-1.16971394587613,0.242116104033662,0.72563381172593
"5222","DPCD",183.359498334797,-0.307294832810788,0.262843988100807,-1.16911493784265,0.242357326256225,0.725670412289674
"5223","GANC",77.0970610040167,0.24535341579173,0.209834437542558,1.16927144402581,0.242294284468288,0.725670412289674
"5224","NAP1L2",3.67292041849386,1.24004159403638,1.06072941090195,1.16904611231809,0.242385053312101,0.725670412289674
"5225","LINC02731",7.87881384178955,-0.691553549699342,0.591687942449142,-1.16878087262828,0.242491928659633,0.725832471068099
"5226","IRF2BPL",301.308442788679,0.292927477584407,0.250647838306483,1.16868144390787,0.242532000882473,0.725832471068099
"5227","TNFRSF11B",171.862150461661,0.339530965530778,0.290577819822284,1.16846828067756,0.24261792660705,0.72595071209762
"5228","CTNNBIP1",106.10032177321,-0.263897986133894,0.226054938662445,-1.16740641764061,0.243046280653546,0.726018579693551
"5229","CENPS",71.5657634404301,-0.328132530111041,0.28127915647754,-1.16657250476803,0.243383052397808,0.726018579693551
"5230","PFDN2",531.137928540577,-0.257727321736915,0.220854335907376,-1.16695613277433,0.243228085348181,0.726018579693551
"5231","FAM71A",26.7816531462998,0.7274657987987,0.622933936984531,1.1678056943248,0.242885150681448,0.726018579693551
"5232","ATAD2B",164.483105683155,0.361438480766509,0.309660098710419,1.16721037767449,0.243125420994764,0.726018579693551
"5233","AC097634.1",6.67349307666047,0.863843406737126,0.74004954620788,1.16727780074129,0.243098200601023,0.726018579693551
"5234","ALG3",140.854685236832,-0.302803619754871,0.25934929999082,-1.16755132851945,0.242987792506865,0.726018579693551
"5235","GLRB",16.4028065977714,0.832707044790671,0.713742860441245,1.16667653148345,0.243341023811648,0.726018579693551
"5236","UBE2D2",915.383400169344,-0.232714623759028,0.199349939749597,-1.16736741456426,0.243062024554752,0.726018579693551
"5237","CEP290",248.365576986459,0.230331077426563,0.19740540691176,1.16679214125842,0.243294321453787,0.726018579693551
"5238","TPP2",196.09206774707,0.252575338339001,0.216498767795788,1.16663637816748,0.243357245839006,0.726018579693551
"5239","PLXDC1",651.243113696376,0.272777454316487,0.233798423701693,1.16672067329474,0.243323191300365,0.726018579693551
"5240","ZNF66",32.5329916961659,0.451013461847692,0.386614405634209,1.16657179679542,0.24338333844842,0.726018579693551
"5241","AC005498.3",4.1882681447338,0.86587308475451,0.741493290895246,1.16774230513818,0.242910726667821,0.726018579693551
"5242","ARHGEF9",85.3132526076905,0.261688866244325,0.224233506368788,1.1670373017935,0.243195305904056,0.726018579693551
"5243","RLIM",210.190820027949,0.269111411960782,0.230487029167617,1.16757725123472,0.242977330763823,0.726018579693551
"5244","STIM2-

AS1",3.5188409377769,0.976649071571252,0.837413578832589,1.16626849176
815,0.243505908125655,0.726107226517681
"5245","KITLG",256.034461662132,-0.287104779916172,0.24616025125057,-1
.1663328196067,0.243479908750033,0.726107226517681
"5246","OXER1",9.71286787047131,0.695278004334164,0.596746710866894,1.
16511409560035,0.243972811934186,0.726115086327434
"5247","INSIG2",226.957339036079,-0.202407879381947,0.173676722437013,
-1.1654289448913,0.243845406566628,0.726115086327434
"5248","NISCH",190.773816153853,0.251953638345474,0.216110596847156,1.
16585508541105,0.243673041079627,0.726115086327434
"5249","AC009126.1",6.98336811295673,0.668151017274822,0.5733680816900
03,1.16530905470958,0.243893915227959,0.726115086327434
"5250","VARS",100.364373771352,-0.312786373488441,0.2684539821073,-1.1
6513963038709,0.24396247741104,0.726115086327434
"5251","RPS10",7726.41033480788,-0.37325112497694,0.320112333449975,-1
.16600045038649,0.243614263380314,0.726115086327434
"5252","POMZP3",21.756210736398,0.417697935624971,0.358397342190302,1.
16546047209017,0.243832651499779,0.726115086327434
"5253","AC073896.2",32.9270604105036,-0.33328162821091,0.2859680134177
93,-1.16545072376326,0.243836595363738,0.726115086327434
"5254","NR2C1",106.887978462225,0.225421514179663,0.193424896266682,1.
16542140402064,0.243848457471957,0.726115086327434
"5255","CAVIN1",4210.09505314964,-0.196718773354163,0.16882185185716,-
1.16524473099967,0.243919943981466,0.726115086327434
"5256","ETS1",177.333161146198,0.309638490484097,0.265795909418247,1.1
6494829119759,0.244039924319301,0.726176639336734
"5257","COX6C",3098.1814867578,-0.25454603272253,0.21853759411854,-1.1
6476999643575,0.244112106873236,0.726253253090623
"5258","THOC3",77.2396111077092,-0.363512130835253,0.312162714257162,-
1.16449567559753,0.244223194818901,0.726307428592436
"5259","GLIDR",31.4967900570326,0.375688841396054,0.322594157290023,1.
16458662659007,0.244186359710645,0.726307428592436
"5260","DDI2",176.367737476265,0.31728083921331,0.272712850121839,1.16
342460236678,0.244657272499618,0.72718353133676
"5261","ADSS",388.989573069787,-0.240571808593362,0.206759588801858,-1
.1635339864402,0.24461291721266,0.72718353133676
"5262","C12orf65",163.99430381534,0.228587497439662,0.196459026668097,
1.16353776823828,0.244611383792933,0.72718353133676
"5263","ANKMY2",149.630842846458,0.226051450702041,0.194352056566823,1
.16310295190686,0.244787734600385,0.72729486496011
"5264","CDK5RAP2",162.066174394658,0.260997330400517,0.224394132213255
,1.16312012184203,0.24478076920535,0.72729486496011
"5265","GPR176",67.4392676312772,-0.399612675735276,0.343710960388401,
-1.16264164309368,0.244974927635134,0.727666433547104
"5266","PRDM15",15.3097009941848,0.469409114506963,0.403770046315114,1
.16256547208215,0.245005846487151,0.727666433547104
"5267","LINC01963",15.5701247302117,0.563636450993123,0.48505940245069
1,1.1619469208396,0.245237621006749,0.727932542031632
"5268","SDHAF2",253.225677660546,-0.267874427021425,0.230551308654312,
-1.16188638696073,0.245281617423702,0.727932542031632

"5269","AC022898.2",2.60149600611341,-1.20104997985023,1.0335760509651
6,-1.16203348435626,0.245221863902361,0.727932542031632
"5270","TNFSF9",315.649562095253,-0.327046901492893,0.281435236891313,
-1.16206806619313,0.245207817641302,0.727932542031632
"5271","CRYZ",104.809374356844,0.261248431476777,0.224922353088644,1.1
6150497222398,0.24543660230226,0.728116172232046
"5272","TRERF1",232.908399589848,0.253966615568902,0.218644881918863,1
.16154841284209,0.245418947083444,0.728116172232046
"5273","NCBP2",312.083550541088,-0.161286405430664,0.138876604848617,-
1.16136483611819,0.245493562757758,0.728147036133385
"5274","RWDD2A",50.392535660349,-0.317966134817749,0.273844830864842,-
1.16111789955489,0.245593956448688,0.728256106254774
"5275","PPIA",4660.17216127095,-0.247541345381099,0.213226608539263,-1
.16093083821439,0.245670026636841,0.728256106254774
"5276","ADGRG2",310.140785123468,0.42888304364185,0.369422762106285,1.
16095456922186,0.245660375292731,0.728256106254774
"5277","ITGAX",12.7701714663077,-0.773155127079313,0.666204179469524,-
1.16053779142447,0.245829916502389,0.728591982963305
"5278","GAPDH",5749.03691477628,-0.33627604084041,0.289936085839513,-1
.15982817339456,0.246118771036007,0.728914918342221
"5279","PTGR2",50.4222316483931,-0.321616823181619,0.277355519309667,-
1.15958328134994,0.246218511099869,0.728914918342221
"5280","PER1",455.477885190926,0.344614068146458,0.297088908322605,1.1
5996948553948,0.246061230064749,0.728914918342221
"5281","GJD3",3.63091356122849,1.02480620638553,0.883603501381894,1.15
980324295095,0.246128923456005,0.728914918342221
"5282","ARMC7",37.7686171374596,0.318488940398054,0.274644774362023,1.
15963954216088,0.246195594591246,0.728914918342221
"5283","MYL9",2509.02583241447,0.373639675099097,0.32216691677533,1.15
977046569205,0.246142271779849,0.728914918342221
"5284","KRCC1",356.911157527079,-0.155104322733598,0.133863207699542,-
1.15867776814173,0.246587556154649,0.728944279533556
"5285","SPICE1",63.0500330710687,0.296966394577369,0.256250322572607,1
.15889178829512,0.246500296539942,0.728944279533556
"5286","ADH5",1285.56529577188,0.279591354266963,0.241241480946984,1.1
5896881900011,0.246468895122557,0.728944279533556
"5287","PDGFRB",1306.03703389501,0.163575983143942,0.141164716330679,1
.15875969148526,0.24655415207375,0.728944279533556
"5288","AP002833.1",2.75764307910398,-1.55009114305599,1.3378492674192
7,-1.15864408704737,0.246601290473916,0.728944279533556
"5289","MIPOL1",50.029521701823,0.378107389790129,0.326241503585577,1.
1589800366738,0.246464322493489,0.728944279533556
"5290","PROCA1",15.3165768800631,0.449204258382168,0.387647494106402,1
.15879572346434,0.246539461109737,0.728944279533556
"5291","PNMA8A",9.18495618822441,0.664436772388407,0.573276564479299,1
.15901610768253,0.246449619371639,0.728944279533556
"5292","AJAP1",23.2404322336601,-0.612260395143678,0.528810399156105,-
1.15780702520364,0.246942795840294,0.72906474445119
"5293","AL592295.5",110.545756489264,-0.512783215667489,0.443305636415
798,-1.15672613552453,0.247384268217724,0.72906474445119

"5294","ANGEL2",150.566076299891,0.251719576227102,0.217615472629014,1
.15671727375851,0.247387889949007,0.72906474445119
"5295","STAT1",321.544974513946,0.27657647883081,0.238962745368488,1.1
5740417362682,0.247107269673047,0.72906474445119
"5296","SF3B1",1006.68758803596,0.182264508933184,0.157561274384808,1.
15678493744627,0.247360237293776,0.72906474445119
"5297","CCDC51",155.717634489079,0.440772238547189,0.380847834307862,1
.15734474202336,0.247131540545312,0.72906474445119
"5298","CEP135",68.8468719895854,0.368865263370875,0.318780213682535,1
.15711467506016,0.247225511783605,0.72906474445119
"5299","YIPF3",742.427381867192,-0.244025981367314,0.210861106576824,-
1.1572830349271,0.24715674245734,0.72906474445119
"5300","NPR2",89.7471926588464,0.282623200937908,0.244187320417671,1.1
5740326096578,0.24710764237592,0.72906474445119
"5301","ARL5B",209.566219108624,0.310918719647572,0.268468927321626,1.
15811808371809,0.246815851204371,0.72906474445119
"5302","RELA",261.793686087061,-0.170217084200553,0.147140872010443,-1
.15683074236826,0.247341519061994,0.72906474445119
"5303","SNRPF",670.348818058111,-0.228613231926384,0.197535946313566,-
1.15732471073131,0.247139721365935,0.72906474445119
"5304","GON7",99.6648687258818,-0.345140850373858,0.298133333980745,-1
.15767279614614,0.246997589556642,0.72906474445119
"5305","BAHCC1",40.0169550152176,0.387970868628297,0.334959595756644,1
.15826169347949,0.246757258723868,0.72906474445119
"5306","ZNF175",35.7276309668204,0.382358058026878,0.330225706386724,1
.15786884737284,0.246917562243085,0.72906474445119
"5307","LPIN3",105.701469724059,-0.262762107038506,0.227012627133348,-
1.15747793572804,0.247077148787112,0.72906474445119
"5308","UBXN10",3.24093425306794,-1.28125838811428,1.10895265337492,-1
.15537699848139,0.247936076957226,0.729203943671727
"5309","LMO4",590.111411740945,-0.238680206699474,0.206480251079762,-1
.1559469026763,0.247702876212615,0.729203943671727
"5310","ATXN7L2",5.96874139536941,0.741389508784945,0.641880329206584,
1.15502761971435,0.248079116210018,0.729203943671727
"5311","NAV1",1072.26159280455,0.296983657981865,0.257064571978281,1.1
5528816630149,0.247972440290164,0.729203943671727
"5312","DALRD3",237.534470032118,-0.279738037709595,0.242219998298001,
-1.15489241051615,0.248134487737911,0.729203943671727
"5313","EEF1AKMT4",10.0200875003329,-0.582860019522149,0.5041929389657
77,-1.15602574823388,0.247670625264981,0.729203943671727
"5314","APOD",5455.26940445441,0.488013035474457,0.42225353137537,1.15
573464568761,0.247789712314164,0.729203943671727
"5315","AC139887.4",8.69768124473721,0.806925572543896,0.6984525846248
76,1.15530472691611,0.247965660941902,0.729203943671727
"5316","SMIM8",163.363551933867,0.262363510820744,0.226979608981032,1.
15589022290839,0.247726062292775,0.729203943671727
"5317","GNA12",251.812879134968,0.211894147641058,0.183467579426751,1.
15494055300194,0.248114771201899,0.729203943671727
"5318","PTP4A3",18.2510551076152,0.529495380216077,0.45836176939342,1.
15519097702409,0.248012228864896,0.729203943671727

"5319","KIAA0586",117.891367873205,0.249992310878494,0.21619002970329,
1.15635448693724,0.247536189826116,0.729203943671727
"5320","TMC3-
AS1",12.799129340252,0.561714903722235,0.485910008121632,1.15600603884
172,0.247678686909382,0.729203943671727
"5321","HSPBP1",79.5286749264421,-0.269431820198317,0.232984641194006,
-1.15643597285008,0.247502874623082,0.729203943671727
"5322","LINC01204",9.91495596645698,-0.616848192154906,0.5340547372551
4,-1.15502803200529,0.248078947379854,0.729203943671727
"5323","LINC00997",15.1300176765416,-0.455401638217226,0.3943793863347
53,-1.15472982107304,0.248201083607711,0.729262624013639
"5324","EIF4E",260.374747529627,-0.262596669201291,0.227474183029984,-
1.15440207633003,0.24833536431868,0.729383116984819
"5325","ADAM9",662.14391207355,0.193187575526561,0.167342598737304,1.1
5444350084361,0.248318389423796,0.729383116984819
"5326","AC099778.1",20.2636944890872,0.407699087558976,0.3532659117383
46,1.15408555994768,0.248465092876939,0.729505235683478
"5327","SENP3",26.8984198675104,-0.410218047587798,0.35545241683409,-1
.15407302963781,0.248470229570709,0.729505235683478
"5328","GRTP1",25.1341444798157,0.379258880453596,0.328761835221728,1.
15359764979354,0.248665162356618,0.72981433469864
"5329","GLIS2",126.531518053271,-0.281422460472766,0.243953899572788,-
1.15358869428032,0.248668835652753,0.72981433469864
"5330","GNG12",758.817382937739,0.160730085722047,0.13942221839746,1.1
5282978258058,0.248980257468666,0.729817428550481
"5331","ADGRL4",3.50857623245241,-1.04227193576422,0.904731734201409,-
1.15202318694415,0.249311545349319,0.729817428550481
"5332","PTGFRN",380.504856316913,-0.28651354365816,0.248539786165805,-
1.15278743929964,0.248997641195925,0.729817428550481
"5333","MDM4",355.140597614611,0.276316069534225,0.239813271409218,1.1
5221341967651,0.249233384522585,0.729817428550481
"5334","LINC01119",6.18332835300352,-0.697806197922257,0.6060894470108
95,-1.15132543779419,0.24959837584883,0.729817428550481
"5335","CALCRL",57.1550168128156,-0.642033062790508,0.556863841193792,
-1.1529444278769,0.248933194931709,0.729817428550481
"5336","CEP44",133.482012901808,0.247590029542223,0.214885797281573,1.
15219354966395,0.249241547703418,0.729817428550481
"5337","NDUFA4",2458.63573670537,-0.301931406448318,0.262133058594364,
-1.15182498562892,0.249392998446133,0.729817428550481
"5338","HOXA2",2.81461001375201,1.14323422232173,0.992001133285674,1.1
5245253655623,0.249135162989339,0.729817428550481
"5339","TMEM225B",34.9509401233887,-0.41963595899254,0.364256020496364
,-1.15203575337124,0.249306381659328,0.729817428550481
"5340","ZNF572",6.05007613745653,0.788015994763268,0.683700900711029,1
.15257416502414,0.249085212305827,0.729817428550481
"5341","LINC00861",2.8363503684213,1.16619417572636,1.0125311251458,1.
15176131060508,0.249419170372268,0.729817428550481
"5342","MICU1",297.542026771553,0.190255964389475,0.165016240476344,1.
15295296899428,0.248929689004104,0.729817428550481
"5343","LINC00863",51.1403750759326,-0.371861497531995,0.3227913505090

63,-1.15201815954965,0.249313611184707,0.729817428550481
"5344","PSMD13",607.877336601316,-0.246633798245,0.214144246543926,-1.
15171806959759,0.249436944533436,0.729817428550481
"5345","CHD4",478.057756875286,0.265200559215016,0.23028068503531,1.15
164048245884,0.249468838847319,0.729817428550481
"5346","ZBTB42",11.0425753237264,-0.548946305529339,0.476799899023672,
-1.15131380407881,0.249603160186478,0.729817428550481
"5347","AC027279.1",47.2587136183345,0.416064899605432,0.3613215891570
14,1.15150855108364,0.249523079388238,0.729817428550481
"5348","PHF12",91.7069420394238,0.279701429149025,0.242853422558717,1.
1517294102841,0.249432282873026,0.729817428550481
"5349","CHD6",301.695423253038,0.219232403745198,0.190192077557009,1.1
5268946299556,0.24903786791118,0.729817428550481
"5350","SAMD11",4.01250323803523,-1.06391032687904,0.928890571393247,-
1.14535593281271,0.252061744939613,0.729826635841038
"5351","CASZ1",19.7972754359631,0.573604995032513,0.501318019507622,1.
14419385043428,0.252543252877638,0.729826635841038
"5352","CSMD2",10.2447136936172,0.637838386133547,0.556834878745155,1.
14547132458896,0.252013967404815,0.729826635841038
"5353","ATP5PB",711.446475893379,-0.230971597822094,0.200952000444781,
-1.14938690488708,0.250396477339002,0.729826635841038
"5354","FAM72B",6.85831840053909,0.843656825845905,0.733795984189517,1
.1497157848005,0.25026095084066,0.729826635841038
"5355","S100A13",5986.96887292363,-0.308898055242048,0.269804886087441
,-1.14489422234532,0.252252977623938,0.729826635841038
"5356","LINC01133",2166.52325399762,-0.229132350071591,0.1994528328037
28,-1.1488046915687,0.250636524397724,0.729826635841038
"5357","ROCK2",810.700388984451,0.208531885145435,0.181254003006217,1.
15049533630594,0.249939913957736,0.729826635841038
"5358","SETD5",430.157351925503,0.268978913827539,0.234685044890622,1.
14612720189691,0.251742523795307,0.729826635841038
"5359","MYL3",5.64925905869461,0.951103101805328,0.827111036156375,1.1
4990981891035,0.250181016314564,0.729826635841038
"5360","SERPINB1",617.074636068237,-0.246032582612362,0.21457635392314
1,-1.1465969018212,0.251548257532819,0.729826635841038
"5361","PBX2",111.551285187696,0.271499887834211,0.236882747293738,1.1
4613618313683,0.251738808204614,0.729826635841038
"5362","NFKBIE",32.4282186239158,-0.386449691614211,0.337559046327323,
-1.14483583189022,0.252277169167164,0.729826635841038
"5363","CCNC",220.788979911556,-0.236208778290362,0.205575994066216,-1
.14900953957824,0.250552047072729,0.729826635841038
"5364","TBRG4",97.0825358557233,-0.29937157415835,0.260751961464052,-1
.1481086181575,0.250923726868575,0.729826635841038
"5365","AC002467.1",21.6399102930695,-0.407977179450695,0.355226494394
337,-1.14849873500088,0.250762734932824,0.729826635841038
"5366","IQUB",6.08415918345087,-0.803454481968067,0.699295282231604,-1
.14894880944151,0.250577089516523,0.729826635841038
"5367","AC078846.1",29.6111981375066,0.368895382520358,0.3220842757251
79,1.14533806932916,0.252069141812845,0.729826635841038
"5368","CTSB",999.873074675283,-0.149155772405263,0.130355668829445,-1

.14422160343802,0.252531745975245,0.729826635841038
"5369","GFRA2",2.76476455252419,-1.24705780818966,1.08990746429545,-1.1441868681905,0.252546147901004,0.729826635841038
"5370","DCTN6",421.1753360982,-0.28822896801191,0.251242940053793,-1.14721220803338,0.251293927269132,0.729826635841038
"5371","AC087623.3",15.2032020969404,0.654252736370079,0.569352987184741,1.14911619170585,0.250508072643678,0.729826635841038
"5372","ZNF250",26.8622153395452,0.353900245796156,0.307831150317315,1.14965702928814,0.250285159350221,0.729826635841038
"5373","SMIM27",101.753021407038,0.277185139437259,0.242134728615767,1.14475581847291,0.252310321869897,0.729826635841038
"5374","BAG1",631.37538477821,-0.266345193159787,0.232510611035776,-1.14551844310798,0.25199445997339,0.729826635841038
"5375","SHB",15.815910778241,0.497113962419175,0.433224143123125,1.14747520494926,0.251185275046504,0.729826635841038
"5376","AL353748.3",3.95274384636486,0.89893850848974,0.782089918240135,1.14940557028601,0.25038878423551,0.729826635841038
"5377","LZTS2",325.195239752773,0.178106962279894,0.154763796188409,1.15083092213031,0.249801800767998,0.729826635841038
"5378","GRK5",625.867670164793,0.25877698833991,0.226111272963973,1.14446743387775,0.252429836136517,0.729826635841038
"5379","RBM4B",74.927199453297,0.292511691829192,0.254732472051446,1.14830939877236,0.250840860480546,0.729826635841038
"5380","NCAM1",10.4481830141219,-0.663664542278662,0.577140768030761,-1.14991797329294,0.250177657415748,0.729826635841038
"5381","ITFG2-AS1",6.02329612689281,0.814363505562841,0.707725073875457,1.15067776404111,0.24986482766169,0.729826635841038
"5382","PLBD1-AS1",12.245908872683,0.495377556657045,0.432344782961663,1.14579283983397,0.251880878524811,0.729826635841038
"5383","AC139768.1",9.43559195475305,0.697203175440744,0.607431385419623,1.14778918603145,0.251055602609292,0.729826635841038
"5384","ALX1",6.7940365533645,0.893440407197629,0.779467654067881,1.1462187077744,0.251704669076954,0.729826635841038
"5385","ULK1",165.177711318199,0.351780905510731,0.307439369722669,1.14422855416358,0.252528864134658,0.729826635841038
"5386","SLC25A30",19.1914909536992,0.514552689812286,0.449381324827099,1.14502464028798,0.252198950466534,0.729826635841038
"5387","ACIN1",321.696527926503,0.247678662112408,0.215947876982848,1.1469372404716,0.251407559997929,0.729826635841038
"5388","AL121603.2",45.3786573610797,-0.346602791905835,0.301888782873384,-1.14811417836351,0.250921431797265,0.729826635841038
"5389","SIX1",184.11610851083,0.297842639418082,0.259945384057151,1.14578929915754,0.251882343894928,0.729826635841038
"5390","CLMN",5.64988032485257,0.845186114236008,0.738113211098639,1.14506298156891,0.252183068677174,0.729826635841038
"5391","SLC24A1",15.5840665500532,0.517025666646499,0.451250270306434,1.14576256385486,0.251893408953808,0.729826635841038
"5392","LRRC28",99.9851504907121,-0.245949545197499,0.213920910257994,

-1.14972185234663,0.250258450976915,0.729826635841038
"5393","CHD9",1007.57329961412,0.197609864373431,0.172578968720659,1.14504024353794,0.252192487178162,0.729826635841038
"5394","TMEM208",447.299559012461,-0.309976695971316,0.270658169113883,-1.14527005405438,0.252097306818121,0.729826635841038
"5395","SNTB2",959.340105581939,0.246173965582702,0.213986036411927,1.1504206989881,0.249970638828324,0.729826635841038
"5396","DERL2",393.192218127536,-0.278619278511662,0.242159871416144,-1.15055924370253,0.249913608216002,0.729826635841038
"5397","UNC13D",7.01550901036755,0.833193841052841,0.725698113229621,1.14812733540787,0.250916001057729,0.729826635841038
"5398","MPPE1",198.761801442057,-0.210669116806649,0.183294808199656,-1.1493457936745,0.250413422258283,0.729826635841038
"5399","PTPN2",145.078936938324,-0.209181711936429,0.182296006819192,-1.14748378522577,0.251181730819116,0.729826635841038
"5400","SERPINB2",24.1318476767156,-0.698520598695655,0.608499043878043,-1.14794033897554,0.250993193946648,0.729826635841038
"5401","TNFSF14",23.0612839952578,0.614456035504888,0.534308624998102,1.15000209009741,0.250143010421613,0.729826635841038
"5402","ZFP30",33.3721477785236,0.430769924875504,0.376240915944641,1.14493109765575,0.252237700777446,0.729826635841038
"5403","BCAT2",80.8840180081151,-0.291598299422605,0.253796637667896,-1.14894469092287,0.250578787876175,0.729826635841038
"5404","PCED1A",113.901691612969,0.21556131064692,0.188290228058944,1.14483535799552,0.252277365511418,0.729826635841038
"5405","PRMT2",559.009409147014,0.147704308388949,0.12851069281758,1.14935422999091,0.250409944973717,0.729826635841038
"5406","LINC00899",47.8240246018308,0.374154249106871,0.326248598816438,1.1468378729111,0.251448633320336,0.729826635841038
"5407","MAOB",21.8452225875179,-0.433050537161354,0.377166778473327,-1.14816723496759,0.250899532502113,0.729826635841038
"5408","SLC25A43",156.510339865349,-0.176884916080776,0.154359321112087,-1.14592960636522,0.251824279882531,0.729826635841038
"5409","LDOC1",32.9106264394031,0.457309644407466,0.398278533253727,1.14821564866046,0.250879550748492,0.729826635841038
"5410","CXorf40B",53.7273020991114,-0.379616044324803,0.331699925932468,-1.14445622276711,0.252434483116383,0.729826635841038
"5411","VAMP7",156.355063747374,0.202285512540175,0.175751538270995,1.15097435009796,0.249742788039949,0.729826635841038
"5412","HR",3.26418797947471,-1.03092780355782,0.898949273158164,-1.14681421337156,0.251458413619349,0.729826635841038
"5413","BOK",36.7199240840405,-0.338703775629481,0.296059977651177,-1.14403769910618,0.252608002883012,0.729870527450639
"5414","FGD2",3.95575237662004,-1.14966504362483,1.00526267344512,-1.14364640605309,0.252770308016617,0.729934936739272
"5415","DNAJC2",218.598650764659,-0.215391423144014,0.188323502354252,-1.14373097596095,0.25273522296216,0.729934936739272
"5416","FAM13C",16.7650602516268,-0.608523575869992,0.531986542065451,-1.14387024436254,0.252677452838657,0.729934936739272
"5417","SLC4A2",124.276353044125,-0.223738538601157,0.19569188286618 9,

-1.14332048587905,0.252905552474437,0.730068951776092
"5418","EPS8",590.97335164951,-0.233616777198226,0.20433378642369,-1.14330958813545,0.252910075493789,0.730068951776092
"5419","TBX21",3.80651322355516,0.987328994166099,0.863844088230156,1.14294814031655,0.253060123387425,0.730367287281663
"5420","FGD5",227.120737115693,0.273452219380764,0.239342853967697,1.14251257076458,0.253241023824153,0.730652600455497
"5421","ZDHHC2",118.83100052029,-0.308204135482237,0.269844344574472,-1.14215525238543,0.253389492272837,0.730652600455497
"5422","CCNJ",17.9169517809246,0.439841194517791,0.38513794936678,1.14203545831033,0.253439281168227,0.730652600455497
"5423","CCDC153",4.15296445237646,-0.81450274170921,0.713135509388515,-1.14214301627977,0.253394577539077,0.730652600455497
"5424","DDX49",181.849247780534,-0.259473567552218,0.227168118620128,-1.14220943118392,0.253366976680621,0.730652600455497
"5425","NRSN2",63.5814677884912,0.338790344779577,0.296542016978039,1.14246995495639,0.25325872782717,0.730652600455497
"5426","UBE2O",54.9985119866618,0.35467679844124,0.310800777719174,1.14117088459061,0.253798817894823,0.73155427789809
"5427","PRICKLE4",4.39966161029299,-0.99660729832045,0.873559680321466,-1.14085771215277,0.253929139566608,0.731759867842847
"5428","AP006623.1",23.968106270569,0.444880038239028,0.389980654207827,1.14077463443082,0.253963718839576,0.731759867842847
"5429","AC004540.2",75.633134384097,0.968923809233911,0.849507256717301,1.14057155082826,0.254048261716536,0.731804576893547
"5430","CDK6",341.691370505661,0.307957473839407,0.270016728012131,1.14051257530079,0.254072816658054,0.731804576893547
"5431","MRPL20",895.931187410961,-0.247286623901797,0.216914778600338,-1.14001740912922,0.254279048283098,0.731883070730031
"5432","TUFT1",37.9837990948977,-0.333271006539562,0.292833534527296,-1.13809030471029,0.255082776122084,0.731883070730031
"5433","DNM3",9.6145201449017,0.797296885010726,0.700331645497001,1.13845617306771,0.25493004954339,0.731883070730031
"5434","HLX-AS1",4.05641707283451,1.20478829681773,1.05708734713501,1.13972445142119,0.254401116987495,0.731883070730031
"5435","DHX57",73.1316729473745,0.298861488733198,0.262513022776813,1.13846347724733,0.254927001164322,0.731883070730031
"5436","HECW2",135.539678136483,0.335359886164026,0.294441468304649,1.13896961625337,0.254715827191253,0.731883070730031
"5437","AC064836.4",24.002726752021,0.533887254172645,0.468732221175741,1.13900267584224,0.254702038130439,0.731883070730031
"5438","AC104083.1",2.61177437088786,1.33574006989865,1.17205939275914,1.13965220376263,0.254431227182664,0.731883070730031
"5439","DYNC1I1",4.77324438884094,1.06582750858038,0.935896071309925,1.13883105320508,0.254773627094733,0.731883070730031
"5440","TSPAN12",74.3631039874891,-0.362057963161669,0.317684133784524,-1.13967908579042,0.254420023442026,0.731883070730031
"5441","MCMDC2",2.95121019561666,1.01807834572118,0.893859846207073,1.13896865380093,0.254716228635219,0.731883070730031

"5442","UBE2W",147.344164933781,0.240495863190975,0.211025857946964,1.
13965115711752,0.254431663404478,0.731883070730031
"5443","CAND1",332.67483416599,0.186951471383963,0.164115794099677,1.1
3914369064574,0.25464322708626,0.731883070730031
"5444","SGCG",49.8554373004192,0.624533180994047,0.548734058696684,1.1
3813453183022,0.255064310755833,0.731883070730031
"5445","LINC00926",8.51344211903355,0.613273005949978,0.53835379062402
2,1.13916353266337,0.254634952615491,0.731883070730031
"5446","KLHL11",9.73064652612068,0.603528253528731,0.52975766349202,1.
13925346459442,0.254597451761407,0.731883070730031
"5447","AP000915.2",3.95476420080673,-1.03175897114953,0.9062014997584
51,-1.13855359037096,0.254889394788079,0.731883070730031
"5448","ABHD3",36.4995407790394,0.390486982222727,0.342671209477148,1.
13953834294552,0.25447868526071,0.731883070730031
"5449","UBXN6",482.547072150437,-0.216930840377382,0.190314119748951,-
1.13985678342491,0.254345972252931,0.731883070730031
"5450","GPR108",286.331208490904,-0.214280797878505,0.188256632410534,
-1.13823770846607,0.255021236855366,0.731883070730031
"5451","AC020910.5",38.0170049261444,-0.484264443520389,0.425417732875
985,-1.13832688695551,0.25498401093832,0.731883070730031
"5452","SFXN5",49.7338236995713,0.369541941663,0.32480422459417,1.1377
3748517196,0.255230115900313,0.732063769506759
"5453","AC003102.1",12.9567297621496,0.678730769270214,0.5965734575921
95,1.13771533183794,0.25523936925322,0.732063769506759
"5454","FAF1",137.549920381633,-0.19669897982309,0.17294786922585,-1.1
3733103913656,0.255399923786457,0.732072410350297
"5455","DCP1A",241.093496814264,0.202652146491915,0.178206395809324,1.
13717661799719,0.255464459518373,0.732072410350297
"5456","HEATR5A",216.338632896743,0.317639786019579,0.279295423891074,
1.13728961826263,0.255417233299038,0.732072410350297
"5457","KIF16B",93.9978196966259,0.277848428405148,0.244293780024678,1
.13735367464976,0.255390464897948,0.732072410350297
"5458","NNAT",3.32508261189802,-1.10427739006514,0.971093814716248,-1.
13714800087343,0.25547642044066,0.732072410350297
"5459","ZC3H11A",12.3089310059277,0.604774617519254,0.531952593506748,
1.13689570255208,0.255581888853607,0.732085361678847
"5460","VCAN",6545.3472886229,-0.283033811431765,0.24897386638775,-1.1
3680128576615,0.255621365737096,0.732085361678847
"5461","VWF",5.621928375271,-1.04782658009302,0.921664701717043,-1.136
88478916567,0.255586451665508,0.732085361678847
"5462","GCFC2",97.5712838313146,-0.215629341812731,0.18979357339052,-1
.13612562301597,0.255903992769748,0.732257010062749
"5463","MOB4",367.590817970482,-0.225808122253864,0.198764983736101,-1
.13605584851741,0.255933191527346,0.732257010062749
"5464","PLXNB1",42.5864863372055,0.359014469417039,0.316348954493647,1
.13486851882183,0.256430412027729,0.732257010062749
"5465","SEPSECS",38.5678751067517,0.320896848991302,0.282628943700956,
1.13539981004506,0.256207839241101,0.732257010062749
"5466","KCTD8",21.337131325817,0.730242047435936,0.643206272662133,1.1
3531549438033,0.256243152475904,0.732257010062749

"5467","AC093726.2",4.91167957575726,0.902875473507622,0.794493761903931,1.13641606366293,0.255782476012171,0.732257010062749
"5468","OSR2",1737.26741611124,0.374167381009108,0.329375665177682,1.1359897544564,0.255960852255164,0.732257010062749
"5469","AC084824.5",4.84366046873313,0.935531411651101,0.823793247140611,1.13563860215938,0.256107846252136,0.732257010062749
"5470","POSTN",663.621792798121,-0.954007777052543,0.840586518873596,-1.13493109350711,0.256404190764108,0.732257010062749
"5471","AL133453.1",52.5094180975507,-0.385084764910869,0.339035882027556,-1.13582303621647,0.256030633981813,0.732257010062749
"5472","RPUSD2",39.9246831867138,-0.339194206332392,0.298554510191368,-1.13612152807531,0.255905706328732,0.732257010062749
"5473","KAT8",188.171317273266,-0.21150274662917,0.186256562182397,-1.13554520791622,0.256146951390214,0.732257010062749
"5474","DDX5",6426.42503938185,0.193909941890247,0.170843763506647,1.13501328880936,0.256369750516804,0.732257010062749
"5475","FARSA",96.7316634228415,-0.287684195771743,0.2534925801449,-1.13488211610493,0.256424714070925,0.732257010062749
"5476","NOSIP",318.277463673022,-0.253744360089476,0.223433623317664,-1.13565879799889,0.256099390590097,0.732257010062749
"5477","CHIC1",56.1806483429824,0.290614802775752,0.25607277972828,1.13489142846079,0.256420811768441,0.732257010062749
"5478","RER1",983.265349497754,-0.242238255608496,0.213720547543333,-1.13343456393391,0.257031806076107,0.732300108788157
"5479","VPS13D",148.336052594794,0.247151665067053,0.218042773131609,1.13350083342535,0.257003991400879,0.732300108788157
"5480","AC093110.1",5.42325439815679,1.08109493150272,0.953475361380219,1.13384674139641,0.256858840589451,0.732300108788157
"5481","FST",228.27965807459,-0.425776401528071,0.375442332242721,-1.13406604680052,0.256766844562227,0.732300108788157
"5482","FOXO3",565.054938860602,0.354640214014126,0.312935409266607,1.13326968924756,0.257101016454972,0.732300108788157
"5483","SERINC1",1312.19335963783,0.1723595391697,0.15207564858216,1.13338026683859,0.257054597246449,0.732300108788157
"5484","YWHAG",507.570132217775,-0.204058611108138,0.17999286253246,-1.13370390490533,0.256918771113748,0.732300108788157
"5485","AC087623.2",7.16982211952954,-0.609789015262832,0.537883159777646,-1.13368303911004,0.256927526678172,0.732300108788157
"5486","RABL6",254.871994926473,-0.194141096379642,0.171270513713816,-1.13353485179616,0.25698971399907,0.732300108788157
"5487","DCAF5",289.683968290102,0.241060794523624,0.21265668226454,1.13356792721778,0.256975832877891,0.732300108788157
"5488","STUB1",1017.61618285113,-0.242452345926917,0.213815006157699,-1.13393512590083,0.256821761581438,0.732300108788157
"5489","EXOSC6",248.845586884429,-0.212302465289788,0.187271966335551,-1.13365854721356,0.256937804066978,0.732300108788157
"5490","SLC46A1",24.6410170898025,0.422874235529622,0.373045897777027,1.13357160083926,0.256974291161405,0.732300108788157
"5491","EIF3G",1429.53234133585,-0.254796052610971,0.224643843019954,-1.13422228352966,0.256701319053996,0.732300108788157

"5492","MRPS22",255.954436796022,-0.24308580751589,0.214577016028382,-1.13286041541251,0.257272875545216,0.732522806030798
"5493","CTBP1",260.225391306973,-0.223388396155907,0.197177393341062,-1.13293107475818,0.257243199129569,0.732522806030798
"5494","CXorf40A",39.4747166530408,-0.447923425501018,0.395454623372817,-1.13267970337708,0.257348784104143,0.732605566688895
"5495","C3orf18",47.1082031484614,0.312028272803777,0.275518470370622,1.13251308481803,0.257418786430428,0.732671486764676
"5496","PRDM2",400.945964807209,0.320603565513431,0.283324803555017,1.1315760621402,0.257812708731331,0.732999257066212
"5497","NBPF3",10.0831741964496,0.877751872405387,0.775693654170162,1.1315702631916,0.257815147897777,0.732999257066212
"5498","PXDN",399.579604647231,0.21022657939118,0.185741380273052,1.13182414754393,0.257708373495012,0.732999257066212
"5499","SLC25A16",72.2479720788722,0.272388992050248,0.240632649483253,1.13197021532693,0.257646956670402,0.732999257066212
"5500","GANAB",475.738175571479,0.161789456032064,0.142923093156292,1.13200360039186,0.257632920745465,0.732999257066212
"5501","MORC2",63.0226956011982,0.280798324811679,0.248138025460554,1.13162150093887,0.257793596718478,0.732999257066212
"5502","NEK11",96.5944514562392,-0.225504394915955,0.199405888474019,-1.1308813227215,0.258105045458707,0.73355677102929
"5503","AGER",9.82274655458283,-0.583474267456928,0.515929149350906,-1.13091936788414,0.258089030634659,0.73355677102929
"5504","PSORS1C1",138.568898816426,-0.287865632659433,0.254616029775,-1.13058723330898,0.25822886342707,0.73366411705523
"5505","RAC1",3713.2179144589,-0.219825320284168,0.194456971608517,-1.13045738841764,0.258283544022129,0.73366411705523
"5506","CCDC130",122.850353655717,-0.196485250436221,0.173799000852618,-1.13053153051692,0.258252320138644,0.73366411705523
"5507","AL627309.5",5.61858066774227,0.861015334447944,0.76922709837771,1.11932527632453,0.263001396326872,0.733673987931018
"5508","TNFRSF14",187.892593141074,0.220890626228552,0.197277310668392,1.11969605364224,0.262843307314333,0.733673987931018
"5509","AC254633.1",2.77632912052472,1.10034736115572,0.976483708105988,1.12684661507562,0.259807337747305,0.733673987931018
"5510","RUNX3",17.5700250293264,0.825936555133866,0.733123763972799,1.12659907606611,0.259912029950436,0.733673987931018
"5511","UQCRH",1454.82850776051,-0.244104407819789,0.217786481164031,-1.12084279297362,0.262354785219207,0.733673987931018
"5512","ACOT11",5.62752397170329,-0.846084871657363,0.750300791443267,-1.12766090787382,0.25946315314855,0.733673987931018
"5513","FPGT",59.6692983052246,0.29634567012849,0.263911373525964,1.12289844188673,0.261480629348395,0.733673987931018
"5514","NOTCH2",886.050013060727,0.222920003009103,0.198996168733382,1.12022258733923,0.26261892091968,0.733673987931018
"5515","NTRK1",3.00449447645957,1.00376051236732,0.891021385883455,1.126527969216,0.25994210872139,0.733673987931018
"5516","FAM78B",38.1521178934415,-0.350156255866502,0.312126134652724,-1.12184215607607,0.261929558550874,0.733673987931018

"5517","DNAH14",11.6215220873075,-0.566742720162432,0.505550753303693,
-1.12104020508101,0.262270749058984,0.733673987931018
"5518","LINC01139",23.5065586500599,0.74919490228789,0.663368942053733
,1.12937892444655,0.258738019609683,0.733673987931018
"5519","CAD",59.5407817240443,-0.246782723365103,0.21984915872031,-1.1
2250929137763,0.261645959243905,0.733673987931018
"5520","PCBP1",1341.90416873874,-0.161638471730802,0.143793570583126,-
1.12410082784167,0.260970254166568,0.733673987931018
"5521","MTHFD2",69.98905256738,-0.46166903141374,0.408699519375904,-1.
12960502649654,0.258642692042846,0.733673987931018
"5522","DNAH7",3.29552044953409,-1.06062130052604,0.946675520541557,-1
.12036413481918,0.262558621936052,0.733673987931018
"5523","C2CD6",16.3483750294242,0.529029591800186,0.470490102065323,1.
1244223618688,0.260833889869641,0.733673987931018
"5524","FARSB",80.9891091362554,-0.257743503843786,0.228883095070961,-
1.12609235629166,0.260126429097671,0.733673987931018
"5525","IQCA1",7.78231636377867,0.703448397686669,0.627677293940269,1.
12071665564122,0.26240849022986,0.733673987931018
"5526","LMCD1-
AS1",14.0483706474342,0.533857918400735,0.476957751595146,1.1192981278
0124,0.263012974271443,0.733673987931018
"5527","ENTPD3-
AS1",78.7802312901669,0.310573600983488,0.276915412212567,1.1215468236
3828,0.262055172211823,0.733673987931018
"5528","ZNF620",11.1114969869438,-0.626008837275479,0.554283849674133,
-1.12940118613146,0.258728632713119,0.733673987931018
"5529","LINC02018",11.695860826932,0.758125397149023,0.677173526869835
,1.11954376104066,0.262908232652097,0.733673987931018
"5530","GATA2",5.22269250305262,-1.1097749061417,0.990438676900403,-1.
12048825639035,0.262505754228164,0.733673987931018
"5531","RNF4",157.486360507836,0.201554441237485,0.179564823729568,1.1
2246060810348,0.261666647329114,0.733673987931018
"5532","JCHAIN",65.427001369719,-0.620315656480799,0.551856206994394,-
1.1240530569716,0.260990518253491,0.733673987931018
"5533","PYURF",885.788264776912,-0.241764866358762,0.215973408711525,-
1.11941959800101,0.262961173995371,0.733673987931018
"5534","AC093599.2",11.5693363946145,0.6169901740078,0.549004888664584
,1.12383366113294,0.261083598491126,0.733673987931018
"5535","AMACR",204.130180898217,-0.288446984216074,0.255561227113866,-
1.12868054154223,0.259032620671953,0.733673987931018
"5536","IL7R",36.6149787447942,0.548529068165964,0.488426924404371,1.1
23052478802,0.261415206995427,0.733673987931018
"5537","TMEM267",80.8028444666928,-0.291437616579818,0.25974305225943,
-1.12202276074253,0.261852762523922,0.733673987931018
"5538","IGIP",73.8555475188856,0.3576064285096,0.317869596414571,1.125
00985480601,0.260584858290284,0.733673987931018
"5539","AL365272.1",7.23396214858701,0.692136581206327,0.6161083988710
86,1.12340065883625,0.261267370134902,0.733673987931018
"5540","TAPBP",589.6983516872,0.168059556303832,0.149813055224834,1.12
179513361912,0.261949555813718,0.733673987931018

"5541","AL583856.2",2.7598710363863,1.15249252862999,1.02933069760035,
1.11965234430175,0.262861940335008,0.733673987931018
"5542","SMPD2",37.3802754408973,-0.324889064523755,0.287659462868231,-
1.12942248200115,0.258719653284568,0.733673987931018
"5543","THEMIS",2.64188674518279,1.13273642519729,1.00697001760222,1.1
2489588110532,0.260633157581984,0.733673987931018
"5544","FGFR1OP",42.0035272152279,0.305116761580096,0.272090740800196,
1.12137870139488,0.262126698184491,0.733673987931018
"5545","CREB5",2632.17308619639,0.333586787907993,0.297664988850639,1.
12067861657516,0.26242468746792,0.733673987931018
"5546","SNHG15",141.549487150521,-0.244525491796917,0.217000875996304,
-1.12684103543011,0.259809697237058,0.733673987931018
"5547","IGFBP3",309.315603324513,-0.350822574661172,0.311720528407999,
-1.12543943272801,0.260402869234937,0.733673987931018
"5548","SEM1",1534.97392924778,-0.247596928099262,0.22096213962623,-1.
1205400550433,0.262483693548902,0.733673987931018
"5549","MOSPD3",140.182660029285,-0.36542671762012,0.324797307150569,-
1.12509158658362,0.260550226156733,0.733673987931018
"5550","PRKAG2",241.424551850691,0.298880569984816,0.265494621473722,1
.12574999947559,0.260271353597251,0.733673987931018
"5551","AC019270.1",11.3424166635004,0.801701315029672,0.7152589767654
01,1.12085460102184,0.262349758140217,0.733673987931018
"5552","RCL1",45.0477171146675,-0.305828315617223,0.272117685152331,-1
.12388254165113,0.261062858598048,0.733673987931018
"5553","BX664615.2",12.6893688026996,0.696256819339588,0.6196098520755
17,1.12370198279986,0.261139474978186,0.733673987931018
"5554","PHF2",134.600886002873,0.235779174032338,0.21003070678846,1.12
259382276807,0.261610040093232,0.733673987931018
"5555","CCDC180",31.9958196527689,0.472110265956761,0.419431411463527,
1.12559587349317,0.260336615549348,0.733673987931018
"5556","TMEFF1",12.6070407630401,-0.614678489652484,0.544379127610236,
-1.12913676971939,0.25884014219369,0.733673987931018
"5557","STOM",722.718706138571,0.218223828626124,0.194110501089799,1.1
2422474518867,0.260917694320308,0.733673987931018
"5558","IDE",89.486196223017,0.287312824326095,0.254919989830232,1.127
0705938653,0.259712635080698,0.733673987931018
"5559","RRP12",23.6692426463874,-0.451252890977924,0.400870043368143,-
1.12568374325619,0.260299407247253,0.733673987931018
"5560","WBP1L",252.870458945378,0.226669630214768,0.201326901943159,1.
12587849923188,0.260216951277619,0.733673987931018
"5561","SLC1A2",29.1111093282414,0.479247462233876,0.427556480820647,1
.12089860341729,0.2623310254424,0.733673987931018
"5562","STX5",290.640331609835,-0.242059617187569,0.214633893223487,-1
.12777909188613,0.259413225482218,0.733673987931018
"5563","ZFPL1",207.803990803143,-0.250351436566071,0.222474806195306,-
1.12530241445089,0.260460906953329,0.733673987931018
"5564","KDM2A",271.932505503012,0.264306991001806,0.235850089801504,1.
12065673252129,0.262434006128152,0.733673987931018
"5565","NARS2",46.0868204462219,0.282524204491803,0.251999582109209,1.
1211296547681,0.262232677434169,0.733673987931018

"5566","MTMR2",95.0261693007775,-0.235431279026297,0.20908312445995,-1
.12601760488515,0.260158067661978,0.733673987931018
"5567","LAG3",7.7742972313605,-0.673481607318766,0.596740635303432,-1.
1286002116754,0.25906652136947,0.733673987931018
"5568","KLRB1",22.9023255424149,0.488128037996446,0.435454580975144,1.
120961999994,0.262304037879095,0.733673987931018
"5569","LARP4",154.478551086111,0.306036303974585,0.271737148071228,1.
12622181452484,0.26007164214572,0.733673987931018
"5570","B4GALNT1",2.57699959380325,-1.08335041519705,0.963682117700708
,-1.12417818624866,0.260937441550077,0.733673987931018
"5571","USP30",39.56107395446,0.300660847797838,0.268590858025264,1.11
940089848314,0.262969147839781,0.733673987931018
"5572","TMEM233",116.388978441046,-0.571521701962662,0.507679159418147
,-1.12575371937206,0.260269778612074,0.733673987931018
"5573","AC156455.1",8.33866463911846,-0.632771520363902,0.564887548233
536,-1.12017254114141,0.262640242802143,0.733673987931018
"5574","CDK2AP1",1344.86747650063,-0.234553134741782,0.208596840187793
,-1.12443282712539,0.26082945232678,0.733673987931018
"5575","FBRSL1",113.369866349749,0.209327884729651,0.185349527760715,1
.12936831972883,0.258742491294383,0.733673987931018
"5576","WASF3-
AS1",3.13103813415487,1.15384562070374,1.02693684096208,1.123579926904
52,0.261191275656714,0.733673987931018
"5577","TMEM260",83.3627665916741,0.256317412001247,0.228940406740437,
1.11958136027883,0.26289220232659,0.733673987931018
"5578","PRKCH",13.4491434061339,0.630884896262677,0.558893174791914,1.
12881123749197,0.258977471119134,0.733673987931018
"5579","TMED10",999.837031558658,-0.198791362345364,0.176589251434862,
-1.12572741959151,0.260280913946429,0.733673987931018
"5580","TTC8",71.5897937349921,-0.327193902160161,0.290360451897585,-1
.12685422557328,0.2598041194859,0.733673987931018
"5581","PPP4R3A",227.647979879681,0.226018148824968,0.201496508078044,
1.12169759655302,0.261991038812051,0.733673987931018
"5582","TMEM121",28.7347625466904,0.522244084919435,0.464021126273454,
1.12547480135995,0.260387889367777,0.733673987931018
"5583","RNF111",85.8166675213762,0.248524633292628,0.220683586024606,1
.12615821488834,0.260098556625612,0.733673987931018
"5584","SNAPC5",191.622873983368,-0.301230503883471,0.267770549311679,
-1.1249575603359,0.260607018650291,0.733673987931018
"5585","AC018553.1",3.95324210511686,0.885708910153363,0.7852744851889
24,1.12789722174696,0.259363327342175,0.733673987931018
"5586","PARD6A",28.0965931396173,-0.404409786090146,0.360661934602862,
-1.12129877674908,0.262160706076885,0.733673987931018
"5587","CCL8",26.5086709687961,-1.44058515246815,1.28250998085883,-1.1
2325453522277,0.261329407020284,0.733673987931018
"5588","DUSP14",135.601150947948,-0.319029156827063,0.28377518219452,-
1.12423205708094,0.260914593192053,0.733673987931018
"5589","UTP18",115.265384347021,-0.233027724284752,0.207083845695907,-
1.1252820011221,0.260469554325073,0.733673987931018
"5590","AC027601.6",6.58008911068456,-0.67166211778037,0.5966418301526

72,-1.12573755951456,0.260276620661157,0.733673987931018
"5591","ARL16",307.816979699154,-0.264694133145538,0.23479478800563,-1
.12734245676353,0.259597718180359,0.733673987931018
"5592","ATP5F1A",897.46071956447,-0.177639644158735,0.158630772443676,
-1.11983092197202,0.26278581952989,0.733673987931018
"5593","CPAMD8",3.54584495344204,1.18400293604981,1.04805089366168,1.1
2971893179074,0.258594677309695,0.733673987931018
"5594","ZNF790",45.8641087101871,-0.298752868251022,0.26473595517872,-
1.12849374029809,0.259111459007199,0.733673987931018
"5595","CAPN12",2.61722241905323,-1.07078173435338,0.948926762045588,-
1.12841346369568,0.25914534436788,0.733673987931018
"5596","EID2",125.323838931325,-0.248310534211169,0.22095248256402,-1.
12381871128885,0.261089941903606,0.733673987931018
"5597","CLDND2",14.0405454274222,0.521105230653378,0.463986314693967,1
.12310474285666,0.261393012047152,0.733673987931018
"5598","ZNF586",22.7435403292313,0.441047969659777,0.391996857887481,1
.12513139017654,0.26053336136871,0.733673987931018
"5599","CRLS1",169.644387148493,-0.179884726557485,0.160500886181983,-
1.12077092430209,0.262385383538649,0.733673987931018
"5600","PHACTR3",2.82890674838817,-1.53195152272396,1.36481634021027,-
1.12245983403741,0.261666976279654,0.733673987931018
"5601","HUNK",271.394725058218,0.355649821597062,0.317751568922059,1.1
192700725399,0.263024939279362,0.733673987931018
"5602","ZNRF3-
AS1",2.56740046129395,1.24313501950831,1.109723482535,1.12022052256528
,0.262619800580598,0.733673987931018
"5603","EIF3D",811.816019334151,-0.243084799329017,0.217025436313548,-
1.12007515551228,0.26268173679161,0.733673987931018
"5604","REPS2",34.7174861063648,0.411154435920955,0.365012813340332,1.
12641096666818,0.259991606986825,0.733673987931018
"5605","PQBP1",229.750877319649,-0.232967737397953,0.208016404039439,-
1.1199488736176,0.262735549618591,0.733673987931018
"5606","AMMECR1",148.269815516557,0.249245465619731,0.220515151019535,
1.13028725902671,0.258355201471893,0.733673987931018
"5607","MT-
ND2",23542.1468089406,-0.246867274444545,0.220527748189459,-1.11943860
340177,0.262953069887473,0.733673987931018
"5608","AFP",4.82579871333898,-0.822370987746764,0.734897670219604,-1.
11902788792489,0.263128241795693,0.733721257396945
"5609","PDCD2",334.30733041604,-0.220957448970772,0.197457908695878,-1
.11901037760452,0.263135711811986,0.733721257396945
"5610","TULP4",128.211443084549,0.306400382631686,0.273879288004992,1.
11874243891747,0.263250034450596,0.733909186953177
"5611","BTBD7",363.100877113078,0.268775277115966,0.240379901785285,1.
11812707767908,0.263512723227031,0.734510602614644
"5612","FUBP1",204.284650308984,0.257767782326869,0.230890078833147,1.
11640908795022,0.264247065195661,0.734586287225857
"5613","LYST",241.273734758018,0.21797767988582,0.195020706816183,1.11
771556694887,0.263688492063625,0.734586287225857
"5614","ACP1",397.09053769515,-0.255879709586812,0.229194968160547,-1.

11642812946737,0.264238918315101,0.734586287225857
"5615","PRKD3",283.739780677047,0.213943949353814,0.191391564984475,1.1178337424179,0.263638007425031,0.734586287225857
"5616","IL1RAP",19.1682857922752,0.459292573026358,0.411117303625719,1.11718132264386,0.263916804807174,0.734586287225857
"5617","AC008467.1",9.32857316357271,0.640975854549995,0.574149558057754,1.11639179296472,0.264254464975734,0.734586287225857
"5618","RTN4IP1",18.6728615420826,-0.40527563335767,0.363042669486761,-1.11633057880115,0.264280657029995,0.734586287225857
"5619","PAFAH1B2",335.304814781141,0.174825240192484,0.156518427902244,1.11696266398532,0.26401028930331,0.734586287225857
"5620","AC093525.6",11.5200609843919,0.854336731486648,0.765325173366956,1.11630554072604,0.264291370730172,0.734586287225857
"5621","PIGL",51.9556216213804,0.263262407422478,0.235584935512146,1.11748404816362,0.263787416372318,0.734586287225857
"5622","SLFN5",605.480704249386,0.231473293370037,0.207168815318936,1.11731726135367,0.263858697595679,0.734586287225857
"5623","ANAPC11",1164.26370797588,-0.248473570329129,0.222393573927032,-1.11726955928436,0.26387908691383,0.734586287225857
"5624","GIPC1",320.911271421725,-0.223720102741529,0.200367927057883,-1.11654647540921,0.264188288089333,0.734586287225857
"5625","LRFN3",50.3171163099039,0.292078934568527,0.261542398678067,1.11675558549896,0.264098843909256,0.734586287225857
"5626","AC006115.2",39.3434986573923,0.349208587188791,0.312486230510913,1.11751671943381,0.26377345490504,0.734586287225857
"5627","MAPRE1",313.434607743576,-0.174049831282625,0.155881703331009,-1.11655074048708,0.264186463547894,0.734586287225857
"5628","DERA",426.893311473408,-0.283122495327622,0.253772839543207,-1.11565325839142,0.264570585463232,0.735231690946153
"5629","GBP2",491.011578847677,0.193894938716193,0.17407196621239,5,1.11387802950195,0.265331516022342,0.735646694865923
"5630","CHI3L2",81.7771220527665,-1.00459531644448,0.901133192504101,-1.11481335367625,0.264930412852481,0.735646694865923
"5631","AL713852.1",2.96871674447325,1.12165338275172,1.00609780972573,1.11485520782268,0.264912473948145,0.735646694865923
"5632","SCFD2",52.2124738085684,-0.312793830645193,0.280734470003876,-1.11419816255864,0.265194183532299,0.735646694865923
"5633","MMAA",35.2379627898154,0.331914013333089,0.298018911153389,1.1137347359888,0.26539300275268,0.735646694865923
"5634","TRIM52-AS1",154.773684364653,-0.283879037798819,0.25478450246153,-1.11419271995039,0.265196517923922,0.735646694865923
"5635","SNX3",1931.8148432926,-0.234252177909359,0.210211048651946,-1.11436663016329,0.265121933014608,0.735646694865923
"5636","ZNF316",54.9266220292267,0.329074834271771,0.29537669914043,1.11408528577036,0.265242600465466,0.735646694865923
"5637","MRPL13",322.906193772216,-0.294328545999195,0.264204346895195,-1.11401855971715,0.265271224544378,0.735646694865923
"5638","TMEM203",296.629054847945,-0.222867291458316,0.200035038607586,-1.11414126749824,0.265218587118472,0.735646694865923

"5639","SLC3A2",439.041332601321,-0.272202094724728,0.244421383069923,
-1.11365908868479,0.26542546669619,0.735646694865923
"5640","RPL36AL",3127.82504367154,-0.262611793605426,0.235770101992068
,-1.11384688468371,0.265344879316995,0.735646694865923
"5641","GSKIP",78.7568895211804,-0.318328185186833,0.285517708632058,-
1.11491573223942,0.26488653434946,0.735646694865923
"5642","ZFP64",57.7136285676445,0.279690164436028,0.251137065320876,1.
11369528061766,0.265409934633369,0.735646694865923
"5643","CCNQ",78.0485263321994,-0.364002457494081,0.326407824045007,-1
.11517687591916,0.264774633269001,0.735646694865923
"5644","RNF220",141.949840733148,0.283411757366969,0.254657328228303,1
.11291420254315,0.265745279696815,0.735750326850592
"5645","AGL",218.350058939451,-0.248313146927791,0.22314580124481,-1.1
1278431206228,0.265801074657407,0.735750326850592
"5646","INKA2",15.3470306675989,-0.69814943378401,0.627351615963578,-1
.11285189360944,0.265772043734842,0.735750326850592
"5647","ARV1",95.3614058250782,-0.323271171310915,0.290520253267922,-1
.11273196162606,0.265823564270938,0.735750326850592
"5648","IRAK2",3.88741464102012,-0.873865931317454,0.785480269927263,-
1.1125243558293,0.265912764090043,0.735750326850592
"5649","SEC24B",123.759202752976,0.223190743517189,0.200546690896585,1
.11291162431735,0.265746387105265,0.735750326850592
"5650","MYBL1",9.71436936816652,0.676586660309511,0.608135531791005,1.
11255900196608,0.265897876613182,0.735750326850592
"5651","CRY2",140.12464125083,-0.283332307193408,0.254686082848836,-1.
11247659873734,0.265933286297084,0.735750326850592
"5652","PLA2G15",71.0126751499552,0.306522221798841,0.275345656580633,
1.11322700929941,0.265610945437127,0.735750326850592
"5653","AC091132.4",21.4089226738785,0.785187489157375,0.7054425968275
8,1.11304235481159,0.265690239295922,0.735750326850592
"5654","METTL26",658.213465804709,-0.302162487924307,0.271660576350118
,-1.11227949223989,0.266017998559724,0.735854527321204
"5655","AL035530.2",9.71784985708429,-0.575582963141733,0.517621599316
479,-1.11197632382766,0.266148330277206,0.735890926800738
"5656","VPS25",312.164925618357,-0.275432924607025,0.24770917760412,-1
.11192054840702,0.266172312846021,0.735890926800738
"5657","ALPK2",46.1093995525627,0.557856811158334,0.501645949318171,1.
11205285703306,0.266115424602482,0.735890926800738
"5658","JUP",55.0408208831894,-0.288602935197851,0.259630038330114,-1.
11159300770468,0.266313180300761,0.736020169624299
"5659","RASIP1",5.1640561141767,-0.813567577948314,0.731832779169655,-
1.11168507493118,0.266273579196571,0.736020169624299
"5660","NDUFAF2",390.246877646573,-0.283475025273468,0.255103537176227
,-1.11121558098052,0.266475566157948,0.736242464829866
"5661","ZNF787",130.256069959294,-0.277161088211736,0.249427889204141,
-1.11118724171577,0.266487761726462,0.736242464829866
"5662","SGMS1",216.006516782378,0.182363164064094,0.164163685933149,1.
11086177815449,0.26662784981464,0.736499394401442
"5663","NPIPB6",2.84323930071957,1.55237634829249,1.39763098319057,1.1
1071975862231,0.266688994636592,0.736538208743829

"5664","GINS4",6.74511042834814,0.801884347814674,0.722062178469164,1.11054750092955,0.266763171084576,0.736605016781351
"5665","CDC25B",73.2486553559468,0.344541996350769,0.310273847200067,1.1104448520555,0.266807379799639,0.736605016781351
"5666","CENPC",292.702860703593,0.217931728723792,0.196298192071283,1.11020751859321,0.266909613635211,0.736647637204918
"5667","IMMP2L",98.3553746709894,-0.277722252718127,0.250157333087608,-1.11019033218133,0.266917017905388,0.736647637204918
"5668","CENPQ",87.3351998333073,0.255557355648992,0.230261361233227,1.10985774721511,0.267060330372201,0.736865854880638
"5669","FN3K",33.6636174024783,0.490244116729528,0.441745665514299,1.10978817677535,0.267090315301684,0.736865854880638
"5670","AC100814.1",5.59824950933791,0.771966146849069,0.696004847541691,1.10913903771745,0.267370206475348,0.737507941670978
"5671","SCAP",138.244077954092,0.2195168369464,0.197992752383631,1.10871147708005,0.267554669153402,0.737822041899019
"5672","CCDC96",13.4923788534772,-0.463999219805268,0.418784568730765,-1.1079663732872,0.267876338644754,0.737822041899019
"5673","UBXN8",50.4489381071033,0.32093434901885,0.289737656607253,1.10767220518348,0.268003407390444,0.737822041899019
"5674","CPB2-AS1",10.4113554481825,0.727320869692227,0.656043965571634,1.10864653569138,0.267582694496966,0.737822041899019
"5675","BORA",11.9194145476617,-0.505297324211124,0.455928264252711,-1.10828251685456,0.267739823549981,0.737822041899019
"5676","FOXN3",964.855645536129,0.250919129216105,0.226466334934439,1.10797540521307,0.2678724378726,0.737822041899019
"5677","ANPEP",534.388708575231,0.156170974278951,0.140979525188847,1.10775642115233,0.267967025261004,0.737822041899019
"5678","TTC23",81.3862857873706,0.227967813718948,0.205828128198129,1.10756394529084,0.268050181718045,0.737822041899019
"5679","NFE2L1",550.997076995658,0.220498561182314,0.19899680849444,1.10805074136892,0.267839902680444,0.737822041899019
"5680","RBBP8",53.2651980794855,-0.365083715559743,0.329608440626412,-1.10762853908083,0.26802227291374,0.737822041899019
"5681","OGFR",177.072098185353,0.171411658821446,0.154649675821658,1.1083867968732,0.267694804507816,0.737822041899019
"5682","Z98885.3",4.19651516729331,0.976545941029534,0.881371196572808,1.10798485907732,0.267868354912525,0.737822041899019
"5683","WSB2",218.430489745668,-0.239927911797984,0.216715608141594,-1.10710951488655,0.268246582250778,0.738232719761071
"5684","AC145343.1",4.38110354963901,1.01751925853956,0.919377180579408,1.10674843800049,0.268402706738238,0.738532430222739
"5685","TTLL7",175.911315587457,0.368814538204347,0.33331583008103,1.10650171674921,0.268509421370024,0.738615012475592
"5686","MAP3K4",172.552391970091,0.234828199958224,0.21224026337314,1.10642625591437,0.268542066351872,0.738615012475592
"5687","LINC00476",64.939342411851,0.303812936874451,0.274607960936819,1.10635152687489,0.268574397439175,0.738615012475592
"5688","TBC1D8",26.7678262007702,0.432449183595529,0.390987826888438,1

.1060425769187,0.268708091224282,0.738852768415572
"5689","PCDHB14",60.4137490348874,0.283414827618294,0.256290790029364,
1.10583305621643,0.268798784387173,0.738972224963155
"5690","IDH1-
AS1",5.22373204547544,0.891435362379494,0.806239285379376,1.1056709571
7747,0.268868965020712,0.739035257104383
"5691","TMEM126A",234.898894042818,-0.293996196392683,0.26599978018202
3,-1.10524977197914,0.269051375582069,0.739121867534698
"5692","ATP2A2",318.365989701854,0.193482559555317,0.17503724606333,1.
10537936300319,0.268995242124655,0.739121867534698
"5693","TANC2",110.869122517237,0.404914423847184,0.36627725256946,1.1
0548613381443,0.268948999478327,0.739121867534698
"5694","COL9A3",2.68137393382499,1.54741532014439,1.40017090086069,1.1
051617478932,0.269089508551315,0.739121867534698
"5695","GMDS",71.7410810292862,-0.309398866957585,0.280119287147779,-1
.10452539740457,0.269365292698788,0.739126982158584
"5696","NUS1",146.725115517487,0.216296383682171,0.195813837302539,1.1
0460213977619,0.269332023497696,0.739126982158584
"5697","KLF14",9.56292551317326,0.62387155337034,0.564809761125829,1.1
0456935469172,0.269346236077598,0.739126982158584
"5698","TGFBR1",333.092677246517,0.272172873873267,0.246395458625291,1
.10461806151702,0.269325121489351,0.739126982158584
"5699","TREH",2.76365589665463,1.23595454068247,1.11924573263503,1.104
27451688619,0.269474073604349,0.739126982158584
"5700","OXA1L",308.356975024754,-0.193443021410051,0.175207901872271,-
1.10407703843787,0.269559720761235,0.739126982158584
"5701","PIK3C3",141.739661140167,0.223206993936707,0.20218745951214,1.
10396062384524,0.269610218964232,0.739126982158584
"5702","ZNF337",52.5724801288131,0.329038867898623,0.297874085706492,1
.10462401292215,0.26932254161118,0.739126982158584
"5703","DOP1B",62.2998002141022,0.318279554806926,0.288307581988505,1.
10395832330076,0.269611216957463,0.739126982158584
"5704","MIAT",11.2316994983559,0.577054033528088,0.522585901606287,1.1
0422809294009,0.269494206167894,0.739126982158584
"5705","SMTN",143.676717929113,0.312818486925254,0.283317606168052,1.1
0412653543212,0.269538251972044,0.739126982158584
"5706","FBXO42",94.828353745672,0.244714347873711,0.221755131467374,1.
10353409300797,0.269795294529171,0.739376469127679
"5707","PLCL1",16.2009300700286,0.51304361675746,0.465041626422618,1.1
0322084649519,0.269931270500564,0.739376469127679
"5708","EDIL3",572.907609898325,-0.288613878928042,0.261635563374265,-
1.10311409965007,0.269977618563932,0.739376469127679
"5709","TRIM56",463.290990403798,0.301067963017487,0.272935044118897,1
.10307551010685,0.269994374974608,0.739376469127679
"5710","CACNB3",22.971807202447,-0.402579646421744,0.364977389752644,-
1.10302626333808,0.270015760018086,0.739376469127679
"5711","AC092376.3",7.41027523224943,0.726320791981046,0.6582726808467
31,1.10337374330465,0.269864894242518,0.739376469127679
"5712","AC010336.8",5.19313921934685,0.891656272482136,0.8084019986733
71,1.10298622955583,0.270033145246631,0.739376469127679

"5713","AC036176.1",110.288865329112,0.385174011889986,0.349280386624771,1.10276450278834,0.270129447089742,0.739510686589106
"5714","CSTB",1677.36980387629,-0.308575451771642,0.279915803750371,-1.10238667355427,0.27029360265004,0.739830581982259
"5715","MGAT4A",14.3943743665035,0.621255908283079,0.563658204208712,1.10218551534298,0.270381027804524,0.73989411032195
"5716","PIGN",72.2652430863822,0.32788692441619,0.297536220690322,1.10200675284323,0.270458735851061,0.73989411032195
"5717","SYMPK",125.269232623226,0.213304406262409,0.193543287144526,1.10210180579979,0.270417414431299,0.73989411032195
"5718","MAP2K4",101.77927832714,0.234490274987037,0.212866499677372,1.10158374071279,0.270642680174506,0.740267841540622
"5719","WRNIP1",108.20985612924,0.197368236867336,0.179225371274608,1.10122933747438,0.270796856331105,0.740560033750392
"5720","XBP1",728.208151403851,-0.304508319298398,0.276621184363731,-1.10081344637003,0.270977858326186,0.740925472766006
"5721","FBXW8",45.2302303101216,0.339931005745454,0.308831902881427,1.10069912652763,0.271027626544876,0.740932018731316
"5722","PPP4R3B",327.775879413572,0.176390249876808,0.160324042453853,1.1002108428471,0.271240267399128,0.74099415347905
"5723","MTO1",72.088047112587,0.265996073977317,0.241782323095452,1.10014690309806,0.271268120744765,0.74099415347905
"5724","AC027373.1",4.60269354330556,0.897021194827299,0.815341061629601,1.10017909932617,0.2712540952232,0.74099415347905
"5725","CBWD6",5.2073852482048,-0.988300297515811,0.898289737666957,-1.10020214645069,0.271244055596264,0.74099415347905
"5726","ZNF410",130.041241117553,-0.219400655235431,0.199436466125775,-1.10010300271298,0.27128724570467,0.74099415347905
"5727","PSMD2",562.227800554638,-0.169837088733398,0.154427643542912,-1.09978424093614,0.271426140212065,0.741120760997368
"5728","VEZF1",336.154859907821,0.212503771921863,0.193224046113542,1.09977912271329,0.271428370779599,0.741120760997368
"5729","GPT2",3.81720285391037,0.959002040593614,0.87216954902607,1.09955918739023,0.271524232432793,0.741253097442641
"5730","ERG28",100.858634373237,-0.363915594340486,0.331089007383163,-1.09914731756507,0.271703813584255,0.741613899556327
"5731","PTER",104.369082706709,0.229856751965132,0.2091946666953,1.09876965601579,0.271868550902227,0.741934066674374
"5732","ARHGEF33",7.55318179354622,0.627125947732114,0.571080617993444,1.09813908574868,0.272143760297192,0.742555549729255
"5733","PPP1R12B",443.292596372712,0.280115853822371,0.25511107108016,1.09801527874247,0.272197817665033,0.74257349874082
"5734","DESI1",142.502754187703,-0.23252439024311,0.211796985176713,-1.0978644953284,0.272263663561705,0.742623595763004
"5735","AL360012.1",52.1103084697603,0.521103001704367,0.474927735956354,1.09722587722747,0.272542663778109,0.742694693798165
"5736","ZNF669",43.855540028234,-0.360192035878467,0.328372812593066,-1.0968996886013,0.272685244897319,0.742694693798165
"5737","GIGYF2",294.686353699047,0.255862744790141,0.233121122866734,1.09755281565114,0.272399806110128,0.742694693798165

"5738","NDUFB4",1178.0846949263,-0.270126873314586,0.246276980821303,-1.09684174466386,0.272710578252399,0.742694693798165
"5739","Z99289.1",3.2811633649898,1.12813030258251,1.02838570379712,1.09699142881616,0.272645138947441,0.742694693798165
"5740","WDYHV1",61.424615515942,-0.279091248943042,0.254373854206373,-1.09716955704345,0.27256727838915,0.742694693798165
"5741","ZWINT",13.5441355091095,-0.540188567096152,0.492501246581052,-1.09682680165004,0.27271711667702,0.742694693798165
"5742","NDUFS8",549.871747026441,-0.297771171047422,0.27146454262068 2,-1.09690631480922,0.272682347991431,0.742694693798165
"5743","AC073655.2",2.66563987228573,-1.08113421528613,0.985617717472031,-1.09691028896993,0.272680610541777,0.742694693798165
"5744","RPL14",12387.1834665693,-0.237710346650724,0.216756946788392,-1.09666772010213,0.272786672268121,0.74275479705317
"5745","LLGL1",40.5283159003544,0.480402282157824,0.438131496625183,1.09647967758137,0.2728689121052,0.74284939692347
"5746","LINC02202",27.6575258056549,0.375147584992582,0.342193654923778,1.09630199039238,0.272946638651968,0.742931679171038
"5747","FRZB",8.42803813094026,0.902175423556219,0.823294589962874,1.09581118903854,0.273161410887443,0.743257561983229
"5748","ZDHHC21",120.541940647666,0.296924239083318,0.270953496394313,1.09584944661947,0.273144665407973,0.743257561983229
"5749","AL445253.1",10.2046936729566,-0.62123728835747,0.567014264043519,-1.09562903043615,0.273241151985912,0.743271853941381
"5750","PTPRE",73.3802834461112,-0.498549663825029,0.455054602164807,-1.095582071807,0.273261711007861,0.743271853941381
"5751","STK40",140.194896485989,-0.229355004840507,0.209412527768466,-1.09523058283356,0.273415630467792,0.74330270476556
"5752","MYSM1",172.185123730526,0.284430193447398,0.259661898461775,1.09538671300006,0.273347252669171,0.74330270476556
"5753","TCP11L2",153.857160253879,-0.343480119266769,0.313586754080391,-1.09532725728177,0.273373290147906,0.74330270476556
"5754","GLRX5",584.509201369403,-0.281355749580117,0.256940303415358,-1.09502380841082,0.273506206108838,0.743419719072336
"5755","TOX2",8.1886899163384,-0.809165479900662,0.739089309338963,-1.09481421213409,0.273598038769668,0.743540108837117
"5756","UBE4B",120.836120662867,0.208017691951299,0.190467376990445,1.09214341709413,0.274770068364475,0.743750899215852
"5757","LINC01715",4.00516448300178,0.917260192515092,0.838910413048659,1.0933946917904,0.274220543168927,0.743750899215852
"5758","HIST2H2AC",111.816268929178,0.347316813012351,0.317501200173431,1.09390708703663,0.273995730199831,0.743750899215852
"5759","SCCPDH",508.352235892169,-0.23584492930546,0.215628421740235,-1.09375622843254,0.274061906195974,0.743750899215852
"5760","AC082651.3",2.6893153441476,-1.19616451166601,1.09518441397691,-1.09220373884104,0.274743559484527,0.743750899215852
"5761","DUBR",61.8400420173748,0.347650954270008,0.318278006128694,1.0922870810289,0.274706936958823,0.743750899215852
"5762","ANAPC13",325.994500879325,-0.27522951639801,0.25162664148508,-1.09380117611406,0.274042188196391,0.743750899215852

"5763","GNB4",244.402661024314,0.225814218394282,0.206462125068447,1.0
9373192937649,0.274072566299138,0.743750899215852
"5764","ART3",11.8771588280249,0.621114813220814,0.568200731238382,1.0
9312568441633,0.274338620353907,0.743750899215852
"5765","COPS4",326.858585340333,-0.244617211964147,0.223708570449365,-
1.09346374827206,0.274190237352287,0.743750899215852
"5766","SLC30A5",164.404290097308,-0.187940378854128,0.17207703298513,
-1.09218746740228,0.274750709927986,0.743750899215852
"5767","LTC4S",44.7541176394624,-0.482612198708818,0.441779939540618,-
1.0924266937305,0.274645595310225,0.743750899215852
"5768","PPP1R3G",34.7000618578057,-0.319148783427086,0.292068967908918
,-1.09271719522977,0.274517987617314,0.743750899215852
"5769","BMP5",48.1082397880525,1.10645952576685,1.0129873646171,1.0922
7376807912,0.274712786759647,0.743750899215852
"5770","CCZ1",271.629567352563,-0.327532292967913,0.299987314926399,-1
.09182047597003,0.274912017159005,0.743750899215852
"5771","NUDCD3",174.984415098085,0.21981227993405,0.201065258655064,1.
09323849084812,0.274289101253299,0.743750899215852
"5772","AC012349.1",3.7730864262278,1.25622430253468,1.15031001699858,
1.09207455726801,0.274800331507195,0.743750899215852
"5773","LERFS",25.4905122201161,1.5180880463808,1.38926718103099,1.092
72576730288,0.274514222804522,0.743750899215852
"5774","MRPL23",142.116650975019,-0.33418825977421,0.305857246090285,-
1.09262822459194,0.274557065170902,0.743750899215852
"5775","STAT6",300.094097522954,0.200432729045988,0.183531240463898,1.
09209052660119,0.274793312956694,0.743750899215852
"5776","POC1B-
AS1",32.5022159562807,0.416233035216881,0.381029768594206,1.0923898065
8271,0.274661801511226,0.743750899215852
"5777","MAPK7",93.7885564278443,0.206690852447649,0.189285076722518,1.
09195535129611,0.27485272661217,0.743750899215852
"5778","LINC00266-1",4.17162717094214,-0.949797582856201,0.86820791327
7056,-1.09397480526431,0.273966028311136,0.743750899215852
"5779","GP1BB",51.5023726880177,0.538354134994526,0.492942230852035,1.
09212419082861,0.274778517869514,0.743750899215852
"5780","LINC01638",61.9294546058697,-0.294925330279171,0.2695092509236
69,-1.09430503505314,0.27382121776529,0.743750899215852
"5781","HORMAD2-
AS1",3.07672023666013,1.25540865529855,1.1497720786016,1.0918761019361
6,0.274887563192959,0.743750899215852
"5782","TOR1B",82.407708950318,-0.223298989895878,0.204554670096886,-1
.09163476829991,0.274993667648834,0.743843127296397
"5783","KIAA0556",34.3243275538297,0.363421250717974,0.333102071670474
,1.09102068592805,0.275263780441302,0.744131143453956
"5784","PSMB3",771.577844871998,-0.330451237103132,0.302825027916957,-
1.09122828907573,0.275172443014333,0.744131143453956
"5785","DIPK1C",16.5999752469817,-0.923337236225977,0.846352928237187,
-1.09096005392117,0.275290460103874,0.744131143453956
"5786","C1GALT1C1",364.882349822546,-0.303973167011651,0.2786043815505
21,-1.09105666364594,0.275247950142462,0.744131143453956

"5787","PRXL2B",18.1478329800703,0.439699691891298,0.403521014191761,1
.08965748109055,0.275864052334657,0.744261605304637
"5788","PEF1",251.200503967393,-0.254887406395868,0.233875174995222,-1
.08984378697344,0.275781962000248,0.744261605304637
"5789","CBLB",727.446991802246,0.302664775943269,0.277729825847945,1.0
897813190182,0.275809484852652,0.744261605304637
"5790","ACTL6A",198.312284265255,-0.24430158458685,0.224089211755841,-
1.09019788446144,0.275625985056125,0.744261605304637
"5791","SLC12A2",64.4404218247235,-0.3209228917988,0.294411400805692,-
1.09004913165916,0.275691502072391,0.744261605304637
"5792","RABGEF1",270.148409574299,-0.261136889836362,0.239673253067383
,-1.08955374241507,0.275909769025338,0.744261605304637
"5793","PTN",7.24143070716782,-0.846235833206134,0.775969666265179,-1.
09055272389596,0.275469741681329,0.744261605304637
"5794","AP000766.1",24.4089761519786,0.408936044617416,0.3751078729305
57,1.09018251582558,0.275632753560285,0.744261605304637
"5795","LINC00517",2.80324612473379,-1.4461897645708,1.32678562949912,
-1.08999504698944,0.275715325878709,0.744261605304637
"5796","C16orf58",139.460745169497,0.212837512546537,0.195269064749846
,1.08997046111322,0.275726156198467,0.744261605304637
"5797","CTDNEP1",699.570814817229,-0.17336266371204,0.15910494544595,-
1.08961203704968,0.275884078474078,0.744261605304637
"5798","MLLT6",253.810132168471,0.259790614258959,0.238416050505851,1.
08965236907397,0.275866305032818,0.744261605304637
"5799","CHST2",44.1234364705491,0.958687242603889,0.880337098769299,1.
08900016135197,0.276153814531284,0.744791457021777
"5800","MCEE",198.662374628631,-0.300932198965844,0.276537601444966,-1
.08821439613785,0.276500470826882,0.745245194275335
"5801","RBSN",161.425553207859,0.206654553838427,0.189874911803275,1.0
8837208600016,0.276430878955815,0.745245194275335
"5802","ZKSCAN7",14.5204026439765,0.457362100118444,0.420297416161069,
1.08818679947148,0.2765126510473,0.745245194275335
"5803","ACE",763.806627991676,0.266763827622906,0.245094517287392,1.08
841205660306,0.276413240980721,0.745245194275335
"5804","DISC1",51.6850239464376,0.439040372850402,0.403744102213644,1.
0874223807685,0.276850184599152,0.745272731066657
"5805","AVL9",91.7661998456747,0.270962070632022,0.249127074328693,1.0
8764601905339,0.276751406760267,0.745272731066657
"5806","UBAP2",114.821811139047,0.234016902493356,0.215123369809176,1.
08782650021213,0.276671708325148,0.745272731066657
"5807","AL365203.2",9.12163820039464,-0.543790989061196,0.499836800168
029,-1.08793708041983,0.276622885087942,0.745272731066657
"5808","ZNF511",251.278056947708,-0.25808343950712,0.237328188599167,-
1.08745379565091,0.276836307643573,0.745272731066657
"5809","RNF135",134.182875202614,-0.256022911802271,0.235347485978414,
-1.08785063387401,0.27666105235942,0.745272731066657
"5810","SEPTIN4",4.13089971308844,0.856141023575858,0.787322543859276,
1.08740824234404,0.276856430146885,0.745272731066657
"5811","VSTM4",142.780151132208,0.278292810270597,0.256024784422192,1.
08697605545752,0.277047391939016,0.745658442596147

"5812","AARS2",42.3449538401117,0.333254871945943,0.306669235562469,1.08669156635394,0.277173142432629,0.745695954556337
"5813","AL161908.1",3.61347098365367,0.920473622491698,0.847181331781897,1.08651310877642,0.277252044484981,0.745695954556337
"5814","GNAS",7008.07047723797,-0.204177226524064,0.187917882233837,-1.08652366713028,0.277247375858445,0.745695954556337
"5815","PRRG1",96.6978140625002,0.228829885856467,0.210546915948707,1.08683561013173,0.277109467028666,0.745695954556337
"5816","PRDM16",10.0814066485406,-0.804408753470236,0.740458802739175,-1.08636530553015,0.277317404817909,0.74574350263963
"5817","NAB1",110.138279875955,-0.264352724899564,0.243372875096307,-1.08620455256139,0.277388503585637,0.745779546802889
"5818","AC005070.3",14.612085119187,0.534007118934027,0.491696149824702,1.08605104824272,0.27745640796634,0.745779546802889
"5819","PPP2R5C",319.229915302305,0.193106896002297,0.177812922304295,1.08601159859366,0.277473860795781,0.745779546802889
"5820","PDHB",296.372711645453,-0.243509020849619,0.22441337336108,-1.085091397195,0.277881177157094,0.746098866403631
"5821","PSMB1",2055.96986603979,-0.294245017483935,0.271204177043634,-1.08495754265833,0.277940460197018,0.746098866403631
"5822","LRWD1",42.0168062989777,-0.344676818009572,0.317549281032196,-1.08542780159727,0.277732224508081,0.746098866403631
"5823","TBCEL",47.9010666064082,0.329535594081494,0.303678708175182,1.08514553444226,0.277857202674415,0.746098866403631
"5824","EIF3J",382.800888878019,-0.18073379878889,0.166593179972065,-1.08488113870685,0.277974302719563,0.746098866403631
"5825","LINC01197",3.09645332427507,1.24099818702777,1.14347607299617,1.08528566214426,0.277795154172756,0.746098866403631
"5826","SLC38A10",319.975493916931,0.164808242432534,0.151871725312813,1.0851805501852,0.277841696830314,0.746098866403631
"5827","AL031668.2",3.60583263605951,-1.10276710946285,1.01587731291371,-1.08553178168722,0.277686195357852,0.746098866403631
"5828","ATPSCKMT",58.0299100542582,0.307133906686418,0.283183353541122,1.08457613361026,0.2781094302534,0.746159519702585
"5829","EPB41L4A",84.7395316923719,0.244153407245918,0.225097278549068,1.08465730380963,0.278073464754641,0.746159519702585
"5830","CDC123",311.343369109397,-0.247683883321676,0.228451854499106,-1.08418416591425,0.278283150794449,0.746159519702585
"5831","TRAF3",69.766745958297,0.262533914686164,0.242113695587252,1.08434144565586,0.278213435359255,0.746159519702585
"5832","DLGAP1-AS2",13.1790559765769,0.510427241282857,0.470793845227308,1.08418418477075,0.278283142435448,0.746159519702585
"5833","OSBPL2",118.24003018281,0.238977694493522,0.220376118362484,1.08440831188633,0.278183799998397,0.746159519702585
"5834","PCDHGA7",9.10431793537853,0.651106404775532,0.600665400605474,1.08397521168892,0.278375789725241,0.746279971083779
"5835","AKIRIN1",270.875913116243,-0.208080248950633,0.192011042486399,-1.08368897046831,0.278502727566935,0.746492315192266
"5836","LRRC29",23.3119843768676,0.394385727915495,0.364009083792931,1

.08345023647774,0.278608627754835,0.746648207348462
"5837","AEBP2",160.08315769995,0.227100192664517,0.209651060548591,1.08322940065396,0.278706612873138,0.746691631881474
"5838","PCCA-DT",28.8732198308746,-0.407184993982971,0.37590985057154,-1.0831985205 0666,0.278720316299491,0.746691631881474
"5839","ADPRH",30.8405519643756,0.441306625781665,0.407458346923707,1.08307175227483,0.278776575987562,0.746714445700542
"5840","RAB4A",489.513461345898,-0.202603970645154,0.187127197618325,-1.08270723456456,0.278938391837345,0.746859138097476
"5841","AC024560.5",35.9157392927658,-0.321471110881017,0.296936065324245,-1.08262736804969,0.278973854524645,0.746859138097476
"5842","FBXL15",201.226539443345,-0.285373919869994,0.263583538601946,-1.08266973492967,0.278955042211143,0.746859138097476
"5843","LST1",26.8787249166112,-0.483873756057117,0.447136161235339,-1.08216198555778,0.279180556735261,0.747156726101897
"5844","C6orf89",466.559730267184,0.149254800818937,0.137910789960658,1.08225615168701,0.279138723924924,0.747156726101897
"5845","AMPD2",58.2995410938056,-0.249884781966221,0.230988684196196,-1.08180529637536,0.279339052696786,0.747167192185084
"5846","MAPK9",178.55014811201,-0.172671640406128,0.159626461515049,-1.08172316022835,0.279375558817031,0.747167192185084
"5847","PSMG4",225.8377946264,-0.271431469261188,0.250891010849492,-1.08187004525251,0.279310276785215,0.747167192185084
"5848","SCYL1",192.164587403011,-0.202476258561493,0.187134814160295,-1.08198070717112,0.279261100713258,0.747167192185084
"5849","AL133243.3",42.2970121740059,0.336038328585457,0.310937622429943,1.080725857358,0.27981907750604,0.747310566727782
"5850","RAD54L2",70.4023142920451,0.281476752884205,0.260510943120639,1.08047957414924,0.279928677756747,0.747310566727782
"5851","AC233280.1",3.72969747656241,-0.831865081071542,0.769687013419736,-1.0807835738004,0.279793396918281,0.747310566727782
"5852","AC010245.2",8.27190652368654,0.597071236463716,0.552444674021818,1.0807801478418,0.279794921233464,0.747310566727782
"5853","TDRD6",10.3297271668943,0.66502147208901,0.615520675250221,1.08042101399545,0.279954742321621,0.747310566727782
"5854","RFC2",99.3021522402584,-0.24307075347628,0.224857036105125,-1.08100132282558,0.279696525206499,0.747310566727782
"5855","CYREN",103.76616990751,-0.263403802431056,0.243680211019569,-1.08094047246989,0.279723593884642,0.747310566727782
"5856","ALG9",64.1755381548699,0.242702488652179,0.224472720210357,1.08121150946422,0.279603039458434,0.747310566727782
"5857","HMBS",38.996363958513,-0.407422782664117,0.376800781551394,-1.08126841188238,0.279577734343164,0.747310566727782
"5858","DDX54",178.302350440333,-0.181726354825607,0.168084985626107,-1.08115757126484,0.279627027791201,0.747310566727782
"5859","KLHDC2",451.028536176032,-0.199057547474541,0.18425315194032,-1.08034812635941,0.279987186215674,0.747310566727782
"5860","MOCS3",28.2063766371747,0.381615000564016,0.353244648719478,1.08031360686533,0.280002552495192,0.747310566727782

"5861","DDAH1",22.4101387589799,-0.501644674919651,0.464457658581589,-1.08006546054516,0.280113031169129,0.747477871947651
"5862","BMPR2",518.809959390468,0.220447594290359,0.204293519823165,1.07907286771101,0.280555245381449,0.747830443950747
"5863","AC112236.2",6.58664956749578,-0.714712415715943,0.662240763556268,-1.07923349791683,0.280483650204087,0.747830443950747
"5864","ZNF622",195.600717541487,-0.230884424127754,0.214082880942999,-1.07848148862136,0.280818938447745,0.747830443950747
"5865","R3HCC1",334.129432971714,-0.272451997468632,0.25262521081347,-1.07848300884666,0.280818260370989,0.747830443950747
"5866","FKBP15",153.020444978037,0.228654716884447,0.211884975574607,1.07914549516483,0.280522872750671,0.747830443950747
"5867","MARCH5",254.235868423053,-0.17463095868739,0.161903210942559,-1.07861331267449,0.280760144171795,0.747830443950747
"5868","AC023794.3",6.86103506067542,0.692184563925726,0.641397700408097,1.07918154911581,0.280506803168988,0.747830443950747
"5869","TSR1",98.7610105693469,-0.28380830157394,0.263046709168798,-1.0789273983725,0.280620093852673,0.747830443950747
"5870","ZNF599",48.8604849009842,0.291669029431024,0.270402162009852,1.07864902877662,0.280744216026747,0.747830443950747
"5871","TMEM91",189.630652607325,-0.208632944525602,0.193407651968948,-1.07872125224445,0.280712008735488,0.747830443950747
"5872","ERCC2",28.9101969588956,0.360348679380691,0.33400781196918,1.078863027952,0.280648792655989,0.747830443950747
"5873","CPXM1",382.545766297484,0.818880308995545,0.758615643501084,1.07944031475061,0.280391487262977,0.747830443950747
"5874","SLC48A1",153.890027785295,0.195105656673898,0.180945797890304,1.07825469808467,0.280920107876212,0.747972503776636
"5875","CAPRIN1",545.276348570162,0.175416027974649,0.162752570593619,1.07780803298432,0.281119433927728,0.747993866388169
"5876","RAB5B",262.150314411201,0.178150725950119,0.165271168230819,1.07792985223722,0.281065062089983,0.747993866388169
"5877","PSMB5",902.429641917146,-0.308693246672555,0.286394679237996,-1.07785957299866,0.281096429095803,0.747993866388169
"5878","CDR2L",99.9789640309371,0.244748115495298,0.227070749604938,1.07784959498797,0.281100882670766,0.747993866388169
"5879","BPGM",101.378065375774,-0.265384986433777,0.246331224685158,-1.07735016855038,0.281323857348352,0.748321127814698
"5880","UBOX5",38.0710648240327,0.292880267242386,0.271860495788549,1.07731822673562,0.281338122221894,0.748321127814698
"5881","ARHGAP29",1102.2809458968,0.251771551813099,0.233772950568533,1.07699180423053,0.281483927169692,0.748351690789263
"5882","CRY1",93.1297643969348,-0.291179075470326,0.270368503122513,-1.07697114163621,0.281493158370411,0.748351690789263
"5883","AL049775.3",6.77938722357256,0.661586695452456,0.61422801695458,1.07710276508175,0.28143435792037,0.748351690789263
"5884","ANKLE2",249.859614287082,-0.198755593232398,0.184610501477565,-1.07662127366331,0.281649496236699,0.74851284981172
"5885","STON2",226.793262159527,0.312168751664263,0.289942493883269,1.07665746915298,0.281633319616605,0.74851284981172

"5886","AL136171.2",7.7943102610768,0.680034795175425,0.631976396953381,1.07604460934573,0.281907306183795,0.74852890570626
"5887","SPCS3",367.680005440412,-0.181194867334523,0.168355273290394,-1.07626487601599,0.281808812434977,0.74852890570626
"5888","WDR41",203.989359059904,-0.186973438106474,0.173771011756023,-1.07597599977715,0.281937990190613,0.74852890570626
"5889","EPHB6",116.804722302044,0.338114349643727,0.314263976277591,1.07589280085055,0.281975201981743,0.74852890570626
"5890","FAM168A",138.192798694087,0.284561937206804,0.264502124349414,1.07583989318321,0.281998867349839,0.74852890570626
"5891","FAR2",88.7691391822683,0.297116174554334,0.276194065210603,1.07575148049535,0.282038416964641,0.74852890570626
"5892","ZNF582",24.8480681563685,0.391306840515013,0.363675708709788,1.07597739179021,0.281937367623185,0.74852890570626
"5893","CRNKL1",170.679203654432,0.255354029988815,0.237360505324033,1.07580673389711,0.282013700040116,0.74852890570626
"5894","STAG1",458.63009078241,0.257399750890354,0.239306590074521,1.0756066132997,0.282103228485085,0.748573887598699
"5895","NEURL2",22.6837014608866,0.477555827976616,0.444037652594092,1.07548498463297,0.28215765121808,0.748591291781301
"5896","SLC25A26",137.281966366001,-0.243104051458491,0.226133611000795,-1.07504607732831,0.282354099442415,0.74871593154794
"5897","AC105285.1",3.49731021257356,1.07008255466849,0.995669652450618,1.07473653739945,0.282492700550468,0.74871593154794
"5898","PAK1IP1",156.452064625424,-0.241224855023184,0.224526663641194,-1.07437063870808,0.282656596608919,0.74871593154794
"5899","MEA1",297.731301494922,-0.207370039930817,0.193018370765021,-1.0743539027343,0.282664094651701,0.74871593154794
"5900","SNX13",215.0815567741,0.216517881313745,0.201485987433338,1.07460515776751,0.282551541675637,0.74871593154794
"5901","TCTN3",167.956681246062,-0.226053906629214,0.210240327480542,-1.07521667863714,0.282277729880348,0.74871593154794
"5902","AC087521.2",2.74916874869077,-0.973272760206855,0.905512787729426,-1.07483049758727,0.282450623603976,0.74871593154794
"5903","HSP90B1",2508.64111452238,-0.166616154194715,0.1550834881478,-1.07436424202636,0.282659462431214,0.74871593154794
"5904","FBXL19",25.2759941455288,0.418217218505074,0.389327524703322,1.07420408773761,0.282731220698346,0.74871593154794
"5905","SPATA20",91.1446728842657,-0.228441335948077,0.212644874260034,-1.07428564522429,0.282694676744159,0.74871593154794
"5906","SF3A2",140.780323345929,-0.308952449403865,0.287425210252851,-1.07489683710095,0.282420918217314,0.74871593154794
"5907","CCDC12",341.171240899941,-0.197607842431815,0.184019541870647,-1.07384161716216,0.282893673808014,0.748973631659579
"5908","NSUN2",59.6746794657002,-0.241654605001576,0.225054919591474,-1.0737583761343,0.282930989942034,0.748973631659579
"5909","GOT2",68.8585089215402,0.271690227207914,0.253049002901033,1.0736664602238,0.28297219881563,0.748973631659579
"5910","ZMYND11",364.668495073449,0.19058400531126,0.177577014723151,1.07324703936705,0.283160290363082,0.749091160568099

"5911","NPIPA9",5.61678504973328,-0.686083209593567,0.639226073217446,
-1.07330291791803,0.283135226435593,0.749091160568099
"5912","PRPS1",132.652732126203,-0.214112690465274,0.199495215304084,-
1.07327230950832,0.283148955434369,0.749091160568099
"5913","MANBAL",333.707067172174,-0.265269075492039,0.247206641717636,
-1.07306613466735,0.283241444193054,0.74917912856069
"5914","TMEM14B",750.82403784461,-0.246623494013502,0.230052441013873,
-1.07203163299028,0.283705824209635,0.750026891588691
"5915","DCAF10",245.203817807862,0.225927066157672,0.210744576345171,1
.07204213781348,0.283701106084001,0.750026891588691
"5916","ATXN1L",100.073504917302,0.223360830586929,0.208321841295266,1
.07219113079145,0.283634193250095,0.750026891588691
"5917","HIF1AN",75.5713708167082,0.261606986357568,0.24412556481675,1.
07160831989857,0.283895994834026,0.750275998513715
"5918","VTN",3.33924765327558,-0.91047729821127,0.849585224756334,-1.0
7167270767026,0.283867063486796,0.750275998513715
"5919","SPEN",291.395628597902,0.265201939990684,0.247533801525954,1.0
7137666999744,0.28400009855814,0.750389648133081
"5920","NHEJ1",68.1656987161836,-0.295865533604588,0.276298641566857,-
1.0708179089364,0.284251312739964,0.750389648133081
"5921","STT3B",395.180043479294,0.191356352812171,0.178709848197042,1.
07076557191848,0.284274850715378,0.750389648133081
"5922","MIER3",52.4534799423216,0.288555798542921,0.269459317718379,1.
07086962509309,0.284228055285517,0.750389648133081
"5923","SLF2",132.46840734718,0.238004852771463,0.222191939386425,1.07
116780846643,0.284093983315417,0.750389648133081
"5924","EPG5",97.2644585615318,0.289227447261073,0.270077191960571,1.0
709065995595,0.284211428157988,0.750389648133081
"5925","CCDC61",107.671265461226,0.193330115087375,0.180508390779478,1
.07103118172252,0.284155409382715,0.750389648133081
"5926","DIP2C",144.978509543495,0.22357056223777,0.2088946020788,1.070
25533457027,0.28450439324803,0.750742147865563
"5927","ENO3",4.6698061412563,-0.864971564109261,0.808184921525743,-1.
07026441730231,0.284500306066669,0.750742147865563
"5928","TGS1",235.9637936082,0.18413986154418,0.172071221439122,1.0701
3746984604,0.284557435353438,0.750755446850164
"5929","POGK",102.245083345261,0.307154231323484,0.287210714862958,1.0
6943862268524,0.284872071689307,0.751205395586033
"5930","PTPN13",235.154419622768,0.198618889430473,0.185719399506973,1
.06945687934456,0.284863849148111,0.751205395586033
"5931","INHBA",16.1154410846153,0.744211750376719,0.695778877957207,1.
06960957561935,0.284795083191794,0.751205395586033
"5932","ZC3H12A",49.6164809739146,-0.375473566180197,0.351213466082035
,-1.06907508521469,0.285035837252535,0.751281431404698
"5933","SULF1",394.000755208435,0.357016450939738,0.333895830077027,1.
06924501230631,0.284959280945108,0.751281431404698
"5934","NTMT1",186.782936609387,-0.255820547502377,0.239296027569592,-
1.06905471896302,0.285045013680018,0.751281431404698
"5935","C1GALT1C1L",45.0751472088283,-0.333863005742981,0.312404699031
357,-1.06868752863884,0.285210492997422,0.751337731258157

"5936","CENPT",163.082582848682,-0.254014474447061,0.237680284565516,-1.06872336892143,0.285194338225969,0.751337731258157
"5937","HCCS",82.3936059461624,-0.236245200311722,0.22102288958793,-1.06887210076826,0.285127304936927,0.751337731258157
"5938","PPA1",1306.5211304162,-0.189551033421873,0.177454000055314,-1.06816996721848,0.285443849388176,0.751572694348666
"5939","APOLD1",76.7141206253231,0.869459983573226,0.813902299880165,1.06826087566191,0.285402851545898,0.751572694348666
"5940","MTSS2",14.9698876092002,0.498916957094727,0.466985690364668,1.06837739868458,0.285350307876022,0.751572694348666
"5941","AC006001.2",7.95319599422062,0.612287867514513,0.573269224736633,1.06806338295206,0.2854919217752,0.7515727413843
"5942","TSNAXIP1",3.58281908764787,0.879585798772831,0.823664629891416,1.06789312889251,0.285568722314278,0.751648404071913
"5943","FBXO28",93.006881986848,0.216706734850282,0.202979397507371,1.0676292151395,0.285687799755039,0.751752724132885
"5944","TIGD5",63.7650224721707,0.243389140137341,0.227979496525552,1.06759223459405,0.28570448799526,0.751752724132885
"5945","DENND6B",25.3575517908317,-0.368959971525286,0.3456380619575,-1.0674749460048,0.285757421269132,0.751765528788768
"5946","IAH1",636.024666800624,-0.291084210778616,0.2728203064892,-1.06694481259275,0.285996757379009,0.752040045370075
"5947","SEPTIN7",2175.33093198246,-0.121778599498147,0.114139869057212,-1.06692429651471,0.286006022369642,0.752040045370075
"5948","AC079949.2",4.86064122730576,-0.693332299086414,0.649813740826033,-1.06697081875348,0.285985013377288,0.752040045370075
"5949","AL353593.1",8.82174106518285,0.6917292208306,0.648864234204441,1.06606156475663,0.286395812662505,0.752053477172865
"5950","AC010913.1",12.6071458213319,-0.515276924066715,0.483224298991455,-1.0663307394561,0.286274158517774,0.752053477172865
"5951","RNF180",5.18416074568953,0.853314078883522,0.800070655011973,1.06654840236673,0.286175810778999,0.752053477172865
"5952","JMJD1C-AS1",7.22501518359508,-0.650773053494981,0.610388083100692,-1.06616277662096,0.286350065624177,0.752053477172865
"5953","PARP11",55.2025405274686,0.249994567056983,0.234349317827687,1.06676037879828,0.286080054332734,0.752053477172865
"5954","CTDSP2",708.852090062444,-0.118884824677961,0.11147021534081,-1.06651650680391,0.286190220885669,0.752053477172865
"5955","ACSF3",82.8085797354332,0.248097199031205,0.232642985615174,1.06642888189887,0.286229811493145,0.752053477172865
"5956","TLE2",86.0693704291141,-0.386985565635653,0.362948714063573,-1.06622657868922,0.286321230082577,0.752053477172865
"5957","YRDC",69.1885267399047,-0.303417016579433,0.285046810401739,-1.06444627867193,0.287126578491709,0.752204302782301
"5958","CHMP3",919.366650918963,-0.188980032149396,0.177508560976193,-1.06462488969612,0.287045711879481,0.752204302782301
"5959","ABI3BP",2533.35029851458,-0.276497029417813,0.259653810572415,-1.06486798251975,0.286935675666368,0.752204302782301
"5960","AL450344.3",3.2320345258828,-0.939529832868766,0.8816490162903

44,-1.06565063365234,0.286581601288329,0.752204302782301
"5961","AP002495.1",23.6462886668834,0.365388581652125,0.343100418900892,1.06496104791312,0.286893557060585,0.752204302782301
"5962","PRAC2",8.93050754505946,-1.02838389721551,0.965814696472725,-1.06478385654236,0.286973752152669,0.752204302782301
"5963","MYL12B",4750.0546002147,-0.275766437441107,0.259068532768681,-1.06445361964255,0.287123254544911,0.752204302782301
"5964","RPS15",13444.0114181422,-0.207203822620703,0.194654576706235,-1.0644693082835,0.287116150910888,0.752204302782301
"5965","PIN1",564.97402226627,-0.249888103100339,0.23461881293421,-1.06508126937975,0.286839154609456,0.752204302782301
"5966","EML2",18.8957526267144,0.408839933916453,0.383737507210194,1.06541561936115,0.286687891643778,0.752204302782301
"5967","NAPB",38.0582947795071,0.370223411704726,0.347413622499374,1.06565600117017,0.286579174021837,0.752204302782301
"5968","BCAS4",6.72847447546612,0.664203074446273,0.623779468388725,1.06480432285142,0.286964488527173,0.752204302782301
"5969","MAGIX",3.89887774428502,0.898871161054461,0.84424671219513,1.06470199773393,0.287010805763733,0.752204302782301
"5970","AC244090.1",46.6535358115419,0.440605764457482,0.41375117504588,1.06490516772218,0.286918846258297,0.752204302782301
"5971","IBA57",54.7109641146565,0.295797205371901,0.277986956121535,1.06406865091389,0.287297601075336,0.752314540333733
"5972","PHF20",418.133190404065,-0.142539651281288,0.133949677207705,-1.06412836710508,0.28727055183228,0.752314540333733
"5973","RUNX1",351.227503991768,-0.331932347439746,0.311956307290773,-1.06403473717989,0.28731296351748,0.752314540333733
"5974","PRRT3",31.7829180806099,0.338866511744351,0.318594637172378,1.06362905148653,0.287496776382851,0.75266983304784
"5975","ADAM22",36.1088348357478,0.480903182799278,0.452284111993709,1.06327675469168,0.287656463534193,0.752709902906941
"5976","AL159169.2",3.04732296511697,1.13672299675039,1.06890374779617,1.06344747980728,0.287579070722563,0.752709902906941
"5977","ZC3H12B",10.9148829017358,0.538805892414107,0.506693896062493,1.06337553422521,0.287611683255467,0.752709902906941
"5978","AL662797.1",3.09220675380003,1.0351960783513,0.973759553227012,1.06309208974709,0.287740191342689,0.752803043258557
"5979","FAM107A",12.3191026030883,-0.549886819848614,0.517384519946631,-1.06282039498463,0.287863408696554,0.75287353043714
"5980","ANKRD29",26.2831119126298,-0.423536893623752,0.398488297580165,-1.06285905055605,0.287845875685949,0.75287353043714
"5981","KIF13B",147.476262911179,-0.364071068559346,0.34273463853895,-1.06225349766616,0.288120619104809,0.752945744312936
"5982","FAM160B1",206.116341631616,0.204078130280294,0.192220569971341,1.06168726016534,0.28837768480864,0.752945744312936
"5983","AP003119.3",7.71185506466033,0.849143543651365,0.799491670204267,1.06210430364385,0.288188336579149,0.752945744312936
"5984","AL138966.2",4.14315337144455,-1.07260322717558,1.01021855320677,-1.06175364110151,0.288347540582538,0.752945744312936
"5985","AC004825.2",9.17397095801586,-0.580070465286174,0.545991977119

386,-1.06241573062407,0.288046995584404,0.752945744312936
"5986","HDC",4.20959420805203,-1.02101342405603,0.96177501143176,-1.06
1592796569,0.28842058530555,0.752945744312936
"5987","B3GNT9",65.9235693770128,0.42373948620377,0.398861000329965,1.
06237382409718,0.288066012120164,0.752945744312936
"5988","H3F3B",11861.582368895,-0.205858052153161,0.193883718073323,-1
.06176039019073,0.28834447587473,0.752945744312936
"5989","ELP2",222.998976667836,0.164167155162872,0.154585479209451,1.0
6198302714085,0.288243390515257,0.752945744312936
"5990","ZNF850",14.9215268480563,0.485447954184936,0.457162509476592,1
.06187175046512,0.28829391122934,0.752945744312936
"5991","SMIM10L2A",2.59399488039747,1.18760641047346,1.11833862607792,
1.06193811317996,0.288263781180308,0.752945744312936
"5992","MEX3A",4.53576465913008,-0.864350968873151,0.814772317279314,-
1.06084970063709,0.288758211223744,0.753396195944411
"5993","CARD9",6.4601309664401,-0.686822534072259,0.64759325019941,-1.
06057704255066,0.288882160299145,0.753396195944411
"5994","HCFC2",194.570578430296,0.18162214917999,0.171238284434706,1.0
6063985503921,0.28885360286129,0.753396195944411
"5995","MYO1D",30.1992314995097,-0.439254951315018,0.414151715325762,-
1.06061362312483,0.2888655288612,0.753396195944411
"5996","PTRH2",85.1893727665812,-0.268587952714975,0.253207094495928,-
1.06074418352956,0.28880617454929,0.753396195944411
"5997","TYMS",13.0937664695029,-0.676084844205902,0.637436130893653,-1
.06063150712537,0.288857398096131,0.753396195944411
"5998","PTGS2",54.0443274467028,-0.576051292913438,0.543237049023448,-
1.06040501830459,0.288960380150354,0.753403019179263
"5999","CAND2",10.027671513283,0.518253023170755,0.488752226036105,1.0
6035941232208,0.288981119696701,0.753403019179263
"6000","TRAM1",1205.35624585911,-0.14885984589716,0.140437885404077,-1
.05996929154017,0.289158570039337,0.753488842954886
"6001","KLHL9",180.592611767404,0.204747364678522,0.193153263258458,1.
06002539757534,0.289133045129091,0.753488842954886
"6002","AP2B1",635.080938441854,0.20917375559349,0.197304517337667,1.0
6015695137638,0.289073201923616,0.753488842954886
"6003","ATP6AP1L",25.5935136668397,0.368980164275858,0.348230291442233
,1.05958663948414,0.289332694456767,0.753816981726443
"6004","SLC35A3",205.206831232415,0.221427089082122,0.209163181545526,
1.05863320420916,0.289766858863584,0.753992258694548
"6005","TRIP12",370.650035693029,0.2329810174297,0.220107757814358,1.0
5848616942525,0.289833852894605,0.753992258694548
"6006","FAM200B",549.74433040378,-0.2355102073608,0.222452769139194,-1
.05869757554439,0.289737532383283,0.753992258694548
"6007","AKR1C3",812.21776782857,-0.35778945597215,0.338014751963829,-1
.05850248811164,0.289826417033577,0.753992258694548
"6008","ATP5F1C",1141.47023320744,-0.28465444981447,0.268905594347337,
-1.05856648503486,0.289797257088453,0.753992258694548
"6009","NUDT13",9.77359012942498,-0.679244254149541,0.641412122006565,
-1.05898256494533,0.289607720092428,0.753992258694548
"6010","LAYN",9.93047601984558,-0.683439895777181,0.645220618231607,-1

.0592344331003,0.289493027091988,0.753992258694548
"6011","AC009560.3",3.63108324213176,1.08971219602535,1.02947503640925,1.05851250150388,0.289821854340617,0.753992258694548
"6012","ATP6V0D1",441.23295928256,-0.230530956741417,0.217726832513333,-1.05880820512694,0.289687136104801,0.753992258694548
"6013","WWP2",64.7938018267994,0.259137894028188,0.244871809043481,1.05825940127789,0.289937196590795,0.754135665172133
"6014","WASHC5",170.08102670167,0.206765145009256,0.19548259211494 3,1.05771640723732,0.290184752831384,0.754158754652182
"6015","FAR1",198.543939524247,0.186857467971721,0.17664217306186,1.05783044180669,0.290132751569719,0.754158754652182
"6016","AACS",106.532916677992,0.29761937740882,0.281379127885283,1.05771661048775,0.29018466014094,0.754158754652182
"6017","ARG2",2.64839823263544,-0.965936410214852,0.913324040070128,-1.05760537097073,0.290235392854954,0.754158754652182
"6018","AC048382.5",10.7738747492306,0.506635027174664,0.478813517545927,1.05810510482522,0.290007527373298,0.754158754652182
"6019","SP2-AS1",12.4436416400647,0.597136593651623,0.564597485562588,1.05763239993287,0.290223065278305,0.754158754652182
"6020","ADAR",449.870645572023,0.229120052864521,0.216991359542776,1.0589482156018,0.291016270262711,0.754306085519409
"6021","CDC42BPA",367.475873518508,0.221152833280658,0.2096968590398,1.05463111986185,0.29159406532075,0.754306085519409
"6022","HS1BP3",125.294033130882,0.249199031449133,0.235680067617769,1.05736150692765,0.290346632173719,0.754306085519409
"6023","PTRHD1",262.48489026457,-0.294554202206191,0.2788445232226,-1.05633848856716,0.290813597686762,0.754306085519409
"6024","AC092958.1",18.8316278445222,0.746841440362243,0.7076118261769,1.05543945526927,0.291224385973069,0.754306085519409
"6025","GRPEL1",141.151187878432,-0.232487455665689,0.220166312415901,-1.05596289057389,0.290985169328091,0.754306085519409
"6026","SRP72",667.929927125094,-0.127137204044288,0.120411452835421,-1.05585640776264,0.291033822627674,0.754306085519409
"6027","AC097534.2",28.4694946935169,-0.359877006206652,0.341235112552084,-1.05463064312212,0.291594283443117,0.754306085519409
"6028","ADAM19",37.9681877124279,0.599696340803281,0.567387330909029,1.05694348134719,0.290537382537675,0.754306085519409
"6029","FAM162B",6.49042887451083,0.727364120440133,0.688543674590535,1.05638051336785,0.290794405161646,0.754306085519409
"6030","AL358852.1",11.1561791735045,-0.506609587764864,0.479769465728875,-1.05594378957655,0.290993896404902,0.754306085519409
"6031","ABCF2",105.118977654332,-0.231781686618629,0.219764698376808,-1.05468115821412,0.291571171921692,0.754306085519409
"6032","MTERF3",59.6640472315081,-0.287678800725134,0.272662029785944,-1.05507466863274,0.291391176307271,0.754306085519409
"6033","NDUFB9",832.495611123515,-0.253795392482353,0.240412552737143,-1.05566614385498,0.291120770155464,0.754306085519409
"6034","LY6E",1444.3659210866,-0.317499946767905,0.301001472569096,-1.05481193848652,0.291511343423141,0.754306085519409

"6035","CPSF1",89.7065529446542,-0.215705494746989,0.204437034992208,-1.05511946382518,0.291370691273491,0.754306085519409
"6036","GBA2",67.6651941264378,0.26503766962531,0.251121239877645,1.05541717520368,0.291234571181787,0.754306085519409
"6037","CKS2",43.0935799892219,-0.412013059391953,0.39045963170005,-1.05520014347721,0.291333798564549,0.754306085519409
"6038","AL512598.1",4.05912594431308,0.870451735813661,0.824362036033339,1.05590953703071,0.291009546531105,0.754306085519409
"6039","TMEM19",132.506724893512,-0.207968235807637,0.197052818283118,-1.05539335909845,0.29124545884737,0.754306085519409
"6040","ANKS1B",16.3111605231308,-0.552158259916057,0.523508233456035,-1.05472698351062,0.291550207127312,0.754306085519409
"6041","OASL",5.65082059695991,-0.751475920271567,0.711352180808886,-1.05640488712223,0.290783274175183,0.754306085519409
"6042","LMO7-AS1",4.9907596069018,1.23706425796326,1.17008321200292,1.05724468591057,0.290399930595654,0.754306085519409
"6043","AJUBA",6.25731207312526,0.673679792842444,0.638194595912092,1.05560247165622,0.291149871220677,0.754306085519409
"6044","ULK2",106.426093614771,0.197958887006398,0.187658334106687,1.05488993040861,0.291475668120067,0.754306085519409
"6045","HAUS5",24.7750874227754,0.373344472206554,0.353367464128782,1.05653324118853,0.290724662272478,0.754306085519409
"6046","RBX1",1236.37571774924,-0.262663203190224,0.248565270502355,-1.05671722626164,0.290640660757411,0.754306085519409
"6047","KYAT3",173.176334962565,-0.258197429588852,0.244891499608473,-1.05433398056549,0.291730036502394,0.754532457565311
"6048","AL356056.2",3.28192107583089,-1.02766112422147,0.975388796722072,-1.0535912732185,0.292070086319356,0.754807827607937
"6049","EDRF1",92.7980957048972,0.268550199196441,0.254851800793656,1.05375044775091,0.291997185579005,0.754807827607937
"6050","BIRC3",40.2808094451829,-0.445479783587698,0.422821303186657,-1.05358878616161,0.292071225469747,0.754807827607937
"6051","PYM1",137.223542127692,-0.252647638935917,0.239800470866658,-1.05357440718456,0.292077811552636,0.754807827607937
"6052","TSR3",405.489320973561,-0.216160547461006,0.205141242914289,-1.05371569553822,0.292013100788095,0.754807827607937
"6053","MFSD2A",2.7916483130955,-1.24814451736105,1.18519890117977,-1.05310974902072,0.292290695226687,0.754923873942382
"6054","GRAMD2B",66.090167279088,-0.314040117460782,0.298218157156042,-1.05305498650929,0.29231579159815,0.754923873942382
"6055","DUSP22",302.390476834432,0.16939398577873,0.160831102477295,1.05324146368171,0.29223033942106,0.754923873942382
"6056","EZR",118.329640316738,-0.405625781375102,0.385123188282075,-1.05323645450819,0.292232634628967,0.754923873942382
"6057","LRRC42",118.592642250469,-0.243720181748834,0.231501252669216,-1.05278126549525,0.292441253199749,0.754979659375753
"6058","ZNF721",202.654454061413,0.210452628256063,0.199918535231697,1.05269192780028,0.292482209472998,0.754979659375753
"6059","PRDX3",636.285490773032,-0.220495622961104,0.209432189639642,-

1.05282584945752,0.292420815418993,0.754979659375753
"6060","ORAI3",621.242602101926,0.276261459476498,0.262580551726157,1.05210175567233,0.292752866741689,0.755553603273929
"6061","TRPV4",11.824637859859,-0.551814747845785,0.524546191870594,-1.05198504230475,0.292806412258298,0.755567115611249
"6062","LINC01089",34.8152606955217,0.300872584568571,0.28607200141489,1.05173726572499,0.292920108478437,0.755735812702534
"6063","LINC00649",14.8964413632539,-0.528870742980282,0.502982835120802,-1.05146877000934,0.293043345456326,0.755929065303801
"6064","ICAM3",76.0751611364808,-0.304137328894436,0.289382309113396,-1.05098798135327,0.293264109816372,0.75612441106628
"6065","ECSIT",172.738172548773,-0.240689954972066,0.229008221967971,-1.05101010305966,0.293253949714488,0.75612441106628
"6066","MX1",107.055791599273,-0.482115873801847,0.458673770381209,-1.05110844555414,0.293208785634704,0.75612441106628
"6067","TMEM189",158.211967957157,0.209251293248991,0.199132759128283,1.05081300618242,0.29334448108899,0.756206969545378
"6068","NEIL2",53.653737372596,-0.281181256306858,0.267648720471626,-1.05056080900139,0.293460348717349,0.756380991090861
"6069","FNDC3B",872.84914561189,0.235711989669037,0.224448269749853,1.05018403542045,0.293633508014096,0.756577934981953
"6070","TSPO",2163.5533647218,-0.316541004662827,0.301396251410595,-1.05024864503573,0.293603809568431,0.756577934981953
"6071","TMEM67",52.9724641644642,-0.2637281282378,0.251180923085756,-1.04995285867215,0.293739787186925,0.756685178841759
"6072","PCK2",133.966684470772,0.277567278230922,0.264379239696701,1.04988303374104,0.293771892962095,0.756685178841759
"6073","MCCC1",48.2206733315739,-0.269671585524252,0.25692018347553,-1.0496317645279,0.293887446999371,0.756858170767357
"6074","KRBA2",34.2652095789811,0.3476813289057,0.331296372828449,1.04945709467739,0.293967792373277,0.756940446611467
"6075","PDP1",229.813997868084,0.223586390241819,0.213073272645351,1.04934038636543,0.294021484563145,0.756954077130467
"6076","HSPE1",886.083031391338,-0.266495606302699,0.254011525275544,-1.04914769522215,0.294110147391694,0.75705771975084
"6077","GSTO2",2.67491914936376,1.12239362157691,1.07023096676222,1.04873962390801,0.294297972113341,0.757416535108219
"6078","TPRG1",66.8271411847744,-0.272369154815925,0.259748310927378,-1.04858874286222,0.294367439133702,0.757470672598076
"6079","AC100830.1",2.52139806552452,-1.0025247758529,0.956251588092205,-1.04839018134654,0.294458875431078,0.757581314647484
"6080","SPEGNB",5.06910150332972,-0.769682101897571,0.734378807241967,-1.04807232222317,0.294605287135343,0.757833337302099
"6081","PREB",119.708934074118,-0.22745565312025,0.217065448890647,-1.04786668851586,0.29470003175632,0.757871263256229
"6082","BCLAF1",1171.0636702616,0.245134489750367,0.233944908471141,1.04782998421445,0.294716945212556,0.757871263256229
"6083","POU3F1",10.28786036486,0.686581006162585,0.655450222462045,1.04749526757899,0.294871213693703,0.75793346629436
"6084","NIT2",241.277301768367,-0.272823337030739,0.260456988501361,-1

.04747942683562,0.294878515918663,0.75793346629436
"6085","GPM6B",13.5859423401389,-0.503035147874216,0.480241875298323,-1.04746206807087,0.294886518056341,0.75793346629436
"6086","AC008063.1",28.5111150671458,0.378368774848809,0.361515502804873,1.04661839371528,0.295275615034291,0.758127352353425
"6087","TUBA4A",26.612422791621,0.454528531868777,0.434403877027287,1.04632706084303,0.29541005564797,0.758127352353425
"6088","FAIM",170.918942431737,-0.241786084283807,0.23107312453222,-1.04636177302434,0.295394034958167,0.758127352353425
"6089","C6orf163",5.63047259117306,0.729580338551721,0.697084404646788,1.04661692857897,0.295276291044343,0.758127352353425
"6090","CASP7",165.789765045996,0.241433532303151,0.230611444653503,1.04692779955439,0.295132879215569,0.758127352353425
"6091","MDP1",217.557426591211,-0.272576430225973,0.260553339580356,-1.04614445036468,0.295494345322276,0.758127352353425
"6092","RSL24D1",941.826559830234,-0.236609083894836,0.226172861824408,-1.04614268036512,0.29549516240067,0.758127352353425
"6093","AC092376.1",3.7434883192935,-0.857268997953394,0.819433477414434,-1.04617277861083,0.295481268463948,0.758127352353425
"6094","DAZAP1",223.974960306068,-0.170355060598141,0.162721651101399,-1.04691084096723,0.295140701392838,0.758127352353425
"6095","NCAPH2",102.475454118384,-0.219418473987471,0.209604081497674,-1.0468234798658,0.295180999050365,0.758127352353425
"6096","IKBKG",114.695928278303,-0.25248646208745,0.241274335875882,-1.04647044689136,0.295343882543545,0.758127352353425
"6097","MRE11",156.223073144023,0.213571582189081,0.204206913634774,1.0458587243087,0.295626263582874,0.758339308255889
"6098","COX5B",1873.82385980579,-0.250417915051683,0.23952359941106,-1.04548326623101,0.295799670603873,0.758535308779239
"6099","CIAO2B",752.426721346971,-0.242291076489792,0.231733873986155,-1.04555744191403,0.29576540683084,0.758535308779239
"6100","COA7",44.0147582092347,0.26631379918564,0.254814733201129,1.04512716293934,0.295964201436654,0.758832804994961
"6101","MPP7",77.9577119066547,0.310200265145531,0.2968467242584,1.04498463279489,0.296030071973315,0.758877286619022
"6102","NCAPG",4.51372774543574,0.735503061884917,0.703938235798267,1.04484033467916,0.296096769575686,0.758923873510935
"6103","UNK",110.475875419822,0.226092423360579,0.216548663841976,1.04407212378631,0.296452021980999,0.75970991705437
"6104","HDAC3",204.446875345039,-0.256274211249162,0.24554798145266,-1.0436828261957,0.296632158071233,0.760047010523277
"6105","SEC24A",133.646169477477,0.188216959201654,0.18037191123241,1.04349373422747,0.296719681271077,0.760146734656779
"6106","ASH1L",675.266436141872,0.242771970920299,0.232952271068065,1.04215326945392,0.297340624652568,0.760499224384837
"6107","IDH1",189.35482283499,0.167129488843895,0.16036120114108,1.04220651663029,0.297315942412486,0.760499224384837
"6108","ALCAM",126.137379334116,-0.378819127524515,0.363376524014199,-1.04249752664185,0.29718107159002,0.760499224384837
"6109","SAP30",129.088040296944,-0.305400368464116,0.292999827700131,-

1.04232268961153,0.297262096246993,0.760499224384837
"6110","LINC02062",4.54916709615738,0.834660008534722,0.800984905973814,1.04204211878371,0.297392151939748,0.760499224384837
"6111","C5orf56",159.573889708396,0.253629500599006,0.243341559355841,1.04227778136377,0.297282910442424,0.760499224384837
"6112","STK19",202.301827359359,-0.215050772491697,0.206354985803724,-1.04213993984252,0.297346803685995,0.760499224384837
"6113","AC005072.1",8.05739916039861,0.803235115980022,0.770160513572068,1.04294507680555,0.296973730872054,0.760499224384837
"6114","SOCS1",889.977968747874,0.431889691499747,0.414196344297747,1.04271729445608,0.29707924562401,0.760499224384837
"6115","COX6B1",1895.93970076721,-0.254480120783387,0.244200657634988,-1.04209433032635,0.297367946941551,0.760499224384837
"6116","MAGEH1",448.967455575638,-0.227470791628645,0.218093951384097,-1.04299449932031,0.296950840377425,0.760499224384837
"6117","SLC66A1",54.140497920289,0.28520249258120 6,0.273899583050665,1.04126661824253,0.297751824987544,0.760688167989156
"6118","AGFG1",329.73070604,0.184681866744113,0.177352309013059,1.04132767017155,0.29772349888946,0.760688167989156
"6119","PJA2",852.178289281158,0.155483965991789,0.149254004464048,1.04174066585424,0.297531929694305,0.760688167989156
"6120","FAM184A",31.2470833003593,-0.503846884300165,0.483884887490002,-1.04125360664539,0.297757862175806,0.760688167989156
"6121","PSMA1",779.595762560729,-0.233275153714475,0.223955970794127,-1.04161167432734,0.297591753923239,0.760688167989156
"6122","ABCA5",45.7731010413302,0.418149447587845,0.401524193404824,1.04140536101211,0.297687455484492,0.760688167989156
"6123","PDE4D",47.7413935858887,-0.463036918797266,0.444791051880145,-1.04102120948701,0.297865704790613,0.760710675150392
"6124","RPS14",19908.4599558712,-0.24207126168585,0.232557955911824,-1.04090724712782,0.297918597927679,0.760710675150392
"6125","GATD1",92.4273443898947,0.19267255276473,0.185062982292661,1.04111881467486,0.297820408465375,0.760710675150392
"6126","NDUFA11",1586.399000336,-0.258074945099157,0.247954577709536,-1.04081540854421,0.297961227363894,0.760710675150392
"6127","KCTD18",109.642858748131,-0.238468349231164,0.229226886714395,-1.04031578777355,0.29819321164561,0.760860175050591
"6128","BACE1",325.419942206141,0.14863331465742,0.142879525460766,1.04027021491077,0.298214378072898,0.760860175050591
"6129","AC087477.2",18.2310337988094,0.475591469367663,0.457141035243557,1.04036048549935,0.298172452652587,0.760860175050591
"6130","NAGLU",372.067900939342,0.315123870220386,0.302894138179307,1.04037625856543,0.298165127387972,0.760860175050591
"6131","PJVK",21.5232433142776,0.385082288696691,0.370256584597538,1.04004170274316,0.298320526237462,0.760875161830854
"6132","AC022075.1",13.5563974752206,0.524424626185664,0.504224997189373,1.04006074492317,0.298311679825998,0.760875161830854
"6133","SRSF9",1057.35565728902,-0.234687182827794,0.225673035713078,-1.03994339459401,0.298366199968582,0.760875161830854
"6134","IRF2BP2",1413.89693280052,0.22815795705111,0.219468219219261,1

.03959451561033,0.298528326002225,0.761164496034365
"6135","ASPSCR1",100.097506721776,-0.251514097570844,0.241999792469525
,-1.03931534405145,0.298658100961938,0.761371263087973
"6136","FKTN",78.514518403343,0.296715316859061,0.285554731038065,1.03
908387642685,0.298765728952126,0.761397425584366
"6137","DCUN1D5",204.129257498755,-0.251151990168772,0.241682539821627
,-1.03918135896012,0.298720398294843,0.761397425584366
"6138","ERI3",267.767460535723,-0.276510441455651,0.266277820097528,-1
.03842836536057,0.299070669457945,0.761474587157002
"6139","LINC00853",3.17017280299199,1.03139610021131,0.993906212245762
,1.03771974408012,0.299400550133913,0.761474587157002
"6140","OSBPL9",271.642275231464,0.155736891670513,0.150007003902934,1
.03819746824147,0.299178131106942,0.761474587157002
"6141","MAP6D1",3.86267813547918,-0.771823732280322,0.743524029578882,
-1.03806158452938,0.299241384682237,0.761474587157002
"6142","SORCS2",39.190263263327,-0.39064276556113,0.376031655651912,-1
.03885606355105,0.298871682833714,0.761474587157002
"6143","TMEM242",249.403525855791,-0.277816944768352,0.267497599244354
,-1.03857733883649,0.299001349501489,0.761474587157002
"6144","ACTB",21263.8572112468,-0.260480535636797,0.250964840457253,-1
.03791644742828,0.299308955545228,0.761474587157002
"6145","HOXC10",229.890839891706,-0.200874092799353,0.193458836072284,
-1.03832989424323,0.299116495673481,0.761474587157002
"6146","CTC1",26.5721119870771,0.37391759144754,0.360305100864855,1.03
778045481457,0.299372278285899,0.761474587157002
"6147","AC127024.5",4.55647948363442,0.759769334110298,0.7321957419790
05,1.03765877148748,0.299428945717107,0.761474587157002
"6148","MEF2B",38.3070208421323,-0.302970585094627,0.291935572518041,-
1.03779947911591,0.299363419392848,0.761474587157002
"6149","ZNF43",137.643714219441,0.199760125866965,0.192400295563879,1.
03825269748945,0.299152424561297,0.761474587157002
"6150","MED12",67.7261140666695,0.226632929152279,0.218260677862007,1.
03835895394573,0.299102971462348,0.761474587157002
"6151","RHEBL1",5.83588645752813,-0.80091847859089,0.772080443798916,-
1.03735107529739,0.299572270507307,0.761555772074713
"6152","CTU2",52.2422797516844,-0.311786656957634,0.300556941804254,-1
.03736302041792,0.29956670562021,0.761555772074713
"6153","C19orf53",1107.20011974121,-0.236960672317421,0.22846802740413
3,-1.03717213743114,0.299655640751137,0.761555772074713
"6154","ELF4",71.7327427307785,-0.220238037378048,0.212326101017711,-1
.03726313591411,0.299613241051836,0.761555772074713
"6155","SLC2A9",5.09190901994704,-0.755809073477289,0.728926167781673,
-1.03688014902446,0.299791716422092,0.761655782619389
"6156","YIPF5",499.198567811243,-0.161688154513441,0.155937386210708,-
1.03687870139726,0.299792391164,0.761655782619389
"6157","MRPL28",236.96995683734,-0.2659908624829,0.25672667629389,-1.0
3608579491133,0.300162118651564,0.762471258033208
"6158","RERE-
AS1",2.59055771889232,1.09004668621709,1.05299715754707,1.035184832555
8,0.300582600736479,0.763043641538474

"6159","SF3B4",202.112686799603,-0.229842950987128,0.22199403778255,-1
.03535641444689,0.300502492660938,0.763043641538474
"6160","MRPL9",253.171241961376,-0.207872703209374,0.200758267834759,-
1.03543782007758,0.300464491014129,0.763043641538474
"6161","BMS1",159.108725102498,-0.188816827420359,0.18238875810712,-1.
03524378026338,0.300555077652883,0.763043641538474
"6162","NR0B1",3.15408194419122,1.51233121318568,1.46138824801434,1.03
485929576932,0.300734626314262,0.763305672761288
"6163","ZNF426",137.887619689224,0.187010450694124,0.180749249657854,1
.03464026018432,0.300836944691878,0.76335549951381
"6164","ZNF473",21.2686964050833,-0.423209150079898,0.409052534708018,
-1.03460830619711,0.300851873337796,0.76335549951381
"6165","TTC17",349.471707609495,0.182581827478603,0.17650892920465,1.0
3440561506615,0.300946580499232,0.763468352058116
"6166","AC090630.1",5.77438837885437,-1.02943554123068,0.9952928312290
36,-1.03430418559278,0.30099398074107,0.763468352058116
"6167","SON",1945.94093770928,0.18911903031068,0.182928071844675,1.033
84367638917,0.301209249446875,0.763890491543558
"6168","PLXND1",120.482344438511,0.255041141125111,0.246906576855721,1
.03294591976034,0.301629207680334,0.764201256384451
"6169","WDR19",85.9174573987027,0.264416427246221,0.256055361196485,1.
03265335281662,0.301766150657093,0.764201256384451
"6170","FCHO2",240.271863516238,0.196432340291987,0.190214720745511,1.
03268737310187,0.301750224520354,0.764201256384451
"6171","ARF3",530.325243908262,0.119516632166417,0.115718108133887,1.0
3282566655976,0.301685490067161,0.764201256384451
"6172","NUDT14",25.4755230208395,0.538533474429163,0.521263398961123,1
.03313118761543,0.301542510014245,0.764201256384451
"6173","RPS17",4519.00676346703,0.229181765683695,0.221827409344785,1.
03315350596499,0.301532067072877,0.764201256384451
"6174","NHLRC4",16.9792518372798,0.435730972216291,0.421684431217623,1
.033310551585,0.301458590929672,0.764201256384451
"6175","ROMO1",794.403790717274,-0.219496723896636,0.212398301775265,-
1.03342033369401,0.301407234807557,0.764201256384451
"6176","STMN3",350.926426450544,-0.254812251751562,0.246757630945429,-
1.03264183067114,0.301771544720612,0.764201256384451
"6177","SPOUT1",61.7909800614336,-0.24101773776633,0.233438060391326,-
1.03246975819752,0.30185210765394,0.764259323245464
"6178","WIPF2",318.793530287271,0.209120107254794,0.202580842644735,1.
03227977791329,0.301941071504714,0.764259323245464
"6179","CTSZ",1132.43606360665,-0.195356809419304,0.189231856742382,-1
.03236745008141,0.301900014267568,0.764259323245464
"6180","CARS",116.004949093307,-0.206087224049511,0.199702441038088,-1
.03197148206218,0.302085477228931,0.764337888759583
"6181","SH2B3",40.1180215704835,-0.44249389286112,0.428857837734893,-1
.03179621293212,0.302167593746835,0.764337888759583
"6182","ARHGAP5",381.45611878719,0.260452487274774,0.252363516730193,1
.03205285236705,0.302047358928539,0.764337888759583
"6183","ZNF254",147.913656155312,0.182000545771013,0.176377574531465,1
.03188030708827,0.302128192375245,0.764337888759583

"6184","AL596244.1",13.7945751353913,0.48304892215342,0.468226077043504,1.03165745317628,0.302232615545707,0.764378736600075
"6185","CGGBP1",429.976342434515,0.163252061723709,0.158300952251693,1.03127656152152,0.302411146414037,0.76452369205939
"6186","MICAL1",56.9100952904019,-0.377868611148084,0.366435574880214,-1.03120067223715,0.302446725485986,0.76452369205939
"6187","ATP5MD",1349.06304644523,-0.272256356576777,0.264066643655891,-1.03101381078467,0.30253434336033,0.76452369205939
"6188","PPM1A",273.409518624331,0.191580687309189,0.185802034158686,1.03110112963332,0.302493398133601,0.76452369205939
"6189","NFIC",2503.18183791962,-0.176346655557146,0.170980842238899,-1.03138254115481,0.302361464783267,0.76452369205939
"6190","CCNL2",320.182538080067,0.235733401926245,0.228667261967871,1.03090140625101,0.302587057099338,0.764533372057133
"6191","PANK4",52.8764018087991,-0.299825694670637,0.291471224653101,-1.02866310397357,0.303638013471399,0.764675342288665
"6192","PARK7",2652.26471677968,-0.254532872913571,0.247625781544819,-1.02789326428639,0.304000038348778,0.764675342288665
"6193","PIK3R3",5.42942792833,0.757354370805336,0.735778269499818,1.02932418936507,0.303327360108188,0.764675342288665
"6194","CNN3",870.818855653626,-0.191898127893017,0.186544073046723,-1.02870128629042,0.303620065311148,0.764675342288665
"6195","PACC1",26.49199176747,-0.422836254409625,0.411920582420303,-1.02649945755365,0.304656219180688,0.764675342288665
"6196","HEATR1",98.9338403332243,0.243133926626109,0.236666267034397,1.02732818526593,0.304265954842959,0.764675342288665
"6197","OST4",3017.31077592112,-0.234205419141887,0.228260669016758,-1.02604369009666,0.304870990685116,0.764675342288665
"6198","UXS1",222.86507856619,-0.193468861807229,0.188387386963588,-1.02697354066822,0.304432923373502,0.764675342288665
"6199","SH3RF3-AS1",6.1870536750149,0.654232757240887,0.635479598389233,1.02951024533154,0.303239967887795,0.764675342288665
"6200","AC012306.3",6.34392654488848,0.621266322161689,0.604009326874174,1.02857074306588,0.303681431991128,0.764675342288665
"6201","NDUFS1",389.476640360667,0.163323272154102,0.159053424707954,1.02684536629116,0.304493283503772,0.764675342288665
"6202","COL4A4",13.3001171484748,-1.66448650066251,1.61727717645561,-1.02919062044168,0.30339010899698,0.764675342288665
"6203","BDH2",587.696422902013,0.244001876296475,0.237817343194647,1.02600539144349,0.304889042752209,0.764675342288665
"6204","CDKN2AIP",220.228120072194,0.232701605004131,0.226712445167444,1.02641742861652,0.304694866300656,0.764675342288665
"6205","MRPL22",350.978729725233,-0.275106109876945,0.267907445604859,-1.02686996718525,0.304481697786143,0.764675342288665
"6206","GABRB2",7.28153351821086,0.886641840751354,0.861284514854218,1.0294412885171,0.30327235559362,0.764675342288665
"6207","MRS2",77.9622956283843,-0.219972287301578,0.21411878422757,-1.02733764389297,0.304261502504619,0.764675342288665
"6208","DSE",631.535388325457,0.199804757579345,0.194245800814404,1.02

861815669443,0.303659142505166,0.764675342288665
"6209","SGK1",303.849669167003,-0.717310183570337,0.698650575368564,-1
.02670806961252,0.304557948339408,0.764675342288665
"6210","PDLIM2",1176.45813662571,-0.31946535047007,0.311364026797498,-
1.02601881712508,0.304882714477522,0.764675342288665
"6211","MRPS28",195.867756592463,-0.264035189180384,0.257055583881141,
-1.0271521248201,0.30434883741884,0.764675342288665
"6212","CENPP",32.4319700748658,-0.411962314527593,0.401185191638353,-
1.02686321208724,0.304484879050237,0.764675342288665
"6213","ZFP37",45.5731449934671,0.287682400977559,0.279441769727655,1.
02948961874216,0.303249655563988,0.764675342288665
"6214","SURF4",558.570910947399,-0.172567968007162,0.167619343781825,-
1.02952299008985,0.303233982168659,0.764675342288665
"6215","BTRC",62.681902267991,0.239353434018083,0.233046366316852,1.02
706357451914,0.304390529205336,0.764675342288665
"6216","CD81",9568.36362249173,-0.25250577805935,0.245925771221788,-1.
02675606873111,0.304535340382396,0.764675342288665
"6217","RCN1",801.693395393011,-0.21056407224871,0.20521431995085,-1.0
2606909839012,0.30485901487945,0.764675342288665
"6218","CLEC2D",48.0963408577729,0.32609309075937,0.316762800591719,1.
02945513220057,0.303265853294016,0.764675342288665
"6219","PRR13",491.210336947881,-0.174295997130427,0.16989943314719,-1
.02587744939342,0.304949353370297,0.764675342288665
"6220","ALDH2",403.011858199641,-0.331227215611507,0.321860993153048,-
1.02910020989715,0.303432587554012,0.764675342288665
"6221","FUT8",213.16303449141,0.180808251039819,0.176232206818541,1.02
596599284483,0.304907614018736,0.764675342288665
"6222","ZNF267",85.1586204093771,-0.213412079688444,0.207333749588354,
-1.02931664580493,0.303330903741729,0.764675342288665
"6223","KIAA0513",39.7787692566373,0.371001449391795,0.36003903590471,
1.03044784702175,0.302799822344108,0.764675342288665
"6224","ACAP1",36.9744883014635,-0.359628974568393,0.349329131263091,-
1.02948463893653,0.303251994456741,0.764675342288665
"6225","DHRS7B",213.546590762819,-0.285879992106107,0.277463550357006,
-1.03033350412431,0.302853476449144,0.764675342288665
"6226","LRRC37B",18.8922409370747,0.388563576681493,0.377933182761447,
1.02812770723749,0.303889758838276,0.764675342288665
"6227","NPLOC4",183.184665225084,0.178581496583465,0.174028575828485,1
.02616191469306,0.30481527000574,0.764675342288665
"6228","AP001269.4",8.8679728600165,-0.576094121454148,0.5616096081892
66,-1.0257910709747,0.30499007577984,0.764675342288665
"6229","RMC1",49.6115054417247,-0.293182386654446,0.285710835870234,-1
.02615074350069,0.30482053483437,0.764675342288665
"6230","RAB11B-
AS1",127.962002670222,-0.287481973918876,0.278950496028199,-1.03058420
046622,0.30273584830381,0.764675342288665
"6231","PLEKHF1",188.434406210864,0.346041158782496,0.33724627762176,1
.02607851218628,0.304854577911671,0.764675342288665
"6232","MARK4",79.470527110656,0.217659692048893,0.21162565911376,1.02
85127661759,0.303708688819147,0.764675342288665

"6233","CDK5RAP1",82.2158863191852,-0.225073960023992,0.218994951173551,-1.02775867122902,0.30406336152363,0.764675342288665
"6234","ARFGEF2",100.421465931064,0.24019751709449,0.233441215055113,1.02894219873634,0.303506837124764,0.764675342288665
"6235","UBE2V1",766.714489062168,-0.182892295464515,0.177828543137996,-1.02847547551795,0.303726221233342,0.764675342288665
"6236","SMDT1",840.988612656996,-0.225174158237589,0.219062805142689,-1.02789772134488,0.303997941547265,0.764675342288665
"6237","SLC9A6",39.9964695862338,0.306384942586263,0.298628221312913,1.02597450850174,0.304903599940952,0.764675342288665
"6238","FAM3A",219.975414379742,-0.269878054872483,0.262631358646672,-1.0275926540652,0.304141481139838,0.764675342288665
"6239","ZNF112",15.1812335057839,0.431090940058519,0.420330658707968,1.0255995634095,0.305080373347006,0.764779137545628
"6240","TSBP1-AS1",5.75499099001364,-0.663224291067917,0.646871846176436,-1.02527926510968,0.305231436563266,0.765035203181005
"6241","PHF13",62.6026415324606,0.355389054274486,0.346740307931753,1.02494300819631,0.30539007977307,0.765187575721053
"6242","HACD3",327.715733410113,0.194033985728803,0.189292954127302,1.02504600143919,0.305341482609671,0.765187575721053
"6243","LINC01355",13.5842490377842,-0.541903652254856,0.528864974964231,-1.02465407600779,0.305526439253961,0.765406617000153
"6244","C2orf76",144.084165125436,-0.242080686659492,0.236439979104456,-1.02385682648256,0.305902904879983,0.765491429171671
"6245","POLK",306.939595027095,0.16337792176221,0.159533419860617,1.02409841088439,0.305788794928794,0.765491429171671
"6246","DOK2",8.27921078089957,-0.708607492012411,0.692077419854594,-1.0238847153275,0.305889730424614,0.765491429171671
"6247","AC023632.2",9.21545405806594,-0.53629652799857,0.523583532168684,-1.02428074041448,0.3057026921092,0.765491429171671
"6248","AC027307.2",23.7488901274096,0.358755703738458,0.350262354405369,1.02424853606523,0.305717899037547,0.765491429171671
"6249","TRPC4AP",229.609361011666,-0.158010734187443,0.154319595843025,-1.02391879219392,0.30587363331069,0.765491429171671
"6250","LINC01684",16.1995634562289,0.423210868319576,0.413305960372569,1.02396507405332,0.305851771743224,0.765491429171671
"6251","NCF2",12.6010602037563,-0.751834011913012,0.734875609267763,-1.02307656211661,0.306271647688214,0.765640431832298
"6252","C2orf69",145.681503611992,-0.196020429127016,0.191609594695987,-1.02301990376853,0.306298435155589,0.765640431832298
"6253","ANXA6",673.873978341293,0.205360237754956,0.200719544375255,1.02312028653785,0.306250976306065,0.765640431832298
"6254","BRPF3",59.3938707760048,0.294549977150754,0.287926022792934,1.02300575089937,0.306305126724724,0.765640431832298
"6255","EYS",2.94671474855363,1.34114374143707,1.31021997780535,1.02360196314784,0.306023317599343,0.765640431832298
"6256","TSTD3",100.199693849191,0.194488777668442,0.19007979755763,1.02319541670111,0.306215459543424,0.765640431832298
"6257","SCAMP2",378.494800107391,-0.21809911735108,0.213088591742486,-

1.02351381445445,0.30606497163913,0.765640431832298
"6258","PSMA2",1079.64263052712,-0.260820559843384,0.254989051537419,-1.02286964193484,0.306369484879845,0.765678929933011
"6259","FGR",9.34855466536911,-0.610265111478436,0.596862953267008,-1.02245433082765,0.306565917166303,0.765680444663204
"6260","AC104695.4",91.2946044395136,0.37520862387331,0.366953697710879,1.02249582498808,0.306546287662913,0.765680444663204
"6261","GCLC",56.2539452796408,-0.311045349476393,0.304191083705837,-1.02253276357431,0.306528813950419,0.765680444663204
"6262","SCN1B",80.8958971288133,0.292924355607295,0.28642964530808,1.02267471403747,0.306461670754593,0.765680444663204
"6263","NUDT4",294.393089630347,0.368599153505869,0.360661110715076,1.02200969983998,0.306776309532551,0.76593637559566
"6264","MEDAG",2587.2342494514,0.214933547807311,0.21032082333393,1.02193184868841,0.306813157306414,0.76593637559566
"6265","TOP2A",5.33874629472383,-0.922892649256326,0.903090309715223,-1.02192730818621,0.30681530646463,0.76593637559566
"6266","METTL5",467.856735095434,-0.247853346389347,0.242712676642097,-1.02118006285609,0.307169136345066,0.765963854023731
"6267","ATF2",198.599948241415,0.191132209475045,0.187111130073807,1.02149032716361,0.307022189577417,0.765963854023731
"6268","PLXNA1",164.107670454956,0.198231194903894,0.1940645720694,1.02147029099677,0.307031677660687,0.765963854023731
"6269","WDFY3",254.347873141974,0.253658095032503,0.248365852426465,1.02130825374879,0.307108417182933,0.765963854023731
"6270","ZFAT",17.189550477162,0.433297796257588,0.424144845563125,1.0215797758483,0.306979833715731,0.765963854023731
"6271","AC109630.1",3.077822964391,-0.851423471527593,0.83335088224057,-1.02168665045201,0.306929231384067,0.765963854023731
"6272","CD99L2",178.114155384067,0.17847269284065,0.174765988784102,1.02120952756504,0.307155179326805,0.765963854023731
"6273","TMEM158",9.17169718878573,-0.5858245444367,0.573912353845508,-1.0207561146077,0.307370001160281,0.766064775561684
"6274","GFER",167.91887315419,-0.250070399810759,0.245003449103293,-1.02068114031052,0.307405532699816,0.766064775561684
"6275","CEP250",49.8240195924877,0.383902687702752,0.376018604084819,1.02096727005602,0.307269945770134,0.766064775561684
"6276","RPS19BP1",853.049381434979,-0.225862116596763,0.221284772120049,-1.02068531165909,0.307403555758411,0.766064775561684
"6277","LRRC1",7.81114402787367,-0.649081384390445,0.636124892920036,-1.02036784225018,0.307554039171217,0.766312756513913
"6278","TRIM59",3.1723419210656,-0.982832316441345,0.963351107760781,-1.02022233485136,0.307623027307361,0.766362559268418
"6279","AC018521.5",87.3047030732693,-0.24588289461286,0.241039938373379,-1.02009192448423,0.307684866324566,0.766394538830421
"6280","PAXBP1",161.864338221566,0.309804107812654,0.303750628673463,1.01992910818202,0.307762083391406,0.766464806407897
"6281","USP18",55.0543152650147,0.351449599699161,0.344683376668878,1.01963025631138,0.307903849897659,0.766695782900714
"6282","NEXN-

AS1",8.97676844848761,0.531064244274992,0.521688189981377,1.0179725254
9257,0.308691013009131,0.767629869680598
"6283","ZNF644",385.548178277838,0.174626778700217,0.171529121474988,1
.01805907474248,0.308649882746086,0.767629869680598
"6284","AC110772.2",6.33154960337902,-0.680387197329606,0.668309391985
218,-1.01807217658353,0.30864365675527,0.767629869680598
"6285","UPK3BL1",197.005174086397,-0.362386130014296,0.35579273071617,
-1.01853157394434,0.308425403777499,0.767629869680598
"6286","BMT2",56.4287017678091,0.248597115565898,0.244124664138194,1.0
1832035875397,0.308525736350331,0.767629869680598
"6287","BHLHE22",12.1490580193899,-0.552886196846945,0.543101368301397
,-1.0180165787027,0.308670077422561,0.767629869680598
"6288","BTAF1",96.0211723147107,0.258899549879104,0.254200133807556,1.
01848707158866,0.308446541730633,0.767629869680598
"6289","RPLP0",10804.0065023162,-0.198159325412408,0.194672728486418,-
1.01791004293769,0.308720708458501,0.767629869680598
"6290","TRMT6",90.2682890154803,-0.237620446799026,0.233363097306274,-
1.01824345640718,0.308562272275187,0.767629869680598
"6291","MBNL2",567.732624198118,0.196310328207866,0.192907419034361,1.
01764011560851,0.308849015783544,0.767704800835129
"6292","ZNF738",45.3840306746592,0.264921446533374,0.260308812670618,1
.01771985287564,0.308811109782389,0.767704800835129
"6293","FBXO6",58.5287543446754,0.34537220848322,0.339568259617984,1.0
1709214186204,0.309109598178276,0.767809673513068
"6294","SLF1",53.4700134921904,0.263688325616561,0.259213287252105,1.0
1726392351216,0.309027893826431,0.767809673513068
"6295","IMPAD1",324.038863496359,-0.140225276134786,0.13790450193436,-
1.01682885016713,0.309234855080487,0.767809673513068
"6296","AL445222.1",5.27850809037819,-0.659562132349632,0.648486410871
606,-1.01707934243856,0.309115686528059,0.767809673513068
"6297","RARA",155.568962754092,-0.257391047305453,0.25312699376055,-1.
01684551094909,0.309226927980293,0.767809673513068
"6298","MKS1",34.1290980334555,0.271603675909148,0.267013297669275,1.0
1719157165557,0.309062304734737,0.767809673513068
"6299","MT-
ND4L",2230.18033326688,-0.320716041313267,0.315364123333385,-1.0169705
9869497,0.309167416269722,0.767809673513068
"6300","CCR1",24.3364217857558,0.48866508502607,0.480819911859249,1.01
631624018333,0.309478817469117,0.768065161736887
"6301","CYP4V2",160.949865562022,0.239162311383989,0.235289937938766,1
.01645787949602,0.309411395487975,0.768065161736887
"6302","FAM118A",62.0385428987935,0.308169739572551,0.303226216718622,
1.01630308522603,0.309485079876334,0.768065161736887
"6303","HDHD3",23.6692354073684,0.396440976212216,0.390252260941904,1.
0158582432178,0.309696895854661,0.768278751638131
"6304","NDUFA7",291.122600264437,-0.258269415369686,0.25424901196766,-
1.01581285752464,0.309718512089413,0.768278751638131
"6305","BCAM",4.40661317892362,0.782179172023181,0.769910855116875,1.0
1593472390312,0.309660471995029,0.768278751638131
"6306","HIST1H1C",72.6672455801868,0.342238004501179,0.337038312043877

,1.01542760057683,0.309902041867239,0.768612105106822
"6307","C1orf112",23.0860909105374,-0.31481085703037,0.310241552133419
,-1.01472821698296,0.310235399682851,0.769209541419703
"6308","CXCL9",20.5737567505493,1.3430597077431,1.32358205683064,1.014
71586201395,0.310241290746514,0.769209541419703
"6309","MRPL47",276.503068451789,-0.260577371977808,0.256837500030833,
-1.01456123792875,0.310315024440207,0.769270404540313
"6310","AC239800.2",2.80566010756832,-0.869730112182885,0.857543257314
385,-1.01421135874436,0.310481909744166,0.769562134453051
"6311","TAF10",752.584941417808,-0.242823846290442,0.23953269735858,-1
.0137398733791,0.31070689242733,0.769997749574306
"6312","ANAPC2",53.0762098292996,-0.275193121813666,0.271579463278811,
-1.01330608173102,0.31091398348317,0.770146530621929
"6313","DCAKD",75.2971623329666,-0.228789925306244,0.225779335780996,-
1.01333421198549,0.310900551408808,0.770146530621929
"6314","PRR12",143.065245653172,0.221877226960594,0.218963981962731,1.
01330467674067,0.310914654370004,0.770146530621929
"6315","GBP3",155.916889845386,-0.261344782476349,0.25808271735455,-1.
01263961087839,0.311232332418093,0.770567307110807
"6316","TLE1",92.1957696231822,0.421559221197523,0.416270466268899,1.0
1270509285952,0.311201044531626,0.770567307110807
"6317","AL021707.6",8.59453735223872,0.750700779707928,0.7412330012839
22,1.01277301254478,0.311168594079991,0.770567307110807
"6318","TMEM198",9.07035188224697,-0.528603820060506,0.522627673658247
,-1.01143480665773,0.311808369106088,0.770651531735022
"6319","SUSD5",5.58710302068899,0.859831178561446,0.849927456143722,1.
01165243262367,0.311704266580464,0.770651531735022
"6320","BBX",630.610970114563,0.166098699423686,0.164203825900576,1.01
153976475711,0.311758158986097,0.770651531735022
"6321","UBTD2",348.279751511799,0.159650491565708,0.157758728879125,1.
01199149295905,0.311542120951234,0.770651531735022
"6322","LEMD2",108.91128973066,0.223800112993911,0.22118976502175,1.01
180139583721,0.311633022455483,0.770651531735022
"6323","SGCE",984.928597630119,0.290722451613276,0.287145417438835,1.0
1245722187157,0.311319490482792,0.770651531735022
"6324","TEK",41.8593999578231,-0.397349595329668,0.392834178521793,-1.
01149446014312,0.311779831267608,0.770651531735022
"6325","AKNA",73.8150917928468,0.310036723213595,0.306301155680237,1.0
1219573437476,0.311444475349959,0.770651531735022
"6326","ZNF771",55.7725439652581,-0.264378409171834,0.261290439712616,
-1.01181814942259,0.311625010447396,0.770651531735022
"6327","CBFB",142.46004116894,0.212467203916087,0.209997854976152,1.01
175892458623,0.311653333940706,0.770651531735022
"6328","CTCF",301.385820339675,0.216906205017704,0.214411645739424,1.0
1163443930331,0.311712872910854,0.770651531735022
"6329","C1orf216",95.220061934796,-0.233191461569111,0.230658677257626
,-1.01098065913495,0.312025686774608,0.770710132114182
"6330","BEND5",76.4249821825515,0.29298103730396,0.28980095391369,1.01
097333651709,0.312029191595077,0.770710132114182
"6331","ITGB5",1299.47825904043,-0.186549174607315,0.184501829345217,-

1.01109661226321,0.311970191652405,0.770710132114182
"6332","FUT11",193.72630527964,0.241926972871789,0.239263284827126,1.0
1113287417494,0.311952838050941,0.770710132114182
"6333","MRPL24",263.595901917961,-0.209581967326659,0.207352953809875,
-1.01074985176641,0.312136170455441,0.770852630021017
"6334","DCTD",351.999464696854,-0.18523682955723,0.183355144824622,-1.
01026251395568,0.312369535703382,0.771217881606554
"6335","ZSWIM1",13.0985988044828,0.461582283636629,0.456905829716727,1
.01023504979746,0.312382690535647,0.771217881606554
"6336","AGAP1",317.778233249169,0.288009460574568,0.285313128425367,1.
00945043140525,0.312758662637151,0.771902396030463
"6337","PARP10",94.7442477492661,0.205601398199773,0.203669949584153,1
.00948322823059,0.312742941144582,0.771902396030463
"6338","TAX1BP1",836.95956357386,-0.140561514362603,0.139305271757508,
-1.00901791144905,0.312966044181885,0.77194473896933
"6339","TLE4",165.56737478401,0.329018251521014,0.326008187875495,1.00
923309216598,0.312862859570534,0.77194473896933
"6340","SLN",3.04746883551398,-1.04940654209415,1.04014923406935,-1.00
889998061968,0.313022604510453,0.77194473896933
"6341","GEMIN2",92.5961090529505,-0.25992166561533,0.257610471914454,-
1.00897166052179,0.312988225600096,0.77194473896933
"6342","CD33",2.73932375050433,-1.36299006978606,1.35094029996479,-1.0
0891954279666,0.313013221908207,0.77194473896933
"6343","TPK1",57.0245566283198,-0.267868746395262,0.265586274343465,-1
.008594088898,0.313169343370682,0.77218485422
"6344","ASAP2",542.370752805483,-0.245739736323971,0.243713181677676,-
1.00831532636989,0.31330310755945,0.772354760921749
"6345","TCAF1",375.812653524176,0.167558188873876,0.166188023389875,1.
00824467044046,0.313337017778037,0.772354760921749
"6346","ISCA1",390.427207855451,-0.215974942496436,0.214273735181225,-
1.00793941130382,0.313483549935639,0.772594188621713
"6347","AC092155.1",2.58947916443998,1.20085459715824,1.19229110204765
,1.00718238616046,0.31384713585385,0.773017698395132
"6348","ZCRB1",733.484854647504,-0.193219045280764,0.191843529824038,-
1.00716998617565,0.31385309365787,0.773017698395132
"6349","LIMA1",2921.60329253296,-0.153571840217675,0.152477067442151,-
1.00717991756983,0.313848321928337,0.773017698395132
"6350","SLC38A5",4.07335490877352,-0.824084213796969,0.818212018657861
,-1.0071768624821,0.313849789798969,0.773017698395132
"6351","KANK2",893.294560243997,0.212700883187148,0.211242910506915,1.
00690187744874,0.313981929694119,0.773091527143581
"6352","TSHZ2",1425.31405877042,-0.250815200397236,0.249078257299102,-
1.00697348342231,0.313947517003779,0.773091527143581
"6353","AL133346.1",3.60183374996756,0.940921101527729,0.9347777200141
09,1.00657202389626,0.314140484098399,0.773267455946261
"6354","ARHGAP28",179.718199880179,0.30856586053508,0.306558661332668,
1.00654752077037,0.314152264391467,0.773267455946261
"6355","CLDN11",123.665823641468,0.68553055637588,0.681291638762655,1.
00622188409786,0.314308847328874,0.773344120138976
"6356","SLC22A5",30.7505794475992,0.373213614848687,0.370923435845657,

1.00617426342396,0.314331750110196,0.773344120138976
"6357","TOMM40",177.510870897395,-0.250711312869426,0.249162074544225,
-1.00621779349058,0.314310814630567,0.773344120138976
"6358","KALRN",4.08001923917886,-0.991804555410358,0.985831271129802,-
1.00605913451468,0.314387124969754,0.773358703134155
"6359","ZNF628",31.2606623964419,0.32608478148001,0.324196080210499,1.
00582579921474,0.31449937457375,0.773513165329998
"6360","GPN1",124.881795369438,-0.201530306455612,0.20043931526092,-1
.00544300001277,0.31468358319923,0.773764659101433
"6361","AC093495.1",12.0525016309433,0.566391551966856,0.5635784912298
88,1.00499142671472,0.314900978101872,0.773764659101433
"6362","HSPA1L",4.87098503441343,0.689913075391944,0.686344340066366,1
.00519962811267,0.314800734222735,0.773764659101433
"6363","AL080276.2",17.1373306022958,0.45282792477532,0.45066869378198
9,1.00479117148167,0.314997415888672,0.773764659101433
"6364","NRGN",2.9806329838175,-1.09394409609622,1.0887226382319,-1.004
79594864749,0.314995115102119,0.773764659101433
"6365","ATN1",333.145642484673,0.175700878446645,0.174802283536445,1.0
0514063599182,0.314829135355776,0.773764659101433
"6366","AKT2",351.366312854904,0.155330081131599,0.154584120821106,1.0
0482559467642,0.314980837179251,0.773764659101433
"6367","STRN4",111.177097604157,-0.184151588882529,0.18319663025971,-1
.00521275212031,0.314794416036983,0.773764659101433
"6368","SLC2A1-
AS1",3.194322796104,0.984329011244314,0.979759420343833,1.004663992818
64,0.315058671948687,0.773793597562417
"6369","D2HGDH",101.686389357849,0.233638210551718,0.232615033473702,1
.00439858534823,0.315186531622124,0.773864576855575
"6370","ADA2",12.1686451363629,-0.461315648038369,0.459249463179676,-1
.00449904686744,0.315138130417804,0.773864576855575
"6371","TNFSF10",719.075672138716,-0.3880648269009,0.386517657458316,-
1.00400284285266,0.315377243318825,0.773943373059343
"6372","KIF3A",129.539953922357,-0.201575377787259,0.200838496105903,-
1.00366902608635,0.315538171404812,0.773943373059343
"6373","RIOX1",19.2256347348704,-0.380989158682705,0.379674605317573,-
1.00346231574807,0.315637850412573,0.773943373059343
"6374","MYO18A",37.4578599205834,0.412412475138982,0.4109215273322,1.0
0362830299123,0.315557807092391,0.773943373059343
"6375","AXIN2",20.1804146448508,0.36367755623597,0.362358149233116,1.0
0364116829067,0.315551603670691,0.773943373059343
"6376","AP001094.2",4.98799593151861,0.740243828203209,0.7377295579518
7,1.00340811917353,0.315663988282963,0.773943373059343
"6377","FAM210A",93.0464131445385,0.205496428874785,0.204654452321338,
1.00411413748344,0.315323601823363,0.773943373059343
"6378","WDR83",62.4928841152501,-0.269722877808985,0.268791776352974,-
1.00346402508531,0.315637026058222,0.773943373059343
"6379","PMM1",313.307094508675,-0.250561207965932,0.249663584360853,-1
.00359533252468,0.315573705238651,0.773943373059343
"6380","FASTKD5",46.9678966554219,0.262712740533064,0.261903117489911,
1.00309130739226,0.315816808396535,0.774196690175832

"6381","SRP9",1040.60835604883,-0.224246700118043,0.223604339971981,-1
.00287275348118,0.315922260292593,0.774333827139345
"6382","KRTCAP2",2003.28656380764,-0.261232247655302,0.260753061505814
,-1.00183770095246,0.316421985370791,0.774392751799585
"6383","GPD1L",43.1128986804032,0.278498354380734,0.278378351614857,1.
00043107793829,0.317101936345792,0.774392751799585
"6384","ANO10",123.364982853315,-0.190806077818942,0.190388837209618,-
1.0021915182394,0.316251103475042,0.774392751799585
"6385","AC046143.1",10.8744767149859,-0.48803495891303,0.4877675816284
13,-1.0005481653449,0.317045300640217,0.774392751799585
"6386","SEMA5A",21.4724246001289,-1.10110300112852,1.10138337578236,-0
.999745434096784,0.317433718535614,0.774392751799585
"6387","ZBED3-
AS1",5.04605911634015,0.757480418542078,0.755738282103216,1.0023052113
1471,0.316196206390034,0.774392751799585
"6388","PPP2CA",292.443420666105,-0.161011642708516,0.160949975326943,
-1.00038314626298,0.317125123026773,0.774392751799585
"6389","C6orf47",113.754595649649,-0.206440214699725,0.206456336350713
,-0.999921912539603,0.317348299097104,0.774392751799585
"6390","RRP36",227.586912548367,-0.225296552510793,0.22478289835405,-1
.00228511225945,0.316205910831499,0.774392751799585
"6391","HACE1",33.0317532871409,0.328676941328563,0.328486781201663,1.
00057889734925,0.317030436530618,0.774392751799585
"6392","MRPL18",665.704977096736,-0.232186161319164,0.231890043271609,
-1.00127697611927,0.316692920763178,0.774392751799585
"6393","LSM8",609.096363737547,-0.18821425354543,0.187873789048077,-1.
00181219796054,0.316434304802238,0.774392751799585
"6394","TMEM209",62.0459525391266,0.246345638421914,0.246325044692844,
1.00008360387835,0.317270050172163,0.774392751799585
"6395","NFIB",2458.35539213683,0.182040335561189,0.18177377481376,1.00
146644227255,0.316601356011072,0.774392751799585
"6396","GPR158",6.70236553895186,0.633330867002694,0.631981119135855,1
.00213574080929,0.316278038082611,0.774392751799585
"6397","GPAM",51.998972668231,0.295204584059963,0.295127016315384,1.00
026282834268,0.317183331048941,0.774392751799585
"6398","STK33",2.84870145925386,1.36564903615413,1.36576645853567,0.99
9914024553165,0.317352116733831,0.774392751799585
"6399","RNF141",102.148561721944,0.21969906272169,0.219646383149359,1.
00023983810512,0.317194454181505,0.774392751799585
"6400","FAU",10220.4799655525,-0.21407417268492,0.213838431605484,-1.0
0110242615262,0.316777292230628,0.774392751799585
"6401","BCDIN3D-
AS1",4.02861212204006,0.734338157495597,0.734521416144163,0.9997505060
51235,0.317431263388013,0.774392751799585
"6402","CERS5",153.916535885256,-0.185385029073433,0.185271948000201,-
1.0006103518339,0.317015223453189,0.774392751799585
"6403","DHX37",42.1341581577013,0.3560718235545,0.35619745342813,0.999
64730271814,0.317481222796607,0.774392751799585
"6404","RCBTB2",57.5553857041024,-0.221145045457454,0.220989405570763,
-1.00070428664347,0.316969794386367,0.774392751799585

"6405","PNMA1",234.641918665911,-0.226697446270392,0.226354668837481,-1.00151433780744,0.316578211921697,0.774392751799585
"6406","GSTZ1",161.324841355326,-0.282643536973137,0.282222279599686,-1.00149264393317,0.316588694702161,0.774392751799585
"6407","BNIP2",415.583627679671,0.145882366134158,0.145599374415085,1.0019436327952,0.316370817485882,0.774392751799585
"6408","SNRNP25",112.50227493129,-0.329280666103064,0.328823461670288,-1.00139042521618,0.316638091266583,0.774392751799585
"6409","NOMO1",83.8514758535456,-0.305456946782764,0.30492444611293,-1.0017463364339,0.316466121214136,0.774392751799585
"6410","LYL1",8.24112240821876,-0.545255593342704,0.543967295839297,-1.00236833631959,0.31616572896922,0.774392751799585
"6411","AC006504.5",21.0896090419859,0.338577496989072,0.338523158434014,1.00016051650738,0.317232833506248,0.774392751799585
"6412","PPIL2",103.086967104059,0.207851981149455,0.207756925814163,1.00045753148743,0.31708914006486,0.774392751799585
"6413","PHYHIP",16.9814030773254,-0.473458954705231,0.473690838729286,-0.999510473910206,0.317547467812995,0.774433556306758
"6414","HSPA9",429.638535557434,-0.133134504866917,0.133216858203915,-0.999381809944267,0.317609768125538,0.774464729261523
"6415","MAP2K1",161.657337424308,0.187778285923653,0.187930601994377,0.999189508951136,0.317702897025055,0.774571053697873
"6416","OCIAD2",4.97044197103188,-0.724798422732823,0.72557857072919,-0.998924791844965,0.317831125391145,0.774762905411082
"6417","CDK11B",144.40414976158,-0.239224495076575,0.239632075680656,-0.998299140034058,0.318134324502173,0.775113790273895
"6418","ZNRD2",186.881742048591,-0.299069070729798,0.299542657984206,-0.998418965573734,0.318076240800815,0.775113790273895
"6419","TAF1D",587.942911680181,0.142596826477309,0.14286591368533,0.99811650518252,0.318222867477537,0.775113790273895
"6420","VPS33A",67.2949466660306,0.225862738486781,0.226267972869789,0.998209051073961,0.318177998401548,0.775113790273895
"6421","ZNF345",50.3035231605481,0.272786644836015,0.273243278799482,0.99832883734424,0.318119928511925,0.775113790273895
"6422","AHCYL1",1015.09843005915,0.160336337464975,0.160719614998714,0.997615241090875,0.318465967649369,0.775118365682855
"6423","BRIX1",201.572908601348,-0.189951327868441,0.190375913295737,-0.997769752381246,0.318391020689708,0.775118365682855
"6424","SART1",161.753442194967,-0.169632230473583,0.169981559262379,-0.997944901845169,0.318306076987195,0.775118365682855
"6425","BCL9L",171.920497621711,-0.214196899357027,0.214711846977243,-0.997601680450032,0.318472545900129,0.775118365682855
"6426","HERC2",307.339156828082,0.254094845242407,0.254650440085526,0.997818205839612,0.318367520324757,0.775118365682855
"6427","C8orf89",3.18505871255144,0.993394971178653,0.995980193501941,0.99740434364041,0.318568283822235,0.775230738910807
"6428","TOMM22",412.787581516443,-0.209995353177257,0.210583053068361,-0.997209177649672,0.318662987107507,0.775340559794869
"6429","PGM3",306.320352652372,-0.162528783048332,0.163011323479994,-0.99703983489392,0.31874517473786,0.775371062624746

"6430","SLC38A1",17.7526030161541,-0.578167667426481,0.579919571829727
,-0.996979056254787,0.318774676002373,0.775371062624746
"6431","HNRNPUL1",666.787393638349,-0.178530027280769,0.17910224785189
8,-0.996805062035836,0.318859140713961,0.775455910553001
"6432","SNRNP27",324.930462674546,0.164378718844457,0.164995593251674,
0.996261267376546,0.319123217834608,0.775927136892408
"6433","SEPTIN2",1540.98595116191,0.170803793964572,0.171556719412725,
0.99561121563335,0.319439083215593,0.775927136892408
"6434","TAPT1-
AS1",6.11909720868259,0.615624828162432,0.618352273198989,0.9955891727
82618,0.319449797599119,0.775927136892408
"6435","TXNDC15",623.050528496411,-0.176130039972568,0.176854702375841
,-0.995902498528238,0.319297521223128,0.775927136892408
"6436","EMC7",792.904522987659,-0.215703408461971,0.21658877695268,-0.
995912214366939,0.319292800082987,0.775927136892408
"6437","ZNF341",9.32781366644139,0.511588715994818,0.513816630502555,0
.995663989105299,0.319413432533362,0.775927136892408
"6438","EFCAB6",4.99571179218305,0.663087307685912,0.66590366476286,0.
995770623851499,0.319361606552835,0.775927136892408
"6439","PPP1R3F",43.4831362395782,-0.301072580244487,0.302229103390359
,-0.996173356129844,0.319165922660251,0.775927136892408
"6440","RABIF",84.5910260890746,-0.237670975908202,0.239207488344542,-
0.993576654113238,0.320429014670516,0.775930182028931
"6441","EFHD1",1114.04911954938,0.238090849423142,0.239278801880313,0.
99503527914786,0.319719106245708,0.775930182028931
"6442","NECTIN3-
AS1",3.65865333908466,0.812786630979938,0.817784230224829,0.9938888534
89486,0.320276981569881,0.775930182028931
"6443","CSTA",27.9651111096768,-0.43079635849981,0.433368881496761,-0.
994063895432301,0.320191761262985,0.775930182028931
"6444","LINC02447",6.30671179624007,0.620010748309097,0.62370059647540
1,0.994083943181783,0.320182001832264,0.775930182028931
"6445","DCLK2",7.81418228478053,0.629691682833784,0.633212757246778,0.
994439350166753,0.320009018697017,0.775930182028931
"6446","TRIML2",3.38057851648312,-3.14246880455635,3.15720539255739,-0
.995332394897151,0.319574627125004,0.775930182028931
"6447","GTF2H2",64.12352224156,0.227447557981844,0.228776636313883,0.9
94190497974559,0.320130133233185,0.775930182028931
"6448","NUDT12",80.0545174995193,0.222570366769698,0.224139198492986,0
.993000636506976,0.32070964428652,0.775930182028931
"6449","BTN3A2",255.071307405066,0.302126328604054,0.304304897312649,0
.992840835859579,0.320787525903114,0.775930182028931
"6450","AL662884.3",7.829108158785,0.632185567576922,0.635645180422747
,0.99455732073116,0.319951613759625,0.775930182028931
"6451","PHF3",699.494768902891,0.240531349268339,0.242205286020296,0.9
93088768707481,0.320666696817322,0.775930182028931
"6452","ZSCAN21",64.2589609821995,-0.261374147791535,0.262991592880306
,-0.993849822075844,0.320295986288885,0.775930182028931
"6453","KCTD9",238.711545079461,-0.219569288609796,0.221176622703649,-
0.992732802977978,0.320840184602372,0.775930182028931

"6454","TMEM64",92.1937118880419,0.271620989423374,0.273434154942146,0
.993368913553774,0.320530205137445,0.775930182028931
"6455","NDUFB6",606.90998116818,-0.251747502960704,0.25356862511555,-0
.992818030408864,0.320798641542602,0.775930182028931
"6456","USP15",300.341592112221,-0.156948800232059,0.158095674972995,-
0.992745691865815,0.320833901847408,0.775930182028931
"6457","RAB21",404.730500788601,0.174692780452008,0.175489056812694,0.
995462529828648,0.319511359582558,0.775930182028931
"6458","ZMYM5",155.281772782448,0.174041127624419,0.174889873622065,0.
995146968889237,0.319664789621452,0.775930182028931
"6459","ACTR10",555.19640699326,-0.226340101002488,0.227650203139418,-
0.994245108860599,0.320103551948951,0.775930182028931
"6460","NUPR1",3552.17411985962,0.274886104631453,0.276744758845769,0.
993283868420605,0.320571636663165,0.775930182028931
"6461","SULT1A2",3.92916156660112,0.963392140905226,0.969752946268947,
0.9934407981041,0.320495187796024,0.775930182028931
"6462","DYNLRB2",11.8939479513163,-0.573406064869602,0.576732581927958
,-0.994232132599072,0.320109867879382,0.775930182028931
"6463","DSEL",239.930186042865,0.22959981727914,0.230956529153079,0.99
412568296331,0.320161683142812,0.775930182028931
"6464","TSHZ1",180.056823121104,0.206456773057534,0.20759730155912,0.9
94506053339708,0.319976559845096,0.775930182028931
"6465","ITM2A",5259.14047120909,-0.304954041109787,0.306507442172615,-
0.994931930357653,0.319769371900711,0.775930182028931
"6466","ARMCX1",462.636552844721,0.223991594660563,0.225593893119106,0
.99289742095236,0.320759946763762,0.775930182028931
"6467","FAM50A",497.708767212262,-0.181167501697452,0.182456635992129,
-0.992934571616611,0.3207418406581,0.775930182028931
"6468","TERC",3.5136398746355,0.876341357019928,0.883153350097464,0.99
2286738110903,0.321057670760614,0.775976196985937
"6469","NPM1",2926.22336629075,-0.22818694305438,0.229917186111442,-0.
992474494463309,0.320966115372108,0.775976196985937
"6470","WAKMAR2",13.2633304568163,-0.4125676199795,0.41569861459644,-0
.992468113900308,0.320969226437821,0.775976196985937
"6471","CLIP1",764.405361177883,0.212222817079333,0.21386462584193,0.9
92323140135334,0.3210399187552,0.775976196985937
"6472","RUSC1-
AS1",6.91942453323207,0.582964070919672,0.58759723696533,0.99211506495
574,0.321141398450113,0.77605863284298
"6473","AL353622.1",6.33973096267435,0.617778021155383,0.6228094866414
41,0.991921340965451,0.321235897804763,0.776167069622508
"6474","TBC1D23",249.045007729429,-0.156912305173125,0.158293467181465
,-0.991274674609557,0.321551475788133,0.776569654312292
"6475","IPO8",98.65677718295,0.270841197625051,0.27321158962640 8,0.991
323969804508,0.321527412242041,0.776569654312292
"6476","BAIAP2",78.6801201108823,-0.392664086863193,0.396098832291294,
-0.991328564620523,0.321525169333504,0.776569654312292
"6477","ANKS1A",152.680428266431,0.233721023524407,0.235805796676445,0
.991158940189677,0.321607976393364,0.77658618971626
"6478","LINC01138",22.3409016644954,0.396128327821766,0.39971684795101

8,0.991022344573045,0.321674669605067,0.776627328283923
"6479","PTAFR",6.36571153295958,-0.641162669400822,0.647136479944897,-0.990768855211835,0.321798460456171,0.776639703998843
"6480","TARBP1",91.5103248457648,0.321668776710502,0.324630603120537,0.990876317939331,0.321745977524023,0.776639703998843
"6481","BAD",703.003249215549,-0.246789808045633,0.249104787471484,-0.990706804757353,0.321828767366784,0.776639703998843
"6482","GPR153",44.4454480256702,0.311915886545592,0.31564955457027,0.988171476973058,0.323068674726777,0.776678549074066
"6483","EXOSC10",148.81019802741,0.190866054654883,0.192874129737292,0.989588675862627,0.322375207256412,0.776678549074066
"6484","RPL5",11090.3846436946,-0.24858783010445,0.251255789362704,-0.989381501357555,0.32247652177613,0.776678549074066
"6485","S100A12",2.92547936042524,1.27172238531503,1.28419230645048,0.990289677743108,0.322032550433624,0.776678549074066
"6486","CENPL",63.8418438900512,0.220290732038374,0.222578919414935,0.9897196581663,0.322311163714794,0.776678549074066
"6487","OBSCN",7.76031414348456,0.543525508853978,0.549507078822777,0.989114662577935,0.322607044510044,0.776678549074066
"6488","HIST3H2BB",4.12013140893178,0.811421328633212,0.82040944575516,0.989044351977598,0.322641442292063,0.776678549074066
"6489","COQ10B",506.374001211561,-0.226424304468431,0.22914036046268,-0.988146758655851,0.323080778581602,0.776678549074066
"6490","SLC26A6",14.7865086562973,0.477540923317684,0.482274713365785,0.990184453140694,0.322083970117846,0.776678549074066
"6491","AC091607.2",73.856664695616,-0.313136887554696,0.316482578805545,-0.989428513684777,0.322453529522476,0.776678549074066
"6492","POLH",34.1180362980436,0.355393312790613,0.359878597919698,0.98753667165813,0.323379614479449,0.776678549074066
"6493","C7orf31",8.36962249055879,-0.698588402487135,0.706202540789306,-0.989218194692899,0.322556398249954,0.776678549074066
"6494","AC018645.3",19.0251699526856,0.461375735564964,0.465924134042388,0.990237898951571,0.322057852298353,0.776678549074066
"6495","CYP7A1",2.5484865946868,-0.903379190740187,0.913986175680566,-0.988394808124443,0.322959329235293,0.776678549074066
"6496","PTCH1",56.7332146509624,0.352881811042297,0.356520679547378,0.989793387273636,0.322275117663452,0.776678549074066
"6497","USP20",34.1949398469964,0.322603324276434,0.326396655109482,0.988378156535412,0.322967481211501,0.776678549074066
"6498","EXD3",43.9184669558696,0.294101011844484,0.297404840177284,0.988891141345144,0.322716405220416,0.776678549074066
"6499","HNRNPF",1022.44443390854,-0.195898086663724,0.19787460441227,-0.990011261149872,0.322168614819606,0.776678549074066
"6500","DGKZ",107.697366747213,-0.224272261715234,0.226668472719211,-0.989428565096722,0.322453504379093,0.776678549074066
"6501","AC090559.2",5.05569447401853,0.760480647269639,0.770230232164189,0.987341986217348,0.323475014191969,0.776678549074066
"6502","ZC3H12C",53.4343107077789,0.278630052919003,0.282185417167979,0.98740060955432,0.323446285668838,0.776678549074066
"6503","COLCA1",28.5674259850647,0.395178367808323,0.399725402864744,0

.988624603230534,0.322846844109582,0.776678549074066
"6504","MLF2",534.862032312391,-0.195941240035305,0.19796921100537,-0.989756129451814,0.322293332628576,0.776678549074066
"6505","RAB3IP",8.80733023801931,0.614884047718692,0.622555439315924,0.987677576786316,0.323310579622819,0.776678549074066
"6506","AC024909.1",3.58584620594837,-0.882099721420196,0.89318018357199,-0.98759437081611,0.323351344275152,0.776678549074066
"6507","LINC00921",30.2514558694324,0.32150469353544,0.325140929406585,0.988816431453951,0.322752963410408,0.776678549074066
"6508","DLX4",2.61873461760768,-1.3278894483096,1.34491633303119,-0.987339818616659,0.323476076462365,0.776678549074066
"6509","PPP1R13L",16.9718653897597,-0.546053674707522,0.552211395330653,-0.988848979439397,0.322737036198879,0.776678549074066
"6510","PPP5D1",28.3462729889776,0.351161603066229,0.355041234649728,0.989072729573714,0.322627558942991,0.776678549074066
"6511","TAB1",75.0073626664058,0.215959372075777,0.21873193207824,0.987324393031596,0.323483636104122,0.776678549074066
"6512","CTPS2",51.848008791133,0.225534308694016,0.228344838889808,0.98769172883672,0.323303646518413,0.776678549074066
"6513","KLHL15",77.9347581852296,0.398913169884571,0.403578794422465,0.988439371437811,0.32293751341461,0.776678549074066
"6514","HNRNPH2",347.428429510143,-0.165989118856835,0.168115106907117,-0.98735397377788,0.323469139520605,0.776678549074066
"6515","TMEM33",180.994161476424,0.158289680008932,0.16037239463219,0.987013259806748,0.323636138254608,0.776925433968083
"6516","ERICH2",3.91457346460399,0.885961947899533,0.897874167728777,0.986732862735793,0.323773615186764,0.777016931336656
"6517","LINC00174",28.0908253390159,0.373485755731247,0.378494110756587,0.98676768043939,0.323756542215595,0.777016931336656
"6518","SLC2A5",19.2792400469035,0.698051927841635,0.707974775877368,0.985984178569871,0.324140876750285,0.77738456098716
"6519","NEPRO",137.838285053693,0.18973597085413,0.192456589035908,0.985863730644889,0.324199986928328,0.77738456098716
"6520","LRRC58",458.761932665313,0.200710761776899,0.203631768575594,0.985655446499689,0.324302219557457,0.77738456098716
"6521","DENND2A",299.649076840889,0.217259073472709,0.220431036682897,0.985610178775543,0.324324441204681,0.77738456098716
"6522","PSMD5",158.593324435654,0.167773914615746,0.170169883549015,0.985920135318306,0.324172305292892,0.77738456098716
"6523","EGFL7",9.80166146697917,-0.6192997843823,0.628324429977304,-0.985636965293025,0.324311291748321,0.77738456098716
"6524","METTL25",47.3989677553603,0.245184109075604,0.248605163262649,0.986239005891319,0.324015842618276,0.77738456098716
"6525","AL136295.2",5.94910125187185,-0.620058486088433,0.628794358295833,-0.986106948810616,0.32408063411267,0.77738456098716
"6526","SLC25A46",160.220233749471,0.164539654489416,0.166977427328975,0.985400584506817,0.324427342664231,0.777512050148417
"6527","SEMA3B-AS1",6.35558000513765,-0.583503484598812,0.592555862971308,-0.984723164619276,0.324760071049408,0.777594536315483

"6528","CLOCK",198.30272183959,0.253574754232478,0.257476888368327,0.984844720780268,0.324700349953288,0.777594536315483
"6529","RARS2",314.778437595247,-0.231266457905964,0.23478726527261,-0.985004266042464,0.324621975488039,0.777594536315483
"6530","TES",289.58761687279,-0.369602607850747,0.375150482792872,-0.985211601219802,0.324520143271317,0.777594536315483
"6531","ERMP1",25.3602433872861,0.341573147086596,0.346806718434196,0.984909256166578,0.324668646329024,0.777594536315483
"6532","FOXO1",594.344535210618,0.314600998663636,0.319454308740236,0.984807498462804,0.324718636688176,0.777594536315483
"6533","RNF181",541.571633378891,-0.257737219151816,0.261822521883177,-0.98439667182954,0.324920513833868,0.777603532996048
"6534","AC069148.1",5.05291401442154,0.759150315228612,0.771250593589268,0.984310834297912,0.324962704070471,0.777603532996048
"6535","MESD",717.101779678366,0.177693051967319,0.180519537168528,0.984342496964358,0.324947141052963,0.777603532996048
"6536","SDF2",526.760435833072,-0.234665714529676,0.238397311253201,-0.984347152642331,0.324944852708019,0.777603532996048
"6537","AC079915.1",7.03465400538131,0.650768711553615,0.661245438364926,0.984156069435856,0.325038781973073,0.777666597836753
"6538","SHANK3",5.9697825817697,-0.575068875077887,0.584532875336341,-0.983809293441352,0.325209289034357,0.777955533878455
"6539","DCTN2",797.66999551237,-0.185501089289994,0.188621303166367,-0.983457786453628,0.325382181664621,0.778166782534734
"6540","CEP170B",40.427017633153,-0.301640870650983,0.306724071589507,-0.983427446981316,0.325397107274755,0.778166782534734
"6541","MKRN1",334.626148613204,-0.1612493078352,0.163984155028056,-0.983322491173691,0.325448744088575,0.778171282303213
"6542","TRIM41",59.0903296058925,0.267804560014851,0.272376291877292,0.98321538254695,0.32550144555436,0.778178325966095
"6543","MESTIT1",2.64393001732076,1.28851018455602,1.31075220531276,0.983031101785227,0.325592131597531,0.77827616356188
"6544","EPRS",556.117405315994,-0.162888261294519,0.165802218261971,-0.982425102643396,0.325890464491041,0.778608227604809
"6545","RPS13",13460.1014601499,-0.235493291879531,0.239754438448203,-0.98222703781314,0.325988010172475,0.778608227604809
"6546","P4HA3",8.55340840027625,-0.713908269720337,0.726888833500856,-0.982142298543779,0.326029749525825,0.778608227604809
"6547","TMBIM4",1965.26832559093,-0.230080791394904,0.234167260088389,-0.982548932365942,0.325829488774577,0.778608227604809
"6548","PCID2",325.034973358498,-0.183450303267504,0.186783200426206,-0.982156333379572,0.32602283625745,0.778608227604809
"6549","ZNF521",378.573090630225,0.165790951655404,0.168769457414224,0.982351630416692,0.325926646885616,0.778608227604809
"6550","TNFAIP3",342.05538316332,-0.303100048273598,0.308723849463627,-0.981783716419061,0.326206411946849,0.778911188221177
"6551","AC090578.1",6.85256271004122,0.70768737618051,0.720978494078966,0.981565167328,0.326314114894698,0.778976483370305
"6552","NFS1",58.1498378096248,-0.270710737473376,0.275805949407692,-0.98152609852957,0.326333370782752,0.778976483370305

"6553","AC068491.3",63.9258573248705,0.46319996249607,0.47199953247086
1,0.981356824807163,0.326416809466845,0.77905675264176
"6554","PTPN7",5.49155085659398,0.771093879180536,0.786285061240989,0.
980679803280916,0.326750666631015,0.779377829206814
"6555","ASAP1",213.073817236675,0.177968508676776,0.18145732448216,0.9
80773353650284,0.326704521274552,0.779377829206814
"6556","ASAP1-
IT2",12.7244282511713,0.467901614816137,0.477053734141391,0.9808153281
89965,0.326683817974421,0.779377829206814
"6557","GADD45G",502.870814020353,-0.274683052415101,0.280065325752097
,-0.980782078886266,0.326700217615094,0.779377829206814
"6558","RAB5C",837.92820475541,-0.199743639404828,0.203732028316258,-0
.980423358347769,0.326877184290856,0.779560713984292
"6559","ZNF776",21.3434178204455,0.434387038621267,0.443119589380164,0
.980293015772305,0.326941501275301,0.779595224873565
"6560","CDC42",2267.00653366796,-0.185158134895844,0.189196176998197,-
0.978656851494463,0.327749558595194,0.779682755792613
"6561","PTCH2",48.7682096424525,0.319204345752942,0.325951543187884,0.
979299998493785,0.327431771202975,0.779682755792613
"6562","NFIA",3084.76634486702,0.208364791797335,0.213485559083586,0.9
76013519095938,0.329057764938522,0.779682755792613
"6563","AL139420.1",3.70872585749767,-0.832962746531365,0.852056557598
547,-0.977590911193741,0.328276694332679,0.779682755792613
"6564","DCAF6",320.760694016081,0.156332501070547,0.159523527287641,0.
979996516680956,0.327087838090717,0.779682755792613
"6565","PRRC2C",1743.20043826298,0.150968902147863,0.15439622742524,0.
977801755039409,0.32817238285728,0.779682755792613
"6566","LMOD1",146.183505426038,0.409831164396562,0.419916719393638,0.
975982011357778,0.329073378751972,0.779682755792613
"6567","AC012306.2",45.2194696495761,-0.297461797387077,0.303887415287
525,-0.978855268177628,0.327651496959679,0.779682755792613
"6568","OSGEPL1",38.4531024784962,0.269631115944487,0.275911714844694,
0.977236925573305,0.328451871168699,0.779682755792613
"6569","METTL21A",110.335157032268,-0.199683167063308,0.20445841577599
6,-0.976644401285395,0.328745229249373,0.779682755792613
"6570","UBE2F",198.396878624292,-0.197100980959581,0.201371967921375,-
0.97879055855748,0.327683475701964,0.779682755792613
"6571","EAF1",57.2810582457443,-0.23874228739714,0.244269199133515,-0.
977373685442203,0.32838418563847,0.779682755792613
"6572","KCNMB3",10.3542370477751,0.594200457171638,0.6065092645363,0.9
79705491598595,0.327231514571267,0.779682755792613
"6573","TAPT1",107.625999367527,0.212384490228579,0.217566156968651,0.
976183489140645,0.328973543734705,0.779682755792613
"6574","SLC39A8",44.6717996000367,0.303794916707943,0.311334298038327,
0.975783646781328,0.329171690313211,0.779682755792613
"6575","DHX29",424.581036372233,-0.135450540775273,0.138472321606712,-
0.978177726809398,0.327986430454641,0.779682755792613
"6576","POC5",49.8531202503368,0.263320181998703,0.269061157006139,0.9
78662936444207,0.327746551003909,0.779682755792613
"6577","CAST",2842.29855223589,-0.212222942701283,0.21693235070969,-0.

978290891178747,0.327930473718485,0.779682755792613
"6578","KLHL3",10.6247574442974,0.62894249724752,0.643468877810473,0.977424890210104,0.328358845576659,0.779682755792613
"6579","MAT2B",211.464748680518,0.227916801474137,0.232699431813037,0.979447176550295,0.327359076722631,0.779682755792613
"6580","ZBTB2",52.5439146794025,-0.280612591199814,0.287303097073689,-0.976712726239222,0.328711392983067,0.779682755792613
"6581","ZNF713",12.7698141002009,0.492004369757531,0.50389817674308,0.976396407975866,0.328868060855933,0.779682755792613
"6582","PHTF2",137.658170002198,0.175702953211651,0.179753268972758,0.977467359652186,0.328337829388798,0.779682755792613
"6583","FZD1",282.280579986967,0.247357864330972,0.25316449920675,0.977063787008162,0.328537574308872,0.779682755792613
"6584","FAM66D",11.0390070224004,0.621612047760955,0.634862844013835,0.979128096126869,0.327516690830745,0.779682755792613
"6585","GKAP1",37.558296155193,0.305170473824704,0.312064895914197,0.977907088622397,0.328120278878851,0.779682755792613
"6586","ZCCHC24",854.654294759716,0.194156996830285,0.198774751485297,0.976768907416529,0.328683572317419,0.779682755792613
"6587","NUTM2B-AS1",184.372683874006,0.27925680788262,0.285115266940786,0.979452313722008,0.327356539549747,0.779682755792613
"6588","AC063960.2",3.3092799460608,0.961297632525233,0.984409419387662,0.976522180296887,0.328805761843918,0.779682755792613
"6589","SOX6",4.57537839183653,1.01503924611238,1.03880038739481,0.977126364631015,0.328506596874822,0.779682755792613
"6590","NDUFA12",752.614422077601,-0.253384277228676,0.259654867249721,-0.975850288933676,0.329138659641835,0.779682755792613
"6591","BDKRB1",33.775011369865,-0.44961135196246,0.459574527191902,-0.978320871501912,0.327915650293177,0.779682755792613
"6592","PTPN9",141.410495451852,0.190350195595693,0.194317352487931,0.979584134708277,0.32729143948726,0.779682755792613
"6593","RHBDL1",8.64670574702374,0.585441294461668,0.598146602300815,0.978758873175447,0.327699134988268,0.779682755792613
"6594","AC004148.1",56.6733187756495,-0.374947194311053,0.383448567235594,-0.977829170191376,0.328158821247919,0.779682755792613
"6595","LRRC75A",184.576042273651,-0.420321428622729,0.428952326118188,-0.979879121827909,0.327145789949667,0.779682755792613
"6596","C17orf67",29.5537334188464,-0.321969279718792,0.329360047354117,-0.977560218081402,0.328291881030946,0.779682755792613
"6597","RNF213-AS1",15.1506731531519,0.454685540302644,0.465740105176891,0.976264520166139,0.328933397281181,0.779682755792613
"6598","IER3IP1",655.96746180418,-0.209424845315362,0.213974238304845,-0.978738594769514,0.327709157068459,0.779682755792613
"6599","MATK",9.82848989718325,-0.528209746241314,0.540925560743399,-0.976492487275681,0.328820469047346,0.779682755792613
"6600","TMEM161A",101.973748062651,-0.275784150124403,0.28165650873161,-0.979150637655589,0.327505554519793,0.779682755792613
"6601","SNRNP70",496.182947433326,0.170983526808693,0.175155284187857,

0.976182520564496,0.328974023630566,0.779682755792613
"6602","HIC2",70.3168130707683,0.316241771050445,0.323924401875997,0.9762826425516,0.328924419062169,0.779682755792613
"6603","FAM104B",224.46809884876,-0.191419685858365,0.19606894530191 7,-0.976287629658063,0.328921948370378,0.779682755792613
"6604","ACSS3",78.28577680397,0.261547700554109,0.268165817247535,0.975320804264486,0.329401153918517,0.77975390453846
"6605","RASL11A",74.7551282355466,0.48421429696558,0.496458627558658,0.975336654630641,0.32939329406332,0.77975390453846
"6606","TRMT61A",79.1695089995487,-0.218504711794021,0.224021851211734,-0.975372315745669,0.329375610928652,0.77975390453846
"6607","BMP7",291.163492640641,-0.300644921808685,0.308210581900945,-0.97545295153204,0.329335628651089,0.77975390453846
"6608","AC244033.2",14.0863440270652,0.410965446324673,0.422091007386558,0.973641795567334,0.330234426299354,0.779759870434104
"6609","ST3GAL5",646.306390171839,0.263154816947708,0.270419630743017,0.973135035443439,0.330486193261504,0.779759870434104
"6610","EIF2AK3",78.7501137037026,0.234354809134399,0.240660841858377,0.973797013775559,0.33015733612519,0.779759870434104
"6611","AC079305.3",7.52071264972755,-0.593677051909638,0.608989648932599,-0.974855735151165,0.329631825761043,0.779759870434104
"6612","OSBPL10",148.539829760882,0.337444998834086,0.346167857685332,0.974801649957995,0.32965865852327,0.779759870434104
"6613","TMEM115",318.343500280228,-0.201283766459056,0.206889314603368,-0.972905569555111,0.330600236627145,0.779759870434104
"6614","FRMD4B",32.3629985940791,-0.419584050909616,0.430265857640627,-0.975173938295766,0.329473987368186,0.779759870434104
"6615","CPEB2",83.4171301708673,-0.233525825920423,0.239681398361447,-0.974317688051281,0.329898824816322,0.779759870434104
"6616","FAM198B-AS1",27.5499353612104,0.550220522077111,0.565062623394754,0.973733705428126,0.330188777237666,0.779759870434104
"6617","LTB",17.2554602159352,0.570541755249761,0.5854666188103,0.974507746332546,0.329804494809575,0.779759870434104
"6618","FOXK1",138.049555265517,0.200357355355953,0.205827835902604,0.973422056726864,0.330343581005029,0.779759870434104
"6619","PTGES2",118.804247739922,-0.263301090376257,0.27031039804449,-0.974069411613684,0.330022076060063,0.779759870434104
"6620","DDB2",84.2979826460955,-0.259507051998777,0.266488565802297,-0.973801826046453,0.330154946264479,0.779759870434104
"6621","AP001160.2",10.8346706392638,-0.477429201582331,0.489983135525544,-0.974378844835654,0.329868469484571,0.779759870434104
"6622","TRPT1",121.517528461005,-0.212703792880366,0.218163394500693,-0.974974712724735,0.329572803550771,0.779759870434104
"6623","RNF169",177.461394893235,0.210293769629793,0.215824300208349,0.974374847627365,0.329870453454234,0.779759870434104
"6624","SLC35F2",33.3645691478949,-0.459783372028509,0.472053409962958,-0.974007098189563,0.330053014835458,0.779759870434104
"6625","PAWR",12.2858519374839,-0.488210098842705,0.501289140818719,-0.973909185515862,0.330101632523347,0.779759870434104

"6626","TAOK3",371.633244229114,0.219491738717041,0.225410878008456,0.
973740667071119,0.330185319750397,0.779759870434104
"6627","VPS36",393.267236522014,0.213668428606558,0.21961080101905,0.9
72941347215537,0.330582453638476,0.779759870434104
"6628","ALKBH1",26.0882624993741,-0.367797586052748,0.378006355362328,
-0.972993127854175,0.330556717598773,0.779759870434104
"6629","AL928654.1",7.9870489228966,0.617562196309116,0.63435954229880
8,0.973520779826499,0.330294537669582,0.779759870434104
"6630","AC027237.3",11.2583333164948,0.477631284112552,0.4907357101705
91,0.973296367502003,0.330406027303333,0.779759870434104
"6631","LDHD",9.617814533956,0.608558642605534,0.625211669988123,0.973
3641769948,0.330372336498894,0.779759870434104
"6632","EXOC7",290.061909861778,0.167102661202963,0.171867849845234,0.
972274112659456,0.330914198900245,0.780382700663424
"6633","METTL14",121.758769378004,0.223660458020976,0.23006968918966,0
.972142218337153,0.330979801498461,0.780419733971947
"6634","USF3",131.517661560852,0.220802505483535,0.227154708190664,0.9
72035786721192,0.331032745395591,0.780426912569647
"6635","TMEM18",264.086264561141,-0.205482152386339,0.211451811308253,
-0.971768229910258,0.331165864441702,0.780623077598827
"6636","XPO5",42.9460062138745,-0.272294435226576,0.280250744272339,-0
.971610034198407,0.331244588728828,0.780690983682772
"6637","HNRNPDL",1263.75379396956,0.195795387299599,0.201653574319143,
0.970949252750299,0.331573549899106,0.780786328451196
"6638","AC073349.1",11.0859583186002,-0.555832631286755,0.572437144624
784,-0.970993298576192,0.331551615708642,0.780786328451196
"6639","BUD31",739.923510787299,-0.238496076079254,0.245637467429993,-
0.970927108859014,0.331584577594172,0.780786328451196
"6640","GDAP1",15.5511897299346,-0.432139296566703,0.444962027419342,-
0.971182415436645,0.331457448881365,0.780786328451196
"6641","PHETA1",46.6338360639196,0.27214733518615,0.280236309660706,0.
97113516630179,0.331480973989749,0.780786328451196
"6642","GPC4",141.121691040736,0.205706811281504,0.211789187134965,0.9
71280989668347,0.331408372738747,0.780786328451196
"6643","TXNDC9",229.523790419468,-0.242184389995633,0.249496260114141,
-0.970693468049726,0.331700945557948,0.780802156078182
"6644","SLC43A3",307.435814841264,0.239819421542134,0.24706862392682,0
.970659154248444,0.331718038233472,0.780802156078182
"6645","AC002558.3",60.4106877830788,0.353813218338745,0.3645255599772
82,0.970612920424002,0.331741069510199,0.780802156078182
"6646","ZSWIM5",6.05201516822706,0.63241926471447,0.65197711593363,0.9
70002242807014,0.332045374189221,0.780841566996555
"6647","NEXN",218.368761653293,0.303767275934671,0.313110029518186,0.9
70161436227731,0.33196602968338,0.780841566996555
"6648","SOS1-
IT1",3.09435216766792,0.929007870083854,0.957860522369428,0.9698780233
53335,0.332107295630504,0.780841566996555
"6649","NEU1",572.883912021679,0.253652082653771,0.261477943907701,0.9
7007066394597,0.332011270515479,0.780841566996555
"6650","MAF1",679.334058180379,-0.186576649257929,0.192259663714935,-0

.970440942487905,0.331826749008877,0.780841566996555
"6651","DGLUCY",94.6922144993096,-0.246586660978157,0.254244648665001,-0.969879453797536,0.332106582534137,0.780841566996555
"6652","CHD2",401.082052224206,0.187051669118515,0.192827603978572,0.970046120260358,0.332023503752841,0.780841566996555
"6653","AK2",218.138782839766,-0.162154619299102,0.167247519506675,-0.969548725011917,0.332271481770756,0.781015921759042
"6654","SMCHD1",387.479304623911,0.187585282387562,0.193480840211515,0.969528983761349,0.332281326303367,0.781015921759042
"6655","DDX20",45.7819487158483,-0.333042842607539,0.343785586985393,-0.968751615004993,0.332669133001027,0.781340327396916
"6656","RCSD1",6.95532264267145,-0.707325476957818,0.729926697570997,-0.969036314621195,0.332527070723466,0.781340327396916
"6657","AC010168.2",8.11618917128516,0.649308739283935,0.670212776314916,0.968809849991343,0.332640071133875,0.781340327396916
"6658","LINC01534",25.5221628836232,0.340963907948244,0.351892618267977,0.968943053214574,0.332573602922143,0.781340327396916
"6659","POLR3F",67.0936327613381,-0.227970649963177,0.235296818333241,-0.968864141802,0.332612978564866,0.781340327396916
"6660","LRFN4",50.1207637785376,-0.374059596371982,0.3863433120139,-0.968205181091691,0.332941907874578,0.781511547224732
"6661","KANSL2",68.4191426004167,0.205426626118081,0.212128419759569,0.968406903473452,0.332841193075004,0.781511547224732
"6662","SNX1",279.165022537335,0.139138321920025,0.143706406360867,0.968212381364746,0.332938312624775,0.781511547224732
"6663","CACTIN",51.9428855836393,0.228904050947492,0.236380175238163,0.968372456433207,0.332858390203819,0.781511547224732
"6664","VPS29",800.794463474674,-0.256575148226757,0.265054179282107,-0.968010196714063,0.333039277259614,0.781622793568481
"6665","TRMT12",39.0396886396501,0.264577121068061,0.273414597470398,0.967677379027672,0.333205518950714,0.781895621363715
"6666","ARFIP2",149.726430022056,-0.219833139058294,0.22724326862389,-0.967391203222569,0.33334850594399,0.782104714087972
"6667","ANKRD52",46.6793422766681,0.271293024793959,0.280464525500332,0.967298892114747,0.333394637392871,0.782104714087972
"6668","ZHX1",320.620052158142,0.151903425074086,0.157075824447762,0.967070684544481,0.333508699266299,0.782137660297634
"6669","TSEN34",277.91399242759,-0.212188608927824,0.219394560098281,-0.967155287864802,0.333466410207455,0.782137660297634
"6670","ARNTL",90.20846560893,1.0168849496269,1.05183863006997,0.966768970597001,0.333659539473398,0.782165278329825
"6671","GPR135",16.89795790396,0.35822577457239,0.370547580070645,0.966747035573931,0.333670507481879,0.782165278329825
"6672","KIAA1671",169.344593349169,0.240586759110317,0.24883620535428,0.966847886013137,0.333620081901089,0.782165278329825
"6673","CLYBL",25.0332464250758,0.356404304814205,0.368734678973457,0.966560307824642,0.333763885004268,0.782266920645399
"6674","JUN",23963.3927839056,0.183379613248912,0.189788264400087,0.966232626809496,0.333927790202549,0.782284015850085
"6675","TCEA1",901.24658864856,-0.152642317717876,0.157971746722786,-0

.96626340396006,0.333912393342686,0.782284015850085
"6676","ADAM12",30.3455665950666,-0.701240793666516,0.72579491097143,-0.966169344902062,0.333959449629069,0.782284015850085
"6677","AC009063.3",4.20709685733133,1.12689281184566,1.16637971547561,0.966145755875183,0.333971251523722,0.782284015850085
"6678","NOL7",862.424936694601,-0.198903612235646,0.205921350023906,-0.965920300214402,0.334084063471666,0.782431080068411
"6679","USP11",226.449446291536,0.166237586821779,0.172121948794828,0.965812832040015,0.334137846302516,0.782439873659432
"6680","UBA6",161.856012190756,0.221113117795908,0.228980954635688,0.965639776232492,0.334224464430986,0.782525542470152
"6681","PID1",293.898801420712,0.285754058398141,0.296040436451198,0.965253469504484,0.334417871424295,0.782560707581314
"6682","YARS2",101.179238278471,-0.231199316853627,0.23953265206603,-0.965210024017494,0.334439627204188,0.782560707581314
"6683","AC087392.1",7.61125267418021,-1.00020752781393,1.03606782973541,-0.96538807509289,0.334350472066773,0.782560707581314
"6684","L3MBTL1",17.7582094660706,0.458357283138515,0.474865262024445,0.965236499263911,0.334426369340346,0.782560707581314
"6685","SLC35F5",238.95043717137,0.168458456489142,0.174565220159,0.96501729460029,0.334536149466702,0.782669465618432
"6686","KLHL29",80.2119857843018,-0.344095426272076,0.356675360922981,-0.964730014940332,0.334680057469872,0.782847271514283
"6687","TALDO1",844.328028861345,-0.247489642338919,0.25655481531419,-0.964665746132382,0.334712257328389,0.782847271514283
"6688","TMCC1",92.9644339998899,0.201056494655054,0.208562707849769,0.964009801790062,0.335041011711571,0.783043526631829
"6689","HYOU1",72.8368112701323,-0.317165691178514,0.328982609205907,-0.964080417332949,0.335005609742744,0.783043526631829
"6690","MPHOSPH8",1118.16953728768,0.139423389372022,0.144627488359722,0.964017220746056,0.335037292223781,0.783043526631829
"6691","OBI1",97.7649577534237,0.203195633622917,0.210849620796794,0.963699307852905,0.335196701457806,0.783043526631829
"6692","AC084781.1",2.73874163375245,-0.925384597926212,0.959976150328681,-0.96396623771265,0.33506285306446,0.783043526631829
"6693","ZNF317",49.0976451027792,0.240975231895431,0.250026384877038,0.963799208687285,0.335146603493799,0.783043526631829
"6694","SS18L1",49.1237278654213,0.279807940519315,0.290301827262188,0.963851806094919,0.335120229045421,0.783043526631829
"6695","SLC2A4RG",629.427745663438,-0.263844791055999,0.273762866841451,-0.963771288999556,0.335160604087525,0.783043526631829
"6696","RPAP2",154.785989450186,0.193373621294009,0.200679898683276,0.963592380516403,0.335250328394735,0.783051842307893
"6697","AL118558.3",15.6115368159854,0.424695767178417,0.44084044949762,0.963377493291277,0.335358116831987,0.783186642862816
"6698","PNO1",88.2187889484604,-0.22847013867118,0.237585835238202,-0.961631986360284,0.336234498112733,0.783266378095263
"6699","ARHGAP25",3.76706805858225,0.807812496162712,0.839208219057875,0.962588875821057,0.335753882272277,0.783266378095263
"6700","WBP1",252.327609524918,0.166020642224119,0.172519732276701,0.9

62328424889054,0.335884654854752,0.783266378095263
"6701","C2orf27A",170.403665389171,-0.180171857914851,0.18711497645406
,-0.962893838479499,0.33560080198695,0.783266378095263
"6702","FGD5-
AS1",789.427211624798,0.136833125688817,0.142063303269438,0.9631841759
2542,0.335455104795678,0.783266378095263
"6703","GLT8D1",896.605065222098,-0.198061551799787,0.205952284673632,
-0.961686597037032,0.336207056961428,0.783266378095263
"6704","SLC25A13",58.5647145726014,0.234940704949225,0.244297815022077
,0.961697937936912,0.336201358488177,0.783266378095263
"6705","LAMTOR4",1465.58854695992,-0.233783254672212,0.243029736819461
,-0.961953289057308,0.336073068410398,0.783266378095263
"6706","AL360020.1",5.2567003672476,0.82311613322021,0.855864812295942
,0.961736154349096,0.336182156307095,0.783266378095263
"6707","RSU1",420.170550559602,0.170865099526956,0.177565029203845,0.9
62267740968309,0.335915128999563,0.783266378095263
"6708","RNH1",2621.11824354432,-0.250966565496904,0.260637286611934,-0
.96289586482141,0.335599784986507,0.783266378095263
"6709","CORO7",53.9086715273232,0.26901503647639,0.279373927584695,0.9
62921052805958,0.335587143558287,0.783266378095263
"6710","INCA1",7.21592536002358,0.582391794027114,0.605640011266634,0.
961613802247149,0.336243635711739,0.783266378095263
"6711","ADA",204.233526993766,-0.280015647605483,0.291174293821428,-0.
961677090139048,0.336211833949808,0.783266378095263
"6712","COL18A1",378.881046953144,0.371455642518546,0.386090275807354,
0.96209530722263,0.336001731187861,0.783266378095263
"6713","PRR14L",183.747397681557,0.210277002721542,0.218495759947806,0
.96238482051905,0.335856335791645,0.783266378095263
"6714","MT-
CO1",35034.2696302487,-0.23277445852592,0.241825810843325,-0.962570776
519512,0.335762968883495,0.783266378095263
"6715","ORC2",66.0025739354509,0.271367052726847,0.282291807275622,0.9
61299781760551,0.3364014576293,0.783517319035332
"6716","ZNF205",94.7438860925227,-0.226054956905473,0.235268166004192,
-0.960839542148021,0.336632853148289,0.783939521030261
"6717","MTCH1",1625.0080787606,-0.173758235769604,0.180869151597512,-0
.960684750466835,0.336710701047824,0.783980038308777
"6718","PLPP5",163.905490142999,-0.226905066894075,0.236218332329279,-
0.960573485794401,0.336766665476988,0.783980038308777
"6719","MICU2",214.767462505428,-0.17337698598056,0.180505892454952,-0
.960505962562021,0.336800631547102,0.783980038308777
"6720","TNS1",648.795142862802,-0.216881171106175,0.22585051920297,-0.
960286351661055,0.336911117192476,0.784003849559639
"6721","NUP210",4.55854725617246,0.791919626593522,0.824604878064834,0
.960362529569293,0.336872789654915,0.784003849559639
"6722","SNAPIN",239.431825950529,-0.213544543501579,0.222465954047427,
-0.959897636543764,0.33710673583399,0.78424632180359
"6723","WDFY3-
AS2",241.090347983121,0.259956988830466,0.27104733448035,0.95908336205
871,0.337516751410484,0.78424632180359

"6724","AL031775.1",5.52891986334553,0.656174340357121,0.683927491753961,0.959420915621236,0.337346742556909,0.78424632180359
"6725","TCP1",578.669848132216,-0.153755592177319,0.160249100434989,-0.959478660160687,0.337317665033347,0.78424632180359
"6726","HSPA14",46.6554029135299,0.269148482323197,0.280604410257951,0.959174098781188,0.33747104644983,0.78424632180359
"6727","TCTN2",35.9658683292575,0.39406390033007,0.410783995556883,0.959297111358619,0.337409090181914,0.78424632180359
"6728","CATSPER2",13.721757547698,0.607454731933177,0.633224536092641,0.959303844543866,0.337405699170374,0.78424632180359
"6729","APOE",282.214602047819,-0.886551688579276,0.924176850305005,-0.959287920149362,0.337413719155213,0.78424632180359
"6730","LZTS3",152.847201923211,0.322534225837316,0.336237832326013,0.959244305157753,0.337435685551528,0.78424632180359
"6731","SLC19A1",5.93049356956405,0.710920849215844,0.741004134156273,0.959402001211933,0.337356267343377,0.78424632180359
"6732","ELOA",165.899569708581,0.181614350526087,0.189408687546833,0.958849104960835,0.337634767356345,0.784277615391556
"6733","RAET1E",2.65009674748506,0.942899678368183,0.983951605414301,0.95827851001998,0.337922337248005,0.784277615391556
"6734","XRCC2",4.96067653607333,0.821714505890289,0.857595353034801,0.95816109891741,0.337981529906591,0.784277615391556
"6735","ZSWIM8",153.725619190216,0.166397224129835,0.173618504827623,0.958407194527115,0.337857468766172,0.784277615391556
"6736","MOB2",219.504941517811,-0.227725889074589,0.237582181891128,-0.958514175019004,0.337803547135734,0.784277615391556
"6737","GAPLINC",4.50212432663681,1.02902081721958,1.07317867662689,0.958853208352868,0.337632699888006,0.784277615391556
"6738","PKNOX1",96.4694981170226,-0.19589467280898,0.204441489334523,-0.958194314894868,0.337964783438141,0.784277615391556
"6739","CDKL5",105.180826864325,0.247992860800433,0.258783906114533,0.958300941213381,0.337911029347954,0.784277615391556
"6740","PLP1",167.762858218677,0.349865821958817,0.364972050977877,0.958609901830604,0.337755302411535,0.784277615391556
"6741","PAM16",170.528983496823,-0.211999203409335,0.221298063956241,-0.957980380032855,0.338072652116925,0.784372686412803
"6742","TBC1D10A",128.071810064186,-0.200384939399013,0.209213518919978,-0.957801104027403,0.338163062386153,0.784466077680129
"6743","AC005944.1",5.15419555019098,-0.771166942413782,0.805229648104717,-0.957698147638864,0.338214991103648,0.784470185505124
"6744","SIDT2",203.41557636231,0.225983065684574,0.236016728363175,0.957487493584941,0.338321255880589,0.784600302783572
"6745","APBA3",53.5680035726074,-0.244121010124226,0.255120484149051,-0.956885178932171,0.338625212743406,0.785188780920217
"6746","NUP205",56.9215394532998,-0.247043249764532,0.25827762306512,-0.956502723049469,0.338818309288249,0.785478375047452
"6747","PPFIBP2",23.5174542666882,-0.419190489090979,0.438317932755392,-0.956361713188021,0.338889520991801,0.785478375047452
"6748","RPL18A",15473.275034945,-0.209368030632298,0.218926484382372,-0.956339436148897,0.338900772047328,0.785478375047452

"6749","ZBTB8OS",264.566432954557,-0.21288430901241,0.222699116323683,
-0.955927946759305,0.33910863855089,0.785751475609163
"6750","Z93943.1",3.32751143321826,0.882752096198174,0.923470423562838
,0.955907275072688,0.339119083143341,0.785751475609163
"6751","MIR4453HG",5.33655371376659,0.718151286926253,0.75141727260193
1,0.955729011178453,0.339209161461182,0.785843769108707
"6752","AC012368.1",3.51857522367973,-0.799953105459211,0.837393772393
371,-0.955289054960189,0.339431540900396,0.785910283481384
"6753","HCP5",7.06792131830035,0.686600048622852,0.718589630244868,0.9
55482823191986,0.339333587656245,0.785910283481384
"6754","EMP2",625.467223397299,-0.269219595355252,0.281824313653998,-0
.955274553372207,0.339438872437132,0.785910283481384
"6755","S1PR4",3.29173974802195,0.819202139755014,0.857466628290128,0.
955374953062119,0.339388115663785,0.785910283481384
"6756","SH2D2A",9.08398164737857,0.503857981419428,0.527708363604014,0
.954803857907995,0.339676896081897,0.786133734643846
"6757","NENF",1626.85469939086,-0.219603591103217,0.230098838454841,-0
.954388090691369,0.33988723233131,0.786133734643846
"6758","SLC25A40",34.5076205861192,0.270006205807413,0.282834094114076
,0.954645183966082,0.339757159231674,0.786133734643846
"6759","AL161457.2",11.8727362277282,0.450964511092531,0.4724657264422
45,0.954491481294057,0.339834919322759,0.786133734643846
"6760","DDX55",93.6405131554489,-0.187045612592256,0.195873672582709,-
0.954929828628574,0.339613184089618,0.786133734643846
"6761","AL133467.1",28.9329696881754,-0.333913200585043,0.349848250350
711,-0.954451537917671,0.33985512904074,0.786133734643846
"6762","TP53I13",414.663751964887,-0.243368358598321,0.254935563811923
,-0.954626945567563,0.339766385667469,0.786133734643846
"6763","NLN",56.2124992397219,0.239407159355146,0.250935199640778,0.95
4059692294527,0.34005342778461,0.786169343762202
"6764","LTO1",57.1630008973774,0.249032340038337,0.261016462889762,0.9
54086716528348,0.340039749427372,0.786169343762202
"6765","TBL1X",144.009440088682,0.228514874059145,0.239499668077117,0.
954134408176153,0.340015611087346,0.786169343762202
"6766","AC007038.2",8.51896667213109,0.591549563836937,0.6203338914679
3,0.953598653842886,0.340286837431167,0.786441904454858
"6767","CHCHD2",3671.55309841849,-0.252816977562812,0.265138251020526,
-0.953528872539932,0.3403221745109,0.786441904454858
"6768","NFIX",1282.96399417464,0.167814331595234,0.175962794544677,0.9
53692125823936,0.340239507134781,0.786441904454858
"6769","DHX15",249.475365351134,0.134179425419499,0.140786140416659,0.
953072688990497,0.340553242665017,0.786589537632753
"6770","INTU",84.848241887005,0.224367576792264,0.235477144958711,0.95
2821034209522,0.34068075498894,0.786589537632753
"6771","LINC02511",377.089605408502,-0.189784087712043,0.1991457264340
66,-0.952991013718178,0.340594623799334,0.786589537632753
"6772","TBP",48.1071748902808,0.270720977994757,0.284001915542466,0.95
3236450809351,0.34047028172637,0.786589537632753
"6773","OTUB1",236.079106934422,-0.147837971486716,0.155140622378127,-
0.952928828185231,0.340626132531697,0.786589537632753

"6774","INPP5K",80.286366102907,-0.204590809879128,0.214746640221121,-0.952707849903792,0.340738114927232,0.786589537632753
"6775","ARL17B",38.0686357081409,0.348401973253302,0.365658844462608,0.952806088323481,0.340688328962866,0.786589537632753
"6776","HIPK1",226.723194918547,0.188043012467493,0.19751120813518 7,0.952062489227378,0.341065291249984,0.786653907159308
"6777","OSBPL11",224.296388093019,0.202205225332,0.212314703844218,0.952384463585548,0.340902035860418,0.786653907159308
"6778","FXR1",675.642574635441,0.154052026360368,0.161877012033368,0.951660920999789,0.341268974429406,0.786653907159308
"6779","LIAS",91.7661154326533,0.210442011920358,0.221116649558627,0.95172395358026,0.34123699793309,0.786653907159308
"6780","AC106791.1",22.395687783302,0.388717059033486,0.40839877385591,0.95180760550136,0.341194564185012,0.786653907159308
"6781","B4GALT1-AS1",48.2500579565663,-0.32443093183354,0.340878112443374,-0.951750552442506,0.34122350487699,0.786653907159308
"6782","AC004241.1",11.1412215846379,0.491956573485503,0.516511593845249,0.952459885407523,0.340863800861532,0.786653907159308
"6783","AL079343.1",39.5502283667466,0.357673249392849,0.375817735388553,0.951719984750202,0.341239011268289,0.786653907159308
"6784","MSRB1",97.5354911671498,-0.268710722826126,0.282318452228916,-0.951800070823014,0.341198386130249,0.786653907159308
"6785","SNHG22",4.98192099900225,0.758960359558782,0.797394503376149,0.951800340164575,0.341198249506998,0.786653907159308
"6786","BPHL",124.088436406387,0.236529984363992,0.248688191418115,0.951110637844155,0.341548215139398,0.787065579015794
"6787","UHRF2",115.995013601136,0.23786775185585,0.250073442769285,0.951191574849893,0.34150713455631,0.787065579015794
"6788","ZNF235",9.12277741298078,0.513915008813306,0.540396012811417,0.950997040373515,0.341605878281562,0.787082489146086
"6789","EIF4B",1304.21064046665,0.177210515381095,0.186376614147846,0.950819480176413,0.3416960219828 1,0.787174220623236
"6790","ARL14EP",209.523791262824,0.175328460934127,0.184436282171637,0.950618060989569,0.34179829683309,0.787293867815836
"6791","NOC2L",128.450490965869,-0.187242127118951,0.19735075520796,-0.948778366323674,0.342733346997288,0.787378138551804
"6792","KCTD3",209.828873899585,0.137004807775693,0.14441726106156,0.948673356416118,0.342786769014625,0.787378138551804
"6793","LINC01816",4.1797831267099,0.803432431720355,0.846902703477475,0.948671468896455,0.342787729307074,0.787378138551804
"6794","LY75",4.69801908074002,-0.984887001947528,1.03744429493086,-0.949339648171825,0.342447894586223,0.787378138551804
"6795","SLC35A5",165.893526717372,0.189979156895823,0.200170411333467,0.949087108480451,0.342576310466043,0.787378138551804
"6796","RAD17",118.450391315542,0.17938999785209,0.188845362662113,0.949930648670781,0.342147492444321,0.787378138551804
"6797","ZNF277",155.874978778302,-0.205283071431913,0.216219954034022,-0.949417792400473,0.342408164653577,0.787378138551804
"6798","RPL8",13201.7820548027,-0.22972721244622,0.241942231769382,-0.

949512661622446,0.34235993539453,0.787378138551804
"6799","AC007066.2",2.56347322990583,0.902002442085136,0.949725422475516,0.949750760313453,0.342238910809176,0.787378138551804
"6800","ZNF22",569.333627771506,0.174059576586181,0.183307573672689,0.94954929083824,0.34234131513374,0.787378138551804
"6801","LIPA",253.182369002186,-0.162140773245947,0.170805423078949,-0.949271810714128,0.342482386760962,0.787378138551804
"6802","HSPB8",356.980828295661,-0.24905608945974,0.262509445745508,-0.948750963046064,0.342747287438839,0.787378138551804
"6803","ATG14",120.413639202047,0.256375972816166,0.269988304304971,0.949581773462939,0.342324803311576,0.787378138551804
"6804","GCHFR",33.4333746058573,-0.313898743811418,0.330821723554264,-0.948845621257787,0.342699134981094,0.787378138551804
"6805","AC022306.2",10.5342429015693,0.477953675109604,0.503092793035913,0.950030852609503,0.342096576076987,0.787378138551804
"6806","CASC3",230.347028496444,0.218633891841835,0.230084408498381,0.950233408985524,0.341993666432822,0.787378138551804
"6807","MYL12A",3306.95091638914,-0.272669262758198,0.287429824338007,-0.948646381377421,0.342800492969262,0.787378138551804
"6808","C19orf44",26.1365421137231,0.387958989220861,0.408433310228261,0.949871079330045,0.342177763555951,0.787378138551804
"6809","RANBP1",609.231520773855,-0.22076978900883,0.232538583521032,-0.949389927753055,0.342422331203837,0.787378138551804
"6810","SNRPD3",875.061406166843,-0.188223929306432,0.198430128658798,-0.948565273724558,0.342841759816994,0.787378138551804
"6811","ASXL2",171.226381592944,0.217230086473618,0.229069722484841,0.948314269198076,0.34296948878989,0.787440223821767
"6812","PHLPP2",53.8107715598502,0.249952029980455,0.263549024490659,0.948408101542089,0.342921736654132,0.787440223821767
"6813","HOXA13",6.93276333514425,0.695034736201034,0.733419758901309,0.947662955307097,0.343301065482952,0.787647444266439
"6814","POP1",23.5473961633156,-0.377842770706301,0.398729608022755,-0.947616537883833,0.343324703894404,0.787647444266439
"6815","NOL8",210.136525358791,0.163442366194698,0.172455419087777,0.947736911134746,0.343263405086251,0.787647444266439
"6816","PRG2",15.2433111280555,-0.463804547408235,0.489481017120892,-0.947543482148327,0.343361910166789,0.787647444266439
"6817","AC026124.1",17.3640127541902,0.460889573496154,0.486344585064461,0.947660542853719,0.34330229401682,0.787647444266439
"6818","TIMM22",156.777371478502,-0.242716847356314,0.256139992722103,-0.947594496184934,0.343335929154487,0.787647444266439
"6819","MYO3B",3.81845119865765,0.969878527135891,1.02408120157658,0.9470718978561,0.343602143477078,0.787736371149443
"6820","FBXL18",38.4227460684665,0.306125785177736,0.323148548311138,0.947322173587448,0.343474635265083,0.787736371149443
"6821","CADPS2",98.3416162158947,-0.226090593186412,0.238721204860897,-0.947090533152071,0.343592648293784,0.787736371149443
"6822","PDXDC1",186.891091707944,0.225905153349305,0.238520435896762,0.947110265415927,0.34358259435859,0.787736371149443
"6823","C2orf68",149.645294023977,0.178817011691024,0.188838975696187,

0.946928519558976,0.343675204171147,0.787788391211599
"6824","PIGC",265.121104255374,-0.206563623937997,0.218184300771368,-0
.946739170543952,0.343771705177285,0.78789411913434
"6825","ID2",941.730359828198,0.225332237422885,0.23815849936126,0.946
14400925109,0.344075139520045,0.788319759779289
"6826","SLC20A1",499.945311171038,0.398567349486663,0.42148366320607,0
.945629414091424,0.344337636165919,0.788319759779289
"6827","EGOT",6.36545684628079,-0.70462613579291,0.745676437784474,-0.
944948908250968,0.344684960578872,0.788319759779289
"6828","MXD4",779.484145667967,0.177735432487081,0.188019567790924,0.9
45302845737422,0.344504286002816,0.788319759779289
"6829","ZFYVE16",325.426821445567,0.166510598287493,0.176157780687531,
0.945235558926858,0.344538629268608,0.788319759779289
"6830","CD24",176.366772680498,-0.298009917533409,0.315341934557921,-0
.945037386008274,0.344639789645463,0.788319759779289
"6831","CALHM6",13.1022418444114,-0.473673423409195,0.501331152854941,
-0.944831416742721,0.344744949866001,0.788319759779289
"6832","ATP6V1H",157.632954816159,0.145149673041333,0.153429243468814,
0.946036555741971,0.344129941346162,0.788319759779289
"6833","CHCHD7",109.818982109243,-0.223655024182976,0.236427361314468,
-0.945977753757088,0.344159933010564,0.788319759779289
"6834","PMPCA",154.24100162452,-0.174629262412602,0.184754399492863,-0
.945196774160433,0.344558426050587,0.788319759779289
"6835","CEP295",76.9899401506555,0.254422369553407,0.269265728793824,0
.944874680833288,0.344722859137863,0.788319759779289
"6836","AL162377.1",25.3148717443858,0.339123796082389,0.3589513803484
52,0.944762479400926,0.344780151271575,0.788319759779289
"6837","COL4A2",314.452445762919,-0.302266384947125,0.319793644231366,
-0.945191971133861,0.344560877694297,0.788319759779289
"6838","SLIRP",645.062367994936,-0.207945386622368,0.220118925138779,-
0.944695629834031,0.344814288788371,0.788319759779289
"6839","PTPN21",145.066333861573,0.201190780647124,0.212742458656531,0
.945701116352818,0.344301052950608,0.788319759779289
"6840","GTF2F1",242.214812058036,-0.181473922100963,0.19193082548745,-
0.945517332299644,0.344394826393298,0.788319759779289
"6841","POLA1",30.889044092809,0.312012557610768,0.329883080047503,0.9
45827708307558,0.34423647051724,0.788319759779289
"6842","POLR2L",2464.49030622406,-0.264405950254075,0.279949827584246,
-0.944476203238621,0.344926356709888,0.788369018301386
"6843","FAM192A",219.538073074603,-0.14488090340363,0.15340142393371,-
0.944456053199597,0.344936649119973,0.788369018301386
"6844","RHOA",4835.4313366767,-0.153907353542949,0.162982354624787,-0.
944319119068259,0.34500659870075,0.788413676750399
"6845","CUTC",801.266688570701,-0.251352642302114,0.266308229148749,-0
.943841063813759,0.345250872899032,0.788856632891286
"6846","ANKRD44",37.3559013814371,0.379226723434444,0.401984914340516,
0.943385460264327,0.345483777440064,0.788896619314048
"6847","MGLL",840.674910300479,-0.233825583068568,0.247902779559881,-0
.943214850126709,0.345571019112567,0.788896619314048
"6848","ZNF451",312.362811572359,0.231777934319483,0.245686528592685,0

.943388860785849,0.345482038722535,0.788896619314048
"6849","RND2",26.5617880414663,0.432476554603569,0.458413602144176,0.943419987061269,0.345466123834507,0.788896619314048
"6850","ZNF792",8.28528188367593,0.532981990015527,0.565041877462992,0.943261041833904,0.345547397548653,0.788896619314048
"6851","L3MBTL2",72.483179138272,0.269632898560708,0.285864207324223,0.9432202131374,0.345568276517698,0.788896619314048
"6852","PARP1",302.890989230683,0.165778143301519,0.175819373374623,0.942888944031733,0.345737710561821,0.789049356357579
"6853","GPN3",167.671936286375,-0.204977195356318,0.217461454060793,-0.942590935214729,0.345890178208923,0.789049356357579
"6854","IMPA2",447.976884491269,-0.363849811873063,0.386005969068654,-0.942601516631857,0.34588476379747,0.789049356357579
"6855","ILF3-DT",288.822816990686,0.210424267263845,0.223186530952814,0.942817948581012,0.345774029457115,0.789049356357579
"6856","GID8",247.4485931769,0.149709823207334,0.158808099804078,0.942708988974941,0.345829774271768,0.789049356357579
"6857","AC244453.2",3.41018441012235,0.862730779192324,0.915577476615496,0.942280474593451,0.34604906202009,0.789103914872304
"6858","UBA6-AS1",28.749381763196,-0.342615714370254,0.363635098767798,-0.942196491843692,0.346092049692457,0.789103914872304
"6859","PDE3A",21.3899010841644,0.434454278186009,0.461031236840956,0.942353236546277,0.346011820610181,0.789103914872304
"6860","EWSR1",446.840907955759,0.17313925001065,0.183770389629571,0.94214987713554,0.346115911510486,0.789103914872304
"6861","PSMC3IP",28.5758262627511,0.300516559591656,0.319017381882726,0.942006851846491,0.34618913192625,0.789155811591102
"6862","AL139246.5",3.23313696643573,-0.7340609911074,0.779395396547227,-0.941833880927881,0.346277695898286,0.789242664507315
"6863","TTC7A",171.897574362591,0.216662180603976,0.23012875545332,0.941482433071797,0.346457687457737,0.789422819323865
"6864","SPAG16",182.113578458634,-0.224582682245112,0.238540105936603,-0.941488146671652,0.34645476080124,0.789422819323865
"6865","ZNF213",43.8030908655305,-0.278856587708164,0.2962534626945,-0.941277057732567,0.346562896575021,0.789547516741927
"6866","MINDY3",130.918408649235,0.190349753579154,0.202279728193962,0.941022391510394,0.346693384539993,0.789729760297915
"6867","CCNG1",458.673131905562,0.167557265079151,0.178102967242963,0.940788734028075,0.346813135385914,0.789887496350035
"6868","AL583785.1",47.2531200760554,-0.446899914190687,0.475230511879209,-0.940385566624302,0.347019822975167,0.790243161230578
"6869","AP003307.1",41.7862877175619,-0.278067650104288,0.295754080195605,-0.940198863597689,0.347115564602746,0.79034611011602
"6870","TYW1B",24.9670665055088,-0.402245417745327,0.427933755122954,-0.93997122902762,0.347232318733866,0.790496865356284
"6871","RECQL5",29.3259114783891,0.290815700498212,0.309426986198816,0.93985241581791,0.347293268140703,0.790520552135146
"6872","ULK4",29.4166026699863,-0.399252371674883,0.424887195880075,-0

.939666752837551,0.347388524112268,0.79062231040685
"6873","FAAP20",354.945381705485,-0.224972496900945,0.23948402625916,-0.93940502176746,0.347522835693228,0.790788484335373
"6874","SVBP",867.681613123547,0.233711356457432,0.248829170704242,0.939244204351028,0.347605378162909,0.790788484335373
"6875","USP4",139.723938601021,0.220216391801068,0.234489679537418,0.939130422436897,0.347663786335679,0.790788484335373
"6876","CPT1B",4.27482465402539,-0.660914670138058,0.703713514514107,-0.939181437483689,0.347637597784397,0.790788484335373
"6877","FOPNL",146.759822925005,0.172041856028944,0.18321449730649,0.939018792498413,0.347721095890926,0.790803830119831
"6878","FAM180A",132.735842120624,0.288411204974094,0.307193347664173,0.938858888602589,0.347803199219434,0.790875550420463
"6879","AC010883.3",11.8712325406444,0.650175100138846,0.692808875307514,0.938462429267027,0.34800681610324,0.790878611429043
"6880","AC110792.3",2.5739623807632,-0.847542478939394,0.9029855581951,-0.938600259159708,0.347936019687923,0.790878611429043
"6881","NDUFA8",393.659735816111,-0.233157252271868,0.248424592372574,-0.93854336257576,0.347965243545867,0.790878611429043
"6882","CPSF6",231.35808529172,0.133831095575559,0.142605096636906,0.938473439811994,0.348001160192979,0.790878611429043
"6883","IGHG4",153.663248104774,0.818563956301147,0.872344933362795,0.938348954633888,0.348065109296993,0.790896165829577
"6884","BUD23",248.595703679356,-0.208918443789036,0.222751231239998,-0.937900287356623,0.348295655508602,0.791214896090843
"6885","TARSL2",60.9349132715738,0.280469102451324,0.299046124129615,0.937879075569496,0.348306557518251,0.791214896090843
"6886","CLSTN2",17.8925548601812,0.537199411406232,0.573587627959985,0.936560318284461,0.348984771862403,0.791357416889782
"6887","GAR1",127.518326356752,-0.210946218024808,0.225308513020933,-0.936254982985082,0.349141920095191,0.791357416889782
"6888","C6orf226",177.3100654787,-0.26313572573687,0.280925582492659,-0.936674130572449,0.348926207097782,0.791357416889782
"6889","CDK14",178.646578474294,0.193511201355194,0.206480820216405,0.93718729493801,0.34866222395175,0.791357416889782
"6890","MTSS1",359.782855854819,0.305584451097748,0.326412275412468,0.936191663477114,0.34917451464388,0.791357416889782
"6891","MRPS2",175.382024166071,-0.2394773290664,0.255435399748641,-0.937526001885623,0.348488055111358,0.791357416889782
"6892","AL157786.1",3.01305257427606,1.04480221413544,1.11455630148233,0.937415375738203,0.348544934866265,0.791357416889782
"6893","H19",1218.35291204775,0.267259338256815,0.285213589971099,0.937049802864922,0.348732940479962,0.791357416889782
"6894","ATP5MG",2142.62836631073,-0.211371570004412,0.225693259210531,-0.936543566891562,0.348993392208978,0.791357416889782
"6895","NAA16",87.600351516386,0.228596811952552,0.244179570269282,0.936183201978997,0.349178870457569,0.791357416889782
"6896","GPR180",70.2005294365246,0.268274404551121,0.286268387097084,0.937142963187687,0.348685024179837,0.791357416889782
"6897","ZNF770",163.585120375756,0.174008023736549,0.185811936255215,0

.936473873764209,0.349029258076053,0.791357416889782
"6898","NME3",690.087506510037,-0.205799840437351,0.21966614293245,-0.936875558927788,0.348822572746205,0.791357416889782
"6899","XPO6",133.955398839737,0.226648061390316,0.242018468994514,0.936490765898753,0.349020564736345,0.791357416889782
"6900","ARHGAP6",247.123903240626,0.196662311948938,0.209905154876213,0.936910349176109,0.348804675236679,0.791357416889782
"6901","TMLHE",51.3031114540802,0.279323731704474,0.298148205733738,0.936862024767391,0.348829535422808,0.791357416889782
"6902","ATP5MC1",712.551138871749,-0.234649387420573,0.250866319395162,-0.935356280533439,0.349604719891691,0.792207739656049
"6903","GDAP2",63.8694895748491,0.235730872558148,0.252347791901666,0.934150724211634,0.350226149722642,0.792260967269073
"6904","AKIRIN2",319.262952832975,-0.194349970088378,0.208033619426387,-0.934223855856862,0.350188432497998,0.792260967269073
"6905","STAG3",4.38890608461594,0.718411169052263,0.768731619472925,0.93454093841598,0.350024928802321,0.792260967269073
"6906","PINX1",62.1534662263818,-0.232765934037545,0.249178988661838,-0.934131466250686,0.350236082333656,0.792260967269073
"6907","SSRP1",279.683351684206,-0.152298744752204,0.162975157705443,-0.934490549949119,0.350050908389627,0.792260967269073
"6908","TMEM87A",510.098085652114,-0.125026193527858,0.133758393773074,-0.934716618532138,0.349934360164706,0.792260967269073
"6909","ADAM10",318.079527267201,0.235112468898005,0.251568505768202,0.934586259834288,0.35000156275924,0.792260967269073
"6910","IRX5",88.0443848838831,-0.332402297720675,0.355445303207987,-0.935171444722036,0.349699952016076,0.792260967269073
"6911","SLC25A11",299.497787337185,-0.236946636245806,0.253490254013235,-0.934736671309796,0.34992402327132,0.792260967269073
"6912","ZNF567",71.4706288129677,-0.1981313201439,0.21200539717227,-0.934557906480573,0.350016180581369,0.792260967269073
"6913","ZNF525",18.7530135802568,0.378898320171787,0.405393458177641,0.934643400204441,0.349972104713232,0.792260967269073
"6914","AL035461.2",2.88083546113708,-0.863076064080519,0.923860043929794,-0.934206506441474,0.350197380125876,0.792260967269073
"6915","GALNT5",37.8520802956486,-1.19594515781901,1.28064398261135,-0.933862317753891,0.350374918809778,0.792448524255021
"6916","TAMM41",61.7565690887988,-0.246333821556798,0.263831892596129,-0.933677195477966,0.350470431796578,0.792448524255021
"6917","NCR3LG1",3.08250218949814,-0.805044192648128,0.862230823414729,-0.933675960991372,0.350471068779676,0.792448524255021
"6918","ATP5F1B",1082.3272187514,-0.193447116185556,0.207210470180659,-0.933577902781152,0.350521668209478,0.792448524255021
"6919","GSTO1",1066.97051683278,-0.227138047640682,0.243347662331754,-0.933389067576193,0.350619122908234,0.792554282740971
"6920","ADK",525.881316117667,-0.162112388005506,0.173720246815299,-0.933180737291176,0.350726658622875,0.79268279492222
"6921","AC083855.2",11.0775244695767,0.637362793669465,0.683264644358475,0.932819806984001,0.350913012750871,0.792808034094987
"6922","TTLL11",24.0249339195154,-0.290891116042816,0.311854309938818,

-0.932778886717602,0.35093414450381,0.792808034094987
"6923","MRPL54",529.99204416319,-0.261416161294288,0.280204723069557,-0.932947019702432,0.350847323615182,0.792808034094987
"6924","AC005696.1",2.79913648233219,1.0662029688128,1.1434469929798,0.932446344569323,0.351105903466651,0.79285248775894
"6925","FZD2",17.8844395662632,0.416855181717326,0.447019499376819,0.932521248622164,0.351067210662985,0.79285248775894
"6926","C1QTNF1",154.771684985061,0.261326745797942,0.280219546639844,0.932578576089894,0.351037599131765,0.79285248775894
"6927","MED8",233.224590306251,-0.220977987694449,0.23736130808343,-0.930977291449616,0.351865310960265,0.793108194548056
"6928","SLC5A6",22.1270910246463,-0.324457654736358,0.348437674689338,-0.931178452575887,0.351761261969276,0.793108194548056
"6929","MPHOSPH10",300.694294121279,-0.182114000111748,0.195634208450766,-0.930890367047331,0.351910277945171,0.793108194548056
"6930","ERCC3",96.4442352183978,0.184757468012125,0.198643982491119,0.930093455110752,0.352322699172947,0.793108194548056
"6931","AC021016.2",5.74573393108016,0.630405377374826,0.677244342831813,0.930838897433774,0.351936905478582,0.793108194548056
"6932","DHX36",644.553102887564,0.150162028981263,0.161374255850652,0.930520349666149,0.352101732868818,0.793108194548056
"6933","TACC3",7.8103898334898,0.536668099681671,0.576309022078536,0.931215856635568,0.351741917165918,0.793108194548056
"6934","TRIP13",4.26678899255715,-0.73508619978504,0.78942392575629,-0.9311678754616,0.351766732412024,0.793108194548056
"6935","AL080317.2",8.54906232246381,0.608147023737712,0.654002878041472,0.929884323382363,0.352430980564147,0.793108194548056
"6936","PIH1D2",12.3249894908851,0.442169104362965,0.475151089193964,0.930586321738314,0.352067592674966,0.793108194548056
"6937","TUBA1A",4426.43391588269,-0.252590359875,0.271216500147211,-0.9313237201199,0.351686135606883,0.793108194548056
"6938","AL139383.1",13.8916869012388,0.507333174610801,0.545012236740278,0.930865658439458,0.351923060654063,0.793108194548056
"6939","AC022467.1",51.192581511002,0.429876855096144,0.461522490135541,0.931432084642064,0.351630100578732,0.793108194548056
"6940","ADAMTS7",92.3765852914975,-0.230345092952894,0.247230628962881,-0.931701277949175,0.351490925856058,0.793108194548056
"6941","ZNF500",52.5426033768743,0.281987032477517,0.303235558667878,0.929927326848784,0.352408713092291,0.793108194548056
"6942","COQ9",142.695814476208,-0.245472253413112,0.263926904524616,-0.930076658365904,0.352331395185989,0.793108194548056
"6943","DHRS13",12.833763281085,0.391112320214686,0.420627393874209,0.929830833442224,0.352458679242267,0.793108194548056
"6944","GJC1",3.14978692202047,-1.4097955813306,1.51468145968203,-0.930753837593385,0.351980913535186,0.793108194548056
"6945","CUEDC1",245.027361577777,-0.155323566154501,0.166869317708787,-0.930809619690329,0.351952052734153,0.793108194548056
"6946","PSMG2",872.607541486062,-0.238958876142913,0.256382693779339,-0.932039805887124,0.351315954213759,0.793108194548056
"6947","DYM",167.71138241345,0.148334330423392,0.159304642134508,0.931

136270957796,0.351783078460611,0.793108194548056
"6948","C19orf12",192.388743903289,0.167264188300864,0.179867460011386
,0.929930229126909,0.352407210306064,0.793108194548056
"6949","EHD2",705.196671457414,0.175896225505361,0.189199698753106,0.9
29685547411438,0.352533919612594,0.793108194548056
"6950","ATRN",147.069596201033,0.162733966353711,0.175043285283567,0.9
29678428338938,0.352537606681463,0.793108194548056
"6951","CRYZL1",141.097708027025,0.157649133114028,0.169559186831796,0
.92975872354482,0.352496022067231,0.793108194548056
"6952","ZRSR2",76.8805262737456,-0.235985878087155,0.253410277250164,-
0.931240361077356,0.351729244211484,0.793108194548056
"6953","ARAP2",4.31452819901617,-0.758360740681602,0.815853757772938,-
0.929530241733179,0.352614360172147,0.793166775937347
"6954","SNRPE",799.319600693212,-0.179755033173191,0.193517579083197,-
0.92882192640032,0.352950142461785,0.793355611159044
"6955","DPH7",91.2039211391313,-0.208056600163107,0.223941315157006,-0
.929067510464685,0.352854100631192,0.793355611159044
"6956","HSPA2",36.6185671232229,0.320572831973004,0.344999848769711,0.
929197021726778,0.352786990628342,0.793355611159044
"6957","SEC11A",2403.25811824076,-0.230074541775383,0.247690614213835,
-0.928878724394279,0.352951940054005,0.793355611159044
"6958","PRKCSH",553.302020199772,0.108762309407156,0.117069653383368,0
.929039304925518,0.352868717217219,0.793355611159044
"6959","PCDHGC3",88.2391100472146,0.209638554357902,0.225735395083128,
0.928691551808711,0.353048960218615,0.793377919273657
"6960","CHMP2A",1108.26558496481,-0.225224818293096,0.242525664945449,
-0.928663852313343,0.353063319574466,0.793377919273657
"6961","PCAT1",6.66335407395813,-0.562415170334951,0.605782527502421,-
0.928411013526143,0.353194407704545,0.793444489586195
"6962","MCTS1",429.337823153952,-0.245020086406382,0.263910576384078,-
0.928420868020824,0.353189297914932,0.793444489586195
"6963","KTI12",54.6868086103279,-0.257494789910533,0.277503889311116,-
0.927896147869299,0.353461442806374,0.793814198804868
"6964","AC245407.2",72.4114108854367,-0.323438236839597,0.348603190622
575,-0.927812038271839,0.353505078379644,0.793814198804868
"6965","SMAD4",296.325822479085,0.174552335068333,0.188135704823862,0.
927800149534369,0.353511246462654,0.793814198804868
"6966","LRRC4",5.5721526317754,0.675453654100104,0.728258819561475,0.9
27491210483152,0.35367155325079,0.794060162624513
"6967","OGG1",101.290855921549,-0.177846826070546,0.191831914433248,-0
.927097175649737,0.353876082440298,0.794164328562252
"6968","DEFB1",384.619278480669,0.942291674422632,1.01617471481503,0.9
27292975001997,0.353774440757456,0.794164328562252
"6969","HAUS1",171.395989360253,-0.206094470191624,0.222289625784379,-
0.92714389825612,0.353851826524778,0.794164328562252
"6970","ZNF676",27.8486867953112,0.43560212718885,0.469899862196757,0.
927010544656117,0.35392105946796,0.794164328562252
"6971","EEF1AKMT3",51.2353842012602,0.259404331455085,0.27988020438988
2,0.926840581743059,0.354009311207057,0.794248404429546
"6972","UBL5",1782.63485861682,-0.203825499315393,0.220065571550413,-0

.926203485076718,0.354340241746364,0.794543881813846
"6973","HOMER3",408.420146363396,-0.254479598925627,0.274757875417235,
-0.926195831654273,0.354344218392044,0.794543881813846
"6974","NXT1",217.050032402435,-0.238604348251988,0.257570108122825,-0
.926366611370162,0.354255489575647,0.794543881813846
"6975","AC145212.1",3.50597922410236,0.902377404228106,0.9741594816193
7,0.92631383387868,0.354282908689208,0.794543881813846
"6976","EIF3A",844.743148885094,-0.175458323918648,0.189506244830659,-
0.925870934097372,0.354513058077296,0.794698795132955
"6977","JMJD7",30.156183714533,0.317521078630638,0.342944466224704,0.9
25867333933276,0.354514929261038,0.794698795132955
"6978","LCK",10.5506224299303,0.630834170130998,0.681987070701479,0.92
499432501284,0.354968859399723,0.79473453146531
"6979","KHDC4",91.0855012132469,0.274441156553481,0.296866683407325,0.
924459267047239,0.355247249650734,0.79473453146531
"6980","FSTL1",20727.2930810866,0.22825721989946,0.246984590943112,0.9
24175953762372,0.355394713111789,0.79473453146531
"6981","LYSMD3",187.006750500551,0.188036349430343,0.203361448519751,0
.924641080200022,0.355152636955887,0.79473453146531
"6982","ZCCHC10",186.878744735603,-0.138677370896992,0.150012245315346
,-0.924440338890162,0.355257100478945,0.79473453146531
"6983","PRDM1",25.1648560785896,0.440219893226703,0.476210902569558,0.
924422122323001,0.355266581135407,0.79473453146531
"6984","Z95118.2",3.29936695418787,0.931144967469405,1.005962213255,0.
925626186749591,0.354640279606756,0.79473453146531
"6985","ANGPTL2",2297.78407351225,-0.226526201229419,0.244794676137355
,-0.925372254020404,0.354772306234784,0.79473453146531
"6986","MED27",100.901418128912,-0.210979033961252,0.228273827364591,-
0.924236634558566,0.355363125744042,0.79473453146531
"6987","P4HA1",360.630085093315,0.177232041214868,0.191496192984452,0.
92551208696487,0.354699599392408,0.79473453146531
"6988","FGFBP3",38.4412315870826,-0.287191311715859,0.310711457576376,
-0.924302289835142,0.355328950907493,0.79473453146531
"6989","POMT2",50.6362492443302,0.251092082992673,0.271420894309243,0.
925102260943077,0.35491271711384,0.79473453146531
"6990","ELOB",2361.84610558091,-0.206055603781218,0.222911750327551,-0
.924381973935586,0.355287476583688,0.79473453146531
"6991","ALKBH7",739.303851180464,-0.232067244899827,0.251078087332839,
-0.924283147784974,0.355338914499272,0.79473453146531
"6992","IFNGR2",755.24510097611,-0.194016796477337,0.209621974121559,-
0.925555621209958,0.354676965463455,0.79473453146531
"6993","FAM199X",90.312962745491,0.214977384805476,0.232489017514415,0
.924677591672245,0.355133638879671,0.79473453146531
"6994","ZNF275",61.0756012473068,0.224789554709392,0.243077105483949,0
.924766461497277,0.355087399773543,0.79473453146531
"6995","L3HYPDH",122.789376196257,-0.190200083143855,0.205849526460186
,-0.923976296737523,0.355498657011027,0.794853323181197
"6996","TUBB2B",34.9790854178665,-0.476568612078624,0.515844138448344,
-0.923861640673361,0.355558357034742,0.794873170958172
"6997","COQ3",36.2949046216584,-0.404719848365174,0.438242396496786,-0

.923506834574691,0.355743140337206,0.795172604669702
"6998","CDK13",294.434671476738,0.214233410067753,0.232006127272944,0.923395483498235,0.355801144552576,0.795188611146369
"6999","HACL1",83.5412018322291,-0.247756089881703,0.268449959202441,-0.922913494260836,0.356052287760394,0.79537930340806
"7000","RNF144B",10.0393030952302,-0.61691879531729,0.668554577591552,-0.922765045659729,0.356129660248453,0.79537930340806
"7001","ERCC5",212.219902799645,0.172739863575556,0.18717536602936 1,0.92287712448474,0.356071242971271,0.79537930340806
"7002","BTBD1",155.196414896755,-0.197591820753366,0.214051136289883,-0.923105684829317,0.355952132386424,0.79537930340806
"7003","NPIPA5",5.50734318383322,1.23233605287028,1.33556875102148,0.922705066233211,0.356160924967277,0.79537930340806
"7004","LLGL2",2.51874176180895,1.03351145842467,1.12016007533606,0.922646219215234,0.356191601091435,0.79537930340806
"7005","KATNAL1",153.473805411404,0.156642877252365,0.16985523624771,0.922214002422173,0.356416960678151,0.795541781790535
"7006","ARHGAP45",13.3777709895375,-0.491983334830656,0.533383895967412,-0.922381306504076,0.356329716993881,0.795541781790535
"7007","RBM41",96.2734488174702,0.242069049955742,0.262484594919312,0.922221930891427,0.356412825933211,0.795541781790535
"7008","NDUFV3",304.111288208818,-0.211155424142687,0.229025780917152,-0.921972291927564,0.356543028681149,0.795709613095487
"7009","FAM13B",132.324357551019,0.240854170595143,0.261289623219375,0.92179003370878,0.356638106972725,0.795808245549068
"7010","HRH1",303.495708145727,-0.233709350135016,0.253817560790356,-0.920776913178404,0.357166910787444,0.795858687035496
"7011","SS18L2",299.078814837035,-0.252532876536556,0.274098967142653,-0.921320058842569,0.356883351555946,0.795858687035496
"7012","SLC25A4",267.65424082282,0.178397405483615,0.193653790431768,0.921218247708255,0.35693649313705,0.795858687035496
"7013","OR2A1-
AS1",17.5430701040481,-0.455854254169025,0.494972462994822,-0.920968918979629,0.357066654400971,0.795858687035496
"7014","RAB1B",410.42377132998,-0.14659310513893,0.159206768477897,-0.920771814794305,0.357169573165099,0.795858687035496
"7015","FGD4",174.060078227833,0.266151980262018,0.289039261046722,0.920816014053522,0.357146492714609,0.795858687035496
"7016","ARHGEF40",211.554569463925,-0.239938822267616,0.260503498831453,-0.921057964073094,0.357020165263751,0.795858687035496
"7017","UBN1",272.959117794158,0.167007136560357,0.181328742248438,0.921018557176898,0.35704073855596,0.795858687035496
"7018","AC015726.1",6.81467088242436,0.562267103804113,0.610194222690493,0.921455960898717,0.356812423563906,0.795858687035496
"7019","SOCS3",5868.26127364298,-0.283854960795849,0.308023382508338,-0.921537055025898,0.356770104346961,0.795858687035496
"7020","PYCARD",259.821392472643,-0.260405263941314,0.282910544999828,-0.92045089355532,0.35733718348246,0.796005348193374
"7021","LRRC3",2.592821643803,1.02799802036064,1.11676500560736,0.920514177287954,0.357304127819197,0.796005348193374

"7022","AL450998.2",32.3193301342295,-0.256088651512021,0.280237372589
882,-0.913827621010411,0.360807430362116,0.796345408051824
"7023","DHDDS",98.801125495241,-0.175239299039886,0.191038461419369,-0
.917298525846058,0.35898622965095,0.796345408051824
"7024","SNHG3",135.73533618106,-0.175603033565214,0.191430890840416,-0
.91731816528819,0.358975941207761,0.796345408051824
"7025","ZMYND12",3.51425778270083,0.831195132824798,0.904930937629608,
0.918517754517317,0.358347868199142,0.796345408051824
"7026","AC118549.1",145.658619675002,0.161950692234748,0.1770296542398
2,0.914822394757406,0.360284876088226,0.796345408051824
"7027","SH3GLB1",976.404724182033,-0.117833830686892,0.12847369896974,
-0.917182517759106,0.359047006166905,0.796345408051824
"7028","FNBP1L",66.5301765800787,0.277883781448762,0.302799464038974,0
.917715565748144,0.358767796294688,0.796345408051824
"7029","TRIM45",12.4279787429322,0.473153370151841,0.518813685408587,0
.911990919783107,0.361773497551765,0.796345408051824
"7030","GON4L",279.092031202105,0.176988303394087,0.193851005061706,0.
913012049319779,0.361236204404894,0.796345408051824
"7031","PEA15",383.842175596875,-0.143045714118203,0.156843560901524,-
0.912027967842531,0.36175399502553,0.796345408051824
"7032","RXRG",6.69580968067159,-0.561287805337818,0.611169158758605,-0
.918383719620105,0.358418010984969,0.796345408051824
"7033","LINC00954",8.66463398729567,0.608349688468737,0.66733339131118
8,0.911612840582489,0.361972560482805,0.796345408051824
"7034","ANKRD36",79.5477655451526,0.312090408077798,0.341302598375848,
0.914409704358942,0.360501604510276,0.796345408051824
"7035","FASTKD1",27.4443594386553,-0.299800039788874,0.328273754726355
,-0.913262286346296,0.361104612240236,0.796345408051824
"7036","NDUFB3",589.96781735248,-0.250711946606194,0.272786012783389,-
0.919079186091835,0.358054155159337,0.796345408051824
"7037","ZFAND2B",164.820842412412,-0.220491404381818,0.24001045828845,
-0.918674152593912,0.358266033290032,0.796345408051824
"7038","CSRNP1",433.684692201847,-0.36485803974442,0.396579751803408,-
0.920011770861381,0.357566608132384,0.796345408051824
"7039","KLHDC8B",110.584878305926,-0.289721155272158,0.318174051611828
,-0.910574428695453,0.362519649008296,0.796345408051824
"7040","THOC7",655.171301160251,-0.207868263730699,0.22798786686928,-0
.911751430394686,0.36189958342843,0.796345408051824
"7041","SUCLG2",415.929618888669,-0.144662407811283,0.157307645782106,
-0.919614600371436,0.357774194219714,0.796345408051824
"7042","UBA3",171.266753610273,-0.163153004380547,0.179048704670848,-0
.911221361140129,0.362178751103586,0.796345408051824
"7043","XXYLT1",102.664781102086,-0.183441983830334,0.201277660685687,
-0.911387697995928,0.362091133173499,0.796345408051824
"7044","STX18",144.249850424796,-0.188914408760872,0.206978935601892,-
0.912722873037833,0.361388311009365,0.796345408051824
"7045","TBC1D19",48.9489248779111,0.253348460755071,0.275844395109297,
0.91844701305128,0.358384887358923,0.796345408051824
"7046","ETFDH",125.680455557979,0.149370906420111,0.163822469737152,0.
911785218839464,0.361881792889734,0.796345408051824

"7047","EXOC3",186.825786294733,0.171666202787582,0.188596482098628,0.
910230142563362,0.362701150809319,0.796345408051824
"7048","MRPS30-
DT",31.2910494327975,0.259732906302025,0.284449783784795,0.91310635869
0469,0.361186606398064,0.796345408051824
"7049","AC112206.2",9.53396660766141,-0.676096489847875,0.740569336602
203,-0.912941511931705,0.361273303339147,0.796345408051824
"7050","BTF3",5056.07987661403,-0.219620089737584,0.24012079854257,-0.
914623352373404,0.360389394941198,0.796345408051824
"7051","ZNF354A",70.2673067342882,-0.232984496798329,0.255257575808136
,-0.91274273079147,0.361377864555207,0.796345408051824
"7052","ZNF354B",50.7461903741723,0.226942796142641,0.24920113480182,0
.910681230738056,0.362463356346982,0.796345408051824
"7053","RIPK1",124.12015930192,-0.188945098728061,0.206792465156912,-0
.913694309822609,0.360877494792267,0.796345408051824
"7054","DEF6",5.71321174776606,-0.674493508749719,0.733579461075009,-0
.919455279951944,0.357857486326268,0.796345408051824
"7055","MAP7",4.0015184473577,-0.773251643898469,0.846359615556598,-0.
913620675757254,0.36091619834522,0.796345408051824
"7056","SF3B5",1158.44335113015,-0.238986830518247,0.261555151995521,-
0.913714865468756,0.360866690794108,0.796345408051824
"7057","RBAKDN",4.56665163081027,-0.701888132970156,0.769969744928103,
-0.911578847861996,0.36199046139289,0.796345408051824
"7058","AC005082.1",5.06128688723087,0.872344317592852,0.9550998363002
74,0.913354064609634,0.361056356334335,0.796345408051824
"7059","FIGNL1",34.4425925591741,0.391888646655481,0.427424471801225,0
.916860574229662,0.35921570592313,0.796345408051824
"7060","DBF4",37.0731113980985,-0.280811392418055,0.306106545725188,-0
.917364872916366,0.358951473396893,0.796345408051824
"7061","ALKBH4",103.433886852009,-0.210724957002219,0.230825268612833,
-0.912919795430507,0.361284725550837,0.796345408051824
"7062","AC073073.2",3.47501244256042,-0.698597016457114,0.760014947445
16,-0.919188522285639,0.357996973534165,0.796345408051824
"7063","AC018638.6",3.43399942265682,-0.840784243875838,0.918596748117
397,-0.915291988131864,0.360038364014341,0.796345408051824
"7064","UBE2H",485.856558321512,0.142094379040575,0.155260609602829,0.
915199157107946,0.360087087068604,0.796345408051824
"7065","PKIA",61.8057731740789,-0.282572775304438,0.309618023239031,-0
.912649633081232,0.361426841568734,0.796345408051824
"7066","ZNF34",55.7124634951996,-0.231263309118066,0.253343985385219,-
0.91284310052367,0.361325066524989,0.796345408051824
"7067","AL359644.1",5.70017724393883,0.638823215609497,0.7004258921102
58,0.912049686919534,0.361742562159017,0.796345408051824
"7068","AL356481.1",12.0528047569769,0.419723233917749,0.4582911563157
3,0.915844061430218,0.359748689749896,0.796345408051824
"7069","SAMD8",192.762427144561,0.223692764870665,0.244899951694042,0.
913404691684579,0.361029738958903,0.796345408051824
"7070","ANXA11",1283.68345352311,-0.154796342203447,0.168677351376982,
-0.917706739759554,0.358772418224775,0.796345408051824
"7071","AL133482.1",25.6974236720909,0.572559296854339,0.6291270250743

32,0.91008536278773,0.362777493239386,0.796345408051824
"7072","CTR9",290.812147799684,0.192612150579228,0.210613478914498,0.914529077492814,0.360438906125888,0.796345408051824
"7073","BTBD10",121.176468378095,-0.158283780394503,0.173346624448443,-0.913105639628885,0.361186984541798,0.796345408051824
"7074","RRAS2",584.863918654582,0.254425947468582,0.278261037126088,0.914342698123755,0.36053680121302,0.796345408051824
"7075","PIK3C2A",206.389719054954,0.177084132875069,0.194136758916524,0.91216178668777,0.361683556729091,0.796345408051824
"7076","AC084117.1",4.3618396139845,0.897446803131507,0.986013287595604,0.910177189721179,0.362729071696068,0.796345408051824
"7077","TRAF6",63.1275403570995,0.223814475067908,0.244414747821812,0.915715917564342,0.359815914113643,0.796345408051824
"7078","NAA40",33.4631938375361,0.317743131276924,0.346937015674173,0.915852494607649,0.35974426597565,0.796345408051824
"7079","B4GAT1",248.367334653584,0.209715581784425,0.227996854770931,0.919817872027783,0.357667942290753,0.796345408051824
"7080","ENDOD1",146.866418343976,-0.228361792789197,0.249838178309457,-0.914038816382743,0.360696449771887,0.796345408051824
"7081","STAT2",251.906909045802,0.23604272214045,0.258492046593503,0.913152745901092,0.361162212582351,0.796345408051824
"7082","MAPKAPK5",107.34420744365,-0.156577431820594,0.172040563822513,-0.910119266884804,0.362759614694211,0.796345408051824
"7083","NFATC4",111.834587437565,0.207171156670669,0.226240582455664,0.915711736691924,0.359818107535197,0.796345408051824
"7084","L2HGDH",23.4489841443087,-0.32160283770305,0.350238531844728,-0.91823945243588,0.358493518152738,0.796345408051824
"7085","ABCD4",204.629493203278,0.170454415463461,0.187123579399408,0.910918955326481,0.362338077371506,0.796345408051824
"7086","PLA2G4B",15.0399520140264,0.372908627316969,0.409486578398694,0.910673626410995,0.362467364214839,0.796345408051824
"7087","CDAN1",20.4932077983181,0.319575718150219,0.350099045462504,0.912815165571326,0.361339760812465,0.796345408051824
"7088","ZSCAN29",64.0183395030361,0.272370835212872,0.29841025726264,0.912739520790503,0.36137955320911,0.796345408051824
"7089","PDIA3",1847.3167513091,-0.191789705290143,0.209369744939014,-0.916033524070098,0.359649311968136,0.796345408051824
"7090","LOXL1-AS1",76.6591864817738,0.259417635088341,0.284055040830158,0.913265380998651,0.361102985043391,0.796345408051824
"7091","NEIL1",24.0096199405229,0.456895018120906,0.497649869404178,0.918105371288319,0.358563703078396,0.796345408051824
"7092","LUC7L",188.886232445124,0.168641383572651,0.18449637973676,0.914063375190721,0.360683545808397,0.796345408051824
"7093","IL32",618.511606381785,0.237582095785826,0.260106307531757,0.913403823384093,0.361030195460782,0.796345408051824
"7094","ZNF213-AS1",24.5373213744877,-0.351643244285466,0.383542337748943,-0.916830320087485,0.359231561780257,0.796345408051824
"7095","AC027277.1",5.91854246506347,-0.772297720710362,0.840026533315

323,-0.919373007971954,0.357900502580563,0.796345408051824
"7096","NPIPA1",81.4673668971826,0.358577758342579,0.392475930126482,0
.913629934521135,0.360911331608678,0.796345408051824
"7097","VPS35L",150.026498894565,0.160175471783405,0.17475961696606,0.
916547395583458,0.359379860632193,0.796345408051824
"7098","AKTIP",161.794244935092,-0.20147527212611,0.220636066932981,-0
.913156561059026,0.361160206336983,0.796345408051824
"7099","ARL2BP",532.597514959042,-0.174515564160195,0.19157239439448,-
0.910964049448782,0.362314316186932,0.796345408051824
"7100","AC009053.2",6.47684316871715,0.608023743575561,0.6615211421529
9,0.919129722138108,0.35802772464952,0.796345408051824
"7101","SPG7",334.587870128408,0.210607266280466,0.229016478984088,0.9
19616209343167,0.357773353117679,0.796345408051824
"7102","NXN",21.6312212248811,0.505341090394517,0.549821813284447,0.91
9099748654537,0.358043400726778,0.796345408051824
"7103","PSMD11",421.29100283575,-0.133574015462861,0.14652767979296,-0
.911595786213212,0.361981541416652,0.796345408051824
"7104","MRPS23",217.378748001411,-0.220967035410531,0.242152708582997,
-0.912511103854929,0.361499726980525,0.796345408051824
"7105","TACO1",109.101690192614,-0.23021534310638,0.251593337821973,-0
.915029567552697,0.36017610809878,0.796345408051824
"7106","GGA3",105.73388889722,0.227302203768876,0.249789280219581,0.90
9975814690938,0.362835264677541,0.796345408051824
"7107","MAFG",285.57188600072,-0.184209068542056,0.201387195538662,-0.
914700996999043,0.360348620822481,0.796345408051824
"7108","PMAIP1",14.1274220058634,-0.487305270932157,0.5344625327872,-0
.911766945366366,0.361891414304185,0.796345408051824
"7109","HSBP1L1",43.8083184068855,-0.318752194300987,0.349215314425371
,-0.91276694100741,0.361365128682396,0.796345408051824
"7110","R3HDM4",100.984440269573,-0.218173413715461,0.239757818212135,
-0.909974136995286,0.362836149474252,0.796345408051824
"7111","AC008760.2",29.4358365354374,-0.502202587953756,0.546081290908
87,-0.91964803833128,0.35775671453134,0.796345408051824
"7112","LINC00663",11.2167508400964,0.434757823666511,0.47397872491544
7,0.917251768513592,0.359010724990748,0.796345408051824
"7113","THAP8",37.5229988468889,0.323816333613258,0.353428560068726,0.
916214392946313,0.359554457915582,0.796345408051824
"7114","SPACA6",52.9250040092322,0.346324534373301,0.378374032527588,0
.915296782022295,0.360035848017654,0.796345408051824
"7115","ZNF343",29.7036904384002,0.321030497597245,0.351327485155876,0
.913764254609373,0.360840732810131,0.796345408051824
"7116","NECAB3",84.7722614932477,-0.275011953822471,0.301822956945024,
-0.911169768549326,0.362205930196686,0.796345408051824
"7117","CTNNBL1",266.804056374437,-0.186256127556238,0.203729982563935
,-0.914230321979173,0.360595834437617,0.796345408051824
"7118","OSER1",268.434796042328,-0.273916669430435,0.299818854324888,-
0.913607218089142,0.360923272249,0.796345408051824
"7119","ZMYND8",204.180903303674,0.198004989180321,0.216809092614649,0
.913268843075003,0.361101164657058,0.796345408051824
"7120","BTG3",259.073034619666,-0.210666779975957,0.231323470659103,-0

.910702140927206,0.362452335754866,0.796345408051824
"7121","EVA1C",58.3369005507204,-0.296069813979165,0.323353918323312,-0.915621544078937,0.359865427555914,0.796345408051824
"7122","C2CD2",181.156265842221,0.170850410259404,0.18649834274098,0.916096131195607,0.35961647679276,0.796345408051824
"7123","CABIN1",124.31478064848,-0.214935441736519,0.236164215430587,-0.91011011699045,0.3627644396215,0.796345408051824
"7124","APOL2",155.069595468483,0.20223209341789,0.222022700837907,0.910862234603361,0.362367966279469,0.796345408051824
"7125","ACSL4",292.751916280154,0.187676563587328,0.204998410254734,0.915502531722651,0.359927873984509,0.796345408051824
"7126","OCRL",110.365966263805,0.178010420737735,0.194249591307527,0.91640049041811,0.359456878207612,0.796345408051824
"7127","EIF5B",842.207624886342,-0.202262057148385,0.222316458543116,-0.90979344702524,0.362931451119995,0.796442808407005
"7128","ACVR1",283.515126383563,-0.175167013281108,0.192577524362693,-0.909592195978203,0.363037615776407,0.796463784133819
"7129","VKORC1",2140.66373429399,-0.239896721148676,0.263743844181956,-0.909582257332885,0.363042859148977,0.796463784133819
"7130","CNKSR2",58.6573634115496,-0.473316156421323,0.520439427846822,-0.90945483969103,0.363110085605065,0.796499542617562
"7131","UBE2J2",243.268464236331,-0.193329362376521,0.212602993872595,-0.909344496307406,0.363168309855494,0.796515547067722
"7132","PLCB3",79.4986463538367,-0.217335230985646,0.239121555220416,-0.90889017004474,0.363408103098195,0.796929715711689
"7133","RCAN3",13.3047554236847,-0.498431134612128,0.548528149098967,-0.908670111881897,0.363524285323075,0.796932532606347
"7134","GUCY1B1",30.9763814400513,-0.32981955333705,0.363036796673648,-0.90850171761939 1,0.363613206693024,0.796932532606347
"7135","JARID2",70.966814830392,0.223788332612266,0.246284346806629,0.908658367914768,0.36353048633796,0.796932532606347
"7136","RPA3",274.198486378288,-0.242739741721221,0.267168197193121,-0.908565256911019,0.363579652877652,0.796932532606347
"7137","PUM1",561.241782763797,0.15780222198461,0.173719600525073,0.908373157131654,0.363681102811911,0.796969657836387
"7138","AC105411.1",9.0768821951085,0.519832096654274,0.572429345664773,0.908115736188512,0.363817077304747,0.797141107634378
"7139","YIPF2",230.694658850377,-0.209272386486228,0.230468055866832,-0.908032072814243,0.363861276688096,0.797141107634378
"7140","LINC01140",338.487653162054,0.25058529014554,0.276072096818541,0.907680613264755,0.364046989545152,0.797387150682675
"7141","WDR37",44.9070089526127,0.310954090063607,0.342601352870206,0.907626567900365,0.364075552623081,0.797387150682675
"7142","IDH3B",326.97673288081,-0.169109280933481,0.186349595679128,-0.907484023870204,0.364150894135393,0.797440490657735
"7143","VAMP3",487.968003901815,-0.169785075694558,0.187308373502445,-0.906446799573227,0.364699411528381,0.797578305587605
"7144","AL358472.4",10.0335944704486,-0.439585999670564,0.48472559662138,-0.90687597835673,0.36447238547449,0.797578305587605
"7145","AC132153.1",2.91569010555132,0.864053572341415,0.9531035932676

83,0.906568371418092,0.364635093752215,0.797578305587605
"7146","FRYL",160.498149910739,0.215161477342856,0.237556850353907,0.905726258882087,0.365080760211104,0.797578305587605
"7147","CDKAL1",80.8223028602701,0.204329194719688,0.225534551996587,0.905977345425905,0.364947843516058,0.797578305587605
"7148","GUCA1B",7.70093325785488,0.526783154108191,0.580904745977947,0.906832243591601,0.364495516152402,0.797578305587605
"7149","TMEM106B",482.782417832587,0.121170922551517,0.133575765500429,0.907132533342121,0.364336715768902,0.797578305587605
"7150","SLC35B4",132.372285397092,-0.194934381845793,0.215203937260211,-0.905812339344379,0.365035188730178,0.797578305587605
"7151","CPQ",1632.51222163321,-0.197979622412871,0.218306535017657,-0.90688820834822,0.364465917374921,0.797578305587605
"7152","DCHS1",154.72752216332,-0.264757940366922,0.291855156885372,-0.907155258767305,0.364324699782382,0.797578305587605
"7153","SNX19",92.2562430687466,0.207507004482352,0.22899170052035,0.906176966286647,0.364842192579153,0.797578305587605
"7154","BAZ2A",214.242532670143,0.218099361919907,0.240561198369083,0.906627350539243,0.364603893308727,0.797578305587605
"7155","CCDC59",447.894110847039,-0.164648155843661,0.181747778263674,-0.905915645388496,0.36498050262023,0.797578305587605
"7156","UCHL3",247.115463162684,-0.227145302270408,0.250602387099086,-0.906397201159126,0.364725653683334,0.797578305587605
"7157","RBBP6",615.358581048304,0.167617847286395,0.184974209984548,0.90616874266092,0.364846544621431,0.797578305587605
"7158","CLIP3",91.7959845506035,0.273106607054275,0.301275372319966,0.906501600018689,0.364670418388892,0.797578305587605
"7159","TCEAL3",207.679174723722,-0.227198801958891,0.250741310912164,-0.906108375729438,0.364878492525109,0.797578305587605
"7160","ETAA1",91.0008151849736,0.19143110649731,0.211398771535577,0.905545028037656,0.365176716454539,0.797676514713546
"7161","WWOX",97.9207563385633,0.192936767827568,0.213111890877949,0.905330843026792,0.365290141235876,0.797812848614593
"7162","SAMM50",204.414619854613,-0.18720138319667,0.206861932165365,-0.904958110161236,0.365487579780506,0.798132609294487
"7163","ASB3",116.801819032201,-0.205005116755364,0.226604767052417,-0.904681394932628,0.365634200362661,0.798341322584394
"7164","WASHC3",527.049352055292,-0.25541075990066,0.282444372296482,-0.904286949759279,0.365843264898918,0.798574830847046
"7165","ZNF564",34.6557319096445,0.283993660565942,0.314045590195881,0.904307111552833,0.365832576900244,0.798574830847046
"7166","BSDC1",257.136550772065,0.17966809888229,0.198819552817715,0.903674192683735,0.36616818742657,0.798838115685808
"7167","PYHIN1",13.0673228477518,0.416775357292107,0.461097299122639,0.90387724691759,0.366060495299989,0.798838115685808
"7168","TAP2",98.6854096780251,-0.179932562497769,0.19910638564545,-0.903700611682919,0.366154174690936,0.798838115685808
"7169","AL138976.2",4.73333748137658,0.683126973906355,0.755936101417658,0.903683489418273,0.366163256365772,0.798838115685808
"7170","AIMP2",102.110919085814,-0.207704882675894,0.229934787185166,-

0.903320829434256,0.366355644772911,0.799135604497674
"7171","HOXA6",22.4746599738006,0.427160419953044,0.472989941673565,0.903106773141172,0.366469229618264,0.799163181108589
"7172","CDON",259.807111419992,-0.163847719932551,0.181427216455149,-0.903104413626149,0.36647048177179,0.799163181108589
"7173","IL15RA",222.241792835587,-0.293089959928796,0.324634003985348,-0.902831977952697,0.366615076578035,0.799255617184341
"7174","MTFP1",12.5224686312037,-0.422822389554711,0.46832322838681,-0.902843087692167,0.366609179405555,0.799255617184341
"7175","KCNIP3",58.8708536095107,0.328701521145594,0.364277119957388,0.902339189417235,0.36687671367707,0.79935761024006
"7176","LSM5",544.730569185849,-0.230024956325227,0.254901957049548,-0.902405611113116,0.366841441501655,0.79935761024006
"7177","EDF1",2297.12436982326,-0.234747002484005,0.26012237155476,-0.902448340298123,0.366818751971295,0.79935761024006
"7178","CORO1B",436.16518666381,-0.202088793366407,0.223980028060112,-0.902262559375027,0.366917409457378,0.79935761024006
"7179","MPRIP-AS1",3.17545720894383,-0.835422345192974,0.925860497829445,-0.902319892847258,0.366886961207119,0.79935761024006
"7180","CDC37L1-DT",8.87527158132453,-0.503427874984257,0.558146724227471,-0.901963324573928,0.367076350498006,0.799518054335947
"7181","AC018557.1",6.55082110626434,0.912355212488504,1.0115571823049,0.901931426565173,0.367093295919849,0.799518054335947
"7182","TMEM81",5.64248292321982,-0.63560487108709,0.705074459165067,-0.901471983313306,0.367337423534098,0.799715660366549
"7183","MORN2",216.615068514854,-0.261387680807184,0.289909919553678,-0.901616892618218,0.367260414285697,0.799715660366549
"7184","DLGAP1",8.12183539263663,0.666627496744471,0.739426166152562,0.901547074284803,0.367297516633197,0.799715660366549
"7185","ATP13A3",264.341739553529,0.258015669649888,0.286310044330545,0.901175752506988,0.367494880972722,0.799811789379397
"7186","INO80",166.985340070795,-0.167705968322118,0.18611243283111,-0.901100296046875,0.367534995541543,0.799811789379397
"7187","APOL3",333.712981365589,0.169575634900622,0.188159313996558,0.901234338597362,0.3674637370016,0.799811789379397
"7188","TOE1",44.7030274538223,0.241476309968815,0.268076936974462,0.900772415166092,0.367709337014125,0.800066614130525
"7189","AC007566.1",24.1507836694683,0.412968342347973,0.458503379952271,0.900687672991553,0.367754404666518,0.800066614130525
"7190","NCSTN",301.842404824671,0.209191808108188,0.232338478263758,0.900375218394547,0.3679206042495,0.8002055695261
"7191","IPPK",32.1403564011994,0.291029443075255,0.323224670175491,0.900393657814683,0.367910794729421,0.8002055695261
"7192","SRGAP2B",326.760141352494,0.284468005092423,0.31613215643317,0.899838878467774,0.368206001036632,0.800489414361216
"7193","MARS2",5.88014556521104,0.588050891446729,0.653469360819266,0.899890533061076,0.368178508638748,0.800489414361216
"7194","APEH",115.368282333983,-0.188833389388183,0.209828537009067,-0

.899941409685488,0.368151431552554,0.800489414361216
"7195","APTR",59.7462243672434,-0.275847156078103,0.306583622363714,-0
.899745243895816,0.368255839918731,0.800489414361216
"7196","RFX5",31.2322223660542,0.316657475838857,0.352224678302721,0.8
99021264962884,0.368641333991639,0.800628284654285
"7197","FLG-
AS1",8.6614856959O623,0.556119169375899,0.619578779283518,0.8975762049
48458,0.369411529549696,0.800628284654285
"7198","CATSPERE",5.57380375439216,0.607092996453143,0.676707326466918
,0.897127861202227,0.369650693317685,0.800628284654285
"7199","EFEMP1",7022.18123067652,0.207580489161367,0.230907869200569,0
.898975378708557,0.368665775314463,0.800628284654285
"7200","PRADC1",151.260489926784,-0.250259059054644,0.278455135595854,
-0.898741043217342,0.36879060988398,0.800628284654285
"7201","RFTN1",485.380982433564,0.236700752686845,0.263482431764283,0.
898354972291293,0.368996333887938,0.800628284654285
"7202","RPL29",13890.9800885572,-0.236583987292481,0.263634222874198,-
0.897394824970713,0.369508273004366,0.800628284654285
"7203","NMD3",195.19474442396,-0.18291143442565,0.203494410251245,-0.8
98852377319936,0.368731297003103,0.800628284654285
"7204","AC016575.1",6.24059284682482,-0.541489496318986,0.602127948711
484,-0.899293077954177,0.368496573421804,0.800628284654285
"7205","GPBP1",574.256506607814,0.112811785996923,0.125487064699257,0.
898991352354027,0.368657266834511,0.800628284654285
"7206","APBB3",38.3137942127935,0.296458240742659,0.330016936576943,0.
898312201239225,0.369019129511102,0.800628284654285
"7207","CCR6",2.89177521565465,0.962810311351678,1.07308963508365,0.89
7231955163399,0.369595157025654,0.800628284654285
"7208","ZNRF2",69.3036396456845,-0.256113422030198,0.285369383043384,-
0.897480378934914,0.36946263873937,0.800628284654285
"7209","FZD4",193.180296007777,0.210601746314295,0.234595580159428,0.8
97722566517121,0.369333475502831,0.800628284654285
"7210","APAF1",90.4976439932661,0.219138322788524,0.243763377430372,0.
898979678976258,0.36866348473317,0.800628284654285
"7211","C12orf76",258.062832080964,-0.211490451510375,0.23566460061005
4,-0.897421381755679,0.369494107308403,0.800628284654285
"7212","GPR137C",3.30731398260334,1.05328173593455,1.17199830019716,0.
898705856277573,0.368809356845973,0.800628284654285
"7213","ANTKMT",322.231550445458,-0.23692726578993,0.263852588429795,-
0.897953160891466,0.369210521239427,0.800628284654285
"7214","AC009060.1",4.54455771160423,0.721182931759964,0.8031190161421
74,0.897977656193731,0.36919746169729,0.800628284654285
"7215","REEP6",11.9660832584424,0.569487383661924,0.634265113425586,0.
897869631495566,0.369255056659363,0.800628284654285
"7216","AP3D1",240.005425472707,0.176049777811351,0.19597054144933,0.8
98348172686301,0.368999957804611,0.800628284654285
"7217","ANGPTL8",4.7971450853137,-0.638160188646649,0.710854085231461,
-0.897737245807425,0.369325647661087,0.800628284654285
"7218","OPA3",51.3300735000159,-0.25968161250027,0.28924802704205,-0.8
97781793555736,0.369301892871441,0.800628284654285

"7219","MACROD2",5.48724375415635,0.736883737533504,0.821337147772894,
0.89717570857669,0.369625165104728,0.800628284654285
"7220","LINC01426",32.3051635769785,0.483887180908202,0.53882372128217
4,0.89804357491306,0.369162318903918,0.800628284654285
"7221","UBE2L3",869.288294392585,-0.190076342329527,0.211356920748782,
-0.89931449443972,0.368485169059102,0.800628284654285
"7222","POLDIP3",187.289534539236,0.162180194353365,0.18080731178585,0
.896978074346093,0.36973061682016,0.800690507763404
"7223","RRAGD",47.5717127336665,0.39293329382774,0.438162419915165,0.8
9677543296346,0.369838759614124,0.800795569794789
"7224","AIF1L",483.462273595454,0.339922805701933,0.3790839644922,0.89
6695290599471,0.369881534283731,0.800795569794789
"7225","PDE4B",66.088320296339,0.303099565807491,0.338152739836577,0.8
96339228107316,0.37007161395436,0.800942306444919
"7226","OPTN",1261.33889008857,-0.140341972850862,0.156561393695588,-0
.896402168747535,0.37003800943723,0.800942306444919
"7227","ANO4",2.9334341676273,-1.057480939523,1.17998275175557,-0.8961
83387383998,0.370154826736089,0.800942306444919
"7228","TCF3",159.935373064109,0.168847008146871,0.188438711779384,0.8
96031428746711,0.370235977829228,0.800942306444919
"7229","ZNF577",50.58436690345,0.241420847311067,0.269412993006006,0.8
96099496232109,0.370199626105028,0.800942306444919
"7230","SBF1",101.737676392472,0.161762521408939,0.180539967454968,0.8
95992857920992,0.370256577723578,0.800942306444919
"7231","PPP2R5B",51.8241073329282,-0.245852274184738,0.274446017861722
,-0.89581286731808,0.370352716460161,0.801039480768486
"7232","PTPN22",4.7250460552571,0.65257491941924,0.729106543825111,0.8
95033688760543,0.370769079450326,0.801075702220726
"7233","LINC02035",14.6958414984304,0.358125710626298,0.40015469014064
4,0.894968169685645,0.370804103558723,0.801075702220726
"7234","AC007938.3",10.9085080388786,0.522418751080206,0.5838725288561
06,0.894747954838195,0.370921837438366,0.801075702220726
"7235","RPL30",20054.1003803698,-0.213869467401355,0.23895794012316,-0
.895008834153516,0.370782365575183,0.801075702220726
"7236","FBXO32",69.3733270911575,0.335148365183005,0.374664494578831,0
.894529292293239,0.371038764363058,0.801075702220726
"7237","TTC39B",83.9640348226036,0.23717915870452,0.265227365288119,0.
894248443960032,0.371188977876829,0.801075702220726
"7238","FAM66C",29.7486535595956,0.324051180315627,0.362279298886642,0
.894478876688518,0.371065726690543,0.801075702220726
"7239","AC138331.1",9.74900327059212,-0.594664463735985,0.664632768364
374,-0.894726369269188,0.370933379020396,0.801075702220726
"7240","OSGEP",171.749575840618,-0.218129508532245,0.24388144995888,-0
.894407953409427,0.371103658605937,0.801075702220726
"7241","ARHGAP5-
AS1",13.4366348619967,-0.396926632598537,0.443825018778995,-0.89433135
9891606,0.371144625839073,0.801075702220726
"7242","CHSY1",122.437808957257,-0.221541270514398,0.247466230637234,-
0.895238392502772,0.370659665526601,0.801075702220726
"7243","MGRN1",125.439874980552,0.212466318302249,0.237577204648835,0.

894304310955662,0.371159094053284,0.801075702220726
"7244","MTHFSD",39.0631175287072,0.233127381383032,0.260329026960395,0
.895510516460768,0.370514246613894,0.801075702220726
"7245","ZNF551",19.8768560396592,0.417282330838726,0.466538225063349,0
.894422596952406,0.371095826598999,0.801075702220726
"7246","Z95331.1",28.1003985607705,0.354633826445328,0.396272534410349
,0.894924062736322,0.37082768268222,0.801075702220726
"7247","RP2",87.030380694104,-0.203520215351233,0.227248699514874,-0.8
95583630558519,0.370475181588686,0.801075702220726
"7248","CHTOP",264.814255491106,0.154432237754033,0.172778739797766,0.
893815048858402,0.371420856034402,0.801096922245626
"7249","PKDCC",837.133860402002,-0.227454696658331,0.254487613730782,-
0.89377511668977,0.37144222535022,0.801096922245626
"7250","AC087672.2",13.6506941081828,-0.491434775271567,0.549784196332
63,-0.893868500676654,0.371392253001925,0.801096922245626
"7251","CRTAM",3.09785896221278,0.842476337268511,0.942831304041161,0.
893559997061501,0.371557357670702,0.801096922245626
"7252","SIVA1",806.62470886876,-0.208184212185953,0.232954918885643,-0
.893667380718201,0.371499883023461,0.801096922245626
"7253","RNF112",32.8067420329365,-0.320393322414948,0.358536048245894,
-0.89361536722024,0.37152772137038,0.801096922245626
"7254","UBA2",399.508741549174,-0.123270289282203,0.137928087100205,-0
.893728694958605,0.371467068451672,0.801096922245626
"7255","AL133338.1",8.62646610257824,-0.445834587859155,0.499004452164
872,-0.89344811639446,0.371617245106369,0.801115604888162
"7256","MOB3A",133.733146456963,0.149993166176702,0.167907502498997,0.
893308303347543,0.371692092589537,0.801166528128494
"7257","S100A11",12000.2807853006,-0.225380976191627,0.252348506710695
,-0.893133782043795,0.371785533900619,0.801179510180323
"7258","TRABD",101.558884978492,-0.193108158341292,0.216220943661393,-
0.893105705077784,0.371800568087518,0.801179510180323
"7259","TATDN2",73.114195571705,-0.19543481530834,0.218919517967414,-0
.892724491278253,0.372004731421694,0.801183857320296
"7260","HCG27",7.66355418983567,-0.502354472526433,0.562956157266499,-
0.892350969151266,0.37220484279765,0.801183857320296
"7261","INMT",124.741863354356,0.434604399890467,0.487040729804908,0.8
92336868960742,0.372212398164276,0.801183857320296
"7262","TERF1",360.927822460325,0.122664586236639,0.137378916033655,0.
892892372266126,0.371914812308669,0.801183857320296
"7263","EMC2",418.672435993136,-0.18743564454308,0.20990621449925,-0.8
92949477414114,0.371884229164033,0.801183857320296
"7264","SYT7",2.49138546426323,-0.833614888651039,0.934007997758796,-0
.892513651544038,0.372117678856869,0.801183857320296
"7265","SLC39A3",194.794725875854,-0.213283095099483,0.239007451730229
,-0.892370064428862,0.372194611043263,0.801183857320296
"7266","AC011447.3",6.39216558351003,0.599807934904884,0.6720513806214
25,0.892503091579486,0.372123336418386,0.801183857320296
"7267","LINC01943",20.6402527653521,-0.358279587160842,0.4016670255239
45,-0.891981577759569,0.372402806774783,0.80130639922988
"7268","CDKL3",40.3112727543616,-0.282090537909828,0.316434419325475,-

0.891466037453017,0.372679203836199,0.80130639922988
"7269","CEP162",150.605463596768,0.184179007569102,0.206496121798376,0
.891924777884862,0.372433252711116,0.80130639922988
"7270","ENTPD4",166.421304724983,0.232522911679345,0.260819033324532,0
.89151051867454,0.372655351072612,0.80130639922988
"7271","PHPT1",1507.49594978361,-0.218088571873538,0.244588079115242,-
0.891656587117566,0.372577029480135,0.80130639922988
"7272","CD44-
AS1",4.03302981445816,2.31692667751208,2.59865343056645,0.891587408409
071,0.372614121686941,0.80130639922988
"7273","USP36",130.509245811412,0.25182037640383,0.282280906435761,0.8
92091426173511,0.372343930073576,0.80130639922988
"7274","NXT2",57.5427651973376,-0.235692060417592,0.264300216177295,-0
.891758863562517,0.372522195136552,0.80130639922988
"7275","AC107464.3",2.53172805277786,0.942763735615699,1.0577390614166
4,0.891300860491098,0.372767787177299,0.801386692983224
"7276","PEX19",135.830977840975,0.205205865904941,0.230425279361287,0.
890552748699049,0.373169158013449,0.801447354601147
"7277","ACTRT3",8.36734429755151,-0.474161759421696,0.532115326034293,
-0.891088334093835,0.372881782901982,0.801447354601147
"7278","CASP8AP2",205.430539113025,0.166583142947624,0.187090355588952
,0.890388723797266,0.373257195071276,0.801447354601147
"7279","TOMM5",875.537139464376,-0.224474155569242,0.252094704625596,-
0.890435822135277,0.373231914672249,0.801447354601147
"7280","RPUSD4",86.8699002864791,-0.22666243048391,0.254461970606467,-
0.890751690493075,0.373062397274245,0.801447354601147
"7281","GNB3",7.83563211608486,0.679582276210534,0.763107422907326,0.8
90546017258523,0.373172770725358,0.801447354601147
"7282","CLPX",169.301073221675,0.148617092466157,0.16684355828659,0.89
0757149945672,0.373059467763416,0.801447354601147
"7283","DDRGK1",391.745473407923,-0.167911562047503,0.188467225110652,
-0.890932425778114,0.372965423331788,0.801447354601147
"7284","ELMO2",104.459924337804,0.188713540210698,0.211789751262985,0.
89104188982387,0.37290669773249,0.801447354601147
"7285","PAQR7",31.9258516567982,-0.375040943243031,0.421290716690247,-
0.890218864041048,0.373348377422177,0.8015330985426
"7286","FEZ2",499.657437273945,0.131955445915884,0.148246488848418,0.8
90108406215331,0.373407679644366,0.801550385621449
"7287","AL513477.2",4.84142307574789,0.628811987866217,0.7069750436580
93,0.889440148569547,0.373766575879319,0.801630824821572
"7288","FAM76A",227.439058252945,-0.205697232916171,0.231333673506787,
-0.889179814585604,0.373906449301366,0.801630824821572
"7289","KLHL12",61.5728351054196,0.234025276113616,0.263070651860403,0
.889590969036716,0.373685557190617,0.801630824821572
"7290","AC025171.4",5.19738184355119,0.751521542938682,0.8448127031011
09,0.889571783402432,0.373695862847119,0.801630824821572
"7291","RASGRP2",8.34690572717523,-0.656148594081382,0.737621188920446
,-0.88954683506543,0.373709264230404,0.801630824821572
"7292","SS18",287.27404487719,0.121100627765349,0.136190941640343,0.88
9197374706127,0.373897013501794,0.801630824821572

"7293","U2AF1L4",68.7403083737676,0.204778248883146,0.230104331477212,
0.889936523873847,0.373499970847953,0.801630824821572
"7294","MED15",181.795203357229,-0.155069168668424,0.174322001371363,-
0.889555922078221,0.373704382967158,0.801630824821572
"7295","RTL3",6.4197857777208,-0.658002056342033,0.739951776274145,-0.
889249917954451,0.373868780659834,0.801630824821572
"7296","MIDN",654.915169128712,-0.28061075279221,0.315634983123227,-0.
889035651294255,0.373983919938712,0.801687021359849
"7297","ZFP90",138.648553077979,0.17094226876426,0.192349076774984,0.8
88708548178962,0.374159735816771,0.801953990430902
"7298","COPG1",273.727317194221,-0.171732964700169,0.193321137033759,-
0.88832999502884,0.374363269576003,0.802040356751891
"7299","C12orf4",64.4460539214741,0.198229631565359,0.223179950679086,
0.888205374014065,0.374430288575754,0.802040356751891
"7300","FASN",60.8585270756158,0.286341317536962,0.322346910080834,0.8
88301728920425,0.374378469943674,0.802040356751891
"7301","ERGIC3",721.556787370544,-0.141959438946758,0.159810357486306,
-0.888299364194358,0.374379741614828,0.802040356751891
"7302","PHF5A",266.080422740667,-0.215491054269206,0.242627276474412,-
0.888156753850929,0.374456437659994,0.802040356751891
"7303","LINC01117",19.8576024733375,0.421179798768335,0.47440317605558
8,0.887809820900068,0.374643059257328,0.80233019947756
"7304","AGO4",78.9446631502009,0.264183886216353,0.298037334369519,0.8
86412055641349,0.375395525186004,0.80240462969418
"7305","SRGAP2C",398.287625780873,0.203172398988031,0.229276520390448,
0.886145684006533,0.375539028192512,0.80240462969418
"7306","RNF2",91.5239624694518,-0.181449743870785,0.204707365919001,-0
.886386003044863,0.375409559066818,0.80240462969418
"7307","AC005674.2",6.01030364230172,-0.553033480267012,0.624170069033
038,-0.886030118560106,0.375601297569763,0.80240462969418
"7308","EXOC1",350.153962360153,0.174744028279721,0.197217040943645,0.
886049336525912,0.375590942036325,0.80240462969418
"7309","NUDT6",42.5417289235247,0.257765494305605,0.290672533584415,0.
886789993973569,0.375191975730993,0.80240462969418
"7310","LINC01060",11.2582269271323,-0.870321893313598,0.9818374303610
04,-0.886421587119158,0.375390390898753,0.80240462969418
"7311","TTYH3",65.1855713447918,0.261637985492735,0.29483015668476,0.8
87419348260514,0.374853170416969,0.80240462969418
"7312","GATAD1",523.807303420257,0.185804415491647,0.209668762971167,0
.886180720764774,0.375520150823788,0.80240462969418
"7313","FAM200A",51.2494933727558,0.242050044725917,0.273075009680774,
0.886386656211691,0.375409207218256,0.80240462969418
"7314","ARF5",624.992344311164,-0.3549701993716,0.400261374105692,-0.8
86846001977367,0.375161816827925,0.80240462969418
"7315","AL160153.1",4.58688507886603,-0.627399459766469,0.707461996382
199,-0.886831325180501,0.375169719769504,0.80240462969418
"7316","TDP1",26.8358526285228,-0.350721847777176,0.395526536831722,-0
.886721408344827,0.375228909409438,0.80240462969418
"7317","PRSS36",25.1718062321365,-0.466260852340756,0.525983509369968,
-0.886455267198874,0.375372248917461,0.80240462969418

"7318","CNN2",250.28577715506,0.223605997481571,0.252183424966598,0.88
6679992989974,0.375251212857392,0.80240462969418
"7319","UCKL1-
AS1",3.91983067462863,0.818540647832085,0.922150758424836,0.8876429806
66489,0.374732826150565,0.80240462969418
"7320","DNAL4",111.242131190084,-0.248632086609462,0.280338428888188,-
0.886899764672033,0.375132868376516,0.80240462969418
"7321","CCDC22",82.8966512460015,-0.237990938481403,0.268380562754539,
-0.886766672067344,0.375204534433368,0.80240462969418
"7322","RPA2",282.231604579958,-0.214423980733933,0.242054735449645,-0
.885849146209081,0.375698822534496,0.802443083652279
"7323","ANKRD11",910.839799323123,0.203550398530333,0.229791092533083,
0.885806304702726,0.375721911866089,0.802443083652279
"7324","SMG8",34.9822084668213,0.268151618018191,0.302773379792995,0.8
85651235922806,0.375805493155133,0.802512003406101
"7325","DEDD",99.54741319643,-0.202639882757285,0.228856735714543,-0.8
85444258936042,0.37591707059218,0.80264068041798
"7326","MAN1A2",439.499043633133,0.139613169779386,0.157715735054857,0
.885220296699153,0.376037827497542,0.802788919200322
"7327","PRR3",36.6323724204936,0.259269579170097,0.292969909471277,0.8
84969994488516,0.37617281483383,0.802967493380797
"7328","RASA2",149.667985836125,0.181962414696931,0.205673102074422,0.
884716634609267,0.376309481617445,0.803008767888522
"7329","AP002813.1",3.26726181946926,0.851013002761317,0.9619785684016
51,0.884648609350304,0.37634618085824,0.803008767888522
"7330","POLR2E",858.151304110267,-0.188915784674392,0.21351417266664,-
0.88479271570111,0.376268438916998,0.803008767888522
"7331","ADPRHL2",189.989059586604,-0.223651616208716,0.253009324330651
,-0.883965904420315,0.376714618592496,0.803189152196431
"7332","LINC00623",279.559317626457,-0.16010183850281,0.18121146534030
5,-0.883508326595936,0.376961685697266,0.803189152196431
"7333","ISG20L2",149.456227061778,-0.204708268391486,0.232129812905256
,-0.881869785829871,0.377847227784654,0.803189152196431
"7334","ACBD3",524.394270967103,-0.15463543141467,0.175429355676574,-0
.88146838833382,0.378064356569186,0.803189152196431
"7335","GTF3C2",85.145450683851,0.173751013087636,0.197113140859855,0.
881478588031686,0.378058838273881,0.803189152196431
"7336","MTA3",94.752184043343,0.184326119898719,0.209121343371907,0.88
1431406888529,0.378084364885335,0.803189152196431
"7337","FAM178B",7.01082737315863,-0.513204958597862,0.582264459198907
,-0.881394958064143,0.378104085672261,0.803189152196431
"7338","DCAF17",39.5308277126767,0.257773791580881,0.291851826984,0.88
3235147933519,0.377109234923281,0.803189152196431
"7339","ICA1L",70.868337077317,0.25154372912837,0.284482488110657,0.88
421516135828,0.376580075457295,0.803189152196431
"7340","DIRC3-
AS1",17.2712608612975,0.569274544384719,0.645689958849073,0.8816530853
28192,0.377964438494735,0.803189152196431
"7341","NCL",1742.74944567351,-0.184619111221446,0.208802380093643,-0.
884181066990946,0.376598477058532,0.803189152196431

"7342","TMPPE",3.7398572784504,0.782232079235588,0.886189385637121,0.882691772112804,0.377402828849272,0.803189152196431
"7343","LRIG1",38.7366866290742,-0.308492151387207,0.349711030418078,-0.882134461181867,0.377704098435362,0.803189152196431
"7344","FAM162A",455.540039983623,-0.23042402306592,0.261442766923201,-0.881355509573563,0.37812543015488,0.803189152196431
"7345","NSD2",68.1452697402316,0.266936305910701,0.302624503390005,0.882071025050768,0.377738399953469,0.803189152196431
"7346","TTC33",103.917699930091,0.184351648607765,0.208863186822335,0.882643090017486,0.377429139374562,0.803189152196431
"7347","DCTN3",913.532463072814,-0.234858699386468,0.266458490269407,-0.881408204140954,0.378096918754426,0.803189152196431
"7348","PGM5",10.8790190099264,0.504073855193708,0.570287346558457,0.883894510785956,0.376753160688709,0.803189152196431
"7349","NR4A3",56.215185745459,-0.543037717411214,0.615836849115395,-0.881788282385583,0.377891309395381,0.803189152196431
"7350","TM9SF3",906.411140573891,0.117558687438779,0.132998339633397,0.883910940263036,0.376744290964195,0.803189152196431
"7351","NEDD8",1689.24306898011,-0.207139037468044,0.234676046524201,-0.882659481169851,0.37742028055343,0.803189152196431
"7352","CDKL1",39.8423082892059,0.331804206151129,0.376032418280638,0.882381917144972,0.377570311094617,0.803189152196431
"7353","CPSF2",147.449754290642,0.185704635390535,0.210182599838264,0.883539529596816,0.376944834602031,0.803189152196431
"7354","COA8",373.031549616225,-0.1994011015814,0.225685070544927,-0.883536961926354,0.376946221247896,0.803189152196431
"7355","MFAP1",201.171031834771,-0.146266512394961,0.165675593880034,-0.882848879364047,0.377317927021282,0.803189152196431
"7356","EME2",13.6136001312764,0.443914539804758,0.502441037897431,0.883515689049626,0.376957709582424,0.803189152196431
"7357","CPPED1",34.5601395261191,-0.267948098532576,0.303325991233922,-0.883366761425786,0.377038143414916,0.803189152196431
"7358","VPS9D1",29.5920906089624,-0.306641047366574,0.347569160512594,-0.882244693155,0.377644497789288,0.803189152196431
"7359","ITGAE",290.635836246649,-0.220006116802145,0.249376703755356,-0.882224014870196,0.37765567776199,0.803189152196431
"7360","AC067852.3",13.0888911016948,0.51469106298688,0.583296152636858,0.882383778223395,0.377569305011168,0.803189152196431
"7361","AC080038.1",39.5861122292808,-0.263315106809047,0.298446438242214,-0.882285975198488,0.3776221787474,0.803189152196431
"7362","BCL2L13",135.000732913165,0.166748659404763,0.188982861173622,0.882348051930314,0.377588618635328,0.803189152196431
"7363","WDR45",452.995718997657,-0.225721795237991,0.255812160836249,-0.882373201102352,0.377575022936436,0.803189152196431
"7364","AL392172.1",39.8338585210543,-0.289382854150298,0.328420687356245,-0.881134670534315,0.378244933731343,0.803268030230967
"7365","USP47",388.554805409323,0.183450817216397,0.208244551156891,0.880939338855427,0.378350653782772,0.803268030230967
"7366","FADD",122.235689766559,-0.207516851391383,0.235571727872309,-0.880907285715827,0.378368003753934,0.803268030230967

"7367","AC107982.3",3.22912257894123,-0.795108859843331,0.902549961584323,-0.880958277863763,0.378340402560197,0.803268030230967
"7368","RBM27",357.408723492676,0.154542336877558,0.175473760679648,0.880714793362731,0.378472207807681,0.80327118063674
"7369","AC010542.2",11.7583551690235,0.492722839978941,0.559415042347924,0.880782250528929,0.378435688449196,0.80327118063674
"7370","TENM3",42.1313043279883,0.480685518007763,0.545902903247795,0.880532994325497,0.378570639350701,0.80337107183785
"7371","AC006213.1",2.65086730216439,-0.776319589729297,0.882020151229922,-0.880160831526092,0.378772188787227,0.803689734450174
"7372","DOCK5",7.64534449568739,-0.681939362977421,0.775039149633223,-0.879877311101174,0.378925777146184,0.803906559219522
"7373","SECISBP2",272.472350968954,0.152360407450237,0.173180031654496,0.87978045733474,0.378978253438515,0.803908840875949
"7374","CCNT1",141.40229825405,0.200932626974809,0.228471347695838,0.879465320274248,0.379149028641637,0.804162029828479
"7375","AMD1",793.986309405382,0.263591709612727,0.299757747162581,0.879349114769541,0.37921201324693,0.804186560973829
"7376","TAF7",787.840301651584,0.107657912188118,0.12249795506334,0.878854770534341,0.379480024947702,0.804427702654116
"7377","HOOK3",745.412581950035,0.175339873652115,0.199468243668488,0.879036534474761,0.379381466994597,0.804427702654116
"7378","RTL6",59.8586934996455,0.236442824233049,0.269018022088709,0.878910722773369,0.379449684259445,0.804427702654116
"7379","AC146507.3",5.48005307082761,0.63573314875455,0.723637611304963,0.878524193357098,0.379659314335522,0.804589658022704
"7380","POMP",1366.54652169095,-0.215792086587878,0.245609560232812,-0.878598074046182,0.379619240433628,0.804589658022704
"7381","SELL",4.83969493908732,0.735499869937375,0.837406706256228,0.878306639345599,0.3797773337303,0.804730727481628
"7382","AL357054.4",6.32032124605968,0.786104876857235,0.895287703037032,0.87804721788379,0.379918094994344,0.804820835467291
"7383","MAPT",13.8500519869747,0.398477758234524,0.453827158112497,0.878038590488555,0.379922776742648,0.804820835467291
"7384","PTPRG-AS1",12.2898404013716,0.446117917195827,0.508424040179669,0.877452445085358,0.380240937866802,0.804949684385055
"7385","MYLK-AS1",8.76691759493632,-0.4634238858028,0.528141931409647,-0.877460883603562,0.380236356257679,0.804949684385055
"7386","WNK1",672.576820208523,0.199327104964654,0.2271195134813,0.877630908543954,0.380144050143507,0.804949684385055
"7387","VPS33B",34.8234382736642,0.304775594505014,0.347229122684103,0.877736268631732,0.380086857327457,0.804949684385055
"7388","AKAP10",95.2110209078147,0.211777890142148,0.241353011075849,0.877461147876848,0.380236212773688,0.804949684385055
"7389","AL021707.2",8.19552635777393,0.511764766150339,0.583408617503936,0.877197817783152,0.380379201171139,0.805133401856356
"7390","DOLK",85.9587998406113,-0.2383863119418,0.271832208711047,-0.876961244115115,0.380507688982575,0.805187424663438

"7391","LIPC",42.660810959941,-0.260803787711058,0.297373364118969,-0.877024707588536,0.380473218105453,0.805187424663438
"7392","STOML2",495.231663810517,-0.21096113919153,0.240619952402222,-0.876740008820572,0.380627870344672,0.805332777623196
"7393","DAGLB",39.480992605184,0.28695688324451,0.327370393800942,0.876551113595796,0.380730502103195,0.805353714611732
"7394","TNFRSF10B",69.4104507089376,-0.256566701030335,0.292738163568212,-0.876437489061968,0.380792245495765,0.805353714611732
"7395","AL139317.5",56.8925311554354,-0.342083005637669,0.39030146906061,-0.876458411650372,0.380780875732315,0.805353714611732
"7396","MZT1",130.32520084507,-0.201815288956212,0.230307008692353,-0.8762880908492,0.38087343760676,0.805416517600018
"7397","ZFAND1",171.393632241219,-0.222616745530331,0.25417117940940 6,-0.875853611914637,0.381109620396664,0.805807011356472
"7398","C1orf43",585.832655410463,-0.188312474778557,0.215180031802061,-0.875139171611339,0.381498185650776,0.805958215515845
"7399","COX7A2L",933.23139956802,-0.210565481777909,0.240551595069332,-0.875344358939792,0.381386564792755,0.805958215515845
"7400","SNRNP200",337.364145559418,0.175978696216727,0.201071059965737,0.875206487928768,0.381461563708677,0.805958215515845
"7401","AL355304.1",5.15232845316493,-0.600261180563597,0.685964475509458,-0.875061613238485,0.38154038222971,0.805958215515845
"7402","COPS5",430.605161644198,-0.199047318431135,0.227346457357881,-0.875524170221844,0.381288764855145,0.805958215515845
"7403","RANGRF",167.495122113688,-0.221107997000659,0.252613277734325,-0.875282562277663,0.381420179751623,0.805958215515845
"7404","PPFIA3",2.94411804404371,0.843661378585543,0.964119528665175,0.875058904525659,0.381541855989726,0.805958215515845
"7405","PIF1",9.26946074258799,0.515848331891477,0.589784293153161,0.874638978826648,0.381770371959791,0.806236489467177
"7406","ZNF23",50.5617584597468,-0.216648152819282,0.247703376674198,-0.874627369752157,0.381776690600634,0.806236489467177
"7407","SCAF11",1076.80524748828,0.145786022435987,0.166777986835067,0.874132283298036,0.382046218257117,0.806696753549522
"7408","AC010538.1",3.47672675348048,0.803579707196231,0.919427476952592,0.874000100431701,0.382118199034904,0.806739826256196
"7409","IPO13",50.7740929001211,-0.207674104040143,0.237769469046307,-0.873426285019366,0.382430769219868,0.806780889785957
"7410","NASP",475.672412313326,0.175609202131067,0.201079879009261,0.873330553988343,0.382482931321793,0.806780889785957
"7411","NAXE",316.441524080472,-0.241589608929282,0.276698737216173,-0.873114244610876,0.38260081043719,0.806780889785957
"7412","ARL8B",274.368381375653,0.137942951882679,0.157905048701726,0.873581642998924,0.38234612675757,0.806780889785957
"7413","SKIL",372.622212759296,-0.335345987236818,0.383825670329651,-0.873693484202879,0.382285200307851,0.806780889785957
"7414","RABEPK",136.618907118985,-0.171277980003997,0.19614479557134,-0.873222149509959,0.382542004220683,0.806780889785957
"7415","SNAPC1",134.72085275558,-0.161743588328672,0.185144841468574,-0.873605697278508,0.382333022481125,0.806780889785957

"7416","H1F0",451.053725203207,0.147884475960686,0.169240043416947,0.873814925681339,0.3822190507683,0.806780889785957
"7417","SUN2",249.365372000713,-0.195213185783738,0.223583153904698,-0.873112228602642,0.382601909177906,0.806780889785957
"7418","LSS",72.320556423058,0.299224278590607,0.342777713266939,0.872939712849959,0.382695938815532,0.806870380570899
"7419","RWDD1",1340.4884138548,-0.179566442147052,0.205915589786887,-0.872039083261712,0.383187056521301,0.807502202701909
"7420","ARHGDIB",68.8130577472047,-0.283156811551176,0.324716851129921,-0.872011448022707,0.383202132254065,0.807502202701909
"7421","ZC3H7A",230.799755124765,0.182540356188748,0.20930275736832,0.872135458146513,0.383134484388749,0.807502202701909
"7422","HNRNPL",319.596055421945,-0.182998676059177,0.209790836616681,-0.87229108292057,0.383049600995728,0.807502202701909
"7423","TICAM2",100.028399607835,-0.193972758612049,0.222471152729542,-0.871900721653838,0.383262539976569,0.80752069584178
"7424","COG1",75.1414756508358,0.208324216071564,0.238961911542413,0.871788372995957,0.383323838713779,0.807541061083446
"7425","ATR",116.19243528941,0.217545205194847,0.249598119654251,0.871581907332458,0.383436504441761,0.807560857725443
"7426","TRIM25",209.151121376237,0.227527823243807,0.261042269930623,0.87161295105301,0.383419562976894,0.807560857725443
"7427","GNL1",333.172071855273,-0.128912442935836,0.147925316704361,-0.871469778181897,0.383497700411713,0.807580992923008
"7428","SLAMF7",3.8372982648022,0.999952557204485,1.14854597857153,0.87062475152118,0.383959077251693,0.807716758042408
"7429","AC133644.2",2.80556303728978,-0.892264925524238,1.02487032678406,-0.870612507949251,0.383965764625933,0.807716758042408
"7430","NGLY1",137.1172229879,-0.166300502548072,0.190894566955097,-0.87116414678889,0.383664533159395,0.807716758042408
"7431","PPRC1",66.1061421613567,-0.217572133465245,0.249954729603804,-0.870446155630309,0.384056632446635,0.807716758042408
"7432","MRPL51",1172.95250552195,-0.208719199296455,0.239819990660763,-0.870316101344939,0.384127682097161,0.807716758042408
"7433","RORA",477.503927229665,-0.221052794335183,0.253783577785397,-0.871028757117249,0.383738451517115,0.807716758042408
"7434","PSMA4",981.175598587029,-0.191914874460309,0.22041961709488,-0.870679647255255,0.383929094368364,0.807716758042408
"7435","ST20-AS1",11.7298803773959,-0.477653324479295,0.548513427295536,-0.870814278575422,0.383855567669413,0.807716758042408
"7436","CMTM3",1273.98859206543,0.18791612721734,0.215918268350417,0.870311385196774,0.384130258716076,0.807716758042408
"7437","AC011481.2",2.72135337833324,0.875491356039652,1.00522395174843,0.870941599149995,0.383786041545177,0.807716758042408
"7438","AP1B1",94.7359324496955,-0.198523003627598,0.228044786670059,-0.870543924842388,0.384003225674215,0.807716758042408
"7439","RCC1",36.7927456349473,-0.278892048310323,0.320495607897973,-0.870189922849444,0.384196622060552,0.807747703861679
"7440","ZNF554",13.9920105677442,0.41952826938294,0.482183177730584,0.

870059945594675,0.384267645461847,0.80778843750313
"7441","C1orf131",204.441381969913,-0.139580683102793,0.16049790399510
7,-0.86967293421507,0.384479167444813,0.80790384450123
"7442","ARHGAP15",20.0520754127006,0.470958803348986,0.541683240682727
,0.869435803026505,0.38460880723671,0.80790384450123
"7443","CYSTM1",664.820752286771,-0.240714186535541,0.276849879063209,
-0.869475498237738,0.384587103973237,0.80790384450123
"7444","AC023794.6",3.43045613576673,0.823761727420593,0.9474554870980
92,0.869446363062022,0.384603033488975,0.80790384450123
"7445","ADSSL1",16.2573652977099,0.568973069067742,0.654393207760507,0
.869466648370185,0.384591942552645,0.80790384450123
"7446","MKNK2",503.313417864457,0.161641344346355,0.185924474688622,0.
869392502611958,0.384632482490803,0.80790384450123
"7447","UCK2",43.3882101840519,-0.224263006839505,0.25821258014403,-0.
868520839358067,0.385109269068318,0.808722615935682
"7448","SLC9B2",59.7935890644043,-0.293462051625933,0.337927012355269,
-0.868418448056502,0.385165299258148,0.808722615935682
"7449","ZNF70",19.1559943315012,0.347777184710914,0.400482108304127,0.
868396309097563,0.385177414712589,0.808722615935682
"7450","EIF2D",190.316577369498,-0.191322008797527,0.220373775030576,-
0.868170492477979,0.385301005235538,0.808873519715949
"7451","CYBRD1",3456.31481609722,0.176894524867686,0.203918001381923,0
.867478710407599,0.385679771709626,0.808933106044759
"7452","LARS2",27.5919421279413,0.318816215335207,0.367882996054989,0.
866623950424585,0.386148086333331,0.808933106044759
"7453","EPHA6",4.55329489776617,-0.6245520404959,0.720038569686134,-0.
867386924520089,0.385730043664068,0.808933106044759
"7454","ETV5",32.6430663277613,0.369468201416804,0.426100304798715,0.8
67092084318825,0.385891557366911,0.808933106044759
"7455","C5orf51",100.202991822797,0.189313354710424,0.218186512936133,
0.86766753894563,0.38557636121206,0.808933106044759
"7456","AGPAT1",203.412979359166,0.154950901599483,0.178742544387012,0
.866894348689501,0.385999900241325,0.808933106044759
"7457","AC093799.1",3.09864235498461,-0.726219355820241,0.837911786711
287,-0.866701444397355,0.386105613838143,0.808933106044759
"7458","COL27A1",8.51795072414558,0.546655799659549,0.630077115715394,
0.867601418976901,0.38561256937731,0.808933106044759
"7459","MACROD1",127.344034714141,-0.212557072171554,0.245274821807691
,-0.866607793678108,0.386156941798604,0.808933106044759
"7460","IVD",146.753382006121,0.194015794434047,0.223639801148731,0.86
7536965412599,0.385647866996863,0.808933106044759
"7461","BECN1",229.90169893451,-0.15141172378468,0.174512695305739,-0.
867625839595299,0.385599196083296,0.808933106044759
"7462","SMIM17",3.91469398104931,0.72444144578737,0.835909307865405,0.
866650770569021,0.386133386564715,0.808933106044759
"7463","MCM8",24.9307114747092,0.400859512401122,0.46233848899573,0.86
7026046807935,0.385927738430009,0.808933106044759
"7464","AL118522.1",3.8844741705685,-0.796427060232301,0.9187415051683
57,-0.866867400408081,0.386014667122992,0.808933106044759
"7465","ZNF41",23.931817678295,0.286137963154151,0.329930123224738,0.8

6726837900534,0.385794978029489,0.808933106044759
"7466","XIAP",390.583090601699,0.198277974884796,0.228478699548759,0.867818204832186,0.385493862348791,0.808933106044759
"7467","GLMP",211.568369961381,0.211725387052056,0.244537099813687,0.865821125765251,0.386588262150251,0.809098437258502
"7468","DTNB",11.4926231115223,0.424905589899429,0.490749083487324,0.865830633610147,0.386583047361098,0.809098437258502
"7469","AC093849.4",3.32895952405658,1.0825853972875,1.24970720543792,0.866271229434215,0.386341439864672,0.809098437258502
"7470","AC073111.4",47.7860654617107,0.253661998010776,0.2928846095818 7,0.866081691260291,0.386445364738025,0.809098437258502
"7471","AL138762.1",22.9718779784314,0.382628457676803,0.44184008117861,0.865988564586854,0.386496432880721,0.809098437258502
"7472","PDCD7",388.691701212583,0.129835179308447,0.149875250342505,0.866288323200385,0.386332068092907,0.809098437258502
"7473","EEF2KMT",53.3865531926976,-0.307679094235972,0.35539351293445,-0.865742010020092,0.3866316566064,0.809098437258502
"7474","NHSL2",24.5457876737039,-0.361135491197274,0.417155722217284,-0.865709067294465,0.38664972634719,0.809098437258502
"7475","RASSF7",2.87640447249908,-0.79495037863145,0.918475701948625,-0.865510515895951,0.386758646654854,0.809218091462463
"7476","MIR4458HG",144.624473202783,0.245844793139702,0.284182912903872,0.865093508359041,0.386987467452734,0.809231105173687
"7477","LINC01023",24.7151797397133,-0.322371595937046,0.372816187060462,-0.864693130625159,0.387207240817414,0.809231105173687
"7478","PON3",18.6426882396347,0.449706752276652,0.520068508778401,0.864706754371606,0.3871997612872,0.809231105173687
"7479","FSBP",12.6958264074116,0.385953014395501,0.446368706745402,0.864650699215883,0.387230536516616,0.809231105173687
"7480","DNM1",793.1446244723,-0.171266132382958,0.198019680178538,-0.864894500529147,0.387096696333391,0.809231105173687
"7481","RALGDS",153.965872267351,-0.160074253353017,0.185103519133794,-0.864782334242465,0.387158269020176,0.809231105173687
"7482","UBALD1",71.0369241868677,-0.213896475538106,0.247344107934414,-0.86477287583023,0.387163461404627,0.809231105173687
"7483","AC138150.2",17.8562757496761,-0.378305630757623,0.437393428732502,-0.864909269107891,0.387088589698261,0.809231105173687
"7484","MAP2K2",1041.92318214034,-0.211038159591635,0.244035384463412,-0.864785080473752,0.387156761429575,0.809231105173687
"7485","CELF2-
AS2",4.74511173706003,0.766900194175024,0.887345932822234,0.864262928141082,0.387443470250017,0.80932703134154
"7486","NAXD",89.8082344183882,0.188025305769192,0.217518378897917,0.864411121128453,0.387362085753523,0.80932703134154
"7487","IRX6",6.70159121754959,-0.593306794567582,0.686546562038825,-0.864190176418113,0.387483427793187,0.80932703134154
"7488","SCPEP1",955.254728280111,0.211292799992727,0.244489151246602,0.864221577584879,0.38746618097284,0.80932703134154
"7489","LSM3",807.195986944248,-0.212666665720833,0.246189398794515,-0.863833563760955,0.387679327061311,0.809356439230248

"7490","CPE",5961.18430512055,0.182190917180662,0.210943934211274,0.86
3693558489272,0.387756253142727,0.809356439230248
"7491","CCIN",7.04438546488313,-0.483293035516966,0.559531306726154,-0
.86374619204909,0.387727332472521,0.809356439230248
"7492","RNLS",24.9212929010609,0.2907937007914,0.336561077212943,0.864
014648394455,0.387579843668198,0.809356439230248
"7493","N6AMT1",87.9615150117292,0.245873173841594,0.284658974556305,0
.863746432814333,0.387727200181769,0.809356439230248
"7494","AC104389.5",5.79942981135816,-0.590093737303001,0.683369647509
136,-0.863505921654374,0.387859365037628,0.809463633465238
"7495","RETSAT",105.744210814218,0.158334638954623,0.18340460331994,0.
863307878256558,0.387968213765111,0.809582770285035
"7496","IL10RB",470.054034596663,-0.135483410579786,0.156973679068552,
-0.863096357196412,0.388084490647901,0.809717373763765
"7497","PYGO2",51.0204451917475,0.224845298219632,0.260579839405018,0.
862865288170495,0.388211537657973,0.809826321091502
"7498","AC144831.1",74.5255074396431,0.305357733639205,0.3539095003702
12,0.862813044916232,0.38824026569975,0.809826321091502
"7499","TLL1",6.61200257018918,-0.794656102742119,0.921542708725769,-0
.862310661478622,0.388516587413779,0.810256967706954
"7500","KIAA0895",2.72844305437711,0.900879674305841,1.04480184108811,
0.862249317408956,0.388550336176608,0.810256967706954
"7501","PHC2",256.303807233173,-0.16188032158684,0.187876137432668,-0.
861633221754173,0.388889383434815,0.810510663574791
"7502","AC011374.2",12.1358776972666,0.443956559793239,0.5151976115232
79,0.861720920018628,0.388841110692037,0.810510663574791
"7503","LINC01503",8.17982969159036,0.656982665618409,0.76289622710220
6,0.86116911092076,0.389144909916087,0.810510663574791
"7504","KAT5",178.665895747154,-0.172373490552083,0.200168185946579,-0
.86114329176209,0.389159128224318,0.810510663574791
"7505","CD248",7022.26564818331,0.206774801329012,0.240035761853122,0.
861433311989309,0.388999435651579,0.810510663574791
"7506","RAB27A",45.0502134109199,-0.258686065459653,0.300334603848154,
-0.861326207986482,0.389058405215981,0.810510663574791
"7507","LRRC49",45.33763120102,0.214409605515645,0.248998821377071,0.8
61086828965164,0.38919022272677,0.810510663574791
"7508","MRPS34",478.73366779382,-0.204740169946097,0.237553363778039,-
0.861870220189338,0.38875893811015,0.810510663574791
"7509","MYO9B",215.754363414905,-0.223281727049176,0.259140495361188,-
0.86162421947202,0.388894338868749,0.810510663574791
"7510","JAG1",1051.14375798591,0.295777161029632,0.34330657206249,0.86
1554031001172,0.388932976426477,0.810510663574791
"7511","GEMIN6",123.981445172713,-0.198579995338543,0.23071703249723,-
0.860707998838044,0.389398886529703,0.810600456409325
"7512","HOXA1",3.08673168874095,0.800423221099846,0.929898707440167,0.
86076388180306,0.389368101298727,0.810600456409325
"7513","C11orf24",145.133094520576,-0.240714460652803,0.27969493237638
3,-0.860632184529092,0.389440654057523,0.810600456409325
"7514","MEG9",16.3786523108401,0.588627962348228,0.683831599416038,0.8
60779119963001,0.38935970704272,0.810600456409325

"7515","MTF2",148.937592517758,0.175836172895024,0.204525383547153,0.8
59727872626065,0.389939066595728,0.81067694013345
"7516","STAM2",231.366046569998,0.147737611215432,0.171774247427552,0.
860068452797284,0.389751309934304,0.81067694013345
"7517","AC026202.2",2.62943831138252,-0.782783244229332,0.910510469045
001,-0.859719103559965,0.389943901574421,0.81067694013345
"7518","TCERG1",216.277045794405,0.211768087526926,0.246242171496547,0
.859999269174312,0.389789445361534,0.81067694013345
"7519","ARMC2",31.2200198869113,-0.287979341202775,0.334881779272163,-
0.859943296493088,0.389820300308102,0.81067694013345
"7520","HOXA7",107.10822050001,0.330633775098163,0.384319356311591,0.8
6030997312063,0.389618196782779,0.81067694013345
"7521","SLC16A12",5.5283691365258,0.748379338020155,0.870303224118369,
0.859906429484132,0.389840624062052,0.81067694013345
"7522","TSPAN11",3.20997790689112,-0.925509547733271,1.07601822887671,
-0.860124413226198,0.389720465061938,0.81067694013345
"7523","SYN3",3.65584619057721,0.786377666383212,0.914323059243423,0.8
60065442332733,0.389752969316684,0.81067694013345
"7524","THRB",153.386404050649,0.202135788035348,0.23522357633212,0.85
9334728207458,0.390155869494287,0.810902033075168
"7525","AC022613.3",13.8550537028567,-0.556965310571414,0.648111633786
524,-0.859366321381091,0.390138444457669,0.810902033075168
"7526","WDR73",156.065443394997,0.196484908031551,0.228694663170428,0.
859158256286575,0.390253210287672,0.810996573066595
"7527","RNF44",74.234758974947,0.212716591128725,0.247626419194898,0.8
59022198924999,0.390328268742726,0.811044788512851
"7528","URM1",300.75140117988,-0.24619935599942,0.286796556412112,-0.8
58445997676639,0.390646237619788,0.81111212940776
"7529","TRIM8",857.393830014462,-0.118651090073132,0.138203170129182,-
0.858526544378295,0.390601779551045,0.81111212940776
"7530","POGLUT3",524.801085089992,0.164741435080177,0.191956738039352,
0.858221684546464,0.39077006433542,0.81111212940776
"7531","SDHD",804.540106382361,-0.228232360409855,0.26586239312344,-0.
858460490513551,0.390638238014652,0.81111212940776
"7532","USP2-
AS1",3.93231188413251,-0.670965298808767,0.781816460678111,-0.85821331
8029661,0.390774683332748,0.81111212940776
"7533","NINJ2",234.784536309993,0.288003555848091,0.335585673159306,0.
858211714274742,0.390775568739608,0.81111212940776
"7534","AMN1",32.1313931661004,0.241217706012351,0.280960239396059,0.8
5854748177487,0.390590223576085,0.81111212940776
"7535","NDUFB10",1363.52469279435,-0.213167084764696,0.248375766432225
,-0.858244295837387,0.390757581230864,0.81111212940776
"7536","TIMM50",146.337971808418,-0.210329395285203,0.245106297335966,
-0.858115020182064,0.390828954218401,0.811115292459632
"7537","FUT10",62.1526266184519,-0.221919246600936,0.25875392666024,-0
.857645908857374,0.391088016708105,0.811545254254314
"7538","AC124016.2",10.8380498787145,-0.420138790660204,0.489991205370
218,-0.857441492940193,0.391200936162933,0.811564218276731
"7539","SF3A1",219.65731653718,0.149031350683721,0.17380864810646,0.85

7444967827133,0.391199016468269,0.811564218276731
"7540","IPP",93.0075203938991,0.211451377171555,0.246902028951927,0.85641814313614,0.391766532662789,0.811579652970681
"7541","AC253572.2",44.037464125692,-0.338198923495033,0.394565262465111,-0.857143179260334,0.391365760308561,0.811579652970681
"7542","TMEM9B-AS1",22.4044836335786,0.4292335919638,0.501050149182805,0.856667925683418,0.391628434274432,0.811579652970681
"7543","SOX5",146.847150391852,0.292106470148614,0.340853754639903,0.856984751296669,0.391453311995678,0.811579652970681
"7544","PWP1",342.737619949307,-0.117867657376186,0.137598155972867,-0.856607826920504,0.391661658645645,0.811579652970681
"7545","ATP6V0A1",137.148383877729,0.194478449393222,0.227032120022131,0.856612048437305,0.391659324810616,0.811579652970681
"7546","COL1A1",27253.3171586126,-0.323672680981123,0.377577292640812,-0.857235557565776,0.391314714981592,0.811579652970681
"7547","BSG",1383.30656865069,-0.218392218034074,0.255013339952074,-0.856395269655766,0.391779180302343,0.811579652970681
"7548","ZNF700",28.2386706326494,0.325213132767733,0.379603960188065,0.856716912559644,0.391601354147727,0.811579652970681
"7549","DHX34",16.7331090123814,0.430102995818715,0.501944383239806,0.856873809489829,0.391514628592424,0.811579652970681
"7550","CD40LG",4.88511272763075,0.599945590096491,0.700459082647797,0.856503406064268,0.39171938964692,0.811579652970681
"7551","UQCRHL",4.28708997881702,0.742339284740778,0.867150502132675,0.856067410345799,0.391960493729544,0.81163274486033
"7552","AC019080.1",22.8298519360339,0.323254441902216,0.37756994373873,0.856144529676603,0.391917840461135,0.81163274486033
"7553","BMP1",366.095776088427,-0.183868293806494,0.214765055619031,-0.856136922631656,0.39192204765111,0.81163274486033
"7554","GNA15",3.92693881886106,-0.748938380511488,0.875086481056433,-0.855844989865853,0.392083526135383,0.811780030282949
"7555","KLF10",351.564462220203,-0.304645523802517,0.356035733176343,-0.855659967286564,0.392185889651636,0.811801226193356
"7556","EIF4G2",1695.73354681444,0.14496690025959,0.16942533695635,0.855638848733345,0.392197574495972,0.811801226193356
"7557","FUOM",124.031178215442,0.273031351919969,0.319146358031571,0.855505146929987,0.392271556279734,0.811846915471092
"7558","TMEM219",772.865398698844,-0.218325875376683,0.255314146191143,-0.855126433978445,0.39248115705042,0.812011859006445
"7559","MVD",76.7311536526085,-0.210527731805165,0.246170001659035,-0.855212781355719,0.392433361643313,0.812011859006445
"7560","NDUFA1",1293.98541734579,-0.182127104444552,0.212994295577293,-0.855079728548224,0.392507011130993,0.812011859006445
"7561","AC091057.6",3.57781724661025,-0.72690371912426,0.850202871715163,-0.854976786490777,0.392563998991269,0.812022344163926
"7562","TMEM65",192.900099096282,0.151054679079465,0.176750837542894,0.854619311451958,0.392761933127858,0.812324336699247
"7563","MYO6",182.361418047922,-0.190488558050268,0.223004054661389,-0.854193249264041,0.392997923006264,0.81259750341327

"7564","RPL18",11276.2535142436,-0.210694912692126,0.246634146794558,-0.854281191110293,0.39294920621351,0.81259750341327
"7565","ELMO3",4.34327709725199,-0.617146572745772,0.722582781690424,-0.854084249422617,0.393058310291477,0.812614933636313
"7566","SUZ12",217.050787470456,0.154697928325624,0.181200204334349,0.853740363560388,0.39324886423811,0.81273170623844
"7567","DCAF15",61.0212868994059,-0.184181672381544,0.215744945050885,-0.853700986310956,0.393270687519986,0.812731706238449
"7568","NCOA5",69.4846589202514,0.196485733523282,0.230156928111554,0.853703319450144,0.393269394449494,0.812731706238449
"7569","PABPC4",494.877984547867,-0.137755834664831,0.161432805624238,-0.853332345505289,0.393475027676662,0.813046562671818
"7570","PSMA6",764.418196904794,-0.220224129193586,0.258180088699118,-0.852986495988988,0.393666792914415,0.813335355506136
"7571","EVI2B",17.7515637569987,-0.487271536778586,0.571323434198474,-0.852882111272389,0.393724682844831,0.813347515479217
"7572","EAF1-AS1",3.36177824337266,0.819093035123806,0.960884078515674,0.8524368895 6643,0.393971652853536,0.813675528238753
"7573","HSPA4L",23.7315758039593,0.41446092518182,0.486276966934044,0.852314531356438,0.394039542895161,0.813675528238753
"7574","CPNE1",583.152293708632,0.195831771582182,0.22976246255465,0.852322739775662,0.394034988259772,0.813675528238753
"7575","AC007620.2",7.05327044103081,0.600619106168748,0.704969528100701,0.851978819264586,0.394225847983734,0.813816580004383
"7576","SDR42E2",37.1964977234649,0.252533823240952,0.296432455952604,0.851910167627965,0.394263953113376,0.813816580004383
"7577","TVP23B",74.6558116633123,-0.259496504537248,0.304596912601721,-0.851934126057788,0.394250654720214,0.813816580004383
"7578","DNAJC16",96.062031045148,0.193127237705203,0.226857671309209,0.85131455590924,0.394594641271902,0.813857517779276
"7579","JAK1",887.829807612165,-0.1140292667461,0.133943558698459,-0.851323257752238,0.394589808735094,0.813857517779276
"7580","VILL",33.5722307083593,-0.246404821858926,0.289487717809849,-0.851175392597409,0.394671929974341,0.813857517779276
"7581","PARP14",296.68985897775,0.16989752063636,0.199497178894962,0.851628687570636,0.394420212079213,0.813857517779276
"7582","C3orf70",10.8664834276,0.519127083485659,0.609916272260723,0.851144832652941,0.394688903622377,0.813857517779276
"7583","TMEM71",32.665986664529,0.428882977676136,0.503861394306355,0.851192376559355,0.394662496908793,0.813857517779276
"7584","CHD3",327.441325585317,0.194190927774883,0.228117670644403,0.851275252926784,0.394616468486028,0.813857517779276
"7585","AC005332.5",51.5353357507608,0.281922803132124,0.331235599646315,0.851124708313824,0.394700081352673,0.813857517779276
"7586","MINDY1",55.952735734799,0.183369402172587,0.215469674283322,0.851021856242626,0.394757211819,0.813868019094274
"7587","MRPL35",150.157267334709,-0.178244425936899,0.209713931430449,-0.849940796594209,0.395358002377611,0.814064269591523
"7588","U73166.1",4.64769497244478,0.663882002263018,0.781462744492879

,0.84953762279971,0.395582204506048,0.814064269591523
"7589","ARHGAP31",22.2822240499763,-0.335398257931869,0.39482080399067
8,-0.849494896271444,0.395605968930891,0.814064269591523
"7590","MSL2",82.5074646872801,0.210172005797236,0.24719779893005,0.85
0217949783239,0.395203923978437,0.814064269591523
"7591","AC010226.1",134.902692809539,0.236762848098734,0.2787564779502
62,0.849353707722549,0.395684503904402,0.814064269591523
"7592","DDAH2",3038.49670588408,-0.184685910658709,0.21742590749535,-0
.849419983047137,0.395647637625199,0.814064269591523
"7593","BTBD9",32.0399577878223,0.272047108080341,0.320290121141736,0.
849377143168127,0.395671467477884,0.814064269591523
"7594","BLVRA",175.414062059411,-0.29179386279873,0.343692552058215,-0
.848996759025798,0.395883095696562,0.814064269591523
"7595","MCU",103.323940802934,0.198354932039617,0.23317757205595,0.850
660422830129,0.394958014180926,0.814064269591523
"7596","PLAAT4",435.776164414524,0.256891280087459,0.302225812250635,0
.849997815125134,0.395326300970894,0.814064269591523
"7597","AP000866.6",9.27666670688502,0.596250085932936,0.7021115189320
72,0.849224189968919,0.395756555376456,0.814064269591523
"7598","SLC16A7",224.831721874212,0.244115834038178,0.287020315740632,
0.850517613738448,0.395037371963658,0.814064269591523
"7599","TGFB3",126.997622328872,0.286119293624812,0.33695753428567,0.8
49125674638403,0.395811365328837,0.814064269591523
"7600","VPS39",112.649970232562,0.196355874355386,0.230921768501901,0.
850313401067554,0.395150867934199,0.814064269591523
"7601","CILP",3650.5113645577,-0.252764760738904,0.297321394287919,-0.
850139833846373,0.395247347473974,0.814064269591523
"7602","MNT",112.370725781647,0.243214573717978,0.28606951652O019,0.85
0193955219823,0.395217261894977,0.814064269591523
"7603","ZNF519",68.6000802539636,0.223058791321022,0.26266945091312,0.
849199594949472,0.395770238622791,0.814064269591523
"7604","EIF3K",1928.62204328808,-0.211229994295039,0.248558932785629,-
0.849818559839148,0.395425969268838,0.814064269591523
"7605","ZSCAN5A",25.2309691605328,-0.286903492409109,0.337939750178061
,-0.848978234309338,0.395893403741248,0.814064269591523
"7606","RBL1",43.5417209583866,0.24557800033417,0.288944782126567,0.84
9913255144366,0.395373315540983,0.814064269591523
"7607","NECTIN3",258.313625897768,-0.136195938395837,0.160478159840798
,-0.848688310801604,0.396054752270926,0.814074954069823
"7608","CRYL1",453.269433178656,-0.258652430147349,0.304731906011614,-
0.848786835394555,0.395999916808738,0.814074954069823
"7609","LZTR1",95.3485160836524,0.164694101514936,0.194049026928848,0.
848724181313852,0.396034787423999,0.814074954069823
"7610","ZNF653",14.420841814817,-0.396545217049232,0.467473681678884,-
0.848272817466602,0.396286052274512,0.814380209316801
"7611","EFNB1",57.1880708283862,0.273043184397459,0.321895882736754,0.
8482344728241,0.396307402372773,0.814380209316801
"7612","ACVRL1",739.198566631021,0.189741402019226,0.223759030606463,0
.847972041642129,0.396453541334666,0.814549888611613
"7613","RPL10",41165.6065199922,-0.207143308102819,0.244301822519001,-

0.847899151823591,0.396494136956535,0.814549888611613
"7614","GOLPH3L",128.523384368785,0.148852612771974,0.175593335704097,
0.84771219918514,0.396598270768924,0.814656810457838
"7615","SLC45A1",25.6614166144903,-0.313959509682354,0.370776339394461
,-0.846762525880431,0.397127499698933,0.814684098134255
"7616","ZRANB2",588.307933145521,0.145365889969525,0.171766146131141,0
.846301167277399,0.397384756852605,0.814684098134255
"7617","RBMS1",1714.03434706691,0.166613630823315,0.197271596849917,0.
844590065087136,0.398339757926682,0.814684098134255
"7618","SSB",987.281729868351,-0.138937544910247,0.164475330913112,-0.
844731815640143,0.398260591568439,0.814684098134255
"7619","CREB1",319.803308111754,0.159863709771811,0.189024489673304,0.
84573014876595,0.397703300346798,0.814684098134255
"7620","C3orf62",27.7871498874574,0.287595488100066,0.339851971151843,
0.846237516661545,0.397420256818945,0.814684098134255
"7621","NPRL2",85.84247542887,-0.188945424788208,0.223096316506762,-0.
846923103647395,0.39703798385604,0.814684098134255
"7622","CPA3",7.62002861304075,0.916181949067041,1.08464924731868,0.84
4680389842062,0.398289311300135,0.814684098134255
"7623","PARL",266.367575061826,-0.191847976531954,0.226489019328112,-0
.847051998816887,0.396966138632968,0.814684098134255
"7624","MAGEF1",224.365397691234,-0.160394638145967,0.18978044106416,-
0.845158949186663,0.398022098793719,0.814684098134255
"7625","PKD2",448.089610890717,0.211490942655213,0.250483086030698,0.8
44332230198143,0.398483780600829,0.814684098134255
"7626","ANAPC10",138.922733026637,-0.239609491813544,0.283490166597239
,-0.84521270945515,0.397992087499203,0.814684098134255
"7627","SLC29A1",223.969757661116,-0.348531461360191,0.411615756825319
,-0.846739843120487,0.3971401454342,0.814684098134255
"7628","TNPO3",181.446573594317,0.138696499725591,0.163794826161121,0.
846769723905431,0.397123486820109,0.814684098134255
"7629","AC106845.1",8.04573422425722,0.622652626297448,0.7363401111921
81,0.845604655828586,0.397773327386612,0.814684098134255
"7630","MALRD1",5.30217667569563,0.641539333875997,0.758173728961125,0
.846164024642551,0.397461248026358,0.814684098134255
"7631","AL158212.3",19.4886731037529,0.361145839839735,0.4277795100347
16,0.844233609530357,0.398538876906981,0.814684098134255
"7632","CTBP2",371.901287107178,0.157524720142144,0.186126236689594,0.
846332698408611,0.397367171649387,0.814684098134255
"7633","UBXN1",1076.19003857114,-0.201503222182983,0.238087978767694,-
0.846339337357276,0.397363469106179,0.814684098134255
"7634","C1RL-
AS1",50.5519302736975,0.311899884364644,0.368920074492201,0.8454402618
0727,0.397865073089251,0.814684098134255
"7635","LINC02422",3.4122437173301,0.758902271644689,0.895556162783266
,0.847408909884696,0.396767239949766,0.814684098134255
"7636","ATP5MC2",1965.32106412784,-0.221219078736788,0.261807231027918
,-0.844969322918353,0.398127967221531,0.814684098134255
"7637","AC127070.1",3.5304684777917,0.725477479835932,0.85859270330407
2,0.844961152178582,0.398132529330332,0.814684098134255

"7638","LCP1",42.601814436313,-0.445805417860713,0.526974563450453,-0.845971416422319,0.397568690065548,0.814684098134255
"7639","PTGDR",3.30778333409723,-0.883465067605772,1.04465347271645,-0.845701556238039,0.397719254774947,0.814684098134255
"7640","ZNF18",31.1358913228206,-0.283895842710914,0.336261373570034,-0.84427134671115,0.398517793773488,0.814684098134255
"7641","SUPT6H",253.314446333322,0.183421836581076,0.216509205697652,0.847178003309562,0.396895912233781,0.814684098134255
"7642","MRPL27",499.816240658901,-0.209445482769846,0.247780579751537,-0.845286111526045,0.397951113507637,0.814684098134255
"7643","AC027575.2",9.19219343015034,-0.430381678936029,0.509368600747247,-0.844931702316665,0.39814897283648,0.814684098134255
"7644","ATP13A1",90.3229616737369,0.186940283765288,0.220594537032903,0.847438410215051,0.396750802756004,0.814684098134255
"7645","NAPA",302.878968134544,-0.18736427704233,0.221428877737365,-0.846160080640255,0.397463447921192,0.814684098134255
"7646","ZSCAN18",215.101743990259,0.181800311676748,0.215027060640381,0.845476430433085,0.397844886857154,0.814684098134255
"7647","CASTOR1",50.2077600062849,-0.271916786880847,0.321003762213817,-0.84708286596257,0.396948934676207,0.814684098134255
"7648","GRAP2",2.66419327463188,1.04362814895517,1.23542533819596,0.844752100097881,0.398249263663744,0.814684098134255
"7649","SRPX2",1025.58069230819,0.131359705007596,0.155455129523671,0.845000775529854,0.398110406041306,0.814684098134255
"7650","RTL8C",797.590720384523,-0.115474876696708,0.136564427290399,-0.845570687681022,0.397792283427123,0.814684098134255
"7651","VMA21",172.502504010982,-0.142236856016966,0.168470247076217,-0.844284723774501,0.398510320392563,0.814684098134255
"7652","MRPS9",145.532949348199,-0.208777688711079,0.247548133157471,-0.843382198234033,0.399014724714296,0.814698471870965
"7653","PPP1R7",443.889796832532,-0.209826530057599,0.248720127461564,-0.843625050369212,0.398878961509706,0.814698471870965
"7654","NSD3",721.326114784474,0.101527586770477,0.120329475600605,0.843746607086237,0.398811017313854,0.814698471870965
"7655","EEF1D",4203.30000954918,-0.172145909943654,0.204058893320005,-0.843608955938496,0.398887958023241,0.814698471870965
"7656","PIGO",47.435110041218,-0.225161395157989,0.266949854615238,-0.84345951595486,0.398971498269633,0.814698471870965
"7657","GSTP1",4193.58829798364,-0.225530612112462,0.267249189212899,-0.843896338008336,0.39872733470814,0.814698471870965
"7658","PSMA3",586.728189676685,-0.197866813242415,0.234605337886481,-0.843402861268902,0.399003172241413,0.814698471870965
"7659","TMED3",523.059373304805,-0.240160370396884,0.284611450371696,-0.843818370916703,0.39877090815141,0.814698471870965
"7660","AC093525.7",5.02419291585914,0.766485780955487,0.908164557747458,0.843994377909461,0.398672547249338,0.814698471870965
"7661","TBX19",13.5479537864548,0.425703217251894,0.505208104441022,0.842629430347134,0.399435726118135,0.814813454609055
"7662","PDK4",2282.51968926,0.262465786481867,0.311444020695642,0.842738241998105,0.399374854377493,0.814813454609055

"7663","MYO7A",3.79784966806232,-0.772613917925011,0.916497025537826,-
0.843007556376542,0.399224217723121,0.814813454609055
"7664","OVCH1-
AS1",3.18545850361213,1.2766508974815,1.51480552035376,0.8427820471524
,0.399350350342315,0.814813454609055
"7665","LINC02185",7.77157002223062,-0.688050983581066,0.8164625701099
71,-0.842722016623972,0.399383930870935,0.814813454609055
"7666","AP001020.2",8.65022710366796,-0.511958459804268,0.607334045653
986,-0.842960251393421,0.399250674526916,0.814813454609055
"7667","SAFB2",233.591979984667,0.212743974060711,0.252315035556708,0.
843168040268832,0.399134469905832,0.814813454609055
"7668","COL16A1",302.360858292724,0.170965497352374,0.202996785277626,
0.842207905502325,0.399671589502752,0.814824010170079
"7669","RGS1",93.5476538405929,-0.432005230740632,0.512809348101026,-0
.842428540627004,0.399548123113176,0.814824010170079
"7670","TPRKB",256.582508014244,-0.217951836941158,0.25887501703867,-0
.84191916019691,0.399833204694994,0.814824010170079
"7671","AC007285.1",7.03884965213939,-0.469049316901738,0.556818431692
28,-0.842373905397143,0.399578694596205,0.814824010170079
"7672","NRP1",292.179650631645,0.216541573773023,0.257081720604028,0.8
42306381271475,0.399616480085207,0.814824010170079
"7673","BFAR",351.653078799236,-0.162493247028576,0.193002975687357,-0
.84192094163251,0.399832207477599,0.814824010170079
"7674","DOK4",67.3932355401999,-0.187145591498958,0.222306817000369,-0
.841834695058618,0.399880488561625,0.814824010170079
"7675","JAM2",52.415517123726,-0.371029725870394,0.440620854793083,-0.
842061200313887,0.399753697744383,0.814824010170079
"7676","OPHN1",207.565382581824,0.221337357624513,0.262938936318726,0.
841782357239839,0.399909789134625,0.814824010170079
"7677","ZNF441",55.8307515539911,0.272424368631908,0.323690592391678,0
.841619667161238,0.40000087707182,0.814903441110234
"7678","SAP25",9.54569480025657,-0.478229413640136,0.568369170662996,-
0.841406322377209,0.40012034478449,0.814934521738432
"7679","PNPLA8",358.873869817181,-0.129346281219492,0.153714711303605,
-0.841469759937406,0.400084819105852,0.814934521738432
"7680","FRMPD1",17.2036209275555,0.508188605418519,0.604139910156966,0
.841177013593562,0.400248775850215,0.815042866373879
"7681","CALHM2",761.142462795412,-0.165657440236419,0.196947408726221,
-0.84112525931581,0.400277765768399,0.815042866373879
"7682","MAPK13",11.2317587124018,0.429441931957967,0.510685894497779,0
.840912068621537,0.400397196851397,0.815179921733383
"7683","FKBP10",659.661070326314,-0.196271589729185,0.233441655330044,
-0.840773637642744,0.400474758427298,0.815231709202517
"7684","NELFE",365.579189890138,-0.191569992702333,0.227890377214138,-
0.840623439410625,0.400558923296164,0.815296923523166
"7685","AL355488.1",10.9226612210808,0.597520614963737,0.7121240767554
31,0.839068126563213,0.401431080895254,0.815703057328019
"7686","PSMD4",659.182360968308,-0.183628048759679,0.218864134563329,-
0.839004751171532,0.401466643448768,0.815703057328019
"7687","OAZ3",6.51649672750027,-0.569923549049947,0.679191179457555,-0

.839120951931566,0.40140143984122,0.815703057328019
"7688","AC012467.2",11.3545331623968,0.384009837979942,0.4575402012477
98,0.839292016160931,0.40130546232271,0.815703057328019
"7689","AC005699.1",5.90594619425976,0.693201291595652,0.8262571337565
13,0.838965575333755,0.401488627577436,0.815703057328019
"7690","PGRMC2",867.779754197136,0.106384698873944,0.126635179813715,0
.84008803107035,0.400859030414363,0.815703057328019
"7691","ZSWIM6",120.451440551333,0.242599816708114,0.288861373071234,0
.839848589407238,0.400993285964577,0.815703057328019
"7692","CREBRF",685.054107421284,0.1948479333492,0.232073017157839,0.8
3959753587673,0.401134081315049,0.815703057328019
"7693","CDCA2",3.59754635551866,-0.82681151163763,0.985026606341306,-0
.839379876964607,0.401256172382021,0.815703057328019
"7694","PABPC1",2804.0848247969,-0.147868647486584,0.176124234469618,-
0.839570136000176,0.40114944945676,0.815703057328019
"7695","RHOD",57.1683674159771,-0.463884185594597,0.552753735941547,-0
.839223971601798,0.401343637841053,0.815703057328019
"7696","HIP1R",2.96088126118823,0.871265419299664,1.03848012763532,0.8
38981311355072,0.401479796978307,0.815703057328019
"7697","AL355073.2",2.96071191059456,-0.893705552132671,1.064205125571
4,-0.839786927029523,0.401027864588907,0.815703057328019
"7698","GEMIN7",62.1912911745488,-0.29779798637263,0.354895005371296,-
0.839115743714314,0.401404362186482,0.815703057328019
"7699","TIAF1",3.187642314576,0.774614489993275,0.92341404268454,0.838
859335235279,0.40154824949233,0.815718226011175
"7700","PMVK",445.109965251067,-0.212810821911666,0.253748184730951,-0
.838669337230175,0.401654889571011,0.815722954537152
"7701","UBAC2-
AS1",10.1628406603228,-0.382715277471542,0.456312389715552,-0.83871331
5915249,0.401630204166386,0.815722954537152
"7702","SMIM19",863.749254658553,-0.248237910223724,0.296069092437271,
-0.838445878224586,0.401780332034231,0.815871772658448
"7703","N4BP2",99.041579935288,0.20771649952539,0.247794657031717,0.83
8260606639322,0.401884355171112,0.815939199190026
"7704","CCDC120",16.833089288925,0.322536089809766,0.384795681263579,0
.838200908987941,0.401917876634268,0.815939199190026
"7705","PDCD6IP",580.765408213775,0.107593225116709,0.128462055307083,
0.837548682056434,0.402284224784503,0.816153233315556
"7706","DIMT1",154.035237694463,-0.184933818199388,0.220765956839288,-
0.837691738559197,0.402203854499755,0.816153233315556
"7707","NAP1L4",463.151143044042,0.122172287037818,0.14584931434038,0.
837661031115273,0.40222110537503,0.816153233315556
"7708","BSCL2",357.507389764381,-0.223721949834744,0.26707133232867,-0
.83768612633954,0.402207007308043,0.816153233315556
"7709","SUMO3",972.851988426651,-0.156168100782546,0.186452678290929,-
0.837574993365722,0.402269442158255,0.816153233315556
"7710","COL5A2",1581.07900388622,-0.212068085073988,0.253292983298104,
-0.837244215424641,0.402455308626011,0.816241765160923
"7711","AC104248.1",4.24379179448646,0.967724299730844,1.1559162545304
2,0.837192396886897,0.402484430493672,0.816241765160923

"7712","TSC2",153.125333396121,0.18796031062816,0.224470063341685,0.83
7351350242413,0.402395103176047,0.816241765160923
"7713","CORO2B",174.683349285424,0.213959911737464,0.255613546184422,0
.837044495220511,0.402567557749581,0.816304499313295
"7714","ELK1",80.1402098000945,-0.189170774270147,0.226038446541073,-0
.836896453523332,0.402650774015401,0.816367397666694
"7715","NOL9",115.871270611721,0.173851791576731,0.208320135538434,0.8
34541467282486,0.403975930259024,0.816429435104359
"7716","PHACTR4",210.273246080375,0.167122588978912,0.20004758685121,0
.835414171245233,0.403484553554137,0.816429435104359
"7717","DPH2",29.1975323395915,-0.272128181585635,0.32626960733005,-0.
834059242638417,0.404247600824327,0.816429435104359
"7718","CCDC18",52.6844067311474,-0.222525697113493,0.26670879352497,-
0.834339558784213,0.404089666009322,0.816429435104359
"7719","RSBN1",225.045392179935,0.156091226436001,0.186673801208623,0.
836171039671264,0.4030586881136,0.816429435104359
"7720","AL358472.2",6.27301231480517,0.601177256453736,0.7192754485581
5,0.835809504771569,0.403262078528145,0.816429435104359
"7721","MPZL1",620.72984377292,0.125810724836373,0.150860902120658,0.8
33951826270732,0.404308130793536,0.816429435104359
"7722","TBCE",25.9413103461667,0.277032661851759,0.331987779908027,0.8
34466443097718,0.404018189402618,0.816429435104359
"7723","RN7SL832P",38.9895093771395,0.369047086601468,0.44139487867798
1,0.83609281491167,0.403102690178557,0.816429435104359
"7724","IL1RL1",3.91571573670964,-0.833913376372265,0.997694951526563,
-0.835840028153197,0.403244904467767,0.816429435104359
"7725","DYNC1LI1",310.087699106695,-0.128023418650517,0.15331743452633
2,-0.835021920670926,0.403705366271393,0.816429435104359
"7726","ATP13A4",2.82159602219034,0.778480801269003,0.933581219855001,
0.83386510430224,0.404357003261953,0.816429435104359
"7727","STK38",321.379565265193,0.225108098703536,0.269298423789113,0.
835905741801954,0.403207931998755,0.816429435104359
"7728","IBTK",240.357507950594,0.155904328031061,0.186828054505609,0.8
34480284257202,0.404010392843681,0.816429435104359
"7729","AL513550.1",12.0711642103373,0.368409696110085,0.4418200085257
45,0.833845658867705,0.404367962289639,0.816429435104359
"7730","PAPOLB",10.8394140184358,0.517626495702293,0.618775122531345,0
.836534108845127,0.402854496423665,0.816429435104359
"7731","SAXO1",4.88577711980992,0.591997864449856,0.709280926442016,0.
834645120685129,0.403917549385352,0.816429435104359
"7732","AL354920.1",6.70713933553997,0.548124082621295,0.6573931775863
23,0.833784257746303,0.404402567804342,0.816429435104359
"7733","ADAMTS13",7.4633848969221,0.488226489380391,0.585479160236635,
0.833892173349198,0.404341748026513,0.816429435104359
"7734","FAM107B",709.991166011931,0.209162293475949,0.250000141830124,
0.836648699255842,0.402790063149248,0.816429435104359
"7735","GHITM",762.552514257246,-0.14191503265956,0.16990721701538,-0.
835250174492084,0.403576864743028,0.816429435104359
"7736","ARHGAP19",39.9586826066639,0.232749265117688,0.279209973628402
,0.833599395082684,0.40450676662454,0.816429435104359

"7737","ERLIN1",167.251680287503,0.151432704811633,0.181582515054204,0
.833960829138361,0.404303057398524,0.816429435104359
"7738","C11orf49",127.412673973389,-0.202204883226622,0.24185357341790
3,-0.836063244255764,0.403119324663166,0.816429435104359
"7739","FGF14",72.3619314091192,0.207752658549384,0.248798299893914,0.
83502442998191,0.403703953455214,0.816429435104359
"7740","LINC00640",16.9941896522957,0.451640336889664,0.54105874114304
9,0.834734387500185,0.403867275540372,0.816429435104359
"7741","ZFHX3",734.205383973767,0.223513246212922,0.267989054435833,0.
834038713571564,0.40425916869604,0.816429435104359
"7742","TUBG2",55.2576403105288,0.21396271310953,0.256674209853043,0.8
33596461569059,0.404508420244481,0.816429435104359
"7743","RPL38",7282.533868633,0.149946731594696,0.179550601430082,0.83
5122413405482,0.403648788209158,0.816429435104359
"7744","LRRC45",46.4794757009642,-0.265088693428219,0.317023137984161,
-0.836180901854119,0.403053140759644,0.816429435104359
"7745","GPS1",201.495563733946,-0.135418733447643,0.162299327673294,-0
.834376428966104,0.404068895479181,0.816429435104359
"7746","CATSPERG",6.03840197365076,0.528568435221823,0.633982896522288
,0.833726648023605,0.404435038107415,0.816429435104359
"7747","SHKBP1",102.593673490675,-0.215152688879153,0.25727489148466,-
0.836275501420168,0.402999932016192,0.816429435104359
"7748","ATP5F1E",4947.15577400151,-0.182417435860771,0.218801286449617
,-0.833712812300015,0.404442836504934,0.816429435104359
"7749","LINC00629",3.45126296779311,0.820979817345784,0.98252983267992
5,0.835577496009968,0.403392633185561,0.816429435104359
"7750","HGS",238.010772601299,-0.150113470841753,0.180113558723609,-0.
833437926081446,0.404597792621782,0.816504448594151
"7751","AC093297.2",23.5098836476387,0.295259562402345,0.3543210206606
37,0.833310882464237,0.404669420378922,0.816543637559842
"7752","GPI",354.790969818628,-0.14046384393315,0.168595166915662,-0.8
33142767392702,0.404764216061959,0.816629558721497
"7753","USF2",349.31575935534,-0.113269321772947,0.135978162798544,-0.
832996412378057,0.404846752630782,0.816690727608078
"7754","GNAQ",375.522208181944,0.157528300259084,0.189159624701963,0.8
32779725098754,0.404968971394497,0.816831920635792
"7755","LACC1",31.8408877149441,-0.29121694559006,0.349772615678735,-0
.832589323852448,0.40507638217523,0.816943213052301
"7756","ENSA",779.436233017576,-0.156989091111275,0.188623962547367,-0
.832286041450603,0.405247507603994,0.817007359706431
"7757","CDK7",69.3145188602055,-0.193014683928613,0.231917662431782,-0
.832255214651395,0.40526490387484,0.817007359706431
"7758","PITPNA-
AS1",136.101837350508,-0.236922785265584,0.284632046441077,-0.83238267
8717909,0.405192975867102,0.817007359706431
"7759","ZNF16",25.1193229790266,-0.325644429171181,0.39134118659337,-0
.832124091016128,0.405338904940711,0.817051227384034
"7760","RHOB",7577.57884904291,0.201124494573937,0.241932020144771,0.8
31326479453133,0.405789219734483,0.817448539107089
"7761","SLC35D2",151.386418155745,-0.153086076816656,0.184170447023055

,-0.831219553903198,0.405849610368705,0.817448539107089
"7762","AP000944.7",8.11114454836131,0.4719600074807,0.56775459090579,
0.831274665217131,0.405818483300579,0.817448539107089
"7763","C11orf1",58.7146082027754,-0.270730485112511,0.325677350010514
,-0.831284352761317,0.405813011888176,0.817448539107089
"7764","LINC00667",269.321941639247,-0.128379040690444,0.1543950963204
29,-0.831496878786925,0.405692990744677,0.817448539107089
"7765","AC011446.2",8.91678544493463,-0.453821932567312,0.545685866090
395,-0.831654181954083,0.405604169606869,0.817448539107089
"7766","SLC35D1",45.626392315712,0.21389141618679,0.257640318701596,0.
83019388139526,0.406429174075106,0.8174735770928
"7767","INO80D",208.216111778331,0.198672815594622,0.239227358592817,0
.830476985421963,0.406269154749024,0.8174735770928
"7768","COPS7B",77.9171117638787,0.19036590190439,0.229142680737259,0.
830774525688074,0.406101016149337,0.8174735770928
"7769","MALINC1",30.3627181385174,-0.303210505184241,0.364911132814158
,-0.830916017403776,0.406021074428188,0.8174735770928
"7770","AC090114.2",63.5245638061707,0.238899252229837,0.2876602262069
3,0.830491081022733,0.406261188453923,0.8174735770928
"7771","LINC01301",4.23049010701008,-0.644803644716792,0.7760474221024
53,-0.830881755872472,0.406040431065145,0.8174735770928
"7772","LAMC3",2.62592708443929,0.967355498048915,1.16485308378723,0.8
30452794015705,0.406282827025089,0.8174735770928
"7773","BCCIP",160.704639157616,-0.152763508593501,0.184012501710537,-
0.830180053928116,0.406436990759182,0.8174735770928
"7774","TMEM218",163.063657633413,-0.222561210778091,0.268072334330318
,-0.830228196930801,0.406409775848585,0.8174735770928
"7775","PRR14",104.120117180449,-0.164653025656861,0.198192980425281,-
0.830771227636568,0.406102879634918,0.8174735770928
"7776","LSR",17.8316071877654,-0.374871237457348,0.451367089061683,-0.
830524082375263,0.406242537714882,0.8174735770928
"7777","TSNAX",359.1227147658,-0.124395788012314,0.149986737201699,-0.
829378585954764,0.406890214898539,0.817758377223681
"7778","POMC",14.387918224912,-0.452851532665521,0.546177451172659,-0.
829128942788165,0.407031447799609,0.817758377223681
"7779","AC109347.1",4.01873029078329,0.806169878005199,0.9721316337450
74,0.829280572734252,0.406945661323826,0.817758377223681
"7780","ECI2",547.939955446502,-0.175034343392954,0.211076908862483,-0
.829244394075287,0.406966128857915,0.817758377223681
"7781","PTPA",117.369073389476,-0.154922988390242,0.186863677475909,-0
.829069568162681,0.407065042650713,0.817758377223681
"7782","ZC2HC1C",9.04476111101108,-0.608791076342201,0.734363380443744
,-0.829005220786383,0.40710145300918,0.817758377223681
"7783","NAA60",161.891424288644,-0.163994905940475,0.19766390059774,-0
.829665434328428,0.406727969657353,0.817758377223681
"7784","MYH11",11.3962469498873,0.516929609236702,0.623112066373936,0.
82959332218498,0.406768753608501,0.817758377223681
"7785","HSPA13",141.655180032622,-0.158339864175232,0.19097273901502,-
0.829122863252113,0.407034887595555,0.817758377223681
"7786","TSR2",265.844967514607,-0.171287915661024,0.206474736579117,-0

.829582923794603,0.406774634753339,0.817758377223681
"7787","SUCLG1",489.030776600386,-0.178690226556948,0.215669580486011,
-0.828536996985248,0.407366451533721,0.817831181001586
"7788","AC114980.1",23.1269523419947,0.390442535755733,0.4713812937607
91,0.828294505793156,0.407503733602645,0.817831181001586
"7789","FAM221A",19.7890980443913,-0.313895514406414,0.378717610206453
,-0.828837914971256,0.407196130523443,0.817831181001586
"7790","ZNHIT1",1060.98496792345,-0.197880743822515,0.238886114239923,
-0.828347618496464,0.407473662433121,0.817831181001586
"7791","NCKAP1L",11.3747248322236,-0.564921553403792,0.681919707341571
,-0.828428255294321,0.407428010294475,0.817831181001586
"7792","GABARAPL2",1466.95949126836,-0.195272183590952,0.2356876437429
19,-0.828521090413841,0.407375455910601,0.817831181001586
"7793","AC005332.4",33.1375998641731,0.352159534853876,0.4250518230680
75,0.828509644569799,0.407381935236575,0.817831181001586
"7794","SHC3",28.5889893807506,0.426328456473996,0.514884624042591,0.8
28007744971484,0.407666113721175,0.818052093738668
"7795","AL365361.1",3.19813117719343,0.821845558487837,0.9932681643772
77,0.827415584192299,0.408001550509192,0.818410189811948
"7796","SERPINB6",727.012670895008,-0.152925253983109,0.18482099491983
8,-0.827423605469913,0.407997005658995,0.818410189811948
"7797","MAP7D2",11.7572397876424,-0.423387472974653,0.511596578873058,
-0.827580735405402,0.407907982032242,0.818410189811948
"7798","AC092053.3",8.6921123470567,0.477701969087521,0.57760313556038
3,0.827041855692251,0.408213338253294,0.818730009012763
"7799","LINC00622",5.53354829528868,0.554947015404632,0.67131559784776
1,0.826655923359733,0.408432110485294,0.818840444534601
"7800","RBM6",349.906272352147,0.187144930314185,0.226385254851022,0.8
26665722718294,0.408426554690336,0.818840444534601
"7801","ZNF609",137.057590309956,0.234551966024367,0.283719188930649,0
.826704626177753,0.408404498624972,0.818840444534601
"7802","THAP10",19.6393348023622,-0.29810260997771,0.36064785684771,-0
.826575298639828,0.408477822778706,0.818840444534601
"7803","ABCF3",96.4761659771594,-0.169778933559531,0.205496617109354,-
0.826188459682451,0.408697193615374,0.818965292523313
"7804","AC008522.1",6.29286262391293,0.754507152267895,0.9131398930410
12,0.826277723728808,0.408646567028188,0.818965292523313
"7805","TSPAN4",2060.19103849968,0.20225146239938,0.244758938451665,0.
826329218776704,0.408617363031298,0.818965292523313
"7806","PIK3CD",13.1351762127935,0.535483542076552,0.649453796818489,0
.824513683190015,0.40964774423143,0.81900270597283
"7807","RBM15",37.8928272800857,0.275595753440425,0.334048816417985,0.
82501640447539,0.409362277393745,0.81900270597283
"7808","ITPKB",47.7444599812926,0.206793668246027,0.250824457363407,0.
824455758500513,0.40968064397389,0.81900270597283
"7809","KCTD6",78.8924422401225,-0.19302102858894,0.233820562308228,-0
.825509213918042,0.409082553823815,0.81900270597283
"7810","PCGF3",123.464075486368,0.16764586501978,0.203283143741054,0.8
24691422685448,0.409546802550726,0.81900270597283
"7811","SLIT2",197.702057738685,-0.200662404197957,0.243051223429061,-

0.825597178104822,0.409032636437222,0.81900270597283
"7812","RFC1",567.315505307242,0.135290234684184,0.163966099191221,0.8
25111015944861,0.409308566153521,0.81900270597283
"7813","OSTC",1458.54007781837,-0.229789249275674,0.27866514111313,-0
.824603020597892,0.409597005948614,0.81900270597283
"7814","LINC00847",59.4586346022271,-0.227886079944488,0.2764065641946
25,-0.824459725146137,0.409678390970492,0.81900270597283
"7815","MYLK4",3.13152683760918,-0.692190318291165,0.839259170264687,-
0.824763485244803,0.409505881051339,0.81900270597283
"7816","EIF3B",386.304905935807,-0.158622841219636,0.192281800865111,-
0.824949841877717,0.409400067710293,0.81900270597283
"7817","YAE1",96.2956982346134,0.19658452385874,0.238450369593881,0.82
4425326719164,0.409697929077104,0.81900270597283
"7818","ZNF202",16.2705541934905,-0.313201481208408,0.379503716739022,
-0.825292263010407,0.409205683312241,0.81900270597283
"7819","MYL6",10393.7311558022,-0.205962726955529,0.249619989668158,-0
.825105101676085,0.409311923580367,0.81900270597283
"7820","RHOT2",139.627051265195,-0.168157370096915,0.203664311145952,-
0.825659484230444,0.40899728152571,0.81900270597283
"7821","LINC01229",2.84571770638807,0.83778862484625,1.0153435400352,0
.825128236712084,0.409298790318752,0.81900270597283
"7822","RPL13",32318.0692971834,-0.192876460971313,0.233995456174114,-
0.824274386028227,0.409783669062308,0.81900270597283
"7823","TIMM13",818.01932555942,-0.208417064198148,0.252515483912475,-
0.825363502344227,0.409165249325086,0.81900270597283
"7824","AC104532.2",17.7826575796261,0.380451788353848,0.4607092649881
33,0.825795826710035,0.408919921860829,0.81900270597283
"7825","LINC00665",40.0310609216351,-0.305216934018878,0.3703108090669
09,-0.824218268939945,0.409815548397914,0.81900270597283
"7826","ZNF460",67.7740198220035,-0.274713480958871,0.333289401931259,
-0.824249074129068,0.409798048216831,0.81900270597283
"7827","PYCR1",101.754785419434,-0.366796428784238,0.445349314385516,-
0.823615119494091,0.410158282278006,0.819582922553725
"7828","KCMF1",287.195436148414,-0.162061831686382,0.196895495352047,-
0.823085522584542,0.410459361051712,0.819870294616702
"7829","POLA2",18.2932761906539,-0.333905285432266,0.405656328243262,-
0.823123570827252,0.410437726038173,0.819870294616702
"7830","EIF1AX",1811.82162690471,-0.107517770976889,0.130614595471433,
-0.823168119832398,0.410412395418481,0.819870294616702
"7831","ZNF276",77.486927237515,-0.22426118691812,0.272514461067929,-0
.822933161195503,0.410546003660856,0.819938640946979
"7832","LRRC8C",188.708450928373,0.251041727097019,0.305144656487292,0
.822697437952592,0.410680072655836,0.819951468135534
"7833","AC025181.2",65.9261360350295,-0.234181980066635,0.284631402182
349,-0.822755248616616,0.410647190090553,0.819951468135534
"7834","LSP1",1685.36980374777,0.178555921993241,0.217050912280005,0.8
22645342134735,0.41070970597019,0.819951468135534
"7835","TMEM38B",60.3819897890827,-0.284224717753104,0.34554406126378,
-0.822542620798029,0.410768139979469,0.819963460022834
"7836","FCER1A",16.3572117109642,-0.616955763010795,0.750582720526931,

-0.821969046366634,0.411094514000537,0.819987016830558
"7837","UTP25",81.4037508906486,0.194287853362157,0.236270155489787,0.822312293143411,0.410899182003529,0.819987016830558
"7838","CALM2",3105.08251387989,-0.139922594983352,0.170225278217079,-0.821984821813108,0.411085535433311,0.819987016830558
"7839","SPIN1",272.111735992774,0.124695837699813,0.151670859318666,0.822147631126841,0.410992879604006,0.819987016830558
"7840","IKZF5",74.9785842306877,0.253705436086588,0.30861719063808,0.822071627189792,0.411036132390907,0.819987016830558
"7841","FKBP2",153.956839402916,-0.256263968661558,0.311677949975337,-0.822207566116999,0.410958773337943,0.819987016830558
"7842","AL021155.5",55.2959068702054,0.337206424091446,0.411386014704112,0.819683732647015,0.412396425938682,0.820053063060066
"7843","NTNG1",7.7015179862744,-0.709999697914911,0.866873141773097,-0.819035293287184,0.412766277643268,0.820053063060066
"7844","LIX1L-AS1",4.19539148616209,0.580990286407613,0.707136065121408,0.821610316690413,0.411298715779815,0.820053063060066
"7845","BX470102.2",14.3598705101117,-0.378997621025694,0.462422918863965,-0.819590910322478,0.412449357149151,0.820053063060066
"7846","AGT",9.89503379014025,0.548559900141336,0.669242215663711,0.819673187527941,0.412402439008266,0.820053063060066
"7847","C2orf42",28.589018801397,0.268159775086511,0.327473521062859,0.818874680970121,0.412857916786123,0.820053063060066
"7848","GCA",43.6838836747118,-0.251696211490435,0.30731877652121,-0.819006942366453,0.412782452700175,0.820053063060066
"7849","AC104162.2",13.1855226541354,0.449923522981261,0.5488016180600,4,0.819829075161434,0.412313553587078,0.820053063060066
"7850","FIP1L1",362.884164315956,0.135758916893297,0.165362875900606,0.820975785247574,0.411660061200676,0.820053063060066
"7851","ZNF330",189.071835774529,-0.136589758182261,0.166485151093075,-0.82043207628709,0.411969835906212,0.820053063060066
"7852","AC012640.2",8.37949454446373,-0.478502855108297,0.582546241085863,-0.821398923141914,0.411419076746275,0.820053063060066
"7853","AL022068.1",3.39826174952555,-0.828355857465882,1.01082104360472,-0.819488140563297,0.412507965504472,0.820053063060066
"7854","IER3-AS1",16.111939706363,-0.341680959992186,0.415930221096269,-0.821486255775346,0.411369349704547,0.820053063060066
"7855","RPS12",23232.0389772618,-0.177644847825234,0.216440934867708,-0.820754391648757,0.411786182105319,0.820053063060066
"7856","SLC37A3",375.457303371622,0.143585896709027,0.174872310874334,0.821089948380734,0.411595035045186,0.820053063060066
"7857","TRBC1",22.2337479353385,0.420422371822499,0.51210118635697 8,0.820975195963379,0.411660396866751,0.820053063060066
"7858","NFX1",138.14609074611,0.193545295414938,0.235940356511786,0.82031449929283,0.412036842824413,0.820053063060066
"7859","FGD3",3.8830621333453,-0.699564872574631,0.852774173870782,-0.820340125216589,0.412022238105588,0.820053063060066
"7860","STXBP1",175.165306692985,0.163117536114566,0.199036968341442,0

.819533865863264,0.412481888307333,0.820053063060066
"7861","RPL27A",13953.7634722709,0.13683628003539,0.16692085492,0.8197
67428707282,0.412348702362272,0.820053063060066
"7862","NRIP3",13.6330956183354,-0.457404866905417,0.557865363242117,-
0.819919817654822,0.412261818441677,0.820053063060066
"7863","COA4",375.638663258461,-0.172643277940141,0.210819262585503,-0
.818916050757558,0.412834311641528,0.820053063060066
"7864","SLC37A4",31.0842761554315,0.260676545116618,0.317989733254076,
0.81976402964034l,0.41235064044982,0.820053063060066
"7865","MCAM",3.49500159006669,0.774256459775826,0.943424507415197,0.8
20687244914955,0.411824437997715,0.820053063060066
"7866","PEX5",61.8224795880098,-0.210369626210386,0.256161586782639,-0
.821237988304978,0.4115107220918,0.820053063060066
"7867","PTGES3",1449.9969199519,-0.149370254322085,0.182329971481299,-
0.819230393711793,0.412654977141873,0.820053063060066
"7868","DCUN1D2",26.9712051895117,0.265731539851145,0.324252693509233,
0.81951991508616,0.412489844349796,0.820053063060066
"7869","ISLR2",6.46815432742175,-0.708191533224899,0.863307725115662,-
0.820323405689458,0.412031766858694,0.820053063060066
"7870","NOMO2",7.00888335129286,0.612809074751121,0.747893004691423,0.
819380674651401,0.412569257298711,0.820053063060066
"7871","COPRS",582.022452805701,-0.228125311589508,0.278451154343688,-
0.819265095622251,0.412635182328438,0.820053063060066
"7872","STARD3",140.623759855486,-0.162726987783644,0.198700426211442,
-0.818956410342483,0.412811283677986,0.820053063060066
"7873","SAP30BP",255.131694732949,-0.124069069533678,0.151087238522505
,-0.821175042624117,0.41154657019738,0.820053063060066
"7874","C18orf25",69.6563124705705,0.233417837729876,0.28454145871599,
0.820329799331131,0.412028122994217,0.820053063060066
"7875","IFFO2",27.8467231759551,0.316027795730572,0.386051814012004,0.
818614974104862,0.413006120927025,0.820139122815981
"7876","CLTC",850.257046302424,0.127863951764285,0.156187012335339,0.8
18659310095234,0.412980817950625,0.820139122815981
"7877","SUMO4",14.9499232226163,0.335886038049034,0.410363920980357,0.
818507721747575,0.413067334677422,0.820156546192063
"7878","LRRC8C-
DT",31.9731748738627,0.257696860122022,0.315082053125561,0.81787222587
1682,0.413430151042971,0.820290779583553
"7879","ABCC5",95.8752554632496,0.175997081069708,0.215367193654216,0.
817195405128794,0.413816767961268,0.820290779583553
"7880","BBS12",20.3006448977997,-0.285954115935934,0.349671956798217,-
0.817778235790719,0.413483827733997,0.820290779583553
"7881","WDR17",6.42687339622316,-0.549415862176756,0.672258478750141,-
0.817268773163303,0.413774847969354,0.820290779583553
"7882","CCDC110",4.57441136577318,-0.559605421620358,0.684786095443004
,-0.817197407109056,0.413815624064689,0.820290779583553
"7883","AL359643.3",16.3413315520615,-0.326478189149533,0.399373468605
757,-0.817475908675889,0.413656511369579,0.820290779583553
"7884","HIST1H1D",6.75275253400798,-0.746086515098006,0.91201020918429
3,-0.818068161501514,0.413318267647845,0.820290779583553

"7885","COG5",122.605670610928,0.149747015303842,0.18318163354393,0.817478326875659,0.413655129969417,0.820290779583553
"7886","AKR1B1",380.14254156455,-0.167312723091909,0.204494293878452,-0.818177954595434,0.413255581304882,0.820290779583553
"7887","ERI1",57.1327809177837,-0.183302840535325,0.22419621886283,-0.817600053493653,0.413585596989055,0.820290779583553
"7888","MAP6",10.035423499571,0.536784766995849,0.656017271259327,0.818247918938792,0.413215638115705,0.820290779583553
"7889","LNX2",11.7267474758476,-0.384237506402179,0.470174115895685,-0.817223861143873,0.413800508866672,0.820290779583553
"7890","CPT1C",7.38487040347129,-0.658270542043828,0.805336165307332,-0.817386043743136,0.413707848780427,0.820290779583553
"7891","FTSJ3",153.191551640466,-0.221978626438678,0.271721432565174,-0.816934550738595,0.41396583161441,0.820482271758886
"7892","MALSU1",298.483900159276,-0.228217165094266,0.279395945208945,-0.816823468656982,0.414029318438579,0.820504123210768
"7893","AGTRAP",593.6350177727,-0.22490550942786,0.276116988936467,-0.814529777012776,0.415341520893475,0.820587438560826
"7894","DIPK1A",156.41272682317,-0.161012594170988,0.197877475526244,-0.813698445175654,0.415817725107124,0.820587438560826
"7895","FLVCR1",38.5210636314242,0.293884011541809,0.361061675728228,0.813944074648941,0.415676989877744,0.820587438560826
"7896","SLC66A3",598.718766527885,-0.161185221946914,0.19798078285201,-0.814145795490663,0.41556143346433,0.820587438560826
"7897","GALM",107.354669779142,0.226138122192079,0.277773540039345,0.814109659833142,0.415582132493303,0.820587438560826
"7898","DDX18",558.021360979457,-0.144260041317285,0.176740191819807,-0.816226574340053,0.414370560526686,0.820587438560826
"7899","LANCL1",105.724954424974,0.155429942054202,0.190451596598113,0.816112570493106,0.414435754983992,0.820587438560826
"7900","RBM5-AS1",10.4884091704902,0.50141472195666,0.614261535963978,0.816288653284753,0.414335062493989,0.820587438560826
"7901","ARF4",1270.44729436687,-0.201778990065467,0.247718585179972,-0.814549259268822,0.415330364916131,0.820587438560826
"7902","TMEM41A",141.904345373047,0.168311153372766,0.206607540995158,0.814641869130567,0.415277336850429,0.820587438560826
"7903","TMEM150C",19.8827140109954,-0.537375113814477,0.65970154469591,-0.81457307192176,0.415316729496708,0.820587438560826
"7904","SPOCK1",539.481161150245,-0.155701889542876,0.191345959957632,-0.813719242242437,0.415805808183265,0.820587438560826
"7905","HLA-F",176.825963396568,-0.325289603472137,0.398484342977672,-0.81631715073524,0.414318767661047,0.820587438560826
"7906","HLA-DOA",4.76698267285191,-0.803386493163375,0.984185270071433,-0.816295993847849,0.414330865126148,0.820587438560826
"7907","AL354740.1",50.9220358348309,-0.228947937120731,0.281331596804714,-0.813801008209026,0.415758957435133,0.820587438560826
"7908","ME1",30.0423234093236,-0.288461756808936,0.354527756163593,-0.

813650699540232,0.415845084588868,0.820587438560826
"7909","HOXA10",467.108766753362,0.134164762882032,0.164561036741468,0
.8152887557023,0.414907043557122,0.820587438560826
"7910","SRRT",126.711120858082,-0.221300916356344,0.271385360900641,-0
.81544898229557,0.414815356202344,0.820587438560826
"7911","ZC3HAV1L",7.78727285373576,0.523291130270226,0.642789496911031
,0.814094089565772,0.415591051556105,0.820587438560826
"7912","CCAR2",73.4384873644821,-0.168181037140366,0.206544784812099,-
0.814259422204082,0.41549635040207,0.820587438560826
"7913","AL158211.1",3.18145742439552,0.761204359820254,0.9356676226942
84,0.813541412952115,0.415907712626066,0.820587438560826
"7914","TMEM179B",709.249259956987,-0.170549877813174,0.20906573379346
5,-0.815771550500283,0.414630807537658,0.820587438560826
"7915","THAP12",380.515074122201,0.141849396959041,0.17391601522751,0.
815620095558653,0.41471745233524,0.820587438560826
"7916","LLPH",330.557040413691,-0.163954220529825,0.201239585003126,-0
.814721519761024,0.415231732392775,0.820587438560826
"7917","DEPDC4",3.20954857734513,-0.66319058894544,0.814207590279939,-
0.8145227296609,0.415345556409031,0.820587438560826
"7918","SAP18",1822.41865210283,-0.200200474666856,0.24563953770084,-0
.81501730763993,0.415062403127011,0.820587438560826
"7919","PSMC1",756.660284236809,-0.168753591836571,0.206953563927303,-
0.815417664881814,0.414833276198403,0.820587438560826
"7920","SLC30A4",106.757479143083,0.163307139594972,0.200193270312253,
0.815747399202044,0.414644623367475,0.820587438560826
"7921","UBE2Q2L",6.25839051942067,0.567260243534848,0.696744389176153,
0.814158323119888,0.415554257598679,0.820587438560826
"7922","ECI1",361.724759081027,-0.190809784536424,0.234295897336623,-0
.814396610036581,0.415417780035309,0.820587438560826
"7923","SNX29",252.831483497968,0.163875845392279,0.201423037002312,0.
813590380877825,0.415879650259252,0.820587438560826
"7924","PRKCA",72.9320620988316,0.282019244322201,0.345515891959807,0.
816226549588079,0.414370574680738,0.820587438560826
"7925","RTL10",57.5763132519963,0.256959180660639,0.315029069107568,0.
815668158460955,0.414689955206385,0.820587438560826
"7926","GGA1",149.524127667031,0.223977624089393,0.27488879730384,0.81
4793568476443,0.415190483007399,0.820587438560826
"7927","PRR34",6.44327696802461,0.506941612791532,0.622825602672073,0.
813938300892945,0.415680297671209,0.820587438560826
"7928","MTOR",66.6731882728053,0.220718178547006,0.271652966214825,0.8
12500528238151,0.416504483592965,0.820644078340296
"7929","HIBCH",210.878963849217,-0.150682995255979,0.185422034597309,-
0.812648807264062,0.416419439857358,0.820644078340296
"7930","UIMC1",104.051553039062,-0.170920879712578,0.210290566722943,-
0.812784341095843,0.416341714945469,0.820644078340296
"7931","COL14A1",2122.46830063957,0.218591074012219,0.26899108630119,0
.812633150852673,0.416428418928794,0.820644078340296
"7932","MTBP",8.83299995119022,0.421190110133481,0.5179409529074,0.813
201017932989,0.41610281590284,0.820644078340296
"7933","ZBTB5",24.5071079853946,0.293754961129932,0.361714285294997,0.

812118771837723,0.416723482747995,0.820644078340296
"7934","MRRF",77.2234696129831,-0.192218992119389,0.236355706057426,-0
.813261483404537,0.416068155129449,0.820644078340296
"7935","RNF121",104.845738756415,-0.173568991476468,0.213700320740956,
-0.812207444867927,0.416672608341552,0.820644078340296
"7936","AC002070.1",6.28861402922427,-0.506187098031647,0.622522056625
441,-0.813123153861534,0.41614745262964,0.820644078340296
"7937","ACADS",213.391678289806,-0.231167452740236,0.28455742097863,-0
.812375414231762,0.41657624926751,0.820644078340296
"7938","RPL27",7657.32272069255,-0.160338345085432,0.197388894144988,-
0.812296688625544,0.416621410190823,0.820644078340296
"7939","G6PC3",148.28559197843,-0.219741740152849,0.270335199466763,-0
.812849161286766,0.416304545381447,0.820644078340296
"7940","TP53INP2",78.154074259089,0.430850440075811,0.530406258329111,
0.81230270817897,0.416617956973609,0.820644078340296
"7941","PIGT",1469.82245099529,-0.192074564007477,0.236271610445124,-0
.812939665690762,0.416252651105401,0.820644078340296
"7942","CLDN5",5.34915324331192,-0.590131575919965,0.726637514898589,-
0.812140254005912,0.41671115744037,0.820644078340296
"7943","CHRD",111.23418709702,-0.200684368978178,0.247232799201031,-0.
811722269968704,0.416951012710684,0.820885427844297
"7944","FKBP8",1251.66685177103,-0.180359734303369,0.22218514818416,-0
.81175423189797,0.41693266885575,0.820885427844297
"7945","CCDC125",44.056029974524,0.265348000395776,0.326938073855633,0
.811615475880446,0.41701230830927,0.820902769283446
"7946","ZKSCAN5",29.0490468402506,0.263544181521172,0.324822204538675,
0.811349032913153,0.417165259236982,0.820919775646545
"7947","CCT2",438.153582802967,-0.151476652705458,0.186690148914719,-0
.811379998280752,0.417147481945836,0.820919775646545
"7948","HEXIM1",348.356559471875,0.176264133899193,0.217254355751845,0
.81132612181331,0.417178412841352,0.820919775646545
"7949","RIN3",33.5922411132299,-0.236204452206098,0.29120997829812,-0.
811113869059419,0.417300281939912,0.821056285009463
"7950","MIA3",429.838469679263,-0.123003835752508,0.151700592344171,-0
.810832929863868,0.417461620978228,0.821157950308358
"7951","CDK4",493.528831681008,-0.198219071022601,0.244450113142451,-0
.810877395287155,0.417436082724294,0.821157950308358
"7952","ZMYM3",54.4492320074609,0.192576013572787,0.23753525707465,0.8
10726019978862,0.417523027328359,0.821157950308358
"7953","TRMT2B",48.0619109334631,0.273697673751415,0.337624000121817,0
.810658228244032,0.417561967954116,0.821157950308358
"7954","TARDBP",275.623883838252,0.160688446850878,0.198283700420479,0
.810396651414732,0.417712241808234,0.821350196364192
"7955","AC017048.3",2.9639520422854,0.727338474261586,0.89806825356424
1,0.80989220070405,0.418002134777643,0.821415503903811
"7956","SSR1",593.975795639975,0.131022278439771,0.161807195621327,0.8
09743212819773,0.418087776388828,0.821415503903811
"7957","NEAT1",14751.5319451634,0.265134330344133,0.327295613229468,0.
810076027991998,0.417896480928279,0.821415503903811
"7958","LGMN",734.415437268113,-0.183655344641335,0.226793284464397,-0

.809791811406859,0.418059839684441,0.821415503903811
"7959","SNRPA1",235.479541992338,-0.188485600575799,0.232713027357409,
-0.809948642395151,0.417969693513666,0.821415503903811
"7960","RPS2",18844.5575703859,-0.18968443284128,0.234160567194274,-0.
81006138272592,0.417904897647762,0.821415503903811
"7961","CNOT3",129.977443930234,0.148751238493287,0.183711734397496,0.
809699167998898,0.418113096328532,0.821415503903811
"7962","ZRANB3",20.0006002796994,0.286412396041966,0.353848375190644,0
.809421255326253,0.418272880138724,0.821419870086596
"7963","DELE1",90.1920293195378,0.206110939648001,0.254628345086262,0.
809457955586896,0.418251777538486,0.821419870086596
"7964","LRRC4C",16.1110307413495,-0.47687115688472,0.589042190534016,-
0.80957045954959,0.418187091818496,0.821419870086596
"7965","SHISAL2A",4.48458508323826,-0.61162346043096,0.756289233627592
,-0.808716339246649,0.418678327575943,0.82195488194327
"7966","AL109615.2",4.05232146085177,-0.664584129356397,0.821820092863
292,-0.808673498162997,0.418702975987342,0.82195488194327
"7967","ENY2",940.589330304689,-0.18734622168388,0.231627589799292,-0.
808825156995405,0.418615723647003,0.82195488194327
"7968","CAMTA1",434.871550654607,-0.184920083426729,0.2288322930729,-0
.808103091322944,0.419031238189489,0.821997432423715
"7969","CYFIP2",10.7346419417308,0.515013198711985,0.63732423148564,0.
808086642981483,0.419040706255269,0.821997432423715
"7970","LINC02848",3.36209401460044,0.755539576767076,0.93491435919644
5,0.808137739393005,0.419011294328612,0.821997432423715
"7971","ZER1",102.873076250932,0.160417593244157,0.198438085826226,0.8
08401232939914,0.418859642431633,0.821997432423715
"7972","ABLIM1",2550.06184984204,0.206397017361999,0.255442935416112,0
.807996576714018,0.419092552822679,0.821997432423715
"7973","DHX38",180.836484236378,0.172232140145989,0.213134730820299,0.
808090448155086,0.419038515893517,0.821997432423715
"7974","SPIRE1",73.0966402958994,-0.205192268915218,0.253925350287078,
-0.808081070610854,0.419043913876002,0.821997432423715
"7975","RANBP2",243.307815208028,0.168760589701047,0.208951910572254,0
.807652771582055,0.419290498634106,0.822108211804724
"7976","PCNP",662.819780506622,-0.102375997505884,0.126740570965572,-0
.807760267497091,0.419228601951621,0.822108211804724
"7977","DINOL",13.3136473920031,-0.418797372294981,0.518554508558567,-
0.807624589860605,0.419306726698612,0.822108211804724
"7978","ACOT9",434.886758918238,-0.137095192296556,0.169780683227244,-
0.807484041709627,0.419387664971597,0.822163835567283
"7979","FAM227A",7.2133944232154,-0.520130150824642,0.644369090625908,
-0.807192893624698,0.419555359319743,0.822389499907355
"7980","AC127035.1",3.25080103392293,-0.802640964435864,0.994874003585
847,-0.806776497870973,0.419795261830191,0.822756628449146
"7981","GOLGA5",201.297011968672,-0.134360617916497,0.166582894947284,
-0.806569113587658,0.419914774411927,0.822887742363431
"7982","RCC2",95.3952725835572,0.165323078685341,0.20504688994668,0.80
6269623149765,0.420087401729541,0.823047041304165
"7983","HYI",330.250967622037,0.219376604861279,0.272161973921142,0.80

6051637929561,0.420213075374675,0.823047041304165
"7984","SPRR2B",101.139057265094,-0.931012875836123,1.15491147800788,-0.806133537993787,0.420165855456815,0.823047041304165
"7985","ZC3H6",356.855705151879,0.157100991675111,0.194921233775594,0.805971666770673,0.420259186179991,0.823047041304165
"7986","PSMD12",390.031109235181,-0.102492730255989,0.127160353803142,-0.806011678881132,0.420236115108213,0.823047041304165
"7987","C9orf64",102.837895146228,-0.17086765684985,0.212054441981454,-0.805772589591847,0.420373985583289,0.823074457683793
"7988","PEMT",139.691495420529,-0.231706499347721,0.287621692885338,-0.805594658119519,0.420476606736718,0.823074457683793
"7989","MRPL12",188.537531081256,-0.222183533754763,0.275864760756761,-0.805407450901893,0.420584593514472,0.823074457683793
"7990","DCXR",95.4144393044243,0.185966807396863,0.23085250981327,0.805565456261602,0.420493450178472,0.823074457683793
"7991","BAX",381.122079004038,-0.211306640278185,0.262362383293626,-0.805399911471678,0.420588942826654,0.823074457683793
"7992","AL022323.4",72.9194089444499,0.335443301187312,0.416330973872392,0.805713055810562,0.420408319778527,0.823074457683793
"7993","ABHD11",71.1108709520265,0.268837839855839,0.333892662499425,0.805162467013788,0.420725932217571,0.823136549897774
"7994","EXOSC3",132.753647820194,-0.186560374567867,0.231682405998798,-0.805241873087397,0.420680117369188,0.823136549897774
"7995","ITGA2B",2.60581334786286,0.876857100203734,1.08921046074458,0.805039183707728,0.420797068676256,0.823172752232227
"7996","CFH",2832.14389465762,-0.212458111033566,0.263977323829314,-0.804834703040403,0.42091507289077,0.82319766662644
"7997","SLC6A2",4.24004593350493,-1.03925412768564,1.29125661487745,-0.804839344644345,0.420912394041532,0.82319766662644
"7998","ABCD3",154.941259849902,-0.125438598540589,0.15592217477695,-0.804494926523644,0.421111198243869,0.823272392566764
"7999","DUSP1",10089.4181501162,-0.25922646473203,0.322193424144408,-0.804567831948812,0.421069111352102,0.823272392566764
"8000","SNU13",1071.29905231923,-0.18020656157952,0.223952711714202,-0.804663449708485,0.421013916810966,0.823272392566764
"8001","PDE5A",83.7564578448043,0.213357630000794,0.265334313114353,0.804108701571673,0.421334199614543,0.823399585401906
"8002","AC069185.1",6.99540796975512,0.562721187026117,0.699690249844262,0.804243287871123,0.421256483334738,0.823399585401906
"8003","MRTFA-AS1",4.15243628821893,0.733516402249247,0.912145676601508,0.804165848795335,0.4213011991685,0.823399585401906
"8004","AL139424.3",2.85614032803521,0.844965346499085,1.05153375694302,0.803555131654103,0.421653944623436,0.823440419809901
"8005","TMEM167B",226.722759186129,0.116488490722294,0.144980439755547,0.803477289203333,0.421698918257985,0.823440419809901
"8006","LBX2",20.2215980707229,0.369233021446503,0.460154142147931,0.802411599997726,0.422314905190733,0.823440419809901
"8007","PLA2R1",142.824752954516,0.203794335836184,0.253744929394691,0.803146436550817,0.421890100436739,0.823440419809901

"8008","TRAT1",2.81404797102095,0.862765933522374,1.07503910266135,0.802543769232695,0.422238480439368,0.823440419809901
"8009","NCBP2AS2",257.668097121323,-0.205579477342843,0.256280231978857,-0.802166736604962,0.422456514959333,0.823440419809901
"8010","NAAA",290.109541841642,-0.17791881191743,0.221410787488004,-0.803568850172078,0.421646019013281,0.823440419809901
"8011","FOXF2",54.8792652170633,0.298012856536593,0.371282720823031,0.802657489354691,0.42217273008099,0.823440419809901
"8012","AL353147.1",2.68275166699137,-0.699035345142008,0.871036914371267,-0.802532399727958,0.422245054355429,0.823440419809901
"8013","BAMBI",53.9550397878452,0.374877630524209,0.4667043288104,0.803244382754599,0.421833497232187,0.823440419809901
"8014","DCLRE1A",13.43335544535,0.337978989455649,0.420682027925766,0.803407245900434,0.421739388323278,0.823440419809901
"8015","RBM4",676.417042691736,-0.163061291879761,0.203060362600931,-0.803018815642621,0.421963859362102,0.823440419809901
"8016","FBXL14",31.8554499288691,-0.276602523381057,0.344766100495335,-0.802290373049017,0.422385009866441,0.823440419809901
"8017","MGP",15330.9990358618,-0.267657085830893,0.333237905282633,-0.803201201267552,0.421858451305886,0.823440419809901
"8018","MED21",299.319498185488,-0.160055868170454,0.199486448034467,-0.80233955613265,0.422356566819587,0.823440419809901
"8019","ATXN3",376.88082296944,0.159839308502442,0.199060914410898,0.802966815336257,0.421993915278497,0.823440419809901
"8020","MYO19",62.3826742985551,-0.248770338659007,0.310125802053287,-0.802159436628438,0.422460737119862,0.823440419809901
"8021","AKAP8",114.242190315441,-0.186450599696898,0.232015578328945,-0.803612417061727,0.421620849659259,0.823440419809901
"8022","TSHZ3",96.7338611379578,0.220888223732729,0.27535607138303,0.802191223252404,0.422442352552384,0.823440419809901
"8023","PYGB",184.931282779162,-0.168543099402471,0.209956403576754,-0.802752840738466,0.422117604730009,0.823440419809901
"8024","CSNK1E",538.26389056215,-0.102272161390348,0.127484525524942,-0.802231964775512,0.422418789386557,0.823440419809901
"8025","AC006064.2",3.48213132464036,0.680106666845082,0.848007195916405,0.802005773205875,0.42254961871011,0.823511032601386
"8026","ANKRD65",170.464528165915,0.250084219563342,0.312053036635849,0.801415753743099,0.422890997981542,0.823855919495899
"8027","SLC16A1-AS1",126.300764469177,0.222502017303471,0.277740083860479,0.801115972209626,0.423064510394638,0.823855919495899
"8028","RGPD5",50.8695719189441,0.416373810964303,0.519675282412796,0.80121919409198,0.423004761256399,0.823855919495899
"8029","ZFYVE28",32.0395494072302,0.262441157699953,0.327611041394053,0.801075435623937,0.423087976015327,0.823855919495899
"8030","HMGB1",4389.31619655971,-0.136789575555969,0.170714358813207,-0.801277505342369,0.422971010452469,0.823855919495899
"8031","CNTROB",34.4275010659345,-0.231279263599088,0.288715536856659,-0.80106275580837,0.423095316201474,0.823855919495899
"8032","LINC00891",76.0455188708312,0.261317449782758,0.32604584904851

9,0.801474548887361,0.422856972464209,0.823855919495899
"8033","ZBED6",6.51499802488638,-0.501404876104137,0.626073434266582,-0.800872307721395,0.423205573179101,0.823865467328994
"8034","NANS",488.136790737685,-0.190364575058005,0.237686366674957,-0.800906580049391,0.42318573050331,0.823865467328994
"8035","KAT2B",108.141689174045,0.191350438145646,0.238962217123231,0.800756037708538,0.423272894174871,0.823893971984441
"8036","OAF",1267.86924607601,0.23866690628722,0.29808925756476,0.800655844618518,0.423330911569298,0.823904362486786
"8037","CDR2",123.229251856136,-0.16064370109714,0.200687462322932,-0.800467050794849,0.423440246384205,0.824014614090949
"8038","RAB11A",518.905962807164,-0.128613975944754,0.160699883886111,-0.800336458462554,0.423515885054666,0.824059273731646
"8039","CCDC127",236.155452144651,-0.14044332954977,0.175540514645467,-0.800062195518904,0.423674763030397,0.824114246499054
"8040","NUDT19",50.2491603307165,-0.215950884830823,0.269933608490334,-0.8000148111922,0.4237022158631,0.824114246499054
"8041","RRBP1",1792.83090463663,0.167615524225292,0.209493984589921,0.800097074641047,0.423654555943752,0.824114246499054
"8042","AC012676.1",3.09180863025876,0.828954969501991,1.03666314520485,0.799637735108434,0.423920717737372,0.824436710446716
"8043","SPARC",9519.35268353488,-0.27532753881584,0.344431859400186,-0.799367222577237,0.424077510501966,0.824639097880237
"8044","DENND2D",4.2101512572464,0.624082024610026,0.780914215024586,0.799168477923505,0.42419272723068,0.824756543016138
"8045","DENND5A",256.367464183076,0.133644037187087,0.167247146145418,0.799081121963579,0.42424337522793,0.824756543016138
"8046","MED6",203.854754598076,0.132724938466152,0.166142654506841,0.798861309036604,0.424370835917213,0.824901798874623
"8047","STK10",42.8131989351332,-0.20965165912104,0.262625240317187,-0.798292117192666,0.424700991430626,0.824928403623322
"8048","PSD3",402.259034221585,-0.213936008643888,0.267955776036476,-0.798400436849571,0.42463814985417,0.824928403623322
"8049","TRIB1",76.9511103221506,-0.733857012710468,0.918992519809698,-0.798545142524591,0.42455420743303,0.824928403623322
"8050","DNAJC25",94.8410182408439,-0.180365453758503,0.225905023956035,-0.79841276037139,0.42463100071554,0.824928403623322
"8051","FBXW2",235.349317618904,-0.127337981663853,0.159504461398829,-0.798334921463128,0.424676157912339,0.824928403623322
"8052","AC015712.2",33.1817728934438,0.305975059560166,0.383221686860054,0.798428351138443,0.424621956278844,0.824928403623322
"8053","TXLNB",3.14388342128084,-0.744578613469573,0.933030586636629,-0.79802165559611,0.424857923268082,0.824947060603093
"8054","POC1B",63.3436012376021,0.224495104551724,0.281288697941979,0.798095004151324,0.424815360371327,0.824947060603093
"8055","KCTD19",2.49698559060574,-0.836067358153021,1.04778946706009,-0.797934493938822,0.424908504908231,0.824947060603093
"8056","MEAK7",75.204531323092,0.22535648372082,0.282432767202512,0.797911963094683,0.424921580576631,0.824947060603093
"8057","HFM1",6.04317082101941,0.535275575293656,0.671386919227241,0.7

97268400626202,0.425295168242081,0.825145192648198
"8058","CC2D2A",115.753544076271,0.206289739844171,0.258662707356083,0
.79752408823351,0.425146718765335,0.825145192648198
"8059","CCNI",4325.43755081116,-0.127967765109079,0.160586464683281,-0
.796877653178714,0.425522090475985,0.825145192648198
"8060","GIMAP5",3.62681103424916,-0.687323987945287,0.86213960794095,-
0.797230496794857,0.425317177375078,0.825145192648198
"8061","TMOD1",46.4592560332892,-0.368389740515919,0.462017319826994,-
0.797350499011305,0.425247499503172,0.825145192648198
"8062","AKIP1",170.111279075124,-0.175970628451101,0.220839051025092,-
0.796827497828304,0.425551222755778,0.825145192648198
"8063","CAPN5",85.6304579734654,0.17705398668445,0.222156866005228,0.7
96977333485983,0.42546419553128,0.825145192648198
"8064","IL34",52.9563731695222,-0.270533446031341,0.339368879569519,-0
.797166335270652,0.425354434742545,0.825145192648198
"8065","PPP4R1",175.419045174117,-0.167944587029128,0.210729177643029,
-0.796968834157475,0.425469131814979,0.825145192648198
"8066","SERPIND1",3.62717643950858,-1.05208211087689,1.31922535430489,
-0.797499917238357,0.425160750889959,0.825145192648198
"8067","ZNF284",22.1870398112886,0.29805014279025,0.3741455785767,0.79
6615434890537,0.425674410455484,0.825179447139782
"8068","ZNF444",79.1500947506528,0.181584067173734,0.227928883416453,0
.796669840399112,0.425642804223666,0.825179447139782
"8069","PCM1",728.310993568575,0.145526179786084,0.182779874107615,0.7
96182733446912,0.425925831971532,0.825359932106897
"8070","CCDC81",6.7919830755296,-0.518973727639783,0.651733244621152,-
0.796297767411664,0.425858982940461,0.825359932106897
"8071","NKAP",317.142756956256,-0.13202759269212,0.165817120773349,-0.
796224129790466,0.425901774845532,0.825359932106897
"8072","ULK3",53.2736045443152,-0.184276973965565,0.231556422495961,-0
.795818885001039,0.426137313450474,0.825462915824302
"8073","HOXB3",60.7815272276823,0.281746808730024,0.353995193451875,0.
795905746579936,0.426086820888448,0.825462915824302
"8074","WWC3",182.378170122426,-0.180673777418967,0.22702071685684,-0.
795847092373076,0.426120916146361,0.825462915824302
"8075","TIFA",80.2654523952208,-0.22890575359326,0.287675128077322,-0.
795709226317728,0.426201062934731,0.8254841639999
"8076","MRPS30",167.822264850384,-0.146917720448974,0.184670455806774,
-0.795566999643399,0.426283753931223,0.825542089089194
"8077","GBP5",7.23950774025754,0.583341797922626,0.734630752657791,0.7
94061228463656,0.427159787431341,0.825592690530316
"8078","AC012358.3",19.965854965403,0.312192821711037,0.39307363419664
6,0.794234958925925,0.427058660337536,0.825592690530316
"8079","LINC01116",208.505807611279,0.345091069997357,0.43419970481719
5,0.794774999081691,0.426744396443012,0.825592690530316
"8080","DUSP19",19.6490683801016,0.307875052954487,0.387637878895511,0
.794233664242794,0.42705941391049,0.825592690530316
"8081","AC137630.3",3.65748299971778,0.694621657238839,0.8741341294635
74,0.794639671219684,0.426823134705151,0.825592690530316
"8082","SERPINI1",20.5942802164434,-0.365545300397353,0.45998879880542

4,-0.794683047384333,0.42679789608422,0.825592690530316
"8083","HINT2",410.962300985115,-0.223231505342165,0.280989643586996,-0.794447448284873,0.426934990973618,0.825592690530316
"8084","XPA",344.999876633534,0.123404243337663,0.155288913106847,0.794675169454976,0.426802479829002,0.825592690530316
"8085","VPS26A",398.213746987835,0.107194047726734,0.134792384310725,0.795252997970787,0.4264663485368,0.825592690530316
"8086","CWF19L1",36.8628424020481,0.208568888264917,0.262664060901946,0.794051868187543,0.427165236368296,0.825592690530316
"8087","RPLP2",11601.72421753,-0.11178553369893,0.140665843116287,-0.794688541457203,0.426794699394722,0.825592690530316
"8088","ZFP36L1",6235.23927022667,0.138960423455104,0.174852629599858,0.794728816907752,0.426771265819775,0.825592690530316
"8089","PLIN1",3.34321670865918,-0.647612838032879,0.815654180843666,-0.793979670849018,0.427207266269939,0.825592690530316
"8090","MRPL38",239.695849091313,-0.177167585437012,0.222877467739282,-0.79491025824225,0.426665706615472,0.825592690530316
"8091","GPX4",9924.05376337254,-0.235223635039278,0.295722060272302,-0.795421331850195,0.426368455303507,0.825592690530316
"8092","ZNF708",158.168952012707,0.176654661563012,0.222343829866198,0.794511193179137,0.426897895348451,0.825592690530316
"8093","MCM5",60.5033164586203,-0.196003753310236,0.246735817984028,-0.794387109709885,0.426970106060288,0.825592690530316
"8094","OLFML3",1627.96836924528,0.190823708269599,0.240409745681303,0.793743646826042,0.427344685190142,0.825697692148522
"8095","AC073957.3",2.49718900750749,0.945380139939546,1.19123369194267,0.793614339767214,0.427419981818058,0.825697692148522
"8096","KAT14",44.3112867165353,0.195246006393143,0.246008028711162,0.793657050202949,0.427395110304218,0.825697692148522
"8097","MARCH1",8.29059866092957,-0.545644093766592,0.687655483571831,-0.793484683540076,0.427495489528386,0.825741565545752
"8098","BAG2",460.315740249731,-0.122754818473881,0.154761881112624,-0.793185102115357,0.427669986043252,0.825941565332226
"8099","IQSEC2",96.1723501355134,0.184882656858254,0.2331064052641,0.793125597079967,0.427704650743331,0.825941565332226
"8100","TIPRL",347.142781800223,-0.140203677163194,0.176901447989491,-0.792552456504048,0.428038617948783,0.826076503543802
"8101","RIF1",402.14065440371,0.178497849858337,0.225135748277756,0.792845433138941,0.427867882427691,0.826076503543802
"8102","NIPSNAP2",368.52647460588,0.123527983290761,0.155846749998148,0.792624698893169,0.427996514178121,0.826076503543802
"8103","MIGA2",35.39759196249,0.225455186810203,0.284424968903176,0.792670164225111,0.427970017648357,0.826076503543802
"8104","VSIR",1669.58163843697,0.211212154191644,0.266470245870867,0.792629411592913,0.427993767641354,0.826076503543802
"8105","DDX19B",159.6885107152,-0.184465218543716,0.232792535978726,-0.792401774258659,0.428126445209785,0.826144059602842
"8106","ZBTB7A",863.97163270715,-0.143748400188423,0.181532604931085,-0.791859953990049,0.428442339403835,0.826651639313593
"8107","UBXN2A",300.000962857557,0.138931216373264,0.175610385413218,0

.791133258129087,0.428866233326857,0.826826160043813
"8108","RAPGEF4",15.1335581301059,0.443537201725005,0.560670987376201,
0.791082848428892,0.428895647195688,0.826826160043813
"8109","GIMAP6",2.83397726128018,-0.801746078567197,1.01318474106759,-
0.791312823880861,0.428761466923425,0.826826160043813
"8110","NPM3",86.060938450438,-0.184116563460957,0.232743559520734,-0.
791070497676026,0.428902853992037,0.826826160043813
"8111","LINC02696",3.99306080302798,-0.731503389897884,0.9245986149571
72,-0.791157782484639,0.428851923882581,0.826826160043813
"8112","B3GNTL1",14.196627730266,-0.352482117712282,0.445476285565351,
-0.791247770383443,0.428799420219819,0.826826160043813
"8113","HCFC1",126.60282890993,0.190138380299851,0.240330763692267,0.7
91152898524946,0.428854773547926,0.826826160043813
"8114","S100A14",3.40530400764479,0.715049623014262,0.905519973497709,
0.789656378591268,0.429728473063992,0.82699602620533
"8115","RAB11FIP5",79.8229812200259,-0.178602174731286,0.2259384726676
82,-0.79049031633484,0.429241475344229,0.82699602620533
"8116","GGCX",207.388881458577,0.174266615460894,0.220751788298361,0.7
89423346484336,0.429864615041205,0.82699602620533
"8117","G3BP2",365.524601271692,0.137248280492808,0.17367822217035,0.7
90244618914797,0.429384922836183,0.82699602620533
"8118","NEK1",170.548871765982,0.137070559421458,0.173702799933355,0.7
89109671657846,0.430047909642126,0.82699602620533
"8119","CLK4",147.808236978746,0.157189256177821,0.199098243788583,0.7
89505990543723,0.429816329876681,0.82699602620533
"8120","ASCC3",153.750836434007,-0.16695974629767,0.211179758714465,-0
.790604872900794,0.429174602389299,0.82699602620533
"8121","ABCB4",4.09559246250718,-0.59595575287985,0.753656492220562,-0
.790752496703021,0.429088435204657,0.82699602620533
"8122","AC006333.2",76.9839447720891,-0.252049397262121,0.319175015738
635,-0.789690247774652,0.429708688100436,0.82699602620533
"8123","HABP4",107.614783402332,-0.182397133421208,0.231051666983392,-
0.78942141297911,0.42986574473807,0.82699602620533
"8124","AL359532.1",6.48903388559728,0.488265544881063,0.6183143883296
16,0.789671976096365,0.429719361586673,0.82699602620533
"8125","CTSW",9.26045484497286,0.539557658520266,0.683359823876635,0.7
89565964617889,0.429781291725942,0.82699602620533
"8126","AP002490.1",4.72434935926056,0.801521269203242,1.0138676758433
9,0.79055806620572,0.429201925294456,0.82699602620533
"8127","AP003486.2",3.21359378858512,0.789447341640481,0.9999160610412
2,0.789513612591065,0.42981187681981,0.82699602620533
"8128","UFM1",512.113749312495,-0.103226576949965,0.130789145179103,-0
.789259512390008,0.429960345162562,0.82699602620533
"8129","COCH",28.2748465070302,-0.377171385661209,0.477971427061315,-0
.789108646054746,0.430048509023526,0.82699602620533
"8130","AC015813.1",22.1874378720426,0.362598054167221,0.4594526276179
94,0.789195735036036,0.429997614342136,0.82699602620533
"8131","ZNF563",38.9230325866811,0.238629879048004,0.302272476694892,0
.789452885877109,0.429847356155943,0.82699602620533
"8132","UBE2S",392.209316430169,0.261774259936727,0.331504528405707,0.

789655155528849,0.429729187535917,0.82699602620533
"8133","GLUD2",3.96243276709499,-0.564760944233445,0.714910760313324,-0.789974043733691,0.42954292713842,0.82699602620533
"8134","PTGER4",161.128342361138,0.275417620428833,0.349113957676887,0.788904638077341,0.4301677446994,0.827078272577891
"8135","TRIAP1",155.607212611813,-0.219613291594293,0.27840566504207,-0.788824794786801,0.430214415593709,0.827078272577891
"8136","RPS16",13007.0148015324,-0.164953167351082,0.209128658180957,-0.788764049776235,0.430249924916478,0.827078272577891
"8137","ZNF124",20.4138835536105,0.322546933603969,0.409004769528905,0.788614113169099,0.430337579683689,0.827145108301941
"8138","NSUN7",10.6097602496828,0.450074924549191,0.570858493007001,0.788417672790359,0.430452436808062,0.827264206399372
"8139","LTV1",180.580511944088,-0.169047244469571,0.214466774863978,-0.788221133911237,0.430567369328319,0.827281775957605
"8140","STX8",440.673034119409,-0.199088497971339,0.252575446079705,-0.78823377751657,0.430559975031769,0.827281775957605
"8141","USP34",429.736253919469,0.15557135306236,0.197455948995996,0.787878784373899,0.43076761190812,0.827387718486227
"8142","SLU7",388.444965529344,-0.128903224914377,0.163612768670667,-0.787855532069402,0.430781214298807,0.827387718486227
"8143","FAM49B",148.739059485215,0.17556796856045,0.222796403772371,0.788019759689773,0.430685147941498,0.827387718486227
"8144","ROBO1",349.683268234523,0.145401827155694,0.184627685436484,0.787540757021058,0.430965379412785,0.827639800345771
"8145","RBMXL1",98.7203660568237,0.158790991038421,0.201754569018494,0.787050284962146,0.431252430394821,0.827853453286553
"8146","CRPPA",18.5566260009857,0.301895143117016,0.383481404860417,0.787248454007574,0.431136437723247,0.827853453286553
"8147","METTL22",30.5076219918755,0.249243969483361,0.316807864729441,0.786735423049613,0.431436763160572,0.827853453286553
"8148","ENKD1",82.8869447750502,-0.155457044329173,0.197537780520113,-0.786973731910208,0.431297243413218,0.827853453286553
"8149","DHX8",129.153529637056,0.193677137188138,0.246095693172496,0.78699929564547,0.431282278482424,0.827853453286553
"8150","TWSG1",240.689459282642,0.120714787140124,0.153425266472634,0.786798614826949,0.431399764501766,0.827853453286553
"8151","AC024075.1",16.9983555868413,0.501149262024669,0.637012841540291,0.786717675601162,0.431447154586873,0.827853453286553
"8152","USP24",109.199088270822,0.219618622689617,0.279499092585078,0.785757909474306,0.432009329669565,0.827856336845819
"8153","ATP1A1",725.988785708625,0.145812807559717,0.18548227197228,0.786128000316435,0.431792501764264,0.827856336845819
"8154","TFB2M",54.5320432715875,-0.212633014666164,0.270408096879808,-0.78634115294512,0.431667649054883,0.827856336845819
"8155","LIMS1",1108.29889237683,-0.119747707003525,0.152368665395253,-0.785907697576092,0.431921564590389,0.827856336845819
"8156","STK36",28.9759381302158,0.299294138080782,0.380942594150714,0.785667296533322,0.432062427365661,0.827856336845819
"8157","AC121247.1",10.8050727831552,-0.415792121041775,0.528653714026

319,-0.786511302219046,0.431568000287725,0.827856336845819
"8158","UNC5C",3.69906080316888,-0.60782635567376,0.773857360290631,-0.785450118928228,0.432189705265097,0.827856336845819
"8159","HEXB",715.794967588544,-0.15561338816434,0.197897611046031,-0.786332828081202,0.431672524894924,0.827856336845819
"8160","CD164",1474.46884764014,0.109462240273526,0.13918800075572,0.786434460436262,0.431613001408232,0.827856336845819
"8161","PRIM1",45.2832692085294,-0.224798195465476,0.286082755750935,-0.785780306385144,0.431996206030115,0.827856336845819
"8162","CSRP2",26.5590144629453,0.467356331847354,0.594911870020343,0.785589186229169,0.43210820175564,0.827856336845819
"8163","ELK3",250.181654682453,-0.17143208431891,0.218158114698898,-0.785815758242689,0.431975433217952,0.827856336845819
"8164","N4BP2L2-IT2",9.43585044961056,0.499334371423371,0.635711783758221,0.785472889099821,0.432176359686,0.827856336845819
"8165","COG8",70.4448756015106,0.208250059417321,0.265126382280707,0.785474676740518,0.432175311961343,0.827856336845819
"8166","LCMT1",195.800014983911,-0.192289649232295,0.244877581080175,-0.785248075320285,0.432308133314666,0.827981778721696
"8167","SCML2",9.63190478861911,0.40020941133523,0.50972721791013,0.785144283595605,0.432368978237035,0.827996916814893
"8168","CRTC1",69.1281091888353,0.184144328306192,0.234572180626701,0.785022025264113,0.432440655038463,0.828032791968849
"8169","MRPS27",133.758316583999,0.144329467023705,0.18387971806033,0.784912379386783,0.432504943345752,0.828054512660982
"8170","SNRPD2",1282.92148531475,-0.150894277459356,0.192299328782053,-0.784684368973409,0.432638649659076,0.828209116360826
"8171","LHFPL2",213.626431054424,0.210902656099227,0.268919742347129,0.7842587318375,0.432888309168539,0.828585626654749
"8172","C1orf53",10.1584653431939,-0.45364799888854,0.578668141076085,-0.783951917665523,0.433068324194867,0.828727344966073
"8173","C1orf35",194.822180754537,-0.146544785972694,0.186920362061577,-0.783995838422452,0.433042552207066,0.828727344966073
"8174","STX3",15.2067865397803,-0.426512839875385,0.544155925820973,-0.783806294550411,0.433153779825503,0.82878946861645
"8175","DNALI1",64.181091157802,-0.216467335130688,0.276227514916384,-0.783655948236056,0.433242017392944,0.828856899330354
"8176","INPP4A",54.7320095850652,0.241060851911967,0.307701248184791,0.783425005046444,0.433377577150068,0.828913453176846
"8177","LINC02588",4.51303864809191,0.634932771282724,0.810402829331003,0.783477979472096,0.433346479887681,0.828913453176846
"8178","ZBTB8A",219.506533710419,0.125204017774923,0.159929215862999,0.7828714540949,0.433702602074181,0.828923939853825
"8179","SIX3",15.292312716051,1.04500714728139,1.33498815422594,0.78278383517741,0.43375406162172,0.828923939853825
"8180","LAMTOR1",997.470819534983,-0.193467612429319,0.247056916668123,-0.783089237243289,0.433574710942114,0.828923939853825
"8181","SART3",124.50134676599,0.145264108725832,0.185473343250982,0.783207474344502,0.43350528643634,0.828923939853825

"8182","CLUAP1",199.097709562545,0.1218372497119,0.15562881041071,0.78
2870789735955,0.433702992246168,0.828923939853825
"8183","SETD6",37.9736654867016,-0.231519911813044,0.295670552781777,-
0.783033378315222,0.433607511499611,0.828923939853825
"8184","ZZEF1",112.885368430587,0.208606151758933,0.266420680337839,0.
782995342157398,0.433629847285312,0.828923939853825
"8185","DNMT1",262.315266479947,-0.175060165834326,0.223705324655864,-
0.782548051118716,0.433892557703911,0.829087306351761
"8186","YWHAZ",1605.86374899775,-0.124241565225107,0.158802824282352,-
0.782363700309291,0.434000860455789,0.829191568346279
"8187","SHOC2",280.859202698431,0.1576849937069,0.201572408342765,0.78
2274692272185,0.434053156652877,0.829191568346279
"8188","SPON2",1745.36568477262,0.225651117286747,0.288516447689384,0.
782108330717015,0.434150911260018,0.8292770215079
"8189","TMEM17",35.4643700510756,0.231027032187307,0.295592853522002,0
.781571778324846,0.434466277906821,0.829676750483843
"8190","SMU1",269.656060362832,-0.125380717026344,0.160411119585013,-0
.781621108004902,0.434437278131663,0.829676750483843
"8191","CASP10",32.3231197841676,-0.259171544030878,0.331723319710418,
-0.781288286446441,0.434632957884635,0.829680834902044
"8192","PRDX5",2262.3575664774,-0.197207207653254,0.252477261779061,-0
.78108898307772,0.43475016105586,0.829680834902044
"8193","MNS1",86.0623969481347,0.183708294000418,0.235213803775696,0.7
81026840480866,0.43478670862258,0.829680834902044
"8194","TSPOAP1",16.9444386842657,0.504634220996815,0.645811483537829,
0.781395552510729,0.434569886108616,0.829680834902044
"8195","PHKA2",29.6240536299685,-0.226056005930833,0.289383117141707,-
0.781165149382701,0.434705368226239,0.829680834902044
"8196","NDP",2.58875550088993,-0.848970719120183,1.08694527138889,-0.7
81061145825104,0.434766532599192,0.829680834902044
"8197","TRMT11",200.679824377698,0.125617365062798,0.160876125046817,0
.780832861471778,0.434900803787859,0.829797312582911
"8198","GNL2",131.393708358232,-0.158567024102267,0.203125832742514,-0
.780634456786546,0.435017519939536,0.829918762119339
"8199","HMGN3-
AS1",45.9904522013872,-0.22370799449276,0.286652607434337,-0.780414999
518205,0.435146641818597,0.830063846571882
"8200","UBL4A",155.179206689033,-0.210466108535469,0.269731643187969,-
0.780279636634255,0.435226296187017,0.83011454541036
"8201","TOGARAM2",3.16979363811491,0.771782815337713,0.989300239716753
,0.780130019536518,0.43531434824511,0.830180337840503
"8202","SDHA",446.146441901005,-0.114391140278093,0.14669499439595,-0.
779788981547223,0.435515092977102,0.830180337840503
"8203","ATG10",102.095698232289,0.166476395387923,0.21349419525826б,0.
779770125302632,0.435526193860698,0.830180337840503
"8204","RASSF8",337.152534972076,-0.14182518394767,0.181858047082902,-
0.779867518774233,0.435468858982603,0.830180337840503
"8205","PARPBP",11.9177783116674,-0.330164575739571,0.423343236432266,
-0.779898076374244,0.4354508708276,0.830180337840503
"8206","EXPH5",10.9932997992727,-0.401268653938667,0.514859962652532,-

0.779374360110178,0.435759222949646,0.830320936517112
"8207","DICER1-
AS1",15.2670252375957,0.30867585811712,0.396006103501786,0.77947247627
6436,0.435701444817851,0.830320936517112
"8208","ZBED4",37.0724052728011,0.247228163492391,0.317204003177095,0.
779397993140595,0.435745305651064,0.830320936517112
"8209","AHR",623.236545304925,0.182689106952086,0.234436344491103,0.77
9269559712059,0.43582094213691,0.83033737788053
"8210","ZMIZ1-
AS1",11.8196936169827,0.382363452496227,0.49085813298009,0.77896937384
9075,0.435997755883647,0.830573069673598
"8211","AP002387.2",10.3151812273036,-0.395805547670629,0.508198743358
906,-0.778840075547173,0.436073927165622,0.830617004124994
"8212","GNB1",960.755336851562,0.12462369824078,0.160157943152993,0.77
8129987107363,0.436492386072002,0.83064792457884
"8213","KAZN",209.857095938676,0.165891618818519,0.213372378466453,0.7
77474666640606,0.436878775131749,0.83064792457884
"8214","FAM43B",123.071003823221,0.297802545290793,0.384008873013426,0
.775509542146389,0.43803862919368,0.83064792457884
"8215","AL139260.1",12.273477658496,0.423602976678232,0.54675218254998
1,0.77476229670013,0.438480132053894,0.83064792457884
"8216","C1orf50",151.803546589739,-0.179515846438857,0.230962247377266
,-0.777251903622267,0.437010165173361,0.83064792457884
"8217","DNTTIP2",232.5443692373,-0.139004608011985,0.17885605164651,-0
.777187054798193,0.43704841857031,0.83064792457884
"8218","AC074117.1",11.6384918687218,-0.366621760894144,0.471030771480
594,-0.778339299875759,0.436369012897748,0.83064792457884
"8219","ARL5A",609.540177704086,-0.118850196129461,0.153087677270688,-
0.776353774832647,0.437540130077006,0.83064792457884
"8220","DCAF1",63.8743002959623,0.217697914993863,0.280383248060308,0.
776429820611246,0.437495242905363,0.83064792457884
"8221","PCCB",57.876844489276,-0.193265366701596,0.248746114122205,-0.
776958335142666,0.43718335242617,0.83064792457884
"8222","NCK1-
DT",24.2125550330221,0.253117071131025,0.326140833705354,0.77609745537
0151,0.437691446038421,0.83064792457884
"8223","TMEM175",114.388894826665,-0.220272066317246,0.283029770623142
,-0.778264653334088,0.436413008771869,0.83064792457884
"8224","MFSD10",557.813728304118,-0.177225874221536,0.228591683695708,
-0.775294496091344,0.438165660900546,0.83064792457884
"8225","MFSD8",86.3304973057075,0.227289601532149,0.292536710578753,0.
776960953319263,0.437181807689242,0.83064792457884
"8226","FRG1-
DT",2.97206336887157,0.703794802253094,0.907990593198239,0.77511243786
5793,0.438273222639194,0.83064792457884
"8227","PSMB9",475.66026028385,0.230191819649968,0.296804879187951,0.7
75566157402013,0.438005189031035,0.83064792457884
"8228","NUDT3",271.79431601333,0.17576524879729,0.22660418277151,0.775
648739787464,0.437956413850072,0.83064792457884
"8229","AL445250.1",10.7362093242918,0.457074758967979,0.5886083830633

22,0.776534572255333,0.437433416001922,0.83064792457884
"8230","SNHG5",1540.47157096035,0.32887004662297,0.424389302381148,0.774925392270157,0.438383746772877,0.83064792457884
"8231","METTL27",97.0010794675263,0.410620992197094,0.528772108602749,0.776555694819339,0.437420949573208,0.83064792457884
"8232","LIMK1",68.6985492856584,-0.189241614590465,0.243359764232164,-0.777620800166166,0.436792595042264,0.83064792457884
"8233","NOS3",2.57510332973825,-0.753917141602223,0.970323980244245,-0.776974656869194,0.437173722579385,0.83064792457884
"8234","TUSC3",76.8285567378908,0.290359280027746,0.373709089444849,0.776966063252782,0.437178792812659,0.83064792457884
"8235","IARS",163.142672640684,-0.170371698561266,0.219789316103259,-0.775159145957868,0.43824562560454,0.83064792457884
"8236","RNASEH2C",695.588147294459,0.182457138165184,0.235397744637859,0.775101471111715,0.438279702387005,0.83064792457884
"8237","ARHGEF25",54.1289943392293,0.280383932936146,0.361412973190722,0.775799303663027,0.437867495175376,0.83064792457884
"8238","ATP2B1-AS1",143.962245279488,-0.210827906888042,0.271345905846396,-0.776971026079855,0.437175864740792,0.83064792457884
"8239","TMEM119",131.880716430508,0.307115176206782,0.395563331177352,0.776399509258572,0.437513134322342,0.83064792457884
"8240","RSRC2",953.306828958917,0.124263641935371,0.160378807387515,0.77481335570179,0.438449956189312,0.83064792457884
"8241","AL355001.2",53.5720410775771,-0.292211127242356,0.376599471587279,-0.775920173256628,0.437796120600924,0.83064792457884
"8242","TSC22D1-AS1",5.95563379812936,0.495637219725026,0.638160704481225,0.77665213393138,0.437356315552595,0.83064792457884
"8243","ESR2",5.98682049363737,1.01441447739467,1.30886765559177,0.775032122660279,0.438320678455223,0.83064792457884
"8244","TRIP11",617.981393272937,0.1387974246199,0.178691633226107,0.776742716567333,0.437310579219564,0.83064792457884
"8245","FSIP1",3.18136434133489,-0.66405883337876,0.856604229741935,-0.775222454340225,0.438208222040239,0.83064792457884
"8246","CLEC16A",46.1597520256083,0.199868136159358,0.257971418386312,0.774768528271824,0.438476449131726,0.83064792457884
"8247","MLKL",24.4335406626309,-0.301813432708344,0.388261305675755,-0.777346153985261,0.436954571665195,0.83064792457884
"8248","RSKR",9.26014645454573,0.418488341014432,0.538336964064393,0.777372480341837,0.436939043810599,0.83064792457884
"8249","FLOT2",149.547565271648,0.169140722848969,0.217359490602402,0.778161203728452,0.436473985089801,0.83064792457884
"8250","AC008764.8",9.70682858885274,-0.459147470445968,0.592452245589427,-0.774994902735435,0.438342671580275,0.83064792457884
"8251","LGALS4",3.10129590011838,-0.716119242016671,0.921016160283288,-0.777531679570536,0.436845151433002,0.83064792457884
"8252","SIRT2",295.173051367625,-0.155678233973755,0.200585401157855,-0.776119463705341,0.437678452427997,0.83064792457884
"8253","ZNF497",4.29637188818413,0.597241834577427,0.767147325240641,0

.778522996726974,0.436260754821482,0.83064792457884
"8254","Z95115.1",6.35025854685239,-0.473570223578065,0.609790072023536,-0.776611895314338,0.437387781325744,0.83064792457884
"8255","MAGEE1",23.8655655038169,0.277360925804738,0.356842243521422,0.777264830160412,0.437002540223235,0.83064792457884
"8256","SEPTIN6",80.2558401931222,0.196093989809301,0.253008015327046,0.77505050405547,0.438309817186307,0.83064792457884
"8257","BOC",205.620972598707,0.199116582789362,0.257035223885413,0.7746665214964,0.438536738408841,0.83065454628972 7
"8258","CERT1",331.390764473427,0.145166358748163,0.1874791375873,0.774306734158975,0.43874942230427,0.830698088752831
"8259","ZNF596",18.5003611505523,0.278008420317134,0.359101231985635,0.774178408633962,0.438825294710735,0.830698088752831
"8260","ASCC1",95.6159856462016,-0.187734833012441,0.242463725344034,-0.774280081468116,0.438765180074987,0.830698088752831
"8261","SAC3D1",82.5537581728208,-0.237828282340807,0.30713139829498,-0.774353529665462,0.438721756358734,0.830698088752831
"8262","IFT52",250.992243501945,-0.185181613787727,0.239188669605734,-0.774207298752782,0.43880821278402,0.830698088752831
"8263","PHF14",449.92645396976,0.106012123407745,0.137001003867843,0.773805449703191,0.439045849292418,0.830873406684224
"8264","AC072061.1",6.98246940721486,-0.493127441465204,0.637393499558857,-0.773662489194665,0.439130407906125,0.830873406684224
"8265","SLC25A15",10.7792484105209,0.403076750480548,0.520957043138887,0.773723583909945,0.439094270317515,0.830873406684224
"8266","MT-
ATP8",447.869035108934,-0.258567753181773,0.334189295687166,-0.773716443101808,0.43909849402491,0.830873406684224
"8267","SP5",26.1863882660857,-0.553431949034238,0.715753050626578,-0.77321633285357,0.439394361633827,0.831071207637327
"8268","MTRNR2L12",46.4496992657859,-0.29604160883311,0.382849007561303,-0.773259439064123,0.439368855287003,0.831071207637327
"8269","PCDHB11",29.9789322477,0.392429641749581,0.507424530366084,0.773375385432116,0.439300252958565,0.831071207637327
"8270","DYNLL2",247.827206469345,0.15969337902026,0.206586836360595,0.773008492862136,0.439517354426424,0.831203315989029
"8271","ERP44",563.096103367624,-0.169592901973745,0.219432111000063,-0.772871851803387,0.439598224826197,0.831255741298721
"8272","PDCD11",89.6679418563746,0.252586748752671,0.326879116743756,0.772722195497965,0.439686808041398,0.831322736674016
"8273","POMGNT1",88.8548502725443,-0.151407017778405,0.196226053176804,-0.771594879106009,0.440354408204069,0.83133832573027
"8274","CD247",15.975024218982,0.462575446960549,0.599815286972261,0.771196495000203,0.440590471417227,0.83133832573027
"8275","SPOPL",120.792348284078,0.182878160568209,0.23706128667203,0.771438319328866,0.440447169326727,0.83133832573027
"8276","ABCF1",334.038660292051,-0.153414796221763,0.19893445208781,-0.771182641375992,0.440598681712162,0.83133832573027
"8277","TBC1D22B",32.0062063293279,0.262815543063677,0.340246019302076,0.772427973155346,0.439860991353827,0.83133832573027

"8278","ZNF484",34.1572216296954,0.216493439109728,0.280720623850776,0
.771206034455131,0.440584817948446,0.83133832573027
"8279","ZNF32",294.414333263158,-0.170112270179898,0.220227740373087,-
0.772437976667749,0.43985506849899,0.83133832573027
"8280","NSMCE4A",251.940932175759,-0.168787673454288,0.218706838252934
,-0.771753068182924,0.440260693107428,0.83133832573027
"8281","AP5B1",57.0673746539414,-0.243355866970278,0.315327236478564,-
0.771756571642746,0.440258617701221,0.83133832573027
"8282","IKZF4",60.5454051900167,-0.188733930933785,0.2443785086887,-0.
772301672297225,0.439935775189503,0.83133832573027
"8283","AL161891.1",5.13351957709009,0.617971310040461,0.8008862557767
6,0.77160933351404,0.440345844582646,0.83133832573027
"8284","AL359397.2",4.31638499589145,0.700781846284388,0.9085535015230
85,0.771315993069872,0.440519655006203,0.83133832573027
"8285","DSG3",7.07756722629141,-0.897594366538688,1.16306344716957,-0.
771750129989101,0.440262433661385,0.83133832573027
"8286","XAB2",133.710408611166,0.161989964763342,0.209770017437588,0.7
722264923372,0.439980293360697,0.83133832573027
"8287","TTI1",49.0014328498133,0.201760989374645,0.261394390845172,0.7
71864265037541,0.440194824205395,0.83133832573027
"8288","YBEY",242.514861008009,0.214043754448158,0.277393717616147,0.7
71624376671532,0.440336932253656,0.83133832573027
"8289","AC004000.1",4.26449325512578,0.675682019804831,0.8758394653708
88,0.771467884835154,0.440429651009071,0.83133832573027
"8290","TEX264",389.538049267947,-0.213362748300683,0.276713886124597,
-0.771059057746787,0.440671926936662,0.831376228864825
"8291","GBF1",150.85232863319,-0.146547611404038,0.190104550593177,-0.
770879029180366,0.440778638289735,0.831477252786329
"8292","ZNF184",25.8447295798597,-0.275073189653067,0.356918534978136,
-0.770689002379541,0.440891292131116,0.831589460797233
"8293","DYNC1I2",1105.15592497462,0.125326873606761,0.162656689827801,
0.770499348901301,0.441003741106123,0.83170125538403
"8294","CAPN10",48.1299561744763,0.179984128682584,0.233934400420691,0
.7693786307568,0.441668570569202,0.831935932097516
"8295","TMEM39A",99.4839467321645,-0.157529639051647,0.204686770339396
,-0.769613193810444,0.441529376226701,0.831935932097516
"8296","AC008494.3",3.95229979323062,-0.58940341863885,0.7666480985210
82,-0.768805687741027,0.442008671874476,0.831935932097516
"8297","HSD17B4",252.889249256734,0.116280953463326,0.151050732952945,
0.769813897556851,0.441410294611769,0.831935932097516
"8298","PCDHB9",14.7722506986714,0.391711733053195,0.509128830936167,0
.76937645101129,0.441669864190999,0.831935932097516
"8299","TAF11",269.376781754043,-0.154497293240568,0.20102959127911,-0
.768530106724755,0.442172311259223,0.831935932097516
"8300","PTPRK",51.2302311820495,0.21692095676669,0.282322940051284,0.7
68343361426055,0.442283219898077,0.831935932097516
"8301","ZDHHC4",199.392773402904,-0.18333015939829,0.238579502863895,-
0.768423763138093,0.442235467109353,0.831935932097516
"8302","AHCYL2",39.4996277461961,0.24027821590217,0.312311977501214,0.
769353189155979,0.441683669627198,0.831935932097516

"8303","TOP1MT",25.6702890269311,0.271577581144861,0.352910822493546,0
.769535995597947,0.441575184394957,0.831935932097516
"8304","ZNF510",49.7765008108238,0.218994885256221,0.284726997758351,0
.769139867242525,0.441810283349086,0.831935932097516
"8305","GAPVD1",181.265374236982,0.157922773522574,0.205567525189224,0
.768228217843293,0.44235161197717,0.831935932097516
"8306","SYT15",2.5717765601714,0.783394392644174,1.01768835969631,0.76
9778277583867,0.441431427323489,0.831935932097516
"8307","NANOS1",10.7664014804506,0.500023724504024,0.649844631658791,0
.769451189013697,0.44162551038464,0.831935932097516
"8308","DNHD1",14.5468898437326,-0.375327041168346,0.487413605370717,-
0.770038088868858,0.441277299099169,0.831935932097516
"8309","CRYAB",1548.14921202626,0.186753414847845,0.2427855353154,0.76
9211454896748,0.441767791355137,0.831935932097516
"8310","C1S",8807.42008237413,0.149309805526309,0.194342241745691,0.76
8282819962992,0.442319179085578,0.831935932097516
"8311","NOP9",34.6424583292884,0.248568548024241,0.323491611846306,0.7
68392560801046,0.442253998685447,0.831935932097516
"8312","TEFM",71.8490641750554,0.17707501005844,0.230062177878474,0.76
9683272980124,0.441487794739705,0.831935932097516
"8313","LIG1",18.7556610376659,0.318696372707529,0.414629311086125,0.7
68629626961735,0.442113212368688,0.831935932097516
"8314","FCGRT",3194.58897609074,0.23047971347267,0.299691105460081,0.7
69057570523626,0.441859134770074,0.831935932097516
"8315","NKILA",8.60719444093531,0.413848849291392,0.538546119227862,0.
768455726474728,0.442216484023918,0.831935932097516
"8316","KLHL22",80.1068250169295,0.174349125349544,0.226949282009561,0
.768229464335556,0.442350871563112,0.831935932097516
"8317","ZBTB18",18.894777139375,0.317542021930152,0.413681923975478,0.
767599461147776,0.442725182640294,0.83202701804728
"8318","AC023481.1",4.97169815176733,0.638131213841462,0.8316136651378
08,0.767340942787076,0.442878831537315,0.83202701804728
"8319","AMIGO3",3.86571880830434,-0.636404874986704,0.828732852782371,
-0.767925240142286,0.442531601190606,0.83202701804728
"8320","COPB2",563.973372500793,-0.13205539276564,0.172023776108938,-0
.767657795641068,0.442690516086248,0.83202701804728
"8321","KIF11",6.64635314718445,-0.468271037779248,0.610145556020906,-
0.767474307004874,0.442799563476717,0.83202701804728
"8322","ADCYAP1",3.88689497261137,-0.818187342978934,1.06551490230483,
-0.767879774566362,0.44255861442702,0.83202701804728
"8323","ZNF471",34.7885717399671,0.304099417012938,0.396259561035489,0
.767424806655209,0.442828984190293,0.83202701804728
"8324","HDAC10",56.4290392333393,-0.217438762596356,0.283229465509709,
-0.767712364266359,0.442658088887261,0.83202701804728
"8325","MAGT1",368.239796378084,0.120987831858486,0.157663652687708,0.
767379353427338,0.44285600046657,0.83202701804728
"8326","AL352979.4",6.69337328721197,-0.512025418890646,0.667383824796
834,-0.767212808980675,0.442954998425146,0.832070162787568
"8327","NOP2",31.5484948988688,-0.288661221126801,0.376552189650189,-0
.766590207309544,0.443325199617595,0.832665560468258

"8328","POLR2J2",15.3567806635219,-0.386773558053385,0.504602337881457
,-0.766491807543403,0.443383724647046,0.832675486729082
"8329","LAMTOR3",250.031726636338,0.12881626714516,0.168129682871374,0
.766172069947398,0.443573924803434,0.832932667057956
"8330","MAPKAPK2",160.740799128532,-0.152257235791484,0.19876704209225
8,-0.766008459897559,0.443671268438869,0.833015442783182
"8331","ACP2",132.793918115374,-0.218907192999295,0.285855659764423,-0
.765796252485256,0.443797544389082,0.833152514013353
"8332","LINC00960",6.51887213490133,-0.626487121664993,0.8183062512891
09,-0.765590047342378,0.443920268294288,0.833282884796287
"8333","PDCD6",877.440118797895,-0.152366255177114,0.199161586627042,-
0.765038367877843,0.444248698030207,0.833534645396692
"8334","SEL1L",411.869600266887,0.118620928807379,0.15505868493843,0.7
65006673792444,0.44426757058995,0.833534645396692
"8335","CAPN3",18.4618643805804,0.328225528632254,0.42903223884951,0.7
65036980699683,0.444249524029723,0.833534645396692
"8336","SCAMP4",158.112317359334,0.159792745646872,0.208839244831736,0
.765147114832842,0.444183947048285,0.833534645396692
"8337","MFAP2",604.183106474986,0.235528227903829,0.308566801939335,0.
763297368425703,0.445286071154242,0.833686152186909
"8338","SFPQ",1335.86099606774,0.19986310424157,0.261777815826288,0.76
3483733756096,0.445174959568955,0.833686152186909
"8339","LINC02245",5.28930266377152,0.534890900105657,0.70037157741657
8,0.763724453351861,0.445031465200022,0.833686152186909
"8340","C2orf81",34.6651293591411,0.259913314578923,0.340264610584951,
0.763856441409244,0.444952797538132,0.833686152186909
"8341","MITF",165.646842747255,0.170172432819102,0.222801866912317,0.7
63783693455643,0.444996155869623,0.833686152186909
"8342","FBXL17",136.135325038445,0.17279582760503,0.226399199027704,0.
763235154307616,0.445323166922771,0.833686152186909
"8343","DEK",1732.18654080822,-0.126570350490046,0.165621981991146,-0.
764212268011693,0.444740756901614,0.833686152186909
"8344","MYOM2",21.5789836286091,0.365040296002608,0.477870622018747,0.
763889385918952,0.44493316315334,0.833686152186909
"8345","UTP23",211.77903309388,0.126651864237632,0.165764589980743,0.7
64046556941657,0.444839498616194,0.833686152186909
"8346","DAGLA",25.8481759286551,0.244869864175873,0.320369825607722,0.
764334979773359,0.444667645102434,0.833686152186909
"8347","ZPR1",129.078665187862,-0.14905748241729,0.195296278408965,-0.
763237700337292,0.445321648793428,0.833686152186909
"8348","MDM2",206.456114353288,0.137679758654359,0.180160538047429,0.7
64205969556516,0.444744509712963,0.833686152186909
"8349","SPSB3",323.739226044522,-0.15712065785497,0.205732351120682,-0
.763713907895818,0.445037750856036,0.833686152186909
"8350","MMP2",7282.95607916772,-0.152498042384983,0.19965996621571,-0.
76378878187441,0.444993123055123,0.833686152186909
"8351","AC005899.6",11.9376487493964,0.512873484028856,0.6711616635182
12,0.764157895044818,0.444773154565755,0.833686152186909
"8352","PCGF2",180.603350196805,-0.137742297714378,0.180427645493809,-
0.763421244773179,0.445212213931881,0.833686152186909

"8353","RPL19",17922.7345831282,-0.182313527831244,0.238717651331137,-0.763720348347211,0.445033911996405,0.833686152186909
"8354","MCOLN1",172.745032344489,-0.155049025503604,0.203164061928415,-0.76317151779647,0.44536111262926,0.833686152186909
"8355","ZFP36",14938.5411205626,-0.216324916511796,0.283316750177759,-0.763544394660989,0.445138796760362,0.833686152186909
"8356","GOLGA7",333.745592861168,0.155671082014345,0.204023131472798,0.76300702224591,0.445459207938709,0.833769987094471
"8357","C8orf37",19.2571740924417,0.287599062012442,0.377034833219176,0.762791754695132,0.445587599232652,0.833910500418653
"8358","CMTR2",103.09749775015,0.15762233995191,0.206716999754738,0.762503036223064,0.44575983171216,0.83413301842285
"8359","RPL32",19264.0293139807,-0.177275180167628,0.23302393651729,-0.760759528901333,0.44680071146884,0.834258772394229
"8360","GRIP2",7.82123168317693,-0.589724939992715,0.775374906433025,-0.760567481743302,0.4469154487633,0.834258772394229
"8361","QRICH1",173.421435404855,0.117074960606738,0.153805311789175,0.761189319437911,0.446543996712046,0.834258772394229
"8362","GXYLT2",367.052959228071,0.195458221290224,0.256723963119465,0.761355577855695,0.44644471274603,0.834258772394229
"8363","ANKRD37",49.1773540147309,-0.195233311296059,0.256167889347544,-0.76213030365873,0.445982238141104,0.834258772394229
"8364","AC058791.1",93.2351259099427,-0.386594929700805,0.507864974478991,-0.761215971031287,0.446528080426207,0.834258772394229
"8365","AC104958.2",4.8380634648205,0.621692617638151,0.816745266338402,0.761183006820838,0.446547766643429,0.834258772394229
"8366","FRAT1",24.112101795437,-0.274269961305554,0.360345238968337,-0.76113108110096,0.446578777670648,0.834258772394229
"8367","SPTY2D1",255.286551821832,0.134532940104082,0.176741153367666,0.761186274620597,0.446545815092273,0.834258772394229
"8368","TMX2",243.830381383131,-0.177433613892621,0.23279178540158,-0.762198775985748,0.445941376533497,0.834258772394229
"8369","RITA1",63.233235235166,-0.189073415629887,0.248560515466963,-0.760673573896806,0.446852062641236,0.834258772394229
"8370","DHRS7",736.941078181321,-0.129809981925281,0.170492712392547,-0.76138141099194,0.446429287189118,0.834258772394229
"8371","DHX58",42.6317683208264,0.228168120757848,0.299391453778758,0.762106325608271,0.445996547808239,0.834258772394229
"8372","CCR10",25.2574309579113,-0.309508630173995,0.406434741460032,-0.761521096996164,0.446345882734749,0.834258772394229
"8373","MRPL34",536.240280997155,-0.199264438844939,0.261863587307901,-0.760947487558255,0.446688433052717,0.834258772394229
"8374","ZNF101",21.4147867459901,0.287475362345703,0.377715139284876,0.761090389148756,0.446603080534809,0.834258772394229
"8375","CSTF1",89.4575914448128,-0.156308042097132,0.205163982064736,-0.761868825726981,0.446138297566508,0.834258772394229
"8376","AL137077.2",2.81705611256597,-0.707866928314672,0.930773943692993,-0.76051433660261,0.446947202934223,0.834258772394229
"8377","DNAJC28",5.24642732241017,0.528536574189058,0.693734809287349,0.761871203683735,0.446136878176369,0.834258772394229

"8378","DGCR6L",401.460646133138,-0.21634440176783,0.284294498047387,-0.76098694576836,0.446664864454671,0.834258772394229
"8379","AKAP17A",133.004263913683,-0.167699356344919,0.220503601299971,-0.760528877334672,0.44693851473313,0.834258772394229
"8380","ADAM23",22.1122442695943,0.383901119206725,0.505028873620605,0.760156773719683,0.447160879780554,0.834348127252488
"8381","FYB1",28.0859954427503,0.445554790257005,0.586133491130062,0.760159241878464,0.447159404628623,0.834348127252488
"8382","JADE2",133.056922958253,0.193750010556409,0.254908343437738,0.760077163200947,0.447208462324655,0.834348127252488
"8383","LMBR1L",113.645888306865,0.201730157795556,0.26536472825287,0.760199590667998,0.447135289637872,0.834348127252488
"8384","ASTN2",11.974720348602,0.401289399211622,0.528308664346511,0.759573761123141,0.447509408216313,0.834810012464591
"8385","NDOR1",8.6420172138326,0.443149161539516,0.583496708961576,0.759471569819424,0.447570514694761,0.834824430509966
"8386","PPP1R8",144.897552258954,0.12873927565843,0.169636031754377,0.758914685323676,0.447903593591591,0.835036526377283
"8387","CD1C",10.4863166897653,-0.567868244768276,0.748476114748814,-0.758699220427162,0.448032503355335,0.835036526377283
"8388","LPGAT1",175.240547825372,0.153271377007277,0.201908062295215,0.759114694405686,0.447783949698592,0.835036526377283
"8389","MAPRE3",54.0873181950938,-0.221233568809554,0.291868387994932,-0.757990854471694,0.448456457748844,0.835036526377283
"8390","GYPC",2827.72987103576,-0.162032286686687,0.213655884693602,-0.758379704444152,0.44822370428926,0.835036526377283
"8391","SATB2-AS1",9.20636655361485,-0.441785204380375,0.582873895538622,-0.757943026376452,0.44848509089317,0.835036526377283
"8392","CD200",101.069349639024,0.192329358061216,0.253565905657637,0.758498495933037,0.448152613095308,0.835036526377283
"8393","MED20",40.0763613569656,0.211519678928173,0.278987526294799,0.758168946609698,0.44834984883534,0.835036526377283
"8394","RTF1",745.057155793393,0.134843956893728,0.17771945822726,0.7587461628739,0.448004416872978,0.835036526377283
"8395","TGM5",5.41050082737384,0.541918991773278,0.714512830954015,0.758445430643575,0.448184369417187,0.835036526377283
"8396","ARRDC4",54.6905235633933,0.265857256745676,0.350759175879648,0.757948116621465,0.448482043477627,0.835036526377283
"8397","VPS4A",254.792000859406,-0.139352187042964,0.183811541787413,-0.758125336896046,0.448375953003959,0.835036526377283
"8398","AC011462.5",7.65597853446824,0.599271260854165,0.78968627154876,0.758872583258734,0.44792878103672,0.835036526377283
"8399","TMEM191B",8.2307325836696,0.689347161729819,0.908800253671063,0.758524394051639,0.448137115123466,0.835036526377283
"8400","TMEM191C",4.85656516993839,-0.535351377428993,0.705379740447581,-0.758954853295474,0.447879563962559,0.835036526377283
"8401","TMEM240",56.5614349151696,0.253910597554563,0.335690848314271,0.756382245240281,0.449420049893812,0.835344521647542
"8402","U2SURP",642.79953905392,0.0925088575551357,0.122187862631719,0

.757103492627274,0.448987861777373,0.835344521647542
"8403","TM4SF1",6.26734000465153,-0.684194530759667,0.904624956193393,
-0.756329488895284,0.449451671973406,0.835344521647542
"8404","MSH5",5.68387253066788,-0.507700752118298,0.67051111925165,-0
.757184695508762,0.44893921790777,0.835344521647542
"8405","DUSP4",6.31440088531404,-0.57018426733475,0.753187618940123,-0
.757028199875493,0.449032967908019,0.835344521647542
"8406","RGS20",6.81810151715694,-0.740725535897517,0.978654778000494,-
0.75688133604263,0.449120958001762,0.835344521647542
"8407","PCGF5",145.633314904427,-0.152969535448402,0.202185605914762,-
0.756579751344374,0.449301676215411,0.835344521647542
"8408","SIPA1",147.112102107458,0.183905296759792,0.243078823906196,0.
75656650712923,0.449309613474888,0.835344521647542
"8409","FCHSD2",64.0680639604616,0.222263728522047,0.293796244084885,0
.756523383116597,0.449335458251104,0.835344521647542
"8410","CCDC32",210.956124340887,-0.197962659196657,0.261341652649753,
-0.757486061596023,0.44875871336044,0.835344521647542
"8411","RNASEK",2267.81424389394,-0.180088334817439,0.23794666237073,-
0.75684329010194,0.449143753948874,0.835344521647542
"8412","AFMID",66.68948370611,-0.217623912697767,0.28730185571623,-0.7
57474789556235,0.448765464056734,0.835344521647542
"8413","PPP6R1",103.316709653316,0.164549535745876,0.217389264706652,0
.756934966259355,0.449088825554853,0.835344521647542
"8414","TNFRSF6B",16.5473391395855,-0.295734754566177,0.39049831644419
4,-0.757326580199073,0.448854230314516,0.835344521647542
"8415","LINC01637",22.3442900088995,-0.367742687444741,0.4861552295120
05,-0.756430590726907,0.449391072784556,0.835344521647542
"8416","LINC01597",5.39266227576148,0.513739755750411,0.67941459493361
4,0.756150603153599,0.44955890523007,0.835444543464626
"8417","FRS3",30.0954073213806,0.223587753464386,0.295760795887711,0.7
55974951965146,0.44966421364499,0.835540964881507
"8418","GLA",197.265736002767,-0.182231163086055,0.241230062548159,-0.
75542476406594,0.449994159102011,0.836054721828872
"8419","HSD17B8",47.0776102609479,-0.259428663167549,0.343711195493863
,-0.75478677031392,0.450376933114942,0.836269171781751
"8420","ELP3",76.9502894385834,0.162970539925288,0.215886519459742,0.7
54889839037303,0.450315082985163,0.836269171781751
"8421","STRN3",290.6989181047,-0.142229320457109,0.188347795984918,-0.
755141942136119,0.450163819647666,0.836269171781751
"8422","AOC3",7.56083973644216,0.460194200758795,0.60951146444889,0.75
5021402550476,0.450236140513746,0.836269171781751
"8423","ST13",1522.84163213778,0.131003274737627,0.173559011625726,0.7
54805374324965,0.450365768746732,0.836269171781751
"8424","KCTD16",26.1367152480871,0.439678215287589,0.582632190818403,0
.754641130744918,0.450464337629681,0.836332174801544
"8425","TCAF2",8.98448131651345,-0.397895361677454,0.527564056966213,-
0.754212415390035,0.450721683751516,0.836512060504771
"8426","EXOSC2",54.578173051746,0.214599614926786,0.284521815988107,0.
754246609109779,0.450701155140698,0.836512060504771
"8427","SVIL",395.089021384581,0.184952759365359,0.245183330781063,0.7

54344754091432,0.450642235561504,0.836512060504771
"8428","AC139887.2",43.6240352037401,0.338475287022326,0.4490398142545
78,0.753775670391738,0.450983935426026,0.836899472005581
"8429","LINC-
PINT",396.29807955339,-0.28325129995762,0.375927237965162,-0.753473734
680193,0.451165288835961,0.83692742673152
"8430","LINS1",65.5205518485616,-0.182512752577518,0.242253981598045,-
0.753394232670854,0.451213047455254,0.83692742673152
"8431","HMGXB4",264.464305637055,0.116839161094264,0.155041854631011,0
.753597545464954,0.451090918649829,0.83692742673152
"8432","APOOL",168.207418770994,0.153018863485982,0.203064789774526,0.
753547001702693,0.451121278244509,0.83692742673152
"8433","S1PR1",6.12621045511832,-0.445623528222432,0.591756046430361,-
0.753052767116716,0.451418205910385,0.837162849638545
"8434","JHY",28.6310036302366,-0.241244495835556,0.320375771139104,-0.
753004807379175,0.45144702518232,0.837162849638545
"8435","CACUL1",273.208626156698,0.127755579646588,0.169688771026156,0
.75288175448507,0.451520973108945,0.837200713624648
"8436","LPXN",28.212912630515,0.26951682162525,0.358124339459187,0.752
578900479802,0.451703000655685,0.837272358562468
"8437","PPHLN1",313.612065635459,-0.13968424284277,0.185594275079065,-
0.75263228234418,0.451670912976544,0.837272358562468
"8438","CHMP1A",248.323438235651,-0.141481245579249,0.188002386665769,
-0.752550263262216,0.451720214932871,0.837272358562468
"8439","AC004846.1",8.2822547830933,-0.428583048545848,0.5696799604691
39,-0.752322493831281,0.451857143891884,0.837426914381925
"8440","KDM4A-
AS1",17.0980115802773,0.407804679484518,0.542593180191524,0.7515846021
88645,0.452300905968992,0.837458294753358
"8441","IFI44L",107.327262662949,-0.370893365554552,0.493688843491782,
-0.751269489768662,0.452490487051082,0.837458294753358
"8442","GNAI3",750.931215682514,-0.142395378515776,0.189524458675026,-
0.751329825771662,0.452454183627526,0.837458294753358
"8443","SCAMP3",127.267797137177,-0.152976154702075,0.203614597873639,
-0.751302491567968,0.452470630074472,0.837458294753358
"8444","NBAS",177.138457135872,0.128401873844153,0.170812147789912,0.7
51713947195838,0.452223101129023,0.837458294753358
"8445","SEPTIN10",713.90671517495,-0.0912303509860101,0.12134002338330
1,-0.75185704141347,0.452137034546668,0.837458294753358
"8446","L3MBTL3",36.2091324079609,0.228702047073997,0.304384338651753,
0.751359442759163,0.452436364056192,0.837458294753358
"8447","AC022034.1",25.8089228137543,0.267847229075642,0.3562211675572
86,0.751912725771885,0.452103544693672,0.837458294753358
"8448","KIFC2",9.33908478200357,-0.477573337791611,0.635436385912906,-
0.751567502867341,0.452311192281604,0.837458294753358
"8449","MARK2",128.755092502111,0.176101362637324,0.234418605944331,0.
751226046788897,0.452516627171396,0.837458294753358
"8450","DLAT",80.0080734887993,0.152207832350185,0.202459231124729,0.7
51794973756538,0.452174365114553,0.837458294753358
"8451","RNF215",56.4732069104578,-0.197230206607225,0.262343546107327,

-0.751801252722779,0.452170588553574,0.837458294753358
"8452","TEP1",36.3973307580875,0.251893831343476,0.335496556380522,0.750808992083295,0.45276761699342 7,0.837823654729911
"8453","PPP4R2",345.048234916621,0.123770513345344,0.164925433420045,0.750463471756445,0.452975615883493,0.837833951180615
"8454","PDS5A",265.047345234699,0.141558626687316,0.188633215613423,0.750443797647071,0.4529874610731,0.837833951180615
"8455","IPMK",188.533484035111,-0.20717591589934,0.276022905869365,-0.750575084509078,0.452908420512036,0.837833951180615
"8456","RAB11FIP3",62.8517255853322,-0.194725831476092,0.25939514541323,-0.750691888107174,0.45283810607034,0.837833951180615
"8457","TMED8",56.9743265462441,0.204315925821636,0.27232593345128,0.750262463924278,0.453096644888561,0.837904616884559
"8458","SUGP2",205.132429311657,0.21215385097038,0.282795486032897,0.750202395188517,0.453132816471202,0.837904616884559
"8459","AL020997.2",12.9258989831325,0.468419216821177,0.625381835452768,0.749013147275133,0.453849281255857,0.838573510509991
"8460","TSTD1",6.68937713485838,-0.561653371848467,0.750969349708961,-0.747904521091487,0.454517750352099,0.838573510509991
"8461","LINC01118",4.96347357534308,-0.698518249613025,0.934364859327968,-0.74758617325937,0.454709807253595,0.838573510509991
"8462","SLC25A12",88.8777282645001,-0.162754462533362,0.217489461893652,-0.748332636975973,0.454259543282871,0.838573510509991
"8463","FGF18",226.050581280369,-0.282130026357957,0.376843379017451,-0.74866653380924,0.454058219415162,0.838573510509991
"8464","PDSS2",92.1840058758989,-0.136397345787336,0.182461692490218,-0.74753962832307,0.454737891301361,0.838573510509991
"8465","ZNF775",55.2562356118568,-0.220111962648106,0.293769770441695,-0.74926689127053,0.453696359081361,0.838573510509991
"8466","AC087854.1",17.2441429707193,0.384249451076657,0.513873969753662,0.747750370116737,0.454610742790323,0.838573510509991
"8467","ZNF79",4.6868048409405,0.561179558620321,0.750404040941758,0.747836536056016,0.454558761383861,0.838573510509991
"8468","ENTPD2",3.09977720761936,-0.716537509739382,0.956512588999033,-0.749114562610432,0.453788158475547,0.838573510509991
"8469","CUEDC2",411.913602844599,-0.192835684625371,0.257832115577025,-0.74791181150497,0.454513352634854,0.838573510509991
"8470","EIF3F",1861.87317935197,-0.15845560680165,0.211826439154519,-0.748044519060546,0.454433305083009,0.838573510509991
"8471","AC090469.1",3.07900765185399,-0.887472542925631,1.18658389722344,-0.747922287671595,0.454507033252196,0.838573510509991
"8472","CKAP4",692.63732100994,-0.151509094067285,0.202457215921996,-0.748351168306388,0.454248368455626,0.838573510509991
"8473","NRL",6.08461951314173,-0.452299405394901,0.603982886619067,-0.74886129295277,0.453940812174409,0.838573510509991
"8474","AC022167.2",7.46039602729498,0.469167154547671,0.626431586444023,0.748951944155509,0.453886170476426,0.838573510509991
"8475","LINC01290",4.22001940227758,-0.624634668817457,0.833565289004927,-0.749353022560618,0.453644457528887,0.838573510509991
"8476","RSL1D1",654.310270184448,-0.125535513105529,0.167551395159946,

-0.749235856769155,0.453715060873766,0.838573510509991
"8477","ZNF830",153.569245514724,-0.143081256914705,0.191419392434351,
-0.747475243208582,0.45477674127619,0.838573510509991
"8478","SLPI",1250.36345428824,-0.280529915295555,0.375307132074327,-0
.747467584069247,0.45478136292492,0.838573510509991
"8479","DOK5",81.7975588524284,-0.224641638845921,0.300519676246721,-0
.747510584503274,0.454755416109271,0.838573510509991
"8480","BCR",70.4150251474528,-0.178162278223757,0.23804263601366 5,-0.
748446921977158,0.454190629213637,0.838573510509991
"8481","RADX",4.55110465981235,-0.538330473062492,0.72001158656907 6,-0
.747669180752616,0.454659725038381,0.838573510509991
"8482","FUNDC2",622.708286311922,-0.144251485224146,0.19253901522823 6,
-0.749206518238137,0.453732741052337,0.838573510509991
"8483","CCL14",3.06387731204027,-0.760677506221834,1.01786485766516,-0
.747326622481815,0.454866426154358,0.838628099431855
"8484","SLC9A3R1",51.3248266968992,0.25242099159569,0.337804072836295,
0.747240817661833,0.454918209436052,0.838628099431855
"8485","SLC9A3R2",31.5438324152609,-0.313031537447722,0.41904286573790
5,-0.747015551491363,0.455054173561467,0.838779879139817
"8486","ST7",132.654474209452,-0.163815007412019,0.219377633588468,-0.
746726112103662,0.455228904366441,0.838832282273743
"8487","ST3GAL1",139.327746563905,0.142655162110183,0.191017076294813,
0.746818896390243,0.455172887582422,0.838832282273743
"8488","AC132192.2",9.08066630467681,-0.401708553177727,0.538041126005
309,-0.746613100303717,0.455297138377353,0.838832282273743
"8489","AC020911.2",12.5973422040183,0.568028203914581,0.7607573007048
99,0.746661521865462,0.455267901810501,0.838832282273743
"8490","EMC3",446.895219594004,-0.165832651822666,0.222174593357128,-0
.746406910515204,0.455421646019981,0.838864037657815
"8491","RAB30-
DT",96.5667544073079,0.203993835500238,0.273278941155007,0.74646745423
5819,0.455385084724787,0.838864037657815
"8492","SMPD4",99.4631950751359,0.17640306189059,0.236558312644841,0.7
45706459935036,0.455844756007671,0.839544510593497
"8493","EYA3",70.8804726753369,0.173703892238055,0.233159638485524,0.7
44999835161607,0.456271819533752,0.839836560845932
"8494","EIF2AK3-
DT",8.8753076505994,0.381453573358098,0.51186140396832,0.7452282403025
39,0.456133753487353,0.839836560845932
"8495","CAMP",3.3216576312085,-0.838857794531638,1.12589058353686,-0.7
45061559975445,0.456234505880584,0.839836560845932
"8496","NDUFC2",1462.56749632142,-0.201638873562137,0.270639037648159,
-0.745047260418782,0.456243150042278,0.839836560845932
"8497","PTPN1",178.671424066224,-0.158676974029363,0.212900665960348,-
0.745309899870937,0.45608439771857,0.839836560845932
"8498","AC099063.4",5.93737087704159,0.465241790720902,0.6246665726976
92,0.744784195369545,0.45640219076105,0.839916061196592
"8499","ORC6",6.62904706087152,-0.454435104404385,0.610184157034742,-0
.744750743140831,0.456422417142573,0.839916061196592
"8500","CAV3",10.6065252040615,-0.490546641724471,0.658835822642835,-0

.744565830917794,0.456534230598086,0.83992416969228
"8501","NDUFA6-
DT",16.7177759661734,0.31151539855127,0.418364709847067,0.744602475350
142,0.456512071074353,0.83992416969228
"8502","PPIP5K2",218.953917784764,0.122160987853954,0.164136149338641,
0.744266198190837,0.456715446387074,0.840158736943523
"8503","HPCAL1",82.1232138154677,-0.178484159813716,0.239850433559675,
-0.744147747263962,0.456787095831022,0.840191718075642
"8504","SHISA3",6.97760689200735,-0.571078677028268,0.767525490915216,
-0.744051740024035,0.456845174011227,0.840199732071447
"8505","LINC01465",4.64257356963618,-0.52457690880801,0.70522400790979
1,-0.743844371326496,0.456970632840133,0.840267944024593
"8506","SELENOO",94.4305887693971,-0.158791977356253,0.213483782410531
,-0.743812834695302,0.456989714314142,0.840267944024593
"8507","C1GALT1",344.148466431808,-0.116061323310817,0.156157270758475
,-0.743233553885085,0.457340292104383,0.840319803631643
"8508","SAMD9",167.686339799926,-0.166827177939255,0.224376898983013,-
0.743513163322064,0.457171055048049,0.840319803631643
"8509","SMARCC2",430.848910193726,0.1331255923653,0.179116736245298,0.
743233687459483,0.457340211248435,0.840319803631643
"8510","FOS",35049.5727263539,-0.140612728781049,0.189176377159271,-0.
743289045347687,0.457306702415229,0.840319803631643
"8511","ALDH3A2",550.647387915329,0.107187473348647,0.144147321122235,
0.743596707272514,0.457120495868501,0.840319803631643
"8512","AC008555.4",7.30145882010225,-0.737210582561244,0.991653992153
768,-0.743415131078231,0.457230386266764,0.840319803631643
"8513","PAPPA",13.0759390877437,-0.460783854419273,0.620290367807646,-
0.742851861536828,0.457571372879438,0.840560851025415
"8514","ZNF799",24.9406396551256,-0.256085093850174,0.34473820731124,-
0.742839315222674,0.457578969669462,0.840560851025415
"8515","KHDRBS3",681.299207261424,-0.19188866991813,0.258353058753956,
-0.742738138435884,0.45764023477572,0.840574664931564
"8516","LAMTOR5",972.428544720338,-0.168193559409574,0.22671350583225,
-0.741877105169132,0.458161798505546,0.840648595255903
"8517","LINC01960",10.7194642619241,0.389671165581695,0.52526037077625
4,0.741862868896468,0.458170424810315,0.840648595255903
"8518","NELFA",108.718201356423,0.136615925953403,0.184164356503548,0.
741815238014154,0.458199286854897,0.840648595255903
"8519","GPAT3",8.77524847671053,-0.487039030623063,0.656577628276302,-
0.741784382604805,0.458217984306686,0.840648595255903
"8520","SMAP1",374.80517165459,-0.137199003442657,0.184857935494628,-0
.742186171643379,0.457974545710304,0.840648595255903
"8521","AP5Z1",88.0483595745252,0.148401968091883,0.200052357133017,0.
741815643757742,0.458199040989314,0.840648595255903
"8522","SRI",1453.961537683,-0.182503773468489,0.24582461983827,-0.742
414545738175,0.457836209256886,0.840648595255903
"8523","IRF5",2.93423524070675,-0.692409517674978,0.933295098669761,-0
.741897732734137,0.458149299632742,0.840648595255903
"8524","CFAP70",16.5186772931288,0.39472245661916,0.532092159576485,0.
741830995843533,0.458189738248431,0.840648595255903

"8525","CNN1",8.0437892425202,-0.545252658698919,0.734520498124898,-0.742324632315712,0.457890671059068,0.840648595255903
"8526","SLC5A3",197.756639724142,0.226397707031395,0.305313242070088,0.741525999646694,0.458374573418578,0.840837242348881
"8527","ANKRD42",53.9498839986412,-0.187294330118616,0.252621517452551,-0.741402917721744,0.458449175922067,0.840875467505704
"8528","AC011365.1",3.87280986444889,-0.636248827559021,0.858282916037888,-0.741304313146708,0.458508947108709,0.840886483674977
"8529","TJP1",414.918537209042,-0.184341491329596,0.248715068687273,-0.741175403253841,0.458587095075419,0.840931195565664
"8530","RAB33B",131.462270264738,-0.15717956264503,0.212246884963117,-0.740550621849379,0.458965956867557,0.840973799508517
"8531","ZFR",497.450098690529,0.119926733075077,0.161988923623342,0.740339094751511,0.459094264718908,0.840973799508517
"8532","ZSCAN26",72.0700194828807,0.171111259181739,0.231070916389507,0.740514045884094,0.45898814163533,0.840973799508517
"8533","KLC4",29.7636021890326,0.230673857438136,0.311382435664999,0.740805617200279,0.458811308812754,0.840973799508517
"8534","MAGI2-
AS3",698.402546920422,0.143180771631154,0.193265150223888,0.74085147511223,0.458783500279495,0.840973799508517
"8535","AL669970.3",10.891544680813,-0.467512189541577,0.631335646725431,-0.740512898275961,0.458988837714719,0.840973799508517
"8536","EID3",57.1853594530697,-0.247230240457408,0.333819976009689,-0.740609484826732,0.458930255408255,0.840973799508517
"8537","MTMR10",57.4891187363276,0.18760661924041,0.253401140109308,0.740354282382009,0.459085051554721,0.840973799508517
"8538","PGBD4",8.48978143316845,0.417621307073359,0.563882274281439,0.740617902212901,0.458925150238648,0.840973799508517
"8539","UHRF1BP1",44.3096587211326,0.298459820242222,0.403357148079166,0.739939335805805,0.459336804888646,0.841122541676434
"8540","AC005562.1",75.6444844959853,0.181340933888148,0.245062487459659,0.739978344984357,0.459313134234233,0.841122541676434
"8541","AP000692.1",5.68552316813129,-0.464009672044928,0.627074218302009,-0.739959734433626,0.459324426976261,0.841122541676434
"8542","AL162231.2",49.4570793683944,0.208699990397915,0.282113968098229,0.739771914892379,0.459438403163454,0.841210094295998
"8543","XPR1",59.6116378344234,0.225867913369671,0.305369976517022,0.739653308245517,0.459510386438614,0.841243409095157
"8544","RNF8",159.567116215067,-0.133453571500958,0.180459882690183,-0.739519329789626,0.459591706577404,0.841293807452083
"8545","PROS1",624.395173475803,0.137634983062854,0.18625560558578,0.738957534351717,0.459932784071137,0.841637524926503
"8546","MRPS7",306.923129268174,-0.189949988131323,0.257036872045251,-0.738998987265464,0.459907612319803,0.841637524926503
"8547","RPS4X",21662.1250995398,-0.187252663680232,0.253405707929449,-0.738944143011824,0.459940915955679,0.841637524926503
"8548","TIPIN",45.7922647791938,0.210222637773073,0.284529712542857,0.738842477624928,0.460002654836097,0.841652026396416
"8549","MAK16",77.0169530355715,-0.171380900184916,0.232046685942233,-

0.738562153943368,0.460172912512204,0.841865054590112
"8550","IGBP1",695.200962606408,-0.19443417005223,0.263336342303444,-0
.738349171069531,0.460302293574559,0.842003259825276
"8551","NCDN",51.2135777447149,0.197543469595714,0.267967269705015,0.7
37192530316015,0.461005275294546,0.842008934439648
"8552","CMC1",310.205410129535,-0.18978204713438,0.257435822176002,-0.
737201394624213,0.460999885475212,0.842008934439648
"8553","TRG-
AS1",3.03054179053628,-0.639356742464743,0.867255205210137,-0.73721868
5599962,0.460989372039213,0.842008934439648
"8554","PLEKHF2",79.2322414370794,-0.184739209567623,0.250386879901745
,-0.737815055006542,0.460626843442609,0.842008934439648
"8555","CH25H",37.7580084244188,-0.667999613532929,0.906023614675901,-
0.737287199486387,0.460947714842543,0.842008934439648
"8556","U47924.2",6.33225646536664,-0.562338485059695,0.76258290918142
8,-0.737412913781821,0.460871284640728,0.842008934439648
"8557","PROSER1",54.8032458995777,0.213846711558374,0.289886839135342,
0.737690307694629,0.460702663231387,0.842008934439648
"8558","AC012146.1",44.8209400778878,-0.209809182850726,0.284353895674
326,-0.737845290823879,0.460608467557898,0.842008934439648
"8559","AC006449.6",16.5686349195939,-0.308361865241647,0.417820158194
014,-0.738025342229801,0.460499049408066,0.842008934439648
"8560","ZNF587B",16.6890293333392,0.339710874189239,0.460578864203876,
0.73757373729348,0.460773519507052,0.842008934439648
"8561","AL121832.2",12.7718608517765,-0.342367658188119,0.463893770850
983,-0.73803029853164,0.460496037643983,0.842008934439648
"8562","AC002470.1",4.81651651556977,0.527232009949852,0.7150289956426
81,0.737357524188186,0.460904958838598,0.842008934439648
"8563","SMIM10",223.311129580261,-0.186072312684938,0.252044013136663,
-0.738253253347645,0.460360567518776,0.842008934439648
"8564","AL121655.1",2.55529079955807,-0.656537866832683,0.890890044392
735,-0.736946013669066,0.461155180422667,0.842128507861637
"8565","PATL1",93.3239130389849,0.172676393779187,0.234325648485177,0.
736907781523159,0.461178431575123,0.842128507861637
"8566","IGSF22",2.89258756932979,-0.661070687680735,0.897326577811932,
-0.736711364654672,0.461297894223159,0.842150001826802
"8567","ALG12",81.6160993999857,-0.178678689267447,0.242533998298855,-
0.736716050206189,0.461295044223905,0.842150001826802
"8568","AC079610.1",2.79051115374476,-0.750893943003722,1.019477089968
91,-0.73654812883203,0.461397188990567,0.842232964030401
"8569","SIX5",123.596490864353,0.172400138927156,0.234101240381095,0.7
36434111355004,0.461466551797869,0.842261275541914
"8570","AL139424.2",4.07593742639552,0.647640334643712,0.879785370659,
0.736134466703623,0.46164886911063,0.84229914989387
"8571","ROPN1L",10.2733819490119,0.378719540124363,0.51444775344095,0.
736167157094668,0.461628976848443,0.84229914989387
"8572","FANCI",15.2538489135611,0.315626106484559,0.428746442720998,0.
736160292039903,0.461633154227998,0.84229914989387
"8573","USP53",973.11436135221,0.190353826407104,0.258624974935516,0.7
36022599729844,0.461716944330941,0.842325091489084

"8574","LOXL3",37.7122760060949,-0.289758182329923,0.393773036313553,-0.735850745502022,0.461821534949465,0.842367334570038
"8575","AC004943.2",9.45146642828471,0.516302765902494,0.701681789432697,0.735807560746189,0.461847819305504,0.842367334570038
"8576","ATP11B",147.599690222554,0.146200852481329,0.198760054050281,0.735564563915563,0.461995734641446,0.842538863082115
"8577","DLEU2L",15.8606392521579,0.324965676338872,0.442061850942989,0.735113594728131,0.462270315526096,0.842702321548557
"8578","AC012645.1",12.7749026349511,-0.318038747305978,0.432668463899696,-0.735063388811502,0.462300889954387,0.842702321548557
"8579","TRAPPC1",697.273545040984,-0.21978603849697,0.298948275661566,-0.735197545497085,0.462219193658946,0.842702321548557
"8580","MAP1LC3A",398.001792643642,0.136749344823495,0.186004536083925,0.735193601739875,0.462221595141452,0.842702321548557
"8581","AC087501.4",4.52214703060936,0.519912774468406,0.707509792716436,0.734848873924749,0.462431538064557,0.842842239287923
"8582","NCKAP5L",233.352753103021,0.156582763543889,0.213125545908275,0.734697301895846,0.46252386388543,0.842912285151261
"8583","PKN2",391.811644917378,0.120659363812336,0.164291423718648,0.73442277802016,0.46269110852628,0.842922415533025
"8584","ABCC6",7.79643216135898,0.521337367520345,0.7097926811814,0.734492452997142,0.462648658144949,0.842922415533025
"8585","SMYD4",176.214300725908,0.17420894127392,0.237162764303467,0.734554354624604,0.462610945599748,0.842922415533025
"8586","AL359232.1",7.85255618897243,0.398564891393787,0.54279983402017,0.734276000863657,0.462780541516164,0.84298714993161
"8587","MIER2",40.5095236520185,0.211187800014481,0.287651331724697,0.734179809800442,0.462839157052936,0.842995739642241
"8588","ILVBL",184.710438864822,-0.182315900983761,0.248358596519808,-0.734083311544324,0.462897963942051,0.843004675832985
"8589","PIK3CD-AS2",5.29954596783955,-0.646829583116508,0.881422563833642,-0.733847316437192,0.46304179899502,0.843075863688874
"8590","STX12",557.259724641257,0.105065677554004,0.143311540731854,0.733127820812346,0.463480473230064,0.843075863688874
"8591","ZCCHC17",349.963307311993,-0.128052708970778,0.17473667876463,-0.732832453243915,0.463660624931275,0.843075863688874
"8592","AC108488.1",31.6933506916078,-0.275486004596046,0.376102892541587,-0.732475102050923,0.46387863405155,0.843075863688874
"8593","AMMECR1L",69.6872531990291,0.193172933168819,0.263947381347759,0.731861525514845,0.464253091595701,0.843075863688874
"8594","CCDC58",83.5704961270888,-0.210285007982322,0.287242519366506,-0.732081756023067,0.464118668511949,0.843075863688874
"8595","SLC41A3",153.103112315882,0.160578088236621,0.219358544495509,0.732034799947846,0.464147327484756,0.843075863688874
"8596","ARRDC3",262.135030080568,-0.198011527018658,0.270192509495254,-0.732853502817537,0.463647785005254,0.843075863688874
"8597","SPATA9",2.53283437680758,0.794708027166687,1.08494017123443,0.732490185391955,0.463869431009127,0.843075863688874
"8598","CHSY3",15.0951321842434,0.372035707297313,0.507459360854982,0.

733133992583163,0.463476709336345,0.843075863688874
"8599","LINC01013",4.84267381242362,-0.62571065844186,0.85291462045903
8,-0.733614647272788,0.463183631329765,0.843075863688874
"8600","PSMG3-
AS1",42.9583017916792,-0.246678403249114,0.336203311382645,-0.73371794
6544438,0.463120658342881,0.843075863688874
"8601","SRRM3",16.8005582769024,-0.433932447037213,0.591566274004225,-
0.73353141669147,0.463234373574584,0.843075863688874
"8602","PLIN2",1407.55859457175,0.179690331017499,0.245530272348798,0.
731845932065896,0.4642621026057,0.843075863688874
"8603","FAM78A",8.0872310970452,-0.42274456853867,0.577260199712149,-0
.732329318303032,0.463967588684093,0.843075863688874
"8604","CFAP300",35.2217666558971,0.238284027791809,0.325040740393951,
0.733089727469261,0.463503705074782,0.843075863688874
"8605","NDUFA9",279.543139961746,-0.196466920119315,0.268026892105398,
-0.733011969717043,0.463551128918157,0.843075863688874
"8606","MAP1LC3B2",8.20824818547634,0.478857336434832,0.65272733020486
3,0.733625381190244,0.463177087529555,0.843075863688874
"8607","TFDP1",192.085943604662,0.13714313692792,0.187294454334382,0.7
32232769065732,0.464026506527745,0.843075863688874
"8608","SHC4",2.70129795179532,0.718957345103736,0.982056661242827,0.7
32093547630816,0.464111471826591,0.843075863688874
"8609","DNAJA3",72.0270037345539,-0.155803891546953,0.212459097958146,
-0.733335936395842,0.463353562012478,0.843075863688874
"8610","WDR83OS",1422.05115643429,-0.172959523913955,0.236036600637023
,-0.732765695858891,0.463701347250809,0.843075863688874
"8611","GMFG",33.1729317804845,-0.275367789031918,0.375737384031733,-0
.732873013798015,0.463635883774992,0.843075863688874
"8612","ADSL",190.445967709062,-0.184248001512263,0.251509852083302,-0
.732567730393475,0.463822118830303,0.843075863688874
"8613","AL031595.3",8.11690206927369,0.517154019109992,0.7066782050831
58,0.731809776203776,0.464284681198995,0.843075863688874
"8614","PBX3",205.794790892514,0.138651042997986,0.189526605367235,0.7
3156506301229,0.464434078959699,0.843249244825829
"8615","WRAP73",116.799795657828,-0.138376143708658,0.189254235832115,
-0.731165371809218,0.464678148542558,0.843527303279628
"8616","KLF11",177.972122707651,0.200197057051072,0.274139631658518,0.
730274042610692,0.465222691491353,0.843527303279628
"8617","ADH1C",205.983735612694,0.474229787194996,0.649180303294351,0.
730505507928159,0.465081247462907,0.843527303279628
"8618","AFAP1L1",41.619508584822,0.31207702162038,0.426852252052646,0.
73111251052247,0.464710433384266,0.843527303279628
"8619","ORC3",112.87362349163,0.136110716991927,0.186378599235755,0.73
0291554663724,0.465211989374562,0.843527303279628
"8620","RNF19A",500.672212885948,0.139298818385101,0.190761294817231,0
.730225796163546,0.465252176983942,0.843527303279628
"8621","TSTD2",96.7265878473478,0.200423068208426,0.274326404975956,0.
730600717149312,0.465023073870095,0.843527303279628
"8622","OSBPL8",880.902089284995,0.129903343059768,0.177784620491272,0
.730678180715554,0.464975745999236,0.843527303279628

"8623","BCL2L2",80.8892989470509,-0.194174261884394,0.265877318781838,
-0.730315255073416,0.465197505593871,0.843527303279628
"8624","KIAA0895L",12.5714171372662,0.327388573017796,0.44799386104515
5,0.7307880787786,0.464908606238343,0.843527303279628
"8625","ZCCHC2",91.9476231961057,-0.217234108847175,0.297404254906712,
-0.730433762339128,0.465125087262844,0.843527303279628
"8626","TP53RK",105.222943097102,-0.208980602887646,0.286209486610504,
-0.730166583094579,0.465288366073744,0.843527303279628
"8627","PLXNB2",109.573014136154,0.133691014240411,0.183058788620709,0
.730317376443552,0.465196209195601,0.843527303279628
"8628","ACAD11",70.0930650112309,0.241022867987553,0.330146593848972,0
.730048022539377,0.465360831107658,0.843560894590145
"8629","AKT3-
IT1",3.45219224987403,0.79775577564904,1.09332993710457,0.729656939388
04,0.465599908310218,0.843798675083639
"8630","RNF216",286.12709945741,0.131325298294498,0.17996772339294,0.7
29715839143909,0.465563897309051,0.843798675083639
"8631","PHTF1",54.9586563841832,-0.184055352075264,0.252820781239978,-
0.72800721195683,0.466609171528693,0.843895289716367
"8632","NCOA7",907.49635501506,0.148630995776685,0.203787682863258,0.7
29342390513447,0.465792248039738,0.843895289716367
"8633","HSPB1",2281.20720854283,-0.134647169092117,0.184723858204515,-
0.728910550054905,0.466056380877512,0.843895289716367
"8634","AC021321.1",8.31486044463335,-0.424713636451461,0.583320749857
786,-0.728096225884311,0.466554684000197,0.843895289716367
"8635","EIF3H",1341.83524681927,-0.148930613558447,0.204394904965398,-
0.72864151669367 6,0.466220975691976,0.843895289716367
"8636","RRAGA",834.619942998175,-0.158229643502507,0.217158468002706,-
0.728636764468862,0.466223883396769,0.843895289716367
"8637","PSMB7",877.75929508826,-0.164452818067931,0.225842268253959,-0
.728175550747673,0.466506130352424,0.843895289716367
"8638","NCOA4",412.374331261366,0.0867073399216251,0.119012063081587,0
.728559254217649,0.466271310379324,0.843895289716367
"8639","SIRT1",197.547632934175,0.236106539726072,0.324073124595746,0.
728559457130533,0.466271186217465,0.843895289716367
"8640","KAZALD1",137.622394007798,-0.298054893706078,0.409417115228444
,-0.72799812860723,0.46661473186018,0.843895289716367
"8641","PRR4",15.2663445035379,0.43850800266575,0.602368774720095,0.72
797266569721,0.466630319061705,0.843895289716367
"8642","ERBB3",30.6788878387823,-0.356547459290953,0.489834244625984,-
0.727894105409468,0.466678411813098,0.843895289716367
"8643","AC092375.2",5.82773582624716,0.655950828987001,0.9002961568599
66,0.728594500808281,0.466249743358827,0.843895289716367
"8644","AC073508.3",30.3347888208065,0.242857528129982,0.3334630993142
37,0.728289062956038,0.466436655983329,0.843895289716367
"8645","CALCOCO2",636.753708914914,0.0990421740531652,0.13606140122060
6,0.727922637608157,0.466660944757372,0.843895289716367
"8646","METTL23",234.792151040775,-0.160568045036846,0.220495822067004
,-0.728213548590742,0.466482873393566,0.843895289716367
"8647","VAV1",3.59450272738856,0.669949599461014,0.920379443738422,0.7

27905869713686,0.466671209809018,0.843895289716367
"8648","ZNF526",38.325924363854,0.258824564448819,0.355382867880719,0.728297810168192,0.466431302550416,0.843895289716367
"8649","PFDN4",358.607519337914,-0.184761816692672,0.25331334673594,-0.729380504712499,0.465768939694109,0.843895289716367
"8650","TMEM177",22.6815424509911,0.279607696921739,0.384254711055144,0.727662378306177,0.466820285688573,0.844054250655407
"8651","CTTNBP2NL",194.225461976818,0.1650043408067,0.226857301778799,0.72734859981536,0.467012433287445,0.844304063878817
"8652","TMCO3",366.84839043216,0.168485765402204,0.231672683366953,0.727257797309381,0.467068045930028,0.844307008592885
"8653","C12orf57",2102.17091504296,-0.163837116162147,0.225318627668619,-0.727135247792764,0.467143108078857,0.844345106940174
"8654","ICOS",2.65838460351491,0.896127535185622,1.23275710948046,0.726929521066229,0.467269131695167,0.844358954319136
"8655","GFPT2",1772.16457694953,0.197020037456485,0.271056989377844,0.7268583551699,0.467312730728033,0.844358954319136
"8656","MATN4",100.814284609547,0.310873535830106,0.427625391894641,0.726976324892091,0.467240459056256,0.844358954319136
"8657","CHCHD3",298.9909965518,-0.160241492686259,0.220497251320256,-0.726727846840683,0.467392691134367,0.844405878403777
"8658","NBN",308.575501302753,-0.122256777339381,0.168255869586409,-0.72661225810369,0.467463516873743,0.844436290587357
"8659","GPRC5A",891.838780503258,-0.445466812054631,0.61322250333293,-0.726435852620331,0.467571618878852,0.844436503379359
"8660","TMSB15B-AS1",9.51064716018223,0.424782465249128,0.584745529547864,0.72643986791584,0.467569158135178,0.844436503379359
"8661","UBE2B",1316.77130574769,-0.148922601307821,0.205090203649672,-0.72613220260009,0.467757729305728,0.84464527445602
"8662","GRWD1",99.6732784516702,-0.18141151856377,0.249853679951307,-0.726071029248496,0.467795228090668,0.84464527445602
"8663","MAGI1",17.5575977951778,0.339256557703984,0.467333484708451,0.725941043825762,0.46787491366548,0.844691636814972
"8664","FUBP3",180.985324482242,0.111732938638707,0.153959470063499,0.72572956111517,0.468004575975018,0.844730706087626
"8665","DNMT3B",4.6937987881511,0.551308251548748,0.759606313156093,0.725781555524616,0.467972695801899,0.844730706087626
"8666","RFX3-AS1",5.38100632596827,-0.518963762475139,0.715279657133054,-0.725539664521177,0.468121020564471,0.844764715872968
"8667","AC025165.5",2.69565645947976,-0.661113447656884,0.911223698693483,-0.725522666503068,0.468131444531395,0.844764715872968
"8668","ABCC9",242.474015592514,-0.218188000710382,0.300842343653312,-0.725256950403964,0.468294410582074,0.844961303819062
"8669","MTURN",551.540956984694,0.181628302172493,0.250579992447057,0.724831621227172,0.468555334128369,0.845139594714299
"8670","GIT1",122.943389617834,0.137549836483449,0.189766907506451,0.724835738174072,0.468552808149747,0.845139594714299
"8671","LRRC37A",3.83519367814257,0.600123894386335,0.82789710240068 9,

0.724877394359914,0.468527250159344,0.845139594714299
"8672","GART",102.615436308438,-0.142750063700714,0.197012553435268,-0.724573440684921,0.468713757478856,0.845327855969708
"8673","KMT5C",56.212164060267,0.186421546132173,0.257342003061101,0.724411654198209,0.468813047135593,0.845409438164495
"8674","RAB5A",373.3092813811,-0.150580801830552,0.207901037004064,-0.72429076834094,0.468887243346317,0.845445755814664
"8675","RPL15",14254.2474177359,-0.17118354811712,0.236501748692836,-0.723815147512715,0.469179228587577,0.845840799391619
"8676","ARGLU1",1551.13390898726,0.125354965166728,0.173200180596338,0.723757704727118,0.469214499713663,0.845840799391619
"8677","AC093424.1",2.92987581324,0.662868458282743,0.916468650638224,0.723285469526017,0.469504518026383,0.845977884377316
"8678","GREM2",4.84758968786281,0.513580905743359,0.710592955086019,0.722749785327086,0.469833622813919,0.845977884377316
"8679","AC010969.2",43.4732239558315,0.200060241779094,0.27729764913308,0.721463894138827,0.470624147400753,0.845977884377316
"8680","RHOQ",1072.45209509035,-0.125191021269578,0.1736649194606,-0.720876856756212,0.470985283100485,0.845977884377316
"8681","CHCHD5",321.894615273953,-0.19281619136764,0.267451234308694,-0.720939620510744,0.470946664576678,0.845977884377316
"8682","AC012076.1",3.7592267082339,-0.693170274616417,0.959857488869866,-0.722159573326405,0.470196375049321,0.845977884377316
"8683","SMARCC1",182.717934546036,0.152289101745803,0.210590630942459,0.723152312447432,0.469586312962677,0.845977884377316
"8684","COMMD2",330.870278584796,-0.126252451280375,0.174956934068071,-0.721620163001109,0.470528039147202,0.845977884377316
"8685","ICE1",203.713880278137,0.176810593677971,0.245105564545818,0.721365073883991,0.47068492928636,0.845977884377316
"8686","CAMK4",4.47541724326299,-0.683619919291125,0.947143566873449,-0.721770113001745,0.470435827299694,0.845977884377316
"8687","TCOF1",86.7018438460042,-0.208610440562529,0.288615261337823,-0.72279767741856,0.469804194475561,0.845977884377316
"8688","ZSCAN16",15.5461039905111,0.309158248480698,0.428568413659738,0.721374321174669,0.470679241323653,0.845977884377316
"8689","UNC5CL",4.2258889384448,0.591903081266217,0.820363537614393,0.721513151337106,0.470593852139137,0.845977884377316
"8690","RNASET2",211.774734869971,0.202936563039884,0.281639475074501,0.72055440021752,0.471183718082532,0.845977884377316
"8691","CDHR3",15.6684255555191,0.304670625695109,0.421308749069391,0.723152857300214,0.469585978257709,0.845977884377316
"8692","NT5C2",94.9960463851058,0.177901198124313,0.246789838868563,0.720861113812148,0.470994970004477,0.845977884377316
"8693","LINC02716",9.10660278046866,0.43499527993094,0.602620941724115,0.72183897009355,0.470393486931036,0.845977884377316
"8694","FXYD2",3.52032633651208,-0.60621362057807,0.840685434170038,-0.721094473554846,0.4708513910083,0.845977884377316
"8695","CDK8",35.1340576975938,0.193603685564199,0.268669019550778,0.720602940703434,0.471153844037389,0.845977884377316
"8696","DHRS1",136.587139725453,-0.191770341680799,0.265741323883158,-

0.721642907766642,0.470514051597271,0.845977884377316
"8697","MYO5A",114.840628159807,-0.194824177749783,0.270336858071603,-0.720671902231625,0.471111403741431,0.845977884377316
"8698","KIF7",52.6314287713903,-0.221623289433572,0.307556621954898,-0.720593456986508,0.471159680670366,0.845977884377316
"8699","MAPK8IP3",47.4704677779533,0.274654177928386,0.380587074206396,0.721659237905274,0.470504009048856,0.845977884377316
"8700","GCSH",361.051311945304,-0.212403498843239,0.294229902982831,-0.721896369777321,0.470358193354664,0.845977884377316
"8701","TMEM101",180.053859056472,-0.187219439349716,0.259266938561232,-0.722110734167133,0.470226399182343,0.845977884377316
"8702","CCDC43",172.011369194924,0.125680151408777,0.174305767646162,0.721032660628342,0.470889420228723,0.845977884377316
"8703","VMP1",2217.03175083576,-0.176294288380324,0.243965726713434,-0.722619077504285,0.469913944265258,0.845977884377316
"8704","RPS6KB1",187.645575640384,0.14324575732487,0.198633079190096,0.721157613369021,0.470812547195632,0.845977884377316
"8705","MALT1",118.437636137705,-0.14011120994064,0.193779134753723,-0.723045905425832,0.46965168169556,0.845977884377316
"8706","TRPM4",19.5650095209864,-0.271052071332353,0.375360738860724,-0.722110874341938,0.47022631300762,0.845977884377316
"8707","AL050403.2",32.9978629100811,0.269175945138648,0.372521310382713,0.722578648888859,0.469938789647889,0.845977884377316
"8708","LINC02246",7.02878867586975,-0.385542289012371,0.534302939976881,-0.721579950559608,0.470552769510307,0.845977884377316
"8709","APEX2",55.5128979258216,-0.166457769254999,0.230236954415525,-0.722984586369139,0.469689353947284,0.845977884377316
"8710","RPS6KA6",11.1659572841509,0.341276046142285,0.472459056889632,0.72233993859495,0.470085503717505,0.845977884377316
"8711","STAG2",665.629742070225,0.142847563275667,0.198227475474144,0.720624438837185,0.471140613432059,0.845977884377316
"8712","LAMB2",1685.63638236042,0.129094256778411,0.179309092800022,0.719953766775151,0.471553461945374,0.846128711426396
"8713","TTC37",449.734234496408,0.122869661589627,0.170643769160379,0.720036027064945,0.47150281389149,0.846128711426396
"8714","B3GALT4",175.537024600948,-0.166865776803528,0.231821519559585,-0.719802791046061,0.471646426228601,0.846128711426396
"8715","AL353135.2",8.41703618638744,-0.419761532283698,0.582865079441721,-0.720169293184889,0.471420767670641,0.846128711426396
"8716","RPS6KA5",221.395875732348,-0.241003019191327,0.334685251908846,-0.720088554296279,0.47147047419325,0.846128711426396
"8717","SRSF1",346.701157678235,0.135583486317756,0.188343449620802,0.719873648861857,0.471602793811283,0.846128711426396
"8718","SAT1",3165.7363057861,-0.202046633266159,0.280659457613553,-0.719899607104502,0.471586809951276,0.846128711426396
"8719","AL353719.1",5.47882604103812,-0.495981597424293,0.689355870835516,-0.719485563854197,0.471841793842838,0.846382114428487
"8720","BCL2L11",67.7975278757259,0.226505394202461,0.314991046532191,0.71908518256665,0.472088436419856,0.846588401978848
"8721","AUTS2",346.646638925205,0.171371363231921,0.238315199810316,0.

719095397055338,0.472082143215379,0.846588401978848
"8722","SYP",28.3376385719705,0.252740358855204,0.351499247658052,0.71
9035276858052,0.472119184274905,0.846588401978848
"8723","TRPM3",8.97345380021316,0.454149986674144,0.63169752185186,0.7
18935837112002,0.472180454280924,0.846601204282203
"8724","LRRTM2",4.9717249654462,0.56355129853348,0.784148895673394,0.7
18678941771035,0.472338761153817,0.846634799123151
"8725","HNRNPUL2",400.734779540963,0.149032472403989,0.207380716638811
,0.718641900845366,0.472361589331753,0.846634799123151
"8726","GLIS2-
AS1",4.16915239045114,0.59402341527016,0.826397830294109,0.71881047298
8236,0.472257703888312,0.846634799123151
"8727","ID2-
AS1",6.2048069623028,0.529378002414169,0.736868152104905,0.7184161792
06513,0.47250071383086,0.846706994586413
"8728","KIAA1324L",13.9581409719065,-0.297632544893756,0.4143493452181
18,-0.718313057154898,0.472564281057851,0.846706994586413
"8729","TMEM107",146.213611719786,0.134771144609089,0.187604350873444,
0.718379632357274,0.472523241760352,0.846706994586413
"8730","NDUFAF3",653.194572715016,-0.186995828491272,0.260480297511746
,-0.717888570757795,0.472825995557847,0.84693177404961
"8731","ZNF786",17.6828141925581,-0.340992148544419,0.475021250850104,
-0.717846092009937,0.472852189961713,0.84693177404961
"8732","CLEC4E",6.499607409158,-0.57327255113718,0.798581745806063,-0.
717863329769124,0.472841560248281,0.84693177404961
"8733","EPS15",416.686968912076,0.113296728042912,0.157906028043379,0.
717494635554939,0.473068945266493,0.847028998737029
"8734","AL080317.1",4.17804335698283,0.657418119163705,0.9160886860379
24,0.717635889606969,0.472981822454924,0.847028998737029
"8735","NDUFB11",1019.39001116753,-0.187620500295816,0.261465323078416
,-0.717573168352986,0.473020506640941,0.847028998737029
"8736","AC073611.1",41.2382377350501,-0.287604809726707,0.400948389591
513,-0.717311297894773,0.473182037615018,0.847134508733846
"8737","GRK2",130.208316771229,-0.126124405689836,0.175885545957951,-0
.717082265077022,0.473323338045582,0.847290489531063
"8738","PDE8B",12.5803507424787,0.359269244623834,0.501182143938233,0.
716843664462457,0.473470565954234,0.847392063648259
"8739","HDX",34.6787378189878,0.196428699057338,0.274029955322437,0.71
6814695773716,0.4734884427252,0.847392063648259
"8740","AC009404.1",33.6421779396224,0.202736876629567,0.2829019540225
7,0.716633002165098,0.473600575528002,0.84749576673432
"8741","AC005921.2",67.0967963949287,-0.306611938849789,0.427950056441
584,-0.716466639587048,0.47370325954076,0.847582539665655
"8742","BTBD19",27.2871050567493,0.308609290423941,0.430921764506799,0
.716160834385222,0.473892043684255,0.847823331414065
"8743","CLPB",50.1672366310251,-0.189507314698603,0.264770692060883,-0
.715741282479356,0.474151114886405,0.848116933686315
"8744","AC090825.1",13.3852769716403,0.384455631983381,0.5372218416277
04,0.715636636847334,0.474215745165185,0.848116933686315
"8745","FIRRE",3.26764607487541,0.828940716107583,1.15833436815811,0.7

15631633572006,0.474218835363607,0.848116933686315
"8746","TSSK3",3.4699322488557,0.581748290391346,0.814395606122337,0.714331322539033,0.475022328085317,0.848119618102541
"8747","MZT2B",2033.54357905831,-0.17189947055319,0.240293721365655,-0.715372293442536,0.474379028078327,0.848119618102541
"8748","LINC02019",4.75999975754707,-0.485657104639271,0.679167935342883,-0.715076609725521,0.474561706253114,0.848119618102541
"8749","AL023806.1",6.25435803226331,-0.518654097820443,0.72507161581511,-0.71531430345316,0.474414852184682,0.848119618102541
"8750","FAM120B",73.8256763119699,0.164774442645772,0.230502576137925,0.714848594781763,0.474702603947401,0.848119618102541
"8751","CAMSAP1",42.7902471320338,0.210349296839224,0.2943339575355,0.714662007063366,0.474817919527526,0.848119618102541
"8752","CPEB3",33.7215301504148,0.265186421751477,0.371225709841313,0.714353598690231,0.475008556840284,0.848119618102541
"8753","ALKBH3",117.908972340042,-0.158041516858705,0.221064667115672,-0.714910794749528,0.474664166328467,0.848119618102541
"8754","AP002360.1",32.6215881839233,0.263145231816925,0.367907217409087,0.715248897996816,0.474455259080074,0.848119618102541
"8755","YAP1",265.00555834874,0.138963396906496,0.194391902717148,0.714862064541322,0.474694279915705,0.848119618102541
"8756","HOXC12",2.70816176909489,0.888987631614726,1.24453541059208,0.714312846423388,0.475033750292727,0.848119618102541
"8757","RAB20",12.1777720906165,-0.370846805057486,0.518435995458163,-0.71531839668994,0.474412323483124,0.848119618102541
"8758","MEX3C",117.362831662955,0.161374908067724,0.225794172312366,0.714699172326185,0.474794949298104,0.848119618102541
"8759","ZNF30",13.995153245184,-0.325104262918506,0.454810983403001,-0.714811811460665,0.474725335684295,0.848119618102541
"8760","PIGA",42.070511409333,0.296861674631893,0.41546834705393,0.714522963631112,0.474903861624929,0.848119618102541
"8761","RTL8B",83.6225575617037,-0.158907063340657,0.222578753552115,-0.713936351986311,0.475266537542337,0.848438379997962
"8762","SPATA41",14.6237610027713,0.296137831804307,0.414927857683272,0.713709205879251,0.475407012694515,0.848592293830428
"8763","LINC02454",7.44054482173975,-0.490350676430329,0.687242878430803,-0.713504194543213,0.475533818479079,0.848624933935737
"8764","SAMSN1",31.6258189727106,-0.354617349005508,0.49698395769061,-0.713538824579665,0.475512397439734,0.848624933935737
"8765","PPM1B",168.93770063696,0.140508419855186,0.196960077902447,0.713385277623516,0.475607380733514,0.848659376460029
"8766","AC006480.2",10.2972707355535,0.460237490736581,0.645256789497031,0.71326253086826,0.475683318677741,0.848698049751297
"8767","AC103591.3",280.872156205624,-0.256817136970787,0.360329134273558,-0.712729314793109,0.476013272853948,0.848996189695033
"8768","LRRC14",36.541134224632,0.194854527009956,0.273352195717233,0.712833224180579,0.475948963871134,0.848996189695033
"8769","AC090517.5",28.4207302136387,0.238875972935053,0.335126281609078,0.712793910964286,0.475973294057363,0.848996189695033
"8770","TNFRSF14-

AS1",3.83514116027574,0.575018119374312,0.806988781680878,0.712547847538359,0.476125593421789,0.849034587761213
"8771","TNFRSF1B",145.099170512463,-0.147233219686263,0.206725252172307,-0.712216906928929,0.476330469080241,0.849034587761213
"8772","PLA2G5",170.111095745436,-0.54296919494219,0.762510758275857,-0.712080700566007,0.476414804488006,0.849034587761213
"8773","SLC3A1",2.8028042030959,0.791513067730771,1.11149098430429,0.712118297771168,0.476391524467219,0.849034587761213
"8774","TRIM44",365.51783522958,0.150690712212918,0.211597889312815,0.712156027181087,0.476368163212413,0.849034587761213
"8775","SPATA13",175.237146835432,-0.219532366906711,0.308203467810353,-0.712296874744432,0.476280958917547,0.849034587761213
"8776","RAD51-AS1",74.2482657622947,0.23674023128593,0.332370670057558,0.712277744738825,0.476292802546929,0.849034587761213
"8777","ATAD3C",2.8433240620273,-0.689017919467808,0.97117200375284,-0.709470533340416,0.478032531052644,0.849045698418044
"8778","TRNAU1AP",171.395885189671,-0.127213812033953,0.179758248336361,-0.707693878925165,0.47913537942696,0.849045698418044
"8779","CCN1",3373.36032084759,0.223452673334965,0.315627428129664,0.707963419589656,0.478967974227929,0.849045698418044
"8780","TMEM79",22.0531262433718,0.26256887226488,0.370769915430867,0.708171999229636,0.478838452371743,0.849045698418044
"8781","CADM3",1049.41498765964,0.1800219654525,0.253437864616592,0.71031992683825,0.47750576550078,0.849045698418044
"8782","FOXN2",68.8246865874915,-0.178010930671781,0.250084875591777,-0.711802064201415,0.476587354299795,0.849045698418044
"8783","RPS27A",16889.4381455885,-0.151467886398192,0.213336294974926,-0.709995860835564,0.477706702964753,0.849045698418044
"8784","AC007364.1",15.0220916782119,0.372721326057998,0.525623982762551,0.709102587174706,0.478260817587306,0.849045698418044
"8785","AC012485.3",7.3590517542623,0.432169626673914,0.60950052003595,0.70905538628322,0.47829010696524,0.849045698418044
"8786","IFT122",69.0170992085224,0.179669455933842,0.252872362795677,0.710514403185359,0.477385202442731,0.849045698418044
"8787","PPM1L",133.296946509804,0.248456408362168,0.349647161516609,0.710591807136311,0.477337221513955,0.849045698418044
"8788","EXOC3-AS1",31.5187519255092,-0.27016206538659,0.380001996344594,-0.710949068650685,0.477115797520948,0.849045698418044
"8789","TARS",304.489219635254,-0.102205454087807,0.144391578042071,-0.707835287027807,0.479047550285814,0.849045698418044
"8790","SCAMP1-AS1",48.8485731647602,0.177692749092009,0.249707115102233,0.711604669411441,0.476709614741669,0.849045698418044
"8791","SGCD",204.716982431411,0.190414904948055,0.268538513385387,0.709078569578527,0.478275721006445,0.849045698418044
"8792","CDYL",229.324084489866,-0.164968776471316,0.232537026688417,-0.709430144612463,0.47805758670614,0.849045698418044
"8793","DDR1",5.90422710313719,-0.450056224581717,0.633278406929641,-0

.710676725523849,0.477284585594524,0.849045698418044
"8794","SNRPC",385.793171888922,-0.185708785442413,0.262030485542889,-0.708729692492275,0.478492235130428,0.849045698418044
"8795","TRAM2",543.771701884674,0.178818364425512,0.251864988886847,0.709977060391858,0.477718361618687,0.849045698418044
"8796","SLC18B1",55.6167454821116,0.227285459108856,0.321132009717738,0.707763325458061,0.479092244807702,0.849045698418044
"8797","TMEM184A",8.59941627557807,0.646159443701271,0.91034417207023,0.709796869717777,0.477830110529385,0.849045698418044
"8798","ZNF138",86.8375910134435,0.159658785285761,0.225544973089252,0.707880043163637,0.47901975390129,0.849045698418044
"8799","MAGI2",128.284300030363,0.128539957942153,0.181618372476141,0.707747548828184,0.479102043799389,0.849045698418044
"8800","VPS50",83.9998779678215,0.150055468305819,0.211682449830848,0.708870614572563,0.478404772035366,0.849045698418044
"8801","GTF2E2",171.676230826034,-0.113212437437287,0.159475327104194,-0.709905660599897,0.477762639941718,0.849045698418044
"8802","ZFPM2",169.827070238573,0.138586292340882,0.195812186633323,0.707751109487372,0.479099832235773,0.849045698418044
"8803","LINC00963",200.981461930131,-0.128434646184307,0.181125704935901,-0.709091215019751,0.478267874214901,0.849045698418044
"8804","AL022344.1",7.90363787113828,0.477526714273562,0.671717908250593,0.710903652274542,0.477143942620339,0.849045698418044
"8805","DNAJC9",133.143846939347,0.177528578249689,0.250195750241536,0.709558727829332,0.477977820991875,0.849045698418044
"8806","ATE1",162.691146646179,0.148484647677831,0.209309026674507,0.709403937503071,0.478073844998912,0.849045698418044
"8807","CD69",61.6800565135908,0.404322957120051,0.56877507183552,0.710866170374243,0.477167171309733,0.849045698418044
"8808","AL355102.1",2.57659948955392,-0.900837787655252,1.27031888742219,-0.709143032174613,0.478235721195279,0.849045698418044
"8809","SPRED1",210.860574311447,0.14325486549598,0.20145980331128,0.711084112767813,0.477032114351999,0.849045698418044
"8810","SHF",28.9715386912622,0.23161451623326,0.326551466150949,0.709274158108372,0.47815436162914,0.849045698418044
"8811","ANKDD1A",65.6027821138095,-0.180729728383487,0.254681126437512,-0.709631416004555,0.477932732601947,0.849045698418044
"8812","AC027682.6",8.12294383843047,0.41393020290698 5,0.582028574207431,0.711185363142435,0.476969377517619,0.849045698418044
"8813","SPAG7",487.060443829661,-0.147365626768824,0.208229896984653,-0.707706380797401,0.47912761410963,0.849045698418044
"8814","STRADA",93.5627564999872,-0.14206507825785,0.19995284903174,-0.710492893428583,0.477398536314748,0.849045698418044
"8815","CHMP6",151.194596536427,-0.190376887498571,0.268657907608057,-0.708621939303981,0.478559117892,0.849045698418044
"8816","AC100847.1",2.91675377853701,-0.629642763808884,0.889498246344022,-0.707862850091964,0.479030431779537,0.849045698418044
"8817","ZNF57",13.9814638220675,-0.339469764694407,0.479030685107216,-0.708659748213055,0.478535649195033,0.849045698418044
"8818","AC011484.1",14.6974346995938,-0.292584180869289,0.411307530175

55,-0.711351384071212,0.476866517279583,0.849045698418044
"8819","RCN3",633.247018317762,-0.236071621892224,0.333398244243791,-0
.708076979912353,0.478897454216563,0.849045698418044
"8820","PLAGL2",45.8492416175069,0.194664638103481,0.274004581215052,0
.7104430051507,0.47742946278182,0.849045698418044
"8821","RBM39",1923.44846405705,0.122022543684081,0.172123063878889,0.
70892616558313,0.478370296777912,0.849045698418044
"8822","GNAS-
AS1",3.27500224129468,-0.55495829641287,0.782232039580037,-0.709454827
126251,0.478042274514105,0.849045698418044
"8823","PCBP3",7.80303392522999,0.684139553094529,0.962900867506831,0.
710498428426928,0.477395105156546,0.849045698418044
"8824","NF2",146.758274339238,-0.137350730644808,0.193329976678312,-0.
710447148469636,0.477426894236575,0.849045698418044
"8825","ARSD",79.2187436501535,0.226034195785965,0.318564604516419,0.7
09539580296704,0.477989698570921,0.849045698418044
"8826","THOC2",487.622937789948,0.12884504809678,0.18102466134615,0.71
1754117580733,0.4766170494264,0.849045698418044
"8827","YTHDF2",434.183411298966,-0.130125849170864,0.184089343616687,
-0.706862475656457,0.479651945074535,0.849146351424978
"8828","SAR1A",679.558580989215,-0.11225048837248,0.158723516551969,-0
.707207670362614,0.47943743258324,0.849146351424978
"8829","TDRD3",164.518495847602,0.1567604775994,0.221706868040507,0.70
7061892059918,0.479528016424127,0.849146351424978
"8830","FCF1",146.699793321811,0.143513636424946,0.203029741591737,0.7
06860163933669,0.479653381812479,0.849146351424978
"8831","AC079322.1",2.81724938048512,-0.646344262353722,0.914304482221
555,-0.706924525605791,0.479613381848392,0.849146351424978
"8832","AC087645.2",11.2931061861545,-0.390982268491045,0.553038607290
65,-0.706971020353311,0.479584487083876,0.849146351424978
"8833","TTC3",1589.56825663484,0.120784871576362,0.170860066763827,0.7
06922769398878,0.479614473284595,0.849146351424978
"8834","LINC01547",7.30315731928387,-0.473531477348464,0.6697824311990
63,-0.706992980542554,0.479570839969379,0.849146351424978
"8835","TTC28-
AS1",58.1241549935337,0.174667697335482,0.24711904233973,0.70681601742
1094,0.479680819363151,0.849146351424978
"8836","INSYN1",23.1703058210331,-0.278990655342819,0.395088250397098,
-0.706147689946258,0.48009629692848,0.84968949688372
"8837","AL008721.2",6.12823004509489,0.53922494968545,0.76353145800548
7,0.706224928955808,0.480048269910369,0.84968949688372
"8838","SP3",435.258748443646,0.10245045631559,0.14523709039716,0.7054
01464842298,0.480560432672536,0.849754785442809
"8839","LINC00242",5.78255964417932,-0.538013742181275,0.7623501960617
17,-0.705730443778518,0.480355784819451,0.849754785442809
"8840","ARMC10",300.874336688264,-0.152906840920645,0.21675827012556,-
0.705425637656507,0.480545393878522,0.849754785442809
"8841","PRDM11",33.5573546119111,0.219487294617014,0.31106917103984,0.
705589994287485,0.480443148391874,0.849754785442809
"8842","AP001267.3",9.78806700608599,-0.366936768388258,0.520190250959

888,-0.705389552593812,0.480567843813404,0.849754785442809
"8843","ZNF286A",68.0835074485938,0.178344755283744,0.252721484555405,
0.705696848835362,0.480376681040391,0.849754785442809
"8844","INAFM1",150.928807373242,-0.140006068194054,0.198318027578027,
-0.705967429708173,0.480208392472775,0.849754785442809
"8845","AMOT",98.9184377116478,0.176132261528717,0.2496390210234,0.70
5547797802587,0.480469397495349,0.849754785442809
"8846","DUSP11",188.177949652731,-0.144722108214645,0.205307146725603,
-0.704905360201948,0.480869134190114,0.849905628966213
"8847","C4orf48",155.411070839998,0.332418267808525,0.472047728042969,
0.704204782822863,0.481305252925432,0.849905628966213
"8848","THAP9-
AS1",67.0215376844981,-0.22366883302197,0.317214713284621,-0.705102328
659272,0.480746557480244,0.849905628966213
"8849","AC016596.1",12.109315123927,0.395361927164736,0.56110617490624
4,0.704611613356059,0.481051969300625,0.849905628966213
"8850","GPSM3",23.1205795793949,-0.283119837275329,0.401739443854686,-
0.704734975880877,0.480975180884456,0.849905628966213
"8851","RECQL4",2.73171453915108,0.756546454701369,1.07416030855693,0.
70431428965919,0.481237069270018,0.849905628966213
"8852","TBC1D10C",10.0941080545148,-0.467983374335857,0.66406136995851
7,-0.704729104126463,0.480978835673925,0.849905628966213
"8853","PRKAG1",261.406130427677,-0.198647120530047,0.281877644273963,
-0.704728184605436,0.480979408017992,0.849905628966213
"8854","AL442128.2",5.22636408814004,-0.46227629257828,0.6564433410655
99,-0.704213545418666,0.481299796763719,0.849905628966213
"8855","ARID4A",375.249379046441,0.119462652614168,0.169557652075945,0
.704554770318831,0.481087354146528,0.849905628966213
"8856","IZUMO4",7.51826728615733,0.454163086102599,0.644894824208953,0
.704243651915935,0.48128105075817,0.849905628966213
"8857","TUBGCP6",56.7768810343355,0.183146444474344,0.260011824609741,
0.704377367257175,0.481197796837632,0.849905628966213
"8858","SEPHS1",176.636741881552,-0.145057339230827,0.206134743158719,
-0.703701554662891,0.481618651968256,0.850173178945617
"8859","DNAH9",4.66183913637984,0.486046342658696,0.690701422529541,0.
703699640401288,0.481619844338758,0.850173178945617
"8860","XPNPEP2",223.471987219861,0.182467405817304,0.259262523505612,
0.703793989775596,0.48156107716101,0.850173178945617
"8861","HADHB",562.022234567283,-0.166666637767687,0.2370973904025,-0.
702945896978245,0.48208946684758,0.85036258761998
"8862","SOWAHC",159.56636649588,0.18956687909774,0.269686170008312,0.7
02916575558534,0.482107740692222,0.85036258761998
"8863","SHISA5",481.703432973361,-0.100648228194242,0.143119694069563,
-0.703245132324854,0.481902997406606,0.85036258761998
"8864","AP000941.1",5.19314097012278,0.553177246998645,0.7869078235002
51,0.702975914685983,0.482070759448932,0.85036258761998
"8865","POLG",93.1480794341205,-0.142235242144083,0.202321216856017,-0
.703016936900421,0.482045194546923,0.85036258761998
"8866","CYTH1",155.069737552784,0.212329701017802,0.301950035934685,0.
703194819502296,0.481934347255474,0.85036258761998

"8867","AC018866.1",2.48101188782389,-0.994875446046712,1.41481842855187,-0.703182419715163,0.481942073715737,0.85036258761998
"8868","ZNF200",37.7930822503938,-0.223004922674796,0.317398221021512,-0.702602938217734,0.482303230913698,0.850611471751267
"8869","AL591623.1",2.47836275046696,0.817136143458768,1.16322338636119,0.702475683552876,0.482382561060601,0.850655457772894
"8870","EFHC1",280.558047184036,-0.115860364328283,0.16496035978677,-0.702352762069903,0.482459196649704,0.850694682705905
"8871","RPF1",169.158903570451,-0.138498754434006,0.197250689995039,-0.702145855294548,0.482588207941251,0.850826239679987
"8872","AC034236.2",20.1832218367456,0.3422312563621,0.487514806467807,0.701991512507424,0.482684456543175,0.850900011309204
"8873","B4GALT7",163.07061582791,-0.192221160950617,0.273987195102284,-0.701569870368787,0.482947446939522,0.851179495887254
"8874","MOK",91.7013036085216,-0.134289447239783,0.191414714680298,-0.701562821145042,0.482951844405594,0.851179495887254
"8875","PROX1",13.0261888013759,-0.331094804210388,0.472026164672727,-0.701433159833308,0.483032733959168,0.851188739378125
"8876","AL121749.1",2.92794464206105,0.666968141321826,0.950981846636934,0.701346869743626,0.483086570338079,0.851188739378125
"8877","SNX5",392.965170624543,-0.100464963838578,0.143256819898161,-0.701292712695959,0.483120360579259,0.851188739378125
"8878","TPRG1L",229.289986952795,0.106965484914749,0.152778033499921,0.700136547541075,0.48384203350813,0.851657962276337
"8879","PTPN14",160.708002786731,-0.206584401319246,0.29486734148678,-0.700601159414965,0.483551954666258,0.851657962276337
"8880","AC007383.2",6.27273242915052,0.428472643298479,0.612295287402819,0.699781056809921,0.48406404666793,0.851657962276337
"8881","SETMAR",48.8495278091195,0.179378471684771,0.256074779616037,0.700492535632496,0.483619765100418,0.851657962276337
"8882","AC018647.1",4.23649367886972,0.575434454334403,0.821605601939202,0.700377958689946,0.483691297498277,0.851657962276337
"8883","DRAM1",258.738379705103,0.129251225344791,0.184559845713441,0.700321485668527,0.483726556709546,0.851657962276337
"8884","LINC00539",9.33193377075593,-0.424006845192358,0.605915817094898,-0.699778472899563,0.484065660589027,0.851657962276337
"8885","AP4S1",66.0406364747051,0.145352114653575,0.207634728920585,0.700037587205217,0.483903831249609,0.851657962276337
"8886","PRORP",77.8189647085018,-0.159795841023898,0.228172960147024,-0.700327685282838,0.483722685881433,0.851657962276337
"8887","UQCRC2",508.960117205924,-0.145218254071281,0.207472718460066,-0.699939033667372,0.483965379212897,0.851657962276337
"8888","JAK3",4.04416483087793,-0.505966314214317,0.72308568545241,-0.699732167838105,0.484094583416665,0.851657962276337
"8889","SAE1",153.695067164609,-0.18314203712252,0.261728163933139,-0.69974142014501,0.484088804213731,0.851657962276337
"8890","RPA4",9.69257046276786,-0.351478915441025,0.501983475612538,-0.700180249981612,0.483814744016565,0.851657962276337
"8891","FHL2",60.0572891277898,-0.328530857601784,0.470750571797077,-0.697887325654491,0.485247661291684,0.85166421993817

"8892","CXCR4",146.134922916234,0.266558997543316,0.381432610527622,0.698836413526874,0.484654269208264,0.851664219993817
"8893","FAM135A",56.0513077230996,0.196374101520589,0.28141492757348,0.697809825561986,0.485296133541234,0.851664219993817
"8894","ENPP3",3.30007271184041,0.632764472667172,0.906066231007267,0.698364480446129,0.4849492837201,0.851664219993817
"8895","AC004130.2",32.9228154882008,-0.301322640832076,0.431269997854856,-0.698686767757693,0.484747805146544,0.851664219993817
"8896","AC002454.1",8.20735095516314,-0.533411897717261,0.762742007365324,-0.699334627654482,0.484342931463404,0.851664219993817
"8897","SRPK2",627.629896952821,0.12922295553934,0.184709661116196,0.699600414826427,0.484176883427025,0.851664219993817
"8898","AP003696.1",3.07301880148366,0.698226902490238,0.998790102660495,0.699072708700615,0.484506593063274,0.851664219993817
"8899","SLC22A18",47.2226582663783,-0.281048609528678,0.402018381786774,-0.699093927694439,0.484493333134409,0.851664219993817
"8900","BEST1",20.4545652156193,0.282758237656644,0.405160709250407,0.697891555624382,0.485245015742468,0.851664219993817
"8901","C11orf98",12.1699548489173,0.415344832832098,0.594840904488306,0.698245244565664,0.485023835769156,0.851664219993817
"8902","CLSTN3",39.9329978100142,-0.252548738447522,0.36167784639433,-0.698269858011079,0.485008445742212,0.851664219993817
"8903","HSPH1",260.970748485061,-0.168717757237752,0.241193803496874,-0.699511159870815,0.484232641178555,0.851664219993817
"8904","MCF2L",94.680855237932,0.193325406301867,0.276649078892363,0.698810952401851,0.484670182968481,0.851664219993817
"8905","AC037198.1",110.956976704036,-0.514608080808132,0.736976636973715,-0.698269191980487,0.485008862187081,0.851664219993817
"8906","UNC119",31.8021323070779,-0.249613126999221,0.357174624811251,-0.698854592851128,0.48464290690611,0.851664219993817
"8907","PHB",772.20583915853,-0.167683605763844,0.240180684483279,-0.698156082470147,0.485079588287145,0.851664219993817
"8908","STXBP4",73.8493926953236,0.159749636192649,0.228655183293416,0.698648654676041,0.484771629252792,0.851664219993817
"8909","UBA52",9021.12374688684,-0.151781857723558,0.217127453985839,-0.699044984580609,0.484523918396946,0.851664219993817
"8910","LINC00662",138.643272830667,0.154662424646287,0.221604938137942,0.697919576819245,0.485227490649446,0.851664219993817
"8911","AP000350.5",4.46106913672875,-0.529783905355148,0.758180930910405,-0.698756568196718,0.484704175234896,0.851664219993817
"8912","PDZD4",2.50542602830367,0.656037302994983,0.940076297823606,0.697855381008744,0.485267640677223,0.851664219993817
"8913","CACYBP",589.425299034348,-0.147842705106519,0.211970329963791,-0.697468863362968,0.485509418676819,0.851942926972451
"8914","COL24A1",13.2923474539591,0.457100881486621,0.655641209818767,0.697181438020001,0.485689253829453,0.851971728341482
"8915","MFSD1",191.079321593796,-0.148236711986629,0.212570182535448,-0.697354211294003,0.485581149408885,0.851971728341482
"8916","AHNAK2",1114.21715546914,-0.139496449093346,0.200065865026655,-0.697252622653852,0.485644711951155,0.851971728341482

"8917","ZP1",3.2028367909825,-0.710998796975382,1.02001010832496,-0.69705073623532,0.485771042726612,0.852019637573648
"8918","ARHGAP24",354.967931322932,0.18861036826092,0.270750760974375,0.696619900834816,0.486040698175429,0.852326042116919
"8919","RMND5B",169.183492052161,-0.142669932745269,0.204885672668402,-0.696339235863376,0.486216407038218,0.852326042116919
"8920","LINC00535",7.14068631077127,0.6138388190147,0.881526329100588,0.696336341582666,0.486218219166698,0.852326042116919
"8921","COG6",108.532622577461,0.180214916407792,0.258722375929618,0.696557132950947,0.486079990704689,0.852326042116919
"8922","CST3",26748.6670913958,-0.19456839620295,0.279351185120646,-0.696501058762002,0.486115094451557,0.852326042116919
"8923","IPO11",46.1340696923022,0.173292008499408,0.248923628220417,0.696165365008905,0.486325275221393,0.85241816703604
"8924","FBXO33",119.279614932921,-0.180715502802994,0.259657048618736,-0.695977651153021,0.486442825910192,0.852528663966315
"8925","RPL17",13950.4875424465,-0.166864193885587,0.2398537550645,-0.69569139678766,0.486622114476152,0.852747324415353
"8926","UNC50",415.933509825065,-0.183465300780233,0.263910775861065,-0.695179271030667,0.486942961261155,0.85300027248431
"8927","PLAC9",14584.211901839,-0.163661819595399,0.235390569015092,-0.695277726207059,0.48688127022143,0.85300027248431
"8928","AL732509.1",14.257216392256,0.298231800295077,0.429040874431102,0.695112792436212,0.486984618479054,0.85300027248431
"8929","MIR222HG",145.696732891981,-0.277578420267027,0.399298835718491,-0.695164612157103,0.486952146727495,0.85300027248431
"8930","TUT7",167.160242874949,0.157316609251062,0.226354626183087,0.695000636407652,0.487054902729687,0.85302784755793
"8931","C5orf30",34.3183311383858,0.218384581343328,0.314351349437405,0.694714947889269,0.487233958458351,0.85315037061001
"8932","GOLGA8A",81.1304766972892,0.309047155666354,0.44480719060866,0.69478902812579,0.487187525140071,0.85315037061001
"8933","AC084757.3",5.49854695430207,-0.462900416337548,0.666596702954103,-0.694423501175673,0.48741665976771,0.853279220815646
"8934","CANT1",126.104398136589,0.127889947192717,0.184166877947145,0.694424255969745,0.487416186556725,0.853279220815646
"8935","CCDC191",60.2956808385157,0.203431956400666,0.293059403646831,0.694166281201555,0.487577935834508,0.853466023105955
"8936","AL137026.3",10.2435469226219,0.444692433271948,0.640794489960936,0.693970438633231,0.48770074778357,0.853489951363438
"8937","MPDU1",164.936397566957,-0.202024838971002,0.29109458715382,-0.694017848103263,0.487671015997539,0.853489951363438
"8938","SLC2A13",82.4376949545371,0.14630246779516,0.210931192770066,0.693602808924722,0.487931331711688,0.853797944503335
"8939","IGHA1",149.723678091107,0.495105761524604,0.714120941998085,0.693307999257542,0.488116284093966,0.85402603011854
"8940","PARP3",43.1480276263128,0.186490138529527,0.269234990972493,0.692666795857082,0.488518681267951,0.854156755173925
"8941","WASHC1",118.565718616724,0.203705687240494,0.293924448005857,0.693054588083926,0.488275294851076,0.854156755173925

"8942","PUS1",23.8300659341426,-0.2589108246682,0.37375580087496,-0.69
2727240786876,0.488480740478702,0.854156755173925
"8943","FMN1",4.73357616901827,-0.587772321639053,0.848368114001218,-0
.692826983874843,0.488418136029866,0.854156755173925
"8944","FBL",456.683994273225,-0.0939537525276201,0.135625367225695,-0
.692744686702091,0.48846979011549,0.854156755173925
"8945","LINC00653",5.31241197111165,-0.464830635311272,0.6709104936123
86,-0.692835541755328,0.488412764817655,0.854156755173925
"8946","CSF3R",18.6684513045506,0.41059872844778,0.593036196189534,0.6
92367061380099,0.488706845621743,0.854160484985682
"8947","FAM86B1",9.80584200809382,0.39850701072532,0.575449884190612,0
.692513842948865,0.488614695656041,0.854160484985682
"8948","HOXC8",171.204956330087,-0.157539554983092,0.227554595666091,-
0.692315417853666,0.488739269829723,0.854160484985682
"8949","SOX12",29.4616890153914,0.22369386548491,0.32304615900503,0.69
2451710844911,0.488653701258022,0.854160484985682
"8950","CYTH2",244.199972492407,-0.149384802234129,0.215832303927842,-
0.692133658935834,0.488853395752271,0.854264481515701
"8951","PLEKHA6",10.9909207134072,-0.484369276704308,0.700112524524222
,-0.691844895980789,0.489034738738976,0.854323048798868
"8952","FAM13A",269.591952029179,0.160165158218381,0.231468597505152,0
.6919519967058,0.488967475311475,0.854323048798868
"8953","AC132217.1",18.84524509253,-0.566541795031815,0.81891579845996
9,-0.69181934955613,0.489050783625081,0.854323048798868
"8954","APMAP",253.03075023935,0.152219569631627,0.220081219250437,0.6
91651791779705,0.489156028294302,0.854411467782319
"8955","UBTF",515.173502448576,0.108042474845112,0.156239604491646,0.6
91517846557844,0.4892401693639,0.85446300936364
"8956","PAPSS1",253.271846573981,-0.140830443576014,0.203700905406001,
-0.691358947547738,0.489339995892158,0.854480042131549
"8957","AL662884.5",45.1442763682217,-0.245998849525853,0.355835013714
202,-0.691328396714308,0.489359190369072,0.854480042131549
"8958","PFDN5",4781.58954646759,-0.163488666864934,0.236566739058854,-
0.691088981973333,0.489509623893114,0.854647300478712
"8959","DFFA",235.284794045823,0.113718150854628,0.164902058552839,0.6
896102562491,0.490439315848128,0.855235733605113
"8960","DARS",352.61714817758,0.0865481221237068,0.125372943300057,0.6
90325359248925,0.489989603821835,0.855235733605113
"8961","ITGA6",5.37813752841919,-0.515696961446041,0.747940261560601,-
0.689489505980093,0.490515274842588,0.855235733605113
"8962","ABHD5",709.649157357999,0.235135623081748,0.341188787945256,0.
689165738703805,0.490718974641131,0.855235733605113
"8963","AMT",29.5772841522361,0.291249492194875,0.422287121374439,0.68
9695416821926,0.490385748661213,0.855235733605113
"8964","DAG1",277.641682753758,0.119564334780655,0.173532878691494,0.6
89001045117313,0.490822609876541,0.855235733605113
"8965","KPNA4",435.761770638232,-0.107814236681651,0.156369212002542,-
0.689485067430656,0.490518067070767,0.855235733605113
"8966","CCDC69",288.111172262999,0.199160087124698,0.28909830658947,0.
688900912199092,0.490885625356337,0.855235733605113

"8967","PDE10A",61.6049012220628,-0.202091036674435,0.29295812711755,-0.689829084664191,0.490301676070628,0.855235733605113
"8968","BORCS7",549.216206135794,-0.151833483046979,0.220384056751887,-0.68894948792923,0.490855055216568,0.855235733605113
"8969","WNT5B",15.3367421550379,0.270670383381753,0.392866251563146,0.688963183538425,0.490846436350618,0.855235733605113
"8970","CACNA1C",14.1370976757119,0.36086392812661,0.523621568498123,0.689169334948629,0.490716711796072,0.855235733605113
"8971","APOBR",7.59313921002254,0.5903201229637,0.855128151781238,0.690329422243974,0.489987049330563,0.855235733605113
"8972","HIRIP3",94.3987460750803,-0.175949631722467,0.254959929159866,-0.690106999567537,0.490126901729973,0.855235733605113
"8973","CDT1",10.3012919570087,-0.35945259090155,0.520783640719167,-0.690214827802905,0.490059100037896,0.855235733605113
"8974","MYO1C",576.094840196421,-0.124017360918551,0.179785211089858,-0.689808467374803,0.490314643148708,0.855235733605113
"8975","NDUFA13",2361.85249449901,-0.174344038048529,0.252871109737605,-0.68945811259119,0.490535024159554,0.855235733605113
"8976","IGFLR1",19.1915838770213,-0.265779099832389,0.38522174059378,-0.689937954754886,0.490233206162273,0.855235733605113
"8977","DGCR11",3.9111377584614,0.650034806242644,0.943031536231544,0.689303359716106,0.490632384138986,0.855235733605113
"8978","KHDRBS1",758.061740031575,0.0944765514079679,0.13716372367323,0.688786720554792,0.490957493555383,0.855265671553375
"8979","POGLUT1",119.604138465804,-0.145254181292722,0.210928865588494,-0.688640603491895,0.491049462732895,0.855330615563256
"8980","AL391988.1",11.4345539139449,-0.32134820331221,0.466802968224941,-0.688402227891062,0.491199521254288,0.855401460017489
"8981","POLR2F",564.479606180771,-0.153336029506767,0.222723176128465,-0.688460142191595,0.491163061674648,0.855401460017489
"8982","WDR53",22.663924184513,-0.282476138057614,0.410421367057978,-0.688258849880274,0.491289790223125,0.85545380900247
"8983","ZDHHC6",132.420842996242,-0.152298058091998,0.221311820509358,-0.688160522747849,0.49135170088241,0.85545380900247
"8984","WIPI1",251.43810131237,-0.132874526561574,0.193115968547657,-0.688055615290993,0.491417759393969,0.85545380900247
"8985","ZNF677",184.382748821526,-0.114758292040651,0.166819203408973,-0.687920153648679,0.491503064430703,0.85545380900247
"8986","AF129075.2",5.07795069674113,-0.484145131623875,0.703773815351636,-0.687927173564955,0.491498643543258,0.85545380900247
"8987","ATG3",334.62192033783,-0.0973625099281004,0.14160891017808,-0.687545083184824,0.491739301180428,0.855484168015784
"8988","PPP3CB-AS1",6.42542519837536,0.433503585315739,0.630433949743778,0.687627285129431,0.491687521374532,0.855484168015784
"8989","STK11",141.622167495243,-0.131687892435462,0.19146514016758,-0.687790437048761,0.491584759060054,0.855484168015784
"8990","C19orf38",3.17584291269096,-0.619328095595563,0.900770934393493,-0.687553374501996,0.49173407826624,0.855484168015784
"8991","AC012510.1",10.4118062415146,0.353620966489906,0.5144176769888

05,0.687419935799759,0.491818138357025,0.855521285118535
"8992","NFXL1",59.9038162832816,0.198378624280857,0.288618922066682,0.
687337555210689,0.491870038093726,0.855521285118535
"8993","ANKRD13C",137.740293732978,0.11267643468961,0.163962783119443,
0.687207380515907,0.491952054087872,0.855543551992124
"8994","COQ7",102.676168399036,0.163556132090186,0.238023221528977,0.6
87143594812131,0.491992244668617,0.855543551992124
"8995","AL603839.3",3.12266878572994,-0.763764221230074,1.112489638822
59,-0.686536031057696,0.492375151343277,0.855662535682299
"8996","MASP1",51.5037668254365,-0.362831842801495,0.528233665950736,-
0.686877543385001,0.492159899369091,0.855662535682299
"8997","PPP2R2D",189.146831191406,-0.109957073606948,0.160159027152687
,-0.686549335131268,0.492366764967218,0.855662535682299
"8998","COG7",76.759442758351,0.200895139340542,0.292592558208319,0.68
6603721470965,0.492332482707996,0.855662535682299
"8999","THOC1",123.461605892355,0.143142925241337,0.208506874210556,0.
686514177450029,0.492388927182909,0.855662535682299
"9000","DHPS",294.936718334883,-0.176312558290956,0.256777602472588,-0
.686635269560857,0.492312597054808,0.855662535682299
"9001","BLOC1S4",245.454616941568,-0.136586086681193,0.199004719232697
,-0.686345968114868,0.49249496807251,0.855687816097944
"9002","AC093627.5",7.29293843975426,-0.462745771054203,0.674244430092
053,-0.686317528779623,0.492512897731054,0.855687816097944
"9003","RBFOX2",706.468238680906,-0.0935019412988611,0.136260503980632
,-0.686199878668816,0.492587074291243,0.855721630780299
"9004","AC011921.1",2.58347458171849,0.625063164809854,0.9111804558518
45,0.685992726024281,0.492717695358231,0.85585348238591
"9005","BMF",9.7487373042926,-0.321830462612739,0.469213582015819,-0.6
85893322248052,0.492780381464603,0.855867314392714
"9006","MIS18A",80.4096175941402,-0.183500752360966,0.267570142131087,
-0.68580429378053,0.492836528311906,0.855869787119499
"9007","BAZ1B",368.72533993294,0.150294033288305,0.219256665403934,0.6
85470760997936,0.493046905151025,0.856045026261327
"9008","MCM3AP",119.264756101845,0.204977682343943,0.298999992615119,0
.685544105038813,0.493000639042958,0.856045026261327
"9009","LINC01703",4.94806705403693,0.467042137325748,0.68160602143612
2,0.685208350040254,0.493212455489463,0.85605992054231
"9010","CHCHD6",83.5391105059287,-0.193482063885634,0.282374406885464,
-0.685196884589166,0.493219689514498,0.85605992054231
"9011","MRPL58",136.563381743558,-0.170494350249826,0.248809432305541,
-0.685240702773908,0.493192043124531,0.85605992054231
"9012","ZFP69",15.4805086635682,-0.254265658953873,0.372897495545211,-
0.68186475369622,0.495324474290326,0.856094799486164
"9013","AC118553.1",6.43496631666632,-0.418891958954629,0.614673841891
572,-0.681486554992399,0.495563671300873,0.856094799486164
"9014","DPH5",131.831725300339,0.146881414734312,0.215052189612666,0.6
8300357694038,0.494604582298228,0.856094799486164
"9015","SSR2",2284.6261514808,-0.169758169004176,0.248929851814585,-0.
681951834087862,0.495269407824763,0.856094799486164
"9016","AC096642.1",21.9387523349138,0.350198319099889,0.5157380741784

86,0.679023590914276,0.497122914482952,0.856094799486164
"9017","SH3BP5L",47.0124833551851,0.204525279351299,0.298919248572122,
0.684215821926076,0.493838892760944,0.856094799486164
"9018","TAF1B",210.09258111158,-0.127382188312905,0.187009224172125,-0
.68115457340041,0.495773688475405,0.856094799486164
"9019","AC019080.6",2.77053895520954,0.722785892116279,1.0594068356545
7,0.682255265673923,0.495077554300308,0.856094799486164
"9020","AC017071.1",4.35968774879032,0.585664878617131,0.8590119422953
16,0.681788983110304,0.495372391507352,0.856094799486164
"9021","CCDC150",13.6936668118169,-0.313583859208924,0.459772582304703
,-0.682041233596448,0.495212878235974,0.856094799486164
"9022","WDR12",67.3314063275437,0.157069045253522,0.230414146774096,0.
681681430817337,0.495440411683987,0.856094799486164
"9023","ZBTB47",185.026378881191,0.146883516471129,0.215971867101829,0
.680104860147712,0.496438067316251,0.856094799486164
"9024","MST1",3.92685245803903,-0.548031401267483,0.802315448422211,-0
.683062257301927,0.494567503446272,0.856094799486164
"9025","ZNF654",86.2461539604919,0.163239368061536,0.238708350728605,0
.683844396575499,0.49407342849317,0.856094799486164
"9026","CFAP44",30.6097406579406,0.296732880713866,0.434583579286975,0
.682798188557234,0.494734374480997,0.856094799486164
"9027","P2RY1",15.5522891052689,0.595747719422082,0.873218519720631,0.
682243569012576,0.495084949121821,0.856094799486164
"9028","CRIPAK",37.2743917677509,-0.19124414962317,0.281199753920036,-
0.680100700506139,0.496440700958205,0.856094799486164
"9029","CASP6",106.799982683353,0.172628506515706,0.252746072138051,0.
683011629242712,0.494599494134479,0.856094799486164
"9030","RAD1",141.14620601785,-0.121577353813722,0.178610229861134,-0.
680685277143676,0.496070654256386,0.856094799486164
"9031","MRPS36",491.858424008155,-0.156093589032268,0.228428852702348,
-0.683335695931826,0.494394743085977,0.856094799486164
"9032","C5orf58",3.33593283429027,0.751897063177181,1.10700584818079,0
.679216884366798,0.497000450617391,0.856094799486164
"9033","KCNMB1",2.84625913135996,-0.626531965176862,0.920706874313435,
-0.680490156700593,0.496194152366226,0.856094799486164
"9034","LMAN2",851.994387306403,-0.170904754234911,0.25089052528639,-0
.681192540211809,0.495749667622265,0.856094799486164
"9035","PRPF4B",672.533461653532,0.126895241883261,0.185959393467181,0
.682381457141376,0.494997777733656,0.856094799486164
"9036","AC019205.1",32.2557121789619,-0.228420526665866,0.333977961824
824,-0.683938920453907,0.494013735942632,0.856094799486164
"9037","ATG5",203.624817054084,-0.128534152876328,0.188280078932608,-0
.68267526551407,0.494812062606598,0.856094799486164
"9038","PEX7",53.8112402489247,-0.204393287076243,0.300476969177881,-0
.680229461963335,0.496359180182724,0.856094799486164
"9039","AC004982.2",10.7845782313472,-0.311224246465676,0.457387288931
095,-0.680439212014399,0.496226399624139,0.856094799486164
"9040","GNG11",1337.65139953601,-0.171024583255735,0.250902583886502,-
0.681637393312375,0.495468264122647,0.856094799486164
"9041","SSBP1",901.922891364376,-0.169822566781136,0.249988261707804,-

0.679322163452745,0.496933756290881,0.856094799486164
"9042","OTUD6B",63.1359751917132,0.206220910503233,0.303528705379573,0
.679411557616428,0.496877128810955,0.856094799486164
"9043","TRIQK",230.135449734139,-0.159942423016033,0.235067797486071,-
0.680409757212749,0.496245044602419,0.856094799486164
"9044","NIPSNAP3B",7.95638651175872,0.376972393639536,0.55243957393465
7,0.682377605490161,0.495000212594674,0.856094799486164
"9045","WHRN",11.9310402687409,0.412784388980397,0.602634444918615,0.6
84966470902842,0.493365079039968,0.856094799486164
"9046","DLG5",116.171516159153,0.163909269204713,0.240430206891734,0.6
81733261904658,0.495407631102743,0.856094799486164
"9047","FAM53B",65.3615997373374,0.155656418091863,0.228584231605235,0
.68095868642716,0.495897632172366,0.856094799486164
"9048","AC069360.1",5.78975900278537,0.559101961671803,0.8230557741801
82,0.679300211736812,0.496947662314687,0.856094799486164
"9049","PLAAT3",527.361276241278,-0.192035233717453,0.281189328467369,
-0.682939266451352,0.494645220412856,0.856094799486164
"9050","ZNHIT2",32.8272522598846,-0.207453623160005,0.305375069480266,
-0.679340404287364,0.496922201202506,0.856094799486164
"9051","MAML2",152.156646340592,-0.174140711449804,0.255380904191459,-
0.681886188793704,0.495310919212612,0.856094799486164
"9052","BRAP",78.0709179750309,0.148475907623294,0.218243262068952,0.6
80322985533383,0.496299973508994,0.856094799486164
"9053","DDX51",72.2769671484249,-0.169126845704446,0.247056152648369,-
0.684568442807257,0.4936162862166,0.856094799486164
"9054","C14orf93",30.1114559828465,-0.25014235802862,0.368306972469449
,-0.679168130734665,0.497031337670908,0.856094799486164
"9055","CHURC1",1104.11331185775,-0.121344117380989,0.178451425699186,
-0.67998401753056,0.496514580835078,0.856094799486164
"9056","TTC9",14.7181822079064,0.287666151947661,0.422256884796254,0.6
81258642085763,0.495707847752033,0.856094799486164
"9057","DCAF4",30.1755643981124,0.224602915002404,0.328648064046037,0.
683414690588111,0.494344839741303,0.856094799486164
"9058","EFCAB11",55.2196592817456,0.187190608221128,0.273405017361953,
0.684664129529532,0.493555889275287,0.856094799486164
"9059","POLR3K",104.821810253121,-0.183050596242928,0.267634313507587,
-0.683957874623347,0.494001766704462,0.856094799486164
"9060","GTF3C1",179.344605600752,0.121657278162813,0.178681004382297,0
.680862963488391,0.49595820502617,0.856094799486164
"9061","C16orf70",36.1964146558765,0.186853559881146,0.273982028395754
,0.681992030554812,0.495243990147326,0.856094799486164
"9062","DUS2",30.0153803671769,-0.249108896615707,0.364848120667242,-0
.682774235372601,0.49474951253077,0.856094799486164
"9063","TERF2IP",772.431508079729,-0.106125414402522,0.15499318116207,
-0.684710215035531,0.493526801764996,0.856094799486164
"9064","CDK10",120.136422049536,-0.12093242399786,0.176838950284878,-0
.683856264714556,0.494065933460959,0.856094799486164
"9065","GGNBP2",518.523631565238,0.105421585465672,0.15494243108555,0.
680391967049132,0.496256306010646,0.856094799486164
"9066","GPATCH8",320.871533361177,0.138735201275266,0.202715216201245,

0.684384743657015,0.493732247214513,0.856094799486164
"9067","RPRD1A",102.49697425077,0.158448333485,0.23218543902551,0.6824
21490985884,0.494972470305125,0.856094799486164
"9068","TXNL1",629.946299331998,-0.178128946938844,0.261326510167886,-
0.681633665196875,0.495470622085452,0.856094799486164
"9069","PLPPR2",126.230723611194,0.134033666915736,0.19717892482755,0.
67975655630012,0.496658618762662,0.856094799486164
"9070","NDUFB7",1581.11280180308,-0.16369412724724,0.240901151447203,-
0.679507450520076,0.496816388473213,0.856094799486164
"9071","ZNF226",144.682584599559,0.17070118563945,0.25024217180967 5,0.
682143958410331,0.49514792697254,0.856094799486164
"9072","AC010332.3",11.3133915156836,-0.298317649360522,0.438869071058
198,-0.679741793244213,0.496667968114461,0.856094799486164
"9073","TMEM86B",9.16740575236047,-0.326053527568596,0.478671668162356
,-0.681163204875548,0.495768227456674,0.856094799486164
"9074","ZNF579",50.8717830532773,0.184950734066558,0.271170009559743,0
.682047156936101,0.495209132879026,0.856094799486164
"9075","ZNF274",50.8782132547253,0.199834161428971,0.293739904691914,0
.680309887206387,0.496308265399025,0.856094799486164
"9076","TM9SF4",183.645506236883,0.111159450611857,0.163277956314606,0
.680798885047737,0.495998755653121,0.856094799486164
"9077","ASXL1",296.442085299728,0.161544689710576,0.237909118837867,0.
679018486133217,0.497126148908781,0.856094799486164
"9078","RPS21",7734.27116719499,-0.155090169615539,0.226655962372223,-
0.684253650300377,0.493815009459649,0.856094799486164
"9079","AL022322.2",4.32628639360053,0.547710406999468,0.8020340094098
41,0.682901723085893,0.494668945075461,0.856094799486164
"9080","XRCC6",834.037168768718,-0.102453134608991,0.150779623790645,-
0.679489257455938,0.496827911992139,0.856094799486164
"9081","PRR34-
AS1",40.5559608099483,-0.23176693956448,0.339858154140608,-0.681951975
377917,0.495269318480739,0.856094799486164
"9082","TCEAL7",139.149781221131,0.199524725057513,0.291859811338236,0
.683632063430221,0.494207532563667,0.856094799486164
"9083","AC009054.2",10.5154019253488,0.332669979877036,0.4901429806154
54,0.678720277620451,0.497315115413497,0.85632592811484
"9084","TMEM25",20.0276528417967,-0.259850975319384,0.382959390298888,
-0.678534021888269,0.497433159937574,0.856434898879751
"9085","IGKC",1002.39205637959,0.532413576445473,0.784893996125318,0.6
78325454231742,0.497565362944439,0.856558188803687
"9086","WDR55",120.261694801178,-0.123190198646271,0.181699379469999,-
0.677989099388263,0.497778604733805,0.856558188803687
"9087","TMEM134",198.265689458739,-0.162143949258092,0.239101621972365
,-0.678138223908964,0.497684056969559,0.856558188803687
"9088","AC012291.3",7.02080117901307,-0.459206979487535,0.677257177574
797,-0.678039295400188,0.497746778446244,0.856558188803687
"9089","TMEM38A",11.927003696322,0.355355227471684,0.523944681823867,0
.67823043118728,0.497625600596048,0.856558188803687
"9090","DYNC2LI1",184.595951582359,-0.148603638022508,0.21931661366019
,-0.677575836788887,0.498040670982976,0.856709763754972

"9091","ZNF485",12.1825096963985,-0.313492520110764,0.46264840012837,-0.677604245521609,0.498022653526296,0.856709763754972
"9092","EXT2",327.848460684327,0.114244818751887,0.168596021172308,0.677624643556249,0.498009716848271,0.856709763754972
"9093","BEX5",2.53799206119914,-0.729704427418633,1.07704705624753,-0.677504685784992,0.498085798070586,0.856709763754972
"9094","PSPH",32.5952993227323,-0.215008423403351,0.317449164894235,-0.677300327676039,0.498215423043716,0.856744278878914
"9095","MAMDC2-AS1",2.76069371131143,0.661916381364893,0.977249671540319,0.677325764992681,0.498199287098251,0.856744278878914
"9096","AC073263.2",10.3177756151039,0.482615377746285,0.712671667421627,0.677191755766491,0.498284297836635,0.856768515629394
"9097","AL157394.3",16.3944506656632,-0.283355341897331,0.418558643866408,-0.676978832117418,0.498419384975743,0.856906582501992
"9098","ZC4H2",25.1302661445808,0.226834395273558,0.335173563211562,0.676766965449422,0.498553820853231,0.857043499466315
"9099","BABAM1",316.099937346775,-0.14566746917689,0.215318156318494,-0.676521997343419,0.498709284635243,0.857216530574261
"9100","MPRIP",284.642211765247,0.126838487846446,0.187514615909469,0.676419207277598,0.498774525832007,0.857234459781603
"9101","SLC35A1",156.996378052694,-0.161883573582103,0.239429486171913,-0.676122127522209,0.498963108841191,0.857275955429664
"9102","FMC1",341.318474938895,-0.174864800695066,0.25861243919365,-0.676165466905972,0.49893559510969,0.857275955429664
"9103","RPS25",10589.2477465471,-0.123447651483366,0.182559550657958,-0.676204838577066,0.498910600954207,0.857275955429664
"9104","HIPK1-AS1",3.06118437218825,0.644023708004801,0.95293357686736,0.675832737599552,0.499146846852775,0.857403260272093
"9105","BROX",217.845645919024,-0.113164748380718,0.167425713306234,-0.675910206060949,0.499097657439263,0.857403260272093
"9106","SDCBP2",22.0766117370014,-0.295489697889673,0.43739458136347,-0.67556780646106,0.499315087125834,0.857538102184205
"9107","MAPK8IP2",3.1193446716514,0.562101828345306,0.832082168805265,0.67553644269579,0.499335006175931,0.857538102184205
"9108","TNFSF4",96.4666100846807,-0.28080773689658,0.416385891293394,-0.674393015633464,0.50006148196116,0.857784523189137
"9109","AL513314.2",3.1718132776226,-0.658589786215814,0.977042736126727,-0.674064461936077,0.500270332054055,0.857784523189137
"9110","TRIM11",83.8576976561563,0.148022825933213,0.219521993745126,0.674296107683286,0.500123078144751,0.857784523189137
"9111","ELOVL2",3.81842084301382,-0.641794382415832,0.951284347609669,-0.674660929750915,0.499891212576391,0.857784523189137
"9112","ZNRD1",191.518046993455,-0.148887781660399,0.220597910635727,-0.674928340124933,0.499721294026176,0.857784523189137
"9113","TBX18",118.969034077721,0.162060533987407,0.240412708785957,0.674093041111616,0.500252163434778,0.857784523189137
"9114","RNF217-AS1",5.28561354828141,0.445596853192708,0.661006564093886,0.6741186508

5415,0.500235882868423,0.857784523189137
"9115","AC016877.3",8.81406570699015,0.389051934185562,0.577214735412195,0.674015942970922,0.500301177783332,0.857784523189137
"9116","UBC",10308.0932275898,-0.147801204730816,0.218934267085463,-0.675093975458492,0.499616060998612,0.857784523189137
"9117","AL354696.2",3.34178526656338,0.587760816662867,0.871988213005568,0.674046745009286,0.500281595399276,0.857784523189137
"9118","AP005205.2",7.8648581435774,-0.478832791936954,0.710369575382961,-0.6740615146402,0.50027220575631,0.857784523189137
"9119","FAM32A",494.223489107702,-0.157488382604011,0.233578080769425,-0.674242985836819,0.500156844915377,0.857784523189137
"9120","OCEL1",129.555446523129,-0.197638211237781,0.292969214301555,-0.674604025235056,0.49992737493861,0.857784523189137
"9121","AP001347.1",28.0730751502693,0.309105623705916,0.458313666323821,0.674441210067513,0.500030850336391,0.857784523189137
"9122","MIR503HG",11.9052149471131,0.369133658435742,0.546758762403871,0.675130759336741,0.499592692709173,0.857784523189137
"9123","ZBED5",182.197816875238,0.144895435371412,0.215015892111871,0.673882446307848,0.500386052934785,0.857804349567814
"9124","TMEM147",530.925377440046,-0.155733411714023,0.231118405374638,-0.673825225912158,0.500422435131505,0.857804349567814
"9125","ST3GAL3",71.472225374842,0.156724753425725,0.232640248762385,0.673678584249627,0.50051568006952,0.857870162880799
"9126","MCOLN2",3.13585682424755,0.693404593613323,1.02958338241061,0.673480754895077,0.500641488288297,0.857984988407895
"9127","TBC1D22A",160.018245053661,0.116724167112367,0.173335376135067,0.673400720124278,0.500692390613737,0.857984988407895
"9128","MED18",41.3920034236991,-0.185056146568752,0.274888860156581,-0.673203513824971,0.500817826048421,0.85810591579725
"9129","PRPF3",85.8787221694705,0.160386309659021,0.238313575174205,0.673005344079877,0.500943891070514,0.858227895316336
"9130","ZNF236-DT",10.7971232846421,0.345600484250979,0.513673231270279,0.67280220812039,0.501073132784964,0.858355289896696
"9131","TOM1L1",3.37045024498943,-0.722164226451176,1.07378502934493,-0.67254078490155,0.501239484746975,0.858546220725298
"9132","ATP6V1F",1144.72912138965,-0.180178028270813,0.26794518166813,-0.672443621300036,0.501301320513093,0.858558109157335
"9133","INPP4B",2.8427425498885,0.65763212594885,0.978339528717761,0.672192124150151,0.501461394262356,0.85863283332027
"9134","HSPA1B",406.149734557507,0.217647159570197,0.323823651496896,0.672116315667829,0.501509650407971,0.85863283332027
"9135","PPIL4",385.057822308744,-0.0987167837053261,0.146852044571141,-0.672219334729818,0.501444073875616,0.85863283332027
"9136","CCDC50",611.118393539737,0.113490467695218,0.168969771143128,0.671661368346673,0.501799300312863,0.858882149410605
"9137","AKAP7",38.549441018507,0.198388510672674,0.295407888812475,0.671574856955195,0.501854389271106,0.858882149410605
"9138","PSD",2.79396036330679,-0.728155409757187,1.08457996413419,-0.671370884431249,0.501984288116679,0.858882149410605

"9139","AC009041.2",28.9942142731747,0.335782334543633,0.500095462258343,0.671436475402715,0.501942514905511,0.858882149410605
"9140","NDEL1",186.694310386754,-0.117853319610871,0.175541498506771,-0.671370135343384,0.501984765202196,0.858882149410605
"9141","CARD8",69.5242899932535,0.15478344615394,0.230458228104577,0.671633412384405,0.501817101836207,0.858882149410605
"9142","ICK",230.94365018613,0.145705567717909,0.217126635930904,0.671062613268125,0.502180642769757,0.858941122941717
"9143","DNLZ",35.431463698243,-0.283589299595818,0.422654984944616,-0.670971145964312,0.502238911080691,0.858941122941717
"9144","MARS",80.2945834504983,-0.168180131555674,0.250637314479907,-0.671009948796577,0.502214191696263,0.858941122941717
"9145","SPESP1",31.7583181373298,-0.275136284280708,0.409977865751875,-0.671100338005136,0.502156611653356,0.858941122941717
"9146","MLST8",101.376436192542,0.178220816869564,0.265657361595667,0.670867224604969,0.502305117460064,0.858960423909404
"9147","MFF",434.693374185145,-0.126977455529772,0.189519817920846,-0.669995660204808,0.502860556702444,0.858994422636136
"9148","ZNF385D",169.667605320277,-0.30821427791259,0.460019523344319,-0.670002602654528,0.502856131066753,0.858994422636136
"9149","GYG1",233.788555513907,-0.136524752544158,0.203673062724896,-0.670313249664066,0.502658122544364,0.858994422636136
"9150","HIST1H1E",46.4532805866085,0.250479772772448,0.373622998395843,0.670407801039784,0.502597863035985,0.858994422636136
"9151","SPTAN1",1470.25187048695,-0.0910640019624441,0.135915784301945,-0.670003137826435,0.502855789909085,0.858994422636136
"9152","BEND7",98.5219942830665,-0.172900014847864,0.258064446280287,-0.66998773887696,0.502865606373269,0.858994422636136
"9153","NPFF",14.2749074609184,0.294406824132178,0.439348149209064,0.670099156357399,0.502794582663097,0.858994422636136
"9154","FGD6",7.79265830606212,0.378572637579514,0.564735817352519,0.670353510344468,0.502632463129707,0.858994422636136
"9155","PSMD9",567.635415434298,-0.154317002088152,0.230332748565458,-0.669974213607303,0.502874228494659,0.858994422636136
"9156","TIGD7",16.9917729102398,0.267380436450653,0.399053865126197,0.670035952079042,0.50283487194106,0.858994422636136
"9157","KLHL17",9.92691969033638,0.315529089190988,0.47587452841289,0.663051015239925,0.507297881645843,0.85907791285644
"9158","MIB2",113.877415541731,-0.129764481117318,0.195711030923114,-0.663041222077546,0.507304153537415,0.85907791285644
"9159","XKR8",48.1054890195078,-0.184716773442082,0.277169724530835,-0.666439214292801,0.505130404525188,0.85907791285644
"9160","MUTYH",46.9690293107462,-0.190076773851043,0.286112277736277,-0.664343296816663,0.506470615676943,0.85907791285644
"9161","NFIA-AS2",3.34298982944069,-0.621385709002975,0.939760163442976,-0.661217333076153,0.508472947031016,0.85907791285644
"9162","ACP6",6.45649304304274,-0.38454039349148,0.578479066139434,-0.664743836035015,0.50621435088512,0.85907791285644
"9163","PI4KB",214.602259388495,0.121516561297015,0.183375344868154,0.

662665754681386,0.507544647000863,0.85907791285644
"9164","SPRR2A",29.503635032402,-0.800106879728231,1.19907758254411,-0
.667268649982285,0.504600547542893,0.85907791285644
"9165","AL596325.2",76.5641861371808,0.371201706099376,0.5549775361549
25,0.668858975214005,0.503585442087819,0.85907791285644
"9166","GORAB",79.686748953305,0.151386161304351,0.226282521772841,0.6
69013939381159,0.503486586056225,0.85907791285644
"9167","CENPO",8.55209058025026,-0.363309695466184,0.547935215759337,-
0.663052282490555,0.507297070056175,0.85907791285644
"9168","HTRA2",228.765880132418,-0.157054836667649,0.237488668404902,-
0.661315075462384,0.508410275489813,0.85907791285644
"9169","KANSL3",93.6623634769737,0.162901639023545,0.243702561407921,0
.668444509086929,0.503849892119822,0.85907791285644
"9170","RBM43",301.61584518722,0.131192133258789,0.197563162537904,0.6
64051595315088,0.506657289073281,0.85907791285644
"9171","METTL8",110.301987942519,-0.122828785595134,0.18429238892077,-
0.666488650532062,0.50509881564063,0.85907791285644
"9172","AC009948.3",7.92660924803923,-0.417089314295984,0.624483732894
543,-0.667894602094332,0.504200873271265,0.85907791285644
"9173","ASNSD1",188.663567955764,-0.156413438579175,0.235895686510793,
-0.663061885076132,0.507290920261517,0.85907791285644
"9174","FBXO36",30.2281154680607,0.211977978019713,0.317308514343939,0
.668050078826263,0.504101626319189,0.85907791285644
"9175","THAP4",149.288302498398,-0.130453346814892,0.195860561113977,-
0.666052144816319,0.505377771062611,0.85907791285644
"9176","AC018809.2",8.03073526262135,0.426772461491809,0.6429264674414
01,0.663796690763407,0.506820443993453,0.85907791285644
"9177","MKRN2",116.95896203105,-0.128882845200952,0.192938626809992,-0
.66799918363614,0.50413411359879,0.85907791285644
"9178","GMPS",210.449273883517,0.100921003878871,0.151157525129397,0.6
6765451334612,0.504354151668123,0.85907791285644
"9179","DNAJC19",545.943386365609,-0.153133254103415,0.230668611988366
,-0.663866890182434,0.506775509202035,0.85907791285644
"9180","IDUA",110.658084988723,-0.205656787372828,0.307433974291739,-0
.668946195184238,0.503529800737585,0.85907791285644
"9181","ACOX3",52.2040120661452,-0.189154742392498,0.28614318240002,-0
.661049271927315,0.508580715801648,0.85907791285644
"9182","COMMD8",239.502937264825,-0.158369707198277,0.238680640510313,
-0.663521376763833,0.506996693255015,0.85907791285644
"9183","AC110792.4",2.53255314508278,-0.615924079407925,0.929208358033
363,-0.662848191240452,0.507427785703958,0.85907791285644
"9184","SLC9B1",14.5692474998602,-0.294304108681255,0.443557642799107,
-0.663508144790437,0.507005164850469,0.85907791285644
"9185","AC099343.3",60.4790662183461,0.214668317891274,0.3219851752790
02,0.666702489346791,0.504962188399703,0.85907791285644
"9186","PRIMPOL",71.0420115949648,0.164920648816644,0.249227800763389,
0.661726534164686,0.50814649628391,0.85907791285644
"9187","AC008875.3",122.177917887283,0.136991731019188,0.2049444164956
68,0.668433584879265,0.503856863299637,0.85907791285644
"9188","AC008875.2",4.1537362555906,0.500612944058546,0.75724437506132

6,0.661098267013213,0.508549296594823,0.85907791285644
"9189","SMIM15",375.501022078614,-0.134269635962458,0.202880020761822,
-0.661817932876142,0.508087911864366,0.85907791285644
"9190","ITK",7.63205977530029,0.524892204193319,0.788410297099148,0.66
5760209023894,0.505564382208164,0.85907791285644
"9191","TBCC",102.565461321724,-0.163364391919682,0.246789221550479,-0
.661959184819049,0.507997379668783,0.85907791285644
"9192","EIF2AK1",296.105697992628,-0.108187567062275,0.163153122499945
,-0.663104483717811,0.507263639237764,0.85907791285644
"9193","AGMO",7.27741374585953,-0.473591310803386,0.712582819645461,-0
.664612305751375,0.506298496372806,0.85907791285644
"9194","MPP6",40.4107538940979,0.197045369795542,0.297355240419198,0.6
62659819002202,0.507548449388221,0.85907791285644
"9195","DDX56",149.248208535144,-0.113292684869141,0.170220651439482,-
0.665563689899398,0.505690021555914,0.85907791285644
"9196","TRIM24",71.2551015068436,0.185364660757688,0.277498076183004,0
.667985390412018,0.504142918242402,0.85907791285644
"9197","ESYT2",510.707867501543,-0.116485349107786,0.175112725708826,-
0.665202078468447,0.505921251280064,0.85907791285644
"9198","INTS9",43.3802422404999,0.163611373938717,0.246159062511826,0.
664657121575028,0.50626982496795,0.85907791285644
"9199","MYORG",3.35148207537837,0.600842341650755,0.902165985071414,0.
665999773426609,0.505411245222765,0.85907791285644
"9200","RPP25L",111.01833437717,0.188356524203958,0.284914736001196,0.
661097866848021,0.50854955320568,0.85907791285644
"9201","FAM27C",6.65230823928036,-0.498142566223369,0.743885970117187,
-0.669649094396678,0.503081509779748,0.85907791285644
"9202","TRMO",52.6653898027832,-0.16649203376057,0.251608168133777,-0.
661711561256025,0.508156093903201,0.85907791285644
"9203","ABITRAM",65.5711066029634,0.143830246497805,0.217079646073378,
0.662569011417988,0.507606622457718,0.85907791285644
"9204","POLE3",246.948594582041,-0.13817156422083,0.208781117082442,-
0.661799104980949,0.508099979811571,0.85907791285644
"9205","SURF2",144.517120231454,-0.171390631494954,0.257659509761878,-
0.665182634451756,0.505933686187201,0.85907791285644
"9206","AL158212.2",26.1124691582715,0.334799704394361,0.5019349050225
58,0.667018175154235,0.504760524039476,0.85907791285644
"9207","PAOX",29.0878104258779,-0.235551859418348,0.356147003716877,-0
.661389417740553,0.50836261060021,0.85907791285644
"9208","CAT",646.175215662672,0.158038223485605,0.23846526523992,0.662
730579762224,0.507503121123025,0.85907791285644
"9209","OSBP",200.31417202948,0.142278771472497,0.214982836548214,0.66
1814560440912,0.508090073452889,0.85907791285644
"9210","INPPL1",105.390550258805,0.130411831384413,0.197103788014649,0
.661640411369065,0.508201702208222,0.85907791285644
"9211","ALG8",94.3448001737998,-0.204552297741394,0.307646742002421,-0
.664893430725114,0.506118657774156,0.85907791285644
"9212","SLC36A4",134.783052500032,-0.149991554334171,0.224128705031368
,-0.669220635139881,0.503354744928246,0.85907791285644
"9213","CWF19L2",335.676215769857,0.153435564651847,0.229887506634968,

0.667437595447426,0.504492658370049,0.85907791285644
"9214","CEP164",189.716729738524,-0.144002458898809,0.215174338087853,
-0.669236211801496,0.503344810075544,0.85907791285644
"9215","HYLS1",28.1883977515709,-0.289999142764528,0.438454752931445,-
0.661411789530473,0.508348267284138,0.85907791285644
"9216","NECAP1",144.140568905674,0.131928767197479,0.198553949473444,0
.664447962618463,0.506403643966043,0.85907791285644
"9217","ALG5",363.33465235358,-0.154249660272109,0.230840718799542,-0.
668208195998805,0.504000704441311,0.85907791285644
"9218","RBM26-
AS1",25.6075680103652,0.260088809602723,0.393267774077384,0.6613529679
9464,0.508385980227672,0.85907791285644
"9219","DCAF11",85.8845783953201,-0.138026583581882,0.206606062276739,
-0.668066474240247,0.504091161078265,0.85907791285644
"9220","HIF1A",256.54991642867,0.140116027865227,0.211870426570384,0.6
61328860914338,0.508401436707636,0.85907791285644
"9221","VTI1B",631.5181865097,-0.12460436062841,0.18764835993448,-0.66
4031173370859,0.506670359382519,0.85907791285644
"9222","AREL1",47.1362031586017,0.199156311877327,0.297566063452095,0.
669284358461088,0.503314102611808,0.85907791285644
"9223","SNW1",508.113338693212,-0.0983084094760755,0.148714320798808,-
0.66105543129955,0.508576765908987,0.85907791285644
"9224","AQR",144.160978395131,0.141329441522946,0.213012174779577,0.66
3480581188339,0.507022812319939,0.85907791285644
"9225","DUOX1",8.67117789424481,0.593592634862739,0.888390085383082,0.
668166658576312,0.504027215612435,0.85907791285644
"9226","CLK3",150.813244480628,0.107324382563602,0.160601253760326,0.6
68266156401044,0.503963712568202,0.85907791285644
"9227","STARD5",61.8906387001237,0.214527686028741,0.323211735699616,0
.66373730385866,0.506858459307401,0.85907791285644
"9228","AC048382.6",9.61241683682641,-0.326272179775424,0.491271251929
249,-0.664138555826613,0.50660163520214,0.85907791285644
"9229","MAN2A2",67.2034110411091,0.199250332373241,0.297824002322364,0
.6690203973472,0.503482466559196,0.85907791285644
"9230","TM2D3",380.136246657523,0.114869048006645,0.172833938771466,0.
664620900403904,0.506292997785291,0.85907791285644
"9231","GSPT1",437.058463776301,-0.0974805801033342,0.147152090854362,
-0.662447808504547,0.507684272808109,0.85907791285644
"9232","MPV17L",38.4769017823747,0.187881311059525,0.282940476449536,0
.6640312245782,0.506670326608934,0.85907791285644
"9233","AC009152.6",4.110137364016,0.646701320215193,0.97506761332978,
0.663237411820867,0.507178514371152,0.85907791285644
"9234","LCAT",78.2424247279865,0.156505683863306,0.234311232317006,0.6
67939314371259,0.504172330599246,0.85907791285644
"9235","IST1",314.238418874832,0.0981699152366914,0.148425962446594,0.
661406627375009,0.508351576898894,0.85907791285644
"9236","ZMYND15",5.08206885772619,-0.487817322467387,0.736081443427037
,-0.662721940382324,0.507508655264296,0.85907791285644
"9237","AC040977.1",11.1812236035325,0.367646339783925,0.5561036248238
96,0.661111209085087,0.508540997368468,0.85907791285644

"9238","MYO15A",2.73554222901337,0.683024889614355,1.02701432920664,0.665058772979324,0.506012902294653,0.85907791285644
"9239","ANKRD13B",69.1061943996802,-0.192184037383235,0.289187818904101,-0.664564773549352,0.506328906543126,0.85907791285644
"9240","TRIM37",84.4940841425154,0.143645290721656,0.215465567057637,0.666673996607685,0.50498039203521,0.85907791285644
"9241","RNF138",71.801176149783,-0.179367650140266,0.269105767767234,-0.666532165506804,0.505071011200882,0.85907791285644
"9242","AC011815.1",2.95125984208748,-0.649687942709843,0.97690753066877,-0.665045485180241,0.506021400948094,0.85907791285644
"9243","LINC01002",6.09317241503967,0.491108528376433,0.739276196456671,0.664309943604706,0.506491958121592,0.85907791285644
"9244","MED26",59.8403008329857,0.20424517857884,0.306483928073059,0.666413993917986,0.505146520300579,0.85907791285644
"9245","LSM14A",436.805255344127,0.0955867544335662,0.143254132002293,0.667253035549692,0.504610519588947,0.85907791285644
"9246","RYR1",4.97942523851318,0.631299832134978,0.954053842020638,0.661702520685748,0.508161888945636,0.85907791285644
"9247","ZNF808",36.7656964235965,0.216123696131373,0.326177375866823,0.662595606323155,0.507589584892044,0.85907791285644
"9248","ZNF331",172.935023820128,-0.155611967705864,0.233308297556857,-0.666979997434258,0.504784910226235,0.85907791285644
"9249","ITPA",401.29160963249,-0.168586377128496,0.251966903583599,-0.669081433834271,0.503443532636572,0.85907791285644
"9250","AL133342.1",3.13509951048308,0.618091590422256,0.929378988377098,0.665058709258729,0.506012943049098,0.85907791285644
"9251","AURKA",7.04483235766792,-0.42408412064057,0.633171681746125,-0.669777459836887,0.502999664395616,0.85907791285644
"9252","TIAM1",126.373159527516,0.195554576861294,0.295688236860394,0.661353927831845,0.508385364824173,0.85907791285644
"9253","ATP5PO",1409.35899666391,-0.170582945535789,0.254841605906226,-0.669368508054995,0.503260435208371,0.85907791285644
"9254","COL6A1",8495.23164557428,-0.139364478860349,0.209729317789863,-0.664496887364044,0.506372340458335,0.85907791285644
"9255","SRPX",734.780951205406,0.255694070823712,0.38533653012331,0.663560422734611,0.506971695023715,0.85907791285644
"9256","Z83745.1",5.10270281633663,0.48082987589375,0.726422092934911,0.661915270157999,0.50802552485766,0.85907791285644
"9257","ARMCX5",34.9468844239799,0.179931226444478,0.272003014565904,0.661504530498069,0.508288810156392,0.85907791285644
"9258","APLN",25.7166326753367,0.364014009153552,0.548787618913821,0.663305797375716,0.507134724363638,0.85907791285644
"9259","ATP6AP1",336.804009755428,0.144106516203608,0.216167794236206,0.666641933007574,0.50500087746026,0.85907791285644
"9260","PAPPA2",4.20337731113978,-0.460666332356925,0.698726214361294,-0.659294474557563,0.50970668988769,0.859103055322692
"9261","DHX9",326.60921620971,0.103559519465632,0.157076680766212,0.659292766822383,0.509707786299267,0.859103055322692
"9262","CLASP2",143.050248925084,0.153671703139407,0.232745731632107,0.660255730843263,0.509089733650858,0.859103055322692

"9263","LARP1B",206.723285753682,-0.153067434989092,0.232248306117666,
-0.659068036050788,0.509852080223588,0.859103055322692
"9264","KIF2A",256.930143050495,-0.109199456385293,0.165522114627155,-
0.659727291614591,0.509428849590487,0.859103055322692
"9265","CDO1",161.905882239594,0.272584021499561,0.412728123790136,0.6
60444505202084,0.50896861993067,0.859103055322692
"9266","ZNF879",13.6891141161597,0.312723602997939,0.473410918593805,0
.660575391726995,0.508884654709727,0.859103055322692
"9267","MRPL14",406.186012180116,-0.164990993401566,0.25005298019082,-
0.659824143170213,0.509366688074116,0.859103055322692
"9268","LINC00271",23.3348693128914,0.251110659783974,0.380784235120948,0.659456554718486,0.50960263571597,0.859103055322692
"9269","GPAT4",143.392335145183,-0.123300780855511,0.187095585928347,-
0.65902560043684,0.509879329453328,0.859103055322692
"9270","SEC61A2",23.9092385990711,0.244894006827258,0.37116362049771,0
.659800673619005,0.50938175099763,0.859103055322692
"9271","PPIF",51.0743791889943,0.220777550721326,0.334682080765143,0.6
59663493834474,0.509469798616982,0.859103055322692
"9272","ST3GAL4",273.037836022254,-0.194204777978323,0.294708811716637
,-0.658971738398683,0.509913917043513,0.859103055322692
"9273","TEX30",91.134222050153,-0.209442606156724,0.317569400120014,-0
.659517592304461,0.509563452954755,0.859103055322692
"9274","AC130456.5",3.15324318584757,-0.598032949140368,0.905926236323
106,-0.660134263875182,0.509167672324953,0.859103055322692
"9275","USP31",37.9333905604494,0.193385551319753,0.292947616372757,0.
660136968220568,0.509165937027203,0.859103055322692
"9276","GAS8",32.5649445439843,0.206726942505609,0.313267743673809,0.6
59904974834767,0.5093148115268,0.859103055322692
"9277","AP000897.1",3.18817714562582,-0.608772076133591,0.923667464296
771,-0.659081433161745,0.50984347767955,0.859103055322692
"9278","VAPA",1035.91937545222,-0.120864676081046,0.183313395179461,-0
.659333574410758,0.509681587075777,0.859103055322692
"9279","GADD45B",6257.22009387744,-0.257243467061054,0.390192974830096
,-0.659272420712001,0.50972084914028,0.859103055322692
"9280","AC119403.1",9.66938690012533,-0.325175115437383,0.493444447291
957,-0.658990322460729,0.509901983120927,0.859103055322692
"9281","AC012313.1",16.7794910574369,0.363497472315092,0.5509804865850
08,0.659728395406631,0.509428141129444,0.859103055322692
"9282","RALGAPB",113.654405145856,0.134652281249897,0.204314542062155,
0.659044040090571,0.509867488683578,0.859103055322692
"9283","FBXO7",370.417417619156,-0.100592140623789,0.152204644618855,-
0.660900597847645,0.508676062646366,0.859103055322692
"9284","PSMD1",401.147261640742,-0.120260080664152,0.182540508145787,-
0.658813114336821,0.510015784858754,0.859105235444861
"9285","SLC25A38",62.6193516300289,0.161042565327227,0.244759191847129
,0.657963298995572,0.510561714549138,0.859105235444861
"9286","HACD2",112.504373303793,0.146348257539284,0.222432829911395,0.
65794360300852,0.510574371065217,0.859105235444861
"9287","SKP1",2828.33625538911,-0.130069360079228,0.197657282449807,-0
.658054985210358,0.510502799729246,0.859105235444861

"9288","AL391422.4",64.876730382593,-0.194087993166367,0.294821589915845,-0.658323541439989,0.510330253981464,0.859105235444861
"9289","SUGT1",505.23224982366,-0.110562075326007,0.167978915692878,-0.658190195298987,0.510415924255602,0.859105235444861
"9290","FGF7",1276.38620423714,0.153479540125268,0.233214657734749,0.658104175852578,0.510471192758586,0.859105235444861
"9291","KLHL25",6.41059424458191,0.434616994037667,0.660196708624781,0.65831439078664,0.510336132718787,0.859105235444861
"9292","PAGR1",190.737821391272,-0.133199020621013,0.202408390268827,-0.658070648376317,0.510492735402658,0.859105235444861
"9293","AC012181.1",5.04380974592835,0.518938000928213,0.787990036186841,0.658559089705503,0.51017894075972,0.859105235444861
"9294","DNTTIP1",202.006217706296,-0.170810887687873,0.259472747833831,-0.658299914398955,0.510345432988307,0.859105235444861
"9295","RPL36A",5540.50459260112,-0.130533238875193,0.198356969835497,-0.65807235804947,0.510491636862895,0.859105235444861
"9296","AC092835.1",4.31745713371044,-0.44814397987256,0.681350360107265,-0.657729130431528,0.510712200396174,0.859117467242558
"9297","COX14",521.956217869402,-0.167483371131783,0.254615710256544,-0.657788833858806,0.510673830444873,0.859117467242558
"9298","ZNF761",53.1833361816257,0.191486986187376,0.291157081841906,0.657675866841359,0.510746432891388,0.859117467242558
"9299","TMEM222",226.395752022979,-0.154373323166463,0.234846742647294,-0.657336446000058,0.510964606742904,0.859310094990668
"9300","LRRC8B",15.7803841215804,-0.281586670559285,0.429306699800411,-0.655910263432175,0.511881864119163,0.859310094990668
"9301","SEMA4F",15.0174319466594,-0.271432309578443,0.413412035478757,-0.656566055857825,0.511459980838099,0.859310094990668
"9302","TIPARP",525.308862718298,0.222730534929066,0.339918535345622,0.655246807010975,0.512308862491093,0.859310094990668
"9303","SNX2",814.58237989279,0.130186125469633,0.198763748896393,0.654979221273864,0.512481132341062,0.859310094990668
"9304","ATP6V0E1",2334.85205416395,-0.161953576159688,0.247313917901641,-0.654850230564452,0.512564186455751,0.859310094990668
"9305","AL133351.1",3.20106284122612,0.560041460250968,0.855084333178279,0.654954650109586,0.51249695260072,0.859310094990668
"9306","CEP57L1",163.141180092711,-0.108551235386542,0.165368787779156,-0.656419127480742,0.511554486771771,0.859310094990668
"9307","TP53TG1",278.400292599524,-0.1841733137006,0.280793936129944,-0.655902033494659,0.511887159742173,0.859310094990668
"9308","SMIM30",220.295616928495,0.179691888200163,0.274230933660258,0.655257544441656,0.512301950437591,0.859310094990668
"9309","AL117336.2",14.1493279499957,0.381488277519657,0.582386383626915,0.655043263793139,0.512439899460508,0.859310094990668
"9310","DUSP5",60.8753276449341,-0.32145616233642,0.490047260474759,-0.655969716114713,0.511843609636131,0.859310094990668
"9311","HPS5",66.5426752095948,0.16207707159509,0.247448827382621,0.654992279856216,0.512472724615022,0.859310094990668
"9312","PAMR1",936.585595147235,-0.168316699264297,0.256637979308874,-0.65585265172978,0.511918935457436,0.859310094990668

"9313","TUT1",26.927427581714,0.20734693958207,0.316632056500634,0.654851381359281,0.512563445454735,0.859310094990668
"9314","C11orf68",232.856753231794,-0.164672413009401,0.251272573197728,-0.655353709773246,0.512240047673742,0.859310094990668
"9315","UBASH3B",6.17610429384317,-0.444660109598992,0.676472369802823,-0.657321909139617,0.510973951872854,0.859310094990668
"9316","TAPBPL",116.21858137763,0.244721028802758,0.372399787380384,0.657145994964789,0.511087046700085,0.859310094990668
"9317","USP5",170.830840631075,-0.137554875900522,0.209860376375261,-0.655459016496538,0.512172264962724,0.859310094990668
"9318","SMIM10L1",262.979592471482,-0.160590749737665,0.244697466742362,-0.656282845407418,0.511642153035267,0.859310094990668
"9319","SARNP",401.064076185089,-0.1391447865369,0.211930592938628,-0.656558284519095,0.511464979219549,0.859310094990668
"9320","PPP2R5E",437.133928458738,0.111407170553758,0.169881212596699,0.655794533432259,0.511956334193201,0.859310094990668
"9321","FOXN3-AS1",26.2164808947056,0.26218139807092,0.399586852261326,0.656131192973927,0.511739715818519,0.859310094990668
"9322","NSMCE3",253.245210823075,-0.150953214932837,0.230427183155831,-0.655101593767918,0.512402346027005,0.859310094990668
"9323","DUT",927.873022224359,-0.15952273347651,0.243245155797889,-0.655810525612507,0.511946043190011,0.859310094990668
"9324","GPRC5B",282.772771479306,0.146600447413467,0.223395682028253,0.656236710049422,0.511671832335475,0.859310094990668
"9325","CFDP1",791.51462236135,0.0986498914171057,0.150460874402114,0.655651456294605,0.512048409448297,0.859310094990668
"9326","TMEM97",15.7521493566631,-0.303673587439757,0.462145913018945,-0.657094607752829,0.511120085890627,0.859310094990668
"9327","JMJD6",97.0818451572499,0.132912212135451,0.202559085186615,0.65616514812456,0.511717870557931,0.859310094990668
"9328","CBX4",95.3287844681716,0.157374569843188,0.239659762264953,0.656658290719674,0.511400659027206,0.859310094990668
"9329","PLTP",3129.1558937277,0.226365193808551,0.344686056517702,0.656728607172205,0.511355436682847,0.859310094990668
"9330","CEP41",54.6596698271378,0.212883619229284,0.325160976813113,0.654702237998379,0.512659484065747,0.859377741777952
"9331","AGRN",15.2540685874006,-0.287694815948643,0.43950060578344,-0.654594810935031,0.51272866590838 7,0.859401600558051
"9332","KLHL23",23.352757885055,0.243593927321008,0.372195218081432,0.654478927957944,0.512803298720712,0.859434589797678
"9333","FAM215B",53.9573233257993,-0.169377323882337,0.258873305164277,-0.654286558341165,0.512927204182268,0.859550141799065
"9334","LINC01128",73.3985649222849,-0.165118093725357,0.252409888189414,-0.654166502389355,0.513004540244487,0.859552697245451
"9335","AC009570.1",4.84558628821242,-0.463285044302407,0.708263936283467,-0.654113559322873,0.513038646341834,0.859552697245451
"9336","TDRD7",49.1487607056048,-0.166100822122663,0.254005050871868,-0.653927241023455,0.513158682594527,0.859661717628364
"9337","SKAP2",303.227534156612,-0.29133547398815,0.445956030821652,-0

.653282955836203,0.513573878425391,0.859712668155102
"9338","TMEM132A",40.8785027715058,0.200434798865204,0.306754948204203
,0.653403637133108,0.513496094633708,0.859712668155102
"9339","NEU3",12.9222920155063,0.312174397328122,0.477528383842622,0.6
5372951198437,0.513286086255331,0.859712668155102
"9340","AC009318.3",5.58201970948025,0.409752891183811,0.6270803747411
5,0.65342962033049,0.513479348254361,0.859712668155102
"9341","NUDT21",275.587360419558,0.0978378316613873,0.149761520515804,
0.653290854182151,0.513568787446538,0.859712668155102
"9342","SYCE1L",23.5603354947263,-0.251034628142536,0.384238776308112,
-0.653329761651221,0.513543709528306,0.859712668155102
"9343","ELOF1",281.162513263943,-0.152747369644988,0.233740186876992,-
0.653492117405439,0.513439069552134,0.859712668155102
"9344","MMP23B",574.891814794616,0.402622760181836,0.616571297628045,0
.653002761774232,0.513754498048478,0.859724743793598
"9345","BTF3L4",437.72812330926,-0.143374697113659,0.219784537387638,-
0.652342056533242,0.514180534884286,0.859724743793598
"9346","SNX17",551.787053435134,-0.149666739950776,0.22940139610486,-0
.652422969049248,0.514128350896659,0.859724743793598
"9347","AC015982.2",8.09076635264221,0.440718370683831,0.6748349890693
03,0.653075756032814,0.513707441064193,0.859724743793598
"9348","RPL35A",13445.2105692747,-0.14080009629472,0.215853251512195,-
0.652295461422619,0.514210587340013,0.859724743793598
"9349","GASK1B",331.17972148467,-0.273735844585261,0.419187457096974,-
0.653015351368052,0.513746381793657,0.859724743793598
"9350","POLR3D",118.776472556472,-0.13321536810071,0.204203845622725,-
0.652364639336084,0.514165969979074,0.859724743793598
"9351","PTGES",1255.04003421979,-0.207920494231821,0.31853624009625,-0
.652737328000716,0.513925633330534,0.859724743793598
"9352","TENM4",11.3936775560226,0.419422022993274,0.642841769496361,0.
652449860751695,0.514111007882392,0.859724743793598
"9353","VPS35",540.398406676259,0.099062488426328,0.151787324703881,0.
652640058183955,0.513988354317698,0.859724743793598
"9354","ZNHIT3",249.77305837534,-0.175969689030802,0.269582900251569,-
0.652747963118548,0.513918775893958,0.859724743793598
"9355","RIOK3",346.621827565705,-0.142173142528756,0.217973812150908,-
0.652248731743638,0.514240727505698,0.859724743793598
"9356","KLHL35",19.0180664428877,0.323526975704338,0.496184076287176,0
.65203014599987,0.514381725276437,0.859868553155567
"9357","AC006994.2",4.47769958164143,0.523138569709512,0.8025482949680
94,0.651846839610206,0.514499981748823,0.859968102729266
"9358","TSNARE1",7.62674659247408,-0.355501329156732,0.545442038684628
,-0.651767381212583,0.514551247144531,0.859968102729266
"9359","AAMP",304.020233423374,-0.120757541063107,0.185321233720147,-0
.651612007102557,0.514651499918864,0.860043750265096
"9360","MIR34AHG",17.206463818493,0.33146420255077,0.509024242145991,0
.65117567122806,0.514933093259886,0.860238579212201
"9361","GAREM2",40.997393772069,0.217116265575202,0.333363055422378,0.
651290723562965,0.514858835415581,0.860238579212201
"9362","SOD2",1814.48763270515,-0.20926925235888,0.321336032412692,-0.

651247389804436,0.514886803525486,0.860238579212201
"9363","FAM120A",602.276901507969,0.111705420482819,0.171578925962984,
0.651043942931067,0.515018121046265,0.860288733649855
"9364","HMGB2",333.948944079659,0.146702949673085,0.225399688883703,0.
650856930635682,0.515138845991287,0.860398499712059
"9365","DNER",19.5828235998041,0.376246364774105,0.578314030544585,0.6
50591797712054,0.515310026540942,0.860581199604762
"9366","TSPYL5",99.9957288519327,0.140211729367826,0.215565997694416,0
.650435276747998,0.515411096674987,0.860581199604762
"9367","AL136295.7",9.33557032406198,0.370202148142481,0.5691636172282
25,0.650431856388382,0.51541330541546,0.860581199604762
"9368","CERS2",537.390553071563,-0.154895269138227,0.238345209905304,-
0.649877835597233,0.515771136195529,0.860647971109627
"9369","SFT2D2",398.904434448129,0.150236391498189,0.231301192107122,0
.649527095513673,0.515997738709762,0.860647971109627
"9370","MDH1",676.883256471163,-0.178118041797529,0.274182106823938,-0
.649634083933509,0.515928611268312,0.860647971109627
"9371","EPM2AIP1",216.426595483105,0.114158470337607,0.175830039898403
,0.649254646154718,0.516173795594603,0.860647971109627
"9372","SAP30L",252.570765474105,0.0902704453175626,0.138964226846014,
0.649594844416981,0.515953964177517,0.860647971109627
"9373","CASTOR2",68.7527273634037,0.183787274077952,0.283062298608833,
0.649282066107751,0.516156075400315,0.860647971109627
"9374","AC016405.1",3.07651072443036,0.57769700978042,0.89025249838226
8,0.648913663067712,0.516394182883448,0.860647971109627
"9375","JRK",32.2899390804778,0.26436464751669,0.406827821487308,0.649
819490098313,0.515808827869535,0.860647971109627
"9376","WDFY4",13.3265873557261,0.345064733399428,0.531901806349736,0.
648737660372864,0.516507957629686,0.860647971109627
"9377","TNNT3",486.756805853194,-0.241631668532504,0.372505154316877,-
0.648666644561263,0.516553868593738,0.860647971109627
"9378","MS4A2",4.66164701533733,-0.859031897624196,1.32154750128287,-0
.650019690393502,0.515679502787419,0.860647971109627
"9379","ST8SIA1",113.966566361798,0.271397226866232,0.417475391599703,
0.650091555878968,0.515633083334985,0.860647971109627
"9380","UBE3A",425.618349341611,0.114988890934139,0.177120887749429,0.
649211351609826,0.516201775414908,0.860647971109627
"9381","AC018362.1",11.8881115789764,0.371890036522056,0.5729533512601
25,0.649075593508861,0.516289516446723,0.860647971109627
"9382","WHAMM",99.8362810664832,0.12845011621315,0.197937087001003,0.6
48944157759072,0.516374471291299,0.860647971109627
"9383","SLX1A",4.01688789984409,0.506082401637559,0.779005966869409,0.
649651508667325,0.515917353240623,0.860647971109627
"9384","RRAD",45.3275421921227,-0.563407782147579,0.868448202175087,-0
.648752315609021,0.516498483439242,0.860647971109627
"9385","CCDC137",125.561881182346,0.182297959440888,0.280608681949117,
0.649651885945369,0.515917109484698,0.860647971109627
"9386","CERS1",8.07163696780192,-0.355718065902378,0.548348346981325,-
0.648708194089792,0.516527007010391,0.860647971109627
"9387","EDEM2",230.475716842535,-0.162403711919485,0.250135815442762,-

0.649262128384199,0.516168960158959,0.860647971109627
"9388","MRPL20-
AS1",62.7988437349988,-0.160872339921115,0.248283601102166,-0.64793783
8854359,0.517025155905928,0.86072092278429
"9389","SYNC",160.189765123824,-0.179896214245742,0.279031665995937,-0
.644716124256525,0.519111165203366,0.86072092278429
"9390","CLCC1",170.000865545658,-0.12939248752492,0.199650354904167,-0
.648095454611281,0.516923213703234,0.86072092278429
"9391","VPS72",176.30248302717,-0.122858610350801,0.190044172932054,-0
.646473966843099,0.517972451443152,0.86072092278429
"9392","PPOX",55.2783892864107,-0.196142686517808,0.302934858246854,-0
.647474799212364,0.517324699383615,0.86072092278429
"9393","USP39",91.4723256078615,0.147113122338218,0.227224196705254,0.
647435988206165,0.517349810566376,0.86072092278429
"9394","AC009951.6",72.0946338997745,0.176952063887371,0.2740204706820
23,0.645762206914493,0.518433367077084,0.86072092278429
"9395","ZCWPW2",47.6117623413081,0.178203843181461,0.276360217499973,0
.644824514879672,0.519040913409208,0.86072092278429
"9396","AC083799.1",16.6041848830346,0.281493027334841,0.4361053955385
75,0.645470178114184,0.51862253799611,0.86072092278429
"9397","PLOD2",171.093336494146,0.195150935774716,0.302821163519132,0.
644442856988053,0.519288301169394,0.86072092278429
"9398","LRPAP1",1129.57093866032,-0.149755922591992,0.232406731231905,
-0.644369987900906,0.519335541280016,0.86072092278429
"9399","AC107068.1",6.37190726097859,-0.378941188048847,0.586451200461
728,-0.646159795990692,0.518175873484694,0.86072092278429
"9400","NDUFC1",920.996225234159,-0.126086328100697,0.194741979921319,
-0.647453251485065,0.517338640941626,0.86072092278429
"9401","FGF10-
AS1",4.44432213837868,-0.711569885024441,1.10403221633116,-0.644519131
324879,0.519238855854595,0.86072092278429
"9402","ZCCHC9",226.969604514406,0.100665580261735,0.156248996517222,0
.644263851324253,0.519404352253077,0.86072092278429
"9403","HMGXB3",114.387724885515,0.139397039522215,0.215030207190782,0
.648267242743886,0.516812116997303,0.86072092278429
"9404","MICA",119.077915111592,-0.200881281406139,0.311023542326728,-0
.64587162728381,0.518362495748055,0.86072092278429
"9405","CUL9",39.3664964596311,0.194559166375039,0.30084779491928,0.64
670298290616,0.517824192188246,0.86072092278429
"9406","CCDC170",4.81371470509946,-0.420996052385739,0.653068221990077
,-0.644643297912812,0.519158369288402,0.86072092278429
"9407","PTPRZ1",39.9450711155022,0.321019808281592,0.49697724508031,0.
645944681490832,0.51831518148934,0.86072092278429
"9408","MRPS33",293.394060530173,-0.172118036081588,0.266342678340798,
-0.646227773760521,0.518131855152809,0.86072092278429
"9409","CASP2",83.3408215140586,0.161296986211193,0.248904246015677,0.
648028263049534,0.516966670368084,0.86072092278429
"9410","LRATD2",6.57451592642696,0.468148751025901,0.722658477007732,0
.647814653699679,0.517104836444931,0.86072092278429
"9411","AL354733.3",15.8852650037754,0.470494133153121,0.7280879393119

13,0.646205091101724,0.518146542869005,0.86072092278429
"9412","DAPK1",9.29743056349414,-0.387872723432336,0.599927396409521,-0.64653277338841,0.517934379465874,0.86072092278429
"9413","TOR1A",208.261739240888,-0.122424328507718,0.189227737322077,-0.64696819948412,0.517652525064919,0.86072092278429
"9414","ZSWIM8-AS1",6.21104843096328,0.60078828359148,0.92803750614849,0.647375003284998,0.517389269810659,0.86072092278429
"9415","TMEM254-AS1",15.8750183610734,-0.32007559360306,0.496555578594162,-0.644591677953255,0.519191829305455,0.86072092278429
"9416","FRA10AC1",293.445803231724,0.102027901218722,0.157754500060938,0.646751130264494,0.517793025589202,0.86072092278429
"9417","RTN3",1085.81220915206,-0.115423538683888,0.178912234711448,-0.645140556597733,0.5188361036318,0.86072092278429
"9418","THYN1",307.844818169005,-0.179760923443301,0.277820414791909,-0.647040008121591,0.517606050449437,0.86072092278429
"9419","AC007406.5",16.5724120687901,0.260374041827129,0.401845965509526,0.647944894748375,0.51702059209530 2,0.86072092278429
"9420","SUOX",38.2434581332901,0.174644022603067,0.270667770814006,0.645233904568109,0.518775617779371,0.86072092278429
"9421","TMTC2",7.82560606502928,-0.36771879520466,0.569427038772878,-0.645769818021109,0.518428437218252,0.86072092278429
"9422","NEK5",14.9471788815936,-0.309184985696278,0.479312089734747,-0.645059852062947,0.51888839996602,0.86072092278429
"9423","PIGH",153.55121769373,-0.134028083167575,0.207794820550524,-0.645002040053196,0.518925863675674,0.86072092278429
"9424","NPTN-IT1",12.4229966834183,-0.329746269144158,0.511246771672601,-0.644984550348075,0.5189371977419,0.86072092278429
"9425","LMF1",116.182185541112,-0.196036652210499,0.303034847218822,-0.6469112513286,0.517689383498127,0.86072092278429
"9426","NLRC3",2.85701762700989,-0.682161816009049,1.05438733396487,-0.646974592765528,0.517648387239815,0.86072092278429
"9427","TVP23A",3.84856454860621,0.756143229903985,1.16803228735407,0.647365007021223,0.517395737869806,0.86072092278429
"9428","ERAL1",102.241683269607,-0.156559772678023,0.24247059686265,-0.645685599424278,0.518482988558007,0.86072092278429
"9429","BAIAP2-DT",20.0708395654545,0.242936098263121,0.377072490918996,0.644268951232799,0.519401045748353,0.86072092278429
"9430","MAST3",20.2205403237556,0.240734647351885,0.372655431269713,0.645997957232644,0.518280678351332,0.86072092278429
"9431","EMC10",312.127897254787,0.118105498165189,0.183194729267743,0.64469921507717,0.519122125082891,0.86072092278429
"9432","PPP2R1A",484.041381196335,0.101820525950279,0.157480921597513,0.646557849150195,0.517918145593589,0.86072092278429
"9433","AC022137.4",19.2400497610989,-0.274616402176962,0.424874501351406,-0.646347100857981,0.518054590748523,0.86072092278429
"9434","AC010327.7",4.67630679485959,0.586888090041722,0.9106097831183

61,0.644500093148503,0.519251197243601,0.86072092278429
"9435","SMIM26",687.036708968019,-0.1429903792068,0.220776455007257,-0
.647670419393679,0.517198140405079,0.86072092278429
"9436","YPEL1",48.6082325054591,0.216203054997563,0.334679944865929,0.
645999434128545,0.51827972188146,0.86072092278429
"9437","PES1",89.2310497288106,-0.120128092446022,0.185679549041839,-0
.646964585307956,0.517654864220091,0.86072092278429
"9438","RAI2",140.535528191232,-0.207730977086413,0.321746490613953,-0
.64563556447818,0.518515399352706,0.86072092278429
"9439","EEFSEC",69.013429929712,-0.13430201448376,0.208534304505884,-0
.644028400037035,0.519557017954691,0.860882695286722
"9440","RIC8B",61.5302636857656,0.192531550524092,0.299063749034147,0.
643780970264333,0.519717475408025,0.860966138690977
"9441","PSMD3",362.276333660642,-0.129631415887654,0.201354679323549,-
0.643796391140009,0.519707474270196,0.860966138690977
"9442","ACY1",50.8557290969561,0.25793196007418,0.400776606229185,0.64
3580378857446,0.519847577085417,0.860978748386188
"9443","FBXO25",168.627511449465,0.136431961266395,0.212010665711635,0
.643514611910904,0.519890236557491,0.860978748386188
"9444","AL136038.5",35.8929162077157,0.242793344356221,0.3772876885363
18,0.643523103810077,0.519884728218866,0.860978748386188
"9445","PDCL",225.977413577391,0.107822964840792,0.167602094139572,0.6
43327074129527,0.520011892118537,0.860997881932449
"9446","PPP1R37",80.3601557984912,-0.159155440603361,0.247377034511079
,-0.643371931909196,0.519982791588481,0.860997881932449
"9447","FXYD7",4.54620251870999,-0.464681693634868,0.722511138614868,-
0.64314813820824,0.520127981346,0.861098933868047
"9448","TMCO1",950.923915865523,-0.114683142734145,0.178400085614418,-
0.642842419829403,0.520326354828317,0.861336175858899
"9449","FLNC",448.672875229584,0.149409002002469,0.232475550336788,0.6
42686948309274,0.520427251606067,0.861412024036288
"9450","PIGV",96.5320332987231,-0.156898508076331,0.244415604988225,-0
.641933268065635,0.520916512412565,0.861927193805001
"9451","OSBPL6",17.2382350209145,-0.279573173643664,0.435497335562641,
-0.641962994520895,0.520897210632085,0.861927193805001
"9452","AC009093.2",9.66517813377432,0.412516090750126,0.6426806343986
92,0.64186793357495,0.52095893625567,0.861927193805001
"9453","BICRA",36.3580938002663,0.218390086331953,0.340149435770734,0.
642041595150994,0.520846175978204,0.861927193805001
"9454","CISH",12.2588462297612,-0.389891381403839,0.607515785950656,-0
.641779835883156,0.521016143814122,0.86193066313231
"9455","BLVRB",1110.36649587764,-0.200682919542152,0.312777278494811,-
0.641616042277449,0.521122514247621,0.862015454556614
"9456","RSBN1L",423.356580183325,0.113414117141936,0.176852216975923,0
.641293160364378,0.521332232149694,0.862121266718693
"9457","STAM-
AS1",10.4419499377237,-0.321642539172688,0.501576669014981,-0.64126295
9468079,0.521351850423619,0.862121266718693
"9458","UBR7",94.2046295321222,-0.128939065363209,0.201068366467222,-0
.641269771215994,0.521347425530485,0.862121266718693

"9459","TMEM183A",245.56491800614,-0.0984606821785001,0.153718229832343,-0.640527036291591,0.521830017961344,0.862217103112376
"9460","IMPDH2",554.132123328329,-0.139627405216957,0.217999211228801,-0.640495001931044,0.521850837471979,0.862217103112376
"9461","HLA-DQA2",8.88983488634844,-1.50206103640753,2.34457146785607,-0.640654830531161,0.521746967221693,0.862217103112376
"9462","AL606970.1",5.74771469520719,-0.444097757984588,0.693001299105776,-0.640832504293478,0.521631512146421,0.862217103112376
"9463","AC027031.2",12.7706783147112,0.337591494247268,0.526922016974863,0.640685876413803,0.521726792193898,0.862217103112376
"9464","ZNF774",3.13273133467305,0.623815228902552,0.973374141817633,0.640879187254418,0.521601179038831,0.862217103112376
"9465","AC092718.4",6.28753597087653,-0.439163589154627,0.685625526798362,-0.640529811084152,0.521828214610871,0.862217103112376
"9466","ABI3",6.23598317275538,-0.405662205534738,0.632784900173118,-0.641074408418653,0.521474340350286,0.862217103112376
"9467","KIAA2013",234.846827573226,-0.0989996468026813,0.155165634239594,-0.638025599468849,0.52345701609286,0.862307568326931
"9468","ZNF436",100.016322415209,0.129189061677184,0.20241055251503,0.638252601319247,0.523309261016148,0.862307568326931
"9469","HENMT1",26.9527563725392,-0.236732837406462,0.370353404842411,-0.63920794114798,0.522687666756671,0.862307568326931
"9470","NDUFS2",366.069786113512,-0.137820805914494,0.21602458552006,-0.637986669816782,0.523482357487518,0.862307568326931
"9471","C1D",402.809268923893,-0.151503094569809,0.2371410911625,-0.638873228705827,0.522905405080323,0.862307568326931
"9472","PAIP2B",31.0494233672995,0.211056715454074,0.329761781096238,0.640027824790523,0.522154509967983,0.862307568326931
"9473","ZEB2-AS1",10.4480234023796,0.31339510854093,0.491209564549215,0.638006934633966,0.523469165957923,0.862307568326931
"9474","CTBP1-DT",65.4346026818823,0.161446271759021,0.252653373316188,0.639003032652867,0.522820959028378,0.862307568326931
"9475","UCHL1",104.472890950533,0.180110478719849,0.281412407987065,0.640023231413904,0.522157496186,0.862307568326931
"9476","UTRN",1041.48827836077,0.179316020376312,0.280442146941548,0.639404676978481,0.522559707201992,0.862307568326931
"9477","AKAP12",1802.77379192796,0.224774469166269,0.352188168840409,0.638222657809166,0.523328749965806,0.862307568326931
"9478","MMP16",51.7349990693875,0.353166630977074,0.552012331008688,0.639780329420785,0.522315422655707,0.862307568326931
"9479","DMRT2",93.7753257326616,-0.271584047909418,0.425140495363912,-0.638810113059089,0.522946468513147,0.862307568326931
"9480","ASPN",1620.83861066231,-0.21268659151489,0.333085003365096,-0.638535477028855,0.523125167699773,0.862307568326931
"9481","IFITM3",6417.64156006585,-0.137144403573083,0.214670288469388,-0.638860666517621,0.522913577986224,0.862307568326931
"9482","WEE1",156.218779707808,-0.217535294935,0.3400978605893,-0.6396

25590581688,0.522416041292314,0.862307568326931
"9483","C11orf91",5.64228174600065,-0.611291023800668,0.95787870738203
,-0.638171638109988,0.523361957363194,0.862307568326931
"9484","SLC35E3",229.59837338381,0.134560458290055,0.210981478474619,0
.63778327492497,0.523614768312076,0.862307568326931
"9485","ABCC4",8.09040288684477,-0.395942329065891,0.619711755907902,-
0.638913697039391,0.52287907700165,0.862307568326931
"9486","AL136419.3",9.34095318909147,0.382926229367678,0.5992712048650
25,0.638986532740089,0.522831692924281,0.862307568326931
"9487","EIF2B2",112.384432417229,-0.146208052838712,0.228764861417223,
-0.639119364455439,0.52274528344875,0.862307568326931
"9488","GOLGA8N",24.1191559989181,0.26322554788251,0.412689842734072,0
.637828995593009,0.523585002491802,0.862307568326931
"9489","SRP14-
AS1",31.5621445637357,-0.235125525191416,0.368001938176156,-0.63892469
2507641,0.522871923635835,0.862307568326931
"9490","AC009152.1",3.75089127810236,-0.629194677156414,0.985863711226
359,-0.638216692623498,0.523332632494443,0.862307568326931
"9491","GNAO1",11.5861824174523,0.369191325939329,0.578720248047062,0.
637944373270496,0.523509891280297,0.862307568326931
"9492","ZSWIM7",211.813558063808,0.184976011457758,0.289706836906937,0
.638493773335347,0.523152306050467,0.862307568326931
"9493","MBD3",195.409743625541,-0.114182503708639,0.178805458694042,-0
.638585111117997,0.523092869648334,0.862307568326931
"9494","ITPKC",66.6870987529331,-0.231883331151149,0.362371615453266,-
0.639904786309221,0.522234502027477,0.862307568326931
"9495","FAM207A",138.224136830896,-0.187407443871237,0.29329157027955,
-0.638979987364145,0.522835951001417,0.862307568326931
"9496","MAOA",179.791136127281,0.258337699783016,0.404819443010759,0.6
38155365912477,0.523372548741439,0.862307568326931
"9497","DLG3",15.8962853781396,0.299280486043365,0.46846056250845,0.63
8859511333927,0.522914329547086,0.862307568326931
"9498","RDH5",127.611237174817,0.131850069551063,0.206861443657819,0.6
37383493122882,0.52387507779995,0.862508615650358
"9499","LINC01551",5.75437935329968,0.60080813208377,0.942678203641429
,0.637341703417916,0.523902292114987,0.862508615650358
"9500","FEM1A",48.8984252946728,0.1745731619507,0.273885708689766,0.63
7394199156413,0.523868105927445,0.862508615650358
"9501","ECE1",377.848840924787,0.169723383116781,0.266456349103836,0.6
36965055205498,0.524147605878287,0.862730852024458
"9502","AC239799.2",23.723983787999,0.266996521960795,0.41911783553791
3,0.63704404661787,0.524096153309407,0.862730852024458
"9503","AC064836.3",18.5680371719079,-0.239249154509302,0.375751633233
824,-0.636721529192717,0.524306247502412,0.862901158680177
"9504","MORN4",51.2661275895247,0.277225257250214,0.435573277162396,0.
636460664107396,0.524476211713639,0.862999258411501
"9505","AC012470.1",4.98176557661973,0.594854895376608,0.9345582514978
92,0.636509168286927,0.524444607132175,0.862999258411501
"9506","SPCS1",1201.80023868202,-0.159441692561588,0.250600329005067,-
0.63623895944033,0.524620683504904,0.863055379196035

"9507","AC079174.2",4.23471727769888,-0.500859647329208,0.787210468661934,-0.636246172107629,0.524615983116364,0.863055379196035
"9508","FMNL1",278.079681691049,0.1078397249733,0.16961569977809,0.63578858038724,0.52491423158816,0.863447473920785
"9509","STOML1",124.905296187266,-0.176820121466954,0.278168730648586,-0.635657793220233,0.524999491807544,0.863496903130717
"9510","TNFRSF4",2.98620755654406,-0.539805439251664,0.849328406386004,-0.635567390885467,0.525058429277916,0.863503031956531
"9511","ARHGAP30",10.8159221393943,-0.450548330999457,0.709003817868006,-0.635466720552039,0.525124064917573,0.863520174041724
"9512","VPS28",1354.77442144753,-0.167502251158851,0.263706741828544,-0.635183803028278,0.525308545639703,0.86373272222508
"9513","TONSL",6.65802240510166,0.440207509415693,0.693660439092734,0.634615273708644,0.525679364172473,0.864069916516813
"9514","GTF3C4",105.39378904346,0.152589600765952,0.240431910267503,0.634647874303297,0.525658097087755,0.864069916516813
"9515","PPP1R12C",205.683230973127,-0.092746888849202,0.14612344775981,-0.634715990288254,0.525613662863771,0.864069916516813
"9516","ENC1",24.2985765015096,-0.419116166531367,0.66070090477275,-0.634350828799793,0.525851891771828,0.864081066118028
"9517","AC073332.1",110.249102941186,-0.180053018021582,0.283821406151185,-0.634388436246674,0.525827354375903,0.864081066118028
"9518","ZNF382",8.0864638904692,0.487419764931746,0.768325819891341,0.634392014836464,0.525825019515797,0.864081066118028
"9519","INTS11",242.27885584697,-0.129394501470458,0.204147685239829,-0.633827913936164,0.526193134023756,0.864194149863932
"9520","AHDC1",218.83773070562,0.160229191162006,0.252881931045571,0.633612652748734,0.526333641410702,0.864194149863932
"9521","AL121987.1",11.332070935598,-0.301586106902103,0.475729885659958,-0.633944000561846,0.526117368782477,0.864194149863932
"9522","GMPR",10.6557939319484,-0.327551675732752,0.51681104293482,-0.633793879234238,0.526215348211449,0.864194149863932
"9523","SRP54",275.576184978118,-0.0966318689437371,0.152444884052087,-0.63388069428896,0.526158685641981,0.864194149863932
"9524","PML",193.916411320136,-0.131114097984507,0.206805513654118,-0.633997109979358,0.526082708182947,0.864194149863932
"9525","MESP2",6.70615301621802,0.654808373502396,1.0335249198593,0.633568055225547,0.526362753938863,0.864194149863932
"9526","KCTD15",4.75203565724588,0.548508677111414,0.865665535075053,0.633626562323356,0.526324561637917,0.864194149863932
"9527","AP005329.3",23.5517697346473,-0.239185610237515,0.377635698067523,-0.633376588763987,0.526487749435894,0.864308638729651
"9528","GUF1",62.4341849210633,0.182603409475907,0.288362793741138,0.633241921077477,0.526575673936896,0.864362252348137
"9529","BBS7",132.772414944673,-0.107988434673078,0.17067911874644,-0.632698571835872,0.526930502679283,0.864853926110189
"9530","AL391001.1",5.54388433896567,0.439858439831735,0.696228365385193,0.631773225137674,0.527535071787478,0.864914059342799
"9531","ST3GAL6",3.77072593627114,-0.567385289943053,0.898011558391674,-0.631824039057172,0.527501863674448,0.864914059342799

"9532","C4orf33",48.7026213159325,-0.173908190605743,0.275217344535191
,-0.631894006896451,0.527456139760947,0.864914059342799
"9533","SLC38A9",63.599125515845,0.14527850836145,0.229742942512323,0.
632352431690726,0.527156609631221,0.864914059342799
"9534","GSAP",20.4733826105035,-0.281572317921292,0.445245863259383,-0
.632397381213306,0.527127244740256,0.864914059342799
"9535","PBK",3.15075624349396,0.59265593611687,0.938300777294869,0.631
626819947334,0.527630757045373,0.864914059342799
"9536","DDX58",88.8633815167846,0.165185130627974,0.261403831435559,0.
631915491524444,0.527442099983549,0.864914059342799
"9537","CLTA",1864.21608047115,-0.148772301204091,0.235530691231968,-0
.631647198188577,0.527617438016999,0.864914059342799
"9538","ALG2",142.5451757167,0.112081207938009,0.177286614776177,0.632
203441187651,0.527253948999886,0.864914059342799
"9539","CHFR",60.8280192694909,0.207188327485187,0.327554517260376,0.6
32530820267978,0.527040075799643,0.864914059342799
"9540","MRM3",69.7563883890641,-0.158588315036988,0.250964628326465,-0
.631915007682636,0.527442416162457,0.864914059342799
"9541","SUPT4H1",517.227511248743,-0.147021555417676,0.232518642626058
,-0.632300076059359,0.527190813881196,0.864914059342799
"9542","FCRLB",7.29820117184626,-0.525042824591989,0.83140750139131,-0
.631510809937801,0.527706583371254,0.864947701103166
"9543","ORC4",166.284677552296,0.110872625521577,0.175914655736504,0.6
30263721106038,0.5285220548251,0.865576881665901
"9544","AC007038.1",5.02060717599601,-0.452474469943888,0.717769762058
863,-0.630389428284082,0.528439825822451,0.865576881665901
"9545","SLC11A1",11.2788327382546,-0.276512672602412,0.438877298544152
,-0.630045512765555,0.52866480720529,0.865576881665901
"9546","ADAMTS3",7.85301916588246,-0.461217780243669,0.731798793203473
,-0.63025217385871,0.528529608568003,0.865576881665901
"9547","TMEM144",15.1525704879399,-0.281303914241302,0.446471222647037
,-0.630060572713979,0.528654954323039,0.865576881665901
"9548","MRPS18A",308.043340367212,-0.159053826795184,0.252221186730418
,-0.630612474935287,0.528293940059642,0.865576881665901
"9549","DLG5-
AS1",4.50194439266637,-0.508716394827272,0.807436313044047,-0.63003903
4173981,0.528669045814297,0.865576881665901
"9550","PTEN",584.596042937257,0.104602463809008,0.16601093679485,0.63
0093810857005,0.528633208795276,0.865576881665901
"9551","M6PR",284.269029429502,-0.114460209486219,0.181646703415092,-0
.630125443150266,0.528612514292188,0.865576881665901
"9552","CDK2",31.9698326372559,0.210927391907708,0.334809157263155,0.6
29992899931116,0.528699229575086,0.865576881665901
"9553","CSNK1D",259.570773961701,0.140095345260801,0.222186779328839,0
.630529618746838,0.528348130534931,0.865576881665901
"9554","EML4",124.54151803045,0.130277542097838,0.206853139725995,0.62
9806935830938,0.528820907225535,0.865594870644414
"9555","GOPC",539.396558827969,0.0964336097120424,0.153108092066432,0.
629840058814139,0.528799233575765,0.865594870644414
"9556","FOXO6",6.15659127611974,-0.418627889014824,0.665245991078796,-

0.629282843683066,0.529163901165722,0.865599278059339
"9557","UBAP2L",236.694476509271,0.116041722025907,0.184573991608075,0
.628700289867008,0.529545288320142,0.865599278059339
"9558","COPS9",1223.72088071709,-0.123673241787678,0.196429971428562,-
0.629604743554403,0.528953219260307,0.865599278059339
"9559","GLB1",196.281468429305,0.124493686582132,0.19797233061038,0.62
8843870243375,0.529451275940214,0.865599278059339
"9560","P4HTM",259.71217157051,-0.136941911625966,0.217840047655995,-0
.628635152716358,0.529587941087294,0.865599278059339
"9561","DENND6A",156.98792058404,0.146888089879581,0.233313603505265,0
.629573619680803,0.528973587820461,0.865599278059339
"9562","HIST1H2AG",2.88984315615995,0.649604566480704,1.03219635584319
,0.629342046019961,0.529125150319779,0.865599278059339
"9563","HLA-
B",6405.53945873794,0.253180288051346,0.402371014501635,0.629220989898
907,0.529204389056638,0.865599278059339
"9564","TPD52L1",14.0762417724431,-0.300615956783341,0.478167749901207
,-0.6286830444869,0.529556580679443,0.865599278059339
"9565","MEST",650.246209424356,0.253390471278568,0.403090614026118,0.6
28619130442342,0.529598432976331,0.865599278059339
"9566","MTMR6",159.567440797259,0.132353475199967,0.210510594195057,0.
62872595892884,0.529528480319565,0.865599278059339
"9567","PRMT5",91.5989617141845,-0.139093615679866,0.220997646474061,-
0.629389579025184,0.529094038671218,0.865599278059339
"9568","AC005838.2",20.3327201845917,0.249544794684549,0.3968666091195
35,0.628787579882759,0.529488132268877,0.865599278059339
"9569","HEXD",90.3695006278958,-0.128249611788216,0.203935419093569,-0
.628873652052432,0.529431776714665,0.865599278059339
"9570","ZNF12",175.318307345573,0.111601073801851,0.177569109269876,0.
628493741173388,0.529680545463377,0.865643023097933
"9571","SH2D4A",265.254312974484,-0.132951235849403,0.211672653559001,
-0.628098309413143,0.529939540507684,0.865794882851789
"9572","HOMER2",8.98716974781167,0.345468255242487,0.549878502101659,0
.628262887023393,0.529831739665764,0.865794882851789
"9573","RPS15A",13239.8493449102,-0.130378227016448,0.207553058913028,
-0.628168178774378,0.529893773650294,0.865794882851789
"9574","UFSP2",303.372492856077,-0.127744197670619,0.203486338376214,-
0.627777759873194,0.530149537328646,0.866047499876751
"9575","RSAD2",16.006879362196,0.363538952236554,0.579574191094719,0.6
27251795926056,0.530494195773233,0.866078934418046
"9576","GREB1",16.2870082556065,0.286300945530961,0.456444621882609,0.
62724136029933,0.530501035276527,0.866078934418046
"9577","AC100810.1",568.824257345956,0.163398700274235,0.2604987192005
32,0.62725337297513,0.53049316218014,0.866078934418046
"9578","AL162171.3",5.16238165054047,0.47305252593842,0.75401670778501
6,0.62737671599884,0.530412326710576,0.866078934418046
"9579","TNFAIP2",1751.44774290497,0.175310191991741,0.279480119097653,
0.627272496368467,0.530480628848579,0.866078934418046
"9580","ZBTB21",145.201581215404,0.1852131249634,0.295188554689492,0.6
27440061686083,0.530370814200465,0.866078934418046

"9581","NMNAT1",57.0685579730015,0.154888722612526,0.247001422084516,0
.627076238287924,0.530609262098756,0.866086918232453
"9582","ANKRA2",103.003524206671,0.136772260336046,0.218114991196805,0
.62706492380726,0.530616678420931,0.866086918232453
"9583","ZUP1",38.9300205251093,0.172339964692192,0.274945339757157,0.6
26815369354538,0.530780267686943,0.866263527770405
"9584","CNNM4",23.9540276407933,0.220289879021031,0.351890384755357,0.
626018466444266,0.531302829043704,0.866499358325531
"9585","RACK1",10387.0732742749,-0.149485681449216,0.238794341089289,-
0.626001775282108,0.531313776900151,0.866499358325531
"9586","ETV1",18.9867315935545,0.289130770941622,0.461893649480143,0.6
25968274876773,0.531335750408457,0.866499358325531
"9587","MCM4",58.0109719773096,-0.158306107823248,0.252986104263563,-0
.625750209815176,0.531478794400053,0.866499358325531
"9588","FNBP4",386.639079398555,0.156537082207953,0.24985717698804,0.6
2650624686857,0.530982940790507,0.866499358325531
"9589","PPP1R12A-
AS1",2.92431713788356,-0.514426613922263,0.82124626431517,-0.626397508
609965,0.531054243288857,0.866499358325531
"9590","ZFP1",44.5230406963209,0.178505010316096,0.285150108343139,0.6
26003655945625,0.531312543353352,0.866499358325531
"9591","CERS4",276.886203859865,-0.143968370841823,0.230020378220379,-
0.625893983636044,0.53138448099104,0.866499358325531
"9592","GADD45GIP1",853.233683549409,-0.158222983727682,0.252849321506
545,-0.625759969553988,0.531472391893451,0.866499358325531
"9593","NOL12",75.1766848648524,-0.191611355645362,0.306073487166579,-
0.626030556972349,0.531294898848544,0.866499358325531
"9594","UBE2E1",327.136677093737,-0.0928999358930626,0.148516497751647
,-0.625519301218725,0.531630284635171,0.866655998717331
"9595","CD34",3588.87553008228,-0.113844336080897,0.182219018838021,-0
.624766485995056,0.532124329644505,0.866695414187293
"9596","LYAR",175.095590845906,-0.109805173024483,0.175600675418762,-0
.625311792011199,0.531766442101362,0.866695414187293
"9597","PLK2",104.720323016797,0.251616083152266,0.402581031261468,0.6
2500729943445,0.531966267329371,0.866695414187293
"9598","BDP1",422.859668322655,0.150586325307557,0.240982138950784,0.6
24885835785164,0.532045989249103,0.866695414187293
"9599","PCDHB4",75.920595054416,-0.20621206251372,0.329750459251555,-0
.625357923630391,0.531736171245324,0.866695414187293
"9600","AL139274.2",9.95553088604563,-0.312405143409253,0.500051975766
703,-0.624745343582054,0.532138207990574,0.866695414187293
"9601","CDKN2B",137.1785235182,-0.209250149164624,0.335057409367466,-0
.624520286119487,0.532285952032213,0.866695414187293
"9602","DEPDC7",4.63722836197315,0.444529759214737,0.711948325450539,0
.624384865198507,0.53237486215456,0.866695414187293
"9603","DNAJC17",109.51449588791,-0.161058038295598,0.257933627192668,
-0.624416599140415,0.532354026673766,0.866695414187293
"9604","KNOP1",230.093266868182,-0.19147513572573,0.30638990063167,-0.
624939449149514,0.532010799700853,0.866695414187293
"9605","CLPP",297.570387790264,-0.15467806091652,0.247648842904765,-0.

624586245193976,0.532242649560249,0.866695414187293
"9606","AP000240.1",3.73230978959607,-0.646059030657009,1.03440279590074,-0.624572007362403,0.532251996619003,0.866695414187293
"9607","VBP1",350.368404654376,-0.134938849565321,0.215885672032374,-0.625047731491351,0.531939731339824,0.866695414187293
"9608","STX6",72.0053050222203,0.147717724754469,0.23691131641558,0.623514853530041,0.532946244469082,0.866698424326907
"9609","ACKR3",6691.78838370591,-0.199583182966769,0.319734807011761,-0.624214751068462,0.532486560633253,0.866698424326907
"9610","ZBTB24",61.3077838076248,-0.170265820005061,0.272990349365462,-0.6237063705762,0.532820438553454,0.866698424326907
"9611","RAB11FIP1",16.1453083734096,-0.340221888726309,0.545299006097393,-0.623918042985656,0.53268141023355,0.866698424326907
"9612","GNE",91.4708177023718,0.161187509732406,0.258470489188319,0.623620554279088,0.532876808689835,0.866698424326907
"9613","SMPD1",140.917614767922,0.187260649125091,0.300017483622808,0.624165788152937,0.532518712325539,0.866698424326907
"9614","SELENOH",915.010692177879,-0.153736969603736,0.246394032479929,-0.623947617790861,0.532661986700353,0.866698424326907
"9615","FKBP11",261.199250642798,0.179199342228197,0.28746886916551,0.623369559105278,0.533041697161159,0.866698424326907
"9616","UGGT2",181.805213574266,0.118172249784614,0.189548624300633,0.623440292540382,0.532995227011076,0.866698424326907
"9617","CDIPT",490.049624707822,-0.11779369121443,0.188889001435818,-0.623613287798841,0.532881581960013,0.866698424326907
"9618","SIRPA",313.16232332727,0.089013244597431,0.14268162449502,0.623859203401052,0.532720054753411,0.866698424326907
"9619","IL13RA1",1010.28599716122,-0.19629733827506,0.314794621059522,-0.623572720570544,0.532908230519223,0.866698424326907
"9620","ZNF100",57.8854546084781,0.21949960274917,0.35222886740853,0.623173234959715,0.533170688095606,0.86681804176874
"9621","NXPE3",44.8154246800875,0.226736658406367,0.364148396315647,0.622649064778058,0.533515161158595,0.867197788455666
"9622","FAM71D",3.77218444197826,-0.555302855105466,0.891831864436753,-0.622654198901241,0.533511786580878,0.867197788455666
"9623","C3orf33",37.1654385961395,0.244266605400917,0.392443690814159,0.622424595218144,0.533662711984838,0.867257358213099
"9624","MRPS11",255.071090786819,-0.156621084206644,0.251628066282213,-0.62243090176986,0.533658566210663,0.867257358213099
"9625","FAM106A",33.6321623679273,-0.355732842028748,0.571704402872283,-0.622232118978831,0.533789249024423,0.867372868025141
"9626","COL3A1",24070.6333721723,-0.247521953150629,0.397900707711775,-0.622069647913079,0.533896071966274,0.867447580178806
"9627","HDAC11",15.0562973240629,-0.273679509903518,0.440003823377632,-0.621993481335352,0.53394615437221,0.867447580178806
"9628","TMEM69",74.3997810608649,-0.139804913528044,0.224978784820045,-0.621413764146117,0.534327418094954,0.867616364099364
"9629","RPP21",360.733969235507,-0.146611943305611,0.235860122415463,-0.621605474482699,0.534201320381226,0.867616364099364
"9630","MARCH8",87.5817286872882,0.144023301873036,0.231686134940707,0

.621631078225268,0.534184480624854,0.867616364099364
"9631","RAB11B",524.349770736656,-0.107485125518537,0.172968452664223,
-0.621414621354064,0.534326854231915,0.867616364099364
"9632","Z82246.1",3.90270927179154,0.686078181367969,1.10396906089439,
0.62146504433026,0.534293687017763,0.867616364099364
"9633","ATOX1",796.123515462658,-0.159675062269852,0.257216298252769,-
0.62078127768147,0.53474354338482,0.867690913837909
"9634","EPC1",600.422919002747,0.102518412355151,0.165101444868547,0.6
20941945340184,0.534637821483263,0.867690913837909
"9635","NECTIN1",62.1299820489463,0.186627727988197,0.30064697838407,0
.620753712514563,0.534761682767494,0.867690913837909
"9636","IGHG2",50.4234950834754,0.666366166465153,1.07294114978453,0.6
21064973227072,0.534556874286435,0.867690913837909
"9637","AC092376.2",47.6451387176668,0.190758874322777,0.3071227770689
74,0.621116011463832,0.534523295079317,0.867690913837909
"9638","THAP7",190.544861915323,-0.135674831532448,0.218485093140308,-
0.620979809571355,0.534612907746227,0.867690913837909
"9639","RIBC1",8.40416464555778,-0.310080684781114,0.499521147730501,-
0.620755870276801,0.534760262831103,0.867690913837909
"9640","TMEM53",27.5700724577898,-0.245979875073981,0.396805719019472,
-0.619900024832835,0.535323609420838,0.868171551369097
"9641","MRPL30",114.205048763034,-0.11941495918803,0.192853371476291,-
0.619200785933427,0.535784094171016,0.868171551369097
"9642","TRAF3IP1",95.9528750266409,-0.149354799168691,0.24114309872461
7,-0.619361698338516,0.535678107412419,0.868171551369097
"9643","CRELD1",132.030799212231,-0.208004047812096,0.336033409382276,
-0.618998117462386,0.535917599057117,0.868171551369097
"9644","RANBP17",11.938811803679,-0.388385462220891,0.626540742227226,
-0.619888597891112,0.535331133045652,0.868171551369097
"9645","EIF4H",786.947874536654,-0.0730418677539128,0.117920258310108,
-0.619417467368725,0.535641376980438,0.868171551369097
"9646","ARHGEF5",34.8879730289967,-0.249471855622871,0.402666741778389
,-0.619549194753636,0.535554624121934,0.868171551369097
"9647","ZNF746",46.702994473929,0.196322885119581,0.317184464849663,0.
618954920168088,0.535946056807457,0.868171551369097
"9648","BET1L",121.630020382878,0.112518014344407,0.181677820570214,0.
619327191350368,0.535700834937196,0.868171551369097
"9649","MSANTD4",104.189853701067,0.153604299882833,0.247936717766008,
0.619530262668873,0.535567091955325,0.868171551369097
"9650","LINC01588",17.9791483654783,-0.284327765614614,0.4593457674689
12,-0.618984185227868,0.535926777327817,0.868171551369097
"9651","DNAAF4",51.3343497395328,-0.157604715643351,0.254451609564284,
-0.619389737456283,0.535659640213893,0.868171551369097
"9652","FLYWCH2",369.41780166558,-0.15237475819861,0.246157155426028,-
0.619014133206455,0.535907048310798,0.868171551369097
"9653","TMEM231",21.1503259024502,-0.210323493017942,0.339182343637548
,-0.620089745127459,0.535198703268793,0.868171551369097
"9654","SCAND1",805.986159754589,-0.167584134060186,0.270390695226571,
-0.619785136909983,0.535399255325523,0.868171551369097
"9655","PLCG1-

AS1",6.36652836619448,0.466804968529695,0.754177652700634,0.618958897625929,0.535943436484172,0.868171551369097
"9656","AC027796.1",14.9966652561236,0.379945277337338,0.613955315321006,0.618848420814932,0.536016220285488,0.868195286377903
"9657","MEF2D",209.743254588778,-0.119523198816102,0.193222112472323,-0.618579298646383,0.536193542883277,0.868243733711129
"9658","C7orf50",338.256896025733,0.174732487527396,0.282487127051315,0.618550265816735,0.536212674163414,0.868243733711129
"9659","TRIM16L",17.6040500986566,-0.280027559225931,0.452663892864193,-0.618621373695348,0.536165817998892,0.868243733711129
"9660","FAM89A",19.8562379949033,0.267466962445864,0.432632551927529,0.618231247866591,0.536422914734555,0.868246199596348
"9661","NSUN3",84.2126569179233,0.130372385368443,0.210959602805352,0.617996922798224,0.536577367058694,0.868246199596348
"9662","NAA15",157.552949854216,-0.123285938455782,0.199505257541321,-0.61795834342986,0.53660279829273,0.868246199596348
"9663","PNRC1",4272.41176818751,0.142752293450622,0.230981874935737,0.61802378862124,0.536559657665169,0.868246199596348
"9664","BNC2",291.205456545737,0.189259797255028,0.30620681032943,0.618078340750863,0.536523698937061,0.868246199596348
"9665","TPM1",3210.32609314534,-0.110455601905711,0.178668825347672,-0.618214183088601,0.536434162005955,0.868246199596348
"9666","MIRLET7BHG",46.4522960386466,0.277951623498081,0.449771045365486,0.617984697685943,0.536585425696587,0.868246199596348
"9667","LIMCH1",552.329235655108,0.181447620861592,0.293682871038665,0.61783521871694,0.536683965247129,0.868287702127351
"9668","CHAC2",7.32118238137124,-0.358299957382459,0.580290908225915,-0.617448855915847,0.536938705555481,0.86834053922942
"9669","DCN",88729.223589137,0.119962914415668,0.194278375029954,0.617479502786515,0.536918496956637,0.86834053922942
"9670","PIEZO2",40.6066664858106,0.320533510519465,0.519012401174138,0.617583529399946,0.5368499044823,0.86834053922942
"9671","PCNT",97.1045233298945,0.204786553219703,0.33160233968223,0.617566671622243,0.536861019768321,0.86834053922942
"9672","TRIM34",68.6507526298735,0.133506715874489,0.216284041569604,0.61727492655312,0.537053402117459,0.868436229230465
"9673","AC009403.1",16.5894245245426,0.245838237104659,0.398371526699831,0.617107952320839,0.537163523750848,0.868524502373955
"9674","AC092821.3",37.8037573005318,-0.215879459174655,0.349891580546688,-0.616989579564488,0.537241598965683,0.868560947676584
"9675","EPHB2",34.6428002185806,-0.236078671247994,0.382949378860839,-0.616474877045781,0.537581148017806,0.868608658375489
"9676","SLC25A44",51.7563626284385,0.17931172567204З,0.290803785552385,0.616607260911126,0.537493804137457,0.868608658375489
"9677","TSC22D2",411.76240449174,-0.243446187921745,0.394929414497182,-0.61642961750949,0.537611010871463,0.868608658375489
"9678","ECD",89.4630641576222,0.117270823182255,0.190290873615616,0.616271400483136,0.53771541115035,0.868608658375489
"9679","SERPINF2",5.93971010937464,0.430766620563643,0.69843594219849,0.616758953165704,0.537393729783721,0.868608658375489

"9680","ZNF493",122.517822194116,0.134679120255297,0.218507509214282,0
.616359230580137,0.537657454774014,0.868608658375489
"9681","ZNF470",14.7906882398157,0.279167568027255,0.452961324280581,0
.616316566255727,0.537685607256033,0.868608658375489
"9682","PKIG",381.702660710138,-0.152949154022721,0.248183692110804,-0
.616273989325758,0.537713702807954,0.868608658375489
"9683","TUBGCP3",103.942469194353,0.154437309816484,0.250716122526223,
0.615984757024674,0.537904580166156,0.868758157704652
"9684","PPP1R9B",99.5225051706276,-0.123870806277495,0.20110110658463,
-0.61596282775981,0.537919053658046,0.868758157704652
"9685","SCMH1",59.6936712432604,0.202012918791483,0.328017911699533,0.
615859413727771,0.537987310389051,0.868778681929248
"9686","ZNF26",61.0049686238655,0.176234552325236,0.286258686172842,0.
615647876686005,0.538126945475164,0.868914456662354
"9687","AL161669.3",82.1558818931851,-0.249546121146866,0.405499049652
847,-0.615404946967214,0.53828732522546,0.869052366538491
"9688","ZNF613",7.023089166405,0.397285743973155,0.645713771447306,0.6
15266022718824,0.538379052390757,0.869052366538491
"9689","TIMM17B",164.161314343526,-0.144328557920913,0.234553921766244
,-0.615332102887416,0.53833542082562,0.869052366538491
"9690","FKBP7",306.906672700555,-0.179295586983999,0.291549931188164,-
0.614973861435362,0.538571982571179,0.869185988838466
"9691","CCDC126",78.1059175970186,-0.165361690498784,0.268929685336011
,-0.614888201323608,0.538628555231612,0.869185988838466
"9692","OXLD1",149.67928282932,0.158717059799906,0.258115254604056,0.6
14907708741876,0.538615671650521,0.869185988838466
"9693","WDSUB1",63.0923835171882,0.154832353984056,0.252261021358085,0
.613778352083459,0.539361804267761,0.869203361025016
"9694","TNK2",16.0021033308131,-0.257547016684597,0.419583210600684,-0
.61381630670085,0.539336720367077,0.869203361025016
"9695","ENTPD7",17.0669377749754,-0.254313738687079,0.414230736226436,
-0.613942222163013,0.539253508038418,0.869203361025016
"9696","CLNS1A",393.95717880713,-0.146697157148256,0.23870682006706,-0
.614549500961241,0.538852272898482,0.869203361025016
"9697","C11orf71",111.591076982118,0.164709808624756,0.268289318283496
,0.613926076813501,0.539264177473609,0.869203361025016
"9698","CNPY2",741.999939780223,0.153441492848493,0.249789774192658,0.
614282523551769,0.539028649091377,0.869203361025016
"9699","PRSS53",18.3886518200543,-0.257254732537462,0.418594708013011,
-0.6145675700455,0.538840336767986,0.869203361025016
"9700","ME2",129.836946496673,-0.134879885480633,0.219699475059135,-0.
613929029390391,0.539262226295325,0.869203361025016
"9701","ELAVL1",285.59204222341,-0.0947859664878817,0.154315963758697,
-0.614233059102675,0.539061330505525,0.869203361025016
"9702","DDX27",320.604913173296,-0.130003624462337,0.211637782669375,-
0.614274175539966,0.53903416459576,0.869203361025016
"9703","AC245060.6",13.1417686061995,0.430596031753277,0.7015485712738
25,0.61377935810122,0.539361139391193,0.869203361025016
"9704","ZNF711",10.8723401698439,0.307221323519979,0.499723875232799,0
.614782160201689,0.538698592286591,0.869203361025016

"9705","DOCK11",271.946387840107,-0.130106478009678,0.211940917208217,
-0.61388088587848,0.539294041852215,0.869203361025016
"9706","AC007920.2",15.8621718970015,-0.310013997254266,0.505326048242
845,-0.613493007796191,0.539550404716231,0.869417713760752
"9707","ZNF782",19.3188875351547,0.248536256497931,0.40517979113216,0.
613397464378631,0.539613562234547,0.869418377564868
"9708","AC009269.4",5.07519390263758,-0.474031558754086,0.772893132362
72,-0.613320961081618,0.53966413624027,0.869418377564868
"9709","KHSRP",353.229641967966,0.102169032205804,0.166605266038856,0.
613240113202516,0.539717584896247,0.869418377564868
"9710","VWA5B1",3.60768211278342,0.666653336380952,1.09364297059732,0.
609571271707476,0.542145846905875,0.87010247566887
"9711","ZMYM1",104.466564915672,0.108714939222736,0.178046869568568,0.
610597307810958,0.541466205433104,0.87010247566887
"9712","NBPF10",47.2361058892892,0.287551374217122,0.470505550137002,0
.611154053620394,0.541097597757056,0.87010247566887
"9713","ACBD6",334.814881501901,-0.123101851223636,0.201618767756998,-
0.610567421838453,0.541485995743788,0.87010247566887
"9714","TBCE.1",26.799210960927,0.237428652715401,0.388715765902301,0.
610802734394559,0.541330182961965,0.87010247566887
"9715","CCDC173",3.73814111923265,-0.549692558224116,0.902407544162124
,-0.609140029668634,0.542431626537633,0.87010247566887
"9716","RNF25",42.2575661332193,0.159376769566626,0.261223018605103,0.
61011763212016,0.541783887391711,0.87010247566887
"9717","AC112715.1",4.90724591194104,-0.531056401924024,0.869401710784
162,-0.61082971811159,0.541312317059118,0.87010247566887
"9718","GOLGA4",1169.87411499791,-0.121646292082411,0.198990882174832,
-0.611315909316558,0.540990460614573,0.87010247566887
"9719","SEC22C",239.011673279506,-0.0892083393488994,0.146311338328475
,-0.609715831787573,0.542050065246744,0.87010247566887
"9720","AC137630.4",2.49752208054075,0.691452097372926,1.1308108103887
5,0.611465765113457,0.540891276015937,0.87010247566887
"9721","KBTBD8",4.0051267479708,0.569717926804795,0.934826511018879,0.
609437066759962,0.542234775085309,0.87010247566887
"9722","AADAT",29.0077334545887,0.238481060237904,0.3916052256078,0.60
8983345070955,0.54253547839445,0.87010247566887
"9723","CASC15",5.39589689229334,-0.384972648169452,0.63089976954719,-
0.610196209844481,0.541731840182988,0.87010247566887
"9724","C6orf136",50.4614930602883,-0.152108303886119,0.24858538562212
2,-0.611895600803102,0.540606832404343,0.87010247566887
"9725","PPP1R18",245.102383005479,-0.0967038332672253,0.15835742119598
2,-0.610668148905668,0.541419296330179,0.87010247566887
"9726","AL024507.2",4.20579611684649,0.455677145616651,0.7461824649582
64,0.610677906565572,0.541412835224366,0.87010247566887
"9727","SAMD5",7.81902427087482,-0.431542370578511,0.706281409640919,-
0.611006271279195,0.541195428579765,0.87010247566887
"9728","AC005014.2",3.90871414392181,0.547615683564725,0.8941336235408
84,0.61245396565683,0.5402374463684,0.87010247566887
"9729","KLHL7",142.290673419442,0.107603164098067,0.176364316415794,0.
610118680948943,0.54178319266681,0.87010247566887

"9730","AP1S1",236.883667234243,-0.191280102625282,0.313524442296032,-0.610096301342508,0.54179801660307,0.87010247566887
"9731","PCMTD1",753.240845716998,0.122848765009908,0.201705199689294,0.609051056686409,0.542490597355548,0.87010247566887
"9732","AC036214.2",4.04897314952777,0.544121546705746,0.893382713374824,0.609057617255973,0.542486248935909,0.87010247566887
"9733","TSTA3",168.326996325972,-0.174451862856809,0.286078140597876,-0.609804938231987,0.541991029895192,0.87010247566887
"9734","TLN1",1034.25877600091,-0.099799669947894,0.163337700927716,-0.611002049012921,0.541198223818788,0.87010247566887
"9735","GAS1RR",78.4284006317159,0.173502516985125,0.284993629849596,0.60879436876077,0.542660746651054,0.87010247566887
"9736","RPARP-AS1",13.1664850378652,-0.258518209642404,0.423172873480875,-0.610904492804376,0.541262810318573,0.87010247566887
"9737","PHRF1",104.989733064696,0.165752395530967,0.272217757414305,0.608896337643022,0.542593151922109,0.87010247566887
"9738","LTBR",344.23886498285,0.112578254612112,0.184247950560206,0.611014962553545,0.541189674775574,0.87010247566887
"9739","FBXL3",482.964583170578,0.0975251568952797,0.159312540378907,0.612162461682723,0.540430274948818,0.87010247566887
"9740","GGACT",31.0363327180922,0.274946054786448,0.450850252226616,0.609838972981763,0.541968481829278,0.87010247566887
"9741","TTC5",149.513052040834,-0.111738761174372,0.18259828627555,-0.611937622490898,0.540579028581082,0.87010247566887
"9742","KLF13",647.17306501587,-0.138616672858793,0.226438321467493,-0.612160839033124,0.540431348420622,0.87010247566887
"9743","RPS27L",1809.65878378518,-0.148264327783836,0.242460833613652,-0.611498053413803,0.540869906647165,0.87010247566887
"9744","UBE2Q2",354.534369027512,-0.0836815815243506,0.137389936801315,-0.609080864818841,0.542470840328425,0.87010247566887
"9745","MRTFB",87.1171180096596,0.153787395865977,0.251955589720431,0.610375011074842,0.54161341763691,0.87010247566887
"9746","DHX40",232.632542491491,0.135149288800139,0.220888122688662,0.611844979055892,0.540640327441797,0.87010247566887
"9747","AC005837.1",2.78462113803817,-0.540262863058931,0.887642513348265,-0.608649152034204,0.542757017431297,0.87010247566887
"9748","PCYT2",40.0612960670048,-0.166471419970493,0.273233312559638,-0.609264728414686,0.54234898225266,0.87010247566887
"9749","ZNF93",26.7335347269662,0.210255653449987,0.345430722794935,0.608676760853161,0.542738713632934,0.87010247566887
"9750","PNKP",106.47553947918,-0.133825964859579,0.219072676171598,-0.610874743478983,0.541282506446025,0.87010247566887
"9751","RDH13",55.7358416973919,0.140004439227468,0.22953289102826,0.609953713388426,0.541892469609367,0.87010247566887
"9752","ZFAS1",2348.61874494492,-0.121249960012257,0.198713540186771,-0.610174625736595,0.541746136512448,0.87010247566887
"9753","SLC16A8",19.2863403404066,0.281013985094744,0.460301715383023,0.610499539114055,0.541530948616005,0.87010247566887
"9754","CBY1",346.938444917283,-0.126794554894772,0.207460164986006,-0

.61117542687448,0.541083449551379,0.87010247566887
"9755","AL136169.1",36.1507992584305,0.226673795676243,0.372338881763995,0.608783575334254,0.542667901812193,0.87010247566887
"9756","TAZ",115.096944584665,0.118680830312141,0.19459483451822 5,0.609886848260741,0.541936765186968,0.87010247566887
"9757","BCL2L10",2.67650088587662,0.613829605243282,1.00898576141671,0.608363000466337,0.542946745238398,0.870241773165514
"9758","PET117",90.7165083554792,0.147584563391479,0.242631477916275,0.60826634968776,0.543010835314722,0.870241773165514
"9759","Z93930.2",33.7743682527871,-0.232339223242018,0.381933815729677,-0.608323258306256,0.542973098197189,0.870241773165514
"9760","SDC3",50.7857822588395,0.209352380345421,0.34424515426651,0.608149098834789,0.543088590559923,0.870277208643156
"9761","PABPN1",499.60219400476,0.0931031912601193,0.153138592683802,0.607966872546343,0.543209445459309,0.870381695213974
"9762","TDP2",154.968225950434,0.0988319493235202,0.162657084444935,0.607609251455495,0.543446663447616,0.870498436293977
"9763","ZC3HC1",44.7010845919831,-0.161722114224606,0.266119699872615,-0.607704406333006,0.543383540076069,0.870498436293977
"9764","PNPLA7",13.6801174584514,-0.309227337435871,0.508927972453582,-0.607605307967376,0.543449279538004,0.870498436293977
"9765","PPDPF",1724.42524796262,-0.222964143014708,0.36709719712217,-0.607370867341452,0.543604817544896,0.87063535385553
"9766","APOBEC3G",78.414626816306,-0.184805314086169,0.30430212137446,-0.607308661705832,0.543646091160685,0.87063535385553
"9767","SAMD4A",226.015196829625,-0.164344012936991,0.270660167506838,-0.607196893620628,0.543720253520082,0.870664970313719
"9768","AL390198.1",3.30771397385419,0.513730760891329,0.846284705028117,0.607042473814129,0.543822725193281,0.870739908069504
"9769","NUP62",170.3032465005,0.10360191586244,0.170715689851859,0.60686815577609,0.543938412706559,0.870835988814678
"9770","TRAF3IP2-
AS1",58.7503617945793,0.157369160970064,0.259434202737632,0.606586021848524,0.544125679002018,0.870995745930145
"9771","ZNF625",27.0411293815237,-0.193661803751034,0.319284145787199,-0.606550016047801,0.544149580146,0.870995745930145
"9772","MED4",518.857201753525,-0.111736472694983,0.184252795141819,-0.60643027211055,0.544229071567182,0.871033839471012
"9773","FHL1",2677.77458077956,-0.144639662519099,0.238543274885326,-0.606345589028368,0.5442852914977,0.8710346832113
"9774","TXNRD1",270.485732114245,0.112447119188555,0.185510549390299,0.606149459198544,0.54441551047226,0.871153937363019
"9775","HNRNPH1",883.075760237879,0.134630292899134,0.22231313173048,0.605588576127624,0.544787990142519,0.871186925151135
"9776","AC067838.1",12.607869016312,0.290662254084806,0.479771530849915,0.605834726312121,0.544624507438441,0.871186925151135
"9777","AC016394.2",3.19351894735143,0.520100911101214,0.858950072113515,0.605507733204404,0.544841687963111,0.871186925151135
"9778","HECTD2",66.5276055660352,-0.148468558670191,0.245221222732184,-0.605447428309823,0.544881745641203,0.871186925151135

"9779","AMPD3",2.78538327655725,0.54331302218035,0.897363344844442,0.6
05454886587253,0.544876791382378,0.871186925151135
"9780","DPF2",192.643124718337,0.111168250425305,0.183592660116835,0.6
05515767103978,0.544836351535495,0.871186925151135
"9781","PMM2",122.015154655963,-0.170238297288445,0.280936761144147,-0
.60596661182798,0.544536924599289,0.871186925151135
"9782","TCP10L",6.15998358506913,-0.398698631300121,0.658380417035777,
-0.605574863686225,0.544797098091891,0.871186925151135
"9783","NIM1K",3.14498640386433,-0.520159130528813,0.859423230564274,-
0.605242111255581,0.545018138977696,0.871226736082872
"9784","QSOX2",30.2840759047773,-0.177455855003803,0.293354003974998,-
0.604920514461183,0.54523181170155,0.871226736082872
"9785","TSPOAP1-
AS1",9.09700848185306,0.375522428126329,0.620599646026768,0.6050961042
76754,0.545115142592424,0.871226736082872
"9786","SMARCB1",319.768174867117,-0.142452308733317,0.235342341591873
,-0.605298255170569,0.54498084055814,0.871226736082872
"9787","IDH3G",443.87843850798,-0.146365897924154,0.241964348914704,-0
.60490687401122,0.545240875497388,0.871226736082872
"9788","DNASE1L1",11.2888476109029,-0.276934324144394,0.45780988824232
7,-0.604911189681005,0.545238007816317,0.871226736082872
"9789","GAK",138.803838258121,0.118514700746131,0.195966477798206,0.60
4770275394599,0.545331646566797,0.87128276149808
"9790","PHYHD1",299.220005810378,0.170968106185222,0.282743038828331,0
.604676624024778,0.545393883118657,0.871293190191603
"9791","MYD88",108.530798263362,0.140277186294302,0.232067301547286,0.
604467692600456,0.545532742389977,0.871426012764707
"9792","SH3D21",4.78689839300985,0.428431993537081,0.709355831632754,0
.603973315551578,0.545861383372044,0.871491383221874
"9793","CDCA8",3.57869874957173,-0.474759458131771,0.787523540416306,-
0.602851132400183,0.546607727482013,0.871491383221874
"9794","CKS1B",639.842519706466,-0.162580643290523,0.269965305989325,-
0.602227914786028,0.547022436642528,0.871491383221874
"9795","EHD3",3.70471307170508,0.554655544533763,0.918502189143873,0.6
03869594530583,0.545930345183176,0.871491383221874
"9796","GPR39",5.65130224616995,-0.434738743469756,0.721480731170235,-
0.602564593464075,0.546798380418888,0.871491383221874
"9797","ATP5MC3",1468.73526074446,-0.182774096265361,0.302705732727004
,-0.603801238314163,0.545975796079932,0.871491383221874
"9798","ZKSCAN3",23.5634719558832,0.200810887777224,0.332835002721129,
0.603334643698748,0.546286090730376,0.871491383221874
"9799","AC007036.3",7.8112165267994,0.407023149304552,0.67512796134332
7,0.602882968281574,0.546586547037943,0.871491383221874
"9800","AC018647.2",38.6682714738767,0.194331154792859,0.3216770565968
16,0.604118791836715,0.545764666688751,0.871491383221874
"9801","DNAJC30",108.003109816932,-0.178850353123472,0.296576425713284
,-0.603049796332686,0.546475562813903,0.871491383221874
"9802","PPP2R2A",624.437433417854,-0.10343858999382,0.171600324997715,
-0.602787844342353,0.546649834298145,0.871491383221874
"9803","DENND1A",63.9788556543272,0.141985385050522,0.235638630823596,

0.602555635950945,0.546804340963993,0.871491383221874
"9804","OLFML2A",1016.10902522918,0.160140871645909,0.265429579781546,
0.603327149060432,0.546291075526157,0.871491383221874
"9805","PANK1",4.21310705401773,-0.479872604916119,0.796538924038279,-
0.60244715033293,0.546876532462808,0.871491383221874
"9806","CMAS",217.732254715216,-0.123492999111749,0.204612268213751,-0
.603546406038273,0.546145253754993,0.871491383221874
"9807","ARHGEF7",174.362423774883,0.128406633317725,0.212977367722747,
0.602912106064173,0.546567161998025,0.871491383221874
"9808","MESP1",26.2469522526189,0.261152923078572,0.433395523985205,0.
602574112157852,0.546792046485915,0.871491383221874
"9809","AL008727.1",4.51672430915142,0.5443441641204,0.902948406900158
,0.602851901571149,0.546607215747,0.871491383221874
"9810","AC026401.3",21.3844505587103,0.24782985870164,0.41060310717054
9,0.603575215028027,0.546126095124282,0.871491383221874
"9811","AC015912.3",24.8081043140757,-0.301424357652562,0.500414611645
758,-0.602349233291252,0.546941695178687,0.871491383221874
"9812","CEP112",98.5746064230131,0.146739217814652,0.243190497208886,0
.60339207123135,0.546247895569571,0.871491383221874
"9813","ABCA7",7.40947478340424,0.35649604639551,0.590422319505577,0.6
03798390775678,0.545977689484317,0.871491383221874
"9814","PLD3",1198.11167653468,0.143372798862013,0.237952671934248,0.6
0252653477928,0.54682370580697,0.871491383221874
"9815","AL109809.5",3.91706249957906,0.48883343554279,0.81119951370866
8,0.602605681194169,0.546771040065346,0.871491383221874
"9816","RPRD1B",86.3509962185021,0.125787930477561,0.208856847025711,0
.602268646055336,0.546995327982453,0.871491383221874
"9817","HSCB",151.986201931779,-0.146922461614623,0.243933594782257,-0
.602305154998314,0.54697103005308,0.871491383221874
"9818","HMGCL",238.069918301471,0.155916606182068,0.259059512942513,0.
601856324097493,0.547269779196931,0.871619078069247
"9819","GPRIN3",8.17247221353168,0.506259097643035,0.840955004860994,0
.60200497614818,0.547170825034443,0.871619078069247
"9820","IL2RB",8.6169026158763,0.502093123690301,0.834144234588985,0.6
01926025344647,0.547223379618906,0.871619078069247
"9821","ZNF35",35.1417578276595,-0.178591628453781,0.296823457141267,-
0.601676262967262,0.547389653499609,0.871658585348385
"9822","APBB1",99.4099059588721,0.121918772270934,0.202668338147796,0.
601567928099477,0.547461782856598,0.871658585348385
"9823","EPN1",391.017744334682,-0.0924177051643478,0.153616218656096,-
0.601614243423381,0.547430945537786,0.871658585348385
"9824","C1orf109",71.9133460474391,0.155349735032997,0.25855546896642,
0.600837165247404,0.547948447551001,0.871685488227386
"9825","EPAS1",56.097508240742,0.195672664851931,0.32550138367475,0.60
1142344290163,0.547745182066095,0.871685488227386
"9826","AC009961.1",8.2476161283383,0.429375584169077,0.71472627955401
4,0.600755277162895,0.548002995716906,0.871685488227386
"9827","KPNA1",191.671948331531,-0.161309077767086,0.268365535168372,-
0.601079708934612,0.54778689750584,0.871685488227386
"9828","ITGA1",4.7138880284213,-0.420665907784687,0.700179724568071,-0

.600797042565304,0.547975174165427,0.871685488227386
"9829","LANCL2",41.758770078949,0.160503689294212,0.267182375055155,0.600727084865081,0.54802177609258,0.871685488227386
"9830","CCNK",244.333704404248,0.101341347484145,0.16855011467229,0.601253506597618,0.547671151316877,0.871685488227386
"9831","CENPN",17.6648688545447,-0.321891308927363,0.535855261785188,-0.600705697756874,0.548036023384904,0.871685488227386
"9832","ZNF460-AS1",3.47869972681864,-0.475992639204899,0.791939892307566,-0.601046422624253,0.547809066983328,0.871685488227386
"9833","PTGIS",2376.57105519982,-0.126535745402978,0.210527503523799,-0.601041399746012,0.547812412378684,0.871685488227386
"9834","DFFB",23.6442103212758,0.210237025533844,0.351249988328886,0.598539594361475,0.549479947874231,0.871686257037941
"9835","ARSJ",9.70268624469884,0.434206111262277,0.724964252514033,0.59893451264188,0.549216555596293,0.871686257037941
"9836","AP3S1",1403.7568640169,0.10968745523178,0.183008213255487,0.599358101369211,0.548934110693911,0.871686257037941
"9837","GNPDA1",129.672836695467,0.136300463123864,0.227751783655036,0.598460573772328,0.549532658439371,0.871686257037941
"9838","HOXA5",33.5438509350341,0.259266726473588,0.43168284781109,0.600595385682422,0.548109512073656,0.871686257037941
"9839","EZH2",23.3120330045822,-0.264272133508703,0.441288302974666,-0.598865031606956,0.54926289172689,0.871686257037941
"9840","AC105046.1",5.92179843037531,-0.520651922193082,0.870004244950144,-0.598447565302302,0.549541335958702,0.871686257037941
"9841","TTI2",60.5069034433709,-0.160391691384203,0.267402616753335,-0.59981346978424,0.548630555370268,0.871686257037941
"9842","SIT1",4.47291050682101,0.479607124926185,0.801355630285326,0.598494734173664,0.549509871489142,0.871686257037941
"9843","POMT1",87.6377759255341,0.120882352434305,0.201372781068059,0.600291418696998,0.548312036722172,0.871686257037941
"9844","MAP3K8",364.403670484883,0.18064724517144,0.301743859192335,0.598677453304173,0.549387995242453,0.871686257037941
"9845","PC",18.9623345934831,0.234322385070035,0.390423495703979,0.600174906603725,0.548389675249857,0.871686257037941
"9846","POLD3",132.411523710684,0.121429676982827,0.202473842795321,0.599730193818561,0.548686062168412,0.871686257037941
"9847","HOXC4",81.1511537074553,-0.139890207517678,0.23356676008195,-0.598930290716861,0.549219371096182,0.871686257037941
"9848","CAMKK2",349.281986767064,-0.139904336328005,0.233285659055699,-0.599712545101634,0.548697826107846,0.871686257037941
"9849","SPTLC2",158.893070447877,-0.139431670313452,0.232444963267852,-0.599848103194999,0.548607471617975,0.871686257037941
"9850","IFI27",5650.4781260977,-0.241751470109298,0.403093139608737,-0.599740968908462,0.548678879979184,0.871686257037941
"9851","ARPP19",334.853218588377,0.0899610014017617,0.150200896030482,0.598937847770929,0.549214331484265,0.871686257037941
"9852","ISLR",3335.85893639425,-0.102202103324415,0.170483298808024,-0.59948454798204,0.548849811199221,0.871686257037941

"9853","TMX3",273.097394163425,-0.0847797839784505,0.141312513463149,-
0.599945340301084,0.548542664010408,0.871686257037941
"9854","SAFB",297.069029234194,0.111601990111984,0.18609285361611,0.59
9711315847773,0.548698645484682,0.871686257037941
"9855","LONP1",107.021403326268,-0.12017704015828,0.200339599641385,-0
.599866628332098,0.548595124498566,0.871686257037941
"9856","STAU1",552.13087647181,-0.0838824547103067,0.140122333122793,-
0.598637296716992,0.549414779111691,0.871686257037941
"9857","RNF185",97.1251268459936,0.114850986735132,0.191908364865358,0
.598467851131506,0.549527803983659,0.871686257037941
"9858","Z95114.1",33.5311812196164,-0.267020583550076,0.44608210208743
8,-0.598590668176454,0.54944588048913,0.871686257037941
"9859","TST",89.4319982441533,-0.206359469122001,0.344135085133785,-0.
59964670281084,0.548741715083771,0.871686257037941
"9860","EIF3L",885.828598530535,-0.115360996860405,0.192499939525893,-
0.599278094032271,0.54898745332788,0.871686257037941
"9861","PIP5K1C",160.416502275038,-0.125664660102282,0.210029657302834
,-0.598318645642938,0.549627337643881,0.871734262321296
"9862","SMYD5",40.640250802157,-0.168655351781068,0.281940716495647,-0
.598194378865713,0.549710241692932,0.871777345373906
"9863","AC011246.1",3.24353995338448,-0.52969425081717,0.8856630995382
22,-0.598076459427235,0.549788916840901,0.871813713818482
"9864","BCL9",46.5106650874245,0.149354019623146,0.249830136725757,0.5
9782227068584,0.549958528928404,0.87188657570336
"9865","ARMC8",151.306117383427,-0.112016162647831,0.187419532371859,-
0.597676033176626,0.55005612026234,0.87188657570336
"9866","DLD",175.944929305507,0.121271720631085,0.202838133019407,0.59
7874368225833,0.549923763791506,0.87188657570336
"9867","RHOBTB2",49.3223635141127,0.198475538004138,0.332219524515288,
0.597422858556299,0.550225096048092,0.87188657570336
"9868","EMX2",368.983245328459,-0.135196367084383,0.226284807774219,-0
.597461086381364,0.550199580095526,0.87188657570336
"9869","HMX3",3.94716851935972,-0.50464865741021,0.844626522093874,-0.
5974814242858,0.550186005378024,0.87188657570336
"9870","MRPL21",383.907594131441,-0.150621204499302,0.25205590659911,-
0.597570620468984,0.550126472530308,0.87188657570336
"9871","EPB41",7.64425168905408,0.463559110510909,0.77649367633681,0.5
96990194044847,0.550513927542045,0.871933298645013
"9872","AL591895.1",43.7729788542162,-0.171393588941514,0.287311047201
507,-0.596543678396418,0.550812083798768,0.871933298645013
"9873","ALAS1",72.134796288624,0.128034194527586,0.21436429338067,0.59
7273886002192,0.550324536420892,0.871933298645013
"9874","SHLD3",11.3162792266601,0.284690601639074,0.477040326015142,0.
596785190084826,0.550650806974583,0.871933298645013
"9875","PCDHGA2",6.89105463845021,0.360315924330175,0.603917540909913,
0.596630996654432,0.550753771676344,0.871933298645013
"9876","TBC1D13",72.799336169877,0.145532299695628,0.243711225927243,0
.597150579100015,0.550406851458577,0.871933298645013
"9877","SF3B2",706.154573027024,-0.0927133955464312,0.155343722360423,
-0.596827436201902,0.550622598224805,0.871933298645013

"9878","DLST",253.147325231369,-0.093941468661633,0.157459646989751,-0
.596606625618483,0.550770046624789,0.871933298645013
"9879","OAZ2",358.19405177367,0.10508960082745,0.176160569153681,0.596
555752131857,0.550804020640419,0.871933298645013
"9880","TEX2",48.2164425621553,0.155800785648443,0.261149551532632,0.5
96596029876678,0.550777122522208,0.871933298645013
"9881","AK9",46.5691329540034,0.174033808650233,0.292155279339226,0.59
5689419146726,0.551382727986546,0.872565539744308
"9882","AC144652.1",16.764707002468,-0.254072588366426,0.4264865394347
31,-0.595734131968565,0.551352852667486,0.872565539744308
"9883","FAM98B",115.441682697954,0.15643952500105,0.26265353464785,0.5
9561172557908,0.551434641613347,0.872565539744308
"9884","AC105114.2",4.37213245001394,0.518162488208981,0.8696507925258
93,0.595828225147686,0.551289985968269,0.872565539744308
"9885","WDR78",20.7570283780336,-0.217907534327184,0.366110316546411,-
0.595196377918948,0.551712211169638,0.872874075109684
"9886","TET3",11.8679477468878,0.340226676889226,0.571875884562338,0.5
9493097378919,0.551889612033348,0.872874075109684
"9887","MREG",10.8816748080997,0.401428609011272,0.674534786925375,0.5
95119209256857,0.551763789193659,0.872874075109684
"9888","HAUS6",91.7764316604299,-0.128404550092971,0.215821216759837,-
0.594957956500903,0.551871575027228,0.872874075109684
"9889","PEX12",27.8664996039935,0.21641779697809,0.363787031511055,0.5
94902451797581,0.551908678309441,0.872874075109684
"9890","AC245297.2",11.7181767783589,-0.278748062478119,0.468853246282
161,-0.594531582512208,0.552156625094905,0.873009819171423
"9891","BACH2",60.7329894463438,0.2342392170956,0.393897945308645,0.59
4669812029759,0.552064204566161,0.873009819171423
"9892","LRCH2",37.0203895821981,0.201536347823458,0.338987971330549,0.
594523596316455,0.552161964913281,0.873009819171423
"9893","NIF3L1",94.76524233352,-0.121095304530899,0.203720995262559,-0
.594417400989178,0.552232972815431,0.873033831480171
"9894","TOM1L2",94.7780794726688,0.135851000228226,0.228581843889111,0
.594321044562619,0.55229740577865,0.873047445560753
"9895","MTMR1",34.4507040574836,-0.169402588239956,0.285086890943017,-
0.594213882229389,0.552369068920574,0.87307248488305
"9896","AC016065.1",25.2692331674512,-0.238091376337379,0.400745962606
499,-0.594120461722945,0.552431546153001,0.873083001397831
"9897","CYP4Z1",7.56647794553416,0.366840766829313,0.620184928054913,0
.591502228181901,0.554183962641762,0.873210957233929
"9898","ATG4C",91.4799759675518,-0.130159095990569,0.219528446026493,-
0.592903099103891,0.553246003859233,0.873210957233929
"9899","GNG12-
AS1",7.04201198414734,-0.338980723081379,0.572388726041085,-0.59222117
3582387,0.553702492646775,0.873210957233929
"9900","CD46",593.525444571069,0.0912335309965052,0.153906866560851,0.
592784019551418,0.553325703787199,0.873210957233929
"9901","CHST10",32.8842274354084,-0.175332508712937,0.296355645685816,
-0.591628711196606,0.554099243478689,0.873210957233929
"9902","RND3",758.432212404511,-0.217000075062421,0.366456938739625,-0

.592157091659285,0.553745399303235,0.873210957233929
"9903","MAIP1",99.2288098180202,-0.159123093517561,0.268193322339401,-0.593314897364183,0.552970430720192,0.873210957233929
"9904","SLC35G2",63.7264510235204,-0.136864914049897,0.231274864007335,-0.591784648268377,0.55399480451836,0.873210957233929
"9905","AC010359.3",28.1270620362536,0.331102527739383,0.558436551116452,0.592909842805647,0.553241490468928,0.873210957233929
"9906","XRCC4",102.423086365088,-0.156321338848218,0.263811436715285,-0.592549514890538,0.553482674172813,0.873210957233929
"9907","PCDHGA11",4.13355309661394,-0.511118479829625,0.862213470110011,-0.592798068631902,0.553316300446836,0.873210957233929
"9908","KIFC1",11.4187491431754,-0.354404734374615,0.599485644367461,-0.591181353055686,0.554398915366807,0.873210957233929
"9909","GJA1",1172.57943340892,0.123853337129677,0.209009129494856,0.592573814498019,0.553466407731323,0.873210957233929
"9910","SMKR1",5.38019297511683,-0.444820551877975,0.752066518490765,-0.591464373085819,0.554209319468917,0.873210957233929
"9911","ABHD17B",46.9059012051022,0.176432320677207,0.297846827702424,0.592359240614371,0.55361005409595,0.873210957233929
"9912","SPACA9",35.902705305791,0.186968080657772,0.315239114066013,0.593099245351324,0.553114735470991,0.873210957233929
"9913","AL450384.2",5.18917144995642,0.409419849389509,0.69168640521499,0.591915420489222,0.553907227163977,0.873210957233929
"9914","ZNF33B",22.5241154702347,0.230255348875512,0.388122144873259,0.593254860401489,0.55301060293533,0.873210957233929
"9915","TYSND1",79.9513223823138,-0.143399252477872,0.241810956608684,-0.593022146262508,0.553166331237,0.873210957233929
"9916","PXMP2",120.457210419092,0.142871108464743,0.241269933517552,0.592162920517194,0.553741496469089,0.873210957233929
"9917","MRPS31",210.443469335083,0.0961150524867466,0.162249494587408,0.592390458479777,0.553589154178562,0.873210957233929
"9918","CIDEB",34.5177671193263,0.191034624589821,0.323102883724735,0.591250137998062,0.554352833225781,0.873210957233929
"9919","IGHG1",110.755711993422,0.61322654500865,1.03738919694092,0.591124861158132,0.554436763243144,0.873210957233929
"9920","MEF2A",426.92005029312,0.142878320872935,0.241563714477969,0.591472610783875,0.554203801487535,0.873210957233929
"9921","METTL9",686.644294369613,-0.1264775391362,0.213485554215439,-0.592440737271456,0.553555494054363,0.873210957233929
"9922","AC087393.2",3.82132938892501,0.577593611763255,0.976276471146142,0.591629142803333,0.55409895439649,0.873210957233929
"9923","CA4",4.35532619918655,-0.595161603903188,1.00537491488196,-0.591979763064873,0.553864139823721,0.873210957233929
"9924","EPB41L3",903.0225930938,0.128013323822497,0.216575185912607,0.591080290583953,0.55446662508249,0.873210957233929
"9925","TAF4B",29.3603166029838,-0.202101580776235,0.341526707077004,-0.591759228746546,0.554011828603062,0.873210957233929
"9926","GET3",512.435440444216,-0.163867451212082,0.276342585441996,-0.592986603747606,0.553190117569137,0.873210957233929
"9927","SYDE1",141.532569376347,0.12955425353204,0.218278095666101,0.5

93528421331923,0.552827568152838,0.873210957233929
"9928","RPS11",14019.8372261565,0.0980587631034228,0.165156322310803,0
.593733026573991,0.552690689826504,0.873210957233929
"9929","AC011468.1",8.23951546201815,0.343833824219155,0.5794505815793
56,0.593379030325586,0.552927519351825,0.873210957233929
"9930","NCAM2",43.3510208756273,0.265815111353987,0.448529341545951,0.
592637062355385,0.553424069974009,0.873210957233929
"9931","AIFM1",94.1871068587084,-0.116660455724416,0.19726416832007,-0
.591392023791817,0.554257783441336,0.873210957233929
"9932","RABGAP1L",360.70551795612,-0.153173685169886,0.259630809448474
,-0.589967290458586,0.555212579058593,0.873857777646814
"9933","AL354953.1",7.14384011241786,0.395389245078949,0.6700174738271
08,0.590117811137827,0.555111668555179,0.873857777646814
"9934","GOLPH3",357.572906347708,0.0885006852037357,0.149994953501895,
0.590024418405634,0.555174278881098,0.873857777646814
"9935","SLC13A4",2.83108878798639,0.615245507428105,1.04281329918977,0
.58998624960588,0.555199868171538,0.873857777646814
"9936","MGST1",3244.67288068265,0.167103747594219,0.283203529653221,0.
590048251866196,0.55515830064251,0.873857777646814
"9937","ADCY6",89.8906564443801,0.122182585391692,0.207068035325907,0.
590060098843295,0.555150358370194,0.873857777646814
"9938","MAP11",17.2133039827989,-0.227700945091412,0.386049832804555,-
0.589822675060423,0.555309539046101,0.873871083155616
"9939","IMP3",542.906936015893,-0.126034315369006,0.213694264547417,-0
.589788011559105,0.555332781041155,0.873871083155616
"9940","CEPT1",115.772161017264,0.124384925835986,0.211147445624068,0.
589090365115969,0.555800656338489,0.874052786635452
"9941","DTYMK",278.913873311922,-0.146548302648745,0.248746324940202,-
0.589147609252018,0.555762258419434,0.874052786635452
"9942","BRAT1",88.8824259464707,0.147026758464398,0.249445596695513,0.
589414126415176,0.555583502496119,0.874052786635452
"9943","TRMT10B",89.947267839831,0.140189691031731,0.237976847714629,0
.589089621019939,0.555801155467817,0.874052786635452
"9944","SELPLG",5.44792018577719,0.441568616488719,0.749623097910266,0
.58905417631832,0.555824931536981,0.874052786635452
"9945","CDC34",148.441377537648,-0.141906081957867,0.240751054547285,-
0.589430780374821,0.555572333438552,0.874052786635452
"9946","HSH2D",3.32585822104685,-0.703371321605442,1.19411286181958,-0
.589032531258103,0.55583945114298,0.874052786635452
"9947","ANKRD39",108.833062076317,-0.132391816724054,0.225038746460057
,-0.588306764086749,0.556326406241537,0.874115430341334
"9948","HDAC4-
AS1",17.626274450267,0.256639194209361,0.436230632166333,0.58831080461
9299,0.556323694661412,0.874115430341334
"9949","SFXN1",135.144127211464,0.136903000183358,0.232571090316618,0.
588650119827794,0.556096005012902,0.874115430341334
"9950","RUFY1",244.677869402821,-0.0924252850098251,0.157008029817664,
-0.588665975346359,0.556085366644643,0.874115430341334
"9951","RPL7A",16773.8140473069,-0.141304248922389,0.240166108070024,-
0.588360489570786,0.556290351878958,0.874115430341334

"9952","UBE2D1",183.579356490829,0.120586835741833,0.204963641858607,0.588332811850692,0.556308925837429,0.874115430341334
"9953","AC012184.3",4.37961708034006,0.450028604563252,0.76454366932201,0.588623806096431,0.556113660609454,0.874115430341334
"9954","POLR3H",97.009677373214,-0.156599718453868,0.266031124174086,-0.588651868987301,0.556094831397534,0.874115430341334
"9955","AC099791.2",6.48377164947579,-0.391553848644832,0.665915218807802,-0.587993542700281,0.556536627079758,0.874270073074268
"9956","CSTF3",114.935509635955,-0.130407910389582,0.2217794736086,-0.588007123777956,0.556527511236401,0.874270073074268
"9957","CCR7",6.67954769602862,0.540050355552846,0.918670733418916,0.587860629393297,0.556625844525999,0.87427267559003
"9958","GAMT",199.155319018188,0.194126620025899,0.330245869475936,0.587824520966628,0.556650083345621,0.87427267559003
"9959","SH3BP1",12.0359677189985,0.289765955775672,0.493028056421459,0.587727112081364,0.556715474426608,0.874287581085666
"9960","NACA",11099.6540662866,-0.149039220165621,0.253657967960124,-0.587559781244681,0.556827813208846,0.874288424715024
"9961","GRAMD4",43.5196748350808,0.151758587936509,0.258282132043384,0.587569053793541,0.556821587726189,0.874288424715024
"9962","EFNB2",12.1488388994996,-0.463293204649621,0.789075019422182,-0.587134547725105,0.557113346541022,0.874572446766725
"9963","ELAC1",76.7734514330934,-0.170183032249281,0.289858838823673,-0.58712383220719,0.557120542655811,0.874572446766725
"9964","TCTEX1D2",133.250178158239,0.166422863772185,0.283510184749428,0.587008413539969,0.557198056102943,0.874597469800461
"9965","LINC00345",4.96161478190406,-0.45952400970692,0.782923374363283,-0.586933568154905,0.557248323948951,0.874597469800461
"9966","CTNND1",305.310215774829,0.10883382872737,0.18553352718311,0.586599254483846,0.557472883497293,0.874629541478515
"9967","TBX3",97.7363050188181,-0.151424845587652,0.258122846510611,-0.586638678577515,0.557446399918169,0.874629541478515
"9968","ESCO1",157.439656971772,0.111063895492889,0.189281580399329,0.586765470039805,0.557361230477444,0.874629541478515
"9969","DMPK",47.261975230048,0.174306922630321,0.297162971316433,0.586570129710778,0.557492448785122,0.874629541478515
"9970","ESD",1386.3329755342,-0.131911146793572,0.224959394185122,-0.586377587259241,0.557621802355393,0.874744733083085
"9971","RB1CC1",789.911099596824,0.156474950550298,0.267154564256423,0.585709441221108,0.558070788444112,0.875116945785485
"9972","NELFB",224.242881792187,-0.109406675747375,0.186789992594753,-0.585720221022417,0.558063543152323,0.875116945785485
"9973","KDM6B",200.148992742445,-0.249164029166724,0.425325991286338,-0.585818958331615,0.557997182219511,0.875116945785485
"9974","OAZ1",6221.94860164409,-0.125063423933703,0.213531249651238,-0.585691434569742,0.558082891129439,0.875116945785485
"9975","PICALM",448.314559687939,0.111130281601717,0.189834510942015,0.585406104771233,0.558274684896202,0.875154462441274
"9976","C16orf54",3.83602007146342,-0.42826146696875,0.731368358014835,-0.58556192960164,0.558169938162775,0.875154462441274

"9977","CLTCL1",6.43555114062773,0.359342205086512,0.613784875459917,0
.585453013675602,0.558243151339248,0.875154462441274
"9978","BNC2-
AS1",28.1542677551714,-0.218613105568543,0.373529229083276,-0.58526371
8464734,0.558370406498234,0.875216792707193
"9979","AL031432.3",2.77418711405574,0.560805751927475,0.9590822970488
34,0.584731626944961,0.558728184613773,0.875390440551127
"9980","UROD",321.443937966765,-0.176016828618115,0.301031517837429,-0
.584712291532117,0.558741187834038,0.875390440551127
"9981","SNX24",186.408507788323,0.0933044984028694,0.159510723031204,0
.58494185613225,0.558586813288311,0.875390440551127
"9982","TRIM5",87.8853728804349,0.13296270220515,0.227409991718523,0.5
84682762618998,0.558761046548715,0.875390440551127
"9983","TLK2",168.997504224811,0.116857834486664,0.199780473274133,0.5
84931212603119,0.558593970248863,0.875390440551127
"9984","CMPK2",9.42436314686725,-0.283198359656327,0.485354533411819,-
0.583487616084623,0.559565091304227,0.875772843790465
"9985","SRGAP3",10.6998940481846,0.300767928367374,0.514874137017981,0
.584158159719081,0.559113908390532,0.875772843790465
"9986","NPHP3",270.104152684789,0.175043615639757,0.29983646526063,0.5
83796955742531,0.559356926649563,0.875772843790465
"9987","HERC6",23.0215510018477,0.222929998277419,0.381874678902335,0.
583777900431134,0.559369748498231,0.875772843790465
"9988","PEX16",142.455217386973,-0.150585090634703,0.257931368077466,-
0.583818446577918,0.559342466168069,0.875772843790465
"9989","CCP110",115.771695117336,0.139938763492134,0.239716558952717,0
.583767613315927,0.559376670503407,0.875772843790465
"9990","SH2B1",112.942320265907,0.122010829671126,0.209065408951971,0.
583601229312667,0.559488632923965,0.875772843790465
"9991","NT5M",7.3904569168488,-0.313834412214597,0.537425153707696,-0.
583959291911541,0.559247700432512,0.875772843790465
"9992","SRRD",94.4901640763709,-0.132647221322788,0.227292390555656,-0
.583597281890997,0.559491289338318,0.875772843790465
"9993","ZMAT5",163.350408096767,-0.161871256348765,0.277385133423408,-
0.583561398374153,0.559515437403819,0.875772843790465
"9994","TLR6",10.6059931421487,0.3667476325497,0.62865512643273,0.5833
84461733081,0.559634515556753,0.875793858645949
"9995","CAPS2",30.9818492237734,0.188135208098677,0.32253958312077,0.5
83293393878521,0.559695808908209,0.875802146205542
"9996","DHX33",71.3686183552333,0.131420275013402,0.225342229926642,0.
583203046566927,0.559756620514583,0.87580967835615
"9997","PLOD1",128.077407127081,0.155924713950076,0.267498467004573,0.
582899467410445,0.559960979187238,0.876041783984035
"9998","VCPKMT",38.4736102116229,-0.188450109423284,0.323387123233764,
-0.582738445300003,0.56006938821486,0.876123747917625
"9999","POFUT2",143.656305525957,-0.123739041638161,0.212374158066491,
-0.582646414068042,0.560131353320268,0.876133049897889
"10000","SUCLA2",331.823932708402,-0.113580045289175,0.19501691030482,
-0.582411264293155,0.560289695972948,0.876293084501691
"10001","MRFAP1",2032.80915003745,0.113225797744555,0.19445898131472,0

.582260572276196,0.56039117876189,0.87636416716688
"10002","POLR3C",96.1069957924051,0.121644687577088,0.209065127183027,
0.581850685555004,0.560667259981644,0.876602711154562
"10003","MUC1",8.64505355558512,-0.353836253310883,0.608425882872412,-
0.581560159210195,0.560862985313009,0.876602711154562
"10004","TRMT9B",108.892529167974,0.242901820168332,0.417553517922512,
0.581726197343184,0.560751122668321,0.876602711154562
"10005","JKAMP",269.331491611123,-0.127429978870369,0.218972684819491,
-0.581944633758383,0.560603974888475,0.876602711154562
"10006","SPNS1",226.484154422301,-0.128481487779595,0.22094525396109,-
0.581508249107815,0.560897960226744,0.876602711154562
"10007","GEMIN4",43.7228701218954,0.157708188326098,0.271231791725353,
0.58145170712801 8,0.560936057112203,0.876602711154562
"10008","COPZ2",1291.12295716928,-0.175690767670696,0.302154252417262,
-0.581460516491673,0.560930121452065,0.876602711154562
"10009","FAM98A",159.886722119589,0.130473699562431,0.224533238063034,
0.581088575963095,0.561180757672424,0.876897497252145
"10010","ARL6IP6",156.500587043581,-0.133136729247658,0.22918966031266
1,-0.580901987751247,0.561306512600875,0.87689945630498
"10011","TRAK2",159.122043439323,0.153561447066567,0.264306481570067,0
.580997659059897,0.561242031269438,0.87689945630498
"10012","SENP1",56.9676294234277,0.146128534247102,0.251582623946572,0
.580837149858709,0.561350214611602,0.87689945630498
"10013","NUDT15",85.256523029031,-0.121311609948257,0.208929809803137,
-0.58063332399796,0.561487607956689,0.877026484414523
"10014","UBE2D3-
AS1",46.6797795477578,-0.159415500750608,0.27466724100383,-0.580395026
971508,0.5616482579747,0.877189809738796
"10015","HSPB11",271.053294855961,-0.149759983254476,0.258186956911322
,-0.580044728231231,0.561884455263397,0.877295885027406
"10016","GLS",499.514446040993,0.154179286704644,0.265756996683746,0.5
80151373730791,0.561812541906462,0.877295885027406
"10017","AC234772.2",6.16996131232804,0.378508000586599,0.652540348983
901,0.580053020745752,0.561878863282762,0.877295885027406
"10018","AC015802.6",16.8536872173653,0.333188010011989,0.574736525496
076,0.579723047398809,0.562101398520889,0.877459414399312
"10019","OTUD5",173.588372224884,0.0985316298199831,0.169942612473693,
0.5797935455137,0.562053850756286,0.877459414399312
"10020","B3GALT6",92.3440857584822,-0.156367184013007,0.26989900473906
8,-0.579354429869719,0.562350045881139,0.877722928470781
"10021","LRP5",100.134598373633,-0.184067763820266,0.317829458017974,-
0.579140036194682,0.562494687472036,0.877722928470781
"10022","AC103703.1",10.7051646441036,-0.274489328013753,0.47392504408
0972,-0.579182998328433,0.562465701449477,0.877722928470781
"10023","SLC1A5",195.675156671217,-0.143979975071554,0.248542120173098
,-0.57929808827284,0.562388055235456,0.877722928470781
"10024","HIST1H2BC",8.79908907878072,0.408885148693263,0.7062303539226
31,0.578968528359314,0.562610408882481,0.877728358595711
"10025","HOXC-
AS1",50.2891770813124,0.216191142210815,0.373389198062557,0.5789967769

08889,0.562591347959824,0.877728358595711
"10026","HS3ST1",27.4533554347546,0.597834433213121,1.03315060506272,0
.57865177669506,0.562824160809183,0.877753753284033
"10027","C5orf15",551.121621848586,-0.112244442478022,0.19402369503477
5,-0.578508941693457,0.562920562251173,0.877753753284033
"10028","TTC9C",115.175744263896,0.142631133340643,0.246504975734879,0
.578613607759568,0.562849920804741,0.877753753284033
"10029","C1R",8297.28916823387,0.145936162341811,0.252120020204532,0.5
78836072690382,0.562699788505471,0.877753753284033
"10030","AC048341.1",35.4755884948419,-0.245539495725165,0.42448234608
9914,-0.57844454071396,0.562964030024468,0.877753753284033
"10031","IRF8",34.6853590388376,-0.227118089875393,0.392610243632662,-
0.578482333456107,0.562938521421903,0.877753753284033
"10032","SH3BGRL",1301.84618785214,-0.121581846343406,0.21021744833804
,-0.578362297252779,0.563019543027201,0.877753753284033
"10033","AL445524.1",29.7599939832308,-0.203825831922592,0.35321261356
9339,-0.577062721126687,0.563897086207338,0.877770883365965
"10034","MAML1",127.594116427204,0.106757078694181,0.184896355644474,0
.577388766382492,0.56367686113973,0.877770883365965
"10035","AL590428.1",7.62157192523266,-0.35529398722875,0.615419894155
954,-0.57731963266484,0.563723553580214,0.877770883365965
"10036","TAGAP",16.4050406454447,-0.249009558134613,0.431525270631125,
-0.577045135202453,0.563908965679142,0.877770883365965
"10037","UBR5-
AS1",10.5747891125397,0.283818170411788,0.491071997530056,0.5779563319
41768,0.563293601711944,0.877770883365965
"10038","UBAP1",213.900221270553,0.136531090432828,0.236507328305429,0
.577280591730795,0.563749922383669,0.877770883365965
"10039","PKN3",7.31530587647246,-0.319323136565484,0.552769612416753,-
0.57767852897951,0.563481177740204,0.877770883365965
"10040","TRAF2",42.3503392555819,-0.148565571193961,0.257269275663437,
-0.577471098368996,0.563621257110136,0.877770883365965
"10041","NPAT",88.530951916851,0.132985138035098,0.230503556248042,0.5
76933129361332,0.563984629600037,0.877770883365965
"10042","MT1M",1899.59083254653,-0.23612537707227,0.408696624635426,-0
.577752207478843,0.563431426163869,0.877770883365965
"10043","CYB5D1",29.8974859887006,-0.233262612817519,0.403434735965519
,-0.578191692540464,0.563134706861641,0.877770883365965
"10044","NR1D1",33.0449306798782,-0.601668858116893,1.04111250954142,-
0.577909546377373,0.563325189800606,0.877770883365965
"10045","TRAPPC8",116.451333860674,0.113570051718272,0.196631017298683
,0.577579535917055,0.563548026367483,0.877770883365965
"10046","AC009831.1",4.85133613348068,-0.441570747616448,0.76493425851
6624,-0.57726627183982,0.563759594391161,0.877770883365965
"10047","PLEKHJ1",421.660521680225,0.13751580342454,0.238175734997631,
0.577371172701146,0.563688743615089,0.877770883365965
"10048","AP000692.2",65.3712759455758,-0.283489014290081,0.49132096846
5986,-0.576993518463496,0.563943834010761,0.877770883365965
"10049","IDS",892.121092984061,-0.139192880499616,0.241198135970036,-0
.577089370694425,0.563879084381352,0.877770883365965

"10050","HEMK1",74.5075834298914,0.14704421144802,0.255007823719466,0.57662627484631,0.564191945760893,0.877993600567842
"10051","EXTL3",41.958252816978,0.171853947267609,0.298113223674475,0.576472070407936,0.56429614276905,0.877993600567842
"10052","TSPAN32",3.61137101994606,0.549049086002581,0.952355589988368,0.576516893242876,0.564265854713711,0.877993600567842
"10053","KCNAB2",31.0160720040958,0.18803513621557,0.326707591457581,0.575545659580988,0.564922319914521,0.878084059015829
"10054","AL031432.4",4.58498961704595,0.405964628175736,0.704636816528242,0.5761331492384,0.564525186703172,0.878084059015829
"10055","GUK1",3208.47626694952,-0.152389073239476,0.264644895887325,-0.575824720626227,0.564733662549906,0.878084059015829
"10056","RAB43",40.7721439327627,-0.148341989661524,0.257805287775763,-0.575403208139588,0.565018634947692,0.878084059015829
"10057","FAM50B",95.9882404291193,-0.159536770728283,0.277225005137259,-0.575477564331883,0.5649683598548,0.878084059015829
"10058","LUC7L2",394.9279782992,-0.0780573827590185,0.135551331359572,-0.575851096231277,0.564715833065206,0.878084059015829
"10059","AF117829.1",120.051926059771,0.175175730381936,0.304120241749074,0.576008125517904,0.564609689399289,0.878084059015829
"10060","PRPF18",219.664650163159,-0.0902165144254589,0.156792116430326,-0.575389352981585,0.565028003192794,0.878084059015829
"10061","PTPN20",10.5126221246247,-0.345731169260831,0.600452253279849,-0.575784614633961,0.564760774074409,0.878084059015829
"10062","ZFYVE27",59.0943846119257,0.149672518089402,0.260012287837129,0.575636326015316,0.564861022150475,0.878084059015829
"10063","MAN2C1",118.335291309369,0.134116153655194,0.232827607973991,0.576032004203626,0.564593549485199,0.878084059015829
"10064","TMEM35A",302.430681832192,0.234143560835731,0.406814387477385,0.575553785812818,0.564916825801099,0.878084059015829
"10065","RSRC1",440.436624230767,-0.0883943146912205,0.153708735625597,-0.575076714615174,0.565239415283805,0.878162591775747
"10066","MFGE8",1292.09500158847,-0.106520377879911,0.185207786694974,-0.575139845795706,0.565196721698902,0.878162591775747
"10067","SPIN2B",97.8231161993759,-0.171081189163258,0.297498601143251,-0.57506552469764,0.565246982826499,0.878162591775747
"10068","VPS8",98.3744960503145,-0.118830242465651,0.206819540990347,-0.574560033818069,0.565588888117063,0.878374774248795
"10069","AC016831.6",10.2856754174511,-0.342254984413899,0.595719468667641,-0.574523752227488,0.565613432178295,0.878374774248795
"10070","TCIM",71.8953360694337,-0.45910139786091,0.799044341772116,-0.574563104774283,0.565586810675471,0.878374774248795
"10071","GDF6",16.7322840445983,0.299499461202601,0.521101788277841,0.574742723858997,0.565465308268501,0.878374774248795
"10072","EXOC6",32.1818410343855,0.188910633766328,0.328855671491441,0.574448459135801,0.56566436868503,0.878374774248795
"10073","AL163051.1",4.15583616705428,0.419531788678811,0.730449222271881,0.574347642364463,0.565732575658562,0.878393475955516
"10074","PLAC8",7.62340941032385,0.450775695466293,0.785384624120429,0.573955335541649,0.565998026036241,0.878432315485996

"10075","MPLKIP",326.740878643394,0.123620311612828,0.215315017657095,
0.574136968976788,0.565875118207161,0.878432315485996
"10076","AC004854.2",10.1447374770974,-0.299212046096922,0.52112659470
864,-0.57416383875824,0.565856937034431,0.878432315485996
"10077","PPP3CC",90.8433655647248,-0.116853684726444,0.203644314782834
,-0.573812653945466,0.566094584896634,0.878432315485996
"10078","MLLT10",186.568357856277,0.106231860562088,0.185062823197136,
0.574031340962125,0.565946593080421,0.878432315485996
"10079","PRKAB1",49.1756510296932,0.141299868891758,0.246230800767866,
0.573851315315211,0.566068420280265,0.878432315485996
"10080","C4orf3",1806.47749553737,-0.121431722688004,0.211683914198806
,-0.573646434815827,0.56620708260231,0.878519719434537
"10081","VDR",14.0532395897981,0.35786073023671,0.624075695667783,0.57
3425199412366,0.566356832160681,0.878664899810838
"10082","PPP1R21",104.494337871957,0.123696229710352,0.215803971484227
,0.573187920776486,0.56651746215838,0.878697441072817
"10083","NUDT1",418.214318294451,-0.135182781133932,0.235895451662897,
-0.573062262035951,0.566602537929626,0.878697441072817
"10084","AP002433.1",9.16366764798828,-0.274759300636599,0.47936988093
799,-0.573167634351524,0.566531196428889,0.878697441072817
"10085","PDZD11",270.601204951386,-0.156190687539833,0.272549648433186
,-0.573072423456536,0.566595658031729,0.878697441072817
"10086","AC103691.1",5.00061887278198,0.426816246721608,0.745015406459
59,0.572895866341736,0.566715203603446,0.878785027201853
"10087","ALG1L9P",7.03221218472752,0.383297366104455,0.669286260154657
,0.5726957042505,0.566850746563019,0.878908067437853
"10088","WDR90",40.7850079437416,0.197562324032063,0.34507199638131,0.
572524940023685,0.566966394571762,0.878913114392146
"10089","ZBTB45",41.2773788063181,0.145371399466888,0.25390255768622,0
.572547991606063,0.566950782505996,0.878913114392146
"10090","THAP5",172.498660545906,0.143688478342356,0.251018530882533,0
.572421796260121,0.567036252916581,0.878934290943045
"10091","CREBZF",310.724994627354,0.11306575521763,0.197586827273861,0
.572233264624052,0.567163954360324,0.879045114081406
"10092","ST7L",89.9189205506226,-0.127315967195067,0.222608846199289,-
0.571926809598072,0.567371560322567,0.879057931969946
"10093","TRMT10C",203.189706560923,-0.111788091952513,0.19547379464659
1,-0.571882753668447,0.567401408717349,0.879057931969946
"10094","PRMT9",41.7470671961701,0.150579048436296,0.263377499627072,0
.571723281789476,0.567509459021774,0.879057931969946
"10095","OCLN",46.4772589109678,0.311056575889518,0.543901189417565,0.
571899054353258,0.567390364729828,0.879057931969946
"10096","PPP6C",197.26913648817,-0.106380047230564,0.18602372396789,-0
.571862797719964,0.567414929347523,0.879057931969946
"10097","AL358781.1",6.20681740071618,0.534599826297192,0.935014220646
675,0.571755824127949,0.567487409133104,0.879057931969946
"10098","TFE3",151.448437048015,0.11989399582199,0.209885137524438,0.5
71236235381505,0.567839518704739,0.879482082842357
"10099","DAAM1",375.475798784812,-0.122387764136101,0.214392647704578,
-0.570858028232128,0.56809588398147,0.879792021533834

"10100","ATMIN",247.785617601635,0.0834615344396031,0.146233866284879,
0.570740120328974,0.568175818413048,0.879828693067334
"10101","MECR",110.476111452664,-0.137522926481714,0.241158285740995,-
0.570260010180261,0.568501359614537,0.880043242893267
"10102","ZYG11B",305.326004742381,0.125511526591834,0.220168586080351,
0.570070094132448,0.568630157793346,0.880043242893267
"10103","LINC01686",10.5325363980813,0.332588157475525,0.5831374842798
26,0.570342614634474,0.568445342881078,0.880043242893267
"10104","CCZ1B",80.7458387311289,-0.277705115248009,0.487166534298213,
-0.570041445166295,0.568649588297576,0.880043242893267
"10105","ADPGK",198.454356763121,-0.0947919552928057,0.166178934900565
,-0.570421006426149,0.568392185329981,0.880043242893267
"10106","ZNF763",12.9480654503883,-0.23334031764777,0.409341750275275,
-0.570037914507506,0.568651982907887,0.880043242893267
"10107","SPHK2",46.3809413900431,0.147894750460052,0.259503080902325,0
.569915200797631,0.568735214425137,0.880054879434706
"10108","TPST2",45.4279669308044,0.2076923351255,0.364461454234789,0.5
69860907682441,0.568772041005499,0.880054879434706
"10109","FBXW11",252.406166343585,0.125272746756649,0.219899994125265,
0.569680537077634,0.568894393106063,0.880157118229185
"10110","CCDC142",40.0087252370898,0.214150950557297,0.376492116322136
,0.568805935830177,0.569487845951947,0.880315929178875
"10111","RNF123",40.2166371722943,0.160036362780237,0.281470123563365,
0.568573178404169,0.569645831184026,0.880315929178875
"10112","FBXO5",43.9536777979081,-0.174805910644597,0.307163470009524,
-0.569097329963021,0.569290090253261,0.880315929178875
"10113","PFKFB3",121.532016379407,0.29240826946224,0.514233935783932,0
.568628884860494,0.569608018253052,0.880315929178875
"10114","SUPV3L1",59.2959718030008,0.141208526545612,0.248109699738385
,0.569137469008696,0.569262852311792,0.880315929178875
"10115","KCTD21-
AS1",13.0861515875009,0.334910682653433,0.588769037560766,0.5688320228
9466,0.569470140533681,0.880315929178875
"10116","ADCK1",12.5042251417409,0.25613490932085,0.450301106944686,0.
568808082793172,0.56948638878774,0.880315929178875
"10117","AL357093.2",3.28115038162413,-0.558169039297139,0.98090958942
1364,-0.569032095635237,0.569334358921062,0.880315929178875
"10118","RANBP3",158.891667174204,-0.108523363198053,0.190804884617041
,-0.568766168727111,0.569514836584817,0.880315929178875
"10119","AC008569.2",5.37595015381834,0.454866908852681,0.800118992471
374,0.568499077178142,0.569696132138907,0.880315929178875
"10120","ZNF492",6.13775161448909,0.368638088633259,0.648072166589731,
0.56882259050423,0.569476542312879,0.880315929178875
"10121","CHEK2",61.8592507666252,0.153505498486398,0.270078861453205,0
.568372873243156,0.569781806023457,0.880315929178875
"10122","BRD1",201.482091964344,0.109656567045242,0.19269317596067,0.5
69073432406468,0.569306307183601,0.880315929178875
"10123","GPR173",18.4354108737853,0.238077242366561,0.418878616380382,
0.568368097717272,0.569785048022874,0.880315929178875
"10124","ZFHX4-

AS1",12.0727272134368,-0.284657894055226,0.500936845880534,-0.56825106
0779652,0.569864504574288,0.880351723779322
"10125","MORN1",14.7935206351181,0.268966131495532,0.473421465061834,0
.568132523227273,0.569944985281906,0.880372624931659
"10126","CCDC28B",135.433070459662,-0.194136534253613,0.34244790616489
2,-0.566908223874889,0.570776536521594,0.880372624931659
"10127","MEMO1",35.3311264370586,0.18444778977513,0.324799760729428,0.
567881544496528,0.570115404407551,0.880372624931659
"10128","STRN",269.357562904706,0.106341351448937,0.187478453216879,0.
567219057039686,0.570565362111724,0.880372624931659
"10129","TRIM38",292.937752914681,0.13472381195937,0.237582898348883,0
.5670602256966,0.570673264592579,0.880372624931659
"10130","HIST1H3D",3.57851743028801,0.666020840805984,1.17383407925051
,0.567389252517901,0.570449750165397,0.880372624931659
"10131","MCM7",75.8531918790783,0.140476623308702,0.247715963956404,0.
567087486268852,0.570654744361866,0.880372624931659
"10132","PRKAR2B",51.4668884853366,0.175159814907843,0.308778042422751
,0.567267716102785,0.570532307397323,0.880372624931659
"10133","RBIS",1024.02856662691,-0.131707382081524,0.232044128637887,-
0.567596270824236,0.570309139918763,0.880372624931659
"10134","GRHPR",654.626520525084,-0.130779223432398,0.230395827738656,
-0.567628436313283,0.570287294071117,0.880372624931659
"10135","LINC01504",46.0375481000247,0.182794819114503,0.3219171108372
57,0.567831944810519,0.570149086371824,0.880372624931659
"10136","PSAP",4246.86386252118,0.074724329673092,0.131774056451914,0.
567064046482928,0.570670668817482,0.880372624931659
"10137","LINC02823",8.50505390742313,-0.324195194805072,0.571106442808
453,-0.567661595990445,0.570264773417885,0.880372624931659
"10138","NMRAL1",120.519000180995,0.164562683934562,0.290282604830871,
0.566905082136914,0.570778671151344,0.880372624931659
"10139","FBXL12",153.174992687489,-0.135207754968858,0.238204909316333
,-0.567611118330493,0.570299055882629,0.880372624931659
"10140","IRF3",259.119964969826,-0.0908038947904744,0.160154990789634,
-0.566975117932769,0.57073108676976,0.880372624931659
"10141","EPHA4",37.6422472605274,0.253143656404065,0.446636536695096,0
.566777761347539,0.570865181483226,0.880419232659269
"10142","ADGRB2",6.54933157359285,-0.349260533053741,0.617020913649461
,-0.566043265840031,0.57136436913281,0.880731091557062
"10143","FBXO48",32.9223319061416,0.206248643468232,0.364297646210682,
0.566154202788765,0.57128895936833,0.880731091557062
"10144","RNF167",259.645041805524,-0.100697397772432,0.177850823535824
,-0.566190224877702,0.571264474250651,0.880731091557062
"10145","NAT9",93.8060509321907,-0.133586922987366,0.236016531169176,-
0.566006636592803,0.571389269026912,0.880731091557062
"10146","SMIM25",5.32647833702613,-0.397636727899721,0.702559363444069
,-0.565983102054802,0.571405267648945,0.880731091557062
"10147","PRDX4",1122.66267933009,-0.197884754380901,0.349578003746853,
-0.566067522155083,0.571347880416437,0.880731091557062
"10148","IDI1",234.227166444655,-0.107398509619761,0.189844688431646,-
0.565717748055037,0.571585668274694,0.880922334629111

"10149","MANEAL",6.32622325890148,0.333789312681567,0.59035479045179,0.565404597506734,0.571798598170614,0.880938448958718
"10150","ITPRIP",45.5692203281505,-0.20275091193734,0.358562364931817,-0.565455083318336,0.571764267280561,0.880938448958718
"10151","PAPLN",64.7188753593358,-0.207237360807827,0.366462649625734,-0.565507456270039,0.571728654150806,0.880938448958718
"10152","RARA-AS1",89.2993646122674,-0.208874253276323,0.36944633494281,-0.565371025560874,0.571821427994176,0.880938448958718
"10153","HIST3H2A",137.838938359171,0.191639996170059,0.339034084724723,0.565252889914181,0.571901766847389,0.880975439130619
"10154","RPS6KC1",111.047709568652,0.109154583945012,0.193255031626691,0.564821433244049,0.572195227108555,0.881082641919515
"10155","TAF1A-AS1",8.39650007778877,-0.405933881830817,0.718750296583511,-0.564777341672584,0.572225220531252,0.881082641919515
"10156","UBE3C",183.565307109427,0.115909058331921,0.205191080954796,0.564883511469274,0.572152999477275,0.881082641919515
"10157","MTG1",160.729130336146,-0.116542527108186,0.206268999782591,-0.565002628756734,0.572071976164334,0.881082641919515
"10158","ANG",410.192524155236,0.140107766346684,0.248124502491397,0.564667193041695,0.572300152728732,0.881082641919515
"10159","FES",98.5805566592316,0.129829318038157,0.229927353891928,0.564653643164096,0.572309370796698,0.881082641919515
"10160","AC253572.1",5.25072212115612,0.390744767984087,0.692866316600622,0.563954053793783,0.572785401695623,0.8810928102361
"10161","MRPS21",892.896920040881,-0.134511715336797,0.238496319917567,-0.563999123270704,0.572754728809327,0.8810928102361
"10162","YOD1",24.2677768742226,-0.240679289581551,0.426812903856715,-0.563898812352565,0.572822998368329,0.8810928102361
"10163","SPRTN",89.3638200408928,-0.107301415733647,0.190122853021598,-0.564379368541547,0.572495976728925,0.8810928102361
"10164","DGKD",55.2189814905889,0.217040311675173,0.384844496018736,0.563968859943383,0.572775325004038,0.8810928102361
"10165","ANKRD10",277.960089192121,-0.119508147345236,0.211729419043933,-0.564438082742006,0.572456027408723,0.8810928102361
"10166","TTYH2",8.89008199412258,-0.279044471611319,0.494442338803218,-0.564362008898221,0.572507788536505,0.8810928102361
"10167","ZSCAN22",16.0134477496328,0.226027247467685,0.400654685587923,0.564144775034919,0.572655608073501,0.8810928102361
"10168","ABHD12",212.632796153857,-0.113367169459102,0.200910822404693,-0.564266116191329,0.572573037711111,0.8810928102361
"10169","IL22RA1",6.03847354420642,-0.431432879347043,0.765555955688407,-0.563554990515472,0.573057025777586,0.881166701374514
"10170","PRKCI",149.733733405352,0.107463956815795,0.190736975357878,0.563414391017586,0.573152739966939,0.881166701374514
"10171","AGFG2",13.4788509562044,-0.250913665641497,0.445295621876729,-0.563476605909584,0.573110385765461,0.881166701374514
"10172","MIR1915HG",3.37403452150132,0.614402317973535,1.09046912062867,0.563429359301184,0.573142549832113,0.881166701374514

"10173","ZBP1",3.65996072188392,0.593290764574103,1.05264681790009,0.5
6361806684378,0.573014088546241,0.881166701374514
"10174","EMC1",137.569778379061,0.10915398048953,0.193932064817865,0.5
62846482308351,0.573539425341388,0.881203394270428
"10175","RBM34",22.7373901932102,-0.216147222331374,0.38452096863861,-
0.562120768333231,0.574033739212232,0.881203394270428
"10176","AC104794.5",6.96839968386274,-0.32028329869449,0.569374435546
118,-0.562517876987731,0.573763227048543,0.881203394270428
"10177","DCK",66.5475370794206,-0.170876407509756,0.303911068517968,-0
.562257927435945,0.573940298999003,0.881203394270428
"10178","SCLT1",85.1152244935526,-0.12791859279263,0.227656973699026,-
0.561891826611669,0.574189722767514,0.881203394270428
"10179","ANKRD55",11.6193919114589,0.353426491234758,0.628965772070618
,0.561916891075396,0.574172644761318,0.881203394270428
"10180","CWC27",225.448158797669,0.094025254395253,0.167188291236902,0
.562391383389529,0.573849388488145,0.881203394270428
"10181","PWWP2A",153.897764365235,0.108669904008384,0.193206630161688,
0.562454321145406,0.573806517507684,0.881203394270428
"10182","ANKRD6",39.8208128984968,0.172530120035744,0.306793160788031,
0.562366252209083,0.573866507386222,0.881203394270428
"10183","LINC01006",12.9948133472858,0.308725697406876,0.5485619576157
48,0.562790935683384,0.573577253318165,0.881203394270428
"10184","PHF20L1",398.868381476007,0.107543266272871,0.191395492882507
,0.561890275749019,0.574190779476338,0.881203394270428
"10185","TOLLIP",171.943036132316,0.13015642905728,0.231265468351899,0
.562800966287071,0.573570422258607,0.881203394270428
"10186","MAP3K11",43.9104416130645,-0.148562911175567,0.26422185172139
2,-0.562265801286636,0.57393493512393,0.881203394270428
"10187","STX2",312.074634510942,0.112069653994127,0.199375590619022,0.
562103182471701,0.574045720175167,0.881203394270428
"10188","STYX",60.3930329780796,-0.121700595081864,0.216508104032016,-
0.56210641918451,0.574043515046204,0.881203394270428
"10189","ZNF649",35.7378862210736,0.162617299140108,0.288785179428032,
0.56310818810781,0.57336121618873,0.881203394270428
"10190","ZNF419",17.7341079804741,-0.202482971219605,0.359782940093184
,-0.562792030014437,0.573576508053011,0.881203394270428
"10191","TMEM164",24.7514140961543,0.194474105093357,0.345949267625778
,0.562146312457944,0.574016336624309,0.881203394270428
"10192","MEIS1-
AS2",9.43301710672637,-0.323338052160714,0.576326229720302,-0.56103303
2138125,0.574775019074932,0.881386013383121
"10193","AC159540.2",11.6023157100859,-0.338557718430157,0.60307868155
1137,-0.561382334987163,0.574536923797232,0.881386013383121
"10194","SPATA5",36.6772327078668,0.161358530049557,0.287767184616542,
0.560725957216323,0.574984369077275,0.881386013383121
"10195","RPP40",25.052966965367,-0.234183669564782,0.41710813662724,-0
.561445939315412,0.574493574206906,0.881386013383121
"10196","AC003986.2",3.87662155250351,-0.465609965557549,0.83049593761
2139,-0.560640870678166,0.575042383667607,0.881386013383121
"10197","C8orf31",26.7975097831236,-0.180684531270964,0.32220695406592

2,-0.560771668615186,0.574953202797728,0.881386013383121
"10198","TMEM30B",57.6757910758457,0.220322397746938,0.392608867289688
,0.561175297103954,0.574678041382181,0.881386013383121
"10199","ERVK13-1",32.4236538239394,0.220332687061855,0.39232646923282
4,0.561605459587534,0.574384859823964,0.881386013383121
"10200","VAC14",57.4150713659348,0.145335073952185,0.258916379033094,0
.561320510100323,0.574579062091109,0.881386013383121
"10201","GNA11",466.625691518151,0.158452429199594,0.282487772560117,0
.560917832880266,0.574853552570984,0.881386013383121
"10202","ZNF773",25.9946944362535,0.175576473620498,0.313130656043295,
0.560713140767097,0.574993107545226,0.881386013383121
"10203","URB1",57.7749722361159,0.150327732388891,0.267871208114964,0.
561194065785428,0.574665247921889,0.881386013383121
"10204","MOSPD1",39.8726293459642,0.169477266622691,0.302224707147888,
0.560765756784273,0.574957233471115,0.881386013383121
"10205","AC135507.1",11.0449025789584,0.252548056380017,0.450555091808
022,0.560526472726284,0.575120388033346,0.881419193419063
"10206","FBLN7",97.8105455627212,0.247986287329742,0.442719904374116,0
.560142620378287,0.575382161424262,0.881733980469867
"10207","AC004067.1",5.10728432453411,0.472099202940888,0.843849857834
042,0.559458769303643,0.57584866253057,0.881888755263449
"10208","APH1B",215.1945228827,0.122740881624513,0.219371378127428,0.5
59511831817985,0.575812458607652,0.881888755263449
"10209","THUMPD1",385.521977170216,0.0826373495428463,0.14770973035213
2,0.559457723914623,0.575849375797871,0.881888755263449
"10210","ARL17A",42.5533328058175,0.193828212966088,0.346483162915135,
0.559415965079845,0.575877868126957,0.881888755263449
"10211","ALKBH6",46.996092995469,-0.140137146737333,0.250438228813724,
-0.559567712170522,0.575774333269204,0.881888755263449
"10212","SRSF6",466.396992326,0.112673307074775,0.201330802766271,0.55
96426653381,0.575723197044225,0.881888755263449
"10213","SERINC3",939.112547835901,0.0941618798644439,0.16828738360607
,0.559530238374015,0.575799900284312,0.881888755263449
"10214","SPDYA",3.8056706386647,-0.459033342049752,0.820734287965302,-
0.559295924126371,0.575959776569907,0.881927834888717
"10215","ATP7B",6.82179634510042,0.330532756671877,0.591082258539333,0
.559199251705983,0.576025743784406,0.881942499538729
"10216","MAP3K6",106.931609456892,0.161627988706869,0.289359485862989,
0.558571592097034,0.576454132147353,0.882179399434367
"10217","CCNYL1",65.3796675821836,-0.16799764997239,0.301339482649044,
-0.557502948155153,0.577183845359459,0.882179399434367
"10218","MAPK10",58.50452586913,0.243175749850165,0.435415396677408,0.
55849138938541,0.576508882688858,0.882179399434367
"10219","AC104596.1",2.90709670938305,-0.512196917774007,0.91845958041
064,-0.557669524820031,0.577070071465317,0.882179399434367
"10220","NFKBIL1",227.710960447106,-0.165961331155325,0.29726496320868
6,-0.55829428858327,0.576643444364486,0.882179399434367
"10221","VEGFA",773.323594583239,0.179647456819235,0.322254480861407,0
.557470780046335,0.577205817787531,0.882179399434367
"10222","YTHDF3-

AS1",23.0005345230728,-0.224407427872395,0.401892025398831,-0.55837740
9080703,0.576586695791047,0.882179399434367
"10223","ACSL5",49.9121394188105,-0.225439976854278,0.404082623085319,
-0.557905645961615,0.576908815953699,0.882179399434367
"10224","MDK",143.79254178407,0.248119409634241,0.444735026135696,0.55
7903909188695,0.576910001980057,0.882179399434367
"10225","AIP",540.595353109003,-0.132461179935081,0.23706309124456,-0.
558759186171377,0.57632608025545,0.882179399434367
"10226","SC5D",111.266347089419,0.12980036061048,0.232846179839241,0.5
57451106563549,0.577219255954953,0.882179399434367
"10227","MYCBP2-
AS1",4.78327581350541,-0.465311457194226,0.834132485091766,-0.55783879
1211969,0.576954471280652,0.882179399434367
"10228","WDR20",116.193816609166,-0.102008520285509,0.182782198211503,
-0.558087829578851,0.57678441080955,0.882179399434367
"10229","SNAP23",276.839136568992,-0.0862700347774625,0.1546753778721,
-0.557748983479443,0.577015803954515,0.882179399434367
"10230","PPCDC",58.0745475639086,0.16614280412731,0.298065903057915,0.
557402918021884,0.577252172238575,0.882179399434367
"10231","ZNF263",77.5044636554364,-0.122884086764738,0.219901173625731
,-0.558815056502995,0.576287945708219,0.882179399434367
"10232","CAMTA2",95.9558730268185,-0.123254111978364,0.221088415971041
,-0.557487878489791,0.577194138645955,0.882179399434367
"10233","AL035071.1",38.8236539987021,-0.181607438475982,0.32548001935
6118,-0.557968009327416,0.576866229342538,0.882179399434367
"10234","CHKB",196.94832748338,-0.121801820147542,0.21835963224016,-0.
557803742834573,0.576978406612392,0.882179399434367
"10235","UCN",6.4280760815548,0.355573609073377,0.638098526498849,0.55
7239351459336,0.577363906707227,0.882263947327897
"10236","FOXP1",1056.07707428522,0.16048341135368,0.288193333829446,0.
556860248018974,0.57762291640421,0.88257350650272
"10237","TFAM",241.648173348834,-0.091913264802361,0.165132171957766,-
0.556604226255006,0.577797865615378,0.882754578316354
"10238","AC104506.1",14.4065706440441,-0.243744791230549,0.43868920888
8983,-0.555620667870662,0.578470199515659,0.882892566207802
"10239","RNF115",535.224787625634,-0.0789023487468777,0.14261717747896
7,-0.553245760024342,0.580095136482524,0.882892566207802
"10240","TSEN15",82.4591603143594,-0.156369986920863,0.282201017052772
,-0.554108516524665,0.579504581976942,0.882892566207802
"10241","KIF21B",4.69435688023944,0.514707303373962,0.93142520427532,0
.552601863264395,0.58053606583636,0.882892566207802
"10242","EXOC8",53.9679395258439,-0.141650411088338,0.255254337118257,
-0.554938312459357,0.578936855170965,0.882892566207802
"10243","REL",96.9986610825084,-0.146909082308783,0.265830933964751,-0
.552641034351039,0.580509237670422,0.882892566207802
"10244","CEP68",190.998327482143,0.0965044671901355,0.17428695666522,0
.553710208937244,0.579777187394094,0.882892566207802
"10245","REV1",235.898846777235,0.117589776228201,0.212426117265796,0.
553556115141284,0.579882666753602,0.882892566207802
"10246","MCM6",99.1748988373216,0.109921292323179,0.198495053605794,0.

553773458463501,0.57973389481332,0.882892566207802
"10247","KYNU",11.7856753563029,-0.335640330232682,0.605387069265586,-0.554422694623884,0.579289598003369,0.882892566207802
"10248","PRRT3-AS1",7.8494859849111,-0.335375168298809,0.603995323113916,-0.555261200649324,0.578716013311645,0.882892566207802
"10249","CNBP",1708.67038630084,-0.0911104441199913,0.164426341459853,-0.554110997733519,0.579502884003039,0.882892566207802
"10250","EIF5A2",94.5625690342689,-0.107105407734864,0.193217238876404,-0.554326354924142,0.579355516796331,0.882892566207802
"10251","RNF168",214.564266292292,-0.130644627454008,0.235310958430454,-0.555199929172105,0.578757917366364,0.882892566207802
"10252","SMAD5-AS1",17.9693339471247,-0.234247802206617,0.422238498463074,-0.554776040221975,0.579047857458109,0.882892566207802
"10253","AL135905.2",3.17968681008662,0.49538228171251,0.895281662424307,0.553325620867827,0.580040460103789,0.882892566207802
"10254","COL12A1",1815.55407142991,-0.170316834438296,0.306444972030589,-0.555782766836505,0.578359367742314,0.882892566207802
"10255","BVES",11.4498872060211,-0.257341007116991,0.463583630898674,-0.555112368006018,0.578817803624334,0.882892566207802
"10256","CRYBG1",43.8947757833383,-0.225184212093336,0.407482328442343,-0.552623258422356,0.580521412281494,0.882892566207802
"10257","AC007036.2",3.75473574763139,0.480791527950949,0.868659256370718,0.553486910344695,0.579930041333215,0.882892566207802
"10258","SPDYE16",2.83848036987637,0.501089759806387,0.905946112213923,0.553112103524391,0.580186649227971,0.882892566207802
"10259","ARPC1A",711.712351940643,-0.128635096476956,0.232413393699337,-0.553475401866749,0.579937919710298,0.882892566207802
"10260","NDUFA5",686.949762537832,-0.122610238664436,0.22160967094946,-0.553271155266498,0.580077749478593,0.882892566207802
"10261","LEPROTL1",200.985118199032,-0.121181106980398,0.217818922507904,-0.556338749568466,0.577979302058017,0.882892566207802
"10262","MED30",237.454349233064,-0.134513066912517,0.24183674121982,-0.556214354502279,0.578064327400965,0.882892566207802
"10263","AK3",748.889428356542,0.0936744336787136,0.168544948005789,0.555783099921193,0.578359140012913,0.882892566207802
"10264","CD72",19.5090281606328,0.344795264761618,0.62310964403309,0.553346057252338,0.580026468810354,0.882892566207802
"10265","OMD",498.782478252182,-0.126462258360282,0.227693451552641,-0.555405776924794,0.578617142088104,0.882892566207802
"10266","ZMIZ1",466.758989150519,0.14666096246551,0.264827726840975,0.553797611054442,0.579717363421668,0.882892566207802
"10267","AL157893.2",10.0821847380522,-0.273487644673011,0.494915962825437,-0.55259410731408,0.58054137793357,0.882892566207802
"10268","IFITM2",2023.14977212196,-0.109848173048339,0.198349899344511,-0.553810077097872,0.579708831046933,0.882892566207802
"10269","ESRRA",220.308188191974,-0.108183991895631,0.194853625848848,-0.555206460358875,0.578753450567467,0.882892566207802
"10270","SIK2",126.82137457519,0.13806353211462,0.249494621143589,0.55

3372780069524,0.580008173896295,0.882892566207802
"10271","DDX6",479.434626188217,0.0996444347244342,0.179939877809916,0
.553765157213766,0.579739576706583,0.882892566207802
"10272","CLEC12A",11.8452277184992,0.420856294428643,0.756820349730629
,0.556084802130964,0.578152884070537,0.882892566207802
"10273","AC011603.2",13.4640294214745,0.264940185671407,0.478936920097
01,0.55318388404415,0.580137501263929,0.882892566207802
"10274","INTS6-
AS1",20.7802018988631,0.195383413868743,0.352748566272041,0.5538886123
15613,0.57965507902645,0.882892566207802
"10275","MIA2",115.676839997142,-0.125065771307682,0.226195238061821,-
0.552910717216336,0.580324548406851,0.882892566207802
"10276","SAMD15",3.1535784552847,-0.478456821678209,0.863220444500522,
-0.554269566628551,0.579394374866188,0.882892566207802
"10277","WDR25",43.2603801682043,-0.159826198935816,0.287972152621291,
-0.555005744413078,0.578890731298012,0.882892566207802
"10278","AC015871.4",4.44688352267761,-0.447935340279329,0.80721963988
4073,-0.554911350204089,0.578955298003196,0.882892566207802
"10279","AC008870.2",3.94027441284148,0.478965939155379,0.864846525847
666,0.553816110535829,0.579704701485684,0.882892566207802
"10280","TERF2",125.027673734388,0.140565350552905,0.254195159021835,0
.552982012300361,0.580275727375841,0.882892566207802
"10281","COX10",35.6106415052396,-0.155740903083747,0.281206340471438,
-0.553831406584398,0.57969423223137,0.882892566207802
"10282","ARL4D",789.248955100799,0.136918979652458,0.247244615010932,0
.553779420621168,0.579729813956729,0.882892566207802
"10283","DMAC2",122.596827522653,-0.100858017114719,0.182516127978159,
-0.552597834678965,0.580538825036008,0.882892566207802
"10284","PRMT1",486.734035776036,-0.146509657230947,0.264702821923694,
-0.553487326527943,0.579929756426927,0.882892566207802
"10285","C12orf81",39.4641882013231,0.258584332573268,0.46805108394968
,0.55247032095554,0.580626163039331,0.882919177170452
"10286","AC015871.7",9.38571050345261,0.330784002322997,0.598808424214
852,0.552403722036335,0.580671781098163,0.882919177170452
"10287","FKBP9",1184.62191914344,0.130375543448482,0.236123272332458,0
.552150333004511,0.580845359550292,0.882925592707413
"10288","AL135999.1",10.6636096365761,-0.295450331782045,0.53502356273
0707,-0.552219289696507,0.580798119915066,0.882925592707413
"10289","ZDHHC15",24.9314765595271,0.176716081763225,0.319964700059752
,0.552298680855181,0.580743734247021,0.882925592707413
"10290","LRP4",15.7304292282259,0.27066120518872,0.490318022048001,0.5
52011537447063,0.580940448618075,0.882984316461292
"10291","INSIG1",238.877859882628,-0.153273868820667,0.27775339204066,
-0.551834372550992,0.581061835168769,0.882997192191949
"10292","RPL23A",13987.6799364805,-0.115821748951166,0.209871996752841
,-0.551868523400794,0.581038435400784,0.882997192191949
"10293","HSPA1A",1329.40933391218,-0.156035970955047,0.282857137732696
,-0.551642331552202,0.5811934276556,0.883046613528189
"10294","BBC3",69.5662252240282,-0.14827735449564,0.268802409100786,-0
.551622118981991,0.581207278750587,0.883046613528189

"10295","TADA2B",80.7139204727602,0.129603085089981,0.235213999438735,
0.551000728694883,0.581633175037767,0.883607854064175
"10296","SLC2A12",5.15176359905555,-0.382304303214867,0.69399962270317
2,-0.55087105339592,0.581722071922059,0.883657071179196
"10297","GFM2",46.7048965221309,0.153662929794646,0.279057996929309,0.
550648723510947,0.581874501493966,0.883802777834868
"10298","POU6F1",28.2601684329262,0.194273756643968,0.35293995443655,0
.550444216365686,0.582014728257725,0.8839299232813
"10299","MORC3",323.920561417034,0.0963070259402767,0.175009185069073,
0.550296979568622,0.582115695578145,0.883997424880299
"10300","AC105429.1",11.2325092749952,0.357143091871672,0.649224882928
952,0.550106906347204,0.582246049982839,0.884109536090447
"10301","ANKRD17",461.296102678324,0.112712335557007,0.204930865711835
,0.550001753837795,0.582318170641484,0.88413320928383
"10302","PDZD8",268.040275430443,0.132875608043892,0.241679927509986,0
.549799933378424,0.582456604362774,0.884257551177809
"10303","SLC6A6",47.8931458635518,-0.190662680251557,0.347004214127376
,-0.549453500819935,0.58269426696807,0.884360828275654
"10304","BRD4",583.517617479286,0.109253740854301,0.198795598988495,0.
549578267377155,0.582608668341138,0.884360828275654
"10305","MAGED1",256.524178559114,-0.154976127186255,0.282038283826674
,-0.549486137426277,0.58267187539561,0.884360828275654
"10306","TTLL7-
IT1",3.09588045780777,0.546725789097567,0.995659123525729,0.5491094051
96385,0.582930371155739,0.884380038178729
"10307","EIF4EBP3",253.131045722954,0.189550351186913,0.34525028167207
1,0.54902301677759,0.582989654323702,0.884380038178729
"10308","AC139795.3",8.05939430312077,0.452111842925386,0.823107240783
36,0.549274530126969,0.582817063687628,0.884380038178729
"10309","OS9",1080.28719083159,-0.0932564174408091,0.169855886706521,-
0.549032590209479,0.582983084515538,0.884380038178729
"10310","IQCH",4.45994319825544,0.377364931459194,0.687131807306012,0.
549188565347748,0.582876050781323,0.884380038178729
"10311","PPIC",3379.54424507169,-0.178268044854493,0.324823221172717,-
0.548815581013228,0.583132016474708,0.88451020634899
"10312","PCGF1",66.7625485556115,-0.148535132228016,0.270723626502937,
-0.548659657624691,0.583239036604613,0.884586746750984
"10313","SRR",46.993271314526,-0.198633072144316,0.362179831478713,-0.
548437695531896,0.583391399087056,0.884732035462188
"10314","GTF2IRD1",14.0199529223938,-0.254403007308492,0.4639861828484
92,-0.548298670763572,0.583486839959715,0.884786823749027
"10315","ZNF48",20.3198283803997,-0.223175676823817,0.407091258777341,
-0.548220262685333,0.583540670522456,0.884786823749027
"10316","GLB1L",48.0270205660955,0.17804092762971,0.324920198674121,0.
547952784579812,0.583724323308223,0.884823135038981
"10317","AC010210.1",3.16003700145845,0.513883105914408,0.937670766524
868,0.54804215323778,0.58366295901804,0.884823135038981
"10318","HDAC2",292.79388935773,0.0877341090964869,0.160140876724658,0
.547855806030927,0.583790916270284,0.884823135038981
"10319","PSTPIP1",5.89881706775908,-0.400288163480344,0.73053598573508

6,-0.547937639345121,0.583734722962992,0.884823135038981
"10320","SLC25A22",17.1762051933393,-0.214939126709529,0.392419701114021,-0.547727665301585,0.583878913018526,0.884870755776139
"10321","TMEM35B",912.42936197779,0.123956833226209,0.227511121184989,0.544838566926231,0.585864550981605,0.884949326849129
"10322","NBPF26",87.1863707568107,0.235739223837927,0.434295434400674,0.54280843215229,0.587261706935699,0.884949326849129
"10323","ARHGEF11",100.499683719473,-0.133708857294981,0.244529788422676,-0.546799873166626,0.584516230237679,0.884949326849129
"10324","AL592435.1",7.64649913945159,-0.326591389095424,0.600199152989493,-0.544138370520395,0.586346257796992,0.884949326849129
"10325","RNPEP",266.329746534499,-0.105938000628493,0.194272240144207,-0.545306939117273,0.585542432398668,0.884949326849129
"10326","AL359921.2",3.37024616951152,0.430501448528073,0.79047967954237,0.544607862377058,0.586023246095617,0.884949326849129
"10327","GEN1",24.9121826960095,-0.227939088031243,0.417479965075138,-0.545988088291179,0.585074125258723,0.884949326849129
"10328","PLEKHB2",206.736010725603,0.117359152573145,0.215473601668262,0.544656754537516,0.585989612900225,0.884949326849129
"10329","ZC3H15",617.623685046373,-0.0812709692492495,0.149634859094345,-0.543128584750484,0.587041272789135,0.884949326849129
"10330","ANKMY1",27.5349549610924,0.201557047813509,0.371403065862216,0.54269085621464,0.587342670923562,0.884949326849129
"10331","H1FX",1475.40870964079,0.130368478377476,0.238732319870444,0.546086422015354,0.58500653273728,0.884949326849129
"10332","EVC2",137.588963071955,0.175723105586057,0.32146442753963,0.546633128060161,0.584630804766543,0.884949326849129
"10333","SLC30A9",170.34191052676,0.10854397403251,0.199933973277328,0.542899099403928,0.587199276071535,0.884949326849129
"10334","NUDT9",179.4075987986,-0.125566959507356,0.230664920345261,-0.544369552680167,0.586187193512001,0.884949326849129
"10335","SUB1",1457.04330997078,-0.133693447285252,0.244582258801474,-0.546619562434289,0.584640126490173,0.884949326849129
"10336","CKMT2-AS1",35.1580499025963,0.144037761782986,0.265356829544385,0.542807818552463,0.587262129453376,0.884949326849129
"10337","ETF1",299.786325602205,-0.0856676705262707,0.157756057097792,-0.543038867110922,0.587103042064645,0.884949326849129
"10338","N4BP3",3.13222520966154,0.502268453129427,0.924886299189758,0.543059675085939,0.587088715811771,0.884949326849129
"10339","AC034213.1",22.8615528262348,0.224784312546759,0.413557648121663,0.543538037726315,0.58675940865459,0.884949326849129
"10340","LINC01011",20.0013211327079,0.278677599159024,0.51113205882066,0.545216435459008,0.585604669030712,0.884949326849129
"10341","TCF19",32.6533703892758,0.149579061000532,0.274935290575293,0.54405187739829,0.586405774309353,0.884949326849129
"10342","SRPK1",150.161947677867,-0.0953000475040695,0.174985204544447,-0.544617744981194,0.58601644772378,0.884949326849129
"10343","PTK7",63.55624187097,-0.179320395329885,0.330532759451648,-0.54251928198396,0.587460827955737,0.884949326849129

"10344","CGAS",45.8623142413903,0.151500794319683,0.277579292407886,0.
545792854378568,0.585208335674373,0.884949326849129
"10345","HDDC2",433.075995081401,-0.12632899391696,0.231358778280153,-
0.546030692485712,0.585044839591246,0.884949326849129
"10346","MRPL32",306.627705065391,-0.12231826134676,0.225108424463428,
-0.543374872079177,0.586871723058049,0.884949326849129
"10347","SEMA3A",45.0583495139022,0.534070719654262,0.982476128081969,
0.543596637504971,0.586719074168335,0.884949326849129
"10348","ASNS",8.62857148584284,-0.299953583022532,0.551855846735416,-
0.543536115086849,0.586760732037587,0.884949326849129
"10349","MCPH1",117.714333644202,0.120381482212605,0.220769000770942,0
.545282543256634,0.585559208392768,0.884949326849129
"10350","COMMD5",233.703337368721,-0.12838425546257,0.234657360358171,
-0.547113694906522,0.584300624187641,0.884949326849129
"10351","APTX",136.528748434364,-0.100234977661282,0.183057065718476,-
0.547561369826791,0.583993120586268,0.884949326849129
"10352","TESK1",234.236407976542,0.116284739425162,0.213755461891434,0
.544008271864526,0.586435780639063,0.884949326849129
"10353","ZMYND19",62.9186431658912,-0.121522859077113,0.22296124981688
,-0.545040266759005,0.585725823731216,0.884949326849129
"10354","TAF3",178.968864688347,-0.108094042461721,0.198990980404503,-
0.543210763834575,0.586984696339307,0.884949326849129
"10355","RPP38",96.6611486530334,-0.116759019043997,0.21439167975341,-
0.54460611147919,0.586024450564834,0.884949326849129
"10356","BUB3",334.055373758477,-0.0831525475270646,0.152064920104322,
-0.546822682509675,0.58450055820767,0.884949326849129
"10357","ECHS1",632.11269269482,-0.13447820854097,0.247798949893264,-0
.542690792672423,0.587342714680776,0.884949326849129
"10358","SVIP",9.12878237183647,-0.34355615712223,0.63177020680103,-0.
543799238115116,0.586579633174886,0.884949326849129
"10359","PACSIN3",12.0462145564191,0.352019335521926,0.643207427820772
,0.547287422837435,0.584181283194894,0.884949326849129
"10360","LINC02739",3.90056990029895,0.451680344473546,0.8327444632308
64,0.542399696926385,0.587543188409786,0.884949326849129
"10361","SSSCA1-
AS1",8.97135807174283,-0.305535904476305,0.558996615614775,-0.54657916
692444,0.584667884983272,0.884949326849129
"10362","SPSB2",71.7836812426331,-0.184974073486968,0.338618296130437,
-0.546261308383985,0.584886328515811,0.884949326849129
"10363","LINC02356",4.58167402188883,0.399883061181013,0.7309008335213
51,0.547109871601113,0.584303250703855,0.884949326849129
"10364","POP5",185.210041678858,-0.123093902648022,0.226604570417779,-
0.543210149826544,0.586985119045709,0.884949326849129
"10365","CLBA1",18.7809003881271,-0.205155267943833,0.376458171804688,
-0.544961653934477,0.585779891080144,0.884949326849129
"10366","APBA2",3.22349132898556,0.539549259784334,0.992461759428858,0
.543647404706891,0.586684131917908,0.884949326849129
"10367","AC124068.2",5.60150623716228,-0.3271954746012,0.6032128212188
44,-0.542421286636569,0.587528318779496,0.884949326849129
"10368","JPT2",45.2569783309494,0.19067031335352,0.351287797282967,0.5

42775225408505,0.587284572914042,0.884949326849129
"10369","POLR2C",278.974522915579,-0.124708224891688,0.229291883556407
,-0.543884166144105,0.586521185557214,0.884949326849129
"10370","FBXL8",45.0823065293677,-0.193586229182263,0.35416916236074,
-0.546592560346139,0.584658681387407,0.884949326849129
"10371","ST3GAL2",123.918011433193,-0.125317361892474,0.22907039334323
7,-0.547069222100209,0.584331176244886,0.884949326849129
"10372","FLII",410.405888359195,-0.0913982365611961,0.167434444828486,
-0.545874755070986,0.585152032617984,0.884949326849129
"10373","TRAF4",15.6717876677296,-0.222491043337196,0.408975424647748,
-0.544020569277063,0.586427318333347,0.884949326849129
"10374","AC090409.1",4.84819412632883,-0.40332172088932,0.741384853889
125,-0.544011276698725,0.586433712895966,0.884949326849129
"10375","CFD",166284.411492254,0.143832294272199,0.263652983861068,0.5
45536379546501,0.585384666910118,0.884949326849129
"10376","WDR18",148.549852316917,-0.136144192297042,0.24917778304192,-
0.546373720140763,0.58480907087945,0.884949326849129
"10377","URI1",499.493704929933,-0.0818534248143035,0.150775481878827,
-0.542882859960522,0.587210457859728,0.884949326849129
"10378","C5AR2",17.3033999979464,-0.436202115573095,0.802456766023788,
-0.543583323167051,0.586728238373089,0.884949326849129
"10379","FTL",33279.1222245548,0.172574247812166,0.318175572542761,0.5
42386854003298,0.587552033887554,0.884949326849129
"10380","ZNF765",69.6588636638884,0.148882663907371,0.274320494424558,
0.542732558935061,0.58731395341437,0.884949326849129
"10381","GMEB2",37.8111292148368,0.16236294003723,0.297059620389734,0.
546566846830997,0.584676351076586,0.884949326849129
"10382","JOSD1",239.942965557903,-0.0984644303751927,0.180955276487017
,-0.544136829202974,0.586347318363602,0.884949326849129
"10383","GPR82",14.0206182614787,0.281321119799539,0.515418255507149,0
.545811322733867,0.585195639287809,0.884949326849129
"10384","SLC25A5-
AS1",7.25261023240858,0.334656392816887,0.611638068816254,0.5471477494
27322,0.584277229816896,0.884949326849129
"10385","CXorf38",100.912740873086,0.121239846301055,0.223601953482297
,0.54221282244144,0.587671903041244,0.88504463780116
"10386","LAT2",3.22704767390289,-0.445491901190677,0.82194571982554,-0
.541996740715717,0.587820751174284,0.885098348740329
"10387","NUP214",136.312271932731,0.120704569998982,0.222672225859411,
0.54207285858449,0.587768315307964,0.885098348740329
"10388","FLNA",1406.93683253006,-0.124728299339039,0.230173788649164,-
0.541887501922091,0.587896007071493,0.885126448844643
"10389","TATDN3",88.376333567018,-0.135854356958677,0.251012623420034,
-0.541225198588296,0.588352371033446,0.885157062801199
"10390","PSME4",115.063132413217,0.156657269796031,0.289574517813918,0
.540991213517964,0.588513638926103,0.885157062801199
"10391","CCT4",569.173767146615,-0.0882072509177588,0.163186202956668,
-0.540531303012065,0.588830679319607,0.885157062801199
"10392","IFIH1",62.0901233390125,-0.153513944415207,0.283742214460332,
-0.541033151190368,0.58848473301582,0.885157062801199

"10393","RCHY1",111.665627173852,0.106796262932851,0.19760910016553,0.540442028446018,0.588892230089551,0.885157062801199
"10394","RWDD4",257.327100069718,-0.104171390421325,0.192376668644592,-0.541497007694718,0.588165059585504,0.885157062801199
"10395","PITX1",7.82132417743474,0.476867683963935,0.880617833428396,0.541514906764275,0.588152725790847,0.885157062801199
"10396","AEBP1",2027.79904091121,0.0923537900185922,0.170607365345071,0.541323581381125,0.588284569447677,0.885157062801199
"10397","ATP5MF",1132.13565835303,-0.136199032610694,0.251632802366075,-0.541261041207783,0.588327669279188,0.885157062801199
"10398","AC073389.1",5.14679750627431,-0.394409001426652,0.728722120799066,-0.541233743521015,0.588346482055729,0.885157062801199
"10399","AC009318.1",20.426342656636,-0.234722458861025,0.434015297442828,-0.540816096215929,0.588634347156088,0.885157062801199
"10400","AL136040.1",4.31309404744204,0.385543846951626,0.713239352896244,0.540553245395185,0.588815551494943,0.885157062801199
"10401","DDX19A",126.842240739116,0.112037453261514,0.20733090253242,0.540379904264366,0.588935063651488,0.885157062801199
"10402","VAMP2",904.868017359809,0.132027154893292,0.244288945234178,0.54045488946186,0.588883362818513,0.885157062801199
"10403","IGFBP4",4824.78203475893,-0.174432565914034,0.322566238831323,-0.540765104699159,0.58866949772107,0.885157062801199
"10404","CCDC159",21.83758012885,0.2181199657459,0.403622348675043,0.540406066368514,0.588917025152344,0.885157062801199
"10405","RAB8A",409.061839410159,-0.0934488961793454,0.172827149951318,-0.540707268537773,0.588709367756301,0.885157062801199
"10406","CTSA",743.386156633633,-0.149904348495017,0.277360752202105,-0.540467053484863,0.588874976159107,0.885157062801199
"10407","STAT4",7.07986493045945,0.347201664807328,0.642883390032976,0.5400694281268,0.589149153193111,0.885393749970237
"10408","KLHDC3",368.073001972957,-0.132883270635618,0.24618167559728,-0.539777261297861,0.589350650296664,0.885611469123734
"10409","ACYP1",231.121830155603,-0.159786474893047,0.296164691355483,-0.539518989119662,0.589528797924955,0.885623897756824
"10410","TTLL5",52.5818027429803,0.163916407055571,0.303812016772169,0.539532335807812,0.589519591211396,0.885623897756824
"10411","EVI2A",9.62733923536035,-0.36101435552408,0.668952615463145,-0.539671042730186,0.589423913354595,0.885623897756824
"10412","PITRM1",91.7606539443996,0.128106491085229,0.23749315637997,0.539411295204939,0.589603088969071,0.885650433295839
"10413","CMTM2",2.64134461589777,0.57643807998514,1.06897103719578,0.539245741865286,0.589717301892759,0.885736925151517
"10414","MTF1",103.987715014267,0.139194482216898,0.258218519617429,0.53905692908133,0.589847573626452,0.885847517910285
"10415","AC119800.1",3.26593603613183,0.441188974498729,0.819056105780135,0.538655375846939,0.590124670114942,0.886051429976115
"10416","CYTIP",20.3943386427235,0.262141763922772,0.486697008563852,0.538613879498256,0.590153308571687,0.886051429976115
"10417","MIR762HG",25.6741684092369,-0.198688057984344,0.368788661649685,-0.538758586274215,0.59005344283226,0.886051429976115

"10418","RILPL1",158.256379031569,-0.133162200382525,0.247286307879238
,-0.538494029550372,0.590236025890051,0.88609055912079
"10419","TTC4",110.227871734919,-0.109963892936259,0.204551281140114,-
0.537585940911003,0.590862938469613,0.88617303884744
"10420","SP100",680.851425939285,0.0779460797837922,0.145005892066532,
0.53753732812477,0.590896507669594,0.88617303884744
"10421","AC096733.2",20.8939430527513,0.256158890875262,0.476522588413
752,0.537558758185974,0.590881709191212,0.88617303884744
"10422","SLCO4C1",4.29261595362132,0.473253541719091,0.879900476338681
,0.537848943653636,0.590681339084208,0.88617303884744
"10423","FARS2",103.276371642676,-0.139033770104051,0.258431699415338,
-0.537990387474112,0.590583684903142,0.88617303884744
"10424","RBM48",84.6956765997624,0.121429062948288,0.225720047885805,0
.537963127713498,0.590602504727401,0.88617303884744
"10425","TAF1C",50.805920192445,-0.140127790802735,0.260475118644141,-
0.537969966314428,0.590597783410274,0.88617303884744
"10426","AC016876.1",121.175663718671,-0.166255212293828,0.30930531410
0663,-0.537511658269539,0.590914234152171,0.88617303884744
"10427","SCGB1B2P",2.97092202946347,0.544783461939493,1.01205432192645
,0.538294684520979,0.590373620471233,0.88617303884744
"10428","ZSWIM9",37.659000328887,0.178437401766352,0.331796089245509,0
.537792359675218,0.590720407290539,0.88617303884744
"10429","ZNF304",18.2115037260791,0.225492908430204,0.419397448455375,
0.537659228163371,0.590812332157751,0.88617303884744
"10430","PIAS3",76.1054343173798,0.124072439437341,0.23089633596694,0.
537351270290863,0.591024996633843,0.886254165613932
"10431","ZDHHC20",145.817810129288,0.125001601440068,0.23281939962305,
0.536903718686902,0.591334122187205,0.886557616721052
"10432","PRPF31",312.101486627112,-0.110786786508484,0.206347539167019
,-0.536894149335177,0.591340732585295,0.886557616721052
"10433","ANXA4",1648.488024446,-0.144624968355675,0.269778089184493,-0
.536088637861061,0.591897292354886,0.886675534622033
"10434","CCDC149",61.5656139436323,-0.11609697482143,0.216562805889557
,-0.536089169811721,0.591896924729763,0.886675534622033
"10435","AC008549.2",10.0954181583985,-0.383044876667863,0.71464617129
9871,-0.535992344254979,0.59196384151097,0.886675534622033
"10436","CDKN2AIPNL",146.100880961802,-0.131915852071342,0.24611015830
0539,-0.536003279922529,0.591956283624986,0.886675534622033
"10437","CDC5L",496.840200230505,-0.0883549695687132,0.164655379272404
,-0.53660542375928,0.591540196860936,0.886675534622033
"10438","TMEM30A",458.890164231583,-0.0715336426964786,0.1335560042072
44,-0.535607838232994,0.592229610435046,0.886675534622033
"10439","GALNT17",69.5242077986792,-0.184403077025157,0.34439027508483
1,-0.535447979707716,0.592340120077925,0.886675534622033
"10440","RPS6",16030.9344204346,-0.124632098400654,0.232468048694213,-
0.536125713192499,0.591871670260963,0.886675534622033
"10441","MRPL11",472.696439094062,-0.132634077608811,0.247599124156079
,-0.535680722057816,0.592179229240446,0.886675534622033
"10442","CARD16",35.1187978247076,-0.172066349519911,0.32130745515345,
-0.535519318833531,0.592290802419304,0.886675534622033

"10443","GLIPR1",340.808697326325,-0.103282916091689,0.192801901111868,-0.535694490023529,0.592169712307663,0.886675534622033
"10444","TBX5",29.9903822197718,0.291255539314098,0.542886982067533,0.536493872453672,0.59161726966709,0.886675534622033
"10445","ZFYVE1",90.7768559687248,0.118758914654781,0.221675845202583,0.535732319171943,0.592143563746961,0.886675534622033
"10446","LAT",14.6347101320905,0.275635563357765,0.51435544387136,0.535885381679175,0.592037768090269,0.886675534622033
"10447","PVR",35.7447356129062,0.247661898210893,0.462615219010209,0.535351817306788,0.592406601368275,0.886675534622033
"10448","ZNF581",169.749351448914,0.159384404135059,0.29767818498624,0.535425208073026,0.59235586279961,0.886675534622033
"10449","A1BG",8.64696812082785,-0.343372529420012,0.641453659652125,-0.535303718753793,0.592439855294133,0.886675534622033
"10450","HUWE1",351.946059739276,0.105022909275438,0.195904055080401,0.536093595573278,0.591893866139752,0.886675534622033
"10451","PGM2L1",63.0906464507426,-0.252545646660393,0.472107296052798,-0.534932734087951,0.592696371954432,0.886833338123056
"10452","HMG20A",119.224845009431,0.100944945077162,0.188773446962315,0.534741229243507,0.592828807549652,0.886833338123056
"10453","REX1BD",855.727125271363,-0.127601406808802,0.238591194625995,-0.534811886116854,0.592779943054826,0.886833338123056
"10454","TP53TG5",2.66559377624881,0.542126423572233,1.01372051696618,0.534788844162578,0.592795878080918,0.886833338123056
"10455","IGLC3",46.167344964057,0.504739007508095,0.943594896110966,0.534910701179479,0.592711608170054,0.886833338123056
"10456","ANKRD36C",257.644976617465,-0.21467052258644,0.401526635084398,-0.534635821958156,0.592901707976336,0.886847127303332
"10457","COL1A2",30807.2055628141,-0.163387494263721,0.305646314279982,-0.534563927749681,0.592951432877937,0.886847127303332
"10458","METTL18",50.1396656273686,0.170431949954977,0.319016868237138,0.534241185730305,0.593174677671951,0.886975772042649
"10459","NDUFA10",590.114841559678,-0.102300951940976,0.19153477165454,-0.534111644884458,0.593264293563821,0.886975772042649
"10460","CELF2",1828.0143385208,0.11424537114078,0.213889611920611,0.534132397150657,0.593249936803471,0.886975772042649
"10461","GLOD4",294.594488052716,-0.116957094648838,0.218960343646309,-0.53414738349955,0.593239569098857,0.886975772042649
"10462","GIGYF1",169.192109704693,0.130449182826627,0.244317356610431,0.53393334242164,0.593387652707986,0.887075405118802
"10463","AC023509.3",10.3779040745088,-0.270089022735153,0.506050397331145,-0.533719614013886,0.593535536884748,0.887185144452917
"10464","RAB40B",179.237605984221,0.127634573858923,0.239166860432307,0.533663291093995,0.59357451096901,0.887185144452917
"10465","RNASEH1",250.913867954427,-0.114641530037183,0.214863272584649,-0.533555728990478,0.593648944571058,0.887211065417742
"10466","SERPINB9P1",2.87880436506312,0.7145214903589,1.33955918637184,0.533400463098731,0.593756397006111,0.887211065417742
"10467","INTS13",52.4132059790779,0.120887179546701,0.226641690190638,0.533384565942028,0.593767399204756,0.887211065417742

"10468","ZFP14",108.444645056985,0.124923641784719,0.234241921083579,0.533310353701145,0.593818761687527,0.887211065417742
"10469","PRSS35",2.58653476534676,-0.639274787215055,1.19906227536499,-0.533145609155674,0.593932789076236,0.887278376940029
"10470","ELANE",93.8006938600727,0.201168873836635,0.377369942210122,0.533081338324034,0.593977276634406,0.887278376940029
"10471","CSGALNACT1",5.3394126388628,-0.467049295574628,0.876410176038709,-0.532911767051413,0.594094659307726,0.887284231433616
"10472","C11orf65",8.44789057414839,0.281148990786362,0.527569033381819,0.532914126866285,0.594093025695398,0.887284231433616
"10473","OSCP1",54.6380455892883,-0.172289519952361,0.324104605272506,-0.531586151969365,0.595012657995337,0.88761810094461
"10474","LRRC34",39.7603500671745,0.145149311646616,0.273023976276259,0.531635769232759,0.594978285992361,0.88761810094461
"10475","EPB41L4A-DT",18.6417966845835,-0.240892586212843,0.452886223612783,-0.531905307896506,0.594791580860543,0.88761810094461
"10476","DNAJB6",711.996142107615,-0.0699375080538922,0.131579310873326,-0.531523592802688,0.595055996701038,0.88761810094461
"10477","COPB1",696.663310538014,-0.0783112649366278,0.147220106225314,-0.531933218529104,0.594772249139792,0.88761810094461
"10478","VWA5A",111.431351475003,-0.104016627825827,0.195649143812741,-0.531648775960823,0.594969275824866,0.88761810094461
"10479","DCP1B",53.422602365798,0.126519699200975,0.237845407372847,0.53194089639344,0.594766931276648,0.88761810094461
"10480","NOL3",49.5477630255526,0.174398448487193,0.327988562847005,0.53172112763134,0.594919156696172,0.88761810094461
"10481","ADAT1",49.1278516676346,0.169955864942623,0.319595906726045,0.531783609757894,0.594875875905763,0.88761810094461
"10482","ZNF407",67.5952551605563,0.163555169716258,0.307168087732758,0.532461464091134,0.594406424940148,0.88761810094461
"10483","DOCK6",32.3525586029308,-0.183113806455281,0.344185360927601,-0.532020902811725,0.594711518307394,0.88761810094461
"10484","ZNF429",121.916371639538,0.131071614835043,0.24653059815561,0.531664693209039,0.594958249533773,0.88761810094461
"10485","INE2",7.33083692664606,0.502133926715929,0.943975635046588,0.531935261963776,0.594770833808427,0.88761810094461
"10486","LRIG2-DT",13.9841749402992,0.217859002017685,0.409961361402507,0.531413500219566,0.595132268320983,0.887627460202785
"10487","AP000851.2",5.32779711227824,-0.345658673806603,0.650666030057302,-0.531238235652415,0.595253699989076,0.887627460202785
"10488","FDX1",374.486796976034,-0.115548040230135,0.217528039975228,-0.531186877072461,0.595289285809912,0.887627460202785
"10489","AC084824.4",5.47814962894641,0.367983245525355,0.692545197663931,0.531349068286984,0.595176908528662,0.887627460202785
"10490","C19orf47",22.1892030484037,-0.170624874732228,0.321266096547486,-0.531101403372042,0.595348511791837,0.88763114627496
"10491","TMEM273",2.93811910245874,-0.633496657507391,1.1932529498888,-0.530898882392393,0.595488852283704,0.887722775980645

"10492","FGF9",12.4662471572792,-0.311431077973518,0.5866614234648,-0.530848902798263,0.595523488848397,0.887722775980645
"10493","TRMT5",87.0515457305016,0.103442768981124,0.194959969725132,0.53058465861974,0.595706629068392,0.88782010458511
"10494","SLC35C2",252.886834677845,-0.104487859632326,0.196957755738178,-0.530508987781243,0.59575907913176,0.88782010458511
"10495","PDXP",26.1988442250033,0.20737929310141,0.390876369922532,0.530549577971445,0.595730944411668,0.88782010458511
"10496","FANCC",15.830207037999,0.232082791810556,0.437748010364778,0.530174406999955,0.595991013824456,0.888081121971655
"10497","RBM45",46.5922026600674,-0.143434255887586,0.270723442128776,-0.529818381296137,0.596237859534223,0.888195077923159
"10498","ZW10",51.72442395636,-0.135758689832066,0.25623247867525,-0.529826236447282,0.59623241276668,0.888195077923159
"10499","SCFD1",426.051444980918,-0.0756708714958725,0.142790759386027,-0.529942356362853,0.596151897776925,0.888195077923159
"10500","ING4",253.306982579349,0.134411252823916,0.253824935788922,0.529543137305039,0.596428728230263,0.888265042699341
"10501","YBX3",3814.55082370057,-0.0930085477458071,0.175706240998344,-0.529341173183962,0.59656879849833,0.888265042699341
"10502","AL442663.3",3.00554109457314,0.468958996449414,0.885752502282534,0.529446990260748,0.596495408217519,0.888265042699341
"10503","EDC3",58.0992824693103,0.126177753101849,0.238359278393403,0.529359519597125,0.596556073901989,0.888265042699341
"10504","AC008440.2",10.3397795554253,-0.327560091786787,0.618528936948146,-0.529579252028184,0.596403682791055,0.888265042699341
"10505","DNPEP",209.830402368154,-0.105840814702431,0.200257473824428,-0.528523668461059,0.597135923961344,0.888371116563746
"10506","TRIP6",486.074047089817,-0.141761779271034,0.267996795477509,-0.528968187916004,0.596827518196184,0.888371116563746
"10507","POLR2K",559.540044495422,-0.118013197751193,0.223141793994488,-0.528870883569701,0.596895021353154,0.888371116563746
"10508","PCGF6",75.7213069760306,-0.128772373997889,0.243582721350369,-0.528659723004995,0.597041522182176,0.888371116563746
"10509","RAB11FIP2",278.141334746175,0.104067320901173,0.196910141052462,0.528501581203209,0.597151249899915,0.888371116563746
"10510","COPS7A",248.000400228616,-0.0982477977478179,0.185829349398571,-0.52869903524816,0.5970142465501,0.888371116563746
"10511","SETD1B",67.2262395455121,0.161330838963449,0.304981299813516,0.528986003607749,0.596815159253672,0.888371116563746
"10512","RPTOR",41.8016513973323,0.150284370486167,0.284304655853644,0.528603269035212,0.597080692083558,0.888371116563746
"10513","AC011477.2",8.04933296213545,0.345327836623074,0.652886680548702,0.528924615727881,0.596857745196013,0.888371116563746
"10514","ITFG2",96.9668141158144,0.125443982268525,0.237496447493847,0.528193089169366,0.597365325497414,0.888605068554266
"10515","WDR60",251.231285931285,-0.133963657735268,0.253683236837103,-0.528074536597348,0.597447603393369,0.888642940282671
"10516","IL2RG",16.612893417241,0.293827329434188,0.556610892662731,0.527886416359135,0.597578173236223,0.888752627369202

"10517","TRAPPC3L",4.8091416959401,0.391006249163616,0.741077132549207
,0.527618829390412,0.597763921411488,0.888795200780449
"10518","IFT74",171.564147231398,-0.0854033428101229,0.161871513627929
,-0.527599581273005,0.597777283696263,0.888795200780449
"10519","R3HDM2",373.569101408112,-0.103232749417537,0.1956083631737,-
0.527752227678869,0.597671318374168,0.888795200780449
"10520","MXD3",11.8737384132534,0.262705625547286,0.498182155884013,0.
52732845294533,0.597965518801842,0.888990562173081
"10521","SFRP2",407.119391351652,0.392503998767373,0.744673745914888,0
.527081827337893,0.598136765884765,0.889160632871184
"10522","TPRN",39.0474270457303,-0.140997301008616,0.267553802163208,-
0.526986721431856,0.598202809618502,0.889174295992527
"10523","UTP15",38.2225485118265,-0.151107590828328,0.286799735055929,
-0.52687493173193,0.598280443195431,0.889205182132143
"10524","SPATS2",167.975896255299,-0.0791465332522824,0.15027607646550
4,-0.526674205993463,0.598419850850887,0.889243369815474
"10525","GLT8D2",271.277270591378,0.173046615129659,0.328557967185183,
0.526685189259543,0.59841222239276,0.889243369815474
"10526","ZMIZ2",100.030318827665,0.111062965591038,0.210928542649862,0
.526543085140453,0.598510924613289,0.889294210616743
"10527","TPR",1129.79265462097,0.0962532556646959,0.182957605708231,0.
526095951529857,0.598821541407268,0.889297709382743
"10528","AC022893.1",2.9923926149231,0.522371917739911,0.9928988859494
97,0.526107869725701,0.598813261073051,0.889297709382743
"10529","R3HCC1L",77.8228082759428,0.12995450199652,0.24703896700591,0
.526048596994864,0.598854442149556,0.889297709382743
"10530","AC040169.1",14.3060140919182,-0.241276899615197,0.45862591613
5442,-0.52608649255648,0.598828113199037,0.889297709382743
"10531","ERBB2",152.189587931154,0.0924637368533466,0.175728213635364,
0.526174681575088,0.598766843562199,0.889297709382743
"10532","RGS9",237.793796877752,0.157522674703343,0.299354425367985,0.
526207937329493,0.598743739745771,0.889297709382743
"10533","NPY1R",7.14579305928969,0.387766863867392,0.737308417200896,0
.525922198663488,0.598942264550839,0.889343683430657
"10534","GPC2",3.92898606619486,-0.456328003520911,0.86830984171517,-0
.525535910798302,0.599210696109694,0.889573354262517
"10535","TRIM13",180.439913306031,0.0998166766671537,0.189930470667019
,0.525543249151158,0.599205596178067,0.889573354262517
"10536","AL158163.1",4.21310339234778,0.532501465157075,1.013585900375
26,0.525363923235244,0.599330227857155,0.889621173706945
"10537","EIF2B1",271.643764965607,-0.0786182696192414,0.14965618975369
3,-0.525325880263509,0.599356669267908,0.889621173706945
"10538","SLC31A1",76.9755254183382,0.105410938500008,0.200728355876454
,0.525142240316495,0.599484313922606,0.889726197546932
"10539","KLC1",460.450316688636,-0.101978548375658,0.19422610229767,-0
.525050686644402,0.599547955747587,0.889736220504058
"10540","SLC25A1",242.623657232059,-0.0809624924923048,0.1542333284632
31,-0.524935131070626,0.599628286413692,0.889771005646123
"10541","SNAP29",241.068195459407,0.101090506788057,0.192646135187338,
0.52474713126090,0.5997589884879,0.889880521767457

"10542","UNC93B1",86.4641444571778,-0.116781117412514,0.222582952496146,-0.524663349564186,0.599817239730238,0.889882529821753
"10543","AC007389.1",3.37700654006618,-0.516930212856248,0.985672175098982,-0.52444435981399,0.599969509720765,0.890023532314894
"10544","AC093673.1",109.752073502596,-0.151953953051625,0.290191175885779,-0.523633954712101,0.600533160208477,0.890023532314894
"10545","COMMD3",499.378923378902,-0.124988268440356,0.238734054321228,-0.52354603869031,0.600594321681519,0.890023532314894
"10546","RPP30",195.942989717497,-0.124869503136716,0.238497024371542,-0.523568390279741,0.600578771846903,0.890023532314894
"10547","TRUB1",79.2254729182628,0.107790546699357,0.205880484409376,0.523558835644783,0.600585418913151,0.890023532314894
"10548","ERO1A",162.185125590984,-0.0855904656501519,0.16344406167,-0.523668249403654,0.600509302819352,0.890023532314894
"10549","AL122035.2",6.42002392730864,0.347378332518784,0.663322375881815,0.523694579211175,0.60049098655919,0.890023532314894
"10550","AC005726.3",4.45458176601818,-0.425713977675024,0.813264646834564,-0.523463031784319,0.600652070561618,0.890023532314894
"10551","NLE1",50.8938891530735,-0.183938992570089,0.351152450480477,-0.523815204246498,0.600407077305129,0.890023532314894
"10552","PSPN",9.66393075826769,0.27297516588463,0.520873127637093,0.524072276723056,0.600228270096462,0.890023532314894
"10553","ZNF585A",154.365730503021,-0.0995541083566909,0.190158753707758,-0.523531556741736,0.600604396762634,0.890023532314894
"10554","TRIM28",342.66662487193,-0.0707337203595724,0.134952748617438,-0.524136937440876,0.600183299012648,0.890023532314894
"10555","TCEAL1",174.768690898083,-0.105427916913533,0.201124150522403,-0.524193224133916,0.600144153246178,0.890023532314894
"10556","FZD4-
DT",4.8834303779564,-0.342748877982649,0.655000104690525,-0.523280646107059,0.600778967234532,0.890076576823492
"10557","NCOR2",478.927700771575,0.12769169673313,0.24403666938632,0.523247989960841,0.600801689355857,0.890076576823492
"10558","MMP19",192.497419959771,-0.16654563774304,0.318418796141027,-0.523039593646594,0.600946700494305,0.890207084270784
"10559","PILRA",8.95764903632226,0.301052772167406,0.575688027592962,0.522944299234697,0.601013015720777,0.890221002545029
"10560","TNFRSF25",3.60587899219092,-0.45271774717162,0.865854813572581,-0.522856418968988,0.6010741743841,0.890227281000693
"10561","LRPPRC",222.245482019618,0.0890171345545577,0.170304415189464,0.52269422642699,0.601187056695945,0.890262222828065
"10562","SUMO1",1082.5768035614,-0.134473415649832,0.257368566833862,-0.522493548082107,0.601326737494998,0.890262222828065
"10563","HOPX",11.2127917640258,0.339797686351166,0.650454171745811,0.522400656512284,0.601391399000592,0.890262222828065
"10564","SCML4",2.96211992061111,0.465514340890116,0.891305280581095,0.522283835889112,0.601472721893209,0.890262222828065
"10565","SORL1",8.27490420320541,0.329705673479678,0.631304204548495,0.522261171562261,0.601488499892935,0.890262222828065
"10566","TEX22",2.48966402224971,0.552323653115697,1.05699363448517,0.

522542080761648,0.60129295531008,0.890262222828065
"10567","TMED1",336.652193615646,-0.137747861083381,0.263758430066235,-0.522250079547372,0.601496221779039,0.890262222828065
"10568","ZBTB17",67.2170554253148,-0.110826313587264,0.212282177340961,-0.522070740819932,0.601621077551284,0.890316253950849
"10569","ACER2",6.83530691545335,0.327824835385825,0.627980995703643,0.522029866554325,0.601649535885202,0.890316253950849
"10570","HIC1",686.226642473918,0.0803434583302137,0.15392872074664,0.52195235522327,0.601703504108726,0.890316253950849
"10571","NDUFA2",901.773970095785,-0.134472408174588,0.257833835094362,-0.521546786616947,0.60198592186605,0.890410666550246
"10572","CCDC90B",470.73698410128,-0.117265579291705,0.224840962866032,-0.521548999777125,0.601984380569491,0.890410666550246
"10573","DDX11-AS1",3.10177789179513,-0.491914298942422,0.942856235780322,-0.521727788685946,0.60185987366541,0.890410666550246
"10574","STK24",431.589143154775,0.122303564663941,0.23450750218988,0.52153369730966,0.601995037602449,0.890410666550246
"10575","PEX14",50.6008021379159,-0.139833575224607,0.268291695898006,-0.521199788746971,0.602227601309123,0.890418682505113
"10576","MGAT1",796.972213284657,-0.0985841167100709,0.189148724762273,-0.52119894984211,0.602228185648279,0.890418682505113
"10577","RMND1",97.9407898857296,0.121339032685907,0.232717496339726,0.521400559022746,0.602087762127685,0.890418682505113
"10578","NECTIN2",200.822890394714,-0.131837876140739,0.252892297066232,-0.521320252416431,0.602143694996407,0.890418682505113
"10579","C17orf75",63.6292250517947,-0.132080232211396,0.253468526328114,-0.52109125391142,0.602303203367214,0.890445420234732
"10580","TMEM44",77.5689569719661,0.137129810323349,0.263367007317678,0.520679532793341,0.602590034618433,0.890532754552802
"10581","GLO1",641.910294316403,0.086158942601553,0.165428608928838,0.520822505607936,0.602490423644438,0.890532754552802
"10582","CDCA4",58.0047030855696,-0.115793291665392,0.222301600984832,-0.520883750510159,0.602447755813022,0.890532754552802
"10583","TNMD",52.0927349383272,-0.419061972907461,0.804771581665047,-0.520721633883288,0.60256070148449,0.890532754552802
"10584","EIF1",18240.0121296565,-0.111936079105344,0.215082699513774,-0.520432742188899,0.602761994210806,0.890636317589822
"10585","ZNF45",24.7861319447663,-0.178271338405974,0.342555776812914,-0.520415507408991,0.602774003944262,0.890636317589822
"10586","CTH",7.36580542910068,-0.29452264707118,0.566028206197642,-0.520332103323382,0.602832124040513,0.890638052143739
"10587","UCHL5",160.262888908587,-0.093796399627065,0.180711825983516,-0.519038525102508,0.603733876146856,0.890919695477303
"10588","COMMD1",466.127054009582,-0.152815107234443,0.294536225551236,-0.518832978688593,0.603877218142896,0.890919695477303
"10589","TENM3-AS1",6.28407933272825,0.48264553229087,0.92923971948349,0.519398301828019,0.603483015298821,0.890919695477303
"10590","AC113410.4",3.21864734399331,-0.450246124239301,0.86685072179

3744,-0.519404452138683,0.603478727290574,0.890919695477303
"10591","PITPNM1",16.8637509511529,-0.202343233351804,0.38932190826595
7,-0.519732460608351,0.603250059012989,0.890919695477303
"10592","MPHOSPH9",48.350551956947,0.140912268672069,0.271387525651048
,0.519228982002861,0.603601070780795,0.890919695477303
"10593","AC073592.1",4.20756832803358,0.546420833490387,1.051929045559
57,0.519446473882392,0.603449430017775,0.890919695477303
"10594","EPSTI1",109.264353580591,0.217097261344735,0.418390788130809,
0.518886331877985,0.603840009735734,0.890919695477303
"10595","LINC00641",17.8335487985133,0.261622014754859,0.5034684592644
24,0.519639333786854,0.603314977612341,0.890919695477303
"10596","ZBTB1",213.113736069337,0.113358835804617,0.218128580835907,0
.519688137016277,0.60328095654584,0.890919695477303
"10597","TUBGCP5",88.8647766742014,0.138178005881485,0.265971334715812
,0.519522173429431,0.603396654424106,0.890919695477303
"10598","ACOT8",95.6494871866635,0.123379718083543,0.237735859926814,0
.518978155510592,0.603775974530709,0.890919695477303
"10599","CEBPB",1681.61081151781,-0.183658205663168,0.353964577494765,
-0.51886040960097,0.603858087751027,0.890919695477303
"10600","USP16",588.137334547644,-0.0911575869593082,0.175570086609001
,-0.519209101732225,0.603614932655646,0.890919695477303
"10601","GAB3",19.608318618747,0.222402535804468,0.428320844847264,0.5
19242849093125,0.603591401788396,0.890919695477303
"10602","KCNK5",20.5606758134229,-0.294088276907139,0.566966182136668,
-0.51870514710214,0.603966371824776,0.89096718122425
"10603","LINC02709",9.31599602457132,0.356896737812836,0.6883579594307
53,0.518475500897784,0.604126549002984,0.891001625643677
"10604","CD63",17274.6130879822,-0.152917500560996,0.294968531270892,-
0.518419710408228,0.604165465494381,0.891001625643677
"10605","NIPA1",40.8056627683904,-0.147880152543461,0.285292921919228,
-0.518344975222794,0.604217598566294,0.891001625643677
"10606","CCDC102B",47.1995428233665,-0.166837785776243,0.3218556493717
81,-0.518362148068204,0.604205619114627,0.891001625643677
"10607","NAIP",73.3769356677548,0.138638872905342,0.267524492238358,0.
518228711492395,0.60429870474702,0.891037215258169
"10608","E2F5",9.63150687377658,-0.274666526136756,0.531072965638226,-
0.517191692871564,0.605022350406771,0.891529649893457
"10609","SPAAR",3.08722231136002,0.513118702394047,0.99184882116693,0.
517335597364881,0.604921908688044,0.891529649893457
"10610","AC116158.3",2.52718886611023,0.528465549551939,1.021824665935
66,0.51717830579871,0.605031694627183,0.891529649893457
"10611","AMDHD2",110.316320282654,0.129414917054069,0.250074427863147,
0.517505600872117,0.604803260148256,0.891529649893457
"10612","BEAN1",2.59721318620496,0.520552114605069,1.00602822522682,0.
517432912468938,0.604853989429697,0.891529649893457
"10613","ADAMTSL5",41.991718047211,0.169529763832967,0.327593132564093
,0.517500969895328,0.604806492052903,0.891529649893457
"10614","LAMA5",7.97438940247813,0.343280517101101,0.66359723761006,0.
517302510687692,0.604945001695057,0.891529649893457
"10615","SLC16A4",107.880886119562,-0.142788699313851,0.27621784075528

4,-0.516942348558707,0.605196404166832,0.891645672307082
"10616","AP003119.2",10.7636208689525,0.23973741261293,0.4637964922969
41,0.516902168504199,0.605224453785933,0.891645672307082
"10617","LRRK1",142.823680663141,0.10840180680726,0.20979257278050l,0.
516709459112631,0.605358991940056,0.891747056128078
"10618","ZNF837",12.20381405591,-0.241312026940434,0.467079406464317,-
0.51664026202l205,0.605407304473653,0.891747056128078
"10619","DIXDC1",258.288925955103,-0.112126358823522,0.217084331844874
,-0.516510601528102,0.605497836439423,0.891796417921893
"10620","RPL22",8163.25540131015,-0.111583334564475,0.21650718393199,-
0.515379363114004,0.606287950447538,0.891797029196942
"10621","GNPAT",145.109057722827,-0.088193790772222,0.170825432684513,
-0.51628021300026,0.605658714014526,0.891797029196942
"10622","SLC30A6",83.6871993907974,0.103273909837172,0.200329169832175
,0.515521079250161,0.606188943482934,0.891797029196942
"10623","MOB1A",587.243791906501,0.0565201765129091,0.109543784696339,
0.515959683788414,0.605882567590731,0.891797029196942
"10624","AC009950.1",22.1890764742437,0.254633514953789,0.494065428092
614,0.515384199086394,0.606284571779092,0.891797029196942
"10625","ZNF467",3.48255497461506,-0.452146772807803,0.876789346623021
,-0.51568461061857,0.606074704759132,0.891797029196942
"10626","MALAT1",218635.026154682,0.0739782269066793,0.143544728450047
,0.515367075513492,0.606296535259021,0.891797029196942
"10627","PACS1",249.83408863443,0.0863295597706324,0.167332230093613,0
.515917105284116,0.605912306669542,0.891797029196942
"10628","RELT",6.63561343878455,-0.387240845448829,0.750953545344583,-
0.515665513332305,0.606088045125543,0.891797029196942
"10629","BICD1",99.6606230025413,0.154688695849483,0.299849933682317,0
.515887043728251,0.605933303643157,0.891797029196942
"10630","C14orf119",394.559662879011,-0.143779702726341,0.278554188473
31,-0.516164210326054,0.605739724549318,0.891797029196942
"10631","C1QTNF1-
AS1",6.88045058138214,0.330285191307451,0.640550529575278,0.5156270677
45067,0.60611490160257,0.891797029196942
"10632","MZF1",69.6347052799591,0.113043554499332,0.219070532711457,0.
516014422844465,0.60584433589943,0.891797029196942
"10633","PACSIN2",158.278887269127,-0.115186340476279,0.22305032274271
5,-0.516414139463698,0.605565192425099,0.891797029196942
"10634","CD3EAP",22.642722983546,0.186928820181387,0.362881331582321,0
.51512382675156,0.606466493767493,0.891963133583184
"10635","METTL17",96.4562976807829,-0.0980231270809832,0.1903782146258
44,-0.514886260876177,0.606632502186353,0.892123397667566
"10636","PPP1R35",140.098266838566,-0.122605213316292,0.23827373143917
,-0.514556147569259,0.606863215444976,0.892200882178225
"10637","AC034111.1",5.59541452547884,-0.390036290248307,0.75796611155
4122,-0.514582755485707,0.606844617956016,0.892200882178225
"10638","LINC02256",47.571352503214,0.20112370599931,0.390922857840675
,0.514484384746006,0.606913375031594,0.892200882178225
"10639","GZMM",11.7400930093543,0.290223840555434,0.563930058807319,0.
514645098311024,0.606801044703193,0.892200882178225

"10640","AL162253.2",38.2615043572938,-0.211168909364044,0.41064356867
3026,-0.514238929995726,0.607084952916325,0.892310269202898
"10641","ZNF592",80.6836429852979,0.123672428046917,0.240507375442867,
0.514214700564541,0.607101890958314,0.892310269202898
"10642","CLIC6",39.4836280291105,0.205999147877212,0.400701491033096,0
.51409628485809,0.607184674732733,0.892348084271749
"10643","TFAP2E",3.43331801780814,0.439889415733389,0.8565966787859,0.
513531544806907,0.607579550738792,0.892436886472162
"10644","LINC02609",120.478712924237,0.138995314773016,0.2707798827098
57,0.513314775758104,0.607731149828816,0.892436886472162
"10645","CADM3-
AS1",11.5686894063403,0.382665890818491,0.746332921645342,0.5127281401
10552,0.60814150253674,0.892436886472162
"10646","RRP15",188.329014032086,-0.111363861221472,0.216962471666018,
-0.513286285717195,0.607751075810936,0.892436886472162
"10647","EDEM1",101.831792170173,-0.149098912012728,0.2908774171494,-0
.512583319371775,0.608242823911283,0.892436886472162
"10648","AC099329.2",3.91878758297479,0.397178172352878,0.775583196665
098,0.512102601062903,0.608579204239127,0.892436886472162
"10649","SLMAP",161.099023180841,0.116444471977926,0.227255645878209,0
.512394187294829,0.608375158287924,0.892436886472162
"10650","AC021148.2",5.05896710835643,0.34492761290205,0.6735504525346
97,0.512103602044988,0.608578503720655,0.892436886472162
"10651","PPARGC1B",5.27116211827789,0.341516259863617,0.66663250354953
9,0.512300642475706,0.608440615676881,0.892436886472162
"10652","SFT2D1",554.523680632292,-0.115526900748593,0.224914439703184
,-0.513648216188574,0.607497962701978,0.892436886472162
"10653","CDNF",9.94054614935212,0.271738280648696,0.530684858101434,0.
512052071017932,0.608614567206654,0.892436886472162
"10654","OSBPL5",93.5095153822153,0.102659377695371,0.200393303819145,
0.51228946146834,0.608448439725175,0.892436886472162
"10655","PGAP2",40.3255512554506,0.152540261363591,0.297642340664766,0
.51249516793512,0.608304501252086,0.892436886472162
"10656","JAM3",270.945921217741,-0.0992281580945326,0.193102351991132,
-0.513863021715496,0.607347762464443,0.892436886472162
"10657","AC092490.1",9.36509348776899,0.371468063402712,0.724856915158
529,0.512470883058998,0.608321493258055,0.892436886472162
"10658","TUBGCP4",139.205768034737,0.107828535564654,0.21049666052555,
0.512257701834493,0.608470664166496,0.892436886472162
"10659","AC015871.1",25.2916292729909,0.212384231891222,0.414595337811
49,0.512268741400537,0.608462938967552,0.892436886472162
"10660","SRCAP",113.792516377934,0.113538621902805,0.221087320211859,0
.513546510917068,0.607569084698025,0.892436886472162
"10661","RTN4RL1",11.6174302423131,-0.359262206933733,0.70022911590957
6,-0.513063794079831,0.607906696855598,0.892436886472162
"10662","PAFAH1B1",603.097734285361,0.0652681285746637,0.1271747320724
38,0.513216167323926,0.607800117973186,0.892436886472162
"10663","HSD17B1",13.8718961477067,-0.230106454630174,0.44824516261568
9,-0.513349554711111,0.607706825766077,0.892436886472162
"10664","AKAP1",62.2404881594852,0.144842795921118,0.282417553338078,0

.512867540310884,0.608043980656093,0.892436886472162
"10665","QPCTL",4.98918623999634,0.339590510593713,0.662527928768559,0
.512567841819001,0.608253652963586,0.892436886472162
"10666","GNB1L",35.0448811787632,0.176888685182409,0.344477458615483,0
.513498578087973,0.607602605177593,0.892436886472162
"10667","PAK2",588.042912389669,-0.0792988107160457,0.154890035274215,
-0.51196844636039,0.608673093131622,0.892439034084426
"10668","TJAP1",68.7360799746861,0.103906062923115,0.202996958450224,0
.511860195918124,0.608748857489046,0.892466453986565
"10669","DTD2",45.7496064975502,0.133399921049527,0.260782695506861,0.
511536706031238,0.60897529269638,0.892714741566349
"10670","FBXO2",3.38135144433922,0.451793173996876,0.88404064713364,0.
511054752359796,0.609312718272553,0.892740928783008
"10671","LINC00884",18.9648185351673,0.267622011568794,0.5233698918083
79,0.511343919009346,0.609110256836667,0.892740928783008
"10672","LINC02664",5.04833867880128,0.396013633141564,0.7744411357410
99,0.51135407827039,0.609103144324886,0.892740928783008
"10673","ITGB7",4.40834457019793,0.429754758541184,0.840971121919176,0
.511022016499738,0.609335640329834,0.892740928783008
"10674","HNRNPA1P48",125.063371608939,-0.161437164701788,0.31586166517
8556,-0.511100847298224,0.609280442672222,0.892740928783008
"10675","JPT1",310.219470432662,0.110440622850763,0.21609468546802,0.5
11075145654644,0.609298438827144,0.892740928783008
"10676","AL391244.2",2.55586253902632,-0.458818715998176,0.89886492595
2635,-0.51044233983422,0.609741600654752,0.892899723411195
"10677","CCDC163",3.86022167095712,-0.44265554512895,0.866921971881135
,-0.510605982414348,0.609626985980114,0.892899723411195
"10678","STRIP1",88.0123822891954,0.104080866804294,0.20386171963334,0
.510546398762313,0.609668717047213,0.892899723411195
"10679","SEMA4A",32.9927246234841,0.351348167575897,0.688231578993729,
0.510508640259732,0.609695162921313,0.892899723411195
"10680","NFKBIB",76.1726612563152,-0.115753311166162,0.22669750256599,
-0.510606909454005,0.609626336712149,0.892899723411195
"10681","RANGAP1",80.3969250943094,0.133906569095592,0.262367368246489
,0.510378138830851,0.609786569421674,0.892899723411195
"10682","TMEM129",140.03278027784,0.109633344797384,0.214897757582485,
0.510165140998753,0.609935771566088,0.893034587838759
"10683","PSD4",5.98521353073328,-0.33427914392792,0.655357779469158,-0
.51007122277344,0.610001565183086,0.893047316246697
"10684","PLRG1",233.787515751556,-0.0963691305662726,0.189076111163822
,-0.509684327507535,0.610272634615332,0.893360539627835
"10685","LMTK2",42.2520802247453,0.155898795196926,0.306015004601943,0
.509448206305164,0.610438093874776,0.893519119157838
"10686","PHF7",37.9206052850733,0.161015632427324,0.316261733269535,0.
509121450650175,0.610667097030734,0.89356110135888
"10687","NIPBL-
DT",92.502467424978,-0.10919081669369,0.214451054967689,-0.50916427858
1615,0.610637079369613,0.89356110135888
"10688","CLDN12",53.3998144791901,0.134473986638288,0.264045707670441,
0.509282986740033,0.610553881451606,0.89356110135888

"10689","RPL39",13697.1208495816,-0.108034100009247,0.21221388581308,-0.509081201709882,0.610695307699813,0.89356110135888
"10690","IL6R-AS1",2.94566369663609,0.436257820301926,0.857944320774093,0.50849199620356,0.61110835072509,0.893647569280657
"10691","AC026304.1",20.0798429071153,-0.213608174831969,0.420055384966969,-0.508523833943389,0.611086028766801,0.893647569280657
"10692","AC244517.1",4.55735007915254,0.37679734782657,0.741202887284121,0.508359255327799,0.611201421410591,0.893647569280657
"10693","PRUNE2",16.3285460430363,-0.27113348526388,0.533435665358863,-0.508277760320877,0.611258564390592,0.893647569280657
"10694","IER5L",370.382953989736,-0.142930970054536,0.281259471278636,-0.508181891279097,0.611325789241288,0.893647569280657
"10695","RIC3",2.60429984371978,0.502077545539109,0.987041319602098,0.508669227486352,0.610984095578041,0.893647569280657
"10696","TYRO3",17.4196185533118,-0.226684090593993,0.446000753257467,-0.508259434403091,0.611271414553567,0.893647569280657
"10697","PNPO",60.3148176748585,0.126085744557598,0.24799300082951,0.508424609306935,0.611155597968482,0.893647569280657
"10698","C19orf25",139.619790011754,-0.113585835874293,0.223490446341727,-0.508235755637695,0.611288018319118,0.893647569280657
"10699","DNAJB1",1720.22450565114,0.13260291005361,0.26066053995316,0.508718772996628,0.610949361700766,0.893647569280657
"10700","AC108134.3",2.80810959348099,-0.547602990606592,1.07788420799432,-0.508035080712005,0.611428741423126,0.893714534192307
"10701","SORT1",62.9468508008329,-0.168944518282816,0.33260774106642,-0.507939225169982,0.61149596509603,0.893729267741512
"10702","PSMB2",611.643400551983,-0.127334006101992,0.251039172206594,-0.507227636957002,0.61199510556321,0.893844679708133
"10703","LURAP1",33.8906711159236,-0.153302803343726,0.30269646394925,-0.506457199214023,0.612535728932469,0.893844679708133
"10704","LEPR",497.786596689879,-0.18874993729939,0.372607715587924,-0.5065647580635,0.612460241204768,0.893844679708133
"10705","BCAS2",321.430667323738,-0.12285992240456,0.243600196229744,-0.504350670919366,0.61401497593365,0.893844679708133
"10706","MBOAT2",68.4380908552549,0.149323939887819,0.295497650220028,0.50533038004407,0.613326808373122,0.893844679708133
"10707","GFPT1",337.753276910417,0.0898567865764424,0.177264809140928,0.506907078804373,0.612220018570871,0.893844679708133
"10708","ZBTB20-AS2",21.5486779408781,0.193765297490345,0.38400728784381,0.504587552435088,0.613848554357264,0.893844679708133
"10709","PARP9",172.473981141113,0.108855759325824,0.215803840586045,0.504419935392304,0.613966311984168,0.893844679708133
"10710","WWTR1",315.94935270284,0.102222782494653,0.201545375486962,0.507194879801475,0.61201808726775,0.893844679708133
"10711","CRMP1",3.41832954509143,-0.498182429812314,0.985758373863598,-0.505379860847369,0.61329206108496,0.893844679708133
"10712","SH3RF1",134.682789910065,0.127463859112101,0.251841712397964,0.506126875879404,0.612767584542411,0.893844679708133

"10713","THBS4",123.946419914639,-0.722017356016811,1.42630707863329,-0.506214521986851,0.612706061519621,0.893844679708133
"10714","UQCRQ",1158.9296716312,-0.112974345096751,0.222846529760059,-0.50696030680124,0.612182669715031,0.893844679708133
"10715","SAR1B",325.407825028277,-0.0710618863665387,0.14061339520655,-0.505370674409463,0.613298512082799,0.893844679708133
"10716","LMBRD1",488.146676842474,0.0795905795231227,0.157258460683654,0.506113179393443,0.612777199011946,0.893844679708133
"10717","ELOVL4",38.240689679672,-0.156703822774089,0.310092577117017,-0.505345288271621,0.613316339161443,0.893844679708133
"10718","COPS6",724.125657329577,-0.112303357180933,0.222051353144343,-0.505753987042496,0.613029363672633,0.893844679708133
"10719","PEX2",319.406310163152,-0.121030034855989,0.238948597617752,-0.506510756131748,0.612498140716035,0.893844679708133
"10720","FANK1",8.49523394768191,0.304125051819876,0.59971663153532,0.507114586836275,0.612074420672614,0.893844679708133
"10721","TM7SF2",15.2865216917343,-0.211767957097095,0.419827819493158,-0.50441620889429,0.613968930110114,0.893844679708133
"10722","CD3D",18.9472745079849,0.322590117266258,0.639378984805577,0.504536628404124,0.61388432945756,0.893844679708133
"10723","H2AFX",220.481937254493,0.147982534372154,0.292152941603234,0.506524197771474,0.612488707038519,0.893844679708133
"10724","MAGOHB",150.904196848644,-0.14839055427283,0.294235485696861,-0.504325825694969,0.614032432147326,0.893844679708133
"10725","MLXIP",216.732509459046,0.116806687378268,0.231494823177794,0.504575807678247,0.613856805190769,0.893844679708133
"10726","AC007950.2",7.53478968327652,0.311811315697119,0.617250937217709,0.505161348320709,0.613445515384079,0.893844679708133
"10727","USP3-AS1",6.35415578581316,0.348550045282568,0.687716084625338,0.506822587219921,0.612279306433331,0.893844679708133
"10728","UBL7",114.683872807236,-0.107121231197049,0.211879853663158,-0.505575350110199,0.613154789642462,0.893844679708133
"10729","SYT17",2.69586637070336,-0.552274550740793,1.08900264152441,-0.507137934916033,0.612058039463888,0.893844679708133
"10730","ZNF423",273.993423339847,0.100466323491534,0.19882038192151,0.505311993270369,0.613339720481207,0.893844679708133
"10731","ZNF232",23.6424003652055,-0.202246404117186,0.399732815087671,-0.505953968459728,0.612888964588106,0.893844679708133
"10732","TRPV2",99.9795600400025,-0.139615045029649,0.276809265167647,-0.504372730967269,0.613999476763714,0.893844679708133
"10733","CDK5RAP3",261.141098994239,-0.0957558627314798,0.189375253907157,-0.505640841429182,0.613108805046353,0.893844679708133
"10734","MTMR4",66.5507427506773,-0.122383576868026,0.241397703867563,-0.506979042912391,0.612169523258447,0.893844679708133
"10735","NACA2",6.77963534318421,0.313671833485722,0.618342995061308,0.507278057633082,0.611959732264835,0.893844679708133
"10736","TRIM65",93.5006002055654,-0.106328049119081,0.210397066697667,-0.505368495806411,0.61330004196893,0.893844679708133
"10737","ZNF414",104.85398729534,-0.151643537472496,0.299740339343474,

-0.505916346810853,0.612915376182838,0.893844679708133
"10738","USE1",279.314792943916,-0.133311063580637,0.263298999588858,-0.506310558675888,0.612638651877684,0.893844679708133
"10739","AURKC",8.45592482487016,0.296682082632039,0.58802645672618,0.504538663589743,0.613882899683322,0.893844679708133
"10740","SNPH",13.7926452693577,0.260223315930513,0.515514320096049,0.504783874640047,0.61371064287813,0.893844679708133
"10741","SLC2A11",53.2791150444436,0.114872434877627,0.226646033458974,0.50683629060034,0.612269690579706,0.893844679708133
"10742","RPL3",16977.015710357,-0.116933726553203,0.231769256323409,-0.504526477791491,0.613891460565867,0.893844679708133
"10743","P2RY8",2.64887983402719,-0.519866751550231,1.02966693670056,-0.504888263399114,0.61363731792933,0.893844679708133
"10744","OFD1",210.191042504483,0.103302380195475,0.203501118686758,0.507625613373084,0.611715924512135,0.893844679708133
"10745","TOMM40L",32.0624376510204,0.155516398224343,0.309425350518436,0.502597469676541,0.615247308637486,0.893872561111145
"10746","MYNN",94.2137181259732,-0.0941772381043732,0.187236387983404,-0.502985766381696,0.614974279454057,0.893872561111145
"10747","CEP120",83.6484243256151,0.142077499278781,0.282106573361326,0.503630587497179,0.614520994025615,0.893872561111145
"10748","LY6G5B",21.2024076522941,-0.209631678853091,0.417236800997059,-0.502428545018416,0.615366103931183,0.893872561111145
"10749","RP9",143.304576097244,-0.107353677110107,0.213472617546812,-0.502892025889764,0.61504018780275,0.893872561111145
"10750","OGDH",422.637425433997,-0.1029427978756,0.204532260867138,-0.503308365336414,0.614747485914856,0.893872561111145
"10751","RMDN1",175.322042262005,-0.0841159629148071,0.1673339848012,-0.502683080276492,0.615187107326407,0.893872561111145
"10752","ADCK5",33.1593253699574,-0.149635004470457,0.297687496689246,-0.502658009270239,0.615204736971373,0.893872561111145
"10753","AL353796.2",4.51229729011743,-0.4001377003714,0.795864200616274,-0.502771327145454,0.615125054904499,0.893872561111145
"10754","STAMBPL1",60.9442738489935,0.157713426706732,0.313311916454347,0.503375130098868,0.614700553545155,0.893872561111145
"10755","SBF2-AS1",38.1600909239263,0.142539863856551,0.282989082598373,0.503693861783523,0.614476522466817,0.893872561111145
"10756","ACAD8",75.6724899639858,-0.11573099952589,0.230274329797128,-0.502578813834131,0.615260427755939,0.893872561111145
"10757","BIN2",9.72745924298586,0.366952414832816,0.728465731098454,0.503733256304986,0.614448835224803,0.893872561111145
"10758","AL137060.3",3.21065066814006,0.508447187271357,1.0101895496716,0.50331859742228,0.614740293155513,0.893872561111145
"10759","GSC",148.021559188396,-0.141276023661943,0.281152031438341,-0.502489784403093,0.615323036521116,0.893872561111145
"10760","CHST14",143.990244756865,-0.127055608547412,0.252594342523305,-0.503002590153772,0.614962451094901,0.893872561111145
"10761","SGF29",114.081802369873,-0.131559602807509,0.261830304993477,-0.502461328190358,0.61534304856558,0.893872561111145

"10762","NDRG4",6.81091528298002,-0.937490790063013,1.86064983205292,-0.503851274921862,0.614365892715762,0.893872561111145
"10763","ATAD5",16.9562967023185,0.210462654197504,0.418177855650104,0.503285028974852,0.614763890610127,0.893872561111145
"10764","NDUFAF8",502.757610669924,-0.136504133947037,0.270804852391767,-0.504068271825351,0.614213401955906,0.893872561111145
"10765","TRMT2A",71.4297569524639,0.122212894367432,0.243168882266902,0.502584431149751,0.615256477547267,0.893872561111145
"10766","ZNF74",22.8920870731324,0.19061996812291 4,0.379266198488571,0.502602048066927,0.615244089051161,0.893872561111145
"10767","SLC25A17",70.6793508123758,-0.109795485732294,0.218376724983833,-0.502780164600522,0.615118840835784,0.893872561111145
"10768","AC123912.4",9.27434551540021,-0.391612878806692,0.77966111993428,-0.502286017340076,0.615466343543452,0.8939351423680 9
"10769","LINC01028",18.533797654079,-0.30800651405779,0.61331015054046,-0.502203516094376,0.615524369888763,0.893936404964273
"10770","AC093690.1",16.6216374062646,0.254737493108331,0.507458521756906,0.501986826876781,0.615676787320058,0.89407474036079
"10771","SLC49A4",49.7503179396094,-0.152882585302946,0.304607022494383,-0.50190105287466,0.615737124635531,0.894079345399657
"10772","MLYCD",109.001872976822,0.104421062537321,0.208124607340202,0.501723769581141,0.615861841960945,0.89417742371604
"10773","AC008264.2",6.0904419730369,0.399545009604074,0.796680779334633,0.501512048449021,0.616010800555266,0.89422767038095
"10774","FARP1",291.438078940753,0.107314105356404,0.213956165165211,0.501570521576413,0.615969659599958,0.89422767038095
"10775","SPTLC1",405.86520473751,-0.0689024365378864,0.137494213289717,-0.501129719493727,0.616279832117917,0.894403628019332
"10776","GBGT1",7.87469869744865,-0.333318274668991,0.665178501297245,-0.501095982535435,0.616303574115367,0.894403628019332
"10777","C18orf21",95.1074902583183,-0.134041172533959,0.267417762588368,-0.50124259225176,0.616200402127127,0.894403628019332
"10778","SILC1",10.8926447645612,0.291823871441539,0.582527966053721,0.500961135683273,0.616398475049065,0.894458354960789
"10779","SYPL1",732.39760304363,-0.110627889392715,0.220888564068155,-0.500831221658813,0.616489910478807,0.894508043407417
"10780","RYK",312.811656852594,-0.0904682843401592,0.180883065738478,-0.500147893727978,0.616970944795209,0.894733314045931
"10781","CAMK1D",630.323830977021,-0.125150634782636,0.250168685579198,-0.500264989172741,0.616888502815115,0.894733314045931
"10782","RHOBTB1",13.5594251338583,0.302391192318062,0.604499729742392,0.500233792407031,0.616910466674495,0.894733314045931
"10783","HSPB6",309.451181850491,0.207016074705156,0.413704467582277,0.500396033707287,0.616796245593746,0.894733314045931
"10784","ATP5PF",1800.31501929442,-0.121073545229324,0.242060097979651,-0.500179691902391,0.61694855655977,0.894733314045931
"10785","CSTF2",18.764574704126,0.195986111520622,0.391875756780897,0.500123082710117,0.616988413809806,0.894733314045931
"10786","FNIP2",251.395416007305,-0.136927673514595,0.273967771397893,-0.499794821908926,0.617219557045203,0.894985524957072

"10787","ZNF565",47.3469216020457,0.121416675493561,0.242987644308785,
0.499682507886149,0.617298651123084,0.895017234037734
"10788","HUS1B",2.68962301449512,0.457866023390066,0.916748161498607,0
.499445804877965,0.617465357275754,0.895175953633
"10789","TRIT1",55.2834394654608,0.140109964254284,0.281294933607779,0
.498089185102941,0.618421182912452,0.895185306465805
"10790","SPRR2E",51.2215199127632,-0.311637066087878,0.627785042916419
,-0.496407280810875,0.61960708923891,0.895185306465805
"10791","TTC31",61.3953312034783,0.113564726607929,0.22861094121836,0.
496759805119985,0.619358443134576,0.895185306465805
"10792","CCDC74A",50.9813402463687,0.181483342394004,0.365427232746563
,0.496633327051105,0.619447646951747,0.895185306465805
"10793","PHOSPHO2",27.8773431518173,0.185886927859678,0.37277943844602
6,0.49865123633044,0.618025103312169,0.895185306465805
"10794","PPIL3",214.758868740284,-0.139559525737955,0.279778644815027,
-0.498821222864323,0.617905335022761,0.895185306465805
"10795","MLH1",105.610637928792,0.107087542892527,0.215859249536834,0.
496098930772265,0.619824613567208,0.895185306465805
"10796","IP6K1",134.284138515309,0.0920765133440737,0.184948753296468,
0.497848791640556,0.618590622948807,0.895185306465805
"10797","QTRT2",56.2117683531223,-0.127803074859026,0.257247815145763,
-0.496809175178456,0.619323624407755,0.895185306465805
"10798","AC095057.3",3.66332048695942,0.436225878815157,0.877224607605
587,0.497279573592731,0.618991914070174,0.895185306465805
"10799","NOA1",171.7379897455,-0.135256875006939,0.271103883936841,-0.
498911609242934,0.617841655160819,0.895185306465805
"10800","SORBS2",144.089392020807,0.198331007270425,0.39902590338535,0
.497037925577708,0.619162307050516,0.895185306465805
"10801","GZMK",22.7485853817588,0.248775305845959,0.499596212905732,0.
497952745476275,0.618517349158857,0.895185306465805
"10802","SPDL1",59.0829337056655,0.125343155307198,0.251803780260812,0
.497781070551724,0.618638359454799,0.895185306465805
"10803","HIST1H4J",9.31383499326648,0.326184659050233,0.65715111855870
2,0.49636171930383,0.619639228329917,0.895185306465805
"10804","SAPCD1",2.71037567636228,0.531546239600543,1.06782714756645,0
.497783036151425,0.618636973883491,0.895185306465805
"10805","MAFK",114.434277025864,-0.145098457025169,0.292436870601188,-
0.496170187866112,0.619774342572055,0.895185306465805
"10806","GAL3ST4",47.5971032241817,-0.149756529747705,0.30095152231302
5,-0.497610141981406,0.618758853934466,0.895185306465805
"10807","TNFRSF10D",16.3490632141196,0.27379061138208,0.55051342745495
7,0.497336845438673,0.618951533041888,0.895185306465805
"10808","AC044849.1",25.3817885075629,0.169078292605833,0.340661149979
967,0.496323964783703,0.619665860922369,0.895185306465805
"10809","WAPL",272.482625055107,0.105782978819885,0.212925863886461,0.
496806620337545,0.619325426213872,0.895185306465805
"10810","ARMH3",63.1417624799855,-0.122157558541693,0.245149226395697,
-0.498298772293564,0.618273473064466,0.895185306465805
"10811","TPH1",6.92220963158852,-0.384977721342611,0.775348293072926,-
0.496522304597892,0.619525954683662,0.895185306465805

"10812","KDM4D",8.55700899402974,-0.316843326101031,0.638396152551116,
-0.496311459326442,0.619674682565437,0.895185306465805
"10813","TBC1D15",232.091730033585,0.0988364515375854,0.19873841122821
,0.497319320038702,0.618963889666091,0.895185306465805
"10814","RPAP1",53.1403714442858,0.139674658465302,0.280702968550658,0
.497588818481254,0.618773886453408,0.895185306465805
"10815","MAPKBP1",44.8418518152731,-0.147716147168006,0.29729697690159
1,-0.496863939578174,0.619285002266968,0.895185306465805
"10816","MINDY2",564.187744367829,0.0970714933226725,0.195061366863612
,0.497645919760961,0.618733631878798,0.895185306465805
"10817","CD276",642.283075484331,0.0874580851743232,0.175280964481686,
0.498959401740746,0.617807985101141,0.895185306465805
"10818","CYLD",153.105566380731,0.1036635506524,0.208962403018424,0.49
6087091050826,0.619832966515887,0.895185306465805
"10819","RIPOR1",132.581166252463,0.0852668061906177,0.170773564111916
,0.499297456453732,0.6175698467518,0.895185306465805
"10820","PIK3R5",9.21770554977851,-0.354116071477606,0.711311798475228
,-0.497835228147054,0.618600183708561,0.895185306465805
"10821","EPOP",6.15042515501305,-0.296235083344616,0.594892717896515,-
0.497963875557386,0.618509504139445,0.895185306465805
"10822","SRP68",207.152719409037,0.0865944525561173,0.174576284865328,
0.496026436940836,0.619875758889045,0.895185306465805
"10823","BTBD2",236.708031572418,-0.0785324281288867,0.158084216270802
,-0.496775895667905,0.619347095021144,0.895185306465805
"10824","RRAS",2059.80730294861,-0.128807942491116,0.2593206455119,-0.
496713025825031,0.619391435487452,0.895185306465805
"10825","NCOA6",157.4033890874,0.118743840020207,0.238794842960172,0.4
97263000105961,0.61900359985951,0.895185306465805
"10826","PARD6B",3.42988754658541,-0.429586017649524,0.862373071746512
,-0.498144053570121,0.618382512012451,0.895185306465805
"10827","CBR1",562.106233870616,-0.109758192082304,0.220567743305254,-
0.497616697879547,0.618754232227022,0.895185306465805
"10828","LINC01422",6.74178473067042,-0.334794565177448,0.674832672110
077,-0.496114931914318,0.619813324805388,0.895185306465805
"10829","BX537318.1",11.9929179018809,-0.224786158394182,0.45248166614
4406,-0.496785119073627,0.619340590109269,0.895185306465805
"10830","ZNF81",12.9615821056135,0.222062369322262,0.446647053477655,0
.497176389261395,0.619064669811946,0.895185306465805
"10831","THAP3",120.392434384197,0.137958757398332,0.278194430451763,0
.495907690079559,0.619959540286387,0.89522363679061
"10832","CST2",5.72523882959786,0.468200711306687,0.944843810746631,0.
495532389566808,0.62022436457292,0.895523362437266
"10833","NVL",61.616374785432,0.115830026520484,0.233839370418253,0.49
5340140171033,0.620360041098653,0.895636577382344
"10834","ERH",1179.33806235357,-0.119802302775763,0.241945806392967,-0
.49516172469293,0.620485966147605,0.895674013540781
"10835","AC025048.2",3.70264451676174,0.416782596828774,0.841745065112
653,0.495141123010914,0.620500507462555,0.895674013540781
"10836","AC016831.1",49.9823841364419,0.272003029508139,0.549573275108
703,0.494934964685714,0.620646028659011,0.895793795942664

"10837","SF1",824.095997639006,-0.0597067925703003,0.120667556260137,-0.494804025380141,0.620738462636969,0.895793795942664
"10838","IRAK3",180.083886624266,0.124116972479783,0.250914441724373,0.494658544270338,0.620841169133597,0.895793795942664
"10839","NSFL1C",362.664334876778,-0.0869027427601876,0.175650097008804,-0.494749187390611,0.620777176195778,0.895793795942664
"10840","HMGN1",592.210911256132,-0.112282747148233,0.227009068120581,-0.494617893804098,0.620869868799136,0.895793795942664
"10841","THG1L",97.8860692168611,-0.113169845283091,0.228912590861644,-0.494380168679719,0.621037716842492,0.895953315322993
"10842","RASAL2-AS1",17.8418265808269,0.32659165229725,0.661839249535479,0.493460689323688,0.621687109477334,0.896299171755396
"10843","LGALS8-AS1",3.61742018543415,0.505096379462403,1.02274349106634,0.493864183809935,0.621402100646723,0.896299171755396
"10844","NUP155",58.4824110621926,0.110580012880198,0.224019353822921,0.493618122689557,0.621575899470328,0.896299171755396
"10845","MTMR9",158.855402745146,0.0999731101924099,0.202592787928963,0.493468258245523,0.621681762637585,0.896299171755396
"10846","OXR1",381.122106842804,-0.0689718935318133,0.139706023637631,-0.493693054429151,0.621522971163975,0.896299171755396
"10847","ZNF140",58.1503292254616,-0.122957247740369,0.249208229927002,-0.493391601779707,0.621735915241323,0.896299171755396
"10848","TPM1-AS",8.65512463106543,0.279838385050103,0.566854515961333,0.493668793615453,0.621540107663496,0.896299171755396
"10849","RNPS1",470.008937258012,-0.0711079319536185,0.143975391120319,-0.493889486253898,0.621384230125904,0.896299171755396
"10850","MTFR1L",207.895651040354,-0.100925527264594,0.205065921132549,-0.49216138257979,0.622605261852442,0.896311821851838
"10851","FAAH",12.5449072069132,0.222485319590544,0.451847249436651,0.492390558685335,0.622443272346026,0.896311821851838
"10852","STUM",2.83806247624137,-0.464593856143976,0.942957917459716,-0.492698398880377,0.622225709139369,0.896311821851838
"10853","CCL20",18.835893218349,-0.381295506236826,0.774545133904541,-0.492283134379383,0.622519201242575,0.896311821851838
"10854","CLEC3B",5983.98184254327,-0.180420943303613,0.36607145644898,-0.492857173443017,0.622113509584656,0.896311821851838
"10855","CCR3",4.26713628707777,-0.467325842148565,0.948061440271926,-0.49292780224721,0.62206360188974,0.896311821851838
"10856","SLAIN2",306.958728139682,0.0799942285469765,0.162531259311756,0.492177497951563,0.622593870359389,0.896311821851838
"10857","SH3D19",1292.88605322541,0.0984171347469363,0.199889743248416,0.492357102208227,0.622466919388572,0.896311821851838
"10858","AL390208.1",6.03693360168564,0.369173042903024,0.748508524834503,0.493211540890131,0.62186312412584,0.896311821851838
"10859","ULBP3",2.57452560218994,-0.478887531181832,0.972186248781826,-0.492588258455507,0.622303546074543,0.896311821851838
"10860","TM2D2",333.818479772582,-0.103946796199428,0.211255765044841,

-0.492042412084538,0.622689361471012,0.896311821851838
"10861","NMT2",217.086465614069,0.104931475856698,0.212867873920763,0.492941813736333,0.622053701303915,0.896311821851838
"10862","VIPAS39",80.320891238112,0.11671565148276,0.237226660179782,0.492000567703086,0.622718942214966,0.896311821851838
"10863","N4BP1",238.835702655623,0.0730227111092874,0.148109339344947,0.493032454484302,0.621989655766102,0.896311821851838
"10864","EXOC3L1",2.57585194763742,-0.531886000973606,1.07824649329861,-0.49328794879401,0.621809142315919,0.896311821851838
"10865","MRM1",10.3807435323644,0.23319340730102,0.473393035002443,0.492599996321906,0.622295250649026,0.896311821851838
"10866","AARSD1",152.701353375613,-0.0998703127540881,0.202832145070278,-0.492379118307331,0.62245135836292,0.896311821851838
"10867","SMC2",136.665197109741,-0.087880907451642,0.178709152197555,-0.491753815464883,0.622893389361163,0.896480409460623
"10868","EPHX1",1117.6417110177,0.143890082345292,0.292731430668224,0.491542988796355,0.623042454873585,0.896597247709038
"10869","GAS2",3.95006591313292,-0.338382598862307,0.688514312824568,-0.491467777153569,0.623095637188767,0.896597247709038
"10870","CIAO2A",158.924535351704,-0.0956356521495001,0.194684654913912,-0.491233642383318,0.623261206975234,0.896597247709038
"10871","FCSK",24.6105745961427,0.187134628878784,0.38086548988642,0.491340470186968,0.623185660699842,0.896597247709038
"10872","ZNF682",22.4470816984212,-0.186295862796735,0.379236347372554,-0.491239471341395,0.623257084761897,0.896597247709038
"10873","TIMM10B",114.408968986187,-0.111969655020533,0.228017970978576,-0.491056273064779,0.623386647415427,0.896695223542471
"10874","AC004837.4",4.15598168371193,0.486776277400992,0.991462832253978,0.490967751453034,0.623449256411295,0.896702811318066
"10875","ASB7",45.0771959871218,-0.123201648983513,0.251094519701693,-0.490658454552809,0.623668035293842,0.896825401006874
"10876","DGCR6",32.8694265101693,0.25672967008515,0.523292999698737,0.490604059738906,0.623706514498195,0.896825401006874
"10877","TSPAN7",6.10215279575227,0.383285094907523,0.781046585635099,0.490732693743048,0.623615519720799,0.896825401006874
"10878","ARHGAP9",6.98073370060559,0.353554302733263,0.7209278713815,0.490415639023296,0.623839812335345,0.896934607917338
"10879","VRK2",84.0956896581612,-0.137023507411591,0.279849748878603,-0.489632411537489,0.62439403694662,0.897059456811207
"10880","RAB28",193.630322579635,-0.10637978851603,0.217263335510333,-0.489635254223419,0.624392025030911,0.897059456811207
"10881","OXCT1-AS1",2.50518511478466,0.445457597563552,0.909106102975905,0.489995167896654,0.624137318154708,0.897059456811207
"10882","ZSCAN25",30.307141515439,0.146730202245931,0.299333034200495,0.490190475093537,0.623999120236595,0.897059456811207
"10883","CHMP7",97.4636132000736,-0.104527639810365,0.213511943621404,-0.489563431616318,0.62444285845931,0.897059456811207
"10884","METTL15",88.5131221867399,0.107142633930647,0.218695523875768,0.489916903793194,0.624192700963651,0.897059456811207

"10885","ANKRD13D",159.107070565762,-0.0869237548155633,0.177512502824705,-0.489676802661057,0.624362619374439,0.897059456811207
"10886","LETMD1",266.956121259795,0.127801418984279,0.260931439444636,0.489789268998362,0.624283025003248,0.897059456811207
"10887","KPNA2",455.531435713329,-0.190403497880619,0.388776837822948,-0.489750106891218,0.624310740206001,0.897059456811207
"10888","FBXO11",232.74198001544,0.0857545935237776,0.175204714492485,0.489453687203467,0.624520535014342,0.897088645079382
"10889","C16orf95",36.5539870451642,-0.195899082074841,0.400593741230574,-0.489021824138,0.624826246075279,0.897392897484005
"10890","GAS7",2561.37748747287,0.125899736930682,0.257467693478129,0.488992367274913,0.624847100613863,0.897392897484005
"10891","AC092803.1",24.6467503860236,-0.236254165055147,0.483629990406467,-0.488501891407906,0.625194386332746,0.89749394086598
"10892","MPV17",253.785171769504,0.106791950645312,0.218574051068373,0.488584761655473,0.625135703486393,0.89749394086598
"10893","PCF11-AS1",9.21746563126566,-0.265466549377946,0.543263494670304,-0.48865155119442,0.625088409589307,0.89749394086598
"10894","P2RX7",14.2059929940154,0.198809891523343,0.406990511207866,0.488487780546321,0.625204378883302,0.89749394086598
"10895","MINK1",99.3918382014137,0.0949643419795643,0.194344972718072,0.488638016468401,0.625097993449612,0.89749394086598
"10896","C9orf116",16.2752652616453,-0.226967710805494,0.464977664880715,-0.488126049804393,0.625460560581098,0.897748575631527
"10897","FAM3B",5.70546649812492,-0.33058862086444,0.677331294111873,-0.488075220705567,0.625496561934575,0.897748575631527
"10898","AL031118.1",3.42139673138775,0.442535442847741,0.907318566502764,0.487739873497224,0.625734104824049,0.898007102169951
"10899","CDH22",3.58838356262118,0.604932130366918,1.24059944600759,0.487612768419064,0.625824149746242,0.898053922564568
"10900","ELAC2",116.160119115432,0.0908783773700678,0.186508410642901,0.487261550601429,0.626072991643807,0.89832858617515
"10901","AGK",56.9183742184456,0.115273159301548,0.236657516291375,0.487088519764666,0.626195601659562,0.898421556146868
"10902","SKAP1",4.74619789699776,0.483272066121296,0.992328836382152,0.487007984050143,0.626252672961199,0.898421556146868
"10903","SRBD1",120.882558491474,0.103326683771887,0.212524301551552,0.486187617216205,0.626834150017342,0.899068083154226
"10904","MTIF2",109.944280400178,-0.117233521234231,0.241157288495187,-0.486128874502464,0.626875795831,0.899068083154226
"10905","ZFP41",16.2926091428214,-0.187595030152797,0.385824403463909,-0.486218674787233,0.626812132144844,0.899068083154226
"10906","ATRIP",25.5319831426896,0.163407547470746,0.336245383052874,0.48597707420432,0.62698342059056,0.899075580647536
"10907","SLC15A3",60.0446475063437,0.130289679328158,0.268142428209486,0.485897290474183,0.627039989570379,0.899075580647536
"10908","WFIKKN2",5.07356893666958,0.479376676497451,0.986618896734032,0.485878263718964,0.627053480415813,0.899075580647536
"10909","IFI6",1259.34869539898,-0.164233437068288,0.338637286719662,-

0.48498332436808,0.627688174449176,0.899078949389607
"10910","FNDC4",117.297104384537,-0.182065545695427,0.374822003525715,
-0.485738681248301,0.627152454627174,0.899078949389607
"10911","VDAC3",665.035942953495,-0.114059429879294,0.234889263014694,
-0.485588095494001,0.627259238498883,0.899078949389607
"10912","AC016405.3",6.38369761130772,-0.311887989089154,0.64254499379
3309,-0.485394784959574,0.627396330954871,0.899078949389607
"10913","BX088651.4",3.92615119190375,-0.455396807900481,0.93897995416
4243,-0.484990979712464,0.627682744083938,0.899078949389607
"10914","LRRC27",31.768959126675,0.142428018327901,0.293531693840471,0
.485221941332535,0.627518919530926,0.899078949389607
"10915","EEF1AKMT1",182.442787664267,-0.141070764334031,0.290808313537
572,-0.485098801399313,0.627606262254494,0.899078949389607
"10916","SRP14",4791.21805804237,-0.099967606089669,0.205968877959198,
-0.4853529673035,0.627425988999014,0.899078949389607
"10917","IFT140",56.7761857976425,0.125354780593701,0.258264391250009,
0.485373844946176,0.627411182019072,0.899078949389607
"10918","HEATR3",32.5703244045686,-0.159644806343303,0.328930464980769
,-0.485345151452108,0.627431532247476,0.899078949389607
"10919","SLC35E1",202.989748366512,0.100663937123137,0.207487796807858
,0.485155940117073,0.627565733313127,0.899078949389607
"10920","NAA35",98.3931304462958,0.126618446658367,0.261142226369262,0
.484863931884095,0.627772868845056,0.89911791838248
"10921","COX7A1",973.775791464006,-0.121975065075367,0.251619527215535
,-0.484759932685528,0.627846647579225,0.899141247883809
"10922","SFSWAP",287.986451543031,0.0972813564186873,0.200756650836671
,0.484573517306942,0.627978903009566,0.899248310114413
"10923","TMEM138",96.8511963707047,-0.103961380754281,0.21458110950666
4,-0.484485241936233,0.628041535579454,0.899255663870975
"10924","EFNA4",58.3018840996024,0.178889865210981,0.370113527883419,0
.483337818625557,0.628855891507781,0.899269099678311
"10925","AC112220.2",13.1942453953033,-0.201650880737987,0.41652435146
3092,-0.48412747065007,0.628295406572519,0.899269099678311
"10926","AC093849.2",43.9199408754667,-0.243132677925487,0.50235879827
6523,-0.483982123453633,0.628398556079705,0.899269099678311
"10927","ODF2",62.8467048262262,-0.140131232206386,0.289642710487849,-
0.483807211893443,0.628522696344934,0.899269099678311
"10928","WDR74",226.564753742415,-0.11229837775255,0.23192131915336,-0
.484208947079553,0.62823758782992,0.899269099678311
"10929","AC068888.1",183.251563572312,-0.0795695903298225,0.1643539666
62101,-0.484135503059755,0.628289706370777,0.899269099678311
"10930","SLC24A4",2.52958993391602,-0.511796089568706,1.05872600333485
,-0.483407499160892,0.628806424541507,0.899269099678311
"10931","ATP5MPL",1251.71087809116,-0.103794859745019,0.21472686133795
3,-0.483380882569967,0.628825319750846,0.899269099678311
"10932","ADGRG1",7.78319954996139,-0.385539552011475,0.797258250495924
,-0.483581765095131,0.628682718563251,0.899269099678311
"10933","PIH1D1",940.579662764169,-0.152979896940474,0.316500626316452
,-0.483347849010313,0.628848770726417,0.899269099678311
"10934","C19orf18",6.65718874461659,-0.308049728736992,0.6366965704645

76,-0.483825016541582,0.628510059342824,0.899269099678311
"10935","STK35",82.2311788372082,-0.135440369566127,0.280006996943029,
-0.483703518286307,0.628596295937214,0.899269099678311
"10936","MIF",2431.73549968252,-0.128065763342012,0.264875187515234,-0
.48349475291885,0.628744484503833,0.899269099678311
"10937","LIMK2",23.3595106837097,-0.158687391760654,0.328300916124092,
-0.483359576434058,0.628840445225115,0.899269099678311
"10938","SIRT5",41.7989812287375,-0.146709720783497,0.304129630332967,
-0.482392066247793,0.629527456044828,0.900064851681242
"10939","CCDC122",41.0687923877487,-0.213730875918737,0.44305642149754
3,-0.48240103415344,0.629521086630034,0.900064851681242
"10940","DUSP28",26.5728682634572,-0.170566509806912,0.353737444736674
,-0.482183925803736,0.62967529460283,0.900111654107327
"10941","AL035587.2",8.38296882548324,-0.250175950099864,0.51882894377
1185,-0.482193511181976,0.629668485948459,0.900111654107327
"10942","CYS1",234.225907802077,-0.200505985983553,0.416434528179357,-
0.481482615911223,0.630173532088682,0.90020643474571
"10943","FAM160B2",113.446001098361,0.117828263603145,0.24474007236186
9,0.481442464513639,0.630202062278183,0.90020643474571
"10944","TMEM268",19.8930557609843,-0.183607027269549,0.38122901980303
5,-0.481618706163585,0.630076835182001,0.90020643474571
"10945","HTRA1",2925.59678237632,-0.111619068128316,0.231655805248529,
-0.481831517274378,0.62992563811389,0.90020643474571
"10946","DDHD1",215.242262513042,0.130381742271814,0.270634191879504,0
.481763746725192,0.629973785742689,0.90020643474571
"10947","NDUFAF1",68.8911916271457,-0.148813258127536,0.30901634104126
3,-0.481570837406508,0.630110846893586,0.90020643474571
"10948","NMT1",498.173989887465,0.101891454506658,0.211527839053938,0.
481692882423276,0.630024133011334,0.90020643474571
"10949","STK4",228.203207308832,0.0843414567181594,0.175116827342205,0
.48162965260525,0.630069057625895,0.90020643474571
"10950","RPP25",67.0536474690165,-0.1577247251857,0.327849016083056,-0
.481089518187674,0.630452877414839,0.900351477585464
"10951","PGAP3",36.8961617324498,-0.131161456707115,0.272652882352796,
-0.481056556509827,0.630476303229923,0.900351477585464
"10952","RGS19",35.4094093958707,-0.173297798999776,0.360179426071131,
-0.481142970574759,0.630414889673035,0.900351477585464
"10953","TWF1",260.92117940176,-0.0792850152400967,0.164906493997893,-
0.480787707736419,0.630667387519491,0.900542129170532
"10954","AC008147.2",39.9068775306991,0.23597859479533,0.4909341624883
94,0.48067258876267,0.630749215893757,0.900576751559098
"10955","PCDHB6",5.02515550660172,0.314232864440455,0.653871990989743,
0.480572449608694,0.630820400059506,0.90059617133096
"10956","HOXA10-
AS",32.6718043426947,0.149085007329828,0.310613237574575,0.47996990886
1447,0.631248789944118,0.900934740891386
"10957","NOTCH1",8.31052711177921,0.298238202853279,0.621200522148146,
0.480099729829516,0.631156480332973,0.900934740891386
"10958","CHUK",51.3381288427003,-0.132957484612535,0.277043927896853,-
0.479914812145014,0.631287968377794,0.900934740891386

"10959","DTX1",148.34555008172,0.166758848703009,0.347407923444425,0.480008766206755,0.631221159702254,0.900934740891386
"10960","SUMF1",223.019426479331,-0.0769840201800522,0.16047981720649,-0.479711539557629,0.631432521371311,0.901058816993368
"10961","COX16",672.9341675459,-0.121080335625818,0.252507166808954,-0.479512471491262,0.631574098070852,0.901178623650043
"10962","BCL7C",793.420461513188,-0.123624772049915,0.258023409198222,-0.479122310778175,0.631851618569879,0.901410135403896
"10963","LRRC37A2",19.830836565941,0.213324155120548,0.445230548355875,0.479131892248411,0.63184480266859,0.901410135403896
"10964","USP37",69.2608611456766,-0.12434138372634,0.259611901514328,-0.478951014961374,0.631973477317668,0.901501749840234
"10965","INPP1",171.30050288853,-0.0989495576097676,0.206704211405637,-0.478701217246071,0.632151199733627,0.901540173034866
"10966","RHEB",876.626942550252,-0.115986007952594,0.242272412274509,-0.478742118690653,0.632122098318236,0.901540173034866
"10967","HNRNPA1L2",25.9681233730493,-0.153485178342959,0.320649189219471,-0.478670096489483,0.632173342562236,0.901540173034866
"10968","LINC00672",16.6937396844985,0.255167266078737,0.533247185874538,0.478515916891819,0.632283048243504,0.901614412338476
"10969","BGLAP",41.0901587453258,0.164000429865292,0.343027618441238,0.478096867565739,0.632581261475913,0.90165201597225
"10970","TMEM178A",2.56527465662091,-0.614336073710578,1.28525143112141,-0.477989021319007,0.63265801909784,0.90165201597225
"10971","TF",7.2095273085298,0.327301413216214,0.685321886447495,0.477587860082577,0.632943573104817,0.90165201597225
"10972","FBXW7",170.049559628835,-0.117398747622621,0.245628689766298,-0.477952098080721,0.632684299456365,0.90165201597225
"10973","ADAP1",13.6509187187017,0.292006044000539,0.611248765275161,0.477720464382597,0.632849176849589,0.90165201597225
"10974","COPG2",54.6379795612538,-0.117802221440908,0.246622150720161,-0.477662777236003,0.632890241529849,0.90165201597225
"10975","AC120053.1",14.7066721949311,0.196166427348288,0.410353091959972,0.478043010255721,0.632619592950758,0.90165201597225
"10976","VAV2",35.4295577994068,0.183797005604698,0.384538014576989,0.477968363691907,0.632672722243103,0.90165201597225
"10977","KNTC1",20.3273542114117,-0.174328979605308,0.364700596620256,-0.478005742850013,0.632646117590379,0.90165201597225
"10978","CIITA",11.3106071047193,-0.312726573342906,0.654503281344207,-0.47780749502223,0.632787226102143,0.90165201597225
"10979","ZCCHC3",100.301771819237,0.118818624363621,0.248756089924594,0.477651117605357,0.632898541592566,0.90165201597225
"10980","NOSTRIN",75.8554621539159,0.175592548128715,0.368251270176727,0.476828085465793,0.633484544680195,0.902340462549932
"10981","AC092747.4",41.8416921517024,0.152727843725444,0.320389278801189,0.476694614429394,0.633579598423712,0.902393672647924
"10982","AC099332.1",2.66422559989748,-0.48337145449739,1.01499238887015,-0.476231605081749,0.633909385537367,0.902413799600314
"10983","FAM102A",122.719078135662,0.0919450638655166,0.193085340174787,0.476188734899734,0.633939924310016,0.902413799600314

"10984","RFXAP",78.8062953751448,0.101382882404826,0.21274616953893,0.476543867391579,0.633686962847975,0.902413799600314
"10985","DRC3",25.4287074100335,0.166562160118558,0.349703905082866,0.476294824557621,0.633864351988673,0.902413799600314
"10986","ZNF615",36.2156169876583,-0.137513936233757,0.288664291235957,-0.476380142638949,0.63380357894358,0.902413799600314
"10987","STS",81.3180936358632,0.114858667892508,0.241166630692776,0.476262688426521,0.633887243562713,0.902413799600314
"10988","AC062004.1",69.0288844102214,-0.230951406838355,0.485083699167894,-0.47610630337512,0.633998646540059,0.902415255905216
"10989","TMEM200C",2.88819314135769,-0.468594491404959,0.984683793956582,-0.475883216806167,0.634157579588991,0.90247721062528
"10990","C18orf54",23.2619247671517,0.199354807638522,0.418855288807835,0.475951511095717,0.634108923053658,0.90247721062528
"10991","AP1AR",72.8109821716086,0.132344792514437,0.278167168691566,0.475774309157176,0.63423517454361,0.902505516318994
"10992","PPIB",3927.47839428769,-0.125841111351717,0.26456649820065,-0.475650213491044,0.63432359561791,0.902549220839166
"10993","PAQR9-AS1",2.59146403422819,-0.539984445453124,1.13546459267954,-0.475562557330683,0.6343860558347,0.90255598228461
"10994","NKAIN1",3.81872976685747,0.390848882246394,0.822787180309704,0.475030350010164,0.634765341020951,0.90275237532206
"10995","SCRIB",72.0126741243202,0.12480418275371,0.262765614534872,0.474963906425236,0.634812699730948,0.90275237532206
"10996","PRRT2",43.8508707931961,-0.163209310006756,0.343557564206379,-0.475056663018819,0.634746586423432,0.90275237532206
"10997","ZNF208",93.3878809331909,0.2253132423934,0.474178186438899,0.475165768559523,0.634668823948273,0.90275237532206
"10998","HMOX1",110.775840575663,0.167929418377423,0.353412107070195,0.475166003138849,0.634668656761574,0.90275237532206
"10999","GXYLT1",107.252328489963,0.102617143345625,0.21615097554383,0.474747537398074,0.634966930514716,0.902889607532518
"11000","SLC38A6",78.2977416727643,-0.11564782405228,0.243657468854161,-0.47463278920253,0.635048731017906,0.902923832101823
"11001","HYAL2",148.343453628506,-0.15605670403701,0.328999941954478,-0.474336570121956,0.635259917196314,0.903059907739533
"11002","SPRR2G",7.38715705408665,-1.40595097551954,2.9638802023495,-0.474361606924945,0.635242066331065,0.903059907739533
"11003","BOLA3-AS1",30.8714446471067,-0.151359495170142,0.319268972677487,-0.474081442680744,0.635441831322966,0.903072261871076
"11004","TSPAN18",219.716849935763,-0.139828197205903,0.294930953539676,-0.474104855823798,0.635425136076705,0.903072261871076
"11005","LMAN1",910.6939093065,-0.0610477391706495,0.128766366659819,-0.474096930388104,0.63543078745869,0.903072261871076
"11006","PARS2",8.22052460947518,0.27497312750223,0.580246155349872,0.473890477286193,0.635578010232477,0.903133087778614
"11007","SLC35B3",178.816919476165,-0.0879146507070354,0.185528950773209,-0.473859472285285,0.635600121303018,0.903133087778614

"11008","C2",539.081161164432,-0.150383023977798,0.317471096118318,-0.473690442426142,0.635720669864544,0.903222318012488
"11009","ZFP36L2",7565.35182791093,-0.0613871061945331,0.129652254145086,-0.473475039823362,0.635874304484803,0.90335800996834
"11010","RYBP",256.622221391295,-0.129980171913435,0.274601660478896,-0.47334080823384,0.635970052289822,0.90335800996834
"11011","AL049828.1",8.95106435766698,0.266282728542823,0.562592586765481,0.473313610607216,0.635989453181675,0.90335800996834
"11012","TGOLN2",1052.28735654554,0.0661207815315449,0.139750232196441,0.473135396573807,0.636116584835493,0.903421164608183
"11013","GCC2",742.76242613492,0.0751273400559373,0.158801591896173,0.473089338456107,0.636149442828,0.903421164608183
"11014","AC025034.1",2.76076280302492,0.504376202778273,1.06655649869097,0.472901532546389,0.636283431510796,0.903529405196009
"11015","C5orf24",466.31025577261,0.0810578862539131,0.171475577608552,0.472708051982504,0.636421481177317,0.903561362165327
"11016","AC022211.2",3.04996036127763,0.427075335745649,0.903320812269988,0.472783677675305,0.636367520240197,0.903561362165327
"11017","ID3",1183.61968080323,-0.29253312862340,0.619509805772151,-0.472200965824571,0.636783350418165,0.903746969196016
"11018","AL391832.2",9.95550426682869,0.255103846837237,0.540050023222696,0.472370772831246,0.636662162278349,0.903746969196016
"11019","AP002840.2",6.29708562738866,0.276726004908772,0.585976926583729,0.472247271786104,0.636750301740687,0.903746969196016
"11020","FGFR1OP2",464.214542176207,-0.0895738204307574,0.189671696611469,-0.472257179278805,0.63674323083288,0.903746969196016
"11021","TNFRSF8",3.1554536721167,0.442177716423864,0.936870048212233,0.471973372686684,0.636945794698043,0.903813484764778
"11022","AC092111.1",5.2245365412269,0.371038985647055,0.786078428704391,0.472012679776239,0.636917738071701,0.903813484764778
"11023","TMEM254",70.7521629905375,0.136290489791506,0.288926486519924,0.47171338091258,0.637131384811608,0.903912813720387
"11024","SMC3",641.593162074942,0.080250201280909,0.170115524645558,0.471739433823653,0.637112786417159,0.903912813720387
"11025","AC098487.1",40.3780439917962,0.179990487496887,0.381758236284874,0.471477679822932,0.637299655160634,0.903925894399582
"11026","PEAK1",186.99433503232,-0.125495162020758,0.266108068562075,-0.471594727280821,0.637216090983354,0.903925894399582
"11027","NDUFV2-AS1",24.9486592181465,-0.192588189482865,0.408495249590784,-0.471457598774509,0.637313992170345,0.903925894399582
"11028","ERVK3-1",239.338898108189,-0.0827350246221637,0.1755208916651,-0.471368529622246,0.637377585371738,0.903934116359629
"11029","DDIT4L",71.6297647097295,-0.152594576841928,0.324212413727226,-0.47066235091884,0.637881874204669,0.904123054296611
"11030","MCCC2",70.3401849717282,-0.119324209281122,0.253375671149256,-0.470937911046842,0.637685074178328,0.904123054296611
"11031","ZNF282",56.2382481739951,0.111883785840996,0.237836337778315,0.470423430187873,0.638052527701588,0.904123054296611
"11032","ADAM32",22.5448356094711,0.166641191985437,0.35427492918500 3,

0.470372522178719,0.638088892156393,0.904123054296611
"11033","TRIM14",5.72426121862949,-0.297892074279209,0.632867655117591
,-0.470702005182835,0.637853552278695,0.904123054296611
"11034","UCK1",134.209030493015,-0.0831493307025852,0.176671498813393,
-0.470643715942041,0.637895183887415,0.904123054296611
"11035","CLEC2B",817.742428452449,-0.159748505425658,0.339524889587586
,-0.470506022753446,0.637993532283048,0.904123054296611
"11036","PROSER3",28.4091011603914,-0.215693010033266,0.45812595857003
4,-0.470815953556783,0.637772170847879,0.904123054296611
"11037","IRF2BP1",101.615480094066,-0.125454777819139,0.26658472701430
2,-0.470600019829374,0.637926393474288,0.904123054296611
"11038","AC010327.5",3.30296616068665,-0.414707465357952,0.88135606262
7742,-0.470533400679757,0.637973976885703,0.904123054296611
"11039","AP3B1",304.680372924618,-0.0964422168928123,0.205118711598934
,-0.470177567619401,0.638228159558252,0.904215261758329
"11040","NUFIP1",75.6373812382724,-0.105811783861684,0.225078509878185
,-0.470110557951315,0.638276031417209,0.904215261758329
"11041","MIR133A1HG",2.79022876088041,-0.521214883954554,1.10887667229
471,-0.470038640885061,0.63832741081034,0.904215261758329
"11042","MCPH1-
AS1",6.00325340050564,0.325575672956361,0.693139826219295,0.4697113924
79352,0.638561227378505,0.904382649298182
"11043","DOLPP1",20.6029041542448,-0.188184575768685,0.400632254858781
,-0.46971898414674,0.638555802780925,0.904382649298182
"11044","KCNK1",110.657532611791,0.168387436808581,0.358605040583161,0
.469562381317201,0.638667706663674,0.904451551269455
"11045","GEMIN5",91.8192295329417,0.105261381755159,0.224534602849876,
0.468798040120063,0.639214000982451,0.904587711217667
"11046","AL022069.1",3.31361660002446,-0.402363228820072,0.85818904064
034,-0.468851511457018,0.639175777270125,0.904587711217667
"11047","LINC00513",31.7586494970172,0.263530125713691,0.5619082340945
52,0.468991393476088,0.639075787855282,0.904587711217667
"11048","SLC39A14",271.305910943001,-0.142246462925622,0.3032762560736
4,-0.469032639637581,0.639046305835896,0.904587711217667
"11049","SLC41A2",144.718294632018,-0.100855385472184,0.21514416944591
5,-0.468780472796117,0.639226559103431,0.904587711217667
"11050","AC009093.1",18.4898600049609,0.32556983801211,0.6937387253582
13,0.469297483492797,0.638857013770033,0.904587711217667
"11051","LRP3",71.7219806891955,-0.134809144509811,0.287304435448957,-
0.469220547532276,0.638911999848236,0.904587711217667
"11052","RCAN1",147.197924210328,-0.100850665769987,0.215097367010561,
-0.468860531263664,0.639169329601832,0.904587711217667
"11053","CENPJ",46.6515538519975,-0.141424494888704,0.301760241846031,
-0.468665103207547,0.639309034407976,0.904622572888876
"11054","ANKS3",99.6461716989885,0.100181839540368,0.213942691084516,0
.468264837805523,0.639595210337528,0.904945638653785
"11055","PCBP2",1831.34152508717,-0.0820691675081469,0.175349929245875
,-0.468030799106111,0.639762564774327,0.90509822471309
"11056","SNX21",872.573777303661,-0.100932888198488,0.215690609488893,
-0.467952167401546,0.639818796191044,0.90509822471309

"11057","EMILIN1",294.327179365354,0.113238661211666,0.242072185049895
,0.467788817572435,0.639935618182947,0.905181610597928
"11058","VAMP5",988.657623464582,-0.115966781432786,0.248366587769551,
-0.466917802729515,0.640558687690918,0.905299134309234
"11059","KLHL6",2.86166511407451,-0.438008799323847,0.938682508244041,
-0.466620817451061,0.640771190332686,0.905299134309234
"11060","Z97989.1",23.91132905578,-0.16410634190981,0.351253474865202,
-0.467202045396954,0.640355330398216,0.905299134309234
"11061","REPS1",93.1547553226876,0.112898247929443,0.24158648062159,0.
467320222716773,0.640270790095992,0.905299134309234
"11062","UBQLN1",248.777808876169,0.0681340306022733,0.145992371130954
,0.466695828518038,0.640717514690164,0.905299134309234
"11063","ABCA2",79.8756101764166,0.106741700056988,0.228460797887218,0
.467221077069344,0.640341715428728,0.905299134309234
"11064","SH3PXD2A",349.630828723307,0.109878443437245,0.23545890212855
5,0.466656569124978,0.640745607285747,0.905299134309234
"11065","FDXACB1",16.3389322558539,-0.179334677400987,0.38365710870792
5,-0.467434783119093,0.640188841678292,0.905299134309234
"11066","KRAS",644.554174880073,-0.0715377267009901,0.153129486386748,
-0.467171466377889,0.640377206417724,0.905299134309234
"11067","CIAPIN1",134.918112887598,-0.0981513919849817,0.2102900206642
47,-0.466742985116218,0.640683771821896,0.905299134309234
"11068","TRAPPC2L",555.229517519233,0.151910565889119,0.32502543516563
4,0.467380547653769,0.640227637351325,0.905299134309234
"11069","RNFT1-
DT",12.5010088932433,-0.348084982419311,0.745805694130835,-0.466723417
585288,0.640697773263753,0.905299134309234
"11070","FGD1",57.0595507291182,0.104303140521509,0.223454813369195,0.
46677508955324,0.64066079994111,0.905299134309234
"11071","ZNF436-
AS1",14.4513852661465,0.20224337022043,0.43349952400752,0.466536545071
088,0.640831495330685,0.905302555051206
"11072","CSTF2T",118.091934310481,0.095154661243207,0.204209198176463,
0.465966577866786,0.64123942394751,0.905492399003503
"11073","PAN3-
AS1",8.7008928338696,-0.229806512742195,0.493029032934499,-0.466111521
616467,0.641135676642534,0.905492399003503
"11074","ASIP",3.80387326587866,-0.40271087813909,0.864289710046538,-0
.465944316423027,0.641255358782787,0.905492399003503
"11075","MB",2.60587052856663,-0.410192676697451,0.880058098716151,-0.
466097269368749,0.641145877753396,0.905492399003503
"11076","RNF113A",75.7417512739444,-0.123435075052439,0.26489478362171
3,-0.465977749221037,0.641231427507336,0.905492399003503
"11077","AKR7A3",3.03646573947197,-0.374891735772383,0.807667245947904
,-0.464166075389622,0.64252876461513,0.905512655664737
"11078","C2orf73",3.82929636506195,0.383701983087552,0.826461655777439
,0.464270762479125,0.642453768592719,0.905512655664737
"11079","ANKAR",73.6984960964466,0.135150819571233,0.29026968421848,0.
465604322184429,0.641498748627606,0.905512655664737
"11080","AC084030.1",4.11463026034274,0.367645673810444,0.792170091993

782,0.464099411889094,0.64257652309452,0.905512655664737
"11081","AC097359.2",4.95412327049839,-0.354484356615635,0.76473340308
0212,-0.463539784175549,0.642977504915782,0.905512655664737
"11082","SLC25A20",85.9129882859442,0.130988432865665,0.28237449413813
,0.463881956709549,0.642732320694135,0.905512655664737
"11083","SELENOT",426.496611555321,-0.0781579793707236,0.1680420738711
63,-0.465109585773424,0.641852981741337,0.905512655664737
"11084","MED12L",3.10305789540533,-0.404059163782798,0.870635228066945
,-0.464096961341575,0.642578278722402,0.905512655664737
"11085","NDUFB5",663.324025872803,-0.123873028159182,0.26718337463281,
-0.463625509369439,0.642916074752571,0.905512655664737
"11086","AC097382.3",46.884949161308,0.158805414336209,0.3424357873607
69,0.463752388616152,0.642825158321292,0.905512655664737
"11087","LARP7",584.618562225476,-0.067896550883799,0.146351107013914,
-0.463929192399917,0.64269847694851,0.905512655664737
"11088","AC025171.3",6.4254028756155,-0.305193415851303,0.656494848512
465,-0.464883184602032,0.642015113016382,0.905512655664737
"11089","RPL10A",10718.1623043224,-0.117213692756093,0.252732079731482
,-0.463786365706435,0.642800812648816,0.905512655664737
"11090","RNF146",411.306862844897,0.0816162056114914,0.175280854411612
,0.465631034749706,0.641479624652282,0.905512655664737
"11091","EFR3A",258.352472183626,-0.0844187750218985,0.182119188632486
,-0.463535861628805,0.642980315847816,0.905512655664737
"11092","SNCG",17.2875381375287,-0.272841107530197,0.58585744503395,-0
.465712452479606,0.641421337795776,0.905512655664737
"11093","PAK1",94.4456577181635,0.118427994651158,0.254596629232616,0.
46515931891209,0.641817368942086,0.905512655664737
"11094","AP000880.1",3.01374946388757,0.44941733048845,0.9677323337726
5,0.464402515865541,0.642359387923214,0.905512655664737
"11095","COMMD6",2409.71243907933,-0.110039809807494,0.237117737095245
,-0.464072452594695,0.642595837454982,0.905512655664737
"11096","BRICD5",5.83476719695777,-0.280498208581879,0.605275276900191
,-0.463422543901677,0.643061522549668,0.905512655664737
"11097","AC020663.3",3.84692475281904,-0.381525039182998,0.82086212979
5721,-0.464785772585153,0.642084877334717,0.905512655664737
"11098","AC009133.1",14.0103561580738,-0.211413434912634,0.45441990079
5491,-0.465238064051643,0.641760982976427,0.905512655664737
"11099","MAPK3",475.061954551194,-0.0907534748627434,0.195212945600695
,-0.464894756766685,0.64200682549956,0.905512655664737
"11100","SEC14L1",189.685258711323,-0.0936445336646803,0.2012372919895
22,-0.465343837312003,0.641685246601257,0.905512655664737
"11101","FN3KRP",167.506851888741,-0.136549690140465,0.294138724815085
,-0.464235677319635,0.642478902588812,0.905512655664737
"11102","ZADH2",210.233023404715,0.077264225757035,0.166435283538308,0
.464229844264069,0.642483081261231,0.905512655664737
"11103","RGL4",2.82205307675229,0.434477309068346,0.936678834638272,0.
463848752636899,0.642756111415649,0.905512655664737
"11104","MICALL1",32.0241171001441,-0.158364664775263,0.34016697986270
3,-0.465549786281965,0.641537792537055,0.905512655664737
"11105","GSPT2",89.4170929366201,0.0989159923902961,0.213448581559505,

0.463418363652701,0.643064518316383,0.905512655664737
"11106","AMER1",15.1904743322307,-0.179287621750549,0.385961570478053,
-0.464521951054564,0.642273836303192,0.905512655664737
"11107","PWP2",3.60137429549853,1.1833202431014,2.55066399183586,0.463
926352859085,0.642700511420902,0.905512655664737
"11108","POLR2G",414.386627503976,-0.10899773175856,0.235362721267934,
-0.463105334487014,0.643288866501092,0.90574701765188
"11109","SOX15",20.7764972535268,0.174655934469948,0.377214040696027,0
.463015465033264,0.643353282000849,0.905756173417345
"11110","ZNF439",16.8118791386014,-0.180497601352991,0.390000628925853
,-0.462813616096264,0.643497970520437,0.905878331137681
"11111","AC124016.1",11.4973842556887,0.242322750276448,0.523690148611
811,0.46272161299729,0.64356392428605,0.905889638721432
"11112","MSH2",65.8650214936014,0.108360436902892,0.234747433805954,0.
461604351306627,0.64436507360628,0.906516002724557
"11113","AC005041.4",13.21481378438,0.193426697923367,0.41893607853731
3,0.461709334270524,0.644289776386279,0.906516002724557
"11114","HNRNPD",839.648302639076,0.0823466096690765,0.178386402649038
,0.461619318772223,0.644354338226703,0.906516002724557
"11115","HOXA3",36.1952204800128,0.195369912679621,0.423345757463346,0
.461490186768994,0.644446960293344,0.906516002724557
"11116","ZNF658",27.139865020182,-0.149580012851501,0.324149035031645,
-0.461454444363527,0.644472598100662,0.906516002724557
"11117","USP35",12.3088804863266,0.234218981304426,0.507306060211769,0
.461691668352343,0.644302446705991,0.906516002724557
"11118","TM9SF2",558.488862991164,0.0639682159155314,0.138506914715842
,0.461841317069025,0.644195119179057,0.906516002724557
"11119","C15orf40",260.868705153102,0.115808688312432,0.25081944822325
5,0.461721326367605,0.644281175493749,0.906516002724557
"11120","BHLHE40-
AS1",7.47090547766975,-0.278256901256521,0.603253277998169,-0.46126048
7767074,0.644611729345454,0.906630166093786
"11121","SLC22A15",9.72409242185176,0.28252452040657,0.613524852119168
,0.460494011661803,0.645161668823599,0.90732204841301
"11122","XCL2",6.79788407464658,0.432190368864536,0.938927024114147,0.
460302406645816,0.64529917398245,0.907433832142197
"11123","ALKBH5",285.476948875986,0.0947935535215334,0.206005747134964
,0.460150043578297,0.645408525850721,0.907506009557249
"11124","VSIG10",72.9321593056863,0.124244475391538,0.270079881686558,
0.460028620479514,0.645495677411483,0.907546961049586
"11125","KHK",9.42562763976618,-0.306089150762414,0.665492477295128,-0
.45994381785727,0.645556547472883,0.907550957525923
"11126","ALG14",107.374590706095,-0.125067688568917,0.272326830566873,
-0.459255844562129,0.646050452206349,0.907665010698724
"11127","IRF1",1586.98126673491,-0.29381902704383,0.640097251514848,-0
.459022478769407,0.646218023921374,0.907665010698724
"11128","AL590062.1",21.3705263671382,-0.161457678729811,0.35122130603
0628,-0.459703542915847,0.64572902614585,0.907665010698724
"11129","ARAP1",377.079348394299,0.0991275456793777,0.215780816008925,
0.459389984303691,0.645954139333824,0.907665010698724

"11130","SYCP3",2.82861405721419,0.468804125697929,1.02007485839141,0.45957815923157,0.64581903898354,0.907665010698724
"11131","TCHP",99.6596565010148,0.10990038195718,0.239299901096396,0.45925794976785,0.646048940614363,0.907665010698724
"11132","AL355075.4",63.6129403033535,-0.270249428499531,0.588000193101875,-0.459607720660576,0.645797816392072,0.907665010698724
"11133","CHTF18",14.2205871510143,-0.251880038351053,0.548275231348146,-0.459404372018974,0.645943809248935,0.907665010698724
"11134","RAB9B",62.2485626985141,0.152307015768682,0.331793219206237,0.459041978413701,0.646204021229638,0.907665010698724
"11135","LINC01285",2.62866529482986,0.457154227525437,0.995858731185187,0.459055298919125,0.646194455848683,0.907665010698724
"11136","ZBTB48",42.3536089631192,0.114121432883432,0.24875488472887,0.458770620757132,0.646398894385808,0.907674511419827
"11137","PSMB8",680.64203237126,-0.130632110739986,0.28471392129022,-0.458818838741741,0.646364265283995,0.907674511419827
"11138","BISPR",11.6075502095002,-0.269941508416651,0.588375094040342,-0.458791527974061,0.64638387918587,0.907674511419827
"11139","ARMH1",4.2484934929119,-0.333073307965118,0.72735869835838,-0.457921667420561,0.647008719520554,0.907747867914073
"11140","IPO9",164.982404603374,0.0981839382782433,0.214802064072589,0.457090292414805,0.647606147492373,0.907747867914073
"11141","PSEN2",24.6213063556152,-0.170802765694621,0.372778496350779,-0.458188354120882,0.646817126006983,0.907747867914073
"11142","AC016949.1",22.1012279885708,0.216272962021138,0.472785486429985,0.457444164909166,0.647351826136779,0.907747867914073
"11143","PSMC2",391.768405324953,-0.0918841312582723,0.200920406801493,-0.457316072175051,0.647443879168918,0.907747867914073
"11144","EXOSC4",86.8651042115481,-0.14760270993055,0.322039481727901,-0.45833731050177,0.646710122698728,0.907747867914073
"11145","DGAT1",83.9606365659401,-0.114927557787448,0.25137556782696,-0.457194622297427,0.64753116333462,0.907747867914073
"11146","RPL35",9205.00521651516,-0.0891854038079294,0.195187910252717,-0.456920737009059,0.647728018281397,0.907747867914073
"11147","HOXC6",286.350601302648,0.0733746832562939,0.160574210881145,0.456951853312267,0.647705652209591,0.907747867914073
"11148","ARL6IP4",1861.73609589723,-0.0951542705325523,0.208115429192059,-0.457218721850457,0.647513842969726,0.907747867914073
"11149","SBNO1",256.113776727408,0.0919376002435166,0.200637686387378,0.458226975693935,0.646789381371017,0.907747867914073
"11150","EID1",4101.96744864747,-0.0927759188814846,0.202597352235111,-0.45793253395444,0.647000912309407,0.907747867914073
"11151","DET1",48.4635767379433,0.117446576075929,0.256924200073346,0.457125393569001,0.647580919129554,0.907747867914073
"11152","TMEM204",121.154884592343,0.162463280693773,0.355013475923474,0.457625672578112,0.64722139603675,0.907747867914073
"11153","SNHG19",183.731164418442,-0.125946045787619,0.275120987577451,-0.457784216669994,0.647107476261452,0.907747867914073
"11154","KCTD5",64.1351418952713,-0.109694415207372,0.239251384880493,-0.45849019959556,0.646600301910143,0.907747867914073

"11155","HSBP1",1199.11370176865,-0.105720808150825,0.231359643131215,
-0.456954405357833,0.647703817840163,0.907747867914073
"11156","AC064805.2",3.0508210271309,0.476050322627645,1.0401055020998
6,0.457694264347753,0.647172109306808,0.907747867914073
"11157","BCKDHA",119.471726012062,-0.0973927543110698,0.21308200454487
8,-0.457067008164726,0.647622882877886,0.907747867914073
"11158","CD37",19.9161672135761,-0.185096361475535,0.404343826646413,-
0.457769722888329,0.647117890234608,0.907747867914073
"11159","SRC",77.5212129153144,0.104617352525649,0.228695291412742,0.4
57453023537942,0.647345460138706,0.907747867914073
"11160","CCT8",543.965491229546,-0.112216064954543,0.245230105404547,-
0.457594979088821,0.647243451395489,0.907747867914073
"11161","FLNB",372.895837656123,0.155122853201595,0.339560812147626,0.
456833791333244,0.647790515605256,0.907754113795019
"11162","ZNF431",152.101403510212,0.111721691537452,0.244624996235545,
0.456705950972717,0.647882412899945,0.907801553283922
"11163","ERCC4",107.869346567685,0.0868785587545439,0.190278821830291,
0.456585540728387,0.647968974006036,0.907841507968682
"11164","RELL2",3.5971681384382,-0.36222775916088,0.793489043485167,-0
.456500013623252,0.648030461040953,0.907846328437881
"11165","F8A3",17.7466335892526,-0.219076648530079,0.480102313534499,-
0.456312419986572,0.64816533402076,0.90795394752214
"11166","GCN1",104.304112798466,0.0935456060839072,0.205088808180686,0
.456122432587799,0.648301939795882,0.907959681655059
"11167","PCTP",60.8475128389032,0.125036770525967,0.274210952417984,0.
455987514077742,0.648398956827266,0.907959681655059
"11168","WBP2",266.078769094109,-0.0770316083112089,0.168957838850881,
-0.455922074022239,0.64844601552924,0.907959681655059
"11169","AP005482.3",4.12451960341974,0.344646194491608,0.755595120980
358,0.456125489593477,0.648299741637274,0.907959681655059
"11170","KDSR",943.064764175155,-0.075050422274654,0.164619258879593,-
0.455903050380928,0.648459695913492,0.907959681655059
"11171","AGGF1",251.87088955571,0.0741814255649197,0.162802557635539,0
.455652703755352,0.648639737591492,0.908112969623102
"11172","TMEM230",1159.85630327788,-0.120986391842409,0.26556018235590
5,-0.455589353679017,0.64868530029599,0.908112969623102
"11173","C6orf52",4.90650574506146,-0.32581286395487,0.715418236229903
,-0.455415933582896,0.648810034395716,0.908125016820208
"11174","ERV3-1",55.2773352131865,0.120740725120288,0.265084638062198,
0.455479902580994,0.648764022921473,0.908125016820208
"11175","SFT2D3",76.5033477621996,-0.109504230692424,0.24053360144972,
-0.455255440538997,0.648925479364305,0.908184157819731
"11176","SUPT3H",38.4297986275728,0.147116223985884,0.323193322160366,
0.455195741677133,0.648968423771951,0.908184157819731
"11177","AC069200.1",2.47353254494254,-0.510460258183187,1.12175913132
845,-0.45505335675643,0.649070853150205,0.908198658493605
"11178","TNXB",13924.1592560961,-0.0737719034463519,0.162179744753663,
-0.454877417388992,0.649197430214565,0.908198658493605
"11179","SLC25A51",31.7954066611879,0.188884011234511,0.41525676604650
5,0.454860767309828,0.649209409400912,0.908198658493605

"11180","PRPSAP2",72.2794793601281,-0.107463762929864,0.236257582906124,-0.454858471029752,0.649211061506298,0.908198658493605
"11181","AL357873.1",9.77842080858243,-0.258757573055358,0.56985785538101,-0.454073889851622,0.649775645359512,0.908257835039338
"11182","TSPAN2",4.73829190254657,-0.366067532270179,0.807130635789011,-0.453541863037239,0.650158605815079,0.908257835039338
"11183","ELF3-AS1",7.3712640727825,0.31901951210523,0.702807251899013,0.453921770504256,0.649885133586182,0.908257835039338
"11184","OXSR1",133.995465378654,0.0972119321562174,0.214431475507522,0.453347307927317,0.650298672427782,0.908257835039338
"11185","PI4K2B",65.2582394860622,-0.10547360182334,0.23262430678863,-0.453407484709571,0.650255347866187,0.908257835039338
"11186","HSD17B11",519.639258479263,-0.118927101593829,0.261757594327671,-0.45434059668563,0.649583700797604,0.908257835039338
"11187","AC107214.1",6.18197958501117,-0.340067878037063,0.749865654948618,-0.453505072265732,0.650185091752238,0.908257835039338
"11188","RFESD",15.4093423746268,0.218031409452657,0.479490921880926,0.454714363720116,0.649314745891973,0.908257835039338
"11189","GLCCI1",31.3418976110724,0.14293301089533,0.315174795720781,0.453503937611677,0.650185908604759,0.908257835039338
"11190","ZNF800",212.947133850971,-0.0792830844315043,0.174513334857176,-0.45430960617646,0.649606002967985,0.908257835039338
"11191","AC009812.1",16.8691375851794,-0.184131522236158,0.405445958764744,-0.454145659256647,0.649723991801773,0.908257835039338
"11192","AL139243.1",4.32112195647161,0.360787943602447,0.794947919957293,0.45385104425687,0.649936041529357,0.908257835039338
"11193","AP000662.1",7.2083222569962,0.302534072587852,0.667106059965134,0.453502210133819,0.650187152239814,0.908257835039338
"11194","LYSMD2",258.548979006447,-0.111717407162742,0.246151145335417,-0.453856946351033,0.649931793207053,0.908257835039338
"11195","AC092868.2",12.6360860904389,0.361512191346165,0.795728072436646,0.45431624680421,0.649601224045836,0.908257835039338
"11196","SAXO2",6.26011466234756,0.282938685941105,0.623478298580876,0.453806791006381,0.649967895343567,0.908257835039338
"11197","C17orf80",68.5353703485586,0.124105796126273,0.273329390033465,0.454052146061124,0.649791295036196,0.908257835039338
"11198","ZNF490",35.8755493991853,0.140181659675984,0.308559497789859,0.454309981316644,0.649605732998285,0.908257835039338
"11199","DDO",5.80593233316655,0.3233559752581,0.713396309483839,0.453262753058053,0.65035955010661,0.908261752269611
"11200","CGREF1",62.0762460715293,-0.243365512237181,0.537137427350278,-0.453078671947539,0.650492092597157,0.908299493089165
"11201","MIR3936HG",14.465840376743,0.202979653009381,0.448175063189274,0.452902603649901,0.650618876028,0.908299493089165
"11202","AL162390.1",35.4376259490537,0.213011239090566,0.470170154974809,0.4530513832848,0.650511741981749,0.908299493089165
"11203","YJU2",104.294118717283,-0.114680639370595,0.253191535108361,-0.45294025853397,0.65059176061247,0.908299493089165
"11204","ZNF502",34.882611919558,0.135525508962249,0.299418550305913,0

.452628966454427,0.650815937031196,0.908334787425623
"11205","TMEM186",66.9306388605091,-0.13733506675423,0.303363261860672
,-0.452708300642236,0.650758801661629,0.908334787425623
"11206","ITGB3",127.678374773031,0.143206369802742,0.316390373266671,0
.452625559760759,0.650818390530149,0.908334787425623
"11207","PLEKHM2",411.063622759356,-0.0980147970012229,0.2167779218514
06,-0.45214381688065,0.651165379577538,0.908429645270387
"11208","SYTL1",6.82493319408567,0.269098990176389,0.594783612278811,0
.452431749330455,0.650957978927378,0.908429645270387
"11209","AL513166.1",6.79116405221395,0.271605226385294,0.600668336061
911,0.452171706213094,0.651145289428171,0.908429645270387
"11210","PPT2",23.7237869388577,-0.163566380117564,0.361770075418945,-
0.452127998503324,0.651176774496567,0.908429645270387
"11211","SCART1",3.51340500791069,0.376832727081756,0.833417828771881,
0.45215342661562,0.651158457152383,0.908429645270387
"11212","MRPS6",590.690544471411,-0.10987225714507,0.243162024596581,-
0.451847928669596,0.651378538973907,0.908630070420256
"11213","IQCE",65.8560710797349,-0.11225743791151,0.248537835949519,-0
.451671422512552,0.651505708494466,0.908645379066653
"11214","AC004522.5",5.69769039626171,0.344686568809214,0.763097034668
106,0.451694284147138,0.651489236525143,0.908645379066653
"11215","SYF2",854.169129407366,-0.0891142009289183,0.197990932555304,
-0.450092333920527,0.652643864223277,0.908666112232494
"11216","NDC1",37.3829626206641,0.124219775900106,0.276022244733798,0.
450035380372718,0.652684929622315,0.908666112232494
"11217","LAMTOR2",612.485845472063,-0.132603375581674,0.29478333543052
3,-0.449833350952523,0.652830608030799,0.908666112232494
"11218","AKT3",303.382519658876,0.117703646155133,0.261633005077968,0.
449880725560817,0.652796446186242,0.908666112232494
"11219","CD28",3.08609218674241,-0.409293861658819,0.908873373750616,-
0.450331007024444,0.65247178444078,0.908666112232494
"11220","CRBN",416.870893869058,0.0917376124606292,0.203783421300693,0
.450172108580244,0.652586345814716,0.908666112232494
"11221","TMEM156",2.7602702637316,-0.445208606935831,0.990624405227685
,-0.449422207434416,0.653127114316489,0.908666112232494
"11222","TMEM232",4.40732397788351,-0.349930801504249,0.77751524993322
2,-0.450062942860997,0.652665056021648,0.908666112232494
"11223","AC113349.2",3.82036834298125,-0.421589412630887,0.93421424797
6585,-0.451277010111982,0.651789912190902,0.908666112232494
"11224","ZNF300",16.9539032954202,-0.169906850501753,0.37822695111662
,-0.449219311321873,0.653273458045177,0.908666112232494
"11225","DNPH1",312.489716270848,0.158039932896424,0.351280705910617,0
.449896422539747,0.652785127250509,0.908666112232494
"11226","CRCP",204.718381558748,-0.101417172695014,0.224612351651445,-
0.451520906794984,0.651614160378885,0.908666112232494
"11227","TSC22D4",207.155311068882,0.0776763753349715,0.17211149964841
,0.4513142671677 9,0.65176306352547,0.908666112232494
"11228","AC015987.1",4.93958667246488,-0.321904866735874,0.71704510564
4185,-0.448932520704787,0.653480335465966,0.908666112232494
"11229","NOL6",40.4690134443418,-0.146210074585864,0.324574114149031,-

0.450467453232363,0.65237341704856,0.908666112232494
"11230","LINC00857",10.7487785264873,-0.255320151846985,0.567913276935062,-0.44957595149898,0.653016231577565,0.908666112232494
"11231","HPS1",189.517489499542,-0.0784514036193131,0.173962684321384,-0.450966849157026,0.652013441941778,0.908666112232494
"11232","PLEKHA1",207.141686424737,-0.0961838664301423,0.214202595678166,-0.44903221702624,0.653408416152567,0.908666112232494
"11233","SERPING1",8179.40116067055,0.0716152085017462,0.159417864156508,0.449229506872819,0.65326610393911,0.908666112232494
"11234","CATSPERZ",6.02361039409022,0.344028544233222,0.763656576140973,0.450501645611068,0.652348767877857,0.908666112232494
"11235","ARHGEF17",36.7412483053447,-0.137391861653724,0.304763384009079,-0.450814857895235,0.652122991868449,0.908666112232494
"11236","EMG1",197.759991519288,-0.115132377966136,0.256103164729255,-0.449554686635173,0.653031567689176,0.908666112232494
"11237","IRAK4",91.1518124601141,-0.0981128378082348,0.218204021898711,-0.449638081619679,0.652971424490545,0.908666112232494
"11238","PIBF1",172.369870462112,-0.0762333570859355,0.1696870046251,-0.449258664529806,0.653245072549254,0.908666112232494
"11239","C15orf48",11.8661696039214,-0.358161607053065,0.796691379026109,-0.449561293723159,0.653026802675228,0.908666112232494
"11240","ABHD17C",7.96600927146022,-0.231182873028143,0.513481621064126,-0.450226188327921,0.65254735489557,0.908666112232494
"11241","SEPTIN9",225.421575959296,-0.0701703450008864,0.155716359136362,-0.450629242746663,0.652256786884716,0.908666112232494
"11242","RNF125",10.5898442995905,0.324820763604155,0.723488705093447,0.448964526076741,0.65345724695758,0.908666112232494
"11243","AC008946.1",4.62784705561218,-0.337245137179742,0.747331566029657,-0.451265746703866,0.651798029063812,0.908666112232494
"11244","TRAPPC2B",120.079021111037,-0.141040796128602,0.313459147933743,-0.449949529494716,0.652746832945018,0.908666112232494
"11245","NDUFAF5",60.220029907225,0.10395232915345,0.231565618098464,0.448910896216244,0.653495935447,0.908666112232494
"11246","DGCR8",100.147067629024,-0.114027675008663,0.253385193197933,-0.45001712045419,0.652698095852704,0.908666112232494
"11247","EIF2S3",753.284086336678,-0.0813152094451601,0.180593917592992,-0.450265493594428,0.652519016814913,0.908666112232494
"11248","CHST7",392.304990841327,0.14587357077389,0.323594362842018,0.450791446095454,0.652139866933064,0.908666112232494
"11249","NR3C1",1160.79762075643,0.0632967507791879,0.141049348010618,0.448756067801343,0.653607633625892,0.908682854173267
"11250","RPL26L1",254.807247630678,-0.109016151503226,0.243082423729587,-0.448474018938118,0.653811132657444,0.908682854173267
"11251","MGAT4B",233.215353106097,-0.110622809227559,0.246543305250381,-0.448695246927165,0.65365151387642,0.908682854173267
"11252","EIF3E",2837.51768368993,-0.104878161200417,0.233817258647385,-0.448547561489385,0.65375806902263,0.908682854173267
"11253","POLD1",20.4860850538414,0.15546567275954,0.34651824503904,0.448650756447254,0.653683613045607,0.908682854173267
"11254","MBOAT7",104.385620996671,0.105031753663071,0.234230971688423,

0.448411040205161,0.653856575502938,0.908682854173267
"11255","TRIM9",4.67150339228134,-0.441559245893227,0.984990106753507,
-0.448288000930883,0.653945359250299,0.90872549255217
"11256","SF3A3",244.060649542909,-0.0725334622238171,0.162059484467993
,-0.447573077638308,0.65446133666788,0.908796528943056
"11257","DVL3",247.11204746982,0.0822806681211737,0.18369708885509,0.4
47914926872255,0.654214595199651,0.908796528943056
"11258","PDGFRL",2229.38648137662,0.0903216795035965,0.201783093699194
,0.447617676227338,0.654429143956927,0.908796528943056
"11259","VMO1",6.92416101406507,-0.276080225502992,0.616278448072972,-
0.447979685751239,0.65416785750192,0.908796528943056
"11260","ZNF20",19.819115082419,-0.169679996769582,0.378892370464731,-
0.447831653515379,0.654274697135871,0.908796528943056
"11261","JOSD2",472.596229131569,-0.118297413665812,0.264262777474504,
-0.447650686170607,0.654405316720967,0.908796528943056
"11262","ZNF468",35.2738443058032,0.132297516820347,0.295545871319605,
0.447637844608154,0.654414585975233,0.908796528943056
"11263","ITSN1",281.036819853751,0.114616632055333,0.255930726007701,0
.44784240580744,0.654266936621697,0.908796528943056
"11264","SPRY4",9.90489600005757,-0.299712944129585,0.669867782543405,
-0.447421046272164,0.654571082677461,0.908868229143776
"11265","NEK7",310.151036839415,0.0827422096511142,0.184980895083885,0
.447301380034908,0.654657470708,0.908907487072625
"11266","FAM167B",3.94503772572267,0.354432556786995,0.793033855151167
,0.446932441136998,0.654923839802144,0.909072561436491
"11267","AC097468.3",8.7919298845209,0.258779526853245,0.5792695807272
11,0.446734189854015,0.655066992799824,0.909072561436491
"11268","CD2AP",149.673784666089,0.0926228398799864,0.207328635287337,
0.446744077351497,0.655059852949601,0.909072561436491
"11269","HIST4H4",7.14727977874006,0.297277115216019,0.665119218734934
,0.446953127863971,0.654908903091168,0.909072561436491
"11270","ZFAND6",425.842743506164,-0.0700525665638398,0.15678366585639
1,-0.446810362426438,0.655011988719525,0.909072561436491
"11271","CD8A",6.32158112927547,-0.298178762477614,0.667939123218788,-
0.446416076124865,0.655296722391055,0.909281761442851
"11272","FTSJ1",81.716038965062,-0.120268595949987,0.269440362220854,-
0.446364438344267,0.655334016303313,0.909281761442851
"11273","SIX4",9.74191065175515,0.2701292109821,0.605653511599748,0.44
6012787523665,0.655588008920499,0.909321617156941
"11274","LINC02308",48.1163069079633,0.182809355902264,0.4098481513901
55,0.446041674903735,0.65556714245548,0.909321617156941
"11275","ZNF821",33.8705599519108,0.135303591195237,0.303369452114868,
0.446002688312882,0.655595304032076,0.909321617156941
"11276","ZNF562",65.3707935535492,0.131838391786065,0.295509081950371,
0.446139898360911,0.655496193888176,0.909321617156941
"11277","NTPCR",313.462496028832,0.134167739504657,0.301100336037034,0
.445591463863912,0.655892377743662,0.909398747143744
"11278","CCDC36",28.8756314308883,-0.168900261099066,0.379568916440316
,-0.44497916921926,0.656334807956289,0.909398747143744
"11279","AC080013.5",2.93546839400144,-0.377684104398275,0.84861667852

8121,-0.445058545223678,0.656277445852835,0.909398747143744
"11280","AL512329.2",5.47201198761476,0.327869423476313,0.736449757229531,0.445202704268291,0.656173272623859,0.909398747143744
"11281","DGKA",43.7360359108771,-0.123531089204228,0.27743299669480З,-0.445264588840966,0.656128555206938,0.909398747143744
"11282","GPR65",9.24903532742535,-0.311086262190932,0.699077317284193,-0.444995502642618,0.656323004229586,0.909398747143744
"11283","OTUD7A",20.5004245011257,0.176730507309199,0.396667055191694,0.44553865766289,0.655930529552216,0.909398747143744
"11284","LCMT2",23.2286568109289,-0.187847227274038,0.42179613448101,-0.445350755774872,0.656066293555327,0.909398747143744
"11285","UNC45A",176.994781120933,-0.06687476684575,0.150060217293471,-0.445652872239707,0.655848012102226,0.909398747143744
"11286","DSC2",3.40369501310344,-0.399903863088535,0.898741151754268,-0.444960000226935,0.656348660982007,0.909398747143744
"11287","AC008736.1",8.75767802204933,0.259893253388782,0.584054528967826,0.444981145592826,0.656333379679592,0.909398747143744
"11288","Z82217.1",24.109479859819,-0.270175323508585,0.607081308482756,-0.445039766063329,0.656291016674672,0.909398747143744
"11289","F8",25.7724765170992,-0.160199513993112,0.360101927144528,-0.444872692749614,0.656411757745685,0.909405606443663
"11290","CNTN3",17.6367103531164,-0.249342332966499,0.561341714045773,-0.444189923405847,0.656905276905235,0.909444855341939
"11291","MED28",277.780708763825,0.0662794965205567,0.149161892641615,0.444346041383398,0.656792418541503,0.909444855341939
"11292","RNF139-AS1",14.046141706045,0.246099437590105,0.553585897733753,0.444555106258264,0.656641296897872,0.909444855341939
"11293","KIF1BP",201.510914738901,0.0888790115176493,0.200087124289643,0.444201553864053,0.656896868926667,0.909444855341939
"11294","XPNPEP1",154.260588460447,-0.0860397057148004,0.193666293491165,-0.444267839094703,0.656848950337049,0.909444855341939
"11295","ATL3",709.975807984223,-0.0670106547941032,0.150753280369365,-0.444505450428133,0.656677189129083,0.909444855341939
"11296","IFI27L2",1558.38838886148,0.117279702838189,0.263974538663537,0.444284147372541,0.65683716105415,0.909444855341939
"11297","BX890604.2",2.73847090339821,-0.48389659057109,1.08836373296224,-0.444609256920067,0.656602156615193,0.909444855341939
"11298","LTN1",288.250979839435,0.0683987361963069,0.15406483692989,0.44396072172804,0.657070981972452,0.909593747393268
"11299","C9orf78",417.404793319508,-0.0664733063439241,0.149907451141598,-0.443428967924585,0.657455486931785,0.909946777155045
"11300","PDE3B",4.16957051896549,-0.369692947033118,0.833823139108789,-0.443370937664617,0.657497453423613,0.909946777155045
"11301","MED16",119.577703944328,0.102247273634676,0.230615610779767,0.443366662338915,0.65750054530877,0.909946777155045
"11302","PRPSAP1",191.656883088268,-0.1085530674902,0.244997757237158,-0.443077800851544,0.657709461456001,0.910155368711012
"11303","AC136469.1",5.33655746675087,-0.296285712530192,0.669042602361669,-0.442850293067028,0.657874022983501,0.910302549717947

"11304","LZTFL1",79.0237634322605,-0.0912307899412155,0.206074602237723,-0.442707587206567,0.657977253823314,0.910364848708124
"11305","SLCO3A1",260.497209658349,0.10849928456201,0.245189463647654,0.442512018860352,0.658118735022815,0.910480054467654
"11306","RELN",2.80640607103432,-0.605250631373656,1.36829151458985,-0.442340411323155,0.658242892194629,0.910547170126651
"11307","METAP2",612.843340176667,-0.0723483171758044,0.163578859633738,-0.442284029475423,0.658283686229031,0.910547170126651
"11308","IGHG3",92.1143349220784,0.514882654111683,1.16460063440326,0.442110916739718,0.658408945076692,0.910559368732821
"11309","ZNF582-
AS1",28.1805556679174,0.177364130199914,0.401109008887934,0.442184359537703,0.65835580304222,0.910559368732821
"11310","SLC9A8",33.034305063822,-0.136527975052076,0.308984005100875,-0.44186097920345,0.658589808809059,0.910728966381404
"11311","MRPL45",158.988435112727,-0.0915469736664891,0.207224626888832,-0.441776515855909,0.658650934021765,0.910732968623499
"11312","CHPF2",122.702741052465,-0.111601951846104,0.252670156108931,-0.441690279393306,0.658713344771884,0.910738747545285
"11313","TAGLN",4427.30681906219,-0.170872983271783,0.387022712914765,-0.441506344640333,0.65884646932811,0.910842285891597
"11314","TIMP4",37.9732269526768,0.215637361504314,0.488760891650228,0.441191930836051,0.659074054392904,0.911076384188176
"11315","ZDHHC18",68.715283879924,0.0966886436460207,0.219210181984696,0.441077338518751,0.659157008665776,0.911110527223397
"11316","KRBOX1",31.6469194035087,-0.187200740439462,0.424600592580656,-0.440886667872235,0.659295045927112,0.911171432004653
"11317","HSPA4",266.905273002798,0.0774340036034048,0.175644850243245,0.440855530328209,0.659317589258098,0.911171432004653
"11318","CORO1C",364.39281891659,-0.10007298144986,0.227200220927511,-0.440461637939114,0.659602790885908,0.911404510067638
"11319","LINC01915",122.354605483532,-0.125926976780889,0.285878597453073,-0.440491096230316,0.659581459610225,0.911404510067638
"11320","C16orf72",152.433615967763,0.111422558108719,0.253046194286633,0.440324970793701,0.659701757631393,0.911460732275175
"11321","FAM131A",53.5873858865246,-0.140135920338722,0.318427460563152,-0.440087422394056,0.659873791142248,0.911537369145447
"11322","SLFN13",8.06050585936406,0.255843158765812,0.581328326414708,0.440100967285909,0.659863981393235,0.911537369145447
"11323","VGLL4",540.177813767974,0.088505957502453,0.201194195665905,0.439903135423561,0.660007264900137,0.911641227858177
"11324","RNF207",11.056138404955,0.216078050135192,0.493149423595228,0.438159389014204,0.661270745417203,0.912058079714743
"11325","RLF",158.353542173299,0.0953385015788419,0.217805466101047,0.437723181541327,0.661586962940508,0.912058079714743
"11326","ACADM",204.431197078532,-0.0716057965630281,0.163481973918391,-0.438004232801673,0.661383215048616,0.912058079714743
"11327","BLZF1",136.070258819244,0.0789695577141267,0.180429844552071,0.437674587096018,0.661622193987861,0.912058079714743
"11328","NUCKS1",3266.29306978134,0.0585601221797261,0.133844850598535

,0.437522414331622,0.661732524322289,0.912058079714743
"11329","DNAJC10",366.894803989112,0.0755641770789895,0.17208796396702
6,0.439102046052844,0.660587596623524,0.912058079714743
"11330","NR1D2",291.330037243871,-0.379701798822317,0.867064330686951,
-0.437916525203488,0.661446795911963,0.912058079714743
"11331","B4GALT4",166.722377662666,-0.0806444956246871,0.1836664378228
12,-0.439081285512202,0.660602638859895,0.912058079714743
"11332","TLR1",13.745847248917,0.215207394837498,0.491930799371974,0.4
37474935727228,0.661766949399163,0.912058079714743
"11333","IGFBP7",1926.60070734057,-0.192221930462553,0.438636544468068
,-0.438225982050036,0.661222475803955,0.912058079714743
"11334","RAB24",60.0083137690839,-0.151430261287197,0.345738308412077,
-0.437990982204701,0.661392820495307,0.912058079714743
"11335","SYTL3",12.8393067360506,-0.23602370520335,0.539131830140498,-
0.437784771753955,0.661542311018736,0.912058079714743
"11336","SMARCD3",85.4498611810493,0.106695988841391,0.243583141221132
,0.438026984570862,0.661366722268449,0.912058079714743
"11337","STKLD1",6.86269219134229,-0.263300175410445,0.599964298501388
,-0.438859738934675,0.660763170962703,0.912058079714743
"11338","GPSM1",58.042299191596,0.129665937998252,0.29598511321735,0.4
38082633916235,0.661326382695198,0.912058079714743
"11339","CASC2",11.3036197223065,-0.245524294121614,0.561122005967703,
-0.437559553021248,0.661705596854732,0.912058079714743
"11340","AC008035.1",3.2710428789654,0.387645834843966,0.8846412459035
17,0.438195524614104,0.661244552542086,0.912058079714743
"11341","C12orf49",127.888749323466,-0.0945567921912545,0.216051229871
431,-0.43765912486369,0.661633404287154,0.912058079714743
"11342","VPS37B",58.0425362921588,0.127099962864599,0.290250482001795,
0.437897508345269,0.661460581907078,0.912058079714743
"11343","RAB11FIP4",3.58439398927433,-0.445022404919253,1.013785102476
67,-0.438971142732386,0.660682446094619,0.912058079714743
"11344","MAPRE2",211.973258948142,-0.0755580087337574,0.17252394416682
8,-0.437956650589288,0.661417707973102,0.912058079714743
"11345","ZNF569",42.6677001030254,-0.12239905507762,0.279417443272192,
-0.438050873432359,0.661349405385825,0.912058079714743
"11346","ZNF350",51.4412696872648,0.116250232749459,0.264932385323079,
0.438792081261393,0.660812198657182,0.912058079714743
"11347","TBC1D20",213.11448971606,0.0853755349619682,0.194607848747805
,0.438705507055922,0.660874936216961,0.912058079714743
"11348","WFDC2",6.01963148131878,-0.316098404773227,0.720812946628292,
-0.438530420758705,0.661001822942875,0.912058079714743
"11349","RPL11",20189.6707963713,-0.106529318303449,0.243557561280367,
-0.437388672079944,0.661829497985013,0.91206391298666
"11350","LYPLAL1-
AS1",9.98251109896154,0.342365038485404,0.783151330520508,0.4371633235
40364,0.661992906250407,0.912149996502734
"11351","SCD",12.3330514251282,0.254316042566062,0.581770300956647,0.4
37141672835261,0.662008606796837,0.912149996502734
"11352","APH1A",511.115257733491,-0.08748155941668,0.200209931737252,-
0.436949149613054,0.662148226327034,0.912166527812243

"11353","AL121944.1",26.6684149767655,0.17228189047627,0.394487752437041,0.436723039972618,0.662312218020194,0.912166527812243
"11354","SLAIN1",2.65651068411659,0.603719749312755,1.38149670974868,0.437004116660244,0.662108362543853,0.912166527812243
"11355","C18orf32",864.463029959679,-0.104493375329498,0.239262625150233,-0.436730873716222,0.662306536130125,0.912166527812243
"11356","RBCK1",425.984863135417,-0.0925513074395563,0.211891574062982,-0.436786162209766,0.662266435403307,0.912166527812243
"11357","PRCP",263.959141695808,-0.0713207227090334,0.163364743551372,-0.436573529628231,0.66242066304578,0.912235552525843
"11358","AIFM2",77.3554688037839,0.11925618215995,0.273294893284251,0.436364473286784,0.662572310711763,0.912364055250218
"11359","AC098818.2",41.9120112727174,0.205777908762266,0.471704563751864,0.436243200883072,0.662660287007658,0.91240486740279
"11360","PFN4",12.5619006929667,-0.204150493916023,0.4685350684196,-0.43572084071452,0.663039283066026,0.912428083176275
"11361","PLCD4",5.4206340871262,0.321671290905284,0.738440722013918,0.435608819118214,0.663120571065831,0.912428083176275
"11362","TAF9",348.719391127256,-0.109010313855048,0.250085528646402,-0.435892130364643,0.662914995124364,0.912428083176275
"11363","PHAX",419.13125743034,-0.0569104589126518,0.130624613874713,-0.435679442216279,0.663069323249859,0.912428083176275
"11364","AL513548.1",7.47131319640187,-0.327086018799265,0.750168055916448,-0.436016991418913,0.662824401665682,0.912428083176275
"11365","CYP21A2",8.31486103330626,-0.279989608972023,0.64272949447779,-0.435625891417215,0.663108182367702,0.912428083176275
"11366","RMI2",7.85266464154582,-0.262516365913973,0.602382291380729,-0.435796950989802,0.662984056228694,0.912428083176275
"11367","PPP1R15A",1888.4532334524,-0.0708152811273018,0.162607466638655,-0.435498335907704,0.66320074662788,0.912428083176275
"11368","RBMX2",256.232031562708,-0.0943276255056787,0.216597980534847,-0.43549632952604,0.663202202656515,0.912428083176275
"11369","ZNF667-AS1",194.891034660218,-0.0980610330744835,0.225299402011791,-0.435247640246073,0.663382686002637,0.912596113033797
"11370","AL034417.2",10.6796384034311,0.270659836398469,0.622152650283869,0.435037665233725,0.663535088132184,0.9127038426242
"11371","RAPGEFL1",10.5321205057044,0.225114271664005,0.517529122786355,0.43497894466692,0.663577710644487,0.9127038426242
"11372","MR1",195.165144136981,0.0727680671771048,0.16744687403347,0.434574055784162,0.663871630169417,0.912789450350096
"11373","CHN2",3.48633633002005,0.431978235774269,0.994479284895107,0.434376303594732,0.664015202504215,0.912789450350096
"11374","NSUN6",78.4672799540072,0.108250431757745,0.249170011876565,0.434444060673606,0.663966008021191,0.912789450350096
"11375","PDCD4-AS1",3.91133606768544,0.403421679763375,0.928835802291752,0.434330458373802,0.664048488880012,0.912789450350096
"11376","CCND2",246.324901533748,0.141948907684753,0.326745602066195,0.434432496679774,0.663974403863576,0.912789450350096

"11377","SNUPN",220.766949074027,-0.0860943812004834,0.198126330992225
,-0.434542853387126,0.663894282959845,0.912789450350096
"11378","AL117335.1",10.9352583563663,0.200380973959523,0.460899437420
779,0.434760725855658,0.663736115010104,0.912789450350096
"11379","TSPYL4",93.9295991752134,0.106603163662331,0.245682100138669,
0.433906921188649,0.664356033578462,0.912889482801845
"11380","GET4",4.89446761632269,-0.298450889888182,0.687631717987467,-
0.434027230101712,0.664268667451338,0.912889482801845
"11381","AL606834.2",3.33072421686138,-0.372221053137415,0.85776877453
5634,-0.433941015559727,0.664331274408058,0.912889482801845
"11382","C15orf41",45.3734933003024,0.114285007166901,0.26343371058404
4,0.433828331664638,0.664413106312878,0.912889482801845
"11383","GSS",121.029458028152,-0.131318611151561,0.302490594162765,-0
.434124609775143,0.664197955458244,0.912889482801845
"11384","AC104695.3",9.42856417423079,0.256713091026082,0.592610926771
539,0.433189938674636,0.66487678768296,0.91290079240002
"11385","STAMBP",185.251102894836,-0.0733240901474623,0.16907407228957
7,-0.433680274890869,0.66452063235339,0.91290079240002
"11386","SCAMP1",327.775815577958,0.0710151434829123,0.163843479377862
,0.43343283329045,0.664700351906705,0.91290079240002
"11387","NCR3",2.55937011703161,-0.437142008338083,1.00991273265743,-0
.432851269423853,0.66512282400889,0.91290079240002
"11388","MAD1L1",99.9772573737577,-0.0982961710109931,0.22713134231720
4,-0.432772377463062,0.665180142595308,0.91290079240002
"11389","GRB10",92.1083984540589,0.134730891514665,0.311302626848784,0
.43279715586888,0.665162139749229,0.91290079240002
"11390","WASHC2A",186.080845194619,0.0793062662065885,0.18300752309780
4,0.433349759966999,0.664760693296851,0.91290079240002
"11391","TOLLIP-
AS1",15.3415815545212,0.210069786920373,0.484775314574461,0.4333343316
06332,0.664771900126702,0.91290079240002
"11392","RBM14",104.667151087724,0.0996023034734648,0.229789244825149,
0.433450675854104,0.6646873920117,0.91290079240002
"11393","CEP95",135.625375097498,0.105722858470793,0.244132737718458,0
.433054818697507,0.664974945273369,0.91290079240002
"11394","ZNF433",18.228700913618,-0.17897583756852,0.413437819445975,-
0.432896627135745,0.665089870460853,0.91290079240002
"11395","RPN2",998.260071650988,-0.0901165759570216,0.208091364258236,
-0.433062545763261,0.664969331809722,0.91290079240002
"11396","PLA2G6",24.2300714438597,0.146902201066924,0.339088835420027,
0.433226298603897,0.664850375078601,0.91290079240002
"11397","FAAP100",68.800791510206,-0.0990181755930649,0.22886738833748
9,-0.432644319980845,0.66527318632852,0.91294837537756
"11398","CHDH",4.46841286564237,0.313605672732567,0.724998721697253,0.
432560311276691,0.665334227994493,0.912952037711341
"11399","ACAP3",176.496098990245,-0.0656867871040302,0.154024389875973
,-0.426470036056782,0.669765377434149,0.912966477922542
"11400","AL691432.2",26.0607646496274,-0.126827987968326,0.29779252547
3875,-0.425893792218275,0.670185238569261,0.912966477922542
"11401","HDAC1",33.3628323615723,0.136929434710663,0.317793884033935,0

.430874984038526,0.666559273804217,0.912966477922542
"11402","AL356055.1",3.39780742984587,-0.43826503859219,1.014642415536
9,-0.431940387944733,0.66578473979197,0.912966477922542
"11403","CYB561D1",49.7610283494769,0.118739618578312,0.27700799466595
2,0.428650511410336,0.668177580472496,0.912966477922542
"11404","AL451085.2",27.4869499579899,0.163373771953372,0.380030039250
665,0.429896995183613,0.667270571355425,0.912966477922542
"11405","LINC00467",114.317251662953,0.126496451143026,0.2964272911906
49,0.426736858927302,0.669571000656923,0.912966477922542
"11406","JMJD4",64.6016128663823,-0.121819005569059,0.284541912886102,
-0.428123239678301,0.668561397910065,0.912966477922542
"11407","SF3B6",1082.9065340982,-0.104732934442166,0.245875422626979,-
0.425959346904949,0.6701374691041,0.912966477922542
"11408","TCF7L1",349.6498079105,0.068644204921251,0.159240876600231,0.
431071508690447,0.666416376309221,0.912966477922542
"11409","STK17B",67.2201214179785,-0.143924052742895,0.338096359866029
,-0.425689447824652,0.670334152153859,0.912966477922542
"11410","CNPPD1",204.604567333664,-0.0829226281651017,0.19452755777395
,-0.426277022721181,0.669905998772707,0.912966477922542
"11411","MLPH",2.49375684294663,-0.437935614803615,1.01823300972538,-0
.430093711970432,0.667127473928136,0.912966477922542
"11412","NCKIPSD",53.8862768023944,0.112610347799997,0.261041500853777
,0.431388677400671,0.666185781340686,0.912966477922542
"11413","AC096992.2",6.98199263291166,-0.286977970667155,0.66979316676
9324,-0.428457596919603,0.66831799885253,0.912966477922542
"11414","GYPE",5.05783916185959,0.293508215730397,0.686342960470186,0.
427640746150185,0.668912695833076,0.912966477922542
"11415","RAPGEF2",89.8009157197754,0.124561135015377,0.291410300246515
,0.42744245797079,0.669057088170383,0.912966477922542
"11416","ERCC8",37.8222266470135,-0.139320154809708,0.32331858349711,-
0.430906733856061,0.666536186978039,0.912966477922542
"11417","SERF1A",6.11363998185963,0.261664632596178,0.611114842150542,
0.428175875544714,0.668523078736593,0.912966477922542
"11418","NREP",70.486421610433,-0.159368478896086,0.372605456453595,-0
.427713754953919,0.668859534316348,0.912966477922542
"11419","CD14",161.790128594353,-0.188271677248975,0.440187872960251,-
0.427707551284532,0.66886405146696,0.912966477922542
"11420","RIOK1",140.44626912308,-0.079838159775969,0.185138268414797,-
0.431235316499201,0.666297277216729,0.912966477922542
"11421","TAP1",148.625569756361,-0.116782800196719,0.27368201678652 6,-
0.426709805664031,0.669590707577702,0.912966477922542
"11422","HCG25",16.7536953887346,0.2104707291639,0.494566820339804,0.4
25565809326413,0.670424258552453,0.912966477922542
"11423","TRAM2-
AS1",63.8482789377016,0.0999830496377448,0.233435415871278,0.428311399
384565,0.668424420679277,0.912966477922542
"11424","ELOVL5",249.864701626301,0.103631960354049,0.241705051071147,
0.428753805080986,0.668102399965681,0.912966477922542
"11425","CAHM",15.2947098186165,0.191456769328302,0.444111718403802,0.
431100467279774,0.666395320890719,0.912966477922542

"11426","TCTE3",12.9277095122123,-0.178611224385379,0.413072402085373,-0.43239689575888,0.665452973895129,0.912966477922542
"11427","FSCN3",5.3168551899012,-0.316727746221673,0.738563386511313,-0.428843010642285,0.668037475924209,0.912966477922542
"11428","KDM7A",108.208604497309,0.12654702817807З,0.29408286769589,0.430310779983738,0.666969586485726,0.912966477922542
"11429","NDUFB2-AS1",4.469523783288,0.330287322088258,0.770647410013197,0.428584223857447,0.668225828471892,0.912966477922542
"11430","WEE2-AS1",8.6885572794366,0.219082831702442,0.514995957377371,0.425406896042693,0.670540079795738,0.912966477922542
"11431","ARMC1",207.684796173315,-0.0644762337506345,0.149243912322056,-0.432019187566593,0.665727467692967,0.912966477922542
"11432","CSPP1",128.549705374257,-0.0804703254377006,0.186333575088322,-0.43186165133985,0.665841968036859,0.912966477922542
"11433","ZC2HC1A",227.56661591017,-0.0948904117856126,0.220842492736416,-0.42967460931021,0.667432355785291,0.912966477922542
"11434","C8orf88",14.0507180908621,0.185971932177407,0.432406720046215,0.430085666007992,0.667133326554329,0.912966477922542
"11435","TAF2",127.006443431643,0.0877745147992102,0.206272110005274,0.425527788497272,0.670451968674219,0.912966477922542
"11436","ZNF7",94.7053979792642,-0.106280497648077,0.247491223722109,-0.42943137962505,0.667609321668038,0.912966477922542
"11437","C8orf33",238.501914295266,0.0975286245564217,0.226082429928881,0.431385245581009,0.666188276248864,0.912966477922542
"11438","NMRK1",79.0736308519562,-0.196467051322062,0.45754454838196,-0.429394366115474,0.667636253095433,0.912966477922542
"11439","KIF27",43.501604857779,0.155542227286944,0.362523664157296,0.42905399747768,0.667883929036724,0.912966477922542
"11440","RMI1",27.2188653552979,0.143319851810897,0.333795921214914,0.42936370009932,0.667658566342613,0.912966477922542
"11441","GOLM1",305.826682076619,0.0648763382974364,0.152394456737539,0.425713242373175,0.670316811488698,0.912966477922542
"11442","HSDL2",184.444766408893,-0.0844146308961781,0.198005408272764,-0.426324874823075,0.669871134679855,0.912966477922542
"11443","AL162424.1",4.51822701838225,0.337137516424674,0.791006270549066,0.426213456172293,0.669952313199958,0.912966477922542
"11444","TOR2A",77.5946956117989,-0.121973495759531,0.286734188749789,-0.425388741717048,0.670553311765526,0.912966477922542
"11445","MTPAP",77.5213984567235,0.0897384455238796,0.207747239270674,0.43195974992938,0.665770667189701,0.912966477922542
"11446","ITGB1",2964.30444954628,0.0802620360935266,0.187264100206942,0.428603432290709,0.668211847297651,0.912966477922542
"11447","AL442003.1",5.33223111969829,-0.320726155156661,0.744020768415269,-0.431071508715803,0.666416376290785,0.912966477922542
"11448","HOGA1",3.01895180053896,0.40494036792131,0.938260890340747,0.431586110100196,0.666042255409368,0.912966477922542
"11449","FBXW4",105.979963813899,0.0980462551338259,0.230159225531766,0.425993157160211,0.670112832213678,0.912966477922542

"11450","PRMT3",56.6340342316194,0.108452814218564,0.253277708237554,0.42819723446346,0.668507529583453,0.912966477922542
"11451","EEF1G",6635.07507072988,0.0840972664297471,0.195586904182341,0.429973912524048,0.667214618036957,0.912966477922542
"11452","ACAT1",550.708741192488,-0.0744936772346416,0.175144528320886,-0.425326888306554,0.670598395036711,0.912966477922542
"11453","FLI1",21.707597542961,-0.187942550278473,0.441682958506108,-0.425514606481866,0.670461576019447,0.912966477922542
"11454","TULP3",376.110578154772,0.0594885884105719,0.138565839441392,0.429316407639801,0.667692977920387,0.912966477922542
"11455","SLC11A2",103.360134023023,0.103242736569449,0.240489499648548,0.429302471502201,0.667703118454226,0.912966477922542
"11456","SMUG1",133.805608789996,-0.0994630784423618,0.232513999303456,-0.427772429790568,0.668816811311582,0.912966477922542
"11457","TSPAN31",324.778522986767,0.0711889633630514,0.167081532400351,0.426073201151116,0.670054507093283,0.912966477922542
"11458","C12orf29",133.600475465907,-0.0893490633760031,0.206711605441991,-0.432240188860982,0.665566852857831,0.912966477922542
"11459","MRPL42",275.172016072705,-0.0773915339442003,0.181776550079963,-0.425750922823411,0.67028935160561,0.912966477922542
"11460","PRDM4",94.0461796432753,0.104302587598364,0.244361565259003,0.426837123456023,0.669497965052091,0.912966477922542
"11461","OAS3",52.041669321864,-0.15243291222326,0.357972670749966,-0.425822764357701,0.670236997822004,0.912966477922542
"11462","ELF1",568.503699927607,0.0786137684816115,0.182873681301651,0.429880166036236,0.667282813921448,0.912966477922542
"11463","TSC22D1",393.486101400461,-0.1127097718571,0.26328258826029,-0.428094286834004,0.668582476093361,0.912966477922542
"11464","ING1",138.606933492954,-0.103634584859865,0.241011570665448,-0.42999837963681,0.667196819887585,0.912966477922542
"11465","REC8",21.7991733827202,0.214788163161388,0.500688711183968,0.428985432192955,0.667933826294278,0.912966477922542
"11466","GMPR2",219.178936367043,0.0887536753312892,0.206479355612693,0.429842853141067,0.667309957952473,0.912966477922542
"11467","LINC00519",10.1542417362558,0.261128730334176,0.613925383666939,0.425342781519262,0.670586810792342,0.912966477922542
"11468","SUSD6",65.2829267904368,0.132499807518941,0.309002807234258,0.428798070492904,0.668070183172382,0.912966477922542
"11469","ACOT4",15.8520010367306,0.254655748804585,0.596600041469693,0.426845006878065,0.669492222670252,0.912966477922542
"11470","SERF2",11914.2522171895,-0.121291575785132,0.284971228523515,-0.425627444614546,0.670379338829217,0.912966477922542
"11471","GABPB1-IT1",247.209785045285,0.0922562822162762,0.216541975239776,0.426043413126353,0.670076212302025,0.912966477922542
"11472","AC116913.1",12.8983838337774,-0.179265805951967,0.415109427816303,-0.431851926117412,0.665849036769934,0.912966477922542
"11473","MORF4L1",2745.87997270519,-0.0617391459809694,0.143581595810543,-0.429993451684677,0.667200404620307,0.912966477922542
"11474","GDPGP1",13.6275980993908,0.186454134588979,0.436771601308532,

0.426891615733205,0.669458272600205,0.912966477922542
"11475","ATXN2L",193.323549212487,-0.0798517604662053,0.18566040905227
,-0.430095790878734,0.667125961735308,0.912966477922542
"11476","ZFP3",16.9126672953576,-0.181532755793928,0.425081842437616,-
0.427053658074255,0.66934024559030 7,0.912966477922542
"11477","CCT6B",27.1269478687964,-0.15756605669719,0.367687017921745,-
0.428533097490881,0.668263042199999,0.912966477922542
"11478","AC006441.4",9.16417758756752,0.267931194259283,0.621609575352
796,0.43102810008552,0.666447938640835,0.912966477922542
"11479","ETFB",932.185219145826,-0.123803489144169,0.289359407123791,-
0.427853686786153,0.668757647293306,0.912966477922542
"11480","ZNF816",15.4733052161369,-0.168021545171287,0.389983857285713
,-0.43084230803992,0.666583034431186,0.912966477922542
"11481","GPCPD1",226.437294566558,0.114888865010802,0.266690119052298,
0.430795356869865,0.666617175954661,0.912966477922542
"11482","ATP9A",114.355489609966,0.121108071597352,0.283338752110055,0
.427432078017732,0.669064647130981,0.912966477922542
"11483","SOD1",2427.57778168402,-0.0912122466982471,0.212742067924353,
-0.428745699372823,0.668108299444418,0.912966477922542
"11484","Z93241.1",31.2225640005352,0.195526914447447,0.45507470670522
2,0.429658936360312,0.667443758348459,0.912966477922542
"11485","ATXN10",514.160421202294,0.0918482233497271,0.215851704137157
,0.425515395937596,0.670461000645082,0.912966477922542
"11486","KLF8",66.8210295426548,0.132286535057898,0.309293680702755,0.
427705263028091,0.668865717645008,0.912966477922542
"11487","SLC16A2",44.7351047848556,0.159603715691852,0.371724553109289
,0.429360165630296,0.667661138116516,0.912966477922542
"11488","KCNE5",3.27755165794894,0.410084158935623,0.962612669006056,0
.426011595462435,0.6700993967228,0.912966477922542
"11489","MOCS1",26.9149261055667,0.130085951819729,0.305991572365774,0
.425129198212779,0.670742493930055,0.913003708012711
"11490","IPO7",244.800938062736,0.102063630768475,0.240034387516907,0.
425204204382118,0.670687819526738,0.913003708012711
"11491","ANKRD10-
IT1",15.8297655240285,0.231616062963947,0.545052904174783,0.4249423518
15585,0.670878699844173,0.913032940947549
"11492","AC006504.1",3.80191160276494,-0.334325371599056,0.78675979705
0481,-0.424939572220674,0.670880726174504,0.913032940947549
"11493","ETV6",95.5472919443547,-0.105347869418233,0.247963954977005,-
0.424851545169143,0.67094489931175,0.913040827045661
"11494","TPD52",3.08757046742535,0.399142326761942,0.939915963145512,0
.424657461318326,0.671086398041279,0.913153929473256
"11495","TRAM1L1",20.1330397766936,0.152118424347777,0.35856745022563,
0.424239356506163,0.671391261062009,0.913330375142195
"11496","RBM25",1027.07873277279,0.103750693462477,0.244544986051874,0
.424260154082523,0.671376095136346,0.913330375142195
"11497","CLN3",232.123511175638,-0.0964667742831601,0.227322179348797,
-0.424361470400757,0.671302215568879,0.913330375142195
"11498","SLC7A14-
AS1",5.7464898901621,0.335381624874762,0.790777396003082,0.42411635255

3728,0.67148096024934,0.913332311600871
"11499","ROGDI",48.2825480589559,0.155557827274376,0.366814840017421,0
.424077246348561,0.671509478970487,0.913332311600871
"11500","CC2D1B",27.6926307879725,-0.146645042166399,0.347778301649,-0
.421662425375815,0.673271434259007,0.913350485257564
"11501","SPR",125.570367476798,-0.090516257781255,0.214211978959617,-0
.422554603252693,0.672620254098996,0.913350485257564
"11502","ZAP70",4.7995506052552,-0.32253447340813,0.762841302105458,-0
.422806778445173,0.672436241686199,0.913350485257564
"11503","BIN1",1309.18946500123,0.107230692901713,0.254254173754744,0.
421746047737054,0.673210389803423,0.913350485257564
"11504","NEB",3.0305523231765,0.365856309800639,0.867594295280411,0.42
1690543368998,0.673250907839419,0.913350485257564
"11505","ZNF639",143.483223667222,0.0712783483805643,0.168466721394304
,0.423100466315447,0.672221962173109,0.913350485257564
"11506","CD38",7.75061234090914,-0.23134395707346,0.545965963675766,-0
.423733295599446,0.671760330006236,0.913350485257564
"11507","ADAMTS12",39.7612482286489,-0.170017118741699,0.4026614024378
07,-0.422233463928689,0.672854618071309,0.913350485257564
"11508","GPX8",799.601436339147,-0.0819055754221317,0.19432155693108,-
0.421495055492896,0.673393621002671,0.913350485257564
"11509","PLPP1",1269.33917219957,-0.112861507088441,0.267380272954489,
-0.422101099087634,0.672951225737406,0.913350485257564
"11510","HARS2",52.5998622804787,0.0954999498474868,0.226319572311246,
0.421969469419775,0.673047302183622,0.913350485257564
"11511","DIAPH1",539.224529553866,-0.134717635833942,0.319215471593942
,-0.422027275687032,0.673005108753109,0.913350485257564
"11512","AC004938.2",3.90498199413432,-0.496427339147067,1.17404988754
367,-0.422833258121326,0.672416920586169,0.913350485257564
"11513","PARP12",39.1859350828325,0.149586946789799,0.355028493157292,
0.42133786350361,0.67350838532452,0.913350485257564
"11514","SOX7",2.74938130440184,-0.406149848153035,0.958681252541575,-
0.42365473099248,0.671817634075823,0.913350485257564
"11515","VPS37A",176.613831749511,0.0675752741728671,0.160038055055354
,0.422245035091773,0.672846173010782,0.913350485257564
"11516","ATP6V1B2",199.205340525612,0.0716024090512201,0.1699265184865
53,0.421372777415471,0.673482894364897,0.913350485257564
"11517","NUP188",94.7610030981933,-0.0891862641842766,0.21166889740475
5,-0.421347988664267,0.67350099281547,0.913350485257564
"11518","RPS24",17024.6274292153,-0.0991752916973896,0.234020215434517
,-0.423789421410649,0.67171939368977,0.913350485257564
"11519","UBQLNL",2.84056569131019,-0.37211657196554,0.87868887263524,-
0.423490707068521,0.671937277280244,0.913350485257564
"11520","OLFML1",569.211702941713,0.0675187365124076,0.159609770083535
,0.423023831668139,0.672277873508836,0.913350485257564
"11521","AC016355.1",5.31341964290245,-0.288140898095465,0.68260018256
9442,-0.422122503704652,0.672935603019856,0.913350485257564
"11522","AKAP13",1453.33886966379,-0.100984473910263,0.239251507802949
,-0.422085005179718,0.672962972388481,0.913350485257564
"11523","WDR24",36.199740474871,-0.120837802835834,0.286609240097698,-

0.42161167865573,0.673308480494202,0.913350485257564
"11524","REXO5",21.3150805057269,-0.159001431352963,0.376295974221032,
-0.422543535529741,0.672628330673374,0.913350485257564
"11525","AC135050.3",7.61905067758255,0.29979617866263,0.7085292969860
46,0.423124604639368,0.672204351637799,0.913350485257564
"11526","CYB5B",228.387631814785,-0.0719597903659043,0.170788152075999
,-0.421339475198975,0.673507208603049,0.913350485257564
"11527","GAN",13.0473679586747,0.195528764922015,0.461406143757006,0.4
2376714650984,0.671735640151586,0.913350485257564
"11528","AC015922.3",101.468330220552,0.0821815040471932,0.19411407859
3624,0.423367046030903,0.67202748426813,0.913350485257564
"11529","CCDC144A",68.8461866449037,-0.357864138937439,0.8481861810724
85,-0.421916964604326,0.67308562691809,0.913350485257564
"11530","AC092329.4",16.9829772073664,-0.155983826038293,0.36905381369
7976,-0.422658756660202,0.672544250907824,0.913350485257564
"11531","SEPTIN5",17.4094991182332,0.191115913666635,0.452895257773749
,0.421987005574057,0.673034502242902,0.913350485257564
"11532","MAFF",931.555070193845,-0.164283762412153,0.387701083731462,-
0.423738207876516,0.6717567471144,0.913350485257564
"11533","XPNPEP3",83.5083709626748,0.108464544770447,0.257055524317578
,0.421949868840185,0.67306160909599,0.913350485257564
"11534","UGP2",2389.99774761722,-0.108534305271596,0.258164301871718,-
0.420407873918707,0.674187516989538,0.91335200076718
"11535","WDR33",375.425356498894,-0.0641765408764377,0.152442955112816
,-0.420987252765754,0.673764390149545,0.91335200076718
"11536","KDM1B",95.4051558466556,0.0999759465841205,0.237567234939085,
0.42083218760115,0.673877603107386,0.91335200076718
"11537","TBPL1",135.239908417741,-0.103451276861322,0.245854624679023,
-0.420782309856418,0.673914050455002,0.91335200076718
"11538","ZNF736",47.9768947435011,0.136420127752454,0.324480464031161,
0.420426321072301,0.674174043238013,0.91335200076718
"11539","AC010998.2",2.6469246879922,-0.494791684009831,1.174940401121
54,-0.421120665812094,0.673666971725068,0.91335200076718
"11540","PANO1",3.86087450145691,-0.328467876438223,0.780192861874657,
-0.421008564022203,0.673748828268009,0.91335200076718
"11541","PGAM5",34.5796522528923,-0.130032806186282,0.309292400054146,
-0.42042030830217,0.674178434937716,0.91335200076718
"11542","MTRF1",25.6308979677413,-0.138282831180031,0.328764411075012,
-0.420613748087472,0.674037152973814,0.91335200076718
"11543","HTR2A",13.7660785069944,0.205200084737388,0.48813381740867,0.
420376702902337,0.674210284453778,0.91335200076718
"11544","NOVA1",6349.76177015241,-0.0920856850250982,0.218929088415883
,-0.420618775199804,0.674033481491951,0.91335200076718
"11545","UACA",552.603752504096,0.0960275061429051,0.228331513696416,0
.420561772610069,0.674075112998882,0.91335200076718
"11546","CDC42-
IT1",10.3275620327416,0.244380849135819,0.581899491146641,0.4199708933
48373,0.674506716973194,0.913386130010401
"11547","DPM3",380.600678396996,-0.0955249908584625,0.227425174764773,
-0.420028217884253,0.674464839939018,0.913386130010401

"11548","BIN3",131.817705789391,-0.0768204787748788,0.182930958789146,
-0.419942470554835,0.674527480922003,0.913386130010401
"11549","ZNF707",12.6398514791344,0.19879126985268,0.473273717538605,0
.420034458888928,0.674460280786934,0.913386130010401
"11550","YES1",239.44674093361,0.0654218420413144,0.155769305620768,0.
419991870545978,0.674491392478843,0.913386130010401
"11551","SMPDL3A",97.0074896162001,0.121572610830308,0.289570362147702
,0.419837893383223,0.674603880719661,0.91340195922723
"11552","AC004918.1",13.3526220375873,0.195224804093908,0.465079424485
861,0.419766589996379,0.674655973976532,0.91340195922723
"11553","FERMT2",1389.54556038412,0.0913645089310068,0.217924292453388
,0.419248849691912,0.675034274550347,0.913835025877905
"11554","EXOSC1",182.292611068077,-0.109640164924269,0.261695467785587
,-0.418960885536235,0.675244718682494,0.914001170937104
"11555","ADRA2A",4.58758481965673,0.351552195167553,0.839194588255627,
0.418916184741252,0.675277388289145,0.914001170937104
"11556","MCMBP",214.931850779398,0.0684536416287967,0.163435857903132,
0.418841021224174,0.67533232297629,0.914001170937104
"11557","SGO2",34.3414480294622,0.146493302709805,0.349923571584979,0.
418643711386069,0.67547653885531,0.914117250817431
"11558","COQ10A",75.0339369232657,0.146045385862659,0.349015897370501,
0.41844909347388,0.675618798847196,0.914221209928725
"11559","CRADD",61.3721809688719,0.119157691382335,0.284808224981757,0
.4183786875887,0.675670266340545,0.914221209928725
"11560","DNAJA4",40.7549164474047,0.131853974880614,0.315229517823509,
0.418279277242167,0.675742939002622,0.914240446885901
"11561","AC092053.4",5.23319467769619,0.267943731638407,0.640834923816
925,0.418116618929704,0.675861854798218,0.914243159405305
"11562","SYNE1",800.241496902204,0.113178726837417,0.270636209107933,0
.418195064180342,0.6758045042484,0.914243159405305
"11563","AL031663.3",4.93615205842932,0.287526846338332,0.687971548504
093,0.417934211034632,0.675995218685062,0.914265411642543
"11564","SSR4",2144.13048815658,-0.114198207486359,0.273208258182592,-
0.417989588770182,0.675954729285527,0.914265411642543
"11565","C3orf67",14.3236578083098,-0.230841156464209,0.55262718501464
5,-0.417715890068078,0.676154853063075,0.914273083656363
"11566","NTHL1",85.8338858433385,-0.13375153793587,0.320219836270031,-
0.417686610216993,0.676176263340994,0.914273083656363
"11567","ANKRD54",68.932325136897,-0.0965056859759767,0.23103043276711
6,-0.417718500632584,0.676152944155165,0.914273083656363
"11568","TAF12",319.519144950378,-0.0992521582857102,0.237795381963048
,-0.417384717341287,0.676397031449355,0.914399654266568
"11569","RNF187",516.27260073675,-0.0889291901050822,0.21323931892905,
-0.417039364746195,0.676649614879244,0.914399654266568
"11570","MXD1",73.5033224808726,0.137346775093961,0.330700262352205,0.
415321034573848,0.677906904809518,0.914399654266568
"11571","TTC30A",14.987958804158,0.178612430845947,0.429518486595824,0
.415843407024344,0.677524593513808,0.914399654266568
"11572","RPL24",11418.5796283354,-0.09361403585579,0.225271204626871,-
0.41556148292831,0.677730916380306,0.914399654266568

"11573","AC024230.1",2.92425495693287,0.379683570960076,0.914098519843
081,0.415363948981402,0.677875493702265,0.914399654266568
"11574","PPAT",45.9774813296735,0.107565989675428,0.25807758126491,0.4
16797108637708,0.676826817477938,0.914399654266568
"11575","ENOPH1",133.870165700002,-0.0899896316330533,0.21653036767847
9,-0.415598202681099,0.677704042087343,0.914399654266568
"11576","AK6",319.938699331785,-0.112575333658905,0.270989089183546,-0
.415423860783765,0.677831642325333,0.914399654266568
"11577","MAP1B",2218.63759243053,-0.0978438106492011,0.235463372586647
,-0.415537285372042,0.677748626206709,0.914399654266568
"11578","SERINC5",84.7199810064993,-0.108936075626933,0.26177272247446
7,-0.41614754431703,0.677302041299213,0.914399654266568
"11579","OSTM1",169.881318015204,0.074963445970965,0.180247292102669,0
.415892217278171,0.677488874753883,0.914399654266568
"11580","SAMD3",5.94076057241099,0.413497283736678,0.991552792226009,0
.417019937797147,0.676663824411296,0.914399654266568
"11581","PMPCB",271.802841638094,-0.0744995875045871,0.178701818823756
,-0.416893280633378,0.676756468595416,0.914399654266568
"11582","FAM180B",473.334376788877,-0.129071135745563,0.31048581070483
2,-0.415707034896569,0.677624392848037,0.914399654266568
"11583","AP001783.1",7.87528118278849,-0.220940667992153,0.53068951811
8525,-0.416327552078781,0.677170334034438,0.914399654266568
"11584","AC004803.1",2.79727279254708,0.37855929929156,0.9110141488441
94,0.415536136043374,0.677749467387452,0.914399654266568
"11585","PAPOLA",673.481048726127,0.0480673441629335,0.115492335696749
,0.41619509963973,0.677267245276709,0.914399654266568
"11586","AC106886.3",3.33856993020194,0.374342567758934,0.896941196442
624,0.417354637342583,0.676419029863788,0.914399654266568
"11587","SLFN11",204.171519396848,0.0986193791784633,0.236381162249106
,0.417204900086475,0.676528541364837,0.914399654266568
"11588","ACLY",391.252883463083,0.0834677727404819,0.200874204103128,0
.415522605867451,0.677759370001454,0.914399654266568
"11589","ZNF442",9.78706760054952,0.219477457568272,0.52731988485793,0
.416213125790627,0.677254055800604,0.914399654266568
"11590","ZNF675",66.7578108901622,0.102815418918802,0.247392718116094,
0.415595979145004,0.677705669427604,0.914399654266568
"11591","SREBF2",119.162773252046,-0.102062225418267,0.244821970055951
,-0.41688344144499,0.67676366573683,0.914399654266568
"11592","PPP2R3B",50.4018612368224,0.118558340787095,0.284870331493055
,0.416183532225733,0.677275709039769,0.914399654266568
"11593","ZXDB",38.5838667975717,0.120693098416538,0.289819082421253,0.
416442897438721,0.677085943851656,0.914399654266568
"11594","BRCC3",93.4585537848305,-0.0989238950147572,0.238184831591876
,-0.415324075650043,0.677904678881981,0.914399654266568
"11595","AC093151.8",2.87074529094639,-1.30415164766065,3.125884524831
36,-0.417210436694237,0.676524492002084,0.914399654266568
"11596","AC090360.1",3.3307700421665,0.330260358819852,0.7955509765469
22,0.415134125349632,0.678043719138205,0.914505326605857
"11597","MCTP1",7.07553447476073,-0.265311395697262,0.639273906043163,
-0.415019904909662,0.678127331756717,0.914539231583604

"11598","PPIH",146.82873144157,-0.116670720396133,0.281333367277587,-0
.414706302082595,0.678356918345142,0.914651189333737
"11599","AK5",6.86744081711193,0.387551420425848,0.934320214237167,0.4
14795071882575,0.678291927521659,0.914651189333737
"11600","HOXD9",14.5208828180436,-0.196145696062432,0.47301994190399,-
0.414666864303671,0.67838579260047,0.914651189333737
"11601","SDHAF4",46.9846112779485,-0.127619994668409,0.308099377648941
,-0.414216982982107,0.678715205311179,0.914840956503039
"11602","CPVL",137.129789848975,0.164221552502756,0.396440749154147,0.
414239840009236,0.678698467427033,0.914840956503039
"11603","YWHAE",1757.94954512359,-0.0630512602555582,0.152199732361082
,-0.414266564582217,0.67867889759364,0.914840956503039
"11604","SUV39H1",9.98825330266019,-0.189345339264616,0.45718460302149
1,-0.414155109365561,0.678760515298035,0.914840956503039
"11605","SOGA3",3.33776264355996,-0.376272166650994,0.909009811694148,
-0.413936309388921,0.6789207516115,0.914978074554404
"11606","WDCP",20.4847126392153,0.168801715809481,0.408177712739748,0.
413549565644971,0.679204015617561,0.91507202114713
"11607","ABTB1",156.074507898319,0.0824121476044658,0.199239674990743,
0.413633216417839,0.679142743162564,0.91507202114713
"11608","NHLRC1",7.25821160990735,0.23542589341771,0.569375993193689,0
.41348054050748,0.679254576694375,0.91507202114713
"11609","HARBI1",12.0994908380501,-0.185037501570638,0.447553998991651
,-0.413441734377374,0.679283002910369,0.91507202114713
"11610","ZNF257",11.0510478317436,0.255832216203127,0.618642753969662,
0.413537885252064,0.679212571431606,0.91507202114713
"11611","MIR181A2HG",3.65840213680014,-0.355568079908321,0.86048215265
084,-0.413219587196483,0.679445738651844,0.915133599079818
"11612","SAV1",627.906564640639,0.0986678119041078,0.238767418734828,0
.413238173059478,0.679432122852549,0.915133599079818
"11613","EED",90.9707248443242,0.0967481279664063,0.234280721058391,0.
412958127878961,0.679637291959866,0.915194036850006
"11614","XRCC3",7.62576543420186,0.244535680386332,0.592212617421615,0
.412918727485063,0.67966615971949,0.915194036850006
"11615","CWC25",218.089903050455,-0.0726042762214307,0.175805994602494
,-0.412979525445605,0.679621614653242,0.915194036850006
"11616","WDR54",76.1128193034177,0.101417981499674,0.245821875792673,0
.41256694983977,0.67992391989598,0.915304708828811
"11617","GPER1",32.5002346864398,-0.15535332220029,0.376528063758652,-
0.412594271591582,0.679903898922025,0.915304708828811
"11618","MRPL43",604.080041382851,-0.0957211747067049,0.23201274650280
7,-0.412568603016588,0.679922708466145,0.915304708828811
"11619","AC002553.1",6.58993466387013,0.330168229490504,0.800609553440
752,0.412396065062616,0.680049146842202,0.915315719157662
"11620","AL118516.1",148.034813120604,-0.115371792003529,0.27971066272
289,-0.412468337389869,0.679996183511855,0.915315719157662
"11621","IGSF8",125.611511145837,-0.117983355348797,0.287202539470697,
-0.410801922455964,0.681217783400617,0.915549207904584
"11622","FOSL2",474.780169279621,0.13276963692569,0.322367840996089,0.
411857573992007,0.68044381894242,0.915549207904584

"11623","EIF2AK2",621.509803709195,0.0901045605791941,0.219119928355192,0.411211163017245,0.680917703550889,0.915549207904584
"11624","CDCA7L",23.8280287246019,0.130960734702034,0.318741670642336,0.410867943429295,0.681169369428826,0.915549207904584
"11625","AC027644.3",121.015905029179,0.118740895237491,0.288236832625973,0.411956009076654,0.680371667134958,0.915549207904584
"11626","GIMAP4",5.64872436282517,0.308163871458939,0.749205512299361,0.411320881120006,0.680837260205653,0.915549207904584
"11627","THEM6",22.9276822167403,-0.147191979673211,0.35782677187276,-0.411349824114198,0.680816040326482,0.915549207904584
"11628","PARD3",133.177246176943,0.0919498648154694,0.223422483059957,0.411551530339021,0.680668164173048,0.915549207904584
"11629","CHORDC1",178.400804412166,0.0854150826203862,0.207507690312164,0.411623696894761,0.680615259948912,0.915549207904584
"11630","CDK17",77.4151564594941,0.105448503882984,0.256490328091156,0.411120780529031,0.680983973096741,0.915549207904584
"11631","RAB2B",110.371827764845,0.0843124767314151,0.205072153852587,0.411135666873728,0.680973058077328,0.915549207904584
"11632","ISCA2",202.021981724924,-0.10338494905799,0.251561764365749,-0.410972427859414,0.681092752426567,0.915549207904584
"11633","ITPK1",83.3930429275047,0.0954505068387166,0.232032219580852,0.411367468755591,0.680803104084698,0.915549207904584
"11634","FAM157C",6.89611661560231,-0.276140888612335,0.671153716373478,-0.411442091246158,0.680748395335547,0.915549207904584
"11635","HDAC5",120.736895243655,0.0960254638863958,0.233689938986157,0.410909704983422,0.681138745858243,0.915549207904584
"11636","CST7",19.2140051579061,0.220482764031662,0.535335380581622,0.411859129863816,0.680442678483169,0.915549207904584
"11637","STK26",9.88027173736205,-0.242658649726455,0.589999594227618,-0.411286129855946,0.680862738816444,0.915549207904584
"11638","LRRC40",97.1288654909948,-0.0814437500773853,0.198631879720196,-0.410023558112181,0.681788666261006,0.915800847691134
"11639","SPSB4",229.187623102401,0.11036156072524,0.268978604891482,0.410298658399864,0.681586876112012,0.915800847691134
"11640","LINC00472",19.0883322346498,0.176321551364543,0.430395758849446,0.409673068888723,0.682045788287344,0.915800847691134
"11641","CALD1",4119.3545483594,-0.062499662364115,0.152299253008679,-0.410374057189588,0.681531573955334,0.915800847691134
"11642","ZNF843",8.85014298012062,-0.214798584993311,0.524322890763886,-0.409668524447543,0.682049122372528,0.915800847691134
"11643","LPCAT2",99.7746760478833,0.143501758453221,0.350090753280173,0.409898739423086,0.681880230131976,0.915800847691134
"11644","VPS4B",648.521359536999,0.0727953535834735,0.177488223403307,0.410141879768893,0.681701872777073,0.915800847691134
"11645","NCLN",95.735666767519,0.111344904787242,0.271762918817082,0.409713382796666,0.682016211764564,0.915800847691134
"11646","SLC17A7",183.326039221783,0.0986666156256584,0.240564331239447,0.410146488123586,0.681698492456952,0.915800847691134
"11647","FIZ1",27.1120774314557,0.121810097829825,0.296854050532874,0.410336654026337,0.681559007545146,0.915800847691134

"11648","P2RY10",5.60208039612529,0.297073972079502,0.725024127349944,
0.409743566969757,0.681994067297907,0.915800847691134
"11649","ARSK",92.685353637065,-0.0835354775749674,0.204094710464709,-
0.409297611803673,0.6823212679201З,0.916087615269193
"11650","EEF1B2",4684.7322230965,-0.0934664953242473,0.228569839325072
,-0.408918760236424,0.682599281058714,0.916303558128769
"11651","CALM3",989.071765780697,-0.0825528584279103,0.201846863292018
,-0.408987571476296,0.682548782014831,0.916303558128769
"11652","LINC00910",32.4272462976851,0.17147223562076,0.41943836191165
4,0.408813907338492,0.682676233008886,0.91632820839847
"11653","EVI5",442.521895116572,-0.0631002841259873,0.154503922552503,
-0.408405709599668,0.682975842330521,0.916573037073051
"11654","FAM72D",6.8675334351863,0.269838642317143,0.660685369047257,0
.40842230653036,0.682963659526797,0.916573037073051
"11655","PIK3C2B",92.6748896705812,0.117671251751142,0.28857560812353,
0.407765758569485,0.683445654656268,0.916666231738358
"11656","COA5",278.75172912641,0.111253953701726,0.272551822493232,0.4
08193761773463,0.683131427679688,0.916666231738358
"11657","RAF1",253.381466581641,0.0816259774609398,0.200439273938676,0
.407235447709281,0.683835068884097,0.916666231738358
"11658","AC106707.1",42.6058504948674,-0.175912641673575,0.43186424213
3372,-0.407333195275862,0.68376328523713,0.916666231738358
"11659","B3GALNT1",65.2845384018155,-0.111524051812692,0.2737988737054
02,-0.407321075881006,0.683772185296964,0.916666231738358
"11660","AC011337.1",3.36682726454086,-0.324440949742519,0.79497974165
5311,-0.408112223170576,0.683191286618694,0.916666231738358
"11661","NFYA",42.0271564885733,0.120746117869867,0.296532483658615,0.
40719356065178,0.683865830685624,0.916666231738358
"11662","HIBADH",345.607448536369,-0.105918043788987,0.259976766356975
,-0.407413498033707,0.683704314809598,0.916666231738358
"11663","EMC4",565.596152071295,-0.0836254663991467,0.205195184042718,
-0.407541077483268,0.68361063065369,0.916666231738358
"11664","CEP89",153.859636688782,-0.0695998828135888,0.170707205563943
,-0.40771496776402,0.683482947367149,0.916666231738358
"11665","SARS2",31.9778937180951,-0.125176824262864,0.306856535319749,
-0.407932730298308,0.68332306254785,0.916666231738358
"11666","MAP3K7CL",21.5668220067408,-0.167687022518973,0.4109918513811
05,-0.408005710953816,0.683269482132413,0.916666231738358
"11667","CRYBB2",3.62890975953234,-0.476078813936145,1.16911685188803,
-0.40721234423002,0.683852035984866,0.916666231738358
"11668","AC007325.4",48.5093491991094,-0.165683325260013,0.40663141156
4626,-0.407453336235146,0.683675060294043,0.916666231738358
"11669","TMEM9",409.415103762149,-0.0834327476436812,0.20520172127664,
-0.406588926859937,0.684309931397182,0.916900661054526
"11670","HMGN3",835.530627003368,-0.100046617097003,0.246080405010375,
-0.406560681224434,0.684330680355874,0.916900661054526
"11671","AC068792.1",3.56480072459243,-0.390303605899236,0.96002335311
8284,-0.406556366187841,0.684333850159174,0.916900661054526
"11672","PRX",15.4761801067549,0.187511957297553,0.461025178761103,0.4
06728235107348,0.684207600451036,0.916900661054526

"11673","LILRB3",5.94532499676891,-0.305232643205061,0.750488633297569,-0.406711880317095,0.684219613805235,0.916900661054526
"11674","ADNP",274.770596892776,0.0756074241051538,0.186140773402174,0.406184108528425,0.684607329615479,0.917188507382738
"11675","WTIP",213.195537496011,0.0833792113509272,0.205384807956268,0.40596581694923,0.684767716976606,0.917324804583651
"11676","DNAJC27-AS1",14.5401585673816,0.260359115973091,0.641602732742618,0.405794898753238,0.684893307182184,0.917364732797109
"11677","ASF1A",123.853280404063,-0.100340719130712,0.247287394043468,-0.405765605314579,0.684914832792317,0.917364732797109
"11678","CDK11A",163.842451021572,-0.12091013930855,0.298194513387915,-0.405474057637206,0.685129083845194,0.917408931260256
"11679","ZNF326",228.11084857076,-0.0764727314833753,0.188596354089946,-0.405483615271288,0.685122059778725,0.917408931260256
"11680","TIMMDC1",464.104228718028,-0.0866744077897723,0.213967250833132,-0.405082588350717,0.685416804610234,0.917408931260256
"11681","CEMIP2",71.3855279363201,-0.308398435690419,0.760757208944681,-0.405383520608668,0.685195622384464,0.917408931260256
"11682","SLTM",581.457297529575,-0.0684581029082727,0.168998055171397,-0.405082193631417,0.685417094742717,0.917408931260256
"11683","CIB2",22.6359828812105,-0.157391250146124,0.388253808282039,-0.405382373047557,0.685196465779265,0.917408931260256
"11684","B4GALT6",11.3438746372667,0.207431034654435,0.511909133111267,0.405210654073929,0.685322674276672,0.917408931260256
"11685","OGFRP1",3.54083442747207,0.375185696618677,0.925785323613551,0.405262091598343,0.685284868256967,0.917408931260256
"11686","KLRG1",10.4441570186311,0.183174184012664,0.452393014240161,0.404900558246514,0.68555060802116,0.917509114277849
"11687","CPLANE2",3.94294066397669,0.336791437204957,0.833914549606483,0.403868043031371,0.686309757226719,0.917685837477305
"11688","TSGA10",25.9933940010113,-0.136130500347167,0.337316746452078,-0.403568757789223,0.686529863717131,0.917685837477305
"11689","DNAJB2",206.401372465927,0.0747646265771114,0.18538844255731,0.403286340538726,0.686737589184594,0.917685837477305
"11690","G3BP1",495.452834759294,0.0633860795365448,0.156843885642002,0.404134845786874,0.686113562101019,0.917685837477305
"11691","UBLCP1",233.582141379405,-0.0748104223471711,0.185449904254791,-0.403399627774347,0.686654260539632,0.917685837477305
"11692","CDC40",375.96324032072,0.0543592526642619,0.134782669560709,0.403310402156541,0.686719890296672,0.917685837477305
"11693","KMT2E",768.330055530013,0.0685997241665533,0.169862109633789,0.403855364297839,0.686319081141434,0.917685837477305
"11694","PCNX3",58.2787629086699,0.0970271983994908,0.239905842885442,0.404438663237654,0.685890173740359,0.917685837477305
"11695","FEZ1",540.051264623369,0.097935632900997,0.242753596639012,0.40343638264043,0.686627226245012,0.917685837477305
"11696","CMC2",172.200745897914,-0.115810931862869,0.287203614296324,-0.403236331640942,0.686774374537238,0.917685837477305
"11697","ZNF207",484.500355099647,0.0600443367600389,0.148572969434785

,0.404140382927427,0.68610949055305,0.917685837477305
"11698","RINL",60.2864017859398,-0.125442267438807,0.310696376548843,-0.403745511396677,0.686399868739045,0.917685837477305
"11699","ZNF227",62.2445579715979,0.116930936161969,0.290003751635705,0.403204908565646,0.686797488981576,0.917685837477305
"11700","BLOC1S3",31.8486327017222,0.11652144983762,0.288002010598976,0.404585542980353,0.685782187069567,0.917685837477305
"11701","IL10RB-DT",6.49386199856124,-0.233738925857621,0.579166604577881,-0.403578044745827,0.68652303331327,0.917685837477305
"11702","IGLC2",271.437525169394,0.371142671699813,0.918568626537414,0.4040445765047,0.686179939832239,0.917685837477305
"11703","CCDC157",4.08611226097936,0.299399278527582,0.740472216502655,0.404335600789566,0.685965949562098,0.917685837477305
"11704","LARGE1",57.3633040055843,0.115094771581099,0.285163713513205,0.40360945704885,0.686499930270704,0.917685837477305
"11705","TXN2",654.817342181023,-0.0923681644591834,0.229074248320383,-0.403223693350277,0.68678367107989,0.917685837477305
"11706","PCDHGB2",6.02662585669146,0.251862489630864,0.624811757782383,0.403101392529469,0.686873636230772,0.917709180817467
"11707","ANAPC4",76.4091112761056,0.080681744907063,0.20019838316543,0.403008973555961,0.686941623074055,0.917721618252176
"11708","MAX",247.440112278489,0.076411410446387,0.189683421890388,0.402836524588547,0.68706848973576,0.917812707504893
"11709","RPE",148.310645757661,-0.0796734803620437,0.197917245088461,-0.402559566380548,0.687272259837869,0.917814681433356
"11710","NCAPG2",37.7338259763782,0.120909238861627,0.300443606012347,0.40243571985572,0.687363386421285,0.917814681433356
"11711","AZIN1-AS1",60.631768211924,-0.105811188293477,0.2628808368764,-0.402506282126708,0.687311465968652,0.917814681433356
"11712","NKIRAS2",128.836157969599,-0.0757762458811727,0.188266408357853,-0.40249477611077,0.687319932114191,0.917814681433356
"11713","SUGP1",65.2205612486931,-0.0920897568745141,0.228683187112702,-0.402695790789069,0.687172030823484,0.917814681433356
"11714","GINS2",11.4970257573461,-0.231884128450558,0.576316307235906,-0.402355660492599,0.68742229671845,0.917814983837848
"11715","BAG5",330.393649528348,-0.0631800016680362,0.157159903807932,-0.402010946413213,0.687675970281708,0.918034231770392
"11716","NUP93",72.5807847802005,-0.0995047614476074,0.247540913970244,-0.40197299045106,0.68770390405511,0.918034231770392
"11717","AL356124.1",2.74612850957318,0.400004425978311,0.995598734662257,0.401772734387823,0.687851289998426,0.918152613772757
"11718","TMCC2",7.87877587972932,-0.254318820388666,0.634037037929139,-0.401110353457126,0.688338878487437,0.918356917071469
"11719","CRTAP",983.835682596631,0.0593539558112877,0.148012472566619,0.401006447511191,0.688415376965851,0.918356917071469
"11720","PCDHGA9",7.90103028341942,-0.21354188867351,0.532368768835446,-0.401116483862551,0.688334365210218,0.918356917071469
"11721","CEP85L",173.645937457669,0.0879523635849183,0.219223550808105

,0.401199429809009,0.688273300511177,0.918356917071469
"11722","AL135910.1",16.2136197982798,0.186796670288548,0.465382167957452,0.40138338584908,0.688137879562486,0.918356917071469
"11723","AC009283.1",15.953025701103,0.162080306459701,0.403844554572466,0.401343300595668,0.688167387842489,0.918356917071469
"11724","AC093227.1",15.861319351793,0.188608356438602,0.470302608826094,0.401036168838997,0.68839349496328,0.918356917071469
"11725","TMEM182",7.68544304903495,-0.238980178449444,0.596452719638747,-0.400669106839159,0.688663758621565,0.918531569575411
"11726","AP1M1",231.55800380361,-0.0869079974621861,0.216865762176618,-0.400745588376496,0.688607442838885,0.918531569575411
"11727","TMEM8A",86.9366760746689,-0.103076217787466,0.257313495913873,-0.400586131020377,0.688724858301511,0.918534730437079
"11728","PINK1-AS",7.72050259471021,-0.207354518366562,0.517928635405052,-0.400353454495517,0.688896201713802,0.918596932187629
"11729","PRKAR2A",329.999052446352,0.0889146098386352,0.222306231843762,0.399964540360366,0.689182634417496,0.918596932187629
"11730","GLYCTK",15.094907292262,-0.197783922773013,0.494389483566905,-0.400056897137147,0.689114610222888,0.918596932187629
"11731","MAML3",22.1505683060624,0.140846508782997,0.351819053742768,0.400337921680548,0.68890764067518,0.918596932187629
"11732","SGK3",97.324619297319,0.0878790623914989,0.219644863382426,0.40009614173627,0.689085705879636,0.918596932187629
"11733","LOXL4",92.2303617500911,0.137971260541122,0.344903525902803,0.400028559232541,0.689135481874353,0.918596932187629
"11734","LGR4",410.219498778349,-0.130394065098657,0.325825906919421,-0.400195510330934,0.689012521176861,0.918596932187629
"11735","AC132872.1",23.688897163442,0.164640791342249,0.411728721105385,0.399876867710931,0.689247210903322,0.91860471909058
"11736","AC007405.3",20.3671788404966,-0.214831074205128,0.537416818918251,-0.399747582588789,0.689342441754243,0.918653356257359
"11737","FAM87B",11.1445654132636,-0.223650187510891,0.5604735576328,-0.399037892983736,0.689865283614911,0.918806702023842
"11738","GADD45A",275.577882964623,-0.153897889302979,0.385676619349241,-0.399033494855493,0.689868524261765,0.918806702023842
"11739","LHX4",3.17176495955828,0.383868947448276,0.963006532003547,0.398615102484956,0.690176831837854,0.918806702023842
"11740","STK11IP",31.2256003939177,-0.116393188537565,0.291727051484537,-0.398979758460056,0.689908118991821,0.918806702023842
"11741","PLCL2",92.4318750252075,0.113184141461053,0.283761704082833,0.398870389599908,0.689988708159698,0.918806702023842
"11742","SNRK-AS1",3.67106004535556,0.42957341107981,1.07782754226074,0.398554865446083,0.69022122391803,0.918806702023842
"11743","MCC",145.448609297625,0.0921744468791838,0.230794190026027,0.399379407552631,0.689613664823514,0.918806702023842
"11744","SIM1",29.8049060898448,-0.203342883395839,0.510111410915411,-0.398624455451671,0.690169939203393,0.918806702023842
"11745","PLBD2",336.963377230124,-0.0656021572000488,0.164424322429766

,-0.398980857762518,0.689907308981381,0.918806702023842
"11746","LINC00944",5.27959692372929,-0.295935709352821,0.740938380998006,-0.399406640204292,0.689593602011846,0.918806702023842
"11747","AC090971.1",2.80817369063864,0.394512782855539,0.989684277263657,0.398624886662152,0.69016962142503,0.918806702023842
"11748","ARMC5",30.5854746900466,-0.124658662131613,0.312235334373258,-0.399245852112918,0.68971206081132,0.918806702023842
"11749","AC012313.6",2.7092366147404,0.356322595927536,0.893752989851683,0.398681291110049,0.69012805492632,0.918806702023842
"11750","CCDC88A",375.177679663093,0.0930406963455998,0.233711449998872,0.398100719267494,0.690555944215636,0.919043885131229
"11751","AC019131.2",7.39460403346797,0.275021267672108,0.690831224192418,0.39810196476514,0.69055502616114,0.919043885131229
"11752","CHD1",347.645012230324,-0.117153121078775,0.294435383886535,-0.397890768196263,0.69071070534839,0.919043885131229
"11753","AP003469.4",17.5794854213612,0.174574887925454,0.438558394808226,0.398065320358974,0.69058203688973,0.919043885131229
"11754","DYNC1LI2",721.805778148041,-0.0824819325351486,0.207338431866453,-0.397813043113374,0.69076800208103,0.919043885131229
"11755","FAAP24",34.7023512653813,-0.149102436595609,0.374785879128118,-0.397833656226519,0.69075280650443,0.919043885131229
"11756","ZNF225",22.643245032148,0.127853247583158,0.32143712507165,0.397755074354462,0.690810736163858,0.919043885131229
"11757","C2orf88",107.090443307081,0.208576022465576,0.524500610051748,0.397665929206446,0.690876455141931,0.919053139271905
"11758","FAM20B",179.713269529925,0.0851849020850724,0.214568232413238,0.397006123073309,0.691362945327291,0.919074922645034
"11759","AC083798.2",16.0331130785293,0.231117416005173,0.581430819967848,0.397497704056956,0.691000479285593,0.919074922645034
"11760","RNF145",327.681517467144,0.0916678329881549,0.230777518725703,0.397213010584056,0.691210388698869,0.919074922645034
"11761","AC008443.8",4.62128963922325,0.306105986870157,0.770640833981481,0.397209664181268,0.691212856200611,0.919074922645034
"11762","USP6NL",42.1376361774175,-0.109844109204541,0.276653766688849,-0.397045413547836,0.691333971989285,0.919074922645034
"11763","AC010198.2",6.62832974607375,-0.286767423970362,0.722239380723086,-0.397053153877124,0.691328264216965,0.919074922645034
"11764","POGLUT2",55.7482554935954,0.11358417425875,0.285929577009343,0.397245277829507,0.69118659630858,0.919074922645034
"11765","PDE8A",159.75986362855,-0.0805356491871171,0.202652999197449,-0.39740664834005,0.691067613654944,0.919074922645034
"11766","AL627309.1",3.03871274593804,-0.374142805468558,0.943009923811565,-0.396753836859219,0.691548995428556,0.919087889913546
"11767","WDR91",40.9018067822952,0.113004682785594,0.284802656667279,0.396782404026558,0.691527927450925,0.919087889913546
"11768","AC004816.2",8.92712578545458,-0.260230429371891,0.655662423831464,-0.396896970015752,0.691443438669604,0.919087889913546
"11769","UBE2Z",290.39647166018,0.0785125507821361,0.197980666615896,0.396566756361413,0.69168697117847,0.919115057708689
"11770","SPOP",629.306121809818,-0.0697410344032141,0.17584952235778,-

0.396594960669385,0.691666169260555,0.919115057708689
"11771","UBE2L6",447.871696591193,0.113518379920946,0.286367873215104,
0.396407525210333,0.691804415521725,0.919193021727957
"11772","GATC",130.871514507462,0.100073731138384,0.252558344942425,0.
396240049645551,0.691927948707766,0.919279061993668
"11773","NEK10",4.12613547355211,0.336787707785278,0.850291434397582,0
.396085029392171,0.692042301923623,0.919352892388131
"11774","MTMR14",120.555520625858,-0.0826404703290644,0.20869185923676
4,-0.395992783960526,0.692110351601773,0.919358546716653
"11775","C14orf132",343.827701433611,-0.0881391255554684,0.22261858149
8909,-0.395919895644023,0.692164123247352,0.919358546716653
"11776","AC004812.2",33.8458768455767,0.150780890460232,0.381207507298
635,0.395534945071561,0.692448137235039,0.919579592965612
"11777","MED24",104.098116850268,0.0843948247042455,0.21334349163159,0
.395581904368482,0.69241348865942,0.919579592965612
"11778","ARHGAP17",180.49050894845,0.0922846823755136,0.23346868971981
9,0.395276482196661,0.692638853704495,0.919676684942551
"11779","AC004076.2",3.27778958084897,-0.354625590285529,0.89703092420
6818,-0.395332625348563,0.692597424729502,0.919676684942551
"11780","ZGRF1",11.8072972867837,0.190158169170735,0.481475953746767,0
.394948424092533,0.692880951669348,0.919712211991784
"11781","INCENP",17.3398345498928,0.184404083341168,0.466938590883307,
0.394921488481668,0.692900830811969,0.919712211991784
"11782","ZNF720",171.711306260869,-0.0648169056190027,0.16406461154896
7,-0.395069387645838,0.692791680202482,0.919712211991784
"11783","APCDD1L",30.8737069755043,-0.21545903096047,0.545363390110068
,-0.39507424749759,0.692788093706377,0.919712211991784
"11784","MFSD3",34.6924493783315,-0.137833209103745,0.34939867095752,-
0.394486930147777,0.693221574756812,0.920059863305884
"11785","DNMBP",182.056892036787,0.0892875979001422,0.226543962683163,
0.394129231442008,0.693485630502725,0.920254137201987
"11786","PPIP5K1",7.36977573136294,-0.237014574498412,0.60125703970128
9,-0.394198419059115,0.693434552803392,0.920254137201987
"11787","TMED4",608.227905695487,0.0566121502493183,0.143760886434011,
0.393793831226162,0.69373325916444,0.920453807178128
"11788","CD226",5.57486558885467,0.346916683973878,0.881022427629662,0
.393766007645499,0.693753803006124,0.920453807178128
"11789","MT1A",518.485914161445,-0.339098396329151,0.8616240788513,-0.
393557242250274,0.693907954372608,0.920502154909889
"11790","AC011471.2",3.0562947884942,-0.339814299041363,0.863283428773
283,-0.393630049778942,0.693854192204022,0.920502154909889
"11791","AL158151.3",4.17506071330449,0.358330020085304,0.910704559258
672,0.393464616425101,0.693976352894432,0.920514812930957
"11792","LINC02193",9.17882649311021,0.202255596329629,0.5141745847634
65,0.393359769858467,0.694053778696215,0.920539441893556
"11793","AL133243.2",18.0257799936218,0.143515383920434,0.365018870782
677,0.393172505335703,0.694192075440326,0.920566733922902
"11794","KCNMB2",3.79556702997337,0.343607562028507,0.8737912769187,0.
393237574126615,0.694144020325509,0.920566733922902
"11795","MRPL19",292.854547102169,-0.0664755929961283,0.16917644359545

2,-0.392936460794092,0.694366411228207,0.920595397618362
"11796","EML3",69.4944641188014,0.0922621494376405,0.234810532250441,0
.392921682657901,0.69437732647744,0.920595397618362
"11797","AC005480.1",4.00881242426131,-0.32353541884294,0.823446170538
009,-0.392904151380721,0.694390275300755,0.920595397618362
"11798","SCYL2",104.539706741753,0.0912461574763369,0.232330942686945,
0.39274216521079,0.694509924556052,0.920675980679492
"11799","SNHG4",3.23195339038836,-0.356001785045473,0.906797880681179,
-0.392592211152994,0.694620693212705,0.920718570262873
"11800","PSMA3-
AS1",292.417184497265,0.0857599931455553,0.218474931144532,0.392539284
467475,0.694659790863293,0.920718570262873
"11801","AC239799.1",3.73335055243624,-0.287331782769114,0.73304144707
5198,-0.391972082773156,0.695078841321389,0.920755773585948
"11802","BRD8",150.222995000215,0.10334789409742,0.263734205780585,0.3
91863822864908,0.695158834686884,0.920755773585948
"11803","ZNF391",12.6475157093819,0.192248052324422,0.490396579319352,
0.392025679688168,0.695039239758021,0.920755773585948
"11804","LINC01003",213.905393287124,-0.123629133947424,0.315294976568
302,-0.39210625964617,0.694979702592782,0.920755773585948
"11805","AC136475.1",20.4056690065453,0.162924629161884,0.415718072133
16,0.391911345893321,0.695123719450892,0.920755773585948
"11806","SLC35C1",121.380148579904,-0.0815276013404264,0.2080126516273
87,-0.391935782283409,0.695105663415031,0.920755773585948
"11807","CEBPA",36.9863615762535,-0.147736319979942,0.37650354024555,-
0.392390254507543,0.694769885651755,0.920755773585948
"11808","BFSP1",3.78691843873882,0.307503732604574,0.783793822967783,0
.392327323326218,0.694816377533305,0.920755773585948
"11809","TENT5C",18.6094087399516,0.178234996003875,0.45517701267577,0
.391572928861491,0.695373793394648,0.920765321553235
"11810","NAGK",313.21905213747,-0.0819306936723099,0.209254885451788,-
0.39153539232988,0.695401533132149,0.920765321553235
"11811","AC055811.3",3.1436997801639,-0.366436891676843,0.935394849403
217,-0.391745680351597,0.695246134192592,0.920765321553235
"11812","ZNF99",9.58484722033171,0.294246396595043,0.751468160682646,0
.391562027495276,0.695381849532158,0.920765321553235
"11813","EPHX2",18.1741027898545,0.196879903195248,0.503266460515772,0
.391204100892152,0.695646377302477,0.921011541607613
"11814","IK",552.864185370194,-0.0676198070719259,0.172938943383688,-0
.391003933231525,0.69579432854667,0.921129447982895
"11815","ABL2",1001.25424158541,-0.151922871085863,0.388985186880518,-
0.390562099046018,0.696120945348339,0.921199386782332
"11816","ERLEC1",687.46783177465,0.0625744373828485,0.160167424488358,
0.390681423408895,0.696032731717991,0.921199386782332
"11817","MAP4K4",353.83447874722,-0.089826527648496,0.230246815973425,
-0.390131465092069,0.69643933685232,0.921199386782332
"11818","CAVIN2",60.6195458316793,0.122618587121729,0.314342736807888,
0.390079275783196,0.69647792693084,0.921199386782332
"11819","LIFR-
AS1",22.0181177646388,0.153989894485488,0.394789089917707,0.3900561044

31829,0.696495060658637,0.921199386782332
"11820","OXCT1",72.9087211962772,0.100873352633314,0.258124764591247,0
.390793005828216,0.695950245240885,0.921199386782332
"11821","NSMAF",121.642391413592,0.0691688048142383,0.177308817892789,
0.390103581064208,0.696459954901237,0.921199386782332
"11822","DNAH10OS",4.58543296123799,0.307869266214214,0.78837156840377
9,0.390512898426257,0.696157319379795,0.921199386782332
"11823","TMEM147-
AS1",6.52420137882054,-0.260001626282008,0.666195994194956,-0.39027797
907461,0.696331004953501,0.921199386782332
"11824","ZNF575",26.5090039162423,-0.126781816430125,0.324486606850147
,-0.390715098107809,0.696007837567318,0.921199386782332
"11825","TOP3B",47.4208350472464,-0.104829883456338,0.268737828307133,
-0.390082349465633,0.696475654151501,0.921199386782332
"11826","AP4B1",31.6786581245545,-0.118714859909197,0.304697127369584,
-0.389615947265565,0.696820558046354,0.921346062290635
"11827","OPN3",31.2609392096154,-0.127400949626041,0.327014982615985,-
0.389587500263402,0.696841596600762,0.921346062290635
"11828","TCF25",1162.60862321917,-0.0529282802098824,0.135843588783882
,-0.389626633716869,0.696812654725929,0.921346062290635
"11829","ZNF486",36.8333271680248,0.107818189264316,0.276746824585773,
0.389591423228415,0.696838695278975,0.921346062290635
"11830","GRAMD1A",114.7349962297,-0.0777606832115096,0.199697370944863
,-0.389392623666436,0.696985727788961,0.921458730567992
"11831","RNPC3",121.457409837947,0.102935808294899,0.264682993356251,0
.388902237312815,0.697348467067302,0.921488434334913
"11832","LRIF1",71.4577194482605,-0.109972010152079,0.282892064155989,
-0.388741941136388,0.697467053317386,0.921488434334913
"11833","RC3H1-
IT1",2.78731534156515,-0.35488839086391,0.914551129776021,-0.388046528
301621,0.697981601533574,0.921488434334913
"11834","MTLN",394.037324397334,-0.127792758799681,0.328445593705506,-
0.389083492818187,0.697214384123856,0.921488434334913
"11835","TRIM26",92.8323907861326,0.0799983102233268,0.20583623444953,
0.38865028034188,0.697534866801834,0.921488434334913
"11836","SH3BGRL2",33.680818019355,0.107703991938312,0.277327787174763
,0.388363506720806,0.697747046377055,0.921488434334913
"11837","SNHG26",57.4114250837881,0.131683202014818,0.339357690060189,
0.388036593458255,0.697988953505974,0.921488434334913
"11838","AC145124.1",36.6839627848185,0.126898816072807,0.326912914630
877,0.388173150687822,0.697887901050634,0.921488434334913
"11839","FNTA",508.709183281351,-0.0703077758927294,0.181082241312988,
-0.388264334387198,0.697820428025037,0.921488434334913
"11840","CEBPD",7651.31904558554,-0.114952339503271,0.295384279508796,
-0.389162008534879,0.6971563054111,0.921488434334913
"11841","RFX3",43.6376352665299,0.135371752906931,0.347844054521263,0.
389173686160148,0.697147667528517,0.921488434334913
"11842","TMEM229B",6.22932218092816,0.232586518426351,0.59943680400516
9,0.388008405343669,0.698009813399343,0.921488434334913
"11843","INTS2",45.8747289035959,0.124276204829359,0.320175834376925,0

.388149858565079,0.697905136860599,0.921488434334913
"11844","AC012615.2",3.5108248599232,0.410073384884562,1.0562407509253
8,0.388238556905983,0.697839502295762,0.921488434334913
"11845","ZNF766",145.5705980493,0.0806253777061448,0.207326008869516,0
.388882119256382,0.697363349891742,0.921488434334913
"11846","TRMU",78.8160075989007,-0.0874756243765059,0.225423069332225,
-0.388050897521876,0.697978368236743,0.921488434334913
"11847","TBC1D25",55.8653914838241,-0.0855287591003758,0.2199074388415
11,-0.388930722630156,0.697327394556054,0.921488434334913
"11848","LDLRAP1",57.3402558416692,-0.0924407499121924,0.2388702655382
51,-0.386991447863525,0.69876253926121,0.921568451045097
"11849","PDCD10",363.518335681451,-0.0637190474769257,0.16472870471572
3,-0.386812047037506,0.698895357918536,0.921568451045097
"11850","CSNK1A1",1115.51606828192,-0.0492782596079499,0.1272476445562
64,-0.387262646627308,0.698561775887943,0.921568451045097
"11851","TRIM39",32.8254078445543,0.114625992702049,0.295946410613033,
0.387320097799493,0.698519248565457,0.921568451045097
"11852","GTF2H5",785.790260031235,-0.109000975390977,0.281071164939274
,-0.387805612911331,0.698159891477117,0.921568451045097
"11853","DNAAF5",69.9358488420232,-0.0903024102742827,0.23332817803174
5,-0.387018880599997,0.698742230361269,0.921568451045097
"11854","CCDC65",3.31786688897401,0.330279070651637,0.853668423957223,
0.386893858766161,0.698834787801153,0.921568451045097
"11855","ZKSCAN2-
DT",11.5853649392681,-0.19762892758477,0.510349019802704,-0.3872426906
22138,0.698576548226887,0.921568451045097
"11856","CNTD1",5.95920221590719,-0.242977379428452,0.627869700392699,
-0.386986948528464,0.698765870212544,0.921568451045097
"11857","YPEL2",366.960473143446,0.131855134231626,0.340279415522407,0
.387490774395501,0.698392913493973,0.921568451045097
"11858","AC027307.3",6.75376908501998,0.251434588756713,0.648617447577
249,0.387646970792853,0.698277304008885,0.921568451045097
"11859","AC011472.4",4.04061202595609,0.330069122005357,0.851562429869
898,0.387604138496088,0.698309005834745,0.921568451045097
"11860","GRIPAP1",107.337455241831,0.088827743795731,0.229609955270044
,0.386863643134553,0.698857158017723,0.921568451045097
"11861","SLC25A14",27.7945125682588,-0.124142593531948,0.3202836424462
46,-0.387602041065157,0.698310558237351,0.921568451045097
"11862","IL1R1",545.708126778919,0.086683609202864,0.224530149707498,0
.386066678866021,0.699447286812813,0.921596930560438
"11863","LINC02427",4.51277008008816,0.366661564243405,0.9488609108411
7,0.386422878268172,0.699183509081727,0.921596930560438
"11864","AC091979.2",3.564470926598,-0.392324449794773,1.0156829415486
,-0.386266652462039,0.699299195118657,0.921596930560438
"11865","UBE2R2",679.642912700803,-0.0667371920305386,0.17281792836097
6,-0.386170536028764,0.699370373315609,0.921596930560438
"11866","DUSP6",91.4483508616959,-0.134529800505551,0.348399058866948,
-0.386137095039992,0.699395138371404,0.921596930560438
"11867","ZKSCAN2",15.3273757230224,0.17089454711814,0.442104040569507,
0.38654825886232,0.699090669139042,0.921596930560438

"11868","DSG2",6.70578574082098,0.388988818376161,1.00633088827023,0.386541666275183,0.69909555060707,0.921596930560438
"11869","HDHD5",73.4861380365219,0.131064037874303,0.338940350046183,0.386687621749506,0.698987481077757,0.921596930560438
"11870","KDM5C",154.441315496828,0.082809870111449,0.214428585853335,0.386188575473279,0.699357014158907,0.921596930560438
"11871","MAST4-AS1",2.56454676996271,0.397065320303307,1.02911797765385,0.385830710302549,0.699622049510149,0.921671904846591
"11872","SH3BGR",18.6799436942435,0.156975602885375,0.406794696744354,0.385884093725108,0.699582511363702,0.921671904846591
"11873","GLMN",38.4795877765795,0.121227103864448,0.314444115542209,0.385528295402861,0.69984604685965,0.921773918754046
"11874","CPEB4",97.8490857479796,0.0922874957248024,0.239352008275368,0.385572264004687,0.699813477891997,0.921773918754046
"11875","PHYKPL",429.213670469118,0.0663812754611996,0.172200869571975,0.385487457910043,0.699876297007948,0.921773918754046
"11876","CDC27",321.76606560124,-0.0501837608096024,0.130219183660244,-0.385379169174777,0.699956513598785,0.921801942799343
"11877","KPNA6",300.806616979149,0.0878900381034656,0.228288200192623,0.384995974515137,0.700240397972136,0.921865305074428
"11878","AC090617.10",4.96849886128045,-0.297976280899912,0.773687842520163,-0.385137602691678,0.700135469848481,0.921865305074428
"11879","ZNF835",18.4074418778091,0.154691977182471,0.401720359361225,0.385073779751783,0.700182753667091,0.921865305074428
"11880","ZNF75D",43.7307254270875,0.117097207159475,0.304151306929852,0.384996560894218,0.700239963529436,0.921865305074428
"11881","MAST4-IT1",6.46286285104641,0.347185378932852,0.90211288867272,0.384858018649603,0.700342610890174,0.921922265324663
"11882","SNAPC3",124.612306913802,0.0733406891580695,0.190648980900549,0.384689646971296,0.700467366549847,0.922008888473288
"11883","AC097500.1",3.27325124557705,-0.342601576089975,0.892806840144589,-0.383735384503204,0.701174583687251,0.92233819267589
"11884","ARL4C",832.086699118722,-0.129741246482747,0.338188461543533,-0.383635934504069,0.701248302372699,0.92233819267589
"11885","SGTB",97.0273765360504,0.109887893124903,0.28612909091007,0.384050055083145,0.700941348309793,0.92233819267589
"11886","FOXC1",183.325928112996,0.129321358296587,0.336856142310611,0.383906784093435,0.701047537976436,0.92233819267589
"11887","FAM126A",353.705762621483,-0.0874265627236957,0.227870596301374,-0.38366759091668,0.701224836314432,0.92233819267589
"11888","SLC20A2",84.2077443621225,-0.0830175583832368,0.216072319325796,-0.384211909430481,0.700821392049239,0.92233819267589
"11889","PIP4K2C",13.9020982241959,-0.190680791517891,0.496416075430078,-0.384114860407556,0.700893317784766,0.92233819267589
"11890","AC073862.1",4.82531337617261,0.271620168505486,0.707898736798119,0.383699185188612,0.70120141660357,0.92233819267589
"11891","DKC1",154.470331838005,-0.0820065737956551,0.213650685177449,-0.38383482705681,0.701100873333823,0.92233819267589

"11892","SULT2B1",5.44639088211134,-0.287191390040912,0.748817831408615,-0.383526377170627,0.701329516515195,0.922367443516453
"11893","AC116036.2",3.66018080431139,-0.317283579518755,0.8278729617594,-0.383251530336807,0.70153327371222,0.922402723905768
"11894","DNAJB12",295.675068821992,-0.0654660845760473,0.170763793667365,-0.383372160866667,0.701443841846065,0.922402723905768
"11895","VWA8",40.7643560337716,0.103818763343709,0.270841104320096,0.383319819952475,0.701482645326185,0.922402723905768
"11896","CNOT11",80.4862402504329,0.0733363713315536,0.191400765837373,0.3831561018615,0.70160402445831,0.922411597987477
"11897","TRIM6",18.9637551061538,-0.189809570258945,0.495478541471155,-0.383083331309102,0.701657978341241,0.922411597987477
"11898","ICMT",228.478115038519,-0.0752990634192211,0.196626387738413,-0.382955025952046,0.701753110762221,0.922459123577167
"11899","FGGY",45.3067601037243,-0.143000208728572,0.373680654500503,-0.382680256540761,0.701956855151192,0.922556118874996
"11900","TRAPPC13",63.6226869795493,0.0868323868051775,0.22688394352449,0.382717196537994,0.701929462510497,0.922556118874996
"11901","TMC8",10.2590050330881,-0.312993472797163,0.818033645113314,-0.382616869937919,0.702003860021185,0.922556118874996
"11902","LINC00562",4.56527462269957,0.31962693544701,0.835703254064038,0.382464629511325,0.702116759850467,0.922626963876768
"11903","CDPF1",35.3719392276218,0.11233807039693,0.293783625206034,0.38238370269325,0.70217677696666,0.922628311497821
"11904","GALE",15.991286839677,-0.171903414478534,0.45023845737708,-0.381805267102191,0.702605811640452,0.922643397861784
"11905","NSMCE2",171.789782212066,-0.0847065751275394,0.221843753315266,-0.381829886402803,0.70258754919063,0.922643397861784
"11906","AC125494.2",2.85556760655213,-0.35388556854482,0.925832624007531,-0.382234930340866,0.70228711467528,0.922643397861784
"11907","GLIPR1L2",20.7961314780098,0.187277148487467,0.490272200875287,0.381986064380398,0.702471701300966,0.922643397861784
"11908","NTN4",469.883957552922,0.0949602812447449,0.248813513504878,0.381652426779799,0.702719191517236,0.922643397861784
"11909","RPS29",14676.9646419008,0.0921742175821421,0.241446840004654,0.381757812942862,0.702641013336105,0.922643397861784
"11910","AP5M1",119.200815920249,0.0813545989178949,0.212951807918268,0.38203291023065З,0.702436953876516,0.922643397861784
"11911","B2M",32781.3251116841,0.103264534299331,0.270248380464099,0.382109724846432,0.702379978776363,0.922643397861784
"11912","MYEF2",7.63768725364689,0.282395046073729,0.739850147942986,0.381692220862394,0.702689670868116,0.922643397861784
"11913","C8orf76",121.725961872365,-0.0858133407732088,0.224904034732631,-0.381555363714239,0.702791198191169,0.922660483481062
"11914","AL022345.4",8.13824535988923,-0.209747361759066,0.549856755806924,-0.381458188053466,0.702863291065824,0.922665903631812
"11915","CETN2",338.897448594188,-0.0886639890346774,0.232475449934302,-0.381390762163204,0.702913314691371,0.922665903631812
"11916","TCEAL8",850.08516102775,0.0772615828193558,0.20264848291685,0.381259122729567,0.70301098239255,0.922716663697506

"11917","Z84485.1",7.80905053478121,0.210835294519372,0.55314360724295
,0.381158331685771,0.703085765954485,0.922734995396759
"11918","SUMF2",449.208039325052,-0.0694411278085388,0.182303902308216
,-0.380908619778949,0.703271056159358,0.922734995396759
"11919","STAU2",115.215140914268,0.0698296086827134,0.183393777664768,
0.380763238381824,0.703378939585688,0.922734995396759
"11920","FAM234A",230.751884903627,-0.0711637458127296,0.1868743546336
23,-0.380810657258189,0.703343750721212,0.922734995396759
"11921","BRWD1-
AS2",2.57619006236341,-0.342410107718173,0.89893454084627,-0.380906609
057233,0.703272548218383,0.922734995396759
"11922","MPST",425.790034574902,-0.0866915924684384,0.22753165073569,-
0.381008937385783,0.703196616781599,0.922734995396759
"11923","ROM1",21.2732006049273,-0.134082146584748,0.352349173351798,-
0.38053770726709,0.703546311670176,0.922877154618934
"11924","AC107294.2",3.12048302379591,0.332906134844952,0.875476623964
444,0.380257023125808,0.703754634210141,0.923050490240209
"11925","POLM",48.3354865148616,-0.105030122539753,0.276306989304217,-
0.38012121091561,0.703855508094932,0.923050490240209
"11926","AL078587.2",2.88288847481344,0.353402593370769,0.929573806660
607,0.380177013206008,0.7038140212953,0.923050490240209
"11927","C7orf61",4.27279424720792,0.283911409483013,0.747217099423225
,0.379958394557838,0.703976299438328,0.923131493520203
"11928","AC239868.1",7.78023089137813,0.223260435371606,0.587875608654
11,0.379774959336621,0.704112471723575,0.923141499552038
"11929","AL513217.1",3.35152446071141,0.430320926133092,1.134161178052
69,0.379417788635593,0.704377642951616,0.923141499552038
"11930","AF213884.3",2.79865217488718,-0.352258722534491,0.92867354498
7545,-0.379313833624081,0.704454828146427,0.923141499552038
"11931","LRSAM1",45.1662790631134,-0.095449877145806,0.251353271761482
,-0.379743921680008,0.704135513322421,0.923141499552038
"11932","UNC79",2.57997060283374,-0.326725301961479,0.860626692553076,
-0.37963649604248,0.704215265590718,0.923141499552038
"11933","FAM227B",7.08605557056102,-0.209340076473728,0.55181416633572
4,-0.379366984838089,0.704415363706164,0.923141499552038
"11934","AC105339.6",3.52961072833075,-0.358964430787873,0.94625643665
215,-0.379352168063328,0.70442636498773,0.923141499552038
"11935","ZNF443",10.1575554560934,-0.217149346910047,0.572482012688677
,-0.379312086837802,0.704456125137696,0.923141499552038
"11936","SCLY",27.4191345969492,0.126603170104974,0.333986049122895,0.
379067240794804,0.704637932169855,0.923286429688761
"11937","AC107223.1",7.33540984155088,0.202055507808653,0.533122165756
828,0.379004139739364,0.704684789718334,0.923286429688761
"11938","SGMS2",349.525147391746,0.06939151225855,0.183157408446441,0.
378862710753201,0.704789816050661,0.923346684790781
"11939","LNCTAM34A",5.14302707456225,-0.321310119898997,0.862987088604
556,-0.372323206386034,0.709652222450375,0.923451265423402
"11940","AL512353.1",8.12656635933815,0.204693905930345,0.545851341887
84,0.374999363787228,0.707660939812529,0.923451265423402
"11941","ALG6",62.3120464911087,0.0927429604332374,0.245606654905115,0

.377607685219551,0.705722054711266,0.923451265423402
"11942","PPP2R5A",169.118974531412,-0.075300249408305,0.20127809481767
2,-0.374110503562327,0.708322104472172,0.923451265423402
"11943","ATF3",2980.46220329909,-0.102641668564577,0.273388847657443,-
0.375442046901588,0.707331739128026,0.923451265423402
"11944","ATRAID",1512.61611402756,-0.106860148337836,0.285721052554329
,-0.374001661349461,0.708403080156457,0.923451265423402
"11945","ALMS1-
IT1",7.24509796179738,0.256023760370348,0.678633740558301,0.3772635297
49819,0.705977772213021,0.923451265423402
"11946","PRPF40A",662.79953071401,0.0599469635699544,0.158933659949026
,0.377182301025352,0.706038132356888,0.923451265423402
"11947","PNKD",566.861845387811,-0.0954499536125678,0.253843060546191,
-0.376019550848423,0.706902361366041,0.923451265423402
"11948","CTDSP1",476.592149788554,-0.0637828001548414,0.17079571077341
1,-0.373444976258566,0.708817290445888,0.923451265423402
"11949","PDE6D",79.1871499213582,-0.0957514517144404,0.253521374686093
,-0.377685912412705,0.705663934270613,0.923451265423402
"11950","THRB-
IT1",12.3011768371336,0.18353447249813,0.489704538691888,0.37478613734
8026,0.707819524864419,0.923451265423402
"11951","EIF1B",997.690045001802,-0.0811419004484075,0.217048112676086
,-0.373842921037053,0.708521184596514,0.923451265423402
"11952","ZNF662",60.0404953427833,0.0988765647371138,0.264191207464809
,0.374261375637509,0.708209865151719,0.923451265423402
"11953","KLHL18",56.7317802872858,0.10752407574076,0.285009691297105,0
.377264630025062,0.705976954623663,0.923451265423402
"11954","TMEM45A",114.934826962063,-0.151072724717726,0.40312353393418
,-0.374755408704053,0.707842380033945,0.923451265423402
"11955","AC107027.3",5.10623549392193,0.257260507685722,0.683474635488
479,0.376400958174342,0.706618833768203,0.923451265423402
"11956","PCYT1A",175.508709162743,0.0719669428507403,0.192626388432077
,0.373608950655881,0.708695273766476,0.923451265423402
"11957","CLINT1",516.462432500375,-0.0568377399709164,0.15209570320200
6,-0.373697210206046,0.708629601136882,0.923451265423402
"11958","TBC1D9B",291.186250784703,0.053038864093523,0.141581249780861
,0.374617854946305,0.70794469218216,0.923451265423402
"11959","SERPINB9",31.5758668974129,-0.167848994537586,0.4475829593211
88,-0.375012030824741,0.707651519227809,0.923451265423402
"11960","ZSCAN12",15.9855304858216,0.150122926307211,0.397905954712756
,0.37728243201483,0.705963726419562,0.923451265423402
"11961","AL645933.2",8.37224724152091,-0.246904907041081,0.65994696271
6124,-0.374128408781369,0.708308783785703,0.923451265423402
"11962","RPS18",26960.8095705323,-0.0973571067708077,0.261354271961249
,-0.372510103011604,0.70951309127118,0.923451265423402
"11963","MTRES1",129.624204510886,-0.0907586449431221,0.24261945999542
8,-0.374078175529829,0.708346155316255,0.923451265423402
"11964","ZDHHC14",85.1377740383269,-0.0945705209223295,0.2539527034019
89,-0.372394227962327,0.70959935082237,0.923451265423402
"11965","DBNL",370.60044173484,-0.0719586815270265,0.19037987621592,-0

.377974200620943,0.705449759670365,0.923451265423402
"11966","SSC4D",2.89981735724508,-0.399315945716514,1.07163154611325,-
0.372624291590528,0.709428090802408,0.923451265423402
"11967","AGPAT5",41.8343412959118,-0.122453815799491,0.328465264757814
,-0.37280598266541,0.709292849823844,0.923451265423402
"11968","WRN",110.369265895846,0.100347117463479,0.267808035908558,0.3
74697932879588,0.707885129770104,0.923451265423402
"11969","HGH1",49.5121176089319,-0.0987397021016705,0.263688015617107,
-0.374456540509021,0.708064684182544,0.923451265423402
"11970","TOPORS",356.867702602965,-0.0807891170865717,0.21530558009033
8,-0.375230019828906,0.707489405943657,0.923451265423402
"11971","CARNMT1",37.1419730117611,0.108737396457724,0.290718614661504
,0.374029700796186,0.708382219245622,0.923451265423402
"11972","AL158152.1",63.7925904175485,0.106532395658317,0.284119376306
535,0.374956460355523,0.707692847796059,0.923451265423402
"11973","WDR31",19.9198106887021,0.14708716202422,0.395003932806108,0.
372368854606872,0.709618239718949,0.923451265423402
"11974","ELP4",79.0885398213257,0.0856784131443588,0.229637832609018,0
.373102341939602,0.709072275717177,0.923451265423402
"11975","HIPK3",395.635319373534,-0.074993463404271,0.201338269631215,
-0.372474957401959,0.709539253926168,0.923451265423402
"11976","NUP160",132.878565747701,0.0762707536623493,0.201792466435728
,0.377966308700836,0.705455622412031,0.923451265423402
"11977","VPS26B",205.090245840241,0.0670087624312505,0.178738397926071
,0.374898528848661,0.707735933219241,0.923451265423402
"11978","FAM234B",17.9318236207767,-0.160039031962642,0.42545851464168
1,-0.376156608588327,0.706800471771457,0.923451265423402
"11979","STK38L",91.3310828718211,-0.0909535695048456,0.24304878923674
6,-0.374219389409303,0.708241099624447,0.923451265423402
"11980","AC023157.3",12.5481069137946,-0.188528501059841,0.50480337247
6335,-0.373469178969638,0.708799280246905,0.923451265423402
"11981","HELB",36.9634612455833,0.172902188171901,0.461798732525381,0.
374410270089681,0.708099103271622,0.923451265423402
"11982","AC025164.1",11.496709663512,-0.190880734676987,0.510290014728
739,-0.374063236919216,0.708357269180703,0.923451265423402
"11983","PPTC7",182.0985520725,-0.102163556696849,0.271733647914883,-0
.375969474081657,0.706939590062492,0.923451265423402
"11984","TBX5-
AS1",17.1760190808901,0.268897295110486,0.715986444012402,0.3755619919
33228,0.707242551589098,0.923451265423402
"11985","UBL3",837.873128014582,-0.0675766256178111,0.181367204253239,
-0.372595618353664,0.709449434440898,0.923451265423402
"11986","USPL1",59.1982389948376,0.0944750412384751,0.252431820560568,
0.374259635844154,0.70821115941227,0.923451265423402
"11987","RBM23",244.814715101404,0.0673702763470792,0.180358769163412,
0.373534797667859,0.708750451585284,0.923451265423402
"11988","BMP4",384.483494385616,-0.101011155759703,0.270988350302488,-
0.372750915849151,0.709333837606985,0.923451265423402
"11989","GPR132",2.81094981083222,0.383217104344757,1.01224378732256,0
.378581828946943,0.704998417883363,0.923451265423402

"11990","RMDN3",101.377701385696,0.0727373262394862,0.193925343581469,
0.375079011830802,0.707601705621627,0.923451265423402
"11991","CSK",173.421598483889,-0.0802991812846051,0.212441139725428,-
0.377983197550102,0.705443076062018,0.923451265423402
"11992","ZNF710",22.0590208872197,0.139445026693733,0.372752186522453,
0.374095798054651,0.708333044782458,0.923451265423402
"11993","ZNF598",86.3677900888567,-0.10105824974887,0.26774389283085,-
0.377443715635803,0.705843884727648,0.923451265423402
"11994","POLR3E",77.9630747930718,-0.0847097377617688,0.22731808941740
6,-0.372648467963423,0.709410094693889,0.923451265423402
"11995","AMFR",227.686060293821,-0.0715196191048694,0.1904310382756,-0
.375567028108953,0.707238806927297,0.923451265423402
"11996","GSE1",101.433378543811,0.087111635748323,0.233463707883536,0.
373127097731948,0.709053851594038,0.923451265423402
"11997","ZNF287",43.8981704686975,0.101483455197642,0.271706044021575,
0.373504592299697,0.708772928074257,0.923451265423402
"11998","MED9",36.395439647513,0.13708806372343,0.362272979465199,0.37
8410953877391,0.705125332124184,0.923451265423402
"11999","AC005726.5",4.53702256466993,0.292828872345384,0.784299757302
095,0.373363461634469,0.708877949913927,0.923451265423402
"12000","GALK1",198.851157623185,-0.112769765329231,0.299821098694081,
-0.376123514390474,0.706825073727406,0.923451265423402
"12001","CCDC40",9.98988506821657,-0.19892726844545,0.527257845909894,
-0.377286502208724,0.705960701974873,0.923451265423402
"12002","AP001033.3",4.52527644227798,-0.28206248023067,0.747463285000
103,-0.377359645471591,0.705906352099575,0.923451265423402
"12003","DPP9",112.846121105126,0.0876791715830096,0.232001030790849,0
.377925784571419,0.705485727211956,0.923451265423402
"12004","KDM4B",126.522046469833,0.0992471835566573,0.265322884919233,
0.374061904184666,0.708358260697022,0.923451265423402
"12005","NACC1",58.2923238131782,0.117378133974196,0.311120958900334,0
.377274917090359,0.70596931055724,0.923451265423402
"12006","PODNL1",5.32290049798142,-0.234916648432737,0.630648810033393
,-0.372499947189781,0.709520651305804,0.923451265423402
"12007","ZNF506",192.235016691326,0.072836421990571,0.192758610074839,
0.377863390705568,0.705532079629747,0.923451265423402
"12008","PPP5C",111.937899249375,-0.0690078475433873,0.184536551114741
,-0.373952190644767,0.708439886122277,0.923451265423402
"12009","NAPSA",3.57756985541388,0.30880325317165,0.828650943972343,0.
372657818612171,0.709403134417459,0.923451265423402
"12010","ZNF836",48.3086249575828,0.103292217272404,0.276841355077369,
0.373109780666754,0.709066739539385,0.923451265423402
"12011","ZNF600",18.7959187999992,-0.168736661589068,0.451444303012337
,-0.373770718698064,0.708574906205322,0.923451265423402
"12012","ZNF28",31.9786846451638,0.118622268476602,0.316952975716666,0
.374258257738025,0.708212184608414,0.923451265423402
"12013","RPS9",14265.3983762676,-0.103375063938004,0.273169560130267,-
0.378428196350673,0.705112525229454,0.923451265423402
"12014","ZNF324B",13.3724055652786,-0.182294900054925,0.48732262379313
9,-0.374074362967205,0.708348991739074,0.923451265423402

"12015","NRIP1",254.932001320554,-0.0982262618132479,0.261025553121492
,-0.376308988291003,0.706687197898934,0.923451265423402
"12016","AC008079.1",4.85791203889454,0.279777211410536,0.743227408495
115,0.376435540741196,0.706593128061833,0.923451265423402
"12017","IFT27",176.243445603855,-0.0944972450747677,0.249822644518248
,-0.378257324339008,0.705239444577582,0.923451265423402
"12018","AL592156.2",2.84868249182851,0.414885542959891,1.109554572959
94,0.373920808467408,0.708463234663931,0.923451265423402
"12019","ZNF280C",22.8750746837069,-0.116866273581867,0.31005653172088
,-0.376919244156007,0.706233619623401,0.923451265423402
"12020","LINC02569",3.18201885656043,-0.308515829131304,0.829007870774
962,-0.372150663470663,0.709780676913468,0.923541579611202
"12021","AL139407.1",3.4406172960249,0.340615428258137,0.9166428103146
21,0.3715901378654,0.710198032992089,0.924007756093193
"12022","H2AFJ",1697.08795811569,0.0960766160233432,0.258630708067214,
0.371481858211418,0.710278665864864,0.924035795552027
"12023","IFT22",247.75041173042,-0.0906027498525666,0.243951794024927,
-0.371396120347075,0.710342514789355,0.924041997114324
"12024","PEX13",98.0543883481337,-0.0741646579813266,0.199980923489955
,-0.370858663351716,0.710742804921544,0.924087479929936
"12025","HAUS3",77.6354924605151,0.0755143837009964,0.203844437657028,
0.370451038885106,0.71104645089374,0.924087479929936
"12026","MANEA-
DT",9.09800736346819,0.224743626026222,0.605818739609794,0.37097503152
6721,0.710656128830407,0.924087479929936
"12027","POR",202.502269827347,-0.0690073313459569,0.186267222265105,-
0.370474904316457,0.711028671888762,0.924087479929936
"12028","INTS8",84.9997897705931,0.0813969672877822,0.219991349822293,
0.370000763000609,0.711381921889798,0.924087479929936
"12029","LRMDA",115.516130984945,0.107216848638872,0.289728011947616,0
.370060347006615,0.711337526549137,0.924087479929936
"12030","NUP98",131.380362126333,0.0789415228836523,0.213313703508778,
0.370072440659696,0.711328515830254,0.924087479929936
"12031","RNF214",115.285641575341,0.0678851780423361,0.183401801738067
,0.370144553646693,0.7112747868433,0.924087479929936
"12032","DDX47",20.564540081571,-0.134983574001894,0.364590697985674,-
0.370233181339141,0.71120875526327,0.924087479929936
"12033","WNT10B",344.771506143956,-0.0802176452079569,0.21644435866952
4,-0.370615550809696,0.710923897813702,0.924087479929936
"12034","TMED2-
DT",5.28559323329626,0.253263437281431,0.683988802276252,0.37027424489
7861,0.711178161816741,0.924087479929936
"12035","LINC02323",5.08584613476727,-0.252919077306169,0.682464729912
21,-0.370596554255198,0.710938048906417,0.924087479929936
"12036","MYBBP1A",42.3980840960812,-0.10044212509442,0.271315508312058
,-0.370204142473475,0.711230390273502,0.924087479929936
"12037","RCVRN",8.85157307596527,0.222876274834196,0.601971301849685,0
.370244020187277,0.711200679987121,0.924087479929936
"12038","ZNF85",39.9464737499016,0.116128757224401,0.313031466481278,0
.370981098257566,0.710651610167337,0.924087479929936

"12039","CLASRP",83.6364682746296,-0.0726622365814717,0.19596848748440
4,-0.370785311017183,0.710797442861119,0.924087479929936
"12040","ASCC2",218.18608564749,-0.0724808362465296,0.195682092187789,
-0.370400967386286,0.711083753115734,0.924087479929936
"12041","AL391069.2",2.89659957101937,0.34879200667844,0.9446098567754
41,0.369244513146508,0.711945479743049,0.924358899483753
"12042","MFAP3L",3.74290990177261,-0.327325383448006,0.886404680631593
,-0.369273076508098,0.711924191446757,0.924358899483753
"12043","KHDC1",6.53268853590555,0.240964656005361,0.651947575173583,0
.369607411978185,0.71167502765295,0.924358899483753
"12044","AC091729.3",27.1684646139599,-0.157230876869711,0.42557841889
9608,-0.369452185278223,0.711790706655888,0.924358899483753
"12045","AC027288.3",14.0167639056456,-0.235099311068326,0.63624612200
0294,-0.369510010260177,0.711747613189122,0.924358899483753
"12046","ARAF",107.629968115748,-0.0736315453751017,0.199358700852552,
-0.369342020489792,0.711872808367042,0.924358899483753
"12047","RTP4",34.8400615876575,0.141682279590545,0.383820502968873,0.
369136819150162,0.71202574618389,0.924386375887445
"12048","HK2",2.51853397530593,-0.396693555422619,1.07624240555423,-0.
368591270308044,0.712432403409299,0.924606655779463
"12049","CCDC66",324.446297977494,-0.0787848420954893,0.21379737985954
8,-0.368502374291239,0.712498675085754,0.924606655779463
"12050","ZDHHC11B",17.4492593770557,-0.286785043576712,0.7775771059770
85,-0.36881878513687,0.712262801774123,0.924606655779463
"12051","EIF4EBP1",164.738738306186,-0.114352707937247,0.3103755563796
33,-0.368433356257531,0.71255012929091 2,0.924606655779463
"12052","PPP1R13B",3.85818747940827,-0.319762348806773,0.8673961476992
64,-0.368646263480568,0.712391407274413,0.924606655779463
"12053","AC104984.3",7.30570708175337,-0.223042686707244,0.60493013452
6478,-0.368708176328223,0.712345253676733,0.924606655779463
"12054","AP000866.1",18.0492602820414,0.145592190345639,0.395300321841
318,0.368307796126924,0.712643740017598,0.924651409812115
"12055","DACT3",5.83193262708828,-0.256468784050994,0.696575606344775,
-0.368185135561657,0.712735193168175,0.924693357208648
"12056","CCDC88C",5.12531136462857,0.338029190219374,0.918624938393249
,0.367973017160426,0.712893353955622,0.924821836087087
"12057","PLA2G12A",156.033535327247,0.0855058267489318,0.2324757031074
37,0.367805433453902,0.713018317290359,0.924843301377982
"12058","MYO1F",21.9088356569313,0.188512555541559,0.512551768782459,0
.367792225143152,0.713028166752923,0.924843301377982
"12059","LGALS8",201.618340092067,0.0730197419494782,0.198753760603019
,0.367387976599467,0.713329638799172,0.924927503798628
"12060","AC100812.1",2.53933357100218,-0.340943225065284,0.92748931117
9877,-0.367598009977672,0.71317299889647,0.924927503798628
"12061","FAM91A1",155.222905157197,0.0806082310243589,0.21938704557309
5,0.367424752969299,0.713302210633116,0.924927503798628
"12062","DNAJC24",86.5121808854108,0.0865667048512138,0.23555729684101
8,0.367497445471364,0.713247996964703,0.924927503798628
"12063","EEF1AKNMT",120.510347626362,0.0806532868793637,0.219737382196
897,0.367043996214963,0.713586200577479,0.924953437512993

"12064","TMA7",2926.96405895103,-0.072207066373936,0.196598957543142,-0.367281023644751,0.713409407434938,0.924953437512993
"12065","AL031123.1",4.8680812979388,-0.322633479755018,0.878894950683807,-0.367089922981124,0.713551943689316,0.924953437512993
"12066","RAB40C",48.6009649614179,-0.0867505996917547,0.236341979147889,-0.36705539999507,0.713577694415873,0.924953437512993
"12067","PRUNE1",52.2389635928279,0.0949376026886324,0.258955782439888,0.366617040925397,0.713904695210979,0.925136252638968
"12068","EEF1AKMT2",176.881659704286,-0.0833311252169212,0.227258682595435,-0.366679610500369,0.713858017242468,0.925136252638968
"12069","PDRG1",104.359815564688,-0.0809511441242492,0.220750173788164,-0.366709310960414,0.713835860568277,0.925136252638968
"12070","PIP4P2",342.185086678182,-0.0713684516783215,0.194903065175056,-0.366174085636984,0.714235178013016,0.925313561469978
"12071","ZBED5-AS1",57.4249481202492,0.120678720500539,0.329499838620557,0.366248193036324,0.714179883773715,0.925313561469978
"12072","DVL2",112.275349593086,0.073987977485195,0.202120226043411,0.366059245695204,0.714320867255181,0.925313561469978
"12073","ARSG",73.2284079977703,-0.0947718292067443,0.258913126369309,-0.366037174459681,0.714337336393127,0.925313561469978
"12074","RPL36",8733.62681135844,-0.075169028214318,0.205349178336218,-0.366054682192318,0.714324272443696,0.925313561469978
"12075","CDC6",13.3960884930455,0.171148739955668,0.467728031962332,0.365915079405486,0.714428443813882,0.925354936749409
"12076","SEC22B",389.880370714284,0.0679891659583303,0.185954249789928,0.365623082210475,0.714646348844855,0.925560524671542
"12077","GNPTAB",232.057810251748,0.0683824838769766,0.187081055380778,0.365523295438938,0.714720820783624,0.925580329308262
"12078","H3F3A",7167.28821473068,-0.0823190259728829,0.225419984007221,-0.365180692987033,0.714976529340411,0.925707318990709
"12079","PCDHB7",8.45548512675042,0.233990655026672,0.64079990477216,0.365154010298844,0.714996445870061,0.925707318990709
"12080","NPIPB15",57.797567417001,-0.107372404068729,0.294023894082798,-0.36518257947598,0.714975121232022,0.925707318990709
"12081","RHBDL2",2.65696087684904,0.392664761256193,1.07722671164765,0.3645145047096,0.715473844562921,0.925965920238803
"12082","SARS",600.654176920837,-0.0632309412855232,0.173649308388225,-0.364130107239811,0.715760855512117,0.925965920238803
"12083","GGH",23.1496624992875,0.190740732554349,0.52399262368722,0.364014155795073,0.715847438721699,0.925965920238803
"12084","LRRC69",9.34624154395588,-0.205865297542861,0.565291073604853,-0.364175744417936,0.71572677834191,0.925965920238803
"12085","AZIN1",608.433122494287,0.0627841699969824,0.172420812091703,0.364133362065308,0.715758425123059,0.925965920238803
"12086","SMC5-AS1",6.15050817021621,-0.223357557024752,0.612762820540799,-0.364508990326185,0.71547796160138 4,0.925965920238803
"12087","PRKCQ-AS1",3.94644592223715,0.32515800533736,0.891325603745436,0.36480272076

8948,0.715258673985403,0.925965920238803
"12088","SDSL",37.4952549587026,0.146710864370158,0.402908452601451,0.
364129527248419,0.715761288593927,0.925965920238803
"12089","INAFM2",8.96488958172589,0.180605251685038,0.495732928250819,
0.364319659624587,0.71561932091549,0.925965920238803
"12090","TANGO6",38.1685425916945,0.107466289215973,0.294675347004658,
0.364693858201425,0.7153399437646,0.925965920238803
"12091","DNAJC5",72.2203205391425,-0.0922347562177137,0.25337163438962
2,-0.364029526982802,0.715835960545309,0.925965920238803
"12092","DNASE1L2",3.24495011944345,-0.34120925417816,0.93790946557114
1,-0.363797644339137,0.71600912183905,0.926021885848237
"12093","AC006141.1",22.9089660075779,0.165288641075493,0.454247783429
804,0.363873302424237,0.715952621602159,0.926021885848237
"12094","WIZ",115.245795131035,-0.0671671425155206,0.184700340843852,-
0.363654675506552,0.716115892901553,0.926083393830022
"12095","ELOA-
AS1",28.1960462388991,-0.120590479671312,0.331857653355329,-0.36338013
7393405,0.716320937249923,0.926110024258908
"12096","AL391684.1",2.62087477664262,0.350639875119871,0.965336562977
137,0.363230699600234,0.716432556490315,0.926110024258908
"12097","ADIPOR2",113.512915600933,0.0766289609904889,0.21093925803491
7,0.363275009613452,0.71639945947811,0.926110024258908
"12098","RPS26",4531.37992083924,-0.22231605056655,0.611857970988602,-
0.363345843492641,0.716346551781597,0.926110024258908
"12099","RCOR1",169.844466092399,-0.0722257540017477,0.198745306222604
,-0.363408602570223,0.716299676505501,0.926110024258908
"12100","SCP2",1362.35417086512,-0.0766638756565682,0.211442814356315,
-0.362574986953102,0.716922398078805,0.926303079256693
"12101","NSL1",263.126611146332,0.0571353390445344,0.157570302268659,0
.362602204996206,0.71690206287615,0.926303079256693
"12102","WASL",534.555367829544,0.0501618916456788,0.138352142366036,0
.362566786374485,0.716928524952006,0.926303079256693
"12103","AATK",8.87986267007238,0.208469215144365,0.575000101930793,0.
362555092502325,0.716937261790426,0.926303079256693
"12104","MISP3",18.6336621540898,0.204156429636845,0.562790523862595,0
.362757404363634,0.716786113827595,0.926303079256693
"12105","WNK3",4.93724614446282,0.260721846704469,0.718998945227498,0.
362617843092905,0.716890379398631,0.926303079256693
"12106","LYRM2",293.00511546079,-0.0613048727285226,0.169217109957123,
-0.362285307579453,0.717138836383861,0.926410456846749
"12107","INIP",131.068532030545,0.0589265448436432,0.162622762220171,0
.362351149612525,0.717089639522269,0.926410456846749
"12108","MRPS16",359.930609406973,-0.0774133997813437,0.21386602444353
7,-0.361971472480341,0.7173733486383,0.926636865931865
"12109","PRNP",563.414364809653,0.0584819975500267,0.161650612471206,0
.361780241076684,0.717516258745322,0.926744924170191
"12110","AC006460.2",6.67916551105103,-0.273265005383779,0.75776927174
352,-0.36061769139178,0.718385262223736,0.926777094047477
"12111","FASTKD2",123.084256784887,0.0718817984106622,0.19958297275579
6,0.360159974661841,0.718727504564024,0.926777094047477

"12112","DBR1",96.5840020087473,-0.06484280111923,0.179410446052899,-0
.361421547885296,0.71778434223007,0.926777094047477
"12113","RGL2",159.784474631608,-0.0765163997162983,0.212332203978225,
-0.36036172696698,0.718576644083693,0.926777094047477
"12114","HOXA4",17.9344944820616,0.191867647484826,0.532131095635263,0
.360564622249284,0.718424940002209,0.926777094047477
"12115","CLDN4",41.6011151958729,0.324370905275224,0.900642528616078,0
.360154994871995,0.718731228345325,0.926777094047477
"12116","LETM2",10.7263671208389,-0.168837059395312,0.468481014943319,
-0.360392532482324,0.718553610194997,0.926777094047477
"12117","AC069544.1",5.39774413151898,0.233234530403135,0.645093729054
348,0.361551383773389,0.717687300250114,0.926777094047477
"12118","AL138963.4",3.1595906452076,0.355558262476609,0.9869685208257
39,0.360252890516846,0.718658025281934,0.926777094047477
"12119","DDX24",1225.46065096198,0.0520983926288872,0.144208763261817,
0.361270642993452,0.717897137333545,0.926777094047477
"12120","MTFMT",49.7097247120871,-0.104261382927164,0.289263127404826,
-0.360437861066367,0.718519717587656,0.926777094047477
"12121","AC135048.3",3.63167895208418,0.349800460573084,0.967691477760
255,0.361479323330103,0.71774115912667 5,0.926777094047477
"12122","RHOT1",132.094826974387,0.0630303046491576,0.174692848091641,
0.360806440204656,0.718244148072289,0.926777094047477
"12123","ADAT3",6.18774112665781,-0.286435381822665,0.795471898174123,
-0.360082339150046,0.718785559513805,0.926777094047477
"12124","ZNF121",62.4535443749553,0.101228450539207,0.280903579436804,
0.360367250364569,0.718572514112049,0.926777094047477
"12125","ERF",89.4683834952243,0.0809885837013956,0.224341038233343,0.
361006547616836,0.718094552393427,0.926777094047477
"12126","CDC42EP5",222.805720218875,-0.116151114276087,0.3221745819598
93,-0.360522278230336,0.718456599558501,0.926777094047477
"12127","ZNF805",77.0352896733952,-0.0927057889122773,0.25703693765112
1,-0.360671076147459,0.718345349239416,0.926777094047477
"12128","ZNF814",14.3784051336744,-0.16654496936121,0.461383005836572,
-0.360969015447878,0.718122609752756,0.926777094047477
"12129","ZNF544",34.8512992243024,0.122942516835383,0.341031949533865,
0.360501463289365,0.71847216254271,0.926777094047477
"12130","USP27X-
AS1",2.98365100336246,-0.308141080052532,0.853588906560134,-0.36099471
0315889,0.718103401385506,0.926777094047477
"12131","SAAL1",42.5020330499286,0.0997644248473949,0.277247233295955,
0.35983920799274,0.718967380717274,0.926858707090188
"12132","GPHN",47.7951994219918,0.0979008410921866,0.272047275490764,0
.35986701545007 8,0.71894658461142,0.926858707090188
"12133","TASOR2",205.460579305376,0.0778551277668041,0.21645463289608,
0.359683351310769,0.719083943663375,0.92689436523351
"12134","AC087294.1",3.95717562687543,0.287205863522117,0.798584353646
36,0.359643739838786,0.719113569548811,0.92689436523351
"12135","CENPH",29.2571084825385,-0.109906725243461,0.305854648525206,
-0.359342994371405,0.719338514382214,0.927107899871268
"12136","SMIM33",3.67915908428772,0.30896377882021,0.8607677662311,0.3

5893976394239,0.719640151759678,0.927127454257853
"12137","SRF",66.8998266991576,-0.118341996548421,0.329710957937724,-0
.358926489094042,0.719650082780712,0.927127454257853
"12138","KIAA0408",86.5933332026168,0.154144505928188,0.42931692633195
1,0.359045955269423,0.71956071087553,0.927127454257853
"12139","AC007032.1",46.5416458414154,0.140815815481186,0.392188822882
906,0.359051067406959,0.719556886602652,0.927127454257853
"12140","BLCAP",207.607410722411,0.072903961079744,0.202951751452019,0
.359218191309768,0.719431868909107,0.927127454257853
"12141","CXXC1",110.772501035397,-0.0733949993346427,0.204546623861227
,-0.35881794550878,0.719731286886725,0.927155697793294
"12142","TMEM59",3337.41607730396,-0.0937936824720677,0.26155680361703
3,-0.358597754579533,0.719896026811181,0.927277210717791
"12143","GLRX",763.644151203741,0.112601960890432,0.314062698840573,0.
358533379819142,0.719944192439011,0.927277210717791
"12144","RABGAP1L-
IT1",2.57794073327051,0.359610442760833,1.00367517145378,0.35829365215
8352,0.720123568060879,0.927314782800693
"12145","FAM155A",16.9354241808907,-0.156691651551739,0.43721905905099
2,-0.358382481980194,0.720057099571789,0.927314782800693
"12146","AL109628.2",11.5016535509793,0.250651121826871,0.700220284434
58,0.357960383894433,0.720372960801591,0.927314782800693
"12147","PIGQ",66.0845182487222,0.0832501837011025,0.232670700992578,0
.357802608347143,0.720491038456455,0.927314782800693
"12148","NPB",22.0693803785466,0.171300240821676,0.478340302731941,0.3
58113752580182,0.720258187589919,0.927314782800693
"12149","ZBTB14",76.9737603931504,0.0866337282650674,0.242075158073203
,0.357879465842875,0.720433518236026,0.927314782800693
"12150","AC010632.2",6.42740345254026,0.27031004821303,0.7549096504235
14,0.358069403486076,0.720291375516105,0.927314782800693
"12151","LINC00237",3.60176884036331,0.366616191349001,1.0236601209211
,0.358142496573098,0.720236677769944,0.927314782800693
"12152","ASB9",3.32828565327024,-0.322398126423045,0.901103900459919,-
0.357781301644011,0.720506984692712,0.927314782800693
"12153","SAMD13",14.2099112366921,-0.151315956864861,0.423236324794096
,-0.357521195607386,0.720701661506441,0.927346710632085
"12154","AC104118.1",4.69480066121548,-0.305923428545882,0.85555638912
107,-0.357572490178191,0.720663268562578,0.927346710632085
"12155","AC130324.1",3.08871026927956,0.320047817125204,0.895212372442
457,0.357510493573721,0.720709671849936,0.927346710632085
"12156","ARL8A",287.812434229174,-0.0674946528929459,0.188870596450257
,-0.357359240461349,0.720822886262689,0.927408830206806
"12157","TXNRD3",40.3740807184617,0.10499842774511,0.293877302630555,0
.357286618616845,0.720877246588032,0.927408830206806
"12158","HSD11B1L",38.8357465938767,-0.142812570985932,0.3998018919979
94,-0.357208342042184,0.720935841282247,0.927408830206806
"12159","ZNF687-
AS1",5.3676515249821,0.24192570375146,0.678102541947586,0.356768613573
727,0.721265034765519,0.927699870634801
"12160","SWSAP1",11.1531128846094,0.177662393481556,0.498005762314799,

0.356747666243371,0.721280717833708,0.927699870634801
"12161","FRRS1",35.3510954027799,0.103864074001606,0.29140056590419,0.356430584406468,0.721518128317644,0.92770579630885
"12162","ABCB10",67.1601102876691,-0.0827815339870113,0.232255371740204,-0.356424625905354,0.721522589916934,0.92770579630885
"12163","ALDH1L2",118.134939921417,-0.10645198010463,0.29857766579233,-0.356530284414076,0.721443476471808,0.92770579630885
"12164","ZDHHC8",157.288092010187,0.06720522768713,0.188516833003557,0.356494571950834,0.721470216398492,0.92770579630885
"12165","PMF1",356.281714989396,-0.0956823278582253,0.268677282870845,-0.356123624728706,0.721747985541994,0.927766786708045
"12166","TAF5L",87.6841336500119,0.103745584079474,0.2913039452101 5,0.356142049516804,0.721734188002404,0.927766786708045
"12167","VMAC",51.9561706526614,0.0932501669961123,0.261799396313903,0.356189388933133,0.721698737943585,0.927766786708045
"12168","ZNF416",13.8650443346976,0.177639991628277,0.498945299471139,0.356030995414864,0.721817353064141,0.927779701012752
"12169","LINC00824",15.5574690769437,0.178434916148555,0.501481951779094,0.355815230270055,0.721978942462244,0.927837717009635
"12170","RPS6KA3",277.969086139454,0.0846036633845905,0.237776108471105,0.35581229724294,0.721981139130899,0.927837717009635
"12171","DPY30",411.237537332766,-0.0817796626779309,0.230061933834778,-0.355468031215725,0.722238990511115,0.927989956873954
"12172","RDX",747.548982616152,0.0529886304731918,0.149088839681696,0.355416479102811,0.722277605180731,0.927989956873954
"12173","ITPRIPL2",460.101119495348,-0.104819001766962,0.294810658862796,-0.35554685224507,0.722179951660766,0.927989956873954
"12174","MRPS25",201.399791492619,0.0803307435829846,0.226315330741165,0.354950516696803,0.722626662426914,0.928143463501657
"12175","CAMK2G",149.311928079927,0.0684181287781895,0.192676123124539,0.355093966334201,0.722519196669051,0.928143463501657
"12176","DDIT3",358.876766849244,0.129911547638095,0.366009770550902,0.354940108409013,0.722634460042179,0.928143463501657
"12177","CELF6",18.089254309909,0.170911725300261,0.481231102971169,0.355155193097527,0.722473330108795,0.928143463501657
"12178","TBC1D10B",131.858961978099,0.0670228664506542,0.188963988989952,0.354685920893731,0.722824899578797,0.928311827016947
"12179","LENG1",142.909355412416,-0.0753832805057609,0.212651494593958,-0.354492126423562,0.722970103647517,0.928422072505721
"12180","AC132192.1",5.92991520262305,0.269293994585439,0.759887400575624,0.354386708322109,0.723049094287053,0.92844727706482
"12181","EGFL8",40.805200967942,-0.0978358373032982,0.276565882855155,-0.353752372828059,0.723524469302057,0.928933633093968
"12182","RNF34",107.880055140905,-0.0662395936035239,0.187264140757973,-0.353722786089272,0.723546644395826,0.928933633093968
"12183","HIST1H4C",960.26251278919,-0.129946901549657,0.367766724894841,-0.35334056278967,0.723833139460966,0.928996414013582
"12184","PACRG",3.53697611780934,0.295350594838075,0.835323713393198,0.353576212553958,0.723656503855814,0.928996414013582
"12185","TSPAN33",9.61122008153672,0.213004450877865,0.602774038021696

,0.353373631646356,0.723808351205228,0.928996414013582
"12186","CYP7B1",158.849040784933,0.0695882249516435,0.196913463393455
,0.353394957116764,0.723792365888926,0.928996414013582
"12187","RPS23",19309.4872482846,-0.0800531522047977,0.226735144782647
,-0.353069006049055,0.724036708039163,0.929028969868941
"12188","SNX10",15.2438837298921,0.169570336262672,0.480220493332646,0
.353109329187274,0.724006479148224,0.929028969868941
"12189","DLGAP4",1249.14932654612,0.0503415153527789,0.142554868217468
,0.353137819719932,0.723985121020378,0.929028969868941
"12190","ZNF2",20.2140521782841,0.134275674432688,0.380903561320406,0.
352518821213484,0.724449205953665,0.92916652900537
"12191","RHOH",8.62470859517398,0.227422862763598,0.645310875552484,0.
352423725338412,0.724520511651785,0.92916652900537
"12192","NNT",165.892050323215,-0.0693487811147107,0.196602695216417,-
0.352735658269449,0.724286624059675,0.92916652900537
"12193","ILRUN",138.887310580163,0.0771065686381456,0.218649611494363,
0.352649008206142,0.724351591774589,0.92916652900537
"12194","CD3E",12.0952606213162,0.203849162712256,0.578230258297644,0.
352539770769527,0.724433497678565,0.92916652900537
"12195","EIF3J-
DT",180.747810769568,0.0687290862059864,0.195047385224888,0.3523712257
24161,0.724559878438296,0.92916652900537
"12196","ABCA3",58.8969725970291,0.0978360715801757,0.277489175514174,
0.352576173102573,0.724406202967411,0.92916652900537
"12197","VANGL1",121.300998298922,0.0718185826115067,0.203953617190417
,0.352131938628257,0.724739316865618,0.929320563727004
"12198","SDE2",102.651314542669,0.0694379519766384,0.197292302466113,0
.351954694170419,0.724872240036044,0.929398423677373
"12199","AL391834.1",2.95530250172754,0.292775310535019,0.832002137639
51,0.351892497975615,0.724918885578022,0.929398423677373
"12200","AC239809.3",25.8850201114686,-0.151121432969432,0.42969970006
5463,-0.351690803941472,0.725070157924254,0.929439986061415
"12201","GSK3B",201.47944591559,-0.0631137720821972,0.179424427293087,
-0.35175685403806,0.725020618573693,0.929439986061415
"12202","NDNF",79.4102879988356,-0.126687802254991,0.360388134749734,-
0.351531557338776,0.725189601894875,0.929494439549518
"12203","DDN-
AS1",6.49119514912616,-0.223834644923029,0.636986035765956,-0.35139647
0809402,0.725290929684292,0.929494439549518
"12204","FAM104A",189.952731161851,-0.0639871146219531,0.1820567722686
39,-0.351467917532533,0.725237337223863,0.929494439549518
"12205","GOLGA1",96.4198710763113,-0.0792444624532848,0.22562773615487
5,-0.351217735034535,0.725425005965824,0.929513935220833
"12206","AQP9",3.15816446970542,-0.364248769899097,1.03705932755661,-0
.351232335721134,0.725414053137805,0.929513935220833
"12207","SLC4A1AP",161.741524774727,-0.0664332686958082,0.189205813314
65,-0.351116424659371,0.725501006343842,0.929535163366731
"12208","CNIH3",28.601559870395,-0.131260354321415,0.374958970284601,-
0.350065913136537,0.726289231682939,0.930204682690851
"12209","ARHGAP22",5.1161789692288,-0.331116342579978,0.94598275900202

8,-0.350023654690379,0.726320945333803,0.930204682690851
"12210","TMEM223",196.868130866721,-0.0845298400558904,0.241312510321168,-0.350291992501292,0.726119574114027,0.930204682690851
"12211","COPZ1",572.14151104574,-0.0919309749023736,0.26253402747296 7,-0.350167846001752,0.72621273616541,0.930204682690851
"12212","TRAFD1",141.902587969534,0.0728342502943226,0.20794756115738,0.3502529670891 4,0.726148859138342,0.930204682690851
"12213","FXR2",88.9297585026138,0.0721541555866461,0.206315054684537,0.349728020075764,0.726542823043992,0.930412654745602
"12214","SMIM3",120.53468421794,0.10367934332843,0.29680311163107,0.349320270797246,0.726848881938526,0.930560582771857
"12215","METTL2B",146.073977530601,-0.0919150482623623,0.263270108698,-0.349128310528405,0.726992983488748,0.930560582771857
"12216","ACO1",194.051460352979,0.0602529750081412,0.172622901612036,0.349043924331417,0.727056333933914,0.930560582771857
"12217","FOLR3",6.68787810616867,-0.306249675404184,0.876724583197775,-0.349311153437908,0.726855725978042,0.930560582771857
"12218","NCAPD2",47.0044482093368,-0.106816837090313,0.30623784393852,-0.348803517281025,0.727236822655901,0.930560582771857
"12219","SNRNP35",220.045512265313,-0.0850254759533804,0.243778602966203,-0.34878153750503,0.727253325014093,0.930560582771857
"12220","PRR11",8.07418226936408,0.188484184156503,0.5399409121001,0.349082982846019,0.727027011675628,0.930560582771857
"12221","TMC6",9.27007159796778,-0.255456951468013,0.732057707696734,-0.348957396093478,0.727121294390691,0.930560582771857
"12222","GZF1",40.8950007877824,-0.110002482315033,0.315274236236075,-0.348910471176794,0.727156523757151,0.930560582771857
"12223","USP27X",18.4180868172954,0.13666113888944,0.39163665401201,0.348948796006334,0.727127750951878,0.930560582771857
"12224","HSPB2",604.249358937771,-0.0831072210311651,0.238380901634965,-0.348632044182918,0.727365567564376,0.930628065830075
"12225","AL645568.1",22.3449017787632,0.130197929302068,0.373774415276274,0.348332908783585,0.727590181915659,0.930718394150901
"12226","SPATA7",108.09064917925,-0.0736909803411611,0.211573137740965,-0.348300266886353,0.727614693432421,0.930718394150901
"12227","DONSON",37.3896626881147,-0.114305412234359,0.328143887737007,-0.348339300246147,0.727585382458827,0.930718394150901
"12228","AP001330.4",8.98006910921386,0.214922194295471,0.618942048731968,0.347241223529382,0.728410103386183,0.931583450565042
"12229","AP001885.2",4.44532174264319,0.257655346902278,0.741996977128917,0.347245817495442,0.728406652388639,0.931583450565042
"12230","ABCC2",158.727949206159,0.286446489136205,0.825129009968442,0.347153579228975,0.7284759430343,0.931591475801836
"12231","FKBP4",148.645182795539,0.0795457001090543,0.22920075224674,0.347056889339619,0.72854858018186,0.931608191811323
"12232","PSMD6-AS2",10.1254965827882,0.181855453191589,0.524326688425185,0.346836156171626,0.728714412523548,0.931667899278042
"12233","LINC00710",18.8668592162374,-0.238942589503269,0.688818944214234,-0.346887366426661,0.728675938168674,0.931667899278042

"12234","COLQ",8.1950145197885,-0.211729083987887,0.611027663846877,-0
.346513090184647,0.72895714831959,0.931749738453611
"12235","MACORIS",2.93244353134994,0.307771274477345,0.887807737642542
,0.346664329930929,0.728843510873735,0.931749738453611
"12236","SYNE3",436.334088694149,0.0822561972280243,0.237341581028241,
0.346573056738157,0.728912090364068,0.931749738453611
"12237","AP000787.1",13.2668607838183,0.176444524624149,0.509420299682
018,0.346363356023084,0.729069660376774,0.931817397098369
"12238","ITPR2",97.2907279299926,0.0956076771297218,0.276284550390205,
0.346047858972542,0.729306747768776,0.931891955482325
"12239","AC026356.1",6.96518050897716,0.215944098128084,0.623897868413
371,0.346120910265729,0.72925184941911,0.931891955482325
"12240","TOB1-
AS1",7.56188893448008,0.214362940388135,0.61945097722468,0.34605311520
9445,0.729302797638614,0.931891955482325
"12241","AC135050.1",3.57174622567693,-0.288845350141964,0.83511699364
9917,-0.345874113852662,0.72943732352179,0.931982659903668
"12242","CCND2-
AS1",11.4582262646971,0.184630404332352,0.534030396104442,0.3457301413
53683,0.72954553001685,0.932044771235381
"12243","ATP6V1G2",10.921850633435,0.191042166822044,0.553599191673046
,0.345091123136742,0.730025866729384,0.932050512347119
"12244","TAF6",111.069893183641,-0.062037000194928,0.179573593527787,-
0.345468389734755,0.729742270448367,0.932050512347119
"12245","CCSER2",460.225556827796,0.0487954053350753,0.141335598555649
,0.345244976026777,0.729910209020716,0.932050512347119
"12246","TRIM68",22.8411433945885,0.112858830042505,0.326802523255747,
0.345342590743047,0.72983683110518,0.932050512347119
"12247","AC103810.2",5.5379886220967,0.270394317238895,0.7823999204451
25,0.345596043881321,0.729646319508541,0.932050512347119
"12248","CFAP53",7.35738138219963,0.200996380019005,0.582272402764953,
0.345193038626873,0.729949251868771,0.932050512347119
"12249","TIMM29",108.107369696411,-0.0770384756042333,0.22324174818436
8,-0.345089913650961,0.730026775975205,0.932050512347119
"12250","ZNF69",17.2807446505562,-0.142801699250593,0.413573608911937,
-0.345287262468916,0.729878421594213,0.932050512347119
"12251","RPS8",19091.4935897321,-0.0690731467569121,0.200419564115097,
-0.344642735163543,0.730362973905501,0.932240398628514
"12252","AUXG01000058.1",111.961393437438,0.257254764905807,0.74655117
9603426,0.344590929509297,0.730401925806035,0.932240398628514
"12253","FAM24B",24.2321961942368,-0.116847392917165,0.339105867975261
,-0.344574965968117,0.730413928695256,0.932240398628514
"12254","ZNF230",30.6410712162278,0.104929874936178,0.304489053910076,
0.344609678373418,0.7303878287338,0.932240398628514
"12255","SNIP1",120.229965927868,-0.087134499023071,0.252949411934236,
-0.344474013032,0.730489836120905,0.932240478105184
"12256","DGKE",19.7590022862724,0.148694970970306,0.431730305289721,0.
344416338506793,0.730533203302886,0.932240478105184
"12257","BST1",241.477353028907,-0.098881059348337,0.287213105897342,-
0.34427767158954,0.730637474586103,0.932297471039133

"12258","HOXC5",71.6849117096007,0.0879514305768265,0.255617208331386,
0.344074763788223,0.73079006109499,0.932416100140777
"12259","SRSF4",609.52283762865,-0.0602438861892385,0.175283841537953,
-0.343693324271389,0.731076932147925,0.932426831550963
"12260","DENND1B",80.1415410383223,0.0702928383107098,0.20450844628468
8,0.343716064483997,0.731059828752158,0.932426831550963
"12261","TRMT44",37.6015966448883,0.0920124735267873,0.267856288503046
,0.343514330169407,0.731211562071404,0.932426831550963
"12262","AS3MT",10.3406208218611,-0.218511880675388,0.636117460876907,
-0.343508697865584,0.731215798527658,0.932426831550963
"12263","CD27",3.81656696636453,-0.273189683674007,0.794390833130924,-
0.343898333515869,0.730922745181616,0.932426831550963
"12264","ZNF10",22.9998254320544,0.122897751061985,0.357639062503732,0
.343636263336593,0.731119849474632,0.932426831550963
"12265","GINS3",15.5121492290854,0.135823254579997,0.394907591475602,0
.34393680322144,0.730893813418432,0.932426831550963
"12266","TINAGL1",3.37382797555108,0.402636461963868,1.17573063542833,
0.342456383997498,0.732007462320297,0.932522539548586
"12267","EFNA1",2.85998346468871,-0.343926601126647,1.0083693170181,-0
.341072060922766,0.733049333648619,0.932522539548586
"12268","CYB5R1",195.759104920797,0.0842985525588463,0.246118326242988
,0.342512294170325,0.731965393484166,0.932522539548586
"12269","MAP3K21",4.720947829982,0.232487047873126,0.677968845860275,0
.342917007606925,0.731660896486238,0.932522539548586
"12270","TSEN2",31.412856742696,0.116956581413459,0.343323112018154,0.
34066037886571,0.73335926942295,0.932522539548586
"12271","CCDC13",7.71698568167782,0.229876794466062,0.670681851009725,
0.342750879750179,0.731785882120291,0.932522539548586
"12272","HYAL3",5.8286314226045,-0.228224665185867,0.666932211180529,-
0.3422006935036,0.732199863358745,0.932522539548586
"12273","POLR2H",253.659542925859,-0.072887313355036,0.213858655640632
,-0.340820029643859,0.733239070808348,0.932522539548586
"12274","SCOC-
AS1",14.5874680628387,0.146461544489714,0.429274831137496,0.3411836284
49911,0.732965347274242,0.932522539548586
"12275","PEX6",53.0505643555654,-0.0974737857344266,0.285241155268062,
-0.341724130386527,0.732558510735199,0.932522539548586
"12276","SERAC1",31.8786646266126,0.1151028737412,0.337130642495143,0.
34141919847247,0.732788024168812,0.932522539548586
"12277","NOM1",97.6635606838941,-0.100428685987038,0.294168361761742,-
0.341398664987564,0.732803479988042,0.932522539548586
"12278","NAIF1",31.9519441258665,0.0938035082932446,0.274802699980801,
0.341348568626866,0.732841188620233,0.932522539548586
"12279","AHNAK",10930.5105455999,0.0574729442402268,0.16860730881042,0
.340868641138497,0.733202473260668,0.932522539548586
"12280","MUS81",114.086249647018,-0.0681292081507924,0.199031779177539
,-0.34230316601863,0.73212275319697,0.932522539548586
"12281","IL18",119.456591946466,-0.142986378038551,0.417375142710628,-
0.34258479580248,0.731910841833668,0.932522539548586
"12282","ARHGEF12",376.67347168437,0.0750981448223166,0.21912784632435

9,0.342713836155511,0.731813752692316,0.932522539548586
"12283","AICDA",16.411312475762,-0.151899253315226,0.445399317106386,-0.341040606667442,0.73307301252158,0.932522539548586
"12284","ATXN7L3B",382.201889609206,-0.0498602232946379,0.146341496775428,-0.340711448176261,0.733320819413493,0.932522539548586
"12285","CHPT1",719.971447585601,-0.0989884416088751,0.288627296780638,-0.342962854563642,0.731626404963681,0.932522539548586
"12286","TMEM263",492.306483830425,0.0687616049951525,0.200657191033905,0.342681987327999,0.731837715148659,0.932522539548586
"12287","LINC00571",3.58398066689226,-0.273295158121514,0.797014321719462,-0.342898679075067,0.7316746855351,0.932522539548586
"12288","AL049840.5",32.7487393747232,-0.108269607499106,0.317648013034438,-0.340847740443345,0.73321820843978,0.932522539548586
"12289","HAUS2",75.3268918399679,0.0816324623900244,0.238700650987946,0.341986760623232,0.732360855718563,0.932522539548586
"12290","RAP1GAP2",7.09058990767042,-0.245024520751979,0.718213530678754,-0.341158318920024,0.732984399631431,0.932522539548586
"12291","AC012101.2",3.42469631491157,0.358844549174103,1.05020400273861,0.341690327058691,0.732583952344281,0.932522539548586
"12292","ZNF44",107.236107553467,0.0693672657923018,0.202245399509364,0.342985630133407,0.731609270678427,0.932522539548586
"12293","ELL",59.6688202433402,-0.1029135730797,0.300971617765458,-0.341937800792559,0.732397701448177,0.932522539548586
"12294","TMEM143",27.6811363249869,0.110658728234762,0.322770311244718,0.342840479373775,0.731718471314583,0.932522539548586
"12295","ESS2",49.8833601263236,0.0870573536418216,0.255573044138363,0.340635898967069,0.733377700540129,0.932522539548586
"12296","TANGO2",73.5110591963993,0.081608448889143,0.239467812208207,0.34079089016852,0.73326100898318,0.932522539548586
"12297","TRAPPC2",131.032041968412,0.0716046234217485,0.209722676729511,0.341425279032178,0.732783447273797,0.932522539548586
"12298","RBBP7",582.588277425322,-0.0586907160047374,0.171864796630462,-0.34149352953841,0.732732075121777,0.932522539548586
"12299","INE1",17.0507002364569,-0.143496774572484,0.419092280317017,-0.342398992565402,0.732050646547259,0.932522539548586
"12300","CUL4B",158.772952365594,0.0661241181006158,0.193706220545803,0.341362904682663,0.732830397489955,0.932522539548586
"12301","AL034417.4",5.8511064199339,0.33721966394776,0.992720691689755,0.339692389582173,0.734088193546106,0.932591937865413
"12302","TRABD2A",13.0504654865623,-0.238338526296698,0.700406660311201,-0.34028592216813,0.733641217753193,0.932591937865413
"12303","SSBP2",407.258815591286,-0.0602068229238692,0.177096183421165,-0.339966800869374,0.733881529481887,0.932591937865413
"12304","OARD1",200.684473548647,-0.0769375047711598,0.226293868015019,-0.339989348567118,0.733864549263082,0.932591937865413
"12305","NOLC1",189.705286893298,-0.074748409400108,0.219764444661577,-0.340129676186772,0.733758874274354,0.932591937865413
"12306","KBTBD3",82.6314247017843,0.0911337772008612,0.268229655473045,0.339760258947279,0.734037078115747,0.932591937865413
"12307","UNG",70.1518598492562,0.0938000080557586,0.275646601516731,0.

340290819983373,0.733637529696062,0.932591937865413
"12308","WDR89",35.9371160136777,0.0930898022524348,0.274038719844918,0.339695800305575,0.734085624750846,0.932591937865413
"12309","TEX9",7.73846756330419,-0.221845262230516,0.652170853597898,-0.340164331181989,0.73373277779192,0.932591937865413
"12310","PRKD2",58.8606723313371,0.0808642100698088,0.23789267896844,0.339918867703098,0.733917627420204,0.932591937865413
"12311","MGME1",63.2156267023558,0.0933883369378763,0.27446803195920З,0.340252146201703,0.733666651238796,0.932591937865413
"12312","SDF4",992.636113107466,-0.0623135318358299,0.184633802738362,-0.337497960349829,0.735741549453014,0.932602421970264
"12313","AL356275.1",3.07360666839395,0.286340586785872,0.844364336484948,0.339119707468811,0.734519552761411,0.932602421970264
"12314","CEBPZ",333.32235859749,-0.0677735239258913,0.200222836195486,-0.338490479975627,0.734993600036253,0.932602421970264
"12315","ELMOD3",107.401006052539,0.0631046850408362,0.186133811116655,0.339028598094339,0.734588186464213,0.932602421970264
"12316","AC009237.14",7.59140515130132,0.21012188674157,0.621508794321747,0.338083529406653,0.735300242151253,0.932602421970264
"12317","AGPS",294.83082582048,-0.0456668168499596,0.135375321507227,-0.337334872719189,0.735864474081982,0.932602421970264
"12318","MON1A",39.7462799388892,0.0958814092315723,0.283164161366532,0.338607148478306,0.734905696713984,0.932602421970264
"12319","HLA-DOB",5.11780242971959,-0.275772482850393,0.81570703216309,-0.338077853906812,0.7353045190066,0.932602421970264
"12320","TOMM6",945.425897471537,-0.0804456061167297,0.238190293996578,-0.337736709447469,0.735561608436227,0.932602421970264
"12321","HIVEP2",224.22067319385,-0.081752762065693,0.241815310695447,-0.3380793458883,0.735303394701482,0.932602421970264
"12322","AL354892.3",5.20503597492712,0.290418678808562,0.85738735271078,0.338725172339378,0.734816775736735,0.932602421970264
"12323","ZCWPW1",67.0539395752232,0.0775825716753471,0.229639610063813,0.337844902513936,0.735480069992087,0.932602421970264
"12324","AC109322.1",6.19563244292976,-0.235823219394317,0.696107949553063,-0.338773920834732,0.734780048914029,0.932602421970264
"12325","NRARP",3.2826965811864,-0.28886242512844,0.85438746180563,-0.338093005857049,0.735293101052694,0.932602421970264
"12326","CCDC3",6.20526242788956,0.239564212454412,0.7075268500797,0.338593810860218,0.734915745702411,0.932602421970264
"12327","NDUFB8",1143.23586761133,-0.0889475547518541,0.263025401871432,-0.338170967971116,0.735234352588081,0.932602421970264
"12328","POLL",81.7183156680317,-0.0746958471168525,0.221006333148587,-0.337980573012055,0.735377827713652,0.932602421970264
"12329","C10orf143",22.4330800209037,-0.151532056102342,0.448960257413486,-0.337517750402534,0.735726633485357,0.932602421970264
"12330","AP001107.2",4.57586449892388,-0.288189308275915,0.848713754684106,-0.339560077452945,0.734187847069538,0.932602421970264
"12331","KLRC3",4.11156526266027,-0.313551326980802,0.923980126556224,-0.339348561694116,0.73434716368807,0.932602421970264

"12332","HOXC11",33.6788695886178,-0.101014612394267,0.299332940689419
,-0.337465740194219,0.735765834331085,0.932602421970264
"12333","TRAC",1246.94709960005,-0.209570017354604,0.618576432009478,-
0.338794054396487,0.734764880586877,0.932602421970264
"12334","C17orf58",5051.56062798142,0.105482434562538,0.31261643946590
7,0.337418066505877,0.73580176728342,0.932602421970264
"12335","ENDOV",133.993030032292,-0.0697741316169367,0.205776565272922
,-0.339077151591072,0.734551610312453,0.932602421970264
"12336","ZNF236",79.9814638462632,0.0784691719712199,0.231301275857604
,0.339250925790516,0.73442070827002,0.932602421970264
"12337","TMEM205",252.118030556202,-0.101640193152035,0.30062382536397
4,-0.338097597650408,0.7352896408572,0.932602421970264
"12338","ZNF831",6.2183491248717,0.238261209457067,0.702744425344526,0
.339043898271064,0.734576660523173,0.932602421970264
"12339","ASMTL",189.471050835684,-0.0821165966878269,0.243345414077504
,-0.337448712559972,0.735778668454631,0.932602421970264
"12340","Z68871.1",7.94200998269626,0.199808846317615,0.59173167058742
6,0.337667994209707,0.735613396421824,0.932602421970264
"12341","ZCCHC18",7.3834145052158,0.193688663154527,0.574220347724972,
0.337307209544055,0.73588532541784,0.932602421970264
"12342","LINC02478",6.63518061523532,-0.246946836795905,0.732627364266
715,-0.337070178975738,0.736063997047608,0.932680358756155
"12343","ZBTB7C",54.873734793131,0.164878811953795,0.489156802384802,0
.33706740078019,0.736066091312482,0.932680358756155
"12344","KIAA1958",5.03360195082885,-0.24673048552115,0.73287668490757
2,-0.336660301251454,0.736372993077299,0.932873101996312
"12345","TRDC",5.18535728927588,0.281707425089971,0.836850172496315,0.
336628269131666,0.73639714304645,0.932873101996312
"12346","EVL",291.715232583666,-0.0563473698423816,0.167333679054768,-
0.336736574254961,0.736315489636645,0.932873101996312
"12347","TXNDC12",430.269198652366,-0.0759818752326454,0.2260768367880
14,-0.336088722366067,0.73680396253986,0.933010604333529
"12348","CIR1",698.447472910659,0.0544970658485982,0.162103144881826,0
.336187591476567,0.736729409480659,0.933010604333529
"12349","RHOF",15.9553577820074,-0.141466626553467,0.420568274426588,-
0.336370180909022,0.736591732944175,0.933010604333529
"12350","E2F1",5.38414508302737,-0.239702003942318,0.71284649515496,-0
.336260338756679,0.73667455538312,0.933010604333529
"12351","FAM122B",59.0997073565588,0.0801253453653636,0.23835011026854
2,0.336166596588056,0.736745240640376,0.933010604333529
"12352","LINC02802",1452.64098923538,0.0749786019244454,0.223568756745
817,0.335371556454514,0.7373448214638,0.933031443937835
"12353","PDE1A",76.702159613238,-0.158189415554931,0.471648589137844,-
0.335396774628533,0.737325800679271,0.933031443937835
"12354","CCNJL",3.41328089093636,0.260655160015407,0.775873782331444,0
.335950467654877,0.736908218786603,0.933031443937835
"12355","SNHG7",682.176154888756,-0.107277413666726,0.3195802289121,-0
.335682260545074,0.737110484411704,0.933031443937835
"12356","GTF2H1",143.887397409787,-0.0640558310822327,0.1910540989752,
-0.335275879585015,0.737416987117365,0.933031443937835

"12357","LDLRAD3",18.3033994884589,-0.12546115645972,0.374115529284723
,-0.33535404611402,0.737358028716623,0.933031443937835
"12358","ASB8",278.955184856057,0.065997196818966,0.196532274098951,0.
335808442259908,0.737015323503867,0.933031443937835
"12359","RFNG",351.126468238571,-0.0924251771804715,0.275177400584954,
-0.335874882835582,0.736965218454674,0.933031443937835
"12360","NARS",481.690669659325,-0.0625480235086307,0.186450393138021,
-0.335467372612775,0.73727255307031,0.933031443937835
"12361","MICAL3",54.4998434394396,-0.156907918069108,0.467701084315893
,-0.335487608070478,0.737257290972744,0.933031443937835
"12362","CYP4F12",31.6662531575213,0.124095446945327,0.370291481849951
,0.335129088914913,0.737527710589705,0.933056458705447
"12363","AL022323.3",4.3049395486548,-0.258164002560899,0.770428412149
505,-0.335091487398055,0.737556074103289,0.933056458705447
"12364","SLC9A3-
AS1",4.88442691949096,0.291923024561016,0.871831751857963,0.3348387162
30395,0.737746753304099,0.933222195218062
"12365","AC069224.1",10.0079874290664,0.153898708489677,0.459790882592
176,0.334714572029001,0.737840408016834,0.933241416816302
"12366","DCUN1D1",209.025079475753,0.0571587886697251,0.17081772536693
7,0.334618603233014,0.737912809796593,0.933241416816302
"12367","COX4I1",3237.98123324793,-0.0798975626747435,0.23879865550559
8,-0.334581291948985,0.737940959192277,0.933241416816302
"12368","CA10",7.04149801323946,-0.711585828363182,2.12739712407931,-0
.334486598815508,0.738012401744127,0.933256303628569
"12369","PLPPR4",6.01869079944211,-0.25314249589067,0.757797955602971,
-0.334050117209999,0.738341740609371,0.933286697198097
"12370","THNSL2",97.8945490907946,-0.149351220720939,0.446935866891911
,-0.334167006464618,0.738253539342535,0.933286697198097
"12371","HBEGF",108.793664729568,0.243999439784909,0.730732428358,0.33
3910786377978,0.738446880143796,0.933286697198097
"12372","SCX",7.23158488959561,-0.275894600073685,0.826272308828761,-0
.333902754728359,0.738452941003895,0.933286697198097
"12373","ANAPC16",795.061883564635,-0.0624357040548493,0.1869885836587
09,-0.333901154996749,0.738454148198623,0.933286697198097
"12374","HAUS8",33.2125103503763,0.0904013114888337,0.27056685346065,0
.334118205288517,0.738290362886407,0.933286697198097
"12375","CASK",160.243531298999,0.0744930850428108,0.222857191787815,0
.334263769749628,0.738180527159404,0.933286697198097
"12376","IL18RAP",2.72076099862698,0.351647334344755,1.05718641612143,
0.332625664672148,0.739416867888086,0.933356713683925
"12377","ABLIM3",59.46542946476,-0.0841742641322698,0.25260108083874,-
0.333230023611841,0.738960656418293,0.933356713683925
"12378","PCOLCE",3552.92671547689,0.136473573590327,0.409847794204878,
0.33298599021398,0.739144858470587,0.933356713683925
"12379","ZNF696",38.8789705780567,0.103386591189339,0.31063395746425,0
.332824498755057,0.739266764189578,0.933356713683925
"12380","COMMD9",222.996447677351,-0.0927294546604717,0.27811412545114
6,-0.333422311829899,0.738815523375083,0.933356713683925
"12381","CCDC15",27.4265187176776,0.11135989281478,0.334695952879245,0

.332719567884817,0.739345977297448,0.933356713683925
"12382","P3H3",287.251640840503,-0.0856403846275048,0.257358010129243,
-0.332767511625136,0.739309783861954,0.933356713683925
"12383","ATP6V0A2",71.5496084425059,0.0843085233684886,0.2533883838907
79,0.332724500128739,0.739342253847245,0.933356713683925
"12384","RCBTB1",40.9076008550968,-0.0927560350751139,0.27868655641131
1,-0.332832829360509,0.739260475471012,0.933356713683925
"12385","PLLP",5.68364203612669,0.238127511626116,0.716037901621117,0.
332562719217785,0.73946438869933,0.933356713683925
"12386","CRK",491.695351534419,0.0627345908859936,0.188362499861319,0.
333052443730475,0.739094696336003,0.933356713683925
"12387","USP14",341.522455414418,-0.0553849849745928,0.165965418326298
,-0.333714008214064,0.738595377968602,0.933356713683925
"12388","CYB5A",1116.17481745234,-0.0947488622263681,0.28452009049974,
-0.333012906258916,0.73912454088277,0.933356713683925
"12389","SNHG17",83.5180270034787,0.100008044401142,0.300595874830197,
0.332699324159506,0.739361259757119,0.933356713683925
"12390","AP000320.1",2.62825012843435,-0.340942518110501,1.02465198232
795,-0.332739821901188,0.739330687171831,0.933356713683925
"12391","TXNRD2",168.725655906206,0.111769013207131,0.335140606361694,
0.333498869088117,0.738757742976186,0.933356713683925
"12392","LINC01948",4.19751660001804,-0.24914065676609,0.7517704359378
18,-0.33140523337459,0.740338412382564,0.933595042353515
"12393","TUBB",3117.46130579914,0.0825612938740468,0.248834136744295,0
.331792474112536,0.740045967473633,0.933595042353515
"12394","PRKAR1B",10.763791657887,-0.164188030111208,0.49484104380058,
-0.331799538797705,0.740040632559415,0.933595042353515
"12395","INTS1",168.777821685014,0.0552066500238608,0.166604226004153,
0.33136404368569,0.740369521119607,0.933595042353515
"12396","GTPBP10",67.5319274121837,0.0855941178038703,0.25818715534124
6,0.331519659414275,0.740251993720996,0.933595042353515
"12397","GSTK1",693.022761985497,-0.080900592318558,0.243892026515043,
-0.331706589487739,0.740110824457304,0.933595042353515
"12398","AC004466.1",3.19651684131231,-0.2771741689456,0.8353082061984
75,-0.331822633716281,0.740023192462647,0.933595042353515
"12399","AL356019.2",10.9510677480002,0.162228610545466,0.489376433227
01,0.331500659882025,0.740266342624305,0.933595042353515
"12400","AL627171.1",6.9872506074506,0.188062232787031,0.5673003341296
86,0.331503828700442,0.740263949450211,0.933595042353515
"12401","AC026461.3",9.09728358843595,0.249989734736604,0.754151031013
866,0.331484973773121,0.74027818921879,0.933595042353515
"12402","ZNF875",56.6446004456086,0.0881615949028934,0.266005994636608
,0.331427098187511,0.740321899035967,0.933595042353515
"12403","PXMP4",108.755590097342,-0.0691776356930346,0.208581921264447
,-0.331656910980933,0.740148340724712,0.933595042353515
"12404","COLGALT1",214.787162283233,-0.0526389372802468,0.158895693515
685,-0.33127982335815,0.740433130302631,0.933599980484775
"12405","CEBPZOS",259.116383957525,-0.0675728469352677,0.2041446932001
89,-0.331004670638213,0.740640957580406,0.933786745389565
"12406","SENP5",170.81723808435,0.0695359619058355,0.210158132183564,0

.330874476202038,0.740739302131693,0.933802785841555
"12407","PUS7L",223.54159725529,0.0565011690811616,0.170786243089186,0
.330829744007287,0.740773092323285,0.933802785841555
"12408","ANKRD35",319.23042651597,-0.0901413456121558,0.2729102459355,
-0.330296670625771,0.741175808368387,0.933934067264065
"12409","SCNM1",140.340795552676,-0.0851124906753147,0.257678756719872
,-0.33030464602446,0.741169782307811,0.933934067264065
"12410","KANSL1-
AS1",68.346897334837,-0.116430661908923,0.352317179363323,-0.330471145
685618,0.741043991487852,0.933934067264065
"12411","CEP76",19.7244019402034,0.118706005975943,0.359269807116769,0
.330409078704912,0.741090882567091,0.933934067264065
"12412","PGLS",1146.26554806306,-0.0873834702130684,0.264538302351294,
-0.330324453723255,0.741154817574711,0.933934067264065
"12413","PAQR8",22.2629310727354,0.124145473674286,0.376303627134583,0
.329907725364246,0.741469686019786,0.934195086466551
"12414","YPEL4",45.1074525499429,0.113092700467582,0.342846022051024,0
.329864409075018,0.741502417096916,0.934195086466551
"12415","ACAD9",111.980411466701,0.0720948946012681,0.218799591500255,
0.329501961621277,0.74177631150214,0.934239129641928
"12416","PAQR3",98.9953321467585,-0.0980298079734645,0.297470822051958
,-0.329544280333962,0.74174433039674,0.934239129641928
"12417","NSMF",77.1224078931187,-0.071794154212769,0.217769837198959,-
0.329679055355937,0.741642481164421,0.934239129641928
"12418","LINC01521",2.74693588478548,-0.28440943994682,0.8629855852144
02,-0.32956453134285,0.741729026458325,0.934239129641928
"12419","USP21",39.1644562987073,-0.095002930916989,0.288927549826879,
-0.328812295587296,0.742297568862562,0.934288059456578
"12420","U91324.1",5.47942710243719,0.266104806388384,0.80973688346709
3,0.328631203322478,0.74243446004256,0.934288059456578
"12421","AC073195.1",2.62870789995429,-0.30446249997754,0.924630072869
262,-0.329280334818387,0.741943806632857,0.934288059456578
"12422","MFSD9",52.725966042556,0.100671188966763,0.306084223899253,0.
328900286608364,0.742231057673311,0.934288059456578
"12423","PRELID2",53.8548286187497,0.0799061805584595,0.24302392059177
2,0.328799652165454,0.74230712600745,0.934288059456578
"12424","RAB32",1683.91896666919,-0.0813587644186787,0.247018634295615
,-0.329362862241859,0.741881434852619,0.934288059456578
"12425","VOPP1",134.161620386284,-0.0612276934347888,0.186290025002411
,-0.3286686293027,0.742406142924933,0.934288059456578
"12426","GOLGA8R",9.45893315830936,0.204849889875039,0.622792897162704
,0.328921365044924,0.742215125061984,0.934288059456578
"12427","SPIRE2",4.27271471084718,0.260246955253019,0.791830365736277,
0.328665035485259,0.742408885030952,0.934288059456578
"12428","GNA13",239.460051738401,0.0529484233475923,0.161142589184723,
0.328581187726205,0.742472269244617,0.934288059456578
"12429","ZNF324",77.497792365976,-0.0853221170472025,0.259428319792265
,-0.328885131413269,0.742242513137341,0.934288059456578
"12430","SUMO2",3864.81479935447,-0.0789244729887107,0.240277189371503
,-0.328472599480437,0.742554358476923,0.934316183956483

"12431","ZNF501",26.3216904371493,-0.105866446640077,0.322967885718896
,-0.327792487492769,0.743068567899537,0.934587245834239
"12432","AC099661.1",6.07145770916307,0.224633031889715,0.685124846546
101,0.327871676267692,0.743008690072625,0.934587245834239
"12433","USO1",442.562668383217,-0.0538987578662579,0.164327637178095,
-0.327995696839743,0.742914916239656,0.934587245834239
"12434","PLBD1",521.042135611732,0.0861535903040793,0.262764866827719,
0.327873323949985,0.743007444210091,0.934587245834239
"12435","SMCR5",14.6035159421172,0.17346832132316,0.529147207595534,0.
327826205700692,0.743043072012427,0.934587245834239
"12436","GNB2",1341.42152661548,-0.0479813272659807,0.146464180745714,
-0.32759768990402,0.743215868827521,0.934622190919228
"12437","AC087627.1",2.65694433112489,-0.280852461174657,0.85730470850
3528,-0.327599345237355,0.743214617067407,0.934622190919228
"12438","LPAR1",538.089306478348,-0.0701679673690496,0.214415832815014
,-0.327251800614868,0.743477444576161,0.934674470712848
"12439","KLRK1",9.22144647874921,0.274489544313276,0.838836232779597,0
.3272266189596,0.743496489139293,0.934674470712848
"12440","CASC4",707.784314444861,0.0552670296381806,0.168884743578603,
0.327247023426116,0.743481057490724,0.934674470712848
"12441","AC016588.2",10.1382953480508,-0.156255919132443,0.47729661196
811,-0.327376971079114,0.743382782087426,0.934674470712848
"12442","CCDC138",18.4659681820384,-0.117384836984238,0.35905657707494
9,-0.326925739504656,0.743724052551175,0.934705680783925
"12443","SMN1",28.1813938197669,0.115582086089864,0.353400371798507,0.
327057058547078,0.743624729591473,0.934705680783925
"12444","MDN1",103.786736622457,0.0923590759638723,0.28253142497963,0.
326898418363661,0.74374471739089,0.934705680783925
"12445","LMO7",34.8012883722351,-0.133001218332324,0.406883702657928,-
0.326877723200774,0.743760370674932,0.934705680783925
"12446","CTBP1-
AS",4.38857947957842,0.254572664496073,0.779273987949279,0.32667927896
0666,0.74391047412236,0.934766941259165
"12447","RAB14",841.977052446723,-0.0385714158486126,0.118079890395795
,-0.326655247725283,0.743928652036626,0.934766941259165
"12448","TMF1",564.341164029347,-0.0555793023346541,0.170206707294401,
-0.326540024292465,0.744015812321318,0.934801358025821
"12449","RASSF1-
AS1",3.60712937487427,-0.249214849425277,0.763825714455718,-0.32627187
6828421,0.744218664045733,0.934906016520102
"12450","HBP1",512.56445266554,0.0792755604716026,0.242959672388292,0.
326291024729843,0.744204178200555,0.934906016520102
"12451","AL050343.2",4.58994883414749,0.258296181486253,0.792113846525
186,0.326084669040108,0.74436029598004,0.934933747922247
"12452","GALNT7",62.7937479624663,-0.106436909287488,0.326335849048087
,-0.326157575387326,0.744305137700799,0.934933747922247
"12453","CUBN",6.6357129262396,-0.227715902955979,0.698961606638532,-0
.325791718448052,0.744581944915435,0.935137044766515
"12454","RAB3D",41.7261085064602,0.0859735006294985,0.263982875236759,
0.325678324976161,0.744667745070797,0.935169707154911

"12455","BABAM2",168.946256125903,-0.0786252584575864,0.24162427228392
7,-0.32540298089422,0.744876099748499,0.935206084937507
"12456","AARS",153.049293078229,0.061358888991084,0.188543677573954,0.
325435940258547,0.744851158196857,0.935206084937507
"12457","HDHD5-
AS1",3.00722207236449,0.301750615524424,0.926869652184343,0.3255588472
58935,0.744758152351442,0.935206084937507
"12458","TRIM62",12.5419309293617,0.147859362048365,0.455664315149476,
0.32449186195294,0.745565682462989,0.935522081961626
"12459","FALEC",5.59501006990886,-0.20082955302571,0.618740365608975,-
0.324578068909485,0.745500427764443,0.935522081961626
"12460","PIGF",232.714442797102,-0.0703860291628796,0.216947425809469,
-0.324438185427907,0.745606314044224,0.935522081961626
"12461","TMCC1-
AS1",5.49388948563142,0.225789451530715,0.695725742430013,0.3245380151
4097,0.745530746396725,0.935522081961626
"12462","PLAA",99.0265695829556,0.0606824921279346,0.186809021963377,0
.32483705278341,0.745304399875087,0.935522081961626
"12463","DGKH",44.4458545487749,-0.134333752187024,0.413637314532902,-
0.324762170788967,0.7453610772285,0.935522081961626
"12464","BTG3-
AS1",4.77104756012844,0.23749912355193,0.730966177330951,0.32491123518
0723,0.745248253398372,0.935522081961626
"12465","PIGP",411.137559924285,0.097339842921854,0.299802078022114,0.
324680347661479,0.745423009824649,0.935522081961626
"12466","AL590560.2",3.69647548952079,0.256049852756878,0.790036920808
398,0.324098590854308,0.74586339373683,0.935740345552556
"12467","PAFAH1B3",16.4350634736694,-0.175831078254275,0.5427365803875
61,-0.323971305064266,0.745959758845861,0.935740345552556
"12468","ARMCX5-
GPRASP2",31.7536779691201,-0.10088598658095,0.311338999831429,-0.32403
9027027045,0.745908487629304,0.935740345552556
"12469","PIDD1",26.4908279762243,-0.100512218440901,0.310470285601563,
-0.323741830063222,0.746133499067212,0.935752552186522
"12470","PRKACA",265.648644209098,0.0578515197900783,0.178707353700159
,0.323722099803142,0.74614843785379,0.935752552186522
"12471","TCEA2",320.85176082578,-0.0688522100929892,0.212689720554843,
-0.323721381143267,0.746148981989649,0.935752552186522
"12472","HOXD8",120.894811163929,0.0978875495878938,0.302542696720986,
0.323549537466339,0.746279097669461,0.935779397794525
"12473","AC010997.4",2.7840192055619,-0.287737610176061,0.889355243204
482,-0.323535069225318,0.746290052985365,0.935779397794525
"12474","NUDT7",78.7951828236623,0.0978737528810348,0.302695657379042,
0.323340459286719,0.746437416127135,0.935819057026848
"12475","ZBTB46",10.6866483399416,-0.162850882997973,0.503659519760242
,-0.323335262431841,0.74644135143286,0.935819057026848
"12476","KNSTRN",18.0333950320812,-0.124229153484887,0.384317356898733
,-0.323246273567658,0.746508739059974,0.935828525079993
"12477","PLEKHH1",3.3060100405626,-0.279487303443547,0.865260084389384
,-0.323009588083312,0.746687980729609,0.935903191105232

"12478","SPPL2B",132.621426735373,-0.0556932539398662,0.1723800327296,
-0.323084136010278,0.746631524183708,0.935903191105232
"12479","UPRT",45.5545931875758,0.0810020234965939,0.250896841250458,0
.32284991350582,0.74680890986158,0.935979754005538
"12480","LIMS2",416.430396615185,-0.0405586042653424,0.125978056398682
,-0.321949753987209,0.747490759922208,0.936011744512999
"12481","RABL3",113.96126987804,0.0569161877329322,0.176726071061992,0
.322058807684165,0.74740814373255,0.936011744512999
"12482","SRD5A3-
AS1",6.20007517855154,-0.184142680894043,0.571062077608828,-0.32245650
3617071,0.747106884428615,0.936011744512999
"12483","CMBL",563.136117937162,-0.0773282067464362,0.239672590267492,
-0.322641010639274,0.74696713132513,0.936011744512999
"12484","ETV7",11.9562554963931,-0.200302474563329,0.62198495720621,-0
.322037490203997,0.747424293062241,0.936011744512999
"12485","CBX3",784.612296281279,0.044875725260912,0.139188626515208,0.
322409426577745,0.747142543819574,0.936011744512999
"12486","WWP1",154.660146199725,0.067762946607949,0.210504341196502,0.
321907596882734,0.747522697804366,0.936011744512999
"12487","MINCR",56.8892204158913,0.108894619559848,0.338387135070523,0
.321804845025073,0.747600543598076,0.936011744512999
"12488","UGCG",253.015712083473,0.0872285842579061,0.270889872966063,0
.322007549794356,0.747446974989618,0.936011744512999
"12489","SFMBT2",30.737188335616,0.123996139807143,0.385333525064021,0
.32178913004401,0.747612449645549,0.936011744512999
"12490","AC004687.1",7.54616642493519,-0.254428018878559,0.78975577207
0778,-0.322160379038998,0.747331198576678,0.936011744512999
"12491","ZNF529-
AS1",20.1376195728294,0.124850209208749,0.387302603649934,0.3223583008
01912,0.747181270615396,0.936011744512999
"12492","CCDC97",118.209425953622,-0.0688092818009255,0.21375252032879
5,-0.321910973003185,0.747520140066791,0.936011744512999
"12493","RBM18",122.712997581639,0.0610676267304031,0.189871110595255,
0.32162674215657,0.74773548213821,0.93607454055922
"12494","LBHD2",58.5665792218367,-0.339435810435869,1.05559425176923,-
0.321558979567156,0.747786824076428,0.93607454055922
"12495","DRG2",158.889873731246,-0.0726707636777022,0.226046470568235,
-0.321485947093191,0.747842160120681,0.93607454055922
"12496","ZNF574",26.8412007258117,0.100896686775833,0.313983927338917,
0.321343476498796,0.747950112539268,0.936134743927189
"12497","TRAF1",13.1066265445073,-0.167241262935122,0.520690876275254,
-0.321191076232173,0.748065594312247,0.93620436065004
"12498","GIN1",44.3712059661429,0.0882656030886606,0.274921836161397,0
.321057084155523,0.74816713189242,0.936256516466431
"12499","TRH",3.68389045130381,-0.424095071651543,1.32240524110154,-0.
320699781330479,0.748437913015309,0.936258111209722
"12500","AC092944.1",64.5402501515772,0.077401549952275,0.241148952936
637,0.32096987778592,0.748233218174583,0.936258111209722
"12501","MYL5",77.9096336820641,0.119985846077634,0.374198677212719,0.
320647435130899,0.748477586068808,0.936258111209722

"12502","VARS2",34.0380258353262,0.094572033832352,0.294825061515223,0.320773387941679,0.748382127875817,0.936258111209722
"12503","ICAM4",4.67535608179354,-0.274965196755988,0.857707714220287,-0.320581466386774,0.748527584563067,0.936258111209722
"12504","AP000254.2",16.8604076543318,0.138436659902691,0.431520009455505,0.32081168165845,0.748353106270839,0.936258111209722
"12505","ATF1",186.157584185467,-0.0559335976839488,0.174562258970747,-0.320422054651127,0.748648408974284,0.936334355566397
"12506","CRTC3",149.527210289791,0.0529981641737604,0.165531527706611,0.320169607011026,0.748839761573743,0.936383194083745
"12507","RPL26",17440.6272558457,-0.0796364556275057,0.24881159044278,-0.320067306694942,0.748917308518865,0.936383194083745
"12508","EFCAB13",54.4868418955559,0.124667124522455,0.389518304725006,0.320054598231188,0.748926942122351,0.936383194083745
"12509","FAM98C",123.873186629521,-0.0644107206423301,0.201242848659237,-0.320064643645531,0.74891932723028,0.936383194083745
"12510","GCLM",152.94041785623,-0.096798172480006,0.302704541650089,-0.319777734263068,0.749136827542509,0.936570742027565
"12511","RAB7A",1033.92273607973,-0.0555908493120073,0.173939982255729,-0.319597878481309,0.749273182820363,0.936666339965668
"12512","CREB3L4",24.8806987032157,0.120732402501027,0.378785666427558,0.318735404218672,0.749927165099573,0.936813077410157
"12513","CDC42EP3",5.4936458795949,0.233177707836924,0.730747013310289,0.319094985801759,0.749654485827217,0.936813077410157
"12514","PPARG",101.579070873805,0.127084440324505,0.398872449471971,0.31860922079913,0.750022860399479,0.936813077410157
"12515","TRAIP",13.4701650738879,-0.141815744457217,0.445637419685528,-0.318231230575952,0.750309544596933,0.936813077410157
"12516","HMGCS1",138.854031305569,-0.0855817552720073,0.269493964983927,-0.317564644822794,0.750815196186807,0.936813077410157
"12517","AC008443.5",18.2470876279904,0.128446885487098,0.402307136289981,0.319275682434115,0.749517471079427,0.936813077410157
"12518","UQCC2",228.775404430338,-0.0923339492493341,0.290557041730555,-0.317782521116659,0.750649910099443,0.936813077410157
"12519","ENPP2",105.253219618016,-0.136051833728522,0.427546521831092,-0.318215274318782,0.750321647274075,0.936813077410157
"12520","ZBTB6",58.9301557784496,0.0826457950524193,0.259385547659988,0.31862143360722,0.750013598251097,0.936813077410157
"12521","AL359091.5",13.4412430802851,-0.155443069196051,0.488494614058223,-0.318208358337239,0.750326893002784,0.936813077410157
"12522","KIF20B",78.4086887502443,0.086135656428997,0.27125274058742,0.317547598754075,0.750828128218435,0.936813077410157
"12523","AC087239.1",30.823537156108,0.135592758931478,0.426296210956973,0.318071696267465,0.750430552694922,0.936813077410157
"12524","EEA1",1456.6653290143,0.0479665733308219,0.150675770986897,0.31834297589221,0.75022478850484,0.936813077410157
"12525","RBM19",74.5028825354285,0.0848651654170628,0.267156473215644,0.317660898856682,0.750742174199381,0.936813077410157
"12526","GMFB",207.60414651887,-0.0525546137138873,0.16544195083289,-0.317661956047483,0.750741372186445,0.936813077410157

"12527","SGPP1",108.252873267287,0.0571333878192391,0.17950042742335,0
.318291096235056,0.750264137582316,0.936813077410157
"12528","ANP32A",408.470157594452,-0.0502458677322909,0.15753389255273
8,-0.318952746726994,0.749762345364506,0.936813077410157
"12529","E4F1",67.3312325469791,-0.0765153796447664,0.240084814102193,
-0.318701455279039,0.749952911003636,0.936813077410157
"12530","NEURL4",27.1434758900133,0.101334501954145,0.31733509406628,0
.319329641911524,0.749476557382355,0.936813077410157
"12531","MBD1",121.383674691782,0.0543929281332122,0.171117589999957,0
.317868713165174,0.750584525940284,0.936813077410157
"12532","TRAPPC5",1039.08303569197,-0.081784704647949,0.25716455720826
1,-0.318024791346798,0.75046613163503,0.936813077410157
"12533","PLA2G4C",95.3933906793039,0.15174430250316,0.477627044373265,
0.317704586226427,0.750709032028265,0.936813077410157
"12534","ARFGAP1",76.2535274672784,0.0759049483714775,0.23876649796028
3,0.317904517676946,0.750557365633181,0.936813077410157
"12535","HDAC8",136.380154778741,0.0688233238854248,0.21658598713355,0
.317764435254009,0.750663630025318,0.936813077410157
"12536","S100A9",59.2982786428637,0.160153131616059,0.504575605956638,
0.317401653439866,0.750938852766564,0.936841516913518
"12537","ANKRD16",78.6683023750745,-0.0807582118109314,0.2544876383460
53,-0.317336481786656,0.75098829829818,0.936841516913518
"12538","GOSR2",157.310848761518,-0.0646953897001291,0.203905848261934
,-0.317280697202085,0.751030622702154,0.936841516913518
"12539","ACTR2",970.764065381939,-0.0453821571809461,0.143166190602532
,-0.316989346366973,0.751251686092181,0.936894414958067
"12540","ZSCAN31",18.499601345739,0.134245623186183,0.423322399674816,
0.317123835850185,0.751149639222957,0.936894414958067
"12541","C9orf92",8.77135498320555,-0.288512756209369,0.91016945527931
2,-0.316987957062161,0.751252740280634,0.936894414958067
"12542","PFKFB2",10.9392189093481,0.145679712778704,0.460411048401659,
0.31641228698667‌9,0.75168959201734,0.937014016038676
"12543","CUL3",313.015071695146,-0.052844474300349,0.167279465526536,-
0.315905327255879,0.752074368274863,0.937014016038676
"12544","REST",348.092063717555,0.0477380592876418,0.151120532935911,0
.315893931553875,0.752083018182357,0.937014016038676
"12545","NSA2",1345.12073579743,-0.0624588363961765,0.197357091827739,
-0.316476270590231,0.751641033626316,0.937014016038676
"12546","EEF1A1",51974.3053290825,-0.0580763638756456,0.18392773999340
7,-0.315756415414702,0.752187402261226,0.937014016038676
"12547","AC008267.5",23.0652516842204,0.139965866913811,0.442451939710
531,0.316341401973246,0.751743389169309,0.937014016038676
"12548","FANCG",48.4656949112885,0.100792416043457,0.318906260516388,0
.316056561198419,0.751959577101461,0.937014016038676
"12549","CHRNA10",3.1825919215248,0.293912410354841,0.929985704643402,
0.316039707801251,0.751972369072154,0.937014016038676
"12550","BAZ1A",232.108678544811,-0.0713890537730319,0.22552168656837,
-0.316550726714211,0.751584528675365,0.937014016038676
"12551","ZNF597",11.1215106593549,-0.144665071789426,0.457352237998919
,-0.316309968050856,0.751767245874513,0.937014016038676

"12552","ALOX12-
AS1",58.2863820129528,0.0910887813146059,0.288449677957818,0.315787425
936827,0.752163862775268,0.937014016038676
"12553","TNFSF13",56.4525807096322,-0.0983529530858307,0.3109464277240
86,-0.316301923150257,0.751773351571982,0.937014016038676
"12554","AP001062.1",4.10376245711586,0.255578786332587,0.809223729809
965,0.315832046092626,0.752129992884788,0.937014016038676
"12555","TBX1",38.647813184052,-0.202564911162295,0.641448544477628,-0
.315792923541913,0.752159689673917,0.937014016038676
"12556","WNT2B",142.371121616977,-0.0891844823299815,0.283124441851014
,-0.315001000079365,0.752760894539528,0.937131289548533
"12557","DDX1",559.425743115843,0.0655923464491314,0.208128958717847,0
.315152426904958,0.752645924164389,0.937131289548533
"12558","AC012020.1",4.64565277944297,0.235033782161759,0.745567372098
676,0.315241507283465,0.752578292710134,0.937131289548533
"12559","VIPR2",3.85036217769948,0.350575668751908,1.11251559101753,0.
315119780416977,0.752670710451215,0.937131289548533
"12560","PSTK",29.0860259679088,-0.111251329352051,0.353168772696527,-
0.315008964418402,0.752754847501715,0.937131289548533
"12561","GTF2A2",721.52186459187,-0.0817185218632429,0.259323901481257
,-0.315121442321618,0.752669448672422,0.937131289548533
"12562","LINC01273",5.43769652223908,0.222702135663007,0.7065586619700
39,0.315192704653942,0.752615344324758,0.937131289548533
"12563","HLCS",24.9068981775089,-0.102753396246248,0.326064538792395,-
0.315132079761886,0.75266137235432,0.937131289548533
"12564","COG2",94.0082407641855,-0.060506577777136,0.192291728256747,-
0.314660325359123,0.753019570870378,0.937149644053637
"12565","C12orf75",755.827214592618,0.0995923744694062,0.3165347340551
49,0.314633320626527,0.753040076910189,0.937149644053637
"12566","MAFG-
DT",14.3328947289918,-0.165172064297331,0.524529298093443,-0.314895783
510468,0.752840783141515,0.937149644053637
"12567","CTIF",128.316257735162,0.0645490142608769,0.205186517093945,0
.314587016608519,0.753075238265096,0.937149644053637
"12568","ACAA2",868.210201796773,-0.0663380466571533,0.21080493685247,
-0.314689246123204,0.752997610085563,0.937149644053637
"12569","HLA-
A",5208.39861752966,0.0882595337744659,0.28066098023095,0.314470268370
897,0.7531638943185,0.937185401156921
"12570","THRB-
AS1",3.71150796075684,0.277836883693195,0.883881641377333,0.3143372038
59386,0.753264944561806,0.937236573822327
"12571","NUSAP1",8.69724318736973,-0.194796145737261,0.619867216506492
,-0.314254634783094,0.753327650297137,0.937240032666234
"12572","PPIE",280.490781039796,-0.0710057012198539,0.226475778070837,
-0.313524482947773,0.753882222940876,0.937280993058616
"12573","YIPF1",68.2274815399801,-0.0784179079695147,0.25011798565156,
-0.313523666701677,0.753882842975841,0.937280993058616
"12574","PARTICL",19.9829827261318,0.114955000758398,0.366771708572368
,0.31342384941808,0.753958667145226,0.937280993058616

"12575","AC084036.1",23.6445906434607,0.150757067722098,0.480213527097292,0.313937569883478,0.753568455169285,0.937280993058616
"12576","NAF1",64.762595580331,-0.0837317386139399,0.26681709166212,-0.313816997600635,0.753660033859304,0.937280993058616
"12577","ZNF239",11.2738999091047,-0.156569325607804,0.498451328051114,-0.314111562747703,0.753436307867864,0.937280993058616
"12578","ARFGAP2",279.373812097001,-0.0503052275488215,0.16048440712246,-0.313458662126816,0.753932222109629,0.937280993058616
"12579","AC103702.1",11.5291585623748,0.170181101147803,0.542834303643748,0.313504691957511,0.753897256571693,0.937280993058616
"12580","C22orf31",5.40170954415585,-0.217250415396744,0.693155616937795,-0.31342228222359,0.753959857651563,0.937280993058616
"12581","PISD",70.1336168840914,0.0822906581520117,0.262343502994583,0.313675228136719,0.753767716951249,0.937280993058616
"12582","ENPP1",266.637340971946,0.149637493876613,0.477573473457513,0.31332873828455,0.754030918590513,0.937281580704539
"12583","S1PR2",262.491534512316,-0.0530546083771732,0.169360757045618,-0.313263882983723,0.754080187340487,0.937281580704539
"12584","DZIP1L",47.4112835431404,-0.136346530200468,0.435397357704452,-0.313154243561165,0.754163479605923,0.937310618327769
"12585","GGT5",87.0093402477543,0.147865129097248,0.472506380798699,0.312937846145706,0.754327883527473,0.937440452790599
"12586","SMIM4",133.3490446918,-0.085349865353388,0.272836520933868,-0.312824196193572,0.7544142314296,0.93747327026529
"12587","MTRR",68.6101847569151,0.0666471704403834,0.213162748347781,0.312658618623394,0.754540037907849,0.937555111851813
"12588","NADK",169.036848060195,0.0485164734318224,0.155737595624539,0.311527048027559,0.755399983812234,0.938070175540513
"12589","AL020997.5",14.5851594691069,0.174393974523199,0.559631479514854,0.311622882033693,0.755327142245807,0.938070175540513
"12590","ZNF672",63.4836180341216,-0.0689666631969952,0.221361098242081,-0.311557286915757,0.755376999585857,0.938070175540513
"12591","HSD17B13",4.55481104124759,0.271149539375513,0.869264303309261,0.311929914001133,0.75509378786325,0.938070175540513
"12592","NRXN3",6.10501754769995,-0.327122418173339,1.04908273727691,-0.31181755885379,0.755179178874961,0.938070175540513
"12593","HYKK",31.4580038724095,-0.113373299275838,0.363784329016229,-0.311649761226467,0.755306712284124,0.938070175540513
"12594","PPM1D",53.771110632843,0.0864720690809284,0.277184310939418,0.311965957913931,0.755066394770698,0.938070175540513
"12595","DOT1L",39.786206033642,-0.106618482859344,0.342294441859456,-0.311481782409778,0.755434390085215,0.938070175540513
"12596","CMTM4",15.7388665220937,0.117697370006781,0.378049814255606,0.311327675794607,0.755551529741097,0.938141149980213
"12597","LINC00310",5.59451783416204,-0.21121458991198,0.678639133614258,-0.311232552692776,0.755623837602313,0.938156451544032
"12598","HECTD3",81.5613519889003,0.073503982296345,0.236956264996605,0.310200628362361,0.756408392719605,0.938190821298075
"12599","PIGM",101.094439685458,0.0792101073117143,0.256641892996545,0.308640597943145,0.75759493486651,0.938190821298075

"12600","IVNS1ABP",134.120397064203,-0.106570299479771,0.3447597618876
3,-0.309114668418021,0.757234301464825,0.938190821298075
"12601","TRAPPC12",220.556679777092,-0.0544874875972911,0.176482031179
329,-0.308742409825988,0.757517480398875,0.938190821298075
"12602","AC017083.1",4.66903849500462,0.212065161221256,0.687375598541
362,0.308514241226,0.7576910654613 8,0.938190821298075
"12603","GMCL1",137.432279563105,0.0569969823180303,0.18338178382929,0
.310810491248623,0.75594469357935,0.938190821298075
"12604","CNOT9",96.3433403590212,0.0719479280677303,0.231283839956137,
0.311080653457567,0.755739308300847,0.938190821298075
"12605","AC137630.2",2.59513874926828,0.294289719113759,0.950908850476
292,0.309482574451122,0.756954465557986,0.938190821298075
"12606","EHHADH",5.6824894785769,0.194118252518031,0.62820423982348,0.
309005002214847,0.757317721698646,0.938190821298075
"12607","AC091978.1",4.13229938153808,0.271297897078349,0.875450318929
044,0.30989525186333,0.756640613653468,0.938190821298075
"12608","PRKAA1",285.247817784157,0.0461802321351148,0.14941729059637,
0.309068863120162,0.757269144021954,0.938190821298075
"12609","KIAA1191",120.482951786804,0.0551725822148665,0.1780666586341
97,0.309842295228371,0.756680886307924,0.938190821298075
"12610","DTNBP1",169.404194744932,-0.0693900142409828,0.22346112833606
,-0.310523869442869,0.756162610783369,0.938190821298075
"12611","BAG6",277.248151452599,-0.0547399704848013,0.176980051760225,
-0.309300228700146,0.757093157016354,0.938190821298075
"12612","Z99289.3",7.87832747540366,-0.192929615077655,0.6213490411427
23,-0.310501187420903,0.756179856645858,0.938190821298075
"12613","AGPAT4",112.140622790579,0.0739641911501066,0.238700277924303
,0.3098621911683,0.756665755693626,0.938190821298075
"12614","YKT6",122.018929506757,0.0695394768146572,0.22434373522362,0.
309968436361025,0.756584959128168,0.938190821298075
"12615","TMEM248",518.081598693535,0.0433648365592808,0.14013855315553
4,0.309442587944747,0.756984878467072,0.938190821298075
"12616","BRI3",2535.09897236805,0.0846709191030444,0.274148917770214,0
.308850094290775,0.757435560951711,0.938190821298075
"12617","ST7-
OT4",13.5848428314432,-0.155493955564257,0.501996867593197,-0.30975084
8266775,0.756750431781775,0.938190821298075
"12618","FAM120AOS",393.151048171394,-0.0430239355892655,0.13849300136
0891,-0.3106578322839,0.756060757053406,0.938190821298075
"12619","C12orf66",21.0303131672073,-0.132059277696797,0.4257729457066
28,-0.310163618962747,0.756436535028438,0.938190821298075
"12620","GTF2H3",123.788636990999,0.0546180641680731,0.176863530645589
,0.308814733985609,0.757462460507829,0.938190821298075
"12621","WBP4",422.101182805561,-0.0416754446058769,0.134866293444985,
-0.30901304945314,0.757311600277263,0.938190821298075
"12622","RUBCNL",5.14825900381771,-0.209861691808948,0.676394690779597
,-0.31026513760341,0.756359340035223,0.938190821298075
"12623","PCCA",94.619146125293,-0.063140974410302,0.203285627722209,-0
.310602255150986,0.756103012572443,0.938190821298075
"12624","TRIP4",109.520555427428,-0.0606793225230294,0.195736804918411

,-0.310004664418235,0.756557409289041,0.938190821298075
"12625","SIAH1",194.756064853365,-0.0660335065857594,0.213288023290422
,-0.309597817857054,0.756866815914381,0.938190821298075
"12626","TOP3A",63.2887336043456,0.0809461301101108,0.262257355563132,
0.308651514983438,0.75758662949603,0.938190821298075
"12627","TCF4-
AS2",6.00892579104947,-0.231065285294637,0.747820761577804,-0.30898484
9266714,0.757333051830629,0.938190821298075
"12628","ZNF555",30.1667569690622,0.105323408164636,0.340677900932252,
0.30915832191176,0.757201096172191,0.938190821298075
"12629","MEIS3",105.984143946922,-0.0655917498155393,0.212600362541013
,-0.308521345079485,0.757685660839916,0.938190821298075
"12630","ZNF83",261.417520074093,0.0637552016971984,0.20634984841406,0
.308966554553836,0.757346968505602,0.938190821298075
"12631","ATF4",1266.86876323264,0.0483481120917018,0.155793228314547,0
.310335132115542,0.756306117473383,0.938190821298075
"12632","DMXL1",174.939030677349,0.0537120483863394,0.174236589411312,
0.308270774627847,0.757876302340712,0.938197353855369
"12633","AC138969.1",14.8334829505035,0.174739043591956,0.566835469508
861,0.308271188010447,0.757875987814725,0.938197353855369
"12634","BACH1",187.897116219189,-0.0887503672449438,0.287772895259028
,-0.308404192010712,0.757774792568053,0.938197353855369
"12635","KLHL28",163.490403240766,-0.0495546430744336,0.16084134844402
1,-0.308096416461471,0.758008967942907,0.938271401214615
"12636","PHF6",147.839441327494,0.0598086364684583,0.194162153145458,0
.308034472730905,0.758056101390529,0.938271401214615
"12637","TRIM27",116.112381017035,-0.063018791789113,0.204809023327014
,-0.307695387465874,0.758314129835442,0.938293749258411
"12638","OGFOD2",42.0952417811276,0.083631224671626,0.271758984710332,
0.307740422127234,0.758279858958029,0.938293749258411
"12639","B3GNT8",39.4207449261179,0.101799639062761,0.33069039819216,0
.307839718417244,0.758204297261718,0.938293749258411
"12640","MRPL40",526.633159907489,-0.0826542404895331,0.26842374218929
,-0.307924477229164,0.758139800006359,0.938293749258411
"12641","ST6GAL1",8.42939746601578,-0.207833481160977,0.67645732462344
5,-0.307238126627824,0.758662127137067,0.938575832022127
"12642","Z97832.2",3.06090373433769,-0.299827160519896,0.9756317626507
69,-0.307315907494927,0.758602928735478,0.938575832022127
"12643","PAXIP1",46.7739640800895,0.0769038272119055,0.250525899450369
,0.306969568338545,0.758866535595806,0.938680213280481
"12644","PSMB10",331.732241863591,-0.0812388460040922,0.26461056741357
3,-0.307012855904277,0.758833586880422,0.938680213280481
"12645","HDLBP",1562.62484706462,0.0538783232853626,0.175816240773255,
0.30644679381382,0.759264484627059,0.938953970711033
"12646","ZFP91",601.674221788191,0.0756344273116434,0.246715020734034,
0.306565960542709,0.759173766321436,0.938953970711033
"12647","FUNDC1",141.224356254396,0.078265902649968,0.255401817207739,
0.306442230934904,0.759267958285322,0.938953970711033
"12648","AL031848.2",4.47233347231118,0.238958957922483,0.780529318858
987,0.306149880790902,0.759490530634679,0.939018385716519

"12649","SEC31A",1211.52703087411,0.0461903065962405,0.150907671869844
,0.306083223098684,0.759541281335524,0.939018385716519
"12650","KIAA0825",38.437475219069,0.0915741914682043,0.29898352792824
8,0.306285072300641,0.759387604001103,0.939018385716519
"12651","SERPINF1",6571.31765413553,-0.0958633569303242,0.313219197984
613,-0.306058369177721,0.759560204456501,0.939018385716519
"12652","RNF103",224.310921236303,0.0723176027007905,0.236598010125078
,0.305656005570629,0.759866573532774,0.939043304559095
"12653","CCNT2-
AS1",3.94703824323345,0.219651715491121,0.718366941616243,0.3057653446
53708,0.759783316463127,0.939043304559095
"12654","C10orf88",44.0995477397254,-0.0774885122607558,0.253530667907
561,-0.305637629168431,0.759880566656005,0.939043304559095
"12655","ARNT2",4.94749477421206,0.309000882630056,1.01068345500124,0.
305734580991708,0.759806741404274,0.939043304559095
"12656","GRIN2D",15.845347966738,0.131924262002595,0.431415960262307,0
.305793651960356,0.759761762104484,0.939043304559095
"12657","PGR",23.4190243847973,0.152938348193745,0.500752042264512,0.3
05417322917194,0.760048329905387,0.939176414610117
"12658","FLAD1",63.4942515589562,-0.068202430186841,0.223744935578254,
-0.304822229877858,0.760501549768581,0.939230186987883
"12659","AC021205.3",2.98025575628887,0.287542606748499,0.942897907430
504,0.304956246569772,0.760399476152259,0.939230186987883
"12660","RNF122",30.8192448430735,0.173195457405384,0.567465046545309,
0.305209031745279,0.760206954051564,0.939230186987883
"12661","FOXRED1",75.2022700939363,-0.0763433588207205,0.2504635797882
22,-0.304808223555985,0.760512217903743,0.939230186987883
"12662","SERPINA3",49.7585804620003,-0.183428365222591,0.6012786077323
75,-0.305063847048145,0.760317525425281,0.939230186987883
"12663","INTS14",74.1672489909198,-0.0673813315864785,0.22089749416313
6,-0.305034386387002,0.760339963000494,0.939230186987883
"12664","BMERB1",357.679969739055,-0.0604836022175668,0.19825632513572
1,-0.305077793488613,0.760306903727634,0.939230186987883
"12665","HOOK2",73.2893871427402,0.073264035074766,0.240436323891934,0
.304712839927195,0.760584869554653,0.939245744953397
"12666","TPSB2",27.5994331370037,0.212943189561273,0.699202968928327,0
.304551323469996,0.760707897968232,0.939323505781079
"12667","SNRNP48",127.90364083178,0.0648408281428905,0.213006841385741
,0.304407256222668,0.76081764028178,0.939364054441359
"12668","IFIT1",427.521090753705,-0.0975430088902896,0.320557844251059
,-0.304291442682321,0.760905864002621,0.939364054441359
"12669","BLOC1S6",671.132276280616,0.0545089506202527,0.17914566057605
,0.30427167727634,0.760920921081687,0.939364054441359
"12670","RSAD1",110.45741710434,0.060044459068381,0.197769255472317,0.
303608662150149,0.761426051485868,0.939913452662903
"12671","ATP5IF1",1101.36220659058,0.0818065385742163,0.26966144104182
,0.303367579206586,0.76160975023151,0.940044338316559
"12672","GPLD1",2.58946396327174,-0.326773828553931,1.0776332525989,-0
.303232874232358,0.76171239766533,0.940044338316559
"12673","OPRL1",4.62234316321753,0.21964052897646,0.724257066989391,0.

303263218251313,0.761689274651585,0.940044338316559
"12674","ANGEL1",26.46580895828,0.114831356052435,0.378939928476601,0.303033139089025,0.76186460688834,0.940157996822917
"12675","SMG7-AS1",3.08423269473353,-0.251513610968321,0.830461438572834,-0.302860071866253,0.761996501131502,0.940172394895605
"12676","CTDSPL2",194.208048592186,0.0581458581430228,0.191969648725982,0.302890892018146,0.761973012638851,0.940172394895605
"12677","AC104041.1",9.78999562008803,0.214084892532546,0.707151403707961,0.302742653708934,0.762085989250838,0.94020863547236
"12678","POT1",50.3852336924384,0.0775857886236804,0.256469435072177,0.302514756200269,0.762259686282348,0.940348753230472
"12679","TXNDC5",597.538223474989,-0.0768244313622791,0.254029269635,-0.302423541478758,0.762329210917902,0.940360348509818
"12680","DBX2",7.72609136005384,0.184832296154296,0.611854679819194,0.302085286344324,0.76258704859387,0.940604214511682
"12681","RHBDD1",61.7392056542516,0.0704237609278905,0.233361885239053,0.301779190958066,0.762820394955089,0.940669477024174
"12682","AC246817.2",4.52965279836245,-0.356034236852193,1.17971811513484,-0.301796024223549,0.762807561854916,0.940669477024174
"12683","CTSD",1273.72481648758,0.0791482642634183,0.262260816333414,0.30179218294965,0.762810490303715,0.940669477024174
"12684","AC022819.1",5.31940111519376,-0.24041340924127,0.796973644731353,-0.301657916583062,0.762912852276189,0.940709319583696
"12685","ATP13A2",38.6572795471225,-0.0956208080363879,0.317125031957051,-0.301523999686464,0.763014951949614,0.940761044421912
"12686","REPIN1",138.609325498613,0.0584108554453935,0.193792255934268,0.301409646963425,0.763102138951377,0.940794375941947
"12687","NBPF1",105.068637440774,0.0916804641098964,0.304440774995296,0.301143840247131,0.763304812027974,0.940808792627481
"12688","MCL1",2772.5697661552,-0.0780079437591658,0.260046562942349,-0.299976830597295,0.764194828739281,0.940808792627481
"12689","KLHL20",215.160768320768,-0.0516214269659272,0.171719410831637,-0.300614978329618,0.763708108437693,0.940808792627481
"12690","AC104113.1",3.91013024265986,-0.264569862875706,0.882831990256694,-0.299683139935583,0.764418860241399,0.940808792627481
"12691","GTF2H2C",73.8975138513941,-0.0655935110814596,0.217762266774417,-0.301216147558791,0.763249677332149,0.940808792627481
"12692","NDST1-AS1",4.25148920395819,-0.393529952554052,1.30905026657889,-0.300622491436112,0.763702378687901,0.940808792627481
"12693","MSX2",50.6181633027449,0.152804203304116,0.508447537924063,0.300530914021138,0.763772219612468,0.940808792627481
"12694","HULC",13.3639086512944,0.154436041030223,0.514915517398684,0.299925008689626,0.764234357809573,0.940808792627481
"12695","C7orf26",81.4418286300735,0.064935223916095,0.216158040755728,0.300406238366473,0.763867305785644,0.940808792627481
"12696","SUGCT",15.233818397169,0.15020976855297,0.498939803565443,0.301057898126317,0.76337034489715,0.940808792627481
"12697","PLOD3",200.476690719383,-0.0642955492783169,0.213622687935082

,-0.300977156966847,0.763431913456928,0.940808792627481
"12698","ZNF251",59.1983629574604,0.0791072661393856,0.263416900281646
,0.300312037894318,0.76393915186435,0.940808792627481
"12699","AL391834.2",22.6669210749216,0.109303801625066,0.364568276539
412,0.299817095065454,0.764316674875943,0.940808792627481
"12700","TWNK",12.5642210233003,-0.143074707123508,0.474964846310935,-
0.301232203256248,0.763237434943443,0.940808792627481
"12701","NEURL1",4.36568823375897,-0.220523450269623,0.735140779933383
,-0.299974448825443,0.764196645510084,0.940808792627481
"12702","BCAT1",284.199790975919,-0.0910149080781082,0.30372819244857,
-0.299659071304418,0.764437221017265,0.940808792627481
"12703","MBD6",111.839958106028,0.0583994186954468,0.194409145424406,0
.300394400520395,0.763876334321076,0.940808792627481
"12704","AC079866.2",21.2744704227786,-0.157124740597445,0.52421815169
9264,-0.299731590919013,0.764381899768899,0.940808792627481
"12705","GOLGA8O",4.64411724630508,0.214491838067771,0.715335251485716
,0.299847991025582,0.764293107006843,0.940808792627481
"12706","MAP2K5",76.8788837288825,-0.0702634768815205,0.23439192986404
7,-0.299769181141496,0.764353224716895,0.940808792627481
"12707","MOSMO",261.353162055077,-0.0431092460525853,0.143273241932504
,-0.300888326885868,0.763499651891101,0.940808792627481
"12708","ADPRM",36.4977276439709,0.109985557435389,0.366602703140592,0
.300012947240079,0.764167279802055,0.940808792627481
"12709","VSIG8",2.85967315503458,-0.263236682028555,0.879241161191194,
-0.299390762907326,0.764641909271571,0.940979364859465
"12710","EXT1",464.85326423676,-0.0571263831293546,0.190919592315327,-
0.299216976301748,0.764774497113652,0.940979364859465
"12711","PPP1R26",14.1031014453304,0.11764326527301,0.393242734392453,
0.299161955159234,0.764816476092936,0.940979364859465
"12712","MSANTD2",70.7236521914455,-0.06040629281373,0.201856505735621
,-0.29925363363243,0.764746529387992,0.940979364859465
"12713","SPATA1",9.46536989406651,0.166228656338321,0.558847600973539,
0.2974489933369,0.766123743997127,0.941028098220905
"12714","SDHC",507.098565539062,-0.0603216770453986,0.201941015903304,
-0.29870938687504,0.765161794195258,0.941028098220905
"12715","GRHL1",55.4665442322774,0.153484402946476,0.516124110979203,0
.297378866209683,0.76617727659757,0.941028098220905
"12716","OXNAD1",33.6048549077518,0.0865013979608951,0.290858819330354
,0.297399948745057,0.766161182808784,0.941028098220905
"12717","ARIH2OS",32.4836830864131,0.104841950118699,0.352000358110799
,0.297846146184026,0.765820592456241,0.941028098220905
"12718","TLR9",2.56243202586236,-0.303448658308339,1.01506985488262,-0
.298943621317006,0.764983063083403,0.941028098220905
"12719","FAM153CP",29.3106665070046,0.144526891950465,0.48487587953450
7,0.298069873240991,0.765649834691648,0.941028098220905
"12720","BTN2A1",92.2324424893751,-0.0664742352126752,0.22272026133089
9,-0.298465145539289,0.765348174322813,0.941028098220905
"12721","SYNCRIP",545.617729907859,0.0552493391662643,0.18542225865132
6,0.297964977711533,0.765729893890872,0.941028098220905
"12722","MED23",94.1996418957321,0.0633061213985298,0.212533605980034,

0.297864053576905,0.765806924372587,0.941028098220905
"12723","GOLGA2",682.495547714103,-0.0587379621411031,0.19754831862115
9,-0.297334659950945,0.766211022685738,0.941028098220905
"12724","EHD1",274.18475922198,0.0631893201431244,0.212047928518896,0.
29799546067008,0.765706628187147,0.941028098220905
"12725","DHRS4-
AS1",215.157042375202,-0.0481549723736451,0.161688778613612,-0.2978250
73493326,0.76583667659385,0.941028098220905
"12726","ENTPD5",71.8377353243176,-0.0823025995561334,0.27683406374698
8,-0.297299394598179,0.766237943800493,0.941028098220905
"12727","WARS",183.359025732867,0.0651413677325383,0.218579443984758,0
.298021472399211,0.765686775255604,0.941028098220905
"12728","NME4",402.135108871712,-0.0953503548115953,0.319625629350939,
-0.298318864495386,0.765459807632779,0.941028098220905
"12729","AC135178.6",3.16062601804018,-0.276091102918141,0.92494449515
3236,-0.298494779270405,0.765325560151012,0.941028098220905
"12730","ALDH3A1",5.16392469592113,0.244559570882684,0.81799149839966,
0.298975687841679,0.764958595897946,0.941028098220905
"12731","NPC1",66.9624378544697,0.0744699998164596,0.24936425800588,0.
298639429772264,0.76521517694647,0.941028098220905
"12732","ENTPD6",99.1512554616269,0.0706167630033679,0.236941538564397
,0.298034542323086,0.765676799955938,0.941028098220905
"12733","CU638689.5",4.31403008233802,-0.209592029857849,0.70396576929
3413,-0.297730428097691,0.765908917762249,0.941028098220905
"12734","NHS",13.1320763924465,-0.131219704684752,0.439453282068964,-0
.298597621269237,0.765247080642814,0.941028098220905
"12735","HDAC6",81.6663042293848,0.0717555401020513,0.241359975706913,
0.297296765513372,0.766239950821179,0.941028098220905
"12736","ATP6V1G1",1241.08896401588,-0.0610396799332551,0.205486080342
142,-0.297050193529516,0.766428188719824,0.941185369941744
"12737","PSPC1",149.916518092825,-0.0572527839878919,0.192826869550438
,-0.296912894563775,0.766533011425726,0.941240189895451
"12738","AL731559.1",4.19622157004878,0.317623421361602,1.070146152857
13,0.296803778169548,0.766616320823433,0.941268586723072
"12739","ARID5B",1966.76558972251,-0.0925988912962952,0.31214757229365
7,-0.296651005855594,0.766732965661123,0.941337905874869
"12740","ATP6V0E2",88.3826809234306,-0.0729373998080443,0.245943173574
678,-0.296562001489737,0.766800924782021,0.941347446121728
"12741","EIF5",1499.8767990957,-0.0480481107185901,0.162131668717143,-
0.296352409734433,0.766960965268641,0.941470017800922
"12742","TMEM63B",79.9881015675376,-0.0633719694825866,0.2138986135549
51,-0.29627106239427,0.767023083309611,0.941472376625515
"12743","STAT5A",123.296746401306,0.0654518946487451,0.220987196638747
,0.296179578022074,0.76709294392784,0.941484237858544
"12744","NDUFAF7",84.126494634843,0.0564552908280533,0.19076817799299,
0.295936625395289,0.767278480110566,0.941502255350329
"12745","RIOK2",118.820631501999,-0.0674072519344228,0.227901193814702
,-0.295774018582936,0.767402665876038,0.941502255350329
"12746","SCIMP",2.8098994576989,-0.317147052050483,1.07228960572715,-0
.295766228038195,0.767408615806009,0.941502255350329

"12747","RBFA",200.767188118693,-0.0509090418222151,0.17210725663806,
-0.295798345805144,0.767384086356139,0.941502255350329
"12748","SIRPB1",3.04228723020001,-0.253552747484552,0.856745530559929
,-0.29594872507691,0.767269239603571,0.941502255350329
"12749","KIAA1217",184.22042489307,-0.104944648073093,0.35530798324017
6,-0.295362482756696,0.767716989914805,0.941806708154958
"12750","TTC25",7.94360382242032,-0.15525849879625,0.525983749104751,-
0.29517736823715,0.767858389686625,0.941906291348926
"12751","VAV3",4.46325133567567,-0.269156875258138,0.913400971218615,-
0.294675486165777,0.768241791290368,0.94214156215 6752
"12752","TGFBRAP1",89.430835785961,-0.0654197423465658,0.2219832724316
45,-0.294705729985625,0.768218685592891,0.94214156215 6752
"12753","IFT80",124.702467327067,0.0628138533146697,0.213209544508415,
0.294610888361007,0.768291143462098,0.94214156215 6752
"12754","MROH6",27.942993077108,-0.122200974344588,0.414756552644258,-
0.294633016803478,0.768274237415065,0.94214156215 6752
"12755","MIR22HG",515.341614790628,-0.0957426602920855,0.3251888315395
39,-0.294421735945887,0.768435659694501,0.942244901420776
"12756","GABARAP",3891.87756019552,-0.0704607010139112,0.2394123895733
12,-0.294306828228432,0.7685234554214,0.942278680055714
"12757","AC007319.1",3.82785576456335,0.247151126564019,0.840948231290
848,0.293895768333616,0.768837551771865,0.942540265012962
"12758","MRI1",108.44093798472,0.070305309728949,0.239239589530602,0.2
93869881096565,0.768857333825791,0.942540265012962
"12759","NUDCD1",76.9487777796326,0.0681833533711238,0.232150667326493
,0.293703025523644,0.768984842196064,0.942597933117119
"12760","MADD",71.5975476082722,0.0773011927047397,0.263255937764162,0
.293635134543442,0.769036725190132,0.942597933117119
"12761","UQCR10",1527.78026806212,-0.0811511407047448,0.27642695952340
6,-0.293571729923374,0.769085180595113,0.942597933117119
"12762","RAMP2",2411.20407933083,-0.0972150751868705,0.331252543111766
,-0.293477219144155,0.769157409783265,0.942612591209079
"12763","DUSP7",31.3284493708923,-0.0947392959988838,0.323495774903499
,-0.292860999582283,0.769628400272539,0.94282041045371
"12764","AL603910.1",3.70291046253523,0.239165385162585,0.816487172860
639,0.292919954057144,0.769583336359807,0.94282041045371
"12765","TPM2",260.742159953648,0.105588278450375,0.360160957883895,0.
293169695768116,0.769392446189526,0.94282041045371
"12766","PSME1",1151.18002044153,-0.0650109442271386,0.221957946655124
,-0.292897574548894,0.769600442827252,0.94282041045371
"12767","NUCB1",1220.34807067985,-0.0638561585011672,0.217906183418752
,-0.293044270241998,0.769488313491107,0.94282041045371
"12768","FAM72A",33.1574575702611,0.078880891855496,0.269808661798947,
0.292358634187495,0.770012432363095,0.942979480189918
"12769","MDC1",65.1701174063705,0.0901072940741556,0.308245206669351,0
.292323423445192,0.770039351251835,0.942979480189918
"12770","NPDC1",800.108071296782,-0.0738435689819936,0.2525404706752,-
0.292402911836519,0.769978582146361,0.942979480189918
"12771","HECTD1",462.691408171732,0.0491889196383137,0.168318494362817
,0.292237165170247,0.770105297550521,0.942979480189918

"12772","ZNF768",63.1761996022687,0.0627451290221036,0.214737709622989
,0.292194273340552,0.770138089891841,0.942979480189918
"12773","RRP1B",167.674931234226,0.0595965794474621,0.204000719456538,
0.292139065029911,0.770180299229285,0.942979480189918
"12774","APOBEC3D",36.2245115335883,0.0897134630794574,0.3069253427920
04,0.292297345873632,0.770059287938481,0.942979480189918
"12775","XPC",266.788988218648,0.0651312985173018,0.223167889144555,0.
291848880082894,0.770402170393532,0.942988538109079
"12776","RAET1E-
AS1",4.01978616040618,-0.230849880061726,0.791085799497078,-0.29181396
0266366,0.770428870841292,0.942988538109079
"12777","STT3A",230.99609039547,0.0530003675030365,0.181613632671843,0
.291830336320637,0.77041634932176,0.942988538109079
"12778","AC090229.1",3.05665671562514,0.266675215818192,0.913742165907
75,0.291849523605235,0.770401678344971,0.942988538109079
"12779","UNC119B",50.8470382420523,0.084348946717003,0.289243192703101
,0.291619470552534,0.770577586864268,0.943022962328415
"12780","TXNDC16",44.1700430316289,0.0865930628227288,0.29689890123611
8,0.291658414572114,0.770547807751603,0.943022962328415
"12781","LINC01176",6.3984643328789,-0.174806567049225,0.5999791049616
25,-0.291354424851855,0.77078026690687,0.943197196966089
"12782","IFI44",107.697189084339,0.0802829479943401,0.276188032218656,
0.29068221149706,0.771294377591372,0.943216038908899
"12783","COLEC11",6.45528063030412,0.277070801328403,0.95447614938436,
0.290285725323901,0.771597658461917,0.943216038908899
"12784","AC092040.1",39.7671932795972,-0.126271405888677,0.43390766096
1507,-0.291009855896227,0.771043781659948,0.943216038908899
"12785","CFI",21.061567574784,0.170249893642119,0.586735573632668,0.29
0164601045147,0.771690316011997,0.943216038908899
"12786","AC026748.3",9.08976975965614,-0.15605723746477,0.537708611493
561,-0.290226405396967,0.771643036563334,0.943216038908899
"12787","CCL28",13.1235774803478,0.159351149046434,0.549498267060104,0
.289993906439389,0.771820899357807,0.943216038908899
"12788","TNIP1",208.801859533042,-0.0591577774087824,0.203934011150172
,-0.290082939452508,0.771752787267255,0.943216038908899
"12789","ECHDC1",388.222248844945,0.0608584039852415,0.209425348809974
,0.29059712365795,0.771359460180353,0.943216038908899
"12790","HSPA14.1",86.5222423934281,-0.067186513659735,0.2307961113067
79,-0.29110765029498,0.770968989104715,0.943216038908899
"12791","SLC29A3",37.6225839094276,-0.090632214644697,0.31178178993275
8,-0.290691174312148,0.771287522143594,0.943216038908899
"12792","NPIPB2",18.8249356167534,-0.134898223550785,0.463587942667528
,-0.290987342713376,0.771060999905286,0.943216038908899
"12793","GGA2",147.179714532143,0.0510145334759267,0.17534335133668,0.
290940791806658,0.771096602733715,0.943216038908899
"12794","ZNF747",50.4473945979543,-0.0723719901857056,0.24950228549943
4,-0.290065439844918,0.771766174688952,0.943216038908899
"12795","EDC4",71.1033552697804,0.0746326909099174,0.25666399388189,0.
290779745850372,0.771219776804605,0.943216038908899
"12796","KEAP1",200.22526898108,-0.0629494372639838,0.216928716198369,

-0.29018489745,0.771674789457922,0.943216038908899
"12797","BX322562.1",3.08110994177247,-0.250150520772276,0.86219730826
08,-0.290131410032899,0.771715707013472,0.943216038908899
"12798","LINC01560",22.3562837665794,0.103338022382638,0.3558169948510
7,0.290424639289338,0.771491396182886,0.943216038908899
"12799","KIF3C",14.1123591693104,0.142457381163651,0.493326004267684,0
.288769251836057,0.772757963624572,0.943255642791564
"12800","C2orf74",185.93267624755,-0.10617079785467,0.367585938128065,
-0.288832588089048,0.772709492728162,0.943255642791564
"12801","NCK2",129.088938354771,-0.0562033741023172,0.19415959899271,
-0.289469974144973,0.772221753972666,0.943255642791564
"12802","NIFK-
AS1",99.8951059466495,0.0591382351776834,0.204104818978494,0.289744433
637869,0.772011760560119,0.943255642791564
"12803","CIP2A",19.9774995274638,-0.105853776844742,0.366344667442859,
-0.288945865060948,0.772622804677291,0.943255642791564
"12804","BOD1L1",929.155061218854,0.0489441891900753,0.169474810695899
,0.288799196701269,0.772735046868533,0.943255642791564
"12805","LMBRD2",76.974108136027,0.079500647425616,0.274794999817032,0
.289308930215434,0.772344979064875,0.943255642791564
"12806","RPL37",18147.2637067935,-0.0636644264608059,0.219829065277751
,-0.289608775711104,0.772115552555484,0.943255642791564
"12807","EMB",25.2340454697262,0.152828329751598,0.527604668489111,0.2
89664475845615,0.772072935841577,0.943255642791564
"12808","IKBKB",95.5218522357599,0.0920219668849671,0.318619820824916,
0.288814319983984,0.772723473120492,0.943255642791564
"12809","PRTFDC1",112.534559578287,0.0935039586393153,0.32359373404171
8,0.288954787447339,0.772615976717301,0.943255642791564
"12810","AL353801.3",2.53835436206399,0.266470509486889,0.922695145745
712,0.288795828953377,0.772737624190559,0.943255642791564
"12811","INPP5A",62.5142277513799,-0.0693304576089053,0.23933016007856
7,-0.289685418612287,0.772056912505246,0.943255642791564
"12812","INTS4",56.5436652904623,0.068696930046159,0.237264848285834,0
.289536905877433,0.772170541879043,0.943255642791564
"12813","SNRPB2",678.434790330358,-0.0522133301147208,0.18081022130463
5,-0.288774217176307,0.772754163644149,0.943255642791564
"12814","AC073352.2",4.48071733547961,-0.20723101892861,0.718020806665
097,-0.288614225388692,0.772876608277625,0.943326842005779
"12815","CACNA1A",24.3665951399923,0.139156914193556,0.482825742920565
,0.288213535077499,0.773183288687663,0.943627517368322
"12816","RAB29",141.305117842237,0.074835489690339,0.260257299889621,0
.28754424841139,0.77369562638352,0.943669897687461
"12817","AL391840.3",32.1030795255472,0.0991155201369698,0.34425549989
2764,0.287912670001916,0.773413588128478,0.943669897687461
"12818","TUBE1",118.963375831004,-0.0559823044941613,0.194748969658782
,-0.287458796789771,0.773761046543734,0.943669897687461
"12819","RIDA",99.8994094138855,0.0759175043673603,0.263753561023857,0
.28783499291027,0.773473049885025,0.943669897687461
"12820","ZNF503-
AS2",17.4774239957884,0.129194030731489,0.448752315248727,0.2878960761

68168,0.773426290584327,0.943669897687461
"12821","MAP4K2",15.5968656875459,-0.130361546114744,0.453484798323788
,-0.28746618761334,0.773755388204217,0.943669897687461
"12822","NGDN",136.805899934761,-0.065045740763317,0.226258847453953,-
0.287483744813801,0.773741946637928,0.943669897687461
"12823","DISP2",3.3240040175061,0.360744407450824,1.25394082704913,0.2
87688541332333,0.77358516207622,0.943669897687461
"12824","ZNF585B",39.2241078237296,-0.0745357250674159,0.2587296343370
83,-0.288083447643681,0.773282862645448,0.943669897687461
"12825","ACD",81.2776156124278,0.0612161107141468,0.213061782886225,0.
287316241725229,0.773870187606176,0.943729413969636
"12826","AL109811.2",18.8860112675928,-0.112202347970416,0.39365527458
3373,-0.285026913685244,0.775623522631518,0.94380010595793
"12827","USP48",310.817091152961,0.048512778546502,0.170401166500852,0
.28469745567299,0.775875940097586,0.94380010595793
"12828","PPT1",288.813245923717,0.0517041007414526,0.181075944641288,0
.285538207981621,0.775231836468544,0.94380010595793
"12829","SELENOF",1336.79080331851,-0.053194129040767,0.18662573543985
8,-0.285031048453172,0.77562035488933,0.94380010595793
"12830","TMED5",370.655376322165,0.0661079278792368,0.230562723646755,
0.286724266757539,0.774323455202059,0.94380010595793
"12831","ZNF670",26.105523234051,-0.115697843127707,0.406235143112913,
-0.284805106326187,0.775793459895281,0.94380010595793
"12832","STEAP3",4.2905738373049,0.250436084675665,0.876577582622288,0
.285697569320087,0.775109766541866,0.94380010595793
"12833","NOP58",242.153768806083,-0.0470565085782031,0.165304646266026
,-0.28466537173114,0.775900522787783,0.94380010595793
"12834","FZD5",7.75233073534891,-0.176934277445014,0.621023401496747,-
0.28490758483268,0.775714944858172,0.94380010595793
"12835","STIMATE",73.6044706360605,0.0632574184363323,0.22112610811003
7,0.286069424261988,0.774824949278829,0.94380010595793
"12836","RUVBL1",117.188457409714,-0.067826223091077,0.237803775274759
,-0.285219286416755,0.775476145347768,0.94380010595793
"12837","PPP2R3A",77.6052183967475,-0.0777455423257748,0.2732114692210
36,-0.284561781199882,0.775979895301344,0.94380010595793
"12838","SRD5A1",21.1614900859018,0.128385037153603,0.450736990899541,
0.284833594192888,0.775771633372252,0.94380010595793
"12839","FAM151B",7.60805772550181,-0.148113064287789,0.51903802833877
9,-0.285360717714338,0.775367799595471,0.94380010595793
"12840","RXRB",131.14693918763,0.0535928646929046,0.187552786561303,0.
285748165492532,0.775071011308595,0.94380010595793
"12841","IPCEF1",2.51908283534233,0.308790526079875,1.07536800492614,0
.287148701342555,0.773998463300902,0.94380010595793
"12842","TMEM60",185.053552947719,0.0751872792127588,0.263225261383245
,0.285638539468631,0.77515498244216,0.94380010595793
"12843","AC105206.1",5.34560869861004,-0.229395003513861,0.80236621698
877,-0.285898133117775,0.774956143652484,0.94380010595793
"12844","AF305872.2",2.55510336188285,0.32228507545554,1.1324964358239
9,0.284579328694355,0.775966450001089,0.94380010595793
"12845","UAP1L1",20.5434427713316,0.142969163185812,0.501293303073291,

0.28520062468281,0.775490441799876,0.94380010595793
"12846","DEAF1",173.446730529149,-0.0763074128697027,0.268081942540894
,-0.284642121533652,0.775918337210526,0.94380010595793
"12847","CBX5",523.963570226757,-0.0612186458840494,0.214432715992856,
-0.285491164912023,0.775267872268434,0.94380010595793
"12848","TESC",2.77388747113365,0.310635036285692,1.08671323139828,0.2
85848213963492,0.774994378666538,0.94380010595793
"12849","SLC15A4",204.227745106207,0.0499013982689396,0.17403357988347
8,0.286734308989969,0.774315765352284,0.94380010595793
"12850","CLN5",335.913077255449,0.0460332773098112,0.161159333608703,0
.285638295213981,0.775155169538809,0.94380010595793
"12851","LINC01852",19.1905803671016,-0.107723701377287,0.376262193779
829,-0.286299562268332,0.774648693360589,0.94380010595793
"12852","LOXL1",2789.50279453059,-0.0855155874504811,0.297993717231821
,-0.286971108803463,0.774134442071045,0.94380010595793
"12853","CCL22",2.51646528704571,-0.354174299846176,1.24322752712484,-
0.284882929406543,0.775733834675234,0.94380010595793
"12854","CENPBD1",31.3733886089355,0.0933332976618379,0.32551016305553
5,0.286729289143314,0.774319609302237,0.94380010595793
"12855","DPH1",3.88302963050892,0.233242271540699,0.81531919712993,0.2
86074794217717,0.774820836455698,0.94380010595793
"12856","AC007114.2",4.40734205041372,0.239658765560086,0.839067223139
503,0.28562522638337,0.775165180152992,0.94380010595793
"12857","EEF2",5112.59267513922,-0.0394079774604415,0.13814767527129,-
0.285259794513756,0.775445113012635,0.94380010595793
"12858","RAB9A",481.543736597692,-0.05767641304066,0.201867678398649,-
0.28571395628160l,0.77509721453271,0.94380010595793
"12859","TSC22D3",4652.35737174831,0.08836959813281,0.309240708593805,
0.285763147208687,0.775059535846391,0.94380010595793
"12860","FNDC5",263.572518321559,0.0904991446988811,0.318971470557212,
0.283721752734775,0.776623623204861,0.94390907606975
"12861","Z99127.4",32.0993793572248,0.0983462765443883,0.3460682285793
26,0.284181754991257,0.776271096726675,0.94390907606975
"12862","UBE2V2",448.890364566873,-0.0566118101975218,0.19950431610683
,-0.283762333077584,0.776592522276925,0.94390907606975
"12863","SNHG6",1568.08717775507,-0.0564817555909852,0.198671123096749
,-0.284297761600108,0.776182201389663,0.94390907606975
"12864","DENND3",18.071164115461,0.143632017149373,0.506403191434443,0
.283631737672346,0.776692612366587,0.94390907606975
"12865","BRD3OS",68.384215065657,-0.0732196749903873,0.258064063780108
,-0.283726737918754,0.776619802521745,0.94390907606975
"12866","AMOTL1",140.537344367152,-0.0746825306850602,0.26337937133542
,-0.283554973597192,0.776751447125058,0.94390907606975
"12867","VRK1",47.5353367946735,-0.0730038989284642,0.257092035175529,
-0.283960173556606,0.77644090171682,0.94390907606975
"12868","HDGFL3",274.163658373585,0.044845697175985,0.157864036710856,
0.284077983246587,0.776350619026241,0.94390907606975
"12869","NDE1",138.021450407397,0.055870346759149,0.197073655373081,0.
283499824740048,0.776793715990649,0.94390907606975
"12870","AC092338.3",39.760284365226,0.0881290431557013,0.310123734799

509,0.284173809568867,0.776277185374982,0.94390907606975
"12871","STAC2",3.36148332200168,-0.223404941699653,0.787487277527434,
-0.283693398071273,0.776645354538537,0.94390907606975
"12872","CLK2",55.048528313659,0.0820714705584514,0.289694676494873,0.
283303343891112,0.776944314193579,0.944018728557145
"12873","STYXL1",51.2877444450692,-0.0772575166824882,0.27279017765131
9,-0.283212237873311,0.777014147776041,0.944030239355028
"12874","HIVEP3",13.4887515730745,-0.124885188467924,0.441458587129463
,-0.282892194441107,0.777259478226002,0.944054124535975
"12875","HIST2H2AA4",25.2832016255101,-0.181335084949716,0.64119367469
7867,-0.282808599188321,0.777323562128878,0.944054124535975
"12876","STK39",104.865163065169,-0.0695217390695551,0.246013470650436
,-0.282593220955529,0.777488677499937,0.944054124535975
"12877","AC025259.3",11.3533079953487,0.221246559573689,0.782073180641
44,0.282897515283963,0.777255399333034,0.944054124535975
"12878","AL512506.1",2.72003602412216,-0.251039865640221,0.88845846545
5998,-0.282556670233735,0.77751669937007,0.944054124535975
"12879","FAH",22.1788167251786,-0.136103775584755,0.481680636513208,-0
.282560197084076,0.777513995472623,0.944054124535975
"12880","AC124283.1",11.8950327061223,0.137835751741685,0.487538006952
054,0.282717962038271,0.777393046043696,0.944054124535975
"12881","AC012615.1",20.6299593355352,-0.141335107454927,0.49999708876
0379,-0.282671860760896,0.777428388716644,0.944054124535975
"12882","IKZF1",13.1551905874673,0.167324677732566,0.592521249406323,0
.282394391593917,0.777641114934711,0.94413189237532
"12883","UBE2L5",3.08897197448686,0.252221641174662,0.893787785625884,
0.282194101587594,0.777794680862661,0.944245036768766
"12884","CUL7",42.5752797644404,0.0804550911923396,0.285396147593523,0
.281906717630009,0.778015038440427,0.944421166046746
"12885","AC113139.1",6.05881142033027,-0.192644244776727,0.68350551547
5547,-0.281847388813966,0.778060532257821,0.944421166046746
"12886","STPG1",11.3175671961459,-0.172914472906451,0.6174822071886,-0
.280031506808481,0.779453332575074,0.944474206192945
"12887","MIER1",525.148250571026,-0.0383202393852731,0.136368988431006
,-0.281004059839166,0.778707285947323,0.944474206192945
"12888","ANP32E",260.399542964668,0.0479878233872418,0.171096838602785
,0.280471712856422,0.779115624761218,0.944474206192945
"12889","YY1AP1",160.994335991881,0.0482582258052721,0.172913283015141
,0.279089176746742,0.780176388968353,0.944474206192945
"12890","AFTPH",151.34137788701,0.059096903631595,0.211558843662868,0.
279340265849483,0.779983707947143,0.944474206192945
"12891","LINC00342",394.507313306502,-0.0975163848305151,0.34824584759
7244,-0.280021672916817,0.779460877204338,0.944474206192945
"12892","FILIP1L",2041.38767545947,-0.0624551776256257,0.2226449534247
38,-0.28051467893135,0.779082665203705,0.944474206192945
"12893","KLHL2",69.7576041782994,-0.0705243105887035,0.252054846787958
,-0.279797478554469,0.779632886294435,0.944474206192945
"12894","U91328.1",6.96051344777937,-0.151789323211586,0.5440401531221
83,-0.279003897672781,0.780241833586706,0.944474206192945
"12895","FYN",1575.90754849807,-0.0448923608652033,0.159637570629747,-

0.281214257321189,0.778546069969116,0.944474206192945
"12896","AL355312.2",15.1764562134169,0.139165608574846,0.498856180601905,0.27896939836835,0.780268309384763,0.944474206192945
"12897","CCN4",6.09864729265841,0.222668007564609,0.790873557271974,0.281546911661425,0.77829095225567,0.944474206192945
"12898","ACBD5",164.433344568846,0.0539745625516095,0.193104994774772,0.279508889009126,0.779854317313097,0.944474206192945
"12899","ALDH18A1",107.969559449867,0.0744952565138839,0.264940481404411,0.281177327522753,0.778574393470038,0.944474206192945
"12900","RRP8",99.8683968213489,-0.0541061090335672,0.19343722426657 7,-0.279708878364607,0.779700866170907,0.944474206192945
"12901","CWC15",673.727442536616,-0.0649364064100772,0.230814366083086,-0.281336068945995,0.778452647995552,0.944474206192945
"12902","DDX10",89.870757383158,0.069498026775904,0.248422690246486,0.279757161903961,0.779663819663938,0.944474206192945
"12903","AP002884.3",5.49423184952506,0.206686311866921,0.73537877895664,0.281061022946799,0.778663595790765,0.944474206192945
"12904","DNM1L",236.268736183337,0.0593285157621375,0.212617560593828,0.279038643828085,0.780215168605487,0.944474206192945
"12905","AC023509.2",4.36904638743237,0.252183226870914,0.898900895028249,0.280546196211084,0.779058488344548,0.944474206192945
"12906","FNTB",30.5633531492233,-0.0882837799896419,0.315102981158489,-0.28017437240696,0.779343727449927,0.944474206192945
"12907","PARP6",82.7764567498212,-0.0679607930085362,0.24205587687475,-0.280764895634829,0.778890730226139,0.944474206192945
"12908","LYSMD4",50.5450402725777,0.0989167312762161,0.352100676258593,0.280933090862821,0.778761719449093,0.944474206192945
"12909","NSMCE1-
DT",19.6977812263797,0.133246365066959,0.475060122624298,0.280483161438363,0.779106842440201,0.944474206192945
"12910","AC020978.5",4.19640795017852,-0.299076065323255,1.0718458962096,-0.279028978308249,0.780222586116888,0.944474206192945
"12911","MOCOS",7.30866366636388,-0.17660141032536,0.632184166011217,-0.27935120779698,0.779975311611108,0.944474206192945
"12912","SAMD1",228.971283601464,-0.0522868715919227,0.186421040128314,-0.280477308548078,0.779111332246998,0.944474206192945
"12913","ZNF14",28.7184370733933,-0.0903699242312718,0.32325359395843,-0.279563556044773,0.779812370633171,0.944474206192945
"12914","KPTN",29.0659075121759,0.0942066758177347,0.33599398831643,0.280382027933825,0.779184423932542,0.944474206192945
"12915","NDRG3",95.0357059567018,-0.0602860069823505,0.215066522755519,-0.280313301251826,0.779237146772042,0.944474206192945
"12916","AL162458.1",18.845101120723,0.11050606214739,0.393868041096787,0.280566206488011,0.779043138601348,0.944474206192945
"12917","CEBPB-
AS1",9.1629472125992,0.149433666777867,0.53483060244976,0.279403732870548,0.779935006696619,0.944474206192945
"12918","ACO2",183.477901358656,-0.0567081831672248,0.203286834755381,-0.278956496299738,0.780278210883571,0.944474206192945
"12919","BTK",2.73910272989895,0.255045757746258,0.913743324803821,0.2

79121883381218,0.78015128975148,0.944474206192945
"12920","TMSB15B",8.17214338779074,0.146198271748353,0.524028516997967
,0.278989152319205,0.780253149580876,0.944474206192945
"12921","LINC00892",3.01376492854679,0.294489487859488,1.0550121131445
5,0.27913375040002,0.780142183005081,0.944474206192945
"12922","PLAG1",2.82883818861916,-0.247085829845511,0.886586110558804,
-0.278693549225327,0.780480013429105,0.944515906532724
"12923","AC026401.1",2.75007282588578,-0.315303864205043,1.13110735099
287,-0.278756798749716,0.780431470313046,0.944515906532724
"12924","KCTD13",50.3265601884653,0.0766777424152258,0.275150600552947
,0.27867554081704,0.780493834784459,0.944515906532724
"12925","APLF",22.790901073387,0.100621927713839,0.361309947816996,0.2
78491993707309,0.780634710147823,0.944613297231099
"12926","AL137127.1",3.18304667563655,0.238876249363525,0.860120048975
018,0.277724312609835,0.781223995751209,0.944741613975791
"12927","MCM3",129.346834649135,0.0524400906030801,0.188757428992536,0
.277817359999927,0.78115256422878,0.944741613975791
"12928","FAM225A",12.5607230028324,0.128147305539328,0.460864437202646
,0.278058568192322,0.780967399784434,0.944741613975791
"12929","TMEM216",118.854168362559,-0.0818436741993125,0.2945331158922
14,-0.277875966345542,0.78110757368899,0.944741613975791
"12930","CALML4",21.2278599360378,-0.0939188053966623,0.33814957838695
2,-0.277743375711647,0.781209361054041,0.944741613975791
"12931","AC103858.1",3.01308872006991,0.310013493808534,1.115010490249
99,0.278036392051369,0.780984422870486,0.944741613975791
"12932","FBXO46",32.9942369508927,0.0945812324771134,0.340032186860939
,0.278153763472379,0.780894326159868,0.944741613975791
"12933","SCAF1",169.98276972479,0.0523940231786639,0.188479402745002,0
.277982752574555,0.78102559861225,0.944741613975791
"12934","MEIS1",66.5700978731043,0.0689120567928129,0.24822309847829,0
.277621451086834,0.781302963630543,0.944764059933639
"12935","HYPK",19.7681507137566,-0.113127697740886,0.40772076780766,-0
.277463663058373,0.781424103551756,0.944786228043708
"12936","ARFRP1",235.94463758126,0.0622494547523351,0.224434353764691,
0.277361525578213,0.781502521240502,0.944786228043708
"12937","CLIC2",180.98153928625,-0.0619530917859167,0.223359484905226,
-0.277369424505094,0.781496456633416,0.944786228043708
"12938","INPP5D",6.00589940133716,-0.228384785206519,0.824385621260872
,-0.27703635206205,0.781752193214724,0.944850943253565
"12939","EXOSC9",101.866517733671,-0.0528806810098066,0.19086469329465
6,-0.277058475808146,0.781735205634055,0.944850943253565
"12940","FTH1",35385.7216346573,-0.0827118442670161,0.298623419940382,
-0.276977084662445,0.781797701831483,0.944850943253565
"12941","AC120049.1",9.87323818206628,-0.155802850403675,0.56218597223
7434,-0.277137563186783,0.78167447973435,0.944850943253565
"12942","HNMT",549.349702262786,-0.0685031879415081,0.247607205234667,
-0.276660721066598,0.782040635020425,0.944925489162504
"12943","ZBED3",85.2480091437407,0.0645226856024901,0.233178665351274,
0.276709215679271,0.782003394997267,0.944925489162504
"12944","TECPR2",91.646468794142,0.0569471589666898,0.205750443985129,

0.276777818135867,0.781950714595436,0.944925489162504
"12945","CHKA",24.8984855065667,0.0974393905318304,0.352365977040466,0
.276528941160061,0.782141834078843,0.94496447244107
"12946","TIMM21",84.1585163099678,-0.0577084781388741,0.20873976474812
1,-0.276461354684906,0.782193737865862,0.94496447244107
"12947","CIAO1",351.596262009832,0.0367906492622634,0.133143211748208,
0.276323882976771,0.782299313788893,0.944978084190475
"12948","MRPS24",1271.65737936653,0.0769178240366883,0.278395920904651
,0.276289335658163,0.782325846169966,0.944978084190475
"12949","SKI",472.118526176534,0.0536013440020933,0.194123306789889,0.
276120085158601,0.782455834410461,0.944989131288001
"12950","HELQ",107.543368802364,0.0665911907161282,0.241150484608947,0
.27613956830364,0.782440870601624,0.944989131288001
"12951","EIF4E3",99.1209249086126,0.0571410535945439,0.207061488139376
,0.275961764343553,0.782577433903411,0.945063011833013
"12952","NT5C3A",200.397640999374,-0.0431570846107563,0.15646470005869
6,-0.275826333956263,0.782681456447407,0.945094648696328
"12953","AL353796.1",3.82426556320972,0.211252761611293,0.766103080377
742,0.275749787492214,0.782740252636976,0.945094648696328
"12954","ANKRD49",128.056591029558,0.0665730137275803,0.24147635818287
7,0.275691642148929,0.782784915550654,0.945094648696328
"12955","PANK2",154.827774058731,0.0490500003434659,0.178024587674565,
0.275523740760636,0.78291388888585,0.945177400399437
"12956","RNF26",85.1866680520285,0.0655092262992289,0.238085051498918,
0.275150522415417,0.783200597666283,0.945450551674952
"12957","MXRA8",3660.75044042618,0.0627567921484645,0.228831272566479,
0.274249194371949,0.783893125125452,0.945689707626402
"12958","IKBKE",28.0856513923365,-0.0832699979860425,0.303897947915559
,-0.274006450379783,0.784079664617707,0.945689707626402
"12959","MAP1LC3C",11.5781438387588,0.199235349583939,0.72745363853244
2,0.273880477092498,0.784176475171477,0.945689707626402
"12960","TLK1",289.885998105301,-0.0472751527212227,0.172357774684453,
-0.274285002853933,0.783865608724672,0.945689707626402
"12961","HMX1",16.3911460258757,-0.221906284195054,0.808564944399507,-
0.274444601799867,0.783742970997169,0.945689707626402
"12962","SCOC",355.436371471331,-0.0619345227937883,0.226323941232866,
-0.273654313619713,0.784350290328866,0.945689707626402
"12963","FEM1C",338.890110872747,0.096673460745524,0.353034231419164,0
.273835940375827,0.784210702466667,0.945689707626402
"12964","STX7",353.544571828507,-0.0421728746128316,0.154043828949095,
-0.273771918684051,0.784259905058971,0.945689707626402
"12965","FSCN1",199.872335022815,-0.111523994936578,0.407533467565862,
-0.27365604008597,0.784348963433685,0.945689707626402
"12966","IL11RA",279.34991222223,0.0850718408600641,0.310928830491672,
0.273605508776847,0.784387800102532,0.945689707626402
"12967","AC067750.1",12.9112549371538,0.111848806723927,0.408597909921
557,0.27373807845811,0.784285912637025,0.945689707626402
"12968","TIMM8B",692.222110407156,-0.0633167948033333,0.23189894838814
,-0.27303614459414,0.784825431399006,0.945689707626402
"12969","AC092794.1",4.16644850935006,-0.20401395037371,0.743772809521

018,-0.274296058906876,0.783857112949041,0.945689707626402
"12970","ALG10",19.0385752966333,-0.100670651709217,0.367077940122012,
-0.274248710439412,0.783893496996784,0.945689707626402
"12971","BCDIN3D",66.3146412053911,0.0800188643148741,0.29283456781473
8,0.273256210535562,0.784656273690956,0.945689707626402
"12972","COQ5",150.502512117057,-0.0718479910033366,0.26159468782499,-
0.274653860904866,0.783582181952154,0.945689707626402
"12973","MLEC",526.231908256049,-0.0409880738031944,0.150015201859734,
-0.273226135052091,0.784679391161108,0.945689707626402
"12974","KDM2B",43.3435459634016,0.0688886489300501,0.252400552027863,
0.272933828300206,0.784904082150298,0.945689707626402
"12975","WDR66",4.99038416011104,0.202033739879119,0.740251915665064,0
.272925656257986,0.784910364111146,0.945689707626402
"12976","NGRN",567.150338562086,-0.042630356272386,0.155424333667985,-
0.274283667597714,0.783866634774075,0.945689707626402
"12977","ALDH1A3",1043.23525974287,0.0819544822450818,0.29998061033000
8,0.273199264962238,0.784700044971483,0.945689707626402
"12978","RNF166",75.4238123638797,-0.0617482059420868,0.22618193844565
9,-0.273002373073755,0.784851391395184,0.945689707626402
"12979","CD40",210.019968155556,-0.0596640714087256,0.218206226910202,
-0.273429737792401,0.784522895965705,0.945689707626402
"12980","FP565260.6",13.4749018168085,-0.132188130850014,0.48322426690
6386,-0.273554413349904,0.784427070879379,0.945689707626402
"12981","DDTL",6.76529568116529,-0.231760242743837,0.848726867854258,-
0.273068111216711,0.784800859039134,0.945689707626402
"12982","FMO1",4.90697201236569,-0.207017908849965,0.760364418990953,-
0.27226143633166,0.785421005886968,0.945796835188087
"12983","UTP3",138.301008768678,-0.0495671145862605,0.182111039191082,
-0.272180724498814,0.785483062164837,0.945796835188087
"12984","CDH23",25.3342487374344,0.101127341222873,0.371541433106957,0
.272183213530754,0.785481148421874,0.945796835188087
"12985","UQCC3",295.571359086246,-0.0706684045081521,0.259212304109654
,-0.272627507983793,0.785139564352809,0.945796835188087
"12986","AMIGO2",53.3820177081216,0.0913902500728041,0.335706372122805
,0.272232693990606,0.785443104630401,0.945796835188087
"12987","FZR1",97.5608012800038,-0.0582040009809659,0.213743655129288,
-0.272307502862529,0.785385587683654,0.945796835188087
"12988","SIRT6",74.5313369685711,-0.0853212663407035,0.312927599355596
,-0.272654973599016,0.785118449496335,0.945796835188087
"12989","ZNF461",12.2924406799775,-0.1778298245273,0.652864271611699,-
0.272384065509205,0.785326723556967,0.945796835188087
"12990","NIPAL3",31.0434768664051,-0.0843108924205039,0.31152257120760
5,-0.270641360250964,0.78666688167494,0.945843702455725
"12991","NRAS",130.073149749609,-0.0480761514887773,0.177693017372387,
-0.27055734772079,0.786731504171773,0.945843702455725
"12992","AC082651.1",7.26203020705826,0.163948341101849,0.604061726237
397,0.271409913889193,0.786075777936047,0.945843702455725
"12993","LINC02580",4.03185606726629,0.236845410176741,0.8740979872709
93,0.270959793553801,0.786421955898815,0.945843702455725
"12994","MRPS5",283.305057297732,-0.0453858548984838,0.167309419943403

,-0.271268975254572,0.786184166293503,0.945843702455725
"12995","AC022382.1",4.71893962541415,0.191178408270073,0.704225084511435,0.271473442901719,0.786026922529613,0.945843702455725
"12996","AC084198.2",14.9576852525891,0.148949314618106,0.550169608961035,0.270733446908105,0.78659605024195,0.945843702455725
"12997","RBP1",2.54956213604816,0.270460652798607,0.998358901669866,0.270905234927271,0.786463918646854,0.945843702455725
"12998","ABCC10",31.8427443327185,-0.0817915851337234,0.301685189192505,-0.271115679734388,0.786302062374874,0.945843702455725
"12999","HSF2",68.6600350035202,0.0581957642821468,0.21461800722197,0.271159745798764,0.786268171669854,0.945843702455725
"13000","AC013643.2",2.91329991348571,-0.259722922210492,0.955884985793745,-0.271709385616956,0.785845484031924,0.945843702455725
"13001","PARG",71.3311828716516,0.0655448964962191,0.241899665913578,0.270959020338771,0.786422550598373,0.945843702455725
"13002","FADS1",65.5574253244007,0.151220652199253,0.55595076602291,0.272003676298598,0.785619192609298,0.945843702455725
"13003","LINC02334",2.53104151647581,-0.244565917011328,0.899829217202857,-0.271791482578847,0.78578235470007,0.945843702455725
"13004","TMEM62",31.6373443966838,0.0856139380342103,0.315602264407915,0.271271621560847,0.78618213112286,0.945843702455725
"13005","AC010931.2",5.34236707878322,0.304129696616606,1.12117941242397,0.271258723846063,0.786192050264581,0.945843702455725
"13006","AC091271.1",57.5668886495657,0.0873507044457797,0.32202737660029,0.271252417629704,0.786196900148283,0.945843702455725
"13007","AC024075.2",58.8618946052084,-0.0710821681516574,0.262474606608936,-0.270815409802913,0.786533007313766,0.945843702455725
"13008","DBP",106.293259793289,-0.141727675845251,0.523804867925852,-0.270573422516022,0.786719139314411,0.945843702455725
"13009","DZANK1",14.8366739972994,0.107155276409217,0.395999200923125,0.270594678371633,0.786702789227732,0.945843702455725
"13010","MMUT",171.94181908396,0.0442365719853863,0.163657498396148,0.270299695515983,0.786929699585944,0.945936553802488
"13011","AC105219.1",7.62216502117908,-0.164189133269065,0.60737659231614,-0.270325092119461,0.786910162984644,0.945936553802488
"13012","STEAP1",81.143278617833,-0.135664915613586,0.503173752678113,-0.269618426818803,0.787453822629696,0.946348377587863
"13013","SYCE1",9.20455494708871,0.23327647451506,0.864808205568099,0.269743595184575,0.787357519147301,0.946348377587863
"13014","RRN3",114.889570290287,0.058261900211158,0.216050248320224,0.269668286262757,0.787415460805509,0.946348377587863
"13015","PDZRN4",17.2964077842622,0.188111420940304,0.698351223354643,0.269365062520661,0.787648768924877,0.946437211584594
"13016","ANKRD9",143.997928672379,0.0675455859607098,0.25074701101814,0.269377432203263,0.787639251001043,0.946437211584594
"13017","XCL1",6.28735489611782,0.271022069904834,1.00735957340053,0.269042035298229,0.787897335313575,0.94647109704023
"13018","AL121821.2",5.9902665873116,0.247701230421541,0.920775049180816,0.269013838550374,0.787919033469552,0.94647109704023
"13019","AC010809.2",3.9973804025049,0.205948754633066,0.7652270354516

63,0.269134185139588,0.787826424679867,0.94647109704023
"13020","SLC37A1",26.0006827132756,-0.0842499866532798,0.3130270701020
85,-0.269146009084148,0.787817326111064,0.94647109704023
"13021","AL355312.3",11.0540696084982,-0.159179618277833,0.59200997308
7134,-0.268879960666481,0.788022058348214,0.946522155945478
"13022","MAP3K2",437.855218550868,0.0581981134081502,0.216572614547374
,0.268723326491585,0.788142599894367,0.946557354042148
"13023","AP001033.2",16.1023329819159,0.127435272597229,0.474293181579
179,0.268684597516093,0.788172405478957,0.946557354042148
"13024","LYRM1",293.158064754343,0.0603278328533855,0.224614818767765,
0.268583494109354,0.78825021550901,0.946578115061495
"13025","CLEC4A",6.3028632083685,-0.174682790270423,0.651081588313139,
-0.268296314019572,0.788471243237653,0.946770844087285
"13026","PAFAH2",45.3370292059713,0.0674062152639117,0.251628936167586
,0.267879427106185,0.788792129931312,0.946811110663075
"13027","CR1",3.2748244637954,-0.333217211678824,1.24546258016119,-0.2
67544940319041,0.789049617415762,0.946811110663075
"13028","C5orf34",11.6453493685117,-0.122334119701459,0.45712820315677
5,-0.267614465387741,0.78899609521225,0.946811110663075
"13029","AL138756.1",3.90093231025595,-0.235213728563455,0.87879007951
5309,-0.267656331183421,0.788963866315799,0.946811110663075
"13030","CNTRL",121.768005803975,0.0626872115312329,0.233961589088916,
0.267938048187085,0.788747005876268,0.946811110663075
"13031","DCLRE1C",129.141122703808,0.0669560396239005,0.25016190616251
7,0.267650821226165,0.788968107940138,0.946811110663075
"13032","PELP1",108.38800893168,-0.0550756980554696,0.205793938364829,
-0.267625463087411,0.788987628988637,0.946811110663075
"13033","PRPF6",339.596615516614,0.0452244266099777,0.168920704789719,
0.267725775039095,0.788910408225326,0.946811110663075
"13034","AIFM3",3.04729093354984,0.236972051171778,0.884125848803794,0
.268029773693866,0.788676400841638,0.946811110663075
"13035","AC079949.6",10.1386382729635,0.255626230512476,0.956562149295
937,0.267234314780933,0.789288757179458,0.946828382001976
"13036","ZC3H13",615.956722595449,0.0498904282113613,0.186707497410371
,0.2672117022794‌5,0.789306166530804,0.946828382001976
"13037","HEXA",802.32238659274,0.0951212827831073,0.355707503714701,0.
267414327192266,0.789150169484733,0.946828382001976
"13038","PSMD10",131.130968087869,0.0500979417082562,0.187382372401653
,0.267356747948902,0.789194497783828,0.946828382001976
"13039","PTPRC",65.7960727381548,-0.145096192253774,0.543222895318724,
-0.267102497895715,0.789390244400988,0.946856616491407
"13040","AL603840.1",4.74005882133866,-0.272193545953455,1.02431226814
766,-0.265732974618845,0.790444866049763,0.946957179911141
"13041","AC093157.1",34.820514022615,-0.0931196066406589,0.34957235988
1535,-0.266381491580787,0.789945418011838,0.946957179911141
"13042","GPR155",55.0090057888534,0.0954433825095017,0.358532124456624
,0.266205943621235,0.790080605788946,0.946957179911141
"13043","GHRLOS",3.57174891503349,-0.225293486097665,0.846087949288995
,-0.266276675240427,0.790026135296442,0.946957179911141
"13044","SACM1L",127.07379538487,0.0588911491831414,0.221427982691346,

0.26596073570896,0.790269448593189,0.946957179911141
"13045","ABRAXAS1",298.991248621899,0.0571899953817879,0.214611938290668,0.266480960179999,0.789868821016053,0.946957179911141
"13046","C5orf22",40.3588659861044,0.0695573720923453,0.261969856152186,0.265516701478575,0.790611445523514,0.946957179911141
"13047","TNF",10.8495405231678,-0.169651018545641,0.637460087756968,-0.26613590686531,0.790134542177116,0.946957179911141
"13048","REV3L",588.929807170678,-0.0570429176437531,0.214232541400212,-0.266266353705762,0.790034083856954,0.946957179911141
"13049","ARHGAP39",6.98789053936952,-0.161392310770526,0.607038793170109,-0.265868199176686,0.790340717269259,0.946957179911141
"13050","ZBTB43",171.231203252227,0.0639200785534944,0.240753790946379,0.265499780095802,0.790624479237831,0.946957179911141
"13051","GTF3C5",104.78425011783,-0.0513551236443784,0.19244456060531,-0.266856716983049,0.789579483280512,0.946957179911141
"13052","SMAD9",43.3397854073336,0.133742861541878,0.503211768737774,0.265778485025004,0.790409813912124,0.946957179911141
"13053","RAP2A",191.774035204105,-0.0446236395052675,0.167848335108802,-0.265856908716679,0.790349412940302,0.946957179911141
"13054","MMP25-
AS1",12.2374777926761,-0.111606043381043,0.420272500958681,-0.26555637860307,0.790580884402907,0.946957179911141
"13055","EFTUD2",90.872195838615,-0.0562870220451709,0.211627996612062,-0.265971529978386,0.790261135306506,0.946957179911141
"13056","AC087289.2",2.64973578149449,-0.241131275444457,0.903711583737035,-0.266823265059112,0.789605240530969,0.946957179911141
"13057","ZNF614",32.0772030997129,0.0835923939909616,0.314024159852351,0.266197333448055,0.790087236562176,0.946957179911141
"13058","EFHC2",3.23317335039449,-0.249110234281422,0.936270363033143,-0.266066559529241,0.79018794862295,0.946957179911141
"13059","BMP8A",2.76216452351386,0.274196257365274,1.03550602315441,0.264794459166933,0.791167805531193,0.947170990596104
"13060","MGAT5",39.7686291276947,-0.0911780562226448,0.344277336072241,-0.26483897331979,0.79113351216668,0.947170990596104
"13061","BBS5",60.1905924546202,-0.0677963813127198,0.256582582828086,-0.264228306401235,0.791604000554703,0.947170990596104
"13062","ATP5ME",1645.07814641316,-0.0694999871020227,0.262851743338738,-0.264407556211099,0.791465889641605,0.947170990596104
"13063","ZBTB49",15.7127616458248,-0.108948156265283,0.412534287910671,-0.264094790319281,0.791706878153411,0.947170990596104
"13064","INTS12",63.5605726759351,-0.0620572817664498,0.234242208367714,-0.264927837723559,0.791065052915664,0.947170990596104
"13065","RAB23",386.314388064369,-0.0445834236974408,0.16839509367957,-0.264754885212251,0.791198293344059,0.947170990596104
"13066","AC102953.2",5.15621329361762,-0.18180369856967,0.688418510387128,-0.264088916591499,0.791711404096092,0.947170990596104
"13067","UBE2D4",236.594380535519,-0.0634605983955847,0.240096903257572,-0.264312440246283,0.791539175112289,0.947170990596104
"13068","ORAI1",232.053559413167,0.0643973369264007,0.243819080648893,0.26411934929381,0.791687954555237,0.947170990596104

"13069","SCARB1",16.6821052973755,0.139480198292123,0.527715777001032,
0.264309320226844,0.791541579073206,0.947170990596104
"13070","LRP10",1022.37436761364,0.0348559812706884,0.131468464438303,
0.265128077821628,0.790910798101339,0.947170990596104
"13071","NPIPB11",9.94897582296146,0.167294645915697,0.632723305534357
,0.264404115436226,0.79146854067659,0.947170990596104
"13072","NSF",174.927284044114,-0.0476428610876837,0.179946619134694,-
0.264761079239961,0.791193521438049,0.947170990596104
"13073","PLS3",690.195600449687,0.0436413044137455,0.165245769359132,0
.264099375027865,0.791703345456566,0.947170990596104
"13074","IQCC",6.48048543125245,0.193453045195308,0.733404361984499,0.
263774058654149,0.791954025027669,0.947306592341119
"13075","UFSP1",5.92620936051747,0.179034727487452,0.678605188731748,0
.263827525135863,0.791912823795378,0.947306592341119
"13076","SLC12A4",210.838032977484,-0.0496605392698431,0.1883686385385
8,-0.263634857984453,0.79206129568891,0.947306592341119
"13077","ZNF24",288.713760951791,0.0355797254146295,0.13497096911715,0
.263610209272096,0.792080290861816,0.947306592341119
"13078","ATG4D",34.7482136383977,-0.0704276335085655,0.267228031458343
,-0.263548824291452,0.792127596843808,0.947306592341119
"13079","C2orf92",4.29138701589315,-0.207940002875731,0.79118284715951
5,-0.262821677216931,0.792688026733361,0.947325354290505
"13080","TRNT1",85.6376977235438,-0.0518659883756188,0.197021442687383
,-0.263250475015124,0.792357528892446,0.947325354290505
"13081","C4orf36",10.2584257397693,0.154378471478059,0.587916508528541
,0.262585706028978,0.792869918485748,0.947325354290505
"13082","ARFIP1",239.948246790904,-0.0388138860519135,0.14785365383070
7,-0.262515568917596,0.792923983878392,0.947325354290505
"13083","CFAP97",471.672783987116,-0.0435561235095791,0.16546956932515
3,-0.263227393938459,0.792375317787138,0.947325354290505
"13084","CCDC91",177.765116081724,-0.060170734189002,0.228397394744147
,-0.263447550513463,0.792205644570664,0.947325354290505
"13085","TMEM116",47.8509352291933,0.101929256198496,0.388213386173451
,0.262559869980771,0.792889834161481,0.947325354290505
"13086","TMEM159",797.212354741307,-0.0373477864990332,0.1419496396868
31,-0.26310589150793,0.792468963118231,0.947325354290505
"13087","NSMCE1",412.997093217288,0.0682918829203127,0.259987675998151
,0.26267353888266,0.792802213683873,0.947325354290505
"13088","RAI1",55.8999696533111,0.073323307710689,0.278632902590667,0.
263153802113623,0.792432036713066,0.947325354290505
"13089","ACTL10",15.469417395594,-0.116823081556507,0.445028695816765,
-0.262506851029236,0.792930704157097,0.947325354290505
"13090","TGIF2",15.3589922586993,0.108517867387405,0.412746395997107,0
.262916571628078,0.79261488322564,0.947325354290505
"13091","MAGED2",921.160253807038,-0.0716376205713445,0.27250030567663
7,-0.26289005582384,0.792635321115266,0.947325354290505
"13092","AC092910.3",4.22016429006973,0.208214365455231,0.793767467307
874,0.262311538367032,0.793081267070119,0.947371492879907
"13093","TMEM241",34.4990211627698,0.0903167997991113,0.34432686814224
7,0.262299600046895,0.793090470350168,0.947371492879907

"13094","NIBAN1",513.104019114698,0.0438229477548387,0.167185246398093,0.262122099281954,0.793227309520867,0.947394673544145
"13095","OTULIN",149.056816564715,0.0684388143176927,0.261099973130117,0.262117278287067,0.793231026218707,0.947394673544145
"13096","CCDC30",53.2515463608056,-0.0773865668373215,0.297215570710402,-0.26037184610602,0.794576957750007,0.947412071439613
"13097","SETDB1",57.1693382296457,0.0669740402857596,0.255943047013421,0.261675560509552,0.793571584044834,0.947412071439613
"13098","TMEM150A",82.5859374016174,0.0790989928778139,0.303194589377794,0.260885238882852,0.794181008432252,0.947412071439613
"13099","SRD5A3",119.996449922884,-0.068450121893197,0.261581075672636,-0.261678417359447,0.793569381329578,0.947412071439613
"13100","GSTCD",35.0578671241365,-0.0873788065556428,0.335402105420682,-0.260519552929004,0.794463034833504,0.947412071439613
"13101","SEC24B-AS1",11.6961724669613,0.119376496881833,0.458148776551626,0.260562732002366,0.79442973262325,0.947412071439613
"13102","ELOVL6",108.13537386633,0.0787188845197187,0.30194254699385,0.260708155586042,0.794317576262052,0.947412071439613
"13103","PLK4",5.16248518733756,-0.183691428605893,0.704722843457232,-0.2606576901988,0.794356496662811,0.947412071439613
"13104","RANBP3L",3.13465737539695,-0.213440352942598,0.815073003676896,-0.261866546897937,0.793424331550156,0.947412071439613
"13105","LCP2",15.7737723470786,-0.13746084025911,0.525793044582852,-0.261435257988563,0.793756870245957,0.947412071439613
"13106","STXBP5-AS1",3.5231721226863,-0.213684481905571,0.818471046346529,-0.26107763110181,0.794032641409614,0.947412071439613
"13107","MIOS",48.992251764118,-0.0635528646630736,0.243205490373836,-0.261313445536881,0.793850798661137,0.947412071439613
"13108","DOCK8",51.2046608328028,0.110558593359074,0.424075863579916,0.260704753215081,0.794320200255174,0.947412071439613
"13109","ITGB1-DT",5.41502296151957,0.208635761103638,0.797634807159333,0.261568024904361,0.793654498355219,0.947412071439613
"13110","AC010864.1",3.77534779276731,0.219719629327352,0.842601373014729,0.260763436144449,0.794274942846414,0.947412071439613
"13111","PTDSS2",91.6945855245027,-0.0674087894699492,0.257766948825617,-0.261510600086873,0.793698776172433,0.947412071439613
"13112","RELA-DT",9.63333853209837,-0.156403805722404,0.598939785634841,-0.261134440345494,0.793988833275098,0.947412071439613
"13113","KTN1",1089.16657625463,-0.0503406505909428,0.192775326713133,-0.261136378027538,0.793987339053752,0.947412071439613
"13114","AC109460.2",3.83747528399832,-0.232560671188362,0.891796791792843,-0.260777649492132,0.794263981342285,0.947412071439613
"13115","COA3",520.180207180784,-0.0723140376242518,0.276405777202656,-0.26162274304141,0.793612308162148,0.947412071439613
"13116","TBC1D17",168.593427054788,0.0513588789368109,0.197253333172043,0.260370144883767,0.794578269889604,0.947412071439613

"13117","ZNFX1",215.899012455815,-0.0537360021732447,0.206100932433042
,-0.260726633008817,0.794303326054613,0.947412071439613
"13118","CYFIP1",470.12428051236,0.0319927939438811,0.122921892535207,
0.260269292019872,0.794656057963734,0.947432592358042
"13119","TMEM132C",16.3193901717005,-0.150458746009152,0.5782886968950
87,-0.260179295941605,0.794725473895118,0.947443129180551
"13120","COMMD4",376.968839736393,-0.0755115331507328,0.29041215345175
9,-0.260015058781885,0.794852157775753,0.947449717827359
"13121","FAM210B",432.825598661733,0.0498009123955655,0.19148207730611
1,0.260081325083767,0.794801042795143,0.947449717827359
"13122","AC233723.1",13.2120821192767,0.166919807653368,0.642525626482
728,0.259787004243098,0.795028075986928,0.947541415697476
"13123","AC092296.2",7.19323634687708,0.191363851296018,0.736699795477
46,0.259758252236237,0.795050255639257,0.947541415697476
"13124","SLC27A5",50.4865160499855,-0.0688710507020757,0.2654645611759
19,-0.259435950309149,0.795298894648832,0.94776552212037
"13125","GPATCH3",36.1109231947261,-0.0720086042357035,0.2795775140277
89,-0.257562216640019,0.79674479278724,0.947804759093111
"13126","PTBP2",82.2733264688203,0.0657516549195941,0.254515942912536,
0.258340024468288,0.796144499276706,0.947804759093111
"13127","AMIGO1",7.52151250254231,0.229451973234622,0.891092760685567,
0.257495048055481,0.796796637543421,0.947804759093111
"13128","CAPZA1",547.546664951073,-0.0390606423089681,0.15159481003882
5,-0.257664772949445,0.796665635385399,0.947804759093111
"13129","WDR3",78.7105108495401,0.0606073035860235,0.233731382748215,0
.25930323465083,0.795401283853735,0.947804759093111
"13130","NBPF20",27.0883330958157,0.126840721282651,0.490198527753643,
0.258753778522967,0.795825222898504,0.947804759093111
"13131","ORMDL1",460.313948506382,0.0478323406134946,0.185983214403178
,0.257186331395492,0.797034935170566,0.947804759093111
"13132","AP1S3",4.17185369261261,-0.234804371676882,0.911480396030758,
-0.257607703576939,0.796709683740979,0.947804759093111
"13133","NBEAL2",24.5523413738568,-0.111507950410942,0.430840873934964
,-0.258814697390513,0.795778217289036,0.947804759093111
"13134","DTX3L",140.128767206452,0.0586716141928281,0.227322197034615,
0.258098922842515,0.796330562871937,0.947804759093111
"13135","CAMLG",1002.04624759338,-0.0538359694139806,0.209081374346101
,-0.257488117161808,0.796801987275345,0.947804759093111
"13136","PAIP2",817.694868145167,0.0418544094070111,0.162036224317547,
0.258302793608594,0.796173230419556,0.947804759093111
"13137","PCDHGB6",29.9111032547987,0.0945425887964482,0.36676285575473
5,0.257775800665244,0.796579941743231,0.947804759093111
"13138","ARMT1",120.002485338885,-0.0531943442809884,0.20619579453999,
-0.257979773058232,0.796422517739558,0.947804759093111
"13139","GARNL3",32.5612712303413,-0.0785904114358411,0.30510994826304
,-0.257580625880108,0.796730583584342,0.947804759093111
"13140","ATG13",142.032486634432,-0.0534032338761931,0.207455003837695
,-0.257420803973346,0.796853944637842,0.947804759093111
"13141","SPINDOC",29.782708490347,-0.076266095480287,0.29648300414559,
-0.257235977826358,0.796996611935947,0.947804759093111

"13142","AP000873.2",49.1250934763585,-0.100933636888846,0.389613426827476,-0.259060981831973,0.795588189299117,0.947804759093111
"13143","AC022364.1",7.12531754596919,-0.146245547001439,0.565767526815564,-0.258490528476574,0.796028357792899,0.947804759093111
"13144","ALOX5AP",22.2113348357737,0.116710174428795,0.453920382350392,0.257115959024513,0.797089258078752,0.947804759093111
"13145","AC123768.4",7.50714041722902,0.155230616394771,0.603357196221315,0.257278138666356,0.7969640673873,0.947804759093111
"13146","AC093249.6",8.76704521656839,-0.138796884828459,0.537877509774592,-0.258045525804983,0.796371772143883,0.947804759093111
"13147","RPAIN",250.523551660659,0.0584686336109504,0.227212451804274,0.257330234970206,0.796923854001196,0.947804759093111
"13148","TMEM106A",42.6637620530155,0.107671175839761,0.416852031944443,0.258295912190998,0.796178540856114,0.947804759093111
"13149","ACBD4",79.1010196356596,0.0652139938492942,0.251957637024105,0.258829200890842,0.795767026351872,0.947804759093111
"13150","PSTPIP2",26.6345924627159,0.0875280104898369,0.340184933413784,0.257295376404493,0.796950761435117,0.947804759093111
"13151","NDUFA3",665.455317367078,0.0751804814471934,0.292115519753271,0.257365584378033,0.796896567931225,0.947804759093111
"13152","ZNF552",18.8808539539214,0.11538783214107,0.447150649026184,0.258051357841846,0.796367271229988,0.947804759093111
"13153","AP001505.1",14.6515398005315,0.127287606158576,0.493854174084207,0.257743303262781,0.796605023702059,0.947804759093111
"13154","TTL",179.705915465368,-0.0757133484061111,0.29471515824466,-0.25690347539999,0.79725328762515,0.947927734412144
"13155","TMEM170A",159.821042611624,0.0621796434380921,0.242231071332295,0.256695572108474,0.797413789989709,0.947991901443269
"13156","SNHG11",77.3499515607524,-0.083835113163834,0.326617744381846,-0.256676541938956,0.79742848180228,0.947991901443269
"13157","C20orf96",39.6280980754926,0.0749050185526816,0.29197593395751,0.256545180068102,0.797529898724951,0.948040405568005
"13158","DCAF8",281.423408604838,0.0559165290047513,0.219084521538772,0.255228112931089,0.798546918936509,0.948156467683416
"13159","TAF1A",45.432137498753,0.0757536826863578,0.29688395809052,0.255162600140424,0.798597515907522,0.948156467683416
"13160","TOPBP1",138.731373063027,0.0459316318066852,0.179592814456309,0.255754284745392,0.798140575557687,0.948156467683416
"13161","GFM1",134.826225330476,-0.0426702183073612,0.1665530549761,-0.256195950974807,0.797799534979089,0.948156467683416
"13162","LXN",251.559700290819,0.0903776603223025,0.353491400789839,0.255671453733707,0.798204539315569,0.948156467683416
"13163","SKIV2L",54.80121718589,-0.0643488002546094,0.251659613560564,-0.25569776311813,0.798184222537805,0.948156467683416
"13164","PSMB8-AS1",62.1750505516014,0.0661275741279157,0.258879024721188,0.25543813060612,0.798384723365091,0.948156467683416
"13165","AL021368.3",4.80363850203081,-0.243338413489403,0.953181986224775,-0.255290612921865,0.798498649605744,0.948156467683416
"13166","AL589843.1",6.14735364910387,-0.195464684130524,0.76379563913

0244,-0.255912280872807,0.798018571557236,0.948156467683416
"13167","CSNK2A2",323.936546593342,0.0497522729802274,0.194756246662554,0.255459189796521,0.798368459940412,0.948156467683416
"13168","TSEN54",28.2746450454224,0.0830763451616601,0.324864344958839,0.255726263749215,0.798162213777698,0.948156467683416
"13169","AC020916.1",906.66345442635,-0.0809658005527558,0.315921971467377,-0.25628417098276,0.797731418923691,0.948156467683416
"13170","AC074212.1",16.6848339875385,0.128374661154011,0.501591131680135,0.255934870148134,0.798001128608908,0.948156467683416
"13171","ZNF530",11.3436109361905,0.129386179834911,0.507026941578108,0.255186005367289,0.798579439438243,0.948156467683416
"13172","ZNF211",21.3018853247552,-0.0945615647248822,0.370088653844706,-0.255510574946082,0.798328776991525,0.948156467683416
"13173","UXT-
AS1",2.73155291394965,0.251546669237355,0.983380619678678,0.25579787134665,0.798106917659689,0.948156467683416
"13174","AC009948.2",5.78692441127566,0.172348788824388,0.675751770490047,0.255047483929494,0.798686424741156,0.948190047286449
"13175","SLC25A34",12.6373807994329,0.127201345792186,0.499752105778163,0.254528884063673,0.799086992421517,0.948481264176779
"13176","HNRNPLL",128.196821779502,0.0505525970677065,0.198619884743344,0.254519315289254,0.799094383861127,0.948481264176779
"13177","PARP2",77.9542326074437,-0.0621135653718445,0.244141880513132,-0.25441585540873,0.799174303025677,0.948481264176779
"13178","PMP22",5437.17778493695,0.0501812935523484,0.197196695722716,0.254473298188067,0.799129930213823,0.948481264176779
"13179","KCND3",7.64522890574326,0.152196092718643,0.602214817815222,0.252727246517772,0.800478991530918,0.948518177706156
"13180","SHC1",219.453117044873,-0.0450714992423167,0.178188915911142,-0.252942215916465,0.800312866345821,0.948518177706156
"13181","FAM228A",4.7052897501911,-0.230937021567995,0.912664984980921,-0.253035917196739,0.800240458202794,0.948518177706156
"13182","ELP6",172.201958637608,-0.054363099748381,0.214221280475045,-0.253770772109234,0.79967265488883,0.948518177706156
"13183","DHX30",165.589836448416,0.0448845559056259,0.176925504111998,0.253691835616944,0.799733641996609,0.948518177706156
"13184","EIF4G1",507.90537382459,0.0505795360687612,0.199588997021318,0.253418459051422,0.799944864847967,0.948518177706156
"13185","RAI14",47.4442097910283,-0.106668032626644,0.421235865722754,-0.253226378156627,0.800093283814944,0.948518177706156
"13186","SLC22A4",24.1615812811973,0.106391159579034,0.420769258305981,0.252849174408237,0.800384766344712,0.948518177706156
"13187","ADAT2",42.4918218668504,-0.0745528650255272,0.294957676946193,-0.25275783901406,0.80045534954938,0.948518177706156
"13188","SLC44A1",725.249333383559,-0.03410000875346,0.134212983914469,-0.254073844116238,0.799438509838002,0.948518177706156
"13189","IGSF9B",9.88609088333147,-0.192391121386251,0.760775010850589,-0.252888329193603,0.800354508363512,0.948518177706156
"13190","TNFSF13B",111.634452000711,0.0653501341356917,0.257372718468043,0.253912436891814,0.799563206352906,0.948518177706156

"13191","PPP1R3E",28.4857617679281,0.110697135351113,0.436870329074159
,0.253386709932233,0.799969396573394,0.948518177706156
"13192","CGRRF1",150.466198393719,-0.0563286068852101,0.22184716721510
4,-0.253907262338823,0.7995672040821,0.948518177706156
"13193","DENND4A",105.300314386392,0.0599363792121479,0.23678859031645
5,0.253121905629178,0.80017401171293,0.948518177706156
"13194","CD2BP2",291.423273780981,-0.0615966918682071,0.24263159881269
1,-0.253869208172506,0.799596603933016,0.948518177706156
"13195","TLCD3A",60.8388764474,0.0690230979057189,0.272201411368721,0.
253573622409404,0.799824976963556,0.948518177706156
"13196","TTC19",168.020486074429,0.0399697328476675,0.157861087307025,
0.253195600825491,0.800117065818978,0.948518177706156
"13197","AC006449.2",8.72970148009122,-0.166260753169043,0.65419126358
369,-0.254147009329135,0.799381987118804,0.948518177706156
"13198","ZNF17",25.4302561933898,0.0873837890693903,0.343923638973199,
0.254079043040714,0.799434493449965,0.948518177706156
"13199","AL121917.1",3.33795279868866,-0.216635805769227,0.85403808553
5081,-0.253660591299624,0.799757781999865,0.948518177706156
"13200","AC099522.2",7.37872128385486,-0.175812573869097,0.69677797887
8432,-0.252322230607938,0.80079200637639,0.948601604160171
"13201","AL451165.2",60.5738379004161,-0.079587609869909,0.31536480429
0617,-0.252366810712862,0.80075755126181,0.948601604160171
"13202","KMT5A",168.348557046114,-0.0460339686636869,0.182344660898818
,-0.252455807791548,0.80068876827305,0.948601604160171
"13203","GDI1",265.269542953752,0.04463583801162,0.176837843139114,0.2
52411119810515,0.800723305987701,0.948601604160171
"13204","ZFP69B",4.7023387573119,0.182998846152015,0.726713289332714,0
.251817118027454,0.801182425334122,0.948624610212037
"13205","PHGDH",531.316553817693,0.0615909170062106,0.245235913954082,
0.251149662433794,0.801698400968359,0.948624610212037
"13206","HIST2H2BE",13.3919901853735,0.107473432548378,0.4272820767009
9,0.251528061692106,0.801405869306324,0.948624610212037
"13207","YPEL5",471.596073239142,-0.0501425453978485,0.199946347976099
,-0.25078000126235,0.801984204265463,0.948624610212037
"13208","GPR1",169.091988270956,0.0889984216882908,0.355170812221082,0
.25057921041353,0.802139456688275,0.948624610212037
"13209","UBP1",108.909338840692,0.0447217471262664,0.177919479018461,0
.251359476618218,0.801536195067814,0.948624610212037
"13210","ACKR2",109.864967639369,-0.0630398793769158,0.252025873054699
,-0.25013257017161,0.802484829016141,0.948624610212037
"13211","STX18-
AS1",18.5227373999742,0.0947135057520587,0.37937620625958,0.2496558935
1498,0.802853470088586,0.948624610212037
"13212","PGGT1B",181.136159969651,0.0454233794957134,0.181256869475019
,0.250602250978266,0.802121641219062,0.948624610212037
"13213","COMMD10",182.548339070496,-0.0652639275199208,0.2616046690762
14,-0.249475392585242,0.802993073142973,0.948624610212037
"13214","BX322234.1",51.2420801812542,0.104793266745825,0.419078411786
402,0.250056466280674,0.802543681521569,0.948624610212037
"13215","POMK",6.06324177027714,0.169732314444437,0.679007977654438,0.

249971016586225,0.802609762639399,0.948624610212037
"13216","AC021733.4",3.70648426634827,0.216781236562853,0.869230119015
433,0.249394529504338,0.803055616317607,0.948624610212037
"13217","PRNCR1",3.13439113808089,0.234055700075381,0.931401905545223,
0.251293988859052,0.801586822285272,0.948624610212037
"13218","EPB41L4B",2.55881634474125,-0.313397897112601,1.2544851702214
,-0.24982192261172,0.802725065395675,0.948624610212037
"13219","LGI1",25.7236727320837,0.159135316211858,0.636856086688639,0.
249876415626785,0.802682922384044,0.948624610212037
"13220","TAF6L",28.7601277776469,0.0988894396716349,0.39437449015777,0
.250750091954665,0.802007329786601,0.948624610212037
"13221","ALDH3B1",33.3384245260267,-0.0807504243872237,0.3235101633759
93,-0.249607071210846,0.802891229621417,0.948624610212037
"13222","USP28",80.7849895188364,0.0662989151087703,0.265784482411922,
0.249446147145709,0.80301569274683,0.948624610212037
"13223","HINFP",32.7574778412473,0.0728972362145855,0.292231416696496,
0.249450374085872,0.803012423450423,0.948624610212037
"13224","PUS3",72.0581121805454,-0.0917813630381139,0.364769491897466,
-0.25161469113188,0.801338902017253,0.948624610212037
"13225","AC009779.2",51.4850318314951,0.0806694944292465,0.32247218988
7885,0.250159539206445,0.802463973646061,0.948624610212037
"13226","CS",129.221951770334,0.0533200377400021,0.213699205444381,0.2
49509761298011,0.80296649120365,0.948624610212037
"13227","ALG11",45.7837130438603,0.0873653616436316,0.348914204717513,
0.250392103452378,0.802284135696474,0.948624610212037
"13228","ABCC1",120.543653496153,-0.0667196075264538,0.26640000189758,
-0.250448975417443,0.802240159172816,0.948624610212037
"13229","CDRT4",22.1099384968176,0.0987986325062643,0.39521276324305,0
.24998846620119,0.802596268151543,0.948624610212037
"13230","AC005332.3",34.8140678085589,0.124840902669242,0.500337097614
32,0.249513584470353,0.802963534245335,0.948624610212037
"13231","AC007773.1",4.37570569249419,-0.202018068925765,0.80576077093
9164,-0.250717180845502,0.802032776465374,0.948624610212037
"13232","ZNF181",95.2095919982911,0.0538494374122314,0.214662590303053
,0.250856180092716,0.801925304483008,0.948624610212037
"13233","ZNF571",49.837089811022,-0.0733508571173938,0.29163771869659,
-0.251513615746341,0.801417036619965,0.948624610212037
"13234","ZNF180",43.1045744598639,-0.064793696235651,0.259560435572562
,-0.249628554108114,0.802874614532327,0.948624610212037
"13235","PTOV1",378.28281388397,-0.0471042848653752,0.188793595440404,
-0.24950149794803,0.80297288234067,0.948624610212037
"13236","BMP2",10.4126244202502,-0.16488593110904,0.660224404550458,-0
.249742254258701,0.802786679174755,0.948624610212037
"13237","KIF3B",216.530327057867,-0.0565046778405209,0.225023053869628
,-0.25110617276245,0.801732023603374,0.948624610212037
"13238","KCNK15",7.86564170600508,0.147170562137326,0.585633038534169,
0.251301672640759,0.801580882071132,0.948624610212037
"13239","MRGBP",93.0225604245704,-0.0709790337461153,0.283651546103885
,-0.250233198870419,0.802407012756472,0.948624610212037
"13240","NAGA",120.422114726574,-0.0546393170469209,0.218233575848601,

-0.250370809507455,0.802300601504119,0.948624610212037
"13241","NCBP1",61.5674594130048,0.065635232165963,0.263316446825286,0
.249263701365045,0.803156807405722,0.948672492094668
"13242","LDHB",911.822176508303,-0.0515089681170526,0.207047985694152,
-0.24877792432688,0.803532568181001,0.94904465838626
"13243","ZFYVE19",90.5933122622265,-0.0536402553665575,0.2157460481473
69,-0.248626826897509,0.80364945511214,0.94911103812987
"13244","LRRC47",143.454072018893,0.0422172005012635,0.170162288802782
,0.248099627704193,0.804057323662992,0.949162694700671
"13245","AC023355.1",5.77141235580802,-0.160370567738558,0.64576872204
2046,-0.248340562595592,0.803870917349175,0.949162694700671
"13246","GAA",417.680497737606,0.0626862244179329,0.252624708604672,0.
248139719840428,0.804026304438033,0.949162694700671
"13247","IGLC1",58.1969890495414,0.371480619352906,1.49639256771737,0.
24825077814946,0.803940380404399,0.949162694700671
"13248","NSDHL",54.6637797575324,0.0693704601062048,0.279493561208231,
0.24820566075015,0.803979228388248,0.949162694700671
"13249","AC240274.1",93.5317061710868,0.0975056061427334,0.39258131675
2539,0.248370469968634,0.80384777941469,0.949162694700671
"13250","CAMKMT",33.8097702498239,-0.0679969244038103,0.27428970699456
7,-0.247901844910124,0.804210352425217,0.949271691466445
"13251","ATF5",107.360190941216,-0.0891378226326158,0.359698906424322,
-0.247812325922013,0.804279617640768,0.949281806648676
"13252","DNM3OS",100.651162714186,-0.0680521640446366,0.27483934316001
6,-0.247607068413838,0.804438441204967,0.949372462580067
"13253","IL20RB",59.899264745078,-0.127866102187021,0.516513500393579,
-0.247556166662804,0.80447782906481,0.949372462580067
"13254","TCTN1",226.784187744115,-0.0645131663961841,0.260690340567988
,-0.247470490297507,0.804544126699891,0.949379066062041
"13255","SNX16",68.5469330207725,0.0672747832067253,0.271975748909718,
0.24735581564317,0.804632865838492,0.949412147998039
"13256","RPL7",10497.1558748192,0.0451794737901832,0.182771576596086,0
.247190917929362,0.804760473696568,0.949491083932885
"13257","PGM5P4-
AS1",3.14197377432695,-0.242945843707509,0.984308565868341,-0.24681878
4405463,0.805048472310899,0.949652265950776
"13258","ITGAV",725.372736863743,0.0410840272360406,0.166533977063725,
0.24670057102113,0.805139964610441,0.949652265950776
"13259","AC108047.1",5.72079459242409,0.17377052094055,0.7043036142476
26,0.246726720444536,0.80511972580189,0.949652265950776
"13260","XYLT2",62.0986385351496,-0.0636163006982294,0.257811741346833
,-0.246754862155974,0.805097945172671,0.949652265950776
"13261","KLHDC4",68.3161794427892,-0.0535775425739696,0.21729383439754
1,-0.246567247167947,0.805243154993251,0.949702356088865
"13262","MMP17",87.4061647825902,-0.0572202648004954,0.232400027167054
,-0.246214535764079,0.805516163942714,0.949952707288798
"13263","RAB27B",20.316573737476,0.171628519953963,0.69747529858057,0.
246071108615953,0.805627187535884,0.950012004302287
"13264","S100A2",9.98031399792001,-0.129969833984674,0.52943947613686,
-0.245485725645205,0.806080359381036,0.950065401670281

"13265","NFKB1",312.714346047242,0.041289479638185,0.168271523146999,0
.245374136193652,0.806166753296177,0.950065401670281
"13266","GALNT12",319.12897036066,-0.0547186179267179,0.22270439135511
1,-0.245700668917062,0.805913954347465,0.950065401670281
"13267","AL359091.4",5.07809808872415,0.184204234469984,0.750476554267
175,0.245449685833098,0.806108261584678,0.950065401670281
"13268","AVPI1",115.96258651903,-0.06980519238526,0.28444054270149,-0.
245412245815176,0.806137248096091,0.950065401670281
"13269","FGF11",3.10211369976285,-0.211995244557711,0.864482355247574,
-0.245227960143847,0.806279928156626,0.950065401670281
"13270","BORCS8",101.529953047729,-0.0690928711738233,0.28154872549267
9,-0.245402890930933,0.806144490801384,0.950065401670281
"13271","HSD17B14",12.3951786551832,-0.169446728039354,0.6907728150981
35,-0.245300226551738,0.806223976345233,0.950065401670281
"13272","VAPB",241.3037499116,-0.0423334236673776,0.172334271133836,-0
.245647156475922,0.805955381850429,0.950065401670281
"13273","KCND1",4.50079615205884,-0.162116613344092,0.659249284470566,
-0.245910943193948,0.805751172465469,0.950065401670281
"13274","BLOC1S1",1774.7485951915,-0.0571649061606214,0.23321558177866
3,-0.245116152722911,0.80636649630218,0.950095826590786
"13275","AL136454.1",17.8682593124408,0.135561508089414,0.554673482906
227,0.244398753982498,0.806922006702326,0.95034049220896
"13276","AC024941.2",5.90466218797026,0.168431238755187,0.688246182466
838,0.244725278608141,0.806669153593586,0.95034049220896
"13277","ADPRHL1",12.1003268405007,-0.106220131614408,0.43445309452719
5,-0.244491598638524,0.806850107896474,0.95034049220896
"13278","PARN",116.161836981401,0.0600156766466794,0.245586276790741,0
.24437715914321,0.80693872995748,0.95034049220896
"13279","AC009113.1",24.3832374101911,-0.0941885680161996,0.3851047600
68787,-0.244579080246569,0.806782363722813,0.95034049220896
"13280","EID2B",50.9021977742799,0.0647445860837391,0.2647426155876,0.
244556721402928,0.806799677868718,0.95034049220896
"13281","DOCK8-
AS1",9.33735631871012,0.167565375744803,0.686161198408076,0.2442070116
08295,0.80707049696229,0.950352550255249
"13282","ENG",351.611152106966,-0.0435889113071206,0.178450380167476,-
0.244263482466176,0.807026763744989,0.950352550255249
"13283","TMA16",228.891832928656,0.05013196400699,0.205646881155001,0.
243776923459415,0.807403593644588,0.950353722713446
"13284","SLC1A3",36.2957012862742,-0.0926703096171024,0.38020931107496
1,-0.243734982068421,0.807436078479301,0.950353722713446
"13285","KIF13A",234.186865141164,0.0581867843584493,0.238659339153081
,0.243806861130739,0.807380406245187,0.950353722713446
"13286","AL450306.1",10.7941517132483,0.188389173288932,0.771887742538
339,0.244062916026387,0.807182092888847,0.950353722713446
"13287","MMS19",75.3324411304466,-0.0480849557588987,0.1972074014433,-
0.243829366479046,0.807362975458467,0.950353722713446
"13288","AF001548.2",13.514635992092,-0.145565171162301,0.596990388163
485,-0.243831683136643,0.807361181171983,0.950353722713446
"13289","MUL1",122.157802359111,0.0508845334093942,0.209159800618478,0

.243280655551069,0.807787988933273,0.950467469631903
"13290","MYOC",442.559013967747,0.199014048106197,0.81913804334011,0.2
42955445329701,0.80803991288376,0.950467469631903
"13291","ZC3H8",87.2233422444803,-0.0530132256737134,0.218303318015586
,-0.242842051855246,0.80812775776896,0.950467469631903
"13292","TMED7",457.260411329515,-0.0297983598422518,0.122620112448358
,-0.243013639828471,0.807994831078654,0.950467469631903
"13293","MAN1A1",553.74889897954,0.0376917636935518,0.155271644724315,
0.242747243132986,0.808201207073829,0.950467469631903
"13294","RAET1G",9.54477123348525,0.166879448548592,0.687303545944902,
0.24280312466476,0.808157914864395,0.950467469631903
"13295","AC037459.2",13.7209524877381,0.132453379742425,0.545464112926
047,0.242826936921481,0.808139467352327,0.950467469631903
"13296","CKAP2",62.8566705699578,0.0764440401154578,0.314237202247547,
0.243268586814993,0.807797337618259,0.950467469631903
"13297","NT5C3B",235.979579636348,0.0628670919924532,0.258694895333144
,0.243016360688114,0.807992723313162,0.950467469631903
"13298","CSE1L",162.299607020601,0.0459174885516335,0.189060338879417,
0.242872137137761,0.808104450740579,0.950467469631903
"13299","DHRSX",155.721305889562,-0.0635560227456595,0.261637313229312
,-0.242916508968871,0.808070076252803,0.950467469631903
"13300","LMAN2L",69.4030218531129,-0.0594751083677859,0.24534418594848
3,-0.24241498993693,0.80845862042443,0.950492723475731
"13301","TARBP2",38.1328231902249,0.0731038646439223,0.30144152229621,
0.242514249818866,0.808381716602003,0.950492723475731
"13302","MRPL10",183.841172993769,0.0679216245527012,0.2801958909784,0
.242407639582185,0.808464315350421,0.950492723475731
"13303","SMAD2",285.227543369595,0.0342314945005017,0.141215682649521,
0.242405757336881,0.808465773682714,0.950492723475731
"13304","AL139011.1",3.28366225792915,0.216893187077086,0.895620662958
29,0.242170816337215,0.808647807277285,0.950597141264823
"13305","NME5",11.9725902120609,0.138981235687916,0.574309551135079,0.
241997082258567,0.808782424070526,0.950597141264823
"13306","SLA",15.6021034798636,0.128823197320787,0.532226149183938,0.2
42045975227469,0.808744539078763,0.950597141264823
"13307","AC138696.2",7.0714135216987,0.155166590864101,0.6413476660729
66,0.241938341826675,0.808827940017101,0.950597141264823
"13308","HACD4",49.528857256648,0.0851217879210787,0.351963671975909,0
.241848220991697,0.808897772819511,0.950597141264823
"13309","SEH1L",78.6191437308656,-0.0716939821344767,0.296476054339332
,-0.241820481233264,0.808919268100609,0.950597141264823
"13310","TMEM14A",257.425655296678,0.0523282685430593,0.21654535861092
6,0.241650381604711,0.809051079874888,0.950680607756818
"13311","IGSF9",5.19816371822173,-0.164447973510133,0.682206985792564,
-0.241052901736389,0.809514115463291,0.950897390213308
"13312","CCNA2",4.56457658720386,-0.187623628101666,0.779217279082185,
-0.240784737631412,0.809721959262202,0.950897390213308
"13313","TLR2",5.63652467946731,-0.201162552880082,0.833560118404033,-
0.241329387573432,0.809299835873295,0.950897390213308
"13314","ZNF777",44.0148781467977,0.0637286744265076,0.264602175599966

,0.240847129400987,0.809673600575962,0.950897390213308
"13315","NIPSNAP3A",97.1715510095473,-0.0634318735545315,0.263231515160818,-0.24097370527909,0.809575496227994,0.950897390213308
"13316","C11orf58",1515.3845911462,-0.035680686624546,0.14801565238647 6,-0.241060226058944,0.809508438847858,0.950897390213308
"13317","CEP126",629.533603388199,0.0659757102395557,0.273743816601376,0.241012604626716,0.809545347363009,0.950897390213308
"13318","CCDC160",5.89834900245498,0.178804388370239,0.742458288641341,0.240827520017915,0.809688799361391,0.950897390213308
"13319","F3",424.567087992774,-0.0917481046036409,0.381999415973404,-0.240178651503563,0.810191762992021,0.95104820209726
"13320","RTKN",29.5369081442713,0.103139064942935,0.430007427735959,0.239854147371302,0.8104433283875,0.95104820209726
"13321","ARL6",39.0522008892557,-0.0787464329874323,0.328200761828264,-0.239933730040022,0.810381631688067,0.95104820209726
"13322","HTT",191.209876598452,0.0574101876364199,0.23943494651923,0.239773635682747,0.810505746510702,0.95104820209726
"13323","AC073475.1",5.56151361117528,0.161641922171294,0.674192922306018,0.239756183761781,0.81051927658276,0.95104820209726
"13324","AC021097.2",10.4960721069303,-0.111756571851077,0.465967573983911,-0.239837658435296,0.810456111631229,0.95104820209726
"13325","AC010186.3",4.91536811439152,0.168993801036549,0.703877736320183,0.240089709215743,0.810260711788058,0.95104820209726
"13326","LINC00621",2.90891659758269,0.254496992792206,1.05856776605282,0.240416344568258,0.810007508594379,0.95104820209726
"13327","CLN6",22.8163267392671,-0.0960923341976668,0.400637085602681,-0.23984882491124,0.810447454682434,0.95104820209726
"13328","RABL2B",99.0976674238028,0.0571833780836256,0.238237185069798,0.240027089250875,0.810309256185216,0.95104820209726
"13329","AL157700.1",12.1118348350477,-0.122452361023761,0.51071524688581,-0.239766409502045,0.810511348795169,0.95104820209726
"13330","MAP4K1",3.72657002145172,0.227878156738394,0.951317665447693,0.239539498755291,0.810687272187632,0.951173963767034
"13331","CDADC1",100.525681091835,0.051082528611532,0.213356012150787,0.239423900440312,0.810776898978851,0.951207763860868
"13332","ZNF584",37.6723892098552,-0.0783642841422944,0.327489859155852,-0.239287666324352,0.810882528500973,0.951260331964838
"13333","CHN1",4.28571999618547,-0.182207082128623,0.762444503241382,-0.238977501121729,0.811123028843673,0.951462008147348
"13334","CCDC106",92.0773966270533,-0.0781918443641661,0.327287085967736,-0.238909042600826,0.811176113595699,0.951462008147348
"13335","EPB41L4A-AS1",512.992404398236,-0.056374547684623,0.236501080601152,-0.238369091343375,0.811594838103863,0.951537138478117
"13336","DPYSL2",2710.72602349253,-0.0594965152618089,0.24939566458526,-0.238562748717988,0.811444653363941,0.951537138478117
"13337","FADS2",35.6433161097944,-0.220053879449186,0.923216264308987,-0.238355722224947,0.811605206348982,0.951537138478117
"13338","GPR162",4.08558305091198,-0.229079429803759,0.960587895344068,-0.238478364045703,0.811510094326176,0.951537138478117

"13339","SSPN",2071.14326082585,0.0697589145447287,0.29252131754559,0.238474635387408,0.81151298595974,0.951537138478117
"13340","CTDP1",49.3463795489115,-0.0660636604376215,0.277114217926569,-0.238398668000237,0.811571900431484,0.951537138478117
"13341","COA6",224.428764878565,-0.0686225978830636,0.288545329356342,-0.237822591119877,0.812018696122476,0.951665223481118
"13342","HIST1H2BK",5.10915244188291,0.172183360154779,0.723202787601556,0.238084480738536,0.811815571272563,0.951665223481118
"13343","DECR1",566.889894367872,-0.0692393166508594,0.291128365849596,-0.237830884148987,0.812012263751772,0.951665223481118
"13344","CHST1",3.81832347950381,-0.23000999448702,0.966887586493769,-0.237887007445309,0.81196873284446,0.951665223481118
"13345","ITGA11",1314.29088992782,-0.0603521100512781,0.25362248035844,-0.237960412523305,0.811911798547562,0.951665223481118
"13346","STK25",337.478897437854,-0.0430203715162443,0.181074037053024,-0.237584427985369,0.812203429400409,0.951724352300927
"13347","QARS",306.87448051173,-0.0427351440170519,0.179920635979463,-0.237522192962512,0.812251704252735,0.951724352300927
"13348","AC009275.1",15.4986612252321,0.124075016023334,0.522301999040395,0.237554166461726,0.81222690275946,0.951724352300927
"13349","C1orf122",822.903692546526,-0.0680937285545826,0.286943408521895,-0.237307171143424,0.812418499214966,0.951768188532608
"13350","MYO1B",206.46169261984,0.0382337344933085,0.161374951724192,0.236924839231892,0.812715099606968,0.951768188532608
"13351","AL122017.1",6.87693333337296,0.14910807191149,0.628925775400231,0.237083735066513,0.812591830233413,0.951768188532608
"13352","HOXA11",38.9914245800451,0.0858610390710391,0.362117767796106,0.237108053530762,0.812572964686889,0.951768188532608
"13353","MAP1LC3B",1115.18394785644,0.0499334079471302,0.210731175703554,0.236953112326266,0.812693165359388,0.951768188532608
"13354","SLC25A10",13.585907296577,0.0996207020957984,0.420281864508897,0.237033073535557,0.81263113230258,0.951768188532608
"13355","AP2A1",359.603562303234,-0.0454559203468626,0.191505988670509,-0.237360307437021,0.812377279972999,0.951768188532608
"13356","SNHG12",95.4251675055896,-0.0607891976679251,0.257554079065443,-0.236024985076936,0.81341328252148,0.951914275594747
"13357","DBI",2756.04972378895,-0.052934139076023,0.223821282901211,-0.23650181247235,0.813043301018466,0.951914275594747
"13358","GALNT3",3.06397275167657,-0.218588163299919,0.9255083909953,-0.236181719611259,0.813291663940748,0.951914275594747
"13359","AC097376.3",78.9476647538205,0.0483208631764796,0.204876525583574,0.235853585660152,0.813546285507136,0.951914275594747
"13360","FAF2",270.909119469123,0.0318320143469756,0.134790704868038,0.236158823994129,0.813309429575321,0.951914275594747
"13361","KATNA1",192.064707164657,-0.0490465096942156,0.207275923226874,-0.236624249120009,0.812948306295034,0.951914275594747
"13362","SPTY2D1OS",16.8015156375524,0.11257042090591,0.476388840200699,0.236299449958746,0.81320031372373,0.951914275594747
"13363","AP001160.3",18.1929886382596,-0.105048055016166,0.445453435198728,-0.235822752089231,0.813570212396099,0.951914275594747

"13364","AP000915.1",2.51906353450919,0.239513292342014,1.01542234407523,0.235875538626387,0.813529250084377,0.951914275594747
"13365","ZNF396",30.6421779715095,-0.0848921477319597,0.359771805673688,-0.235961090872575,0.813462862691158,0.951914275594747
"13366","REXO1",88.2349976676678,-0.0607096246995637,0.257274418235402,-0.235972255290518,0.813454199351729,0.951914275594747
"13367","TFIP11",65.1402123024576,-0.0562327879672394,0.237766531846338,-0.236504219204329,0.813041433684716,0.951914275594747
"13368","DHRS3",277.637670382439,0.0624726257412402,0.265651442299537,0.235167651266876,0.814078612521772,0.951988125410277
"13369","CCDC85A",24.4008945031413,-0.244417486849539,1.03849157805552,-0.235358179126679,0.813930742633196,0.951988125410277
"13370","LINC02227",5.0503270026789,0.187717713953841,0.798302781425882,0.235146010162398,0.814095408740262,0.951988125410277
"13371","PTK2B",26.7999028337535,0.079235887036868,0.337010551447118,0.235113965116612,0.81412027988251,0.951988125410277
"13372","DDX50",299.407581734725,-0.0434411252932631,0.184376123647264,-0.235611447045995,0.813734190050039,0.951988125410277
"13373","BRI3BP",45.957037485954,-0.0608189665663901,0.258499387161938,-0.235277024189963,0.813993726687422,0.951988125410277
"13374","FOXO3B",8.41315967155671,0.132409533065656,0.56274823915776,0.235290888273284,0.813982966737082,0.951988125410277
"13375","SNX11",55.9565176790329,-0.0627162352989309,0.266616092851505,-0.235230494259255,0.814029838941589,0.951988125410277
"13376","GTF2IRD2",131.240061061956,0.0700808548956386,0.298333736316011,0.234907576196495,0.814280469138328,0.952033081955853
"13377","MRPL50",188.749229974831,0.0462004999801876,0.196651317841285,0.234936132070447,0.814258304965113,0.952033081955853
"13378","SLC35G1",21.3394761803867,-0.0863717638314227,0.367814447584969,-0.23482428272878,0.814345119763287,0.952033959869531
"13379","SIK3",199.788482655661,0.0494253263278238,0.210544736003612,0.234749760388101,0.814402963497088,0.952033959869531
"13380","RNF10",345.874911641184,-0.0318160983059756,0.13563079175747,-0.234578725772449,0.814535723227929,0.9521179903800З
"13381","BX284668.5",166.765361918258,-0.0718285346075,0.306557488858733,-0.23430689909063,0.814746730287859,0.952135166392108
"13382","SDAD1",176.675073116474,-0.0429416742204146,0.183380024836473,-0.234167675888949,0.814854808322146,0.952135166392108
"13383","LSMEM1",24.7050789658731,-0.129194500033162,0.551428829303966,-0.234290434535743,0.814759511427212,0.952135166392108
"13384","PHYH",461.731714648859,0.0673606815712304,0.287547696338432,0.234259159189889,0.814783790054355,0.952135166392108
"13385","TBL3",66.4976992382285,0.0644693817154361,0.275229701198519,0.234238461309578,0.814799857636156,0.952135166392108
"13386","FAT4",292.093167177272,0.0734342016976748,0.313992898949929,0.233872173362064,0.815084216618476,0.95226093582677
"13387","AXIN1",64.6583242064355,0.0592666906031611,0.253414845775344,0.233872212268503,0.815084186413088,0.95226093582677
"13388","ZNF514",69.2601702960475,0.052425095326885,0.224379914548219,0.233644332347844,0.815261107891051,0.952279001029349

"13389","LFNG",10.9157557962948,-0.119945415496473,0.513101823548101,-0.233765326864462,0.815167168960096,0.952279001029349
"13390","AC027702.1",2.96637373719714,-0.203352652057578,0.870453205326582,-0.233616983444024,0.815282341674103,0.952279001029349
"13391","AL445426.1",69.43968059431,0.0853310983877127,0.365590884329368,0.233405979320962,0.815446170561482,0.952399231392845
"13392","PAX3",10.3619282732173,-0.195675431317534,0.838769585075054,-0.233288658529535,0.815537264853963,0.95243070648731
"13393","CRYM",19.7493701953642,-0.105354154700032,0.45174804154601,-0.233214413812355,0.815594913809753,0.95243070648731
"13394","AL078459.1",12.9257455954067,-0.135033812547945,0.579842095395068,-0.232880319694522,0.815854340938368,0.952457651685434
"13395","ATIC",113.541492785722,0.0535943341713865,0.230364521642473,0.232650122463585,0.816033102934128,0.952457651685434
"13396","ARF4-AS1",14.8127966120733,-0.10292298213243,0.442724959259022,-0.232476123109684,0.816168230251967,0.952457651685434
"13397","LOX",1297.53929626792,0.0846189880801113,0.3634225700253,0.232839110884667,0.81588634135392,0.952457651685434
"13398","NEDD9",429.41614365199,0.0570230740291397,0.245448376721129,0.23232206621569,0.81628787488437,0.952457651685434
"13399","SHLD2",143.669496438069,0.0432647155526919,0.185754223614242,0.232913764817215,0.815828369586008,0.952457651685434
"13400","FAM76B",118.252864103741,0.0494447345493548,0.212660736029306,0.232505235675198,0.816145621149824,0.952457651685434
"13401","TLCD5",70.1678981425718,0.0597333344497694,0.25647299288255,0.232903019450176,0.815836713731684,0.952457651685434
"13402","PARP4",278.358723428265,-0.0367764801248073,0.158095150471039,-0.232622442973318,0.816054598368174,0.952457651685434
"13403","ZNF557",46.5239801354549,-0.075594729523305,0.325386931883043,-0.232322573884057,0.816287480608741,0.952457651685434
"13404","ZSWIM4",12.5897710913197,0.107539836862511,0.462661972219328,0.232437164322491,0.816198486265236,0.952457651685434
"13405","TRAPPC4",300.317828213107,-0.0552347459106271,0.237878545020121,-0.232197258083763,0.816384807299546,0.952499693111891
"13406","OPLAH",13.6970652492745,0.110487332527252,0.476442924414208,0.231900458303789,0.81661532855611,0.952585531341296
"13407","NUP85",117.067726735048,0.047463291886569,0.204685058664084,0.231884497072465,0.816627725925777,0.952585531341296
"13408","ZNF784",16.8174143086295,-0.0928560328803293,0.400490529438223,-0.231855752021354,0.816650052829296,0.952585531341296
"13409","GET1",232.094431030968,-0.0653006258655478,0.281724604128939,-0.2317888636935,0.816702007017611,0.952585531341296
"13410","ZNF77",25.9104573186319,0.0767430654941595,0.331321081366966,0.231627475008631,0.816827365808852,0.952660701062672
"13411","RDH14",388.623735486736,0.0468491256925108,0.202370716069939,0.231501506751203,0.816925215011006,0.952703777702791
"13412","IFT46",67.0189184268663,-0.0609818609216164,0.263735993778315,-0.231223126005604,0.817141464795386,0.952884917193546
"13413","FAM219A",56.6156614097547,0.0551534260997901,0.23869479901533

4,0.231062538133673,0.817266217875342,0.9529593415023
"13414","ELMOD2",108.513468013754,-0.0483490989007969,0.20938694518780
4,-0.230907895702052,0.817386356600777,0.953028374626223
"13415","FBXO38",84.8421802761528,0.0588257997133632,0.254870557589999
,0.230806572048208,0.817465075321578,0.953049107568355
"13416","BX005266.2",2.74777809464025,-0.238148125052553,1.03258810804
077,-0.230632256170773,0.817600506277909,0.953135950967986
"13417","AC108519.1",8.36843254724826,-0.152268531855077,0.66089253350
2252,-0.230398323685341,0.817782263626078,0.953230276870049
"13418","ZBED8",25.4431817947962,0.0745551001797628,0.323776349143208,
0.230267282885405,0.817884082019642,0.953230276870049
"13419","APOM",31.002090772965,0.0833118016057308,0.361876441924789,0.
230221677771016,0.817919517807107,0.953230276870049
"13420","MFSD14B",204.549834517039,0.0442045738668693,0.19201485033328
9,0.230214349515891,0.817925211994633,0.953230276870049
"13421","ZYX",1035.65916869882,0.0498148730081046,0.21676764905584,0.2
29807691438644,0.818241207766514,0.953527493440747
"13422","UBE2G1",160.852171919224,0.0397980703057637,0.173555888013042
,0.22930982498717,0.818628117684999,0.953799193138953
"13423","NFKBID",11.4291857070334,-0.127809838939381,0.557458800016146
,-0.229272260005007,0.818657312576554,0.953799193138953
"13424","ABCB7",152.697572824129,-0.0533120401320318,0.23239418293732,
-0.229403505105852,0.818555312120095,0.953799193138953
"13425","ZNF781",18.6759274095249,0.112912968940311,0.493022062683931,
0.229022142184938,0.818851706476396,0.953954613727437
"13426","EIPR1",64.7798825548128,0.0573132121522901,0.250462013906348,
0.228829958117803,0.819001081289793,0.95397664868913
"13427","USP46-
AS1",17.1459916715593,0.099340189269093,0.434343355392914,0.2287135005
88096,0.81909160094841,0.95397664868913
"13428","H2AFZ",1326.57207493986,-0.0519687451745818,0.22701892014574,
-0.228918123393501,0.818932554129335,0.95397664868913
"13429","CALHM5",82.3815123674721,0.0465373004518077,0.203500549209537
,0.228683905928381,0.819114604555392,0.95397664868913
"13430","PRKAG2-
AS1",109.559346311244,0.0650956527249749,0.285489299727288,0.228014334
642865,0.819635096600361,0.954014505531714
"13431","TTF1",180.569082731374,-0.0537727831699623,0.235784516920499,
-0.228059008590854,0.819600366776393,0.954014505531714
"13432","MXI1",235.823815019706,-0.0706584207257713,0.309465897197638,
-0.22832377126403,0.819394545780065,0.954014505531714
"13433","LRMP",5.52419369025389,0.213952948089422,0.937633615730194,0.
228183956398367,0.819503233399692,0.954014505531714
"13434","ZNF641",45.8228089934942,0.0835140509413345,0.366078616440765
,0.228131464638137,0.819544039716212,0.954014505531714
"13435","RND1",18.8766853079677,0.0962796357107116,0.421396283010065,0
.228477657712069,0.819274923371892,0.954014505531714
"13436","KARS",226.214080869777,-0.0353870114056881,0.155137832527928,
-0.228100462853364,0.819568140273809,0.954014505531714
"13437","TMX4",591.178520458896,-0.033019749568189,0.144618528079868,-

0.228323092529011,0.819395073398653,0.954014505531714
"13438","CNOT10",79.7608404612904,-0.051994932801318,0.228276129334451
,-0.227772097559703,0.819823419476334,0.954162693898635
"13439","PRKDC",645.351388572155,0.0387923234339517,0.170422722275223,
0.227624127323259,0.819938461395878,0.954190217022255
"13440","VSIG10L2",5.02047588694455,-0.251849212566447,1.1066173239179
1,-0.227584736948443,0.819969086750582,0.954190217022255
"13441","OGFOD1",101.934956911542,0.0478070306411026,0.210188585855793
,0.227448271971829,0.820075188100312,0.954242685952599
"13442","AC099850.1",16.5099122580273,0.0826192969152236,0.36351249165
687,0.227280489148115,0.820205643550761,0.954323483494561
"13443","DRAM2",271.416713779047,-0.0616912584618068,0.27217865469133,
-0.226657224578353,0.820690291282254,0.954333125720111
"13444","PIK3CB",33.2536582854481,-0.082797035359631,0.365515614371366
,-0.226521199380305,0.820796072974095,0.954333125720111
"13445","KIAA1586",165.162020438909,0.0544585139896172,0.2399602569822
35,0.226948056626098,0.820464133056071,0.954333125720111
"13446","WIPI2",424.005099761117,-0.0347215029956139,0.152966964776953
,-0.226986938299015,0.820433898831967,0.954333125720111
"13447","LY96",311.849232029527,0.0835930159764055,0.368964099025063,0
.226561381438705,0.820764824555913,0.954333125720111
"13448","AC068733.3",3.09465839463426,0.225254242111553,0.993390908214
753,0.226752872659529,0.820615911311727,0.954333125720111
"13449","MIR17HG",10.7453608057355,0.183638891272994,0.809216090858214
,0.226934305122672,0.820474826230732,0.954333125720111
"13450","METRNL",2944.80318406172,0.0530330964645782,0.234157072005192
,0.22648513670944,0.820824118106581,0.954333125720111
"13451","AC005261.1",322.753406320674,0.0919470628336022,0.40492072427
5584,0.227074232859033,0.820366019924111,0.954333125720111
"13452","ATP6V1E1",413.777033777617,0.0359286742892386,0.1584427619193
54,0.226761221869675,0.820609418691324,0.954333125720111
"13453","GABBR1",51.8799427205127,-0.0761736745924766,0.33671328440009
4,-0.226227114051029,0.82102478323709,0.954424528751902
"13454","AP001160.1",7.56001499848947,0.162211564415231,0.716931810910
751,0.226258009404222,0.821000755194501,0.954424528751902
"13455","NPHP1",71.951544472347,-0.0633625205269163,0.28024327730971,-
0.226098271241995,0.821124989129324,0.954470072834086
"13456","PPIG",970.304644626915,0.0306680872067355,0.135761195899443,0
.225897297114642,0.821281300084656,0.954543171009248
"13457","KBTBD2",158.634195064592,0.0554786798505619,0.245633865063865
,0.225859247201673,0.82131089483367,0.954543171009248
"13458","AC021546.1",6.91512451542697,0.188250444755791,0.834633470152
758,0.225548640795985,0.821552490140975,0.954543171009248
"13459","PLPP6",25.6235721846766,0.080715183688529,0.35777841643804,0.
225601042377321,0.821511730051167,0.954543171009248
"13460","FZD8",43.9410499912893,0.0891339300219077,0.395190728695866,0
.225546612179012,0.821554068091783,0.954543171009248
"13461","UBALD2",187.864324087688,-0.0538333225320196,0.23856633130984
6,-0.225653478579514,0.821470943514175,0.954543171009248
"13462","PSME3",117.415594597049,-0.050901602544435,0.225838349427086,

-0.225389543775731,0.821676245251402,0.954614208567221
"13463","PALLD",938.250107056591,-0.0449445763446394,0.199712158513856
,-0.225046770707859,0.821942889575814,0.954811102660062
"13464","ERAP2",236.652288230928,0.210558865396023,0.935755938012925,0
.225014725360059,0.821967818811706,0.954811102660062
"13465","SSBP3",246.559621854797,-0.035972787677338,0.16025165425586,-
0.224476857005815,0.822386272882244,0.954833168017166
"13466","AL359504.2",15.0461526898597,0.106749393239268,0.475283971572
401,0.224601290226777,0.822289461076874,0.954833168017166
"13467","SELENOI",36.1792951104331,-0.06657427872074,0.296259133071356
,-0.224716375932637,0.82219992425392,0.954833168017166
"13468","PPP1R2",353.054399634712,-0.0465017309166826,0.20724240273007
2,-0.224383284038885,0.822459076507079,0.954833168017166
"13469","KIAA0232",221.606088065243,-0.0540765009737125,0.241009420558
433,-0.224375050769443,0.82246548240321,0.954833168017166
"13470","AC012531.1",2.97585591941256,0.230270671075673,1.026332986227
58,0.224362535517896,0.822475219918623,0.954833168017166
"13471","AC090061.1",3.50878213603112,0.193352792853775,0.860775708268
882,0.224626219114187,0.822270066174916,0.954833168017166
"13472","P2RX4",85.6337873769402,0.0704096505489375,0.313068947147498,
0.224901419289486,0.822055965146248,0.954833168017166
"13473","ATG7",83.1254194010707,0.0463572677590795,0.207000644027138,0
.223947456670724,0.822798188292511,0.954860008128207
"13474","HAND2-
AS1",76.0404921551808,-0.0912199427334387,0.407340087320923,-0.2239404
9977598,0.822803601633238,0.954860008128207
"13475","AC073140.1",2.92892505912764,0.225069830805037,1.004635361559
75,0.224031364430178,0.82273289814707,0.954860008128207
"13476","AP001437.1",3.13236978234525,-0.206971640797552,0.92382008933
8096,-0.224038904529392,0.822727031119572,0.954860008128207
"13477","PARVB",118.237494739303,-0.0657248157751399,0.293311850081618
,-0.224078283086248,0.822696390427051,0.954860008128207
"13478","TMCO6",27.8874101208549,0.0661466672754423,0.295548045958583,
0.223810199999467,0.822904992835987,0.954906817625378
"13479","ALDH9A1",687.277219061424,0.0502894264610227,0.22498127960684
8,0.223527159899272,0.823125247268471,0.955091539971725
"13480","DIRAS3",5.21314105632125,0.199532314904648,0.895387936761027,
0.222844542251078,0.823656499838196,0.955218191293582
"13481","SMG5",64.6859277418867,-0.0513943231609062,0.23024550725339,-
0.223215313836051,0.823367933885145,0.955218191293582
"13482","CHD7",26.3360708296542,0.128477107998724,0.576094829576277,0.
223013818911064,0.823524751397213,0.955218191293582
"13483","IMMP1L",69.442140053167,-0.0730399225088755,0.327496377941203
,-0.223025130745075,0.82351594754621,0.955218191293582
"13484","MBIP",190.344601645698,0.0512178639413919,0.229843934760355,0
.222837570174709,0.82366192632898,0.955218191293582
"13485","ITGB4",4.12379372878211,0.190309558663082,0.853688199870251,0
.222926308097038,0.823592860658344,0.955218191293582
"13486","TGIF1",224.100486891326,0.0595766412698693,0.266806421239817,
0.223295380197463,0.823305622573255,0.955218191293582

"13487","ARHGEF2",154.98337611125,0.0427786143305248,0.192739068682422
,0.221950923717555,0.824352087627226,0.955301921023918
"13488","SEMA4C",72.2012373004953,-0.0462491326086327,0.20861220922635
3,-0.221699069197098,0.824548154757375,0.955301921023918
"13489","XRCC5",1022.81020051727,-0.0319492175862933,0.144511208073655
,-0.221084703478565,0.825026480434003,0.955301921023918
"13490","MBD4",246.887821163829,-0.0356772439845953,0.161229272734788,
-0.22128267019651,0.824872342718469,0.955301921023918
"13491","DGKQ",29.8566660971263,-0.0711645075818693,0.3206109159851,-0
.221965329418717,0.824340873212447,0.955301921023918
"13492","NOP14",103.420005006814,-0.057133494258088,0.258259568122775,
-0.221225082475655,0.824917180062921,0.955301921023918
"13493","B3GAT2",17.8033729120834,0.129089387413729,0.581575112643287,
0.221965116125776,0.824341039254468,0.955301921023918
"13494","CNPY4",290.859767987909,-0.0588172271490685,0.265844973203636
,-0.22124633932429,0.824900629582008,0.955301921023918
"13495","DMRT3",23.2572290498469,0.155086473915248,0.702437415614079,0
.220783333102593,0.825261141621109,0.955301921023918
"13496","ZNF33A",128.014757808414,0.0636244416620232,0.288127221747121
,0.220820654418638,0.825232080636886,0.955301921023918
"13497","AL442125.1",13.0583205399068,-0.128288183746478,0.57770009267
6581,-0.222067099127676,0.824261649507997,0.955301921023918
"13498","AC027807.2",10.3911564062469,0.129258283869404,0.581279773157
211,0.222368452917844,0.824027067971535,0.955301921023918
"13499","AC021739.2",4.08599302879184,0.176479744005163,0.798606092822
475,0.220984720241038,0.825104330365804,0.955301921023918
"13500","MRPL46",136.40493976156,-0.0506224487923334,0.228635080317786
,-0.221411555575688,0.824771995651141,0.955301921023918
"13501","C17orf97",44.2600005794126,-0.134008334715554,0.6061483843748
42,-0.221081732080776,0.825028794028169,0.955301921023918
"13502","RNMT",379.558700677849,0.032364039117264,0.145707965792175,0.
222115784413772,0.824223750566081,0.955301921023918
"13503","TTC39C",93.9906678552906,-0.0642130510755913,0.29069950273953
8,-0.22089150641969,0.825176910977561,0.955301921023918
"13504","EVI5L",26.6457747653365,0.0803455709693954,0.363179022064487,
0.221228556959793,0.82491447484083,0.955301921023918
"13505","ZNF730",2.70660838896392,0.194256405047227,0.8748604981293,0.
22204272048241,0.824280627158754,0.955301921023918
"13506","AKT1S1",166.641038722693,-0.0483813133189255,0.2179783388197,
-0.221954684033737,0.824349160328185,0.955301921023918
"13507","AC020915.2",70.8255053116519,-0.0572077775568165,0.2584828413
73872,-0.221321373800865,0.824842208718954,0.955301921023918
"13508","TTPAL",65.1089677768544,-0.0527857300734505,0.237812754598375
,-0.221963410510074,0.824342367024333,0.955301921023918
"13509","MXRA5",237.168362884712,-0.0761927669154282,0.344466479599486
,-0.221190656937123,0.824943983788035,0.955301921023918
"13510","SH2D1A",36.6070287609755,0.152554666287843,0.689774963379894,
0.221165850294602,0.824963298367014,0.955301921023918
"13511","UTP14A",91.3702394657149,-0.0444684821058257,0.20079644637507
9,-0.221460503453136,0.824733886757032,0.955301921023918

"13512","NT5C",545.9864257266,0.0575182977571091,0.260644992605591,0.220676780252387,0.82534411243251,0.955327258617855
"13513","C1orf74",4.99307541426154,0.148139056510438,0.672742798006274,0.220201623784691,0.825714132067947,0.955470447364236
"13514","GNRH1",14.4745460204995,0.0981651560895732,0.445775317823656,0.220212183502735,0.825705908454663,0.955470447364236
"13515","UNC13B",41.94459484272,0.0643773207256085,0.292249091792251,0.220282363687796,0.825651254573891,0.955470447364236
"13516","ZNF865",50.7522238701179,-0.0516729339070948,0.234590479645042,-0.220268674096582,0.825661915483816,0.955470447364236
"13517","ESF1",319.289707720967,0.0327726286083583,0.148881442198074,0.220125679362759,0.825773276024449,0.955470447364236
"13518","RTL8A",380.577380282254,-0.0461507862181895,0.20985796477887,-0.21991438955781,0.825937829387341,0.955590150289837
"13519","ATP8B2",240.168982577703,0.044633356642075,0.2032674879322,0.219579417722535,0.82619872248778,0.955751214467586
"13520","CCDC144NL",2.94855160787202,0.254276615289133,1.15802025727661,0.219578728171071,0.82619925956533,0.955751214467586
"13521","TMEM200B",56.6697348121329,0.0560136678271154,0.25561535277248,0.219132642932338,0.826546723277126,0.955807807284273
"13522","TCAIM",45.7100888130088,0.0596686082992239,0.272261514312951,0.219159172936348,0.826526057635381,0.955807807284273
"13523","H1FX-AS1",22.5603105277677,0.0711686118322437,0.324717121108058,0.219171109886012,0.826516759344835,0.955807807284273
"13524","AP001816.1",66.9217196936223,-0.0713995547343449,0.325621989710323,-0.219271293065504,0.82643872258586,0.955807807284273
"13525","TRIM21",58.3985221798457,-0.0541420245269991,0.247089889578527,-0.219118736988194,0.826557555410167,0.955807807284273
"13526","SMARCD2",369.198724261118,0.0444540974067888,0.202944885155143,0.219045172647763,0.82661485941989,0.955807807284273
"13527","LAMP1",1816.98308925532,0.0409526162312337,0.187094157826458,0.218887733892899,0.826737501723411,0.955878947804698
"13528","PEX10",88.7583639059359,-0.0599729411509609,0.27533853401227,-0.217815284613553,0.827573035077725,0.955928611075882
"13529","LAMTOR5-AS1",4.83562380020492,-0.155407574572598,0.712507163291709,-0.218113701277937,0.827340522319807,0.955928611075882
"13530","ADI1",1889.48619917996,0.051465297825612,0.235794696305199,0.218263169749155,0.827224068939311,0.955928611075882
"13531","GRK4",68.1397924025625,0.0511511633503961,0.235163402683868,0.217513281261536,0.82780835780755,0.955928611075882
"13532","UBE2D3",1380.57709028463,-0.0323321884118876,0.148568289881761,-0.217625096429523,0.827721228989296,0.955928611075882
"13533","ZC3H12D",3.74701020801584,-0.190386124666759,0.874531884863888,-0.217700609848423,0.827662388485222,0.955928611075882
"13534","EBLN3P",342.233437486519,-0.0305784383959573,0.140441884336767,-0.217730191675818,0.827639338413457,0.955928611075882
"13535","PTPDC1",46.349621023958,0.0576279882327651,0.265183122866252,0.217313936158111,0.827963697117063,0.955928611075882

"13536","ASAH2B",4.11567246454662,-0.167968382320205,0.771847708100376
,-0.217618554226971,0.827726326757864,0.955928611075882
"13537","MIR210HG",7.3393900239841,0.136022252274995,0.623179645276296
,0.218271333645192,0.827217708421052,0.955928611075882
"13538","CHID1",490.689415513248,0.0657928287891247,0.300797045306018,
0.218728308059642,0.826861696236458,0.955928611075882
"13539","RPS3",12891.0704087497,0.0526470256582283,0.242229012122371,0
.21734401340675,0.827940259044507,0.955928611075882
"13540","PCSK7",343.380737935636,0.0591112513934696,0.272173671313281,
0.217182106954903,0.828066428481087,0.955928611075882
"13541","LOH12CR2",17.0225116614058,0.103668508542963,0.47737191839820
5,0.217165075169935,0.828079701160795,0.955928611075882
"13542","WBP11",293.087905886059,0.0402611709345961,0.185511093404082,
0.217028373860634,0.828186232741573,0.955928611075882
"13543","MFSD5",115.612133912816,-0.0407112646904182,0.18735644879827,
-0.217293106010206,0.82797992936039,0.955928611075882
"13544","AC023794.2",33.1812520260985,0.0797360171268703,0.36512253640
0672,0.218381527234383,0.827131857342329,0.955928611075882
"13545","MORN3",3.70309722439059,-0.183115110759894,0.838493481792184,
-0.218385848830341,0.827128490458292,0.955928611075882
"13546","CKLF",501.311823448836,-0.056541443645254,0.260465101651916,-
0.217078769042985,0.828146959315726,0.955928611075882
"13547","DDX28",18.4493043058822,-0.0958067029545353,0.439769309208217
,-0.21785672839933,0.827540743051495,0.955928611075882
"13548","RFXANK",186.515760585338,0.0591430013003835,0.271548231784268
,0.217799250290717,0.827585528724366,0.955928611075882
"13549","ATP5MGL",3.80828055687925,0.177600552701963,0.816172471845278
,0.217601743293825,0.827739426086977,0.955928611075882
"13550","WDR44",76.9855912959217,0.0466216489044105,0.21420434214019,0
.217650344706355,0.827701555274554,0.955928611075882
"13551","BRD9",219.339314263212,0.052876298390668,0.243886279811946,0.
216807187478687,0.828358610387693,0.956057019147187
"13552","FAM167A",7.44193626596513,0.160019724369258,0.739264868990023
,0.216457904442118,0.828630834698652,0.956159528898253
"13553","SFXN2",5.1460868262844,0.154944297926984,0.715308817337798,0.
216611754491785,0.828510924465615,0.956159528898253
"13554","ZDHHC24",181.097483729397,0.0453537932626388,0.20945347769031
1,0.216533970993296,0.828571548174464,0.956159528898253
"13555","SLC4A7",247.503751750358,-0.0677300768940674,0.31342766525830
3,-0.216094762529177,0.828913882167639,0.956223436000119
"13556","DDX46",533.628557284955,0.0392951164471326,0.181928924917596,
0.215991582783942,0.82899430868079,0.956223436000119
"13557","HNRNPA1",3431.1154555862,0.0306186075631256,0.141725624737944
,0.216041436541489,0.828955448466759,0.956223436000119
"13558","CYBA",1497.27732183288,0.0619540336158833,0.286850618480547,0
.215980129114083,0.829003236694067,0.956223436000119
"13559","MC1R",6.25173336129435,-0.131978082940109,0.611246735703096,-
0.215916217185682,0.829053055764809,0.956223436000119
"13560","TMEM99",153.243487455264,-0.0746420764703331,0.34529318116457
9,-0.216170143350604,0.828855125483834,0.956223436000119

"13561","ORC5",39.2792009743776,-0.0574872259359528,0.266479382123017,
-0.215728607136347,0.829199300954151,0.956303533636721
"13562","MAPK8",106.820338046392,0.0450611438544124,0.209011383482179,
0.215591816597178,0.829305935211947,0.956303533636721
"13563","MILR1",11.0884494746013,-0.15378292650024l,0.713299313722657,
-0.215593823717087,0.829304370550726,0.956303533636721
"13564","AC002310.1",5.19202433904273,-0.138964466393622,0.64487712743
9493,-0.215489835940353,0.829385435677637,0.956324698761298
"13565","SPRR2D",4.57176308813384,-0.287128183764696,1.33518057927254,
-0.215048202634235,0.829729737332867,0.956374830699841
"13566","RNF150",3.33337353880415,-0.246412277759217,1.14555131533865,
-0.215103657478994,0.829686502395617,0.956374830699841
"13567","HOMER1",19.7397955263866,0.106967482722706,0.497426260512228,
0.215041889048148,0.829734659703717,0.956374830699841
"13568","AC091057.2",3.89694084470109,0.186426302818001,0.866582738450
774,0.21512810554164,0.829667441818028,0.956374830699841
"13569","TASP1",181.054089482742,0.0594544917235223,0.276463749292229,
0.215053481245664,0.829725621882426,0.956374830699841
"13570","TGFB2",121.48810103945,0.0702593951070871,0.327464213627128,0
.214555948965737,0.830113541653815,0.956588177572244
"13571","EGLN1",244.490345147463,-0.0378860714562505,0.176632688355686
,-0.214490714085488,0.830164407556782,0.956588177572244
"13572","FNIP1",408.93079067776,0.0472491889829817,0.22014380692026,0.
214628744928066,0.83005678095292,0.956588177572244
"13573","GDPD3",22.0625657904861,-0.0790418106195199,0.368285665026549
,-0.214620926431719,0.830062877173428,0.956588177572244
"13574","USP1",314.337232019389,-0.0327022183378287,0.152626058205991,
-0.214263663244793,0.830341452535266,0.956693386719374
"13575","TMEM98",624.566429110871,-0.0475779720173063,0.22210203826310
8,-0.214216728443276,0.830378051452398,0.956693386719374
"13576","AC104463.2",2.8246985261717,-0.206998631597163,0.968359087958
165,-0.213762264609537,0.830732453226111,0.956755695859966
"13577","ABCE1",257.040088347238,-0.0360457734951144,0.169127618470678
,-0.213127659580707,0.831227391006727,0.956755695859966
"13578","SYNGAP1",72.3082131104236,-0.0601448367887032,0.2812937675663
8,-0.213815035110972,0.830691299757992,0.956755695859966
"13579","AL109615.4",4.55155046261101,0.178258505099243,0.835323286491
803,0.213400617439859,0.831014498948325,0.956755695859966
"13580","HYMAI",470.32161124467,0.0522639286513955,0.244636532633793,0
.213639099968898,0.830828505893344,0.956755695859966
"13581","GIMAP1",4.53525008557274,-0.182513459478401,0.853897980851871
,-0.213741528345483,0.830748624682953,0.956755695859966
"13582","PTPN3",6.19372507493299,0.237520708666212,1.11402455535742,0.
21320958099528З,0.831163495525302,0.956755695859966
"13583","RTKN2",4.50972291499303,0.154268992479166,0.721272714523188,0
.213884414830732,0.830637194173977,0.956755695859966
"13584","NUP37",120.765106396926,-0.0536454850770498,0.251306647445622
,-0.213466239840144,0.830963318947933,0.956755695859966
"13585","XPO4",56.5548026656055,0.0528219185117671,0.247778438772576,0
.213182062060896,0.831184959087354,0.956755695859966

"13586","EGLN3",24.4905930087627,0.129760722845269,0.606324233889895,0.214012100444649,0.830537620997124,0.956755695859966
"13587","ZNF578",11.4825923733917,-0.0969482399466374,0.454312585315,-0.213395452999432,0.831018526811654,0.956755695859966
"13588","SLC25A53",24.7040450220763,-0.0755144607191557,0.354040702337578,-0.21329316155054,0.83109830712955,0.956755695859966
"13589","IPO9-AS1",10.1165487653992,-0.102907545363931,0.483512316106927,-0.212833348677666,0.831456951136434,0.956894132378987
"13590","AC010894.2",3.44829985945271,-0.181340899428525,0.853042668648963,-0.21258127652129,0.831653576817619,0.956894132378987
"13591","DDX23",196.663016768459,0.0407318063160052,0.191518115135695,0.212678608951147,0.8315776526436,0.956894132378987
"13592","TSPAN8",70.5620820652385,-0.108942708309012,0.512288266129186,-0.212658995944168,0.831592951645716,0.956894132378987
"13593","ZWILCH",38.2306147656586,-0.061656126894988,0.289910527784354,-0.21267294901705,0.831582067632864,0.956894132378987
"13594","MED7",103.408235308918,0.0590134116261724,0.277794608494662,0.212435410269334,0.831767362746684,0.95693601149961
"13595","TOP1",542.288181519277,0.0397446212347211,0.187141175452884,0.212377747112781,0.831812345034348,0.95693601149961
"13596","BBS4",120.513927734401,-0.0393093295061201,0.185191145047228,-0.21226354800115,0.831901431919954,0.956968107916157
"13597","PORCN",45.8121820096489,0.0626527488281205,0.295382189653385,0.212107401944715,0.832023245170279,0.957037843234769
"13598","IARS2",336.707543003514,-0.0344741133417863,0.16334539541196,-0.211050414092432,0.83284793246592,0.957197430501391
"13599","AC009228.1",2.78289347706369,0.200041185068341,0.946777458355371,0.211286383408229,0.832663807557467,0.957197430501391
"13600","RMDN2",58.6746130293647,0.0556716659589952,0.262788239574666,0.211849914018612,0.832224126432659,0.957197430501391
"13601","PDLIM5",617.694030010491,0.0461669306488362,0.218134900361922,0.211643944055892,0.832384823433714,0.957197430501391
"13602","SNX9",1253.73226457292,-0.0372357015790412,0.176278076598115,-0.211232742594148,0.832705662235883,0.957197430501391
"13603","AC003077.1",2.54939880885438,0.282812473722139,1.33939531446015,0.211149367680241,0.832770718681543,0.957197430501391
"13604","C2CD3",42.8526449407958,0.0586378223602538,0.277422734751986,0.211366319392229,0.832601436250333,0.957197430501391
"13605","AP000866.2",3.88964686484481,0.208915103340455,0.990173924092008,0.210988290296607,0.832896408676051,0.957197430501391
"13606","AC005520.3",3.09532194830096,-0.198600597043221,0.939667786256108,-0.211351926657505,0.832612666329902,0.957197430501391
"13607","AC145285.2",3.01644255477476,-0.187604152422276,0.889121025765096,-0.210999568096865,0.832887608377539,0.957197430501391
"13608","ABHD8",80.9695688415692,-0.0654496605649148,0.309130713004289,-0.211721636872771,0.832324206962918,0.957197430501391
"13609","ZNF383",62.6409858234504,0.0509443749377228,0.241088149477231,0.211310157916054,0.832645257014212,0.957197430501391
"13610","TVP23C",20.0275848651124,-0.0729560337879216,0.34591630976557

9,-0.210906602921853,0.832960151632612,0.957200350590305
"13611","B3GNT5",5.197946549224,0.183245349627548,0.86993401662245,0.2
10642814427473,0.833166000712847,0.957312753403297
"13612","FKBP14",248.989991324895,0.0662819383620891,0.314692616121062
,0.210624383816462,0.833180383588598,0.957312753403297
"13613","CRTC2",79.3614020154127,-0.0406950585796383,0.193412917820989
,-0.210405070344386,0.833351535666546,0.957374403858769
"13614","MTAP",220.826419895208,0.0363222506001643,0.172635294659216,0
.210398752305343,0.833356466376809,0.957374403858769
"13615","RNF227",16.4256943112284,-0.0792006721299825,0.37685409227025
7,-0.21016269626491,0.83354069341311,0.95751571391708
"13616","KCNJ16",10.9912637918289,-0.1984990611492,0.945974953954796,-
0.209835429912118,0.833796119534234,0.957738785951486
"13617","AC078785.1",3.69229489583335,0.164090917278411,0.782780059956
103,0.209625826809657,0.833959720598841,0.957786020720067
"13618","SMNDC1",191.526186728511,-0.0372695725805751,0.17776425439342
3,-0.209657294194203,0.833935158969224,0.957786020720067
"13619","PPM1F",101.674858248909,0.0423045070134238,0.201996089016498,
0.209432307424371,0.834110774256237,0.957889162887697
"13620","NFE2L2",1129.23162450109,0.0352492373713402,0.168428446799764
,0.209283158760267,0.83422719804328,0.957952524037952
"13621","CARD6",198.378506240651,0.0474606783589061,0.227181538274651,
0.208910806394526,0.834517868011418,0.958074927495161
"13622","TAF8",73.3248085857229,0.0444624261491763,0.212806161873877,0
.208933922578463,0.834499822132468,0.958074927495161
"13623","DHX32",92.4009141758456,0.0426199197844758,0.204086404325471,
0.208832724185325,0.834578824309084,0.958074927495161
"13624","CRLF3",50.135192876537,-0.0644283265565398,0.308299745439454,
-0.208979499690157,0.834464242162161,0.958074927495161
"13625","USP19",83.5534752511262,0.0453985006107017,0.217665742253484,
0.208569801295753,0.834784087154314,0.958130471213884
"13626","JAK2",65.0330989414528,0.053437449613272,0.256251290679129,0.
208535338384637,0.834810993045498,0.958130471213884
"13627","CHCHD1",398.25637680081,-0.046934505328488,0.224988793464416,
-0.2086081915716,0.834754115317827,0.958130471213884
"13628","AP001462.1",7.09602325465611,0.120743337553564,0.579485073960
344,0.208363153736427,0.834945423975986,0.958144136105688
"13629","SDHAF1",216.137093561808,-0.0585181776675635,0.28074943603601
7,-0.208435601844116,0.834888860467755,0.958144136105688
"13630","CNRIP1",392.622026523607,0.0383182252461581,0.184107580781869
,0.208129535369635,0.835127826195873,0.958242114238298
"13631","LIN7B",19.3457213721049,0.0849429544212753,0.408189512050223,
0.208096856763003,0.835153341379938,0.958242114238298
"13632","GJC2",68.9077534952573,0.0776420132852978,0.374063718987167,0
.20756360305545,0.83556972593475,0.95826092921999
"13633","KIAA1143",238.403884969839,-0.0381786314644904,0.183850466085
636,-0.207661325409461,0.835493417192064,0.95826092921999
"13634","SPATA24",6.40357537823358,-0.133154988517548,0.64193450462599
9,-0.207427685469448,0.835675862874226,0.95826092921999
"13635","MAPKAP1",205.269767443467,-0.0336213190470063,0.1620111029196

74,-0.207524783432132,0.835600039558058,0.95826092921999
"13636","CYB561A3",172.31092795313,0.0439164582162398,0.211857348679156,0.207292588574534,0.835781361908018,0.95826092921999
"13637","AC016957.2",5.00759890725072,0.15359086870484,0.739086311374329,0.20781816212964,0.835375906023455,0.95826092921999
"13638","BCKDK",159.802722244798,-0.0509347191826195,0.245132835761243,-0.20778415516813 7,0.835397504952784,0.95826092921999
"13639","PWWP3A",355.703465214848,-0.0474607525437356,0.228751534379875,-0.20747730795510 2,0.835637112731119,0.95826092921999
"13640","RPS19",10624.3517823313,-0.0423635050555115,0.204367107600524,-0.207291210180062,0.835782438330555,0.95826092921999
"13641","SHROOM4",4.53340040111191,-0.157481189776627,0.758893384581621,-0.2075142476877,0.835608266794605,0.95826092921999
"13642","KLF9",1822.32354524888,0.0769191476357812,0.371455129476856,0.207075206483516,0.835951124790597,0.958384077974266
"13643","POU2F2",5.97469898377171,0.139570035799248,0.67469102304167,0.206865114597246,0.836115201705404,0.958501924406107
"13644","SAP130",50.6763083153771,0.0521129492657374,0.252261746239428,0.206582845170173,0.836335658839373,0.958543873973896
"13645","CCDC74B",24.825456757369,0.0951209470911429,0.460350188099412,0.206627366622476,0.836300885987643,0.958543873973896
"13646","AC009118.3",9.67607753827716,-0.12502952900343,0.605073869949151,-0.206635148554568,0.836294808052296,0.958543873973896
"13647","MZT2A",535.889415429209,0.0519352061718095,0.251871942116293,0.20619687026445 5,0.836637132487863,0.958648883263971
"13648","AC091488.1",2.98912120578155,-0.203835981949796,0.989596558574814,-0.205978871069796,0.836807415853963,0.958648883263971
"13649","ITGA9",102.246456412964,0.0596047029142223,0.289492903707729,0.205893485300762,0.836874114405746,0.958648883263971
"13650","IP6K2",417.330054189068,-0.0564712220513726,0.275048957067604,-0.205313347316174,0.837327316536733,0.958648883263971
"13651","CLCN3",240.072523344441,0.0358403845717814,0.173752171105928,0.206273017158049,0.836577654487262,0.958648883263971
"13652","FAM114A2",137.643109918311,-0.0332598137816546,0.161903469572959,-0.205429901344187,0.837236260525972,0.958648883263971
"13653","CTTNBP2",11.2398089006782,-0.108947742043185,0.529733450139736,-0.205665211465212,0.837052434820785,0.958648883263971
"13654","PIWIL4",20.6966172890859,0.0814340949731194,0.39670276586907,0.20527735619568 7,0.837355434476865,0.958648883263971
"13655","TNFRSF1A",859.156019898198,-0.0473479416761665,0.230589460302364,-0.205334370504536,0.837310892338654,0.958648883263971
"13656","LINC01252",10.3064119538636,-0.125935701630082,0.613703104557608,-0.205206231962707,0.837411000652176,0.958648883263971
"13657","NFYB",351.612782040972,0.0468075483175299,0.2282702993282,0.205053169226503,0.83753058445773,0.958648883263971
"13658","DPH6-
DT",3.4118590568724,0.195462555896468,0.952911991610026,0.205121309855926,0.837477347550079,0.958648883263971
"13659","CNTNAP1",51.7320167216146,-0.0636303764363982,0.309439539290687,-0.205631046963989,0.837079123786206,0.958648883263971

"13660","DLX3",10.55509089967,-0.151923534413624,0.740213791585346,-0.205242777344426,0.837382449282155,0.958648883263971
"13661","AC090220.1",2.53682911225773,-0.24220806092626,1.17587983473174,-0.205980282825002,0.836806313080197,0.958648883263971
"13662","ZNF561-AS1",36.6451335669804,0.0560776543733351,0.27206809606362,0.206116245104394,0.836700109460401,0.958648883263971
"13663","ZNF428",634.060320737652,-0.0517938793257838,0.251370057337605,-0.20604633612436,0.83675471678417,0.958648883263971
"13664","ZIK1",7.21065605103129,0.11754691861487,0.571159808232208,0.205803904477607,0.83694409115786,0.958648883263971
"13665","USP42",89.8012552688535,0.0439491141726155,0.215004084059293,0.204410601616737,0.838032646197069,0.958732396058968
"13666","PRKRIP1",138.133949130917,0.0427299815098792,0.208918941994948,0.204528996278913,0.837940135124952,0.958732396058968
"13667","TRIM35",47.4010086669052,-0.0519185962250452,0.25377984028916,-0.204581247138814,0.837899308126208,0.958732396058968
"13668","CASP4",616.879150293021,0.0362324764336914,0.177217450240456,0.20445208067563,0.838000235087382,0.958732396058968
"13669","DMXL2",72.4708352655356,0.052056700001777,0.254627494674046,0.204442572348347,0.838007664726329,0.958732396058968
"13670","MON1B",152.379524546331,0.0498212132065247,0.243545469487075,0.2045663723963,0.837910930686555,0.958732396058968
"13671","COASY",129.446803249509,0.053365096035095,0.260759079507188,0.204652877805638,0.837843339131813,0.958732396058968
"13672","AC005253.1",13.8813990153113,0.123556593577311,0.605088086558322,0.204196044050524,0.838200302622388,0.958854061806184
"13673","MARCKSL1",81.5405036983836,0.0876228738165091,0.429823080418209,0.203858000671471,0.838464466420332,0.958945326928561
"13674","QRSL1",70.7989639977578,0.0452728618473109,0.222083693053073,0.203854957673509,0.838466844452003,0.958945326928561
"13675","IGHA2",16.1580494249468,-0.181601841639849,0.890310624503948,-0.20397582219243,0.838372392794424,0.958945326928561
"13676","IDH2",80.3938909783285,-0.0598048458423756,0.293477349062256,-0.203780107846378,0.838525338304028,0.958945326928561
"13677","SSU72",712.292318775296,-0.0453082979263555,0.223852621326041,-0.202402355880229,0.839602186791529,0.958949363123395
"13678","SELENON",356.598842127216,-0.0465029617608888,0.232273468974807,-0.200207806626131,0.841318061974426,0.958949363123395
"13679","ADAMTSL4",1220.64810687574,0.0306458308713811,0.152077999302779,0.201513900839575,0.840296761076249,0.958949363123395
"13680","INTS7",29.1871243183101,-0.0681588266881372,0.334832226899049,-0.203561130657494,0.838696470331472,0.958949363123395
"13681","CHML",38.9018475797153,0.0691941554409841,0.345706477918686,0.200152903866787,0.84136099907075,0.958949363123395
"13682","RBKS",22.6117985894659,0.0819703208390487,0.408489886697047,0.200666708059388,0.840959193291264,0.958949363123395
"13683","AC016747.1",65.7120785277643,0.0636697662576722,0.314611824331681,0.202375630327702,0.839623078411627,0.958949363123395
"13684","IL18R1",8.53368541981403,0.125138711024296,0.624530102331825,

0.200372585015617,0.84118919868866,0.958949363123395
"13685","CCDC115",319.108206088206,-0.0510365697960058,0.2529325850600
46,-0.201779338885458,0.840089234495907,0.958949363123395
"13686","HOXD3",6.53857904563408,-0.120314930683243,0.597375172068764,
-0.201405977865771,0.840381141325488,0.958949363123395
"13687","SP140L",79.0631027257978,0.0478482485846828,0.235035229016971
,0.203579049765462,0.838682466151084,0.958949363123395
"13688","ZNF860",2.73715758313364,0.184325062549811,0.921600103008291,
0.200005470863269,0.841476302441912,0.958949363123395
"13689","AC092903.2",35.6723391386505,0.0856181510797209,0.42665982465
4122,0.200670759542754,0.840956025109795,0.958949363123395
"13690","ZXDC",59.6187513465847,-0.0568892072182044,0.281582530070197,
-0.202033866248813,0.839890248618383,0.958949363123395
"13691","TIPARP-
AS1",11.6555449600919,-0.127108381277667,0.632968698780424,-0.20081305
9986337,0.84084475055525,0.958949363123395
"13692","CXCL6",5.31708589321676,0.182926687415324,0.900813409440973,0
.20306834411895,0.839081613946977,0.958949363123395
"13693","STBD1",36.8938321373034,-0.0678107436817227,0.335553179800008
,-0.20208642851228,0.839849157461664,0.958949363123395
"13694","HIST1H3H",2.48950708507423,0.182909177010548,0.91402121151963
1,0.200114805548602,0.841390794410779,0.958949363123395
"13695","DAXX",232.154543276816,-0.0337324019696558,0.167256445454351,
-0.201680729720293,0.840166328487993,0.958949363123395
"13696","NUP43",54.8577238787241,0.06065820517234,0.302613240036738,0.
200447955168703,0.841130257634767,0.958949363123395
"13697","CLIP2",33.2448319197067,0.0647808701675823,0.318614798830883,
0.203320342951073,0.838884656218932,0.958949363123395
"13698","NAPEPLD",64.5531222427089,-0.052869610223167,0.26443708969156
,-0.199932658027791,0.841533248648887,0.958949363123395
"13699","TTC26",40.1182893721473,-0.0594830282296924,0.297078234919208
,-0.200226813135163,0.841303197907823,0.958949363123395
"13700","LACTB2",110.742989542158,-0.046811219184804,0.231604647278274
,-0.20211692526428 5,0.839825316473535,0.958949363123395
"13701","IMPA1",91.1268104902779,0.0377908703110696,0.188800382737235,
0.200163102230918,0.841353023331324,0.958949363123395
"13702","MYC",2599.53712067317,0.0770617814099785,0.383752947762038,0.
200810917178319,0.840846426141403,0.958949363123395
"13703","LURAP1L",311.069078115158,-0.0595279725899065,0.2964912355586
28,-0.200774813723407,0.840874657639957,0.958949363123395
"13704","NUDT5",287.315710267513,0.0370560438448236,0.182280947326953,
0.203290823249657,0.838907727761908,0.958949363123395
"13705","PCBD1",893.139946824652,-0.0671803923259941,0.330547003635255
,-0.203240058409741,0.838947404069159,0.958949363123395
"13706","EMX2OS",433.003010274832,0.0458031557870285,0.228017479698663
,0.200875633953852,0.840795820650503,0.958949363123395
"13707","MEN1",55.5237204690662,0.0452017303266784,0.222545312074842,0
.20311248035401,0.839047117135761,0.958949363123395
"13708","LIPT2",5.53052457217778,0.13279052779815,0.659523760392116,0.
201343053537904,0.840430339949648,0.958949363123395

"13709","RPAP3",144.791052667664,0.0365281327003278,0.181923862188055,
0.200788023412611,0.840864328152663,0.958949363123395
"13710","CEP83",76.0649722966645,-0.042352398818311,0.211550193753452,
-0.200200236486996,0.841323982227928,0.958949363123395
"13711","PEBP1",2815.3006555605,0.0434063593025496,0.216546945929565,0
.200447801820618,0.841130377555309,0.958949363123395
"13712","CIT",11.1463289997243,-0.130000273415256,0.639979723234101,-0
.203131862925761,0.839031967846187,0.958949363123395
"13713","DHRS4L2",354.780575571849,0.0501923446576291,0.25099794569482
4,0.199971137288332,0.841503154291995,0.958949363123395
"13714","EMC9",52.5372694395623,0.0656226050343344,0.327310031962316,0
.200490662143499,0.841096860272378,0.958949363123395
"13715","NUBPL",69.1368616874622,0.045809016643775,0.228510366750432,0
.200468001934483,0.841114580789816,0.958949363123395
"13716","SOCS4",89.5010599883522,0.0486227474429608,0.242330890714891,
0.200646097158809,0.840975310655932,0.958949363123395
"13717","GOLGA8Q",2.90529765543168,0.209916429591365,1.04663320579812,
0.200563510147082,0.841039892926816,0.958949363123395
"13718","UBL7-
AS1",60.4862006872584,-0.0465498714293633,0.232775946786262,-0.1999771
54306694,0.841498448445523,0.958949363123395
"13719","BNC1",4.14679545811967,0.159242691656882,0.791656213086146,0.
201151319252709,0.840580255016553,0.958949363123395
"13720","RALBP1",729.372312718573,0.0283735330916082,0.140757668638155
,0.2015771742039,0.84024729125553,0.958949363123395
"13721","TLE5",1890.15419384591,-0.035130838052963,0.17509402043181,-0
.200639850329124,0.840980195580923,0.958949363123395
"13722","KLHL26",21.4666436411329,-0.073594695536512,0.363880447032939
,-0.202249656821627,0.839721554627858,0.958949363123395
"13723","ADNP-
AS1",12.7233323346832,0.0888232194185635,0.437899518387426,0.202839271
770969,0.839260661495521,0.958949363123395
"13724","AL021707.1",2.54584750096261,0.282767445208568,1.408910903281
44,0.200699309338856,0.840933699795606,0.958949363123395
"13725","MMGT1",148.847191714463,-0.0381645990438392,0.19047207396912,
-0.200368475275943,0.841192412617606,0.958949363123395
"13726","PPP3R1",151.908329230389,-0.0382314858191498,0.19167486084068
7,-0.199460094304836,0.841902856254803,0.9592204387115
"13727","TMEM87B",106.133049388754,-0.0425190502965858,0.2131324130547
43,-0.199495936292265,0.841874821837425,0.9592204387115
"13728","ZNF446",44.2517378865484,-0.0526263114808894,0.26393223917925
1,-0.199393267167896,0.841955126766718,0.9592204387115
"13729","GSDMD",282.115376873506,-0.0391357614941905,0.196363224969164
,-0.199302906643218,0.842025805612368,0.959231087462848
"13730","LSG1",125.097305164387,-0.0398445483152242,0.200073940928946,
-0.199149115223229,0.842146102218804,0.959298254821711
"13731","KCTD21",34.6507296570799,0.0664456073971363,0.333920835022028
,0.198986108167683,0.842273611374921,0.959370207516757
"13732","HEXIM2",57.6417111778873,0.0693501803500046,0.348648366768673
,0.198911530814708,0.842331949464201,0.959370207516757

"13733","C3orf38",143.83121372106,-0.041607859971397,0.209459398348773
,-0.198644034592877,0.84254120533875,0.959444408254227
"13734","TENT5A",1010.46598074284,-0.0376102306051765,0.18945038544126
5,-0.198522850811707,0.842636008163036,0.959444408254227
"13735","NCAPD3",45.3317767577418,-0.0586319668382565,0.29541796885322
2,-0.198471227277944,0.842676394266785,0.959444408254227
"13736","ERC1",317.999025594784,-0.0469269464347263,0.236320420218859,
-0.198573387738845,0.842596472533894,0.959444408254227
"13737","VPS26C",114.884548071561,-0.0401393571601619,0.20227843815909
5,-0.198436163169263,0.842703825843243,0.959444408254227
"13738","CCDC107",793.27017271639,-0.0499254463367434,0.25180228228933
7,-0.198272413906781,0.842831933733942,0.959520413713704
"13739","RAB42",40.02868527226,-0.0861854917891742,0.435595314455783,-
0.197856792598541,0.843157110319792,0.959657598165487
"13740","AL137186.2",12.1011458241256,-0.107424492844915,0.54308363203
7021,-0.197804696197495,0.843197871738499,0.959657598165487
"13741","ZNF214",22.9900773776703,-0.0792904605455156,0.40049551182173
5,-0.197980896676836,0.843060010136122,0.959657598165487
"13742","SLC25A21",10.0049025357592,-0.102804159375487,0.5196200271001
67,-0.197844875127704,0.843166434785174,0.959657598165487
"13743","THUMPD3",163.332983818274,-0.0397388931241648,0.2010363785170
16,-0.197670160083993,0.843303137821264,0.959707565707966
"13744","AC027020.2",8.11873298945363,0.133685195460186,0.677264410165
899,0.19738996092743,0.843522385096954,0.959887231003809
"13745","SLC7A2",28.5021088295994,-0.114373191044744,0.580342745495993
,-0.197078695188986,0.84376595524057,0.959987338140255
"13746","MYL6B",330.344334308874,0.0601618241595111,0.305191480034709,
0.197128124784706,0.84372727483644,0.959987338140255
"13747","HYDIN",9.31959988048292,0.121999075225526,0.619151947102616,0
.19704221697651,0.843794497277116,0.959987338140255
"13748","MAGI3",156.818456465385,-0.0484238025184109,0.246277897193478
,-0.196622608322698,0.844122877085874,0.959991729084303
"13749","UBQLN4",47.0257950266994,0.0546701016175352,0.27796000961763,
0.196683334745674,0.844075352301115,0.959991729084303
"13750","VPS41",165.085324915481,-0.0388798156987721,0.19743661726674,
-0.196923023889966,0.843887775990785,0.959991729084303
"13751","API5",211.831157971883,-0.0321157293958688,0.163248704377121,
-0.196728847058278,0.844039734522938,0.959991729084303
"13752","CLP1",38.9120253473934,-0.0531312647622507,0.269927408115504,
-0.196835383013479,0.843956361100549,0.959991729084303
"13753","TBKBP1",18.7356457163044,0.0924812484703945,0.470482810549922
,0.196566689359592,0.84416664003174,0.959991729084303
"13754","SNX6",977.127256625696,-0.0264197655584846,0.134546721918109,
-0.196361272737398,0.844327406019896,0.96010474263132
"13755","GSEC",33.2498757926435,0.085859052045432,0.437671620884565,0.
196172308069472,0.844475301879507,0.960148616679108
"13756","F7",5.96648967557412,0.117453372928048,0.598778074641811,0.19
6155099697511,0.844488770526714,0.960148616679108
"13757","RAD52",22.4406499799044,-0.0825559903997105,0.421371833895421
,-0.195921947692877,0.844671258398537,0.960165703234272

"13758","SETD3",150.681071465408,-0.0351109930494195,0.179228621398774
,-0.195900592078424,0.844687973836339,0.960165703234272
"13759","LINC00526",32.4354425523153,-0.0708926069821632,0.36165193086
8838,-0.196024411681834,0.84459105889593,0.960165703234272
"13760","C21orf91",65.3048587056758,-0.0456379284923919,0.233069097214
971,-0.195812868534423,0.844756637424167,0.960173968700143
"13761","FBXO22",117.711887211213,0.0407289905905805,0.208198950318076
,0.195625340705882,0.844903424476845,0.960271023822241
"13762","PER3",223.475247191548,-0.179005052109534,0.916796298566608,-
0.195250626981593,0.845196747060499,0.960426637664689
"13763","SOBP",485.58976880305,0.0458345766557996,0.234790134005179,0.
195215087933757,0.84522456782716,0.960426637664689
"13764","TCFL5",63.8075685869518,-0.0520170354394757,0.266287805411361
,-0.19534141024265,0.845125680756628,0.960426637664689
"13765","TATDN1",325.659665966842,-0.0442045056574972,0.22692980593050
3,-0.194793740188694,0.845554423027064,0.960661860826913
"13766","COPS2",414.501208172717,-0.039871632981608,0.204652855003372,
-0.194825686555661,0.845529412628036,0.960661860826913
"13767","SLC45A4",9.90705926528902,0.0930899486441621,0.47846575151513
7,0.194559272736613,0.84573798933534,0.960758462290438
"13768","C10orf25",12.6932455768493,-0.0935011344509308,0.481054101752
968,-0.194367190946322,0.845888377835069,0.960758462290438
"13769","ERGIC2",577.559908241244,-0.0296059182890576,0.15240109649197
9,-0.194263158012225,0.845969831715552,0.960758462290438
"13770","PHKB",334.647217374968,-0.0328490575057928,0.169013282076064,
-0.194357846331918,0.845895694249901,0.960758462290438
"13771","SLC47A1",7.63658343411382,0.1678340316373,0.863961304053365,0
.194261051796983,0.845971480819993,0.960758462290438
"13772","FLT3LG",111.051084039744,0.0428192432019616,0.220474118119509
,0.194214375669943,0.846008027024547,0.960758462290438
"13773","AL050341.2",44.4561819806934,-0.0664050898601773,0.3426369968
78914,-0.193805953429029,0.84632782523743,0.960772823307934
"13774","HHIP",5.52277279695396,0.196517737968728,1.01292917968358,0.1
94009356142862,0.846168555953027,0.960772823307934
"13775","MSH3",127.196899362877,0.046322250878267,0.238971215950959,0.
193840294505482,0.846300934896537,0.960772823307934
"13776","SNURF",3.00415536820937,0.174754703228788,0.901293612083471,0
.193893200712715,0.84625950771765,0.960772823307934
"13777","MYH9",503.060053502859,0.0421033195311919,0.217152754279406,0
.193888029055429,0.846263557264789,0.960772823307934
"13778","MKNK1",184.432394148251,0.038687337146953,0.200268607649936,0
.193177241310716,0.846820161824559,0.961020461425446
"13779","AC245014.3",11.3363021655032,0.122917498139002,0.637152414860
976,0.192916946200106,0.847024013180843,0.961020461425446
"13780","TOMM20",1327.5896654532,-0.037443114139715,0.194660857521942,
-0.192350504443321,0.847467660101007,0.961020461425446
"13781","CAPN10-
DT",4.25736658428325,0.153894838260533,0.799304807913702,0.19253585958
3055,0.847322481441874,0.961020461425446
"13782","MRPL1",221.395891349349,0.0406269419540804,0.211053664790319,

0.192495790084683,0.847353865274684,0.961020461425446
"13783","BMP6",4.12611495920625,-0.212850573809353,1.10629804839233,-0
.192398941784872,0.847429721250511,0.961020461425446
"13784","RBM28",169.86827238501,0.0480630840846577,0.248946539618038,0
.193065885384073,0.846907369495399,0.961020461425446
"13785","PREX2",10.0679007049823,-0.130736176505397,0.679538653453091,
-0.192389609981799,0.847437030416096,0.961020461425446
"13786","CNTLN",267.867294417537,0.0316332156660218,0.163942675726835,
0.192952905799402,0.846995850639579,0.961020461425446
"13787","CHMP5",852.384950510635,-0.0375088571398348,0.19435086904367,
-0.192995572000281,0.846962435931085,0.961020461425446
"13788","SLC38A2",890.687681656592,0.0540713895815619,0.28047291161165
3,0.192786495033894,0.847126180484863,0.961020461425446
"13789","GIHCG",118.66077452244,0.0563410648392271,0.291854207158105,0
.193045237852972,0.846923539681383,0.961020461425446
"13790","ANAPC7",121.588840810089,0.0368477798536216,0.191293630690468
,0.192624185764162,0.847253302142221,0.961020461425446
"13791","MRPS26",405.163785765036,-0.0458518142174836,0.23717504674767
2,-0.19332478203858,0.846704619151147,0.961020461425446
"13792","THOC5",99.1128009494944,0.0402521126993486,0.208963028109624,
0.19262791635194,0.847250380277393,0.961020461425446
"13793","MTHFD1",66.2808687808782,-0.0478306326254283,0.24896088545274
7,-0.192121073711825,0.847647367956324,0.961154559184869
"13794","LINC01409",14.2369153463045,0.0873432391416161,0.455165814015
687,0.191893231987334,0.847825839018061,0.961287235192292
"13795","PDCD2L",29.4824703019114,0.0798488512268304,0.417089090282891
,0.191443154681109,0.848178412708283,0.961617279793951
"13796","ACTR5",25.6982880723797,-0.0735663304916874,0.384446418403065
,-0.191356524524982,0.848246279013892,0.96162451462578
"13797","LINC01068",4.26228399470811,-0.1934890774031,1.01256146916239
,-0.191088722310516,0.848456083127058,0.961653245895151
"13798","YPEL3",905.230660538189,-0.0504342148664373,0.263894778233358
,-0.191114864811152,0.848435601851806,0.961653245895151
"13799","GNL3L",166.549529441686,0.053381634035849,0.279281696752769,0
.191139035090812,0.848416665799073,0.961653245895151
"13800","MAMSTR",18.1205792903606,0.0958998475099967,0.502538820759564
,0.19083072500757,0.848658215939325,0.961812644731236
"13801","C12orf45",180.484153299366,-0.0391246616119272,0.205333316287
163,-0.190542198993223,0.84888427882902,0.961930756845072
"13802","TCEAL4",886.181027870309,0.0293128786674953,0.153840498932163
,0.190540715032529,0.848885441558547,0.961930756845072
"13803","FAM228B",97.7384034963578,0.0403713621852841,0.21238177816171
9,0.190088634414498,0.849239676129621,0.961997047771752
"13804","AC109454.2",6.40285297330421,0.119099548279892,0.626228139655
779,0.190185558166322,0.849163727499293,0.961997047771752
"13805","MBOAT1",134.080584944396,-0.0557143305873659,0.29297838955660
9,-0.190165324724747,0.849179582136998,0.961997047771752
"13806","AC079630.1",19.1878629247213,-0.0646428350812699,0.3399681024
1515,-0.19014382414716,0.849196429751354,0.961997047771752
"13807","TRAPPC6B",150.718988438801,-0.0359516317113643,0.189145881651

247,-0.190073563312645,0.849251485843004,0.961997047771752
"13808","SMAP2",112.400022808442,-0.0479362086914649,0.252833556525251
,-0.189595911833315,0.849625790990899,0.962026282376709
"13809","TMEM42",182.436300272424,0.0537683333482401,0.283522478788035
,0.189643987235446,0.849588115813968,0.962026282376709
"13810","HNRNPH3",834.781108706304,0.0287511401675704,0.15150628162899
2,0.189768634398777,0.849490435361964,0.962026282376709
"13811","DOCK1",372.041368803128,0.0389303223721489,0.20505253646144 1,
0.189855356309965,0.849422476610412,0.962026282376709
"13812","GLCE",125.882803480129,0.0337902146160839,0.178371576645207,0
.189437214446419,0.849750159566938,0.962026282376709
"13813","MTG2",99.1391896564912,-0.0367844028273837,0.19420242014909,-
0.18941269011552,0.849769379222137,0.962026282376709
"13814","EP300",318.444780767674,0.0387738258301078,0.204301631546558,
0.18978715704123 8,0.849475920185961,0.962026282376709
"13815","UBA1",493.406255163926,-0.025245308055769,0.133181376661836,-
0.189555842479914,0.849657192342245,0.962026282376709
"13816","MX2",213.050595248634,-0.0473693766563396,0.250291591995108,-
0.18925676359622,0.849891580523134,0.962094985479287
"13817","TM2D1",474.391958594644,-0.0349166651938746,0.184962786176496
,-0.188776704307191,0.85026783081445,0.962131634803187
"13818","ACAA1",360.068359910138,-0.0467272662192697,0.247727472945357
,-0.188623674490785,0.850387776323278,0.962131634803187
"13819","FIG4",87.661700524934,-0.0349609679271473,0.185268727914937,-
0.188704096587736,0.850324740663633,0.962131634803187
"13820","TSPYL1",360.040693986139,0.0342599912947624,0.181167370324513
,0.189106853145767,0.850009070366098,0.962131634803187
"13821","MEPCE",134.942265079924,0.0430512501680781,0.228076957300225,
0.18875756094645,0.85028283528345,0.962131634803187
"13822","PTPRN2",87.2463226155398,0.0525832525362873,0.278215306516086
,0.189002011408912,0.850091240327546,0.962131634803187
"13823","MT1E",3539.85141473156,-0.0703382761344702,0.372974131465791,
-0.188587545892366,0.850416094598418,0.962131634803187
"13824","AC104564.3",3.25516436323414,-0.164222083896028,0.86932187763
7282,-0.188908260703578,0.850164719045575,0.962131634803187
"13825","PLK3",236.536027662664,-0.0588622600360309,0.312625262636499,
-0.18828376037055,0.850654215034117,0.96219222702926
"13826","REP15",5.48064787958103,0.133562852220517,0.709229272544815,0
.188321121802086,0.850624928768009,0.96219222702926
"13827","EIF2AK4",416.688310186953,-0.0343221943139554,0.1822011477711
84,-0.188375291450188,0.850582467518777,0.96219222702926
"13828","AC092683.1",66.9430321758743,0.0764905617377336,0.40652353885
0806,0.188157768069134,0.850752977331243,0.962234348095215
"13829","RAD23B",608.389077852131,0.024223330437922,0.128830653806894,
0.188024586712341,0.850857377499325,0.962282839257317
"13830","ATXN7L3",74.7682514751568,0.0467285838115765,0.24866353750146
4,0.187918921612307,0.850940209677869,0.96230693270874
"13831","EXOSC7",273.175600063324,-0.045903223123799,0.24470804331818,
-0.18758363027779,0.851203059586904,0.962424910538115
"13832","SYTL2",62.017234148326,-0.051000756180758,0.271930995684309,-

0.187550360165511,0.851229142421595,0.962424910538115
"13833","CU638689.4",17.4244601388668,-0.0937781346203811,0.4996945633
20613,-0.187670912401365,0.85113463362433,0.962424910538115
"13834","ABHD13",135.491184830325,0.032750086980158,0.174860022386377,
0.187293164745183,0.85143078190606,0.962473930115816
"13835","METTL3",92.3593654455004,0.042623739424442,0.22767975509472,0
.187209176356982,0.851496630343591,0.962473930115816
"13836","GSDMB",6.48578515570161,0.133475062394526,0.713079866220409,0
.187181084079675,0.851518655435585,0.962473930115816
"13837","U2AF1L5",13.4867251843498,-0.180637575962495,0.96476454759694
3,-0.187234881725734,0.851476476752828,0.962473930115816
"13838","UBE2Q1",375.671853196365,-0.0627295002535557,0.33586845299526
,-0.186768062597534,0.851842488613894,0.962489669280878
"13839","TMEM128",192.499146103031,0.0460687883490884,0.24627331091399
2,0.187063665884516,0.851610715674619,0.962489669280878
"13840","MDFIC",247.79761786798,-0.041504534659943,0.222315083501178,-
0.186692391745534,0.851901821729872,0.962489669280878
"13841","GCC1",38.2947145217215,0.0610380898904584,0.32687394343421,0.
186732809746714,0.851870130083995,0.962489669280878
"13842","AC007216.3",6.25427107123759,0.134563292478871,0.719574691525
702,0.18700392615749,0.851657554622205,0.962489669280878
"13843","PIGS",110.505677611077,0.0402536577770641,0.215437748604948,0
.186845889532934,0.851781465799019,0.962489669280878
"13844","ALDH7A1",239.078127538743,-0.0316355024087216,0.1695607188765
9,-0.186573297272622,0.851995204775611,0.962525643072129
"13845","APOBEC3A",2.74481044884006,-0.201703748589497,1.0836101263358
7,-0.186140516489578,0.85233456953541,0.962839484834511
"13846","SCNN1D",9.2656554739431,0.0891343833183144,0.480015893473497,
0.185690483440703,0.852687491643162,0.962912846753011
"13847","RIT1",140.174439851642,-0.0334926858716913,0.180615055615886,
-0.185436843885929,0.852886412271306,0.962912846753011
"13848","ATP6V1E2",57.5023130591284,-0.0517774202624203,0.278951791505
24,-0.185614223816332,0.852747298413906,0.962912846753011
"13849","BZW1",1558.7730575561,0.0294300768535306,0.15881892252176,0.1
85305858938177,0.852989142847049,0.962912846753011
"13850","KIF9-
AS1",7.27702741919824,-0.111044422389445,0.599477348841998,-0.18523539
3136952,0.853044409712334,0.962912846753011
"13851","H2AFV",839.269495998565,0.0338978169462277,0.183227437607369,
0.185004044093363,0.853225863602252,0.962912846753011
"13852","AC083843.3",18.5858378568238,0.104861485959213,0.565377199978
47,0.185471727482478,0.852859053731899,0.962912846753011
"13853","DAB2IP",43.4075295948741,-0.0636108644659693,0.34343997672650
3,-0.185216831984081,0.853058967486127,0.962912846753011
"13854","LDB1",57.3116643099837,0.0472941581236381,0.254399837455572,0
.185904828386132,0.852519395705675,0.962912846753011
"13855","HOXC-
AS2",14.0245757485316,-0.0815786010865889,0.44125121239719,-0.18488017
4364612,0.853323021483167,0.962912846753011
"13856","MOAP1",128.818158980979,0.037575650889096,0.202436130100869,0

.18561731480627,0.852744874282085,0.962912846753011
"13857","NLGN2",83.2057144335098,-0.0442801640585385,0.239373176206771,-0.184983817987564,0.853241727903911,0.962912846753011
"13858","WDR45B",343.355434496068,-0.0368481683154298,0.199024469514384,-0.185143909215478,0.853116162325816,0.962912846753011
"13859","SNRPA",177.417033664884,0.0469817568492028,0.253788569101461,0.185121642852323,0.853133626450102,0.962912846753011
"13860","SASH3",5.1698240370642,-0.153893342016262,0.832057087840822,-0.184955268412668,0.853264120801297,0.962912846753011
"13861","PARD6G-AS1",10.7152176156326,0.0849217519910193,0.459664415031068,0.184747283483494,0.853427257626919,0.962960991940337
"13862","PPARGC1A",6.00911050788196,0.130259860238641,0.705697940751436,0.184583024431018,0.853556101683139,0.963036894410929
"13863","NCF1",9.97441717104204,-0.144125360549466,0.781438219938519,-0.184436026895133,0.853671409196404,0.96309751423442
"13864","KAT2A",34.0772109061777,0.0640001174546046,0.347320715761948,0.184268068531996,0.853803162586411,0.96317667793216
"13865","MTERF4",154.445455074101,0.0353145867478091,0.191865536694982,0.184059041327211,0.853967137745608,0.963221269699745
"13866","ARHGAP21",1018.88460828917,0.0314763931288133,0.171083943529908,0.183982157994334,0.854027451849512,0.963221269699745
"13867","TNFAIP1",85.1010998024488,-0.047694172567587,0.259116150507505,-0.184064839162565,0.85396258944404,0.963221269699745
"13868","SRRM1",918.474028459574,-0.0229650031784108,0.125250570620217,-0.183352483463289,0.854521456463669,0.963323285561702
"13869","RTCA",174.113654433337,-0.0349722010391892,0.190798851938302,-0.183293561171417,0.854567686257153,0.963323285561702
"13870","AL353807.5",73.2144534253332,-0.0611369974294061,0.332665836948231,-0.183779007758227,0.854186825013533,0.963323285561702
"13871","AL353708.3",2.69126666357882,0.183171652221073,1.00006199042315,0.183160298036693,0.85467224491395,0.963323285561702
"13872","IGFBP2",3.37983453795728,-0.154981748087286,0.843710395554606,-0.183690693991521,0.854256109803639,0.963323285561702
"13873","SMIM20",443.946847471404,-0.049518300669374,0.26997657963607,-0.183417023565989,0.854470819569664,0.963323285561702
"13874","LCA5",178.153800469832,0.0373800107208204,0.203752166393774,0.183458224677617,0.854438494302046,0.963323285561702
"13875","PTBP1",408.778282076393,0.0421616769229877,0.230178084421701,0.18316981405469,0.854664778532938,0.963323285561702
"13876","LINC00323",2.47611516973374,-0.179951723252171,0.981686844035188,-0.183308683767713,0.854555821183881,0.963323285561702
"13877","IQCB1",71.4441638932988,-0.0406204122631968,0.222432234037863,-0.18261927026405,0.855096763128161,0.963524018395133
"13878","COX7C",3142.30747368255,-0.0369918641905429,0.202534329158785,-0.182644909355301,0.855076644430874,0.963524018395133
"13879","ZNF143",94.981764390665,-0.036478392816323,0.19959320244802,-0.182763703219859,0.854983429494023,0.963524018395133
"13880","DEPDC5",38.2854254554935,-0.0558054079604386,0.305428478943635,-0.182711868105584,0.855024103121117,0.963524018395133

"13881","C4orf47",2.48122225201318,-0.184736494111422,1.01284193411486
,-0.182394199814472,0.855273377338924,0.963582706984428
"13882","STK32C",69.0952707233098,-0.0471907115256139,0.25883828192437
9,-0.182317357288752,0.855333677817443,0.963582706984428
"13883","CRACR2A",50.9102603296379,-0.0634878233149741,0.3479701281029
09,-0.182451935346008,0.855228071209022,0.963582706984428
"13884","NUP54",252.4861998675,-0.0329265148850319,0.180831799261875,-
0.182083654641675,0.855517076009394,0.963668786897511
"13885","CCDC82",412.202547985071,0.0297879117188206,0.163683835671691
,0.18198444334215,0.85559493445389,0.963668786897511
"13886","B9D1",49.0855949929388,0.0530703075669456,0.291563100710858,0
.182019972477845,0.855567051951735,0.963668786897511
"13887","CREB3L2",372.299411657587,-0.0428451339841367,0.2355885534955
51,-0.181864243183384,0.85568926629l807,0.963697801890976
"13888","MTRNR2L8",7.12728513375099,-0.118908469512998,0.6543640558274
52,-0.181716077547439,0.855805547983617,0.963697801890976
"13889","ZNF155",18.237544031188,0.0701125311555237,0.385790532548908,
0.181737303640636,0.855788889367145,0.963697801890976
"13890","PIGK",253.97727995844,-0.0418521772912364,0.23161405755481,-0
.180697915027598,0.856604695414825,0.9637072742732
"13891","C1orf52",238.365084829658,0.0379552576517041,0.20938966137206
5,0.181266149450719,0.856158674682183,0.9637072742732
"13892","CDC7",20.0540174681478,-0.075046907210817,0.4160203988883,-0.
180392373574371,0.856844541121612,0.9637072742732
"13893","USF1",31.0787256952817,-0.0634890653038042,0.350687786536174,
-0.18104156386768,0.856334951772918,0.9637072742732
"13894","MRPS14",280.059462706824,-0.0415695928316765,0.23001191151536
2,-0.180728000379668,0.856581079555967,0.9637072742732
"13895","TOR1AIP2",385.577401451481,0.0307212223987392,0.1698075023259
59,0.180917933412432,0.856431992303121,0.9637072742732
"13896","THADA",132.616341239181,0.0363004820309854,0.200591914459972,
0.180966825750243,0.856393615266805,0.9637072742732
"13897","LHCGR",22.3665455935812,0.111837282195029,0.619036470715507,0
.180663478624713,0.856631726840916,0.9637072742732
"13898","WDR75",97.6695104977616,0.0352206489358521,0.194503839022595,
0.181079453818701,0.856305211470024,0.9637072742732
"13899","PNPLA2",202.302624214536,-0.0307051680614308,0.17023357524870
4,-0.180370811202031,0.856861467777693,0.9637072742732
"13900","AC020765.2",8.69018505025853,-0.0926490727367046,0.5131127384
87917,-0.180562799921379,0.856710757224829,0.9637072742732
"13901","KLF2",7376.12230144634,-0.0409508175970238,0.226968654223709,
-0.180424991887475,0.856818935577799,0.9637072742732
"13902","ZNF845",34.4389072022347,-0.0551247165788309,0.30411964023385
6,-0.181259969058369,0.856163525572486,0.9637072742732
"13903","ZNF547",4.11299238515009,0.131047436266634,0.721658188893806,
0.181592114221707,0.855902837895482,0.9637072742732
"13904","ZNF749",56.0318708260566,-0.0818923093920299,0.45260058039653
3,-0.180937261106211,0.856416821386303,0.9637072742732
"13905","RAC2",20.1904390263967,-0.0922512455935233,0.51097225853147,-
0.180540614589631,0.856728172374989,0.9637072742732

"13906","MAFIP",4.01195283202646,0.148733980469372,0.823223101723276,0
.180672748563571,0.856624450231985,0.9637072742732
"13907","CBARP",3.86007038967387,-0.143997582711716,0.798894886493054,
-0.180245968707884,0.856959471551379,0.963748194079497
"13908","SMOC1",7.43301110978596,-0.121892265797355,0.677399652872347,
-0.179941435281964,0.857198545431768,0.96394774594139
"13909","TMUB2",181.633810862548,0.0356619792153629,0.198641221026732,
0.179529601313534,0.85752187640746,0.964242012151317
"13910","LINC01358",45.0504634750248,-0.0612061438298106,0.34123237420
089,-0.179367927715374,0.857648812936898,0.964315415839905
"13911","CTNNB1",676.832249007984,0.0404488571194644,0.225885587409766
,0.179067897085831,0.85788438896106,0.964316768331594
"13912","AC083862.3",27.0955305122722,-0.0641736779646723,0.3579629584
48322,-0.179274632891763,0.857722064179485,0.964316768331594
"13913","AL606834.1",33.259513619879,0.0617346665689265,0.344785687039
245,0.179052289261357,0.857896644153823,0.964316768331594
"13914","AC010624.5",4.7603784656754,0.166519411915762,0.9299511442311
67,0.179062537799694,0.857888597044289,0.964316768331594
"13915","MRPS18B",382.95062260092,-0.0351734971913248,0.19654571209957
6,-0.17895835434713,0.857970402144315,0.964330369352287
"13916","IFT172",42.9811020017763,-0.0577740119083867,0.32316215858223
8,-0.178777156836216,0.858112682502689,0.964420979760136
"13917","ZNF852",10.2779119849717,0.0900643222317861,0.504145696912656
,0.178647408444289,0.858214566688506,0.964466179708863
"13918","DR1",250.117375527279,0.0231015924584783,0.130003085642555,0.
177700339528836,0.858958318586969,0.964700765393226
"13919","TRAF3IP3",6.95408855958539,-0.129407460614242,0.7285588363924
96,-0.17762115309041,0.859020510938652,0.964700765393226
"13920","CFLAR-
AS1",3.07653063197551,0.193538799805133,1.09218374122714,0.17720351667
9052,0.859348533469209,0.964700765393226
"13921","ANKZF1",78.6010827197641,0.0523873240410864,0.294656726945428
,0.177791033600902,0.858887089313378,0.964700765393226
"13922","PLA1A",2.7974996327101,0.169617273869717,0.956620613214315,0.
177308821832503,0.859265821763956,0.964700765393226
"13923","RNF139",408.730483342628,0.0326057638048896,0.183414460138757
,0.177770955355552,0.858902858258612,0.964700765393226
"13924","PIP5KL1",9.9557879542934,0.114436643071343,0.64510528096649,0
.17739219697581,0.859200336033155,0.964700765393226
"13925","STIM1",361.350188373381,-0.030412329549836,0.170747988241447,
-0.178112374049358,0.858634724329866,0.964700765393226
"13926","DOCK9-
DT",4.58024968286728,0.135513408715425,0.762894733989979,0.17763054675
5361,0.859013133188779,0.964700765393226
"13927","ALDH1A2",3.72608913299338,0.173627159524677,0.9741210920522,0
.178239811191125,0.858534645464983,0.964700765393226
"13928","EIF3C",34.3133789326353,-0.0745691923383603,0.420774508144115
,-0.177218892530487,0.859336456443671,0.964700765393226
"13929","AC005332.2",5.50401510680775,0.123804483502022,0.698034319004
485,0.177361599754276,0.859224368040245,0.964700765393226

"13930","ZNF606",25.919849993116,0.0697531763220624,0.393345919943233,
0.177332909242148,0.85924690257709,0.964700765393226
"13931","TRAPPC10",101.084646551934,-0.0465538699620007,0.261384152883
152,-0.178105173739481,0.858640378941006,0.964700765393226
"13932","C22orf39",204.300251863473,0.0319978757710867,0.1803738805979
2,0.177397501595116,0.85919616963384,0.964700765393226
"13933","TRIB2",6.60413494250907,-0.133371546187537,0.753787878417791,
-0.176935116637171,0.859559354680438,0.96472969552939
"13934","B3GNT10",3.20052202370064,0.159557928436162,0.901784649720343
,0.176935733476549,0.85955887015776,0.96472969552939
"13935","SIRT3",57.2878871044719,-0.0441215219097723,0.249360901446093
,-0.176938411971976,0.859556766220421,0.96472969552939
"13936","BPNT1",50.2375369337267,-0.0439092102343602,0.248386069409775
,-0.176778071083854,0.85968271449798,0.964757043932867
"13937","SNHG1",72.8771578880533,0.0414620140443467,0.23465378739318,0
.176694416505939,0.859748426852357,0.964757043932867
"13938","BCL11B",12.835980245689,0.125234710545927,0.709029395261669,0
.176628375893653,0.859800303768376,0.964757043932867
"13939","NAA38",877.144065674052,-0.0456011002410939,0.258231522510358
,-0.176589983274659,0.859830462620219,0.964757043932867
"13940","TSN",240.504027299325,0.0284710088651524,0.161691671000016,0.
176082099276157,0.860229443904535,0.964777742364888
"13941","LAPTM4B",201.384546585475,0.0499130021330197,0.28323175582726
3,0.176226715776393,0.86011583307052,0.964777742364888
"13942","AL132656.3",3.36158086231172,0.152329469178069,0.865409318445
649,0.176020139754985,0.860278120237523,0.964777742364888
"13943","BIRC2",409.712199005569,-0.0263127141434659,0.14972048450718,
-0.175745585048545,0.86049382093558,0.964777742364888
"13944","IQGAP1",1378.97258911956,-0.0285142773427478,0.16169029063894
2,-0.176351203464782,0.860018037765431,0.964777742364888
"13945","ZNF689",54.4533010894051,0.060660217450693,0.344925116477706,
0.175864889371179,0.86040008960844,0.964777742364888
"13946","OSBPL7",8.81599060502877,0.0863052709540975,0.490948713674455
,0.175792844650014,0.860456691239687,0.964777742364888
"13947","STAP2",10.1835301875936,-0.118310857725629,0.673357968684029,
-0.175702766177772,0.860527462019833,0.964777742364888
"13948","NOP53",2559.36928392984,0.0362682525434202,0.206316221459344,
0.175789631502955,0.860459215651819,0.964777742364888
"13949","NONO",491.104999183282,0.0227642519028991,0.129070198825827,0
.176371091932834,0.860002413938928,0.964777742364888
"13950","AC134312.6",2.56721314127853,0.23965891696051,1.3608116283252
6,0.176114689184025,0.8602038409952,0.964777742364888
"13951","IGF2",66.8159900927719,0.187896982904922,1.07150194366699,0.1
75358508694707,0.860797940628623,0.964942645601466
"13952","SNAI1",5.30725582651098,0.127230669720206,0.725241857762938,0
.175432055332077,0.860740154623429,0.964942645601466
"13953","KDM1A",198.865610920596,0.0299373047079885,0.170976988387968,
0.175095520106233,0.861004578331603,0.964996246526074
"13954","PDIK1L",42.0098223844911,0.0459055181889858,0.262813638686354
,0.174669466997373,0.861339360709974,0.964996246526074

"13955","EIF2B4",116.331038013876,-0.0332247212166642,0.189648874456242,-0.17519071131809,0.860929782757266,0.964996246526074
"13956","R3HDM1",85.3681100179039,-0.0352199822710501,0.2014219416663,-0.174856730998054,0.8611922100492,0.964996246526074
"13957","TPCN2",26.9683318275482,-0.0653970352569118,0.374344790447686,-0.17469732964282,0.861317466145218,0.964996246526074
"13958","AQP11",7.23364196051356,0.0973001872751521,0.556522501916502,0.174836034374313,0.861208473065664,0.964996246526074
"13959","ARF6",654.340644499367,-0.0327738165639305,0.187475033595748,-0.174816965946519,0.86122345672855,0.964996246526074
"13960","MAPK1",410.30997948795,0.0270020401260522,0.154380485029897,0.174905786316341,0.861153663540674,0.964996246526074
"13961","AL109627.1",2.59414034771911,0.184078362082554,1.05512571025353,0.174461071599064,0.861503121924458,0.964998588899507
"13962","LINC02777",3.74473802747201,0.139135514831914,0.80053500087686,0.17380316248448,0.862020158920398,0.964998588899507
"13963","ITPR1",26.3284411282217,-0.0738827611103873,0.42420526726103,-0.174167476956208,0.861733844487041,0.964998588899507
"13964","FAM241A",41.9497471730481,-0.0632848744993934,0.362978952448096,-0.174348606365662,0.861591501797249,0.964998588899507
"13965","LINC02104",20.3133128835371,0.075793562837428,0.435799297875256,0.173918506080575,0.861929508523288,0.964998588899507
"13966","RING1",177.052420420265,0.0315091959565912,0.181218751002158,0.173873817043447,0.861964630144399,0.964998588899507
"13967","EPHA1",2.85866155021672,-0.161904450672191,0.930502197929728,-0.173996849263131,0.861867938386375,0.964998588899507
"13968","CLDN23",5.8471846047894,-0.137230623557858,0.788168983213567,-0.174113199682552,0.861776499799467,0.964998588899507
"13969","AC011978.2",5.71426163188031,0.1326941565717,0.762358994087505,0.174057311057931,0.861820421858321,0.964998588899507
"13970","ZNF169",17.6736794855746,0.0727439793630145,0.417338771557548,0.174304388474445,0.861626250535752,0.964998588899507
"13971","SPRYD3",777.74026837579,0.0255258307070811,0.146485340224335,0.174255189413422,0.861664914053565,0.964998588899507
"13972","METTL6",74.3802753507294,0.0457418493445414,0.263565336411293,0.173550323298817,0.862218875680763,0.965151962184879
"13973","LINC00240",4.62003947516865,-0.125060935996675,0.721184510491005,-0.173410457625511,0.862328805637854,0.965167054035245
"13974","FAM131B",10.0639744111568,0.0930021454483644,0.536418373411033,0.17337613709421,0.862355780888012,0.965167054035245
"13975","SLC9A1",73.8910945526792,-0.0392532312120255,0.226750501469251,-0.173111992951198,0.862563398262911,0.9653303433869
"13976","ABHD14A",455.664326995957,0.0444887636955488,0.257698516071252,0.172638804343165,0.862935348377261,0.965332154253656
"13977","DNAH1",7.08837120063471,0.0984093309540366,0.568979008045595,0.172957753383673,0.862684635000081,0.965332154253656
"13978","SSH3",66.8772970156226,-0.0448294987268386,0.259512559918148,-0.172745006025828,0.862851865850233,0.965332154253656
"13979","PPME1",105.687384223489,0.034875593201187,0.201994266224302,0.172656352346456,0.862921554218491,0.965332154253656

"13980","ATP2A3",7.18286906114302,0.130500941778708,0.755503027097799,
0.172733843675011,0.862860640227274,0.965332154253656
"13981","AC008982.2",9.08702822080837,-0.108820479293867,0.62991145355
9183,-0.17275520024124,0.862843852509125,0.965332154253656
"13982","HECA",176.552713461137,-0.0425577238228947,0.246916963071504,
-0.172356420124002,0.863157331100129,0.965511418853241
"13983","ADAL",131.018840705409,-0.0547143447707039,0.317749975159858,
-0.172193073321808,0.863285743237365,0.965530445734709
"13984","CNDP2",197.713298363499,-0.0289424541770169,0.168096392299798
,-0.172177723632512,0.863297810303975,0.965530445734709
"13985","RRAGC",438.816053567306,-0.0405961625264775,0.236221295582418
,-0.171856489172093,0.863550354170779,0.965546488126253
"13986","ZNF131",237.049699214937,0.0324725219586197,0.188963614174398
,0.17184536875258,0.863559096925194,0.965546488126253
"13987","PRKN",21.5156544259376,0.0601273672305569,0.349807361940717,0
.171887083499251,0.863526301325493,0.965546488126253
"13988","NPIPA3",3.3702086251377,0.175649259706983,1.02117976883153,0.
172006207984287,0.863432648475448,0.965546488126253
"13989","PGAP1",52.4335510625238,-0.0481548521910903,0.280710197220888
,-0.171546501223814,0.863794069622168,0.965615449682266
"13990","FBXL13",7.01375636605684,-0.0992960677127451,0.57864414278418
7,-0.171601266427713,0.863751011758605,0.965615449682266
"13991","AL139339.1",16.6720646421598,0.0961842070236924,0.56073840765
0111,0.17153133388307,0.863805994661419,0.965615449682266
"13992","ABHD6",28.2970212317942,0.0667934812445504,0.389580959064599,
0.171449552886066,0.863870293982151,0.965618310311667
"13993","TXLNA",301.312851975101,0.0343005283060866,0.202190315994476,
0.169644763337843,0.865289516700383,0.965717289553274
"13994","RAVER2",16.287915888348,-0.0678942607526515,0.397869124015915
,-0.170644708660367,0.864503141060725,0.965717289553274
"13995","LEPROT",1817.89300000378,-0.0200664917871294,0.11802371248289
9,-0.170020848903875,0.864993740308105,0.965717289553274
"13996","NEMP2",23.2732917035667,0.0603802062492419,0.354913847673799,
0.170126374738518,0.864910751817461,0.965717289553274
"13997","LRTM1",6.24718278973968,0.148530551357534,0.872224925305467,0
.170289276364713,0.864782644312658,0.965717289553274
"13998","MAEA",231.935630863541,0.0283023574657677,0.166925218971351,0
.169551117800991,0.865363168139767,0.965717289553274
"13999","LSM6",396.17929595698,-0.0441974037798045,0.25901894632052,-0
.170633864462999,0.864511668421396,0.965717289553274
"14000","SLC17A5",235.935191305106,-0.0293900509565968,0.1719127129283
75,-0.170959148139566,0.864255887686698,0.965717289553274
"14001","TBC1D32",31.4369704500367,0.0539831795062197,0.31757229527945
2,0.169987055888224,0.865020316402879,0.965717289553274
"14002","ZNF212",30.8085498715928,0.0527757414187311,0.309319326826646
,0.170618958602314,0.864523389706533,0.965717289553274
"14003","COL5A1",1608.09961716745,0.0578322198131117,0.33909534575931,
0.170548550831957,0.864578755550566,0.965717289553274
"14004","ARRDC1-
AS1",52.8676277265589,-0.0501176625612536,0.295443916335412,-0.1696351

14450473,0.865297105416354,0.965717289553274
"14005","AGAP5",20.34079529272,0.0909048601027761,0.535542923866361,0.
169743368928278,0.86521196548459,0.965717289553274
"14006","AC022400.4",2.66717610200683,-0.149803475349099,0.87652034402
3822,-0.170907014732139,0.864296880860031,0.965717289553274
"14007","BCO2",27.8957545897832,-0.0562880828663264,0.331350126817179,
-0.169874939861976,0.865108489743654,0.965717289553274
"14008","RPL23",10193.1991585581,0.0288215562572799,0.169874684134781,
0.16966363413169,0.865274675118892,0.965717289553274
"14009","HOXB-
AS1",119.590781737385,0.0659680913181301,0.38552536693775,0.1711121938
41143,0.864135547980791,0.965717289553274
"14010","CASKIN2",38.708241997563,-0.0441817381848804,0.25894346248610
3,-0.170623107301856,0.864520127356513,0.965717289553274
"14011","IMPACT",154.319530518252,-0.037498409551798,0.220307429896634
,-0.170209463972195,0.864845409150531,0.965717289553274
"14012","FUZ",92.500693537029,0.0512347741527823,0.300802515946498,0.1
70326946872663,0.864753020345747,0.965717289553274
"14013","LRP5L",11.989268338893,-0.0873257139132311,0.515060325308443,
-0.169544633166875,0.86536826829411,0.965717289553274
"14014","TTC38",54.068990647437,0.0383560872916639,0.226093700760485,0
.169646863944683,0.865287864603911,0.965717289553274
"14015","ZFX",291.70907080701,0.0298936302650424,0.176331384590264,0.1
69530967697584,0.86537901618217,0.965717289553274
"14016","AL592148.3",25.0734696408392,-0.0613410658846506,0.3627667128
22859,-0.169092322190553,0.865724023976423,0.965765672369066
"14017","CAMK1",184.032585169618,0.0457325073992507,0.270438611616396,
0.169104948165168,0.865714092912222,0.965765672369066
"14018","C5",29.2137725934167,-0.0547103141879591,0.323706509658202,-0
.169012091371678,0.865787130682388,0.965765672369066
"14019","PGR-
AS1",4.13444209204637,-0.138865383968521,0.819965564930508,-0.16935514
1127529,0.865517305954556,0.965765672369066
"14020","CLCN7",104.653902506007,0.0323056238488733,0.191095801095709,
0.169054598079281,0.865753696312785,0.965765672369066
"14021","SNHG29",3222.98829684106,-0.0471272394357323,0.27890107623887
2,-0.168974749295585,0.865816502921945,0.965765672369066
"14022","DDT",552.802411219317,-0.0386860464253709,0.229011401755318,-
0.168926289821605,0.865854620074107,0.965765672369066
"14023","POLE2",7.23029240055248,-0.110973062956348,0.659529101146309,
-0.16826105590105,0.866377909891748,0.966280432910714
"14024","GAS5",1624.72993838851,0.0381122233145482,0.226813623678877,0
.168033219064952,0.866557145584131,0.966342513863516
"14025","TACC1",1941.43642023611,0.0397276433823016,0.23635410510217,0
.168085269198638,0.866516197949103,0.966342513863516
"14026","RWDD3",175.867171472736,-0.040222937743643,0.239586912746604,
-0.167884536273416,0.866674115722532,0.966404047476145
"14027","GORAB-
AS1",4.47154430148447,-0.140951764374515,0.840687591240604,-0.16766247
7528082,0.866848816843917,0.966434992893998

"14028","GSTA4",282.345570942962,0.0400620727086932,0.238903235825207,
0.16769162866427,0.866825882288587,0.966434992893998
"14029","NOMO3",7.89079915707866,-0.0998895430169464,0.595951183252116
,-0.167613633170166,0.866887245224418,0.966434992893998
"14030","AC011445.2",3.77421812768638,-0.13898797505173,0.829775394341
753,-0.167500718868612,0.86697608194467,0.966465140528484
"14031","AC078909.1",6.1529487346173,-0.118101017541266,0.706543145670
663,-0.167153298797008,0.867249429506935,0.966700953423737
"14032","LRRC17",1274.84236156831,-0.0657000939764926,0.39409929824728
9,-0.16670949242662,0.867598636188791,0.966711897567727
"14033","HGSNAT",537.258705592283,0.0336910879475848,0.202278398575366
,0.166558012051059,0.867717833634411,0.966711897567727
"14034","LINC00545",17.463392973301,0.113128053420574,0.67837411041491
6,0.166763518365082,0.867556124783945,0.966711897567727
"14035","DOCK9",184.658943460975,0.0415324525325937,0.24942068859988,0
.166515667829063,0.867751154152465,0.966711897567727
"14036","AC020659.1",3.52980911805129,-0.133289158294089,0.79838638563
5226,-0.166948185355189,0.867410818712123,0.966711897567727
"14037","AC008915.2",32.7506462241409,0.0630939450196898,0.37834457270
4399,0.166763182483881,0.867556389077694,0.966711897567727
"14038","USP25",178.9237899879,-0.0322450903362151,0.193503453725375,-
0.16663831944818,0.867654640674069,0.966711897567727
"14039","UPF3B",109.541592254609,-0.0350130726811529,0.210273071144683
,-0.166512395004025,0.867753729536657,0.966711897567727
"14040","CENPI",2.8511248105231,0.138895920245812,0.834743805366852,0.
166393472287908,0.867847310707305,0.966747289135488
"14041","RBM22",225.619924497955,-0.0250701703236743,0.151058436468495
,-0.165963390789517,0.868185760489081,0.966900166433275
"14042","RPF2",196.870510620188,-0.0363757909800709,0.2195446598064,-0
.165687432398256,0.868402936841783,0.966900166433275
"14043","UMAD1",83.7078798461,-0.0349127019757816,0.210286097748295,-0
.166024774579111,0.868137453443723,0.966900166433275
"14044","RALGPS1",32.8900131436151,-0.0550977898510543,0.3325552194301
24,-0.165680123576083,0.868408688943048,0.966900166433275
"14045","TOX4",344.247148943421,0.0229521126700891,0.138541840572965,0
.165669176727885,0.868417304212801,0.966900166433275
"14046","RAMAC",122.426087482949,-0.0445288274023211,0.268722830027343
,-0.165705412516645,0.868388786375044,0.966900166433275
"14047","RPGRIP1L",45.7789278791423,-0.0471721877767469,0.284015017673
427,-0.166090470015172,0.868085753818265,0.966900166433275
"14048","ABHD14B",304.075511825685,0.0430329516284966,0.26011232701223
2,0.165439877928095,0.868597768057275,0.96698805438635
"14049","ITGB2-
AS1",4.71407770135027,-0.132713497087952,0.802703222824175,-0.16533320
5740749,0.868681724049119,0.96698805438635
"14050","L1CAM",3.21083867885828,-0.141690567840438,0.856995967012345,
-0.165333996068149,0.86868110201911,0.96698805438635
"14051","EVC",51.4160813218325,-0.0670772397455748,0.40668747967392,-0
.164935590835884,0.868994678581917,0.96706110523845
"14052","TECR",370.078777255527,0.0384702492174043,0.23308214531316,0.

165050176476268,0.868904488468351,0.96706110523845
"14053","ZNF607",12.1101468375511,0.078877798060252,0.478213371022365,
0.164942686340242,0.868989093676026,0.96706110523845
"14054","FPR1",5.3273551184407,0.140182848320248,0.849575658300613,0.1
65003371919404,0.8689413280294,0.96706110523845
"14055","EIF4G3",323.080199603752,-0.0307226464758862,0.18656109976925
8,-0.164678738032122,0.869196853067944,0.967203306972014
"14056","COLEC12",438.964220771815,0.0559424159780325,0.33983559090658
5,0.164616118720214,0.869246143401447,0.967203306972014
"14057","AC067852.2",31.2107500185456,-0.0490481088001308,0.2982775596
46055,-0.164437810401603,0.869386500273312,0.967290664030348
"14058","GALK2",139.234475032896,0.0385036048777276,0.23431863622409,0
.164321564422664,0.869478006476106,0.967323660640652
"14059","COL5A3",189.545586956529,0.073451721833014,0.447537899606272,
0.1641240259152,0.869633508325131,0.967427844811512
"14060","AC017083.2",3.9236014334347,-0.125487179540495,0.765885486738
356,-0.163845877371174,0.869852474751552,0.967602610605567
"14061","MOB3B",19.2107448055907,0.0992859903529005,0.606950557526511,
0.16358167748872,0.870060469637215,0.96769632663391
"14062","XPOT",153.337953094497,-0.0348643713927283,0.213103493518457,
-0.163603002546313,0.870043680867147,0.96769632663391
"14063","AL512343.2",3.69060146575347,0.128265147074123,0.784754876133
588,0.163446129453916,0.870167185127752,0.967724951255466
"14064","CRYGS",5.7221687980615,0.110118926904049,0.674051909128417,0.
163368615106273,0.870228212391595,0.967724951255466
"14065","CUL1",183.414073541577,-0.0256159802216262,0.156880344313013,
-0.163283554315232,0.870295181880867,0.967724951255466
"14066","ATP6V0C",1343.19936784128,-0.0402020080238514,0.2462835834409
33,-0.16323462352696,0.870333706161086,0.967724951255466
"14067","ETV3",72.7520343446442,-0.0526294693276389,0.3230371093635,-0
.162920815603316,0.870580781316346,0.96779580290061
"14068","DUOXA1",8.37674122291996,-0.148205331585493,0.909693280048715
,-0.162917914022137,0.870583065921271,0.96779580290061
"14069","DCAF12L2",22.3979234529053,-0.0751176578644725,0.460870244004
408,-0.162990904363429,0.870525596172632,0.96779580290061
"14070","MTX2",232.950629648941,0.0440214754889119,0.270756225331585,0
.162587122179741,0.870843527081862,0.967814355037979
"14071","AL662844.4",82.6135248300178,-0.0719701844517249,0.4423519701
43931,-0.162698912425568,0.870755503339153,0.967814355037979
"14072","Z94721.3",5.61808574683712,-0.1154316446587,0.709988741329049
,-0.162582359323925,0.870847277394468,0.967814355037979
"14073","DIABLO",4.98657144283633,-0.111687409742365,0.686708566384385
,-0.162641643354493,0.870800596858624,0.967814355037979
"14074","AC020656.1",3.28133620503447,-0.140832001342946,0.86724907681
1941,-0.162389335553579,0.870999268372536,0.967839554609724
"14075","THAP11",187.365628616284,0.0391556150507106,0.241039682428639
,0.16244468402958,0.870955685330541,0.967839554609724
"14076","NUP88",76.7944719259049,-0.0373339666295847,0.230228305876961
,-0.162160627848849,0.871179363797679,0.967839554609724
"14077","TADA2A",60.4118636679469,0.043407823011687,0.267564323859448,

0.16223322446564,0.8711221970128,0.967839554609724
"14078","UBE2G2",316.123512449565,-0.0407252399150793,0.25104355508024
1,-0.162223801770423,0.871129616951281,0.967839554609724
"14079","SFMBT1",56.4876435163973,-0.0393048386542242,0.24290196040168
3,-0.161813591743872,0.871452649439181,0.968028461317311
"14080","AL355353.1",4.12303258378725,-0.121368224152729,0.75037763037
6239,-0.16174286018078,0.871508351350461,0.968028461317311
"14081","DCPS",154.075605301966,-0.0418688554018433,0.258914955442918,
-0.161708910673853,0.871535087200068,0.968028461317311
"14082","VCAN-
AS1",4.18197190340141,-0.138599823455894,0.857559105142147,-0.16162130
7061885,0.871604077303992,0.968036342070334
"14083","SMN2",19.730162153239,-0.0846409925697771,0.524723219483224,-
0.161305978899002,0.871852414447553,0.968037183157726
"14084","ZBTB9",9.502005941602,0.0839504702669056,0.520180011756272,0.
16138734355337,0.871788334388025,0.968037183157726
"14085","FBRS",118.052557024388,0.0286358918114159,0.177489057469285,0
.16133891418276,0.871826475626751,0.968037183157726
"14086","AC132938.3",15.52650897889,0.0681601261281054,0.4221146390427
7,0.161473021363751,0.87172085834173,0.968037183157726
"14087","ABHD1",4.30522526920387,-0.120376044202936,0.74751129650663,-
0.161035752590621,0.872065241613218,0.968086364333258
"14088","RMND5A",141.092548275903,-0.0313442602942696,0.19485974594535
5,-0.160855491944753,0.87220721799744,0.968086364333258
"14089","TOMM7",3601.52526441188,-0.0274051523796687,0.170359700692177
,-0.160866403664251,0.872198623622436,0.968086364333258
"14090","AC107375.1",23.4130168015863,0.071649572507084,0.445814712267
805,0.160716034117877,0.87231706020454,0.968086364333258
"14091","MAPKAPK5-
AS1",144.216422138596,0.0416509874070059,0.258993264703611,0.160818805
286967,0.872236113544261,0.968086364333258
"14092","TPSAB1",11.1058660468032,0.1319723011511,0.819834748198556,0.
160974271267577,0.872113664897518,0.968086364333258
"14093","INSR",229.173548793715,0.0500493044066824,0.311446331693461,0
.16069961118034,0.872329995687251,0.968086364333258
"14094","AL162595.1",8.13608729635553,-0.0882013102714813,0.5515586835
52295,-0.159912830495975,0.872949741315358,0.968209781737857
"14095","AC010136.1",2.9917452330615,0.165516726957069,1.0363605525553
1,0.159709597735037,0.8731098400572,0.968209781737857
"14096","RIOX2",113.758914146054,0.0309997814746848,0.193773434963873,
0.159979521859972,0.872897205626482,0.968209781737857
"14097","LEKR1",2.53788817073946,-0.172835534265783,1.07824378919542,-
0.16029355883863,0.87264983257355,0.968209781737857
"14098","NOP14-
AS1",43.7909492171857,0.0409345596619209,0.256119475632266,0.159826032
600092,0.873018116633244,0.968209781737857
"14099","AC090198.1",7.89994863067353,0.107925239463158,0.674134574313
618,0.160094502752725,0.872806631491291,0.968209781737857
"14100","AP002748.4",20.4472457775623,-0.0671286941184587,0.4197535231
05884,-0.159924075494955,0.872940883101163,0.968209781737857

"14101","CEP128",19.8080489281493,0.0640994520500541,0.40120659258105,
0.159766696847348,0.873064859106621,0.968209781737857
"14102","SEC14L5",10.7303285647393,-0.0788913901271815,0.4928926223044
41,-0.160057965076323,0.872835413209621,0.968209781737857
"14103","IL4R",291.719944887765,0.0278708224030942,0.17421419248077,0.
159980206010889,0.872896666693945,0.968209781737857
"14104","BST2",518.767489864392,0.11033383440459,0.691248219548573,0.1
59615361435065,0.87318407745604,0.968209781737857
"14105","ENOX2",129.981393522277,-0.0456727918361151,0.286018948155798
,-0.159684496885977,0.873129613875341,0.968209781737857
"14106","CEP78",50.6393092974062,0.0559520046962072,0.350718378101658,
0.159535422691733,0.873247052412272,0.968210966945125
"14107","PABPC1L",6.79620465311027,-0.0986317385520356,0.6190623895843
46,-0.159324391550033,0.873413304528972,0.96832665221756
"14108","CHCHD4",48.1268966740385,-0.04585566490053,0.288294624740668,
-0.159058341589889,0.873622908900787,0.968421737558176
"14109","TIGD2",6.27362446115529,0.0921261971838033,0.579164828025985,
0.159067320261495,0.873615835013914,0.968421737558176
"14110","CCS",406.626220562212,0.0392841228807972,0.24711367284473,0.1
58971870834039,0.873691035792384,0.968428617986739
"14111","LIX1L",216.19992734456,0.0257565656247347,0.16219208712861 7,0
.158802849637849,0.873824203617783,0.968438955823564
"14112","TMEM104",30.1240340484543,0.0484360894041218,0.30492550455385
9,0.15884564813622,0.873790483349361,0.968438955823564
"14113","SLC36A1",40.9051637969307,-0.0471427528218636,0.2974495735580
92,-0.158489898835429,0.874070779628232,0.968643590546698
"14114","STON1",8.37908845375964,0.0923040349808043,0.583417652394916,
0.158212619384927,0.874289260163652,0.968817063125941
"14115","ARMC9",37.7253079729855,0.0453211088592656,0.286948130553536,
0.157941816076094,0.874502647117164,0.968916222790624
"14116","TAF5",26.2023136336586,-0.0530387729485891,0.335646736136342,
-0.15801962968305,0.874441330799246,0.968916222790624
"14117","LINC00346",15.4785022341176,-0.117652426752817,0.745772985691
231,-0.157759035269653,0.874646679466639,0.96900715923059
"14118","LINC01135",5.01354018060897,0.108013423253098,0.6880351565796
58,0.156988232679928,0.875254122069097,0.969238127137049
"14119","MAP3K20",733.216256822651,-0.0300161800942791,0.1915240535010
61,-0.156722769519458,0.875463341350684,0.969238127137049
"14120","NAA80",45.3772324211441,0.0435345211983213,0.277806385139579,
0.156708137490966,0.875474873533574,0.969238127137049
"14121","AC008393.1",16.2437826435164,-0.103461692487035,0.66014906056
2321,-0.156724743952381,0.875461785210197,0.969238127137049
"14122","PHACTR1",46.2877576210511,-0.0499139232343876,0.3176719018620
73,-0.157124136386665,0.875147015756131,0.969238127137049
"14123","HLA-
E",5224.11524011336,-0.0298654679288993,0.189773060850329,-0.157374644
193855,0.874949595438894,0.969238127137049
"14124","AC067930.3",2.87981087212163,-0.22693268181199,1.445265901376
28,-0.157017944999525,0.875230705463223,0.969238127137049
"14125","MMP27",81.9880397201413,0.0615692775361352,0.392491389984914,

0.156867842472931,0.875349004033072,0.969238127137049
"14126","NOP56",345.699597174006,-0.029579197601849,0.188650972100785,
-0.156793242422555,0.875407798745144,0.969238127137049
"14127","MTM1",29.6360743399112,0.0477344491523082,0.3039046405816,0.1
57070484547245,0.875189298735191,0.969238127137049
"14128","ITIH4",27.1755499577635,0.0853431669309857,0.545909820412592,
0.156331986968991,0.875771344343373,0.969429105069739
"14129","SPTLC3",31.7546225763876,-0.0437641283110928,0.27982688787719
5,-0.15639715197883,0.875719981944061,0.969429105069739
"14130","ODR4",208.075976869937,0.0302119247624398,0.193925307132405,0
.155791552991133,0.876197329063318,0.9695599584853
"14131","EXOG",105.205206779653,0.0344415259679795,0.220699234774122,0
.156056390513675,0.875988572455492,0.9695599584853
"14132","TMEM154",20.1863774410757,-0.0848884509909755,0.5441182559916
05,-0.156011032631637,0.876024324925352,0.9695599584853
"14133","AP000654.1",2.64960891704892,-0.158231429794366,1.01489720327
422,-0.155908824345841,0.876104889549441,0.9695599584853
"14134","FBLN1",6879.3695865375,-0.0430137470003718,0.276102989570964,
-0.155788776743095,0.876199517470027,0.9695599584853
"14135","HNRNPK",1962.96047333866,0.0186133445678429,0.11964178666350 4
,0.155575615233776,0.876367547096267,0.969677285927529
"14136","ADIPOR1",188.448744671912,-0.0293393204083245,0.1887487911554
07,-0.155441103642184,0.876473581923597,0.969726006033182
"14137","PHF23",76.9453191598667,-0.0382314748168827,0.246265176669861
,-0.155245152131822,0.876628053504124,0.969759708360766
"14138","AC002377.1",3.74119970713901,-0.147139601996502,0.94765144706
2428,-0.155267638172887,0.876610327174338,0.969759708360766
"14139","CCDC92",181.906706381032,0.0330101799329014,0.21275431822379,
0.155156333410723,0.876698072212071,0.969768572699398
"14140","MFSD14A",192.556630884522,-0.0283159038138044,0.1827641312876
23,-0.154931405929112,0.876875394230154,0.969896122048063
"14141","SFI1",41.8111455371737,-0.0542966452787611,0.350710112213986,
-0.154819160861925,0.876963885159272,0.969925405833464
"14142","RAB7B",188.8006054083,-0.0318833392994671,0.2062717409977,-0.
15456959419285,0.877160642177967,0.970037485519591
"14143","AC146944.4",21.6605602473137,0.0684599616288213,0.44301110541
3592,0.154533285491585,0.87718926839537,0.970037485519591
"14144","LTB4R",20.0714691911345,-0.0663823069183535,0.429873848134927
,-0.154422761948333,0.877276407476538,0.970064224965449
"14145","DIO2",75.1303573393172,-0.164122805663107,1.063348401525,-0.1
54345278958177,0.877337497579046,0.970064224965449
"14146","MVB12A",426.655947850134,0.0377184858667382,0.244841001935789
,0.154052979560303,0.877567962478382,0.970250454768973
"14147","KLHDC9",26.2938009719313,-0.0534473736164317,0.34815377686275
3,-0.153516569884869,0.877990924311589,0.970443678885742
"14148","NDUFS4",910.055187732035,-0.0379973656411427,0.24750723411690
8,-0.153520222456185,0.877988044122096,0.970443678885742
"14149","NEDD1",120.533304866957,0.0334066052055573,0.21751136457242,0
.153585562166958,0.877936521582113,0.970443678885742
"14150","USP7",306.311092736936,-0.0299804721364517,0.195022376868859,

-0.153728370137811,0.877823914536978,0.970443678885742
"14151","C1orf159",24.2948637194707,-0.0582860085557365,0.380854658087965,-0.153040030672998,0.878366707191889,0.970647454846804
"14152","NRDC",337.14203763579,0.0260551562507036,0.17026292534078,0.153028947426777,0.878375447395524,0.970647454846804
"14153","AC087500.1",12.0751944151611,-0.0681716291952119,0.445659501287092,-0.152967969937425,0.878423534264812,0.970647454846804
"14154","ZNF177",16.9895620252371,-0.0592600514035026,0.386863620282974,-0.153180729064564,0.878255754292133,0.970647454846804
"14155","RETREG2",278.757742921682,-0.0258757011784837,0.169365568425778,-0.152780174972951,0.878571632256117,0.970648038902898
"14156","GUCY1A1",12.0963158998174,-0.102584118795053,0.671938992005616,-0.15266879882779,0.878659467201282,0.970648038902898
"14157","NLRX1",40.5075912025184,0.0404143447970685,0.264918850368252,0.152553677252073,0.878750257487683,0.970648038902898
"14158","AC009318.2",6.03986821906718,0.101916410498243,0.668173831902969,0.152529784364621,0.878769100741761,0.970648038902898
"14159","TIMELESS",7.91663651194222,-0.088359150079291,0.579855630443699,-0.152381291894466,0.878886211665502,0.970648038902898
"14160","SPAG9",803.74752773916,-0.0267843592612308,0.175462453774186,-0.152650089435666,0.878674222198869,0.970648038902898
"14161","AC005523.2",25.7386590774288,-0.0569460127401942,0.373774832282004,-0.152353791164917,0.878907900839607,0.970648038902898
"14162","PTOV1-AS1",4.33834899218329,0.104970641605055,0.689065229663411,0.152337742620289,0.878920557988672,0.970648038902898
"14163","JAZF1-AS1",2.48662695974516,0.162090085136863,1.06480379818657,0.152225307059303,0.879009234414673,0.970677428951881
"14164","BNIP3",364.775722652543,-0.0320422410143065,0.210774584484785,-0.152021369619255,0.879170081023561,0.970733749407646
"14165","GRN",1324.14307306458,0.0447818295772916,0.294610997054293,0.152003251830545,0.879184370866963,0.970733749407646
"14166","APPL1",617.067494511421,0.0207238186640063,0.136676512198402,0.151626774276499,0.879481314748694,0.970769122967199
"14167","SEPTIN8",347.709921204329,-0.0305880108916285,0.201748121003532,-0.15161484894867,0.879490721039869,0.970769122967199
"14168","AC246817.1",5.30988293634143,0.143217507570487,0.943311441727322,0.151824202734399,0.879325592398654,0.970769122967199
"14169","CENPV",36.6899430384226,0.0671758977489405,0.442923815581786,0.151664677729519,0.87945141791283,0.970769122967199
"14170","AC145207.5",14.9838943403567,0.0706081550680469,0.465847759362871,0.151569163206057,0.879526756550205,0.970769122967199
"14171","AHCTF1",180.617333220263,0.0290753328349134,0.191935560056731,0.1514848672456,0.879593247269171,0.970774002349152
"14172","ZBTB12",3.13291089543787,0.129532475604061,0.856166638566574,0.151293533021713,0.879744170463906,0.970805195366613
"14173","TDG",226.107951134234,0.0254467657764597,0.168196760797258,0.15129165184776,0.879745654343415,0.970805195366613
"14174","ZNF106",687.240907986673,-0.0291166754855697,0.19279505579342

4,-0.151023973958998,0.879956804316154,0.970862553771483
"14175","AC008735.1",4.29732678239073,0.128952598288235,0.854049106287249,0.15098967651734,0.87998385947983,0.970862553771483
"14176","IFNAR1",659.092317034578,-0.0284422398122898,0.188223532212858,-0.151108841056734,0.879889858431979,0.970862553771483
"14177","AL031293.1",6.16440285715934,0.100660376645942,0.668032111138645,0.150681943229298,0.880226617800342,0.970970560716684
"14178","AL590560.3",6.24943041565958,0.129298036732356,0.85838466315429,0.150629481492862,0.880268003862012,0.970970560716684
"14179","AC017002.3",7.91856968683904,-0.0949713981638139,0.630137404559034,-0.150715379656401,0.880200240612452,0.970970560716684
"14180","SPAG5",6.80247552960811,0.0977632169930477,0.649740965946304,0.150464911583129,0.880397832041521,0.970992049496969
"14181","AC007114.1",13.5131359301367,0.088535943967023,0.588484403737201,0.150447392326408,0.880411653063716,0.970992049496969
"14182","LINC02767",30.1621950666339,0.0468787707849915,0.311831520254945,0.150333650513151,0.880501385389093,0.971022540367043
"14183","DMAC2L",119.929248022211,0.0321992261681983,0.215120845017005,0.149679712189923,0.881017315167433,0.971523007066111
"14184","LYG2",4.60658285410743,-0.13340820859163,0.8935096696693,-0.149308074797899,0.881310543927626,0.971572348419544
"14185","RPS3A",17265.5960445045,-0.0355612879720904,0.237996842643642,-0.149419158578238,0.881222895068389,0.971572348419544
"14186","ADAMTS6",199.432239099921,-0.0739107320163066,0.494463770220464,-0.149476536942944,0.881177622167986,0.971572348419544
"14187","DPP7",981.526967036464,0.0417863770314634,0.279836736129292,0.149324129524427,0.881297876115574,0.971572348419544
"14188","RAB15",3.22685719056353,-0.138042498880981,0.925991596995863,-0.149075325660431,0.881494195580757,0.971637833454299
"14189","SNRPN",447.828097424101,0.0339259805948363,0.227501626840576,0.149124123049064,0.881455691274605,0.971637833454299
"14190","SEMA4B",6.14548927662704,-0.097440682508642,0.65398200440032,-0.148995969083265,0.881556813667789,0.97163837672757
"14191","VPS52",77.5608099060728,0.0319198937510267,0.214883461770971,0.14854513924877,0.881912565099475,0.971961984226326
"14192","SEC24D",334.998995197771,0.0331792516362449,0.223705882806264,0.148316401965071,0.882093071621571,0.972039752164243
"14193","SIL1",328.416034386933,0.0384219009534401,0.259223066688087,0.148219452243699,0.882169580704557,0.972039752164243
"14194","LENG8-
AS1",15.6680430701048,-0.0581286907893207,0.392073827881063,-0.148259553828098,0.882137933905213,0.972039752164243
"14195","DENND1C",2.73623835695577,-0.140774051026947,0.95109581798414,-0.148012480304371,0.882332918872418,0.972151239955239
"14196","WDPCP",55.8368691996931,-0.033762408765445,0.228867264362496,-0.147519606438646,0.882721905328802,0.97227592942609
"14197","NCK1",422.613601474145,0.0271709081350927,0.184547525165404,0.147229869979239,0.882950584663332,0.97227592942609
"14198","POLR2J3",282.577930832418,0.0359577320345366,0.243327982634534,0.14777475095638,0.882520536353982,0.97227592942609

"14199","CDKN2B-
AS1",7.88575877532131,-0.0843938256260445,0.571601470780466,-0.1476445
2147195,0.882623317068906,0.97227592942609
"14200","AL157938.3",20.8927307282401,-0.0578538160455497,0.3921229335
36924,-0.147539996000009,0.882705812895211,0.97227592942609
"14201","JMJD1C",928.994763058288,-0.0465348000618091,0.31611770025685
5,-0.147207195370579,0.882968481388352,0.97227592942609
"14202","LINC01309",4.47180275635079,0.154801309610155,1.0519238202859
,0.147160190334012,0.883005581941699,0.97227592942609
"14203","DPH6",93.6653484084125,-0.0367031727230793,0.249258721645755,
-0.147249301772644,0.882935247490793,0.97227592942609
"14204","MED11",210.045594755932,-0.0372716616080687,0.253067809115764
,-0.147279346742276,0.882911533611433,0.97227592942609
"14205","LIN7C",198.922123720832,0.0279597408114658,0.190100420334587,
0.147078795313841,0.883069826734101,0.972278218241559
"14206","COX19",146.521013180538,0.0300277817490103,0.204370492266564,
0.146928166664317,0.883188719405828,0.972340670949398
"14207","MYH3",2.48984391473343,-0.142978095935874,0.976335832738562,-
0.14644356085429,0.883571241366103,0.972693335325252
"14208","ZC3H10",41.9069119140108,0.0414152551310841,0.283167271779103
,0.14625720999068,0.883718344022891,0.972786803245919
"14209","ENO2",51.3087120952769,0.0403816012166283,0.276335469518503,0
.146132529736377,0.883816767038116,0.972826675802388
"14210","AC233263.6",9.3910553939058,-0.0888046160973562,0.60937645891
8924,-0.145730303160876,0.884134298276498,0.973025391369956
"14211","AC093388.1",2.62944148273323,0.124018926590814,0.856947828861
555,0.14472167664579,0.884930623846757,0.973025391369956
"14212","AC124045.1",3.87303650548795,0.119828973721573,0.825908512639
863,0.145087466574914,0.884641813804452,0.973025391369956
"14213","AL033523.1",2.50462473218074,0.141932001894383,0.982198541153
405,0.144504390861456,0.885102189437405,0.973025391369956
"14214","PPARD",55.7580450883002,0.0345956807511911,0.237393099736812,
0.145731618945731,0.884133259521139,0.973025391369956
"14215","WIPF3",4.87483235108202,0.101519183401003,0.701608960405092,0
.144694821660186,0.884951827847877,0.973025391369956
"14216","AC124242.3",2.99229862271302,-0.149796879635523,1.03403775809
57,-0.144865966897951,0.88481669741188,0.973025391369956
"14217","AC090197.1",5.10890044037814,0.0980590905163176,0.67608877605
4395,0.145038778913893,0.884680254344124,0.973025391369956
"14218","FZD3",15.4132167688095,0.0591650328827076,0.407531384677216,0
.145179083396409,0.884569479990536,0.973025391369956
"14219","ATG2A",71.6357984169152,-0.0438957831730701,0.303807653105205
,-0.144485442431793,0.885117151088258,0.973025391369956
"14220","PRDM10",20.3810197822469,-0.0546907567470378,0.37689502726532
5,-0.145108724686189,0.884625029898499,0.973025391369956
"14221","PRICKLE1",125.830028285451,0.0302532721604207,0.2087973049771
68,0.144893020356412,0.884795337231368,0.973025391369956
"14222","ZFYVE21",559.78315226143,-0.0327726668288496,0.22623735640623
1,-0.144859661328446,0.884821676014838,0.973025391369956
"14223","SLFN12L",4.78225500698153,0.107613513379807,0.744335374113908

,0.144576648003483,0.885045135685466,0.973025391369956
"14224","DDX52",196.657451585115,0.033274037047719,0.230032102959115,0.144649536389418,0.884987584111448,0.973025391369956
"14225","AC027514.1",3.63260951232352,-0.117560978443355,0.810654298049082,-0.145019866947325,0.884695186048585,0.973025391369956
"14226","ZNF335",92.1286573501712,-0.0351165928324978,0.241623338328061,-0.145336096568696,0.884445516329763,0.973025391369956
"14227","FGF16",80.3639316788814,0.047183883263962,0.326515587433093,0.144507291780153,0.88509989888009,0.973025391369956
"14228","LEAP2",17.6932440661537,0.0878125636663133,0.608729684180722,0.14425543217019,0.885298770101146,0.973052144633954
"14229","NRG2",4.85828974881737,-0.106748779301028,0.74099879548821,-0.144060665079349,0.88545256537279,0.973052144633954
"14230","FBXO3",179.328947819725,0.0230721437567139,0.159910526194136,0.144281582368778,0.885278121269666,0.973052144633954
"14231","PPP1R12A",726.741652006303,-0.0224476869296969,0.15569315032398,-0.144179027034817,0.885359101896644,0.973052144633954
"14232","PJA1",89.8870805133214,0.0388571351983418,0.269712439752548,0.144068754240597,0.885446177787072,0.973052144633954
"14233","CAB39",191.806393871727,-0.0265950463875719,0.185548823648713,-0.143331797338271,0.886028144443001,0.973205996143587
"14234","MFAP3",19.3829766401135,0.0580480946770248,0.404839739215967,0.143385367230607,0.885985838820122,0.973205996143587
"14235","SIMC1",38.2462776441967,-0.0437706507127765,0.305186531475679,-0.143422616001862,0.885956422628661,0.973205996143587
"14236","VKORC1L1",269.140862580446,-0.0233638901125906,0.162943584908283,-0.14338637587814,0.885985042266437,0.973205996143587
"14237","MSRB2",494.507970909996,0.0356400437116165,0.248281124875059,0.143547133232748,0.885858089715068,0.973205996143587
"14238","AP002784.1",5.00440715562587,-0.107728446310279,0.751318444791165,-0.143385866615085,0.885985444443942,0.973205996143587
"14239","CBX6",440.872380648105,-0.0259601813505955,0.180921712293015,-0.143488479196743,0.885904409368661,0.973205996143587
"14240","ARL15",41.4915886441758,-0.0424625775345845,0.296726412240293,-0.14310346427873,0.886208469005254,0.973267358699682
"14241","SETDB2",48.92025249148,-0.0352857969696243,0.246568109808038,-0.143107707631354,0.886205117791104,0.973267358699682
"14242","HDDC3",145.190490945384,-0.0403353214481324,0.282925444436426,-0.142565196030631,0.88663358618625,0.973665867711904
"14243","AL592295.4",8.92404848660124,-0.0797761855828539,0.560109618619248,-0.142429594013247,0.886740688024981,0.973667614643647
"14244","HOXD-AS2",184.705276505556,-0.0485238307497083,0.341865701359678,-0.141938283240225,0.887128754777662,0.973667614643647
"14245","BEND3",4.09846404178032,0.12207915954394,0.86010096008854,0.141935848474548,0.887130677969272,0.973667614643647
"14246","ANLN",3.25155570445603,0.128482043823064,0.903861589887772,0.142147918730585,0.886963168762871,0.973667614643647
"14247","UNC5B",11.2064768763401,-0.085352025071607,0.60019823953778,-0.142206390237562,0.886916984410338,0.973667614643647

"14248","ZNF841",32.3323326110712,0.0449739128969969,0.316076001299437
,0.142288287348936,0.886852297736327,0.973667614643647
"14249","AL022238.2",3.22601963838565,-0.151219492045862,1.06543144642
004,-0.141932634477774,0.887133216667006,0.973667614643647
"14250","ZNF630",7.7357001056163,0.0923234716453517,0.649500306319837,
0.142145385840493,0.886965169402195,0.973667614643647
"14251","FAM229B",286.8285326945,-0.0483385935538131,0.340999113932653
,-0.141755774659754,0.887272917913251,0.973752609372201
"14252","GMNN",102.979760100586,-0.0364460929076149,0.257315479181361,
-0.141639721883684,0.88736458969191,0.973784885123595
"14253","IL6",241.112314598286,0.124950230521547,0.88273598818955,0.14
1548812094785,0.887436401711287,0.973795363977024
"14254","FAM41C",2.54591826757144,0.127260566910043,0.907909251786993,
0.140168818259713,0.888526608070274,0.973938232297386
"14255","PTH2R",32.3811993420763,0.081094728866627,0.574827271838682,0
.141076690058966,0.8878093581006,0.973938232297386
"14256","MRPL44",126.346616255637,-0.0347699144695861,0.24695063205404
6,-0.140797025625659,0.888030292865653,0.973938232297386
"14257","COL7A1",5.54191122692953,0.0946724167250731,0.672600441864019
,0.140755805129568,0.888062857773374,0.973938232297386
"14258","MUSTN1",144.297561890414,-0.112900403755905,0.802680631040994
,-0.140654202169404,0.888143126682991,0.973938232297386
"14259","YTHDC2",134.495357947554,-0.0361905227113606,0.25669872345651
8,-0.140984428064329,0.887882244068433,0.973938232297386
"14260","NAPRT",149.975328897673,-0.0586967457267277,0.418309731433475
,-0.140318862593954,0.888408061569988,0.973938232297386
"14261","SMC5",298.473127582008,0.033357222002027,0.237996968587945,0.
140158180164807,0.888535013073594,0.973938232297386
"14262","RFK",140.14586104842,0.0323657845686448,0.23111154593022,0.14
0043996669976,0.888625228573127,0.973938232297386
"14263","ABRAXAS2",94.5609291541995,-0.0309860285641452,0.219572800453
106,-0.141119612721626,0.887775449989315,0.973938232297386
"14264","RAB6A",456.866773380429,-0.0212627130614865,0.151123432668434
,-0.140697658106649,0.888108795252464,0.973938232297386
"14265","KLF5",21.0006571700588,0.0798990723058177,0.567188119472286,0
.140868733957538,0.887973642488633,0.973938232297386
"14266","LINC02352",5.49560310501409,0.0993447523095414,0.707769779886
48,0.140363088581538,0.888373120133277,0.973938232297386
"14267","AC009107.2",109.18531634316,-0.0486460720841141,0.34681768031
2071,-0.140264106605931,0.888451322699495,0.973938232297386
"14268","STAT3",1196.89673163045,0.0200074922893281,0.142540564163598,
0.140363498676524,0.888372796132301,0.973938232297386
"14269","DNASE2",359.120730144923,-0.033211879625504,0.236187539767221
,-0.140616561137123,0.88817286434226,0.973938232297386
"14270","MORC2-
AS1",18.7803352533027,0.0530750144280941,0.378820884454931,0.140105829
974135,0.888576374376751,0.973938232297386
"14271","EXTL2",205.938145661225,-0.0237382551005849,0.169755832566139
,-0.139837640579072,0.888788272589323,0.973980422035945
"14272","CYP39A1",8.27244506716564,-0.0971479713554046,0.6944784927847

72,-0.139886220184953,0.888749888932009,0.973980422035945
"14273","AKR7A2",593.073012120608,-0.0394350599629035,0.282966044404092,-0.139363223053675,0.889163132463439,0.974008698251893
"14274","OTUD3",47.0788567287191,0.0435772332556431,0.313222792930164,0.139125358177108,0.889351090161226,0.974008698251893
"14275","DMAP1",171.346995626625,0.0340001963043089,0.244277379919336,0.13918683881224,0.889302508375242,0.974008698251893
"14276","AMY2B",8.07879845184535,0.0790944506247914,0.569762640461014,0.138820001537471,0.889592388079255,0.974008698251893
"14277","PEX11B",75.2402512579679,0.0350569305954679,0.251443955780403,0.139422443011852,0.889116338599463,0.974008698251893
"14278","AL162258.2",6.38124344188904,-0.094174363970999,0.683886459798978,-0.137704676882593,0.890473822999861,0.974008698251893
"14279","OSBPL10-AS1",4.90209735433919,0.109721653228588,0.79740504457602,0.137598393658177,0.89055782512801,0.974008698251893
"14280","RHOA-IT1",2.79264928039489,0.150446967903547,1.08798724552489,0.138280084185146,0.890019064809658,0.974008698251893
"14281","CDV3",918.449387085815,-0.0257813694410221,0.186229462831543,-0.13843872526412 8,0.889893693317195,0.974008698251893
"14282","ING2",158.722091601384,-0.0311222719360601,0.225957671421104,-0.137734964873396,0.890449884774624,0.974008698251893
"14283","LINC00265",9.34981546590945,-0.0843825728080543,0.608137231219046,-0.138755807860842,0.889643116296905,0.974008698251893
"14284","TRIM3",60.320060179519,-0.0345539643785184,0.248986337440317,-0.138778556019368,0.889625139808935,0.974008698251893
"14285","DYRK4",12.9083868893276,-0.17673559251173,1.27912699287351,-0.138168917938867,0.890106919344676,0.974008698251893
"14286","ETFRF1",313.032362879896,-0.0335613635243078,0.243194758621998,-0.138002001829624,0.890238835455361,0.974008698251893
"14287","IFNG",7.04686343192903,0.0906269603969189,0.657445116038434,0.137847187827647,0.8903611898195,0.974008698251893
"14288","SUDS3",228.074652305021,0.0227893180030674,0.164850984314707,0.13824192859875,0.89004921896614,0.974008698251893
"14289","DHRS12",104.899857373301,0.0471751316720428,0.340372886552917,0.138598382937616,0.889767521206365,0.974008698251893
"14290","UPF3A",652.949033986798,-0.0262506745613921,0.188525826650694,-0.139241795290097,0.889259082307741,0.974008698251893
"14291","LEO1",195.003624562201,-0.0216132653138255,0.155754815954473,-0.138764667926178,0.88963611471868,0.974008698251893
"14292","ARID3B",19.5544687475327,0.0656622210812894,0.476690415077714,0.137746048597568,0.890441124737305,0.974008698251893
"14293","ZSCAN32",44.5167780155104,0.0412893786483445,0.296897121722013,0.139069649476104,0.889395111343618,0.974008698251893
"14294","ZNF764",29.5586581241366,-0.0461545643525305,0.33445342655234,-0.13799997455044,0.890240437660834,0.974008698251893
"14295","CFAP20",238.546615398231,-0.0348107336858692,0.249224092339724,-0.139676438818835,0.888915642989802,0.974008698251893
"14296","DLG4",38.7176452321913,0.0402373606608652,0.292184861583589,0

.137711996585949,0.890468037836169,0.974008698251893
"14297","SOCS6",87.7509474916345,0.0315774318365134,0.22701991158202,0
.139095428310502,0.889374740789061,0.974008698251893
"14298","SMIM7",638.146693654933,0.0303619590250155,0.220186923994198,
0.13789174431546,0.890325975161173,0.974008698251893
"14299","BX842570.1",3.20612265500224,0.137489664655485,0.991128100140
758,0.138720377957157,0.889671114510795,0.974008698251893
"14300","JADE3",10.3652554587812,0.0770723686451989,0.559866345004769,
0.137662085483174,0.890507485438385,0.974008698251893
"14301","PLCD1",98.1690481386907,-0.0253972557252504,0.185192844541484
,-0.137139508754407,0.890920524004506,0.974208924342076
"14302","DDX39B",703.920034266643,-0.0224991854269537,0.16444954051167
,-0.136815130993675,0.891176923309596,0.974208924342076
"14303","NOXA1",4.83684873789555,-0.105679024829246,0.771271106399569,
-0.137019297044038,0.891015542133681,0.974208924342076
"14304","SPPL2A",928.768238893576,-0.0197001805229855,0.14397649176959
2,-0.136829146764543,0.891165844528292,0.974208924342076
"14305","STX1B",7.93525966209866,-0.0840079914860664,0.61252682220948,
-0.137149898486137,0.890912311792649,0.974208924342076
"14306","ARRB2",57.3600820904889,-0.0399497069350482,0.291456860457395
,-0.137069022401304,0.890976237865977,0.974208924342076
"14307","INTS6L",17.8438546038209,0.0661231489003009,0.482860976835017
,0.13694034530128,0.891077948277305,0.974208924342076
"14308","AC021851.1",3.58669317236467,0.123287467687874,0.903183050824
317,0.136503300826286,0.891423415294425,0.974285723917912
"14309","NDUFAF4",95.5786915313495,0.0334844845695358,0.24513968600013
8,0.136593487231263,0.891352124685265,0.974285723917912
"14310","AC016590.3",2.86573407153104,-0.117079561382801,0.85778960978
9116,-0.136489833925109,0.891434060694714,0.974285723917912
"14311","SLC31A2",186.634757649657,0.0398301962105592,0.29210179588868
3,0.136357245217821,0.891538871289907,0.97430377368697
"14312","AAMDC",483.799520586304,-0.0340006356519098,0.249433671100494
,-0.136311330791468,0.891575166816363,0.97430377368697
"14313","ZNF132",6.76638123575389,0.103484854903901,0.76059664568813,0
.136057469475528,0.891775849238055,0.974454990713559
"14314","QDPR",253.512241997097,-0.0357420224444923,0.262968747732098,
-0.135917377075183,0.891886598037016,0.97450792184567
"14315","WARS2-
IT1",6.22965527977731,0.1153218825383,0.850718109965825,0.135558278573
537,0.892170489783694,0.974681926530942
"14316","VCP",740.734007098284,-0.0225423058609705,0.166274131784746,-
0.135573138280783,0.892158741901858,0.974681926530942
"14317","MSC-
AS1",77.480882643535,0.0316267579664936,0.23376211750262,0.13529462474
2347,0.89237893483838,0.974802531706081
"14318","LIN52",42.7807224120367,0.0341955705669442,0.252959234010077,
0.135182139923708,0.892467867742927,0.974802531706081
"14319","LYRM9",101.39479251982,-0.0378318498991575,0.279696734521595,
-0.135260248797207,0.892406113052624,0.974802531706081
"14320","TAS2R31",2.74136056784329,0.135093006372337,1.00070712024573,

0.134997546873819,0.892613813888081,0.97481299286466
"14321","PHLDA1",307.522596478353,0.058384014089157,0.432687225051261,
0.134933528675916,0.892664429910976,0.97481299286466
"14322","EFL1",57.6823439476868,-0.0328431347834806,0.243283912129702,
-0.134999205232983,0.89261250271132,0.97481299286466
"14323","MFN2",82.8460759319458,0.0335654135065759,0.249558476223684,0
.134499192391648,0.893007849665588,0.974973700176005
"14324","NAMPT",1219.7464101545,-0.0562349455587967,0.418157178369767,
-0.13448279371416,0.893020816117819,0.974973700176005
"14325","ACTR3B",20.7484319300578,-0.0478149713131137,0.35585165076057
,-0.13436770972094,0.893111813961485,0.974973700176005
"14326","CBWD5",40.6042273641955,-0.0478015818718251,0.355790421701974
,-0.134353200525071,0.893123286599848,0.974973700176005
"14327","FXN",57.7283733245717,0.0408152520124855,0.303681994188181,0.
134401290802884,0.893085260983107,0.974973700176005
"14328","CMIP",185.868225161995,-0.0291190539785479,0.217030472427726,
-0.134170347844793,0.893267872867417,0.974996750736008
"14329","PITPNB",381.490118911183,-0.0265349614230397,0.19777293753639
4,-0.134168818816056,0.893269081924313,0.974996750736008
"14330","ZNF432",43.0323028832039,0.0374133456504476,0.27941155026699
,0.133900497723506,0.893481256704421,0.975160282962815
"14331","CASP3",183.478971184939,0.0341630883735913,0.255566161118002,
0.133676102595669,0.893658702902246,0.975225514179371
"14332","FANCF",42.5799305576987,-0.039515365755095,0.295723298098423,
-0.133622768341855,0.893700879113463,0.975225514179371
"14333","PDK2",139.819107539826,-0.0441428958836888,0.330439683516394,
-0.133588361464155,0.893728087898525,0.975225514179371
"14334","ARHGEF10L",142.10466434057,-0.0267299404587634,0.200581843645
408,-0.133262014013676,0.893986168027843,0.975226341544407
"14335","UPF2",263.768636809273,-0.0288752022090016,0.216613252397321,
-0.13330302688977,0.893953733854469,0.975226341544407
"14336","NAB2",67.5871262987253,0.0370090826847008,0.277438773589835,0
.133395495538828,0.89388060761231,0.975226341544407
"14337","AL138995.1",10.472491498901,0.0684623470229871,0.514007974721
469,0.133193161176314,0.894040619249598,0.975226341544407
"14338","HIRA",51.1535647953485,-0.0320022591624306,0.239712265562778,
-0.133502802150312,0.89379574831376,0.975226341544407
"14339","HMGN2",1530.31279099936,0.0275431050506437,0.207765258353828,
0.132568386403358,0.894534735692049,0.975496787901848
"14340","NSUN5",105.331715133285,0.0259920037700992,0.196021323736025,
0.132597838208162,0.894511442183973,0.975496787901848
"14341","ATG16L2",24.6059139179886,0.052737132333899,0.397823307581181
,0.132564209609909,0.894538039136081,0.975496787901848
"14342","HOXC-
AS3",23.5769108454624,-0.0543391064813847,0.409868936849469,-0.1325767
86372424,0.894528092125665,0.975496787901848
"14343","GATB",45.9993231986899,0.0326557415810254,0.246670400934795,0
.132386137360913,0.894678878949771,0.975512820752136
"14344","AC116351.2",2.58157790374746,-0.123919212860899,0.93571105846
4513,-0.132433203326942,0.89464165350089,0.975512820752136

"14345","MAPK1IP1L",333.771524003883,-0.0204751344526585,0.154816412059522,-0.132254288678299,0.894783162042698,0.975512820752136
"14346","ZNF568",53.3312254062033,0.0339174711055642,0.256503257877518,0.132230176669959,0.894802233152822,0.975512820752136
"14347","HPGD",79.3850184755321,0.0536103704235069,0.406395590566965,0.131916712847978,0.895050169214959,0.975715107445596
"14348","DCTN1",325.405359968909,0.0268132894814115,0.204744208233271,0.130959941249534,0.895806996667682,0.975941248101216
"14349","GORASP2",336.253927253893,0.0212579991893441,0.16225742780454,0.131014027998472,0.895764210320032,0.975941248101216
"14350","PTGR1",325.283328221966,-0.0379507862441327,0.288874065018082,-0.131374847519652,0.895478784964334,0.975941248101216
"14351","SPRYD4",37.3884470471322,0.0399817218396354,0.305333358298176,0.13094449313524,0.895819217246871,0.975941248101216
"14352","AL049840.6",5.50109286594063,0.0873857512029543,0.666336080719524,0.131143658180108,0.895661665142804,0.975941248101216
"14353","PLCG2",6.34388127623907,0.0885837279747092,0.676332580380523,0.130976579488259,0.895793834641375,0.975941248101216
"14354","RPS28",13628.5663745232,-0.0322820219168227,0.245837314859587,-0.13131457254673,0.895526464386591,0.975941248101216
"14355","PMEPA1",747.954308161408,0.0321382113552453,0.244757077250073,0.131306566152565,0.895532797727633,0.975941248101216
"14356","C1QTNF6",82.324306286201,-0.0472038333842874,0.359619348919547,-0.13126054959531,0.895569198581529,0.975941248101216
"14357","GCNA",7.27092464367121,-0.0737474232083245,0.563784015837731,-0.130807935550891,0.895927245278318,0.975990953273866
"14358","LINC02606",13.8372201534051,-0.0590706346229175,0.453556640485722,-0.13023871629276,0.896377564252917,0.976053325834769
"14359","PATJ",16.3001007226096,0.0716695666540731,0.550081291045721,0.130289046038681,0.89633774619283,0.976053325834769
"14360","MRFAP1L1",222.576600946244,0.0196898321815196,0.151337560042847,0.130105389408584,0.896483046235123,0.976053325834769
"14361","TNS3",107.421090482083,0.0562169425572328,0.430433319691571,0.130605461950565,0.896087421730614,0.976053325834769
"14362","HOTAIR",13.0966193041668,0.0759375168746351,0.583665632106769,0.1301044856805,0.896483761228673,0.976053325834769
"14363","AC090517.2",7.0151844897163,0.0746697002046484,0.572555106432338,0.130414870753532,0.89623820190822,0.976053325834769
"14364","SPN",7.41501166317164,-0.0886732864614549,0.681240862176304,-0.130164368264959,0.896436384702296,0.976053325834769
"14365","MIEF2",44.8257882801914,0.0391163954502791,0.299677089879251,0.130528481393223,0.896148322004254,0.976053325834769
"14366","RGP1",54.3854246078338,0.0402436020491391,0.30972787410319,0.129932128858804,0.896620124595637,0.976101350064988
"14367","Z69706.1",6.01642302063286,-0.0767386013128337,0.590882469147894,-0.129871176282311,0.896668349113196,0.976101350064988
"14368","MAP3K10",32.8941257739613,0.0412048175179189,0.317418966282915,0.129812084011367,0.896715102156889,0.976101350064988
"14369","ISY1",200.444340614478,-0.0258552764375589,0.19969781171631,-0.12947200680540 7,0.896984173828366,0.976161086925367

"14370","MYO1G",9.21958698465252,0.0829928760301172,0.640832216720263,
0.129507964588405,0.896955723196361,0.976161086925367
"14371","TRIM66",25.9495926439372,-0.0512499319932192,0.39597501912202
9,-0.129427184843257,0.897019638189986,0.976161086925367
"14372","NADSYN1",92.6139371874799,-0.0325909004601125,0.2517996497522
58,-0.12943187368282,0.897015928242322,0.976161086925367
"14373","DNAJC11",67.8375785318926,0.0332092269527717,0.25683059117187
4,0.12930401632159,0.897117093550271,0.976199216804163
"14374","HSP90AA1",5630.989642596,-0.0221404187575551,0.17168720554984
2,-0.128957884116342,0.897390974093981,0.976429305331144
"14375","OCIAD1",1011.4569924134,-0.0266316324804322,0.207510763009554
,-0.128338559861621,0.897881051044692,0.976710209023954
"14376","CDC42SE2",122.807435924853,0.0244999180223985,0.1910323323024
83,0.128250112047028,0.897951043793007,0.976710209023954
"14377","TPST1",654.198614678608,-0.036753344616024,0.286604745318508,
-0.128237041487849,0.897961387183851,0.976710209023954
"14378","AAAS",90.0990356639831,-0.0287865056427659,0.224362489244044,
-0.128303557959968,0.897908749534528,0.976710209023954
"14379","EIF3CL",3.63097739589075,-0.118608938878344,0.92333410753686,
-0.128457226815494,0.897787145775484,0.976710209023954
"14380","ATP8B3",4.47929197184932,0.101135666553855,0.791161650792299,
0.127831861481878,0.89828203509072,0.976923094973844
"14381","TBC1D8B",134.637186137394,0.0280693690542046,0.21956557865172
4,0.127840480400292,0.898275214151302,0.976923094973844
"14382","PLEKHA5",581.805865025816,0.0252002618729254,0.19739673028780
7,0.127663015674997,0.898415659754778,0.977000481057205
"14383","LRRC24",13.9542981591727,0.0575184213849216,0.4512800725446,0
.12745615169887,0.898579375944631,0.97710683757313
"14384","YY2",13.4684419701178,0.065487291552788,0.51434655233073,0.12
7321338611168,0.898686071978201,0.97710683757313
"14385","LONRF3",43.9168827951394,-0.0565658625574927,0.44434169701508
8,-0.127302620792691,0.898700886092677,0.97710683757313
"14386","RRM2B",111.297058741445,0.0305144651338916,0.240434504485909,
0.126913835429473,0.899008596103502,0.977305514913379
"14387","ZNF583",44.1691677114286,0.031170054082811,0.245598577812906,
0.126914635908665,0.899007962536617,0.977305514913379
"14388","TMTC3",178.679323971768,0.0236780257697279,0.186786235216209,
0.126765367599599,0.899126107204433,0.977365326430173
"14389","GOLGA6L10",15.3722994265645,-0.0688095271986978,0.54330560964
3956,-0.126649763921618,0.89921760812338,0.97739685808949
"14390","FMO2",12.2736803890132,0.100233815865712,0.793464515978079,0.
126324257540561,0.899475255328308,0.977520320569676
"14391","AL590064.1",3.97152469522609,0.112873574804997,0.894181056321
767,0.126231230249167,0.899548890921991,0.977520320569676
"14392","CDC42BPB",569.756660009103,0.0334435842714968,0.2647107647263
44,0.126340099187393,0.899462715986013,0.977520320569676
"14393","PINLYP",46.8435563509046,-0.0344075112580358,0.27266343428792
9,-0.126190412542453,0.899581200381032,0.977520320569676
"14394","RPS20",9653.82309227711,0.0213345027050576,0.169907055431214,
0.125565725631063,0.900075695148557,0.977785918741643

"14395","FAM118B",49.1941122897608,-0.0451319918678717,0.358875338909605,-0.125759524198568,0.899922282336507,0.977785918741643
"14396","ARIH1",249.59331904637,0.0308924790785426,0.24593306342099,0.125613362631362,0.900037984894177,0.977785918741643
"14397","PPP1R3D",41.5268982064198,-0.0360032417980194,0.286631699274636,-0.125608025522407,0.900042209828586,0.977785918741643
"14398","NFYC",132.375076248249,-0.0233124664390759,0.187239607554963,-0.124506063345773,0.900914599881269,0.977808190780777
"14399","RAP1A",970.590367025654,-0.0189691188364346,0.154268662168081,-0.122961582539473,0.902137519740069,0.977808190780777
"14400","AL078645.1",3.9498867066494,0.105075264550114,0.852156158128778,0.123305175404523,0.901865442729689,0.977808190780777
"14401","CDC73",289.378396594543,0.0199597368520988,0.159890583580325,0.124833723194659,0.900655188959228,0.977808190780777
"14402","ZC3H11B",5.99063787788357,0.082703620975537,0.672621903884365,0.122957073651522,0.902141090218846,0.977808190780777
"14403","THUMPD2",97.7291138648157,0.0283924591925626,0.22916651673358,0.1238944484441,0.901398848581912,0.977808190780777
"14404","NFU1",260.738876865477,-0.0332678020383032,0.269019365857133,-0.123663223769439,0.901581931270399,0.977808190780777
"14405","XYLB",20.8984039547232,-0.0596821805226544,0.483998005173495,-0.123310798566743,0.901860990075365,0.977808190780777
"14406","REELD1",2.57769691849933,0.17400598480909,1.40960182555848,0.123443359432476,0.901756023748857,0.977808190780777
"14407","AL021368.1",3.11454862592291,-0.134070026242247,1.08900196416246,-0.123112749705056,0.902017815281993,0.977808190780777
"14408","CYTH3",142.349558861164,0.0322658652263984,0.257552814033639,0.125278635946816,0.900302964966714,0.977808190780777
"14409","ZNF789",21.6262067612771,-0.0451142861052682,0.36098500702951,-0.124975512075991,0.900542936855839,0.977808190780777
"14410","AC026979.2",35.8495037342261,-0.0467856153659965,0.376712504966517,-0.124194484518519,0.901161289147026,0.977808190780777
"14411","FAM122A",106.990480765331,0.0245997269103393,0.199727126065168,0.123166679433983,0.901975110588351,0.977808190780777
"14412","MYOF",1066.56361839289,0.0218831787290081,0.174720698607959,0.125246630212428,0.900328302285762,0.977808190780777
"14413","AL121929.2",2.69880401649177,0.114583098687755,0.920974637059525,0.124415042583142,0.900986663626205,0.977808190780777
"14414","PGGHG",17.6794072773067,0.0680926450055834,0.546562288047949,0.12458350401155,0.900853288530961,0.977808190780777
"14415","ERP29",1087.31634092861,0.0314985804724078,0.256223468148595,0.1229340181054,0.902159347376382,0.977808190780777
"14416","OAS2",38.5246637871839,0.0541305306845479,0.439651536577272,0.123121440916502,0.902010933056487,0.977808190780777
"14417","DENR",369.872503501837,-0.0186636812829124,0.150448533141676,-0.124053594230374,0.901272840689364,0.977808190780777
"14418","OTUB2",3.60397636081707,0.119236130419724,0.957967455104136,0.124467830075461,0.900944870157073,0.977808190780777
"14419","AC135050.6",24.8478817090999,0.0448786561793809,0.36173828192464,0.124063883812912,0.901264693725373,0.977808190780777

"14420","C16orf86",72.8233836833436,0.0369179204740406,0.296712067820786,0.124423387107865,0.900980056992977,0.977808190780777
"14421","ZFPM1",188.090912889289,-0.0376048250398596,0.300315408663939,-0.125217767570296,0.900351151464943,0.977808190780777
"14422","TMEM199",83.7424980553171,-0.0388532047199099,0.314961321611058,-0.12335865407591,0.901823096212652,0.977808190780777
"14423","GRB2",342.305437039254,-0.021883454165261,0.177485401736929,-0.123297206142604,0.901871753128873,0.977808190780777
"14424","AC132938.6",4.80987334738091,-0.0934427853363832,0.748054996238162,-0.124914325559338,0.900591377047723,0.977808190780777
"14425","NAPG",199.289759021929,-0.0223894231179022,0.179995043883253,-0.124389108915822,0.901007196202864,0.977808190780777
"14426","AC006305.1",2.78767931096507,0.113016025058946,0.915290637838712,0.12347556108059,0.901730525617661,0.977808190780777
"14427","CLPTM1",322.664173400318,0.0219045091840797,0.176577162635841,0.124050635184652,0.90127518356952,0.977808190780777
"14428","AL110114.1",5.04596711236863,0.088557447803535,0.716346288203852,0.123623796565739,0.901613150080962,0.977808190780777
"14429","TOM1",140.189534772013,-0.0248732147463598,0.200965942263362,-0.123768308531422,0.901498724964835,0.977808190780777
"14430","YIPF6",345.872627103198,-0.0197707258486402,0.157667023091542,-0.12539544072676,0.900210497399906,0.977808190780777
"14431","NCOA3",238.459548311086,-0.0235222509999811,0.191776065190542,-0.122654779555573,0.902380474077566,0.977980085550076
"14432","TRHDE",4.69608445897214,-0.125338131603277,1.02395597310453,-0.122405781982271,0.902577659587534,0.978126011360105
"14433","FDX2",171.141107423261,-0.0367718914316882,0.300670366306786,-0.122299686142559,0.902661680555385,0.978149288705482
"14434","PLEKHH3",135.759133129837,0.0212477555051241,0.174243456267409,0.121942918031398,0.902944225563338,0.978369665351561
"14435","NOL11",157.44295809958,0.0192533801553002,0.157963599073695,0.121884916956836,0.902990161083746,0.978369665351561
"14436","OSGIN1",29.0423845798882,-0.0583615306896924,0.479292543522104,-0.121765989224076,0.9030843501351,0.978403937109516
"14437","PLXNA2",186.93495477109,0.0444870264799408,0.366868692412227,0.121261441491316,0.903483959824692,0.97845157614579
"14438","CCNH",210.064924059758,0.0246617573311251,0.203101588749522,0.12142572336812,0.903353843321543,0.97845157614579
"14439","SUPT16H",506.156311009458,-0.0199801602071711,0.164413174497721,-0.121524082654629,0.903275940861305,0.97845157614579
"14440","C17orf100",11.995801027709,-0.0640582604956165,0.527279292896836,-0.121488291610476,0.903304287953854,0.97845157614579
"14441","CD7",10.3347897531989,0.0631086870328268,0.520275510881898,0.12129859221291,0.903454535037502,0.97845157614579
"14442","RAB33A",8.8388371649326,-0.0744220093410756,0.613857938172313,-0.121236534893819,0.903503686873241,0.97845157614579
"14443","CFAP54",3.25645317993351,-0.0982781738896721,0.811231663276905,-0.121146866349725,0.903574708537539,0.97846073817954
"14444","GAS1",2517.88750487772,0.0298390038437061,0.246732770429334,0.12093652493661,0.903741311762458,0.978468832384504

"14445","AC010997.5",3.03307518470611,0.101370530379213,0.838462658826308,0.120900471013358,0.903769869093769,0.978468832384504
"14446","EBPL",316.068343817135,-0.0441328223886724,0.364763780172879,-0.120990144272975,0.903698841575809,0.978468832384504
"14447","SIGMAR1",175.647472148683,-0.0275670012204568,0.228473761727232,-0.120657186243417,0.903962571590551,0.97852261458438
"14448","LINC01001",7.69270417748278,0.0774467689117205,0.642595490062241,0.120521805878561,0.90406980695296,0.97852261458438
"14449","GUCD1",164.130857488382,0.0218820325709652,0.181490134253344,0.120568716646712,0.904032648535923,0.97852261458438
"14450","MAMLD1",17.2169639671968,-0.0522858274668493,0.432979357463621,-0.120758245319449,0.903882523408609,0.97852261458438
"14451","OAT",695.285252525298,0.0191450895443437,0.159223893348094,0.120240054063298,0.904292989371106,0.978696446873165
"14452","CCDC14",196.88696736653,-0.0257265292977099,0.214563311762475,-0.119901809337235,0.90456093117397,0.978849237567165
"14453","NFE2L3",2.77554033968453,-0.116569888974111,0.972193036030448,-0.119904056760246,0.904559150833676,0.978849237567165
"14454","HOXA9",66.1631439323056,-0.03402067138436,0.283920080367327,-0.119824815984643,0.904621923260601,0.978849237567165
"14455","TMEM127",168.686990067948,-0.0231415270060526,0.193513543721832,-0.119586084575649,0.904811043510547,0.978971226432194
"14456","PLEKHA3",374.234805381361,0.0154468266073715,0.1300015288399,0.118820345769892,0.905417689281277,0.978971226432194
"14457","LINC02158",7.49886126981059,-0.0756234305638902,0.634581953006742,-0.119170471529446,0.905140300215127,0.978971226432194
"14458","GASK1A",91.996762851621,0.0304450648761184,0.255994024060309,0.118928810888749,0.90533175595667,0.978971226432194
"14459","SH3BP2",46.9750887739809,0.0311214373936213,0.262284239541182,0.118655384891073,0.905548384449779,0.978971226432194
"14460","PIWIL2",9.1382020965965,0.0680403043975928,0.569743163524962,0.119422765824222,0.904940425839835,0.978971226432194
"14461","SCUBE2",5.32813036455356,0.0969221774278511,0.811545754109969,0.119429098035448,0.90493540936797,0.978971226432194
"14462","TMBIM6",2907.88789908769,0.0168693572636484,0.141640142127512,0.119100115336383,0.90519603936944,0.978971226432194
"14463","AC108134.2",29.9051045658177,0.0574598006752033,0.483478060776225,0.118846759215819,0.905396762680029,0.978971226432194
"14464","PALB2",36.9635370904087,0.0365698170600145,0.307541463066507,0.118910200580356,0.905346500209449,0.978971226432194
"14465","ZNF527",22.0560926400044,-0.042558624697122,0.356598236233961,-0.119346144688163,0.905001126543432,0.978971226432194
"14466","ZNF580",249.658065036224,0.0292518645358951,0.246477833478379,0.118679493904514,0.905529283206253,0.978971226432194
"14467","MMP24OS",491.8064846144,-0.0236762410269646,0.19942020909066,-0.1187253846284,0.905492924760752,0.978971226432194
"14468","ZEB2",1236.49692052916,0.0247719260349721,0.209104480720357,0.118466739448307,0.905697847554648,0.979036129158853
"14469","TSPAN3",611.112969597508,-0.0181212521307471,0.153023206156148,-0.118421594906696,0.905733615907893,0.979036129158853

"14470","IDO1",17.2598777687062,-0.052948241972554,0.447853547999398,-0.1182266886331,0.905888043779876,0.979135383878179
"14471","TMPO-AS1",6.74733593631495,0.0686937858947172,0.582137168851047,0.118002748442084,0.906065480158386,0.979259492065314
"14472","ATP2B4",870.260086084904,-0.021942226125074,0.186224790673153,-0.117826539276496,0.906205100716611,0.979302475013977
"14473","AC142472.1",29.6437006960881,0.0490048371139226,0.41601970887952,0.117794508452277,0.906230480874507,0.979302475013977
"14474","MTA2",46.6898972083296,-0.0331425269888267,0.282117897178337,-0.117477576999931,0.906481611954984,0.979506177350832
"14475","PDIA4",390.354353622423,0.024809704714435,0.211488768485848,0.117309798019346,0.90661456094604,0.979582157733753
"14476","CDC42-AS1",4.73583926513214,-0.101189446729741,0.864667037555699,-0.117027066298018,0.906838605042164,0.979621203402365
"14477","KIF15",4.42696199327338,-0.0839059320441461,0.716176556802538,-0.11715816616332,0.906734717117605,0.979621203402365
"14478","AC007541.1",52.8955361342876,0.0327921299097063,0.280107682419532,0.117069726993749,0.906804799102885,0.979621203402365
"14479","PGBD2",20.4469399842988,-0.0493972122435215,0.42277327090226,-0.116840906564648,0.906986126988594,0.979654844039939
"14480","CEL",2.51631012605276,-0.106306994532772,0.909931385278997,-0.116829682163537,0.906995021847718,0.979654844039939
"14481","TTC21A",14.9531021263808,0.0569191475750613,0.489138108963259,0.116366209322154,0.907362314477503,0.979819571805928
"14482","TMEM41B",132.10051646384,0.0256636669037973,0.220627934331146,0.116321022456195,0.907398125194185,0.979819571805928
"14483","PPFIBP1",485.208210590379,-0.0262721144798366,0.225602167381531,-0.116453289366702,0.907293303831672,0.979819571805928
"14484","SPC24",3.39411970233761,0.101265757629177,0.869356854606616,0.116483532731791,0.907269336229079,0.979819571805928
"14485","CIART",4.05639677614567,-0.13079336256068,1.12708256288985,-0.116045946292812,0.90761612787506,0.979919663120663
"14486","TEC",7.95646330693579,-0.0853110877036041,0.734991862937943,-0.116070792080057,0.907596436867044,0.979919663120663
"14487","ZC3H14",246.029080014369,-0.0195875279123421,0.168930813582923,-0.115950000458189,0.907692168268492,0.979934114151944
"14488","TRIM32",44.2693014274255,-0.0346228928389162,0.299008990340126,-0.11579214658239,0.907817274766906,0.980001530739537
"14489","RABL2A",56.3042882065122,0.03196292396294,0.276606336167057,0.115553838736492,0.908006149089192,0.980137771533919
"14490","JRKL",84.6357481264071,-0.0230945119130352,0.200245981618596,-0.115330713387412,0.908182995019415,0.980261010497146
"14491","OSBPL1A",683.96944100432,-0.0181907766600511,0.157863156217738,-0.115231299664127,0.908261790367216,0.980278407379977
"14492","GTF2B",298.084235792719,0.0256818997295435,0.224767447068629,0.114259871989835,0.909031791628137,0.980707403222469
"14493","UBE2T",10.4027652492493,-0.0702913322525004,0.615213454548725,-0.114255193433734,0.909035500288756,0.980707403222469

"14494","CSNK1G3",369.04169408269,-0.0270971827733352,0.237089260459785,-0.114291059497111,0.909007069547107,0.980707403222469
"14495","PHF11",295.049917154516,0.0156063457402081,0.136385210359659,0.114428431785623,0.908898176710792,0.980707403222469
"14496","RIN2",212.140816377165,-0.0330099096349943,0.288894952463227,-0.11426267352039,0.909029570874259,0.980707403222469
"14497","PDK3",66.2600043300697,-0.0386151691383557,0.337557288410165,-0.114395898012531,0.908923965562583,0.980707403222469
"14498","PRKAB2",114.263553692351,0.0234164944858538,0.206421191430969,0.113440361057526,0.909681442717732,0.980912795514627
"14499","TDRKH",5.8365841002337,0.0796845700575281,0.703237397198509,0.113311053102363,0.90978395457063,0.980912795514627
"14500","RGS5",15.2328647775506,-0.0735621168056274,0.657412765336829,-0.111896392471079,0.910905557031779,0.980912795514627
"14501","FCMR",4.27049320808627,-0.0963648242914417,0.854277077863649,-0.112802774168339,0.910186918915386,0.980912795514627
"14502","MRPL53",30.4854269373606,-0.0467906760584398,0.418270227854752,-0.111867096777178,0.910928785774548,0.980912795514627
"14503","BCS1L",77.2681344561367,-0.0317441612861431,0.28304708486503,-0.112151521720424,0.910703266642025,0.980912795514627
"14504","GTPBP8",129.820411556021,-0.0261134008212658,0.230814823486209,-0.113135718178109,0.909922957759765,0.980912795514627
"14505","PLSCR1",429.799924706787,-0.026835415399339,0.236994922156411,-0.113232026893928,0.909846605152944,0.980912795514627
"14506","HLA-DRB5",40.2247437226204,-0.195162455147069,1.73328720315483,-0.112596720723401,0.910350285000862,0.980912795514627
"14507","AL009176.1",13.0947354399767,0.0784392446194819,0.688479113170698,0.113931189950325,0.909292340535043,0.980912795514627
"14508","FAM71F2",10.7981622322001,-0.0654754553722053,0.580541829923836,-0.112783355130147,0.910202314818533,0.980912795514627
"14509","MKLN1-AS",33.0842314424308,0.0362997552512843,0.32236004106889,0.112606249617417,0.910342730090294,0.980912795514627
"14510","AC009630.1",5.6944578058718,0.0750439141818383,0.670357168117001,0.111946165046065,0.91086609221383,0.980912795514627
"14511","DCAF12",103.963522639482,0.0198949103014598,0.177126750369509,0.112320190258989,0.91056953358768,0.980912795514627
"14512","INPP5E",60.4335200680061,0.0266199926354503,0.234529152663263,0.113503981629402,0.909631006599219,0.980912795514627
"14513","AL135791.1",8.48876621376866,-0.0592555468168295,0.530389110556732,-0.11172089629561,0.911044710207511,0.980912795514627
"14514","FRAT2",54.8733833678894,-0.0300653932129232,0.266735697485818,-0.112716046244698,0.910255679261123,0.980912795514627
"14515","C1QTNF4",148.56317121879,-0.0561645412881446,0.502473732910602,-0.111776074269214,0.91100095858798,0.980912795514627
"14516","AP001160.4",5.70977536350617,-0.0757046141489494,0.673303146194974,-0.112437636117962,0.910476415207447,0.980912795514627
"14517","AP000759.1",3.6088626690728,0.11222400364866,0.986245365311145,0.113789131585176,0.909404954369709,0.980912795514627

"14518","EAPP",530.408700292684,-0.0253459366965364,0.226551844030686,
-0.111876982529012,0.91092094729028,0.980912795514627
"14519","GNB5",147.008643371161,0.0301154259631028,0.268501994953362,0
.112160902075732,0.910695829127075,0.980912795514627
"14520","DAPK2",6.15984051433567,0.0675203501276792,0.60153123023758,0
.11224745571566,0.910627202672563,0.980912795514627
"14521","AC022558.1",2.75118228083687,0.107814749082749,0.948580640690
482,0.11365902323736,0.909508096672151,0.980912795514627
"14522","RHBDF1",308.847860497375,-0.0203468917223417,0.17886610469008
2,-0.113754876909722,0.909432109328606,0.980912795514627
"14523","OGFOD3",187.895448280886,0.0305438534970346,0.272916976876993
,0.111916282550649,0.910889786104073,0.980912795514627
"14524","PEPD",427.26015010472,-0.0308880568809848,0.27380756920798,-0
.112809360860008,0.9101816968278,0.980912795514627
"14525","CIC",122.129288439359,-0.0226347538869572,0.201982298359477,-
0.112063057360963,0.910773408825734,0.980912795514627
"14526","NIPSNAP1",71.5230529269487,-0.0364316168716727,0.324865921123
134,-0.112143547546386,0.9107095892273,0.980912795514627
"14527","SNX25",14.6856766897245,-0.0470522907363988,0.422849127899686
,-0.111274418301653,0.91139874064421,0.980988022760007
"14528","PEX1",101.490601629992,-0.024308111082726,0.218110936261827,-
0.111448382641143,0.911260795196838,0.980988022760007
"14529","EXOC5",254.151176650868,0.0170625380780271,0.153388674445691,
0.111237274457759,0.911428194291922,0.980988022760007
"14530","CNEP1R1",78.196607275069,-0.0259372178698606,0.23282462420678
7,-0.111402382622656,0.911297270763784,0.980988022760007
"14531","NLK",24.8497697036892,0.0399790585160178,0.359310893216771,0.
111265923941523,0.911405476336208,0.980988022760007
"14532","OVGP1",3.91251213006397,-0.093738496550222,0.844043596557647,
-0.111058832662822,0.911569693507069,0.981037028488085
"14533","ZNF888",5.51487536505386,-0.0727832587274154,0.65557715747342
4,-0.111021651529043,0.911599177430776,0.981037028488085
"14534","AC010618.3",21.2425392824789,0.0462759081725272,0.41744670055
2425,0.110854650692623,0.911731607381313,0.981112036565552
"14535","NIT1",151.141212582188,0.0215354256658186,0.1944722127247,0.1
10737803432641,0.911824267518128,0.981144241072138
"14536","URB2",16.4788713492703,0.0492082233623275,0.444983041889999,0
.110584491384937,0.911945846131049,0.981207555963786
"14537","ANKRD23",7.21465483432981,-0.0823789017714187,0.7461821911292
89,-0.110400519807025,0.912091740903741,0.981229524538073
"14538","KLRF1",3.78014690887068,-0.112526284813975,1.01857914179303,-
0.110473776849477,0.912033645598765,0.981229524538073
"14539","C9orf170",5.06202257008629,-0.0884637322888274,0.803264354493
026,-0.110130284997721,0.912306050281186,0.981392573519345
"14540","FAHD2B",16.1158412925643,-0.0588271168584604,0.53641509094918
3,-0.10966715487882,0.912673349866257,0.981399404702236
"14541","FKBP5",389.603200486607,0.0755802006462573,0.69079666392117,0
.109410199257827,0.912877144476223,0.981399404702236
"14542","AC004908.2",5.66628199161539,-0.0800785533625625,0.7299784092
90702,-0.109699892960358,0.912647385310108,0.981399404702236

"14543","GSR",75.4641630112144,-0.0255715441687487,0.232426822560114,-0.110019764014693,0.912393700647087,0.981399404702236
"14544","EDRF1-DT",40.8297040788626,0.0432796303594859,0.39430050259704,0.109763061610185,0.912597286548986,0.981399404702236
"14545","CAPN15",73.3107816631939,-0.0273800662116897,0.249747199020525,-0.109631124269143,0.912701925832585,0.981399404702236
"14546","C17orf53",2.56176424656403,-0.0976540727593695,0.889320250527911,-0.109807544246745,0.912562007786124,0.981399404702236
"14547","YJEFN3",22.1942808180235,0.0518860178444403,0.47417020108832,0.109424881035019,0.912865500026751,0.981399404702236
"14548","CCN5",7616.95163258576,-0.0307545768144256,0.280764546154789,-0.109538676573039,0.912775246847041,0.981399404702236
"14549","MROH1",53.3567480029241,0.033466356273372,0.306873180174687,0.109055982847121,0.913158087184701,0.981566493716063
"14550","MIEF1",107.489974599917,0.0237317288063418,0.217585427597934,0.109068557891637,0.913148113245474,0.981566493716063
"14551","SZRD1",340.760860736372,-0.0150621081619258,0.138601318591153,-0.108672185192957,0.91346250416836,0.981666038556463
"14552","HSD11B1-AS1",35.3151377544842,0.0385962059195183,0.354732763528954,0.108803611867016,0.913358258973647,0.981666038556463
"14553","GPR137B",120.449079937296,0.0285395193961867,0.264811360380614,0.107773017574347,0.914175747054013,0.981666038556463
"14554","CCDC71",47.3424069091657,0.03254058646199,0.301333681617102,0.107988547073004,0.914004777191435,0.981666038556463
"14555","MFN1",178.160118871157,-0.0176698784647823,0.163709515080655,-0.107934340017298,0.914047776837716,0.981666038556463
"14556","CLCN2",17.563673092173,0.0434036530841351,0.40501819445812,0.107164699458022,0.914658319866989,0.981666038556463
"14557","AC024560.4",2.78443320844509,0.104723540261943,0.973493378337904,0.107574989817335,0.914332837072627,0.981666038556463
"14558","UVSSA",71.2296060312783,-0.0336517972662986,0.314109331821475,-0.10713402582017,0.914682653797116,0.981666038556463
"14559","CNPY3",218.023387542976,-0.0218347512909149,0.202488476559205,-0.10783206857962,0.914128904150737,0.981666038556463
"14560","CBLL1",87.0016920236661,0.0207150921870385,0.193091052644969,0.107281471115737,0.914565683607498,0.981666038556463
"14561","NUB1",417.365234701378,-0.0188231184816201,0.173760141930287,-0.108328171653842,0.913735376357146,0.981666038556463
"14562","C11orf74",194.790782843407,-0.0351716036242431,0.32334265884976,-0.108775018271206,0.913380938755488,0.981666038556463
"14563","ESYT1",94.1389318657521,0.0255575880406619,0.237799110857809,0.107475540797728,0.914411728530181,0.981666038556463
"14564","NEK3",75.7800473528914,0.0338535667965147,0.314810153348045,0.107536451529526,0.914363408834443,0.981666038556463
"14565","IPO5",511.655306932051,-0.0172211489352653,0.15996812199734,-0.107653629487203,0.914270453966406,0.981666038556463
"14566","AC005225.2",3.29398836431313,-0.0888796864929663,0.820686892170515,-0.108299142268376,0.913758402986221,0.981666038556463

"14567","C16orf87",96.3870194652485,-0.0261536743276218,0.243451285648
639,-0.107428778853804,0.914448824390143,0.981666038556463
"14568","AC007611.1",5.57573400018777,-0.0730758264184994,0.6744650108
21504,-0.108346356365458,0.913720951954711,0.981666038556463
"14569","ANKRD12",1449.35402480576,-0.0201347510512087,0.1878582178417
28,-0.107180570978121,0.914645728744832,0.981666038556463
"14570","TYK2",89.7894311882228,0.0254831501224326,0.237056895433408,0
.107498033650707,0.91439388520398,0.981666038556463
"14571","CARM1",199.030778510213,-0.0198100433732484,0.183441278933377
,-0.10799119744713,0.914002674793534,0.981666038556463
"14572","CC2D1A",77.0196430808464,-0.0286798014458228,0.26531947121334
6,-0.10809535129354,0.913920055678486,0.981666038556463
"14573","CEBPA-
DT",4.30780962404306,-0.0924679610261111,0.863223943603555,-0.10711931
9049586,0.914694320964408,0.981666038556463
"14574","NUDT16",199.524469599344,0.023625679670998,0.220838895087949,
0.106981515468954,0.914803644105447,0.981716000673061
"14575","HADH",143.152213252963,-0.0237013143908011,0.221918603998524,
-0.106801836185662,0.914946190743825,0.981801607082911
"14576","TNFRSF18",2.74811319943866,-0.100067399430101,0.9404071837016
88,-0.106408586795572,0.915258180481879,0.981817043895488
"14577","ZMPSTE24",259.82534819675,0.0191793823258234,0.18037278538391
3,0.106331907471525,0.915319016586289,0.981817043895488
"14578","DENND2C",64.2306209989,0.0346955320657297,0.326608203904212,0
.106229824147054,0.915400008573146,0.981817043895488
"14579","ATG16L1",40.5953931609965,-0.0290740431864375,0.2725962463529
31,-0.106656065794814,0.915061838134982,0.981817043895488
"14580","TPRA1",131.523944159789,0.0261758470068932,0.245539797489071,
0.10660531316948,0.915102103309625,0.981817043895488
"14581","LTBP3",982.296503385639,-0.0215217310615122,0.202445219942597
,-0.106308911949685,0.915337260948766,0.981817043895488
"14582","TMEM126B",434.819429788691,0.0278460702202207,0.2614088685667
78,0.106523050931255,0.915167367458012,0.981817043895488
"14583","IKZF2",27.0783335653151,-0.036621335939795,0.345242682803283,
-0.106074184230174,0.915523493566382,0.981882153149435
"14584","CANX",1169.10153991149,-0.0144455875543855,0.136543779870134,
-0.105794548591848,0.915745362110572,0.981918103894786
"14585","DACT1",25.996059123303,-0.0423558591364364,0.400183314094917,
-0.105841142408027,0.915708393182746,0.981918103894786
"14586","AC023043.1",2.89969377893346,-0.124251686179443,1.17348655118
779,-0.105882496952075,0.915675581402898,0.981918103894786
"14587","ZMYM6",70.8142990512512,0.0264227053255654,0.251557569303987,
0.105036415317067,0.916346913282821,0.981955235470005
"14588","SLC8A1",14.3689796979597,-0.0582297966737803,0.55472355056393
8,-0.104970839284871,0.916398947734559,0.981955235470005
"14589","SCRG1",5.72491261051807,0.0719065058765062,0.686049142458425,
0.104812471040825,0.916524614099332,0.981955235470005
"14590","FOXP4",27.1336319797214,0.0426317160545825,0.406218341341443,
0.104947787226448,0.916417239582385,0.981955235470005
"14591","SNX14",250.030067863662,0.0153197976504765,0.145504371359437,

0.105287542273436,0.916147647856345,0.981955235470005
"14592","AC064807.4",3.3076017827054,-0.0941077096583822,0.90204849254
4604,-0.104326663628595,0.916910118849358,0.981955235470005
"14593","AC022387.2",6.57572280447221,-0.0602551009634013,0.5702304217
93458,-0.105667987291682,0.915845780552334,0.981955235470005
"14594","VPS37C",66.421218358144,0.0281566896148792,0.268918926351967,
0.10470326502057 8,0.916611271093786,0.981955235470005
"14595","NKAPD1",237.43528144402,0.0176367312172738,0.168022567298144,
0.104966442906319,0.916402436265052,0.981955235470005
"14596","NANOG",3.82379520496352,0.089598041174094,0.856914502308914,0
.10455890398946,0.916725825759285,0.981955235470005
"14597","AC008115.3",3.48971731012267,-0.0929521483837051,0.8895267433
75105,-0.104496181903446,0.916775598092824,0.981955235470005
"14598","TPT1",46182.9679859408,-0.0245969910339453,0.235469703539132,
-0.104459260211612,0.91680489699606,0.981955235470005
"14599","LINC00909",77.8140465229124,0.0226296613249405,0.216851465417
539,0.104355584046287,0.916887168963742,0.981955235470005
"14600","CHAF1A",43.4305865945252,-0.0340838605873558,0.32402355185719
4,-0.105189454260342,0.916225478578895,0.981955235470005
"14601","TRAPPC6A",167.415174710206,-0.034382390603576,0.3283566580596
52,-0.104710502314011,0.916605528137082,0.981955235470005
"14602","TFPT",64.5804095113381,-0.0324469389112317,0.307973201513287,
-0.105356371112153,0.916093034137375,0.981955235470005
"14603","SLX4IP",44.4612231263222,-0.0330764026565685,0.31651826294011
9,-0.104500771454146,0.916771956100717,0.981955235470005
"14604","LAS1L",94.0597082720175,0.0224616820912599,0.214165001877372,
0.1048802647228 1,0.916470819059242,0.981955235470005
"14605","SAP30L-
AS1",12.8314901056498,0.0558773605499847,0.537191850629541,0.104017513
45353,0.917155450235641,0.982039632599988
"14606","UVRAG",197.14631337198,-0.021050934062795,0.202281380086034,-
0.104067581770708,0.91711571713388,0.982039632599988
"14607","AC092756.1",3.73390831341019,-0.0939506056987795,0.9039779886
27814,-0.103930191753221,0.917224747287923,0.982039632599988
"14608","NR2F6",218.218863616891,-0.0256717730519546,0.247055720262998
,-0.103910862798992,0.91724008651027,0.982039632599988
"14609","EFCAB7",62.9898448272412,-0.0278842541967326,0.26907732175549
5,-0.103629150219023,0.917463653671854,0.982041075538657
"14610","AC053503.5",2.49685284093879,0.104679397144797,1.010490273138
62,0.103592681619447,0.917492595637523,0.982041075538657
"14611","ZNF451-
AS1",4.29618493374596,0.0856468709869616,0.826659677651288,0.103605961
803172,0.917482056294427,0.982041075538657
"14612","UTP20",92.9367698703295,0.0271257270260774,0.261748868844344,
0.1036326427917,0.917460881925464,0.982041075538657
"14613","ASB1",133.638892569735,-0.0204069189834051,0.197297826810933,
-0.103432051499283,0.917620075138232,0.982060692313905
"14614","AC067817.2",2.82903634857718,0.100544008195841,0.972272486389
453,0.103411347748009,0.917636506232443,0.982060692313905
"14615","RNF149",280.269492578279,-0.0186059924637741,0.18060622666433

7,-0.103019662208845,0.917947365794936,0.982326158127458
"14616","PLA2G4A",63.0368379273714,0.0350720557586301,0.340986138681904,0.102854784344615,0.918078224164934,0.98239897550216
"14617","AC096586.1",4.70626209769521,-0.0808824014591018,0.787288587361396,-0.102735391770608,0.918172983677602,0.98243315760537
"14618","VPS13A",131.334511427579,0.0311424634642445,0.303430872045489,0.102634459223964,0.918253092740198,0.982451660313086
"14619","C1QTNF7",350.482224491803,0.0844845678550367,0.825221488598669,0.102378051253249,0.918456604682981,0.982483344220027
"14620","USP38",81.4231965940344,-0.0225114516964038,0.219924925752123,-0.102359710339411,0.918471162138172,0.982483344220027
"14621","LRRC61",3.39513843674784,-0.114114111076365,1.11375001822104,-0.102459357314881,0.918392071185862,0.982483344220027
"14622","PSMB4",733.136544666033,-0.0229181382348577,0.224465624167842,-0.102100882127594,0.918676600838595,0.982510311136447
"14623","LINC00303",2.87124298733533,-0.104819130444969,1.03768496397922,-0.101012478819216,0.919540554180919,0.982510311136447
"14624","AL121658.1",9.08454585761595,0.0519438905639997,0.509571885038902,0.101936335361269,0.918807208721156,0.982510311136447
"14625","CRIPT",263.606951094428,0.0161729737686099,0.159173790538586,0.101605758799149,0.919069608249011,0.982510311136447
"14626","SHQ1",43.5851742733987,-0.0257128857466526,0.254534559646484,-0.10101923205385,0.919535193300917,0.982510311136447
"14627","ATXN1",230.959170929322,0.0218480385177302,0.216354608280194,0.10098254292525,0.919564318057245,0.982510311136447
"14628","BCKDHB",81.073219020115,0.020736813323084,0.204209939692111,0.101546542515752,0.919116612890479,0.982510311136447
"14629","POT1-AS1",4.044755880339,0.0791411563155408,0.781420303491183,0.101278602516416,0.919329301562299,0.982510311136447
"14630","CTSL",1742.80499278051,-0.0354664524916028,0.348321762517772,-0.10182094921443,0.918898797001539,0.982510311136447
"14631","MARVELD1",435.587499110198,0.0218503898147923,0.213715278460421,0.102240653883989,0.918565659675965,0.982510311136447
"14632","ANO6",423.107595345123,0.0180204553248959,0.178119200010798,0.101170762746539,0.919414905556383,0.982510311136447
"14633","AL356966.1",7.61253956347155,0.07182347618889,0.707473365258617,0.101521102723966,0.919136806549447,0.982510311136447
"14634","PSEN1",207.944042016709,0.0171505519855723,0.168276041423752,0.101919155219393,0.91882084546891,0.982510311136447
"14635","DTWD1",475.460773947842,0.0229717122552078,0.227347446056024,0.101042314984031,0.919516869543638,0.982510311136447
"14636","GOSR1",320.345901275132,-0.0146886827629867,0.143898269633543,-0.102076854714053,0.918695672298813,0.982510311136447
"14637","NOL4L",29.4270425652014,-0.0422118113172843,0.417823635781123,-0.101027820597964,0.919528375513413,0.982510311136447
"14638","SETD4",66.7004904398767,0.0216506449504621,0.214167508647799,0.101092108168783,0.919477342721658,0.982510311136447
"14639","XRCC1",94.9155812516618,0.0232225562442505,0.230288722881135,0.1008410483749,0.919676641012863,0.982563198677585

"14640","HADHA",932.687186708627,-0.0123569605848492,0.122666693182218
,-0.100736069949268,0.919759977322062,0.982573617035983
"14641","GTPBP1",86.3953935987982,0.0210109110171409,0.208709741339681
,0.100670485633658,0.91981204136981,0.982573617035983
"14642","CYP27A1",256.059594535943,0.0210459229653375,0.20956145619025
6,0.100428405814428,0.92000421918279,0.982711787188829
"14643","ZNF513",33.0849129916909,-0.0316085292547771,0.31548950589685
5,-0.100188845156425,0.920194401730613,0.982780691277437
"14644","KLRC1",3.34278778528559,0.109295973732257,1.09067383250061,0.
100209586473411,0.920177935421747,0.982780691277437
"14645","GPR89B",27.8148651051045,0.0340941890650067,0.340808326701557
,0.100039190341915,0.920313212094798,0.98279868581205
"14646","KCTD20",353.640158246676,-0.0176087283725832,0.17631980826269
2,-0.0998681234178102,0.920449023626505,0.98279868581205
"14647","IFITM10",10.4335627234299,-0.0470074425926989,0.4707758065609
73,-0.099851015998654,0.920462605484329,0.98279868581205
"14648","AC008124.1",132.397817343437,-0.0240028908485047,0.2403360962
10186,-0.0998721841080125,0.920445799782031,0.98279868581205
"14649","NUF2",2.78456536870473,0.0865705639870455,0.871530436477027,0
.0993316588425623,0.920874942587964,0.982849071693088
"14650","GOLGB1",844.688275753348,0.0236221914731844,0.237320221632522
,0.0995372046709196,0.920711749504546,0.982849071693088
"14651","STX1A",3.58959008598242,-0.0859357406779348,0.862972908895886
,-0.0995810410640626,0.920676946037699,0.982849071693088
"14652","ARRB1",153.898002626649,0.0159387818090608,0.160253636387943,
0.0994597200307904,0.920773268032681,0.982849071693088
"14653","THAP2",73.300423999457,-0.0234035362393974,0.235707756804153,
-0.0992904796885541,0.920907637172729,0.982849071693088
"14654","JSRP1",5.91795283611514,-0.0726995664329145,0.730937794445814
,-0.0994606750195948,0.920772509821387,0.982849071693088
"14655","ECH1",748.210148791852,-0.0272037233578674,0.274127415480288,
-0.099237514460948,0.920949689620345,0.982849071693088
"14656","AC092620.1",5.2920527589067,-0.0722386418630274,0.73136713085
6971,-0.0987720651027653,0.921319248828031,0.982877098354064
"14657","ZBTB20-
AS4",5.78119958294137,-0.0670613286172952,0.676732431596903,-0.0990957
806751612,0.921062222133377,0.982877098354064
"14658","ASTE1",70.2943459228608,-0.0209158973472109,0.211850448913427
,-0.0987295398923518,0.921353014000571,0.982877098354064
"14659","USP45",49.2460131608137,0.0258921016362189,0.261809587607387,
0.0988966900442431,0.921220296985934,0.982877098354064
"14660","AC022706.1",3.51792298032616,-0.105256939425922,1.06448184549
709,-0.0988809155094319,0.921232821853442,0.982877098354064
"14661","KRI1",69.2066249267026,0.0301566841709109,0.304810391784736,0
.0989358794309358,0.921189180978536,0.982877098354064
"14662","GLE1",74.7880556547044,-0.0202809507557198,0.20607598366991,-
0.0984149166465006,0.921602830408365,0.983076542599019
"14663","EMC1-
AS1",27.8433251548586,0.037754234873689,0.390211079721098,0.0967533646
165909,0.9229222597478,0.983150029922579

"14664","P3H1",288.945346766129,0.0249301670774329,0.2550564401466,0.0
977437270868508,0.922135792581732,0.983150029922579
"14665","AC012360.3",11.4768094214823,0.0574077396256588,0.58581645161
1795,0.0979961205727648,0.92193537387627,0.983150029922579
"14666","PECR",13.3884648026321,-0.0435525626825608,0.443514298852362,
-0.0981987791493025,0.921774451877978,0.983150029922579
"14667","AC008771.1",47.8973525931932,0.0289415661878317,0.29816112137
4641,0.097066867921611,0.92267329214959,0.983150029922579
"14668","REEP2",3.88093054712174,0.085904510437273,0.87529669819062,0.
0981433045672988,0.921818501413343,0.983150029922579
"14669","TWISTNB",236.05019948153,-0.0223536622989505,0.23089211910325
1,-0.0968143147794243,0.922873855788268,0.983150029922579
"14670","DNAJA1",1756.18110254879,-0.0219088451958199,0.22356401620872
,-0.0979980838032797,0.921933814948482,0.983150029922579
"14671","PRPF19",165.410189927538,-0.0195635437468595,0.20166238743046
6,-0.0970113663541005,0.922717367982069,0.983150029922579
"14672","RAP1B",3147.23242482497,0.0174384159279263,0.179664222472187,
0.0970611493372079,0.92267783347645,0.983150029922579
"14673","AC084018.2",2.7213162823507,-0.084582422480441 9,0.87287740591
7933,-0.0969006894977348,0.922805261245503,0.983150029922579
"14674","BRCA2",21.4787489586405,0.0404974892868239,0.418303048141323,
0.0968137561195629,0.922874299450254,0.983150029922579
"14675","MTA1",171.0960561648,0.0176501273906076,0.181609459135284,0.0
971872691799589,0.92257767790631,0.983150029922579
"14676","TAOK2",70.8121477335937,0.0296084902689488,0.30624511654963,0
.0966823262442145,0.922978675683327,0.983150029922579
"14677","ADCY7",115.520680328809,-0.0263636801065008,0.272438720395184
,-0.0967692113230423,0.922909674957601,0.983150029922579
"14678","PFAS",27.2415579765309,0.0371186902793814,0.380600843848964,0
.09752655801812,0.922308244507018,0.983150029922579
"14679","SNHG20",34.7156493347778,0.0268332280741997,0.277587637596224
,0.0966657892497039,0.922991808782176,0.983150029922579
"14680","ZGLP1",4.04066122500259,-0.0679190475226929,0.69621633272938,
-0.0975545162182989,0.922286042956866,0.983150029922579
"14681","UQCC1",78.5538619187955,0.0208633596510907,0.214418393098936,
0.0973020987125112,0.922486489379601,0.983150029922579
"14682","TIMP3",10596.2839988365,-0.0205701924019875,0.212209178952622
,-0.0969335657557965,0.922779152693064,0.983150029922579
"14683","SMC1A",292.10759207302,-0.0261647814430797,0.269607415350286,
-0.0970477069745475,0.922688508534309,0.983150029922579
"14684","MATN1-
AS1",6.27928616651106,0.0623204148994516,0.646235922366371,0.096436011
590393,0.923174292280667,0.983217556815876
"14685","TMEM74",2.64601157158353,0.104697933354269,1.08576674912428,0
.0964276475022953,0.923180934900328,0.983217556815876
"14686","TMEM50B",342.70277025138,0.0134291633295042,0.139594900671888
,0.0962009590956969,0.923360969101453,0.983342336697993
"14687","C1orf56",167.098753790524,-0.0272882067002509,0.2846860635938
47,-0.095853679508464,0.923636783508631,0.983379731491279
"14688","AC108865.2",3.39905557628386,0.0937068244351267,0.97854983641

3645,0.0957609116553116,0.923710462617543,0.983379731491279
"14689","LRRC20",20.7101564833241,0.0335990516135789,0.350028202323531,0.0959895556716407,0.923528867624365,0.983379731491279
"14690","USP12",176.47662162037,-0.0186428627004946,0.194606248671381,-0.0957978627499034,0.923681114832619,0.983379731491279
"14691","RAVER1",55.8414178780299,-0.0253819423575883,0.264484026813379,-0.0959677703920391,0.923546169885416,0.983379731491279
"14692","ARIH2",233.636892208101,0.0185338549972613,0.193775660434439,0.0956459389982671,0.923801778379261,0.983410006387942
"14693","MOGS",109.876682034384,-0.0198515828458826,0.207843682528139,-0.0955120819859171,0.923908094096437,0.983456243903102
"14694","MAP3K5",28.5488946277469,-0.0322806269672822,0.339522425760668,-0.0950765679025782,0.924254009853748,0.983590443817845
"14695","NUP107",142.864817918343,0.0235756170988288,0.248473047641693,0.0948819895058628,0.924408562264023,0.983590443817845
"14696","AC015712.1",12.7530936483574,0.0463928964713288,0.487768931243103,0.0951124466929335,0.924225511865766,0.983590443817845
"14697","SAT2",850.651967588615,0.0250758675108429,0.263362788559696,0.0952141631244881,0.924144720550251,0.983590443817845
"14698","PET100",718.672738917951,-0.0250085432686669,0.26358553227582,-0.0948782850588992,0.924411504710902,0.983590443817845
"14699","HELZ2",131.532562003886,-0.0294460906607199,0.310214144293654,-0.094921818370879,0.924376926211822,0.983590443817845
"14700","AOAH",5.98889418847578,-0.0797418346702582,0.841905656017831,-0.0947158795053508,0.924540504662188,0.983660781831062
"14701","ATAD2",49.2060266712733,0.0307119028528274,0.325215003176849,0.0944356888606597,0.924763066859275,0.983763730491026
"14702","SNX12",91.564901940359,-0.0213200816408498,0.225590209045351,-0.0945080095943517,0.924705620189049,0.983763730491026
"14703","GPSM2",436.370089726403,0.0309699255101612,0.330215127705336,0.0937871190983015,0.925278264051411,0.983843364590664
"14704","VNN2",3.16018283420928,-0.101110908463684,1.07589128094404,-0.0939787413975179,0.925126043892541,0.983843364590664
"14705","HOXA11-AS",24.2513457045376,-0.0356119730597578,0.379542237409243,-0.0938287482912187,0.925245194586147,0.983843364590664
"14706","COL15A1",36.3620449309517,0.050169370074151,0.534423275710275,0.0938757205278408,0.925207880860331,0.983843364590664
"14707","YY1",1008.2465431573,-0.0133369320439699,0.141851874705729,-0.0940201324207898,0.925093164216139,0.983843364590664
"14708","SPATC1L",225.805198832523,-0.0230956047705283,0.245648696438057,-0.0940188370849021,0.925094193186703,0.983843364590664
"14709","CSF2RB",3.87021924608007,-0.101521141340394,1.08213575854234,-0.0938155314977714,0.925255693749804,0.983843364590664
"14710","MRPS35",194.330117015321,-0.0192305401382873,0.205347368299465,-0.0936488268514972,0.925388121795549,0.983893285172154
"14711","LARS",332.232888130833,-0.0171755156956919,0.184049751147264,-0.0933199615246921,0.925649374277325,0.983903507795118
"14712","LINC00900",9.33984059186923,-0.0460081011719047,0.492213662717713,-0.0934718083969371,0.925528745262291,0.983903507795118

"14713","COG3",87.4602404763404,-0.0184722505366632,0.1978275940933,-0
.093375500123361,0.925605253535928,0.983903507795118
"14714","ZNF420",81.7106936006402,0.0208633382768239,0.223063582673036
,0.0935309028341265,0.925481800380295,0.983903507795118
"14715","ARSB",32.4252946180403,-0.0379003346419276,0.406896477318581,
-0.0931449072542681,0.925788441654732,0.983917588167981
"14716","ACTR6",138.116427555557,-0.0200344354882373,0.215016999491678
,-0.093176053686921,0.925763698003831,0.983917588167981
"14717","KRTCAP3",4.85959713380427,0.0729913142203426,0.78453155338800
8,0.0930380861102767,0.925873304088391,0.983921435422729
"14718","KCTD17",88.3732499683715,-0.021209215101903,0.228100291457504
,-0.0929819728259943,0.92591788277185,0.983921435422729
"14719","PDE4DIP",618.742578778794,0.0220197120987504,0.23709983651022
9,0.092871055597714,0.926006000629535,0.983948219977303
"14720","CENPE",17.6140089497408,-0.0411879856069395,0.446892118993276
,-0.092165388147199,0.92656663722144,0.984020078810583
"14721","SQSTM1",1840.34176361309,0.0166078947509845,0.180222069635629
,0.0921523916830062,0.926576962956743,0.984020078810583
"14722","TRDMT1",53.0967913265884,0.0291378651662522,0.315825830420435
,0.0922592845792985,0.926492036552202,0.984020078810583
"14723","IGHMBP2",35.3140087417365,0.0282693360757625,0.30623825241524
,0.0923115771880486,0.926450490380336,0.984020078810583
"14724","NDRG2",99.2137507632524,0.0287638195291817,0.311736621993336,
0.0922696196079155,0.926483825416505,0.984020078810583
"14725","AAR2",85.4788376759808,0.0182012098248636,0.197211692731354,0
.0922927518788536,0.926465446955311,0.984020078810583
"14726","ZSWIM3",11.2352815565437,-0.0489429521158379,0.53083222585759
5,-0.0922004161235074,0.926538807435915,0.984020078810583
"14727","DRG1",183.921850684814,-0.0170049521304663,0.183700878327325,
-0.0925687034558773,0.926246207983746,0.984020078810583
"14728","CTBS",229.33943065301,-0.0186489093508915,0.203247538348465,-
0.0917546628236066,0.926892965869322,0.984021584726866
"14729","RCOR2",3.11641664376844,-0.0922802525004979,1.00259840650974,
-0.0920410923270317,0.926665391182429,0.984021584726866
"14730","TMEM251",135.498448926306,0.0264777046348336,0.28844084456503
,0.0917959614033238,0.926860152848815,0.984021584726866
"14731","NPRL3",134.937586604949,0.0220525928145355,0.239969186259908,
0.0918976021806839,0.92677939658433,0.984021584726866
"14732","NELFCD",220.237852338796,-0.0193021012168522,0.21023230388823
6,-0.0918132031084699,0.926846453808595,0.984021584726866
"14733","LRP12",89.4818481614611,-0.02251612131125,0.245877610942119,-
0.0915745082481318,0.927036105799268,0.984106746399278
"14734","THAP1",87.7789508480416,0.0229365502852211,0.250812811445956,
0.0914488783606789,0.927135925107599,0.984145912086524
"14735","SGTA",137.127278518198,-0.0185281128821264,0.202970769343763,
-0.0912846364135622,0.927266425382335,0.984217637799777
"14736","AC006483.2",7.18983189143523,0.0618882154810224,0.67918256127
9422,0.0911216203261158,0.927395953571302,0.984288322058575
"14737","CLDND1",350.900169818507,-0.0166443984629721,0.18323396372813
2,-0.0908368630155688,0.92762218668513,0.984328075173047

"14738","SRSF2",1132.98274616842,-0.017091904825229,0.187956284913014,
-0.0909355323400815,0.927543816420591,0.984328075173047
"14739","ZNF558",33.403815009835,0.0259693749957617,0.285750669449307,
0.0908812393889029,0.927586957293966,0.984328075173047
"14740","ATP1B1",87.5460707166059,-0.0310775418018317,0.34277767505677
9,-0.0906638444195172,0.927759700244198,0.984407171765215
"14741","HSPG2",2066.7108826798,-0.0160995250431942,0.17855037970806,-
0.0901679686681029,0.928153737913968,0.984480332049135
"14742","STARD7-
AS1",11.0445588725574,0.0456087563172444,0.506263862669688,0.090088903
594136,0.928216567012218,0.984480332049135
"14743","ST8SIA4",4.37835697542053,0.0791950400764319,0.87995289718467
9,0.089999181012766,0.928287865645799,0.984480332049135
"14744","AC083805.1",6.4423620551265,0.0572710961881902,0.633057586696
504,0.0904674351776574,0.927915770775583,0.984480332049135
"14745","CTSG",23.7163387414273,-0.0716099750766898,0.79304440122782,-
0.0902975608500869,0.928050758394468,0.984480332049135
"14746","VPS16",123.240694518144,0.0192701721906767,0.213673340223757,
0.0901851965738784,0.928140047809952,0.984480332049135
"14747","UCKL1",138.918231386836,-0.0154995757285719,0.172325950300264
,-0.0899433643137621,0.928332221039683,0.984480332049135
"14748","SMIM11B",4.4687721078043,0.0656817810258039,0.728073145989012
,0.0902131624928729,0.928117824821001,0.984480332049135
"14749","DHX16",97.7740829013935,-0.0225862742018747,0.25134572397918,
-0.0898613823394329,0.928397369379877,0.984482667102941
"14750","FOXK2",101.972980611352,0.0193926671291751,0.216212932856809,
0.089692447500438,0.928531617773996,0.984558271321037
"14751","AKAP8L",158.201574310936,-0.0143780686194778,0.16058943841571
1,-0.0895330898552491,0.928658257270429,0.984559052583345
"14752","GRIA3",23.7944159724864,-0.0425682774764816,0.475242249723926
,-0.0895717447285254,0.928627538567531,0.984559052583345
"14753","TMEM201",13.4426930637112,0.0422267621320206,0.47325621175722
8,0.0892260071457492,0.928902297091958,0.984560916356221
"14754","AC007384.1",2.68628935790289,-0.0918265088494795,1.0292833544
9281,-0.0892140229885752,0.928911821083912,0.984560916356221
"14755","CCDC134",7.55147370428637,-0.0511661278150215,0.5727422215236
1,-0.0893353517380109,0.928815399752175,0.984560916356221
"14756","ETNPPL",2.77426751791931,0.118770220295716,1.32969268221938,0
.0893215566904354,0.928826362780138,0.984560916356221
"14757","AC016727.1",16.3506357218551,-0.0368534049033128,0.4162739489
92173,-0.0885316147996708,0.929454158005792,0.984935499099573
"14758","CMA1",5.34600278386465,0.0704031658609002,0.794963389871506,0
.0885615196346084,0.92943039075607,0.984935499099573
"14759","TM6SF2",9.01155761311758,0.0497602547251915,0.561257997265509
,0.0886584333187717,0.929353367799756,0.984935499099573
"14760","NECAP2",271.814049409871,0.0216374748914975,0.245017575433023
,0.0883098890079917,0.929630379373618,0.985055496843048
"14761","SURF6",104.497147544345,0.0210365562864279,0.239307533647608,
0.0879059508314738,0.92995142683779,0.985328928645961
"14762","RAD51C",131.488312195758,0.0192913128343508,0.219674500559006

,0.0878177156896232,0.930021557079805,0.985336482368795
"14763","RRNAD1",80.2393169024278,0.0204410778774845,0.233594004494832
,0.087506860125499,0.93026863275951,0.985531491997475
"14764","ATG4B",142.722503083422,-0.0155501862893249,0.177909840617562
,-0.0874048688669888,0.930349699396191,0.985550616266352
"14765","SEC61A1",413.396057960148,0.0165418502113204,0.18975552887459
9,0.0871745361488367,0.930532779496438,0.985676852641703
"14766","POLRMT",46.2285913698762,-0.024976936347378,0.286773563530941
,-0.0870963698321627,0.930594910876431,0.985676852641703
"14767","PEBP4",19.1137611317693,-0.0328827619198978,0.38031200100797,
-0.0864625934305152,0.931098690795894,0.986143666557038
"14768","CBWD3",12.8348864255953,0.0395745940688581,0.459248791954103,
0.0861724511031771,0.931329330062397,0.986321148576374
"14769","TMEM117",27.5951252725976,-0.0262626228962,0.305061022186071,
-0.0860897361059164,0.9313950827335,0.986323995798763
"14770","CCL13",7.49090115625994,0.0528181579983991,0.614923124446678,
0.0858939205545832,0.931550744345911,0.986422047499665
"14771","IRF2",301.002748417093,-0.0142099195872804,0.165695045662547,
-0.0857594717479975,0.931657624595192,0.986444023064256
"14772","FKBPL",29.7677734198374,0.0338371521849437,0.395075001553834,
0.085647413913464,0.931746706054528,0.986444023064256
"14773","LINC02697",2.73051366859371,0.0946053181676018,1.106991239892
07,0.0854616683116895,0.931894368199698,0.986444023064256
"14774","IFT43",254.634573429906,-0.0203582203530578,0.237950879494953
,-0.0855563988511739,0.931819059987815,0.986444023064256
"14775","ATPAF2",68.9647638553019,0.0230332687448404,0.269530318382265
,0.0854570605751787,0.931898031240918,0.986444023064256
"14776","TYMSOS",3.86572507284321,-0.0646447534491408,0.75703720196907
1,-0.0853917790050454,0.931949928695489,0.986444023064256
"14777","CD53",35.715428541081,-0.0409599071973073,0.481573052175435,-
0.0850544003911286,0.932218142097091,0.986514740451575
"14778","TENT2",143.115686353092,-0.0168485115683,0.198198241512133,-0
.0850083806988196,0.932254727995512,0.986514740451575
"14779","FUT8-
AS1",4.63860888094347,-0.0618501213810524,0.727134816945738,-0.0850600
465548439,0.93221365337698,0.986514740451575
"14780","LIMD2",30.6624547469305,-0.0342473427141652,0.403332076033021
,-0.0849110317508231,0.932332121394803,0.986514740451575
"14781","AC245452.1",61.9551702014185,-0.0268526259488561,0.3161202994
08227,-0.084944326571637,0.932305651603134,0.986514740451575
"14782","MELTF-
AS1",16.4590778690773,-0.04036115717944,0.475957469451228,-0.084799923
8797025,0.932420454058994,0.986527851635734
"14783","CMTM1",7.0233194149815,0.0567729364309791,0.669991785950346,0
.0847367648701095,0.932470666926538,0.986527851635734
"14784","AC004832.6",3.024787491991,-0.105722434088408,1.2495918498672
2,-0.0846055726913083,0.932574968589423,0.986571462982858
"14785","HNRNPA3",1189.93919765762,0.01484160212144,0.175991764280792,
0.0843312309646516,0.932793082116943,0.986735461907946
"14786","TIGD6",29.2374055829327,-0.0254712402547381,0.302412770685138

,-0.0842267348598777,0.93287616236371,0.98675660620644
"14787","MINPP1",98.4951542374674,0.0184099745713452,0.219215440619305
,0.0839811945697584,0.933071383501257,0.986764467641601
"14788","MCTP2",3.31203212328077,-0.110743999215862,1.31751265769334,-
0.0840553588378791,0.9330124174672,0.986764467641601
"14789","AC087741.1",10.341280910522,-0.0431072942590923,0.51330842834
1069,-0.0839793229158698,0.933072871608161,0.986764467641601
"14790","HIVEP1",324.307834358009,-0.0213633180799212,0.25521340873946
2,-0.0837076632667456,0.93328886412686,0.986859430393082
"14791","KCNC1",6.31567092685897,-0.0576025418948218,0.687980360675102
,-0.0837270148791711,0.933273477786958,0.986859430393082
"14792","CPT2",88.991286994432,-0.0209483710366869,0.250933307181264,-
0.0834818273907127,0.933468426589329,0.98695060620194
"14793","PAXIP1-
AS1",78.3813969541678,-0.0209244397485593,0.25077083355186,-0.08344048
4095341,0.933501299075786,0.98695060620194
"14794","GPX7",194.379568669243,-0.0220752487983355,0.267028248809908,
-0.0826700878904029,0.934113869838624,0.987230750707143
"14795","AC092802.1",12.5849770303191,-0.0374706269394742,0.4601549329
22935,-0.0814304580013046,0.935099627552017,0.987230750707143
"14796","IRS1",79.1192014183157,-0.0278475492708326,0.342271980776504,
-0.081360879168828,0.935154959829049,0.987230750707143
"14797","AC096921.2",25.9221163168787,0.0374708016259961,0.45893633278
7259,0.0816470585329878,0.934927378888635,0.987230750707143
"14798","EBLN2",17.0640829436882,0.0431476001646761,0.520648524441277,
0.0828727983258552,0.933952683412048,0.987230750707143
"14799","SEC62",2150.84442382144,-0.0122724449604853,0.149732313251654
,-0.0819625683593031,0.934676479389727,0.987230750707143
"14800","CTNNA1",863.80954987895,0.0122982092281798,0.149422650114179,
0.0823048528371188,0.934404295509787,0.987230750707143
"14801","PURA",786.42732119586,0.0151044904245954,0.185275376093146,0.
0815245433208655,0.935024807091722,0.987230750707143
"14802","AC016394.1",15.6082156889948,0.0363916484417255,0.44030327764
3214,0.0826513230528671,0.934128790949178,0.987230750707143
"14803","ADAM8",9.28191917205834,-0.0499061297243719,0.611282289223716
,-0.0816417072834697,0.934931634361269,0.987230750707143
"14804","XRRA1",199.886012281804,0.0290031569919396,0.35642330058434,0
.0813727860787726,0.935145490886234,0.987230750707143
"14805","WNT11",630.510612283476,0.0360625909035566,0.4373857446385,0.
082450311528471,0.934288629391351,0.987230750707143
"14806","PHLDB1",579.447893589205,0.019267534275924,0.232521274594698,
0.0828635328509565,0.933960050851348,0.987230750707143
"14807","ISCU",1267.16840646439,-0.0181505296618253,0.221712124120896,
-0.0818653004827472,0.934753827990466,0.987230750707143
"14808","C12orf43",127.687674665411,-0.0153821293538683,0.187108540605
226,-0.0822096591855876,0.934479992528207,0.987230750707143
"14809","FURIN",101.754530223677,-0.0152836680720663,0.187269794295522
,-0.0816130979881779,0.934954385354572,0.987230750707143
"14810","ACOX1",178.575814734833,-0.0165066404923192,0.202850852553646
,-0.0813732862569746,0.935145093120867,0.987230750707143

"14811","AC068473.5",11.4777888709315,0.0511554905904749,0.623724613160542,0.0820161486513405,0.934633871951922,0.987230750707143
"14812","LIN37",88.6305061688108,-0.0231204847914241,0.281414905418112,-0.0821579964183944,0.934521074480504,0.987230750707143
"14813","ZNF223",23.9998684908356,0.0275228851732415,0.33432123062977,0.0823246705612917,0.934388536732709,0.987230750707143
"14814","DPM1",282.729116231482,-0.0161374674910214,0.195891450551415,-0.0823796416106778,0.934344824657685,0.987230750707143
"14815","APOBEC3C",317.524193412674,0.0136368320947037,0.167232173410635,0.081544309426744,0.935009088355944,0.987230750707143
"14816","MRPL55",332.207163796586,-0.0206405340290453,0.258196644030142,-0.0799411398493456,0.93628406964433,0.9872924768119
"14817","CCSAP",18.5603328847705,-0.032502570575983,0.407252890322167,-0.0798093060807281,0.936388922754644,0.9872924768119
"14818","RFC4",56.9412594602432,-0.0226188931773946,0.281817863690669,-0.0802606792954109,0.936029930661637,0.9872924768119
"14819","GRSF1",228.823519100316,-0.0139452896591839,0.176024257660276,-0.079223681125235,0.936854708974771,0.9872924768119
"14820","ZMAT2",425.73958561262,-0.0120062116664536,0.151275816946557,-0.0793663647554133,0.936741221259094,0.9872924768119
"14821","AL022069.3",14.4524587984133,0.0375648561300292,0.469509214457809,0.0800087729341143,0.936230278549314,0.9872924768119
"14822","ASB6",52.9459118600465,0.0196853197888739,0.246209184420707,0.0799536371284869,0.936274130073867,0.9872924768119
"14823","DNAJC1",548.713109767762,-0.0130370396469416,0.161999344800555,-0.0804758788561286,0.93585877988507,0.9872924768119
"14824","CCAR1",327.971234923116,0.0134838330379026,0.169086412297697,0.0797452193506994,0.936439894107389,0.9872924768119
"14825","TIAL1",432.327681041146,0.0105044997302179,0.130406474639144,0.0805519799479706,0.93579825648835,0.9872924768119
"14826","ASCL2",2.49705625988728,-0.103463829629514,1.28155812787035,-0.0807328418270397,0.935654418053759,0.9872924768119
"14827","PPFIA1",327.65160069755,0.0150398220818869,0.189336128431018,0.0794345073310533,0.936687022465851,0.9872924768119
"14828","AP004609.3",13.8292919631703,-0.0373362337916118,0.461515390898801,-0.0808992170746451,0.935522102610176,0.9872924768119
"14829","SCNN1A",4.1755250369887,-0.0546359323211439,0.687313495040412,-0.0794920116008074,0.936641285326549,0.9872924768119
"14830","MFAP5",37467.4178492312,-0.0233736007157994,0.29112445150241,-0.0802873155970752,0.936008746332861,0.9872924768119
"14831","IFT88",96.6406758122104,-0.0186040797554791,0.234759549265839,-0.0792473823265528,0.93683585742271,0.9872924768119
"14832","MNAT1",279.244425913736,-0.0119181866264421,0.147519045691937,-0.0807908332821698,0.9356082982203,0.9872924768119
"14833","AC104938.1",3.4801418898708,0.0817711963270142,1.01498855150672,0.0805636637040162,0.935788964399555,0.9872924768119
"14834","CIAO3",64.1719528416206,0.0176492122325214,0.2174532667863,0.0811632425364572,0.935312131246258,0.9872924768119
"14835","AC007952.4",186.492443837934,-0.0278768590479274,0.350046543214054,-0.079637578454476,0.936525506833301,0.9872924768119

"14836","AFG3L2",185.68531642873,-0.0171446060711909,0.214350688810379,-0.0799839093885882,0.936250053413302,0.9872924768119
"14837","ZNF576",120.443027015409,-0.0184696041064209,0.232108065816265,-0.0795732972116589,0.936576633591779,0.9872924768119
"14838","AL121894.2",3.43118302340163,-0.0654880911072207,0.822421633628859,-0.0796283663140775,0.93653283378941,0.9872924768119
"14839","TTLL1",43.001488026408,0.0231897961884496,0.292452970953321,0.0792941036394905,0.936798696150019,0.9872924768119
"14840","TIMM8A",35.9850198122619,-0.0235673155069642,0.29420295703054,-0.0801056377707235,0.936153238867399,0.9872924768119
"14841","NKRF",44.5426871015994,-0.020030811434973,0.249480305225441,-0.0802901512280592,0.936006491107315,0.9872924768119
"14842","UTP4",61.6490168396723,-0.0183083410267738,0.231596118958737,-0.0790528835676896,0.936990559634674,0.987369111486747
"14843","RANBP9",147.873855636714,-0.0149659695009033,0.189534384021128,-0.0789617650549096,0.937063035134101,0.987378957724001
"14844","SLC16A1",235.13723310646,-0.0229866225550064,0.292111918806969,-0.0786911490941122,0.93727828564916,0.987473412678876
"14845","RFPL4B",14.4565981436697,-0.0662988987615278,0.843374474288697,-0.0786114600129964,0.937341671978323,0.987473412678876
"14846","PYROXD1",141.66658373083,0.0174178013335015,0.221569701416332,0.0786109347178894,0.93734208981014,0.987473412678876
"14847","TEPSIN",41.708975941624,0.0212822495748465,0.272123053499522,0.0782081830302696,0.937662452840764,0.987744376805385
"14848","AL358115.1",3.30114105798321,-0.073524512999558,0.947560357668117,-0.0775934877441443,0.938151422791395,0.987913929421272
"14849","SH3PXD2B",540.469225869894,-0.0191448680572824,0.246899946677303,-0.0775409971323514,0.938193178435876,0.987913929421272
"14850","GFOD1",7.57786594245146,0.0436774859656706,0.560798448995769,0.0778844628473645,0.93791995871654,0.987913929421272
"14851","KIN",224.926252696634,0.0129798412246922,0.166930656314858,0.0777558868528629,0.938022237349359,0.987913929421272
"14852","PIGB",34.8517488889448,-0.0227556709050111,0.293510214840206,-0.077529400185952,0.938202403688884,0.987913929421272
"14853","SRRM2-AS1",3.81038989227621,-0.062883188820871,0.810800142622995,-0.0775569533293858,0.938180485456849,0.987913929421272
"14854","PVT1",14.9366790602304,0.0522581425610493,0.676759475515555,0.0772181911767678,0.93844997038697,0.988108087845174
"14855","AL604028.1",11.0390512212501,-0.0364435810670539,0.480298913373911,-0.0758768759459646,0.939517054135011,0.988420375830879
"14856","SEC16B",20.4519838619222,-0.0334876039500734,0.442186558223462,-0.075731845139332,0.939632439978753,0.988420375830879
"14857","ARID4B",670.46180134422,0.0179507630396351,0.236666071098602,0.0758484854052286,0.939539641418969,0.988420375830879
"14858","ACOXL-AS1",4.01590504586576,0.0612187505445089,0.807692497039934,0.0757946257627327,0.939582491847374,0.988420375830879
"14859","EPC2",190.25952732393,-0.0155314441907783,0.206309091516753,-0.0752824031000938,0.939990022039635,0.988420375830879

"14860","SP140",2.60275184190187,-0.0730453590784457,0.96061777277754,
-0.0760399829655885,0.939387288456348,0.988420375830879
"14861","IL17RC",127.46360473428,0.0185359049136167,0.243325729909913,
0.0761773320087412,0.939278016714008,0.988420375830879
"14862","LNP1",15.2239022655675,0.0359398632875613,0.471063880483083,0
.0762950945224339,0.939184328474674,0.988420375830879
"14863","IQCG",40.820726988976,-0.0203829436337158,0.269020564444226,-
0.0757672324263586,0.93960428589872,0.988420375830879
"14864","AIMP1",372.221887899882,-0.0102300463953349,0.135242326258585
,-0.0756423427365109,0.939703648311961,0.988420375830879
"14865","WWC2-
AS2",14.6272131667143,-0.0303678326159262,0.40281999043224,-0.07538809
72573889,0.939905929272345,0.988420375830879
"14866","ZNF92",82.8730635445962,0.0179764266854986,0.235163338408418,
0.0764423009435175,0.939067216750774,0.988420375830879
"14867","TACC2",209.735751321004,0.0192383415139343,0.253137140125673,
0.075999679479602,0.93941935320026,0.988420375830879
"14868","ZNF195",81.0010785211795,0.0164645740989011,0.216386198861161
,0.0760888364671778,0.939348421602299,0.988420375830879
"14869","ARCN1",382.002026198965,-0.0134910351108075,0.178554488738721
,-0.0755569641855876,0.939771576147688,0.988420375830879
"14870","SLC2A3",1261.67441665187,-0.0188329589667508,0.24976366423054
1,-0.0754031176823514,0.939893978721924,0.988420375830879
"14871","TM7SF3",296.71172333515,-0.0136010167294971,0.17850144210366,
-0.0761955565692216,0.939263517758376,0.988420375830879
"14872","PIGBOS1",228.346531816942,-0.0148579597447076,0.1956612889342
47,-0.0759371453885325,0.939469104416607,0.988420375830879
"14873","AC068870.2",8.43729716742088,0.0419198691634414,0.55702560464
5783,0.0752566288045207,0.940010528779315,0.988420375830879
"14874","ZNF699",40.5866239953672,-0.0237068416875583,0.31320365317513
8,-0.0756914596851839,0.939664570686305,0.988420375830879
"14875","LINC00115",4.47569631850723,-0.0532109748202155,0.71036898808
0394,-0.0749061061407054,0.940289418211305,0.988560319708082
"14876","SDC1",4.66117924030894,0.0753396508412911,1.01017742591831,0.
0745806121858273,0.940548400389111,0.988560319708082
"14877","AC093827.4",44.2329661110661,-0.0259859202972216,0.3474075369
56579,-0.0747995294657911,0.940374216202441,0.988560319708082
"14878","AC124852.1",59.9323831549054,0.0206613330394485,0.27663291892
6483,0.0746886275126907,0.940462456329403,0.988560319708082
"14879","E2F3",73.6602488376163,0.0246087212314936,0.330170957591971,0
.0745332703123615,0.940586068898719,0.988560319708082
"14880","GABARAPL1",810.813819299888,0.0184075445525571,0.246934992309
6,0.0745440910597162,0.940577459142688,0.988560319708082
"14881","CCNF",9.64389702158517,-0.0399581003975081,0.533912447335623,
-0.0748401738841462,0.940341877292126,0.988560319708082
"14882","AL807776.1",3.389811277043,-0.0650704608826588,0.874394614269
377,-0.0744177283582999,0.940678002725988,0.988590509517166
"14883","PIGU",76.4150416694782,0.0181952923284641,0.244905172356152,0
.0742952553978878,0.940775452243722,0.988626491506538
"14884","SPRR2F",248.676956712574,0.0812970015257744,1.09697031258131,

0.0741104846624995,0.940922472650882,0.98862798505046
"14885","PRICKLE2",80.0165800187611,0.0199602721821508,0.2691460469775
11,0.0741614911543494,0.940881887044112,0.98862798505046
"14886","MTPN",966.666313883962,-0.00867355640639129,0.117122944771891
,-0.0740551428525295,0.940966508021812,0.98862798505046
"14887","AC008033.3",4.65741793359921,0.0546071712645648,0.73890032430
1942,0.0739032985486284,0.941087331166626,0.988688510744007
"14888","DNAJB4",221.227299235646,-0.019234144001685,0.274074250225069
,-0.0701785884149642,0.94405151626955,0.988849568307481
"14889","HCN3",15.0231569721237,0.029838294930177,0.425791448410208,0.
0700772527057207,0.944132171894171,0.988849568307481
"14890","IFI16",1332.35664522692,0.0152790430722234,0.212949065346494,
0.0717497540896107,0.942801060125473,0.988849568307481
"14891","LINC01142",2.90255383101246,-0.0724226239580868,1.01982399385
858,-0.0710148264741941,0.943385955463119,0.988849568307481
"14892","RASAL2",87.9099625021148,0.0201709131146686,0.282497291931207
,0.0714021468198024,0.943077701057623,0.988849568307481
"14893","AL160408.2",3.26216169878807,0.0726506210294292,0.99567954620
1792,0.0729658666852892,0.941833279521245,0.988849568307481
"14894","AC010896.1",7.4527642776351,-0.0417726931558351,0.58775550939
5553,-0.0710715467368295,0.943340813304416,0.988849568307481
"14895","AC009506.1",15.8248944293841,0.0292637256062008,0.42073775652
7321,0.0695533622837591,0.944549158496033,0.988849568307481
"14896","BTD",192.101496490693,0.0182213063546808,0.254999612202889,0.
0714562120203665,0.943034673175825,0.988849568307481
"14897","ZNF619",22.9453621734238,0.0287491382590483,0.394618746133394
,0.0728529461429341,0.941923137927034,0.988849568307481
"14898","CACNA2D3",16.926166464996,-0.0299875740905918,0.4116053766374
59,-0.0728551564014306,0.941921379069857,0.988849568307481
"14899","GPR27",2.4719606248322,-0.0929298903640579,1.30963179142223,-
0.07095879236647,0.9434305517078,0.988849568307481
"14900","MBNL1",979.547596335253,-0.0142996813319264,0.201927565346787
,-0.0708158953304291,0.943544280941693,0.988849568307481
"14901","SSR3",1974.53905856241,0.0131020375919835,0.185447219761721,0
.070651032724126,0.943675493615726,0.988849568307481
"14902","FBXO45",76.8967917055236,-0.0173831690367867,0.25010300109291
7,-0.0695040401787447,0.944588416835228,0.988849568307481
"14903","SNCAIP",65.8295613868708,0.0194935448824839,0.276205641997295
,0.0705762009114743,0.943735052090728,0.988849568307481
"14904","RNF14",117.432352727966,0.012900384050845,0.176396196767294,0
.0731330056274599,0.941700277278385,0.988849568307481
"14905","CDKN1A",1588.48769337496,0.0309998939714797,0.445315809773846
,0.0696132795896535,0.944501466999873,0.988849568307481
"14906","EPM2A",18.0123462561792,0.0274359128165152,0.378483401782907,
0.0724890779550014,0.942212697132319,0.988849568307481
"14907","AFDN-
DT",8.887070727157,-0.043168534731418,0.622552629048042,-0.06934118131
89639,0.944718046652838,0.988849568307481
"14908","MRM2",112.738841293416,-0.0140988635200139,0.203062367801476,
-0.069431198270069,0.944646396203642,0.988849568307481

"14909","COA1",226.360362033402,-0.0149797353488889,0.208486443510498,
-0.0718499250918184,0.942721340979209,0.988849568307481
"14910","VPS37D",22.8087577728038,0.0318880690703147,0.444219283372346
,0.07178452233823,0.942773390425228,0.988849568307481
"14911","MAN1B1",302.789155734387,-0.0162234247397619,0.22671011058470
1,-0.0715602171333274,0.942951900997401,0.988849568307481
"14912","DNA2",6.36137238370658,0.0434842271842632,0.605985679038729,0
.0717578462468651,0.94279462011803,0.988849568307481
"14913","GOT1",54.6179539858574,0.0221486526080291,0.310711634413202,0
.0712836281456218,0.943172024926985,0.988849568307481
"14914","MGMT",637.557528966387,-0.0233999016846458,0.327050624767095,
-0.0715482555684147,0.942961420544067,0.988849568307481
"14915","APIP",122.168352453276,0.0156229994898604,0.213639574424342,0
.0731278347279854,0.94170439204152,0.988849568307481
"14916","AP001107.9",7.12709402470952,-0.0460816511106282,0.6454151387
53101,-0.0713984664190786,0.943080630116837,0.988849568307481
"14917","MIR100HG",257.547011666056,-0.0135337705310841,0.187521574615
041,-0.0721718050782546,0.942465182932523,0.988849568307481
"14918","CCDC77",34.637844640286,0.0225837705585368,0.31142307266686,0
.0725179748730289,0.942189701249008,0.988849568307481
"14919","PCED1B-
AS1",18.9365486697867,-0.0352197542735197,0.505674194430116,-0.0696491
034374645,0.944472952913967,0.988849568307481
"14920","LTA4H",160.97283971625,0.0153846852544292,0.209907939891194,0
.0732925360632089,0.941573331085938,0.988849568307481
"14921","ALKBH2",115.55169641893,-0.0169709068654996,0.244556596781226
,-0.0693945985872603,0.944675528259565,0.988849568307481
"14922","FGF14-
AS2",64.6974725657111,-0.0173529687519762,0.242249440626544,-0.0716326
473534684,0.942894257973008,0.988849568307481
"14923","ZBTB25",89.1192615593936,-0.0178722941227564,0.24412681993850
3,-0.073209056371842,0.941639759785977,0.988849568307481
"14924","AC123768.3",6.96626643751624,0.0427162023836852,0.58844236430
9012,0.0725919902688267,0.942130800744433,0.988849568307481
"14925","LRRC57",78.6617969508756,-0.0169638383443976,0.23425595482442
6,-0.0724158254893113,0.942270990940228,0.988849568307481
"14926","GOLGA6L9",15.9045047606324,-0.0402199856271179,0.573374316368
447,-0.0701461235338501,0.944077355817723,0.988849568307481
"14927","UBE2I",1047.52459339151,-0.0127635574863512,0.182711627246033
,-0.0698562958402435,0.944308038972725,0.988849568307481
"14928","ZNF646",42.2393471750059,0.0209501868007924,0.295104620284115
,0.0709924052718063,0.943403799955971,0.988849568307481
"14929","ITFG1",359.261018931448,-0.0112238552217288,0.161094082057996
,-0.0696726725050521,0.944454193091418,0.988849568307481
"14930","CLUH",41.221030090756,0.0205091948314808,0.290321531097079,0.
0706430375796785,0.943681856918578,0.988849568307481
"14931","NCOR1",706.987807454315,0.0106202542140179,0.151606850854106,
0.0700512816814452,0.944152842973969,0.988849568307481
"14932","AC005726.1",9.37724092891193,0.0392539997629321,0.53997599696
8123,0.0726958234872233,0.942048172131761,0.988849568307481

"14933","CDK3",14.2045617665739,-0.0290582423974125,0.414408035028119,
-0.0701198817137818,0.944098242330988,0.988849568307481
"14934","AC145207.2",3.00045730456958,-0.0676634026140051,0.9495706167
83188,-0.0712568411638778,0.943193343633721,0.988849568307481
"14935","ROCK1",787.689425019337,-0.0153118961897313,0.211537823645674
,-0.0723837275331844,0.942296534370197,0.988849568307481
"14936","AC015961.2",3.42595771855608,-0.0648384615425856,0.9267433205
33484,-0.0699637754122264,0.944222491989041,0.988849568307481
"14937","RAB3A",12.3704790633694,0.0374497821002574,0.528897219352515,
0.0708072962571142,0.943551124829408,0.988849568307481
"14938","RTN2",184.085744636095,-0.0222935146822829,0.311641842671064,
-0.0715356913924219,0.94297141968427,0.988849568307481
"14939","RNF114",242.175349796612,-0.013488058656579,0.185278875131061
,-0.072798686018781,0.94196631658924,0.988849568307481
"14940","ADORA2A",4.80711792598807,-0.0547187723105292,0.7830971072149
8,-0.0698748237049834,0.944293291911113,0.988849568307481
"14941","MED14OS",33.668179827442,-0.0252323333127571,0.35279138926640
9,-0.0715219647657074,0.94298234396567,0.988849568307481
"14942","SLC6A8",42.9132198462538,0.0202987927443609,0.286059124369702
,0.0709601303195165,0.943429486859983,0.988849568307481
"14943","GPATCH11",155.495565139359,0.0144132449285558,0.2095231857381
44,0.0687906919598344,0.945156227277086,0.988853263102626
"14944","NPR3",10.6251827657218,-0.0506406048579893,0.732709398781885,
-0.069114173971534,0.944898738809984,0.988853263102626
"14945","AL031963.3",18.7932319291507,-0.0370841673644484,0.5391650830
95812,-0.0687807288103974,0.945164157936135,0.988853263102626
"14946","PDP2",12.1378331540089,0.0335460386817062,0.486601560223267,0
.0689394392124725,0.945037825228754,0.988853263102626
"14947","TEKT3",43.1382925037285,-0.030853587595954,0.448278978862281,
-0.0688267553260237,0.945127520911656,0.988853263102626
"14948","METTL4",69.8118737266059,0.0147658516542788,0.213696843497473
,0.0690971912013916,0.944912256783327,0.988853263102626
"14949","C19orf57",2.69092224758998,-0.073307098007379,1.063663761564,
-0.0689194279774929,0.945053753998286,0.988853263102626
"14950","RHBDD3",76.028743840463,-0.0180115319913372,0.262615554085359
,-0.0685851683616683,0.945319824997471,0.988949970766586
"14951","TMEM181",153.539573370025,0.0147665255917404,0.21587786167587
9,0.0684022227990705,0.945465452438884,0.988970015793482
"14952","LBHD1",2.79106013533564,0.0661755913157449,0.966842048065994,
0.0684450903310609,0.945431329069859,0.988970015793482
"14953","LINC02614",69.7352619429491,0.0239651755177968,0.350847018158
272,0.0683066244758159,0.945541550878791,0.988983471928328
"14954","SNAP47",98.0795143709636,-0.0189931088724806,0.27968401600103
1,-0.067909168153573,0.945857940472264,0.98904982208907
"14955","MTMR12",94.4225339163689,-0.0141833894034373,0.20831484690849
6,-0.0680863107643381,0.945716927495706,0.98904982208907
"14956","GATA3",4.0912972715933,0.0720543387381048,1.0606692124982,0.0
679329030097846,0.945839046428877,0.98904982208907
"14957","TCN2",108.991358505315,0.0257609147624167,0.378662627324238,0
.0680313104687734,0.945760709864855,0.98904982208907

"14958","CLCA4-
AS1",4.1767237859931,0.0594204586088674,0.878428677725817,0.067644033164653,0.946069002254851,0.989080593134127
"14959","BOLA1",151.958360022891,-0.0178094902176639,0.263835357600738,-0.067502287713139,0.94618184084982,0.989080593134127
"14960","NUP35",69.4089020609581,-0.016174446598382,0.240442102772193,-0.0672696104879206,0.946367069388281,0.989080593134127
"14961","BRPF1",46.4336259981459,-0.0182487798088556,0.271301626973842,-0.0672638052797784,0.946371690806873,0.989080593134127
"14962","AC100800.1",2.62132127268057,0.067252085400815,1.0014124008644,0.067157232467627,0.946456531767605,0.989080593134127
"14963","TGDS",71.4280708413361,0.014741087070416,0.21944614053955,0.0671740548007465,0.946443139729066,0.989080593134127
"14964","CDC16",208.480257559457,0.0105674338084371,0.156422582983183,0.0675569576138069,0.946138319926504,0.989080593134127
"14965","GNPTG",399.54513156602,-0.0139435843599487,0.206871715321937,-0.0674020821949946,0.946261611608661,0.989080593134127
"14966","KATNAL2",18.052682135085,-0.0263995214304291,0.391109638781084,-0.0674990304833824,0.946184433829173,0.989080593134127
"14967","AC016831.7",17.4245565511601,-0.0516342433135422,0.770829025258372,-0.0669853386699275,0.946593375029666,0.989091420728486
"14968","INO80E",153.644442090024,-0.0130072602356061,0.193973260887551,-0.067056975668139,0.946536345203064,0.989091420728486
"14969","ATAD3B",28.7307561312514,0.0225392053991008,0.338206857131362,0.0666432537479462,0.946865710753373,0.989241841352508
"14970","AC008443.1",43.3783086310454,0.0180003006838979,0.270412175822769,0.0665661619308722,0.946927084839411,0.989241841352508
"14971","RNF31",64.716194124079,0.0167593584919064,0.251296491067464,0.0666915738485463,0.946827242473173,0.989241841352508
"14972","PFN2",170.957635204011,0.0125967348483578,0.190718902559632,0.0660486961664388,0.947339056130847,0.989415863262357
"14973","KIT",6.34185117588996,-0.0467977203608755,0.706116382743464,-0.0662747976177142,0.947159047687935,0.989415863262357
"14974","RET",2.76563367295747,0.0734721293747535,1.11269419808307,0.066030837135064,0.947353274537404,0.989415863262357
"14975","NR2E3",2.49821863797168,0.0700677734153761,1.05867366673174,0.0661844868888485,0.947230947361976,0.989415863262357
"14976","DNAAF1",4.25043266335957,-0.0479195004857421,0.727212763012508,-0.0658947462462479,0.947461623381998,0.989415863262357
"14977","PRDX2",1198.18854960285,0.0177332186823201,0.269173875745923,0.0658801625275802,0.947473234276235,0.989415863262357
"14978","WDR77",54.5039696297172,-0.016111585063009,0.246292981192189,-0.0654163386427837,0.947842515587069,0.989669333318763
"14979","CCDC88B",5.43180388648647,0.0492757402195442,0.752948032653236,0.0654437465569921,0.947820693995611,0.989669333318763
"14980","NPHP4",40.2161210286167,0.0183001761368542,0.282735746417098,0.0647253711946891,0.948392661882045,0.98972345040359
"14981","MEAF6",560.165044699468,-0.0127588362918213,0.197638446511097,-0.0645564489958937,0.948527161003799,0.98972345040359
"14982","WDR92",92.8244542566391,-0.0133556531660331,0.20589438212858,

-0.0648665253901515,0.948280273309251,0.98972345040359
"14983","MITD1",239.645957427631,-0.0116052330629193,0.179217798463258
,-0.0647549136437952,0.948369139763666,0.98972345040359
"14984","CTLA4",4.08064878834984,-0.058000923775629,0.890696594058454,
-0.0651186095944839,0.948079563565456,0.98972345040359
"14985","MSR1",41.2636694136202,0.0227953802071969,0.351896593007396,0
.0647786328716111,0.948350254209512,0.98972345040359
"14986","IFFO1",203.533563054526,-0.0107163176108174,0.164375630233679
,-0.0651940777083743,0.948019476403518,0.98972345040359
"14987","MAPK6",248.722237530635,-0.0166017638274133,0.256226406070566
,-0.0647933368071406,0.948338546761559,0.98972345040359
"14988","SOX8",8.46904533701377,-0.0487724209274161,0.755459182836337,
-0.0645599683417737,0.948524358817569,0.98972345040359
"14989","NAGPA",61.912880420446,0.0197838092737352,0.306062557383224,0
.0646397567963979,0.948460829538263,0.98972345040359
"14990","C10orf55",7.65323936997101,0.0383425494962709,0.5954427592501
19,0.0643933424340541,0.94865703099482,0.989792926268111
"14991","AL049597.2",66.7551478395381,-0.0176402084181779,0.2747964025
12598,-0.0641937385529244,0.948815963024455,0.989892713074677
"14992","ADAMTS4",66.7051815960968,-0.0647865625775249,1.0178739968837
1,-0.0636489022962307,0.949249792251469,0.990015120753066
"14993","CCDC25",476.256067287928,-0.012152558531886,0.190448516295073
,-0.0638102032417905,0.949121353806054,0.990015120753066
"14994","CYHR1",219.002423826549,-0.0135167997719549,0.211933939495646
,-0.0637783632207367,0.949146706825018,0.990015120753066
"14995","RNF40",137.721304012011,0.0114605730958371,0.179344191405628,
0.0639026723197095,0.949047724426814,0.990015120753066
"14996","TCEAL9",1056.90870067123,0.0140386532281526,0.220495515979796
,0.0636686563251426,0.94923406272059,0.990015120753066
"14997","RABGGTA",50.465087539404,0.0181078577121465,0.286145295866925
,0.0632820387883215,0.949541918064092,0.990046562483257
"14998","EARS2",26.4687504105175,-0.0206842347740627,0.326278782955349
,-0.0633943604506253,0.949452477928721,0.990046562483257
"14999","CDC42EP4",395.924281619311,0.0205343870966535,0.3252505408101
71,0.0631340598097214,0.949659752581446,0.990046562483257
"15000","FECH",107.675229517697,0.0130116266710685,0.205999535054497,0
.0631633788281431,0.949636405986068,0.990046562483257
"15001","SMOX",19.0696817882158,0.0249041984396646,0.393150808193327,0
.0633451538713824,0.94949166034662,0.990046562483257
"15002","TMEM184B",143.685106771752,-0.0117683607752491,0.186226435391
866,-0.0631938250360942,0.949612161860771,0.990046562483257
"15003","C15orf39",69.2670654357646,-0.0216487862745595,0.343342074869
25,-0.063053111922865,0.949724211224312,0.99004776801628
"15004","DIS3L2",77.9135327618143,0.0141358196196958,0.224892538241617
,0.0628558854385322,0.949881263661801,0.990079504409902
"15005","AL132639.2",5.57625222494187,0.04045455597335,0.6434460810864
27,0.0628717108744287,0.949868661715993,0.990079504409902
"15006","NFYC-
AS1",5.68608004047763,-0.039386414200174,0.650157703076859,-0.06057978
55101593,0.951693872776723,0.990160002825552

"15007","AKNAD1",22.8979250891842,0.0214552634137586,0.354591779718073
,0.0605069396442782,0.951751888941828,0.990160002825552
"15008","CSDE1",1425.74771517035,0.00751356138231919,0.122262715633816
,0.0614542327427336,0.950997462454225,0.990160002825552
"15009","PRCC",152.00261056025,0.0126238914965039,0.208021044253473,0.
060685646213379,0.951609563273209,0.990160002825552
"15010","LIN9",19.8218855558198,0.0216718487377627,0.348067805774254,0
.0622632957666254,0.950353157474954,0.990160002825552
"15011","WDR43",198.086719620549,-0.0134595442504128,0.218428634581036
,-0.0616198708389554,0.950865552370446,0.990160002825552
"15012","INO80B",68.1671998414754,-0.0124606316776587,0.20227385180974
1,-0.0616027804195829,0.950879162695577,0.990160002825552
"15013","CYP20A1",242.966824130524,-0.00948734168037926,0.154849997619
381,-0.0612679485065219,0.95114581621738,0.990160002825552
"15014","RPN1",504.814099582877,0.00845202796966285,0.13649433954234,0
.0619221866489276,0.95062479900901,0.990160002825552
"15015","TBC1D14",89.5300984371532,0.0118232587018008,0.19284149012864
8,0.0613107619833954,0.951111720104432,0.990160002825552
"15016","CCDC112",95.0700006652596,-0.0118702551436215,0.1895113723179
75,-0.0626361098990135,0.950056274318211,0.990160002825552
"15017","AL513548.4",2.54605930916634,-0.0701049100324432,1.1337291680
2876,-0.0618356764643679,0.950693692127313,0.990160002825552
"15018","EHMT2",181.617766473413,-0.0106615856902846,0.173930053779506
,-0.0612981221968714,0.951121786261678,0.990160002825552
"15019","GTPBP2",81.1933783005796,-0.0133438400284132,0.21975980313357
1,-0.0607201127692252,0.951582113560948,0.990160002825552
"15020","PRIM2",75.4924776410605,0.0136151817129618,0.222908427802788,
0.0610797081436844,0.951295729528204,0.990160002825552
"15021","AC002456.1",8.20413417605116,0.039274581267697,0.648909317264
595,0.0605239903677985,0.951738309320671,0.990160002825552
"15022","ING3",175.277866526465,-0.0112460296008598,0.180685571311101,
-0.0622408835373834,0.950371005230207,0.990160002825552
"15023","SND1-
IT1",24.5971735638101,-0.0296316822361953,0.477487685586089,-0.0620574
794506461,0.950517058180375,0.990160002825552
"15024","NUDT18",69.9317618286591,0.0196432875463382,0.317030520498309
,0.0619602412899013,0.950594493990155,0.990160002825552
"15025","TBC1D31",14.95204269463,0.027461680084761,0.44164191308499,0.
0621808738507938,0.950418793468234,0.990160002825552
"15026","DUSP8",60.719713647267,0.0227728944699858,0.373686664869153,0
.0609411483226457,0.951406078703993,0.990160002825552
"15027","RTN4RL2",13.6694812229897,-0.0326168788038038,0.5395368561158
18,-0.0604534767812085,0.951794468189217,0.990160002825552
"15028","MS4A4E",3.28127379610602,-0.0580277810236329,0.93561562549956
8,-0.0620209618588288,0.950546138985154,0.990160002825552
"15029","CDKN3",7.34230318720899,-0.0429786281390641,0.693899739998092
,-0.0619378069505866,0.950612359688783,0.990160002825552
"15030","KDM8",8.71137477547094,0.0306159803417607,0.501581773340803,0
.0610388613961027,0.951328259820593,0.990160002825552
"15031","AC026471.1",14.1970665121393,0.0295625427091557,0.48811452926

2087,0.0605647669489519,0.951705833892027,0.990160002825552
"15032","LENG9",69.1933140250771,0.0162869055878537,0.263902834416998,
0.0617155386899652,0.950789365472802,0.990160002825552
"15033","OSER1-
DT",98.0319954685905,0.0179481501019064,0.295541992296524,0.0607296105
789889,0.951574549364561,0.990160002825552
"15034","RRP1",91.7023272554419,-0.0150855666426067,0.243967277434248,
-0.0618343853374862,0.950694720329935,0.990160002825552
"15035","ADGRL1",30.1559108568585,0.0193830220364977,0.32182421589736,
0.0602286002078835,0.951973567384059,0.990280451871413
"15036","LINC00957",5.60179067472303,0.0394368108173315,0.656813760690
27,0.0600426074750412,0.952121700026146,0.990368674408681
"15037","DENND4B",60.1342421474559,-0.0167379672601307,0.2792079226527
6,-0.0599480383690509,0.952197019579603,0.990370246824586
"15038","NARF",190.397942365607,-0.0124110791147114,0.207259977043576,
-0.0598816968512061,0.952249857528652,0.990370246824586
"15039","CD48",28.4888003139144,0.0245617990599932,0.413762235937808,0
.0593621092662624,0.95266369223807 7,0.990603028163256
"15040","MEF2C",46.5413540010028,-0.0194539530640931,0.327654074232452
,-0.0593734508251272,0.95265465891652,0.990603028163256
"15041","CTU1",86.8290204782082,0.0170031295637383,0.28599549775638,0.
0594524378779632,0.952591747500059,0.990603028163256
"15042","TOX",2.98939858851481,0.0579496267293849,0.982370426212657,0.
0589895880241415,0.95296040122400 7,0.990845677113646
"15043","RAD18",50.4290309563789,0.0151917161139978,0.259289157745508,
0.0585898625537921,0.953278785322936,0.99104494831499
"15044","LRCH1",64.1840471041591,-0.0167991213122907,0.286382164224862
,-0.0586598029167081,0.953223076801003,0.99104494831499
"15045","G0S2",346.649183262184,-0.0224199871265956,0.384962383085464,
-0.0582394231532441,0.953557918862637,0.991159822757447
"15046","ANKRD53",6.17542286502031,-0.0366371684859434,0.6277006712758
85,-0.0583672603240546,0.953456092772658,0.991159822757447
"15047","DYRK1B",56.9727537639436,0.0178429965718688,0.307354076639889
,0.0580535542815478,0.953705970271859,0.991159822757447
"15048","ZNF813",34.2249629682475,0.0181862460415099,0.313243136664808
,0.0580579234237796,0.953702490070628,0.991159822757447
"15049","IL17RA",154.534977788633,0.0174345685919965,0.300319872736023
,0.0580533297152843,0.953706149148135,0.991159822757447
"15050","FAM110A",7.4420149655957,0.0304290120013761,0.525246100435461
,0.0579328660910543,0.953802103721062,0.99119368120913
"15051","AC068338.3",2.7513666316842,-0.0538484006541231,0.93118981604
2518,-0.0578275231606102,0.953886014545492,0.991215020097767
"15052","AL078581.2",2.87877402457968,0.0549345879754481,0.95287746557
0708,0.0576512615318761,0.954026416743318,0.991295054336002
"15053","FBXL22",4.42001514885027,-0.0415594524513994,0.72492944525703
4,-0.057328961767671,0.954283150130843,0.991480190720098
"15054","THSD4",20.6766333576352,-0.0274910347910401,0.480038341562847
,-0.0572684146469183,0.954331380505137,0.991480190720098
"15055","TBK1",170.62420446981,-0.00950670821290615,0.166822274688216,
-0.0569870434309433,0.954555516212793,0.991521478956801

"15056","RAB35",151.008518907459,-0.0105239249518414,0.184695817757153
,-0.0569797685710389,0.95456131129492,0.991521478956801
"15057","MADCAM1",3.92210109360394,0.0447247924537073,0.78394683921255
3,0.0570507976008065,0.954504730368502,0.991521478956801
"15058","AL021408.1",3.09594453501829,-0.0462411470574363,0.8190594478
41824,-0.0564563990797971,0.954978228588044,0.991527129219137
"15059","TYW1",71.4036618556906,-0.0144719064383547,0.255194356528708,
-0.0567093513947934,0.954776724669816,0.991527129219137
"15060","ARHGAP1",455.114172788417,-0.0102830234349095,0.1825293643621
19,-0.0563362693495665,0.955073925939021,0.991527129219137
"15061","AP001267.1",5.26455205137501,0.0498317828301342,0.87855994088
3746,0.0567198440438888,0.954768366200615,0.991527129219137
"15062","IFI27L1",250.46625838823,0.0164755257871277,0.291438678875593
,0.0565317062604467,0.954918237960109,0.991527129219137
"15063","AC139530.1",39.0967871053876,0.017331760776339,0.307392441073
699,0.0563831716739697,0.955036562686321,0.991527129219137
"15064","GCDH",81.6811033519949,0.0123741875090474,0.218262284559097,0
.0566941170529942,0.954788860391544,0.991527129219137
"15065","EGLN2",274.294299204508,0.0127950723445331,0.227072703979748,
0.0563479102520146,0.955064652573202,0.991527129219137
"15066","SLC33A1",148.150690334161,-0.0105558927857407,0.1888261307495
77,-0.0559027118960565,0.955419310429773,0.991637058186198
"15067","RPL7L1",657.428646953275,-0.00832047639346133,0.1489780820501
19,-0.0558503390496204,0.955461032732083,0.991637058186198
"15068","BAG3",326.736029006262,-0.0161415131696538,0.289246436982273,
-0.0558054001911287,0.955496832919821,0.991637058186198
"15069","AL356215.1",3.82266258438739,-0.0448927869442349,0.8012320878
64875,-0.0560296918011176,0.955318153670575,0.991637058186198
"15070","Z82215.1",5.07564261107176,-0.0426043983399493,0.759866242631
831,-0.0560682866926514,0.955287407734458,0.991637058186198
"15071","PEX3",108.67494231992,0.0111657305677111,0.200374677534974,0.
0557242596972476,0.955561473082667,0.991638341119562
"15072","ZNF710-
AS1",5.07881481244646,-0.0388726662597019,0.700991108293075,-0.0554538
649632197,0.955776883783338,0.991796076325066
"15073","STX10",360.532518025834,-0.014643219157845,0.265036507421051,
-0.0552498193563274,0.955939439410545,0.991898947281956
"15074","HOXD4",9.24600779879247,0.0332032258039685,0.602325142894372,
0.0551250868333604,0.956038810125051,0.991936247204179
"15075","FBLIM1",11.2135100806183,0.0326036170063072,0.599616236038923
,0.0543741397359206,0.956637081867959,0.991959175838745
"15076","DNAJC8",1048.06386394907,0.00887181970476059,0.1642618692017,
0.0540102200704092,0.956927021689583,0.991959175838745
"15077","CTSK",2537.56514053695,0.0155163153509588,0.283349530902979,0
.0547603354115721,0.95632940071712,0.991959175838745
"15078","COP1",127.682285945904,0.00958933994847736,0.177636884470554,
0.0539828199366272,0.956948851985613,0.991959175838745
"15079","CASP8",68.8680608066561,0.014357268203517,0.265591819769259,0
.0540576446066386,0.956889237583089,0.991959175838745
"15080","MCM2",14.3427983868047,-0.0217216176421147,0.401893446588687,

-0.0540482006524118,0.956896761768551,0.991959175838745
"15081","AC025449.2",12.8340957790421,0.0249730442838259,0.45840478916
1854,0.0544781487328841,0.956554217516673,0.991959175838745
"15082","BOD1",421.875229513315,0.0133909636570344,0.246042182071599,0
.054425479177134,0.956596179487268,0.991959175838745
"15083","ZNF322",261.700651727282,-0.00859181085110626,0.1573502561144
04,-0.0546030941624871,0.956454673653213,0.991959175838745
"15084","CPSF4",85.3323377665059,0.0119610839370504,0.218464408315529,
0.0547507213155514,0.956337060165135,0.991959175838745
"15085","AC016831.5",88.288667709095,0.0252510945606492,0.463802773804
972,0.0544436040205039,0.956581739364336,0.991959175838745
"15086","CSKMT",164.380061269765,-0.01357328599559,0.250520741368577,-
0.0541802883124173,0.956791525261634,0.991959175838745
"15087","CBL",62.914144184086,0.0184159779159612,0.340407201178364,0.0
540998482176988,0.956855613176852,0.991959175838745
"15088","NAP1L1",1719.33118913424,0.00934189285921494,0.17031099366649
6,0.0548519661479299,0.956256399686796,0.991959175838745
"15089","PIGG",90.8941227537563,-0.0120036279805504,0.222736314824111,
-0.0538916520641428,0.957021487689784,0.991968723405674
"15090","EXO5",27.2237282950522,-0.0183482689008679,0.341157712257427,
-0.0537823658725406,0.957108559178224,0.991993231646616
"15091","RUNDC1",66.399070178311,0.0130882037829942,0.245353865730937,
0.0533441922506621,0.957457669980122,0.992157818756318
"15092","SHFL",202.970151105246,-0.0105369178713935,0.197481752053066,
-0.0533564127411736,0.95744793330581,0.992157818756318
"15093","SRXN1",37.4400261841614,-0.0152344359754063,0.285250908024452
,-0.0534071427884829,0.957407514214751,0.992157818756318
"15094","RNF20",158.139370587073,0.0109345295121551,0.205662489164373,
0.0531673498486923,0.957598569851927,0.992237982003351
"15095","SMIM11A",9.21265422205395,0.0301404442081391,0.56774658830144
,0.0530878473410329,0.95766191421615,0.992237982003351
"15096","AL359715.3",2.99169431431425,-0.0464115448226445,0.8789391069
85544,-0.0528040503076715,0.957888034326237,0.992355626975591
"15097","AC090510.1",6.14498224347983,0.0456146122427753,0.86414106883
6305,0.0527860714966391,0.957902359363843,0.992355626975591
"15098","ZNF692",31.3569343680973,0.0152027098854264,0.289177520571459
,0.0525722395550787,0.958072735998668,0.99240066170072
"15099","AC103769.1",2.940955819234,0.0514605077519299,0.9777160718040
87,0.0526333863541537,0.958024015364762,0.99240066170072
"15100","DEGS2",4.95786432627332,-0.0355265854750993,0.676938831694098
,-0.0524812343623292,0.958145247536089,0.992410044467843
"15101","RAB1A",852.888707545238,0.00802633427100059,0.154106798556939
,0.0520829343426729,0.958462610930565,0.992499532803672
"15102","B3GAT3",221.823077661427,0.0131283815278711,0.252205851873617
,0.0520542304246369,0.95848548231884,0.992499532803672
"15103","SBNO1-
AS1",11.5823093780383,-0.0291825711336271,0.560282805520865,-0.0520854
305112891,0.958460621975776,0.992499532803672
"15104","ZNF665",39.2686303780989,0.0152845826716783,0.292581035139962
,0.0522405106139795,0.958337054183153,0.992499532803672

"15105","CCDC28A",130.782057366351,-0.0126454154994255,0.244388839867848,-0.0517430153777214,0.958733461811586,0.99262487374111
"15106","CINP",234.734109854279,-0.0122600449676518,0.236896240479172,-0.0517528051219948,0.958725661177221,0.99262487374111
"15107","NOL10",107.257661693789,-0.01153439877403,0.2247159176302,-0.0513288016962439,0.959063517913166,0.992821367814647
"15108","PPFIA2",6.44779063089133,-0.104860355447581,2.04942493023339,-0.0511657460103406,0.959193446800267,0.992821367814647
"15109","ANKRD13A",142.721345302013,-0.00934308208668028,0.182502360213798,-0.0511943082584524,0.959170687251132,0.992821367814647
"15110","AC087482.1",3.70110364428319,-0.0492825534033227,0.964310597828267,-0.0511065143474648,0.95924064507974,0.992821367814647
"15111","HNRNPM",838.977150191019,0.00901029558866681,0.175923976865076,0.0512169844567418,0.959152617967151,0.992821367814647
"15112","KIAA1841",70.4525550372049,0.0157404330623711,0.310217040531072,0.0507400658436574,0.95953264982542,0.992919904712157
"15113","PACS2",209.013984595841,0.00737323466671991,0.144957916750877,0.0508646566671586,0.959433368929727,0.992919904712157
"15114","NTN1",86.5475693968134,0.0375198619075171,0.740498906365595,0.0506683555978042,0.959589792821244,0.992919904712157
"15115","ZNF540",11.9649627711872,-0.0242152227716232,0.477652271605634,-0.0506963416927201,0.959567491808906,0.992919904712157
"15116","TXNDC11",102.284575118089,-0.0125280081070047,0.247754416671979,-0.0505662352069851,0.959671168790468,0.992938414916838
"15117","PCAT6",14.1997417604592,-0.0221901124339342,0.439619841389604,-0.0504756845455223,0.959743325621041,0.992947384581139
"15118","CUL2",172.592740424056,-0.00801557204040533,0.159262414921381,-0.0503293388108057,0.959859944399148,0.992976357648771
"15119","AVP",5.30117122945615,-0.0505048699961809,1.00444847903443,-0.0502811951537133,0.959898308906123,0.992976357648771
"15120","SKIDA1",11.2186854886521,-0.021569755648793,0.431466609157382,-0.0499917147491829,0.960128990740152,0.993000771566288
"15121","TUBGCP2",260.223839209462,0.00780197448176242,0.156015612173937,0.0500076522666479,0.960116290326272,0.993000771566288
"15122","DPEP2",3.70461409456859,-0.0403784487282276,0.808654480159193,-0.0499328819896956,0.960175873938425,0.993000771566288
"15123","COMT",1687.15206974826,-0.0132393782899461,0.264126946616688,-0.0501250571345894,0.960022732126701,0.993000771566288
"15124","ATPAF1",150.503817214916,0.00988297102750132,0.199336634867537,0.0495793010355007,0.960457641723514,0.993009506519109
"15125","FAM189B",54.4647153408625,0.0123236718742254,0.252076963638962,0.0488885287109221,0.961008130694239,0.993009506519109
"15126","EFCAB2",81.9828147787646,0.0108817749981746,0.22179928065265,0.0490613628960143,0.960870394249188,0.993009506519109
"15127","AC007390.2",15.0181352397731,0.0233310552319166,0.469474348890683,0.0496961235199439,0.960364545627401,0.993009506519109
"15128","RPL37A",15356.6429411522,0.0110155997136424,0.224906853742952,0.0489784972326022,0.960936432092009,0.993009506519109
"15129","CXCL1",124.139067573415,0.0639917258274001,1.29873237197489,0.0492724499737329,0.960702174577961,0.993009506519109

"15130","HBS1L",178.236344913242,0.00736259728767146,0.149634042120596
,0.0492040259243794,0.960756702931057,0.993009506519109
"15131","FBH1",261.021053917145,-0.00825347111980965,0.168672077209124
,-0.0489320535821515,0.960973444384881,0.993009506519109
"15132","NTAN1",762.331663995443,-0.0139841541726336,0.282764236391222
,-0.0494551728008687,0.960556560378922,0.993009506519109
"15133","SCO1",152.517358348391,0.00936948128362325,0.189193555512937,
0.0495232581163821,0.960502302641116,0.993009506519109
"15134","PCSK4",2.5183349423502,0.0524133927477789,1.07214352920927,0.
048886544870009,0.961009711679874,0.993009506519109
"15135","MPND",62.5384719782922,0.0129225139730548,0.261855507064786,0
.049349788812567,0.960640542090452,0.993009506519109
"15136","TPD52L2",329.236892860041,0.0111880159169603,0.22677107996508
4,0.0493361671985815,0.960651397343095,0.993009506519109
"15137","LINC01719",11.1092770570009,0.0224338250270017,0.463471828140
67,0.0484038590155532,0.961394383862173,0.993239431221344
"15138","FCGR3A",13.2095458907726,0.0287313936925381,0.594993500041328
,0.0482885841451082,0.961486252473859,0.993239431221344
"15139","CTSF",924.747848976355,-0.0130703263951958,0.269437097114783,
-0.0485097506436827,0.961309993701853,0.993239431221344
"15140","C14orf28",79.1242875890238,0.0114831666325441,0.2375462569771
82,0.0483407601478104,0.961444670607629,0.993239431221344
"15141","HIF1A-
AS3",3.00500924836254,-0.048477062606454,1.01617622339901,-0.047705369
885848,0.961951054427513,0.993653952265128
"15142","ELF2",515.516059247261,-0.00854186247852061,0.17936524687481,
-0.0476227286352886,0.96201691774672,0.993656359368558
"15143","TANK",345.645373451683,0.00808058505257363,0.172700696500079,
0.0467895336633452,0.962680970825246,0.993744596638922
"15144","AC093788.1",3.83926700842016,-0.0460695763183519,0.9887816436
79614,-0.046592264948316,0.962838197013908,0.993744596638922
"15145","ATG12",526.262502087184,-0.0071968862086717,0.1522402301495,-
0.0472732220754288,0.962295469903094,0.993744596638922
"15146","UFL1",388.672111247946,-0.00632094192460571,0.135853499690543
,-0.0465276340985255,0.962889709106126,0.993744596638922
"15147","CCDC146",108.801694268442,-0.0137220525695416,0.2905859585778
05,-0.0472220083747355,0.962336286940045,0.993744596638922
"15148","LYN",36.2435833482184,0.0205435562204544,0.438081883851904,0.
0468943295253891,0.962597447507241,0.993744596638922
"15149","CD59",2943.18509661632,0.013459877770962,0.286756692832005,0.
0469383212577618,0.962562385792322,0.993744596638922
"15150","AP002381.2",3.4729408356286,0.0422268320109413,0.906136621644
811,0.0466009550902948,0.962831270807118,0.993744596638922
"15151","KLHL42",176.09738623331,-0.0115025587567067,0.245841159176562
,-0.0467885800540244,0.962681730863001,0.993744596638922
"15152","AC026356.2",24.6794415061393,-0.0204192969273502,0.4393216031
71786,-0.0464791550880454,0.962928347957984,0.993744596638922
"15153","IFI35",259.694025503814,-0.0111201876940069,0.234894947171652
,-0.0473411106875819,0.962241363206028,0.993744596638922
"15154","AC010487.2",6.20119288617486,0.0317758815052349,0.67527012071

1143,0.0470565489729812,0.962468157844637,0.993744596638922
"15155","SNTA1",80.2707657784744,-0.0161779577053994,0.345702192442604
,-0.0467973824264517,0.96267471527076,0.993744596638922
"15156","CPTP",59.8401608466312,-0.0133974959703012,0.298351845462166,
-0.044905021283001,0.964183014478794,0.994061064781013
"15157","GMEB1",94.6137508529744,0.0107457561098618,0.247693483838627,
0.04338328139816,0.965396004664885,0.994061064781013
"15158","HHLA3",58.917326081712,-0.0138033115302915,0.308922337752512,
-0.0446821412485548,0.96436066869891,0.994061064781013
"15159","NGF",15.8773420503048,-0.0283391032885644,0.61982503310917,-0
.0457211338277346,0.963532519063417,0.994061064781013
"15160","CCDC121",57.7083363168803,-0.0133496712167194,0.2915069512457
03,-0.0457953786682339,0.963473342236671,0.994061064781013
"15161","KLF7-
IT1",3.84908507321021,0.0389940998118327,0.876724379191392,0.044477033
7603674,0.964524158261371,0.994061064781013
"15162","UMPS",113.206029954862,0.00893018896957793,0.195502321652837,
0.0456781735075029,0.9635667606649,0.994061064781013
"15163","SNX4",214.956778725419,0.00670830316633547,0.154169662724453,
0.0435124722191628,0.965293022549964,0.994061064781013
"15164","AC069243.1",7.39047580503816,-0.024284747298737,0.54633362210
0777,-0.0444503986508401,0.964545389006835,0.994061064781013
"15165","LNX1",60.8552017096814,-0.0149787182682917,0.341702755208021,
-0.0438355209022911,0.965035512701031,0.994061064781013
"15166","PREP",76.3485430644455,0.00915682000718543,0.203247333114327,
0.0450525960999189,0.964065385857953,0.994061064781013
"15167","AFG1L",18.6376161050843,-0.0180516623617672,0.413636234382478
,-0.0436413951710898,0.96519025453911,0.994061064781013
"15168","STK17A",450.134735846793,-0.0117300906804714,0.26038352810574
,-0.0450492808274256,0.964068028379715,0.994061064781013
"15169","EPHA1-
AS1",9.90435650976144,-0.0264219979374312,0.595656803222513,-0.0443577
539859997,0.96461923591584,0.994061064781013
"15170","UBXN2B",50.84085064516,-0.012400298622041 7,0.280283017038748,
-0.0442420620166486,0.964711454219159,0.994061064781013
"15171","PLAAT5",20.6845895613586,0.0190140095346021,0.41665944423573,
0.0456344139024117,0.963601639406916,0.994061064781013
"15172","PTPN11",243.851333436804,0.00787158945082366,0.17320090390412
6,0.0454477388592665,0.963750430166237,0.994061064781013
"15173","PAN3",123.742241631475,-0.00992083496522327,0.217713548719821
,-0.0455682938593342,0.963654340744161,0.994061064781013
"15174","AL132800.1",5.91711679216596,0.0269163429563959,0.61609562848
7429,0.0436885796811739,0.965152642620132,0.994061064781013
"15175","NEMF",400.204171523858,0.0072989256153931,0.166959457688381,0
.0437167544531444,0.965130183862068,0.994061064781013
"15176","POLR2M",185.333187704491,-0.0080956345412543,0.18412648117638
8,-0.0439677904532314,0.964930078522087,0.994061064781013
"15177","MTHFS",127.906712091494,-0.0116781280389197,0.262642596040545
,-0.0444639529724907,0.964534584904967,0.994061064781013
"15178","SULT1A3",9.04782689748873,0.0255741541238373,0.58272934838360

3,0.0438868476330838,0.964994599268616,0.994061064781013
"15179","FOXF1",9.07369407473613,0.033495299246652,0.762931585835953,0
.0439034113523441,0.964981396058734,0.994061064781013
"15180","SLC7A5",8.22839983915265,0.0309046741850938,0.696414648922398
,0.0443768295697317,0.964604030774789,0.994061064781013
"15181","AC134312.5",4.10812431152836,-0.0368606841082277,0.8138014802
09618,-0.0452944422007356,0.963872617375063,0.994061064781013
"15182","METTL16",113.197936321701,-0.00974909106515192,0.220866969398
507,-0.0441401042976316,0.964792725315023,0.994061064781013
"15183","SKA2",185.425111714594,0.0099042063945819,0.224790110924975,0
.0440597958416751,0.964856739913866,0.994061064781013
"15184","ILF3",402.253243185229,0.00815360381604545,0.187799486059755,
0.0434165395609821,0.965369493469621,0.994061064781013
"15185","ZNF627",18.7287983673805,-0.0171099392455776,0.38417618095345
7,-0.0445366998107841,0.964476598768658,0.994061064781013
"15186","ZNF222",21.7350137227369,0.0172392623045157,0.391302330384154
,0.0440561196954574,0.964859670208793,0.994061064781013
"15187","MED25",109.92197946392,0.0104802637662106,0.241545092071772,0
.0433884359906454,0.965391895764104,0.994061064781013
"15188","ZNF334",4.89436695883406,0.034085022058394,0.770735245646283,
0.0442240344540268,0.964725824068411,0.994061064781013
"15189","URB1-
AS1",40.0326463362608,-0.0200476033634112,0.461739347428258,-0.0434175
763340725,0.965368667023689,0.994061064781013
"15190","NEK4",51.682933605296,-0.0121490944933223,0.284409822300214,-
0.0427168597591474,0.965927239692448,0.994119113645054
"15191","SHOX2",202.554333756937,-0.00894522739736019,0.20961450261672
,-0.0426746588890203,0.965960880438867,0.994119113645054
"15192","UBE2K",606.535349848729,-0.00640880427902384,0.14895538320149
4,-0.0430249927278865,0.965681610981242,0.994119113645054
"15193","PHF1",158.772380616004,-0.00818896358191979,0.190010199499059
,-0.0430974947845384,0.965623816317948,0.994119113645054
"15194","AC083880.1",6.25504076340562,0.0271729993360332,0.63655265326
2418,0.0426877481332736,0.965950446241206,0.994119113645054
"15195","RPL12",16182.2895996746,0.0108381726667449,0.252177473477175,
0.0429783537653133,0.965718789099667,0.994119113645054
"15196","MRPL16",272.057871069989,-0.00985133147127006,0.2291142146850
75,-0.0429974695581897,0.965703550989883,0.994119113645054
"15197","ARHGAP8",5.34170929927091,0.029503072827052,0.689471124789602
,0.0427908751596452,0.965868237896282,0.994119113645054
"15198","HCG17",3.60292985963309,-0.0359155446785086,0.85201514286164,
-0.0421536459526764,0.966376214858922,0.994481115962202
"15199","GORASP1",70.3929009615428,-0.0100129409891025,0.2388065235404
82,-0.0419290932284984,0.96655522374331,0.994583430379897
"15200","RBPMS2",9.84219367357951,0.0228567506174967,0.54590605325492,
0.0418693848167009,0.966602822364095,0.994583430379897
"15201","TOR3A",165.912668685476,-0.0106540839328954,0.257141960021104
,-0.0414326931785889,0.966950949778915,0.994643624649471
"15202","GLI2",97.5114708511811,-0.0111119908789851,0.272513155193806,
-0.0407759796809901,0.967474488866209,0.994643624649471

"15203","SLC35A4",132.751236096166,0.00781860535166886,0.193613340618718,0.0403825755326747,0.967788121627904,0.994643624649471
"15204","TCF21",9.00299362011791,0.0334774911982626,0.805912779131929,0.0415398440937022,0.966865529258826,0.994643624649471
"15205","DUS4L",27.61782877833,-0.0123593279730087,0.305632606017041,-0.0404385125463989,0.967743526775117,0.994643624649471
"15206","VPS13B-DT",39.7102462381064,0.0129991058223428,0.317979448616939,0.0408803332381471,0.967391296141602,0.994643624649471
"15207","ALDH1B1",29.9810930913297,0.0141103925041509,0.340262465537743,0.0414691420102749,0.966921892792127,0.994643624649471
"15208","SLK",560.992301919029,0.00865308443380475,0.20784272018091 6,0.0416328482723509,0.966791386800227,0.994643624649471
"15209","AP003168.2",2.56730519048311,-0.0394819995221643,0.969700858622657,-0.0407156487189706,0.967522586066759,0.994643624649471
"15210","CUL5",309.876482707632,-0.00646778325293964,0.159310572267601,-0.0405985815057861,0.967615915020369,0.994643624649471
"15211","C11orf45",2.50914589646875,0.0473070139287362,1.15582380123026,0.040929260911899,0.967352290152235,0.994643624649471
"15212","LINC00937",7.48644164792778,0.0263488661826045,0.646953136958588,0.040727627207241,0.967513036536952,0.994643624649471
"15213","AC025159.1",53.5504110781926,-0.0112280793594502,0.276373171290161,-0.040626517063995,0.967593644043996,0.994643624649471
"15214","TIMM9",277.849468131708,-0.00952255740462641,0.233495491749196,-0.0407826178282485,0.967469196793059,0.994643624649471
"15215","ZNF174",44.7213252035918,-0.00999960892482914,0.24775987210913,-0.0403600826869359,0.967806053703046,0.994643624649471
"15216","ASPA",202.398000757902,0.0111351574810538,0.272083849998706,0.0409254627979822,0.967355318071671,0.994643624649471
"15217","COX11",329.842650998912,-0.00671547536998775,0.16437191136994,-0.0408553706896656,0.967411196747882,0.994643624649471
"15218","ZNF430",177.513647876058,0.00748422364090206,0.183327137109003,0.040824417808107,0.967435872986894,0.994643624649471
"15219","CYP2R1",21.8412695150208,-0.0142771933957336,0.356204056539634,-0.0400815014136286,0.968028149682878,0.994806509037402
"15220","KIAA1328",110.785257052058,0.00851721503935752,0.21335076589174,0.0399211833328003,0.968155962706054,0.994872487301096
"15221","ANKRD45",3.12241461958129,0.0335139982048,0.845504652908934,0.0396378637178354,0.968381840265623,0.994908165200222
"15222","TIGD1",10.3911544858073,0.0233866105469125,0.587860800211538,0.0397825650876823,0.96826647627147,0.994908165200222
"15223","ABCB9",3.58748642257653,0.0339424630802119,0.855606350059752,0.0396706535404295,0.968355698334081,0.994908165200222
"15224","PBX4",15.7563141643314,0.0187924273709118,0.475054410071989,0.0395584736663407,0.968445134719193,0.994908165200222
"15225","MRAS",60.7078297499737,0.0108472961034654,0.275283855513741,0.039404040179 6244,0.968568258815335,0.994917060516394
"15226","ADO",76.6910265723145,0.0109992516105159,0.279253639599684,0.0393880331382023,0.968581020679196,0.994917060516394
"15227","PRPF38A",226.735235201467,0.00651039987425385,0.1658429383936

37,0.0392564189787875,0.968685952428831,0.994925311986311
"15228","CRIM1-
DT",55.5918289232182,-0.0136125892920422,0.347097209712747,-0.03921837
71898016,0.968716282028615,0.994925311986311
"15229","AL162591.2",2.73130552781002,0.0350112623872157,0.90140537978
8615,0.0388407515333734,0.969017354307765,0.99492692733966
"15230","BNIP1",62.2772249926077,0.0108585211150746,0.279700196329801,
0.0388220003330676,0.969032304325351,0.99492692733966
"15231","VIRMA",223.675221155477,-0.0065153752115477,0.167846520579143
,-0.0388174576932952,0.969035926097509,0.99492692733966
"15232","TMEM202-
AS1",9.31397741736123,0.0193752620051572,0.496638634259326,0.039012796
5659638,0.968880186197036,0.99492692733966
"15233","HMGN5",51.678981299288,-0.00926974429102988,0.238168671896234
,-0.0389209219551283,0.968953435897647,0.99492692733966
"15234","MIATNB",28.2090228172426,-0.0123821875259459,0.32023869810034
6,-0.0386654942060313,0.969157084460149,0.994986005051643
"15235","BET1",187.220132063623,0.00962939728893084,0.249721602630598,
0.0385605297559106,0.969240771563776,0.995006607630946
"15236","KIF1B",450.799512922943,-0.00968982129674492,0.25373568824733
3,-0.0381886417463656,0.969537276864676,0.995033468729627
"15237","AC097662.1",11.2301666445064,-0.0177764287805746,0.4660235431
70598,-0.0381449157260004,0.969572139779522,0.995033468729627
"15238","AC022098.1",20.8101990196829,0.0135882260195758,0.35510359087
6708,0.0382655269298407,0.969475976169867,0.995033468729627
"15239","CNFN",28.5289835715809,-0.0173201933110762,0.454255675268367,
-0.0381287329010115,0.969585042419407,0.995033468729627
"15240","RAP2C-
AS1",46.092810989497,0.0103086790954963,0.269623010793004,0.0382336769
594585,0.96950137018657,0.995033468729627
"15241","NDUFAF6",42.7040727849663,-0.0117978975593728,0.3115642855217
44,-0.037866655799833,0.969793998788925,0.995182608822176
"15242","ZKSCAN4",35.140389032102,0.0103266375880807,0.27735252221091,
0.0372328959036037,0.970299309650792,0.995635822263377
"15243","NFKBIA",3063.65044489728,-0.00917721090976406,0.2473231765741
64,-0.0371061500862299,0.970400368347431,0.99567419543094
"15244","WDR63",2.52104393186975,0.0363650358785725,0.985476303270403,
0.0369009744403711,0.970563962786921,0.995711405574775
"15245","RDH11",363.173946930278,-0.00567415305730176,0.15357710708445
7,-0.036946607245189,0.970527577887923,0.995711405574775
"15246","RRAGB",101.25889612554,-0.00690518143049869,0.187831937502645
,-0.0367625523236774,0.970674332768434,0.995759318148911
"15247","RCN2",544.610657524103,-0.00750684366174037,0.20466332375512,
-0.0366789883209477,0.970740962258783,0.995762356511272
"15248","GTF3C3",91.1796355870745,-0.00761021732488437,0.2095993704021
98,-0.0363083978271557,0.971036453858618,0.995842027518281
"15249","GALNT15",1335.72398468063,-0.0213964097245963,0.5927925009519
46,-0.0360942651775057,0.971207195075458,0.995842027518281
"15250","P2RY12",2.634967562746,0.0371618453827535,1.01987329706786,0.
0364377079874469,0.970933347505602,0.995842027518281

"15251","MPZL3",3.09272466869444,-0.0305892503151104,0.841084920494571
,-0.0363688012586451,0.970988290703295,0.995842027518281
"15252","CPNE8",129.388626843036,0.0083946360288057,0.231950253410928,
0.0361915363547096,0.971129634580531,0.995842027518281
"15253","CHMP4A",449.779579056481,-0.00774334496067374,0.2149579861797
57,-0.0360225972446469,0.971264340649863,0.995842027518281
"15254","MT-
ND6",254.172935882452,0.0116944550747162,0.324569493739999,0.036030666
1601544,0.971257906763441,0.995842027518281
"15255","AL645728.1",9.32938575435385,0.0193397109780514,0.56643769427
1419,0.0341426977294072,0.972763360453523,0.995852761476966
"15256","COPA",701.946975624131,0.0057806456343064 6,0.165250738527096,
0.0349810577903022,0.97209484532185,0.995852761476966
"15257","PPP1R15B",263.300879073638,0.0114990127498479,0.3296923419683
22,0.0348780098475953,0.972177015542223,0.995852761476966
"15258","AGBL5",26.8798917594933,0.0121264407526056,0.343459543898559,
0.0353067514588767,0.97183513980319,0.995852761476966
"15259","FBXL2",54.6560489970054,0.0095593690158809,0.281730625626737,
0.0339308834267313,0.972932265947957,0.995852761476966
"15260","ZBTB11-
AS1",19.6575832255088,-0.0136435446976779,0.399089148526475,-0.0341867
092805025,0.972728264804682,0.995852761476966
"15261","GNPDA2",196.969614776805,0.00720911004752352,0.20315097990970
3,0.0354864645532492,0.971691839300157,0.995852761476966
"15262","SH2B2",5.91014461718549,-0.0225896200306414,0.635491207402742
,-0.0355467074406354,0.971643802737307,0.995852761476966
"15263","REEP4",42.2570349262157,0.00906803518740998,0.265831721037229
,0.0341119380035914,0.97278788887588,0.995852761476966
"15264","NFIL3",724.399696831759,-0.015531924966 0618,0.458324756508366
,-0.0338884704469991,0.972966087159136,0.995852761476966
"15265","CDC42EP2",458.154914486525,-0.00962690469815721,0.27512029197
2623,-0.0349916199533372,0.972086423091203,0.995852761476966
"15266","UCP3",3.08951893185205,-0.0308805878482843,0.888360746373645,
-0.0347613151237728,0.9722700680347,0.995852761476966
"15267","NT5DC3",35.5479571625358,0.0103559372711354,0.297786043497806
,0.0347764359588319,0.972258010644,0.995852761476966
"15268","GOLGA3",333.503304909338,-0.0083019024563949,0.24581021098983
7,-0.0337736273158243,0.973057666297379,0.995852761476966
"15269","AL139089.1",4.709629186692,-0.0237967096797672,0.700469133903
175,-0.0339725314478405,0.972899054782008,0.995852761476966
"15270","PLK1",3.51310541907368,-0.0313963284269349,0.904444900409507,
-0.0347133677382885,0.972308301438547,0.995852761476966
"15271","NOB1",248.402555245173,-0.00821625815863623,0.229730696929291
,-0.0357647378798712,0.971469950163576,0.995852761476966
"15272","MPP2",18.7491998280151,-0.013081359727866,0.369054135826046,-
0.0354456391569391,0.971724392773575,0.995852761476966
"15273","FAM20A",244.017687068558,0.00847263206598206,0.23883082487936
9,0.0354754545199997,0.971700618507895,0.995852761476966
"15274","AC100786.1",4.37096636552206,0.0269875453616579,0.76636684128
0778,0.0352149178539032,0.971908366882,0.995852761476966

"15275","LGALS3BP",2554.28429641849,0.00994344338818827,0.294207714763
159,0.0337973577483952,0.973038742954807,0.995852761476966
"15276","SH2D3A",6.02129124601425,-0.0242538270094006,0.70829043530766
3,-0.0342427707623433,0.972683560388108,0.995852761476966
"15277","PDE4A",172.900561992748,0.00851450195513951,0.246563444851075
,0.0345327019594583,0.972452365499339,0.995852761476966
"15278","AC008622.2",7.82238552822936,0.0204413643767697,0.57239272352
7855,0.0357121317873898,0.97151189695604,0.995852761476966
"15279","CLEC11A",258.911081881931,0.0131504433662601,0.37814415808133
6,0.0347762700684948,0.972258142925324,0.995852761476966
"15280","NRSN2-
AS1",17.4485901723354,-0.0130299114191598,0.362918204049298,-0.0359031
629545643,0.971359573822078,0.995852761476966
"15281","AL353194.1",14.0325518368947,0.0143780899870111,0.41649487099
5945,0.034521649576691,0.972461178770079,0.995852761476966
"15282","AP000547.3",36.6737891220296,0.0155314276758464,0.45661954733
9119,0.0340139351597045,0.972866038480892,0.995852761476966
"15283","THORLNC",9.59639055148215,-0.0202010906529027,0.6020869763704
54,-0.0335517814630047,0.97323457341586,0.995965081723387
"15284","AC003991.1",3.89021238417955,0.0353196513000962,1.05506483023
196,0.0334762853315193,0.973294776794133,0.995965081723387
"15285","PPP2R1B",60.9621236466489,-0.00925935395246859,0.278741270776
377,-0.0332184535382168,0.973500382447356,0.995979981780379
"15286","KRR1",442.602725727896,-0.00491170253229889,0.147740691475246
,-0.0332454280757299,0.973478871761411,0.995979981780379
"15287","AC087190.1",20.1653185724722,0.0182235997826935,0.54842412562
9655,0.0332290264615377,0.973491951129675,0.995979981780379
"15288","PDPN",444.575222736501,-0.0102156963511237,0.310722152098347,
-0.0328772708419266,0.973772458235936,0.996012457926426
"15289","HIST2H2BF",7.45364436475513,0.020034343909486,0.6053284749350
69,0.0330966487437006,0.973597515202591,0.996012457926426
"15290","RAD50",453.980236885744,-0.00515425060356137,0.15685862409964
8,-0.0328592108540173,0.97378686023996,0.996012457926426
"15291","LRCH4",53.3857865851473,0.00819654813792149,0.24825085232987,
0.033017200388219,0.973660871192882,0.996012457926426
"15292","SREK1IP1",401.478598381795,0.00658101699272486,0.201568801214
754,0.0326489861182106,0.97395450535909,0.996079757345344
"15293","NDUFB1",1040.08327797368,-0.00746453257144428,0.2288542339215
8,-0.0326169738856661,0.973980033828794,0.996079757345344
"15294","UBIAD1",65.345344022082,0.00696832787560667,0.220371917890323
,0.0316207615848528,0.974774486317156,0.996403090527436
"15295","MMRN2",6.29629034648089,-0.0212620212474678,0.671104936101776
,-0.0316821112521863,0.974725560878008,0.996403090527436
"15296","ATF7",292.80017912412,-0.00414963442492915,0.131394810901969,
-0.0315814178386779,0.974805862414341,0.996403090527436
"15297","SLC7A1",72.777840896105,-0.0114351129030302,0.35700108445972,
-0.0320310312791792,0.974447304205913,0.996403090527436
"15298","DHODH",28.5503086754617,0.0114175728011299,0.361206128608668,
0.031609576629027,0.974783406161832,0.996403090527436
"15299","DHRS11",13.7561733698336,-0.0139545435889131,0.43597632578130

1,-0.0320075718880046,0.974466012499273,0.996403090527436
"15300","CIRBP",2789.28421314147,-0.00655842845791097,0.207218417908537,-0.0316498336591188,0.974751301762312,0.996403090527436
"15301","AL022328.4",5.89195832235183,-0.0202804894102856,0.640763931152586,-0.0316504853414669,0.974750782055397,0.996403090527436
"15302","PNPT1",159.066802911383,-0.0066474285238497,0.211131191854154,-0.0314848245087425,0.9748828944335,0.996416708204152
"15303","NRN1L",4.17245596984654,-0.0222284519032479,0.708432472550205,-0.031376952306026,0.97496892149495,0.996439517230675
"15304","PRMT6",40.2021277678717,0.0092644851906431,0.30787809082208,0.0300914078228352,0.975994153193671,0.99656493081127
"15305","AC008269.1",6.51397961958141,0.0193009934792784,0.641127915740811,0.0301047466588261,0.975983515161932,0.99656493081127
"15306","ZNF142",28.3868884557571,0.0114024039116143,0.372424165117123,0.0306167133596939,0.975575213064608,0.99656493081127
"15307","ATG9A",114.663254270508,-0.00635539485817559,0.213479060363019,-0.0297705772517844,0.976250024313274,0.99656493081127
"15308","AC108673.3",18.5098366857548,0.01408316497611,0.476808810748627,0.0295362934967546,0.976436873535059,0.99656493081127
"15309","NKX3-2",7.5179888159394,0.0237045586673339,0.765495613765629,0.0309662893438753,0.975296423979026,0.99656493081127
"15310","ZSCAN16-AS1",294.884419764264,-0.00765693157854893,0.256782645855441,-0.0298187268576534,0.976211623534523,0.99656493081127
"15311","AC064807.1",25.3075894291426,0.0119495705361572,0.393356017274934,0.0303785121146505,0.975765181794023,0.99656493081127
"15312","ZCCHC7",234.678440234953,-0.00457490392450679,0.150719978048073,-0.030353666340421,0.975784996715912,0.99656493081127
"15313","AL731563.3",5.20310832607755,-0.0220419005296321,0.744160651848379,-0.0296198145855783,0.976370262491889,0.99656493081127
"15314","ZBTB16",1382.89240243957,-0.0192327175000022,0.64775013233211,-0.0296915686157534,0.976313036228689,0.99656493081127
"15315","AP000821.2",3.3831510593272,-0.0265718549554508,0.865475028471039,-0.0307020469468577,0.975507158705897,0.99656493081127
"15316","MANSC1",123.299782105866,0.00594076161665144,0.196416121625986,0.0302457943241736,0.975871026632211,0.99656493081127
"15317","YAF2",156.397446735423,0.00499068220640231,0.167452576131656,0.0298035558585762,0.976223722862903,0.99656493081127
"15318","CES4A",5.58733715468674,-0.0208876077899777,0.703295280437952,-0.0296996274125012,0.976306609073635,0.99656493081127
"15319","SLC7A6",63.7854307495698,-0.00783628176898077,0.259137578989652,-0.0302398509684837,0.975875766575816,0.99656493081127
"15320","UBB",4547.62991039165,0.00809332094173096,0.263770487924446,0.0306831935802052,0.975522194432295,0.99656493081127
"15321","TAF15",240.604521051672,0.00674075295446865,0.219191168417264,0.0307528492281067,0.975466643481307,0.99656493081127
"15322","QRICH2",2.76065899150439,0.028094772818261,0.910800074918205,0.0308462565956465,0.975392150525762,0.99656493081127
"15323","DMWD",153.047800887348,0.00615841227034611,0.20678677879873,0.0297814604305057,0.9762413446416,0.99656493081127

"15324","AL121672.3",3.2041111511106,-0.0301324301157246,0.97811750571
318,-0.030806554365627,0.975423813274511,0.99656493081127
"15325","PGRMC1",1403.16806996697,-0.00595436706658256,0.2020802826087
52,-0.0294653540153189,0.976493450427284,0.99656493081127
"15326","B4GALT3",67.0452483075394,0.00884144133309556,0.3059683422859
79,0.0288965886700518,0.97694706633419,0.996682697864201
"15327","E2F6",65.8416430902854,-0.00786008866702674,0.272801565745862
,-0.0288124763710084,0.977014150306614,0.996682697864201
"15328","DMAC1",426.475408735167,0.00663089356398328,0.228054224404019
,0.0290759514817671,0.976804015624635,0.996682697864201
"15329","TSPAN14",326.449706174533,-0.00598674849088661,0.207913972311
59,-0.0287943538585977,0.977028603982575,0.996682697864201
"15330","TELO2",79.4896993528701,-0.00737480321526036,0.25258452075521
7,-0.0291973680461891,0.976707180335156,0.996682697864201
"15331","AMZ2",134.516945102816,0.00625811614342283,0.216461285501071,
0.0289110181016266,0.976935558121671,0.996682697864201
"15332","AL022311.1",33.8583198461959,0.00862360992488334,0.2998333437
29709,0.0287613439439787,0.977054931179918,0.996682697864201
"15333","GPBP1L1",201.087189434683,-0.00387792208630057,0.145711339842
059,-0.0266137288319768,0.978767823114238,0.997007489963646
"15334","AIDA",758.989563437319,-0.00406651148005117,0.157123484697853
,-0.0258809909153367,0.979352262020328,0.997007489963646
"15335","LDAH",39.5862009888876,-0.00829973631191765,0.297388418575464
,-0.0279087408705244,0.977734936953642,0.997007489963646
"15336","HSPBAP1",32.8625901561849,0.00786879391402312,0.3136711255245
17,0.02508612771056,0.979986265184692,0.997007489963646
"15337","TM4SF19-
AS1",4.40358320207388,0.0199289191475023,0.719874675763148,0.027683873
0663437,0.977914286203331,0.997007489963646
"15338","PACRGL",80.4067687370252,-0.00567018106297661,0.2056405223671
8,-0.027573267164009,0.978002503268953,0.997007489963646
"15339","STIM2",64.4202042217414,0.00688757852137354,0.246699177583127
,0.0279189358831678,0.977726805678946,0.997007489963646
"15340","RPL9",15464.0974734986,-0.00563956808870146,0.20151043962069,
-0.0279864809948159,0.977672933527935,0.997007489963646
"15341","AC108471.3",14.2951263698892,0.0160334923322124,0.60455579452
1223,0.0265211126541431,0.978841694057901,0.997007489963646
"15342","PRDM8",157.136548671313,0.00556861759566316,0.206693174023507
,0.0269414682994313,0.978506418589884,0.997007489963646
"15343","DAAM2",42.8242598215146,0.00802239163511311,0.300352870979873
,0.0267098883021854,0.978691126222529,0.997007489963646
"15344","SNX8",60.460548346278,-0.00711811377979377,0.279111856104092,
-0.0255027281146349,0.979653972475444,0.997007489963646
"15345","C7orf25",71.2552918193169,0.00608038924821487,0.2360227855827
21,0.0257618739360392,0.979447271939943,0.997007489963646
"15346","RINT1",56.2910489633714,-0.00656814464665616,0.25618040122464
1,-0.025638747598403,0.979545480104813,0.997007489963646
"15347","AGBL3",12.5609354936751,-0.0129155311222788,0.489947082023908
,-0.0263610736672344,0.97896934206538,0.997007489963646
"15348","CDK20",14.8446013051714,-0.0115828915259047,0.454380092078248

,-0.025491635148289,0.979662820505478,0.997007489963646
"15349","AK8",7.89303728888796,0.0141868271816289,0.561872531233142,0.0252491915746318,0.97985620024408,0.997007489963646
"15350","ASB13",52.3327941906876,-0.00740477000679501,0.29303469008278,-0.0252692607987922,0.979840192427512,0.997007489963646
"15351","PPP3CB",349.457350311395,0.00358006866946573,0.143172227819842,0.0250053290640321,0.980050712960428,0.997007489963646
"15352","VCL",630.741644481427,-0.00486510015683699,0.190416487288606,-0.0255497841920756,0.979616439387846,0.997007489963646
"15353","PI4K2A",118.674887736258,0.00541450667192043,0.200267986478975,0.0270363065366359,0.978430776178658,0.997007489963646
"15354","ZNF408",28.2864929300688,-0.00717504202614107,0.286484404019165,-0.0250451400686408,0.980018958319428,0.997007489963646
"15355","CCDC15-DT",4.67064452047852,-0.0191967204873984,0.72350463185933,-0.0265329614242619,0.978832243432869,0.997007489963646
"15356","RAD51AP1",6.81386990773666,-0.018388150904982,0.662426258279915,-0.0277587892616598,0.977854534690848,0.997007489963646
"15357","AC048344.4",2.57391520122245,-0.0284214467960381,1.02459674620214,-0.0277391538684731,0.977870195437326,0.997007489963646
"15358","HMGA2-AS1",8.9159326996726,0.0185998387700116,0.733059961617708,0.0253728749950628,0.979757546758714,0.997007489963646
"15359","RFC5",45.3253119639147,-0.00641390508550588,0.25319049762403,-0.0253323293950395,0.979789887071869,0.997007489963646
"15360","RPL21",16767.1383983815,0.00473055567086337,0.187604856368815,0.0252155288643674,0.979883050652061,0.997007489963646
"15361","AL136418.1",4.11498898694747,-0.0243462179434964,0.944499196987868,-0.0257768540419513,0.979435323512613,0.997007489963646
"15362","IGHM",27.7247347405979,0.0305062753833152,1.21582738026586,0.025090959356948,0.979982411301711,0.997007489963646
"15363","NDN",1083.64337372993,0.00621635419961001,0.238608083190986,0.0260525717170878,0.97921540649467,0.997007489963646
"15364","ALPK3",4.60099733074063,0.0287112098214397,1.05578629113093,0.0271941491025472,0.97830488232174,0.997007489963646
"15365","LITAF",520.316102384941,0.00458360771031396,0.169483578451224,0.0270445535325599,0.978424198433151,0.997007489963646
"15366","AC140912.1",4.09660155074964,0.0235027410767986,0.870960336820262,0.0269848580735644,0.978471811141314,0.997007489963646
"15367","AC138028.1",3.55809034082649,-0.0280579256501645,1.0413529242315,-0.0269437238781183,0.978504619551557,0.997007489963646
"15368","SLFN12",58.0800368154326,0.00825876086923522,0.304826102224196,0.0270933519438601,0.978385277196143,0.997007489963646
"15369","AC060780.1",61.62951313887,0.00686304547496128,0.251769880823364,0.0272591997601819,0.978252998640391,0.997007489963646
"15370","HLF",96.0555516285032,0.015079265137058,0.556199262101012,0.0271112641899181,0.978370990539554,0.997007489963646
"15371","PLK5",2.90147228119625,-0.039267863097553,1.47618301947003,-0.0266009448555035,0.978778019641735,0.997007489963646
"15372","TIMM44",182.065652169125,0.00493424594784879,0.18825859772843

3,0.0262099367964408,0.979089890188822,0.997007489963646
"15373","ZNF543",18.6160128516477,-0.00931286182274736,0.369597809377875,-0.0251972863108232,0.97989760148062,0.997007489963646
"15374","TFAP2C",3.73771269196367,0.034934457345366,1.23875854485346,0.0282011837500572,0.977501693104931,0.997007489963646
"15375","FMO5",31.3376860861292,-0.00842623597691134,0.3387323304986,-0.0248757948924104,0.980154034136471,0.997009895412599
"15376","CHAF1B",4.82307249787891,-0.0172042236246176,0.692531230492064,-0.0248425238705747,0.980180572369828,0.997009895412599
"15377","PASK",6.87528563103417,-0.0151664457237585,0.623553823880141,-0.0243225927625355,0.980595292042012,0.997047679002016
"15378","ZNF165",4.92514430517936,-0.0174042154684713,0.724365759908117,-0.0240268334476203,0.980831204886454,0.997047679002016
"15379","SLC35B2",211.171746840116,-0.00495502561586798,0.205709382405286,-0.024087504215562,0.980782810623482,0.997047679002016
"15380","AL356776.2",3.54304728606258,-0.0198059392716545,0.802106718190202,-0.0246923991814241,0.9803003178082,0.997047679002016
"15381","HNRNPA2B1",2935.41059505687,-0.00275728628090809,0.115255467037538,-0.0239232580612428,0.980913822340302,0.997047679002016
"15382","CD274",2.53052906769123,-0.0236467774296184,0.963206233764337,-0.0245500668503812,0.980413848160991,0.997047679002016
"15383","AKR1E2",8.59427209050275,0.0119128377793272,0.4952517806901,0.0240541038797022,0.980809452516466,0.997047679002016
"15384","ANKRD2",13.5290452137806,-0.0144907040179867,0.607290404136818,-0.0238612431865827,0.980963288930565,0.997047679002016
"15385","SPATA5L1",43.9818798656558,-0.00681612973786746,0.282695711080991,-0.0241111890654566,0.980763918334341,0.997047679002016
"15386","AC105020.1",8.12111714497317,0.0167909646507446,0.695129944841472,0.024155145056474,0.98072885673935,0.997047679002016
"15387","GATAD2A",153.874635952771,0.00516700027376933,0.216764888666401,0.0238368875400355,0.98098271639917,0.997047679002016
"15388","TAF4",43.4448303472468,0.00691250727909104,0.28523845100972,0.0242341355263337,0.980665849907203,0.997047679002016
"15389","AL136366.1",7.39350035994494,-0.0143625368980474,0.606403887692574,-0.023684770479784,0.98110405399582,0.99709236569055
"15390","AC068790.7",6.36334702984427,-0.0149170443568343,0.631491649360239,-0.0236219186301936,0.981154188489615,0.99709236569055
"15391","DIAPH2",291.000979454917,-0.00472596211022103,0.200800797110883,-0.0235355744509886,0.981223062029062,0.997097569367457
"15392","RNGTT",66.9297935079843,0.00540148112844254,0.230310287233357,0.0234530606223838,0.981288880371131,0.997099667944678
"15393","GPANK1",143.805708924782,-0.00459911246078859,0.197483250532098,-0.0232886204191837,0.98142004884354,0.99712582151449
"15394","COQ6",73.7466284927673,0.00549398516166562,0.236189304722338,0.0232609396438349,0.981442128925451,0.99712582151449
"15395","GLI4",102.41703959301,-0.00546576059418623,0.236530198073347,-0.0231080878412461,0.981564054245554,0.997174235216091
"15396","PUM3",218.061261848971,0.00473188870909291,0.205365168769516,0.0230413401524948,0.981617297019625,0.997174235216091
"15397","TMBIM1",464.41872173282,0.00384007024923673,0.17031761274495,

0.0225465246215447,0.982012000138233,0.997394943302288
"15398","HHIPL1",13.4371714756199,-0.0106329444924144,0.46946428510412
8,-0.0226491020292545,0.981930176103216,0.997394943302288
"15399","KCNJ14",5.31621805235119,0.014646957812159,0.650134285616854,
0.0225291268837819,0.982025877999484,0.997394943302288
"15400","CNKSR3",5.69704991009788,-0.0150965201827879,0.67347424419217
9,-0.0224158834773198,0.982116210352656,0.997421917526983
"15401","SRSF10",783.562435379735,0.0030249812614442,0.139921587164113
,0.0216191176983737,0.982751783377301,0.99743576138539
"15402","WASF2",2142.25848943177,0.00234440702763496,0.110228886021585
,0.0212685359731921,0.983031442815321,0.99743576138539
"15403","POLR2B",236.291183862923,0.00324995294833386,0.15319878571643
7,0.0212139602356206,0.983074978131247,0.99743576138539
"15404","RPL34",19274.6913902574,0.00428623197495735,0.20153635607534,
0.0212677854181061,0.983032041536209,0.99743576138539
"15405","FGF2",246.292155621873,-0.00360318299860125,0.163294577379587
,-0.0220655398141327,0.982395675024015,0.99743576138539
"15406","FNDC1-
IT1",4.53782052669677,0.0202636592202884,0.955852621330088,0.021199564
4183003,0.983086461749091,0.99743576138539
"15407","FAM133B",880.577883905927,0.00620178416796962,0.2803018088833
26,0.0221253804699886,0.982347940742191,0.99743576138539
"15408","WDHD1",16.754784012073,-0.00908695492475186,0.425430918246073
,-0.0213594135617005,0.982958949458763,0.99743576138539
"15409","VASH1-
AS1",14.2747666183952,-0.0101839107551435,0.469146351912911,-0.0217073
216356034,0.982681423329355,0.99743576138539
"15410","CERNA1",4.32202322638215,0.018335851066048,0.828473755255422,
0.0221320843898005,0.982342593097556,0.99743576138539
"15411","RGS11",15.2174382870922,0.0121875026502922,0.569932589490128,
0.0213841125688134,0.982939247002406,0.99743576138539
"15412","EPOR",29.0867083315872,0.00806247132333919,0.370038586418427,
0.0217881907975468,0.982616914330663,0.99743576138539
"15413","ARRDC2",62.9419985661291,-0.00641366436455136,0.2941357812993
35,-0.0218051144142314,0.982603414445436,0.99743576138539
"15414","NR2C2AP",81.9944909314286,-0.0050732104509442,0.2357068689964
49,-0.0215233882344796,0.982828146669816,0.99743576138539
"15415","APOO",27.2736040341446,0.00801328839255809,0.377192183675266,
0.0212445770070806,0.983050554986153,0.99743576138539
"15416","TCHH",18.6004768643909,0.0122316266224041,0.588994818288761,0
.0207669511557697,0.983431561207863,0.997442331369801
"15417","GPATCH2",212.540466561061,-0.00414587302613557,0.202455061554
049,-0.0204779914827112,0.983662068647846,0.997442331369801
"15418","WDR27",90.5437844434901,-0.00453842730809508,0.22103902782889
8,-0.0205322442496815,0.983618790302065,0.997442331369801
"15419","ZC3HAV1",669.212051980737,0.00737963887522152,0.3538131802692
11,0.0208574447950369,0.983359373365953,0.997442331369801
"15420","SERPINH1",981.673976421932,0.00598872266191986,0.283771308062
906,0.021104045728937,0.983162657589701,0.997442331369801
"15421","CCDC89",46.4161673920436,0.00629952945095029,0.30042924506085

,0.0209684295204828,0.983270839729332,0.997442331369801
"15422","AP001528.1",112.338971957586,0.00623765792983711,0.3033696597
13362,0.0205612451018693,0.983595655853691,0.997442331369801
"15423","DNAJC14",68.6267979800494,-0.0054070313892645,0.2646076014444
22,-0.0204341498874143,0.983697041861801,0.997442331369801
"15424","AC000403.1",8.94854719676053,0.0116678829499021,0.56944442239
0627,0.0204899415836199,0.983652535846989,0.997442331369801
"15425","BOLA2",158.839182717258,-0.00619897401339744,0.30399090698620
2,-0.0203919718351273,0.983730688067723,0.997442331369801
"15426","MEOX2",117.112041489135,0.007633870309204,0.37855106767589,0.
020166025038767,0.983910930462667,0.997560414393628
"15427","LDLRAD2",13.4526824291528,-0.0100538242679468,0.5179733953120
94,-0.0194099240596886,0.984514093645881,0.997581066251003
"15428","KIFAP3",399.355502663465,-0.00306266179765098,0.1564681910632
82,-0.0195737023406395,0.984383442305852,0.997581066251003
"15429","B3GNT7",2.51381396739537,0.0251036279454439,1.2980940691221,0
.0193388357150584,0.984570803294049,0.997581066251003
"15430","CCDC174",207.987045181336,0.00319527504814439,0.1639183756344
9,0.0194930863350507,0.984447752302025,0.997581066251003
"15431","CPOX",67.6141379391235,0.00447725235976674,0.226820795942985,
0.0197391616635195,0.984251450368512,0.997581066251003
"15432","IRAK1BP1",64.4734370499522,0.00460819131717162,0.234669641129
951,0.0196369300050183,0.984333003623086,0.997581066251003
"15433","UQCRB",3020.85689330079,0.00389507312825784,0.200301817392072
,0.0194460199062178,0.984485298761998,0.997581066251003
"15434","AP003392.5",4.73486794018489,-0.0144921058771012,0.7379281004
01227,-0.0196389131532212,0.984331421604834,0.997581066251003
"15435","SIRT4",8.27160103285441,0.0109614856140259,0.553567775268863,
0.019801524047714,0.98420170210841,0.997581066251003
"15436","RNASE4",1764.31526131257,0.00463090419635424,0.24143172694057
4,0.0191810092858844,0.984696707211188,0.997581066251003
"15437","ALG1",69.5449343186201,0.00552957857227285,0.288134942794949,
0.0191909336598858,0.984688790163669,0.997581066251003
"15438","MHENCR",25.6825988295739,0.00733409335785686,0.36856294969523
1,0.0198991606831927,0.984123814691162,0.997581066251003
"15439","TIGIT",4.89552363793778,0.0140430316610117,0.735451747834436,
0.0190944296513835,0.984765775115517,0.9975864189913
"15440","TARS2",65.7223118298277,-0.00424468621720142,0.22483570160405
8,-0.0188790578494355,0.984937585979398,0.997608785895642
"15441","LMOD3",3.22309942144992,0.0158999489881342,0.848013649027769,
0.0187496380587308,0.985040829757474,0.997608785895642
"15442","AC093323.1",100.782829164409,0.0042004060401053,0.2259696663
02157,0.0185883623622682,0.985169486723114,0.997608785895642
"15443","FBXL5",280.938612027246,-0.00256157025570019,0.14022802862592
5,-0.0182671772597865,0.985425711847939,0.997608785895642
"15444","AP003059.1",2.46894584701526,0.0177102491459806,0.95667126662
9521,0.0185123665398419,0.985230112184505,0.997608785895642
"15445","DNAL1",83.4134627381027,0.00538500175286112,0.286854716510775
,0.0187725752546894,0.985022531743494,0.997608785895642
"15446","TXNL4B",41.7877952143929,0.00501497589455297,0.26990974889267

4,0.0185801954732176,0.985176001832621,0.997608785895642
"15447","PAK4",59.2490801342181,0.00439921173520435,0.240652035293904,
0.0182803844971919,0.985415175756434,0.997608785895642
"15448","MYH7B",3.63989586057482,0.0152082539003597,0.829417247985518,
0.0183360714252054,0.98537075146228,0.997608785895642
"15449","PIN4",359.21846870642,0.00431579668174038,0.234718478930293,0
.0183871193329525,0.985330027990332,0.997608785895642
"15450","CSGALNACT2",210.013455554144,-0.00336166937656459,0.186522885
764869,-0.0180228252569624,0.985620644445359,0.997713543487803
"15451","AL365295.1",31.7475059349074,-0.00801801870437266,0.447994817
544455,-0.0178975702181578,0.985720567389612,0.997713543487803
"15452","MMP15",2.70930312439575,-0.016051044428132,0.893594435498971,
-0.0179623370407061,0.985668899247746,0.997713543487803
"15453","SLC35E2A",76.8820783444863,-0.00702551668976132,0.41364738451
8671,-0.016984313095407,0.986449130304674,0.997785003279571
"15454","AK4",28.3143407245156,0.00647503988484622,0.365537405062449,0
.0177137545848147,0.985867207797886,0.997785003279571
"15455","SLC25A24",219.9274988289,-0.00332030467751311,0.1927695218163
39,-0.0172242201268545,0.986257740186918,0.997785003279571
"15456","ZBTB7B",64.4243530523873,0.00404746027064336,0.23732310065681
5,0.0170546409491601,0.986393024922386,0.997785003279571
"15457","AC006059.1",15.5137816499488,0.00786813178572813,0.4581508401
64704,0.0171736709746064,0.98629806661026,0.997785003279571
"15458","SEMA6A",21.4441157782901,0.0102384144270115,0.604771109696141
,0.0169294039726131,0.986492935147826,0.997785003279571
"15459","HIP1",87.0951229753811,-0.00476026758173264,0.270235757402393
,-0.0176152394764117,0.985945799219282,0.997785003279571
"15460","MASTL",33.2480104940056,0.0058124253216472,0.337492371615357,
0.0172223902241905,0.986259200021461,0.997785003279571
"15461","INTS5",60.9910243500835,-0.00473881255036877,0.27282231642097
5,-0.0173695928270641,0.986141766903874,0.997785003279571
"15462","ZNF266",44.2735432043362,-0.00529650847688734,0.3060354553275
99,-0.0173068459378919,0.986191824153458,0.997785003279571
"15463","SLA2",2.81785019583596,0.0178988791623613,1.0428009024646,0.0
171642344382886,0.986305594767265,0.997785003279571
"15464","C9orf85",133.013120946055,0.00394560620024544,0.2350499020735
86,0.0167862490706769,0.986607140004905,0.997835984847175
"15465","TP53I3",488.357973166074,0.00437647475706981,0.26302100666071
2,0.016639259398453,0.986724404416799,0.997890053997978
"15466","TMEM100",252.325571792005,-0.00588092688606375,0.358386496176
349,-0.0164094544543608,0.986907737201665,0.998010927830987
"15467","COPS8",416.097292594381,0.00385089602580812,0.24253940547657,
0.0158774035841368,0.987332197058663,0.998159024234913
"15468","KRBA1",14.4598124975317,0.00610010867488682,0.38770520971136,
0.0157338836881461,0.987446695064998,0.998159024234913
"15469","ASH2L",154.019327732627,-0.00282973741756383,0.17767132710416
2,-0.0159268097091708,0.987292781658233,0.998159024234913
"15470","TCP11L1",44.6990230608446,0.00425961146960749,0.2719034179175
18,0.0156658989512947,0.987500932352098,0.998159024234913
"15471","TIRAP",18.7177624281368,-0.00571514481786983,0.35669171688259

,-0.0160226451789209,0.987216325772656,0.998159024234913
"15472","RAB12",140.333303135117,-0.0041443060486954,0.263350181645286
,-0.0157368642117646,0.987444317245615,0.998159024234913
"15473","ZXDA",16.9914200683495,-0.0058275791406203,0.365027363600726
,-0.0159647733888146,0.987262494875104,0.998159024234913
"15474","ERMARD",46.7815428153935,-0.00422492365252268,0.2733247315236
1,-0.015457524201968З,0.987667171217155,0.998262540896749
"15475","NPR1",595.219106759951,0.00300203527367932,0.198764460656464,
0.0151034810939764,0.987949623768026,0.99835483992461
"15476","RPS10-
NUDT3",7.71315462359463,-0.0155989393115723,1.04951690363537,-0.014862
9710084134,0.988141501512348,0.99835483992461
"15477","ITGB8",35.699154937947,-0.00781580636748655,0.516465915493263
,-0.0151332471960359,0.987925876568802,0.99835483992461
"15478","STAM",220.946670711384,0.00222824035452756,0.14957214795426,0
.0148974283314362,0.988114011589885,0.99835483992461
"15479","EHBP1L1",139.051634771697,-0.00298599231340347,0.200496037952
604,-0.0148930240412498,0.988117525315213,0.99835483992461
"15480","BTG1",2472.69127223741,-0.00392760935050236,0.261523359333001
,-0.0150181970762363,0.988017662851991,0.99835483992461
"15481","AC015917.2",3.56057332076161,0.0137083011128773,0.92730347245
955,0.0147829718317757,0.988205324608241,0.99835483992461
"15482","HERC3",119.381049191836,-0.00356346663294574,0.24458218748342
8,-0.0145696081534441,0.988375545859433,0.998357887838119
"15483","AC009812.4",4.80255171293074,-0.00946739174268075,0.662096202
970012,-0.0142991180136847,0.988591343283181,0.998357887838119
"15484","CACFD1",10.0676854316019,-0.00735096861650443,0.5113364002572
71,-0.0143759932068319,0.988530012057591,0.998357887838119
"15485","SMARCD1",159.533110343115,0.00267083300870459,0.1841095339520
02,0.0145067610100023,0.988425685325879,0.998357887838119
"15486","KIAA0100",184.647759980747,0.00222248003594531,0.153213607258
327,0.0145057614380039,0.988426482785034,0.998357887838119
"15487","KCNJ15",49.8106578013985,0.00474295893338,0.331547503317368,0
.014305518472989,0.98858623697781,0.998357887838119
"15488","UBXN4",1306.16544920898,0.00168349345838592,0.122358110685966
,0.0137587402171208,0.989022459952508,0.99836619076567
"15489","ZDHHC3",337.781122822052,-0.00265764527623255,0.1871629090498
89,-0.0141996365077022,0.988670709982952,0.99836619076567
"15490","NADK2",120.117073300655,0.00318313704784572,0.230577573256423
,0.0138050591950058,0.988985506264857,0.99836619076567
"15491","ERICD",2.73902981362844,-0.0124482663584041,0.895961454845843
,-0.0138937521152023,0.988914746339619,0.99836619076567
"15492","ZNF839",29.4249616012522,0.00524358556063768,0.37411813049102
,0.014015855242716,0.988817331624975,0.99836619076567
"15493","CCL4",257.687026904406,0.00860429289353377,0.626736356861969,
0.0137287278762875,0.989046404074379,0.99836619076567
"15494","COIL",156.347896367623,-0.0030353144591799,0.216981259963355,
-0.0139888323060366,0.988838890695338,0.99836619076567
"15495","PRDM16-
DT",4.50497819005754,0.0148785549468673,1.10598509353812,0.01345276264

01092,0.989266572140566,0.998515046403864
"15496","THRAP3",892.816718318709,-0.00182806166018756,0.136923755966947,-0.0133509459134969,0.989347802839488,0.998515046403864
"15497","SMARCAL1",85.0628948655095,0.00279588624581363,0.210794793730679,0.0132635450635739,0.98941753245399,0.998515046403864
"15498","PPP3CA",446.817441002736,0.00327958797827879,0.251871542477975,0.013020875427265,0.989611138089421,0.998515046403864
"15499","MANEA",81.1163777285108,-0.00330954351779524,0.251698376133938,-0.013148847317291,0.989509040034884,0.998515046403864
"15500","LINC00645",24.3456318750358,0.00909683305619125,0.703555859603542,0.0129297950290932,0.989683803615672,0.998515046403864
"15501","LRFN5",12.8386518162191,0.00728707026607357,0.573684169453308,0.012702233483308,0.989865356548628,0.998515046403864
"15502","FKBP3",520.755483342168,0.00236701061337869,0.186232814782889,0.0127099545595021,0.989859196518412,0.998515046403864
"15503","TENT4B",164.253008167926,0.00243772195160726,0.185572253008307,0.0131362416098819,0.989519097065608,0.998515046403864
"15504","SCARF2",19.5242844639705,0.007539288094199,0.595349407944739,0.0126636358306395,0.989896150542961,0.998515046403864
"15505","TTLL12",37.1397211928593,-0.00336696623697138,0.26537468182716,-0.0126875940605529,0.989877036176836,0.998515046403864
"15506","LYSMD1",30.6005132853763,-0.00537684486874324,0.443284797724392,-0.0121295494371685,0.990322257083055,0.998731596095216
"15507","POLR2D",87.1298424708438,0.0024808209148111,0.203632679306246,0.0121828231267348,0.990279753969178,0.998731596095216
"15508","KLHDC1",68.4991535926508,0.00274533495350387,0.223580805256133,0.0122789384820349,0.990203070747064,0.998731596095216
"15509","RCCD1",46.8906733268424,-0.00328855563761444,0.274175275277355,-0.0119943551959153,0.990430118634067,0.998731596095216
"15510","ANKRD40",166.571119733497,-0.00250006095673362,0.207448989475829,-0.0120514491926456,0.990384567507898,0.998731596095216
"15511","AL137796.1",10.9630823409118,-0.00669742420363564,0.578491874705361,-0.0115773868164471,0.990762788158153,0.998745085839092
"15512","RBPMS",269.439116004859,0.002699937567991,0.228993065476334,0.01179047742068,0.990592778060104,0.998745085839092
"15513","AC005899.8",2.55313097236111,-0.0122692612893816,1.03193133559317,-0.0118896101573745,0.990513687123998,0.998745085839092
"15514","GTPBP3",63.3547760476302,-0.00282404843387274,0.243741238832509,-0.0115862561764254,0.990755711907378,0.998745085839092
"15515","BCOR",19.8630525095886,-0.00533360993923374,0.454853124379863,-0.0117260048427841,0.99064421617865,0.998745085839092
"15516","TMCO4",73.7415050586117,0.00211351513408005,0.200981231164356,0.0105159826210423,0.991609614468327,0.998850940514322
"15517","DARS2",28.6108856113236,0.00287609503850603,0.294221055337282,0.00977528625614167,0.992200574232937,0.998850940514322
"15518","AC104794.3",82.3975644382376,0.00267742518907688,0.252107853436401,0.010620157811753,0.991526499333527,0.998850940514322
"15519","AC026329.1",4.73004446672875,0.00654176738071719,0.749844544204428,0.00872416480359658,0.99303921189624,0.998850940514322
"15520","GOLIM4",1135.95376869851,-0.00241256252855361,0.2487791907666

73,-0.00969760582112479,0.992262551314964,0.998850940514322
"15521","SMIM14",2668.45049522787,-0.00235607224998184,0.212092918073656,-0.0111086794947279,0.991136738432448,0.998850940514322
"15522","PFDN1",586.915540370194,-0.00218444186283918,0.206857888031549,-0.0105601090856444,0.991574408598368,0.998850940514322
"15523","PRR7",6.54148671495064,-0.0067139528011497,0.612905278808979,-0.0109543073510421,0.991259902088033,0.998850940514322
"15524","ZNF311",3.3105708663965,-0.00771422213555269,0.819444515763413,-0.00941396517660008,0.992488853472884,0.998850940514322
"15525","CCHCR1",50.0655292685284,-0.00304125482109895,0.297342144116787,-0.0102281324099972,0.991839273353116,0.998850940514322
"15526","SMIM29",119.511715633102,-0.00239018428246814,0.264119195350377,-0.00904964245138392,0.992779528562045,0.998850940514322
"15527","CYB5R4",82.2690436687796,-0.00244806843622394,0.229648999683625,-0.010660043978404,0.991494676578374,0.998850940514322
"15528","FAXC",3.47411248257249,-0.0119083727920458,1.28303805102063,-0.00928138708167926,0.992594630866379,0.998850940514322
"15529","AL606970.4",53.8576621703062,-0.00415777775358398,0.371832104099514,-0.0111818686653028,0.991078345550052,0.998850940514322
"15530","AC005076.1",12.6775764056531,0.00510396833927398,0.470553375830883,0.0108467362076866,0.991345726343433,0.998850940514322
"15531","SDCBP",3831.5314788775,-0.0025352053729633,0.269006671189528,-0.00942432156701843,0.992480590635412,0.998850940514322
"15532","KYAT1",11.0789223967865,0.00421785148123057,0.477105493518054,0.00884050076667366,0.992946392807095,0.998850940514322
"15533","SH3GLB2",430.556422044039,0.00148171960780357,0.164632203204255,0.00900018088177584,0.992818991577639,0.998850940514322
"15534","ZNF384",90.3054626048304,0.00227383407924335,0.207487856047805,0.0109588778955789,0.991256255539997,0.998850940514322
"15535","CUL4A",195.756472471554,-0.00166241545759338,0.189802083382149,-0.00875867866132038,0.993011674874135,0.998850940514322
"15536","PRKD1",55.5216878507678,-0.00234824310248311,0.262570482969548,-0.00894328667840189,0.992864384757174,0.998850940514322
"15537","GCH1",14.9814389884899,-0.00724897497050208,0.697552676138642,-0.0103920108379906,0.991708524235059,0.998850940514322
"15538","CORO1A",32.05462474525,0.00462843844599894,0.479951361056564,0.0096435572884092,0.99230567378829,0.998850940514322
"15539","CHRNB1",63.2246311504147,0.0031476404731335,0.314027030014703,0.0100234698681388,0.992002562063793,0.998850940514322
"15540","TMEM220-AS1",17.6214850648774,-0.00442925877011778,0.486788883495085,-0.00909893163195561,0.992740203104971,0.998850940514322
"15541","AC005332.1",3.16726455727704,-0.00789790152892313,0.858433922777026,-0.0092003604696486,0.992659277988826,0.998850940514322
"15542","PRCD",16.7596121135674,-0.00570255230740016,0.614987812166768,-0.00927262653760328,0.992601620468463,0.998850940514322
"15543","PRELID3A",11.61532656204,0.00430684242651396,0.476834182066799,0.00903215958186199,0.992793477303633,0.998850940514322
"15544","GMIP",12.7342900400482,0.00514863508801657,0.456465411165208,0.0112793542776303,0.991000568190159,0.998850940514322

"15545","ZFP28",10.4914550763054,0.00562167338494342,0.508871895356653
,0.0110473253410927,0.991185688960618,0.998850940514322
"15546","PDE9A",10.337275671513,-0.00627680780803828,0.690068316551979
,-0.00909592232751854,0.992742604083162,0.998850940514322
"15547","AJ011932.1",13.107881342312,0.00526089150926598,0.53667312101
7797,0.00980278553784982,0.992178634031867,0.998850940514322
"15548","SGSM3",152.744634650666,-0.00185688433495095,0.18238836035642
7,-0.0101809366086859,0.991876928193715,0.998850940514322
"15549","TEF",66.7741104479972,-0.00787646049608506,0.879459877251051,
-0.00895602027997537,0.992854225219954,0.998850940514322
"15550","TYW3",250.313994826417,-0.00166516856004432,0.19469030397996,
-0.00855290954918702,0.993175848721212,0.998859898012974
"15551","TTC13",29.1371017462136,-0.00257499961056649,0.29996405415890
6,-0.00858436060876274,0.993150755327571,0.998859898012974
"15552","GPR89A",49.3736747514902,0.00199968042788129,0.25674627234551
9,0.00778854707261417,0.993785701367815,0.998882349733647
"15553","THEM4",81.467613549229,0.00200975206146279,0.243119738624579,
0.00826651127889789,0.993404353398098,0.998882349733647
"15554","PYCR2",144.849431389896,0.0016893063718118,0.231831434018425,
0.0072867873977674,0.994186036288448,0.998882349733647
"15555","AC079807.1",12.2741288649846,-0.00435266757699804,0.516926704
78431,-0.00842027996757143,0.993281668005869,0.998882349733647
"15556","FANCD2",6.66425780888668,0.00447940505564445,0.61220386651514
8,0.00731685195185486,0.994162048884387,0.998882349733647
"15557","THAP9",16.9096138365732,0.00281580249603188,0.396635794478113
,0.00709921427978246,0.994335694111448,0.998882349733647
"15558","KLKB1",2.72482765482431,-0.0077058749234595,0.923075539635204
,-0.00834804367853235,0.993339302199901,0.998882349733647
"15559","RAPGEF6",84.9972895597188,0.00197418900023642,0.2695005126915
74,0.00732536268862593,0.994155258480897,0.998882349733647
"15560","C6orf62",505.363186339945,-0.00111442600668427,0.159112407936
491,-0.00700401697854443,0.994411648679852,0.998882349733647
"15561","FAM185A",25.5966306458284,-0.00250646802858368,0.350200746505
219,-0.00715723211214322,0.99428940375482,0.998882349733647
"15562","DDX31",23.881952996541,-0.00230500566827032,0.329057038069017
,-0.00700488183385053,0.994410958642084,0.998882349733647
"15563","AL133330.1",125.740890370203,-0.0079289553760213,1.0740726320
4632,-0.00738214077842678,0.994109957344565,0.998882349733647
"15564","BORCS5",107.910228022139,0.00151634919520945,0.19992382642684
2,0.00758463471968574,0.99394839507935,0.998882349733647
"15565","MIS18BP1",173.890123484189,0.00158664799782518,0.220086149255
858,0.00720921331573959,0.994247929825037,0.998882349733647
"15566","NUDT16L1",164.460756948266,0.00172836090154661,0.218787098710
777,0.00789973865795165,0.993696986048023,0.998882349733647
"15567","CHTF8",9.81268312889798,0.00401924030990508,0.491632939302454
,0.00817528686260876,0.993477137491866,0.998882349733647
"15568","PITPNM3",2.84320201052175,0.00890930663900083,1.1436644364330
5,0.00779014049504579,0.993784430039227,0.998882349733647
"15569","GID4",80.9109058310667,-0.00153520540981733,0.206760652070558
,-0.00742503660364466,0.994075732365905,0.998882349733647

"15570","BACE2",293.742110266573,0.00117344091839276,0.162052239921866
,0.00724112742260483,0.994222466716534,0.998882349733647
"15571","HESX1",3.15972445552087,-0.00575474609203718,0.83242572610527
6,-0.00691322470169475,0.994484088681885,0.998890960566739
"15572","SLC35F6",398.82359388985,0.00151343014372898,0.22236348328213
6,0.00680610917489868,0.994569552496122,0.998912650978638
"15573","MSH6",117.831583914061,-0.00143771723461117,0.217344075968503
,-0.00661493637774382,0.99472208288484,0.998937548241871
"15574","TBCA",1276.17104110411,0.00138636411308671,0.208244301568328,
0.00665739279608486,0.994688208310045,0.998937548241871
"15575","BUD13",60.6466379086359,-0.00146825273945836,0.23229894542734
,-0.00632053123081271,0.994956979292254,0.999045143562587
"15576","AC007336.1",7.10690557158111,0.00401839882237884,0.6305203317
21546,0.00637314709805974,0.994914998749675,0.999045143562587
"15577","CERCAM",807.483260699802,-0.00183261829750094,0.2965328055342
11,-0.00618015363999754,0.995068982216738,0.999093463559721
"15578","RPS27",26709.7817930072,-0.00129670907392719,0.22812380671217
2,-0.00568423389305997,0.995464661959933,0.999189804322473
"15579","BARD1",33.6364640925303,-0.00177615547093571,0.30547405193749
1,-0.00581442338447511,0.995360787491615,0.999189804322473
"15580","ACOT13",498.058553999274,0.00169079729350822,0.29391454107196
2,0.00575268337300211,0.995410048069668,0.999189804322473
"15581","PPP2CB",513.57153511711,0.000784760998049069,0.13866175952323
,0.00565953440045305,0.99548436898675,0.999189804322473
"15582","KIZ",144.136842739799,0.00121712304266414,0.207683407485636,0
.0058604731952326,0.995324045684596,0.999189804322473
"15583","RCAN2",42.6216093978547,-0.00242011521872196,0.43456047505863
5,-0.00556911030253136,0.995556515841316,0.999198094574741
"15584","FBXO8",177.759815988526,-0.00114847018639426,0.21322121647683
3,-0.00538628474863353,0.995702387339423,0.999242328651699
"15585","PROCR",3948.92448416868,-0.00132860406986926,0.24816460613672
2,-0.00535372102634685,0.995728369056057,0.999242328651699
"15586","AL161729.1",7.7052071302333,0.00323435960115865,0.62509708001
5205,0.0051741716679113,0.995871626732012,0.999257858605804
"15587","ABCD1",34.0363610339715,0.00176015452241298,0.33684675067548,
0.00522538667475147,0.995830763621267,0.999257858605804
"15588","AL606760.1",5.04702197149019,-0.00268346223779305,0.679749380
617157,-0.00394772296130182,0.996850180980254,0.999319719817203
"15589","RO60",455.697134077452,-0.000595285381120341,0.12713576670125
5,-0.00468228097069764,0.996264093954992,0.999319719817203
"15590","LYPLAL1",238.436397071696,0.00102800154839749,0.2875243413772
67,0.00357535484986513,0.997147285643687,0.999319719817203
"15591","OPA1",171.197318012874,-0.000633955974780986,0.17668004568623
7,-0.00358815831362653,0.997137070023156,0.999319719817203
"15592","AC008608.2",28.1498741732049,-0.00189738522405682,0.410000384
349625,-0.00462776450092016,0.996307591333252,0.999319719817203
"15593","SAYSD1",178.112867394951,-0.00125174693296483,0.2916318631898
85,-0.00429221594400954,0.9965753176822,0.999319719817203
"15594","OTUD6B-
AS1",441.284550506629,-0.000767414479078753,0.175413996289703,-0.00437

487598088431,0.996509365134286,0.999319719817203
"15595","DSCC1",45.9591636170165,-0.00133950666316814,0.32630299137499
5,-0.00410510077619469,0.996724612669528,0.999319719817203
"15596","POLR1E",145.831752798837,-0.00131179652571913,0.2645351829152
17,-0.00495887356556107,0.996043407558788,0.999319719817203
"15597","ACCS",71.080853811231,0.00183696184625495,0.375539354208046,0
.0048915295445635,0.996097139661684,0.999319719817203
"15598","CIPC",27.60959637946,0.00139340617805866,0.338161746312994,0.
0041205316486891,0.996712300718735,0.999319719817203
"15599","TMEM256",531.703388618105,0.000869751775068186,0.219651658268
163,0.0039596868146852,0.99684063528096,0.999319719817203
"15600","KCTD1",19.4896405195776,0.00141741211705244,0.38300628459884,
0.00370075420181952,0.997047232099022,0.999319719817203
"15601","ATP8B1",30.3244812679869,-0.00158258615717791,0.4127164831392
52,-0.00383456009592895,0.996940471199799,0.999319719817203
"15602","AC104971.1",2.64541790156442,0.00549257906509868,1.1998804191
7763,0.004577605382429,0.996347612095559,0.999319719817203
"15603","ZNF573",43.2917524000176,0.00100029402391566,0.27748620104463
8,0.00360484240351379,0.997123758131517,0.999319719817203
"15604","TEAD2",77.5225318527797,0.00127028150056493,0.277074130553632
,0.00458462685789802,0.996342009827475,0.999319719817203
"15605","TSKS",42.2280284614327,-0.00152914658974091,0.342470088894258
,-0.00446505151640574,0.99643741616956,0.999319719817203
"15606","RBBP9",65.5871286873379,-0.000975360752608234,0.2326370342924
81,-0.00419262889752097,0.996654775933963,0.999319719817203
"15607","DENND10",288.419536947913,0.000683384569600824,0.197238612781
147,0.0034647605758569,0.997235526560697,0.999344117089082
"15608","BCAR3",6.72852627246617,-0.00203024948388581,0.63103939614089
7,-0.00321731019695719,0.997432962295139,0.999421688895211
"15609","ASH1L-
AS1",14.5805185364566,0.00135776802836146,0.445551657305204,0.00304738
63268146,0.997568541262349,0.999421688895211
"15610","ACOX2",85.4292775894774,0.00111121896612752,0.354309407103859
,0.00313629540691757,0.997497602419113,0.999421688895211
"15611","KIAA0753",39.0732472985049,0.000941460205805806,0.30295482894
8288,0.00310759267008253,0.997520503778081,0.999421688895211
"15612","AC004865.2",3.13383614127065,0.00164935486866763,0.7974548749
6387,0.00206827360450001,0.998349757600009,0.999424365579023
"15613","C1orf198",47.8196253900971,0.000652301380512842,0.29261713605
124,0.00222919747392585,0.99822135922568,0.999424365579023
"15614","COX18",50.5654754534047,-0.000595096249927462,0.2430452489943
79,-0.00244849982622464,0.998046381743567,0.999424365579023
"15615","DDX41",118.151556950598,-0.000551950001851887,0.2085656169528
13,-0.00264640936466898,0.99788847329104,0.999424365579023
"15616","PELI3",42.9602670037588,0.000566673859903214,0.26516089027491
2,0.0021370944233733,0.998294846652568,0.999424365579023
"15617","CBLN3",18.8494961637536,0.000879865055504315,0.43930062891696
4,0.00200287684011175,0.998401936560528,0.999424365579023
"15618","GABPB1",44.449625686538,0.000653879238166318,0.25658558046098
,0.00254838653439356,0.997966683930068,0.999424365579023

"15619","SCAMP5",2.87011023771992,-0.00211481892015068,0.848063674815369,-0.00249370298829401,0.998010314948573,0.999424365579023
"15620","GFOD2",70.9918708674327,0.000478498551903508,0.237772760932859,0.00201241954724421,0.998394322597183,0.999424365579023
"15621","PGS1",77.6673591322666,0.000555394082505909,0.224660757810806,0.00247214550470636,0.998027515278878,0.999424365579023
"15622","GGT7",90.9421156043974,-0.00048831216731545,0.218085908394893,-0.00223908170367089,0.998213472771053,0.999424365579023
"15623","B4GALT5",161.01824877335,0.000395214089776475,0.165067792999575,0.0023942531889155,0.998089664171062,0.999424365579023
"15624","AL050309.1",38.3711093408422,-0.000762458831773969,0.333787762759172,-0.00228426238718669,0.998177423893431,0.999424365579023
"15625","UGDH-AS1",6.60532288387766,-0.00108119685038382,0.594286226119192,-0.00181932005633758,0.998548393416674,0.999455739984623
"15626","EFEMP2",1218.79326052238,-0.000552351311995144,0.306281335068926,-0.0018034115982642,0.998561086508934,0.999455739984623
"15627","KIF23",18.3260209408795,-0.000702380500481937,0.437278809817117,-0.00160625322954865,0.998718395898504,0.999549223257989
"15628","TNNI3K",14.2751507175723,-0.000688710646181824,0.459014676434053,-0.0015004109488061,0.998802845718271,0.999569779052583
"15629","CEP70",26.5976904713588,-0.000438872109511428,0.331390099737817,-0.00132433681591166,0.998943332410157,0.999623386731364
"15630","NUBP1",154.514612064682,0.000238312846860923,0.194728032751367,0.00122382403547005,0.99902352994071,0.999623386731364
"15631","MPP3",4.77930597509952,0.000751746841546678,0.675511987542534,0.00111285492398363,0.999112070421015,0.999623386731364
"15632","TMEM187",100.405323269086,0.000353079800059864,0.313989545050251,0.00112449540319362,0.999102782668185,0.999623386731364
"15633","SEMA3D",208.173686129053,-0.000353229060782899,0.355577119025764,-0.00099339648667693,0.999207384410888,0.999631983267133
"15634","MARCH9",80.9203629214098,-0.00023160287057689,0.245895597176318,-0.000941874816940381,0.999248492736468,0.999631983267133
"15635","HIST1H2BJ",3.51668414200851,0.000571548575558373,0.813216139310435,0.000702824929228553,0.999439226886188,0.99970945718378
"15636","ATAD1",227.454205428712,-9.53044709061103e-05,0.181752080162966,-0.000524365227735807,0.999581617099741,0.99970945718378
"15637","CBX8",9.28531082668989,-0.000289084935847451,0.534435278965742,-0.000540916640845452,0.999568410984635,0.99970945718378
"15638","KMT2B",134.363476481115,-0.000165855174343273,0.26165503213388,-0.000633869614471661,0.999494245254919,0.99970945718378
"15639","PALM",1001.74115275314,-4.11965807018126e-05,0.121939667696685,-0.000337843963986237,0.999730439522303,0.99979436499321
"15640","AC127070.2",3.67474936180411,5.84560459765139e-05,0.829164957213774,7.04998992877635e-05,0.999943749218867,0.999943749218867
"15641","AL627309.4",0,NA,NA,NA,NA,NA
"15642","LINC02593",0.39105863032616,-2.36094068777937,2.89444947621853,-0.815678666073604,0.414683943869914,NA
"15643","PLEKHN1",0.697247977452859,1.30340710451565,1.84743374188958,0.705523058803998,0.480484787210143,NA

"15644","AL645608.7",1.6218265776459,-1.38234442387203,1.2581302974440
7,-1.09872914330122,0.271886226753809,NA
"15645","AL390719.2",0,NA,NA,NA,NA,NA
"15646","TTLL10",0,NA,NA,NA,NA,NA
"15647","C1QTNF12",2.37424360407974,-1.26177337395704,0.97389359920871
1,-1.29559674176135,0.195114452251315,NA
"15648","AL162741.1",0.244438889809347,0.987541826011378,3.31639350116
213,0.297775829576715,0.765874263356537,NA
"15649","LINC01786",1.01352575587411,-0.210687700817314,1.377807217365
28,-0.152915225121408,0.878465129206843,NA
"15650","TAS1R3",0.182244833681627,-0.893803921161021,3.34011187015073
,-0.267597001510217,0.789009539265076,NA
"15651","AL691432.4",1.3095909430023,0.0259675874410671,1.484238559715
88,0.017495561795698,0.9860412734796,NA
"15652","FO704657.1",1.64418439329524,1.77788802098719,1.3896831594424
1,1.27934774837492,0.200774625221897,NA
"15653","AL109917.1",0.951754277049571,-1.75881745381838,1.66520413777
197,-1.05621732130191,0.290868938965974,NA
"15654","CALML6",0.37277280500713,1.32395835315804,3.07648417780955,0.
430347850545649,0.666942624170393,NA
"15655","TMEM52",1.35233489315869,0.375166184854776,1.2296058102558,0.
305110940209959,0.760281659155462,NA
"15656","CFAP74",0.0814810698691117,-0.172465128888762,3.3401119144486
9,-0.0516345359994407,0.958819900285368,NA
"15657","PRKCZ",1.51740590691604,0.562569849683605,1.27451641757842,0.
441398668486742,0.658924406002777,NA
"15658","AL590822.2",0.160441014831806,-0.819797003458808,3.3393523523
1193,-0.24549580785964,0.806072553724142,NA
"15659","PRKCZ-
AS1",2.33483351104882,-0.639074898721821,1.14225432908245,-0.559485643
82783,0.575830326227777,NA
"15660","AL590822.1",0.594133409909803,1.03802105907226,1.853508821270
55,0.560030277255825,0.575458786011955,NA
"15661","AL590822.3",1.64287072299464,0.0412363262480165,1.13217544457
56,0.0364222050969087,0.970945708817282,NA
"15662","PLCH2",0.703099203890974,-0.350471317539936,2.03125356098427,
-0.172539423079269,0.863013470927434,NA
"15663","AL139246.1",0,NA,NA,NA,NA,NA
"15664","HES5",0.40608427471248,0.298735989724419,2.28392316074513,0.1
30799492232899,0.895933924685244,NA
"15665","AL139246.3",0.482201607145061,0.71305380742368,2.094810270441
74,0.340390639422117,0.733562366952099,NA
"15666","MMEL1",0.103280568920944,0.158353271622336,3.33696113528856,0
.047454335007903,0.96215112468834,NA
"15667","TTC34",1.99310316339299,0.856049728383267,1.14324232678862,0.
748791142808647,0.453983099024113,NA
"15668","AL354743.1",1.6670462085148,-2.11611873250354,1.5317509543227
2,-1.38150312655702,0.167124317160038,NA
"15669","AL513320.1",0.504284308494148,1.79267362885552,2.069318666522
1,0.866311051003306,0.386319607637693,NA

"15670","TP73",0.26043840714347,-0.221777593548505,2.87432468312725,-0
.077158156435971,0.938497728695292,NA
"15671","AL365330.1",0.0543178333807489,-0.0984569371683306,3.33935238
193321,-0.0294838417475822,0.976478705757268,NA
"15672","LINC01134",0.108635666761498,0.317965188909593,3.335651221681
85,0.095323272062381,0.92405805838549,NA
"15673","AL365255.1",0,NA,NA,NA,NA,NA
"15674","CHD5",0.227126937438121,0.554229032074634,3.33087194591576,0.
166391575861755,0.867848803034167,NA
"15675","AL031847.1",0.777937700665424,-0.46927278228989,1.57525291407
81,-0.297903135487629,0.765777094776798,NA
"15676","LINC00337",0.160441014831806,-0.819797003458808,3.33935235231
193,-0.24549580785964,0.806072553724142,NA
"15677","HES2",0.265793504984024,-0.111760333164042,2.80521572110529,-
0.0398401920833407,0.968220533004581,NA
"15678","ESPN",0.378691776364091,-0.566961236770659,2.53220200834996,-
0.223900476700161,0.822834744793225,NA
"15679","PLEKHG5",1.46002419711034,-3.63294624005321,1.52424364460729,
-2.38344194703152,0.0171515894573111,NA
"15680","CAMTA1-
DT",0.673893822109934,-0.388988330583385,2.01558496207667,-0.192990292
099921,0.846966570953452,NA
"15681","Z98884.2",0.447445350206982,0.442973996145131,2.1933643399307
3,0.20196097295861,0.839947234512298,NA
"15682","Z98884.1",0,NA,NA,NA,NA,NA
"15683","UTS2",2.422299213627,-0.611502402746877,1.06198518650267,-0.5
75810670919694,0.564743160036041,NA
"15684","TNFRSF9",0.605051107037864,-0.576570240092695,2.0191926862534
9,-0.28554493289221,0.775226685110988,NA
"15685","AL034417.3",49.5947612190552,1.41297289247627,1.3791796617743
6,1.02450241374531,NA,NA
"15686","ENO1-
AS1",1.21545427718508,0.463927262791155,1.31974082872339,0.35152906744
5706,0.725191469508787,NA
"15687","CA6",0,NA,NA,NA,NA,NA
"15688","BX323043.1",1.25921001946624,-0.522311851934936,1.35760371070
437,-0.384730719146268,0.700436933214379,NA
"15689","PIK3CD-AS1",0,NA,NA,NA,NA,NA
"15690","AL354956.1",0.484899149824861,-0.309078067968908,2.3798856301
5317,-0.129870975332968,0.896668508101121,NA
"15691","AL139423.2",0.471570147045456,1.70931642166734,2.881174101929
06,0.593270785171532,0.552999947138789,NA
"15692","C1orf127",0.212246362902114,-2.18436561798774,3.3278803578472
2,-0.656383458268551,0.51157743097293,NA
"15693","MASP2",0,NA,NA,NA,NA,NA
"15694","AL109811.1",1.23205539990566,0.478137443107886,1.449824710903
21,0.329789828737306,0.741558773315222,NA
"15695","DISP3",0,NA,NA,NA,NA,NA
"15696","DRAXIN",0.985187720437975,-0.122501348031625,1.48253106076905
,-0.0826298694666659,0.934145850082407,NA

"15697","AL096840.2",1.41357214741288,-0.667060241281481,1.23354237343035,-0.540767999259284,0.58866750235491,NA
"15698","AADACL4",0,NA,NA,NA,NA,NA
"15699","KAZN-AS1",0.37277280500713,1.32395835315804,3.07648417780955,0.430347850545649,0.666942624170393,NA
"15700","TMEM51-AS1",1.46485586094173,-0.0511140342321834,1.2727511005999,-0.0401602750200657,0.967965348004347,NA
"15701","TMEM51",1.69378272241854,0.0213211601473706,1.23978185873573,0.0171975093821045,0.986279049121527,NA
"15702","AL031283.1",1.19656243986539,-1.69059510785338,1.46539581581444,-1.15367813228932,0.248632152434956,NA
"15703","CTRC",0.524122021915971,-0.43626830241685,2.42986386151119,-0.179544339634536,0.85751030493431,NA
"15704","CELA2A",0.0814810698691117,-0.172465128888762,3.34011191444869,-0.0516345359994407,0.958819900285368,NA
"15705","AL121992.3",0.103280568920944,0.158353271622336,3.33696113528856,0.047454335007903,0.96215112468834,NA
"15706","AGMAT",1.15622191004295,1.03448772236963,1.45486129839391,0.711055908567812,0.477049591064323,NA
"15707","AL121992.1",0.904864747807437,-0.565997251050964,1.59394681380659,-0.355091679438962,0.722520909861052,NA
"15708","AL121992.2",0.319073466028949,-0.315882173113154,2.64758835416815,-0.119309398160728,0.905030238029845,NA
"15709","AL450998.3",0.308608007307233,1.09138513794226,3.21893777835761,0.339051330932875,0.73457106136617,NA
"15710","HSPB7",1.94353030305097,0.532889005815595,1.27906084536067,0.416625219784076,0.676952559531832,NA
"15711","CLCNKB",0.258366485912196,-0.234297029349722,3.33313776462557,-0.0702932329519363,0.943960268507764,NA
"15712","FAM131C",0.600955652244462,-3.32160226945334,2.51303051565943,-1.32175166547142,0.186250858188948,NA
"15713","AL451042.2",0.297718850854272,0.964807331370471,2.68623467910202,0.359167178830777,0.719470028199226,NA
"15714","SPATA21",1.86620330473299,0.436418664518556,1.18533355071004,0.368182157888918,0.71273741331001,NA
"15715","AL137802.2",1.69878647878725,-0.615542915595511,1.14129005665813,-0.539339593825881,0.589652553491464,NA
"15716","AL137798.1",0.655485420821475,2.14949994066725,2.25741183291789,0.952196630372422,0.340997269611689,NA
"15717","BX284668.2",0.228364956691709,0.894097963350471,3.31920276856316,0.26937129958394,0.787643969776493,NA
"15718","BX284668.6",1.58833871730227,0.598058705409203,1.25293968787732,0.477324416486806,0.633131126907221,NA
"15719","PADI2",0.108639986559486,0.158353243860761,3.33696113552764,0.0474543266850853,0.962151131321515,NA
"15720","PADI4",0.545577589266514,1.84504665440883,2.39714391115998,0.769685393446406,0.441486536595641,NA
"15721","PADI6",0,NA,NA,NA,NA,NA

"15722","IGSF21",1.3010745554193,-1.92517548348074,1.50682730748736,-1
.27763511711968,0.201378112382669,NA
"15723","PAX7",0,NA,NA,NA,NA,NA
"15724","HTR6",0.447897588613326,-0.193327973477887,2.89366921944241,-
0.06681066798476,0.946732430563418,NA
"15725","PLA2G2D",0,NA,NA,NA,NA,NA
"15726","Z98257.1",0.0543178333807489,-0.0984569371683306,3.3393523819
3321,-0.0294838417475822,0.976478705757268,NA
"15727","PLA2G2C",0.0814810698691117,-0.172465128888762,3.340111914448
69,-0.0516345359994407,0.958819900285368,NA
"15728","UBXN10-
AS1",1.46049959583197,-1.10541711808645,1.36176973759022,-0.8117503918
41278,0.416934872747709,NA
"15729","KIF17",0.464096519397402,1.61543785725421,2.90255333716689,0.
556557509751396,0.577829791433073,NA
"15730","AL031005.1",0,NA,NA,NA,NA,NA
"15731","AL031728.1",0.357892230471124,-1.49336789435882,3.04455030095
008,-0.490505246010486,0.623776418525306,NA
"15732","LINC02596",0,NA,NA,NA,NA,NA
"15733","RAP1GAP",0.254285854128495,0.774189186800036,2.84671923849372
,0.271958392078624,0.785654012208812,NA
"15734","LINC01635",0.626405722212542,-1.52887744794794,1.978802232761
35,-0.772627715208532,0.439742737240506,NA
"15735","TEX46",0.276085353180328,0.94030842345474,2.73997410419095,0.
343181500152313,0.731461921261812,NA
"15736","AL109936.6",0.161919947604411,-0.172465230976139,3.3401119155
193,-0.0516345665469491,0.958819875944452,NA
"15737","AL109936.2",1.31498508044788,1.44504039930407,1.4283140694064
1,1.01171054059883,0.311676474201292,NA
"15738","E2F2",0.0814810698691117,-0.172465128888762,3.34011191444869,
-0.0516345359994407,0.958819900285368,NA
"15739","MDS2",0.631498697719982,-0.160484934811068,1.80803955335488,-
0.08876184954764,0.929271177621879,NA
"15740","CNR2",0.129994601734164,-1.12621966659706,3.34272090207402,-0
.33691705038799,0.736179431667661,NA
"15741","MYOM3",0.311794551538636,-2.55129968274416,3.07846480580536,-
0.828757138276498,0.407241846346242,NA
"15742","IFNLR1",0.290439936363958,-1.49465207780988,2.72633900900541,
-0.548226787964694,0.583536190545527,NA
"15743","RCAN3AS",0.108639986559486,0.158353243860761,3.33696113552764
,0.0474543266850853,0.962151131321515,NA
"15744","NCMAP-
DT",0.63646830854882,-1.95731465137301,2.29643072750179,-0.85232906350
3827,0.394031479411486,NA
"15745","AL031432.1",0.145645867569009,-0.172465240332149,3.3401119156
1742,-0.051634569346539,0.958819873713678,NA
"15746","AL031432.5",0.276085353180328,0.94030842345474,2.739974104190
95,0.343181500152313,0.731461921261812,NA
"15747","RHD",0.950077342886343,1.13350639446873,1.77239218764498,0.63
9534749910425,0.522475114906059,NA

"15748","RHCE",1.92052132542065,-0.201658317687211,1.07222580113387,-0
.188074487177943,0.850818260479321,NA
"15749","AL606491.1",1.54390069980709,-1.37407884819642,1.144650491732
2,-1.20043529280018,0.229970329011013,NA
"15750","AUNIP",0.189612912069306,-1.5929471954674,3.34910741318451,-0
.47563335508333,0.634335608031733,NA
"15751","SLC30A2",0,NA,NA,NA,NA,NA
"15752","TRIM63",0.514724261271966,0.77578372518459,1.92821799959079,0
.402331959015645,0.687439737404259,NA
"15753","FAM110D",0.0814767500711233,0.132145805215161,3.3371886294311
4,0.0395979430259797,0.968413667381931,NA
"15754","CNKSR1",1.25147426948507,0.0479922299042071,1.4173543800912,0
.0338604307986257,0.97298844672942,NA
"15755","CRYBG2",0.0271589166903744,-0.404880044879902,3.3427209427763
7,-0.121122897128116,0.903593693408407,NA
"15756","ZNF683",1.39891480734416,0.436932034786417,1.88178165521657,0
.232190612324856,0.816389968816546,NA
"15757","AL513365.2",0.0814810698691117,-0.172465128888762,3.340111914
44869,-0.0516345359994407,0.958819900285368,NA
"15758","AL512408.1",0.133282098141432,-1.12621966659706,3.34272090207
402,-0.33691705038799,0.736179431667661,NA
"15759","SFN",0.911732262964136,0.289357512644088,1.50935782930529,0.1
9170902156268,0.847970138882496,NA
"15760","TRNP1",1.12585602124669,-0.594683484448953,1.56148633904782,-
0.380844500254537,0.703318636710143,NA
"15761","TENT5B",1.09786324275495,-0.290648534973506,1.54163002571179,
-0.188533260332232,0.850458645004493,NA
"15762","FCN3",0.0271589166903744,-0.404880044879902,3.34272094277637,
-0.121122897128116,0.903593693408407,NA
"15763","GPR3",0.0271589166903744,-0.404880044879902,3.34272094277637,
-0.121122897128116,0.903593693408407,NA
"15764","BX293535.1",0.0271589166903744,-0.404880044879902,3.342720942
77637,-0.121122897128116,0.903593693408407,NA
"15765","LINC02574",1.66714686284003,-0.775729113282867,1.253736426127
81,-0.618733808092922,0.536091734147014,NA
"15766","AL445490.1",0.189612912069306,-1.5929471954674,3.349107413184
51,-0.47563335508333,0.634335608031733,NA
"15767","AL020997.3",1.3991091782924,-0.620807190203631,1.457762061240
17,-0.425863182140634,0.670207544479887,NA
"15768","SMPDL3B",0.0271589166903744,-0.404880044879902,3.342720942776
37,-0.121122897128116,0.903593693408407,NA
"15769","MATN1",0.70601860409832,1.26301056672102,1.98834570930567,0.6
35206725274179,0.525293597602338,NA
"15770","AL137857.1",0,NA,NA,NA,NA,NA
"15771","LINC01778",0.460168526435215,0.146911777211049,2.401216053809
1,0.0611822401312033,0.951214073555063,NA
"15772","LINC01226",0,NA,NA,NA,NA,NA
"15773","AC114488.2",0.478895986458187,0.538851362430089,2.52180921983
105,0.213676497885986,0.830799340033851,NA
"15774","AC114488.3",0.836580163883382,-2.17689301867458,1.65770579336

416,-1.31319624229386,0.189116829884053,NA
"15775","SPOCD1",0.760989474892841,-2.22759066220126,1.71523464551864,
-1.29870899472631,0.194043816072961,NA
"15776","AL354919.1",0.696824677192944,-2.22722807279096,1.74211949086
006,-1.27845884537542,0.201087686818221,NA
"15777","AL136115.2",1.83900973108009,-1.18688122570662,1.247291383582
19,-0.951566924400556,0.341316662569022,NA
"15778","AL136115.1",0,NA,NA,NA,NA,NA
"15779","AL445248.1",1.14240573460815,1.31057519284713,1.4897117571466
2,0.879750855532882,0.37899429286867,NA
"15780","AL049795.2",2.13339026541312,-0.126283569167861,1.01438558374
178,-0.124492669446304,0.900925204163897,NA
"15781","AL049795.1",1.32218810678098,2.28279354851201,1.6240200048172
4,1.40564373698642,0.159829941682977,NA
"15782","DCDC2B",0.0761216522305695,-0.172465129916519,3.3401119144594
7,-0.0516345363069754,0.958819900040317,NA
"15783","HPCA",2.28772238012164,-0.438040262168586,0.992537343750212,-
0.44133378449368,0.658971371232159,NA
"15784","AL031602.2",0.818352450363005,-2.59285109856702,2.14436183862
714,-1.20914812596507,0.226605942743922,NA
"15785","AL020995.1",0,NA,NA,NA,NA,NA
"15786","AL662907.3",0.108639986559486,0.158353243860761,3.33696113552
764,0.0474543266850853,0.962151131321515,NA
"15787","AL513327.1",0.454253874876242,1.61402870923758,2.890807350345
1,0.558331467174696,0.576618061226143,NA
"15788","GJA4",0.45920205781989,0.691131733812388,2.40789192570582,0.2
8702772181513,0.774091093958518,NA
"15789","DLGAP3",0.190116736630609,0.75633002596515,3.32364755018387,
0.227560237524184,0.819988134779865,NA
"15790","AL590434.1",0.0814810698691117,-0.172465128888762,3.340111914
44869,-0.0516345359994407,0.958819900285368,NA
"15791","AL139286.3",1.12740764280658,1.42977535012389,1.5765469390838
1,0.906903127765279,0.364458027010718,NA
"15792","AL139286.2",0.412677391806621,0.539874968279704,2.48131238184
931,0.217576381042897,0.827759188820758,NA
"15793","TEKT2",0.41040150021721,-0.196503808306448,2.61757426265859,-
0.0750709582951298,0.940158254526615,NA
"15794","AL591845.1",0.524485798317905,-1.9223889106764,2.039085642066
26,-0.94277006861193,0.345798524618682,NA
"15795","AL929472.2",0.736325779693656,1.36037481521203,2.065470892922
56,0.658626960018376,0.51013534608595,NA
"15796","AL442071.2",0.265797824782013,-0.180783873541719,3.3313926647
1278,-0.0542667561997844,0.956722635218719,NA
"15797","PABPC4-
AS1",0.535734944745354,1.86684482059919,2.76670470305506,0.67475391158
94,0.499832126353361,NA
"15798","HEYL",1.47712905441261,-2.8646776349924,1.35168609315871,-2.1
1933647130899,0.0340620415098403,NA
"15799","HPCAL4",0.290439936363958,-1.49465207780988,2.72633900900541,
-0.548226787964694,0.583536190545527,NA

"15800","MYCL",0.726202994741546,0.431427801109218,1.94526680152386,0.221783356797768,0.824482536180973,NA
"15801","COL9A2",2.14810905973595,-0.384660433427752,1.04075149443368,-0.369598732728281,0.711681495482187,NA
"15802","AL031985.3",2.03419781942458,0.579750291127702,1.05267580612867,0.55073963679264,0.58181216900942,NA
"15803","AL603839.2",0,NA,NA,NA,NA,NA
"15804","RIMS3",1.62752670494763,-0.38703496413534,1.29399246350574,-0.299101405186525,0.764862674151993,NA
"15805","KCNQ4",0.91708736080469,0.352897015530175,1.48837608242204,0.237102046786391,0.812577624531698,NA
"15806","SLFNL1-AS1",1.44972530027216,1.30191666979231,1.71076020040198,0.761016458932348,0.446647236548001,NA
"15807","SLFNL1",0.88276046284111,0.840426695024599,1.55660436634007,0.5399102772663,0.589258909957729,NA
"15808","AC093151.2",1.06666879030709,0.0972235276867874,1.43538517579629,0.0677334065630514,0.945997855775129,NA
"15809","EDN2",0,NA,NA,NA,NA,NA
"15810","RIMKLA",0.209403750372001,-0.564164464581827,3.01325682585908,-0.187227474186832,0.851482284420997,NA
"15811","AC098484.4",0.884830871522366,1.25548661127732,1.91977056707071,0.653977424600797,0.513126350200164,NA
"15812","AC098484.2",0.938331790574812,1.72787303375754,1.60618902838259,1.07575945497366,0.282034849589874,NA
"15813","TMEM269",0.990637139766051,1.82565559164045,1.67079841506648,1.09268453643332,0.274532331524878,NA
"15814","CFAP57",0.841358620745354,0.250567432632994,2.58083591540345,0.097087703692245,0.922656745760448,NA
"15815","TIE1",0.900139184835398,-0.89699701272551,1.80947033237649,-0.495723525650421,0.620089486559718,NA
"15816","AL139289.2",1.65498566314807,-0.631504104865567,1.30752708604858,-0.482975925779104,0.629112829948347,NA
"15817","CDC20",0.972977610653397,-0.553602596115188,1.7300007674455,-0.320001358688777,0.74896730058454,NA
"15818","AL139289.1",0,NA,NA,NA,NA,NA
"15819","SZT2-AS1",0.199963700949758,0.396207878749762,3.33505440362465,0.118801024150955,0.905432997276861,NA
"15820","HYI-AS1",0.23863458829365,-0.32004419838901,2.8922201657631,-0.110656927912183,0.911888402687771,NA
"15821","AL451062.1",1.69735450794999,0.363788740752786,1.35622971143184,0.268235342203729,0.788518172297287,NA
"15822","ARTN",0.887047862603953,0.869130797630151,1.86648484761986,0.465651140291047,0.641465230913549,NA
"15823","AL357079.1",2.15297284585484,-0.871115460860905,1.10122921684517,-0.791039183791815,0.428921126293848,NA
"15824","AL139220.2",2.16251153423111,-1.40468575314927,1.08461600508221,-1.29509959890625,0.195285873270801,NA

"15825","ERI3-IT1",0,NA,NA,NA,NA,NA
"15826","KIF2C",1.58115337332353,1.15068067379526,1.26825381507627,0.9
0729525913238,0.364250680558456,NA
"15827","AL592166.1",0.0543178333807489,-0.0984569371683306,3.33935238
193321,-0.0294838417475822,0.976478705757268,NA
"15828","BEST4",1.39080608976611,-0.384172373918309,1.28777882924839,-
0.298321703380177,0.765457641112229,NA
"15829","TCTEX1D4",1.47124073768304,-0.248032891754116,1.1879461059848
7,-0.208791367305744,0.834611110712479,NA
"15830","LINC01144",1.14538473721627,0.543633286484266,1.5735085262393
,0.345491160307567,0.729725154690599,NA
"15831","HPDL",0.699662317835405,-0.317418169099841,1.78563928723589,-
0.177761640533343,0.858910173903105,NA
"15832","TSPAN1",1.81691352650762,0.321971809704723,1.07638346332187,0
.299123705144142,0.764845659765255,NA
"15833","RAD54L",0.263276699875595,-1.39514324907608,2.84772410622012,
-0.489915173323408,0.62419392553726,NA
"15834","TMEM275",0,NA,NA,NA,NA,NA
"15835","MKNK1-
AS1",0.145645867569009,-0.172465240332149,3.34011191561742,-0.05163456
9346539,0.958819873713678,NA
"15836","TEX38",0.157602722099681,0.512552377050138,3.33247271335336,0
.153805423521194,0.877763157373649,NA
"15837","CYP4A22-
AS1",0.45055514377408,0.702428225244874,2.10390376053722,0.33386899078
7635,0.738478420196678,NA
"15838","CYP4A22",0.628723665469994,1.16143720877039,2.02804233781724,
0.572688837463044,0.566855396793604,NA
"15839","PDZK1IP1",0.696824677192944,-2.22722807279096,1.7421194908600
6,-1.27845884537542,0.201087686818221,NA
"15840","TAL1",0.801709889193704,-2.93827302190526,2.06850031897142,-1
.42048468397933,0.155466624401805,NA
"15841","STIL",1.93262177742794,-0.0803415922025091,0.985102559585368,
-0.0815565764404521,0.934999333187342,NA
"15842","FOXD2-
AS1",2.09134957593617,0.507767898951871,1.10354440011919,0.46012457577
3327,0.645426804991832,NA
"15843","FOXD2",1.69815604118312,0.15363784738416,1.14110833592629,0.1
3463914209289,0.892897192447556,NA
"15844","TRABD2B",2.23237031053854,0.0900278860487678,0.99772182838456
4,0.0902334533409319,0.928101700826979,NA
"15845","SLC5A9",0.145645867569009,-0.172465240332149,3.34011191561742
,-0.051634569346539,0.958819873713678,NA
"15846","AL109659.2",2.43171823547074,0.957895026106532,1.072954894636
01,0.892763555015507,0.37198380720247,NA
"15847","AGBL4",0.269081001391292,-0.111451794566982,2.79490872673425,
-0.0398767206602876,0.968191410559562,NA
"15848","DMRTA2",0.0271589166903744,-0.404880044879902,3.3427209427763
7,-0.121122897128116,0.903593693408407,NA
"15849","LINC01562",0.236562667062376,-0.3201857164476,2.9019980438058

1,-0.11033285054448,0.912145405328295,NA
"15850","TTC39A",1.10325223151705,-0.47759229984635,1.46974126550452,-0.324949915373307,0.745218978033957,NA
"15851","TTC39A-AS1",0.189612912069306,-1.5929471954674,3.34910741318451,-0.47563335508333,0.634335608031733,NA
"15852","AC104170.2",0.292956741472387,-0.0141756301256079,2.76371736584978,-0.00512918951147857,0.995907516823941,NA
"15853","AC104170.1",0.227126937438121,0.554229032074634,3.33087194591576,0.166391575861755,0.867848803034167,NA
"15854","AL050343.3",1.74150508763847,-0.265197903008879,1.0948407707642,-0.24222508888098,0.80860575568793,NA
"15855","RAB3B",0.889169454277758,0.843482762270959,1.94522242790578,0.4336176419573,0.664566121518711,NA
"15856","AL513218.1",1.32202666231128,0.610173325818225,1.3812672633599,0.441748922894177,0.658670903244553,NA
"15857","AL513218.2",2.17455816296809,-0.114291606240552,1.02985982962824,-0.110977827227041,0.911633929415335,NA
"15858","ORC1",1.0133806860238,-0.245429126425888,1.39488455573895,-0.175949418477768,0.860333680586796,NA
"15859","AL445183.1",0.813861868951197,0.0425125318987411,1.65858644584935,0.0256317854309793,0.979551033285727,NA
"15860","AL606760.2",1.01190298851469,0.743073076757746,1.50889000505837,0.492463383193397,0.622391801320712,NA
"15861","AL606760.3",0.67231834377567,1.36010682665718,1.74457003235606,0.779622945156485,0.435612846079642,NA
"15862","LINC01771",0,NA,NA,NA,NA,NA
"15863","LINC02812",0,NA,NA,NA,NA,NA
"15864","GLIS1",0.103280568920944,0.158353271622336,3.33696113528856,0.047454335007903,0.96215112468834,NA
"15865","DIO1",0.184316754912901,-0.893803915826854,3.34011187009479,-0.267596999917697,0.789009540491034,NA
"15866","SSBP3-AS1",1.31684554979928,-1.01076200651163,1.42520190400844,-0.709206185922725,0.478196535313086,NA
"15867","FAM151A",0,NA,NA,NA,NA,NA
"15868","MROH7",0.373929666982207,-1.65984287015097,2.53449598679645,-0.654900571473771,0.51253177228147,NA
"15869","TTC22",0.0814810698691117,-0.172465128888762,3.34011191444869,-0.0516345359994407,0.958819900285368,NA
"15870","LEXM",0,NA,NA,NA,NA,NA
"15871","TMEM61",0.184757318992067,0.733466356403203,3.32442169734661,0.220629758549771,0.82538072801744,NA
"15872","MIR4422HG",0.0761216522305695,-0.172465129916519,3.34011191445947,-0.0516345363069754,0.958819900040317,NA
"15873","LINC01767",0,NA,NA,NA,NA,NA
"15874","DAB1",0.330407506359065,1.19000212515397,2.31685257342759,0.513628764644898,0.607511564783461,NA
"15875","TACSTD2",1.60310201485051,-1.7199251339629,1.26105980254837,-1.36387277628487,0.172607610295725,NA

"15876","AL136985.3",0,NA,NA,NA,NA,NA
"15877","AL136985.2",0.102835685043789,-1.5929471954674,3.34910741318451,-0.47563335508333,0.634335608031733,NA
"15878","HOOK1",0.330407506359065,1.19000212515397,2.31685257342759,0.513628764644898,0.607511564783461,NA
"15879","AC099792.1",0,NA,NA,NA,NA,NA
"15880","NFIA-AS1",0.823606831280477,2.51059194018793,1.85284966383401,1.35498955430248,0.175420885101773,NA
"15881","AC099791.4",0.585320494486105,0.987631437567534,2.12287111767557,0.465233819116131,0.641764022537237,NA
"15882","L1TD1",0.413688996624478,-0.19715538477177,2.61211023162031,-0.0754774367425807,0.939834849190878,NA
"15883","ANGPTL3",0.714094973763055,2.21259848960938,1.78444315218508,1.23993778501714,0.21499840677635,NA
"15884","AC103923.1",0.99192585757914,0.277514461327882,1.46686795584433,0.189188440733334,0.849945127026774,NA
"15885","AL357078.2",2.32753088847791,0.752949625117997,1.22215015738608,0.616086019027644,0.537837748962355,NA
"15886","DNAJC6",0.575375385239061,-2.19102768029138,2.00483726574193,-1.09287058741925,0.274450623844728,NA
"15887","SGIP1",0.653264824876074,-1.9108159768798,1.932706352441,-0.988673718832893,0.322822805246474,NA
"15888","AL139147.1",0.377217163389475,-1.67447570333794,2.53023072741686,-0.661787751288369,0.508107257158806,NA
"15889","TCTEX1D1",0.21677182875968,-1.12621966659706,3.34272090207402,-0.33691705038799,0.736179431667661,NA
"15890","IL23R",0.108639986559486,0.158353243860761,3.33696113552764,0.0474543266850853,0.962151131321515,NA
"15891","IL12RB2",0.296244237879655,-0.0142838044026472,2.75474048780207,-0.00518517242037703,0.995862849519269,NA
"15892","DEPDC1",0.320886349461601,-1.16345308374614,2.67501533014526,-0.434933239684636,0.663610886506367,NA
"15893","LRRC7",0.233724374330251,0.915053379377898,3.31855872315749,0.275738191098591,0.782749160064026,NA
"15894","AL353771.1",0.108639986559486,0.158353243860761,3.33696113552764,0.0474543266850853,0.962151131321515,NA
"15895","AL354872.2",0.0271589166903744,-0.404880044879902,3.34272094277637,-0.121122897128116,0.903593693408407,NA
"15896","AL031429.1",0.540166343733733,-0.357554486247571,2.39557666228147,-0.149256123536889,0.881351535728395,NA
"15897","AL031429.2",0,NA,NA,NA,NA,NA
"15898","ZRANB2-AS1",0.443898766197801,0.0229149734454248,2.18991473319919,0.0104638656008076,0.991651195546299,NA
"15899","NEGR1-IT1",0.0543178333807489,-0.0984569371683306,3.33935238193321,-0.0294838417475822,0.976478705757268,NA
"15900","LRRIQ3",0.372361570312236,0.410659335162911,2.22865170218333,0.184263577283342,0.853806685761018,NA

"15901","AC105271.1",0.784618192937045,0.392202340273901,1.93544758742
07,0.202641674630195,0.839415114073643,NA
"15902","ERICH3",0.729241407256331,-1.9054123904284,1.90933947217602,-
0.997943224971332,0.318306890164358,NA
"15903","AC135803.1",2.38604175032649,0.403763818367908,0.966877097065
094,0.417595803637828,0.676242665415527,NA
"15904","LHX8",0.291291735138019,0.586693126276678,3.32965205071425,0.
176202533279964,0.860134830656811,NA
"15905","SLC44A5",0.727377539070986,0.394516738201056,2.09725224231711
,0.188111248728566,0.850789443301463,NA
"15906","ST6GALNAC3",0.35504961794101,0.00448761101616321,2.3055378097
7927,0.00194644867550138,0.99844695963408,NA
"15907","AC093433.2",0.152243304461139,0.487043191987424,3.33313782850
767,0.146121527835375,0.88382545206217,NA
"15908","AC093575.1",0,NA,NA,NA,NA,NA
"15909","AC096531.2",0.501707277634783,-2.16562391875308,2.39457499266
342,-0.904387595038033,0.365789913620539,NA
"15910","LINC01781",0.0761216522305695,-0.172465129916519,3.3401119144
5947,-0.0516345363069754,0.958819900040317,NA
"15911","LINC01725",0.302799385993547,-0.316556827499144,2.68241739040
323,-0.118011771259639,0.906058330944223,NA
"15912","DNASE2B",1.03855684538027,-2.05301650219511,1.42468118273337,
-1.44103573983916,0.149574586092254,NA
"15913","AL359762.1",1.03811196150311,-3.2011774678427,1.5564667800257
8,-2.05669501522523,0.0397155719690571,NA
"15914","LPAR3",0,NA,NA,NA,NA,NA
"15915","AL360219.1",0.494339199247104,-1.31516429006975,2.11851708109
479,-0.620794753937086,0.534734675386869,NA
"15916","AC092807.2",0.387567952269927,0.234844999070202,2.22701245362
968,0.105452934799463,0.916016414275287,NA
"15917","LINC02795",1.03929116706509,1.15999421010353,1.59751334798146
,0.726124893772713,0.467762209471953,NA
"15918","AL121989.1",0.0814810698691117,-0.172465128888762,3.340111914
44869,-0.0516345359994407,0.958819900285368,NA
"15919","LINC02801",0.145645867569009,-0.172465240332149,3.34011191561
742,-0.051634569346539,0.958819873713678,NA
"15920","PKN2-
AS1",0.296239918081667,0.0549847168141913,2.72269295886329,0.020194975
2120222,0.983887836269458,NA
"15921","GBP7",0.133282098141432,-1.12621966659706,3.34272090207402,-0
.33691705038799,0.736179431667661,NA
"15922","GBP6",0.30756581517342,0.581813708968262,3.25425745403676,0.1
78785396418636,0.858106212497307,NA
"15923","AC099568.1",1.857492209241,0.89983398038734,1.24479729321666,
0.722875913444587,0.469756122837248,NA
"15924","AC099568.2",0.495165746695601,0.573128650888931,2.13539207191
423,0.268395044838375,0.788395253288003,NA
"15925","LINC01763",0.0543178333807489,-0.0984569371683306,3.339352381
93321,-0.0294838417475822,0.976478705757268,NA
"15926","BRDT",0,NA,NA,NA,NA,NA

"15927","EPHX4",0.633511678267107,-0.971442268484683,2.06062487867907,
-0.471430913280738,0.637333044681601,NA
"15928","AC104836.1",0.471316770490088,0.590230300083222,2.12445759519
075,0.277826350320834,0.781145662549889,NA
"15929","GFI1",1.90980456504038,1.25000664124434,1.19439085447761,1.04
656414318498,0.295300646778138,NA
"15930","AC093577.1",0,NA,NA,NA,NA,NA
"15931","AL049796.1",1.77574714150267,0.401390143107005,1.104928421723
65,0.363272529890082,0.7164013116737,NA
"15932","ABCA4",0.671529528197226,2.22728670326219,2.12457109494863,1.
04834651500144,0.294478986054842,NA
"15933","AC118469.2",0.396682194270486,1.55030794993893,2.403180466205
49,0.645106754045316,0.518858007268818,NA
"15934","SLC44A3",1.74477224298747,-0.0314098037981653,1.2408041633984
9,-0.0253140702817565,0.979804451066017,NA
"15935","AC092802.4",0.37443349154351,0.406187043897745,2.538480100562
4,0.160011907837195,0.872871693985419,NA
"15936","DPYD-
AS1",0.495170066493589,0.483943710070004,2.2324130173484,0.21678054477
7875,0.828379374457492,NA
"15937","DPYD-
IT1",0.804651733478643,-1.12025283029287,1.53907306241473,-0.727875016
235578,0.46669009816951,NA
"15938","LRRC39",0.502297231362251,-0.62810443533012,2.09107372887437,
-0.300374121991495,0.763891800504591,NA
"15939","AL445928.2",0.4933653262993,1.81970042119372,2.01146347076771
,0.904664910717569,0.365642935862377,NA
"15940","GPR88",0.179402221151514,0.662828673469361,3.32688171260342,0
.199234217122397,0.842079534527828,NA
"15941","AC093157.2",1.14752730059398,0.747736662285333,1.512385074281
87,0.494408914105676,0.621017419817493,NA
"15942","AL109741.1",0,NA,NA,NA,NA,NA
"15943","COL11A1",0.324603204843368,-0.228543376077218,2.7193229244371
9,-0.0840442207225235,0.933021273062582,NA
"15944","VAV3-AS1",0,NA,NA,NA,NA,NA
"15945","LINC02785",0.312094365565477,-1.12559151541374,3.145866405770
12,-0.35780016384332,0.72049286795132,NA
"15946","AL160171.1",0.502297231362251,-0.62810443533012,2.09107372887
437,-0.300374121991495,0.763891800504591,NA
"15947","FNDC7",0,NA,NA,NA,NA,NA
"15948","SPATA42",0.364159540143581,1.43618542166959,2.9519758747263,0
.486516652783535,0.62660090131027,NA
"15949","AL356488.2",1.3090105075596,0.558540803806463,1.4736836190441
5,0.379009983274931,0.704680450381687,NA
"15950","KIAA1324",1.29381882391247,-0.890757965414298,1.2821567454580
1,-0.694734063186713,0.487221976806037,NA
"15951","CELSR2",1.63949388589418,-1.59724122659092,1.27050151167077,-
1.25717381043528,0.208690704502783,NA
"15952","GNAT2",0.106123181451057,-1.5929471954674,3.34910741318451,-0
.47563335508333,0.634335608031733,NA

"15953","AC000032.1",2.44781701169479,2.55605028573156,1.2178382821022
2,2.09884212320814,0.0358308202058027,NA
"15954","ALX3",1.24639244927901,-0.431938216639708,1.34756984422004,-0
.320531227744791,0.748565661735455,NA
"15955","UBL4B",0,NA,NA,NA,NA,NA
"15956","RBM15-
AS1",0.361057101105673,-0.901416986870427,3.00395421674134,-0.30007680
6046757,0.764118570516335,NA
"15957","KCNA3",1.27714882854949,0.192712411713489,1.41877154377407,0.
135830474299516,0.891955299312008,NA
"15958","AL360270.2",1.51825264698622,0.0722781624247695,1.70042567844
671,0.0425059226880141,0.966095390396982,NA
"15959","AL360270.1",0.269085321189281,-0.180783873541719,3.3313926647
1278,-0.0542667561997844,0.956722635218719,NA
"15960","AL360270.3",1.79285932201228,0.186082860782972,1.265913970797
93,0.146994870959265,0.883136068726246,NA
"15961","AL355816.1",0.106123181451057,-1.5929471954674,3.349107413184
51,-0.47563335508333,0.634335608031733,NA
"15962","AL355816.2",1.88737589726951,0.182062984952578,1.054179562401
57,0.17270585718605,0.862882639610781,NA
"15963","AL390195.1",1.65457545890163,-0.220836213852522,1.19816201469
667,-0.18431248123688,0.853768323052667,NA
"15964","TMIGD3",1.22544070648977,-0.541599703874523,1.43419552808827,
-0.377633100415853,0.705703171800108,NA
"15965","ADORA3",0.425196647480006,-0.899163251063593,2.1666734891418,
-0.414997116810499,0.678144013769305,NA
"15966","LINC01160",1.19536211230553,-0.654988262624799,1.440538476858
87,-0.454682934990404,0.649337359585928,NA
"15967","INKA2-
AS1",0.945810054367046,-0.529203314967987,1.46572196793714,-0.36105300
0872185,0.718059826558094,NA
"15968","AL049557.1",0.135798903249861,0.396207859892014,3.33505440376
505,0.118801018491549,0.90543300176066,NA
"15969","LINC01750",1.25680402602044,0.0741884820964529,1.721974952457
76,0.0430833688902177,0.965635076692032,NA
"15970","AL354760.1",1.53079817017758,-0.207106427832441,1.28565067547
914,-0.161090747107691,0.872021927806214,NA
"15971","AL603832.1",0.427009530912658,-1.5600192257504,2.479724775425
93,-0.629109827514001,0.529277157045906,NA
"15972","TAFA3",0.926708147798304,-1.74403282622447,1.81190832493044,-
0.962539220239758,0.335778811842826,NA
"15973","AL390242.1",0.320052397680624,-0.315960993051199,2.6515825393
8631,-0.119159403246155,0.905149068946699,NA
"15974","AL137856.1",0.152243304461139,0.487043191987424,3.33313782850
767,0.146121527835375,0.88382545206217,NA
"15975","AP4B1-
AS1",1.24457546695641,-0.0016475928797659,1.49785373933455,-0.00109996
913350023,0.999122351788003,NA
"15976","BCL2L15",1.69726101856356,-0.936379829658943,1.26901229101493
,-0.737880819822514,0.460586875254455,NA

"15977","AMPD1",0,NA,NA,NA,NA,NA
"15978","LINC01765",0,NA,NA,NA,NA,NA
"15979","CASQ2",0.516041016107056,1.89801139531455,2.7007320330553,0.702776644289051,0.48219495455876,NA
"15980","MAB21L3",0.718768594005425,0.509776513711723,1.63416070089915,0.311950050831128,0.755078483991569,NA
"15981","LINC01779",0.769414232676362,1.54172659686653,2.24280277095633,0.687410688461534,0.49182396402981,NA
"15982","LINC01762",0.145645867569009,-0.172465240332149,3.34011191561742,-0.051634569346539,0.958819873713678,NA
"15983","AL390066.1",1.11698397783194,0.157897242676719,1.49157861345022,0.105859148993482,0.915694106267933,NA
"15984","AL157904.1",0.996105417566036,-1.55521875674767,1.65261959739037,-0.94106275830415,0.346672698985756,NA
"15985","CD101",1.75259187067891,-1.85641429567239,1.12654337425204,-1.6478853261242,0.0993762051176202,NA
"15986","AL445231.1",1.42807715497505,-1.32454471397532,1.40964775118214,-0.939628153816829,0.347408329755035,NA
"15987","AL358072.1",0.318039417133499,0.181467551941473,2.67646341466774,0.0678012450859525,0.945943852609915,NA
"15988","AL157902.1",0,NA,NA,NA,NA,NA
"15989","AL139420.2",0.876778231002012,1.6430499461704,1.76915574578825,0.928719786305946,0.353034323899153,NA
"15990","AL359915.1",0.243401017473523,0.554229042268252,3.33087194552902,0.166391578941419,0.867848800610731,NA
"15991","HAO2",0.64297274919654,-1.63181093836966,2.01214757994403,-0.810979748520758,0.417377300707104,NA
"15992","HSD3B2",0.0761216522305695,-0.172465129916519,3.34011191445947,-0.0516345363069754,0.958819900040317,NA
"15993","ADAM30",0,NA,NA,NA,NA,NA
"15994","AC245008.1",0.184761638790056,0.686691032805108,3.12643007489066,0.219640617687291,0.826151055409304,NA
"15995","FCGR1B",1.53822712640586,-0.654135519217636,1.18972288586339,-0.549821750081679,0.582441638997535,NA
"15996","AL592494.1",0,NA,NA,NA,NA,NA
"15997","AL592494.2",0.211475671603275,-0.564205700181922,3.00373162390721,-0.187834923630098,0.851006057908311,NA
"15998","FAM72C",2.28655985690331,0.218647423195754,1.02443515392132,0.213432175144339,0.830989886480753,NA
"15999","AC246785.3",0.735368481462511,-1.1019227232958,1.96925774257978,-0.559562468370573,0.575777910891866,NA
"16000","FP700111.1",0.319607513803469,-1.8692453277473,2.64644018618841,-0.706324419309668,0.479986410923902,NA
"16001","PDZK1",1.15964887143575,-0.107424470670033,1.43200968151614,-0.0750165812819765,0.940201519110482,NA
"16002","CD160",2.25856850098198,0.367471082325289,1.02811593121458,0.357421834608838,0.720776033197497,NA
"16003","ITGA10",1.84033978562705,0.950294183681241,1.26007568774418,0.754156431176354,0.450755295690393,NA
"16004","ANKRD34A",0.787702855616397,-0.123871898651002,1.822912289597

69,-0.0679527475665545,0.945823249267172,NA
"16005","LINC00624",1.76195621739343,0.796714894531251,1.3786851412541
3,0.577880235806788,0.563344979777984,NA
"16006","AC239803.1",1.5200114784505,1.51713171081892,1.42862905498254
,1.06194936014196,0.288258675036693,NA
"16007","LINC02804",0.685489984773164,-0.495748302102427,1.80452250977
685,-0.274725474144256,0.783527158395024,NA
"16008","LINC02805",0.106123181451057,-1.5929471954674,3.3491074131845
1,-0.47563335508333,0.634335608031733,NA
"16009","LINC01731",0.0814767500711233,0.132145805215161,3.33718862943
114,0.0395979430259797,0.968413667381931,NA
"16010","AC245100.6",0.898178147017942,1.44576790794854,1.601604697210
24,0.902699592768964,0.366685352648382,NA
"16011","PPIAL4G",1.34064882769221,0.415414718238623,1.40254951133388,
0.296185421535348,0.767088481563196,NA
"16012","NUDT4B",1.7123906185351,0.148337955312104,1.21494570993822,0.
122094307670462,0.902824329973428,NA
"16013","AC239802.2",0,NA,NA,NA,NA,NA
"16014","AC239804.1",0.59992524838915,1.93981579919947,2.0230274685756
,0.958867751096469,0.33762537268336,NA
"16015","AC243772.2",0.518007437881245,0.849777506979123,1.86735886653
9,0.455069200787375,0.649059454885824,NA
"16016","HIST2H3D",0.255523873382083,1.02024589536627,3.31544774637337
,0.307724920859415,0.758291655197173,NA
"16017","HIST2H4A",0.443894446399813,0.145797320919682,2.4222085898601
6,0.0601918932704715,0.952002802242453,NA
"16018","HIST2H2AB",2.27119477717278,-0.260919192500516,1.089662957941
47,-0.239449446821088,0.810757091901001,NA
"16019","SV2A",0.184316754912901,-0.893803915826854,3.34011187009479,-
0.267596999917697,0.789009540491034,NA
"16020","AC242988.2",0.469252203788004,-1.66305111152443,2.39529207475
765,-0.694299926530962,0.487494137063531,NA
"16021","AL356356.1",1.19221968574857,-0.530015957548776,1.27026288901
277,-0.417249029419961,0.676496266324163,NA
"16022","HORMAD1",0.67051755927663,-1.23808122745013,2.68535337542693,
-0.461049647610456,0.644762985825986,NA
"16023","ANXA9",0.76619683225462,2.51475396100308,2.01081481904596,1.2
5061439630538,0.211075195662889,NA
"16024","BNIPL",1.39408112313703,-0.628828217521281,1.35687920273043,-
0.463437140355531,0.64305106207695,NA
"16025","AL592424.1",0.145645867569009,-0.172465240332149,3.3401119156
1742,-0.051634569346539,0.958819873713678,NA
"16026","TNFAIP8L2",2.13705434917972,-1.66505957996001,1.0410296689948
2,-1.59943527984917,0.109723918468302,NA
"16027","TMOD4",0.46039778829524,0.669994755852884,2.13264108274409,0.
314161985002557,0.753398013595703,NA
"16028","CGN",0.184316754912901,-0.893803915826854,3.34011187009479,-0
.267596999917697,0.789009540491034,NA
"16029","AL391335.1",0.106123181451057,-1.5929471954674,3.349107413184
51,-0.47563335508333,0.634335608031733,NA

"16030","AL589765.5",0.0761216522305695,-0.172465129916519,3.34011191445947,-0.0516345363069754,0.958819900040317,NA
"16031","TDRKH-AS1",1.98716387242179,0.100409711094282,1.06869322108047,0.0939555984015369,0.925144427988084,NA
"16032","LINGO4",0,NA,NA,NA,NA,NA
"16033","RORC",0.522088069664946,-0.056896090419036,2.34497412020546,-0.0242629928956533,0.980642831825987,NA
"16034","C2CD4D",0.748765803427924,-0.0831726269760398,1.69018177688966,-0.0492092792108417,0.960752516486273,NA
"16035","C2CD4D-AS1",0.297889172030148,1.04151653687855,3.24529295162179,0.320931438980899,0.748262348259367,NA
"16036","AL450992.2",0.53689180672043,-0.520251997651278,2.43518870965169,-0.213639294396076,0.83082835426302,NA
"16037","AL450992.1",0.619360366712056,-1.2548037876338,2.10337888334853,-0.59656574360782,0.550797348113676,NA
"16038","AL589986.2",0.102835685043789,-1.5929471954674,3.34910741318451,-0.47563335508333,0.634335608031733,NA
"16039","HRNR",1.87907393345308,0.742431053803741,1.16970034995505,0.634719014859036,0.525611689884571,NA
"16040","FLG",0.0814810698691117,-0.172465128888762,3.34011191444869,-0.0516345359994407,0.958819900285368,NA
"16041","FLG2",0.391327096429932,1.48510245450094,2.96763503455182,0.500432983574488,0.61677023345353,NA
"16042","LOR",1.37011812917992,-0.792968120535672,1.30625139434876,-0.607056286382767,0.543813558898728,NA
"16043","S100A7",1.15401842218475,-2.87722962669851,1.72618325961094,-1.66681585554654,0.0955510264697732,NA
"16044","BX470102.1",0.941248091902925,-1.16768853628403,1.51796271300056,-0.769247179975758,0.441746587039868,NA
"16045","S100A5",1.7163246582873,0.56247789817333,1.22142763208859,0.460508574880952,0.645151218039171,NA
"16046","AL358472.5",0.346681586394467,1.18876916460876,2.29108047590777,0.518868358012498,0.603852544558385,NA
"16047","AL358472.3",0.890534220862198,-0.528356407492854,1.85182606204247,-0.285316433504615,0.775401723766491,NA
"16048","NUP210L",0,NA,NA,NA,NA,NA
"16049","C1orf189",0.491051702839836,-1.30223814088097,2.11996939217282,-0.614272142649318,0.539035507724315,NA
"16050","UBE2Q1-AS1",0,NA,NA,NA,NA,NA
"16051","CHRNB2",0,NA,NA,NA,NA,NA
"16052","AL451085.1",0.930684829687867,1.08412894463255,1.52167934363187,0.712455583477265,0.476182706549349,NA
"16053","DCST2",1.39357689219772,-0.968446490698825,1.23001510744683,-0.787345199937464,0.431079816789953,NA
"16054","DCST1-AS1",2.03779201745877,0.760868534211364,1.07247481236495,0.709451192176299,0.478044529496585,NA
"16055","TRIM46",0.190121056428598,0.711879596672421,3.3251624461104,0

.214088667308612,0.830477913101807,NA
"16056","AC234582.2",0,NA,NA,NA,NA,NA
"16057","ASH1L-
IT1",0.998793828917129,0.631140765502258,1.78404777030126,0.3537689831
00537,0.723512020098288,NA
"16058","AL355388.2",0.744727460422461,0.672268291464694,1.84035774826
721,0.365292178706921,0.714893316112058,NA
"16059","PAQR6",1.83797259765015,-0.262020831511663,1.11361678478332,-
0.235288148573161,0.813985093022535,NA
"16060","TSACC",0.103280568920944,0.158353271622336,3.33696113528856,0
.047454335007903,0.96215112468834,NA
"16061","C1orf61",1.85478819394058,1.36167776075651,1.15070994952247,1
.18333708796173,0.236675579689853,NA
"16062","IQGAP3",0.187604251320169,-0.893803915826854,3.34011187009479
,-0.267596999917697,0.789009540491034,NA
"16063","TTC24",0,NA,NA,NA,NA,NA
"16064","HAPLN2",0.373840503084841,1.3304658453771,2.20658475434634,0.
602952523240481,0.546540273467799,NA
"16065","BCAN",1.31192161040093,-3.40859706503315,1.63375420205354,-2.
08635856039343,0.0369461557506058,NA
"16066","INSRR",0.303248589668691,1.05043806006203,2.75265656400703,0.
381608833371102,0.702751531154593,NA
"16067","LRRC71",0,NA,NA,NA,NA,NA
"16068","FCRL5",0.704845317700564,1.36328265694793,1.78919707516519,0.
761952205193528,0.446088530328062,NA
"16069","FCRL3",0.849923702754803,2.49338453076324,1.84951494286103,1.
34812889205761,0.177616930235295,NA
"16070","AL356276.1",0,NA,NA,NA,NA,NA
"16071","FCRL2",0,NA,NA,NA,NA,NA
"16072","FCRL1",0,NA,NA,NA,NA,NA
"16073","KIRREL1-
IT1",0.60736041069934,2.13693080909106,2.19101780424582,0.975314214676
872,0.329404421588848,NA
"16074","AL138899.1",0.565329559068936,0.048367919401856,2.39066809577
271,0.0202319675773406,0.983858326661504,NA
"16075","CD1A",0.0814810698691117,-0.172465128888762,3.34011191444869,
-0.0516345359994407,0.958819900285368,NA
"16076","CD1B",0.714161970869348,-1.66763767101093,1.88086381511997,-0
.886633927243991,0.375276021646786,NA
"16077","CD1E",2.13268639354063,-0.772150352949114,1.10799346592569,-0
.696890709823827,0.485871192162145,NA
"16078","SPTA1",0.145645867569009,-0.172465240332149,3.34011191561742,
-0.051634569346539,0.958819873713678,NA
"16079","AIM2",1.06536470332339,-0.200978963157245,1.32925879906097,-0
.151196263134931,0.879820897995089,NA
"16080","FCRL6",1.90537801663819,0.711166036409279,1.09427787251406,0.
649895291015435,0.515759860128994,NA
"16081","CFAP45",2.23235922444193,0.436558211263022,1.30002862017824,0
.33580661570602,0.73701670098473,NA
"16082","KCNJ10",1.32635630104907,0.986228755433043,1.65323247669243,0

.596545718365125,0.550810721449889,NA
"16083","KCNJ9",1.39841146376777,-2.08110369057687,1.36828381086744,-1
.52095908323108,0.12827010559666,NA
"16084","ATP1A2",0.380233473261219,1.50452154122864,2.92165695493815,0
.514954891841673,0.606584541536873,NA
"16085","ATP1A4",0,NA,NA,NA,NA,NA
"16086","AL121987.2",1.46547867514229,-0.410104220941477,1.33363709631
762,-0.307508108520557,0.758456652127068,NA
"16087","NHLH1",0.921934314285951,-0.560407968382482,1.50339850552365,
-0.372760759255435,0.70932651082054,NA
"16088","SLAMF6",1.62552693524941,2.65672773089173,1.37333597339828,1.
93450676480696,0.0530508511329782,NA
"16089","SLAMF1",1.54030373153786,0.919294667026644,1.45783124913146,0
.630590589668274,0.528308253416858,NA
"16090","AL121985.1",0.103280568920944,0.158353271622336,3.33696113528
856,0.047454335007903,0.96215112468834,NA
"16091","LY9",1.3140872192585,-0.137710816628769,1.26811582918941,-0.1
08594825061678,0.913523865482641,NA
"16092","CD244",1.36966718658818,0.278720402377572,1.43620158448974,0.
194067744658977,0.846122837481997,NA
"16093","ITLN1",0.0271589166903744,-0.404880044879902,3.34272094277637
,-0.121122897128116,0.903593693408407,NA
"16094","F11R",1.54989597785637,0.438683939407694,1.31598763252348,0.3
33349591262111,0.738870409457503,NA
"16095","NECTIN4",0.390089077176344,1.43589461022523,3.02189611913533,
0.475163458178732,0.634670470577488,NA
"16096","AL590714.1",0.416735529709438,-0.0651937170305982,2.453080709
1645,-0.0265762625693643,0.978797706296763,NA
"16097","NR1I3",0.737621879171039,-0.625022008009999,1.72671196715864,
-0.361972361284142,0.717372684444929,NA
"16098","MPZ",1.14154097644012,0.340209013193943,1.42726469587084,0.23
836434417399,0.811598519705271,NA
"16099","CFAP126",0.145645867569009,-0.172465240332149,3.3401119156174
2,-0.051634569346539,0.958819873713678,NA
"16100","FCRLA",0.129994601734164,-1.12621966659706,3.34272090207402,-
0.33691705038799,0.736179431667661,NA
"16101","NOS1AP",2.07798247718398,-1.04189901242829,1.04595138443148,-
0.996125659315045,0.319189094004335,NA
"16102","C1orf226",0.724301344882859,1.04266737606744,1.97549752818275
,0.527799888986236,0.597638234209277,NA
"16103","SH2D1B",0.692884143371903,1.3122643914245,1.92174966003207,0.
682848769908253,0.494702408646936,NA
"16104","AL596325.1",2.03068597458798,-1.33678773231048,1.018756997938
1,-1.31217526359677,0.189461006068024,NA
"16105","RGS4",0.518210619530211,-1.03230712426938,2.09062669745542,-0
.493778791558456,0.621462412833205,NA
"16106","AL499616.1",0,NA,NA,NA,NA,NA
"16107","AL357568.1",1.4356413585798,1.74961209298075,1.59240607937027
,1.09872231439398,0.271889206309524,NA
"16108","AL356441.1",2.34716937575669,-1.62517726366427,1.036072425098

75,-1.5685942645461,0.116742509724487,NA
"16109","AL451074.5",0.312094365565477,-1.12559151541374,3.14586640577012,-0.35780016384332,0.72049286795132,NA
"16110","TMCO1-AS1",0.6816133233476,0.926191098362454,1.93044293537068,0.479781650828547,0.631382661633782,NA
"16111","LINC01675",0.0271589166903744,-0.404880044879902,3.34272094277637,-0.121122897128116,0.903593693408407,NA
"16112","ILDR2",0.74058282925074,-1.52742401147007,1.99264988702888,-0.766529043266864,0.443361577512112,NA
"16113","GPA33",0,NA,NA,NA,NA,NA
"16114","DUSP27",0,NA,NA,NA,NA,NA
"16115","AL451050.2",0.539221303003598,0.018000024250349,2.12347165061346,0.00847669628419524,0.993236655904305,NA
"16116","AL136984.1",1.79277947246874,-0.881595739742445,1.14031547466852,-0.773115650297315,0.439453939753183,NA
"16117","AL359962.3",0.206116253964733,-0.564101255590599,3.02806587112849,-0.186290945969538,0.852216607256885,NA
"16118","AL359962.2",0,NA,NA,NA,NA,NA
"16119","AL031733.2",0.102835685043789,-1.5929471954674,3.34910741318451,-0.47563335508333,0.634335608031733,NA
"16120","ADCY10",0.512507922280043,-2.5013792633973,2.33217867824942,-1.07255043823438,0.283472872034156,NA
"16121","Z99943.1",1.15989107002156,1.32363640107467,1.42349057088344,0.929852594845917,0.352447410375515,NA
"16122","AL023755.1",0.23863458829365,-0.32004419838901,2.8922201657631,-0.110656927912183,0.911888402687771,NA
"16123","CCDC181",0.672706582793995,-3.30529800121583,2.49750844408952,-1.32343816856275,0.185689705766506,NA
"16124","F5",1.40612714156507,-1.99517007876108,1.35506507867973,-1.4723795263804,0.140918418709609,NA
"16125","SELP",0.108639986559486,0.158353243860761,3.33696113552764,0.0474543266850853,0.962151131321515,NA
"16126","SELE",0.189078864294786,0.158353043331628,3.33696113725457,0.0474542665671888,0.962151179234678,NA
"16127","LINC01681",1.36527514549712,-0.504893472093243,1.75705936996838,-0.287351401280384,0.773843268812144,NA
"16128","MROH9",0.0761216522305695,-0.172465129916519,3.34011191445947,-0.0516345363069754,0.958819900040317,NA
"16129","AL021026.1",0,NA,NA,NA,NA,NA
"16130","MYOCOS",0.0814810698691117,-0.172465128888762,3.34011191444869,-0.0516345359994407,0.958819900285368,NA
"16131","FASLG",0.739248966328448,-1.52318205137722,1.71017246332163,-0.890659909479993,0.373111648570308,NA
"16132","GAS5-AS1",1.34300039299682,1.13927667619475,1.37474605625051,0.828717908311028,0.407264049771905,NA
"16133","SERPINC1",0.190121056428598,0.711879596672421,3.3251624461104,0.214088667308612,0.830477913101807,NA
"16134","RABGAP1L-

DT",0.712200982855182,0.337998533131799,1.83793766752915,0.18390097722
2036,0.854091138171987,NA
"16135","GPR52",0.91572800084517,1.73413758653999,1.62366766483846,1.0
6803727394086,0.285503698479953,NA
"16136","Z99127.1",0,NA,NA,NA,NA,NA
"16137","TNN",1.01220653916569,-2.3997027974519,1.60067377857609,-1.49
918292507209,0.133826183428573,NA
"16138","TNR",0.145645867569009,-0.172465240332149,3.34011191561742,-0
.051634569346539,0.958819873713678,NA
"16139","LINC02803",0.680219731031987,-3.38661515833317,2.106625010931
14,-1.60760227414004,0.107922313844627,NA
"16140","AL590723.1",2.34819934010694,1.0519644964375,1.18398843176798
,0.888492208379659,0.374276045189508,NA
"16141","AL591043.2",1.37867587932836,0.522383761322656,1.296293815428
55,0.402982529967526,0.686961076429761,NA
"16142","ASTN1",0.996332246981235,-1.37811610417227,1.75616472855638,-
0.784730544785013,0.432611570027214,NA
"16143","CLEC20A",0,NA,NA,NA,NA,NA
"16144","TEX35",0.996306303389682,0.343801353279112,1.84741408220256,0
.186098696871044,0.852367363810678,NA
"16145","AL449106.1",0.662652117027893,-0.228057037555362,2.0097024370
0152,-0.11347801214573,0.909651594234941,NA
"16146","AXDND1",0.227126937438121,0.554229032074634,3.33087194591576,
0.166391575861755,0.867848803034167,NA
"16147","AL160286.2",0.394572304058963,1.39660610374938,2.558085761144
67,0.545957498752676,0.585095152600352,NA
"16148","AL353708.1",2.22986279395235,0.614197783960389,1.093880476297
27,0.56148527857396,0.574466763272681,NA
"16149","AL162431.1",1.35558157316142,0.159796019948634,1.490449746775
79,0.107213289340558,0.914619772829907,NA
"16150","KIAA1614-
AS1",0.830106123244711,0.169139208742674,1.60549581919428,0.1053501396
39452,0.9160979786168,NA
"16151","LINC01732",0,NA,NA,NA,NA,NA
"16152","TEDDM1",0,NA,NA,NA,NA,NA
"16153","RGSL1",0.145645867569009,-0.172465240332149,3.34011191561742,
-0.051634569346539,0.958819873713678,NA
"16154","AL355999.1",1.26914130885896,-0.436933195935459,1.41568737664
011,-0.308636781781896,0.757597838098907,NA
"16155","SHCBP1L",1.50875035327425,0.657857458089835,1.2391307792526,0
.530902362450098,0.595486440588782,NA
"16156","NMNAT2",1.31285303235195,2.04584381995344,1.76794800446617,1.
15718551381899,0.247196574907575,NA
"16157","AL713852.2",0.845572331710197,2.58080690158898,1.715208415860
08,1.50466082006417,0.13241130096444,NA
"16158","AL078644.2",0.401088583672581,-1.30424181033257,2.20856889191
588,-0.590537073625615,0.554830638229451,NA
"16159","OCLM",0,NA,NA,NA,NA,NA
"16160","AL096803.4",0.517566873802079,-0.283105925827492,2.1723282407
2134,-0.130323733089933,0.896310303903651,NA

"16161","PACERR",0.33324579909119,-0.0718179676319118,2.61596981774167,-0.0274536682896101,0.978097893252759,NA
"16162","ERVMER61-1",0.442538170974784,-0.240457067282785,2.50816062752167,-0.095869883549038,0.923623913823757,NA
"16163","BRINP3",1.77611103843111,2.74795901517388,1.70112703048133,1.61537555158145,0.10622939140076,NA
"16164","RGS18",1.56866541893332,-1.87692338902901,1.31081572216643,-1.43187433388955,0.152179788793584,NA
"16165","AL390957.1",2.08285556958626,0.136180186809044,1.21709951427079,0.111889114416937,0.910911327840276,NA
"16166","AL136987.1",0.563863414585544,-1.3133416503556,2.05799440787007,-0.638165801293331,0.523365756464518,NA
"16167","RGS13",2.03629023744616,-0.35994494508275,1.24304178623885,-0.289567856099076,0.772146860986123,NA
"16168","B3GALT2",1.29957095449497,0.881854996380096,1.54425967109866,0.571053568829331,0.567963331135882,NA
"16169","LINC01031",0.312094365565477,-1.12559151541374,3.14586640577012,-0.35780016384332,0.72049286795132,NA
"16170","AL138778.1",0.133282098141432,-1.12621966659706,3.34272090207402,-0.33691705038799,0.736179431667661,NA
"16171","AL136456.1",0.129994601734164,-1.12621966659706,3.34272090207402,-0.33691705038799,0.736179431667661,NA
"16172","CFHR3",1.06361485288965,1.90643206058819,1.51174112457223,1.26108368000351,0.207278699846595,NA
"16173","CFHR1",0,NA,NA,NA,NA,NA
"16174","ASPM",1.85083154635197,-1.22247354015528,1.3669302256895,-0.894320366307396,0.371150506158716,NA
"16175","CRB1",1.07134364581295,0.338299617091691,1.38742101776549,0.243833423856112,0.807359832958991,NA
"16176","LHX9",1.6780392668618,3.50246512316124,1.61587907995107,2.16752922085438,0.0301945240819776,NA
"16177","AL450352.1",0.162962139738223,0.5405559663786,3.33139278619782,0.162261252596259,0.871100126217942,NA
"16178","AL157402.2",0.0814810698691117,-0.172465128888762,3.34011191444869,-0.0516345359994407,0.958819900285368,NA
"16179","MIR181A1HG",2.29940822126829,-1.54398758648384,0.982020122996594,-1.57225656616121,0.115891061613825,NA
"16180","NR5A2",0.189612912069306,-1.5929471954674,3.34910741318451,-0.47563335508333,0.634335608031733,NA
"16181","LINC00862",0.406358837413758,1.49204554244713,2.44879071898036,0.609298920843835,0.542326322264659,NA
"16182","AL359834.1",0,NA,NA,NA,NA,NA
"16183","KIF14",0.265200516525355,0.743208915143452,2.80940224896213,0.264543432830957,0.791361201874853,NA
"16184","DDX59-
AS1",0.233720054532262,0.948343271444607,3.31755240507268,0.285856304784982,0.774988181555882,NA
"16185","INAVA",0.341322168755925,1.18021435095547,2.62154099087253,0.450198701856903,0.652567172228299,NA
"16186","AL358473.1",0,NA,NA,NA,NA,NA

"16187","ASCL5",0,NA,NA,NA,NA,NA
"16188","IGFN1",0.187604251320169,-0.893803915826854,3.34011187009479,-0.267596999917697,0.789009540491034,NA
[truncated: 536,093 more chars]
