# Supplementary material for: PPARγ agonist treatment reduces fibroadipose tissue in secondary lymphedema by exhausting fibroadipogenic PDGFRα+ mesenchymal cells
Source: JCI Insight. 2023 Dec 22;8(24):e165324. doi: 10.1172/jci.insight.165324 (PMC10807713; doi:10.1172/jci.insight.165324)
Supplement: Supplemental data set 3 [file jciinsight-8-165324-s185.pdf]

"","gene","baseMean","log2FoldChange","lfcSE","stat","pvalue","padj"
"1","PLP2",778.307732717723,-1.44200099295344,0.124039592311809,-11.62
53283816716,3.06412848245448e-31,4.99115888507011e-27
"2","EDN1",52.2479335932514,2.81623467625312,0.356776531498389,7.89355
360462053,2.93701330770366e-15,2.39205048845925e-11
"3","ANOS1",128.702620464656,3.14272950791524,0.452111824425005,6.9512
2166272063,3.62136330481535e-12,1.96627956240457e-08
"4","C1QC",24.0265718869416,-2.80703731893298,0.41354443477801,-6.7877
5261584597,1.13893723403742e-11,4.63803715130887e-08
"5","PDGFD",1151.2237945395,1.95988200691736,0.296673606225369,6.60618
931307467,3.94338563914436e-11,1.28467617352045e-07
"6","IL17RD",208.570346459364,1.33158000180269,0.205809577969808,6.469
96128624309,9.80280293466667e-11,2.66129761671309e-07
"7","C1QA",68.7963158597336,-2.53419209567682,0.395487475821442,-6.407
7682622268,1.47665121057281e-10,3.43616736700293e-07
"8","ADAMTSL1",415.182116300722,2.04094099669432,0.323668831697891,6.3
0564576140378,2.8699476078989e-10,5.84357207313315e-07
"9","GSTM1",15.0917206017108,7.01900805144277,1.12587642808415,6.23426
148408374,4.53914342994602e-10,8.2153452589323e-07
"10","CAV1",2605.92540008243,-1.25867775062535,0.203518608962191,-6.18
458310541608,6.22668037036296e-10,1.01426396552842e-06
"11","STEAP4",474.867665578542,1.95657935788617,0.326201195094928,5.99
807538202547,1.99669833953601e-09,2.9567472047911e-06
"12","RNASE1",58.7892540870326,-2.46763617642454,0.416205239829789,-5.
92889262382594,3.04984430239359e-09,4.13990948680743e-06
"13","S100A3",21.8369713990364,-2.1405823837191,0.36346397919109,-5.88
939346474743,3.87615585905855e-09,4.54266796368837e-06
"14","IGSF10",737.59972761125,1.8976707607762,0.322909860076063,5.8767
8171341438,4.18319230494969e-09,4.54266796368837e-06
"15","VEGFC",492.17541530033,1.97379949890932,0.335543104411504,5.8824
0220990712,4.04354250555618e-09,4.54266796368837e-06
"16","VEGFD",1045.19724404356,1.3888554306256,0.24288053322594,5.71826
573409905,1.07616753638159e-08,1.09560581250748e-05
"17","TMCC3",74.3681329750924,1.73172474010475,0.309094230538242,5.602
57866052436,2.11186144672065e-08,2.02353594739016e-05
"18","AQP7",17.37522254922,-2.99284698375541,0.53998214253707,-5.54249
251594455,2.98196153097164e-08,2.69850952099983e-05
"19","PLCXD3",18.8642185148099,-3.46558900558359,0.628097472199604,-5.
51759744144021,3.43665540416583e-08,2.94629894097143e-05
"20","TRIL",189.462232176806,1.5544686092246,0.283645394406684,5.48032
381232969,4.24548104508991e-08,3.45773203717348e-05
"21","ADIRF",2359.57881991521,-1.69877993831992,0.315028590412028,-5.3
9246274789877,6.94985107036167e-08,5.30333767394734e-05
"22","PTGDR",45.3592451556828,-1.91025204040241,0.354601360164735,-5.3
8704092819886,7.16271280169694e-08,5.30333767394734e-05
"23","VGLL3",1236.78296434711,1.88338003951101,0.354352847607876,5.314
98491468352,1.06666163200331e-07,7.28065373914385e-05
"24","CD9",1365.25235782675,-1.96010500738881,0.368860045913115,-5.313
95316219886,1.07272201939623e-07,7.28065373914385e-05
"25","KANK4",21.6859175933527,3.23909559494164,0.614896600073146,5.267

70776510445,1.38137779228386e-07,8.8073763564808e-05
"26","ARHGAP20",418.076204041938,1.65196214251963,0.314207622777458,5.2575495397502,1.45987575434331e-07,8.8073763564808e-05
"27","MXRA5",700.427528629408,1.53648529561549,0.292099578240346,5.26014212300996,1.43944100602767e-07,8.8073763564808e-05
"28","LARP6",785.628517188574,-0.826959798261675,0.158317013305252,-5.22344238939886,1.75627123251551e-07,0.00010217107895159
"29","CX3CL1",19.2169036828719,-3.05282399464471,0.585348867721696,-5.21539232923941,1.83428582828008e-07,0.000103029937437428
"30","STAC",92.7146277170093,1.36292300468801,0.261900829184027,5.20396597801659,1.95079736054992e-07,0.000105921794019992
"31","MARCKS",8172.13674232938,1.47237068559801,0.283607889825951,5.19157166784605,2.0852629009853e-07,0.000107538735315434
"32","KLHL13",82.6019519327339,1.67416294204465,0.322627881901977,5.18914525358134,2.11261558726374e-07,0.000107538735315434
"33","AFF3",191.28519561057,-2.08769876505563,0.403380762798419,-5.175504033887,2.27296519466409e-07,0.000108895088399657
"34","HRH2",77.3748114288823,2.14506016362575,0.414177506094393,5.17908416575592,2.22977857938017e-07,0.000108895088399657
"35","DBNDD2",621.339723906807,-1.87803651783552,0.363582381752897,-5.16536722373938,2.39967323480887e-07,0.000111680792348005
"36","FOLR2",19.9592978208,-2.73580067637588,0.531512464291431,-5.14719947353075,2.64404122215479e-07,0.000119635520743554
"37","EYA2",58.8977066361252,2.48458062847557,0.485254551973515,5.12015934393786,3.05277600669143e-07,0.000134396401008099
"38","SPP1",39.5544697202032,-2.94867413729484,0.576980468311058,-5.11052678425358,3.21261759549426e-07,0.00013771138950791
"39","CD36",200.594876552515,-2.3860679406357,0.472571250988124,-5.04911785396709,4.43854863863111e-07,0.000185383381473493
"40","MUC12",26.2374562112521,-2.66205489661811,0.528526946293173,-5.03674394520174,4.735175280969e-07,0.00019282817537926
"41","PERP",231.77085866832,-2.09643507603397,0.419433284694701,-4.99825634381862,5.78510462921476e-07,0.000229837973915315
"42","IL33",566.953818870294,0.867776614401496,0.17432111017104,4.97803515334462,6.42329920807184e-07,0.000249116954286386
"43","MIR497HG",142.245689946571,1.18284068361193,0.238079292763321,4.96826359774098,6.75550912005265e-07,0.00025590811175939
"44","MAF",1542.07454908656,0.762112686167013,0.153661046372672,4.95969996402779,7.06021396147191e-07,0.000261372330041855
"45","ELMO1",63.4689185676376,1.16828937857966,0.235840474950721,4.95372721253118,7.28052541464782e-07,0.000263538841064885
"46","FBN1",8627.46519125782,1.58495870689684,0.32040460134585,4.94674140208744,7.54661239683666e-07,0.000267232107243635
"47","LILRB5",17.901533189528,-3.17921104310019,0.645458121245269,-4.92551094866791,8.41401990387741e-07,0.000291608447264381
"48","ROR1",165.720829398551,1.45822670484935,0.299908126444319,4.86224472186845,1.1606200743929e-06,0.000393861258162206
"49","PLEKHA2",305.543680625701,1.06906680655346,0.22146726566929,4.82720009804909,1.38465979734095e-06,0.000460300478344626
"50","SUCNR1",36.9792655852218,-2.40263072361727,0.498836516987044,-4.

81646920744511,1.46120701721287e-06,0.000476032022067609
"51","PILRA",73.3653819547093,2.15915162794022,0.449232353192151,4.806
31373185333,1.53738583747126e-06,0.000491028978560185
"52","DYNLT3",166.905581295399,-1.29486810620026,0.270572619365647,-4.
78565831692823,1.70427782481559e-06,0.000533865028623484
"53","CRIM1",417.204901549217,1.15179847423057,0.24119083870102,4.7754
6527237025,1.79292231298011e-06,0.00055103606709685
"54","AC097518.2",17.1047977709086,2.55668750450533,0.536925991218717,
4.76171305974991,1.91956490960065e-06,0.000579033200231203
"55","LYVE1",14.9033806349254,-3.12656716943836,0.660006309372173,-4.7
3717769821396,2.16715129276847e-06,0.000641831407416465
"56","AIF1",67.09052575409,-1.86481652966704,0.394540313510166,-4.7265
5509667958,2.28360850440383e-06,0.000652591209267262
"57","FXYD5",503.758450887065,-1.66321787455642,0.3518389025972,-4.727
21425140568,2.27621055208651e-06,0.000652591209267262
"58","CRIP2",331.613252156743,-1.47113399375246,0.312240258007285,-4.7
1154489539956,2.45845920843932e-06,0.000682836623059742
"59","EMILIN2",482.312913888941,0.926742798211327,0.19679894636846,4.7
0908414558387,2.48832337194285e-06,0.000682836623059742
"60","SLITRK2",92.4195637876191,-1.83064506555601,0.38892853586687,-4.
70689316091385,2.51520642050368e-06,0.000682836623059742
"61","FKBP1B",47.9116246545746,-1.69290799944169,0.361429408403417,-4.
68392432956678,2.81433942246361e-06,0.00073665228229959
"62","FREM1",133.519833067424,1.33000611566323,0.284103840029841,4.681
40844391095,2.84910637760907e-06,0.00073665228229959
"63","MS4A7",32.1400548150748,-2.29816587464703,0.490559608830371,-4.6
8478413892756,2.80255135289317e-06,0.00073665228229959
"64","C1QB",62.7875304128331,-2.28310262005671,0.48903689727532,-4.668
56924861307,3.03304547592752e-06,0.000748564814505808
"65","EHBP1",497.718482060413,0.943066301302933,0.201902657948038,4.67
089592027879,2.99888868336583e-06,0.000748564814505808
"66","VSIG4",20.9256990232087,-2.38242767954014,0.510047595254043,-4.6
7099090694371,2.99750209785221e-06,0.000748564814505808
"67","CDA",57.2306363777451,-2.3805628417121,0.510377053638052,-4.6643
2184743231,3.09636445456554e-06,0.00075278627761818
"68","TNFAIP6",539.442905730364,-1.79571967773053,0.387217386819334,-4
.63749753718669,3.52652811184802e-06,0.000844759064910182
"69","SYNE2",632.803833871252,1.1757904520892,0.255157582120311,4.6080
9528887444,4.06374431063391e-06,0.000959338131535012
"70","ADD3",3499.75729515481,0.657702354838831,0.143015683391987,4.598
81279618934,4.24905378365202e-06,0.000988754815455825
"71","CD163",31.7194795982955,-2.32590468505392,0.511551907424905,-4.5
4676182669763,5.44775873237161e-06,0.00124983861960002
"72","ANKRD33B",108.543774436085,1.12023659624192,0.247676093568052,4.
52299041099872,6.0972003482452e-06,0.00137940689545231
"73","ACP5",18.7633521532904,-2.49777892260855,0.55442223568329,-4.505
19254432536,6.63127872530421e-06,0.00147968355008877
"74","PLXNA4",304.096415496186,1.46486243463244,0.325745510926508,4.49
695355882565,6.8934037510765e-06,0.0014971553826838
"75","MBP",773.399639049995,0.677595730939899,0.150635101767094,4.4982

5919052765,6.85121405565928e-06,0.0014971553826838
"76","SEPTIN11",1450.03360220322,1.17969854823696,0.263433091177744,4.47817145128891,7.52851270288191e-06,0.0015965243569736
"77","SYNPO",19.5887462170752,-1.83135438953819,0.408999098791222,-4.47764896047613,7.5469565649805e-06,0.0015965243569736
"78","APBB2",276.29695316406,1.1314704723367,0.254417820551865,4.44729252802495,8.69593803047105e-06,0.00181600172536337
"79","RERG",204.317745676089,-2.04462822326405,0.461272642570866,-4.43258072247355,9.3111824432962e-06,0.00191987152935255
"80","GPR34",7.23567418618554,-3.62178814670275,0.81759893120957,-4.42978581361966,9.43267247172931e-06,0.00192061002364998
"81","DUXAP8",331.238726555918,1.04115429619818,0.235362756675296,4.42361532005059,9.70627832576383e-06,0.00195192058825144
"82","MYADM",2865.67994471316,-1.0400549630821,0.235634680138766,-4.41384503533014,1.01550661853046e-05,0.00201726674502959
"83","VEPH1",14.903469021436,2.9523300666078,0.671379756987694,4.39740703510951,1.09551782675538e-05,0.00214998673253233
"84","PRR16",58.5003613647614,-1.46208786059639,0.333247432426644,-4.38739422521502,1.1471675141163e-05,0.00219837783969888
"85","ENPP5",21.4050958980617,2.23171980354218,0.508463057472853,4.38914837713915,1.13795410944882e-05,0.00219837783969888
"86","ABCA1",838.39365629264,1.7704435879426,0.404747963072546,4.37418776490611,1.21885438091218e-05,0.00228689956851699
"87","PLXNC1",208.675728339442,1.70732684490868,0.390359851763503,4.37372551812283,1.22143939137442e-05,0.00228689956851699
"88","VCAM1",503.397361652831,1.8179033217197,0.416174935125325,4.3681230374248,1.2531889159345e-05,0.0023188821482422З
"89","SEC11C",64.0100880092773,-1.23738191013533,0.283430745768256,-4.36572929581557,1.26699313152163e-05,0.00231888214824223
"90","PHACTR2",864.875149827591,0.973642912243625,0.223556056385287,4.35525177884515,1.32914055528597e-05,0.00240559672278368
"91","SLC6A6",79.2776541969739,-1.42902423472347,0.328708043388302,-4.34739661370369,1.37762972094543e-05,0.00246595720049232
"92","A2M",253.36728319332,-1.67508863747475,0.386182683994951,-4.3375550144984,1.44076478451109e-05,0.00255093669292403
"93","STARD10",112.885648688834,-1.09832443786232,0.253584734083105,-4.33119305006104,1.48303582316128e-05,0.00259754521757787
"94","SAA1",9.19295635927053,-2.92410713534375,0.678183500623837,-4.31167542804265,1.62022151760871e-05,0.00275685558221605
"95","LMO7",96.1466123107061,1.81634402474685,0.42132227948376,4.31105619900375,1.62476601321592e-05,0.00275685558221605
"96","SYCE1L",74.0408744474988,-1.47580928805212,0.342034107633311,-4.31480152159068,1.59746361693549e-05,0.00275685558221605
"97","KCTD16",53.4414612629339,2.60491473287885,0.605476111546774,4.30225847593596,1.69065919255329e-05,0.00283908738015469
"98","TRIO",582.018022532065,1.06589085170049,0.248989687955095,4.28086343837953,1.86169579173573e-05,0.00309440436240646
"99","PCSK6",32.1688301431204,1.65030954262881,0.385865484256004,4.27690376559803,1.89510676447821e-05,0.00311812061480663
"100","FAM107A",65.3722343043947,-1.78527884418323,0.4179435289023,-4.

27157910273702,1.94093653957514e-05,0.00316159152931394
"101","SERTAD4-
AS1",61.5407128220949,-2.21414139085979,0.519800766606112,-4.259596239
76007,2.04796507013948e-05,0.00330290128985169
"102","TAGLN2",1648.17039518265,-0.973149177706298,0.230827647256894,-
4.21591256190926,2.487701425722e-05,0.00397276161995937
"103","LINC00702",51.6988740216457,-1.92704348348789,0.457667445416371
,-4.21057582921313,2.54720606475287e-05,0.00401966774880107
"104","ATP23",55.7145001125729,-1.24300066144533,0.29532832561787,-4.2
0887721773652,2.56642793219541e-05,0.00401966774880107
"105","CRIP1",6070.91206390113,-1.0372184033575,0.246883848102622,-4.2
012404267385,2.65456492056047e-05,0.00411811504676281
"106","GPX3",7674.26859959696,0.912354699810065,0.218034431682766,4.18
445239482871,2.85854340881789e-05,0.00439271826285233
"107","HAS2",229.316818365854,-1.41202477873143,0.337941939868815,-4.1
7830583348005,2.93688567127145e-05,0.00447092810274211
"108","RAB13",756.053187638605,-1.03362841921428,0.248094655572549,-4.
16626636647569,3.09628901230761e-05,0.00457844942474298
"109","FAM171A1",128.605363997765,0.845465143050287,0.20297047729728,4
.16545871255936,3.10727156147965e-05,0.00457844942474298
"110","MS4A4A",19.6624693806117,-2.39706598032372,0.575270759340705,-4
.16684829083074,3.08839882761648e-05,0.00457844942474298
"111","ABCG1",30.5755705737343,3.16936591114405,0.761038068920481,4.16
453005516497,3.1199452768523e-05,0.00457844942474298
"112","MAP4K3-
DT",282.852747592652,1.09708684758254,0.264859972612252,4.142139096225
96,3.44081550039078e-05,0.00500245200739488
"113","LINC00968",24.1716499469,2.33950648438526,0.565073304064204,4.1
4018228707446,3.47029944647075e-05,0.00500245200739488
"114","MAN2B1",296.007818151782,-0.582116846075463,0.140678294614826,-
4.1379293633697,3.50454219288841e-05,0.0050074989280666
"115","PLAT",327.000456010085,0.937059816609134,0.227025778898312,4.12
754807474466,3.66651794901111e-05,0.00519338355403843
"116","LAMC1",3238.96489834939,1.02744705761723,0.249250581695032,4.12
214507436676,3.75360675741756e-05,0.00520758783913557
"117","STAB1",20.0391028797416,-2.4205068796467,0.587360340955235,-4.1
2099134189106,3.77245604406653e-05,0.00520758783913557
"118","TBC1D12",249.277316992805,0.802541455321435,0.194717173386479,4
.12157511001115,3.7629074378741e-05,0.00520758783913557
"119","SLC4A4",51.2991805505825,2.54556365738727,0.618975533521333,4.1
1254325822158,3.91324115135563e-05,0.00535653656423797
"120","KCNB2",7.82962740079384,4.65078513156949,1.13219812445736,4.107
74848598032,3.99534837509179e-05,0.00539074137520767
"121","SLCO2B1",15.5975977124015,-2.65129286248716,0.645519331001642,-
4.10722457896526,4.00441835840216e-05,0.00539074137520767
"122","TEAD1",661.601150280507,1.00988314931007,0.246133188683746,4.10
299462137006,4.0783673448974e-05,0.00544114241675241
"123","FGF9",17.5457717864328,2.30388150544307,0.561746598232304,4.101
28252256959,4.10866546295381e-05,0.00544114241675241
"124","SIGLEC1",7.10188413919588,-3.30780784889798,0.810152723072478,-

4.082943566928,4.44688245499418e-05,0.00584155389591937
"125","F13A1",14.4375569231504,-2.74795655765988,0.674210692219316,-4.07581278281773,4.58539006995513e-05,0.00597531350795993
"126","FBXL7",309.138941597541,0.977580207866598,0.240210734856032,4.0696774374072,4.70782730993521e-05,0.00599107805090114
"127","SESN1",211.940888579354,0.742893937500765,0.182485150837683,4.07098294897185,4.6815176480287e-05,0.00599107805090114
"128","LAMA4",2097.07046505578,0.870595215169635,0.213763338378604,4.07270592690544,4.64700825231207e-05,0.00599107805090114
"129","MAP4K3",201.446380397627,0.940304159143749,0.231312725137958,4.06507752041285,4.80164995980308e-05,0.00602074593738379
"130","PFKP",171.543219465824,-1.07739019915511,0.265046395411755,-4.06491172038527,4.80506459487932e-05,0.00602074593738379
"131","MUC20",40.930559660812,1.97893902841476,0.487508522643266,4.05929114363986,4.92219112165747e-05,0.00609340774841862
"132","NTM",329.004473822504,1.2874817163024,0.317227147655853,4.05854834876597,4.93787109577787e-05,0.00609340774841862
"133","ANTXR2",1151.77772189316,0.688556462930941,0.169795038752159,4.05522133032397,5.00868536204397e-05,0.00613432149340859
"134","TNMD",156.542524802196,-2.11219306723363,0.522439707604583,-4.04294129349041,5.27848403591109e-05,0.00641650943738475
"135","NBL1",1212.95327071247,-0.915144232484169,0.226764357593173,-4.03566169832556,5.44486246614309e-05,0.00652142387580918
"136","ATP11A",197.420249585295,0.787976762069766,0.195210372315346,4.03655170943917,5.42425744841271e-05,0.00652142387580918
"137","MAN1C1",690.899061402261,0.794701517289433,0.197299517571672,4.02789386953644,5.62787362096383e-05,0.00665006737513618
"138","FCGR2B",20.0096715169111,-2.23663365032423,0.555729695146171,-4.0246790298581,5.70530769098024e-05,0.00665006737513618
"139","CFH",10848.2834247665,-1.13269502325196,0.281466939289291,-4.02425601426596,5.71557144403625e-05,0.00665006737513618
"140","CEP152",85.2046318063543,1.3886634202351,0.344783626769717,4.02763737143062,5.63401501776379e-05,0.00665006737513618
"141","EVA1C",55.1496999618752,-1.21599029706468,0.302358133695914,-4.02168872456271,5.77823848782337e-05,0.00667529976795424
"142","SERPINA5",252.136108904053,-1.3674280003835,0.340847437419249,-4.01184767806115,6.02453689750034e-05,0.00691082264249177
"143","TNFRSF12A",63.8796884992627,-1.77585244048112,0.443513200530841,-4.00405768837457,6.2265170385431e-05,0.00709256895390409
"144","PARP16",89.3855596898345,-1.14932608623329,0.287403569717574,-3.99899725449725,6.36114179199268e-05,0.00719559990623394
"145","CCN3",96.9234380910493,-1.92706884525445,0.482857546966376,-3.99096764120504,6.58042592291289e-05,0.00739231433505711
"146","RBMS3",1586.7356931567,0.811873021013877,0.203551884304305,3.98853110001255,6.64836879746276e-05,0.00741748488642951
"147","RDH10",282.281857820384,1.49098334256311,0.374701663803346,3.97912122254579,6.91704675175524e-05,0.00766474656730212
"148","PRDM6",60.5703501142261,1.00203839312266,0.252041329138954,3.97569079859208,7.01752547012521e-05,0.00772354543127497
"149","NLGN1",65.294141254428,1.13595344983656,0.285986015435408,3.972

05943132252,7.12539363227663e-05,0.00776888244504309
"150","PLAGL1",1876.60292135548,1.05366228904076,0.26533249412084,3.97
110158909107,7.15410624812121e-05,0.00776888244504309
"151","LRRC6",46.7012882906077,1.39451070245124,0.352761975997553,3.95
312079344095,7.71384705930899e-05,0.00828573286331398
"152","PRG2",48.4344042035865,-1.06418509732921,0.269346022638172,-3.9
5099614579715,7.78265779413739e-05,0.00828573286331398
"153","MS4A6A",47.5485833841613,-1.99455971346264,0.504812658059163,-3
.9510889468007,7.77964017640094e-05,0.00828573286331398
"154","FAM43A",413.640951064101,1.16136684385782,0.294986825633267,3.9
3701258137423,8.25022830222702e-05,0.00867102690572597
"155","DDIT4L",26.8565701239487,-1.76619809339159,0.448792431666373,-3
.93544536130804,8.30425561601848e-05,0.00867102690572597
"156","AC105446.1",8.93729702135717,-2.36469876274458,0.60067489697935
1,-3.9367364520077,8.25972323130197e-05,0.00867102690572597
"157","GPC1",72.6314738553857,-0.869669854558608,0.221218494907528,-3.
93127100391015,8.44979573095727e-05,0.00872338622688433
"158","RGCC",150.54359842292,-1.5017288213531 9,0.382028108066435,-3.93
093803739708,8.4615079123809e-05,0.00872338622688433
"159","MME",481.501741927313,1.11508376868799,0.286519184938535,3.8918
2933396661,9.9491222426033e-05,0.0100758556135757
"160","SEMA3C",1239.67624542903,1.47316318663089,0.378550467306151,3.8
9158993017297,9.95894624461719e-05,0.0100758556135757
"161","LHFPL6",5714.04921174353,0.520983773391831,0.133870717016757,3.
89169330680898,9.95470303242277e-05,0.0100758556135757
"162","LMNA",5332.73847397292,-0.867862087294331,0.223217670624686,-3.
88796319245504,0.000101088951419951,0.010111387295944
"163","MEG8",349.73826539647,1.55516274769197,0.400017225924116,3.8877
3944446829,0.000101182155395596,0.010111387295944
"164","SNX18",349.744046725685,0.573796765935392,0.147768664047394,3.8
8307473464981,0.000103143849049654,0.0102445741290842
"165","SCARA5",3637.93425644729,0.566209398459784,0.146062559443261,3.
87648553207594,0.000105976108378325,0.0104620898749972
"166","SEMA3E",73.7939443774907,-3.3332375209035,0.864657625497519,-3.
85497961575898,0.000115739272363276,0.0113570904067795
"167","MLIP",4.06037328783096,-4.51250845890865,1.17104546641643,-3.85
340158714552,0.000116488096682092,0.0113621233943389
"168","ANGPT1",905.96154685769,1.28272795316172,0.33318214436492,3.849
93006034804,0.000118151557997437,0.0114557781441681
"169","CPB1",846.156402663003,1.36283387175536,0.354424422078715,3.845
20305841873,0.000120452649837645,0.0114861750383318
"170","NT5DC1",97.0076628216657,-0.821088713242556,0.213708839303829,-
3.84209055609168,0.000121990808621241,0.0114861750383318
"171","STK3",318.894791647316,0.807416181054828,0.210141675831461,3.84
224679783366,0.000121913156543649,0.0114861750383318
"172","GAB2",108.465370594599,0.888751684302722,0.231202225967025,3.84
404466948982,0.000121022961982546,0.0114861750383318
"173","METTL21C",6.1383713521027,4.19659298756497,1.09165719007378,3.8
4424068812423,0.000120926277085262,0.0114861750383318
"174","AC022893.2",3.31255509196392,4.82856020702066,1.25968725823024,

3.83314205607223,0.000126516843226023,0.0117761877674782
"175","LAYN",37.4957501726326,-1.72023792725979,0.448721448617566,-3.83364319347681,0.000126259246602701,0.0117761877674782
"176","WASF1",150.343252403081,0.711544755345064,0.185869063559142,3.82820433761241,0.000129081571716165,0.0119466461459353
"177","WLS",226.967267671324,0.87700063045541,0.229299411237863,3.82469639028272,0.000130933339584172,0.012049565923653
"178","FABP4",492.740998841554,-2.62027734350359,0.685607065552814,-3.82183538524479,0.000132462105044951,0.0121217709498719
"179","DLGAP2",15.4069028984315,2.13763601270422,0.560677545161893,3.81259429978953,0.000137515736021762,0.0125139319779803
"180","CKB",1009.98819301168,-0.757875350812789,0.198944852881289,-3.80947453445813,0.000139262448664116,0.0126024779238321
"181","ERLIN2",216.536328630223,0.720407096125653,0.189197299666818,3.80770284456656,0.000140263675037329,0.0126229558159284
"182","PROSER2",33.7328554590319,1.53778505157391,0.404022397464729,3.80618763024928,0.000141125335560321,0.0126307175326487
"183","CYP11A1",45.8237662972514,1.7553293452802,0.462027319929816,3.79918950582154,0.00014517005987926,0.0129217218872856
"184","VIM",80652.4632410054,-0.778583215823626,0.205893838590553,-3.78147894639986,0.000155899408803393,0.0138013340760786
"185","CCND1",535.050472092492,-1.04745594434084,0.277129874871608,-3.77965726295701,0.000157044379689219,0.013827545409501
"186","SLC25A33",85.1048257776933,-1.60157708973022,0.42431534630149,-3.77449720753735,0.000160330704111611,0.0140197875518386
"187","MRC1",22.6150850371824,-2.49201238157747,0.660391607009151,-3.77353733016618,0.000160949123469447,0.0140197875518386
"188","S100A6",32784.7792158107,-0.80683036224232,0.214034126960398,-3.76963418731633,0.000163486992693649,0.0141651043829088
"189","LINC02518",11.7318254019391,2.31683550096242,0.615895155432058,3.76173684843669,0.00016873752949724,0.0145426752274103
"190","CXXC5",261.376056272016,-0.648473182944924,0.172496758733703,-3.75933546638997,0.00017036527415763,0.0146056839513349
"191","KCP",108.890340809158,2.86800217686795,0.763568968150467,3.75604863017795,0.000172617165662299,0.0146445886014229
"192","PHKG2",75.6783789526141,-0.967364055571539,0.257475898985363,-3.75710526454568,0.000171890204273001,0.0146445886014229
"193","IL7",52.4932522866184,-1.48741197176421,0.397491868326351,-3.74199346021088,0.000182566267510997,0.0153289790282816
"194","SCN2B",13.8126773120077,2.01819092456777,0.539307251355527,3.74219133804547,0.0001824225303977,0.0153289790282816
"195","GALNT2",346.005645535054,0.754425107885253,0.201961557953094,3.7354886520556,0.000187351028550899,0.0154911721018558
"196","CAV2",291.977213091326,-0.625752910164479,0.167492773585904,-3.73599945100641,0.000186971075259399,0.0154911721018558
"197","ADAMTS15",85.9451952608,1.99904265817887,0.5349878759653,3.73661301122369,0.000186515641438926,0.0154911721018558
"198","NT5DC2",92.0801111613984,-0.899873939621439,0.241161588203898,-3.73141488378577,0.000190407344515167,0.0156643698727654
"199","CYP26B1",643.244595788274,-1.49392775646916,0.400563165331579,-

3.72956848199588,0.000191807972126258,0.0157003017988171
"200","CEBPG",165.299595793295,-0.713228283341912,0.191578011401854,-3
.7229130740158,0.000196937339296477,0.0160395615990015
"201","PLEKHO1",149.332255254309,-1.07188764756121,0.288425890944195,-
3.7163364358597,0.000202132391096596,0.0162996758345171
"202","MID1IP1",484.668808857503,-1.33282637479532,0.35852009393588,-3
.71757789127907,0.000201141980929968,0.0162996758345171
"203","THBD",40.4942689481586,-1.56766654562267,0.422234666250439,-3.7
1278502436567,0.000204990999892537,0.0164487605775839
"204","RNF24",384.437042456787,-0.863101339526206,0.23294798620786,-3.
705124708638,0.000211286752490871,0.016870832898646
"205","AC068338.3",10.0940099869244,-1.80212993804571,0.48710416990646
1,-3.6996807857173,0.000215870819023357,0.0170695134518032
"206","CMC4",199.922567327509,-0.820397788711195,0.221730685218483,-3.
69997408298637,0.000215621486082622,0.0170695134518032
"207","IFRD2",124.158083543472,-0.95576608782628,0.258424152084875,-3.
69843948452765,0.000216929053546024,0.0170703253778318
"208","NID1",1519.69106758363,1.12420581971036,0.304268426527274,3.694
78303267062,0.000220074628919911,0.0172345943772905
"209","CYP1B1-
AS1",29.0216230443646,1.33819820291124,0.362552077477331,3.69105098561
989,0.000223329361554969,0.0173879178389999
"210","KCNS2",10.7971311069367,4.06515773997296,1.10163941214951,3.690
09831632754,0.000224167398010312,0.0173879178389999
"211","IL17RE",31.7935166760363,-1.25318892423322,0.340153611899273,-3
.684185263346,0.000229435326782655,0.0177121897533775
"212","MAST4",308.285726153051,0.944357657678275,0.256452371109775,3.6
8239004221973,0.000231057537487722,0.0177367036545812
"213","ST8SIA4",13.1292026287764,-1.84466086091153,0.501557948203747,-
3.67786188518774,0.000235197249026309,0.0177367036545812
"214","KCNQ1OT1",3163.63424047387,1.42118882160369,0.386148461873149,3
.68042077575479,0.000232849397480705,0.0177367036545812
"215","LMO7DN",9.57968163594628,2.5615455833287,0.69630643210829,3.678
76191459671,0.00023436892652554,0.0177367036545812
"216","NID2",884.716260689027,1.21282755040435,0.32956001213032,3.6801
4172157741,0.00023310436467308,0.0177367036545812
"217","MAP7D3",1355.24273770508,0.687729437827417,0.187129709620681,3.
67514831942757,0.000237711277488561,0.0178436820230929
"218","C16orf89",329.03149190558,1.23079722608987,0.33565516380556,3.6
6685026422788,0.000245556486236902,0.0183480257078573
"219","EBF1",3026.04552452881,0.513308477167997,0.140087631055176,3.66
419557031285,0.000248117153339168,0.0184139752623629
"220","PXN",522.963575464932,0.7790997128726,0.212659896846827,3.66359
489694366,0.000248700015821711,0.0184139752623629
"221","LINC01625",16.4775777465755,1.89882228864804,0.519677941330455,
3.65384430939432,0.000258342955133071,0.0189556234061378
"222","KRT222",927.23642276844,1.2582611499646,0.344268250514031,3.654
88582837912,0.000257296453664452,0.0189556234061378
"223","TSHR",15.0917042227778,2.88969513221403,0.792444736395193,3.646
55729225885,0.000265777241112289,0.0194136568631304

"224","TNFSF14",67.6664576401371,1.55374689469425,0.426412785610122,3.64376244598556,0.000268681417382239,0.0195249481793913
"225","NFATC2",50.3118434816798,-1.54638253954526,0.424504914114928,-3.64279066773442,0.000269698160744248,0.0195249481793913
"226","PDE1B",23.478577654093,-1.66501225018839,0.457694627501264,-3.63782345289555,0.000274951784425705,0.0197299102048912
"227","AC009271.1",15.6093460356894,1.88313482135859,0.517535178522138,3.63866051914776,0.000274059785319211,0.0197299102048912
"228","IGFBP6",11432.2368695788,-0.868178608387487,0.239401606976714,-3.62645271830582,0.00028734140393216,0.0205285268800481
"229","UBXN11",59.0737971330689,-0.966503056604405,0.267069423016962,-3.61892067495507,0.000295834295267125,0.0209249075485172
"230","NLRP3",37.2614283724296,-1.46143337180924,0.404293329173416,-3.61478477717445,0.000300597235333847,0.0209249075485172
"231","CAPG",295.437785022066,-1.65448315290065,0.457353281968961,-3.61751673843447,0.000297443100329211,0.0209249075485172
"232","MAN2A1",212.846148507588,0.939670280254107,0.259670879887194,3.61869717799053,0.000296089859239868,0.0209249075485172
"233","SUSD1",55.0726165025983,1.46092061438521,0.403951401670513,3.61657518291476,0.000298526638389609,0.0209249075485172
"234","C20orf194",141.483652445535,0.704217440911757,0.194761917632932,3.61578613247687,0.000299437520252387,0.0209249075485172
"235","GAB1",225.625214992469,0.654316312496335,0.18137141134021,3.60760446015933,0.000309037131785447,0.0214208759134176
"236","ARRB2",63.5876203589488,-0.990779287944026,0.274764718474417,-3.60591888742176,0.000311050292278624,0.0214690602157903
"237","HPSE2",40.1792069707819,-2.25877470391305,0.62685862563687,-3.6033239578034,0.000314173557142227,0.021593135326117
"238","KCTD21",39.4169164132753,0.982036933309293,0.2726631270658,3.60164919942514,0.000316204864195975,0.0216414329112951
"239","PHLDB2",311.192573638846,0.771026297305917,0.21421983716715,3.59922921939441,0.000319161774430717,0.0216617755987581
"240","TM4SF19",3.30795559429711,-4.2412236916118,1.1781455746628,-3.59991480070338,0.00031832146353496,0.0216617755987581
"241","DEPTOR",394.094863206145,1.1627511126795,0.323470364938409,3.59461403180133,0.000324872833292994,0.021957898678463
"242","TM6SF1",6.10025410356053,-2.67512925806604,0.745139159502739,-3.59010692694135,0.00033054233995523,0.0222487775848378
"243","RANBP3L",24.3030751040322,-1.25016559075836,0.348872324728288,-3.58344730190915,0.000339089200255618,0.0227301398475875
"244","MYO10",46.4980417400268,2.45011688957715,0.684521922583936,3.57931106184071,0.000344501213262689,0.0229982797657211
"245","HMGA1",62.6067164569564,-1.32033117488847,0.369856283619547,-3.56984924513716,0.000357186726184376,0.0236512787919402
"246","TYROBP",82.7499070572975,-1.43147182053531,0.400897019271916,-3.57067214701437,0.000356066349193523,0.0236512787919402
"247","HS6ST1",136.575817827165,1.06415375494296,0.298239048131871,3.56812349559414,0.00035954704098623,0.0237111811766182
"248","CYP27C1",41.1124912241833,1.44342988951873,0.404965498805779,3.56432805702048,0.000364789484904752,0.023768223678454

"249","ECHDC3",16.3173001615342,-2.30755947756727,0.647160218988032,-3.56566953570727,0.000362928456764778,0.023768223678454
"250","TBC1D2B",604.964929593768,0.692954552794276,0.194389979747304,3.56476477694517,0.000364182647720427,0.023768223678454
"251","DOCK7",223.041790953258,0.744833170386864,0.209057637830503,3.56281252441372,0.000366902710229504,0.0238106703064876
"252","HLA-F-AS1",160.798816177189,-1.04840512777482,0.294543722823499,-3.55942105207605,0.000371673239610077,0.0239295865612986
"253","RNASE6",9.39078792592459,-2.16127411649908,0.60715264751007,-3.55968820256727,0.00037129536533837,0.0239295865612986
"254","EPB41L2",1760.69453721251,0.510351502634164,0.14371627656332,3.55110440402559,0.000383618209187332,0.0245772695555104
"255","TMEM64",505.284671917796,1.3965238605816,0.393350600149563,3.5503285365539,0.000384750674483095,0.0245772695555104
"256","SHTN1",65.8936191716977,-1.007147933159,0.284985270310345,-3.53403504701219,0.000409266806248368,0.0260411992460143
"257","KCNIP4",5.91433375627616,-3.36619645874731,0.953798829163791,-3.52925203493749,0.000416736039132845,0.0264132814841825
"258","LINC01615",4.66669249829966,-3.30183399180063,0.936107323028685,-3.52719598551784,0.000419985762886216,0.0264136991955737
"259","CDH13",28.9909980424039,-2.30178946943415,0.652417145919161,-3.52809469191872,0.00041856239671188,0.0264136991955737
"260","ADAMTS5",1493.28376485555,0.963725205790186,0.273541131704735,3.52314549473476,0.000426457181980656,0.0267175424510881
"261","PLEKHG1",36.265163593096,1.39979097575687,0.398777745732187,3.5102033419311,0.000447764134255401,0.0276273862988115
"262","PTPRD",391.04742641256,1.04410881594209,0.297394560364937,3.5108537784311,0.000446670017811481,0.0276273862988115
"263","CCL18",6.32960101001153,-3.27556438999259,0.932711180817319,-3.5118742622151,0.000444958462340464,0.0276273862988115
"264","MMP9",10.8971688566777,-2.42799241551467,0.691521781551303,-3.51108595606043,0.000446280070367977,0.0276273862988115
"265","SPRED2",197.294643320453,0.598316803000058,0.170748291771752,3.50408661071618,0.000458176315959984,0.0280572707168127
"266","LUZP2",35.1031208250941,1.98059379573528,0.565211454821455,3.50416428902866,0.000458042683600349,0.0280572707168127
"267","SEPTIN1",237.694243218821,-0.908805639891956,0.25948711540196,-3.50231508984238,0.000461233810277435,0.028138717361832
"268","SERGEF",111.25996663281,-0.858746839459076,0.245413767504125,-3.49917956189904,0.000466692180112107,0.0283654810516646
"269","AL359555.4",16.4837678160654,-1.46745961262707,0.419504928583144,-3.49807478444494,0.000468629707010555,0.0283773579832525
"270","IL10",15.1930558494007,-2.6292304931144,0.752099430127157,-3.495854919967,0.00047254555063401,0.0284310290305996
"271","SSC5D",591.2655770238,-0.780546782909924,0.223294428299939,-3.49559453342678,0.000473006867658695,0.0284310290305996
"272","CSF1",1029.98189917293,0.573825730611277,0.164424329106633,3.48990769023688,0.000483187418780262,0.028830182653889
"273","PI16",2710.32166477831,1.18724471946132,0.340131769173211,3.490

54345128437,0.000482039220099803,0.028830182653889
"274","AIF1L",84.1044032050817,1.61431568999772,0.462717240360642,3.48
877359473255,0.000485241956536153,0.0288471030292606
"275","KL",16.2574760648813,1.7256416043701,0.494909746774346,3.486780
3990874,0.000488872603237101,0.0289572575786514
"276","IL6ST",2884.36533627089,0.867369108574189,0.249036881135311,3.4
8289419872277,0.000496024355149024,0.0292744229022553
"277","RECK",767.5386638902,0.727591488793804,0.209249943246443,3.4771
4067447506,0.000506791842369853,0.0298019217341608
"278","SNAI2",866.206739483429,0.869642496851934,0.250399997390727,3.4
7301320253185,0.000514650038055545,0.0301551599636215
"279","LIFR",341.578791642172,0.854368146085118,0.246123126550072,3.47
130380659821,0.000517937655051249,0.0302390195811104
"280","AC007262.2",77.5909210707459,2.89343293920172,0.835824502820846
,3.46177089740322,0.000536633755558653,0.0311299002347321
"281","SLC7A4",34.7688875685547,1.782046130508,0.514807482703492,3.461
57775553232,0.000537018967767187,0.0311299002347321
"282","FZD7",255.795596705311,0.646018738532345,0.186696243255166,3.46
026640530417,0.000539641213772475,0.0311709777699995
"283","FSTL1",9677.53588657504,1.04219970684641,0.301729263020029,3.45
408892864736,0.000552155269993466,0.0317811208230515
"284","GALNT16",379.294648560039,0.888962273264441,0.257612003463905,3
.45077970479353,0.000558969623720601,0.0320600570450172
"285","SMOC2",2929.25744473938,1.10526744826717,0.320585698117115,3.44
765051828168,0.000565485221755011,0.0323199606216399
"286","IL1RN",12.8304071692223,-2.64516442228396,0.767942228751096,-3.
44448361250532,0.000572151317725742,0.0325654015299183
"287","MAOA",126.123997206996,-1.59628367740733,0.463535224340034,-3.4
4371601894995,0.000573778024377589,0.0325654015299183
"288","ANGPTL1",817.95471970898,-0.802415135274091,0.233250070986293,-
3.4401495865836,0.00058139275586346,0.0328830090286802
"289","APBB1IP",380.227840863848,1.24144986426435,0.361232428000568,3.
43670658566236,0.000588833113165844,0.0331885902434548
"290","FLRT2",704.099521471393,0.883412036482525,0.257232100557799,3.4
3429935286799,0.000594087714190252,0.033369292332569
"291","SNX33",219.184248982535,0.539180712742131,0.15712620120742,3.43
151370426354,0.000600222802882207,0.0335980386121934
"292","RPL39L",63.6904225709941,-0.792539714275311,0.231397109340027,-
3.42501994314334,0.000614754292620124,0.0342936050427712
"293","SERTAD4",53.6787152021417,-1.99366852167922,0.582279823333486,-
3.42390108979854,0.000617290840192557,0.0343175784842886
"294","AGTR1",1204.4201248147,0.690813649155369,0.202087893742059,3.41
838215225851,0.000629945909651985,0.0343182007415564
"295","IRF4",127.485284115645,1.78356460957652,0.521384747259618,3.420
82237532049,0.000624320936290862,0.0343182007415564
"296","PLPP7",45.2496724538331,0.91835889087976,0.268584687860312,3.41
925259476217,0.000627934059896349,0.0343182007415564
"297","PTMS",2441.49448512248,-0.638747299040083,0.186906230364238,-3.
4174746224099,0.000632049863249242,0.0343182007415564
"298","FPR3",7.7514714344964,-2.53331099863703,0.740387254903704,-3.42

160265706697,0.00062253218737406,0.0343182007415564
"299","SNTA1",267.734956001479,-0.730807431220579,0.213829088165705,-3
.41771756822088,0.000631485994880435,0.0343182007415564
"300","TOX2",11.8819778353713,-2.48641601523257,0.726627909145664,-3.4
218559236955,0.000621952614685489,0.0343182007415564
"301","TET1",238.498309024688,0.579110656131564,0.169653307033429,3.41
349465128584,0.000641354174586499,0.0347077014944833
"302","SLC43A2",88.1844449850664,1.56020856955401,0.457415565854421,3.
41092145965704,0.000647437381215714,0.034920885770274
"303","TRIM47",275.980234710616,-1.00118888420876,0.294242535757689,-3
.40259739004306,0.000667485612380855,0.0358834097032071
"304","NATD1",106.695819526025,0.767827423414977,0.226233345486292,3.3
9396220201103,0.000688892000758412,0.036912374343269
"305","KANK1",279.587475833878,0.815576723424361,0.240429023797235,3.3
9217250290122,0.00069340769893979,0.0370325180591156
"306","CTHRC1",2802.63473860686,-1.07909175880519,0.318400619425866,-3
.38910068941131,0.000701222565509509,0.0373274979398183
"307","FCER1G",61.9925159669981,-1.62058159174112,0.478984509242525,-3
.38336952546533,0.000716022204550977,0.0379911585991233
"308","CCDC18-
AS1",302.306481729672,0.731358184966707,0.216610798979931,3.3763699151
2352,0.000734490952880224,0.0382234510865011
"309","S100A4",21672.378472587,-0.723594162060633,0.214264128931968,-3
.37711293844424,0.000732509669143629,0.0382234510865011
"310","NT5E",1090.00171144361,0.704051398341971,0.208512651483972,3.37
654043210941,0.000734035826979771,0.0382234510865011
"311","ADGRA2",305.655985895886,0.566850398436975,0.167849066459276,3.
37714358735803,0.00073242805002778,0.0382234510865011
"312","TUSC1",388.800325818709,-0.778770315492167,0.230712819975719,-3
.37549649635477,0.000736826302483967,0.0382234510865011
"313","MPEG1",13.2863864569153,-2.21490582100858,0.655975193909917,-3.
37650850454681,0.000734121024715058,0.0382234510865011
"314","ARNTL2",8.13973591806331,-1.94051997562372,0.574403251695898,-3
.37832345115462,0.000729292446489555,0.0382234510865011
"315","MMP23B",831.192608378076,1.32344563184481,0.392232640579493,3.3
7413436548656,0.000740482140775246,0.0382911542574222
"316","SGMS1",198.542916938767,0.492316578716402,0.146025645644169,3.3
7143915060005,0.000747765555046804,0.0385454212853082
"317","PTMA",6890.41938582811,-0.698896350965549,0.207859095228379,-3.
36235636067624,0.000772803203043158,0.0397103828844479
"318","NEGR1",2489.55946370355,0.612280995583544,0.182379553794394,3.3
5718002838082,0.000787418241152939,0.0402076982136057
"319","MEG3",3942.7167125979,1.37197371124531,0.408648623902061,3.3573
4327977114,0.000786953420185394,0.0402076982136057
"320","MAZ",598.424971139223,-0.630449245702798,0.187848822042538,-3.3
5615224438317,0.000790350478198838,0.0402313091855653
"321","HRCT1",273.696332009238,-1.60895592103982,0.479745182101196,-3.
35377192115384,0.000797180428166831,0.0404525607302477
"322","CPZ",11.2877996506693,2.07466797242132,0.620229046757126,3.3450
0291991925,0.000822817041412677,0.04140092691929

"323","AC022424.1",5.81183704172582,4.08295753911349,1.22054430614892,
3.34519404051466,0.000822250230002158,0.04140092691929
"324","ABCA6",2177.66583689006,0.724988024848589,0.216752425320959,3.3
4477468372063,0.000823494402470991,0.04140092691929
"325","SAMD9L",202.752714635556,0.982194980335682,0.293791771637588,3.
34316708347873,0.000828280124566445,0.0415133998432702
"326","PCSK5",1269.68305509325,1.50280053691737,0.45003510386082,3.339
29625494756,0.000839909384297615,0.0419671287141836
"327","RASL12",91.3069933856979,-0.857953004340492,0.257024839695831,-
3.33801591066378,0.000843789175790447,0.0420320546925095
"328","PLEKHG3",40.2608122868165,1.02559535346175,0.307569024695526,3.
33452094038738,0.000854464665684897,0.0424340699370161
"329","PRSS12",37.1753584251445,1.58295800730095,0.47515701368925,3.33
144194802142,0.000863973180136012,0.0427758636207766
"330","RHOBTB3",1943.48737214397,0.808578498425735,0.242818767019571,3
.3299670711225,0.000868562551716313,0.04280083955384
"331","AL513534.2",49.4218626122697,0.969537826177747,0.29118816513582
7,3.32959214096321,0.000869732819222853,0.04280083955384
"332","KREMEN1",332.188296464343,0.664638607015663,0.199708396237243,3
.32804538786696,0.000874576164743947,0.042909551649139
"333","CDKN2C",468.613842524131,-0.705153622531142,0.212118428062741,-
3.32433928052008,0.00088628301376366,0.0430945194364067
"334","CAMK2D",764.2885399613,0.606155411727552,0.182294453303674,3.32
514457100783,0.000883726977946928,0.0430945194364067
"335","CYGB",1639.51045672485,-0.712054350729908,0.214133409799508,-3.
32528376303633,0.000883285868163738,0.0430945194364067
"336","C3AR1",8.87322146744015,-2.05855452655079,0.619678493125078,-3.
32197187636667,0.000893837025918859,0.043332474152358
"337","TREM2",6.60915472831592,-2.85236524288392,0.859540250471772,-3.
31847780405671,0.000905095154829536,0.043748056311627
"338","PLAUR",232.20911898481,-0.736595453006315,0.222551182475766,-3.
30978000122073,0.000933693265350543,0.0449968331340089
"339","SMARCE1",1095.94711484622,0.576830889808292,0.17436587381269,3.
30816390383789,0.000939098303653722,0.0451238120006356
"340","LINC01341",19.1426619848734,1.23835824609577,0.374963455747971,
3.30261050006997,0.000957893389314644,0.0457569660367925
"341","STXBP6",188.242034209324,-1.27147158959299,0.384920937245937,-3
.30320194762648,0.000955875227614473,0.0457569660367925
"342","HFE",66.7865093393118,0.979307281762852,0.296687479225808,3.300
80421431436,0.000964081329115328,0.0459178969881859
"343","CTSO",543.916664604655,0.569825676799544,0.17290442514492,3.295
61071859176,0.000982079961962504,0.0466387769691173
"344","PTPRQ",16.0563876023601,2.14273927345206,0.650749504284426,3.29
272517204335,0.000992214107264772,0.046983068584988
"345","ZDBF2",69.9777655418281,0.829356950217989,0.25237160513734,3.28
625302266733,0.00101529765318797,0.047936763689214
"346","IER3",750.702449847448,-1.27635036186198,0.388760722640308,-3.2
8312580857838,0.00102662837949138,0.0483316464553038
"347","FBLL1",7.67745745715158,2.97475917793905,0.908022733056612,3.27
608447414674,0.0010525707313672,0.0489743497620188

"348","GDF10",1074.90365077987,-1.05479957309183,0.321771443859852,-3.27810187392281,0.00104507671274803,0.0489743497620188
"349","LINC02762",292.366852031241,-0.913312008843323,0.278778143011322,-3.27612487470451,0.00105242016912034,0.0489743497620188
"350","TAFA2",31.0056216408614,1.64441956832081,0.502059264087002,3.27534951737459,0.00105531320317199,0.0489743497620188
"351","WDR81",122.425615787306,0.698295307940961,0.213083102206473,3.27710316167787,0.00104878042865166,0.0489743497620188
"352","CEP128",51.3855735578899,1.58636393703056,0.484754058856799,3.27251295383002,0.00106595987795874,0.0493278990115621
"353","UCP2",72.822496505114,-1.57985862892367,0.483556941185007,-3.26716151577116,0.00108631684147374,0.0501275213336141
"354","SLC30A1",180.862791593765,0.679904536250568,0.20826837762876,3.26455962250065,0.00109634386083373,0.0504311262467386
"355","IQSEC3",15.8440273187852,2.18466569193489,0.669515598763358,3.26305420810228,0.00110218435409411,0.0504311262467386
"356","GPR183",79.2340753862429,-1.77566313329883,0.544149638265945,-3.26318903557003,0.00110166009893084,0.0504311262467386
"357","SCIN",330.75818765336,2.17472970178218,0.666911336426612,3.26089778805476,0.0011106006645958,0.0506406599974536
"358","CABLES1",120.768267948641,0.730814057509805,0.224156116305224,3.26029050447451,0.00111298153840557,0.0506406599974536
"359","LINC02066",13.746033694712,1.60467914399472,0.493090829379836,3.25432769863706,0.00113661085146436,0.0515689543988269
"360","ARHGAP12",496.625122618397,0.414059962254153,0.127294781559889,3.25276462381412,0.00114288124120428,0.0515689543988269
"361","SELENOM",4518.72782607582,-0.580329760123921,0.178381747432695,-3.25330236123448,0.00114072046894371,0.0515689543988269
"362","FRMD6",842.376856169154,0.840423030043947,0.258475120471856,3.25146586065894,0.00114811563069834,0.0516620317912852
"363","PKN1",364.30030070342,-0.72698023941113,0.223842907892677,-3.24772514016698,0.00116331588684944,0.0522017974680181
"364","STK17A",235.347640363228,0.805652713558746,0.248941174272113,3.23631763975734,0.00121082564938277,0.0541768953542299
"365","ABCA8",5023.0622913417,0.826904835576133,0.255566634821997,3.23557430003364,0.00121398285986211,0.0541768953542299
"366","PDCD4",868.472842414897,0.463576470431534,0.143584055572476,3.2286068852369,0.00124394763367854,0.0552194161891607
"367","GRK5",255.665939283554,0.691730513883258,0.214253142445435,3.22856647976319,0.00124412338028252,0.0552194161891607
"368","BAALC",2.62714933655286,-3.85457767314967,1.1951579396333,-3.22516175086644,0.00125901513642232,0.0556392339601349
"369","VPS51",517.909870750356,-0.558829801508083,0.173325262007942,-3.22416822010906,0.00126339160404301,0.0556392339601349
"370","EPB41L3",495.028158302717,0.434184368759328,0.134669688110933,3.22406901545412,0.00126382936737982,0.0556392339601349
"371","AC116351.1",3.56101419077771,4.17617657098539,1.2973836886609,3.21892174804189,0.00128673594280893,0.056303676733997
"372","ATP6AP1L",28.2525661541079,1.09995232491683,0.341774945140842,3.21835272174078,0.00128929163372711,0.056303676733997

"373","AKT1",297.09622366726,-0.589030495339711,0.182965312944942,-3.2
1935609465474,0.00128478829209157,0.056303676733997
"374","PTGER2",37.2320697011357,-1.27570481548873,0.396841243333582,-3
.21464776385749,0.00130604675998762,0.0568828761321881
"375","FAM43B",42.1510931942864,-1.54843248936765,0.482283333845015,-3
.21062823594324,0.00132445159133621,0.0571934204649138
"376","ARHGAP29",674.646777153928,0.808287830067074,0.251787066834739,
3.21020392440409,0.00132640835312985,0.0571934204649138
"377","DOCK4",101.834715923351,0.987281704354495,0.307349950565835,3.2
1223967186881,0.00131704450929899,0.0571934204649138
"378","LRRC23",65.4430317989728,-0.75687525043176,0.235784644574044,-3
.21002774289687,0.00132722161800831,0.0571934204649138
"379","CXCL12",12938.3449448183,0.54022714758426,0.168334656353203,3.2
0924496053116,0.00133084055682317,0.0571980523221442
"380","SGIP1",24.3795417998387,-2.40378854938237,0.749388068840408,-3.
20766856230038,0.00133815616474905,0.0573611204410454
"381","NR2F1-
AS1",39.9849366338208,1.43194719024959,0.446692839486028,3.20566407981
201,0.00134751196922268,0.0574597446771419
"382","HIST1H2BD",16.3515458549008,2.80052743498002,0.873528343132902,
3.20599492505984,0.00134596362204164,0.0574597446771419
"383","ZNF727",85.176191793487,0.754380443100844,0.235484743944842,3.2
0352151253392,0.00135757897153431,0.0577378691052803
"384","AC087645.2",20.4599784018534,-1.49913784480453,0.46831757932776
,-3.2011137548081,0.00136897474979273,0.0580709106754524
"385","VEGFB",1543.06527648093,-0.654580185726731,0.204570418007025,-3
.19977928433549,0.00137532865878777,0.0581889052545297
"386","CCDC85B",785.095918704176,-0.876817943995062,0.274175847399306,
-3.19801307194677,0.00138378007568561,0.0583948022094374
"387","NMUR1",22.5884070542435,1.64624765480037,0.515295409712284,3.19
476483541655,0.00139944821096226,0.058500986613816
"388","ZDHHC14",278.904470834898,0.831858632101755,0.260493908808758,3
.19338995643259,0.00140612919415406,0.058500986613816
"389","FXYD6",25.3467503733117,-1.79250394315231,0.560852799440601,-3.
19603280030013,0.00139331271508762,0.058500986613816
"390","OLR1",11.2413369740015,-2.55067504292309,0.798823932486815,-3.1
9303783874201,0.00140784497222763,0.058500986613816
"391","AL161431.1",44.1561802142763,-1.01808662928171,0.31859117378647
9,-3.19558956132299,0.00139545465784437,0.058500986613816
"392","DGCR2",136.231259751246,0.525683935831887,0.164587244076628,3.1
9395308415968,0.00140338922585603,0.058500986613816
"393","FGFRL1",55.0658160085987,-0.753879827294918,0.236207692781771,-
3.19159726940569,0.00141488461513801,0.0586439071144605
"394","AC117453.1",12.3902713420285,2.21164275604872,0.694583365007124
,3.18412859776168,0.00145190494945932,0.0600255830501088
"395","ADGRD1",357.586096531771,0.980376277275501,0.308236258209098,3.
18060011165346,0.00146970348414082,0.0606075950713161
"396","KIFC3",47.7462900760929,-0.900699769969352,0.283276009475795,-3
.17958365636435,0.00147486790770055,0.0606669781528641
"397","DYNC1I1",9.09674619732305,-1.71868182318452,0.541280087417788,-

3.17521716230798,0.00149724404033453,0.0609715204325227
"398","BAALC-
AS1",10.1479483964237,-1.65051431885086,0.519680996442384,-3.176014382
18811,0.00149313548425915,0.0609715204325227
"399","LMO2",57.6994632044644,-1.44215975778552,0.454102693041116,-3.1
7584497930942,0.00149400764962372,0.0609715204325227
"400","FZD10-
AS1",32.8375932494709,-1.93102544598156,0.607741654143629,-3.177378797
08538,0.00148612792324997,0.0609715204325227
"401","ALPL",710.46840724878,2.20849784690693,0.695928751279985,3.1734
5395321712,0.00150636794158477,0.0611900932680157
"402","SCRN1",279.751252165209,0.476024796353001,0.150139754748399,3.1
7054465121987,0.00152153446397261,0.0616524250837061
"403","PELI3",74.1806346115958,-0.621243981882135,0.196081303647111,-3
.16829789646948,0.00153334317008021,0.0619767416809842
"404","AC096642.1",18.452057608041,1.48158133944199,0.467888829284717,
3.16652428250307,0.00154272465313995,0.0622015887994966
"405","IGSF3",60.9370207741062,1.32064297077759,0.417576005509009,3.16
264094046252,0.00156345034683166,0.0628815869124469
"406","EMCN",152.91891397605,-1.3772169808929,0.435624178772977,-3.161
47966068392,0.00156969780328929,0.0629445327804593
"407","TTC28",342.963728962672,0.557916473023914,0.176504737734812,3.1
6091499969905,0.00157274386651402,0.0629445327804593
"408","ABRACL",90.1342164721261,-0.995646291618164,0.315248627229887,-
3.15828906335606,0.00158698111329159,0.06335866508433
"409","SREBF1",204.839594986677,0.986556659738291,0.312904363388443,3.
1529015736785,0.00161656303412104,0.0643818955080628
"410","CSF2",4.88936062546442,-3.86519339015885,1.22676968371962,-3.15
070827185703,0.00162875084898411,0.0647090794612248
"411","CCL3",94.7608587155072,-1.79075326977601,0.568752415807309,-3.1
4856380387263,0.00164074901187645,0.0650271548770208
"412","ZNF610",5.23584766253594,3.02343877934337,0.960603906937146,3.1
4743543879965,0.00164709474944216,0.065120209644814
"413","FHDC1",54.5635082828298,0.82668882262996,0.263267688365353,3.14
010742359964,0.00168885893211317,0.0664488481767907
"414","ACADVL",971.658601546962,-0.543454394610367,0.173055353478268,-
3.14035008849705,0.00168746047185249,0.0664488481767907
"415","GASAL1",55.4920026972956,-1.57238263145471,0.500889373952505,-3
.13918145048094,0.0016942050494875,0.0664985687978359
"416","FCGR2A",37.934961471196,-1.58730035683606,0.506657144299086,-3.
13288853161627,0.00173095165237022,0.0677756122028632
"417","ARHGAP28",90.443116359863,1.13037296733754,0.360888759215787,3.
13219222952206,0.00173506232970679,0.0677756122028632
"418","NMB",133.722423927923,-0.717651973174869,0.229267336101509,-3.1
3019719851033,0.00174688991822809,0.0680743776986062
"419","AC025171.2",71.4900502612791,0.831246022886953,0.26580424995275
9,3.12728642613761,0.00176427952700554,0.0685879456214636
"420","TMC3-
AS1",16.533376502711,1.55215966159946,0.496477145006463,3.126346655049
45,0.00176992782609345,0.0686437008553243

"421","MT-
CYB",66934.6345749963,-0.540766011981236,0.173031308934162,-3.12524950
144714,0.00177654307050572,0.0687366035046737
"422","CAB39L",887.976103716475,-0.979251399245507,0.313415471912897,-
3.12445136568642,0.0017813696714086,0.0687600250653428
"423","DTNA",13.9583416966553,-1.95242814320499,0.625117878986531,-3.1
2329595558897,0.00178837819057876,0.06886735779276
"424","PRIMA1",49.0731995068612,1.07920417154815,0.345723832055203,3.1
2157876167423,0.00179884122993358,0.0691068980999719
"425","AQP3",92.4232935987528,-1.27583318899016,0.409063787284143,-3.1
1891012758835,0.00181521319761544,0.0695717830022539
"426","SBF2",435.846773060998,0.574657342878535,0.184443094378914,3.11
563490524605,0.0018354936930762,0.0700195708817756
"427","AL161772.1",106.40268844065,-0.817887923003102,0.26245977001470
6,-3.11624110223549,0.00183172443208964,0.0700195708817756
"428","FMO4",65.0103641837861,0.849090850882284,0.272794823657817,3.11
256217950584,0.00185470939038391,0.0705872926634663
"429","CLEC10A",28.3794489815109,-1.59133938483213,0.511576840950553,-
3.1106556385064,0.00186672493078214,0.0708789799475765
"430","KCNIP4-
IT1",7.12651904690921,-2.92059986818046,0.939179237234719,-3.109736408
54728,0.00187254368169513,0.0709345675142603
"431","MIR155HG",14.9723464949045,-1.81081776260782,0.582945787298635,
-3.10632275258241,0.00189429834287438,0.0715921710141084
"432","COCH",136.98484352324,-1.2203228938856,0.393234070971586,-3.103
29898645476,0.0019137619236473,0.0721603425330809
"433","MED12L",6.37016315327248,2.38373931452445,0.768855130768618,3.1
003751150642,0.00193275698486497,0.0725407339319483
"434","MYZAP",12.5374479676873,-1.80815342718032,0.583099232717792,-3.
10093604265714,0.0019290995279268,0.0725407339319483
"435","RGPD2",10.9879988202215,3.13248685915985,1.0111732415526,3.0978
7356946876,0.00194914563621575,0.0729876626857893
"436","ECM2",1930.99912826007,0.448680938528651,0.145025326658903,3.09
381091472348,0.00197603381861157,0.0738248047508347
"437","LINC01278",95.225575101963,0.693944445665006,0.224388150902177,
3.09260735415362,0.00198406456419864,0.0739552120966401
"438","TUBB6",569.254818079239,-0.626340141649935,0.202598398468983,-3
.09153550266502,0.00199124168997525,0.0740532782831207
"439","PRICKLE4",3.36058045360653,-3.15742996329929,1.02178997618002,-
3.09009682704404,0.00200091253183169,0.0742434264943199
"440","IL6R",178.039311733128,0.902668100420313,0.292234965590582,3.08
88435906227,0.00200937195292202,0.0743878630480609
"441","CXCL3",119.376220095077,-2.54624555025018,0.824625957686419,-3.
08775818480655,0.00201672501694374,0.0744907795940965
"442","NECAB1",124.426985710868,2.69465057879746,0.873320511754382,3.0
8552306115458,0.00203194466282441,0.0748831371329114
"443","FCGR1A",4.21901380517165,-2.51331118862226,0.816629082378381,-3
.07766554345872,0.00208628923837409,0.0765395617204405
"444","DHRS4",141.716783156878,-1.20440507427262,0.391266617589662,-3.
07822088603462,0.00208240500079649,0.0765395617204405

"445","JUNB",25133.0700826602,-0.614666197364017,0.199783581235141,-3.07666022184561,0.0020933376767347,0.0766255672277113
"446","GTF2A1",231.378493234856,0.56666470028698,0.184447683095763,3.07222455048554,0.00212469826157323,0.0774255256885153
"447","MID1",208.249248469586,1.21049018693629,0.393925285421093,3.07289283459506,0.0021199460327134,0.0774255256885153
"448","TXN",2315.38903207811,-0.835015727748758,0.271882103131975,-3.0712419763188,0.00213170317761762,0.0775073952236906
"449","EPB41L4A",71.1297277097182,0.770353158803549,0.250940030575396,3.06986955025533,0.0021415228549445,0.0776910151095569
"450","PARD6A",31.4842433486454,-0.989366881095412,0.322483512382493,-3.06796113012419,0.00215524650003975,0.0780151338647723
"451","SERTAD3",232.358782873007,-0.746584854640643,0.243474825161961,-3.06637392241275,0.00216672163833571,0.0782566048045462
"452","A1BG",68.5716044554845,-1.26008972610723,0.411298173738615,-3.06368908632215,0.00218625994662304,0.0787875846693423
"453","NIPAL2",211.602198088974,0.750787243651654,0.245393322583666,3.05952597139506,0.00221687565483254,0.0797145420343646
"454","ALCAM",66.1643374920905,-1.01601708243263,0.332437025645091,-3.05626932036543,0.00224109854319007,0.0804080488326499
"455","CNTNAP2",13.0328234174794,6.80062169203669,2.22618634735749,3.05483038295923,0.00225187837675779,0.0805008124855772
"456","C11orf96",3206.21255122136,-1.06671235869539,0.349289594940798,-3.05394828287452,0.00225851011761979,0.0805008124855772
"457","MBNL2",878.037780323211,0.609321152833369,0.199480549025553,3.05453918093697,0.00225406569529005,0.0805008124855772
"458","ZNF528",46.0031728313866,0.868872746355833,0.284607926876944,3.05287612994528,0.00226659479011261,0.080612581956647
"459","FADS2",47.1389747371978,1.59664597595069,0.523155540602643,3.05195272157771,0.00227357908192945,0.0806848140861629
"460","MDFI",29.941170098646,1.93085510343683,0.632901269335397,3.05079985929623,0.00228232654343102,0.0808191675346692
"461","PLXNB2",224.491636296817,0.621591952197021,0.204128375293867,3.0451031185751,0.00232600544590447,0.0821872076102773
"462","MAP3K13",165.502588101537,-0.562659216789614,0.185132436741016,-3.03922546850461,0.0023718726818476,0.0836235441673909
"463","ZNF442",6.29827761269337,2.20547455743467,0.725822976523098,3.0385846532436,0.00237692313521407,0.0836235441673909
"464","TMEM131",237.435577232934,0.528539273450575,0.174319105247302,3.03202149128033,0.00242921907789273,0.0837607104268547
"465","SRSF8",248.829246857899,-0.492861004831601,0.162449513318728,-3.03393340344825,0.00241387709649098,0.0837607104268547
"466","GNS",676.646714179238,0.789407624825902,0.260219692919083,3.03361984625573,0.00241638711506197,0.0837607104268547
"467","CMKLR1",67.1918384396126,-1.02741889157509,0.338802727342372,-3.03249888108736,0.00242537996558697,0.0837607104268547
"468","GPRC5B",411.1268149531,0.683224149328359,0.225364101284836,3.03164588074672,0.00243224360193396,0.0837607104268547
"469","ANKFY1",244.317983148921,0.536930515483772,0.176839595610845,3.03625731346586,0.00239534850590501,0.0837607104268547

"470","NPLOC4",202.371835753819,0.597547828631139,0.197065986560702,3.
03222204430026,0.00242760557729315,0.0837607104268547
"471","ALPK2",53.0186418902291,2.60882817268609,0.85929937952169,3.035
99447975656,0.00239743753620943,0.0837607104268547
"472","JUND",15418.9139318243,-0.578356053700753,0.190728220529061,-3.
03235699518641,0.00242652041484922,0.0837607104268547
"473","TMEM59L",6.79829247024782,2.46250415387229,0.811843769636125,3.
03322418176098,0.00241955780991365,0.0837607104268547
"474","ANPEP",658.865812020832,0.763923926144952,0.25205661892452,3.03
076320472946,0.00243936473353213,0.0838287176044407
"475","LAMB1",513.936379746774,1.46642653473921,0.484938398952718,3.02
394394402699,0.00249502663840612,0.0855155101099485
"476","ZNRF1",221.647030873813,-0.729292239717651,0.241210451688313,-3
.02346865408646,0.00249894915662935,0.0855155101099485
"477","ARSI",7.06904955322089,-1.71431244570954,0.567907583194975,-3.0
1864686515548,0.00253906301268805,0.0865246807817482
"478","RFX2",121.87529235859,-0.985525592070828,0.326450347152393,-3.0
1891421059132,0.00253682355822492,0.0865246807817482
"479","PRELP",2168.80830781755,0.629851117093954,0.208762066311978,3.0
1707646518833,0.00255225422122063,0.0866052956610023
"480","ST3GAL5",757.460200914883,0.652614195515842,0.21635041949923,3.
01646836195835,0.00255737904186519,0.0866052956610023
"481","ENPEP",74.4453141020923,1.54665981934329,0.5126108820026,3.0172
2002720856,0.00255104571626343,0.0866052956610023
"482","ZDHHC7",278.764071946107,0.646192744495179,0.214456437388193,3.
01316552846343,0.00258537857206318,0.0873718497102431
"483","LASP1",556.996215385266,0.57991527511133,0.192588266414812,3.01
116618321006,0.00260246378678074,0.0877671482875186
"484","MERTK",7.32446932767202,-2.23822989669742,0.744002115311648,-3.
00836496380103,0.00262657499932269,0.088397273066048
"485","AC068888.1",269.632026659846,-0.607940855991008,0.2022420560202
87,-3.00600610948112,0.0026470367926826,0.0889022315793954
"486","SHKBP1",83.2110124145548,-0.737942521344792,0.24559749237319,-3
.00468263830428,0.00265858089949617,0.0891062227816731
"487","ATP1B3",474.013490082926,-0.680191690795447,0.226478740350234,-
3.00333572035758,0.00267037675142378,0.0893177965173347
"488","LSAMP",349.94350683871,2.44028301977732,0.813490240957677,2.999
76926201907,0.00270184196276918,0.0901850486302197
"489","DPYS",12.820246787802,2.61741385264673,0.874333418706091,2.9936
1067145322,0.0027569749990718,0.0918371487932116
"490","GNG4",20.8843969071975,1.57244361358278,0.525691581242244,2.991
19040458474,0.00277892162009902,0.0923792944281488
"491","MIR4435-2HG",326.849941558154,0.72461047149016,0.24263142703235
7,2.98646585214836,0.00282222355621909,0.0936276975707796
"492","TLR5",163.048380744166,0.779288563097878,0.261270290792893,2.98
269106959281,0.0028572623943276,0.0945974535390291
"493","RGS22",44.7668353685003,-0.851033572279693,0.285463608655145,-2
.98123314663126,0.00287090132716914,0.0948562103818623
"494","TGFBR2",2651.44840361666,0.484390841299104,0.162603478663425,2.
97896973226354,0.00289219346245864,0.0953662739068598

"495","KCTD12",1111.45584583489,1.09109800742871,0.366527550471427,2.97685127905212,0.00291225242599187,0.0958336964989528
"496","GALNT8",61.5257092778451,1.10606577247716,0.371803839776003,2.97486376994794,0.0029311868826251,0.0961558030594053
"497","AL110292.1",37.2098105475223,-1.38340197809147,0.465170314420149,-2.97396874909338,0.00293975013343875,0.0961558030594053
"498","CRAMP1",65.3870942521276,0.820938361097721,0.276003957136408,2.97437170689547,0.00293589195071796,0.0961558030594053
"499","AC097534.2",52.7415234259525,-0.905994866385245,0.304746129985974,-2.97294953811865,0.00294952938936663,0.096282333113012
"500","ZDHHC12",133.417126947046,-0.620404688102428,0.209024116689124,-2.96810099202638,0.00299645859955392,0.0976062593243294
"501","LINC02254",35.6299687684235,1.1479201605927,0.386827359457895,2.96752577739436,0.0030020710861003 7,0.0976062593243294
"502","TKT",674.098485148563,-0.466010933709337,0.157154428180803,-2.96530577664156,0.00302382213065546,0.0980769299860522
"503","SVEP1",4854.65075596261,1.0309386860041,0.347777627883608,2.96436171664593,0.00303311529471808,0.0980769299860522
"504","NPAS3",18.9996843628144,1.22008692513993,0.411606094706078,2.96421005624608,0.00303461063987785,0.0980769299860522
"505","C1orf162",34.3983725206484,-1.48961497349001,0.50303531197015,-2.9612532918532,0.00306389849219204,0.0984375592491444
"506","DAB2",2341.70825444826,0.636415605433854,0.214902188469571,2.96141984391176,0.00306224190521688,0.0984375592491444
"507","AP000662.1",14.6279266955692,1.4211308102671,0.479760430230494,2.96216761683396,0.00305481434727188,0.0984375592491444
"508","JAG1",364.878617770304,0.630129878360585,0.212910854665441,2.95959489407313,0.00308043815585323,0.098774128190341
"509","SNX7",377.304197020061,-0.605114392425106,0.204556635191179,-2.95817533300528,0.00309466046412042,0.0990352147348869
"510","MYO1B",288.123966745286,0.668123180403577,0.225980802182876,2.95654840566012,0.00311103395044508,0.0993639843505882
"511","AC108463.3",42.4502500487445,1.27276469030344,0.43073587925845,2.95486109142943,0.00312809857559249,0.0997134984301881
"512","RIPOR3",268.050234592724,0.802694715757409,0.271766916309495,2.95361454093728,0.0031407603051041,0.0999215715035951
"513","FZD1",244.225957263869,0.862304770248289,0.292157266238283,2.95150889570891,0.00316225437210886,0.100409281612634
"514","HECW1",8.55085984457955,2.02141394150132,0.685471691302101,2.94893861723378,0.00318867302182567,0.100854941461201
"515","CNTNAP4",17.4990566769985,-1.36751742164773,0.463658002771081,-2.94940972327593,0.00318381574582761,0.100854941461201
"516","USP46",147.492504980837,0.545916630258694,0.185262955811309,2.9467122980306 6,0.00321171868700869,0.10138698777652
"517","PTX3",661.867460589655,2.82581369545353,0.959859055943924,2.94398815946433,0.00324012395356817,0.101799338495479
"518","ULBP2",8.00043952366318,2.20494807119321,0.749049084735263,2.94366299369087,0.003243529785693,0.101799338495479
"519","TP53INP1",123.511526603951,0.697082160900515,0.236746616008405,2.94442291363425,0.00323557536794888,0.101799338495479

"520","SIGIRR",379.118151794088,-0.605672357374145,0.206305458113465,-2.93580384597015,0.00332684619097281,0.103814171656621
"521","LINC01970",3.02330060947503,-3.09315100597537,1.05344126767228,-2.93623489120576,0.0033222265505126,0.103814171656621
"522","JAM2",82.1147533232013,-1.0627622001643,0.361890060533556,-2.9366990588175,0.0033172584613726,0.103814171656621
"523","EFHD2",154.603870031933,-0.609631724741096,0.207821703448944,-2.93343628034916,0.0033523245776056,0.104409206586267
"524","MCC",170.681155606034,0.79457622154185,0.27107111242037,2.93124639671952,0.00337604890483571,0.104747544020702
"525","MRPS6",2061.49720057492,-1.00054206395279,0.341336385841408,-2.93124936413213,0.00337601665389357,0.104747544020702
"526","CUL7",74.9278105077352,0.886668379034263,0.302817534204048,2.92806155153749,0.00341082515218946,0.105625343923981
"527","TNK1",4.26238397496953,-2.63497613245951,0.900443837003798,-2.92630814291241,0.00343011002526594,0.10597217238643
"528","SERPINB8",43.1121950116657,-0.797720962143949,0.272644797000337,-2.92586167394554,0.00343503634477469,0.10597217238643
"529","BCL2A1",35.995892477599,-1.94112290928384,0.665438415206017,-2.91705868631415,0.00353349282552233,0.108803524829742
"530","CISD1",259.779085042002,-0.705387917457071,0.242474552617364,-2.9091214308588,0.00362446050632783,0.111394032429385
"531","RTCA-AS1",91.1597943312072,0.842055712966172,0.289597358381051,2.90767746526955,0.00364123675488295,0.111698880414856
"532","NDRG1",1260.02946173658,0.54772529605006,0.188472755936171,2.90612451295378,0.00365935801486846,0.112043764481565
"533","CAMK2N1",665.364887680503,-0.952317290055791,0.327979304204198,-2.90358957973422,0.00368911416927354,0.112321459258498
"534","CD24",28.9831290042379,1.84355770324276,0.634914165699174,2.90363296779276,0.00368860301626924,0.112321459258498
"535","INSYN1",32.9628982239146,-1.30408600132427,0.449005751891063,-2.90438595904817,0.0036797423057462,0.112321459258498
"536","MT-CO2",49155.0935113061,-0.602940453681484,0.207904916978946,-2.90007789350428,0.00373069937827963,0.113375675695517
"537","FAM198B-AS1",8.36414241600413,2.1796809250945,0.751901632907764,2.89889106460004,0.00374484979682494,0.113426741524153
"538","KCNK15-AS1",48.069706354392,-1.34273664413274,0.463209241534379,-2.89876911713792,0.00374630652219253,0.113426741524153
"539","STXBP5",148.927080935068,0.8285795738398,0.285899047018285,2.89815437470419,0.00375365778339552,0.113438463142356
"540","SPI1",17.1739975810022,-1.87914346901883,0.648945149652252,-2.89568921198626,0.00378326877636144,0.113700489110981
"541","SNHG9",484.715926070857,-1.10828190162304,0.382727341378595,-2.89574791712287,0.00378256116278123,0.113700489110981
"542","CENPB",168.167952825892,-0.684843751132852,0.236484075631923,-2.89594024165408,0.00378024378471083,0.113700489110981

"543","CACHD1",222.592894784354,0.683444267114434,0.23621239846753,2.8
9334629150883,0.00381160801375614,0.114341220876747
"544","AC087854.1",25.4992531156215,1.27727739256245,0.442067723520259
,2.88932515224426,0.00386069642670857,0.115600889879882
"545","SH3BGRL3",2675.63246330252,-0.808560051383174,0.280095444006362
,-2.88673046522245,0.00389267543110006,0.116344568985668
"546","BOLA3-
AS1",57.27942904034,-0.74138887967384,0.257107228507541,-2.88357851304
868,0.00393184619868172,0.116447457769491
"547","GJA1",817.082245609173,0.928616210782888,0.322036184140377,2.88
35772391903,0.00393186210161583,0.116447457769491
"548","PTHLH",28.9391670258457,-1.30999145147896,0.454052745462104,-2.
88510853545382,0.00391278741203528,0.116447457769491
"549","UBAC2",292.371191069522,-0.536422459442873,0.185902546021009,-2
.88550356584278,0.00390788034760134,0.116447457769491
"550","ISYNA1",73.1771809228933,-0.66420687469968,0.23026152597439,-2.
88457601368261,0.00391941125056982,0.116447457769491
"551","FIGN",88.8978552109413,1.31478334691193,0.456050995890318,2.882
97440145957,0.00393939452700899,0.116458797550725
"552","PARVA",858.01097672304,0.447129654933114,0.155224817707423,2.88
052942523592,0.00397007891832444,0.117153288950338
"553","BCAN",4.43593813940938,-2.13061527054216,0.739866112294685,-2.8
7973085283509,0.00398014790090469,0.117238027410193
"554","KLF3-
AS1",81.5298918671571,0.62754907387766,0.218106393705233,2.87726124492
152,0.00401143342259442,0.117561051229667
"555","TPMT",66.3054158344096,-0.582518832596708,0.202463384684817,-2.
87715644734251,0.00401276594534318,0.117561051229667
"556","MYH10",418.767881713308,0.629647593969443,0.218794109378573,2.8
7780871138529,0.00400447880271593,0.117561051229667
"557","AL592183.1",40.4950203557275,-1.40383472410409,0.48837763566367
2,-2.87448609762069,0.00404685585843001,0.118346921145362
"558","A4GALT",429.438694627128,-0.725908015469702,0.252617894337404,-
2.87354154927821,0.00405897684111925,0.118488662661275
"559","LRRFIP2",319.509874217408,-0.447949036145221,0.156115525395403,
-2.86934329568231,0.00411325093836249,0.119292038263095
"560","LST1",33.6424530919183,-1.02615978997152,0.357653136923341,-2.8
6914802089786,0.00411579136250595,0.119292038263095
"561","AC005162.3",11.2498094954193,1.68427446866492,0.587025979334987
,2.86916512719412,0.00411556876153488,0.119292038263095
"562","OXT",40.7244066682093,-1.01935688349737,0.355186468652122,-2.86
992037553026,0.00410575174132327,0.119292038263095
"563","RUNX1T1",1420.20167640804,0.650469524927372,0.226803634259897,2
.86798545821357,0.00413094519610482,0.119518590229754
"564","CLDN5",7.4830968510021,-2.14623008853191,0.748599042681954,-2.8
6699550248255,0.00414388901463772,0.119680510920982
"565","CISD2",230.079564091435,-0.471093013199993,0.164411087320759,-2
.86533603588978,0.00416566935766534,0.119884431390478
"566","FZD8",237.648320334262,1.01262736784127,0.353380801113289,2.865
5415479593,0.0041629664078805,0.119884431390478

"567","AGA",134.314178453407,-0.682770555888499,0.238644592366471,-2.8
6103510294511,0.00422260295528155,0.120733340248216
"568","LINC01554",11.1377471730416,2.37230560603192,0.829386624702872,
2.8603133151343,0.00423222648914797,0.120733340248216
"569","KCND2",220.441601375998,0.677472368802527,0.236842220829081,2.8
6043749476336,0.00423056939747397,0.120733340248216
"570","LURAP1L",267.432913313887,0.812882400676211,0.284180008451227,2
.86044892850274,0.00423041685168276,0.120733340248216
"571","LINC01894",38.418452403036,1.00532743508806,0.351367672138436,2
.86118363983117,0.00422062499024897,0.120733340248216
"572","USP24",102.638816860172,0.580004169066694,0.202898329116181,2.8
5859509830946,0.00425521541585008,0.121176929910458
"573","ADAMTS16",53.6531750963011,2.75639127005971,0.964484973922893,2
.85788928245146,0.00426469166866884,0.121234838727656
"574","S100A10",23053.5729524811,-0.762293780468174,0.266854646346802,
-2.85658799988629,0.00428221281428105,0.121520844132098
"575","FAM20A",163.578617586404,0.743732533872466,0.26089232585468,2.8
5072598987343,0.00436195410929804,0.123568470411054
"576","ARHGEF10",438.23322528799,0.660677560646696,0.23214636709809,2.
84595261560797,0.00442787831841877,0.125042743772691
"577","FZD6",57.6962995636416,-1.10867802675219,0.389577540521009,-2.8
4584687625852,0.00442934883398875,0.125042743772691
"578","LILRB4",4.98236955580768,-2.1591017156142,0.758851693893651,-2.
84522223905951,0.00443804468748937,0.125071470440336
"579","ALDH5A1",27.1955529196809,1.02058374266133,0.359354406029897,2.
8400479458054,0.00451067536960946,0.126898775640015
"580","PINK1",547.228818457152,-0.657147507878561,0.231455981769423,-2
.83918999567362,0.00452282181893916,0.12702111139431
"581","ZIC1",39.9367241645596,-1.33219765183208,0.469769065903326,-2.8
3585648465461,0.00457029781468818,0.127981227647283
"582","LRAT",6.36769618635784,3.41198687255109,1.20323109183245,2.8356
870892979,0.00457272235807715,0.127981227647283
"583","HIST1H2AC",158.507723999054,1.25647102326116,0.443194372964992,
2.8350337908293,0.00458208388544724,0.128023266569554
"584","KAZN",166.582920517142,0.834586141376534,0.294576826602663,2.83
316970653043,0.00460889103224427,0.128373005408438
"585","RGS3",42.4182958524577,-0.89179139386335,0.314798734018583,-2.8
3289383816488,0.00461287030060572,0.128373005408438
"586","TYMP",1025.40037447539,-0.657091261382044,0.231981022323408,-2.
83252162095391,0.00461824428567406,0.128373005408438
"587","HELLPAR",849.291310687628,-0.615342631740711,0.217291719353234,
-2.83187336163693,0.0046276172391514,0.128414407510285
"588","ERLIN1",187.057875104628,0.402205860375285,0.142144124438629,2.
8295637400682,0.00466115142582018,0.129124992474804
"589","AC008969.1",33.7567886052349,0.829315751771594,0.29340110575537
2,2.82655973513286,0.0047050967548172,0.130121088351812
"590","STAMBPL1",87.8804091912355,0.814429868275833,0.28824711758589,2
.82545711158119,0.00472132080730945,0.130335842839715
"591","IL4R",217.399477024228,0.53293931723778,0.188656377509251,2.824
92075949909,0.0047292310090449,0.130335842839715

"592","ZNF358",530.134085285713,-0.436905816593648,0.154689572482966,-2.82440380163154,0.0047368665333115,0.130335842839715
"593","COL6A6",207.007718079743,1.33564207375774,0.473560321674317,2.82042648555404,0.00479598611115034,0.131739996230233
"594","H19",4874.49458717758,1.06400763935491,0.377339155641117,2.81976472212939,0.00480588722533118,0.131789725611818
"595","KLF7",362.36422535435,0.567599439379942,0.201398846821143,2.81828544869481,0.00482808660594686,0.132175970965157
"596","RAI2",287.742374114064,-0.790307600344231,0.280566315799909,-2.81682994657083,0.00485001976914149,0.132553644328097
"597","NNT-AS1",267.443003637906,0.469200422821508,0.166656613345954,2.81537235997661,0.00487207464571925,0.132711076762744
"598","NHSL1",255.413784987451,0.581660425631705,0.20658572866706,2.8155886148802,0.00486879675122285,0.132711076762744
"599","KDM4A",145.577584019767,0.462811174224669,0.16456017055845,2.81241306844833,0.00491713128000869,0.133714776995094
"600","C9orf72",53.2432070851283,-0.826493063260345,0.29395859507681,-2.81159686126676,0.00492962456250574,0.133831090831093
"601","DIP2B",87.7964691941321,0.676430487619882,0.240901193687769,2.80791671168139,0.00498631211291372,0.13514482197546
"602","GPR88",9.26603712213533,2.24850876793415,0.801325012817153,2.80598849651436,0.00501624830907803,0.135505254903104
"603","ZMAT3",250.40174885215,0.625979609930909,0.223067953383521,2.80622832834559,0.0050125160099678З,0.135505254903104
"604","EMX2",573.822500805224,-0.744068024907606,0.265421487395311,-2.80334509541578,0.00505755210431695,0.136394811634468
"605","KCNAB1",24.6354966526027,3.15573225227923,1.12668967825748,2.800888579329,0.00509621113440704,0.136983800607849
"606","WDR34",131.44428083311,-0.480363721684398,0.171473264543501,-2.80139135954068,0.00508827703957218,0.136983800607849
"607","KLK1",2.63637341299594,-3.82474815254585,1.36637186510794,-2.79920002029881,0.00512293925505277,0.137475383073401
"608","SESN3",455.995160050942,0.743051705697389,0.265606870846796,2.79756206354311,0.0051489873867687,0.137947130827427
"609","KHDRBS3",316.770665970605,-0.816484565571418,0.292030699418171,-2.79588607361536,0.00517576418993463,0.138436819195148
"610","AKR1C1",1910.08596849221,-0.951173224102415,0.340311316525727,-2.79500909288894,0.00518982554537558,0.138585357882988
"611","PXDN",443.539508982679,0.812349074977326,0.290850990832285,2.79300776199093,0.00522204388758525,0.139053349478791
"612","LINC01474",5.90229011518952,2.15402287407264,0.771260507065017,2.79286032973429,0.00522442445091903,0.139053349478791
"613","AL512625.1",22.8645564791085,1.09439679972701,0.392059147399894,2.79140738581146,0.00524793738306892,0.139451308373262
"614","BTNL9",5.58700189553281,-3.03729437140957,1.0889975871771,-2.78907355459147,0.00528590584033681,0.139886455909661
"615","CLEC7A",19.2736836951029,-1.52506899293514,0.546714197825472,-2.78951781205065,0.00527865927394167,0.139886455909661
"616","POC1B-

AS1",38.112499789177,0.811566526752393,0.29100733474623,2.788818115048
93,0.00529007654492915,0.139886455909661
"617","HMGN5",61.0007052432083,-0.636419349169287,0.228307838979958,-2
.78754926687014,0.00531083782624061,0.140207840116099
"618","BZW2",240.613690765377,-0.484241632910054,0.173779744433382,-2.
7865251758135,0.00532764795203439,0.140424041247068
"619","KCNS3",46.6619390723086,-0.958757166568226,0.344197082503309,-2
.78548894021788,0.00534470632373567,0.140427524105156
"620","NR2F1",45.2464828653267,1.56049824758824,0.560228028782662,2.78
546978625667,0.00534502209743978,0.140427524105156
"621","CD58",139.400785874003,-0.610655868868733,0.219375707050667,-2.
7836075246367,0.00537580405775097,0.140835400897443
"622","SPON1",210.483467416766,1.90825322059426,0.685562967083884,2.78
348351969946,0.00537785945100874,0.140835400897443
"623","ZNF626",166.324353443464,0.465868149028567,0.167414720214802,2.
78271915654032,0.00539054451576355,0.140835400897443
"624","ZNF91",300.267565914338,0.548363085047118,0.197079706140074,2.7
8244318396421,0.00539513107987012,0.140835400897443
"625","SET",1201.53583625542,-0.463195890926089,0.166542756926662,-2.7
8124308420124,0.00541511730731413,0.140905504502939
"626","CRHR1",3.48419762190355,3.47653652388785,1.24990063982046,2.781
45031143214,0.00541166141788104,0.140905504502939
"627","PDZRN3",204.280987790968,1.08389086474843,0.390032919244185,2.7
7897277708974,0.00545310949047249,0.140993175381439
"628","PLD1",151.257026239062,0.755036072890321,0.271637414583981,2.77
957318231245,0.00544303874836453,0.140993175381439
"629","C5AR1",41.4119938808624,-1.1631730169094,0.41848834865637,-2.77
946332471135,0.00544488016057264,0.140993175381439
"630","ZNF415",27.6039199685774,1.0984007958826,0.395190844463932,2.77
941863094668,0.00544562946987442,0.140993175381439
"631","LINC00623",227.722708047701,-0.51301264653643,0.184707991733609
,-2.77742528475059,0.00547914354431219,0.141441789529796
"632","KCNB1",19.0458933006576,1.25165683212145,0.450999375689968,2.77
529615247601,0.00551514611296451,0.142145909864049
"633","LINC01068",2.50809674174418,3.61136997486425,1.30180060716974,2
.7741344987661,0.00553487899406529,0.142429137337014
"634","NEK6",173.570488186658,0.707413887709523,0.255078237118418,2.77
332121979936,0.00554873196747701,0.142560402236961
"635","PODN",3571.63015592499,0.513564813067944,0.185306933379709,2.77
142794228648,0.00558110235297954,0.142643907283341
"636","FMO3",123.065123984178,1.19359704154539,0.430731936275777,2.771
09018631296,0.00558689502654823,0.142643907283341
"637","LRRN2",17.3110569468918,2.48229104798642,0.895783574579714,2.77
10834608137,0.00558701042708403,0.142643907283341
"638","HPRT1",61.7627862958281,-0.835097085535989,0.301280475206717,-2
.77182610311207,0.005574280667882,0.142643907283341
"639","NCKAP5",17.2565188951352,1.26473868121372,0.456552324935406,2.7
7019437233762,0.00560228494001621,0.142810045990491
"640","RHOBTB1",48.7637886353527,1.13520465289254,0.409952848869477,2.
76911029164229,0.00562096038531501,0.143062224556869

"641","SNHG18",159.617089194902,-0.621925783073832,0.224836419674922,-
2.76612563023838,0.00567266764118321,0.144153015923921
"642","SHOX",12.2708361697315,2.67257286808324,0.966483653215293,2.765
25408287263,0.00568784733922056,0.144313621975956
"643","TMEM25",59.1788589142874,-0.769408221877341,0.278916370101377,-
2.75856243790096,0.00580562132375001,0.147072730548311
"644","DPYD",394.971820415709,0.422170532639985,0.153274286501104,2.75
434674841526,0.00588094247641516,0.148749490680631
"645","ZEB1",1166.76042299529,0.50867330177214,0.184834697024047,2.752
04444815878,0.0059224481907701,0.149567067565045
"646","FMNL2",264.56186650846,0.602027160250799,0.218947043150021,2.74
964736490319,0.00596594299926298,0.150432268599063
"647","BTK",3.27106984122385,-2.60265749339797,0.947117142179487,-2.74
797844689917,0.00599639507029468,0.150966428593555
"648","LINC00882",107.191151002054,0.926669519924713,0.337521370035465
,2.74551362429983,0.00604162588193618,0.151870438257498
"649","PHYHIP",51.9880970066775,-0.902462900310846,0.329017783049202,-
2.74290007046789,0.00608992151245043,0.152613433102008
"650","ADAP2",7.33505047161066,-1.78069743590902,0.649179149038517,-2.
74299850595381,0.00608809624847727,0.152613433102008
"651","BDKRB2",212.744688915365,1.02252170245725,0.373114137032233,2.7
4050645893622,0.00613445762654569,0.153493364483568
"652","PCBP4",102.63057705842,-0.636034871694489,0.232221605818373,-2.
73891341614418,0.00616426051260571,0.154002514554961
"653","LVRN",162.315636011501,0.832360484694244,0.303980530143841,2.73
820327999421,0.00617758781287471,0.154099123865109
"654","MBLAC1",29.3490150814497,-0.984807436991649,0.359973567013613,-
2.73577708819494,0.00622331672515772,0.155002455865587
"655","TBC1D1",61.3001598685397,-0.756150752582183,0.276467315773816,-
2.73504573394421,0.00623716096086259,0.155110099070978
"656","SGCD",374.680735734971,0.752287222992455,0.275342228473959,2.73
218978128378,0.00629148889701446,0.156222656468702
"657","IER2",3660.42047067752,-0.611965873586392,0.224035921185159,-2.
73155246868033,0.00630367028380036,0.156286887751635
"658","KCNMA1",16.9640152391103,-1.11283972128535,0.407661937506776,-2
.72981021503105,0.00633707957107633,0.156704040065653
"659","FAM160B1",234.958944759018,0.481515257329674,0.176400406971962,
2.72967203191435,0.00633973616571094,0.156704040065653
"660","TMEM265",104.778444756811,-1.02813439068417,0.376921484262068,-
2.72771501124973,0.00637746799570984,0.157397842700178
"661","CCDC74B",41.8279731337555,-0.798307721339818,0.292775087649154,
-2.72669279257963,0.00639725685420633,0.157409240027442
"662","MRC2",1981.67976466091,0.669033923974126,0.245329438601465,2.72
70837441607,0.00638968201144953,0.157409240027442
"663","PLGLB1",8.20608739346994,1.98038475854206,0.726736724309119,2.7
2503740666847,0.00642942023459798,0.157724135845431
"664","ROR2",29.8886255756046,1.58757616912065,0.582524335542589,2.725
33879231313,0.00642355364052686,0.157724135845431
"665","PIM3",186.662003149339,-0.658831686497323,0.24193476173071,-2.7
2317909912693,0.0064656995629815,0.158375609295347

"666","GTF3A",673.169848561457,-0.737352261147833,0.270829593137244,-2
.72256902433176,0.00647765001057856,0.158430091625096
"667","GDF7",2.43405712095081,4.38459340801951,1.61144239600152,2.7209
1228262271,0.00651020339744423,0.158786647557673
"668","AL356218.2",7.76534950694452,-2.16955306147638,0.79754949494268
,-2.72027388297989,0.00652278656335211,0.158786647557673
"669","ECPAS",225.213825520001,0.455219277566087,0.167369467535104,2.7
198466020728,0.00653122069271537,0.158786647557673
"670","PCNX4",277.874617800885,0.583180409610175,0.214415445789172,2.7
1986193654909,0.00653091783474559,0.158786647557673
"671","FLNB",299.382264244783,0.801683327768049,0.295023807752032,2.71
735130082066,0.0065806718232115,0.159039411466309
"672","ITPR3",128.527703438366,0.671389154276735,0.247061663467573,2.7
1749629162865,0.00657778925119746,0.159039411466309
"673","ESR1",56.7680794280004,1.20940834275793,0.445048218638673,2.717
47710047533,0.00657817072659616,0.159039411466309
"674","NRK",48.882277465353,2.74889772555889,1.01157710064784,2.717437
67607869,0.00657895445413027,0.159039411466309
"675","SLC37A3",229.94931182624,0.564511361016343,0.207808500091398,2.
71649793328021,0.00659766071129339,0.159213770853716
"676","FAM131C",4.53814588839418,-2.45996121476276,0.906528394461744,-
2.71360635782774,0.00665551997432555,0.159825360588931
"677","RALGPS2",229.216983955878,0.750949103412661,0.276768886597033,2
.71327139638358,0.0066622518165562,0.159825360588931
"678","C5orf38",193.9401596614,-0.800121383178827,0.294798225809043,-2
.71413228822181,0.00664496249823843,0.159825360588931
"679","GLI1",37.2944908705596,-1.23677205166224,0.455616843211902,-2.7
1450028700328,0.00663758428238118,0.159825360588931
"680","CPNE2",93.0857477816672,-0.656582457135446,0.242135353971508,-2
.71163399464874,0.00669524745885838,0.160380714496094
"681","SCUBE2",19.3313972608526,1.51993142299145,0.560803721214705,2.7
1027342632333,0.00672277623615443,0.160678293571201
"682","GALC",177.361094501914,0.526828877656506,0.194398538648713,2.71
004546288545,0.00672739862579405,0.160678293571201
"683","OSBPL10",124.60472694024,0.875057491817884,0.323053914146847,2.
70870419301008,0.00675465330596805,0.160871189974771
"684","CDS1",19.407064360068,1.53808949247409,0.567838120068091,2.7086
760083836,0.00675522708224836,0.160871189974771
"685","AFAP1",193.764445264771,0.57928215389002,0.214125647241435,2.70
533754995195,0.00682350136532146,0.162023343643909
"686","FRAS1",6.46210806774433,3.41405499901215,1.26186040754972,2.705
57264384066,0.00681867327996767,0.162023343643909
"687","EVI2B",23.0473152557372,-1.00122977901828,0.370387289523,-2.703
19691668605,0.00686760473550769,0.162151850760598
"688","TLE2",234.186217247844,-0.623842140430625,0.23070879256994,-2.7
0402412271091,0.00685053157459385,0.162151850760598
"689","C1QTNF6",99.5469076845929,1.20701650251574,0.446387242621786,2.
70396728953659,0.00685170336456426,0.162151850760598
"690","BEX5",16.3012208123648,-1.86330026257524,0.689308964896968,-2.7
0314236063033,0.00686873209066317,0.162151850760598

"691","RIPOR2",63.9050982453185,0.918425452823804,0.340004729467785,2.70121375741282,0.00690869204420093,0.162859167450056
"692","FAM87B",8.43232880930266,-1.6328948177414,0.605291256990323,-2.69770098094694,0.00698201244251713,0.16418884522248
"693","PPP1R9A",25.0575067379055,1.14916970166706,0.42600556075132,2.69754624714367,0.00698525813365946,0.16418884522248
"694","NDRG2",360.571801155054,-0.518009962197897,0.192136294753598,-2.69605470877957,0.00701661414961853,0.16468822461547
"695","PDGFC",18.7862632759608,-1.55371668774792,0.576679364702833,-2.6942470683836,0.00705478484059467,0.16534588527834
"696","NRN1",317.76178233041,-0.851782722177663,0.316224956642834,-2.6935974036348,0.00706854884364186,0.165430448439773
"697","TRIM7",29.5735397179019,-1.02718965076459,0.381660714561515,-2.69136856787767,0.00711595311192146,0.165935920594774
"698","SDSL",28.7077581082128,1.3379907522167,0.497182566576116,2.69114575241621,0.00712070774729859,0.165935920594774
"699","TEDC1",39.0865791458,-1.04552389626263,0.388429216173068,-2.69167161668083,0.00710949096032534,0.165935920594774
"700","TPP1",478.434586374958,0.608988323334211,0.22633953238154,2.6905963661162,0.0071324432308469,0.165971953981807
"701","REEP2",13.0819134805165,-1.22086810771041,0.453996440808639,-2.68915788312316,0.00716325306939657,0.16645111162254
"702","CERNA1",4.45011309805054,-2.19143708600632,0.815087390579629,-2.68859156862669,0.00717541529220096,0.166496210391256
"703","CMPK1",883.862433163935,-0.514829105874521,0.191671358294157,-2.68599915217596,0.00723132723062211,0.167348098238078
"704","YPEL1",34.8460400255777,0.908305728066638,0.338170919392743,2.68593683246831,0.00723267611023433,0.167348098238078
"705","AL731577.1",7.51823627432331,3.1543420258776,1.17464532694422,2.68535697841957,0.00724523759685799,0.167400957752085
"706","AL512308.1",13.9681921752228,1.40395532467123,0.52310660056148,2.68387996474196,0.00727732291624301,0.167904126037794
"707","SV2A",5.64422940656608,-2.24424693633327,0.836787207401565,-2.6819804563005,0.0073187735320698,0.167909157836458
"708","CYP2U1",195.925890499818,0.436409129035924,0.162707550701849,2.68216888001477,0.00731465235169983,0.167909157836458
"709","ZNF608",22.1930409098092,1.29763180486831,0.483728579546662,2.68256179133434,0.00730606534057998,0.167909157836458
"710","HLA-DQA1",78.7985481332012,-1.19472181354051,0.44524984839453,-2.68326158413845,0.0072907938839115,0.167909157836458
"711","ADCY3",653.296643891385,0.643409541193415,0.240067917920317,2.68011463908715,0.00735969504808428,0.168610510039726
"712","PXDNL",14.1714662035036,3.984072503144,1.48680350507308,2.67962275415014,0.0073705173112372,0.168621287194863
"713","MSANTD3",146.154395194587,-0.541148794097248,0.202055557151843,-2.67821782150034,0.00740150676190744,0.169092768085148
"714","FOS",52751.2326275285,-0.338423571132646,0.126499741302223,-2.67529061837456,0.00746644956648568,0.169897058015536
"715","AC018926.2",63.9855226233788,-0.914998831465014,0.3420080089045

36,-2.67537252825098,0.00746462538987983,0.169897058015536
"716","SMS",386.625848366169,-0.599853562595061,0.224225806036667,-2.6
7522089985025,0.00746800255013346,0.169897058015536
"717","RGL1",336.78615607783,0.412102936278913,0.154147408426094,2.673
43408810208,0.00750790285073219,0.170566568389926
"718","ARHGEF4",58.0722155358056,0.784351593930558,0.293519798666436,2
.67222721429404,0.00753496093543999,0.170705116380225
"719","SYS1",348.962906708332,-0.411445486357475,0.153954960994509,-2.
67250554122871,0.00752871310617787,0.170705116380225
"720","DDX59",136.423152049766,-0.547309271363114,0.204946780768823,-2
.670494600159,0.00757395897878586,0.171054471480944
"721","FGF12",42.2276303696435,1.36009747437143,0.509921987496248,2.66
726579304729,0.00764711676677955,0.171054471480944
"722","RELL1",187.677398066847,0.584144898952156,0.218760577002546,2.6
702475690826,0.00757953392094505,0.171054471480944
"723","PCDHA10",7.27834175767486,1.788088003001,0.669925347084411,2.66
908545972019,0.00760580953775708,0.171054471480944
"724","CSF1R",12.2765359420746,-1.66456157966818,0.624064760237814,-2.
66728981625859,0.0076465701221153,0.171054471480944
"725","FAM184A",117.388867699377,-0.69651183463438,0.261096621055536,-
2.66764017021204,0.00763860184839119,0.171054471480944
"726","DENND2A",195.739222000074,0.610878735213483,0.229059291232257,2
.666902232724,0.00765539380622555,0.171054471480944
"727","GSN",86464.4150577974,-0.76788388953817,0.287687706153475,-2.66
915781631809,0.00760417115417917,0.171054471480944
"728","LINC00963",340.310823514429,-0.48012542158436,0.179934596953217
,-2.66833299273287,0.00762286655094289,0.171054471480944
"729","ICAM4",43.4294271479184,1.44659086261809,0.54235115025584,2.667
25877125124,0.00764727655333864,0.171054471480944
"730","HTR2B",42.8179003702118,1.43934050773205,0.539827726398911,2.66
62960002696,0.00766921352981686,0.171128519434502
"731","DUSP10",23.2923688728021,-0.913814140517946,0.342895476100594,-
2.66499328282145,0.00769898605838016,0.171552847602093
"732","POLR3G",44.3390051755523,0.766779059016195,0.287771286609101,2.
66454331858954,0.00770929366104315,0.171552847602093
"733","AC092053.2",4.00168552004718,-2.11418477227576,0.79459314821213
4,-2.66071357025008,0.00779752583006269,0.173279533759742
"734","ADGRG4",20.0630391262772,1.40809916540015,0.529845186083515,2.6
5756715807588,0.00787069070855632,0.174667140261136
"735","SLC44A3",29.8100290299565,-0.989996692953973,0.372648857362416,
-2.6566476010717,0.00789218937880799,0.174668305423102
"736","BOP1",62.488858963032,-0.643796844199727,0.242313170686752,-2.6
5687928714361,0.00788676775167753,0.174668305423102
"737","SLITRK4",110.227772270317,-1.00847297297579,0.37993972797356,-2
.65429724434074,0.00794737848280339,0.175651082912326
"738","PRKACB",148.156859392763,0.489524588201122,0.18449419675712,2.6
5333325820303,0.00797011376732553,0.175754979093942
"739","AL160006.1",22.5205023107157,1.03018705054416,0.388444577985333
,2.65208245636281,0.00799970031091974,0.175754979093942
"740","WEE2-

AS1",10.0557902350674,1.38100464226189,0.520724397997658,2.65208361193036,0.00799967293172725,0.175754979093942
"741","PTPRD-AS1",69.8693163848984,0.995025629340793,0.375222075559739,2.65183125981183,0.00800565397462562,0.175754979093942
"742","ITGA7",22.3348415919275,-1.38768148461183,0.523554530219694,-2.65050038632944,0.00803726353887421,0.175754979093942
"743","AGAP2-AS1",40.7662123710697,-0.96202534193827,0.362966422126824,-2.6504527231506,0.00803839765639308,0.175754979093942
"744","APPL2",332.265615994049,0.488473562707506,0.184272866207252,2.65081654592662,0.00802974433202006,0.175754979093942
"745","SULT1A1",405.501879443861,1.13692231275557,0.428838683237065,2.65116547829495,0.0080214530042532,0.175754979093942
"746","RNF150",40.8915309779672,1.67969869621572,0.633985073204405,2.64942940647777,0.00806278146566881,0.176051806024503
"747","CDK6",143.48761267927,0.810728353257312,0.306213705456896,2.64759002882526,0.00810677709053242,0.176775491335586
"748","SLC35E2B",205.436859554723,0.602562038210901,0.227730525472491,2.64594321275427,0.00814634908280823,0.177400909371475
"749","PLD5",17.7901999962499,1.30701133953291,0.49441298582866,2.64356191482773,0.00820387600022277,0.177726938587526
"750","GBE1",193.949859236977,-0.45363207648341,0.171581546622195,-2.64382787900998,0.00819743290559135,0.177726938587526
"751","OGN",4940.8335982494,0.603858889476864,0.228468995804062,2.64306711443132,0.00821587480854609,0.177726938587526
"752","ENTPD1",51.7131963349621,2.31032569814647,0.87408063061035,2.64314940434411,0.00821387820692191,0.177726938587526
"753","CYBB",12.2795778010783,-1.5959551351415,0.603566968137349,-2.64420556357935,0.00818829111890365,0.177726938587526
"754","ENO1",1461.42301117691,-0.50846640642427,0.192498033169607,-2.64141091756645,0.00825615158474154,0.178070909542251
"755","NPR3",181.44444552151,2.05683257820629,0.77939617152703,2.63900780289496,0.00831490670519357,0.178070909542251
"756","RBM12B",133.922841929257,0.561932135928793,0.212892486708633,2.63951135437607,0.00830256419888725,0.178070909542251
"757","EMP2",1284.8885634701,-0.622432973677503,0.235608140959058,-2.64181437510542,0.00824632372432796,0.178070909542251
"758","PYCARD-AS1",18.4323912808616,2.04132435950222,0.77318407683325,2.64015312868692,0.00828685748402373,0.178070909542251
"759","CES2",203.568507365181,0.404808320691714,0.153404383005837,2.63883151680426,0.00831923151584831,0.178070909542251
"760","ZNF532",229.741281786355,0.460309826673852,0.174384811574441,2.63962109152697,0.00829987661744594,0.178070909542251
"761","NAPB",52.16989146565,0.732184810703492,0.277340300175193,2.6400231421145,0.00829003661259891,0.178070909542251
"762","FBLN5",4831.73341567616,0.57037855712438,0.216190400532938,2.63831583510795,0.0083318942441507,0.17810790727424
"763","SMAD1",80.4259103334945,0.528196874986053,0.200319268868944,2.6

3677517379329,0.00836982844570321,0.178450439204267
"764","PDLIM4",435.903533893152,-0.665408372923682,0.252318961438631,-
2.63717149567264,0.00836005546790101,0.178450439204267
"765","KIAA1109",393.63957583567,0.564922359866659,0.214320358790974,2
.63587819213025,0.00839198503808108,0.178688946778173
"766","ALG13",268.061994284255,-0.429241738682048,0.162877920770413,-2
.6353586578938,0.0084048421766O387,0.178729078609269
"767","TXNIP",16840.8089607998,0.634877497798353,0.241006327857142,2.6
3427729654751,0.00843165960749581,0.179065584545631
"768","TLE1",44.5651511420935,1.41047569267577,0.535899426941523,2.631
97835594939,0.00848892706700712,0.180047048169894
"769","LGI2",5.4737441301336,-1.83928741516712,0.69909449773681,-2.630
95678927738,0.00851448609992366,0.18012008322293
"770","RTN1",7.99890361627377,-1.61222287390602,0.612692224106737,-2.6
3137479222383,0.00850401959173745,0.18012008322293
"771","DUSP2",94.266534468168,-1.03476059856157,0.393846634967382,-2.6
2731862276103,0.00860607082481127,0.181352689818213
"772","SERP2",58.5117228606898,-0.732719049725109,0.278931780583699,-2
.62687546106007,0.00861728663019812,0.181352689818213
"773","SNHG14",312.755427733346,0.828760099148438,0.315473736312609,2.
62703358078342,0.00861328334183226,0.181352689818213
"774","TMEM102",4.2226006616856,-2.53481090795401,0.964535664319568,-2
.6280115932698,0.00858855886180144,0.181352689818213
"775","GBA",174.539713856875,0.569718062110009,0.217183212683444,2.623
21408303506,0.0087104520644O425,0.182849959404198
"776","CFAP36",496.641279938071,-0.551881499164767,0.210411422768927,-
2.6228685301503,0.00871929113316577,0.182849959404198
"777","PFKL",330.314116566061,-0.349598096209635,0.133294049029286,-2.
62275847088136,0.00872210807643575,0.182849959404198
"778","PAFAH1B3",32.8160854012853,-1.10050265352371,0.419840687820132,
-2.62123868755471,0.0087610898422457,0.183431095681671
"779","EDEM3",252.958978368139,0.578482057523248,0.221016910855289,2.6
1736559109727,0.00886113783979106,0.184705116623714
"780","IFIT2",251.95959866516,0.861050050630161,0.328985988332312,2.61
728487281472,0.008863233729328,0.184705116623714
"781","DHCR7",53.0010700811647,-0.709985242487355,0.271208080752639,-2
.61786168213369,0.00884826631381395,0.184705116623714
"782","HELZ",364.909057398274,0.646913560049502,0.247184491161054,2.61
712843314269,0.00886729702251483,0.184705116623714
"783","KLHDC8B",224.378134509103,-0.596567877401674,0.22803453762022,-
2.61612948471529,0.00889328251729312,0.184854152973254
"784","HOXC10",309.321843849414,-0.36671919675328,0.140184193061545,-2
.61598107992303,0.00889714874645659,0.184854152973254
"785","VILL",59.2462607009091,-0.586106003949008,0.224175081964991,-2.
61450112479735,0.008935786629236,0.185190618127591
"786","NUDT2",264.580035129409,-0.615629982258767,0.235468490560872,-2
.6144898656817,0.00893608114975056,0.185190618127591
"787","KPNA5",144.22663849475,0.496265926293827,0.189936783165446,2.61
279525757552,0.00898050837275952,0.185546915475904
"788","CCT6A",380.122148370349,-0.482357189149168,0.184632119505173,-2

.61253128893238,0.00898744651669766,0.185546915475904
"789","LGALS2",13.4724713197371,-1.59581204673109,0.610763948686824,-2
.61281310097325,0.00898003955006284,0.185546915475904
"790","ZFP36",24115.0685608668,-0.576586431815443,0.220786657055041,-2
.61150940689185,0.00901435078899945,0.185866784812673
"791","CDH11",1026.5915224049,0.754042108956436,0.288901668667181,2.61
00303000503,0.00905342033398171,0.18620096441948
"792","MSL1",395.477846078578,0.507496993529066,0.194433861268904,2.61
012660149351,0.00905087200665071,0.18620096441948
"793","LINC02130",2.64920759983624,-2.83116692228944,1.08555457823712,
-2.60803738388456,0.00910630085609596,0.187052376601447
"794","TRIP10",760.282206921298,-0.384857053120007,0.147641032875206,-
2.60670794307778,0.00914172971311145,0.187543621280696
"795","C6orf120",283.424211590309,0.553319799334545,0.212350381390004,
2.60569251494921,0.00916887313375492,0.187863867265074
"796","TBC1D4",202.225214114803,0.574659916716755,0.22058908460902,2.6
0511492549735,0.0091843447599843,0.187944462054503
"797","HDAC9",8.86217715426405,-1.61872731379816,0.621725403838681,-2.
60360490950466,0.00922490301871828,0.188537572486703
"798","RCAN3",34.6201588265475,-0.950462291185616,0.36528556294083,-2.
60197058852713,0.00926898010004706,0.189201023621136
"799","PTCD2",52.1262800384619,0.722757937397304,0.277828654395214,2.6
0145210352987,0.00928300267938528,0.189250100931798
"800","UBE2E3",1114.86629332388,-0.699894179392902,0.269210566501687,-
2.5998020378168,0.00932775526011607,0.189614145621774
"801","TBCEL",55.9891385005299,0.663109781665158,0.255090607444804,2.5
9950685094762,0.00933578149601958,0.189614145621774
"802","ZNF134",43.1777113190792,0.825751738851391,0.317612705401101,2.
5998699825581,0.00932590869031705,0.189614145621774
"803","ZNF365",2.3766017004833,3.58376432443706,1.37898505926651,2.598
84202541199,0.00935388090938686,0.189745163303864
"804","NAT14",75.0390634029368,-0.578474423956493,0.222684221657544,-2
.59773422495152,0.00938410955823197,0.190121592778657
"805","TNFSF12",712.552829947809,-0.584665234545635,0.225224912191514,
-2.59591724936929,0.00943387825028159,0.19089247555135
"806","APCDD1L",12.008763033483,2.07143177051459,0.798335738184865,2.5
9468751232947,0.00946769537834218,0.191216226768609
"807","CR381653.1",3.79383019090589,2.6639832895786,1.02678809707854,2
.59448205248801,0.00947335594586946,0.191216226768609
"808","SOD3",7614.05846377822,-0.717521011584869,0.276647713670887,-2.
59362711538063,0.00949694250773137,0.191455069936184
"809","CMTM8",201.54180651476,0.900897875272262,0.347519998464155,2.59
236268201464,0.00953192253066088,0.191826998955514
"810","PHLDA2",39.4578814697612,-1.33639718289192,0.515563582419481,-2
.59210935074266,0.00953894463465936,0.191826998955514
"811","ATP8B4",402.705857173981,0.854336224665434,0.329809617099248,2.
59039209401933,0.00958666711460991,0.192548977348805
"812","AL592295.5",126.834715714245,-0.938807011090971,0.3627872801811
24,-2.58776165091087,0.0096601798277492,0.193786538441141
"813","PHF21A",207.645441156562,0.615992116618048,0.238364142938283,2.

58424823895404,0.00975915249234072,0.194933159487332
"814","HOMEZ",37.6134432637003,0.684032981170767,0.264641850833038,2.58474983838562,0.00974496738092706,0.194933159487332
"815","PTPRS",146.963933979158,0.642488550672908,0.248629873527588,2.58411646821522,0.00976288198802277,0.194933159487332
"816","YDJC",53.4881553107423,-0.837277586370921,0.32401952760949,-2.58403434060929,0.0097652070809541 8,0.194933159487332
"817","AKAP12",1326.27386758625,0.572122906680008,0.221730139671937,2.58026674915056,0.00987240238981817,0.196591152234411
"818","LDLR",137.226805892082,-1.28275228924249,0.497138025498924,-2.58027393489993,0.00987219694731228,0.196591152234411
"819","KIRREL1",400.626119490599,0.661189379006895,0.256321223749746,2.57953426304032,0.0098933643597786,0.19676802448893
"820","FTX",883.054943246333,0.812396759624293,0.315028909673085,2.57880065822321,0.00991439807982484,0.196945890636911
"821","TCEA3",286.28994431583,0.660594296919918,0.256283867202849,2.57758829742122,0.00994924589434519,0.1971572583613
"822","AUP1",499.358739049845,-0.419410207296801,0.162708206335167,-2.57768318355649,0.00994651457917415,0.1971572583613
"823","ABCB5",52.3999089965453,1.18841485251469,0.461210488442547,2.57672989295587,0.00997398560602425,0.19716778099 0933
"824","CPSF7",198.340574576449,0.394790030602323,0.153202684442355,2.5769132704125,0.00996869596408929,0.197167780990933
"825","XG",964.184223587999,-0.628100273042412,0.243928156364437,-2.5749396150234,0.0100257588358147,0.197951012941316
"826","ROBO2",100.919812212632,0.851251094352621,0.330724268545701,2.57389969625102,0.0100559421271693,0.198232370512762
"827","MDGA2",8.15075457562747,2.24238964136133,0.871301070077228,2.57361056742714,0.0100643483586503,0.198232370512762
"828","DDX39A",108.061847196487,-0.616899543047968,0.239776522460424,-2.57281045165624,0.0100876438320011,0.198451244419644
"829","IQCN",100.348530275423,1.23432225328899,0.47989168646921,2.57208509355618,0.0101088042098406,0.19862763784571
"830","AL138756.1",10.4241407457897,1.53400631287507,0.596634604839894,2.57109845863989,0.0101376500796621,0.198954436322429
"831","GNB4",472.223832069443,0.59873703146949,0.233129031699816,2.56826456621002,0.0102209115122004,0.200283999746109
"832","RTN4RL1",9.4759681182412,2.19893024552859,0.85629563519,2.56795685410761,0.0102299888138475,0.200283999746109
"833","AC025280.2",12.0026625429831,2.30543519530208,0.897953257011571,2.5674334129314,0.0102454464668317,0.200345831330399
"834","DCP1B",74.1801798901166,0.511297156832358,0.199183473189426,2.56696576601066,0.0102592740503175,0.200375677464773
"835","MT-CO3",73937.9011507634,-0.39871744513662,0.155432561428967,-2.56521182866071,0.0103112833196562,0.20115029220824
"836","AC114964.2",2.82488407556005,3.03732450033208,1.18495592530318,2.56323837492518,0.0103700823178774,0.202055347937686
"837","AL354822.1",19.3611401056381,1.45447917040408,0.567887387378253,2.56121055464699,0.0104308117972727,0.20299581047285

"838","BCL7A",131.756929788225,0.934068123684235,0.364766643180314,2.5
6072790960357,0.0104453126648578,0.203035439138268
"839","TRABD",115.626635157799,-0.568952937876391,0.222283463160057,-2
.55958283980267,0.0104797875832013,0.20337795432081
"840","AL683813.1",30.1679819662229,1.13875544831948,0.444945627767658
,2.55931371667308,0.010487904821013,0.20337795432081
"841","AC098818.2",86.2981955562164,-0.964615967122296,0.3770206366008
58,-2.5585229917892,0.0105117868755177,0.203598687770877
"842","MTX3",125.352232434673,0.603199176749311,0.235963165959827,2.55
632769756957,0.0105783445542079,0.204401725318496
"843","TMEM140",338.106389574534,0.603285064557297,0.235990406029028,2
.55639657013467,0.0105762507703587,0.204401725318496
"844","AC096921.2",25.7132466165677,0.949797904776679,0.37178261745069
5,2.55471305057085,0.0106275369321746,0.205108944417289
"845","FNDC4",179.454326770742,-0.776074465903845,0.303894592933597,-2
.55376200810991,0.010656606820927,0.205426589948023
"846","LINC01871",16.7661560754751,1.82624242555586,0.715881562743613,
2.55103989346617,0.0107402031278045,0.206367974842846
"847","NRG2",11.9041020018711,-1.31205428256656,0.51432139503854,-2.55
103967134839,0.0107402099727769,0.206367974842846
"848","ECSIT",215.245624425933,-0.543863896654639,0.213201820922743,-2
.5509345759843,0.0107434491169951,0.206367974842846
"849","MAPK8IP1",57.6996781614516,-0.685854195541226,0.269206526852918
,-2.54768784233803,0.0108439455244422,0.207564075966673
"850","EGLN3",15.8816896762448,-1.46566615216347,0.575247042632724,-2.
54788993865241,0.0108376657194421,0.207564075966673
"851","DANT2",20.1198036345819,1.35723958648927,0.532640022034485,2.54
81366970982,0.01083000249659,0.207564075966673
"852","LINC01816",5.20062221572611,1.86172189176567,0.731307609616902,
2.54574390760263,0.0109045154231893,0.208178919305977
"853","ACVR2A",380.687242044327,0.733762379538043,0.288319101461734,2.
5449662398987,0.0109288303850875,0.208178919305977
"854","HOXC12",5.20594714925767,2.35386066601743,0.925185965710098,2.5
4420273680957,0.0109527493305434,0.208178919305977
"855","ACTN1",301.411117427509,-0.963293361728935,0.378587313927564,-2
.54444173455121,0.0109452570371582,0.208178919305977
"856","C17orf49",457.752175825565,-0.761484088952192,0.299089080490515
,-2.54601100014529,0.0108961754678727,0.208178919305977
"857","MXRA7",1669.091993381,-0.554692791540306,0.217962259665712,-2.5
4490292214367,0.0109308122304612,0.208178919305977
"858","ESAM",4.38736067000441,-2.69018471566843,1.05759958055991,-2.54
367036931331,0.0109694547995673,0.20825343733118
"859","NHLRC3",304.822801627465,0.540684549122026,0.212614464250019,2.
54302806269201,0.0109896402469268,0.208393771806973
"860","NDUFA4L2",17.7278187334944,-2.1066421723502,0.829024160114308,-
2.54111071028344,0.0110500923677693,0.209296458812319
"861","AMH",2.32435798385971,-3.10692389694824,1.22328395905149,-2.539
82231513712,0.0110908799069984,0.209825020679556
"862","OSCAR",11.6893159549128,-1.29800937419573,0.511168652084043,-2.
53929768365826,0.0111075267782451,0.209896175975447

"863","IMPDH1",84.8795798540332,-0.598599568281659,0.235991933050495,-
2.53652555214071,0.0111958571936479,0.210173694313708
"864","PNMA2",18.8995599795286,1.96660759125752,0.775064880607436,2.53
734576351369,0.0111696574503553,0.210173694313708
"865","ANGPTL5",295.271421272649,-0.882160210646798,0.347799073402541,
-2.53640759308692,0.0111996296067468,0.210173694313708
"866","USP30",51.6537807252423,0.705360931804814,0.27802020936698 3,2.5
3708510403194,0.0111779777017654,0.210173694313708
"867","SIPA1L1",43.2478919627209,-0.704892703509127,0.277864733540122,
-2.53681960473529,0.0111864580984763,0.210173694313708
"868","MARCH2",353.011130256676,-0.748961853503801,0.295276359883079,-
2.53647753514832,0.0111973926744586,0.210173694313708
"869","AC013275.1",8.21679849774788,2.05081427738104,0.809325114700591
,2.53398077006387,0.01127749203219,0.211148353692347
"870","PMS2",72.6586844716724,0.607553750400248,0.239751957751815,2.53
409296882227,0.0112738816662004,0.211148353692347
"871","SERPINB7",8.65503967652449,2.53516479583532,1.00115562964103,2.
53223846600585,0.0113336883284304,0.211713817869039
"872","CDC25B",81.7313549769583,0.536249330613955,0.211743885057132,2.
53253750619181,0.0113240254442114,0.211713817869039
"873","ERCC6",102.924583238616,0.544661656525535,0.21517663751012,2.53
123044782182,0.0113663143665338,0.212080062676367
"874","RBP7",9.06086431258305,-1.85599923547495,0.733463437995747,-2.5
3045910583714,0.011391336304889,0.212303749508396
"875","ZNF706",1070.55941047594,-0.546479895564506,0.216027496989108,-
2.5296774863436,0.0114167415088752,0.212534059929221
"876","STMN1",58.4479492784814,-1.09040213324578,0.431321514872458,-2.
52804948431153,0.0114698184602993,0.21321092288530 5
"877","RASSF1",249.777772959313,-0.581798477273852,0.230268161212839,-
2.52661277273193,0.0115168406659106,0.213210922885305
"878","EPHB3",69.6486127942212,0.828034889585616,0.327774472139502,2.5
2623361478011,0.0115292786432438,0.213210922885305
"879","AC009549.1",4.33551517361005,-1.91245628046866,0.75691000581177
3,-2.52666270201778,0.01151520366273,0.213210922885305
"880","IQCH-
AS1",149.891013581985,0.79396272996693,0.314296073300948,2.52616178633
16,0.011531636261400 6,0.213210922885305
"881","ABCA10",1153.82376165213,0.880548377831792,0.348472599278108,2.
52687981682326,0.0115080876437973,0.213210922885305
"882","PCDHGA8",20.5236433305825,1.14381470738026,0.452880777501371,2.
52564198836369,0.0115487102940102,0.21328451471557
"883","LRRC17",325.492975984197,1.15896813502708,0.459047598644022,2.5
2472322794097,0.0115789440977984,0.213397912677376
"884","TTPA",14.2954891516843,1.62273030041601,0.642950442904056,2.523
88083455782,0.0116067265759517,0.213397912677376
"885","COL5A1",1390.99362958716,0.955825014667732,0.378717619367251,2.
52384617400346,0.0116078709610173,0.213397912677376
"886","TMEM238",30.4465664193815,-1.05128503358832,0.416527539324435,-
2.52392683396971,0.011605207971215,0.213397912677376
"887","ZNRF3",85.2246318042652,0.53964629900265,0.213851032665009,2.52

346828667407,0.0116203541374445,0.213397912677376
"888","IGSF6",12.5299942262615,-1.25303012437838,0.496830559208237,-2.52204720735223,0.0116674049304938,0.214020674451367
"889","UGGT1",188.977996236068,0.452010169305306,0.179391849205997,2.51968063937097,0.011746135263926,0.214739390924905
"890","NEK9",148.981538169511,0.417979790087898,0.165872372801733,2.5198879296645,0.011739220405913,0.214739390924905
"891","CNN2",424.441503299598,0.718636970258061,0.285125407576552,2.52042417533456,0.0117213488955921,0.214739390924905
"892","RAB30",372.80204741448,0.429067380931758,0.170318648072237,2.51920377356318,0.011762056428161,0.214789391433088
"893","PTGS1",98.6841977253256,1.09447616361555,0.434775714723841,2.51733509152124,0.0118246309144703,0.215690272078171
"894","SOX13",90.3815220509983,0.642562764443813,0.255317090128191,2.51672445476012,0.0118451425348681,0.215822736857344
"895","PLEKHO2",86.3783089462297,0.577965226348665,0.229686170755873,2.51632575198865,0.0118585521981882,0.215825650007025
"896","PLEKHA8",64.3762265261681,0.676450867842883,0.268979569638809,2.51487824428909,0.0119073497885912,0.21647189810978
"897","MON2",234.441110676958,0.516689480181696,0.205622757596746,2.51280299039173,0.0119776202170532,0.217506639593735
"898","SCARB2",1018.85940785929,0.388563292065642,0.154875697031482,2.50887195030126,0.0121117380796993,0.219188897244904
"899","ABCB4",26.2142759435673,-0.994539111108013,0.396376505903679,-2.50907684056756,0.0121047149640263,0.219188897244904
"900","RECQL",383.783486920454,-0.378997844174755,0.151084721792674,-2.50851204329472,0.0121240835175676,0.219188897244904
"901","ENOX1",8.63792134399731,1.6220484960316,0.646421688771729,2.50927300894509,0.0120979941948159,0.219188897244904
"902","SPAG17",23.2250607898986,-3.00902166520816,1.20006885392243,-2.5073741855504,0.0121631874174013,0.219362895095732
"903","TSPAN9",117.040854141345,0.59523896766967,0.237425385277915,2.50705697275344,0.0121741087339027,0.219362895095732
"904","EBP",144.046462265091,-0.632593223349652,0.252254267984119,-2.50776023892479,0.0121499077027891,0.219362895095732
"905","MAP1LC3C",14.7723097040238,1.43822019709821,0.573890244128011,2.50608929462374,0.0122074786219397,0.21972112626826
"906","LINC01426",36.9640445501628,1.3346915798963,0.53266524983894,2.50568547563382,0.012221428065545,0.219729405915743
"907","FNDC3B",1018.055433727,0.591542943277887,0.236349563348192,2.5028307008396,0.012320446336568,0.220535989424567
"908","CCDC112",131.270044060758,-0.623903601012055,0.249214134904706,-2.50348400683863,0.0122977238280831,0.220535989424567
"909","AGTPBP1",23.5666634317814,-0.925278098265059,0.369674074946109,-2.50295641748734,0.0123160709227002,0.220535989424567
"910","TMEM245",283.461917831467,0.591520709637832,0.236265115803569,2.50363117562232,0.012292610305783,0.220535989424567
"911","HLA-DPA1",432.935570846282,-0.722619462443006,0.288920320115646,-2.50110294130148,0.0123807185443417,0.220886664149816

"912","LINC00517",10.0065694730036,-1.98224628353117,0.792452750533078
,-2.50140627589181,0.0123701179739121,0.220886664149816
"913","DNASE1",94.5229464147161,0.817730570836913,0.326938964874248,2.
50117195774276,0.0123783059343796,0.220886664149816
"914","TGFBR3",1919.16830640705,0.4900044404138,0.195995260841431,2.50
008310563303,0.0124164175531678,0.221039372156885
"915","PTPRT",6.29683617310197,-2.81094088617117,1.12427668700888,-2.5
0022162573665,0.0124115633585863,0.221039372156885
"916","POLG2",210.325597981012,1.24109988647911,0.496551251083843,2.49
943965254364,0.0124389882933878,0.221199432653923
"917","VENTX",6.10038080683754,-3.10119212061047,1.2410188647382,-2.49
890812196854,0.0124576604669707,0.221289892417106
"918","HSPA12A",178.888438619228,0.65557761091994,0.262476450092215,2.
49766259292831,0.0125015120041276,0.221826937946879
"919","MARC2",137.925655393845,0.530576531389302,0.212510257104737,2.4
9671022292257,0.0125351344033383,0.221854261394714
"920","NRBP2",201.591651796692,0.509859138161734,0.204232687048944,2.4
9646197936742,0.0125439115197085,0.221854261394714
"921","CCDC34",144.970032254911,-0.539205658880543,0.215972705982948,-
2.49663797296273,0.0125376883749542,0.221854261394714
"922","TARS",285.876663096442,0.356945795282173,0.143138201001747,2.49
371441574718,0.0126414208019451,0.223112330890746
"923","ADAMTS2",497.732289569459,0.578495670748234,0.232002229763629,2
.49349185711544,0.0126493485652219,0.223112330890746
"924","LRRC1",12.0016640238232,-1.42106700911071,0.569953956752528,-2.
49330141895608,0.012656135658607,0.223112330890746
"925","TMEM37",727.050592336889,1.02149661039443,0.409772285435927,2.4
9283967388799,0.0126726053409514,0.223161155025684
"926","FEZ2",655.662121681566,0.418049546293073,0.167744929402483,2.49
217396783431,0.0126963833658334,0.223338432663132
"927","NCF4",11.7987452429693,-1.58990181024437,0.638175450625567,-2.4
9132399042596,0.0127268006829241,0.223631991719688
"928","NRM",50.7781856764446,-0.685903373820931,0.275589839434721,-2.4
8885581278261,0.0128154928404309,0.224222946163027
"929","TWIST1",759.244861748978,-0.779684773809757,0.313196230706185,-
2.48944494654916,0.0127942732001326,0.224222946163027
"930","ZNF469",45.6568185727411,0.73004160936931,0.293248684177389,2.4
8949662439986,0.0127924133321241,0.224222946163027
"931","ZC3H7B",190.941186368549,0.479440301491375,0.192630578081778,2.
488910671741,0.0128135155958497,0.224222946163027
"932","ABCC9",452.819842802752,0.635961221919503,0.255766844440657,2.4
8648812675584,0.0129010874528269,0.225478340685726
"933","SMYD2",58.4300018729438,-0.603136528312199,0.242697091903377,-2
.48514114273903,0.0129500078852567,0.226090759317198
"934","SDC4",135.364672652596,-0.662070027960289,0.266514933809803,-2.
48417609661143,0.0129851577412304,0.226461707116597
"935","EREG",29.5170299359029,-1.47062414774846,0.592103025473327,-2.4
8373016937862,0.0130014282288343,0.226502956598377
"936","TAF9B",65.3511728220364,0.571260977442831,0.230139988317174,2.4
8223258209057,0.0130562025499523,0.227214191598475

"937","TREM1",6.84165175936859,-1.69728104895371,0.684424108093906,-2.47986742267243,0.0131431241008266,0.228482762516931
"938","EN1",159.966290505475,0.77377842346535,0.312247911478403,2.47808998882246,0.0132087826331188,0.229260442101468
"939","PKM",916.247927025365,-0.62560619225918,0.25247487307395,-2.47789486788232,0.0132160080504192,0.229260442101468
"940","ETAA1",116.961626236705,0.424661270475851,0.171413342562099,2.47741082536796,0.0132339474542676,0.229327414981451
"941","PDGFB",3.41605628347039,-2.97575335067867,1.20221354200721,-2.4752286068167,0.0133150916137812,0.230488339316559
"942","AC091563.1",47.2681291446336,1.0674568281682,0.431523311629003,2.47369446655974,0.0133724004379541,0.231234639844835
"943","LPCAT3",136.864551633676,0.70321509729781,0.284797682330521,2.46917422762485,0.0135425264250617,0.23392811552262
"944","IL1RAPL1",4.89909654989712,3.39944302959367,1.37736613663691,2.4680750739916,0.013584182649711,0.234399100827481
"945","MLF1",256.647380115875,-0.587484502875425,0.238317534898861,-2.46513334876825,0.0136962268917661,0.235832811670167
"946","AC024560.1",4.63186274003839,-2.14274170353197,0.869160492557539,-2.46530039259708,0.0136898427455106,0.235832811670167
"947","PPL",1027.15927041022,-1.04382203847935,0.423618252765006,-2.46406294267588,0.0137371985375284,0.236288518455966
"948","NGF-AS1",12.8420029603392,1.38040658489726,0.560714261202779,2.46187172399748,0.0138214089654431,0.236753755455489
"949","HSD11B1",135.145076681883,-0.772854621405015,0.31393453840195,-2.46183368462466,0.0138228748678484,0.236753755455489
"950","FBLN2",8199.79272702921,0.395778990016987,0.160789724225667,2.46146942488385,0.013836919098387,0.236753755455489
"951","PRDM10",23.6773289350088,0.986139570810293,0.400622899098401,2.46151573719224,0.0138351328035517,0.236753755455489
"952","RFX7",67.6488841192981,0.667303055598817,0.270969531657744,2.46264977289651,0.0137914558256726,0.236753755455489
"953","DANCR",227.342673345735,-0.732919278430513,0.29832476723197,-2.4567832072106,0.0140187256496124,0.238113683114219
"954","HPGDS",5.27500398252076,-1.98605491647314,0.808298088519197,-2.45708228768869,0.0140070598424529,0.238113683114219
"955","HSPB2",855.248551846434,-0.51049414361475,0.207758066366026,-2.45715679079996,0.0140041551389521,0.238113683114219
"956","PRICKLE1",537.592199382586,0.592291847459367,0.240945875625381,2.45819458798396,0.0139637490847622,0.238113683114219
"957","SLC38A4",6.66303107161091,2.01669013998408,0.820327413998836,2.45839661770336,0.0139558951485631,0.238113683114219
"958","FRY",6.38414852921246,-1.67750082056987,0.682573208845575,-2.45761304257312,0.0139863785357123,0.238113683114219
"959","LIG4",46.1945747508381,0.799340284905926,0.325145774734494,2.45840588135782,0.0139555351161026,0.238113683114219
"960","BBS2",169.006222565638,0.452492420428785,0.18430710470328,2.45510025865396,0.0140845300670662,0.238982198190042
"961","SERINC2",5.46551511033796,-2.3614063325619,0.962053950635504,-2

.45454668212945,0.0141062347832892,0.239101413511964
"962","ZNF667",62.4358145412102,0.693866870617258,0.282834199848319,2.
4532636823601,0.014156652359816,0.239706559552019
"963","EDA2R",15.4478073355018,1.21288110469796,0.494604755032686,2.45
222289587128,0.0141976685141881,0.240151425158473
"964","PM20D2",91.0981377256931,0.651307096620199,0.265700412369953,2.
4512837251955,0.014234770070198,0.240279968573528
"965","SYNJ2",63.7672635711081,-1.14373587942598,0.466585352236688,-2.
45128972425563,0.0142345328085732,0.240279968573528
"966","GCK",7.25559059082716,-1.43145092183122,0.584317646080313,-2.44
978212010812,0.0142942680993393,0.241034506283786
"967","RNF130",763.073427377095,-0.368366800646751,0.150524539575129,-
2.44722090953744,0.0143962571522874,0.242503239662471
"968","ADM",4222.63057928262,0.951690493452459,0.389221712911603,2.445
11151840236,0.0144807358683222,0.243171862432063
"969","DHH",3.58037915901004,4.18211536979886,1.71015764230186,2.44545
605992777,0.0144669075728627,0.243171862432063
"970","CSPG4",6.68152169705173,-1.58305111552299,0.647299380768516,-2.
4456243317327,0.0144601581581828,0.243171862432063
"971","MIR3945HG",4.72293772474137,2.10340301916564,0.860595645934076,
2.44412463519106,0.0145204093018915,0.243481226621489
"972","ZNF177",17.9398787273114,1.04992287258902,0.42960784347971,2.44
390992511898,0.0145290534886173,0.243481226621489
"973","KCNMB1",19.7314468339486,-0.997578548634707,0.409321455086142,-
2.43715186741132,0.0148034607497617,0.246972147166676
"974","NOC4L",124.574655781044,-0.540644115262979,0.221812502207308,-2
.43739243677838,0.014793614709563,0.246972147166676
"975","CIB2",71.8377120143171,-0.689331266417059,0.282767733427357,-2.
43780030366919,0.014776934690785,0.246972147166676
"976","HYKK",75.3299305755152,-0.524633189859104,0.215318514330741,-2.
43654472301085,0.0148283356823015,0.246972147166676
"977","SNHG16",697.540376862321,-0.365056485997729,0.149809714218994,-
2.43680116406927,0.0148178247068696,0.246972147166676
"978","CEP131",111.290952265079,0.800767721465351,0.328597710060995,2.
43692422968106,0.0148127828401664,0.246972147166676
"979","STK38",281.536662992583,0.451304304989412,0.185414102751884,2.4
3403440348513,0.0149315758127784,0.248437628615269
"980","S100B",2.88037047670657,-2.47902039380024,1.01879117499312,-2.4
3329590464601,0.0149620678400403,0.248690941884098
"981","SWAP70",592.281980201158,0.484322555023679,0.199123948343531,2.
43226673161443,0.0150046530611288,0.2491445399722
"982","PDPR",146.159562728437,0.645733117255655,0.265618447380727,2.43
105523589665,0.0150549192360164,0.249724622643046
"983","PRR15",3.25194402970552,2.61831730034291,1.07790215417297,2.429
08624888299,0.0151369309439252,0.249813037634851
"984","EBF2",355.130848823758,0.558498811244965,0.229829491260316,2.43
005720537571,0.0150964398878558,0.249813037634851
"985","AC132192.2",11.6360630104383,-1.41735193274795,0.58332107106182
6,-2.42979724728257,0.0151072713594857,0.249813037634851
"986","LGALS3",10259.1058443201,-0.629089259869871,0.258970316661271,-

2.42919446514293,0.0151324133531177,0.249813037634851
"987","ZNF714",57.8117445383973,0.814439690949539,0.335171311379504,2.42992065042039,0.0151021287638781,0.249813037634851
"988","PLSCR4",1278.74096874381,0.554679974781166,0.228385570793939,2.42869973288122,0.015153076120907,0.249826373414427
"989","AC093627.6",17.5795412186993,-3.5607638183653,1.46693652447547,-2.42734689535289,0.0152097050490252,0.250506456565795
"990","AOX1",623.159203797172,0.389042369286321,0.160313884192935,2.42675405966782,0.0152345794444554,0.250662691485589
"991","WASHC4",382.612966448352,0.395761928974041,0.163124371047944,2.42613612197604,0.0152605451971511,0.250836549663365
"992","LY86",5.17590825985768,-1.63362366100609,0.673721043251308,-2.42477755054584,0.0153177694379198,0.251523333038585
"993","IL17D",234.225607729025,-0.745154892183979,0.307409279919526,-2.42398307682529,0.0153513208426284,0.251820408062009
"994","H2AFY",451.307003166854,-0.37826880778083,0.156077546677708,-2.42359529498458,0.0153677207319204,0.25183581790971
"995","NUTM2A-AS1",294.070463753739,-0.468058289514985,0.193222687531905,-2.42237749352129,0.0154193237338504,0.252427501809738
"996","RBM24",6.23924442475541,-1.65074775033718,0.681776559914058,-2.4212445064777,0.0154674697055466,0.252961459873142
"997","DBT",169.950671007105,0.473991647166807,0.195828334902824,2.42044465833821,0.0155015386556332,0.2532643562303
"998","CADM1",3.67808987099688,-2.20883016268764,0.912870061968105,-2.41965451022186,0.0155352592647,0.253307145308006
"999","TUBA1C",209.568501449548,-0.776880348283279,0.321023619691957,-2.42000993269201,0.0155200831647058,0.253307145308006
"1000","AC027796.1",10.765442947937,1.5410407335695,0.63718871905145,2.41849971208462,0.0155846578959754,0.253858492467543
"1001","TBCK",119.030943291351,0.556386266466834,0.230227880691827,2.41667631563524,0.0156629384827247,0.25486407707606
"1002","P4HA1",454.121264358297,0.530104193907726,0.219435933036603,2.4157583790951,0.0157024773410023,0.25486407707606
"1003","DDX11",34.9340701833281,1.13106159520442,0.468230669466422,2.41560766724942,0.0157089774316633,0.25486407707606
"1004","CCL4L2",77.3577377110049,-1.3533936891653,0.560175195677083,-2.41601859491379,0.0156912599938971,0.25486407707606
"1005","ZNF75D",45.6817003853542,0.638536366628581,0.264541506929115,2.41374737008541,0.0157894058120582,0.255914060967778
"1006","DDI2",206.200670148177,0.69101334660057,0.286544085926025,2.41154286736514,0.0158851843497896,0.256549497357012
"1007","AMOTL2",948.579081143685,0.474857468883612,0.196833689118316,2.41248066329833,0.0158443778729064,0.256549497357012
"1008","PCDH18",1378.38690017843,0.405944228176693,0.168303180559699,2.41198191755325,0.0158660683915251,0.256549497357012
"1009","RND2",12.6757496220154,-1.14527459427076,0.47494268442979,-2.41139537846712,0.015891610463087,0.256549497357012
"1010","PLGLB2",14.4679181134767,1.25462036417814,0.52046817083831,2.4105611725638,0.0159279999867865,0.256628280697097

"1011","DTX2",89.5495803678722,0.597964853940425,0.248031189739385,2.4
1084540443775,0.0159155930733362,0.256628280697097
"1012","CORT",4.90835689247071,-1.68063293435413,0.697512577082993,-2.
40946613662876,0.0159758785771397,0.257145342038566
"1013","SIDT1",2.66886999199965,-2.78948187865756,1.15827314920863,-2.
40831092438206,0.016026525390188 5,0.257451747614182
"1014","CYP7B1",491.768621763604,0.54641499127056,0.226866461046805,2.
40853138339311,0.0160168491422497,0.257451747614182
"1015","CALCOCO1",559.032163954801,0.461015681784978,0.191593620004796
,2.40621624965089,0.0161187202239454,0.258645662986171
"1016","LINC01260",8.75821989247046,-1.28371415488355,0.53356882719039
,-2.40590171214311,0.0161326044320676,0.258645662986171
"1017","MAB21L3",4.05917272607312,2.65188160398724,1.10314849294649,2.
40392079665006,0.0162202871434009,0.259285826573953
"1018","BMPER",276.42738993927,0.808187089303178,0.336169769749155,2.4
0410400348085,0.0162121601862305,0.259285826573953
"1019","KIF5B",782.89170186636,-0.376702860220781,0.156669333204703,-2
.40444541707842,0.0161970248089818,0.259285826573953
"1020","ABTB2",7.02780599804755,-1.60169414880548,0.666549994619375,-2
.40296176091053,0.0162628879071635,0.25945757210557
"1021","ZFYVE26",53.4544353630381,0.587127056245538,0.244306622843592,
2.40323839530713,0.0162505896146047,0.25945757210557
"1022","CCND3",112.642058965801,-0.518972814802263,0.216089148906637,-
2.40166069156249,0.0163208390998434,0.260127346474901
"1023","CPPED1",113.940080869563,-0.472249472199705,0.196677780600195,
-2.40113281103009,0.0163444032269524,0.260248273864934
"1024","CTNND2",23.630870982645,1.03770195912198,0.432687203406751,2.3
9827281914431,0.0164725911755599,0.261777597715801
"1025","AC110048.2",10.6502791806608,1.59012260736915,0.66293313227634
2,2.39861688902028,0.0164571230022775,0.261777597715801
"1026","LGALSL",77.1319565497946,-0.731256738386603,0.30501191824704,-
2.39746939263643,0.0165087601456305,0.262096680323758
"1027","TGFB1I1",532.939586397354,-0.544646360562702,0.227413340053037
,-2.39496223236368,0.0166220773274547,0.263638770775958
"1028","AC130895.1",13.1451381591146,1.38684807540643,0.57963707317385
5,2.39261451620514,0.0167288067243768,0.264393251370251
"1029","C16orf74",59.0576222032049,0.785086168480545,0.328144640593262
,2.39250035307956,0.0167340119937976,0.264393251370251
"1030","RAD23A",826.820816258839,-0.399647094938887,0.167042460621697,
-2.39248807429851,0.0167345719296905,0.264393251370251
"1031","LYL1",9.58112682666783,-1.29162488297115,0.539628438173221,-2.
39354487569934,0.016686439940055,0.264393251370251
"1032","ASPH",1584.85220632405,0.517710758174589,0.21643002765045,2.39
204681436685,0.0167547051467096,0.264454837339877
"1033","PTP4A2",1232.4779218769,-0.359106622667793,0.150172677502447,-
2.39129133634806,0.0167892244596565,0.264522735204506
"1034","LDHA",3358.41146712088,-0.490596134746115,0.205163741517568,-2
.3912418983844,0.0167914855547584,0.264522735204506
"1035","TMSB15A",7.08240370474376,-1.85559007565292,0.776137103991308,
-2.39080191645328,0.0168116203508048,0.264584042409913

"1036","WDR19",107.727922438206,0.667098325598293,0.279223816019453,2.
38911685653568,0.0168889295281151,0.265032536689274
"1037","CREB3L1",271.516451412652,0.928003757147862,0.388376126503508,
2.38944593608917,0.0168738071536663,0.265032536689274
"1038","HSDL1",93.2655038879859,0.551791960427749,0.230924108645108,2.
38949481570052,0.0168715619759636,0.265032536689274
"1039","RNF126",148.358206105608,-0.618221573531234,0.258875210505393,
-2.38810650245075,0.0169354332382533,0.265506517822818
"1040","PRPH2",11.3901097020814,1.95083007319417,0.817297984035422,2.3
8692632467036,0.0169898957155767,0.266104241645219
"1041","ZNF436",204.67087293535,0.432801006703705,0.181550756535819,2.
38391188757352,0.0171297032409729,0.268013198665562
"1042","CLIP4",177.132195666903,0.545200228319827,0.228825444817762,2.
38260316178573,0.0171907145980615,0.268013198665562
"1043","DCT",5.56649093321102,2.4637061541094,1.03378960352721,2.38317
946485766,0.0171638245328497,0.268013198665562
"1044","AC099518.5",86.5991350828628,-0.977696687756721,0.410360385446
556,-2.38253184866465,0.0171940446071283,0.268013198665562
"1045","AP001269.4",8.02225579284946,-1.41526158267859,0.5939912142671
29,-2.38263049803649,0.0171894382654015,0.268013198665562
"1046","ENOSF1",220.837299815894,0.600450168163271,0.252105443561662,2
.38174217771862,0.0172309566081342,0.268331789856499
"1047","ANXA5",4271.12006300484,-0.456148499921275,0.191707043279178,-
2.37940397034343,0.0173406605682256,0.269267893227671
"1048","IGF2R",396.915396186185,0.549737435344041,0.230976720708065,2.
38005559027251,0.0173100264832448,0.269267893227671
"1049","APPBP2",329.70457322113,0.348993656759329,0.146654118774143,2.
37970579808129,0.0173264650770441,0.269267893227671
"1050","SOWAHC",166.58619765419,0.541220230610527,0.227597768786975,2.
37796808595736,0.017408332511891,0.270061265034469
"1051","CGNL1",13.4125353626425,-1.21275865339981,0.510238925278851,-2
.37684463751375,0.017461440990558,0.270627414172407
"1052","AKR1C2",1358.03669581573,-0.796851267215472,0.335345572241316,
-2.37620929923015,0.0174915380085539,0.270836181198987
"1053","ABCB9",8.20848669054826,-1.38895149639367,0.584677762040566,-2
.37558461526932,0.0175211746477429,0.271037430044714
"1054","TPPP",29.3689870540123,-1.52083833543914,0.640366466389332,-2.
37494999389069,0.0175513278132045,0.27124627964828
"1055","MYO5B",3.45546906246099,2.39854153753256,1.01083640583208,2.37
282860381169,0.0176524531964679,0.27255053091684
"1056","ADAMTS1",1342.47532430993,1.10554056703007,0.466002431266406,2
.37239227277346,0.0176733160400011,0.272614247135964
"1057","SCX",12.4032475656358,-1.57982107933149,0.666070577295849,-2.3
7185237298026,0.017699160857581,0.272754617984046
"1058","MAPKAPK3",54.2809072407503,-0.558056606112059,0.23540001604709
6,-2.37067361117091,0.0177557029115114,0.273271771543607
"1059","HPGD",247.239894020941,0.931676424777071,0.39309504933511,2.37
010470203817,0.0177830485503237,0.273271771543607
"1060","CBLN3",32.9203197526113,0.846300573906505,0.357027468987121,2.
37040745438283,0.0177684916249107,0.273271771543607

"1061","APOC1",233.251312915363,-1.11209394942189,0.469369542119054,-2.36933556532266,0.0178200771560749,0.273582692549768
"1062","HBA1",59.814067380458,3.21730584681194,1.35833676425702,2.3685627389845,0.0178573514188762,0.273896795915325
"1063","POU3F3",6.00303068195599,-1.84122618599985,0.777485853421242,-2.36817966255943,0.0178758529245355,0.273922641851137
"1064","SRGAP2C",229.523487818253,0.384985523360064,0.162648157895007,2.36698360647023,0.0179337271630299,0.27392997235559
"1065","LRCH3",272.01212290251,0.482700169606199,0.203948057415829,2.36677993270621,0.0179435987785263,0.27392997235559
"1066","ISLR",3602.84858869726,0.528880996451086,0.223436669585697,2.36702864141214,0.0179315450616948,0.27392997235559
"1067","FSTL3",505.139528615942,-0.519178484043484,0.219270507883652,-2.36775337027526,0.0178964613747213,0.27392997235559
"1068","VAMP1",27.0792732287965,0.855730313073415,0.361671140285747,2.36604533167154,0.0179792428173785,0.274217121959062
"1069","SRRM3",93.4759602981623,-0.793463579571135,0.335705265690686,-2.36357203971361,0.0180997073141175,0.275054655958862
"1070","PECAM1",10.3089371401745,-1.35995009723704,0.575277825463698,-2.363988384466,0.0180793794231219,0.275054655958862
"1071","BCLAF3",164.78049732384,0.473688712812428,0.200415675931589,2.36353124879373,0.0181016999931181,0.275054655958862
"1072","SPIN4",25.2319508110358,0.829615656446007,0.350854316979606,2.36455878208342,0.0180515624156462,0.275054655958862
"1073","AC074387.1",5.27664854123453,-2.64230928934904,1.11826520324247,-2.36286462431946,0.0181342925311955,0.275293095098457
"1074","PPP1CC",897.42579435285,-0.364406065204007,0.154306679934471,-2.36157025320458,0.0181977235871899,0.275485798802729
"1075","FBXO31",85.0296066788518,0.483075987724996,0.20454185405461,2.36174640128187,0.0181890799919199,0.275485798802729
"1076","PQBP1",334.548308767403,-0.499049375381091,0.211309840743644,-2.36169491030248,0.0181916062850307,0.275485798802729
"1077","LGI1",11.5501067767177,1.37894344803874,0.584115008563114,2.36073962802437,0.0182385308917414,0.275591307695339
"1078","ALDH6A1",243.535207340426,0.533631570172804,0.226041903621598,2.36076391864989,0.0182373363953406,0.275591307695339
"1079","TUG1",454.826531839052,0.560819844694352,0.237606081517271,2.36029246858139,0.0182605322877219,0.275668035620669
"1080","MSN",610.283621136019,-0.390084154242937,0.16541177286228,-2.35826112913811,0.0183607722169265,0.276924646890292
"1081","DPT",7006.48561594823,-0.55688459581509,0.236308841357799,-2.35659653111287,0.0184432734117517,0.277398412376754
"1082","SLC25A3",3110.45389696988,-0.518380605098052,0.219915343057601,-2.3571825316539,0.0184141929859843,0.277398412376754
"1083","PEX11A",58.0506372878443,-0.633501821501087,0.268819503360577,-2.35660662110274,0.0184427723535511,0.277398412376754
"1084","AC068491.3",32.7450137080853,1.09019455988126,0.462690169296801,2.35620860831805,0.0184625462831104,0.277432118455337
"1085","TRMT61A",88.3701191048939,-0.487889430829961,0.20710369539163,-2.35577366163056,0.018484176365203,0.277501150979531

"1086","SLC1A3",241.161103719719,0.693257058461194,0.294587107934793,2
.35331771074872,0.0186067284822716,0.277855077652425
"1087","AC011346.1",16.6873833220511,1.01191452014684,0.43006942095430
2,2.35290971839257,0.018627156043738,0.277855077652425
"1088","TMEM30B",45.6605301167733,-0.664572486890629,0.282217260210996
,-2.35482580474976,0.0185313905287274,0.277855077652425
"1089","MYLPF",7.63277048316651,-1.92928848413152,0.819409298124376,-2
.35448693168072,0.0185482958928041,0.277855077652425
"1090","IFI30",107.316337560479,-0.770849298093169,0.327569524813305,-
2.35323874689658,0.018610680552766,0.277855077652425
"1091","GMFG",36.0066139791029,-0.786154182371228,0.33402558164642,-2.
35357477261549,0.0185938678506059,0.277855077652425
"1092","LINC00649",20.865343839669,-1.15918467831832,0.492627088678566
,-2.35306726925583,0.0186192653853801,0.277855077652425
"1093","ACOT7",28.640928490506,-0.793190540882751,0.33742947778964,-2.
35068538196073,0.0187388706700322,0.278397749636052
"1094","CSF3R",28.4321836970148,1.28091728876416,0.544882715991455,2.3
5081284682233,0.0187324531055451,0.278397749636052
"1095","CDC42SE1",149.602977029806,-0.444945000263183,0.18933226550636
3,-2.35007487536894,0.0187696349548675,0.278397749636052
"1096","EPHA4",46.4027536884587,0.94968494156942,0.404133287041962,2.3
4993001571487,0.0187769411181335,0.278397749636052
"1097","P2RY14",7.07824708260559,-1.96116406705596,0.834251604564845,-
2.35080646692783,0.0187327742729399,0.278397749636052
"1098","TNKS",179.839832614197,0.406655350097457,0.172993280517144,2.3
50700263512,0.0187381213187847,0.278397749636052
"1099","BATF2",5.81762361721633,1.74781057384204,0.743810436072374,2.3
4980646825985,0.0187831743415815,0.278397749636052
"1100","ZNF772",7.64835182523908,1.4419475036667,0.613758749587749,2.3
4937180876888,0.0188051181948766,0.27846960934213
"1101","OSR2",1343.15992494255,0.872681482161455,0.371689928779629,2.3
4787497478539,0.0188808577561214,0.278758361328961
"1102","TIGD7",20.2203732249089,0.922724814112154,0.392973852948139,2.
34805651111329,0.0188716578537161,0.278758361328961
"1103","ZNF512B",71.5579018347714,0.625095228863907,0.266266037245433,
2.34763410058084,0.018893070839657,0.278758361328961
"1104","SYNGR1",158.248690351162,0.503149160990603,0.214305883254177,2
.3478084378759,0.0188842306973534,0.278758361328961
"1105","AC016877.3",4.52637751298339,1.85866269852905,0.79208770916972
5,2.34653647192345,0.0189488115947687,0.278924225039263
"1106","AC015912.3",43.0709458912127,-1.16770427946106,0.4975466440315
87,-2.34692424010588,0.0189291031997752,0.278924225039263
"1107","TGM2",9.74515016181443,-1.82421935915807,0.777454100023093,-2.
34640136196321,0.0189556827993409,0.278924225039263
"1108","SNHG10",34.8013805147828,-0.654487722292682,0.279150122806317,
-2.34457257519025,0.0190489028444757,0.280042940824607
"1109","NCF1",12.6570631351536,-1.09925993185928,0.468929640892105,-2.
34418948174831,0.0190684812344315,0.280077989925748
"1110","CDK19",110.770664147238,0.555378391822403,0.236996315134442,2.
34340517702712,0.0191086188292486,0.280414677576244

"1111","BICC1",1380.71781227794,0.640494121984922,0.273448142958221,2.34228733483402,0.0191659531886369,0.281002890629799
"1112","HSPB7",12.0532427973716,-1.11712019568855,0.477437441278731,-2.33982528202342,0.0192927629747012,0.282607748286788
"1113","SRGAP1",553.930212892405,0.506191363740062,0.216448849254873,2.33861887223069,0.019355167205231,0.283267132619952
"1114","CCNB1IP1",262.272362544611,-0.511219811835239,0.218711921348495,-2.33741173632992,0.0194177854662798,0.283673818349984
"1115","GABRE",160.4380433073,0.85773650596967,0.366934945282587,2.33757105175442,0.019409511105406,0.283673818349984
"1116","ITGB3BP",130.522411536239,-0.574336183903666,0.24612922692312,-2.33347413098187,0.0196232745094514,0.283907068867766
"1117","LBX2-AS1",40.1584578929777,0.599896833867089,0.256954845150874,2.33463912118432,0.0195622810468449,0.283907068867766
"1118","CCRL2",3.96530437158824,-1.90544291538403,0.816356972930299,-2.33408052918869,0.0195915056267438,0.283907068867766
"1119","PCDHGB7",43.3657103080678,0.618392775529788,0.265024878512194,2.33333858693321,0.0196303817403529,0.283907068867766
"1120","PPP1R11",161.181329010428,-0.52779163528286,0.226219049471337,-2.33309987163453,0.0196429042061497,0.283907068867766
"1121","ANGPT2",43.7599836724633,-0.801902438666797,0.34352544223496,-2.33433201759281,0.0195783434663337,0.283907068867766
"1122","SLC39A4",94.6546367931901,-0.601372331587678,0.257501631884462,-2.33541172996335,0.0195219222371763,0.283907068867766
"1123","MDK",553.301842613502,0.758840428807446,0.324849649822155,2.33597428602089,0.0194925817440677,0.283907068867766
"1124","LRRC32",233.282832061977,-0.486188948994338,0.208320795538098,-2.33384740941729,0.0196037133293571,0.283907068867766
"1125","MYO9A",439.551424727514,0.521206007376495,0.223085076833791,2.33635532584198,0.019472730244266,0.283907068867766
"1126","AC104564.5",11.4212180545169,1.12117925760554,0.479834353097512,2.33659647411216,0.0194601759746539,0.283907068867766
"1127","MT-ATP6",92139.0805951486,-0.442933685414599,0.189677794579829,-2.33518998043908,0.019533498330952,0.283907068867766
"1128","HDGF",482.577956058484,-0.398001234092313,0.170690781754448,-2.33170901205942,0.0197160043985981,0.284710989050324
"1129","LPCAT2",56.2516943989527,-0.585976578893694,0.251378238448655,-2.33105531532866,0.0197504430960895,0.284955684315503
"1130","TPST1",634.176056496824,0.645161399901486,0.276813522051981,2.33067154783116,0.01977068557677,0.284995307398236
"1131","AC004540.1",32.8658859211976,0.996988259904187,0.427917355027482,2.32986170855388,0.0198134614097899,0.285359392488124
"1132","UBAC1",296.751374450344,-0.42156203094597,0.181011075624088,-2.32892948396949,0.0198628016826717,0.285817293824239
"1133","LAIR1",6.24126825917207,-1.5043284809281,0.646041985699283,-2.32853052004011,0.0198839505843873,0.285869083026554
"1134","CACNB2",6.44822159307538,1.65765134771865,0.712456271709459,2.32667100219541,0.0199827823573835,0.286783632310049

"1135","MEOX1",3.13586378965142,-2.83170118603883,1.21708735356497,-2.32662115643919,0.0199854375070389,0.286783632310049
"1136","ZNF790-AS1",25.6565395017091,0.848116417037924,0.364571022410591,2.32634072623235,0.0200003810119845,0.286783632310049
"1137","UHRF1BP1",47.4042312357723,0.809342767517439,0.347972514371572,2.32588130984738,0.0200248833712057,0.286882432043597
"1138","CACNB1",24.1124720778405,-0.737629556550031,0.317217847106055,-2.32530913149859,0.0200554363713031,0.287067665247941
"1139","AL139384.1",18.4825586325193,1.08518263137386,0.466828310229428,2.32458616496615,0.0200940992870434,0.287183088891765
"1140","HSD11B1L",70.8945795204234,-0.687031645698521,0.295561161445517,-2.32449907267388,0.0200987612091971,0.287183088891765
"1141","SPSB1",330.289016217493,-0.621268041397635,0.267352160368957,-2.3237816389449,0.0201372003074239,0.287480154082057
"1142","ACSL1",218.001785922659,0.640477098944364,0.275658103281473,2.32344738398775,0.0201551311183766,0.287484177572011
"1143","HSD17B12",498.269301814775,-0.329987982712588,0.14209400237447,-2.32232168281775,0.0202156208717167,0.287842874457512
"1144","DCLK1",1868.81533598934,0.502012899789511,0.216162275084214,2.32238904588662,0.0202119966543823,0.287842874457512
"1145","ST6GALNAC4",117.122876335196,-0.568392980700967,0.244851677388694,-2.32137670757576,0.020266521521854,0.288315606174218
"1146","PGM1",237.388275862477,-0.471758258945026,0.203308876551857,-2.32040168115679,0.0203191580358773,0.288789406216644
"1147","SPINT2",6.81022107580023,-1.71705294131665,0.740180583820462,-2.31977571264304,0.020353013588109,0.288789406216644
"1148","MBTPS2",84.8235335271485,0.647381493483423,0.279067387059116,2.31980347222116,0.0203515111672562,0.288789406216644
"1149","SPATA18",9.11692428049598,1.30915429631514,0.564433693801168,2.31941202428697,0.0203727062909006,0.288817243492149
"1150","DNAJC18",136.08202294357,0.596312014071475,0.257426100037045,2.31643960727239,0.0205342783006131,0.290620218139397
"1151","AC108863.2",46.1480015248108,0.801107296649403,0.345839137253867,2.31641595861761,0.0205355682410489,0.290620218139397
"1152","IGF1R",278.884156584063,0.529321007272047,0.228545977812471,2.31603729078256,0.020556232698251,0.290660134046711
"1153","TPRG1",56.4645018304244,-0.691906380266086,0.298906393115944,-2.31479284552371,0.0206242716658673,0.290787812297802
"1154","PLCE1",103.766672261403,0.639198133941059,0.276274166431874,2.31363700123109,0.0206876422562179,0.290787812297802
"1155","BORCS7",740.695314668314,-0.411533935016023,0.177769821128473,-2.31498199415192,0.0206139174903579,0.290787812297802
"1156","CARD16",27.2107740578006,-0.857231075942242,0.370546500291252,-2.31342375455834,0.0206993522990403,0.290787812297802
"1157","ADAMTSL3",166.709337893166,0.91423457449357,0.395213949127575,2.31326494551045,0.0207080767551998,0.290787812297802
"1158","HBA2",217.207323779651,3.48161884273053,1.50455528065798,2.31405179157521,0.0206648813612404,0.290787812297802
"1159","ZMYND15",9.32924693664619,-1.29249100786707,0.558555405439881,

-2.31398889936297,0.0206683310565552,0.290787812297802
"1160","BCL3",155.359015928437,-0.652151773116643,0.281905010028556,-2.31337418604438,0.0207020750890215,0.290787812297802
"1161","Z83843.1",125.901924135093,0.760446288633439,0.328816247624627,2.31267856782295,0.020740318229193,0.290989701666946
"1162","DYSF",4.10614042026036,-1.82193210864043,0.788192793107378,-2.31153104237053,0.0208035405675283,0.291625535546014
"1163","DMD",191.562171527813,0.619629071866463,0.268172899332059,2.31055812652874,0.0208572744020029,0.292127379823066
"1164","VPS13B",188.251336795073,0.446206486152415,0.193158157409602,2.31005768607645,0.020884960654684,0.292263852323151
"1165","CP",791.922396194716,-0.797579501899066,0.345335730035007,-2.30957712316132,0.0209115773489884,0.292286911536498
"1166","UPP1",126.317737881478,-0.87891526441076,0.380595341907276,-2.30931692439076,0.0209260011793195,0.292286911536498
"1167","TOB2",422.728022713222,0.425354174187682,0.184211236635639,2.30905661324565,0.0209404399142423,0.292286911536498
"1168","MSRB3",214.082338790185,-0.40151257660901,0.173921295470022,-2.30858777542982,0.0209664669445361,0.292399640461942
"1169","CEP192",88.6607166597481,0.507470160795707,0.219893136715641,2.30780354664707,0.0210100655840451,0.292757021641156
"1170","KLHL24",389.415166068744,0.564769994199685,0.244897058652405,2.30615262309578,0.0211021057745086,0.293788205949548
"1171","ILF2",499.32147838602,-0.562319937218247,0.243869409019695,-2.30582400424332,0.0211204683782552,0.293792749285567
"1172","SYBU",28.3210338368331,-0.961667334162266,0.417202033147788,-2.30503990334488,0.0211643387075003,0.294151803077195
"1173","HLA-DQB1",85.0942639888379,-1.22312877693538,0.530746902579772,-2.30454246834073,0.0211922112590682,0.294288089683684
"1174","KIDINS220",807.098002948178,0.381876660615902,0.165743859543375,2.30401694317951,0.0212216925036851,0.294446464388864
"1175","HBB",464.372443768858,3.26101054968932,1.41571794421364,2.30343237720324,0.0212545278216942,0.29465106696815
"1176","ZNF678",65.3252935030003,0.547718701986265,0.237943035246424,2.30189003607113,0.0213413742627867,0.295002070160187
"1177","SASH1",1419.29721038565,0.615887102076714,0.267606731330942,2.30146341616146,0.0213654509558487,0.295002070160187
"1178","FAM92A",328.159566855138,-0.508659010736112,0.220974021207022,-2.30189507326551,0.0213410901252187,0.295002070160187
"1179","SQLE",73.3764234758106,-0.768148832157454,0.333730044861974,-2.30170715518032,0.0213516924216248,0.295002070160187
"1180","AC008764.7",10.2089420158895,1.8331208190113,0.796532594358887,2.30137577795764,0.0213703998274309,0.295002070160187
"1181","LINC00886",11.6885637113101,1.15715483699907,0.503270080181539,2.29927206596836,0.0214894950119142,0.296394906222752
"1182","EXOC4",204.963028758371,0.373542464038425,0.162511886808676,2.29855471728166,0.0215302374145495,0.296705615267003
"1183","SOS1",283.752317083766,0.491646502147472,0.214015598204407,2.29724611791099,0.0216047336393057,0.297480563187363

"1184","ISM1",841.537286231891,0.691430364431411,0.301092849405286,2.2
9640247450948,0.0216528796241155,0.29789168597738
"1185","AKIRIN1",337.196319966466,-0.497977074196973,0.216957183176527
,-2.29527811389308,0.0217171910528446,0.298460802871029
"1186","AC137936.1",9.06520869500011,-1.41418831434023,0.6161936032151
45,-2.29503894062086,0.021730892762296,0.298460802871029
"1187","CD209",3.32756558075072,-2.24523930609778,0.979274141132414,-2
.29275869931726,0.0218619010025372,0.300007165484691
"1188","SEC14L2",17.1575790999467,-0.911621578857909,0.398287762463795
,-2.28885159116777,0.0220879767294764,0.302854421672088
"1189","NOTCH4",30.8091581042329,-0.851509781435308,0.372165762922768,
-2.28798526427595,0.0221383792729857,0.303290210241938
"1190","AP001267.3",13.7660418510292,-1.04898259201213,0.4587939399723
39,-2.28639155973894,0.0222313616668395,0.304308109404326
"1191","LAPTM5",56.6401290892999,-0.95328953533068,0.417132625088608,-
2.28533918949202,0.0222929466994561,0.304894885631772
"1192","AP1S2",170.194268591624,-0.68076786993406,0.298027347037157,-2
.2842463173327,0.0223570588827326,0.305515211527544
"1193","CDC42EP3",32.7401191119215,1.13970340412741,0.499098779142904,
2.28352272486943,0.0223995958205827,0.305637340266315
"1194","RIN1",29.0513809218235,-0.747043184792405,0.327217973293608,-2
.2830139104923,0.0224295489814191,0.305637340266315
"1195","CPD",208.317789127621,0.398862255672696,0.174707881293934,2.28
30238265075,0.0224289649075303,0.305637340266315
"1196","RNF185",119.39773979775,0.400357241543217,0.175378465573931,2.
2828187042978,0.0224410497242626,0.305637340266315
"1197","LINC02427",7.40652015836339,2.0557788810502,0.900813067548692,
2.28213705496572,0.0224812499030525,0.305702335283047
"1198","SLC37A4",41.2831560001735,-0.629948338379127,0.276038720005055
,-2.28210136015553,0.0224833567234999,0.305702335283047
"1199","MYLK",170.93306581054,-1.75025260095147,0.767074503120152,-2.2
8172438769917,0.0225056173082569,0.305749791771641
"1200","TTN-
AS1",93.4981232276651,0.634067443212542,0.278110550278879,2.2799115048
9157,0.0226129375780364,0.306441048426485
"1201","RGS14",35.4068648723433,-0.853356998841597,0.374282423432916,-
2.27998149369295,0.0226087860939671,0.306441048426485
"1202","AL117339.4",5.21918631978163,1.86112761817407,0.81610602568227
7,2.28049733687255,0.022578208558564,0.306441048426485
"1203","METAP1D",51.6081987111635,0.739063460897124,0.324789461584126,
2.27551552101605,0.0228750226736565,0.30922094965244
"1204","SORBS2",21.5416143058647,1.35272460189751,0.594390985915704,2.
27581614450888,0.0228570160476921,0.30922094965244
"1205","CDC14B",231.670865397198,0.401756032922713,0.176521665830275,2
.27595876706148,0.0228484776066483,0.30922094965244
"1206","CACNB3",32.2479133496038,-0.81045382495031,0.356234921233355,-
2.27505439990094,0.0229026666704374,0.309337924871273
"1207","IFT57",620.66896826936,-0.396736723036661,0.174492738784285,-2
.27365749314716,0.0229865877736857,0.310214190758547
"1208","CEP70",53.8342706183277,-0.540979998670519,0.238155710332466,-

2.27153906121046,0.0231143651373191,0.311620124664637
"1209","GANC",76.4435438059659,0.551954003891814,0.24301273493354,2.27
129662172958,0.0231290276087879,0.311620124664637
"1210","IRX1",137.626785462116,-0.640216882030257,0.282039601397319,-2
.26995386058698,0.0232103826493974,0.312457787583499
"1211","CHMP3",895.420821853668,-0.407517469529317,0.179602847906927,-
2.26899224749765,0.0232687973167431,0.312469447232011
"1212","WDR86",219.204779989856,0.959300783893014,0.422766194684146,2.
26910475803232,0.0232619561044322,0.312469447232011
"1213","SFMBT2",56.1174564434007,1.05998960308499,0.467034302524001,2.
2696183071703,0.0232307518786556,0.312469447232011
"1214","DAAM2",45.5681059069376,0.799066850172476,0.352230077685757,2.
2685934586352,0.0232930597762567,0.312537603538258
"1215","NUDT10",3.60268385824096,-2.01330225610191,0.887814220729855,-
2.26770669932141,0.0233470892652319,0.313004721844743
"1216","TJP2",105.272662228926,-0.952832820716749,0.420380123526383,-2
.26659817482296,0.023414783695261,0.313396394093761
"1217","ZFP30",35.9285209721557,0.659249037814095,0.29081997738409,2.2
6686297050156,0.0233985979097518,0.313396394093761
"1218","SNED1",657.549050367584,0.798938442643938,0.352598320559766,2.
26586003409088,0.0234599542786323,0.313666080265315
"1219","C19orf44",43.2360209958448,0.735026357260075,0.324423305562812
,2.26563981272846,0.0234734453829836,0.313666080265315
"1220","CDO1",581.11292945808,-0.698630982174992,0.308694121356647,-2.
26318201041423,0.0236244717079991,0.315425425943932
"1221","AC093418.1",3.2323997435599,2.28425943507322,1.00962697027347,
2.26247862064788,0.0236678482758903,0.315487381805219
"1222","LRRC4C",68.3583839196431,-1.22117891541911,0.539726760438732,-
2.26258730329851,0.0236611415350178,0.315487381805219
"1223","SLC39A9",143.413095792755,0.547220573939286,0.241997057885636,
2.26126953244983,0.0237425716932329,0.316224652748218
"1224","MBD2",652.152784556587,-0.398292191950783,0.176190969892107,-2
.26057097134253,0.0237858370048566,0.316542074323619
"1225","SMURF1",74.3171600757609,0.63099912671919,0.279186748481612,2.
26013279695741,0.0238130101814162,0.316644998240889
"1226","CAPN1",471.031541775639,-0.465568169932774,0.206256782672707,-
2.25722598743115,0.0239939573620545,0.318790841329939
"1227","HMG20B",368.006482354607,-0.524186745690402,0.232300081570866,
-2.25650693768909,0.0240389013120711,0.319127680091545
"1228","YRDC",68.032411682903,-0.547664511788137,0.242760227201701,-2.
25598945140672,0.0240712917661626,0.319297452425915
"1229","ARID1A",365.449516467415,0.38948962058409,0.172804770940503,2.
25392862977257,0.0242006582409682,0.319751593874221
"1230","RSPH9",5.92404593467558,-1.57036767423124,0.696697812794813,-2
.25401550771587,0.024195192401115,0.319751593874221
"1231","DKK3",58.7941640208122,-1.87787968457709,0.833176192070609,-2.
25388063467127,0.0242036782642836,0.319751593874221
"1232","ANO8",48.0675785050487,0.594506364789171,0.263633590246887,2.2
5504786485072,0.0241303245452741,0.319751593874221
"1233","GPCPD1",168.805660032802,0.55560889655169,0.246466521084303,2.

254297638914,0.0241774497761136,0.319751593874221
"1234","NR1H2",401.466048543756,-0.348632466573557,0.154737794296816,-2.25305309641944,0.0242558013335311,0.3201805088508
"1235","UBAP1L",19.5112929259373,1.01804791518311,0.452063424712412,2.25200239508596,0.024322120660921,0.32079597040141
"1236","SLC2A8",66.8140992450629,-0.590635836372485,0.262424838781049,-2.2506857167788,0.0244054500521002,0.321634608332249
"1237","FAM53C",420.046489903139,0.636708300991128,0.283129932892132,2.24882016001361,0.0245239402116715,0.322934892569052
"1238","PHLDA3",664.004666641802,-0.534015336154194,0.237517935893975,-2.2483158341043,0.0245560577541715,0.323096627429483
"1239","SLC30A4",80.9627692270569,0.447693407371948,0.199194336035802,2.24752076932289,0.0246067647560873,0.323502494844153
"1240","HCLS1",34.4963404696622,-0.939850669905841,0.418249653368868,-2.24710448014874,0.0246333506775906,0.323590846118768
"1241","ADAM10",184.082700082006,0.592973322388476,0.264108871388547,2.2451851740948,0.0247562474734962,0.324943203139227
"1242","DNMT3A",107.234589734266,0.604523255434228,0.269383764487464,2.24409684297199,0.0248261710540973,0.325143962670243
"1243","AL139339.1",18.9759897389351,-1.0218633074383,0.455372727545828,-2.24401516741133,0.0248314254749697,0.325143962670243
"1244","ATRN",137.407461707981,0.448872434184861,0.199981227817784,2.24457284857686,0.0247955674168985,0.325143962670243
"1245","GIPC3",3.44056345233525,-2.13805954875691,0.953076682696409,-2.24332374044446,0.0248759455382801,0.325332543301391
"1246","FBXO27",28.9288868532269,0.893570932730622,0.39835163135915,2.24317126474872,0.0248857725430372,0.325332543301391
"1247","HES4",23.6411731377136,-1.13533899136876,0.506474649219797,-2.24165018548845,0.0249839897582028,0.326164348952514
"1248","ZNF528-AS1",53.6415397892927,0.892481531051375,0.398150937354223,2.24156581667761,0.0249894473259708,0.326164348952514
"1249","USB1",76.013001599374,-0.488955052440399,0.218202178915518,-2.24083487557523,0.0250367729615156,0.326520412145819
"1250","AP000926.2",4.69808510644206,-1.53067250415825,0.683285651049906,-2.24016485902533,0.0250802221123618,0.326825390390609
"1251","CCND2-AS1",21.558465784051,-1.03400263962514,0.461660973125789,-2.23974453076286,0.0251075128166696,0.326919485428243
"1252","ELOVL6",45.4205239089179,0.769383056989596,0.343769508425104,2.23807824176826,0.0252159532789509,0.327255427551103
"1253","KLF10",536.57044071324,-0.584086972264964,0.260975116432587,-2.23809449823865,0.0252148933685736,0.327255427551103
"1254","ORMDL3",97.5072195194133,0.420966686902014,0.188045483362865,2.23864290369414,0.0251791603063636,0.327255427551103
"1255","FCGBP",4.08135892036938,-1.79014437114193,0.799955435041419,-2.23780512354321,0.02523376616147,0.327255427551103
"1256","PPP1R15A",2980.22548422483,-0.366223918577016,0.163578249743882,-2.23883015712921,0.025166969269554,0.327255427551103
"1257","NKD1",7.66367293401309,1.82563345336031,0.816311461135483,2.23

644226533444,0.0253228151937636,0.328149034758326
"1258","PUDP",43.7189384411099,0.611512956135329,0.273473572973593,2.2
3609524491194,0.0253455328703922,0.32818234095852
"1259","DENND5A",219.833218984878,0.419459231639127,0.18761972690106,
2.23568831790519,0.0253721947941375,0.32826662510064
"1260","KLF7-
IT1",3.36792644780117,2.35379568778102,1.05516934604594,2.230727888941
66,0.0256991591034742,0.328437405779435
"1261","TFRC",94.1440782737965,-0.862091507276662,0.386525531214095,-2
.2303610956016,0.0257234801694401,0.328437405779435
"1262","CCNG2",643.635862931968,0.432179132081505,0.193517948889779,2.
23327672994126,0.0255307010807604,0.328437405779435
"1263","CACNA2D1",238.509069694582,0.381661824582169,0.171075643071092
,2.23095361636938,0.0256842016217672,0.328437405779435
"1264","OR2A1-
AS1",31.4158611195673,-1.0148856064696,0.45500151417921,-2.23051039357
612,0.0257135782266078,0.328437405779435
"1265","SH2D3C",9.21533083489322,-1.3905373092134,0.623478131046353,-2
.23029043036317,0.0257281680750542,0.328437405779435
"1266","PAPSS2",299.454877950346,0.54573520338158,0.244565397187292,2.
23144896889746,0.0256514042372808,0.328437405779435
"1267","FTH1",43594.9747388138,-0.547792438100727,0.245371930053741,-2
.23249838716576,0.0255820417050877,0.328437405779435
"1268","ACER3",130.321135089357,0.536095795069487,0.239985752556178,2.
23386509140368,0.0254919510833324,0.328437405779435
"1269","PDZRN4",43.491524241573,2.41868092153978,1.08242561294583,2.23
450082168448,0.0254501385406155,0.328437405779435
"1270","KCTD10",195.430890719426,0.458861172539485,0.205717112385616,2
.23054449490498,0.0257113169745655,0.328437405779435
"1271","TPT1-
AS1",269.783976692715,0.475556012063156,0.212836118477145,2.2343764557
7164,0.0254583135246601,0.328437405779435
"1272","LINC02175",16.5718591124038,1.08263310481509,0.484853788668646
,2.23290635263029,0.025555120580912,0.328437405779435
"1273","SNHG30",143.058694656541,-0.747703151921266,0.334807948990547,
-2.23322998804421,0.0255337817266532,0.328437405779435
"1274","AC005332.2",4.26347971704117,1.72226905677098,0.77124344445065
1,2.23310689920708,0.0255418957541116,0.328437405779435
"1275","KIAA0355",205.759689466381,0.437904988523374,0.196269754139634
,2.23113841683335,0.0256719617088949,0.328437405779435
"1276","ZNF606",26.2912677401018,0.6658167887631,0.298470037203077,2.2
3076592545899,0.0256966381443192,0.328437405779435
"1277","PCGF1",94.6983778394427,-0.509262874290226,0.228698184417723,-
2.22679019331454,0.0259612991117557,0.330400955554803
"1278","AC087071.1",12.8716565191226,1.23675394813695,0.55548003567102
8,2.22645976221797,0.0259834013178036,0.330400955554803
"1279","AC092111.1",5.98114281216923,1.66537190625863,0.74771608950479
5,2.22727841440672,0.0259286722427215,0.330400955554803
"1280","SLC7A8",110.942568503185,0.748565996800474,0.336142570308945,2
.22693006753793,0.0259519479625559,0.330400955554803

"1281","SGSM2",100.811123244625,0.510493695763968,0.22927434892824,2.2
2656262312077,0.0259765193115174,0.330400955554803
"1282","LAMC2",52.3600045214774,0.542839750804234,0.243963625409256,2.
2250847842321,0.0260755470023619,0.330885797221243
"1283","KIF9",57.6245030115152,-0.541157606181437,0.243269190640968,-2
.22452175203769,0.0261133606739231,0.330885797221243
"1284","KLF12",129.12368600293,0.551704108165142,0.247967113718602,2.2
2490837551639,0.0260873896510759,0.330885797221243
"1285","DOCK9-
DT",8.87190786950336,-1.24567948411152,0.559710712130532,-2.2255773511
4602,0.026042504660554,0.330885797221243
"1286","SLC16A3",11.9866115833845,-1.06862886686867,0.480417177744167,
-2.22437688820057,0.0261230975030093,0.330885797221243
"1287","ZNF667-
AS1",209.683269610103,-0.515417746302456,0.231752587482022,-2.22399996
436906,0.0261484466409077,0.330949531727852
"1288","CCNI",5260.57257725067,-0.332423968547435,0.149534358368573,-2
.22306078799679,0.0262117012766236,0.331492548210343
"1289","C8orf82",250.985156761493,-0.378822766491697,0.170466820739966
,-2.22226686018602,0.0262652763575596,0.3318350197117
"1290","NPR2",145.218597352608,0.579943403542361,0.260994059569767,2.2
2205595214835,0.0262795245520347,0.3318350197117
"1291","RPUSD1",57.5785619617907,-0.620733204544567,0.279410905325126,
-2.22157830175696,0.0263118176033681,0.33198543527596
"1292","FAM107B",220.347926788343,0.502277676595954,0.22622145099041,2
.22029199440175,0.0263989530119933,0.332809652522721
"1293","MEIS2",415.011306979157,0.3234537654024,0.145699156831408,2.22
001123710466,0.0264180048322106,0.332809652522721
"1294","C3orf14",155.063335192207,-0.496042416519041,0.223484332092731
,-2.21958475511033,0.0264469680668353,0.332867549007985
"1295","RSRC1",548.808159536436,-0.320981846238506,0.144746686745198,-
2.21754192414463,0.0265860814819442,0.332867549007985
"1296","GTF2I",932.614475985752,0.408362827732704,0.18413299267318,2.2
1776022756286,0.0265711852777284,0.332867549007985
"1297","HGF",113.641051057394,-0.665942932089962,0.300199967099436,-2.
21833112949469,0.0265322631536486,0.332867549007985
"1298","CCDC71L",1629.26311209077,-0.932903210913296,0.420651331408156
,-2.2177588450519,0.0265712795923877,0.332867549007985
"1299","RIMKLB",152.206769246809,0.444834335338002,0.200576825118259,2
.21777533409321,0.0265701547316797,0.332867549007985
"1300","ITGAM",12.7138846087458,-1.38090285001861,0.622322220893,-2.21
895153934417,0.0264900216037226,0.332867549007985
"1301","FBXL20",207.224872120515,0.441553585105082,0.199042754052088,2
.21838562879576,0.0265285501558743,0.332867549007985
"1302","PCYOX1",1213.91770461559,0.466078833640479,0.21057668372584,2.
21334492211535,0.0268738747727173,0.333674667264685
"1303","USP40",128.498498662209,0.592442389383131,0.267661775718932,2.
21339930885479,0.0268701282769131,0.333674667264685
"1304","R3HCC1",282.701748573792,-0.599660086231364,0.27057991355045,-
2.21620325900339,0.0266775847903618,0.333674667264685

"1305","HTRA1",3517.50888384386,-0.429546799694603,0.194126004441869,-2.21272158219911,0.0269168465090427,0.333674667264685
"1306","LRRK2",234.512899423436,0.54951868451606,0.248086056532305,2.2150325262012 6,0.0267578320574072,0.333674667264685
"1307","SSH1",107.955810798043,0.659064937808513,0.297841115681746,2.2128071079103 4,0.0269109470381243,0.333674667264685
"1308","UBE2L5",12.0223267020303,1.01230453831565,0.457000889923341,2.21510408543286,0.0267529210928538,0.333674667264685
"1309","ZFYVE1",103.395344058953,0.516732530833768,0.233322069751026,2.21467489717181,0.0267823870796329,0.333674667264685
"1310","AP3S2",153.037093502604,0.417003187610198,0.188432979083787,2.2130053329188,0.0268972779837185,0.333674667264685
"1311","SOX15",40.8817748438783,-0.700634470871566,0.316173366477177,-2.21598194268568,0.026692738849525,0.333674667264685
"1312","STXBP4",82.9122790643799,0.464226485855658,0.209568274700536,2.21515630893568,0.0267493375913661,0.333674667264685
"1313","ZGPAT",127.866892870575,-0.426122230838061,0.192483915874086,-2.21380695058599,0.0268420617418043,0.333674667264685
"1314","ARFGAP3",316.98931101495,-0.352058992600412,0.159065095105339,-2.21330136801707,0.0268768753742499,0.333674667264685
"1315","ID1",407.954708074001,-0.789703301338797,0.357037985434461,-2.21181872393171,0.0269791928352424,0.334186046149827
"1316","AC003681.1",67.4371638971169,1.04443760889045,0.472269166350528,2.21153037993433,0.0269991305011463,0.334186046149827
"1317","NDNF",130.540085733768,-0.49703703811201,0.224875376850673,-2.2102777328176,0.0270858930282308,0.334497431036279
"1318","RASA3",243.817333294795,0.463308707786965,0.20958831819877,2.21056551132573,0.0270659392621328,0.334497431036279
"1319","RBM3",2022.2497008286,-0.532728205729397,0.240995550294671,-2.2105312943663,0.0270683111066693,0.334497431036279
"1320","OMA1",105.354432693552,0.528001127172907,0.239069937933431,2.20856345108488,0.0272050198796543,0.334699825392514
"1321","LRRTM4",11.9678474335001,4.12321889974606,1.86645844493306,2.20911368851501,0.0271667342485506,0.334699825392514
"1322","SLC12A7",79.7278631353869,0.52953874474643,0.239754637508731,2.20866945577704,0.0271976404327632,0.334699825392514
"1323","SLC5A3",1571.12301039913,-1.00365102414449,0.454403806995172,-2.20872054479761,0.0271940845211626,0.334699825392514
"1324","ASMTL-AS1",2.55788874307241,2.98059639537555,1.34896370229916,2.20954529042957,0.027136735851449,0.334699825392514
"1325","ANO2",4.4974728164237,1.94428632358206,0.880900637232171,2.20715735850878,0.0273030676778847,0.335652580683067
"1326","LYZ",165.202984481333,-1.02724940343348,0.465620885879886,-2.20619270867174,0.0273705095867386,0.335974552116342
"1327","C14orf39",3.89055820848707,2.16739547812041,0.982394875922097,2.20623654626256,0.027367441639016,0.335974552116342
"1328","LOX",1263.01967662586,0.827624188381461,0.375243313985075,2.20556678170255,0.0274143470898749,0.336006245106826
"1329","SHMT1",77.2075614469359,-0.529661909645325,0.240142814545708,-

2.20561215061677,0.027411167591747,0.336006245106826
"1330","MEAK7",71.2471178309549,0.510674240873533,0.231705301093301,2.20398168908487,0.0275256318647668,0.337116554470065
"1331","ZNF740",74.9483974507353,0.502570721508733,0.228080449775933,2.20348005277287,0.027560931378589,0.337264688545603
"1332","STX16",445.105570130966,0.45303683344157,0.205624728712032,2.20322154966111,0.0275791371565316,0.337264688545603
"1333","IGFBP7",9218.78710380591,-0.703474087151694,0.31937405719281,-2.20266509225888,0.0276183623676687,0.337491001205518
"1334","SLC39A8",193.197750209879,0.966232795839353,0.439004924109763,2.20096118010232,0.0277387721435095,0.338454576363765
"1335","C1QTNF3",83.4828138680919,0.786242300288256,0.357179759434995,2.20125099342688,0.0277182601110023,0.338454576363765
"1336","DNASE1L3",282.396178421783,1.59848993025746,0.726451379692872,2.20040869209068,0.0277779116885112,0.338678445729161
"1337","LINC01137",12.0374826632127,-1.21473576030291,0.552424344919414,-2.19891786354946,0.0278837631756805,0.339323052415729
"1338","ZBTB37",123.414174487367,0.60616016236982,0.275679706262658,2.19878412737531,0.0278932756574782,0.339323052415729
"1339","CDK5R1",7.26150376480243,1.7007926881756,0.773344321511178,2.19926964078836,0.0278587550666636,0.339323052415729
"1340","CRISPLD1",124.266450927452,0.686745333421543,0.312524802243722,2.19741066466137,0.0279911303085852,0.340259344475033
"1341","XPO1",374.505761229629,0.372580655714763,0.16967346398038,2.19586874090014,0.028101339703434,0.341344312027767
"1342","HIVEP3",41.7542946615689,-0.731950798551535,0.333472134848196,-2.19493841332421,0.0281680158394628,0.341700896082261
"1343","ZSCAN31",23.1716914246261,0.740714356125172,0.337474687746477,2.19487381726722,0.0281726504658651,0.341700896082261
"1344","KDM5B",466.3126960358,0.393114714599941,0.179280167985488,2.19273954848012,0.0283261495911614,0.342034581683045
"1345","SRGAP2",147.946701649417,0.544297926039611,0.248182615403407,2.19313478164007,0.0282976697122085,0.342034581683045
"1346","IL1A",2.93585515719599,-2.82127149725242,1.28629563770632,-2.19333053347146,0.0282835732818541,0.342034581683045
"1347","MPDZ",340.309238564272,0.543271269302471,0.247745628256749,2.1928591560835,0.0283175282508355,0.342034581683045
"1348","MCU",102.818501067555,0.511802750238742,0.233327478353669,2.19349539904155,0.0282717057192522,0.342034581683045
"1349","SULF2",216.363849769184,0.939959568686085,0.428624431125556,2.19296778351569,0.0283097003226125,0.342034581683045
"1350","DLEU2",124.827925768939,0.650571658348997,0.296774769996278,2.1921393734284,0.0283694444837966,0.342303615701158
"1351","COL1A1",29928.5156603993,0.898688343419291,0.410046632518778,2.19167351259283,0.0284030896268244,0.342455904464354
"1352","NBR1",369.77888336514,0.340446255046449,0.155357011958383,2.19138003978635,0.0284243023009269,0.342458180606359
"1353","SLCO4C1",7.50911070416085,1.72921431427925,0.789567811422589,2.19007701335198,0.0285186519961756,0.343340962576278
"1354","LINC02656",3.09086280462128,2.20934867751466,1.00911899171764,

2.18938370563623,0.028568962965665,0.343692642354296
"1355","COL27A1",30.347727486426,0.978891328664884,0.447215726164453,2
.18885712508446,0.0286072261775493,0.343898972107824
"1356","DLG1",237.654886471651,0.464914764527064,0.212487124398503,2.1
8796675724762,0.0286720238729269,0.344169931367801
"1357","GATD3B",321.496249986091,-0.503285791209945,0.230006989719402,
-2.18813259468302,0.0286599452648171,0.344169931367801
"1358","MBNL1-
AS1",24.0900334882492,-0.670250558201459,0.30667999391232,-2.185504667
75177,0.0288518640805795,0.344804118861746
"1359","AC079140.6",5.27956812472667,1.60187123801877,0.73270054597990
9,2.18625637282205,0.0287968540267697,0.344804118861746
"1360","IQGAP2",20.5214272118159,-1.13995834163998,0.521376346102473,-
2.1864404669711,0.0287833957235881,0.344804118861746
"1361","GCNT1",8.13165752339375,-1.28496413134806,0.587754910205232,-2
.18622440925144,0.0287991912929788,0.344804118861746
"1362","CSAD",333.965253428559,0.519304167960356,0.237612140948791,2.1
8551192664972,0.0288513324394528,0.344804118861746
"1363","SLC12A6",128.410557227061,0.495991979819651,0.226929825664694,
2.18566236662305,0.0288403161259542,0.344804118861746
"1364","FOXP2",153.212725882958,0.618857745646157,0.283335530737644,2.
18418686860418,0.0289485194458684,0.345199438692351
"1365","GFRA1",669.317381926639,0.6598792102582,0.302061169837631,2.18
458801113996,0.0289190677573402,0.345199438692351
"1366","DSEL",379.640274863103,0.423385055039175,0.193839907361494,2.1
8419963567977,0.0289475816957806,0.345199438692351
"1367","NPR1",262.360983231721,-0.502918251207911,0.230326738621973,-2
.18349920732969,0.0289990672698397,0.345527721838067
"1368","ADAMTS9",15.2911346905947,1.19318797855213,0.546811168149133,2
.18208414175387,0.029103323369257,0.345527721838067
"1369","B3GALNT1",67.3248509962866,0.526896680838816,0.241372723153922
,2.18291724911608,0.0290419045289504,0.345527721838067
"1370","TMEM74",13.4984234326889,-1.30190239720309,0.596601948559916,-
2.18219601921455,0.0290950689794622,0.345527721838067
"1371","NAV2-
AS3",7.95818271826684,-1.94181056373607,0.889715238998004,-2.182507929
07957,0.029072066710882,0.345527721838067
"1372","AP001056.2",4.69943665903201,-1.59681937994972,0.7315578196656
45,-2.18276578696068,0.0290530624081039,0.345527721838067
"1373","ARAP1",333.204308028273,0.406177682676122,0.186224854273592,2.
18111424632604,0.0291749673779941,0.346126033226617
"1374","PGP",125.445409714899,-0.60222005766646,0.276161595017557,-2.1
8067996611974,0.0292070958477484,0.346255010381349
"1375","ZKSCAN1",579.68062560141,0.548761552554754,0.251732246062936,2
.1799414303782,0.0292618033504264,0.346651283472797
"1376","TMEM161B-
AS1",190.242130774995,0.393089424779964,0.18059851578608,2.17659277579
878,0.0295109646651314,0.34792528052964
"1377","YTHDF3",258.629861696063,0.439116000544874,0.201737956868541,2
.17666525110601,0.0295055527792117,0.34792528052964

"1378","PGM5-
AS1",15.035902457622,-1.10107925677204,0.505715284305961,-2.1772710672
2344,0.029460348578213,0.34792528052964
"1379","MFSD13A",4.31598774452239,-1.74274843866319,0.800409771593014,
-2.17732029332262,0.0294566780911715,0.34792528052964
"1380","PIGW",12.650737969019,-1.04995013380949,0.482405874331563,-2.1
7648704063635,0.0295188616668895,0.34792528052964
"1381","RAMP2",1549.6682393849,-0.751665912581412,0.345245746702921,-2
.17719094227744,0.029466323843316,0.34792528052964
"1382","STK26",18.5381460305864,-0.833686055948598,0.382839965673155,-
2.1776359071674,0.0294331540557937,0.34792528052964
"1383","TTC32",137.02135581523,-1.03422579166629,0.475280788775508,-2.
17603112957884,0.0295529329406698,0.3480749997618
"1384","SH3GLB2",266.583096821161,-0.438251272520602,0.201538718569582
,-2.1745264415249,0.0296656218474213,0.348529499803365
"1385","ESR2",6.49131441515373,2.31367326982521,1.0639767723776,2.1745
5242434944,0.0296636728141334,0.348529499803365
"1386","AC108134.3",3.00559539661776,-2.59891964717108,1.1954066692757
7,-2.17408829477723,0.0296985048638388,0.348529499803365
"1387","TPGS1",444.459944381263,-0.643197026018602,0.295745776729907,-
2.17483080614202,0.0296427976449604,0.348529499803365
"1388","APOL1",381.917650571117,0.646207414524236,0.297229690707721,2.
17410115720801,0.0296975390890134,0.348529499803365
"1389","RBL2",369.800820560957,0.36574320616776,0.168331451560865,2.17
275620673607,0.0297986709558724,0.349453240604899
"1390","ST5",347.247674883968,0.400902325152502,0.184602629765483,2.17
170430162237,0.0298779738697799,0.349517744087402
"1391","MAP2K3",179.665742865677,-0.393570181100325,0.181195933615327,
-2.17206961131844,0.0298504127061104,0.349517744087402
"1392","ALYREF",162.705246536228,-0.655628452930248,0.30191796955909,-
2.17154498583739,0.0298900004612776,0.349517744087402
"1393","C21orf58",22.1942140691452,0.868555052865274,0.399876326365336
,2.17205919830258,0.029851198023996,0.349517744087402
"1394","MFSD1",213.42335530489,-0.411408166250715,0.189503054776489,-2
.17098435028371,0.0299323553827605,0.349761934598125
"1395","KLHL8",153.652951079318,0.439280262667356,0.202370107863273,2.
17067761294147,0.0299555506185857,0.349782053065336
"1396","ZNF90",29.4753532851985,0.831515287570499,0.383121473658451,2.
17036982978351,0.0299788404664564,0.349803246674863
"1397","IRX5",102.480777617607,-0.685166484080538,0.315830635226785,-2
.16941109461579,0.0300514874132244,0.350399913009315
"1398","ALDH2",931.442175966251,-0.535811234376965,0.247026086867839,-
2.1690471689479,0.030079103012174,0.350471036455867
"1399","DDX17",1938.43513314873,0.395326549864175,0.182320289490348,2.
16830804168454,0.0301352569927278,0.350874339638701
"1400","EMILIN1",1013.56745771032,0.522202320272865,0.240909973912142,
2.16762432784666,0.0301872812187268,0.351229016979886
"1401","GABPB2",206.136446451422,0.678978088497338,0.31337586853531,2.
16665722115369,0.0302610007700477,0.351584480416054
"1402","TMEM200A",11.2082049717335,-1.08929150686293,0.502738679581556

,-2.1667151367179,0.0302565816954408,0.351584480416054
"1403","AL354953.1",7.10199642031911,-1.30081486429548,0.600939047825531,-2.16463694446619,0.0304154994243489,0.351934672517375
"1404","TRIM61",18.1554667467835,1.17093121509672,0.540738578119352,2.16542940059711,0.0303548165468844,0.351934672517375
"1405","AL157392.3",35.38479632099,0.848746482382072,0.392167222479427,2.16424635648023,0.0304454472912999,0.351934672517375
"1406","FAM149B1",90.6742805486316,0.606613300506253,0.280228803496661,2.16470717120084,0.0304101175583011,0.351934672517375
"1407","PELI2",120.188334915264,-0.77024056309524,0.355698530336625,-2.1654308280844,0.0303547073299755,0.351934672517375
"1408","AC018618.1",2.41838205641801,3.59645981822606,1.66169846607533,2.16432757907055,0.0304392175611121,0.351934672517375
"1409","AC015813.1",31.4665052096287,1.04410679977409,0.482488226883269,2.16400471886892,0.0304639872459635,0.351934672517375
"1410","AC093227.1",17.1653835353967,0.945223584650735,0.436526858049662,2.16532744141759,0.0303626183067204,0.351934672517375
"1411","SLC25A45",78.2114156878045,0.701720324422504,0.324376155197277,2.16329194726332,0.0305187320637023,0.352317240670196
"1412","DISP1",44.7329347617985,0.74513794715064,0.344544090220575,2.16267806733706,0.0305659491574825,0.35261242622545
"1413","GDPD5",146.614230751126,0.555289087396319,0.256920820420287,2.16132381364789,0.0306703346487717,0.353566228658063
"1414","MN1",141.577224297492,0.66863792329123,0.309410112169047,2.16100863221276,0.0306946726118654,0.353596550335697
"1415","TMEM192",153.102221487458,0.572894577621916,0.265164697828578,2.16052356257573,0.03073216155657,0.353778218795031
"1416","ECRG4",24.4238690342531,-1.49071034480216,0.69009848007698,-2.16014146942604,0.0307617195675444,0.353868396917889
"1417","C9orf16",839.706937713728,-0.591183607137063,0.273750938223517,-2.15956741910548,0.0308061729096194,0.354129675740855
"1418","AC008014.1",18.0332337225884,1.04194324569648,0.482815544434992,2.15805654500166,0.0309234357656479,0.354751216828678
"1419","TGFB1",207.199388362209,-0.523916551787271,0.24277549193904,-2.15802899873775,0.0309255772543878,0.354751216828678
"1420","KIAA1755",73.4876334413194,-0.720917857752754,0.334016635641384,-2.15832919928804,0.0309022460688293,0.354751216828678
"1421","CLCN6",89.1698094564359,0.519383856851255,0.241116772093713,2.15407602026702,0.0312342107356165,0.355724254011014
"1422","THEMIS2",16.6129019785051,-0.944990139385202,0.438877865267354,-2.15319617180861,0.0313032644198131,0.355724254011014
"1423","POLR3GL",452.948794926974,-0.408885843268292,0.189752274840882,-2.15484027061687,0.031174335726722,0.355724254011014
"1424","BOLA3",241.485585728838,-0.543307762867154,0.252409906180936,-2.15248193340595,0.0313594167138181,0.355724254011014
"1425","RNF175",14.1494892599343,-1.28309060059495,0.595892512036256,-2.1532249099933,0.0313010068757011,0.355724254011014
"1426","NIM1K",4.79818441955648,-1.6342209386557,0.759162562678236,-2.15266270888064,0.0313451962698771,0.355724254011014
"1427","FANCE",14.1674817097087,-0.831221835191762,0.386121953575046,-

2.15274430136801,0.0313387797259156,0.355724254011014
"1428","LINC00242",7.90906417982252,-1.30376564266217,0.60555386548920
3,-2.1530134922166,0.0313176181836848,0.355724254011014
"1429","GIMAP2",28.3612235652879,0.765262130959903,0.355315806287203,2
.15375200714077,0.0312596252362488,0.355724254011014
"1430","ASAP1",415.631654830476,0.587120146211202,0.272517336204739,2.
15443228085168,0.0312062873191557,0.355724254011014
"1431","THOC6",80.5247126058538,-0.553441288101742,0.256886612206648,-
2.15441857147672,0.0312073614524427,0.355724254011014
"1432","CRNDE",592.073949191857,-0.386525927123483,0.179572627514042,-
2.15247686952321,0.031359815136584,0.355724254011014
"1433","AC139099.2",6.44575842928493,-1.38622544526266,0.6438761175566
88,-2.15293813120909,0.0313235412023278,0.355724254011014
"1434","TASP1",245.258906625441,0.565740318029899,0.262613466748837,2.
15427001910367,0.031219002606182,0.355724254011014
"1435","MAGT1",442.428774047506,0.384515746583079,0.178577717219135,2.
15321235241928,0.0313019933257414,0.355724254011014
"1436","LINC01285",3.7832049076602,1.97890859774528,0.918809986686483,
2.15377349660929,0.0312579391253551,0.355724254011014
"1437","CLCN4",88.9001630330737,0.751115710815141,0.349041388568008,2.
15193881131604,0.0314021739345227,0.355956862365651
"1438","LINC00578",7.59018513829277,1.35191941801829,0.628384711825192
,2.15141997024646,0.0314430663334168,0.355999440237786
"1439","ZNF99",7.50662378040407,1.49010418018721,0.692641110847332,2.1
5133661119871,0.0314496405244137,0.355999440237786
"1440","C14orf180",11.3835197336034,-1.48232546546686,0.68934431947376
6,-2.15034116274207,0.0315282386761241,0.356641305413463
"1441","NOTCH2",738.276696539298,0.343524356858059,0.159967595944629,2
.14746214587714,0.0317565077051821,0.35748566275723
"1442","GPR1",24.644571950242,1.61205634386775,0.750427826664737,2.148
18305849946,0.0316992160086677,0.35748566275723
"1443","LIMK1",68.4007798466887,-0.532934500342314,0.248158658277792,-
2.1475555358046,0.0317490809055177,0.35748566275723
"1444","AOPEP",45.536084640441,-0.707419513573584,0.329253445718961,-2
.1485561435169,0.0316696013631625,0.35748566275723
"1445","EIF4G2",1363.17432926337,0.368951295137839,0.171780845256541,2
.14780230349222,0.0317294639622813,0.35748566275723
"1446","AJUBA",9.49915471226594,1.19313604628824,0.555314686384931,2.1
4857642979963,0.0316679917642375,0.35748566275723
"1447","APOL6",747.309072704422,0.549620720644594,0.255831571009091,2.
14836940756253,0.0316844210740881,0.35748566275723
"1448","BATF3",16.323231012702,-1.26577221919944,0.58995507940591,-2.1
4553999683176,0.031909697081786,0.357903403003321
"1449","ELL2",209.381774521228,1.44809250502346,0.674646547384605,2.14
64461807998,0.0318373982520541,0.357903403003321
"1450","AL353135.2",12.7202658843029,-1.10519627215661,0.5149992039559
85,-2.14601549607651,0.0318717423983709,0.357903403003321
"1451","PILRB",119.289089203532,0.448020906817209,0.208834181736081,2.
14534279346762,0.031925449356242,0.357903403003321
"1452","PAAF1",129.81130579737,0.494006114523894,0.2302576897383,2.145

44893195688,0.0319169703632734,0.357903403003321
"1453","EMD",291.028809543483,-0.280441553294076,0.130702159782196,-2.14565355126042,0.0319006295675651,0.357903403003321
"1454","PHKG1",79.0328190099375,-0.703551637987037,0.328020824692964,-2.14483833044923,0.0319657753577517,0.35810901980909
"1455","LYPD3",5.55756145438855,-1.4505423673983,0.676611681416195,-2.14383287672808,0.0320462800435139,0.358764161944191
"1456","TRIM69",195.414708568974,0.520035237293339,0.242673226275316,2.14294442479349,0.0321175611895036,0.358822327994393
"1457","AC012615.2",4.73895498376475,2.37973532590272,1.11032745525401,2.14327342320678,0.0320911495801589,0.358822327994393
"1458","ADGRE5",197.266934244789,-0.410677177148058,0.191619973114869,-2.143185652687,0.0320981938739723,0.358822327994393
"1459","APP",2533.90900840006,0.295446958434335,0.137888629787863,2.14264917193585,0.0321412796041231,0.358841194977081
"1460","ANXA1",9063.07857481949,-0.407209941947997,0.19011867530928,-2.1418723925230,0.0322037519830653,0.35904648600421
"1461","USP9X",349.100018521362,0.338093546204458,0.157839280803554,2.14201144660084,0.0321925609374115,0.35904648600421
"1462","PIP5K1A",119.235752537098,0.385014649990422,0.179980063399915,2.1392072139397,0.0324188902079205,0.359445437522075
"1463","POMGNT2",112.987629161268,0.45439341343327,0.212259367985864,2.14074609636797,0.032294519001448,0.359445437522075
"1464","NQO2",477.212077887747,-0.514031990375218,0.240281972592361,-2.1392865425126,0.0324124689218316,0.359445437522075
"1465","TCF19",48.5198416297693,-0.632017884551499,0.29546746836225,-2.13904389560962,0.0324321135131872,0.359445437522075
"1466","AC003991.1",3.28959267505323,2.31733018203153,1.08366215130271,2.1384249502908,0.0324822692634596,0.359445437522075
"1467","PEG10",196.082689471418,0.71392900511606,0.333615617900485,2.13997476979336,0.0323568057812485,0.359445437522075
"1468","RAD23B",608.650101436464,0.381380103415849,0.178126307505491,2.14106556609608,0.0322687510302282,0.359445437522075
"1469","CDKN1B",412.278051133223,0.404509508554757,0.18900063352685,2.14025477590418,0.032334182614336,0.359445437522075
"1470","SLC44A2",262.425732410852,0.579820768900493,0.270960890739594,2.13986884718993,0.0323653673603438,0.359445437522075
"1471","G6PD",184.232596813782,-0.422842475299943,0.197729682809767,-2.13848760232298,0.0324771892843819,0.359445437522075
"1472","MT-
ND4",55028.9311282035,-0.408728041267589,0.19110810372924,-2.13872689483995,0.0324577931284627,0.359445437522075
"1473","C1GALT1",434.653005738941,0.329197563933275,0.153983501631365,2.13787555449526,0.0325268447715479,0.35969434791836
"1474","NCOA2",295.197151421172,0.434379329917909,0.203230273051155,2.13737512328476,0.0325674931029652,0.359899521814247
"1475","PMAIP1",14.6096333797922,-1.06440309291799,0.498291934973991,-2.13610339283044,0.0326709872823854,0.360798448706967
"1476","MMP14",974.823780145986,0.611858380330546,0.286585895473281,2.13499125391393,0.0327617245387543,0.361367601177749

"1477","CSF2RA",3.72059137495473,-1.92848417460105,0.903301753642753,-2.13492796490656,0.0327668946491212,0.361367601177749
"1478","PITHD1",468.397264934564,-0.368650318569713,0.172786684167999,-2.13355745753693,0.0328790235649369,0.361885181788643
"1479","REEP5",1249.74014662833,-0.411210292387942,0.192736177212549,-2.13353973465221,0.0328804757227081,0.361885181788643
"1480","UXT",992.92321052474,-0.520329484613895,0.243819437799263,-2.13407712408181,0.032836468115504,0.361885181788643
"1481","TMEM70",266.848080182903,-0.559163249121275,0.262170504761531,-2.13282287277086,0.0329392591915988,0.362287368650879
"1482","AC008440.2",8.52562674458285,-1.41712401427228,0.664638245114837,-2.13217344124913,0.032992590945573,0.362443267143651
"1483","AC011503.1",2.5340280532359,-3.29704945599262,1.54637983453392,-2.13210841370442,0.0329979351202673,0.362443267143651
"1484","VAMP8",45.0730787126888,-0.721305927597861,0.338363411998131,-2.13174918451835,0.0330274710964013,0.362523232270405
"1485","TFCP2",120.974113956361,0.486522895405929,0.228360915055549,2.13049985058775,0.0331303681342564,0.362682161055502
"1486","SERPINF2",25.8285407692337,0.939834219555273,0.441058779109796,2.13085934136074,0.0331007318506942,0.362682161055502
"1487","PIEZO2",10.3430283548805,1.51336783778888,0.710427239058604,2.13022214603464,0.0331532775377029,0.362682161055502
"1488","PLEKHG2",173.647333428967,0.996729446965073,0.467846065213456,2.13046452899056,0.0331332812583068,0.362682161055502
"1489","HCK",4.99454478955755,-1.6759261375566,0.786426090256461,-2.13106629894497,0.0330836806393396,0.362682161055502
"1490","PPM1G",442.734236513738,-0.327120530062331,0.153676623679629,-2.12862907988064,0.0332849606191296,0.362854818340579
"1491","FSIP2",22.5592103123928,1.21558281113337,0.570942987722887,2.12907915023446,0.0332477124546488,0.362854818340579
"1492","GLB1",223.108284785628,0.474163103214465,0.222777594449878,2.12841468364605,0.0333027167670922,0.362854818340579
"1493","HNRNPA0",1464.33393401751,-0.308335226978041,0.144810753790806,-2.12922879624992,0.0332353355409599,0.362854818340579
"1494","UBN1",229.480438132449,0.458553602790451,0.215433285615269,2.12851789119234,0.0332941681779735,0.362854818340579
"1495","LINC00205",65.3895800784981,0.705223141548915,0.331173160604383,2.12946949040768,0.0332154364939546,0.362854818340579
"1496","ETHE1",213.577866159677,-0.506746470317915,0.238215589703302,-2.12725989490893,0.0333984950314091,0.36360473162104
"1497","PLP1",192.428901536449,0.569887573238318,0.267924311991944,2.12704688500032,0.0334161878099758,0.36360473162104
"1498","FAN1",83.8482207129718,0.465266505888105,0.218768969228384,2.12674817424582,0.0334410124780353,0.363631944095271
"1499","AC005726.1",7.649645453505,1.32916595120182,0.625116939733204,2.12626768964075,0.0334809767509325,0.363823635954596
"1500","PSTPIP1",9.19398348421094,-1.11569121526775,0.52482274091685,-2.1258438864876,0.0335162604527596,0.363964244343334
"1501","TM9SF4",198.27790787386,0.42028661125219,0.197749900560249,2.12534423563029,0.0335578997012943,0.364173636398656

"1502","TRPV1",41.6800804571099,0.668813049937681,0.314779668842543,2.
12470218421962,0.0336114710971273,0.364512152264385
"1503","RILPL2",215.487247971456,-0.43855448149652,0.206623348956163,-
2.1224826899382 2,0.0337972247959051,0.366053995812974
"1504","ST20-
AS1",16.3108458934502,-0.815219421305495,0.384090607333596,-2.12246643
302435,0.033798588599835,0.366053995812974
"1505","LAGE3",188.518895828421,-0.512773542980299,0.24173137366481,-2
.12125358494558,0.0339004680822783,0.366913438267263
"1506","ITGB1BP1",1446.31925797786,-0.523424577397802,0.24694368633465
3,-2.11961109501082,0.0340388560372003,0.36694832957641
"1507","NCOA1",480.966755613007,0.410418781184513,0.193551620841114,2.
12046160812792,0.033967135902906,0.36694832957641
"1508","MAP3K5",62.6651117531865,0.724890825469501,0.341779812892205,2
.12092931801718,0.0339277510119437,0.36694832957641
"1509","KLF4",5446.22648998894,0.818210878148717,0.385904866523166,2.1
202398547612,0.0339858229543135,0.36694832957641
"1510","ZEB1-
AS1",103.050257988541,0.523730928331415,0.247026451576532,2.1201410820
1346,0.033994149315861,0.36694832957641
"1511","EHD4",179.20647537717,0.519771007385596,0.245204849731048,2.11
974195435247,0.0340278128092635,0.36694832957641
"1512","PIK3CD-
AS2",14.0420207859635,-1.19212882986921,0.562666286309227,-2.118713807
59082,0.0341146607338141,0.367522294109192
"1513","USP49",40.1932857061058,0.933006948851205,0.440446134497312,2.
11832248208072,0.0341477658826716,0.367635795414962
"1514","NINL",52.6686723270279,0.632297599787065,0.298541985938637,2.1
1795201200621,0.0341791320123972,0.367730436822945
"1515","EFEMP1",9274.25506871775,0.681350717010896,0.321976145221914,2
.116152786851,0.0343318151942883,0.367864771051936
"1516","AC097448.1",77.2461705977455,-0.728108707445805,0.344072128089
756,-2.11615137642264,0.0343319351122155,0.367864771051936
"1517","EIF5A2",86.099871328042,-0.417763607900577,0.197385423313927,-
2.11648662239944,0.0343034417791755,0.367864771051936
"1518","NOD1",42.2205111433159,0.651387572566451,0.307886113321255,2.1
1567701296998,0.0343722869139325,0.367864771051936
"1519","AP4E1",50.9317088488712,0.668965085726324,0.316189189431208,2.
11571144139913,0.034369356890947,0.367864771051936
"1520","TMOD3",710.849122935865,-0.450863009418481,0.212970886327288,-
2.11701710592314,0.0342583960417666,0.367864771051936
"1521","ZNF880",56.6430664583497,0.631201112769684,0.298271618890533,2
.11619568471695,0.034328168088095,0.367864771051936
"1522","SLC25A5",806.53543272568,-0.423708133666707,0.200133791157688,
-2.11712440570749,0.0342492908844024,0.367864771051936
"1523","NCSTN",366.373754944855,0.496722840597599,0.234861875375024,2.
11495731184314,0.0344335857832545,0.368122104201739
"1524","FILIP1L",1733.56118165858,-0.559407433250616,0.264545384038301
,-2.11459910852054,0.0344641297137732,0.368122104201739
"1525","PRUNE2",13.6200489596282,-1.40322960931203,0.663565150409145,-

2.11468249718482,0.0344570171115906,0.368122104201739
"1526","ZNF595",90.2639801158972,0.527622747619922,0.249729670648981,2
.11277557147603,0.0346199814844057,0.369216745426684
"1527","AC008592.5",3.08379538560606,2.57718966555252,1.21991094695392
,2.11260475364016,0.0346346115177097,0.369216745426684
"1528","PTPN6",12.3138557666393,-1.11378503745455,0.527114288247655,-2
.11298585958887,0.0346019781902278,0.369216745426684
"1529","BCL7B",208.905787462883,-0.560050199717763,0.265242236985696,-
2.11146688431815,0.0347322014134246,0.37001493055806
"1530","IDH1-
AS1",5.17165091733964,1.57335981104703,0.745559472040858,2.11030758785
774,0.0348318704417084,0.370591990610704
"1531","MBOAT1",175.300576274804,-0.56552377652345,0.267972673459104,-
2.1103785293605 9,0.0348257643288977,0.370591990610704
"1532","GAS2L1",128.029188820555,-0.54069086288279,0.256285934777691,-
2.10971727087481,0.034882715967145,0.370890705214637
"1533","MYOZ3",3.4611007231,-1.74985976132747,0.82991610537561,-2.1084
7789311849,0.0349896730100948,0.371785247006807
"1534","OLA1",271.439917583465,-0.396880063195643,0.188396307083674,-2
.10662336931781,0.0351502394061888,0.372439007671807
"1535","AC010680.2",5.06031289415796,1.60706015607284,0.76289982724466
6,2.10651529687324,0.0351596157814609,0.372439007671807
"1536","COX15",123.557167095333,0.448083412211941,0.212719979797656,2.
10644723000711,0.0351655223647394,0.372439007671807
"1537","ZMYM2",267.703274587617,0.441604048513021,0.20961507042566,2.1
0673806809914,0.0351402904640957,0.372439007671807
"1538","PEAK1",327.288991050351,0.706368367297798,0.335170400304809,2.
10749029942804,0.0350751016734457,0.372439007671807
"1539","MEX3B",101.5113147216,0.538615630241967,0.255794064181868,2.10
566117694981,0.0352337944431459,0.372919608631841
"1540","BTBD8",102.806026311505,0.510171929540188,0.242407529781701,2.
10460430003812,0.0353257670160679,0.373650272029045
"1541","AVPR1A",6.06831229013871,1.46258479109153,0.695183794103386,2.
10388217259566,0.0353887264466196,0.374073306352359
"1542","SHISA3",2.52148454410761,-2.50404015064577,1.19048741770225,-2
.10337388989696,0.0354330989961137,0.374202537079368
"1543","SLC2A10",246.342045036276,0.357336629364361,0.169900114717087,
2.10321593931462,0.0354468975820164,0.374202537079368
"1544","TRMT112",1427.48558265641,-0.460375138411868,0.218933281582512
,-2.10281020356588,0.0354823637335618,0.374334341227972
"1545","TTF2",77.6186565219444,0.445513457378096,0.211914155008536,2.1
0232986730004,0.0355243899958627,0.374535138280004
"1546","IL1B",127.067889511051,-1.79790702850171,0.856869016930118,-2.
09822854249419,0.0358849613949211,0.377847534687699
"1547","ABCD4",231.661036898504,0.317904300232451,0.151500793651613,2.
09836722679813,0.0358727180587967,0.377847534687699
"1548","LHFPL2",156.675796041507,0.508910274480716,0.242610296198976,2
.09764499880638,0.0359365168831873,0.377982785355398
"1549","WDR86-
AS1",91.0164755627979,1.14139207646294,0.544152830276788,2.09755791563

625,0.035944216005618,0.377982785355398
"1550","TUFM",889.825327536795,-0.475342274313187,0.22671821648418,-2.09662144350168,0.0360270995030207,0.378609950841744
"1551","GRIA1",104.327041821638,0.829064339949207,0.395629686023745,2.09555644896538,0.0361215559944541,0.379113418552618
"1552","SH2D3A",7.96960886115588,-1.1644762932255,0.555650327957594,-2.095699821695,0.0361088276958706,0.379113418552618
"1553","ZDHHC16",93.4979696220939,-0.499021983738544,0.238222906127497,-2.09476910449353,0.0361915228095467,0.379603164871027
"1554","TMEM131L",34.3678743605909,0.58102189223372,0.277421061714454,2.09436835344448,0.0362271796140766,0.379671624933063
"1555","ITGB8",43.5325901985304,0.688116039321915,0.328627283036294,2.09391025895412,0.0362679752222878,0.379671624933063
"1556","LRRC61",20.843788919169,-1.1494059558894,0.54888261486206,-2.09408337004491,0.0362525542124671,0.379671624933063
"1557","NOTCH2NLA",50.172568714289,0.729993141621625,0.348872107225211,2.0924376770264,0.0363993813679025,0.379857907367902
"1558","MYLIP",433.959005834174,0.62421819733117,0.298325812763406,2.09240424604565,0.0364023692922398,0.379857907367902
"1559","ZNF462",234.824321232284,0.603680155421907,0.288402229156416,2.09318824333532,0.0363323538209327,0.379857907367902
"1560","PSD",5.26503721944525,-1.80901456169943,0.864534933812732,-2.09247132874249,0.0363963739262223,0.379857907367902
"1561","ARHGEF17",82.1939482676287,0.570761404432251,0.272734286674352,2.0927379956219,0.0363725494906656,0.379857907367902
"1562","KYNU",3.31276125114563,-1.98509364504899,0.949365495830099,-2.09096881419023,0.0365308596281322,0.380752159946622
"1563","PTPN20",16.3727554482824,-0.970264399937403,0.464036036322216,-2.090924678237,0.0365348165017233,0.380752159946622
"1564","IQSEC1",214.89124654957,0.628333597838855,0.300629788649285,2.09005767745747,0.0366126188758492,0.381137097478532
"1565","NET1",130.284231933757,0.851448782644205,0.407393389665767,2.08999164994491,0.0366185497915098,0.381137097478532
"1566","TRIP12",367.061365154776,0.400034912339485,0.191511995705476,2.08882431027816,0.0367235412117136,0.381791876601636
"1567","C19orf12",257.198969579056,0.317844290063767,0.152168083690732,2.08877106390955,0.0367283363395398,0.381791876601636
"1568","FUT11",166.176910692595,0.481640100597809,0.230656980188799,2.08812280557724,0.0367867583484929,0.382155297664924
"1569","IL16",129.941541365097,-0.729850158944929,0.349661073583681,-2.08730743592555,0.0368603530548351,0.382432032426885
"1570","AC087392.1",10.4933257037173,-1.71361396018688,0.820904891077144,-2.08746954587928,0.0368457111424596,0.382432032426885
"1571","DIRC3",18.5322326010999,1.60308076774842,0.768351912493266,2.08638872589839,0.0369434253995037,0.382590677671227
"1572","DOK2",13.4880781888426,-1.28789557164864,0.61737010146391,-2.08609968087988,0.0369695946132059,0.382590677671227
"1573","WBP1L",356.010632293961,0.546118898566394,0.261774522300949,2.08621868074139,0.0369588188343729,0.382590677671227
"1574","PPP1R1A",24.4523103144396,-1.19014722588985,0.570333647544435,

-2.08675611374853,0.0369101860454492,0.382590677671227
"1575","DBF4",45.6809046537864,-0.523670101894475,0.251133924712149,-2
.08522246643781,0.037049111481417,0.383170144076699
"1576","OGA",547.566453053463,0.507583262253291,0.243456162223686,2.08
490620084173,0.0370778157079566,0.383223692935854
"1577","ARL15",45.9103984028771,0.516979923441494,0.2481522057544,2.08
331786481542,0.037222259397378,0.384001196156633
"1578","LTBP2",1309.93037567537,0.68395854036944,0.328301208190628,2.0
8332629702752,0.0372214913068313,0.384001196156633
"1579","FOXRED2",95.8633068593361,0.515216357744386,0.247307648436671,
2.08330134955902,0.0372237638118561,0.384001196156633
"1580","PCBP1",1602.04824940826,-0.235000882197505,0.112843156507693,-
2.08254438700928,0.0372927729318525,0.384226425228935
"1581","PNP",28.3989448129797,-0.994413440392653,0.477480050193106,-2.
08262824800845,0.0372851223139225,0.384226425228935
"1582","VOPP1",142.559786193439,-0.399607430560317,0.191932065996642,-
2.08202536916009,0.0373401525340233,0.384369089031545
"1583","PCNX1",226.122186557383,0.506655120938039,0.243364716989981,2.
08187582491219,0.0373538134898972,0.384369089031545
"1584","AL357568.2",21.4886786595741,0.985735577182795,0.4735487799738
95,2.08159247551463,0.0373797092921112,0.384392730214141
"1585","PTPRM",140.083364966922,0.391275878691514,0.188052056287709,2.
0806785440989,0.0374633392487872,0.385009673831858
"1586","AC005229.4",19.9345409122955,-0.804798305847763,0.387330020841
784,-2.0778102975305,0.0377268350085951,0.387473149719423
"1587","OGT",425.32568809075,0.46134203440305,0.222076417961983,2.0774
0217820888,0.0377644553318753,0.387615131002468
"1588","TRAK1",117.463090072793,0.565936559835102,0.272546011244428,2.
0764808013556,0.0378495049577141,0.387773248705909
"1589","TSPAN5",37.7923534515415,-1.21143480731466,0.583621130053701,-
2.075721294058,0.0379197353304268,0.387773248705909
"1590","TMUB1",140.202072818918,-0.556992602622009,0.268232261422974,-
2.07653098723904,0.0378448682523463,0.387773248705909
"1591","PTGDS",4793.17164879404,1.82750134530225,0.880469360782549,2.0
7559902331864,0.0379310518533493,0.387773248705909
"1592","GANAB",536.627897908855,0.303414675527743,0.146149638482239,2.
07605491658206,0.0378888721736197,0.387773248705909
"1593","DACT1",97.550384785842,-0.665400538823898,0.320525598320222,-2
.07596691905751,0.0378970106799598,0.387773248705909
"1594","CD320",192.682626332833,-0.550628034339357,0.265340513881858,-
2.07517512604397,0.0379703070591151,0.387773248705909
"1595","APMAP",269.751064045728,0.389792576021051,0.187829536201089,2.
07524643836495,0.037963700731825,0.387773248705909
"1596","ZKSCAN8",94.6722251075927,0.571177506255966,0.275316997593366,
2.07461766345272,0.0380219837365285,0.387814710760371
"1597","ADA",428.837380783222,-0.51675431691838,0.249068869578939,-2.0
7474469929531,0.0380102022787695,0.387814710760371
"1598","CCNQ",88.7705830562425,-0.747358611153583,0.360291071520897,-2
.07431898880746,0.0380496954191497,0.387854498549768
"1599","CD93",10.3669456026316,-1.49505076117879,0.720870168229696,-2.

073952879546,0.0380836872608727,0.387958212503037
"1600","ACE",254.434345508218,0.573236465345004,0.276449734086479,2.07
356490046644,0.0381197378108882,0.388082755750974
"1601","LRRC37A3",15.4755753218313,0.893413599376795,0.430951362298085
,2.07311932978374,0.0381611754988951,0.388261953592444
"1602","EMX2OS",689.882659992837,-0.408835731268624,0.197259918033287,
-2.07257376635244,0.0382119645784379,0.38853601187152
"1603","PRXL2B",16.884220748272,0.913725857395091,0.441494763025867,2.
06961879034012,0.0384880570245203,0.389145465919507
"1604","TPRKB",333.003836479379,-0.50140823971225,0.24228907955833,-2.
06946281122645,0.0385026776202224,0.389145465919507
"1605","COQ10B",478.639662274734,-0.4118416055591,0.198883498152652,-2
.07076810989615,0.0383804717565509,0.389145465919507
"1606","PARD3B",256.297900986832,0.460755506058865,0.222535953976237,2
.07047669298448,0.0384077264303595,0.389145465919507
"1607","ZNF518B",77.0771486221571,0.486717273395605,0.235010998155616,
2.07104040753581,0.0383550200660644,0.389145465919507
"1608","MSRA",70.8412203054845,-0.560645341234212,0.270754099724846,-2
.07068089385892,0.0383886268842439,0.389145465919507
"1609","PIWIL4",26.6662481783752,0.678344546482522,0.327572942053588,2
.07081983704121,0.0383756357102797,0.389145465919507
"1610","SRPRA",377.318934183778,-0.2963908630198,0.1431694690948,-2.07
020997489028,0.038432685572426,0.389145465919507
"1611","CIT",6.92682999165511,1.47073330222442,0.710713377835892,2.069
37613402293,0.0385108042889217,0.389145465919507
"1612","ARHGAP4",15.7789585169409,-1.06504172392356,0.514630082835123,
-2.06952869536152,0.0384965014400729,0.389145465919507
"1613","STK32B",8.81511947132558,-1.12667048985351,0.544857119533962,-
2.06782741651094,0.0386562546370719,0.389752471085505
"1614","THOC3",98.7118726091745,-0.521450917855414,0.252179242505326,-
2.06777890469871,0.0386608182335397,0.389752471085505
"1615","PRXL2C",295.690390879769,-0.367545741606156,0.177733111009787,
-2.06796437376216,0.0386433732838938,0.389752471085505
"1616","SCG5",31.8491463749577,-0.805635460596778,0.389625476048522,-2
.06771761633074,0.0386665843989303,0.389752471085505
"1617","AVEN",142.725812483365,-0.415952899348371,0.20121893034117,-2.
06716584092319,0.0387185297451362,0.390034713060312
"1618","AC009509.1",11.942189430799,6.6732278002441,3.22863061945002,2
.06689107141679,0.0387444192566549,0.390054292504111
"1619","AC006942.1",16.7507350545545,-0.894038249637549,0.432670174240
785,-2.06632743106533,0.0387975729805059,0.390107201407075
"1620","BCORL1",82.9138490172341,0.604140702663309,0.292359760786276,2
.06642904973833,0.0387879853244823,0.390107201407075
"1621","HSP90AB1",4083.22365746634,-0.331809640039884,0.16065319486842
4,-2.06537840913551,0.0388872098327617,0.39045608423092
"1622","RRS1",57.6113408172646,-0.632287642553984,0.306104139049566,-2
.06559651403995,0.0388665938716525,0.39045608423092
"1623","AC009271.2",4.86666378416583,1.84296712050028,0.89239205901192
3,2.06519892449609,0.0389041822522428,0.39045608423092
"1624","CDK9",158.601181102403,-0.412002106192328,0.199534386602706,-2

.0648175645668,0.0389402652857043,0.390577574654457
"1625","AL512329.2",6.2739720912017,1.41732562871797,0.686533097662902
,2.06446802571185,0.0389733624780914,0.390668985480388
"1626","YWHAQ",1818.54408518737,-0.323358575409648,0.156753456849826,-
2.06284813048451,0.0391270595229467,0.391542103392824
"1627","RARB",3.5379197697511,1.80450954346531,0.874897279521206,2.062
53875249542,0.0391564720401373,0.391542103392824
"1628","SUSD5",7.12781822547306,1.62792797193385,0.789284722390599,2.0
6253576909883,0.0391567557625658,0.391542103392824
"1629","TNNI2",3.73911458130355,-2.15023967045123,1.04264935243469,-2.
06228456904539,0.0391806512695871,0.391542103392824
"1630","PRTN3",5.72435518899055,-1.29132209553449,0.626144157602105,-2
.06233992580841,0.0391753843715421,0.391542103392824
"1631","AC023481.1",5.04758683869484,1.89132266609367,0.91760246324292
7,2.06115691909707,0.0392880720512028,0.392374865507076
"1632","DENND5B",124.455820362085,0.49635534453413,0.240924566209706,2
.06021059762785,0.039378412231257,0.392962890850949
"1633","PLD6",21.3468103614102,0.878693693728943,0.426548542964561,2.0
6000866307483,0.0393977126435556,0.392962890850949
"1634","ARMCX2",215.057949644199,0.484345914777583,0.23514418579913,2.
05978265263736,0.0394193236939316,0.392962890850949
"1635","HSPA4L",9.63957366267761,1.11393148348923,0.541405366524074,2.
05748142217519,0.0396399401435175,0.394437987170285
"1636","MESTIT1",2.66159748776705,2.4105143952586,1.17152045868636,2.0
5759479263516,0.0396290469518041,0.394437987170285
"1637","RTTN",37.9174861971436,0.609184592554824,0.296014799007189,2.0
5795316517276,0.0395946294625504,0.394437987170285
"1638","STX3",42.3917434573422,-0.889369538313322,0.432469745427051,-2
.05648961046997,0.0397353467044144,0.394912243652273
"1639","ZNF624",66.7137781884614,0.549853299267099,0.267375706597862,2
.05648189307673,0.0397360898364587,0.394912243652273
"1640","PANTR1",3.53894716548455,-1.91956420391549,0.933958564390373,-
2.05529910758777,0.0398501234750555,0.39556286489042
"1641","COQ8B",97.790429081594,-0.379009396849382,0.184400029452801,-2
.05536516438786,0.0398437475541741,0.39556286489042
"1642","SEPTIN4",40.0432211357847,-0.777313948125479,0.37839361824799,
-2.05424698155466,0.0399517933305619,0.396330549063047
"1643","MBD5",204.437936463805,0.403890643292252,0.196680538573584,2.0
5353639064367,0.0400205841349491,0.396771329868646
"1644","COL24A1",28.7863129766581,1.11366763529847,0.542530814601719,2
.05272697020174,0.0400990648066683,0.39711842192883
"1645","AC104211.1",8.23891790297037,1.18762115035613,0.57857313445062
6,2.05267247931209,0.0401043528806511,0.39711842192883
"1646","CARF",74.1941581564909,0.503412477321882,0.245352581855019,2.0
517920517313,0.0401898760898723,0.397723506456822
"1647","ARHGDIA",756.644593333446,-0.363886471449786,0.177384978251974
,-2.0513939513688,0.0402285976241714,0.397864982817321
"1648","ARHGEF26",133.016196466397,-0.672460977195139,0.32786093695019
1,-2.05105549764625,0.0402615424625041,0.397949190031389
"1649","AC073896.2",31.2089376292445,-0.629932401996981,0.307282340941

627,-2.05001172558968,0.0403632865947822,0.398712901966287
"1650","NECTIN3",315.69082641106,-0.324876094758529,0.158500600106271,
-2.04968368915138,0.0403953077102834,0.398754675407585
"1651","LINC02517",5.2886286208395,2.68502108272017,1.31090742668424,2
.04821563145123,0.0405388754665701,0.398754675407585
"1652","AL662797.1",5.18942063826719,1.46105913457479,0.71333003711321
2,2.04822320462991,0.0405381337438962,0.398754675407585
"1653","RPS6KA2",61.6647061593123,0.812154126779279,0.396515147091797,
2.04822976558638,0.0405374911683092,0.398754675407585
"1654","CTNND1",324.593143233432,0.340301180566488,0.166087262934151,2
.04893002963996,0.0404689574133154,0.398754675407585
"1655","IGF1",4225.66185422901,-0.511765988970434,0.249726855778289,-2
.04930297694849,0.0404324977480063,0.398754675407585
"1656","ARID3A",74.7869794554734,0.610908591267534,0.298249721062851,2
.04831236418422,0.0405294022567885,0.398754675407585
"1657","AC107204.1",11.8785901109878,0.975997221241832,0.4765766302796
38,2.04793344707052,0.0405665210141832,0.398785794085715
"1658","RBM17",516.105202539902,-0.289614766965427,0.141454304947773,-
2.04740864601015,0.0406179781725304,0.399050812094299
"1659","NUDT11",24.5415780985345,-0.941200768846697,0.459846672632335,
-2.04677085833613,0.0406805882284771,0.399425016066102
"1660","TSPAN33",4.88923543835589,-1.70096072251611,0.831823960861782,
-2.044856607345,0.0408689969995627,0.400069165941031
"1661","TOP1MT",48.9643468778841,-0.533300459019634,0.260718229293208,
-2.04550506677412,0.0408050901967719,0.400069165941031
"1662","NINJ1",137.482760619418,-0.536870408410688,0.262439530222805,-
2.04569185120434,0.0407866979850942,0.400069165941031
"1663","CIZ1",383.089398164689,-0.293053779028242,0.143293274850258,-2
.04513281823228,0.040841765574813,0.400069165941031
"1664","AL137026.3",43.9318378305724,0.894641206935132,0.4374984209420
04,2.04490156789326,0.0408645633245858,0.400069165941031
"1665","TM4SF1",9.49310586522803,-1.16841419455152,0.571523838523867,-
2.04438400604479,0.0409156260941471,0.400285065133671
"1666","AC004160.1",42.241506721341,0.745616951883513,0.36479006610506
9,2.04396177737131,0.0409573233080188,0.400452484612435
"1667","LRIG3",320.373249007028,0.459627319703662,0.224913741564179,2.
04357153327827,0.0409958938961194,0.400589151574019
"1668","MPZL1",1244.24513652744,0.334839033906179,0.163981745839524,2.
04192870488072,0.041158603924187,0.401786124342605
"1669","CTNNBL1",313.925153799626,-0.417814452665922,0.204626762419829
,-2.04183679458653,0.0411677230970476,0.401786124342605
"1670","AC092164.1",26.6673060316234,0.710352096229981,0.3480553265842
92,2.0409171817629,0.0412590596887459,0.402195585439846
"1671","DPY19L3",117.772514022808,0.447702828135564,0.219350636063237,
2.04103729157409,0.0412471205613065,0.402195585439846
"1672","AC026904.2",8.1115274929823,1.21683727690805,0.596320217005337
,2.040576928649,0.0412928973420073,0.402284691868395
"1673","AL445423.1",11.3412952137961,0.968143461099988,0.4746348310719
85,2.03976488390747,0.0413737489528669,0.402831438549461
"1674","LINC01779",3.14902842989327,-1.8073640104467,0.88644703606834,

-2.03888550235658,0.0414614561538072,0.402962803871936
"1675","AL591846.2",27.8430573576205,-1.33473851700071,0.654504922337037,-2.03931012808088,0.0414190854636516,0.402962803871936
"1676","CAMKK1",25.4097559862642,-1.20803658152456,0.592489661928298,-2.03891588182809,0.0414584235617072,0.402962803871936
"1677","ASAP1-IT2",18.3517479935486,0.94132303486034,0.461826168731382,2.03826265940303,0.0415236720767453,0.403326830326836
"1678","ACAP3",141.524311831223,-0.353528384429041,0.173549811241061,-2.03704274813638,0.0416457583527351,0.404271607751908
"1679","HECW2",105.308269303958,0.598215695150517,0.293764099443522,2.03638121977368,0.0417120897740303,0.404575796626091
"1680","RRAS2",192.021635567179,0.571093314123062,0.280465334186777,2.03623494425425,0.0417267688827941,0.404575796626091
"1681","DMTF1",164.784464929797,0.575982938424765,0.283032038130343,2.03504501550284,0.0418463437262673,0.405225342887133
"1682","TRIM34",81.3270050761816,0.449480461790902,0.220893816676365,2.03482591117271,0.0418683929080389,0.405225342887133
"1683","SLC46A3",57.0647807509884,0.649563453054112,0.319152397135681,2.03527674829891,0.0418230343961744,0.405225342887133
"1684","AC123768.3",11.2768501386045,1.07377557070411,0.527931380312611,2.03393018628346,0.0419586349172139,0.405382647918197
"1685","TPPP3",1261.1748923947,-0.737777219184628,0.362735968087834,-2.03392352590185,0.0419593065498238,0.405382647918197
"1686","AC011825.4",2.36135909557177,-2.09691286935882,1.0308502898507,-2.03415849032988,0.04193561824697,0.405382647918197
"1687","STOX2",19.5406495562881,1.23503155547799,0.607600849275578,2.03263632193813,0.0420892790530234,0.406156555980271
"1688","BIRC3",57.2458352148741,-0.806657143167131,0.39683967608421,-2.03270285654592,0.0420825525321733,0.406156555980271
"1689","STX1A",8.69475239976896,-1.04992149075895,0.516693432992157,-2.03200084173488,0.0421535707330406,0.40629557021923
"1690","PAWR",240.198581433955,0.853250502833285,0.419881712854775,2.03212113485971,0.0421413942777813,0.40629557021923
"1691","CKAP4",487.310946844066,0.479440451462256,0.236032425000259,2.03124825524175,0.0422298175369914,0.406638645678759
"1692","CARD8",93.353628832393,0.460600395545155,0.226767524878834,2.0311567795753,0.0422390931603205,0.406638645678759
"1693","PRG4",268.224088768513,-1.70448354387946,0.839782729295629,-2.02967206209289,0.0423898845504933,0.407608517971066
"1694","AL591686.2",2.50524459407919,2.67779446990871,1.31918778703011,2.02988118616323,0.0423686179073642,0.407608517971066
"1695","OSMR",304.397868935061,0.439474331940593,0.216901330712236,2.02614862019286,0.0427495569748375,0.409439725043474
"1696","MED20",38.1622215015455,0.675168568120591,0.333172748221461,2.02648197286473,0.0427154182343025,0.409439725043474
"1697","SGCZ",4.74358675368347,2.1019330879123,1.03677465876319,2.02737699088806,0.0426238731985942,0.409439725043474
"1698","PRF1",13.5245995851463,1.22376419467825,0.603783240893842,2.0268270329375,0.042680104818191,0.409439725043474

"1699","ARL3",422.894273992185,-0.442950561905646,0.218514744054771,-2
.02709690744996,0.0426525030917006,0.409439725043474
"1700","TPCN1",268.796018761313,0.510107125084029,0.251770097492787,2.
02608304228283,0.0427562755417121,0.409439725043474
"1701","GINS3",18.2749682885417,-0.713537791856568,0.352049160576548,-
2.02681293342104,0.042681547277526,0.409439725043474
"1702","BBIP1",246.42045628868,-0.335021603241381,0.16546146559504,-2.
02477115766237,0.0428908680053834,0.410487279047997
"1703","TWF2",430.677192207978,-0.466556271408569,0.230457952553746,-2
.02447460041442,0.0429213428030627,0.410537729253722
"1704","ACP6",11.0407790415794,-1.01924361591268,0.503836501963443,-2.
0229650133341,0.0430767547709776,0.410559816091582
"1705","PPM1B",221.523885432627,0.38422553892939,0.189979786500482,2.0
2245484115445,0.0431293843990174,0.410559816091582
"1706","GTDC1",95.1775365116115,0.452854307168736,0.223766105920105,2.
0237841888817,0.0429923617464521,0.410559816091582
"1707","PLK2",284.728986407016,-0.640326941288089,0.316640785649218,-2
.02225035532049,0.0431504945146288,0.410559816091582
"1708","BTN3A1",127.61176875915,0.582808941609603,0.288056346321731,2.
02324631639486,0.0430477586231349,0.410559816091582
"1709","HERC4",239.191257140579,0.36155467194564,0.178705608965969,2.0
2318592033947,0.0430539827347001,0.410559816091582
"1710","PBLD",41.5451088427338,0.67863100482267,0.335515320579745,2.02
265280658435,0.0431089557335156,0.410559816091582
"1711","VSIG10L",10.1613923894841,-1.01867970575371,0.503318870744821,
-2.02392511976801,0.0429778568769444,0.410559816091582
"1712","RTCB",374.547705212747,-0.430079189218705,0.212610839343399,-2
.02284695618957,0.0430889287781645,0.410559816091582
"1713","FAM86C1",9.51174044557122,-0.994974103066148,0.492245875694906
,-2.02129495074293,0.0432492415445613,0.411259133403012
"1714","SLC3A1",3.38401787376015,2.15169609748918,1.06548785853879,2.0
1944684798192,0.0434407964840421,0.411878424871107
"1715","MLPH",17.4041395976085,1.14441835215065,0.566644363304117,2.01
964128872211,0.0434206091179224,0.411878424871107
"1716","USP53",2534.9552128398,0.687404477641412,0.340236289589508,2.0
2037377750257,0.0433446312927773,0.411878424871107
"1717","CD83",85.6786216641518,-0.930606880104338,0.460741906803881,-2
.01980081768525,0.043404052306606,0.411878424871107
"1718","EPS8L1",2.79542769484664,-2.08061784306756,1.03007515505445,-2
.01986994139041,0.0433968799168698,0.411878424871107
"1719","NPAT",108.952526630821,0.414054918025196,0.20514227297548,2.01
837930339539,0.0435517731875862,0.41245048456546
"1720","GZMH",18.7254280715712,1.04920581569416,0.519807376968542,2.01
845118438489,0.0435442932800725,0.41245048456546
"1721","TNFRSF25",4.17764066342078,-1.59868843199976,0.793378454961541
,-2.01503887835883,0.0439005756072356,0.413217581776535
"1722","ACOT11",8.34106209836697,-1.35423800052478,0.672209105032321,-
2.01460823780372,0.0439457136517137,0.413217581776535
"1723","SLAMF8",3.7000068332918,-2.02555556948664,1.00534678226907,-2.
01478296366051,0.0439273948548734,0.413217581776535

"1724","BAZ2B",472.573499596267,0.472841260647491,0.234828171058932,2.01356276172175,0.0440554593908524,0.413217581776535
"1725","C4orf46",66.7509429728825,0.750203606943122,0.372355556804952,2.01475066836748,0.043930780307047,0.413217581776535
"1726","DHFR",73.9387984502877,0.493890984871418,0.244950262898314,2.01629089525185,0.0437695664237284,0.413217581776535
"1727","FER",222.694157379535,0.362651000496358,0.180129763255479,2.01327639553941,0.0440855601625033,0.413217581776535
"1728","GFPT2",525.372086260703,0.653412371320237,0.324050262015895,2.01639204750338,0.0437589964321117,0.413217581776535
"1729","CCDC170",56.1084398453834,0.821907090320953,0.407933808345428,2.01480503333272,0.0439250814611578,0.413217581776535
"1730","FOCAD",76.9554750409701,0.535778064094598,0.266091033367527,2.01351416210473,0.0440605666134292,0.413217581776535
"1731","LINGO2",6.28227293138278,5.74717870692006,2.85514887985737,2.01291734643524,0.0441233253728377,0.413217581776535
"1732","RAB21",457.368048656181,0.300636101665008,0.149218122593727,2.01474255565823,0.0439316307810513,0.413217581776535
"1733","DIO2",9.2044578416384,-1.83460810872427,0.91148020259573,-2.01277888812027,0.0441378958764352,0.413217581776535
"1734","GOLGA8B",46.7789814543954,-1.17736806152283,0.584076283816299,-2.01577789433602,0.0438232060971936,0.413217581776535
"1735","CD300E",4.98805305665826,-1.48310253792073,0.735509980487875,-2.01642748197239,0.0437552941866202,0.413217581776535
"1736","LINC01415",6.23782639905867,1.27049869317108,0.630535523778657,2.01495180724675,0.0439096988991784,0.413217581776535
"1737","LGALS4",4.54569266919018,1.62068029853372,0.805203893388778,2.01275765286347,0.0441401309037492,0.413217581776535
"1738","GSTT2B",17.5958127979045,-1.53722877806968,0.763215863110444,-2.01414678647374,0.0439941246430498,0.413217581776535
"1739","MCM5",85.4260029008901,-0.444033552195428,0.220357464941703,-2.01506017648596,0.0438983442378096,0.413217581776535
"1740","SRPX2",698.515981863738,0.397604344839009,0.197485720686061,2.013332120711,0.044079701369628,0.413217581776535
"1741","PIP4P2",615.63100093817,-0.425823726391086,0.211589726136871,-2.01249717633092,0.0441675540352312,0.413236810844274
"1742","PTPRG",447.797297318386,0.403780704603324,0.200713832695889,2.01172335349259,0.0442491074189427,0.413762176089069
"1743","TRMT61B",86.5596076668633,-0.443748280750151,0.220625130697571,-2.0113224606241,0.0442914075716309,0.413920102085081
"1744","AADAC",1149.58891080677,-1.28715246404267,0.640280836718465,-2.01029359341679,0.0444001245523284,0.414698181670227
"1745","AC009088.1",8.74487116964234,1.82552635292028,0.908326385763814,2.00976915515362,0.0444556267897719,0.414978627380283
"1746","POU5F2",41.8304407451398,0.820976167543049,0.408659600510275,2.00894868618756,0.0445425759152612,0.415314263928844
"1747","RAET1G",25.9634237760249,1.00454644282816,0.499996625825929,2.00910644380605,0.0445258464330127,0.415314263928844
"1748","TEX35",4.62881030827204,1.57086255360153,0.782159204906134,2.00836676695513,0.0446043316857974,0.41550571997898

"1749","NCLN",112.978569965063,0.544187642998704,0.270972734800104,2.0
0827453507509,0.0446141263578634,0.41550571997898
"1750","TMEM183A",265.970382157323,-0.32481892724024,0.161855098513302
,-2.00685014079766,0.0447656221071502,0.416130926109062
"1751","CKMT2",3.87355828327363,-1.80166861665133,0.897795599526364,-2
.00676926641411,0.044774236748046,0.416130926109062
"1752","DDO",6.26693720245154,1.64037933307473,0.817366166324151,2.006
90877682376,0.0447593771433046,0.416130926109062
"1753","PIGN",81.8013161187687,0.576357689010011,0.287219123851586,2.0
0668284646614,0.0447834436410575,0.416130926109062
"1754","COBLL1",272.754005338931,-0.435850072300953,0.217304446841634,
-2.00571170372132,0.0448870157918007,0.416618005830565
"1755","ABHD8",103.771168559317,-0.562806933744393,0.280590076687594,-
2.00579771169533,0.0448778349152259,0.416618005830565
"1756","NCEH1",44.6737296495152,-0.731614092816624,0.364845537814441,-
2.00527077074661,0.0449341078325081,0.416816430867842
"1757","AC091979.2",3.11117494423329,2.22718277760453,1.11079634409965
,2.00503250612492,0.0449595720446189,0.416816430867842
"1758","BEND6",50.9486835111673,-0.733420640903289,0.36588895729838,-2
.0044896853916,0.0450176307280948,0.416999790001574
"1759","RHBDF2",7.1981342368106,-1.28236511140588,0.63981712751988,-2.
0042681826551,0.0450413402359194,0.416999790001574
"1760","CAPS",57.3765522587208,-0.603692049458208,0.301227316775707,-2
.00410791398349,0.0450585018540899,0.416999790001574
"1761","PLCXD1",17.6578796744787,0.982582299100504,0.49033722690107,2.
00389088405631,0.0450817502727468,0.416999790001574
"1762","PRKAR2B",81.8563006578241,-0.600522957727873,0.299764004903016
,-2.00331910404707,0.0451430482301145,0.417329802849226
"1763","AFF4",751.571961022071,0.489643685738664,0.244505863440425,2.0
0258463682188,0.045221890241176,0.417597800927172
"1764","LRRC59",258.666485798396,-0.395228932310365,0.197360718803998,
-2.00257140684045,0.0452233114884604,0.417597800927172
"1765","GPATCH4",114.879933120898,-0.553013294102319,0.276233476199615
,-2.00197782582548,0.045287116426137,0.417950050688581
"1766","AC092140.2",5.30229313759878,1.76342112145991,0.88109070855370
5,2.00140701103809,0.0453485457440027,0.418127104295774
"1767","GNRH2",2.80733772820687,2.01470054549938,1.00668457073623,2.00
132256325927,0.0453576397133423,0.418127104295774
"1768","HNRNPAB",555.623432723118,-0.3405301927057,0.170180586201125,-
2.00099318204986,0.045393124640403,0.418217538047249
"1769","FCGR3A",14.6153134990974,-0.997680864694327,0.499090066866672,
-1.9989996414031,0.0456083926648203,0.418370309354678
"1770","AC079848.1",9.33934175560007,-1.03306347949454,0.5167913273647
22,-1.9989953870984,0.0456088529746182,0.418370309354678
"1771","SH3TC1",4.44482919953843,-1.69287966257473,0.847026176077104,-
1.99861552144126,0.0456499696883809,0.418370309354678
"1772","COL25A1",8.18004760739091,1.09243451679392,0.546341055055066,1
.99954681546642,0.0455492218202588,0.418370309354678
"1773","AL158151.4",2.35366270645189,2.3005637336512,1.15034312073059,
1.99989350324457,0.0455117648466197,0.418370309354678

"1774","ALOX5",9.90122188522923,-1.50983864027007,0.755417738933009,-1
.99868041542504,0.0456429433429546,0.418370309354678
"1775","AC090559.1",3.06488663176056,-1.74760256270973,0.8741080405867
31,-1.99929812055805,0.0455761074095751,0.418370309354678
"1776","PPP1R14B",1014.03505017084,-0.602380552815392,0.30124127531191
1,-1.99966140825714,0.045536838068876,0.418370309354678
"1777","BTBD6",431.256527752945,-0.426436050422694,0.213382069591389,-
1.99846243519565,0.0456665485930762,0.418370309354678
"1778","CRKL",131.718567293364,0.369826826860542,0.184946759108441,1.9
9963940240607,0.0455392159650859,0.418370309354678
"1779","AC008875.3",186.515035193994,-0.355853637751985,0.178109017692
637,-1.99795407532976,0.0457216392512181,0.418404371777017
"1780","RREB1",215.377413723964,0.466514247819108,0.233474931017672,1.
99813421417735,0.0457021113089201,0.418404371777017
"1781","AMPD2",73.144476245642,-0.461150010063598,0.230982107585589,-1
.99647502953328,0.0458822411078888,0.418760603858512
"1782","CDK14",326.708366704409,0.325731752544878,0.163158591906015,1.
99641188821065,0.0458891078572929,0.418760603858512
"1783","GABPB1-
AS1",503.926310560422,0.782922316837069,0.392041338044832,1.9970402119
8789,0.0458208148868579,0.418760603858512
"1784","AC011511.5",5.61327277118091,2.00587929374431,1.00470414826385
,1.99648752044123,0.045880882798375,0.418760603858512
"1785","MTFP1",10.5794322461163,-0.936727935709061,0.469160237914511,-
1.99660555181095,0.0458680492841511,0.418760603858512
"1786","ARHGEF3",120.448849274793,0.623285224919892,0.312316837779523,
1.99568242734288,0.0459685010245187,0.418781271358158
"1787","WFS1",99.4731438534595,0.432167433722172,0.216507172697128,1.9
9608829739203,0.0459243126157188,0.418781271358158
"1788","C11orf80",21.5915103806276,-0.83241693859046,0.417076124305933
,-1.99583934461774,0.0459514126795879,0.418781271358158
"1789","RANGAP1",65.5619510364362,-0.467149205013876,0.234218586399709
,-1.99450100094386,0.0460973306948592,0.419720189876222
"1790","ALDH16A1",205.588675040349,-0.414552303919167,0.20801603207363
,-1.99288631643752,0.0462738970283003,0.421092462957533
"1791","SETD9",53.0475230003907,-0.662550346326083,0.332574169035749,-
1.99218823352112,0.0463504087687275,0.421168412582326
"1792","SLC25A27",41.8946339105572,0.791251807097178,0.397194321760155
,1.99210251443366,0.046359811146179,0.421168412582326
"1793","KLHL36",217.025994488904,0.308620005702237,0.154906435096907,1
.99229945166042,0.0463382118375603,0.421168412582326
"1794","RIPK2",143.625095798335,-0.513369050780423,0.257778546934453,-
1.99151192713861,0.0464246352913896,0.421522232029791
"1795","AMPH",103.892217562907,1.5214450369005,0.764163949653883,1.990
99295064838,0.0464816622507283,0.42180490050257
"1796","ADORA2B",4.40957914853955,-1.99561532687353,1.0024759959531,-1
.99068639541459,0.0465153753108964,0.421875806480619
"1797","KATNB1",54.3250674915129,-0.465905085368771,0.234091472162061,
-1.99026936379051,0.046561270928261,0.422057062966301
"1798","CCDC47",459.716154759793,-0.24629420868687,0.123792871636339,-

1.98956697127439,0.046638657551478,0.422523410932161
"1799","KLHL33",23.9448014045441,1.02118583508463,0.513417401395758,1.98899731935161,0.0467014988497421,0.422622619313027
"1800","HSPA13",113.153788621413,-0.339127546041157,0.17049352240344,-1.98909343452167,0.046690890885599,0.422622619313027
"1801","DHRS11",25.2246077454371,-0.797889025873292,0.401307961050282,-1.98822127471655,0.046787223060472,0.423163285081637
"1802","DCBLD2",227.859768236048,0.400135171703824,0.201394236390833,1.9868253375797,0.046941755755469,0.423329326885136
"1803","GNG11",1552.81324534494,-0.511163502189234,0.257301966566751,-1.98662881986417,0.0469635450547813,0.423329326885136
"1804","ZNF395",238.779139463316,0.518554540783405,0.260989492037455,1.98687899936212,0.0469358073753163,0.423329326885136
"1805","BEGAIN",7.97087020070268,1.34334084896791,0.675919640456539,1.98742686047793,0.0468751135409543,0.423329326885136
"1806","KLF16",26.1790437596425,-0.745613655221636,0.375356844543031,-1.98641283903951,0.046987502179859,0.423329326885136
"1807","B9D2",47.2552291656119,-0.619486357312125,0.311763970585342,-1.98703639855827,0.0469183634157749,0.423329326885136
"1808","UFD1",418.356684894688,-0.478660134078572,0.240849292769867,-1.9873844285519,0.0468798119272147,0.423329326885136
"1809","PDE1C",45.7291052315095,1.20736294692283,0.60798447974017,1.98584501275231,0.0470505359219594,0.423662896424985
"1810","TIAM1",36.8274892029117,0.579942926225777,0.292140629441153,1.98514984832877,0.0471278021589659,0.424124181970936
"1811","DDTL",11.21217818205,-1.2751319333007,0.642585233283686,-1.98437789611913,0.0472137282525275,0.424662848981458
"1812","LSM10",300.706060285766,-0.495842789322433,0.250053391047297,-1.98294767067824,0.0473732750436042,0.425295158818115
"1813","ANKRD36B",119.701360932999,-0.835382892632027,0.421482081504098,-1.98201282875629,0.0474778048470681,0.425295158818115
"1814","TRANK1",129.32994525818,0.494870682394858,0.249741564161587,1.98153112420914,0.0475317425399936,0.425295158818115
"1815","AC006427.2",3.54935368866314,1.720248122706,0.867732682598295,1.98246321384943,0.0474274206354272,0.425295158818115
"1816","RRP36",215.815134467388,-0.484623368825123,0.2444271407095,-1.98269049590158,0.0474020118517689,0.425295158818115
"1817","STMN2",19.9133583782382,2.5664134258257,1.29507652935725,1.98166932042188,0.0475162630884753,0.425295158818115
"1818","AC012146.1",48.6572551508234,-0.557872510125755,0.281291237694457,-1.98325591190909,0.0473388513534336,0.425295158818115
"1819","USP22",461.730023721804,0.337594285405263,0.170380685410537,1.9814117110271,0.0475451215057884,0.425295158818115
"1820","KXD1",376.364255116764,-0.347431582034453,0.175295744200627,-1.98197385577607,0.0474821668373257,0.425295158818115
"1821","ZCCHC12",6.62054053161997,-1.51018228341642,0.76206175301287,-1.98170591483669,0.0475121648330843,0.425295158818115
"1822","PDE7B",119.476769151986,0.610771714548174,0.308557779349785,1.97944033637796,0.0477664508577851,0.42591619189558
"1823","ADAM9",510.037454700192,0.417177861475432,0.210784922178781,1.

97916367624054,0.0477975811151772,0.42591619189558
"1824","RNF170",236.621933096904,0.371498268190034,0.187696130487223,1
.97925373967858,0.0477874451563333,0.42591619189558
"1825","FAM89B",565.307707861854,-0.520739257255799,0.263096079432935,
-1.97927410540733,0.0477851533980443,0.42591619189558
"1826","AP003120.1",8.14005405055804,1.2836713555958,0.648351986933866
,1.97989885350154,0.0477148952773065,0.42591619189558
"1827","JHY",65.6120207858266,0.45761832323502,0.231158420003926,1.979
67404011174,0.0477401674057369,0.42591619189558
"1828","LCTL",3.35912902613466,2.07432515527743,1.04756913881499,1.980
1319821467,0.047688700277305,0.42591619189558
"1829","ANP32B",776.902989982466,-0.490709861398425,0.247972979806014,
-1.97888440015643,0.0478290230160573,0.425963343853776
"1830","PATZ1",96.8387854507247,0.440726159705222,0.222771422520093,1.
9783783517631,0.0478860400418414,0.426238090842379
"1831","TIPARP-
AS1",10.7479630312203,-0.91178956719336,0.4610607361121,-1.97759101085
474,0.0479748641722557,0.426562534116743
"1832","CDKL3",36.3490332741589,0.628182627813616,0.317619178415162,1.
9777855699649,0.0479529020514004,0.426562534116743
"1833","FERMT2",1000.66329265673,0.422277337342692,0.213584197423088,1
.97710009653105,0.0480303168817481,0.426822603211562
"1834","PLEKHM1",153.376346884803,0.323221562655216,0.163516942728542,
1.97668545694255,0.0480771956947148,0.427006238097715
"1835","HDAC7",620.089673630302,0.387394279100615,0.19602864651761,1.9
7621258924427,0.0481307046455752,0.427248527505054
"1836","FERMT3",34.7537296788037,-0.606127383477974,0.306779569533184,
-1.97577493312314,0.0481802736877339,0.427455598093408
"1837","CXCL8",400.352908546744,-1.87344114694595,0.948370005794647,-1
.97543272720459,0.048219061887536,0.427566847624428
"1838","VASH2",74.7376326441745,0.556475373747011,0.281852963424677,1.
97434636480494,0.048342372265702,0.42782599579895
"1839","TRIM59",4.57105241583722,-1.65294504239719,0.837154836994885,-
1.97447947422812,0.0483272491111745,0.42782599579895
"1840","LINC01088",47.9517680593542,-1.18953702053073,0.60238460851891
5,-1.97471350314784,0.048300669678641,0.42782599579895
"1841","PCDHA3",2.44025741713478,2.34272635198884,1.18664130359601,1.9
7424979637016,0.0483533463236459,0.42782599579895
"1842","TMEM39B",112.981487756363,-0.438934267417377,0.222385912133609
,-1.97375032980356,0.0484101392178034,0.428095959673616
"1843","MAVS",263.133918683066,0.542929204255974,0.275237140833659,1.9
725869939337,0.0485426360097416,0.429034724884797
"1844","XPR1",70.8342238356665,0.650518246718182,0.329949202311688,1.9
7157090291635,0.0486586115478348,0.429073665411248
"1845","AC092155.1",8.96390315239397,1.25709385774108,0.63756639327073
9,1.97170658775182,0.0486431111794757,0.429073665411248
"1846","PABPC4L",21.1537362771612,0.858652225360752,0.435555716795748,
1.97139468556996,0.0486787484609237,0.429073665411248
"1847","AL021368.1",3.69559370762625,1.81143219307907,0.91865305835371
5,1.97183493442592,0.048628452924205,0.429073665411248

"1848","PPP1R16A",45.299971750185,-0.560413026203701,0.284171856982615
,-1.97209193110909,0.0485991129191778,0.429073665411248
"1849","CTSC",300.794831619777,0.67455832811585,0.342241453939821,1.97
100123421771,0.0487237346439744,0.429237919748891
"1850","ROCK2",768.063271604904,0.370487977266367,0.188035082338216,1.
97031305360334,0.0488025033069027,0.429699446684399
"1851","WDR76",12.586429289805,-1.0327801117173,0.52436847444045,-1.96
956941932745,0.0488877393022725,0.429985089359998
"1852","FLT3LG",171.435074247587,-0.466877518208215,0.237020276018071,
-1.96977881408179,0.0488637256662446,0.429985089359998
"1853","TIFA",119.800314603491,-0.700173235190008,0.35576334340241,-1.
9680870673571,0.0490580206165607,0.431017312741725
"1854","AGPAT2",331.499134267124,-0.4486869627814,0.227957935119535,-1
.9682884149048,0.0490348621678756,0.431017312741725
"1855","TTI1",61.9660409926851,0.413168872306228,0.209959368471059,1.9
6785156725779,0.049085118845481,0.431022911522393
"1856","FTO",226.900629784081,0.370226068482996,0.188209687779845,1.96
709357977397,0.0491724232946523,0.431556898193206
"1857","FAM174C",289.754564434524,-0.515286447616744,0.262071240482156
,-1.96620753451896,0.0492746424981622,0.43222113713116
"1858","PCYOX1L",35.171526922695,0.569723265771918,0.290122003587112,1
.9637368373573,0.0495606182921405,0.433429134037353
"1859","AC083837.1",5.02405083910573,-1.29190240842544,0.6577784954941
64,-1.9640386806122,0.0495256063589251,0.433429134037353
"1860","AL391834.2",14.3044733987406,0.839285636051318,0.4274942379520
13,1.96326771577569,0.0496150746740438,0.433429134037353
"1861","BBS1",102.143816324539,0.365319993875341,0.186068385425222,1.9
6336413109877,0.049603878533967,0.433429134037353
"1862","ULK2",136.078704032178,0.417224053978771,0.212524572655975,1.9
6318029846908,0.0496252277598173,0.433429134037353
"1863","ZNF471",50.6001911417414,0.673196958829968,0.342633868451715,1
.96477062198198,0.0494407918767166,0.433429134037353
"1864","SLC25A6",3652.91634758054,-0.448417199030378,0.22833569096175,
-1.96385066715433,0.0495474123086394,0.433429134037353
"1865","CITED1",3.39053790585469,-1.94817091958464,0.991821958305435,-
1.96423451131608,0.0495029023185578,0.433429134037353
"1866","IKBIP",491.025771822113,-0.353191276791438,0.179968618780389,-
1.96251590518916,0.049702450704793,0.43387096437855
"1867","MNT",125.283593754934,0.509242927738712,0.259599112722617,1.96
165126451276,0.0498030994572129,0.434516704369866
"1868","GPD1L",92.7632555385733,0.485786366593731,0.247890233315189,1.
95968336508063,0.0500328106695323,0.43473253088424
"1869","SLC9A9",217.738556911151,0.377553190283803,0.192525354165105,1
.96105698348708,0.0498723759752743,0.43473253088424
"1870","DENND4C",201.386973764801,0.36450251319907,0.185953865907345,1
.96017711931136,0.0499750918498988,0.43473253088424
"1871","TNFAIP2",1238.75666923794,0.533261201936673,0.27212409805541,1
.95962505984343,0.0500396301156402,0.43473253088424
"1872","TLCD3A",61.4795533561572,0.561874473865894,0.286727646578616,1
.95961038487385,0.0500413466393241,0.43473253088424

"1873","HPS4",169.800216080063,0.406083620570969,0.207116526623539,1.9
6065290969792,0.0499195257916051,0.43473253088424
"1874","NIPSNAP1",98.043425358863,-0.427396185486309,0.217995662160913
,-1.96057197308279,0.0499289744654197,0.43473253088424
"1875","PAK3",119.185891551183,0.444810456314815,0.226904330268355,1.9
6034362054151,0.0499556408011397,0.43473253088424
"1876","RAB3GAP2",234.253494481846,0.380904540377655,0.194534604981548
,1.95802973159344,0.0502265240684106,0.43518077156933
"1877","HLA-
DPB1",512.641439512507,-0.440134698996643,0.22471096012285,-1.95867036
817439,0.0501514028107608,0.43518077156933
"1878","SLC43A1",205.270059059372,0.330178002965556,0.168557202998808,
1.95884837367579,0.0501305465543461,0.43518077156933
"1879","GCOM1",3.80317491468878,-1.68552079224656,0.860807761552033,-1
.95806876695393,0.0502219440723828,0.43518077156933
"1880","IFNAR2",112.9163671639,-0.44313201009423,0.22627417575673,-1.9
5838525811556,0.050184823272408,0.43518077156933
"1881","EDIL3",50.1570397029344,-1.11262654406178,0.568376337662068,-1
.95755254104773,0.050282540859436,0.43538161634682
"1882","FGD6",41.8115384544158,0.686518742351737,0.350734041483901,1.9
5737698983305,0.0503031617634426,0.43538161634682
"1883","LEPR",1492.59485821961,0.821567046742311,0.419975498351141,1.9
562261369243,0.0504385208612231,0.435396431536017
"1884","SRGAP3",20.5182861089737,0.928488089180171,0.474526476664133,1
.95666234623479,0.0503871796466476,0.435396431536017
"1885","KAT2B",119.838377651532,0.453397371534839,0.231739003208778,1.
95650005073322,0.0504062764778323,0.435396431536017
"1886","BMPR1A",227.27878280949,0.347048634235137,0.1773920277321,1.95
639363658019,0.0504188012080583,0.435396431536017
"1887","CLCF1",31.4618516404432,-0.842345545729731,0.43057568294244,-1
.9563240078338,0.0504269977818133,0.435396431536017
"1888","CERS2",572.613862827512,-0.354657055136003,0.181388792032255,-
1.95523136331895,0.05055576828919,0.435946484734048
"1889","SERPINB2",4.57841650106877,2.16203189357795,1.10563989050293,1
.95545757000003,0.0505290867539758,0.435946484734048
"1890","TFPI",1883.43503536839,0.324690178172639,0.166104948126486,1.9
5472911454386,0.0506150517508619,0.436226760830577
"1891","VLDLR-
AS1",12.4458312104923,1.0220665536357,0.523051275178539,1.954046576574
78,0.0506957091814834,0.436690855027596
"1892","WDTC1",162.641105581419,0.368785530985472,0.188775482693125,1.
95356688127225,0.0507524605302743,0.436748332124951
"1893","LINC02615",11.3289108088444,1.38833389866498,0.710677071387432
,1.95353692212776,0.0507560066739844,0.436748332124951
"1894","SLFN13",16.9849320989685,0.826051998871524,0.4229557043807,1.9
5304612354394,0.0508141300902448,0.437017616177401
"1895","AC073195.1",3.19704296183275,-1.56962055669081,0.8039390777373
95,-1.95241231600328,0.0508892719910209,0.437432903146037
"1896","GASK1B",130.68851096975,1.06853698137959,0.547499653552039,1.9
5166695439384,0.0509777583873265,0.437769344893615

"1897","SMURF2",360.875183898804,0.430378178963363,0.220522437460215,1
.95162988365303,0.0509821626412418,0.437769344893615
"1898","RNF38",154.766222727881,0.452055307301786,0.231835994293445,1.
94989267598197,0.0511889122685179,0.43844700393335
"1899","TIMM10",334.249558155062,-0.518802480928399,0.266003568536193,
-1.95035910150885,0.0511333329286516,0.43844700393335
"1900","KLF13",553.871110059413,-0.551550868219361,0.282870385129556,-
1.9498360281397,0.0511956658776618,0.43844700393335
"1901","EMP3",2313.01630918977,-0.49525411993081,0.253995143734211,-1.
94985664942107,0.0511932073027118,0.43844700393335
"1902","LILRA6",2.83517243182487,-2.00768122989256,1.0293966767281,-1.
95034749507246,0.0511347153401813,0.43844700393335
"1903","HEBP1",986.740172011432,-0.46370827083172,0.237853640902621,-1
.94955296489056,0.051229424123537,0.438505564660165
"1904","HLA-
DMA",71.9387249555781,-0.784933057192664,0.402902790392294,-1.94819464
126421,0.0513916777819032,0.439663361023856
"1905","CLIC4",542.017956363122,0.306824397601044,0.157611652859253,1.
94671137593511,0.0515693470065241,0.440258434690393
"1906","CLASP1",143.98319656109,0.397770552268075,0.204303833997231,1.
9469558866598,0.0515400235654731,0.440258434690393
"1907","RBM5",497.186421248965,0.437991622389259,0.22492133162712,1.94
731028498165,0.0514975464055228,0.440258434690393
"1908","DOCK2",12.8785062384037,-1.10000178211268,0.565002295306136,-1
.9468978997982,0.0515469764940219,0.440258434690393
"1909","RCBTB2",98.515290721586,-0.350035547404633,0.179893823871631,-
1.94578968789063,0.0516800078433201,0.440741176837613
"1910","ANKRD29",33.450494504747,-0.746277702833125,0.383504691338326,
-1.94594152219839,0.0516617644679753,0.440741176837613
"1911","MMP2",7432.12722410026,-0.483341049133904,0.248461972015004,-1
.94533209735901,0.0517350214102716,0.440869582533277
"1912","CTU2",57.9856723793138,-0.46126195315295,0.237126552415287,-1.
94521426830821,0.0517491952731061,0.440869582533277
"1913","CCDC191",62.0436969182641,0.479130167297987,0.246354044935123,
1.94488451538989,0.0517888791004597,0.440977026485828
"1914","STAT1",438.191766527013,0.625708442481413,0.321940651224105,1.
9435521426148,0.0519494817133122,0.44177586172768
"1915","LPP",1687.62146051845,0.357783924023056,0.184155730358202,1.94
283351013367,0.0520362776320205,0.44177586172768
"1916","GRK6",61.2737017269355,-0.512915346396304,0.263993023443673,-1
.94291250467739,0.052026730797174,0.44177586172768
"1917","ANKMY2",161.398931405462,0.475324211680288,0.244623819109891,1
.94308229431558,0.0520062159381944,0.44177586172768
"1918","CBWD1",155.799883330101,-0.552803225308908,0.284437278146027,-
1.94349780349503,0.0519560405181201,0.44177586172768
"1919","AC004846.1",45.2332153928599,-0.486784143123543,0.250563466412
873,-1.94275785729046,0.0520454219814242,0.44177586172768
"1920","ARID5A",67.1006978366465,-0.682940045472598,0.351608993318229,
-1.94232814987896,0.0520973873000739,0.441986636318179
"1921","RFPL4B",14.1626351738466,1.65114074746902,0.850378179976199,1.

9416546500702,0.0521789221970899,0.441987760617991
"1922","FZD2",47.2574176660833,0.794516072857212,0.40917982782898,1.94172835223267,0.0521699945056376,0.441987760617991
"1923","MPP1",76.2851479008225,-0.519728682085193,0.267655800101666,-1.94178000957865,0.0521637379087466,0.441987760617991
"1924","TMEM61",4.98663613907486,1.55943203231417,0.803278535146253,1.94133412519263,0.0522177628838506,0.442086870901789
"1925","NCF2",14.5390573883356,-1.21540754049073,0.626326483978539,-1.9405335261735,0.052314883812079,0.442219586100132
"1926","HMCES",126.922560223188,-0.398966471375262,0.205581630301289,-1.94067179441354,0.052298099664007,0.442219586100132
"1927","BAHD1",41.1240079181306,0.565225594975338,0.291254669758253,1.94065762256958,0.0522998197530667,0.442219586100132
"1928","LINC01376",7.83747071022213,1.13007872004095,0.583044902433235,1.9382361724196,0.0525944154988036,0.443891416611405
"1929","GSAP",23.602720687651,-0.7507894796541,0.387323313773386,-1.93840508163516,0.0525738209461071,0.443891416611405
"1930","TFEC",4.11945361984149,-1.71835917227857,0.886434627701665,-1.93850636987626,0.052561474433135,0.443891416611405
"1931","CACNB4",229.264626388669,0.677280176458506,0.349474611297735,1.93799536379339,0.0526237881739286,0.443909314119691
"1932","MT-
ND1",55521.4744072841,-0.32214151955023,0.166290877041399,-1.93721703368027,0.0527188189283808,0.444480766834573
"1933","AP003117.1",12.0993047807096,-0.921066119160099,0.475727361275833,-1.93612180869717,0.0528527840723542,0.445173246881305
"1934","MED16",127.834789501156,0.450138729921778,0.232497819517949,1.93609871634528,0.052855611729906З,0.445173246881305
"1935","HERPUD1",1305.74258665689,-0.385486379738631,0.199141992657215,-1.93573627839595,0.052900008808484,0.445316921695812
"1936","MTHFD2",59.9610050001503,-0.863011986119414,0.445991892915935,-1.93503962701422,0.0529854331043369,0.445805640411438
"1937","MFSD12",99.1127322071142,-0.497864183617643,0.257321864441073,-1.93479160699791,0.053015873449916,0.445831472702985
"1938","SEMA3B",129.824023483019,0.85030120785499,0.439557657894318,1.93444748961563,0.0530581323544276,0.445956613994464
"1939","KCTD7",62.735244457089,0.69047488250154,0.357001837971006,1.93409335488524,0.0531016508077978,0.446092207327601
"1940","ZDHHC11",12.4362958073455,-1.57630890350621,0.815776022294633,-1.93228148465597,0.0533247724466443,0.447311567815405
"1941","URGCP",105.659511260109,0.38085385146902,0.197126956957364,1.93202318621189,0.0533566441319499,0.447311567815405
"1942","LINC02649",3.64071766339326,1.75891852360008,0.910274539789821,1.93229454050885,0.0533231618950984,0.447311567815405
"1943","AL121672.3",2.81666935168146,-1.72587682363913,0.893216035119897,-1.93220537448979,0.0533341620979522,0.447311567815405
"1944","AC245297.3",555.880654004242,-0.342916466987265,0.177674038688349,-1.93003136259407,0.0536029518942269,0.447391912364971
"1945","PGAP1",74.3655953015738,0.479611719361347,0.24844733561256,1.9304361553278,0.053552818734928,0.447391912364971

"1946","CHMP2B",592.803586898747,-0.304644277523318,0.157784511088716,
-1.93076161545432,0.0535125392376854,0.447391912364971
"1947","FAM200B",594.988169998896,-0.45217232999574,0.234154206093793,
-1.93108779696495,0.0534721958480183,0.447391912364971
"1948","HINT3",85.4147669421492,0.4478954630695,0.232100500340445,1.92
974794286323,0.0536380764530474,0.447391912364971
"1949","SCAI",102.716565568719,0.465029068967828,0.240961415230015,1.9
2989017981956,0.0536204464491364,0.447391912364971
"1950","FBXW5",414.429912006322,-0.345738797814233,0.179124984155005,-
1.93015396174466,0.053587763981412,0.447391912364971
"1951","NANOS1",28.7566768974283,1.11659538104378,0.578445309093024,1.
93033872604923,0.05356488167055,0.447391912364971
"1952","AMBRA1",51.0472854949528,0.440088116991269,0.228057394047573,1
.9297252730138,0.0536408867854865,0.447391912364971
"1953","FAM50A",487.063767225234,-0.368472951309356,0.190912899947176,
-1.93005790290394,0.0535996637043965,0.447391912364971
"1954","AC005899.7",10.0230710958173,1.16182765278883,0.60214511787122
4,1.92948114716309,0.0536711583332556,0.447415300967452
"1955","ATXN7",233.176223235807,0.585286460992726,0.303710038327042,1.
92712254167403,0.0539643601319695,0.448048549735982
"1956","ACKR4",981.141046135461,0.575454206839105,0.298432890013056,1.
92825330617523,0.0538236266843655,0.448048549735982
"1957","CCDC96",15.4840472741447,-0.809292714217654,0.419853565719319,
-1.92755946428875,0.0539099449554845,0.448048549735982
"1958","ZFHX4",1831.87273149943,0.445155524166998,0.231024087093952,1.
92687926945887,0.05399467758191,0.448048549735982
"1959","PCOTH",8.71582615177932,-0.96490944682394,0.50072289983771,-1.
92703279026519,0.0539755436177641,0.448048549735982
"1960","PYGO1",88.7506770334592,0.536642335238516,0.278413099590235,1.
92750389988237,0.0539168625097565,0.448048549735982
"1961","ZNF844",90.3689522619195,0.812322646449463,0.421261508403193,1
.9283096847101,0.0538166179132335,0.448048549735982
"1962","RAB4B",68.4292207729911,-0.494075655103282,0.256337469063492,-
1.92744219917731,0.0539245448777153,0.448048549735982
"1963","ZNF784",16.1676377723286,0.801896589040927,0.415918406798283,1
.92801418723899,0.0538533615371005,0.448048549735982
"1964","MBNL3",78.3413612342952,0.575529508546688,0.298728639930737,1.
92659635406946,0.0540299533839527,0.448112989140125
"1965","EVA1B",970.683573013232,-0.379659747630411,0.197132940417146,-
1.9259071914974,0.0541159633309,0.44859792707228
"1966","INTU",121.878794047718,0.483413448295305,0.251104565181989,1.9
2514798743284,0.0542108469486734,0.448927547507342
"1967","KRT24",23.7077075759872,0.845557657770584,0.439195115650466,1.
92524376442183,0.0541988693093032,0.448927547507342
"1968","FUCA1",207.855007327906,-0.344929930530422,0.179299774700436,-
1.92376109287762,0.0543845363439191,0.449908437026967
"1969","PPIF",128.285409865591,0.538696006361393,0.279994529535329,1.9
2395189740099,0.0543606132034591,0.449908437026967
"1970","AC108488.3",5.49282921566684,1.31700927121436,0.68481836927229
8,1.92315120374741,0.0544610633805837,0.450084353833753

"1971","VIM-
AS1",35.2000925245305,-0.693301593359549,0.360492707082607,-1.92320560
094071,0.0544542341390188,0.450084353833753
"1972","NOG",2.6831702554659,-1.75341512936761,0.91192087810296,-1.922
77112134463,0.0545088003876184,0.45025043078799
"1973","CARD19",190.825098017353,-0.453745467052332,0.236013296564993,
-1.92254196545821,0.0545375984198641,0.450259980061412
"1974","ITGA8",8.2617411369612,-1.45025996050647,0.754438299918289,-1.
92230426353426,0.0545674838419684,0.450278492554115
"1975","OSR1",366.957458729502,-0.704785889412961,0.366767356054568,-1
.92161564484518,0.0546541385509083,0.450765196382655
"1976","ZNF697",30.327816347346,0.646176804592545,0.336402595379025,1.
92084369582374,0.0547514158355022,0.451110679081687
"1977","NOSTRIN",66.9068702419857,0.739607740613573,0.385012399622774,
1.92099719733241,0.0547320608242699,0.451110679081687
"1978","MALL",58.252862902866,0.619414782466728,0.322654230284352,1.91
974790450088,0.0548897500847062,0.451842479171371
"1979","ADAM33",465.171444971953,0.432649034782856,0.22537316859064,1.
91970072341976,0.0548957128295256,0.451842479171371
"1980","LRTOMT",43.0224524102872,-0.543061369887916,0.282974621158868,
-1.91911687226195,0.0549695446420284,0.452221673067677
"1981","RCAN2",127.766532003558,-0.523268583694149,0.272701734319054,-
1.91883115448741,0.0550057056910542,0.452290731954357
"1982","POLR2J",687.749191719776,-0.397607239782091,0.207266857593204,
-1.91833486742228,0.0550685639799145,0.452579131518076
"1983","AC112206.2",9.05646044706751,-1.20169588763195,0.6265813586373
06,-1.91786089877523,0.0551286513758837,0.452782752585485
"1984","ADAMTS14",3.51581377858911,2.74381040499445,1.43078112375241,1
.91770100922106,0.0551489337055438,0.452782752585485
"1985","AC068228.3",3.71767547967061,1.68181454340469,0.87718717418264
6,1.91728127462852,0.0552022075302223,0.452991817863875
"1986","MED13",382.388883773894,0.383106867485108,0.199866294188044,1.
91681578447971,0.0552613389294463,0.453248715922332
"1987","DRD4",6.98134646981323,1.15651584126683,0.603689594915198,1.91
574585848094,0.055397452204852,0.453679788318167
"1988","ART5",8.46083440247613,-1.48529686350898,0.775286351316303,-1.
91580421993396,0.0553900204109635,0.453679788318167
"1989","RPP25",99.1447373882403,-0.579032323239652,0.302205174093139,-
1.9160238568953,0.0553620591124245,0.453679788318167
"1990","CD300A",5.20662163332761,-1.39484990711685,0.728192578127884,-
1.91549591277473,0.0554292898909238,0.453712413584551
"1991","CMTR2",104.765117219491,0.411978821871485,0.215221259751515,1.
914210623742,0.0555932488606062,0.454825932039385
"1992","CD52",76.7609561783146,-0.813384274455821,0.424993571535328,-1
.91387430053917,0.0556362188519892,0.454948980361472
"1993","DSTN",5408.73501760552,-0.292940177362956,0.153114324750648,-1
.91321209063894,0.0557209063079305,0.455412866457541
"1994","RHOB",4711.80478655847,0.619124461021406,0.323686574178606,1.9
1272826990897,0.0557828481345006,0.455462061785905
"1995","AC092053.4",10.7478841997402,-0.851411029150805,0.445119512703

721,-1.91276950313683,0.0557775669578208,0.455462061785905
"1996","TNFRSF19",38.9876477718699,0.775057729517494,0.405258833995067
,1.91250051695833,0.0558120263747866,0.455471992795039
"1997","IL18",35.0028896159086,-0.821958742078982,0.429864283375602,-1
.91213546662768,0.0558588207892855,0.455625604324823
"1998","ANKRD35",569.310616654241,-0.478237862494066,0.250261820411915
,-1.91095014695776,0.0560109877939288,0.456157381959922
"1999","ALMS1",76.439069002929,0.537185077050735,0.281152095946089,1.9
1065649090427,0.0560487395952425,0.456157381959922
"2000","AC002429.2",2.75631958433446,2.04232228306453,1.06970832754534
,1.90923285392296,0.0562320598548421,0.456157381959922
"2001","RABGAP1",223.93007608934,0.416570582101038,0.218151685376472,1
.9095455594677,0.0561917503584792,0.456157381959922
"2002","AK1",351.861106953619,-0.597737473113562,0.313000030145121,-1.
90970420302012,0.056171309516984,0.456157381959922
"2003","NOVA1",6043.04927537857,-0.418383848157354,0.219113799218614,-
1.90943632783221,0.0562058281929955,0.456157381959922
"2004","AC109630.1",6.18558166599776,-1.34726181912499,0.7051951260426
34,-1.91048090006728,0.0560713233036492,0.456157381959922
"2005","MIR762HG",31.0673675062237,-0.635187360553206,0.33246938576217
3,-1.91051383301673,0.0560670870376811,0.456157381959922
"2006","RAB34",1054.69796813809,-0.392609360284464,0.205492637708748,-
1.91057628468872,0.05605905442013,0.456157381959922
"2007","PIP4K2B",190.309999245843,0.356968633080606,0.186966071251785,
1.90926958399784,0.0562273238949085,0.456157381959922
"2008","HOXB5",12.1047883352718,-0.837499576190136,0.438597605256625,-
1.90949418362672,0.0561983713399658,0.456157381959922
"2009","AC079922.2",24.8506865918466,-0.792880990926485,0.415364571408
557,-1.90887968186049,0.0562776146600589,0.456299684020756
"2010","SDF2L1",209.536169169026,-0.643969374949742,0.337404686129115,
-1.90859641677684,0.0563141745353789,0.45636894975462
"2011","SLC1A1",7.70458465914697,1.54572074743507,0.810135975657755,1.
90797692471327,0.0563941987739309,0.456790205782477
"2012","KCNJ12",111.392309777284,0.485573401353928,0.254581620523065,1
.90733879514266,0.0564767295104909,0.457231335485281
"2013","ATP7A",101.781382056917,0.38583098611402,0.202336975861567,1.9
0687334567062,0.0565369905289436,0.457491822516623
"2014","FAXDC2",452.803492220943,0.376028779185398,0.197280480973354,1
.90606175192865,0.0566421943224431,0.457940279933006
"2015","DDX54",196.55549894887,-0.295271422327816,0.154915818705952,-1
.90601208317063,0.0566486379805395,0.457940279933006
"2016","Z95331.1",55.4034249429427,1.03761075943329,0.54461200251088,1
.90522932775901,0.0567502674809909,0.458534279264812
"2017","ZMYM4",258.582612651013,0.290956619326816,0.152887311246488,1.
9030789210344,0.0570302479832699,0.458546302210372
"2018","CHIT1",2.39196457841175,-2.51747770852066,1.32379041236315,-1.
90171924876431,0.057207868026782,0.458546302210372
"2019","CCDC39",28.3581003928865,0.919818703743711,0.48370698295146,1.
90160311131174,0.0572230608848366,0.458546302210372
"2020","ARL9",5.80868128623952,-1.28497433853541,0.674824169948853,-1.

90416169982886,0.0568891281224574,0.458546302210372
"2021","SLC38A9",64.8483361206761,0.450417626327979,0.23697612527142,1
.90068778368325,0.0573429196146189,0.458546302210372
"2022","ZBED3",141.256444601269,-0.327795849038587,0.172230574565093,-
1.90323843409522,0.0570094401819671,0.458546302210372
"2023","SMAD5",622.36962393291,0.306374827412758,0.160979413315825,1.9
0319259526485,0.0570154190159119,0.458546302210372
"2024","UBR2",263.775621864626,0.417563167182073,0.21965160351256,1.90
102489808683,0.0572987514799214,0.458546302210372
"2025","TRAM2",464.793273302167,0.586122359756867,0.30773600665086,1.9
0462717098246,0.0568285520655528,0.458546302210372
"2026","PNISR",2320.52305549094,0.338521301489835,0.178041232051864,1.
90136463103795,0.0572542688949042,0.458546302210372
"2027","CPED1",522.221566027911,0.340506342850396,0.179144175052078,1.
90073912674754,0.0573361909097194,0.458546302210372
"2028","AL353746.1",4.78413212489954,5.3544028505476,2.81650593003991,
1.90107991374679,0.0572915461112716,0.458546302210372
"2029","GNA14",17.3825468988309,0.763273297119772,0.401357570456949,1.
90172891531803,0.0572066036197513,0.458546302210372
"2030","SURF2",171.866006942167,-0.526930245456685,0.277062884758295,-
1.90184349634694,0.0571916179332656,0.458546302210372
"2031","AL731569.1",3.80573432383994,1.56549427996395,0.82255028785052
9,1.90322014725065,0.0570118253026076,0.458546302210372
"2032","UBXN1",1237.35065819377,-0.502337975978867,0.264231197995477,-
1.90113044859852,0.0572849282554449,0.458546302210372
"2033","TMTC2",21.0043618007778,0.959147306052273,0.504448127601998,1.
90137945523118,0.0572523285585285,0.458546302210372
"2034","AC012645.1",13.4531696835691,-0.810711314972047,0.425956193788
915,-1.90327392063654,0.0570048119722821,0.458546302210372
"2035","ZNF423",174.639155824513,0.489822973069637,0.257563842702091,1
.90175363098689,0.0572033708602029,0.458546302210372
"2036","ZNF43",170.935531201921,0.330750918651519,0.173644165035575,1.
90476264252102,0.0568109319905677,0.458546302210372
"2037","LRCH2",62.146078897795,0.55747554155412,0.293244269954261,1.90
10620110022,0.0572938907404947,0.458546302210372
"2038","DNAJB9",253.775721041066,-0.391031391234515,0.205856791461962,
-1.89953116658174,0.0574946728144239,0.458858758194097
"2039","MARK3",355.852671393425,0.406096048914526,0.213719588051409,1.
90013490395107,0.0574154182354121,0.458858758194097
"2040","NF1",434.558801414625,0.389966032160476,0.20529056615017,1.899
58086956229,0.0574881447073688,0.458858758194097
"2041","MED1",227.304176025465,0.434900632308342,0.228936495866778,1.8
9965619357352,0.0574782526482767,0.458858758194097
"2042","GARS-
DT",241.968210432281,0.449306237488381,0.236650590977663,1.89860602347
192,0.0576162958531588,0.459604232689571
"2043","BORCS6",69.5819141357281,-0.466564940159031,0.245853456520892,
-1.8977359389673,0.0577308757686205,0.460067629841027
"2044","AC005332.6",125.332084195372,0.416497438863657,0.2194486451459
3,1.89792668160103,0.0577057410057678,0.460067629841027

"2045","RFTN1",685.438280218209,0.471669559814084,0.248631353343715,1.
89706387979973,0.0578195076716247,0.460337237139443
"2046","AC108734.4",95.7732266916913,0.497061192757601,0.2620178552004
26,1.89705084173513,0.0578212282636933,0.460337237139443
"2047","KLHDC9",19.445748464168,-0.74515050117572,0.393259790479425,-1
.89480470471518,0.0581182796886359,0.460669694580536
"2048","LPIN1",167.761688600935,0.399182527044299,0.210570441225745,1.
89571966853766,0.0579971230932075,0.460669694580536
"2049","AC091607.2",29.1497762170866,-0.912486599237374,0.481513755070
532,-1.89503745143005,0.0580874401833023,0.460669694580536
"2050","SNX13",225.125701524893,0.303716378901213,0.160302139258104,1.
89464956803974,0.0581388432304849,0.460669694580536
"2051","RASSF4",233.396126253546,0.498558568139228,0.26300241495244,1.
89564254848909,0.0580073269778544,0.460669694580536
"2052","NAALADL1",149.810996692143,-0.410900386030582,0.21670518965295
2,-1.89612619194136,0.057943359943247,0.460669694580536
"2053","ZDHHC17",241.787530866169,0.366626524603179,0.193507026095852,
1.89464192592973,0.0581398563570299,0.460669694580536
"2054","AHSA1",412.488510811099,-0.394458432422434,0.208105045239099,-
1.89547750737723,0.0580291688604411,0.460669694580536
"2055","TEX22",5.89557569333046,1.32236664348043,0.697289587053168,1.8
9643824894749,0.0579021181801148,0.460669694580536
"2056","ZNF587",70.0098249930754,0.57258750855144,0.302221250089506,1.
89459711513291,0.0581457972900474,0.460669694580536
"2057","VIT",1497.11748329682,0.379969878955839,0.200653590306992,1.89
366100239971,0.0582700207707104,0.461429444984979
"2058","SDS",4.14015981261152,-1.7763202299769,0.938550512577971,-1.89
262080854634,0.0584083144994215,0.462299822585558
"2059","FLNC",194.230649440121,0.618573616196506,0.326903160314222,1.8
9222280874228,0.0584613006212221,0.462303458290041
"2060","METTL2A",111.315724170216,-0.380355745181039,0.201013398920776
,-1.89219100429692,0.0584655365017794,0.462303458290041
"2061","TAB1",74.5981883991734,0.529877672472963,0.280066211124568,1.8
9197286722061,0.0584945959968394,0.4623088181429
"2062","UBXN10-
AS1",3.55523271271448,-1.64155894041966,0.867865194738044,-1.891490695
06716,0.058558871933231,0.462520808838944
"2063","MTHFD2L",39.6012058669278,-0.531692625928109,0.28115301512901,
-1.89111479271932,0.0586090222597112,0.462520808838944
"2064","SLC3A2",505.987513828265,-0.536245509225814,0.283589708703251,
-1.89092020185734,0.0586349972528958,0.462520808838944
"2065","GAL",201.490786074348,1.62208017399029,0.857646370602098,1.891
31584950512,0.0585821942000829,0.462520808838944
"2066","SLC30A6",97.3463194543708,0.412689784422189,0.218471191063808,
1.88898949290598,0.0588932366191567,0.462943966767991
"2067","HEBP2",650.791333085151,-0.475252996339031,0.251613759014025,-
1.88881958681973,0.0589160073122994,0.462943966767991
"2068","ACVR1B",113.717054082028,0.519206403737435,0.274789412137144,1
.88947019355428,0.0588288530350555,0.462943966767991
"2069","TRAFD1",114.338480788175,0.381961033380704,0.202189666631583,1

.88912242521617,0.0588754262202728,0.462943966767991
"2070","AL161421.1",25.0817913449179,-0.77266522831374,0.408939265626666,-1.8894376091024,0.058833215450317,0.462943966767991
"2071","ERBB2",148.226362263585,0.378911826989659,0.200495959880976,1.88987262992531,0.0587749968829394,0.462943966767991
"2072","NEDD4L",12.7181463173888,0.943072025500198,0.4991476572721,1.88936482373612,0.0588429609449767,0.462943966767991
"2073","ZNF507",124.765894484127,0.374737999891257,0.198367550975371,1.88910937322498,0.0588771747406132,0.462943966767991
"2074","WDFY3",190.059202236052,0.465720757304815,0.246635110310635,1.88829869647611,0.0589858622244378,0.463046125192225
"2075","C22orf39",246.746126934215,-0.329834715670551,0.174667086481679,-1.88836215405211,0.0589773484588158,0.463046125192225
"2076","COL1A2",39536.8612256395,0.611217233533161,0.323977127073363,1.88660612881709,0.0592133218216616,0.464607803060234
"2077","SH3RF3",52.7754887279402,0.756365954164794,0.401214479554968,1.88519107038152,0.0594040459830745,0.464835416583955
"2078","ITGA4",54.4983335855313,-0.674889041518256,0.357967118808662,-1.88533808290641,0.0593842076679673,0.464835416583955
"2079","PPIL1",40.6544826059541,-0.623188846043168,0.330583004125351,-1.88512064524305,0.0594135513124068,0.464835416583955
"2080","SKIDA1",13.6408034562818,-0.800319398678746,0.424321053761139,-1.8861175790944,0.0592791119237969,0.464835416583955
"2081","HELLS",24.979387710266,-0.852055377027069,0.451789047958152,-1.88595845976769,0.0593005526744604,0.464835416583955
"2082","PSMC5",1056.34193043032,-0.475323278128814,0.252113799816693,-1.88535208494899,0.0593823184767277,0.464835416583955
"2083","SECISBP2L",385.185791541092,0.427420969601787,0.226789809402872,1.88465685793893,0.0594761805308888,0.465102018563441
"2084","TMOD1",13.7148182550266,-1.35416936207415,0.718610593597617,-1.88442721849494,0.0595072110047736,0.465121381985008
"2085","DCP1A",303.185271596698,0.335818968472661,0.17827555276188,1.88370734668936,0.059604572137993,0.465658933120272
"2086","CHL1",1535.63467424336,0.45984905569514,0.244182532363926,1.88321847285042,0.0596707666040139,0.465952596938055
"2087","AC007383.2",6.67225732878939,-1.19213089457853,0.633122749774505,-1.88293801636906,0.0597087684745935,0.466025936599259
"2088","BTN3A2",256.234341590088,0.590393463848014,0.31360562367808,1.88259845892961,0.059754805393627,0.466161889395015
"2089","NUMBL",368.134727959818,-0.399400931057853,0.212193615580947,-1.88224763485162,0.0598024007543258,0.466309863995794
"2090","AC018647.1",14.5284987759936,0.865670839663875,0.46018130229865,1.88115170116597,0.0599512857123765,0.466557550470178
"2091","FKTN",90.4145864027177,0.501283562986071,0.266486339172881,1.88108540400965,0.0599603021755566,0.466557550470178
"2092","MRPL23",112.12595111901,-0.759901323194086,0.403901460218388,-1.88140276289967,0.059917151318227,0.466557550470178
"2093","SH3GL1",374.791108456589,-0.294440950752015,0.156537603755932,-1.88095986962402,0.0599773780271688,0.466557550470178
"2094","AC010618.3",21.4192481206204,0.712113430720049,0.3784775669313

09,1.88152084281733,0.0599011027272751,0.466557550470178
"2095","SPATA17",10.4437792241872,-0.973500628060359,0.517800894221924
,-1.8800674910444,0.0600988803450375,0.467150054191842
"2096","HSPD1",853.255840887097,-0.282062253409413,0.150101917367516,-
1.8791382439093,0.0602256193629362,0.467150054191842
"2097","SEPTIN8",252.34503188337,0.364725025729539,0.194073228931456,1
.87931652262227,0.0602012869579481,0.467150054191842
"2098","DOK3",2.50862712590932,-2.04565735174643,1.08856073392152,-1.8
79231252791,0.0602129240104593,0.467150054191842
"2099","LINC00957",8.58181777829095,-1.25683573707862,0.66873382211665
,-1.87942600704797,0.0601863479947252,0.467150054191842
"2100","ZNF688",226.795797284225,-0.397091361700717,0.211225745645376,
-1.87993826456832,0.0601164921556121,0.467150054191842
"2101","ACADSB",104.661636697745,0.41328578242254,0.219998711789017,1.
87858273833389,0.060301489939835,0.467293515523298
"2102","INAFM1",320.76579076621,-0.345306502737545,0.183809009819807,-
1.87861576032676,0.0602969776043401,0.467293515523298
"2103","ENHO",13.1991151525806,-1.13909310154436,0.606580312127785,-1.
87789329585823,0.0603957637043254,0.467561250034231
"2104","SPA17",38.2920461282639,-0.610572335521381,0.325201605342396,-
1.87751943868335,0.0604469356732586,0.467561250034231
"2105","CDRT4",22.2200663691913,0.720435730422311,0.383668991289848,1.
87775334149444,0.0604149158515874,0.467561250034231
"2106","NBR2",25.802285290203,0.732153268051561,0.389963703039799,1.87
74908083608,0.060450855950156,0.467561250034231
"2107","ADCY9",116.626840446952,0.387814709246852,0.206599244571977,1.
87713517564069,0.0604995693985109,0.467715940167225
"2108","MUSTN1",262.500187357471,-0.693839481315876,0.369939311849034,
-1.87554947282548,0.0607171700822496,0.467972594353512
"2109","LINC00598",6.45063851544217,1.34586955746685,0.717633305989955
,1.87542794660325,0.0607338734399488,0.467972594353512
"2110","KHNYN",134.750070547796,0.428702700681238,0.228576612498264,1.
87553177902001,0.0607196017976449,0.467972594353512
"2111","GPATCH2L",263.507156992667,0.443467971568708,0.236454469135696
,1.87548991224273,0.0607253560020526,0.467972594353512
"2112","SMAD6",133.887107333126,0.458583252208188,0.244468528342569,1.
87583757842884,0.0606775861752682,0.467972594353512
"2113","ZNF286A",99.4777121439882,-0.38335886976864,0.204392188629503,
-1.87560430924074,0.0607096342536271,0.467972594353512
"2114","ACSS1",60.8788848610311,0.653514890965205,0.348245261325899,1.
87659377898505,0.0605737906668937,0.467972594353512
"2115","SETD7",606.936730712372,0.309756924743534,0.165212602797874,1.
87489888481752,0.0608066355229157,0.468311719164432
"2116","AL158071.1",80.0496940153761,0.789920642799026,0.4213941080360
05,1.87454126133993,0.0608558605255936,0.46846933464149
"2117","ASAP3",241.579947748758,0.354545817033797,0.189197015047083,1.
87395037361221,0.0609372654706372,0.468874405881535
"2118","RIPK3",19.6924392258559,-0.817957677530785,0.436617480496939,-
1.87339654060534,0.0610136473721699,0.469240463666324
"2119","HEATR1",126.791772901541,0.392715855326488,0.209673365760437,1

.87298875039373,0.0610699384572611,0.46945173550275
"2120","IDS",593.50655358198,-0.490904164278156,0.262233677704539,-1.87201037096106,0.0612051687243709,0.470269336486452
"2121","ABHD12",184.736139526085,-0.430280263504899,0.22987876947241,-1.87177034439686,0.0612383827252132,0.47030269505469
"2122","ZNF341",13.0332992611513,0.819378727629517,0.437858217000713,1.87133344954032,0.0612988769546338,0.470375763302013
"2123","SLC35E4",25.9659149441296,-0.695609473124985,0.371728325815774,-1.87128455061486,0.0613056507759944,0.470375763302013
"2124","TSR2",296.302583261232,-0.413269727727302,0.22089885847284,-1.87085497220039,0.0613651856308448,0.470610879821484
"2125","C9orf40",59.6339593738879,0.47323606837149,0.253051347111866,1.87011874772707,0.0614673295938586,0.471172391413818
"2126","DEXI",411.409080253945,-0.448053320492446,0.239679945710467,-1.86938176727432,0.0615697193899642,0.471735258298742
"2127","METRN",218.431107216025,-0.586674421392324,0.313947652712218,-1.86870141032748,0.0616643677023439,0.472238310062755
"2128","ENPP4",13.4746067093228,1.03638920502552,0.554699740621128,1.86837874462501,0.0617092976455036,0.472360314543049
"2129","TRMT1L",115.615914266171,0.358439894665981,0.191913606372015,1.86771486108788,0.0618018260804822,0.472624387335669
"2130","AC087284.1",5.05675290989601,1.92784923459257,1.03211867684337,1.86785616600671,0.0617821221646834,0.472624387335669
"2131","HEYL",3.27581393561461,-1.77543829610807,0.950897197582925,-1.86711907514402,0.0618849611863294,0.472855568434693
"2132","CDKN1C",3714.38847914463,0.519448005904801,0.278213789954498,1.86708216724181,0.0618901143043014,0.472855568434693
"2133","AL589743.4",2.56189899828717,2.97275453292827,1.59245651789536,1.86677281264618,0.0619333206643829,0.472963835115862
"2134","AREG",96.4310629325912,-0.883589222474939,0.473497501399468,-1.86609057041147,0.0620286950305214,0.473470203070367
"2135","BDNF",12.3094573118249,-0.948698121818364,0.50849955616326,-1.86568131735748,0.0620859650442155,0.473685379206195
"2136","CAMSAP2",189.428396151268,0.373906799172449,0.200505863001848,1.86481728551251,0.0622070195459743,0.473721431222242
"2137","ZNF518A",199.721312879885,0.364079163288486,0.195184496501047,1.86530779757158,0.0621382728006813,0.473721431222242
"2138","AC007114.2",5.29474149378485,-1.40788385599779,0.754880147053588,-1.86504289653527,0.0621753916691857,0.473721431222242
"2139","EML2-AS1",6.69476104390425,-1.36222287633056,0.73042533099597,-1.864972117647,0.0621853125619637,0.473721431222242
"2140","FAM117B",67.6786414981709,0.596931664704249,0.320223576955249,1.86410904025243,0.0623063932669784,0.473876240578563
"2141","LINC00924",7.84013829526435,1.03507605045911,0.555280427258839,1.86406003101677,0.0623132745961915,0.473876240578563
"2142","ZNF668",48.8317548323221,-0.471492379070237,0.252939713123073,-1.86405042232661,0.0623146238148004,0.473876240578563
"2143","ANKS1B",18.6112803468706,-0.793805296789955,0.42610462825562,-1.86293516697911,0.0624713883253507,0.474846684289145

"2144","ZCWPW2",57.23450420122,0.502736663137982,0.26994220410713,1.86238630154499,0.062548658610593,0.474990722661049
"2145","NPC2",2045.38463132964,-0.370008000599265,0.198662502025648,-1.86249542226894,0.0625332901023715,0.474990722661049
"2146","STIM1",188.662441242712,0.319251803018385,0.17146855131252,1.86186796689333,0.0626217032696739,0.475323823187194
"2147","HABP4",152.795609380659,-0.386836918611217,0.207815315450204,-1.8614456676265,0.0626812665831508,0.475554332264994
"2148","GTF2H1",163.572656064062,0.299769359995246,0.161075045778644,1.86105401085654,0.0627365497823534,0.475752169182847
"2149","VPS52",83.3792003491401,0.444006816694895,0.238756196118246,1.85966615281053,0.0629327735915286,0.476353136167476
"2150","AC006517.2",2.92893881254227,-1.80048959593595,0.967965738933005,-1.86007574805348,0.0628748098222252,0.476353136167476
"2151","AXL",1152.4943004604,-0.308040931845385,0.165620853636732,-1.85991633952711,0.0628973632319955,0.476353136167476
"2152","PSMG1",133.372983409464,-0.579117772499592,0.311375057521913,-1.85987206910058,0.06290362787938,0.476353136167476
"2153","MELTF",87.0202239297459,1.07538885696934,0.579152869989996,1.85683074830987,0.0633352376125425,0.476644536416743
"2154","CRMP1",3.45314460688046,-2.35178831848628,1.26581361296133,-1.85792623369277,0.0631794904161952,0.476644536416743
"2155","CTNNAL1",133.856278117729,-0.4276601926642,0.230086886535152,-1.8586899892657,0.063071093244678,0.476644536416743
"2156","MRPL49",100.749285310236,-0.409375706520853,0.220483837080078,-1.856715267397,0.063351674218322,0.476644536416743
"2157","STRAP",504.361555547001,-0.411646388017268,0.22159924851673,-1.85761635372238,0.0632235144891762,0.476644536416743
"2158","LINC02381",302.352220368611,0.403604203986751,0.217135139713258,1.85876963314063,0.0630597985228021,0.476644536416743
"2159","LINC00173",8.77456565694702,1.5157718958043,0.81537929219578,1.85897766881274,0.0630303037636354,0.476644536416743
"2160","SLC25A30",26.7613011645501,0.640997783296087,0.345110352865434,1.85737048446653,0.0632584627136992,0.476644536416743
"2161","PATL2",15.3293502639153,0.948343697836851,0.510356456377642,1.85819868835972,0.0631408042203127,0.476644536416743
"2162","NR2F2-AS1",77.526603577848,0.578127240374034,0.311295067216125,1.85716800958061,0.0632872547831313,0.476644536416743
"2163","GLIS2",202.133035327422,-0.513916818826026,0.276666139568217,-1.85753420938348,0.0632351888331947,0.476644536416743
"2164","RAB31",270.997106892783,-0.445245550783061,0.239623552749167,-1.85810428764127,0.0631542060850392,0.476644536416743
"2165","NCOA3",304.178820833197,0.462056166510645,0.24881148336461,1.85705322062465,0.0633035826620049,0.476644536416743
"2166","TP53BP1",235.783643558343,0.310970399022266,0.167506808012802,1.8564642399402,0.0633874155661321,0.476693265076974
"2167","IL6R-AS1",4.78622224476489,1.44223408723433,0.777755096474204,1.85435504540235,0.063688381790468,0.478735602669558

"2168","PGF",17.9728450850149,-0.827538654482997,0.446442710124275,-1.85362788038948,0.0637924160182613,0.479296431974843
"2169","DIP2A",170.482830864772,0.497101632855604,0.268249819544615,1.85312942129651,0.063863810743233,0.479611624341412
"2170","TRPC4AP",198.903157525588,0.285394861802784,0.154101842177937,1.85198864445271,0.0640274535500533,0.480397600588125
"2171","MRTFA",120.786036962854,0.379780478956355,0.205058894359443,1.85205562598346,0.0640178355863009,0.480397600588125
"2172","FABP5",217.626228328201,-1.28126414975183,0.691909060220963,-1.85178114208052,0.0640572566554081,0.480399932624283
"2173","CFL2",452.667756620133,0.357293907759633,0.193067667995298,1.85061492413289,0.0642249711294944,0.481324527727096
"2174","MEI1",4.49849380760836,-1.23332415878649,0.666476884127827,-1.85051303077144,0.0642396416771261,0.481324527727096
"2175","KPNA3",202.33163016983,-0.322606744528045,0.174357508342092,-1.85026012126238,0.0642760673988742,0.481376028441499
"2176","NACC2",58.4041137450238,0.504514881829123,0.2729120899974,1.84863514780137,0.0645105138949583,0.482909816560191
"2177","NYNRIN",157.775649259384,0.459512387351652,0.248694588249265,1.84769757390573,0.0646461051474658,0.483418956238966
"2178","AL049840.2",40.6524338871761,-0.535761407872506,0.28998039053797,-1.84757806167018,0.0646634058074745,0.483418956238966
"2179","ACTG1",12712.0631999699,-0.461807576359502,0.249956827357629,-1.8475493597891,0.0646675612772243,0.483418956238966
"2180","AC008124.1",141.413642598102,-0.423470614474434,0.229248569278697,-1.84721159136056,0.0647164800823806,0.483562726633898
"2181","AQP5",8.36617853754598,-1.17046669444413,0.633965571698539,-1.8462622367776,0.0648541381019305,0.484369122211071
"2182","C3",17982.4391647811,0.399870353959726,0.216620592032733,1.84594802464256,0.0648997525690475,0.484487657927229
"2183","ATAD3A",54.7321332234275,-0.53908910167328,0.292306471672214,-1.84425989130272,0.0651452737670684,0.485966231244949
"2184","DNA2",5.65270097136106,1.27931039025334,0.693726550259174,1.8441133466427,0.0651666232332307,0.485966231244949
"2185","TMEM123",652.891477458696,0.309972026375613,0.168100241132938,1.84397133690296,0.0651873175314761,0.485966231244949
"2186","TNFRSF21",21.791795696793,-0.91849904278403,0.498311947591362,-1.8432209928413,0.0652967510408272,0.486427784821962
"2187","MAPK11",13.6778700862298,-0.932540617274913,0.505952853672954,-1.84313738030165,0.0653089548410357,0.486427784821962
"2188","TLR3",186.406336413158,0.299485931837991,0.162539068253903,1.84254736449062,0.0653951250067413,0.486846979540589
"2189","RUFY3",542.633333705923,0.386787696614838,0.209970777470799,1.84210251194898,0.0654601564527892,0.486850680624715
"2190","LGALS9",45.9070718491132,-0.710786757402712,0.385892236220353,-1.84193070159835,0.0654852870801615,0.486850680624715
"2191","GRAMD1A",183.824348144928,-0.312881956838398,0.169858776976553,-1.84201230226442,0.0654733503935585,0.486850680624715
"2192","PCNP",822.03840234212,-0.265273817677666,0.144035928086946,-1.8417197792313,0.0655161494822085,0.486857919213364

"2193","MBD3",169.859536022979,-0.315725872122719,0.171495180757759,-1
.84101891801081,0.0656187864370031,0.487398272810006
"2194","LINC00899",69.5920593199708,0.519876601842656,0.28245670551285
9,1.84055323062241,0.0656870568548497,0.487682985008499
"2195","GNG10",705.87066646937,-0.404621962320866,0.219984181217302,-1
.83932299168902,0.0658676932881895,0.488356329527227
"2196","ZDHHC13",38.8690460029594,-0.542035132919557,0.294671643733208
,-1.83945467589785,0.065848338502581,0.488356329527227
"2197","STX4",423.739959571195,-0.323560077018216,0.175909378526993,-1
.83935660353985,0.0658627526134435,0.488356329527227
"2198","HAGH",198.475606654474,-0.45463811948393,0.24724560225536,-1.8
3881175372483,0.0659428788667255,0.488691334786211
"2199","AC127502.2",74.7184113903923,-0.583833642698803,0.317550172077
857,-1.83855558596787,0.0659805789523427,0.488748363144479
"2200","ZNF689",77.4459056286962,-0.397420686620056,0.216261839577457,
-1.83768290973829,0.0661091436362222,0.489255720440901
"2201","MRPL4",325.64603773391,-0.44134976883535,0.240140449230181,-1.
83788183227851,0.0660798197582055,0.489255720440901
"2202","SIDT2",251.687966426264,0.336542424076694,0.183155689168495,1.
83746639596376,0.0661410728862583,0.489269725814833
"2203","AGPS",460.643693372094,-0.490680293336873,0.267260563939028,-1
.83596220147472,0.0663632471341097,0.490467755248418
"2204","ATP2C1",189.010489695497,0.365751422170173,0.199209231559804,1
.83601643009386,0.0663552267279711,0.490467755248418
"2205","MVK",42.758178305417,-0.448593428655995,0.244391180301096,-1.8
3555490056276,0.0664235124164251,0.490690518708004
"2206","ATP6V1A",173.780215164583,0.359290293549058,0.19581266599781,1
.83486748274535,0.0665253267523977,0.491219876459568
"2207","SOCS2",94.2223478002544,0.633375214375541,0.345227660285005,1.
83465952250945,0.0665561533225144,0.491224821690275
"2208","NUDT17",17.8914230484993,0.819252513484801,0.446913927232462,1
.83313265388274,0.0667828461036341,0.491251459279799
"2209","AC110772.2",10.0383747604768,-0.956882726638003,0.521678542758
66,-1.83423822950042,0.0666186389081647,0.491251459279799
"2210","GHR",427.647581927096,0.430396965464567,0.234745015913589,1.83
346583010307,0.0667333255829409,0.491251459279799
"2211","CLTB",1023.91518330771,-0.468772978457797,0.255739404661999,-1
.83301036098585,0.0668010302845328,0.491251459279799
"2212","EEF1D",6259.42227329259,-0.393335404616346,0.214463781091509,-
1.83404117289397,0.0666478826459892,0.491251459279799
"2213","CACNA1C",91.8825496055481,0.743255865059451,0.405408016308359,
1.83335265993882,0.0667501428554382,0.491251459279799
"2214","AC009831.1",3.67415726614308,-1.55325352876007,0.8471504222964
39,-1.83350381216778,0.0667276821677088,0.491251459279799
"2215","IGFLR1",28.8808728183388,-0.539730954262004,0.294339461685089,
-1.83370232170723,0.0666981938049039,0.491251459279799
"2216","AL136084.3",2.85069428505562,-1.57175940981628,0.8575893243653
3,-1.83276466387857,0.0668375762151563,0.49129841108695
"2217","PRRC2B",528.460594197668,0.343210941261581,0.187322287177988,1
.83219491087824,0.0669223868145505,0.491699936320349

"2218","NABP2",181.647204875899,-0.382574349816373,0.20890876340029,-1
.83129871427807,0.0670559694585147,0.492070950382881
"2219","PAGR1",257.204081281502,-0.344134203842002,0.187937729536732,-
1.83110759446916,0.0670844852111263,0.492070950382881
"2220","MYBBP1A",52.7405065260322,-0.472347855093334,0.257885637118982
,-1.83161753547137,0.0670084224131884,0.492070950382881
"2221","CFAP410",218.365037507726,-0.383490903756797,0.209438190725199
,-1.83104572489346,0.0670937185094468,0.492070950382881
"2222","TBC1D9",129.64086941387,0.408514577552979,0.223147106308896,1.
83069628062971,0.0671458885513194,0.492231943569956
"2223","MRE11",194.26068573995,0.310633171185537,0.169700904851438,1.8
3047445420209,0.067179023297885,0.492253311065789
"2224","RAP2B",262.815546345812,-0.318855799552541,0.174231202045733,-
1.83007289055404,0.0672390400575956,0.492386985023834
"2225","SPPL3",346.465224939674,0.281208723362272,0.153670339902251,1.
82994794923436,0.0672577224923587,0.492386985023834
"2226","MMS19",93.2106796939752,0.366568994960862,0.200380217656913,1.
82936718627831,0.067344619878535,0.492409612817929
"2227","SLPI",637.925386708223,0.725662501957864,0.396684024289008,1.8
2932121670011,0.0673515020786019,0.492409612817929
"2228","DYRK1A",180.087187073386,0.393238116895362,0.214949237348145,1
.82944643929324,0.067332756105668,0.492409612817929
"2229","FBXO10",26.3224074990506,0.713080221580144,0.389865647531918,1
.82904091728617,0.0673934788051324,0.492495458168148
"2230","CMPK2",16.9668121302831,-0.889588892160464,0.486601398993573,-
1.82816755973242,0.0675244080461406,0.493230978772908
"2231","EXT2",373.904290357936,0.267779492314723,0.146508927950114,1.8
2773497875776,0.0675893358068225,0.493483949330942
"2232","COL15A1",560.034367180443,-0.522170202457133,0.285757577276512
,-1.82731883239568,0.0676518452977003,0.493719044827169
"2233","AC002398.2",10.9885605037227,0.914159198665021,0.5004325604326
97,1.826738048129,0.0677391646045448,0.494134909199924
"2234","ANKRD50",135.703866915654,0.409884713952876,0.224474168338216,
1.82597720257638,0.0678536958875743,0.494748814822157
"2235","KRIT1",326.378376336563,0.286235237093607,0.156836974209677,1.
82504947277888,0.0679935639800537,0.494756389392886
"2236","SGPL1",90.7384588896683,0.439983619325992,0.241091793795583,1.
82496306655317,0.0680066029744411,0.494756389392886
"2237","FOLH1",54.4057759989776,0.880122559781372,0.482179274846897,1.
82530151272228,0.0679555420369052,0.494756389392886
"2238","KCNE3",42.2119366467901,0.595015833736031,0.325977460943704,1.
82532814389517,0.0679515255646093,0.494756389392886
"2239","PAQR4",7.41144235339635,-1.60048584207647,0.876877519555747,-1
.82521025614538,0.0679693066935172,0.494756389392886
"2240","SUPT7L",130.804376767317,0.329687225797794,0.180721729129361,1
.82428105013207,0.0681095937678665,0.494842628405342
"2241","HLA-
DMB",25.5281002653196,-0.81823703292615,0.44848033141847,-1.8244658140
0393,0.0680816800546187,0.494842628405342
"2242","MORC2",68.8414277135662,0.420313237085535,0.230368027562348,1.

82452939122283,0.0680720771260652,0.494842628405342
"2243","EBNA1BP2",315.8424899444,-0.395338272914336,0.216780670920112,-1.82367861136488,0.068200674246843,0.495143806213173
"2244","FAM120AOS",420.054438126682,-0.271210312531894,0.148722088535332,-1.82360478663841,0.0682118424177273,0.495143806213173
"2245","ABCF2",96.6880287764493,-0.384130462564574,0.210668604709715,-1.82338732007019,0.068244749399692,0.495162014686674
"2246","GNG12",1127.15356199662,0.346431683107476,0.190092236183633,1.82243993790896,0.068388259331783,0.495541083743511
"2247","AL161908.1",4.08166966589352,1.50961139085222,0.8282114118681,1.82273676650647,0.068343268921397,0.495541083743511
"2248","NCAPD2",44.5797689797962,0.458192283387022,0.251400567220963,1.82255866982314,0.0683702601748069,0.495541083743511
"2249","WDFY3-AS2",237.100684363481,0.4513298075194,0.247801146730821,1.82133865590891,0.0685553938011529,0.495879396829745
"2250","RASA1",149.941501583583,0.332504340713024,0.182586191938691,1.82108152419693,0.0685944653288283,0.495879396829745
"2251","STARD9",197.559735539948,0.601909487596837,0.330520103172839,1.82109796595967,0.0685919664321043,0.495879396829745
"2252","LRP3",107.56654473474,-0.534929412097016,0.293767260446014,-1.82092930057916,0.0686176045462733,0.495879396829745
"2253","FXYD7",6.68660675196461,-1.13169445484548,0.621159584300546,-1.82190613080504,0.0684692299164464,0.495879396829745
"2254","PNMA6A",32.5408752595682,0.654186946887724,0.359181352350661,1.82132770147002,0.068557057970529,0.495879396829745
"2255","TCP1",674.836050289239,-0.316109392601842,0.17362937751362,-1.82059854805991,0.0686679037096155,0.496022830831897
"2256","SLC11A1",12.6631786647673,-0.915278898615473,0.503001456887247,-1.81963468710319,0.068814655628504,0.496389470573351
"2257","CC2D2B",3.46804589908705,1.95975560410076,1.07685490700981,1.81988826103098,0.0687760229639259,0.496389470573351
"2258","AC092747.4",45.0970411470622,0.558759059902339,0.307100788634991,1.81946475092404,0.0688405558367733,0.496389470573351
"2259","LINC02384",45.2489093783521,0.977757412547114,0.537323226054359,1.81968201845084,0.0688074432181372,0.496389470573351
"2260","PLPP3",6529.79112984845,0.437797925118641,0.240680802904131,1.81899810801706,0.06891171872739,0.49668273732321
"2261","NDST1",169.423173241709,0.491422780375251,0.270245244980035,1.8184326625675,0.0689980299540655,0.497084878337803
"2262","MFSD9",41.6404210772102,0.573681689595483,0.315529469126975,1.8181556581158,0.0690403451136788,0.497169841537009
"2263","INPP5B",70.5528309450179,0.566862753724046,0.311950064982177,1.8171586332461,0.0691928269842278,0.497398649971434
"2264","NFIA-AS2",10.9731220354091,-0.816496942477113,0.449300598703953,-1.81726208429806,0.0691769926524937,0.497398649971434
"2265","KIF5C",6.52749788727499,-1.10144081534704,0.606269738311391,-1.81675044249252,0.0692553341601825,0.497398649971434
"2266","ZNF219",75.2481883095363,-0.444885092155677,0.244824967458235,

-1.81715572874147,0.0691932715938611,0.497398649971434
"2267","C15orf56",3.52649077828955,-1.62824837369503,0.896150274641706,-1.81693675689162,0.0692267976626755,0.497398649971434
"2268","KCTD2",79.379540675689,0.451937871944324,0.2487475040832,1.81685389612256,0.0692394876851445,0.497398649971434
"2269","ZNF544",30.2422173607814,0.713228503491571,0.392667798209662,1.81636616688071,0.0693142215314867,0.497602183572669
"2270","FRMD8",66.7787947738814,-0.415785545126742,0.229025657981482,-1.81545399232238,0.0694541701566083,0.498334387611312
"2271","PUS3",85.9124010002776,-0.519932798050668,0.286416581077461,-1.81530271779221,0.0694774015756209,0.498334387611312
"2272","ZNF117",83.1792948506625,0.574482145043017,0.316626549313787,1.81438399997749,0.0696186273063889,0.498803650274087
"2273","AC079781.5",23.0944794350443,1.02656414069723,0.565882268899723,1.81409490474623,0.0696631158813526,0.498803650274087
"2274","ATP6V0E2",61.351790696854,-0.48159125470606,0.26550311814522,-1.81388172790743,0.0696959364002592,0.498803650274087
"2275","MSC",73.3477803074283,-0.41143752083494,0.226788419461377,-1.81419105001968,0.069648317592001,0.498803650274087
"2276","DUSP6",106.63810895623,-0.620246291639739,0.341906892609205,-1.81407952003083,0.0696654840737216,0.498803650274087
"2277","PDE7A",118.586083839802,0.533342934936509,0.294136044386287,1.81325255818043,0.0697928768481294,0.498839917059754
"2278","MAP1A",856.833558153292,-0.368950012357401,0.203457754052657,-1.81339862948606,0.0697703607912912,0.498839917059754
"2279","KIF1C",83.3641183536692,-0.489420038168033,0.269867548151385,-1.81355647064866,0.069746037181748,0.498839917059754
"2280","AUTS2",312.566536220651,0.437471164503965,0.241348021475404,1.81261549951653,0.0698911455453275,0.499323188503439
"2281","LINC00892",4.29392439553309,1.75267521921131,0.967115693342973,1.81227047733342,0.0699444139077503,0.499484681342983
"2282","IDNK",79.3305073905083,-0.51839051462016,0.286246348009847,-1.81099433485987,0.0701417286102701,0.500674240724229
"2283","CNIH3",41.2174914716279,-0.597352900531502,0.330151394087299,-1.80933023827714,0.0703997139654376,0.50185599158994
"2284","ZNF284",24.5014808292559,0.669134133970937,0.369812486820434,1.80938761620519,0.0703908057094618,0.50185599158994
"2285","REM1",68.4364086301201,-0.738079280045762,0.407887975829795,-1.80951467996632,0.070371081615408,0.50185599158994
"2286","LINC01358",25.6856056296064,-0.665706522938548,0.36836008386558,-1.80721677536992,0.0707284863271221,0.501934135950405
"2287","LINC00622",6.43388176052637,1.17821160316172,0.651747456137329,1.80777322882786,0.0706418019980154,0.501934135950405
"2288","FLG-
AS1",16.4197998584571,0.922623995048413,0.510858982832299,1.80602480538408,0.0709144650192897,0.501934135950405
"2289","ARF1",1423.59254582009,-0.265751349144272,0.147143733164193,-1.80606637761275,0.0709079719154075,0.501934135950405
"2290","TMEM178A",5.70064515381895,1.2616373472346,0.697662013190973,1.808379019325,0.0705475310314196,0.501934135950405

"2291","EPHA3",15.8437626329805,1.20208961058675,0.665668617116937,1.8
0583788941876,0.0709436651669882,0.501934135950405
"2292","SEL1L3",20.7639713178606,0.939184475169108,0.519543819658807,1
.80770984011683,0.070651672292666,0.501934135950405
"2293","PCDHGA12",27.028984367538,0.697177458401731,0.385438019234432,
1.80879265565572,0.0704832217184275,0.501934135950405
"2294","CDCA7L",51.3750299446615,0.494745770238316,0.27374745883422,1.
80730726175592,0.0707143844198656,0.501934135950405
"2295","AC007036.1",3.87473221426127,1.57239641026311,0.87038007856849
3,1.80656295908015,0.0708304492862028,0.501934135950405
"2296","AQP1",67.4733548993697,-0.908532034271019,0.503256799226085,-1
.80530503645092,0.0710269619599536,0.501934135950405
"2297","AVL9",102.832172783735,0.460536014912393,0.255046633265069,1.8
0569337072471,0.0709662487395388,0.501934135950405
"2298","AC018638.7",7.17691631565964,1.04209416507446,0.57718050502056
1,1.80549092703215,0.0709978940172384,0.501934135950405
"2299","ATG101",225.897559384209,-0.407750612975003,0.225681525311384,
-1.80675229136461,0.0708009104359829,0.501934135950405
"2300","GLIPR1",277.869643389153,0.466255574306607,0.257917862275367,1
.80776767531056,0.0706426666943592,0.501934135950405
"2301","ZNF268",101.162189352644,0.372535158871427,0.20623626515586,1.
80635136400424,0.0708634734438091,0.501934135950405
"2302","HAUS4",248.200664034135,-0.3749461973856,0.207550608942274,-1.
80652901620676,0.0708357459829017,0.501934135950405
"2303","AC079414.3",14.7530180869977,0.912923811769046,0.5048441755706
25,1.80832790778891,0.0705554808392012,0.501934135950405
"2304","BCAS3",74.2294017378123,0.394872161265163,0.218562560115537,1.
80667796468172,0.0708125053769783,0.501934135950405
"2305","BGN",1096.25933231534,-0.774597082766971,0.428743747456825,-1.
80666677324542,0.0708142513726742,0.501934135950405
"2306","MYO15A",4.3302029864324,1.32511816568591,0.734099035446212,1.8
0509454678748,0.0710598882706299,0.501949054657541
"2307","C8orf88",57.0643741685838,-0.490354720531839,0.271721248935022
,-1.80462412289699,0.0711335206040323,0.502251372830118
"2308","PRKG2",57.6404558361781,1.03788496262073,0.575816432067171,1.8
0245804881729,0.0714733692949009,0.502601804686434
"2309","SRP19",372.504986964942,-0.41181058414076,0.228478065612481,-1
.80240752230119,0.0714813125642253,0.502601804686434
"2310","AC083862.2",26.0170070218394,0.607686546691296,0.3368955326007
88,1.80378333307075,0.0712652796555309,0.502601804686434
"2311","KBTBD11",21.7908531921271,0.828910841487081,0.459794353561054,
1.80278603916569,0.0714218235469534,0.502601804686434
"2312","AL583785.1",116.944672549951,1.00274015916334,0.55621497617747
2,1.80279244916159,0.071420816479345,0.502601804686434
"2313","GSN-
AS1",7.63894505765305,1.22976417670886,0.681863329060191,1.80353470306
702,0.0713042804333118,0.502601804686434
"2314","CRAT",255.92695180629,-0.287579436678943,0.159508514927678,-1.
80290962403689,0.0714024093105318,0.502601804686434
"2315","Z69720.1",2.78592135490137,2.17067751818493,1.20436532664953,1

.80234142427831,0.0714917049210183,0.502601804686434
"2316","CMIP",96.9513870729079,0.374888455832239,0.207864717591037,1.8
0352134877364,0.0713063757190011,0.502601804686434
"2317","TLE5",1821.28378319295,-0.342950150329563,0.190184039974917,-1
.80325410257766,0.0713483171995048,0.502601804686434
"2318","NFKBIZ",2832.67610804904,0.594455713927716,0.329933823242721,1
.80174226481289,0.0715859651224335,0.502919119309099
"2319","ABO",5.98360154438828,-1.39381485659455,0.773627244269569,-1.8
0166206260037,0.071598590317257,0.502919119309099
"2320","POLH",48.0230465681437,0.525335363114174,0.291644489365926,1.8
0128677986107,0.0716576904593516,0.503114701169497
"2321","GIMAP4",6.91697003762197,-1.26903592894216,0.704592079805941,-
1.80109309388161,0.071688208079956,0.503114701169497
"2322","UTP18",137.349431031147,-0.451265239689054,0.250676264899695,-
1.80019133390879,0.0718304317431323,0.503895737581345
"2323","AL096803.2",20.0372346570646,0.646633934387311,0.3595452683044
56,1.7984771081447,0.0721014331222877,0.505046989074149
"2324","TBC1D5",398.612068194239,0.329050651626287,0.183031465699845,1
.79778187519899,0.0722115806712317,0.505046989074149
"2325","SSBP2",583.237892627319,-0.335812751461953,0.186784210741315,-
1.79786476666935,0.0721984407309603,0.505046989074149
"2326","GEM",482.185357165402,-0.657162607732258,0.365331416593306,-1.
79881219595145,0.0720483934603147,0.505046989074149
"2327","PAOX",50.5269845510288,-0.487724526912163,0.271283423109163,-1
.79784124412167,0.0722021693217952,0.505046989074149
"2328","CYB5R2",38.8970689839845,-0.512533895466702,0.284947476832382,
-1.79869603045545,0.0720667771957999,0.505046989074149
"2329","MATN4",32.7459474666945,1.15963102565774,0.644972633524637,1.7
9795384390281,0.0721843224073828,0.505046989074149
"2330","AAR2",88.3363712083865,0.366867705473942,0.204161955146286,1.7
9694451501052,0.0723444286948878,0.50575896953263
"2331","SLC39A6",380.145881974387,0.341416326995823,0.190021439386109,
1.79672529636033,0.0723792409835557,0.505785266572775
"2332","SLC6A9",9.17705853647607,-0.944218478127901,0.525997170160767,
-1.79510182124993,0.0726374786624805,0.506686030808065
"2333","ANXA11",1228.6798822485,-0.282146558277445,0.157128388847825,-
1.79564342475819,0.0725512449285535,0.506686030808065
"2334","SLF2",156.402374017184,0.371136387415619,0.206768428589209,1.7
9493740871322,0.0726636729061109,0.506686030808065
"2335","LRRN4CL",978.272980884691,0.397456128646421,0.221419835281325,
1.79503398212465,0.072648285870918,0.506686030808065
"2336","SLC9A6",50.1603631731018,0.427475997356942,0.238143631464828,1
.79503434430527,0.0726482281697257,0.506686030808065
"2337","FABP3",40.5575869932217,-0.626334570334638,0.349376012001756,-
1.79272345215129,0.0730171546312527,0.507214936442656
"2338","ODF2L",668.590405668892,0.27522904278839,0.153619140355339,1.7
916324889708,0.0731918553016972,0.507214936442656
"2339","INHBB",415.883029198168,0.829186964710881,0.462784835405033,1.
79173322303262,0.0731757100028688,0.507214936442656
"2340","ARPC4",566.428630068829,-0.330114421977844,0.184034442393466,-

1.79376435021907,0.0728507893186006,0.507214936442656
"2341","GTF2E1",40.1124668494721,0.48056914886431,0.268153223489164,1.79214384452003,0.0731099271872499,0.507214936442656
"2342","ZP3",3.29362921080404,-1.65505439221949,0.923144750233733,-1.79284385444476,0.0729978950124037,0.507214936442656
"2343","LINC01151",9.70752479162021,-1.2997292026215,0.724625258665335,-1.79365704835549,0.0728679248750547,0.507214936442656
"2344","ARHGAP21",1056.15129707529,0.335476730644763,0.187126116757559,1.79278412045181,0.073007449576015,0.507214936442656
"2345","RHNO1",41.1965474324,0.534151604094578,0.297993504652401,1.79249411733872,0.0730538506593887,0.507214936442656
"2346","DLST",253.619477205898,0.305004019370957,0.170197874901628,1.79205539168598,0.0731240935111104,0.507214936442656
"2347","UBR1",155.86502220694,0.426394756345834,0.237787881407772,1.79317277996446,0.0729453010891382,0.507214936442656
"2348","TLCD2",153.615049832531,0.47722425404436,0.266376480109128,1.79154050631217,0.0732066005019759,0.507214936442656
"2349","ZNF431",157.763992237407,0.391636696940173,0.218553490103847,1.79194894922101,0.0731411439741833,0.507214936442656
"2350","TCEANC",19.7849337163131,0.620878433337922,0.34617935090636,1.79351666040262,0.0728903490906691,0.507214936442656
"2351","PGK1",839.089244750019,-0.421937660564677,0.235419107889398,-1.7922829813921,0.0730876480134871,0.507214936442656
"2352","NUP153",154.800360026778,0.393851202973966,0.219981407732028,1.7903840466997,0.0733921931840862,0.50788043535904
"2353","UTRN",669.697278148983,0.484617183390795,0.270681544025221,1.7903591659195,0.0733961903637535,0.50788043535904
"2354","C1RL",400.900097114683,0.399396762779098,0.223066507792803,1.79048287764508,0.0733763174239679,0.50788043535904
"2355","ALG3",170.191093281259,-0.454144819648922,0.253700264450687,-1.79008413977115,0.0734403860880566,0.507970466661722
"2356","TAX1BP3",1000.14750274903,-0.457410864118675,0.25556420796254,-1.78980800075776,0.0734847825418948,0.50806180934844
"2357","CEP63",240.829769281929,-0.287323856513377,0.160581490314959,-1.78927132853127,0.0735711291995854,0.508442988346223
"2358","LRRC36",10.7112935960847,-1.04514302278673,0.584259265080969,-1.78883431594686,0.0736415026210907,0.508569488861658
"2359","TMEM35A",38.6956391472719,0.648207631352022,0.362376191456963,1.78876992096487,0.0736518769859813,0.508569488861658
"2360","FAP",283.490358855778,-0.777655194839174,0.434866365014129,-1.78826245808619,0.0737336737066866,0.508918563986533
"2361","GNA12",247.306291367831,0.289427274815433,0.161873343117173,1.78798602192289,0.0737782630292379,0.509010642305487
"2362","MLH3",236.64486700926,0.341908016011672,0.191270360123317,1.78756403130749,0.0738463728876304,0.509264846725915
"2363","AL132800.1",5.7340515683831,1.40732239998701,0.787606216173029,1.78683505930817,0.0739641510541176,0.50986121731719
"2364","TRRAP",137.687429625404,0.36573111000446,0.204749082639414,1.78624053055492,0.0740603212132728,0.510308194688241
"2365","CIC",136.306740855797,-0.336883667880802,0.188639408824523,-1.

78586049426279,0.0741218488959191,0.510516193093288
"2366","FNDC10",31.8150070959766,0.980507527373008,0.549306723201845,1.78499094578297,0.0742627855184353,0.511054716227204
"2367","IQCE",66.7330406447361,-0.439473263939465,0.246203175815149,-1.78500241714763,0.0742609248124618,0.511054716227204
"2368","ID4",809.659109429706,-0.499440091259484,0.279834966893676,-1.7847656010343,0.0742991895281492,0.511089315128388
"2369","AC007098.1",21.7402038655394,0.792430333514022,0.444250308839807,1.78374740038676,0.074464720118825,0.511173454559136
"2370","ABCF3",113.971699767001,-0.353907282586248,0.19837933549959,-1.78399268096641,0.0744248544600833,0.511173454559136
"2371","ZSWIM8",160.079535486899,0.393444472308084,0.220592738939494,1.78357852665314,0.0744921774488325,0.511173454559136
"2372","HEXD-IT1",7.96098020605162,1.45373682820685,0.814758226012317,1.78425547824401,0.0743821611508606,0.511173454559136
"2373","YIPF2",293.362783131485,-0.393674806002892,0.220727612769819,-1.78353220543108,0.0744997103028663,0.511173454559136
"2374","MED29",370.085504157658,0.329120203816299,0.184514134385152,1.78371269449363,0.0744703623055627,0.511173454559136
"2375","FHAD1",4.99730433543396,-1.30970966903871,0.734958337428177,-1.78201892861267,0.0747461450278437,0.511214538867736
"2376","C1orf220",15.4881915576244,0.713766374405502,0.400882665243237,1.78048700103414,0.0749962948117713,0.511214538867736
"2377","LYST",259.595528766161,0.356186360007024,0.199736844978348,1.78327819309269,0.0745410293855202,0.511214538867736
"2378","PIGX",141.608453050122,-0.376688093470137,0.211451024617766,-1.78144368962537,0.0748399962732315,0.511214538867736
"2379","CLINT1",618.901472957279,0.241721106038831,0.135687078089427,1.78146002878418,0.0748373291834948,0.511214538867736
"2380","AKR1B1",426.12829719918,-0.343959493021102,0.19296196787373,-1.78252479911576,0.0746636908362278,0.511214538867736
"2381","SDC2",1485.63785278886,-0.298731143849149,0.167679436804075,-1.78156099246803,0.0748208503014522,0.511214538867736
"2382","PTBP3",237.553309514943,0.3949987851762,0.221619156650347,1.78233141550754,0.0746952025548198,0.511214538867736
"2383","VWF",3.64848297526499,-1.68644937757529,0.946958187819281,-1.78091218732577,0.0749267973279785,0.511214538867736
"2384","MLF2",594.528900268885,-0.353508400799564,0.198276213191008,-1.78290877715631,0.0746011540819538,0.511214538867736
"2385","LINC00377",33.4136257047149,1.22314276142281,0.686894720501141,1.78068447014768,0.0749640115767806,0.511214538867736
"2386","LINC02328",32.9616307882315,0.719404219521208,0.403898048403862,1.78115299730755,0.0748874598276678,0.511214538867736
"2387","KIAA0513",26.4748504812617,0.70727393353457,0.396853529494895,1.78220396435624,0.0747159765662221,0.511214538867736
"2388","PTRH2",110.688549335893,-0.41218855545858,0.231275225539828,-1.78224258346944,0.074709681311928,0.511214538867736
"2389","AXIN2",21.7256889273732,0.937604814193792,0.526456865720363,1.78097176662488,0.0749170631843335,0.511214538867736

"2390","AC010331.1",5.22821595514072,-1.20376733234563,0.676115623270794,-1.78041638281075,0.0750078425866468,0.511214538867736
"2391","AL596325.2",21.6233070909091,1.04103043393468,0.584795801929535,1.7801605799833,0.0750496846467943,0.511285785533933
"2392","AC079610.1",4.64768092583659,-1.21755408872613,0.684144665681329,-1.77967343721607,0.0751294200447118,0.511312010972257
"2393","MRPS30-DT",52.1457549971807,0.598884223235747,0.336468904788994,1.77990956879453,0.0750907614570503,0.511312010972257
"2394","FGD2",5.94621010364741,-1.27238117634934,0.714996907538916,-1.77956178961527,0.0751477042339974,0.511312010972257
"2395","OTUD4",138.961830642935,0.34509524309389,0.194003762351694,1.77880696183765,0.0752714154308475,0.51166211339263
"2396","IRF7",141.328992510358,-0.602329333605932,0.338639729928875,-1.7786729682676,0.0752933934435591,0.51166211339263
"2397","PROCR",1178.40196546588,0.673680505342418,0.378688709612022,1.77898228345024,0.0752426665832066,0.51166211339263
"2398","METTL7A",1975.34722465172,0.416509052141381,0.234322725339562,1.77750174054953,0.0754857247155568,0.512541463064487
"2399","LINC01578",1186.69612789082,-0.355394218108754,0.199923348908526,-1.77765238552183,0.0754609643445079,0.512541463064487
"2400","RGS18",2.88677655271286,-1.53556679804365,0.864547496150575,-1.77615088226015,0.0757080515174994,0.512805421207259
"2401","WDR26",262.961369504945,0.354961223613965,0.200002698973652,1.77478216761828,0.0759338618670213,0.512805421207259
"2402","EXOC6B",102.084151653161,0.417728342556815,0.235123688860944,1.77663231034057,0.0756287559706867,0.512805421207259
"2403","VDAC1",726.262125441065,-0.367509193323708,0.207035761326369,-1.77510006469062,0.075881366320807,0.512805421207259
"2404","PANK3",159.048876364704,0.446911703777265,0.251713661987741,1.77547654842442,0.0758192344411674,0.512805421207259
"2405","SOBP",375.029194093437,-0.277022441436621,0.156082719584759,-1.77484376344549,0.0759236880013368,0.512805421207259
"2406","PENK",16.1087357890185,1.0846178111883,0.610808119764126,1.77570954951801,0.075780802594295,0.512805421207259
"2407","USP3-AS1",5.00150142248964,1.3153482094335,0.740729160188533,1.77574784432506,0.0757744876638229,0.512805421207259
"2408","AC243829.1",11.4734733418759,-1.10490538356242,0.622326710285387,-1.77544264981931,0.0758248270961109,0.512805421207259
"2409","AC091271.1",77.9492331218958,-0.591146516075405,0.33285943491688,-1.77596442841713,0.075738780368057,0.512805421207259
"2410","SELENOW",2076.23614295152,-0.474568958698695,0.267345415604562,-1.77511537882753,0.0758788381872186,0.512805421207259
"2411","ATF5",151.609768231428,-0.455687155699667,0.256584709369956,-1.77597159557406,0.0757375989842013,0.512805421207259
"2412","AC011043.1",23.6791930776221,0.77981571828967,0.438925963037704,1.77664522939757,0.0756266290196924,0.512805421207259
"2413","CCND2",330.919294440568,-0.504231996526048,0.284166151644425,-1.77442666414749,0.075992602591219,0.512989433737409

"2414","ZNF619",26.0500177349003,0.608149239968295,0.342794963954717,1
.77409035696519,0.0760482055826025,0.513152121265539
"2415","NFATC4",238.686812633855,0.495511776891017,0.279343672448644,1
.77384285295424,0.0760891475929123,0.513215786807846
"2416","REPIN1",170.389805807426,-0.335306229820744,0.189149382287174,
-1.77270592040141,0.0762774495651141,0.514272920515788
"2417","ATM",352.683595314359,0.394505461090582,0.222582289284376,1.77
240274758138,0.0763277260378147,0.514398977836145
"2418","EIF1B-
AS1",33.1973110870466,0.648160827155812,0.36579610466224,1.77191834165
182,0.0764081132750707,0.514672675832096
"2419","AC008883.3",4.46609372424438,1.33849041274072,0.75553182377165
5,1.77158707367071,0.0764631269883769,0.514672675832096
"2420","ZNF217",280.52609355541,0.270481516932787,0.152675014205104,1.
77161612422987,0.0764583012668355,0.514672675832096
"2421","ARMC9",42.0405892968598,0.550200228236582,0.310661371699228,1.
77106096334779,0.0765505645723559,0.515048387575012
"2422","SLIT3",5176.2668911386,0.528005029103605,0.298234900881956,1.7
7043339844586,0.0766549701383457,0.515325137673757
"2423","FDXR",44.8937875539787,0.657263762441476,0.371232751885676,1.7
7048969710487,0.0766455992085948,0.515325137673757
"2424","PHC3",268.251632368227,0.386943377022585,0.218644903358377,1.7
6973426354399,0.0767714192098201,0.51546976401845
"2425","AC129507.1",7.23822063723125,1.49397937735392,0.84410559294985
6,1.76989631372182,0.0767444150317143,0.51546976401845
"2426","ARSA",247.722828653245,0.571216929258718,0.322723818460491,1.7
6998689462596,0.0767293239277794,0.51546976401845
"2427","KDM2A",288.55227723195,0.419285851286023,0.237002474945217,1.7
6912013844134,0.076873827844149,0.515944697879416
"2428","MMAA",50.4451649360514,0.445558948074618,0.251903996356865,1.7
6876490456074,0.0769331158028177,0.515956748299528
"2429","HIST1H1A",5.27428903057213,-1.27691925469083,0.721948530119078
,-1.76871231316201,0.0769418963883822,0.515956748299528
"2430","CORO6",26.3728148213244,0.54657027289785,0.309051664831453,1.7
6854013453036,0.0769706488039691,0.515956748299528
"2431","HIST2H2BE",17.4270424016819,0.875543144229185,0.49535125255345
8,1.76751979472324,0.0771412169255012,0.516256331182503
"2432","AL358472.4",18.6229399944942,-0.8188548034799,0.46353845795188
6,-1.76653045595818,0.0773068966647073,0.516256331182503
"2433","JAGN1",410.88238927198,-0.380532683132391,0.215430930081778,-1
.76637905702742,0.0773322762652898,0.516256331182503
"2434","RBMS3-
AS3",23.6331824316738,0.743801120757851,0.42089335406471,1.76719616400
38,0.0771953819098233,0.516256331182503
"2435","ACTRT3",15.6566685886829,-0.675539848212654,0.382401338518928,
-1.76657291741989,0.0772997799009206,0.516256331182503
"2436","PAM",1755.03486467026,0.429886344518965,0.243178892039667,1.76
777820193721,0.077097990420362,0.516256331182503
"2437","ZNF300",17.8938258412494,-0.62103560520359,0.351512241514983,-
1.76675384768106,0.0772694610228769,0.516256331182503

"2438","CNTN1",123.081109230228,1.47753772709262,0.83624710782918,1.76
686736881897,0.0772504429859183,0.516256331182503
"2439","SPRYD3",303.275687704095,-0.312126933714432,0.176637340982361,
-1.76704954897165,0.0772199305724451,0.516256331182503
"2440","RNF213",603.681364936972,0.415459748230654,0.235203865538705,1
.76638146349804,0.0773318728060298,0.516256331182503
"2441","FAM172A",309.697943837027,0.27656635647324,0.1566996973815,1.7
6494505793405,0.0775730000048507,0.517428961247253
"2442","CCZ1",276.942276343845,-0.572379186433491,0.324332807136966,-1
.76478966616465,0.0775991220531252,0.517428961247253
"2443","RNF6",144.603253424079,-0.294768362145847,0.167029785535441,-1
.76476525549572,0.0776032262463651,0.517428961247253
"2444","SORT1",35.7069692857694,-0.69532006183374,0.394093111762011,-1
.76435477069094,0.0776722680074152,0.517677403262188
"2445","PTAR1",166.97865944815,0.374782566778631,0.212485827217218,1.7
6380030464574,0.0777656062082439,0.518087509008624
"2446","ZNF514",70.3578882991783,0.364292876162807,0.20658694078019,1.
76338772812565,0.0778351181406298,0.518338609727195
"2447","CSNK1G1",164.99648519295,0.430239311341941,0.244024419317883,1
.76309941662634,0.0778837236165265,0.518450336734614
"2448","KCNK6",32.098337285437,-0.650251685855545,0.368859544803196,-1
.76287070516905,0.0779222988976807,0.518495231513203
"2449","RAB4A",574.341989428701,-0.330266043346163,0.187385604491606,-
1.76249421209385,0.0779858334309653,0.518706100758266
"2450","GRAMD4",58.3782311433631,0.547747309310934,0.310826303549413,1
.76222958950402,0.0780305146626379,0.518791450342738
"2451","ZNF280D",489.496148827323,0.274150428742229,0.155633314286053,
1.76151507149904,0.0781512643657802,0.519382270605546
"2452","AC046134.2",26.2124294311534,0.598999476462048,0.3400996958658
06,1.7612467277783,0.0781966522869299,0.519471969454242
"2453","COL5A2",2101.88579308568,0.6322789507247,0.359092156128732,1.7
607707100626,0.0782772191860713,0.519795199071307
"2454","PCED1B",16.7915698113657,0.741905979108667,0.421461907014995,1
.76031562226636,0.0783543068220778,0.519883219480581
"2455","CHERP",128.921301866687,0.395363311952315,0.224575094478881,1.
76049491538562,0.0783239288634826,0.519883219480581
"2456","SLC25A46",179.941664250746,0.311642142146272,0.177112987683439
,1.75956685177307,0.0784812760346688,0.520265673378968
"2457","VASH1",30.8452555864419,0.620886801629398,0.352894741615481,1.
75941074890236,0.0785077675219782,0.520265673378968
"2458","MIR503HG",16.8635408802757,0.981226793272907,0.557659269936928
,1.75954538222575,0.078484919099486,0.520265673378968
"2459","CPT1A",213.347862435125,0.415435066761656,0.236204251487855,1.
75879589018751,0.0786121831069234,0.52047028973293
"2460","MPRIP-
AS1",4.7078915671035,-1.40442767012888,0.798490243831693,-1.7588538882
9486,0.0786023290175862,0.52047028973293
"2461","MX2",518.530635286822,0.655280546361412,0.372601212475296,1.75
866455723049,0.078634500769399,0.52047028973293
"2462","MEG9",14.8207660580298,1.38058100171639,0.785102273573478,1.75

847281072379,0.0786670938809695,0.520474529742938
"2463","WDFY1",238.765695479413,0.354841185106018,0.201825713400425,1.75815647633564,0.0787208884926602,0.520618981996323
"2464","APOO",34.955432951937,-0.595861416132139,0.338996629491335,-1.75772076856997,0.0787950322829724,0.520897841257036
"2465","SMARCA2",691.385520445016,0.283444890030012,0.161300176299331,1.75725096235489,0.0788750422075556,0.521215238344371
"2466","ODF3B",196.33574823383,-0.680733606857368,0.387480191237707,-1.75682169631159,0.0789482057473285,0.521487154670817
"2467","BMPR2",516.936126569527,0.383560946456985,0.218437848889333,1.75592713628722,0.0791008507797353,0.521516429054865
"2468","SEMA3D",527.539335018846,0.47397983449093,0.26989583874013,1.7561583635504,0.0790613718686626,0.521516429054865
"2469","RSU1",375.676155944526,0.353089625301474,0.201012483493716,1.75655570820562,0.0789935681056175,0.521516429054865
"2470","CEP295",88.047649960888,0.519855186515938,0.296001984236743,1.75625574894848,0.0790447494331737,0.521516429054865
"2471","PEG3",106.805369707806,0.444683258091364,0.25325701171535,1.75585763679139,0.0791127200070337,0.521516429054865
"2472","MINDY1",95.5826916449159,0.384994480305279,0.21932681196765,1.7553461742838,0.0792001128969634,0.521881326447669
"2473","HCST",43.7301358068531,-0.782220027958085,0.445675139952291,-1.75513498025001,0.0792362222244662,0.521908137409757
"2474","ANXA4",1188.13816747061,-0.360333082189256,0.205461335910435,-1.75377562202912,0.0794689617882597,0.522638158740322
"2475","CD8B",7.93436695746187,-0.976299568053038,0.556696335432264,-1.75373809007555,0.0794753956166601,0.522638158740322
"2476","MTREX",308.365992474344,0.278300572184609,0.158656206967274,1.75411083817234,0.0794115169152713,0.522638158740322
"2477","AL080317.1",4.60449616690647,1.32089407658766,0.752969822616962,1.75424570402684,0.0793884149299242,0.522638158740322
"2478","RXRA",445.748416771333,0.328482020102352,0.187380791347412,1.75301864049305,0.0795988074865347,0.523238488760356
"2479","RCOR3",222.589923574471,0.28799426032385,0.164400232305911,1.75178742927783,0.0798103665610209,0.52359979747838
"2480","TMEM167A",554.280385975014,-0.302443948527909,0.172597970292968,-1.75230304281412,0.0797217131118145,0.52359979747838
"2481","ADHFE1",18.5384692432757,0.712926313308448,0.406910546476467,1.75204678149004,0.0797657641056054,0.52359979747838
"2482","MAFTRR",6.83145669118383,1.03018176992396,0.588014178135035,1.75196756852244,0.0797793847157178,0.52359979747838
"2483","CXCL16",100.079098997265,-0.538882317654832,0.307622768752111,-1.75176343363932,0.0798144942684522,0.52359979747838
"2484","ZNF672",64.5304049132675,-0.380117763840775,0.217109715439519,-1.75080955300071,0.079978720523622,0.523622740598585
"2485","RN7SL832P",59.8269616703038,-0.629812752781678,0.359690177661382,-1.75098679890723,0.079948183968041,0.523622740598585
"2486","CCDC36",51.3070227339777,-0.510989902395714,0.291782437494973,-1.75127025047393,0.0798993696160763,0.523622740598585
"2487","FAM225B",18.3527695332083,0.867372597088358,0.495399948207598,

1.75085322521044,0.0799711956400539,0.523622740598585
"2488","SSBP4",559.322462214151,-0.410380989560062,0.234391868034252,-1.75083288085785,0.0799747009758076,0.523622740598585
"2489","CCT3",621.720253243037,-0.411687519502118,0.23520531800974,-1.75033253068313,0.0800609507093681,0.523950512697829
"2490","SLC39A1",747.019724895939,-0.335746761546603,0.191858568510942,-1.74997011680224,0.0801234703468278,0.524149079710634
"2491","PCCA-DT",42.781970588817,-0.445997399311174,0.254892558879097,-1.74974664334052,0.080162041286859,0.524190883388858
"2492","AL031280.1",8.8418011895417,1.07145574108265,0.612537895853451,1.74920727082491,0.0802551977348662,0.524398334004712
"2493","METTL14",149.241049768581,0.293915887718726,0.16807380634767,1.74873107300697,0.0803375161991968,0.524398334004712
"2494","TNKS2",253.149278913154,0.317050024854201,0.181299127497341,1.74876751604252,0.0803312140105855,0.524398334004712
"2495","AC083805.3",2.86395196766155,-1.64217594205763,0.939120629669597,-1.74863152844953,0.0803547327445369,0.524398334004712
"2496","SLC23A2",105.166630314985,0.390719064558174,0.223394484575809,1.74900944980843,0.0802893859598947,0.524398334004712
"2497","DUSP23",179.58390222872,-0.535723645913294,0.306424952319879,-1.74830294288191,0.0804115839317549,0.524559187290491
"2498","CALR",2178.95211835591,-0.386681604057789,0.221219277406209,-1.74795618443213,0.0804716147819143,0.524740645789673
"2499","PIK3AP1",6.61901659122535,-0.992771873483976,0.5680620220356,-1.74764697344572,0.0805251761103717,0.52487978938049
"2500","DCAF5",355.577535086137,0.397446127871982,0.227764206646975,1.74498940690891,0.0809867129086973,0.527466040211824
"2501","ZNF681",29.2837159833035,0.626284811031044,0.358884974041284,1.74508507274255,0.0809700615405598,0.527466040211824
"2502","NBEAL1",502.549295104473,0.401584359883019,0.230244077535349,1.74416803325316,0.0811297936904536,0.527787738764122
"2503","PRIMPOL",90.1504956312757,0.330778343844569,0.189644482449415,1.74420230724508,0.0811238191659911,0.527787738764122
"2504","ZNF32",334.952791464724,-0.365730244817483,0.209689931438206,-1.74414785826405,0.0811333106922071,0.527787738764122
"2505","BRMS1L",127.104017046775,-0.341149468112603,0.195633785445499,-1.74381673050866,0.0811910521720866,0.527952514503441
"2506","PPIP5K2",244.735046834694,0.291889748743054,0.167410766697657,1.74355421996368,0.0812368520024629,0.528039538016009
"2507","STARD13",50.9952386907914,0.651852099324868,0.373917964989873,1.74330243625102,0.0812808000378042,0.528114460237652
"2508","THBS2",1494.4474673737,0.470845357896314,0.270117655132058,1.74311211781445,0.0813140323112765,0.528119725804778
"2509","ANKIB1",385.824448207077,0.443648380551579,0.254553115670921,1.74285189706778,0.0813594883560648,0.528204346684711
"2510","PTGFR",1187.09772807277,-0.609663948199528,0.350025793249932,-1.74176863521656,0.081548937044821,0.528329466827216
"2511","CLK4",211.180425404627,0.295397463891627,0.16954113847284,1.74233502589667,0.081449837933676,0.528329466827216

"2512","FAM131B",5.941984933254,1.3860775463395,0.795500520327125,1.74
239678155021,0.0814390387051054,0.528329466827216
"2513","HMCN2",182.286021920051,-1.22614754248231,0.703856173010147,-1
.74204274893052,0.0815009642551446,0.528329466827216
"2514","SSRP1",274.817292658688,-0.282632616269409,0.162261235970708,-
1.74183694940196,0.0815369791991842,0.528329466827216
"2515","MBTPS1",558.420094736315,0.222298124728965,0.127638041300673,1
.74162908223657,0.0815733690877555,0.528329466827216
"2516","MGA",174.264153209751,0.408023885093442,0.234339547940594,1.74
116528208408,0.0816546109245676,0.528645452047012
"2517","OR2L2",2.50241312306317,2.27056874339446,1.30426087882124,1.74
088541660971,0.0817036654802246,0.52875288319721
"2518","GNA15",5.19376185411729,-1.51080592974117,0.868343563363331,-1
.73987116791585,0.0818816423948072,0.529694230726376
"2519","ZNF511",365.072423606914,-0.369803682373522,0.212657119829779,
-1.73896685269476,0.082040593631097,0.530163475656611
"2520","DICER1",411.284929355854,0.386616311854637,0.222303619946606,1
.73913637550075,0.0820107776103516,0.530163475656611
"2521","KSR1",61.3679592315518,0.616129266137746,0.354320658698562,1.7
3890302755933,0.0820518216053974,0.530163475656611
"2522","EPHA2",47.6607046985153,0.595873783642292,0.342843316889325,1.
7380352898483,0.0822045957567195,0.530243576119443
"2523","WDR46",258.153659392564,-0.333068470519376,0.191641743955426,-
1.7379745333399,0.082215301199916,0.530243576119443
"2524","RBM33",292.327658724862,0.384745774610849,0.221384398214842,1.
73790826143707,0.0822269797579785,0.530243576119443
"2525","TANC2",92.0748980210269,0.636313381413524,0.366130158122469,1.
73794309836853,0.0822208405613835,0.530243576119443
"2526","PKIG",546.168216415587,-0.379814917362724,0.218485419990701,-1
.73839937410418,0.0821404669141396,0.530243576119443
"2527","EXOC7",388.792735275039,0.252044550232252,0.145061086132936,1.
73750629442602,0.0822978439595917,0.530490534332327
"2528","MDGA1",68.5677421300251,0.472183971349239,0.271833016380895,1.
73703686783805,0.0823806635238551,0.530814330751612
"2529","TMCC1",119.688760079633,0.325694390629311,0.187575352074471,1.
73633895406474,0.0825039193150804,0.530830502948144
"2530","COQ2",68.6152160079357,-0.505365457663738,0.290984203995772,-1
.73674533092896,0.0824321325361988,0.530830502948144
"2531","AC100793.4",54.7302918411186,-0.558785948535209,0.321783396875
543,-1.73652821730679,0.0824704795182454,0.530830502948144
"2532","TST",90.9950730657589,0.506879878899119,0.291933639028639,1.73
628459051748,0.082513526518798,0.530830502948144
"2533","BEST4",2.60102501704146,-1.6826736072164,0.969551055553302,-1.
73551830775547,0.0826490416114076,0.531486678377067
"2534","LAT2",4.65464968783612,-1.2305311421013,0.709251928448539,-1.7
3497045653868,0.0827460382076396,0.531486678377067
"2535","OSGIN2",158.147587965963,-0.475984448697183,0.274343705480555,
-1.73499314614645,0.0827420192011347,0.531486678377067
"2536","THRA",483.572549794829,0.290468780064322,0.167403430497701,1.7
351423396805,0.0827155965150557,0.531486678377067

"2537","MAN2B2",298.248054865084,0.366286279955133,0.211241875842231,1
.73396623417934,0.0829240744717317,0.532340215718407
"2538","SLC22A16",2.43314007461069,2.36265975615872,1.36266491532725,1
.73385234299608,0.0829442855603976,0.532340215718407
"2539","TIAM2",52.789405175861,0.517453927060949,0.298500361344384,1.7
3351189502902,0.083004725135828,0.532518301590194
"2540","ATF2",232.741875151019,0.280337263711078,0.161823513730908,1.7
3236421115688,0.083208735903594,0.53270431585028
"2541","RC3H2",183.409850556722,0.363082141273634,0.209529922792559,1.
7328414788426,0.0831238481467109,0.53270431585028
"2542","TBC1D13",95.1681015159769,0.41625969472158,0.240300696785336,1
.73224505917031,0.0832299394584667,0.53270431585028
"2543","HIF1AN",90.9042821908972,0.422340364922924,0.243786043049085,1
.73242224879087,0.0831984094681205,0.53270431585028
"2544","KANSL1",417.865669393821,0.376556065332881,0.217308446474967,1
.73281835768983,0.0831279589020301,0.53270431585028
"2545","ASPSCR1",113.807354846256,-0.346351234671709,0.199925123463273
,-1.73240475570012,0.0832015218439425,0.53270431585028
"2546","ZBTB44",474.040223569159,0.318308352958386,0.183821821355433,1
.73161352994601,0.0833423956073798,0.533005214781551
"2547","NAE1",323.05422738895,-0.330585849945336,0.190908276589195,-1.
73164755269728,0.0833363340549875,0.533005214781551
"2548","AGO3",354.056441618953,0.410110026671069,0.236892209280273,1.7
3120943030193,0.0834144180155726,0.533256458028125
"2549","DLG5",128.079310655466,0.434622511312835,0.251160723611919,1.7
3045572198777,0.0835488856364632,0.533798087774288
"2550","ACSM5",13.0849487916304,-1.0488917677628,0.606167144108119,-1.
7303672393958,0.0835646831496369,0.533798087774288
"2551","SPATS2L",552.701772245322,-0.312945699400832,0.180906954931479
,-1.72987102413705,0.0836533213025535,0.534154821911915
"2552","ANKRA2",115.790712179299,0.398944474694136,0.230691393431693,1
.72934268920727,0.083747780611678,0.534412812663314
"2553","EHD1",150.262396397381,0.386343306283785,0.223436754914112,1.7
2909468915396,0.0837921495206644,0.534412812663314
"2554","CLDN7",6.56680753760178,-1.07919259515936,0.624073298087184,-1
.72927218400008,0.0837603925406583,0.534412812663314
"2555","ECM1",1538.47103922941,-0.560097518931203,0.324331987859439,-1
.72692654408773,0.0841808565661911,0.534797961235057
"2556","CSRP1",574.431154258651,-0.39342021614234,0.2276741587889,-1.7
2799679258778,0.0839887994173808,0.534797961235057
"2557","RPL22L1",761.752522232306,-0.417140035351582,0.241508244993548
,-1.72722896215293,0.0841265513516601,0.534797961235057
"2558","PCDHGB3",3.30647041740581,1.56244399272064,0.904522297607787,1
.72736923882681,0.084101371496609,0.534797961235057
"2559","BBS10",73.8273569934968,0.443266977383303,0.256675153608439,1.
72695709402209,0.0841753694262532,0.534797961235057
"2560","AC132938.3",11.498088842326,-0.84030997778918,0.48623552675786
3,-1.72819535296449,0.0839532066002709,0.534797961235057
"2561","PSMC4",372.057810600899,-0.509131908385153,0.294786737113499,-
1.72711945378033,0.0841462124953103,0.534797961235057

"2562","ZNF787",138.707339042339,-0.441055241738201,0.255175374512167,
-1.72843967636528,0.0839094273180273,0.534797961235057
"2563","TP53TG5",3.6656177293516,-1.40741530724663,0.814355479902727,-
1.72825669130972,0.0839422139022377,0.534797961235057
"2564","MT-
ND3",69155.8526199325,-0.681315013631942,0.394505921014506,-1.72700833
457678,0.0841661666491071,0.534797961235057
"2565","AKNAD1",23.3392367256958,-0.639027967081775,0.37037708737874,-
1.72534422041156,0.0844654571968747,0.53485047204193
"2566","KDM3B",155.669488879231,0.324426323892843,0.188030382194529,1.
72539309927692,0.0844566540853328,0.53485047204193
"2567","MLLT3",137.376504809189,0.334728815360353,0.194039448168596,1.
72505549010589,0.0845174728366338,0.53485047204193
"2568","NCS1",76.0560389436587,-0.436566794327049,0.252993361606625,-1
.72560572955214,0.0844183678899675,0.53485047204193
"2569","PLAAT3",779.661911138693,-0.426884692212345,0.247426532248197,
-1.72529877185576,0.0844736431738011,0.53485047204193
"2570","BHLHE41",171.431308086083,-0.706313160214518,0.40941518457597,
-1.72517577955992,0.0844957991781803,0.53485047204193
"2571","C16orf91",107.725452052661,-0.394078596532275,0.22831797160365
7,-1.7260077853896,0.0843460121231773,0.53485047204193
"2572","AC116407.2",22.7632491305151,-0.790815265827781,0.458221285635
376,-1.72583703686141,0.0843767346587769,0.53485047204193
"2573","DACT3",73.9038918029879,-0.640231757026906,0.3710245923507,-1.
72557768467742,0.0844234168443725,0.53485047204193
"2574","AHCY",169.435229599534,-0.288431344729661,0.167110239631419,-1
.72599444154846,0.0843484127350849,0.53485047204193
"2575","MAP2K4",106.692203650049,0.318173245127401,0.184467136457838,1
.72482346306776,0.0845592919905148,0.534907303779998
"2576","CADM3",143.522266379207,-1.20910093047044,0.701181894447297,-1
.72437557222368,0.0846400644829033,0.535002720357785
"2577","ART3",6.30878171712368,1.46519853132328,0.849666651314297,1.72
443925986368,0.0846285752688291,0.535002720357785
"2578","NDC1",41.3135254942809,0.425516544068506,0.247002058484394,1.7
2272468771911,0.0849383236606556,0.536159508235339
"2579","AHCTF1",266.346739980438,-0.429166431062268,0.249149705407339,
-1.72252433676619,0.0849745780927649,0.536159508235339
"2580","AC072022.2",32.0872578924943,0.624964249195728,0.3628986463529
37,1.72214544054242,0.0850431753311027,0.536159508235339
"2581","RADIL",68.7709629779097,0.366680638509911,0.212920454883008,1.
72214848362684,0.0850426242177774,0.536159508235339
"2582","ORAI2",128.925202434955,-0.4514417141033,0.262179084146133,-1.
72188302348206,0.0850907108509073,0.536159508235339
"2583","ARL2",1185.09791097392,-0.459999980436804,0.267173869893785,-1
.7217251845013,0.0851193129287609,0.536159508235339
"2584","BRMS1",173.454334661937,-0.408979422462794,0.237508329789153,-
1.72195822700561,0.0850770859180369,0.536159508235339
"2585","SULT1A2",14.3798455330795,1.18394797571371,0.687629801308015,1
.72178107095067,0.0851091848294233,0.536159508235339
"2586","CA11",48.8746484154135,-0.626797424658716,0.363836572114846,-1

.72274442070344,0.0849347535626706,0.536159508235339
"2587","ALDOC",22.671922438529,-0.625761812103124,0.363491873327899,-1
.72152903000017,0.085154869014226,0.536176135049373
"2588","DHRS7",861.879928302218,-0.352720733744841,0.204909955610913,-
1.72134503027561,0.0851882327710719,0.536178950389486
"2589","AC022467.1",10.4621265505365,1.11239940325752,0.64644483023276
9,1.72079557486286,0.0852879257056769,0.536599081429035
"2590","ZNF862",31.0160160011675,0.593838774313289,0.345172895037279,1
.72040963485604,0.0853580068609965,0.536832653960916
"2591","AC245407.2",63.2974959044989,-0.546074519280608,0.317571626296
736,-1.71953182860978,0.0855175772195259,0.537628643507857
"2592","FAM181B",3.2056623496876,1.94322694625218,1.13037556526537,1.7
1909850669498,0.0855964366766023,0.537916804407861
"2593","LYRM7",195.635599052445,0.387627372790711,0.225638708052044,1.
71791168340365,0.0858127254426434,0.53867478675348
"2594","AL590617.2",8.55399389590521,-1.0810292170464,0.62933950405118
8,-1.71772026082518,0.085847651955804,0.53867478675348
"2595","RNF215",78.1501864607112,-0.371654722439618,0.216326198034596,
-1.7180291884027,0.0857912914454167,0.53867478675348
"2596","GTPBP6",324.940548828083,-0.401490546751222,0.23373578688626,-
1.7177106213442,0.0858493306164917,0.53867478675348
"2597","ADA2",24.0611267183872,-0.679432880089901,0.395644654920719,-1
.7172805739687,0.0859279196804034,0.538960294060104
"2598","ITGA3",3.22587317855601,-1.766211732678,1.02860999010508,-1.71
708592145558,0.0859634741360235,0.538975762202343
"2599","VPS4A",266.99615923975,-0.268714465001269,0.156537588438693,-1
.71661303640505,0.0860498989588404,0.539310043917103
"2600","TCFL5",81.156734064028,-0.453513973162568,0.26423911787476,-1.
71630141975238,0.0861068886105467,0.539314405328654
"2601","ETS2",55.9643824389703,-0.59909121348322,0.349070474149079,-1.
71624717027016,0.0861168130799822,0.539314405328654
"2602","TCF7L2",1428.03632636303,0.267678876326073,0.155991046815204,1
.71598871724465,0.0861641074949747,0.53940264389191
"2603","SNRNP25",193.560543278977,-0.593779324105731,0.346063904203796
,-1.71580831428191,0.086197131932632,0.53940264389191
"2604","FAM76A",235.198903299235,-0.346703592482144,0.202127046308071,
-1.71527560915186,0.0862947082294322,0.539598657331755
"2605","C11orf65",10.9798665761009,0.823063361114473,0.479835376589227
,1.71530362551628,0.0862895742134538,0.539598657331755
"2606","TCP11L2",182.227841549486,-0.502857528315285,0.293292272970892
,-1.71452702528304,0.0864319780765544,0.540249612773981
"2607","ANP32E",281.523969926878,-0.262312846790655,0.153028056846632,
-1.71414871361499,0.086501417159798,0.540476250140372
"2608","BCL2L13",166.828643873801,0.313623778865503,0.183006921442151,
1.71372632463324,0.0865789998547564,0.540753576930263
"2609","PRR5",145.396753312974,-0.470751061285658,0.274755632359855,-1
.71334453544196,0.0866491736830453,0.540984434696483
"2610","C1orf198",116.374131920396,0.577375434772726,0.33703042637883,
1.71312555064017,0.0866894443696214,0.541028490167342
"2611","UEVLD",92.4004862455429,0.426260758938036,0.24888303514545,1.7

1269511676007,0.0867686439879799,0.541315374155574
"2612","KIAA0319L",156.080828396946,0.414069061258579,0.24180127042345
3,1.71243542489848,0.0868164554082998,0.54140629484908
"2613","SGMS2",187.937850806122,0.375679565436862,0.219459327692074,1.
7118414122,0.086925898027974,0.541700661717751
"2614","CCDC110",10.626611128656,-0.905233923376752,0.528814275794207,
-1.7118182409452,0.0869301694229357,0.541700661717751
"2615","LINC00853",6.6028282236248,1.11874260001025,0.653783861396436,
1.71118111973688,0.0870476828993039,0.5418111218065
"2616","EIF2A",600.399779748323,-0.346267882841036,0.202328429071836,-
1.71141487347828,0.087004553434175,0.5418111218065
"2617","ZNF185",24.28751837281,0.659824704657307,0.385563086505249,1.7
113274785664,0.0870206764863717,0.5418111218065
"2618","ENSA",776.447813794048,-0.361347555560081,0.211377638158266,-1
.70948809300975,0.0873605754406656,0.541891853709184
"2619","ZBTB47",131.528617000595,-0.313841375015002,0.183562972397669,
-1.70972048946287,0.0873175720055774,0.541891853709184
"2620","MAP3K1",61.4770656610487,0.484042917163152,0.283041663361312,1
.71014723208878,0.0872386505724915,0.541891853709184
"2621","PSMG4",244.554994991559,-0.42660701467847,0.249582996405332,-1
.70927916093148,0.0873992515342985,0.541891853709184
"2622","CLIC1",2972.19901778619,-0.435349822526811,0.254736902302629,-
1.70901749448776,0.0874477089423151,0.541891853709184
"2623","AC100814.1",9.66913440632828,1.02175694946019,0.59733833175566
9,1.71051629393527,0.0871704430046458,0.541891853709184
"2624","AL158151.1",24.4182947842238,1.00230027360334,0.58604533031349
9,1.71027772385315,0.0872145290279548,0.541891853709184
"2625","ITGB1",3071.67877007757,0.320670467660883,0.187618520085597,1.
70916212064024,0.0874209232783392,0.541891853709184
"2626","DDB1",417.738063198066,0.236288283339399,0.138279557939178,1.7
0877233671322,0.0874931288142399,0.541891853709184
"2627","N4BP2L1",260.687724565805,0.362140188773964,0.211762790770119,
1.71012191262198,0.0872433315270672,0.541891853709184
"2628","CDH5",28.3038219515101,0.909134009817247,0.531434301027508,1.7
1071759587116,0.0871332578566544,0.541891853709184
"2629","SMAD4",303.39709990076,0.284938576900756,0.166735433281422,1.7
0892635892112,0.0874645912175026,0.541891853709184
"2630","SLC27A1",271.599862395221,0.350044258294072,0.204776887805648,
1.70939338928862,0.0873781046416443,0.541891853709184
"2631","MEGF6",354.827440247308,0.482182174639723,0.282254446434988,1.
70832445947236,0.0875761552473244,0.541993918246074
"2632","PHIP",830.219666301308,0.347711725185355,0.203521211954783,1.7
0847904179446,0.0875474919586974,0.541993918246074
"2633","TSSC4",261.890382246944,-0.356402742587775,0.208689199100235,-
1.70781594890588,0.0876704987826813,0.542371726042953
"2634","DOCK10",23.1940434153775,0.640419375983111,0.375118397649304,1
.70724597885981,0.0877763422718445,0.542690185501958
"2635","ZBED1",247.345549016755,0.390948137234968,0.22900229960664,1.7
0717996241306,0.0877886081894321,0.542690185501958
"2636","LINC00926",16.3861564510375,-0.674475753961929,0.3951726735935

1,-1.70678743504345,0.0878615686933168,0.542861410677236
"2637","ZNF200",41.5812389467939,0.470057900392342,0.275423626049836,1
.70667239820337,0.0878829602772344,0.542861410677236
"2638","MAMDC2",101.507987197399,-1.58607575222283,0.929585280797044,-
1.70621866007054,0.0879673757510213,0.54317687020788
"2639","POU6F1",38.6930116424767,-0.470942287953405,0.276052035709321,
-1.70599099819463,0.0880097556146629,0.543232629483609
"2640","GRK4",75.7098475324993,0.344922294817402,0.202354242705649,1.7
0454688869132,0.088278964035514,0.543493009816134
"2641","FAT4",233.472259276738,0.449271412838908,0.263577754845224,1.7
0451187393536,0.0882854996607214,0.543493009816134
"2642","ZFAND5",2093.83167018138,0.576379681606349,0.338031924036504,1
.70510428341705,0.088174976900954,0.543493009816134
"2643","MDP1",245.288693407611,-0.436093000554164,0.25581306839756,-1.
70473308219121,0.0882442169268345,0.543493009816134
"2644","NEO1",106.147544062839,0.356091362489212,0.208878196670129,1.7
0477995389614,0.0882354715478781,0.543493009816134
"2645","PPARA",131.585624002075,0.407567329462242,0.239074171199238,1.
70477357473546,0.0882366617382532,0.543493009816134
"2646","MED14",67.2615846281825,0.354817205545031,0.208101546096637,1.
70501955511788,0.0881907773795441,0.543493009816134
"2647","LINC00894",13.4144861567083,0.977106357434783,0.57351851288826
,1.70370499901396,0.0884362137482011,0.544215143839988
"2648","PEAR1",132.4374708976,0.723611899410387,0.425138902173993,1.70
205995195951,0.0887441302863549,0.54438061679304
"2649","IGSF8",185.41201111434,-0.427536776518617,0.25134541249495,-1.
70099295735985,0.0889443096512976,0.54438061679304
"2650","TBX19",38.401167266635,0.719499889639061,0.422493427797596,1.7
0298480946727,0.0885709112037817,0.54438061679304
"2651","KLHL2",50.3120125206745,0.507731535163467,0.298496267609086,1.
70096443493357,0.0889496657456839,0.54438061679304
"2652","RASA4",18.7708563647644,-0.684396127181425,0.402202861675262,-
1.70161923843795,0.0888267687069029,0.54438061679304
"2653","C2CD5",109.390885864089,0.394897245380231,0.232140559119191,1.
70111266587186,0.0889218329778501,0.54438061679304
"2654","APEX1",678.549386568157,-0.39008993823355,0.229330716181879,-1
.70099297960669,0.0889443054737687,0.54438061679304
"2655","BAG5",268.68225861535,-0.298861871179808,0.175656860128253,-1.
70139595437149,0.088868660477526,0.54438061679304
"2656","NOX5",2.79698163414636,1.69867644617846,0.998167786791726,1.70
179449653277,0.0887938985476257,0.54438061679304
"2657","PSMD7",676.362299688673,-0.311774618447728,0.18309991841805,-1
.70275673054037,0.0886136033921557,0.54438061679304
"2658","USP32",84.1124007606986,0.380261254858278,0.223298875531573,1.
7029250772224,0.0885820903809642,0.54438061679304
"2659","POLR2I",664.668522669956,-0.412886308070081,0.242583934537681,
-1.70203483943388,0.0887488374838991,0.54438061679304
"2660","ARFGEF2",111.479481675823,0.366135614976482,0.215261708247145,
1.70088594928419,0.0889644055438069,0.54438061679304
"2661","HEPH",63.6611710198522,-0.546040645640779,0.320965556051615,-1

.70124374826366,0.0888972259749517,0.54438061679304
"2662","ENOX2",100.433432549938,-0.512256726988552,0.301110457890102,-1.70122529312985,0.0889006900712393,0.54438061679304
"2663","ALG14",145.298455587698,-0.372377991426612,0.219160669618988,-1.69910957141167,0.0892985402989893,0.544584022062987
"2664","PARM1",3.08148509993146,-1.54442791917078,0.908390816209793,-1.70018002341197,0.0890970686267072,0.544584022062987
"2665","GMDS-DT",89.4321591837654,0.39715921077906,0.233679614003486,1.69958861183815,0.0892083339340497,0.544584022062987
"2666","ZNF703",441.029222739834,0.53110406871888,0.312487546372537,1.69960075172313,0.0892060488703136,0.544584022062987
"2667","AC016405.2",3.54913625337357,-1.44780322430834,0.851602366263178,-1.70009300310106,0.0891134331882666,0.544584022062987
"2668","SF1",1001.50522867127,-0.231679561286328,0.136334631740535,-1.69934490106115,0.089254217050036,0.544584022062987
"2669","DDHD1",202.299686393612,0.340513464723477,0.200372636768762,1.69940102707958,0.0892436485920458,0.544584022062987
"2670","NFIC",2714.48585912486,-0.299695407006612,0.176316584320312,-1.69975733231175,0.0891765802774639,0.544584022062987
"2671","OSER1",379.285704898121,-0.397848993193737,0.234144887187472,-1.6991572951802,0.0892895503155953,0.544584022062987
"2672","CDC42BPA",330.076877481544,0.363748884675989,0.214129936498968,1.6987297088081,0.0893701230619854,0.544612770129697
"2673","SIAE",54.0049222874344,0.458165480017262,0.269705114949912,1.69876451954702,0.0893635612702414,0.544612770129697
"2674","TMEM63A",85.1010671469993,0.440889822868034,0.259627425551902,1.69816352001648,0.0894769036124519,0.544698836085856
"2675","XRN1",359.548479390966,0.263920322776183,0.155435075477838,1.69794573049126,0.0895180050464631,0.544698836085856
"2676","SLC25A10",13.9102395118629,-0.691336489206736,0.407097944889917,-1.69820677771704,0.0894687417897631,0.544698836085856
"2677","EIF3G",2012.28246009299,-0.433533756684366,0.255304525661695,-1.6981044717509,0.0894880457520865,0.544698836085856
"2678","AGPAT3",117.702260668124,0.395265297204961,0.232824430316569,1.6976968296133,0.0895649964574218,0.544781264859949
"2679","AL392172.1",36.7546534105665,-0.48300289429255,0.284576676287032,-1.69726802840785,0.0896459988574602,0.54486704305566
"2680","AC079630.1",23.9113997247088,-0.56564655679604,0.333264396022445,-1.69729069035608,0.089641716441769,0.54486704305566
"2681","TRIR",1803.76444976117,-0.389831344274933,0.229753395404188,-1.69673812040572,0.0897461822430754,0.545272496291479
"2682","SETD3",168.557987114453,-0.30204294010213,0.178053115927727,-1.69636424798504,0.0898169200595253,0.545498810905894
"2683","ELP5",246.118991690813,-0.473509810726555,0.279222363764444,-1.69581621021594,0.0899206916091221,0.545925510853891
"2684","DPYSL3",730.762680925815,-0.425995411102617,0.251289300647569,-1.69523895368738,0.0900301001044444,0.546040680836742
"2685","ZFAND1",311.021971627197,-0.426463247309673,0.25153229861104,-1.69546117800641,0.0899879688417832,0.546040680836742

"2686","AP001830.1",3.49182399363859,1.53760067132443,0.907134324760024,1.6950088089007,0.0900737497334597,0.546040680836742
"2687","NPIPB13",6.39853953418599,1.27493597174578,0.752099010406157,1.69517038861317,0.0900431024854107,0.546040680836742
"2688","HDAC3",222.2617317814,-0.368788741887059,0.217667854088038,-1.69427287934717,0.0902134416348502,0.546480755221299
"2689","LRP6",644.613520131041,0.481619030692948,0.284248433129417,1.69435949176778,0.0901969920833895,0.546480755221299
"2690","VLDLR",137.188392879556,1.22388741564079,0.722642253162307,1.69362836214602,0.0903359250749652,0.547019287563609
"2691","PBX1",853.251569525726,0.370849890558504,0.219056468431872,1.69294197616374,0.0904665123108758,0.547199784267307
"2692","TMEM17",27.6600861329737,0.559150044089594,0.330282700436983,1.69294378225019,0.0904661684976869,0.547199784267307
"2693","OCEL1",202.045993911992,-0.464435807647438,0.274307555642741,-1.69312072560014,0.0904324900139761,0.547199784267307
"2694","AMOT",127.837163880443,0.374399955530162,0.221208002348971,1.69252446364721,0.0905460197274522,0.547477399903663
"2695","AC093635.1",2.67781211733921,-1.65086693486276,0.975522426006458,-1.69229009077832,0.0905906762670241,0.547544165385364
"2696","YIPF4",568.497281115726,0.220198191709126,0.130154408404515,1.69182276965033,0.0906797707884252,0.547879371799947
"2697","DBN1",908.282789417306,-0.449358710848409,0.265695323447094,-1.69125562700348,0.0907879908862755,0.548126606207021
"2698","WIPF2",307.404340272144,0.307764852609076,0.181968994063847,1.69130380806024,0.0907787931165214,0.548126606207021
"2699","TOLLIP-AS1",20.7721528513619,-0.683043518426373,0.403969538990599,-1.69082926433785,0.0908694162401189,0.548225079623246
"2700","TRMT1",82.1750089594377,-0.378290085609373,0.223732027207011,-1.6908177623553,0.0908716136646058,0.548225079623246
"2701","RAB8B",452.866569987798,0.312390519367739,0.184795039105331,1.6904702684669,0.0909380216386831,0.548422596990933
"2702","KDM2B",56.5160742837065,0.438402381524784,0.259382825219469,1.69017505748055,0.0909944687214039,0.548559918950018
"2703","BTN3A3",141.782201871704,0.493373951191967,0.291975992763723,1.68977574670402,0.0910708654772185,0.548817361360863
"2704","LRIG2",105.460258811121,0.350906376303684,0.207761785701074,1.68898421391394,0.0912224551945163,0.549527578647735
"2705","TPI1",1466.55109082606,-0.316820234860885,0.187634850405623,-1.68849355104339,0.0913165258762709,0.549890902032745
"2706","KCTD3",282.357394060108,0.310346588553936,0.183881884776215,1.68774966023232,0.0914592945532619,0.550129535680452
"2707","TTC30B",21.2062010601637,-0.628462335799548,0.372350417075709,-1.68782498146568,0.0914448306269913,0.550129535680452
"2708","PLPP1",1515.19789080056,0.585079857931558,0.346742166122102,1.6873628739042,0.0915335980489153,0.550129535680452
"2709","UBTD2",312.576026820757,0.299235432574418,0.177284040582123,1.6878870291531,0.0914329169991493,0.550129535680452
"2710","SMIM13",84.7049431583671,-0.339362001753315,0.201144181821342,

-1.68715793159128,0.0915729881032516,0.550129535680452
"2711","ENDOG",219.222807390627,-0.410581717455108,0.243371704028372,-1.68705609838374,0.0915925655820115,0.550129535680452
"2712","REPS2",40.369348796497,0.639266585710565,0.37882664763309,1.68749107198425,0.0915089652135886,0.550129535680452
"2713","SPG7",345.59802701381,0.377005491533951,0.223514837020313,1.68671349320622,0.0916584562746853,0.550132153574668
"2714","FN3K",39.8511127601089,0.530263705945672,0.31437889489317,1.6867026208164,0.0916605479035943,0.550132153574668
"2715","GIPC2",5.64327900001519,-1.11058053528566,0.658723893546916,-1.68595757063815,0.0918039719588717,0.550410052979292
"2716","SLC35G2",94.0335017874003,0.38887294285892,0.230699826032256,1.68562304335916,0.0918684279449276,0.550410052979292
"2717","EYS",4.61228281951985,1.91612744588997,1.13700954456635,1.68523426654328,0.0919433822921392,0.550410052979292
"2718","SGSH",107.062289215823,0.446001442932601,0.264648900357793,1.68525711737183,0.091938975400912,0.550410052979292
"2719","PTTG1IP",859.162727389048,0.347667222456779,0.206275782997806,1.68544856504303,0.091902060471353 6,0.550410052979292
"2720","ASCC2",176.488166533711,-0.288495458956768,0.17110053251112,-1.6861166632431,0.0917733310954748,0.550410052979292
"2721","FAM120C",61.5826123274254,0.369811805569922,0.219409444223131,1.68548718073337,0.091894616039493,0.550410052979292
"2722","ZBTB11",156.130330106747,0.29032321769986,0.172311690777624,1.68487243314521,0.092013186210211,0.55062556582591
"2723","NUMA1",757.362300900706,0.307500742939374,0.18256755170349,1.68431213581036,0.0921213612535606,0.551070456650477
"2724","NBPF14",124.549249630054,0.7421402303677,0.4408529269395,1.68341908381964,0.0922939914669372,0.551319542011542
"2725","MLLT11",21.2118377373684,-0.639584195107811,0.379936778541962,-1.68339637337103,0.0922983848649686,0.551319542011542
"2726","MTDH",2030.33534961145,-0.216601839415305,0.12863316400966 1,-1.68387243743019,0.0922063241152282,0.551319542011542
"2727","JMJD8",273.160814691802,-0.367905793968179,0.218532023691249,-1.6835326363333,0.0922720269437809,0.551319542011542
"2728","S100PBP",143.828636907842,0.308574378262337,0.183360478020329,1.682883801318,0.0923975880065562,0.551608845985494
"2729","GPR27",2.51275610411154,-1.64015705672183,0.974718602798344,-1.68269801357342,0.0924335664834229,0.551608845985494
"2730","EXOC1",334.791162907903,0.303774804828562,0.180536636620428,1.68262138098451,0.0924484099417028,0.551608845985494
"2731","CENPL",61.0477586465363,-0.387720398315356,0.23048694428942,-1.68217943758454,0.0925340501514745,0.551823539747261
"2732","AC006449.2",14.1499582359227,-0.85772825871537,0.509919356182614,-1.68208609521423,0.0925521462698457,0.551823539747261
"2733","ZNF687-AS1",8.74016619082077,0.847748346280946,0.504192552426872,1.68139799408065,0.0926856349063865,0.552362393129055
"2734","AC005303.1",2.35309950590325,1.75857844851956,1.04598172822688,1.68127071540786,0.0927103433491826,0.552362393129055

"2735","PRKCI",154.734187422852,0.373998618603353,0.222491685430012,1.
68095548326007,0.0927715617231729,0.552525034335928
"2736","C2CD6",18.4542076966425,0.676633253348066,0.402619470740075,1.
68057757391691,0.0928449948336513,0.55255831963659
"2737","ZNF426",150.263633580589,0.302056888183418,0.179724098679725,1
.68066992908777,0.0928270446158607,0.55255831963659
"2738","PTPRJ",16.2140027200668,0.765985324572868,0.455966066798159,1.
67991738936122,0.0929733899436555,0.552701440937038
"2739","RAB20",22.8459270441199,-0.706432531936536,0.42054926212805,-1
.67978545096446,0.09299066885232,0.552701440937038
"2740","AL627171.2",202.819234200004,0.62756557284537,0.37360516111984
,1.67975616547778,0.0930047669966494,0.552701440937038
"2741","PYGB",125.299257781675,-0.360336470042292,0.214472541710564,-1
.68010537464779,0.09293681537238,0.552701440937038
"2742","LRRC8D",40.9977247488261,0.73701861107677,0.438819174217584,1.
67954969695861,0.0930449618763606,0.552738652080247
"2743","RTRAF",1120.22501823768,-0.443621557136422,0.264318609825941,-
1.67835914931815,0.0932770073732211,0.553915119614436
"2744","AC005082.1",3.99788688700274,1.49843019181487,0.89311484615715
7,1.67775756753146,0.0933944360619595,0.554235349337432
"2745","RAD9A",76.2919177053088,-0.358501437283857,0.213713568387055,-
1.67748561773381,0.0934475595427902,0.554235349337432
"2746","MEF2B",34.8649399331626,-0.460923233833698,0.274745870998218,-
1.67763479814657,0.09341841519711,0.554235349337432
"2747","NBDY",413.63334675045,-0.388237135778083,0.231453669743119,-1.
67738595896523,0.0934670332512693,0.554235349337432
"2748","RNF207",33.5224955909064,0.60113385560995,0.358502590483757,1.
67679082820236,0.093583391884217,0.554261117398937
"2749","POGZ",332.002315806109,0.352361888964865,0.210153006388105,1.6
766921160011,0.0936027031032567,0.554261117398937
"2750","ID2",1854.26169491723,0.341893455800771,0.203868948118776,1.67
702565278152,0.0935374656368486,0.554261117398937
"2751","BRWD1",308.966296783014,0.338752270225275,0.202039006257815,1.
67666767175149,0.093607485662992,0.554261117398937
"2752","PYURF",893.301889338788,-0.37581171349083,0.224253290439258,-1
.67583589411199,0.0937703412476697,0.555003798635434
"2753","BNC2",413.992171617117,0.470214824493322,0.280611493864832,1.6
7567913208794,0.0938010594661029,0.555003798635434
"2754","CHAC2",4.4812466148475,-1.19296685072365,0.712092467442848,-1.
67529766886545,0.0938758426026206,0.555244589743677
"2755","SEC61A1",392.488924522994,0.282640504132221,0.168765878384383,
1.67474910709424,0.0939834680249323,0.555276282429497
"2756","SH3D19",1146.84759136246,0.286627958184909,0.171137803254689,1
.67483719396787,0.0939661790998604,0.555276282429497
"2757","N6AMT1",67.149176487824,0.47987457230638,0.286513671083022,1.6
7487495620176,0.0939587682382375,0.555276282429497
"2758","JTB",1322.7969390838,-0.349362837319022,0.208695026527636,-1.6
7403528072462,0.0941236657613522,0.555500141879227
"2759","KLHL5",58.7800716757149,-0.465711506153975,0.27814501573372,-1
.67434783947314,0.0940622576206244,0.555500141879227

"2760","CHP1",336.320164466665,-0.343738517239507,0.205317955687743,-1
.67417660130164,0.0940958966526919,0.555500141879227
"2761","ZSCAN30",81.7238703646585,0.434188097234641,0.2594133850544,1.
67373050987169,0.0941835747690724,0.555652390225795
"2762","AL158206.1",40.0034472811418,0.563155893143953,0.3365914796540
02,1.67311392945195,0.0943048698755687,0.555897036651673
"2763","CNOT2",484.159193138943,0.212702966010724,0.127150348473814,1.
67284611142476,0.0943575946435169,0.555897036651673
"2764","COPE",1089.42374684978,-0.441984807365424,0.264214450467779,-1
.67282601910271,0.0943615511290979,0.555897036651673
"2765","GYG2",2.97261129576307,1.50005161198145,0.896561885915292,1.67
311552671019,0.0943045554975819,0.555897036651673
"2766","SPAST",111.995027767174,0.292515527390216,0.174946690072783,1.
67202664576575,0.0945190678895848,0.556146084994666
"2767","AC017048.3",5.57106581659225,1.11689957859184,0.6683757018715,
1.67106550322586,0.0947087402755247,0.556146084994666
"2768","TRERF1",126.536903596248,0.395443121064323,0.236578357742494,1
.67151012813584,0.0946209598783852,0.556146084994666
"2769","NFKBIE",40.0539130409439,-0.514984724735836,0.308031841669989,
-1.67185548722449,0.0945528221098187,0.556146084994666
"2770","RRP12",33.1089673078868,-0.527315981091735,0.31561833642927,-1
.67073937166482,0.0947731685150533,0.556146084994666
"2771","TNKS1BP1",388.323524738747,0.328077039205546,0.196307213276337
,1.67124291425665,0.0946737069522098,0.556146084994666
"2772","ST3GAL4",285.88265147138,-0.519296688717331,0.310560933264921,
-1.67212496194539,0.0944996832978645,0.556146084994666
"2773","AC091814.1",2.3186903746755,-1.72046918399586,1.02977826912866
,-1.67071809104267,0.0947773737842139,0.556146084994666
"2774","HSD3B7",240.515612245728,-0.52930733427478,0.316676997907603,-
1.67144231432059,0.0946343438548822,0.556146084994666
"2775","DUS1L",214.723424352679,-0.309571853404354,0.185293825656338,-
1.67070787333472,0.0947793929612126,0.556146084994666
"2776","MEX3D",153.910943031146,-0.336554526088703,0.201380016082331,-
1.67124093361432,0.0946740980119515,0.556146084994666
"2777","AC005072.1",5.33632515757008,1.11894842222632,0.66996332143728
1,1.67016370362758,0.0948869791161201,0.556576882543205
"2778","ANKRD13C",146.300496367292,0.309341426615507,0.185337458503724
,1.66907126661225,0.0951032569256165,0.557514754351247
"2779","AC124947.1",4.19096741623313,1.32966448967901,0.79667838395512
5,1.66901037665647,0.0951153233680468,0.557514754351247
"2780","GJC1",3.0109734417673,-2.22171920743388,1.33137676448932,-1.66
873815638962,0.09516928371911,0.557630382194454
"2781","AC087500.1",16.8975122324074,0.674005278687927,0.4040235530549
27,1.66823264037851,0.095269553725956,0.55801717390942
"2782","NEXN-
AS1",14.2641048897992,0.749718754945125,0.449457059550872,1.6680542423
6588,0.0953049594838519,0.558023898286292
"2783","LINC01750",11.2801683735248,1.07248631328369,0.643818692065378
,1.66582040332371,0.0957491910295851,0.558326885182273
"2784","SHE",61.1664677487206,0.42664679184824,0.256125431736435,1.665

77285572828,0.0957586645507439,0.558326885182273
"2785","AL590560.3",12.2079983918516,1.16702809615766,0.70018056248909
8,1.66675306153736,0.0955635171540132,0.558326885182273
"2786","TENT2",166.67761683366,0.267657446061386,0.160506350881454,1.6
6758165388153,0.0953988026841699,0.558326885182273
"2787","PUS1",31.818340930722,-0.506449088977144,0.303847416118381,-1.
66678754569309,0.0955566575687039,0.558326885182273
"2788","ODF3L1",10.3354828876333,-0.893392357694842,0.53594794017226,-
1.66693869073868,0.0955265964600743,0.558326885182273
"2789","UQCRC2",612.212237970205,-0.304054800762062,0.182485525418309,
-1.66618585263177,0.0956764028900437,0.558326885182273
"2790","WDR7",62.9765763266096,0.394813913238973,0.236827858683178,1.6
6709235743733,0.0954960415855201,0.558326885182273
"2791","EPS15L1",140.844294436983,0.331968803194621,0.199169466136303,
1.6667655421009,0.0955610344758564,0.558326885182273
"2792","ZNF615",43.8160922467532,0.483916967800104,0.290514165650508,1
.66572589228665,0.0957680224199932,0.558326885182273
"2793","OPRL1",5.28681775545898,1.29713088742692,0.778678914361588,1.6
6580969832784,0.095751323858638,0.558326885182273
"2794","SPIN3",57.9483889613196,0.419342157936795,0.251733259289847,1.
66581944364357,0.0957493822316284,0.558326885182273
"2795","GPAT2",41.6544209273129,-1.00855820970658,0.605623242599091,-1
.66532282575261,0.0958483669979275,0.558595366736759
"2796","RNF112",13.4084486742567,-0.721266018565222,0.433365685136009,
-1.66433578685137,0.0960453445674557,0.559543139363121
"2797","SNX15",198.328082595942,-0.334580436430409,0.201077843527396,-
1.66393487497703,0.0961254446670788,0.559609495418887
"2798","ZNF543",19.3801740225401,0.638851052670923,0.383903379881116,1
.66409332699482,0.0960937803942353,0.559609495418887
"2799","SMG7-
AS1",5.21868350539815,-1.09853427159094,0.660671492422622,-1.662754158
74645,0.0963616562089718,0.559830086930712
"2800","EBLN2",19.3670003744938,0.932753480302533,0.561021325768633,1.
66259897344295,0.096392736748253,0.559830086930712
"2801","PCDH7",165.690150449195,0.801101892084446,0.481853289449564,1.
66254316329265,0.0964039163755079,0.559830086930712
"2802","MDN1",118.460395756417,0.424325676045589,0.255119945951374,1.6
6323991040068,0.0962644216114251,0.559830086930712
"2803","PABPC1",2893.3587099542,-0.266723744079233,0.160397636798904,-
1.66289073456634,0.0963343094144024,0.559830086930712
"2804","LRRC10B",11.9487587175977,1.25799401053695,0.756234854683524,1
.66349646904784,0.0962130970679902,0.559830086930712
"2805","ZNF676",36.1627969493815,0.605963001965886,0.364390201857834,1
.66295086661606,0.0963222710470855,0.559830086930712
"2806","TTLL3",307.754669201999,0.403400581385267,0.242688453146857,1.
66221580035849,0.0964695130716004,0.559835342208287
"2807","EXOSC6",274.210629130681,-0.281121409328883,0.16912654983494,-
1.66219561389531,0.0964735591858715,0.559835342208287
"2808","COL8A2",122.951640172702,-0.473654812101335,0.285257897565453,
-1.66044416699332,0.0968251311798476,0.559921629638707

"2809","ZNF385B",47.0609123226797,0.942281994852837,0.567475556171202,
1.66048032308296,0.0968178631402202,0.559921629638707
"2810","COL3A1",21581.136643309,0.71363745029683,0.429633538735992,1.6
6103757261687,0.0967059009010097,0.559921629638707
"2811","YEATS2",72.8648929475222,0.437646659910374,0.263631881874066,1
.66006727562425,0.0969009192676969,0.559921629638707
"2812","GALNT10",126.921367897825,0.448571986314326,0.270120295391374,
1.66063784901611,0.0967862026192436,0.559921629638707
"2813","AC006001.2",7.93822003181733,1.06240666701429,0.63938478063764
,1.66160768786955,0.096591460889543,0.559921629638707
"2814","HSPA8",1852.24709426897,-0.422047722050982,0.254225401268388,-
1.66013199289013,0.0968879020713884,0.559921629638707
"2815","MAPK1IP1L",308.24426628167,0.210680495194049,0.126899852866587
,1.66021071289612,0.0968720702564968,0.559921629638707
"2816","FUT8",254.63374183012,0.379256259986057,0.228281177731036,1.66
13558058339,0.0966420081485129,0.559921629638707
"2817","CBX8",20.8697987926345,0.884488280292927,0.532398603439553,1.6
613271984162,0.0966477503742751,0.559921629638707
"2818","METTL4",59.9652146034211,0.36311156127413,0.218604416153753,1.
66104403407266,0.0967046032768551,0.559921629638707
"2819","ZNF133",53.8466635370958,0.437100099772305,0.263183916970274,1
.66081615018168,0.09675037655686,0.559921629638707
"2820","PEF1",245.953971645739,-0.36760951916644,0.221476038043641,-1.
65981621494423,0.0969514307163249,0.559985369437442
"2821","SLC12A8",2.49874432452703,-1.53650007341431,0.92578607650787,-
1.65967075159533,0.0969807064388866,0.559985369437442
"2822","AZIN2",45.9708996065167,-0.570551586245854,0.344128315041819,-
1.65796175817884,0.0973251853964981,0.56088703040417
"2823","AGO4",85.9318653478313,0.483935010871313,0.292170529590317,1.6
5634436693492,0.0976521004913075,0.56088703040417
"2824","PTRHD1",292.50971398591,-0.421958229990407,0.254504013546889,-
1.65796296926636,0.0973249409337191,0.56088703040417
"2825","SKIL",449.895883594167,-0.503905521100262,0.303854994145694,-1
.65837498415,0.0972418025946506,0.56088703040417
"2826","PTGER4",454.497241537957,0.432069591745556,0.260624424412279,1
.65782463681175,0.0973528670577815,0.56088703040417
"2827","PTTG1",27.5610055599376,-0.583175636705442,0.351929222734688,-
1.6570821603669,0.0975028653560709,0.56088703040417
"2828","C6orf163",5.84373536605599,1.10079817066298,0.663780139849259,
1.65837768347961,0.0972412580982443,0.56088703040417
"2829","CLDN23",47.7732452803282,-0.717616725172552,0.432739749132164,
-1.65831016589461,0.0972548781682933,0.56088703040417
"2830","KLHL9",169.916849247931,0.347054284104907,0.209522325485844,1.
65640717904477,0.0976393882401833,0.56088703040417
"2831","ASPN",3083.89459863875,0.545789697153723,0.329256189832155,1.6
5764445440479,0.0973892513244788,0.56088703040417
"2832","SHMT2",196.732793714761,-0.378383243104907,0.228286198673611,-
1.6574950448314,0.0974194298700733,0.56088703040417
"2833","CDYL2",18.099373158554,0.793422454555189,0.478861016312693,1.6
5689506459446,0.0975406923758712,0.56088703040417

"2834","AC004687.1",9.46170169936007,-1.02165780748741,0.6165181604925
7,-1.65714146469124,0.0974908776564274,0.56088703040417
"2835","GATA6",90.0190552722438,0.729657753624524,0.440524636378802,1.
65633813269208,0.0976533622829042,0.56088703040417
"2836","ZNF558",38.9245988885756,0.489818351225651,0.295664719110747,1
.65666824468895,0.0975865665480167,0.56088703040417
"2837","ADRB2",3.76465805418705,-1.33503307368514,0.806306886500988,-1
.65573815136144,0.0977748576399832,0.560937146278719
"2838","CLEC12A",15.8696726653269,1.14436844818674,0.69102332228614,1.
65604895128688,0.0977119060318536,0.560937146278719
"2839","AC005225.2",4.83854123407496,-1.08086992149582,0.6527669301347
65,-1.65582824680269,0.0977566057394466,0.560937146278719
"2840","EGFL6",9.14989573808842,1.65084850963654,0.997121084827191,1.6
5561488444771,0.0977998339635067,0.560937146278719
"2841","ATXN7L1",67.6965422756414,0.409778052837864,0.247662627958497,
1.65458170340716,0.0980093774423894,0.561867464891333
"2842","CD99L2",186.498090243791,0.349071953221393,0.210986528600843,1
.6544750773249,0.09803102309664,0.561867464891333
"2843","SPOCK1",736.775726072844,-0.470461930366386,0.284449685347282,
-1.65393725006939,0.098140263072375,0.562295724652098
"2844","LINC02593",8.14394996215136,-0.884119831321495,0.5351990317102
99,-1.65194587235364,0.0985455855652012,0.563043934193216
"2845","ACTR3",600.434596177798,-0.250231835433019,0.15143169416789,-1
.65244030853667,0.0984448241244599,0.563043934193216
"2846","RICTOR",301.015732322073,0.390802887782054,0.23653806597294,1.
6521775730878,0.0984983568876478,0.563043934193216
"2847","PARP8",73.2236055023062,0.408852277608612,0.247437119401472,1.
65234819495793,0.098463589766252,0.563043934193216
"2848","MBLAC2",43.7507438559627,0.472881270757076,0.286054282578618,1
.65311725625752,0.0983070020032763,0.563043934193216
"2849","HCG25",14.4275318612429,0.772922210370853,0.467888409160783,1.
65193707567406,0.0985473789910282,0.563043934193216
"2850","HOXB7",36.0260552564381,-0.870031899808535,0.526617851315209,-
1.65211243340054,0.0985116327973267,0.563043934193216
"2851","CLCN5",50.2520787378954,0.509367208166007,0.308301116721629,1.
6521743858162,0.0984990064419542,0.563043934193216
"2852","LRWD1",46.7541268416647,-0.460092999674395,0.278600911056073,-
1.65144111672267,0.0986485349418785,0.56342425864946
"2853","SUZ12",251.862919041565,0.234603134179702,0.14207598644783,1.6
5125113712196,0.0986873051971625,0.563448129813032
"2854","NCK1",368.919835145988,0.27196786347311,0.164842066698665,1.64
986928955625,0.0989696731376783,0.564071730489728
"2855","UBE2H",612.134374772801,0.322977199635444,0.195726707870644,1.
65014372922933,0.0989135426353184,0.564071730489728
"2856","ZNF189",65.4058067687994,0.417197297952876,0.252851241041434,1
.64997132794183,0.0989488004977451,0.564071730489728
"2857","FAM182B",2.94506291424531,1.70150221443222,1.03098791403403,1.
65036097055164,0.0988691287984706,0.564071730489728
"2858","SLC2A11",52.9801541557074,0.419221156673291,0.254077499721372,
1.64997355977219,0.0989483440010631,0.564071730489728

"2859","GCA",70.470121118625,-0.383420690404254,0.232483520772837,-1.64923814440551,0.0990988562421264,0.564303304428835
"2860","AC109454.2",8.87836481149037,1.0092461052451,0.612203615448767,1.64854646359003,0.0992405843326461,0.564303304428835
"2861","PCDHB15",21.1753578739414,-0.806138492344631,0.489017241214461,-1.64848685159363,0.0992528066295421,0.564303304428835
"2862","AL031775.1",5.23986199299862,1.39433956354622,0.845344481008345,1.64943356805621,0.09905884245646,0.564303304428835
"2863","LZTS1",3.1142158026325,-1.4016873912218,0.850057388352268,-1.64893266081576,0.0991614310885238,0.564303304428835
"2864","VEZT",228.947925377792,0.271836185863209,0.164850353443375,1.64898758289034,0.0991501786005134,0.564303304428835
"2865","ZNF616",51.2654031641863,0.416714888390595,0.252766087812988,1.64861865765358,0.0992257839321894,0.564303304428835
"2866","AC104232.3",8.87322308126829,1.10236677995863,0.668949148837497,1.64790818834934,0.0993715128649651,0.564419870319288
"2867","SNAPC2",201.623131505438,-0.449591263173185,0.272822388110445,-1.64792657335431,0.0993677396470896,0.564419870319288
"2868","ZNF540",16.3923491717428,-0.592937500700193,0.359818332394433,-1.64788018652205,0.0993772599960536,0.564419870319288
"2869","MTA3",151.592791513512,0.288216920410521,0.174937480916022,1.64754241859055,0.0994466048162835,0.564616851116222
"2870","LCOR",195.213633751391,0.366376741819754,0.222573610490199,1.64609245908732,0.0997447247492371,0.566112132906036
"2871","ZNF618",181.799963195333,0.452394245825715,0.274858181957308,1.64591878838805,0.0997804801871128,0.566117813224619
"2872","ZNF148",572.676204601803,0.261930251621987,0.159191252479279,1.64538093357914,0.099891278947598,0.566332709660374
"2873","NUP88",76.7910192442384,0.376261129596385,0.228723558426849,1.64504755078267,0.0999600054622163,0.566332709660374
"2874","AC005838.2",32.7530598808676,0.659739888111408,0.401081004548172,1.64490434757593,0.0999895382282462,0.566332709660374
"2875","CEP295NL",10.809038868483,1.44378246715168,0.877737223810275,1.64489146408101,0.099992195529697,0.566332709660374
"2876","MAOB",91.8755813516109,-0.408350130761413,0.24820430224827,-1.64521777850956,0.0999249084846395,0.566332709660374
"2877","COX20",413.015246726418,-0.318399332592734,0.193611549009771,-1.64452654927453,0.100067484881759,0.566562134598181
"2878","TYW3",326.699086538982,-0.260485079205288,0.15850243348106,-1.64341375387409,0.10029735634859,0.566685488914758
"2879","WARS2-AS1",52.7868364506491,0.517795032714798,0.315066712061898,1.64344569861469,0.100290751621829,0.566685488914758
"2880","LMTK2",48.969593865776,0.468960356321314,0.285290980396253,1.64379664463964,0.100218214691592,0.566685488914758
"2881","SFRP1",3739.4811980077,0.999825959842608,0.608375223311243,1.64343635561092,0.100292683296334,0.566685488914758
"2882","NR4A1",835.045235608391,-1.21640895280699,0.740221070413144,-1.64330495500225,0.100319853633461,0.566685488914758
"2883","ZBTB4",413.917043980292,0.277844904888795,0.169083338645669,1.

64324236269694,0.100332798209231,0.566685488914758
"2884","ZNF317",48.2645452681213,0.414440936353033,0.25217702806751,1.64345237759755,0.100289370754243,0.566685488914758
"2885","AC104695.4",88.9469486184585,0.501314148532454,0.305114448629567,1.64303641071121,0.100375400081015,0.566729598585667
"2886","DNAJC6",2.63709547445062,1.76790614337152,1.07611923616946,1.64285339760725,0.100413269065558,0.566746964590742
"2887","FBXO28",102.420503408771,0.35208570569077,0.214368007243823,1.64243587565894,0.100499705136141,0.567021722920878
"2888","ADTRP",4.21259753775195,-1.1574650232799,0.704856257470614,-1.64212917316429,0.100563236958122,0.567021722920878
"2889","ZPR1",145.161221737973,-0.402662814591042,0.245210023877819,-1.64211400587636,0.100566379613139,0.567021722920878
"2890","DIPK2A",193.786932031906,0.491761181434225,0.299631396028517,1.6412204727285,0.100751657441643,0.567261729652415
"2891","RUBCN",52.5069162883076,0.429763326591192,0.261884886783321,1.64103905295906,0.100789308788051,0.567261729652415
"2892","CDK1",3.6848141446086,-1.71670571470455,1.04587691470457,-1.64140320009785,0.10071374605013,0.567261729652415
"2893","AEBP2",222.747767983839,0.320735552413065,0.195453568786486,1.64098079356862,0.100801402155435,0.567261729652415
"2894","R3HDM4",109.804933733761,-0.417241894206087,0.254273868291674,-1.64091535244776,0.100814987670803,0.567261729652415
"2895","PRRG1",169.550648534916,0.344686646681712,0.21005933592764,1.64090134418233,0.100817895963149,0.567261729652415
"2896","HOXB2",255.657559394543,-0.40453372023218,0.246655509822386,-1.64007574987269,0.100989417847025,0.568030603352968
"2897","SFRP2",4530.99039235673,0.694496687663284,0.423737153448747,1.63897992425459,0.101217440804561,0.568920253024672
"2898","AC005089.1",7.37340229870985,1.34519848457317,0.820735882585644,1.63901507551401,0.101210120055214,0.568920253024672
"2899","CANT1",121.57843004594,0.284369242027587,0.173530415559393,1.63872852554921,0.101269810453933,0.56901826232636
"2900","AC067930.3",2.93826069561379,2.35192758866616,1.43560957274858,1.63827800629889,0.101363713515716,0.569311146683109
"2901","TSC22D1-
AS1",9.80185049343198,1.00770137925841,0.615215439272692,1.63796503619889,0.101428987610964,0.569311146683109
"2902","SLC7A6OS",163.227699825026,0.269319398264135,0.164432823461421,1.63786884269686,0.101449056774942,0.569311146683109
"2903","CSNK1G2",212.750150188624,-0.292904841402677,0.178839540206636,-1.63780806562266,0.101461738524223,0.569311146683109
"2904","GAPLINC",10.9506280745525,0.956396823466227,0.58422681180784,1.63703000981201,0.101624199267847,0.57002637116872
"2905","SNCA",3.34407746363205,-1.6919710785339,1.03379815774724,-1.63665514960957,0.101702545274918,0.570267940079865
"2906","GAGE10",2.82237007849115,-1.56926547171515,0.958922098844775,-1.63648900531718,0.101737284908348,0.570267940079865
"2907","AC106897.1",18.1333634393321,-0.840613915344598,0.513851345787542,-1.63590875500433,0.101858685392596,0.57075202145167

"2908","PDCL3",101.34489185034,-0.4748592032991,0.290360167433596,-1.6354144147809,0.101962202640558,0.571135597940872
"2909","CENPA",49.9658516816709,2.22842294952242,1.36281085015573,1.63516672124218,0.102014102354674,0.571229877365169
"2910","ATP6V0B",562.257223461498,-0.414855078447946,0.253886411896262,-1.63401843899175,0.102254978937322,0.571433318823876
"2911","CNKSR3",20.3428089764868,0.768453831542838,0.470340903138093,1.63382309813106,0.10229600084047,0.571433318823876
"2912","MTERF3",72.8089450956495,-0.402186496356512,0.246130308195392,-1.63403889307786,0.10225068430271,0.571433318823876
"2913","SURF1",394.507675710414,-0.339234945054125,0.207587738479519,-1.63417621647048,0.102221854964035,0.571433318823876
"2914","AGAP9",34.3144646697383,0.59029841961232,0.361080398789468,1.63481158653672,0.102088551111359,0.571433318823876
"2915","TM9SF3",967.82923875058,0.217605731423633,0.13318114075644,1.63390800069499,0.102278169553249,0.571433318823876
"2916","PRPS2",102.50467385386,-0.437708711761762,0.267836708641952,-1.63423719616753,0.10220905510905,0.571433318823876
"2917","SCARA3",451.626142497358,-0.387101198707972,0.237002362008498,-1.63332211302641,0.102401268401086,0.571480620697652
"2918","AC002558.3",62.8334015888105,0.574603146880355,0.351808921805726,1.63328190749426,0.102409720167994,0.571480620697652
"2919","PLXNA3",73.6711853437712,0.455828930466718,0.279037923483088,1.63357340384718,0.102348456119003,0.571480620697652
"2920","CRB1",2.91472299350431,1.73501760213004,1.06241628006336,1.63308642260881,0.102450821743558,0.571514190198908
"2921","AL139243.1",10.2755185557591,-0.767155644186267,0.469829397188793,-1.63283874695052,0.102502915510788,0.571609034835752
"2922","TCERG1",243.132858419527,0.430707488452725,0.263855442986821,1.6323615824527,0.102603337209333,0.571973223751825
"2923","MAGEF1",295.947178090654,-0.290546096442515,0.178020683773197,-1.63209179003423,0.102660151024309,0.572013886043132
"2924","GALNT17",32.7015183568257,1.00148934072501,0.613722822885893,1.63182678463176,0.102715981133045,0.572013886043132
"2925","VPS39",124.452036679172,0.333839425299567,0.204561345982374,1.63197706632382,0.102684317519568,0.572013886043132
"2926","N4BP2L2",1217.05475028602,0.228801182450172,0.14024525641538,1.63143615904203,0.102798320354262,0.572081257345602
"2927","PIAS1",367.785467770724,0.3302069420755,0.20238844084301,1.63155040228625,0.102774233809347,0.572081257345602
"2928","TUT4",407.347060312075,0.295591725805953,0.181334040643479,1.63009507071601,0.103081405373333,0.572166762541078
"2929","KANSL1L",142.241838763867,0.420052793190313,0.25752961259987,1.63108540780885,0.102872299261773,0.572166762541078
"2930","MRPL22",395.532621307698,-0.43122394420863,0.264569404450329,-1.62990858714198,0.103120818496215,0.572166762541078
"2931","HSPA1L",4.72779609261442,1.19460472092325,0.732583448355523,1.63067391654133,0.102959143158945,0.572166762541078
"2932","ANXA7",798.343455244713,-0.280821306328838,0.172187670952158,-1.63090251918712,0.102910890117042,0.572166762541078

"2933","NLRX1",46.3712551697696,0.422502382189486,0.259185016466115,1.
63011885467045,0.103076379518967,0.572166762541078
"2934","GZMB",26.5808443881027,0.769059272548496,0.471785233054874,1.6
3010458714176,0.103079394407233,0.572166762541078
"2935","MEGF8",104.161355043756,0.396141655726865,0.243051669907415,1.
62986601111511,0.103129818578219,0.572166762541078
"2936","CYTH2",276.477730788907,-0.356768638852589,0.218836288630494,-
1.63029925742797,0.103038264448299,0.572166762541078
"2937","GRB10",78.5321536801962,0.445131285796286,0.273246860685726,1.
62904446433239,0.103303606443748,0.572935800259523
"2938","AL627309.5",6.94027244229656,1.08958266856884,0.669483558018597,1.6274972783403,0.103631526178653,0.573320382234838
"2939","PDGFRL",3095.96724288328,-0.229628964202822,0.141051127850064,
-1.62798389281166,0.103528301173022,0.573320382234838
"2940","PEBP4",19.2987714324233,0.662918891315242,0.407473479700966,1.
62690070480597,0.103758188277664,0.573320382234838
"2941","CLU",4872.07517332225,-0.715202719368452,0.439695921980733,-1.
62658483650843,0.103825301963169,0.573320382234838
"2942","AC021321.1",9.49095308580655,-0.785019505871315,0.482241306550804,-1.62785621058907,0.10355537835398,0.573320382234838
"2943","AL162231.1",35.5853380065876,-0.507162631763885,0.311823917773546,-1.62643916279763,0.103856265406753,0.573320382234838
"2944","CYP2E1",3.12738734727671,1.5269951714973,0.938676190879281,1.6
2675391826752,0.103789372257935,0.573320382234838
"2945","INTS4",58.9343294095833,0.397968492297344,0.244597110053646,1.
62703677165302,0.103729288286237,0.573320382234838
"2946","CDADC1",208.479847765535,-0.327853623186782,0.201481905224499,
-1.62721125165793,0.10369223887262,0.573320382234838
"2947","FAM124A",13.6533614278975,0.840266464073058,0.516230501729451,
1.62769627377312,0.103589303609539,0.573320382234838
"2948","CREBBP",507.186593881652,0.373823722261864,0.229848085015519,1
.626394765206,0.103865703724907,0.573320382234838
"2949","DPEP2",5.2130323800583,-1.16861264019482,0.718507808446631,-1.
62644389727829,0.103855258961411,0.573320382234838
"2950","TIAF1",3.95668826009985,1.69354096029419,1.0408829473815,1.627
02344634865,0.103732118238862,0.573320382234838
"2951","CRYZL1",172.308872430813,-0.268437614313434,0.16489082971345,-
1.6279717603455,0.10353087382627,0.573320382234838
"2952","UTS2B",6.59406559967075,1.16496129867116,0.716521406997387,1.6
2585693503978,0.103980093175673,0.573416241477712
"2953","FAM114A2",118.104197020285,0.321852838888344,0.197938899941749
,1.62602115593783,0.103945154925581,0.573416241477712
"2954","RBFADN",4.06423912228392,1.35660667598048,0.834415569610985,1.
62581658994324,0.103988678084914,0.573416241477712
"2955","AC010999.2",2.88895942713338,-1.52089090015403,0.93577695070996,-1.62527074320452,0.104104882495413,0.573862751596545
"2956","LINC02728",2.52878443082401,2.00987634404719,1.23732375779921,
1.62437384021632,0.104296046680643,0.574722024486127
"2957","TMEM9B-
AS1",28.8279221225347,0.786277917827339,0.484186560099296,1.6239152067

0481,0.104393906631768,0.575066738290451
"2958","ECI1",504.285403203645,-0.340977614926082,0.210015594372672,-1
.62358236275073,0.104464972159039,0.575263668525553
"2959","NBPF9",61.0233060178757,0.765155306757675,0.471471654698871,1.
6229083957261,0.104608988544716,0.57529468227408
"2960","RPS6KC1",111.345653692377,0.351140711373719,0.216347788934265,
1.62303813273732,0.104581253494858,0.57529468227408
"2961","AC051619.5",2.30131198471853,2.1779208855747,1.34193769390889,
1.62296721782268,0.104596412891481,0.57529468227408
"2962","ELMO2",59.3056041500786,0.431346341265788,0.26578821821979,1.6
2289489035625,0.104611876044928,0.57529468227408
"2963","SCAF11",1203.71096961864,0.249192780919119,0.15362115296968,1.
62212544367703,0.104776491260673,0.576005489755351
"2964","HDAC4",206.156769138741,0.33756363531476,0.208125927944046,1.6
2192014541078,0.104820447455076,0.576052722198288
"2965","DDAH2",2789.40626717723,-0.360004376209979,0.222079640875957,-
1.62105979093806,0.105004816295941,0.57669066536951
"2966","APOBR",11.4803981495717,1.29948563809279,0.801633023295062,1.6
2104803610927,0.105007337067098,0.57669066536951
"2967","HRAS",187.078996947732,-0.404492999165463,0.249562387203012,-1
.62080914395333,0.105058576844047,0.57677760640805
"2968","QPCT",24.0084246920264,-0.978347834750135,0.603779753952918,-1
.62037204517862,0.105152381165074,0.576903717345197
"2969","ZNF461",10.125094687369,0.770554210212508,0.475511872434024,1.
6204731256619,0.105130682710634,0.576903717345197
"2970","COA6-
AS1",28.5536262255711,-0.623233605943192,0.384800981184636,-1.61962582
3262,0.105312678982335,0.577214935646956
"2971","TSC22D1",1040.7229279445,-0.530830424039401,0.327751103351271,
-1.61961445319828,0.10531512291347,0.577214935646956
"2972","ZNF559",113.162899886767,0.341310540908713,0.210735849032881,1
.61961309608722,0.105315414619851,0.577214935646956
"2973","TIA1",263.929817618821,0.288170888667762,0.17794485276106,1.61
943930491044,0.10535277573783,0.577225484020689
"2974","MGAT4C",29.615111317171,1.8718263063411,1.15606644127398,1.619
13384863794,0.105418467319143,0.577321738255576
"2975","ZNF598",81.1727224788167,-0.371939526316152,0.229730131521122,
-1.61902804762011,0.105441228516811,0.577321738255576
"2976","AC120053.1",20.7797455973998,0.654297847105535,0.4042493897362
18,1.61854999343964,0.105544121946627,0.577690928221979
"2977","SEC63",953.062487935871,0.186442401400964,0.115203523312103,1.
61837412642203,0.105581994514887,0.577704100991937
"2978","STAT2",409.633111690758,0.55355168934982,0.342162444460643,1.6
178037604986,0.105704895727183,0.577918066607394
"2979","AC069503.1",4.62438816853129,1.26879814369003,0.78434195300956
5,1.61765941350145,0.105736017292838,0.577918066607394
"2980","HP",238.822960293874,1.70361312943876,1.05332323916114,1.61736
973618422,0.105798494359583,0.577918066607394
"2981","TOMM34",39.8306775646817,-0.4474194444517,0.276616191412746,-1
.61747380790192,0.105776044995038,0.577918066607394

"2982","WDR13",479.61280731324,-0.283210152765315,0.175097451393161,-1
.61744303250536,0.10578268317839,0.577918066607394
"2983","ZNF112",13.5680374869865,0.664294403511235,0.410912149139766,1
.61663364030954,0.1059573858992,0.578591974157582
"2984","RXYLT1",195.824087389984,-0.309652803056814,0.191614805434162,
-1.61601710449879,0.106090615135564,0.578793443577237
"2985","TMEM233",33.9651012648193,-1.07334201309445,0.664093710055034,
-1.61625083454789,0.106040092020258,0.578793443577237
"2986","MAU2",169.731114704409,0.383332219877108,0.237214989965005,1.6
1596963131908,0.106100879275685,0.578793443577237
"2987","PIK3CA",272.149654559432,0.321741020509128,0.199206443952582,1
.61511351804319,0.10628611405647,0.579128866556303
"2988","FRMPD1",19.4023731064757,0.804282730879474,0.497902993877385,1
.6153402184152,0.10623703860541,0.579128866556303
"2989","HEXIM1",310.742029891264,0.460172677571739,0.284875761460952,1
.61534514278013,0.106235972792314,0.579128866556303
"2990","SECTM1",73.8267400554136,0.525810269574989,0.325573422217205,1
.61502823539508,0.106304580453272,0.579128866556303
"2991","FOXP1-
IT1",7.26084068000896,1.03839865597111,0.643113752654494,1.61464228013
327,0.106388183809301,0.57939054699756
"2992","FAM215B",38.0071844097515,-0.436358923026,0.270305885822441,-1
.61431528469431,0.106459056417005,0.579582743976135
"2993","YBX1",5016.26677692761,-0.318398486909269,0.197365051478523,-1
.61324654250612,0.106690955436493,0.57991185438058
"2994","ERICH2",19.2025029778594,-0.793722215571433,0.492012259861405,
-1.61321633691611,0.106697515352606,0.57991185438058
"2995","TRPS1",123.02326527726,0.412593843127509,0.255699573792206,1.6
1358830993908,0.106616754168067,0.57991185438058
"2996","NAV2",118.105888673713,-1.16074883437917,0.719415738200188,-1.
6134604412229,0.106644511007501,0.57991185438058
"2997","MED13L",781.96249941692,0.435058922889359,0.269621215064314,1.
61359306531418,0.106615722015213,0.57991185438058
"2998","ABCA9",1917.03870935886,0.490487115542795,0.304121358440242,1.
61280062031281,0.106787831319823,0.580209134212341
"2999","OTULIN",134.279889360424,0.416878410680352,0.258577538163379,1
.61219885393507,0.106918674598826,0.580726338959747
"3000","LIN7A",5.36834376637912,-1.13315889283016,0.703085377263151,-1
.61169458144775,0.107028417385571,0.580934985269434
"3001","SLC7A6",128.686137522444,0.576711991024578,0.357812777870031,1
.61177025163159,0.107011943899779,0.580934985269434
"3002","PRKAG2",264.684748827975,0.387704803384284,0.240639415697322,1
.61114421866758,0.107148292451976,0.581391917305207
"3003","AC006064.4",3.09414677232413,-1.40495209888853,0.8721899541402
15,-1.61083270016966,0.107216191734338,0.581566615771105
"3004","LATS2",445.016893591623,0.307675695210203,0.191025562694929,1.
61065195081543,0.107255603899071,0.581585451039494
"3005","LCA5L",6.33519776546908,-0.936634006357058,0.581642595771379,-
1.61032567622543,0.107326776709725,0.581585451039494
"3006","CHIC1",62.0919313637636,0.457342524582896,0.284000989442462,1.

61035539165103,0.107320293105123,0.581585451039494
"3007","AC012020.1",6.17418483951732,1.20650951504457,0.74939067558742
1,1.6099873595289,0.107400615859648,0.581664248863342
"3008","COPS3",242.586995465904,-0.379829940980233,0.235929205586217,-
1.60993184390404,0.107412736238009,0.581664248863342
"3009","CCDC138",24.3589099858081,-0.488263964275495,0.303387299765042
,-1.60937509465172,0.107534347731697,0.582083943278032
"3010","AC106791.1",30.6945388584243,0.578538934102117,0.3595449703672
25,1.609086433642,0.107597443256809,0.582083943278032
"3011","LMTK3",7.75386891173526,-0.964830174859781,0.59960829275398,-1
.60910078549506,0.107594305536273,0.582083943278032
"3012","EFCAB2",116.250837828293,-0.266415666386734,0.165651767867968,
-1.60828749258553,0.107772228666011,0.582449181400352
"3013","AC011389.1",6.81028981359479,1.6213881652749,1.00811865580044,
1.60833068205402,0.10776277430358,0.582449181400352
"3014","SRGN",284.636966741268,-0.498332156269564,0.309811042056766,-1
.60850353480382,0.107724942658386,0.582449181400352
"3015","PRKD1",36.5905897915634,0.443907650813381,0.276059353891009,1.
60801524946204,0.107831838987571,0.582578051498685
"3016","SLC19A2",277.097012486613,0.723419497434558,0.450020153463453,
1.60752688933365,0.107938835673248,0.582746493329681
"3017","RBM43",405.453364206976,0.320346893501172,0.19929715731277,1.6
0738315498616,0.10797034298416,0.582746493329681
"3018","MSX1",1151.11632313399,-0.348688380699297,0.216905135144825,-1
.60756166729977,0.107931213257303,0.582746493329681
"3019","QRICH1",180.724960597263,0.271068331452066,0.168687081750781,1
.60692999510504,0.108069725632477,0.582944300332002
"3020","ZNF135",39.0452154534396,0.582248896152212,0.362345280005268,1
.60688969411647,0.108078567561093,0.582944300332002
"3021","CD1C",11.0662256548874,-1.2035312212376,0.749302285980856,-1.6
0620252167274,0.10822941948972,0.582980994584093
"3022","SLC49A3",115.480819566367,-0.462891618014515,0.288240656360562
,-1.60592063541335,0.108291348913443,0.582980994584093
"3023","ZNF622",238.509460900656,-0.277248512842423,0.172636627352868,
-1.60596576226973,0.10828143281618,0.582980994584093
"3024","ANAPC15",72.2571734913781,-0.526081668243036,0.327516184298726
,-1.60627686039234,0.10821309220502,0.582980994584093
"3025","VPS35L",161.986792822464,0.272072383972126,0.169375046849839,1
.60633097396769,0.108201208268743,0.582980994584093
"3026","PHF12",107.893599163434,0.301333129609311,0.18764352595692,1.6
0588076818857,0.108300109866257,0.582980994584093
"3027","CETN3",255.104865882304,0.34248792802575,0.213329484962835,1.6
0544112355316,0.108396760414425,0.583308500294207
"3028","GZMM",12.4251572086105,0.901600527648968,0.561787605078781,1.6
0487792806061,0.108520671829787,0.583782438386856
"3029","C6orf226",152.103906685407,-0.425328227484546,0.26505306298610
1,-1.60469085960666,0.108561854468952,0.583811174461791
"3030","MATN2",209.804675920004,-0.768812023857027,0.4791805761294,-1.
60443069305341,0.1086191500227,0.58392651310883
"3031","RASA2",174.548130055658,0.3171169370268,0.197710959572798,1.60

394212699189,0.108726809831774,0.584067174491454
"3032","SPATA13",113.65439391548,0.440259480193416,0.274533963873338,1
.60366125189719,0.108788741322799,0.584067174491454
"3033","VTN",6.96426674853383,-1.13604933366288,0.708260897692783,-1.6
0399838161849,0.108714409333594,0.584067174491454
"3034","NGFR",82.2212937985156,0.879430769918665,0.548365405261723,1.6
0373131032752,0.108773291193387,0.584067174491454
"3035","AC007563.2",17.3930704234057,0.650447415528021,0.4060342664803
76,1.60195202529651,0.109166218664485,0.58435037215743
"3036","HYAL1",16.0668513432312,-0.780989260228993,0.487484924635834,-
1.60207879415434,0.109138186646033,0.58435037215743
"3037","PRRC1",298.745037846648,0.272876181848371,0.170390042013417,1.
6014796324006,0.109270727634965,0.58435037215743
"3038","EXT1",288.457951474464,0.356113030723769,0.222174380560295,1.6
0285371259142,0.108966954978359,0.58435037215743
"3039","AL135791.1",12.8687640286049,-0.738583779901174,0.461065023989
048,-1.6019080638802,0.109175941050477,0.58435037215743
"3040","ADRB1",11.1966362225375,-1.01607306412389,0.633844133254004,-1
.60303300262103,0.108927368020487,0.58435037215743
"3041","DNAJC4",301.60717940407,-0.436069142908866,0.272269991508056,-
1.60160559925666,0.109242851858115,0.58435037215743
"3042","TMEM135",258.897667533621,0.289753901227709,0.180866599282619,
1.60203101278498,0.10914875172887,0.58435037215743
"3043","ITGAE",365.139959977445,-0.381395592711795,0.238126471202962,-
1.60165138627835,0.109232720833956,0.58435037215743
"3044","FLCN",206.763184854214,0.406188750542124,0.253634310506464,1.6
0147398721819,0.109271977014644,0.58435037215743
"3045","SNHG25",174.411974437694,-0.727556000182796,0.454105197917646,
-1.60217501037005,0.109116914441938,0.58435037215743
"3046","MFNG",3.48018302126949,-1.51253101841217,0.944458624586375,-1.
60147938621937,0.109270782119033,0.58435037215743
"3047","MAPK13",79.4212062207196,0.472525544102418,0.295098829834078,1
.60124506209699,0.109322651749273,0.584429496010473
"3048","SPOCK2",13.2712441870812,1.03700536837396,0.647778614623276,1.
60086385219284,0.109407077387201,0.584688938175891
"3049","NPL",28.0135043559001,-0.46681910015814,0.291843356702145,-1.5
9955362847123,0.109697642570467,0.58489849421615
"3050","EEF1E1",137.620675485,-0.420283758584529,0.262629236969089,-1.
60029311067905,0.109533574482738,0.58489849421615
"3051","GALNT11",500.105989151796,0.240238867608432,0.150188472450086,
1.59958260237498,0.109691210508772,0.58489849421615
"3052","PTDSS1",91.0247919713579,-0.313554649009746,0.196017900144416,
-1.59962252824224,0.10968234765437,0.58489849421615
"3053","IST1",317.573601427874,0.266463507184321,0.166539394956393,1.6
0000285370373,0.109597950330163,0.58489849421615
"3054","AC130371.2",10.2818335522136,0.791671582573925,0.4949190299012
97,1.59959818625647,0.109687751088324,0.58489849421615
"3055","HOMER3",411.593103464499,-0.410769921572776,0.256737132691394,
-1.59996303326537,0.109606784403765,0.58489849421615
"3056","BHLHE40",202.29881902063,-0.679359320383092,0.424827811849667,

-1.59914040802841,0.109789407888449,0.585099368401443
"3057","TRAPPC11",132.919978133385,0.360201008656934,0.225282770429669
,1.5988839624528,0.109846388161925,0.585099368401443
"3058","OXA1L",384.832464907378,-0.276397443987635,0.172890105558182,-
1.59868861838724,0.109889807820068,0.585099368401443
"3059","NIP7",109.990166064425,-0.369135493417185,0.230915243425142,-1
.5985756849216,0.109914916035878,0.585099368401443
"3060","SOX12",34.2215012178483,0.523900478152636,0.327680596247451,1.
59881446796748,0.109861833336706,0.585099368401443
"3061","ING2",225.072868427374,0.422148781379884,0.264203986800385,1.5
9781381989073,0.110084418059715,0.585618904563911
"3062","SEC61A2",24.4451244031921,-0.565294315006047,0.35377391403926,
-1.59789711047863,0.110065877269965,0.585618904563911
"3063","UST",308.441352611432,0.343646624571544,0.215108316035199,1.59
755155405202,0.110142815514627,0.585738270296363
"3064","NR1H3",174.384646844437,0.498396526844263,0.312128873722224,1.
59676521079497,0.110318053444353,0.586478711669409
"3065","AC147067.1",47.9536424109094,-0.567068748294276,0.355241501966
683,-1.5962908194985,0.110423878817388,0.586756060636839
"3066","PTPN11",257.848868590048,-0.309769768835718,0.194065990068131,
-1.59620842748885,0.110442266677669,0.586756060636839
"3067","ZNF197",88.6024150814785,0.329464026710942,0.206539558364224,1
.59516186303616,0.110676044527635,0.587614761835283
"3068","PPP5D1",30.9354288543937,0.494496302285869,0.309984975307939,1
.5952266776628,0.110661555124482,0.587614761835283
"3069","ANO7",6.47733098604659,-1.00359500102243,0.629559153086877,-1.
59412343717277,0.110908390466885,0.588081631612985
"3070","AL691447.2",14.3011364805982,0.945435912459949,0.5929933511866
3,1.59434487851854,0.110858811081741,0.588081631612985
"3071","PLD2",79.7269208050449,-0.322945228117951,0.202574874011507,-1
.59420179671249,0.110890844236355,0.588081631612985
"3072","AL034397.3",2.4491393326007,-1.49293320931098,0.93629883358740
5,-1.59450504022401,0.1108229627462,0.588081631612985
"3073","FKBP14",293.287115585186,0.447846432303866,0.280990631098923,1
.59381268532829,0.110977995454741,0.588134306658807
"3074","CYC1",558.101331372148,-0.38709092336178,0.242928668505343,-1.
5934345079295,0.111062749541561,0.588134306658807
"3075","AP003307.1",35.775515181532,-0.518695489635674,0.3254949971573
2,-1.59355902292094,0.111034838599528,0.588134306658807
"3076","ZNF548",63.4577812776212,0.342671659303797,0.215040671664095,1
.59352022411401,0.111043535040676,0.588134306658807
"3077","AP003392.5",5.90444904616918,1.08342535259363,0.68018197008078
2,1.59284632679246,0.111194669509004,0.588641524742337
"3078","CCDC173",2.91651023164486,-1.31294935304429,0.824574363528309,
-1.59227525268461,0.111322870941846,0.588950153282882
"3079","TCTA",95.7365986123183,0.443177179598985,0.278331379596747,1.5
9226451663866,0.111325282212413,0.588950153282882
"3080","VPS8",98.5363534655398,0.377728259669778,0.237456323859336,1.5
9072731157725,0.111670957661789,0.589629896062522
"3081","ZNF718",31.6744575769564,0.580909739991245,0.365092902775831,1

.59112854721234,0.111580649152206,0.589629896062522
"3082","N4BP2",119.659557964527,0.402625091995331,0.253083552110437,1.
59087814533139,0.111637001842304,0.589629896062522
"3083","SND1",318.995023483681,-0.290189757665989,0.182364244204721,-1
.59126455370398,0.111550050436594,0.589629896062522
"3084","ZNF438",136.188348065927,0.288593905119062,0.181347106154125,1
.59138963526547,0.111521915454849,0.589629896062522
"3085","ZWILCH",42.9969836753315,0.475913056370261,0.299149674918407,1
.59088608904578,0.111635213772591,0.589629896062522
"3086","KCNN4",2.57989738908714,-1.50639337632245,0.947133844006432,-1
.59047571349611,0.111727615761971,0.589737891492788
"3087","TNFRSF4",4.28508951635674,-1.18187146308961,0.743375724980987,
-1.58987094059311,0.111863899099981,0.589883798135184
"3088","UBE3C",176.111597502788,0.291476460139569,0.183305671450075,1.
59011152155734,0.111809669370304,0.589883798135184
"3089","PKN3",6.0642920416272,-1.01708859587752,0.639720119843067,-1.5
8989621293609,0.111858201446679,0.589883798135184
"3090","STXBP3",291.228529440969,-0.23752616615975,0.149445365440801,-
1.58938462533882,0.111973583538595,0.589891386525136
"3091","CCL19",26.7342209227482,-1.93850350565504,1.21941817326936,-1.
58969543684736,0.111903472759834,0.589891386525136
"3092","MPI",103.025236403858,0.370915108574394,0.233370521624469,1.58
93828663213,0.111973980424564,0.589891386525136
"3093","NPIPA2",2.9219953160356,2.34992556744793,1.47872908215451,1.58
915219549485,0.11202603614589,0.589909641836862
"3094","UPF1",243.664406600485,0.391057817215915,0.246095879944238,1.5
8904658340694,0.112049876103091,0.589909641836862
"3095","CLSTN1",451.852886464285,0.270054647097445,0.170083024250407,1
.58778131026088,0.112335799124675,0.590460739574647
"3096","LINC02019",5.27698094357587,-1.29957333031334,0.81819458382959
6,-1.58834262166663,0.11220888456451,0.590460739574647
"3097","SAMD8",220.533314776079,0.414888660161902,0.261289907871927,1.
58784800967231,0.112320712220728,0.590460739574647
"3098","SLC38A1",34.4710116094218,-0.754546417488298,0.475101139752564
,-1.588180609041,0.112245504597417,0.590460739574647
"3099","GPR108",297.234399027883,-0.281642771774987,0.177354999650258,
-1.58801709751845,0.112282472983874,0.590460739574647
"3100","CD33",4.14499910864776,-1.60564091872933,1.01154776622802,-1.5
8731102211479,0.112442220114486,0.590829459175761
"3101","BRINP3",8.73971812804814,1.54324532572174,0.972463307853006,1.
58694452866186,0.112525208612958,0.591074854271679
"3102","AC093157.1",45.7567082529587,0.505510209699965,0.3187612836694
46,1.58585824439136,0.112771469691436,0.591286825101233
"3103","INTS3",130.836125257621,0.34457458729908,0.217370075963862,1.5
8519789704801,0.112921378177909,0.591286825101233
"3104","SCN9A",2.61920157659644,-2.12789558437015,1.34277358957138,-1.
58470169572622,0.113034126429287,0.591286825101233
"3105","ACADL",97.5401883099821,0.491837564394661,0.310305946683542,1.
58500850419167,0.112964402089043,0.591286825101233
"3106","TRIM23",209.147372300348,0.31111773549033,0.196286076004319,1.

58502193239363,0.112961351211874,0.591286825101233
"3107","RCL1",79.6373309256081,-0.34801361505111,0.219448256359206,-1.
58585728054936,0.112771688383274,0.591286825101233
"3108","LCNL1",4.00092580903467,1.37577993530008,0.867448419560098,1.5
8600777207914,0.112737546514248,0.591286825101233
"3109","DHTKD1",72.6776437299993,0.464154663426398,0.292827756824411,1
.5850774136303,0.112948746605908,0.591286825101233
"3110","TKFC",66.9899363313462,0.616318948669074,0.388693462116885,1.5
8561696744912,0.112826224882891,0.591286825101233
"3111","PLCB2",11.070213905683,-1.00887241956954,0.636669581784131,-1.
58460911033694,0.113055173757383,0.591286825101233
"3112","POLDIP2",214.845614687709,-0.29969732415293,0.189084804332418,
-1.5849889429827,0.112968846496886,0.591286825101233
"3113","MTCL1",10.6531171095606,-1.06454860293084,0.671783209807042,-1
.58466092541464,0.113043394315957,0.591286825101233
"3114","MPV17L2",21.5438177500295,-0.553084670267251,0.348708675380149
,-1.58609380642538,0.112718031645279,0.591286825101233
"3115","ZNF429",117.466616731004,0.394829120922877,0.249177848991007,1
.58452736678502,0.113073758990137,0.591286825101233
"3116","NDUFB2-
AS1",6.75639078762302,0.996559722973358,0.629076252312068,1.5841636356
6525,0.113156486172531,0.591529526079705
"3117","DMC1",13.0913470134713,-0.726282119879198,0.458587792667936,-1
.5837362692406,0.113253747525479,0.591848024845215
"3118","COX5A",537.119163736994,-0.449932829553315,0.28418991416398,-1
.58321181410294,0.113373194618241,0.591902553569398
"3119","NCBP3",279.997753218115,0.250699513918719,0.158332922831158,1.
583369456181,0.113337280475512,0.591902553569398
"3120","LINC01638",33.5673538600491,-0.598971526307083,0.3782841892995
48,-1.58339032729909,0.113332526272838,0.591902553569398
"3121","AL356512.1",12.155285171027,-0.818487084758646,0.5171649022695
13,-1.58264236642282,0.11350300132722,0.591948191269827
"3122","NICN1",46.6329879184984,0.395441740710267,0.249999427053701,1.
58177058791948,0.113701951699573,0.591948191269827
"3123","TNIP2",245.463326355189,-0.319744696384735,0.20203211787133,-1
.58264289734553,0.113502880247852,0.591948191269827
"3124","KDM7A-
DT",126.794024825274,-0.468672431454186,0.29617895870235,-1.5823961077
7073,0.113559172714161,0.591948191269827
"3125","ERMP1",26.9429163314347,0.501889470053639,0.317146298702916,1.
58251719192781,0.113531550786413,0.591948191269827
"3126","ACO1",261.038596811181,0.341376043962399,0.215783110080647,1.5
8203319914525,0.113641991766275,0.591948191269827
"3127","SOCS7",61.7896519698394,0.523247478581303,0.330805042919802,1.
58173972791629,0.113708999354367,0.591948191269827
"3128","PRPF31",352.71574697898,-0.338645916925326,0.214091436385687,-
1.58178170338048,0.113699413289875,0.591948191269827
"3129","HMGB3",14.8659700843563,-0.747037871330442,0.472140533162581,-
1.58223625988324,0.11359564559727,0.591948191269827
"3130","AL133245.1",7.43009699240432,1.10884925578604,0.70139485848913

,1.58092013701755,0.113896299394454,0.59197728807794
"3131","WDR1",262.61408121953,-0.323420191825608,0.204526997670636,-1.58130806939451,0.113807615456393,0.59197728807794
"3132","NDUFB2",1385.55552189994,-0.387657535140173,0.245181112142584,-1.58110684690399,0.113853609474686,0.59197728807794
"3133","MARCH8",103.546599248615,0.375708478482588,0.237604649140571,1.5812336999362,0.11382461259807,0.59197728807794
"3134","PIP4P1",138.549527298775,-0.34996828582298,0.22135585483728,-1.58102113937869,0.113873204340902,0.59197728807794
"3135","TCF4-AS2",9.49608218052382,1.0720595178312,0.678239175449717,1.58065112815159,0.1139578285651,0.592108156139366
"3136","AC010210.1",3.99014965789351,1.26914060759144,0.803229467227394,1.5800473704884,0.11409601858485,0.592575630077594
"3137","GATM",5.05508224430022,-1.14453908490142,0.724419233802621,-1.57994022176013,0.11412055691285,0.592575630077594
"3138","CBFB",158.475746092074,0.323099140681563,0.20455143098153,1.57954964739766,0.114210038243525,0.592851278823702
"3139","AC107021.2",2.29839686582198,1.73039053068428,1.09576719975146,1.57915890444317,0.114299613455606,0.593127239113848
"3140","AC011365.1",28.6449574026064,0.725254775694031,0.459358718746728,1.57884186387656,0.1143723335162,0.593315586192794
"3141","RGS4",2.91340451510367,-1.63107953779603,1.0334394354646,-1.57830200960229,0.114496244474125,0.593354287044882
"3142","MOSPD3",191.025930489557,-0.50928282672415,0.322636514980038,-1.57850337168333,0.114450014156492,0.593354287044882
"3143","DCHS1",151.093831255102,-0.371224660657987,0.235247712078129,-1.57801602990595,0.114561927236549,0.593354287044882
"3144","TCF4",4760.15918182061,0.365181606374031,0.231406515090546,1.57809561338902,0.114543645818592,0.593354287044882
"3145","AC012313.1",18.0103736987258,0.755959551439338,0.479039514548959,1.57807347511011,0.114548731054494,0.593354287044882
"3146","WDR6",151.508190211731,0.358145188170475,0.227096430804259,1.57706216210492,0.11478122248594,0.594112276159348
"3147","HAUS7",52.7585226249194,-0.475413568105534,0.301442877172036,-1.57712656064589,0.114766406802487,0.594112276159348
"3148","WDR17",14.8398658883742,-1.0762509858031,0.682738411515522,-1.57637386098443,0.114939668709154,0.594365797969335
"3149","HLA-C",4620.82448372331,-0.460810341682379,0.292309590065138,-1.57644619726535,0.1149230088835,0.594365797969335
"3150","CEMIP",10.0409940127007,1.15690944144599,0.733843199378362,1.57650768233052,0.114908849703467,0.594365797969335
"3151","AC005070.3",12.0883968354764,0.830192209712355,0.526826003382432,1.57583757138446,0.115063241250686,0.594708047707879
"3152","CDC26",645.853348022069,-0.359320832971228,0.228028765034607,-1.57576976271698,0.115078873250367,0.594708047707879
"3153","RXRG",10.4830366882227,-0.796368984480469,0.505485134199077,-1.5754548068803,0.115151502232526,0.594724458165365
"3154","NABP1",1085.12042760242,0.975848161446929,0.619413352251782,1.

57543933772074,0.115155070357515,0.594724458165365
"3155","TOM1L2",109.198739160466,0.347570137229213,0.22066682659072,1
.57509011474892,0.115235645466634,0.594951958480506
"3156","LRRC8B",14.9736392910565,-0.610035395147928,0.387702558511935,
-1.57346239212179,0.115611789863949,0.595850230110259
"3157","CENPF",32.1680379962007,-0.564405784510276,0.359002035325199,-
1.5721520464334,0.115915293546464,0.595850230110259
"3158","HNRNPLL",162.842555835509,-0.306002230569872,0.194662060589117
,-1.5719664614861,0.115958329513753,0.595850230110259
"3159","FAHD2B",113.42318950354,-0.499492903189003,0.317575042027138,-
1.57283425045196,0.115757202662046,0.595850230110259
"3160","FAM168B",204.88773564488,0.275060925531401,0.174917677475438,1
.57251645174642,0.11583082680156,0.595850230110259
"3161","U73169.1",2.47471475280979,2.46732873314328,1.56916140957593,1
.57238682909623,0.115860866930112,0.595850230110259
"3162","LNPEP",354.716453356169,0.246596906559352,0.156789778819165,1.
57278687690329,0.115768175315644,0.595850230110259
"3163","BVES",16.5485156203062,-0.719769586820734,0.457874647616389,-1
.57197955940064,0.115955291778987,0.595850230110259
"3164","HOXA10-
AS",33.1368008315138,-0.455224827098877,0.289533595835729,-1.572269448
67965,0.115888075196657,0.595850230110259
"3165","LINC00621",3.20779238893578,-1.46510765444547,0.93084860897926
,-1.57394837389515,0.115499385196106,0.595850230110259
"3166","GABPB1",68.4810011193815,0.366830131675199,0.233142799389013,1
.57341394474345,0.1156230001644,0.595850230110259
"3167","AC015712.2",36.7555424397991,0.521683815321117,0.3317588568584
09,1.57247893925486,0.115839519714872,0.595850230110259
"3168","GALNS",102.784024319167,0.439424920250422,0.279529631126319,1.
57201552651084,0.115946950426055,0.595850230110259
"3169","USF2",388.783920129657,-0.272091214455926,0.173040247469064,-1
.57241577283672,0.115854158669565,0.595850230110259
"3170","SPECC1L",332.06311903104,0.33828897430676,0.215066034253137,1.
57295397890951,0.115729474820313,0.595850230110259
"3171","LDAH",36.9611959376836,0.417453657578111,0.265649211959659,1.5
7144700147465,0.116078855751701,0.596069897248145
"3172","PDCD4-
AS1",5.81583464000781,1.23891772144778,0.788462110736134,1.57130914038
607,0.116110859105431,0.596069897248145
"3173","CD27-
AS1",212.370026236865,0.378645767773288,0.240962488177077,1.5713888524
2184,0.116092353745774,0.596069897248145
"3174","NR2C2",133.046872178612,0.3607094062706,0.229591738766045,1.57
109052881979,0.116161622254291,0.596142616540687
"3175","MED15",157.153652224652,0.294632992078183,0.187556304559441,1.
57090422937399,0.116204896062888,0.596176866761694
"3176","WAPL",251.451305605989,0.288500494843502,0.183722213694334,1.5
703081790833,0.116343432280238,0.596467915442936
"3177","LINC01220",14.5786064327106,1.41041130410219,0.898082041413149
,1.57047044597716,0.116305704759353,0.596467915442936

"3178","RASGRP4",2.57787457370793,-1.77641914998794,1.13134200073805,-1.57018757266067,0.116371479850061,0.596467915442936
"3179","SFXN4",80.8158825865574,-0.431025846453399,0.274581512672459,-1.56975552453729,0.116471998200854,0.596607666255885
"3180","GLCE",187.131754231646,0.405855212841482,0.258545340269523,1.56976417528312,0.116469984888719,0.596607666255885
"3181","SMIM10",298.76952476281,-0.377762260107947,0.24076474460813,-1.56900986779769,0.116645639733587,0.597309281867461
"3182","TMTC4",42.8756893519102,0.439476253307341,0.280141818914677,1.56876347490695,0.116703062068407,0.597415517923408
"3183","PUM2",280.123923817809,0.287592967011744,0.183380144683995,1.56828847260062,0.116813824921296,0.59761785000748
"3184","TEAD3",86.9968318812152,0.499737831191727,0.318653590631123,1.56827930355955,0.116815963805256,0.59761785000748
"3185","AC023509.2",5.96256684162004,1.27899853615893,0.815845045568888,1.56769786506098,0.116951660169052,0.598124204864578
"3186","VANGL1",124.245736773497,0.382441549421868,0.244057186480917,1.56701613640774,0.117110919900601,0.598258458091457
"3187","FBXW4",113.446536661224,-0.315357194997193,0.201231535664756,-1.56713605526803,0.117082893127011,0.598258458091457
"3188","FCHSD2",57.9139960541759,0.429394467855659,0.27403085114578,1.56695666221622,0.117124821833977,0.598258458091457
"3189","ALDOA",96.6699325489711,-0.504392329457925,0.321865690582205,-1.56708945444157,0.117093783789071,0.598258458091457
"3190","AASS",335.603504458043,0.459728736261713,0.293438439009357,1.56669568517931,0.117185839836529,0.598312576722284
"3191","B4GALT1-AS1",34.2600045732065,-0.573833992480729,0.366293264465212,-1.56659717267399,0.117208879140574,0.598312576722284
"3192","DPY19L4",220.342047303525,0.266918434829109,0.170401018688434,1.56641337524601,0.11725187369554,0.598344539670004
"3193","TNFAIP8L1",7.44331723673135,-0.860566684650982,0.549474933861115,-1.56616186038524,0.117310729018348,0.598457395859655
"3194","AC012065.4",2.82745629088036,1.52258812875166,0.973827280219505,1.56350942274739,0.117932821109928,0.599175365587668
"3195","SH3RF3-AS1",5.73600740093445,1.16510592343682,0.744840146037475,1.56423620509065,0.117762107623629,0.599175365587668
"3196","SORCS2",43.1453479307413,0.926394629781606,0.592290201847822,1.5640890679796,0.117796652911399,0.599175365587668
"3197","SEC24B",135.01181159162,0.278122964520876,0.177917860478026,1.56320992042969,0.118003227503545,0.599175365587668
"3198","EDNRA",5.41795204623106,1.25923732424942,0.804944551455555,1.56437772262994,0.1177288892159,0.599175365587668
"3199","MIR3936HG",25.2189736265893,-0.574231011881546,0.366832181348778,-1.56537796048918,0.117494313014401,0.599175365587668
"3200","CGAS",59.0733644762819,0.341718212124083,0.218432734270965,1.56440935130255,0.117721466023935,0.599175365587668
"3201","MAN1B1-DT",17.2351684597531,-0.712467058772572,0.455501051087965,-1.564139220

03219,0.117784877170463,0.599175365587668
"3202","FBXL15",229.22132690599,-0.398747050028737,0.255045460528316,-1.56343519779866,0.117950266686852,0.599175365587668
"3203","CSRNP2",63.3459934984391,0.45224607897324,0.289040863779536,1.56464408893473,0.117666385006077,0.599175365587668
"3204","LONP2",374.924406213871,0.30936490355256,0.197881823867701,1.5633821111301,0.117962745236861,0.599175365587668
"3205","CBFA2T3",2.76914860660266,-1.52722688280142,0.976217317846669,-1.56443330279181,0.117715844897332,0.599175365587668
"3206","GPS2",410.50416893096,-0.317418957372171,0.203051875614766,-1.56324070590898,0.117995988995712,0.599175365587668
"3207","STMN3",289.54653871051,-0.333224897297523,0.212942149257594,-1.56486115341319,0.117615468980791,0.599175365587668
"3208","RRP7A",213.360925083076,-0.50054617121871,0.320082308060785,-1.5638045546824,0.117863474340847,0.599175365587668
"3209","PLEK",28.8208266612039,-0.99730773293928,0.638146221504021,-1.56282008626293,0.118094918312194,0.599267328469573
"3210","MIER2",38.6985270272231,0.441833427216675,0.282693696765247,1.56294049804577,0.118066590937618,0.599267328469573
"3211","PDLIM5",361.604291727174,0.258666001656341,0.165657525635841,1.56145035164268,0.118417529495421,0.600596276987928
"3212","NCKAP1L",12.9334300521713,-0.931974767858022,0.596885920383858,-1.56139512766303,0.11843055078183,0.600596276987928
"3213","CPLANE1",179.251016004042,0.38855868550291,0.248878627251839,1.56123765947057,0.118467686441819,0.600597617320508
"3214","AC092683.1",133.884366883284,-0.595096325557373,0.381331188033382,-1.5605760667687,0.11862380936538,0.600641352425449
"3215","PCDHB7",24.410950101713,-0.518687648024157,0.332364552522795,-1.56059857793825,0.118618494520307,0.600641352425449
"3216","MFSD4B",55.3148399240489,0.580267904794089,0.371813351914145,1.56064299952326,0.118608007214819,0.600641352425449
"3217","POGLUT3",581.243945017964,0.280782182023756,0.17991277346134,1.56065729309704,0.118604632859607,0.600641352425449
"3218","PTGER3",72.4380741052743,-1.48537666080015,0.952223191217428,-1.55990389070558,0.118782594920766,0.600884996479613
"3219","ANXA2",10723.8261109352,-0.351098467332329,0.225070005630756,-1.55995227506384,0.118771159708152,0.600884996479613
"3220","AC144831.1",21.9466445031388,0.567943328215905,0.364035982825306,1.56012964380076,0.118729247565876,0.600884996479613
"3221","DDX41",122.09620014244,-0.274605136708942,0.176079138786943,-1.55955520114859,0.118865030120854,0.60092876338876
"3222","CEP83",129.259658223095,0.325486542504534,0.208693395156638,1.55963988347708,0.118845005884769,0.60092876338876
"3223","Z99572.1",15.7509356212053,0.790990789776262,0.507245327214968,1.55938506938881,0.118905267978785,0.600945674870132
"3224","B3GNT9",91.47951343965,0.420018176541168,0.269454348986734,1.55877304679111,0.119050105724552,0.601491058358322
"3225","KCND3",55.2602155000601,0.670489607994323,0.430196259032385,1.55856680274816,0.11909894539125,0.601551231466068
"3226","EPB41L4A-

AS1",1043.85452253368,-0.40012691622122,0.256782466806277,-1.558233010
21205,0.119178022478949,0.601764044686795
"3227","RBM26",371.736120899412,0.279123481435293,0.179165455954347,1.
55790902854854,0.119254814667466,0.601965192475475
"3228","AC027279.1",51.18787508063,0.472199058213801,0.303193045664427
,1.55742047835896,0.11937068724617,0.602363421484777
"3229","PER2",324.929190587708,-0.61205626862885,0.393136910529445,-1.
55685272035277,0.11950545685974,0.602856731739953
"3230","IRAK1",169.189490074281,-0.347374222719904,0.223183055012521,-
1.55645428682036,0.119600104815688,0.603147401654099
"3231","AC023794.1",3.97663190098281,-1.17948661908263,0.7579438210177
35,-1.55616628353651,0.119668556604378,0.603156440382462
"3232","LAMA5",14.077733698091,-0.89025019381983,0.572090530743886,-1.
55613516738731,0.119675954037456,0.603156440382462
"3233","SDR39U1",149.275511305521,-0.36747713108395,0.236218696376888,
-1.55566488478811,0.119787800829176,0.603533401703201
"3234","STARD4",9.18172253866211,-1.04521490089066,0.672034302717149,-
1.55529992541255,0.119874655124346,0.603784247779986
"3235","FOXJ3",173.655733967988,0.36572728073191,0.235195527103904,1.5
5499250022021,0.119947855500673,0.603966188021783
"3236","LGALS1",12853.4369481085,-0.468149931856778,0.301116442023125,
-1.55471394624418,0.120014211636252,0.604113564073829
"3237","CCNB1",27.5675310663576,-0.603310426265142,0.388180194196645,-
1.55420197960825,0.120136245471243,0.604507266510752
"3238","ZDHHC8",153.355058626035,-0.297668783087055,0.191540871361751,
-1.55407449580235,0.120166647980957,0.604507266510752
"3239","DVL3",239.273352794828,0.307953865707106,0.198188617565676,1.5
5384234215699,0.12022202776861 9,0.604553834350159
"3240","ZNF354C",75.2211153852507,0.367222086020931,0.236351475289068,
1.55371184195827,0.120253167102404,0.604553834350159
"3241","AL358334.2",2.94042636951498,1.67362111953005,1.07727501651055
,1.55356904586087,0.120287247659701,0.604553834350159
"3242","CHIC2",324.691217676898,-0.325326168155394,0.209429280785709,-
1.55339390430449,0.1203290582993,0.604577430794974
"3243","WTAP",660.189885252707,-0.295200522139313,0.19007195256898,-1.
55309880363427,0.120399531893375,0.604744981502063
"3244","N4BP3",3.05668018307366,1.85000687147353,1.19165170860168,1.55
247280570292,0.120549134704956,0.605309758079231
"3245","LARP4B",135.420766454684,0.334337389971412,0.21540295417271,1.
55214858243467,0.120626675701647,0.605398719832833
"3246","SLC35E3",209.005933019822,0.247020689053012,0.159153797366197,
1.55208793720857,0.120641183901859,0.605398719832833
"3247","UBE3B",111.567773900675,0.398531496503003,0.256826378458075,1.
5517545311961,0.120720969251508,0.605612524834559
"3248","HAVCR2",5.52476797076048,-0.982560879436348,0.63327719685345,-
1.55154943888454,0.120770069146029,0.605672307980192
"3249","DHCR24",35.3229242218158,-0.631025590936932,0.406844950848481,
-1.55102229884117,0.120896340221718,0.606118955331351
"3250","LINC00310",56.4268972079555,-0.751274189970255,0.4845504913899
38,-1.55045594488041,0.121032119689504,0.606612983883795

"3251","AKR1A1",733.585382805199,-0.374716039779696,0.241802863466242,
-1.54967577475364,0.121219355455824,0.606707165477787
"3252","WDR82",396.329572315088,0.215691831372423,0.139214226183795,1.
54935193970521,0.121297140278663,0.606707165477787
"3253","RIOX2",115.689186626568,0.304954073189886,0.196757018502993,1.
54990188156997,0.121165067984446,0.606707165477787
"3254","ADRA1B",39.4773991555635,-0.836360207393139,0.539833948951609,
-1.54929160905386,0.121311635948257,0.606707165477787
"3255","XPO5",39.2096466176061,0.428869134561087,0.276703575610144,1.5
499226333285,0.121160086511416,0.606707165477787
"3256","TNFRSF11B",42.6914678587804,1.01860583354818,0.65712537996405,
1.55009358123392,0.121119056452346,0.606707165477787
"3257","TAOK1",449.290397982559,0.327555780342988,0.211387242673725,1.
54955320954997,0.121248790892402,0.606707165477787
"3258","FLYWCH1",134.071414780548,0.330440892769298,0.213320365090682,
1.54903584863465,0.121373102654457,0.606828259404065
"3259","FOXP4",52.282844395243,-0.464183823745905,0.299801109192923,-1
.54830589184779,0.121548666590157,0.607180099005416
"3260","C9orf116",21.6284951852466,-0.524605739916624,0.33880867417224
7,-1.54838343852413,0.121530006200503,0.607180099005416
"3261","LINC00294",69.1468668640384,0.505694270789781,0.32661716145788
3,1.54827832234097,0.121555301298831,0.607180099005416
"3262","TMEM158",8.40489444843221,-1.02923319684836,0.664963129312984,
-1.54780491049441,0.121669273886281,0.607563090844152
"3263","RAVER2",34.5682431334851,-0.468022423015924,0.302688145065547,
-1.54621986571219,0.122051477340084,0.607608959166452
"3264","RABGGTB",199.689551095488,-0.358658643397131,0.231935926991929
,-1.54636949975246,0.122015355863515,0.607608959166452
"3265","MRPL9",297.962565722526,-0.298643059645127,0.193052169575486,-
1.54695521061396,0.121874046337527,0.607608959166452
"3266","GGCX",255.35042971429,0.425181949932688,0.27496997934736,1.546
28498333475,0.122035756997498,0.607608959166452
"3267","PARP9",169.082688285689,0.358153936291975,0.231436743381122,1.
5475240925862,0.121736919491774,0.607608959166452
"3268","C5orf49",6.72671688465033,-1.07729616350283,0.696476539822595,
-1.54678026021843,0.121916241738494,0.607608959166452
"3269","HPS5",74.7961649448747,0.361686054019582,0.233809329617944,1.5
4692738142911,0.121880757554121,0.607608959166452
"3270","DICER1-
AS1",16.1763619013977,0.71423101783173,0.46188024912254,1.546355400969
4,0.122018758936081,0.607608959166452
"3271","SALL1",2.30293374320011,-1.85875603539993,1.20187986627478,-1.
54654062153578,0.121974057515152,0.607608959166452
"3272","PLEKHA4",236.977104864423,-0.459343922192291,0.29688355090431,
-1.5472191732857,0.121810404125588,0.607608959166452
"3273","TPBG",375.826121974191,-0.386314858699977,0.249971292814054,-1
.5454368953772,0.122240621750719,0.607649959618442
"3274","EGR2",77.2252681086559,-1.26641102982625,0.819219408522463,-1.
54587527669826,0.122134692500767,0.607649959618442
"3275","BCDIN3D-

AS1",4.8220862587003,1.12859746569831,0.730226512697822,1.545544356543
15,0.122214648490753,0.607649959618442
"3276","UBFD1",150.751862046493,0.31448243584973,0.203444372218485,1.5
4579078507022,0.122155103240525,0.607649959618442
"3277","SLC25A39",171.455960927583,-0.388361945235562,0.25129966920853
9,-1.54541367467254,0.122246234739372,0.607649959618442
"3278","TRNP1",6.07651529601978,-1.40439149344179,0.909239235912167,-1
.54457863010374,0.122448218485902,0.607689769432359
"3279","MGST2",178.929876473109,-0.49800467380138,0.322331185764016,-1
.54500928174532,0.122344018292849,0.607689769432359
"3280","NSD1",267.728375660465,0.292443324610195,0.189298389974609,1.5
4488014741922,0.122375256274436,0.607689769432359
"3281","AC083864.5",2.74519781146465,-1.89742190253017,1.2285379600435
7,-1.5444552502577,0.12247808417008,0.607689769432359
"3282","ESCO2",6.16500376725283,-1.49584049138292,0.968439164810805,-1
.5445890105809,0.122445706016979,0.607689769432359
"3283","CEACAM19",3.81246342831762,-1.35388101927008,0.876311509604939
,-1.54497687686476,0.122351856545129,0.607689769432359
"3284","MAP4",685.458850845182,-0.279191980030645,0.180829415562053,-1
.54395223345085,0.122599904791499,0.6078343415892
"3285","IL1RAP",32.5946071595392,0.572043011398642,0.370503941870345,1
.54395931258087,0.122598189711109,0.6078343415892
"3286","NLRC5",37.2475094565155,0.647210616528387,0.419212417353524,1.
54387272355674,0.122619169160913,0.6078343415892
"3287","BMP2K",102.716966678223,0.344418157504534,0.22316322559797,1.5
4334638505811,0.122746754819939,0.608281682160628
"3288","CAPN2",975.125340506921,-0.27448373916558,0.177934605348915,-1
.54261021136018,0.122925378948504,0.608466972042147
"3289","MATN3",3.91580784066453,1.33512812874888,0.865622360786471,1.5
4239098853203,0.122978610074073,0.608466972042147
"3290","STON1",60.747524005424,0.642730508657212,0.416590775378809,1.5
4283423120153,0.122871001617931,0.608466972042147
"3291","COX17",453.994743702744,-0.391670258892338,0.253954399139371,-
1.54228578130433,0.123004162616089,0.608466972042147
"3292","PARG",72.2790431620943,0.365123039805578,0.236729656101916,1.5
4236290381965,0.122985430832762,0.608466972042147
"3293","ESRRA",163.288991132672,-0.32169965818137,0.208588570791961,-1
.54226886430044,0.123008271774498,0.608466972042147
"3294","LETM1",84.1317089449687,-0.33657674857222,0.218314029843675,-1
.54170920125119,0.123144274983574,0.608585283740122
"3295","AQP9",4.73525278739054,-1.28119606699549,0.830953243086587,-1.
54183893938059,0.123112736987736,0.608585283740122
"3296","KIF23",10.1695163125684,0.841719443448519,0.545862210622027,1.
54199984367731,0.123073631582382,0.608585283740122
"3297","FAF1",168.053623370522,-0.267837065510683,0.173902722913035,-1
.54015452446148,0.123522691783524,0.609161709494952
"3298","HMCN1",540.644570150551,0.614109169694696,0.398576270323846,1.
5407569778194,0.123375943765088,0.609161709494952
"3299","EIF2B5",131.592038551957,-0.289431480593332,0.187906818866613,
-1.54029258937531,0.123489049345367,0.609161709494952

"3300","SSR1",666.343592736297,0.235330629224698,0.152752246193902,1.5
4060339594596,0.123413340923823,0.609161709494952
"3301","PTK2",264.522351284696,0.275933029966792,0.179134940007291,1.5
4036409622579,0.123471627999961,0.609161709494952
"3302","DUS3L",75.880201437512,-0.40192677879968,0.260936255098443,-1.
54032554291102,0.123481020576508,0.609161709494952
"3303","LGI4",158.745228601634,0.675691953760223,0.438517545497483,1.5
4085500271984,0.123352079345474,0.609161709494952
"3304","ERI2",39.6817737490599,0.396936905221691,0.257761725904886,1.5
3993733487089,0.123575629103874,0.609238323993038
"3305","HRH1",72.9541069644603,-0.454954784027723,0.295481461004106,-1
.53970669591823,0.123631863929787,0.609331144191317
"3306","TMOD2",253.464877472645,0.359356536005779,0.233596128676471,1.
53836683014334,0.123958947724393,0.610758408796925
"3307","AP003555.1",10.9516542378499,-0.700029982320022,0.455129440250
641,-1.53808987160776,0.12402664210864,0.610835798297086
"3308","C12orf76",287.438831917052,-0.366274788851122,0.23819788121248
8,-1.53769121281302,0.124124133189603,0.610835798297086
"3309","FARP1",289.376871615613,0.373128691530765,0.242637251959794,1.
53780463847568,0.12409638912374,0.610835798297086
"3310","PHLPP2",52.5031520798592,0.348338749934234,0.226533929257451,1
.53768908293801,0.124124654206112,0.610835798297086
"3311","ZNF215",10.6492910343972,-0.90375165457676,0.587818289076823,-
1.53746773683431,0.124178809867789,0.610917739032444
"3312","ARHGEF7",222.889310533034,0.40706574386497,0.26483025074572,1.
53708174469774,0.124273292765282,0.611197966743262
"3313","YARS",107.522421823681,-0.33152488622855,0.215711643732498,-1.
53688915670993,0.124320455291465,0.611245365602981
"3314","USP6NL",37.6749796652313,-0.420600179700403,0.273777748188306,
-1.53628328994478,0.12446891597409,0.611606085158959
"3315","NDUFV1",590.946008053812,-0.319620661850951,0.208035271619003,
-1.53637726604509,0.124445879154579,0.611606085158959
"3316","ERCC1",442.934995533098,-0.358719937155569,0.233524867783863,-
1.53611022483301,0.124511348971596,0.611630085463912
"3317","AARS",163.677602900604,0.279833219832368,0.18223425386371,1.53
556871937836,0.124644190959566,0.612098048399268
"3318","CCDC180",33.9450293511655,0.571127548171159,0.372057512618044,
1.53505178312978,0.124771108734495,0.612394510199693
"3319","CSTF3",97.2437024432181,0.328976654530382,0.214336091043146,1.
53486355437993,0.124817347526735,0.612394510199693
"3320","BCAM",21.366758515597,-0.64873770419094,0.422658430077336,-1.5
3489829617793,0.124808812124905,0.612394510199693
"3321","GJA9",3.66242335426947,1.96143316846013,1.27873578511979,1.533
88463143415,0.125058037504318,0.613021478455561
"3322","MCFD2",637.056920842959,0.268435063998314,0.174987635358747,1.
53402303795915,0.125023985235818,0.613021478455561
"3323","FASN",60.3381339220734,0.375587610305186,0.244835195881424,1.5
3404255851796,0.125019183158772,0.613021478455561
"3324","HMBOX1",159.503633263135,0.422600281510367,0.275597458072321,1
.53339687697508,0.125178097766415,0.61342540147928

"3325","APLP2",2233.72639306514,0.234854964059353,0.153209479038254,1.53290100282055,0.125300248768215,0.613486557371403
"3326","POLR3K",121.03131614783,-0.439628808424248,0.286767485231414,-1.53304970425597,0.12526360869867,0.613486557371403
"3327","ATP5MGL",3.89435596960133,1.34111074670007,0.874891802549427,1.53288754425643,0.125303565373851,0.613486557371403
"3328","CREB3L4",39.8550276471635,0.472198289841457,0.308297266357036,1.53163307421159,0.12561300604942,0.613581872167263
"3329","LANCL1",121.618039309313,0.346916340156377,0.226556959177953,1.53125439807782,0.125706531164281,0.613581872167263
"3330","RAMP1",2.52240537475234,1.50721636635907,0.984515577303396,1.53092180672992,0.125788719041966,0.613581872167263
"3331","TOP2B",277.266964802526,0.2509135273542,0.163860839020339,1.53125987181755,0.125705178878268,0.613581872167263
"3332","MRFAP1L1",242.295103624901,0.243162789571597,0.158844307694246,1.53082469936319,0.125812723496756,0.613581872167263
"3333","SRFBP1",210.644298842493,-0.362316419135921,0.236558037799942,-1.53161745212958,0.125616863305616,0.613581872167263
"3334","CSNK2B",936.486752610241,-0.338838961086617,0.221258103729677,-1.53141943899418,0.125665762828719,0.613581872167263
"3335","MICAL1",57.3045997235956,-0.466686043537953,0.304686453439876,-1.53169278866559,0.125598262773906,0.613581872167263
"3336","FGF7",3556.52019857667,0.279554315314708,0.182458275064372,1.53215476369093,0.125484248430112,0.613581872167263
"3337","SMG1",327.930695098763,0.363232887995887,0.237256844251899,1.53096906072913,0.12577703938069,0.613581872167263
"3338","LSM12",256.149368509831,-0.291973372178255,0.190669773671133,-1.53130392173145,0.125694296770822,0.613581872167263
"3339","C19orf47",24.4684671918879,-0.585855878797239,0.382556398295821,-1.53142355325139,0.125664746658312,0.613581872167263
"3340","TMEM160",503.682449876973,-0.320429433074021,0.209261463500352,-1.53123956850031,0.125710194864599,0.613581872167263
"3341","AL020997.2",13.3279593928297,-0.850673789420001,0.555932594270734,-1.53017433801647,0.125973581564457,0.614182481323986
"3342","B9D1",69.1286271349618,-0.364141829381849,0.238023369447103,-1.52985746831373,0.126052013013134,0.614380981439541
"3343","SLC24A4",3.80033235337204,1.34071643307742,0.876527842463021,1.52957654979908,0.126121577641966,0.614536158603046
"3344","CAPZA1",484.163152093872,0.255358187469773,0.167027236458753,1.52884160023107,0.126303716683855,0.614747268441636
"3345","RPRD2",296.010102951169,0.247686675685694,0.162115744745739,1.52783849634201,0.12655264162571,0.614747268441636
"3346","LRRFIP1",372.106057068876,-0.500904357625933,0.328134308745905,-1.52652235464294,0.126879827521381,0.614747268441636
"3347","JCHAIN",83.1750405226377,-0.762728805776039,0.499360741184674,-1.52741043271955,0.126658983830403,0.614747268441636
"3348","IL15",9.20319128063302,-0.818132003519736,0.53584808010144,-1.52679842272619,0.12681114393907,0.614747268441636
"3349","HIST1H1D",4.98967940945348,-1.72619223513302,1.13007791405498,-1.52749842613865,0.126637118284326,0.614747268441636

"3350","ZNF282",79.9863629412425,0.335976568240328,0.220004328937142,1
.52713616983564,0.126727154462649,0.614747268441636
"3351","STAR",2.79940624498585,1.69848913288568,1.11135616761104,1.528
3031510382,0.126437287935081,0.614747268441636
"3352","PRRX2",789.226655298714,-0.38221391438195,0.249973813907252,-1
.52901581332741,0.12626052377239,0.614747268441636
"3353","ZNF487",52.736560145163,-0.411276761700602,0.269099286371915,-
1.52834579104824,0.126426706364977,0.614747268441636
"3354","CERS5",179.920404599622,-0.258640058585046,0.169431854480753,-
1.52651376789615,0.12688196430111,0.614747268441636
"3355","SF3B3",231.426666146157,0.233344089388182,0.152709076880611,1.
52803025304523,0.12650502679318,0.614747268441636
"3356","SPATA33",74.8983739922435,-0.355713940680964,0.232978384095046
,-1.52681091880115,0.126808035698018,0.614747268441636
"3357","TMEM205",435.850277424133,-0.300455211191031,0.196547338180571
,-1.52866588768045,0.126347293011936,0.614747268441636
"3358","ZNF136",110.078859019881,0.340923070819816,0.223129840780778,1
.52791338723164,0.126534043915793,0.614747268441636
"3359","ZNF45",30.3372628634317,0.457184684724253,0.299380602633867,1.
52710189204668,0.126735676538643,0.614747268441636
"3360","IDH3B",375.352776073676,-0.35179009720962,0.230451291466581,-1
.52652690714313,0.126878694660148,0.614747268441636
"3361","AC245060.5",76.8171363385266,0.666190673244234,0.4359251361669
15,1.52822266479525,0.126457263328053,0.614747268441636
"3362","PDXP",14.6436319260349,-0.825876573047799,0.54069360941061,-1.
52743912388396,0.126651854020049,0.614747268441636
"3363","LINC00629",5.82402748384155,1.09697423663179,0.718737753246852
,1.52625103061065,0.126947359029872,0.614881216544035
"3364","ACSL3",290.405141576562,0.366107949630689,0.239955694491222,1.
52573144974512,0.127076758784845,0.615325007088684
"3365","LINC00877",2.68680913714947,-1.82676636569451,1.19760306085737
,-1.52535211824419,0.127171294744103,0.615496391447166
"3366","AL353719.1",5.07493886380384,-1.11499004756994,0.7310265329505
69,-1.52523882145511,0.12719954087081,0.615496391447166
"3367","MFAP5",14228.1456830758,0.525807229782109,0.344761180675344,1.
52513467076577,0.127225511081258,0.615496391447166
"3368","RPL14",17251.925130506,-0.356516017765808,0.233831964990774,-1
.52466758674279,0.127342030284648,0.615511671010869
"3369","NXF1",162.572588972972,0.436431975903744,0.286222361127028,1.5
2480041805696,0.127308885619664,0.615511671010869
"3370","ROBO3",564.411888273534,-0.35707082848117,0.23416626704145,-1.
5248602328275,0.127293962561923,0.615511671010869
"3371","BTG3",320.357490967565,-0.31282679030351,0.205215339974849,-1.
52438307166438,0.127413046352839,0.615672237330581
"3372","CYSLTR1",20.6058765272293,0.918634713003665,0.602711237673485,
1.52417054068823,0.127466115010712,0.615746010501036
"3373","LAMTOR5-
AS1",4.58326960460494,1.13038027548767,0.742200423657807,1.52301216687
102,0.127755661489727,0.61612202528 7407
"3374","HULC",20.3913176995658,0.597252532300873,0.392115745864899,1.5

2315365704965,0.127720267282262,0.616122025287407
"3375","CFL1",3103.47571783549,-0.386712306820142,0.253939692734686,-1
.52285096770664,0.127795995257003,0.616122025287407
"3376","NRGN",2.76668392653222,-1.49997469111373,0.98509408497663,-1.5
2267150314816,0.127840910871759,0.616122025287407
"3377","TIGAR",14.0357795825298,-0.726234850909932,0.477002205505688,-
1.52249788895635,0.127884373963824,0.616122025287407
"3378","NUMB",334.340508162901,0.300214865674204,0.197109012563462,1.5
230905059582,0.127736063781404,0.616122025287407
"3379","CCL13",22.5896094513158,-0.814395578559886,0.534565213193119,-
1.52347283074268,0.127640452998711,0.616122025287407
"3380","AC080038.1",49.7760751730542,-0.409316899717001,0.268633627838
255,-1.5236994080408,0.127583817414877,0.616122025287407
"3381","ZNF524",227.581178403549,-0.484130483889389,0.317984317853569,
-1.52249798718794,0.127884349368986,0.616122025287407
"3382","NCL",1855.26689950995,-0.239620766898174,0.157511222846009,-1.
52129329306547,0.128186252597505,0.616441414888806
"3383","ZBTB20",2495.15868577923,0.348805838454567,0.229223882813369,1
.52168192150623,0.128088799607485,0.616441414888806
"3384","LINC02482",52.3332195722687,0.535062657791249,0.35181243364519
5,1.52087478048278,0.128291263826696,0.616441414888806
"3385","YME1L1",680.245128201095,0.191328781934308,0.125783433637293,1
.52109682810871,0.128235540345046,0.616441414888806
"3386","AP2A2",319.736795189762,0.283672977148358,0.18644770154252,1.5
2146137925796,0.128144096006275,0.616441414888806
"3387","ZNF44",83.5556363795236,0.33729507203267,0.22169431497596,1.52
144213562376,0.12814892182902,0.616441414888806
"3388","PAK4",59.4424940367264,0.332985285655608,0.2189341304158,1.520
93821563226,0.128275342675714,0.616441414888806
"3389","SMIM25",6.39745026870225,-1.07459608392121,0.706486350447712,-
1.5210429518422,0.128249059017828,0.616441414888806
"3390","ZMYM3",67.3984627413292,0.331049587476133,0.217518378147949,1.
52193846926793,0.128024498917087,0.616441414888806
"3391","SIN3B",139.714574916525,0.372439318339631,0.244961155801959,1.
52040153925765,0.128410087861683,0.616830410256252
"3392","SHPRH",350.295035996691,0.314318550221098,0.206801592123572,1.
51990391850214,0.128535125494323,0.617249015087569
"3393","DYRK3",113.271495082021,-0.7293168866773,0.480176051425341,-1.
51885310504848,0.128799475244468,0.617665702362172
"3394","NBAS",189.532655795639,0.26077292663323,0.171705389174198,1.51
872301671715,0.128832230510788,0.617665702362172
"3395","DNER",27.713564355064,0.666687896757746,0.438998974201828,1.51
865479405708,0.128849411052039,0.617665702362172
"3396","CHL1-
AS2",62.824047522551,0.636966637332738,0.419351601221924,1.51893216927
447,0.12877957062706,0.617665702362172
"3397","ZFAT",20.8964713663124,-0.617178468287171,0.406231659253879,-1
.51927712729415,0.128692754542093,0.617665702362172
"3398","AC008555.4",8.55548994608779,-1.26517627920313,0.8330573939706
18,-1.51871442275171,0.128834394634736,0.617665702362172

"3399","FRMD4A",84.8223115779326,0.45763976651443,0.301381882725143,1.51847139043787,0.128895606488265,0.617705364544675
"3400","H6PD",594.049151171362,0.385795479685169,0.254401267741616,1.51648410839291,0.129396985677397,0.617748454707536
"3401","DNAJB11",307.930198932106,-0.362511934516491,0.238974981736226,-1.51694512907892,0.129280538244017,0.617748454707536
"3402","AC108519.1",24.3800057207701,-0.708158516628176,0.466831020849927,-1.51694828535362,0.129279741293404,0.617748454707536
"3403","RGS17",8.76782655684263,-0.894670803760303,0.589366642008825,-1.51802077007763,0.129009162736122,0.617748454707536
"3404","GGCT",232.698676171796,-0.353350711489249,0.232828708925125,-1.51764236086058,0.129104581812394,0.617748454707536
"3405","AC090204.1",3.3430877047348,2.20115712551655,1.4516331379366,1.5163315496127,0.129435537842521,0.617748454707536
"3406","SLC52A2",159.483884106477,-0.374024298500752,0.246652495287575,-1.51640184326809,0.129417773269171,0.617748454707536
"3407","PRR5L",12.4465988028133,1.06994850509547,0.705593889667193,1.51638006049079,0.129423277998018,0.617748454707536
"3408","PHB2",1052.45362656852,-0.376596817770991,0.248159440826957,-1.51755990630876,0.129125380695782,0.617748454707536
"3409","CREBL2",427.433617331099,0.251686143048192,0.16585821162486,1.51747773343571,0.129146111116045,0.617748454707536
"3410","POLR1D",903.046671619269,-0.290618060023062,0.191495853418425,-1.51762064209324,0.129110060045931,0.617748454707536
"3411","GLG1",1344.0276903905,0.293575752746828,0.193439444436275,1.5176623030653,0.12909955184917,0.617748454707536
"3412","DUSP14",194.737400830352,-0.402992799276988,0.265704796683059,-1.51669373043984,0.129344027960162,0.617748454707536
"3413","CD37",21.5833674934953,-0.540744564148021,0.356551704067265,-1.51659509120172,0.129368945516988,0.617748454707536
"3414","ACOX1",196.321912872143,0.358854807998996,0.236711051789811,1.51600360560116,0.129518440696537,0.617951984857385
"3415","ASPHD2",13.1249109153267,-0.693438656018088,0.457454771551946,-1.5158627675159,0.129554056620294,0.617951984857385
"3416","CCL26",104.167620649563,0.741306162037618,0.489260131647552,1.5151575084226,0.12973252089306,0.618260103226174
"3417","ZC3HAV1L",9.63654683102564,0.840139060111244,0.554444200428583,1.51528153682881,0.129701121951973,0.618260103226174
"3418","DENND11",64.3148655378425,0.471633304940535,0.311255726757503,1.51525984711594,0.12970661247818,0.618260103226174
"3419","RAB6B",30.1001966497131,0.68717808741614,0.453646402946569,1.51478791180248,0.129826122700501,0.618264071201056
"3420","KMT2C",549.301995978244,0.403694594633414,0.266506997757078,1.51476170618748,0.129832761366153,0.618264071201056
"3421","AC022509.1",3.52406348861537,1.70257023183657,1.12402787824155,1.51470463036922,0.129847221289141,0.618264071201056
"3422","ADNP2",120.562293546594,0.37584401457468,0.24817221225002,1.51444841937435,0.129912146689873,0.618392448109685
"3423","TSC1",177.900311730109,0.411127228658553,0.271518235291432,1.51417906873648,0.129980428923549,0.618536724141308

"3424","ACKR1",4.8000993808778,-1.59197484229703,1.05165846517967,-1.5
1377552219394,0.130082782868864,0.618843005301087
"3425","BAG1",727.805571745045,-0.324322716217511,0.21427973985669,-1.
51354820775131,0.130140465539809,0.618936654942467
"3426","ADAM11",2.44579423637227,-1.45420262283162,0.960967101974917,-
1.51326993384376,0.13021106563744,0.619091860717113
"3427","ADAMTSL4",630.021564219259,0.365792720622038,0.241798427229944
,1.51280024776248,0.130330405883317,0.619215386699703
"3428","CD164",1549.0913765947,0.245090323734332,0.161990608515441,1.5
1299094423101,0.130281959140854,0.619215386699703
"3429","RIC1",118.494490281309,0.266893384091672,0.176448335947488,1.5
1258657475297,0.1303847064588,0.619215386699703
"3430","TMEM38B",78.43037949213,-0.464299087078471,0.306990927396917,-
1.51241957218549,0.130427158927293,0.619215386699703
"3431","AC007906.2",25.8755993808682,-0.687516901412712,0.454573537866
574,-1.51244373933291,0.130421014914629,0.619215386699703
"3432","AL445250.1",26.0741986337957,0.647010151625466,0.4278609178850
17,1.51219736269379,0.130483661761105,0.619303137070701
"3433","ABHD17A",563.909347012401,-0.305086286538956,0.201848444054676
,-1.51146216641786,0.130670740868847,0.619829847994367
"3434","XIST",3371.57425443108,0.416749739970251,0.275719787868903,1.5
114973908525,0.130661772865182,0.619829847994367
"3435","MPP3",25.6562677995561,0.682666805715863,0.451744781556666,1.5
1117806687985,0.130743088959832,0.619992482115489
"3436","PRELID1",858.15783792828,-0.402107427317493,0.26621140266938,-
1.51048160704404,0.13092057916069,0.620504396470659
"3437","ZNF468",36.1326504412558,0.458955360311639,0.303852281800485,1
.51045553316923,0.130927227618003,0.620504396470659
"3438","SLC30A7",227.37761108405,0.240873046495712,0.159516329408966,1
.51002124602657,0.131038003003278,0.620515050653791
"3439","TMEM243",544.276123248003,-0.367465317079815,0.243354725470586
,-1.5099986917009,0.131043758011483,0.620515050653791
"3440","RRBP1",1714.27501428467,0.326817886454579,0.216424564282387,1.
51007759926989,0.13102362465025,0.620515050653791
"3441","IFFO2",30.2863341777103,0.547722478515923,0.362825345780176,1.
50960368366264,0.131144580855511,0.620811995802216
"3442","CTNNBIP1",131.479104833627,-0.26603129754758,0.176491591984402
,-1.50733128165726,0.131725763199005,0.620924623092357
"3443","RSRP1",1410.2877822874,0.262731667490597,0.174308579886812,1.5
0727903159559,0.131739149947523,0.620924623092357
"3444","LINC01719",18.9235833835811,0.666419139732674,0.44200627847533
,1.50771419363417,0.131627691249263,0.620924623092357
"3445","SEC16B",22.4260798043384,0.618585494138924,0.410432975925361,1
.50715349502379,0.131771317406181,0.620924623092357
"3446","AC074117.1",14.2856918036551,0.690819122603721,0.4577029046433
07,1.50931775961108,0.13121759834819,0.620924623092357
"3447","CLHC1",26.9024475265383,0.788253074846753,0.522424916899725,1.
50883514424342,0.131340917137688,0.620924623092357
"3448","LIMD1-
AS1",6.78532462643976,1.09876652480624,0.729119350580316,1.50697759417

813,0.131816400433014,0.620924623092357
"3449","NIPBL-
DT",114.598057629062,-0.378291934655864,0.250702383019524,-1.508928356
00371,0.131317092494406,0.620924623092357
"3450","MEF2C",100.803744771467,-0.435472612667558,0.288937189348608,-
1.50715321087364,0.131771390223648,0.620924623092357
"3451","PHF1",209.146283689047,-0.282620388770698,0.187535245038306,-1
.50702545920352,0.131804131541351,0.620924623092357
"3452","PDE10A",60.934271931251,0.581322000850571,0.385223407197438,1.
50905160483311,0.13128559561159,0.620924623092357
"3453","HSPA5",1491.01257436932,-0.317288542188375,0.210377908747489,-
1.5081837445642,0.131507506470405,0.620924623092357
"3454","TMEM9B",377.679262886508,-0.32268362006605,0.21393527213388,-1
.50832360109424,0.131471725679257,0.620924623092357
"3455","DTX3",102.828588465192,0.326199002811463,0.2163331544833,1.507
85488054557,0.131591672540974,0.620924623092357
"3456","AC068338.2",8.62335793080776,-0.774643255920932,0.513600381691
69,-1.50826067023047,0.131487824930877,0.620924623092357
"3457","PNKP",141.857449406436,-0.272014412962087,0.180365249064977,-1
.50813094192048,0.131521017422481,0.620924623092357
"3458","MAP3K7CL",20.1112013252312,-0.502976904532339,0.33376487816502
6,-1.50697972566079,0.131815854066835,0.620924623092357
"3459","MEST",983.816731821708,0.981402771091835,0.65142831186907,1.50
653994186407,0.131928621692838,0.621273581600067
"3460","HSF1",233.264839368201,-0.231167640272854,0.153486984121036,-1
.5061058212634,0.132040010486037,0.621618419308397
"3461","STARD4-
AS1",22.9054924467956,0.641623384402592,0.426091088035089,1.5058361989
2504,0.132109228181352,0.62176458186826
"3462","AL137009.1",3.8619113787318,-1.23620310379574,0.82110925816678
3,-1.50552815170504,0.132188344750895,0.621957235022338
"3463","NECAB2",4.14440728362283,1.68038151869652,1.11642307978103,1.5
0514759962335,0.132286133608484,0.622237606222524
"3464","FHIT",78.0347828132572,0.614908573797001,0.408706375820793,1.5
0452405485992,0.132446484388519,0.622812004677997
"3465","CCDC93",185.298627981317,0.245628241799906,0.1633599475512,1.5
0360137525707,0.132684036832324,0.623748997391549
"3466","CEP350",381.868886065745,0.319959917879981,0.213197909667361,1
.50076479820648,0.133416406499253,0.623954907056867
"3467","C2orf68",165.11069644962,0.306906465213216,0.204625695586309,1
.49984323490676,0.133655014988245,0.623954907056867
"3468","NPAS2",57.9357305673348,0.699289278111302,0.466025031239706,1.
50054016680407,0.133474537003765,0.623954907056867
"3469","FASTKD1",24.5957835712819,-0.494416510817805,0.329008170945559
,-1.50274842535633,0.132903930061766,0.623954907056867
"3470","AC107068.1",7.60800392868828,0.956593015225822,0.6373141436411
67,1.50097565662127,0.133361858020855,0.623954907056867
"3471","SLC39A7",511.512936026427,-0.310648668268751,0.206826085809018
,-1.50198011558175,0.13310244230072,0.623954907056867
"3472","AC073349.1",23.8060903818023,-0.642016989687559,0.428133097194

196,-1.49957336607487,0.133724951103843,0.623954907056867
"3473","ST7-
AS1",36.3730473412077,-0.59992631626202,0.400093782876096,-1.499464230
48471,0.133753241471758,0.623954907056867
"3474","MICU3",204.145325954068,0.392281168931499,0.260968185111879,1.
50317621576487,0.132793609076502,0.623954907056867
"3475","PSIP1",832.639648527023,-0.249793948237137,0.166319010023386,-
1.50189655531267,0.133123826398174,0.623954907056867
"3476","CDKN2B-
AS1",3.63173479786497,1.25677287183451,0.837108125252462,1.50132681062
615,0.133271053776523,0.623954907056867
"3477","MIR181A2HG",2.32558564328937,1.55911823055443,1.03951316327296
,1.49985424489043,0.133652162367894,0.623954907056867
"3478","HOGA1",2.59234789426345,1.61460762889445,1.07646841191659,1.49
991175869224,0.133637261654531,0.623954907056867
"3479","FAM204A",660.715351297802,-0.284390286826735,0.189280888142531
,-1.50247755923771,0.132973819212117,0.623954907056867
"3480","ANKRD13D",153.713468829063,-0.293650243386239,0.19562041997574
5,-1.50112265080838,0.133323841249641,0.623954907056867
"3481","AC074032.1",59.2951691987937,-0.393662632675755,0.262364882638
237,-1.50043949753198,0.133500594731902,0.623954907056867
"3482","AC138331.1",8.23345427076735,-0.788738864963119,0.526025752624
376,-1.49943013441462,0.133762080879279,0.623954907056867
"3483","FAM177A1",413.306391433117,-0.211178125291657,0.14066165326033
8,-1.50131980107475,0.133272865894866,0.623954907056867
"3484","KNSTRN",22.1369821668741,-0.547571071328339,0.364583986449528,
-1.50190653369287,0.133121249013235,0.623954907056867
"3485","SHISA6",2.38254365992467,1.61888707480977,1.07797791873537,1.5
017812950278,0.133153600575555,0.623954907056867
"3486","ZNF121",64.7864088189995,0.344870055984498,0.229979026302715,1
.49957177195174,0.13372536430263,0.623954907056867
"3487","TNPO2",172.797268614118,0.325746156649652,0.216930427556098,1.
50161579599254,0.133196361502063,0.623954907056867
"3488","SNRPB",777.630264101351,-0.355466668317119,0.236888944408408,-
1.50056250706355,0.133468754875086,0.623954907056867
"3489","HSF2BP",3.15165868119635,1.39531364825229,0.928487210715793,1.
50278176387223,0.132895329990028,0.623954907056867
"3490","AL035681.1",23.8819219684788,0.587984140897223,0.3913743017344
25,1.50235755973628,0.133004790698111,0.623954907056867
"3491","CFP",7.85196027468642,-0.875692737102646,0.58385864202092,-1.4
9983690242487,0.133656655717442,0.623954907056867
"3492","CCL17",3.50813773220377,-1.91821143544802,1.27672278758781,-1.
5024494385913,0.132981076559724,0.623954907056867
"3493","SLC8B1",147.025872971105,0.324275259642776,0.216347492501625,1
.49886303692815,0.133909167202049,0.624462188535407
"3494","AL356488.2",2.94229133787342,1.41184456401996,0.94242772080234
7,1.49809320423848,0.134109036480332,0.625036364872141
"3495","SMIM33",4.50611015467163,1.48694188348749,0.992477680567622,1.
49821191206746,0.134078201697678,0.625036364872141
"3496","ADSS",255.103204502407,-0.311684291332334,0.20813221338403,-1.

49753027782027,0.134255333301804,0.625360344338888
"3497","DCTN5",127.539196348322,0.329630435179894,0.220113784179543,1.4975456280876,0.134251342342709,0.625360344338888
"3498","SLC45A4",18.5725086116651,0.675872072519758,0.451420683564375,1.49721113171673,0.134338329777189,0.625380387002384
"3499","AC010997.4",2.31503595325423,1.62565613068717,1.08568363377284,1.49735713067525,0.134300356668165,0.625380387002384
"3500","NFATC3",133.761321420029,0.304252933365624,0.203232149121772,1.49707088509566,0.134374814568626,0.625380387002384
"3501","PLPP5",188.365874828349,-0.380376143233381,0.25411546902337,-1.49688649677762,0.134422794364665,0.625424022128589
"3502","AL110114.1",4.01402908838952,1.19347856359734,0.797385477689528,1.4967397789279,0.134460981367445,0.625424022128589
"3503","PCDHB12",40.1421900894782,0.471655364629647,0.315166765074132,1.49652633747313,0.134516549838485,0.625503876768222
"3504","DGCR6",35.0850189914112,0.617474320561401,0.412739675166102,1.49603819965431,0.134643700936529,0.625916451071666
"3505","Z99127.4",36.2057256126662,-0.557567941315559,0.372772153387413,-1.49573388529398,0.134723016341623,0.626106480225019
"3506","CCDC89",47.6184762611164,-0.445147617366858,0.297705328569057,-1.49526251178134,0.134845944736219,0.626141845441352
"3507","APAF1",87.6625640728472,0.365069578544497,0.244135413149296,1.49535691620964,0.134821318286445,0.626141845441352
"3508","VPS53",164.772077164726,0.450387122173364,0.301197318011083,1.4953224854306,0.134830299537072,0.626141845441352
"3509","ITPKB",53.9275635713792,0.376353404217073,0.251811328000037,1.49458488307968,0.135022813806103,0.626585024150879
"3510","FH",209.575383350565,-0.453781421102037,0.303643456905533,-1.49445479815892,0.13505678800379,0.626585024150879
"3511","ASB7",45.6117055588323,0.418760131841856,0.280174259506242,1.49464170113217,0.135007976743179,0.626585024150879
"3512","AC005046.1",3.91768172312147,1.43532649139852,0.960624349258047,1.49416001427313,0.13513380097285,0.626763805252495
"3513","GNB5",105.039267633222,-0.415522129682524,0.278153407305321,-1.49385957090368,0.135212327406587,0.626949502170765
"3514","SLC26A7",13.0488979198112,-1.20865113322223,0.809199174803857,-1.49363861315751,0.135270101297791,0.627038895856492
"3515","MIEF1",101.067500297961,0.350807818211682,0.234950098453287,1.49311628520739,0.135406750381371,0.627493757314978
"3516","UQCRC1",395.292261379966,-0.254916763785305,0.170803132483486,-1.49245953559986,0.135578717558452,0.62792908801302
"3517","AL360178.1",17.3843918462707,0.741637474472867,0.496970816396552,1.49231594694101,0.135616338119578,0.62792908801302
"3518","ANO1",6.61829366439819,-1.23393862820713,0.826847232432211,-1.49234172868601,0.135609582648878,0.62792908801302
"3519","AC080013.1",13.8959896055522,0.759084695870282,0.508770522681828,1.49199818391404,0.135699621432609,0.628136156156797
"3520","PAQR7",56.3580970199528,-0.528354826942794,0.354362416031597,-1.4910013111991,0.135961150659433,0.628275512933762
"3521","AC118549.1",175.813022030499,0.251018694487975,0.1683015746047

34,1.49148155670859,0.135835109867107,0.628275512933762
"3522","C1orf21",1403.50145640969,0.274524268877019,0.184067787790169,1.49143026149674,0.135848568028198,0.628275512933762
"3523","UBA5",204.572937280155,-0.278775014062462,0.186953699509193,-1.491144678037,0.135923514474374,0.628275512933762
"3524","RPP38",106.251074158268,-0.336692771493943,0.225718801167137,-1.49164699507966,0.135791711344491,0.628275512933762
"3525","RHOT2",160.165030226959,-0.315728123698011,0.211718965637018,-1.49126046761116,0.135893123653453,0.628275512933762
"3526","KCTD17",91.3788712067096,-0.375566463556671,0.251949769842956,-1.49064023273674,0.136055975428188,0.628535389605718
"3527","SNF8",688.160662226959,-0.406103716405548,0.272469217066918,-1.49045723688416,0.136104052451288,0.628579220408002
"3528","BNIP2",417.992469477976,0.192107333820967,0.128906216995363,1.49028757726928,0.13614863746813,0.62860690354829
"3529","CAMTA1",406.077911799467,-0.360011525259004,0.241633832172269,-1.48990529191434,0.136249139937174,0.628714515704427
"3530","FOXN2",146.99217470194,-0.342397468155052,0.229791974411534,-1.4900323174118,0.136215738699263,0.628714515704427
"3531","AC110792.4",3.37495065213596,-1.25439790212932,0.842103210512385,-1.48960113970599,0.136329142173752,0.628727462306977
"3532","RASIP1",6.79146252983486,-1.14426233188518,0.768098525950852,-1.48973379485224,0.136294244961694,0.628727462306977
"3533","HPF1",447.355265658339,-0.343232105403897,0.230532311088568,-1.48886767231527,0.136522217857561,0.629439684880217
"3534","ACTL10",10.8460108709389,-0.718794377016329,0.482872354332089,-1.48858051318876,0.13659786619396,0.629610255357504
"3535","EMSY",100.189669387566,0.350253086023868,0.235367449605755,1.48811182944179,0.136721404307875,0.629823233815319
"3536","TRPM7",230.510440594945,0.344905018297343,0.231752119634332,1.48824968177874,0.136685059521769,0.629823233815319
"3537","ITGB5",977.629535534305,-0.277188981290412,0.186332260427444,-1.48760596074209,0.136854840548215,0.630259682694339
"3538","RASSF8",405.473862115505,-0.301848488710395,0.202964161168213,-1.48720092736091,0.136961751219644,0.630395582259618
"3539","ZNF585B",39.5675554218253,0.395202624687739,0.265731062069391,1.4872278069793,0.13695465420886,0.630395582259618
"3540","PPM1K",111.306458095836,0.418406371608273,0.281443371183451,1.4866449682183,0.137108604433639,0.630715068517241
"3541","GLUD1",584.535520007511,-0.2259607313457,0.151993055095255,-1.48665168421076,0.13710682972078,0.630715068517241
"3542","NDUFS5",1923.9138641522,-0.341932696015341,0.230113930092638,-1.48592784399314,0.137298207756484,0.630931029261943
"3543","DNAJC3",677.76251873137,-0.195413722456304,0.131513671364311,-1.48588143292708,0.137310485526035,0.630931029261943
"3544","CALM1",4737.56353184367,-0.258266274319367,0.173768571982815,-1.48626573477803,0.137208846292195,0.630931029261943
"3545","SYNRG",227.045572711375,0.282176934047061,0.18990527792724,1.48588252589364,0.137310196378542,0.630931029261943
"3546","CBX4",119.746491106198,0.4838171853163,0.325741552731715,1.485

27929967466,0.137469853036806,0.631485176569804
"3547","PTAFR",6.7281910770954,-1.00695933717249,0.678232763145254,-1.48468105920273,0.137628331451439,0.631584533106315
"3548","MKLN1",457.625625984623,0.290261772701406,0.195501799395006,1.48470128458992,0.137622971294168,0.631584533106315
"3549","ZC3H4",123.944908210737,0.344591990373037,0.232131960338782,1.48446594717128,0.137685350669809,0.631584533106315
"3550","SLX4IP",39.9677608644664,0.401672574866114,0.270552671658439,1.48463725160773,0.137639941946639,0.631584533106315
"3551","KLHL15",83.1247933034772,0.654586582628328,0.440938188338866,1.48453139224419,0.13766800137767,0.631584533106315
"3552","CLPTM1L",321.958583972765,-0.265628009170537,0.178980151259702,-1.48411992782992,0.137777107374929,0.631827506202203
"3553","SRM",563.936667971959,-0.445109117624198,0.300113760990537,-1.48313464919135,0.138038639784578,0.631831239986184
"3554","RHOU",227.250604685813,0.476973746433962,0.32155527755765,1.48333359681353,0.13798580031177,0.631831239986184
"3555","EPDR1",241.065497720111,0.415129266110609,0.279834163955227,1.4834831467434,0.137946090878795,0.631831239986184
"3556","TRGC2",4.38209742966686,1.31915034841986,0.889697433178139,1.48269546390355,0.138155340365413,0.631831239986184
"3557","ADAM28",3.23163464275348,-1.84174290229886,1.24201790013592,-1.48286341291644,0.138110703876754,0.631831239986184
"3558","DUSP16",95.2185754976202,0.429472643624738,0.289656490957055,1.48269642501611,0.138155084895013,0.631831239986184
"3559","ST8SIA1",151.238528073918,0.644311695581388,0.43442538753363,1.48313545679121,0.138038425258659,0.631831239986184
"3560","SYNGR2",9.50150619364462,-0.861772736128436,0.581235762772395,-1.48265607748898,0.138165809861304,0.631831239986184
"3561","COLEC12",1316.75251580021,0.334049498592827,0.225228941615036,1.48315530054653,0.138033154164799,0.631831239986184
"3562","AC016590.3",4.33111730590792,1.13480997708375,0.76478269443464,1.48383323176873,0.137853168556807,0.631831239986184
"3563","MAF1",774.791357855853,-0.284750578789559,0.192112180733847,-1.4822099135091,0.138284449590655,0.631929367384018
"3564","CEP83-DT",4.07167988287835,-1.11900933028792,0.754889124120616,-1.4823492543908,0.138247388947205,0.631929367384018
"3565","HTATSF1",363.466936657041,-0.307112832098497,0.207209375185786,-1.48213772578165,0.138303652447911,0.631929367384018
"3566","MXD4",800.550431785808,0.294689375622949,0.198879506737181,1.48174832318134,0.13840727395044,0.632127200629836
"3567","C11orf54",215.026800652275,0.341186536056775,0.230269531941482,1.48168336983236,0.138424564101334,0.632127200629836
"3568","UHMK1",200.953505517766,0.360677279055202,0.243456679601266,1.48148442526169,0.138477532181607,0.63219185025398
"3569","SUCLG2-AS1",77.8292052820756,0.441485582125095,0.298153254033207,1.48073373727434,0.138677540052974,0.632396739704785
"3570","DNAJC13",204.611200420409,0.254108356127685,0.171609923199944,

1.48073229909684,0.138677923444019,0.632396739704785
"3571","BICRAL",190.006413401235,0.329412052356962,0.222477464475983,1
.48065357151049,0.138698911982093,0.632396739704785
"3572","KDM6A",118.888966064069,0.302280761444419,0.204162714075541,1.
48058749519059,0.13871652961908,0.632396739704785
"3573","IGBP1",893.089918124375,-0.37303005073206,0.251923086461216,-1
.48072991630915,0.138678558652156,0.632396739704785
"3574","SYT11",57.7241507417903,0.464895971168029,0.31409061049148,1.4
8013329796957,0.138837676865336,0.632418042074794
"3575","IPCEF1",4.83400142874075,1.12479491580101,0.759854960606815,1.
48027580803415,0.138799656630456,0.632418042074794
"3576","TTC17",350.14106213658,0.252122142221267,0.170318726893217,1.4
8029607090321,0.138794251354437,0.632418042074794
"3577","CYSTM1",1073.22314532436,-0.36924361146146,0.249518927915607,-
1.47982205015864,0.138920742502866,0.632442698331242
"3578","RAP2C",148.675319210686,0.276005767652959,0.186508651544191,1.
47985503818606,0.138911936864153,0.632442698331242
"3579","AC004951.1",28.6496596819778,0.611898548351329,0.4135969466409
74,1.4794561548891,0.139018441359952,0.632710643004264
"3580","EPSTI1",421.621440950348,0.481512704450675,0.325551562398683,1
.47906740456984,0.139122300777787,0.63300646853893
"3581","FOXO3",1009.89392104316,0.465786351165705,0.314953837967302,1.
47890355669856,0.139166092639998,0.633028897797524
"3582","KRT27",66.8618891843532,1.53678170067032,1.03957741917103,1.47
827537644648,0.139334085656892,0.633616114256034
"3583","ANAPC1",91.8505836206444,0.377841223997456,0.255841758665073,1
.47685517004319,0.139714464104886,0.634708404615904
"3584","AC093495.1",11.9275808471885,0.859240514399114,0.5817494718563
34,1.47699406010171,0.139677229515061,0.634708404615904
"3585","MYBL1",11.4426883812615,0.932747895076383,0.631664255109521,1.
47665138169748,0.1397691108943,0.634708404615904
"3586","AC005840.2",2.8407099698032,1.35271294685408,0.916063935398107
,1.47665779055718,0.139767392078397,0.634708404615904
"3587","YJU2",114.607740074001,-0.363888711037163,0.246342694270119,-1
.47716461458424,0.139631516559205,0.634708404615904
"3588","CD82",36.5863073772426,-0.574982204073433,0.389577791716297,-1
.47591114354935,0.139967747578253,0.635054075970206
"3589","PLTP",3574.40362489299,-0.376812627997586,0.255330016765946,-1
.47578664181501,0.140001177899749,0.635054075970206
"3590","TMSB4X",26532.6694717452,-0.407096714873453,0.275785878490003,
-1.47613328536765,0.139908114891447,0.635054075970206
"3591","PPP1R3F",40.7850968863673,-0.430252739340724,0.291488212691476
,-1.47605536212925,0.139929030707892,0.635054075970206
"3592","SNAPC3",159.879634485435,0.253455583944328,0.171775116385845,1
.47550814854353,0.140075979174947,0.635216487967902
"3593","TIMELESS",5.64245741723289,0.996227151417506,0.675312498495021
,1.47520911228159,0.140156332377665,0.635403979432172
"3594","DSTYK",227.173217538395,0.370138491265703,0.25102710202252,1.4
744961332203,0.140348058056778,0.635742357532496
"3595","DENND1A",73.4529689159857,0.323146062671267,0.219138672809616,

1.47461905526831,0.140314988967177,0.635742357532496
"3596","RAB11FIP4",5.47093279706884,1.02798628104136,0.697144956753328
,1.47456604409618,0.140329249556482,0.635742357532496
"3597","PYCARD",427.847270857168,-0.408130195018666,0.276866750139249,
-1.47410331798021,0.140453775318563,0.636044355341696
"3598","IVNS1ABP",182.953926818077,-0.394153982286029,0.26762152458852
,-1.47280373987876,0.140803963626416,0.63713729368531
"3599","WDR11",263.171118848013,0.35448282652613,0.240660705074701,1.4
7295681867174,0.140762679698667,0.63713729368531
"3600","MYO7A",3.59916358549821,-1.31421142762983,0.892338551393653,-1
.47277221809738,0.14081246591363,0.63713729368531
"3601","ZNF205",122.303559115842,-0.343477781014698,0.233258359675483,
-1.47252077692974,0.140880300611593,0.637267208181682
"3602","RPS13",18356.3848590779,-0.375650308167454,0.255337269468132,-
1.47119262671656,0.141239030639327,0.638535267855677
"3603","INO80C",64.8178190601679,-0.381270129264929,0.259146224755536,
-1.47125480845649,0.141222219858099,0.638535267855677
"3604","LMNB2",59.7155812285393,-0.476124467331863,0.323700461023815,-
1.47087979370173,0.14132362811565,0.638563267233238
"3605","SNHG11",79.7776285133666,-0.477933254494753,0.324914225141307,
-1.47095207754261,0.141304077389948,0.638563267233238
"3606","ZC2HC1B",12.6193429797307,1.16092347067308,0.789470422189885,1
.47050913883871,0.141423912389215,0.638839187162487
"3607","POFUT1",145.301450206644,0.309903118753546,0.210781662647334,1
.47025654348336,0.141492285841378,0.638970846706463
"3608","RHOQ",674.931557627183,-0.30371569347071,0.206624232864674,-1.
46989387091699,0.141590499814969,0.639060030891114
"3609","PCDHB5",25.015662728976,0.649457795378785,0.441803648880696,1.
47001455742654,0.14155781134859,0.639060030891114
"3610","LINC02035",23.9874702526871,0.493094277504558,0.33554004312200
9,1.46955419364138,0.141682534016604,0.639298281605669
"3611","SAMD3",6.41311491960797,-0.995179532774765,0.67756594745762,-1
.4687567114447,0.141898789243619,0.640096753804847
"3612","SLFN12L",9.02251956095015,0.757562508617554,0.515856471227391,
1.46855288412892,0.141954102264284,0.640168984436027
"3613","CTLA4",6.74485190200615,-1.09042948983676,0.742593420485612,-1
.46840715222562,0.141993659970451,0.640170143165976
"3614","PALMD",99.3100261817877,-0.475416349189303,0.323883489909422,-
1.46786225294243,0.14214164333789,0.640315904102693
"3615","SLC27A2",2.54850023617975,1.48205517836392,1.00987391872747,1.
46756456512061,0.142222539200905,0.640315904102693
"3616","SLC16A11",6.17947027364437,1.15852459807097,0.789203602686143,
1.46796668708531,0.142113272015407,0.640315904102693
"3617","ZNF347",69.9945335305148,0.422251630392214,0.287678166140951,1
.46779172036757,0.142160807179886,0.640315904102693
"3618","PDHA1",266.399208622314,-0.307753638241018,0.209691036903671,-
1.46765280378863,0.142198556895053,0.640315904102693
"3619","LINC01686",17.9984875491066,-0.635067710825573,0.4330019375689
09,-1.4666625151637,0.142467884725071,0.640670978283666
"3620","MYO6",140.488766663511,-0.348266096817676,0.237509342574399,-1

.46632588445898,0.142559526986221,0.640670978283666
"3621","POLD2",299.803496564084,-0.379154998940791,0.258553370337951,-1.46644771423866,0.14252635557665,0.640670978283666
"3622","FGFR2",23.10429658152,-1.66989923390136,1.13825939775176,-1.46706386716391,0.142358682253928,0.640670978283666
"3623","DMAC2L",124.309485834227,0.300221073455402,0.204752578449347,1.46626272415745,0.14257672639685,0.640670978283666
"3624","HSD17B14",48.2138357757047,-0.872879578154223,0.595211646782827,-1.46650285301408,0.142511344521114,0.640670978283666
"3625","MT-
ND2",36940.0183651626,-0.324145926663731,0.220968812886929,-1.46693066061589,0.14239491883293,0.640670978283666
"3626","F2R",74.2374672446323,-0.754593858363912,0.514924522139848,-1.4654455671058,0.142799393044009,0.640964263789987
"3627","PLAU",419.47598988983,0.510126623351547,0.348075763275586,1.46556203325096,0.142767640952892,0.640964263789987
"3628","LINC02666",3.48523974999488,1.30400404123806,0.889626550315385,1.46578813410613,0.142706014692432,0.640964263789987
"3629","NCAPH2",98.6384144589179,-0.317282329908432,0.21647409749598,-1.46568265477732,0.142734761695562,0.640964263789987
"3630","HPCA",2.4596384334226,-1.39784885859325,0.954338549112977,-1.46473058213199,0.14299443772496,0.641662919036329
"3631","AC233280.1",3.26145633590208,-1.1462333291425,0.782981762166161,-1.46393362467523,0.143212084994179,0.64246258674475
"3632","LRP11",339.810450065443,0.310429494758397,0.212110822834673,1.46352501305583,0.143323774417679,0.642786608339639
"3633","TBX15",394.634361196304,-0.271489431443957,0.185608221937401,-1.46270153665671,0.143549065517284,0.643257662475595
"3634","LINC02084",2.55589599130581,-1.56283179907764,1.06826245211238,-1.46296614281191,0.143476643544632,0.643257662475595
"3635","LRBA",85.5939053163611,0.343445048017935,0.23482186484767,1.46257695483651,0.143583172923675,0.643257662475595
"3636","CALML4",29.7564477201829,0.475016325399597,0.324783313491022,1.46256382538177,0.143586767804117,0.643257662475595
"3637","C15orf61",967.12200428033,-0.336148691357161,0.229877260304148,-1.46229640510074,0.14366000322801,0.643408796420414
"3638","CCNL2",337.676013675881,0.374042296206675,0.256371302192482,1.45898660656584,0.144568794061361,0.643410132914073
"3639","MFSD6",21.9082925637133,-0.79224379397305,0.542581591562267,-1.46013762039351,0.144252255095003,0.643410132914073
"3640","CRYBG3",354.579698580286,0.318465085629083,0.21824643551866,1.45919948187128,0.144510211388524,0.643410132914073
"3641","GMPS",212.302681906975,-0.236029873319482,0.161452370192922,-1.46191643416226,0.14376411091707,0.643410132914073
"3642","LDB2",390.908165134652,0.275554888806644,0.188551071193075,1.46143369572522,0.143896459156922,0.643410132914073
"3643","RAB24",87.013337311273,-0.428529249329474,0.293634194699186,-1.45939831622295,0.144455509179923,0.643410132914073
"3644","AL603910.1",3.55138378232392,1.22005372231324,0.835069370092776,1.46102080378985,0.14400973229109,0.643410132914073

"3645","PAXIP1-
AS2",119.32912117013,0.40337953635046,0.275909196116351,1.46200105697222,0.143740920229218,0.643410132914073
"3646","SULF1",175.54588804591,0.32589449923015,0.22322811688529,1.45991689477726,0.14431291538166,0.643410132914073
"3647","STRBP",38.7029208722287,0.453717322504583,0.310940853426065,1.45917565191371,0.144516768419992,0.643410132914073
"3648","DDX21",734.479047566678,-0.258094513420892,0.176807160680578,-1.45975147402071,0.144358389484404,0.643410132914073
"3649","RBP4",4.96932094833849,-0.965192999598388,0.660985217529291,-1.46023386605558,0.144225810776119,0.643410132914073
"3650","ELOVL3",3.51772511174376,1.41265397236242,0.967030045592239,1.46081704369099,0.144065657201999,0.643410132914073
"3651","KLC2",45.5765858076494,-0.375091209757548,0.257019927900905,-1.45938570919748,0.144458977083862,0.643410132914073
"3652","KDM5A",538.090850138828,0.326780164576902,0.223755386991749,1.46043484793934,0.144170601266974,0.643410132914073
"3653","PRDM4",89.4402722907037,0.346231138640241,0.237133003064207,1.4600714964441,0.144270425369488,0.643410132914073
"3654","CPEB1",69.3586982875258,0.430361668207551,0.294939216547371,1.45915376478404,0.144522791065417,0.643410132914073
"3655","DECR2",51.6653137491576,0.455327181820836,0.311762147848393,1.46049539677362,0.14415397174283,0.643410132914073
"3656","AKTIP",147.240836679055,-0.280262640707488,0.19194952912935,-1.46008506495802,0.144266696719221,0.643410132914073
"3657","HYDIN",8.35459990889415,1.04655571875793,0.71650376463718,1.46064231677538,0.144113626794582,0.643410132914073
"3658","ZNF98",8.80712352439927,0.991658994933936,0.679109009076637,1.46023536969752,0.14422539766712,0.643410132914073
"3659","RABAC1",2402.27938944108,-0.364797369957833,0.250023031336892,-1.45905506387645,0.144549952821122,0.643410132914073
"3660","DIDO1",222.323768730731,0.282742302451448,0.193746017335184,1.45934510727154,0.144470146177335,0.643410132914073
"3661","CEPT1",158.112966702921,0.233967940254451,0.160561126072949,1.45718920872634,0.145064158573102,0.64432517614076
"3662","SUCO",192.176918360292,0.252325831600933,0.173181878687564,1.45699904350935,0.14511664424732,0.64432517614076
"3663","NRBP1",486.067187584606,-0.204586785114325,0.14032034574749,-1.4579980117957,0.144841091037839,0.64432517614076
"3664","SLC19A3",27.7016781181297,0.69424357561334,0.476839833278858,1.4559261352802,0.14541303991463,0.64432517614076
"3665","AC107294.2",17.6132340096329,0.74835878280826,0.513947071353322,1.45610087987793,0.145364734365249,0.64432517614076
"3666","AC093599.2",4.07732871593351,1.37279060219866,0.942799748205143,1.45607866868029,0.145370873639236,0.64432517614076
"3667","AMACR",72.6194914286673,0.361735279303868,0.248468811078303,1.45585789111322,0.145431908338596,0.64432517614076
"3668","ZBED8",28.9247105543065,0.478835838433428,0.3287094581029,1.45671451377445,0.145195201726747,0.64432517614076
"3669","PI4K2A",103.655030305319,0.335223790491736,0.230086710499391,1

.45694546966294,0.145131433275669,0.64432517614076
"3670","LDLRAD3",28.7657223732296,-0.554663537298273,0.380529169678361
,-1.45761108870339,0.144947771448248,0.64432517614076
"3671","HARBI1",7.8213511625993,-0.963013001607004,0.661477204586547,-
1.4558521365962,0.1454334994565,0.64432517614076
"3672","LARP4",174.696685337767,0.431118423864637,0.296089712743877,1.
45603985991084,0.145381601032571,0.64432517614076
"3673","AL138976.2",3.46489892258094,1.47011587874623,1.00902192888883
,1.45697118829238,0.145124333519283,0.64432517614076
"3674","AC007448.4",5.55171192179964,0.978485176118249,0.6718135274187
32,1.45648328916183,0.145259065869464,0.64432517614076
"3675","DLGAP1",3.76923526323373,1.19279003987381,0.819334297136938,1.
45580386911895,0.145446845887996,0.64432517614076
"3676","STXBP2",9.6674755876131,-0.962349170150733,0.660478318353077,-
1.45704884385967,0.145102897925559,0.64432517614076
"3677","ZNF546",50.5005619405999,0.376669139801098,0.258691999549393,1
.45605252755093,0.14537809941822,0.64432517614076
"3678","BMP1",268.587325342276,0.404732519446715,0.278150652371383,1.4
5508384034391,0.145646052281576,0.644856902858004
"3679","VSIR",1018.44000045819,0.469768409945476,0.322840142406646,1.4
5511151879546,0.145638390778359,0.644856902858004
"3680","ORC3",139.306442213863,0.283618817602025,0.19498001630264,1.45
460454348205,0.14577877246202,0.645269137128762
"3681","BTBD3",42.9479916099404,0.413473482564767,0.284415575517784,1.
45376525815097,0.146011398586906,0.646123246830238
"3682","PDK4",5227.86011510303,0.477387779708107,0.328432296424028,1.4
5353482256741,0.146075318490249,0.64623054396732
"3683","AL445426.1",114.758164299517,0.368366477409879,0.2535615073347
55,1.45276970973184,0.146287704674338,0.646341782976134
"3684","HIST2H3PS2",14.7097304686549,0.906270893770965,0.6236385847522
9,1.45319888141773,0.146168542662242,0.646341782976134
"3685","HIST2H2BF",7.60466888854356,0.767438971888012,0.52824244046348
8,1.45281581543249,0.146274899595932,0.646341782976134
"3686","RCSD1",11.0635157364648,-0.801313528741289,0.551557178973563,-
1.45282041334775,0.146273622649784,0.646341782976134
"3687","AC093525.7",8.44908667101386,1.03723789957434,0.71399243284338
8,1.4527295414654,0.146298861429984,0.646341782976134
"3688","SETX",413.047044571567,0.265536618118479,0.182820705830569,1.4
5244280133438,0.146378522554751,0.646478765803473
"3689","AC004825.2",18.0679393896704,-0.624275463811473,0.429843420742
117,-1.45233225329743,0.146409243480202,0.646478765803473
"3690","NUDC",918.889912712814,-0.325448327890807,0.224166934720729,-1
.45181236606664,0.146553784535623,0.646796260576649
"3691","AL139420.1",9.48902917771444,-0.954708332624749,0.657860223999
561,-1.4512327965665,0.14671504733808,0.646796260576649
"3692","EDEM1",119.36634958828,-0.302999585490077,0.208810487150579,-1
.45107455868141,0.146759099950353,0.646796260576649
"3693","COL21A1",56.3346788234278,1.45868911234862,1.00482421326829,1.
45168587011264,0.14658896993551,0.646796260576649
"3694","SPAG1",32.366934164659,-0.565563218064791,0.389673095469996,-1

.45137866750243,0.146674446580961,0.646796260576649
"3695","RHOG",266.767206327856,-0.331422840660555,0.228395019499891,-1.45109486794529,0.146753445390036,0.646796260576649
"3696","VPS11",142.28092138872,0.248959037914408,0.17151126968570 6,1.45156081212986,0.146623761709449,0.646796260576649
"3697","MDM2",227.284589570118,0.287402195764022,0.198098290696443,1.45080603549691,0.146833878571924,0.646950783894528
"3698","ST3GAL3",72.4937857808705,0.312605219835024,0.215969185170286,1.44745288356088,0.147770123508237,0.647051911177278
"3699","UBE2T",10.2126636283727,0.881404266403603,0.608068399854225,1.44951499965284,0.147193814527925,0.647051911177278
"3700","FLVCR1-
DT",26.2533552045551,-0.594210405888616,0.410770932388688,-1.44657364734452,0.148016371366358,0.647051911177278
"3701","KCNK1",75.8767655667023,0.478663967975046,0.331029441900188,1.44598608881252,0.148181103678308,0.647051911177278
"3702","TTC27",83.4200751423623,0.388012528006366,0.267878268299317,1.44846586649132,0.147486805442994,0.647051911177278
"3703","FAM126B",149.652064951053,0.309178574834562,0.213511566554984,1.44806475744227,0.147598940698474,0.647051911177278
"3704","SMARCAL1",122.792945667476,0.294000358915895,0.202993766016735,1.44832210705258,0.147526987775785,0.647051911177278
"3705","COL8A1",528.944915719079,1.45272187392242,1.00474967062714,1.44585454107755,0.1482180045679,0.647051911177278
"3706","LRRC58",462.853103603931,0.258221321675427,0.178035719464289,1.45039053091378,0.146949646157694,0.647051911177278
"3707","CFI",84.254638174041,0.846918710386145,0.585896252362976,1.44550969044509,0.14831477307841,0.647051911177278
"3708","TMEM184C",105.630240238232,0.290469647628043,0.201022746318616,1.44495910511369,0.148469372806923,0.647051911177278
"3709","NNT",153.802015303301,0.28123866137545,0.194549554175032,1.44558882474964,0.148292562941858,0.647051911177278
"3710","PRRT1",7.53484394451369,-0.804699738821693,0.556833513713534,-1.44513524959217,0.148419899536359,0.647051911177278
"3711","CCDC136",53.1356921642302,0.545768216961356,0.377676554622802,1.44506777103607,0.148438850585999,0.647051911177278
"3712","EIF3H",1600.4094313236,-0.296092646486668,0.204634391200876,-1.44693492012304,0.147915151710412,0.647051911177278
"3713","LY6K",20.886760763494,0.933769508673005,0.643907058662611,1.45016193891776,0.147013366050875,0.647051911177278
"3714","DAB2IP",75.7330013395667,-0.441653860954307,0.304955506937311,-1.44825671583985,0.147545268102257,0.647051911177278
"3715","SLC16A9",7.41642250550202,1.14728413671647,0.793213677469486,1.44637462679229,0.148072154582128,0.647051911177278
"3716","CTBP2",317.726786204196,0.27905097357996,0.193092598536382,1.4451665972447,0.148411096317478,0.647051911177278
"3717","IFITM10",8.61868314486977,-0.853856552479827,0.590146295193001,-1.44685573634006,0.147937332511964,0.647051911177278
"3718","LRFN4",57.5735011391246,-0.406812804923338,0.281059480750792,-1.44742601756974,0.147777643231843,0.647051911177278

"3719","NTF3",24.3499284344084,0.942888621323368,0.652550377460394,1.4
4492847432395,0.148477977287571,0.647051911177278
"3720","MTUS2",2.83288128627197,-1.36711921851695,0.945358864366627,-1
.44613783193634,0.14813854643087,0.647051911177278
"3721","EFS",234.330549736308,0.239831420296001,0.16600975758463,1.444
68267278649,0.148547039071931,0.647051911177278
"3722","CYP46A1",51.174770746307,0.437435760036102,0.30237997814726,1.
44664260747803,0.147997046364844,0.647051911177278
"3723","GOLGA8N",30.9380036220341,0.513921425631505,0.35578356961262,1
.44447768116742,0.148604653429566,0.647051911177278
"3724","KIF22",168.885673496265,-0.307622178111589,0.212346859523817,-
1.44867778502316,0.147427587155814,0.647051911177278
"3725","ARL17B",36.8702432778267,0.724468349139728,0.500506399941796,1
.44747070012287,0.147765136858437,0.647051911177278
"3726","CLTC",719.13884723203,0.246551404861345,0.170458803159293,1.44
639877959805,0.148065383966934,0.647051911177278
"3727","SLC16A5",36.9741356220928,0.409352932592439,0.282229779528545,
1.45042430772631,0.146940232680997,0.647051911177278
"3728","TBC1D16",249.814155632892,0.27625573954666,0.190610053035607,1
.44932407890917,0.147247099723495,0.647051911177278
"3729","TTC39C",192.63744267229,-0.323016172552297,0.222768428869953,-
1.45000875658582,0.147056077363358,0.647051911177278
"3730","LINC01915",41.4646789592658,-0.446538225462939,0.3091337028996
51,-1.44448250473644,0.148603297535054,0.647051911177278
"3731","LPAR2",16.4674325160734,-0.728299158723315,0.502587303102484,-
1.44909979664728,0.147309714818649,0.647051911177278
"3732","ZNF28",33.7171602513116,0.473774924746343,0.326944104844648,1.
44910068028743,0.147309468084163,0.647051911177278
"3733","C20orf27",136.971588077165,-0.41147952303288,0.284101062530929
,-1.44835615666902,0.147517469780048,0.647051911177278
"3734","BMP2",12.2729088012713,1.00507221376692,0.693790627965233,1.44
866790246882,0.147430348322102,0.647051911177278
"3735","MKKS",116.705706509041,-0.3335086127317,0.230845793848175,-1.4
4472466737274,0.148535238299243,0.647051911177278
"3736","EIF6",486.01652166202,-0.404830845450777,0.279295222699213,-1.
44947286078989,0.147205574043465,0.647051911177278
"3737","PIGA",24.2416963058897,0.502181340488457,0.347034727510208,1.4
4706365294143,0.147879096756873,0.647051911177278
"3738","SCML1",186.871735294078,0.440500854013068,0.304645989862789,1.
44594338567026,0.148193081708371,0.647051911177278
"3739","RPGR",79.8356764246526,-0.44125112281283,0.304921222373669,-1.
44709876005972,0.147869265266393,0.647051911177278
"3740","RTL3",4.13752746853771,1.36538572465774,0.943423963136508,1.44
72663171692,0.147822349009517,0.647051911177278
"3741","AC004000.1",4.41585405369745,1.363143656013,0.941797528627312,
1.44738504251525,0.14778911261003,0.647051911177278
"3742","SEMA6C",77.3302881223447,0.314095271320745,0.217542479305899,1
.44383419883285,0.148785619589323,0.647256086985708
"3743","UBAP2L",232.16698148221,0.265502498672952,0.184126523207888,1.
44195683515506,0.149314551040939,0.647256086985708

"3744","PELI1",91.0347453013723,-0.573121663852691,0.39734772275628,-1
.44236805958549,0.14919856944561,0.647256086985708
"3745","IL1RL1",4.60792677750047,-1.36353183994677,0.945489459905371,-
1.44214388184003,0.149261787931131,0.647256086985708
"3746","LY6G5C",5.87799539270838,-0.995449009126979,0.689559665267814,
-1.44360098083806,0.148851248875132,0.647256086985708
"3747","BRD2",1206.10948774325,0.41572050350834,0.288018851230562,1.44
33794931553,0.148913597623463,0.647256086985708
"3748","AFG1L",18.3550760464787,0.55717712573474,0.386391433749517,1.4
4200175539072,0.149301878406761,0.647256086985708
"3749","PLPBP",338.901845543125,-0.287617219473026,0.199361965615421,-
1.4426885217808,0.149108234079836,0.647256086985708
"3750","IER5L",622.597286350091,-0.309830585641365,0.214874745279297,-
1.44191252089045,0.149327053526447,0.647256086985708
"3751","RPL6",16226.9941803359,-0.367859145705685,0.255051523946515,-1
.44229346295859,0.14921960354551,0.647256086985708
"3752","LINC01550",39.3404330326098,0.643209050611023,0.44560674295253
7,1.44344550611868,0.148895012899273,0.647256086985708
"3753","FAHD1",160.533316455967,-0.30513698062399,0.211502369659967,-1
.44271187653623,0.149101652220117,0.647256086985708
"3754","ZNF500",72.3621251213955,0.457744908727497,0.317282622041616,1
.44270400245072,0.149103871277718,0.647256086985708
"3755","MT1G",399.529473389631,-0.960058025575551,0.66487878889475,-1.
44395947293113,0.148750375644564,0.647256086985708
"3756","WSB1",2644.40255696475,0.439980279558433,0.305114953320547,1.4
42014803831,0.149298197410951,0.647256086985708
"3757","PHLDB3",22.7707093450063,-0.600677969728948,0.416246322282403,
-1.4430829477009,0.148997106337273,0.647256086985708
"3758","TNRC6B",1299.23391729809,0.282454652775167,0.195594082542324,1
.44408587981719,0.148714819468799,0.647256086985708
"3759","AC027117.1",3.56790695981822,1.30059333149331,0.90210200389202
2,1.4417364398727,0.149376739563541,0.647299204775346
"3760","OSMR-
AS1",43.5677416531814,-0.488793277954132,0.339136692294408,-1.44128691
78125,0.149503641625874,0.647332487624633
"3761","EBF3",603.462285650081,0.360120208637964,0.249859967720902,1.4
412881420053,0.149503295919168,0.647332487624633
"3762","AC124283.1",13.9175442689812,0.720729695890804,0.5000407074431
2,1.44134204508297,0.149488074529951,0.647332487624633
"3763","SLC38A7",69.2979165985004,0.352675780469673,0.244736862405033,
1.44104070389692,0.149573183760244,0.647387435535111
"3764","TRMT6",100.115284750329,-0.330479157624151,0.229346425001932,-
1.44096058014144,0.149595819716014,0.647387435535111
"3765","MAN2A2",91.21260460204,0.340767964210232,0.236612534273495,1.4
4019405082043,0.149812505894223,0.64812288763606
"3766","CDIP1",109.572927442638,0.338925941503861,0.235352495027003,1.
44007796248336,0.149845343166395,0.64812288763606
"3767","AL356599.1",84.330612710969,0.351717580208694,0.24426209278242
3,1.43991880280002,0.149890372717859,0.648145548500452
"3768","NOP16",62.7496279314711,-0.394429514081215,0.273957930712548,-

1.43974482890613,0.149939605321151,0.648186367058446
"3769","KIF1B",384.009064091288,0.389455237275918,0.270542098980371,1.4395365406852,0.149998564704953,0.64826920150676
"3770","AC007405.3",74.6628030204941,-0.50287002758932,0.349441376061267,-1.43906835892597,0.150131155741938,0.648480161974785
"3771","AC008914.1",7.93342454660875,1.10197278138882,0.765987759937675,1.43862975235856,0.150255452033429,0.648480161974785
"3772","LINCADL",2.87421715676191,1.51374887567951,1.05239790268976,1.43838074155279,0.15032605387788,0.648480161974785
"3773","EYA4",5.48185115208307,1.25473636127821,0.872045091432219,1.43884344239295,0.150194884822148,0.648480161974785
"3774","DLL1",38.0374257831123,0.687059636440401,0.477414050939413,1.43912738866497,0.150114433349764,0.648480161974785
"3775","THEM6",34.9534029266529,-0.52496305227802,0.364946518884058,-1.43846570692951,0.150301960866785,0.648480161974785
"3776","GYS1",37.0059112598205,0.424039216327496,0.294799026229792,1.43840100746114,0.150320306954909,0.648480161974785
"3777","MNDA",14.4754679518952,-0.652618007935244,0.453762540516619,-1.43823685223603,0.150366862236197,0.648484463586288
"3778","C1orf216",100.391428913586,-0.330857086466524,0.230102316815329,-1.43786942715599,0.150471105753134,0.648732264602519
"3779","HLTF",259.264093696747,0.199250426832036,0.138588813048972,1.43770931035847,0.150516550309769,0.648732264602519
"3780","AC114296.1",10.2529598615153,-0.682539737742715,0.474772826183036,-1.43761331757345,0.15054380012263,0.648732264602519
"3781","IQCA1",18.4222588457158,0.872144598475708,0.606924606283963,1.43699001399139,0.150720831026613,0.649244349513784
"3782","AP000487.1",11.9023625628538,0.658263068248907,0.458108791557111,1.43691428844112,0.150742349429746,0.649244349513784
"3783","AC092803.1",31.1690441046645,-0.529241014236027,0.368430776338146,-1.43647341163022,0.150867676833442,0.649348738048013
"3784","AC005520.2",38.3232093912049,0.486313681486213,0.338539049065808,1.43650690467817,0.150858153027493,0.649348738048013
"3785","UBE3A",451.538136454753,0.230403446322777,0.160418123009472,1.43626818466871,0.150926043480249,0.649348738048013
"3786","ZNF227",61.4427522684641,0.336836981516964,0.234501323131861,1.43639693379282,0.150889425156126,0.649348738048013
"3787","CSNK1A1",1041.88672123382,0.188778803547965,0.131474658926881,1.43585695592452,0.151043048918773,0.649642841739923
"3788","PPIL6",32.2450391775382,-0.564885903742838,0.39348212373811,-1.43560754012502,0.151114047968111,0.649642841739923
"3789","ZBTB2",52.2046875139739,-0.489100025491167,0.340673282565287,-1.43568647886978,0.151091574404932,0.649642841739923
"3790","POLN",13.0036502273699,0.996891634605969,0.694598502462666,1.43520556274097,0.151228528925598,0.64966965230283
"3791","AACS",91.1649645346445,0.37795064661418,0.263401243030737,1.43488558468981,0.151319704146168,0.64966965230283
"3792","CDC37",702.678476997949,-0.250860262917718,0.174818987864217,-1.43497148669321,0.151295222937072,0.64966965230283
"3793","ZNF66",33.4530930531812,0.559446810432579,0.38982350230069,1.4

351284802758,0.15125048913787,0.64966965230283
"3794","TCEAL7",162.532538937002,-0.416330507430769,0.290063367585959,-1.43530881164231,0.151199117899479,0.64966965230283
"3795","DNAJC9",142.459710849179,-0.326991697816474,0.227924139207998,-1.43465145443884,0.15138644424045,0.649784924962499
"3796","AL157392.4",4.12048156417742,1.13174788436998,0.788954166674099,1.43449129515464,0.15143211142511,0.649809711012545
"3797","AL391001.1",5.90274276496209,1.08631589172883,0.757900083812162,1.43332335611414,0.151765450284653,0.64989996971775
"3798","OPN3",43.5263025129576,0.423706285647151,0.295616061533204,1.43329927152676,0.151772330088177,0.64989996971775
"3799","FAM49A",150.112625686625,0.360665697883115,0.251540620987729,1.43382685654064,0.151621678900924,0.64989996971775
"3800","LINC01094",2.30845927817103,-1.37177358166923,0.956694735574963,-1.43386759711269,0.151610050224364,0.64989996971775
"3801","GCLC",67.9356933452492,-0.419260197723114,0.292502799331814,-1.43335447961818,0.151756560154307,0.64989996971775
"3802","VMO1",18.0781791023413,-0.558353486809411,0.389518701918393,-1.43344461783093,0.151730815276064,0.64989996971775
"3803","AC233296.1",2.72633664669618,1.41649459946349,0.988152573951427,1.43347761955343,0.151721390301683,0.64989996971775
"3804","XIAP",385.633298531386,0.297490311018059,0.207510846387058,1.43361330840109,0.151682643557579,0.64989996971775
"3805","CSRNP1",514.46014263757,-0.546844237502641,0.381679550470474,-1.432731297311,0.151934641742907,0.649935585448012
"3806","DLG5-AS1",5.96742549375291,-0.888456455092306,0.620091892946661,-1.43278192345071,0.151920168799519,0.649935585448012
"3807","PIAS4",73.5957313978012,0.327083614875632,0.228296884963827,1.43271168560823,0.151940248596355,0.649935585448012
"3808","ZSWIM1",14.9362439843534,0.580908556714531,0.405348594934986,1.4331085983108,0.151826804601181,0.649935585448012
"3809","CYBC1",229.173998438569,-0.26845301040801,0.187398040460809,-1.4325283751521,0.151992663437026,0.649989103367215
"3810","SNHG6",2077.46733055713,-0.333449467588326,0.232868495715389,-1.43192176581871,0.152166212322221,0.650508980341984
"3811","PTDSS2",104.090939160818,-0.31320380979108,0.218762666801205,-1.43170594128703,0.152227995363756,0.650508980341984
"3812","PIAS2",151.775947999688,0.297058013930492,0.207497291658535,1.43162357231796,0.152251579762818,0.650508980341984
"3813","CPAMD8",4.91719800973139,1.10765478401051,0.773747590963495,1.43154537338362,0.152273972745042,0.650508980341984
"3814","PYM1",140.22565775452,-0.315771188751579,0.220662563224825,-1.43101387084791,0.152426239804604,0.650988731037546
"3815","UBAC2-AS1",11.5162842219267,-0.636505905600188,0.444955137238937,-1.4304945652496,0.152575124533384,0.651400605233141
"3816","PRKCSH",603.579886946864,0.224795739731946,0.157156015567782,1.43039856870761,0.152602658823112,0.651400605233141
"3817","AL513218.2",2.71676681175721,-1.57896405072707,1.1040379571328

4,-1.43017188904229,0.152667691416858,0.651507473274612
"3818","SLC26A11",87.6907021849012,0.314687239824944,0.220085282309712
,1.42984227078894,0.152762293898109,0.651740441410764
"3819","STK38L",87.4912927206182,-0.347910811510632,0.243351964207928,
-1.42966099592838,0.152814339912963,0.65179177345961
"3820","LARP1",459.878548524869,0.258494779172242,0.180860105306051,1.
42925261894998,0.152931638884393,0.652121326122481
"3821","PSMD2",563.232104525702,-0.270284859449271,0.189216586924038,-
1.42844168073795,0.153164769400316,0.652273299812742
"3822","HMOX2",265.442155997284,-0.309857354318884,0.216860412902963,-
1.42883318430982,0.153052185262237,0.652273299812742
"3823","NUDT19",54.0643894101247,0.353886357636658,0.247740778529794,1
.42845420821223,0.1531611659166 7,0.652273299812742
"3824","YWHAB",2363.75040933378,-0.280631110646224,0.19643706486646,-1
.42860570044152,0.15311759481761,0.652273299812742
"3825","LANCL3",14.4582122649348,-0.866513662589055,0.606618686734628,
-1.42843219560778,0.153167497807339,0.652273299812742
"3826","C1orf167",56.1857233813205,0.412606455439275,0.288938710874485
,1.42800683989523,0.153289889800101,0.65245336162891
"3827","HFM1",6.19421226776218,1.00841776102215,0.706167852757931,1.42
801425621938,0.153287755187622,0.65245336162891
"3828","DROSHA",95.3563585849151,0.289613134620893,0.202959076440274,1
.42695335286529,0.153593340726244,0.653206395430848
"3829","FCF1",174.20872801738,0.354975545055375,0.248785131652769,1.42
683585107014,0.153627214739737,0.653206395430848
"3830","FAM214A",214.652135433298,0.409912765211058,0.287266279681036,
1.42694355100154,0.153596166240123,0.653206395430848
"3831","PCGF2",220.262943658316,-0.29650820900112,0.207803087493555,-1
.42687104690067,0.153617067711381,0.653206395430848
"3832","PIGS",109.648077446446,0.323041874364013,0.226454929626825,1.4
2651729815003,0.15371907736366,0.653426422540882
"3833","PDLIM3",276.451601461798,-1.00903166238554,0.707462480215332,-
1.42626879955305,0.153790767078106,0.653560606557598
"3834","AP1S3",6.26290446352377,1.08674773573355,0.762336852668097,1.4
2554794764289,0.153998870472764,0.653960790509036
"3835","LINC00472",45.1652681330435,-0.515339852755212,0.3615087079206
32,-1.42552542017426,0.154005377395338,0.653960790509036
"3836","CARD10",2.5189613007233,-1.46631726323035,1.02849538580672,-1.
42569163018678,0.153957373558618,0.653960790509036
"3837","BACE1-
AS",32.8944156691692,0.512406256984173,0.359530801059716,1.42520823104
406,0.154097017736378,0.654075830232719
"3838","RLIM",269.665344177887,0.36019005754298,0.252737693103361,1.42
515369638859,0.154112777729337,0.654075830232719
"3839","FAM120B",83.5704304883719,0.283892184290599,0.199246330344905,
1.42483017779634,0.15420629669209,0.654302257571622
"3840","LINC00467",114.398129861868,0.340567950063062,0.23909173152833
7,1.42442378866915,0.154323831999342,0.654630442561794
"3841","ZFAND2A",126.504826515554,-0.451597752032623,0.31710067280936,
-1.42414630669712,0.154404124053777,0.654800514634724

"3842","KLRD1",32.1483864185417,0.615833078427056,0.432579093151071,1.42363116520748,0.154553269308148,0.655255843863833
"3843","AC106739.1",40.5301778426003,-0.587086826549872,0.41246580367399,-1.42335878834188,0.154632172865896,0.655255843863833
"3844","GMEB2",40.3161290317156,0.406937811238803,0.285882800234027,1.42344279161139,0.154607835095913,0.655255843863833
"3845","DCUN1D3",302.792106428107,0.497858193843115,0.349818900747965,1.42318837769663,0.154681553873416,0.655294624458796
"3846","PUM1",638.170763788772,0.244348037186492,0.171846458931661,1.42189742346487,0.155056031292468,0.655445250091819
"3847","AC099661.1",3.79189332022729,1.24567323421443,0.87612316809151,1.42180149958587,0.155083884152409,0.655445250091819
"3848","LINC02447",19.6843469337424,0.598652760252695,0.420951676468765,1.42214129012292,0.154985238250623,0.655445250091819
"3849","SEMA6A-AS2",3.7405950151215,1.29458482650973,0.910211850440526,1.42228957564458,0.154942203846348,0.655445250091819
"3850","TNF",10.0535140722966,-0.805842852004607,0.566988252377121,-1.42126904503943,0.155238558964932,0.655445250091819
"3851","MACC1",3.05966875537372,1.415194867193,0.995730208870396,1.42126336490129,0.155240209641736,0.655445250091819
"3852","FAM66D",18.8388193850663,0.846929262957057,0.595789785996136,1.42152363612784,0.155164587193388,0.655445250091819
"3853","MPG",753.133975161412,-0.34324694964104,0.241296574079757,-1.42251066327856,0.154878058165729,0.655445250091819
"3854","PLA2G15",77.5620878576428,0.309940789845289,0.218023139557113,1.42159584746323,0.155143610968855,0.655445250091819
"3855","PIP5K1C",120.516273397397,-0.320052050439322,0.225172411055351,-1.42136440667524,0.155210848382226,0.655445250091819
"3856","PITPNB",402.080347094405,-0.230588874378403,0.162165194335073,-1.42193813736597,0.155044210581678,0.655445250091819
"3857","CYB5R3",1710.36354931657,-0.259977048319407,0.182823671990604,-1.42200977307123,0.155023413819515,0.655445250091819
"3858","BCAP31",664.377551925398,-0.244972055314707,0.172214883648926,-1.42247899905157,0.154887243888746,0.655445250091819
"3859","RET",3.42031351862522,-1.71726337278554,1.20840158739265,-1.42110320832237,0.155286757436835,0.655447508349195
"3860","MGAT5B",2.98125580036635,1.37288254645164,0.966148751823364,1.42098465051129,0.155321221820117,0.655447508349195
"3861","GADD45G",92.9063112669117,-0.534697060432228,0.376362377336643,-1.42069742522101,0.155404741385032,0.655630104227089
"3862","RPS6KA5",224.822352702012,-0.48358212284614,0.340426656027277,-1.42051779519696,0.155456991650488,0.655680719056136
"3863","ZBTB16",1810.12004383475,-0.625244735428695,0.440284162606913,-1.42009363163697,0.155580424049616,0.655851136370127
"3864","LINC00944",5.10744986493952,1.18996057094915,0.838043713547737,1.41992661207567,0.155629047465082,0.655851136370127
"3865","WASF3-AS1",6.98330326185036,0.859188649918537,0.605136735849297,1.41982563447034,0.155658450071024,0.655851136370127

"3866","AP5S1",61.5666173756465,0.379425925091851,0.267156381834627,1.42023904683183,0.155538099575792,0.655851136370127
"3867","SLC4A3",11.677253654543,-0.69761884892295,0.492060311028456,-1.41775069699252,0.156263563060767,0.656702058487315
"3868","PPARG",237.280826426048,-0.442048641875037,0.311649575169859,-1.41841567290476,0.156069442478498,0.656702058487315
"3869","AC107294.3",2.88425467380257,1.32971746936503,0.937753979506376,1.41798115329244,0.156196267248153,0.656702058487315
"3870","WDR36",153.024263727622,0.326422347387661,0.23010095398272,1.41860492856616,0.156014228192781,0.656702058487315
"3871","TCF7",120.978939198778,0.743606481967038,0.524459084754495,1.41785413501823,0.156233355282481,0.656702058487315
"3872","FAM71F2",14.8733116240275,0.98112012799097,0.691627773494723,1.41856669958968,0.156025380089201,0.656702058487315
"3873","RPL30",25577.8360731836,-0.37775325254381,0.266378760302163,-1.41810575330898,0.156159891810055,0.656702058487315
"3874","LMBR1L",159.806257901753,0.372705035200796,0.262801526288912,1.41819965988729,0.15613248118981,0.656702058487315
"3875","LENG8",399.654969845247,0.324706472959834,0.228985100814475,1.41802445576105,0.156183624897817,0.656702058487315
"3876","EIF3D",987.711510301915,-0.341669513042119,0.240807979441926,-1.41884630996839,0.155943828019621,0.656702058487315
"3877","WDR27",111.775899650393,0.376210828315375,0.26539459287154,1.41755272496254,0.156321390663648,0.656702754382173
"3878","ADPGK",228.637716524032,0.263709985353444,0.186042189025141,1.41747410485375,0.156344360089267,0.656702754382173
"3879","MIR34AHG",7.89632677242934,0.909062421469425,0.643235552522372,1.41326519951306,0.15757776228219,0.656788333599731
"3880","PINK1-AS",7.38513366768717,-0.984707464108226,0.695744432286083,-1.41532927668952,0.156971974940207,0.656788333599731
"3881","DCLRE1B",24.8132999525603,0.516403335343298,0.365027420924098,1.41469737817499,0.157157243437312,0.656788333599731
"3882","SMG5",65.1787503505808,0.305634071594664,0.215994859547309,1.41500622855204,0.157066669837158,0.656788333599731
"3883","SLC5A6",27.0481032839582,-0.476781895932362,0.337282126284908,-1.41359965078498,0.157479483885252,0.656788333599731
"3884","CBWD2",70.6008588714463,-0.619610900273927,0.437860634666889,-1.41508701905872,0.157042983707249,0.656788333599731
"3885","STAM2",222.774804055918,0.223322736672012,0.157900866320898,1.41432242821366,0.157267254628723,0.656788333599731
"3886","AMT",37.0784514683372,0.532881385944364,0.377033965602225,1.41335114223253,0.157552503606805,0.656788333599731
"3887","RASSF1-AS1",3.29531272037634,-1.15340879971722,0.81477966555037,-1.41560822942005,0.156890240529406,0.656788333599731
"3888","EFCC1",2.64117174784631,-1.49470895933474,1.05632234749982,-1.41501215312971,0.157064932779731,0.656788333599731
"3889","QDPR",282.593788289705,-0.362732901181622,0.256469883831763,-1.41432941662486,0.157265203678982,0.656788333599731

"3890","NOCT",25.5187698907107,-1.06097188037535,0.750269401728088,-1.4141212182339,0.157326314163818,0.656788333599731
"3891","ARFIP1",285.257785633137,0.211990147827554,0.149940856971108,1.41382510484385,0.1574132604959,0.656788333599731
"3892","NUP155",64.0938636974596,0.356176487558969,0.25149712753445,1.41622487322516,0.156709675609706,0.656788333599731
"3893","AGGF1",243.557816242489,0.233625958397797,0.165212344280802,1.41409505091651,0.157333996079422,0.656788333599731
"3894","KLC4",40.3134144179836,0.406832326306416,0.287787415171629,1.41365572244982,0.157463011791996,0.656788333599731
"3895","GUSB",635.994686934289,-0.317685920545125,0.22452289833858,-1.41493773194597,0.157086753767156,0.656788333599731
"3896","LPL",57.2704547514935,0.602602984293781,0.425378530983448,1.41662763962394,0.156591823123861,0.656788333599731
"3897","SEC16A",148.737283532879,0.291992978269253,0.206205154593884,1.41603142193184,0.156766304820871,0.656788333599731
"3898","ARFIP2",160.120175900665,-0.322247833743551,0.227861725733598,-1.41422537157602,0.157295740779088,0.656788333599731
"3899","C12orf57",3170.32813595922,-0.341608238230553,0.241604793248754,-1.41391333192151,0.157387351001437,0.656788333599731
"3900","AC025031.4",3.31143255352837,1.34317950538004,0.94883776891295,1.41560501635477,0.156891181788338,0.656788333599731
"3901","PAN2",55.7387287815026,0.39550219775544,0.279455564490933,1.41525969781959,0.156992366892613,0.656788333599731
"3902","TMED2",1207.10402947967,-0.235818943862789,0.166908296071123,-1.41286532433536,0.157695326460099,0.656788333599731
"3903","AL590807.1",23.9526744179145,1.51856731937807,1.0747929241024,1.412892925999,0.157687209376035,0.656788333599731
"3904","GAS6-DT",30.7104514769953,0.513538695810944,0.363452903102948,1.41294426712966,0.157672111842848,0.656788333599731
"3905","NRL",5.10387960245366,-0.960001267512137,0.678017966197883,-1.41589355352267,0.156806672661273,0.656788333599731
"3906","SEC14L5",14.1635340202708,0.663309640822927,0.469362395440835,1.41321428232429,0.157592728356521,0.656788333599731
"3907","MPHOSPH6",120.76779805494,-0.347996757456089,0.245904547059381,-1.41517007968159,0.157018634848514,0.656788333599731
"3908","AC011815.1",2.54464566548129,1.33746378231929,0.944184763631249,1.41652760543977,0.156621087605114,0.656788333599731
"3909","LRRC25",2.85584287931525,-1.28354668687989,0.905744544248354,-1.41711776795196,0.156448498553834,0.656788333599731
"3910","ARHGEF1",153.945082246061,-0.248364700937192,0.175507716224245,-1.4151212623601,0.157032945086307,0.656788333599731
"3911","PSMD10",141.375334041812,0.226444674725282,0.160110073973589,1.41430622761836,0.157272009236122,0.656788333599731
"3912","BX284668.6",4.28761350127855,-1.33658117806389,0.946428050847151,-1.41223749324369,0.157880044229692,0.656885834088748
"3913","COL12A1",2986.38241540719,0.567808468054671,0.402038868712768,1.41232232065685,0.157855077100214,0.656885834088748
"3914","TGIF2",33.0076910208612,0.446507671437833,0.316135068682227,1.

41239525655616,0.157833612374196,0.656885834088748
"3915","TCEAL3",209.452662598061,-0.374008728258729,0.264789000600175,
-1.41247834090916,0.157809163695209,0.656885834088748
"3916","CFAP57",11.6001045583697,-0.783662092196808,0.555545488444431,
-1.41061732746876,0.158357479619618,0.657299460504262
"3917","DAP3",278.440139654478,-0.262846106797759,0.186472003673693,-1
.40957410023712,0.15866547907842,0.657299460504262
"3918","AL713852.1",4.34343911205002,1.33145051680588,0.94468324814688
6,1.40941476353866,0.158712561086561,0.657299460504262
"3919","AC108488.1",53.0734343703284,-0.337163752416374,0.239232540821
064,-1.40935573086839,0.158730007189284,0.657299460504262
"3920","ADCK2",64.1489674603267,-0.443448910136585,0.314317285050676,-
1.41083208346334,0.158294131892582,0.657299460504262
"3921","NCBP1",59.6071398451529,0.363743168665949,0.25803853418456,1.4
0964670185959,0.158644029710532,0.657299460504262
"3922","NCOA4",379.354301928264,0.199876306505209,0.141665815042438,1.
4109000569074,0.158274085399818,0.657299460504262
"3923","CKAP5",160.016558825695,0.240499878507346,0.170369551464644,1.
41163650687463,0.158057017352073,0.657299460504262
"3924","GPRC5D-
AS1",3.48568912202297,-1.77764233897586,1.25934514486705,-1.4115608784
6238,0.158079298373276,0.657299460504262
"3925","THAP2",91.1245480328604,0.407617886928007,0.289072913421673,1.
41008675667032,0.158514067223546,0.657299460504262
"3926","AC027288.3",6.23706819900588,1.26165101933427,0.89518547097000
1,1.4093738786524,0.15872464376553,0.657299460504262
"3927","PNN",809.646113574345,0.210611391436131,0.149252249623881,1.41
111033144811,0.158212084131984,0.657299460504262
"3928","TLN2",416.512024897512,0.345853778125208,0.245050002896132,1.4
1136002463874,0.158138483823816,0.657299460504262
"3929","PLEKHG4",62.3450897239399,-0.335017186699159,0.237718671833762
,-1.40930110417846,0.158746152472452,0.657299460504262
"3930","AC005921.2",93.9771898564874,-0.665136175853975,0.471840666887
642,-1.40966267329467,0.158639311416513,0.657299460504262
"3931","RPS15",19575.4615565757,-0.307836597131153,0.218402195732515,-
1.40949405796347,0.158689129250792,0.657299460504262
"3932","RFX1",133.11770206755,-0.337147725474202,0.239216399362276,-1.
40938383143045,0.158721702364578,0.657299460504262
"3933","NOL4L",30.707264872684,0.518197250549314,0.367177593709066,1.4
1129867243454,0.158156565778846,0.657299460504262
"3934","WDR45",501.226660929152,-0.33668070590224,0.238830810871446,-1
.40970381783556,0.15862715695293,0.657299460504262
"3935","MAPK1",383.283917101423,0.223096100920874,0.158371333589585,1.
40868991795587,0.158926877408246,0.657880535223106
"3936","SAMD5",2.38384667084354,2.0270623810917,1.43923451899236,1.408
4309084741,0.159003512271069,0.658030541510019
"3937","MCM2",19.0266742366423,0.533217936498168,0.378689088728042,1.4
0806258318444,0.159112539285611,0.658314491344505
"3938","RGS10",368.868901624153,-0.427283557837587,0.303535993511829,-
1.40768662356656,0.159223884447379,0.658394430888959

"3939","FRS2",210.078491582832,0.23335847928753,0.165786074067206,1.40758794488933,0.159253119141906,0.658394430888959
"3940","TCEAL5",4.04979582625366,-1.42499759520939,1.01232139492918,-1.40765334245364,0.159233743906521,0.658394430888959
"3941","NSUN4",68.3114247330431,0.313645321634998,0.222884349507728,1.40721105958193,0.159364813130866,0.658689023366831
"3942","RAD1",152.063730865776,0.264278452254711,0.187859971275929,1.40678426840882,0.159491368799445,0.659044877314603
"3943","ZNF213-AS1",28.1787618192299,-0.557948471514102,0.396770238206377,-1.40622561318193,0.159657140758136,0.659562557902429
"3944","TYSND1",107.694530511826,-0.322438000273018,0.229348968392411,-1.40588380463667,0.159758631187395,0.659814488694593
"3945","SIKE1",240.942697788273,0.21525969554363,0.153130434710003,1.40572771148522,0.159804994880992,0.659838672146129
"3946","ATP1A1-AS1",69.0638994669329,0.680173143519826,0.483970237178134,1.40540283527699,0.159901524123615,0.659868968760624
"3947","NBPF12",107.315490432632,0.540845976034227,0.384863213098806,1.40529403077913,0.159933862647611,0.659868968760624
"3948","IDH3G",583.017056143278,-0.364464270340445,0.259301003475775,-1.40556444230843,0.15985350092611,0.659868968760624
"3949","AC107029.2",6.83229325595031,1.09569043214603,0.779892185506247,1.40492551728132,0.160043427787185,0.660050100054657
"3950","KIF16B",113.189524306,0.279104936971663,0.198669044383375,1.40487380828726,0.16005880626287,0.660050100054657
"3951","TRIM52-AS1",157.78357959,-0.330003561237775,0.23492282264586,-1.40473180732741,0.160101043701577,0.660057175615033
"3952","ICAM2",92.8534022778711,-0.330007046694562,0.235002627360239,-1.4042696049891,0.160238581708945,0.660434107902138
"3953","RAB8A",369.510874895102,-0.26347742243656,0.187641653306781,-1.4041521047877,0.160273560595319,0.660434107902138
"3954","ERBIN",294.611370681186,0.306602418310025,0.218397894084002,1.4038707634795,0.160357337146472,0.660533965987846
"3955","CLBA1",24.7769923115565,-0.440273783661869,0.313635143884026,-1.40377694351967,0.160385281790846,0.660533965987846
"3956","PLCG1",62.6885029694834,0.34210959563139,0.243726433547205,1.40366225629413,0.16041944683209,0.660533965987846
"3957","CACNA2D3",16.195022226282,-0.695605014165772,0.495683357017142,-1.40332533727114,0.160519845992698,0.660780331406384
"3958","ZNF530",13.5617401648741,-0.565769918610115,0.403248497593356,-1.40303044397365,0.160607760790792,0.660975193411118
"3959","AC254633.1",94.3679879715712,0.790057753073384,0.565224463947387,1.39777699563076,0.162180044495708,0.661082909875897
"3960","EIF3I",1154.55999876149,-0.262726913641496,0.188189683725319,-1.39607500496664,0.162691909773399,0.661082909875897
"3961","TRAPPC3",342.010754086128,-0.27459926597646,0.196352206706392,-1.3985035899651,0.161961895378801,0.661082909875897
"3962","ZNHIT6",202.847285586612,0.287067289978926,0.205601287291295,1

.39623294076078,0.162644360130709,0.661082909875897
"3963","SETDB1",69.720842450068,0.314965240959994,0.22494036558463,1.4
0021663137865,0.161448456969666,0.661082909875897
"3964","SFT2D2",836.736916838062,0.456337612580487,0.326441906899833,1
.39791369592909,0.162138985343288,0.661082909875897
"3965","NR4A2",195.286618730896,-0.662097895652733,0.473173403150409,-
1.39927115777103,0.161731685237297,0.661082909875897
"3966","SLC25A12",149.971706967895,-0.340528431974647,0.24312504800779
2,-1.40063080610161,0.161324503868316,0.661082909875897
"3967","CFLAR",948.056999364187,0.333918608318091,0.238475403786457,1.
40022242552569,0.161446722416984,0.661082909875897
"3968","SLC23A3",4.38278138096297,1.04945025005627,0.748209458007963,1
.40261558955742,0.160731500482735,0.661082909875897
"3969","THRB-
AS1",5.04946186000039,-1.14923422805913,0.823373616706414,-1.395762755
49876,0.162785949407801,0.661082909875897
"3970","PRKCD",48.6854927924574,-0.395612780409033,0.282696821661047,-
1.39942422445546,0.161685806794984,0.661082909875897
"3971","MAGI1",36.3312359598666,0.531953084968261,0.380827800000365,1.
39683364756394,0.162463601427941,0.661082909875897
"3972","HEG1",424.831359365782,0.305228415966438,0.218645008473468,1.3
9599992745079,0.162714516996662,0.661082909875897
"3973","SRPRB",236.763314261812,-0.279026323027205,0.199531473856706,-
1.39840756765816,0.161990711937399,0.661082909875897
"3974","NELFA",138.371047395838,0.242111354837656,0.17282254878518,1.4
0092456996803,0.161236630581492,0.661082909875897
"3975","RASGEF1B",35.9688050801491,-0.69299180534794,0.494902570093721
,-1.40025905546764,0.161435757096891,0.661082909875897
"3976","PIK3R1",3861.24074996955,0.351770933441584,0.251394462551794,1
.39927876640922,0.161729404479755,0.661082909875897
"3977","AC114980.1",22.6671446462585,0.62540540135457,0.44628049865656
7,1.40137291061837,0.161102588594999,0.661082909875897
"3978","FGF1",5.48341883983331,-1.36312050799143,0.975300944002634,-1.
39764091932198,0.162220924019853,0.661082909875897
"3979","RBM22",300.568634724082,-0.238591879267289,0.170884795352891,-
1.39621479356649,0.162649823163369,0.661082909875897
"3980","ECI2",634.731387023222,-0.279722985000715,0.199623007583992,-1
.40125623988017,0.161137461952511,0.661082909875897
"3981","PIM1",440.521961128268,-1.13149786468544,0.810377632333483,-1.
39625998983621,0.162636217532038,0.661082909875897
"3982","LATS1",205.492755351616,0.267379596111456,0.191135146446322,1.
39890334709606,0.161841968747812,0.661082909875897
"3983","MTRF1L",128.837169244752,-0.294445045490524,0.210058055694616,
-1.40173174752503,0.160995366517609,0.661082909875897
"3984","AC018645.3",26.228442382603,0.518766627687774,0.37091650912465
8,1.39860754354675,0.161930702984887,0.661082909875897
"3985","CLN8",124.05098784071,0.356146588802872,0.254296095761056,1.40
051929518225,0.161357869485002,0.661082909875897
"3986","RDH10-
AS1",2.94043670462764,1.39291204575612,0.998132979380432,1.39551750571

426,0.162859839605947,0.661082909875897
"3987","PLEC",455.565341407386,-0.367661074863756,0.262422241147423,-1
.40102863711621,0.161205509754528,0.661082909875897
"3988","TNC",686.990632050033,1.46223764445244,1.04489524602979,1.3994
1075433963,0.161689843777787,0.661082909875897
"3989","C9orf50",2.63030692918351,-1.41201531792087,1.01123295819584,-
1.39633039694441,0.162615024281907,0.661082909875897
"3990","WDR37",53.7506934863109,0.359064633518536,0.256039602897165,1.
40237927826638,0.160802017854624,0.661082909875897
"3991","DDIT4",1559.56249648571,1.03691213660051,0.741934211506424,1.3
975796243378,0.162239340564513,0.661082909875897
"3992","SVIP",18.9561924689752,-0.649760288381999,0.465139616633313,-1
.39691452877089,0.162439275043619,0.661082909875897
"3993","GPR137",96.7928757315357,-0.356793005223751,0.255556000979361,
-1.39614410875277,0.162671103444356,0.661082909875897
"3994","NDUFS8",545.735647226273,-0.376320530425996,0.269424076890182,
-1.3967590972925,0.162486026111052,0.661082909875897
"3995","MGP",100650.196972881,-0.422643545308159,0.302586986239284,-1.
39676709352574,0.162483620736426,0.661082909875897
"3996","AC022506.2",4.62147871912092,-1.13427823661492,0.8128097647025
09,-1.39550271892965,0.162864295457821,0.661082909875897
"3997","KERA",6.55096811314798,1.26279161855415,0.900820603908755,1.40
182364066137,0.160967917108648,0.661082909875897
"3998","TMED8",78.7531643021659,0.310264352420353,0.222193962321113,1.
39636716128209,0.162603958673228,0.661082909875897
"3999","AC012409.3",4.14526746999667,1.09816454009563,0.78456883019846
6,1.39970452282413,0.16160181886728,0.661082909875897
"4000","LIG3",53.3670636372912,0.345053825554473,0.246372564495976,1.4
0053672883739,0.161352652749452,0.661082909875897
"4001","EPOP",7.02804718283071,-0.854928294748032,0.612483443854879,-1
.39583902769231,0.162762974859235,0.661082909875897
"4002","MBTD1",115.93762871257,0.327463020538578,0.23453097149331,1.39
624638252914,0.16264031370904,0.661082909875897
"4003","ERN1",85.9896565376224,-0.358505896512348,0.255963033758107,-1
.4006159063232,0.16132896178745,0.661082909875897
"4004","AC139530.1",41.9860792578754,-0.452694372768743,0.323934606407
642,-1.39748691190798,0.162267199717907,0.661082909875897
"4005","OGFOD3",271.407921923444,0.302467340789935,0.215808066624695,1
.40155715919529,0.16104752755797,0.661082909875897
"4006","PPAN",70.1577300389226,-0.454174099337169,0.324840516891938,-1
.39814486100038,0.162069570705294,0.661082909875897
"4007","AL117335.1",13.7980201529263,-0.591956443795379,0.423868638495
29,-1.39655636212387,0.162547020555251,0.661082909875897
"4008","CTSZ",1405.00947600111,-0.231361220142965,0.16572023696349,-1.
39609515640468,0.162685842207475,0.661082909875897
"4009","AF165147.1",12.2021353295513,0.676861309453936,0.4836912454995
,1.39936646724907,0.161703117119359,0.661082909875897
"4010","RWDD2B",115.972948457455,0.243877141706681,0.174718123398909,1
.39583196615425,0.162765101818029,0.661082909875897
"4011","PHF5A",286.343898694578,-0.329768431630066,0.236213351869377,-

1.39606177644193,0.162695892953947,0.661082909875897
"4012","SH3KBP1",413.978555572109,0.285520847616489,0.204601582802237,
1.39549676843149,0.162866088607771,0.661082909875897
"4013","FP236383.5",3.30451159113247,1.86285228983349,1.32947715150806
,1.40119165471961,0.161156769171058,0.661082909875897
"4014","PRKCE",97.993599055497,0.378202206490347,0.271045693884006,1.3
9534482570381,0.162911880759006,0.661104042272908
"4015","RAB33B",114.719519943742,-0.291956025495619,0.209343914746626,
-1.39462389364879,0.163129285956449,0.661110272464323
"4016","NXNL2",3.69546688238114,-1.1861215680122,0.850213099666981,-1.
3950873827712,0.162989490483567,0.661110272464323
"4017","USP20",40.5320182001943,0.421334990668196,0.30204334015592,1.3
949487859944,0.16303128393869,0.661110272464323
"4018","ABI1",305.142855883585,0.250560865747694,0.179678989951359,1.3
9449173114521,0.163169164768442,0.661110272464323
"4019","ATG14",202.342106051001,0.401551286137758,0.287900407091872,1.
39475761842052,0.163088943224995,0.661110272464323
"4020","CRTC3",205.338503814399,0.238965510612902,0.171343603743416,1.
39465673297468,0.163119378133884,0.661110272464323
"4021","PLEKHH3",235.253482336725,-0.250934767277441,0.179959242676565
,-1.39439777332492,0.163197520141141,0.661110272464323
"4022","PRAC2",9.23042039533659,-1.34517820260471,0.964872565011393,-1
.39415115672693,0.163271963823919,0.661247394014873
"4023","HTRA4",7.66065064259425,1.07227121114999,0.769443374847404,1.3
9356741015886,0.163448275574735,0.661796907988282
"4024","LURAP1",44.1074817540906,-0.410633527314138,0.294731039310033,
-1.39324832659442,0.163544710548431,0.66185833294991
"4025","KCTD20",342.636785337204,0.239979831732875,0.172228694332833,1
.39337891785391,0.163505237445091,0.66185833294991
"4026","MRPS15",596.069171855217,-0.353710272005922,0.253987338178993,
-1.39262954815744,0.163731842968004,0.662179629705059
"4027","PTPMT1",354.934589520454,-0.313839874377566,0.225327197134922,
-1.39281843633658,0.163674701860172,0.662179629705059
"4028","BRCA2",25.9604499824276,0.624266787867308,0.448279910990307,1.
39258256406856,0.163746058594879,0.662179629705059
"4029","NPHP3",309.046071449538,0.405077497612744,0.290959221250489,1.
39221398748524,0.163857608345799,0.662301881475118
"4030","TEX9",20.6642386788749,-0.483838480448711,0.347508005781987,-1
.39230887461123,0.163828885272738,0.662301881475118
"4031","MOSPD2",148.194961314052,0.257032671540484,0.18464176470768,1.
39206138950964,0.163903808901637,0.662324272686374
"4032","FAM136A",216.676734711897,-0.2555208884506,0.183591672916584,-
1.3917890958306,0.163986272906697,0.662493154607438
"4033","TUBA1A",3413.1974087826,-0.41866786148375,0.300864525066337,-1
.39154944037167,0.164058878305143,0.662622134567935
"4034","IL20RB",7.64525775231894,-0.955369454085938,0.686770385674161,
-1.39110461664434,0.164193705145389,0.662837983423358
"4035","NIN",211.259667313364,0.295327798473028,0.212283294924458,1.39
119660158903,0.164165817505998,0.662837983423358
"4036","PTPN21",157.924651318552,0.312791269178625,0.22491184532755,1.

39072830389654,0.164307831353744,0.663134357017131
"4037","VDAC2",1250.06255043446,-0.328526218385628,0.236309287284072,-1.39023828543268,0.164456531201861,0.66337398299425
"4038","AP002490.1",4.73603100427861,1.19478948568864,0.859480348088868,1.3901300807463,0.16448938039866,0.66337398299425
"4039","CRYAB",1780.83191564593,-0.349789328655249,0.251588653006527,-1.39032235546082,0.164431012308787,0.66337398299425
"4040","LTO1",51.9374486334413,0.350233975741396,0.251967947760507,1.3899941593932,0.164530650930548,0.663376181437548
"4041","DEGS1",496.559906587848,-0.310062973560856,0.223097190579414,-1.38981119733324,0.16458621699869,0.663436003140723
"4042","PTCD3",230.077971610156,0.280662118897611,0.201978821651622,1.38956211647627,0.164661886237258,0.663496637587587
"4043","AC018521.5",94.4725468292779,-0.400322116475327,0.288106497687821,-1.38949353689724,0.164682724892051,0.663496637587587
"4044","AC116366.1",57.6214152103369,0.73963124151722,0.532423398859749,1.38917869331294,0.164778419044012,0.663718018745774
"4045","MAP3K12",219.396054906494,0.296348485788945,0.213372320692751,1.38887970485955,0.164869332911582,0.663920040493637
"4046","GEMIN4",84.0218910406979,0.353018410939876,0.25420742787217,1.38870218661507,0.164923329015405,0.6639733332506
"4047","EIF4EBP2",804.522816373649,0.254942532105814,0.183650558273668,1.38819361347058,0.165078096504535,0.664432200138961
"4048","ACTR1B",152.686238275618,-0.26931124734701,0.194173538516519,-1.38696162929584,0.165453463490637,0.665778524406863
"4049","METTL9",771.740140682675,-0.289872792878306,0.209062924161013,-1.38653371486881,0.165583992740709,0.666139209126551
"4050","ATP11C",168.448102863027,0.254442037547164,0.183529138977489,1.38638495753185,0.165629387209737,0.666157305743064
"4051","SNRNP40",219.805517401348,-0.395960219434757,0.285726363334637,-1.38580218784718,0.16580731415044,0.666379309202199
"4052","TRMT9B",14.1796645104985,0.738405772892018,0.532744404305933,1.38604134914194,0.165734277799655,0.666379309202199
"4053","APOBEC3F",47.4624963388714,0.382239033499043,0.275819896283703,1.38582835629044,0.165799321512245,0.666379309202199
"4054","AC024337.2",11.9950363866101,0.763459610842793,0.551062284359997,1.38543252280361,0.165920252064301,0.666668718765514
"4055","C1orf109",85.3465606979877,0.2877184807241,0.207773121205427,1.38477238564285,0.16612207724739,0.666799059842718
"4056","INKA1",19.6561283975513,-0.697896968346993,0.5040532407074,-1.38456994615796,0.166184006492899,0.666799059842718
"4057","PTPN13",294.683362313797,0.292171304635226,0.210964667331748,1.38493003748243,0.166073861230626,0.666799059842718
"4058","C6orf62",463.243250127291,0.281944691489702,0.20361402688381,1.38470171137586,0.166143695583159,0.666799059842718
"4059","C8orf48",34.456415728766,-0.471188459897578,0.340337408002199,-1.38447449154497,0.166213213499668,0.666799059842718
"4060","SCRIB",76.0045542526606,0.273546562031877,0.19759364942765,1.38438944178738,0.166239240101988,0.666799059842718
"4061","SDK2",5.11750991099,1.27429610387796,0.920182779377372,1.38482

93322118,0.166104659574409,0.666799059842718
"4062","AC092645.2",6.38370771011306,0.908187931702906,0.656138780968741,1.38413999910512,0.166315591275369,0.666941079833699
"4063","TMEM120B",48.4438522705059,0.407121002736831,0.294193787798035,1.38385315945666,0.166403421767244,0.667129051726962
"4064","PGBD2",28.2915705415533,-0.463387633756186,0.334896105744396,-1.38367579021614,0.166457749794632,0.667182649213769
"4065","AC004160.2",3.23340360045251,1.4272356517925,1.03161808245113,1.38349227884934,0.166513973184962,0.667243815303774
"4066","ZNF493",144.612740935971,0.35361841485139,0.255647732816542,1.38322531146855,0.166595790946486,0.667407486160186
"4067","GRID1",34.1298232632506,0.438760415924134,0.31724308972118,1.38304168046577,0.166652086065716,0.667468854173701
"4068","CIB1",782.727060420896,-0.325357786710335,0.235287274981099,-1.3828108074968,0.166722884281005,0.667588265008185
"4069","EEPD1",48.4687545293705,-0.412024428120753,0.298034745360269,-1.38247111967664,0.166827092151025,0.667841362508738
"4070","AC103858.1",7.76625335026151,1.19364913223939,0.863710534958031,1.38200135800983,0.16697128413302,0.668254360501908
"4071","LSM14B",83.8681477216055,0.31271155907678,0.226333989386392,1.38163764057075,0.167082990464568,0.668537173096867
"4072","PDS5A",284.054569374108,0.252536062121019,0.182841158351868,1.3811773257038,0.167224444678295,0.668774608240794
"4073","PCDHGB5",3.97437963646046,1.05253339420623,0.762003447865422,1.38127117030069,0.167195599044912,0.668774608240794
"4074","KLHL29",96.1930948457578,0.436366185091927,0.31610528387519,1.38044571651077,0.167449452445216,0.669181582649686
"4075","GAP43",2.72676883628969,-1.68344554999218,1.21938284915112,-1.38057177953923,0.167410665316634,0.669181582649686
"4076","IFT52",298.275170599429,-0.296844534438354,0.215005311183047,-1.38063814705318,0.167390248042696,0.669181582649686
"4077","TDRD6",12.2122364955207,0.644337477156589,0.467000882570514,1.37973503092748,0.167668242816134,0.669414280705048
"4078","GGH",98.9246969915185,0.55110795473147,0.399428594026361,1.37974086726275,0.167666445178603,0.669414280705048
"4079","CCDC6",305.709081632532,0.296036257012328,0.214512815633428,1.3800399576975,0.167574342331078,0.669414280705048
"4080","RANBP10",65.1775431742442,0.392005280714659,0.284118905104213,1.37972262201586,0.167672064907397,0.669414280705048
"4081","NR2C2AP",109.748800114731,-0.397618231003642,0.288217485582442,-1.37957705862335,0.167716905036899,0.669429224735615
"4082","CD84",11.5761067997313,-0.699410260503327,0.507051049241009,-1.37936853015146,0.16778115695914,0.669481322509422
"4083","ARHGEF35",59.4469854654847,-0.410959046204454,0.297964727866368,-1.37922045051172,0.167826794572827,0.669481322509422
"4084","AC105046.1",7.09226508126418,1.33004863584254,0.964408142035716,1.37913459858915,0.167853258096168,0.669481322509422
"4085","DENND6A",185.674999047247,0.30176510340019,0.218877011343488,1.37869711189826,0.167988160350085,0.66971661512483
"4086","IL10RA",18.5671207473329,-0.642771570102532,0.466223594016456,

-1.37867662287345,0.167994480287314,0.66971661512483
"4087","TTC14",350.344276673268,0.280844248111836,0.203734176929031,1.37848372985386,0.16805398780963,0.669789921074396
"4088","PRDM16",10.4607579053578,-0.984968070431997,0.715098900950794,-1.37738719654356,0.168392569220475,0.669889341274728
"4089","PDE6D",81.1432559948257,-0.316170688969279,0.229765762237318,-1.37605657992994,0.168804116894509,0.669889341274728
"4090","PDCD6IP",452.667708620005,0.186308336609321,0.135274861587616,1.37725763991007,0.168432606789379,0.669889341274728
"4091","GSK3B",206.372322678809,0.216924535961166,0.157577332058072,1.37662272312887,0.168628921780686,0.669889341274728
"4092","SLC9B2",115.884743728959,-0.389616984780199,0.282855043929081,-1.37744400583461,0.168375015399152,0.669889341274728
"4093","NELFE",434.471593980248,-0.2554551492608,0.185397650880659,-1.37787694745516,0.168241283458526,0.669889341274728
"4094","MRPL2",285.69231413634,-0.319315180364544,0.232046531545496,-1.37608253929854,0.16879608068514,0.669889341274728
"4095","GPNMB",4362.63581337244,-0.255464589471721,0.18555708829426,-1.37674390032786,0.168591440781871,0.669889341274728
"4096","SUSD3",3.98892451313092,-1.23323064218627,0.89609196713509,-1.37623222550365,0.168749748113051,0.669889341274728
"4097","ADAMTS13",6.56511219888537,0.845213134096359,0.613303427489719,1.37813208961812,0.168162509590736,0.669889341274728
"4098","EED",99.204701850352,0.299498397999425,0.217586180148251,1.37645873370893,0.168679654871328,0.669889341274728
"4099","RNF111",99.9553110443066,0.282211895231986,0.204927399723611,1.37713109917273,0.168471719237121,0.669889341274728
"4100","STUB1",1067.64617682038,-0.302796706533239,0.220054482680454,-1.37600789970244,0.168819187545752,0.669889341274728
"4101","SDR42E2",19.756448201653,0.56680052361867,0.411817127159511,1.37634033710096,0.168716290120917,0.669889341274728
"4102","EIF5A",871.748048598503,-0.325675168094249,0.236482166899674,-1.37716586567145,0.168460972588833,0.669889341274728
"4103","POLR2A",457.506145370465,0.401127573172773,0.291325421989911,1.37690549088663,0.168541469361571,0.669889341274728
"4104","ZNF397",202.984715028798,0.316764716923158,0.229898749557159,1.3778444534097,0.168251317820985,0.669889341274728
"4105","ZNF607",13.1574804415886,0.733378734865736,0.532468100871888,1.3773195683739,0.168413467818672,0.669889341274728
"4106","ZBTB34",44.755870506317,0.380121646722938,0.276287600845468,1.37581869602446,0.168877771687607,0.669958602781158
"4107","EPB41L5",51.1191825373736,-0.303731777324765,0.220872859891091,-1.37514304597917,0.169087101301287,0.670053303788811
"4108","LMX1B",30.8506255189006,0.482126191727971,0.350617386591562,1.37507782034097,0.169107319778734,0.670053303788811
"4109","PDS5B",366.293793743861,0.239669375257304,0.174256525724037,1.37538249578589,0.169012892806799,0.670053303788811
"4110","ZNF600",31.2169044315931,-0.468516781436002,0.340621368679285,-1.37547677426292,0.168983681430535,0.670053303788811
"4111","KLHL4",8.87244540567095,-1.15466939591838,0.839556192975515,-1

.37533306951861,0.169028208624658,0.670053303788811
"4112","PCDHGA6",28.2753912824955,0.57112797009017,0.415428091645659,1
.37479381287801,0.169195376830179,0.670066029311488
"4113","AL441992.1",11.0638464758418,-0.688874748706144,0.501120278504
971,-1.37466947209024,0.169233939749982,0.670066029311488
"4114","AC004477.1",2.41518844101073,-1.38654485127785,1.0086041837312
3,-1.3747165376099,0.16921934212439,0.670066029311488
"4115","PDIA4",465.154140909792,0.327483022295976,0.238285146161672,1.
37433250696116,0.169338478897321,0.670317006745676
"4116","PCBP3",21.2031809022283,0.748408387319864,0.544765346931646,1.
37381790441559,0.169498221241483,0.670786327940357
"4117","C1GALT1C1L",63.1600156560149,-0.464345463329268,0.338091639783
859,-1.37343077641944,0.169618467538724,0.671099154174952
"4118","ADGRL4",13.2288850683323,-0.847987299400488,0.61796026989941,-
1.37223595222153,0.16998999668462,0.671639927949168
"4119","STRADB",88.6365514380481,0.317389385480752,0.231278841214149,1
.37232348542801,0.169962757649524,0.671639927949168
"4120","SYNPO2",409.076065065275,0.449837948300684,0.327690228057407,1
.37275362456606,0.169828952271029,0.671639927949168
"4121","PDE5A",115.881064278275,-0.424465681740511,0.309407673915299,-
1.37186539806608,0.170105343886586,0.671639927949168
"4122","PTPN12",586.177485237294,0.207836287104246,0.1515064491007,1.3
7179828540569,0.170126241188426,0.671639927949168
"4123","PAXIP1",54.1550601719274,0.383219708748788,0.279203212730681,1
.37254763296166,0.169893021178744,0.671639927949168
"4124","BIN3",119.092308211004,-0.304623434420376,0.222037189482684,-1
.37194780356438,0.170079687386644,0.671639927949168
"4125","AC092490.1",6.01526800005415,0.861913146601202,0.6280817186431
82,1.37229459323089,0.169971748110831,0.671639927949168
"4126","SLC35B1",195.062593860317,-0.383568463949008,0.279577556791547
,-1.37195727851287,0.17007673759932,0.671639927949168
"4127","ALDH1A1",1656.11779135931,0.551466316081368,0.402174318027746,
1.37121216189474,0.170308828030647,0.672197843419239
"4128","KIAA2026",344.538645406848,0.282224184364139,0.206043509688279
,1.36973100871322,0.170770885273011,0.673858272822692
"4129","PEPD",545.491011514142,-0.357614565743289,0.261181377934464,-1
.36921923213462,0.170930756037044,0.674325765339647
"4130","LMO3",762.600238483686,0.258616206672973,0.188901510880201,1.3
6905314027363,0.170982664545437,0.674367221012258
"4131","ARL14EPL",2.62000920401458,1.30796848424645,0.955686689632505,
1.36861640790394,0.17111921236996,0.674742399006119
"4132","SRSF7",1804.58389289776,0.232982782486357,0.170262815219951,1.
36837149195133,0.171195823004956,0.67476171027025
"4133","SPAG16",286.763084050434,-0.321748296097306,0.235138386864246,
-1.36833589950186,0.17120695859457,0.67476171027025
"4134","ATXN1",252.359444017178,0.303101240240269,0.22160553133915,1.3
677512398208,0.171389955088117,0.674992983179481
"4135","ZNF311",4.26152857801943,1.03249755855364,0.754862548330399,1.
36779544943289,0.171376112514056,0.674992983179481
"4136","MSS51",12.4941950280751,0.647209597757086,0.473189404811527,1.

36776012137227,0.171387174097772,0.674992983179481
"4137","PRRT2",89.8459936243562,-0.491020041080707,0.359113969216826,-1.36730977675847,0.171528228526378,0.675251135349315
"4138","HOOK2",106.991155130711,0.26350809019106,0.192724691074311,1.36727727372232,0.171538412307414,0.675251135349315
"4139","TMEM8B",111.636358880843,0.273718621853044,0.200219981839956,1.36708943501872,0.171597274389836,0.675319643038423
"4140","TRAF7",208.672587402571,-0.283544221717896,0.207464252356398,-1.36671363137203,0.171715083492741,0.675620047104653
"4141","DRAP1",883.048350844147,-0.372801728031622,0.272811653723575,-1.36651687324677,0.171776788507781,0.675699615552581
"4142","B3GNT5",9.48036854718977,-0.898921126257521,0.657937710017253,-1.36627086815551,0.171853961131299,0.675839974135137
"4143","SH3BP2",79.6412350258975,0.408568316641853,0.299085041631185,1.36606068432428,0.171919917056533,0.675936164357679
"4144","UBXN7",316.460427470973,0.318561164662032,0.23330737544232,1.36541403398878,0.172122955452535,0.675970370673801
"4145","MRPL41",687.89381971504,-0.323885760900088,0.23717606162784,-1.36559212037304,0.17206702113736,0.675970370673801
"4146","KRT25",10.2685938288274,1.28967815359378,0.944561631919769,1.365372157847,0.172136110108351,0.675970370673801
"4147","ABCC3",14.3868854631218,-0.778622349495934,0.570060729556271,-1.36585859913909,0.171983349477015,0.675970370673801
"4148","HAS1",102.017084446486,-1.27802295193671,0.935754352027742,-1.36576757475643,0.17201192679613,0.675970370673801
"4149","PRMT6",56.7908703594762,0.406437491485393,0.297817964519399,1.36471784749881,0.172341747937486,0.676512989717331
"4150","HMGCLL1",71.0837467280717,-0.626507259789674,0.459108356132771,-1.36461741856969,0.172373327181756,0.676512989717331
"4151","AC036214.2",4.58613745023755,-0.929681247064679,0.681316684286822,-1.36453615257908,0.172398883928826,0.676512989717331
"4152","AL359397.2",3.89420304571998,1.39253651842299,1.02082630646686,1.36412679571576,0.172527662672927,0.6768552739112
"4153","ITGBL1",455.821171802529,-0.58919284875402,0.431980697407845,-1.36393327824494,0.172588565979882,0.676931170538477
"4154","TAGLN",4387.54262547958,0.515214462747207,0.377866932029773,1.36348121276358,0.172730901482963,0.67732634912277
"4155","SPRTN",85.4509206509107,-0.363372658214505,0.266594347602114,-1.36301711376428,0.172877117106319,0.67773654886759
"4156","P2RY13",2.43657887984763,-1.65475899296191,1.21462648725733,-1.36236037195139,0.173084183466027,0.677895711584064
"4157","TENT5A",1131.64624131753,-0.3046095125477,0.223547864491618,-1.36261428057221,0.173004105780365,0.677895711584064
"4158","HSDL2",262.795793141024,-0.221779529061475,0.162770980434506,-1.3625249935182,0.173032261968152,0.677895711584064
"4159","SMIM26",972.663593814035,-0.355270038660917,0.260764707526999,-1.36241611079265,0.173066602180379,0.677895711584064
"4160","ZNF792",10.1051583380669,0.615334135963514,0.451876329323155,1.36173128803891,0.173282703276085,0.678510085015421
"4161","DENND2D",26.019655585557,0.55967372818843,0.412114768800795,1.

35805307297531,0.174446844132053,0.67855368941677
"4162","MOV10",69.8193071703434,0.365483400391046,0.268851591353084,1.
35942435211795,0.174012158739839,0.67855368941677
"4163","NGF",7.71098763884079,0.99275867907049,0.731256099837193,1.357
60738172511,0.174588299414122,0.67855368941677
"4164","ETNK2",59.4642178299231,-0.322234153241706,0.236796110940253,-
1.36080846920248,0.17357422478053,0.67855368941677
"4165","PSME4",100.73517144883,0.403329533926121,0.297070973065877,1.3
576874568512,0.174562878539802,0.67855368941677
"4166","DNAH7",4.80930420941064,-0.931564896012732,0.685178105605716,-
1.35959524741265,0.173958042936438,0.67855368941677
"4167","KBTBD8",2.63152485123055,-1.40801031835424,1.03586743230999,-1
.35925725091518,0.17406508526185,0.67855368941677
"4168","PLOD2",152.947755533346,0.38027651979427,0.279485552253899,1.3
6063033214972,0.17363054103573,0.67855368941677
"4169","AP2M1",1394.73079186672,-0.229516953715959,0.168836293687454,-
1.35940530737328,0.174018190238421,0.67855368941677
"4170","AFF1",473.947938093311,0.371257796798549,0.273568539535534,1.3
5709243990143,0.174751840328034,0.67855368941677
"4171","NKD2",9.72586670785267,0.822419061309446,0.604282729302666,1.3
6098389285179,0.173518779680516,0.67855368941677
"4172","EMB",18.8810005630338,-0.809490832799041,0.59556795596876,-1.3
5919138141392,0.174085951632595,0.67855368941677
"4173","PAQR8",44.8617894624771,0.38624063304052,0.284600567750186,1.3
5713233495567,0.174739165930789,0.67855368941677
"4174","ATG5",219.927488899974,-0.264510705957844,0.194558791947922,-1
.35954126415756,0.173975135946882,0.67855368941677
"4175","ALDH8A1",2.28612405403259,1.48447059103543,1.09310404905173,1.
35803228642617,0.17445343954757,0.67855368941677
"4176","CPVL",444.29162269687,-0.48207313435541,0.354223223386807,-1.3
6093034710204,0.173535702160765,0.67855368941677
"4177","RHBDD2",148.519861586635,-0.366849997436628,0.269795097089738,
-1.35973559710245,0.17391360912447,0.67855368941677
"4178","CASTOR3",61.2041811594731,0.407949055650128,0.300254331025742,
1.35867833864869,0.174248539062042,0.67855368941677
"4179","AC022034.1",53.7427493697333,0.392382635397497,0.2883251475630
14,1.36090326741874,0.173544260833485,0.67855368941677
"4180","FBXL6",45.7639164376449,-0.347395567069777,0.255688989553634,-
1.35866455445046,0.174252908950471,0.67855368941677
"4181","CD274",5.66340870994804,1.00439530268576,0.737716893384273,1.3
6149153109141,0.173358408072972,0.67855368941677
"4182","SNX32",4.24099825526532,1.00324022347195,0.73730764815956,1.36
068061409129,0.173614643517876,0.67855368941677
"4183","BANF1",738.736039690967,-0.376223730039864,0.277053845781018,-
1.35794444209675,0.174481313947796,0.67855368941677
"4184","CDK2AP2",117.220086921578,-0.276014102319594,0.203188617048419
,-1.35841321393423,0.174332603672271,0.67855368941677
"4185","UNC93B1",110.081727019677,-0.339900204022188,0.249754077313338
,-1.36093955974042,0.173532790531086,0.67855368941677
"4186","SC5D",153.006175380249,0.253354409233518,0.186438032551039,1.3

5892020403165,0.174171875829819,0.67855368941677
"4187","BCL2L2",80.5265900077892,-0.34406154678141,0.2529738934018,-1.36006740519637,0.173808594525712,0.67855368941677
"4188","DHRS1",150.369142238862,-0.293327126235823,0.215843862905403,-1.35897830166419,0.174153464577319,0.67855368941677
"4189","AL139317.5",60.7610092898553,-0.641068568122634,0.471675460945307,-1.35913063367307,0.174105197170159,0.67855368941677
"4190","BRD7",479.946247799155,-0.243762314731596,0.17957391324896,-1.3574483638592,0.174638789865948,0.67855368941677
"4191","XAF1",322.048376086569,0.381593363252579,0.281033434695833,1.35782193910694,0.174520191643251,0.67855368941677
"4192","AC017100.1",4.84444100594048,-1.1174912385221,0.822677678565051,-1.35835852562728,0.17434994777384,0.67855368941677
"4193","REEP6",8.32928802047782,0.9785241043789,0.72011161849414,1.35885059933506,0.174193935607259,0.67855368941677
"4194","STAP2",24.8930534366987,-0.607261041389667,0.446509534628388,-1.36001808314142,0.173824201562015,0.67855368941677
"4195","XBP1",738.132058108841,-0.36794801466695,0.271082739510898,-1.35732734341855,0.174677222894852,0.67855368941677
"4196","TMEM173",508.986655434575,-0.301516066267356,0.222241633766573,-1.35670378748227,0.174875348562213,0.678709686140074
"4197","TAB2",233.503998727772,0.255032159624215,0.187975379759972,1.35673171640812,0.174866470972967,0.678709686140074
"4198","AC022079.1",2.56493313122393,-1.41956542854475,1.04656798994146,-1.35640057997966,0.174971748845011,0.678804795454834
"4199","AL118506.1",9.82742406245483,0.817429458225566,0.602662053184052,1.3563645726603,0.174983199466809,0.678804795454834
"4200","AC007878.1",17.2236973841026,0.603216683869304,0.444825767047656,1.35607405990193,0.175075605357379,0.679001556111035
"4201","RTL10",46.2279346927939,0.35662716584097,0.263014697834586,1.35592105223435,0.175124288459809,0.679028691911885
"4202","SLC45A1",11.4064340574114,-0.715969815050274,0.528826988127471,-1.35388289766651,0.17577374061216,0.679274489443388
"4203","KIF2C",2.30084596634476,1.40914717110973,1.0415591746115,1.35292089538297,0.176080903183903,0.679274489443388
"4204","ZFYVE9",133.600616452939,0.300046799771833,0.221638672810213,1.35376554988109,0.175811187765094,0.679274489443388
"4205","NES",89.1579111636893,-0.346185673446389,0.255502479558565,-1.35492099350463,0.17544273022409,0.679274489443388
"4206","PRRX1",6440.19055975357,0.211767442848256,0.156525800122448,1.35292356073307,0.176080051598263,0.679274489443388
"4207","RGS1",113.343425701919,-0.702300678668947,0.518913623859868,-1.35340574303095,0.175926043741083,0.679274489443388
"4208","TARBP1",96.451748652221,0.337186155090636,0.249261267441245,1.35274187823873,0.176138106613068,0.679274489443388
"4209","PJVK",31.1362424330147,0.377085331029142,0.278761921741718,1.35271463431266,0.176146813396088,0.679274489443388
"4210","TADA2B",80.2283614377355,0.271159110859814,0.200348090442756,1.35343995673016,0.175915119786263,0.679274489443388
"4211","UBE2QL1",17.1713182693115,0.850978930889085,0.628056104482261,

1.35494094367666,0.175436373409197,0.679274489443388
"4212","RAET1E",2.82474837421786,1.34821684821452,0.99571679161164,1.35401638254221,0.175731151143219,0.679274489443388
"4213","PRRT4",5.43296321368099,-1.0825828151953,0.799076640812376,-1.35479221879732,0.175483766434553,0.679274489443388
"4214","UBN2",207.85560127394,0.323135568208438,0.238628152228983,1.35413850038265,0.17569219516391,0.679274489443388
"4215","PKIA",32.63637149345,-0.441432491902876,0.325877235587314,-1.35459751003258,0.175545827230776,0.679274489443388
"4216","CDK5RAP2",161.071484067608,0.311593352775408,0.229990614979827,1.35480898993526,0.175478421625936,0.679274489443388
"4217","SYT15",3.19415750044016,-1.23640973358097,0.912772870689647,-1.35456450699154,0.175556348128126,0.679274489443388
"4218","KAT5",195.879113557479,-0.272889134773081,0.201729598859186,-1.35274712444933,0.176136430033358,0.679274489443388
"4219","AP000821.2",22.5988186030799,-0.550051585893637,0.406085169595983,-1.35452271364868,0.175569671922883,0.679274489443388
"4220","AC020656.1",5.28654154125983,-0.894557387641183,0.660674774667872,-1.35400566502768,0.175734570371908,0.679274489443388
"4221","CEP170B",46.6933717900284,-0.345314584565116,0.255242957401128,-1.35288584680687,0.176092101572237,0.679274489443388
"4222","TMEM100",158.470272669073,-0.55058819259791,0.40652549319589,-1.35437556023726,0.175616590705315,0.679274489443388
"4223","ZSCAN5A",23.8279599175942,-0.500883759759133,0.37021377635684,-1.3529581872619,0.176068988619798,0.679274489443388
"4224","TTLL1",54.5555387307521,0.308383747568241,0.227735732961386,1.35412982213261,0.175694963340641,0.679274489443388
"4225","DIPK1A",96.6819689846023,0.386961036007239,0.286160661419865,1.35225098407036,0.176295038821559,0.67968232148422
"4226","AC005306.1",6.24641774632182,0.895742840864298,0.662471499020482,1.35212283424831,0.176336023733336,0.67968232148422
"4227","STRN",256.253923758297,0.244787808618817,0.18107882455231,1.35183011721011,0.176429667218184,0.679739203852227
"4228","LINC02604",18.1423107545356,0.608737469022278,0.45031095123549,1.35181582271566,0.176434241137407,0.679739203852227
"4229","C1RL-AS1",72.75582517341,0.471553930696558,0.348960711166102,1.35130951883034,0.176596304094307,0.680121441535851
"4230","AC026401.3",16.4949132980912,0.553827243726892,0.409864415090016,1.35124500526658,0.176616962225837,0.680121441535851
"4231","NOB1",304.288052616096,-0.272689793558042,0.201852548041084,-1.35093560227211,0.176716062356772,0.680342221633055
"4232","PREPL",231.320133923492,0.289650406945696,0.21449728353637,1.35036864882526,0.17689776204732,0.680561747807061
"4233","SMO",93.0688026218703,0.331680205453888,0.245576165845415,1.35062050631849,0.17681702849362,0.680561747807061
"4234","FBH1",282.9295053909,-0.194686851780702,0.144173333663115,-1.35036658190633,0.176898424716993,0.680561747807061
"4235","CDHR4",6.68592041294097,-0.852579118588755,0.63154730739325,-1.34998456743934,0.177020933177228,0.680843449547149

"4236","LTC4S",78.4182482644876,-0.469399634649887,0.34780859896322,-1
.34959180436918,0.17714695450169,0.680843449547149
"4237","FKBP9",1271.60818040281,0.305775143652402,0.226673690309006,1.
34896618674873,0.177347827148907,0.680843449547149
"4238","PYROXD2",77.2197653399694,0.379887063594071,0.281500316699133,
1.34950847675277,0.177173699457302,0.680843449547149
"4239","NOX4",324.115382481044,0.398035964356376,0.295054124282462,1.3
4902694657922,0.177328311011807,0.680843449547149
"4240","DZIP1",170.631331208824,0.321949465756013,0.238585189679187,1.
34941094285409,0.177205007902942,0.680843449547149
"4241","AC090360.1",3.61062909526926,1.07304661808072,0.79526068864038
3,1.34930172383506,0.177240072170922,0.680843449547149
"4242","PPP1R12C",237.613659470181,-0.248754659875812,0.18439739346475
5,-1.34901397032685,0.177332478866724,0.680843449547149
"4243","PRRG3",244.618494048264,0.396225296302592,0.293717975453841,1.
34899914004364,0.177337242308919,0.680843449547149
"4244","NSUN7",15.3864795408016,0.565381438202162,0.41931385427618,1.3
4834905271166,0.177546142057164,0.681123200181147
"4245","MYO1G",8.72648963865139,0.815871210735756,0.60506143235735,1.3
484105366906,0.17752637688815,0.681123200181147
"4246","LINC01465",2.99832674108814,-1.179375415088,0.874562979656504,
-1.34853114357896,0.177487610319419,0.681123200181147
"4247","PLCB1",92.3108759583128,0.499732184430485,0.370683213477642,1.
34813815749071,0.177613950700285,0.68122289685824
"4248","ANKHD1",506.499735773834,0.29422238185188,0.218268058564171,1.
34798643368781,0.177662746022573,0.68124963982149
"4249","FOSL2",405.186703755821,0.436141068137361,0.323671641520167,1.
34748001427918,0.177825685924231,0.681422895083257
"4250","LPXN",24.5104750205916,-0.444087104782367,0.32960627593225,-1.
34732599834855,0.177875262440543,0.681422895083257
"4251","RHOT1",140.761170937225,-0.222287563087708,0.164973125117138,-
1.34741681670802,0.177846027480504,0.681422895083257
"4252","RAB22A",391.414180267201,-0.195644534238903,0.145180960539313,
-1.34759085152853,0.177790014651495,0.681422895083257
"4253","GAREM1",99.298647980686,0.33601165856997,0.249454694650304,1.3
4698470614476,0.177985158377762,0.681683575080029
"4254","DOCK11",266.661573770917,-0.294998639366405,0.219057801674699,
-1.34667031765651,0.178086436044681,0.681911132282982
"4255","FGFBP2",116.167317959444,-1.11980043641104,0.831810795663946,-
1.34622012872197,0.178231535387959,0.682070702221983
"4256","HEXB",989.731723001004,-0.188708373617455,0.140183628754547,-1
.34615129665299,0.178253728243538,0.682070702221983
"4257","PITX1",6.58865990843726,-1.23693873980877,0.918789900931543,-1
.34626941214163,0.178215646676822,0.682070702221983
"4258","PPP1R12B",294.837452907385,0.360322100534997,0.267780136574771
,1.3455893523095,0.178434987435172,0.682479088829206
"4259","RFTN2",200.065775765652,0.285585185604704,0.212257186018824,1.
34546768927473,0.178474248776793,0.682479088829206
"4260","FGFR1",2626.1701499726,0.2377888069827,0.176748503466016,1.345
35117593468,0.178511854308162,0.682479088829206

"4261","SLC27A4",31.5538829199856,-0.460035375952059,0.342056187033643
,-1.34491172325093,0.178653743923367,0.682479088829206
"4262","AC103691.1",9.48311043923498,0.741284626769872,0.5510212152371
01,1.34529235222078,0.178530842330826,0.682479088829206
"4263","STAG2",673.422196628169,0.270571432764986,0.201175847298558,1.
34494988537784,0.178641418885467,0.682479088829206
"4264","SMARCA1",595.815392221851,0.315448792278701,0.234543572644567,
1.3449475025979,0.178642188421886,0.682479088829206
"4265","FHOD1",62.7701116960708,0.391430778479111,0.291134931175731,1.
34449953119105,0.178786907922742,0.682827653728849
"4266","AP003068.2",10.3346986274347,-0.796229392653929,0.592539181849
784,-1.34375821387586,0.179026585888293,0.683582760791001
"4267","EXOC3L4",34.2163796287422,0.441373240440598,0.328496352731906,
1.34361686749324,0.179072312207132,0.683597115899221
"4268","MGST3",2941.57150023299,-0.356883459629653,0.265936677795781,-
1.34198660593825,0.179600338578558,0.683740013053474
"4269","ZIC4",23.0218711796672,-0.757850214686263,0.564672503949182,-1
.34210575047668,0.179561709626819,0.683740013053474
"4270","NUDT12",86.7745776411909,0.292351197165733,0.217630319973274,1
.34333854401186,0.179162376746402,0.683740013053474
"4271","UBLCP1",203.348355857169,-0.260293509216301,0.193906563389975,
-1.34236564593645,0.179477467948796,0.683740013053474
"4272","CTTNBP2",16.5308097940399,-0.988794663962985,0.736668501322727
,-1.34225185709387,0.179514347481518,0.683740013053474
"4273","HNRNPF",1118.84552255409,-0.272748695532886,0.20334618890602,-
1.34130222454743,0.179822347850307,0.683740013053474
"4274","SFR1",104.050767654258,-0.347636423614463,0.259134703928673,-1
.3415278553742,0.179749132020767,0.683740013053474
"4275","CCNDBP1",588.25282426242,-0.252035421751575,0.187922072120349,
-1.34116987380901,0.179865305171228,0.683740013053474
"4276","AC093525.6",15.5245083627112,0.720345421989595,0.5370687341101
99,1.34125369108117,0.179838099560662,0.683740013053474
"4277","AP1G1",177.103776309425,0.223117013469465,0.166239439326061,1.
34214248059297,0.179549802261981,0.683740013053474
"4278","DNAAF1",3.72817521034677,1.10568816375248,0.823882498325471,1.
34204594223057,0.179581099838867,0.683740013053474
"4279","P2RX1",2.45538830759902,-1.3985554629573,1.04222771379507,-1.3
4189049518242,0.179631504035867,0.683740013053474
"4280","TMEM221",129.750165081762,0.691859625450304,0.515749833180509,
1.34146359521586,0.179769981794058,0.683740013053474
"4281","ZFP82",56.1616102676379,0.417331216651259,0.311038867127145,1.
34173333546982,0.179682474259203,0.683740013053474
"4282","CD3EAP",38.1349756166884,0.382654480522089,0.284967520250299,1
.34280033102014,0.179336636195823,0.683740013053474
"4283","SH3BP1",20.5287432516122,-0.450629028597474,0.335909089231489,
-1.34152079548799,0.179751422575028,0.683740013053474
"4284","PORCN",51.7551956116319,-0.356162192575555,0.265362090938724,-
1.3421743524692,0.179539470342958,0.683740013053474
"4285","DKC1",174.241672597816,-0.248007878961654,0.184671396470309,-1
.34296855767552,0.179282155221695,0.683740013053474

"4286","MTR",375.415186059965,0.343666420296507,0.256293251353353,1.34091092325601,0.179949375282,0.683805317189942
"4287","ZC2HC1A",420.187950102261,-0.302235982416088,0.225404853359202,-1.340858361796,0.179966443292607,0.683805317189942
"4288","SNHG7",1279.37784611806,-0.415553647522531,0.309966344514587,-1.34064118533028,0.1800369786354,0.683913793141799
"4289","ADAM17",258.834428733114,-0.406296316512808,0.303435458418069,-1.33898760095802,0.180574709396847,0.684026842600906
"4290","RHBDD1",70.6056169812383,0.297353192987556,0.222092477744157,1.33887106851992,0.180612649642489,0.684026842600906
"4291","VHL",125.019936287138,0.306502591175073,0.22887033398661,1.33919755276368,0.180506368922229,0.684026842600906
"4292","SERPINI1",78.827129261398,-0.559607189233786,0.417910178077504,-1.33906092406776,0.180550840135499,0.684026842600906
"4293","MAT2B",234.365241330627,0.306847971143918,0.22917338033768,1.33893373956341,0.18059224467576,0.684026842600906
"4294","HLA-DOB",2.8644792029946,1.32413774989186,0.988163244930261,1.33999898972696,0.180245673379108,0.684026842600906
"4295","ZNF394",156.400673753425,-0.231800866048284,0.173036169139713,-1.339609326771,0.180372389993584,0.684026842600906
"4296","TCAF1",581.618183764564,0.271393800850341,0.202625239981832,1.33938793051979,0.180444416518216,0.684026842600906
"4297","ISG20",67.9233976643575,-0.387350694941691,0.289183084195072,-1.3394652595945,0.18041925673391,0.684026842600906
"4298","AC105411.1",2.39411119972665,1.71169085347352,1.27761135111948,1.33975864567397,0.180323824349756,0.684026842600906
"4299","PRPF8",425.166506619998,0.233447155211479,0.174192356711345,1.34016876296315,0.180190484586932,0.684026842600906
"4300","IL27RA",7.57384595820998,-1.16505554660511,0.869900009556884,-1.33929823405632,0.180473603420117,0.684026842600906
"4301","LINC01534",27.7295636949549,0.450576367933052,0.336390860399807,1.33944295453667,0.180426513639103,0.684026842600906
"4302","TRNT1",80.2856577110104,-0.377661252767443,0.282284652287115,-1.33787384368074,0.180937564939827,0.684371408123424
"4303","RABGEF1",158.395985944748,0.314073520647675,0.234839976492528,1.33739376633632,0.181094138163258,0.684371408123424
"4304","SGCG",59.1871592782757,0.605753866722118,0.452707416327879,1.33806923605465,0.180873868132643,0.684371408123424
"4305","PROSER1",57.0011431700268,0.312037192472645,0.233204131353734,1.33804315841786,0.180882368331624,0.684371408123424
"4306","CYP19A1",45.6509180971816,-0.681551906904839,0.509614518759623,-1.33738714619768,0.181096297969128,0.684371408123424
"4307","ARRDC4",100.946333364129,0.367743776614111,0.274966447670502,1.33741327252693,0.181087774422062,0.684371408123424
"4308","MLST8",114.909367278732,-0.317825470379651,0.23764313445678,-1.33740649022391,0.181089987076226,0.684371408123424
"4309","ZDHHC1",145.080204810259,-0.326548591505596,0.244020787799432,-1.33819989046997,0.180831284823189,0.684371408123424
"4310","MRPS23",243.726766477723,-0.316890196779491,0.236962163407924,

-1.33730293571794,0.181123773124199,0.684371408123424
"4311","UBQLN2",232.96208562317,0.229696714386356,0.171741822313121,1.33745357591217,0.181074626285639,0.684371408123424
"4312","SH3BP5",2341.34773493124,0.223923573182812,0.167563724016714,1.33634875028486,0.181435309166394,0.684424647206585
"4313","HACL1",101.332050006364,-0.389158201144859,0.291235871239647,-1.33623031904829,0.181474004007934,0.684424647206585
"4314","ATP5MG",2517.23679692016,-0.326568117449097,0.244347120995958,-1.33649259347934,0.181388319753396,0.684424647206585
"4315","KLRC2",2.6860997779439,2.06650662785764,1.54635905306995,1.33636921111888,0.181428624652392,0.684424647206585
"4316","RPL41",42577.5279486082,-0.322277872476267,0.241053121103002,-1.33695789127973,0.181236382276625,0.684424647206585
"4317","SPRY2",1011.67010333325,0.298949716986546,0.223630851797847,1.33679997452581,0.181287937525577,0.684424647206585
"4318","ACOT4",16.5754241510146,0.554938603733785,0.415232372144437,1.33645313073219,0.181401210192846,0.684424647206585
"4319","ATP8B1",31.2051219460791,-0.427392037809579,0.319754339871483,-1.33662622994064,0.18134467267903,0.684424647206585
"4320","RAB5A",371.017571474441,-0.195902791657426,0.146653224882845,-1.33582327844426,0.181607042406017,0.684609376012872
"4321","ZNF888",9.82917943466537,-0.717907448517706,0.537416862217605,-1.33584838695854,0.18159883376843,0.684609376012872
"4322","NUCB2",1234.30983933823,-0.250836242294262,0.187833216234422,-1.33542004616058,0.181738907425863,0.684845539685781
"4323","GPD1",5.03883832854927,-1.33656361127257,1.00089040213263,-1.33537459088898,0.181753776662879,0.684845539685781
"4324","RCHY1",149.624932474936,0.277590294996422,0.20791371574646,1.3351225723604,0.181836232832349,0.684997779048595
"4325","TMPO-AS1",5.6343157023935,-0.803432078903582,0.602036888180716,-1.33452300793637,0.182032511642682,0.685103415468496
"4326","UBL7",122.241524683129,-0.301316894713703,0.225755319223405,-1.33470562620729,0.181972711443979,0.685103415468496
"4327","ZNF426-DT",4.56854005835665,0.990342595826583,0.742019148433137,1.33465908247491,0.181987951275665,0.685103415468496
"4328","HS6ST3",2.44103394968386,-2.10071670691171,1.57409369099852,-1.33455633481329,0.182021597332747,0.685103415468496
"4329","ACYP2",124.884398972064,0.323448758404184,0.242430762238491,1.33419024639287,0.182141515251992,0.685197030471062
"4330","MCTP1",4.34351065880096,-1.10077787743974,0.825005571985966,-1.33426720354135,0.182116301885861,0.685197030471062
"4331","NDUFAF2",435.175630971446,-0.333908968658322,0.250371294583464,-1.33365515888647,0.182316896833378,0.685281720500816
"4332","ISOC1",87.3058241623345,-0.383759191700015,0.287760081608894,-1.3336081556357,0.182332308714503,0.685281720500816
"4333","SBNO1",280.701847525925,0.242724708540479,0.182004498437978,1.33361928207062,0.182328660383976,0.685281720500816
"4334","YLPM1",215.061434324177,0.305000949388468,0.228669526009846,1.

33380671535277,0.182267209610647,0.685281720500816
"4335","RBM8A",817.196268785644,-0.286011414428029,0.21453322477417,-1
.33318004579058,0.182472725992897,0.685512762331201
"4336","IDH3A",116.11477496638,-0.271002732815709,0.203297405794374,-1
.33303586318172,0.182520035007086,0.685512762331201
"4337","LINC00667",311.533408384752,-0.243535478647414,0.1826864452364
94,-1.333079081659,0.182505853260958,0.685512762331201
"4338","AC127024.5",9.00576516921647,0.686086047675678,0.5147718229038
62,1.33279642969854,0.182598617706717,0.685649811854475
"4339","CCDC163",4.35238731811082,-0.992697530008697,0.744902241157425
,-1.33265477690905,0.182645120333461,0.685666366700104
"4340","AL139383.1",2.67818101039606,1.32932651599016,0.99763120058054
2,1.33248290071181,0.182701556670646,0.685720197375152
"4341","ADRM1",391.422282341715,-0.385380102064644,0.289336298570941,-
1.3319452276402,0.182878187563159,0.686225016635867
"4342","MIIP",68.7617600438592,-0.388100049088968,0.291507519881145,-1
.33135518852895,0.183072166842372,0.686267687874239
"4343","LINC01873",4.68049766248206,1.19893055556601,0.900408631992448
,1.33154049502278,0.183011229683643,0.686267687874239
"4344","AC008269.1",8.80939692559737,0.823646106879117,0.6187337826763
64,1.33118011322478,0.18312975333632,0.686267687874239
"4345","BRK1",1818.28784364993,-0.276964341872421,0.207971611948756,-1
.33174109330203,0.18294528084009,0.686267687874239
"4346","HPS6",51.8420790420368,-0.312603926641646,0.234799959599761,-1
.33136277865852,0.183069670569074,0.686267687874239
"4347","AC010998.2",2.71213657200439,1.25195647431137,0.94051320150915
1,1.3311418407551,0.183142343863301,0.686267687874239
"4348","SLC25A44",56.0450481770011,0.554092318067044,0.417165030816642
,1.32823289857818,0.184101179287847,0.686815896384413
"4349","TSTD1",8.4468173993765,-0.893063058251638,0.672329994895525,-1
.32831059900936,0.184075519719554,0.686815896384413
"4350","STX6",94.9430481583891,0.338257357044965,0.254511728401594,1.3
2904428086406,0.18383336126349,0.686815896384413
"4351","ENAH",604.037884443349,-0.27307121670243,0.205379310482826,-1
.32959457337974,0.183651887049685,0.686815896384413
"4352","GREM2",13.5212458629543,0.736852751640644,0.554649822317437,1.
32850083420549,0.184012708170698,0.686815896384413
"4353","CD86",7.3160989703348,-1.00020452791097,0.752765258467214,-1.3
2870707921297,0.183944628460291,0.686815896384413
"4354","DTX3L",166.919157264177,0.285034929568114,0.214324346418816,1.
32992324171665,0.183543562867007,0.686815896384413
"4355","LINC02362",4.05809636260651,1.04459425843428,0.786584869123412
,1.32801214393864,0.184174095119842,0.686815896384413
"4356","PCDHB2",54.1162809738185,0.415704932164601,0.313007231258832,1
.32810009050828,0.184145043577075,0.686815896384413
"4357","TCEA1",1061.01212146405,-0.206830913087976,0.155488456403917,-
1.33020108290666,0.183452027477915,0.686815896384413
"4358","GNAQ",446.156929659721,0.244351864058743,0.18395433322242,1.32
832893783097,0.184069463945802,0.686815896384413
"4359","ZMYND19",51.2835296186301,-0.367173119743904,0.276339289955677

,-1.32870399935817,0.183945644956774,0.686815896384413
"4360","UBE4A",218.167429693934,0.238573360554685,0.179643011755387,1.32804142072357,0.184164423691968,0.686815896384413
"4361","DTX1",146.17283384422,-0.49243736188029,0.370334415740578,-1.3297099619962,0.183613851289439,0.686815896384413
"4362","AKAP5",3.63778406991923,1.16257642756673,0.87400259580004,1.33017502825897,0.183460609801429,0.686815896384413
"4363","AC023449.2",2.96050663966135,1.29625818808274,0.974882983543119,1.32965515858284,0.183631915510979,0.686815896384413
"4364","PTPN9",159.125112947258,0.225118312871515,0.169261615162752,1.33000215468259,0.183517561377986,0.686815896384413
"4365","PCYT2",47.3860510491745,-0.401624947246288,0.302168548068788,-1.32914212883221,0.183801083479133,0.686815896384413
"4366","SAMD4B",674.839861263565,0.245221480900057,0.184555767599776,1.32871209656173,0.183942972508867,0.686815896384413
"4367","TFF3",3.06422834204548,1.94693960229379,1.46331927179255,1.33049542900423,0.183355091408146,0.686815896384413
"4368","CHST7",275.565226605817,-0.525184629646853,0.395356629003766,-1.3283820002468,0.184051942713865,0.686815896384413
"4369","ZNF813",29.2831544354907,0.420606549421802,0.316788460975529,1.32772054931095,0.18427044235602,0.687012649769249
"4370","GRK3",34.8154760846863,-0.3921022100597,0.29534727017381,-1.32759720389137,0.184311208759999,0.687012649769249
"4371","CLEC2D",70.3852046961443,0.448346356065302,0.337818829253962,1.32717988827156,0.184449183592356,0.68721243173282
"4372","L3HYPDH",132.240033969049,-0.281331646563541,0.211967686938703,-1.3272383665011,0.184429844643188,0.68721243173282
"4373","RBMS2",185.493724239769,0.238731473529974,0.179957401385558,1.32659991582393,0.184641063721612,0.687727451133492
"4374","CTSA",857.118093016631,0.296371457632682,0.22342247995601,1.32650688368975,0.184671856544778,0.687727451133492
"4375","DIAPH1",208.705321391588,-0.481213871558513,0.362839583483538,-1.32624414056066,0.184758842746865,0.687894123315129
"4376","SLC66A2",230.640797228553,-0.260706597821794,0.196611524775834,-1.32599855536972,0.184840175892713,0.688039676671938
"4377","UAP1",792.874362810664,-0.328535077892363,0.247797577264404,-1.3258203793567,0.184899200981633,0.68810214411465
"4378","RBL1",58.5605594565448,0.312647416312974,0.235854156815976,1.32559637927822,0.184973426180335,0.688221137289053
"4379","CLK1",778.029908295623,0.217481759510251,0.164355900090714,1.32323670394683,0.185756673534311,0.688530068576319
"4380","CLASP2",149.029031817257,0.301475844976549,0.227841105283876,1.32318461412363,0.18577399133685,0.688530068576319
"4381","GNAI2",1799.28863721805,-0.209490737477865,0.158325683979804,-1.32316331887495,0.185781071507113,0.688530068576319
"4382","STAG1",455.382046677165,0.314433673876789,0.237458746439897,1.32416126418142,0.185449492464908,0.688530068576319
"4383","TMEM165",786.366816964493,-0.231732431982299,0.175123584565541,-1.3232508491486,0.185751971021201,0.688530068576319
"4384","AC016575.1",5.65589256659213,0.863543079544998,0.6522748352350

21,1.32389451945338,0.185538078643425,0.688530068576319
"4385","NPM1",3752.61990840399,-0.331129222808484,0.249922997480643,-1.32492498148007,0.185196034214623,0.688530068576319
"4386","SOGA3",5.44556011699272,-0.808259474838228,0.610364324882319,-1.32422463418724,0.185428451824622,0.688530068576319
"4387","JAZF1",304.233721512134,0.220618885174463,0.166658135327434,1.32378107279919,0.185575763868576,0.688530068576319
"4388","PBX3",235.970237643339,0.213803878279584,0.161542608352143,1.32351384232646,0.185664556070806,0.688530068576319
"4389","AL356056.2",6.71778729869871,0.806444894342013,0.60912729104388,1.32393492493167,0.185524657934867,0.688530068576319
"4390","AL157893.2",11.0921065509176,-0.608837498869764,0.460020823611505,-1.32349986700588,0.185669200490385,0.688530068576319
"4391","PICALM",346.112986278365,0.198473619588513,0.149770199543545,1.32518765544415,0.185108918689845,0.688530068576319
"4392","TINF2",256.847280735375,-0.251602637171163,0.190116096832903,-1.32341575154629,0.185697156403429,0.688530068576319
"4393","SPTSSA",248.739580935271,-0.276296914988841,0.208702407986543,-1.32387986154264,0.185542947456435,0.688530068576319
"4394","ISOC2",266.315962889417,-0.40478808347571,0.305484213447011,-1.32507038222427,0.185147808474646,0.688530068576319
"4395","AP000320.1",3.29099161775471,-1.11026795905647,0.839170345920283,-1.32305432914087,0.185817311158542,0.688530068576319
"4396","COL4A5",3.87681446747827,-1.30003173792368,0.982047656698226,-1.32379699605879,0.185570474066387,0.688530068576319
"4397","AURKAIP1",901.341489254564,-0.321893409255027,0.24347057385761,-1.32210395759481,0.186133536128192,0.68860530774293
"4398","CYB5RL",31.726793813603,0.490811998516858,0.371207896302644,1.32220247307644,0.186100737791782,0.68860530774293
"4399","PNO1",82.0699315752988,-0.300522902722753,0.227293703715703,-1.32217873970959,0.186108638849109,0.68860530774293
"4400","IL20RA",17.2058262453237,-0.534119764028532,0.403959342642964,-1.32221168728015,0.186097670364961,0.68860530774293
"4401","AC007336.1",9.25625042009765,0.962279957419974,0.727526974635656,1.3226725481923,0.185944296521092,0.68860530774293
"4402","ACSF2",72.8268023878499,0.311279352000238,0.235372443802694,1.32249700504947,0.186002706003311,0.68860530774293
"4403","SERTAD1",1483.89718474249,-0.5289662315221,0.399921216693736,-1.32267609079413,0.18594311791035,0.68860530774293
"4404","C15orf48",10.9095325072772,-1.15159806146229,0.871204066368368,-1.32184651784598,0.186219264608193,0.688766031154145
"4405","GBP4",145.767319060379,0.293218462741634,0.221913821489753,1.3213168101617,0.18639575120312,0.688793192229497
"4406","CYP1B1",301.270520088361,0.928097065902933,0.702293480762651,1.32152311152749,0.186327001574633,0.688793192229497
"4407","HOXC-AS2",33.0184564738879,-0.377542846237703,0.285709645902597,-1.3214214208449,0.186360887503645,0.688793192229497
"4408","GON7",103.548208383568,-0.36865157608991,0.278942361107861,-1.32160484562386,0.186299768989917,0.688793192229497

"4409","S100A13",7596.46543850198,-0.318604026629638,0.241207328730079,-1.32087208256499,0.186544019780011,0.689011992236128
"4410","SYK",6.25131412856695,-1.06854977632717,0.808971702222472,-1.32087410893554,0.186543344006928,0.689011992236128
"4411","ATP7B",11.1022608584416,0.683709627098895,0.517664327875205,1.32075862732369,0.186581858785288,0.689011992236128
"4412","RPL5",16602.1285340145,-0.34018356462513,0.257699027607142,-1.32008090128976,0.186808008585191,0.689118106587093
"4413","SH2D2A",11.81059313021,0.81553677877003,0.618397086031186,1.3187914322237,0.18723884937969,0.689118106587093
"4414","ZNF669",50.3800570995475,-0.429712400635146,0.325905222360055,-1.31851953007493,0.187329791636543,0.689118106587093
"4415","WDR5B",32.0684450969984,0.378108694788733,0.286433685824305,1.32005666058656,0.186816101192966,0.689118106587093
"4416","MRPL3",411.81290318795,-0.272881989093424,0.206840865742041,-1.31928469799457,0.187073951550673,0.689118106587093
"4417","LXN",269.500395042517,0.450632351244214,0.341590465929262,1.31921817553752,0.187096183625571,0.689118106587093
"4418","IGF2BP2",108.504577251227,-0.421459103439404,0.319409558038196,-1.31949433832849,0.187003901663289,0.689118106587093
"4419","AC136424.2",2.82613904511131,1.6819265590426,1.27417536362562,1.32001183436536,0.186831066829916,0.689118106587093
"4420","ZSWIM8-AS1",9.60291240305358,0.802766200792484,0.608829359873143,1.31854055290591,0.187322759033254,0.689118106587093
"4421","PDLIM1",1532.78168433023,0.327439546815883,0.248287753198169,1.31879056698596,0.187239138721321,0.689118106587093
"4422","SLC25A28",207.492581633761,-0.276662242737593,0.209780805846464,-1.31881580691457,0.187230698445963,0.689118106587093
"4423","TTC9C",144.098265661732,0.298850859995315,0.226384451429975,1.32010329378895,0.186800533218905,0.689118106587093
"4424","MPHOSPH9",48.2392183848817,0.323746942012264,0.245523321829401,1.31859955135837,0.187303023783383,0.689118106587093
"4425","DNAJA2",516.275257177918,-0.251801828907429,0.190721674188062,-1.32025806704663,0.186748870763022,0.689118106587093
"4426","TMEM208",483.447714247088,-0.32100354306448,0.243214648781501,-1.31983638597716,0.186889650367901,0.689118106587093
"4427","TOMM40",191.433004404353,-0.28058186632498,0.212682819653423,-1.3192502656406,0.187085458739396,0.689118106587093
"4428","SBF1",94.4645957385932,0.291365983018225,0.220870691504589,1.3191699678821,0.187112296038768,0.689118106587093
"4429","SNHG15",148.659633752117,-0.400309931480559,0.303726858792927,-1.3179931899058,0.187505927641777,0.689299042057529
"4430","AC009560.3",6.96858853743016,0.969037331983186,0.735192399288827,1.31807310973368,0.187479175168283,0.689299042057529
"4431","SOGA1",130.420369690436,0.332012525199056,0.251889831305478,1.31808625810071,0.187474774135699,0.689299042057529
"4432","PRDM2",510.820610789434,0.354243727634795,0.268950619432838,1.31713296805868,0.187794057926181,0.689554605706629
"4433","RIT1",121.01983315037,-0.326177338439176,0.247728309006986,-1.

31667365650156,0.187948037543773,0.689554605706629
"4434","CCL20",18.6398815278682,-0.666936004519787,0.506763792621171,-1.31606877648094,0.188150959650368,0.689554605706629
"4435","AC112715.1",10.2724151578774,-0.9032610851816,0.686111183004849,-1.3164937513855,0.188008374325851,0.689554605706629
"4436","SLC25A26",178.850142137338,-0.300312985264542,0.228102736143021,-1.3165689738866,0.187983144379507,0.689554605706629
"4437","SLC7A14-AS1",3.56154153408003,1.17045966606017,0.888754662700673,1.31696599205846,0.187850024189732,0.689554605706629
"4438","DLX5",4.43444422152786,1.04219386383464,0.791657312492903,1.31647096210456,0.188016018440624,0.689554605706629
"4439","PPP2R5B",41.6219577814651,-0.36985501597257,0.28102010530976,-1.31611585429053,0.188135160424991,0.689554605706629
"4440","RBM26-AS1",29.5411773535681,-0.49044115666975,0.372642779194203,-1.31611608771884,0.188135082089323,0.689554605706629
"4441","KAT8",221.815595486877,-0.255991283307858,0.194519635765463,-1.31601769816448,0.188168102545642,0.689554605706629
"4442","AC005332.7",10.4342826416087,0.662538832638758,0.502992989256001,1.31719297642448,0.187773947598819,0.689554605706629
"4443","QTRT1",194.308293226141,-0.291998644330013,0.221795495784376,-1.31652197578389,0.18799890744989,0.689554605706629
"4444","CASS4",5.73232736295231,-0.845967679708519,0.642139059199712,-1.31742130865367,0.187697442200167,0.689554605706629
"4445","NAA10",295.679948719263,-0.359197153222821,0.272634836852879,-1.31750277172632,0.187670152608956,0.689554605706629
"4446","CDH12",5.78469238662149,-1.39497867821875,1.06037953584698,-1.31554658597264,0.188326271468876,0.689944989137508
"4447","WNT10B",23.867862453699,0.619106922631127,0.470643362206875,1.31544811283027,0.188359344753791,0.689944989137508
"4448","OSBPL10-AS1",3.89269238902005,1.15251625532239,0.876519002152855,1.31487880181906,0.188550638096372,0.690025015491305
"4449","SNRK",152.893697327115,0.299888809215458,0.228019253415642,1.315190733779,0.188445808565328,0.690025015491305
"4450","SERP1",1135.90708178147,-0.216840661398323,0.164912608293682,-1.31488224970747,0.188549479145428,0.690025015491305
"4451","ENTPD1-AS1",85.4207963844763,0.278992154864598,0.212155776643604,1.31503444911269,0.188498325055163,0.690025015491305
"4452","DISC1",21.2360490362368,0.585333471906108,0.445358440218158,1.31429747153637,0.188746117836144,0.690074557642296
"4453","STBD1",56.1921711646885,-0.421211773717156,0.32044418534887,-1.31446221518602,0.18869070550853,0.690074557642296
"4454","ZNF525",25.6895922929165,-0.431053664558501,0.327994846209289,-1.31420865156964,0.188775997842352,0.690074557642296
"4455","ZNF71",48.9550516373387,-0.327875406145276,0.249455988591325,-1.31436173569849,0.188724500845368,0.690074557642296
"4456","UBE2M",620.294196139661,-0.371389171730752,0.282551958220584,-

1.31441018519084,0.188708204754069,0.690074557642296
"4457","DYNC2LI1",300.475510269597,-0.285040436378467,0.2169990039591,
-1.31355642734743,0.188995520049382,0.690567076286312
"4458","ATP6V0D1",454.450099749838,-0.307454135605031,0.23405595700667
7,-1.31359243976115,0.188983394273129,0.690567076286312
"4459","PCDHGA11",4.41874524157425,0.98065957465976,0.746690153929183,
1.31334204622814,0.189067716404555,0.690572499814868
"4460","BRAP",98.6756151498764,0.273300322298281,0.208101934301954,1.3
1330024977915,0.189081794411831,0.690572499814868
"4461","INKA2",5.43266367524201,-0.863425018127648,0.658395786631383,-
1.31140726544633,0.189720205514533,0.690899995309699
"4462","CD34",2616.9769859913,-0.284636267689052,0.216889674158408,-1.
31235508925688,0.189400352617373,0.690899995309699
"4463","FAM193A",136.729844996188,0.310833697540514,0.236923559995026,
1.31195773669381,0.189534394911883,0.690899995309699
"4464","FAM13B",143.509947304835,0.250425690985014,0.19095575648317,1.
31143305442633,0.189711497494026,0.690899995309699
"4465","FBXO38",74.3100977552208,0.337049305986995,0.257028316243937,1
.31133141636859,0.189745818754914,0.690899995309699
"4466","MAP3K7",157.423574026691,0.244408515565542,0.186300794380033,1
.31190270218051,0.189552965679297,0.690899995309699
"4467","PCMT1",714.45757698262,-0.346549252435721,0.264334458745037,-1
.31102563805343,0.18984910192748,0.690899995309699
"4468","STIP1",183.796500683979,-0.326147320202749,0.248773212518997,-
1.3110226655848,0.189850106145633,0.690899995309699
"4469","AAGAB",55.8450084550351,-0.35992127394006,0.274259701430806,-1
.31233743806458,0.1894063055598,0.690899995309699
"4470","FUS",1968.6019351441,0.347526042123997,0.2649148583693,1.31184
05070339,0.18957395433447,0.690899995309699
"4471","AC090617.10",11.1399981568264,1.17397648792705,0.8951242296238
81,1.31152352832672,0.189680950007479,0.690899995309699
"4472","NMT1",621.482668788737,0.245294664032883,0.186992471394628,1.3
1178898382072,0.189591342887743,0.690899995309699
"4473","EPOR",46.0895219293757,-0.361583450868635,0.275502594297874,-1
.3124502576469,0.189368259034229,0.690899995309699
"4474","ZNF529-
AS1",19.7057272129577,0.442961396307254,0.337631848317581,1.3119656765
6319,0.189531715804396,0.690899995309699
"4475","ADGRG2",6.41311245025224,1.06424449130811,0.811381382706744,1.
31164519422152,0.18963987661199,0.690899995309699
"4476","TSPAN7",9.71744101099441,-0.744702478198703,0.567937713612313,
-1.31123970173084,0.189776792989978,0.690899995309699
"4477","DDAH1",30.9859203623,0.534293910791067,0.407681444862755,1.310
56715365335,0.190004042451186,0.690911755186788
"4478","SLC4A5",10.1300326317084,0.61895084035385,0.472228379035034,1.
31070233775156,0.189958348566736,0.690911755186788
"4479","SERINC1",1495.21630769108,0.200654772664052,0.153111848421522,
1.31051107234787,0.190023000996796,0.690911755186788
"4480","LINC00174",34.8404050607174,0.418845785317404,0.31955944259640
5,1.31069757136357,0.189959959526689,0.690911755186788

"4481","YBEY",239.012527357355,0.406111054095598,0.309927117542021,1.3
1034372634572,0.190079581372265,0.690963245028528
"4482","WDR63",2.79566163116824,-1.13568585199117,0.866793859671904,-1
.3102144637031,0.190123294100263,0.690967946809277
"4483","RABL3",123.840729708107,0.25567178315099,0.195229924137431,1.3
0959321057263,0.190333486488121,0.691577551060674
"4484","ITGA6",3.54769981762651,-1.14457448652778,0.874319868365378,-1
.30910268420146,0.190499570153929,0.691808753766608
"4485","TOMM70",244.204557459621,0.187350846429442,0.143119784674812,1
.30904924748964,0.190517669335174,0.691808753766608
"4486","QPRT",123.142107685611,0.958439448839511,0.732175875520705,1.3
0902899273743,0.190524530013936,0.691808753766608
"4487","OTUD5",193.451741529625,0.21667718832101,0.165545111663309,1.3
0887095453289,0.190578066876472,0.691848926086663
"4488","WDR47",70.3411964033482,-0.388501678706775,0.296851170673119,-
1.30874228262545,0.190621663821372,0.691853004007648
"4489","ADAMTS19",2.94670732096471,-3.99220059072094,3.05071232013741,
-1.30861260315136,0.190665609581321,0.691858345838749
"4490","UGDH",623.836805089357,0.226436380062102,0.173083974431669,1.3
0824578535141,0.190789957127896,0.691884586778504
"4491","TMEM176B",3810.39041060557,0.392198136627483,0.299853719612659
,1.30796488745949,0.190885219042458,0.691884586778504
"4492","MTUS1",462.977719414231,0.362938222506127,0.277415399997582,1.
3082843364474,0.190776885887298,0.691884586778504
"4493","STX17",313.717800356888,0.216221176450243,0.165285621634254,1.
30816688295307,0.19081671204747,0.691884586778504
"4494","PPFIA3",6.4408416568704,-0.855041143612918,0.653660875163707,-
1.30808065175811,0.190845955238314,0.691884586778504
"4495","PAQR3",78.498610977346,-0.426266106415072,0.326083447094048,-1
.3072301284037,0.19113456608523,0.692190984030715
"4496","DCP2",226.949993349133,0.234988002430609,0.179752221099586,1.3
0728844958428,0.191114765518622,0.692190984030715
"4497","C1QTNF2",178.090046848823,-0.440106491297733,0.33665792826203,
-1.30728093519065,0.191117316638285,0.692190984030715
"4498","NOL8",231.4953666212,0.219892865630693,0.168214776256604,1.307
21492204262,0.19113972903003,0.692190984030715
"4499","UCK2",27.2204635503259,0.421014010606836,0.32211091701785,1.30
704669840018,0.191196852066139,0.692243948278582
"4500","MAGED1",202.290897044479,0.483230855814885,0.369748451107729,1
.30691786366427,0.191240608461093,0.692248504716164
"4501","SP140L",84.9961202492706,0.297864207130312,0.227969432067752,1
.3065971364169,0.191349569691398,0.692489033704327
"4502","CRABP2",926.929507072612,-0.489840025706585,0.37506195212645,-
1.3060243059297,0.191544292031312,0.693039754086639
"4503","MEIS3",149.98362736345,-0.306618555809727,0.234955897085947,-1
.30500472476997,0.191891238950359,0.694140881914813
"4504","POLR1A",94.2661440398034,0.338286095124154,0.259268930349215,1
.30476912396911,0.191971475774012,0.694148059226021
"4505","YIF1B",130.725771143409,-0.327768488767828,0.251211979128207,-
1.30474864258185,0.191978452134153,0.694148059226021

"4506","SNRPG",652.226285795363,-0.271283545657657,0.20801456698576,-1
.30415648090755,0.192180234578888,0.694415226498561
"4507","DACT2",4.19957557415179,1.88597260808509,1.4461097508441,1.304
16976096333,0.192175707616011,0.694415226498561
"4508","DUS2",30.0934711299579,0.439352856957343,0.336858006336576,1.3
0426722444696,0.192142486241136,0.694415226498561
"4509","MCPH1-
AS1",5.93978731549992,0.8249112328227,0.632848100984511,1.303490097448
98,0.192407494944232,0.695018433291579
"4510","ZNF433",17.7368077233248,0.506069799898482,0.388263988321124,1
.30341678631273,0.192432508695747,0.695018433291579
"4511","TMEM43",793.413844288598,-0.223543655133929,0.171541519769703,
-1.30314605719967,0.192524902151919,0.695043912046234
"4512","ZBED5-
AS1",55.208425046219,-0.349736960126266,0.268362161748248,-1.303227540
90182,0.19249709025829,0.695043912046234
"4513","ZFR",477.609299474825,0.194842328341109,0.149591076287155,1.30
249967562964,0.19274562877672,0.695133468368539
"4514","ZNF879",15.346796151406,0.565727779726772,0.434337201179659,1.
30250823137014,0.192742705945233,0.695133468368539
"4515","CARNMT1",58.4806940003351,0.42546448652243,0.326617103642881,1
.30263994682786,0.192697713122078,0.695133468368539
"4516","ZNF256",14.1044353069962,-0.560410108736007,0.430225747634709,
-1.30259546718676,0.192712906106698,0.695133468368539
"4517","TSPAN6",191.595868726102,0.312909528468883,0.240247125857605,1
.30244858227501,0.192763084082552,0.695133468368539
"4518","AC006449.6",13.7144523810849,-0.527672414942905,0.405191273211
234,-1.30227981160843,0.192820750396442,0.695187517310236
"4519","PLA2G4A",42.9296858894627,0.372357657204344,0.286182099841051,
1.30112140979871,0.19321690043713,0.695893531223142
"4520","SAP30",208.227027724062,-0.319114797291286,0.245257509160474,-
1.30114180146258,0.193209921733985,0.695893531223142
"4521","NSMCE4A",231.359517757982,-0.301072062622861,0.231359923623255
,-1.30131466983506,0.193150767886192,0.695893531223142
"4522","LRP1",6201.3707283171,0.23789497586903,0.182790082200527,1.301
4654460741,0.193099184611626,0.695893531223142
"4523","AURKC",10.1568235595604,0.604471116164178,0.464590884138422,1.
30108260149177,0.193230182437367,0.695893531223142
"4524","SCAF8",165.200879001727,0.233591981272167,0.179630488711516,1.
30040274870772,0.193462968346896,0.696090847912598
"4525","POM121C",164.927705853083,0.291861139930104,0.224478541181504,
1.30017389811045,0.193541374559283,0.696090847912598
"4526","ZKSCAN5",29.2855839645375,0.39306617688254,0.302211214810157,1
.30063398583489,0.193383768184787,0.696090847912598
"4527","PLGRKT",226.508740238445,-0.335886844619938,0.258321757064774,
-1.30026540712834,0.193510019971451,0.696090847912598
"4528","WNK1",555.867704711633,0.275459184077377,0.211846105984564,1.3
0027966668148,0.193505134423791,0.696090847912598
"4529","IREB2",319.654340335872,0.264488739219506,0.203363711414651,1.
3005699855675,0.193405686292825,0.696090847912598

"4530","SLC2A6",10.5626730609639,-0.65612712007045,0.504762332232587,-1.29987338232702,0.193644369327597,0.69630753465281
"4531","BCL10",213.729802189828,-0.280895511595619,0.216161512296154,-1.29947051448629,0.19378250600829,0.696343092955889
"4532","ASL",107.491767602238,-0.269049961284258,0.207043520426765,-1.29948505864701,0.193777517799048,0.696343092955889
"4533","SAE1",163.202287134199,-0.264349519295599,0.203423917552488,-1.29950068053031,0.193772160068421,0.696343092955889
"4534","SLC16A14",7.47141699632843,1.0207712190365,0.785633996294549,1.29929614025231,0.193842318461813,0.69637800424999
"4535","AC007608.3",2.36137077304463,-1.43345659100965,1.1034948040157,-1.29901526114413,0.193938691862691,0.69637800424999
"4536","EIF3K",2490.04227056571,-0.356858622039354,0.27472984610565,-1.2989437700268,0.193963227041697,0.69637800424999
"4537","PSMA7",2213.88611613979,-0.307575122405883,0.236785134096525,-1.29896297577744,0.193956635558032,0.69637800424999
"4538","PXMP2",127.427615579241,-0.363060459753367,0.27958067490353,-1.29858925291829,0.194084928140781,0.696661391468747
"4539","PDE11A",7.40522504089636,-0.998332751217509,0.769031621155473,-1.29816866271053,0.194229383954701,0.697026313117014
"4540","GAS5-AS1",2.60267843907865,1.21421158042753,0.935958932584001,1.29729151371559,0.194530903200583,0.697800898972539
"4541","IPO9",194.306500189895,0.266438631992095,0.205368556176982,1.29736818991163,0.194504532130373,0.697800898972539
"4542","PITPNA-AS1",201.922046521479,-0.289988878328732,0.223559102814535,-1.2971463683557,0.194580829898085,0.697826318408171
"4543","HDLBP",1399.38374669042,0.235313195337867,0.181495997783952,1.29652002364249,0.194796386034409,0.698394909517282
"4544","HDAC11",52.4260164309186,-0.324829431354616,0.250555594481421,-1.29643655344005,0.194825125474033,0.698394909517282
"4545","IL18R1",13.7516410443794,0.740954347907207,0.571904251423854,1.29559160657133,0.195116222364274,0.698663077478664
"4546","AC058791.1",168.304481677697,-0.636678812373789,0.491432138446477,-1.29555794699644,0.195127825196265,0.698663077478664
"4547","AC246817.1",11.9290249012369,1.03182238007264,0.796379873237357,1.29564095571399,0.195099212077481,0.698663077478664
"4548","AC044849.1",33.0691601579774,-0.428527042408408,0.33068997195035,-1.29585738533598,0.195024623221297,0.698663077478664
"4549","GPAA1",569.855220725051,-0.329342523488488,0.254225794153349,-1.29547249359689,0.195157284211917,0.698663077478664
"4550","STAU1",716.788955278293,-0.186183321183032,0.143716815115942,-1.29548738630779,0.195152149897092,0.698663077478664
"4551","SMPD2",35.8612586234807,-0.469276472298708,0.362339637799788,-1.29512872273171,0.195275827970575,0.698780307955339
"4552","RPGRIP1L",60.7249370577751,0.360294811465302,0.278182256614987,1.29517538555293,0.195259733963778,0.698780307955339
"4553","PNRC2",1106.34651123937,0.157664774502144,0.121838000567954,1.29405254327207,0.195647272202639,0.699034528824036

"4554","CZIB",812.639676614891,-0.334280631813736,0.258308723453289,-1
.29411282493596,0.195626452253363,0.699034528824036
"4555","MANBA",173.067558139055,0.331108453380015,0.255787404011006,1.
29446739044964,0.195504026060125,0.699034528824036
"4556","MOB3B",109.490502604472,-0.721612291380014,0.557596524402976,-
1.29414775702315,0.195614388228732,0.699034528824036
"4557","CARS",122.633255671956,-0.242318377373744,0.187158322978064,-1
.29472402572311,0.195415448767569,0.699034528824036
"4558","CAPRIN2",101.749212474019,0.304376072591645,0.235134720445746,
1.29447523536565,0.195501317962681,0.699034528824036
"4559","SIRT7",94.7525523008691,-0.276974150926532,0.213999360384741,-
1.2942756017054,0.195570240882398,0.699034528824036
"4560","KLHL12",69.0202612033517,0.2910568783118,0.224999256879748,1.2
9359039824454,0.195806940801239,0.699188789201939
"4561","APOB",2.60462496085303,1.26072756778741,0.974644046435068,1.29
352615695827,0.195829143375306,0.699188789201939
"4562","RTN4RL2",44.9083820345828,-0.4164221577452,0.32188082469644,-1
.29371533125007,0.195763767709776,0.699188789201939
"4563","VASH1-
AS1",10.1067721231335,0.63187243175198,0.48852438619573,1.293430685564
21,0.195862142865028,0.699188789201939
"4564","EPB41",18.682826065233,-0.636240108835599,0.492872430228335,-1
.29088191956861,0.196744625817421,0.699751545016115
"4565","FAM72D",3.90302159484146,1.00789934475183,0.781129890880578,1.
29030953304785,0.196943208289604,0.699751545016115
"4566","AC245297.2",14.7005619614416,-0.501715908975905,0.388489149421
447,-1.29145411068258,0.196546257739224,0.699751545016115
"4567","TSEN15",76.8873072353029,-0.404000415782108,0.313113219125277,
-1.29026943324442,0.196957125922701,0.699751545016115
"4568","RBBP5",122.172760878606,0.261224771679143,0.202499749793093,1.
29000046640084,0.197050496163077,0.699751545016115
"4569","AC009236.1",12.8722546914274,0.912777072525873,0.7074671271091
8,1.29020421946052,0.196979761524546,0.699751545016115
"4570","CASP10",22.7270813520216,0.471332487457678,0.365144624010065,1
.29081042541841,0.196769421817621,0.699751545016115
"4571","SATB1",155.253565972165,0.353726253467681,0.274056697199791,1.
29070465010315,0.196806111600262,0.699751545016115
"4572","LPCAT1",105.156044835607,0.358261470258198,0.277122860115511,1
.29278930691198,0.196083939727999,0.699751545016115
"4573","CEP120",104.498216825897,0.390926335382926,0.302851287571073,1
.29081945967023,0.196766288381668,0.699751545016115
"4574","MCPH1",123.553771791928,0.262806345293918,0.203365766094395,1.
29228409648816,0.196258777428029,0.699751545016115
"4575","AL162586.1",2.64144296755544,1.38002928522144,1.06904824474598
,1.29089523508769,0.196740007907562,0.699751545016115
"4576","POP5",209.647212129822,-0.350549489173935,0.271727279159003,-1
.290078383955,0.197023444213981,0.699751545016115
"4577","COMMD6",2741.83955356794,-0.355577861057914,0.275126345936471,
-1.29241661625536,0.196212905389607,0.699751545016115
"4578","GDPGP1",15.1254479554271,0.583925898707175,0.452655869893688,1

.28999961680452,0.19705079114672,0.699751545016115
"4579","LINC02185",3.75399279908709,-1.35753402065782,1.05016524974479
,-1.29268609962834,0.196119647297294,0.699751545016115
"4580","FBXL19",24.8752476807051,0.412095314055853,0.318948810485701,1
.29204217262421,0.196342540222657,0.699751545016115
"4581","E2F4",155.828325746792,-0.26344042524753,0.204047390703512,-1.
29107470739637,0.196677773471743,0.699751545016115
"4582","FOXO3B",13.6397088713064,-0.519749153540295,0.402665660598958,
-1.29077099042212,0.196783099862371,0.699751545016115
"4583","AATF",218.690204644067,-0.212165663967252,0.164319409725368,-1
.29117834784004,0.196641841322061,0.699751545016115
"4584","TMEM241",34.1155358502466,0.39692186006446,0.307531549702833,1
.29067037332594,0.196818002098295,0.699751545016115
"4585","ZNF492",5.41316981975925,1.05424240044252,0.815598614591278,1.
29259954784356,0.196149596084073,0.699751545016115
"4586","ZNF829",37.6073906649086,0.409726944664194,0.317123885016133,1
.29200909809537,0.196353993855871,0.699751545016115
"4587","RINL",47.5122951928503,-0.41488102446812,0.321366819889063,-1.
29098898452347,0.196707497231531,0.699751545016115
"4588","ADAMTS9-
AS2",35.8461577383436,0.560179690397629,0.43429097605341,1.28987181702
052,0.197095167485753,0.699756578721758
"4589","HP1BP3",1403.37685560588,0.21529311908192,0.166927249699307,1.
28974220488109,0.197140180607115,0.699759760641971
"4590","ORC5",52.0751531919725,0.309903945247171,0.240306057683524,1.2
8962186069943,0.197181981788118,0.699759760641971
"4591","CCNY",479.09327207742,-0.175196065646764,0.135890535942936,-1.
28924405537949,0.197313253460529,0.700073096410055
"4592","INTS10",312.462048832339,-0.214068430966338,0.166078039170436,
-1.28896289982477,0.197410984856015,0.700267319756017
"4593","HDAC1",105.912095351468,-0.271118454327404,0.210440418799383,-
1.28833831387623,0.197628221527822,0.70040603127485
"4594","SLC10A7",59.4977640465181,0.295532614186126,0.229401438588812,
1.2882770744775,0.19764953056029,0.70040603127485
"4595","STX7",350.679837853686,0.176942532391138,0.137352961014653,1.2
8823238380905,0.197665082311406,0.70040603127485
"4596","TMEM126A",256.868072632047,-0.338186809064298,0.26251695953381
8,-1.28824747042955,0.197659832270418,0.70040603127485
"4597","ZNF480",123.503121299272,-0.318681307522847,0.247332308378762,
-1.28847423780488,0.197580931064071,0.70040603127485
"4598","CNTNAP3B",3.13462138654491,1.87799787625091,1.4580359252846,1.
28803264973347,0.197734598024716,0.700499971123227
"4599","BAMBI",27.2740818239747,0.827490867212669,0.642538620611171,1.
28784611643355,0.19779953547453,0.70057765456504
"4600","AC009951.6",74.8845881398733,-0.381558599581327,0.296479965805
63,-1.28696250535685,0.198107357182837,0.701058166663314
"4601","EFHC1",280.45240183196,-0.202186652293593,0.157099266943517,-1
.2869993363259,0.198094519462436,0.701058166663314
"4602","HOTAIRM1",56.2818271206873,-0.555197809189755,0.43137211859196
6,-1.28705075098958,0.198076599499608,0.701058166663314

"4603","ZNF334",8.75921946399309,0.793290292306806,0.616328474453507,1
.28712257373831,0.198051568529315,0.701058166663314
"4604","LINC00570",3.36815684183447,1.2411700710123,0.965197500533557,
1.28592342015617,0.198469789200392,0.701117146037324
"4605","MAATS1",12.2310955736418,-0.866869285438844,0.673718471551426,
-1.28669365921144,0.19820108402885,0.701117146037324
"4606","TFB1M",61.3312425393467,-0.332743327607612,0.258758979976153,-
1.28591992300432,0.198471009822011,0.701117146037324
"4607","CELF2-
AS2",4.58545762099195,1.27306082712884,0.989494438664625,1.28657704114
73,0.198241750240682,0.701117146037324
"4608","C12orf10",433.870378910838,-0.355964505045514,0.27675291527604
5,-1.28621772489898,0.198367086782324,0.701117146037324
"4609","DENR",417.027620978534,0.188703376923984,0.14673653848513,1.28
600128415259,0.198442613512906,0.701117146037324
"4610","LINC02289",12.5273591865949,0.670249930218364,0.52126904838109
6,1.28580419708394,0.198511405090806,0.701117146037324
"4611","HUNK",38.0984010457988,0.483444828651278,0.375922041068578,1.2
8602416415133,0.198434628571887,0.701117146037324
"4612","VAMP7",161.066844976374,0.216563562684758,0.16839380536152,1.2
8605421214768,0.198424142412519,0.701117146037324
"4613","TET2",119.18409811976,0.322553911663394,0.251023624226203,1.28
495440482022,0.198808217588137,0.701984748800417
"4614","ARMC10",307.449590241972,-0.279426179929205,0.217476959403938,
-1.28485417809343,0.198843245807915,0.701984748800417
"4615","BAK1",42.9992256634476,-0.348031550091597,0.270940237728798,-1
.28453253384964,0.198955687662796,0.70202731949238
"4616","CCDC77",45.2341873968308,0.381584449245841,0.297069759831118,1
.28449442131966,0.198969014293068,0.70202731949238
"4617","UACA",830.985714373491,0.28049149804585,0.218395768078683,1.28
432661728498,0.1990276973059,0.70202731949238
"4618","PFN1",1667.3347132067,-0.317696864460158,0.247359145602094,-1.
28435463215583,0.199017899301833,0.70202731949238
"4619","SMPD4",115.613730475197,0.286001358250277,0.222815179603,1.283
58112207552,0.199288558541319,0.702543494374492
"4620","CDK5",31.8948054586377,-0.503399062031725,0.392232484549356,-1
.28342011909108,0.199344928965571,0.702543494374492
"4621","DYNLL1",3815.02271886624,-0.35963677677883,0.280218513422948,-
1.28341547596469,0.19934655479151,0.702543494374492
"4622","AREL1",37.5396042136971,0.447289871376261,0.348439388874193,1.
28369491411822,0.199248724655099,0.702543494374492
"4623","AC007405.1",7.64602043054912,0.728155530830383,0.5677825936628
82,1.28245483210907,0.199683139923642,0.702685656482016
"4624","PSMD6",245.439797950494,-0.262030085868167,0.2042892296296,-1.
28264268431213,0.199617288668448,0.702685656482016
"4625","CTTN",851.402214116005,-0.220412439510258,0.171869130765369,-1
.28244344128999,0.199687133465179,0.702685656482016
"4626","RSL24D1",1281.3433563187,-0.308503719465508,0.240583324585579,
-1.28231547218381,0.199732002548452,0.702685656482016
"4627","MCRIP2",70.3579639143899,-0.404241115912097,0.315217130931177,

-1.28242115115361,0.199694948399939,0.702685656482016
"4628","AC060766.4",4.45767160458991,0.998049277890807,0.7779020836080
33,1.28300116289919,0.199491668661044,0.702685656482016
"4629","TSPOAP1-
AS1",3.56905439329071,-1.04879428923345,0.817397433558984,-1.283089775
15008,0.19946062558O652,0.702685656482016
"4630","HMOX1",93.771430269283,0.428423067914816,0.33394176741136,1.28
292747336116,0.199517486631075,0.702685656482016
"4631","GLS",643.883183985693,0.337150998835263,0.263051531126842,1.28
16918319806,0.199950771373843,0.702696464920933
"4632","IL6",504.334334577336,1.20878746472028,0.942846558392765,1.282
06170342378,0.199821001832073,0.702696464920933
"4633","MEDAG",1356.169213723,0.345517832052694,0.26957525610875,1.281
71196807955,0.19994370503191,0.702696464920933
"4634","RAMP2-
AS1",131.078333455174,-0.404638764582671,0.315654740966299,-1.28190301
639053,0.199876669704319,0.702696464920933
"4635","ELANE",158.031450847485,-0.47071142755742,0.367201067385028,-1
.28189013967069,0.199881187391813,0.702696464920933
"4636","SMIM10L1",264.784375096619,-0.316583789081545,0.24706072677861
9,-1.28140070341986,0.200052957221432,0.702903930150973
"4637","NAT1",18.7237060219369,-0.504234734683357,0.39354506691842,-1.
2812630040866,0.200101302837736,0.702922174234176
"4638","RPS7",12816.9471582762,-0.355028805594451,0.277133313624831,-1
.28107588709119,0.200167012444683,0.702940483851168
"4639","AC008079.1",7.81890481315259,0.735395274772599,0.5740779842968
41,1.28100239843433,0.200192823659253,0.702940483851168
"4640","CCDC167",126.134777811625,-0.369301114443598,0.288348916252136
,-1.28074389612297,0.200283636007907,0.703107790287241
"4641","LARP1B",134.158426096437,-0.267229784229266,0.208711231868882,
-1.28038046556664,0.200411360704325,0.703253049227217
"4642","KIAA1549L",12.668315468757,-0.932211395675759,0.72802690248222
5,-1.28046284072383,0.200382405419321,0.703253049227217
"4643","EFCAB14",534.01571259717,0.21309482735984,0.166577356219183,1.
27925446889342,0.200807460488808,0.70332831889811
"4644","NBPF15",52.2938297173888,0.462842525196699,0.361672611037079,1
.27972788392663,0.200640854401011,0.70332831889811
"4645","AC008780.1",2.4182545307218,2.37766782245145,1.8582144827301,1
.27954433922943,0.200705436197211,0.70332831889811
"4646","OLFML1",854.954398569916,0.161514782183458,0.126236721981801,1
.27945957125489,0.200735267665929,0.70332831889811
"4647","FDXACB1",12.0934119773713,0.63644072999762,0.497419725973406,1
.27948430020977,0.200726564740288,0.70332831889811
"4648","CHEK1",21.7387957266715,-0.520404161404165,0.406815296388377,-
1.27921483293329,0.200821413910928,0.70332831889811
"4649","LAMA1",7.28365146820139,0.988909775831686,0.772689366813104,1.
27982837386564,0.200605502568461,0.70332831889811
"4650","CRTC1",95.4948325639018,0.331034797029866,0.258762038389021,1.
27930201466488,0.200790723434298,0.70332831889811
"4651","TP53RK",100.385068525632,-0.332061085868012,0.259572020678472,

-1.27926378582741,0.200804180663066,0.70332831889811
"4652","P2RY12",10.9942946994234,0.754346488847299,0.589958431088697,1
.27864345875224,0.201022638789052,0.703729119911783
"4653","NRARP",5.26550297756635,-0.954886453947792,0.746867490553579,-
1.27852191456349,0.201065462831938,0.703729119911783
"4654","AC005261.1",392.602647862715,0.533345632884504,0.4171217012822
83,1.27863314530252,0.20102627230055,0.703729119911783
"4655","FAAP20",454.463199102004,-0.272137718469515,0.212890845370812,
-1.27829695070029,0.201144742610056,0.703767017300718
"4656","HSD17B7",61.0482648813801,-0.351005372470015,0.274628762786834
,-1.27810855974494,0.201211151229559,0.703767017300718
"4657","CLDN11",148.545983168795,1.11349071234286,0.871511023155858,1.
27765533970042,0.201370978755524,0.703767017300718
"4658","LIN54",43.6825708713071,0.333079394718284,0.260664344648241,1.
27780957218282,0.201316578457972,0.703767017300718
"4659","TBXAS1",6.00488780442574,-1.05545086621234,0.82606203361054,-1
.27768959626335,0.201358894984138,0.703767017300718
"4660","PLAG1",8.91865519785921,-0.768410703085125,0.601172210557554,-
1.2781873306693,0.201183382195662,0.703767017300718
"4661","PABPC5",84.363059118568,0.302055349291358,0.236417860216297,1.
27763337767718,0.201378725989235,0.703767017300718
"4662","UFC1",824.337718302249,-0.272346746605692,0.213257500905866,-1
.27707933108486,0.201574241127941,0.704299187844923
"4663","ZNF853",66.4510190633302,-0.288462938583833,0.225927070239494,
-1.27679670381264,0.201674029560412,0.704496733328234
"4664","Z97200.1",188.598306858986,0.403204492961515,0.316002703346263
,1.27595266968239,0.201972250741024,0.704551962243327
"4665","NMI",250.557997795621,-0.273108003738348,0.214115449710038,-1.
27551750286212,0.202126133052577,0.704551962243327
"4666","TMEM45A",434.269374523056,0.6260829058884,0.491012178752711,1.
27508630738814,0.202278695301254,0.704551962243327
"4667","PPA2",126.619275974637,-0.273679134082926,0.214643710330059,-1
.27503915051639,0.202295385070418,0.704551962243327
"4668","FBXL18",55.4727861871237,0.366821298124194,0.287629898018655,1
.27532395154694,0.202194603505274,0.704551962243327
"4669","TUB",94.9241256968581,0.34887555918576,0.273526906365133,1.275
47071628867,0.202142682658752,0.704551962243327
"4670","PPP6R3",220.489006097939,0.235754421181308,0.184794491746683,1
.27576541353019,0.202038457196877,0.704551962243327
"4671","LINC00545",6.62441051243305,1.02287254287131,0.80211822319127,
1.27521419324168,0.202233438966081,0.704551962243327
"4672","GSC",407.324852633865,-0.335182957269986,0.262678597606922,-1.
27601928868054,0.201948700681868,0.704551962243327
"4673","IGDCC4",151.954949506143,0.456374654668976,0.357770423851888,1
.27560755233895,0.202094283031988,0.704551962243327
"4674","SPSB3",405.312536997036,-0.26777389726695,0.209992419883706,-1
.27515982441292,0.202252678143346,0.704551962243327
"4675","TCF3",156.553841324691,0.211254957418331,0.165484606664235,1.2
7658373595414,0.201749246836895,0.704551962243327
"4676","PRMT2",608.124094158589,0.171056280281601,0.134071290384763,1.

27586062452816,0.202004792333023,0.704551962243327
"4677","WDR44",77.6815779614488,0.287537806135157,0.225440263430266,1.27545009822125,0.202149976109389,0.704551962243327
"4678","TRGC1",3.05946751939096,1.32271249005181,1.03776484790603,1.27457823679491,0.202458564586512,0.704969550780184
"4679","SLC36A4",93.5687654416909,-0.27426883003379,0.215232686599624,-1.27428985981105,0.2025607088512,0.705174478836758
"4680","AC020916.1",1277.29884067284,-0.501673156473712,0.393813790362074,-1.27388417762738,0.202704466636986,0.705373436669488
"4681","CHMP4B",608.398284108736,-0.228665197169493,0.179485608268106,-1.2740029653404,0.202662365257296,0.705373436669488
"4682","USP8",557.398807501489,0.198758696820894,0.156040770185905,1.27376131625148,0.202748018529002,0.705374300260339
"4683","ZDHHC2",133.806759633813,-0.313876828834808,0.24644716303395,-1.27360698727771,0.202802734711471,0.705413996522559
"4684","SBDS",1186.04231957473,-0.239841200533759,0.188366526921412,-1.27326868766775,0.20292271393736,0.705536817110481
"4685","TBRG1",282.115006761557,-0.179268290918471,0.140807876355922,-1.27314107390791,0.20296798606297,0.705536817110481
"4686","PTGES3",1725.88629246089,-0.2630638278894,0.206615891607247,-1.27320229747601,0.202946265535238,0.705536817110481
"4687","AC091182.1",2.853682916874,1.29350374468867,1.01618271761918,1.27290468757353,0.203051865668062,0.705677798136776
"4688","INTS11",293.849898581221,-0.250540860158965,0.197429293030753,-1.269015637512,0.204435486833654,0.706267156952998
"4689","GSTM2",90.3462677233543,0.31093586773941,0.24458284780071,1.27129056896404,0.203625296235192,0.706267156952998
"4690","ADAR",462.914569075297,0.230978192151637,0.181879154562503,1.26995417758147,0.204100952921887,0.706267156952998
"4691","TAF1A-AS1",5.80612230318482,1.06744585295411,0.840783910241262,1.26958406310107,0.204232829201266,0.706267156952998
"4692","PTPN23",159.069757760769,0.275438929753086,0.216515109250356,1.27214645992487,0.203321086414788,0.706267156952998
"4693","TMEM115",321.960998622569,-0.278744456613555,0.219370971343247,-1.27065333624934,0.203852003300668,0.706267156952998
"4694","SHQ1",40.8666530352331,-0.374941581178329,0.295114811725299,-1.27049394432745,0.203908738569757,0.706267156952998
"4695","PDE4D",48.0882168734992,-0.504394058629185,0.397383084097663,-1.26928920433166,0.204337935316384,0.706267156952998
"4696","ZKSCAN4",40.1934092102096,0.356760418407339,0.280737024628212,1.27079931433984,0.203800052740703,0.706267156952998
"4697","TSPYL4",110.733013214042,-0.244347262490236,0.192422615515293,-1.26984690357676,0.204139169572825,0.706267156952998
"4698","TXLNB",10.1409584378728,-1.04500103032501,0.822976727527224,-1.26978199427934,0.20416229621169,0.706267156952998
"4699","USP42",112.218326605893,0.249100492463131,0.196193130964464,1.26966979546419,0.2042022762048,0.706267156952998
"4700","SAP25",12.9007892025339,-0.762812020809281,0.600601455234141,-1.27008020736797,0.204056061124448,0.706267156952998

"4701","KLHDC10",214.189104678563,0.301115751789247,0.237216149446037,
1.26937290101215,0.204308096583703,0.706267156952998
"4702","GLT8D2",912.60033199216,0.293220551744186,0.230746586105957,1.
27074708533084,0.203818638849324,0.706267156952998
"4703","RALGAPA1",253.425162785642,0.28318341288187,0.223136887260066,
1.26910174449024,0.204404778191354,0.706267156952998
"4704","LINC00648",27.1698725497423,0.490947544032889,0.38588688958519
2,1.27225764150897,0.203281593377852,0.706267156952998
"4705","MFAP1",215.512103556085,-0.289799830578676,0.22809637733679,-1
.27051483220525,0.20390130291286,0.706267156952998
"4706","TSPAN3",899.170181576071,0.231228017766119,0.182002168268574,1
.27046847829255,0.20391780422381,0.706267156952998
"4707","LRRK1",136.472185149581,0.251598755085488,0.197817326393647,1.
27187420673566,0.203417817613187,0.706267156952998
"4708","ACSM3",4.07408796260556,0.993838235570566,0.782917366936052,1.
26940374238977,0.204297102112712,0.706267156952998
"4709","AC092376.1",2.43264025786564,-1.36148733533964,1.0703976211141
9,-1.27194540466415,0.203392517848509,0.706267156952998
"4710","MFAP4",6797.68302664919,-0.267195962617876,0.210461564066945,-
1.26957130534716,0.204237376048767,0.706267156952998
"4711","TLCD1",5.43498628139407,-0.940019167009778,0.739194451263004,-
1.27168049679437,0.203486662897063,0.706267156952998
"4712","CDK12",320.15861806461,0.265029666420481,0.208539260062993,1.2
7088619351783,0.203769138826216,0.706267156952998
"4713","ZNF516",113.288718702966,0.374837867245184,0.295090148340027,1
.27024866588655,0.203996067437508,0.706267156952998
"4714","ZNF345",51.9278850648155,0.306647576505922,0.241251065628687,1
.2710724228588,0.203702884989395,0.706267156952998
"4715","QPCTL",7.13365641815246,0.720284456518266,0.567588126893735,1.
26902664518407,0.20443156094078,0.706267156952998
"4716","GLRX5",641.686217435825,-0.368009457490509,0.290057606040655,-
1.26874610362373,0.204531633369695,0.706449485996386
"4717","AL050341.2",57.7612431989341,-0.387941351417206,0.306185394022
453,-1.26701455716322,0.205150084795429,0.706488315271194
"4718","LINC01138",27.28611829151,0.392189136002207,0.309303089349361,
1.26797678234383,0.204806242010252,0.706488315271194
"4719","HIST1H1C",132.37913124156,0.382461011019319,0.301785222444627,
1.26732849249931,0.205037856646668,0.706488315271194
"4720","TBC1D32",41.7078834692396,0.505124505166837,0.398603568187099,
1.26723528207289,0.205071173605568,0.706488315271194
"4721","AC006483.2",2.65522544051828,-1.07149244070215,0.8455952683551
16,-1.2671457383938,0.205103183635645,0.706488315271194
"4722","ADK",413.454870928967,0.236614419789515,0.186657721990246,1.26
763799143483,0.204927258158659,0.706488315271194
"4723","RNF141",138.802003818798,0.210420566984502,0.165945027724495,1
.26801369025558,0.204793061639922,0.706488315271194
"4724","ELP4",84.1500744645857,0.250603677169839,0.197765803443042,1.2
6717396439073,0.205093093028222,0.706488315271194
"4725","IRAK3",212.33452183213,0.294588258820955,0.232413557653078,1.2
6751753123062,0.204970299083349,0.706488315271194

"4726","MIR193BHG",128.419379160883,-0.250030567820025,0.197322307971496,-1.26711759248297,0.205113245972197,0.706488315271194
"4727","RECQL5",29.4351616697186,0.4064176320688,0.320424902246524,1.26837093253169,0.204665516928948,0.706488315271194
"4728","GTF2F1",280.669677453349,-0.241064027681211,0.190203471542914,-1.26740077731347,0.205012022008493,0.706488315271194
"4729","TRIM28",406.128739933591,-0.165706155302395,0.130728923767114,-1.2675554156446,0.204956762118528,0.706488315271194
"4730","ZMYND8",210.359217736092,0.30257334032135,0.23879026400606,1.26710919970201,0.205116246512564,0.706488315271194
"4731","HSPB6",1243.04414111434,0.396934741155993,0.31339060831382,1.26658148210528,0.205304977259781,0.706872283784522
"4732","SRSF11",1535.62921518199,0.248167586086212,0.196235809415201,1.26463965382145,0.206000531440144,0.707429551652486
"4733","F11R",2.67354549286124,-1.16777030025051,0.922877716129974,-1.26535756562362,0.20574317948012,0.707429551652486
"4734","DENND1B",121.238936376239,0.303421760871645,0.240004230541796,1.26423505196841,0.206145673247517,0.707429551652486
"4735","TIMM17A",182.364313229104,-0.251601250440747,0.199117762408357,-1.26358014170908,0.206380764951784,0.707429551652486
"4736","PARP1",276.145949276631,0.219005738433418,0.17323425529891,1.26421727651688,0.206152051493454,0.707429551652486
"4737","PFN2",204.37688966101,-0.221374029681023,0.175199665573941,-1.26355280962334,0.20639058052121,0.707429551652486
"4738","SLC2A9",3.56450327606446,-0.945487564613023,0.74705359880696,-1.26562212687679,0.205648400355641,0.707429551652486
"4739","RPS14",25671.4305320238,-0.313734544923952,0.248295463150928,-1.26355327214838,0.206390414415202,0.707429551652486
"4740","SMIM3",183.487092501439,-0.348718869705179,0.276002106421898,-1.26346452288348,0.20642228859993,0.707429551652486
"4741","PSMG3",199.704641679702,-0.27546111753226,0.21788381826329,-1.26425688574722,0.206137838975402,0.707429551652486
"4742","LUC7L2",466.631001118182,-0.165369366070277,0.130878537749257,-1.26353311180095,0.206397654664398,0.707429551652486
"4743","RNF139",350.449112661783,0.215912482864277,0.17075203738289,1.26447968746704,0.206057906893836,0.707429551652486
"4744","CH25H",206.153672377079,1.30378463723837,1.03003494653206,1.2657673816097,0.205596376310295,0.707429551652486
"4745","AC104389.5",7.89573664647965,0.81370011458258,0.642904957401942,1.26566159618809,0.205634263186651,0.707429551652486
"4746","NCAM1",2.96807854308846,-1.3350094996547,1.05621875690948,-1.26395170595244,0.206247361464524,0.707429551652486
"4747","ASIC1",62.7027575006176,0.342121762627267,0.270349324976182,1.26548036566175,0.20569918239455,0.707429551652486
"4748","AC023794.2",23.0213197922811,-0.534315014375863,0.422530660590667,-1.26455915324329,0.2060294032935,0.707429551652486
"4749","TMEM202-AS1",14.3667933358959,-0.489898226755647,0.387322987739224,-1.26483127070548,0.205931819104829,0.707429551652486
"4750","RPS17",6298.74995474255,0.291812314224534,0.230681382140099,1.

26500158581202,0.20587075940007,0.707429551652486
"4751","STX10",372.700806743288,-0.305760692810501,0.241595473795141,-1.26558949142304,0.205660090298073,0.707429551652486
"4752","ZNF470",19.4855983210659,0.483022179625151,0.382299607602903,1.26346501544639,0.206422111686728,0.707429551652486
"4753","ZNF324",73.9458188632608,-0.369228488716454,0.291859521791123,-1.26508974745975,0.205839157715966,0.707429551652486
"4754","MPP6",88.9354765107374,0.317526171178263,0.251448076582611,1.26279021694542,0.206664581470712,0.707813155503875
"4755","CIPC",30.2716481631779,0.40006851802536,0.316792018218931,1.26287436241174,0.206634334894512,0.707813155503875
"4756","JPT1",222.441467800986,0.333573009530245,0.264133494003342,1.2628955323857,0.206626725730535,0.707813155503875
"4757","HCN3",13.434878124392,0.58927055755343,0.466780973288149,1.26241340430486,0.206800068386611,0.707879423030201
"4758","AL512625.3",5.93275384490163,-0.896834082529541,0.710350614041571,-1.26252313266397,0.206760607757946,0.707879423030201
"4759","ORAI3",346.634919606195,0.383734167999273,0.303978235116471,1.26237382703483,0.206814302547776,0.707879423030201
"4760","PPWD1",180.867631400213,0.193272846482928,0.153120470521928,1.26222735486729,0.206866988174711,0.707911002180225
"4761","BCL2L1",153.184358136913,0.254366946785393,0.201547252812733,1.26207101925491,0.206923232402604,0.707954743248481
"4762","AC093525.4",12.3706000394339,0.663490657121975,0.525877194118005,1.26168364885032,0.207062642900197,0.70828294628335
"4763","AC073508.3",27.2446100269232,0.488451209392943,0.387203197451185,1.2614854748314,0.207133989976993,0.70837824117893
"4764","CHST10",29.7363981194744,-0.365988539799606,0.290344719784549,-1.26053106828045,0.207477847527411,0.708515880356612
"4765","AL024507.2",5.12261221872512,0.992934651964816,0.787788267303202,1.26040802227721,0.207522209170692,0.708515880356612
"4766","SLAIN1",2.72541946452329,-1.13453774921512,0.900069153991649,-1.26050064507116,0.207488815334084,0.708515880356612
"4767","HIF1A-AS3",4.82762390722791,1.21764350649073,0.965528252774318,1.26111639197713,0.207266915605085,0.708515880356612
"4768","JDP2",1190.98070365004,-0.233872514552084,0.185509315995021,-1.2607049586575,0.20741516674852,0.708515880356612
"4769","ZNF785",68.7730867744654,0.331125017376086,0.262703865781231,1.26044973259675,0.207507170623174,0.708515880356612
"4770","CTNS",54.9240756730294,0.318331372477593,0.252415825695433,1.26113872456513,0.207258870738109,0.708515880356612
"4771","ATF4",1554.69749674685,-0.223984892755315,0.177654668638436,-1.26078810352668,0.207385201083172,0.708515880356612
"4772","MTFR1L",243.254533611575,-0.213278342054728,0.169295852469165,-1.25979661606639,0.207742740837206,0.708981651684885
"4773","ATP6V0A2",60.7720003570401,-0.318374936055794,0.252745147039185,-1.25966785034426,0.207789207756378,0.708981651684885
"4774","TUBG1",61.204531176404,-0.405019674553087,0.321516570273527,-1.25971633190949,0.207771711618,0.708981651684885

"4775","FTCDNL1",3.957165759602,-1.03707811316547,0.823840819191835,-1
.2588331252909,0.208090612842801,0.709267209164341
"4776","TIMP4",79.1629959430094,-0.552568564417746,0.438841238081646,-
1.2591536903716,0.207974824746442,0.709267209164341
"4777","NDUFS6",904.163276927585,-0.346404099058399,0.275107125046052,
-1.2591607687384,0.207972268567899,0.709267209164341
"4778","UNKL",150.81277254938,0.303543424863534,0.241108767072176,1.25
894810275675,0.208049077611312,0.709267209164341
"4779","ZNF865",64.5296470171699,-0.291948031234945,0.231906525162178,
-1.25890390980063,0.208065041460101,0.709267209164341
"4780","NAB1",130.906319537369,-0.306762057877396,0.243768024937593,-1
.25841794860474,0.208240644108418,0.709467095816303
"4781","LINC00887",5.97169649387941,-0.924391081281445,0.7346473156286
59,-1.25827871635305,0.208290975642417,0.709467095816303
"4782","PAPPA",70.5702953144251,0.609598700228847,0.484504941061647,1.
25818882031026,0.208323477149572,0.709467095816303
"4783","APCDD1L-
DT",4.25953060547757,1.27197537491753,1.01067675664129,1.2585382681052
2,0.208197156510953,0.709467095816303
"4784","PPP1R8",152.946161159042,0.225723684140453,0.179475440497412,1
.25768563941041,0.20850546780895,0.70949353773553
"4785","PPP2R5D",144.480764763947,0.225014712084366,0.178893449438448,
1.25781415021452,0.208458977018959,0.70949353773553
"4786","MAD2L1BP",80.6389582523929,-0.324597540699748,0.25808986385118
6,-1.25769193666168,0.208503189504884,0.70949353773553
"4787","SHPK",65.0229510504643,0.373510772188878,0.29691145372366,1.25
798707831767,0.208396429436291,0.70949353773553
"4788","ARPC5",1034.70824896644,-0.253998383700034,0.202002300405234,-
1.25740342159714,0.208607590882207,0.709692783600726
"4789","MARCH7",438.052629872296,0.18730464144591,0.14898301244234,1.2
572214668998,0.208673452055866,0.709733696550068
"4790","CCT5",511.225697573461,-0.320502928429628,0.25496881830101,-1.
25702793998618,0.208743518499003,0.709733696550068
"4791","AC026741.1",2.80228948795716,1.18756044466637,0.94489713074260
3,1.25681453147503,0.208820802841005,0.709733696550068
"4792","SOX8",4.57975031785355,-0.858656523654528,0.683225685587662,-1
.25676850529408,0.208837473605775,0.709733696550068
"4793","MCOLN1",201.246907136225,-0.286388677803491,0.22787530374245,-
1.25677804088491,0.208834019718584,0.709733696550068
"4794","TAF1B",166.243709191043,-0.237615205741011,0.189150189049293,-
1.25622505023819,0.209034386917031,0.709810533352412
"4795","DAPP1",5.32878387230897,-1.02180372755303,0.813239413162806,-1
.25646114909642,0.208948823290873,0.709810533352412
"4796","ATXN2L",204.805024117173,-0.258675217237786,0.205894365735235,
-1.25634917844436,0.208989398955018,0.709810533352412
"4797","TOB1",368.295071253749,0.366860111583177,0.292011467646902,1.2
5632090595422,0.208999645176794,0.709810533352412
"4798","ANAPC16",892.634037151379,-0.227156295098505,0.180861909621106
,-1.25596536923879,0.209128526161784,0.709982193132408
"4799","MR1",210.177495152484,0.275010709288534,0.219091040046264,1.25

523485228087,0.209393517008885,0.710302013361821
"4800","RIF1",494.266385374149,0.284962690684705,0.226987440838111,1.2
5541170750475,0.209329341488649,0.710302013361821
"4801","ZNF141",142.078525864466,0.269828030208925,0.214962767248533,1
.25523146944307,0.209394744679355,0.710302013361821
"4802","UBR5",318.285411049372,0.299126233379795,0.23830490535084,1.25
522482610844,0.209397155636532,0.710302013361821
"4803","SELENOK",1037.70346996354,-0.278582929236368,0.221965626238365
,-1.25507239097103,0.209452481960158,0.710341761117845
"4804","DDX47",21.9331100061831,-0.504066390449271,0.401728851601849,-
1.25474281580564,0.209572137432663,0.710599614204964
"4805","MICOS13",888.6543463356,-0.357021268744342,0.284601463358206,-
1.25446041117149,0.209674706557542,0.710799437068846
"4806","COL5A3",230.710667802682,0.584124762946406,0.465696554463534,1
.25430338134946,0.209731755378748,0.710844894582695
"4807","TOR1AIP1",478.353353310952,0.183162324357906,0.146144660221348
,1.25329467447179,0.210098485840213,0.71093377878344
"4808","GRPEL1",149.854090181054,-0.285835988938604,0.228093825729398,
-1.2531509260479,0.210150785489733,0.71093377878344
"4809","C4orf3",1476.77768866535,-0.307570769234372,0.245359806209267,
-1.25354993544478,0.210005638062168,0.71093377878344
"4810","HAND2",108.051243858282,-0.485491460245249,0.387196317494389,-
1.253863836792,0.209891501353673,0.71093377878344
"4811","UBE2D4",176.439613997875,-0.304603119438975,0.242985275132859,
-1.25358674212841,0.209992252571875,0.71093377878344
"4812","TIGD5",76.9143859338598,0.310366011896699,0.247649488050794,1.
25324713707077,0.210115780217858,0.71093377878344
"4813","AL359095.1",2.52602767644631,1.59092487734557,1.26871370797293
,1.25396680696975,0.209854070465884,0.71093377878344
"4814","NSUN6",91.0616646064009,-0.27975944161388,0.223187683826467,-1
.25347168274482,0.210034098281703,0.71093377878344
"4815","DLG2",42.3252831301981,0.424366079209137,0.338394233402991,1.2
5405824721535,0.209820834897348,0.71093377878344
"4816","ZFAND2B",159.37356003144,-0.25699399148167,0.205172911235296,-
1.25257271992863,0.21036124803275,0.71095345383013
"4817","ARHGEF37",5.2350054861794,-0.861016797289672,0.687172884832514
,-1.25298424354961,0.21021144098618,0.71095345383013
"4818","BNC1",4.1094017980227,1.06024663126954,0.846239681239277,1.252
89165088177,0.210245140784587,0.71095345383013
"4819","SYT17",3.62344689833666,1.01522230903975,0.810472118297617,1.2
5263076436511,0.210340113423437,0.71095345383013
"4820","CXorf40B",47.1296383204301,-0.350617197783705,0.27992597576938
1,-1.25253541340716,0.210374832553332,0.71095345383013
"4821","CPLANE2",6.58201807630075,-0.708616292264656,0.565890645689409
,-1.25221418247932,0.210491829472016,0.711009551727404
"4822","ACVR2B",63.0673632136052,0.392634823997263,0.31357520284121,1.
25212331982796,0.210524931494986,0.711009551727404
"4823","AC007036.3",18.4699620083679,0.525707511149592,0.4198906503171
01,1.25201051929253,0.210566030912456,0.711009551727404
"4824","MTFR1",81.3115240357881,-0.261665470473052,0.208936731967057,-

1.25236701086293,0.210436161309509,0.711009551727404
"4825","WASF3",225.358607250246,-0.251612392591405,0.201034924818128,-1.25158547858803,0.21072094868496,0.711385188213329
"4826","QSOX1",227.696559169324,-0.264526093710722,0.2114690335768,-1.25089753916454,0.2109718618162,0.711407207290709
"4827","TBL1XR1",885.968691174293,0.231987944622151,0.185464364635015,1.25084915950669,0.21098951552713,0.711407207290709
"4828","DMAC1",617.357430719135,0.302775602150723,0.24196293517453,1.25133050618818,0.210813919943641,0.711407207290709
"4829","AC138649.1",4.23909323712911,-1.0411509083618,0.832219336483796,-1.25105349361475,0.210914961394325,0.711407207290709
"4830","SHF",58.9066163333889,-0.268291166009314,0.214418907449033,-1.25124770572337,0.210844118076881,0.711407207290709
"4831","THOP1",54.108489252488,-0.327783467729467,0.261977101059759,-1.25119129268744,0.21086469425069,0.711407207290709
"4832","AC073263.2",8.1621501923966,-0.964373684870943,0.771072898980132,-1.2506906754815,0.211047353744391,0.711454955534434
"4833","PLEKHG5",2.41861015175732,-1.64606088489265,1.3166273030967,-1.25021020073115,0.211222771478594,0.711468010494401
"4834","CSDE1",1567.43092383932,-0.163115700313476,0.130434234198555,-1.25055896035063,0.211095431466945,0.711468010494401
"4835","SMIM30",308.124819814249,0.288012954965439,0.230421229988086,1.24994105352329,0.211321081167145,0.711468010494401
"4836","LYPLA1",210.586248678364,-0.18937475430793,0.151532943422079,-1.2497266273014,0.211399426943457,0.711468010494401
"4837","RND1",28.1437756002216,0.396795325875821,0.317506548214153,1.24972328321301,0.211400648952846,0.711468010494401
"4838","MRPL57",576.709576677337,-0.260691661614125,0.208582706262522,-1.24982394890408,0.211363865553479,0.711468010494401
"4839","TMEM87A",606.921554256082,-0.157786128034034,0.126202666694268,-1.2502598571572,0.211204637405815,0.711468010494401
"4840","DENND6B",33.7364197378462,-0.347296143633318,0.27788134359602,-1.24980014541103,0.211372562968935,0.711468010494401
"4841","LINC01127",2.43776117306252,2.06993077543521,1.65678690537784,1.24936451918851,0.211531779520809,0.711755157765088
"4842","AC124852.1",106.268121311594,0.40970803713746,0.327963000412711,1.24925078933258,0.211573360789401,0.711755157765088
"4843","ZNF526",38.2298765707594,0.380367140348387,0.30453408417661,1.24901336208987,0.211660186641054,0.711900223042768
"4844","LIMCH1",274.626371985974,0.325818350558,0.260998002428388,1.24835572504965,0.211900815389118,0.712297449211336
"4845","SPARC",7825.98107027045,0.441529185318358,0.353762995787358,1.24809318831005,0.211996932517439,0.712297449211336
"4846","PLAC9",10573.5896896715,-0.297421511985088,0.238293887806372,-1.24812900038276,0.211983819531155,0.712297449211336
"4847","NME3",792.111605308832,-0.293002263463502,0.234705622226575,-1.24838195473924,0.211891214187807,0.712297449211336
"4848","ZNF439",9.97598995906396,0.869821495264984,0.69679353243545,1.24832027677519,0.211913791490903,0.712297449211336
"4849","S100A9",60.5033291796696,-0.467205646700163,0.374580928493843,

-1.24727558495505,0.212296466483832,0.712735562340726
"4850","PLCL1",44.3695865046941,0.41144592191636,0.329876031740215,1.2
4727437681918,0.212296909317865,0.712735562340726
"4851","CSRP2",115.712954742173,-0.454118535574703,0.36412790173346,-1
.24714017633045,0.212346103753425,0.712735562340726
"4852","DDX51",90.9257090008982,-0.287928088469162,0.230861281926165,-
1.24719089345284,0.212327511197434,0.712735562340726
"4853","SNAPC5",181.44854267689,-0.313451278516748,0.251251243972978,-
1.24756110083363,0.212191831282015,0.712735562340726
"4854","VWCE",29.3253006152417,0.527264899109644,0.42285336196716,1.24
692138347145,0.212426325224514,0.712857933988898
"4855","SUPT20H",165.964012039547,0.242960944857938,0.194882377910751,
1.2467055639541,0.212505477949978,0.712976669480369
"4856","ZFAND3",295.79354571317,0.2204106973604,0.176873670566905,1.24
614758462327,0.212710217968541,0.713076093947226
"4857","FAM225A",20.437888418042,0.513305066747243,0.411861589402878,1
.24630477799942,0.212652524409256,0.713076093947226
"4858","SPHK1",31.6235869605417,-1.22388706683449,0.982017439082758,-1
.24629870929547,0.21265475155247,0.713076093947226
"4859","AL118522.1",13.9640142928487,-0.676686911010836,0.543012532583
408,-1.24617181078946,0.212701325675529,0.713076093947226
"4860","SLC41A3",205.364632910173,0.214483630735519,0.172151513833197,
1.24590034650139,0.212800982874785,0.713233582314273
"4861","CCDC40",12.9400662936217,-0.565674288884431,0.454171835765053,
-1.24550719427935,0.212945372673718,0.713570700572349
"4862","AC253572.1",8.74316029784073,-0.629702694398169,0.505785534847
094,-1.24499941380992,0.213131965709305,0.713722022538199
"4863","AC010883.3",16.8318201181955,0.87438337196453,0.70236820924252
,1.24490738683563,0.213165795300407,0.713722022538199
"4864","WDR73",163.344114835593,0.292107540622799,0.234602116905684,1.
24511894639141,0.21308803070138,0.713722022538199
"4865","AC009120.2",52.5831271794584,0.416391980202484,0.3344721814788
44,1.24492260719991,0.213160199948377,0.713722022538199
"4866","MYOC",6852.23768787842,-0.591965135262412,0.475615035818053,-1
.24463082678671,0.213267483529715,0.713915749941538
"4867","ENTPD7",20.5427543621806,-0.485083918516819,0.390038964063315,
-1.2436806658067,0.213617114535905,0.71493921895939
"4868","KLHL21",160.01914726763,0.444267826196741,0.357398593241925,1.
2430598066065,0.213845795579388,0.715410590304508
"4869","AL353194.1",19.108727089033,-0.468529016931032,0.3768910222528
68,-1.24314188788684,0.213815552457913,0.715410590304508
"4870","DDX60",88.8319979499744,0.29843432992381,0.240108091250336,1.2
4291658964822,0.21389857173491,0.715440212523602
"4871","LEMD3",145.159685411361,0.284461444858282,0.228902772515013,1.
24271734122236,0.213972011390805,0.715458174884194
"4872","LUC7L3",934.942700478587,0.340535826692863,0.2740418915464,1.2
4264149824409,0.213999970629955,0.715458174884194
"4873","RPRD1B",107.95943631535,0.24264273115458,0.195278896991058,1.2
4254456007958,0.214035710369616,0.715458174884194
"4874","SDHB",365.187676584291,-0.267931040900435,0.215713231762489,-1

.24207049660932,0.214210552922509,0.715477153487637
"4875","ZNF142",35.0257899065784,0.345388748919676,0.278039720311898,1
.24222808357104,0.214152420784414,0.715477153487637
"4876","NADSYN1",88.0576183290804,-0.243130262630412,0.19574873444818,
-1.24205279444488,0.214217083771822,0.715477153487637
"4877","MCAT",99.5054209558851,-0.321067507185405,0.258440811007864,-1
.24232510311862,0.214116636974286,0.715477153487637
"4878","CCT7",747.778997359917,-0.292633214719298,0.235746384765272,-1
.2413052060614 5,0.214493022220032,0.715850504169042
"4879","AC139887.2",45.4361439011334,0.442590659243,0.35661860305521,1
.24107563500965,0.214577809403584,0.715850504169042
"4880","DNM1",529.377914014776,-0.28869034148762,0.232613785121362,-1.
24107150974307,0.214579333203994,0.715850504169042
"4881","PSTK",31.7403824767391,-0.393999371203668,0.317537120379687,-1
.24079783406914,0.214680441578106,0.715850504169042
"4882","PACS1",236.285139539826,0.228469440045292,0.184031187307201,1.
24147131466316,0.214431688606151,0.715850504169042
"4883","GOLGA8R",13.2769965124927,0.616952693306899,0.497183178123917,
1.24089615347591,0.214644113917612,0.715850504169042
"4884","NUDT21",320.423443463045,0.177135771525077,0.142683250298026,1
.24146156717827,0.214435287399028,0.715850504169042
"4885","CCDC102A",191.685761760956,-0.293848799937052,0.23678947848395
4,-1.2409706791806,0.214616580653691,0.715850504169042
"4886","HEATR3",30.1080484273985,-0.366379666346354,0.295313514915252,
-1.24064645822761,0.214736381522123,0.715890486822322
"4887","AC105345.1",7.71449649774599,0.69344893947895,0.55912725322675
,1.24023455389989,0.21488865124406,0.716056142053857
"4888","SIRT3",75.0048558092781,-0.25394846820114,0.204739135312666,-1
.2403513759757,0.214845457425939,0.716056142053857
"4889","SOX18",2.28650724689545,-1.44007943375644,1.16123548002644,-1.
240126966947,0.214928436002017,0.716056142053857
"4890","HDAC10",62.9604457812282,-0.26885317648694,0.216810702928851,-
1.24003645970913,0.21496190893507,0.716056142053857
"4891","GSTM3",1201.43379964081,0.377369338029759,0.30443190406561,1.2
3958538178847,0.215128790303279,0.716460934840812
"4892","AGAP1-
IT1",2.61749011356792,1.51689688982095,1.22407806033839,1.239215813901
29,0.215265585676082,0.716460934840812
"4893","CNBP",2151.71923866323,-0.199800472857079,0.161306484893381,-1
.23863881225322,0.215479287849784,0.716460934840812
"4894","FBXO45",77.1184587283637,-0.319891562477727,0.258202954649641,
-1.23891518945549,0.215376907885958,0.716460934840812
"4895","LMLN",58.3118737048464,0.36947004147108,0.298257467387067,1.23
876208266597,0.215433619766005,0.716460934840812
"4896","CLOCK",197.165041723293,0.306816553149374,0.247658689085592,1.
23886851812955,0.21539419414836,0.716460934840812
"4897","NAP1L5",80.1463724499901,0.308707314465181,0.249088859247359,1
.2393461329341,0.215217340997908,0.716460934840812
"4898","TPM2",798.599046036135,-0.306711486073661,0.24750113340597,-1.
23923265260596,0.215259351475334,0.716460934840812

"4899","BTG3-
AS1",7.59550298185222,-0.715215386840917,0.577409870146055,-1.23866151
900037,0.215470875139909,0.716460934840812
"4900","CENPH",25.4716655538566,-0.454901978424275,0.367322102614219,-
1.23842800415971,0.215557402244945,0.716519395910673
"4901","UBXN2B",44.9393745546853,-0.367914404010474,0.297099551237271,
-1.23835395401405,0.215584846175837,0.716519395910673
"4902","ZBTB22",68.8870459067092,-0.350156711771334,0.282936181857834,
-1.23758195036108,0.215871110506511,0.716947770382022
"4903","TAF2",137.875796267509,0.26685546208503,0.215655943761799,1.23
741297100433,0.215933805727436,0.716947770382022
"4904","SIVA1",936.38314815216,-0.295560818045026,0.238808331117864,-1
.2376486894804,0.215846352372422,0.716947770382022
"4905","MOCOS",7.0187634335337,-0.7170903592904,0.579386299397795,-1.2
3767227501881,0.215837603361489,0.716947770382022
"4906","CFAP298",353.917798837943,-0.210239282317905,0.169887836365576
,-1.23751815795392,0.215894777413726,0.716947770382022
"4907","USP33",654.381401793941,0.26439244073531,0.213703018862183,1.2
3719562850825,0.216014464059894,0.717069412078991
"4908","RHOC",859.303330561661,-0.313990029255708,0.254140900037471,-1
.23549585764988,0.216646017173011,0.717213994864119
"4909","PYGO2",57.0522824253918,0.318862422401863,0.258174734395482,1.
23506439601257,0.216806538812138,0.717213994864119
"4910","GUK1",2436.25159689305,-0.342321010299744,0.27711997265078,-1.
23528090388176,0.216725978203389,0.717213994864119
"4911","PLA2R1",325.117881006969,0.343253618578955,0.277855650808434,1
.23536670058803,0.216694059989454,0.717213994864119
"4912","SMARCAD1",140.303027183417,0.224324254935184,0.181533474645013
,1.23571840055311,0.216563255417375,0.717213994864119
"4913","CUTA",1016.92890316426,-0.377018952636077,0.30514590724473,-1.
23553665208986,0.216630844374751,0.717213994864119
"4914","STAG3",6.9026762969576,0.771605248350263,0.624330972851601,1.2
358913491445,0.216498953066323,0.717213994864119
"4915","FGFBP3",27.396539123549,-0.404548174244186,0.327410842182481,-
1.2355979769867,0.216608037061046,0.717213994864119
"4916","QSER1",229.329696303363,0.288760924832318,0.233634624626596,1.
2359509010868,0.216476814812701,0.717213994864119
"4917","TRAF6",70.4452815926506,0.305528103627319,0.247228331596388,1.
23581347515668,0.216527904943233,0.717213994864119
"4918","PSMC3",761.41219633137,-0.291916297206571,0.236154134511486,-1
.23612613351206,0.216411682142028,0.717213994864119
"4919","SINHCAF",32.1413434495484,-0.461074680598936,0.373041234790197
,-1.2359885117211,0.216462834013156,0.717213994864119
"4920","MARS",69.6803828414718,-0.327409113007156,0.264802615139937,-1
.23642703767912,0.216299871070736,0.717213994864119
"4921","AC020765.2",11.9975223080836,-0.579318732638059,0.468402756925
54,-1.23679616328593,0.21616276683119,0.717213994864119
"4922","DCAKD",94.1772487524492,-0.292591972308145,0.236886956839216,-
1.23515442222822,0.216773038259538,0.717213994864119
"4923","MAP2K2",1349.32177957384,-0.282940550271719,0.228837724519141,

-1.23642441763597,0.216300844456451,0.717213994864119
"4924","IL10RB",466.993073000364,0.180010582309156,0.145741135916276,1
.23513914707353,0.216778722187236,0.717213994864119
"4925","SNAP47",124.506372481342,-0.295905987082285,0.239671499553718,
-1.23463151702759,0.21696767374755,0.717601307141898
"4926","JAML",8.41742748165684,-0.72776258220398,0.589527497076433,-1.
23448454196467,0.217022403354096,0.717636607437041
"4927","AK5",3.50142511602815,1.1290528052031,0.914989763952035,1.2339
5129616148,0.217221053298973,0.717639756054273
"4928","ACP1",480.01712958108,-0.279682930268948,0.226689226839875,-1.
23377248300603,0.217287695798372,0.717639756054273
"4929","FBXO48",45.4270481913851,0.346029519485453,0.280422871792689,1
.23395612231396,0.217219254827121,0.717639756054273
"4930","VIL1",2.52654154054331,1.15307097009784,0.93422626419502,1.234
25235865253,0.217108882483927,0.717639756054273
"4931","NIPBL",576.931421440417,0.269899314882796,0.218743119796603,1.
23386424740472,0.217253493967497,0.717639756054273
"4932","TRMT5",100.129988852344,0.26295310239014,0.21309750034657,1.23
395676609293,0.217219014922882,0.717639756054273
"4933","APCDD1",33.7228967269513,0.781996702121998,0.633907169665681,1
.23361390995849,0.217346807242828,0.717689467500188
"4934","ATXN3",415.759445926068,0.254098283443037,0.206115066818487,1.
23279820037031,0.217651062943751,0.718440230332372
"4935","AC015908.2",19.4568088367871,-0.614605854144314,0.498557651359
716,-1.23276787041198,0.217662381772377,0.718440230332372
"4936","RPS10",10984.9656028496,-0.403993325273165,0.327790992332625,-
1.23247232145786,0.217772699759477,0.718467993569767
"4937","YIF1A",569.970702704857,-0.335990080523402,0.272632728020817,-
1.23239085403476,0.217803115737462,0.718467993569767
"4938","AC145285.6",2.64146284551157,-1.14189633328121,0.9264600250124
25,-1.23253707926135,0.217748524520178,0.718467993569767
"4939","FAM89A",96.3247211275499,0.343343415603671,0.278724498819468,1
.23183795130279,0.218009623457695,0.718858047874979
"4940","RNF4",160.119953782002,0.203233271933459,0.164971434156482,1.2
3193007912317,0.217975204190557,0.718858047874979
"4941","CNBD2",33.2775902719484,-0.41584047562967,0.337655625624547,-1
.23155204318159,0.218116464577642,0.719064782737343
"4942","UPF3A",557.215424900868,-0.302159887505519,0.245446463351053,-
1.23106229920841,0.218299564628319,0.719522785963312
"4943","TADA3",393.514049266783,-0.266304172732554,0.216362626130549,-
1.23082335195853,0.218388939659008,0.719671745520044
"4944","AL662884.5",33.3183068820673,-0.513308529916674,0.417111995636
547,-1.23062519248175,0.218463078508227,0.719770446161106
"4945","IDO1",14.8333345418185,0.625480227141174,0.508387511399158,1.2
3032177840042,0.218576632069122,0.719853368332779
"4946","BMF",45.6817550403433,0.407854751912384,0.331481600746486,1.23
039936754833,0.218547590081013,0.719853368332779
"4947","SLC25A38",105.110267993888,-0.265440154288567,0.21582487412678
1,-1.22988675592898,0.218739514406827,0.720098615637189
"4948","TOMM22",455.679940636639,-0.270472971753308,0.219910599134614,

-1.22992239945535,0.218726165379081,0.720098615637189
"4949","SPG11",192.881290652316,0.26077748863637,0.212080435567224,1.2
2961596122199,0.218840950141496,0.720286974510979
"4950","C5orf58",30.318641667043,0.616291642048074,0.501434888465891,1
.22905616706005,0.219050747944264,0.720551821610766
"4951","RPL36",12043.1057240412,-0.296149606392291,0.240943113139555,-
1.22912667033052,0.219024317014986,0.720551821610766
"4952","ZNF264",229.474842411417,0.319643550183214,0.260091893017494,1
.22896391146538,0.219085337048889,0.720551821610766
"4953","FITM2",48.3370481380372,0.326943753525476,0.26603953944405,1.2
289291817626,0.219098359164965,0.720551821610766
"4954","ZNF329",36.8757214750403,0.334878738597919,0.27256513414896 9,1
.22861913224342,0.219214638773482,0.720788706294155
"4955","PNPLA2",244.848611455835,0.196845304986729,0.160248875446438,1
.22837245776819,0.219307182128986,0.720947465125944
"4956","SEPTIN10",725.034373744618,0.184940345952081,0.150689015225586
,1.22729812571421,0.219710559820265,0.721178874631733
"4957","SPEGNB",6.08426334913986,-0.753425427121214,0.613918517796299,
-1.2272401064325,0.219732359369762,0.721178874631733
"4958","DNAH1",7.85029207787203,0.708523702089042,0.577551958225163,1.
22677049570806,0.219908863054566,0.721178874631733
"4959","CD14",156.760138768528,-0.623541554799691,0.508230768213763,-1
.22688666998891,0.219865189360841,0.721178874631733
"4960","AL441883.1",2.94922907792684,-1.0357438566362,0.84371517279901
8,-1.22759894574389,0.219597557780085,0.721178874631733
"4961","HIST1H2BC",15.868176041851,0.579972416224753,0.472737570009984
,1.22683800276865,0.219883484195473,0.721178874631733
"4962","IKBKB",91.8830394488521,0.337422667842212,0.27502044049533,1.2
2690032506126,0.219860056383479,0.721178874631733
"4963","TRMO",64.6054448950578,-0.304335593390225,0.247994917785376,-1
.2271847992208,0.219753141359961,0.721178874631733
"4964","FNBP1",378.273894895536,0.264726680007795,0.215608805297243,1.
22781015201507,0.219518243779668,0.721178874631733
"4965","ZFPL1",189.111887151606,-0.31005471795542,0.252616816693482,-1
.22737164537875,0.219682938550985,0.721178874631733
"4966","AC004494.1",5.16553454668138,0.909513176645741,0.7408410228973
78,1.22767658449676,0.219568399815547,0.721178874631733
"4967","ARVCF",30.4876273182901,0.470167562288315,0.383218695316316,1.
22689098427265,0.219863567601024,0.721178874631733
"4968","AL590399.1",13.0236697075056,0.555058795974607,0.4525111854926
35,1.22661895168477,0.219965842741844,0.721214649780589
"4969","ZNF30",12.1715745641357,0.561374128015621,0.457721390816037,1.
22645377576694,0.220027960008531,0.721214649780589
"4970","RBM42",340.19680858676,-0.256950361493362,0.209517955386883,-1
.2263882635686,0.220052600491714,0.721214649780589
"4971","LIPE",11.1407015740506,0.709486250305333,0.578618114309212,1.2
2617358973001,0.220133357604257,0.721334190709263
"4972","ZNF562",51.2876957592104,0.415626646119002,0.339077242935424,1
.2257580087678,0.220289753413198,0.7217014869967
"4973","CDK7",65.9799684708184,-0.316617432991963,0.258342204720031,-1

.22557378239876,0.220359108914641,0.721783536117151
"4974","SPAG8",18.7067158476351,0.435381252230506,0.355465213715798,1.22482098228212,0.220642677520412,0.722452706025918
"4975","AP1M1",236.335729611122,0.255872500605589,0.20891030839467,1.22479595464576,0.220652109551166,0.722452706025918
"4976","AL049828.1",4.87632695626213,0.872785379169985,0.712687049619042,1.22464043599013,0.220710725511209,0.722499398684099
"4977","TTC7A",129.157959617989,0.291173390302861,0.237793542250473,1.22447980524282,0.220771279972049,0.722552417011193
"4978","KDM4A-AS1",15.3932506315124,0.726186596316444,0.593252971884033,1.22407578340526,0.220923640487055,0.722905821593742
"4979","CC2D1B",27.4144406308602,-0.407421640988781,0.333462159319241,-1.22179272700845,0.221786019623878,0.723112985118764
"4980","NOTCH2NLC",43.027925801723,0.419522824055678,0.343260303954785,1.22217110228667,0.221642929416373,0.723112985118764
"4981","FAM20B",144.164709689528,0.221919095344273,0.181518626961711,1.22256927048641,0.22149242555471,0.723112985118764
"4982","TSNAX",383.365253623153,-0.237909307596688,0.194717614673507,-1.22181708108741,0.221776807647801,0.723112985118764
"4983","PPP1CB",812.501762260078,-0.299894942952728,0.245208648724385,-1.22301943472561,0.221322355892754,0.723112985118764
"4984","SLC2A12",26.5248716046792,0.509063562226135,0.416204753997285,1.22310847566497,0.221288727787436,0.723112985118764
"4985","TRIM73",49.5205133962181,0.329914496914251,0.269902178006127,1.22234840545363,0.221575901427932,0.723112985118764
"4986","ZYX",539.582630395223,0.304102858709454,0.248754081096723,1.22250399820057,0.221517092849522,0.723112985118764
"4987","FCN1",18.3943061149815,-0.681976913116817,0.558171344633028,-1.22180566894774,0.221781124276923,0.723112985118764
"4988","CACUL1",287.643774706711,0.171774469354045,0.140492386517582,1.22266034204315,0.221458011649617,0.723112985118764
"4989","SLC35C1",114.938007349717,0.245534625879459,0.200890472386867,1.22223131322335,0.221620165543424,0.723112985118764
"4990","NCKAP5L",200.520074663092,0.434490107671817,0.355535224271887,1.22207330809943,0.221679905911558,0.723112985118764
"4991","KATNAL1",209.21624453695,0.200633846561909,0.164147961327098,1.22227437331437,0.221603886896464,0.723112985118764
"4992","HCFC1R1",865.783667135123,-0.314128671655402,0.257004901198529,-1.22226724156029,0.221606582960381,0.723112985118764
"4993","AC108134.2",58.4800361077966,0.551811910947505,0.451020618219295,1.22347380287436,0.221150792939059,0.723112985118764
"4994","TVP23B",76.3055285538333,-0.347390195372843,0.284010889577757,-1.22315801302307,0.221270020591214,0.723112985118764
"4995","ICAM5",3.09451540498531,1.50616392400658,1.23116490981926,1.22336488962123,0.221191908346235,0.723112985118764
"4996","CCDC124",409.180639544982,-0.286947513012485,0.234591992183078,-1.22317693090115,0.221262876777834,0.723112985118764
"4997","AC067852.5",2.65446538561867,-1.04930872686567,0.859130968048713,-1.2213606142599,0.221949512663168,0.723460688498195

"4998","POLR2E",750.15880446298,-0.276684317847221,0.226553449603975,-1.22127611974515,0.221981491872674,0.723460688498195
"4999","ZNF260",87.5737134156069,0.268235020535825,0.21965726594071,1.22115250495846,0.222028283141436,0.723468434505072
"5000","MFAP2",494.317635204259,-0.405154311508164,0.331856569763216,-1.22087175130282,0.222134581618127,0.72367003999553 4
"5001","AAK1",355.650629666576,0.266450548987885,0.218290689366053,1.22062260081589,0.222228945075468,0.723818776080441
"5002","AC090630.1",5.66311916521258,-1.14276906804988,0.936299505469391,-1.22051657762757,0.222269109089224,0.723818776080441
"5003","ZBTB24",72.823925359159,-0.269517713971887,0.220892812339957,-1.22012894451765,0.222415997638216,0.723862974131649
"5004","SRPK2",465.760502532372,0.188474688708766,0.154460077146655,1.22021620207929,0.222382926454622,0.723862974131649
"5005","CLEC14A",111.713326885723,-0.419152961883404,0.343516690101972,-1.22018223265653,0.222395800668309,0.723862974131649
"5006","SP5",125.051935229824,-0.544652191019065,0.446483777528955,-1.21987005672058,0.222514138519437,0.723977668151613
"5007","PCMTD2",391.459069648249,0.250450074229717,0.205320351735737,1.21980150585397,0.222540130421458,0.723977668151613
"5008","ZNF48",22.7836696127111,-0.400122652899218,0.328117754154556,-1.21944834692104,0.222674069406383,0.724268753306822
"5009","LYPLAL1-AS1",22.0617022214513,0.915107869618923,0.751552801961132,1.21762285661234,0.223367324547563,0.724342457531716
"5010","GCC2",960.218110373495,0.19357557594579,0.158924742833683,1.21803296638567,0.223211445385738,0.724342457531716
"5011","AC018816.1",3.3981161049017,-1.20149097150383,0.986811600492857,-1.21754848737464,0.223395599987968,0.724342457531716
"5012","DPH3",187.253693605305,-0.338923753700484,0.278073029807666,-1.2188300099974,0.22290871868775,0.724342457531716
"5013","KIF9-AS1",12.0909995266332,0.569195050737469,0.467337614285671,1.2179525750511,0.22324199529893,0.724342457531716
"5014","GMPPB",76.947447733815,-0.371175575721535,0.304508353736962,-1.21893396738193,0.222869256101187,0.724342457531716
"5015","RBP1",40.6814661831931,-0.572796164381156,0.469942285992612,-1.2188649148082,0.222895468141949,0.724342457531716
"5016","NPY5R",29.690750352182,0.710607959308035,0.583589694045868,1.21764994577198,0.223357025791895,0.724342457531716
"5017","EFNA5",224.895251937307,0.248974455343986,0.204214542114081,1.21918083191598,0.222775565496152,0.724342457531716
"5018","KLHL3",17.9707775065549,0.640010931034714,0.525399228860459,1.21814212103592,0.223169969769176,0.724342457531716
"5019","AL359715.2",2.96039385493817,1.16581593099803,0.956971131620487,1.21823521366198,0.223134601615215,0.724342457531716
"5020","AL133482.1",90.0369896179454,0.568804699608575,0.467184849304178,1.21751529497532,0.223408220678945,0.724342457531716
"5021","SGPP1",112.821203109717,0.234161826361303,0.192302904366074,1.21767181381486,0.223348712247993,0.724342457531716

"5022","NDUFAB1",753.512570900116,-0.323193925895879,0.26531975973865,
-1.21812987549151,0.223174622447983,0.724342457531716
"5023","MIR99AHG",1146.45910800288,0.215473599553785,0.176885046190862
,1.21815610869268,0.223164655261942,0.724342457531716
"5024","HSD17B10",385.731660505094,-0.348961397423732,0.28644740560751
3,-1.21823898765512,0.223133167867984,0.724342457531716
"5025","DDR2",2041.40012972907,0.277300373998027,0.22781051933641,1.21
724130565075,0.223512418667978,0.724399481858846
"5026","AC010245.2",8.47618394683031,0.652971711621079,0.5364384197251
11,1.21723517110442,0.223514752030362,0.724399481858846
"5027","CHCHD6",105.08657401778,-0.3257542291519,0.267780999540449,-1.
21649493321386,0.223796440006008,0.724915624565439
"5028","ADD1",850.70422155046,0.181920299075348,0.149540346794266,1.21
652987287524,0.22378313846296,0.724915624565439
"5029","ARHGDIB",275.361552336815,-0.343007158145142,0.281970236126608
,-1.21646583290843,0.223807518935453,0.724915624565439
"5030","EPAS1",198.111816783155,0.354621693081969,0.291597473572781,1.
21613431260905,0.223933761447862,0.72503618370587
"5031","MT1H",16.3602495025837,-0.870534698089277,0.715769393498576,-1
.2162223000822,0.223900250966552,0.72503618370587
"5032","SCO2",162.921330825317,-0.339797534395456,0.279479003018738,-1
.2158249125165,0.224051626539294,0.725273637658696
"5033","TARDBP",289.267957373382,-0.214395450000927,0.176438074377053,
-1.21513143213498,0.224315967031007,0.725408534240237
"5034","ACADM",285.703493108061,-0.158526999480131,0.130424238143595,-
1.21547192252405,0.224186151238424,0.725408534240237
"5035","ICE2",209.439825748522,0.207684666867838,0.170878192346725,1.2
1539597309427,0.224215103158565,0.725408534240237
"5036","RGL3",6.80005659837585,-1.297208227787,1.06737091382058,-1.215
33031394281,0.224240134578943,0.725408534240237
"5037","F8A1",149.890794737715,-0.357869095138719,0.294487890823923,-1
.2152251630363,0.224280225711634,0.725408534240237
"5038","BECN1",229.045156282142,-0.200076806844857,0.16469599122557,-1
.21482499577557,0.224432845271847,0.725498435529495
"5039","PIK3IP1",406.839100384037,0.280656719008714,0.231015164459268,
1.21488439802487,0.224410185192148,0.725498435529495
"5040","NCK2",171.631755419655,-0.219231447563842,0.180487086834303,-1
.21466555535415,0.224493674836081,0.725547551137103
"5041","NPIPA5",8.2175262184262,1.3269817791698,1.09256920819984,1.214
55169083172,0.22453712353626,0.725547551137103
"5042","POT1",60.4976530105455,0.269240635671343,0.221743215189429,1.2
1420010727877,0.224671319564019,0.725581874725776
"5043","ILK",726.962526110465,-0.246982320424467,0.203427797590777,-1.
21410310365403,0.22470835499073,0.725581874725776
"5044","IMPA2",241.622171798299,-0.440875519844313,0.363142328454201,-
1.21405709359468,0.22472592289223,0.725581874725776
"5045","USP16",601.884161853147,-0.191619909263614,0.157834236132861,-
1.21405795066105,0.224725595631834,0.725581874725776
"5046","AC008467.1",16.6198376173837,0.613428320813877,0.5053790965150
64,1.21379836452257,0.224824731017761,0.72575704390573

"5047","PICK1",25.7673322788874,-0.399790453327716,0.329434335077458,-1.21356644028533,0.224913328807976,0.725899190202717
"5048","UBA6",153.702830361707,0.244988881019664,0.201901305217597,1.21340909983534,0.224973448911852,0.725949387742703
"5049","CDC14A",57.6287652805627,0.322860436868682,0.266531917740563,1.2113387379854,0.225765607927788,0.72688236301921
"5050","MMADHC",371.275724859826,-0.204176175292454,0.168524939546759,-1.21154872294616,0.225685173153003,0.72688236301921
"5051","DENND6A-AS1",3.38241517574845,-1.08600363192949,0.896638152404155,-1.21119498319092,0.225820685025267,0.72688236301921
"5052","NOA1",244.946985133122,-0.304695091935435,0.251391954184768,-1.21203199570775,0.225500133168813,0.72688236301921
"5053","IFT22",284.664798570855,-0.281387566419588,0.232278858209259,-1.21142134324634,0.225733963527198,0.72688236301921
"5054","ARFGEF1",205.319672516342,0.249447918430547,0.205918602325027,1.21139088753532,0.225745630124063,0.72688236301921
"5055","COQ10A",74.8203413500077,-0.314635219859947,0.259627404253321,-1.21187214718272,0.225561325470023,0.72688236301921
"5056","ACYP1",127.502490868115,-0.327590663083973,0.270210700783312,-1.21235266454779,0.225377412293967,0.72688236301921
"5057","ARL4D",280.726465202152,0.45423528556076,0.374855692480982,1.21176040452902,0.225604109196038,0.72688236301921
"5058","CBX1",455.246088089113,0.203299742274959,0.167730570795679,1.21206135119285,0.225488896757753,0.72688236301921
"5059","HOXB13",3.1508243051868,-1.33228060836941,1.09957229366716,-1.21163530223752,0.225652014898104,0.72688236301921
"5060","TXNL4A",437.796493072824,-0.270254327778516,0.223101976455952,-1.21134887315475,0.225761725179483,0.72688236301921
"5061","ALG12",98.0741780338318,-0.24675142474919,0.203735261606099,-1.21113754587194,0.225842693795827,0.72688236301921
"5062","RALGAPA2",64.397250084702,0.425214154392844,0.351156402211121,1.21089677339044,0.22593496939991,0.727035700623297
"5063","PDAP1",959.388036026529,-0.251788128888899,0.2079594431356,-1.21075592958152,0.225988960000012,0.727065804748212
"5064","CCNT2",162.594854906733,0.247207453248137,0.204244864116418,1.21034844287311,0.226145216465604,0.727259705268068
"5065","GOLGA6L4",74.5918887955823,0.406759819620575,0.336095838890014,1.21024949598881,0.226183170660448,0.727259705268068
"5066","FAM104B",152.60736430716,-0.315157925314427,0.260359869327284,-1.21047043896868,0.226098427276179,0.727259705268068
"5067","S100A12",2.49578477445006,1.47587520361185,1.21975725971643,1.20997451899156,0.22628867061913,0.727320801870025
"5068","GPR107",168.018768402612,0.210375419723008,0.173870787169327,1.20995265017194,0.226297062499116,0.727320801870025
"5069","FKRP",53.1833426228577,0.3024272323014,0.249970671399871,1.20985086213421,0.226336125279585,0.727320801870025
"5070","BAZ2A",262.255613203384,0.304046147063641,0.251333009196445,1.2097342407817,0.226380886494327,0.727321155839467
"5071","STARD5",49.5266093837486,0.424409395144022,0.351014372890024,1

.20909406543587,0.226626708931469,0.727680359113878
"5072","AC005632.3",11.8111351201741,0.654887592951789,0.5415847989980
6,1.20920600829887,0.226583709997195,0.727680359113878
"5073","FBXO46",30.3535971320895,0.340493422414127,0.281573230254596,1
.20925352920182,0.226565458264695,0.727680359113878
"5074","KLF6",4587.83818769637,-0.394715402476094,0.326522954449799,-1
.20884426989582,0.226722680133519,0.72770162299406
"5075","ERG28",170.265824056112,-0.292049446770096,0.241579283969506,-
1.20891759413841,0.226694506026119,0.727701622994067
"5076","GPC3",11898.4224950767,0.21655110273648,0.179156529920161,1.20
872570390252,0.226768243215658,0.727704474732044
"5077","HNRNPR",797.104640143696,-0.210983159068369,0.174744555500602,
-1.20738044435177,0.227285663159354,0.727990897081676
"5078","NBPF11",77.0213999996005,0.363626547695346,0.301188364350296,1
.20730609391149,0.227314284692944,0.727990897081676
"5079","UCN",7.25635263963172,0.799730398131291,0.662312113299256,1.20
748266877907,0.227246315604695,0.727990897081676
"5080","TMEM129",156.863187059884,-0.258060476718588,0.213704316808347
,-1.20755855835153,0.227217107830273,0.727990897081676
"5081","PRR3",52.6741857320384,0.274540216182581,0.227236195440443,1.2
0817115270941,0.226981435388072,0.727990897081676
"5082","FIS1",1559.36358107356,-0.305881327003964,0.253361636541273,-1
.20729140835864,0.227319938265867,0.727990897081676
"5083","GPAT4",157.524648333546,-0.242796332898907,0.200990568115398,-
1.20799863981431,0.227047785541788,0.727990897081676
"5084","BLOC1S2",267.017437853259,-0.350069434996075,0.289841032687295
,-1.20779805312714,0.227124950538882,0.727990897081676
"5085","FAM111A",186.815848614228,0.233280411179895,0.193066675198483,
1.20828936915224,0.226935976167568,0.727990897081676
"5086","PCBP2",2560.1831163541,-0.209677415957382,0.173686796857332,-1
.20721563038331,0.227349112496439,0.727990897081676
"5087","AC130650.2",9.08686367510081,0.756608353099313,0.6263353206007
28,1.20799247338253,0.227050157467989,0.727990897081676
"5088","MAPK14",274.700215012843,0.228183438732791,0.189071456989506,1
.20686349153937,0.227484719624333,0.728138848096041
"5089","KMT2A",845.376879146009,0.269088043736037,0.222947874362327,1.
2069549642744,0.227449488332668,0.728138848096041
"5090","SHB",9.08411395764903,-0.689956090948127,0.571786585430007,-1.
20666715262173,0.227560353911502,0.728237839855492
"5091","OLFML3",2945.0320993519,0.283762719164633,0.235223202085776,1.
20635514119545,0.227680584790413,0.72827014462805
"5092","RANBP9",224.915362183494,0.297326863044103,0.246479826289258,1
.20629289431246,0.227704576498905,0.72827014462805
"5093","AUXG01000058.1",154.044938521697,0.977414279213889,0.810206655
876125,1.20637651162832,0.227672348436836,0.72827014462805
"5094","CHSY3",22.8744474719847,0.63462459348214,0.526338595350554,1.2
0573448173502,0.227919885071526,0.728815667045562
"5095","ANKRD9",136.590850700341,-0.391442986475,0.32470242443183,-1.2
0554377491931,0.227993449606411,0.728907811705364
"5096","CSF2RB",2.9395852188856,-1.23967181794218,1.02844291401254,-1.

20538709640724,0.228053900490474,0.728958003353478
"5097","GFRA2",2.39393010699268,-1.50137028707904,1.24583477668601,-1.
20511187773452,0.22816011507615,0.729011399465558
"5098","ZW10",51.5159696620689,0.299205249306445,0.248279013017066,1.2
0511695962751,0.22815815351219,0.729011399465558
"5099","JARID2",69.8910311033794,0.298191922256129,0.247532631163517,1
.20465702180149,0.228335733943542,0.72935450230534
"5100","ICOSLG",32.4518538994931,-0.512408584055984,0.425375895703821,
-1.20460183388662,0.228357048422692,0.72935450230534
"5101","PITX3",3.67666031358675,1.18223272019693,0.981545487910334,1.2
0446045013548,0.228411659614743,0.729385909324554
"5102","LINC02817",2.47155497064339,1.42078218080804,1.18013891444248,
1.20391096626048,0.228623992757988,0.729553962040463
"5103","ZRANB3",24.8027056365499,0.417607826217783,0.347085941852365,1
.20318277366421,0.22890559953545,0.729553962040463
"5104","STK16",176.086331402279,-0.303094202812653,0.251956432226044,-
1.2029627508804,0.228990735342914,0.729553962040463
"5105","ZNF717",42.8999973613051,0.404603505644808,0.336297508627899,1
.20311181398756,0.228933054272505,0.729553962040463
"5106","AC034206.1",2.58015106781512,1.27752052209693,1.06226857283559
,1.2026342064199,0.229117904574551,0.729553962040463
"5107","TNRC18",346.985227290105,0.252717360090316,0.209995907039786,1
.20343945581014,0.22880630720198,0.729553962040463
"5108","HIPK2",103.96267093121,0.403450547500467,0.335385683191645,1.2
0294505019145,0.228997585442338,0.729553962040463
"5109","OLFML2A",189.84478223553,0.284502498136828,0.236545429656972,1
.20273935771831,0.229077198344636,0.729553962040463
"5110","SLC24A1",16.7667313945317,0.522492103224204,0.434097790298579,
1.20362765003901,0.22873352752868,0.729553962040463
"5111","RSL1D1",966.89798987034,-0.238030273614071,0.197928401599203,-
1.20260797182646,0.229128061327563,0.729553962040463
"5112","PSMD8",1171.37717869111,-0.311708147851628,0.259105751130224,-
1.20301516462661,0.228970452277532,0.729553962040463
"5113","NCOA6",155.612417229908,0.244950220410471,0.203488132420096,1.
20375678668463,0.228683596517238,0.729553962040463
"5114","ITGB2",48.9904698385419,-0.547481961537844,0.455232843510107,-
1.20264161372111,0.229115036887386,0.729553962040463
"5115","SMDT1",1095.23368869908,-0.333118414671506,0.277001466719509,-
1.20258718705205,0.229136108404384,0.729553962040463
"5116","RAB33A",7.97547869295439,-0.85192368551666,0.707719229809342,-
1.20375941423291,0.228682580649599,0.729553962040463
"5117","DESI2",495.407466319076,-0.201485326740199,0.167580279634677,-
1.2023212228756,0.229239097407418,0.729559866898135
"5118","NOXA1",9.71650951991728,-0.580221564610645,0.482603695001549,-
1.20227335724975,0.22925763585613,0.729559866898135
"5119","ZDHHC5",141.869360596489,0.201733851080664,0.167854945469425,1
.20183442028766,0.229427686735656,0.729559866898135
"5120","RFC3",55.6180009275451,0.366330688098599,0.304802764634881,1.2
0186143500838,0.229417218229293,0.729559866898135
"5121","AC110597.1",6.49421852838258,0.874607017986908,0.7275306298321

29,1.20215834512523,0.22930218463417,0.729559866898135
"5122","ZNF441",54.2475380101314,0.467214815352808,0.388771264426298,1
.20177301694936,0.229451482480149,0.729559866898135
"5123","C19orf48",117.400483062309,-0.372799498031731,0.31016313689719
8,-1.20194650389835,0.229384255303469,0.729559866898135
"5124","CXCL9",44.4512932921039,-0.79638463410644,0.662817715175494,-1
.20151380367916,0.22955195521199,0.729736884943033
"5125","SLC25A34",8.74193157911945,0.966214603488711,0.804713536569629
,1.2006938613305,0.229869976232492,0.730462747337313
"5126","OSTF1",258.351856904554,-0.348239131519,0.290017698480427,-1.2
0075131050149,0.229847683920676,0.730462747337313
"5127","TAFA5",21.3945748852557,0.668866589197074,0.557154805820844,1.
20050402905822,0.229943648809406,0.730554338883638
"5128","ACAD8",80.4572176873906,-0.298850962837238,0.248991228215248,-
1.20024695239018,0.23004344523319,0.730728876638736
"5129","PLCL2",117.131342414075,0.277681819732681,0.23145905034946,1.1
9970171532905,0.230255206624152,0.730974936632865
"5130","AP000842.1",3.66800616893188,1.50904648674776,1.25764934098474
,1.19989446785395,0.230180328780868,0.730974936632865
"5131","ARID2",149.007498235019,0.277037049576106,0.23092177068612,1.1
9970087165436,0.230255534401328,0.730974936632865
"5132","TDP2",156.202382617516,-0.224959609539013,0.187605482224281,-1
.19910999866238,0.230485176739122,0.731276401227807
"5133","ZFP36L1",8764.82606650932,0.310983280443823,0.259336881249849,
1.19914791504035,0.230470435680928,0.731276401227807
"5134","RASD1",3099.05959629907,0.459514996604766,0.383144315181606,1.
19932615047926,0.23040150621911,0.731276401227807
"5135","PCDHGA10",53.2085011887992,0.393124881387578,0.327907280456134
,1.19889037181707,0.230570576036619,0.731404890566795
"5136","PMM1",371.552955922982,-0.283223468448474,0.236271903470597,-1
.19871835917942,0.23063747681776,0.731474661192463
"5137","RGS5",12.3114228273019,0.609776619239083,0.509246643646115,1.1
9740920602479,0.231147097161862,0.731768818775101
"5138","CYS1",314.490647866482,-0.402330455915163,0.335902589679611,-1
.19775931557691,0.231010730032363,0.731768818775101
"5139","PSMD14",310.411567457417,-0.30329059352094,0.253304753878337,-
1.19733478696026,0.231176090641108,0.731768818775101
"5140","TYW5",62.2610951295312,0.347992741941746,0.290809718584939,1.1
9663381139755,0.231449315735158,0.731768818775101
"5141","IRS1",210.381648566945,0.314469998888334,0.262798077112727,1.1
9662214557773,0.231453864758552,0.731768818775101
"5142","TMEM108",13.9322851765363,1.02054864539816,0.852474634461706,1
.19716013138922,0.231244146156689,0.731768818775101
"5143","AC104825.1",29.5355360983619,0.400415346031035,0.3346326573377
86,1.19658179574161,0.231469599448767,0.731768818775101
"5144","WWC2",195.79540393164,0.242860085059308,0.202909198579745,1.19
689046509078,0.231349251080887,0.731768818775101
"5145","AC011379.1",29.5022982881075,-0.526823306718786,0.439776683964
458,-1.19793369209488,0.230942831977827,0.731768818775101
"5146","LYRM2",294.801096917767,-0.189494928192942,0.158339520567556,-

1.19676330655614,0.231398824062637,0.731768818775101
"5147","NACAD",45.7235662419019,-0.369643934624191,0.308639261263965,-1.19765687978384,0.23105062269835,0.731768818775101
"5148","DEPP1",6651.37156165358,0.526910717849306,0.439975260099042,1.19759169579375,0.231076010544671,0.731768818775101
"5149","NAT10",84.9822748398821,0.292854501572523,0.244758818626822,1.19650234960086,0.231500582226677,0.731768818775101
"5150","LAMTOR1",1132.65477702804,-0.289381078225596,0.241761109784526,-1.19697116911613,0.231317792386014,0.731768818775101
"5151","SPART-AS1",8.9891297652597,0.699245989997382,0.584456311196345,1.19640420781165,0.231538860087597,0.731768818775101
"5152","PPP1R3E",33.2272787104225,0.385245009089476,0.321831499303361,1.19703947538815,0.231291168750631,0.731768818775101
"5153","NUFIP2",664.148525373378,0.300167357461729,0.250763367093927,1.19701438427925,0.23130094822237,0.731768818775101
"5154","VMA21",187.786427010726,-0.212194643624678,0.177203454249747,-1.19746335940842,0.231126000764609,0.731768818775101
"5155","UBE4B",145.059494741658,0.269180442959632,0.225922761588833,1.19147110749968,0.233468692508369,0.731989755720869
"5156","ZMYM6",80.4715399107311,0.292524194397706,0.244933192570919,1.1943019699668,0.232359867320672,0.731989755720869
"5157","ADAMTSL4-AS1",52.3220046677959,0.580916288019773,0.487582223881164,1.19142220443491,0.233487880336933,0.731989755720869
"5158","TPM3",981.187339844299,-0.1702154833258,0.142560861722455,-1.19398466920875,0.232483965075814,0.731989755720869
"5159","BX284613.2",6.91085421693459,0.715155988833197,0.60048390337631,1.19096612717198,0.233666882718765,0.731989755720869
"5160","NENF",2080.57328690315,-0.26245954332808,0.219828403912832,-1.19392916773463,0.23250567678409,0.731989755720869
"5161","LRATD1",4.99564589750429,-0.828646873896606,0.696065451499611,-1.19047263746729,0.233860678382586,0.731989755720869
"5162","HTRA2",252.13197283874,-0.295117860131179,0.246975570756459,-1.19492733320654,0.232115422589272,0.731989755720869
"5163","CNNM4",26.5200209358473,0.462565182989164,0.387262479339375,1.19444874643742,0.232302478287747,0.731989755720869
"5164","THUMPD3-AS1",226.361341029011,-0.351119681966403,0.294474452059191,-1.19236042213885,0.233119951983571,0.731989755720869
"5165","STT3B",318.998389364817,0.225739397088761,0.189348449383852,1.19219036555793,0.23318661028231,0.731989755720869
"5166","PTH1R",199.213107687865,0.334288110829041,0.280382593219962,1.1922570049375,0.233160487555949,0.731989755720869
"5167","SEMA3G",2.69178026258731,-1.18677501928313,0.994333707397678,-1.19353795456568,0.232658756888577,0.731989755720869
"5168","ALG1L",12.9366544244455,0.653135422436857,0.54796661055591,1.19192558425093,0.233290425417322,0.731989755720869
"5169","C3orf70",21.3670546632632,-0.669011293362319,0.560555127772161,-1.19347992769453,0.232681468653072,0.731989755720869

"5170","RPL35A",19413.011552994,-0.299219523065495,0.250542312782819,-1.19428738300533,0.232365571315864,0.731989755720869
"5171","NOP14-AS1",59.877753948913,-0.318410620725067,0.266390325059758,-1.19527847212034,0.231978247917996,0.731989755720869
"5172","LRPAP1",1328.59661718043,-0.222987066550782,0.187219239800555,-1.19104781532246,0.233634814393245,0.731989755720869
"5173","DCLK2",13.9977693875866,0.507253533054807,0.426114848132778,1.19041506128589,0.233883296230677,0.731989755720869
"5174","PURA",857.624136679498,-0.229290069036462,0.192375062247072,-1.1918908114087,0.23330406154555,0.731989755720869
"5175","MRNIP",203.75777424521,0.281250921477506,0.235656811319956,1.19347673382394,0.232682718782288,0.731989755720869
"5176","PPIL4",459.468186388799,-0.217118143867049,0.181726224351659,-1.19475405732804,0.23218313513675,0.731989755720869
"5177","AC004889.1",184.864000644193,0.32489698443532,0.272265650206391,1.19330875631587,0.232748474433395,0.731989755720869
"5178","ATP6V1B2",307.309905390366,-0.180209300152519,0.151284735819852,-1.19119287994204,0.233577874050465,0.731989755720869
"5179","ZBTB10",229.188909060447,0.260102710706231,0.217968388159512,1.19330473974916,0.232750046900255,0.731989755720869
"5180","ZBTB6",57.8061595531726,0.309253611498693,0.259110162798629,1.1935217366948,0.232665104418036,0.731989755720869
"5181","PGAM1",832.347780127332,-0.294987853999491,0.247780803334388,-1.19051940275371,0.23384230855626,0.731989755720869
"5182","SHOC2",277.213802084334,0.200509356585117,0.168128016479463,1.19259931083294,0.233026335894477,0.731989755720869
"5183","EI24",556.978997419027,-0.205258950705807,0.172191204290218,-1.19204085685965,0.233245225444649,0.731989755720869
"5184","PDE3A",61.6450790535015,0.373674440911545,0.312437323315704,1.19599808673935,0.231697305697735,0.731989755720869
"5185","DDX11-AS1",2.85453181422962,-1.08098765110872,0.90628980236684,-1.19276157393103,0.232962763176916,0.731989755720869
"5186","ATP5F1B",1205.83959668238,-0.235214733615994,0.197613054632018,-1.19027932670741,0.233936623444358,0.731989755720869
"5187","ZNF664",140.106525303768,0.223499793739787,0.187676914971017,1.19087525375352,0.233702560571841,0.731989755720869
"5188","SPART",900.302043862852,0.210288177113013,0.175995803428077,1.19484767827973,0.232146548308492,0.731989755720869
"5189","PPIP5K1",9.39422694606018,-0.628983618145608,0.526698188523279,-1.19420121779631,0.232399266853409,0.731989755720869
"5190","VPS33B",32.5742023735256,0.343869229694583,0.287599204693893,1.19565431365007,0.231831486788938,0.731989755720869
"5191","NAGPA",81.4514484264079,0.283978477406544,0.238146071141149,1.19245501740077,0.233082878649542,0.731989755720869
"5192","DYNC1LI2",754.402517514864,-0.275716859761371,0.231040945210996,-1.1933679526353,0.232725300228516,0.731989755720869
"5193","AC002553.2",5.58682162299765,0.777449528888041,0.651394171618742,1.19351624985536,0.232667251945685,0.731989755720869

"5194","UNC119",66.3535798347999,-0.265706369315944,0.222870105697869,-1.19220282363102,0.233181726543181,0.731989755720869
"5195","MILR1",45.6273388183905,0.502284415254261,0.420258691296824,1.19517912575305,0.232017052374956,0.731989755720869
"5196","AC068473.5",13.1433013754263,0.594886370556685,0.498913429279393,1.19236391655344,0.23311858239451,0.731989755720869
"5197","NDUFS7",664.916769069047,-0.296242361376996,0.248468273873683,-1.19227439688175,0.233153670231934,0.731989755720869
"5198","SMIM17",6.43776785015859,0.795205376413592,0.667452126509417,1.19140436419356,0.233494880493363,0.731989755720869
"5199","ZNF343",40.2813974957036,0.426213294220328,0.357911842285964,1.19083317137015,0.233719083863074,0.731989755720869
"5200","L3MBTL1",24.484702124496,0.437709693674496,0.367679292849887,1.19046599084164,0.233863289320102,0.731989755720869
"5201","L3MBTL2",85.7316271738667,-0.253731056380333,0.212385849291775,-1.19467025334516,0.232215888990112,0.731989755720869
"5202","ASMTL",228.946702719632,-0.2246491237011,0.188632500477968,-1.19093540684596,0.233678943407557,0.731989755720869
"5203","ZNF630",10.0423584026998,0.586623250508233,0.491232749407647,1.19418595607808,0.232405235421825,0.731989755720869
"5204","RTL5",58.8587372525666,0.401505919574043,0.33626539499042,1.19401498208129,0.23247210754338,0.731989755720869
"5205","PHKA1",7.11557742723339,-0.674049368978837,0.566305895272786,-1.19025666977059,0.233945525709549,0.731989755720869
"5206","ARMCX6",276.141929714961,0.334367368412695,0.280017152657728,1.19409602318684,0.232440408678322,0.731989755720869
"5207","PIK3R4",76.2895237186768,0.27228236599525,0.228805002954193,1.19001928489195,0.234038812380018,0.732141005350126
"5208","ZBTB5",28.4787681987923,0.395309985030253,0.332249088129241,1.18980006011779,0.234124985965977,0.732269949385521
"5209","CISH",40.7312507702915,-0.627781124473173,0.527815632172562,-1.18939471703242,0.234284378752214,0.73248718723509
"5210","DCAF10",252.451365933735,0.239314243645044,0.201204741389468,1.18940658153681,0.234279712189278,0.73248718723509
"5211","SMG7",109.562137025414,0.32079191471215,0.270094221044692,1.18770373342815,0.234950152383031,0.732846869553319
"5212","BEND3",3.16217739457591,1.12740298657644,0.949118703133254,1.18784192414988,0.23489569375614,0.732846869553319
"5213","ZNF655",308.445360887383,0.217694447568546,0.183132088208809,1.18872912823628,0.234546274517903,0.732846869553319
"5214","PAXX",249.461493507274,-0.269163201164284,0.226610634983518,-1.18777832816125,0.234920754749699,0.732846869553319
"5215","CELF2",2058.31072046919,0.301412750776747,0.253564098786328,1.18870436398309,0.234556022751566,0.732846869553319
"5216","ZWINT",16.1054023997775,-0.496672722812836,0.417930947471104,-1.18840857758488,0.234672478687937,0.732846869553319
"5217","AL354950.1",3.28140083604181,1.22880957801626,1.03457308179365,1.18774555383352,0.234933670725134,0.732846869553319
"5218","INTS6",1062.24886221055,0.286036076512796,0.240658308526913,1.18855683090122,0.234614103818104,0.732846869553319

"5219","DENND4A",130.443988936974,0.374049571701199,0.314885478965851,
1.18789082599063,0.234876424508784,0.732846869553319
"5220","NR2F2",976.401826832919,0.28233648413874,0.237683524493479,1.1
8786729008845,0.234885698439609,0.732846869553319
"5221","RPL19",25509.7744483294,-0.299383467490322,0.252103102619346,-
1.18754376435567,0.235013204652903,0.732846869553319
"5222","NXT1",228.331554673061,-0.279918150905864,0.235526219050577,-1
.18847978808574,0.23464443820876,0.732846869553319
"5223","DOP1B",37.6822326371644,0.331749719738775,0.27936748671277,1.1
8750296837463,0.235029286423141,0.732846869553319
"5224","MSL3",111.530264779467,-0.22829068170137,0.192195196227798,-1.
18780638737084,0.234909697346126,0.732846869553319
"5225","AL662844.4",85.4353797642652,-0.50242661819204,0.4233451365066
78,-1.18680144134386,0.235305950183852,0.733008167917356
"5226","SCYL1",239.722446975777,-0.222858679051176,0.187790813371042,-
1.18673898392914,0.235330592911653,0.733008167917356
"5227","CDR2",120.224084979751,-0.253074154988089,0.2132414235277,-1.1
8679640569561,0.235307936943657,0.733008167917356
"5228","AC130343.2",3.21148494636952,-0.926177414452203,0.780473066007
554,-1.1866872218794,0.235351017140879,0.733008167917356
"5229","RABEP1",330.987350646969,0.245653945797999,0.206970184162656,1
.18690499692913,0.235265096095843,0.733008167917356
"5230","SPATA20",196.230331678111,-0.252752649214642,0.212964889894318
,-1.18682778809253,0.235295555556628,0.733008167917356
"5231","RASL11B",4.20446131360117,-0.946410698086447,0.797839500933477
,-1.18621689823472,0.235536654570666,0.733411133969367
"5232","DNAJC2",232.31144509528,-0.198923018188337,0.167707417704828,-
1.18613130480877,0.235570449562756,0.733411133969367
"5233","GSTO2",4.12701164854783,0.968151631588845,0.816321536569179,1.
18599300424901,0.235625062249658,0.733440978212246
"5234","EGFR",1458.10727451571,0.235076524102485,0.19823173073698,1.18
586728385271,0.235674715003719,0.733455374989603
"5235","HAS2-
AS1",54.9232653720825,-0.415457293090444,0.350381069837733,-1.18572984
916922,0.235729002737414,0.733484188269289
"5236","TLR9",2.81051922350853,-1.13158406160253,0.954555696515772,-1.
18545629734643,0.23583708409612,0.733540244957362
"5237","MTHFS",207.877489678087,-0.285491798575651,0.24081499652335,-1
.18552333823599,0.235810592744691,0.733540244957362
"5238","ANAPC7",120.116966337503,0.199867189714426,0.168636451803838,1
.18519565358809,0.2359400980423,0.733720553075796
"5239","UTP23",278.95055193918,0.190163594041724,0.160508435827475,1.1
8475763009818,0.236113289273834,0.734101208810997
"5240","NIPSNAP3B",27.7347390264156,-0.424287671079652,0.3581730270560
71,-1.18458856203382,0.236180161575607,0.734101208810997
"5241","TPP2",227.782584886935,0.237789637967648,0.200743573558603,1.1
8454421106651,0.236197706143928,0.734101208810997
"5242","MTCH2",147.906183348564,-0.262103888341195,0.221385459238592,-
1.18392549015028,0.23644255886022,0.734441808023289
"5243","IRF9",432.699764859414,-0.368660105839399,0.311343594672317,-1

.18409407531703,0.236375825156544,0.734441808023289
"5244","RPS5",13037.8383433673,-0.25516358557627,0.215522297871452,-1.
18393125953242,0.236440274855309,0.734441808023289
"5245","AC073225.1",2.57253982857433,1.65986869539804,1.40272110919209
,1.18332053643511,0.23668213682275,0.734568209116761
"5246","HIVEP1",217.43227749418,0.236944618048034,0.200248413594424,1.
18325340907785,0.236708731638153,0.734568209116761
"5247","CCM2",256.973648165117,-0.286373764319615,0.241972897989107,-1
.18349520421294,0.236612946063881,0.734568209116761
"5248","AC067838.1",12.9144878951304,0.583636853489816,0.4932270128860
61,1.18330269478699,0.236689205200255,0.734568209116761
"5249","AC092757.3",2.90838984454237,1.11787350261507,0.94451764352088
6,1.18353903739476,0.23659558476779,0.734568209116761
"5250","ESCO1",153.265287187655,0.227021715326318,0.191891897326674,1.
1830708773484,0.236781058553806,0.734652697672943
"5251","LINC01170",3.06332399800205,-1.19437767124389,1.00999555654656
,-1.18255735235884,0.236984623017566,0.734795216792755
"5252","ZNF419",15.6813370100604,0.494133821030481,0.417912412743628,1
.18238608369264,0.237052542467059,0.734795216792755
"5253","PCNA",204.863500689081,-0.267942717893844,0.226592133769516,-1
.18248905395096,0.237011706242768,0.734795216792755
"5254","SRRD",118.065865441233,-0.29215076884068,0.247084810042995,-1.
18239064874058,0.237050731942639,0.734795216792755
"5255","PPP2R3B",60.3101665201203,-0.326858883702054,0.27638660551823,
-1.18261477646208,0.236961853609583,0.734795216792755
"5256","WDR33",447.283068575619,-0.182851001325633,0.154660847853578,-
1.18227078063573,0.237098275567143,0.734797148157001
"5257","CDKN2AIPNL",132.152370517795,-0.359602974360739,0.304202209084
154,-1.18211822144019,0.23715879529649,0.734844895679005
"5258","NAPA",329.01923273992,-0.220318769990339,0.186396627208524,-1.
1819890375155,0.237210050667765,0.73486392455824
"5259","MRPL28",276.308507850518,-0.302916506910273,0.256351920909527,
-1.18164321076876,0.237347300367323,0.73514930132788
"5260","HLX",59.1625844108823,0.334655133037155,0.283351692511626,1.18
10592344474,0.237579192828268,0.735179623919404
"5261","RNF212",155.724032253532,-0.219353183313614,0.185761705173185,
-1.18083101740002,0.237669859523375,0.735179623919404
"5262","RANBP17",11.525007581872,-0.691964384853708,0.586001758709895,
-1.18082305141386,0.237673024713585,0.735179623919404
"5263","AC068587.4",48.581329997598,0.476817156457307,0.40366487533534
5,1.18122032802876,0.237515207839636,0.735179623919404
"5264","AP3M2",71.4049456382357,0.295230209640667,0.249931192031655,1.
18124595509981,0.237505030109274,0.735179623919404
"5265","C11orf53",20.9051333432001,-0.769936072228734,0.65201058936179
4,-1.18086436752871,0.237656608568152,0.735179623919404
"5266","AC022150.4",9.41294918541701,0.58449607879844,0.49492231765924
7,1.18098549599225,0.237608485172019,0.735179623919404
"5267","COLGALT2",21.6016116470729,-0.731212616996097,0.61933031592663
7,-1.18065045128957,0.237741612639255,0.735252160296339
"5268","POU2F1",197.602079643282,0.274944071999493,0.233033144650817,1

.17984964075165,0.238060022033938,0.735386977340478
"5269","VPS54",102.46070061042,0.280984060294423,0.238141291584211,1.17990483055334,0.238038068421843,0.735386977340478
"5270","ZNF638",581.272701653856,0.260935461874065,0.221059233280618,1.18038707545333,0.237846300002988,0.735386977340478
"5271","AHCYL2",44.6261277056713,0.355763656965628,0.301495348178917,1.17999716783194,0.238001341339833,0.735386977340478
"5272","AL049840.4",12.9538642753328,0.770056235399173,0.652730500251944,1.17974605921118,0.238101228957805,0.735386977340478
"5273","GABRB3",7.86816117938594,0.703202549230476,0.595990807411846,1.17988824741141,0.238044664777735,0.735386977340478
"5274","SPIN2B",107.351717038218,-0.375010685334208,0.317736696108789,-1.18025613637592,0.237898358147525,0.735386977340478
"5275","SPATA6",432.123965047004,0.235780231564169,0.199894775508331,1.17952173069347,0.238190488841793,0.735523198624449
"5276","TRO",20.9783457532455,0.51589771548729,0.43745728289422,1.1793099250151,0.238274787592347,0.735644051382057
"5277","SBF2-AS1",41.0249360890627,0.33220183744297,0.281827825084275,1.17874037932071,0.238501571476893,0.736204680270441
"5278","PRDX6",2279.05421758575,-0.29160138129606,0.24746631404585,-1.17834777804155,0.238657987632137,0.736408403208919
"5279","RECQL4",5.55712738038508,-0.772361625290363,0.655412553696249,-1.17843581258029,0.238622907523687,0.736408403208919
"5280","UPK3BL1",238.331062274424,-0.378385429527665,0.321148725691437,-1.17822491343535,0.238706952951424,0.736419991785179
"5281","AASDHPPT",226.641258913959,0.205349256821796,0.17432243586062,1.17798524216346,0.238802489716221,0.736575223440166
"5282","KAT14",47.3007462746414,0.29076308039987,0.246878685665646,1.17775691982441,0.238893527707386,0.736716522685652
"5283","PPCS",574.865076242811,-0.274774166148105,0.233353005441493,-1.17750429495539,0.238994284274597,0.736748277166712
"5284","OTUB2",4.57375235791082,0.934771959305127,0.793809608281499,1.17757702798382,0.238965272457432,0.736748277166712
"5285","EVI5",338.740989280125,0.163328152637021,0.138753941873519,1.17710639735124,0.239153042016904,0.736958740335481
"5286","AP000525.1",28.1550221905885,0.482988372073992,0.410287221507081,1.17719574667684,0.239117385924829,0.736958740335481
"5287","TRABD2B",29.2587050081727,0.498929603783979,0.423926367822427,1.17692514939993,0.239225383054741,0.737042228972701
"5288","TDP1",29.0351397599259,0.362468359289299,0.308018620633238,1.17677417860037,0.239285651430612,0.737088497759689
"5289","LBX2",18.6472216958974,0.538253281156616,0.457725259610046,1.17593091020403,0.239622485789907,0.73719247131374
"5290","AC007620.2",14.184582630088,0.822630497288193,0.699508813347896,1.17601162643116,0.239590230112534,0.73719247131374
"5291","FRG1",569.096951410523,-0.230566935145694,0.196011465904093,-1.1762931014378,0.239477771516991,0.73719247131374
"5292","AL031118.1",3.50716914091964,-1.00754425067307,0.856551154343562,-1.17628030219073,0.239482884432498,0.73719247131374

"5293","MTURN",1048.96276281241,0.210319718891194,0.178760620382752,1.17654390794163,0.239377597370124,0.73719247131374
"5294","SFXN3",245.003704907872,-0.234849484686631,0.199719506034689,-1.17589658290983,0.239636204531049,0.73719247131374
"5295","ADAMTSL5",14.7498250140294,0.48634367605081,0.413547418438202,1.17602880435702,0.239583365895005,0.73719247131374
"5296","ZNF776",15.3103317449446,0.50227213193745,0.427209699545841,1.17570395164578,0.239713198979885,0.737213571905385
"5297","SREBF2",84.6808415991455,0.27155618061087 1,0.230983281811878,1.17565296709238,0.239733580353786,0.737213571905385
"5298","LINC01142",8.63271524704646,-0.654101774233254,0.556696988747983,-1.17496912585127,0.240007067989674,0.737223413655061
"5299","FLVCR1",36.6359726138503,-0.326928435653228,0.278245027333442,-1.17496595998981,0.240008334619239,0.737223413655061
"5300","NCKAP1",509.547070276104,0.21329987971066,0.18146519375686,1.1754313612144,0.239822182797444,0.737223413655061
"5301","UROS",305.003412405259,-0.271744046522947,0.2312538548294,-1.1750898021718,0.239958790111289,0.737223413655061
"5302","WDR59",108.618395520336,0.246297632097118,0.209608061669409,1.17503892806172,0.239979142019307,0.737223413655061
"5303","Z93943.1",4.55191197969032,0.868138893177097,0.738850448047435,1.17498594671132,0.240000338210523,0.737223413655061
"5304","SCML2",16.5184317128661,0.436867165624736,0.371916186429615,1.17463875347467,0.240139272088674,0.737486539037031
"5305","ISG15",342.779144095973,-0.447213868278398,0.380862441525813,-1.17421362549368,0.240309469748805,0.737862762739866
"5306","RHBDL2",3.76156762867857,1.28352478248448,1.0931928172885,1.17410649080924,0.240352373939329,0.737862762739866
"5307","PCDHGA4",3.95594571505218,0.900674972532251,0.767377555339472,1.17370512893593,0.240513155177558,0.738017539106262
"5308","BACE1",398.0515393243,0.199631657391675,0.170095965523843,1.17364134285531,0.240538714170001,0.738017539106262
"5309","PXN-AS1",42.9343237902976,0.33038880538536,0.281465336114586,1.17381703177423,0.240468320491137,0.738017539106262
"5310","PDPN",253.007586829915,0.548095752585341,0.467155273683201,1.17326247494538,0.240690565443814,0.738288366626954
"5311","ADGRL2",201.154274996284,0.267688612644925,0.228202609699132,1.17303046182448,0.240783590256044,0.738288366626954
"5312","FCER1A",15.3997010306349,-1.02633361881238,0.875023556286089,-1.17292113045333,0.240827435046635,0.738288366626954
"5313","RABIF",81.4996200954616,-0.303139347102737,0.258478164495919,-1.1727851274939,0.24088198370201,0.738288366626954
"5314","LTBP1",125.81046289706,0.388981899908516,0.331566385375415,1.17316446137353,0.240729860538109,0.738288366626954
"5315","ANKRD17",461.744844318873,0.238729611625345,0.203584789978948,1.17262989857952,0.240944254219957,0.738288366626954
"5316","GIPC1",270.090476720047,-0.260292219214146,0.221953895639076,-1.1727310235519,0.240903686364402,0.738288366626954
"5317","ZNF292",723.447081309818,0.240860322826467,0.205423907375524,1

.17250385266095,0.240994826236085,0.738304443212259
"5318","TOR1AIP2",411.368014813283,0.266114986416792,0.227081853113501
,1.17189014783925,0.241241163063281,0.73838360066057
"5319","RNF7",1043.31370724405,-0.279765959455234,0.238674067127602,-1
.17216739473276,0.24112985615229,0.73838360066057
"5320","ARHGEF26-
AS1",5.79285583458912,0.81523202139656,0.695639197374967,1.17191789144
845,0.241230023147816,0.73838360066057
"5321","PSMG3-
AS1",45.2572034357365,0.400850217922526,0.34215776657681,1.17153622416
033,0.241383306127911,0.73838360066057
"5322","SYPL1",913.117096592463,-0.271146446012777,0.231385879747028,-
1.17183661470276,0.241262659293715,0.73838360066057
"5323","HCFC2",204.59120118579,0.245802235113847,0.209735749715607,1.1
7196155375106,0.241212492119103,0.73838360066057
"5324","CCDC113",39.827783566322,-0.344608387654207,0.294105403058281,
-1.17171729614882,0.241310576510483,0.73838360066057
"5325","MANBAL",337.155467261449,-0.291859773228702,0.249118096583443,
-1.17157194612292,0.241368956774847,0.73838360066057
"5326","CSGALNACT1",98.3013217096414,-0.63687244011301,0.5437348520610
43,-1.17129229016482,0.241481309378344,0.738444131901931
"5327","FKBP8",1663.0943250514,-0.24792567202414,0.211674091897757,-1.
17126129986608,0.241493762087396,0.738444131901931
"5328","CEP135",62.3342468916657,0.339678547339444,0.290056236306538,1
.17107824215323,0.241567328664377,0.738530446061193
"5329","FAM162A",548.861103878006,-0.34206873383329,0.292292264776983,
-1.17029690845321,0.241881505591261,0.738622060870807
"5330","CREB5",388.931200003622,0.377546479244522,0.322521211976603,1.
17060976216321,0.24175567155768,0.738622060870807
"5331","RUSC2",109.975684878354,0.230220956712752,0.196734001438642,1.
17021437590469,0.241914708990469,0.738622060870807
"5332","DLGAP1-
AS1",109.900606971509,0.323165714902719,0.276158187851118,1.1702195666
0921,0.241912620640637,0.738622060870807
"5333","POLR3F",84.8737756258889,-0.326432449449259,0.27893635054965,-
1.17027575934803,0.241890013712883,0.738622060870807
"5334","FNDC11",4.52699010109497,-0.889483599289613,0.759816537904816,
-1.17065575032409,0.241737178375866,0.738622060870807
"5335","URB1",71.5968038769139,0.284016136334813,0.242637863910336,1.1
7053510015967,0.24178569744883,0.738622060870807
"5336","GSTCD",36.6164141856247,0.33295695938452,0.284595264674948,1.1
6993148064079,0.242028543993343,0.738709922955677
"5337","CHCHD3",317.811393306907,-0.241030825469855,0.206023786642175,
-1.16991746146517,0.242034186187884,0.738709922955677
"5338","UBR7",112.581869556497,-0.252970295391193,0.216257844077508,-1
.1697624031641,0.242096597534537,0.738761985245424
"5339","DGKZ",97.3775423926529,-0.237231160812367,0.202857721056293,-1
.16944605104055,0.242223965152294,0.739012206099592
"5340","RPS6KA3",297.838481113666,0.284919658096125,0.243661448673058,
1.16932596292008,0.242272326584533,0.73902133478192

"5341","ZBTB25",105.848605829397,0.245164400607844,0.209688078748492,1
.16918616485539,0.242328634085729,0.73905469399409
"5342","LHX9",10.3135842671485,1.13389055557822,0.970060001132736,1.16
888703199202,0.242449148961812,0.739064776968769
"5343","ATPSCKMT",55.2478613217251,0.308207442261428,0.263672038003818
,1.16890453987755,0.242442094210399,0.739064776968769
"5344","CDK2AP1",1350.09984454839,-0.260309213055491,0.222728743321224
,-1.16872752557162,0.242513428258215,0.739064776968769
"5345","RHBDL1",11.5831178615394,0.703771959487676,0.602156641795067,1
.16875229905243,0.242503444037533,0.739064776968769
"5346","FUBP1",220.376360370015,0.241514965679554,0.206893704104568,1.
16733840077361,0.243073736666502,0.739331095831084
"5347","AC104463.2",2.49775332649783,1.2549320794408,1.07474299157763,
1.16765783938602,0.242944809475056,0.739331095831084
"5348","ST3GAL6",14.8635115447818,-0.676253559875809,0.579300548206355
,-1.16736219561615,0.243064131278941,0.739331095831084
"5349","RETREG1",84.3445398687727,0.592088287953487,0.506881768738713,
1.16809939609151,0.242766673908997,0.739331095831084
"5350","TTC9",4.83332755859716,1.02950493603101,0.881869844852749,1.16
741142929421,0.243044257711784,0.739331095831084
"5351","TLNRD1",221.83856112236,0.340646536716783,0.291644480019225,1.
16801983255204,0.242798765144552,0.739331095831084
"5352","BCL7C",1009.27709731287,-0.297296538344854,0.254604855618139,-
1.1676781953866,0.242936595310201,0.739331095831084
"5353","LDHD",19.7094691849859,0.433977655352037,0.371787584863977,1.1
6727312320236,0.243100088972391,0.739331095831084
"5354","PEMT",183.327385029814,-0.289846667186693,0.248247116294641,-1
.16757314853429,0.242978986487401,0.739331095831084
"5355","TEN1",176.734832524761,-0.286315280783132,0.245229327036196,-1
.16754094725739,0.242991982206777,0.739331095831084
"5356","MT-
ND5",12014.8330904902,-0.271951510879152,0.232978387066687,-1.16728214
279082,0.243096447679463,0.739331095831084
"5357","MTMR11",56.9557892640871,-0.338398988051683,0.289937871774723,
-1.16714310545334,0.243152582623372,0.739352700831082
"5358","LINC02352",13.9110039531868,-0.579570963058878,0.4966346496219
18,-1.16699663122599,0.243211729991784,0.73939452591194
"5359","GBP1",298.633858901441,0.457188746077134,0.391961819614046,1.1
664114288665,0.24344813990216,0.739423224103352
"5360","NKTR",836.204635546476,0.318444183023932,0.272990830365458,1.1
6650138979989,0.243411787006088,0.739423224103352
"5361","FAIM",194.961828465211,-0.321124920213386,0.275308298843913,-1
.16641932539581,0.243444948789773,0.739423224103352
"5362","RACK1",14709.0980621172,-0.291751296993997,0.250034763184004,-
1.16684293527334,0.243273804417187,0.739423224103352
"5363","PSMD3",433.318702330781,-0.248461739817116,0.213008161724833,-
1.16644234570731,0.243435646085546,0.739423224103352
"5364","SREK1",607.603071272726,0.205791709536864,0.176526207504423,1.
16578559323384,0.243701143460566,0.73945532616142
"5365","RMRP",10.9146714611193,0.671705451559698,0.576034787183494,1.1

6608487283204,0.243580132102154,0.73945532616142
"5366","LINC02397",5.43862561455509,-1.32525204832401,1.1368451816736,
-1.16572781385504,0.243724510962373,0.73945532616142
"5367","AC027277.1",10.5135639974719,-0.938285019113779,0.804903247217
082,-1.1657155894557,0.243731085159351,0.73945532616142
"5368","MTMR4",72.8922784792664,-0.325023432869652,0.278742596598582,-
1.16603431565833,0.24360057153479,0.73945532616142
"5369","NUCB1",1777.24318453973,0.249722698044801,0.214134225990213,1.
16619702847602,0.24353479372398,0.73945532616142
"5370","ETV5",80.8018702049971,0.341373415967237,0.293015215243316,1.1
6503648345962,0.244004225263394,0.739475723322445
"5371","ZBTB49",18.0265025783774,0.444851296176717,0.381748417532204,1
.16529964695712,0.243897721977215,0.739475723322445
"5372","CXCL14",52462.7423404782,-0.336685618637357,0.288950521249455,
-1.1652016309972,0.243937385599143,0.739475723322445
"5373","LINC00861",3.46318479229955,1.24336458701918,1.06683327747475,
1.16547225632321,0.243827884032574,0.739475723322445
"5374","SH2B3",49.0297389151023,-0.519865505206168,0.446149360852727,-
1.16522750186742,0.24392691612511,0.739475723322445
"5375","AC022167.2",6.83168274534135,0.732253216967385,0.6285318032160
92,1.16502174308534,0.244010191715768,0.739475723322445
"5376","TMEM54",365.731337061932,-0.271364878649755,0.233019412418476,
-1.16455910618475,0.244197505049917,0.739768116004855
"5377","PSMD6-
AS2",15.2861493313293,0.6116515269266,0.525212059854453,1.164580126161
04,0.244188992251819,0.739768116004855
"5378","SIAH2",103.01740825672,-0.233023017437146,0.2001445382189,-1.1
6427367696782,0.244313120541064,0.739843171684959
"5379","MIB1",262.35272656563,0.269942630401095,0.231834755154717,1.16
4375161183,0.244272009108724,0.739843171684959
"5380","XYLT1",151.478619932813,-0.31491952644529,0.270572180067069,-1
.16390209210432,0.244463691674618,0.740023986933258
"5381","ATRX",1219.10589908538,0.218131278327846,0.187404015721429,1.1
6396266903956,0.244439140650523,0.740023986933258
"5382","NDE1",98.7186348056426,0.275142790465291,0.236493340435109,1.1
6342722361261,0.244656209517333,0.740194093021514
"5383","IRX3",245.340205754074,0.306829988706405,0.263723353957888,1.1
6345399109174,0.24464535480492,0.740194093021514
"5384","AC006946.2",10.4311064278096,0.75668938308733,0.65028890787227
1,1.16362031387433,0.244577915387922,0.740194093021514
"5385","MGLL",630.145215500073,-0.280011998723639,0.240801001500359,-1
.16283568996378,0.244896173737286,0.740370039719128
"5386","PEX6",65.1131912956704,-0.365479879564663,0.314278755264253,-1
.16291627557631,0.244863473298358,0.740370039719128
"5387","PSMB7",924.836265812939,-0.25991977281115,0.223501444645615,-1
.16294448665994,0.244852026385788,0.740370039719128
"5388","LSM4",353.5584362969,-0.3128303168384,0.269000434778955,-1.162
93610118311,0.244855428832671,0.740370039719128
"5389","TCEANC2",39.1294787885415,0.383634983870595,0.330159572768842,
1.16196837987549,0.245248309215357,0.741297032623668

"5390","PAPOLB",5.86652111018962,0.836221220916492,0.719956867664859,1
.16148794250513,0.245443523796124,0.741444842973346
"5391","CHCHD7",147.040806656398,-0.235936526984118,0.203148384626083,
-1.16139996593321,0.245479282838434,0.741444842973346
"5392","PSMF1",460.31402578582,-0.213612256335392,0.183917394830739,-1
.16145760183251,0.245455855682426,0.741444842973346
"5393","FAM217B",183.486831922069,0.251806810363425,0.216793718095191,
1.16150418275893,0.245436923166392,0.741444842973346
"5394","ARPC1B",1532.14553218669,-0.295738810712532,0.254684668890008,
-1.16119596833783,0.245562213957307,0.741503411412257
"5395","SNCG",272.361202510127,-0.753445784069658,0.648891055142351,-1
.16112832516141,0.245589717267427,0.741503411412257
"5396","FZD5",17.7735760182962,-0.551348644628344,0.475308814360038,-1
.15997984462099,0.24605701231559,0.741657301620597
"5397","MOCS2",345.755799106915,-0.28714781260522,0.247420620451946,-1
.16056540510127,0.245818680986445,0.741657301620597
"5398","NAIP",80.7038564605451,0.303848683559702,0.26205773887423,1.15
947227838034,0.24626372991506,0.741657301620597
"5399","AL359715.1",7.6267751445414,0.646750881424685,0.55726425312557
9,1.16058203589624,0.245811914384296,0.741657301620597
"5400","AL139274.2",11.8971667118553,-0.575136140452441,0.496069535330
079,-1.15938613337695,0.246298826455688,0.741657301620597
"5401","LRP4",45.1092023182709,0.368638055389353,0.317957454526147,1.1
5939428417785,0.246295505568372,0.741657301620597
"5402","PGR-
AS1",11.9228853665056,-0.553500423371767,0.477453701309929,-1.15927559
437323,0.246343866548977,0.741657301620597
"5403","ATXN2",367.685164008613,0.202721167281263,0.174756234383666,1.
16002251934659,0.246039637633142,0.741657301620597
"5404","LINC01089",34.9240971659394,0.367542527112053,0.31706198988956
9,1.15921346245277,0.246369185282648,0.741657301620597
"5405","POMT2",39.5985115062612,0.391845965337417,0.337732873220263,1.
16022453367121,0.245957400745317,0.741657301620597
"5406","SERPINA3",474.433357276595,-0.519243679582735,0.44770446438767
9,-1.15979115886838,0.246133844560371,0.741657301620597
"5407","SQOR",351.699305355102,0.254312591386894,0.219373880052624,1.1
5926559408936,0.246347941537668,0.741657301620597
"5408","PRC1",20.7266140079564,-0.458358898819026,0.395358240863997,-1
.15935081514262,0.246313216554704,0.741657301620597
"5409","RFFL",56.2979908914957,0.313881487759768,0.270626721878397,1.1
5983183619542,0.246117279459014,0.741657301620597
"5410","BTBD2",267.728270051365,-0.189992665956503,0.163687965149484,-
1.16070027373727,0.245763810501359,0.741657301620597
"5411","IFNAR1",513.193994222967,0.198507060531088,0.17118162986472,1.
1596282888997,0.246200178221001,0.741657301620597
"5412","LINC02777",3.88564687257871,-0.86918717350344,0.74995794400839
7,-1.15898122081057,0.246463839810585,0.741791899548397
"5413","AC083855.2",6.04335897589076,-0.930036243978778,0.802530024854
147,-1.1588803099894,0.246504975888973,0.741791899548397
"5414","DPH2",31.8160180309434,-0.417049908434319,0.359934805252421,-1

.1586818011163,0.246585911643055,0.74189839578015
"5415","PKDCC",933.789601418239,0.311851045683808,0.269204253731773,1.158417972082,0.246693508455642,0.741941359352925
"5416","RNF208",28.7548632956737,-0.462691465719979,0.399453312138745,-1.1583117517355,0.246736837351267,0.741941359352925
"5417","COQ9",171.033166014198,-0.28226168206124,0.243680521432859,-1.15832681414797,0.246730692839631,0.741941359352925
"5418","RELCH",82.5223116512937,0.25856715149933,0.223272353291033,1.15807957271938,0.246831565267948,0.742089214959322
"5419","DIS3L",147.769167960811,0.225240768041197,0.194556032419911,1.15771670114581,0.246979666135822,0.742260476325905
"5420","TYMSOS",4.48905756867965,1.00770711977775,0.870352133505555,1.15781541859266,0.246939369853283,0.742260476325905
"5421","TSPYL1",451.673671516158,-0.206254113708262,0.17821097606645,-1.15735920570547,0.247125633665883,0.742562155835374
"5422","HAX1",519.602475907277,-0.250946361384675,0.216867716212427,-1.15714024091473,0.247215068131648,0.742693885060202
"5423","MTA1",210.404441567461,-0.173379103656381,0.149850789043543,-1.15701161644201,0.247267614363562,0.742714764958152
"5424","C8orf31",28.0038702599361,0.397308073802784,0.343455293090086,1.1567970614987,0.247355282689889,0.74280636608793
"5425","KCNA1",19.8834502432488,-0.77814830925032,0.672723292857182,-1.15671378932841,0.247389314017252,0.74280636608793
"5426","CTPS1",25.1291929566346,-0.369997619795749,0.319942329789337,-1.1564509767725,0.247496740669159,0.742962422974775
"5427","KTN1",1248.649101783,-0.268010459291614,0.231770081620253,-1.15636348495894,0.247532510865253,0.742962422974775
"5428","AL096701.3",2.73052144014147,1.01086167580813,0.87439465118592,1.15607028752649,0.247652408234136,0.743185349617879
"5429","ZNF691",28.5568467703319,0.428614160237481,0.371079852637161,1.15504562479326,0.248071743334998,0.743269625227406
"5430","SIPA1L2",166.367234476194,0.406291171170679,0.351680663875006,1.15528436136905,0.247973997917473,0.743269625227406
"5431","CREB3",247.016019092666,-0.238939408906944,0.206773415836813,-1.15556155001819,0.247860542965027,0.743269625227406
"5432","STN1",105.217655430091,-0.294519463142908,0.25498535966593,-1.15504460149702,0.248072162359061,0.743269625227406
"5433","HOXC11",33.47181427845,-0.316176271072051,0.273558503384948,-1.15579032331205,0.247766932073157,0.743269625227406
"5434","MT1F",255.968087588169,-0.844342974586882,0.730589648630303,-1.15570070855759,0.247803598252099,0.743269625227406
"5435","C16orf70",42.6805873694314,0.332088478484399,0.287423137695832,1.15539925263719,0.247926967833161,0.743269625227406
"5436","PPP1R9B",119.176384351172,-0.236696177632316,0.204932074603449,-1.15499820167405,0.248091162892836,0.743269625227406
"5437","PISD",63.4435918526353,0.276754078066838,0.239600200885121,1.15506613535575,0.248063344679769,0.743269625227406
"5438","LINC02591",47.6545196915366,0.293558855432357,0.254236454641305,1.15466861684541,0.248226155810411,0.743383069376603
"5439","FOXF2",73.3581905585929,0.276909775030608,0.239927317630063,1.

15414025283093,0.248442672865162,0.743383069376603
"5440","SMAGP",14.4586581427578,-0.548410404972035,0.475141577299378,-
1.15420420180676,0.248416460338981,0.743383069376603
"5441","AC091057.3",5.44344827706568,0.811572629760797,0.7031068831773
13,1.15426636999105,0.248390979609445,0.743383069376603
"5442","ANP32A",439.855057634292,-0.223603749921904,0.19374074228011,-
1.15413901738137,0.248443179291913,0.743383069376603
"5443","SUSD2",29.9931304646552,0.517213696543345,0.448075538560218,1.
15430022849559,0.248377102871768,0.743383069376603
"5444","ZNF275",88.4270999684213,0.282575965449541,0.244839774377959,1
.15412606537257,0.248448488531293,0.743383069376603
"5445","EIF4G3",311.36824804569,0.210796348513218,0.182729386455871,1.
15359851308933,0.24866480825931,0.7434919989152
"5446","CGGBP1",473.451645251389,0.158820884996112,0.137675089241236,1
.15359202504547,0.248667469463442,0.7434919989152
"5447","MYOF",850.045378369391,0.248881678115222,0.215729402413734,1.1
5367527713217,0.248633323426149,0.7434919989152
"5448","CASP1",60.1206345850647,0.39641029423416,0.34354604582559,1.15
387820366708,0.248550106373951,0.7434919989152
"5449","ETF1",299.008064364175,-0.196763447796341,0.170614698204707,-1
.15326199833182,0.24880286295822,0.743759686176275
"5450","AC004263.2",4.32618901099239,0.898944068051168,0.7795543641885
65,1.1531512224768,0.248848320318049,0.743759686176275
"5451","AC013400.1",3.42947550318816,-1.03341421771546,0.8967016382670
15,-1.1524616144481,0.249131434616194,0.743787012181669
"5452","SEMA3A",107.868790691468,0.571642280659648,0.495819527675485,1
.15292409586939,0.248941540881031,0.743787012181669
"5453","RAP2A",169.908297838388,0.217132658299732,0.188388734914177,1.
15257771861279,0.249083753014427,0.743787012181669
"5454","RGS11",51.0664500080813,-0.40061986238774,0.347608866910241,-1
.15250185056754,0.249114909743839,0.743787012181669
"5455","C17orf58",918.683781939393,0.46980589108042,0.407571872448139,
1.15269458674482,0.249035764110316,0.743787012181669
"5456","CSNK2A1",435.983672336828,0.229573944216088,0.199157186531896,
1.15272739193532,0.249022294661305,0.743787012181669
"5457","KAT6A",319.891107303173,0.256933841549473,0.223010555620521,1.
15211515811241,0.249273755062856,0.74380293024709
"5458","TMEM132C",82.63814819318,-0.524653768264716,0.455371129914451,
-1.15214543434776,0.249261315653202,0.74380293024709
"5459","USP36",144.054730892006,0.306541484447769,0.266039172217496,1.
15224191194356,0.249221679394199,0.74380293024709
"5460","ARF4-
AS1",18.4670821773738,-0.420493022717539,0.365047948818866,-1.15188435
951515,0.249368596117634,0.743813415520993
"5461","EHD2",684.221862413793,0.257778780246011,0.223770134024477,1.1
5198027372953,0.249329179448227,0.743813415520993
"5462","RHOBTB2",53.3346018783721,0.337354345277391,0.292982581485968,
1.15144847030283,0.249547782945963,0.743865122123838
"5463","ADAM32",31.3889789028261,0.414185605728071,0.359793540349173,1
.15117576965421,0.249659931404692,0.743865122123838

"5464","TMEM250",141.565526235493,-0.229316980998858,0.199172357553276
,-1.15134943330436,0.249588507955009,0.743865122123838
"5465","RHOF",14.935825671059,-0.528980959555195,0.459361057553239,-1.
1515581280938,0.249502695977873,0.743865122123838
"5466","LDOC1",104.940137494533,-0.25686805608187З,0.223125825980449,-
1.15122512131062,0.249639632869114,0.743865122123838
"5467","TMEM185A",173.003369579184,0.184338157189172,0.160063166707348
,1.15165881683577,0.249461301743432,0.743865122123838
"5468","AC018638.6",5.53572719450738,1.01131883316426,0.87884789596720
9,1.15073249626576,0.2498423032395,0.744272362375313
"5469","SOAT1",170.587670364569,0.219000172088691,0.190347210937376,1.
15052997630074,0.249925655105907,0.744293340670594
"5470","PTPN4",93.2336343904847,0.255349540339751,0.221947862821713,1.
15049335052561,0.249940731381187,0.744293340670594
"5471","AC234582.1",3.77869205434947,-0.867238880410999,0.754012000345
197,-1.15016588597259,0.250075553914777,0.744308576929591
"5472","SEMA6A",165.706736675279,0.454856979208607,0.395462098627617,1
.15019108224811,0.250065178390876,0.744308576929591
"5473","PCOLCE",5053.39663521436,0.408355787262946,0.355061685906952,1
.15009814764964,0.250103449234119,0.744308576929591
"5474","SLC2A4RG",790.995283671674,-0.315823432508924,0.27462023109697
2,-1.15003702111591,0.250128623617937,0.744308576929591
"5475","PLEKHA6",129.669348479028,-0.566675447963853,0.493641959390312
,-1.14794829974288,0.250989907371738,0.744450966626067
"5476","AC079148.1",4.00740417335857,0.924773346859368,0.8048452574337
41,1.14900763633499,0.250552831860218,0.744450966626067
"5477","WDCP",30.469443623166,0.322181343350705,0.280414266432776,1.14
894776021655,0.250577522186216,0.744450966626067
"5478","RAB3GAP1",281.024793942594,0.217915894976358,0.189602471010174
,1.14933045869779,0.250419743121992,0.744450966626067
"5479","ARHGAP31",27.3390824088467,-0.428524932044516,0.37310580824921
5,-1.14853460484936,0.250747935920492,0.744450966626067
"5480","AC021148.2",3.67531784831169,1.00893707993646,0.87792002310189
7,1.14923575426796,0.250458781436793,0.744450966626067
"5481","AC099343.3",77.5184539063973,-0.408242986697279,0.355301336086
465,-1.14900492971389,0.250553947917241,0.744450966626067
"5482","PCDHGA7",8.53069094829538,0.638594392136265,0.556314761586907,
1.14790121749538,0.251009345549162,0.744450966626067
"5483","ZDHHC21",134.600226538658,0.309188520344449,0.269016636566748,
1.14932862253571,0.250420499969886,0.744450966626067
"5484","EIF5AL1",22.9474533252473,-0.47950600928902,0.417477509711007,
-1.14857926028387,0.250729513044607,0.744450966626067
"5485","FAU",13881.4213243245,-0.294518425818867,0.25654948965299,-1.1
4799848644109,0.250969188660688,0.744450966626067
"5486","ANKRD49",120.606824205875,0.325809833898433,0.283701701692681,
1.14842396769042,0.250793584035808,0.744450966626067
"5487","CD4",12.629561398835,-0.631150625866246,0.54987124952349,-1.14
781528660244,0.251044825322426,0.744450966626067
"5488","SUCLA2",378.481727313467,-0.265998995996472,0.231604986145964,
-1.14850289029975,0.250761020528062,0.744450966626067

"5489","TMEM229B",4.17217775728818,0.898721511138869,0.782763821048994
,1.14813879611155,0.25091127057151,0.744450966626067
"5490","AL049775.1",21.4455171360126,0.478458699519158,0.4164865787407
88,1.14879740174518,0.250639531016334,0.744450966626067
"5491","SLC9A5",3.84999436697184,-0.966349198000339,0.840484178297752,
-1.14975299113602,0.25024562189181,0.744450966626067
"5492","ACD",95.9079464090299,-0.216850954427828,0.188641232780959,-1.
14954165232595,0.25033270184914,0.744450966626067
"5493","PSMD11",477.682723967375,-0.185114380627581,0.161256679580265,
-1.14794860659053,0.250989780691361,0.744450966626067
"5494","DYNLT1",730.437791425272,-0.320276943285851,0.279117797640074,
-1.14746155921899,0.251190911716558,0.744748591363491
"5495","DNAH5",4.68370391543078,-0.923631449770848,0.805186913579376,-
1.14710191409463,0.251339502890686,0.744782456355536
"5496","CYP3A5",51.6083229870634,0.368296674651357,0.32102414977411,1.
147255354186,0.251276100000623,0.744782456355536
"5497","GNAS",8166.42798147976,-0.217048400707899,0.189196496440909,-1
.14721152236394,0.251294210583399,0.744782456355536
"5498","IQCC",8.14288856380286,0.677847755440955,0.591106888913576,1.1
4674311559252,0.251487805371444,0.744950875012812
"5499","IL18BP",20.2525376389793,0.591103031975479,0.515430765339813,1
.14681363962777,0.251458650794878,0.744950875012812
"5500","DNAJB12",334.109990063068,-0.248991699306048,0.217160702837309
,-1.14657806892708,0.251556044739708,0.744990278007783
"5501","AL137060.3",3.30145151521206,-0.909953568758751,0.793686636807
862,-1.1464897184341,0.251592578999375,0.744990278007783
"5502","RIOX1",21.1192178979518,-0.455965606324621,0.39774685800805,-1
.14637135943231,0.251641528029294,0.744999790997667
"5503","TRIM33",219.320548602576,0.228868777059254,0.199873986437468,1
.1450653541193,0.252182085938301,0.745212964906341
"5504","PHOSPHO2",48.3810408538655,-0.383445681415755,0.33477324834049
1,-1.14538925471656,0.252047947481064,0.745212964906341
"5505","POC5",55.2497845580496,0.292596439575938,0.255462861688439,1.1
4535802833363,0.252060877240687,0.745212964906341
"5506","AL589863.1",3.41315494662801,1.25701315214064,1.09718193727304
,1.14567430381223,0.251929939937524,0.745212964906341
"5507","MZT1",151.819831677528,-0.284761142203009,0.248604332656765,-1
.14543917702417,0.252027277349602,0.745212964906341
"5508","ZNF266",42.9256107136662,0.344504507585246,0.300797074266632,1
.14530538046348,0.252082677940608,0.745212964906341
"5509","ADGRE2",2.5357619248126,1.29241446270832,1.12793563957401,1.14
582287974909,0.251868446226075,0.745212964906341
"5510","IRGQ",83.5798265720356,0.294898027085749,0.257581842884052,1.1
4487117486187,0.252262526152223,0.745212964906341
"5511","SIX5",143.04881423242,0.250097117290993,0.218281473208133,1.14
575512807046,0.251896486495283,0.745212964906341
"5512","CCDC8",206.404416262295,0.255078139536664,0.222766336821232,1.
14504796001275,0.252189290848158,0.745212964906341
"5513","ACSL4",284.46677475474,0.189518102958554,0.165527805906093,1.1
4493212739176,0.252237274183679,0.745212964906341

"5514","GPC4",322.311389527477,0.260847328243611,0.22777268232193,1.14
520901095125,0.252122586527604,0.745212964906341
"5515","PAIP2B",57.2382712611251,0.282146564592611,0.246493186939737,1
.14464244669607,0.252357301451548,0.745222640200194
"5516","PRPF4",116.371719816995,-0.202027856595887,0.176495177281942,-
1.14466502545369,0.252347944663301,0.745222640200194
"5517","MICOS10",1332.08483742925,-0.283167495386646,0.247553385229355
,-1.14386436333438,0.25267989217021,0.745545403225882
"5518","BSDC1",295.825851287047,0.208683068147074,0.182582132285282,1.
14295449141217,0.253057486320914,0.745545403225882
"5519","LINC01121",8.67932682526549,0.634858815307302,0.55510265248004
2,1.14367822324562,0.252757107794036,0.745545403225882
"5520","TMSB10",22897.6552868051,-0.276916575160935,0.242220507487552,
-1.14324166039148,0.252938269618777,0.745545403225882
"5521","EGFLAM",37.9585591170319,-0.508610918285397,0.44474610738068,-
1.14359835835517,0.252790242813564,0.745545403225882
"5522","NOL7",987.837496442652,-0.257465565360159,0.225052739966835,-1
.14402324272124,0.25261399798002,0.745545403225882
"5523","LRATD2",5.73325081454132,0.85542168311086,0.747785324856815,1.
1439401853395,0.252648443993841,0.745545403225882
"5524","HACD1",8.49603721082358,-0.718219262255429,0.627969167973026,-
1.14371739710361,0.252740856119638,0.745545403225882
"5525","AIP",702.21091560103,-0.272028574426859,0.237921332312533,-1.1
4335512407741,0.252891176547729,0.745545403225882
"5526","MAGEL2",6.86134789101057,0.916137448339159,0.801542789953857,1
.14296761173773,0.253052038633112,0.745545403225882
"5527","TSR3",411.523569483475,-0.245947092727767,0.215187245607728,-1
.14294456454967,0.253061608105832,0.745545403225882
"5528","NPEPPS",609.347553216094,0.29090541581836,0.254411766852435,1.
14344324328006,0.252854606911887,0.745545403225882
"5529","GEMIN8",106.061989345689,-0.288653615233604,0.252407142938955,
-1.1436031955063,0.252788235849394,0.745545403225882
"5530","ZC3H12C",71.769007288695,0.339221189942356,0.296865708250901,1
.14267556175824,0.253173319977781,0.745604810905456
"5531","CENPX",428.614488346874,-0.325869086783311,0.28515403246027,-1
.14278267072627,0.253128835494415,0.745604810905456
"5532","NSRP1",539.433849335772,-0.231494064709503,0.202623307438495,-
1.1424848781514,0.253252528144933,0.745669193140562
"5533","SLFN5",735.594436117217,0.31943353247413,0.279625162564482,1.1
4236333220001,0.253303026189879,0.745669193140562
"5534","AC020910.5",46.7186302755046,-0.468620717981428,0.410262494472
07,-1.14224606025577,0.253351755182573,0.745669193140562
"5535","ZNF566",107.231275783826,0.263454479382789,0.230658889810705,1
.14218220506913,0.253378291118731,0.745669193140562
"5536","PLOD1",170.432472835106,0.311584304320746,0.273629213788879,1.
13870993526719,0.254824157452141,0.74593635468369
"5537","VAV3",3.43535454255801,-1.12916892822512,0.991037710718477,-1.
13938038483571,0.254544533632167,0.74593635468369
"5538","TMEM167B",217.95704496414,0.186219506766088,0.16394431953349,1
.13587044245255,0.256010790243193,0.74593635468369

"5539","TSPAN2",3.81802809737127,-0.933278921622775,0.821137844990162,-1.13656790673673,0.255718962719988,0.74593635468369
"5540","CEP170",354.421045456883,0.188069447880124,0.165187165260584,1.138523368831,0.254902006534388,0.74593635468369
"5541","RBSN",169.690371062624,0.247890733538168,0.218081406321682,1.13668899022283,0.255668323478051,0.74593635468369
"5542","SMARCC1",190.356124479649,0.249588543070946,0.219085838835505,1.13922718327013,0.254608410453871,0.74593635468369
"5543","NIT2",309.338689745258,-0.298548988115641,0.26284507792827,-1.135836327881,0.256025070126748,0.74593635468369
"5544","PARP15",14.4780745159716,0.503409911532873,0.442550408562276,1.13751993398518,0.255320996201755,0.74593635468369
"5545","AC106707.1",49.9804150546174,-0.439205871500835,0.38608069829334,-1.1376012150888,0.255287039026519,0.74593635468369
"5546","ADH1C",461.476521630261,-0.414447386347447,0.363120616893141,-1.14134909191733,0.253724680571306,0.74593635468369
"5547","AC098487.1",113.433410059573,0.323559830691339,0.284388252964082,1.13773978819091,0.255229153952527,0.74593635468369
"5548","PRMT9",46.3219074754607,-0.27500818522548,0.241680929419945,-1.13789774760269,0.255163181914505,0.74593635468369
"5549","GALNT7",64.076318795326,-0.260814155755287,0.229222164025776,-1.13782258737405,0.255194571243168,0.74593635468369
"5550","SMN2",24.2112023228624,-0.518938867170965,0.456412903416691,-1.13699429460957,0.255540670757407,0.74593635468369
"5551","SLC22A4",35.8655673880359,0.368949272935433,0.324447196782789,1.13716276976323,0.255470247530497,0.74593635468369
"5552","DCTN4",191.446528302018,0.258263426830086,0.227130179827524,1.13707225973318,0.255508079375191,0.74593635468369
"5553","ZNF391",24.2895158509205,0.427105548890472,0.374594772256521,1.14018021745907,0.25421122743687 8,0.74593635468369
"5554","RPP21",410.72626713669,-0.285027741748654,0.250673076936695,-1.13704967933447,0.255517518252455,0.74593635468369
"5555","CD2AP",161.335698742317,0.239188830528332,0.210540853145179,1.13606849670832,0.255927898426678,0.74593635468369
"5556","Z95118.2",8.02440147698951,0.685851098807362,0.600714709205178,1.14172516220691,0.253568278322085,0.74593635468369
"5557","RAET1E-AS1",7.50389421146523,-0.691149259373347,0.607742848625183,-1.13723964162942,0.255438119292989,0.74593635468369
"5558","UHRF2",131.276845723428,0.265576452963614,0.232747311596558,1.14105057172029,0.253848878651587,0.74593635468369
"5559","SLC25A25",137.19617550393,-0.577361641882706,0.505845338090238,-1.14137978233123,0.25371191435139,0.74593635468369
"5560","PPP2R2D",200.266992735759,-0.162582571154767,0.142636170274079,-1.13984111352935,0.254352501699751,0.74593635468369
"5561","DEAF1",178.251385332332,-0.271967294614274,0.238276392498986,-1.1413942093127 5,0.253705913348042,0.74593635468369
"5562","PCF11",551.06558008086,0.216910881523895,0.190806934522307,1.13680816720284,0.255618488375952,0.74593635468369
"5563","YAP1",359.23774353241,0.224252130529288,0.197151218369254,1.13

746256494989,0.255344965413885,0.74593635468369
"5564","FOXJ2",125.346020033711,0.227042271614525,0.199692455822903,1.13695968472578,0.255555139485101,0.74593635468369
"5565","STAT6",437.614060230544,0.248046507256985,0.217803872583669,1.13885260309914,0.254764637217494,0.74593635468369
"5566","NUDT4",433.59894661204,0.497390639069445,0.437908593372247,1.13583210422782,0.256026838127515,0.74593635468369
"5567","POLE",46.1179943386923,0.334717011580259,0.29437399084628,1.13704682474834,0.255518711520924,0.74593635468369
"5568","ZNF84",133.621138241849,0.206879644248237,0.181544395773728,1.13955401028235,0.254472154626956,0.74593635468369
"5569","HMGB1",5512.08316347714,-0.241195850122097,0.212135743262005,-1.13698826238915,0.25554319249765,0.74593635468369
"5570","ALOX5AP",22.162211856379,-0.445392482395349,0.39055304108987,-1.1404148362344,0.254113514751823,0.74593635468369
"5571","NDFIP2",195.157341856046,-0.215278528012229,0.189008964807665,-1.13898580541561,0.254709074672696,0.74593635468369
"5572","RAB11FIP3",71.7704164442206,-0.266474626175649,0.23369004867704,-1.14029085827235,0.254165145126914,0.74593635468369
"5573","CIITA",16.5043319801864,-0.499788908149928,0.439497313903014,-1.13718307789298,0.255461759569517,0.74593635468369
"5574","HSD11B2",47.465034576796,-0.69861617913865,0.614177815215298,-1.13748195039209,0.255336865856137,0.74593635468369
"5575","PSKH1",82.1800696562393,0.277608480156358,0.244046625856693,1.13752230411648,0.25532000597681,0.74593635468369
"5576","C1QBP",689.777642850085,-0.260720391661848,0.228477547951617,-1.14112040329258,0.253819821726646,0.74593635468369
"5577","RANGRF",200.023212739866,-0.258006552653713,0.226672039912519,-1.13823722040569,0.255021440597576,0.74593635468369
"5578","GAS7",769.306481288389,-0.249113844598693,0.219210832996972,-1.13641210697892,0.25578413117016,0.74593635468369
"5579","EZH1",376.091667445356,0.228478213352532,0.201157748531261,1.13581611954175,0.256033529316502,0.74593635468369
"5580","GRN",1545.52945018621,0.309189232029247,0.27175877480165,1.13773412562268,0.255231519154681,0.74593635468369
"5581","GPX4",7673.33191496648,-0.335137867150243,0.293899772773484,-1.1403134612443,0.254155731620229,0.74593635468369
"5582","AC005944.1",4.00810958088148,-0.833186468249025,0.733114240960606,-1.13650291004754,0.255746148352387,0.74593635468369
"5583","MPND",74.8941613045521,-0.245721813158332,0.216302587601099,-1.1360095867715,0.255952552104184,0.74593635468369
"5584","HSPBP1",90.2755162009604,-0.28836168858916,0.252684729510995,-1.14119159138429,0.253790202737066,0.74593635468369
"5585","EPN1",422.308316214114,-0.195990293472644,0.172499988202387,-1.13617569203945,0.255883041651705,0.74593635468369
"5586","BID",98.3513427280579,-0.335636767486811,0.294287355223389,-1.14050692810785,0.254075167999509,0.74593635468369
"5587","NUP50-DT",11.2274331834702,-0.524083494017361,0.459794052933416,-1.1398222544937,0.254360360175459,0.74593635468369

"5588","CCDC22",94.2959690734483,-0.302503471389916,0.265482610595855,
-1.13944740377146,0.254516593842139,0.74593635468369
"5589","MAGEE1",28.9026257063048,0.335480838863281,0.29386622605286,1.
14161073686275,0.253615859077827,0.74593635468369
"5590","CXorf40A",44.7801351683088,-0.479050176744998,0.42151874562989
3,-1.1364860559858,0.255753198090529,0.74593635468369
"5591","SPRY3",5.93280038823492,0.706380064062644,0.621487771083417,1.
13659527496613,0.255707516235848,0.74593635468369
"5592","RPUSD2",43.479011571428,-0.334045998910759,0.294261817884186,-
1.13519994307325,0.256291553368324,0.746228068814365
"5593","ZNF594",22.9004265685469,0.446902524893168,0.393698354410721,1
.13513942815962,0.256316903739725,0.746228068814365
"5594","AC145207.2",2.52898215258448,1.04937217845593,0.92418885937814
1,1.13545209705516,0.256185942001862,0.746228068814365
"5595","ZNF335",97.5963273493252,0.317985020649965,0.280101111146484,1
.13525083620132,0.256270235018183,0.746228068814365
"5596","LERFS",38.6245253745045,1.73641531785264,1.53013347895661,1.13
481297006631,0.256453690709651,0.746359508302572
"5597","GLYR1",190.913407725916,0.249333162889097,0.219697929398914,1.
13489081836713,0.256421067424288,0.746359508302572
"5598","NBPF3",10.9719922811295,0.688236914676171,0.606602882560175,1.
13457574050994,0.256553122439122,0.746382177426479
"5599","ZNF398",64.8351916000902,0.297889130730445,0.262542141184184,1
.13463358448602,0.256528875410092,0.746382177426479
"5600","SLC2A1",5.37629931959857,0.943843565687463,0.833938455814397,1
.1317904326234,0.257722550981694,0.746523483265199
"5601","LMO4",925.59593870019,-0.187838363829177,0.165849715531883,-1.
13258176673247,0.257389929138273,0.746523483265199
"5602","C2orf49",242.395309372818,0.21691001152702,0.191395284918918,1
.13330906568002,0.257084486011874,0.746523483265199
"5603","RNPEPL1",199.629783982942,-0.192009278582873,0.169581192206694
,-1.13225574183275,0.257526931243046,0.746523483265199
"5604","SEPTIN2",1436.5420907883,0.225393007949168,0.198872781867006,1
.13335271842226,0.257066161212569,0.746523483265199
"5605","AZI2",651.55318212941,0.204216455783433,0.180208603681083,1.13
32225632514,0.2571208011773,0.746523483265199
"5606","ANKRD31",3.86671459990258,1.04149771576269,0.91941680601913,1.
13278081164531,0.257306311399127,0.746523483265199
"5607","LAMA2",1797.77548282295,0.293925262996835,0.259426221156751,1.
13298209289044,0.257221773358757,0.746523483265199
"5608","MINDY4",102.528637116185,-0.280280016223386,0.247248855555537,
-1.13359479700576,0.256964556525464,0.746523483265199
"5609","TECPR1",36.9472684489074,0.415590187179756,0.366451580792907,1
.13409303974219,0.256755522923185,0.746523483265199
"5610","SRRT",125.62500021882,-0.300049236014504,0.264730089449411,-1.
13341568628844,0.25703972979643,0.746523483265199
"5611","AL450326.1",44.3039973034311,-0.343568755764604,0.303045466331
844,-1.13372016391886,0.256911948759619,0.746523483265199
"5612","CRTAM",5.06674320714383,0.942673345660037,0.832417877748235,1.
13245206627476,0.257444425774806,0.746523483265199

"5613","NUP58",133.346882256384,0.263115382883158,0.232097043156385,1.
13364383839166,0.256943976379552,0.746523483265199
"5614","ARHGAP23",140.113411910144,0.2323666413246,0.205310698684627,1
.13178048106267,0.257726735823416,0.746523483265199
"5615","CNTNAP1",56.8676210776286,0.349511362652325,0.308741593770642,
1.13205142975316,0.257612812997655,0.746523483265199
"5616","ARL16",343.389296583791,-0.268310969591869,0.236934402477779,-
1.1324272321198,0.257454861330476,0.746523483265199
"5617","ZNF781",20.4962191310378,0.679721968964405,0.600603696158517,1
.13173124526527,0.257747441210846,0.746523483265199
"5618","LENG1",131.866901202661,-0.321984048064923,0.284094230446148,-
1.13337059875968,0.257058655563367,0.746523483265199
"5619","PTPRH",9.49875297914379,0.774081508225664,0.683121978890561,1.
1331526903626,0.257150137583163,0.746523483265199
"5620","FAM210B",573.175201153389,0.200501375378798,0.176931785219734,
1.13321286579341,0.257124872554179,0.746523483265199
"5621","LINC01684",28.7720192427659,0.368446717883155,0.32540803615534
2,1.13226066029687,0.257524864031456,0.746523483265199
"5622","THAP7",226.407701403414,-0.225434958480565,0.19897469063708,-1
.1329830832191,0.257221357468784,0.746523483265199
"5623","WBP2NL",5.23491240631912,0.730845560234019,0.645601706333411,1
.1320378385998,0.25761852668859,0.746523483265199
"5624","SH3BGRL",1476.11188621014,-0.241408301012205,0.21322577488704,
-1.13217222983523,0.257562032772266,0.746523483265199
"5625","NBPF19",166.597424581372,0.506129128631782,0.447382228150244,1
.13131254838717,0.257923564624764,0.746886231455458
"5626","AC010642.2",256.652318873064,-0.263001649057358,0.232494731012
948,-1.13121552437552,0.25796438935284,0.746886231455458
"5627","IFI6",1373.19217138293,0.421876553067786,0.373138942970504,1.1
3061517972177,0.258217095615739,0.746965854242239
"5628","PSMA5",493.363645569263,-0.285340747121966,0.252334904329931,-
1.13080173303682,0.25813855036424,0.746965854242239
"5629","GNPDA1",228.527899768398,0.209193345613427,0.185004893063489,1
.13074493409014,0.258162462886428,0.746965854242239
"5630","TCTE3",9.6543461677544,-0.530562739523326,0.469189491956143,-1
.13080695245604,0.258136353050575,0.746965854242239
"5631","TMEM254",72.2739060889514,0.386144071906761,0.341537410688855,
1.13060549100006,0.258221175347661,0.746965854242239
"5632","B4GALNT1",4.49391861761774,-0.881413942128033,0.77986501295241
8,-1.13021346962492,0.258386285397329,0.747310760446927
"5633","LINC01002",9.26010744885072,0.563466459898756,0.49865972979332
8,1.12996182814339,0.258492309344275,0.747484684343849
"5634","VPS13D",256.034032114506,0.289836376773287,0.256647656695887,1
.12931627938737,0.258764435888943,0.747767616382783
"5635","AC099791.2",11.9562758018565,-0.540189389253536,0.478321405246
691,-1.12934395853545,0.258752763866416,0.747767616382783
"5636","HIST2H4B",4.74022751242976,0.859148255974959,0.760783426001854
,1.12929412840924,0.2587737769998,0.747767616382783
"5637","AL162377.1",37.6809409768538,-0.336502345111871,0.297946727494
503,-1.12940440038255,0.258727277406523,0.747767616382783

"5638","ARL10",62.573082980231,0.354045881363638,0.313639461869911,1.12883078950852,0.25896922148678,0.748071620278637
"5639","VPS37C",53.8274248675761,0.29146342452062,0.258200265440078,1.12882697476646,0.258970831036358,0.748071620278637
"5640","RNF217-AS1",10.200358669696,0.537221559206864,0.476142920686665,1.12827795156991,0.25920255202499,0.748608221619692
"5641","AHI1",613.973922164974,-0.25973675996524,0.230289118415383,-1.12787248373907,0.259373776144571,0.748837192417392
"5642","TEP1",38.402271999499,0.343396466612856,0.304452657505489,1.12791417038843,0.259356168768893,0.748837192417392
"5643","RMDN2",75.5743608974326,0.256926693311222,0.227891738175744,1.12740679134707,0.259570528997387,0.748897176172761
"5644","RCN2",543.228251605094,-0.210154005953708,0.18638883867373,-1.12750316729843,0.259529802130874,0.748897176172761
"5645","SAMD1",192.605814544962,-0.263783702052303,0.233977737586248,-1.12738803603085,0.259578455195003,0.748897176172761
"5646","CYP4F12",41.7052771365581,0.51451452849808,0.456278623051655,1.12763233363188,0.25947522553674,0.748897176172761
"5647","CDKN2AIP",230.076589765386,0.249368914198426,0.221272941363566,1.12697428190597,0.259753354754404,0.749136401486276
"5648","SUV39H1",10.3136657991682,0.679682401674621,0.603087050883743,1.12700546410114,0.259740170769543,0.749136401486276
"5649","FGGY",47.1406166463091,0.367603494145814,0.326406898938844,1.12621239116238,0.260075629859742,0.749806004582693
"5650","ENPP2",316.254933410734,-0.405971319397275,0.360476403528025,-1.12620775014394,0.260077593829714,0.749806004582693
"5651","AC000403.1",7.86488918103161,-0.640208038492137,0.568533391393277,-1.12606937109395,0.260136157308743,0.749842128190075
"5652","ANKEF1",2.2916624743252,-1.47429751931975,1.3094517865831,-1.12588911972605,0.26021245528254,0.749929349627972
"5653","AC009950.1",32.2245787539941,0.511321643203487,0.454291429428823,1.12553662710822,0.260361705407835,0.749984155291494
"5654","KIAA1958",8.97953675343524,0.693377230705318,0.616072488876006,1.12547994469019,0.26038571103508,0.749984155291494
"5655","STXBP1",263.533644508534,0.208135557301713,0.1849421018667,1.12540927782755,0.260415641373239,0.749984155291494
"5656","LINC00327",4.6539765665596,0.771363403111163,0.685353175204215,1.12549767188475,0.260378203208695,0.749984155291494
"5657","ERO1A",166.689848039719,0.196773898273971,0.174888158827911,1.1251413451473,0.260529143564238,0.749998453098601
"5658","YES1",299.814536733191,0.165518586471681,0.147132720954561,1.12496109225628,0.260605521918922,0.749998453098601
"5659","DTD1",355.372626188761,-0.293487447495176,0.260907472480629,-1.12487175896032,0.260643380756367,0.749998453098601
"5660","DGCR11",5.54684770793211,1.03315199474098,0.918476362502103,1.12485420084897,0.260650822210767,0.749998453098601
"5661","H1F0",451.732596305482,-0.154368675595579,0.137208311406953,-1.12506796427025,0.260560235291409,0.749998453098601
"5662","S100A1",5.63855811998637,-0.902765747334016,0.80284773814673,-

1.1244544942207,0.260820265076911,0.750324983956155
"5663","AL591848.4",3.89118276507929,0.860324912364838,0.765187633733369,1.12433196047261,0.260872224602908,0.750324983956155
"5664","PARTICL",18.0014250206129,-0.513260173203272,0.4566195383458,-1.124043388644,0.26099461962647,0.750324983956155
"5665","ADCY1",36.1058013426216,0.343771976168208,0.305834031544186,1.12404749213967,0.260992878889126,0.750324983956155
"5666","AC004839.2",3.14906714734577,-0.960346886121814,0.854357043903935,-1.1240580188039,0.260988413426425,0.750324983956155
"5667","LINC01354",54.8797704403179,0.387568119898178,0.344896587605039,1.12372268623894,0.261130689116812,0.75058369419865
"5668","PCBD2",61.7313736035196,0.267785465645565,0.23836741900168,1.12341471316463,0.261261403896468,0.750694480167503
"5669","NNAT",29.1331646947976,-0.839075553863231,0.746877879662978,-1.12344410875022,0.261248925406744,0.750694480167503
"5670","SLC25A37",356.828699479259,0.165037111390308,0.146983124055338,1.12283034158516,0.261509556425131,0.750845464691339
"5671","RANBP6",72.5013889247199,0.264230583928966,0.235342680212619,1.12274825667086,0.261544426708739,0.750845464691339
"5672","USP47",380.030771247124,0.220330141621688,0.196202863706428,1.12297107931799,0.261449777465566,0.750845464691339
"5673","CHFR",49.1804115184061,0.318406090098779,0.283545601086899,1.12294491213495,0.261460891374569,0.750845464691339
"5674","ZNF737",92.2864154984686,0.272674487655944,0.242832173214335,1.122892753652,0.261483045464521,0.750845464691339
"5675","SUOX",49.6862860521021,0.305167059272977,0.271830634585223,1.12263674673247,0.261591802110382,0.750849139132338
"5676","ITPR1",37.5907406941476,-0.439020851731104,0.391247684733121,-1.1221046637773,0.261817941156495,0.750924229923017
"5677","NGLY1",158.347174153667,-0.1918218521479,0.171008776322649,-1.12170764724953,0.261986763991191,0.750924229923017
"5678","MANF",442.652739904475,-0.261329530686006,0.232943351696888,-1.12185872136868,0.261922514072194,0.750924229923017
"5679","AC108865.2",3.05419222127667,-0.947900178179619,0.844720363407188,-1.12214671179022,0.261800065536946,0.750924229923017
"5680","CCDC152",138.625088587375,0.403379273388188,0.359444562046665,1.1222294506039,0.261764893743964,0.750924229923017
"5681","AC007529.1",9.38942950398566,0.72110299884488,0.642781946455055,1.12184700087139,0.261927498262381,0.750924229923017
"5682","ADAL",57.3679470193462,0.295616988184703,0.263536663367296,1.12173002574862,0.261977246010545,0.750924229923017
"5683","PABPC1L",20.0772414963917,0.548717347981609,0.489114514792941,1.12185864738424,0.261922545534179,0.750924229923017
"5684","RDH13",75.5382070428904,0.253228677217782,0.225834015702355,1.12130440770948,0.262158310006561,0.75128372830698
"5685","ZC3H12A",68.336248759914,-0.450749325653885,0.402236263215662,-1.12060837590919,0.262454598134396,0.751289460097468
"5686","RNF187",550.527727459237,-0.235663324488752,0.210248899460986,-1.12087780289419,0.262339880525015,0.751289460097468
"5687","RNF168",213.510734368731,-0.264470788328437,0.235925930303999,

-1.12099076175161,0.262291794794336,0.751289460097468
"5688","FAM66B",22.9534286633013,0.424587839662421,0.378915362242859,1.12053477364766,0.262485942799265,0.751289460097468
"5689","MPP7",9.33100483939969,0.607407576108063,0.542118607370959,1.12043299722496,0.26252929012676,0.751289460097468
"5690","H2AFY2",31.0634491786024,0.49606396872021,0.44249250463544,1.12106750628218,0.262259128701984,0.751289460097468
"5691","RPL13",48908.6399169794,-0.262764988734095,0.234467452444217,-1.12068854757831,0.262420458726986,0.751289460097468
"5692","RELB",64.2059293460905,-0.274889859186526,0.245280895832023,-1.12071451082264,0.262409403486728,0.751289460097468
"5693","PIFO",39.6402477000332,0.415187426512245,0.370779905127242,1.11976787514891,0.262812692231872,0.751440397360885
"5694","ACIN1",351.666664139566,0.238581229117301,0.213057260646206,1.11979863250697,0.262799582161945,0.751440397360885
"5695","RABGGTA",63.5247128204374,-0.277100886484997,0.247372555060288,-1.12017635269791,0.262638618869301,0.751440397360885
"5696","PITPNA",175.854111629979,-0.185543206829022,0.165673827204078,-1.11993070939605,0.262743290610935,0.751440397360885
"5697","FAM182A",44.8508057330918,-0.352295899854684,0.314540069231459,-1.1200350426433,0.262698829366286,0.751440397360885
"5698","MARC1",14.5755458467281,-0.46233912236765,0.413135340648149,-1.11909845728111,0.263098137934038,0.751478754076344
"5699","WDR48",192.872552787415,0.203207045038346,0.181516386560404,1.11949697153499,0.262928182163703,0.751478754076344
"5700","DST",1506.88334087841,0.304251972354602,0.271789934911004,1.11943796761359,0.262953340991625,0.751478754076344
"5701","RAB23",104.236021520907,0.290210748630413,0.25932807708189,1.11908726542854,0.263102912057056,0.751478754076344
"5702","COPRS",1080.56603986406,-0.286345205337501,0.255835006281994,-1.11925732720829,0.263030375032534,0.751478754076344
"5703","AC087741.1",20.0552908756698,0.543565415984505,0.485677689535496,1.11918959362612,0.263059264011479,0.751478754076344
"5704","LRIG2-DT",20.2624111168819,0.44437633019331,0.39732729573559,1.11841379880689,0.26339030364699,0.751833142510739
"5705","FAM72B",5.11331392814942,1.12614707678662,1.00695249479293,1.11837160403301,0.263408316830402,0.751833142510739
"5706","AC008771.1",48.0654894221659,0.317669907226281,0.284039732504855,1.11839954370064,0.263396389135974,0.751833142510739
"5707","TTC23",85.1341939003559,0.230469386582275,0.206077279015839,1.11836388602822,0.263411611781496,0.751833142510739
"5708","PRPF38A",246.845191287437,-0.242695676240245,0.217075636600605,-1.11802356100781,0.263556930755967,0.751977725898554
"5709","LINC01886",39.4717078026143,-0.483667875631416,0.432595298152349,-1.11806087051154,0.26354099687795,0.751977725898554
"5710","PID1",833.605714587905,0.338548577926741,0.302837675505051,1.1179209368918,0.263600762163469,0.751977725898554
"5711","ILKAP",152.524742805547,-0.209142563678312,0.187187804665656,-1.11728733638321,0.263871488312991,0.752372201083636

"5712","TUBB",3169.10419068631,0.27201884257152,0.243505899294385,1.11
709343945982,0.263954375306946,0.752372201083636
"5713","PTK2B",45.3314946203342,0.450355599155065,0.403015769201842,1.
11746396436789,0.263795999066548,0.752372201083636
"5714","HSBP1L1",64.9833733719932,-0.452373971006178,0.404969489009151
,-1.11705692227089,0.263969987672232,0.752372201083636
"5715","SUN2",310.248235181622,-0.266756970138318,0.238763048013375,-1
.11724562220941,0.263889318758624,0.752372201083636
"5716","SAR1B",304.607343841496,-0.165224335645778,0.147925214776259,-
1.11694504480311,0.264017823135969,0.75237689311788
"5717","TSTD3",135.324553759721,0.231541655363244,0.207340488832654,1.
11672185527701,0.26411327016976,0.752517239425437
"5718","RTN4",5040.9436748182,-0.181751640242697,0.162773764948046,-1.
11659050400848,0.264169453680433,0.752545685729376
"5719","RGS2",159.03113380768,-0.385157825449718,0.345132080045861,-1.
11597225444398,0.264434011226863,0.753134397490028
"5720","AC004938.2",3.32919468365875,-0.996008353412095,0.892653776608
031,-1.11578349805094,0.264514819082844,0.753134397490028
"5721","FIZ1",26.7879595006265,-0.367747346857222,0.329560417783727,-1
.11587231661587,0.264476793149603,0.753134397490028
"5722","TPRN",36.1390765172386,-0.398420366615712,0.357113022889502,-1
.11567022505083,0.264563320172653,0.75314084625871
"5723","ZCCHC8",128.305778071373,0.220088516800388,0.197400220305332,1
.11493551759954,0.264878055105,0.753641683773858
"5724","ZNF8",36.3768356501894,0.354359252479096,0.317821927866658,1.1
1496162287382,0.264866867674583,0.753641683773858
"5725","XPNPEP2",89.3985772873122,0.443324743837785,0.397606236130912,
1.11498438292054,0.26485711410908,0.753641683773858
"5726","TMEM219",999.781269952033,-0.267831354031244,0.24024954083258,
-1.11480485291701,0.264934056424268,0.753669375671483
"5727","PRKCZ",3.55238535106681,-0.923739158208117,0.828912389969365,-
1.11439902381271,0.26510804194023,0.754032170931282
"5728","AC105942.1",34.3025989260319,-0.347419737735764,0.311938665662
769,-1.11374374509684,0.265389136687772,0.754032170931282
"5729","COL11A1",11.1931093760438,1.16134995268136,1.04279281160491,1.
11369194317132,0.265411366899449,0.754032170931282
"5730","AL139420.2",2.58266371628893,-1.12799767701106,1.0126432283503
2,-1.11391420535015,0.265315994647648,0.754032170931282
"5731","FAM241A",109.764815977283,-0.454287333548439,0.407845934481958
,-1.11386995710885,0.265334979596301,0.754032170931282
"5732","DEFB1",36.6336970254151,2.78514947129077,2.49992584178735,1.11
409283616969,0.265239361636342,0.754032170931282
"5733","SARAF",1279.20707248785,-0.20598798583133,0.184967524795856,-1
.11364406297091,0.265431915287616,0.754032170931282
"5734","SLC7A5",7.11754845842088,0.806439216524533,0.723988618051456,1
.11388383244889,0.265329026205465,0.754032170931282
"5735","MATN1-
AS1",6.17230383845651,-0.669855925693457,0.6021413377663,-1.1124563016
6889,0.265942008696042,0.754064478420299
"5736","AL513166.1",16.4061087155284,0.446710516838056,0.4038729694207

65,1.10606688404705,0.268697570997447,0.754064478420299
"5737","GPR89B",32.1353677358041,0.352807690268361,0.319377909562279,1
.10467154961312,0.269301935511328,0.754064478420299
"5738","PDE4DIP",852.773012885167,0.289912662341625,0.261739954385115,
1.10763625302333,0.268018940112077,0.754064478420299
"5739","OTUD7B",95.303986325956,0.284106946130357,0.257147493873739,1.
10484042387695,0.269228741018065,0.754064478420299
"5740","NMNAT2",3.02352309659785,1.08305474856501,0.974253878005752,1.
11167609697584,0.266277440728398,0.754064478420299
"5741","YOD1",23.8935564328276,0.413499759252866,0.372703819432327,1.1
0945940903604,0.267232045850212,0.754064478420299
"5742","ARHGEF33",10.6999489414787,0.674512526896567,0.611815653956748
,1.10247673876002,0.270254465790177,0.754064478420299
"5743","RPS27A",25689.3337993684,-0.276152205918247,0.248991386886995,
-1.10908336778565,0.267394219226673,0.754064478420299
"5744","TEX261",226.24309856802,0.204991924086052,0.185145929337883,1.
10719109417713,0.268211317172625,0.754064478420299
"5745","MOGS",100.021899330995,0.294396789545619,0.264572814168603,1.1
1272501851989,0.265826547109992,0.754064478420299
"5746","EIF2AK3",86.8657191087149,0.232612294589268,0.209915259461466,
1.10812475084485,0.267807943081924,0.754064478420299
"5747","AC159540.2",24.6078044563935,-0.48702779855872,0.4411008813459
5,-1.10411885161651,0.26954158466754,0.754064478420299
"5748","DDX18",665.726015549679,-0.231313254125826,0.208425943880477,-
1.10981027514729,0.26708079063026,0.754064478420299
"5749","GULP1",465.033832346784,0.317206360035055,0.286242751265748,1.
10817255155768,0.267787302637282,0.754064478420299
"5750","CCNYL1",47.7598876702139,-0.341055649246176,0.308972182963438,
-1.10383933587489,0.269662838039571,0.754064478420299
"5751","LARS2",27.7783157928268,0.403972726619471,0.365327968411676,1.
10578100104355,0.268821320256407,0.754064478420299
"5752","PTPRG-
AS1",13.201314613623,0.693197244729991,0.623886064242887,1.11109589468
266,0.266527074781747,0.754064478420299
"5753","KLF15",41.1019653638187,0.658547691552407,0.59215870801539,1.1
1211349700407,0.266089354258711,0.754064478420299
"5754","AC107027.3",5.15715581590985,0.741396724876663,0.6702493541025
49,1.10615059953229,0.26866134074973,0.754064478420299
"5755","SHOX2",413.200831468164,-0.194728754062827,0.175467031020202,-
1.10977402951787,0.267096413067145,0.754064478420299
"5756","EHHADH",7.90487439152479,0.674597491602436,0.607964108673041,1
.10960084975219,0.267171064946844,0.754064478420299
"5757","ST6GAL1",7.10130184045038,-0.631640531890765,0.572941758097288
,-1.10245155456711,0.270265408917118,0.754064478420299
"5758","PIGZ",70.1857325036364,-0.279065138575655,0.252615120332288,-1
.10470481026067,0.269287518402668,0.754064478420299
"5759","AC073648.7",2.42417589923042,3.58055475625667,3.23988686586593
,1.10514808216913,0.269095429020852,0.754064478420299
"5760","PRDM8",158.825909418993,0.205722972933255,0.184766985850398,1.
11341846048095,0.265528750168498,0.754064478420299

"5761","DAP",256.885130299823,-0.25038209796724,0.225882914911849,-1.108459655149,0.267663353728882,0.754064478420299
"5762","C6",50.9912707334936,-0.455983227712394,0.411855923879542,-1.10714257407587,0.268232291047696,0.754064478420299
"5763","AC146944.4",18.0528841764209,0.504231400078806,0.456957888598075,1.10345266524617,0.269830636671921,0.754064478420299
"5764","HARS",209.359270880123,-0.22358624963666,0.202618773983378,-1.10348239327024,0.269817733430846,0.754064478420299
"5765","PCDHGA2",16.0595856091782,-0.448363618011278,0.406175733869299,-1.10386608707539,0.269651231802945,0.754064478420299
"5766","AC008429.1",5.51820462110777,0.767516493472908,0.691610353187313,1.1097527530289,0.267105583861558,0.754064478420299
"5767","SIRT5",50.1666221174579,-0.294288992065002,0.264351396368429,-1.1132492436501,0.265601398718409,0.754064478420299
"5768","TFEB",73.5957188418355,-0.316312366301297,0.285399190865498,-1.1083155678965,0.267725554421504,0.754064478420299
"5769","MYMX",12.2609081272093,0.713420782350323,0.642964843580409,1.10957976858824,0.267180153295795,0.754064478420299
"5770","ASCC3",153.839679335083,-0.209789592045917,0.189492462814095,-1.10711312170624,0.268245023028241,0.754064478420299
"5771","RWDD1",1589.39540316139,-0.270062379610549,0.244604398620637,-1.10407818147782,0.269559224966712,0.754064478420299
"5772","AKAP7",75.3740681273268,-0.273539555488441,0.248060660289343,-1.1027123574104,0.270152098637252,0.754064478420299
"5773","TYW1B",39.6127998329329,-0.381003095174187,0.344429385571142,-1.10618637995245,0.268645856782685,0.754064478420299
"5774","DNAJC30",153.125199303568,-0.273693775841232,0.247773461227061,-1.10461295768241,0.269327333967181,0.754064478420299
"5775","POR",299.037060859911,-0.245487746631126,0.222209514254227,-1.10475803637403,0.269264448196308,0.754064478420299
"5776","LAMTOR4",1610.17641947499,-0.263323555094205,0.236670596944525,-1.11261626283018,0.265873272825106,0.754064478420299
"5777","AC002467.1",21.7342784959871,-0.394099479736691,0.35660295760063,-1.10514921802206,0.269094936926233,0.754064478420299
"5778","LSM8",629.799611940979,-0.219865411047916,0.19876532503495,-1.10615576941932,0.268659103444652,0.754064478420299
"5779","KLF14",11.7204885265328,0.570706851495353,0.516809860065096,1.10428785438317,0.269468289754243,0.754064478420299
"5780","AC016831.6",11.2941489233116,0.768569854390287,0.691788876737421,1.1109890318199,0.266573070454699,0.754064478420299
"5781","XPO7",129.776937000538,0.274367494539299,0.248689690000383,1.10325238870528,0.269917576336217,0.754064478420299
"5782","AC016885.2",16.9460143630439,0.662558779104858,0.601035216621786,1.10236265826298,0.270304038891038,0.754064478420299
"5783","TMEM65",180.516946665698,0.269519099729113,0.243038988398252,1.10895417029744,0.267449953177005,0.754064478420299
"5784","LY6E-
DT",22.0812291858309,0.500463742820058,0.453499373494464,1.10355994312342,0.269784075451373,0.754064478420299
"5785","GLIS3",109.426166644823,-0.277758724339791,0.251324064069951,-

1.1051815725154,0.26908091999068,0.754064478420299
"5786","TEX10",56.3549862225397,0.284203265649536,0.257130094737139,1.
10528977924608,0.269034045235184,0.754064478420299
"5787","RALGPS1",26.5802223826499,0.404103328982976,0.366258934503488,
1.10332688410944,0.269885235779427,0.754064478420299
"5788","TMEM86A",22.8839840126576,0.3760047476044,0.338197381686738,1.
11179082974889,0.266228095616443,0.754064478420299
"5789","CD44",3585.17742235315,-0.256437252359666,0.230349005695864,-1
.11325530398966,0.265598796643506,0.754064478420299
"5790","DTX4",53.495213095198,0.480808362522049,0.435941777353481,1.10
291875543781,0.270062448561191,0.754064478420299
"5791","CDC42BPG",2.7075129513077,1.44333947928703,1.29775749225372,1.
11217965444414,0.266060913849272,0.754064478420299
"5792","ZNHIT2",35.8699936672372,-0.478596715544878,0.431777014804279,
-1.10843490768452,0.267674036198005,0.754064478420299
"5793","FIBP",446.516448953311,-0.299859527071193,0.269503686029163,-1
.1126360885422,0.265864754499248,0.754064478420299
"5794","AP001992.2",2.32655381479465,1.14907785133402,1.03922011625274
,1.10571170954369,0.268851320136592,0.754064478420299
"5795","TENM4",8.53879882053628,1.09420275967808,0.98571778985468,1.11
005682451911,0.266974540644881,0.754064478420299
"5796","C1R",14206.4412004865,0.254347876141986,0.230684446721128,1.10
257921484176,0.270209940644542,0.754064478420299
"5797","HOXC9",467.138390778239,-0.23551074968118,0.211822477462125,-1
.11183077689803,0.266210916330968,0.754064478420299
"5798","AC009779.2",80.5543427489553,-0.364447356790174,0.328491349874
669,-1.10945800225553,0.267232652419148,0.754064478420299
"5799","SPRYD4",51.6980989773445,0.292082275511356,0.263596850090529,1
.10806436196428,0.267834020686032,0.754064478420299
"5800","RASSF3",30.3523530052367,-0.432087318836421,0.388864499948128,
-1.1115136222015,0.266503202721967,0.754064478420299
"5801","AC025034.1",3.73722259573299,0.928520847199481,0.8361198905039
95,1.11051161172567,0.266778627238285,0.754064478420299
"5802","RPL36AL",3296.04105764643,-0.301681749093435,0.272565926168781
,-1.10682121325255,0.268371234737806,0.754064478420299
"5803","ATP10A",41.8437950848122,0.348499369157299,0.313865378329459,1
.11034664292117,0.266849681129233,0.754064478420299
"5804","IVD",141.103620022008,0.247345421212364,0.222457052395806,1.11
187943267483,0.266189992927745,0.754064478420299
"5805","NEDD4",120.571076104004,0.263037755427519,0.238209809468173,1.
10422721891587,0.269494585213823,0.754064478420299
"5806","AC092868.2",11.6759589928815,0.879903592395447,0.7966378744444
85,1.10452141508967,0.269367019181033,0.754064478420299
"5807","SPATA41",24.7509796594065,0.398269418551749,0.360539760933372,
1.10464770243565,0.269312272597913,0.754064478420299
"5808","CAPN15",64.6937219498644,0.246640483296999,0.223679149443614,1
.10265299161992,0.270177888261488,0.754064478420299
"5809","NTAN1",1096.19514628446,-0.270580769809057,0.2437916337878,-1.
10988537877627,0.267048421783922,0.754064478420299
"5810","SH2B1",111.653494578094,0.213466126323606,0.193162955041077,1.

10510903231011,0.269112347278098,0.754064478420299
"5811","NPIPB11",11.3426719729409,0.651743498793341,0.589137642765345,
1.10626694253338,0.268610995591164,0.754064478420299
"5812","POLR2C",350.516807925785,-0.254489006822321,0.229119247209592,
-1.11072731741965,0.266685740043183,0.754064478420299
"5813","RFWD3",45.3695807671463,0.2975133925241,0.269221750762378,1.10
508676093817,0.26912199662323,0.754064478420299
"5814","SMCR8",75.7228577536232,0.330214594465092,0.299187801541393,1.
10370340222379,0.269721819309643,0.754064478420299
"5815","MPP2",15.7480578509466,-0.504245149681824,0.457409349102293,-1
.10239362328612,0.270290582580964,0.754064478420299
"5816","DBF4B",17.253150482731,0.468859203676106,0.422626950398626,1.1
0939258188308,0.267260861110188,0.754064478420299
"5817","NME1",283.514885080068,-0.383417497274839,0.347044524453285,-1
.10480779916887,0.26924288034991,0.754064478420299
"5818","TTYH2",14.7235023626913,0.483655692928775,0.43583225909496,1.1
0972899053669,0.26711582645267,0.754064478420299
"5819","SEC14L1",240.940704171278,-0.199495208242367,0.179423581980658
,-1.11186726984345,0.266195223193894,0.754064478420299
"5820","TSPAN10",33.8588335285131,0.345083630750679,0.311395614535946,
1.10818397768683,0.267782368973478,0.754064478420299
"5821","NDUFV2",1125.37777875877,-0.28707566258649,0.259430752637203,-
1.1065598803082,0.268484261192995,0.754064478420299
"5822","POLRMT",52.3313278381156,-0.262259681747283,0.237604022442022,
-1.10376785313589,0.269693853117398,0.754064478420299
"5823","LSM7",783.981005627805,-0.293899362024336,0.265677939399726,-1
.10622418514828,0.268629497260363,0.754064478420299
"5824","BABAM1",359.463422061546,-0.239495391194236,0.215664607379256,
-1.1104992798984,0.266783938242726,0.754064478420299
"5825","ANKRD27",77.65863866732,0.249942762036455,0.225943772561369,1.
10621664497775,0.268632760079677,0.754064478420299
"5826","SARS2",37.4446837110126,-0.286060754620423,0.258792889595659,-
1.10536558816345,0.269001208432077,0.754064478420299
"5827","AC007193.1",5.1496460309295,1.04607359010311,0.9470389239483,1
.10457296279009,0.2693446719147,0.754064478420299
"5828","ZNF331",223.480320924844,-0.210918408697347,0.190900181432198,
-1.10486227469752,0.269219271302505,0.754064478420299
"5829","ZFAS1",3620.88439539186,-0.249196995994934,0.224374983050529,-
1.11062736409796,0.26672877918015,0.754064478420299
"5830","APOBEC3D",37.7274401257488,0.416603091620922,0.374751975888243
,1.11167683808333,0.26627712196726,0.754064478420299
"5831","FBLN1",16964.7779828067,0.236710347946436,0.212761543591166,1.
11256171557623,0.265896710596634,0.754064478420299
"5832","ATP6AP2",1754.34433059603,-0.205063280792691,0.184399762933255
,-1.11205826694536,0.266113098700895,0.754064478420299
"5833","AL050309.1",80.8217206679474,-0.239199260152358,0.216965003018
669,-1.10247854181246,0.270253682332942,0.754064478420299
"5834","ZXDB",49.1464290051632,0.256900774309571,0.231183903212038,1.1
1123988625603,0.266465106889321,0.754064478420299
"5835","AMMECR1",84.0355896214084,0.23817342484366,0.214594613735451,1

.10987606211438,0.267052437017162,0.754064478420299
"5836","ZNF449",41.7650934926956,0.283820595326253,0.257124858884055,1
.10382401980913,0.269669483199691,0.754064478420299
"5837","FHL1",4699.22055047605,0.279054733365326,0.252184483388778,1.1
0654997331903,0.268488546607278,0.754064478420299
"5838","CD40LG",4.04182382901473,0.840048589064092,0.755487710929965,1
.11192885987521,0.266168738948852,0.754064478420299
"5839","AC245140.2",5.93007225287779,-0.748229856262324,0.676080999569
329,-1.106716291005,0.268416609665016,0.754064478420299
"5840","SERBP1",1882.44077754193,-0.139210937299596,0.126317368483725,
-1.1020728104982,0.270430018809534,0.754286742532278
"5841","HOMER2",8.21252303669706,0.731302008733837,0.663752019502867,1
.10176991895492,0.270561711104107,0.754524860841432
"5842","FOPNL",190.009483248409,0.169194954625738,0.153588321142866,1.
10161341283465,0.270629774631849,0.754585484248236
"5843","ZSWIM5",8.84099557624593,0.574498804632154,0.521608969527376,1
.10139748009453,0.270723701689103,0.754718188741022
"5844","CAPN7",201.277884019741,0.193018516568733,0.175329044353731,1.
10089299397146,0.270943231616386,0.754813459859638
"5845","NME5",13.4677203093636,0.650823176095682,0.591030864542149,1.1
0116614062085,0.270824355201606,0.754813459859638
"5846","FAM78A",6.3029464307542,0.77885980673313,0.707471831998048,1.1
0090574847831,0.270937679919523,0.754813459859638
"5847","SUFU",55.3755859928291,0.247426261695883,0.224727003110235,1.1
0100814887169,0.270893110583017,0.754813459859638
"5848","UQCRFS1",611.442745525095,-0.277025293992539,0.251748378737148
,-1.10040547383934,0.271155494573039,0.755275624333144
"5849","AC096992.2",4.63628828433465,0.790038437815725,0.7182470798654
81,1.09995356745995,0.271352353269292,0.755565552547607
"5850","OSBPL1A",1194.99599620631,0.190845289307118,0.173493560017134,
1.10001367940268,0.271326161762298,0.755565552547607
"5851","UBA6-
AS1",45.2094148244678,0.371283766621788,0.337641335832039,1.0996395500
7714,0.27148920262976,0.755817402430934
"5852","AC103702.1",18.02615508681,0.492745018450602,0.448163281734232
,1.09947654913596,0.271560257376389,0.755886027410116
"5853","GTF2B",376.925897930856,-0.243556799839814,0.22156773611127,-1
.099243076246,0.271662054170987,0.755940557952874
"5854","DGUOK",513.505538066721,-0.273232971674845,0.248570152928459,-
1.09921874551642,0.271672664144891,0.755940557952874
"5855","ITM2B",15771.3239276636,-0.223643924278009,0.203534155069575,-
1.0988029218072,0.271854037499086,0.756186888118616
"5856","AL121832.2",12.0054806014921,-0.626881855917926,0.570473750714
796,-1.09887940528806,0.271820670829768,0.756186888118616
"5857","FHL2",406.457382991958,0.477415657094063,0.434755408407443,1.0
9812471072617,0.272150036429819,0.756836007545621
"5858","ENTPD2",2.65871446550392,-0.973211066991084,0.88630420860641,-
1.09805533759263,0.272180326122061,0.756836007545621
"5859","COA7",51.1315241794317,0.268048888663403,0.244220510843066,1.0
9756910972825,0.272392687658114,0.75690967063511

"5860","ZNF445",99.385180775828,0.287516858562909,0.261926453423025,1.09770072783963,0.272335191848208,0.75690967063511
"5861","BNIP1",68.8572740220258,0.342139308397151,0.311717143839962,1.0975954167372,0.272381195089276,0.75690967063511
"5862","SNX30",55.4193949189394,0.297569555184633,0.271042782512178,1.09786931947271,0.272261556728126,0.75690967063511
"5863","DLEC1",2.33899849449051,1.22375034813834,1.11546970149193,1.09707179540743,0.272610008506189,0.756942984066919
"5864","CERCAM",661.022641444834,0.366774781957491,0.334252899509997,1.09729723360716,0.272511479815591,0.756942984066919
"5865","MESD",761.5794545847,0.180185044507196,0.164251051339921,1.09700999194397,0.272637024220064,0.756942984066919
"5866","NUBP2",293.836603021362,-0.244858597000077,0.223198543229226,-1.09704388504277,0.272622208541036,0.756942984066919
"5867","SMOX",46.9397886294,-0.345309957846062,0.314745229801319,-1.09710942422872,0.272593560980945,0.756942984066919
"5868","ZNF713",22.3962667524668,0.442040856592621,0.403023125778016,1.09681263510446,0.27272330569284,0.757047080858962
"5869","FGFR1OP2",551.664820369533,-0.198421014446437,0.180923597996036,-1.09671163211547,0.27276746992211,0.757047080858962
"5870","ISY1",233.312846413548,-0.169614947513019,0.154937954695486,-1.09472819520807,0.273635732385705,0.757392089181096
"5871","CXCL11",2.99366717893577,-1.19414420606564,1.09026764255873,-1.0952762050822,0.273395648821733,0.757392089181096
"5872","TRIM41",54.5669666261552,0.326953038226044,0.298530245522946,1.09520908895951,0.273425044707163,0.757392089181096
"5873","SDK1",193.839686737696,0.296584483830613,0.270739588092302,1.09546034963125,0.273315007285234,0.757392089181096
"5874","AC072061.1",6.8445183816763,-0.675310674092349,0.616702825486007,-1.09503418207976,0.273501661529537,0.757392089181096
"5875","BNC2-AS1",21.7760393616362,-0.573631958283985,0.52338997541336,-1.09599339924489,0.273081663121424,0.757392089181096
"5876","BCCIP",182.668446274565,-0.216639857612283,0.197841668341119,-1.09501633012289,0.273509482288646,0.757392089181096
"5877","BAD",802.553720515452,-0.274621276580767,0.250544892953148,-1.09609608618971,0.273036727240838,0.757392089181096
"5878","PKNOX2",32.4366978899791,0.415692315526243,0.379614333633469,1.09503851329178,0.273499764093045,0.757392089181096
"5879","ANO6",609.564360797369,0.212442936191239,0.194045785355044,1.0948082989926,0.273600629863327,0.757392089181096
"5880","LINC01257",4.96799100901801,0.97476750390565,0.890390272222147,1.09476432337128,0.273619900172629,0.757392089181096
"5881","ITGA11",498.779171738493,-0.423280130699437,0.386525312135978,-1.09509032761748,0.273477065742154,0.757392089181096
"5882","TCF25",1263.89269326514,-0.143638295290775,0.131135657099828,-1.09534125551702,0.273367159739642,0.757392089181096
"5883","CAMTA2",99.2164837735016,-0.219772675615532,0.200518152125114,-1.09602384266141,0.27306834053312,0.757392089181096
"5884","ATP5F1D",1768.7276848602,-0.276691063213889,0.252487332955444,

-1.09586116647965,0.273139535723891,0.757392089181096
"5885","UBE2A",442.800419285704,-0.248135305314305,0.226597581170333,-1.09504834090785,0.273495458800962,0.757392089181096
"5886","PAQR6",2.72453738441489,1.14981848630689,1.05095700975075,1.09406805001432,0.273925133836388,0.757481742237872
"5887","MACROD1",172.229690610667,-0.243402523398605,0.222484960281906,-1.09401787469227,0.273947138776064,0.757481742237872
"5888","CDH24",4.43783522961567,0.835386354654978,0.76338149394914,1.09432356073153,0.273813095536915,0.757481742237872
"5889","ZNF382",3.72360899871267,1.08480605129851,0.991530156708807,1.09407267540839,0.273923105379725,0.757481742237872
"5890","CLTCL1",7.68110843596267,0.680715404592055,0.622144021056283,1.09414441279421,0.273891646416995,0.757481742237872
"5891","AC234775.3",3.14029007039959,0.940184690658377,0.859276475773214,1.09415853589191,0.273885453312378,0.757481742237872
"5892","FYCO1",139.158787171128,0.239379534344349,0.218846111422955,1.0938258522753,0.274031363477649,0.757586028460188
"5893","BCAP29",506.769351925156,-0.172008308338584,0.157309002671909,-1.09344224053936,0.274199675893665,0.757789775727109
"5894","PTOV1",412.091968566977,-0.191551446649455,0.175176893993095,-1.09347438627954,0.27418556900763,0.757789775727109
"5895","NORAD",1485.713599699,0.149533651520065,0.136767772654204,1.09333981696212,0.274244626920702,0.757789775727109
"5896","AC010680.4",4.10637158280292,0.813979303445477,0.744572596550164,1.09321684308138,0.274298603588488,0.757810372091737
"5897","C6orf47",117.946038372128,-0.222382595843515,0.203453761144872,-1.09303752652262,0.274377323635909,0.757899308920693
"5898","TMEM184A",5.70593494897646,0.888738566269743,0.813266761228973,1.09280079875233,0.274481270862336,0.758057887601999
"5899","WDR83",82.7072207110592,-0.281384991657002,0.257553870510355,-1.09252868574417,0.274600788980902,0.758259408664166
"5900","TMEM104",19.9661960533124,0.3754753592766,0.343734522445143,1.09234113758976,0.274683185011671,0.758358372992392
"5901","SETD2",314.548018779188,0.228347133810774,0.20907955837979,1.09215427648831,0.274765295986899,0.758456516917574
"5902","CSMD2",14.7542007518981,0.513951369933893,0.470706423749889,1.09187243683545,0.274889174377664,0.758669901971835
"5903","NDUFB4",1445.91661977827,-0.262653814167166,0.24063487421311,-1.09150352801546,0.27505138032962,0.758988977501133
"5904","SELENOT",385.330580619907,-0.199154570571235,0.182479798273173,-1.091378730445,0.275106267507464,0.75901185491685
"5905","NPIPB5",74.1500500996107,0.468617081209245,0.429542027223114,1.09096910548833,0.275286477066448,0.75938042759278
"5906","PON3",41.6198250662056,0.339876848314358,0.31157265089352,1.09084300993578,0.275341967498321,0.759404894781604
"5907","BBS12",22.0641772209598,-0.37928311460978,0.347829288094341,-1.09042891899002,0.275524248756838,0.759650387271518
"5908","CNOT3",126.216078900775,0.188767391743324,0.173097812497914,1.09052442095765,0.275482201850433,0.759650387271518
"5909","AL080276.2",22.7707493559072,-0.404266099816335,0.370850514790

119,-1.0901052680084,0.275666776005278,0.759914725731931
"5910","CCNA2",3.83926416737754,0.820636299612001,0.75304914007198,1.0
8975132689689,0.275822699793241,0.760099953454175
"5911","TMEM109",856.249048559057,-0.192748332323058,0.176875377516184
,-1.08974089570732,0.275827296019868,0.760099953454175
"5912","SLC66A3",708.873707483466,-0.253066594727242,0.232334913875922
,-1.08923187869384,0.276051644329242,0.760589518687251
"5913","LINC00484",3.09165405420379,0.965400676768117,0.88655205517306
5,1.08893851312505,0.276181001180288,0.760688591177835
"5914","SOCS2-
AS1",7.87772771464906,0.652744960179206,0.599419027763256,1.0889626954
5352,0.276170336641815,0.760688591177835
"5915","MARCH3",246.94503798815,0.306004238396583,0.28112490000421,1.0
8849923429764,0.2763747744203,0.761056982841071
"5916","KMT2B",155.404111524173,-0.223075500877764,0.204952854432828,-
1.08842348888034,0.276408196358756,0.761056982841071
"5917","MMAB",146.749138733348,-0.280884735371476,0.258163033915829,-1
.08801299361494,0.276589371387466,0.76142712025189
"5918","MAP3K10",37.1126676493515,-0.288918093487266,0.265612131863018
,-1.08774434157348,0.276707986709173,0.761624940098972
"5919","RAPGEF4",13.6005475872151,0.60561026457485,0.557008213684056,1
.08725553716585,0.276923892684858,0.76182534738124
"5920","RNF13",840.388145052919,-0.173384050517334,0.159442235070583,-
1.08744116915182,0.276841885113983,0.76182534738124
"5921","KIAA0825",34.9729595156214,0.308396096265554,0.283656117567365
,1.08721820953611,0.276940385097483,0.76182534738124
"5922","PTPRO",12.9170743879819,0.717579607041708,0.660052108371727,1.
0871559956257,0.276967874466923,0.76182534738124
"5923","AL139220.2",5.30527469827823,-0.911120799289701,0.838260384648
344,-1.08691859471794,0.277072787726884,0.761886125503572
"5924","AC012640.2",5.68084294793465,-0.709244529385515,0.652542309294
969,-1.08689431977493,0.277083516942916,0.761886125503572
"5925","TAF1",179.920812283054,0.264773757229253,0.24363438231766,1.08
676679666678,0.277139885184355,0.761912504602187
"5926","SPAAR",10.5293201858319,-0.58890757462681,0.542032530987773,-1
.08648012980626,0.277266627263603,0.762132313786168
"5927","DHRS3",2680.71229677829,0.298270701902025,0.274608608128572,1.
08616661340192,0.277405285350923,0.762384797212955
"5928","HS2ST1",128.987613722365,0.246483925665279,0.227209078069632,1
.08483308747787,0.277995588066137,0.762887911416174
"5929","SLC1A4",15.2110041592573,-0.481975226278817,0.444335414253931,
-1.08471035802556,0.278049958807156,0.762887911416174
"5930","WNT5A",102.87444956397,0.547010092292257,0.504315388419915,1.0
8465873707743,0.27807282972008,0.762887911416174
"5931","NCBP2",299.888906932124,-0.161100887711354,0.148520838673439,-
1.08470224885799,0.278053551528629,0.762887911416174
"5932","CCDC183",16.4578087317991,0.519311128776292,0.478584112045535,
1.08509897363012,0.277877821875652,0.762887911416174
"5933","DLEU1",24.656623932593,-0.478059376074624,0.440529205717487,-1
.08519337621671,0.277836017291175,0.762887911416174

"5934","SLC7A7",52.3643164755057,-0.279972911665373,0.257929843705887,
-1.08546148690193,0.277717312373424,0.762887911416174
"5935","AC012291.3",7.0472364371675,0.658199443547224,0.60668185112403
,1.08491698297509,0.277958425436749,0.762887911416174
"5936","PDE8A",152.312860441878,0.224481843864007,0.206974025526521,1.
08458944687841,0.278103531093943,0.762887911416174
"5937","LILRB2",12.5584652058379,-0.807588808253393,0.74455400484692,-
1.08466115687529,0.278071757588221,0.762887911416174
"5938","ANKRD54",78.2400615467795,-0.218395213023613,0.201173153111071
,-1.08560814227052,0.277652395918542,0.762887911416174
"5939","TSPAN12",17.3349721484045,0.456213165294449,0.420706203872091,
1.08439847355603,0.278188160247979,0.762991571355334
"5940","NSD2",47.757304639078,0.314905036975347,0.290443094443474,1.08
422284089331,0.278266006715143,0.763076596529118
"5941","FGFR1OP",51.4233134974471,0.287505731652025,0.26522232214025,1
.08401785088056,0.278356884124207,0.763079870341445
"5942","SEC31B",28.777803443263,0.420321791241591,0.387747579042129,1.
08400880872018,0.27836089321437,0.763079870341445
"5943","CDC7",9.07522948027986,0.559521636585854,0.517367849448193,1.0
8147739984736,0.27948480851453,0.763108650468337
"5944","COQ8A",222.394181145946,0.194856016739908,0.180053891225852,1.
08220941748762,0.279159484815687,0.763108650468337
"5945","CCDC74A",91.6660566459586,-0.363974316456646,0.336465546360033
,-1.08175805931457,0.279360047220305,0.763108650468337
"5946","AC064836.4",47.2710705152491,-0.425817491651743,0.393516078060
096,-1.08208410124151,0.27921515965651б,0.763108650468337
"5947","FAM184B",8.9180736901562,0.986510228819133,0.911241344786131,1
.08260038294319,0.278985837265451,0.763108650468337
"5948","FUCA2",623.297043617963,-0.229447003827114,0.212046903763417,-
1.08205778888953,0.279226850528538,0.763108650468337
"5949","AL035530.2",16.4276372797409,-0.428576486774198,0.396318795145
549,-1.08139329253058,0.279522204038137,0.763108650468337
"5950","TAF6",129.450498714625,-0.220762746641581,0.203795674107546,-1
.08325531250031,0.278695114542094,0.763108650468337
"5951","GAL3ST4",66.1848720634493,0.291895078697182,0.269830347014972,
1.08177260981318,0.279353580133215,0.763108650468337
"5952","MTBP",17.0152758227099,0.408035350039287,0.376695811648954,1.0
8319587694152,0.278721489433725,0.763108650468337
"5953","NFIB",4673.30178897337,0.201263847076245,0.185801071192359,1.0
8322221063988,0.278709803484996,0.763108650468337
"5954","CELF1",425.080259215836,0.262791263491518,0.242865566433932,1.
08204414215717,0.279232914055316,0.763108650468337
"5955","TMX2",281.856183016904,-0.263271325596351,0.243190937197936,-1
.08257046347937,0.278999123417329,0.763108650468337
"5956","GAPDH",4529.31564740975,-0.32563377338483,0.301137046855262,-1
.0813474356124,0.279542594225831,0.763108650468337
"5957","NAA25",98.3665585955342,0.2259916768213,0.208848995996283,1.08
208170091142,0.279216226136202,0.763108650468337
"5958","OSGEP",182.670170807233,-0.225992964406145,0.208879970702493,-
1.08192740379127,0.279284786996142,0.763108650468337

"5959","LINC02126",4.39390602522282,0.813833639385121,0.751539815097875,1.08288825559978,0.278858025418699,0.763108650468337
"5960","METTL23",254.668672096865,-0.270367931027952,0.249515121299507,-1.08357333062558,0.278554020909512,0.763108650468337
"5961","CBX2",2.3958016264088,-1.04921624327031,0.968984027015455,-1.08280034966312,0.278897050326225,0.763108650468337
"5962","KCTD1",73.5077701477242,0.403404545614513,0.372667747475322,1.0824777522268,0.279040295864616,0.763108650468337
"5963","XAB2",196.584901517472,-0.183247978110306,0.169431441166752,-1.08154647595753,0.279454098661335,0.763108650468337
"5964","NKG7",58.3361173750688,0.459045649833703,0.423975120683027,1.08271836586585,0.278933449510613,0.763108650468337
"5965","AC010332.3",8.23832270842864,-0.631462583532373,0.582701290410576,-1.08368145724791,0.27850605994444,0.763108650468337
"5966","PTPRA",1165.08518156212,0.214724444412224,0.198280310556252,1.08293377093187,0.278837820829753,0.763108650468337
"5967","OCRL",94.7771871930178,0.240597663534001,0.222177101883853,1.08290936146866,0.278848656245244,0.763108650468337
"5968","TRMT13",134.048141129544,0.258164634733679,0.23877184967891,1.08121889192904,0.279599756309304,0.763136801360968
"5969","UBR4",121.848461111342,0.305108786485543,0.282287050746857,1.08084584708475,0.279765690587381,0.76320605157894
"5970","TGFBI",178.490727366251,-0.43667183553731,0.403999917910012,-1.08087109966833,0.279754455860474,0.76320605157894
"5971","ARPC5L",193.71642024057,-0.25808405980624,0.238755339373994,-1.08095618084573,0.279716605998714,0.76320605157894
"5972","CYP20A1",256.396997809255,-0.203242064681829,0.188372759711902,-1.07893553713747,0.280616465420902,0.763353641489825
"5973","RPSA",8919.44244117809,-0.234466505617913,0.217238461476121,-1.07930476042193,0.280451891461811,0.763353641489825
"5974","RPL29",19569.0405699255,-0.293296332677463,0.271520314507735,-1.08020032758583,0.280052982551842,0.763353641489825
"5975","MTRNR2L12",66.8302968832723,-0.373376938327472,0.345990440653629,-1.07915391425875,0.28051912022616,0.763353641489825
"5976","LCORL",91.4046154094349,0.236823132966528,0.219391411192778,1.07945489606443,0.280384990228005,0.763353641489825
"5977","TLR1",21.2151150276313,0.398982704605229,0.369659588386737,1.07932464662005,0.28044302944418,0.763353641489825
"5978","BTF3",6353.90026056965,-0.267217090862678,0.247455282112234,-1.07986012091462,0.280204474018089,0.763353641489825
"5979","ARMT1",149.870715276536,0.231302178365907,0.214339713025478,1.07913822922032,0.280526111325496,0.763353641489825
"5980","FAM180A",172.58164893852,0.317766543311036,0.294353690253801,1.07953986592473,0.280347132014474,0.763353641489825
"5981","PYCR3",27.5881157432838,-0.511930401836235,0.473897153788696,-1.08025633355987,0.280028048849684,0.763353641489825
"5982","ELP1",81.6483582741499,0.253389395385519,0.234769530568453,1.07931124951343,0.280448999664258,0.763353641489825
"5983","FBXO3",200.151112671446,-0.159059103511521,0.147252916955777,-1.08017624913529,0.280063702673991,0.763353641489825

"5984","MTMR10",75.274638403043,0.229828076814119,0.213003399617896,1.07898783412098,0.280593151091192,0.763353641489825
"5985","MTFMT",61.6968007791315,-0.287747381801807,0.266419302637956,-1.08005455668065,0.28011788642161,0.763353641489825
"5986","FAM222B",84.0528251228053,0.282045963867438,0.261051219697344,1.08042385013345,0.279953479945699,0.763353641489825
"5987","TIMM17B",138.259586005335,-0.27106936866461,0.250971479302378,-1.08008037175419,0.280106391626308,0.763353641489825
"5988","CTAGE9",3.25818989633348,-1.0380852303474,0.96193622056916,-1.0791622318065,0.280515416840503,0.763353641489825
"5989","MAP3K2-DT",25.4472160631731,-0.375503823656742,0.348216606837814,-1.07836276697635,0.280871896129655,0.763475496549179
"5990","ANXA6",1029.43049990052,0.187290434085217,0.173658745363616,1.0784969895588,0.280812024507977,0.763475496549179
"5991","EIF3F",2303.03181598761,-0.250840860742194,0.23262415581116,-1.07830960145782,0.280895613654566,0.763475496549179
"5992","STX5",300.402659149752,-0.229171256124449,0.212484748600563,-1.07853037751549,0.280797132748252,0.763475496549179
"5993","STAT5B",190.912960844039,0.266351978657019,0.2469839737086,1.07841806355934,0.280847229363949,0.763475496549179
"5994","CDH26",2.7840744679727,1.12919942751734,1.04742943304619,1.07806730638965,0.281003720467828,0.763580502224056
"5995","AC024132.3",7.85323185512115,-1.15123018299016,1.06791874886026,-1.07801289584888,0.281028001156192,0.763580502224056
"5996","C16orf54",5.21127252577582,-0.711443004393675,0.660074565581271,-1.07782217569187,0.28111312121766,0.763684394848976
"5997","CD46",694.872421130635,0.188514355093254,0.174990361300742,1.07728422121073,0.281353309265833,0.763827342438525
"5998","COX7A2L",1029.168236721,-0.252545310222635,0.234424214852749,-1.07730044177078,0.281346065010115,0.763827342438525
"5999","PGGT1B",181.0325926255,0.225023503284461,0.208870181785894,1.0773366564842,0.281329891632984,0.763827342438525
"6000","ZSWIM9",47.6686104035319,-0.343398135360221,0.318738434250284,-1.0773665754114,0.281316530410167,0.763827342438525
"6001","CHMP1B",344.296416245135,-0.209293345758759,0.19431298968024,-1.07709395086335,0.28143829525638,0.763930743447955
"6002","C2orf27A",108.061871567641,0.204880160798409,0.190252329133963,1.0768864787676,0.28153098441723,0.764020490936817
"6003","ARHGEF9",96.3042803296182,0.278228206189601,0.258381898472845,1.07680997714645,0.28156516711239,0.764020490936817
"6004","PGRMC2",765.312770928329,0.16234253907525,0.150884043081495,1.075942397617,0.281953018788383,0.764216382275356
"6005","B4GALT1",631.337239567999,-0.317121943329001,0.294836531736749,-1.07558565236465,0.2821126069453,0.764216382275356
"6006","AL365295.1",54.6749783827623,0.428357533863278,0.398202863260267,1.07572690551776,0.282049410748351,0.764216382275356
"6007","ADGRG1",4.44490379033422,1.03199799024418,0.95952320058254,1.07553208678815,0.282136574519456,0.764216382275356
"6008","PSMG2",1150.12011568149,-0.263096108229351,0.244628058721644,-

1.07549440405249,0.282153436245564,0.764216382275356
"6009","CEP76",31.6625463061481,0.465392272554508,0.432468214779035,1.07613058405297,0.281868859227197,0.764216382275356
"6010","C5AR2",21.9616071905624,0.90323827217232,0.839748377604308,1.07560585558872,0.282103567500738,0.764216382275356
"6011","RPL28",23016.6602733871,-0.228831217551246,0.212604836070424,-1.07632178919697,0.28178336711222,0.764216382275356
"6012","UBE2V1",914.075223091913,-0.182072828900318,0.169173283773097,-1.07625048612595,0.281815246259974,0.764216382275356
"6013","HSCB",186.240763975317,-0.302156563914727,0.280864646097603,-1.07580846544041,0.282012925479419,0.764216382275356
"6014","C22orf31",4.51947511551861,0.772235530629327,0.717399659185471,1.07643699121089,0.281731866196848,0.764216382275356
"6015","LINC01128",79.0418038228327,-0.291703778602061,0.271939043249473,-1.07268075637987,0.28341437738566,0.764457049443572
"6016","AL354743.1",3.00486223261763,-0.998803846584893,0.930227616559861,-1.0737198388913,0.282948266938561,0.764457049443572
"6017","ADGRB2",6.65392583982942,0.706987684112885,0.659174659748397,1.0725346820564,0.283479944911786,0.764457049443572
"6018","ELOVL1",323.048357723887,-0.201042081980802,0.187096038392876,-1.07453949163072,0.282580954746943,0.764457049443572
"6019","HIPK1-AS1",6.12176247959361,0.673177631700161,0.6270487002503,1.07356514961509,0.283017624347845,0.764457049443572
"6020","TARS2",74.7588646364459,0.225529198306747,0.210141762490493,1.0732240732822,0.283170592073316,0.764457049443572
"6021","PLCD1",78.7555410918417,0.266217413356806,0.247869997781762,1.0740203160497,0.282813576120482,0.764457049443572
"6022","ZKSCAN7",19.2894583572208,0.413813134327138,0.385731784358022,1.072800197204,0.283360772316612,0.764457049443572
"6023","NISCH",231.850364966695,0.24289105955158,0.226066958456066,1.07442087605555,0.282634090106828,0.764457049443572
"6024","AC092040.1",142.438557052831,-0.33831165151176,0.314881749234118,-1.07440857507503,0.282639600875525,0.764457049443572
"6025","MSL2",100.041941449824,0.238370057920481,0.222062241277076,1.07343804398991,0.283074622807472,0.764457049443572
"6026","SNHG8",1280.0197507949,-0.29092682174006,0.27087694333408,-1.07401840171111,0.282814434097499,0.764457049443572
"6027","FGF18",174.136866123071,-0.38975946697227,0.363282926483086,-1.0728813235059,0.283324366730706,0.764457049443572
"6028","LYRM4",264.397400143438,-0.168621428068251,0.156848927126073,-1.07505630518413,0.282349520554864,0.764457049443572
"6029","CHRAC1",237.763340088193,-0.186054869024142,0.173439254667854,-1.07273794147955,0.28338871184291,0.764457049443572
"6030","WNT5B",90.94088342164,0.25422002186666,0.236656967290853,1.07421313125433,0.282727168357523,0.764457049443572
"6031","AC023157.3",42.4893642281023,-0.399089911860374,0.371757904415034,-1.07352098535295,0.28303742821445,0.764457049443572
"6032","STYX",65.6516934280601,0.245026436557984,0.228470516986504,1.07246414018688,0.283511612306112,0.764457049443572

"6033","ZSCAN29",56.1732451573064,0.289938784447533,0.270305876589351,
1.07263219026498,0.283436175833244,0.764457049443572
"6034","GFER",211.039908409014,-0.222029051169519,0.206808607685459,-1
.07359676008848,0.283003450350455,0.764457049443572
"6035","RPA1",193.886574542175,0.186062983523933,0.173409576749764,1.0
7296832742075,0.283285327075773,0.764457049443572
"6036","SNX11",58.652835769732,-0.271992112954765,0.253080744816301,-1
.07472464233575,0.282498027662323,0.764457049443572
"6037","BPTF",1135.61007070183,0.245309399069109,0.228299704876319,1.0
7450598414924,0.28259596416436,0.764457049443572
"6038","AC023043.1",9.81907151694038,-0.617049100759718,0.575365546664
341,-1.07244708053347,0.283519271022185,0.764457049443572
"6039","AC012615.1",15.8967182833982,-0.465246488837407,0.433224620927
227,-1.07391516170444,0.28260707288383,0.764457049443572
"6040","RPL18A",21774.6624633656,-0.222390484071612,0.207383299934983,
-1.072364477474,0.283556356604952,0.764457049443572
"6041","ZNF850",15.3519151589556,0.463815606492946,0.432287571049384,1
.07293301393567,0.28330117220234,0.764457049443572
"6042","AL031651.2",7.49737683872477,-0.771227836841754,0.717348299887
298,-1.07510931156165,0.282325791046274,0.764457049443572
"6043","NDUFV2-
AS1",23.3570521341539,-0.386426780894434,0.360430528210199,-1.07212555
721438,0.28366364106517,0.764493224571566
"6044","MOB3A",153.099566769635,0.175754404719831,0.163929087979072,1.
07213678113227,0.283658600475606,0.764493224571566
"6045","AL023806.1",8.87901955015096,-0.559876302288567,0.522335149694
131,-1.07187177163248,0.283777630734486,0.764547440792929
"6046","ZNF383",80.3842445764773,0.267998214291961,0.250019786650929,1
.07190801928862,0.283761347933536,0.764547440792929
"6047","LINC01277",9.89202972842794,-0.651358250625046,0.6079183643800
48,-1.07145677576182,0.283964096015437,0.764923294194717
"6048","PACC1",25.6891875878183,-0.500570123059516,0.467918883467092,-
1.06977970059787,0.284718481537517,0.765076250166987
"6049","SFXN5",53.6956337466698,0.312108419087597,0.29174626931169,1.0
697940365234,0.284712027180966,0.765076250166987
"6050","PAX8-
AS1",123.801957015569,-0.841096092291845,0.785545841986016,-1.07071547
876237,0.284297380775102,0.765076250166987
"6051","ITGAV",578.023771933758,0.197291229583703,0.184380701502497,1.
0700210378635,0.284609839258064,0.765076250166987
"6052","LRTM1",11.8969933126006,-0.579455366664608,0.541666385841885,-
1.06976430845711,0.284725411536146,0.765076250166987
"6053","PTER",161.397100451846,0.242085134002802,0.226056146161403,1.0
7090710920089,0.284211198980852,0.765076250166987
"6054","UNC5B",18.707135478318,-0.444767239358368,0.415725060234807,-1
.06985910136656,0.284682734767302,0.765076250166987
"6055","ATP5MD",1447.80040153036,-0.294529563167993,0.275292914322781,
-1.06987702132666,0.284674667498438,0.765076250166987
"6056","GSTP1",4546.82064752215,-0.276196523947336,0.257908220518898,-
1.07091012218084,0.28420984409715,0.765076250166987

"6057","AC016957.2",4.38385871565091,0.788829742067777,0.7373759942214
6,1.06977952665877,0.284718559849424,0.765076250166987
"6058","DCTN2",785.99541803524,-0.225175742640651,0.210476679939811,-1
.06983701332159,0.284692678651661,0.765076250166987
"6059","TGFB3",158.014438561002,-0.260164713883885,0.243112889144343,-
1.07013953394062,0.284556506401141,0.765076250166987
"6060","TNFSF13",105.301331021119,-0.417087121233922,0.389611406028811
,-1.07052081838456,0.284384943327756,0.765076250166987
"6061","STX8",497.521347703374,-0.287136085489068,0.268138857938019,-1
.07084846895052,0.284237569333015,0.765076250166987
"6062","COASY",145.83338743973,-0.24609822307555,0.23001484867307,-1.0
6992320059015,0.284653879072663,0.765076250166987
"6063","PHLPP1",66.6157382572774,0.271655647131427,0.254015309030375,1
.06944596437273,0.284868765077777,0.765335199464277
"6064","AL137186.2",59.9795565648156,0.330739943042103,0.3093603063797
05,1.06910917859047,0.28502047623823,0.765364084643015
"6065","VDR",68.7196085445635,0.570865974005619,0.533940181187251,1.06
915717175707,0.284998853522581,0.765364084643015
"6066","CDKL5",88.4365419667429,0.254117212599345,0.237671924326495,1.
06919323062433,0.284982608384867,0.765364084643015
"6067","NUF2",6.57928585064962,0.825804934170392,0.772882611166663,1.0
6847394706402,0.285306776317329,0.765375836533756
"6068","LOXL3",49.1547687923363,-0.308808089109443,0.288895371346778,-
1.0689270917351,0.285102523263472,0.765375836533756
"6069","FST",1720.90454996688,-0.346577279393737,0.324307270319043,-1.
06866947217306,0.28521863206491,0.765375836533756
"6070","CRPPA",29.3065580778119,0.352557400863672,0.329917557472638,1.
06862272976458,0.285239702234148,0.765375836533756
"6071","AP002387.2",17.9410930924606,-0.448870584933006,0.420086424084
873,-1.06851961691177,0.28528618634341,0.765375836533756
"6072","LMF2",460.825077265133,-0.218196876631218,0.204197587950931,-1
.06855756143237,0.28526908005058,0.765375836533756
"6073","LRRC8C",27.365500084861,0.471025071608613,0.440897943009087,1.
06833129770103,0.285371095388102,0.765422323855885
"6074","MTF2",176.761917844132,0.186079950541245,0.1743781784617,1.067
10571347157,0.285924102124958,0.765859264793809
"6075","MAPKAPK2",141.219189232739,-0.294286888032287,0.27582627648529
5,-1.06692840066663,0.286004168932454,0.765859264793809
"6076","AC099778.1",24.1386867349447,-0.346584591636303,0.324832179210
123,-1.06696507864176,0.28598760549974,0.765859264793809
"6077","ACAP2",470.197027231098,0.152342541859076,0.142716361134876,1.
06744973489832,0.285768800107581,0.765859264793809
"6078","AC010623.1",4.34943047663325,0.966504180488874,0.9052335172506
99,1.06768492556955,0.285662660508781,0.765859264793809
"6079","AC007938.3",13.0010848946406,0.85781737053464,0.80388294653596
5,1.06709238481931,0.285930120242322,0.765859264793809
"6080","RPUSD4",101.970524391795,-0.250877236723886,0.23512990007905,-
1.06697292279519,0.285984063237082,0.765859264793809
"6081","TM9SF2",598.669991996927,0.123510651523562,0.115690232252754,1
.06759792178239,0.285701921490374,0.765859264793809

"6082","DAAM1",561.361055952788,-0.26752469609407,0.2505468759504,-1.0
6776304864815,0.285627410107785,0.765859264793809
"6083","MYEF2",3.502625577578,1.24079015120591,1.16240569052049,1.0674
329636586,0.28577636984688,0.765859264793809
"6084","ACACA",64.7826927707449,0.232731981146861,0.218168514455217,1.
06675329264633,0.286083255021495,0.765945125089602
"6085","SH3BGRL2",43.837083929904,0.336497978032224,0.315578144385695,
1.06629050211082,0.286292341662096,0.766198961818629
"6086","FEN1",25.0580930085824,-0.365413295281055,0.342714903173585,-1
.06623112066992,0.286319177395174,0.766198961818629
"6087","CARHSP1",1564.67988586855,0.239588435927684,0.224674189395858,
1.06638166391934,0.286251147013085,0.766198961818629
"6088","GSTA4",280.678791336344,0.286199789694344,0.268476243719963,1.
06601532310199,0.286416715202302,0.766208223670602
"6089","SMPDL3A",224.039900461098,0.336636407020791,0.315761884803451,
1.06610842923722,0.286374629639019,0.766208223670602
"6090","TSG101",366.301286870062,-0.234628050484641,0.220195433604125,
-1.06554457848778,0.286629563761562,0.766567407011062
"6091","CCR10",20.6394267619319,0.398140263971112,0.373661609214448,1.
06551022142233,0.286645102590974,0.766567407011062
"6092","FAM160A2",74.6731367576994,0.219232178936292,0.205801802795629
,1.06525878762102,0.286758837037643,0.766619841868729
"6093","BRF1",80.4167463500691,0.236891920743256,0.22236421138059,1.06
533294756592,0.286725288100508,0.766619841868729
"6094","SLC35B2",238.437080384003,0.207850671735173,0.195158784307585,
1.06503364669245,0.286860703928661,0.766766328568093
"6095","PARP14",371.212439012812,0.25539261528778,0.239879475750108,1.
06467055795066,0.287025037925069,0.766830386034578
"6096","NDFIP1",2401.97734612276,-0.235010569295011,0.220752591219834,
-1.06458804400161,0.287062392606063,0.766830386034578
"6097","RCE1",70.5035883963665,-0.258100401330902,0.242434831665343,-1
.06461765233134,0.287048988316578,0.766830386034578
"6098","ARHGAP5-
AS1",15.0709701553031,-0.506902502433064,0.476219486288957,-1.06443040
872437,0.2871337643961,0.766830386034578
"6099","AC103740.1",10.1792312076577,0.634180962989003,0.5958349734549
39,1.06435672836006,0.287167128418622,0.766830386034578
"6100","DPEP1",4.12352527300149,-1.15977315621463,1.08949479343041,-1.
06450545996914,0.287099782300035,0.766830386034578
"6101","CEP290",252.008973652567,0.195545214717693,0.183882233094277,1
.06342636494651,0.287588641713038,0.76783009094635
"6102","ABAT",27.7265102705316,0.313681772052511,0.295170130639653,1.0
627151581115,0.287911144643458,0.768061682786978
"6103","AC009107.2",116.222505733292,-0.353179869322649,0.332323214888
478,-1.06276014885439,0.28789073597485,0.768061682786978
"6104","IKZF3",8.15601268584987,0.865261811611921,0.814133776401033,1.
06280053314691,0.287872417712022,0.768061682786978
"6105","HIF3A",109.614254766552,-0.421568748771892,0.396664221523451,-
1.06278490949547,0.28787950448685,0.768061682786978
"6106","NAP1L3",39.3472099135816,-0.302851571952258,0.284897416449218,

-1.0630197203148,0.287773008409938,0.768061682786978
"6107","ZBTB40",79.3150530749619,0.347847226960471,0.328564957995076,1
.05868632212959,0.289742659102604,0.768216598166307
"6108","GPX7",236.879956605351,0.26675903538766,0.251672218062493,1.05
994629618364,0.289169031997968,0.768216598166307
"6109","MAGOH",522.774574208556,-0.285222365457879,0.270013063574822,-
1.05632802236194,0.290818377685059,0.768216598166307
"6110","ATP5PB",806.79979510786,-0.229854378444033,0.217354852386577,-
1.05750746265938,0.290280050579847,0.768216598166307
"6111","LIX1L",259.053310146569,0.200577253110391,0.189286730540951,1.
05964772352068,0.289304893653187,0.768216598166307
"6112","RNASEL",105.707149190259,0.191208539618702,0.180899064576182,1
.05699020648157,0.290516057082835,0.768216598166307
"6113","BIRC6",312.106898833776,0.224511662726377,0.213540668051065,1.
05137660556858,0.29308565607954,0.768216598166307
"6114","DHX57",81.4656264778902,0.286422267984361,0.27228704176506,1.0
5191295967546,0.292839485461615,0.768216598166307
"6115","MAT2A",661.828008636627,0.320980503375585,0.303675870746475,1.
05698389070746,0.290518939554572,0.768216598166307
"6116","PSD4",5.46637961003764,-0.875525149407604,0.83094812199734,-1.
05364598129558,0.292045029051951,0.768216598166307
"6117","RALB",248.27421161697,-0.164051719698342,0.155651193933415,-1.
05397019806042,0.291896561679155,0.768216598166307
"6118","KLHL23",38.9549706754323,0.373738971353949,0.35539947258046,1.
05160249293656,0.292981963556093,0.768216598166307
"6119","AC010136.1",3.10529151639892,-1.38910232616548,1.3121553738542
7,-1.0586416470522,0.289763012334699,0.768216598166307
"6120","ARPC2",1991.50260499633,-0.20984112472558,0.197958471279697,-1
.06002599115394,0.289132775094234,0.768216598166307
"6121","GLB1L",73.4370804285935,0.318618355982436,0.301901406104969,1.
05537221602623,0.291255124750294,0.768216598166307
"6122","RPL32",29200.8522972782,-0.269845636197381,0.254980812883494,-
1.05829781129719,0.289919690476852,0.768216598166307
"6123","ZNF501",33.2098365878848,0.34425671692562,0.325391691076308,1.
05797636008133,0.290066220067914,0.768216598166307
"6124","STIMATE",96.7990662306907,-0.220586948024378,0.209071088638693
,-1.05508107056154,0.291388248618455,0.768216598166307
"6125","THOC7",817.38814938835,-0.251657981843112,0.238950360703633,-1
.05318100839881,0.292258040896024,0.768216598166307
"6126","AC097634.1",4.79583160423029,0.758489101523406,0.7140106552011
12,1.06229381312211,0.288102322218363,0.768216598166307
"6127","DZIP3",152.969362580191,0.188268869646975,0.178729721119866,1.
05337192083857,0.29217056809633,0.768216598166307
"6128","CXCL1",17.9597646832713,-0.920745215310556,0.872907308649767,-
1.05480296268201,0.291515449351365,0.768216598166307
"6129","AC093827.4",47.1298245454299,-0.356168211305138,0.337362800017
716,-1.05574239746182,0.291085921393529,0.768216598166307
"6130","ADH1B",7877.59300232251,0.277904253286915,0.263721093945487,1.
05378090591555,0.291983237361993,0.768216598166307
"6131","PLK4",4.40638241770573,0.818932872462124,0.7789538944332,1.051

32393369445,0.293109838394229,0.768216598166307
"6132","ABHD18",55.0660849650818,0.296496005676257,0.281533988607047,1
.05314462080844,0.292274715060481,0.768216598166307
"6133","OXCT1-
AS1",3.74793548655594,-0.786457617064358,0.746392414594573,-1.05367846
951064,0.292030149558081,0.768216598166307
"6134","AC025171.1",44.5975620957157,0.265730907523275,0.2502779320653
46,1.06174326010451,0.28835225455369,0.768216598166307
"6135","TRAPPC13",58.7053906388922,-0.2549970946029,0.240588859329009,
-1.05988737514312,0.289195839855858,0.768216598166307
"6136","RAD17",134.232133299524,0.178706865920089,0.169516338461065,1.
0542161749272,0.291783956251358,0.768216598166307
"6137","NSA2",1594.46739633512,-0.262597844392729,0.248302387507516,-1
.05757277257264,0.290250261051532,0.768216598166307
"6138","FAM174A",181.408091761124,-0.259612408226931,0.246295417903698
,-1.0540691760999,0.291851247141566,0.768216598166307
"6139","CCDC69",178.497118610339,0.361985215257066,0.343635012316772,1
.05340027145831,0.292157579829429,0.768216598166307
"6140","LTB",22.6901355491156,0.614329259280748,0.582299048563561,1.05
500646239454,0.291422369172179,0.768216598166307
"6141","SNHG32",1319.58410660051,0.255860235326041,0.241765217323101,1
.05830043775116,0.289918493445328,0.768216598166307
"6142","TAP1",165.114283004934,0.318947470671555,0.301229767867378,1.0
588179014631,0.289682719310306,0.768216598166307
"6143","GNMT",2.49674834657025,1.02015920103419,0.966080844096311,1.05
597705126683,0.290978699546303,0.768216598166307
"6144","THEMIS",2.95722026448679,1.06773700185906,1.01506199371682,1.0
5189339022473,0.292848464818084,0.768216598166307
"6145","C7orf61",6.21611355756704,0.670262413243358,0.635928932944869,
1.05398949240994,0.291887727888878,0.768216598166307
"6146","SERPINE1",118.377442087628,-1.01273409970668,0.961444077376846
,-1.05334685972561,0.292182049664769,0.768216598166307
"6147","COG5",134.616153991995,0.194814712726247,0.185154824731844,1.0
5217194857543,0.292720666953321,0.768216598166307
"6148","SORBS3",654.12342048574,-0.12836903392549,0.121488821079191,-1
.05663247684175,0.290679352518198,0.768216598166307
"6149","PEX2",380.157133829665,0.221390043760649,0.209868689918133,1.0
5489791663068,0.291472015203643,0.768216598166307
"6150","MMP16",41.3740122807588,-0.374069136550206,0.352195971533223,-
1.06210509711898,0.288187976401407,0.768216598166307
"6151","NBN",373.367801235122,-0.141332436135067,0.133990247176356,-1.
05479644312505,0.291518431708174,0.768216598166307
"6152","RPL8",20041.2908126328,-0.244098342794825,0.231745797453877,-1
.05330213309869,0.292202541599987,0.768216598166307
"6153","CD72",12.9228231018819,-0.52679548643405,0.500393260546279,-1.
05276295260042,0.292449648310964,0.768216598166307
"6154","AL360020.1",7.41346733161071,0.639780806049217,0.6058838161086
21,1.05594635314458,0.290992725123108,0.768216598166307
"6155","MEGF9",143.29195924787,0.261844381559621,0.249037226705455,1.0
5142666830816,0.293062672892354,0.768216598166307

"6156","MAPKAP1",213.80065019625,-0.1965050645684,0.186725022818863,-1
.05237670667752,0.292626751277884,0.768216598166307
"6157","ZBTB43",179.010456915548,0.27962908501514,0.263379266949434,1.
06169740790123,0.288373076485915,0.768216598166307
"6158","SPTAN1",1044.84835634607,0.174062217755997,0.164097603118963,1
.06072370618241,0.288815483251223,0.768216598166307
"6159","TACR2",2.38844326156243,-1.16774662236115,1.10412919381064,-1.
0576177397601,0.290229751558767,0.768216598166307
"6160","ADIRF-
AS1",28.0773473769763,-0.483907656184242,0.458195897570983,-1.05611520
912685,0.290915582626427,0.768216598166307
"6161","IFIT5",162.588508714929,0.24225921023939,0.228613920413709,1.0
5968704705728,0.289286997524791,0.768216598166307
"6162","DNMBP",170.743751393786,0.215156930540273,0.204066697094473,1.
05434612116384,0.291724480120001,0.768216598166307
"6163","AL121929.3",7.32992527880238,-0.568045455831756,0.534774684276
062,-1.06221455976498,0.288138291541575,0.768216598166307
"6164","NAA40",27.9474919895802,0.332439745615208,0.313556890617645,1.
06022146399133,0.289043858488233,0.768216598166307
"6165","CD248",2656.24552261749,0.348040667950717,0.329264356381052,1.
0570250353729,0.290500161784791,0.768216598166307
"6166","AP000766.1",27.7469857635198,0.448185175018931,0.4235989053894
93,1.0580413908455,0.290036572570727,0.768216598166307
"6167","PPP2R1B",107.920327539241,-0.223941443473891,0.212143781601531
,-1.05561163180602,0.291145684498815,0.768216598166307
"6168","FMNL3",47.1002227753355,-0.377987995849903,0.356875108074687,-
1.05916043819676,0.289526718918231,0.768216598166307
"6169","LGR5",11.2866250746622,0.698331749772229,0.662159319975725,1.0
5462798559995,0.291595499339204,0.768216598166307
"6170","NAV3",192.925624904356,0.214592057233478,0.202883094651097,1.0
5771285479708,0.290186372891548,0.768216598166307
"6171","NR2C1",136.874027277777,0.184271692312016,0.173824668544602,1.
06010092730154,0.289098685991539,0.768216598166307
"6172","TMEM119",610.957214278659,-0.232382987424425,0.221032428380581
,-1.05135246048286,0.293096741223462,0.768216598166307
"6173","ATP2A2",326.856441448345,0.247424894182789,0.234217755051686,1
.05638829186194,0.29079085285411,0.768216598166307
"6174","IQCD",13.9395848344835,-0.481452072909921,0.453665859515005,-1
.06124819139933,0.288577123688972,0.768216598166307
"6175","KBTBD7",106.487747402761,0.233822193855016,0.220775237966599,1
.05909610157635,0.289556015220182,0.768216598166307
"6176","LPAR6",172.531710343174,0.219133752590962,0.208281738629493,1.
05210257045517,0.292752492960416,0.768216598166307
"6177","FGF14-
AS2",74.3428674373027,-0.27578856305043,0.262143958574026,-1.052050043
61201,0.292776590282238,0.768216598166307
"6178","TOGARAM1",168.884816534655,0.21345427511086,0.202919041798853,
1.05191840656556,0.292836986212914,0.768216598166307
"6179","MOK",109.96619877825,-0.228679520162627,0.216296079755781,-1.0
5725226467732,0.29039647265935,0.768216598166307

"6180","AC010809.2",4.78659194557294,0.788745650216935,0.749363648401476,1.05255392612047,0.292545482997784,0.768216598166307
"6181","MYO5A",96.6469360157861,-0.247630557317629,0.233458946653698,-1.06070279536108,0.288824989212831,0.768216598166307
"6182","ALPK3",13.1091907404108,-0.603020849021613,0.573104000541685,-1.05220143019705,0.292707143475051,0.768216598166307
"6183","WDR90",56.7372513766716,0.375363940972395,0.356918156053966,1.05168071336679,0.29294606257993,0.768216598166307
"6184","ITPRIPL2",585.413431565359,-0.290096307985282,0.273138994138539,-1.06208309399478,0.288197964269497,0.768216598166307
"6185","AC009093.6",2.42820986404172,-1.10442573117813,1.04393226403038,-1.0579476937653,0.29007928967674,0.768216598166307
"6186","AC093249.6",7.51402569316901,-0.625599806243886,0.594157619071523,-1.05291893289444,0.292378148035597,0.768216598166307
"6187","CES1",226.032774320739,0.524480194910959,0.494315474693422,1.06102321647172,0.28867935041183,0.768216598166307
"6188","ARL2BP",541.51692635703,-0.191115865053683,0.180706372921454,-1.05760445502801,0.290235810611317,0.768216598166307
"6189","BCO1",2.68367151120166,1.12357856837168,1.06573235294434,1.05427837042531,0.291755488480203,0.768216598166307
"6190","AC007952.4",317.350637924644,-0.365072648261131,0.347060072521518,-1.05190045518272,0.29284522307184,0.768216598166307
"6191","AC005899.6",19.4947501425778,0.467445697954972,0.443552683126554,1.05386736623934,0.291943645578203,0.768216598166307
"6192","RETREG3",206.569330706778,0.197471906681699,0.187738223126703,1.05184710600157,0.292869702866052,0.768216598166307
"6193","BRCA1",58.4831716045132,0.255249101806705,0.242312410144101,1.05338848165024,0.292162981046039,0.768216598166307
"6194","DDX42",442.730218319721,0.201643015346317,0.190135279401247,1.06052393843641,0.288906305348958,0.768216598166307
"6195","LLGL2",3.14886332493915,-0.833350971326364,0.791115592864579,-1.05338711414959,0.292163607537865,0.768216598166307
"6196","NAPG",167.90382606574,0.170699788266451,0.160975161721757,1.06041072697602,0.288957784174385,0.768216598166307
"6197","SLC14A2",14.0712946976828,-0.80219345696265,0.757642008672405,-1.05880276935582,0.289689612181938,0.768216598166307
"6198","AC023421.1",5.13053772483945,-0.701220083708911,0.661281034595972,-1.06039648352737,0.288964261306265,0.768216598166307
"6199","MALT1",180.708836976297,0.250590793241432,0.236028210648807,1.06169848321349,0.288372588164535,0.768216598166307
"6200","DOHH",90.9064014292509,-0.309750657339292,0.292456384311452,-1.0591345375091,0.289538512806642,0.768216598166307
"6201","PDE4A",241.097137227284,-0.230031171704641,0.218284247219472,-1.05381480631242,0.2919677133124,0.768216598166307
"6202","CDKN2D",79.1875704617708,-0.256739646629366,0.24258797848591,-1.05833623014537,0.289902181050819,0.768216598166307
"6203","C19orf53",1300.24687665382,-0.246273792356145,0.234228280324064,-1.05142637778587,0.293062806264069,0.768216598166307
"6204","NFKBIB",78.312684074455,-0.263034951581904,0.247824618457206,-1.06137539207924,0.288519335565178,0.768216598166307

"6205","ZNF285",4.43244083568298,0.790356913323224,0.75032626560609,1.05335098816619,0.292180158228591,0.768216598166307
"6206","ZNF320",2.35774367922986,1.10425928147169,1.04615650261723,1.05553927993479,0.29117875462002,0.768216598166307
"6207","UBE2S",439.525534215463,0.348413930334815,0.330367757289249,1.05462449844876,0.291597094820617,0.768216598166307
"6208","AL133230.1",2.98120318074371,1.16451021665381,1.09887130684756,1.05973302733197,0.289266072862872,0.768216598166307
"6209","LINC01637",22.5519788108193,-0.570114266217274,0.541404866860216,-1.05302759748643,0.292328343886326,0.768216598166307
"6210","LINC01422",8.88091340574113,0.584317182927729,0.552204043706818,1.05815448037168,0.28998501996283,0.768216598166307
"6211","CDC42EP1",316.86969665255,-0.190245820733536,0.179601563841241,-1.05926594771582,0.289478678460261,0.768216598166307
"6212","PARVB",149.370331586255,-0.363482367077368,0.342262845167759,-1.06199773714616,0.288236712473749,0.768216598166307
"6213","SELENOO",102.635702263416,-0.201102666551171,0.190497440739516,-1.05567122461323,0.291118448104169,0.768216598166307
"6214","AL022157.1",2.45909414444119,1.07893659888207,1.01999852238258,1.057782511647,0.290154607588029,0.768216598166307
"6215","PLS3",1120.42312466987,0.183971561162669,0.17415214940903,1.05638409739392,0.290792768393543,0.768216598166307
"6216","ZNF273",75.5828566711348,0.204970942690815,0.195015276842366,1.05105069720511,0.29323530616803,0.768326310951998
"6217","KIAA0930",513.441083262154,-0.248305719136401,0.236250486481523,-1.05102733473449,0.293246035679819,0.768326310951998
"6218","SNX3",2308.73837054777,-0.229095226634155,0.218059000393988,-1.05061119339365,0.293437198026459,0.768703525032644
"6219","HEATR5B",79.6624894973058,0.2612610637538,0.248908082075327,1.04962868853224,0.293888861781408,0.768748069222201
"6220","MRPS18C",498.809272571971,-0.275601114635952,0.262479212281827,-1.04999215838867,0.293721718059434,0.768748069222201
"6221","PCDHB16",61.0970434040519,0.391838670574189,0.373313755758304,1.04962290976462,0.293891519696283,0.768748069222201
"6222","SLC25A13",121.798901211903,0.224920601650639,0.214284797085292,1.049633967085,0.293886433951911,0.768748069222201
"6223","ISCU",1666.89509179616,-0.242863348044643,0.231420740946332,-1.0494450369985,0.293973339258708,0.768748069222201
"6224","GAS6-AS1",3.90696846288592,0.908937507867366,0.865622193417316,1.05003951467447,0.293699945692769,0.768748069222201
"6225","SLX4",26.6955515687378,0.410757885916752,0.391097169068916,1.05027067031613,0.293593685892061,0.768748069222201
"6226","PSMB6",1082.96046262013,-0.300519796218999,0.286174729937152,-1.05012694966113,0.293659749722128,0.768748069222201
"6227","HOXB-AS1",172.788842347194,0.266032703739229,0.253477703309556,1.04953098543086,0.293933801995593,0.768748069222201
"6228","ATP5F1A",995.188077941891,-0.180031745755554,0.17152370127364,-1.04960273372565,0.29390079968954,0.768748069222201

"6229","LBP",19.9822290581395,0.635301822868599,0.604927925681413,1.05
021077040372,0.293621218771457,0.768748069222201
"6230","ZNF593",230.137812355618,-0.245779091926087,0.234313165402919,
-1.04893419669122,0.294208405241732,0.769120819223673
"6231","AC087286.2",5.41270553927407,-0.637080596103018,0.607362328754
178,-1.04893004709363,0.294210315217798,0.769120819223673
"6232","WNT3",10.6404973109587,0.656115407989833,0.625668817682819,1.0
4866247037814,0.294333492934473,0.769319362389221
"6233","METTL5",464.641071819583,-0.27832993844983,0.265696505871918,-
1.04754835799008,0.294846741096668,0.769459833830442
"6234","GMDS",56.6044065720974,-0.288128594681875,0.274825417225188,-1
.04840592107893,0.294451626691142,0.769459833830442
"6235","AC067817.1",6.36998024656523,0.877255797833896,0.8374917924768
86,1.04747987468559,0.29487830946828,0.769459833830442
"6236","SEC61B",1922.02949940254,-0.266266199442391,0.254008842257919,
-1.04825563187295,0.294520845416015,0.769459833830442
"6237","SYT7",8.71450830700788,0.9482175941202,0.905379132237043,1.047
31549508692,0.294954091867965,0.769459833830442
"6238","COX6A1",1587.19369663982,-0.254687812634205,0.243146289335178,
-1.04746740462536,0.294884057966974,0.769459833830442
"6239","KLHL25",9.28243232863794,0.601420289417595,0.574232707210037,1
.04734593112895,0.294940059237915,0.769459833830442
"6240","AC026471.4",7.3264431493321,-0.708633498803027,0.6762696868851
32,-1.04785636935312,0.294704786792866,0.769459833830442
"6241","RSAD1",134.375629877886,0.204278857695466,0.195026154174392,1.
04744339834954,0.294895124688306,0.769459833830442
"6242","MX1",163.295993453351,-0.418185117728268,0.398952671941204,-1.
0482073367086,0.294543091043324,0.769459833830442
"6243","PDXK",253.092166943952,-0.162656105790441,0.155198715599429,-1
.0480505921857,0.294615298168064,0.769459833830442
"6244","GPKOW",86.7572636740399,-0.281812234050834,0.268979166434655,-
1.04771026613801,0.294772116195594,0.769459833830442
"6245","HACD3",476.978481366706,0.190761338527589,0.182189190075068,1.
0470508071801,0.295076145895264,0.769531754801146
"6246","METTL22",46.0258813503925,0.251897129937395,0.240576036209878,
1.0470582769002,0.295072700961877,0.769531754801146
"6247","ACTR5",31.6378677769632,-0.33229484073971,0.317430651654549,-1
.04682657143437,0.295179572918315,0.769678255685358
"6248","RPH3AL",17.3878913803776,0.458987054617879,0.438521068053502,1
.04667047504746,0.295251585517562,0.76974280993847
"6249","PGD",302.832422727887,0.256467856927272,0.245684603607786,1.04
38906352337,0.296535991399558,0.769992713150933
"6250","RBBP4",447.969179185851,0.160709144416131,0.153892094891256,1.
04429759390625,0.296347725744056,0.769992713150933
"6251","AGL",348.749376527139,-0.215928846060703,0.20693071766337,-1.0
4348377321133,0.296724292310699,0.769992713150933
"6252","SCNM1",155.372574501531,-0.290241426838746,0.27778957959956,-1
.0448247456119,0.296103975770024,0.769992713150933
"6253","AL133243.2",22.9795200993903,0.343061458278452,0.3283882998153
5,1.04468234243228,0.296169808424975,0.769992713150933

"6254","IL1R1",1111.55552845444,0.21956655174306,0.21041207649772,1.04350736610614,0.296713371035448,0.769992713150933
"6255","CTDSP1",583.966644369245,-0.137797172804448,0.132044309569165,-1.04356767250367,0.296685456108455,0.769992713150933
"6256","CCDC66",339.82523102054,-0.233542716328866,0.22342729132469,-1.04527390071375,0.295896396587224,0.769992713150933
"6257","UMPS",124.48255286958,0.232063167764179,0.222025562063396,1.04520923450209,0.295926276382666,0.769992713150933
"6258","GPR78",3.25576279935601,1.14063600102411,1.09252537670586,1.04403616185403,0.296468659246068,0.769992713150933
"6259","TMEM156",2.70644125917191,1.26393521554905,1.21075025586998,1.04392727519257,0.296519037885841,0.769992713150933
"6260","PKIB",3.49348827403651,-0.923048396665548,0.884387915244471,-1.04371439359886,0.296617548450064,0.769992713150933
"6261","HOXA3",58.825943113806,0.463812636813138,0.444240093901973,1.04405847914188,0.296458334403806,0.769992713150933
"6262","MDH2",668.129007398128,-0.25221198471547,0.241218283621157,-1.0455757371675,0.295756956162313,0.769992713150933
"6263","SYCE1",4.40365600621456,0.907220932235471,0.869073910646356,1.04389387498785,0.296534492321074,0.769992713150933
"6264","ATF7",401.820462559743,-0.140893970026341,0.134995424736017,-1.04369440891688,0.296626797448679,0.769992713150933
"6265","GGACT",36.1066105456941,0.394447540618111,0.378079524591004,1.04329252171171,0.296812833560974,0.769992713150933
"6266","TMEM251",130.616470940837,-0.310777538969252,0.297213142424177,-1.0456386162282,0.295727913244125,0.769992713150933
"6267","EDC3",66.8549273282364,0.233409673925258,0.223482608090719,1.04441985852657,0.29629117978242,0.769992713150933
"6268","HAGHL",32.2399014117022,-0.416682461493368,0.399108457187167,-1.04403315437127,0.296470050642543,0.769992713150933
"6269","YWHAE",1705.1247107628,-0.178586038311762,0.170787247339191,-1.04566377814546,0.295716291857607,0.769992713150933
"6270","PROCA1",15.4526906056268,0.45486448342087,0.435976926257585,1.04332237791898,0.296799010252366,0.769992713150933
"6271","TBCD",152.464381162629,0.22802317515198,0.218034720277085,1.04581130409965,0.295648161065859,0.769992713150933
"6272","C18orf25",78.8916620233421,-0.269968830342073,0.258574762637582,-1.04406488703022,0.296455369910774,0.769992713150933
"6273","PBX4",26.4455160716447,-0.349571316961792,0.334548929772495,-1.04490340829819,0.296067614394661,0.769992713150933
"6274","HNRNPL",374.441024022016,-0.211635274809259,0.202519343368203,-1.04501264565372,0.296017125010644,0.769992713150933
"6275","TEAD2",108.468478851869,-0.246123856859311,0.235761646007795,-1.04395206356497,0.296507568535165,0.769992713150933
"6276","TAF4",44.176046866809,0.266949903040474,0.25537261288912,1.04533489327762,0.295868216095407,0.769992713150933
"6277","HMGXB4",311.918725783179,0.140498427840457,0.134350628660848,1.04575936295116,0.29567214745339,0.769992713150933
"6278","USP27X-AS1",4.30926754417908,-0.755291829590539,0.723736669976987,-1.04360033

272123,0.296670338916884,0.769992713150933
"6279","UPF3B",179.650630997242,-0.202488035297161,0.193916017369092,-1.04420479568613,0.296390648658877,0.769992713150933
"6280","SESN2",111.641147134688,0.279285726088376,0.268128721056717,1.04161063010217,0.297592238251147,0.770119944748387
"6281","ZRANB2-AS2",10.4799379487073,0.608894716874837,0.584553824075659,1.04164012242614,0.297578559454278,0.770119944748387
"6282","IMP4",242.181760399502,-0.245868890011421,0.236021873134566,-1.04172078098558,0.297541151457643,0.770119944748387
"6283","PIKFYVE",120.96466330324,0.266279752783427,0.255464619482213,1.04233515123595,0.297256320672483,0.770119944748387
"6284","THUMPD3",197.459921586099,-0.196005084467891,0.188215464782718,-1.04138671439228,0.297696106015271,0.770119944748387
"6285","NKIRAS1",118.129479720711,0.258268912429089,0.247908560042313,1.04179102321037,0.297508576931039,0.770119944748387
"6286","BCHE",87.2468583251969,0.463377487953113,0.444769799065025,1.04183667354934,0.297487408062602,0.770119944748387
"6287","KLF3",487.488073404934,0.180182500375102,0.172900013862599,1.04211964099835,0.297356213520291,0.770119944748387
"6288","LINC01948",4.63998627124714,0.760152359896763,0.729227753446938,1.04240733612188,0.297222866703614,0.770119944748387
"6289","AC083880.1",7.70302002179082,-0.612014816779014,0.587675976734421,-1.04141540748328,0.297682794800115,0.770119944748387
"6290","RFX3-AS1",7.17658321709641,0.702655399605897,0.674753963430598,1.04135053321279,0.297712891649616,0.770119944748387
"6291","WASHC2C",205.874463357239,0.217800455127018,0.208903743359103,1.04258761295924,0.297139328687585,0.770119944748387
"6292","SCART1",4.42488153383905,0.98390652609654,0.943784690205192,1.04251164095767,0.297174531242299,0.770119944748387
"6293","INCA1",9.23965903403824,-0.54454070917411,0.522564396829696,-1.04205474478885,0.297386298469046,0.770119944748387
"6294","LINC00526",33.6603334716601,-0.368227019837301,0.353095473383887,-1.04285398028018,0.297015926161773,0.770119944748387
"6295","SHC2",262.78368360276,-0.281897516965344,0.270638980217098,-1.04159983435947,0.297597245515756,0.770119944748387
"6296","NAPA-AS1",32.3615590195878,0.405496226948774,0.389183892083747,1.04191420867315,0.29745145596342,0.770119944748387
"6297","LINC01560",23.1842279283141,-0.333747949075499,0.320150459881877,-1.0424721838558,0.2971928152724,0.770119944748387
"6298","SVBP",689.196082497322,0.276334735939764,0.265434776403188,1.04106454958268,0.297845591079801,0.770265147061921
"6299","CLCC1",173.485719108443,0.216790088236727,0.20828738340584,1.04082198687147,0.297958173714603,0.770265147061921
"6300","ASNS",10.2985114028295,-0.475287858237654,0.456618495155861,-1.0408861298433,0.297928399742617,0.770265147061921
"6301","ZFP90",162.863111715738,0.18310279619362,0.175911990678825,1.04087729032595,0.297932502763717,0.770265147061921

"6302","AC037459.2",10.6457036025812,0.5694139317492,0.547215094169307
,1.04056693211943,0.29807658504391,0.770326748180271
"6303","AL122035.2",7.86736888413913,0.70029354044768,0.67294686156444
,1.04063720398319,0.298043957598984,0.770326748180271
"6304","UBXN10",8.67775084367441,-0.631697264677421,0.607203965113468,
-1.04033784522368,0.298182967369472,0.770479434562393
"6305","AC078881.1",4.32990878842573,0.79386758645016,0.76345241271148
2,1.03983899092106,0.298414710778467,0.770771998431894
"6306","ZNF346",89.2576071045487,0.250533648556413,0.24094672674645,1.
03978855384099,0.298438148082138,0.770771998431894
"6307","TMED2-
DT",6.64965527467742,0.616927567943983,0.593260520986357,1.03989317697
776,0.298389532754989,0.770771998431894
"6308","METTL15",131.97924782398,0.207897867861082,0.199966925740142,1
.03966126944036,0.298497300570568,0.770802556910903
"6309","MLLT1",193.805064808822,0.180487492497454,0.173628666235711,1.
03950284483803,0.29857093568467,0.770870497918465
"6310","YPEL4",60.6898014392258,-0.318412193303674,0.306402628568744,-
1.03919537110706,0.298713882840084,0.770995157278106
"6311","RNF214",140.376513820537,-0.168991300080008,0.162602642006892,
-1.03929000165228,0.2986698834277,0.770995157278106
"6312","MIR29B2CHG",30.768312877474,0.387251945059501,0.37287402749633
4,1.03855971857227,0.299009547958514,0.771513785315419
"6313","ADCY4",5.04439230625555,-0.756940085687598,0.72877343651563,-1
.03864939055221,0.298967826429371,0.771513785315419
"6314","AC006042.4",2.31492562487775,1.19016063065298,1.1461334641057,
1.03841364721135,0.299077518651575,0.771535630873242
"6315","LINC01232",15.3135505741998,0.478745521660003,0.4610775417597,
1.03831889064228,0.299121616789835,0.771535630873242
"6316","AKAP10",98.1417509547503,0.219343681394037,0.211265687751504,1
.03823618368183,0.299160110786138,0.771535630873242
"6317","SPPL2A",862.381114923998,0.168302861074019,0.162157049028485,1
.03790036931699,0.299316441589435,0.771694447143131
"6318","SNHG19",248.539010436132,-0.289179470970731,0.278598213146496,
-1.0379803506445,0.299279203142282,0.771694447143131
"6319","UBR3",175.461317641087,0.213734625308433,0.205982281281959,1.0
3763597518304,0.299439562659335,0.771807096986542
"6320","RPS25",15303.8712204208,-0.216696101258306,0.208842969450606,-
1.03760304609899,0.29945489919301,0.771807096986542
"6321","RDH14",461.534466723136,-0.230839266578523,0.222502738033717,-
1.03746708296031,0.29951822880047,0.771848193154699
"6322","AC011484.1",16.7476248985858,0.426095530021907,0.4107674950575
46,1.03731559860211,0.299588798482827,0.771907930795123
"6323","NAXE",373.751183663727,-0.272729375477209,0.26388560133043,-1.
03351366691548,0.301363578064829,0.77207393851146
"6324","AC017071.1",5.38483906337319,0.677396550380943,0.6539425178190
78,1.03586558745268,0.300264853966933,0.77207393851146
"6325","IDH1",208.893459048089,0.172510530027783,0.166522844511848,1.0
3595714169721,0.300222137516472,0.77207393851146
"6326","AAMP",386.37822469969,-0.164737072387065,0.159030923829789,-1.

03588074834668,0.300257780070949,0.77207393851146
"6327","COL6A3",10557.6914872173,0.221992551649685,0.214782419616288,1
.0335694701935,0.301337478016293,0.77207393851146
"6328","PXK",626.150732886221,0.233669726695943,0.225464452489843,1.03
639276220924,0.300018945614681,0.77207393851146
"6329","PRICKLE2",261.080999727808,0.277197330397312,0.268041964849448
,1.0341564633471,0.301063023517279,0.77207393851146
"6330","MASP1",318.125167590474,-0.511371650506855,0.493825756608066,-
1.0355305361537,0.300421213344708,0.77207393851146
"6331","AC090018.2",2.62664358816077,0.964921705544172,0.9313115807673
38,1.03608902269758,0.300160612938446,0.77207393851146
"6332","HSD17B11",757.788830549483,-0.237795591947188,0.22982319255474
1,-1.03468927267011,0.300814047411663,0.77207393851146
"6333","ENC1",16.4320367484859,-0.493350783356737,0.476679645485724,-1
.03497346284636,0.300681304492842,0.77207393851146
"6334","VCAN",3324.21330650613,0.338973589507335,0.327431565194052,1.0
3525018825366,0.300552085820068,0.77207393851146
"6335","APC",329.43084716882,0.212974075447902,0.20560622224763,1.0358
3477737069,0.300279229933458,0.77207393851146
"6336","C5orf56",275.951813630486,0.201929799308323,0.195306276777179,
1.03391351594245,0.301176595795384,0.77207393851146
"6337","SIL1",471.384971446441,0.215801937572658,0.208713977851506,1.0
3396015827074,0.301154789375493,0.77207393851146
"6338","STK32A",30.1646112206106,-0.57667148002485,0.557278203553343,-
1.03479999100602,0.30076232713605,0.77207393851146
"6339","HIGD2A",1232.81473458535,-0.307794985458901,0.29733436004012,-
1.03518135414074,0.300584224884488,0.77207393851146
"6340","KIF13A",232.464821614083,-0.18115687950801,0.17494233802673,-1
.03552337045096,0.300424557976959,0.77207393851146
"6341","HIST1H2AG",3.27991882422492,0.910606879912203,0.87856490814472
2,1.03647080764373,0.299982551598317,0.77207393851146
"6342","LSM2",576.499014419549,-0.272115321981134,0.263315095949953,-1
.03342089445891,0.301406972496431,0.77207393851146
"6343","RNF5",293.317940696591,-0.23179110372716,0.224243796076231,-1.
03365670659786,0.301296679228739,0.77207393851146
"6344","IFNGR1",918.932328015731,-0.228475327732284,0.220626091660926,
-1.03557709794099,0.30039948097191,0.77207393851146
"6345","NTMT1",232.257452045627,-0.229153644020893,0.221089899959796,-
1.03647269306541,0.299981672428053,0.77207393851146
"6346","TASOR2",217.590608163912,0.206282024020681,0.199596428047202,1
.03349556922882,0.301372042953561,0.77207393851146
"6347","HK1",122.189147821953,-0.238974312461307,0.231189428168314,-1.
03367318460308,0.301288973195865,0.77207393851146
"6348","C12orf4",74.6524027799039,0.2307142974886,0.222516385272867,1.
03684183618964,0.299809574451632,0.77207393851146
"6349","ITPR2",139.898754318005,0.30084269964492,0.290461963587386,1.0
3573871060198,0.300324057578006,0.77207393851146
"6350","DBX2",65.1235244075623,0.328360635552459,0.317705286400781,1.0
3353846979504,0.301351977192012,0.77207393851146
"6351","LINC02356",8.29648518616289,-0.555073343910558,0.5363354882151

7,-1.03493681866502,0.300698418499459,0.77207393851146
"6352","C12orf65",166.981467729832,0.215756036297318,0.208711945713713
,1.0337502990522,0.301252911929505,0.77207393851146
"6353","RB1",124.839301309592,0.198708727887497,0.191915759556751,1.03
539557327879,0.300484212197622,0.77207393851146
"6354","CCL5",68.6077866462251,0.399355947284305,0.386404324232004,1.0
3351831809347,0.301361402580079,0.77207393851146
"6355","TEX2",57.4392782220657,0.246483033035993,0.238430458985727,1.0
3377326070051,0.301242174858885,0.77207393851146
"6356","AL121894.1",7.56071477540661,-0.755271455982037,0.729087133162
017,-1.03591384572439,0.300242337607241,0.77207393851146
"6357","DYNLRB1",1418.87906768108,-0.282639294376451,0.273307369132218
,-1.0341444333311,0.301068646599392,0.77207393851146
"6358","AF129075.2",4.24244962166142,-0.795876620764708,0.769919395712
625,-1.03371421111954,0.301269787482293,0.77207393851146
"6359","C1GALT1C1",334.969343199516,-0.27488994747243,0.26545319243696
,-1.03554960084992,0.300412314910853,0.77207393851146
"6360","IL17RC",193.254673879059,-0.262248719522544,0.25387730186028,-
1.0329742659187,0.301615941779657,0.772244903434271
"6361","AP001372.2",33.4462294914412,-0.317285132554205,0.307153155767
476,-1.03298672534037,0.301610110937792,0.772244903434271
"6362","GLIPR1L2",22.9375538361476,0.57964215670667,0.56103500644487,1
.03316575623277,0.30152633517306,0.772244903434271
"6363","ATP5PD",1451.3893491061,-0.259906344561968,0.251647005699634,-
1.03282112910254,0.30168761388075,0.772307015952151
"6364","AC093151.2",2.66574893668381,1.06483882693843,1.03148692288925
,1.03233381181,0.301915766769155,0.77276962993444
"6365","AL031293.1",9.97920290814413,0.785532883052683,0.7613760814633
01,1.03172781779926,0.302199642038646,0.772788924931569
"6366","DCAF8",337.158978266223,0.197569972175838,0.191471022482543,1.
0318531212411,0.302140929624371,0.772788924931569
"6367","MFAP3L",3.00373146182412,-0.960857881219047,0.931256106089637,
-1.03178693265562,0.302171942125539,0.772788924931569
"6368","HEATR5A",136.832933357458,0.261635247034875,0.253492501721111,
1.03212223343286,0.302014859571601,0.772788924931569
"6369","N4BP1",282.4604883753,0.140284110215802,0.135929144541261,1.03
203849836058,0.302054082897562,0.772788924931569
"6370","ROMO1",950.803469678903,-0.262842581389762,0.254763997666628,-
1.03171006813021,0.302207959470446,0.772788924931569
"6371","BSG",1192.94465472206,-0.241543868831024,0.234168086544074,-1.
03149781166086,0.3023074338986,0.772921957428079
"6372","SDF4",1272.61588979157,0.19182063966932,0.185991449838678,1.03
134117098231,0.302380857841103,0.772988354264553
"6373","NKX3-2",3.27081925208207,-1.08014690820682,1.04747586837603,-1
.03119025537213,0.302451609428248,0.773047899886511
"6374","HEMK1",88.2903399355682,0.268250955141968,0.260279907215166,1.
03062490690152,0.302716751481119,0.773195003030782
"6375","TSPAN11",25.4448533712584,0.522841629822113,0.507281121076388,
1.03067433046337,0.302693566224178,0.773195003030782
"6376","GCH1",50.882139112217,0.664296744557102,0.644492727338004,1.03

072806934672,0.302668357932131,0.773195003030782
"6377","CNOT1",252.340395605306,0.18357423651489,0.178070663770322,1.0
30906678439,0.302584584493648,0.773195003030782
"6378","MATK",8.19098180244804,-0.718677807459482,0.697411893350784,-1
.03049261750688,0.302778816024925,0.773195003030782
"6379","RBBP9",81.727416211418,0.224773924758332,0.218129625099479,1.0
3046032677048,0.302793966746477,0.773195003030782
"6380","PAFAH2",37.494993484267,0.299233920678719,0.29043462952623,1.
03029697652252,0.302870617943392,0.773238557794223
"6381","NME7",162.669280340499,-0.238149124149655,0.231358474696831,-1
.02935120255146,0.30331467072426,0.773238557794223
"6382","UNC50",471.774269162683,-0.259555067233918,0.252107377830698,-
1.02954173522133,0.303225178449453,0.773238557794223
"6383","AC092903.2",20.7239949785635,0.42206619165841,0.40988347415839
6,1.02972239250443,0.303140340808218,0.773238557794223
"6384","ABRAXAS1",301.051298900498,-0.214468044903876,0.20834837009238
5,-1.02937231910563,0.303304751515852,0.773238557794223
"6385","HSD17B13",3.26108576601888,-0.775675390318718,0.75300922560067
3,-1.0301007795754,0.302962699325144,0.773238557794223
"6386","IL7R",38.7711937231027,0.573345019870597,0.556582784584756,1.0
3011633803648,0.302955396571871,0.773238557794223
"6387","LINC00539",8.13181003796315,-0.620565977191186,0.6026181862160
34,-1.02978302246046,0.303111872188364,0.773238557794223
"6388","DIO3OS",858.522632977072,0.24537720196824,0.238277746067046,1.
02979487601497,0.303106306593967,0.773238557794223
"6389","LRRC75A",227.255564971108,-0.484595132009535,0.470670405145297
,-1.0295848787432,0.303204916582959,0.773238557794223
"6390","KCNK15",69.1116095383881,-0.434170950002825,0.421807038234665,
-1.029311772084,0.303333193216593,0.773238557794223
"6391","HES1",419.694191110699,-0.325123706882969,0.316082083609869,-1
.0286053014136,0.303665185757233,0.773417573284381
"6392","IRX2",105.83115440053,-0.501083207587311,0.487046937602732,-1.
0288191319992,0.303564674646046,0.773417573284381
"6393","PSMB8-
AS1",65.5721018630711,0.245717930301546,0.238882333918848,1.0286149095
6891,0.303660668967428,0.773417573284381
"6394","AL590064.1",3.54054931884636,1.13077218888524,1.09937820884019
,1.02855612362753,0.303688304912941,0.773417573284381
"6395","AC087164.1",2.54286201953781,-0.917105368700599,0.891386502450
567,-1.02885265390414,0.303548919672373,0.773417573284381
"6396","GEMIN7-
AS1",6.14583063643697,-0.615186333650904,0.597934694826706,-1.02885204
517058,0.303549205766463,0.773417573284381
"6397","SSU72",728.761629236788,-0.232440821078442,0.226081164127646,-
1.02812997259341,0.303888693368209,0.773436819175339
"6398","ODC1-
DT",5.49336237979996,0.796005864220447,0.774526383149454,1.02773240723
402,0.304075719196935,0.773436819175339
"6399","CASP8",74.5645755378136,0.309135044271801,0.300747073597844,1.
02789044818828,0.304001363173401,0.773436819175339

"6400","GRAMD2B",175.798868709217,0.264121168859627,0.256982856549547,
1.02777738719977,0.304054555532529,0.773436819175339
"6401","KIF3A",141.116085053487,-0.219304183700607,0.213318578191191,-
1.02805946655078,0.303921855800852,0.773436819175339
"6402","MCUR1",260.869312629017,-0.215185594602526,0.209329963822412,-
1.0279732087714,0.303962430313397,0.773436819175339
"6403","AP001462.1",6.31348216413615,0.714796104350838,0.6954082381412
79,1.02787983395391,0.304006356641514,0.773436819175339
"6404","DNM1L",249.29919523917,-0.246928923464848,0.240166965074037,-1
.0281552393716,0.30387680975445,0.773436819175339
"6405","CHST2",5.18286596659394,-0.723321329478949,0.703917449154449,-
1.02756556233662,0.304154230456797,0.773515731445865
"6406","TMEM92",4.82208319554592,0.969793527530877,0.943964020802366,1
.02736280849619,0.304249657303972,0.773637631567966
"6407","MECR",138.748473696485,-0.200852310410128,0.195990750575128,-1
.02480504728276,0.305455184403256,0.773716967958752
"6408","TMEM42",228.176375895091,0.228032496223654,0.222457621602512,1
.02506038939454,0.305334694089042,0.773716967958752
"6409","TMCC1-
AS1",12.6050159865891,-0.494731976462178,0.483059671473485,-1.02416327
77025,0.305758160549948,0.773716967958752
"6410","FXR1",748.834862653377,0.137969075226912,0.13478098607956,1.02
365384940477,0.305998800873841,0.773716967958752
"6411","FRG1-
DT",3.18152831919135,-0.854152038125174,0.834647033830923,-1.023369165
05259,0.306133332831614,0.773716967958752
"6412","UIMC1",107.239265004146,-0.235479350507119,0.229954675988258,-
1.02402505839517,0.305823439251641,0.773716967958752
"6413","TMEM14C",1521.56617870458,-0.242687809228766,0.236500780001275
,-1.02616071383552,0.304815835950414,0.773716967958752
"6414","DXO",45.6452695226533,-0.303692060051425,0.296754254648948,-1.
02337895849441,0.306128704139389,0.773716967958752
"6415","GJB7",2.53670979333934,1.25911995726123,1.22694247880601,1.026
22574326918,0.30478518963718,0.773716967958752
"6416","AMD1",677.807385271933,0.313564659564794,0.305652531992488,1.0
2588602005267,0.304945312998827,0.773716967958752
"6417","SEC61G",1288.15520187321,-0.263426024513605,0.25658179642435,-
1.02667464404971,0.304573692696749,0.773716967958752
"6418","CASTOR2",78.0751007355429,0.224201822283832,0.218701738869919,
1.02514878684702,0.305292988627591,0.773716967958752
"6419","NRF1",43.4999286906455,0.292010745922285,0.284610495320254,1.0
2600132716014,0.304890958493473,0.773716967958752
"6420","SLC20A2",93.1372476546244,0.184653753250068,0.179982228589767,
1.0259554773652,0.304912570813691,0.773716967958752
"6421","TRUB2",142.067414589504,-0.208978986118452,0.20403020485192,-1
.02425514040984,0.305714780418032,0.773716967958752
"6422","CACFD1",24.8019440122882,-0.361608344560034,0.352457622460828,
-1.02596261654186,0.304909205537223,0.773716967958752
"6423","HTATIP2",216.756869961823,0.381728690767188,0.372676062618028,
1.02429087633256,0.305697906021569,0.773716967958752

"6424","REXO2",1322.55480601609,-0.277717810329437,0.27040104406596,-1
.02705894235265,0.304392710254254,0.773716967958752
"6425","AC135279.4",4.36575828526435,-0.834362971422258,0.814348260548
69,-1.02457758166031,0.305562546974143,0.773716967958752
"6426","ATP2B1-
AS1",114.750984269762,-0.327191803556228,0.319471341584489,-1.02416636
789218,0.305756701209876,0.773716967958752
"6427","AL359317.2",3.25620467674878,-0.915077681206694,0.894020998813
129,-1.02355278278868,0.306046556965732,0.773716967958752
"6428","CCNK",228.316459266609,0.170477348456738,0.166572002808964,1.0
2344539047329,0.30609730749696,0.773716967958752
"6429","RPLP1",44752.7729865523,-0.232805990592532,0.227096932941346,-
1.02513929878859,0.305297464863496,0.773716967958752
"6430","USP31",47.9196327050352,0.289664495556145,0.282852053379659,1.
02408482489375,0.305795211385103,0.773716967958752
"6431","AC106738.1",2.80898761123276,-1.07112701420985,1.0431062497314
8,-1.02686280950343,0.30448506864484,0.773716967958752
"6432","AC010542.4",3.86543459948453,1.3175239045742,1.28599902853283,
1.02451391901698,0.305592599887114,0.773716967958752
"6433","IFT20",445.783633931829,-0.218897702618063,0.213702643737785,-
1.02430975485101,0.305688991890619,0.773716967958752
"6434","COX11",352.103495791507,-0.186923925823123,0.182120755402136,-
1.02637354765184,0.304715541717819,0.773716967958752
"6435","KDM4B",151.229606750484,0.28257836605168,0.276002733218472,1.0
2382452070865,0.305918166325665,0.773716967958752
"6436","POP4",182.012461338712,-0.236552387446389,0.230812669299237,-1
.02486743108417,0.30542574395153,0.773716967958752
"6437","THAP8",37.0785461348713,0.392567046863268,0.383285123619951,1.
02421675841643,0.305732904969163,0.773716967958752
"6438","ACTN4",534.686547685332,-0.188658324598521,0.184264057277661,-
1.02384766397626,0.30590723325286,0.773716967958752
"6439","AC008982.2",12.4023433450828,-0.548329081171659,0.535773291562
069,-1.02343489271924,0.306102268734037,0.773716967958752
"6440","RPS16",17820.9596425372,-0.212778409546812,0.207727738140937,-
1.0243138997761,0.305687034747135,0.773716967958752
"6441","CPT1C",15.8349008141366,-0.444936624490229,0.433586760958016,-
1.02617668377866,0.304808309632373,0.773716967958752
"6442","CBR1",710.411075987945,-0.233924975806851,0.228437350612181,-1
.0240224515823,0.305824670495192,0.773716967958752
"6443","PRR34-
AS1",38.0512968747624,-0.428676869604227,0.41780916431325,-1.026011170
21653,0.304886318882561,0.773716967958752
"6444","FTSJ1",86.3768218882295,-0.26681779475751,0.260093845774626,-1
.0258520110803,0.304961345683198,0.773716967958752
"6445","FUNDC2",777.38561135463,-0.19326646607574,0.188636221147722,-1
.02454589526787,0.305577504769578,0.773716967958752
"6446","CLEC2B",1570.17826887224,-0.234451123520278,0.229150188760514,
-1.02313301502581,0.306244958894443,0.773879015735586
"6447","USP25",157.572559235963,0.191742219273362,0.187429753802248,1.
02300843587334,0.306303857244019,0.773907791321209

"6448","RRAGC",490.438497770583,0.231936472275269,0.22678002369582,1.0
2273766664019,0.30643189701519,0.774059775948613
"6449","VARS",94.525305080367,-0.280761147288561,0.274534632835351,-1.
02268025126339,0.306459051819793,0.774059775948613
"6450","ZNF182",35.0718552819697,0.30153692841869,0.294893168676804,1.
02252937825483,0.306530415340468,0.774119989997035
"6451","LDB3",2.40399039990245,0.975692810958045,0.954398283008332,1.0
2231199314671,0.306633258704237,0.774259673079107
"6452","HSPG2",2925.41831221039,0.157062618119759,0.153690039609009,1.
02194402785847,0.306807392582897,0.77433343715976
"6453","PCDHGB2",5.15826539697635,0.762874258565935,0.746589264549518,
1.02181252100677,0.306869642051448,0.77433343715976
"6454","PTPRN2",139.46317333473,0.27229594092874,0.266500081022608,1.0
2174806057804,0.306900157793813,0.77433343715976
"6455","PSMA1",876.759635253654,-0.225867464372875,0.220999157649592,-
1.0220286211724,0.306767354308542,0.77433343715976
"6456","LIF",8.46300477851435,-0.738643031372839,0.722654267080407,-1.
02212505346025,0.306721716833576,0.77433343715976
"6457","SLC20A1",356.438549198468,0.426232126621644,0.417202895906299,
1.02164230115357,0.306950228947137,0.774339829536924
"6458","AL021368.2",94.1025175318846,0.354761620117523,0.3474666143482
43,1.02099483941202,0.307256883703452,0.774393838564991
"6459","ARPC1A",619.849482775151,-0.279953985032757,0.274134141135523,
-1.02122991274683,0.307145523398653,0.774393838564991
"6460","ARC",40.1555668631299,-0.593310975652095,0.581067764309584,-1.
02107019541354,0.307221182709972,0.774393838564991
"6461","PHF19",187.487254040762,-0.176295274058849,0.172657113266678,-
1.02107159515955,0.30722051958608,0.774393838564991
"6462","CLN6",51.58063869795,-0.272309513758693,0.266652327783938,-1.0
212155881847,0.307152308548581,0.774393838564991
"6463","ZNF490",35.2720542820684,0.418316936075216,0.409636312941523,1
.02119104888763,0.307163932367324,0.774393838564991
"6464","PSMD9",627.802845943515,-0.263306369270597,0.257983292125272,-
1.02063341816237,0.307428150418656,0.774647611362271
"6465","CCL2",1593.76478589925,-0.770385409701666,0.754849363259845,-1
.0205816513837,0.307452686319423,0.774647611362271
"6466","MED19",211.46107719718,-0.207031905747684,0.202890370192895,-1
.02041267681089,0.307532784221569,0.774729588955325
"6467","MTX1",307.364065205365,-0.25364272175467,0.248814791122788,-1.
01940371233597,0.308011344462296,0.774846606200458
"6468","DHFR2",77.1260300295131,0.217809322170742,0.213571141711751,1.
01984434987341,0.307802285842069,0.774846606200458
"6469","SLC66A1L",335.169789472396,0.473759872525468,0.464895678117144
,1.01906706133347,0.30817113040374,0.774846606200458
"6470","SCAMP1",301.325372497382,0.1435240097624,0.140752256270863,1.0
1969242671466,0.307874354534303,0.774846606200458
"6471","Z97989.1",32.1853146376439,-0.351229295387549,0.34427278642959
5,-1.02020638642426,0.307630589416865,0.774846606200458
"6472","AC004837.4",5.29284872642924,-0.684091854829068,0.671378494383
715,-1.01893620446842,0.308233254291193,0.774846606200458

"6473","ZC3HAV1",796.735504520132,0.472513361792251,0.463460781789866,1.01953256965438,0.307950198933825,0.774846606200458
"6474","AL161729.1",13.7727605590812,-0.567272157731565,0.556631947026825,-1.01911534320222,0.308148210829629,0.774846606200458
"6475","NDST2",77.4715513152557,0.252832430969047,0.248083625913035,1.01914195279327,0.308135579644538,0.774846606200458
"6476","SLC22A17",169.581927648219,0.189276254390519,0.185717517389268,1.0191620965 6083,0.308126017919435,0.774846606200458
"6477","EXOC5",226.578708259203,0.19838820338987,0.19470610623397,1.01891105126141,0.308245196646754,0.774846606200458
"6478","SCO1",152.556931208426,0.185558229802111,0.181908463421061,1.02006375246325,0.307698226236426,0.774846606200458
"6479","AP001469.3",3.53723543191924,1.01653988026955,0.997083375954193,1.0195134176184,0.307959286472582,0.774846606200458
"6480","EFNB1",108.827362598159,0.337975003359291,0.331610295900384,1.0191933348801,0.308111190286023,0.774846606200458
"6481","CACNA2D3-AS1",4.40321622109473,-0.808806661252798,0.793973801485359,-1.01868180000359,0.308354055722947,0.774895357761929
"6482","AC021546.1",24.444192124769,-0.495473175616167,0.486456536628247,-1.01853534346648,0.308423613354894,0.774895357761929
"6483","HYOU1",76.9009808925986,-0.339142997386376,0.332986317351141,-1.01848928834137,0.30844548878359,0.774895357761929
"6484","PIF1",7.16563931336783,0.644676392052947,0.632985452933762,1.01846952258539,0.308454877508033,0.774895357761929
"6485","DYRK2",228.459125158257,-0.271402547639189,0.266509969129751,-1.01835795683521,0.308507874729105,0.774908985576312
"6486","RNF19B",84.6040439616807,-0.385831416466675,0.37891406695456,-1.01825572106022,0.30855644520739,0.774911491825959
"6487","BACH2",41.266436465782,0.391350208925632,0.384390655898164,1.01810541676984,0.30862786140163,0.774971363399283
"6488","AL139260.1",22.8335461607474,0.414019301916833,0.406954343887832,1.01736056669529,0.308981933791317,0.775290634091663
"6489","AC021016.2",5.64874299603375,0.613080076057786,0.602632051648347,1.01733731948186,0.308992988920319,0.775290634091663
"6490","SYTL3",8.36211516745087,0.617759532893643,0.607185674258945,1.01741453904953,0.308956268439908,0.775290634091663
"6491","ARHGAP32",124.096967940064,-0.265036300575684,0.260459944872299,-1.01757028592488,0.308882214382046,0.775290634091663
"6492","SLC25A18",19.8018375882222,0.596393417387144,0.58612034261486,1.0175272448768,0.308902678237252,0.775290634091663
"6493","STK32C",75.8646320785425,-0.22106702873309,0.217425408147217,-1.01674882718126,0.309272931409102,0.775873522212053
"6494","ACOT2",129.964128207932,-0.413162653526819,0.40644175926263 7,-1.01653593438916,0.309374244587722,0.775888694394058
"6495","RPS21",11048.8864992598,-0.289431610018684,0.284718287143829,-1.01655433840283,0.309365485467966,0.775888694394058
"6496","ATP6V1C1",212.357820689797,0.171527446736724,0.168758632359656,1.01640694960817,0.309435637596908,0.775923199017247
"6497","RPS6KA1",5.01475079801236,-0.907767981701047,0.89363998555477,

-1.01580949417511,0.309720114020507,0.776014040884135
"6498","CYP4X1",93.0382346411344,0.351537380182499,0.346127218727938,1
.01563055767311,0.309805347649919,0.776014040884135
"6499","MZT2A",1019.09504651286,-0.274103357814318,0.26981997709305,-1
.0158749576937,0.309688935358441,0.776014040884135
"6500","ALS2CL",3.70893406118002,1.00898899257321,0.993130119155814,1.
01596857562922,0.309644351043542,0.776014040884135
"6501","BX255925.3",22.2234909455354,0.374037621452651,0.368253271601361,1.01570753146642,0.309768680477856,0.776014040884135
"6502","LINC01303",10.520634404379,-0.698696375978623,0.68761513639222
,-1.01611546779576,0.309574404120719,0.776014040884135
"6503","SAFB",301.103610164788,0.163569318230986,0.161047550280729,1.0
156585303276,0.309792022287941,0.776014040884135
"6504","DCUN1D2",27.9757701655701,0.346816373673661,0.341625201069677,
1.01519552008379,0.310012635678271,0.776413871857836
"6505","RPL22",11162.9713781511,-0.254508221023972,0.251353453191115,-
1.01255112190744,0.311274616549922,0.776457174439699
"6506","PLA2G5",131.215938916573,-0.713465374108504,0.704258779352716,
-1.01307274403345,0.311025415980708,0.776457174439699
"6507","TMEM222",240.169495312387,-0.251947514120798,0.249090119906982
,-1.01147132698352,0.311790897815941,0.776457174439699
"6508","ECHDC2",543.842785955507,0.218000529481699,0.215631152605325,1
.01098810096661,0.312022124921491,0.776457174439699
"6509","LINC00607",5.00960265690368,-0.653773490993445,0.646459236962717,-1.01131433138011,0.311866008943756,0.776457174439699
"6510","IGFBP5",7764.2777992957,0.442813658254735,0.437211303692741,1.
01281383741609,0.311149089976615,0.776457174439699
"6511","HIGD1A",280.920532222305,-0.298713388162805,0.294947717490185,
-1.01276724805557,0.311171348132669,0.776457174439699
"6512","TUSC2",178.588124631095,-0.233508924952858,0.230652675738068,-
1.01238333440378,0.311354803599318,0.776457174439699
"6513","GYG1",212.528620622125,-0.22540246516434,0.222313378952879,-1.
01389518807195,0.310632767714016,0.776457174439699
"6514","TAPT1-
AS1",9.15460212356341,0.540669639485314,0.53482609153069,1.01092607119
802,0.312051814792398,0.776457174439699
"6515","MARCH1",8.09039709865792,-0.789978663811081,0.781396526689049,
-1.01098307559466,0.312024530191551,0.776457174439699
"6516","CPE",6336.98354799624,0.27987383295066,0.276523762224,1.012114
94701112,0.311483096497613,0.776457174439699
"6517","HOMER1",20.747691600343,0.486214197099972,0.479416318244284,1.
01417948992763,0.310497113469225,0.776457174439699
"6518","GNL1",330.107537138057,-0.185324867375453,0.182709439073255,-1
.01431468628804,0.310432618429801,0.776457174439699
"6519","CUL9",44.444677949646,0.289298008278322,0.286117338178459,1.01
111666325471,0.311960595915432,0.776457174439699
"6520","Z99289.1",4.19804212971243,0.953824486285827,0.940778847941403
,1.01386684912503,0.31064629174752,0.776457174439699
"6521","RAMP3",2.35884126862175,-1.00439824524394,0.992318158844786,-1
.01217360207659,0.311455055586074,0.776457174439699

"6522","POM121",163.108891991694,0.225792094215299,0.223295292735632,1
.01118161269357,0.311929514612041,0.776457174439699
"6523","CASD1",179.088389781698,0.150300215134268,0.148440516984824,1.
01252823816044,0.311285552081508,0.776457174439699
"6524","SDHAF3",179.998516795855,-0.268049420588645,0.264652385709355,
-1.01283583697984,0.311138580008033,0.776457174439699
"6525","CYREN",95.1992321319638,-0.318151835389018,0.314731348307309,-
1.01086795802231,0.312079631718136,0.776457174439699
"6526","AC087672.2",15.1336769170633,-0.485251444983307,0.479991192395
93,-1.01095906064676,0.312036024521466,0.776457174439699
"6527","S1PR3",127.82640393476,-0.439419011160055,0.433827410113713,-1
.01288899897975,0.311113183609797,0.776457174439699
"6528","GDI2",1185.61725880118,-0.186388454173674,0.183840956054623,-1
.01385707610384,0.310650955760619,0.776457174439699
"6529","ANKRD42",79.9696176472213,-0.275615897582507,0.272433099237546
,-1.0116828621554,0.311689712280091,0.776457174439699
"6530","ETNK1",278.936308219379,0.159939384847424,0.157786571520735,1.
01364383106839,0.31075273494036,0.776457174439699
"6531","MFSD5",128.907963474387,0.23748011976531,0.234716114927032,1.0
1177594831585,0.311645192393752,0.776457174439699
"6532","ATP5MC2",2540.964645165,-0.249815143252683,0.247045493687638,-
1.01121109121928,0.311915408450754,0.776457174439699
"6533","NEMP1",39.4424301960811,0.29190472002797,0.287661562997465,1.0
1475051788738,0.310224766411521,0.776457174439699
"6534","CAPS2",44.7966718413053,0.288077111452793,0.284742142751109,1.
01171224136147,0.311675660770602,0.776457174439699
"6535","ULK1",150.868415056787,0.308140336456495,0.304226076875728,1.0
1286628556291,0.311124034030876,0.776457174439699
"6536","AL139089.1",6.27179082031391,-0.602576180013135,0.593933044459
568,-1.0145523735953,0.310319251816925,0.776457174439699
"6537","AC018362.1",13.8005729613015,0.606024587231401,0.5975190158253
26,1.01423481291943,0.310470720724237,0.776457174439699
"6538","LACTB",185.34029932402,-0.411965489967412,0.406275350105504,-1
.01400562416703,0.310580068635867,0.776457174439699
"6539","CSNK2A2",411.019294101586,-0.202871288689512,0.200426349437212
,-1.01219869173472,0.311443061616546,0.776457174439699
"6540","KIAA0895L",20.5423582470834,0.41004789571801,0.404387791549331
,1.01399672365725,0.310584315657783,0.776457174439699
"6541","LCAT",68.5608665367059,0.24064496830792,0.237614689048185,1.01
275291217001,0.311178197340708,0.776457174439699
"6542","IRF8",48.3005599736422,-0.457004755558335,0.45130441138241,-1.
01263081864958,0.311236533583021,0.776457174439699
"6543","CTXN1",24.3966819845814,-0.41810272472375,0.413245157041681,-1
.01175468750038,0.311655360335932,0.776457174439699
"6544","ICAM3",83.5960879627079,-0.238921534032693,0.235803909964112,-
1.01322125688694,0.310954489295904,0.776457174439699
"6545","ZNF709",26.6338606324313,0.384018601899889,0.379246022489847,1
.01258438883211,0.31125871962693,0.776457174439699
"6546","ZNF805",67.5438657701786,-0.20871302556954,0.205866624865593,-
1.01382643109734,0.310665580883943,0.776457174439699

"6547","UCKL1-
AS1",4.94598392744034,0.831055103931967,0.819007094715406,1.0147105065
3199,0.310243844356211,0.776457174439699
"6548","CAPN10",46.2901301530136,0.262840299380617,0.260110996216209,1
.01049284038011,0.312259227901003,0.776592501772956
"6549","UBE3D",64.5449723078944,0.248452199081142,0.245836505857334,1.
01063997071829,0.312188777758185,0.776592501772956
"6550","MED21",321.489152785567,-0.247910612743534,0.245345374458057,-
1.0104556211469,0.31227705117643,0.776592501772956
"6551","RUFY2",235.272531211909,0.175973707029385,0.174238821028034,1.
00995694295401,0.312515919216318,0.776854769492162
"6552","NDUFAF5",67.7553186091233,0.215907715357318,0.213748677862676,
1.01010082268686,0.312446988131321,0.776854769492162
"6553","APOOL",207.062024453564,0.200211803244387,0.198241920306841,1.
00993676279213,0.312525588095165,0.776854769492162
"6554","SNHG4",6.73339260486166,-0.669235849724851,0.66276691408079,-1
.00976049876152,0.312610049489964,0.776946154431191
"6555","MRPL47",269.485411258013,-0.267676875959257,0.265426954272353,
-1.00847661343616,0.313225709382329,0.777838496296898
"6556","DSE",1194.43025539199,0.254049499089756,0.251903641879182,1.00
851856366413,0.313205580437478,0.777838496296898
"6557","DIP2C",198.737073776737,0.291499509947052,0.289124264074966,1.
00821531143256,0.31335110888945,0.777838496296898
"6558","CGRRF1",160.924511857637,-0.251272904454588,0.249195438425575,
-1.00833669364952,0.313292853140406,0.777838496296898
"6559","SIAH1",243.094935325383,-0.177685243093532,0.17622001475621,-1
.00831476685182,0.313303376081992,0.777838496296898
"6560","AC011447.3",10.4832051787821,0.466737582844948,0.4629129807083
8,1.00826203259782,0.313328684855742,0.777838496296898
"6561","ZNF550",18.9580323263909,0.442622044761773,0.43889955703344,1.
00848141144979,0.313223407112096,0.777838496296898
"6562","AL008721.2",8.82355882184973,-0.656418612501062,0.650981737132
199,-1.00835180936537,0.313285599056778,0.777838496296898
"6563","ARHGAP22",24.6478663296395,-0.686323610302635,0.68083161519704
2,-1.00806659823517,0.31342249149046,0.777897145190933
"6564","AC137630.4",3.66086921876756,0.805900096254166,0.7995652362817
09,1.00792288069191,0.313491486337285,0.777949850845223
"6565","COG6",129.051474756859,0.23735823897162,0.235525831586084,1.00
77800696985,0.313560055875102,0.778001485171292
"6566","AL359258.2",11.0232529897804,-0.540749143966893,0.536735434364
892,-1.00747800377061,0.313705122890003,0.77809243941066
"6567","RAB32",668.327988512674,0.305082268632053,0.30280888900962,1.0
0750763833211,0.31369088895285,0.77809243941066
"6568","RARG",400.381437336348,-0.237151616418447,0.23540833330815,-1.
0074053585351,0.313740017315318,0.77809243941066
"6569","PDGFRA",3614.56528735573,0.223827340959617,0.222309514414183,1
.00682753749624,0.314017658916342,0.778662451832592
"6570","AJM1",5.42151172155835,-0.675404314734451,0.671032645776388,-1
.00651483796739,0.314167977655155,0.778916619181859
"6571","KIAA2013",268.514578118534,-0.160349910834839,0.15937654166036

9,-1.0061073553506,0.314363930865774,0.779133256024267
"6572","PEA15",538.02262961557,0.181006253208053,0.179907381433497,1.0
0610798604148,0.314363627512459,0.779133256024267
"6573","EIF3M",863.814168137879,-0.236322984804498,0.234951829583053,-
1.00583589931553,0.314494515216377,0.779133256024267
"6574","FKBP4",153.636207521492,-0.247857924482854,0.246403462295636,-
1.00590276684292,0.31446234517753,0.779133256024267
"6575","GGA3",108.723484248235,0.259423061800645,0.25787851994298,1.00
598941648186,0.314420661162319,0.779133256024267
"6576","CASP6",125.82881696521,0.221366560531936,0.220190862731605,1.0
0533944862991,0.314733425838736,0.779483621412216
"6577","TNPO1",443.882892553151,0.184287522307587,0.183307040816802,1.
00534884795705,0.314728901423565,0.779483621412216
"6578","RNF19A",743.924718026285,0.210252905911659,0.209156145909911,1
.00524373786377,0.314779499149706,0.779483621412216
"6579","FGR",8.95523865678426,-0.556918383456252,0.554279280353002,-1.
00476132375284,0.315011791451577,0.779680005355895
"6580","MIGA1",330.387540725573,0.219427105791483,0.21839502936579,1.0
0472573221419,0.315028933969403,0.779680005355895
"6581","AC012076.1",4.52328809558605,-0.99728202225114,0.9931271140833
02,-1.00418366199947,0.315290095620558,0.779680005355895
"6582","NDUFC1",1018.09492833009,-0.241843645448048,0.240829627354624,
-1.00421052054335,0.315277152211232,0.779680005355895
"6583","SPDYE16",5.20149533069069,0.764363141751388,0.761212031581681,
1.00413959585368,0.315311332306974,0.779680005355895
"6584","GSTO1",1016.83102681144,-0.2382994946241,0.237308170512909,-1.
00417737033263,0.315293127690173,0.779680005355895
"6585","SEL1L",456.303780874344,0.16817338481696,0.16735733689218,1.00
487608096504,0.3149565232883,0.779680005355895
"6586","RPAP1",52.799295366396,0.250165272331867,0.249035271098124,1.0
0453751482174,0.31511959829308,0.779680005355895
"6587","NR1D1",88.9720806932836,-0.960752063975789,0.95639448863231,-1
.00455625308936,0.315110571300573,0.779680005355895
"6588","TRIOBP",949.674624146982,0.213736163093578,0.212866521421988,1
.00408538489651,0.315337459345855,0.779680005355895
"6589","DAGLB",40.1815165983725,0.30820196332607,0.306989132727031,1.0
0395072811948,0.315402363502409,0.779722127650741
"6590","LINC01807",4.65233766277583,0.809816966798525,0.80695279792883
4,1.00354936357745,0.315595872046468,0.779845442925502
"6591","ZNF140",68.7826976795397,0.22387808611164,0.223081698958026,1.
0035699349491,0.315585952144432,0.779845442925502
"6592","WARS",131.442757281274,0.263973934885539,0.263037150598581,1.0
0356141436609,0.31559006090485,0.779845442925502
"6593","MAD2L2",167.700787899958,-0.25401696864906,0.253371687153532,-
1.00254677822443,0.316079585758223,0.779857319293601
"6594","SLC9A1",86.6257848152955,0.26443657093607,0.263869139979341,1.
00215042561162,0.31627094672807,0.779857319293601
"6595","LINC01359",35.3550631341207,0.377537162292779,0.37626030972767
4,1.00339353509284,0.3156710221217,0.779857319293601
"6596","WDR3",85.8499855147045,0.243302611818376,0.242695567159789,1.0

0250125977038,0.316101558425188,0.779857319293601
"6597","PDK1",27.3047199782299,0.364115071946594,0.363033479365798,1.0
0297931910491,0.315870839669946,0.779857319293601
"6598","NDUFAF4",108.521724645027,-0.273097098246378,0.272495152828246
,-1.00220901330495,0.316242655516383,0.779857319293601
"6599","HOXA6",37.1549202396913,0.421551922593401,0.420348713347007,1.
0028624073494,0.31592725285689,0.779857319293601
"6600","LSM1",301.19563011807,-0.219638838222809,0.218942455624322,-1.
00318066496743,0.31577370017375,0.779857319293601
"6601","AKR1E2",8.10717187521141,0.54967846513146,0.548345066036572,1.
00243167884144,0.316135148467276,0.779857319293601
"6602","FRAT2",58.4365642191647,-0.307365761401359,0.306578224496766,-
1.00256879596027,0.316068957717152,0.779857319293601
"6603","MOB2",240.251036103515,-0.236945075172128,0.236300801335744,-1
.00272649873696,0.315992840871972,0.779857319293601
"6604","RPS6KA4",68.3096468148225,-0.275668591172899,0.274761035443703
,-1.00330307289653,0.315714653945203,0.779857319293601
"6605","VRK1",59.2102486834628,-0.264079033700243,0.2634861810398,-1.0
0225003322035,0.316222848537805,0.779857319293601
"6606","SYMPK",137.078545501618,0.188604428473785,0.188113180092078,1.
00261145115651,0.316048368566482,0.779857319293601
"6607","PPP2R3C",209.119126741012,-0.222287186647693,0.221832845511061
,-1.00204812382758,0.316320350870783,0.779861086020006
"6608","PHF8",64.8899990940353,0.224349960789128,0.223937780897687,1.0
0184060005323,0.316420584956081,0.779990149568644
"6609","ZNF589",39.7390354201504,0.31316430485695,0.31273008540491,1.0
0138847994582,0.316639031355511,0.780410528332564
"6610","SAMD13",5.92201599490017,-0.686534195619694,0.685986967708132,
-1.00079772348065,0.316924610386895,0.780442072942766
"6611","ORC4",254.457021499312,0.250809291821803,0.250641797091278,1.0
0066826336417,0.316987215580413,0.780442072942766
"6612","PPM1L",40.2532661655248,-0.420714306379528,0.420428211722707,-
1.00068048396574,0.316981305513063,0.780442072942766
"6613","YTHDF3-
AS1",23.5618121361004,0.397288513598926,0.39684624521112,1.00111445778
596,0.31677147607857,0.780442072942766
"6614","NDUFS3",572.152691066686,-0.263910972110198,0.263616866839597,
-1.00111565422246,0.316770897718981,0.780442072942766
"6615","PPP1R14B-
AS1",4.30240154495079,0.824483805744657,0.823920613004916,1.0006835521
9041,0.316979821684658,0.780442072942766
"6616","WDR61",397.535293784349,-0.233558867992111,0.233393171113327,-
1.00070994741617,0.316967056840955,0.780442072942766
"6617","AC096861.2",2.3485250488045,-0.977284759697769,0.9767900710682
29,-1.0005064431388,0.317065481098243,0.780516793351863
"6618","RSAD2",25.7455935878026,-0.549335125750612,0.549175550205856,-
1.0002905729228,0.317169908011824,0.780655882684284
"6619","CD44-
AS1",3.70847912059491,3.19811943085644,3.19755094506837,1.000177787875
11,0.317224476589352,0.780672231328593

"6620","LINC01140",441.612544955474,-0.308112247766498,0.308648119259169,-0.998263810925018,0.318151451104358,0.78071548741939
"6621","ZNF644",450.788350131059,0.140275797344611,0.140381715954854,0.999245495686372,0.317675781522054,0.78071548741939
"6622","PNPT1",148.719923243721,-0.213944034464099,0.214111128545398,-0.999219591795931,0.317688327109133,0.78071548741939
"6623","AC110769.2",8.14959289834037,-0.628410015734754,0.628762307434035,-0.999439706077931,0.317581733277139,0.78071548741939
"6624","AL359643.2",2.90495564793982,0.998554958643871,1.00029424506554,0.998261225204238,0.318152704615993,0.78071548741939
"6625","TMEM176A",2641.28175361383,0.301830529724777,0.301862733953312,0.999893314990846,0.317362139914871,0.78071548741939
"6626","AL591926.2",3.2583659865968,0.858228835269741,0.859556258250363,0.998455688074073,0.318058441489702,0.78071548741939
"6627","SHC3",85.9092594475841,-0.351283351143117,0.35132360496238,-0.999885422389233,0.317365959894495,0.78071548741939
"6628","SLC44A1",749.21616966147,0.185694772411181,0.18585932065788,0.99911466239026,0.317739149090777,0.78071548741939
"6629","LINC02664",7.45687433167025,0.580843895832521,0.581838600356011,0.998290411597166,0.31813855575752,0.78071548741939
"6630","ZNF143",113.766448333947,-0.192388173422262,0.192655995913274,-0.998609840873405,0.317983731103566,0.78071548741939
"6631","CAPRIN1",509.006123972769,0.15198754216316,0.152239393068329,0.998345691610475,0.318111758471847,0.78071548741939
"6632","HOXC-
AS1",63.9005915331867,-0.356031479457397,0.35641512041545,-0.99892361200163,0.317831696980491,0.78071548741939
"6633","SCAMP5",4.41953657636084,-0.87509402095509,0.875799386793601,-0.999194603411205,0.317700429612746,0.78071548741939
"6634","RNF166",89.7111010130576,-0.221883856489484,0.222130110048043,-0.998891399466258,0.317847302992402,0.78071548741939
"6635","C17orf67",24.6647337178767,-0.458697227432202,0.458869001016276,-0.99962565877474,0.317491701005585,0.78071548741939
"6636","AP000894.4",4.79219162774358,-0.755268937278788,0.755641244078335,-0.999507296878692,0.317549006065317,0.78071548741939
"6637","EVI5L",17.8711840668779,0.387527924015473,0.388119087092837,0.998476851314394,0.318048184012415,0.78071548741939
"6638","TMEM184B",117.459523930723,0.22372670100406,0.223788534018906,0.999723698914617,0.317444239883144,0.78071548741939
"6639","EFNA1",9.13772025164405,-0.505966360963321,0.506899083796389,-0.998159943738541,0.318201806622118,0.780718365426673
"6640","NUP98",151.523231058779,0.214636480046902,0.215086812933547,0.997906273841235,0.318324809457759,0.780800707993207
"6641","NFAT5",520.678883705142,0.24236390284011,0.242875636397614,0.99789302226813,0.318331235912756,0.780800707993207
"6642","ISG20L2",144.60575685023,-0.184898393026311,0.185325260583555,-0.997696657455654,0.318426474538627,0.780836737242832
"6643","PRKD3",243.310410215004,0.187075709390218,0.187569412141876,0.997367893058785,0.318585969878154,0.780836737242832
"6644","ANTXR1",764.269263338191,0.239974588486033,0.240564179627614,0

.997549131618457,0.318498038116483,0.780836737242832
"6645","CTDSPL",521.495239842583,-0.159029074169292,0.159464435820195,
-0.997269851119697,0.318633543646209,0.780836737242832
"6646","TF",7.56094233507079,0.685590107217959,0.687260957161443,0.997
568827494021,0.318488483199015,0.780836737242832
"6647","CCDC7",50.5797841824573,0.251958758771269,0.252634919147619,0.
997323567230409,0.318607477920658,0.780836737242832
"6648","TNK2",26.1822217512766,-0.391516574877422,0.392944728586598,-0
.996365509942548,0.319072584557426,0.780855368067295
"6649","MFAP3",19.8068005086427,0.402354546427557,0.403615251213717,0.
996876468908523,0.318824474796022,0.780855368067295
"6650","SAP30L-
AS1",8.78768027551581,0.491606820639945,0.493274583852776,0.9966189962
60045,0.318949481719187,0.780855368067295
"6651","AL355297.4",4.14752767934546,-0.768348969008701,0.770771654511
049,-0.996856805140448,0.318834020725774,0.780855368067295
"6652","RAC1",3254.04016974656,-0.21157731316457,0.21232827916394,-0.9
96463184261996,0.319025146417488,0.780855368067295
"6653","IGF2",683.247028586683,0.688359311214089,0.690371416456014,0.9
97085474291136,0.318723023065476,0.780855368067295
"6654","LINC01252",17.5151233811692,0.568494824841175,0.57047470345848
2,0.996529419086763,0.318992980334108,0.780855368067295
"6655","ABHD3",55.8468996754341,0.259117831826545,0.259891799243979,0.
997021962910389,0.318753849411128,0.780855368067295
"6656","NRIP1",423.29092526772,0.301725724690585,0.302723820607381,0.9
96702948863444,0.318908717916663,0.780855368067295
"6657","SOCS3",9351.77448592022,-0.302645921732627,0.303781510567822,-
0.996261823726295,0.319122947587364,0.780861302876758
"6658","CIAO2B",834.454609667408,-0.25460104350277,0.2555841018874,-0.
996153679444965,0.319175481555842,0.780872547170788
"6659","PTGES",815.131399260901,-0.257529842771133,0.258650659309705,-
0.995666678207728,0.319412125524275,0.781102986712074
"6660","DHODH",37.4046246758601,-0.31411167458541,0.315468926028553,-0
.995697669940967,0.319397062580836,0.781102986712074
"6661","THAP7-
AS1",53.5964988978334,-0.343526410500696,0.345022501201164,-0.99566378
8027565,0.319413530265156,0.781102986712074
"6662","HIST1H3A",7.47840844973005,0.614315887795995,0.61715535251654,
0.995399108653979,0.319542191954664,0.781208834840137
"6663","KMT5B",336.468702378171,0.156377085866781,0.15715002284394,0.9
95081534426971,0.319696610783004,0.781208834840137
"6664","SLC9A3R2",266.663908751891,-0.252372250816779,0.25360944631833
3,-0.995121650555549,0.319677101829387,0.781208834840137
"6665","ANKRD11",966.344205994739,0.229021170658859,0.23014565300707,0
.995114040462995,0.319680802648652,0.781208834840137
"6666","AC010776.2",5.48417042365059,-0.88605647005012,0.8904119428447
39,-0.995108474420611,0.319683509455669,0.781208834840137
"6667","NME2",5844.28165042158,-0.244104265180452,0.245419433832604,-0
.994641138920364,0.31991083159386,0.781615049622376
"6668","RGS12",111.999164908334,0.240708684290483,0.242030566628609,0.

994538366138876,0.319960836684141,0.781619986314933
"6669","ATL2",124.065592571638,0.185202339565188,0.186268616722952,0.994275594157929,0.320088714173316,0.781623942503249
"6670","CD96",5.48820140193084,0.78769351679965,0.792336087641354,0.99414065456046,0.320154395259481,0.781623942503249
"6671","SNRPD2",1507.62584591261,-0.209114494723162,0.210301974993397,-0.994353451648409,0.320050821488806,0.781623942503249
"6672","TFIP11",73.7926600787078,0.201278584031209,0.202451211391611,0.994207852092654,0.320121686139159,0.781623942503249
"6673","ASXL2",178.986556132312,0.258643020436184,0.260376034172546,0.993344188754279,0.320542249982795,0.781826271849427
"6674","CBWD6",6.53107220554431,0.60454953164402,0.608512943388177,0.993486725652722,0.320472816277639,0.781826271849427
"6675","NDUFA8",368.555903584968,-0.265688089756588,0.26738529225724,-0.993652595898885,0.320392028624593,0.781826271849427
"6676","ATP5F1C",1253.1452129477,-0.270968503301221,0.272828636967972,-0.993182043910701,0.320621247219238,0.781826271849427
"6677","LRRC18",2.57698350949865,1.14919841997962,1.15706126828496,0.993204466763462,0.32061032201017,0.781826271849427
"6678","RAB1B",428.183100668957,-0.186871070855202,0.188065409563669,-0.993649344070036,0.320393612310659,0.781826271849427
"6679","SPCS2",1827.7955532436,-0.228947244524962,0.230409555656742,-0.993653426709618,0.320391624008983,0.781826271849427
"6680","CENPBD1",32.7285331763081,-0.279010090256675,0.280848025113307,-0.993455767204022,0.320487896167057,0.781826271849427
"6681","SLMAP",189.793825998538,0.24279093593126,0.244531395613676,0.992882469434865,0.320767233867868,0.782065180732479
"6682","GMFB",217.929471347469,-0.162157802614536,0.163355782612808,-0.992666436540472,0.320872536537876,0.782204841015483
"6683","METTL8",135.504007428476,-0.183117048739282,0.184534259686743,-0.992320065933191,0.321041417912226,0.782265318829056
"6684","ZNF654",99.9440538482318,0.208021907152061,0.209599945313997,0.99247119001118,0.320967726570371,0.782265318829056
"6685","MAPKAPK5",118.744498965045,0.187086173438238,0.188517354898743,0.992408224371312,0.320998428623817,0.782265318829056
"6686","TPRG1-AS1",5.87141308366038,-0.680586998776795,0.685927450299891,-0.992214261842472,0.321093016833586,0.782274027999145
"6687","FAM193B",74.1150868933686,0.262977558842521,0.265335074638933,0.991114948524538,0.321629454467635,0.78264511976765
"6688","INPP5A",77.0304499802055,-0.222665537393311,0.224542537721353,-0.99164078064188,0.321372788473685,0.78264511976765
"6689","GIT2",166.646183563499,0.190844002560307,0.19252176400165,0.99128534142598,0.321546268661082,0.78264511976765
"6690","BBOF1",95.5196877677541,-0.189989173119865,0.19165696894227,-0.991298016286025,0.321540081356414,0.78264511976765
"6691","HEXA",879.612241966348,0.281965245038436,0.28433125706872,0.991678677699119,0.321354295560237,0.78264511976765
"6692","RFNG",423.522286999329,-0.223742020887472,0.225696177632921,-0.991341648910743,0.321518782438144,0.78264511976765

"6693","ZNF443",10.0583295787946,0.519890115951681,0.524380266404035,0
.991437224586758,0.321472131152214,0.78264511976765
"6694","PJA1",134.676163184619,0.208005579188929,0.209870400599119,0.9
91114414396376,0.321629715251068,0.78264511976765
"6695","ARHGAP35",226.530218471452,0.203572606599974,0.20548733255412,
0.990682024383955,0.32184087120827,0.783041964318374
"6696","AC025175.2",2.63684655134227,1.0874985623683,1.09907019840557,
0.98947143134801,0.32243254077426,0.783375126110978
"6697","MAK",5.9226853776657,-0.627269379409311,0.633948759222126,-0.9
89463849064062,0.322436248801555,0.783375126110978
"6698","ZNF277",199.227294053298,-0.217856810444396,0.220125525646358,
-0.989693538742043,0.322323934093606,0.783375126110978
"6699","ZNF251",69.2441478656315,0.25477151482531,0.257349499632726,0.
989982553643606,0.322182646537626,0.783375126110978
"6700","GAS1RR",128.966432250167,0.252439754104962,0.255051544367861,0
.989759755153134,0.322291560031063,0.783375126110978
"6701","RABEPK",142.432301774953,-0.195582627687459,0.197592976239776,
-0.989825809648831,0.322259267244953,0.783375126110978
"6702","CAND1",322.882794925846,0.160896355076353,0.162646366854562,0.
989240388137447,0.322545542245786,0.783375126110978
"6703","MINDY2",453.796836889472,0.206092983068299,0.208133561299187,0
.990195823210105,0.322078413693462,0.783375126110978
"6704","TNFSF9",174.26382623653,-0.352553202597763,0.356122969402156,-
0.989976027633414,0.322185836390866,0.783375126110978
"6705","ZNF846",56.4262735325464,-0.241538876831592,0.244101496474068,
-0.989501827397653,0.322417676220946,0.783375126110978
"6706","TMEM147-
AS1",9.03964911368871,0.48167537072534,0.486828602442504,0.98941468991
0763,0.322460290185454,0.783375126110978
"6707","ZMAT1",74.7817762808048,0.414466792249253,0.418982911888718,0.
989221231913477,0.322554912568379,0.783375126110978
"6708","BCL6",674.553401115517,0.277810199575274,0.280907707414015,0.9
88973218758372,0.322676244954986,0.783460399901691
"6709","AC004522.5",4.7565964556167,0.820917405262308,0.83008752030270
8,0.988952833507175,0.322686219101261,0.783460399901691
"6710","AC145212.1",6.9793572271774,0.9111619108177,0.921562461265134,
0.988714220810213,0.322802983065538,0.783627092571467
"6711","OXCT1",109.77910722212,-0.244715692488136,0.247555851712224,-0
.988527198188031,0.322894520936548,0.783732506561679
"6712","ITPK1",64.8101303262066,-0.238537088687875,0.24134829604142,-0
.98835207291846,0.322980251014736,0.783772107103033
"6713","MAPK8IP3",46.865138887918,0.367510206557857,0.371898977512679,
0.98819902387424,0.323055186143395,0.783772107103033
"6714","TP53I13",503.139936062017,-0.215776561936043,0.218346885208117
,-0.988228257666127,0.32304087196068,0.783772107103033
"6715","PRR14L",171.624073620347,0.200063878204112,0.202534396933242,0
.987801979483295,0.323249637928718,0.784127081492314
"6716","EXD2",70.235909126111,0.229359685737426,0.232278032017835,0.98
7435978103236,0.323428954021081,0.784445240031177
"6717","SERPIND1",3.94819167162733,-1.19881319751755,1.2142612789879,-

0.987277794542518,0.323506473401283,0.78451644264307
"6718","AP4B1",41.7813081945259,0.277831537555782,0.281512061304263,0.
986925875461856,0.323678978172545,0.784584356466159
"6719","AP002761.3",2.3840145991995,0.987961804358289,1.0008743924678,
0.987098692696416,0.323594258603769,0.784584356466159
"6720","B3GLCT",64.5312541215667,-0.241359397611266,0.244538969114017,
-0.986997689921279,0.32364377107463,0.784584356466159
"6721","SULT1C4",4.82583022344488,0.700434948011536,0.710102555112816,
0.986385618483313,0.32394391960132,0.78465810293568
"6722","PCDHB3",17.7789311528954,0.381539076674173,0.386887284384343,0
.98617631561947,0.324046599450446,0.78465810293568
"6723","AL512274.1",6.89416417056734,0.772985536955557,0.7834717557094
26,0.986615702892348,0.323831069194533,0.78465810293568
"6724","SSSCA1-
AS1",11.932400619603,-0.46555285627303,0.471922745875956,-0.9865022619
51578,0.323886705818581,0.78465810293568
"6725","AC009269.4",4.41370395429869,-0.763018237346351,0.773712315087
142,-0.986178224732552,0.324045662781505,0.78465810293568
"6726","RPL18",17217.1716542074,-0.255313607631581,0.258843405844965,-
0.986363190509482,0.323954921308137,0.78465810293568
"6727","LIME1",22.1151371984151,0.342108214617644,0.346742355599014,0.
986635203612883,0.323821505776766,0.78465810293568
"6728","UTP15",45.7925204220679,-0.230709784217178,0.234000674750972,-
0.985936405793292,0.324164320542794,0.7847764023128
"6729","CNOT6",77.5704297726176,0.251512104405023,0.255114217783916,0.
985880389536172,0.324191811109511,0.7847764023128
"6730","F7",6.4907333078872,0.69480626428072,0.70486504528358,0.985729
493794357,0.324265872333521,0.78483904820813
"6731","FCRL6",2.92732940685871,0.897236969081549,0.910874757129149,0.
98502781206652,0.324610409863172,0.784870464887625
"6732","TRIM17",4.21167113206931,0.792940611907431,0.80453237050205,0.
985591930145228,0.324333399625415,0.784870464887625
"6733","TMEM30A",516.500603974426,0.139658266558672,0.141810205225994,
0.984825219991098,0.324709930298471,0.784870464887625
"6734","GCHFR",155.808505156392,-0.25339325114238,0.257299058404,-0.98
4819970637097,0.324712509231856,0.784870464887625
"6735","AC005736.1",25.3216514310945,0.482909825866697,0.4903374097291
25,0.984852096301338,0.324696726556205,0.784870464887625
"6736","LINC01572",6.73730896207024,-0.745840005347652,0.7570238521981
81,-0.98522655948283,0.324512797357707,0.784870464887625
"6737","METRNL",1325.60131279889,0.266165284532111,0.270243880712625,0
.98490772050135,0.324669400715815,0.784870464887625
"6738","TBXA2R",41.4634933137325,-0.389274528717911,0.395079685862892,
-0.985306363873652,0.324473607727633,0.784870464887625
"6739","AC008555.1",2.54724838702121,-1.15912432463233,1.1766090551109
,-0.985139728100318,0.324555441240686,0.784870464887625
"6740","CD2",18.067322637942,0.556583501240927,0.565479480656791,0.984
268254251186,0.324983634026816,0.784968199548456
"6741","CACYBP",619.839391384725,-0.255261337667485,0.259323974165237,
-0.984333741179042,0.324951444698841,0.784968199548456

"6742","ARHGAP25",8.11844465415995,0.573868297177888,0.58314231488821,
0.984096476154196,0.325068079386431,0.784968199548456
"6743","GATAD1",588.989978526151,0.197965758077068,0.201145620331374,0
.984191242896231,0.325021490705903,0.784968199548456
"6744","IGF2-
AS",11.7157863863569,0.581986490553867,0.591418832727745,0.98405133274
1006,0.32509027405942,0.784968199548456
"6745","DHPS",331.002604443213,-0.223070585537403,0.22656822466838 5,-0
.984562534591504,0.324839000279798,0.784968199548456
"6746","ZNF529",50.3582874427747,0.308102291439576,0.313073724412071,0
.984120567825262,0.325056235165806,0.784968199548456
"6747","ABCC10",28.1236291546249,0.322897784596233,0.328252356603121,0
.983687635749825,0.325269120961695,0.785118358709212
"6748","ACAD10",49.9195942550206,0.248858808398318,0.252973186803295,0
.98373591107829,0.32524537803048,0.785118358709212
"6749","RIMBP2",2.59051257583831,1.15825555071436,1.17753074872346,0.9
83630832545138,0.325297059544998,0.785118358709212
"6750","HSPE1",956.425576320381,-0.286446394601162,0.291279140130868,-
0.983408542309158,0.325406407719926,0.785149603814232
"6751","ZP1",11.8321503531938,-0.55553859658575,0.564890580031502,-0.9
83444610732877,0.325388663452089,0.785149603814232
"6752","EIF1",20831.1473743447,-0.200943361220765,0.204371653732038,-0
.983225205410494,0.325496612105526,0.785250935217257
"6753","ULBP3",4.75308473371126,-0.77811814299145,0.791625442533106,-0
.982937259446293,0.325638318479902,0.785360167266674
"6754","EGR3",129.345782079352,-0.480200075576206,0.488492444293957,-0
.983024571178912,0.325595345662795,0.785360167266674
"6755","MPC2",669.051236216161,-0.241584931748289,0.245951455653557,-0
.982246399421932,0.325978473865139,0.785366567192613
"6756","AL590428.1",9.6275267620409,-0.512898614632937,0.5221195291192
1,-0.982339456825474,0.325932642187931,0.785366567192613
"6757","RESF1",239.075444353553,0.203193214491519,0.206786306625444,0.
98262412926387,0.325792464251416,0.785366567192613
"6758","KMT2D",82.610916295073,0.288759703819625,0.293861742084649,0.9
82637963591888,0.325785652973185,0.785366567192613
"6759","DEPDC4",4.35412211944643,0.774186573112701,0.787970008482912,0
.982507664984929,0.325849808662364,0.785366567192613
"6760","AL132639.2",6.03199740921252,0.590784469964211,0.6013427636071
83,0.982442137360003,0.325882075886744,0.785366567192613
"6761","NDUFB11",1219.99116403302,-0.237994903845345,0.242205350335843
,-0.982616211885244,0.325796362384012,0.785366567192613
"6762","DNPEP",374.74490986468,-0.172166548307746,0.17535262860299 2,-0
.981830438923961,0.326183389676334,0.785408496560068
"6763","CBLL1",93.9931335222944,0.206436406549133,0.210202154867867,0.
982085110777762,0.326057920015866,0.785408496560068
"6764","DERA",347.573710822053,-0.246087477047612,0.250650414602267,-0
.981795611382111,0.326200550665659,0.785408496560068
"6765","WIPI1",265.071453050715,0.180217522220258,0.183559164637352,0.
981795284241481,0.326200711864338,0.785408496560068
"6766","TUBA8",8.36840610927943,-0.6005369669038,0.611718123096783,-0.

981721718270534,0.326236962841514,0.785408496560068
"6767","SEC24D",254.662712019181,0.193499687683338,0.19719161611376,0
.981277457425208,0.326455936141822,0.785819527680529
"6768","AL136454.1",22.1046031185644,0.524443085552021,0.5346995959948
23,0.980818181798474,0.326682410506582,0.785841932100016
"6769","FAM114A1",1001.65165615763,-0.162020843196565,0.16521371447550
6,-0.980674296385884,0.326753383135285,0.785841932100016
"6770","SLC37A2",2.55276418324111,-1.18793493103445,1.21121568914645,-
0.980779015397001,0.326701728654792,0.785841932100016
"6771","AC083805.1",9.64960895649261,-0.541221962756075,0.551814078921
219,-0.980804918595316,0.326688952268398,0.785841932100016
"6772","GDE1",238.594869276915,-0.169630091460197,0.172952516347907,-0
.980789959245076,0.326696330716836,0.785841932100016
"6773","TBCB",661.073427851296,-0.26386463985115,0.269065236417764,-0.
980671614676598,0.326754706004875,0.785841932100016
"6774","MED17",127.668105269274,0.178509155451429,0.182061625414498,0.
980487541210393,0.326845516552645,0.785944289803077
"6775","NANS",320.254668044398,-0.241349938063891,0.246203821870705,-0
.980285099679064,0.326945407702263,0.78606844960327
"6776","SLC2A5",8.55632496453964,0.688724505688791,0.703076562577953,0
.979586779515821,0.327290133429687,0.786322564040153
"6777","PRR7",5.37491367052103,-0.615317179438351,0.628084929795748,-0
.979671936466381,0.327248083074261,0.786322564040153
"6778","CTR9",312.712690716553,0.152655598164827,0.155837479196218,0.9
79582055306578,0.327292466338771,0.786322564040153
"6779","PSME2",595.367164371303,-0.318983925828922,0.32552996961632,-0
.979891117874297,0.327139867806819,0.786322564040153
"6780","AC004148.1",64.2270043648638,-0.374438341882882,0.382207304930
842,-0.979673431282622,0.327247344967906,0.786322564040153
"6781","FAM47E",2.34637645393221,1.05963878148378,1.08199515746925,0.9
79337822511363,0.327413088088071,0.786496356269959
"6782","ITGA2",9.45266754667379,-0.558682845615079,0.570632766728069,-
0.979058473663352,0.327551088337511,0.786711836910899
"6783","HAUS1",193.969987891936,-0.233168563658988,0.238201042754742,-
0.978872976215579,0.327642746206095,0.786815965347351
"6784","STARD7",187.55401632741,0.188204591204297,0.192295143136924,0.
978727741814498,0.32771452094385,0.786872321883015
"6785","SYF2",1000.5181874805,-0.211371750356848,0.216081279107216,-0.
978204827508308,0.327973029324027,0.786886652616415
"6786","PRDX1",2809.53744075218,-0.279472541556003,0.285790148463813,-
0.97789424533432,0.328126631611994,0.786886652616415
"6787","DCAF6",311.739288786525,0.150269675938095,0.15367568710928,0.9
77836369335613,0.328155260066505,0.786886652616415
"6788","LONRF2",12.8863640566744,0.413946843510684,0.423315631373024,0
.977868079588858,0.328139574338798,0.786886652616415
"6789","PAK1IP1",176.40794010014,-0.231225468722774,0.236372009752536,
-0.978226943895981,0.327962093168234,0.786886652616415
"6790","TRA2A",662.06062659954,0.192778012473528,0.197101339018673,0.9
78065463346575,0.328041947837448,0.786886652616415
"6791","NUDT8",106.3569868226,-0.258048159040393,0.263855499369118,-0.

977990451809381,0.328079046511733,0.786886652616415
"6792","TMEM94",70.6681700611005,0.237290077785231,0.242481369265682,0
.978590967643526,0.327782123970819,0.786886652616415
"6793","SDCBP2",21.4974011133909,-0.433326755568575,0.442895087211086,
-0.978395940892541,0.327878535005331,0.786886652616415
"6794","SARS",670.09791930402,-0.171191236646667,0.175091617640177,-0.
977723770868771,0.328210961718999,0.786904379664524
"6795","KCTD6",92.0267963300278,-0.214115026856387,0.219140224651557,-
0.977068574228395,0.328535204452377,0.787532215424956
"6796","RNF44",92.0310782516916,0.271884997406951,0.278285777801832,0.
976999254344076,0.328569521519308,0.787532215424956
"6797","DNAJC11",77.7932773990926,0.235048209158034,0.240851257473747,
0.975906090852171,0.329111003501497,0.787952441445445
"6798","ELOA-
AS1",37.7229510373017,-0.391453173587966,0.402717800236534,-0.97202848
5848028,0.331036377380707,0.787952441445445
"6799","ELOA",197.334396415223,0.166820134954983,0.171539548358331,0.9
72487898863478,0.330807882165103,0.787952441445445
"6800","AL020996.1",2.92999365453556,0.922169397434384,0.9473574031649
49,0.973412351403582,0.330348402637934,0.787952441445445
"6801","CYP4B1",264.005265761265,0.359960441228438,0.368950605986529,0
.975633148144447,0.329246291710373,0.787952441445445
"6802","AL162595.1",9.31318611583727,0.539951929516074,0.5549712607247
5,0.972936740563715,0.330584743301746,0.787952441445445
"6803","CHCHD5",362.787214544752,-0.24392156606239,0.250270196643034,-
0.974632894104848,0.329742390821301,0.787952441445445
"6804","RBMS1",1476.75199053577,0.172829192673513,0.177652321392359,0.
972850741937709,0.330627489439694,0.787952441445445
"6805","HAT1",289.08369148923,-0.207084279605199,0.212795913955233,-0.
973159097635511,0.33047423595092,0.787952441445445
"6806","STK11IP",27.8253728818752,0.315933964596567,0.32447495910664,0
.973677492606554,0.330216696091445,0.787952441445445
"6807","PXYLP1",83.1108788250986,0.250892925182614,0.257994700566697,0
.97247317340828,0.330815204484192,0.787952441445445
"6808","USO1",461.80519152331,0.151735643437586,0.156106251258757,0.97
2002352334201,0.33104937830176,0.787952441445445
"6809","MARCH6",499.114336193811,0.173052367910323,0.177876441327116,0
.972879638355693,0.330613125901142,0.787952441445445
"6810","CMYA5",180.985277082907,0.361101464336115,0.371008628213865,0.
973296675267512,0.33040587438657,0.787952441445445
"6811","SLC26A2",174.84236814118,0.204166794478383,0.209341242458798,0
.975282233354311,0.329420280909749,0.787952441445445
"6812","GPLD1",7.74914187857966,-0.607959941753775,0.625218080166917,-
0.972396610141962,0.330853277707993,0.787952441445445
"6813","C6orf89",528.757808359195,0.143301454300018,0.147022445075261,
0.974691001953222,0.329713557652195,0.787952441445445
"6814","AARS2",43.8909454450693,0.30605908286032,0.314898213403716,0.9
71930197863447,0.331085275479409,0.787952441445445
"6815","SMAP1",427.072930204816,-0.176747720419414,0.18135126523239,-0
.974615314610151,0.329751114104876,0.787952441445445

"6816","CEP162",159.555747622053,0.201195633564491,0.206037329667978,0
.976500879179083,0.328816312423699,0.787952441445445
"6817","GNAI1",272.915453982389,-0.260228689503382,0.266521947517983,-
0.976387467999512,0.328872489400471,0.787952441445445
"6818","KIF13B",128.078914764831,-0.397761693871583,0.409554213081459,
-0.971206451226201,0.33144548199597,0.787952441445445
"6819","CEBPD",11032.447790826,-0.243829433745487,0.249961134373617,-0
.975469383896437,0.329327481248045,0.787952441445445
"6820","COX6C",2218.58118410237,-0.268933354268478,0.276152030818919,-
0.973859773802736,0.330126169240658,0.787952441445445
"6821","HAUS6",88.4631382074771,0.222246929430761,0.228085253795579,0.
974402885466454,0.329856537349135,0.787952441445445
"6822","MIR1915HG",6.35381189818817,0.713757046685801,0.73197915686930
3,0.975105698007251,0.329507832492331,0.787952441445445
"6823","ZNF239",13.8757639696975,0.455874170494463,0.468187608637776,0
.973699777789635,0.330205627672756,0.787952441445445
"6824","BTAF1",115.005839898264,0.272720503000911,0.27992233885319,0.9
74272021726513,0.329921492597061,0.787952441445445
"6825","MARVELD1",385.605590958531,0.181054257760303,0.186320641637541
,0.971734833934915,0.331182482559295,0.787952441445445
"6826","PPRC1",80.8637006368676,-0.279592605537953,0.287143100934233,-
0.97370476472632,0.330203150835562,0.787952441445445
"6827","CNNM2",47.1222554995883,0.269750321864932,0.277404824182371,0.
972406744042756,0.330848238179856,0.787952441445445
"6828","SLC5A12",2.44714759039383,1.15927531329901,1.18962897833242,0.
974484763244447,0.329815900881259,0.787952441445445
"6829","AL356215.1",6.44704081879491,-0.760156034210809,0.779810357151
686,-0.974796022185874,0.329661450656523,0.787952441445445
"6830","TCIRG1",198.288948003535,-0.142141494543625,0.14585613666084,-
0.974532150636537,0.329792383694985,0.787952441445445
"6831","LOH12CR2",33.1644239063664,-0.295302146208781,0.30273823512815
,-0.97543723237925,0.329343422555757,0.787952441445445
"6832","LDHB",1107.16087772627,-0.234388253712057,0.241288868532799,-0
.971401022920359,0.331348619357652,0.787952441445445
"6833","LINC02361",3.28729712132507,-0.800332379196258,0.8216196481428
05,-0.974091090695477,0.330011312780478,0.787952441445445
"6834","AL121603.2",59.1856633894302,-0.322054095482858,0.331594846019
647,-0.971227687488773,0.331434909165277,0.787952441445445
"6835","PTGR2",58.7763016631985,-0.25347762429051,0.2598481322073,-0.9
75483726349407,0.329320370159939,0.787952441445445
"6836","ZC3H14",280.500163463939,-0.151650025616095,0.155760122012432,
-0.973612652948431,0.330248901477511,0.787952441445445
"6837","CCDC32",290.507746355706,-0.214204569821307,0.219786091911177,
-0.974604753006274,0.329756355047841,0.787952441445445
"6838","CCDC154",2.40474806459489,1.02262947719925,1.0526736171895,0.9
71459206823799,0.331319657516054,0.787952441445445
"6839","SETD6",47.8054470339755,-0.248069614837836,0.255035010127103,-
0.972688474081282,0.330708155401274,0.787952441445445
"6840","ENKD1",100.012945502486,0.190302360850242,0.195947642754011,0.
971189845285069,0.331453749695143,0.787952441445445

"6841","TXNL4B",72.5093472978514,0.236105654568635,0.241924654645405,0
.975947056387044,0.329090701420172,0.787952441445445
"6842","AC233723.1",26.2747416260407,-0.659327304730789,0.677710389502
199,-0.972874718971163,0.330615571150421,0.787952441445445
"6843","MINK1",120.332302044563,0.208507689739666,0.214008628365693,0.
974295715700641,0.32990973129238,0.787952441445445
"6844","ZNF287",51.7017671014455,0.264262656783165,0.271903972925166,0
.971897004446846,0.33110179020073,0.787952441445445
"6845","GAA",410.183344752336,0.246547221695513,0.252569661516948,0.97
6155331621131,0.328987494993361,0.787952441445445
"6846","AP005136.4",2.48967069241679,-1.16964917717859,1.2040518262370
7,-0.971427601114149,0.331335389491491,0.787952441445445
"6847","P2RY11",8.92253454108036,0.568813656184101,0.585368629196496,0
.971718721867417,0.331190500252155,0.787952441445445
"6848","KCTD15",16.8444691713272,0.561471603741415,0.577920449134311,0
.971537872699373,0.331280503092693,0.787952441445445
"6849","AR",694.919580080399,0.226722914964962,0.23309162731606,0.9726
77215288983,0.330713752800412,0.787952441445445
"6850","OPHN1",198.360106758466,0.206011114686956,0.211378338091547,0.
974608451116377,0.329754519943137,0.787952441445445
"6851","NHSL2",139.868761512787,0.357443483855705,0.36706814120892,0.9
73779643960609,0.330165962382432,0.787952441445445
"6852","MAFIP",7.40515725581285,0.691712083948775,0.709701109937408,0.
97465267316516,0.32973257624962,0.787952441445445
"6853","AL136366.1",5.75808807312653,-0.592475892254217,0.610114560313
916,-0.971089580208309,0.331503672101292,0.787956123574777
"6854","AL357497.1",4.5439011216545,0.818251797576618,0.84296094389531
7,0.970687673613302,0.331703831890766,0.787977306824879
"6855","AC012101.2",2.87240967250675,1.13984604351098,1.17403510229513
,0.970879014846051,0.331608529310118,0.787977306824879
"6856","GCDH",107.587172403348,-0.268935049233692,0.277021965547674,-0
.970807671160681,0.331644061856887,0.787977306824879
"6857","EIF3L",1196.72990406772,-0.201498501890983,0.207584216483558,-
0.970683153586212,0.331706083424286,0.787977306824879
"6858","MAFG-
DT",14.5217860216366,-0.411538191860908,0.424079747694133,-0.970426421
206347,0.331833984173757,0.788166195422328
"6859","TMUB2",207.431596201039,0.155625739728403,0.160387695404439,0.
970309719433102,0.331892134022944,0.788189381994421
"6860","HIST2H2AB",3.6890421113924,0.839582265929089,0.865710712600612
,0.969818501387106,0.332136969037165,0.788317067509683
"6861","CAPN10-
DT",5.94206236171448,-0.742894078756527,0.766247557861852,-0.969522279
235068,0.332284669747774,0.788317067509683
"6862","TNFAIP8",66.5213283363457,-0.236244457087361,0.243663768683021
,-0.969551026663664,0.332270333999318,0.788317067509683
"6863","P4HA2",586.419411021364,-0.201498929774176,0.207749771712269,-
0.969911678426535,0.332090518369718,0.788317067509683
"6864","FAM8A1",320.152955894455,0.206582330176558,0.213014569778515,0
.969803757514592,0.332144319545027,0.788317067509683

"6865","ZKSCAN3",33.057248028582,0.284730928955294,0.293675937269328,0
.969541228344388,0.332275220173733,0.788317067509683
"6866","HNRNPA1L2",33.3978133000378,0.350349281845292,0.36128844946244
8,0.96972178979585,0.332185186190843,0.788317067509683
"6867","ABCD3",133.243690626682,-0.161926095794082,0.167057170458551,-
0.96928551674624,0.332402753503892,0.78842851231199
"6868","QARS",356.49283336622,-0.141890477486982,0.146394448894487,-0.
969234001415235,0.332428450031233,0.78842851231199
"6869","APLF",27.9713163928505,0.295903184649333,0.305435755366405,0.9
68790259327573,0.332649847569762,0.788838749026621
"6870","SAMD11",69.7764338999381,-0.448637026332075,0.463301678038103,
-0.968347510054082,0.332870844639078,0.788998187324403
"6871","PON2",185.202730188106,0.2180307322732,0.22514855838942,0.9683
86090645496,0.332851583474816,0.788998187324403
"6872","EIF4A1",3524.77673987142,-0.200269869527807,0.206785603332371,
-0.968490389565027,0.332799516371369,0.788998187324403
"6873","AC009283.1",16.8455719655958,0.397516874332419,0.4105445112504
61,0.968267419095771,0.332910831940611,0.788998187324403
"6874","APOL2",203.448070038374,0.223144525521789,0.230496946539202,0.
968101872377025,0.332993494877372,0.789079289795971
"6875","SMPD1",190.031527533546,0.293658465679908,0.30340857169526,0.9
67864764133476,0.33311191392156,0.789245085944479
"6876","FLAD1",57.5062674485656,-0.258022371361928,0.266616938443107,-
0.967764362116802,0.333162065923191,0.789249111667081
"6877","AC026124.1",16.1101430767143,0.484752729039742,0.5011527371403
9,0.967275429454446,0.333406363252831,0.789712992733076
"6878","PSMD4",726.555887311553,-0.213823366041492,0.221093094052556,-
0.967119153846856,0.333484471411093,0.789783157140926
"6879","FIGNL1",50.285385078711,0.285242144460782,0.295115514137806,0.
966544050705469,0.333772015549662,0.78999433339025
"6880","AC002456.1",19.2772856915737,-0.440538364077111,0.455720160699
088,-0.966686142217875,0.333700956758529,0.78999433339025
"6881","AL451123.1",4.25811352639384,-0.8372909966173,0.86635204741382
6,-0.966455841036819,0.333816133386033,0.78999433339025
"6882","CCDC59",545.336590283277,-0.192130909209201,0.198764497083099,
-0.966625891589059,0.333731086408293,0.78999433339025
"6883","LINC00653",5.65894455510009,-0.657993044964935,0.6807892623544
06,-0.966515016246534,0.333786536643526,0.78999433339025
"6884","GPR150",6.71284418315772,-0.618317496003203,0.639965889985577,
-0.966172581506084,0.333957830335281,0.790114303840787
"6885","ASCL2",6.11963118180931,0.781368706347684,0.808735866652409,0.
966160570548199,0.333963839520156,0.790114303840787
"6886","PLK3",362.821511544744,-0.301721612435637,0.312343988900196,-0
.965991417020823,0.334048475881969,0.790199771077751
"6887","HMGN2",1995.8839609261,0.179840678702716,0.186250408999222,0.9
65585416263256,0.334251675744577,0.790338788141958
"6888","MYCBP",48.6167271092691,-0.340893880081518,0.353907997867499,-
0.963227398464013,0.335433418337059,0.790338788141958
"6889","C2orf69",145.776151539453,-0.221832318144144,0.229952118484558
,-0.964689169232597,0.334700521691825,0.790338788141958

"6890","DGKD",41.3770660150393,0.31681146367067,0.328711642075665,0.96
3797514654938,0.335147452971742,0.790338788141958
"6891","NBEAL2",21.7054809868601,-0.407171122197665,0.422209320184572,
-0.964382126902519,0.334854379639643,0.790338788141958
"6892","TSPAN17",61.7185108094231,-0.211511385240693,0.219474336288906
,-0.963718076642315,0.33518728897489,0.790338788141958
"6893","ZSCAN16",9.9562556482994,-0.544473515946761,0.564355078120199,
-0.964771182285342,0.334659432919459,0.790338788141958
"6894","PFDN6",254.046829810457,-0.264577514206241,0.274742674880158,-
0.963001158526426,0.335546941874904,0.790338788141958
"6895","GRIK2",2.65443738961971,1.09127539872269,1.13129112858652,0.96
462826512763,0.334731036924916,0.790338788141958
"6896","PACRG",6.2311965583402,-0.647212561879193,0.670992373150709,-0
.964560236117356,0.334765124121568,0.790338788141958
"6897","HOXA11-
AS",34.020144539213,0.339125574213829,0.351777184492433,0.964035159651
248,0.335028298677606,0.790338788141958
"6898","LSMEM1",41.3338026570135,-0.547237866682885,0.56777791968373,-
0.963823790449113,0.335134277046723,0.790338788141958
"6899","ZNF596",19.5139006789682,0.356474598848717,0.370329997426343,0
.962586345492086,0.335755152594409,0.790338788141958
"6900","SLC35G5",2.28735708329724,1.06945312680636,1.11035634659687,0.
963162078628292,0.33546619223317,0.790338788141958
"6901","AL132656.3",5.07392814121632,-0.69514196085456,0.7198850375572
,-0.965629127691553,0.334229794725975,0.790338788141958
"6902","DPYSL4",4.91339230033365,-0.867372985513635,0.900276714676834,
-0.963451538147346,0.335320973065818,0.790338788141958
"6903","ITFG2-
AS1",7.86373768169242,-0.559060720310129,0.579367338081279,-0.96495035
8026048,0.33456967667418,0.790338788141958
"6904","RPAP3",152.950047121595,-0.199882392782909,0.207471538054308,-
0.963420788496723,0.335336397959224,0.790338788141958
"6905","AC048341.1",27.950759081506,0.423917522889398,0.43975791403029
7,0.963979292616419,0.335056307741568,0.790338788141958
"6906","CHD8",259.529955615336,0.188918510928605,0.195708352631385,0.9
65306326421493,0.334391404003031,0.790338788141958
"6907","TTC7B",64.434811667696,0.256378616298684,0.266344472191685,0.9
62582831883109,0.335756916565925,0.790338788141958
"6908","AMN",6.47127774275449,-0.591148254166135,0.612959060978064,-0.
964417188356549,0.334836808150582,0.790338788141958
"6909","AC103858.3",6.28934587347857,0.737141559306581,0.7644388759373
65,0.964291040801252,0.334900031362678,0.790338788141958
"6910","LITAF",561.035653560045,-0.191889407814091,0.198760879938546,-
0.965428447858654,0.334330258428581,0.790338788141958
"6911","TK2",387.21802629168,0.178471607164681,0.18501803007275,0.9646
17378611722,0.33473649166518,0.790338788141958
"6912","KDM6B",231.885179191578,-0.372984670709048,0.386514645525108,-
0.964994923290221,0.334547354572953,0.790338788141958
"6913","TUBD1",72.3160078738459,-0.218280650723433,0.226672924997166,-
0.962976282792318,0.33555942562497,0.790338788141958

"6914","WDR18",147.230566705058,-0.247221624061626,0.256731989178982,-0.962956057218384,0.335569575938199,0.790338788141958
"6915","ZNF254",139.474369800187,0.179913102507293,0.186875889085926,0.962741118649972,0.335677456230153,0.790338788141958
"6916","PLD3",1377.22100297259,0.178587463279536,0.185047122061444,0.96509181710071,0.334498825166357,0.790338788141958
"6917","DKKL1",2.51242177865553,0.992507663775538,1.02798375470706,0.965489638557925,0.334299623271596,0.790338788141958
"6918","AL391097.1",6.64280368502787,0.787561906315173,0.817879580386401,0.962931371807933,0.335581964712296,0.790338788141958
"6919","PARVG",11.8330231120573,-0.661042769742026,0.686109297073895,-0.963465693529045,0.335313872480079,0.790338788141958
"6920","LPAR4",45.0605947818879,0.32829115260275,0.341028623628284,0.962649847716542,0.335723272979213,0.790338788141958
"6921","CAMLG",1368.76689299908,-0.191129066695863,0.198634403983253,-0.962215320524119,0.335941454838794,0.790634701676387
"6922","TMEM72-AS1",11.4283687098203,0.470967316702857,0.489549374087466,0.962042526518909,0.336028242354081,0.790634701676387
"6923","PLAAT5",28.5305790791672,-0.375911213704162,0.390732713195143,-0.962067421051641,0.336015737936712,0.790634701676387
"6924","SLC40A1",826.730418272947,0.302905241464873,0.314982938981093,0.961656026338158,0.33622241816049,0.790634758108303
"6925","SUN1",589.757391566725,0.149159446459911,0.155060258613826,0.961945038614887,0.336077212994209,0.790634758108303
"6926","ELOC",678.482229427231,-0.25681550675228,0.267006786688744,-0.961831382404732,0.336134311181742,0.790634758108303
"6927","MCM3AP-AS1",9.77033868574771,0.481697761774819,0.500900670384958,0.961663240347869,0.336218793216088,0.790634758108303
"6928","SPATA1",10.3264438492263,-0.5008304742565,0.521167871479304,-0.960977262153408,0.33656360067594,0.790742395092741
"6929","MAP3K21",7.50885690914903,0.536450720666545,0.558177138678447,0.961076123498462,0.336513893922469,0.790742395092741
"6930","NREP",140.36268120946,0.250668892319885,0.260871862734783,0.960888957866372,0.336608003411693,0.790742395092741
"6931","RPL26L1",249.841814735004,-0.250716175497042,0.260918203532658,-0.96089951602653,0.336602694171073,0.790742395092741
"6932","CD81",12393.6954371349,-0.211531434430586,0.220017886542163,-0.96142835364455,0.336336833596366,0.790742395092741
"6933","PLAAT4",893.867030499138,0.276226518417437,0.287396746583254,0.96113307370868,0.336485261921601,0.790742395092741
"6934","RELA",261.063098605469,-0.16622796229263,0.172982361828957,-0.960953247111951,0.336575675974183,0.790742395092741
"6935","PITPNM2",41.6855739820211,0.271816058360202,0.282918360695779,0.960757929219321,0.336673896514962,0.790783143523031
"6936","CTBS",316.637717672909,0.147115739905903,0.153164147758895,0.960510289506435,0.336798454907257,0.790961653976977
"6937","EZH2",19.6518756859515,0.542316698468184,0.564853531386043,0.96010145698734,0.337004155266919,0.791330645112129

"6938","GNG12-
AS1",10.737273899575,0.504300132235183,0.526578589892975,0.95769205568
6653,0.338218063897766,0.791422181452672
"6939","C1orf43",629.569475128429,-0.228888602555279,0.238955409221469
,-0.957871610025532,0.338127503816371,0.791422181452672
"6940","SLC8A1",13.0424600812391,0.417249035845881,0.435105849053992,0
.958959841043426,0.337578976528012,0.791422181452672
"6941","WIPF1",483.666740728053,0.188658569927575,0.19692291615019 7,0.
958032582575006,0.338046328891851,0.791422181452672
"6942","RETREG2",352.309486332521,-0.137715716366197,0.143649329891141
,-0.958693761193031,0.337713042322493,0.791422181452672
"6943","ZNF660",43.0606477064246,0.271543650783788,0.283051639644388,0
.959343147154853,0.337385905765075,0.791422181452672
"6944","UFSP2",327.394096954516,-0.212094848536465,0.22099635891162,-0
.959721008893569,0.337195646803351,0.791422181452672
"6945","UBE2D2",957.521419900439,-0.213088267696973,0.222378435821347,
-0.958223610620965,0.337950013881515,0.791422181452672
"6946","CHCHD2",4068.7056121671,-0.262853999771876,0.274515508717363,-
0.957519671657265,0.33830502221305,0.791422181452672
"6947","INTS8",88.1362717096965,0.210435526047419,0.219548637737905,0.
958491604482809,0.337814922956302,0.791422181452672
"6948","AP002851.1",3.50008368260465,-0.780891639001941,0.814083909838
529,-0.95922745747036,0.337444171021706,0.791422181452672
"6949","FAM53B",53.5391672639896,0.268863499457606,0.280757274656565,0
.957636804910904,0.3382459333294,0.791422181452672
"6950","PPP1R32",11.2676038301074,0.430084162012365,0.448995927177019,
0.957879873691599,0.338123336326098,0.791422181452672
"6951","HSP90B1",3424.79080341559,0.180892324383629,0.188694398581674,
0.958652327484603,0.337733922001865,0.791422181452672
"6952","AC084018.2",3.62061549512543,-0.782318415102528,0.816400976209
92,-0.958252669827004,0.33793536397701,0.791422181452672
"6953","AL512506.1",2.91976893051657,-0.875895327411606,0.914756968640
125,-0.957516977119845,0.338306381573758,0.791422181452672
"6954","ANKRD10",380.418694483267,-0.225837929611249,0.235648018155677
,-0.958369738811268,0.337876348966009,0.791422181452672
"6955","PRTG",33.9527772161136,0.273664698558576,0.285299650256082,0.9
59218485942542,0.337448689658163,0.791422181452672
"6956","AC027237.3",4.13575729697566,0.794405879761098,0.8284712042681
24,0.958881703634926,0.337618342943254,0.791422181452672
"6957","GOLGA6L9",21.6419673266659,-0.51127971328978,0.533745497079876
,-0.957909183472261,0.338108555231758,0.791422181452672
"6958","MRPS34",538.959737796889,-0.22071892440052,0.230005167057019,-
0.959625938950335,0.337243509428154,0.791422181452672
"6959","VPS35",560.793806769703,0.138584432502413,0.144411638523426,0.
959648639953155,0.337232080292155,0.791422181452672
"6960","GPS1",228.093672891245,-0.153802199295696,0.160566535109696,-0
.957872069610404,0.338127272039987,0.791422181452672
"6961","LDLRAD4",89.4037033120777,-0.299755963965362,0.312595412571172
,-0.958926305091291,0.337595871917952,0.791422181452672
"6962","MIATNB",27.1424177394516,0.347510356871795,0.3621093858294,0.9

5968337323219,0.337214593821044,0.791422181452672
"6963","TIMP3",10348.1360388822,-0.193704303082026,0.202257887621628,-0.957709513136003,0.338209258369771,0.791422181452672
"6964","LLPH",327.950969866823,-0.231294981545034,0.241680969529055,-0.957026041379015,0.338554111097386,0.791887983294849
"6965","KIFC2",11.5109502939248,-0.486256538214306,0.5083045618409,-0.956624383722342,0.338756876879167,0.792218737157399
"6966","MTERF2",166.458090882571,0.188782199354395,0.197356704022252,0.956553263744768,0.338792787957422,0.792218737157399
"6967","XRN2",515.078693167294,-0.122334192872369,0.12793120412812 5,-0.956249835261846,0.338946027544726,0.79246330453223
"6968","BICRA",38.3689323617335,0.249917854902016,0.261401546282815,0.95606877027279,0.339037491445145,0.792563389516355
"6969","POLR2K",632.589370471136,-0.254884893871686,0.266668353455954,-0.955812306066478,0.339167069909393,0.79275253289627
"6970","RIOK2",146.105517372745,-0.216936938014117,0.227011831572077,-0.955619522171199,0.339264494830452,0.792866478664739
"6971","MON1A",34.5793433723601,-0.26942790252421,0.282010138430223,-0.955383746215471,0.339383670542355,0.793031216391396
"6972","LRCH4",53.7801813447989,-0.23311732865796,0.244066944668004,-0.955136833359642,0.339508504310881,0.793087032989351
"6973","LIN52",50.6545238813509,0.255353225621223,0.267372248391911,0.955047605564994,0.339553623185446,0.793087032989351
"6974","SMCHD1",375.256734440473,0.180299710838586,0.188753230048773,0.955213909674541,0.339469533041747,0.793087032989351
"6975","HCP5",7.69362622484109,0.582984241623,0.610720861392038,0.954583801663795,0.339788211932559,0.793443781393672
"6976","POT1-AS1",2.99395348347098,0.844921019602401,0.885214248915228,0.954481946757858,0.339839743338872,0.793443781393672
"6977","ZNF730",4.24859497573738,0.858083111932232,0.899027760412859,0.954456747295741,0.33985249326439,0.793443781393672
"6978","DHX15",264.543772175101,0.160965829214629,0.16867167784245,0.954314507768052,0.33992446655853,0.793454667143257
"6979","SLC38A10",417.978944346009,0.169893807754944,0.178038162673131,0.954255004680436,0.33995457805837,0.793454667143257
"6980","SSNA1",431.880991355271,-0.245111018621202,0.257059415933101,-0.953518927643527,0.340327210773346,0.793928054874875
"6981","COTL1",571.81199692489,-0.198579896087427,0.208221901561277,-0.95369360570837,0.340238757817211,0.793928054874875
"6982","HS3ST3A1",3.08241981782471,-0.859896921179774,0.901861183950228,-0.953469266094094,0.340352360930153,0.793928054874875
"6983","SMAD7",273.266326461089,-0.321755572093073,0.337401314726215,-0.953628685039837,0.340271630430808,0.793928054874875
"6984","HIST1H4J",8.23219633955654,-0.572444780037439,0.600504573759871,-0.953272972515856,0.340451781774004,0.794046259065972
"6985","AC003992.1",2.50378196374823,1.21623230014781,1.27603557927976,0.953133533184311,0.340522417781382,0.794097303255682
"6986","MMUT",200.558504467369,0.158405491854931,0.166337553698553,0.952313463392659,0.34093803182967,0.794611475243025

"6987","NPFF",17.7998400510022,0.383047043721817,0.402188251321804,0.952407342737936,0.340890437012346,0.794611475243025
"6988","TRPV2",156.987721854055,-0.219003797943939,0.229963976042702,-0.952339586889346,0.340924787349064,0.794611475243025
"6989","RAD51D",76.6584668066899,0.194784092830747,0.20448213237933,0.952572679892673,0.340806624962711,0.794611475243025
"6990","AIDA",771.637712058851,0.171899151522052,0.180564279558719,0.952010840361985,0.341091484191585,0.794741694463843
"6991","SENP2",166.116050063042,0.225790714733804,0.237163242476,0.952047679802883,0.341072801494123,0.794741694463843
"6992","CD2BP2-DT",3.64995933352655,-0.727341974463328,0.764293823358165,-0.951652299461904,0.341273348294036,0.795051712008232
"6993","ZCCHC14",205.281034316841,0.229084077540394,0.240797243122776,0.951356728879118,0.341423318386993,0.795193256429961
"6994","CARM1",199.127711800737,0.193183725052821,0.20308536805778,0.951243936972645,0.341480559195014,0.795193256429961
"6995","UQCC1",77.9226841893378,0.242796673011641,0.255234502862417,0.951269010610685,0.341467834033884,0.795193256429961
"6996","NUP93",58.9407496859209,-0.244923304283747,0.257503863897766,-0.95114419091197,0.341531184479085,0.795197464834164
"6997","MRPL54",554.989910820219,-0.247707551996937,0.260459523598175,-0.951040486348615,0.341583823961195,0.795206361083879
"6998","EID3",94.2191926623956,-0.348643392092739,0.366631759649921,-0.950936144827284,0.341636791997186,0.795216019554468
"6999","TMCO4",89.7592042163135,-0.182468522641407,0.19192263991976,-0.950739958129452,0.341736398643552,0.795220599643546
"7000","TNFAIP1",73.8090743266938,0.225231289085135,0.236877231082409,0.950835536433543,0.341687869921652,0.795220599643546
"7001","SMARCA5",518.349633152479,0.161500801611692,0.169914447322808,0.950483046946961,0.341866864031412,0.795296964896839
"7002","NUDT18",69.8591608057822,-0.293250743193443,0.308501909340284,-0.950563786851581,0.341825859023707,0.795296964896839
"7003","KIF20B",82.7952455378479,-0.267485370357196,0.281449423306093,-0.950385213851688,0.3419165542968,0.795298979428898
"7004","NIFK",401.426738194307,-0.204908219758522,0.215677140948642,-0.950069250998258,0.342077066087884,0.795449158860114
"7005","AC108673.3",18.1473892460223,-0.472572011982342,0.497560722557024,-0.949777565949614,0.342225287328362,0.795449158860114
"7006","AL442125.1",9.40948035485469,0.547807267225739,0.576749886999073,0.949817727882137,0.342204876400087,0.795449158860114
"7007","FANCM",57.5664100382614,0.265871149334367,0.279902881504662,0.949869282892461,0.342178676470999,0.795449158860114
"7008","AL133467.1",102.989768866164,-0.263119516518023,0.276997208775895,-0.949899523106384,0.342163309183169,0.795449158860114
"7009","USP48",318.731512200002,0.158874630614173,0.167318332038803,0.949535108784902,0.342348524425878,0.795622073672867
"7010","ANKRD37",51.7722599261227,-0.260147189636124,0.274083253913762,-0.949153900945647,0.342542343649676,0.795849730716915
"7011","FAM155A",8.49482567826633,-0.706611178562166,0.744467145357191

,-0.949150251920302,0.342544199279041,0.795849730716915
"7012","SLC25A16",77.7960027572576,0.199310310791573,0.210071763530817
,0.948772492988257,0.342736334816994,0.796182566719054
"7013","RTL8B",107.855189301065,-0.189463529331968,0.199800387733904,-
0.948264072361546,0.342995036162523,0.796669919300063
"7014","PANK1",5.79180557916511,0.591771254093068,0.624187518864419,0.
94806646433699,0.343095619373389,0.796789926429017
"7015","AC114284.1",3.20212141389713,-0.792247852261424,0.835880426751
348,-0.947800459140427,0.343231046755009,0.796990808352436
"7016","PLLP",5.3549075777858,0.71060501572036,0.750103888273302,0.947
342130642908,0.343464469026555,0.797142905011097
"7017","ABR",147.406506438348,0.241130492291034,0.254546716698621,0.94
7293665455245,0.343489157804531,0.797142905011097
"7018","CIRBP",4251.85573156909,-0.218175152119193,0.230315666630401,-
0.947287500286769,0.343492298500392,0.797142905011097
"7019","SDCBP2-
AS1",24.0652456801997,0.353492759321341,0.373112824874846,0.9474151938
89179,0.343427251829689,0.797142905011097
"7020","ADAMTS4",79.8036102232562,1.04004059715711,1.09806842902587,0.
947154630499449,0.34355999025503,0.797186421832505
"7021","RANBP2",264.200248998319,0.185991158753848,0.196421744563355,0
.946896990286418,0.343691271744547,0.797377456978626
"7022","CD53",43.6728603550929,-0.463690687822025,0.490099427038782,-0
.946115547662805,0.344089654529767,0.797514882371224
"7023","TRIM52",93.3604671405602,0.248175577160826,0.262299624498289,0
.946153002069759,0.344070553390266,0.797514882371224
"7024","COL14A1",2915.48757501624,0.335625108868842,0.354695669055387,
0.946234020174723,0.344029237787695,0.797514882371224
"7025","MIPEP",46.4112415429649,-0.241425310826288,0.255087722112441,-
0.946440341491107,0.343924037465908,0.797514882371224
"7026","ARL6IP1",885.644687139955,0.142660790984395,0.150714180972861,
0.946565147775204,0.34386041047711,0.797514882371224
"7027","DEF8",175.489735830039,-0.228363318210165,0.241245957484509,-0
.946599564159863,0.343842866120838,0.797514882371224
"7028","RNFT1-
DT",19.1183591950599,-0.513206326880203,0.54243916518623,-0.9461085404
92294,0.344093228148135,0.797514882371224
"7029","EIF2D",248.940126254439,-0.187394078354448,0.198179110646804,-
0.945579368798476,0.344363171247862,0.797741077661155
"7030","COPS9",1224.83367245558,-0.218837410597966,0.231443386270708,-
0.945533221424623,0.344386718528654,0.797741077661155
"7031","MTHFD1L",41.5219980998511,0.322376541300143,0.340915194309333,
0.945620924738341,0.344341967683524,0.797741077661155
"7032","SCARF1",14.8402520169265,0.433372007203776,0.458279349908265,0
.945650305409844,0.344326976948947,0.797741077661155
"7033","ANKRD26",214.76682015739,0.213804019324471,0.226180710398027,0
.945279634802741,0.344516132643882,0.7979273829996
"7034","OSBPL6",8.29689393490883,-0.521558316654503,0.552125398345663,
-0.944637428774788,0.344844011598717,0.798462891501344
"7035","PLEKHA3",368.12595225895,-0.13579749547046,0.143756617438233,-

0.944634743710545,0.34484538287875,0.798462891501344
"7036","OBSL1",215.260082370849,0.186942015960658,0.19794605728175,0.944408888602264,0.344960741024106,0.798502985724266
"7037","OPA3",35.9136544254195,-0.277676035334535,0.294008621976284,-0.944448613336699,0.344940449366589,0.798502985724266
"7038","MRTO4",195.626087301557,-0.211497820267745,0.223972177637649,-0.944303986765334,0.345014329236888,0.798513556257412
"7039","DDX39B",875.217553076366,-0.154129971707393,0.163274052363785,-0.943995506181116,0.345171944682902,0.798525674741594
"7040","FBRSL1",122.211520570417,0.17291821040129,0.183155967956796,0.944103609236907,0.345116705150921,0.798525674741594
"7041","NLE1",92.2954240481967,0.234478345481642,0.248411768060322,0.943909973800854,0.345215654830272,0.798525674741594
"7042","VRK3",131.203813011375,0.185907171535253,0.196945599087645,0.943951895327806,0.345194230969131,0.798525674741594
"7043","HIST3H2BB",2.36608143537415,0.97387058661113,1.03226879433909,0.943427324309117,0.345462372362231,0.798619055056662
"7044","HOXA5",51.9436390185521,0.346157330770801,0.366933240733203,0.943379591554891,0.345486778177601,0.798619055056662
"7045","AC069113.2",3.0711464394196,-0.981940432746472,1.04059331895408,-0.943635150121313,0.345356123581013,0.798619055056662
"7046","TRUB1",83.8295338023736,0.201331478644793,0.213388201271962,0.943498644464402,0.345425908327821,0.798619055056662
"7047","PFKM",123.458802738107,-0.191581098214505,0.203113012143773,-0.943224149907709,0.345566263298012,0.798619055056662
"7048","TIPIN",57.3906268581855,-0.274185683134787,0.290737253761312,-0.943070348184161,0.345644921313994,0.798619055056662
"7049","SCAMP2",362.011196111399,-0.17850868756596,0.18928037620127,-0.943091360808287,0.345634174263931,0.798619055056662
"7050","AURKA",8.80394697147367,-0.559869648837897,0.593670995156358,-0.943063840756514,0.345648249625482,0.798619055056662
"7051","APOE",2429.54330526058,-0.724706465029639,0.768764615224732,-0.942689674677322,0.345839656255391,0.798834679629051
"7052","CRYBB2",4.10279216570646,-1.11292522819733,1.18052942624235,-0.942734000066214,0.345816977843165,0.798834679629051
"7053","ANKRD28",682.242186625282,-0.308057890564566,0.326854491282184,-0.942492450864349,0.345940574313499,0.798954489577851
"7054","TTN",12.885947618389,0.678616331414193,0.72027055524033,0.942168642709213,0.346106305388373,0.798961547091831
"7055","ESPNL",10.3305740714598,0.534297275278353,0.567044885281189,0.942248645825283,0.346065353592383,0.798961547091831
"7056","UCK1",152.591082718738,-0.198208918052255,0.210389824002037,-0.942103160133523,0.346139826743634,0.798961547091831
"7057","RNF165",3.91682277207689,-0.756116896291181,0.802529511258425,-0.942167092529138,0.346107098921178,0.798961547091831
"7058","TAPBP",631.896440005791,0.14297523235254,0.151782045917797,0.941977237742417,0.346204293813753,0.798997129772205
"7059","ACKR2",38.286791594189,0.31803042551223,0.337695422823317,0.941767059953977,0.346311913206225,0.799019086999462
"7060","STAU2",133.655844025031,0.165989383916143,0.176238418483462,0.

941845627897069,0.346271680799024,0.799019086999462
"7061","NOP58",315.854333517427,0.129503296434535,0.137577510558443,0.94131152619978,0.346545237742443,0.799217807955069
"7062","CPEB4",95.6286911474326,0.244972845352764,0.260235907317943,0.941349131553427,0.346525972478516,0.799217807955069
"7063","KIAA1586",130.333417066574,-0.177513708165289,0.188571786006018,-0.941358789270965,0.346521024929288,0.799217807955069
"7064","TRPM4",21.8928369929733,-0.363489902102349,0.386223645328555,-0.941138396105017,0.34663394127711,0.799309211418863
"7065","TMEM127",146.895851785951,0.198414193763143,0.210915684656761,0.940727542790556,0.346844500608285,0.799538287010643
"7066","RPLP2",16014.7631705536,-0.139962732025152,0.148787315703947,-0.940689946336863,0.34686377258208,0.799538287010643
"7067","AJ011932.1",12.5665585045329,-0.430624693500519,0.457791291343631,-0.940657241942332,0.346880537436565,0.799538287010643
"7068","LY75",11.1086342978167,-0.57808613205074,0.614676505966383,-0.94047214500558,0.346975431139263,0.799643859341746
"7069","ESRG",50.1256341009348,0.287460306835642,0.305758249737486,0.940155521829568,0.347137792738489,0.799791726438084
"7070","MIF",3222.85599756975,-0.2480918923947,0.263858383191263,-0.940246390484646,0.347091191140068,0.799791726438084
"7071","APEH",127.175424094668,-0.168562501296545,0.179372963568198,-0.939731930294264,0.347355082370465,0.799839827075559
"7072","PRKG1",133.528563409366,0.265121266422521,0.282114428354127,0.939765002340557,0.347338114298473,0.799839827075559
"7073","MANSC1",127.501730683509,0.223334645234208,0.23761764380385,0.939890833268966,0.347273559815946,0.799839827075559
"7074","AC022467.2",4.12645702341904,0.911599500276112,0.970005604471085,0.939787869342446,0.34732638237441,0.799839827075559
"7075","ZNF385D",115.796297587774,-0.321354113146254,0.342027941576941,-0.939555147642706,0.347445792104969,0.799935619448459
"7076","TBC1D8-AS1",4.92738209069505,0.714210431265479,0.760240084999359,0.939453792766111,0.347497805553496,0.799942305633252
"7077","USP28",79.8003118933641,0.224480054063626,0.239023566465339,0.939154483314004,0.347651434538923,0.800182876530241
"7078","GMEB1",103.221351233451,-0.203416665515101,0.216659140428089,-0.938878761879962,0.347792994530689,0.800375917518015
"7079","HOXA1",4.28318550460243,0.694765255379209,0.7400569375578,0.938799732993444,0.34783357603966,0.800375917518015
"7080","DDX56",161.937502211412,-0.181843960818545,0.193789553793877,-0.938357910725994,0.348060508226244,0.800558827802497
"7081","NINJ2",248.471278168875,0.331119747938409,0.352869169888856,0.938364062926502,0.348057347639147,0.800558827802497
"7082","NTN5",3.99275716815707,0.727810765272511,0.775618056267016,0.938362328457647,0.348058238690815,0.800558827802497
"7083","ALG10B",58.7181535041548,0.227513480533774,0.242532751522098,0.938073225599156,0.348206780265741,0.800669147903537
"7084","TAF4B",52.3281787986994,0.322529384787433,0.343802931162549,0.938122847576716,0.348181281539087,0.800669147903537

"7085","PSMB9",579.015635961517,0.269465642808021,0.287285445211839,0.
937971788335195,0.348258908464945,0.800675985883626
"7086","AL358781.1",8.24266291862511,0.684319819600369,0.7296655795203
7,0.937854050961527,0.348319419444088,0.800702091917126
"7087","PRKAR2A-
AS1",4.31691543867112,-0.723934325626594,0.772266252701323,-0.93741546
1434877,0.348544890802014,0.800881326883058
"7088","SNURF",3.9237691345523,-0.820227564834418,0.874940599685634,-0
.937466572164014,0.348518610879212,0.800881326883058
"7089","AQR",170.500604051398,0.172839984202704,0.184360363152141,0.93
7511628028581,0.348495445268187,0.800881326883058
"7090","SEM1",1656.36916681229,-0.225943303210631,0.241062916347895,-0
.937279390101443,0.348614861587709,0.800929122764767
"7091","GTPBP8",130.180464112433,0.225492239208034,0.240615779656196,0
.937146514373368,0.348683197737386,0.800973150182524
"7092","GMPPA",146.479426334193,-0.258013903678569,0.275370199085603,-
0.936971046741196,0.348773451353298,0.801067505512389
"7093","AC008875.2",7.32323594901991,-0.513747546633161,0.548459588135
76,-0.936709937699172,0.348907782997454,0.801263058965957
"7094","KIAA0100",173.39813435877,-0.155097426482839,0.165634240354769
,-0.9363850502809,0.349074972306847,0.801421032263034
"7095","AC099850.1",19.1510040253989,-0.388154744469462,0.414517246621
174,-0.93640191725049,0.349066291195841,0.801421032263034
"7096","H2AFZ",1071.74320100214,-0.247390612473067,0.264229965822879,-
0.936270084668975,0.349134146590359,0.801443928101798
"7097","TNFAIP8L3",25.9084670259864,-0.406056841252763,0.4337693070701
03,-0.93611243265568,0.349215302471792,0.801517269545304
"7098","SEC24A",126.238323987173,0.197059220482968,0.210556925338629,0
.93589522247272,0.349327137244049,0.801660994444676
"7099","NKAPL",14.9146276906535,0.433270182242635,0.463285783432978,0.
935211477960912,0.349679324473653,0.801957496494304
"7100","TP53TG1",303.817561566714,-0.266059473792451,0.284497761717148
,-0.935190042222447,0.349690369364645,0.801957496494304
"7101","PPP1R3C",5.71854212273128,-0.564013642481693,0.603283749304332
,-0.93490607551103,0.349836705786273,0.801957496494304
"7102","EXOSC8",287.488174696062,-0.226614856252181,0.242238775632508,
-0.93550198831904,0.349529659128449,0.801957496494304
"7103","EPG5",107.256232922203,0.227127165601626,0.242914977869702,0.9
35006838991442,0.34978477494534,0.801957496494304
"7104","RBFA",211.533354879626,-0.190770658504675,0.204031257625551,-0
.935007021594638,0.349784680840908,0.801957496494304
"7105","ASIP",23.7173445602103,-0.472041129671338,0.504639884751379,-0
.935401944901556,0.349581194980561,0.801957496494304
"7106","KDM5C",178.080992463201,0.204066871016119,0.218281379255126,0.
934879886284788,0.349850203823963,0.801957496494304
"7107","ZNF3",92.8340582012781,0.205188628368146,0.219576918878977,0.9
34472664138433,0.350060130357129,0.802325800392186
"7108","FNDC3A",505.107316002556,0.125823575444509,0.134663016256869,0
.934358808691027,0.350118838116302,0.802347461181267
"7109","SELENOH",1054.56335936628,-0.226546668496383,0.242497258110974

,-0.934223629005767,0.350188549491159,0.802394328690601
"7110","VPS28",1545.32189448572,-0.235857749346331,0.252493235776903,-0.934115120433286,0.350244513098796,0.802409686901026
"7111","TFAM",330.389489201179,-0.144652739826421,0.154911148260249,-0.933778759314378,0.350418028358028,0.80269431358795
"7112","SP110",296.79055525542,0.166104128100227,0.17791650083525,0.933607210800748,0.350506544369561,0.802784181838553
"7113","NEK11",112.463564117678,0.199733322209941,0.213995724524642,0.933351928659404,0.350638291705101,0.802860153722855
"7114","CCNT1",170.993200904074,0.191363342807731,0.205011782203997,0.933426073128398,0.350600023606764,0.802860153722855
"7115","MMS22L",48.6777662858272,0.266578720562057,0.285690195115582,0.933104198602989,0.350766171536887,0.803040079854443
"7116","ZDHHC20",120.819275323932,0.240614757039398,0.257940632783882,0.932829986662085,0.350907755959204,0.803224392602635
"7117","Z95114.1",17.840097673459,-0.532331867400869,0.570707810500688,-0.932757284211423,0.350945300641718,0.803224392602635
"7118","MRPL38",314.361755157756,-0.242378858725581,0.25996549685598,-0.932350106675339,0.351155620550338,0.80359284955668
"7119","MCM6",113.91145343406,0.175530557696683,0.188350107318659,0.931937656927973,0.351368745137725,0.803645229232755
"7120","AC008966.1",15.3272227313981,0.386271948240099,0.414488725024249,0.931923897851506,0.35137585625856,0.803645229232755
"7121","MCM7",114.183285070754,-0.203988467229027,0.218886464347218,-0.931937330329579,0.351368913932928,0.803645229232755
"7122","NNMT",8209.36284729286,-0.26455580361097,0.283808058686812,-0.932164522864775,0.351251506876336,0.803645229232755
"7123","WNT9A",30.4647651539869,0.294532753532994,0.316255743496362,0.931311951134197,0.351692221660936,0.804157261093039
"7124","TTC25",8.94837586700132,-0.442312077036146,0.474940552669271,-0.931299874374285,0.351698466942526,0.804157261093039
"7125","FBLN7",196.910363975085,0.24891595955238,0.267430958140682,0.930767182987984,0.351974008696428,0.804674333706122
"7126","LHCGR",8.49828580296226,-0.709634355717654,0.762553097373278,-0.930603204107477,0.35205885647618,0.804682129302908
"7127","TXNDC9",210.518151292194,-0.257186703582531,0.276474203248171,-0.930237615520581,0.352248070012434,0.804682129302908
"7128","TRIM2",231.404018573441,0.173327536738829,0.186377492666626,0.929981052212463,0.352380895004147,0.804682129302908
"7129","PHTF2",167.418330462822,0.206117608721303,0.221566548277796,0.930274043276948,0.352229213624953,0.804682129302908
"7130","CEP41",50.6514584727773,0.292549061246912,0.314662855848351,0.929722259267563,0.352514906407713,0.804682129302908
"7131","NMRK1",107.959693710426,-0.360679496485521,0.387987844477276,-0.9296154547611,0.352570222655464,0.804682129302908
"7132","FAM168A",154.982345646326,0.251265209335843,0.270189018459219,0.929960850254793,0.352391355074414,0.804682129302908
"7133","ENO2",54.9555994796311,-0.228100261526582,0.245356776948795,-0.929667663405055,0.35254318204013,0.804682129302908
"7134","SUGT1",546.590033484009,-0.17418112484145,0.187264333128304,-0

.930135076614459,0.352301151467754,0.804682129302908
"7135","SPIRE1",67.7442631510672,-0.247963512438484,0.266662503360337,
-0.929877689265577,0.352434415831825,0.804682129302908
"7136","A1BG-
AS1",12.6941961342165,-0.529283789287103,0.568911001708846,-0.93034549
8148718,0.35219222773087,0.804682129302908
"7137","CBFA2T2",143.600563240987,0.221707813737558,0.238490213634491,
0.929630655945265,0.352562349314705,0.804682129302908
"7138","AC098484.4",2.39053210826437,-0.971850316854637,1.045776277887
36,-0.929309965624711,0.35272847207609,0.804902468559417
"7139","PSMD12",455.999002473533,-0.147957243104472,0.15922421588934,-
0.929238321432848,0.352765591690446,0.804902468559417
"7140","PIGC",282.022719577717,-0.216529850823238,0.233047300183621,-0
.929124047575884,0.352824803300127,0.804914012623129
"7141","AC012360.3",17.6727941837583,-0.437920464296451,0.471369893651
467,-0.929037832484591,0.352869480271457,0.804914012623129
"7142","LMOD3",3.396821651869,-0.730520376689095,0.786675165420296,-0.
928617565165922,0.353087315551517,0.805298135398861
"7143","MAST2",95.5081656397161,-0.206575400266276,0.223134683270972,-
0.925787946714734,0.354556192318915,0.805601355631197
"7144","PREB",193.157338684511,0.267349718956896,0.288590839254562,0.9
26397108263961,0.35423964635312,0.805601355631197
"7145","GNLY",53.5305595524237,0.834538954971613,0.899900275184637,0.9
27368262889336,0.353735362865965,0.805601355631197
"7146","AC009570.1",5.94912000853212,-0.667563772357347,0.720483573959
562,-0.926549607076559,0.354160429504147,0.805601355631197
"7147","SCOC",386.266897589147,-0.223911347132391,0.241237975939461,-0
.928176197219388,0.353316179367171,0.805601355631197
"7148","C5orf34",11.33110216524,-0.447085589349638,0.482343394669509,-
0.926903103246537,0.353976845852914,0.805601355631197
"7149","PELO",109.193992444975,-0.225105101402678,0.243047664036182,-0
.92617677398935,0.354354120696392,0.805601355631197
"7150","ZNF451",297.58361741531,0.228915341660429,0.246727578140891,0.
927806057941803,0.353508181069991,0.805601355631197
"7151","MRAP2",12.8959609375376,-0.894962985195365,0.966196058003482,-
0.926274722176666,0.354303228977477,0.805601355631197
"7152","HYMAI",620.745628100604,0.258143937047944,0.278606446316535,0.
926554071023384,0.354158110836327,0.805601355631197
"7153","AKR1B10",10.2689571591268,-0.767787873052333,0.829247329194999
,-0.925885252832435,0.354505615982637,0.805601355631197
"7154","ARMC1",233.266185599928,-0.171532403575576,0.18494400900769,-0
.927482887907138,0.353675872425559,0.805601355631197
"7155","PUF60",438.555781464475,-0.209955473990883,0.22673948174562,-0
.925976686435376,0.354458096127576,0.805601355631197
"7156","AUH",152.751717253388,0.20538733588196,0.221632946342638,0.926
700381289144,0.354082119517036,0.805601355631197
"7157","AL158152.1",97.9714627137216,-0.304157093585305,0.328383064241
709,-0.926226491879703,0.354328287799575,0.805601355631197
"7158","TGFBR1",192.227233025573,0.205343178155755,0.221796130795167,0
.92581947854353,0.35453980267177,0.805601355631197

"7159","SMC2",183.08542170744,-0.171419795679483,0.184977776336458,-0.
926704813272732,0.354079817773162,0.805601355631197
"7160","AL158212.3",17.3367471861672,0.43037655256073,0.46497094261107
8,0.925598813000914,0.354654510481387,0.805601355631197
"7161","TRPT1",129.4533449359,-0.241530404655611,0.260828251264141,-0.
926013204033685,0.354439118332455,0.805601355631197
"7162","PRMT5",117.075985671424,-0.205252231507818,0.221698793114765,-
0.925815736856837,0.354541747504596,0.805601355631197
"7163","SNHG21",35.7580797444019,-0.397110717489805,0.428413188078289,
-0.926933923932417,0.353960842379838,0.805601355631197
"7164","KARS",256.081805163348,0.136945312522537,0.147691556864021,0.9
27238600704993,0.35380266522619,0.805601355631197
"7165","ARMC7",35.4947309076656,0.250662317445499,0.27080905237327,0.9
25605385967668,0.354651093340118,0.805601355631197
"7166","GATAD2A",133.590654366923,-0.173545037199478,0.187168021594475
,-0.927215214014959,0.353814805166115,0.805601355631197
"7167","ZNF253",64.999743491854,0.323096992912374,0.348962561561719,0.
925878671529726,0.354509036571264,0.805601355631197
"7168","COX6B1",2090.18451543487,-0.241491195025289,0.260350184819031,
-0.927562986725548,0.353634304853302,0.805601355631197
"7169","BCAT2",94.118585048134,-0.242111280609499,0.260992331435937,-0
.927656683541016,0.353585684470296,0.805601355631197
"7170","LPIN3",135.903028015676,-0.226556483119286,0.244689129937955,-
0.925895168194571,0.354500462577996,0.805601355631197
"7171","PKNOX1",100.124939597933,-0.230421443312826,0.248829082899936,
-0.926022957716272,0.354434049559238,0.805601355631197
"7172","AC105285.1",6.60112908852493,0.581215288532905,0.6280429361372
51,0.925438779882857,0.354737714604861,0.805668027154557
"7173","ZHX2",174.125524807814,0.211864814129964,0.228955889221849,0.9
25352105377276,0.35478278339859,0.805668027154557
"7174","EIF4E3",118.931388994708,-0.229990520048131,0.248622541637711,
-0.925059001219887,0.354935217749989,0.805901834670974
"7175","MAP4K2",15.2823860040522,-0.484050156210369,0.523330544659097,
-0.924941532938202,0.354996320950984,0.805928233027259
"7176","SEPHS1",182.121564773101,-0.179614493987589,0.194281192388015,
-0.924507883546782,0.355221948834007,0.806103416628188
"7177","CPNE8",147.834966936547,0.24278536706293,0.262588247305702,0.9
24585808976752,0.355181397545523,0.806103416628188
"7178","TBX2-
AS1",15.4264562216291,0.439591224972287,0.475411966598618,0.9246532604
49849,0.355146299114559,0.806103416628188
"7179","GTF2H5",831.24729256837,0.226129255047881,0.244635647394287,0.
924351203336368,0.355303491835645,0.806160709490508
"7180","AP002813.1",5.84086461935361,0.608405073078982,0.6582552761711
23,0.92426919327999,0.355346178042964,0.806160709490508
"7181","ERI3",297.464387209832,-0.241505496027191,0.261423560823546,-0
.923809220815413,0.355585653526416,0.806306395143452
"7182","TMEM161B",85.5271649633441,0.209795760592198,0.227123093079661
,0.923709508123921,0.355637580372978,0.806306395143452
"7183","ARHGEF7-

AS2",4.88190760172876,-0.572807700363626,0.619999899587609,-0.92388353
731126,0.355546955232077,0.806306395143452
"7184","ICAM1",267.333645120963,-0.343241848342952,0.371606376638003,-
0.923670501696793,0.355657894843496,0.806306395143452
"7185","RGN",53.4750168743216,-0.224383519599679,0.2428273762610 5,-0.9
24045398235731,0.355462679752313,0.806306395143452
"7186","BCAR3",20.3834858474957,0.408147259793897,0.442284038965312,0.
922817067395706,0.356102544935792,0.806449859053121
"7187","LINC01305",3.56481285028274,1.06227028543166,1.1509397448065,0
.922959077766714,0.356028531433485,0.806449859053121
"7188","DCUN1D4",143.53276486583,0.227043679247163,0.246091424080136,0
.922598908498452,0.356216264711597,0.806449859053121
"7189","BCKDHB",90.6415196736959,-0.191001169384157,0.207010731569408,
-0.922663129278962,0.356182785942116,0.806449859053121
"7190","LINC01004",18.2906548203104,-0.465213336588017,0.5040869677725
19,-0.922883086312906,0.35606813574027,0.806449859053121
"7191","KIF27",54.3280700827717,0.25437505818455,0.275568395954453,0.9
23092277340083,0.355959118792221,0.806449859053121
"7192","USP54",28.9858735737476,0.324193880908516,0.351199308119107,0.
923105124109665,0.355952424565797,0.806449859053121
"7193","GAS2L3",7.79861635166905,-0.537369499313452,0.582394463721028,
-0.922689916865103,0.356168821960947,0.806449859053121
"7194","NOP53",3650.72186291734,-0.233342482303307,0.2528909217065,-0.
922700114059926,0.356163506401487,0.806449859053121
"7195","Z98885.3",6.89373923477585,-0.580446550787472,0.62902604738911
9,-0.922770294166219,0.356126924508034,0.806449859053121
"7196","CNNM3",125.681308253951,0.23782317271564,0.258029714415008,0.9
21689090168632,0.356690772867568,0.806515269189314
"7197","AC012603.1",4.08428253525152,1.09673377946126,1.1896868196579,
0.921867638893934,0.356597620873364,0.806515269189314
"7198","PC",15.620204806821,0.345898437415824,0.375197329663957,0.9219
10712226084,0.356575151057682,0.806515269189314
"7199","LINC00987",2.41368623363057,-0.844983785943616,0.9164004836445
7,-0.922068245297159,0.356492979292069,0.806515269189314
"7200","OIP5-
AS1",492.868374707322,0.161957955576289,0.175613605777827,0.9222403632
05033,0.356403213486399,0.806515269189314
"7201","GALK2",172.217724001563,0.160919728443042,0.174483694709243,0.
92226227047287,0.35639178906436,0.806515269189314
"7202","ACLY",247.718921200685,0.161486892332878,0.17513471911662,0.92
20724088714,0.356490807667143,0.806515269189314
"7203","AC092068.2",16.0110009101337,-0.512758831930262,0.556276088327
591,-0.921770399068993,0.356648350690381,0.806515269189314
"7204","IGKV4-1",2.91854319420389,-1.35717842039422,1.47138564250986,-
0.922381176751986,0.356329784650196,0.806515269189314
"7205","TINAGL1",2.61931369604389,-0.92918690358306,1.00835424936352,-
0.921488558380719,0.356795412088659,0.806527958300328
"7206","WDR83OS",1693.10221498333,-0.2280626779933,0.24749330434453,-0
.921490294847811,0.356794505902112,0.806527958300328
"7207","TCOF1",100.293232548623,-0.233777790859583,0.25376130963007,-0

.921250726520844,0.356919539877594,0.806696598455131
"7208","USP10",142.056059356697,0.173862271463897,0.188772300928608,0.
921015798444126,0.35704217884492,0.806861827303677
"7209","AC019069.1",2.59800874741345,0.871776637439073,0.9472797673969
95,0.920294792988776,0.357418729379758,0.806957370511985
"7210","MST1",7.14299796534479,-0.696560274426182,0.756611175168676,-0
.920631755499637,0.357242717057144,0.806957370511985
"7211","GMNN",104.909114154901,-0.209837621874085,0.227949741615398,-0
.920543363581119,0.357288883281949,0.806957370511985
"7212","AC007036.2",5.9667764848886,0.680236203886908,0.73888379005486
1,0.920626779261732,0.357245315997192,0.806957370511985
"7213","TPK1",58.8817429118075,-0.238371292466174,0.259058109556925,-0
.920146035473923,0.357496450217623,0.806957370511985
"7214","ACTA2",384.530978294757,0.249029497823122,0.270685996517114,0.
919994019001189,0.357575884743456,0.806957370511985
"7215","FZD4",530.136235940138,0.199873932255498,0.217251231204605,0.9
20012886220465,0.357566025282896,0.806957370511985
"7216","C15orf39",34.569477481936,0.277129352101546,0.301076303896224,
0.920462183556855,0.357331286115097,0.806957370511985
"7217","HDDC3",156.901926870087,0.222677080302905,0.242008023003027,0.
920122719650991,0.357508632921573,0.806957370511985
"7218","GCNA",7.38710243425093,0.565597994693534,0.61478950612551,0.91
9986416583477,0.35757985759442,0.806957370511985
"7219","ADAM22",80.250709461617,-0.323954980454151,0.352234986262663,-
0.919712672189176,0.357722928715696,0.807056618538777
"7220","AC005670.3",142.477901205762,0.212938163238868,0.2315196303128
55,0.919741289112816,0.357707970539301,0.807056618538777
"7221","UTP25",93.8177071624832,0.203676296644537,0.221503529244119,0.
919517162275394,0.357825133034694,0.807069732624293
"7222","ZNF107",74.9472958668511,0.230196601149769,0.250372295030888,0
.919417226739762,0.357877382205493,0.807069732624293
"7223","DCLRE1A",11.5166390710658,0.448423859692307,0.487675971850276,
0.919511900475549,0.357827883935172,0.807069732624293
"7224","BHLHE22",113.946732934262,0.332796826285498,0.362043283559922,
0.91921834045132,0.357981379959209,0.807192510818875
"7225","MRPS18B",409.900057055254,-0.196412491059195,0.213743030508752
,-0.918918809149907,0.358138040929966,0.807415819384298
"7226","TWF1",241.878903843474,-0.156191013590541,0.169987273087216,-0
.918839456353913,0.35817955128436,0.807415819384298
"7227","WRAP73",116.680527645212,-0.202762556166358,0.220716995278922,
-0.91865402530569,0.358276564184956,0.80742557714171
"7228","CPNE1",597.40614987637,0.225949731562988,0.245960676109984,0.9
18641691576551,0.358283017470703,0.80742557714171
"7229","ARSJ",35.853508209876,0.414281391912931,0.451117895337442,0.91
8343954418041,0.358438822465617,0.807664957690196
"7230","ZMPSTE24",272.289924063316,-0.141376018263286,0.15403343936169
8,-0.917826796889922,0.358709550781002,0.808051427557979
"7231","GPC6",190.730574715428,0.386008062022129,0.420534704213129,0.9
17898233260906,0.35867214669364,0.808051427557979
"7232","ZNF570",69.2703689483528,-0.261484056870232,0.284933968321751,

-0.917700540972218,0.358775664383425,0.808088605799449
"7233","LINC01091",69.8131597225771,0.257170880125638,0.280340637158137,0.917351414809587,0.358958523324331,0.808276940341447
"7234","TRIP11",649.178245841845,0.159066234739724,0.173387778600144,0.917401653241969,0.358932206745515,0.808276940341447
"7235","KLHL18",50.7614253324255,-0.233231135310811,0.254377918117329,-0.916868637957934,0.359211479887675,0.808459863369562
"7236","AC002074.1",32.8978598287794,-0.374384311649613,0.408276154784431,-0.916987943729624,0.359148957817979,0.808459863369562
"7237","AGPAT5",40.5295368067102,-0.293227698795007,0.319745282078706,-0.917066537741216,0.359107774442163,0.808459863369562
"7238","NDUFA1",1347.55372780211,-0.216037139583159,0.23563811044496,-0.91681748412942,0.359238289095027,0.808459863369562
"7239","SLFN11",199.342927006049,0.187676935036937,0.204741704981584,0.91665220358411,0.359324919561875,0.808543115726396
"7240","IARS2",379.434200726112,0.145797268435484,0.159087213703989,0.916461260719339,0.359425017067928,0.808544966581892
"7241","NMT2",217.41994865574,0.183636749714245,0.200356120448239,0.916551734498605,0.359377586038487,0.808544966581892
"7242","ARHGAP10",543.062421604222,0.158026421131266,0.172485280776387,0.916173370968008,0.359575969917583,0.8087728492112
"7243","AC109347.1",4.85989401559873,-0.669363950352766,0.730786287488319,-0.915950342545892,0.359692940585417,0.808820012243869
"7244","SCARF2",48.4871469416668,-0.45068695255233,0.492046384396406,-0.915944038701125,0.35969624708052,0.808820012243869
"7245","ZSCAN20",13.9958170936297,-0.401980024762998,0.438987238747469,-0.915698656548512,0.359824969857784,0.808930881918904
"7246","AC068790.7",4.82201471933816,0.699149413325741,0.763546366870358,0.915660716442711,0.359844875092662,0.808930881918904
"7247","GPR146",91.8980221102811,0.323048967739174,0.35297332768261,0.915222036350734,0.360075078343229,0.809083401953079
"7248","PEX16",162.600549078416,-0.22735986395989,0.248439229771825,-0.915152828998483,0.360111404270356,0.809083401953079
"7249","SIPA1",97.5167268358959,0.186949688590067,0.204247078190259,0.915311446540844,0.360028151648275,0.809083401953079
"7250","TFAP4",65.3933652332329,-0.247006134014577,0.269831152258182,-0.915409996019418,0.359976432671753,0.809083401953079
"7251","ADAM15",169.089764667587,-0.178534511316415,0.195176106369362,-0.914735490104237,0.360330508122123,0.809139367984423
"7252","GPR171",3.10427455725832,1.13658888664389,1.24254692725805,0.914725119599316,0.360335953725666,0.809139367984423
"7253","ACAT2",126.527384343142,-0.237928416203994,0.26013502769151,-0.914634289412763,0.360383651314485,0.809139367984423
"7254","EPYC",2.66650878666047,-0.802884628024875,0.877822276303954,-0.914632323305121,0.360384683818957,0.809139367984423
"7255","HM13",761.21593654442,-0.177442257034864,0.193978222669361,-0.914753494454461,0.360321054071263,0.809139367984423
"7256","XRCC5",1135.84712051856,-0.145103279320523,0.15870229954151,-0.914311133107237,0.360553382275475,0.809388903888692
"7257","SIK3",184.174829792173,0.188996122338393,0.206739716813745,0.9

14174234400554,0.360625300527822,0.809388903888692
"7258","TSFM",152.11281556933,-0.237409423756242,0.259708816915617,-0.914136942194688,0.360644893144092,0.809388903888692
"7259","BRD8",177.813007206989,0.23820546683896,0.260621462576381,0.913990215863931,0.360721986853723,0.809450398658258
"7260","CCN1",4476.88596812035,0.392247645714346,0.42942838154312,0.913418075220908,0.361022702719941,0.809565048105213
"7261","HS3ST1",5.18191762865172,0.71824586928379,0.786569976987619,0.913136644287525,0.361170679880937,0.809565048105213
"7262","HOXA13",16.099578697915,-0.486228801616762,0.53230554377285,-0.913439296856637,0.36101154586748,0.809565048105213
"7263","AC007529.2",2.41426974396164,1.00060124183164,1.09556549897068,0.913319416111347,0.36107457362231,0.809565048105213
"7264","PA2G4",998.308168378716,-0.186507418351079,0.204145927195412,-0.913598526864321,0.360927840760399,0.809565048105213
"7265","PCID2",317.727936828607,-0.164704654423021,0.180318170152538,-0.913411301166663,0.361026264089624,0.809565048105213
"7266","SIX4",9.91029866611829,-0.482737825457495,0.528638630575197,-0.913171678226091,0.361152256866803,0.809565048105213
"7267","NFE2L1",821.899523019034,0.228935999489491,0.250650534310653,0.913367290914091,0.361049402436152,0.809565048105213
"7268","AP002748.4",16.5491860447258,-0.453402173879798,0.496614523218301,-0.912986134480189,0.361249833961824,0.809631060182189
"7269","KLRK1",10.8543997686053,0.795402431434242,0.871565119563237,0.912613886880694,0.361445648100945,0.809958475982432
"7270","FAM189B",66.199490481203,0.258531383609711,0.28346328630755,0.912045390348053,0.361744823845272,0.810071684852301
"7271","ELF2",534.890967548386,-0.162522200828946,0.178141065769052,-0.912323052112226,0.361598682785603,0.810071684852301
"7272","KIF2A",206.37106049714,-0.187753082559488,0.205840234545462,-0.912130142943559,0.361700212287358,0.810071684852301
"7273","WEE1",221.493164729573,0.215296132606089,0.236048003519733,0.912086225665072,0.361723328767921,0.810071684852301
"7274","CBX6",463.176187495499,-0.163978734322178,0.179734251769914,-0.91233992801825,0.361589801723118,0.810071684852301
"7275","AL109627.1",3.76229502127542,0.898085871573537,0.985154213224784,0.911619581500607,0.361969010713119,0.81023955139563
"7276","C4orf48",110.600022727371,0.42033789875075,0.461044280145245,0.911708304066432,0.361922291413933,0.81023955139563
"7277","AP000759.1",5.14629426271566,0.686147216421987,0.752519850719462,0.911799490426706,0.361874278674036,0.81023955139563
"7278","MTF1",118.274044416435,0.214168340560733,0.235017565856943,0.911286523540538,0.36214442523022,0.810405051341423
"7279","GNL3",340.797398543187,-0.188033284694021,0.206327645244081,-0.911333449628532,0.362119707038745,0.810405051341423
"7280","GAR1",146.848059174351,-0.166113329890974,0.182345260901745,-0.910982435570306,0.362304628374569,0.810405051341423
"7281","LINC00824",18.0121620580275,-0.407874467075214,0.447842295979107,-0.910754680246287,0.362424646142098,0.810405051341423
"7282","NELFB",240.373712852155,-0.133965971683728,0.147077791872951,-

0.910851121557838,0.362373822464622,0.810405051341423
"7283","AICDA",5.23928542695829,-0.587511861120693,0.645097146762025,-0.910733932198626,0.362435580754472,0.810405051341423
"7284","CCL22",4.32787558273028,-0.872292760226994,0.957494833017737,-0.911015631779222,0.362287137405268,0.810405051341423
"7285","RTF2",686.588151251417,-0.187541625924794,0.205942014262104,-0.910652576633093,0.362478458665182,0.810405051341423
"7286","TBL1X",131.063926885818,0.17599971928249,0.193272623319523,0.910629328973939,0.362490711773197,0.810405051341423
"7287","LINC02550",17.6998293195608,0.63246022441744,0.694653298741902,0.910468899468122,0.362575276178118,0.81048286999662
"7288","AC015987.1",3.75499958655004,-0.768095974633977,0.843967436735608,-0.910101434250716,0.362769018254548,0.810693447434261
"7289","TUSC3",323.849165925579,0.265453526800399,0.29164704512528,0.910187609431702,0.362723577502046,0.810693447434261
"7290","UTP6",173.626038058507,-0.197337696643402,0.216923439255187,-0.90971126643099,0.36297480096668,0.810923468487893
"7291","SAFB2",307.348447411808,0.208019874068728,0.228687978876627,0.909623125319375,0.363021298558151,0.810923468487893
"7292","HAR1B",4.59981931847026,-0.682231552282453,0.750014347761489,-0.909624668272892,0.363020484562879,0.810923468487893
"7293","DOP1A",75.0265345326102,0.215074927204107,0.236509779810407,0.909370121508367,0.363154787834118,0.81095913880475
"7294","PYGM",37.6782954821705,0.335888209640323,0.36942642046601,0.909215451392511,0.36323640964574,0.81095913880475
"7295","AC008735.1",5.43580343041809,0.636650902896293,0.700021505270814,0.909473349179458,0.363100319383366,0.81095913880475
"7296","SUMO3",1076.08685031557,-0.170492598605754,0.187515472699428,-0.909218829525817,0.363234626829941,0.81095913880475
"7297","DVL1",151.142338330697,-0.171341490218963,0.189882180949451,-0.902356868676249,0.366867325183887,0.811033701295812
"7298","MRPL20",1098.32482094605,-0.222779965421617,0.24524625606017,-0.908392931254204,0.363670659070292,0.811033701295812
"7299","B4GALT2",210.323935057135,-0.260124273352492,0.287611514312534,-0.904429274934475,0.365767820853945,0.811033701295812
"7300","PATJ",30.743466664739,-0.35108201493318,0.388362451902554,-0.90400607271187,0.365992181319034,0.811033701295812
"7301","AC119800.1",2.75370400814267,0.829309995171962,0.918926575853637,0.902476886579946,0.366803594160596,0.811033701295812
"7302","C1orf54",222.397500307736,0.296446466402336,0.32806648571387,0.903617038958646,0.366198503120105,0.811033701295812
"7303","VPS72",192.686749998678,-0.20463497123309,0.225255731874761,-0.90845622231209,0.363637233035036,0.811033701295812
"7304","ST3GAL5-AS1",3.29272499873581,0.766803097595129,0.846199962415489,0.906172455274377,0.364844579857434,0.811033701295812
"7305","COX5B",2032.15912076677,-0.21268525352092,0.234917984185201,-0.905359605645376,0.36527490829512,0.811033701295812
"7306","SLC35F5",290.875525817324,0.144402329726548,0.159637834197337,0.904562069841442,0.365697437391881,0.811033701295812

"7307","ABCB11",20.070191785153,0.771296131286377,0.849935523889928,0.907476049190602,0.364155109432646,0.811033701295812
"7308","PMS1",111.162684465332,0.176142870278858,0.194964846802498,0.903459639866737,0.366281999430869,0.811033701295812
"7309","LMCD1-AS1",16.3753951470552,0.373090752746152,0.413460505473309,0.902361284348202,0.366864980283984,0.811033701295812
"7310","WNT5A-AS1",3.58521657312773,0.860685217253383,0.953474142061463,0.902683333805503,0.366693984223998,0.811033701295812
"7311","ECT2",21.9480090221377,0.303422310415855,0.334874886925684,0.906076634176488,0.36489529176221,0.811033701295812
"7312","TMEM33",176.894987472658,-0.148985597264513,0.165274486975959,-0.901443410840442,0.367352609009792,0.811033701295812
"7313","HPSE",15.0638394497735,-0.37991115012994,0.418705658918437,-0.907346585931732,0.36422354605687,0.811033701295812
"7314","ARHGAP24",328.261081319282,-0.175094535308883,0.19396108710099,-0.90273022246837,0.366669092265485,0.811033701295812
"7315","NPY1R",398.380142822681,0.654192255550642,0.720836528370438,0.907545927270839,0.364118173954258,0.811033701295812
"7316","AC116351.2",5.87657870276583,0.631327256662403,0.694802396379814,0.908642888901735,0.363538659621856,0.811033701295812
"7317","MED10",599.328263428662,-0.186152572914718,0.205613910854389,-0.905350091057542,0.365279947262262,0.811033701295812
"7318","C7",461.08409885429,-0.585341647518884,0.647052442839903,-0.904627830396295,0.365662586357292,0.811033701295812
"7319","LMNB1",9.28991077302772,-0.459879682301133,0.509126977278156,-0.903271095080633,0.366382033390459,0.811033701295812
"7320","SPATA24",8.76325579887283,-0.505995393500683,0.556848484434697,-0.9086769698482,0.363520664230539,0.811033701295812
"7321","PPP1R3G",52.1044562244531,-0.302575170258311,0.33487486972128,-0.903546959227329,0.366235677211303,0.811033701295812
"7322","FILIP1",537.472056315456,0.188861887282744,0.208843557573133,0.904322304587288,0.36582452302667,0.811033701295812
"7323","AL391840.3",37.7334658317818,0.318619314572319,0.350819767683655,0.908213686691758,0.363765334294368,0.811033701295812
"7324","USP45",52.4657398115578,-0.232900711222222,0.257855097788682,-0.903223218076882,0.366407437634159,0.811033701295812
"7325","TCF21",20.3799775970647,-1.00802947527435,1.11051783721978,-0.907711196965541,0.36403082662188,0.811033701295812
"7326","REPS1",81.3918381007814,0.198865659812801,0.220211338231629,0.903067305297536,0.366490174866421,0.811033701295812
"7327","OSBPL3",9.33430216135022,-0.549055088488379,0.60523671809449,-0.907174122245935,0.364314725974306,0.811033701295812
"7328","ERV3-1",60.5778321011665,0.236296105253251,0.261091253601698,0.905032635117404,0.365448098194487,0.811033701295812
"7329","PARP12",42.3714099918145,-0.251516354752267,0.279004947978177,-0.901476323537961,0.367335116859468,0.811033701295812
"7330","BAG4",159.988436506734,0.192045760146312,0.211358425934563,0.908625995377963,0.363547579931972,0.811033701295812

"7331","C8orf37",28.8593258209646,0.3360141905281,0.371434648886004,0.904638787834856,0.365656779459522,0.811033701295812
"7332","DSCC1",53.0899836157704,0.287766420361501,0.316858122218678,0.908186977649576,0.363779443068634,0.811033701295812
"7333","RFX3",55.821585276554,0.260565405469911,0.287809579213314,0.905339586618797,0.365285510509171,0.811033701295812
"7334","PLPP6",24.9477050416189,-0.294065725645195,0.324656840746015,-0.905774001156032,0.36505548474389,0.811033701295812
"7335","STOM",1161.44525835228,0.189287923692408,0.209592675146421,0.903122800260895,0.366460724375591,0.811033701295812
"7336","ATAD1",282.362134196046,-0.1616012369069,0.178821038386944,-0.903703716098651,0.366152528119227,0.811033701295812
"7337","UBTD1",61.5066039995487,-0.337034692814169,0.371790931612458,-0.906516711831698,0.364662423471778,0.811033701295812
"7338","BTRC",86.3083461936743,0.190483372911383,0.211293303710523,0.901511640768083,0.367316347346597,0.811033701295812
"7339","CD151",1303.95728216706,-0.249789196601308,0.275644553870994,-0.906200369618816,0.364829807429962,0.811033701295812
"7340","KBTBD3",91.0692254280071,-0.268636978251643,0.296533110778836,-0.90592574146636,0.364975158432771,0.811033701295812
"7341","SLC35F2",31.5869268572911,-0.364369277908036,0.402156858134967,-0.90603770776862,0.364915894247931,0.811033701295812
"7342","DIXDC1",461.731133065025,-0.221311923165239,0.244287827620026,-0.905947403607337,0.364963692111058,0.811033701295812
"7343","MCAM",3.26502452973956,0.830833543828652,0.916795092669539,0.906236901213572,0.36481047526986,0.811033701295812
"7344","ATF7IP",365.573808351145,0.197910742058132,0.21799137465473,0.90788336176877,0.363939849062735,0.811033701295812
"7345","ETFBKMT",72.5381830018158,-0.187945440155329,0.207598557544093,-0.905331146703225,0.365289980404042,0.811033701295812
"7346","METTL1",44.9309877476375,-0.304444204664015,0.337280915083369,-0.902642844730073,0.366715479657477,0.811033701295812
"7347","USP15",289.066830688433,-0.142958530787172,0.158567420831193,-0.901563070382299,0.367289015897952,0.811033701295812
"7348","HELB",49.2055698841966,0.451176304303133,0.499583290661884,0.903105273407727,0.366470025500373,0.811033701295812
"7349","DCN",119694.014610918,0.17662926739711,0.194689312495481,0.907236587017125,0.364281699793441,0.811033701295812
"7350","PAN3-AS1",7.39357769295809,-0.523209557189805,0.579923755173614,-0.902204044104332,0.36694848695287,0.811033701295812
"7351","SLC25A21",10.9138021260421,-0.527351252694087,0.5840521366856,-0.902918112219773,0.3665693571057,0.811033701295812
"7352","MPP5",89.6314575439852,0.234690274476084,0.260282427030366,0.901675449832439,0.36722929804053,0.811033701295812
"7353","PLEKHH1",3.84280192869817,0.709330106517776,0.786440870972829,0.901949698570895,0.367083589083603,0.811033701295812
"7354","RDH11",353.017890663394,-0.156136370515958,0.173128395168742,-0.901853045907213,0.367134936743825,0.811033701295812
"7355","ELMSAN1",441.850824003481,0.258939159536872,0.285311356269598,

0.907566957454695,0.364107058482037,0.811033701295812
"7356","CCDC85C",16.7150872714892,0.633421959827888,0.702585913828606,
0.901558012138584,0.367291703964753,0.811033701295812
"7357","ALG1",78.408209110209,0.202794431995772,0.22484595432388,0.901
92608804362,0.367096131990888,0.811033701295812
"7358","TVP23A",9.21630865090226,0.69178888350946,0.763411060994276,0.
906181373123499,0.364839860441095,0.811033701295812
"7359","RRN3",120.59897387493,0.185515717565377,0.204637093072088,0.90
6559582040313,0.364639743548678,0.811033701295812
"7360","TNRC6A",885.604090227656,0.197865697168257,0.218646994590117,0
.904955028259971,0.365489212557352,0.811033701295812
"7361","AC133552.5",21.8946368712596,-0.416960612066903,0.458852222730
089,-0.908703481016309,0.363506666212279,0.811033701295812
"7362","COG4",162.451584386496,0.160225957908896,0.177016169100419,0.9
05148714510944,0.365386607339369,0.811033701295812
"7363","LINC01229",3.22460080377902,0.881313976523216,0.97193625325172
1,0.906761090117466,0.364533150145039,0.811033701295812
"7364","FLII",292.750023043758,-0.167769587635142,0.185182708798768,-0
.905967888273258,0.364952849263823,0.811033701295812
"7365","LRRC37B",27.3882824485476,0.3059285582363,0.33929426454761,0.9
0166144907932,0.36723673763822,0.811033701295812
"7366","CNP",302.663069944821,0.187898819817426,0.207477091128353,0.90
5636467118121,0.365128300262234,0.811033701295812
"7367","KAT2A",46.4646582506979,0.282436042533084,0.310786307251583,0.
908778913172808,0.363466839532714,0.811033701295812
"7368","CAVIN1",4149.2310830011,-0.185482235890006,0.204631020552331,-
0.90642286486849,0.36471207505677,0.811033701295812
"7369","AC005332.4",20.0980752830685,0.365698163698697,0.4040580033877
08,0.90506353204888,0.365431730500642,0.811033701295812
"7370","CHAF1A",41.3125404149543,-0.29298952864378,0.324217832023836,-
0.903681104814245,0.366164521174471,0.811033701295812
"7371","ZNF430",183.876361998135,0.181828713733703,0.200568845512751,0
.906565091247649,0.364636829038513,0.811033701295812
"7372","PNMA8A",7.03437863432316,0.627321777247556,0.691789142245251,0
.906810672413184,0.364506925171141,0.811033701295812
"7373","AP2A1",404.663841951104,-0.153184447656162,0.169545012057559,-
0.903503121661621,0.366258932231062,0.811033701295812
"7374","MRPL39",131.884191451781,-0.270832974403056,0.299948639812165,
-0.902931163724088,0.366562429767809,0.811033701295812
"7375","FAM118A",90.7175565136227,0.275856657710412,0.30383123770329,0
.907927242095506,0.363916663525014,0.811033701295812
"7376","ARMCX5",49.9917668786519,-0.210755912922989,0.233678667781989,
-0.901904803392726,0.367107439523533,0.811033701295812
"7377","NKAP",365.530643260982,-0.165349761467424,0.182948445301125,-0
.903805228818781,0.366098688657581,0.811033701295812
"7378","ZFP92",3.58655692972468,-0.735369638026057,0.812613435468033,-
0.904943981885449,0.365495064912212,0.811033701295812
"7379","TMEM198",16.854912079541,-0.36792641239295,0.408203500081003,-
0.901330861494179,0.367412429665922,0.811055843180405
"7380","RBX1",1257.92207217658,-0.251282438034153,0.278821373500404,-0

.901230902349705,0.367465563642321,0.811063220348206
"7381","AVPI1",74.8799498510527,-0.278658712609457,0.309274786431956,-0.901006887190157,0.367584657838179,0.811216161973458
"7382","CEP104",89.86650635862,0.214102184793822,0.238256380338035,0.898620991765496,0.368854573554746,0.811278316997808
"7383","UQCRH",1773.34237214809,-0.220802767992406,0.245629864900043,-0.898924762598636,0.368692737165332,0.811278316997808
"7384","THEM4",111.58493498714,-0.194429703954535,0.216130902419385,-0.899592338615507,0.368337235853221,0.811278316997808
"7385","AC012306.2",55.1733432350585,-0.263645270987246,0.29334818104552,-0.898745204581086,0.368788392824211,0.811278316997808
"7386","PDIA5",104.848473556137,0.349072049842334,0.3877516412827,0.900191869242214,0.368018152359163,0.811278316997808
"7387","AC096586.2",3.84989530675628,0.742463704272512,0.826487858331518,0.898335888165822,0.369006505017315,0.811278316997808
"7388","DDX60L",112.066922743486,0.195988806689086,0.217984151771266,0.899096586135032,0.368601216339621,0.811278316997808
"7389","MSH3",145.49758672204,0.184911556140335,0.205786168321351,0.898561636327186,0.36888620080885,0.811278316997808
"7390","ERGIC1",684.215926353406,0.151420445360273,0.168526258299931,0.898497639998549,0.368920302828461,0.811278316997808
"7391","AL359643.3",18.0579311497181,-0.350261126914372,0.388961511874433,-0.900503304880833,0.36785246734892,0.811278316997808
"7392","TNPO3",194.769171662188,0.152382163139971,0.169571758596451,0.898629373200121,0.368850107684344,0.811278316997808
"7393","DNAJC1",577.340896117782,-0.14082404869447,0.156752022875709,-0.898387440946338,0.368979029702611,0.811278316997808
"7394","DDX50",323.214522239125,-0.160252804222258,0.178147372730607,-0.899551880928331,0.368358774531829,0.811278316997808
"7395","C11orf21",2.65391665388969,0.846299047185686,0.941717578780355,0.898676064093177,0.36882523004382,0.811278316997808
"7396","TSPAN18",31.690808174243,0.437119474666648,0.485984572834059,0.89945133878952,0.368412304090801,0.811278316997808
"7397","RPS6KB2",119.282132909434,-0.194952786216157,0.217055358963267,-0.898170803740206,0.369094496156983,0.811278316997808
"7398","KLHL35",20.6133257327593,0.324485713120518,0.361052143204272,0.898722578519452,0.3688004474655,0.811278316997808
"7399","AP000787.1",19.1944354679079,0.416730298541053,0.463932066016933,0.898257156740364,0.369048467783633,0.811278316997808
"7400","LRMP",7.56975735357836,0.720279417343497,0.800645123257476,0.89962381137475,0.368320481071577,0.811278316997808
"7401","IGHG4",176.51762730986,0.81631319215436,0.9078443675595,0.899177459622075,0.368558144431553,0.811278316997808
"7402","FLYWCH2",332.905415278503,-0.264343128423187,0.294242725257745,-0.898384584331297,0.368980552116721,0.811278316997808
"7403","NSMCE1-DT",12.325356672136,0.422212156314111,0.469216400538458,0.899823952934284,0.368213945170376,0.811278316997808
"7404","ITGAX",14.4604077172075,-0.60547786371245,0.673603974294474,-0.898863259152562,0.368725500060589,0.811278316997808

"7405","CYB561",213.444221422518,-0.17878018966274,0.198806920674715,-0.899265423235732,0.368511299992971,0.811278316997808
"7406","CDK3",21.0839067350697,0.389122370126171,0.432394262684184,0.899924915077752,0.368160210024221,0.811278316997808
"7407","DOT1L",37.6800689210185,-0.253640237320326,0.282403803778972,-0.898147383024774,0.369106980617027,0.811278316997808
"7408","ZNF841",31.4139518179254,0.305828055535276,0.340137169179611,0.899131536470754,0.368582601973138,0.811278316997808
"7409","LINC01315",91.0348545268843,-0.378521654815399,0.420522086791997,-0.900123124811343,0.368054730931561,0.811278316997808
"7410","CNKSR2",109.902031243408,-0.48980228632763,0.545126045111074,-0.898511987677692,0.368912657143785,0.811278316997808
"7411","BEX3",1358.04527945763,0.239163510563366,0.265911806968859,0.899409143541247,0.368434770671045,0.811278316997808
"7412","EDF1",2894.13895846083,-0.234988774895321,0.261671518739569,-0.898029621363551,0.369169757690209,0.811306824475959
"7413","LINC01117",25.0744363245846,0.391394576281925,0.436047934878672,0.897595298532553,0.369401346435028,0.811706263601804
"7414","ALG6",82.6384930899715,0.19822387451729,0.220888671093469,0.89739267086908,0.36950942204187,0.811724743848957
"7415","PIGU",80.5524913992328,0.20427104604022,0.227611068983905,0.897456555835011,0.369475345567748,0.811724743848957
"7416","HIF1A",303.812266598554,0.233869674551857,0.260672749873984,0.897177302441145,0.369624314742315,0.811867646013695
"7417","ENTR1",118.979668442119,-0.166878729295565,0.186052673135662,-0.896943464896548,0.369749085311131,0.811922735323943
"7418","CA12",190.964282696142,-0.437831039127523,0.488127507756678,-0.896960388771559,0.369740054225227,0.811922735323943
"7419","TMEM214",216.761976599909,-0.251068985296363,0.280072510702497,-0.896442798568894,0.370016317853779,0.811968691428179
"7420","SHARPIN",412.609277834027,-0.194477780529933,0.216900146154154,-0.896623556868029,0.369919823591079,0.811968691428179
"7421","PMEL",2.84347751491124,-0.931270430127478,1.03885729373017,-0.896437302551547,0.370019252039497,0.811968691428179
"7422","C12orf75",182.078776343951,0.396640823503481,0.442435604894628,0.896493905814714,0.369989033670202,0.811968691428179
"7423","FBRS",136.610898970974,-0.164976740716087,0.184035864679615,-0.896437990515014,0.370018884752278,0.811968691428179
"7424","MTFR2",6.6855793165232,0.80837895457661,0.901874883386417,0.896331597062841,0.370075688359621,0.811983147587537
"7425","TRAF5",99.1873443722656,0.210213272941148,0.234588384372835,0.896094124622353,0.370202494755018,0.812117078335471
"7426","ITGB7",7.25373886657855,0.725826507644905,0.81004660131986,0.896030557331233,0.370236443226669,0.812117078335471
"7427","RNF114",362.587139496035,-0.169670252499135,0.189383896374443,-0.895906440554318,0.370302734067707,0.812153121748874
"7428","MFSD11",177.817207068421,-0.202426001390399,0.225999846601454,-0.895690879593265,0.370417882818621,0.81219305100724
"7429","SNAI1",20.9177887152383,0.567867900401326,0.634003552402726,0.895685675970171,0.370420662774436,0.81219305100724

"7430","SSR2",2728.07682925519,-0.219423239684785,0.245190018057381,-0
.894910981381934,0.370834676019291,0.812593325105453
"7431","AC017002.3",10.3275266424392,-0.47845105261119,0.5345065539566
33,-0.895126634218986,0.370719397720702,0.812593325105453
"7432","MCMDC2",4.97926327183716,0.585386277580464,0.654152513352724,0
.894877365188412,0.370852647727543,0.812593325105453
"7433","TRAF2",42.214534784684,0.220647812523968,0.246536819554111,0.8
94989287697609,0.370792814415255,0.812593325105453
"7434","LYRM9",157.801657260048,-0.23764389417713,0.265530080322868,-0
.894979182351657,0.370798216445439,0.812593325105453
"7435","TUBB3",74.9065676497653,-0.519860004600568,0.581017402972059,-
0.894740849312509,0.370925636665354,0.812643940234291
"7436","NFYC",190.083811126325,-0.161878774553171,0.181372628882489,-0
.892520418050796,0.372114053690131,0.813387791271946
"7437","P3H1",231.786069555852,0.233831766980642,0.261898004485082,0.8
92835237291628,0.371945412987804,0.813387791271946
"7438","AL512353.1",9.91523051514679,0.502329558280821,0.5619296090421
93,0.893936803111407,0.371355705125438,0.813387791271946
"7439","CHD1L",14.9528843525872,-0.431480251891204,0.483429794545435,-
0.892539634006053,0.372103758830964,0.813387791271946
"7440","CD48",36.3258572754657,-0.316171697676002,0.354097535241085,-0
.892894375728253,0.371913739311199,0.813387791271946
"7441","TRIB2",20.0630399731218,-0.441812669776294,0.494388621188749,-
0.8936546086234,0.371506718706229,0.813387791271946
"7442","SLC30A9",157.666627265035,0.151172896814985,0.169173092672902,
0.893598943108997,0.37153651205176,0.813387791271946
"7443","TICAM2",89.7148885546868,0.200010355517103,0.224073537338337,0
.892610336289287,0.372065881929649,0.813387791271946
"7444","PPP1R18",240.873186240766,-0.192844739147604,0.215882525008267
,-0.893285545646733,0.371704276576371,0.813387791271946
"7445","SOD2",4124.33872211329,-0.333260344786975,0.372949797846966,-0
.893579636484272,0.371546845706559,0.813387791271946
"7446","AL022069.3",15.9433720163244,0.401165056125531,0.4490156504953
52,0.893432234896419,0.371625746633077,0.813387791271946
"7447","ZNF467",9.42769713135237,-0.543520284981731,0.60836344105029,-
0.8934137857518,0.371635622798457,0.813387791271946
"7448","RASGEF1A",2.58889508725064,-0.80555975883351,0.902433251412099
,-0.892653010705219,0.372043021382857,0.813387791271946
"7449","AFAP1L2",7.33886169651441,-0.735708586458111,0.823070486222663
,-0.893858544041004,0.371397580873757,0.813387791271946
"7450","BUB3",391.064235730062,-0.140615056175323,0.15743128325043,-0.
893183700673031,0.371758805234064,0.813387791271946
"7451","PPFIBP1",540.305008478828,-0.208296790786497,0.233237759613411
,-0.893066333391931,0.371821650818178,0.813387791271946
"7452","SELENOS",977.374726758185,-0.186196869448581,0.208538503049654
,-0.892865666175072,0.371929115516625,0.813387791271946
"7453","MZT2B",2710.99526195626,-0.224152515567194,0.251184163531974,-
0.892383151928528,0.372187598515762,0.813439392489365
"7454","RPL39",24429.008859175,-0.203582101949992,0.22823026552244,-0.
892003089441157,0.37239127646037,0.813775355817409

"7455","EIF5B",871.503123288973,-0.236425835874097,0.265090692340499,-0.891867736987223,0.372463829393146,0.813824723941643
"7456","KBTBD2",142.262669000179,0.190070530656345,0.213164261127092,0.891662277960477,0.372573978268552,0.813956214058
"7457","ABCB10",55.7680052643062,0.252142051257368,0.282811937563395,0.891553777502224,0.372632154716954,0.813974140832032
"7458","TACC3",7.49023983203943,0.531890623241924,0.597138370799318,0.890732616177294,0.373072632554104,0.814059933610368
"7459","TTC37",462.233193561447,0.160524553611343,0.180310039055425,0.890269640294406,0.373321118796086,0.814059933610368
"7460","AC008494.3",4.39838732198667,0.753776583590298,0.846037644055097,0.89094922535292,0.372956410273497,0.814059933610368
"7461","CARMN",14.5047840963574,0.810389846461625,0.91019691067799,0.890345635053825,0.373280324217885,0.814059933610368
"7462","FAM200A",64.8294942383547,0.212435213013773,0.238397925575304,0.891095056725525,0.372878176660313,0.814059933610368
"7463","LINC00865",7.46636706943681,0.622120180742067,0.698357625654484,0.890833231983443,0.37301864406266,0.814059933610368
"7464","ERC1",277.693528703029,-0.184863966892807,0.207607986009593,-0.890447282140028,0.373225763585986,0.814059933610368
"7465","ZBTB1",197.175185173826,0.161291907315844,0.181065632070595,0.890792501433729,0.373040498703266,0.814059933610368
"7466","EEF2K",360.871168292682,0.219568236711898,0.246484867888523,0.890798038000456,0.37303752792154,0.814059933610368
"7467","TM6SF2",10.6420484484544,0.48557646590732,0.54480843261404,0.891279276969851,0.372779363244243,0.814059933610368
"7468","AC008736.1",13.5291458276357,-0.393277452342237,0.441303748360659,-0.891171792225132,0.372837014734613,0.814059933610368
"7469","FKBP1A",1901.92907194157,-0.241484012168269,0.271192555154029,-0.890452217728151,0.373223114459101,0.814059933610368
"7470","MIS18A",113.834661646918,-0.19760451276051,0.221944503644466,-0.890332986470587,0.373287113884325,0.814059933610368
"7471","C5orf30",37.8960700425664,-0.245600489622965,0.275942838752269,-0.890041179301833,0.373443775055606,0.814074614348973
"7472","ATAD5",26.8331018278015,0.290669616227055,0.326585813042972,0.890025238753432,0.37345233415666,0.814074614348973
"7473","DHX58",59.2943484045125,0.222876531557431,0.250429303009508,0.889977845559746,0.373477782124739,0.814074614348973
"7474","ANKRD65",272.636848804448,-0.20736722162782,0.233943187026753,-0.886399917276095,0.37540206377884,0.814452011601825
"7475","PANK4",53.6791230237803,-0.217676022569165,0.24526312187199,-0.887520394047567,0.37479879125587,0.814452011601825
"7476","CDC42",2087.70603028329,-0.218173028452884,0.246241839270977,-0.886011203858804,0.375611489862368,0.814452011601825
"7477","MKNK1",168.059640372059,0.168041447165778,0.189289834618334,0.887746811679563,0.374676959241612,0.814452011601825
"7478","MAN1A2",460.923895322289,0.131329599884506,0.147647003705258,0.889483677885355,0.373743191411407,0.814452011601825
"7479","ELK4",244.999591050713,0.17863808217868,0.201330467382179,0.887287873025086,0.37492393289877,0.814452011601825

"7480","INTS7",29.4356022761639,0.328883218864337,0.370436622741916,0.
887825875395347,0.374634421981187,0.814452011601825
"7481","RRP15",201.724026860506,-0.218124738480219,0.24583866001379,-0
.887267846594931,0.374934712230328,0.814452011601825
"7482","RAB11FIP5",90.7964030949894,-0.187674601286826,0.2116403368434
84,-0.886761966484764,0.375207068391996,0.814452011601825
"7483","AC009404.1",23.4889324538441,0.308253857827045,0.3475343670962
9,0.886973741338332,0.375093037995285,0.814452011601825
"7484","PECR",40.281915329087,-0.229824197335663,0.258574389986454,-0.
888812683064641,0.374103758420693,0.814452011601825
"7485","AC139887.4",9.8411781773178,0.563113595817224,0.63465690524471
3,0.887272463536967,0.37493222711996,0.814452011601825
"7486","LINC02511",444.903881563231,-0.230115788676793,0.2595807510070
79,-0.886490187673883,0.37535343936157,0.814452011601825
"7487","C5orf63",25.4593474140076,0.268476362384333,0.302097769342524,
0.888706867874718,0.37416063910176,0.814452011601825
"7488","GRPEL2",42.9957240147916,0.220247056923314,0.248294653590445,0
.887039063219644,0.375057869680199,0.814452011601825
"7489","MEA1",319.895676813761,-0.165302303720943,0.185953783955059,-0
.888942941655293,0.374033745598594,0.814452011601825
"7490","GOPC",617.631054704584,0.120327583299399,0.135813508181659,0.8
85976549095934,0.375630164218362,0.814452011601825
"7491","ZDHHC4",252.992667090641,-0.191503719828021,0.215612208512703,
-0.888185883114029,0.374440771111414,0.814452011601825
"7492","ELP3",103.848702358946,0.174963628455362,0.197353762712706,0.8
86548227155222,0.375322178278033,0.814452011601825
"7493","RAB11FIP1",30.5551642944054,-0.36700952390904,0.41283870643163
5,-0.888990102408956,0.374008399116038,0.814452011601825
"7494","OMD",1099.38902586124,-0.132718951916744,0.149780039872032,-0.
886092379399386,0.375567749174736,0.814452011601825
"7495","NOTCH1",19.7643438083666,0.367506683684818,0.413639951911768,0
.888469989386349,0.374287991909863,0.814452011601825
"7496","BMI1",371.322523020715,0.14242517152805,0.160320835296791,0.88
837593232588,0.374338567173355,0.814452011601825
"7497","GHITM",853.081831987688,-0.129533843348392,0.146069771965587,-
0.886794314835441,0.375189649002096,0.814452011601825
"7498","ZRANB1",198.84006471374,0.199554284824825,0.225246327236121,0.
885938018494912,0.375650927814139,0.814452011601825
"7499","FAM118B",34.9089416771043,-0.302087932383285,0.339791826001359
,-0.889038255976403,0.373982520143503,0.814452011601825
"7500","RIC8B",66.3876058278993,0.264184794491459,0.297361285456615,0.
888430362028426,0.374309299354526,0.814452011601825
"7501","ANAPC5",606.58273217708,-0.129403791045986,0.14578206713876,-0
.887652326419648,0.374727797400278,0.814452011601825
"7502","GMPR2",269.598932065171,-0.168246759844483,0.189854287543399,-
0.886188887390932,0.375515750831613,0.814452011601825
"7503","FANCI",18.292296404949,0.346216680892778,0.389778513262436,0.8
88239523505164,0.374411922839435,0.814452011601825
"7504","ZC3H7A",250.742245967186,0.173602437054693,0.195739142322364,0
.886907110121012,0.375128913336462,0.814452011601825

"7505","HIRIP3",125.491471357804,-0.202765143963128,0.227983769184834,
-0.889384120142077,0.373796676351849,0.814452011601825
"7506","AC087501.4",4.06892858352779,0.666649162057219,0.7519655396794
72,0.886542170990151,0.375325440159695,0.814452011601825
"7507","TAF15",275.549695799611,-0.197997256159088,0.222700758947844,-
0.889073109110773,0.373963789858813,0.814452011601825
"7508","AC138150.2",7.49276734767511,-0.505933449204321,0.570184169947
198,-0.887315846125951,0.374908876550452,0.814452011601825
"7509","AP3D1",247.897877921279,0.171097931209946,0.192960170944115,0.
886700765099858,0.375240026332166,0.814452011601825
"7510","ZNF506",200.447173969763,-0.162448219385372,0.182906800517241,
-0.88814750969338,0.374461409516329,0.814452011601825
"7511","SLC17A9",3.49612522758529,-0.951576267982141,1.07324101057023,
-0.886638004521051,0.375273825781103,0.814452011601825
"7512","RANBP1",684.491482062531,-0.219496693803167,0.246889335704598,
-0.889048905967303,0.373976796712561,0.814452011601825
"7513","P2RX6",3.14416768111744,0.788914383096283,0.890360687784314,0.
886061563499077,0.375584353666522,0.814452011601825
"7514","AL353807.5",70.3098950087406,-0.335770970250758,0.379104155521
44,-0.885695831502879,0.375781455186761,0.814518180111397
"7515","MINCR",63.5443034030896,0.246755306313217,0.278573713887072,0.
885781012393891,0.375735543509282,0.814518180111397
"7516","DCAF1",60.8810802079444,0.234482439352003,0.264901828954093,0.
885167310009924,0.376066400587033,0.814531137607674
"7517","EIF4E",248.558302310014,-0.229683617579366,0.25951909209046,-0
.885035531410173,0.376137468051134,0.814531137607674
"7518","MYORG",16.1638119320962,0.458492317531073,0.517872227420618,0.
885338686368064,0.375973990672364,0.814531137607674
"7519","THNSL1",26.0413338699626,0.298932047778131,0.337757319612425,0
.88504979883531,0.376129773308167,0.814531137607674
"7520","GXYLT1",112.171909515665,0.236557464857372,0.267284936396256,0
.885038521238138,0.376135855561683,0.814531137607674
"7521","INPP5K",73.0463296629002,-0.178141976241287,0.20123849899552,-
0.885228110577653,0.376033613974396,0.814531137607674
"7522","MAPRE1",282.449851350057,-0.168508665654614,0.190312086295924,
-0.885433337074521,0.37592295893227,0.814531137607674
"7523","BBC3",35.341232359472,0.346084847035942,0.391345190278269,0.88
4346749706711,0.376509059065535,0.815134827050297
"7524","SEC23B",60.3937297081174,-0.206068402351515,0.233021199388898,
-0.884333283374787,0.376516326277024,0.815134827050297
"7525","CCDC17",15.5001596453421,0.398086092799299,0.451263041942804,0
.882159751185108,0.377690424027074,0.815538345980213
"7526","SEPSECS",47.3082268836415,0.249076503971109,0.282450872270749,
0.881840094770009,0.377863285999792,0.815538345980213
"7527","AC124016.1",12.6405754607231,-0.54057637990531,0.6126661518420
94,-0.882334332131728,0.377596035716447,0.815538345980213
"7528","AL021368.5",2.40039306155908,0.941359542910653,1.0664098516206
7,0.882737102887817,0.377378330581296,0.815538345980213
"7529","RBAK",57.3045477092247,0.268398455582885,0.303904462032201,0.8
8316720915551,0.377145935536455,0.815538345980213

"7530","CAPZA2",639.539463685022,-0.179783581092509,0.203569219114907,
-0.8831570012116,0.377151450070868,0.815538345980213
"7531","LACTB2",133.351972288161,-0.169906750140874,0.192687213560612,
-0.881774908678241,0.377898542945176,0.815538345980213
"7532","RBM18",125.908469720283,0.158385856669388,0.179415652813881,0.
882787282967401,0.377351212730022,0.815538345980213
"7533","WAC-
AS1",233.84473041951,0.193999382084482,0.220009438040528,0.88177754469
2177,0.377897117175354,0.815538345980213
"7534","ANKRD2",12.4564345739716,-0.69931660038387,0.79214620491493,-0
.882812536429397,0.377337565943666,0.815538345980213
"7535","TRIM8",504.043240001972,-0.132738359224465,0.150514958739186,-
0.881894798605873,0.377833700114984,0.815538345980213
"7536","ADAM8",10.8668294625736,0.53562352214184,0.607359560132257,0.8
81888682258009,0.377837007994288,0.815538345980213
"7537","TAF1D",706.523739744519,0.137637845283838,0.155840660235882,0.
883195984125756,0.377130390992568,0.815538345980213
"7538","PFDN5",6318.4368999252,-0.219800890601827,0.249089886789644,-0
.882415956081943,0.377551910190871,0.815538345980213
"7539","MAPKAPK5-
AS1",185.097899498191,-0.236757112434493,0.268026508081002,-0.88333469
0026036,0.377055466136991,0.815538345980213
"7540","ATG2B",110.490375759076,0.202509751957224,0.229542402410596,0.
882232432136797,0.377651126837158,0.815538345980213
"7541","RAD51-
AS1",85.4659594411581,0.266889326691925,0.301954132670438,0.8838737338
40286,0.376764377650376,0.815538345980213
"7542","THSD4",3.43744527626999,0.743097401286836,0.84263481280585,0.8
81873606446939,0.377845161459486,0.815538345980213
"7543","AC106886.3",3.55413218106384,0.791561708785723,0.8965623891768
11,0.882885249639464,0.37729827395537,0.815538345980213
"7544","AC055811.3",3.67411328473592,-0.722873031140817,0.819020201321
209,-0.882607083408576,0.377448600085861,0.815538345980213
"7545","ZNHIT3",337.460395548561,-0.242268593166474,0.274260999210582,
-0.883350508689923,0.377046921942248,0.815538345980213
"7546","SP2",89.6648697748788,-0.164896791992207,0.186727265975398,-0.
883088986125538,0.377188194442856,0.815538345980213
"7547","TLK2",200.022385822472,0.19113422051693,0.216763408690455,0.88
1764231664557,0.377904317972782,0.815538345980213
"7548","CBX7",217.608631062809,0.235171883215352,0.26622756850485,0.88
3349100681392,0.37704768245038,0.815538345980213
"7549","SLC17A5",193.440444993556,0.1487385302691,0.1687590312508,0.88
1366343280637,0.378119568262957,0.815567899467928
"7550","CORO1A",40.3404882181315,-0.411114962299292,0.466499573904169,
-0.881276179651443,0.378168355619207,0.815567899467928
"7551","THAP11",173.712194485568,-0.220459271204399,0.250130866955572,
-0.881375712992497,0.378114498553309,0.815567899467928
"7552","AL121894.2",2.37699854539689,-0.901908558453188,1.023237542637
35,-0.881426375471486,0.378087087116608,0.815567899467928
"7553","RTEL1",15.4635878373439,0.342620981469532,0.388632889239466,0.

881605728583657,0.377990056173534,0.815567899467928
"7554","COL16A1",613.581353896201,0.206627045380676,0.234536879515625,
0.881000232489707,0.378317694155136,0.815615955382397
"7555","PRELID2",89.9898200565445,-0.238263839559672,0.27063056908585,
-0.880402536803187,0.378641282743059,0.815615955382397
"7556","COQ3",46.327294054497,-0.366856840190238,0.416648530186855,-0.
880494742236851,0.37859135220473,0.815615955382397
"7557","RNF217",176.137320866506,0.242283898750745,0.275020341469621,0
.880967194848413,0.378335576073449,0.815615955382397
"7558","KCNQ1",2.81474351574552,-0.894683556445704,1.01598050870872,-0
.88061094556117,0.378528432244608,0.815615955382397
"7559","TRDV3",2.9605369207323,0.82810579088055,0.940135783945107,0.88
0836369620521,0.378406391507105,0.815615955382397
"7560","PYGL",200.546129715299,-0.153472419532739,0.174287757285709,-0
.880569134188538,0.378551070871758,0.815615955382397
"7561","DNAH9",4.71835333049183,0.672711657880963,0.763952785286928,0.
880567059688517,0.378552194124525,0.815615955382397
"7562","BCL2",140.418486376179,0.261777933745595,0.297186456185014,0.8
80854185301851,0.378396747434499,0.815615955382397
"7563","GOLGA5",189.415721411116,-0.163938297193731,0.186250815373796,
-0.880201769128986,0.378750015282099,0.815742297888418
"7564","MYOM2",22.6360612424228,0.40941038743327,0.465197891432941,0.8
80077908720029,0.378817105669536,0.815778931022088
"7565","AC022087.1",2.5065127329114,1.14847675496438,1.30511594477627,
0.879980632802134,0.378869801395103,0.815784559805002
"7566","INSRR",2.39901792765797,0.827026595514783,0.940075556838109,0.
879744813593963,0.378997566682108,0.815904843406664
"7567","EXD3",54.0400590565115,0.208498732579688,0.237038069345798,0.8
79600197365443,0.379075931911206,0.815904843406664
"7568","SMG6",294.162153903023,0.164064009295614,0.186505670792931,0.8
79673034058931,0.379036461621912,0.815904843406664
"7569","NTRK2",5127.67321707885,0.210027185415646,0.238843372683866,0.
879351112218799,0.379210930554065,0.816087574024991
"7570","AC079921.1",2.48608887517192,-0.931041006274241,1.059068886131
97,-0.87911279281811,0.37934012208347,0.816149946984235
"7571","ADCY6",94.9947286978237,0.167289240949566,0.190285402460171,0.
879149103329568,0.379320436623186,0.816149946984235
"7572","PFDN2",538.99082098568,-0.227728346673321,0.259075231244501,-0
.879004702917368,0.37939872589873,0.816168231136345
"7573","MACF1",888.62925970928,0.207522491780944,0.236188235044149,0.8
78631790199683,0.379600953192643,0.81641886224038
"7574","GORAB-
AS1",8.51510046106283,0.484884171651993,0.55187958418094,0.87860501738
1868,0.379615474406571,0.81641886224038
"7575","SMYD5",44.3211081020015,0.22724792311825,0.258703408477875,0.8
78411013041154,0.379720709940076,0.816537378774112
"7576","CHST12",373.067859960736,-0.140856319808539,0.160379221972406,
-0.878270377398228,0.3797970074202,0.816593644913891
"7577","GCFC2",114.09913060564,-0.164656131098326,0.187556747253022,-0
.877900334218304,0.379997807824514,0.816795352115846

"7578","MRPS9",174.762401085631,-0.21362316249033,0.243401761865901,-0
.877656598919866,0.380130104100512,0.816795352115846
"7579","HLA-
DRA",995.972249519846,-0.390759703358944,0.445241435494006,-0.87763553
0317135,0.380141541186704,0.816795352115846
"7580","ATP2B1",646.481479356605,0.218324677882843,0.248757360556499,0
.87766117711824,0.380127618854538,0.816795352115846
"7581","ZNF747",53.9753089896021,0.212732285232632,0.242294283711997,0
.877991349913544,0.379948412974735,0.816795352115846
"7582","ZNF362",119.621409509372,0.171314990866746,0.195246528120687,0
.877429127758175,0.38025359795294,0.816864896846643
"7583","AP006248.6",2.63973963441597,-0.888819064567257,1.013024849939
87,-0.87739117615922,0.380274204235257,0.816864896846643
"7584","LRRC69",7.1602962478576,0.576007413929773,0.656576656154808,0.
87728890226332,0.380329738507241,0.816876464997949
"7585","MRPL18",732.858564143274,-0.228335705556267,0.260347911124886,
-0.877040666735894,0.380464550022557,0.817031683955901
"7586","PDIA3",1985.22061654316,-0.188286131295983,0.214700492082881,-
0.876971121348426,0.380502323929613,0.817031683955901
"7587","RPL15",19892.9218010118,-0.194041031286397,0.221354362796195,-
0.876608117568723,0.380699528550484,0.817131983207121
"7588","RBPMS-
AS1",23.105497068151,0.289469522783665,0.330189186477857,0.87667777940
1104,0.380661679352627,0.817131983207121
"7589","AC093462.1",6.26167465272548,-0.59065245086292,0.6736804356654
29,-0.87675464447695,0.380619919115902,0.817131983207121
"7590","ZNF696",39.8737366219843,0.238524424596547,0.272205152962248,0
.876267116918349,0.380884836972378,0.817422017054423
"7591","FUT4",83.2665513699386,0.216051342139505,0.246629379146325,0.8
7601624302562,0.381021203582079,0.817606953648859
"7592","ATP6V1D",329.021542323296,-0.201156895555028,0.229711136405926
,-0.875695008532629,0.38119585960773,0.817873993302201
"7593","UBAP2",150.656824272077,0.173387056179147,0.198038459775675,0.
875522140373887,0.381289868810097,0.81796795377949
"7594","AC092835.1",9.48627070998598,-0.440375280398753,0.503140619162
837,-0.87525288880767,0.381436321600875,0.818012254831881
"7595","SLC6A16",6.77131206629255,-0.498980693789962,0.570128642583318
,-0.875207201534419,0.381461175498985,0.818012254831881
"7596","CST7",22.9665673821722,0.314445522898158,0.359272784948964,0.8
75227782540853,0.381449979298807,0.818012254831881
"7597","HECA",185.909373220663,-0.227098469959776,0.259533058210248,-0
.875027141150562,0.381559138109034,0.818114624280381
"7598","CTTNBP2NL",154.134996900196,0.175166551652515,0.20021647839577
7,0.874885788902228,0.381636052210169,0.818148798038581
"7599","AC009315.1",4.49073612511801,0.654376442220344,0.7481026730637
54,0.874714749434638,0.381729132758194,0.818148798038581
"7600","CCDC81",2.66538804827955,-0.765033756013856,0.874695496582616,
-0.874628666779237,0.381775984645543,0.818148798038581
"7601","RGS9",219.503798971289,-0.237468274579798,0.271488398269708,-0
.874690322287317,0.381742427272517,0.818148798038581

"7602","FAM161B",46.7936955186479,0.241278023536176,0.275919601866304,
0.874450462758666,0.381872986296524,0.818249023123398
"7603","ARNT",323.599723207542,0.16566918378477,0.189511253382487,0.87
4191800369779,0.382013810681815,0.818335476354036
"7604","CD69",74.7259157814256,0.464451794862261,0.531267746057328,0.8
74232998914531,0.381991378695032,0.818335476354036
"7605","FAAH",13.6189207406803,-0.367195279222306,0.420121870793324,-0
.87402086096809,0.382106893240395,0.818346623921486
"7606","CPSF3",77.8010057912793,-0.223177590299967,0.255433473369389,-
0.873721002013013,0.38227021065864,0.818346623921486
"7607","JRK",45.3157906628289,0.339599140470732,0.388627006710029,0.87
3843388666299,0.38220354790991,0.818346623921486
"7608","RAB27A",68.5254972834686,0.229445918161367,0.262547736262481,0
.873920763620597,0.382161406247969,0.818346623921486
"7609","CLUH",43.6536006905686,0.256553600049376,0.293628510831174,0.8
7373531719774,0.382262412956898,0.818346623921486
"7610","AC004130.2",51.9175656832966,-0.31220849886255,0.3574721154422
93,-0.873378608779766,0.382456746592673,0.818530803474978
"7611","VPS37B",73.2891808535084,0.222747849429116,0.255034847174748,0
.87340162294171,0.382444206721483,0.818530803474978
"7612","FAR2",16.7039782914333,-0.527421898684716,0.604003426983993,-0
.873210109615311,0.382548565470677,0.818619756036765
"7613","RNF41",175.338133989341,-0.141444088085044,0.162024003099677,-
0.872982307430263,0.382672721341018,0.818777874415322
"7614","MRRF",75.5289180405068,-0.183344472411959,0.210069491917424,-0
.872780101186847,0.382782947696884,0.818798612611235
"7615","AC069544.1",5.80858033823965,-0.60767433295967,0.6962449509267
,-0.872788136058807,0.382778567368688,0.818798612611235
"7616","GVQW3",57.6652712054176,0.259218124884616,0.297043919110308,0.
872659254096209,0.382848832964645,0.818801838241997
"7617","DHRS7B",259.954674284652,-0.200879656201703,0.23021004063367,-
0.872592940120106,0.38288498998645,0.818801838241997
"7618","AC016747.1",84.771630499427,0.247642820170846,0.28391677246243
3,0.872237374435543,0.383078894248304,0.818844860793344
"7619","AC007285.1",4.62883713196415,0.593021992768326,0.6799250213156
72,0.872187335628293,0.383106187249437,0.818844860793344
"7620","C11orf1",79.2098092892542,-0.274116524519427,0.314204962387134
,-0.872413097606288,0.382983057821417,0.818844860793344
"7621","EML1",249.739518349285,-0.187533025880594,0.214993232781501,-0
.872274087208993,0.383058870512456,0.818844860793344
"7622","HHIP-
AS1",52.6872030235459,-0.761070482805758,0.872897737872885,-0.87188962
6682236,0.383268593255195,0.818920788412911
"7623","GFOD1",9.1850705574402,-0.446909132549753,0.512632407645767,-0
.871792586430804,0.383321539706251,0.818920788412911
"7624","JAZF1-
AS1",2.57561686616218,0.910967306293603,1.04486499653087,0.87185168353
6311,0.383289295006632,0.818920788412911
"7625","AC090559.2",6.98165372116608,0.609448089709238,0.6991058997873
24,0.871753606849319,0.38334280874507,0.818920788412911

"7626","AL606970.4",13.1295812144357,-0.506059844272075,0.580581712525
864,-0.87164275648715,0.383403297712418,0.818942606404088
"7627","LINC01409",13.3685392953731,0.410573101948132,0.47115045263122
7,0.87142673779726,0.383521191860339,0.819087019039343
"7628","ARV1",111.797304589616,-0.218188162081284,0.250615422904305,-0
.870609476275516,0.383967420521106,0.819395429433812
"7629","RNASEH1-
AS1",63.1691265146785,-0.322761175910903,0.370705168300009,-0.87066813
0663058,0.383935384367492,0.819395429433812
"7630","SEMA5A",57.9992342796403,0.747746269670908,0.858474510326156,0
.871017439279379,0.38374463108336,0.819395429433812
"7631","TRAPPC3L",4.11988779039343,-0.692485225858143,0.79517202486103
2,-0.87086215838537,0.383829420970204,0.819395429433812
"7632","PTGES2",122.516524738125,-0.277050667129739,0.318102019420974,
-0.870949098764106,0.383781946460832,0.819395429433812
"7633","FANCF",44.6389542387705,0.222602830565367,0.255661454129967,0.
870693751323988,0.383921391257146,0.819395429433812
"7634","TET3",12.1717732883465,-0.384863407000811,0.442324116366754,-0
.870093654766272,0.38424922500591,0.8196223825218
"7635","NSUN3",94.0283585206926,0.159284357020857,0.183095077441171,0.
869954338734399,0.384325358076095,0.8196223825218
"7636","FGF10-
AS1",23.4059146013844,0.357240214024778,0.410484759321394,0.8702886183
04273,0.384142697321766,0.8196223825218
"7637","ZNF267",91.4705036461478,-0.207626835906867,0.238639289921017,
-0.870044643426427,0.384276007545892,0.8196223825218
"7638","POLR3H",107.343067431787,-0.209836903222793,0.241189400303632,
-0.870008810331758,0.384295589477671,0.8196223825218
"7639","OMG",34.4926057775366,-0.5123562345432,0.589205932762374,-0.86
9570732496057,0.384535037920547,0.819870394129838
"7640","MEX3C",109.995819215292,0.210597852116634,0.242189702553621,0.
869557416752709,0.384542317585608,0.819870394129838
"7641","CMSS1",225.533123645845,-0.22509180722934,0.258977098136389,-0
.869157191308078,0.38476115861753,0.820229618207166
"7642","CCDC24",9.14356779636037,-0.467323070542321,0.538291430150716,
-0.868159967569008,0.38530676615992,0.820245823704994
"7643","SHISA4",163.850846099978,-0.242467237458145,0.279345505463461,
-0.867983313552399,0.385403467530093,0.820245823704994
"7644","U91324.1",2.76420979336249,-0.842823453757971,0.97014332962085
,-0.868761788103374,0.384977437644173,0.820245823704994
"7645","ACAD11",92.9418029992671,0.244236648630373,0.281376693967882,0
.86800596448209,0.385391067459102,0.820245823704994
"7646","MRPS22",304.050761649599,-0.221005171164013,0.254436887223681,
-0.868605073641399,0.385063178430442,0.820245823704994
"7647","NEIL2",70.2154457159477,-0.215615210154893,0.248204027621532,-
0.868701496188726,0.385010422855638,0.820245823704994
"7648","ATP6V1H",191.648881188154,0.128512028249042,0.147882917178858,
0.869011990706212,0.384840572316259,0.820245823704994
"7649","LINC00535",14.4712527915318,-0.700789877519347,0.8071767614053
6,-0.868198777550552,0.385285523351608,0.820245823704994

"7650","TMEM71",4.73208639504042,-0.638767093338602,0.7357274946839,-0
.868211529342184,0.385278543760497,0.820245823704994
"7651","EIF2B1",299.924308604415,-0.144475251215425,0.166390899692666,
-0.868288178513846,0.385236592077913,0.820245823704994
"7652","PSMC6",619.154572385754,-0.135901668573267,0.156398203385842,-
0.868946481680421,0.384876404057318,0.820245823704994
"7653","AC026401.1",5.48779688765111,-0.572170088945276,0.659222435454
956,-0.867946929855927,0.385423386005158,0.820245823704994
"7654","SMIM10L2A",6.425929230029,0.49885228430188,0.574446197711088,0
.868405581392278,0.385172340459003,0.820245823704994
"7655","FBXO9",325.532915865532,-0.137188906126431,0.158126108785048,-
0.867591741683354,0.385617868964899,0.820552510459731
"7656","THAP10",16.937011401168,0.361884940492125,0.417338745952047,0.
867125192669522,0.385873418527023,0.820886074212014
"7657","SF3A1",233.247405663762,0.151031341868225,0.174175515319992,0.
867121544556667,0.385875417167499,0.820886074212014
"7658","SLC25A35",25.5886354166595,0.342594205044104,0.395267319270296
,0.866740528097714,0.386084194128215,0.821196873706534
"7659","ZNF160",132.400950947996,0.198453056334479,0.228983172232149,0
.866670919089558,0.386122343650214,0.821196873706534
"7660","COIL",184.563167500325,0.187297098492353,0.216160557388001,0.8
66472129585424,0.386231303780734,0.821321371708144
"7661","CUL1",211.365495591351,-0.144189199358631,0.16647005609988,-0.
866156969828375,0.3864040870227,0.821581539422108
"7662","ZNF613",10.2858907089388,-0.40689454584979,0.469934568789573,-
0.8658536163829,0.386570442125148,0.821827973345934
"7663","LINC00240",6.33510303805633,0.621894147750138,0.71850959907812
6,0.865533527385091,0.386746022171127,0.822093952126514
"7664","PXDC1",891.987972566921,-0.2303209496676,0.266143193408953,-0.
865402367490538,0.386817982027357,0.822139628032831
"7665","AC103591.3",312.22092763058,-0.350256703621389,0.4054511725064
26,-0.863869011541304,0.387659851707458,0.822166664466098
"7666","STK25",362.251657707555,-0.16749313055196,0.193919615509133,-0
.86372453922317,0.387739229936065,0.822166664466098
"7667","CCDC12",343.075650812627,-0.181321354766958,0.209896594459391,
-0.863860393895238,0.387664586270805,0.822166664466098
"7668","HIST1H4H",23.6491863831185,0.401765655809782,0.464830340337486
,0.864327521129715,0.387407995846829,0.822166664466098
"7669","AL356776.2",5.04911717037129,0.60118784521056,0.69524992429812
8,0.864707530630045,0.387199335118647,0.822166664466098
"7670","PRKN",31.8410195908171,0.296264178944176,0.342524455307976,0.8
64943143045931,0.38706999631264,0.822166664466098
"7671","ZNF12",202.676664467442,0.1354215117704,0.15668373373852,0.864
298472720829,0.38742394895818,0.822166664466098
"7672","RASSF8-
AS1",240.911028031109,-0.165269997384055,0.191248681426515,-0.86416280
703905,0.387498460570614,0.822166664466098
"7673","CCDC91",207.33101399011,0.133184353985865,0.154072323895013,0.
864427501441589,0.38735309066715,0.822166664466098
"7674","AC009318.2",5.73376528220668,0.560886445849796,0.6492985050539

67,0.863834494433615,0.387678815733115,0.822166664466098
"7675","MLKL",32.4928379104444,-0.310868581479279,0.359691472855082,-0
.864264529297103,0.387442590872833,0.822166664466098
"7676","OSGIN1",33.5021341452544,-0.300273535258642,0.347242562481116,
-0.864737125291117,0.387183087761296,0.822166664466098
"7677","FLOT2",138.00062320797,0.208771232583654,0.24129929634964,0.86
5196193034674,0.386931114551122,0.822166664466098
"7678","CASKIN2",43.5563647711448,0.231267302704497,0.267645540160702,
0.864080539379201,0.387543648662137,0.822166664466098
"7679","DNAJB1",2480.54989593259,0.226117958859593,0.261775965246527,0
.863784261655372,0.387706415107167,0.822166664466098
"7680","ZNF428",687.896618252263,-0.241260523022525,0.279259536187189,
-0.863929398138105,0.387626676099617,0.822166664466098
"7681","PPM1F",104.766297449224,0.198874106938007,0.230062310423409,0.
864435841629153,0.387348510784865,0.822166664466098
"7682","RPS19BP1",960.003353973352,-0.204169502549363,0.23621929876979
4,-0.864321855211053,0.387411107484313,0.822166664466098
"7683","HHAT",17.4884828536305,0.405190709160231,0.469330118043472,0.8
63338391427716,0.38795144188533,0.822509571374482
"7684","CCNC",259.923513172514,-0.177532965723461,0.205688155120675,-0
.863117108611841,0.388073082287115,0.822553342534133
"7685","ZCCHC7",294.798458617054,-0.126844543377709,0.146949564738369,
-0.863184206115517,0.388036195959431,0.822553342534133
"7686","BLOC1S4",272.186020973183,-0.18139380127917,0.210269353217789,
-0.862673511395118,0.388317000132269,0.822749169505011
"7687","FAM234A",227.285903005881,0.193384409181403,0.224159491987999,
0.862708991113153,0.388297487701065,0.822749169505011
"7688","FAAP100",76.8844410505697,-0.211910441847551,0.2456413377665,-
0.86268233097227,0.38831214966048,0.822749169505011
"7689","ZNF124",20.7595383766153,-0.392427131199123,0.455064578131356,
-0.862354817442739,0.388492295882658,0.822799506908414
"7690","LTV1",217.284369045727,-0.148865606914019,0.172613808923733,-0
.862420033728547,0.388456420123628,0.822799506908414
"7691","ASGR1",5.09823582805517,0.627178258714612,0.727249956903348,0.
862397106746016,0.388469032125243,0.822799506908414
"7692","CFAP97",533.846110202018,-0.143341479702816,0.166262910230465,
-0.862137439457324,0.38861189101729,0.822887454346177
"7693","C6orf52",6.27072051190235,0.592761672358498,0.687648920292513,
0.862012074571932,0.388680873373999,0.822887454346177
"7694","INPP5E",66.2566227183633,0.229101501568925,0.265777803519816,0
.862003893985239,0.388685375022376,0.822887454346177
"7695","AC106845.1",15.9355051913033,0.613621983987202,0.7122200150693
02,0.861562397860293,0.388928370490375,0.822974048703262
"7696","TEAD4",2.99953209810816,0.849227939067345,0.985448659418839,0.
861767816060728,0.388815298672651,0.822974048703262
"7697","ARPC3",1734.53850225857,-0.219916698079045,0.255234791008239,-
0.861625083360781,0.388893863327257,0.822974048703262
"7698","TBC1D8B",135.208997791793,0.164706333130404,0.191159654740556,
0.861616607091831,0.388898529241421,0.822974048703262
"7699","BSN",2.80576270413462,-0.766016212787299,0.889345320048302,-0.

861325961377629,0.389058541000719,0.823142560639136
"7700","TXNRD3",50.3232247856165,-0.204094960180488,0.237223685940059,
-0.860348153565317,0.389597156284626,0.823588267764196
"7701","LAMTOR3",266.973508397548,0.146134828424101,0.169882879208219,
0.860209275385479,0.389673692655084,0.823588267764196
"7702","AC027644.3",199.435250551495,-0.244287990665284,0.283847195277
8,-0.860632039806492,0.389440733790531,0.823588267764196
"7703","CHST15",7.1868107685782,0.876208247984716,1.01849697942838,0.8
60295382001504,0.389626237846383,0.823588267764196
"7704","MS4A2",4.8709866992609,-0.787770131130804,0.915540033297119,-0
.860443129170246,0.389544820079729,0.823588267764196
"7705","MRPL48",120.751292963955,-0.188108192704091,0.21860170769023,-
0.860506510638288,0.389509896169342,0.823588267764196
"7706","SETD1B",85.838178980906,0.215544825887265,0.250496540318587,0.
86047027081943,0.389529864488885,0.823588267764196
"7707","KDELR3",410.634309699937,-0.257148829844742,0.298811798235188,
-0.860571206905111,0.389474249754241,0.823588267764196
"7708","RGS16",588.999754393291,-0.535262885884328,0.622319498487292,-
0.860109456935581,0.389728708686035,0.823597682380232
"7709","CEP85",11.4838202667525,-0.426175578197468,0.495669520270042,-
0.859797830549044,0.389900495370895,0.823639328401654
"7710","AC010896.1",10.1350087575626,0.416237480672187,0.4847070136223
77,0.858740370933579,0.390483772056373,0.823639328401654
"7711","PLEKHM3",96.8549834814378,0.233954459772122,0.272065171644403,
0.859920651945509,0.389832783539371,0.823639328401654
"7712","HIST1H2BE",2.36437576484223,1.01954950308007,1.18710479939069,
0.858853829588917,0.390421164816903,0.823639328401654
"7713","INMT",669.750259085228,0.303226336711396,0.353003982299517,0.8
58988430487775,0.390346899058575,0.823639328401654
"7714","AC004854.2",8.0245004346507,-0.554635508230138,0.6452740934425
22,-0.859534752543947,0.390045555184607,0.823639328401654
"7715","GTF3C5",123.729169347023,0.152838441361309,0.177921187214329,0
.859023277408748,0.390327673743555,0.823639328401654
"7716","AL356419.1",2.43534941929188,0.95159127330513,1.10789629742444
,0.858917279096721,0.390386155616818,0.823639328401654
"7717","KCNC1",3.11352160010942,-0.89491176859926,1.04171938305787,-0.
85907182217569,0.390300892180558,0.823639328401654
"7718","RITA1",52.4357177138238,-0.233832593360393,0.272122415606061,-
0.859291921393577,0.39017948010565,0.823639328401654
"7719","PCDH9",256.299159747878,-0.374552326940806,0.435961104034082,-
0.859141614871049,0.390262390348593,0.823639328401654
"7720","C18orf54",26.6150116535383,0.276038302582938,0.321315370703366
,0.859088384034305,0.390291755458233,0.823639328401654
"7721","LRRC8E",14.4945318565397,-0.383158347543505,0.446045224683898,
-0.859012329557031,0.390333713695772,0.823639328401654
"7722","CHD6",337.053359730022,0.164206392946156,0.191070956332785,0.8
59400068423593,0.390119831989396,0.823639328401654
"7723","PRR34",7.665612842193,0.51869440705193,0.60404717907779,0.8586
98500742657,0.390506877846766,0.823639328401654
"7724","PIK3C2A",246.954913589653,0.200145514822746,0.233312796357193,

0.857841995585747,0.390979716902807,0.824529856114685
"7725","NAA80",57.4014111232058,-0.26695615737445,0.311288748915175,-0
.857583701000366,0.391122378264085,0.824599089509824
"7726","ATP11B",167.653112914089,0.21910495621 5215,0.255513718833048,0
.857507601610924,0.391164415534558,0.824599089509824
"7727","CD74",897.840048593461,-0.348772920312378,0.406671494853061,-0
.857628146370074,0.39109782792969,0.824599089509824
"7728","DPM3",431.049504427675,-0.199927378753024,0.233187194597763,-0
.857368600783969,0.391241206614043,0.82465424618739
"7729","RFLNB",542.281341890148,0.417817536966534,0.487400862220065,0.
857235941404402,0.391314502892952,0.824702023240173
"7730","RALGDS",280.819198254161,-0.173499846718776,0.202467096937116,
-0.856928603923546,0.391484343444568,0.824953230319348
"7731","SERPINE2",210.283171717279,0.377432933722704,0.440563041901068
,0.856705846441517,0.391607471439468,0.824999237231957
"7732","COX10-
AS1",40.0332799751166,0.234785132355503,0.27404133532352,0.85675079665
7471,0.391582623557802,0.824999237231957
"7733","TIMP1",7265.07808962525,-0.245724063661231,0.286901000461782,-
0.856476844854931,0.391734075326426,0.825154111426902
"7734","SLC6A8",54.78660254176,-0.218688256455581,0.255360702364662,-0
.856389626244403,0.391782300802729,0.825154111426902
"7735","ZNF547",5.07540484041564,0.568962966143596,0.664514210421086,0
.856208877434026,0.391882253067733,0.825257921166168
"7736","BCL6B",2.27775558247967,-0.896033119525195,1.04710531236636,-0
.855723974411177,0.392150475954428,0.825502597935084
"7737","MKS1",37.1582497242992,0.222947463626134,0.260508656616867,0.8
55815950692285,0.39209959095046,0.825502597935084
"7738","AL390198.1",21.9064148266146,0.502061915688311,0.5866574979975
88,0.855800731094339,0.392108010771931,0.825502597935084
"7739","USP21",40.3250702387102,0.302522422600446,0.353846163846069,0.
854954648404921,0.392576255105404,0.825720812938654
"7740","SPTBN1",4239.84058293532,0.174455971215894,0.204067004167884,0
.854895537508702,0.392608981288592,0.825720812938654
"7741","AC010207.1",10.4555798931701,0.653564396241987,0.7644252086381
84,0.854974939152393,0.39256502170813,0.825720812938654
"7742","MAGI2-
AS3",781.525556644777,0.146702042643649,0.171517292424572,0.8553192542
27177,0.392374431136961,0.825720812938654
"7743","MAP11",12.9439438553562,-0.406658685632384,0.475431722427,-0.8
55346133733902,0.392359554740852,0.825720812938654
"7744","SNX1",354.271860688511,0.107325646058742,0.125497885880954,0.8
55198837058902,0.392441079917234,0.825720812938654
"7745","SLC39A3",213.050901303864,-0.240180671987572,0.28086929956842,
-0.855133232277897,0.392477393895682,0.825720812938654
"7746","ZSCAN12",13.2740533533467,-0.374977056055209,0.438754518203341
,-0.854639759815364,0.392750609226277,0.82590434510389
"7747","MARCH5",230.318354994254,-0.170462910766468,0.199475689807785,
-0.854554812823188,0.392797652496767,0.82590434510389
"7748","LPIN2",379.635607022446,0.120348593198531,0.140915502268149,0.

854047931288061,0.393078432244215,0.826281401835852
"7749","AC024075.1",16.8987900320251,0.408905342513982,0.478770306111453,0.854074150577736,0.393063905463329,0.826281401835852
"7750","ZNF621",120.642090322839,0.252010232307637,0.295158445921126,0.853813386641089,0.393208396027762,0.826447943599511
"7751","PMVK",462.939294716567,-0.214324515267126,0.251049657076929,-0.853713634834602,0.393263677496427,0.82645749487025
"7752","COX7B",987.692082035872,-0.20926511052787,0.245163686283622,-0.853573029921643,0.393341607349248,0.826514633915364
"7753","CCDC88B",7.3171447244068,-0.529129741961025,0.620184105598023,-0.853181720048764,0.393558538913876,0.826757162802183
"7754","TYMS",16.5983284750423,0.459220526539854,0.538221915570842,0.853217814538082,0.393538526076647,0.826757162802183
"7755","ERRFI1",1752.94388930452,0.280068154868083,0.32831939055487,0.853035680880007,0.393639517624198,0.826820645078087
"7756","GATB",53.3261495260056,0.219834250972548,0.257756419631512,0.852875948877715,0.393728100561799,0.826900081234032
"7757","COX14",647.385540838595,-0.211469017012812,0.248030001307612,-0.852594508317339,0.393884209054804,0.82712129448159
"7758","MPHOSPH10",344.143949535268,-0.193476644433006,0.226982793171383,-0.85238463114216,0.394000647398367,0.827259157704563
"7759","IGFBP3",1843.80069307839,-0.262792225717325,0.308400191439519,-0.852114340431146,0.394150633426014,0.827467414341583
"7760","SS18L2",314.688586506401,-0.248179911924176,0.291462410137671,-0.851498866721609,0.394492292584942,0.827544746157903
"7761","ELOVL4",49.8585848118046,-0.232683281264253,0.273260750730768,-0.851506411520863,0.394488103263556,0.827544746157903
"7762","AC123768.4",7.97599194115618,0.467929072503246,0.549442831574836,0.85164287458633,0.394412335497207,0.827544746157903
"7763","ZNF319",31.3991410537621,0.263063417984894,0.308891662459884,0.85163651194068,0.394415868004278,0.827544746157903
"7764","TRIM16L",15.8163799934865,-0.356303364577636,0.418299488746959,-0.851790102935492,0.394330600445799,0.827544746157903
"7765","UBA52",11993.6474633605,-0.186566879829393,0.218990184097268,-0.851941746149342,0.394246425160469,0.827544746157903
"7766","LRRC37A2",17.8390569122598,-0.348381884231934,0.409303053507424,-0.851158771591268,0.394681161582565,0.827834334408757
"7767","FARSA",107.76680442141,-0.183389672638307,0.215514470881503,-0.8509390199563,0.394803227903587,0.82787716005684
"7768","TSPYL2",220.031563300376,-0.238675097639774,0.28047531045055,-0.850966515578043,0.394787953550952,0.82787716005684
"7769","TLCD4",93.0407034086826,-0.321570153752087,0.378612435329112,-0.849338594683396,0.395692910966649,0.828077487848364
"7770","FBXO11",303.434225813082,0.164396283150473,0.193577808349939,0.849251701689315,0.395741249785049,0.828077487848364
"7771","PPP4R3B",367.170487436187,0.145877041434344,0.171885324743455,0.848688168417348,0.396054831520514,0.828077487848364
"7772","EGOT",3.2249654960817,-0.827046621544516,0.972447171443104,-0.850479744125519,0.395058417398547,0.828077487848364
"7773","ZNF662",68.6061963523194,-0.215445207221694,0.253559064545244,

-0.849684500958751,0.395500517694628,0.828077487848364
"7774","BOC",1036.31270346447,0.263677811454935,0.310620075961919,0.84
8875626079176,0.395950502839629,0.828077487848364
"7775","ETV7",27.9039276985613,0.356310965380563,0.41999316399296,0.84
8373249681119,0.396230135429308,0.828077487848364
"7776","AL451064.1",2.62282402446129,0.767327486262926,0.9033161396944
88,0.849456189858896,0.395627498118623,0.828077487848364
"7777","VNN2",2.43778399444851,0.934892743470391,1.09937343774576,0.85
0386876171366,0.395110030155833,0.828077487848364
"7778","AC004982.2",19.3561732068589,-0.343565048838015,0.405030850361
796,-0.848244148639847,0.396302014863194,0.828077487848364
"7779","ZMIZ2",95.2706696393673,-0.195469383360714,0.23047465089733,-0
.848116626273971,0.396373023067941,0.828077487848364
"7780","PPIA",4148.58435195311,-0.213513503519765,0.25111819059847,-0.
850251043187733,0.39518552871333,0.828077487848364
"7781","MRPS17",195.738592005568,-0.222534450025183,0.26213000578848,-
0.848946877927253,0.395910852312317,0.828077487848364
"7782","CHPF2",115.612282876816,0.21215602799322,0.249896104082378,0.8
48976932922824,0.395894127901495,0.828077487848364
"7783","PTP4A3",25.1849744123928,-0.354140496401601,0.417450452868715,
-0.848341387505874,0.396247874563221,0.828077487848364
"7784","NUP188",62.3437311815489,-0.185690972708551,0.218844740046843,
-0.84850553259267,0.396156492594926,0.828077487848364
"7785","RAB5B",279.428887379314,0.139960731501995,0.165012431787428,0.
848182951950518,0.396336090021589,0.828077487848364
"7786","LACC1",32.5013081067197,-0.251226583256188,0.295832995283246,-
0.849217589862317,0.395760227265987,0.828077487848364
"7787","EIF2S1",303.848724002222,-0.155661409131863,0.183052233500082,
-0.850366073964309,0.395121591852954,0.828077487848364
"7788","AC027020.2",8.78095178100069,0.562296013996803,0.6627433285582
96,0.848437079283767,0.396194600005246,0.828077487848364
"7789","CNEP1R1",79.5291108754105,-0.169727012709668,0.199818714024709
,-0.84940498960813,0.395655977686505,0.828077487848364
"7790","PRKAR1A",920.673656543409,0.125551736862345,0.147666146228657,
0.850240492278654,0.395191393474379,0.828077487848364
"7791","FBF1",21.8190406157099,0.291295525135848,0.343073118178589,0.8
49077090861465,0.395838397068956,0.828077487848364
"7792","SCAMP4",175.137156510253,0.164199975760671,0.193567966638319,0
.848280728533345,0.396281647519957,0.828077487848364
"7793","AC006504.1",4.00316332019683,0.755330421873892,0.8896517743714
62,0.849018058113279,0.395871244058537,0.828077487848364
"7794","PRMT1",560.455412114845,-0.223878586040354,0.263675573416353,-
0.84906835752602,0.395843256365901,0.828077487848364
"7795","ZIK1",6.73076807109018,0.552896521192726,0.651015823111118,0.8
49282769427181,0.39572396630488,0.828077487848364
"7796","ZNF831",4.45353115203248,0.744480384341621,0.87694164024166,0.
848950888153131,0.395908620756289,0.828077487848364
"7797","AC007325.4",65.0359969389946,-0.252888100229975,0.297526260980
858,-0.849969005748521,0.395342318339308,0.828077487848364
"7798","CCDC115",367.466121330066,-0.216592535230166,0.255531389521692

,-0.84761616033,0.396651771441057,0.828145534743296
"7799","NUDCD1",96.7009849859774,-0.181248112197071,0.213751835834832,
-0.847937101869492,0.396473000554144,0.828145534743296
"7800","DPM2",235.587484904727,-0.255105832323719,0.300889845619218,-0
.847837958102983,0.396528220432256,0.828145534743296
"7801","PGBD4",11.854213420768,0.388339649872438,0.458197644802,0.8475
37420320549,0.396695638639165,0.828145534743296
"7802","SPECC1",70.5822869201273,-0.399222521832541,0.471115723507133,
-0.847397999923679,0.396773318939782,0.828145534743296
"7803","HOXB4",68.9488125108377,-0.2157958443217,0.254678050312173,-0.
847328005131133,0.396812321116792,0.828145534743296
"7804","CTIF",114.475662914957,0.207612097610259,0.244970331349118,0.8
47498945961672,0.396717074325699,0.828145534743296
"7805","COL6A1",12568.8648985765,0.246823518604065,0.291272065942785,0
.847398523456583,0.396773027227896,0.828145534743296
"7806","SEMA6A-
AS1",2.90776554516805,0.701907570549692,0.828571434842558,0.8471298201
139,0.396922765409622,0.828269911062944
"7807","ZNF100",73.7537924147323,0.225042979962893,0.265856975749955,0
.846481380930743,0.39728425635663,0.828918054539918
"7808","VCPIP1",155.669267962043,0.202321553563071,0.239041128755536,0
.846388044669849,0.397336305662881,0.828920476811305
"7809","AC093799.1",3.14816942167896,0.762444471830358,0.9012080507196
28,0.846024923125726,0.397538840832707,0.829024475524767
"7810","FRAT1",25.2040929852174,-0.280964864674227,0.332081264573137,-
0.846072617301624,0.397512235313628,0.829024475524767
"7811","PSMB3",850.64109776843,-0.249362177199753,0.294721897086132,-0
.846093146336111,0.397500783813621,0.829024475524767
"7812","SDHD",970.586644394258,-0.215375040580877,0.254602595078052,-0
.845926336747867,0.397593839247369,0.82903303219411
"7813","MAPRE3",56.6995891041634,-0.228424962625264,0.270125337152119,
-0.845625830710686,0.39776151094699,0.82917451443974
"7814","SAC3D1",97.741670377677,-0.25564108204257,0.302319241099994,-0
.845599774306181,0.397776051503326,0.82917451443974
"7815","FAM210A",106.105984836136,0.170739455840311,0.201931621433551,
0.845531049709794,0.397814404220429,0.82917451443974
"7816","NUS1",153.471625636918,0.163232961727041,0.193095975566805,0.8
45346264974683,0.397917536964811,0.829283362285032
"7817","LINC02193",10.5038233056383,0.414064995670772,0.49017226344305
6,0.844733630504319,0.398259578045225,0.829783866305792
"7818","URI1",563.884248653868,-0.126699575321873,0.149978069420605,-0
.844787346652339,0.398229580601463,0.829783866305792
"7819","AC009275.1",17.2532827717729,0.398538532458541,0.4718453244573
59,0.84463808752768,0.398312936781081,0.82978890231833
"7820","DNAJC17",137.372762744594,-0.221301351056836,0.262038920331154
,-0.844536188659169,0.398369849893798,0.829801340782619
"7821","DNAH10OS",6.60056132000083,-0.541222858189756,0.64104953838243
5,-0.844276184264056,0.398515091161937,0.829997739411429
"7822","AL445248.1",2.40303950760566,0.853734728812019,1.0136877494657
6,0.842206812957892,0.399672200942627,0.830166404593957

"7823","ANKRD53",14.7912445130154,-0.421991394247648,0.500209455430422
,-0.843629382984237,0.39887653967233,0.830166404593957
"7824","GPBAR1",3.65840298974206,-0.63850953506012,0.757639982147764,-
0.842761139994312,0.399362045422548,0.830166404593957
"7825","RNF25",49.8163068759721,0.215068512092507,0.255222817487405,0.
842669610067759,0.399413248008137,0.830166404593957
"7826","AC018809.2",10.8127796684072,0.511007745663055,0.6066212156166
95,0.842383570682672,0.399573286223072,0.830166404593957
"7827","EXOC3-
AS1",33.5471500651378,0.29040338020965,0.344820017859789,0.84218828713
0517,0.399682568963196,0.830166404593957
"7828","HBEGF",37.5668727959042,-0.444480482282836,0.527109284981978,-
0.843241610320017,0.399093331197441,0.830166404593957
"7829","AC092171.3",2.38207841353249,1.01648400686339,1.20711278096341
,0.842078737706781,0.399743881848807,0.830166404593957
"7830","ZNF783",63.8128010556146,0.234321243937957,0.277643564452684,0
.843964254672615,0.398689380480304,0.830166404593957
"7831","EYA1",9.19981648980763,0.863443434838692,1.02499596334592,0.84
2387156355358,0.39957127981079,0.830166404593957
"7832","PCBD1",870.13076165168,-0.269134820851406,0.319322393828884,-0
.842831026112212,0.39932295321864,0.830166404593957
"7833","IGSF22",4.98504612847905,-0.567131441334786,0.673291589712575,
-0.84232663826514,0.399605144349404,0.830166404593957
"7834","TMEM258",1895.17717763983,-0.203746552810939,0.241709721870439
,-0.842939006483779,0.399262556755996,0.830166404593957
"7835","MARCH9",99.0045913862099,-0.219869831484567,0.260676144027021,
-0.843459735470755,0.398971375547684,0.830166404593957
"7836","ARL6IP4",2027.06971726222,-0.197404712977475,0.23438498286052,
-0.842224235393734,0.399662450585189,0.830166404593957
"7837","GPALPP1",179.15162064699,0.173939840482828,0.206159047222679,0
.843716746007997,0.39882770753483,0.830166404593957
"7838","MYCBP2",1436.10953473931,0.178577021459642,0.211934852952106,0
.842603370668805,0.399450305330103,0.830166404593957
"7839","ZZEF1",121.951205589592,0.226317702904086,0.268532045070725,0.
842795886220875,0.399342609134937,0.830166404593957
"7840","MIEN1",289.996308783275,-0.215234286711002,0.255497560679434,-
0.842412295986851,0.39955721275573,0.830166404593957
"7841","NT5C",620.979055665008,-0.219706658938797,0.260943032510839,-0
.841971739290147,0.399803772440904,0.830166404593957
"7842","LRRC45",59.0204561495757,-0.204225973010706,0.242162252158014,
-0.843343548347268,0.399036333976415,0.830166404593957
"7843","PDCD5",951.280516892583,-0.221149129725643,0.262186705757196,-
0.843479569595124,0.398960287230731,0.830166404593957
"7844","GSK3A",179.962589067295,-0.152467671630507,0.181090012088848,-
0.841944124205487,0.399819230403315,0.830166404593957
"7845","TSPO",2541.06572424982,-0.246694289295521,0.29271414421839,-0.
842782264431558,0.399350228801427,0.830166404593957
"7846","CNST",138.786503619937,-0.22423726647455,0.266391717928556,-0.
841757650043342,0.399923621550791,0.830277322386036
"7847","VPS45",113.596248037889,0.184003976787882,0.218719073829736,0.

841279974197045,0.400191106699414,0.830508921081537
"7848","FAM72A",12.4091848025553,0.47609365921456,0.566218550437456,0.
840830203896946,0.400443063796083,0.830508921081537
"7849","AC027031.2",17.2874791899186,-0.390957683396941,0.464870228135
146,-0.841003918373717,0.400345739281253,0.830508921081537
"7850","GTF3C4",107.081034490904,0.189382937019376,0.225225989464075,0
.840857387151515,0.400427833286188,0.830508921081537
"7851","G2E3",106.672740005029,0.15526420955521,0.184563628035198,0.84
1250311386379,0.400207720591569,0.830508921081537
"7852","MIA2",108.106286717484,-0.143100205242215,0.170061605422631,-0
.841460980487556,0.400089735573962,0.830508921081537
"7853","NDUFA11",1829.03524398547,-0.199302503459156,0.236953618669261
,-0.841103438632611,0.400289988900911,0.830508921081537
"7854","RRAS",1660.80070019693,0.219218561850134,0.260658618010048,0.8
41017893533385,0.400337910237162,0.830508921081537
"7855","AC093702.1",7.42159884464351,0.459158883887546,0.5462680046736
82,0.840537757948736,0.40060694039936,0.83063846723759
"7856","ARPP19",404.428008948914,-0.124540777838384,0.148168155873447,
-0.840536734119553,0.400607514188625,0.83063846723759
"7857","MED28",374.786871307119,0.121135607066873,0.144169656930788,0.
840229557631722,0.400779688808148,0.830889697212157
"7858","FAM229A",34.5108607813347,0.284123116634631,0.338947849770109,
0.83825024064126,0.401890175772587,0.830950408893098
"7859","MYSM1",205.605821989537,0.193654658925208,0.230951371594321,0.
838508373379019,0.401745246962727,0.830950408893098
"7860","SF3B1",1115.41824920985,0.121022754738214,0.144086448914571,0.
839931552549875,0.400946765262189,0.830950408893098
"7861","STX18-
AS1",32.8786834874886,-0.279157744179116,0.33298429793882,-0.838351075
132096,0.401833558442772,0.830950408893098
"7862","AC108471.3",7.70432987036805,0.57106463623205,0.67988340244148
2,0.839944958475732,0.40093924833566,0.830950408893098
"7863","CASP3",144.419404063604,0.161302813254143,0.192127012902769,0.
839563426386972,0.401153212822731,0.830950408893098
"7864","AC025181.2",69.9722740691506,-0.2502557330573,0.29815849829084
1,-0.839337917556812,0.401279711170594,0.830950408893098
"7865","CRIP3",2.74257185798087,0.850806959655516,1.01531902789502,0.8
37970072736077,0.402047511736068,0.830950408893098
"7866","IMMP2L",177.967533292816,-0.213763361834782,0.254564515536664,
-0.839721755344154,0.401064413086201,0.830950408893098
"7867","BRAF",174.20800480305,0.221903169220612,0.264709813935677,0.83
8288410699174,0.401868743190316,0.830950408893098
"7868","PPDPFL",41.2297592694935,-0.449714345059276,0.536606441390268,
-0.838071089668869,0.401990778650847,0.830950408893098
"7869","NEURL1",3.5721497669147,0.819945814912337,0.977570793038925,0.
838758502965716,0.401604841450018,0.830950408893098
"7870","DCUN1D5",228.526526184144,-0.206269626889883,0.245827943138503
,-0.839081286921346,0.4014236963111,0.830950408893098
"7871","NAA16",92.6259687122172,0.202389943726688,0.241150739554979,0.
839267356592723,0.4013192970021,0.830950408893098

"7872","DAD1",2431.52782684336,-0.234826043924636,0.280014919783142,-0
.838619756784735,0.401682720357067,0.830950408893098
"7873","DISP2",9.35236189938934,0.92864816650674,1.10684464263163,0.83
9004979324639,0.401466515419232,0.830950408893098
"7874","MAP2K1",172.912885062838,0.186334846573053,0.222358877879881,0
.837991486329194,0.40203548504222,0.830950408893098
"7875","ZNF774",3.52781176829375,-0.630204789489885,0.750695024152988,
-0.839495093498119,0.401191541376361,0.830950408893098
"7876","NDUFB10",1517.43258103916,-0.214911240269265,0.256068211311551
,-0.83927340753667,0.401315902233687,0.830950408893098
"7877","ARHGAP17",130.315622291836,0.194825976514618,0.232416172827664
,0.838263422653815,0.401882773962293,0.830950408893098
"7878","METTL16",143.155670144409,0.182548372184476,0.21773537410632,0
.838395565873174,0.401808578959047,0.830950408893098
"7879","MYO1D",254.349332416788,-0.237106322663895,0.282975327924484,-
0.837904577770011,0.402084297556351,0.830950408893098
"7880","AC139100.2",4.01231886507789,0.882740866753708,1.0511276357243
8,0.839803689630302,0.401018464390199,0.830950408893098
"7881","EXOSC5",89.8980118465495,-0.260094524039418,0.30980731803097,-
0.839536411510518,0.401168365455669,0.830950408893098
"7882","DDX3X",2198.24275556785,0.208853396689545,0.249078221953924,0.
838505249680882,0.401747000577796,0.830950408893098
"7883","FRZB",131.723490865857,0.455498478059335,0.543826107480853,0.8
37581116083824,0.402266002244909,0.830981083227437
"7884","CIDEC",6.4042991404566,-0.629046598341489,0.750996978500837,-0
.837615351791709,0.402246767996984,0.830981083227437
"7885","RNF180",20.7001605953167,0.457448723991343,0.546051382094012,0
.837739339175567,0.402177114229045,0.830981083227437
"7886","ARID4A",457.151346347325,0.166085482167742,0.198326885712224,0
.837433016564053,0.402349213622027,0.830981083227437
"7887","SEPHS2",355.614562796782,-0.279918283235182,0.334261072512967,
-0.837424116217788,0.402354214710221,0.830981083227437
"7888","DNAH14",30.6412003282854,-0.375748563261461,0.448809499284477,
-0.837211698639412,0.402473582817183,0.831122235105108
"7889","AL713852.2",2.7810520755702,1.21135722854535,1.44712458123668,
0.837078745155549,0.40254830686066,0.831171171308568
"7890","SCAND1",817.188294838179,-0.227082471322331,0.271338232502342,
-0.836898174017446,0.402649806842874,0.831275374355332
"7891","AL391832.2",14.0373850223179,0.379729136644654,0.4538128092281
34,0.836752795256078,0.402731536152565,0.831338739372593
"7892","AL031595.3",11.6511705729024,0.540730676610385,0.6464009561652
16,0.836525180622068,0.402859516951696,0.831497550890291
"7893","GPN2",93.5110460400626,0.168084550735158,0.200985162440295,0.8
36303280771238,0.402984307962158,0.831649739312757
"7894","KBTBD6",72.9274605866906,0.202499222757498,0.242164368210218,0
.836205690598183,0.403039197604534,0.831657650086174
"7895","HIRA",53.9204161728016,0.198423304629593,0.237391785671942,0.8
35847390708791,0.403240761972226,0.83196817881768
"7896","ARL5A",759.723331216358,-0.137105521765509,0.164056647157357,-
0.835720613222107,0.403312096086539,0.832009971270724

"7897","COG7",87.9914698743058,0.179388821805428,0.214735489299258,0.8
3539438399691,0.403495690813649,0.832283311088201
"7898","PEX11B",74.8315348493031,0.212063530159068,0.253915708828812,0
.83517294434918,0.403620340753638,0.832435012729299
"7899","GRIN2D",11.687319252154,-0.475754659620637,0.569710404846227,-
0.835081570520112,0.403671782446179,0.832435708857553
"7900","PPIE",305.252117714014,-0.176449085075819,0.211347609170754,-0
.834876182267386,0.403787426374692,0.83246340820369
"7901","NKAIN2",3.09178403461717,0.697154928387129,0.834961705215634,0
.834954374592646,0.403743397820318,0.83246340820369
"7902","SSC4D",3.34941659997626,-0.726295149133079,0.87018947839089,-0
.834640233154861,0.403920302083476,0.832631966671443
"7903","TEKT4",5.10315023656856,-0.607458583324197,0.727892751948365,-
0.834544074931644,0.403974461486641,0.832638239042881
"7904","CDK11A",147.860913816493,-0.238264435751172,0.285746430614356,
-0.833831713099276,0.404375821933492,0.832886321890258
"7905","SLC27A3",489.511356021987,-0.229082498419474,0.274822928326375
,-0.833563996332287,0.404526721149759,0.832886321890258
"7906","NDUFA10",721.5185252576,-0.162710323784246,0.195269968537663,-
0.833258309010596,0.404699063744642,0.832886321890258
"7907","USP4",146.873744469891,-0.181383669090158,0.217509593017657,-0
.833911123521957,0.404331068529719,0.832886321890258
"7908","IP6K1",143.352094753402,0.171083956838963,0.205365293263611,0.
833071421758477,0.404804450008704,0.832886321890258
"7909","POMZP3",26.7386426272871,0.297499112078395,0.357075904472251,0
.83315370304275,0.404758049331702,0.832886321890258
"7910","MTPAP",93.553352585557,0.168607294157572,0.202174290184415,0.8
33970006788575,0.404297885547314,0.832886321890258
"7911","AP004609.3",19.9261771391766,-0.306724446930553,0.367692374498
739,-0.834187674815663,0.404175235398868,0.832886321890258
"7912","ARHGAP11A",8.69423333964525,0.46221946766453,0.55465162837196,
0.833350961253388,0.404646822960284,0.832886321890258
"7913","TCF12",373.021733068554,0.151538224728245,0.181870339336619,0.
833221212876093,0.404719981057251,0.832886321890258
"7914","MMP28",82.8178111710333,0.381699168346399,0.458099043649715,0.
833224110893942,0.404718346935266,0.832886321890258
"7915","SS18L1",51.719533590879,0.227073105644326,0.272577003915582,0.
833060391677983,0.404810670415936,0.832886321890258
"7916","CHM",251.565981302436,0.138563898426809,0.166125010929483,0.83
4094141824477,0.404227936025222,0.832886321890258
"7917","RADX",5.49135682583307,-0.627911397619105,0.753305883017625,-0
.833541077767494,0.404539640820365,0.832886321890258
"7918","ZNF728",10.1272221295884,0.490956788421315,0.589512339362668,0
.832818510554159,0.404947093499249,0.833061784037544
"7919","GNB1",826.39740924594,0.151017827588078,0.181503146740051,0.83
2039721076387,0.405386524313607,0.833274133918237
"7920","TMEM9",502.471987667767,-0.150927886512958,0.181289774569079,-
0.832522887028293,0.405113865092748,0.833274133918237
"7921","AC079807.1",12.4485272353013,0.380757033601617,0.4577398547689
77,0.831819710769529,0.405510716407997,0.833274133918237

"7922","TMEM170B",23.2482039036102,0.29933748436955,0.359722965277292,
0.832133372799275,0.405333666398729,0.833274133918237
"7923","AL354892.3",6.36466499108998,0.57575576298222,0.69208768366171
2,0.831911586601281,0.405458851291809,0.833274133918237
"7924","NKX3-1",3.17062664726626,-0.708460274383524,0.851477524206773,
-0.832036377053542,0.405388411787775,0.833274133918237
"7925","FZD10",3.91818944609972,-0.680931848401555,0.818579859153183,-
0.831845348731125,0.405496243042522,0.833274133918237
"7926","DOK4",66.5787764424603,-0.186553106828401,0.224193878278969,-0
.832106158564551,0.405349025944683,0.833274133918237
"7927","ZNF350",55.3124405713071,0.251229351040585,0.301960851216655,0
.831993121056375,0.405412827338708,0.833274133918237
"7928","GZMK",23.5956802341529,-0.433520710681093,0.521417101042057,-0
.831427871879726,0.405731959119314,0.833291144590739
"7929","SLC29A4",4.68860053546654,-0.679176418044736,0.816836199982732
,-0.831471986744826,0.405707047085869,0.833291144590739
"7930","SWI5",380.459006422036,-0.1818186372695,0.218702338047425,-0.8
31352050886963,0.405774778003176,0.833291144590739
"7931","STARD3",136.190961524227,-0.172865857771074,0.207858850288319,
-0.831650216150494,0.405606408752688,0.833291144590739
"7932","SLC37A1",27.0234027030249,0.33767311122306,0.406125410150232,0
.831450342144683,0.405719269858497,0.833291144590739
"7933","ASS1",220.048256222821,-0.28895222679596,0.34762512739664,-0.8
31217895437882,0.405850547097975,0.833341681794897
"7934","CENPW",61.1588442721057,-0.326431297257886,0.392762592882064,-
0.831116056298937,0.405908070040898,0.833354745764581
"7935","FAM86B1",20.441483401022,0.305278493076707,0.36743710168219,0.
830831975538369,0.406068556296605,0.833474132247405
"7936","WASHC3",621.746111022213,-0.225785500398894,0.271734421872898,
-0.83090503898142,0.406027276815304,0.833474132247405
"7937","INIP",140.412031606984,0.165055704373175,0.198709191435612,0.8
30639504799447,0.406177310666103,0.833592316169857
"7938","UTP11",229.626806824693,-0.180220200795785,0.217068215841618,-
0.830246842436303,0.406399235963894,0.833732639120387
"7939","RBPJ",2845.90346931532,0.208732545849468,0.251391067425264,0.8
30310113988128,0.406363471195653,0.833732639120387
"7940","CCDC125",49.5499160335898,0.231382604855095,0.27864580475187,0
.830382517551762,0.406322546787358,0.833732639120387
"7941","HEIH",167.210600163836,0.16864348120037,0.203326091064953,0.82
9423712013901,0.406864688442605,0.833791859711203
"7942","AL121933.2",28.1179574301325,-0.488221772746764,0.588463484561
353,-0.829655170720898,0.406733774223807,0.833791859711203
"7943","KATNA1",226.185428191824,-0.167221613076935,0.201481729993241,
-0.829959188272525,0.406561858547265,0.833791859711203
"7944","AC067930.8",8.37173735794689,0.509316494908996,0.6140019445432
73,0.829503064990866,0.406819803164731,0.833791859711203
"7945","AP002807.1",10.55852420304,-0.410122503797771,0.49447658508069
5,-0.829407329228423,0.406873955581699,0.833791859711203
"7946","EMC8",145.336698542546,-0.184912553285941,0.222902098520196,-0
.829568472049117,0.406782808488056,0.833791859711203

"7947","ZNF396",27.0152626880925,0.291574886684672,0.351288080421081,0
.830016453547664,0.406529481065388,0.833791859711203
"7948","ZNF765",68.8559806591461,0.252597021493505,0.304560858409045,0
.829381105677902,0.406888789541675,0.833791859711203
"7949","IFT27",170.494278782822,-0.197027507416177,0.23745316247149,-0
.829753141063483,0.406678369272681,0.833791859711203
"7950","MDM4",443.93363568025,0.198810736372879,0.239816640111229,0.82
9011432570603,0.407097938044724,0.834024296528734
"7951","RAN",1118.33239315223,-0.218978742819745,0.264148182031529,-0.
828999621105127,0.40710462162809,0.834024296528734
"7952","GRAMD1B",4.03479589282461,-0.653274207655536,0.788276171181179
,-0.828737733726808,0.40725282886769,0.834223004203445
"7953","NKRF",49.6422595763587,0.204527292960455,0.246862061805903,0.8
28508404508369,0.407382637221018,0.834383978082882
"7954","SQSTM1",2736.25354863043,-0.148284345863137,0.178998050334209,
-0.828413189899408,0.407436539264329,0.834389462921379
"7955","GPD2",81.6293952447698,0.211752582680044,0.255923317878589,0.8
27406367013808,0.40800677299322,0.834452665878826
"7956","JMY",165.113555700081,0.22414255283003,0.270834271973823,0.827
600403732118,0.407896839548877,0.834452665878826
"7957","HSPA1A",1568.86758340475,-0.298218831448231,0.360229344820009,
-0.827858240136538,0.407750787066757,0.834452665878826
"7958","ATP6V1F",1407.23253590073,-0.210316497990408,0.254146752035361
,-0.827539586109467,0.407931294482291,0.834452665878826
"7959","AC009812.4",6.10473712104512,0.559121555057798,0.6752007417437
81,0.828081962134409,0.40762408408733,0.834452665878826
"7960","MACORIS",8.31858341303089,-0.485085641487685,0.586076728390666
,-0.827682823748526,0.407850149005268,0.834452665878826
"7961","FZD4-
DT",10.8645545082687,-0.392619675514118,0.474323526202233,-0.827746577
652824,0.407814034909253,0.834452665878826
"7962","CPB2-
AS1",12.1403495612649,0.500920281943782,0.605051845724033,0.8278964612
43512,0.407729139217781,0.834452665878826
"7963","MT1M",4068.70485924929,-0.534867901606876,0.646472472394333,-0
.827363769451607,0.408030909431202,0.834452665878826
"7964","PGPEP1",102.215959862409,0.212016705060615,0.256253071476982,0
.82737234655804,0.408026049441559,0.834452665878826
"7965","AC008532.1",5.0981925729107,0.555012339303545,0.67010818257286
2,0.828242892323132,0.407532957208834,0.834452665878826
"7966","TAS2R30",4.39725682384687,0.65919748686619,0.796982978668612,0
.827116142389143,0.408171235602549,0.83463485522595
"7967","AL355999.1",3.35949957015539,-0.684913395055187,0.828386360477
904,-0.826804288110266,0.408347999171516,0.834786716679823
"7968","RPL23A",17558.2751083423,-0.188995152518081,0.228577905555447,
-0.826830362535785,0.408333218059192,0.834786716679823
"7969","FGF2",247.456424828762,-0.150773565364629,0.182401138188027,-0
.826604301170562,0.408461378684604,0.834834689684733
"7970","PRDM15",16.9752336882943,0.330794144308566,0.400195148095587,0
.826582095966731,0.408473968738862,0.834834689684733

"7971","ZNF592",96.1677660059744,-0.166284454132723,0.201206015603799,
-0.826438780340241,0.40855523234531,0.834896020533529
"7972","CCSAP",18.8869267631876,0.339346467099956,0.410696519376861,0.
826270618545384,0.408650596634661,0.834986147589312
"7973","PI4KA",125.395585939332,0.168545997295967,0.20401141634726,0.8
26159635150394,0.408713542402311,0.835010020342562
"7974","FRYL",144.942215728217,0.166773292956922,0.202015433101824,0.8
25547288126554,0.409060947256481,0.835103972091964
"7975","MALINC1",30.3367353591235,-0.239974638585303,0.290664659865723
,-0.825606520917141,0.409027334847347,0.835103972091964
"7976","CYHR1",373.696515276095,-0.158424461327107,0.191904881488079,-
0.825536380829109,0.409067136921959,0.835103972091964
"7977","GLRX3",307.39344831086,-0.2075486747205,0.251371464896837,-0.8
25665215443919,0.408994029504306,0.835103972091964
"7978","UBE2Z",290.72231019973,0.153319822168083,0.185677640673206,0.8
25731206041805,0.408956586040808,0.835103972091964
"7979","ZNF175",36.2289761242571,0.253496107263525,0.306906417691914,0
.82597199879344,0.408819976085496,0.835103972091964
"7980","FAM178B",4.04185661204296,-0.64352360022142,0.78022524616832,-
0.824792075598373,0.409489646385429,0.835469046143713
"7981","PPP4R2",360.339449554572,0.117869762943773,0.142894334425748,0
.824873592206844,0.409443360325712,0.835469046143713
"7982","C5orf24",416.149400826832,0.155471741643163,0.188490219885715,
0.824826570510811,0.409470059401013,0.835469046143713
"7983","ZFHX4-
AS1",49.8773672120249,0.329023972464027,0.39892835276413,0.82476958627
848,0.409502416625416,0.835469046143713
"7984","B3GNT10",4.07270881128509,0.645081414964495,0.781991381213014,
0.824921387194644,0.409416223234017,0.835469046143713
"7985","CRIM1-
DT",73.8975444047487,0.27420421244214,0.332593441017694,0.824442633634
355,0.409688098780597,0.835677780449691
"7986","AL133346.1",3.41964362601921,0.648650236817693,0.7869630601948
99,0.824244833877016,0.40980045704367,0.835677780449691
"7987","AC009996.1",11.8709895900146,0.557901237315346,0.6768772009267
2,0.824228141458329,0.40980993985095,0.835677780449691
"7988","AP005205.2",4.75719342733723,0.831635532106344,1.0088798302873
,0.824315748159535,0.409760172756056,0.835677780449691
"7989","AC092756.1",3.27142110424687,0.827115412357102,1.0038233148882
6,0.823965134192141,0.409959369046471,0.835877852346722
"7990","HADH",234.750730433263,-0.169617157022047,0.205946247663102,-0
.823599162144073,0.410167352201467,0.836021197487779
"7991","AL589843.1",11.1738914451848,-0.41748390500618,0.5069147951349
7,-0.823578062847865,0.410179344914654,0.836021197487779
"7992","BAIAP2L2",4.67971134807408,0.548107327893507,0.665525696584318
,0.823570495784854,0.410183646038574,0.836021197487779
"7993","SLC50A1",113.204320792144,-0.209057664624131,0.254050464927506
,-0.822898177666338,0.410565899107691,0.836067999820626
"7994","C1orf74",6.86789306917148,-0.437249367361498,0.531283680829684
,-0.823005454785782,0.410504891462393,0.836067999820626

"7995","AC104695.3",7.88302641009424,0.492567222063083,0.598494614401182,0.823010283151698,0.410502145735731,0.836067999820626
"7996","LRRC15",2.64175452849473,1.35391718868751,1.6451698726874,0.822964978367779,0.410527909446935,0.836067999820626
"7997","SERF1A",5.45695729470404,0.520874398499532,0.632896671696539,0.823000691571468,0.410507600150257,0.836067999820626
"7998","COL6A2",19674.4180790047,-0.173031974126361,0.210207528823445,-0.823148319638404,0.410423653718716,0.836067999820626
"7999","HDHD5",94.6894350293881,-0.21326614515068,0.259057036528856,-0.823240117343519,0.410371459507885,0.836067999820626
"8000","DNLZ",23.8296257716461,-0.429001701254844,0.521488657102577,-0.822648192653707,0.410708084495775,0.836184882666558
"8001","F8",39.4083884660296,-0.253688225975478,0.308391754891017,-0.822616759209821,0.410725965143049,0.836184882666558
"8002","B3GAT3",238.41997692705,-0.222564488166264,0.270612390675382,-0.822447514730563,0.410822246372387,0.836276377300651
"8003","AC026461.3",7.60574340631915,0.593861987409367,0.722209635952529,0.822284774179337,0.410914840232284,0.836318712262729
"8004","GALK1",269.791342402638,-0.186477828316338,0.226795078120422,-0.822230490457663,0.41094572858683,0.836318712262729
"8005","CD8A",7.64130583531281,-0.590481026400272,0.718361501240778,-0.821983117664815,0.411086505340664,0.836500697750665
"8006","SEC24C",96.9868746329614,0.197148747582067,0.239887594814494,0.821838026824701,0.411169088037938,0.836564236204094
"8007","NPHP1",86.6438156640069,0.216723642009323,0.264631761224194,0.818963079135903,0.412807478738594,0.83659015622167
"8008","PAX8",8.71317923729099,-0.718838590176019,0.876894168622636,-0.819755240595476,0.412355651832003,0.83659015622167
"8009","AC093388.1",5.26565623112546,-0.529432925211229,0.644670977741553,-0.821245167676025,0.41150663349609,0.83659015622167
"8010","ANAPC10",185.146821539324,-0.206266536871049,0.251250046873547,-0.820961187620642,0.411668376299488,0.83659015622167
"8011","SHROOM1",37.4793773604555,0.251543852404867,0.307075065892784,0.81916078621862,0.41269468464486,0.83659015622167
"8012","CCDC126",127.298053832523,0.19629435762108,0.239514729162972,0.81955025608265,0.412472541182135,0.83659015622167
"8013","TNS3",84.0580904325713,-0.322103141480286,0.393108586616994,-0.81937447424447,0.412572793785375,0.83659015622167
"8014","ABCB8",74.9837255849141,-0.208253725726028,0.254258070496798,-0.819064367628994,0.412749690242863,0.83659015622167
"8015","EFR3A",269.00147257118,-0.137878318171507,0.168285184935037,-0.81931346615409,0.412607591539954,0.83659015622167
"8016","NDUFB6",616.320956594574,-0.225051321099984,0.274519017060032,-0.819802298253056,0.412328820678976,0.83659015622167
"8017","CCL27",5.21805699931762,-0.522377623240605,0.636108929230637,-0.821207814001938,0.411527906385164,0.83659015622167
"8018","BX088651.4",3.60729557447673,0.655125379932851,0.799859457684838,0.819050614002973,0.412757536868315,0.83659015622167
"8019","TRDMT1",76.8064189944795,0.214502154201104,0.261146355347962,0.821386742753091,0.411426012520856,0.83659015622167

"8020","PLEKHA1",279.688521763549,-0.168628146617628,0.205666911082249
,-0.819908976754125,0.412267998977192,0.836590156221673
"8021","OTUB1",270.636146577491,-0.148604018784983,0.181128053553033,-
0.820436237622754,0.411967464486904,0.836590156221673
"8022","COA4",433.177730410175,-0.167896959086335,0.20504199962621 8,-0
.818841795302443,0.412876681556021,0.836590156221673
"8023","AP002784.1",9.56519221787908,0.657007818169894,0.8000122520663
61,0.821247195243448,0.411505478816651,0.836590156221673
"8024","DDN-
AS1",4.96673861480675,0.694995570399147,0.846443973461408,0.8210768724
0901,0.411602482676149,0.836590156221673
"8025","RNASE4",2883.05204169686,-0.17023173692932,0.207587769180409,-
0.820047046131011,0.412189287973224,0.836590156221673
"8026","PSME1",1540.75516509036,-0.180860519402276,0.220400056582363,-
0.820601057035974,0.41187354540029,0.836590156221673
"8027","NGRN",617.204110064814,-0.14522331393291,0.177274464813893,-0.
819200408165768,0.412672082079588,0.836590156221673
"8028","DDX19A",133.450717455101,0.163408485823905,0.199102383601311,0
.820725914317126,0.411802406400761,0.836590156221673
"8029","TXNDC17",382.802023095798,-0.193306678515625,0.235691338401523
,-0.820168784422227,0.41211989443615,0.836590156221673
"8030","LINC02076",4.26074527698916,-0.745829568613501,0.9095097957535
14,-0.820034673728383,0.412196340906086,0.836590156221673
"8031","TK1",3.25500400933596,-0.786570304946463,0.960496186541881,-0.
8189207994447,0.412831602144166,0.836590156221673
"8032","AC061992.2",2.77246729771526,-0.859454313674923,1.045953213271
29,-0.821694797405826,0.411250620911992,0.836590156221673
"8033","C19orf25",164.809762584387,-0.207045680753209,0.25266973099720
2,-0.819432070220955,0.412539943826316,0.836590156221673
"8034","HDGFL2",292.983950555232,-0.156464296717384,0.190511809900541,
-0.821283976038379,0.411484532854334,0.836590156221673
"8035","NOSIP",351.201102149912,-0.198884227318464,0.242623591638975,-
0.819723366449889,0.412373826296922,0.836590156221673
"8036","ZNF649",30.269424836444,0.24616783239249,0.299915276437494,0.8
20791242502093,0.411765187754056,0.836590156221673
"8037","UPRT",47.256224366201,-0.212429096761772,0.259328114800283,-0.
819151818249133,0.412699800575917,0.836590156221673
"8038","AL359851.1",5.81378183361624,0.671261069679037,0.8177670429554
35,0.820846322264443,0.411733809369379,0.836590156221673
"8039","DNASE1L1",24.6440008569321,-0.341020936693902,0.41543768624348
6,-0.820871451931857,0.411719493722765,0.836590156221673
"8040","AC046143.1",5.04333254714987,0.625083995670843,0.7636308145772
91,0.818568323512272,0.41303274586072,0.836802288224536
"8041","SGPP2",2.34533835093802,0.844525471677505,1.03195258497461,0.8
18376235472371,0.413142387148706,0.836816257680337
"8042","MED30",290.680425638875,-0.197876519223995,0.241776940122949,-
0.818425938897937,0.413114015444062,0.836816257680337
"8043","LINC00471",2.43757980632368,0.860832295763075,1.05329421313162
,0.817276203581976,0.413770602621786,0.836859852696794
"8044","RAB28",188.728525507996,-0.169379922830112,0.207193299806659,-

0.817497105302962,0.413644402858243,0.836859852696794
"8045","SYNGAP1",104.450781521856,-0.221631621332119,0.271052817322266
,-0.817669498961939,0.413545931276566,0.836859852696794
"8046","PLA2G7",22.7791224561673,0.549657209652181,0.672046782519898,0
.817885337671275,0.413422663367835,0.836859852696794
"8047","MIR210HG",4.55769418539057,-0.622865150851937,0.76132436222679
6,-0.818133743980188,0.413280822668813,0.836859852696794
"8048","AQP11",6.60499953752244,0.547911371614627,0.670285108265091,0.
81743032160269,0.413682553562919,0.836859852696794
"8049","TMEM106C",317.362384730734,0.297086190725563,0.36327447336975,
0.817800898504588,0.413470884932398,0.836859852696794
"8050","AL442128.2",7.14199830908352,-0.571872182611998,0.699694579354
649,-0.817316868653542,0.413747369210952,0.836859852696794
"8051","SRSF5",2587.84388858775,0.143132766836589,0.175020822601207,0.
817804217288611,0.41346898957657,0.836859852696794
"8052","GNA13",232.134901842132,0.142382570790676,0.174234364269059,0.
817189946357561,0.413819887017381,0.836859852696794
"8053","AP005329.3",32.0636419589733,-0.310482432478912,0.379585351597
938,-0.81795156523263,0.413384844426839,0.836859852696794
"8054","FCGRT",4148.08264841631,0.215725834700386,0.264020716431679,0.
817079195966085,0.413883171055643,0.836859852696794
"8055","FP565260.1",27.3188542566408,-0.376680169078257,0.460853598812
639,-0.81735321162458,0.413726605824275,0.836859852696794
"8056","PRPS1",120.749721627019,-0.177784238078,0.217571515057243,-0.8
17130119405682,0.413854072109545,0.836859852696794
"8057","TIMM13",815.902705408026,-0.204082058136856,0.249911062412982,
-0.816618744950182,0.414146339421743,0.837288038083749
"8058","CPSF4",119.866144852652,0.147688311540033,0.180908048719136,0.
816372254223592,0.414287260579415,0.837296726432858
"8059","PRR12",131.395050609819,0.176759115427524,0.216525945679666,0.
816341500657966,0.414304844683457,0.837296726432858
"8060","MCM3AP",125.491943748752,0.248983822445598,0.304987649255218,0
.816373459888026,0.414286571220251,0.837296726432858
"8061","GATAD2B",226.239543428387,0.165793980384895,0.203173108774707,
0.816023249261493,0.414486838631998,0.837401876165752
"8062","MEIS1-
AS2",15.7145127356302,-0.329877955158688,0.404270311385305,-0.81598362
7460305,0.414509499902984,0.837401876165752
"8063","MAP2",3.81512749082193,-0.765484348459042,0.9383373464389,-0.8
15788001366614,0.414621396921164,0.837401876165752
"8064","TAF9",347.334002305127,-0.203692233965858,0.249735291813533,-0
.815632554320541,0.4147103244853,0.837401876165752
"8065","PDSS1",23.0786909105528,-0.276569878486568,0.339098681746718,-
0.815602930279586,0.414727272974932,0.837401876165752
"8066","PSMC1",809.830695024253,-0.191491563801318,0.234774664382585,-
0.815639814904671,0.414706170626478,0.837401876165752
"8067","PCLAF",3.84477114571845,-0.655791811239436,0.804003946972462,-
0.815657452564592,0.41469608003266,0.837401876165752
"8068","MGME1",70.8917867560361,0.167976680513786,0.205972034618304,0.
815531491083592,0.414768146412014,0.837401876165752

"8069","PLXND1",304.371446472899,0.194778488285928,0.238882690134241,0
.815372968951711,0.414858852247572,0.83748120513827
"8070","ZBTB17",72.5109356713597,-0.185182939413958,0.227196236985737,
-0.81507925426416,0.415026945800195,0.837580243200999
"8071","AKR7A3",4.20581019726652,0.572837908980957,0.702895921997766,0
.814968320420527,0.415090443950762,0.837580243200999
"8072","NBPF10",42.9793612671132,0.417128333314267,0.511931903048425,0
.814812147534415,0.415179846503707,0.837580243200999
"8073","MEMO1",30.7024592365794,0.229854174010275,0.282006102216886,0.
815068086127791,0.415033338143145,0.837580243200999
"8074","TRBC1",24.0525468751839,0.397396047198257,0.487753180620493,0.
814748243553658,0.415216432184177,0.837580243200999
"8075","DCAF13",207.094901150779,-0.197720111752753,0.242643777894704,
-0.81485753918056,0.415153860480647,0.837580243200999
"8076","AL109659.2",2.64984466421788,0.792222391817647,0.9741848287454
67,0.813215694231097,0.416094402815155,0.83765080596917
"8077","LRIF1",80.4282996911351,-0.178721685339873,0.219684364220019,-
0.813538487249272,0.415909389312965,0.83765080596917
"8078","SLIT2",426.033846822729,0.243796934622467,0.299542653043127,0.
813897226807852,0.415703829524765,0.83765080596917
"8079","MTMR9",127.74045796501,0.168539061458784,0.20724027638642,0.81
325437505462,0.416072229778234,0.83765080596917
"8080","ENY2",1181.28402748344,-0.187338033188401,0.230304890663602,-0
.813434889066418,0.4159687628214,0.83765080596917
"8081","PALM2-
AKAP2",207.961527877307,-0.272909934862976,0.335506612822216,-0.813426
395883262,0.415973630600531,0.83765080596917
"8082","GPAM",68.7899128937334,0.215989344650107,0.265300771780151,0.8
14130102980224,0.415570422287212,0.83765080596917
"8083","AP003119.3",7.47926121485099,0.506568968714287,0.6226000813045
79,0.813634600967022,0.415854309726541,0.83765080596917
"8084","SP1",299.93856824054,0.160506816480112,0.197094069400785,0.814
366545721503,0.415434997751985,0.83765080596917
"8085","CLYBL",41.6145497825829,0.230596494556249,0.283165758247569,0.
814351622114708,0.415443544600297,0.83765080596917
"8086","NEIL1",54.3125804849492,-0.305106018359748,0.374710220967439,-
0.814245252163165,0.415504466383666,0.83765080596917
"8087","AC015871.7",9.88354122444267,-0.479751952097561,0.589424616691
37,-0.813932670119146,0.415683523564931,0.83765080596917
"8088","HNRNPM",862.535865893766,-0.151646542167275,0.186180982907706,
-0.81451144901544,0.415352016077972,0.83765080596917
"8089","ZNF816",20.6165619322731,0.275532618482099,0.338682032341726,0
.813543655023569,0.415906427723213,0.83765080596917
"8090","OSBPL2",113.627685132521,0.188067604688283,0.231193835805473,0
.813462885085414,0.415952717433938,0.83765080596917
"8091","RHBDD3",80.8128976114597,0.210509001932636,0.258544926447749,0
.814206663518414,0.415526568751573,0.83765080596917
"8092","CERK",148.203510203549,0.228589683051299,0.281112373929514,0.8
13161227504754,0.416125625999295,0.83765080596917
"8093","CFAP300",44.3940097738456,-0.271340541156165,0.333819713038696

,-0.812835583273991,0.416312331200922,0.837819565472179
"8094","LETMD1",483.663702264052,-0.181125534110487,0.222815769492525,
-0.81289369474616,0.41627900988578,0.837819565472179
"8095","NSL1",291.137450142623,0.141827450995731,0.17452582432452,0.81
2644498570089,0.416421910914846,0.837833066562738
"8096","ABCC5",114.56432714172,0.183215820907503,0.225443885389323,0.8
12689244559037,0.416396249288609,0.837833066562738
"8097","PDLIM7",272.737097484454,-0.244994761045513,0.301562464146354,
-0.812417957052548,0.416551845791623,0.837887505075296
"8098","STOML2",618.133244315735,-0.193851787993236,0.238603987300968,
-0.812441527847216,0.41653832544057,0.837887505075296
"8099","EMC1",165.595814451735,0.150625920949983,0.185872946925636,0.8
10370327911383,0.417727366220592,0.838029094522996
"8100","F3",1803.68886414524,-0.254468537510908,0.313538868346322,-0.8
11601250119435,0.417020473736557,0.838029094522996
"8101","GLUL",2634.59556004791,0.214138671714883,0.264243041398858,0.8
10385282356987,0.417718773965951,0.838029094522996
"8102","TOMM20",1682.92398858626,-0.153692903705391,0.189284786968817,
-0.811966487992034,0.416810861157025,0.838029094522996
"8103","FAM228B",144.129078532544,-0.174009428422464,0.214472384917507
,-0.811337219425211,0.417172041506193,0.838029094522996
"8104","AC015982.2",10.6733125016564,-0.387916736074812,0.478055904840
406,-0.811446385552571,0.41710937036054,0.838029094522996
"8105","KCMF1",313.06225942575,-0.134104229523267,0.165445314784082,-0
.810565289795497,0.417615356720988,0.838029094522996
"8106","TCF7L1",685.81514230759,0.162197841773504,0.200068696033535,0.
810710745804615,0.417531800864585,0.838029094522996
"8107","ALDH7A1",290.325404864755,0.126116342382031,0.155402020027614,
0.811548925552063,0.417050508262537,0.838029094522996
"8108","IK",628.666135736616,-0.165927521066725,0.204763410529784,-0.8
10337748513862,0.417746085461574,0.838029094522996
"8109","SRI",1849.7319431083,-0.201218347864524,0.248055853061746,-0.8
11181616482304,0.417261381142967,0.838029094522996
"8110","TSC22D4",268.876725376666,0.136700041268718,0.168667918312473,
0.810468538631446,0.417670939986529,0.838029094522996
"8111","AF117829.1",164.59450137197,0.247066738694557,0.30434874621234
9,0.811788258599147,0.416913140492875,0.838029094522996
"8112","DAPK1",19.6131519838837,0.500528675935795,0.61693123866664,0.8
11320037898513,0.417181905748723,0.838029094522996
"8113","TRIM29",8.30787095436851,-0.491285513635567,0.604943164800961,
-0.812118463719167,0.416723659531341,0.838029094522996
"8114","SENCR",7.58951258435828,-0.473554412343586,0.583955180897374,-
0.810943078912095,0.417398359701376,0.838029094522996
"8115","C12orf73",70.3849544405355,0.203136995389188,0.250348570412732
,0.811416638226815,0.417126447442622,0.838029094522996
"8116","CENPN",13.7405687560814,0.332876593212259,0.410490324198098,0.
810924335092528,0.417409124333789,0.838029094522996
"8117","AC005726.3",3.48967678526643,0.630113500028628,0.7759441855677
9,0.812060341128206,0.416757008220491,0.838029094522996
"8118","STAT3",1399.01968544205,-0.114179829764832,0.14090641966307,-0

.810323830793901,0.417754082357832,0.838029094522996
"8119","AC011472.3",3.8809928431191,-0.811942828219154,1.0015451686329
2,-0.810690175189433,0.417543616865693,0.838029094522996
"8120","GPRASP1",73.3419491806733,0.201956946806852,0.248991742042741,
0.811098975210929,0.417308834324198,0.838029094522996
"8121","AL137796.1",7.70980080415354,-0.477849349375302,0.590299982739
9,-0.809502563692017,0.41822612878243,0.83804851507992
"8122","HADHA",1034.27177993073,-0.135072636472653,0.16679757253458,-0
.809799773582735,0.418055262764141,0.83804851507992
"8123","INPP4A",59.9857557592846,0.213605692421902,0.263769290874643,0
.809820171687156,0.418043537398162,0.83804851507992
"8124","SOX4",1466.34135536161,0.249970036580202,0.308713824238656,0.8
09714424667161,0.418104325662306,0.83804851507992
"8125","KRT8",3.9279335398292,-0.650069973616011,0.803049852040246,-0.
809501392677465,0.418226802080218,0.83804851507992
"8126","MT1A",1403.85243399472,-0.635199958226749,0.784641074787439,-0
.809542067879669,0.418203415448717,0.83804851507992
"8127","VPS4B",608.771287893165,0.159213915890202,0.196636523237564,0.
809686386174805,0.418120444353901,0.83804851507992
"8128","OSM",5.77880461064996,-0.796265220606186,0.983086708901878,-0.
809964384012093,0.417960645892789,0.83804851507992
"8129","SYTL4",106.822031095166,0.23805368932976,0.293821370711942,0.8
10198688927717,0.417825990896537,0.83804851507992
"8130","NHP2",622.369912175295,-0.201341373214128,0.248815826666285,-0
.809198417607777,0.418401024983973,0.838107980131225
"8131","GOLGA1",94.6057480367655,0.201392736276031,0.248884547129677,0
.809181359785665,0.418410835191057,0.838107980131225
"8132","AC092338.3",40.8502032605796,-0.271312208751891,0.335261356777
582,-0.809255833596964,0.41836800519001,0.838107980131225
"8133","R3HCC1L",79.8909024229682,-0.194039024646745,0.239860810192614
,-0.808965101430816,0.418535220326188,0.838254051874249
"8134","SLC35D2",156.615101067711,-0.193619401872782,0.239397283133593
,-0.808778609925722,0.418642501963011,0.838365836547269
"8135","PHF20",397.584952540632,-0.102335879314703,0.126549205856567,-
0.808664729438859,0.418708021135875,0.838393971270101
"8136","SLC7A2",63.4222734343573,0.316194396360239,0.391198881757001,0
.808270194792243,0.418935056498312,0.838745468940635
"8137","PCDHB6",7.78605558870454,0.441213147111766,0.5460644784294,0.8
07987269893092,0.419097910501029,0.8389683991829
"8138","KIF3C",40.9939135863109,-0.299798531214357,0.37131293708875,-0
.807401254491439,0.419435344422769,0.839144785112097
"8139","TASOR",333.972122658706,0.144514143978221,0.178949716591615,0.
807568442860514,0.419339059292941,0.839144785112097
"8140","COPS4",325.347614846225,-0.187221437848498,0.231792698379634,-
0.807710679228832,0.41925715445535,0.839144785112097
"8141","RPARP-
AS1",21.3081251150185,-0.298823267547013,0.370097766591322,-0.80741710
5753537,0.419426214995335,0.839144785112097
"8142","COG8",79.5054321809909,0.189603014547245,0.234835381522344,0.8
07386916392769,0.419443602454582,0.839144785112097

"8143","COG2",105.478137123926,-0.17000607881549,0.210870185576976,-0.806212022578371,0.420120607677085,0.839164644816734
"8144","CCDC71",55.506674186933,0.205312269817384,0.254455117268629,0.806870272530755,0.419741227406042,0.839164644816734
"8145","TRMT10A",83.7867009941399,0.210297790115186,0.260762988529348,0.806471007642704,0.419971318423953,0.839164644816734
"8146","ZDHHC11B",9.68684441376674,-0.630347002355013,0.781987240538685,-0.806083487910606,0.420194711728182,0.839164644816734
"8147","HOXA2",2.64418924201235,0.947733892875632,1.17463184762297,0.806834834925938,0.419761646627077,0.839164644816734
"8148","AP3M1",95.417869410544,0.153405394372755,0.190263495117214,0.806278652025435,0.42008219684508,0.839164644816734
"8149","CHID1",644.459178083097,0.227982217387168,0.282508826836991,0.806991483911103,0.419671389572376,0.839164644816734
"8150","IGHMBP2",37.491491049945,0.223438764485695,0.277209432306212,0.806028722135541,0.420226288155817,0.839164644816734
"8151","SYTL2",75.1405627921805,-0.250052947173487,0.309966929552368,-0.806708468979563,0.419834463698972,0.839164644816734
"8152","SRSF9",1150.74219407201,-0.196579670215881,0.243650675992088,-0.806809459548818,0.419776268330458,0.839164644816734
"8153","MORN3",3.47723738015201,0.637806868637354,0.790892071754971,0.806439830939354,0.419989288260486,0.839164644816734
"8154","KRBA2",37.3240329999808,0.238401869331194,0.295721498547783,0.806170232810019,0.420144699876526,0.839164644816734
"8155","TMEM11",272.416840162865,-0.183970640103707,0.228157250051179,-0.806332650233292,0.420051069238278,0.839164644816734
"8156","TSEN54",37.2174896057347,-0.225806564811495,0.279856784782715,-0.806864714703325,0.419744429799075,0.839164644816734
"8157","GTPBP1",86.0199410906774,0.163766332429456,0.20295399133055,0.806913583496518,0.419716272331014,0.839164644816734
"8158","NTRK1",3.50178876506707,0.833116429736372,1.03403719556843,0.8056929028345,0.420419942732978,0.839289675267154
"8159","ZNF554",24.7903188789755,-0.243700801776172,0.302488879391098,-0.805652102869814,0.420443474134691,0.839289675267154
"8160","SCAF4",109.439621266891,0.192513485349737,0.238934792493043,0.805715581816501,0.420406862952317,0.839289675267154
"8161","PRDX2",1550.75731676029,-0.208483224523591,0.258820821131899,-0.805511796198748,0.420524401986789,0.839348362205957
"8162","AC012467.2",9.40666576711991,0.37670826816868,0.467928784914429,0.805054701299407,0.420788114386813,0.83936420156151
"8163","AL954642.1",16.4006463751841,1.62245895412785,2.01531125355256,0.805066190777135,0.420781484557078,0.83936420156151
"8164","TCP11L1",46.7084683112564,0.237781565832259,0.29534407023085,0.805100185849005,0.420761868573033,0.83936420156151
"8165","AC022211.2",3.40279910670765,-0.645706854672384,0.801774717835756,-0.805346988759325,0.420619473383382,0.83936420156151
"8166","ENDOV",128.916776829934,0.155693507526919,0.193395721190268,0.805051459094812,0.42078998526314,0.83936420156151
"8167","NIPAL3",46.351916738601,-0.245590812337209,0.305407278598782,-0.804141975476118,0.42131498495882,0.839598166555332

"8168","LMX1A",10.9176178161549,0.43047441319928,0.535441216129038,0.8
03962041456927,0.421418897443805,0.839598166555332
"8169","BTG2",2643.48740629446,0.178093399534238,0.221347220921775,0.8
04588369316714,0.421057255972509,0.839598166555332
"8170","RRAGD",48.2876890788843,-0.339434347704859,0.422163084857707,-
0.804036070134526,0.421376143819978,0.839598166555332
"8171","COPG2",47.6699952065354,-0.19745042338465,0.24538302219866,-0.
804662122160987,0.421014683097435,0.839598166555332
"8172","AC144652.1",19.7423073189311,-0.275075279759666,0.341954410605
075,-0.80442091468547,0.421153926371496,0.839598166555332
"8173","ENGASE",48.975214588313,0.254826876302102,0.316959113941686,0.
803973967282684,0.421412009775216,0.839598166555332
"8174","ZNF708",150.423553691586,0.16766168984326,0.208490977107847,0
.804189089188241,0.421287779119472,0.839598166555332
"8175","BLOC1S3",30.9475254772463,0.277798699747495,0.345539925535664,
0.803955430958798,0.421422715314408,0.839598166555332
"8176","DDRGK1",420.259609967761,-0.14314917008636,0.178023247589032,-
0.804103801189053,0.421337029478117,0.839598166555332
"8177","LGALS8-
AS1",4.87435009230697,-0.654376407131141,0.814554864155049,-0.80335461
2350067,0.421769800769651,0.840103470498883
"8178","RUNDC3B",2.39891176682337,0.843469251768341,1.04995583826238,0
.803337836726771,0.42177949424396,0.840103470498883
"8179","AC062004.1",235.266052114423,0.37575189150156,0.46786602468904
5,0.803118567438817,0.421906206836895,0.840132168321468
"8180","GCN1",101.845498535462,0.173375041166573,0.215881082024989,0.8
03104373668572,0.421914409983258,0.840132168321468
"8181","DPM1",298.48302782552,0.159800469540583,0.19901524374418,0.802
955927064537,0.422000208803871,0.840132168321468
"8182","ITSN1",334.808401043432,0.179031593732249,0.222964097342453,0.
802961534462978,0.421996967666976,0.840132168321468
"8183","TLR6",8.207563715987,-0.413103823698212,0.514699658416002,-0.8
02611419967806,0.42219936561752,0.840323248600169
"8184","LINC01504",62.2279149017531,0.234833340202303,0.29258325474513
,0.802620575148318,0.422194072368055,0.840323248600169
"8185","C5",25.0497853572921,0.262264112539695,0.326893085267949,0.802
293240080993,0.42238335180367,0.84052264805008
"8186","COMMD7",320.151119365179,-0.184776750326037,0.230320369821612,
-0.802259697955291,0.422402750134321,0.84052264805008
"8187","LAMB3",15.4495943933314,-0.494852969828002,0.617131717722352,-
0.801859563553718,0.422634199080406,0.840629007324877
"8188","ZNF704",536.43808975463,0.188684659227162,0.235323281144321,0.
801810421432311,0.42266262938l223,0.840629007324877
"8189","LMF1",122.032622614978,-0.206508101962138,0.257489252676049,-0
.802006685001136,0.4225490912796,0.840629007324877
"8190","TRIM37",100.013753599171,0.16668141980384,0.207837318603876,0.
801980226282289,0.42256439655605,0.840629007324877
"8191","DHRS9",7.48922141658905,-0.680864065641282,0.84937518133853,-0
.801605792823271,0.422781025669753,0.840659195206861
"8192","ZNF181",107.383916951517,0.155195453514669,0.193588167917896,0

.801678404128963,0.422739011192485,0.840659195206861
"8193","PPP1R35",168.797081885628,-0.229220786906661,0.286073708452659
,-0.801264779439155,0.422978376125772,0.840948952607433
"8194","TOP2A",6.76325750146116,0.608904698375643,0.760184678951813,0.
800995751736586,0.423134105255924,0.841155899501312
"8195","ANKRD23",11.0690219854262,-0.370801380842391,0.463436975471369
,-0.80011177456275,0.423646039768979,0.841191905874753
"8196","TMEM39A",141.022145547881,0.157724449833884,0.197130014271798,
0.800103679881125,0.423650729286561,0.841191905874753
"8197","FAM135A",65.287138603177,0.223423862227981,0.279192668889745,0
.800249745512527,0.4235661132918,0.841191905874753
"8198","GSDMD",358.874616380368,-0.123475504394616,0.154210280849064,-
0.800695671616535,0.423307848955941,0.841191905874753
"8199","ALAD",102.307153451361,0.180971005225452,0.226193180620496,0.8
00072772879404,0.42366863501728,0.841191905874753
"8200","QSOX2",33.92827015905,0.251039874335132,0.31358014928533,0.800
560478420806,0.423386138246935,0.841191905874753
"8201","SARNP",419.516532545494,-0.179283867996143,0.223866446302926,-
0.800851896105698,0.423217391166539,0.841191905874753
"8202","MORF4L1",2952.67594217243,-0.121420772217482,0.151650160535636
,-0.800663657648743,0.423326387221153,0.841191905874753
"8203","AC009152.6",6.88896971203371,-0.517798844466343,0.647069416777
066,-0.800221477079545,0.423582488457004,0.841191905874753
"8204","WAS",12.7745762424971,-0.488867601964646,0.6109358700745,-0.80
0194629110632,0.423598041127433,0.841191905874753
"8205","TLL1",3.52511637034199,-0.840287464154315,1.05055135763151,-0.
799853770165752,0.423795525217789,0.841301912791491
"8206","PCDHB10",31.8871730295698,0.306310318185926,0.382984184229983,
0.799798871072927,0.423827337243967,0.841301912791491
"8207","SDC1",3.85558908704489,0.76471957516638,0.956270194264378,0.79
9689857273706,0.42389051091367,0.841324787653561
"8208","NASP",686.588412998967,0.225805446228729,0.28267607154619,0.79
8813443931109,0.424398593937637,0.841651347574904
"8209","MORN2",233.801118786373,-0.198448746643335,0.248495085841104,-
0.798602298196871,0.424521054629249,0.841651347574904
"8210","AC009961.1",4.31577461189543,0.606772043223273,0.7598363307376
53,0.79855623991316,0.424547770341189,0.841651347574904
"8211","ADAM19",40.4285073280706,0.328019764948653,0.410568113638218,0
.798941159950128,0.424324530990167,0.841651347574904
"8212","SLC29A1",524.722224835746,-0.195756288207231,0.245178698139124
,-0.798422904163361,0.424625116137602,0.841651347574904
"8213","DGAT1",119.094089499464,-0.15572805701983,0.194959264903572,-0
.798772282491185,0.424422465204732,0.841651347574904
"8214","AL356481.1",16.0329758268599,-0.314998269765625,0.394290947315
28,-0.798898052086772,0.424349528539761,0.841651347574904
"8215","SFTA1P",16.0655330598533,-0.343464800680824,0.429872247414299,
-0.798992730390902,0.424294627260439,0.841651347574904
"8216","AC022306.2",15.4225024040941,0.342243808971731,0.4286960339864
88,0.798336774402067,0.424675082922103,0.841651347574904
"8217","FAM192A",191.579324339526,-0.151575567828333,0.189801768054052

,-0.798599345951124,0.424522767024568,0.841651347574904
"8218","RPAIN",314.079883689568,-0.172704871882691,0.216080611503258,-0.799261306607726,0.424138910084724,0.841651347574904
"8219","TMEM47",279.896711901617,0.156621088231963,0.196181269785472,0.798348835254411,0.42466808580729,0.841651347574904
"8220","PWWP2A",160.299123873392,0.141265305681824,0.176983112131463,0.798185227847568,0.424763008539678,0.841674946236539
"8221","PIK3R2",119.779898807653,0.176027962154841,0.220548241258072,0.798138135904984,0.424790332924709,0.841674946236539
"8222","FBLIM1",47.9756438110386,-0.205550353513756,0.25762963074812,-0.797852145022554,0.424956296851244,0.841761241220905
"8223","ZNF385A",185.530445559038,-0.318111318033126,0.398737698041942,-0.797795943536958,0.424988915697754,0.841761241220905
"8224","AXIN1",60.6671843565026,-0.20550662337201,0.257576217555749,-0.797847818879204,0.424958807654727,0.841761241220905
"8225","CYB561D2",160.324604794629,-0.19921009804712,0.249753079058557,-0.797628196609394,0.425086283232249,0.841851728579952
"8226","PHF3",844.458904230848,0.173877395713525,0.218108584935658,0.797205647658568,0.425331606566187,0.841922393548988
"8227","RELN",20.7051345176903,-0.55524936798248,0.696410281795325,-0.797302082546892,0.425275611150048,0.841922393548988
"8228","DCTN6",436.98515484679,-0.209241067235357,0.262597065076483,-0.796814188210422,0.425558953721932,0.841922393548988
"8229","CLTA",1886.87793722168,-0.205370028712736,0.257703475445748,-0.796923783652932,0.425495297036898,0.841922393548988
"8230","SSH3",96.962198351293,-0.189533724020741,0.237746906130511,-0.797207951537744,0.425330268756644,0.841922393548988
"8231","CINP",276.624256637626,0.169228903349924,0.212334779838868,0.796990975658085,0.42545627243035,0.841922393548988
"8232","AC105020.1",5.92751400495989,0.492881141198359,0.618299888586281,0.797155474708741,0.425360741451295,0.841922393548988
"8233","MCRIP1",828.739837785196,-0.221558492496177,0.278084699235626,-0.796730251988609,0.425607710435794,0.841922393548988
"8234","SLC19A1",11.7526542805503,0.354667555741701,0.445183804612082,0.796676680659454,0.425638830552883,0.841922393548988
"8235","ACOT9",343.010617423854,-0.135126457444228,0.169546902087391,-0.796985705905608,0.425459332987066,0.841922393548988
"8236","HILPDA",327.183293710924,0.295685552619333,0.371392407246412,0.796154005440266,0.425942527469484,0.84204742439184
"8237","RRP8",116.539125444352,-0.18478484679305,0.232157756314468,-0.79594517851366,0.426063900290843,0.84204742439184
"8238","TAF6L",40.4045089955105,-0.235597524574999,0.295957557539076,-0.796051726247581,0.426001970894367,0.84204742439184
"8239","RHBDF1",206.762781493976,0.186157080407101,0.233774317403716,0.796311085300349,0.425851243987338,0.84204742439184
"8240","DNAJA3",80.4034561699848,0.174286124519413,0.218832452643987,0.796436371359211,0.425778444953535,0.84204742439184
"8241","CABIN1",241.45119003095,-0.175037122467712,0.219854711872805,-0.79614906124449,0.425945400864702,0.84204742439184
"8242","EMID1",21.0365217580282,0.477033352079437,0.599281403293875,0.

796008935797912,0.4260268416202,0.84204742439184
"8243","AC130324.1",3.27365143444945,-0.602243704472279,0.757026999609
571,-0.795537946180097,0.426300646849101,0.842413106456994
"8244","CILP2",9.48577782202056,-0.431176353179173,0.542089532752541,-
0.79539693561285,0.426382641929872,0.842472932362408
"8245","FBXO2",7.7505631376985,-0.465559930325772,0.588825321083143,-0
.790658814517988,0.429143115804057,0.842751966175549
"8246","GPBP1L1",224.026285118067,0.125315471187976,0.158115893027373,
0.792554554691607,0.42803739506476,0.842751966175549
"8247","SRGAP2B",182.506723463204,0.201700101489721,0.254559977458286,
0.792348048988861,0.428157762273367,0.842751966175549
"8248","AL451085.2",31.0120781658938,-0.307697543423121,0.389626241053
926,-0.7897248978683,0.429688447509299,0.842751966175549
"8249","GPR161",102.779216679979,0.217591033702388,0.275504907717286,0
.789790045866161,0.429650393296829,0.842751966175549
"8250","RNF2",90.8315222125333,-0.181380182889751,0.229204424826198,-0
.79134677712827,0.428741658808016,0.842751966175549
"8251","NVL",69.0789486550126,0.194081766347002,0.245816352607345,0.78
9539688016685,0.429796642880239,0.842751966175549
"8252","IBA57",55.990183167684,0.193846013707038,0.244463548636224,0.7
92944448317291,0.427810189153086,0.842751966175549
"8253","RNF144A-
AS1",2.39442717583306,0.736852207674056,0.93406718075634,0.78886425179
5472,0.430191351373743,0.842751966175549
"8254","EML4",131.938631987468,0.175047402431707,0.221308874886559,0.7
90964223741297,0.428964868878792,0.842751966175549
"8255","INO80B",83.8509530715914,-0.172621250132338,0.217253084743865,
-0.794562941814395,0.42686778219309,0.842751966175549
"8256","RMND5A",155.329151147005,0.164215121377713,0.208185430943914,0
.788792571282059,0.430233252070133,0.842751966175549
"8257","LINC02158",4.83625088908597,-0.673411116364175,0.8528236389865
8,-0.789625293647344,0.429746632056737,0.842751966175549
"8258","AC007920.2",61.0886934899188,-0.321908318034824,0.405990823968
163,-0.792895550910448,0.427838679688737,0.842751966175549
"8259","TCTEX1D2",127.553813686952,0.249781543959527,0.315287756000104
,0.792233568243748,0.428224498833344,0.842751966175549
"8260","LIAS",134.632369218036,0.168433553105335,0.213252912683778,0.7
89830023822917,0.42962704236424,0.842751966175549
"8261","ANXA2R",67.8844967452911,-0.295000900768864,0.371362923058984,
-0.79437359642392,0.426977970583567,0.842751966175549
"8262","BDP1",427.488396165677,0.196050107200502,0.247979710284848,0.7
90589306581995,0.429183688948374,0.842751966175549
"8263","TXNDC5",618.538247031004,0.167041314320629,0.210422715268924,0
.793836892120454,0.427290392437648,0.842751966175549
"8264","HDAC2-
AS2",2.67346551555216,0.803320001339042,1.01261888801496,0.79330931987
0374,0.427597628175522,0.842751966175549
"8265","CITED2",1747.92451522482,0.284839205260515,0.360760270801593,0
.789552587449872,0.429789106812944,0.842751966175549
"8266","QKI",1141.57486097377,0.116407795442501,0.146511944684525,0.79

4527679590596,0.426888301572272,0.842751966175549
"8267","PLOD3",221.673083048903,0.129891965350354,0.164076838472195,0.791653267821624,0.428562878111827,0.842751966175549
"8268","AC099552.5",2.42499660355568,-0.895906963624611,1.1298421773826,-0.79294876891574,0.427807671768732,0.842751966175549
"8269","AC064807.4",3.15141453641929,0.674238509461644,0.850532231328411,0.792725407253032,0.427937824105549,0.842751966175549
"8270","ANKRD46",91.2205093558577,0.213702303248355,0.269405285836187,0.793237232094613,0.427639619031777,0.842751966175549
"8271","DMRT3",19.4608671218436,0.460516015813219,0.583523203011763,0.789199149984677,0.429995618722095,0.842751966175549
"8272","TOMM5",965.127590787472,-0.187891746918728,0.238203451228811,-0.788786837258059,0.430236603984386,0.842751966175549
"8273","ODF2",71.0504626099321,-0.172579233976679,0.218063078827775,-0.791418863314229,0.428699605935603,0.842751966175549
"8274","AL391121.1",65.3672545459658,-0.270821672220327,0.342041849470309,-0.791779347000157,0.428489346794464,0.842751966175549
"8275","SH3PXD2A",468.10871453752,0.238843724712928,0.300924809577992,0.793699014208487,0.42737067427922,0.842751966175549
"8276","SLC22A18",64.4726532562227,0.233006848033191,0.294836833306097,0.790290837886205,0.429357936316877,0.842751966175549
"8277","TRIM44",437.47219670884,0.146533157353511,0.185478240128997,0.790028831692597,0.429510930592617,0.842751966175549
"8278","SDHAF2",229.921320987325,-0.201692835185119,0.253943848195587,-0.794241863381452,0.427054641598213,0.842751966175549
"8279","CATSPERZ",10.0535320812335,-0.547305847666607,0.688460940124308,-0.794970078575243,0.426630907591785,0.842751966175549
"8280","WNT11",1788.70995880481,-0.281810709947417,0.355385727184752,-0.79297137839448,0.42779449856113,0.842751966175549
"8281","UBE2N",403.856781933225,-0.171082804711681,0.215913968092622,-0.792365617764437,0.428147521089929,0.842751966175549
"8282","ACTR6",177.296526624045,-0.200435933448614,0.252875786564022,-0.79262604052392 4,0.427995732281689,0.842751966175549
"8283","RFC5",57.8770743763853,-0.181916822672221,0.230077782060738,-0.790675314421262,0.429133484814651,0.842751966175549
"8284","DEGS2",4.50159553301015,0.686617160177264,0.868070606256067,0.790969254377361,0.428961933193586,0.842751966175549
"8285","AL049840.3",2.82415137765539,0.709229879219155,0.892298634833293,0.794834656842952,0.426709688257826,0.842751966175549
"8286","NIPA2",170.260606912386,-0.166519192388098,0.210573052592451,-0.790790608475356,0.429066191210588,0.842751966175549
"8287","AC090510.1",8.24288875849233,0.64613763752039,0.81642232565232,0.791425733004211,0.428695598494137,0.842751966175549
"8288","EID1",5558.32500292641,-0.155451631748691,0.196490652449148,-0.791140086365796,0.428862249166847,0.842751966175549
"8289","HDC",3.97611399776373,-0.679115014168076,0.859925073827944,-0.789737425779441,0.429681129560722,0.842751966175549
"8290","FBXO22",128.537017237249,-0.152271466973096,0.191773705198501,-0.794016399774323,0.42718588411255,0.842751966175549
"8291","VPS33B-

DT",2.49263493949811,-0.832866447690598,1.05123131711545,-0.7922770508
54952,0.42819914992856,0.842751966175549
"8292","AC022819.1",5.7953397072159,0.474004253886923,0.59932074734536
1,0.790902460804974,0.429000912298691,0.842751966175549
"8293","PMM2",116.597658704367,0.183669019415447,0.23184967541661,0.79
2190108031909,0.428249835552618,0.842751966175549
"8294","RABEP2",80.9653002603084,-0.159833097743216,0.202455987480756,
-0.78947083626464,0.429836868537804,0.842751966175549
"8295","CD19",3.57797235045218,0.624277301517139,0.786411324029395,0.7
93830508846798,0.427294109017138,0.842751966175549
"8296","ATMIN",269.72009819793,0.114948870712957,0.145045549083255,0.7
92501882611901,0.428068094482151,0.842751966175549
"8297","C17orf97",56.7308758394314,-0.494147190647749,0.62629839896472
7,-0.788996413633782,0.430114102654864,0.842751966175549
"8298","DVL2",135.659975366571,0.146999203259816,0.185415864742294,0.7
92808120621863,0.42788962452495,0.842751966175549
"8299","TRIM16",34.7498627247158,-0.226801788035105,0.28638876764848,-
0.791936743529991,0.428397561017189,0.842751966175549
"8300","ZNF207",509.550364281766,0.154253704535007,0.1941963042073,0.7
94318435485492,0.427010074271628,0.842751966175549
"8301","AP2B1",681.1532469891,0.172163751581917,0.217455115456294,0.79
172086258196,0.428523454894584,0.842751966175549
"8302","SLC9A3R1",44.539074282824,-0.294004926666608,0.372590501133734
,-0.789083258354675,0.430063346219124,0.842751966175549
"8303","SNRPD1",550.425178532698,-0.210613455093711,0.266605840183968,
-0.789980650642835,0.429539068584489,0.842751966175549
"8304","ELAC1",80.6491030845041,-0.222788608659452,0.28056715655985,-0
.794065176377576,0.427157489227726,0.842751966175549
"8305","AC090337.1",5.89373453920963,0.603912488498094,0.7641516716367
1,0.790304478696742,0.429349971853829,0.842751966175549
"8306","PIN1",522.090704380428,-0.209630485843167,0.265693609841579,-0
.788993329452522,0.430115905269924,0.842751966175549
"8307","ZNF799",29.7140942658413,-0.22562091619333,0.285839912539203,-
0.789326144795771,0.429921409597773,0.842751966175549
"8308","ZNF675",69.8509867280021,0.166897649632226,0.210121587311041,0
.794290828315367,0.427026142183781,0.842751966175549
"8309","AJ009632.2",2.41242241732337,1.06336712419366,1.34792583226168
,0.788891420241901,0.43017547073355,0.842751966175549
"8310","TCN2",142.876832730447,0.28800498698609,0.362973310657585,0.79
3460506681116,0.42750957023031,0.842751966175549
"8311","LIMK2",39.2479418301335,0.199952435849877,0.252807374191356,0.
790928019759932,0.428985996470684,0.842751966175549
"8312","ARHGAP8",6.96844995216847,0.525133178590711,0.662696278049543,
0.792419085461427,0.428116354599129,0.842751966175549
"8313","MAGIX",4.70136369917199,-0.525293828253631,0.662952699625526,-
0.792354912424895,0.428153761425087,0.842751966175549
"8314","SNX12",100.272754335367,0.160448118722967,0.202178889588938,0.
793594816200563,0.427431351232616,0.842751966175549
"8315","BEX4",366.128845028208,-0.180653851685845,0.228117898682636,-0
.791931947160255,0.42840035785093,0.842751966175549

"8316","RBM41",101.774249479661,0.225788928274951,0.286256019248276,0.788765696064263,0.430248962533972,0.842751966175549
"8317","TRADD",308.014004928376,-0.184726823409176,0.234259109019149,-0.788557696572115,0.430370564197425,0.842888796466498
"8318","SNX27",431.56471436317,0.129624275669321,0.164486433252228,0.788054510675367,0.430664821647512,0.843021690058534
"8319","KIAA0408",257.777026464269,0.260922791961415,0.331138126133551,0.787957566251923,0.43072152706479,0.843021690058534
"8320","APTX",151.963743976101,-0.152262787762493,0.193248788196191,-0.787910698865093,0.430748942621228,0.843021690058534
"8321","SCARB1",41.7096999874809,0.300751787717517,0.381662143510486,0.788005289052866,0.430693612165443,0.843021690058534
"8322","ALG5",413.104803401825,-0.196273432532142,0.248985074155885,-0.788293969819488,0.430524774078432,0.843021690058534
"8323","SPAG5",9.08403164663602,0.415314700227373,0.526896905239203,0.788227632574206,0.430563568738512,0.843021690058534
"8324","BOK",54.2177793556133,-0.273242008996552,0.346916179790123,-0.787631205791144,0.430912455968483,0.843240388667783
"8325","NPC1",63.5285790118713,0.177726650685627,0.225732217163457,0.78733400539335,0.431086368219971,0.843479381613827
"8326","UBE2J2",262.265522723371,-0.153662732590448,0.195372453896943,-0.786511760104642,0.431567732142743,0.84361055908714
"8327","SRP9",1119.795483407,-0.204988898859841,0.260610139805089,-0.786572997555479,0.431531871401637,0.84361055908714
"8328","POLE4",674.78277256917,-0.186957040876155,0.237701348957647,-0.786520739978915,0.431562473407114,0.84361055908714
"8329","EGFL8",64.4957228864173,-0.216356116504205,0.274971741297905,-0.786830368397038,0.431381173544612,0.84361055908714
"8330","CTDNEP1",743.770395884772,-0.118410011265682,0.150542944150058,-0.786553045937866,0.431543554908497,0.84361055908714
"8331","ALDH3A2",621.374017246213,0.134323276543713,0.170756982230772,0.786634167393401,0.431496051979183,0.84361055908714
"8332","DYM",221.258322179515,-0.134199523108121,0.17050055643799,-0.78709140844903,0.431228358431785,0.84361055908714
"8333","ZNF575",22.2317874792909,-0.281061526007847,0.357238562506127,-0.78676144041148,0.43142152982465,0.84361055908714
"8334","MARCKSL1",339.323710791252,-0.256016590867352,0.325766508325661,-0.785889845408597,0.431932024137254,0.843773473706248
"8335","CD247",19.5458691749818,0.465945023895589,0.59330407696245,0.785339325967717,0.432254644335452,0.843773473706248
"8336","NEB",6.54982410845698,0.683882236992087,0.870272377201166,0.785825512687742,0.431969717758455,0.843773473706248
"8337","STK17B",369.994619748763,0.275271684542114,0.350345686359102,0.785714496452978,0.432034768515766,0.843773473706248
"8338","EIF4H",884.705721106735,-0.0949197001543273,0.120778239056894,-0.7859006795886,0.43192567639679,0.843773473706248
"8339","MRPS28",211.787802312276,-0.213915215803864,0.272354905881444,-0.785428171787664,0.432202568692275,0.843773473706248
"8340","AC109322.1",3.44902683992719,-0.744204591740257,0.947467073306219,-0.78546749824596,0.432179519239509,0.843773473706248

"8341","NACA",13855.7203120311,-0.213364586698764,0.271535429676138,-0
.78577070753988,0.432001830517879,0.843773473706248
"8342","AC009093.1",39.6381237307535,-0.324972262681356,0.413711025231
599,-0.785505444287916,0.432157279531256,0.843773473706248
"8343","POLI",125.012526907043,0.166904404422694,0.212533534645712,0.7
85308561780234,0.432272677148913,0.843773473706248
"8344","HAUS8",40.9701885676161,-0.278419454848631,0.354316132068349,-
0.785793898864653,0.431988241526469,0.843773473706248
"8345","ZNF611",57.0730519705333,0.189729516619248,0.241371267523984,0
.786048474474682,0.431839088847136,0.843773473706248
"8346","RCC1",41.7217647370588,-0.249833625142836,0.318479652307214,-0
.784457102150563,0.432771943735094,0.844325884105227
"8347","ZYG11B",345.526831557899,0.18148738424053,0.231607969679633,0.
783597319606788,0.433276429086238,0.844325884105227
"8348","TOR3A",173.359906275854,-0.185791952551064,0.236964906343569,-
0.784048386817624,0.433011718827091,0.844325884105227
"8349","AL353708.1",2.73007724340328,-0.733124195425323,0.936182024565
958,-0.783100055531638,0.433568358571283,0.844325884105227
"8350","TLK1",443.155362110523,0.12585269889564,0.160607953005252,0.78
3601910993315,0.433273734144669,0.844325884105227
"8351","CNPPD1",215.983901936755,0.141445089781052,0.180475118055572,0
.783737344543517,0.433194244979918,0.844325884105227
"8352","AC006059.1",12.0588947027167,-0.384577617169408,0.490175126641
478,-0.784571872923072,0.432704626653767,0.844325884105227
"8353","CCNL1",4597.6561129905,0.241875353040507,0.308791955848299,0.7
83295511620561,0.433453598339854,0.844325884105227
"8354","CLCN3",264.575785059128,0.112972468177507,0.144273211268392,0.
78304535668333,0.433600477639213,0.844325884105227
"8355","COX7C",3745.66951331972,-0.171623818691014,0.218800109908322,-
0.784386345888606,0.432813447783215,0.844325884105227
"8356","FBXL4",149.967126195975,0.179997278326053,0.229730148572516,0.
78351613597305,0.433324081903209,0.844325884105227
"8357","STEAP1",362.089913626407,-0.206344594526152,0.263540565128158,
-0.782970904026894,0.433644198319377,0.844325884105227
"8358","PIK3CG",4.0305790442192,-0.814000641768902,1.0392803207277,-0.
783234922796327,0.433489170627739,0.844325884105227
"8359","ZC3HC1",55.8528988659241,0.215452183641537,0.27503308057072,0.
783368252264248,0.43341089383062,0.844325884105227
"8360","AL353150.1",8.0574597598871,0.531088114855678,0.67819317692995
1,0.783092683503262,0.433572687333219,0.844325884105227
"8361","IFIT3",691.030480799296,0.300419232167714,0.383634920349929,0.
783086252663572,0.433576463461457,0.844325884105227
"8362","NPM3",114.112955022744,-0.220839680667815,0.281863563533508,-0
.783498505089899,0.433334431203115,0.844325884105227
"8363","COX8A",1311.02332794151,-0.188832497794983,0.241150530870029,-
0.783048235945025,0.433598786907485,0.844325884105227
"8364","IMP3",560.702877844562,-0.181554189660577,0.231621559083397,-0
.783839770265977,0.433134134495685,0.844325884105227
"8365","CHTF8",11.9088855539149,-0.384715937692597,0.491058791647658,-
0.783441706443648,0.433367772888777,0.844325884105227

"8366","DCXR",147.301839189804,0.147294714402186,0.187853132182077,0.784095067733131,0.432984329324299,0.844325884105227
"8367","UMAD1",87.3399823313639,0.167885295373824,0.214480284669958,0.782753974950034,0.433771599665906,0.844372082571456
"8368","GABARAPL2",1849.95328639908,-0.200215148003484,0.255770946969058,-0.782790814891518,0.433749962229981,0.844372082571456
"8369","GPATCH11",178.282310183556,0.16429679517015,0.209953772394475,0.782537952504414,0.433898490051921,0.844518162797914
"8370","EWSR1",451.464122910857,0.166819830986351,0.213214411624777,0.782404105403185,0.433977121860302,0.84457029127628
"8371","ZBED5",209.739938276074,0.144252786851371,0.184414901920574,0.782218710901679,0.434086050073677,0.844681360608066
"8372","CENPS",59.4396903694204,-0.230151258636591,0.294422279237417,-0.781704629258036,0.434388180472678,0.844749829969157
"8373","DHDDS",93.2662796153919,0.152074335727326,0.194488808119549,0.781918184381329,0.434262657461625,0.844749829969157
"8374","HECTD3",119.213585342002,0.156082038375777,0.199687166206739,0.781632797644007,0.434430406227741,0.844749829969157
"8375","UQCR11",1592.42629438485,-0.174543707111978,0.223307496968491,-0.781629410035465,0.434432397670307,0.844749829969157
"8376","PREX1",14.2693621322227,-0.444154584158612,0.568177075812956,-0.781718592787485,0.434379972376408,0.844749829969157
"8377","EFHC2",7.35611454769778,-0.575036247866336,0.735404784281966,-0.781931611211626,0.434254766165935,0.844749829969157
"8378","GLMN",53.6089270213382,-0.213882407341207,0.27377008207043,-0.781248285874363,0.434656479324403,0.844921674067984
"8379","E2F3",52.5091435002616,0.243545658557773,0.311697576555931,0.781352429007645,0.434595241823764,0.844921674067984
"8380","RPS6KL1",2.33812028297024,-0.801259609956254,1.02566254510498,-0.781211728731155,0.434677976575885,0.844921674067984
"8381","CSTB",2162.74188412834,-0.182926912624462,0.234183548419619,-0.781126231363982,0.434728255286621,0.844921674067984
"8382","FAM151A",2.34373997220276,1.17525271106764,1.5051031445906,0.780845296411439,0.434893489248659,0.844956150957682
"8383","MRPL53",33.2309256737216,-0.325866319600274,0.417332453073087,-0.780831486266431,0.434901612722034,0.844956150957682
"8384","TTC5",161.655325628447,-0.163231457304272,0.209009868352132,-0.780974882148939,0.434817267938001,0.844956150957682
"8385","ARNTL",99.4780600903068,0.86535496231169,1.10859504151753,0.78058707634767,0.435045395254377,0.845134698067805
"8386","CDC40",441.00913967623,0.112121092101133,0.143704498332489,0.780219780188915,0.435261521430195,0.845453723178685
"8387","NDUFB8",1352.17707034798,-0.204250451173519,0.261917696045995,-0.779826847353038,0.435492801443679,0.84578110630965
"8388","PLA2G4B",19.0372608113966,0.342817270797567,0.439646281269127,0.779757012405419,0.43553391366722,0.84578110630965
"8389","URM1",287.600503103892,-0.235520597446416,0.30209473630764,-0.779624962437518,0.435611658339485,0.845831243615672
"8390","PIGQ",69.2111706727796,-0.183878433030211,0.235892592652012,-0.779500665802883,0.435684845534936,0.84587252072927

"8391","CNTN3",309.365008598006,0.364939792098654,0.468353405060174,0.779197478134629,0.435863395439372,0.845950601456598
"8392","LY6G5B",27.8023374754668,-0.30934487834939,0.397087578430655,-0.779034387255233,0.435959458553701,0.845950601456598
"8393","ARHGAP27",23.9617525537269,0.37639532048448,0.483102111867473,0.77912166235310,0.435908050618739,0.845950601456598
"8394","CBARP",2.62595989969655,0.6812957889388,0.874253835943733,0.77928830384068,0.435809902950054,0.845950601456598
"8395","ARHGAP6",336.167893414509,-0.18534253182446,0.237926262243499,-0.778991482809816,0.435984731980364,0.845950601456598
"8396","KIF15",4.36156870737569,-0.572958778223726,0.7356059074094,-0.778893660929843,0.436042358401089,0.845961645544943
"8397","EEF1AKNMT",117.435586077444,0.144505532769552,0.185582976316277,0.778657265003018,0.436181636283421,0.846131079364136
"8398","CASP7",198.936834259927,0.162540421266831,0.208805909652571,0.778428261619987,0.436316583134587,0.846210586837788
"8399","HIPK3",493.116033683768,-0.142247567983347,0.182746666441557,-0.778386663643129,0.436341098524774,0.846210586837788
"8400","ALKBH3",139.185618110217,-0.181585998327695,0.233304090790622,-0.778323250622548,0.436378471940415,0.846210586837788
"8401","ZNF576",131.572948697862,-0.161334335018311,0.207327655512289,-0.778161189445119,0.436473993509168,0.846295069666805
"8402","PEX13",116.798334726111,-0.173947578785818,0.223668081816588,-0.777704075490116,0.436743489029453,0.846449062900718
"8403","GASK1A",52.5628229790985,0.296531104496525,0.381248611901289,0.777789335462029,0.43669321599896,0.846449062900718
"8404","OSTN-AS1",6.03628382975506,-0.565448245742602,0.727094167361718,-0.777682274352917,0.436756344474282,0.846449062900718
"8405","MOXD1",61.6578413429505,-0.824859836761006,1.06079591598924,-0.777585796031071,0.43681323732233,0.846449062900718
"8406","GNG2",176.651673566228,-0.260765350247088,0.335344689811652,-0.77760393460695,0.436802540756982,0.846449062900718
"8407","WWC2-AS2",17.9419964574521,0.326763881891217,0.420688644139789,0.776735684319155,0.437314728990792,0.847108895267515
"8408","FBXO4",97.2726434571486,-0.168864351758426,0.217498931888352,-0.776391636925871,0.437517781072233,0.847108895267515
"8409","KIFC1",4.52468057928988,0.659524661881186,0.849363890708888,0.776492465827268,0.437458267609441,0.847108895267515
"8410","CTSB",1194.7929785741,0.152096911609575,0.19588456349275,0.776461957479385,0.437476274429251,0.847108895267515
"8411","MRPS2",230.56433434865,-0.193233263122763,0.248856638463936,-0.776484261442621,0.437463110008554,0.847108895267515
"8412","MAMDC4",7.73567580685412,-0.504668170385158,0.649648687793842,-0.776832432462804,0.437257639328469,0.847108895267515
"8413","ROGDI",56.4383912522746,-0.288362619358604,0.37134234217633,-0.776541176717403,0.43742951806038,0.847108895267515
"8414","CLEC3B",2000.35793252451,0.31031425229522,0.399743858136503,0.776282726998784,0.437582069530742,0.847132675372742

"8415","SEPTIN7",2544.04078459884,-0.117149363459608,0.150988398992338
,-0.775883208520893,0.437817947897004,0.847303556941707
"8416","AC092139.1",2.87948538111198,-0.682259240548176,0.879253084098
88,-0.775953195828028,0.437776621641767,0.847303556941707
"8417","SF3A2",170.737854429945,-0.255799650691322,0.329694418438651,-
0.775868914926507,0.437826388285245,0.847303556941707
"8418","PCYT1A",167.055505286238,0.143725587501073,0.185315731920716,0
.775571431585547,0.438002073875578,0.847542858322558
"8419","AC009974.1",3.27780754757569,-0.620907370223816,0.800892154640
896,-0.77526963727372,0.438180346832556,0.847754485504479
"8420","RPS15A",19021.0783797675,-0.180657725819137,0.233050442796846,
-0.775187224066423,0.438229036400385,0.847754485504479
"8421","CATSPERG",6.20890395980143,0.472802061600422,0.60997115729022,
0.775121997080701,0.438267574586115,0.847754485504479
"8422","RBMS3-
AS2",2.8322726322757,0.754345108574851,0.973717736799353,0.77470613922
9231,0.438513322491746,0.847907362708214
"8423","POLK",360.809891794945,0.12855625759975,0.166013620754695,0.77
4371747422505,0.438710986119763,0.847907362708214
"8424","LINC01048",13.1399324986018,0.608303475348198,0.78546320675795
4,0.774451903175716,0.438663600278967,0.847907362708214
"8425","TM9SF1",456.511636588617,-0.146783091082083,0.189541609395802,
-0.774410914574276,0.438687831226945,0.847907362708214
"8426","BCL2L10",2.31280167522323,-0.858078880092551,1.10783736040865,
-0.774553116511641,0.438603769985979,0.847907362708214
"8427","ERCC2",43.4717403302498,0.21667769297234,0.279789970955101,0.7
74429806160245,0.438676663123191,0.847907362708214
"8428","PTGIR",133.338187960981,-0.558515163746279,0.720918037744278,-
0.774727686789263,0.438500587186384,0.847907362708214
"8429","FDPS",509.980673771505,-0.191235993366651,0.247063835962837,-0
.774034745398416,0.438910244474679,0.847990626526871
"8430","MANEA-
DT",10.6483807452197,0.399358187129821,0.515885079255796,0.77412238343
0678,0.438858421949838,0.847990626526871
"8431","DMAC2",123.806665226063,-0.201205366402508,0.259942620614226,-
0.774037616174961,0.438908546858178,0.847990626526871
"8432","PAIP1",529.850657781217,-0.143940204691718,0.185997831749093,-
0.773881089570388,0.439001113431737,0.848065599702273
"8433","CYCS",539.292501922129,-0.199382665088281,0.257797213342898,-0
.773408922861709,0.439280410915936,0.848155296795421
"8434","FAM160B2",103.759952586993,0.175438662357524,0.226955083918112
,0.773010497622623,0.439516167981469,0.848155296795421
"8435","SLC25A51",37.3089677758035,-0.230224799351361,0.29780396799026
3,-0.773074989245571,0.4394780019289,0.848155296795421
"8436","PLCB3",60.0676310195772,-0.18917632319388,0.244657862419513,-0
.77322805538741,0.439387425217059,0.848155296795421
"8437","ERGIC2",573.998228021518,-0.142531917645583,0.184382984099987,
-0.773020993999596,0.439509956110566,0.848155296795421
"8438","PARPBP",20.9663010302383,-0.292168371779402,0.377721746985989,
-0.773501589756863,0.439225588266405,0.848155296795421

"8439","SPNS1",265.390835126378,0.150908280824711,0.195128117486101,0.
7733804987662,0.439297227678772,0.848155296795421
"8440","ATP5PO",1543.5034221222,-0.214400481557014,0.277248879611973,-
0.773314149572331,0.439336483793307,0.848155296795421
"8441","SGSM3",189.852495692091,-0.130253791148097,0.16846912208802 4,-
0.773161215145649,0.439426976534167,0.848155296795421
"8442","PTPRK",107.792372046609,-0.19805619172415,0.256274848391182,-0
.772827271062646,0.439624611586188,0.848264072272851
"8443","EIF1B",1291.80920076936,-0.159036819002719,0.205944684336302,-
0.77223075465748,0.439977769338339,0.848785915857437
"8444","NDUFB9",990.446789551206,-0.19606130857681,0.253901479984844,-
0.772194429857256,0.43999928009701,0.848785915857437
"8445","PLXNA1",243.629494876675,0.170822906775326,0.221258870970949,0
.772049979400622,0.440084826494175,0.848850413115882
"8446","ACY1",70.5857641485789,0.282918909771115,0.366522373641062,0.7
7190079001338,0.440173189394665,0.848889564210191
"8447","NRN1L",4.49834245439273,0.56209246173284,0.728250223918936,0.7
71839737594654,0.440209352869021,0.848889564210191
"8448","CBWD3",24.1038800196295,-0.298572210527242,0.386927431399547,-
0.771649116340197,0.440322275444925,0.848909798673747
"8449","BAIAP2",57.46868269927,-0.208504326949141,0.270207201924247,-0
.77164607554611,0.440324076922738,0.848909798673747
"8450","DDOST",1009.73647963138,-0.183027811954301,0.237220070668263,-
0.771552809333127,0.440379333297089,0.848915853263465
"8451","AC079915.1",8.22673460192983,0.413199970277345,0.5357630925451
51,0.77123634686076,0.440566853860691,0.849176840910755
"8452","PPP2R1A",530.074164371411,0.123014920168946,0.159600296766252,
0.77076874330072,0.440844017205378,0.84961052960937
"8453","FASTKD3",43.7098377934893,-0.23459281801982,0.304475255744152,
-0.770482374492022,0.441013806341421,0.849642693293393
"8454","RBM7",460.246676866588,-0.176821904202682,0.229500280909564,-0
.770464870465059,0.441024185759481,0.849642693293393
"8455","SUSD6",93.9766977297896,0.285759624695168,0.37092914377102,0.7
70388710334315,0.441069348301856,0.849642693293393
"8456","AC015726.1",9.03981071102923,0.405409860770553,0.5262155134218
8,0.770425520399901,0.441047519802743,0.849642693293393
"8457","BROX",232.23939954389,-0.153974970682945,0.199973125510566,-0.
769978317285486,0.441312754751466,0.84978865781811
"8458","SELENOP",10570.8190375739,0.178802910705304,0.232274132129188,
0.769792611283358,0.441422923318696,0.84978865781811
"8459","DELE1",126.546265507514,0.167548416553162,0.217628348186828,0.
769883234188436,0.441369160053544,0.84978865781811
"8460","CREB3L2",303.826428479841,-0.146031812874422,0.189717427130175
,-0.769733255839603,0.441458138771984,0.84978865781811
"8461","AP002884.3",4.42547617433243,0.572965705096822,0.7441052889126
66,0.770006225777639,0.441296199628164,0.84978865781811
"8462","TMBIM4",2257.63634445157,-0.19579522955333,0.254270737059257,-
0.770026593770797,0.441284117700948,0.84978865781811
"8463","TANC1",38.0237822316169,0.335315236804689,0.435766173675843,0.
769484317647203,0.441605850774102,0.849843710099861

"8464","SERPINB1",789.781450584034,-0.140802666063115,0.18301773731403
9,-0.769338907417005,0.441692145670106,0.849843710099861
"8465","ABCA7",4.71277222123295,0.57240076827407,0.743916207919716,0.7
6944252884976,0.441630649690717,0.849843710099861
"8466","AL121890.5",2.48572118938263,0.707043322321536,0.9190337319973
65,0.769333374505086,0.44169542941282,0.849843710099861
"8467","TRIM45",16.7516272560488,0.333622560787319,0.434320721622956,0
.768147924281966,0.442399307695242,0.849925382304816
"8468","C2orf88",36.4065588233698,0.317311540644708,0.412588872929316,
0.769074401817593,0.441849143444324,0.849925382304816
"8469","MOB4",378.148087757477,-0.186556848523962,0.242765780159495,-0
.768464354413524,0.442211359954839,0.849925382304816
"8470","AC006059.5",2.50801740046105,-0.777438690322001,1.011008812829
15,-0.76897320820227,0.441909215544236,0.849925382304816
"8471","GATA2",5.72296018586005,-0.700708854558615,0.911849047874868,-
0.768448304235957,0.442220892063847,0.849925382304816
"8472","SRD5A3-
AS1",4.88324091674171,0.628608543889044,0.818040925791365,0.7684316567
4252,0.442230779039559,0.849925382304816
"8473","BMP5",60.0866265118477,0.651247520861719,0.847695697860061,0.7
68256253400531,0.442334959080309,0.849925382304816
"8474","AC026471.2",6.93224515439926,-0.453261851508557,0.590069497163
72,-0.768149944518816,0.442398107604693,0.849925382304816
"8475","HOXB3",85.9950719417523,0.264895143992952,0.344803224121438,0.
768250194492548,0.44233855799283,0.849925382304816
"8476","UBA2",458.247713124136,-0.118953901423241,0.154863794800031,-0
.768119505122815,0.442416189855886,0.849925382304816
"8477","IL4I1",3.37604414644287,-0.726995987838603,0.945175084481936,-
0.769165416836057,0.44179511771183,0.849925382304816
"8478","E2F1",9.3278361940834,-0.419230539000184,0.545573517260695,-0.
768421717214439,0.442236682203117,0.849925382304816
"8479","TANGO2",55.2344715372365,-0.186480802810798,0.242550720153896,
-0.768832195973191,0.441992933173694,0.849925382304816
"8480","PRKAB2",133.914358184349,0.150260152492151,0.195707959501908,0
.767777421391417,0.442619430712193,0.849952050510245
"8481","TP53BP2",143.248205815325,0.192900021229384,0.251925165568392,
0.765703659633067,0.443852649038723,0.849952050510245
"8482","SDCCAG8",337.76825075945,0.122541389453021,0.159561385648377,0
.767989002822175,0.442493718373244,0.849952050510245
"8483","TMA7",3132.24438379598,-0.179757942833902,0.23452929007802,-0.
766462657069837,0.443401063262687,0.849952050510245
"8484","NDUFAF3",807.551023051964,-0.195790095233737,0.255706327213646
,-0.765683420379943,0.443864694516382,0.849952050510245
"8485","ANK2",1381.69948302619,-0.213329081939909,0.278038666084781,-0
.767264082164957,0.442924519083743,0.849952050510245
"8486","MRPL36",416.120553837226,-0.221508711625612,0.289909362964435,
-0.764061944604339,0.444830329093243,0.849952050510245
"8487","PHACTR1",109.121413191813,0.182624755890928,0.238875891901,0.7
64517316660044,0.444559021254185,0.849952050510245
"8488","PRDM1",31.4406875995652,0.344422071048943,0.449340529253286,0.

766505686948166,0.443375469354196,0.849952050510245
"8489","INTS1",186.466491323095,0.144288144088032,0.188627949880149,0.
764935123239743,0.444310177763834,0.849952050510245
"8490","SH2D4A",133.627717544701,0.141237791410363,0.184729261480185,0
.764566426989766,0.44452976726624,0.849952050510245
"8491","TRPA1",3.87424518730397,0.628301461744374,0.822130961456082,0.
764235251074336,0.444727063046398,0.849952050510245
"8492","TUBB4B",1423.71334321884,-0.186230058770952,0.243445314499914,
-0.764976968866729,0.444285259100106,0.849952050510245
"8493","UBE2D1",275.364111948556,0.187667026075391,0.24492159414973,0.
766233074412634,0.443537631889327,0.849952050510245
"8494","FRA10AC1",368.992160436098,-0.119846796419681,0.15683716887979
,-0.764147920264615,0.444779098098781,0.849952050510245
"8495","SPTY2D1",295.415629744149,0.138631482127608,0.180639703845193,
0.767447461309027,0.442815519175365,0.849952050510245
"8496","PATL1",98.1527522286149,0.182181831915493,0.238294396641036,0.
764524195631547,0.444554923529749,0.849952050510245
"8497","PRDX5",2550.83810786607,-0.186188840410469,0.243630622396899,-
0.764225935880706,0.444732613231273,0.849952050510245
"8498","CTSF",1681.89661353862,-0.16260321813367,0.212529212592233,-0.
765086437531989,0.444220075347808,0.849952050510245
"8499","CLNS1A",425.995107235203,-0.190803379072934,0.249087560556571,
-0.766009264559799,0.443670789656346,0.849952050510245
"8500","RSF1",914.387266871272,0.114893995846757,0.149986067207987,0.7
66031125327346,0.443657782381924,0.849952050510245
"8501","AKAP3",3.18227563079461,-0.651036444225239,0.851762707180419,-
0.764340160395561,0.444664558633023,0.849952050510245
"8502","BCAT1",81.7009850686036,-0.221142184002815,0.288520362024026,-
0.766469938036469,0.443396732528927,0.849952050510245
"8503","AC011603.2",15.2746128020319,0.31067125010318,0.40536698147926
8,0.76639505509175,0.443441274210645,0.849952050510245
"8504","RNF10",354.812198411165,0.111884166878542,0.146201311936996,0.
765274711944841,0.444107979024391,0.849952050510245
"8505","CAMKK2",324.091252738857,-0.18336406784251,0.239221972634388,-
0.766501780013127,0.443377793140423,0.849952050510245
"8506","SNAPC1",164.653450771803,-0.129384103293895,0.169181194615338,
-0.764766459937064,0.444410623205992,0.849952050510245
"8507","NME4",485.757555117079,-0.219166225603516,0.286227951746401,-0
.765705181014948,0.443851743589363,0.849952050510245
"8508","SLC46A1",37.6402977207777,0.221700562852913,0.290080342673795,
0.764272962481374,0.444704594214495,0.849952050510245
"8509","SLFN12",69.1555214129206,0.220503052265859,0.288522806221055,0
.764248258756078,0.444719312866904,0.849952050510245
"8510","RAB5C",868.205742342742,-0.166537512291914,0.217465214829177,-
0.765812189424098,0.443788060255935,0.849952050510245
"8511","PPM1D",68.1493655958201,0.205132638631323,0.268240601326612,0.
764733741338255,0.444430109882504,0.849952050510245
"8512","EIF4A3",634.115517061829,0.210445074968742,0.274684559234304,0
.766133617249425,0.443596802040904,0.849952050510245
"8513","MIR133A1HG",2.44858488881529,0.734235037163484,0.9566547217417

97,0.767502653231722,0.442782716296555,0.849952050510245
"8514","NDUFA13",2760.25239955064,-0.186696183705329,0.243304259741879
,-0.767336272301171,0.442881607694025,0.849952050510245
"8515","AC011477.2",8.2740428809596,0.424876776231552,0.55532825435557
5,0.765091228294509,0.444217222785131,0.849952050510245
"8516","TRIB3",8.69508789800057,0.619801986566532,0.80832478928183,0.7
6677344897118,0.443216225110933,0.849952050510245
"8517","PRNP",1047.08237645147,0.149283606179525,0.194729151711262,0.7
66621766015178,0.443306430445153,0.849952050510245
"8518","ATP5PF",2020.30534840124,-0.199009154676963,0.260418424198665,
-0.764189996500192,0.444754027027206,0.849952050510245
"8519","ATP6V1E1",425.746693390687,0.115383813304228,0.150898844073584
,0.764643453782604,0.444483886242286,0.849952050510245
"8520","MMP11",44.3985964531849,0.282240746437631,0.368572559676992,0.
765767117022982,0.443814883313147,0.849952050510245
"8521","MTMR3",184.380312464852,0.162174942337797,0.211899406543691,0.
765339294635346,0.44406953098634,0.849952050510245
"8522","AL022322.1",11.945440303644,0.434945572782848,0.56853857752378
1,0.765023852342992,0.444257341393634,0.849952050510245
"8523","AL021707.2",12.144638605791,0.375777153190948,0.49014486166294
1,0.766665495412986,0.443280423645897,0.849952050510245
"8524","KCND1",9.18891239955608,0.411481019685757,0.536037460943188,0.
767634819704079,0.442704169856206,0.849952050510245
"8525","NEXMIF",6.00555659764815,0.531553952652398,0.693186193692561,0
.766827091319926,0.443184326700948,0.849952050510245
"8526","FOXD2-
AS1",4.90114908027836,0.558784494121353,0.731523350489589,0.7638641934
63371,0.444948177397188,0.849987453488548
"8527","TBX18",151.770503094586,0.169837332148594,0.222342158679554,0.
763855730992376,0.444953220940319,0.849987453488548
"8528","VIRMA",224.570844905187,0.125605562159473,0.16447319069587,0.7
63684109416541,0.445055512617615,0.850083166630901
"8529","LINC02802",908.686193641809,0.206471261393747,0.27042735515855
1,0.763499910253878,0.445165315836663,0.850093532199696
"8530","ZNF83",331.272739769394,0.173364716911498,0.227063277387599,0.
763508388084979,0.445160261765926,0.850093532199696
"8531","SELENOI",29.7454213023704,-0.252604502856368,0.331053759940706
,-0.763031668637781,0.445444509520223,0.850120997283055
"8532","HERPUD2",212.016502668366,-0.121169430525672,0.158745575585473
,-0.763293276545095,0.445288510923438,0.850120997283055
"8533","LUM",20818.8630290068,-0.225317554252844,0.295292962163825,-0.
763030559894754,0.445445170737603,0.850120997283055
"8534","CDC16",234.840803723152,0.106060207303381,0.138976798938387,0.
763150454705754,0.445373672709574,0.850120997283055
"8535","TRIM9",12.5307012379226,-0.493680820864662,0.646798072941662,-
0.763268849301581,0.445303075738658,0.850120997283055
"8536","CRLS1",182.584945039743,-0.117802707630453,0.154404102262295,-
0.76295063346397,0.445492837670094,0.850120997283055
"8537","TMEM87B",130.10291653669,0.164210936411596,0.215313447071272,0
.762659920433304,0.445666239103764,0.850252678468166

"8538","BIRC2",436.581652487517,0.0911037787456741,0.119444479182963,0
.762729088601263,0.445624978920477,0.850252678468166
"8539","MED31",206.894882983439,-0.201970549144442,0.264873444136171,-
0.762517170428793,0.445751399192526,0.850315557026239
"8540","SDHAF1",268.466815328483,-0.207190841116353,0.271773076719953,
-0.762367058639338,0.445840961086363,0.850386816760628
"8541","KLRF1",6.17919072972987,0.450468816706012,0.590990805558612,0.
762226438159598,0.445924869434459,0.850447277627666
"8542","PCBP2-
OT1",19.6483414416985,0.428487937709927,0.562317062575955,0.7620041542
88043,0.446057524736488,0.850600681389915
"8543","AC024451.4",2.43485815863864,-0.723469980521425,0.949903915108
971,-0.761624380123151,0.446284219606687,0.850933355164851
"8544","AL109936.2",2.44967232588943,0.81356795371903,1.06851961609934
,0.761397302830041,0.446419797959592,0.850992637678617
"8545","AC015961.2",8.60546774031747,0.427202250589421,0.5610448491472
62,0.761440464588045,0.446394026074802,0.850992637678617
"8546","CXCR2",2.51829960041374,0.7612683759304,0.99995473558622,0.761
302835856973,0.446476207142638,0.851000577831317
"8547","GET3",581.309770866785,-0.216385456011295,0.284338295213746,-0
.761014114713713,0.446648636710997,0.851141354969576
"8548","ZNF578",10.8862388897548,0.378151628525964,0.496911368652832,0
.761004179781929,0.446654570709063,0.851141354969576
"8549","BAP1",120.523167741726,-0.137594520887229,0.180839877871578,-0
.760863823326295,0.446738408482932,0.85117829099215
"8550","FAM174B",24.3570488856631,0.249036502008399,0.327336433908884,
0.760796771182887,0.446778463256362,0.85117829099215
"8551","C3orf18",43.1489634949386,0.209381474979401,0.275277430219471,
0.760619840182563,0.446884165900867,0.851280105058967
"8552","NAPEPLD",58.5290212191259,0.228972160282648,0.301176124103805,
0.760260000569401,0.447099186239514,0.851424143990694
"8553","ANKRD20A1",3.50032836689366,0.69984233750526,0.920365904013914
,0.76039576700212,0.447018052783463,0.851424143990694
"8554","IMMP1L",89.7583046423528,-0.195274863908425,0.256862578213187,
-0.76023088013371,0.447116589581705,0.851424143990694
"8555","RNASEK",2401.41130286873,-0.179794243762609,0.236585303927976,
-0.759955249871922,0.447281334486941,0.851638300111956
"8556","PIK3C2B",38.3744788307225,-0.301142511655889,0.396663767488154
,-0.759188351290195,0.447739893293604,0.851714950585019
"8557","NUDT9",245.365173594361,-0.15885598983284,0.20911050300423,-0.
759674849185489,0.447448966100352,0.851714950585019
"8558","ZFP2",11.2044061358031,0.348356851388476,0.458819514867308,0.7
59245934622511,0.447705452680947,0.851714950585019
"8559","STMP1",648.845241608503,-0.122179682665518,0.160854021088232,-
0.75956871851217,0.447512423392939,0.851714950585019
"8560","SNRPF",718.339678291392,-0.167582428849644,0.220670277315719,-
0.759424562692145,0.447598624745758,0.851714950585019
"8561","ARG2",2.87508534222433,-0.64219534484407,0.845738478652312,-0.
759330881890831,0.447654648436914,0.851714950585019
"8562","EVL",337.067493290905,-0.123413667990143,0.162513943949125,-0.

759403562495399,0.447611183093393,0.851714950585019
"8563","ZNF155",17.9600053173258,-0.30262238317497,0.398442968966383,-
0.759512418954249,0.447546087910538,0.851714950585019
"8564","ZBTB7C",35.0282298956491,0.272267384751486,0.358800955399952,0
.758825696124449,0.447956832083635,0.85202812211704
"8565","NHLRC1",12.0812669952438,-0.363941262279547,0.479780314988608,
-0.758558137776429,0.448116922634757,0.852097937528496
"8566","KIAA1328",138.981594828976,0.142901413699219,0.188406056288896
,0.758475690824384,0.448166260397855,0.852097937528496
"8567","REXO1",91.1801099182827,-0.187089326902163,0.246653290071722,-
0.758511377844426,0.448144904254037,0.852097937528496
"8568","AL157700.1",10.6350682194461,0.390270365141787,0.5145870516234
52,0.758414662612551,0.448202782782501,0.852097937528496
"8569","AL590004.3",6.25680184742548,-0.518586435792081,0.684058326882
747,-0.758102657934564,0.448389528656291,0.852154594829346
"8570","PARP10",145.620674163392,0.127051533962397,0.167575239692514,0
.758176053905845,0.448345594588417,0.852154594829346
"8571","NLK",22.436265551213,0.26469007699209,0.349117203993564,0.7581
69674723247,0.448349413004012,0.852154594829346
"8572","NUDCD2",426.366606242528,-0.186565268719618,0.246158324836048,
-0.75790761431237,0.448506291627241,0.852177648934577
"8573","FAM110B",146.3883814956,0.175801158333522,0.231954425587456,0.
757912498924226,0.448503367240942,0.852177648934577
"8574","KHDC1",17.690773984633,-0.324230455427827,0.428006440261098,-0
.757536394148732,0.448728570470995,0.852301735587923
"8575","TMEM242",297.715205592434,-0.150797880539004,0.199050257874888
,-0.757586963960568,0.448698286650503,0.852301735587923
"8576","NOMO1",56.795613442786,-0.256515648815952,0.338563378344582,-0
.757659171733797,0.448655046909917,0.852301735587923
"8577","EFEMP2",1688.10433308598,0.200220968513289,0.264336427457936,0
.757447508989846,0.448781802315904,0.852303460175325
"8578","C2orf76",154.507589523797,-0.222199085548689,0.293493387792139
,-0.757083787202924,0.448999666588879,0.852617809403854
"8579","AL844908.2",2.88152399398075,0.714432385209663,0.9440587773986
76,0.756766847905656,0.449189557725285,0.852878972582721
"8580","LRR1",40.2055841697589,-0.263201504634775,0.347837999093137,-0
.756678411562217,0.449242551639883,0.852880177582991
"8581","PDE4B",50.9601186496031,0.409027964979456,0.540942408951087,0.
756139578282614,0.449565514575802,0.853134592858161
"8582","RPP14",108.98432492934,0.171825573193325,0.227225495068059,0.7
5618967467475,0.449535482525092,0.853134592858161
"8583","LINC02018",9.99049663295525,0.426006325924241,0.56342194987166
,0.756105306194192,0.449586060844401,0.853134592858161
"8584","ZC3H18",178.154880585373,0.154256402479109,0.203977623827517,0
.756241785665411,0.449504243959389,0.853134592858161
"8585","HNRNPA1P48",132.125016418813,-0.248141221841763,0.328264634892
378,-0.755918230189849,0.449698223121411,0.853148655535135
"8586","ANKRD13B",87.7679676616151,-0.216902474946584,0.28692231023153
3,-0.755962388465206,0.449671746394471,0.853148655535135
"8587","SPIDR",121.151289420176,0.129530253174185,0.171482966071045,0.

755353468288625,0.450036924930774,0.853691798089832
"8588","ECE1",326.521074924117,-0.189323835986499,0.251434976902065,-0
.752973346505571,0.45146593076084,0.853752719282704
"8589","LINC02609",188.859846829351,-0.156586140319853,0.2075703719345
95,-0.754376161012004,0.450623381901374,0.853752719282704
"8590","S100A14",4.64708581895907,0.529862219539215,0.703672366528217,
0.752995633683119,0.451452537825675,0.853752719282704
"8591","ZBTB18",14.6893451561822,0.343794865998437,0.455924812514099,0
.75406044277927,0.450812928741108,0.853752719282704
"8592","USF3",159.956088659641,0.184820985100246,0.245346440979257,0.7
53306159089024,0.451265958548055,0.853752719282704
"8593","IQCB1",84.0580008117249,-0.164699460631709,0.218891992538536,-
0.752423415409833,0.451796469612128,0.853752719282704
"8594","JADE1",445.230416546251,0.271290410602248,0.359807704092118,0.
75398722016467,0.450856895637408,0.853752719282704
"8595","MSMO1",100.070634742299,-0.170400152444618,0.226191919523826,-
0.753343235263847,0.451243684234125,0.853752719282704
"8596","SAPCD1",4.73752361741269,0.560918309725126,0.745156650063625,0
.752752202744527,0.451598833883301,0.853752719282704
"8597","C7orf50",300.137315131869,0.190255791866614,0.252461881682941,
0.753602051122908,0.451088212339379,0.853752719282704
"8598","HIBADH",458.230996262911,-0.154627958217229,0.204768186484105,
-0.755136629728526,0.450167006826401,0.853752719282704
"8599","ZNF736",52.320227206558,0.249138046553639,0.330858276725995,0.
753005332128857,0.451446709851698,0.853752719282704
"8600","AC006333.2",66.3502300994029,-0.229646030104746,0.304731872344
74,-0.753600298970204,0.451089264763753,0.853752719282704
"8601","ARF5",574.222434313687,-0.317005728556941,0.42135048471093,-0.
752356387520058,0.451836766416243,0.853752719282704
"8602","TACC1",2443.06584522278,0.175629754070419,0.233424726542789,0.
752404240423196,0.451807997302661,0.853752719282704
"8603","RBM4",802.5520388295,-0.154301654511163,0.204367282357483,-0.7
55021316187272,0.450236192331961,0.853752719282704
"8604","CORO1B",417.970474991318,-0.175031051080829,0.232602664962487,
-0.752489448515378,0.451756772854135,0.853752719282704
"8605","CEP164",203.434149049499,-0.144342181308503,0.191410706367179,
-0.754096696302945,0.450791161024466,0.853752719282704
"8606","AC008115.3",3.58472376412586,-0.609115096017849,0.809115444201
48,-0.752816054103364,0.451560458179753,0.853752719282704
"8607","AC026124.2",16.4956511638741,-0.296181566707656,0.393367161192
046,-0.752939228099563,0.451486433819262,0.853752719282704
"8608","NTN4",127.330250279985,-0.198902694926579,0.263619196125115,-0
.754507630135476,0.450544465476326,0.853752719282704
"8609","TCHP",95.4015190503856,-0.180949311727259,0.240231985967324,-0
.753227389760958,0.451313282990417,0.853752719282704
"8610","LCP1",51.5799682572201,-0.405379226058088,0.537159067550602,-0
.754672592434457,0.450445455314893,0.853752719282704
"8611","SERPINA1",10.5903956736468,-0.512007688328469,0.67973210770785
4,-0.753249232340997,0.451300159734091,0.853752719282704
"8612","RBBP6",852.903647162656,0.193510401560474,0.257004781925048,0.

752944751109374,0.45148311479516,0.853752719282704
"8613","DNAJC7",465.090336908662,-0.0997275804874887,0.132557837456941
,-0.75233258478499,0.451851076980551,0.853752719282704
"8614","TGIF1",273.56096699756,0.162558092706967,0.215945021570532,0.7
52775366270121,0.451584912015062,0.853752719282704
"8615","LINC00663",13.2149127460631,0.303899384657809,0.40297144799459
,0.754146196139158,0.450761440780453,0.853752719282704
"8616","BCAS4",9.12314190552807,0.394410389479003,0.522943376648585,0.
754212419720624,0.450721681151561,0.853752719282704
"8617","NPEPL1",271.756361168933,-0.125231138696734,0.16631650518443,-
0.752968796198935,0.451468665184138,0.853752719282704
"8618","KRTAP13-4",25.572977639594,-0.344550862960098,0.45718086802046
4,-0.753642348272271,0.451064008381502,0.853752719282704
"8619","URB1-
AS1",39.3454898845703,-0.289702412102399,0.38365942639572,-0.755103073
640083,0.450187139095847,0.853752719282704
"8620","KLHL22",95.7590292909445,0.152645034234816,0.202800611515835,0
.752685275916425,0.45163905997377,0.853752719282704
"8621","ELK1",76.9409972465794,-0.170103364074784,0.225444690193346,-0
.754523710134402,0.450534813739356,0.853752719282704
"8622","ZNF746",50.9615946500006,0.198021601129637,0.263266382551608,0
.752171998606084,0.451947630516424,0.853753990945456
"8623","SHC4",7.24736675346466,0.436091617217912,0.579922328657516,0.7
51982801261399,0.452061401676257,0.853753990945456
"8624","AKAP1",73.2274521249134,-0.20516017005716,0.272807886806763,-0
.752031667627264,0.452032015024418,0.853753990945456
"8625","SLC24A3",6.75371828181931,0.481408078807414,0.640103309817333,
0.752078721393886,0.452003719431703,0.853753990945456
"8626","VGLL4",634.097918586683,-0.128618038223438,0.171060334419851,-
0.751886979875521,0.452119028693211,0.853763837048888
"8627","NAGLU",452.746475334348,0.215387792581452,0.286522092607625,0.
751731884341684,0.452212312003553,0.853841005010534
"8628","MTX2",280.684130837431,0.199691295343648,0.265759249555467,0.7
51399229481832,0.452412426312451,0.854119843788075
"8629","KCNA2",8.69582870259906,0.588317562569325,0.783603974835761,0.
750784301078403,0.452782478877924,0.854225231941794
"8630","DAG1",215.761167174644,0.14310662242764,0.190609680398526,0.75
0783602010313,0.452782899661455,0.854225231941794
"8631","ANKRD6",62.225473581045,0.186486020796487,0.248256364598611,0.
751183241960399,0.452542384143455,0.854225231941794
"8632","AP005482.3",6.22503047765431,0.507765300276419,0.6761124574747
84,0.751007165542955,0.452648343409226,0.854225231941794
"8633","CRELD2",332.419182078207,-0.207487749686136,0.276348641741305,
-0.750818778694667,0.452761726362165,0.854225231941794
"8634","TSIX",67.9787955250663,0.587380082064298,0.782236513688662,0.7
50898317561894,0.452713852955504,0.854225231941794
"8635","RNASEH1",250.670074836516,-0.158533665098912,0.21125900994173,
-0.750423213394016,0.452999854420057,0.854430084659973
"8636","POLR2B",239.558283309515,0.121259276226253,0.16158308139697,0.
750445375703344,0.452986510966422,0.854430084659973

"8637","ASB16-
AS1",41.2851623035748,0.215418844538376,0.28709426964044,0.75034184697
6496,0.453048845307151,0.854430084659973
"8638","NOC2L",143.548634023723,-0.170412446901685,0.227341643916167,-
0.749587466537917,0.453503201722098,0.854830204027037
"8639","TIE1",6.09680126339872,-0.4690071600375,0.626814808898983,-0.7
48238799369344,0.454316131941548,0.854830204027037
"8640","CYB561D1",42.4924483948888,0.184821428565743,0.24692957607001З
,0.748478296959209,0.454171711072053,0.854830204027037
"8641","CHI3L2",985.754807373097,-0.723674068375552,0.970145654012505,
-0.745943730595966,0.455701406948563,0.854830204027037
"8642","HIPK1",263.313082643168,0.177323241795867,0.237174464363621,0.
747648960741431,0.454671924409113,0.854830204027037
"8643","NEK7",346.411275334128,0.149295558335728,0.200279907945296,0.7
45434526445392,0.456009078099918,0.854830204027037
"8644","ATF3",3912.98196407954,-0.254557886460199,0.341382450250984,-0
.745667758471614,0.455868140227504,0.854830204027037
"8645","NTPCR",404.087526679263,0.193919046563216,0.259410421511522,0.
747537610221273,0.454739108995499,0.854830204027037
"8646","TTC31",75.8341182229889,0.15989014583376,0.214814136939801,0.7
44318544912934,0.456683784539789,0.854830204027037
"8647","MDH1B",4.53097668509911,0.591303988685293,0.791353572943475,0.
747205811538718,0.45493933661469,0.854830204027037
"8648","FBXL2",34.768993856804,0.274475713006904,0.36754436979175,0.74
6782526317629,0.455194844873493,0.854830204027037
"8649","MLH1",122.535755818805,0.162667393149761,0.218037171943175,0.7
46053490329417,0.455635103267351,0.854830204027037
"8650","H1FX-
AS1",31.4121845705074,0.230050493607445,0.308776005314021,0.7450400602
64678,0.456247502610421,0.854830204027037
"8651","DNAJC19",653.901555128225,-0.167447609666913,0.223876560287813
,-0.747946142515523,0.45449264383314,0.854830204027037
"8652","PI4K2B",55.7856450224938,-0.167517958160985,0.224860891173936,
-0.744984853908656,0.456280876203426,0.854830204027037
"8653","DNAJC21",429.186230241108,-0.129496404890353,0.173130711741768
,-0.74796899745611,0.454478857804026,0.854830204027037
"8654","CCL28",29.6141672682399,-0.347545458932591,0.465681058046791,-
0.74631650338174,0.455476244384186,0.854830204027037
"8655","ZCCHC9",276.87876818516,0.14844629754234,0.198070603708477,0.7
49461529186965,0.453579077669015,0.854830204027037
"8656","LINC01366",5.78165754889587,0.509737550116064,0.68146446023407
3,0.748003131287252,0.45445826882201,0.854830204027037
"8657","VTA1",187.980467607545,0.124414949947314,0.167170993909725,0.7
44237663709175,0.456732705950956,0.854830204027037
"8658","AL139393.3",57.1293028464518,0.219908912076719,0.2942378589390
51,0.747384829639722,0.454831299975394,0.854830204027037
"8659","AFDN",190.693673081121,0.174821514348063,0.234039074777949,0.7
46975754001463,0.455078196550234,0.854830204027037
"8660","AC008267.5",35.5496680093182,0.307795195946218,0.4124978294628
47,0.746174098290475,0.455562252647276,0.854830204027037

"8661","AC005076.1",10.9949063700684,-0.347699514383252,0.466811651905619,-0.744838979412516,0.456369067510748,0.854830204027037
"8662","SMIM19",934.454522480429,-0.233923019411287,0.312441039112501,-0.748694922010734,0.454041105004289,0.854830204027037
"8663","TMEM68",83.9641886652423,0.15669974951218,0.209733077525561,0.747138941367428,0.454979696177567,0.854830204027037
"8664","AC234917.3",3.96509846693533,-0.583722536676929,0.78436356738883,-0.744198941595617,0.456756128262502,0.854830204027037
"8665","CDKN2B",14.6927504947204,0.438822209582363,0.588850447126417,0.745218436572156,0.456139679151066,0.854830204027037
"8666","HINT2",382.884292866637,-0.197303304982378,0.265067318946827,-0.744351683060395,0.456663741611727,0.854830204027037
"8667","TUT7",170.648578448952,0.144134609976271,0.192789908866508,0.747625281964696,0.454686210796677,0.854830204027037
"8668","PDCL",278.713946433063,-0.144093147044759,0.193172304354579,-0.745930673272226,0.455709294980572,0.854830204027037
"8669","GOLGA2",700.54949912899,-0.137855556888655,0.185054761899284,-0.744944661103522,0.456305174601012,0.854830204027037
"8670","RABL6",251.29245801697,-0.162329015419983,0.217790879098372,-0.745343496899436,0.456064092248137,0.854830204027037
"8671","TSPAN15",113.299036444643,-0.293552601684756,0.394055355000134,-0.744952702608638,0.456300313083438,0.854830204027037
"8672","EXOC6",24.8202677531667,0.276476737857485,0.371296816514034,0.744624584862377,0.456498701361331,0.854830204027037
"8673","AL162274.2",6.06881791613968,0.510863184167612,0.681412351065462,0.749712245997334,0.453428030456769,0.854830204027037
"8674","FAR1",202.349830555446,0.117640312623606,0.156936434849542,0.749604849481828,0.453492729241326,0.854830204027037
"8675","API5",196.322489222919,-0.121885969852077,0.163772621769268,-0.744238985340278,0.456731906531934,0.854830204027037
"8676","FAM180B",322.182027778084,-0.194474968115702,0.259596044249324,-0.749144574517949,0.453770071260961,0.854830204027037
"8677","NKAPD1",270.498658373244,0.140324483271535,0.188566539608231,0.744164280487275,0.456777094717375,0.854830204027037
"8678","GSEC",32.5623714544465,0.325618187482096,0.436629174137204,0.745754536731381,0.455815707958831,0.854830204027037
"8679","SLC16A7",166.969934002613,-0.204642885248827,0.272982573476012,-0.749655491348828,0.453462220458724,0.854830204027037
"8680","TXNRD1",477.282682114362,0.152986110020771,0.20425134845404,0.74900905760823,0.453851746185727,0.854830204027037
"8681","SART3",150.316416449083,0.152427117233757,0.203749996352802,0.74810856423194,0.454394676672526,0.854830204027037
"8682","PEBP1",3658.14047038969,-0.155237945550897,0.208130288579222,-0.745869073697113,0.45574650880474,0.854830204027037
"8683","SIRT4",11.7689588159326,0.335968104610341,0.449965689177044,0.746652717510092,0.455273217733987,0.854830204027037
"8684","CAPN3",28.0437650993629,0.296352963016826,0.395624486256492,0.749076392669724,0.453811162862697,0.854830204027037
"8685","LIPC",22.2960934303772,-0.308965883204972,0.413254218716393,-0.747641207788875,0.454676602058926,0.854830204027037

"8686","HERC1",269.110882892992,0.153357007936045,0.204986723001164,0.74813141890743,0.454380892478166,0.854830204027037
"8687","CLPX",206.884861108353,0.116039677234444,0.155098369278918,0.748168261045778,0.45435867261092,0.854830204027037
"8688","ETFA",393.006398827843,-0.144367653557645,0.193358571632688,-0.746631775041717,0.455285862588851,0.854830204027037
"8689","BTBD1",142.36989896379,-0.192769232404131,0.257963189693197,-0.747274185256422,0.454898071669849,0.854830204027037
"8690","ELOB",2496.92346672794,-0.182594507140026,0.245234844736696,-0.744569995083995,0.456531712424119,0.854830204027037
"8691","OGFOD1",97.0041404649368,0.202521535558658,0.270798940884937,0.747866793336942,0.454540508840393,0.854830204027037
"8692","ST3GAL2",114.441227567198,0.145653457005282,0.194737486335871,0.74794771025270 5,0.454491698171417,0.854830204027037
"8693","SLC25A11",329.143388642063,-0.176421186934,0.23598073569146,-0.747608428361995,0.45469637942789,0.854830204027037
"8694","FGF11",9.78883870610614,0.491657155776921,0.659568301549374,0.745422656943916,0.456016251279149,0.854830204027037
"8695","SAT2",1178.34253897723,-0.18676331937597,0.249999786939597,-0.747053914174312,0.455031017342319,0.854830204027037
"8696","PIK3R5",13.4252040210031,-0.527127808929648,0.703754037310736,-0.749022784926348,0.453843472466833,0.854830204027037
"8697","TEKT3",58.4011601584595,-0.265914348735213,0.357023477428894,-0.744809138744028,0.456387109456841,0.854830204027037
"8698","AC060780.1",85.2357342966628,-0.175684048956985,0.234980263594557,-0.74765448923028,0.454668588879036,0.854830204027037
"8699","MRO",8.29993685809559,0.674521931510416,0.90213212863732,0.747697493635759,0.454642643292572,0.854830204027037
"8700","TBC1D10A",96.5934175313898,-0.190186658338642,0.255548984162143,-0.744227800248154,0.45673867211744,0.854830204027037
"8701","MICALL1",13.3741004481324,0.315744402015529,0.42398491103967,0.744706695437061,0.456449050685611,0.854830204027037
"8702","AL033543.1",4.30749639732705,0.628761687994512,0.841813850214884,0.746912975872294,0.455116092811365,0.854830204027037
"8703","TXLNG",290.446451397481,0.149931360053989,0.20131413487105,0.744763203786095,0.456414882943957,0.854830204027037
"8704","HCFC1",149.216961117371,0.16721676384566,0.224404042502961,0.745159320574419,0.456175411520756,0.854830204027037
"8705","C1QTNF12",4.2186751275931,0.637931681210937,0.858814238241511,0.742805199081413,0.457599627342323,0.85486050601256
"8706","NKAIN1",3.24981826828955,0.735391548140023,0.989529683440615,0.74317280264201,0.457377067194219,0.85486050601256
"8707","PKN2",494.040586734579,0.117293030816453,0.157906378139535,0.742801096437071,0.457602111570743,0.85486050601256
"8708","STK39",59.6877149021459,0.168197244756704,0.226189341115363,0.743612603172661,0.457110876333476,0.85486050601256
"8709","CD28",3.23170624085345,-0.707756425060535,0.952459889334831,-0.743082656798084,0.457431639038798,0.85486050601256
"8710","TXNDC15",574.904967085523,-0.148530565094912,0.199749053983219,-0.743585824979126,0.457127081443629,0.85486050601256

"8711","AC019205.1",50.2175919747636,0.243483102946798,0.327824578446013,0.74272375823979,0.45764894272227,0.85486050601256
"8712","CDK13",311.229346325844,0.193382665222863,0.260429531534917,0.742552751537454,0.457752503183506,0.85486050601256
"8713","PUS7",34.9132628336521,-0.277809211702013,0.373561892236769,-0.743676529847787,0.457072191727133,0.85486050601256
"8714","PRDM11",42.6227274833098,-0.192096489355836,0.258273973210841,-0.743770217988705,0.457015500581571,0.85486050601256
"8715","AP000873.2",65.9425617848906,-0.255649463576511,0.343924645328234,-0.743329874869899,0.457281988670046,0.85486050601256
"8716","C11orf52",18.0872926969707,-0.323743857101267,0.436017846274137,-0.742501390408044,0.457783609691917,0.85486050601256
"8717","ZNF384",103.337011651123,0.163794644419053,0.22039550673378,0.743185044225532,0.457369656754101,0.85486050601256
"8718","ANG",714.535739099815,-0.161192232670507,0.2166429537866,-0.744045582157668,0.456848899264308,0.85486050601256
"8719","PSEN1",200.985992988166,0.132205157586322,0.178010700984515,0.742680956005125,0.457674862221843,0.85486050601256
"8720","BDKRB1",17.1619858457057,0.348663987345998,0.468657575181612,0.743963195753073,0.456898741254852,0.85486050601256
"8721","SENP3",28.9352715484289,-0.297133124107819,0.400199210496698,-0.742463044190014,0.457806834583848,0.85486050601256
"8722","CCL8",42.5587927150579,-0.800465313155215,1.07734644516844,-0.742997126639298,0.457483420052693,0.85486050601256
"8723","LILRB3",7.86219378351186,-0.448701957672148,0.604390956543477,-0.742403493656296,0.457842903459609,0.85486050601256
"8724","CHMP2A",1185.39816894755,-0.177389740624459,0.238816095493283,-0.742788044742351,0.457610014668069,0.85486050601256
"8725","COA8",427.350272573259,-0.149182338959154,0.201002815961348,-0.74219029343271,0.457972048749451,0.854905650020605
"8726","CHSY1",101.034311081092,0.174185314984232,0.234675995497757,0.742237460694597,0.457943475584883,0.854905650020605
"8727","IMMT",230.763098344079,-0.120261156343314,0.162123822186877,-0.741785844431249,0.458217098474087,0.85518427645305
"8728","YTHDC2",130.277611962274,0.171454960466975,0.231143009387414,0.741770044966418,0.458226672630765,0.85518427645305
"8729","CPEB3",33.5150128889363,0.23742422276482,0.32018282687884,0.741527036534858,0.45837394496811,0.85518427645305
"8730","AC009152.1",2.85782833687266,-0.847283308599669,1.14264466320338,-0.741510756479911,0.45838381224824,0.85518427645305
"8731","VASP",121.761369250618,-0.175877949215261,0.237152170434587,-0.741624876942769,0.458314646773655,0.85518427645305
"8732","LYPLA2",94.0130716480203,-0.175219327452476,0.23644981132861,-0.741042365261025,0.45866775341514,0.855308478406168
"8733","TFB2M",73.7357755036493,-0.184636223301583,0.249609815078742,-0.739699371370223,0.459482429628162,0.855308478406168
"8734","DYNC1I2",1137.53476372517,0.142917990992371,0.193062512894603,0.740267951813067,0.459137423032435,0.855308478406168
"8735","CFLAR-AS1",3.19619059864034,0.857203376936713,1.15944289923027,0.73932349536

6431,0.45971058557591,0.855308478406168
"8736","U73166.1",4.50948566089158,-0.47613599579856,0.643960059350648
,-0.739387464928621,0.459671751707474,0.855308478406168
"8737","COX18",54.6846321833147,0.178136577731388,0.24080018753755,0.7
39769264937179,0.459440011372424,0.855308478406168
"8738","TMEM14B",854.222785095537,-0.179026230949429,0.241594397050045
,-0.741019796549107,0.458681437173858,0.855308478406168
"8739","U91328.1",8.91962442065687,-0.361814640265625,0.48880872338484
6,-0.740196774231387,0.45918060463666,0.855308478406168
"8740","SIM1",71.6962988103903,0.636064130717314,0.859852068072909,0.7
39736699293931,0.459459775118529,0.855308478406168
"8741","FBXO30",86.2586834358397,0.16271197974116,0.219898510489886,0.
73994125462093,0.459335640541314,0.855308478406168
"8742","FPGS",100.022897221006,-0.15449645379116,0.208783068779878,-0.
739985549087064,0.459308762880716,0.855308478406168
"8743","REXO4",203.779571833797,0.125207230494052,0.169216617906021,0.
739922780891347,0.459346850568388,0.855308478406168
"8744","AP001453.1",2.74873029628098,0.703005587392986,0.9508188524491
59,0.739368582755963,0.45968321427751,0.855308478406168
"8745","CACNA1C-
AS2",2.45472050059742,-0.721938599018525,0.976029144389439,-0.73966910
021948,0.459500801805173,0.855308478406168
"8746","ACVRL1",635.054842220306,-0.162126500534491,0.21923369045165,-
0.739514534469994,0.459594617314557,0.855308478406168
"8747","LINC00345",8.65129447450962,-0.43722997443012,0.59100677803345
6,-0.739805346877711,0.459418114178522,0.855308478406168
"8748","TTBK2",157.657137509017,0.17427324469607,0.235291145293182,0.7
40670646483187,0.458893161407252,0.855308478406168
"8749","RPS2",27175.8836695845,-0.152632543293073,0.206201852512908,-0
.740209369765573,0.459172963085035,0.855308478406168
"8750","LYRM1",316.148182162363,0.165176475684911,0.223181215215474,0.
740100261240352,0.459239160214655,0.855308478406168
"8751","BANP",74.5259271794404,0.169359778805031,0.228586165914709,0.7
40901262013482,0.458753310251442,0.855308478406168
"8752","SP2-
AS1",21.3149159435475,-0.343149821384601,0.463422080966094,-0.74046929
4577501,0.459015285887904,0.855308478406168
"8753","S1PR2",250.257489583102,-0.11792226401122,0.159392684093586,-0
.739822311681404,0.45940781888117,0.855308478406168
"8754","ZNF565",46.2007697330846,0.217756304535225,0.294443600919274,0
.73955183218577,0.459571978043216,0.855308478406168
"8755","RBBP7",674.753983051329,-0.153174422808667,0.206724644185506,-
0.740958696105985,0.458718484464954,0.855308478406168
"8756","GLMP",291.889090869673,0.19711407313934,0.266844793501833,0.73
8684351126325,0.460098690372366,0.855511334925668
"8757","AC097662.1",13.3040871958561,0.316139826598268,0.4279779935836
12,0.738682435400748,0.460099853925665,0.855511334925668
"8758","KPNA4",458.572822142911,-0.137000617831337,0.185561477434646,-
0.738303120482475,0.460330270677105,0.855511334925668
"8759","AL391422.4",87.0707215537104,0.25014360903325,0.33872210013109

6,0.738492141305325,0.460215440961305,0.855511334925668
"8760","AC068733.3",3.41045613166177,0.567592127474822,0.768426545456088,0.738642009221501,0.4601244079382,0.855511334925668
"8761","GLB1L2",4.37733498442667,-0.613822936142192,0.831288475646183,-0.738399429470078,0.460271761185648,0.855511334925668
"8762","CLEC4E",9.5585003941405,-0.477094707355203,0.646225344044072,-0.738279164926509,0.460344824766621,0.855511334925668
"8763","TUBGCP3",106.215715595545,0.16093127454678,0.217884454375663,0.73860833719377,0.460144860180596,0.855511334925668
"8764","LINC00638",3.342657978891,0.711378081841322,0.962725247020274,0.738921186541128,0.459954856448394,0.855511334925668
"8765","MZF1",79.1208767411219,0.146509849880608,0.198417975548281,0.738390004614061,0.460277486776357,0.855511334925668
"8766","HECTD1",442.85321468223,0.133599184248071,0.181006975581393,0.738088594756921,0.460460613978308,0.855628900421247
"8767","NEDD8",1735.58249235518,-0.189434463605939,0.256801280879493,-0.737669465499407,0.460715331526041,0.856004566582375
"8768","ZFHX2",17.1462413398283,-0.274355724877848,0.372015991022891,-0.737483687525052,0.460828259624348,0.85611673369309
"8769","CD99",6635.02169928952,-0.180977879700902,0.245447567226584,-0.737338250062319,0.460916676898838,0.856183344737732
"8770","DAB1",6.33689431949769,-0.679361007119716,0.922183914647688,-0.736687114499562,0.461312644602142,0.856192622238758
"8771","AC093110.1",6.59560745591748,0.569717392299647,0.77317654906938,0.736852912811928,0.461211801518214,0.856192622238758
"8772","ATP5MC3",1728.80740107364,-0.220635025815774,0.299873330132705,-0.735760748440468,0.461876312509351,0.856192622238758
"8773","TIGD1",13.8513905369152,0.3967957770598,0.539430029500093,0.735583403518568,0.46198426578414,0.856192622238758
"8774","PPARGC1A",10.200327317576,0.498059557463449,0.67676748189392,0.735938960999781,0.461767845275664,0.856192622238758
"8775","AK6",355.8791222554,-0.218221289959258,0.29615322030527,-0.736852666110871,0.461211951559417,0.856192622238758
"8776","RPS12",35332.5155697425,-0.167014643133689,0.226980940257316,-0.735809107779506,0.461846877690884,0.856192622238758
"8777","NDUFA4",2696.3942403068,-0.200675913610608,0.272522501166667,-0.736364567151396,0.461508862041686,0.856192622238758
"8778","BLVRA",243.952892350333,-0.20989757016063,0.285227024815267,-0.735896503133162,0.461793685526953,0.856192622238758
"8779","NDUFA5",763.576634063742,-0.17148941305873,0.232901909042377,-0.736316047231399,0.461538382530506,0.856192622238758
"8780","AC124242.3",2.67797337288431,0.693288432915111,0.941099296250504,0.736679366010884,0.461317357746909,0.856192622238758
"8781","C9orf24",3.00758629429523,-0.600170769380591,0.815366627319634,-0.736074729172496,0.461685220925948,0.856192622238758
"8782","RPL35",12843.4350620793,-0.162738229076089,0.22125731746034,-0.735515692516066,0.462025486492644,0.856192622238758
"8783","IFITM1",727.868747642405,-0.521565680235139,0.708938657770499,-0.735699308421664,0.461913710639413,0.856192622238758
"8784","WDR66",45.7643208898504,0.36933120561579,0.501176880999349,0.7

36927858442595,0.461166221571569,0.856192622238758
"8785","SPRYD7",117.017732236376,-0.180715207555762,0.245447280512185,
-0.736268933917934,0.461567048221054,0.856192622238758
"8786","SOCS1",487.317136264456,-0.308198373901436,0.418972672003321,-
0.73560495587405,0.461971145697824,0.856192622238758
"8787","ZSWIM7",303.381578077602,-0.210159832356932,0.285709548960403,
-0.735571608025107,0.461991446428066,0.856192622238758
"8788","SSH2",208.894159991845,0.205884067781589,0.279531234176824,0.7
36533319390534,0.461406198020899,0.856192622238758
"8789","DGKE",29.6334331512803,0.255934328395078,0.347360238566677,0.7
36797998098882,0.461245200789475,0.856192622238758
"8790","AC011444.2",2.62193674700841,-0.809245551754708,1.098921254345
14,-0.736399945451001,0.461487337842186,0.856192622238758
"8791","ZNF41",22.3702510904196,0.275177555620521,0.374188193090481,0.
735398819903387,0.462096640348782,0.856227070258367
"8792","ATG4A",43.0315668681004,-0.215205098623377,0.292766625957855,-
0.735073876399959,0.462294503123438,0.856496264942866
"8793","AC012313.6",5.84006930908997,-0.43999556925346,0.5987011493853
51,-0.734916860783006,0.462390129130398,0.856576005163773
"8794","MTHFR",150.96130739918,0.170389545843111,0.232210967875253,0.7
33770447632977,0.463088654618677,0.856820292899006
"8795","TSSK3",7.89453648859816,0.444360103376324,0.605165128912194,0.
73427909531912,0.462778655929805,0.856820292899006
"8796","TDRKH",4.32892266218691,-0.50226425936791,0.683993742367649,-0
.734311190668673,0.462759099089465,0.856820292899006
"8797","MFF",452.830011683657,-0.118481447651921,0.161527525111847,-0.
733506240313409,0.463249723181841,0.856820292899006
"8798","RRP9",69.2232365020793,-0.218176924526661,0.29755462366744,-0.
733233185347861,0.46341621833386,0.856820292899006
"8799","KPNA1",114.536985686656,-0.158236247138829,0.215729164918936,-
0.733494922665136,0.463256623450847,0.856820292899006
"8800","UTP3",150.23574270636,-0.15819493992393,0.215717198151568,-0.7
33344124990806,0.463348568911401,0.856820292899006
"8801","RPS23",30659.2643250169,-0.179754011646353,0.244992332294328,-
0.73371280628652,0.463123791818393,0.856820292899006
"8802","TMEM248",582.978036602905,0.0959361934551124,0.130836769029282
,0.733251013204409,0.463405346788397,0.856820292899006
"8803","TMEM168",150.823135908657,0.144097546862563,0.196371577414224,
0.733800424481009,0.463070381821412,0.856820292899006
"8804","SIN3A",186.674506409554,0.142126390718476,0.193814395819218,0.
733311837429482,0.463368256841075,0.856820292899006
"8805","RGMA",300.467911855039,-0.155417900298418,0.211757192661892,-0
.73394390218693,0.46298292859155,0.856820292899006
"8806","EXOC3L1",7.15863772689958,-0.439710946264093,0.598717452772057
,-0.734421460787947,0.462691911087973,0.856820292899006
"8807","KRT10",1821.41122580029,-0.194569301642722,0.265094552007941,-
0.733961902155172,0.462971957813362,0.856820292899006
"8808","FBL",574.354386411393,-0.135633758232292,0.184790626193516,-0.
733986138941126,0.462957185993894,0.856820292899006
"8809","Z93241.1",47.6472404667393,-0.28464884400453,0.388046673058977

,-0.733542802366116,0.463227432016501,0.856820292899006
"8810","KANTR",70.6521682669772,0.267780736175533,0.364774757800811,0.734098866352364,0.46288848442308,0.856820292899006
"8811","LINC01960",8.5211159187732,0.491426905021067,0.670476370323878,0.732951863439542,0.46358778911784,0.857024862313809
"8812","DCK",63.4994352560854,-0.213067778759931,0.290731869640883,-0.732866950648089,0.463639582132841,0.857024862313809
"8813","ZC3H12D",5.82933482685157,-0.568199165962745,0.775388383278134,-0.732792982480021,0.463684702042581,0.857024862313809
"8814","SMIM20",501.403075814676,-0.185619257496198,0.253340369946821,-0.732687244181263,0.463749205690046,0.857046835884407
"8815","NAAA",261.862795134837,-0.216025992139444,0.294876597547539,-0.732597954317542,0.463803679172967,0.857050269999826
"8816","LNCTAM34A",6.37822303229336,0.518313437868603,0.708236017405821,0.731837163220135,0.464267963049965,0.857224091575285
"8817","HSPB11",297.864340353806,-0.18573122170913,0.253967511406192,-0.731318823737553,0.4645844353953,0.857224091575285
"8818","RBM15",41.3308178118192,0.217281549588997,0.296882580749972,0.731877057387836,0.464243610626456,0.857224091575285
"8819","ZC3H15",712.324946740714,-0.126246121832157,0.172781793232152,-0.730667968369392,0.46498198527725,0.857224091575285
"8820","SUCLG2",510.624200293854,-0.112266314719993,0.153674191200984,-0.730547620538082,0.46505551582361,0.857224091575285
"8821","MAD2L1",21.5017827830981,-0.260019171178693,0.355420288255254,-0.731582241562851,0.464423590556515,0.857224091575285
"8822","RGS7BP",2.53014521385045,0.934533978334661,1.27694839502802,0.731849448241922,0.464260463881713,0.857224091575285
"8823","CDC23",80.1617932229081,0.187579472145117,0.256709572743095,0.73070696250521,0.464958161888173,0.857224091575285
"8824","HUS1",69.2686341199669,-0.163292471419997,0.223354097178503,-0.731092348350765,0.464722747687201,0.857224091575285
"8825","AC034111.1",4.31852355202841,-0.578342306617115,0.791526943908101,-0.730666607205557,0.464982816889826,0.857224091575285
"8826","ADGRB1",3.28012004440293,0.612664845925167,0.838269288555506,0.73086877246917,0.464859311667907,0.857224091575285
"8827","CENPP",26.7279769834968,0.243146628603045,0.33273382009549,0.730754176215886,0.464929317674825,0.857224091575285
"8828","ECD",107.019735736306,0.148664085535346,0.203419739672135,0.73082428369517,0.464886488835966,0.857224091575285
"8829","TMEM116",65.9412600596503,0.244595043448831,0.334477463511082,0.731275108586583,0.464611131182418,0.857224091575285
"8830","TDRD3",177.911912142738,0.141907736629987,0.194234089216896,0.73060160140851,0.465022533599643,0.857224091575285
"8831","STK24",488.583209811278,0.143065517848975,0.195779398356808,0.730748582587009,0.464932734931569,0.857224091575285
"8832","ERCC5",244.788135624339,0.140002678360464,0.191486629723595,0.731135529214513,0.464696374647104,0.857224091575285
"8833","METTL26",677.397745325485,-0.18711014387189,0.256009700495386,-0.73087130491472,0.464857764681791,0.857224091575285
"8834","MYCBPAP",7.9205930025835,0.387506435080408,0.529336060728242,0

.73206128172581,0.464131164587013,0.857224091575285
"8835","ZNF564",30.8086167691121,0.274230206909426,0.374909963783313,0
.731456171882174,0.464500565753084,0.857224091575285
"8836","MED26",59.8573270382965,0.250422297350977,0.342279829531928,0.
731630308725562,0.464394243741223,0.857224091575285
"8837","UBOX5",41.023205300418,0.211178803872668,0.288632678597763,0.7
31652441084005,0.464380731448851,0.857224091575285
"8838","IPO8",102.024268932762,0.196739245178142,0.269428131677249,0.7
30210479334127,0.465261537978761,0.857312804540276
"8839","RNF31",66.0945984055037,0.150627418810887,0.206277898578491,0.
730215984596,0.465258173384753,0.857312804540276
"8840","KDELR1",1836.50584960496,-0.159310370634996,0.21812471448939,-
0.730363686700645,0.465167908865376,0.857312804540276
"8841","VSTM4",257.355900377177,0.187516730502296,0.25684334025207,0.7
30082120557474,0.465339989519832,0.857360376573752
"8842","BOD1",567.901315029704,-0.158383751776432,0.217083930111753,-0
.729596850835054,0.46563664773053,0.857573080082481
"8843","CDAN1",28.1732538069832,0.21349531869453,0.29258055923261,0.72
9697554938348,0.465575076010579,0.857573080082481
"8844","TMEM170A",166.573760513195,-0.154841471393132,0.21222778715545
,-0.729600366985475,0.46563449783695,0.857573080082481
"8845","ZNF567",80.4760407366608,-0.177158397385473,0.24283282545674,-
0.729548804006455,0.465666025743112,0.857573080082481
"8846","SNRNP27",369.945759937961,0.128622396117341,0.176406140948796,
0.729126522611676,0.465924272056982,0.857660805462332
"8847","AC008035.1",8.29218367181032,-0.39346657606668,0.5396232658366
02,-0.72915050365123,0.465909604314975,0.857660805462332
"8848","SMAD3",472.496471619602,0.155676529194019,0.213463569744455,0.
729288512228974,0.465825197854685,0.857660805462332
"8849","SYAP1",482.834583786408,-0.112425750804656,0.154162823761024,-
0.729266291715914,0.465838787413406,0.857660805462332
"8850","MORC2-
AS1",19.7752885931606,-0.250522839019435,0.343745382692397,-0.72880350
292186,0.46612186852899,0.857927583781776
"8851","SH3BP5L",48.6788553203754,0.208789191813024,0.28652136670501,0
.728703741065094,0.466182903905564,0.857942980648258
"8852","ARRDC1",72.0617129645612,-0.192526695688994,0.264289212273386,
-0.728469747338156,0.466326081192536,0.858109527400047
"8853","PTPN22",5.47766495217375,0.563134505353648,0.773671511436783,0
.727872872438915,0.466691410607944,0.858116562851831
"8854","PRPF3",110.212096980838,-0.155515853245365,0.213733077026727,-
0.7276171541l002,0.466847976789335,0.858116562851831
"8855","CKS1B",852.753340602704,-0.193336464655959,0.265811293225865,-
0.727344810333838,0.467014754108999,0.858116562851831
"8856","DUSP12",126.592723630489,-0.200768054194354,0.275816417656262,
-0.727904654481308,0.466671953760787,0.858116562851831
"8857","C1orf229",5.12142122016426,0.482511510422969,0.663075211633664
,0.727687450770738,0.466804934030726,0.858116562851831
"8858","SLC35F6",363.960453999505,-0.160082151642094,0.220050678408752
,-0.727478564482023,0.466932841890702,0.858116562851831

"8859","RCC1L",240.882116992543,-0.144498749180516,0.198496281509728,-0.727967033344321,0.466633766963501,0.858116562851831
"8860","ZNF248",75.5858064517467,0.198099479738335,0.272060437221536,0.728145120111765,0.466524756190701,0.858116562851831
"8861","SPATA7",135.654759184829,-0.155409353330275,0.213393373341465,-0.728276379424369,0.466444418566948,0.858116562851831
"8862","NDC80",8.81214619566751,-0.387686847614095,0.532795216239502,-0.727647012956328,0.466829693900656,0.858116562851831
"8863","DEDD2",65.5022681520805,-0.192645677754713,0.264849897802917,-0.727376824959419,0.466995147344864,0.858116562851831
"8864","DHX35",36.59832315108,0.196730053123496,0.27040844805414,0.727529241557229,0.466901808885389,0.858116562851831
"8865","SH2D1A",6.01498064917778,0.477177749743905,0.655521978567812,0.727935546549402,0.466653042183944,0.858116562851831
"8866","CSNK1G3",407.546109627774,0.223047087934009,0.306774067576859,0.727072824948369,0.46718134493274,0.858238433028261
"8867","MTIF3",555.064629543777,-0.155678324690334,0.214119007050354,-0.72706448079933,0.46718645623805,0.858238433028261
"8868","TNFRSF18",4.7497990146309,-0.60102611154872,0.829617699669559,-0.724461534256214,0.468782434105404,0.858304210466563
"8869","AC253572.2",54.7916618759537,-0.270573310389838,0.372829895479804,-0.725728579360917,0.468005177945452,0.858304210466563
"8870","AL645568.1",23.7065745034078,0.242539101004312,0.334270469784584,0.72557740790149,0.468097875046423,0.858304210466563
"8871","ZNF670",31.162281677079,-0.230318660183678,0.317750437271565,-0.724841363434021,0.468549356748369,0.858304210466563
"8872","DCUN1D1",219.264289197993,0.139474496120594,0.192640702729708,0.724013638572991,0.469057362026428,0.858304210466563
"8873","NCBP2AS2",283.237419591288,-0.201556059807525,0.27758639849407,-0.72610207452881,0.467776197369043,0.858304210466563
"8874","PPIC",1722.90909268536,0.245627846902237,0.338922272939753,0.72473208907666,0.468616404998247,0.858304210466563
"8875","LSM11",32.8212494005112,0.256754584255831,0.353290793085892,0.7267514163422,0.46737824990929,0.858304210466563
"8876","DUSP22",269.935249699835,0.125121093922327,0.172200381234098,0.726601724256531,0.467469971669606,0.858304210466563
"8877","RPL7L1",693.491926597319,0.11831347260489,0.163148435168241,0.725189135175482,0.468336007296956,0.858304210466563
"8878","ARID1B",762.728597082327,0.152342143332444,0.210399262153474,0.724062155794629,0.469027576814412,0.858304210466563
"8879","TBL2",150.612337975984,-0.130177815459662,0.179199160782128,-0.72644210436863,0.467567787545007,0.858304210466563
"8880","COPS6",835.218219053032,-0.167229807440037,0.230271531909389,-0.726228752870077,0.467698548136134,0.858304210466563
"8881","DPYSL2",2825.69782040052,0.16845561941217,0.232483324178598,0.724592269176084,0.468702203039759,0.858304210466563
"8882","AC022893.1",3.42699163639941,0.660295780342649,0.911914593226825,0.724076339217449,0.469018869665648,0.858304210466563
"8883","PIP4K2A",227.598616261971,0.125787131878529,0.173568328661795,0.724712468273116,0.468628444437503,0.858304210466563

"8884","POLR2L",2569.96280092506,-0.220751388394107,0.304059910081087,
-0.726012805618594,0.467830920266442,0.858304210466563
"8885","LINC02709",23.3115452363652,0.472982713121389,0.65166188565649
5,0.725809999835879,0.467955255790417,0.858304210466563
"8886","MCRS1",151.862483249926,-0.185308704105357,0.255945056192114,-
0.72401751712783,0.469054980903991,0.858304210466563
"8887","KITLG",339.069063932428,-0.167572153766429,0.231170555056285,-
0.724885371865933,0.468522355679678,0.858304210466563
"8888","FICD",41.3227346368773,-0.225304615545563,0.310569948419812,-0
.72545530142861,0.468172757159349,0.858304210466563
"8889","CLN5",371.089267766436,-0.178185342597374,0.245261574586188,-0
.726511451694025,0.467525289759366,0.858304210466563
"8890","TPSAB1",13.4035109017299,0.706072712279252,0.973990898686975,0
.724927423070483,0.468496556259023,0.858304210466563
"8891","ABCC6",3.0134983362866,0.633698149622637,0.871826532796156,0.7
26862656485363,0.46731009550862,0.858304210466563
"8892","PRKCB",6.71036927264042,-0.434821361345897,0.599761571728015,-
0.724990365910078,0.468457940789754,0.858304210466563
"8893","YPEL3",1260.75786410248,-0.153908164839305,0.21240164588001,-0
.724609097079458,0.468691876430407,0.858304210466563
"8894","MTHFSD",46.4468952791687,0.186618285219616,0.2576699099402,0.7
24253310225189,0.468910235362871,0.858304210466563
"8895","DCAF15",61.6354276182428,-0.192741859760397,0.26625005371599,-
0.72391294225238,0.469119183853141,0.858304210466563
"8896","HAUS5",38.0488038526891,0.20364308327137,0.280855924300361,0.7
25080248097541,0.468402801069423,0.858304210466563
"8897","SLC35C2",250.587084010819,-0.129130541804294,0.17770357924645,
-0.726662582441336,0.46743268045288,0.858304210466563
"8898","KCNJ15",35.0394513088743,-0.329260885320395,0.454245708052254,
-0.724851945728277,0.468542863976342,0.858304210466563
"8899","C2CD2",117.470933135795,0.191790725560001,0.264154537498008,0.
72605501073949,0.467805047586297,0.858304210466563
"8900","RPS4X",31690.1419704371,-0.183275726714883,0.253154754287232,-
0.723967152941306,0.469085901006356,0.858304210466563
"8901","ARMCX5-
GPRASP2",40.7315440228855,-0.189756640038003,0.26150886159341,-0.72562
2217472053,0.468070397124351,0.858304210466563
"8902","SLC25A43",161.693307148039,0.111773793028879,0.153894558313173
,0.726301139260693,0.467654181108704,0.858304210466563
"8903","ARHGEF6",145.701114364711,0.160908689251285,0.221852809441565,
0.725294800892155,0.468271194621979,0.858304210466563
"8904","TTC34",3.21827490369601,0.592032550846722,0.819024121634547,0.
722851177649307,0.469771321298522,0.858385858586662
"8905","PDIK1L",41.2256988047916,-0.238521745299861,0.329891799942646,
-0.723030233977716,0.469661309518095,0.858385858586662
"8906","EXOC8",55.409434127572,-0.189013060033321,0.261456472479306,-0
.722923621821149,0.469726810057141,0.858385858586662
"8907","INPP5D",8.62778751448331,-0.476742120624227,0.65961680305659,-
0.722756179671375,0.469829693610922,0.858385858586662
"8908","ZBED3-

AS1",11.5786003866787,0.386450172987295,0.53410737194134,0.72354397877
4624,0.469345744998346,0.858385858586662
"8909","PFKFB3",161.624866041105,0.383091865017358,0.529890520909182,0
.722964178260922,0.469701892334956,0.858385858586662
"8910","MRVI1",21.1772184057817,-0.432553244953804,0.598044219919092,-
0.723279701645345,0.469508060922518,0.858385858586662
"8911","PRMT7",95.5297454087527,0.173763763103195,0.240421622651721,0.
722745987597431,0.469835956455184,0.858385858586662
"8912","P3H4",170.613789298754,0.196081663112806,0.271210958717673,0.7
22985767389013,0.469688628354824,0.858385858586662
"8913","CUEDC1",218.79945837498,-0.14172725031026,0.196101223991806,-0
.722724965328017,0.469848874403504,0.858385858586662
"8914","CDR2L",40.202190415226,0.214449819618478,0.296306145810634,0.7
23744082431319,0.469222864316187,0.858385858586662
"8915","RETN",2.53170538595417,-2.2264762102695,3.07901459650402,-0.72
3113236552202,0.469610317765528,0.858385858586662
"8916","PRAF2",186.750710705244,-0.190975508380012,0.264038134374815,-
0.723287599468164,0.469503209722228,0.858385858586662
"8917","MAP3K14",32.2006864619029,-0.286746972998291,0.396904873846778
,-0.722457676619479,0.470013137422408,0.858589659692005
"8918","LINC00685",42.395058896262,0.340177516238532,0.471180109567383
,0.721969177669381,0.470313427774729,0.859041873180372
"8919","TNIK",16.9160043681949,0.329140005395844,0.455966149889156,0.7
21851842457729,0.470385571921061,0.859077315957188
"8920","AL034417.3",55.5654451111931,0.674611504133958,0.9374080792954
02,0.719656165798172,0.471736721345584,0.859285647381337
"8921","SMIM12",170.600523730539,-0.183871898531714,0.255343683382445,
-0.72009573957746,0.471466050491035,0.859285647381337
"8922","FAM78B",17.0245178903274,-0.312728087360942,0.434466794864693,
-0.719797441501453,0.471649720430691,0.859285647381337
"8923","SELL",6.95109100567007,-0.442437540123823,0.614547028940855,-0
.719940898398523,0.471561385337746,0.859285647381337
"8924","ATOH8",219.748503823194,0.195763682590795,0.271666233051919,0.
720603662779769,0.471153399646326,0.859285647381337
"8925","LINC01116",275.487127338631,0.271545905215534,0.37646930917443
,0.72129626133672,0.470727256735129,0.859285647381337
"8926","RPE",164.553144730544,-0.137209691379596,0.190551030530545,-0.
720067957636163,0.471483154894262,0.859285647381337
"8927","GAK",131.381263104394,0.168490452230397,0.234153998453276,0.71
9571108515655,0.47178910589531,0.859285647381337
"8928","CARD6",297.271122924206,0.156934427181744,0.217975962835113,0.
719962078114349,0.471548344477846,0.859285647381337
"8929","TUBB2A",387.381389066428,-0.214338346061905,0.298047437239193,
-0.719141718000718,0.472053605198035,0.859285647381337
"8930","RXRB",145.243671660823,0.136122844854032,0.188988649737219,0.7
20269947657203,0.471358804258645,0.859285647381337
"8931","AC073111.4",59.1818870334812,0.195921092987146,0.2717179815827
77,0.721045739578557,0.470881373513451,0.859285647381337
"8932","UBE2W",160.114742272908,0.124576592624035,0.172916231041994,0.
720444760294254,0.471251199374005,0.859285647381337

"8933","RMDN1",181.647291387565,-0.134843542355893,0.187088403719367,-0.720747730351895,0.471064740042795,0.859285647381337
"8934","RNF139-AS1",10.4617797626693,-0.360526907374629,0.499948246821348,-0.7211284561289,0.470830484601857,0.859285647381337
"8935","C9orf170",4.59022387537605,0.555628438952927,0.771260395497955,0.720416142454964,0.471268813988112,0.859285647381337
"8936","AL137026.1",80.3389347807008,0.198807345789201,0.276558748016273,0.718861172229139,0.472226462082883,0.859285647381337
"8937","AL355334.2",2.33172168206536,0.758725953173653,1.05467387650167,0.719393899932682,0.471898254312693,0.859285647381337
"8938","SART1",180.791864022768,-0.159565032722499,0.221464416505535,-0.720499641614033,0.471217420297155,0.859285647381337
"8939","U47924.2",10.1884699095952,-0.373016203331516,0.518758790253403,-0.719055195477854,0.472106911902572,0.859285647381337
"8940","FGD4",220.50165041355,0.218465977294356,0.303827280437866,0.719046614179972,0.472112199038654,0.859285647381337
"8941","FKBP11",426.395164149105,0.214171967514522,0.296964952060162,0.721202842385027,0.470784723249691,0.859285647381337
"8942","CKAP2",52.6137982009958,0.244460758984115,0.339791546843983,0.719443321220586,0.471867812766111,0.859285647381337
"8943","CIAO2A",185.112395056465,0.146336719314278,0.202830820175144,0.721471811768629,0.470619277650711,0.859285647381337
"8944","AC027682.6",12.7405081545414,-0.430293620230614,0.598319439217217,-0.719170382953908,0.47203594539277,0.859285647381337
"8945","ZNF232",28.3420237901277,0.238749143723918,0.330905008920531,0.721503565336648,0.470599747850744,0.859285647381337
"8946","CISD3",293.965746813223,-0.175899393757363,0.244197869388159,-0.720315022395901,0.471331057473376,0.859285647381337
"8947","AP000919.4",5.30430653382819,0.591897115037136,0.821321555287705,0.720664289432653,0.471116088705757,0.859285647381337
"8948","SIRT2",311.986447195204,-0.138264013375306,0.192319401667617,-0.71892909491402,0.472184608658782,0.859285647381337
"8949","ZNF81",11.8558654792821,-0.317366886457824,0.440058338313255,-0.721192757474594,0.470790927197323,0.859285647381337
"8950","TMSB15B.1",35.1237716787543,-0.196841154193416,0.27350428400363,-0.719700442391619,0.471709453821791,0.859285647381337
"8951","TMEM255A",30.5779437948556,0.359637173672773,0.500303209530569,0.718838430019703,0.472240476110119,0.859285647381337
"8952","LAMP2",1199.84052359969,0.140795983297672,0.195820573294612,0.719005061260054,0.472137801215049,0.859285647381337
"8953","SLC25A15",10.2461688657624,0.405387829630608,0.564032412601622,0.718731442685608,0.472306406086131,0.859309622331842
"8954","MED9",50.7441240117461,0.19347920487024,0.269239437509229,0.718613909834837,0.472378840485416,0.859345424689183
"8955","MRTFA-AS1",2.89349474907562,0.679077945303356,0.945155707166018,0.718482616308293,0.472459762695277,0.859396658240465
"8956","PRPF40B",46.3164503412299,0.188166095585961,0.261939051347136,0.718358315105116,0.472536382251119,0.859440054766467

"8957","ZNF583",44.6990608550719,0.208427765846974,0.290203365198161,0
.718212780560459,0.47262609878368,0.859507259471627
"8958","ESD",1607.71043170291,-0.175416499115902,0.244369195583882,-0.
717833926231051,0.472859692103182,0.859767822922872
"8959","GDF15",73.2365455245221,-0.414954332479154,0.578084446557771,-
0.717809197168368,0.472874941713181,0.859767822922872
"8960","HNRNPA3",1167.25759409704,-0.154134141837219,0.214766592300516
,-0.717682113340721,0.472953314453207,0.859814345884854
"8961","PRRT3",37.2997455850508,-0.208753058372404,0.291002098447411,-
0.717359288768598,0.473152432873054,0.860080345839659
"8962","TAF5",32.521273495795,0.23721609121607,0.330764898148684,0.717
174320926393,0.473266541948835,0.860095805177348
"8963","ATP1B2",7.39666780170516,-0.498635615660458,0.695264590793339,
-0.717188279488654,0.473257930201496,0.860095805177348
"8964","TUBGCP6",69.7755391557896,0.171013918367624,0.238511294036384,
0.717005536607993,0.473370680406892,0.860189091158842
"8965","ZNF571",44.4714594084616,0.188532147719623,0.262988012024863,0
.716884949500279,0.473445089323658,0.860228339095712
"8966","CPXM2",282.406767405681,0.265690361888481,0.370704003191288,0.
716718351032699,0.473547900377549,0.86031917792214
"8967","FXN",79.2549557535429,-0.206252886084843,0.287935765341747,-0.
716315619353659,0.473796484393411,0.860578828533037
"8968","SPATA2L",50.9584631444489,-0.188802498192693,0.263571685585878
,-0.716323142878624,0.473791839879621,0.860578828533037
"8969","EPRS",510.288459169635,0.106249817076224,0.148373557505274,0.7
16096714688851,0.473931632237968,0.860728326181766
"8970","SETD5",493.10668988941,0.162755261982518,0.227386963175314,0.7
15763382868322,0.474137466065257,0.86100615214459
"8971","ZBP1",3.38683221648131,-0.597763050171544,0.835357693053846,-0
.71557735703167,0.474252359170704,0.861118791498338
"8972","AKT3-
IT1",4.87024362505895,0.549828661153353,0.768480703747159,0.7154749084
42534,0.474315639904991,0.861137701561792
"8973","IRF1",3156.40888350052,-0.321806198049576,0.44992434699915,-0.
715245129977783,0.474457586985512,0.861203435971362
"8974","AL109615.2",2.86560390826021,-0.658107712734456,0.920014396755
765,-0.715323276521686,0.474409308852112,0.861203435971362
"8975","CNIH1",595.556145478543,-0.133098307132823,0.186111392975287,-
0.71515400000524,0.47451388955625,0.861209665401866
"8976","APH1A",630.128236625451,-0.124727478743385,0.174458285681514,-
0.714941558987251,0.474645155631279,0.861267790819345
"8977","GRHL1",41.9084634999127,-0.465420195607368,0.651000136306291,-
0.71493102635602 3,0.474651664201931,0.861267790819345
"8978","CDON",559.420204591891,0.168213915215217,0.235332029116113,0.7
14793969384508,0.474736362130173,0.861325529376073
"8979","TMEM234",69.8655745961263,-0.204881097216182,0.286729359227092
,-0.714545234462422,0.474890095553576,0.861430016078132
"8980","MRPL37",248.836586900824,-0.151696912929769,0.212355315710992,
-0.714354205930132,0.475008181444146,0.861430016078132
"8981","MTCH1",1848.97735780127,-0.126033441358876,0.176471087338996,-

0.71418748112981,0.475111256949226,0.861430016078132
"8982","ADD3-
AS1",23.3022545837989,0.272260224367601,0.381066028500576,0.7144699448
51537,0.474936634534229,0.861430016078132
"8983","OAF",1856.86049991611,0.239474218540308,0.335273554816306,0.71
426515780976,0.475063232794273,0.861430016078132
"8984","TRMU",71.4812724455707,-0.1686674661112,0.236099106080975,-0.7
14392650234568,0.474984415479369,0.861430016078132
"8985","SFN",2.41215165224545,-0.652535014221121,0.91401180613586,-0.7
13924054197751,0.475274142344674,0.861535072220604
"8986","AGBL3",15.5275199168635,0.326561882137613,0.457473910012511,0.
713837171891795,0.475327871204283,0.861535072220604
"8987","SAP18",2090.69882311123,-0.189659327716798,0.265686654263857,-
0.713845895806438,0.475322476100455,0.861535072220604
"8988","SPEN",322.286799132293,0.15894333014392,0.222811456434256,0.71
3353490379513,0.475627045446009,0.861723984202783
"8989","MMACHC",22.9606570757666,0.303646368962651,0.42566402911402,0.
713347495193952,0.475630754329647,0.861723984202783
"8990","GTF3C6",502.54063385988,-0.182900228306496,0.256520473614814,-
0.713004407520063,0.475843029774527,0.861723984202783
"8991","MAP7",5.8398957507725,-0.557716921279191,0.782011345018223,-0.
713182647326165,0.475732742634768,0.861723984202783
"8992","WNT2",26.8836253038325,-0.429991607345195,0.602893487170396,-0
.71321322338927,0.47571382488824,0.861723984202783
"8993","ABCC2",325.819654733267,0.545767433342035,0.765371341570482,0.
713075344867451,0.475799135110206,0.861723984202783
"8994","AMPD3",22.4634011501089,0.292387846471089,0.409763379506013,0.
713552896853726,0.47550369292973,0.861723984202783
"8995","TSPOAP1",4.74417959685776,0.638504294617192,0.89553734632167,0
.712984553061672,0.475855315728653,0.861723984202783
"8996","TAB3",50.8685588890452,0.204801481648836,0.287302367491067,0.7
12843000345979,0.475942913698045,0.861786807606431
"8997","GXYLT2",331.735258363514,0.152046265423108,0.213331870822455,0
.712721755248881,0.476017951602657,0.861826877143012
"8998","PRKAG2-
AS1",135.925463455553,-0.195136738524506,0.27389914698397,-0.712440110
432057,0.476192285012642,0.861855125618991
"8999","UBXN8",74.4861987081848,0.20056741469535,0.281503405153214,0.7
12486637901189,0.476163482878407,0.861855125618991
"9000","KIZ",254.309235199689,-0.123321654381052,0.173072042347027,-0.
712545207814559,0.476127227406534,0.861855125618991
"9001","GNL2",142.689056324352,-0.14598490303802,0.204979651477507,-0.
712192171202123,0.476345784185645,0.862037160159978
"9002","RUNX2",31.2607644667686,0.235725832641472,0.331073269391219,0.
712005028599641,0.476461662119766,0.862055316479936
"9003","ACCS",113.507024938087,0.237198494556047,0.333137061433197,0.7
1201472911957,0.476455655218023,0.862055316479936
"9004","FBXO42",143.029247234053,0.158113356311981,0.22213915594009,0.
711776164102397,0.476603395069176,0.862180917826933
"9005","SNHG5",2260.8939440956,0.256256424478701,0.360137772651213,0.7

11551089440653,0.476742803540253,0.862180917826933
"9006","RBM14",110.188216742046,0.158218173831895,0.222340043468113,0.
711604492667944,0.476709724219012,0.862180917826933
"9007","ZNF93",22.5738188634416,0.310368396756713,0.436134500018535,0.
711634591493044,0.476691080792861,0.862180917826933
"9008","SLC25A20",59.1013507225799,0.253489315201901,0.356343591647847
,0.711362070606307,0.476859896725947,0.86229694246991
"9009","C17orf75",62.4787188748726,0.169612922069805,0.238516688476368
,0.71111553306095,0.4770126452026,0.862477409002681
"9010","PHKB",287.283893046676,0.128098260411931,0.180168476758853,0.7
10991527021595,0.477089486356495,0.86252060413551
"9011","BAG6",286.434311081389,-0.110459594899247,0.155424212801392,-0
.710697470544029,0.477271727458067,0.862658585060414
"9012","MAGEH1",423.891659694496,-0.175038154043774,0.246261152398951,
-0.710782648171021,0.477218934820732,0.862658585060414
"9013","AL442003.1",5.02793028826812,0.520487515878101,0.7324718286941
59,0.710590490293694,0.477338037772337,0.862682713555264
"9014","AK4",93.4274157907103,0.207490932477936,0.292045911158143,0.71
0473677426629,0.477410448463286,0.862717860552304
"9015","AC135178.6",6.19142129147114,-0.483538306012921,0.680779470480
617,-0.710271573952653,0.477535743822836,0.862848555865799
"9016","AC246817.2",7.44633822000922,-0.632333933389284,0.890508522318
693,-0.710081843734434,0.477653384671384,0.862965392958316
"9017","S1PR1",14.607080999071,0.319633269923361,0.450847556038473,0.7
08960857483466,0.478348767485705,0.862983729769519
"9018","PRPF38B",412.211523183232,-0.136132396077967,0.191922682980918
,-0.709308529682768,0.478133036387189,0.862983729769519
"9019","TADA1",30.5233225069901,0.229238643761302,0.323195063888544,0.
709288814634735,0.478145268180217,0.862983729769519
"9020","SPR",126.68957717354,-0.19213296374678,0.27100968890296,-0.708
952379247136,0.47835402890781,0.862983729769519
"9021","AC008063.1",6.87980157567125,0.725612540039385,1.0223900345053
,0.70972184347482,0.477876643794402,0.862983729769519
"9022","LINC01060",7.99390886389238,-0.842319688784783,1.1876182553348
7,-0.7092512135116,0.478168597492119,0.862983729769519
"9023","AC040970.1",6.50320826632206,-0.507997169818231,0.716715984364
943,-0.708784484928641,0.478458227242221,0.862983729769519
"9024","SCNN1A",6.35084511266033,-0.457916134363327,0.64512662108366,-
0.709808151451161,0.477823113513554,0.862983729769519
"9025","TRPV4",8.55157494778234,0.369386223340811,0.521070305579324,0.
708899009184814,0.478387150015888,0.862983729769519
"9026","UNC119B",62.4603864475154,0.178016030615515,0.250932210566097,
0.709418811614163,0.478064617402638,0.862983729769519
"9027","AL133445.2",3.17633842065254,-0.59424805312834,0.8380204662365
9,-0.709109236671759,0.478256691480622,0.862983729769519
"9028","BBS4",157.494468931111,0.116461059604009,0.164307680104361,0.7
08798636375598,0.478449444112658,0.862983729769519
"9029","AC087393.2",4.58687530348933,0.670676217645718,0.9450821860750
63,0.709648565518989,0.477922095117313,0.862983729769519
"9030","AC090409.1",8.73606641060149,-0.370169570804266,0.521910879257

602,-0.709258200041389,0.47816426270931,0.862983729769519
"9031","DNTTIP1",170.850287436885,0.170363537242853,0.240070439165453,
0.709639795032995,0.477927535249918,0.862983729769519
"9032","NOP56",418.170382932043,0.140460608221236,0.19820865908063,0.7
08650211715003,0.478541568618428,0.863038486628163
"9033","UBIAD1",66.8826544630452,-0.157356090425464,0.222130238795043,
-0.708395629874845,0.478699605532324,0.863070684549602
"9034","ING5",122.175029893095,0.144689786617407,0.204277851577745,0.7
08298944304985,0.478759632557059,0.863070684549602
"9035","NHLRC2",189.06720247282,0.160790157142834,0.227014944667827,0.
708280053448049,0.478771361384382,0.863070684549602
"9036","AC090517.5",39.4727756135406,-0.20451034651975,0.2886513381821
56,-0.708503025857072,0.478632933791057,0.863070684549602
"9037","RBP5",43.2460298575658,-0.407239573021506,0.575043471400116,-0
.708189194862015,0.478827775240487,0.86307686520884
"9038","ALDH1L2",269.247898923403,0.157461388684762,0.222436692540151,
0.707893049867839,0.479011676082387,0.863312811651471
"9039","TAF13",116.096251381968,-0.144806186770701,0.204635777787078,-
0.707628882576784,0.479175751868896,0.863512979554426
"9040","SWT1",57.6470265524121,0.156959614500411,0.221865870522797,0.7
07452724164185,0.479285181895035,0.863614638040733
"9041","SEC13",404.139941779151,-0.149468024262755,0.211338415088242,-
0.707244937936843,0.479414276726265,0.863617763692766
"9042","DND1",2.93709140052097,-0.592230501074313,0.837437198386269,-0
.707193927157206,0.47944597194878,0.863617763692766
"9043","ABCD2",6.18185107864939,0.594582063567202,0.840736516120537,0.
707215699766222,0.479432443537194,0.863617763692766
"9044","FAM3C",1006.449811583,-0.137744461038347,0.194812996214306,-0.
707059917536609,0.47952924341913,0.863672251885692
"9045","RSPH3",25.3885919548089,-0.334958558483264,0.473837861849552,-
0.706905432115125,0.479625248021163,0.863749658929433
"9046","FECH",101.954466328869,-0.142623705328592,0.201810786218648,-0
.706719933066752,0.479740539822148,0.863791961799588
"9047","VAPB",222.43723176309,0.115675329232989,0.163684478337031,0.70
6696996613266,0.479754796390256,0.863791961799588
"9048","ZNF226",119.010477722079,0.185286469983509,0.262273155081746,0
.706463724530856,0.47989980400423,0.863957549450143
"9049","CEP97",91.1717945403374,0.144779059746224,0.205210696297917,0.
705514197642211,0.480490299655142,0.864041402510741
"9050","STYXL1",76.6265337781774,0.159901948596297,0.226435281216776,0
.706170645038376,0.480082023237473,0.864041402510741
"9051","FASTK",276.398699920033,-0.143299397628355,0.203063732890365,-
0.705686808711053,0.480382926143564,0.864041402510741
"9052","SLC25A25-
AS1",3.80419595373556,-0.601224443998065,0.852051819401356,-0.70561957
654228,0.480424746669416,0.864041402510741
"9053","CEP57",413.570517150957,0.0935810698801199,0.132546789863121,0
.706022906905252,0.480173892237923,0.864041402510741
"9054","COL4A2",1102.97879601664,-0.192132763394211,0.272252522127808,
-0.705715274527429,0.480365220107372,0.864041402510741

"9055","CHMP1A",239.772100892169,-0.131194097245272,0.185972057049051,
-0.705450589335953,0.480529870792854,0.864041402510741
"9056","RSKR",10.5840010523828,0.350531488979217,0.496478195793464,0.7
06036019203227,0.480165738141639,0.864041402510741
"9057","ARSG",76.1611553773619,-0.163673436263575,0.231958454048305,-0
.7056153091514,0.480427401187869,0.864041402510741
"9058","GK",25.2761974647583,-0.25376146692601 8,0.359465039402795,-0.7
05941994658533,0.480224210507849,0.864041402510741
"9059","PCDH19",8.02623968718598,0.509041308271363,0.720847231267126,0
.706170858666621,0.480081890402519,0.864041402510741
"9060","PIK3CD",16.0591371277652,0.329728575169776,0.467835641758875,0
.70479575675365,0.480937349588954,0.864045319796157
"9061","RPL11",29720.5608990231,-0.185161174316981,0.262892451744313,-
0.704322901203217,0.481231707576193,0.864045319796157
"9062","RPS27",36189.8296511289,-0.201208595474583,0.285297866729815,-
0.705257973993664,0.480649708869496,0.864045319796157
"9063","LAPTM4A",9252.17643062036,-0.165842448314251,0.235261668131618
,-0.704927622214555,0.480855279320426,0.864045319796157
"9064","RAB10",490.282791685534,-0.0989203542521632,0.140269785716675,
-0.705214980879547,0.480676459802967,0.864045319796157
"9065","HHIP",23.1005831999595,-0.946847001069052,1.34391440574959,-0.
704544126484702,0.481093980101143,0.864045319796157
"9066","HOXA9",60.0348054994352,-0.258024359109674,0.366215310133982,-
0.704570103896732,0.481077808839804,0.864045319796157
"9067","TRIM32",64.7594590885841,0.200069294047583,0.284224397036961,0
.703913162041347,0.481486854183173,0.864045319796157
"9068","CHUK",45.0246890920306,-0.175201813500481,0.248663053866699,-0
.704575170199597,0.481074655037894,0.864045319796157
"9069","EIF3A",859.317802273273,-0.135695982453965,0.192629858029246,-
0.704438989065562,0.481159432472143,0.864045319796157
"9070","KLRB1",29.5641880676701,0.298423423072274,0.423650176666204,0.
704410005020258,0.481177477085802,0.864045319796157
"9071","NAA60",155.069239923545,0.145526968392699,0.206726411681115,0.
703959243568647,0.481458155314043,0.864045319796157
"9072","NQO1",168.028024381581,0.252541732093313,0.358703629824697,0.7
04040079596444,0.481407814123463,0.864045319796157
"9073","MPDU1",138.328838892063,0.182304674699123,0.258561870903801,0.
705071765074558,0.480765576585881,0.864045319796157
"9074","CLPP",331.185323887486,-0.176116616141832,0.249762165331322,-0
.70513728894128,0.480724802900631,0.864045319796157
"9075","AC008622.2",7.87316669749081,0.449870127433173,0.6387162284320
58,0.704334894601832,0.481224240336349,0.864045319796157
"9076","AP001505.1",14.0858486976505,-0.342971942989622,0.487122889417
414,-0.704076836544896,0.481384924429307,0.864045319796157
"9077","TCEAL4",1063.48217087874,-0.130619858018064,0.185557643064192,
-0.703931435326956,0.481475473749498,0.864045319796157
"9078","KATNBL1",315.759083580666,-0.0978607141037411,0.13905352885394
6,-0.703762895557496,0.481580444314032,0.864118071979651
"9079","UBA7",153.092186241893,0.12193795380184,0.173611205983206,0.70
2362230083441,0.482453293566787,0.86420889283321

"9080","AC107223.1",11.56198413973,0.357742653199005,0.509373089667631
,0.702319499116913,0.482479935626816,0.86420889283321
"9081","AC093726.2",3.26364755029571,0.595316705471882,0.8462838389990
64,0.703448037216437,0.481776578911605,0.86420889283321
"9082","SNTB1",26.0401486071164,0.311513284833245,0.443048506929255,0.
703113270807132,0.481985162488386,0.86420889283321
"9083","UBQLN1",272.102819605107,-0.0995739906318563,0.14177022352452,
-0.702361808822533,0.482453556212184,0.86420889283321
"9084","LINC02739",4.96447563309962,0.50153911420581,0.7135139528366,0
.70291423484001,0.482109199502985,0.86420889283321
"9085","MSANTD2",77.0374912777199,-0.150906237248716,0.214830923145349
,-0.702441878661094,0.482403636113144,0.86420889283321
"9086","MFGE8",1305.77926041483,0.10775357418989,0.153148128346847,0.7
03590539127266,0.481687804863035,0.86420889283321
"9087","WWP2",80.0731241632666,0.170560101800072,0.242840498468682,0.7
02354437894833,0.482458151809565,0.86420889283321
"9088","RNF157",3.40765848281667,0.6254344751163,0.889950839462459,0.7
02774184126923,0.48219648796147,0.86420889283321
"9089","ERF",123.453187089615,-0.160739232404431,0.228591871978267,-0.
703171250199624,0.481949033636275,0.86420889283321
"9090","AC011481.2",4.50843452727738,0.510346726417966,0.7260958128272
61,0.702864163932835,0.48214040587104,0.86420889283321
"9091","CCN5",20299.3252421207,-0.193579645233804,0.275450776260733,-0
.702774005075113,0.482196599563377,0.86420889283321
"9092","Z82217.1",17.1328525272075,-0.42382082436412,0.603410773493431
,-0.702375302168406,0.482445143494936,0.86420889283321
"9093","ZRSR2",113.190309494927,-0.164112047737932,0.23353019779099,-0
.702744438579256,0.482215028373524,0.86420889283321
"9094","RP2",80.38346740965,-0.14081577430208,0.200311449673781,-0.702
98415058853,0.4820656268038З,0.86420889283321
"9095","FAM66C",37.16881150368,0.210493758434595,0.299852558714093,0.7
01990869570333,0.48268485750239,0.864480884426215
"9096","APRT",1570.02689349452,-0.208710109703093,0.297402641026558,-0
.701776248464635,0.482818713229362,0.864574345380368
"9097","PALM",748.66600581521,-0.114824046065117,0.163628320271539,-0.
701736996838741,0.482843196017264,0.864574345380368
"9098","LCA5",293.138731947757,0.134219188424703,0.191319305849397,0.7
01545449523835,0.482962681310767,0.864598210338617
"9099","SMC5-
AS1",6.10616463438432,0.464433672364765,0.661945493134448,0.7016192075
96046,0.482916669863999,0.864598210338617
"9100","BBS9",86.2652507362094,0.149309181460976,0.212881932907387,0.7
01370846373946,0.483071610993759,0.864671269996156
"9101","SAP30BP",279.926761808534,-0.122467034316391,0.17462613948326,
-0.701309865056776,0.483109658557003,0.864671269996156
"9102","SUGP1",75.3398369062219,-0.171600850638654,0.244726793203205,-
0.701193557078844,0.483182230132346,0.864706146629948
"9103","MED8",266.911012629049,-0.153909508036174,0.219595593553911,-0
.700877032846242,0.483379758697149,0.864739781980593
"9104","IL1R2",9.79263073453013,-0.470732747291419,0.671497574092753,-

0.701019282053873,0.483290981929522,0.864739781980593
"9105","HPS3",95.3815128387658,0.17773586675178,0.253610145193409,0.70
0823173364118,0.483413374345588,0.864739781980593
"9106","ACTR1A",222.916979609142,-0.143298876311741,0.204459402831604,
-0.700867137080333,0.48338593490698,0.864739781980593
"9107","FOXD2",4.4573329356898,0.57057230968567,0.815915599466309,0.69
9303102010652,0.484362628860787,0.86480046034814
"9108","CD47",1063.93756553728,-0.13712503723379,0.196106219251327,-0.
699238595070013,0.484402934508958,0.86480046034814
"9109","AC092944.1",113.634909280728,-0.161022010859179,0.230045818616
173,-0.699956260138949,0.483954620751636,0.86480046034814
"9110","TBCCD1",45.9288485586673,-0.174256365973046,0.249151053218277,
-0.699400479035438,0.484301788555736,0.86480046034814
"9111","EXOC2",62.3432219936844,0.195531322562508,0.279180573019434,0.
700375819304936,0.483692633208515,0.86480046034814
"9112","RIPK1",119.796154084905,0.123651259333948,0.176525595583727,0.
700472126577812,0.48363250641154,0.86480046034814
"9113","DTNBP1",212.600017794745,-0.142583386884657,0.203670718327997,
-0.700068169126976,0.483884733305835,0.86480046034814
"9114","BEND7",78.5735680838972,-0.222702106785227,0.317994679214834,-
0.70033280850832,0.483719487126541,0.86480046034814
"9115","FEM1B",234.117868297898,0.128580176215065,0.183839103530183,0.
699416901768968,0.484291528185186,0.86480046034814
"9116","TEPSIN",39.8807356320192,0.183105516085407,0.261724406339442,0
.699611926325009,0.484169692424013,0.86480046034814
"9117","CCDC57",46.2437292199232,0.226131471751887,0.322938123406458,0
.700231578008124,0.483782693880938,0.86480046034814
"9118","NARS",502.494284471827,-0.14078901695203,0.201277762354487,-0.
699476262579244,0.484254442533953,0.86480046034814
"9119","ANGPTL4",1218.34260213788,-0.266912260280952,0.381227884876695
,-0.700138344725971,0.483840911260014,0.86480046034814
"9120","PODNL1",13.7264927856961,0.359271137101125,0.513328474545473,0
.699885463044383,0.48399883654334,0.86480046034814
"9121","TMEM143",33.2567724305934,0.216807853804464,0.310041569316728,
0.699286403053202,0.484373062638093,0.86480046034814
"9122","TBC1D20",210.139872217902,0.146010222285549,0.208493007927628,
0.700312320959136,0.483732278872868,0.86480046034814
"9123","LINC01273",4.71990160013424,-0.545924021362677,0.7798097512703
19,-0.700073345419649,0.483881500830457,0.86480046034814
"9124","AP000317.1",2.36340905929084,-0.651703581595339,0.931411074196
483,-0.699694903410458,0.484117860010997,0.86480046034814
"9125","RASSF5",77.8812918045345,0.187099629606637,0.267791237704024,0
.698677190526408,0.484753791718379,0.864902938676674
"9126","STAM",226.932518137515,0.12767137301258,0.182752231775068,0.69
8603632757375,0.484799772821281,0.864902938676674
"9127","RNF121",115.559671492049,-0.139129808803101,0.199101504840274,
-0.698788333692982,0.484684320393909,0.864902938676674
"9128","KNTC1",28.0613198761502,0.212818426074949,0.304656504099365,0.
698552051938261,0.484832017499951,0.864902938676674
"9129","ZNF724",18.5159784400898,0.259912544895858,0.371828044617671,0

.699012752421918,0.484544061312458,0.864902938676674
"9130","FAM98C",154.751705001413,-0.135489784694437,0.193904264264471,-0.698745771313408,0.484710923865149,0.864902938676674
"9131","ZNFX1",231.418287810627,0.184533687927692,0.264118171421018,0.698678500365412,0.484752972956912,0.864902938676674
"9132","RAI1",64.8292710175395,-0.19684705406521,0.281829377839521,-0.69846179832004,0.484888440471495,0.86490887065705
"9133","PER1",480.277369746415,0.202282211354961,0.289666444228914,0.69832807832965,0.484972043428615,0.864963277719119
"9134","CREM",177.763884335782,-0.162867467443814,0.233255336981286,-0.698236831583757,0.485029096207463,0.864970324953292
"9135","NSUN5",116.851631773571,-0.153030592923608,0.219249056898455,-0.697976060140976,0.485192165736203,0.865166413538808
"9136","AC087623.3",10.2079065675866,0.334560871559002,0.479450157970727,0.697801149915208,0.485301559867148,0.865172059612123
"9137","EIF3E",3329.61828031006,-0.165901256244496,0.237735825902819,-0.697838685500908,0.485278082854351,0.865172059612123
"9138","AL158071.3",3.32785352453734,0.577028711308228,0.827152260964298,0.697608818279146,0.485421865287306,0.865291832311767
"9139","TRNAU1AP",207.765801111949,-0.11839016037925,0.169988337652675,-0.696460486725553,0.486140494345177,0.865637809658674
"9140","ANAPC13",368.952624561534,-0.154878907847483,0.222374967093243,-0.696476360950023,0.486130556291384,0.865637809658674
"9141","LIFR-AS1",39.5315235342646,0.238026995167514,0.342012036835331,0.695960871348271,0.486453334553662,0.865637809658674
"9142","WRNIP1",125.335626299632,-0.120922376580445,0.173737418464172,-0.69600652323139,0.486424744558094,0.865637809658674
"9143","EZR",32.7394103707753,-0.357324906272559,0.513182895143687,-0.696291535929917,0.486246272750976,0.865637809658674
"9144","CRCP",228.158550079648,-0.145020822536579,0.208110586050909,-0.696845005765844,0.48589979721375,0.865637809658674
"9145","PHYHIPL",17.3913654029384,0.481885400263756,0.691888081297423,0.696478828425732,0.486129011538774,0.865637809658674
"9146","MBD6",124.90023605413,0.142933310889516,0.205308472845648,0.696188076938131,0.486311053536044,0.865637809658674
"9147","LINC01481",3.82764641295884,-0.516152211680703,0.741631306221463,-0.695968748016378,0.486448401638497,0.865637809658674
"9148","UCHL3",192.758042204056,-0.196663390803052,0.28258659361897,-0.695940271916177,0.486466235472742,0.865637809658674
"9149","RHOJ",135.874797532662,0.213567670411424,0.306866968962173,0.69596174242445,0.486452789024091,0.865637809658674
"9150","UNC45A",194.816451827186,-0.115242683778962,0.165459294550491,-0.696501723230751,0.486114678470634,0.865637809658674
"9151","ZNF213",45.7471368970479,-0.201748654494557,0.289583810648156,-0.696684852799595,0.486000040226052,0.865637809658674
"9152","BAIAP2-DT",34.7403149335662,0.240288905283885,0.344907343150992,0.696676687392801,0.486005151420615,0.865637809658674
"9153","ZNF561",87.9024045153651,0.150470099159988,0.216132211337111,0

.696194695964558,0.486306908896937,0.865637809658674
"9154","RPL10",61070.3689336937,-0.179518146968707,0.257484254990016,-0.69720048309621,0.48567733666644,0.865637809658674
"9155","AKAP9",1236.48177470759,0.161957700598915,0.23274736880922,0.69585190770371,0.486521577909984,0.865641723929626
"9156","ZNF786",16.2337607647556,-0.253061374818351,0.363913395043287,-0.695389008113454,0.486811547163467,0.865684857177479
"9157","ARGLU1",1592.93634299435,0.112454286091295,0.161646874386256,0.695678691705381,0.486630072827807,0.865684857177479
"9158","LGMN",895.349371646426,-0.114824809527512,0.165091965484343,-0.695520276778106,0.486729308449389,0.865684857177479
"9159","ZKSCAN2-DT",13.7399455676949,-0.395950481683029,0.569326697637132,-0.695471481183539,0.486759877602186,0.865684857177479
"9160","RBM38",46.5534980745,0.221272494239966,0.318162871443297,0.695469252072679,0.486761274106116,0.865684857177479
"9161","ASH1L-AS1",16.1563671496032,-0.278653618808934,0.400943439877473,-0.694994832423469,0.487058540032174,0.865733056244532
"9162","NUP133",110.003813473864,0.133763478535093,0.192448795523188,0.695060097266114,0.48701764001215,0.865733056244532
"9163","CDKAL1",84.9825048348326,0.14724105515323,0.211959105454847,0.694667279507822,0.487263838151505,0.865733056244532
"9164","BEST1",39.4281409332035,-0.259700932160562,0.373722005978555,-0.694904040987793,0.487115440083764,0.865733056244532
"9165","YARS2",112.547944922073,-0.18087231244324,0.260356021819597,-0.694711461556161,0.487236143742635,0.865733056244532
"9166","GDPD1",13.3165268693468,-0.343937602139622,0.495100752735571,-0.694682042471698,0.48725458426331,0.865733056244532
"9167","AC027097.2",24.334384372769,0.267002247119451,0.384262290311605,0.694843740464187,0.487153233109161,0.865733056244532
"9168","DUSP15",6.14456493782301,0.444659780955751,0.639884987933256,0.694905786728868,0.48711434597362,0.865733056244532
"9169","CYTH4",8.11540318300844,-0.409116783827496,0.58910453572011,-0.694472303336452,0.487386064226294,0.865855774913525
"9170","AP001160.4",6.16717087614187,0.431032379725595,0.620943306229047,0.694157381844134,0.487583516217427,0.866112093311414
"9171","RUNX1",368.607257869453,0.18611839650094,0.268181619487011,0.694001314694704,0.487681384442888,0.866191480884332
"9172","LINC01003",203.058132691361,-0.240614174095477,0.346748893702688,-0.693914756370602,0.487735668915119,0.866193448643521
"9173","RASSF2",153.018182954191,-0.287170843983455,0.413892685727793,-0.693829231310263,0.487789308585408,0.866194270963448
"9174","VRK2",97.5996859826445,-0.160830754196216,0.231925598953664,-0.693458397528374,0.488021925209602,0.866229610955564
"9175","ITFG2",120.053826526282,0.155668097084095,0.224473446935918,0.69348111863108,0.488007670995038,0.866229610955564
"9176","AC010186.3",7.72829845450905,0.420935686055807,0.60699711271893,0.69347230363305,0.488013201108126,0.866229610955564
"9177","MYH3",4.10746050606492,0.599339147490041,0.864083833944414,0.6

93612267636287,0.487925398301543,0.866229610955564
"9178","TBKBP1",34.5003296919407,0.197990296907761,0.285743124204049,0
.692896101907168,0.488374756149611,0.866761429823601
"9179","ZSWIM6",80.3715323765471,0.203390971819045,0.293623907504208,0
.692692136508434,0.488502774954466,0.866894182507168
"9180","AL109809.5",6.62007644940804,0.490622689709502,0.7084989767185
52,0.692481860710434,0.488634773415534,0.867033967774035
"9181","TRIM21",67.1203441113398,-0.161337536475477,0.233021164633171,
-0.692372886941232,0.488703188136265,0.867060911834399
"9182","SLC25A1",238.085635378318,-0.116791128769827,0.168741397116275
,-0.692130862762441,0.488855151573969,0.86723606665088
"9183","PLSCR1",517.863713509189,0.146759216151407,0.212121756210505,0
.691863101518764,0.489023304600294,0.867345449546405
"9184","CKLF",470.984005215232,-0.160386115944889,0.231802061889669,-0
.691909789919073,0.488993982222643,0.867345449546405
"9185","CNTRL",116.747670094193,0.176074069737942,0.254551867880436,0.
691702132080386,0.489124407779892,0.867367676610466
"9186","SLC2A3",1872.33916683698,-0.181133906403873,0.261877724099641,
-0.691673593187917,0.48914233392742,0.867367676610466
"9187","MRPS36",517.552965921145,-0.173580081459446,0.251188296926707,
-0.691035703427273,0.489543104246601,0.867606095644967
"9188","GABBR1",129.717722253459,-0.205226421071471,0.296860522492396,
-0.6913227105727,0.489362762902738,0.867606095644967
"9189","SMUG1",130.036467379147,0.156936698787839,0.227096575325827,0.
691057091295515,0.489529663915875,0.867606095644967
"9190","GADD45GIP1",1038.07123536584,-0.169959434818201,0.245940673273
731,-0.691058671003298,0.48952867122103,0.867606095644967
"9191","GLUD2",3.72087885720617,-0.578038718798187,0.836324980261916,-
0.691165195875364,0.489461733055331,0.867606095644967
"9192","ECH1",988.470794578548,-0.18301295414268,0.264945193226529,-0.
690757782445231,0.489717770377278,0.867821231687933
"9193","TUBGCP5",88.3118316931376,-0.167104439427314,0.241946803606058
,-0.690666034585838,0.489775438890856,0.867829013824992
"9194","ZBTB41",161.487670305617,0.117457911705501,0.170144356002964,0
.690342685851152,0.489978710268932,0.867908626393267
"9195","TAMM41",80.3335046906261,-0.180917350741878,0.262080299276214,
-0.690312668451295,0.489997582841952,0.867908626393267
"9196","CXCL2",490.89228414249,-0.602385053765135,0.872508129519244,-0
.690406236211291,0.489938756106265,0.867908626393267
"9197","MAP9",140.526357436453,-0.133746500138558,0.193832757741276,-0
.690009788320094,0.490188031853992,0.867908626393267
"9198","RMND5B",160.523992926347,-0.164098732814185,0.237911421557919,
-0.689747182962527,0.490353188574942,0.867908626393267
"9199","ACP2",145.712677278056,0.17552527826874,0.25446030633899,0.689
794336861746,0.490323530527579,0.867908626393267
"9200","CAPN5",91.0032901206941,-0.154070213940544,0.223210074840298,-
0.690247579777827,0.490038506808468,0.867908626393267
"9201","ALDH3A1",5.98667319267347,0.423476190896586,0.61375264307719,0
.689978602411212,0.490207643606552,0.867908626393267
"9202","TPM4",1004.39718089384,-0.131523650294082,0.190633868894308,-0

.68992803354897,0.490239445533155,0.867908626393267
"9203","AP000547.3",17.8111500277714,-0.317951672669567,0.460886690706296,-0.689869503895448,0.490276255255902,0.867908626393267
"9204","TCHH",15.788201251332,-0.523928188432583,0.760077147974834,-0.689309223186815,0.49062869505286,0.868023768805557
"9205","MLEC",690.929544390899,0.121056713947413,0.175583794668513,0.689452658065385,0.490538455599458,0.868023768805557
"9206","SMIM11B",2.32244230982034,0.689914478569846,1.00088429836502,0.689304927349589,0.490631397838588,0.868023768805557
"9207","PHF6",126.211690766704,0.156272304116335,0.226666719793133,0.689436474216225,0.490548636928145,0.868023768805557
"9208","RFX8",18.8291698852262,0.267158399687957,0.387754233836023,0.688988994510724,0.490830193313918,0.86816177331942
"9209","AC095057.3",5.10927622698657,0.483929084577674,0.702354741993829,0.689009492843897,0.490817293766297,0.86816177331942
"9210","GHDC",138.397154956394,0.160065450736843,0.232340266469908,0.688926862178554,0.490869294141559,0.86816177331942
"9211","CLK2",71.1139262842869,0.189941046190503,0.275823546826788,0.688632454972317,0.491054591856342,0.868231324162667
"9212","AOC3",33.8272647739884,0.381875858617863,0.554465748366843,0.688727590013744,0.490994710456818,0.868231324162667
"9213","CRNKL1",203.899837747624,0.151190978062191,0.219559558402857,0.688610321326933,0.491068524127365,0.868231324162667
"9214","TXNDC12",431.027965060796,0.155915524767105,0.226484457615889,0.68841600173524,0.491190849886521,0.868353348578418
"9215","DENND2C",52.5177232806489,0.195557361340615,0.284555242127165,0.687238653130214,0.491932350245082,0.869192542915951
"9216","GLI2",95.7152583511777,0.18990857530734,0.276285916591581,0.68736248901196,0.4918543294992,0.869192542915951
"9217","TWIST2",831.499375543421,0.140882747612204,0.204975518786611,0.687314994718317,0.491884251707113,0.869192542915951
"9218","AL135905.2",4.85816080705717,0.4422053354942,0.643299640704871,0.687401807048532,0.491829559210515,0.869192542915951
"9219","ZCRB1",795.161786628961,-0.135182643846626,0.196623962112241,-0.68751866453316,0.491755943167967,0.869192542915951
"9220","AF213884.3",3.1554374510947,-0.543898961317302,0.791763651502802,-0.686946111108989,0.492116687869837,0.869423940207352
"9221","USP3",181.276640653825,0.136151970354699,0.19835406468233,0.686408773990845,0.492455373171994,0.869927944214142
"9222","VWA1",171.019636909878,0.20325939880039,0.297717384411103,0.682725999364952,0.494779997708051,0.870057494892725
"9223","RER1",1037.35706727913,-0.137764873490888,0.201167906469019,-0.684825307918111,0.49345416329433,0.870057494892725
"9224","TPRG1L",257.700237148518,-0.127458998855397,0.18644739074795,-0.683619107481656,0.494215715832701,0.870057494892725
"9225","CAPZB",979.022857523497,-0.148106734421256,0.216679443563792,-0.683529235562448,0.494272482956966,0.870057494892725
"9226","SELENON",307.713036193575,-0.133789033648395,0.196053590667116,-0.682410524556822,0.49497940267374,0.870057494892725
"9227","ZBTB8OS",243.955591873668,-0.170471165284138,0.249680971844969

,-0.682755934601163,0.494761078507881,0.870057494892725
"9228","ZFP69",13.8507977483527,0.291024220972578,0.424578615279923,0.685442484616675,0.493064742778483,0.870057494892725
"9229","HHLA3",89.1963614858704,0.170473640343763,0.249308166473892,0.683786827984291,0.494109785388509,0.870057494892725
"9230","HIST2H2AA4",27.7937848242167,0.484645093053976,0.7077100946275,0.684807376259153,0.493465480110132,0.870057494892725
"9231","SNAPIN",237.012185686422,-0.153595386600907,0.224936319877325,-0.682839421773568,0.494708316292925,0.870057494892725
"9232","ZBTB7B",46.6383106354747,-0.17774657178926,0.259982957256807,-0.683685475635564,0.494173796976641,0.870057494892725
"9233","RUSC1-AS1",6.84347296568512,-0.393600963993405,0.578403737816891,-0.680495194375819,0.496190963650579,0.870057494892725
"9234","CD1E",2.62140039022271,-0.615568715758578,0.897924531174964,-0.685546161605682,0.492999341774839,0.870057494892725
"9235","AL356275.1",2.32431135977712,0.676873405163013,0.993863290241881,0.681052828702707,0.495838063402937,0.870057494892725
"9236","PPP2R5A",186.946724825207,-0.115098300210653,0.168112141827683,-0.684651917222195,0.493563597397334,0.870057494892725
"9237","AL390728.6",84.6442629071153,0.291521481754505,0.426005516828767,0.684313865051853,0.493776993582955,0.870057494892725
"9238","AC022007.1",8.92671279033214,0.337344427688686,0.491914121322355,0.685779108722968,0.492852412185948,0.870057494892725
"9239","ITGA9-AS1",74.765155209503,0.145053304465179,0.212103585475465,0.683879549419297,0.494051228753375,0.870057494892725
"9240","TCAIM",41.6428386298192,-0.181631359101288,0.267090513944365,-0.680036727695698,0.496481205741595,0.870057494892725
"9241","SMIM4",178.931516315393,-0.17448660159423,0.254557582594934,-0.685450418783586,0.49305973762245,0.870057494892725
"9242","POPDC2",7.76017017796511,-0.379305506175586,0.55358504858636,-0.685180185310611,0.493230225878385,0.870057494892725
"9243","USP13",76.245276940661,0.160485830290952,0.235384347702347,0.681803322342795,0.495363323206025,0.870057494892725
"9244","LPP-AS2",34.4925193175664,0.268937031363175,0.394419077824461,0.681856042173668,0.495329983319569,0.870057494892725
"9245","SMIM14",1191.14047698372,0.111680537841306,0.164021046746201,0.680891507869206,0.495940141914309,0.870057494892725
"9246","BASP1",2407.99939430735,0.150054985631451,0.219133990260206,0.684763625457062,0.493493092180309,0.870057494892725
"9247","AC026801.2",3.95231248571307,-0.697639898800453,1.01686856845975,-0.686066931793525,0.492670902430066,0.870057494892725
"9248","SRA1",225.771688262618,-0.216005383983974,0.316358221718137,-0.682787325111551,0.494741239983396,0.870057494892725
"9249","NUP43",63.0625140160494,0.181762360277788,0.266202022032478,0.68279857113787,0.49473413269784,0.870057494892725
"9250","FBXO5",50.4507052221369,-0.177821461252831,0.260423003499724,-0.682817795905726,0.494721983118101,0.870057494892725

"9251","MPC1",580.051832649095,-0.160046210729751,0.235230043488295,-0
.680381673855849,0.496262821800016,0.870057494892725
"9252","ZNF316",67.5813247474906,0.239680751211978,0.352638122785487,0
.67967906963302 9,0.49670769163474,0.870057494892725
"9253","LMBR1",82.4525568278513,0.159794853506556,0.235009718937661,0.
679949979213172,0.496536133898485,0.870057494892725
"9254","LONRF1",144.330249419943,0.151048279248258,0.221672873989672,0
.681401727372812,0.495617330170584,0.870057494892725
"9255","PPP2CB",464.736758491381,0.0953907588415083,0.139700486070091,
0.682823385407899,0.494718450720335,0.870057494892725
"9256","AGO2",363.882200113895,0.167333686656431,0.246159351068364,0.6
79777899682381,0.496645102296844,0.870057494892725
"9257","EXOSC3",165.753754743512,-0.194109292933027,0.284763985270973,
-0.681649727399054,0.495460463087939,0.870057494892725
"9258","MED22",76.7684648744238,0.158518181724094,0.232137068919397,0.
682864578509583,0.494692418331089,0.870057494892725
"9259","SNAPC4",60.1689991647123,0.154263725152346,0.22518756011321,0.
685045501957532,0.493315208376717,0.870057494892725
"9260","CLIC3",39.6249708877789,-0.367398618665205,0.537656957339485,-
0.683332771295702,0.494396590719939,0.870057494892725
"9261","MINDY3",116.426513232424,0.123981177759897,0.180938277397724,0
.685212546195361,0.493209807976689,0.870057494892725
"9262","LINC00863",56.6354523766636,-0.272784397137993,0.3985612300884
73,-0.684422810210214,0.493708216316726,0.870057494892725
"9263","CUEDC2",485.798439192015,-0.150015400734952,0.219846229292148,
-0.682365129563355,0.495008099424343,0.870057494892725
"9264","TMEM179B",782.061570537795,-0.145324359330713,0.21361409924768
1,-0.680312581625115,0.496306559692421,0.870057494892725
"9265","AP003486.1",19.7279489764888,0.313531785389843,0.4603279812014
88,0.681105208011693,0.495804921912686,0.870057494892725
"9266","VPS26B",209.984314489247,0.111984592900224,0.164589731866192,0
.680386264868972,0.496259915594749,0.870057494892725
"9267","AC021054.1",34.0009906372806,0.204389113118319,0.2992092185498
55,0.68309764688705,0.494545142250878,0.870057494892725
"9268","PLEKHG6",2.74675157956212,-0.636509629909102,0.932606046709058
,-0.68250643683385,0.494918774005713,0.870057494892725
"9269","GNPTAB",197.611974953493,0.137496919084058,0.201305983106409,0
.683024503108674,0.494591359334358,0.870057494892725
"9270","CRY1",79.0882170317888,-0.186490906430291,0.274133288710973,-0
.680292814153316,0.496319073598538,0.870057494892725
"9271","RAB35",132.986553452451,0.116969587006491,0.170648761509071,0.
685440585516779,0.493065940802603,0.870057494892725
"9272","MLXIP",316.371722865327,0.172514666184059,0.253622538401831,0.
680202427083717,0.496376295771144,0.870057494892725
"9273","EP400",157.892945372811,0.158539489502635,0.232847447380385,0.
680872782958367,0.495951991147634,0.870057494892725
"9274","RCBTB1",43.4778236002262,-0.173407071128462,0.25508251152107,-
0.679807761396214,0.496626191619768,0.870057494892725
"9275","ADPRHL1",11.4567733398393,-0.355444673424946,0.523002242421961
,-0.679623612661628,0.49674281452649,0.870057494892725

"9276","METTL17",123.944951956684,-0.13953923634957,0.203505134291272,
-0.685679193478485,0.492915430077833,0.870057494892725
"9277","LINC02256",61.4675604541145,-0.269876236920368,0.3970876204944
08,-0.679639009104209,0.496733063273686,0.870057494892725
"9278","MYO1E",188.968942659764,-0.238003913033211,0.347759706876763,-
0.684391861181184,0.493727753970631,0.870057494892725
"9279","AC007950.2",9.76802843056245,-0.42507308147706,0.6229927999649
17,-0.682308176757416,0.495044103781096,0.870057494892725
"9280","PPCDC",68.5952670209816,-0.179717001758724,0.263102408176893,-
0.68306863097161,0.494563476151658,0.870057494892725
"9281","PAM16",205.2216944627,-0.171830290586766,0.250951724092763,-0.
684714525106232,0.493524081450654,0.870057494892725
"9282","SNX29",376.017627477599,0.222689491360978,0.327416998088262,0.
680140287954592,0.496415636801301,0.870057494892725
"9283","ZNF646",44.098870512267,0.178692870439401,0.262932579558339,0.
67961479227702,0.496748400914871,0.870057494892725
"9284","CCDC144NL-
AS1",27.6052666128881,-0.325281198807583,0.475579976251098,-0.68396739
7811216,0.493995753030581,0.870057494892725
"9285","AC127496.5",4.62741380676021,0.52170718585128,0.76093031401631
5,0.685617560821863,0.492954304778428,0.870057494892725
"9286","AC036176.1",106.605006382847,0.247380092143943,0.3626744647622
72,0.682099558087435,0.495176000030996,0.870057494892725
"9287","AC011471.2",3.2202263803762,-0.615501149786244,0.9021380164734
99,-0.682269385112787,0.495068627837647,0.870057494892725
"9288","RAB3D",50.2134045007117,0.161111532588286,0.236608655099773,0.
6809198611959,0.495922200050567,0.870057494892725
"9289","ZNF433-
AS1",37.2138951204176,-0.252632551221553,0.371140258744684,-0.68069293
2844413,0.496065809047378,0.870057494892725
"9290","CCDC130",137.997064404926,-0.128155974887364,0.187728241542716
,-0.682667529585329,0.494816951979604,0.870057494892725
"9291","PROSER3",32.1815101742558,-0.308368453685338,0.452629653568729
,-0.681282039862009,0.495693045416209,0.870057494892725
"9292","SNRNP70",626.031190430667,0.128699832289943,0.189305513247408,
0.679852530875538,0.496597840946575,0.870057494892725
"9293","ZNF417",19.6360716870714,0.299677293732613,0.440470948468783,0
.680356547405422,0.496278727518174,0.870057494892725
"9294","ESF1",422.342671871056,-0.127547738665698,0.18742634696662,-0.
680521926239181,0.496174043268502,0.870057494892725
"9295","SNU13",1106.9685271085,-0.162164775012225,0.2373024366729,-0.6
83367508930193,0.494374645516202,0.870057494892725
"9296","NUP50",146.247963663865,0.138707325103754,0.203510085806381,0.
681574697166213,0.495507919050646,0.870057494892725
"9297","INE1",15.8738504309754,-0.324643854274696,0.476368126680501,-0
.681497850280051,0.49555652654636,0.870057494892725
"9298","BRWD3",88.2308477557,0.219094847770727,0.319645272901829,0.685
431214989425,0.493071852108887,0.870057494892725
"9299","TCEAL9",1231.76128974417,-0.14984797336448,0.219926606495387,-
0.681354456163189,0.495647233565893,0.870057494892725

"9300","MT-
ND4L",3388.00940913037,-0.243343249436351,0.355753834877249,-0.6840214
37239927,0.493961629117321,0.870057494892725
"9301","RDH5",68.3018482987004,-0.188326734221628,0.27719365861919,-0
.679404915557508,0.496881336160405,0.870103212719505
"9302","MRPL34",632.990709179753,-0.185170917923821,0.272531965457969,
-0.679446602209234,0.496854930459203,0.870103212719505
"9303","GLRX2",160.00877757495,-0.145271102943726,0.214295913818674,-0
.677899547196437,0.497835387109962,0.870366502019644
"9304","STAT4",9.96585253535059,0.351775833581512,0.518982144180343,0.
677818760291051,0.497886614636813,0.870366502019644
"9305","NR1D2",321.246628182244,-0.539853584647629,0.795228076415253,-
0.678866353765064,0.497222546212068,0.870366502019644
"9306","CYB561D2.1",3.6416385691383,0.572744467438133,0.84413130077775
7,0.678501634651414,0.497453687733091,0.870366502019644
"9307","CD200",64.2840870800027,-0.178696826157331,0.263323563571852,-
0.678620719442644,0.497378211200429,0.870366502019644
"9308","BTN2A1",108.888513775637,0.143810584406291,0.2120212706041,0.6
78283758966916,0.497591794210943,0.870366502019644
"9309","NCOA7",903.292409516304,0.11253613104036,0.165869194117601,0.6
78463120527202,0.497478099481672,0.870366502019644
"9310","SLC15A4",220.010582632594,0.107493341095536,0.158454255529124,
0.678387214887885,0.497526213293019,0.870366502019644
"9311","OLFM4",36.7285093858645,0.348476664072742,0.513894793662531,0.
678108959986045,0.497702610095737,0.870366502019644
"9312","ACOT1",5.05418834544504,-0.703573971672074,1.03773104881076,-0
.677992599795846,0.497776385299679,0.870366502019644
"9313","AFMID",113.931951248804,0.150950868990664,0.222665788459413,0.
677925738098645,0.497818779882692,0.870366502019644
"9314","MAFG",308.491058890814,-0.127331843506987,0.18771998030772,-0.
678307355979145,0.49757683561193,0.870366502019644
"9315","NOTCH3",122.698340771741,0.297045369133082,0.437919978784741,0
.678309699314026,0.497575350140037,0.870366502019644
"9316","DGCR6L",481.5078547676,-0.172754837181181,0.254496534241417,-0
.678810175926816,0.497258145309849,0.870366502019644
"9317","RPL36A",8646.32020416412,-0.172782075401619,0.254860934041802,
-0.677946488940042,0.497805622319309,0.870366502019644
"9318","CHRDL1",2424.63717988869,0.142713796929807,0.210313953379554,0
.67857502860141 4,0.497407169561617,0.870366502019644
"9319","GOLGA8Q",3.00623528427243,0.581459624291032,0.858141397835266,
0.677580205031261,0.498037900522937,0.870537542828428
"9320","HINT1",2198.28207090885,-0.167493928601202,0.24727367855562,-0
.677362546549924,0.498175955521876,0.87058317669599
"9321","AL662884.3",14.4097533250884,0.293044226010524,0.4326699253635
69,0.677292801814874,0.498220197063031,0.87058317669599
"9322","TBC1D25",55.6481810612658,-0.155974843193462,0.230293824212888
,-0.677286261264546,0.498224346071583,0.87058317669599
"9323","CASP9",34.0660293539208,-0.203105097004639,0.300018817675741,-
0.676974526391721,0.498422116898396,0.870776543069166
"9324","RNPS1",502.048578744448,-0.0826181554407785,0.122045899810796,

-0.676943310417301,0.498441923235122,0.870776543069166
"9325","C10orf25",11.1431713256994,0.314675087304484,0.464979722332369
,0.676750129502536,0.498564504603041,0.87089728852321
"9326","HIKESHI",474.661675225955,-0.198020971433526,0.29265241184516,
-0.676642198794854,0.498632998128604,0.870923537048771
"9327","R3HDM1",92.3189877837719,-0.151519579201664,0.224105007654729,
-0.676109743317764,0.498970971022545,0.871420408168353
"9328","PUSL1",61.7398599060598,-0.20014744584603,0.296143912046066,-0
.675845214791708,0.499138924154519,0.871620276109881
"9329","MAML3",55.9069538966393,0.237762723904212,0.3519876869562,0.67
5485912476815,0.499367098681279,0.871870726023915
"9330","PLEKHF1",419.981185685414,0.211465172338986,0.313072647988343,
0.675450805740333,0.49938939614483,0.871870726023915
"9331","GATD1",97.3056562508457,0.162176074663229,0.240130845354159,0.
675365442636253,0.499443615315645,0.871871937614033
"9332","SFRP4",1632.88152185441,0.595148914211133,0.881473746057098,0.
675174861274408,0.499564676122718,0.871935923625617
"9333","AL121839.2",38.521111146171,0.319386906403314,0.47306822654775
4,0.675139205044609,0.499587327349615,0.871935923625617
"9334","ZNF354A",77.2939175018735,-0.142172990378138,0.210743278828681
,-0.674626451521212,0.499913123048355,0.872411062924219
"9335","SERPINB9",99.210068177526,0.276833609711179,0.410672004556888,
0.674099053841959,0.500248341010489,0.872528667600369
"9336","ARMC2",29.0133997464054,0.248600997893173,0.36865052097437,0.6
74354120634639,0.500086203737047,0.872528667600369
"9337","AC124798.1",2.88839298282681,-0.639967739278359,0.949286831593
638,-0.674156343456274,0.500211921525749,0.872528667600369
"9338","SF3B2",757.163219719464,-0.11602853583171,0.17211646960199з,-0
.674128025632976,0.500229923225963,0.872528667600369
"9339","SUMO2",4550.7594871499,-0.160681840287968,0.238282908055797,-0
.674332211231626,0.500100129703681,0.872528667600369
"9340","SGCA",69.094707650439,0.866075908765507,1.28495213519259,0.674
014140328816,0.500302323824951,0.872529395373086
"9341","ZNF485",19.7711524598675,-0.255323492991007,0.379001007055189,
-0.673674972462085,0.500517976810747,0.87253181980632
"9342","ATE1",177.99794216087,-0.110471483729555,0.16396639949148,-0.6
7374464568453,0.500473672487843,0.87253181980632
"9343","VPS36",464.411316957332,0.144030398800933,0.213762457752193,0.
673787157555528,0.500446640747151,0.87253181980632
"9344","GABPA",248.826197931346,0.117783357087719,0.174786779440855,0.
673868798684369,0.500394730316803,0.87253181980632
"9345","AC010168.2",10.6607783287824,0.465795545651303,0.6916215620688
03,0.673483261941688,0.500639893844353,0.872562997350273
"9346","RYR1",6.33294444119696,0.65746800244218,0.976349430300192,0.67
3394157909256,0.500696564321505,0.872562997350273
"9347","NUP62",188.669577807055,0.117324311938241,0.174227324955365,0.
673397883875552,0.500694194525747,0.872562997350273
"9348","GRSF1",243.71304921482,0.106422923225294,0.158196378174068,0.6
72726673351483,0.501121194971077,0.872649470267679
"9349","PAG1",12.8015320852027,0.361883848171297,0.537904559476034,0.6

72765905765519,0.50109623138625,0.872649470267679
"9350","NUSAP1",8.95991712100646,0.376092548060397,0.558691983852553,0
.673166179093869,0.50084157512742,0.872649470267679
"9351","SEMA4B",32.1074145542538,-0.245846422837803,0.365428835767387,
-0.67276142103437,0.501099084987301,0.872649470267679
"9352","AC027307.3",7.20522591717972,-0.535942574235884,0.796258399577
459,-0.67307619551679,0.500898817234054,0.872649470267679
"9353","RASAL3",7.49366071661309,-0.381449044790477,0.566859098850087,
-0.672916859876243,0.501000185431547,0.872649470267679
"9354","HIC2",85.5366890153415,0.213428870976146,0.317241451726247,0.6
72764765810988,0.501096956730017,0.872649470267679
"9355","C1orf174",168.751539561534,-0.162180406894325,0.24132197663362
1,-0.672049886034828,0.501551938436694,0.872719919926631
"9356","MAP4K4",524.135454353411,0.199361381273715,0.296863218247864,0
.67155972521749,0.501864025228921,0.872719919926631
"9357","INO80D",235.526126713186,0.159264305929974,0.237150463378397,0
.671574930367527,0.501854342522045,0.872719919926631
"9358","IP6K2",490.954836868218,-0.134509596260927,0.200391977093041,-
0.671232442596614,0.502072464217107,0.872719919926631
"9359","ZCCHC4",41.6506909692928,-0.17256954812102,0.256743333282428,-
0.672148117400916,0.501489406607907,0.872719919926631
"9360","ABT1",158.981244748435,-0.154269161258241,0.229818963608901,-0
.671263845401253,0.502052462486092,0.872719919926631
"9361","AOAH",8.39840709536204,-0.555291899885236,0.82694770516949,-0.
671495786751623,0.501904742609143,0.872719919926631
"9362","VIPR2",68.5183133302073,0.382010097420929,0.568914064164143,0.
671472409426516,0.50191963022535,0.872719919926631
"9363","LINC01235",31.6331414733391,0.301442810949793,0.44858338989232
8,0.671988347633975,0.501591114470787,0.872719919926631
"9364","SIRT1",208.549531397417,0.187661566969147,0.279300629078464,0.
671898117767674,0.501648558741764,0.872719919926631
"9365","RIN3",60.3239461060064,0.239660940159113,0.356871030443839,0.6
71561768017561,0.501862724359041,0.872719919926631
"9366","FAH",81.7345311014322,-0.184195525938735,0.274030257531114,-0.
672172217762563,0.501474065502583,0.872719919926631
"9367","AP001020.3",2.66201200156717,-0.689371963824122,1.025177483746
03,-0.6724415769503,0.501302621598552,0.872719919926631
"9368","RHPN2",3.16232513317746,0.541023354237806,0.805549364901399,0.
671620359733048,0.501825413489926,0.872719919926631
"9369","ZNF551",18.7319364539606,0.30328548135599,0.45162402273162,0.6
71544174115422,0.501873928341855,0.872719919926631
"9370","CD40",298.285954018927,-0.135577665958862,0.20169019668922б,-0
.6722075152109,0.501451597330913,0.872719919926631
"9371","MT-
ATP8",692.788386154301,-0.268325686014874,0.399742322371447,-0.6712466
28135465,0.502063428814848,0.872719919926631
"9372","AC108471.2",15.9779155449155,0.268853977652235,0.4006452675249
1,0.671052423289935,0.502187134014185,0.872826101787992
"9373","TALDO1",931.025704785987,-0.182834303039696,0.272508782434693,
-0.670929947307339,0.50226515743215,0.872868574566552

"9374","ITK",9.21217019739059,0.508384740399823,0.757843535762532,0.670830740659803,0.502328361802677,0.87288528754041
"9375","FMN2",8.62897353499648,-0.425714566506885,0.634911167287788,-0.670510440579352,0.50253245310O531,0.872965994695221
"9376","PDCD11",108.573354715907,0.166812743411834,0.248774389740248,0.670538247871928,0.50251473289563,0.872965994695221
"9377","ARF3",424.832505800989,0.0825875623518589,0.123172082881589,0.670505527062118,0.502535584275099,0.872965994695221
"9378","PRUNE1",62.7589850180763,0.170598159981783,0.254619764001436,0.670011460621814,0.502850484367066,0.873063116485878
"9379","AL732292.2",2.9524631577124,0.613502113724184,0.916860029011197,0.66913388555702,0.503410076029196,0.873063116485878
"9380","ORC2",76.3641895446703,0.16616216131399,0.248296074678178,0.669209779209582,0.5033616689422,0.873063116485878
"9381","PAX3",7.76492007452758,-0.563077550989564,0.84122025398594,-0.669358052568924,0.503267103154397,0.873063116485878
"9382","CHCHD4",63.5457117363369,-0.202655190251267,0.303152804881929,-0.668491885899577,0.503819659643964,0.873063116485878
"9383","ACOX2",125.597949458945,0.18573508155969,0.277612740573954,0.669043795236809,0.503467541337622,0.873063116485878
"9384","TMEM128",223.976867117194,0.153180596969362,0.229007360694638,0.668889403837176,0.503566029998675,0.873063116485878
"9385","TMEM154",16.7571088738133,0.318567811493798,0.476498702720459,0.668559661705286,0.503776411661873,0.873063116485878
"9386","AADAT",28.26604542539,-0.205519616460259,0.307212212502384,-0.668982573271446,0.503506594558868,0.873063116485878
"9387","SMIM8",186.072633956971,0.143744968946574,0.21468588890082,0.669559465144825,0.503138661268494,0.873063116485878
"9388","SLC18B1",90.8838266031613,-0.174726378620455,0.261168813958948,-0.66901700846993,0.503484628301055,0.873063116485878
"9389","RNASET2",408.655599254068,-0.156575200691553,0.233907316414461,-0.669389923717124,0.503246777597905,0.873063116485878
"9390","LFNG",18.0313867916189,0.369859821331769,0.552975717072426,0.668853640246425,0.503588845597216,0.873063116485878
"9391","TBRG4",108.619054357722,-0.186765711966309,0.279216907420544,-0.668891127302012,0.503564930517667,0.873063116485878
"9392","RNF169",213.95008417866,0.135951356085404,0.202916008869944,0.669988320993148,0.502865235286197,0.873063116485878
"9393","CCDC90B",477.12204547284,0.162838924099788,0.243103238205306,0.669834451000881,0.502963329271626,0.873063116485878
"9394","MYO16",4.56148933946506,-0.650210285561521,0.970227192653911,-0.670162916979237,0.502753940475466,0.873063116485878
"9395","CCDC88C",6.77863078559346,0.52419533686107,0.782853155192099,0.669595994324684,0.503115368268904,0.873063116485878
"9396","AC137932.3",17.4425427027192,0.273090535275818,0.408125686569934,0.669133417136739,0.503410374808341,0.873063116485878
"9397","EMC6",260.435376918014,-0.174609991952641,0.260521127598269,-0.670233518342108,0.502708939900343,0.873063116485878
"9398","RNF152",26.4097829272909,-0.361948559427669,0.541446301009776,-0.66848468398925,0.50382425532367,0.873063116485878

"9399","SEPTIN5",25.1261443070557,-0.274755267262663,0.41092335157718,-0.668628994210514,0.503732172371942,0.873063116485878
"9400","JOSD1",229.06530645064,0.110767515926536,0.165628243765705,0.668771903922531,0.503640991827307,0.873063116485878
"9401","SEMA6D",6.76234271393556,0.628055192757646,0.939948459466678,0.668180458654085,0.504018407660672,0.873306652737441
"9402","BUD13",77.2647947991761,-0.152621723459297,0.228452129399549,-0.668068727835627,0.504089722610673,0.873337320953547
"9403","NADK",160.101700904117,0.148901259700562,0.222999612992531,0.66771981216644,0.504312460907179,0.873351655934194
"9404","ARL5B",260.574612747438,0.185257099267779,0.277418364484498,0.667789602220552,0.50426790467744,0.873351655934194
"9405","AC069287.2",2.42770925542477,-0.611728842279472,0.915923233714699,-0.667882219559483,0.504208777976507,0.873351655934194
"9406","LRFN3",56.949254059133,-0.153200382625539,0.229353026930848,-0.667967563697035,0.504154297708757,0.873351655934194
"9407","EVA1A",5.49928502988663,-0.512747469067228,0.76821134026813 8,-0.667456261304679,0.504480739033685,0.873460367490432
"9408","LSG1",149.572488589605,-0.138449843655975,0.207455424299953,-0.667371528718354,0.504534847369069,0.873460367490432
"9409","AP002360.1",45.6554035005409,-0.201550022744673,0.302006616031751,-0.66736956094857,0.504536103979218,0.873460367490432
"9410","TRIM39",38.8724808386183,0.204925770837081,0.307169094712466,0.667143193650088,0.504680672271117,0.873524967657446
"9411","WNK3",8.74419106565716,-0.383027941783768,0.574110422450063,-0.667167720364951,0.504665007358103,0.873524967657446
"9412","CCHCR1",88.962868277799,0.156583985120207,0.234786934987653,0.666919499283216,0.504823554958293,0.873592284899061
"9413","LINC00900",16.0004128419816,0.337310633984308,0.505905388909672,0.66674647350818,0.504934088177583,0.873592284899061
"9414","TMEM88",9.39483470403737,-0.548390066447476,0.822434783017316,-0.666788513534855,0.504907230777733,0.873592284899061
"9415","CTC1",26.8508025630871,-0.193248153225698,0.289769887025192,-0.666902124335982,0.504834653935788,0.873592284899061
"9416","ADGRL1",43.8522361309981,-0.193867952843514,0.290844622574032,-0.666568806147229,0.505047599828799,0.873695874427709
"9417","AL161945.1",2.31615988618181,0.813002292282252,1.22010515855864,0.666337886188992,0.505195154648268,0.873775761300382
"9418","PIR",133.760594567174,0.175427667064395,0.263274991533094,0.666328640038504,0.505201063289766,0.873775761300382
"9419","AHCYL1",899.230047232608,0.0924553778029667,0.138866633853205,0.665785403142274,0.505548276198034,0.873937997592906
"9420","TFR2",2.82435098158263,-0.775262486260007,1.16447361470328,-0.665762174832573,0.505563125502975,0.873937997592906
"9421","AMDHD2",143.889725377982,0.147200300496206,0.221018831871171,0.666007956199894,0.505406014972133,0.873937997592906
"9422","EIF3CL",3.22113352824381,0.715864855558484,1.0747609263935,0.666068925636015,0.505367045532637,0.873937997592906
"9423","HOXB6",7.92087680130448,-0.371381084890773,0.557802224986709,-0.665793480654584,0.505543112490688,0.873937997592906

"9424","SNHG12",117.322439015335,0.187149887161323,0.283579377879402,0.659955912735348,0.50928212176915,0.874047078777767
"9425","DPH5",149.609467753643,0.150605057882475,0.227472163664712,0.6620812650486,0.507919141962743,0.874047078777767
"9426","PHGDH",469.672232538398,-0.181119718431789,0.273571576169376,-0.662056054828054,0.507935297949558,0.874047078777767
"9427","BOLA1",177.023349556948,-0.164796175950721,0.24968660187093,-0.660012090019588,0.509246070874281,0.874047078777767
"9428","KRTCAP2",2305.0330280107,-0.18516240101532,0.280052014470628,-0.661171466183973,0.50850235786719,0.874047078777767
"9429","ARHGEF2",143.081434494592,-0.139799155667067,0.212140962949566,-0.658991803013087,0.5099010323772,0.874047078777767
"9430","LINC01118",13.5006306509419,0.368378474867818,0.557116097981048,0.661223892475548,0.508468741076172,0.874047078777767
"9431","CDK15",7.66012590424333,-0.402198423129492,0.608477813391867,-0.660991106458751,0.508618017044891,0.874047078777767
"9432","AC022382.1",2.83784454506177,-0.557909165640085,0.8454794595896,-0.659873116149855,0.509335257621533,0.874047078777767
"9433","NME6",141.167686051887,-0.136160868015641,0.205190478939458,-0.66358277791152,0.506957382958023,0.874047078777767
"9434","ATG3",338.841858815705,-0.101648179667789,0.154012381627697,-0.660000050603134,0.509253796869245,0.874047078777767
"9435","CD200R1",3.35442358638951,-0.53396549236695,0.808785066993747,-0.660206913008049,0.509121056620094,0.874047078777767
"9436","FBXL5",324.513217606369,0.089483772846102,0.134974458493269,0.662968192982706,0.507350925266795,0.874047078777767
"9437","GPX8",827.545639442029,0.131234189043203,0.19739830635915,0.664819224965551,0.506166124713182,0.874047078777767
"9438","SMIM15",408.056167715645,-0.135446680895014,0.204110699535749,-0.663594222170072,0.506950056281786,0.874047078777767
"9439","MEF2C-AS1",4.30106595295482,-0.504649423854531,0.763694613508676,-0.660800030441485,0.508740563295575,0.874047078777767
"9440","LINC02062",6.57427944078769,0.480898249127418,0.72914033441472,0.65954141669235,0.509548159440694,0.874047078777767
"9441","C5orf15",598.156082502227,0.137753436284851,0.20706340014387,0.665271777577003,0.505876678378327,0.874047078777767
"9442","TIGD6",31.6062969157446,-0.230987434877444,0.348941056294135,-0.661966915933037,0.507992424832771,0.874047078777767
"9443","MDC1",70.9256195163467,0.187621788599838,0.28411940449889,0.660362458983583,0.509021257230798,0.874047078777767
"9444","CFB",1394.37561970735,-0.418414740787098,0.629800953726183,-0.664360284486027,0.506459745589496,0.874047078777767
"9445","ME1",72.4068205898725,-0.147270980458084,0.22200896591394,-0.663356003897486,0.507102576382957,0.874047078777767
"9446","AL009176.1",18.2735738090397,0.443980705189509,0.668337945434648,0.664305697772061,0.506494675028738,0.874047078777767
"9447","AC093627.4",4.26019496504152,-0.557787185276248,0.83969346333833,-0.664274773628319,0.506514463611009,0.874047078777767
"9448","PRKAR1B",13.9282431541674,0.300460589624225,0.453040086177061,

0.663209722035052,0.507196245814068,0.874047078777767
"9449","LINC00997",12.9397797610428,-0.314884984579682,0.473861319465247,-0.664508732080158,0.506364762009603,0.874047078777767
"9450","AC008080.4",2.5389994056168,0.663551492704999,1.00607552066099,0.659544416972843,0.509546233497151,0.874047078777767
"9451","RFC2",113.498261920767,-0.17947917912788,0.270920604076693,-0.662478882843009,0.50766436400196,0.874047078777767
"9452","TGS1",293.53965939843,0.118299356059216,0.178872176679936,0.661362534156974,0.508379846861531,0.874047078777767
"9453","LYN",34.4345872852153,-0.328853661784005,0.498690939992354,-0.659433800399596,0.509617243136353,0.874047078777767
"9454","VPS13B-DT",45.3940789087813,-0.209581311210282,0.314911989566141,-0.665523441959214,0.505715755048457,0.874047078777767
"9455","AL159169.2",5.36665289583586,0.47511916138466,0.715748135536596,0.663807752748756,0.506813363055065,0.874047078777767
"9456","GRHPR",748.965518490924,-0.156921332587612,0.237129023977021,-0.661755064630208,0.50812820854763,0.874047078777767
"9457","CKS2",46.7375756404696,-0.207122751995389,0.314271377332418,-0.659057002751818,0.509859164975272,0.874047078777767
"9458","MFSD14C",129.852677362933,0.127064274446759,0.191428239767228,0.663769747876624,0.506837690746343,0.874047078777767
"9459","TAF3",217.569803536288,-0.162632664317237,0.246591425617865,-0.659522787176159,0.509560118206689,0.874047078777767
"9460","ATG13",137.249728324481,0.119692754523362,0.180523581652366,0.663031131045549,0.50731061623795,0.874047078777767
"9461","FADS1",44.724819000222,0.347740446466979,0.527310703921837,0.659460245886691,0.509600266144911,0.874047078777767
"9462","MAP3K11",51.5620542743392,0.193951752974239,0.293943031892826,0.659827694248441,0.509364408987378,0.874047078777767
"9463","MIR100HG",427.066783401141,0.144996786497215,0.219310766582805,0.661147597796886,0.50851766314236,0.874047078777767
"9464","AC092828.1",14.8206931004176,0.328580961387961,0.498549263499738,0.659074208798091,0.509848116573731,0.874047078777767
"9465","TMTC1",61.0202704620543,0.264487131292501,0.399167154161835,0.662597432015335,0.507588415304962,0.874047078777767
"9466","R3HDM2",401.641667388451,-0.118314482332799,0.179274361784701,-0.659963204749203,0.509277442157646,0.874047078777767
"9467","PPP1R12A-AS1",5.68626223272961,-0.429415106288484,0.646850523473626,-0.663855235027869,0.506782969545292,0.874047078777767
"9468","CHPT1",811.01543888383,-0.179248455402023,0.269868584418832,-0.664206453626451,0.506558183513267,0.874047078777767
"9469","NUP37",150.837364583952,0.18046891572317,0.273047592014521,0.660943077328337,0.508648818982873,0.874047078777767
"9470","SLC41A2",161.79057929741,0.129478649418813,0.195364854596207,0.662753030407785,0.507488740025584,0.874047078777767
"9471","HCAR1",2.56965984943475,-0.684935455037512,1.03657627430012,-0.660767057880011,0.508761711752545,0.874047078777767
"9472","MTMR6",145.10661878921,0.12607449598472,0.191143247208917,0.65

9581218932218,0.509522609850679,0.874047078777767
"9473","TNFSF13B",516.464241452489,0.156993730138184,0.23675803984347,
0.663097777976107,0.507267933678021,0.874047078777767
"9474","ZNF770",183.181078966484,0.133168976954179,0.201340865878372,0
.661410570443385,0.508349048876902,0.874047078777767
"9475","ZNF75A",74.6955896295878,0.192297064052997,0.290668127902729,0
.661569142239595,0.50824738910224,0.874047078777767
"9476","NPIPA1",107.93368458666,0.257328659757727,0.389748020019803,0.
660243661390898,0.50909747767509,0.874047078777767
"9477","ZKSCAN2",19.4983668148586,0.257905177612254,0.388614515216127,
0.663652971039491,0.506912445813738,0.874047078777767
"9478","NSMCE1",424.803291313411,0.161848063188649,0.244699081978741,0
.661416715910322,0.508345108842833,0.874047078777767
"9479","MIS12",46.873038793775,0.177478409360563,0.266773122102968,0.6
6527845069812,0.505872410994969,0.874047078777767
"9480","ELAC2",108.483454784326,0.125853505666059,0.189578612356898,0.
6638591985742,0.506780432514018,0.874047078777767
"9481","MAPK7",85.8352752723734,0.134297503974288,0.203645195598666,0.
659468069352127,0.509595243836019,0.874047078777767
"9482","MYO18A",47.8999303427053,0.292191351537415,0.441392640809919,0
.661976037935903,0.507986578614974,0.874047078777767
"9483","AC022706.1",13.7902165701899,-0.411007298637171,0.620079901015
661,-0.662829577227001,0.507439708422038,0.874047078777767
"9484","AC008105.3",44.9499435762511,0.191099789579685,0.2875220686472
43,0.664643901870863,0.50627828232697,0.874047078777767
"9485","AMZ2",148.457307560281,0.116357635053017,0.174941448971528,0.6
65123306895407,0.505971628552362,0.874047078777767
"9486","ZBTB7A",852.921944003799,-0.106180539112107,0.160409590644381,
-0.661933857480526,0.508013612023176,0.874047078777767
"9487","SWSAP1",19.6697860329484,-0.220129482180427,0.332492791925635,
-0.662057907798677,0.507934110462905,0.874047078777767
"9488","MRI1",125.406456672973,0.162611954907549,0.246660365079652,0.6
59254496988349,0.509732356878208,0.874047078777767
"9489","ZNF208",90.9350548163869,-0.346902507089407,0.525482312842417,
-0.660160196854117,0.509151032033655,0.874047078777767
"9490","AC008738.2",2.46180126409942,-0.692731139729594,1.042243303868
74,-0.664653960508279,0.506271847266383,0.874047078777767
"9491","SCN1B",357.652803209922,-0.167127324004855,0.253334525989372,-
0.659710015254166,0.509439938366361,0.874047078777767
"9492","SUPT5H",287.165174601472,0.135055579504505,0.20495139678587,0.
658963937901867,0.509918926246247,0.874047078777767
"9493","DYRK1B",75.6546328871058,0.144409924410833,0.218854343367706,0
.659844909580818,0.509353360239816,0.874047078777767
"9494","ZNF780A",67.9284585400355,0.143965175945079,0.218102587625817,
0.660080091264528,0.509202433925556,0.874047078777767
"9495","AL078587.2",3.46341774076616,0.534400571262224,0.8042422583064
22,0.664477109655451,0.506384994702128,0.874047078777767
"9496","CEBPB-
AS1",18.486614533331,0.28453147347519,0.43175517773061,0.6590111437012
59,0.509888612747656,0.874047078777767

"9497","C21orf62-
AS1",39.7736197160908,0.184278677409117,0.27899958516023,0.66049803372
749,0.508934279884658,0.874047078777767
"9498","SETD4",79.7114756938691,0.158341236113896,0.239312867968058,0.
661649486123873,0.508195885014368,0.874047078777767
"9499","PACSIN2",119.480369066138,-0.133693314825583,0.202162949352266
,-0.661314623940436,0.508410564992322,0.874047078777767
"9500","CA5B",101.692742333237,-0.257241072432469,0.390317121083198,-0
.659056594080681,0.509859427394014,0.874047078777767
"9501","EIF1AX",1937.10566794272,-0.0873060873409106,0.131433906073913
,-0.66425848511124,0.506524886913833,0.874047078777767
"9502","ZC4H2",39.1394254940005,-0.163672433422092,0.246938285018403,-
0.662807038648926,0.507454145113943,0.874047078777767
"9503","MORC4",256.034132478767,-0.12403798109433,0.186570472148278,-0
.66483179072276,0.506158086654457,0.874047078777767
"9504","GARS",255.742261283151,-0.105867922363444,0.160679041148972,-0
.658878230828434,0.509973965988396,0.87404944570549
"9505","ZNF721",237.689798334899,0.144024506064143,0.218685855953825,0
.658590860556399,0.510158533363819,0.874273787476406
"9506","NFYB",523.220515999906,-0.126506583424395,0.192171989869491,-0
.658298764093088,0.510346171999298,0.874503344802921
"9507","RNF216",281.430802053892,0.117731131330883,0.178926382669366,0
.657986427571364,0.510546852482742,0.874663197317141
"9508","AC104024.4",2.69706566079681,0.738072303705382,1.1215820154128
9,0.658063604411199,0.510497261471305,0.874663197317141
"9509","RPA2",342.248784197125,-0.15761921301751,0.239870990076717,-0.
657099939292783,0.511116657947698,0.874683576390787
"9510","FAM102B",259.029798318564,0.16690935001201,0.253765143084503,0
.657731585919307,0.51071062228375,0.874683576390787
"9511","TDRD10",19.6995559762182,0.250211484756543,0.38112636937448,0.
656505308638707,0.511499053006252,0.874683576390787
"9512","PTPN14",106.529390111362,0.204539646540442,0.311584065451375,0
.656450920377257,0.511534036461351,0.874683576390787
"9513","VAX2",5.33145462400655,-0.488988391806193,0.74436509850951,-0.
656920095777363,0.511232295954298,0.874683576390787
"9514","MOB1A",560.69141311266,0.092082434089246,0.140107003549766,0.6
57229344402747,0.511033459903969,0.874683576390787
"9515","LNPK",424.982407845748,0.108462158139413,0.165148577653103,0.6
5675502435897,0.511338447640759,0.874683576390787
"9516","RBM6",394.534469557412,0.153055135169704,0.233062328431061,0.6
56713318707693,0.511365268915417,0.874683576390787
"9517","PIGY-
DT",3.28389659065921,-0.697631362130696,1.06284420520195,-0.6563815832
23799,0.511578637109299,0.874683576390787
"9518","TBC1D7",53.2864816278587,-0.152124410248096,0.231734096008429,
-0.656461059759471,0.511527514543481,0.874683576390787
"9519","AL390208.1",5.33932322780909,0.447944453306451,0.6810043686148
14,0.657770308019589,0.510685736393233,0.874683576390787
"9520","DCTN3",985.736931545627,-0.179056410299611,0.272674637514888,-
0.656666905039287,0.511395118821375,0.874683576390787

"9521","SPTLC1",444.141413409229,0.0937578984669543,0.142854597463627,
0.656316983363638,0.511620192352459,0.874683576390787
"9522","UAP1L1",60.1431738447833,0.204248846184251,0.310790094982678,0
.657192264108786,0.511057299104221,0.874683576390787
"9523","SPINDOC",35.6907698218646,-0.187766643458138,0.285547066496697
,-0.657568105187697,0.510815694944813,0.874683576390787
"9524","ATF7IP2",27.2035720430376,-0.343425793490329,0.52275489477561,
-0.656953759634797,0.511210649311703,0.874683576390787
"9525","TNRC6C-
AS1",3.08095839523032,0.577519940599619,0.879281476134004,0.6568089471
63131,0.511303770495562,0.874683576390787
"9526","NDUFAF8",597.91762723819,-0.181806065426849,0.276827529355964,
-0.656748502758447,0.511342641691507,0.874683576390787
"9527","RBBP8",57.3198745266983,0.201404455176506,0.306879798862111,0.
65629753383344,0.511632704024275,0.874683576390787
"9528","RBMX2",307.258049272946,-0.126809820996031,0.193036378476415,-
0.656921881755694,0.511231147516704,0.874683576390787
"9529","RBM47",60.707198300682,0.255572525786335,0.389535506200995,0.6
56095584915597,0.511762625010522,0.874813873312666
"9530","SLC35A4",150.050441289197,-0.10494316996466,0.159986069510672,
-0.655951923099529,0.511855058291909,0.874880067630315
"9531","GALE",15.3201911580543,-0.253869105637167,0.388294727539869,-0
.65380518361816,0.513237326458097,0.875107651685197
"9532","MED18",42.0592482309107,-0.195776081760434,0.300754472594543,-
0.650949859769386,0.51507885417056,0.875107651685197
"9533","SZT2",89.3555393281466,0.189358022428876,0.28987191181226,0.65
3247226490562,0.51359690855555,0.875107651685197
"9534","MOB3C",76.2876134316439,0.142782345734374,0.218497575276187,0.
653473364882394,0.513451155181688,0.875107651685197
"9535","ATP1A1",813.781082397659,0.139529388155249,0.213586836211442,0
.653267732366805,0.51358369097939,0.875107651685197
"9536","AL513217.1",5.16821008306495,0.503251450071637,0.7744774212617
16,0.649794863292181,0.515824737418515,0.875107651685197
"9537","TRAF3IP3",9.22015285438163,-0.397920015996702,0.61053803063536
7,-0.651753037534124,0.514560501758936,0.875107651685197
"9538","SMC6",161.439604269598,-0.110447149176122,0.169495537353687,-0
.651622755976466,0.514644564045109,0.875107651685197
"9539","WDR43",224.927070947742,-0.142760096448746,0.218379774146681,-
0.653723986145609,0.513289646981001,0.875107651685197
"9540","LINC01126",2.36366838069612,-0.713848805158036,1.0948888921018
,-0.651982872698343,0.514412221354571,0.875107651685197
"9541","PGM5P4-
AS1",5.68686555834994,-0.481827506221756,0.739080396888213,-0.65192840
7586533,0.514447358045684,0.875107651685197
"9542","AC009948.3",9.5054401466615,0.348562163341479,0.53671378002939
3,0.649437700895979,0.516055502113971,0.875107651685197
"9543","FARP2",90.7827352116278,0.162013147428182,0.248516050776339,0.
651922267885994,0.514451318985376,0.875107651685197
"9544","OXSM",29.8257937988835,0.230171283501892,0.352981967125711,0.6
52076607131374,0.51435175404631,0.875107651685197

"9545","CRTAP",1503.31102728804,-0.0968790897603152,0.148340283359165,-0.653086859256896,0.513700283379412,0.875107651685197
"9546","RFT1",128.969376700647,-0.121064364696623,0.186455294953547,-0.649294324018983,0.516148153805029,0.875107651685197
"9547","INTS12",76.5084180127399,-0.15635709881263,0.240106626477623,-0.651198599165698,0.51491829451376,0.875107651685197
"9548","LINC00603",24.7969246440267,0.276552048814595,0.42488788487088,0.650882406069631,0.515122399575009,0.875107651685197
"9549","TMEM267",80.7764639308047,-0.176121332783673,0.271327090607738,-0.649110755543001,0.516266790334134,0.875107651685197
"9550","RELL2",2.94412059903998,-0.615568913028347,0.941337795852757,-0.653929881218361,0.513156981537041,0.875107651685197
"9551","PDGFRB",1813.71769647255,-0.126947550659007,0.193913659183845,-0.654660178108706,0.51268656959074 9,0.875107651685197
"9552","THG1L",109.443686977222,-0.132624912131148,0.203492297161673,-0.651744139611232,0.514566242787592,0.875107651685197
"9553","ZFP62",73.6575798130201,0.148921617616729,0.228971033850474,0.650395008977336,0.515437100408694,0.875107651685197
"9554","OARD1",217.374670902629,-0.133288645880864,0.205098131378517,-0.649877426893153,0.515771400216769,0.875107651685197
"9555","L3MBTL3",42.745041239006,0.210771434271077,0.324515546379607,0.649495645501447,0.516018060129902,0.875107651685197
"9556","ENPP1",180.938254005898,-0.280469967213367,0.431163552512938,-0.650495538360587,0.515372182782589,0.875107651685197
"9557","HBS1L",205.436909286151,0.117979829892151,0.180062199189273,0.655217088446953,0.512327993606653,0.875107651685197
"9558","AL596202.1",2.69058923380315,-0.803978896152912,1.23191497947183,-0.652625310634351,0.513997864098979,0.875107651685197
"9559","AGMO",13.2910323496107,0.278130552160703,0.425302579816115,0.653959240691549,0.513138065649807,0.875107651685197
"9560","GSDME",56.7437242730702,0.276591648522053,0.42565249287367,0.64980624606407,0.515817383824013,0.875107651685197
"9561","PNPLA8",364.432060220689,-0.10205060895961,0.156325645150303,-0.652807854152728,0.513880159536217,0.875107651685197
"9562","AC145124.1",93.2853655319915,0.152861794471457,0.234157525528885,0.652816065279954,0.513874865308414,0.875107651685197
"9563","AC027702.1",3.50894709965502,-0.580040958873844,0.888998046662608,-0.652465954285703,0.514100628975494,0.875107651685197
"9564","RBIS",1265.8094650683,-0.15705905150194,0.239835242759786,-0.654862270009446,0.512556434240549,0.875107651685197
"9565","TONSL",6.76930459147476,0.379931671817156,0.583707630637956,0.650893789758949,0.515115050587435,0.875107651685197
"9566","VCP",815.393743178606,0.101552465142334,0.154908323392366,0.655564936204962,0.512104092411377,0.875107651685197
"9567","GLIPR2",414.513797847031,-0.167932422307687,0.258604388760489,-0.649379630069697,0.516093027071594,0.875107651685197
"9568","AL158071.5",3.67607500555553,0.488136181147397,0.746637658532985,0.653779213476187,0.513254060314897,0.875107651685197
"9569","GTPBP4",182.349161856484,-0.123100395351175,0.189346754880165,-0.650132057605552,0.515606923359967,0.875107651685197

"9570","LINC00710",24.113998422956,0.379081402340293,0.580293463113145
,0.653258095148282,0.513589902867928,0.875107651685197
"9571","PHYH",525.354027478253,-0.158418383139081,0.243037849609551,-0
.651825974405165,0.514513443416801,0.875107651685197
"9572","MRPL43",712.832149643123,-0.15809816559673,0.243008694770802,-
0.65058645636463,0.515313475434559,0.875107651685197
"9573","SLC15A3",60.6098312914771,-0.163227404928496,0.249223203414178
,-0.654944654800991,0.512503388200008,0.875107651685197
"9574","EIF1AD",72.0827502077929,-0.143886478036883,0.221141984649229,
-0.650652015559655,0.515271144868477,0.875107651685197
"9575","EXPH5",16.1717470828345,-0.286983512699107,0.440857398534263,-
0.650966760801233,0.51506794384148,0.875107651685197
"9576","PTS",141.71992803887,-0.165759874105799,0.254602901159055,-0.6
51052573836329,0.515012549759041,0.875107651685197
"9577","THYN1",412.642222722619,-0.178858605967909,0.274350039014861,-
0.651935777411064,0.514442603530089,0.875107651685197
"9578","CHD4",487.416604013812,0.18070451006259,0.276590416557936,0.65
3328890824887,0.513544270814886,0.875107651685197
"9579","PRH1",121.809503331701,0.130488723978911,0.200843593405851,0.6
49703193246639,0.515883960858905,0.875107651685197
"9580","AC012673.1",4.18980223905978,-0.461268874330049,0.707898772429
613,-0.651602873595763,0.514657393490689,0.875107651685197
"9581","ESYT1",155.896144658245,0.145699830892135,0.223413876714589,0.
652152109057513,0.51430305106496,0.875107651685197
"9582","DDIT3",443.093587946224,-0.2131841389011,0.325931357539617,-0.
654076798594588,0.513062328418541,0.875107651685197
"9583","PPTC7",155.292269334919,-0.149485530713833,0.23030340427426,-0
.649080855686422,0.51628611533518,0.875107651685197
"9584","OAS2",38.3030857953073,0.333712105677075,0.513716375129287,0.6
49603792740868,0.515948182549832,0.875107651685197
"9585","SACS",148.5413564402,0.150367600726618,0.230020985863839,0.653
712530454191,0.513297028810742,0.875107651685197
"9586","STK24-
AS1",3.21004380049919,-0.535357524801111,0.824668430774763,-0.64917911
8325351,0.516222607222271,0.875107651685197
"9587","MCF2L",53.0509351557113,-0.279531025037702,0.429234544079784,-
0.651231427882803,0.514897105733591,0.875107651685197
"9588","TMEM260",121.139441229312,0.147725998611138,0.227173704312311,
0.650277720558929,0.515512845676357,0.875107651685197
"9589","ATP5MPL",1429.02410457028,-0.144400472353528,0.221113213802069
,-0.653061252516503,0.513716790822898,0.875107651685197
"9590","IGHA1",179.987562680609,0.438704164988275,0.674394389490746,0.
650515739491179,0.515359138258613,0.875107651685197
"9591","HERC2",277.993332228366,0.154904950290496,0.2364138774177,0.65
5227823266936,0.512321083099176,0.875107651685197
"9592","SEC11A",2606.30569293212,-0.163107574391152,0.251222021692336,
-0.649256674603569,0.516172484695056,0.875107651685197
"9593","CHD2",495.438386299371,0.136374558558434,0.209766312018231,0.6
50126120092063,0.515610758343621,0.875107651685197
"9594","CLUAP1",234.075230246799,-0.0995226048783529,0.153279827652666

,-0.64928703536823,0.516152864040502,0.875107651685197
"9595","RMI2",11.1683762472017,-0.356560894268527,0.544202048620299,-0.655199470807775,0.512339335013245,0.875107651685197
"9596","AC067852.3",12.7931253491279,0.326808020737215,0.499943960456595,0.653689306374946,0.51331199413548,0.875107651685197
"9597","C17orf82",7.47186801327111,-0.393313634799609,0.603633956075373,-0.651576391356119,0.514674481866052,0.875107651685197
"9598","PRKCA",74.6938664907554,0.187460466846555,0.286089714059049,0.655250635148187,0.512306398181901,0.875107651685197
"9599","DLGAP1-AS2",14.4799170321979,0.338828458861096,0.518486726041752,0.653494953376707,0.513437241837735,0.875107651685197
"9600","TMEM259",447.728554930421,-0.103150676801713,0.158067946409853,-0.652571752493414,0.514032401196469,0.875107651685197
"9601","SGTA",154.283102803021,-0.112427601051504,0.171701922454984,-0.654783589164414,0.512607098050408,0.875107651685197
"9602","GNA11",467.921796841974,0.197407327339815,0.303052482779397,0.651396502445141,0.514790567814362,0.875107651685197
"9603","VMAC",57.4895456232827,-0.172836179327495,0.266044343808057,-0.649651771782042,0.515917183244582,0.875107651685197
"9604","CCDC151",2.90208271355606,-0.569347152152653,0.872429752720757,-0.652599421760994,0.514014558451225,0.875107651685197
"9605","AC010327.7",3.76168062104454,0.527034419814479,0.80758700314323,0.652603890061622,0.514011677062568,0.875107651685197
"9606","ZNF586",19.1604856855004,0.282714105065798,0.431830250229738,0.654688051416942,0.512668619788719,0.875107651685197
"9607","AL117332.1",14.5148103943198,0.29833334671966,0.45859518577108,0.650537458691469,0.515345113659117,0.875107651685197
"9608","GPM6B",12.2149985575484,-0.326110341722838,0.499246144770006,-0.653205528253146,0.51362378674501,0.875107651685197
"9609","EDA",41.9138148142427,-0.199995198910942,0.307414840513022,-0.650571060841385,0.515323416349428,0.875107651685197
"9610","MAMLD1",10.4503887381414,0.309329956115483,0.471892898084424,0.655508818571248,0.51214021052269,0.875107651685197
"9611","TRAF3IP1",116.496387579055,-0.181178363039412,0.279202019070423,-0.648914945682084,0.516393353801077,0.875188537067031
"9612","SRRM2",2565.31350935991,0.125192955172836,0.192948652699717,0.648840784432279,0.516441292791964,0.875188537067031
"9613","TSBP1-AS1",13.8225184623486,-0.4168557952059,0.642644408311202,-0.648657001935722,0.516560102611294,0.87527901578662
"9614","LINC00265",6.9655456920153,-0.424462092596103,0.654436251673436,-0.648591962182299,0.516602152236022,0.87527901578662
"9615","EID2",126.764891369866,-0.156513445394209,0.241374639830196,-0.648425391765739,0.516709851746286,0.875370439427483
"9616","BLVRB",1551.57600834632,-0.183920610186821,0.283690779809806,-0.648313668530668,0.516782095207513,0.875401783364723
"9617","ZDHHC18",72.9014967881997,0.147439248878834,0.22802268157335,0.646599048224092,0.517891474173591,0.876453619085655
"9618","WARS2",164.766392722034,0.112410976152056,0.17406092741961,0.6

45813956173324,0.518399848573435,0.876453619085655
"9619","LYPLAL1",246.078766337603,0.154152811176388,0.23886590670495,0.645352923331999,0.518698503535231,0.876453619085655
"9620","ALAS1",84.4956734238254,-0.133053592487269,0.206222599031832,-0.645194043290722,0.518801445890369,0.876453619085655
"9621","CLDND1",386.517770363136,-0.113683802327947,0.17565308739022,-0.647206399938728,0.517498369812884,0.876453619085655
"9622","EEF1AKMT4",9.46385814885643,-0.340767178830828,0.528729028092379,-0.644502496979019,0.519249638965857,0.876453619085655
"9623","BBS7",126.924678707582,-0.136852342853984,0.212314823886883,-0.644572716820263,0.519204120176493,0.876453619085655
"9624","SENP6",502.893319591648,0.0995403788909318,0.154264981387231,0.64525583185382,0.518761410279834,0.876453619085655
"9625","TBPL1",148.810539960147,-0.173093233642377,0.268587746177302,-0.644456927413632,0.519279179745382,0.876453619085655
"9626","POLM",71.1971737764745,-0.158763970315891,0.245776550715706,-0.645968746219145,0.518299596225811,0.876453619085655
"9627","KIAA1324L",43.3618594323215,-0.218535780459145,0.337852517775194,-0.646837803365306,0.517736923071758,0.876453619085655
"9628","TDRP",13.5405983253063,0.318012491977896,0.492720252063839,0.645422002943554,0.51865374841805,0.876453619085655
"9629","AC090136.3",14.8435015857473,0.353120059939157,0.546804252671537,0.645788795924516,0.518416144974004,0.876453619085655
"9630","OTUD6B",56.5249362047531,0.191496211382596,0.297043190458287,0.64467463834855,0.519138054985891,0.876453619085655
"9631","FAM214B",46.1712507964155,-0.199488281814235,0.308919206515591,-0.645761990859468,0.518433507020396,0.876453619085655
"9632","NUP214",158.949876212167,0.110589184133895,0.171444545569757,0.645043467357779,0.518899017545972,0.876453619085655
"9633","TWNK",13.2440830757692,0.296655291634696,0.460125522200353,0.644726878474529,0.519104194795152,0.876453619085655
"9634","AL158163.1",7.00779946481382,-0.377900631854386,0.584194236745096,-0.646874974254971,0.517712863739126,0.876453619085655
"9635","COPB1",596.199503205586,0.115829252298188,0.179572845522997,0.645026545972701,0.518909983021829,0.876453619085655
"9636","IRS2",376.420149705166,0.225122993395215,0.348015417913581,0.646876493992336,0.517711880082034,0.876453619085655
"9637","LINC00641",35.2804263208294,0.352304187149312,0.544678590870818,0.646811152584603,0.517754173485695,0.876453619085655
"9638","IQCK",141.696853893975,-0.169530562419146,0.261980500014171,-0.647111378175001,0.517559861825518,0.876453619085655
"9639","CTF1",65.3899501956735,0.191679812354731,0.296801264870232,0.645818717917315,0.518396764401321,0.876453619085655
"9640","TP53",114.391696931222,-0.150688674672101,0.233742803125545,-0.644677280571349,0.519136342363713,0.876453619085655
"9641","FTSJ3",195.157279598803,-0.14471385119605,0.223919072320154,-0.646277468446912,0.518099677074081,0.876453619085655
"9642","SEH1L",69.9378000332708,-0.158811724574914,0.246431389014518,-0.644446006695835,0.519286259303558,0.876453619085655
"9643","DOCK6",47.8364632163176,-0.211774925630796,0.327742755206474,-

0.646162034908691,0.518174423664715,0.876453619085655
"9644","ZNF257",9.93550122668904,0.390478379927965,0.604278287422267,0.646189658069081,0.518156536353327,0.876453619085655
"9645","CCDC106",80.0668731839836,-0.202264145019258,0.313536969282068,-0.645104612328169,0.518859395091202,0.876453619085655
"9646","ZHX3",314.985923676133,0.161082022562078,0.24993609609963,0.644492832671383,0.519255903846278,0.876453619085655
"9647","TMEM191C",6.77374846291785,-0.409412001123665,0.634912497672485,-0.644832166045749,0.519035954603546,0.876453619085655
"9648","EIF4ENIF1",80.9748901441131,0.149634937519334,0.232067772769346,0.64478982037742,0.519063399608213,0.876453619085655
"9649","TTLL12",49.5315873819613,0.154057955351133,0.238953401377527,0.644719658573656,0.519108874410071,0.876453619085655
"9650","AL118516.1",142.845432244039,-0.236887954559434,0.367486983619108,-0.644615905103628,0.51917612514705,0.876453619085655
"9651","PRICKLE3",26.1482788724581,-0.193355488817384,0.299058506744366,-0.646547362662597,0.517924934440084,0.876453619085655
"9652","CPOX",82.1923650776875,-0.132273212404854,0.205319286692317,-0.644231793981792,0.51942513674795,0.876597187369183
"9653","AGK",58.4972352054728,0.168137302983487,0.261026100727739,0.644139810213312,0.519484777142491,0.876607016976488
"9654","MIB2",137.454638596261,-0.122479460909137,0.190332593942658,-0.643502294441682,0.519898226436361,0.87695817963253
"9655","HMGCL",260.376602129258,0.156434960510436,0.243067835331098,0.6435856076858,0.519844185503883,0.87695817963253
"9656","PBRM1",374.222256547856,0.113157827607354,0.175873710274211,0.643403880153127,0.519962066357111,0.87695817963253
"9657","SNX14",213.44931325246,0.101894121212745,0.158305707674454,0.643654121570175,0.519799746311185,0.87695817963253
"9658","AC105114.2",3.50753357088068,0.541301952983023,0.841255766039201,0.643445162380972,0.519935286682791,0.87695817963253
"9659","CNIH4",585.993911652821,-0.157368985598416,0.244696116832245,-0.643120077407284,0.520146187715869,0.877172096517195
"9660","AC021097.2",17.3683032949412,-0.31134679217807,0.484177719464649,-0.643042378162968,0.520196602145995,0.877172096517195
"9661","XCL2",8.16787031957832,0.510583231128287,0.794292809294848,0.642814872743931,0.520344231400743,0.877330212740575
"9662","PTBP2",126.64090799801,-0.15003979585637,0.233569816121115,-0.642376649295163,0.520628657346596,0.877523809720427
"9663","TYW1",84.8184303026086,0.136919593589492,0.213160536688848,0.642330872854549,0.520658372853628,0.877523809720427
"9664","CDK2",49.6088873496361,-0.196605514689593,0.306093241510732,-0.642305964415422,0.520674542387374,0.877523809720427
"9665","MHENCR",20.6540972335436,0.27410095161743,0.426731713027113,0.642326181180762,0.520661418475422,0.877523809720427
"9666","TRAT1",3.95858786661102,0.479821611957549,0.74744553411209,0.641948597000504,0.52090655908646,0.877539849578973
"9667","NDST1-AS1",4.62359315487477,0.893200674567675,1.39158916676484,0.641856588064842,0.520966303451481,0.877539849578973

"9668","EHMT2",152.980911740389,-0.113551493136151,0.176867241951035,-0.64201540027173,0.520863183782163,0.877539849578973
"9669","GABARAP",4560.18194111899,-0.147419361022406,0.229634744490421,-0.641973240371538,0.520890557951136,0.877539849578973
"9670","AC015802.6",24.3226741811755,0.361197572447822,0.562794102193325,0.641793457039014,0.521007298500721,0.877539849578973
"9671","CUL4B",183.142559317832,0.12864158966504 6,0.200354415175814,0.642070151297446,0.520827635377216,0.877539849578973
"9672","CCDC14",260.223733261736,0.123160892258169,0.191940379050646,0.641662233175394,0.52109251593841 5,0.877577367261578
"9673","KIAA0586",143.055021642126,-0.132969310028405,0.207248614867046,-0.641593238698879,0.521137324177128,0.877577367261578
"9674","CHML",51.9526602549797,-0.187746887414332,0.292731455796039,-0.641362189464001,0.521287392795158,0.877581184149831
"9675","AC104596.1",3.86930878232625,-0.5181075145215,0.807850411311601,-0.641340905775262,0.52130121786689,0.877581184149831
"9676","HOOK3",679.609977645174,0.125695372610114,0.195959727636489,0.641434717868577,0.521240282529517,0.877581184149831
"9677","ADCY2",11.4784067424732,-0.432760528523541,0.675020997864678,-0.641106765408055,0.521453318967862,0.877655828959238
"9678","KCTD11",51.9793819155095,-0.167026713641822,0.260509681177311,-0.641153575893932,0.521422908346495,0.877655828959238
"9679","PLEKHH2",729.006528178364,0.273760686163889,0.428290097423233,0.639194526819425,0.522696392197306,0.877760189715722
"9680","ZEB2-
AS1",9.79119392716719,0.32388503867018,0.507693387278533,0.637954022616586,0.523503609777768,0.877760189715722
"9681","TMEM237",249.396813376399,-0.145509624359751,0.227166834718206,-0.640540792586433,0.521821077713377,0.877760189715722
"9682","ADAM23",14.9404798759621,0.467577302849116,0.730282145974236,0.640269388244927,0.521997478578804,0.877760189715722
"9683","TTC21A",18.1303744569607,0.238262476673804,0.372756750059899,0.639190240379329,0.522699180356998,0.877760189715722
"9684","INPP4B",4.16081410307707,0.457478625410092,0.716381693861776,0.638596197152913,0.523085655848494,0.877760189715722
"9685","CBR4",154.465189706777,0.16082959426172,0.251244541948922,0.640131694062497,0.522086985453729,0.877760189715722
"9686","LINC01011",18.1793629170649,0.250667380588198,0.392406671565176,0.638794900169183,0.522956366361691,0.877760189715722
"9687","LINC00271",23.2864854351772,0.240634606947933,0.377202428279206,0.637945540397782,0.523509131505203,0.877760189715722
"9688","GLE1",76.7510805145088,0.137618566997997,0.214867831453128,0.640480085210043,0.52186053217154,0.877760189715722
"9689","TMEM273",8.98801289437895,-0.357089067191487,0.557210726810031,-0.640851028184224,0.52161947580105,0.877760189715722
"9690","NOLC1",242.916049736881,-0.148745955340397,0.232567136507102,-0.639582864433878,0.522443825643552,0.877760189715722
"9691","ITPRIP",54.1447989396481,-0.238644928227865,0.373052077128041,-0.639709420907356,0.522361529545751,0.877760189715722
"9692","ZNRD2",236.156369577242,-0.185485790834612,0.290374352893518,-

0.638781589993352,0.52296502634053,0.877760189715722
"9693","PPP1CA",421.165876235067,-0.133363359515537,0.208585105370417,
-0.639371441593125,0.522581322801089,0.877760189715722
"9694","PPFIA1",336.922556488941,0.134966810243613,0.210742323667611,0
.640435238137006,0.521889679811988,0.877760189715722
"9695","ARCN1",368.742839930614,0.113718208149566,0.178196509878632,0.
638161814880766,0.523368351172151,0.877760189715722
"9696","TECTA",4.37305489501048,0.45785843366906,0.717477704745213,0.6
38150050713635,0.523376008364463,0.877760189715722
"9697","FAIM2",22.7469846519335,-0.300241445674369,0.469411689827442,-
0.639612204341862,0.522424746137435,0.877760189715722
"9698","AC023509.3",15.527200436584,-0.253929877879004,0.3979469325921
26,-0.638099849708526,0.523408684400614,0.877760189715722
"9699","GATC",165.64325344277,0.160929056261163,0.251543962176505,0.63
976513237969,0.522325304061791,0.877760189715722
"9700","AC127070.1",5.13213275990855,0.522741568511748,0.8192583141658
41,0.638066845917818,0.52343016727103,0.877760189715722
"9701","AL356019.2",8.91569910901264,-0.375600113842199,0.588544669797
816,-0.638184547608306,0.523353554803773,0.877760189715722
"9702","MIPOL1",82.9354689633801,-0.166783672541057,0.260929415759632,
-0.6391907637378,0.52269883993261,0.877760189715722
"9703","SRP14",6051.7685995681,-0.144973349048721,0.227190542230424,-0
.638113486703526,0.523399807922162,0.877760189715722
"9704","INO80",185.685257790498,-0.122668542261865,0.192258644332105,-
0.63803915131103,0.523448194629631,0.877760189715722
"9705","DUOX1",7.09800004014221,0.505634162209217,0.79079820244754,0.6
39397207333383,0.522564565262824,0.877760189715722
"9706","MESP1",30.0544495305121,0.283579851623025,0.443143128005249,0.
639928352041749,0.52221918054871,0.877760189715722
"9707","AC145285.2",2.49916581684375,0.603016519515602,0.9438426894366
1,0.638895153042452,0.52289114135785,0.877760189715722
"9708","TRPV3",12.0276228052487,0.315304943654135,0.493934130550627,0.
638354234202524,0.523243115298094,0.877760189715722
"9709","MAP1S",127.981474263639,0.1250215745113,0.195096054255786,0.64
0820620325758,0.521639234116687,0.877760189715722
"9710","AKT2",341.126803513485,0.109754692398583,0.171674990268856,0.6
39316724158242,0.522616910876244,0.877760189715722
"9711","ZNF222",27.1174368557382,0.222167511722382,0.347926813872732,0
.638546679542924,0.523117877874946,0.877760189715722
"9712","BLCAP",331.129182015097,0.155600526125934,0.24290464476065,0.6
40582753282704,0.521793807846044,0.877760189715722
"9713","CHADL",50.6686770421478,0.201005676666273,0.314486129459481,0.
639155936739557,0.52272149379266,0.877760189715722
"9714","ZC3H12B",12.7746624775678,0.299598054396004,0.468025752045263,
0.64013155918615,0.522087073132763,0.877760189715722
"9715","CDR1",14.5034988236527,0.309124158835265,0.483547592883851,0.6
39283833451979,0.522638303503835,0.877760189715722
"9716","ID3",2597.50214419206,-0.209768700837605,0.329139879722172,-0.
637323866723995,0.523913907957492,0.877769595445416
"9717","SNIP1",121.58936345368,-0.138088514784931,0.216494558443711,-0

.637838270751893,0.523578964131426,0.877769595445416
"9718","GGPS1",234.740621944509,-0.125698586572118,0.197363844808029,-0.636887605702967,0.524198056608249,0.877769595445416
"9719","AC007365.1",8.28999929834612,-0.342491195936112,0.537463120636446,-0.637236645242832,0.523970711360102,0.877769595445416
"9720","HAND2-AS1",141.237493185593,-0.223038479897309,0.35026072056537,-0.636778453311275,0.524269162876079,0.877769595445416
"9721","FAM149A",113.588026230561,0.141292130420306,0.221841063658123,0.636907018432122,0.524185410891674,0.877769595445416
"9722","AC107375.1",30.8093714613293,-0.278283315201864,0.436700815490675,-0.637240200454368,0.523968395950029,0.877769595445416
"9723","KCTD21-AS1",22.652155223763,0.289859568941203,0.454656631376864,0.637535117575222,0.523776342925777,0.877769595445416
"9724","ZNF446",46.6264585401829,-0.192104494862637,0.301580220705402,-0.636993017689624,0.524129391682833,0.877769595445416
"9725","TNFRSF6B",16.2289352309419,-0.282758454631227,0.443813074795725,-0.637111591994835,0.524052158388352,0.877769595445416
"9726","ZBTB46",10.2981167073355,-0.338561469641868,0.531619658954688,-0.636849040360122,0.52422317906246,0.877769595445416
"9727","POLR2F",703.946751516797,-0.146161000360387,0.229318245082358,-0.637371877269922,0.523882642209471,0.877769595445416
"9728","SLC38A5",9.47046413386171,-0.403823291193719,0.633428951509638,-0.637519472754277,0.523786530085264,0.877769595445416
"9729","LAS1L",112.787709406297,-0.115611783023631,0.181321592529794,-0.637606263052396,0.523730017673883,0.877769595445416
"9730","KCTD9",221.906304782665,0.144734523524684,0.227414718193856,0.636434284791133,0.524493400482231,0.877784136489783
"9731","NXPE2",3.66583590630708,-0.594120923165251,0.933277777208151,-0.636596024971827,0.524388015082887,0.877784136489783
"9732","ZNF605",90.8611737597223,0.15516106650103,0.243770792963939,0.636503924914363,0.524448023586498,0.877784136489783
"9733","PIH1D1",951.700743808241,-0.160131798214314,0.251525801044227,-0.636641638947241,0.524358296247686,0.877784136489783
"9734","EMP1",4769.8682574532,-0.176981381258553,0.278183230878155,-0.636204348838232,0.524643239022186,0.877944701092295
"9735","RPL24",16046.9761139846,-0.158898599396037,0.249893731839548,-0.635864686266171,0.52486462139062,0.878160850183194
"9736","Z84485.1",7.21816960752974,0.410283265378585,0.64534498790404,0.635758041154249,0.524934139494982,0.878160850183194
"9737","TAZ",119.277916870098,-0.122876430885846,0.193271353832428,-0.635771563914142,0.52492532423429,0.878160850183194
"9738","TMEM255B",4.67474156193295,-0.407028550745671,0.640328026893713,-0.635656309970067,0.525000458779719,0.878181605366898
"9739","METTL18",52.9975293783005,0.223927450702478,0.352443063379856,0.635357803768529,0.525195081855591,0.878245976144806
"9740","FOXO1",537.289232057889,0.147992064778178,0.232960593465765,0.635266516866622,0.525254607379379,0.878245976144806
"9741","GPR180",74.9756380577084,0.156944728855268,0.247017684911581,0

.635358269637438,0.52519477808493,0.878245976144806
"9742","ZDHHC15",31.218679543409,-0.198378501523404,0.312271305891762,
-0.635276113368433,0.52524834961832,0.878245976144806
"9743","PTPA",132.543726198457,-0.125743454495953,0.197977303308721,-0
.635140758028569,0.525336616699899,0.8782929435928
"9744","DIABLO",7.05640845680924,-0.358448701165832,0.56448519522194,-
0.635001066812566,0.525427719223168,0.878355102465742
"9745","MIS18BP1",259.484447048137,0.136697293921846,0.215327476676479
,0.634834420723874,0.525536411496628,0.878358158878973
"9746","NDEL1",162.336962497,-0.117252081492211,0.184721600815465,-0.6
3475024563773,0.525591317735487,0.878358158878973
"9747","CFD",221561.54062549,0.169095182948978,0.266371134030097,0.634
810463095719,0.525552038425839,0.878358158878973
"9748","ARHGAP15",53.2703139068602,-0.212492817463284,0.33527092911286
2,-0.633794340671131,0.52621504703194,0.878618875334833
"9749","DOCK1",331.293951238722,0.102496589835341,0.161589192112924,0.
634303498242092,0.525882773951231,0.878618875334833
"9750","MS4A4E",3.95004665778157,-0.489102406351394,0.771097332037462,
-0.63429399380626,0.525888975504681,0.878618875334833
"9751","ZFC3H1",291.961685271501,0.138078556323562,0.217717684472266,0
.63420919002632,0.52594431081473,0.878618875334833
"9752","VPS29",851.354774405907,-0.169503082976389,0.267403710834249,-
0.633884557725739,0.52615616412121,0.878618875334833
"9753","LRCH1",64.8607536606796,0.211296739206481,0.333420693444653,0.
633724130987558,0.526260873851832,0.878618875334833
"9754","C15orf65",19.097002126842,-0.270034538121183,0.426073595612179
,-0.633774401657534,0.526228061291265,0.878618875334833
"9755","MVP",607.139783851631,-0.105145428391622,0.165888900935772,-0.
633830399734409,0.526191511581174,0.878618875334833
"9756","Z93930.2",29.0476424100651,-0.214388239501804,0.33832013782914
4,-0.633684535828819,0.526286719052242,0.878618875334833
"9757","PIM2",14.9079499663696,0.332703624108054,0.524834744692456,0.6
33920729282153,0.52613255659798,0.878618875334833
"9758","G0S2",358.469303476214,-0.308969897717828,0.488198250569207,-0
.632877928910211,0.526813361858304,0.878867559536041
"9759","PPM1M",148.220905944842,0.118940800456479,0.187881348250968,0.
633063375176554,0.526692258074652,0.878867559536041
"9760","MLXIPL",148.477160757517,0.301477776686616,0.476149416878292,0
.633157924802577,0.526630518882191,0.878867559536041
"9761","TMEM209",57.8476449218673,0.150788785116871,0.23809394853378,0
.63331632763223,0.526527092987709,0.878867559536041
"9762","GPN3",214.592020793187,-0.14428507770711,0.227938246672468,-0.
633000735126467,0.526733162927482,0.878867559536041
"9763","SPPL2B",158.508177606304,0.132736344887374,0.209609465121037,0
.633255491638828,0.526566813380819,0.878867559536041
"9764","RUVBL2",192.822344300704,-0.172878080494829,0.273142526035236,
-0.632922610053506,0.526784181995711,0.878867559536041
"9765","CSKMT",197.853622685788,-0.154780269231003,0.244728348763801,-
0.632457457474161,0.527087999067248,0.878974985946373
"9766","CCDC168",3.17377954081784,-0.568587846356175,0.89914320780156,

-0.63236628094694,0.527147562017933,0.878974985946373
"9767","TERF2IP",925.582232061658,-0.099329745954811,0.157058609104002
,-0.632437448169658,0.527101070264776,0.878974985946373
"9768","KAT7",128.069145885672,0.13352527468514,0.211087195704922,0.63
2559801835611,0.527021144589487,0.878974985946373
"9769","APLN",6.90319748695081,0.488184343169229,0.771645657614616,0.6
32653522185754,0.526959927376837,0.878974985946373
"9770","CENPE",19.0779720815273,-0.243758282416951,0.385548865864978,-
0.632237062531826,0.527231982500613,0.87902576898183
"9771","ALKBH7",671.829907936455,-0.155157023484663,0.245466283515011,
-0.632090979106606,0.527327429473576,0.879094923620415
"9772","FSD1L",26.2079204461793,0.215098853323513,0.340361529821163,0.
631971696203543,0.527405372244359,0.879134886255462
"9773","LINC02848",3.25620393588221,-0.479905570520369,0.7596629449290
25,-0.631734868370086,0.527560139559572,0.879165985619303
"9774","SNPH",7.42953829488388,0.379236947911091,0.600347826659504,0.6
31695379029298,0.527585948150819,0.879165985619303
"9775","RBM39",2000.85289823504,0.0998330874180157,0.157999330930834,0
.631857659332233,0.527479892605779,0.879165985619303
"9776","MAEA",231.019415711122,0.092994932391751,0.147263998514664,0.6
31484499468422,0.527723781162138,0.879215779006859
"9777","PANK2",174.265014834344,0.110611984105412,0.175158902126614,0.
631495075399909,0.52771686818924,0.879215779006859
"9778","CYB5R1",295.538371821845,-0.16013594943155,0.253628049383988,-
0.63138107090477,0.527791389825706,0.879238489350678
"9779","AKR1C3",1072.43140878596,-0.220010379021014,0.348525591663688,
-0.631260327170791,0.527870322568526,0.8792800577072
"9780","THORLNC",13.4538375407645,0.304318664997984,0.482549137850372,
0.630648033801578,0.528270684344438,0.879299382373558
"9781","PCDHGC3",85.2626237553049,-0.193278395383829,0.306614392812661
,-0.630363087690801,0.528457055489976,0.879299382373558
"9782","PURB",226.897043718581,0.153195358379014,0.242932623675591,0.6
30608421632119,0.528296590977719,0.879299382373558
"9783","CRYGN",7.48823000520218,0.403047280857685,0.639418028588089,0.
630334558673081,0.528475716952368,0.879299382373558
"9784","BICD2",194.177063224719,0.117138309162381,0.185802395053139,0.
630445636230251,0.528403060552339,0.879299382373558
"9785","LINC00476",69.7355302104977,-0.166963507767388,0.2648015361136
8,-0.630523184335719,0.528352338954594,0.879299382373558
"9786","PPA1",1237.07757056526,-0.151328856418606,0.239990779984667,-0
.630561125841061,0.528327523600377,0.879299382373558
"9787","SLIRP",708.960228044765,-0.18018451781276,0.285648028878495,-0
.630792092352945,0.528176474510395,0.879299382373558
"9788","LCMT1",214.323998819499,-0.126538221771431,0.200716058280133,-
0.630433971530201,0.528410690253405,0.879299382373558
"9789","NANP",35.5605293282864,-0.176993852305382,0.280656177506172,-0
.63064299484906,0.528273979818372,0.879299382373558
"9790","GPR173",17.5396862907761,0.286754226423315,0.454563809873462,0
.630833823975427,0.528149184919938,0.879299382373558
"9791","ACAD9",135.578740996894,-0.180391237600621,0.28630983457207,-0

.630056029581524,0.52865792663075,0.879377794220885
"9792","NEK1",186.200307167951,0.108324245181886,0.171981837634031,0.629858633167967,0.528787079855096,0.879377794220885
"9793","PAPLN",131.019562985548,0.303505455612689,0.481932603297764,0.629767427096371,0.528846759910847,0.879377794220885
"9794","PLIN1",12.0763810811836,-0.459655098225631,0.729448876814828,-0.630140250859986,0.528602826922751,0.879377794220885
"9795","U2AF1L5",13.2450940727586,0.522088542346691,0.828715974870287,0.629996956953086,0.528696575195256,0.879377794220885
"9796","FAM3B",12.9001475210655,-0.370970818111333,0.589046342854201,-0.629782058086989,0.528837185993496,0.879377794220885
"9797","BDNF-AS",57.1287670383806,0.151505770093499,0.240608828831825,0.629676686549996,0.52890613875526,0.879386760659837
"9798","AMIGO2",49.8423095170672,0.231693053933243,0.368132834133027,0.629373509914954,0.529104556238073,0.879537107517294
"9799","NPAS1",3.16779734969883,-0.600428439464977,0.953955747485809,-0.629409111531045,0.529081254383123,0.879537107517294
"9800","AP000866.1",16.098226248875,0.278085217529093,0.441992763687043,0.629162376346039,0.529242757429915,0.879677068956724
"9801","TNFRSF1A",1251.32733589532,0.166768502713848,0.265150856364025,0.628957058637184,0.529377169356139,0.879731020797221
"9802","AC068790.9",4.88669044194093,0.439102802400241,0.69815460598372,0.62894779843432,0.529383231988112,0.879731020797221
"9803","LUZP1",282.311054701534,-0.120996951331245,0.192438137708493,-0.628757650495102,0.529507729180999,0.879732374669047
"9804","CCDC142",45.4517383654829,0.214405450843109,0.340976047947941,0.628799155053389,0.529480553275137,0.879732374669047
"9805","AL646090.2",3.91324865388121,0.481120957412763,0.765565556118455,0.628451676760546,0.529708093238014,0.879732374669047
"9806","SCML4",3.21574549510432,0.5467846860576,0.869883183952649,0.628572544158254,0.529628939740922,0.879732374669047
"9807","ING3",164.620588361385,-0.122784961257525,0.195374964201475,-0.62845801026434,0.529703945411328,0.879732374669047
"9808","PGRMC1",1263.95548082912,0.136814022293598,0.217642988893423,0.628616722225748,0.529600009961447,0.879732374669047
"9809","CAP1",815.840165611694,-0.107446811281806,0.171070096491368,-0.628086459793559,0.529947302619124,0.879770853277917
"9810","FAM220A",3.00107647866824,-0.510522455635039,0.812680464366316,-0.628195801449609,0.529875680393017,0.879770853277917
"9811","LRRC14",44.1951949904003,-0.170056262705946,0.27072575029725,-0.628149566560362,0.529905965100404,0.879770853277917
"9812","BSCL2",395.907999921993,-0.153928284103457,0.245031008688514,-0.628199201918696,0.529873453057611,0.879770853277917
"9813","AC007773.1",4.85419202402365,-0.48614346593199,0.774117933302471,-0.627996646270743,0.530006136982956,0.879778861236663
"9814","ADGRA3",93.2512131663467,0.174058721010985,0.277293018054134,0.627706828799435,0.530196011052133,0.879946834451497
"9815","CEBPA-DT",18.7718262036351,0.229076510088103,0.365007002482804,0.62759483661

9321,0.530269392042231,0.879946834451497
"9816","TCEA2",382.475829613145,-0.13621952458563,0.217040808835294,-0
.627621714628803,0.530251780208724,0.879946834451497
"9817","KANSL2",67.1151000437512,-0.160575439891394,0.25592374573117,-
0.627434704945541,0.530374324584681,0.880031310294374
"9818","PPP2R3A",96.7085248748934,-0.124318065611897,0.198192642998889
,-0.627258730348499,0.530489650981816,0.880043377619188
"9819","CYP51A1",210.19401705906,-0.107140056163636,0.170792615787673,
-0.627310821779499,0.530455511108208,0.880043377619188
"9820","FAXC",5.8250873506735,-0.636422788333129,1.0149355168624,-0.62
7057362521498,0.530621634659296,0.880172689100333
"9821","MEX3A",7.83511449719754,0.414715729726808,0.661752559228419,0.
626693050058397,0.530860460430758,0.880248204612254
"9822","RPL37",26616.0241474949,-0.163045201763706,0.260130430238382,-
0.626782501433195,0.530801815349434,0.880248204612254
"9823","NEAT1",14880.5018542801,0.248669404690973,0.396731682642934,0.
626794923547309,0.530793671565875,0.880248204612254
"9824","AP003059.1",3.50051138395951,0.561367870392433,0.8958119144644
66,0.626658187202199,0.530883317705862,0.880248204612254
"9825","AC099332.1",2.65465154577653,0.60130021060438,0.95973391081445
3,0.626528044730754,0.530968647950967,0.880287838018458
"9826","GNPDA2",216.809151313365,-0.130539465316114,0.208377411664468,
-0.626456890280942,0.531015304584036,0.880287838018458
"9827","ZNF483",71.5441694260215,-0.163123086706674,0.260459923439728,
-0.626288622650314,0.531125647505336,0.880381161312143
"9828","SRD5A1",33.3526927543977,-0.17528045182199,0.27993854718264,-0
.626139035106274,0.531223750566181,0.880392934759446
"9829","AL391834.1",3.94191438163954,0.509753024603883,0.8142604004906
06,0.62603194788393,0.531293986551441,0.880392934759446
"9830","SRR",49.907585089494,0.154712247619227,0.247132100085681,0.626
0305624627,0.531294895247428,0.880392934759446
"9831","DARS2",29.3448060717345,0.203449418233815,0.325776655643089,0.
624505822346913,0.532295447811837,0.880476918589223
"9832","TMEM18",389.679001750253,-0.139074693971055,0.22254623089408,-
0.624924957894466,0.532020311032428,0.880476918589223
"9833","SERTAD2",246.692625956411,0.120023304203517,0.192023583271505,
0.625044602119594,0.531941785155893,0.880476918589223
"9834","DUSP11",211.435826375633,-0.145088934576346,0.232052324261519,
-0.625242324282141,0.531812027399812,0.880476918589223
"9835","SETMAR",114.040425841669,-0.131377123877599,0.210334022869218,
-0.624611853495938,0.532225838058914,0.880476918589223
"9836","EIF4A2",2799.67141806733,0.129770254528243,0.20771018546061,0.
624765965330347,0.532124671418099,0.880476918589223
"9837","AC113349.2",2.36286906398281,-0.655724078844752,1.049043690218
34,-0.625068417034449,0.531926155456896,0.880476918589223
"9838","RNF14",126.359345492659,0.126922774097686,0.20314500993614,0.6
24789031921509,0.532109530203238,0.880476918589223
"9839","GCC1",38.1352883478082,-0.194595851980586,0.311591886868938,-0
.62452156227815,0.532285114210558,0.880476918589223
"9840","TSPYL5",110.765853756875,-0.149160815319058,0.238952480881923,

-0.6242279417587,0.532477899082317,0.880476918589223
"9841","APOLD1",58.3996286861289,-0.372610039761312,0.596375328342183,
-0.624791171018261,0.532108126082955,0.880476918589223
"9842","TSPAN31",353.54235065011,0.113943942188433,0.18217732505045,0.
625456225997823,0.53167166963166,0.880476918589223
"9843","METTL25",59.7436285863441,-0.150050897347851,0.24002498267865,
-0.625146997922054,0.531874584568554,0.880476918589223
"9844","BMP4",373.873016186045,0.179451553137142,0.286843097808159,0.6
25608754431871,0.531571595231072,0.880476918589223
"9845","RPL4",6128.38221901103,-0.0930756384801303,0.14889010086276,-0
.625129796680863,0.5318858731439,0.880476918589223
"9846","TLE3",275.91099350444,-0.131246688944715,0.210188238019718,-0.
624424516715356,0.532348828314264,0.880476918589223
"9847","MIR9-3HG",2.36142502472279,0.710413353690553,1.13781325823154,
0.624367266377893,0.532386417149442,0.880476918589223
"9848","MRPL45",203.224786402633,-0.123847026519518,0.198412326790768,
-0.624190182750685,0.532502693393542,0.880476918589223
"9849","AP000915.2",10.6660207785816,0.404740394372081,0.6471298202541
6,0.62543925763322,0.531682803255929,0.880476918589223
"9850","FAM71E1",2.47611382518558,-0.644711635466912,1.03260355422745,
-0.624355429368297,0.532394189138555,0.880476918589223
"9851","PET117",93.17698285389,-0.144323477432055,0.230621617360219,-0
.625802034883092,0.531444796976685,0.880476918589223
"9852","P2RY8",3.29475148303505,0.59073601766449,0.946477922573615,0.6
2414135985147,0.532534753633803,0.880476918589223
"9853","C12orf45",228.57922066642,0.128116445802249,0.205344272237929,
0.623910491419999,0.532686369853201,0.880638209534029
"9854","LINC00884",11.832606415825,0.400418856933121,0.64238476332712,
0.623331809520545,0.5330664985607,0.880871373820976
"9855","PDZD2",9.91428437181767,0.374332452393019,0.60066002887302,0.6
23201868610029,0.533151873933391,0.880871373820976
"9856","SLF1",58.1590726745259,0.15930333873883,0.255600718289621,0.62
3250747512858,0.533119758105152,0.880871373820976
"9857","DUSP4",12.2758882481217,-0.475074085023426,0.762031133318404,-
0.623431332726039,0.533001113278292,0.880871373820976
"9858","ANK1",11.3901922571969,-0.326597745974138,0.523826661413597,-0
.623484389077834,0.532966257693199,0.880871373820976
"9859","EML2",33.2717729384688,0.187424601901697,0.300595278087777,0.6
23511463965733,0.532948471180304,0.880871373820976
"9860","CITED4",170.641365205768,0.286665188750246,0.460054708314452,0
.623111085637032,0.533211525379746,0.880880581836784
"9861","CCDC9",54.0882325401964,-0.175687049038476,0.282003463440682,-
0.622996068540949,0.533287105368261,0.880916099720475
"9862","SRRM1",1106.00917106905,-0.0840886801292924,0.135947787625625,
-0.618536583771829,0.536221690111742,0.880927189549189
"9863","PTPRU",54.0062866248668,-0.176489790970982,0.284036805249657,-
0.621362399903966,0.534361205541361,0.880927189549189
"9864","AL356055.1",5.63941058796746,-0.46935800349085,0.7582579006759
82,-0.61899520344255,0.535919518747145,0.880927189549189
"9865","EPHX1",3339.8476073765,-0.157681027138224,0.254784511804926,-0

.61887995475546,0.535995444738932,0.880927189549189
"9866","B3GALNT2",98.3836450937422,-0.141971300847608,0.229487414785094,-0.618645257651966,0.536150080253059,0.880927189549189
"9867","LGALS8",207.876086287127,0.160606069354128,0.259187354889845,0.61965241098427,0.535486653051227,0.880927189549189
"9868","SMYD3",105.161252088278,-0.134736909550167,0.217772097187486,-0.618706029331976,0.536110037297676,0.880927189549189
"9869","EPC2",227.911956824657,0.109085988256748,0.175882321987199,0.620221447068952,0.53511200330071,0.880927189549189
"9870","CWC22",306.853998845359,0.107962079099028,0.174009334223779,0.620438435562238,0.534969174265948,0.880927189549189
"9871","SP100",857.375009503425,0.0793787334738872,0.128301210369763,0.618690449179071,0.536120303043926,0.880927189549189
"9872","FANCD2",7.2674030445815,-0.342003267378423,0.549107349737503,-0.622834983982487,0.533392966293353,0.880927189549189
"9873","NEK10",3.74848667993971,-0.459136669550491,0.737282098885311,-0.622742190871927,0.533453952531355,0.880927189549189
"9874","PRKAR2A",325.64640612942,0.125170366343216,0.201762791939572,0.620383793958923,0.535005139373354,0.880927189549189
"9875","SEMA3B-AS1",2.88945699650422,0.536031752927175,0.861476570942987,0.622224412139759,0.533794315942539,0.880927189549189
"9876","RAD54L2",105.457528212489,0.17685946894079,0.284759903665391,0.621082767146215,0.534545167144832,0.880927189549189
"9877","NMNAT3",101.100720753942,0.159368874231488,0.257238284965612,0.619537928628286,0.535562043476011,0.880927189549189
"9878","AC069243.1",4.06935564043046,-0.480753030825378,0.774936473062704,-0.620377343868389,0.535009384903882,0.880927189549189
"9879","UNC5C",10.301098055577,0.481412817048407,0.775490980014992,0.62078454740905,0.5347413917464,0.880927189549189
"9880","SLC30A5",181.707239238443,-0.106448052998604,0.17103175851863,-0.622387642626086,0.533687004049848,0.880927189549189
"9881","FAM151B",6.68670439389645,0.436746091269149,0.703474119903244,0.620841732357147,0.534703761999125,0.880927189549189
"9882","SIMC1",44.3482153283584,0.163783688749407,0.263804652482354,0.620852161659136,0.53469689928831 8,0.880927189549189
"9883","AC005532.1",2.44919252538777,0.652522408787961,1.05448157003528,0.618808737232011,0.536042365647554,0.880927189549189
"9884","FAM185A",28.1530767401689,-0.200252365537637,0.323788863345211,-0.618465883813105,0.536268279917107,0.880927189549189
"9885","NCALD",300.755902755153,-0.199936159109631,0.323144540237576,-0.61872052352374,0.536100487178924,0.880927189549189
"9886","LYNX1",15.9756294406339,-0.322727396321414,0.519758743050286,-0.620917686593278,0.534653783452635,0.880927189549189
"9887","RPS6",21934.7804043846,-0.142109881874777,0.229462151260182,-0.619317308298231,0.535707344354911,0.880927189549189
"9888","FBP1",19.3179169045172,-0.324302455548371,0.523369635329137,-0.619643238080527,0.535492693515208,0.880927189549189
"9889","POLE3",253.458680293849,-0.125417283205753,0.202730854894813,-0.618639344616909,0.536153976480432,0.880927189549189

"9890","AL596244.1",15.1497335157341,-0.24504032307835,0.393568574100816,-0.622611507126026,0.533539847535539,0.880927189549189
"9891","NIBAN2",89.2115718050364,-0.158376237015743,0.255801208363142,-0.619137954934551,0.535825481379405,0.880927189549189
"9892","AL359091.5",10.3691638809445,0.360974530596579,0.581835179142658,0.620406849803203,0.534989963866228,0.880927189549189
"9893","ALDH18A1",87.8431667744829,0.145112425025163,0.233887153197649,0.620437775402456,0.53496960877589,0.880927189549189
"9894","AL139407.1",3.25089083567584,-0.528564208337485,0.853200078487481,-0.619507922777624,0.535581804207382,0.880927189549189
"9895","AC136475.1",33.0025893185665,-0.216681266354891,0.349971554991548,-0.619139650821406,0.535824364266,0.880927189549189
"9896","LINC00937",6.1409782982275,0.415883658402671,0.668602501305606,0.622019297849708,0.533929178771658,0.880927189549189
"9897","PCED1B-AS1",18.1758021705934,0.273995603038818,0.442065730406933,0.619807382007643,0.535384608016998,0.880927189549189
"9898","TBC1D15",324.986073980301,0.104701555606501,0.168792382664206,0.620297870993345,0.535061696341654,0.880927189549189
"9899","AL356966.1",8.02013462342543,0.387987237615206,0.624941075410359,0.620838112393941,0.534706144025168,0.880927189549189
"9900","PPP1R36",4.14266438036688,0.521971958023158,0.841138931772301,0.620553797127604,0.534893247279441,0.880927189549189
"9901","PAPOLA",719.363615481299,-0.0773148478538483,0.124305938551307,-0.621972278677068,0.533960096344233,0.880927189549189
"9902","DIO3",132.896346448229,0.254366966606162,0.411187676764658,0.618615247926675,0.536169854460975,0.880927189549189
"9903","STOML1",112.022767423018,0.166449075004323,0.268903904981149,0.618990918023268,0.535922341890245,0.880927189549189
"9904","ZNF597",13.4338917332428,0.269789913379824,0.435152116397925,0.619989891381143,0.535264442149208,0.880927189549189
"9905","AC012645.4",2.31116862003472,0.752851440331924,1.21495714973465,0.619652668817455,0.535486483265566,0.880927189549189
"9906","DCTPP1",234.797818222199,-0.155063502940845,0.250620115161686,-0.618719302881202,0.536101291448214,0.880927189549189
"9907","UTP4",75.5038916624908,-0.136112877352672,0.21888198041242,-0.621855107013407,0.534037146793809,0.880927189549189
"9908","CDC6",33.7231865978647,-0.202956115199861,0.326152930428008,-0.622272855048467,0.533762467199035,0.880927189549189
"9909","VAT1",1413.47587116279,0.0951219139119351,0.15355795677488,0.619452849658491,0.535618074260359,0.880927189549189
"9910","ZNF85",54.3010121706073,0.178906065163353,0.28831099608117,0.62053153572049,0.534907898543321,0.880927189549189
"9911","NFKBID",16.6532400697946,-0.25874890251914,0.416937938077278,-0.620593327899995,0.53486723074462,0.880927189549189
"9912","ZNF234",72.1942246244748,0.176203536862261,0.283053965583472,0.622508631875353,0.533607469660559,0.880927189549189
"9913","AC010624.5",2.69866316929406,0.633120769544529,1.02187389895504,0.619568393117734,0.535541981078093,0.880927189549189
"9914","SPACA6",42.9820127624162,-0.170106265402039,0.275036938750571,

-0.618485161210679,0.536255576310209,0.880927189549189
"9915","MCTS1",439.106683968557,-0.167853821499113,0.270866417700611,-0.619692256146135,0.535460414946422,0.880927189549189
"9916","RTL8C",779.723857457512,-0.0951109661057497,0.153603397937963,-0.61919832101737,0.535785717797726,0.880927189549189
"9917","ERCC3",104.751481928149,0.111518603451466,0.180356779531438,0.618322215229104,0.536362960807955,0.880993876031137
"9918","ASH1L",848.47813053731,0.122535795935662,0.198218278624197,0.618186157130229,0.536452633969298,0.881052324533766
"9919","TSLP",14.3822380625219,0.454077191947421,0.734739264727087,0.618011332382085,0.536567868523205,0.881152738217007
"9920","SKP1",3046.44048586091,-0.116093143888088,0.187924914139861,-0.617763453129411,0.5367312778032,0.881332236304066
"9921","ZNRF2",88.5963983060193,-0.166632833595598,0.269819961611584,-0.617570444381993,0.536858532164446,0.881452336501024
"9922","AMFR",245.174218978183,-0.104218047722707,0.168808787260539,-0.617373357240328,0.536988491142421,0.881576852672736
"9923","GOT2",71.2637376750373,0.18104018220871,0.293420311450711,0.61699948893661,0.537235062814369,0.88189276813295
"9924","MEAF6",556.753100299025,-0.126345352636053,0.204823751609095,-0.616849128304135,0.537334243914234,0.881966696807633
"9925","ASNSD1",233.963844523823,-0.132002006179492,0.214119787766779,-0.616486722484845,0.537573332374609,0.882270227813602
"9926","SCOC-AS1",16.9523434935577,-0.290757129797713,0.472168821968401,-0.61579061613088,0.538032721519458,0.882603183755463
"9927","WHAMM",113.899676753883,0.111886610719126,0.181676759907054,0.615855383904733,0.537989970295476,0.882603183755463
"9928","RPL27",9331.12715747191,-0.13369437490587,0.21713007121845,-0.615734035159795,0.538070070281088,0.882603183755463
"9929","RNMT",433.453742191398,0.0854454182229836,0.138712918792081,0.61598745788818,0.537902797587955,0.882603183755463
"9930","S1PR4",3.40682076254939,0.512645594191651,0.832640340999968,0.615686712435748,0.538101308728314,0.882603183755463
"9931","ZSWIM4",22.862939784842,-0.258363539450368,0.419422596562518,-0.615998140223847,0.537895747242699,0.882603183755463
"9932","C3orf67",13.1532434527886,-0.309126460664459,0.502238442748215,-0.615497409901441,0.538226279302046,0.882646037608605
"9933","SIRT6",80.1390729850869,0.15342193827743,0.249270807432147,0.615482975555377,0.538235808924199,0.882646037608605
"9934","AC092718.4",4.35750674644257,0.454553777201761,0.738635278073386,0.615396787420434,0.538292712492208,0.882650492629914
"9935","ETV1",93.8716181334929,-0.175904319508036,0.285882224371082,-0.615303451954775,0.538354338311237,0.882662689154679
"9936","BIVM",128.876950440071,0.14896197924419,0.242177548650236,0.615094091398736,0.538492583908818,0.882800493084817
"9937","FOXO6",9.34326357746154,-0.319571777264752,0.52042955419426,-0.614053861256054,0.539179735752309,0.882808272743848
"9938","AL391069.2",3.42442865151559,0.643376172779137,1.04735858555228,0.614284526478463,0.539027325768399,0.882808272743848

"9939","POMC",15.7015403845489,-0.391258267235396,0.636848267110047,-0
.614366541359823,0.538973140372317,0.882808272743848
"9940","ARIH2",255.332534076293,0.128263517491838,0.208838363832364,0.
614176031348321,0.539099010266396,0.882808272743848
"9941","PCOLCE-
AS1",5.15842605091323,0.432160607888898,0.703034154296513,0.6147078420
69689,0.538747679957354,0.882808272743848
"9942","TTC26",38.7770677128903,0.184310093386441,0.300105423707192,0.
614151157648753,0.539115445397595,0.882808272743848
"9943","AL022345.4",12.6997029502889,-0.327279827839316,0.532994953461
011,-0.614039261936945,0.539189382848418,0.882808272743848
"9944","ME3",70.2581688875399,-0.173566625533272,0.282416056665194,-0.
614577753059687,0.538833610099978,0.882808272743848
"9945","INTS6-
AS1",26.931852816905,-0.187241412962229,0.304937316388078,-0.614032467
984129,0.539193872259587,0.882808272743848
"9946","FES",132.492578009597,-0.1121653077251,0.18257963985915,-0.614
336340084959,0.538993093360954,0.882808272743848
"9947","C17orf53",3.9426590071537,0.476780196144103,0.776089192163877,
0.614336858389631,0.538992750930988,0.882808272743848
"9948","EGLN2",337.261226325831,-0.11869823396868,0.193313194750444,-0
.614020342077079,0.539201885046875,0.882808272743848
"9949","MYH9",491.019069362642,0.112787047838118,0.183500479883035,0.6
1464170507897,0.538791365810211,0.882808272743848
"9950","AP001033.3",2.87470129675074,0.664981845237889,1.0832491638820
2,0.613877090709956,0.539296549922779,0.88287452278313
"9951","HNRNPH3",876.138212507184,-0.0919290912970278,0.14983256399076
9,-0.613545472683037,0.539515725177814,0.883144573150579
"9952","AL603840.1",17.9580647334393,-0.457119577486383,0.745209715150
964,-0.613410652320575,0.539604844326546,0.883201699079091
"9953","PAPSS1",254.285427558592,0.147435215778522,0.240460320976245,0
.613137399051743,0.53978549319971,0.883319861234687
"9954","SBNO1-
AS1",15.8201247433461,0.33343838849591,0.543799764042245,0.61316390801
1566,0.539767966690132,0.883319861234687
"9955","AL133243.3",42.3064147786386,0.198231548548095,0.3235182607567
81,0.612736814559979,0.540050375983998,0.883487051762915
"9956","HLA-
E",7555.19205060424,-0.114268234710986,0.18648275121117,-0.61275498119
175,0.540038362059261,0.883487051762915
"9957","SECISBP2",273.985440899675,-0.110230508086247,0.17986103225761
6,-0.612864869630925,0.539965693682552,0.883487051762915
"9958","BMS1",174.439887446309,-0.118697832021706,0.193773651824796,-0
.612559194214024,0.540167846601964,0.883501762556421
"9959","CTSW",11.3752518790276,0.44147360593702,0.720699839505794,0.61
2562375814808,0.540165742312737,0.883501762556421
"9960","APPL1",725.775453982273,0.104844669017395,0.171266390553567,0.
612173052042006,0.540423268870862,0.883830785806975
"9961","TAF11",284.734318669794,-0.131535474411336,0.214942751101885,-
0.611955852137513,0.540566967082473,0.88397704314892

"9962","AL606491.1",2.42430165009108,-0.582016538323203,0.952056346770
185,-0.6113257269884,0.540983962343927,0.88426072544214
"9963","SLC39A10",243.656335786631,0.120821659795741,0.197627806007697
,0.611359617032008,0.540961530983734,0.88426072544214
"9964","LINC02268",3.10945030488753,-0.739962579929089,1.2106686924812
5,-0.611201548800727,0.541066158173111,0.88426072544214
"9965","LRP5",188.341168604654,-0.148331952582316,0.24266977919197,-0.
6112502062524,0.541033950166423,0.88426072544214
"9966","ARHGEF12",360.11839898417,0.154386516592629,0.252560398172672,
0.611285528965144,0.541010569472826,0.88426072544214
"9967","ZNF677",234.390270791138,-0.110154158310839,0.180132406331743,
-0.611517719404536,0.540856891309467,0.88426072544214
"9968","LRRC4",5.09614686055465,0.423717541539598,0.693719281272918,0.
610791069209019,0.541337906565473,0.884527350791953
"9969","MLLT10",237.994787912776,0.127244927233442,0.208313830519669,0
.610832832923342,0.541310254763085,0.884527350791953
"9970","WDR60",284.32886236273,-0.156008552538059,0.255501560700577,-0
.61059725862452,0.54146623800377,0.884559577860135
"9971","NUBP1",165.666345644223,-0.131229784760385,0.21489723277522,-0
.610662980931212,0.541422718357828,0.884559577860135
"9972","AC009498.1",3.61796178848513,-0.542492600242685,0.888581876318
357,-0.610515040538957,0.541520683195436,0.884559808320342
"9973","PCDHA4",2.93748709721037,0.53466784249101,0.876123810817197,0.
610265165596062,0.541686168297173,0.884571851876085
"9974","SERPINH1",1257.17640050926,0.185573100024314,0.304089624333803
,0.610257914688363,0.541690970744917,0.884571851876085
"9975","TSKS",49.0918274569794,0.200816669032747,0.329057661458424,0.6
10278053222353,0.541677632569779,0.884571851876085
"9976","AC079322.1",4.86881522355875,0.408152398810327,0.6691662973945
21,0.609941654861455,0.541900457786584,0.88482523625558
"9977","BTBD19",37.9440869010781,0.272810745314598,0.448225459872481,0
.60864625001938,0.542758941395791,0.884849673033837
"9978","CD160",5.5181457849516,0.391034017933945,0.642092820885953,0.6
0899920574474,0.542524965356891,0.884849673033837
"9979","KIF21B",5.42765715216624,0.480633959890106,0.78862195791266,0.
609460534376011,0.542219224221968,0.884849673033837
"9980","NOL10",134.712600187727,0.123850978206969,0.203722880362722,0.
607938479892177,0.543228277051114,0.884849673033837
"9981","EIF2AK2",693.048376134725,-0.102836353639283,0.168962258066315
,-0.608635057415731,0.542766361848716,0.884849673033837
"9982","AC008273.1",2.44594231470901,-0.618522147794486,1.017131661112
78,-0.608104310820293,0.543118293325924,0.884849673033837
"9983","PPP1R7",458.716033940172,-0.144836600315992,0.238336261357243,
-0.607698549482978,0.543387425258831,0.884849673033837
"9984","MTMR12",88.836715869837,0.148615581269298,0.243996888156815,0.
609088019080733,0.54246609848975,0.884849673033837
"9985","FCHSD1",46.612338001893,-0.14977087456212,0.245885908389236,-0
.609107189359684,0.54245339254709,0.884849673033837
"9986","B3GALT4",156.63907654285,-0.121854827453093,0.20027931254206,-
0.60842443438362,0.54290600976284,0.884849673033837

"9987","THSD7A",21.0180434624677,0.258866599794111,0.426052018312995,0.607593882125297,0.543456859420158,0.884849673033837
"9988","MRPL15",266.255980861723,-0.162137471512299,0.266695912194139,-0.6079488439788,0.543221402973151,0.884849673033837
"9989","RPL7A",24656.9746254343,-0.151458379723901,0.249097473023241,-0.60802856763533,0.543168526955928,0.884849673033837
"9990","ZNF37A",332.952716767088,0.132864489878487,0.218410541283628,0.608324530023254,0.542972254913942,0.884849673033837
"9991","AC022387.2",6.7252852596183,-0.410265672442221,0.6748517165298,-0.607934546794775,0.543230885726307,0.884849673033837
"9992","PSMD13",654.827305150279,-0.134641273332404,0.221581465326904,-0.607637796481603,0.543427727006294,0.884849673033837
"9993","AC079601.1",5.56422532288444,0.364003640345472,0.598482231362126,0.608211273903675,0.543047358291031,0.884849673033837
"9994","TEX30",117.727899714786,-0.170025341468041,0.279486011928017,-0.608350093427331,0.542955303804162,0.884849673033837
"9995","ING1",195.103740263603,-0.153064717380096,0.251067259269602,-0.60965622449294,0.542089558429974,0.884849673033837
"9996","AP5M1",112.657916611212,0.126218071056744,0.207340951059622,0.608746465238549,0.542692503136531,0.884849673033837
"9997","LEO1",195.238606056524,-0.127169634529057,0.208604117437647,-0.609621881346946,0.5421123133463,0.884849673033837
"9998","CMTM4",27.4244312963353,0.2597647845929,0.427464652358644,0.607687169359109,0.543394974383774,0.884849673033837
"9999","SPATA22",12.4769564860499,-0.495462480647118,0.813897458260829,-0.608752952375406,0.542688202591292,0.884849673033837
"10000","RPL13A",29799.4870438713,0.110591495018361,0.181720133110566,0.608581411015544,0.542801928947698,0.884849673033837
"10001","TFPT",78.1851351981772,-0.169005055559411,0.277370553792856,-0.609311454472657,0.542318016103239,0.884849673033837
"10002","CDC42EP5",400.36347127432,0.137677293336354,0.226613330381664,0.60754278269719,0.543490759328598,0.884849673033837
"10003","DSN1",73.8558922644837,-0.129193490133436,0.21243997501997,-0.608141147264265,0.543093863867879,0.884849673033837
"10004","LRRC75B",69.7338712003582,0.142593634361267,0.234551080755633,0.607942772644172,0.54322542983771,0.884849673033837
"10005","AL672277.1",3.29481814378003,-0.517963148372788,0.851371793808979,-0.608386549964801,0.542931129906628,0.884849673033837
"10006","FHL3",166.001104654084,-0.16979558654421,0.279655337620544,-0.607160185065377,0.543744612135406,0.88486151319995
"10007","UBE2F",202.270193096277,0.13564332000769,0.223371427340291,0.607254569766644,0.543681982583324,0.88486151319995
"10008","RTP4",80.3774409841302,0.204874571300574,0.337382431025216,0.607247302943471,0.543686804401231,0.88486151319995
"10009","BAHCC1",61.9492783046536,0.151841728236615,0.249972866854243,0.607432839201515,0.543563700590439,0.88486151319995
"10010","MKNK2",402.446260080227,0.121725389310216,0.200495611793725,0.607122461290824,0.54376964498321,0.88486151319995
"10011","MAMDC2-AS1",4.33886699202359,0.453289030287954,0.746766168607379,0.6070026326

08917,0.543849165037968,0.884902512166963
"10012","LEPROT",2607.84260373636,0.0818389793232602,0.134870273726414
,0.606797755072933,0.543985138147044,0.884946960479097
"10013","SNX9",1612.97015556712,-0.117400429747348,0.193475199525969,-
0.60679833919277,0.543984750454399,0.884946960479097
"10014","EPHB2",73.9974550257457,-0.168577734028069,0.278361999588293,
-0.605606132580601,0.544776329070193,0.885214609756308
"10015","RC3H1",221.859210549729,0.143327621189941,0.236766605965502,0
.605354038866547,0.544943782700361,0.885214609756308
"10016","SCAANT1",3.35093904411935,-0.752450674977097,1.24188511490187
,-0.605893947796091,0.544585178632312,0.885214609756308
"10017","AC084036.1",9.29595825569753,-0.313012293473369,0.51717006002
3848,-0.605240553675777,0.545019173751981,0.885214609756308
"10018","HNRNPDL",1331.31072698681,0.143872929055512,0.237554635276126
,0.605641430184171,0.544752884623802,0.885214609756308
"10019","DHX29",431.333837939268,-0.110821145006121,0.183078164957692,
-0.605321475839188,0.544965414604356,0.885214609756308
"10020","RAD50",520.866163626909,0.0990748364877417,0.163676406999375,
0.605309209213763,0.544973563541523,0.885214609756308
"10021","UQCRQ",1256.79462863156,-0.148723444674556,0.24547031272746,-
0.605871410770882,0.544600145235405,0.885214609756308
"10022","TRBC2",33.4196486152076,0.323006282003813,0.533506141674172,0
.605440606532104,0.544886277116317,0.885214609756308
"10023","NUDT13",10.6224761975965,-0.388766007881901,0.641910923201009
,-0.605638561100109,0.544754790232687,0.885214609756308
"10024","MRPL17",258.693127288334,-0.151374846803188,0.2496685866699,-
0.606303134976804,0.544313477243519,0.885214609756308
"10025","ROM1",51.1210640204408,-0.1432560828199,0.236626320246422,-0.
605410601283546,0.544906208805465,0.885214609756308
"10026","DPF2",220.12400690087,0.104791006750691,0.173102371578674,0.6
05370139039744,0.544933087371966,0.885214609756308
"10027","NPTN-
IT1",10.9683688450995,-0.271625867129473,0.448778712945261,-0.60525568
4581912,0.545009121613254,0.885214609756308
"10028","AC087294.1",2.93690487476775,0.580453104897252,0.957571390394
051,0.606172146244249,0.544400446781304,0.885214609756308
"10029","GID8",281.95228797296,0.0856819611879256,0.141383167775136,0.
606026605120343,0.544497086430618,0.885214609756308
"10030","RNPEP",191.068253286466,-0.0988065930979489,0.163561225502909
,-0.604095455962402,0.545780180455162,0.885270310016397
"10031","KDM3A",156.672498258823,0.16406971162412,0.27131779646529,0.6
04714153518896,0.545368942256114,0.885270310016397
"10032","PACRGL",86.9221075102119,0.121039614533074,0.200306627943509,
0.604271639814188,0.545663058255771,0.885270310016397
"10033","DMXL1",187.591086557284,0.105595465824397,0.174798537773815,0
.604098107279564,0.545778417839733,0.885270310016397
"10034","AL080250.1",12.2761483056842,0.300133515617244,0.496715775642
423,0.604235923912562,0.545686800201462,0.885270310016397
"10035","AC093627.5",7.82769376004794,0.347168734650303,0.574279750768
163,0.60452894984705,0.545492027995962,0.885270310016397

"10036","FKBP15",151.643757512156,0.136394591688766,0.225723068440576,
0.604256324491148,0.545673238968287,0.885270310016397
"10037","CD59",3149.71199063735,-0.124536617134971,0.206046823330701,-
0.604409304263293,0.545571551377689,0.885270310016397
"10038","NECTIN1",31.9575159355021,0.180439225273932,0.298718638769062
,0.604044079798546,0.545814336269549,0.885270310016397
"10039","ASB8",323.352677744286,-0.11129849738239,0.184186426301297,-0
.604270898878969,0.545663550783106,0.885270310016397
"10040","CDK4",606.84183785087,-0.124902841149039,0.206435653310948,-0
.605044909373778,0.54514915731555,0.885270310016397
"10041","UBL5",1845.19234653885,-0.149025898167196,0.246637939248402,-
0.604229416696125,0.545691125892346,0.885270310016397
"10042","NR2F6",228.4923034954,-0.147895151518234,0.244835368966535,-0
.604059585600349,0.545804027616929,0.885270310016397
"10043","CASTOR1",53.7792232673809,-0.183118545941875,0.30292304307090
3,-0.6045051709685,0.545507832357363,0.885270310016397
"10044","CDK11B",128.735793017775,-0.182370163612608,0.302647724244734
,-0.602582306104292,0.546786594095936,0.885300289419778
"10045","TNFRSF14-
AS1",4.78772617156223,0.44323758998364,0.742977664108331,0.59656919904
2588,0.550795040507823,0.885300289419778
"10046","RUNX3",21.2410536734806,0.369040094648597,0.612086182709694,0
.602921786299543,0.546560721921381,0.885300289419778
"10047","KPNA6",311.667876513063,0.11953991508757,0.198764952936913,0.
601413444982483,0.547564645849725,0.885300289419778
"10048","TMEM35B",1323.25235549755,0.138610454582052,0.230841575596783
,0.600457063350524,0.548201667767736,0.885300289419778
"10049","RFX5",36.4567814006454,0.185029000115316,0.306429369184064,0.
603822670809904,0.545961545145596,0.885300289419778
"10050","AL592435.1",8.24282230845959,0.314599163031858,0.526166462958
996,0.597908048457993,0.54990128925896,0.885300289419778
"10051","ALDH9A1",683.570789946413,0.136655316053516,0.226297914647443
,0.603873510131172,0.545927741708553,0.885300289419778
"10052","PLXNA2",21.9419441054398,-0.227948149214995,0.378069390362595
,-0.602926751082325,0.54655741896032,0.885300289419778
"10053","GPATCH2",231.911074979377,-0.106486779088713,0.17802210023651
6,-0.59816606447872,0.549729132377269,0.885300289419778
"10054","AL390728.5",5.04282850919136,0.492187718558919,0.819275263044
596,0.600759892017059,0.547999921549579,0.885300289419778
"10055","WDR35",67.9669491139871,0.133618155309919,0.222951182379824,0
.599315751025194,0.548962346271969,0.885300289419778
"10056","ZNF513",34.0854306628981,0.217014307070939,0.362770842365375,
0.598213201634234,0.549697683767327,0.885300289419778
"10057","CALM2",3786.03813592487,-0.128340141659775,0.21465962050197,-
0.597877427341288,0.549921722451087,0.885300289419778
"10058","RETSAT",109.931728892799,-0.114994315165243,0.192537073029363
,-0.597258041560163,0.550335113233442,0.885300289419778
"10059","RPL31",6825.39533239197,0.1124099192509,0.188648534399689,0.5
95869560336669,0.55126236970449,0.885300289419778
"10060","IL1RL2",2.9692352183243,-0.560647041104813,0.93512323704556,-

0.599543481430457,0.548810523599724,0.885300289419778
"10061","RGPD8",7.96961636111421,0.399470905085898,0.664445142108018,0
.601209761002297,0.547700283975605,0.885300289419778
"10062","AC012306.3",7.86585378442018,-0.300229299971936,0.50335885512
4431,-0.596451809510172,0.550873438161384,0.885300289419778
"10063","CXCR4",177.167635800365,0.261226673067653,0.43652104760813,0.
598428585514984,0.549553996867129,0.885300289419778
"10064","TEX41",16.0810792254059,-0.24062826434685,0.400007767993625,-
0.601558978601349,0.547467741642834,0.885300289419778
"10065","PRKRA",288.255309987581,-0.137483467622969,0.22913784702011,-
0.600003314209822,0.54850402674984,0.885300289419778
"10066","SESTD1",1407.88582939364,-0.141046356066655,0.236564023601378
,-0.596229104998337,0.551022184660576,0.885300289419778
"10067","KCTD18",111.689013665282,-0.142090049402002,0.238279258575561
,-0.596317322168198,0.550963261196176,0.885300289419778
"10068","ALS2",70.7448478959566,0.167438312449455,0.277685794528355,0.
602977594636579,0.546523594428242,0.885300289419778
"10069","AC006994.2",7.39840252963892,-0.345731010145289,0.57656048493
3512,-0.599643956149991,0.548743545983175,0.885300289419778
"10070","AC079834.2",3.37217984141934,0.469646954003343,0.781741518386
951,0.600770130480488,0.54799310126703,0.885300289419778
"10071","HDAC4-
AS1",22.6080240917766,-0.259943311897359,0.435180657169236,-0.59732276
1512975,0.550291910650707,0.885300289419778
"10072","POC1A",6.20976170769687,-0.403790233484656,0.671854512616973,
-0.601008441413653,0.54783436391827,0.885300289419778
"10073","SAMMSON",2.383204186273,0.668826862212379,1.10833044918789,0.
603454378342174,0.546206456488995,0.885300289419778
"10074","TNFSF10",1460.59627453983,-0.162667658425154,0.27274159702851
2,-0.596416755630235,0.550896849677082,0.885300289419778
"10075","MCCC1",75.8075269583669,-0.137373011658249,0.228476927544578,
-0.601255510280995,0.547669816969481,0.885300289419778
"10076","BDH1",3.46145771133808,-0.51884529909044,0.868504127424685,-0
.59740107468336,0.550239636405329,0.885300289419778
"10077","DGKQ",31.4331200524541,0.17741042296026,0.29739635368051,0.59
65453872069,0.550810942606618,0.885300289419778
"10078","ITGA1",30.1216427599489,-0.277979066532094,0.463081695806954,
-0.600280833920018,0.548319089727162,0.885300289419778
"10079","TMCO6",30.7609778059593,0.179270114744179,0.300724987605801,0
.596126434891309,0.551090765671253,0.885300289419778
"10080","AC008443.1",54.2579503396796,-0.163303652877177,0.27337074280
592,-0.597370630086465,0.550259957962568,0.885300289419778
"10081","MRS2",79.8871422200773,0.127422378009568,0.212461969543493,0.
599742053993733,0.548678156712826,0.885300289419778
"10082","TRIM38",438.040281116934,0.143945594344482,0.239532558297281,
0.600943752146765,0.547877450766716,0.885300289419778
"10083","HLA-
B",7136.2360912615,0.26554501184153,0.441075106552344,0.60204035071749
5,0.547147278322635,0.885300289419778
"10084","SKIV2L",61.4594189971949,0.133255694096965,0.223409525353092,

0.59646379842739,0.550865431207903,0.885300289419778
"10085","PBX2",127.081597697867,0.152943604614162,0.25346732574015,0.6
03405603335859,0.546238895533881,0.885300289419778
"10086","SF3B5",1288.77808406299,-0.153733437223831,0.25539546156267,-
0.601942713794493,0.547212270536496,0.885300289419778
"10087","ERMARD",64.5034696835934,-0.14269467449994,0.236404468774984,
-0.603603964169394,0.5461069766262,0.885300289419778
"10088","PSMB1",2274.90675010406,-0.162788021361596,0.27177279984962,-
0.598985702217704,0.54918241907024,0.885300289419778
"10089","TMEM60",211.393809885422,0.135826549847757,0.225661051751693,
0.60190515285382,0.547237274069503,0.885300289419778
"10090","BMT2",59.9151570018477,0.156810990235571,0.260582727074781,0.
601770470344988,0.547326934047502,0.885300289419778
"10091","GIMAP1",7.91833036049241,-0.325728782624346,0.54267642839123,
-0.60022651728208,0.548355283496903,0.885300289419778
"10092","LINC01301",4.06960333745171,0.497272993718663,0.8308412741766
9,0.598517441507017,0.549494724651006,0.885300289419778
"10093","GALT",160.820581315513,0.12173949827186,0.204333339594124,0.5
95788717170073,0.551316382097954,0.885300289419778
"10094","AL359091.4",6.02093150870398,0.3707622959649,0.62231397935104
6,0.595780117861941,0.551322127563032,0.885300289419778
"10095","SARDH",16.1302879881513,0.296494893091498,0.492774519733322,0
.601684708154054,0.547384030891612,0.885300289419778
"10096","AL355574.1",4.38754105450449,0.402486266379563,0.673976263182
653,0.597181664052887,0.550386099744891,0.885300289419778
"10097","NPDC1",653.660753904629,0.151179177195632,0.252628795866092,0
.598424168857477,0.549556943121836,0.885300289419778
"10098","PRKCQ",2.87189026968397,0.629891315646126,1.04365024952181,0.
603546366165043,0.546145280271826,0.885300289419778
"10099","PRPF18",257.479289520494,-0.119890258656861,0.199140338378635
,-0.60203904258166,0.547148149060633,0.885300289419778
"10100","LINC00857",6.25108260855631,0.375259992061905,0.6291713063195
57,0.596435324199782,0.550884448182715,0.885300289419778
"10101","RRM1",145.92699751024,-0.117385865009998,0.19546401210725,-0.
600549757187982,0.548139910697526,0.885300289419778
"10102","TAF10",643.388164277378,-0.155771649699143,0.259803975867078,
-0.599573771645586,0.548790331365193,0.885300289419778
"10103","AP001160.3",21.4538765745883,-0.217944803368502,0.36306642799
8822,-0.600289056109614,0.548313610992421,0.885300289419778
"10104","CLPB",53.3355271369405,-0.186929560028694,0.313702001394346,-
0.595882586651751,0.551253666893739,0.885300289419778
"10105","DYNC2H1",287.253883984076,0.137171684581875,0.228639083719883
,0.599948540512571,0.548540531164108,0.885300289419778
"10106","IGSF9B",4.47697186332582,-0.506683972820675,0.84373963712324,
-0.600521713722294,0.548158594233654,0.885300289419778
"10107","JAM3",300.735115313288,-0.121991964561963,0.20274235566661,-0
.601709318020195,0.547367646380818,0.885300289419778
"10108","CD27",4.10730321109573,-0.43826744105311,0.73138099992111,-0.
599232740665104,0.549017692541368,0.885300289419778
"10109","RHEBL1",8.61333606453991,-0.348256192627414,0.578678832746203

,-0.601812564967539,0.547298910303971,0.885300289419778
"10110","CCDC169",2.29780862158225,0.650597592786187,1.07933493895224,
0.602776366544522,0.546657470879679,0.885300289419778
"10111","LRFN5",61.2463302771703,0.270416470335024,0.450462209074159,0
.60030889359357,0.548300392688962,0.885300289419778
"10112","FBXO34",102.57035807759,0.121737831933434,0.202080263622723,0
.602423164692193,0.546892494287849,0.885300289419778
"10113","HSPA2",46.1556194783625,-0.19147063199568,0.319932536873246,-
0.598471896190848,0.549525105684123,0.885300289419778
"10114","MOAP1",140.216105714224,0.139152297580797,0.231208939509172,0
.601846528409327,0.547276300266348,0.885300289419778
"10115","AC091057.2",3.99289460370101,-0.534911431747678,0.88882632085
2912,-0.601817722088135,0.547295477091808,0.885300289419778
"10116","FAM98B",106.044231105585,0.150485088851452,0.251549872782882,
0.598231623759711,0.549685393274364,0.885300289419778
"10117","CD276",619.01077523103,0.106269314119427,0.176558585127206,0.
601892646811046,0.547245599206185,0.885300289419778
"10118","PRSS27",3.7142753033501,0.493482876942221,0.828092732483296,0
.595927071431188,0.551223947362112,0.885300289419778
"10119","AC010654.1",6.52407531262525,0.372591160551907,0.622900617453
901,0.598155066975,0.549736469735976,0.885300289419778
"10120","AC026471.1",17.910960086843,0.234624849244853,0.3935895366632
35,0.596115565555807,0.551098026355635,0.885300289419778
"10121","LLGL1",50.158297328967,0.263680320601223,0.440134306793087,0.
599090587876356,0.549112477786311,0.885300289419778
"10122","JUP",158.585330640238,-0.17100527375127,0.286251322374611,-0.
597395576490924,0.550243306383293,0.885300289419778
"10123","ATP8B3",8.76750396697234,-0.342906985223899,0.57412171511344
,-0.597272279027795,0.550325609137411,0.885300289419778
"10124","CD70",46.7660544806381,0.600995099321387,1.00412348528356,0.5
98527081708153,0.549488294254691,0.885300289419778
"10125","ZNF791",323.188602292196,0.14988228626988,0.249888360989555,0
.599796988048374,0.54864154089356,0.885300289419778
"10126","ZNF146",181.221775714628,0.102860726131607,0.172575640191826,
0.596032707844936,0.551153376610776,0.885300289419778
"10127","MRPS12",297.489646004833,-0.145224687912163,0.243341105651542
,-0.596794723699993,0.550644441088,0.885300289419778
"10128","ZNF780B",45.2378486510499,0.185610029236497,0.311478618899746
,0.59589974391224,0.551242204323755,0.885300289419778
"10129","IRF2BP1",142.865620377895,-0.136145726152948,0.22587826154621
1,-0.602739392542627,0.546682071339406,0.885300289419778
"10130","SLC17A7",99.2643339465502,-0.156061110344016,0.25956962202227
5,-0.601230256176217,0.547686634988176,0.885300289419778
"10131","ZNF582",27.3566670371664,0.202389933681154,0.33690412775849,0
.60073450280264,0.548016834582516,0.885300289419778
"10132","ZNF460-
AS1",5.3046435908839,-0.395523566690317,0.662520523830942,-0.596998209
811299,0.550508575821976,0.885300289419778
"10133","ITCH",185.880599860163,0.116188238695985,0.192846896096051,0.
602489545064366,0.546848320427009,0.885300289419778

"10134","RAB5IF",406.993668561234,-0.128921950684561,0.214884417341598
,-0.599959514419401,0.548533217410888,0.885300289419778
"10135","AP001627.1",9.00686401953329,0.345508227590402,0.575105387520
989,0.600773762665877,0.547990681721599,0.885300289419778
"10136","KIAA1671",71.1870408465223,0.191660762461699,0.31801378550129
6,0.602680673605322,0.546721140805428,0.885300289419778
"10137","Z95115.1",10.5154872595091,-0.33669689912599,0.56154390547900
7,-0.599591404769644,0.548778576841175,0.885300289419778
"10138","MIAT",15.7151139489714,-0.26998210292402,0.448226934953661,-0
.602333509814468,0.546952159337958,0.885300289419778
"10139","XRCC6",936.715694380125,-0.100850437561259,0.169122631274162,
-0.596315447562852,0.550964513281208,0.885300289419778
"10140","SAMM50",268.608232868237,-0.133506386866518,0.223275949089427
,-0.597943430140996,0.549877679850187,0.885300289419778
"10141","PPP6R2",157.340388681551,0.159980001572274,0.26706351118047,0
.599033543987843,0.549150516024174,0.885300289419778
"10142","ASB9",4.36305181964267,0.502140361835207,0.841152464103839,0.
596967117453773,0.550529334752635,0.885300289419778
"10143","AL732509.1",16.8700093185125,0.252968643920006,0.422915388741
601,0.598154265969661,0.549737004155821,0.885300289419778
"10144","USP27X",25.2030959810754,-0.222227384589482,0.37214826776386,
-0.597147437833816,0.550408948528603,0.885300289419778
"10145","CCT4",720.76332979416,-0.0999932052354367,0.167875662283242,-
0.595638485504389,0.551416760778536,0.885364969573344
"10146","WDR45B",337.260926506032,0.111336608529156,0.186973811874527,
0.595466324470459,0.55153180281495,0.885462402528357
"10147","RPP25L",160.642718596903,0.163026877903957,0.273852472299117,
0.595309133181353,0.551636852017753,0.885543774762706
"10148","HOXD3",9.65219177802807,0.289974381702424,0.487462617294812,0
.594864860225889,0.551933807879264,0.885620389843187
"10149","AC008443.5",27.1966037581906,0.213191444933597,0.358306178113
788,0.594997959722296,0.55184483479523,0.885620389843187
"10150","LINC01013",3.56690960617975,-0.456342336437954,0.767041595147
908,-0.594938187608924,0.551884789816914,0.885620389843187
"10151","MYOM1",33.8970563078586,0.274448432177061,0.461291434682522,0
.594956705333034,0.551872411383114,0.885620389843187
"10152","AP001432.1",3.26585554224982,-0.482327508531306,0.81086474197
3671,-0.594831028609415,0.551956424439071,0.885620389843187
"10153","DNTTIP2",258.009645367061,-0.09680712276219,0.162885326107854
,-0.59432684990967,0.55229352367351,0.885798119143402
"10154","MCM3",158.686075603832,-0.115407459255162,0.194129805614334,-
0.59448603932791,0.552187077021577,0.885798119143402
"10155","RDX",858.097091437738,0.0846002649067368,0.142362685058229,0.
59425870530704,0.552339093630028,0.885798119143402
"10156","CLEC16A",40.0769967077631,-0.1615478055964,0.271719950292559,
-0.594537888817008,0.552152408521679,0.885798119143402
"10157","ZNF875",65.0943683036101,0.137223658065471,0.230878023871412,
0.5943556505053,0.552274264568143,0.885798119143402
"10158","ACAP1",36.5600397355588,0.212154007912026,0.357116685525264,0
.59407475626233,0.552462114050485,0.885908188203225

"10159","CDPF1",38.8477944101799,-0.195901302856833,0.329927950432446,-0.593769950681838,0.552665989835014,0.886147879557294
"10160","VPS33A",72.1342057361313,0.128510135999333,0.216496360342756,0.593590283900734,0.552786181121159,0.886198863649645
"10161","KCNJ14",9.4039290564449,0.382536517749821,0.64447851507579,0.59355976778347,0.552806596693722,0.886198863649645
"10162","RABGAP1L-IT1",3.18770700291427,0.620170009577594,1.04554689182419,0.593153702074104,0.553078293708099,0.886414869931451
"10163","RPN1",484.624120860441,0.120019891980878,0.202325488743711,0.59320203660997 3,0.553045949822723,0.886414869931451
"10164","COMTD1",121.555850453254,-0.170118439875586,0.286822306152698,-0.5931143994952,0.553104594387824,0.886414869931451
"10165","RAP1A",646.989035076385,-0.0913395696567793,0.154119542475581,-0.592654041074973,0.553412704785507,0.886734167642251
"10166","PDPK1",217.540415129661,0.0909293966193814,0.153423630575903,0.592668784320001,0.553402836068203,0.886734167642251
"10167","ATF6",264.984057875325,0.10688059534949,0.18063448805635,0.591695398257214,0.55405457859496,0.88673764874092
"10168","MTMR14",130.672720654941,-0.117305957711387,0.198260743394727,-0.591675163236106,0.554068131185775,0.88673764874092
"10169","AC078785.1",9.71574083440918,-0.29975055065078,0.506417990232265,-0.591903440304919,0.553915249937897,0.88673764874092
"10170","AC069224.1",8.50864409560866,-0.35506855765253,0.599383296779103,-0.592389810594584,0.553589587924567,0.88673764874092
"10171","HIST1H4C",1376.30310495497,-0.223014108129007,0.376616503592488,-0.592151714016006,0.553749000029102,0.88673764874092
"10172","SLC35A1",171.77619690657,-0.121242703883355,0.204861278654662,-0.591828307816701,0.553965565232846,0.88673764874092
"10173","HIP1",115.632610076139,0.18370081225249,0.310430851906076,0.591760809612023,0.554010769850745,0.88673764874092
"10174","AC007566.1",23.7862166638348,0.251835228391673,0.425541045429202,0.591800088608776,0.553984463864173,0.88673764874092
"10175","FOXN3-AS1",38.6089031665919,-0.18867368240051,0.318457115926127,-0.59246181970786,0.55354138030175,0.88673764874092
"10176","PLSCR3",204.674454074958,-0.121454447814309,0.20506172920716,-0.592282374111904,0.553661516774715,0.88673764874092
"10177","DNASE2",445.234128314949,-0.111802547070063,0.188952848149726,-0.591695484692937,0.554054520704191,0.88673764874092
"10178","EIF2S3",910.170894301631,-0.0945152061685979,0.159579927201134,-0.592275029988396,0.553666433841091,0.88673764874092
"10179","HMGXB3",138.155088511425,-0.107090338498365,0.181144115593194,-0.591188613263398,0.554394051337139,0.887016166151502
"10180","PTCH1",77.2980830575594,0.210462124543716,0.356000238532542,0.591185346985314,0.554396239601632,0.887016166151502
"10181","EEF1AKMT2",218.166014417347,-0.155978138871058,0.263845841666632,-0.591171488190955,0.554405524439097,0.887016166151502
"10182","VAMP4",173.621496920902,0.101960519009067,0.172529440426039,0.590974611389733,0.554537432230672,0.887140074013495

"10183","AC005041.4",15.2688074516161,-0.259213321116088,0.43902862031
178,-0.590424653709377,0.554905986120379,0.887293352638065
"10184","SUMF1",257.08730467462,0.0996710694858419,0.168897496991216,0
.590127570043418,0.555105126400881,0.887293352638065
"10185","AC027607.1",2.56183986611196,-0.513705569692698,0.87044209214
4036,-0.590166277951196,0.555079177847391,0.887293352638065
"10186","IKZF1",19.6106142897234,0.313696183868685,0.531671395757952,0
.590018922160519,0.555177963661806,0.887293352638065
"10187","MALRD1",7.5429900857702,-0.324860731295449,0.550037694195738,
-0.590615397314649,0.554778145818732,0.887293352638065
"10188","AL136441.1",5.04115210232084,0.474996354782377,0.804732203642
316,0.59025394116513,0.555020413401025,0.887293352638065
"10189","EIF3J",380.751294476613,-0.0797137350735087,0.135058633546374
,-0.590215767629086,0.555046002400662,0.887293352638065
"10190","ZNF69",19.075980630507,0.229245289908168,0.388502589779821,0.
590074032809124,0.555141017041313,0.887293352638065
"10191","BMP7",3.24896571733011,0.593588214576833,1.0056716406769,0.59
0240582082337,0.555029368376036,0.887293352638065
"10192","ZNRF3-
AS1",3.97722304758164,0.431606613275357,0.731294319129743,0.5901954958
28518,0.555059591501863,0.887293352638065
"10193","PODXL",10.070194008505,-0.403212500738123,0.68365321575123,-0
.589790980936226,0.555330790045128,0.887450528700587
"10194","DHX33",83.7810362457876,0.140099079098874,0.237591460610311,0
.58966378142967,0.55541608166669,0.887499760081293
"10195","EVC2",117.169959015721,0.165221143321207,0.280279397135835,0.
589487293784687,0.555534433267633,0.887545121930054
"10196","NFKBIL1",273.367437934471,-0.143762383470015,0.24388871295408
9,-0.589458945142235,0.555553444852283,0.887545121930054
"10197","EYA3",90.6046694309259,0.153733614450058,0.260890608950889,0.
58926465413325,0.555683751703401,0.887594386637865
"10198","MRPL33",736.570314615901,-0.143538964432074,0.243595840435952
,-0.589250473962072,0.555693262627107,0.887594386637865
"10199","PARK7",2879.01651839521,-0.155240407714289,0.263722387066215,
-0.588650851530899,0.556095514069531,0.887637714459908
"10200","IAH1",716.610313269199,-0.149039459856823,0.253087717266839,-
0.588884602802297,0.555938687187478,0.887637714459908
"10201","VARS2",39.7029030717593,0.189418501614327,0.321546750353742,0
.589085417302282,0.555803975267604,0.887637714459908
"10202","SLC4A2",130.955395800019,-0.116662392904532,0.198216582041722
,-0.588560208751738,0.556156333340157,0.887637714459908
"10203","AL121749.1",5.58486373196778,0.474407741290176,0.805748982312
199,0.588778579563083,0.556009816934602,0.887637714459908
"10204","MIF4GD",124.260189868428,-0.161645301024016,0.274462184156024
,-0.588952906284987,0.555892865534408,0.887637714459908
"10205","DNMT1",244.419074799195,-0.108748781037362,0.184767856011068,
-0.58856980529583,0.556149894120852,0.887637714459908
"10206","TNNC2",68.0855331381905,-0.261916280237717,0.444693054205629,
-0.588982170422219,0.555873234139853,0.887637714459908
"10207","TXK",2.40541138010696,0.654689478867055,1.11255016491473,0.58

8458390024358,0.556224655290689,0.887659783484867
"10208","ERMAP",54.8216898548775,0.153852541243058,0.26149274913979,0.588362552113484,0.556288967760356,0.887675450220263
"10209","FLI1",37.9149433111189,-0.222340678276213,0.378044755442322,-0.588133217232622,0.556442878705398,0.88771954214071
"10210","PPM1N",9.57191657318872,-0.439099203625443,0.746610936179771,-0.588123187522819,0.556449610305856,0.88771954214071
"10211","CPXM1",1253.43027536694,0.24828973209717,0.422205607542531,0.58807776983909,0.55648009360911,0.88771954214071
"10212","LMAN2L",82.1008415488885,0.128190538184459,0.21803711746154,0.587929888621238,0.556579353717745,0.887790941314958
"10213","NBPF26",113.306432832216,0.256524092668527,0.436984751975166,0.587032136725688,0.557182123538134,0.888033389584876
"10214","SLC12A2",62.4070165625592,-0.194465506943718,0.331345551963308,-0.58689638593746,0.557273297184504,0.888033389584876
"10215","FSCN3",5.55328618063369,-0.421333408492288,0.717906717785826,-0.586891580833465,0.557276524546418,0.888033389584876
"10216","TAS2R4",5.6516724678797,0.376487490435981,0.641272034019243,0.587094821641144,0.557140025223021,0.888033389584876
"10217","ZC3H3",53.7174818145149,0.147236682424594,0.250632994411735,0.587459295892687,0.556895280093765,0.888033389584876
"10218","PKP3",8.04571620423885,-0.407652764617368,0.694077608701174,-0.587330234410253,0.556981939125289,0.888033389584876
"10219","RNH1",2577.46054692022,-0.142300078052575,0.242272564447223,-0.587355313538087,0.556965099094788,0.888033389584876
"10220","SAMD4A",257.384161647584,0.169327934766149,0.288505527969269,0.586914004587759,0.557261463645239,0.888033389584876
"10221","RUNDC1",63.2689934558589,-0.139161383992361,0.236972258388604,-0.587247574626032,0.557037444936242,0.888033389584876
"10222","PCIF1",106.167289314723,-0.164293800296254,0.27961481179048,-0.587571878772154,0.55681969107478,0.888033389584876
"10223","EME1",2.67667139442175,-0.497520926433296,0.848391891642777,-0.586428195901219,0.55758780112121,0.888442501463699
"10224","LINC01135",5.41685440866791,0.363716010943329,0.620906020554223,0.585782709303858,0.558021544593666,0.88851934962321
"10225","IPO9-AS1",10.7303323158187,-0.332854410403838,0.568325008813592,-0.585676162832758,0.55809315571582,0.88851934962321
"10226","FAM71A",34.2382287283406,0.382348566309415,0.65246901639677,0.586002640280021,0.557873740562109,0.88851934962321
"10227","ARHGAP26",166.119146783927,0.178671508961532,0.304836890869315,0.586121674617554,0.557793751794257,0.88851934962321
"10228","MICB",11.4360432134752,0.309336428300652,0.528215096396457,0.58562587554006,0.558126955942334,0.88851934962321
"10229","AC016394.2",3.49258433478809,0.450376732067115,0.768501911180924,0.586045038424225,0.557845249191104,0.88851934962321
"10230","TESPA1",3.65600490499774,-0.523127167239165,0.892979374727994,-0.585822228423262,0.557994984470247,0.88851934962321
"10231","ELOF1",314.644803236046,-0.14038559678371,0.239507721239053,-0.586142259036364,0.557779920029692,0.88851934962321

"10232","DBP",190.691755532865,-0.282645382048862,0.482455197278254,-0
.585847937059009,0.557977706466514,0.88851934962321
"10233","ARHGAP33",30.2900147745483,-0.169647205519995,0.2897633356154
08,-0.585468155105591,0.558232973005751,0.88860127990723
"10234","NIFK-
AS1",89.5748191824916,0.116898870302121,0.199742525040687,0.5852477847
58446,0.558381118698007,0.888602094481099
"10235","IQCG",39.8698998489449,0.170932849756858,0.292284081749662,0.
584817512926551,0.558670427378972,0.888602094481099
"10236","PDCD2",374.571938732811,-0.130513858308194,0.223069257465493,
-0.585082228681302,0.558492427637749,0.888602094481099
"10237","PRSS23",1183.38586683913,0.208113880689199,0.355877021015139,
0.584791566748407,0.558687875549347,0.888602094481099
"10238","AP000866.2",5.14214496675661,-0.372197423579739,0.63652088686
6094,-0.584737172431607,0.55872445525664,0.888602094481099
"10239","CHST11",49.6923667874039,0.188921650929529,0.322874570643422,
0.585123971061107,0.558464361809955,0.888602094481099
"10240","LINC01197",16.0355213788663,0.290630959395027,0.4969220191855
4,0.584862308720741,0.558640303925847,0.888602094481099
"10241","ATP9B",116.692025133462,0.138089279573066,0.235935192868633,0
.585284788988445,0.558356240979483,0.888602094481099
"10242","RFXANK",233.440533229387,-0.138332729171991,0.236497058481755
,-0.584923677529218,0.558599037037586,0.888602094481099
"10243","DPP9",99.795533351462,-0.128097202721142,0.219139131455445,-0
.584547368926607,0.558852105583828,0.888718339144291
"10244","ARHGAP5",350.027894395844,0.128193004146063,0.219354012675171
,0.584411484351994,0.558943502008111,0.888776913726095
"10245","RASSF9",90.6122410936846,-0.134608365743247,0.23056620127067,
-0.583816556812788,0.559343737722052,0.889326514763739
"10246","SLBP",327.656307606875,-0.121571943495925,0.208326331791015,-
0.583564940882659,0.559513053425263,0.889338289935229
"10247","TMEM150C",197.584322140192,-0.166517990192803,0.2854665382366
42,-0.583318770814272,0.559678728597583,0.889338289935229
"10248","GGNBP2",616.962577543451,0.0933859713335004,0.160054538603442
,0.583463437827763,0.559581363218233,0.889338289935229
"10249","ZNF519",93.7040622388713,0.167112888722557,0.286411435403574,
0.58347142629652,0.559575986970832,0.889338289935229
"10250","SMAD2",291.512870864824,0.0893601871397041,0.153104161142406,
0.58365616239063,0.559451666381216,0.889338289935229
"10251","ILVBL",224.161447057055,-0.140226411945981,0.240379584948233,
-0.583354081321753,0.559654962774676,0.889338289935229
"10252","NDUFS2",423.881556033052,-0.121820669780608,0.208988891417106
,-0.582905000139338,0.559957254427668,0.889433809592616
"10253","TLN1",1243.42464503329,0.10766909401578,0.184647063862844,0.5
83107533709589,0.559820912467291,0.889433809592616
"10254","AKAP17A",143.179643325028,0.111544654924147,0.191356134853936
,0.58291653418528,0.559949489482893,0.889433809592616
"10255","CXorf56",82.4996897578877,0.162208779810203,0.278254544926077
,0.582951052437602,0.559926251433841,0.889433809592616
"10256","PRRC2C",1583.95481156894,0.087674163865736,0.15049214349107,0

.582582996240852,0.560174054805722,0.889691417582918
"10257","MMRN2",11.2058643497836,0.315190792296157,0.541116561260859,0
.582482250333881,0.560241893862848,0.889712411926677
"10258","AC099063.4",11.507448684276,-0.292709505184813,0.502993319617
414,-0.581935174422304,0.560610346700045,0.889777057137
"10259","PPAT",65.7930654531956,0.184569013306444,0.317054255197654,0.
582137001099013,0.560474403801287,0.889777057137
"10260","RASL11A",146.35003588788,0.211120433975003,0.362746854904664,
0.582004864054546,0.560563404565938,0.889777057137
"10261","LINC02588",5.08679570703737,0.398555393845164,0.6848168500157
76,0.581988299259843,0.560574562248493,0.889777057137
"10262","MRM1",13.1198044906605,-0.254412511026257,0.437121611838856,-
0.582017690582742,0.56055476497164,0.889777057137
"10263","GGT5",1391.68866335692,-0.128390242014208,0.220599575376064,-
0.582005843825111,0.560562744617334,0.889777057137
"10264","LHX4",3.76630779369674,0.54415407227343,0.935525869930412,0.5
8165582563089,0.560798531939269,0.889916604766867
"10265","LINC01119",13.8488460285742,-0.263881232171147,0.453872710630
982,-0.581399202882884,0.560971434572177,0.889916604766867
"10266","AC010864.1",3.29448398247741,-0.472817759108173,0.81304675419
9657,-0.581538216179958,0.560877769504665,0.889916604766867
"10267","ATL3",643.951842264098,0.083929741010334,0.144349649732744,0.
581433631226164,0.56094823656726,0.889916604766867
"10268","NKILA",7.06569955893041,-0.394189921362086,0.677874067754285,
-0.581509073902163,0.560897404505635,0.889916604766867
"10269","CCNG1",523.972505095712,-0.0951843822921595,0.163758870322723
,-0.581247184378944,0.561073871018343,0.889992432078858
"10270","AL357873.1",8.00244678275606,-0.381529212392176,0.65687764663
6696,-0.580822340881378,0.561360196383547,0.889999599389775
"10271","TRPC1",106.528853915386,0.108692570593406,0.187174309398693,0
.580702399504434,0.561441044256699,0.889999599389775
"10272","ILRUN",145.400531179165,-0.126974274605572,0.218618111698891,
-0.580804003926525,0.561372556255487,0.889999599389775
"10273","GNB3",7.88210069039458,-0.355232156121598,0.61187753286188,-0
.580560875409337,0.561536447453139,0.889999599389775
"10274","AC023794.3",6.4006254970823,-0.350190659722394,0.602735962000
676,-0.581001768270136,0.561239261790571,0.889999599389775
"10275","AC127070.2",4.66073460588743,0.405236645180643,0.697846521091
986,0.58069594521519,0.561445395004732,0.889999599389775
"10276","PABPN1",522.880039511743,-0.0980397067318902,0.16888520985327
1,-0.58051090925646,0.561570132146118,0.889999599389775
"10277","RAB11A",552.154915266913,0.0928853178846699,0.159918516348992
,0.580829037220211,0.561355682804851,0.889999599389775
"10278","AC100793.2",14.6763845630355,-0.227193200628153,0.39128330228
2021,-0.580636074432845,0.56148575385677,0.889999599389775
"10279","SSR4",2426.89931983059,0.139999665493979,0.24125835467738,0.5
8028939839697,0.561719475507267,0.890149677647424
"10280","EOGT",32.787389432016,0.285460405130187,0.492091155502216,0.5
80096597832272,0.561849477918717,0.890182486705377
"10281","EMC9",38.7774821293985,-0.196557533410909,0.338807963338646,-

0.580144372859518,0.56181726260828,0.890182486705377
"10282","ADPRHL2",176.067641371074,-0.142012600448372,0.24509116618053
4,-0.57942765813014,0.562300646244234,0.890291137895832
"10283","PDZK1",2.49821980937584,0.602264468745299,1.0386510722092,0.5
79852546114731,0.56201405903448,0.890291137895832
"10284","AC078795.2",3.93382166848245,0.453504098475155,0.782633336929
54,0.579459214265471,0.562279359185151,0.890291137895832
"10285","GLTP",318.123216340077,-0.101084717833468,0.174411457950861,-
0.57957613003813,0.562200493827528,0.890291137895832
"10286","MYDGF",1249.52351751557,-0.150722358745344,0.26011418698062,-
0.57944689789863,0.56228766748287,0.890291137895832
"10287","PGLS",1162.3598465046,-0.138362059995971,0.238749973750096,-0
.579527016580106,0.562233622585485,0.890291137895832
"10288","LTN1",323.711562720308,0.0907561676337991,0.156617766362861,0
.579475558497814,0.562268333886664,0.890291137895832
"10289","VAC14",73.7171646568624,-0.138196837725958,0.238567637633896,
-0.579277386893665,0.562402021179961,0.890365100884476
"10290","ATP9A",134.684650173039,0.135822203424017,0.234672623871767,0
.578773106053628,0.562742280100047,0.890817201219599
"10291","GUCY1A1",7.20789908298187,0.690813974507598,1.19390289418183,
0.578618225882604,0.562846804029749,0.890896083066813
"10292","LCK",10.8916663193214,0.321110998587362,0.555236648403165,0.5
783317789106,0.563040143085068,0.891115516003952
"10293","EFNA4",70.5257975537111,0.186038546832445,0.321858578631905,0
.578013323812038,0.563255123812061,0.891261384230777
"10294","PIGL",80.1795293346742,0.129789166428542,0.224512607990279,0.
578092997049684,0.563201334775779,0.891261384230777
"10295","PPP6R1",98.9537937771878,0.116566759921218,0.201689307217138,
0.577952106284558,0.563296454702919,0.891261384230777
"10296","PARP11",61.6293053934421,0.13181204268285,0.228113062542256,0
.577836451862256,0.563374542545091,0.891298360869948
"10297","HSPA6",8.59436176155234,0.469999206669446,0.813786219719106,0
.577546283385918,0.563570482193216,0.89134863427957
"10298","CRYL1",329.768414633925,-0.131621404327171,0.227867083460079,
-0.577623596741345,0.563518272238487,0.89134863427957
"10299","KRI1",75.2679883004621,-0.130708751856201,0.2262866267606,-0.
577624730755674,0.563517506452355,0.89134863427957
"10300","NUP210",3.51432517547069,-0.435283024283431,0.755024949490813
,-0.576514755673947,0.564267299111628,0.891931104825746
"10301","CTNNB1",831.3573859473,0.137523499481928,0.238427178257396,0.
576794560448407,0.56407824458507,0.891931104825746
"10302","HTRA3",1130.22812920273,-0.191273959941044,0.331659875929705,
-0.576717214902367,0.564130501277303,0.891931104825746
"10303","RNF144B",11.2985199706333,-0.333717703074626,0.57881000369730
6,-0.576558285003564,0.564237885827228,0.891931104825746
"10304","ZNF609",170.545750083017,0.166172397496481,0.288217095728266,
0.576552883084875,0.56424153592765,0.891931104825746
"10305","AC009065.4",6.13101322767748,-0.432831959578361,0.75045368786
6599,-0.576760387185013,0.564101332656281,0.891931104825746
"10306","VPS13C",550.798430126151,0.10326158939977,0.179182474447898,0

.576292908767685,0.564417215164459,0.892081507647377
"10307","AL354740.1",52.2648135724926,-0.200700811892947,0.34831461874
9609,-0.576205536860409,0.564476263175986,0.892088275043527
"10308","AKR7A2",769.126134640712,-0.149371435100923,0.260140104557824
,-0.574196106189852,0.565835103965601,0.892272360404576
"10309","IKBKE",30.6133423920436,-0.157004514096739,0.27317390633398 5,
-0.574741988368332,0.5654658057 6181,0.892272360404576
"10310","H3F3A",7834.2874634977,-0.125756730882918,0.218920377143679,-
0.574440499891808,0.565669753319542,0.892272360404576
"10311","ZNF512",86.3242283085725,0.142005161534284,0.24702423579905,0
.574863276370188,0.565383768175495,0.892272360404576
"10312","TLR2",7.38159599085863,-0.307302616140007,0.5351624842601,-0.
574223016706552,0.565816895848254,0.892272360404576
"10313","CMBL",915.345884779371,-0.113558483383801,0.197504159269736,-
0.574967554119765,0.565313240669622,0.892272360404576
"10314","RFESD",19.2777616405593,-0.199809310458434,0.347159348453522,
-0.57555503358477,0.564915982189658,0.892272360404576
"10315","PAIP2",960.86177438813,0.0943546347988683,0.16426708026167,0.
574397710415048,0.565698701929726,0.892272360404576
"10316","RPF2",274.70409054173,-0.12732674692249,0.221400489676068,-0.
575096952625455,0.565225728800201,0.892272360404576
"10317","FNDC1",227.61723299894,-0.249655609054749,0.433788532013165,-
0.575523764761886,0.564937123043606,0.892272360404576
"10318","YWHAG",482.84869230896,-0.103000956383799,0.179170995580292,-
0.574875169109839,0.565375724392967,0.892272360404576
"10319","AC008264.2",5.36298183995918,0.545618902327888,0.949465604335
237,0.574658944817596,0.56552197861,0.892272360404576
"10320","ZNF782",23.5660287456789,0.214694721240899,0.372818151210871,
0.575869818954883,0.564703176969585,0.892272360404576
"10321","ANAPC2",64.2952630154193,-0.150709885331651,0.262425523210728
,-0.574295836349083,0.565767626239458,0.892272360404576
"10322","AS3MT",39.976381112447,-0.169177488675199,0.293913626335852,-
0.575602740112096,0.564883728535323,0.892272360404576
"10323","CLEC4A",8.87512364598156,-0.341738105853917,0.59493187754843,
-0.574415523441333,0.565686650694416,0.892272360404576
"10324","ABHD4",86.4125300736227,0.171999298964119,0.299240330023086,0
.57478649001239,0.565435704779215,0.892272360404576
"10325","EIF2B2",109.837155382971,0.14395175814237,0.250388871740976,0
.574912763260845,0.565350297545795,0.892272360404576
"10326","SNW1",562.055166824642,-0.107835449356687,0.18758748923951,-0
.5748541642828,0.565389931271132,0.892272360404576
"10327","CYB5D2",278.985953509019,-0.153548490166923,0.267104473676671
,-0.574863041615668,0.565383926954796,0.892272360404576
"10328","AC120049.1",25.8392428142237,-0.225314446901696,0.39123862168
0862,-0.575900318669171,0.564682560213301,0.892272360404576
"10329","AC008760.2",97.0774487065125,0.216943049421667,0.377180167009
475,0.575170882238399,0.565175733320277,0.892272360404576
"10330","PWP2",4.93475041843514,1.56057030835735,2.71795766367803,0.57
4170204787347,0.565852629564692,0.892272360404576
"10331","MXRA8",4235.01830624053,-0.11389962454192,0.199543460146142,-

0.570801089940518,0.568134484029955,0.892272985007405
"10332","COPA",692.051375185247,0.105771255617963,0.184507643972585,0.
573262187628819,0.566467183540987,0.892272985007405
"10333","PTPN18",218.649635644903,-0.0971826602754101,0.16958779958039
5,-0.573052191937543,0.566609356036398,0.892272985007405
"10334","PPIG",1226.2417831001,0.0994831012689913,0.173925276167016,0.
571987599855584,0.567330375464628,0.892272985007405
"10335","LINC02104",12.4817536742714,-0.357172054743327,0.626202761202
14,-0.570377642630724,0.568421590068596,0.892272985007405
"10336","APBB3",42.777597150684,0.159709723988218,0.279635318107777,0.
571135738750506,0.56790763429615,0.892272985007405
"10337","AL031963.3",19.6022579644841,0.265919295873392,0.463486815219
389,0.57373648427846,0.566146135469941,0.892272985007405
"10338","HIST1H2BK",7.59383058413205,0.314217823676503,0.5505571175123
73,0.570727021196744,0.568184699164967,0.892272985007405
"10339","NT5C3A",279.242788169416,-0.112638249875554,0.197466894959969
,-0.570415865901919,0.568395671051699,0.892272985007405
"10340","AC006480.2",12.2729831349146,0.290786683262563,0.507001655122
61,0.573541881618199,0.566277850079113,0.892272985007405
"10341","BUD31",791.574116702712,-0.137535900955114,0.24018227123054,-
0.572631361384286,0.566894320663801,0.892272985007405
"10342","WDYHV1",79.9752072355899,-0.151554314334508,0.264159556281314
,-0.573722626082518,0.566155514746822,0.892272985007405
"10343","NSMCE2",202.476571664528,-0.134651578057996,0.235810581129175
,-0.571015844213689,0.567988902704807,0.892272985007405
"10344","UBE2R2",823.552374319004,-0.0888997001932249,0.15529161156364
1,-0.572469428954265,0.567003991207977,0.892272985007405
"10345","UNC13B",37.9914741455811,-0.175151760282612,0.306292896481532
,-0.57184401693486,0.567427653917469,0.892272985007405
"10346","KLF9",2733.56239987914,-0.161584761916471,0.282917560337841,-
0.571137266006102,0.567906599108627,0.892272985007405
"10347","MFSD14B",196.550560014276,0.106408335836537,0.186232265586585
,0.57137432926232,0.567745926459164,0.892272985007405
"10348","KYAT1",13.7630926085533,-0.239879712370801,0.418326559364719,
-0.573426924494319,0.566355664415748,0.892272985007405
"10349","SAAL1",37.8856570605499,-0.179980495939918,0.314526359193227,
-0.572227066760241,0.567168152702184,0.892272985007405
"10350","DDIAS",2.75770015330906,-0.586594234236936,1.02387066846916,-
0.572918291637342,0.566700018924178,0.892272985007405
"10351","CACNA2D4",27.0912640739092,0.210053695485132,0.36753280874645
4,0.571523658531501,0.567644727811022,0.892272985007405
"10352","TCTN1",294.917706832635,-0.110134729880787,0.193067379576819,
-0.570447116038917,0.568374480903534,0.892272985007405
"10353","RBM19",89.0216505426172,0.145935125109687,0.255351133135231,0
.57150764642349,0.567655578610518,0.892272985007405
"10354","ARF6",595.256060129763,-0.100405805902698,0.176033558053451,-
0.570378778983783,0.568420819504886,0.892272985007405
"10355","AL928654.1",15.0492395933617,0.261600199421151,0.457250842758
581,0.572115291997987,0.567243869839001,0.892272985007405
"10356","GOLGA8O",9.84236153468686,0.308445219252747,0.539472911596292

,0.571752932580178,0.567489368358193,0.892272985007405
"10357","AC090825.1",20.1546961734825,-0.216804273732108,0.379862812588986,-0.570743612027881,0.568173451174943,0.892272985007405
"10358","ZNF629",59.4423675452802,0.172938625862299,0.301670970047666,0.573269034918984,0.566462548037156,0.892272985007405
"10359","AC009063.3",4.54937156557795,-0.541299849015141,0.944791790566417,-0.572930305301048,0.566691884278654,0.892272985007405
"10360","TMEM99",173.559403771029,0.157474903363936,0.274312205303656,0.574071806938431,0.565919210622888,0.892272985007405
"10361","PNPO",67.7363686352928,0.153209363451744,0.268559499687781,0.570485734557372,0.568348294912085,0.892272985007405
"10362","SKAP1",6.76670290058586,0.427385929359796,0.747368751017991,0.571854160048375,0.567420781623502,0.892272985007405
"10363","SNHG20",35.4073824297269,-0.164975899691359,0.287839342687347,-0.573152711339244,0.566541299675565,0.892272985007405
"10364","PYCR1",85.5833559896619,0.25491734167941,0.444602243594385,0.57336044824815,0.566400664446396,0.892272985007405
"10365","PARD6G-AS1",16.609522821656,-0.20676771614979,0.361411464824178,-0.572111668483953,0.567246324518036,0.892272985007405
"10366","IZUMO4",11.4533997544784,-0.329857736354931,0.577448453455834,-0.57123321463733,0.567841566077331,0.892272985007405
"10367","AC092069.1",4.57416937441366,-0.572406423497988,1.00071687873164,-0.571996371464712,0.567324432895802,0.892272985007405
"10368","CD79A",3.43546392780529,0.513040136344136,0.896743711516502,0.572114562673123,0.567244363905522,0.892272985007405
"10369","ZNF577",35.2770475286295,0.168845426970741,0.295750850336562,0.570904282366716,0.568064527943194,0.892272985007405
"10370","NRSN2-AS1",21.4007098305895,-0.193046488231388,0.337982096836389,-0.571173710200508,0.567881897175082,0.892272985007405
"10371","NAA20",416.392144010575,-0.133996533755116,0.234699482911645,-0.570928116639952,0.568048370826889,0.892272985007405
"10372","RBM12",137.598408789082,0.0954723880130455,0.166878821843184,0.572106076484414,0.567250112719235,0.892272985007405
"10373","AP000704.1",3.69119218563443,-0.448699491889035,0.78379129469412,-0.572473176120364,0.567001453283026,0.892272985007405
"10374","MAPK12",6.01857479671456,-0.429158309514689,0.750113058156128,-0.572124834847714,0.567237405245598,0.892272985007405
"10375","BCOR",45.2546075096362,0.21683964963223,0.377899614174561,0.573802251970722,0.566101624675919,0.892272985007405
"10376","FOXO4",25.0103642810195,0.177196709413349,0.310540496033525,0.570607414094614,0.568265791653127,0.892272985007405
"10377","MORF4L2",1100.00192637951,-0.112347333704299,0.196973134499249,-0.57036881699584,0.568427574769589,0.892272985007405
"10378","AP1B1",89.6250252832975,-0.177939297631912,0.312055201131641,-0.570217374960041,0.568530272959197,0.892348199675501
"10379","STPG1",20.8851776664575,-0.202476944863534,0.355311161798056,-0.569858103637659,0.568773942997312,0.892489041110834
"10380","ANGEL1",24.020652399615,0.207996340216524,0.364954889793473,0

.569923423506475,0.568729637127671,0.892489041110834
"10381","TFE3",168.988630021853,0.126481521909418,0.221958652064186,0.569842719502738,0.568784378155293,0.892489041110834
"10382","TRMT10B",127.908520553757,0.135898167521519,0.23855239568646,0.569678485644453,0.568895784741928,0.892515780684761
"10383","ZNF337",54.4645068947837,0.141860273534854,0.2490279414667 6,0.569656050237996,0.568911004410945,0.892515780684761
"10384","AC004918.1",19.7214715666123,0.272281150816784,0.478866712752541,0.568594858581218,0.569631114775503,0.893043429122057
"10385","SUV39H2",18.6808121897177,-0.21181309308204,0.372484488885266,-0.568649432130537,0.569594071294096,0.893043429122057
"10386","DUOXA1",3.20675662523667,0.618900706032399,1.08845622870264,0.568604129143606,0.56962482201403,0.893043429122057
"10387","RCVRN",15.1753062992504,-0.299775564687293,0.526844578844408,-0.569001896811441,0.569354852701622,0.893043429122057
"10388","DCAF7",298.787844961877,0.117172880800987,0.206035358665819,0.568702777813184,0.569557862376153,0.893043429122057
"10389","TMEM38A",19.0103163619586,0.227757650574895,0.400561249336518,0.568596315674939,0.56963012571322,0.893043429122057
"10390","ETFB",1165.30884326601,-0.149126652751878,0.26226784917533,-0.568604398979092,0.569624638853,0.893043429122057
"10391","PIK3R3",6.94357237574422,-0.490516539498063,0.86556342609632,-0.566702017101492,0.570916650194651,0.893047526820344
"10392","CCDC13",6.32438453339201,-0.385970059004305,0.680005033380403,-0.567598826563962,0.570307404121438,0.893047526820344
"10393","AC096586.1",6.95530590944189,-0.423686721007104,0.747203076958817,-0.567030214505468,0.570693653864093,0.893047526820344
"10394","SRP72",774.396114529127,-0.095506041862480 7,0.168463509268278,-0.566924209743176,0.570765675104984,0.893047526820344
"10395","AC096751.2",3.67383033349369,0.513115079829023,0.905677270167805,0.566553999675792,0.571017235382107,0.893047526820344
"10396","AL121944.1",31.676385021127,0.214860273506947,0.378846573471254,0.567143240964934,0.570616866744345,0.893047526820344
"10397","MICA",177.642562749625,-0.175368887554918,0.308805107532316,-0.567895035662797,0.570106243042293,0.893047526820344
"10398","WDR91",44.7380694947681,-0.139586516314259,0.246279902491033,-0.56677997230952,0.570863679153262,0.893047526820344
"10399","BNIP3L",1161.06238655447,0.0803063665767448,0.141307416774202,0.568309635898787,0.569824737177978,0.893047526820344
"10400","HSPA14.1",95.6025277400567,-0.168022922928417,0.296130575899631,-0.567394712342591,0.570446041550374,0.893047526820344
"10401","TNKS2-
AS1",5.01227434422048,-0.412543075845256,0.727241593615035,-0.56727101346686,0.570530067489386,0.893047526820344
"10402","ZDHHC6",154.378439121648,-0.095274624128627,0.167654832163477,-0.568278425972992,0.569845925830356,0.893047526820344
"10403","VTI1A",107.739276107391,0.10736331702532,0.188880054295964,0.568420617123966,0.569749394237347,0.893047526820344
"10404","PCNX3",49.9333492410232,-0.159427376104579,0.28119863655049,-0.566956433574148,0.570743781288289,0.893047526820344

"10405","CD3E",20.1288149253032,0.264010292050953,0.46515969133928,0.567569153059713,0.570327557811261,0.893047526820344
"10406","USP44",53.0539603811181,0.256763758913007,0.45314275921777,0.566628846406464,0.570966372259289,0.893047526820344
"10407","SOS2",212.036077769357,0.11299851698625,0.199205557072414,0.567245807029235,0.570547190394003,0.893047526820344
"10408","NHLRC4",28.6130928747867,0.197453579693488,0.348241693695177,0.567001548833272,0.570713129328903,0.893047526820344
"10409","VASN",636.702255138352,-0.148036918514815,0.260709180748016,-0.567823956525328,0.570154511114546,0.893047526820344
"10410","AC009159.3",2.55049566712325,0.545941771833777,0.963723243515725,0.56649227411196,0.571059183458819,0.893047526820344
"10411","ZNF726",8.91710307205574,0.334231537979696,0.58837664034194,0.568057116926761,0.569996184979087,0.893047526820344
"10412","MAP1LC3A",634.906846238497,-0.119095024057001,0.209743921141135,-0.567811564735948,0.570162926269565,0.893047526820344
"10413","TSHZ2",2120.06311362392,-0.130776777360779,0.230561103046731,-0.567210928611287,0.570570883944442,0.893047526820344
"10414","CSNK1E",615.254214617493,-0.0853311796942661,0.150456799470391,-0.567147380474878,0.570614054566673,0.893047526820344
"10415","USP51",89.0582953060753,0.143779702171925,0.253801468932521,0.566504609987706,0.57105080000358,0.893047526820344
"10416","TMSB15B-
AS1",22.297419519618,-0.246681757129719,0.434130420433126,-0.568220390737899,0.569885327385253,0.893047526820344
"10417","CDYL",230.192281840358,-0.134175324427807,0.237114025079561,-0.56586835967542,0.571483271805675,0.893281932102739
"10418","FMC1",350.017263424102,-0.169099688585018,0.298745536791464,-0.566032518514431,0.571371674915194,0.893281932102739
"10419","WDR5",75.7444660398308,-0.124286894913586,0.21962927033755,-0.565894039180517,0.571465813926278,0.893281932102739
"10420","MAP4K1",4.61563304229619,-0.40230734997287,0.710931402986592,-0.565887718959656,0.571470110623148,0.893281932102739
"10421","SPATA2",43.116006135512,-0.152633262567673,0.269663633917124,-0.566013519696844,0.571384589978951,0.893281932102739
"10422","SDE2",111.957787547634,0.10982632448757,0.194253251287408,0.565377020769015,0.57181735106007,0.893461182869782
"10423","KRBA1",18.5058955815462,-0.204147643654997,0.361022103753565,-0.565471314726891,0.571753229957916,0.893461182869782
"10424","CENPV",60.9948599070057,-0.236733250325758,0.418706923437778,-0.565391296571044,0.571807643111773,0.893461182869782
"10425","AC245452.1",64.291609443981,-0.191726928327619,0.3390018387928,-0.565563092549487,0.57169082314331,0.893461182869782
"10426","UBL4A",159.463838123312,-0.146505822800992,0.259243258982328,-0.565128765068405,0.571986184471059,0.893639263269622
"10427","TRIT1",56.3621595051239,-0.149257251866674,0.26433117891377,-0.564660031707286,0.572305024621319,0.893798385681114
"10428","AL022068.1",2.9824987518889,-0.532283826372862,0.942421289024004,-0.56480454396792,0.572206716000643,0.893798385681114
"10429","CASC15",11.6547767877286,0.289067314386813,0.511738886415613,

0.564872676398573,0.5721603697387,0.893798385681114
"10430","AC073316.1",8.10761613292545,0.467375556724669,0.827716792887794,0.564656366453624,0.572307518119837,0.893798385681114
"10431","BABAM2",166.417196229172,0.131935249243474,0.233707210481994,0.564532215207963,0.572391982155957,0.893800302815994
"10432","USP37",82.8991066480519,0.146578044082162,0.259811520022409,0.56417068831097,0.57263797410447,0.893800302815994
"10433","ITGA9",54.9235832387529,-0.184122162389213,0.326341193588473,-0.564201412529602,0.57261706625891,0.893800302815994
"10434","ZSCAN21",66.0201569187921,0.158913756034042,0.281516866015985,0.564491066851456,0.572419977996248,0.893800302815994
"10435","DNAJB6",744.59087834705,-0.0835683589959653,0.148083667183906,-0.564332046775835,0.572528175565402,0.893800302815994
"10436","KIF3B",170.400720629067,-0.134726563506765,0.238785979102647,-0.56421471651336,0.572608013529894,0.893800302815994
"10437","ZNF684",57.4433624897866,0.139941664808403,0.248268516940981,0.563670603637877,0.572978326937265,0.893817656337973
"10438","AC079848.2",2.74208126093176,0.582498561036083,1.03308559253862,0.563843465868785,0.572860667706281,0.893817656337973
"10439","EBAG9",277.569879144522,-0.104286887374875,0.184976799538355,-0.563783607647785,0.572901409098268,0.893817656337973
"10440","PHF2",140.241334383842,0.128264826270277,0.227481818358443,0.563846496374361,0.572858605085231,0.893817656337973
"10441","DOLK",87.3010323663748,-0.145488047217256,0.258003742332419,-0.563898980309384,0.572822884057125,0.893817656337973
"10442","TRAF3",74.2984997044523,-0.130788429206996,0.23202240896683,-0.563688782430033,0.572965952944683,0.893817656337973
"10443","MRPS35",206.358512885048,-0.119396855458899,0.211885461049773,-0.563497159584969,0.573096393710165,0.893830635498361
"10444","ZMYM5",175.382691828097,0.118814522657906,0.210836002646231,0.563540008189535,0.573067224750197,0.893830635498361
"10445","AC007390.2",16.0152128480562,-0.256941567745792,0.45624468592556,-0.563166159896301,0.573321743721538,0.893925326264011
"10446","RIC3",2.81132513932996,-0.53757420686604,0.954519797426557,-0.563188116491007,0.573306794010973,0.893925326264011
"10447","LINC00923",2.82021145714394,-0.590238058454862,1.04779591785295,-0.563313951121633,0.573221119841572,0.893925326264011
"10448","OPA1",161.368362502115,0.0933286876714427,0.165750799881286,0.563066288297171,0.573389746193602,0.893945786346437
"10449","RNF11",410.851341260116,0.0850059338351703,0.151023493121061,0.562865631554616,0.57352638472881,0.893950154673019
"10450","SEC62",2644.02821298149,-0.0963088642300937,0.171142014609576,-0.562742377725316,0.573610322890293,0.893950154673019
"10451","AC025774.1",2.32288508563766,0.569590792276553,1.01237874047863,0.562626188700153,0.573689455119273,0.893950154673019
"10452","TRIM27",121.149907463873,0.10147963779602,0.180434706063086,0.562417508306519,0.573831592931493,0.893950154673019
"10453","AL353796.1",5.80726753081905,0.388548436676861,0.690849170434862,0.56242151442736,0.573828864098082,0.893950154673019
"10454","PRMT3",61.3632091753832,-0.125745590347098,0.223433950238783,

-0.562786408299699,0.573580336577683,0.893950154673019
"10455","AL606834.1",43.3257520009412,0.196258933938067,0.348935315827129,0.562450761032451,0.573808942490626,0.893950154673019
"10456","CLTRN",2.85836911192869,0.497647564471115,0.884448428069645,0.562664309955592,0.573663491512419,0.893950154673019
"10457","AL121655.1",2.43568364880253,0.511905672189986,0.910454141213891,0.562253109758469,0.573943580939866,0.893989741667745
"10458","NELL2",7.44094324368149,0.316611329818937,0.563145838976072,0.562219069920874,0.573966770112424,0.893989741667745
"10459","ZBTB8A",184.998770170818,0.1019564125489,0.182028520934868,0.560112294629815,0.575402844885341,0.894003905030267
"10460","LRRC41",297.410591755823,0.0938544466509257,0.167413960903255,0.560613022620988,0.575061371921359,0.894003905030267
"10461","AMY2B",19.4780749952239,-0.250869907009926,0.44701950490599,-0.561205728735896,0.574657298070973,0.894003905030267
"10462","RUSC1",181.731372604739,-0.104059194991489,0.185376828977675,-0.561338736698432,0.57456663914648,0.894003905030267
"10463","THADA",119.018922807681,0.112416630525243,0.200596279075944,0.560412341859456,0.575198215266725,0.894003905030267
"10464","USP39",92.1569379027032,0.122358613607921,0.21802751367646,0.561207214377052,0.574656285412799,0.894003905030267
"10465","DNAJC10",358.495996957198,0.0919803165129205,0.164204132128036,0.560158354853092,0.575371429976057,0.894003905030267
"10466","AC107214.1",6.87913195481258,0.328225775794913,0.58553254620548,0.560559405146592,0.575097931938866,0.894003905030267
"10467","AC139795.2",2.94432035162395,-0.482233789539128,0.858041166561483,-0.562017078354915,0.574104383226316,0.894003905030267
"10468","HLA-
DOA",8.04246283391188,-0.38631245287198,0.68949047275135,-0.560286861296916,0.575283787741522,0.894003905030267
"10469","CCN6",6.71799741164651,-0.337109681155924,0.600270071774167,-0.561596682905675,0.57439084095198,0.894003905030267
"10470","TAX1BP1",841.102242188621,-0.0841777879963032,0.150264946561554,-0.560195773681796,0.575345909439208,0.894003905030267
"10471","AGAP3",166.275337245753,-0.114878332410287,0.205029103768759,-0.560302563385596,0.575273079247017,0.894003905030267
"10472","INVS",62.20899501571,0.140436169452518,0.250226779555233,0.561235570797565,0.574636956976416,0.894003905030267
"10473","AC073389.1",7.01541034329398,-0.368317887168917,0.65698853814567,-0.560615392482314,0.57505975601511,0.894003905030267
"10474","IDE",79.8345871456387,0.147587700794718,0.262839600823423,0.561512421767329,0.574448264683322,0.894003905030267
"10475","TMEM138",123.857240953333,-0.129647493192287,0.231388100610232,-0.560303199906874,0.575272645154869,0.894003905030267
"10476","KLRC3",4.97976249538701,0.5861449110556,1.04364005960021,0.561635120905705,0.574364646464396,0.894003905030267
"10477","MMP19",288.827000628432,0.242585649047316,0.431599750067246,0.562061606869605,0.574074045431178,0.894003905030267
"10478","NT5M",11.0502558547702,-0.244894444839109,0.436399972006058,-0.561169707947894,0.574681851193484,0.894003905030267

"10479","RNFT1",150.333647234305,-0.153735181446781,0.274312843232249,
-0.560437417494957,0.575181115457586,0.894003905030267
"10480","MYL12A",2391.08865069881,-0.15260904095752,0.27240194839287,-
0.560234762849129,0.575319318462925,0.894003905030267
"10481","SNHG22",3.2956036703593,0.599727956933705,1.06943047470699,0.
560791908513759,0.57493940338036,0.894003905030267
"10482","CHEK2",148.003394539682,0.170362873358222,0.303762095591372,0
.560843093429927,0.574904506571239,0.894003905030267
"10483","HDAC6",76.3524370605796,-0.142422780660977,0.254035509093058,
-0.560641231493369,0.575042137646981,0.894003905030267
"10484","BRCC3",111.263664534744,-0.106282211234663,0.189419171446777,
-0.561095323260487,0.574732556161963,0.894003905030267
"10485","MAMSTR",17.6301516239371,-0.286428877214966,0.51146679784005,
-0.560014605883647,0.575469475188584,0.894022153681148
"10486","ADO",73.8134679907936,-0.116293186692057,0.207747885491085,-0
.55978036270818,0.575629259657281,0.894099838901253
"10487","DIS3",311.231285523749,0.116896136531977,0.208798999909493,0.
559850078700796,0.575581702045899,0.894099838901253
"10488","CORO1C",147.732922019558,0.1123847303812,0.200801302842757,0.
559681280898892,0.575696852710583,0.894119568440378
"10489","RRP1B",205.815192598175,-0.0891012800216242,0.159257587524199
,-0.559479026442525,0.575834841198618,0.894248615529058
"10490","OLFM1",165.279882789103,-0.159784191154155,0.285650904684751,
-0.559368755826261,0.57591008011442,0.894280199712467
"10491","ZNF665",38.2923831821222,0.190520624178703,0.340710127333805,
0.559186853850233,0.576034204076897,0.894302435208595
"10492","ATP6AP1",400.773306918489,-0.0900516302695443,0.1610186877508
25,-0.559261980875776,0.57598293831974,0.894302435208595
"10493","CYTH3",228.1821139625,0.143541617847738,0.256819883780564,0.5
58919409722906,0.576216722135239,0.894483652948233
"10494","DDX28",24.4530851598214,0.192651931325128,0.344726213902718,0
.558854892826614,0.576260755972666,0.894483652948233
"10495","CCT2",482.101300930336,-0.112784038054751,0.20186428810534,-0
.558712187843231,0.57635816009858,0.894549601700407
"10496","RRAGA",884.193299994372,-0.124618593771981,0.223112863022657,
-0.558545088273666,0.576472224764059,0.894641393786371
"10497","ARHGEF19",25.7577976839427,-0.217085798517555,0.3896588084509
54,-0.557117647052702,0.577447051195363,0.89470882421331
"10498","SLC1A7",29.4336315901538,-0.267209252461369,0.47932375713223,
-0.557471330150769,0.577205442035363,0.89470882421331
"10499","NDUFAF7",97.330178592439,-0.112062406380305,0.201225342718007
,-0.556900064706795,0.577595710418569,0.89470882421331
"10500","BBS5",76.7811156118202,0.131458946513421,0.235503756585856,0.
558203182909719,0.576705647655108,0.89470882421331
"10501","CAVIN2",60.9165272189803,0.247956181589616,0.445167605417519,
0.556995115035516,0.577530766775409,0.89470882421331
"10502","AC093772.1",40.2170875120817,0.296361457473992,0.532020365196
867,0.557049084698718,0.577493893245996,0.89470882421331
"10503","SCAMP1-
AS1",55.9453531755445,-0.159609846847256,0.286049644336468,-0.55797953

2599992,0.576858360507896,0.89470882421331
"10504","MEOX2",319.322208961086,-0.184974134132413,0.332295539700032,-0.556655482945669,0.577762838012344,0.89470882421331
"10505","DLC1",1080.3981621418,0.130948740383183,0.23488939924359,0.557491060920053,0.577191964889989,0.89470882421331
"10506","TMEM268",31.8645333096631,-0.168476439271088,0.301786897683191,-0.558262935085904,0.576664850914759,0.89470882421331
"10507","MASTL",32.1822800277466,0.169179210466772,0.303643848684617,0.557163305628139,0.57741585808236,0.89470882421331
"10508","TIMM23",98.7201359992131,0.144669337364048,0.259465019629889,0.557567789177939,0.577139556893799,0.89470882421331
"10509","ENDOD1",89.521140912511,-0.143911590986136,0.257978264751187,-0.557843859927251,0.576951009778771,0.89470882421331
"10510","PRKAG1",259.378910093088,-0.152242262122905,0.272773079522086,-0.558127885602355,0.576757060016665,0.89470882421331
"10511","TBX5",37.9834685141853,-0.291146657130497,0.522545796174657,-0.557169647640192,0.577411525397185,0.89470882421331
"10512","EEF1AKMT1",212.511024644474,-0.161827336210553,0.28983440541139,-0.558344120605198,0.576609422402115,0.89470882421331
"10513","PML",311.874501340285,0.175235818884583,0.314194280109672,0.557730773531,0.577028240505835,0.89470882421331
"10514","CRABP1",3.20055781582416,0.605329158261333,1.08737975583736,0.556686065757391,0.577741938922403,0.89470882421331
"10515","MRPL46",162.920878548417,-0.1694678679789,0.304496314350913,-0.556551458890891,0.577833926620666,0.89470882421331
"10516","SNX20",3.02949119594823,0.49967015180833,0.897339095675303,0.5568353749619,0.577639911997244,0.89470882421331
"10517","RBCK1",518.746469928327,-0.120869023478205,0.217078412445928,-0.55679891020169,0.577664828548774,0.89470882421331
"10518","DLGAP4",461.450956439641,0.0839273677733419,0.150543776880851,0.557494766720027,0.577189433651699,0.89470882421331
"10519","CDH22",5.31083136800796,-0.603896874683476,1.08235208143697,-0.557948642628119,0.576879454289412,0.89470882421331
"10520","BCR",62.5534060801907,0.137638224832744,0.247281318815582,0.556605834569301,0.577796766516463,0.89470882421331
"10521","AC124016.2",15.3460437789998,-0.227444434450758,0.408817848550614,-0.556346635199806,0.577973912343936,0.894755470268996
"10522","AL078581.2",3.54859120180039,-0.420052791851006,0.755013475646533,-0.556351383650876,0.577970666858248,0.894755470268996
"10523","ZNF70",24.9585926057743,0.213628309848466,0.384039996377652,0.556265784458532,0.578029173768702,0.894755983228964
"10524","ACTB",11007.2534018969,-0.173403858868994,0.311811923514008,-0.556116831309063,0.578130989648758,0.89482855286855
"10525","TMEM97",59.8464862970637,-0.190543634222868,0.34268192813005,-0.556036424980529,0.578185954335796,0.894828599541642
"10526","PFN4",16.9077855599407,0.255461090110992,0.459723527274913,0.555684177456133,0.578426774977068,0.894861215462196
"10527","TTC39B",89.159978281417,0.116571996029317,0.209734128770358,0.55580842618587,0.578341824614816,0.894861215462196
"10528","PMP22",5500.45611602664,0.0889391824272824,0.160035727882748,

0.555745792542303,0.578384647262773,0.894861215462196
"10529","SEZ6",2.36764985798083,0.50147043676842,0.90240152437106,0.555706548831382,0.578411478963703,0.894861215462196
"10530","IPP",98.750157335644,0.116440738441899,0.209797615470497,0.555014594330665,0.578884678027841,0.894925897828949
"10531","SCLY",24.4595017139415,0.188143905150585,0.338962177272468,0.555058699069392,0.57885451195967,0.894925897828949
"10532","NUDT16",187.847484524536,-0.133485757538523,0.240343403531311,-0.555395969172637,0.578623849054132,0.894925897828949
"10533","ANKDD1A",42.4843348050714,0.149386266685787,0.269131119894212,0.555068721686689,0.578847656012649,0.894925897828949
"10534","AC008915.2",36.017041115576,-0.194260627035041,0.349928751672361,-0.555143371633914,0.578796598785598,0.894925897828949
"10535","COLGALT1",238.419388477118,0.0930124297599631,0.167595900794214,0.554980338535668,0.578908108872468,0.894925897828949
"10536","SNRPA",268.350257022726,-0.108234948604363,0.194993766521095,-0.555068762121964,0.578847628356156,0.894925897828949
"10537","ELF4",46.5731799643742,0.184187524666367,0.331761449207366,0.555180612775904,0.57877112830020,0.894925897828949
"10538","PBXIP1",257.649493476319,0.0964384045157861,0.173862593860444,0.554681730983463,0.579112374263145,0.895100883484563
"10539","BBOX1-AS1",9.9842703621765,-0.391334591347739,0.705546966444949,-0.554654204410464,0.579131205785733,0.895100883484563
"10540","PLEKHJ1",494.267678444674,0.130433550378028,0.235255042329278,0.554434664127049,0.579281408321289,0.895248089197862
"10541","CNN3",1550.60468913301,0.142378699770675,0.256893223669883,0.554232991188731,0.579419402695524,0.895301410237916
"10542","CD68",505.598649844245,0.123478117135718,0.222794770746991,0.554223587572174,0.579425837481006,0.895301410237916
"10543","TMEM200B",97.4415400170074,0.131587080801707,0.237545120252333,0.553945627937666,0.579616057171962,0.895360411377467
"10544","CLDND2",20.8970038508154,-0.274654306863337,0.495831385800732,-0.553926828209532,0.579628923689324,0.895360411377467
"10545","ITM2A",8141.36962153505,-0.167802816512743,0.302861812927311,-0.554057359991493,0.579539590617567,0.895360411377467
"10546","Z94721.3",4.5316051036298,0.375732265403148,0.678416453961888,0.553837194261589,0.579690270926569,0.895370265799628
"10547","ZNF432",45.5576476699451,0.14364617715156,0.259416473251067,0.553728047225964,0.57976497741703,0.895400750653835
"10548","NCDN",49.20499190372,0.135235411261303,0.245029718756044,0.551914322670165,0.581007054962497,0.895464520499948
"10549","GDAP2",64.0439095117557,0.120668632050888,0.218529944687499,0.552183510701225,0.580822630534045,0.895464520499948
"10550","AC008074.2",3.37287326350258,-0.446695921721184,0.810114779400535,-0.551398311794445,0.581360657722335,0.895464520499948
"10551","ALMS1-IT1",4.7684286273585,-0.363369620138308,0.658610405442253,-0.551721650820727,0.581139073926754,0.895464520499948
"10552","AC064836.3",23.2940610129501,-0.2047053046125,0.3702117710411

66,-0.552941101890943,0.580303741531597,0.895464520499948
"10553","THAP4",154.605967445145,-0.0993453515229669,0.179731632656652
,-0.552742720101753,0.580439596099406,0.895464520499948
"10554","DYNC1LI1",327.164545507762,-0.101349070725524,0.1837129680196
99,-0.551670749310723,0.581173954041469,0.895464520499948
"10555","ANAPC4",103.302200080212,-0.113284954360581,0.205229220770469
,-0.551992323194954,0.580953612908367,0.895464520499948
"10556","MAML1",133.02565426735,0.108756447340414,0.196523615987123,0.
553401415876353,0.579988569615917,0.895464520499948
"10557","STARD3NL",169.86843952446,-0.0960244592372251,0.1738379385012
44,-0.552379187564616,0.580688586823551,0.895464520499948
"10558","AP4M1",103.084474818243,-0.112669524253425,0.204016724345575,
-0.552256314352831,0.580772756458247,0.895464520499948
"10559","PCAT1",8.40912171501677,-0.491633274072356,0.889230475423919,
-0.552874971854717,0.580349026631055,0.895464520499948
"10560","MED27",95.1459197187856,-0.143992374243415,0.261167932622799,
-0.551340177170146,0.581400501492262,0.895464520499948
"10561","AL596094.1",2.34297029301616,-0.515046907076797,0.93321008560
6915,-0.551908852058576,0.581010803237989,0.895464520499948
"10562","REEP3",327.294854557889,0.0914186570176837,0.165658191307928,
0.551851111592504,0.581050365715687,0.895464520499948
"10563","CWF19L1",48.7270659105627,0.138833603238746,0.251274489498427
,0.552517700924889,0.58059371034782,0.895464520499948
"10564","B4GAT1",281.582655958455,0.12868606044175,0.232831473400784,0
.552700451369979,0.580468544231576,0.895464520499948
"10565","AC068792.1",2.81553323838716,-0.508368656066614,0.91922671788
2691,-0.55303946912854,0.580236383825397,0.895464520499948
"10566","OBI1",109.46290987471,0.119377159409152,0.216171340206564,0.5
52233979282731,0.580788056864639,0.895464520499948
"10567","PCCA",112.798737843939,0.110003188764971,0.199284280143934,0.
551991299491966,0.580954314283497,0.895464520499948
"10568","F10",1951.58190506185,0.148863671901032,0.268965299655643,0.5
53467945833989,0.579943023925312,0.895464520499948
"10569","CHURC1",1229.39522653957,-0.103917822743183,0.18846687323836,
-0.551385084060664,0.581369723511662,0.895464520499948
"10570","C16orf58",179.98376589311,-0.110992187117843,0.20087560298001
6,-0.552541898922813,0.580577136368466,0.895464520499948
"10571","KANSL1-
AS1",54.3786007504015,0.155898215940331,0.28275779137118,0.55134896613
9296,0.58139447770811,0.895464520499948
"10572","AC011921.1",4.49571835062368,0.43947540905714,0.7950993384318
9,0.55273019082607,0.580448176819297,0.895464520499948
"10573","UBALD2",287.98501288508,-0.137496170862624,0.249229564665449,
-0.551684833407268,0.58116430285642,0.895464520499948
"10574","SLC35E1",241.873590355842,0.107013499823921,0.193851633757739
,0.552038163153469,0.580922206737427,0.895464520499948
"10575","SPHK2",48.4800361141615,-0.164734627584981,0.29874074525853,-
0.551430061682478,0.581338897817611,0.895464520499948
"10576","ADNP-
AS1",15.7275424426276,-0.208372220673071,0.376549890786944,-0.55337214

475776,0.580008608838845,0.895464520499948
"10577","CDC5L",466.791704834423,-0.0833266580632308,0.151268578164274
,-0.55085239164964,0.581734865714706,0.895640758754901
"10578","NFIL3",997.728062183362,-0.221938010858716,0.402870286637493,
-0.550891982407276,0.581707723934055,0.895640758754901
"10579","KCNRG",5.5168614766652,-0.357035094937086,0.647930928672592,-
0.551038820864037,0.581607062750549,0.895640758754901
"10580","MAPK3",596.037564156885,-0.0934626809743439,0.169622038340791
,-0.55100552904904,0.581629884351545,0.895640758754901
"10581","SENP5",162.636258434477,-0.119066760054801,0.216273236110043,
-0.550538578866126,0.581950023676202,0.895887339160917
"10582","MARK4",99.9642802799697,0.105093175558034,0.190974193354712,0
.550300402959871,0.582113347901974,0.896054084669746
"10583","TRIM22",434.02479422203,0.119453546619552,0.21719421546636,0.
549984935662587,0.582329706063388,0.896217742069777
"10584","DDX27",372.861694994454,-0.125122180402038,0.227500303933289,
-0.549986871396569,0.582328378356946,0.896217742069777
"10585","SF3A3",297.729342311087,-0.108685809730176,0.197896712447479,
-0.549204725970478,0.582864961513729,0.896224821435821
"10586","IFT80",111.061183576285,0.12101342659687,0.220093381568821,0.
549827649220024,0.582437592460977,0.896224821435821
"10587","PPP3CB",356.33842437921,0.0735126509700017,0.133771747930596,
0.549537941360695,0.582636334180682,0.896224821435821
"10588","AL133215.2",3.33200571432066,-0.487923353882977,0.88839848769
6288,-0.549216776750954,0.582856692445365,0.896224821435821
"10589","PCK2",55.2825182339394,-0.158206447345233,0.287977361787066,-
0.549371125436634,0.582750785486721,0.896224821435821
"10590","GNAO1",11.3966375602342,-0.263562645249419,0.479628884854369,
-0.549513704391356,0.58265296235423,0.896224821435821
"10591","TVP23C",23.4457014942596,0.187182495851769,0.340522427237458,
0.549692122690175,0.582530560716286,0.896224821435821
"10592","EPB41L1",159.385394295949,-0.106280182803872,0.19352654955479
2,-0.549176239892507,0.58288450845915,0.896224821435821
"10593","TTPAL",93.774143200873,-0.13637861002779,0.248253894132669,-0
.549351342520767,0.58276435911068,0.896224821435821
"10594","BACE2",359.839395366108,0.0908117756852454,0.165225496733232,
0.549623257189339,0.582577803607788,0.896224821435821
"10595","DNAJC16",92.8407170149406,0.113654794674459,0.207043645714833
,0.548941235467803,0.583045778501167,0.896388172345966
"10596","PTPRF",71.8397852706458,0.210393371681478,0.384189537829538,0
.547629102213678,0.583946602423673,0.896419395615795
"10597","PDE1A",113.943475991541,-0.199018973334528,0.362772264356854,
-0.548605813863311,0.583275995126472,0.896419395615795
"10598","ARL8B",305.225015916076,0.0860848295696441,0.156942436670407,
0.548512125821202,0.583340305486446,0.896419395615795
"10599","GUCY1B1",21.3046119356868,0.276511865424166,0.504846713967699
,0.5477144998152,0.583887954365753,0.896419395615795
"10600","LINC01184",138.759763890899,-0.096703336718911,0.176282421507
562,-0.548570503467713,0.583300232882332,0.896419395615795
"10601","MTO1",87.1992052004591,0.114151758632421,0.20824491736655,0.5

481610791561,0.583581304126992,0.896419395615795
"10602","AKR1C4",12.2728555454262,-0.404182256999363,0.737105131943188
,-0.548337325957615,0.58346030233607,0.896419395615795
"10603","MICAL2",12.4575797557421,-0.256112199080098,0.467139708683747
,-0.548256109080818,0.583516060122631,0.896419395615795
"10604","AKAP11",241.971397675156,0.103816231614033,0.189306739945159,
0.54840219447077,0.583415769968012,0.896419395615795
"10605","RSPRY1",206.147388467021,0.0831847247366516,0.151842783767364
,0.547834560673608,0.583805505446041,0.896419395615795
"10606","UBE2G1",168.980080868815,0.143032493456295,0.261164526254921,
0.547671980982141,0.583917154456384,0.896419395615795
"10607","RAPGEFL1",11.0387010964721,0.284259967772734,0.51853833786662
2,0.548194698471555,0.583558221969527,0.896419395615795
"10608","AC090912.2",2.30411969584817,0.64850030989496,1.1838669701143
8,0.547781403034078,0.583842009513242,0.896419395615795
"10609","TRAPPC6A",207.778478444935,0.163326132137302,0.29823801565345
4,0.547636865741098,0.583941270593773,0.896419395615795
"10610","AP2S1",1095.40181061113,-0.134076679228263,0.244728874464521,
-0.547858030735563,0.583789388576596,0.896419395615795
"10611","AC010487.2",3.23736253566834,-0.466658754147562,0.85082384682
4854,-0.548478696135589,0.583363253453141,0.896419395615795
"10612","GJC2",30.3673754390594,0.2340788752923,0.427507810940064,0.54
7542920391499,0.584005791837362,0.896425776784658
"10613","AC010969.2",34.6753652878971,-0.203051509996657,0.37092193206
9532,-0.547423844321488,0.584087577516175,0.896466837855552
"10614","AC090061.1",3.17122177822645,-0.442301516122898,0.80810717467
2396,-0.547330267550471,0.584151853110917,0.896481018967753
"10615","Z82195.3",3.2408538454373,0.630494643870138,1.15216201899745,
0.547227415479952,0.584222503479716,0.896504979668497
"10616","SYTL1",8.47173264353932,0.341024765025465,0.623348633783393,0
.547085124668721,0.584320251366946,0.896506333826922
"10617","RPL34",27523.9417489615,-0.136462249716007,0.249480431870028,
-0.546985784388492,0.584388498531172,0.896506333826922
"10618","VAV2",41.0801251196143,0.169455834223228,0.309789580603357,0.
547003013765635,0.584376661615305,0.896506333826922
"10619","AC119674.1",22.2023965216674,0.267446595859409,0.489387413897
623,0.546492591072964,0.584727379183878,0.896659374537526
"10620","LINC02614",33.0834647178453,0.174989095640052,0.3202634184947
78,0.546391144085366,0.584797096286476,0.896659374537526
"10621","FAM120A",604.137864477868,0.090640245906885,0.165791470192709
,0.546712359818806,0.584576361438178,0.896659374537526
"10622","AP001160.2",5.79083769776437,-0.373114723623467,0.68281575630
5143,-0.546435433245517,0.584766659107264,0.896659374537526
"10623","ZC3H10",34.1256831822288,-0.168429700228352,0.308321277087293
,-0.546279847500327,0.584873586743275,0.896659374537526
"10624","NDUFA12",808.998508162161,-0.15312778805877,0.280278829821053
,-0.546340899726663,0.584831626994243,0.896659374537526
"10625","HDHD2",135.547451938013,0.134578971016412,0.246175443120447,0
.546679105399503,0.584599211600131,0.896659374537526
"10626","UBA1",413.4562038552,0.0808499317135206,0.148031445648963,0.5

46167277898814,0.584950956832921,0.896693594565353
"10627","ZADH2",196.437366190702,0.1026203105325,0.187956805054861,0.545978159729558,0.585080950143628,0.896808468701379
"10628","TRAM2-AS1",69.7071670589263,-0.128735361758525,0.235859646642415,-0.545813425870597,0.585194193458628,0.896897649345841
"10629","AL445222.1",8.04174184281524,0.324510613184229,0.594652408035011,0.54571478867218,0.58526200469253З,0.89691718829962
"10630","PPP1R1C",16.3641333491964,-0.33023117099004 8,0.605361708466829,-0.545510504498887,0.585402457858785,0.896962077177094
"10631","AL606834.2",4.58985700763705,0.390766084638311,0.716397830038491,0.545459615109828,0.585437448691595,0.896962077177094
"10632","CEBPB",3182.79947421205,-0.185712735087588,0.340487471553081,-0.545431919243572,0.585456492390378,0.896962077177094
"10633","SNRPC",431.152771579312,-0.139154354210117,0.255257218652117,-0.545153453230119,0.585647981819347,0.89701297073421
"10634","PRKAB1",51.2114211210639,0.134572471978945,0.246857028252178,0.545143368741649,0.585654917045756,0.89701297073421
"10635","AC016588.2",11.2937757461448,0.24282461753309,0.445315466851788,0.545286736276574,0.585556325015695,0.89701297073421
"10636","WDR70",190.796064263005,0.132924000690133,0.243917272984115,0.54495525906765,0.58578428936385,0.897042426384108
"10637","PLA2G4C",64.8190502520591,0.278674544355652,0.511339245172486,0.544989548497592,0.585760705839945,0.897042426384108
"10638","KIAA1614",55.530042762214,0.136201362702793,0.250050482856224,0.544695459680867,0.585962988047014,0.897231727044352
"10639","RPL17",20293.3737459024,-0.129528810095115,0.237948560640381,-0.544356350576442,0.586196276646351,0.89750457282568
"10640","PHACTR4",247.04303867775,-0.124454138825643,0.2287800175809,-0.543990424258247,0.586448062339673,0.897536144603495
"10641","MANEAL",9.57512923528429,0.324185083260433,0.596217368942637,0.5437363957299,0.58662288315171,0.897536144603495
"10642","FMOD",503.385753175032,0.170786760107924,0.313883293606197,0.54410911184778,0.586366390577423,0.897536144603495
"10643","EML3",107.735767049239,0.0997091635656426,0.183375044497551,0.543744454644014,0.586617336687505,0.897536144603495
"10644","TMBIM6",3205.56832419319,0.0809519518913579,0.148858571897029,0.543817872627149,0.586566808640029,0.897536144603495
"10645","RSRC2",1099.50340199157,0.078167672733114,0.143753613950436,0.54376144421708,0.586605643869365,0.897536144603495
"10646","AC006141.1",24.8815350948397,0.232239847843898,0.427158197721365,0.543685803252189,0.586657703455916,0.897536144603495
"10647","LIG1",23.0094559439102,-0.200371984483932,0.368182680352089,-0.544218930375319,0.586290826525996,0.897536144603495
"10648","CDC37L1-DT",7.39412384783905,0.323423313222521,0.59521294170856,0.543374128079496,0.586872235209984,0.897780037503328
"10649","LDLRAD2",21.2857493151503,0.262900632806226,0.485950775945451,0.541002599069287,0.588505791269661,0.897861741292143
"10650","C2orf42",37.3759295242066,-0.154082401105561,0.28387256982579

1,-0.542787213291229,0.587276318077402,0.897861741292143
"10651","ACKR3",2544.27130139485,0.209930396789583,0.386614007236406,0
.542997389800249,0.587131599600391,0.897861741292143
"10652","RPUSD3",132.742158997158,-0.119647104457505,0.221034453721672
,-0.541305223882273,0.588297220446292,0.897861741292143
"10653","SCAP",135.566787818222,0.108961882115671,0.201417231389346,0.
540975969950874,0.588524145851569,0.897861741292143
"10654","SMC4",132.528048971867,-0.163239817848889,0.300976573442163,-
0.542367188190007,0.587565578704937,0.897861741292143
"10655","HNRNPD",1024.75146182577,-0.0895789624267155,0.16538011428172
2,-0.541654979595185,0.588056209285888,0.897861741292143
"10656","SLCO6A1",4.0515362178905,-0.561309174648983,1.03628730402486,
-0.541654010879902,0.588056876749313,0.897861741292143
"10657","RING1",232.360839456125,-0.0930504126611778,0.17178037418636,
-0.541682442490374,0.588037286969651,0.897861741292143
"10658","SKAP2",375.539932337694,0.240973124194814,0.445323722627498,0
.541118992657802,0.588425568073111,0.897861741292143
"10659","SAXO1",7.26595819785071,0.346809564066453,0.640491465969061,0
.541474137429344,0.588180819079015,0.897861741292143
"10660","CAMK2G",187.43156837429,-0.117229063695156,0.216003698610281,
-0.542717853672794,0.587324079742014,0.897861741292143
"10661","CYP2R1",25.4714242739883,0.168179505823873,0.310059979432637,
0.542409588401626,0.587536375764075,0.897861741292143
"10662","ARFGAP2",300.833580946454,-0.118503963215106,0.21831549548076
4,-0.54281059140645,0.587260220099613,0.897861741292143
"10663","AC073611.1",17.5588047720733,-0.281924669644955,0.52021143549
6202,-0.541942468788758,0.587858139189914,0.897861741292143
"10664","MED4",542.867640655023,-0.108917949646704,0.201046498633761,-
0.541755018798492,0.587987282240211,0.897861741292143
"10665","AL157813.1",2.4361727975711,-0.519165347225228,0.957065531311
446,-0.542455380786546,0.587504837234601,0.897861741292143
"10666","HNRNPC",1273.38987744497,0.0875496294971056,0.161385438441265
,0.542487787886568,0.587482518008306,0.897861741292143
"10667","NOP9",38.5678316249324,0.173604954289195,0.319590819177243,0.
543210079488909,0.586985167468813,0.897861741292143
"10668","EFCAB11",75.9634753469895,0.126876340693184,0.234503410416984
,0.541042624785618,0.588478203347618,0.897861741292143
"10669","ZNF839",37.9315252339375,-0.15155290087296,0.279482014587673,
-0.54226351952039,0.587636982828447,0.897861741292143
"10670","ZNF830",169.6715909883,-0.137049451838504,0.252968018298451,-
0.541765922666212,0.58797976969734,0.897861741292143
"10671","MLLT6",352.297705690988,0.131096070804608,0.241814084867637,0
.542135793605101,0.587724962439439,0.897861741292143
"10672","PCTP",63.6561097373464,-0.141259338928285,0.260875320386754,-
0.541482186658609,0.58817527247589,0.897861741292143
"10673","TMC6",11.7758275475813,-0.262445852124815,0.485021264169483,-
0.541101744423947,0.588437455938741,0.897861741292143
"10674","ARHGEF18",58.7028160042622,0.145404406552644,0.26773125297354
7,0.543098368000433,0.587062076241069,0.897861741292143
"10675","MRGBP",104.168195623174,0.143935083455842,0.265688083465183,0

.541744596063917,0.587994463333626,0.897861741292143
"10676","OFD1",203.853352239629,0.104161874350841,0.192404155152227,0.541370191659475,0.588252448721285,0.897861741292143
"10677","LINC00632",471.88683559264,0.14286565765595,0.263346338923899,0.542501020670103,0.587473404516699,0.897861741292143
"10678","GTF3C3",106.089891551247,0.112915613136103,0.208866265770229,0.540612016592091,0.588775033518309,0.898160378439758
"10679","PDCD10",399.058262469928,-0.111718455463557,0.206724731454799,-0.540421335547771,0.588906497329657,0.898276798857832
"10680","CD302",1470.49739162698,0.0964555732787223,0.178559169076296,0.540188296001244,0.589067183332137,0.898291896749632
"10681","AC096733.2",15.0825847993137,0.264371539107079,0.489308043791522,0.540296736302416,0.588992408791531,0.898291896749632
"10682","VCL",534.124719345309,0.104377886404174,0.193232606898642,0.540167045714618,0.589081836888671,0.898291896749632
"10683","SDC3",161.160530811551,0.143264673117333,0.265269017857199,0.540073146402854,0.589146589034792,0.898306542056325
"10684","CNN1",3.55847038500857,-0.455836812555632,0.844213059017478,-0.539954704190609,0.589228270434356,0.898346995236356
"10685","TAF12",370.966306198686,-0.143824591034746,0.266820589557917,-0.539031081795609,0.589865408041526,0.898516463889817
"10686","GNG5",1224.55975163395,-0.141405113515124,0.26266435470673,-0.538349079276502,0.590336073933628,0.898516463889817
"10687","PIGM",128.237338732728,0.1113083106322,0.206675635688214,0.53856522691488,0.590186886676173,0.898516463889817
"10688","GAS5",2746.492769963,-0.122299001587617,0.226661786718268,-0.539566035185413,0.589496345251387,0.898516463889817
"10689","CYBRD1",4547.32322735212,0.11878142387337,0.220522960684272,0.538635176603821,0.590138610421758,0.898516463889817
"10690","SCRN3",66.4772701121976,0.137526176047346,0.255414490836246,0.538443122773008,0.590271162039832,0.898516463889817
"10691","AC010680.3",8.56203063662741,0.357990840839698,0.664273582190996,0.53892078570839,0.589941514229102,0.898516463889817
"10692","SPON2",2554.74082968225,-0.124311180118186,0.230496163690567,-0.539319952782682,0.589666103562133,0.898516463889817
"10693","DMGDH",41.0778097971376,0.138841826605881,0.258015260289465,0.538114786118137,0.590497805018141,0.898516463889817
"10694","AC244517.1",4.50997358761663,-0.362923655904828,0.673875557783581,-0.538561833431838,0.590189228757873,0.898516463889817
"10695","TES",42.3415423640355,-0.281602190054536,0.522384396079803,-0.539070830154576,0.589837982102726,0.898516463889817
"10696","CPNE3",971.089565375864,0.0743713316287592,0.137942331963343,0.539147994457009,0.589784741244446,0.898516463889817
"10697","LGR4",153.965709005693,0.216364695798423,0.401134860795987,0.539381432391796,0.589623690150549,0.898516463889817
"10698","AP001160.1",10.1603334561578,-0.320249876192872,0.593867213634995,-0.539261755557541,0.589706253825911,0.898516463889817
"10699","POC1B",61.6335988951724,0.136804219107749,0.253913663102161,0.538782424846143,0.590036991988692,0.898516463889817
"10700","KLHDC2",492.460350515084,-0.100937918217261,0.187465045114243

,-0.538435942315264,0.590276118110467,0.898516463889817
"10701","AC135050.3",4.47100606956181,-0.49123785452786,0.912816538751
163,-0.538156172323444,0.590469234899454,0.898516463889817
"10702","TMEM220-
AS1",26.8617875638202,0.183961215707717,0.341678515889094,0.5384043981
49015,0.590297890642446,0.898516463889817
"10703","AC003102.1",6.23603083630092,0.337530213208532,0.625663540346
755,0.539475599011997,0.589558729425725,0.898516463889817
"10704","TBX1",32.743336494332,-0.608760467272672,1.13126908128639,-0.
538121723065601,0.590493016194233,0.898516463889817
"10705","ZMAT5",154.613992185016,-0.168921651126466,0.313375462563989,
-0.53903917602347,0.589859823063991,0.898516463889817
"10706","SCG2",6.33364043144299,0.996168642336856,1.85198558781497,0.5
37892221673369,0.590651458732103,0.898664569960864
"10707","G3BP2",353.141704791328,0.0842418569991088,0.156653560735879,
0.537758967005814,0.590743463698011,0.898664569960864
"10708","LINC02156",2.78476061590036,0.609311919611407,1.1331101097190
1,0.537734077549185,0.590760649219776,0.898664569960864
"10709","ARMCX3",670.708779036116,-0.0579138142667755,0.10776142388528
1,-0.537426215975285,0.590973238636407,0.898904013834013
"10710","RCOR1",158.842823551818,0.130639572843744,0.243122456084343,0
.537340626397846,0.591032347547521,0.89890998218502
"10711","PHF7",42.9648011961364,0.139238052973384,0.259180590320599,0.
537224075310386,0.59111284311983,0.898948473679293
"10712","AL357078.2",2.79093099464857,0.528100898635174,0.983689353662
668,0.536857389651361,0.591366126067819,0.899001863296912
"10713","TRAM1L1",30.6184317078625,0.188295022108141,0.350790292838442
,0.536773753300126,0.591423903682836,0.899001863296912
"10714","STX11",43.2551367062384,0.184444832773201,0.343597572831358,0
.536804818652572,0.591402442831496,0.899001863296912
"10715","C9orf78",433.947964136472,-0.0999433234455927,0.1861674197406
91,-0.536846477137632,0.591373664496988,0.899001863296912
"10716","GRIA3",83.1336322915689,-0.204205222337076,0.380233474562993,
-0.537052195553723,0.591231560427524,0.899001863296912
"10717","HCG17",4.61516809654354,0.456641258670505,0.851064954995199,0
.536552769551039,0.591576576026922,0.89906613611705
"10718","PTN",50.6101723871895,0.590522748546087,1.10054171629121,0.53
6574615759349,0.591561482201875,0.89906613611705
"10719","FADD",101.961148922658,-0.130542255167166,0.24335449719715,-0
.536428365494347,0.591662531754855,0.899112881775803
"10720","AC017083.2",2.58727373876153,-0.477795427656965,0.89165121916
5186,-0.535854622735002,0.592059027745097,0.899360407011643
"10721","CNOT6L",188.20842409774,0.122157700414424,0.228149736722623,0
.535427750955239,0.592354104818495,0.899360407011643
"10722","AL162426.1",5.01504313007449,0.372409432728091,0.695676984882
18,0.535319466966644,0.592428967348352,0.899360407011643
"10723","PCGF6",85.0819456830626,-0.121464651154429,0.226608038370935,
-0.53601210278165,0.59195018598075,0.899360407011643
"10724","AL354811.1",5.57722908977015,-0.351210057583765,0.65601967497
3125,-0.53536512848971,0.59239739855891,0.899360407011643

"10725","ATL1",250.156714669872,-0.0883838949752115,0.165056303735595,
-0.535477246096545,0.592319887617608,0.899360407011643
"10726","PSMA4",1048.60296223543,-0.115035771484763,0.214894014262321,
-0.535313986662925,0.592432756291665,0.899360407011643
"10727","AC103810.2",6.81722978036753,0.373212857483287,0.696569739392
289,0.535786779668164,0.592105920066191,0.899360407011643
"10728","C19orf38",5.37511295020566,0.362371144144688,0.67634456209368
,0.535778897996812,0.592111367894228,0.899360407011643
"10729","AL121761.1",2.69463096561217,-0.571907173791868,1.06725201597
063,-0.535868909342597,0.592049153229089,0.899360407011643
"10730","BRWD1-
AS2",2.98963026143025,0.535453708262613,0.999550721821683,0.5356943840
59618,0.592169785553613,0.899360407011643
"10731","KCNAB2",33.16875133162,-0.150976543725377,0.282182121889455,-
0.535032278850474,0.592627536914023,0.899491796793116
"10732","PCNX2",178.347558378853,0.134203686669529,0.250996192021987,0
.534684154322841,0.592868280459828,0.899491796793116
"10733","FAM50B",125.998071837975,-0.148941672177811,0.278671636050659
,-0.534470153793244,0.593016293557693,0.899491796793116
"10734","EXTL3",36.3546030053619,0.153274381563482,0.286574751772355,0
.534849565830689,0.592753885478868,0.899491796793116
"10735","PTPDC1",54.4834916086105,0.130964323059741,0.245008085761335,
0.534530616215316,0.592974473111399,0.899491796793116
"10736","OVCH1-
AS1",3.74707126760075,0.560462625228154,1.04779974042832,0.53489479296
7829,0.592722609141749,0.899491796793116
"10737","AC073578.1",10.6340911231468,-0.2622848210799,0.4905084009121
5,-0.534720344426629,0.592843251310844,0.899491796793116
"10738","TPD52L2",267.742125167337,0.10520289744736,0.196799375271494,
0.534569265284647,0.592947741160227,0.899491796793116
"10739","UTP14A",96.8437196490142,0.120505084505685,0.225306021378816,
0.534850705579124,0.592753097288882,0.899491796793116
"10740","FXYD2",7.24385895302195,-0.295375349773767,0.552792318113563,
-0.534333311254673,0.593110949338581,0.899551606496848
"10741","SOS1-
IT1",2.65544752512449,0.536547084223678,1.00479760415852,0.53398523444
2328,0.593351749831373,0.899749269503186
"10742","SAV1",1256.64921810314,0.109206197163407,0.204495790090901,0.
534026627711326,0.593323111509026,0.899749269503186
"10743","SLCO1C1",2.29329264618175,0.557682879962784,1.04453629844967,
0.533904739156038,0.593407443064405,0.899749961842697
"10744","OGFOD2",55.3330394973513,0.130796781643063,0.245056222801502,
0.533741931332262,0.593520094169853,0.899837007998206
"10745","TUBA1B",2739.16760065667,-0.171782978434138,0.321976052036773
,-0.533527190446198,0.593668694127,0.899978535005556
"10746","COQ4",250.359699312765,-0.109939905226217,0.206123179803671,-
0.533369926327225,0.593777531158846,0.900059762241433
"10747","AAMDC",732.388465885069,-0.124363337601507,0.2332276545288,-0
.533227236078687,0.593876290021202,0.900125699093269
"10748","STX1B",5.98682790938014,-0.43199771016165,0.810305714201123,-

0.533129290082268,0.593944084813809,0.900144696458144
"10749","RBFOX2",753.218983168524,-0.0682901224387485,0.128125173531579,-0.53299535568564,0.594036795248419,0.900201447371987
"10750","AC019080.6",3.12241356987331,-0.430791220251934,0.808471592947038,-0.532846452503811,0.594139874962296,0.900273899838217
"10751","PEX10",102.044486044005,-0.123266992782705,0.232656530001341,-0.529823911591882,0.596234024820751,0.900315403972393
"10752","DDX20",50.5949939200687,-0.155569163121203,0.293465474375007,-0.530110615064747,0.596035239693094,0.900315403972393
"10753","FCGR3B",2.93108341832939,0.534836727837883,1.00689729848291,0.531173068637407,0.595298853695188,0.900315403972393
"10754","MBOAT2",89.183797126031,-0.155161847218396,0.291362945728668,-0.532538023427629,0.594353414083274,0.900315403972393
"10755","TBC1D8",28.2800349604375,0.217158361080987,0.410765696617741,0.528667225304051,0.597036316891078,0.900315403972393
"10756","AC114763.1",2.84651296201641,0.458550557281182,0.86880657491391,0.52779360852169,0.597642593755146,0.900315403972393
"10757","AC108047.1",8.18500067315493,0.275732322557245,0.520080717968538,0.530172169493746,0.595992565024318,0.900315403972393
"10758","NDUFS1",397.171371778503,0.0997103779987767,0.188698991063297,0.528409703925391,0.597215003758806,0.900315403972393
"10759","ZNF620",12.0477820168814,-0.233683132801107,0.442896571191337,-0.527624614867821,0.597759905086956,0.900315403972393
"10760","ARL6IP5",3375.69489126449,-0.0971123304229512,0.182457467232639,-0.532246401837476,0.594555348898511,0.900315403972393
"10761","CLCN2",19.2254282211748,-0.192437096122429,0.362361405194878,-0.531063996782263,0.595374432212907,0.900315403972393
"10762","ATP13A3",200.124387694836,0.13903325712346,0.262863928953723,0.52917214609298,0.596862879586449,0.900315403972393
"10763","AC092535.2",3.40863798823641,-0.414193741371895,0.781557210378983,-0.529959593324011,0.596139946470313,0.900315403972393
"10764","ARAP2",10.8295450588808,-0.386588295009141,0.725953295513106,-0.532525022475308,0.594362415989394,0.900315403972393
"10765","ABLIM3",50.9581708623817,-0.210148513583901,0.395642670381475,-0.531157353126946,0.595309743081248,0.900315403972393
"10766","DEK",1931.4577713138,-0.106887103992571,0.20095086974565,-0.53190665025666,0.594790651096056,0.900315403972393
"10767","RNGTT",64.8058038130672,0.130298980014058,0.245734732795921,0.53024242251611,0.595943861466213,0.900315403972393
"10768","AL355881.1",2.81891089517007,-0.514419242554839,0.969444675813962,-0.53063290292757,0.59567319037693,0.900315403972393
"10769","PHF10",273.047485578553,0.102208455485505,0.19247828679326,0.531012911577308,0.595409831888337,0.900315403972393
"10770","HSPB1",2379.9970906317,-0.113766013537828,0.215345361871636,-0.528295629629778,0.597294164487715,0.900315403972393
"10771","FGL2",1956.65316711859,-0.122870329466176,0.232837418178753,-0.527708692302397,0.597701539245456,0.900315403972393
"10772","MRPS33",377.143898889392,-0.141601666721601,0.266731724657979,-0.530876733553804,0.595504201628307,0.900315403972393
"10773","OR2A42",3.07195368961231,-0.430950753460531,0.812858097795029

,-0.530167263670664,0.595995966099347,0.900315403972393
"10774","FBXO25",171.542407556205,0.120341356669472,0.227189135587669,
0.529696793634897,0.596322171398216,0.900315403972393
"10775","AC069185.1",5.4917362794316,-0.322914500744175,0.607583892076
207,-0.531473110060122,0.595090970351884,0.900315403972393
"10776","TNFRSF10D",49.2622734964538,0.345443531424638,0.6536899172680
69,0.528451674562049,0.597185879851399,0.900315403972393
"10777","WRN",126.544256899326,0.143090803924758,0.269095400117372,0.5
31747491270182,0.594900894672336,0.900315403972393
"10778","BX005266.2",2.29948221166288,0.533110992272784,1.009825691369
94,0.527923776181175,0.597552241628451,0.900315403972393
"10779","WNK2",8.69114039340092,-0.295502520340318,0.559294119657179,-
0.52834905634525,0.59725708895306,0.900315403972393
"10780","FANCC",14.4891281485119,0.256258541908769,0.482287497807082,0
.531339798510128,0.595183331009726,0.900315403972393
"10781","TDRD7",37.08358823131,-0.145743527055483,0.274773864952971,-0
.530412625234309,0.595825874378558,0.900315403972393
"10782","DDX31",27.368772571536,-0.195065513796234,0.369400400068489,-
0.528059833611626,0.597457807923528,0.900315403972393
"10783","ARID5B",3772.23633470159,-0.123998746818107,0.233516976020984
,-0.531005278207115,0.595415121541973,0.900315403972393
"10784","TACC2",96.6155895825671,-0.156082267474266,0.29548958546287,-
0.528215798975691,0.597349565017934,0.900315403972393
"10785","TOLLIP",185.744849135853,0.112580119096397,0.212535042879053,
0.529701443918887,0.596318946677457,0.900315403972393
"10786","TP53I11",217.731888175706,-0.129883292520568,0.24393140610403
9,-0.532458261914709,0.594408642223222,0.900315403972393
"10787","NDUFC2",1633.58659384229,-0.138988441249835,0.26279200264607,
-0.528891442092419,0.596880758954764,0.900315403972393
"10788","CLMP",809.444573767515,0.104018906694037,0.19700687399758,0.5
27996331210832,0.597501882341918,0.900315403972393
"10789","STT3A",279.225029155803,0.0880468928210316,0.166769120155108,
0.527956810824098,0.597529312571541,0.900315403972393
"10790","RAD51AP1",9.87482751710739,0.240068860599152,0.45462451881529
7,0.528059641888092,0.597457940988765,0.900315403972393
"10791","AC087239.1",29.8534496153651,-0.18613328514005,0.350647922385
122,-0.530826716080231,0.595538864851904,0.900315403972393
"10792","TSPAN8",405.495652785172,0.347275823454815,0.658156445178583,
0.527649354494411,0.597742730779285,0.900315403972393
"10793","C12orf29",169.088566590577,-0.12912703967644,0.24288992364789
5,-0.531627816160992,0.594983795364711,0.900315403972393
"10794","TDG",239.755031641143,0.111668793490823,0.211587439949429,0.5
2776664587233,0.597661309876693,0.900315403972393
"10795","TMEM263",474.105089611795,0.118042328875065,0.221656183771935
,0.532546969213005,0.594347220025518,0.900315403972393
"10796","ORAI1",61.960894931772,0.14975869444577,0.282884470305243,0.5
29398783482794,0.596528841904479,0.900315403972393
"10797","NGDN",160.199708192533,-0.124975921900096,0.236358033491839,-
0.528756818855539,0.596974156139726,0.900315403972393
"10798","ZFHX2-

AS1",30.4278093129952,0.225912179468129,0.425889043256939,0.5304484420
1788,0.595801047001196,0.900315403972393
"10799","PRORP",75.93286293389,-0.121115273822046,0.229517842549103,-0
.527694372153811,0.597711479988108,0.900315403972393
"10800","DYNC1H1",1068.50489714141,0.0883558435360047,0.16704264932893
1,0.528941823486164,0.596845807706622,0.900315403972393
"10801","HYPK",27.8919726487655,-0.214241581243743,0.405726703608202,-
0.528044073358873,0.597468746332305,0.900315403972393
"10802","DTWD1",652.392315312112,0.094425475914729,0.178638253252481,0
.52858485904064,0.597093465887483,0.900315403972393
"10803","ZNF764",26.9796875842865,0.180678332132058,0.340326802986321,
0.530896569258225,0.595490455298073,0.900315403972393
"10804","DDX19B",186.889037752171,-0.110719813674464,0.208645450000422
,-0.530660091913051,0.595654345751682,0.900315403972393
"10805","GPATCH8",388.656535012808,0.121033908839465,0.229028220493156
,0.528467228094634,0.597175087240325,0.900315403972393
"10806","DHX40",292.169758500002,0.122704594191885,0.231425439804635,0
.53021221130862,0.595964805443151,0.900315403972393
"10807","RAB11B",523.275664167499,-0.102484682849203,0.192752539877858
,-0.531690440572894,0.594940413863909,0.900315403972393
"10808","CACNA1A",37.9774328966858,0.213073437242692,0.402259923384564
,0.52969094074776,0.596326230070587,0.900315403972393
"10809","PVR",35.1315268234333,-0.194535447545507,0.366989005277493,-0
.530085219851237,0.596052846180876,0.900315403972393
"10810","ZNF324B",17.7448352293759,-0.217941672554271,0.41298334262502
3,-0.527725092176794,0.597690154893645,0.900315403972393
"10811","NSFL1C",383.326223661123,-0.0842701502100316,0.15959204031195
7,-0.52803479450045,0.597475186369721,0.900315403972393
"10812","EIF2S2",1065.85934225087,-0.0904890405362873,0.17025979414644
7,-0.531476271247304,0.595088780303533,0.900315403972393
"10813","SFI1",42.2618817815859,0.195491533202951,0.3696819186602,0.52
8810102239921,0.596937189080752,0.900315403972393
"10814","SLC25A5-
AS1",9.6236336135456,-0.250711067969344,0.47149789618815,-0.5317331635
96766,0.594910819382569,0.900315403972393
"10815","ZNF280C",31.0150667364248,0.155102418615817,0.291531575613336
,0.532026139156646,0.594707891662843,0.900315403972393
"10816","MTERF1",46.4018129430445,0.124789737176155,0.236577810149704,
0.527478621503808,0.59786125859247,0.900346666223978
"10817","CCAR2",73.060797268725,-0.121208220906037,0.229828382058328,-
0.52738578160149,0.597925715236534,0.900346666223978
"10818","POLR2G",453.95174847981,-0.119337942295203,0.226329061961246,
-0.527276264307748,0.598001754673535,0.900346666223978
"10819","LRP5L",16.2960306365996,-0.257496988350073,0.488304208222221,
-0.527329037952688,0.597965112622306,0.900346666223978
"10820","IGFBP4",11081.1032404495,-0.168343404005237,0.31932209682964,
-0.527189961723974,0.598061678896083,0.90035366797951
"10821","ELL",84.6547406160799,0.164824539604193,0.312705168577687,0.5
27092469733977,0.598129375765521,0.900372368713111
"10822","DNAJC25",92.6543218782147,0.121612463579219,0.230811573962232

,0.526890664499861,0.598269517126569,0.900416905153347
"10823","AP000254.2",11.2157919723231,-0.23108125848985,0.438520262063
224,-0.526956855773596,0.598223549703093,0.900416905153347
"10824","FRMD4B",71.5656984191433,-0.143041217064285,0.27165560168509
,-0.526553533875208,0.598503666913238,0.900436522614735
"10825","SSBP1",1029.06865713679,-0.141416002171179,0.268563759201833,
-0.526563980901465,0.598496410439832,0.900436522614735
"10826","DSCAML1",3.46792933440606,0.465970412156401,0.884596097799213
,0.52676064626069,0.598359814714936,0.900436522614735
"10827","EMG1",225.285048339539,-0.137440510243738,0.260944790141316,-
0.526703407909797,0.598399568675661,0.900436522614735
"10828","TMEM134",189.583352471602,0.107026652218184,0.203351528773934
,0.526313487110122,0.59867041367819,0.90060420838604
"10829","FUBP3",185.267252415338,0.0959820195054598,0.182398554430668,
0.526221382647766,0.598734398975913,0.900617289215869
"10830","CHTOP",252.890834739628,0.0865204471135761,0.164586581375126,
0.525683481549316,0.599108142713747,0.9008616199578
"10831","ZBTB11-
AS1",19.2333108474286,0.198207715706646,0.377000002464851,0.5257499055
98809,0.599061984320727,0.9008616199578
"10832","SEC31A",1215.24721839942,0.101024640985507,0.19221172050839,0
.525590430793202,0.599172806844288,0.9008616199578
"10833","UFSP1",7.36403407048095,0.381589203553773,0.72622651019286,0.
525441027280093,0.599276639034796,0.9008616199578
"10834","AL157786.1",3.70466647539644,0.433265720508635,0.824591896120
537,0.525430485731212,0.599283965489761,0.9008616199578
"10835","FKBP3",538.664522706148,-0.100629291338799,0.191436041594966,
-0.525654889750107,0.599128011789418,0.9008616199578
"10836","SLC25A29",100.287332581919,0.1588191025806,0.302215974569912,
0.525515247189092,0.599225056794117,0.9008616199578
"10837","PSEN2",41.456950875797,-0.135086601373547,0.257385905082869,-
0.524840710799817,0.59969392808749,0.900959987526423
"10838","ZDHHC3",310.038706230361,0.0923504540411486,0.17596534997326,
0.524821813244383,0.599707066199133,0.900959987526423
"10839","CEP19",35.118021689518,-0.153963009955939,0.293319447820511,-
0.5248987446961,0.599653582106331,0.900959987526423
"10840","RBAKDN",4.31251431474949,-0.402623341301726,0.767687048218685
,-0.524462855321005,0.59995664857651,0.900959987526423
"10841","AC005014.2",3.2069426811458,-0.435525820402619,0.829438411101
462,-0.525085183629556,0.599523975453522,0.900959987526423
"10842","ZNF517",16.4747887905123,0.206698815683699,0.393895796727124,
0.524755068221487,0.599753470257703,0.900959987526423
"10843","CCAR1",373.778178171422,0.0924280081234361,0.176307614450451,
0.524242860477315,0.600109633563817,0.900959987526423
"10844","CCDC186",493.40734686888,-0.110304585263838,0.210349848043554
,-0.52438633205477,0.600009861053478,0.900959987526423
"10845","H2AFX",244.418995166058,0.192352147817265,0.366283596220471,0
.525145405915163,0.599482113470549,0.900959987526423
"10846","USP2-
AS1",2.44060393532509,0.534076930536121,1.01895266721642,0.52414302226

1391,0.600179067140353,0.900959987526423
"10847","AL136038.5",39.913192681303,-0.168258468650417,0.320503270039938,-0.524982065329474,0.599595658578737,0.900959987526423
"10848","RILP",185.574032265676,0.163524723298369,0.311880938708715,0.524317786060966,0.600057528136136,0.900959987526423
"10849","GMIP",13.7574401847714,-0.27866480977208,0.531397103421781,-0.524400317535977,0.600000135719777,0.900959987526423
"10850","ARFGAP1",83.6616553232097,0.128099994197075,0.244165535315507,0.524644045407744,0.599830661772878,0.900959987526423
"10851","RIBC1",7.38914855882222,0.289380364573614,0.552029087657904,0.524212167517059,0.600130978931052,0.900959987526423
"10852","FAM41C",3.4526353501892,-0.403235441858038,0.769679132900273,-0.523900706958993,0.600347603071599,0.901129939774537
"10853","CATSPER2",12.6120017199528,0.280750120443235,0.536163467773366,0.523627843592482,0.600537411504948,0.901248746637561
"10854","CHKB",198.321503076427,-0.11135183448361,0.212651310934836,-0.523635777245373,0.600531892337178,0.901248746637561
"10855","BGLAP",43.6365665354363,-0.172983155612804,0.330720448243904,-0.523049471332446,0.600939826817814,0.901271415066327
"10856","MSH2",91.6774928863596,0.124865975073898,0.238682382286359,0.523147011848114,0.600871952305111,0.901271415066327
"10857","GZMA",38.5706425726569,0.247716152782932,0.473424024345858,0.523243730871511,0.600804652855546,0.901271415066327
"10858","MAP1B",2094.6170938167,-0.137505026614463,0.262793954070803,-0.523242732507521,0.600805347524103,0.901271415066327
"10859","MRM2",119.929364264489,-0.109597555958934,0.209527355661488,-0.523070391514892,0.600925269014477,0.901271415066327
"10860","MARF1",242.301631748986,0.11792616038765,0.225386434180771,0.523217649794605,0.600822800368033,0.901271415066327
"10861","ZNF738",54.7968575503688,0.171765734790501,0.328156861374657,0.523425699743014,0.600678043719599,0.901271415066327
"10862","ST6GALNAC6",530.412320872728,-0.0940019541196058,0.179786341497596,-0.522853701435727,0.601076065646456,0.901392748417891
"10863","SUCLG1",586.869853859168,-0.107751879990417,0.206117027235475,-0.522770396194962,0.601134043081826,0.901396706964915
"10864","ZSWIM3",13.4017826315548,0.211769302581626,0.405226924235761,0.522594353721714,0.60125657058311,0.901497448290527
"10865","ZNF572",6.52208908596115,0.371659279414203,0.711347544548196,0.522472147774486,0.601341633898095,0.9015420041018
"10866","CDC73",302.631898605609,0.0746464750445946,0.14292047702533,0.522293772020959,0.601465804813697,0.901622825697603
"10867","ANKMY1",20.8149419899236,0.185355089935594,0.355021992039028,0.522094670448521,0.60160441704788,0.901622825697603
"10868","ALDH3B1",17.4339304462739,-0.23555876694771,0.451231037354971,-0.522035825213863,0.601645387185139,0.901622825697603
"10869","C14orf93",47.6497417075014,-0.135278256635975,0.259128912339451,-0.522050030676487,0.601635496723514,0.901622825697603
"10870","COMT",1485.61302601897,0.146475143193795,0.280605244088703,0.52199716961630,0.601672301266679,0.901622825697603
"10871","RIC8A",285.893888402433,-0.105840380369392,0.202837134217719,

-0.521799821209199,0.601809714179765,0.901745785509539
"10872","ZFYVE16",365.340300842933,0.085117512803553,0.163207124222149
,0.521530620732557,0.601997180220351,0.901943714919913
"10873","MYL3",46.4073455748381,0.157953812537386,0.303082385978327,0.
521158008003411,0.602256703995296,0.902152428745749
"10874","ABCG2",3.94874039825839,0.430428855849884,0.826256289580134,0
.52093867396593,0.60240949313701,0.902152428745749
"10875","ZNF658",32.7424121768585,0.211520994117086,0.405995145977924,
0.520993899095995,0.602371021402007,0.902152428745749
"10876","ERP44",681.026050452195,-0.100569392812862,0.193144693220924,
-0.520694569111604,0.602579558275753,0.902152428745749
"10877","C11orf58",1780.28142396627,-0.0868674863767089,0.166751225076
267,-0.520940618798922,0.602408138280302,0.902152428745749
"10878","SMARCD1",147.033558443902,-0.110361162608445,0.21192046725722
7,-0.520766889752511,0.602529171035218,0.902152428745749
"10879","AC002070.1",5.74779201985554,-0.445126652101725,0.85485530548
3761,-0.520704087868799,0.602572926258327,0.902152428745749
"10880","AC124068.2",3.9020721369919,0.445412263819146,0.8551293724615
62,0.52087120167208,0.602456498171944,0.902152428745749
"10881","MRPL1",250.532642129073,0.117965993139445,0.226721414440524,0
.520312531705696,0.602845762878126,0.902229032849799
"10882","AHR",612.014194522117,0.121474148157842,0.233468010533116,0.5
20303179354036,0.602852280283456,0.902229032849799
"10883","PGAM5",34.8967552537665,-0.182593856228501,0.350883787417497,
-0.520382710105789,0.602796858428017,0.902229032849799
"10884","HNRNPH2",384.324261891302,-0.0949753222108103,0.1824611318016
55,-0.520523583697013,0.602698694788234,0.902229032849799
"10885","ZCCHC17",374.912976572432,0.0974968023464502,0.18754334435106
9,0.519862769237721,0.603159226292775,0.902293454415661
"10886","SYNC",217.532967617761,0.144807667252074,0.27841496288897,0.5
20114528865399,0.602983752566874,0.902293454415661
"10887","SP4",50.2430623219052,0.148034190622437,0.284852386918812,0.5
1968737992225,0.603281484314699,0.902293454415661
"10888","TARBP2",47.6922933193131,-0.140551848560714,0.270487019318885
,-0.519625115152064,0.603324889682638,0.902293454415661
"10889","HTR2A",12.2730463108638,-0.334799258711263,0.643905876866917,
-0.519950618156043,0.6030979939416,0.902293454415661
"10890","ZNF18",28.0747619270354,0.153918609380281,0.296115446970493,0
.519792570617293,0.603208158063951,0.902293454415661
"10891","RARA",270.069515894072,0.109177650008508,0.210116369271104,0.
519605637520039,0.603338468014941,0.902293454415661
"10892","AC125437.1",2.6980253662289,-0.467524663994429,0.899413921529
49,-0.519810348498258,0.60319576586806,0.902293454415661
"10893","NFYC-
AS1",3.91114168534172,0.374486050238029,0.720892168493796,0.5194758198
30954,0.603428970602037,0.902345956314751
"10894","AL034417.2",20.0922132027245,-0.289382632968302,0.55749826124
0271,-0.519073606300601,0.603709413083356,0.902354225591829
"10895","PYCR2",168.962868888182,-0.108580499440865,0.209106302537387,
-0.519259812465248,0.60357957397159,0.902354225591829

"10896","TM4SF19-
AS1",4.75109066448148,0.366814181958456,0.706674882775145,0.5190706375
72344,0.603711483240208,0.902354225591829
"10897","C1QTNF1",145.38386302341,-0.188044925330802,0.362216845615076
,-0.519150137844873,0.60365604712394,0.902354225591829
"10898","GREB1L",11.1414484578878,0.362350603098362,0.697776508210876,
0.519293210411227,0.603556287350346,0.902354225591829
"10899","AC145343.1",5.30624076792386,0.422004615246613,0.813494126718
194,0.518755577190297,0.603931199623393,0.90243420884923
"10900","AP000915.1",8.21793507183382,-0.342159011748868,0.65956285665
552,-0.518766343944641,0.603923690526045,0.90243420884923
"10901","RTL6",51.9445748198936,0.149249446134434,0.28769146232317,0.5
18783021676112,0.603912058996835,0.90243420884923
"10902","PRKX",36.2880211617699,0.194888993275698,0.375757805723754,0.
518655874361196,0.604000737729024,0.902455330844622
"10903","FNDC5",85.7509098422876,0.255201626467903,0.492387325555038,0
.518294467023963,0.604252832747091,0.902536500221627
"10904","B3GNT2",43.7482656189217,0.154356250771434,0.298063214680882,
0.517864141459704,0.60455306237474,0.902536500221627
"10905","GALNT13",22.651097357229,-0.318673240936374,0.614693959984167
,-0.518425853646882,0.604161180241233,0.902536500221627
"10906","SH3BP4",99.9435318889361,-0.158704985202726,0.306399179818424
,-0.517968048402664,0.604480562428113,0.902536500221627
"10907","RNF146",499.306738105278,-0.0854596654234792,0.16499600255206
2,-0.517949914553314,0.6044932148445,0.902536500221627
"10908","CPSF2",117.685155901795,0.109759384539573,0.211832669108544,0
.518141913622074,0.604359258577168,0.902536500221627
"10909","WDR20",144.055386699041,-0.0881027953767306,0.170105850584424
,-0.517929248606325,0.604507634110296,0.902536500221627
"10910","ABCC1",87.6241732647302,0.107065458192386,0.206744681226152,0
.51786318060231,0.60455373282081,0.902536500221627
"10911","ZNF236",91.2828213390539,0.144338067124452,0.278505278972584,
0.518259717219439,0.604277074513301,0.902536500221627
"10912","TAOK3",383.966043379672,-0.102030836776868,0.197150076679233,
-0.517528770444629,0.604787090490357,0.902787076421942
"10913","ZNF627",21.7365295539588,0.170264081705824,0.329035727473006,
0.517463811645781,0.604832424641945,0.902787076421942
"10914","RNF181",628.800151341199,-0.141235027558492,0.273173664766121
,-0.51701553178419,0.605145316594254,0.902805653652392
"10915","BYSL",43.4629441462849,-0.174689190076573,0.337865111028288,-
0.517038262828941,0.605129448953838,0.902805653652392
"10916","SLC31A2",215.002067217966,-0.141930880538352,0.27440727763328
9,-0.517227100397914,0.604997636150017,0.902805653652392
"10917","ARHGAP1",408.675432474359,-0.0882596170448218,0.1707110197556
46,-0.517011831873276,0.605147899372905,0.902805653652392
"10918","IFT43",252.855374803009,-0.129642604617776,0.250629781142457,
-0.517267357561501,0.604969537423245,0.902805653652392
"10919","DHX8",132.030205039163,-0.135692972667809,0.262477689506433,-
0.516969548623232,0.60517741618457,0.902805653652392
"10920","ARMH1",8.41347604738537,-0.275137918562134,0.533235890687869,

-0.515977869020048,0.605869866262486,0.903137271277513
"10921","DHX9",293.384211984723,-0.0818381406107904,0.158619932626517,
-0.515938566204567,0.60589731714626,0.903137271277513
"10922","PRADC1",138.070039269489,-0.167967690915342,0.325245162153452
,-0.516434094832421,0.605551258034489,0.903137271277513
"10923","MAIP1",132.117710469423,-0.135729471283557,0.263297540969224,
-0.515498438701414,0.606204760335387,0.903137271277513
"10924","CAMK1",253.491451747262,-0.119896896194175,0.232601594064575,
-0.515460337562818,0.606231378485379,0.903137271277513
"10925","DALRD3",278.358422846452,-0.115054572840216,0.22319646026249,
-0.515485651989759,0.606213693306962,0.903137271277513
"10926","TMED9",940.042228469162,-0.128841841648134,0.249633553012503,
-0.516123894778205,0.605767880071439,0.903137271277513
"10927","AL645933.2",13.8441050831986,-0.283330401846665,0.54943886358
0704,-0.515672298825378,0.606083305119556,0.903137271277513
"10928","MSR1",55.4104597251991,-0.202983517652019,0.393274108852263,-
0.516137505833803,0.605758374333864,0.903137271277513
"10929","MTSS1",226.012169859269,-0.216881783338751,0.420164496387661,
-0.516183031177978,0.60572658066781,0.903137271277513
"10930","IPPK",31.3313519363541,-0.151917356402773,0.294664338068364,-
0.515560713585664,0.606161255091596,0.903137271277513
"10931","AL353801.3",4.59467662250744,0.425537850321554,0.825470688917
152,0.515509340349531,0.606197144341472,0.903137271277513
"10932","KLHDC1",99.9569947172141,0.117145823325419,0.226805388356638,
0.516503704670431,0.605502652155372,0.903137271277513
"10933","AC011472.4",4.65518020809124,-0.380552976998416,0.73762151173
9034,-0.515919032921389,0.605910960291625,0.903137271277513
"10934","AL022328.2",3.1769296362445,0.461356936301266,0.8939407710438
79,0.516093404893623,0.605789173948164,0.903137271277513
"10935","LAMB2",1982.30083137976,0.0778350065725849,0.151052935130613,
0.515282980137278,0.6063552904961,0.903156668516001
"10936","LRRN3",106.461591973247,0.442394264744026,0.858423714412688,0
.515356527687147,0.606303904609405,0.903156668516001
"10937","DCTN1",287.350635785178,-0.109305311285294,0.212660918723557,
-0.51398871001485,0.607259884087667,0.903278904208995
"10938","RPIA",199.298573668648,0.0936913878578814,0.182077617744271,0
.514568396811253,0.606854653858248,0.903278904208995
"10939","PLXNB1",45.312857104638,0.148045380686889,0.287610952709872,0
.514741804135079,0.606733456895891,0.903278904208995
"10940","NFKB1",370.190873428489,0.0942423218677111,0.183214851716878,
0.514381454257562,0.606985322908402,0.903278904208995
"10941","STK19",237.825089461664,-0.0975839475609892,0.189509476028447
,-0.514929119145162,0.606602551798208,0.903278904208995
"10942","TMEM63B",70.2145764757409,0.128354609985865,0.249520995689076
,0.514404046967677,0.606969530395049,0.903278904208995
"10943","IFRD1",849.014041420395,0.178676092588506,0.347779956633017,0
.513761903700068,0.60741846598221,0.903278904208995
"10944","FUT10",79.2454446680874,-0.116495736152127,0.226407356747463,
-0.514540418764162,0.606874209145514,0.903278904208995
"10945","MROH1",55.931136068203,0.146472473862894,0.284895734311959,0.

514126595179228,0.607163484472629,0.903278904208995
"10946","APBA1",27.2984366882072,-0.16568210364804,0.322377436549289,-0.513938275027846,0.60729514639217,0.903278904208995
"10947","BRD3",323.920722275757,0.092967358297596,0.180922784025885,0.51385102654785,0.607356149507311,0.903278904208995
"10948","HSPA14",62.2202060789896,0.109321346770992,0.212545184890291,0.514344029141004,0.607011483814873,0.903278904208995
"10949","CYB561A3",228.628512356837,-0.122814203309477,0.238454579193974,-0.51504233520956,0.606523436871426,0.903278904208995
"10950","HDAC5",209.986961759986,-0.111374718074174,0.216437994481653,-0.514580253531292,0.606846366675467,0.903278904208995
"10951","AP000897.1",3.77743236619982,0.400372996019757,0.779165204020463,0.513848660019528,0.60735780419429,0.903278904208995
"10952","AC027307.2",26.7380279665094,-0.164216166728826,0.3196499383777,-0.513737520370763,0.607435515790124,0.903278904208995
"10953","ZNF573",41.4580578120169,0.148249391723328,0.288365024237939,0.514103234659286,0.607179816025408,0.903278904208995
"10954","AC074212.1",20.4840707971317,0.183780180880093,0.357717108478935,0.513758432358893,0.607420893271018,0.903278904208995
"10955","KLHDC4",73.80445352341,-0.114259131049283,0.222479217613037,-0.513572154177639,0.607551152141795,0.903368390437034
"10956","TNFSF4",10.7548249948932,0.329950896113843,0.642639724762558,0.513430594779608,0.607650148812371,0.903433121030003
"10957","FAAP24",31.6930199110851,0.189966684179239,0.370069651379844,0.513326838531417,0.607722713192855,0.903458544784012
"10958","HLA-DRB5",92.3669354474246,1.00970777516457,1.96762039424431,0.513161877218882,0.607838090736345,0.903491279364883
"10959","CD79B",4.70902023987142,0.499811551850635,0.974031874701578,0.513136751303715,0.60785566520718,0.903491279364883
"10960","TMEFF1",12.9237670896043,0.235898903190128,0.459893243361292,0.51294274615990,0.607991370887789,0.903610532882409
"10961","C11orf95",149.969233669265,-0.11056970404004,0.215706864459321,-0.512592421744147,0.608236455369241,0.903644985544469
"10962","DCAF4",38.712297382438,0.131626220428684,0.256745392813571,0.512672180740008,0.608180652669811,0.903644985544469
"10963","MSI2",75.6033873822885,0.132249845013194,0.257886810767056,0.512821282406149,0.608076341083977,0.903644985544469
"10964","SLC13A3",11.4643789128249,-0.242158782542337,0.472391926713832,-0.512622610269615,0.608215333957183,0.903644985544469
"10965","TRHDE",40.2478640569034,0.504074893689532,0.983584197158068,0.512487792245939,0.608309661962436,0.903671325463395
"10966","CLDN12",78.2399108665912,0.103733328202464,0.202588276463497,0.512040133877911,0.608622921433267,0.903806980328788
"10967","CUTC",747.329169598951,-0.14337431265165,0.280005766818777,-0.512040570737399,0.608622615695552,0.903806980328788
"10968","TMC5",10.1365458111889,0.471340873456209,0.920175755305717,0.512229180934692,0.608490622546801,0.903806980328788
"10969","LINC01521",2.65654594639025,0.47004786015584,0.917885866866402,0.512098374235296,0.608582162308834,0.903806980328788

"10970","STX18",178.708548541898,-0.0965767822148111,0.188693517405693
,-0.511818230655852,0.608778230056074,0.903904142565259
"10971","USP7",324.022455737618,-0.0933544544226849,0.182408418841608,
-0.511788079824034,0.608799333788659,0.903904142565259
"10972","DDX49",192.235938899561,-0.110215087329636,0.215415983476393,
-0.511638391687469,0.608904111122575,0.90397731189169
"10973","VAMP3",434.845640492179,-0.0931522549968898,0.184027865210518
,-0.506185597981744,0.612726364369693,0.904071152014923
"10974","CROCC",245.425504772595,-0.118239716368979,0.231563788193319,
-0.510614018243078,0.609621357961228,0.904071152014923
"10975","TRIM62",11.9058211850749,0.246652533474382,0.483812752079228,
0.509809905618178,0.610184645286314,0.904071152014923
"10976","AC245014.3",10.8819302936204,0.274406735565823,0.539513362838
204,0.508618978633371,0.611019323427793,0.904071152014923
"10977","SF3B4",199.47607179165,-0.122244278779606,0.239596287534167,-
0.510209402815448,0.609904765418291,0.904071152014923
"10978","VANGL2",36.6564853698674,-0.15570246374406,0.306549643888331,
-0.507919245212951,0.611509977489922,0.904071152014923
"10979","NIT1",184.962808517222,0.0898252178431097,0.177448093803971,0
.50620559464753,0.612712327924075,0.904071152014923
"10980","RGS13",2.75907702244664,-0.634850095720208,1.25232711582417,-
0.506936316956139,0.612199502710427,0.904071152014923
"10981","LPGAT1",196.255044756464,0.0998291926161987,0.197180783748171
,0.506282563232406,0.612658301975332,0.904071152014923
"10982","TATDN3",103.360251497825,-0.109645529511632,0.216686344371363
,-0.506010334106328,0.61284939505134,0.904071152014923
"10983","RBM34",17.7920417429219,0.186707949375786,0.367276354653716,0
.508358207682122,0.611202155987766,0.904071152014923
"10984","AC104794.5",8.15962616441489,-0.319867980513047,0.62621289200
8968,-0.510797501288852,0.609492859011701,0.904071152014923
"10985","PROM2",2.96266176158176,-0.469696249808521,0.924892707077136,
-0.507838634918924,0.611566512748949,0.904071152014923
"10986","STARD7-
AS1",13.0780765033822,0.203675922991091,0.401182602786385,0.5076888219
39123,0.611671588797464,0.904071152014923
"10987","CERS6",25.9026505264184,0.201840134911103,0.396841552716886,0
.508616432753199,0.611021108286214,0.904071152014923
"10988","TNS1",461.783468092956,-0.113055952121816,0.222302507337237,-
0.508568047549314,0.61105503048651,0.904071152014923
"10989","DNAJB2",224.609845593619,-0.0942168096778872,0.18616445657763
7,-0.506094511325773,0.612790303470883,0.904071152014923
"10990","ARL4C",165.898811098669,0.238181531353313,0.470467120201672,0
.50626605160253,0.612669891675461,0.904071152014923
"10991","GALNT15",1001.6638856826,-0.368721572143662,0.721932656926306
,-0.510742336707039,0.609531491237975,0.904071152014923
"10992","MYD88",107.795289884441,0.102517077406974,0.202495671002581,0
.506267995258363,0.612668527396375,0.904071152014923
"10993","DUSP7",52.8871797969807,-0.129907809348094,0.255436452148926,
-0.508571929555121,0.611052308834884,0.904071152014923
"10994","MELTF-

AS1",28.1301343153382,0.189889541545466,0.37527134367475,0.50600597340
0528,0.612852456292515,0.904071152014923
"10995","METAP1",91.7251924725279,0.117365820559698,0.230332138246368,
0.509550345224344,0.610366518740254,0.904071152014923
"10996","PLRG1",293.160484982285,-0.102931428719652,0.202101838730645,
-0.509304761233942,0.610538621104024,0.904071152014923
"10997","MIER3",57.5911707665537,0.13092793173853,0.258202628869267,0.
50707435595019,0.612102647454995,0.904071152014923
"10998","CDC42SE2",128.222452530712,-0.103037429122586,0.2022398809991
22,-0.509481258659528,0.610414931613371,0.904071152014923
"10999","GM2A",135.882338081609,0.111920658292357,0.219966901915261,0.
50880681283347,0.610887643537938,0.904071152014923
"11000","HMGN4",336.304409273726,-0.103441562969479,0.203921072232922,
-0.507262745516201,0.61197047458918,0.904071152014923
"11001","SUMO4",16.1632109896521,-0.207443645688361,0.408881587722401,
-0.507344062235445,0.611913427222363,0.904071152014923
"11002","TOMM7",5056.77244086037,-0.124448413143656,0.244328576993592,
-0.50934857753835,0.610507913576705,0.904071152014923
"11003","HOXA7",117.083720414685,0.192564690367401,0.380385779441644,0
.506235250566046,0.612691511530948,0.904071152014923
"11004","MPLKIP",375.336104383209,-0.119863960619853,0.236545412372399
,-0.506727056837393,0.612346343293876,0.904071152014923
"11005","ZNF680",152.423978256611,0.107883318695564,0.21229493707248,0
.508176597064729,0.611329501721489,0.904071152014923
"11006","PRKRIP1",169.844868217049,0.0989387935053075,0.19365161503578
8,0.51091127480152,0.609413185890016,0.904071152014923
"11007","EIF4EBP1",260.843095184867,0.151906378197816,0.29757342322570
4,0.510483686853305,0.609712640447257,0.904071152014923
"11008","POLB",42.3600913841571,-0.132231313569432,0.260417439708935,-
0.507766736810043,0.611616939752968,0.904071152014923
"11009","RAB2A",1519.1010451523,-0.0785517637154544,0.154898036524701,
-0.507119169989791,0.61207120507834,0.904071152014923
"11010","TSNARE1",12.7401951282418,-0.257497033878211,0.50613593243235
,-0.508750747335308,0.610926946557165,0.904071152014923
"11011","DPP7",1082.30103137799,-0.111699773715828,0.220066801733869,-
0.50757212280891З,0.611753444943069,0.904071152014923
"11012","AKR1C8P",8.1333118473897,0.29148322928466,0.574890209458115,0
.507024166508956,0.61213786216317,0.904071152014923
"11013","UPF2",304.096817766486,-0.10268871560644,0.202509689496955,-0
.507080504945342,0.612098333161557,0.904071152014923
"11014","OPTN",944.199950587994,-0.0744991217919808,0.146749066947336,
-0.507663342205213,0.611689460606064,0.904071152014923
"11015","PLXDC2",1107.16379735659,0.164469462401781,0.322122806968428,
0.510579998819834,0.609645184156763,0.904071152014923
"11016","AL158211.1",3.09426462998511,-0.404698380471098,0.79771077987
2187,-0.507324697976303,0.611927011914972,0.904071152014923
"11017","JMJD1C-
AS1",12.2190894854139,-0.246673422137668,0.486158914998366,-0.50739257
1703631,0.611879396750927,0.904071152014923
"11018","KAT6B",542.330956820923,0.106597558283547,0.208570889187396,0

.511085505263254,0.609291185055539,0.904071152014923
"11019","TMEM216",136.308962540881,-0.130307452756323,0.257345972105483,-0.506351242610129,0.612610096181415,0.904071152014923
"11020","FADS3",118.036105538565,-0.150310514565945,0.295786643198307,-0.508172082892773,0.611332667217747,0.904071152014923
"11021","CWF19L2",423.989797991958,0.134895051456557,0.265286297849533,0.508488574608053,0.611110749682971,0.904071152014923
"11022","COPS7A",274.691799405178,0.102471852938607,0.20042219390792,0.51127996825384,0.609155029691521,0.904071152014923
"11023","OSBPL8",1042.71340944782,0.0884028014914803,0.174116051770259,0.507723444178055,0.611647304690358,0.904071152014923
"11024","ATP8A2",2.47118247237647,0.579839408893688,1.13392483425296,0.511356124655026,0.60910171165283,0.904071152014923
"11025","FGF14",78.2679263972637,-0.148581937575011,0.291117308852701,-0.510385102694771,0.609781691601525,0.904071152014923
"11026","AL136295.2",8.74324048308987,-0.262792295130759,0.516785615123871,-0.508513177302292,0.611093500271319,0.904071152014923
"11027","PPP2R5E",438.303468098308,0.0794315197161247,0.156678600415202,0.506971082876854,0.612175108514368,0.904071152014923
"11028","RAD51B",101.063117620639,0.126767495942569,0.249835866193915,0.507403111786106,0.611872002759283,0.904071152014923
"11029","NOP10",850.254232343904,-0.13351226705333,0.262780457239386,-0.508075328188136,0.61140051676778,0.904071152014923
"11030","FAM219B",154.435011695857,-0.0942064031635452,0.184835653531274,-0.509676576806139,0.610278065515558,0.904071152014923
"11031","MT1X",4492.29440702428,-0.218501990283997,0.428813127547165,-0.509550608988677,0.610366333908896,0.904071152014923
"11032","SDF2",578.895076748944,-0.119757333672198,0.234776847570187,-0.510090048961904,0.609988376403672,0.904071152014923
"11033","PEX12",30.2083702334052,0.1530097399382,0.302238139712557,0.50625556418432,0.612677252963462,0.904071152014923
"11034","TBX2",16.6033335899205,0.239870900475876,0.471259487335113,0.508999621062914,0.61075248972713,0.904071152014923
"11035","PRPSAP1",212.603517495948,-0.124335442395384,0.244144070011063,-0.509270785850955,0.610562432346969,0.904071152014923
"11036","AC104532.2",19.7968363297123,-0.182038462920939,0.356128347373586,-0.511159710434331,0.609239227906295,0.904071152014923
"11037","LINC01480",5.68318924365063,0.373248460044741,0.733275951879097,0.509015001907881,0.610741708705545,0.904071152014923
"11038","ZNF132",6.8602074428701,-0.303906507254417,0.598028531693603,-0.50818061538596,0.61132668393932,0.904071152014923
"11039","ATP5F1E",4374.05651351219,-0.137765378622116,0.270888235769157,-0.508569071783224,0.611054312401568,0.904071152014923
"11040","DGCR8",95.3169026166929,0.125302408699673,0.245748172841637,0.509881344185698,0.610134592646323,0.904071152014923
"11041","YIPF6",390.213332402995,-0.0878628585818317,0.173457332459176,-0.506538739735955,0.612478501198017,0.904071152014923
"11042","RAP2C-AS1",46.6048506165167,-0.131802949569935,0.259555524914975,-0.507802519761856,0.611591842519829,0.904071152014923

"11043","DHX36",779.810030725421,0.0839868908345331,0.166026997916049,
0.505862852962029,0.612952931432599,0.904093964408479
"11044","CCDC120",20.7175149215517,0.175171257596395,0.346307461492368
,0.505825825529478,0.612978927062879,0.904093964408479
"11045","HYI",578.460782664576,-0.123091258198442,0.243990131493766,-0
.50449277372346,0.613915138941456,0.904158713011274
"11046","MRPS14",277.911406717907,0.118710823309259,0.23534608137403,0
.504409602302212,0.613973571716025,0.904158713011274
"11047","MEIS1",118.79533566494,0.120275475870312,0.238011149886295,0.
505335468223953,0.61332323522103,0.904158713011274
"11048","AC012459.1",6.12382917137322,-0.340263116634768,0.67428862802
614,-0.5046253228841,0.613822020492214,0.904158713011274
"11049","PARL",309.261805064909,-0.122074648741044,0.241505554897582,-
0.505473461232865,0.613226333736982,0.904158713011274
"11050","AIMP1",457.999334538862,-0.0658525987813429,0.130454890169212
,-0.504792106266969,0.613704860662902,0.904158713011274
"11051","CRYBG1",52.0536776484198,-0.20202757535243,0.400065978667942,
-0.50498564268099,0.613568920074632,0.904158713011274
"11052","SLC22A3",7.28582187051945,0.4050925092064,0.803029231973855,0
.504455495612131,0.613941328685824,0.904158713011274
"11053","ZNF33B",62.1290537801933,-0.15162080679826,0.3006316900644,-0
.504340732561427,0.614021958581295,0.904158713011274
"11054","SEMA4G",4.91303666778921,-0.331200739447998,0.656089747471941
,-0.504810112830124,0.613692212235664,0.904158713011274
"11055","ATG16L2",37.8842473821007,-0.162366951717386,0.32177879601677
3,-0.504591830559657,0.613845548935243,0.904158713011274
"11056","KRR1",568.453773708713,-0.0911018995021421,0.180363622993146,
-0.505101294763877,0.613487692027702,0.904158713011274
"11057","COL4A1",1213.02918720177,0.186226166991294,0.368567245463616,
0.505270528739043,0.613368839380193,0.904158713011274
"11058","CMC2",184.894890941754,0.136804904114561,0.270694122151451,0.
505385573307793,0.613288049635528,0.904158713011274
"11059","AC005899.8",6.39673984619047,-0.397221787578919,0.78738274239
8391,-0.504483736040455,0.613921488322181,0.904158713011274
"11060","PARD6B",6.39931820019331,0.324210855557837,0.642465992281331,
0.504635045983676,0.613815190064531,0.904158713011274
"11061","TCEAL1",209.072194472973,-0.107179240917686,0.212446708777522
,-0.504499417921913,0.613910471110255,0.904158713011274
"11062","LONRF3",41.975494381074,-0.254944289588697,0.504952773483789,
-0.504887393388843,0.613637929027615,0.904158713011274
"11063","DNAAF2",91.3190848559406,-0.123022242271761,0.24409510774284,
-0.503993068150174,0.614266248128569,0.904436673213981
"11064","MLX",214.834857616018,-0.114095225242608,0.226482854401409,-0
.503769813146163,0.614423142851616,0.904585915935463
"11065","SNX17",599.890686025973,-0.113246645362126,0.22484807979629,-
0.503658494503162,0.614501379752854,0.904597493715913
"11066","HOXA10",438.537546761074,-0.103154429259871,0.204833811889831
,-0.503600593613678,0.614542075355166,0.904597493715913
"11067","TMEM107",156.295259745467,0.106748488705438,0.212013290795294
,0.50349904152239,0.614613454041864,0.904620814393053

"11068","SAMSN1",32.297097667372,-0.236248813002234,0.46943417866655,-0.50326291467168,0.614779436416311,0.90478336102144
"11069","FAM98A",147.320221227168,0.123772271196268,0.246256624765847,0.502614990820885,0.615234987570802,0.904981188705051
"11070","LINC01943",31.4200396929842,-0.20951699539697,0.416933798132167,-0.502518616470027,0.615302760446217,0.904981188705051
"11071","TRMT44",46.4965666113141,0.126652317507871,0.252023345531987,0.5025420055452б,0.61528631235667,0.904981188705051
"11072","FLOT1",718.165085093749,-0.0892278712252629,0.177443799789378,-0.502851445534723,0.615068720558148,0.904981188705051
"11073","SYNE3",602.216079618526,0.130208361407076,0.259082527876033,0.502574845453795,0.615263218407068,0.904981188705051
"11074","ANKRD12",1508.09358534604,-0.111328561001035,0.221372371535151,-0.502901785932022,0.615033325433674,0.904981188705051
"11075","DAPK3",175.220352160583,-0.103378939624737,0.205674340643877,-0.502634112262632,0.615221541280724,0.904981188705051
"11076","FMO1",190.06024769063,0.262362538267182,0.522817762018602,0.501824072032668,0.615791278655967,0.905049868239486
"11077","CLSTN2",128.681831273575,0.182442259521728,0.3633869383593,0.502060586837431,0.615624903302711,0.905049868239486
"11078","PGM2",82.0726518985725,-0.130354417189262,0.259483438532244,-0.502361221689546,0.615413451484895,0.905049868239486
"11079","AC093849.4",5.27942012674465,0.354522495158901,0.70655711681127,0.50176056078648,0.615835958741046,0.905049868239486
"11080","AC113370.1",4.48383449993768,0.397093726921535,0.791394501231905,0.501764576710362,0.615833133501268,0.905049868239486
"11081","ZMIZ1-AS1",12.8611401122029,-0.242657083835935,0.483330735572759,-0.502051837337387,0.615631057750193,0.905049868239486
"11082","LOXL1",621.660697654042,-0.168390297894023,0.335611799294739,-0.501741292314161,0.615849514369603,0.905049868239486
"11083","GGA2",157.738619402339,0.095056197224615,0.189413834065854,0.501844005710621,0.615777255631174,0.905049868239486
"11084","WIZ",128.646727082747,0.0863771564355442,0.172006623577347,0.502173431691731,0.615545530021259,0.905049868239486
"11085","AC007541.1",60.9020537587257,-0.143447994030646,0.285948892783711,-0.501656056906466,0.615909480193478,0.905056339456163
"11086","BUD23",283.381597657091,-0.122294454334634,0.243891799957494,-0.501429135198261,0.616069139341522,0.905209291965908
"11087","C1S",15101.3186980173,0.101246632692536,0.202019327242764,0.501173001981486,0.616249373151404,0.905229149541277
"11088","IL34",124.450897064106,0.206571837023879,0.412113651623479,0.5012496824847,0.616195412790318,0.905229149541277
"11089","NAP1L2",6.99541331240137,0.341764693472931,0.681813870262333,0.501258053523368,0.616189522184203,0.905229149541277
"11090","AL360012.1",85.1562740458489,0.207963289983011,0.415460800159218,0.500560558067844,0.616680426662746,0.905326134648026
"11091","MUTYH",63.3185933648474,-0.131747472498723,0.263266194862881,-0.500434446463366,0.616769203612078,0.905326134648026
"11092","AC008280.3",13.4823218212949,0.225596838206612,0.450867552799

321,0.500361662323978,0.616820442952606,0.905326134648026
"11093","ATG9A",135.776909367485,-0.0945608693153911,0.188966894696188
,-0.500409711803866,0.616786616367317,0.905326134648026
"11094","PAICS",123.871750363503,-0.114438748846871,0.228495083549832,
-0.500836810442415,0.616485976887007,0.905326134648026
"11095","PVT1",19.5625909421505,-0.205096777652426,0.409792928841125,-
0.500488815735376,0.616730929380436,0.905326134648026
"11096","PLEKHA7",3.37483447005547,0.435367673535966,0.870301801891301
,0.50024907749225,0.616899705276932,0.905326134648026
"11097","SIX1",235.506135510432,-0.112156661865985,0.223870346572546,-
0.500989360954244,0.616378610468834,0.905326134648026
"11098","CCDC9B",17.5270425234482,0.259994381021551,0.519537590644797,
0.500434204768268,0.616769373760035,0.905326134648026
"11099","AC116667.1",11.5268135097372,0.273875262362217,0.547485659667
297,0.500241892232665,0.616904764017347,0.905326134648026
"11100","PLPPR2",119.195562066624,-0.101867167922987,0.203648542218433
,-0.500210641398672,0.616926766197623,0.905326134648026
"11101","SSPN",3033.78980960362,-0.124408148566339,0.248827657848931,-
0.499977171516319,0.617091151778844,0.905404230888632
"11102","AC018529.1",3.32143273788413,-0.431570858781867,0.86305545642
3445,-0.500049974274334,0.617039889473167,0.905404230888632
"11103","TBC1D23",295.594739192095,0.0747905530005247,0.14975796892221
9,0.499409504140438,0.617490925049359,0.90550141142681
"11104","CEP44",145.972633063048,0.0840096136178807,0.168091045338223,
0.499786371420569,0.617225507915995,0.90550141142681
"11105","CNOT8",200.410453067285,0.0923601312257975,0.184937612462166,
0.499412369372361,0.617488906957778,0.90550141142681
"11106","DDHD2",128.741283525378,0.106835429683147,0.213888088162647,0
.499492190523044,0.617432687052947,0.90550141142681
"11107","ERVK3-1",258.54275907042,-0.107248837084417,0.214617244538554
,-0.499721433452429,0.617271238355279,0.90550141142681
"11108","KRBOX4",119.571751221163,0.115275998572794,0.230807696167539,
0.499446077782074,0.617465165062394,0.90550141142681
"11109","MRPS10",346.524106982836,-0.0973480673787222,0.19502307335856
5,-0.499161795075089,0.617665406860036,0.905512718238064
"11110","TMEM62",35.380637354353,-0.141247782192555,0.28295062606122,-
0.499195863811214,0.617641408175209,0.905512718238064
"11111","LRRC4B",15.0278018262626,0.298604684123576,0.5980874753631,0.
499265904109249,0.617592071699885,0.905512718238064
"11112","CCDC25",510.056273970086,-0.0846063104649848,0.16955663976489
4,-0.49898553416899,0.617789575011029,0.905613245802254
"11113","CALCRL",23.7027667271672,-0.204911522613198,0.410976860324894
,-0.49859625296472,0.61806384539907,0.905933769252718
"11114","CREG1",1067.14700281403,0.0763655708137584,0.153213957134213,
0.498424374920774,0.618184960041891,0.906029765531974
"11115","AC083798.2",23.5480636746449,-0.256661000495941,0.51539306759
4843,-0.497990789231385,0.618490534260167,0.906345285315887
"11116","NDUFB5",833.474057514229,-0.119112260316759,0.239370100087034
,-0.497607095762799,0.618761001430437,0.906345285315887
"11117","TAF7",791.315311388732,0.0622185451114807,0.125043944034388,0

.497573437817749,0.618784729524444,0.906345285315887
"11118","FAM24B",32.9424667808876,-0.15144886218602,0.304427521348157,
-0.497487420044445,0.618845371924814,0.906345285315887
"11119","CUL4A",184.492175541996,-0.0861284404870897,0.173007102040775
,-0.497831820030085,0.618602586063471,0.906345285315887
"11120","SORD",67.932846052614,-0.121170611442983,0.243447802498443,-0
.49772727541362,0.618676280723426,0.906345285315887
"11121","DNAAF4",42.9051798034166,0.136814082661943,0.274868401512233,
0.497743945499877,0.618664529537686,0.906345285315887
"11122","AEN",20.1616708412056,-0.174096705752204,0.349915963023195,-0
.49753862112505,0.618809274927613,0.906345285315887
"11123","EPHB4",45.1674095382956,-0.124813484099567,0.250975198575289,
-0.497314016715976,0.61896762889818,0.906442839802432
"11124","GPR157",24.4847904191129,-0.188656907298299,0.379779229378069
,-0.496754147422033,0.619362433337101,0.90649984782785
"11125","ACBD3",525.540025896731,-0.0950579575774795,0.19135249853004,
-0.496768833998562,0.619352075363977,0.90649984782785
"11126","PAPOLG",84.8503045319531,0.128344378280182,0.258431885875194,
0.496627487918284,0.619451765373675,0.90649984782785
"11127","ZNF623",74.7446075919649,0.115271265111773,0.232059968371357,
0.496730504277706,0.619379108250039,0.90649984782785
"11128","RNF34",112.728789893882,-0.0901346925908961,0.181473092362626
,-0.496683510582305,0.619412252298734,0.90649984782785
"11129","NEMF",434.291141852647,-0.0888849469612108,0.178949257719067,
-0.496704753594181,0.619397269779976,0.90649984782785
"11130","NSF",123.885308089985,-0.111492892488737,0.224381973301876,-0
.496888813517731,0.619267460473501,0.90649984782785
"11131","MBOAT7",132.079763624578,0.108641717222581,0.218542751338294,
0.497118831703593,0.619105255529743,0.90649984782785
"11132","AC097468.3",10.340938642918,0.24016889513575,0.48408960067520
4,0.49612488018905,0.619806306370034,0.906906049662981
"11133","UBE2D3-
AS1",49.5944974055469,-0.138816732966149,0.279829489902013,-0.49607613
9133005,0.619840693160904,0.906906049662981
"11134","AC009318.3",4.05106686086174,-0.361860559671983,0.72977108069
4907,-0.495854890998709,0.619996794062153,0.907029002696086
"11135","TMEM191B",7.59051168156735,0.38182210923994,0.770114423260148
,0.495799192571361,0.620036094605004,0.907029002696086
"11136","AC080013.5",4.70746246105854,0.355365167518781,0.716998905556
573,0.495628605238842,0.620156466959348,0.907123625206611
"11137","AL050403.2",31.2351130847374,0.209521911600227,0.422850735053
971,0.495498515743349,0.620248269471804,0.907176444412877
"11138","RWDD3",196.594893736563,-0.113665427183663,0.229529282300301,
-0.495211007695961,0.620451181256315,0.907216102274053
"11139","HTT",172.599704298186,-0.120387096892869,0.243090659784612,-0
.495235386664125,0.620433974425595,0.907216102274053
"11140","TMEM67",68.186115379912,0.145954903687679,0.294698298026223,0
.495268906082015,0.620410316546525,0.907216102274053
"11141","ERP29",1355.33890392705,-0.111139967166743,0.224459687967868,
-0.495144442964087,0.62049816412519,0.907216102274053

"11142","RPS9",21843.3097257875,-0.130512872058709,0.263639130753562,-0.495043629091264,0.620569323748424,0.907238710692702
"11143","INPP1",175.313889976824,-0.0864882041269965,0.174809625418454,-0.49475653254198,0.620771990734151,0.907453554434945
"11144","CNNM1",6.16642224180639,0.41966527843242,0.848645012221748,0.494512160430588,0.620944520398236,0.907624308396165
"11145","COQ6",86.198519132885,-0.0926687990861602,0.187426943843098,-0.494426239824604,0.621005186339176,0.907631537037132
"11146","ENTPD3-AS1",79.2641062398223,-0.120199051677,0.243357190887672,-0.493920279234654,0.621362482069405,0.907778589739108
"11147","NEK4",60.126466424975,-0.134173894153711,0.271666039672774,-0.493892774802937,0.621381907517494,0.907778589739108
"11148","RPS10-NUDT3",10.5927558903764,0.444120042926763,0.899224898622642,0.49389206594149,0.621382408165801,0.907778589739108
"11149","ACAT1",493.618658738312,0.0869099943239889,0.175892882570445,0.494107510513857,0.621230253974645,0.907778589739108
"11150","ANAPC11",1168.21388791768,-0.112390985315313,0.227563166832006,-0.493889177585067,0.621384448130091,0.907778589739108
"11151","MRPL52",340.479119441901,-0.108284326586935,0.219319115987751,-0.493729541537014,0.621497199071657,0.907861884645164
"11152","TSC22D2",498.559363446347,-0.225386441317117,0.456765712129454,-0.493439930651448,0.621701773926517,0.908079285822188
"11153","HMGA2-AS1",20.4325601578812,-0.264987380909563,0.53713602088159,-0.493333849542701,0.621776714639976,0.908107316844845
"11154","WFDC3",11.7537920773755,-0.343701078256613,0.69699969922978,-0.49311510268429,0.621931260150198,0.90825159553403
"11155","YIPF5",485.423984390512,-0.0848495405577691,0.172177203039725,-0.492803571319442,0.622151387053493,0.908476093616869
"11156","CYBA",2254.91595306479,0.145510202030483,0.295308467685214,0.492739687321093,0.622196531425489,0.908476093616869
"11157","ZMYM1",125.712393023873,0.0886363510146049,0.180780817176265,0.490297324677886,0.623923519865033,0.908526837595638
"11158","ATAD2B",137.140956701697,0.120468215013012,0.245760661908285,0.490185101543914,0.624002922343317,0.908526837595638
"11159","UBXN2A",283.522420924151,0.0644639764294093,0.131298926678601,0.490971084532984,0.623446898962656,0.908526837595638
"11160","KHK",27.9987176447928,-0.217756629995603,0.443272980133613,-0.491247244372906,0.623251587726803,0.908526837595638
"11161","AC068193.1",2.92757618395886,0.431776237436261,0.878974040080013,0.49122751952601,0.623265537045517,0.908526837595638
"11162","CEP68",226.406172982818,0.0949345808262363,0.19331634399853,0.491084089749587,0.623366973971558,0.908526837595638
"11163","CCDC150",9.4079037453924,0.242427327616616,0.493151125496367,0.491588308497943,0.623010410163897,0.908526837595638
"11164","NAA15",182.271804138142,-0.0808809310051319,0.164533994524763,-0.491575806195838,0.623019250235562,0.908526837595638
"11165","GPBP1",674.871612206099,0.0638645054162642,0.130206126004927,

0.490487716483075,0.623788819833765,0.908526837595638
"11166","FOXC1",87.4423401582067,0.155870282889508,0.317397119426244,0
.491089154089593,0.623363392228977,0.908526837595638
"11167","SRSF3",2765.73338686643,0.0961992422626312,0.195949500950603,
0.490938950065926,0.623469627457733,0.908526837595638
"11168","CASP8AP2",226.829349761189,0.105681139466175,0.21484439574841
3,0.491896188858143,0.622792732646105,0.908526837595638
"11169","MDFIC",420.948049745427,0.127968456734285,0.260820883068661,0
.490637311049198,0.623682992207954,0.908526837595638
"11170","AC016831.5",166.643805601291,-0.252075608208993,0.51364991666
8171,-0.490753721608875,0.623600645304628,0.908526837595638
"11171","VPS13A",115.552897072348,-0.130851707117109,0.266393565573031
,-0.491196950780842,0.623287155385,0.908526837595638
"11172","ZNF79",11.3027816806161,-0.241150008199179,0.489729077301047,
-0.492415131909635,0.622425904235899,0.908526837595638
"11173","CUBN",23.1625787959577,-0.191800191019154,0.391170721941144,-
0.490323483484054,0.6239050120627,0.908526837595638
"11174","PDE3B",3.39637070836922,0.419274691412147,0.853030028257178,0
.491512229960727,0.62306420427514,0.908526837595638
"11175","AP001107.4",2.40839376952597,0.466808426114768,0.950986539924
935,0.490867542827278,0.623520134606921,0.908526837595638
"11176","SENP1",56.1092375922264,0.118697541449716,0.241983442542349,0
.490519269428703,0.623766497699426,0.908526837595638
"11177","JKAMP",286.881960742973,-0.107966732774572,0.220300863454323,
-0.490087651412942,0.624071875858407,0.908526837595638
"11178","AL121612.2",4.75154979777463,0.365027768509965,0.741724415414
088,0.492133952886232,0.622624651322089,0.908526837595638
"11179","UBALD1",62.3441348173546,-0.139229842710544,0.283741352689055
,-0.49069281368769,0.62364372997334,0.908526837595638
"11180","NPIPA9",7.84629940644864,-0.317904700911586,0.648593289763781
,-0.490144911963809,0.624031359184835,0.908526837595638
"11181","GTF3C1",164.894768929725,0.0753760432870227,0.153599291184281
,0.490731713055824,0.623616213430222,0.908526837595638
"11182","ADCY7",114.071466129005,0.12763792793127 9,0.260259592270093,0
.490425451058183,0.623832870527939,0.908526837595638
"11183","GAN",12.9509101187824,0.206588874458836,0.41995895178346,0.49
1926350376639,0.622771409627207,0.908526837595638
"11184","GID4",98.3281024933822,0.110566785880032,0.224677965784856,0.
492112279429782,0.622639972014708,0.908526837595638
"11185","VMP1",1925.48789311942,-0.176136695328945,0.3589298718373,-0.
490727323494509,0.623619318481442,0.908526837595638
"11186","HEATR6",63.4164214999481,0.152372865604151,0.310419376904481,
0.49086132162116,0.623524535020358,0.908526837595638
"11187","IER3IP1",647.636257939305,-0.120656796648572,0.24536870376911
8,-0.491736699893499,0.622905490391545,0.908526837595638
"11188","LINC02246",15.5601607628539,0.217549898769599,0.4420787929014
63,0.492106615976237,0.622643975465416,0.908526837595638
"11189","HDHD5-
AS1",4.52007443642908,0.413118680562495,0.842134480421751,0.4905614128
94056,0.623736683839803,0.908526837595638

"11190","FOXP1",1401.73667423756,0.138431853260782,0.282550056452767,0
.48993744683227,0.624178163682244,0.908600367133161
"11191","NMRAL1",166.060045823637,-0.0839967505119174,0.17147841851239
8,-0.489838611999122,0.624248105492285,0.9086209802845
"11192","CTDSPL2",206.808242024214,0.0872658420521636,0.17820115326527
8,0.489704137448852,0.624343273669299,0.908678304574625
"11193","SMTN",107.780093764192,0.120568303021338,0.246247662429031,0.
489622122021507,0.624401319392136,0.908681594887742
"11194","PPP1R16B",3.77662172221577,0.530409561208817,1.08355657149634
,0.489507954786657,0.624482124164632,0.908718002547588
"11195","HSF4",49.3225030282453,0.195124431531502,0.398786791253645,0.
489295121631535,0.624632774314834,0.908774853591847
"11196","UCKL1",144.915937499563,-0.0807101756503639,0.164928071586225
,-0.489365908872391,0.624582667088235,0.908774853591847
"11197","AL050343.3",2.40346416956608,-0.481690526797799,0.98496033236
7499,-0.489045610232834,0.624809406482642,0.908950649477159
"11198","GPC2",6.78834215490969,0.294859996866635,0.603048700013862,0.
488948897261293,0.624877876573871,0.908969077648847
"11199","PRR13",474.582124334699,-0.0845093658581143,0.172873467321171
,-0.488850991234588,0.624947194612969,0.908988735873797
"11200","CSRNP3",2.75646895024818,-0.455551769106529,0.932082059936376
,-0.488746419105658,0.625021235955661,0.909015260043014
"11201","SLC66A1",45.7506467757536,0.159146251576684,0.32575908832767,
0.488539713177869,0.625167603336387,0.909039383030996
"11202","LMBRD2",80.0842027921655,0.122472884423249,0.250714048591905,
0.488496297319986,0.625198347755636,0.909039383030996
"11203","GPER1",26.5714322779468,-0.173980130807265,0.356168237788204,
-0.488477388909459,0.62521173772369,0.909039383030996
"11204","PDE4C",15.8346745746571,0.860739794535286,1.76233851006992,0.
488407754592583,0.625261050247362,0.909039383030996
"11205","AC118553.1",12.9902520023493,-0.210577336493479,0.43133448853
2602,-0.488199627184605,0.625408448603786,0.909076721938473
"11206","LSM5",567.105795802785,-0.119720547890689,0.245206877120349,-
0.488243026854135,0.625377711194946,0.909076721938473
"11207","AC079174.2",5.57551345908724,0.338419545664672,0.693290762161
807,0.48813508578914,0.625454160646109,0.909076721938473
"11208","MRPS21",1126.05849895895,-0.113997817688878,0.233815062629243
,-0.487555491108981,0.625864728477396,0.909158269217274
"11209","REV1",254.312510856101,-0.0942438638296519,0.193254883375867,
-0.487666144230643,0.625786336094136,0.909158269217274
"11210","SPICE1",70.4359303159116,0.105563797370741,0.216593804700402,
0.487381425875774,0.625988053832907,0.909158269217274
"11211","ACTL6A",180.90634346319,-0.131216128165676,0.269093669498205,
-0.487622501154943,0.625817254596663,0.909158269217274
"11212","PTGR1",452.816628786767,-0.146512515928314,0.300632975880333,
-0.487346790548464,0.626012594237887,0.909158269217274
"11213","RELA-
DT",13.6457666673037,-0.200517700137678,0.411281532747539,-0.487543651
177655,0.625873116746416,0.909158269217274
"11214","MAP3K3",228.438360294442,0.101560336778863,0.208353353734187,

0.487442774299813,0.625944587231807,0.909158269217274
"11215","AL022311.1",44.9878933718076,-0.121704339644767,0.24960313649
2714,-0.487591387491718,0.625839297163406,0.909158269217274
"11216","MIRLET7BHG",60.8574527377076,-0.17327696784758,0.355401799567
304,-0.487552308566086,0.625866983217639,0.909158269217274
"11217","TMEM246",6.10948643676203,0.3637506846787,0.746639223633488,0
.487184001542971,0.626127941792062,0.909163669446506
"11218","ZNF263",79.5609309488639,-0.0948639802725131,0.19470472689257
3,-0.487219708460667,0.626102640125475,0.909163669446506
"11219","SNRNP48",137.972189689883,-0.11880632745041,0.244022798208596
,-0.486865687643055,0.626353516194108,0.909183477774368
"11220","RSBN1L",539.444016242462,-0.104573896624058,0.214788716320609
,-0.486868669897742,0.62635140264528,0.909183477774368
"11221","PAFAH1B2",390.052900035798,0.076738365274503,0.15765855441391
7,0.486737719749947,0.626444211000567,0.909183477774368
"11222","NOMO2",4.38237304786679,-0.380707738310068,0.782122562663749,
-0.486762249913179,0.626426825276976,0.909183477774368
"11223","AC103703.1",9.21948507513659,0.26184286258495,0.5380050596985
25,0.486692193437132,0.626476478269968,0.909183477774368
"11224","YY2",15.5662418769936,-0.199935194498586,0.410637304281047,-0
.486889993710233,0.626336290370528,0.909183477774368
"11225","PTBP1",459.307546909566,-0.0839216253097478,0.172468570569267
,-0.486590832362951,0.626548321601671,0.909206735908207
"11226","KIAA1522",39.1514396521738,0.182561037504789,0.37710756149695
,0.48410866326865,0.628308753322624,0.909421355300803
"11227","AL358472.2",7.43922253207149,0.271899049115803,0.561341325370
741,0.484373832509527,0.62812058589876,0.909421355300803
"11228","SEMA4A",184.124452846488,0.461939287646902,0.953369251702311,
0.484533444750893,0.628007334698241,0.909421355300803
"11229","AL359541.1",20.5965540199214,0.214147191002284,0.442668110559
709,0.483764666787306,0.628552893540673,0.909421355300803
"11230","PTPN7",8.79934010616313,0.291082191324834,0.600392701780385,0
.484819669629009,0.627804268758791,0.909421355300803
"11231","TMCC2",8.87544590344589,-0.278006342449941,0.5751419295746,-0
.483369979051199,0.628833060266472,0.909421355300803
"11232","ORMDL1",481.294583689415,0.0902324455830556,0.186676948151506
,0.483361478085787,0.628839095214052,0.909421355300803
"11233","ROBO1",246.257615287102,0.10138114023903,0.209729864747315,0.
483389146134124,0.628819453392239,0.909421355300803
"11234","RFC1",673.277453168794,0.0881864743139134,0.181546836309298,0
.485750542982043,0.627144043526854,0.909421355300803
"11235","SNX25",17.3697160292169,0.219037682870679,0.451497340605312,0
.485136152910714,0.627579768406877,0.909421355300803
"11236","AL451165.2",61.9890527041925,-0.153311471713263,0.31530523281
7643,-0.48623192943306,0.626802735532413,0.909421355300803
"11237","HACE1",41.866297121435,0.133485317604606,0.275160976946634,0.
485117181534412,0.627593224957714,0.909421355300803
"11238","ATAD2",72.00318278964,0.139133496430147,0.287286241607085,0.4
84302678930362,0.628171075000304,0.909421355300803
"11239","COMMD5",212.596844037321,-0.115825007596258,0.239309383226411

,-0.483996933319893,0.62838804553182,0.909421355300803
"11240","SMU1",308.239285551451,-0.0718664654394149,0.148251871079532,
-0.484759247327548,0.627847133795735,0.909421355300803
"11241","AL354920.1",10.4085637715576,0.282865753238168,0.583974163753
111,0.484380595573328,0.628115787059744,0.909421355300803
"11242","WHRN",15.1024991140637,-0.200549009326181,0.414521214238529,-
0.483808795394434,0.62852157243708,0.909421355300803
"11243","CHST3",97.8390165571985,0.095088002235159,0.196591939956337,0
.483682099359099,0.628611499111806,0.909421355300803
"11244","GBF1",158.978553522661,0.0946933433552844,0.19530919948465,0.
484838111083071,0.627791186196907,0.909421355300803
"11245","NAALAD2",106.101022613592,0.153648800910235,0.31677969285553,
0.485033619185646,0.627652497882425,0.909421355300803
"11246","MTMR2",99.6229690287392,-0.0878899518412055,0.181849950213865
,-0.483310288168034,0.62887543613674,0.909421355300803
"11247","RAD52",26.7872310720922,0.172085462319455,0.354010181389293,0
.48610314439013,0.626894037641729,0.909421355300803
"11248","BTG1",3471.46350176643,-0.119755936203941,0.247779776089027,-
0.483316023987822,0.628871364099075,0.909421355300803
"11249","ARL1",844.338933632544,-0.0992942538059551,0.204703460958755,
-0.485063874059078,0.627631037044535,0.909421355300803
"11250","AL162311.3",2.35356909506034,0.487424000097139,1.003496082572
85,0.485725862374511,0.627161544485544,0.909421355300803
"11251","CCNB2",2.76651962580334,-0.435994359620003,0.9003432126834,-0
.484253508526551,0.628205966313937,0.909421355300803
"11252","HDGFL3",307.764673799492,-0.0986637001265712,0.20380136175428
,-0.484116981737975,0.628302850065241,0.909421355300803
"11253","CCNF",9.45632699989789,-0.248961398398113,0.51298224224061,-0
.485321669831486,0.627448186280347,0.909421355300803
"11254","XPO6",124.027365290282,0.102547970812308,0.211368838430719,0.
485161254486056,0.627561963846566,0.909421355300803
"11255","SNHG29",4946.31991312301,-0.128228392757648,0.264630928961259
,-0.48455557806858,0.627991630925013,0.909421355300803
"11256","SCRN2",239.851231171041,0.126463445754427,0.260761911843661,0
.48497667799831,0.627692889108468,0.909421355300803
"11257","UNK",128.460782519459,-0.0981725666633326,0.202036447568422,-
0.485915129893013,0.627027340714401,0.909421355300803
"11258","PRCD",59.2584754222176,-0.138253678958474,0.284961512996188,-
0.485166145788688,0.627558494468333,0.909421355300803
"11259","FZR1",93.9989537392619,0.100119709180986,0.207149558414263,0.
483320891183188,0.628867908733878,0.909421355300803
"11260","SMARCA4",166.155269657021,0.139997451429256,0.288421852651721
,0.485391277193921,0.627398818719875,0.909421355300803
"11261","TMEM147",632.118962300278,-0.109155543813763,0.22588537318154
2,-0.483234227503681,0.628929435045954,0.909421355300803
"11262","ZNF527",32.5337058361934,-0.143071386215699,0.29473512593411,
-0.485423601147844,0.62737589420127,0.909421355300803
"11263","ZNF460",68.0539815105678,-0.173478617874696,0.357097416677219
,-0.485801940234991,0.627107598533399,0.909421355300803
"11264","CHAF1B",9.55962724125642,-0.253471622129076,0.524088392814101

,-0.483642884682213,0.628639334134343,0.909421355300803
"11265","DHRSX",193.570538245137,-0.114867318197665,0.237515928019291,
-0.483619431991676,0.628655981372431,0.909421355300803
"11266","TAGAP",18.2334904039586,-0.236524752560081,0.489886988955996,
-0.482814930570298,0.629227148544643,0.909613807570782
"11267","SLC12A9",71.8471673508816,-0.101528506001902,0.210235844883154,-0.482926715272223,0.629147772132471,0.909613807570782
"11268","TMEM218",192.618282989085,-0.123518886338999,0.255884616778951,-0.482713216190335,0.629299377915424,0.909613807570782
"11269","ODF3L2",4.11285437265725,-0.491631366528324,1.01875984149646,
-0.482578274587429,0.629395208060553,0.909613807570782
"11270","AC011446.2",7.9839742629638,-0.296975088083967,0.615379132036008,-0.482588818215874,0.629387720171321,0.909613807570782
"11271","IGLC3",52.2341807569075,0.48790394573707,1.0110428755241,0.4825749308447,0.629397582732537,0.909613807570782
"11272","ZGRF1",11.2765288955971,0.226265663473787,0.469086189870837,0.482354135251967,0.629554396669353,0.909663632057498
"11273","HGH1",51.5928729502157,-0.163728514900123,0.339466144998594,-0.482311763079646,0.629584492238123,0.909663632057498
"11274","ILF3-
DT",276.738340668961,-0.151047900085143,0.313188627397175,-0.482290501224327,0.629599594070614,0.909663632057498
"11275","AC002451.1",77.206956166429,0.11822905795645,0.24527702863918,0.482022546556421,0.629789929703931,0.909857930372268
"11276","DUSP8",16.8751438665284,-0.247718292125351,0.514158918889267,
-0.481793241398,0.629952831040844,0.909944928727963
"11277","UQCC3",330.485444502113,-0.135869971207809,0.28201632785443,-0.481780513353617,0.629961873734744,0.909944928727963
"11278","AL450306.1",15.0465581944051,-0.29573790548908,0.613984544808387,-0.481669950798801,0.63004042571033,0.909977699449864
"11279","TGFB2",54.5068430129812,0.233348185628466,0.484554713594497,0.481572419133961,0.630109723031994,0.909997098897787
"11280","AC135803.1",4.38428549570769,0.34913676302298,0.727179691414591,0.480124468745545,0.631138890300851,0.910012859574498
"11281","OLFML2B",863.608622553377,-0.102625932709074,0.213369456230052,-0.480977617520029,0.630532406561728,0.910012859574498
"11282","OR2C3",3.96402825002271,0.515618896838141,1.07295202997947,0.480560996606724,0.630828541672656,0.910012859574498
"11283","ZNF692",37.6821660692639,-0.123525236518905,0.257088894600004,-0.480476749923772,0.630888431618997,0.910012859574498
"11284","FAM13A-
AS1",24.5893241916439,0.202310701371023,0.421324274920497,0.48017812742738,0.631100738258452,0.910012859574498
"11285","AC108075.1",12.3998675222806,-0.386330442929323,0.804138122103078,-0.480427966676851,0.630923112146629,0.910012859574498
"11286","MGARP",2.60798280511291,0.45107847987543,0.93738647246929,0.481208650992356,0.630368213006683,0.910012859574498
"11287","RPL10A",16309.6158770271,-0.117686100724301,0.245186974942918,-0.479985124624583,0.631237970429876,0.910012859574498
"11288","IFT74",194.046826727944,-0.091674525045929,0.190470213842562,

-0.481306358597912,0.630298778520988,0.910012859574498
"11289","LINC02680",3.13736335990563,0.602216409737705,1.2544892217781
6,0.480049090325463,0.631192487028638,0.910012859574498
"11290","AL117336.2",13.2029235633354,0.267298673600283,0.556383798613
619,0.480421382265857,0.630927793136498,0.910012859574498
"11291","EDRF1",96.7345995930487,0.0979498452111268,0.203684173817902,
0.480890799590035,0.630594112054926,0.910012859574498
"11292","ACADS",264.05874450847,-0.13275764203912,0.276491971364094,-0
.480150079527265,0.631120680568966,0.910012859574498
"11293","SGF29",161.653240525724,-0.123776031650958,0.25751456603759,-
0.480656428704268,0.630760703079484,0.910012859574498
"11294","CIAPIN1",144.162121429596,-0.107797965722974,0.22457541229530
8,-0.480007871837829,0.631221795654205,0.910012859574498
"11295","PGAP3",37.2646683206332,0.1349743299688,0.280870027504859,0.4
80557968993203,0.630830693924431,0.910012859574498
"11296","AC006115.2",58.2623849909963,0.131890328481513,0.274777161052
65,0.479990141743409,0.631234402910815,0.910012859574498
"11297","ZBTB21",103.616514117045,-0.173127957907177,0.359999161118906
,-0.480912114820161,0.630578962102555,0.910012859574498
"11298","ZBED4",31.242733291145,-0.144136264373014,0.299878111399938,-
0.480649500225725,0.630765628133557,0.910012859574498
"11299","NR0B1",26.8151233585027,-0.537902978448569,1.11838356182178,-
0.480964667946619,0.630541610255253,0.910012859574498
"11300","LIN9",23.8721571719916,-0.155791551399153,0.324850418801705,-
0.479579346007406,0.631526535578355,0.91007135730913
"11301","IGFBP2",26.6620432695947,0.540206612601161,1.12591731415037,0
.479792437519097,0.631374990811163,0.91007135730913
"11302","SLC25A36",775.120670092561,0.0653362892481884,0.1362938428470
13,0.479378142720117,0.631669639986311,0.91007135730913
"11303","C14orf132",224.945380590862,-0.117106160161692,0.244137990934
345,-0.479672007267335,0.631460635559378,0.91007135730913
"11304","EEF2",5917.25962732621,-0.0560606357340576,0.116922097282893,
-0.479469980754954,0.631604319124202,0.91007135730913
"11305","OSER1-
DT",126.050980957792,0.104466311600726,0.217886631343567,0.47945259861
3092,0.631616682152532,0.91007135730913
"11306","LINC00891",69.149372739599,0.138650054323547,0.28916497692900
5,0.479484257727338,0.631594164721147,0.91007135730913
"11307","ZNF436-
AS1",17.8675438851767,-0.179174188676777,0.374317554667418,-0.47866894
4169541,0.6321741624594,0.910084549409603
"11308","GTF3C2",80.2331868613776,0.104391543940828,0.218076109936058,
0.478693168047781,0.632156926812706,0.910084549409603
"11309","PIGG",100.939887593815,0.100942190336907,0.211078624029555,0.
478220808956823,0.632493053312814,0.910084549409603
"11310","SREK1IP1",453.639196834611,0.0907537293454411,0.1895511100777
35,0.47878236802846,0.632093461433694,0.910084549409603
"11311","TJAP1",89.2812570177985,0.0898608140343731,0.187738937195045,
0.478647718885375,0.632189264726454,0.910084549409603
"11312","BCLAF1",1563.33394232132,0.102326322661526,0.213946836167636,

0.478279204752293,0.632451495272806,0.910084549409603
"11313","HBP1",704.372723008683,0.0933932338549278,0.195147373362624,0
.478577970308543,0.632238893488039,0.910084549409603
"11314","NUDT5",306.437843750744,-0.0883086789122877,0.184538519386122
,-0.478537918295062,0.632267392765671,0.910084549409603
"11315","AL731559.1",2.44868178999281,-0.518000672229803,1.08211610113
103,-0.47869232487012,0.632157526742692,0.910084549409603
"11316","PGR",72.1724268662168,-0.158736079247508,0.331198094035926,-0
.479278359706652,0.631740615065912,0.910084549409603
"11317","MPHOSPH8",1185.1987978227,-0.0772631881067543,0.1615721619639
57,-0.478196164287198,0.63251059232388,0.910084549409603
"11318","GPR65",10.6751875034367,-0.281996637662242,0.589402003029664,
-0.478445333087967,0.632333274478714,0.910084549409603
"11319","NDUFAF1",90.2493415316759,-0.138213235676184,0.28917815855693
2,-0.477951849357853,0.632684476488081,0.910084549409603
"11320","MAPK6",200.264938372458,-0.115280902504208,0.240861168035288,
-0.478619710452114,0.632209193591461,0.910084549409603
"11321","ELMO3",3.19196483857397,-0.39465585069367,0.825317012362951,-
0.478186981222812,0.632517127739977,0.910084549409603
"11322","AC005726.5",4.8344679647926,-0.313423850322265,0.655657164397
288,-0.478030085449275,0.632628791976877,0.910084549409603
"11323","IL3RA",2.52246769661626,0.497144377008027,1.03995137470301,0.
478045790506314,0.632617614158895,0.910084549409603
"11324","TMEM31",5.1650553504396,0.339868851494182,0.70932239126931,0.
479145809687464,0.63183490237621,0.910084549409603
"11325","AC048382.5",15.1336697427208,-0.23105325114156,0.483544181467
041,-0.477832760680855,0.632769241810019,0.910087235913949
"11326","NPIPA3",5.51897802742655,0.412817205003321,0.864009861746902,
0.47779223742732,0.632798086681895,0.910087235913949
"11327","FBXW7",189.250898153895,0.0962504814731593,0.201558636981464,
0.47753092060257‌9,0.632984108117485,0.910120556435659
"11328","CALHM5",79.3540241792365,0.10392197128052,0.217632716350123,0
.477510794440172,0.632998436124346,0.910120556435659
"11329","RIDA",129.195105587737,0.123296085716994,0.258241041394526,0.
477445742362189,0.633044748260545,0.910120556435659
"11330","AC005052.2",4.15452143943639,-0.386171364904929,0.80861872099
7251,-0.477569161926739,0.632956884133701,0.910120556435659
"11331","TBX21",4.05056683793368,0.552666428521811,1.15837867757054,0.
47710341982547,0.63328847959081,0.910390613719416
"11332","DNAJC27-
AS1",20.9515421907387,-0.19186664867225,0.402311423183047,-0.476910765
183401,0.633425665900905,0.910458496143066
"11333","AL022323.4",28.1384143315754,0.202028849135456,0.423647034620
751,0.476880121009963,0.63344748829206,0.910458496143066
"11334","MRGPRF",414.492490813127,-0.101955588108786,0.213858598577377
,-0.476742991803983,0.633545144927602,0.910489526964213
"11335","FP565260.6",12.30496535154,0.218643047961117,0.45866653437259
8,0.476692829269078,0.633580869797984,0.910489526964213
"11336","AL590822.3",5.97178283183271,0.412499860110424,0.870469894349
676,0.473881822666136,0.635584182201372,0.910653979665393

"11337","MRPL35",152.214462708166,0.10387045908555,0.219587516617672,0
.473025337166143,0.636195102719445,0.910653979665393
"11338","GALNT5",35.2565361540955,-0.622439775674094,1.31097596002557,
-0.474791143890964,0.63493584593887,0.910653979665393
"11339","PPIL3",233.3492964649,-0.133775862597955,0.282523622255757,-0
.47350328276923,0.635854159479598,0.910653979665393
"11340","EXOG",108.662931020269,-0.109058751578652,0.229641668847469,-
0.474908374103006,0.634852282413461,0.910653979665393
"11341","AC121247.1",25.0651707086022,-0.189351460160727,0.40005298167
6118,-0.473315957719884,0.635987778905184,0.910653979665393
"11342","RYBP",285.973591038758,-0.0944040181282338,0.199072151263161,
-0.474220113306745,0.635342951946209,0.910653979665393
"11343","KIT",22.8438947484449,0.242730517008042,0.511291179119596,0.4
74740278965903,0.634972104698528,0.910653979665393
"11344","BANK1",17.7167249100317,-0.310936642730932,0.65749476084036,-
0.472911209715977,0.636276527117696,0.910653979665393
"11345","SDHA",478.369551662458,0.0736381056328202,0.155414459811844,0
.473817595363852,0.635629986148231,0.910653979665393
"11346","IPO11",50.2460329512073,0.113846092741565,0.24079419456073,0.
47279417574518,0.636360029741466,0.910653979665393
"11347","PCDHB8",3.09939633168627,0.499966904398878,1.05298625872191,0
.474808574430712,0.634923420883606,0.910653979665393
"11348","PCDHB11",67.0715986198216,-0.217910754909921,0.45901191647926
2,-0.474738774934978,0.634973176851367,0.910653979665393
"11349","MED7",112.703486181004,0.133767541015052,0.282510902047044,0.
473495146721018,0.635859962696945,0.910653979665393
"11350","BLOC1S5",176.415829799052,0.0934838521288688,0.19752381126945
4,0.473278900037738,0.636014213646259,0.910653979665393
"11351","GLI3",475.795830215871,0.104680321255561,0.220602103792063,0.
474520956310694,0.635128457576539,0.910653979665393
"11352","GSTK1",871.120684289439,-0.112744548770042,0.237639077285024,
-0.474436065221783,0.635188979934299,0.910653979665393
"11353","INSIG1",282.744693625273,0.154009518627337,0.325876972407778,
0.472600188621556,0.636498447883258,0.910653979665393
"11354","SLC39A14",159.456901696419,0.159308728037388,0.33480395241074
1,0.475826903745586,0.634197701240168,0.910653979665393
"11355","RPL7",14477.0113267133,0.0829445358717953,0.174926984936136,0
.474166612441628,0.635381100078469,0.910653979665393
"11356","PGM5P3-
AS1",2.43664368288596,-0.502959006387811,1.06026922536687,-0.474369145
453392,0.635236691514806,0.910653979665393
"11357","ANK3",4.92949206472484,0.437889045743596,0.925995096466978,0.
472884842926608,0.636295339167575,0.910653979665393
"11358","RNLS",51.7568943442842,-0.118126814582601,0.24835776499397,-0
.475631654140022,0.634336820039737,0.910653979665393
"11359","CALHM2",714.718423516538,-0.0811345780412045,0.17168271485047
8,-0.472584430598422,0.63650969246672,0.910653979665393
"11360","PWWP2B",14.361167573855,0.218579551521942,0.458824177036757,0
.476390657819305,0.633796089031315,0.910653979665393
"11361","ZNF214",29.8707589828475,0.132959297786331,0.281340688218367,

0.472591784104588,0.636504445153822,0.910653979665393
"11362","AC026894.1",3.02407429234502,0.434986530896227,0.917752550681
093,0.473969296596789,0.635521802074649,0.910653979665393
"11363","TMEM126B",474.901791739902,-0.109952406543001,0.2315113999285
,-0.474933012270489,0.634834720534599,0.910653979665393
"11364","FAM76B",120.255893849183,-0.110004109059698,0.231233217828146
,-0.475727968900446,0.634268192453338,0.910653979665393
"11365","FOXRED1",87.0627960238736,-0.104086327061136,0.22029965300563
6,-0.472476128042165,0.636586977086799,0.910653979665393
"11366","DYRK4",38.2096572684116,-0.29992308430056,0.631384848237999,-
0.475024202968369,0.634769722358909,0.910653979665393
"11367","AC048341.2",11.3216833287106,-0.219484815446537,0.46295101393
379,-0.474099437825029,0.635428999481017,0.910653979665393
"11368","AL356515.1",2.55671319347056,-0.496045778053521,1.04570671802
08,-0.474364149627329,0.635240253434237,0.910653979665393
"11369","NUFIP1",94.7217416217024,-0.10025121735844,0.211956712860608,
-0.472979675922645,0.636227679273742,0.910653979665393
"11370","LINC00640",12.526087931405,0.302767924875005,0.64034657021080
1,0.472818843669819,0.636342429027779,0.910653979665393
"11371","TJP1",310.209198456461,-0.114633536447467,0.240748658571303,-
0.476154414017287,0.633964373398875,0.910653979665393
"11372","TMEM199",83.7860462457427,-0.140801179560846,0.29561631314659
,-0.476297055673734,0.6338627627077,0.910653979665393
"11373","ACAA2",742.276356526174,-0.0939153148324345,0.198498662627617
,-0.473128199400614,0.636121719272172,0.910653979665393
"11374","KDSR",1179.37366718312,-0.0790631433656368,0.167336239549196,
-0.472480698613958,0.636583715450382,0.910653979665393
"11375","FEM1A",51.1213893922104,0.108646538700002,0.229982832290455,0
.472411517059621,0.636633085317271,0.910653979665393
"11376","VAV1",2.89046639303651,-0.500824052186297,1.05685722356248,-0
.473880521437047,0.63558511016405,0.910653979665393
"11377","ZNF440",20.4798021827321,-0.198747524357365,0.419657066611772
,-0.47359508553499,0.635788680664451,0.910653979665393
"11378","ARMC6",62.0357231479825,-0.136985243425031,0.289991874261405,
-0.472376144241717,0.636658328960004,0.910653979665393
"11379","TMEM161A",147.823739783843,-0.130103811312195,0.2733084469919
51,-0.476032895229273,0.634050942672759,0.910653979665393
"11380","MYH14",4.60597749068012,-0.390643400506201,0.826366962796448,
-0.472723884295003,0.636410184242874,0.910653979665393
"11381","SNHG17",100.636475555078,-0.126783836196943,0.266576769469696
,-0.475599717294027,0.634359576837355,0.910653979665393
"11382","CABLES2",9.39640095269888,-0.251643274840003,0.53179816877870
2,-0.473193195489771,0.636075351937105,0.910653979665393
"11383","SLC25A17",87.0979336692209,-0.0947722024984915,0.199532841527
457,-0.474970444830006,0.634808039313826,0.910653979665393
"11384","USP11",251.050852888538,0.0880555554047338,0.185434900613778,
0.474859668343313,0.634887000074545,0.910653979665393
"11385","ZXDA",20.9160147974776,-0.163314398446516,0.344679321410304,-
0.473815480947021,0.635631494076402,0.910653979665393
"11386","RAB9B",58.7250273524965,0.122083263685125,0.258291245330881,0

.472657381510287,0.636457636982108,0.910653979665393
"11387","AL121601.1",8.0023013305449,-0.266420704770554,0.561497477989822,-0.474482460231787,0.635155902716489,0.910653979665393
"11388","AC244090.1",63.3913880932567,0.172822715179949,0.363028596067549,0.476058131651401,0.63403296398118,0.910653979665393
"11389","CU638689.4",20.5682189407741,0.20447276583455,0.433025148711822,0.472196052452894,0.6367868571459,0.910757846698531
"11390","ATP13A2",30.1923997767066,-0.137970654253809,0.292324087455231,-0.471978397178573,0.636942208285678,0.910900055378877
"11391","ATG7",106.481521451646,0.0888427739729587,0.1882820765078,0.471859964691214,0.637026746008741,0.910940976712877
"11392","UBE2D3",1587.53793505869,-0.0657117227242516,0.139320667762544,-0.471658109163314,0.637170842418054,0.91102620951445
"11393","LINC01588",9.66459690189393,0.275780674303554,0.584772551701657,0.471603315684101,0.637209959610593,0.91102620951445
"11394","CNTROB",40.2727702774333,0.127593858427955,0.270588857277257,0.471541436376357,0.637254136607996,0.91102620951445
"11395","AC012557.2",5.17859012055184,0.426951681834259,0.905949292212509,0.471275473698487,0.637444027882611,0.91113774747103
"11396","NFKB2",120.093675718711,-0.112086129141441,0.23782290132928 3,-0.471300823070232,0.637425927984041,0.91113774747103
"11397","ZNF568",61.5518640702818,0.112398734400189,0.238545266597268,0.471184090145666,0.637509279146008,0.911151061508232
"11398","FBXL17",127.84927374165,-0.0992918937812762,0.211383707489324,-0.469723494590004,0.638552579869697,0.91157868147263
"11399","UNC5CL",6.47562760326155,0.35615755669299,0.758267449570917,0.469699123830952,0.638569993936289,0.91157868147263
"11400","AL356124.1",9.43406150852034,0.312501458618046,0.664949607401178,0.469962618429681,0.638381725086951,0.91157868147263
"11401","AIMP2",96.7659959366681,-0.126925062251961,0.270142115006198,-0.46984551908557,0.638465390561294,0.91157868147263
"11402","LEPROTL1",203.655197087969,-0.0901493275847215,0.191973985700095,-0.469591373310103,0.638646989197718,0.91157868147263
"11403","C8orf37-
AS1",2.50483975774619,0.527731453461582,1.12381304662923,0.46959007554189,0.638647916567355,0.91157868147263
"11404","DNAJB5",7.38359078963042,-0.308127880609599,0.655898409302608,-0.469779887006007,0.638512285570365,0.91157868147263
"11405","AL450384.2",9.23334197440456,-0.271800781539301,0.577983101515721,-0.470257315181919,0.638171189684172,0.91157868147263
"11406","NCAPD3",41.398328842317,0.14247827883493,0.303292266487823,0.469772211750907,0.638517769738441,0.91157868147263
"11407","HOXC8",285.610753395375,-0.0908028656272542,0.19327047233457,-0.469822754249112,0.638481656181234,0.91157868147263
"11408","NRDE2",150.708591592198,0.11693346723934,0.24888640386175,0.4698266575634,0.638478867226077,0.91157868147263
"11409","AC090517.2",12.7825802213426,0.219744365402613,0.467172304204736,0.470371131646346,0.638089885449482,0.91157868147263
"11410","NLRP1",81.0634860705254,0.164813441173439,0.350771540963876,0.469859786003597,0.638455196846616,0.91157868147263

"11411","ZNF584",42.9666393455291,-0.127018335092008,0.270020147913559
,-0.470403175738836,0.638066995693925,0.91157868147263
"11412","SMARCB1",347.006232275536,-0.114301940169503,0.24284107188501
2,-0.470686195223293,0.637864844027576,0.91157868147263
"11413","AC132872.1",32.1376470421044,0.173447899260324,0.369423121571
431,0.469510133860928,0.638705043025682,0.911580342227752
"11414","LAMA3",8.15268553712501,-0.265866984385433,0.566377289595611,
-0.469416746168019,0.638771780742011,0.911595718986036
"11415","AK2",253.417876643722,0.0828668090732556,0.17668271867166 2,0.
469014794974098,0.639059060812568,0.911742915891101
"11416","MCM4",68.3913835824186,-0.113986512797391,0.243074532621004,-
0.468936468038453,0.63911504846226,0.911742915891101
"11417","JAKMIP3",6.69340570481215,0.33636900873406,0.717060352522905,
0.469094417995053,0.639002148836525,0.911742915891101
"11418","DTD2",58.7755997677763,0.1175116882536,0.250505428043832,0.46
909837112607,0.638999323320926,0.911742915891101
"11419","HELZ2",176.977525644778,-0.178934141076166,0.381619621266202,
-0.468880872745663,0.639154788910337,0.911742915891101
"11420","C11orf91",5.23375873922565,-0.386353263571602,0.8243066974326
61,-0.468700866770725,0.6392834673081,0.911770882207671
"11421","C11orf71",110.104092741243,-0.125754909881805,0.2683523961180
56,-0.468618546735398,0.639342317918596,0.911770882207671
"11422","DYNLRB2",18.3089730989484,0.167562375206243,0.357518307861953
,0.468681943054349,0.639296995675922,0.911770882207671
"11423","ARMC8",157.841980591022,-0.0748531265002059,0.159791097558194
,-0.468443659528311,0.639467352354208,0.911789539784462
"11424","SERPINB6",712.016351692619,-0.0968106797818019,0.206647134745
178,-0.468483049141629,0.639439190120496,0.911789539784462
"11425","PHF14",555.159715403671,0.0753807090636148,0.161043582898688,
0.468076453012327,0.639729917521208,0.912019394772136
"11426","STRN3",255.470759526035,-0.0742160975868254,0.15856823231609,
-0.468038878297406,0.639756787268207,0.912019394772136
"11427","NDUFA6-
DT",23.616267669553,0.203864895193302,0.435624287919333,0.467983307742
135,0.639796526739591,0.912019394772136
"11428","SEC24B-
AS1",14.5133979406979,0.188013757494589,0.401896075408168,0.4678168536
57108,0.639915567136994,0.912109264358986
"11429","RBM25",1266.74642018109,0.110266107160959,0.235833923149131,0
.467558295636849,0.640100494465672,0.912213206854884
"11430","U2AF1L4",72.4997568997425,-0.110963757492832,0.23731421557960
5,-0.467581586808105,0.640083835106098,0.912213206854884
"11431","DNMT3B",3.70954448806457,0.417418654197588,0.892980735073189,
0.46744418754272,0.640182114612977,0.912249712617513
"11432","AL355488.1",7.85597690278351,-0.279558419828335,0.59838355195
4406,-0.467189345220566,0.640364415978937,0.912278219900228
"11433","LMCD1",489.884277519222,0.370168291160003,0.792370046180594,0
.467165932059521,0.640381165664344,0.912278219900228
"11434","FARS2",113.771237695653,-0.116099507824029,0.248552229898209,
-0.467103062690591,0.64042614307564,0.912278219900228

"11435","ANGPTL8",2.76362992203137,-0.411236324636487,0.88029889537319
,-0.467155334168799,0.640388747414858,0.912278219900228
"11436","AL592295.4",10.6570639993305,-0.271475495053288,0.58150876597
1398,-0.46684678020252,0.640609503942604,0.912304111888165
"11437","ALKAL2",84.9550188414484,0.483293306941514,1.03523775481859,0
.466842814311968,0.640612341566507,0.912304111888165
"11438","AC099522.2",7.02645806730129,-0.287652949612593,0.61616602865
8,-0.466843247166834,0.640612031855406,0.912304111888165
"11439","EPS15",612.481068844705,0.0849447427819248,0.182570420332132,
0.465271113619575,0.641737318235301,0.91243215786163
"11440","HIST2H2AC",132.847575324771,0.133203124593159,0.2863165300618
65,0.465230297965605,0.641766543840671,0.91243215786163
"11441","PFKFB2",13.0373219862538,0.189428474329707,0.406912125468455,
0.465526737773245,0.641554293962708,0.91243215786163
"11442","BCS1L",95.5406202975631,-0.10505895426191,0.225479295799859,-
0.465936146772265,0.641261206694698,0.91243215786163
"11443","RYK",330.272838021811,0.0928141361005594,0.199212222936532,0.
465905830136384,0.641282907818871,0.91243215786163
"11444","GUF1",71.429411026928,0.113798973671669,0.24450122375747,0.46
5433145580286,0.641621302486392,0.91243215786163
"11445","YTHDC1",557.210984883576,0.104350620050456,0.22424537075359,0
.465341245171659,0.641687102595865,0.91243215786163
"11446","VDAC3",688.01623310941,-0.123628549240561,0.265225584446513,-
0.466126031915644,0.641125290899789,0.91243215786163
"11447","COPS5",455.674821194131,-0.115667084814844,0.248142496022846,
-0.466131705244857,0.641121230235028,0.91243215786163
"11448","TMEM141",295.762924383162,-0.114968370166673,0.24638795634981
2,-0.466615218819563,0.640775196618786,0.91243215786163
"11449","ABRAXAS2",113.974416208707,-0.101769242313113,0.2184186011535
8,-0.46593670033421,0.641260810449212,0.91243215786163
"11450","AP002433.1",10.8822121618429,-0.245362636821527,0.52648830050
3986,-0.466036256810743,0.641189548534129,0.91243215786163
"11451","CASC1",6.18259365770139,-0.273642941659066,0.587552756115033,
-0.465733398083986,0.641406343219782,0.91243215786163
"11452","CCDC102B",190.092332862994,-0.194457855948915,0.4179781722536
41,-0.465234476002524,0.641763552177252,0.91243215786163
"11453","KHSRP",446.045326124733,0.0808527881764461,0.173750372963318,
0.465338789192216,0.641688861100172,0.91243215786163
"11454","RNASEH2A",25.8071565637144,-0.183456959977375,0.3933091049497
53,-0.466444731811668,0.640897199252371,0.91243215786163
"11455","SPTBN4",2.65614040462633,-0.407525864095705,0.873692768439094
,-0.466440697253081,0.640900086550451,0.91243215786163
"11456","NECAB3",142.45929831714,-0.154741792471139,0.332397443690706,
-0.465532438375566,0.641550212638126,0.91243215786163
"11457","PES1",95.1962611651852,-0.0897638182147838,0.192759848822975,
-0.465676948611949,0.641446754700905,0.91243215786163
"11458","AC022898.2",3.15222768175161,0.495725505377096,1.065811746501
58,0.465115445578699,0.641848785621731,0.912469442223109
"11459","AC025171.3",5.3399258631508,-0.332843714211161,0.716004575948
608,-0.464862551709518,0.64202988957107,0.912567615289978

"11460","ZNF552",22.8437082195465,-0.170433111825074,0.366607847563039
,-0.46489215372229,0.642008689690909,0.912567615289978
"11461","NANOG",3.63245326570779,0.363505236023389,0.782178745701455,0
.464734228615991,0.642121793256099,0.912618610099345
"11462","SYCP2L",3.89517379554561,-0.395016554084155,0.850902117977394
,-0.464232660535755,0.642481063744866,0.912864271070441
"11463","C6orf136",53.920881883664,-0.123687791563592,0.26640430502180
1,-0.464286009017271,0.642442846540245,0.912864271070441
"11464","SLC43A3",724.735147788501,0.0956297459251203,0.20602139269680
1,0.464173864050406,0.642523184827607,0.912864271070441
"11465","BORCS5",88.8948444279274,0.0981545295993767,0.211565630012588
,0.463943645258148,0.642688121815462,0.912864271070441
"11466","SUDS3",242.199167114554,-0.0858303332746278,0.185032169372995
,-0.463867086277347,0.642742975315052,0.912864271070441
"11467","MGAT2",170.47106053878,0.0958955184590436,0.206668649144073,0
.464006122148662,0.642643359391462,0.912864271070441
"11468","GALNT1",603.635252580529,0.0587063456943811,0.12655072816484,
0.463895755841978,0.642722433722099,0.912864271070441
"11469","CSTF2",20.2730790467192,-0.198207652895852,0.427149732577516,
-0.464023825321915,0.642630675946425,0.912864271070441
"11470","AC106820.4",2.62657495934454,0.427380416290507,0.921819046160
181,0.463627235812442,0.642914837618879,0.913028752395285
"11471","THAP9-
AS1",79.8922095223359,-0.138694329318151,0.299294606747301,-0.46340403
7999431,0.643074784809961,0.913176285395298
"11472","SCNN1D",12.7615387197399,0.207093925990219,0.447142965800495,
0.463149242702433,0.643257395496068,0.913214686166573
"11473","CENPQ",91.8211794733358,0.122415231948725,0.264320601837497,0
.463131632940158,0.643270017132743,0.913214686166573
"11474","HOXA-
AS2",6.34874929975699,0.323092963000824,0.697433093923127,0.4632601547
24513,0.643177902680793,0.913214686166573
"11475","PEX14",58.8943726629972,-0.104303661477841,0.225310481281653,
-0.462933019735794,0.643412378464447,0.913257601325146
"11476","MZB1",7.20908779448433,0.370531774153914,0.800352380752611,0.
462960794600854,0.643392469298917,0.913257601325146
"11477","RBMXL1",99.7523051435268,-0.100717863334404,0.217921037322092
,-0.462175954061476,0.643955145560722,0.913297955567067
"11478","TMEM79",26.1566647189678,-0.149830116114877,0.324229486852997
,-0.462111319883772,0.644001492887429,0.913297955567067
"11479","FCRLB",5.32404463525901,0.368485435819603,0.796472434159019,0
.462646816155892,0.643617545563827,0.913297955567067
"11480","ACAA1",400.092791536353,-0.100883793822566,0.218261122506171,
-0.462216049583976,0.643926394900105,0.913297955567067
"11481","TIGIT",5.08351241849972,-0.353992215150271,0.765807563812811,
-0.462246955864226,0.643904233787379,0.913297955567067
"11482","NCAPG",5.0704140784458,0.295342244236088,0.639067511717815,0.
462145608751425,0.643976905145288,0.913297955567067
"11483","GRIN3A",7.52601172112015,0.335366520104875,0.725572828733979,
0.462209314935403,0.64393122397055,0.913297955567067

"11484","SLC2A13",126.055466861188,0.101078102876095,0.218520831800651
,0.462555913059608,0.643682715789224,0.913297955567067
"11485","TRAC",67.8496909188467,0.274484337923953,0.593267211744718,0.
462665612543684,0.643604070404055,0.913297955567067
"11486","PSME3",113.495541809242,-0.0903930542205817,0.195538923616812
,-0.462276525556214,0.643883031361438,0.913297955567067
"11487","CDKN3",12.0807991825284,-0.263214786885454,0.56979626570628,-
0.461945440374536,0.644120446690858,0.913310434449029
"11488","TMEM50B",351.353349841764,-0.087008776852574,0.18835405720688
6,-0.461942674040753,0.644122430532902,0.913310434449029
"11489","AC009126.1",7.72231955024788,0.261926902477006,0.567155256515
92,0.461825751357827,0.644206282506111,0.913349824679436
"11490","SUB1",1915.92102978,0.11456271553801,0.248201500911862,0.4615
71405157184,0.644388704415975,0.913449447935934
"11491","RPL26",24776.5979135862,-0.122804384228,0.266025307719518,-0.
461626697402332,0.644349045948868,0.913449447935934
"11492","C10orf55",6.12631591084395,-0.282309615821814,0.6119529872270
83,-0.461325660163915,0.644564977720424,0.913546497538079
"11493","AC092376.3",7.42480834493936,0.270799341816367,0.587010285209
189,0.461319586112301,0.644569334901169,0.913546497538079
"11494","RAB36",15.6782852539993,0.191325587677014,0.414849597213022,0
.461192656235774,0.644660390001627,0.913596058181355
"11495","SFT2D3",93.3140250517818,-0.102854027080523,0.223232705258615
,-0.460748020597459,0.644979398253658,0.913730163346133
"11496","GDF6",22.7303807807759,-0.288532982918274,0.626195630760407,-
0.460771312900891,0.644962685336662,0.913730163346133
"11497","ANKRD10-
IT1",17.5889435072198,0.247047040874076,0.536041867642245,0.4608726589
97142,0.644889968601685,0.913730163346133
"11498","BCKDHA",144.936417419437,-0.0972085310658591,0.21096136701939
6,-0.460788306595119,0.644950491971333,0.913730163346133
"11499","CA2",3.47775581529395,-0.380695797549143,0.826423143280142,-0
.460654811817261,0.645046280106677,0.913745443660985
"11500","ZC2HC1C",7.21166852561738,-0.272544672857965,0.59182643369104
3,-0.460514531529432,0.645146943479101,0.913808570637484
"11501","CATSPERE",5.3642106185606,0.337043210056939,0.733393698135663
,0.459566547836075,0.645827375056684,0.913818807531126
"11502","TP53I3",572.019702282998,0.132295361396186,0.287524890358294,
0.460117943985052,0.645431564895133,0.913818807531126
"11503","SOCS5",295.717591697996,-0.114096863290054,0.248159301145406,
-0.4597726652329,0.645679405512769,0.913818807531126
"11504","ABCB6",167.871783582795,0.108861047384385,0.236675329608416,0
.459959420208678,0.645545348158781,0.913818807531126
"11505","TUBA4A",31.6151594932967,0.179109855661957,0.389505869177441,
0.459838656706769,0.645632033880782,0.913818807531126
"11506","COL7A1",6.64806126770688,0.279410160617758,0.607299786164213,
0.460086051375961,0.645454455716492,0.913818807531126
"11507","P2RY1",32.3806075995492,0.18344930663415,0.399061146397744,0.
459702249367335,0.645729954756716,0.913818807531126
"11508","AC016773.1",2.49000485609111,-0.408193504206079,0.88811477321

2956,-0.459617964386908,0.645790462335189,0.913818807531126
"11509","AL133351.1",3.57205826046685,-0.414187260003985,0.900486479002819,-0.459959443769381,0.64554533124705,0.913818807531126
"11510","ADAMTSL2",83.054899614696,-0.349854719981546,0.761159630260235,-0.459633835102281,0.645779068684147,0.913818807531126
"11511","CPNE8-AS1",3.80408648832216,-0.351618665079182,0.764964141865779,-0.45965378745933,0.645764744923889,0.913818807531126
"11512","THOC1",146.869672080149,-0.0937089100916155,0.203668175416821,-0.460105806416901,0.645440276557474,0.913818807531126
"11513","KIAA0040",70.5998654539888,-0.188526083928951,0.410792279687042,-0.458932879830599,0.646282366517319,0.913901753807707
"11514","COMMD2",335.760095261494,-0.103259601646737,0.225016960758036,-0.458896970694462,0.646308154251116,0.913901753807707
"11515","AC008906.1",8.73735006571835,0.336730266983929,0.733841269353102,0.458859812123628,0.646334839699478,0.913901753807707
"11516","COX7A2",1500.12573653723,-0.104450275786221,0.227558803529823,-0.459003449508524,0.646231688951688,0.913901753807707
"11517","AC012085.2",6.11627694485672,0.304805577626739,0.664074117995938,0.458993310184396,0.646238970111999,0.913901753807707
"11518","PRSS53",12.1451776926396,-0.211866737418119,0.461286762450715,-0.459295073399717,0.646022285129654,0.913901753807707
"11519","SARM1",28.0705899901102,-0.154084761992044,0.335649240425662,-0.459064831478952,0.646187610606327,0.913901753807707
"11520","RAB40B",204.084020626758,-0.106621388757213,0.232236526074074,-0.459106887963028,0.64615741059475,0.913901753807707
"11521","B3GALT6",91.3804741587863,0.115440275595686,0.252889971292369,0.456484197478216,0.648041831826356,0.913928326417711
"11522","ATAD3B",36.2928923426425,-0.156010955711412,0.345747607891482,-0.451227867237706,0.651825326860245,0.913928326417711
"11523","TMEM201",27.1464470935085,-0.14735660852891,0.321795286323588,-0.457920344988312,0.647009669642895,0.913928326417711
"11524","GPATCH3",36.2425653325525,-0.149149189007878,0.327376124231438,-0.455589696279859,0.648685053886738,0.913928326417711
"11525","BTF3L4",484.086113968229,-0.103863790322064,0.227006248135922,-0.457537143470496,0.647285011060961,0.913928326417711
"11526","AC093424.1",3.1813750920783,0.378528716327509,0.832586775146247,0.45464175942624,0.64936698684049,0.913928326417711
"11527","LIX1L-AS1",3.70947494725464,-0.392812233578746,0.857876056601755,-0.457889260990411,0.647032002597952,0.913928326417711
"11528","LINC01133",435.064847056121,0.144157786731168,0.316645758451415,0.455265175305631,0.648918476765486,0.913928326417711
"11529","AC012358.3",20.4029400005545,0.178907734242599,0.396173875773845,0.451588923911601,0.651565150733914,0.913928326417711
"11530","VAMP5",1408.9765145372,-0.111541735288185,0.246298660621364,-0.452871871112848,0.650641006979624,0.913928326417711
"11531","RABL2A",60.6974385817491,-0.116379282635612,0.257240736616328,-0.452413891230574,0.650970841511269,0.913928326417711
"11532","AC092620.1",9.00580778858948,0.272318857376014,0.602784536741

885,0.451768153921012,0.651436013993679,0.913928326417711
"11533","CHROMR",79.7308022899118,-0.127719642627999,0.278499931411222
,-0.458598470673997,0.646522535059101,0.913928326417711
"11534","METTL21A",76.0743010692773,0.109215608524123,0.23925696078785
5,0.456478290807021,0.648046078361998,0.913928326417711
"11535","CAND2",11.9785234496757,-0.199511059975866,0.442037871083544,
-0.451343816960332,0.651741769289625,0.913928326417711
"11536","PFKFB4",2.9199616090246,0.442033316648244,0.966457968709835,0
.457374589438517,0.647401825439015,0.913928326417711
"11537","RBM15B",192.865208774205,-0.0803414279222856,0.17691944822063
7,-0.4541130369234,0.649747470394017,0.913928326417711
"11538","CHDH",10.7411127659627,0.272669344544749,0.596461097111714,0.
457145228523898,0.647566663269115,0.913928326417711
"11539","TFG",704.540087938102,-0.091602327095349,0.200525813610168,-0
.456810649193667,0.647807150799008,0.913928326417711
"11540","STIM2-
AS1",5.43483704791499,0.294237742697985,0.641775383274181,0.4584746476
20256,0.646611472598544,0.913928326417711
"11541","SCLT1",111.793170159705,-0.0839073793454058,0.185490332300048
,-0.452354461307868,0.651013647618065,0.913928326417711
"11542","DCTD",359.059656442715,-0.0834359461911559,0.183179972421485,
-0.455486181639854,0.648759506606668,0.913928326417711
"11543","KLKB1",3.49682013406234,-0.383612547133466,0.844803328381652,
-0.454085032865973,0.64976762544827,0.913928326417711
"11544","FAT1",206.169789459878,-0.146448104741384,0.325100764787258,-
0.450469886889433,0.652371662620821,0.913928326417711
"11545","PDCD6",884.777322679815,-0.105076425413734,0.230619813315448,
-0.455626183644542,0.648658811251759,0.913928326417711
"11546","GFM2",54.4729881101402,0.110169906803394,0.243073079398839,0.
453237796122316,0.650377519007057,0.913928326417711
"11547","LYSMD3",147.135275196742,0.0871262926382309,0.19281733488458,
0.451859230864199,0.651370396290862,0.913928326417711
"11548","GIN1",53.8726082203046,-0.135399157197936,0.296317148003047,-
0.456939998614401,0.647714173204361,0.913928326417711
"11549","FEM1C",226.836810002528,0.176534961819032,0.386859065616579,0
.456328874024624,0.648153503705791,0.913928326417711
"11550","FNIP1",450.541247392143,0.0987580615564772,0.218374324370128,
0.452242093210051,0.651094587109085,0.913928326417711
"11551","PKD2L2",2.37270238816354,-0.436133054256715,0.958919974901369
,-0.454816945805696,0.649240937970738,0.913928326417711
"11552","CTNNA1",745.229807594336,0.0603388137250083,0.13217091632663,
0.456521112223318,0.648015292639822,0.913928326417711
"11553","HARS2",50.0201865562109,-0.127610642243736,0.281018312195718,
-0.454100806622382,0.649756272743404,0.913928326417711
"11554","RGL2",169.351754149171,0.107902808475559,0.23939068698465,0.4
50739374345325,0.652177400531774,0.913928326417711
"11555","UQCC2",258.747030904057,-0.142540661795068,0.313846455554197,
-0.454173240680276,0.649704141469751,0.913928326417711
"11556","AL590999.1",3.42717606766131,0.438065220383996,0.967136910532
908,0.452950575676627,0.650584331282181,0.913928326417711

"11557","YIPF3",798.471401773807,-0.0950502563868063,0.21095996204824,
-0.450560644133369,0.652306236934549,0.913928326417711
"11558","HMGN3",1158.12000244824,-0.0962863021621766,0.213818132413139
,-0.45031869409528,0.652480661428455,0.913928326417711
"11559","MANEA",92.8688417092535,0.102190855308397,0.225873027203703,0
.452426111136484,0.650962039916114,0.913928326417711
"11560","AIG1",423.900151520631,-0.0986581404111634,0.217835429307023,
-0.452902178148955,0.650619182435382,0.913928326417711
"11561","CCR6",2.36105196495298,0.437821002924499,0.965849253181121,0.
453301590783957,0.650331587493414,0.913928326417711
"11562","MICALL2",54.920213936233,0.178708774921423,0.394260703725433,
0.45327564536048,0.650350267776673,0.913928326417711
"11563","AC102953.2",7.09471929263833,0.290950768579718,0.643341950215
186,0.452249023217592,0.651089595259365,0.913928326417711
"11564","FAM126A",378.444038141177,-0.13732672280223,0.300912692227188
,-0.456367332949015,0.648125852409075,0.913928326417711
"11565","PSMA2",1095.01441251306,-0.119667907492811,0.265777117526343,
-0.450256623318785,0.652525412010435,0.913928326417711
"11566","ZNF212",39.004125720007,-0.121940155387214,0.267659346749266,
-0.455579664480926,0.648692269086565,0.913928326417711
"11567","ERICH1",351.506339985684,0.0835440599789664,0.184193614821684
,0.453566536819663,0.650140843231819,0.913928326417711
"11568","ASH2L",156.266200444465,0.0831303953754763,0.184580566098867,
0.450374582397527,0.652440369236744,0.913928326417711
"11569","POMK",5.63911839202707,0.309985064604535,0.682500894691597,0.
454189975449934,0.649692097588495,0.913928326417711
"11570","AP003352.1",2.72012212032493,-0.486268649295794,1.07867403317
095,-0.450802220450529,0.652132100829745,0.913928326417711
"11571","FAM49B",75.8231642140508,-0.105384648724819,0.232139299069777
,-0.453971598721601,0.649849268787083,0.913928326417711
"11572","NAPRT",239.476817065716,-0.130755725378267,0.289787443973479,
-0.451212528691317,0.651836380699878,0.913928326417711
"11573","LINC01230",15.2074560277532,0.204413524050556,0.4483770769274
92,0.455896464313701,0.648464432149573,0.913928326417711
"11574","PDCD1LG2",89.8463255563949,0.115327176871162,0.25543813410374
4,0.451487704746242,0.651638084618656,0.913928326417711
"11575","DNAJA1",2309.86537867788,0.129640731024695,0.286553460033549,
0.452413769526695,0.650970929170804,0.913928326417711
"11576","NFX1",184.475889000524,0.110894783129251,0.246102511769781,0.
450604028101055,0.652274962956136,0.913928326417711
"11577","GALNT12",122.995546270442,0.119228420247172,0.263977197148128
,0.451661816002493,0.651512630103622,0.913928326417711
"11578","HDHD3",71.8897572526097,0.122862936506603,0.269673077916183,0
.455599563204415,0.648677957302153,0.913928326417711
"11579","PPP1R26",12.7023714062547,0.194451704326667,0.42948596244485,
0.452754504989523,0.650725526884164,0.913928326417711
"11580","IDI1",233.755299449289,0.0847567247529566,0.187131201795981,0
.452926737708671,0.650601496953498,0.913928326417711
"11581","WAC",834.941419276637,0.07843486293421,0.17326660034241,0.452
68310672228,0.650776945747151,0.913928326417711

"11582","AL359532.1",4.32662195388486,0.361863292091044,0.790456526505576,0.457790251528136,0.647103140156712,0.913928326417711
"11583","CSTF2T",159.864503171638,-0.0928546291816893,0.205931494943904,-0.450900573547447,0.65206121013437,0.913928326417711
"11584","AGAP5",21.6346151976006,-0.202400715636964,0.444833034203014,-0.455003788105791,0.64910651358256,0.913928326417711
"11585","ACSL5",151.950607126134,-0.10625658052412,0.23408153189737,-0.453929789602995,0.649879361633868,0.913928326417711
"11586","MTA2",45.5336652628907,-0.146938380223872,0.322510462174609,-0.45560810410026,0.64867181446248,0.913928326417711
"11587","PGM2L1",53.7203127840436,0.16707949748458,0.365085355711634,0.457645027034579,0.647207488703236,0.913928326417711
"11588","CREBZF",322.417484544128,-0.0943904243283079,0.207150001691339,-0.455662194340472,0.648632911876754,0.913928326417711
"11589","AP001528.1",357.586220666827,-0.140255364400248,0.308901864616126,-0.4540450559421,0.649796398046949,0.913928326417711
"11590","LINC02731",4.13431364748246,0.331067256998294,0.732499496773166,0.451969262035979,0.651291126341397,0.913928326417711
"11591","ALG10",22.374258359763,-0.148675989462337,0.329498575267265,-0.451218914502869,0.651831778707121,0.913928326417711
"11592","CBX5",516.618786482529,-0.101039078754294,0.220610004614904,-0.457998624906734,0.646953429189873,0.913928326417711
"11593","IFT81",97.7411058841342,-0.104129656909133,0.227266299917617,-0.458183447994179,0.64682065046355,0.913928326417711
"11594","SCFD1",456.590757251687,0.0682591421104811,0.149075684569127,0.457882466263834,0.647036884455161,0.913928326417711
"11595","PSMA6",776.861724316537,-0.117910203214536,0.257297432626719,-0.458264204235559,0.646762637913838,0.913928326417711
"11596","GPR135",24.0978774124132,-0.162518181392977,0.360603102460449,-0.450684368171242,0.652217050235935,0.913928326417711
"11597","EMC7",858.975277592011,-0.103789416482301,0.229965764917275,-0.451325511515318,0.651754960565798,0.913928326417711
"11598","ZFYVE19",123.922986443416,-0.0998487955105593,0.220010490330683,-0.453836520978992,0.64994649542075,0.913928326417711
"11599","IL32",469.545030669128,0.14504116029072,0.321995183750053,0.450445123437984,0.652389514727092,0.913928326417711
"11600","COQ7",123.763122025995,-0.103140083632375,0.226148339991489,-0.456072698284042,0.648337701981379,0.913928326417711
"11601","CCP110",135.891399240592,0.118007575203128,0.257585210191587,0.458130244028203,0.646858871564404,0.913928326417711
"11602","ZNF19",14.7143451558118,0.215464718364514,0.473196718813304,0.455338572306381,0.648865680428022,0.913928326417711
"11603","NPIPB15",62.5768769322642,0.160226862686064,0.354006647823969,0.452609756542589,0.650829772048882,0.913928326417711
"11604","TMEM220",223.043409473663,-0.109780773585162,0.243813004276514,-0.450266276447899,0.6525184524029,0.913928326417711
"11605","AC107993.1",3.6927890394973,0.349638027359954,0.768589404415184,0.454908726755076,0.649174904397365,0.913928326417711
"11606","TACO1",113.991474225442,-0.107782340758205,0.238959066230125,-0.451049388745132,0.651953953471347,0.913928326417711

"11607","SLC25A19",41.9752092183822,-0.138869964527707,0.30688360632342,-0.452516725123969,0.650896775168134,0.913928326417711
"11608","ZBTB14",79.0323916628163,-0.108233327506554,0.24023678189138,-0.450527711262342,0.652329977492647,0.913928326417711
"11609","AC007996.1",2.59934748643024,-0.385362625198367,0.854093670467955,-0.451194802775236,0.651849155110854,0.913928326417711
"11610","AC027097.1",27.3914668114496,0.144036732231219,0.319695337276209,0.450543737854941,0.652318424242356,0.913928326417711
"11611","TSHZ1",238.129518516124,0.0890445974139055,0.19640852079774,0.453364228049979,0.650286490585265,0.913928326417711
"11612","RANBP3",155.669691493867,-0.0766419873801663,0.168529611995716,-0.454768669271692,0.649275672560118,0.913928326417711
"11613","AC136469.1",8.94444528303617,0.327267852812939,0.714862236078552,0.457805485163406,0.647092194693705,0.913928326417711
"11614","BRD4",673.679615548478,0.0946661282564188,0.207484660451301,0.456256033822019,0.648205875872566,0.913928326417711
"11615","ETV2",14.4798624526263,-0.216247718595997,0.472949862676267,-0.457231803329687,0.64750444138176,0.913928326417711
"11616","RTN2",238.513140829889,0.140814396663347,0.30761622208305,0.457759983234338,0.647124888343187,0.913928326417711
"11617","ZNF701",55.776976442702,0.114438300785969,0.254028227339778,0.450494427270483,0.652353971523669,0.913928326417711
"11618","ZNF761",57.7686846731618,0.153620046968179,0.336078768356299,0.45709536404072,0.647602502321729,0.913928326417711
"11619","ZNF582-AS1",25.5548728429308,0.191571172816257,0.41995433356339,0.45617143938185,0.648266701530521,0.913928326417711
"11620","SRXN1",36.6047328797865,-0.12476085197919,0.273363903557689,-0.456391097564428,0.648108766307148,0.913928326417711
"11621","OGFR",245.620719413258,-0.0653691207825854,0.142843493154278,-0.457627570840651,0.647220032016236,0.913928326417711
"11622","AP001347.1",44.1277315074217,0.217704218847142,0.482727943661578,0.450987397157531,0.651998632245774,0.913928326417711
"11623","SOD1",2649.20311235957,-0.107790294304631,0.238416035059168,-0.452110086797981,0.65118967746327,0.913928326417711
"11624","GART",102.227531281628,0.0969346852566235,0.212881394740893,0.455345970344692,0.648860358927975,0.913928326417711
"11625","APOBEC3C",384.045392540204,0.0778771509908454,0.171004752404293,0.455409278958089,0.648814820995964,0.913928326417711
"11626","NDUFA6",553.461914359146,-0.12372802039908,0.269997308446756,-0.458256495632732,0.646768175420446,0.913928326417711
"11627","RABL2B",95.1112355945683,-0.0916070724629302,0.203073959842049,-0.451102014921964,0.651916025546003,0.913928326417711
"11628","NHS",19.3684361486648,-0.196356537536486,0.432183449961276,-0.454336087034523,0.64958694612674,0.913928326417711
"11629","FGD1",53.4322029067469,0.138195897868717,0.303827723768513,0.454849531683977,0.649217493116795,0.913928326417711
"11630","MT-CO1",57075.4049542352,-0.106525623310497,0.234020322245122,-0.455198173767652,0.648966674223554,0.913928326417711

"11631","LINC00672",11.5969588174095,-0.2967096549829,0.65927668239462
4,-0.450053312829435,0.652671999612061,0.914055042703196
"11632","LIPT1",85.9489418622958,0.114247995964465,0.25417651236898,0.
449482900287085,0.65308334072109,0.914361594153449
"11633","CCR1",12.3629188113318,0.261491402316126,0.582102064725157,0.
449219163033875,0.653273565006361,0.914361594153449
"11634","TEX264",486.850028404162,-0.121722265210243,0.27079095732862,
-0.449506388289496,0.653066400755045,0.914361594153449
"11635","ZNF454",14.1940163962857,-0.188800909044128,0.420389566527376
,-0.449109407266493,0.653352734573381,0.914361594153449
"11636","LINC01239",48.0731992738066,0.231278840988167,0.5149979247644
9,0.449086937765685,0.653368942864894,0.914361594153449
"11637","AC005906.2",7.42613947255135,-0.40560536465164,0.903253527636
505,-0.449049300380776,0.653396092820071,0.914361594153449
"11638","CYFIP1",388.370560488919,0.056404589502116,0.125583808367749,
0.449139027038785,0.653331368709829,0.914361594153449
"11639","AC099518.6",3.15720263281303,0.357502532913933,0.795789794502
321,0.449242419774322,0.653256789841727,0.914361594153449
"11640","PPP1R3D",46.0857820160674,0.124880511813916,0.277966914908704
,0.449263941555535,0.653241266265127,0.914361594153449
"11641","ADIPOR2",141.774923772806,0.0771481971740623,0.17188882527541
4,0.448826135442192,0.653557083623435,0.914429765945896
"11642","SEC23A",416.574645017398,-0.0743619875621698,0.16565623701443
6,-0.448893376442504,0.653508574383587,0.914429765945896
"11643","CHI3L1",1143.09532985837,-0.499751269767151,1.11389964631517,
-0.448650173667215,0.653684033516329,0.914460592089311
"11644","PDP1",216.882246051222,-0.085395186470799,0.190342350428456,-
0.448639970445761,0.653691395069552,0.914460592089311
"11645","EAF2",20.450333920456,-0.183466261432687,0.409525157406501,-0
.447997535962305,0.654154974908123,0.914794847723079
"11646","APBB1",115.236084220807,0.0934855530993923,0.208625881525689,
0.448101416831549,0.654080005567754,0.914794847723079
"11647","USP5",120.465721530468,-0.122553550439438,0.273467583153089,-
0.448146537247275,0.654047443894854,0.914794847723079
"11648","VSIG10",174.694247181081,-0.118985722071681,0.265549205645108
,-0.448074102811285,0.654099717368129,0.914794847723079
"11649","CLEC2L",13.2763547191483,0.268081739257341,0.598572899437313,
0.447868153585554,0.654248353201274,0.914799624503066
"11650","MYL12B",4139.07954210893,-0.127277865114908,0.284205671556495
,-0.447837175162098,0.654270711858353,0.914799624503066
"11651","TENT5C",44.3411150283849,0.17647257848178,0.396537725332988,0
.445033516883139,0.656295532690389,0.914882150791078
"11652","GPN1",127.86008659181,-0.109025345982316,0.244854914809312,-0
.445265091236674,0.656128192183888,0.914882150791078
"11653","AASDH",169.679275778974,0.0747001344728744,0.167343728413247,
0.446387415776982,0.655317421402618,0.914882150791078
"11654","RAPGEF6",109.380357577303,0.114068775467387,0.255211432167865
,0.44695793796713,0.654905430008579,0.914882150791078
"11655","TBC1D9B",340.430729060456,-0.0674846069781665,0.1512515355746
62,-0.446174689875161,0.655471064098165,0.914882150791078

"11656","AC019270.1",16.0658457413226,0.284629864472934,0.637913311888
917,0.446188940045976,0.655460771361843,0.914882150791078
"11657","AC009630.1",9.51746901186532,-0.259004989461188,0.57962829405
9395,-0.446846698333619,0.654985751284456,0.914882150791078
"11658","TTLL11",20.3572417768394,0.188557020631225,0.422459412066884,
0.446331683578096,0.655357672943275,0.914882150791078
"11659","POLR3A",87.6810873156309,0.111576091199971,0.250194028552423,
0.445958250264923,0.655627404011729,0.914882150791078
"11660","FAS",196.178533653133,0.071716416478412,0.160936458261856,0.4
45619452875768,0.655872156402609,0.914882150791078
"11661","LBHD1",4.68065792428048,0.304211641851413,0.680643871160519,0
.446946861260534,0.654913427835117,0.914882150791078
"11662","SMCO4",98.9621913119752,-0.114969963364156,0.257619922966946,
-0.446277454166101,0.65539684008528,0.914882150791078
"11663","ART4",5.3066851273987,0.509905151130757,1.14387575144643,0.44
5769700499363,0.655763610670778,0.914882150791078
"11664","PHETA1",40.6612452737455,-0.118631129102203,0.266556224194219
,-0.445051056154537,0.656282857839623,0.914882150791078
"11665","RPLP0",15579.396374585,-0.0729454153112427,0.163776608907246,
-0.445395809560051,0.656033739987637,0.914882150791078
"11666","LTB4R",22.3303628493666,0.158454517377376,0.354449402015249,0
.447044109755781,0.654843211872398,0.914882150791078
"11667","WDR93",3.67492848361791,-0.390523348347059,0.877196569028022,
-0.445194796851267,0.65617898656422,0.914882150791078
"11668","RCCD1",56.3485386810482,-0.104735237459267,0.234784888323751,
-0.446090198594234,0.655532092533767,0.914882150791078
"11669","NPRL3",188.230521071525,0.0845845829313722,0.189647090438094,
0.446010443587495,0.655589702047485,0.914882150791078
"11670","AC020663.3",5.80563829683915,-0.306500773399458,0.68678974369
0855,-0.446280359040165,0.65539474201868,0.914882150791078
"11671","AC133919.1",2.50226715382163,0.426918049589696,0.954913905283
299,0.44707491139009,0.654820972921534,0.914882150791078
"11672","AC016876.1",122.971742855431,-0.131481443406095,0.29528107521
3589,-0.445275550798469,0.656120634291381,0.914882150791078
"11673","AATK",9.65209276299747,-0.25447022518068,0.569229826336926,-0
.447043027977349,0.654843992927993,0.914882150791078
"11674","AC145207.5",18.5692067295483,-0.190060572906805,0.42504086820
163,-0.447158349057024,0.654760732001516,0.914882150791078
"11675","PSPN",8.4639084312702,0.217028938390949,0.485011842783957,0.4
47471420790899,0.654534718285687,0.914882150791078
"11676","PET100",767.445359769143,-0.128208577901635,0.287889461784061
,-0.445339600508897,0.656074353882555,0.914882150791078
"11677","TIMM44",180.911818470296,-0.0927867288034252,0.20754154622568
,-0.447075443403169,0.654820588807829,0.914882150791078
"11678","AC092329.4",21.9089412180128,-0.150265687772246,0.33633487578
618,-0.446774029666121,0.655038224308226,0.914882150791078
"11679","CLPTM1",350.332543821958,0.06935082852837,0.155289956501017,0
.446589271392549,0.655171643333174,0.914882150791078
"11680","IRF3",277.05697219898,0.0639890016381592,0.143344015771932,0.
44640162544329,0.655307158896486,0.914882150791078

"11681","STK4",248.415714342208,0.0813716832161701,0.182525342709105,0.445810329724207,0.655734259515432,0.914882150791078
"11682","SLC9A8",37.0634789023242,0.139724443634584,0.313924010899517,0.445090017912993,0.656254702264979,0.914882150791078
"11683","AP001437.1",2.72182225301152,-0.388827871581672,0.872758893569129,-0.445515794163458,0.655947048418196,0.914882150791078
"11684","PBDC1",361.575546397393,-0.111394600283658,0.24889353113453,-0.447559242604211,0.654471323375705,0.914882150791078
"11685","MTMR1",41.7987479215536,0.12307647109504,0.275257458653657,0.447132192882379,0.654779616180626,0.914882150791078
"11686","LRRC40",121.253450260626,0.0798202322903774,0.179418466959876,0.444883035971029,0.656404282613075,0.914913273178784
"11687","MAPK9",171.784930189137,-0.0718371469900715,0.161504311889043,-0.444800179944579,0.656464164322075,0.914913273178784
"11688","AC107959.1",46.8614940894241,0.105877540697066,0.238050379714414,0.444769467807975,0.656486361158673,0.914913273178784
"11689","ZMYND12",3.99048866544679,0.319960189952698,0.721186780330571,0.443657868778512,0.657289960279782,0.915073448593191
"11690","ZBED6",7.88909610294918,-0.238710137582527,0.537116300930205,-0.444429143500425,0.656732346852954,0.915073448593191
"11691","RAPH1",96.7507327005522,0.124648875649288,0.281024073456247,0.443552305381748,0.657366294978356,0.915073448593191
"11692","NADK2",174.118489604169,-0.0935353338517478,0.210660715231885,-0.444009381382707,0.657035801276393,0.915073448593191
"11693","LRRC24",15.2832649599163,-0.181764207280506,0.409752250656722,-0.443595384745752,0.657335143121353,0.915073448593191
"11694","POMT1",84.560397316568,0.103822936287456,0.233695860389198,0.444265192008744,0.65685086392823,0.915073448593191
"11695","ZFAND4",21.6667284291443,-0.171570515362508,0.386816106796922,-0.443545427265731,0.657371268785783,0.915073448593191
"11696","TSPAN14",306.601389232202,0.11032793720489,0.248654278125366,0.443700136738709,0.65725939659674,0.915073448593191
"11697","CCDC84-DT",6.10934378098619,0.293303139841234,0.660742137718602,0.443899553393016,0.657115207462366,0.915073448593191
"11698","ZBTB39",23.4340441447527,0.199294329129068,0.449128063605795,0.443736086160029,0.657233402258525,0.915073448593191
"11699","RAMAC",130.737094099352,-0.129682873803842,0.291854198920502,-0.444341298783802,0.656795846872052,0.915073448593191
"11700","PLXDC1",230.349457509059,0.137346908464779,0.309147538406388,0.444276248074893,0.656842871459727,0.915073448593191
"11701","ALKBH6",58.7664052928235,-0.105490737608928,0.237524430838435,-0.444125841020046,0.656951604605901,0.915073448593191
"11702","PPP1R37",89.128850518056,0.104379484381884,0.235341974895864,0.443522599094659,0.657387776747346,0.915073448593191
"11703","AL034417.4",9.26899310753631,0.345664657044038,0.780677121767974,0.442775441223669,0.657928168640521,0.915278156886621
"11704","HDDC2",529.950368349129,-0.121704702406831,0.274855427442882,-0.442795339859617,0.657913774368079,0.915278156886621
"11705","SGK3",124.369715937409,0.0937717475876675,0.211685909208475,0

.442975859556707,0.657783195850119,0.915278156886621
"11706","FRMD3",2.31131871814253,-0.39328173737205,0.88789264201652,-0
.442938390027488,0.657810298491434,0.915278156886621
"11707","LINC02308",3.29092788897763,0.393235968621295,0.8874811525952
85,0.443092191277915,0.657699053092762,0.915278156886621
"11708","AC090527.3",3.20023287733383,-0.41605123305094,0.939444769939
767,-0.442869284457898,0.657860285446468,0.915278156886621
"11709","NEURL2",26.0489593402342,0.139749338629411,0.31543996040415,0
.443029914315105,0.657744097493124,0.915278156886621
"11710","HUS1B",3.58970528665804,-0.370103095227997,0.836356891612337,
-0.442518139014205,0.65811430729703,0.915458919860062
"11711","FAM234B",30.7729051049271,-0.166236924075002,0.37594185721978
5,-0.442187856665867,0.658353272619233,0.91559058460892
"11712","FIGNL2",7.1044796307483,0.251291262299278,0.56833392506203,0.
442154253367598,0.658377587176885,0.91559058460892
"11713","NXT2",52.2137627184308,-0.119994238933213,0.271360739802877,-
0.44219454524033,0.658348432966906,0.91559058460892
"11714","AC022098.1",15.3911533375681,-0.179265729718624,0.40553520844
0589,-0.442047264916793,0.658455003913967,0.915620075017467
"11715","AL024508.2",3.46457132699345,0.387077841686633,0.876256692068
103,0.441740240263466,0.658677186976542,0.915772678274231
"11716","ZNF22",738.231470306592,0.0890538447825269,0.201576708455132,
0.441786382291032,0.658643793671526,0.915772678274231
"11717","AL355312.2",19.117898826311,0.212017141768968,0.4801831489213
74,0.441533906896188,0.658826520190159,0.915823962056452
"11718","PRCP",541.543136339037,0.072670092007597,0.164580676093092,0.
441546928428539,0.65881709548925,0.915823962056452
"11719","AC007389.1",4.96393004516011,0.302236193698957,0.685193479499
749,0.441096132321072,0.659143403398126,0.915846305235155
"11720","HELQ",122.557761977657,-0.100028708653942,0.226669766068492,-
0.441297092192331,0.658997930956605,0.915846305235155
"11721","AP000866.6",8.59669109818394,0.262878878191075,0.595749343982
804,0.441257520207463,0.659026575622149,0.915846305235155
"11722","NOP2",37.1765166708828,0.147644060366359,0.334799776882248,0.
440992110990225,0.659218708257842,0.915846305235155
"11723","PEX5",73.1575596950321,-0.108107139550529,0.245059567256535,-
0.441146374168526,0.659107032717917,0.915846305235155
"11724","ZNF699",45.3901670200749,-0.13874531557368,0.314638062586076,
-0.440967994886896,0.659236167283577,0.915846305235155
"11725","CSE1L",144.325051268989,0.0923436266625943,0.209241388374161,
0.441325816943382,0.658977138507346,0.915846305235155
"11726","LRIG1",50.5308840515719,-0.128221682790279,0.291095716807158,
-0.44047945533744,0.65958988895594,0.916194340149177
"11727","LRRC29",23.0437563551119,0.171241876937228,0.388773784158339,
0.440466625875899,0.65959917901218,0.916194340149177
"11728","C3orf33",53.4498727683751,-0.132739389367344,0.30142160695198
6,-0.440377817335729,0.659663488398585,0.916205539096569
"11729","HLA-
DQA2",12.1478886217238,-1.3530862842033,3.07465606837198,-0.4400772815
2819,0.659881135598628,0.916351561616884

"11730","H3F3B",12884.5010145813,-0.0949045273394854,0.21561961131919,
-0.440147938115863,0.659829963704369,0.916351561616884
"11731","ABHD5",787.964837966638,-0.162576023918526,0.369694071903391,
-0.439758265750637,0.660112197438815,0.916594287279929
"11732","DTNB",17.7361944503613,0.183807522825375,0.418146189480484,0.
439577180061697,0.660243371497269,0.916620974614271
"11733","ERLEC1",760.010397206213,0.0672806734481473,0.153057986723963
,0.439576365064089,0.660243961885275,0.916620974614271
"11734","CENPJ",49.8845395744616,0.134486296379929,0.306185629674279,0
.439231248452108,0.660493984945951,0.916889937002267
"11735","PSRC1",11.1104032729284,-0.241927916936869,0.551522655785697,
-0.438654540115346,0.660911871444233,0.917001147696346
"11736","ADI1",712.063010400592,0.115668413292927,0.263533667335009,0.
43891323056605,0.660724409629114,0.917001147696346
"11737","TMEM217",6.14670937078199,-0.549556177494429,1.25237724037194
,-0.438810415726827,0.660798912561844,0.917001147696346
"11738","GLO1",635.62732073791,0.0840086217486878,0.19146712429297,0.4
38762644286356,0.660833530424794,0.917001147696346
"11739","AC073332.1",57.3703678004333,-0.133925089225718,0.30528093614
6866,-0.43869457069959,0.660882861614392,0.917001147696346
"11740","DUSP3",151.155510278348,0.0914659396718716,0.208376848276476,
0.43894482726082,0.660701514325198,0.917001147696346
"11741","NDUFV3",375.060644499805,-0.100619967140403,0.229450580554278
,-0.438525659413622,0.661005273671606,0.917052627786116
"11742","MYLK-
AS1",10.8464965372996,-0.257329288664784,0.587058941561144,-0.43833637
5527265,0.661142460773307,0.917086736228937
"11743","DAXX",215.222476951754,-0.091563698922577,0.208881416004551,-
0.438352538363594,0.661130746005296,0.917086736228937
"11744","FBXO21",263.032786648818,-0.0896044751683959,0.20458534639142
1,-0.437980905030026,0.661400125544191,0.917366029035194
"11745","TCAP",3.55475985837746,-0.381002390752653,0.870164655553287,-
0.437850915135591,0.661494359462802,0.917418613988045
"11746","AL353622.1",5.78491797990879,0.285571390123825,0.652502909351
425,0.437655351464529,0.661636140057197,0.917521582627589
"11747","IFI44L",192.678291823754,-0.157410577608736,0.359878867715956
,-0.437398779783247,0.661822168906101,0.917521582627589
"11748","ATP1B1",157.098951668668,-0.207885953639462,0.475816833990766
,-0.436903318228325,0.662181465380468,0.917521582627589
"11749","LINC01703",7.23744870160772,0.289043945057825,0.6622577900154
43,0.436452314212996,0.662508589976366,0.917521582627589
"11750","ACTR8",72.9154772379909,0.111686304808665,0.255981340903377,0
.436306429267525,0.662614417796482,0.917521582627589
"11751","SNX4",225.925579056389,0.0707202976317396,0.16190151626824,0.
436810594871573,0.662248714709977,0.917521582627589
"11752","THPO",4.75370979123093,0.387648490973103,0.887102220152795,0.
436982888968911,0.662123757401135,0.917521582627589
"11753","PPP1R2",417.408302081417,-0.0763944948820907,0.17480573341034
7,-0.43702511005608,0.662093137744033,0.917521582627589
"11754","HIST1H1E",54.9204737640797,0.166847573130356,0.38214972564209

,0.436602624403347,0.662399559043973,0.917521582627589
"11755","RALA",452.91210634219,0.0652861623063558,0.149425273060568,0.436915128004434,0.662172900317487,0.917521582627589
"11756","RAB6A",382.129752896505,0.0721934480942626,0.165507229024222,0.436195134918831,0.662695157444508,0.917521582627589
"11757","RPL21",23223.9809569491,-0.0962477499194561,0.219996455681383,-0.43749682067083,0.661751081302503,0.917521582627589
"11758","PARP2",89.1967859537113,0.128439431340949,0.29432168615107,0.436391327532092,0.662552830099427,0.917521582627589
"11759","ZNF106",1537.19213696018,0.0843506366886125,0.192905711924803,0.437263551436432,0.661920225439127,0.917521582627589
"11760","DPP8",186.213742985645,0.0817609135692776,0.18742840779061,0.436224767275508,0.662673659958856,0.917521582627589
"11761","GCSH",592.352267495349,-0.121824538927338,0.278496222017952,-0.437436953523501,0.66179448948259,0.917521582627589
"11762","BRIP1",3.17057999691202,0.379513416737874,0.869099896345808,0.436674101945662,0.662347713684896,0.917521582627589
"11763","HNRNPUL1",666.714868345557,-0.0752420900034893,0.172310449975403,-0.436665855229499,0.662353695256794,0.917521582627589
"11764","DNAJC28",6.72049295171122,0.244694328461291,0.560925910016747,0.436232885826198,0.662667770215025,0.917521582627589
"11765","TENM1",86.2452249069237,0.25073332070314,0.57332119058247,0.437334821774868,0.661868545298114,0.917521582627589
"11766","PGS1",69.4102976954602,0.0919108482029788,0.210776398947064,0.436058537208721,0.662794259038083,0.917580799377132
"11767","BX284668.5",168.988711181707,0.11210141902255,0.25717980789466,0.435887327003784,0.662918480321366,0.917661592937347
"11768","TAP2",108.334399487019,0.0831158838626431,0.190710264486015,0.435822812613938,0.66296529103608,0.917661592937347
"11769","MTRR",91.9590692093343,0.0830223892070904,0.190708444064636,0.435336723626942,0.663318032531168,0.917892314986068
"11770","CAST",2804.05726061688,-0.092964099135323,0.213571868573524,-0.435282510548993,0.663357378108908,0.917892314986068
"11771","HOXA-AS3",5.95435651123132,0.311476800620554,0.71536946228699,0.435406900966646,0.663267102140967,0.917892314986068
"11772","SERPING1",9082.9538472758,-0.0588904178757269,0.135255331047266,-0.435401824236764,0.663270786466573,0.917892314986068
"11773","KRBOX1",35.1422880021395,0.189538380698539,0.435644772291559,0.435075531152453,0.663507603610703,0.917900083591247
"11774","NOP14",95.400740686849,-0.11400284013665,0.262050279002842,-0.435041857503282,0.663532045201481,0.917900083591247
"11775","SOCS6",99.3143151108561,0.0788430686175142,0.181197730105402,0.435121723498696,0.663474076065087,0.917900083591247
"11776","NMNAT1",67.5890046863255,0.101672989474259,0.23389432843792,0.434696258576639,0.663782914486246,0.918153383825122
"11777","TRIM56",578.628682525401,0.131326324459798,0.302184371000557,0.434590061772442,0.663860010017976,0.918153383825122
"11778","VTI1B",676.200833712379,-0.0799592525915105,0.184001898193342,-0.434556672385481,0.663884250395499,0.918153383825122

"11779","NDUFA7",258.67056435919,-0.117116074384639,0.269559320525795,-0.434472360874761,0.663945461316109,0.918160083146116
"11780","PRPF19",183.960817935047,-0.0784249938606693,0.180565549187183,-0.434329772283251,0.664048987027872,0.918225292843549
"11781","DPY30",455.660893286263,-0.105867844881807,0.243798559527191,-0.434243110735032,0.664111910330873,0.918234352548985
"11782","JUN",42628.6914147406,-0.0688972739793145,0.15897668990811,-0.433379723902526,0.664738928351656,0.918303821105468
"11783","ZRANB2",781.535019013518,-0.083454794337189,0.192336090214802,-0.433900856797006,0.664360437549251,0.918303821105468
"11784","AL359504.2",13.5501459210049,0.182760062118129,0.421665109428456,0.433424672878082,0.664706279232258,0.918303821105468
"11785","ZBTB38",728.009671952389,0.0803543272238229,0.185288843931759,0.433670616744832,0.664527646809369,0.918303821105468
"11786","TBC1D19",50.056528081494,-0.111918640365319,0.258133290054702,-0.433569185677684,0.66460131528466,0.918303821105468
"11787","NUB1",475.247099240391,-0.0695633362588597,0.160492574872561,-0.433436477133578,0.664697705203542,0.918303821105468
"11788","GSR",79.6989360338587,0.138070893044596,0.318782700012254,0.433119153076025,0.664928208998589,0.918303821105468
"11789","MINPP1",107.366366019398,-0.0916268498057811,0.211489927346568,-0.433244509350237,0.66483714657232,0.918303821105468
"11790","C11orf98",10.7161244093343,-0.242510904275846,0.559958590206317,-0.433087211299843,0.664951413219902,0.918303821105468
"11791","IRF2BPL",337.54990364458,0.0923623211412815,0.213262517590046,0.433092144766055,0.664947829264185,0.918303821105468
"11792","CDC42BPB",288.633339377886,0.100297791257701,0.231064246684337,0.434068847504219,0.664238446718501,0.918303821105468
"11793","WFIKKN2",4.23464824383939,0.316712372860758,0.730543311477475,0.433529905598928,0.664629844924222,0.918303821105468
"11794","AKT1S1",189.672464424102,-0.0858900834383376,0.198197229169133,-0.433356630657247,0.664755702599123,0.918303821105468
"11795","ZNF836",55.6809192621542,-0.122765398673989,0.282997930795979,-0.433803167142215,0.664431381527641,0.918303821105468
"11796","ANKRD39",129.26859726038,-0.111976755315931,0.258736834892929,-0.432782426832537,0.665172841163981,0.918340929138407
"11797","CXCR6",3.60174752884059,0.379422780954264,0.876673655774775,0.43279820085268,0.665161380516472,0.918340929138407
"11798","GPSM1",68.8615080462662,0.101666054571662,0.234872604253928,0.432856164279374,0.66511926773891,0.918340929138407
"11799","C18orf32",881.987158065744,-0.114982644526475,0.265708488993446,-0.432739823112355,0.665203795377498,0.918340929138407
"11800","CPT2",89.3489030829887,0.0965161802181051,0.223545071757575,0.431752663833148,0.665921186801222,0.918652489141069
"11801","MRFAP1",2062.11201198446,0.0888228113396626,0.20549241233138,0.432243752126603,0.665564263345625,0.918652489141069
"11802","AC097382.3",52.9232433389912,0.160051829892335,0.370699331092063,0.431756457236731,0.66591842946161,0.918652489141069
"11803","AC008443.8",4.12593827441033,0.314796846426557,0.728828569482379,0.43192166115295,0.665798350837801,0.918652489141069

"11804","TM7SF3",311.978634051357,-0.0586444565725409,0.135835689584448,-0.431730841518512,0.665937049037863,0.918652489141069
"11805","AC010198.2",8.30593318803436,0.300378537471643,0.695375911935336,0.431965692679293,0.665766347926961,0.918652489141069
"11806","DAZAP2",694.938615348512,0.0559158703569853,0.129441240748679,0.431978788472452,0.665756829784741,0.918652489141069
"11807","CS",133.550548635084,0.10918235560314,0.252843433175298,0.431818039456232,0.665873667365259,0.918652489141069
"11808","AC007216.1",2.71652453465123,-0.439612807683047,1.0176025656382,-0.432008352305343,0.665735342718641,0.918652489141069
"11809","CHMP7",97.9786806026594,-0.0831729589453564,0.192699266495231,-0.431620526938616,0.666017236951464,0.91868530550448
"11810","KCNMB2",10.2769134827619,0.364941921116831,0.845748982185694,0.431501460603241,0.66610379082012,0.918726896585008
"11811","CDC42-AS1",5.37602074205245,0.323925345103838,0.756210693887604,0.428353298521302,0.668393920286224,0.918839319439747
"11812","SH3D21",17.9412011261175,-0.18010007184471,0.421005204409197,-0.427785856228183,0.668807035248361,0.918839319439747
"11813","AL139289.2",2.42884230761596,-0.459348359553488,1.0706495860216,-0.429037068290821,0.667896248837388,0.918839319439747
"11814","AC244033.2",17.1770337374935,-0.166721702298912,0.389690362805689,-0.427831217324828,0.668774007323525,0.918839319439747
"11815","TRAK2",170.489078497936,-0.105528997836443,0.246911914189672,-0.427395325101154,0.669091411861526,0.918839319439747
"11816","ASB1",110.010417199587,0.0831496715433789,0.194532440405172,0.427433446936639,0.669063650245548,0.918839319439747
"11817","TSEN2",37.4906179373665,0.147471671269912,0.345357546479623,0.427011579081314,0.669370893941148,0.918839319439747
"11818","ZNF639",154.648064400337,0.0672556966327274,0.156746791668761,0.429072237566767,0.667870655359251,0.918839319439747
"11819","TENM3",29.6179738016088,0.158452142482234,0.369724386328775,0.428568275021306,0.668237437182187,0.918839319439747
"11820","CERT1",486.729395265515,0.0821920567353778,0.192390089557328,0.4272156477732,0.669222265086711,0.918839319439747
"11821","AL133338.1",9.44997745227846,-0.213305364789741,0.49479567857879,-0.431097873373554,0.66639720687591,0.918839319439747
"11822","ASF1A",167.514043753069,-0.108308570973273,0.25322769505847,-0.427712185858123,0.668860676839355,0.918839319439747
"11823","SOD2-OT1",3.61782001253972,-0.391687660696015,0.913369676800875,-0.428838038578115,0.668041094540198,0.918839319439747
"11824","GTF2IRD2B",137.017572260279,0.0789583289125761,0.183372972257187,0.430588695491253,0.666767462299041,0.918839319439747
"11825","AP1S1",291.599835752666,-0.121710072579938,0.282419900638887,-0.430954307060541,0.666501594793983,0.918839319439747
"11826","AC073073.2",3.96146711524231,-0.308791737274187,0.721018889813618,-0.428271355489742,0.668453571064221,0.918839319439747
"11827","AC083862.3",46.6091148352626,0.127304225671824,0.295874639229421,0.430264067252862,0.667003562395966,0.918839319439747

"11828","PSD3",125.792330628511,0.117503394586392,0.272709345497908,0.
430874102872626,0.666559914547017,0.918839319439747
"11829","AC124067.2",7.43443942043371,-0.377052803940474,0.87967539682
1788,-0.428627201923281,0.668194546338782,0.918839319439747
"11830","TERF1",443.591012869557,0.0684950839029797,0.159289528043358,
0.43000368413632,0.667192961252068,0.918839319439747
"11831","PLEKHF2",81.1869939991464,-0.100165132954207,0.23393631790638
8,-0.42817264908089,0.668525427593517,0.918839319439747
"11832","CDC37L1",204.282421895873,-0.0874905101692328,0.2029715018128
97,-0.431048247600213,0.666433289339643,0.918839319439747
"11833","PIGO",48.4132638519418,-0.115944875938253,0.271457322545166,-
0.427120089637524,0.66929186109864,0.918839319439747
"11834","TMEM203",284.587796956049,-0.0858656786404022,0.1994178951924
47,-0.430581611332012,0.666772614211819,0.918839319439747
"11835","COMMD3",501.529215954797,-0.10123496854416,0.235613564903139,
-0.429665280883884,0.667439142499098,0.918839319439747
"11836","MSRB2",642.703574771957,-0.103279828746544,0.240909949190056,
-0.428707195754153,0.668136323343709,0.918839319439747
"11837","OTUD1",227.350109987649,-0.139922282179691,0.325835138101184,
-0.429426620453195,0.667612784468651,0.918839319439747
"11838","ZNF503-
AS2",21.6875146082931,-0.157832738371492,0.369066714197156,-0.42765367
967369,0.668903278139269,0.918839319439747
"11839","PAMR1",275.441651494287,0.179731761942873,0.420568845461862,0
.427353960908574,0.669121535183785,0.918839319439747
"11840","INTS5",72.0957400604176,-0.102655399088041,0.239628666332701,
-0.428393650305235,0.668364546803015,0.918839319439747
"11841","KDM4D",10.124430569895,0.194678729568216,0.455758132126091,0.
427153605927005,0.669267450503191,0.918839319439747
"11842","SCYL2",99.7504277123486,0.0900876603427813,0.210644393517794,
0.427676516038731,0.668886649704752,0.918839319439747
"11843","TRIM13",177.986745381645,0.090267098507029,0.211312792740686,
0.427172900117793,0.669253398310034,0.918839319439747
"11844","DHRS12",255.187970814463,-0.1119209560237,0.259870861108422,-
0.430679128650001,0.666701696701729,0.918839319439747
"11845","UNC79",3.27222959365355,-0.362328538724939,0.841573382342573,
-0.430537070595525,0.666805006558176,0.918839319439747
"11846","NIPA1",34.0984447157731,0.121235893579559,0.283944220707173,0
.426970808835684,0.669400589587542,0.918839319439747
"11847","NSMCE3",266.952784150501,-0.104701280416497,0.24478223128466
8
,-0.427732355682039,0.668845990421683,0.918839319439747
"11848","ZFAND6",409.273649342413,0.0826862471989833,0.192319203370495
,0.429942750125122,0.66723728681972,0.918839319439747
"11849","SRCAP",112.352172981427,0.10960026379277,0.256137921787327,0.
427895498753096,0.668727204390111,0.918839319439747
"11850","AC007611.1",10.6803210261745,-0.1879534409865,0.4389188654317
94,-0.428219098765777,0.668491612661676,0.918839319439747
"11851","RIPOR1",134.090108213155,0.0863342782396815,0.201476156105994
,0.428508662803068,0.668280827945633,0.918839319439747
"11852","CFDP1",969.569071836234,0.0699933412362046,0.163709574829705,

0.427545800598491,0.668981833122569,0.918839319439747
"11853","SHBG",5.09600888999886,-0.326108892127195,0.760504579934907,-
0.428805954271974,0.668064445346166,0.918839319439747
"11854","AC006441.4",3.90916884471213,0.380558317514371,0.885866052287
296,0.429589006748564,0.667494635272317,0.918839319439747
"11855","GJD3",7.20063321518962,0.264561269860064,0.615429512586018,0.
429880700306985,0.667282425258393,0.918839319439747
"11856","C17orf113",8.51403753762552,-0.229031028319709,0.535673983853
205,-0.427556751351346,0.668973858878885,0.918839319439747
"11857","RAB37",3.50197666950162,0.390772953329295,0.911275537445765,0
.428819755685093,0.668054400703725,0.918839319439747
"11858","SBNO2",82.1037945151242,0.108891326126751,0.253518353606225,0
.429520484721534,0.667544489597842,0.918839319439747
"11859","FBXL12",180.003329559094,-0.0962291905193404,0.22369206627496
9,-0.430185978974564,0.667060360436168,0.918839319439747
"11860","AC006504.5",22.1190188508008,-0.151965738865819,0.35309316482
3917,-0.430384255502657,0.666916146384205,0.918839319439747
"11861","PDCD2L",44.6570984664792,-0.135258098377719,0.31418230534569,
-0.430508326141716,0.666825911353794,0.918839319439747
"11862","AC021092.1",5.77107780438962,0.268592195001275,0.625230748170
437,0.429588909034361,0.667494706364844,0.918839319439747
"11863","KPTN",40.7915221558716,0.120117628832885,0.278829035094015,0.
430793115904821,0.666618805536443,0.918839319439747
"11864","MORC3",301.965020681043,0.0657678334736678,0.15260468265206,0
.430968646116968,0.666491168490616,0.918839319439747
"11865","TOP3B",53.73648406987,0.113294244296047,0.264321184024912,0.4
28623398892497,0.66819731439998,0.918839319439747
"11866","TPST2",66.9107844654466,0.119237838141765,0.278885350608223,0
.427551457549557,0.668977713774981,0.918839319439747
"11867","RNF113A",90.246319863353,-0.111472021399066,0.260116240988307
,-0.428546948762332,0.668252960091658,0.918839319439747
"11868","RPP30",257.344768625183,-0.0977315824955736,0.22898618510348,
-0.426801217075205,0.669524119947125,0.918854022227544
"11869","CCDC157",7.05073130300134,-0.268220326591213,0.62842674652068
6,-0.426812397906721,0.669515975572473,0.918854022227544
"11870","JPX",728.108869983097,0.0770202714886983,0.180629590522222,0.
426398970766768,0.669817151247439,0.919178734344527
"11871","ATP10D",152.054874929466,0.0872541359956654,0.204722003210931
,0.426207904510219,0.669956358186531,0.91921488531843
"11872","ZNF510",54.3578496041295,0.106188129587314,0.249109284772944,
0.426271263570532,0.669910194823653,0.91921488531843
"11873","ZNF276",97.0906927965111,0.102477912640902,0.240541519789196,
0.426030037270534,0.670085958782851,0.919315268475858
"11874","AL122017.1",8.1258367391261,0.253630595396644,0.5955340858725
43,0.425887621570878,0.67018973516658,0.919326919138348
"11875","CDC42EP2",195.436699956278,0.147173040429909,0.34558737150409
4,0.425863479297203,0.670207327937128,0.919326919138348
"11876","CRYGS",5.69220553051342,0.294958683389081,0.693570968311294,0
.425275417895946,0.670635911325377,0.919390308667821
"11877","CALHM6",29.5404275417241,0.164073410229776,0.385514803618415,

0.425595615757927,0.670402535514185,0.919390308667821
"11878","RPS24",24549.0868821134,-0.102344954708391,0.24048908964399,-0.425570053343782,0.670421165479969,0.919390308667821
"11879","ANO9",2.3347924844835,0.506275344001972,1.19029328075698,0.425336639454104,0.670591287611313,0.919390308667821
"11880","C12orf43",145.484535345377,-0.083755277547479,0.196981270055589,-0.425194118830906,0.670695171107137,0.919390308667821
"11881","UBC",12639.8742992268,-0.0786861656060599,0.185001408980936,-0.425327385556119,0.670598032600277,0.919390308667821
"11882","NEK5",18.6515414189861,0.172977220858076,0.406971710834541,0.425034999369777,0.670811160958645,0.919390308667821
"11883","SLC47A1",8.28159867281525,0.377132548242834,0.887014934793679,0.425170460439379,0.670712416353478,0.919390308667821
"11884","ZNF549",14.1792600266485,0.168024262519053,0.395250601789772,0.425108176327137,0.670757817769764,0.919390308667821
"11885","PIGT",1305.56214723534,0.0720840266593203,0.169603797427793,0.42501422581655,0.670826304382745,0.919390308667821
"11886","TRMT2B",29.0325504313383,-0.150672898546657,0.35456764845448,-0.42494824105759,0.670874406582707,0.919390308667821
"11887","DERL2",394.600624777321,-0.0945222187202667,0.222574137541911,-0.424677456977534,0.671071819471423,0.919583483416339
"11888","IMPAD1",388.468162471971,0.0664910614658226,0.156698815223728,0.424323957847987,0.671329569244047,0.919746529796929
"11889","PRDX3",739.115623418195,-0.100883732800052,0.237723560237734,-0.424374145747962,0.671292972965739,0.919746529796929
"11890","DHX34",23.3832698370222,0.151741423714897,0.357642886509626,0.424281956774815,0.671360196407728,0.919746529796929
"11891","FKBP7",402.92646763367,-0.104260966621284,0.245980528625036,-0.423858616794077,0.67166892619836,0.919819267897528
"11892","COL4A4",38.6611542715311,-0.487636183737416,1.1518743324435,-0.423341479189818,0.67204613509485,0.919819267897528
"11893","GYPE",7.95087595996651,0.265008393397931,0.625337840006804,0.423784355341502,0.671723088673624,0.919819267897528
"11894","ATP6V0E1",2765.83129312039,-0.0986041977661625,0.232692662037597,-0.423752931883305,0.671746007837384,0.919819267897528
"11895","NUP205",59.024890973725,-0.104288384161842,0.246370726801158,-0.423298601728814,0.672077414347474,0.919819267897528
"11896","TRIQK",255.282406182954,0.0983143087525669,0.232210677386371,0.423384100417499,0.67201504332799,0.919819267897528
"11897","ABL1",351.667639243698,0.0760060041185765,0.179564324282872,0.423280094317859,0.672090915741689,0.919819267897528
"11898","NRXN2",68.7359956041335,-0.205300388111364,0.484800131965217,-0.423474282647459,0.671949258126773,0.919819267897528
"11899","TAPBPL",183.204156638016,0.141285978527196,0.33341106680087,0.42375911478541,0.671741498222216,0.919819267897528
"11900","STON2",42.1309507974842,0.15203539968544,0.358846089451812,0.42367857461592,0.671800242625033,0.919819267897528
"11901","GFOD2",86.6516197197799,-0.0879426933007607,0.207678836696102,-0.423455248015704,0.671963143104096,0.919819267897528
"11902","MVB12A",511.525271054414,-0.101461448513359,0.239670361098973

,-0.423337487573025,0.672049046968816,0.919819267897528
"11903","NUAK2",3.66617362418615,0.340472807906311,0.805164745124207,0.422861047963282,0.672396643746198,0.91994133903699
"11904","GYPC",2177.25712860145,-0.0876116409427878,0.207262222581559,-0.422709164514108,0.672507468312639,0.91994133903699
"11905","JADE2",159.178809058004,0.0975439736067837,0.230767673174616,0.422693405297607,0.672518967723769,0.91994133903699
"11906","IFITM3",9692.08116647037,0.0921206170296924,0.21793265272617,0.422702224184096,0.672512532622747,0.91994133903699
"11907","DPH1",7.18016920622135,-0.242758638867032,0.573893059279812,-0.423003266796211,0.672292877598226,0.91994133903699
"11908","COX10",42.6401560159129,0.110089056815732,0.260373747758924,0.422811661172775,0.67243267895006,0.91994133903699
"11909","MTSS2",25.3232658272561,0.153934260235545,0.364270719484698,0.422582030346284,0.672600239579236,0.919975254220016
"11910","IGHA2",19.3896002661777,-0.482155274388562,1.14206962610585,-0.422176777463718,0.67289599053513,0.920302501244898
"11911","E4F1",80.1113035570787,0.0866709573602542,0.205338578864096,0.422088035476362,0.672960760623902,0.920313813265279
"11912","BARD1",51.3194880187151,0.116721364969121,0.276653592256493,0.421904389590957,0.67309480589836,0.920317166309339
"11913","PPIB",4686.51070204205,0.103725541498852,0.245836657096118,0.421928701456014,0.673077059787831,0.920317166309339
"11914","ZNF24",338.981829770832,-0.0671736652710227,0.159234972725782,-0.421852461938132,0.67313271038182,0.920317166309339
"11915","DDX6",576.353162815145,0.0745408866511895,0.176777410883479,0.421665224525334,0.673269390840622,0.920426781989332
"11916","FGD5",136.10813026205,-0.0939033947786037,0.22275871691011,-0.421547565370906,0.673355285752452,0.920466956161605
"11917","UBXN6",471.363327911868,-0.0665782514981857,0.157973503849805,-0.421452014899193,0.673425043760022,0.920485066527398
"11918","SPTLC3",62.6813195324169,-0.124494028346383,0.295630151097052,-0.421114111278566,0.673671757728333,0.920745029504683
"11919","GLYCTK",16.9876416060439,-0.186820496853316,0.443896764542033,-0.420864741030626,0.673853853238094,0.920916638593449
"11920","SLC35D1",63.7096275439973,0.0950466284072824,0.227618601583973,0.417569687828074,0.676261762999647,0.920965630421165
"11921","DIRAS3",6.43493649814806,-0.349098544571868,0.836422732476553,-0.417370942965918,0.676407105033128,0.920965630421165
"11922","XCL1",7.97217032710229,-0.31304602743228,0.749066049328591,-0.417915119384829,0.676009177751078,0.920965630421165
"11923","HADHB",689.843084205018,-0.0928870496167326,0.222042547634985,-0.418329957956657,0.675705889137024,0.920965630421165
"11924","WDR54",82.5560588460455,0.110196014416454,0.263511977278397,0.418182184941193,0.675813920004163,0.920965630421165
"11925","STEAP3",7.67657539880375,0.323481568105202,0.775260674707832,0.417255226091676,0.676491734288075,0.920965630421165
"11926","CYP27A1",394.132260932568,-0.0708493850733849,0.168844757885993,-0.419612583537973,0.67476849426093,0.920965630421165
"11927","LIMD1",49.5542024327321,0.104231937840542,0.248645466151555,0

.419199028455281,0.675070682080415,0.920965630421165
"11928","SHISA5",519.422111593834,-0.056991842167391,0.136228453228144
,-0.418354909102182,0.675687649023922,0.920965630421165
"11929","ABTB1",286.200876862038,-0.0661539600593571,0.157310914692358
,-0.420530007016548,0.674098313246781,0.920965630421165
"11930","SSR3",1093.67792853522,-0.100734334214401,0.240510954198244,-
0.418834703600941,0.675336940410276,0.920965630421165
"11931","CPEB2",75.5391552323508,0.118651430101201,0.283050670363955,0
.419187949453132,0.675078778311662,0.920965630421165
"11932","CENPU",10.236253150286,0.202080374876992,0.484067633549422,0.
41746309992932,0.676339709185416,0.920965630421165
"11933","ARHGEF28",11.3306696700018,0.369812896154684,0.88370718615989
1,0.418478996149946,0.67559694011366,0.920965630421165
"11934","PCSK1",4.25421465531537,-0.442359876873294,1.05366327566733,-
0.419830402263123,0.674609353553896,0.920965630421165
"11935","AC139795.3",9.50117312905167,0.25444284993526,0.6082603538150
54,0.418312402475971,0.675718722888485,0.920965630421165
"11936","POLR1C",70.4467506834365,-0.10594764593861,0.253247113107796,
-0.41835677666151,0.675686283783907,0.920965630421165
"11937","ICK",332.369269499269,0.0944607395746104,0.225252786323583,0.
419354366782015,0.67495716904688,0.920965630421165
"11938","TRAF3IP2",136.518995243053,-0.0985643116978901,0.234902245774
332,-0.41959714507191,0.67477977432265,0.920965630421165
"11939","SGK1",485.013478466048,0.296118637472185,0.705842545696869,0.
419525061612475,0.674832442819496,0.920965630421165
"11940","RPA3",317.947805288703,-0.109018020586855,0.260533401163713,-
0.418441628213155,0.675624255964828,0.920965630421165
"11941","C7orf25",73.8677942460584,-0.125629094443244,0.29931343892250
2,-0.419724202479835,0.674686942442075,0.920965630421165
"11942","FZD3",30.52136828525,0.16656121290815 7,0.397449787998226,0.41
9074856592703,0.675161425626498,0.920965630421165
"11943","WASHC5",194.348713029563,0.0794355509359805,0.189676016473843
,0.418795968055007,0.675365251771552,0.920965630421165
"11944","DENND3",19.173048545069,-0.261355295044,0.626343532873173,-0.
417271483342547,0.676479844340394,0.920965630421165
"11945","NOL6",45.1029953978344,0.104124285183365,0.249185001703966,0.
417859359397021,0.676049947906241,0.920965630421165
"11946","DPCD",190.215521301069,-0.0924240190242459,0.221579842995365,
-0.417113839304324,0.676595142718092,0.920965630421165
"11947","CUL5",341.241239750165,0.0678152281385762,0.162261493330847,0
.417937902249566,0.675992519826232,0.920965630421165
"11948","CLSTN3",145.738461229381,-0.15404110941604,0.367651789808406,
-0.418986425977461,0.675226052708171,0.920965630421165
"11949","GDF11",108.68515963862,0.174040576514383,0.415224543711326,0.
419148095049459,0.675107903128913,0.920965630421165
"11950","ALG11",47.6283812922129,0.125032366840525,0.298295037366894,0
.419156711235324,0.675101606547895,0.920965630421165
"11951","PIBF1",192.792705894864,0.079387252417843,0.189224550187353,0
.419539918785597,0.674821587147812,0.920965630421165
"11952","AC009754.1",2.450721986343,0.390820545103294,0.93623000926375

1,0.417440737037082,0.676356063284655,0.920965630421165
"11953","AC015871.1",40.1083959636142,0.163814574360787,0.390984664182086,0.418979539014597,0.675231085955972,0.920965630421165
"11954","AC104041.1",12.1292894093306,0.268699222251662,0.643415383268537,0.417613922885516,0.676229415561734,0.920965630421165
"11955","BFAR",429.192689654379,-0.0708220695734068,0.169344022440297,-0.418214168724943,0.675790537388958,0.920965630421165
"11956","PDP2",23.9350728172471,0.164734290112391,0.392763331837777,0.419423802475611,0.674906431505523,0.920965630421165
"11957","MPRIP",312.208797389604,0.0929123344379834,0.220887709671376,0.420631526200407,0.674024169010091,0.920965630421165
"11958","SS18",295.341164560368,0.0646963452282886,0.154354313723238,0.419141802180478,0.675112501878732,0.920965630421165
"11959","NDUFB7",1632.41150805592,-0.109110171422647,0.260742715145224,-0.418459136478183,0.675611457421744,0.920965630421165
"11960","SCGB1B2P",4.43093056948137,0.33866674412399,0.808494671067165,0.418885561332111,0.675299769781825,0.920965630421165
"11961","ZNF420",73.2310428307723,0.0891963985395282,0.213447498443715,0.417884487707166,0.676031574665842,0.920965630421165
"11962","ZNF235",14.1247224563886,0.168919779006829,0.404984045878553,0.417102304957181,0.676603579056424,0.920965630421165
"11963","CDS2",159.40029510266,-0.0694263725712805,0.165404779916332,-0.41973619266867,0.674678182312941,0.920965630421165
"11964","RALY",682.461051283305,0.0995097460631793,0.238493359718807,0.417243256502006,0.676500488451628,0.920965630421165
"11965","IL10RB-
DT",5.0471761936632,-0.259722352514893,0.622456985245424,-0.41725349489409,0.676493000426251,0.920965630421165
"11966","POFUT2",141.359099746212,-0.0893971728261528,0.213564805143612,-0.418595061887831,0.67551209912685,0.920965630421165
"11967","CBY1",351.729169655002,0.0950204643678695,0.22650689937623,0.41950362054994,0.674848109252521,0.920965630421165
"11968","PNKD",509.769035006911,-0.10935987501247,0.262455362681293,-0.416679902804152,0.67691255622142,0.921078324976251
"11969","HINFP",41.0353850697758,0.108190128077992,0.259610521480703,0.416740151596796,0.676868482330834,0.921078324976251
"11970","KCTD13",43.5400041822748,-0.107190497865868,0.257155060801339,-0.416832153844625,0.676801181922218,0.921078324976251
"11971","PCSK4",2.59010414171118,-0.428323442238122,1.02753595500357,-0.416845211257482,0.676791630525364,0.921078324976251
"11972","AC009961.4",6.36184937529635,-0.271451934435868,0.652027871159014,-0.41631952627017,0.677176206111578,0.92136010869959
"11973","AC009948.2",5.63216413823747,-0.275270832751346,0.661339649944101,-0.416232162663486,0.67724012690117,0.9213701183574
"11974","ZNF653",9.95182105782134,-0.201857123556882,0.485198797571771,-0.416029727540747,0.677388250287504,0.921494672534922
"11975","CCDC146",471.068392239741,-0.164288979701816,0.395032612185687,-0.415887130920198,0.677492596855968,0.921559658470719
"11976","TMX1",300.9592153134,-0.0662172901186357,0.15925420262746 7,-0.415796186387205,0.677559149701229,0.921573228914773

"11977","PSMB2",731.80038911933,-0.106324071760604,0.255819108349426,-0.415622087210837,0.677686561801737,0.921588172897398
"11978","FAM228A",4.4348582400231,0.332926608879032,0.801029490699573,0.415623410554177,0.677685593296239,0.921588172897398
"11979","BISPR",15.1338051855694,-0.181556564183778,0.436907451530365,-0.415549250871863,0.677739868815638,0.921588172897398
"11980","AL445223.1",3.62797272016284,-0.318110354484441,0.766004557335955,-0.415285198290176,0.677933135528353,0.921774027096941
"11981","LRP10",1129.44883402765,-0.0553456219504686,0.133322303648531,-0.415126504987288,0.678049297337282,0.921855020810199
"11982","MRPL20-AS1",47.7739637002812,-0.125887742868277,0.303331160660302,-0.415017509557012,0.678129085265584,0.921886552319405
"11983","PIGV",109.580947519773,-0.109448462545342,0.263973167389562,-0.414619651033781,0.678420360279953,0.9218978266956
"11984","TIGD4",2.91986604477209,-0.350905638787716,0.846087016014593,-0.414739420586574,0.678332671093413,0.9218978266956
"11985","TRAP1",161.151084572846,-0.0765409955954879,0.184601619782019,-0.414627973935814,0.678414266532789,0.9218978266956
"11986","SULT1A3",12.4921256901614,0.230786189356876,0.556534586517905,0.414684360950228,0.678372982423219,0.9218978266956
"11987","EDEM2",106.976143356661,0.122101803314647,0.294379186130817,0.414777297673441,0.678304940319719,0.9218978266956
"11988","GALM",157.467285625291,0.0741565114904657,0.178979181313946,0.414330376002718,0.678632170731065,0.922108727814341
"11989","BRD1",226.34089280373,0.0651736616674276,0.157361739771369,0.41416459783756,0.678753566824202,0.922196751188541
"11990","CCDC122",74.0343135454561,-0.156747792842396,0.37870860312335,-0.413900797472355,0.678946759832515,0.92238229949223
"11991","SCCPDH",379.107318045428,0.0985762328588717,0.238358971414454,0.413562083583039,0.679194846352855,0.922512220513187
"11992","AC026329.1",11.704819971981,0.200477572850246,0.484786029372534,0.413538263694866,0.679212294223995,0.922512220513187
"11993","LINC01597",6.61391568809978,-0.301267845727008,0.728391701354139,-0.413606916672619,0.679162006951628,0.922512220513187
"11994","AL021408.1",2.7083593424545,-0.38859530475695,0.940283999946859,-0.413274398776233,0.679405584590499,0.922697812855981
"11995","AC012063.1",34.0421851566422,0.120036548415189,0.290664209272578,0.41297326807313,0.679626199213809,0.922722079348883
"11996","KIAA1549",12.7541081666268,0.246389088363413,0.596526367615368,0.41303972756202,0.679577507248359,0.922722079348883
"11997","EXOSC4",107.148604537887,-0.115425638706809,0.279796117833635,-0.412534811420937,0.679947470740855,0.922722079348883
"11998","MEN1",60.0117954020629,-0.0897869377072216,0.217671903458116,-0.412487492785205,0.679982146127961,0.922722079348883
"11999","GPR132",5.19374791782361,-0.275126816927953,0.666503541873632,-0.412791230117898,0.679759577317661,0.922722079348883
"12000","ABHD17C",14.258938016904,-0.177545819098635,0.429866776943351,-0.413025217629304,0.679588137928415,0.922722079348883
"12001","CACTIN",59.5718216880538,-0.102428897874106,0.248310679505531

,-0.412502990520084,0.679970789218048,0.922722079348883
"12002","GTPBP3",86.179484199321,-0.0817913916465795,0.198103119018489
,-0.412872811149157,0.679699802152082,0.922722079348883
"12003","PEX26",131.133164187696,0.0983497830522274,0.23843707952483,0
.412476881734267,0.679989922064215,0.922722079348883
"12004","ARMCX4",60.0312231471862,0.109495083783962,0.265378376875596,
0.412599869940763,0.67989979656275,0.922722079348883
"12005","LINC02606",9.47869888662501,0.200270234056806,0.4869267857497
96,0.41129434633262,0.680856714703098,0.922866953024263
"12006","THRAP3",1018.10155841123,-0.0551484093635624,0.13398267268681
5,-0.411608518158701,0.680626387123109,0.922866953024263
"12007","KCNT2",86.1628169071556,-0.172200825381025,0.418584718493063,
-0.411388227455987,0.680787884877899,0.922866953024263
"12008","CCDC85A",38.1656692199614,0.243675974705647,0.591332615526416
,0.412079375139356,0.680281245332807,0.922866953024263
"12009","TMEM150A",92.7889463804493,-0.0918736692139521,0.223345947058
319,-0.411351405405009,0.680814880992756,0.922866953024263
"12010","SUMO1",1088.33986473357,-0.109869986691251,0.266871557907171,
-0.41169612660435,0.680562164390748,0.922866953024263
"12011","RAD18",61.4212429399206,-0.107574843362534,0.26156333373925,-
0.411276465338884,0.680869824622515,0.922866953024263
"12012","ASTE1",64.7014245651383,0.105240848261563,0.255670242447548,0
.411627286985318,0.680612628148178,0.922866953024263
"12013","AC093849.2",50.9572147303101,-0.151114735559064,0.36740828779
4477,-0.411299201948312,0.680853154697779,0.922866953024263
"12014","PRRC2A",329.097203182936,0.0671241320543941,0.163220119523406
,0.411249129398955,0.68088986687001,0.922866953024263
"12015","GAPVD1",178.922371365363,0.0880860719347824,0.21403132425122,
0.411556916927688,0.680664215291083,0.922866953024263
"12016","CNTD1",9.49289031793759,-0.190856530062569,0.463347143185036,
-0.411908291374425,0.680406643307461,0.922866953024263
"12017","ZNF302",330.980829149854,0.0907728970543991,0.220585807991549
,0.411508328123616,0.680699835823791,0.922866953024263
"12018","Z68871.1",9.24901259997017,0.202842175553602,0.49276095295099
6,0.411644174196112,0.680600248631355,0.922866953024263
"12019","SLC25A21-
AS1",49.9223629527943,-0.138562623907334,0.337019825230652,-0.41114086
9272315,0.680969243571602,0.922897745947069
"12020","C1orf159",24.3572838192827,0.15035931320427,0.365813033046592
,0.411027764516851,0.681052176145742,0.922933352515639
"12021","NUP35",83.0273914463842,-0.108962877465976,0.265333420642048,
-0.410663975922485,0.681318945486421,0.923074002846146
"12022","EFCAB1",3.96640192881122,0.322634582125329,0.786074557842175,
0.410437634581358,0.681484943676063,0.923074002846146
"12023","HNRNPUL2",358.0225110103,0.0928041348568728,0.226063602723466
,0.410522232410832,0.681422898028252,0.923074002846146
"12024","ERP27",3.89740901896923,0.309403115806172,0.753165978813614,0
.410803361423127,0.681216728174735,0.923074002846146
"12025","ANKRD52",50.3841845495286,0.140101224782896,0.341358461907373
,0.410422592133991,0.681495976317008,0.923074002846146

"12026","CACNA1G",2.33989044031032,-0.397961404866139,0.96913125181145
,-0.410637263138806,0.6813385357742,0.923074002846146
"12027","TBCE.1",31.0174706873768,0.121937575130799,0.297636134231499,
0.40968673190721,0.682035764283116,0.923114297001054
"12028","OXER1",14.4147881365609,0.174814013223548,0.426563085432158,0
.409819834846799,0.681938114974132,0.923114297001054
"12029","TWISTNB",265.005413836409,-0.088674397030972,0.21626424480842
9,-0.410028005829264,0.681785403613364,0.923114297001054
"12030","PURG",10.2667257706475,-0.223328661967137,0.544952593935446,-
0.409813008420311,0.681943122967082,0.923114297001054
"12031","DNAJC3-
DT",20.2019138802927,-0.13913160816264,0.339333175737517,-0.4100147527
87574,0.681795125473471,0.923114297001054
"12032","AC005520.5",2.94332313184681,0.390285392802659,0.951785433362
333,0.41005606843962,0.681764818266421,0.923114297001054
"12033","UBE2O",65.9018279682654,0.108359297282078,0.264358398382853,0
.409895422066934,0.68188266372546,0.923114297001054
"12034","AC027601.4",3.14307012596325,-0.400191990607414,0.97663434101
5669,-0.409766453830844,0.681977276650032,0.923114297001054
"12035","GUCD1",189.161842434498,0.0763995922377896,0.186479936614451,
0.409693362325334,0.682030899833647,0.923114297001054
"12036","ZNF184",29.28548292273,-0.133816611570078,0.326755560376468,-
0.409531245362445,0.682149841787883,0.923115292255781
"12037","ZNF778",20.490664037491,0.165950307230311,0.40520091882446,0.
409550668620778,0.682135590917551,0.923115292255781
"12038","GPT2",3.06866466553569,-0.327546597785233,0.800139074045024,-
0.40936208268064,0.682273961463627,0.923206559086311
"12039","MPZ",4.80532228305867,0.305827490328644,0.747921447552786,0.4
08903222830843,0.682610683812972,0.923228423332529
"12040","RAF1",240.489963915632,0.0775119217108035,0.189443627592456,0
.409155603151522,0.682425473265267,0.923228423332529
"12041","QTRT2",62.1384059040097,-0.117252422085198,0.286659763915745,
-0.409029926221739,0.682517699512441,0.923228423332529
"12042","SPACA9",61.2119980152381,0.089911550730633,0.220023631296863,
0.408644972363542,0.682800221983362,0.923228423332529
"12043","AVIL",36.6306361850625,0.158008047127565,0.386567920806775,0.
408745885581502,0.682726156205416,0.923228423332529
"12044","CTSG",30.8545260745595,-0.291168359572585,0.711751458577583,-
0.409087127344253,0.682475722685327,0.923228423332529
"12045","TBC1D10B",139.188591471932,0.0722262524507737,0.1767232295278
71,0.408696992714151,0.682762040996668,0.923228423332529
"12046","SNTB2",1263.48225538278,0.0738985585404461,0.18081427862809,0
.408698688516992,0.682760796354634,0.923228423332529
"12047","TMEM189",137.801137837905,0.102123233268126,0.249645363038763
,0.409073223011431,0.682485926236948,0.923228423332529
"12048","LRRC49",59.012420569245,-0.0938875099300701,0.229826327885679
,-0.408515033041696,0.682895596118727,0.923280740801621
"12049","TMEM91",234.214523167218,0.0810408903465294,0.198533127312538
,0.408198326614539,0.68312807660753,0.923518403175372
"12050","LINC-

PINT",644.186605792765,-0.15921928457787,0.390305801681046,-0.40793471
1429123,0.68332160803434,0.923703375375217
"12051","AARSD1",179.427891925303,-0.0889344770840847,0.21814766001505
4,-0.407680179003284,0.683508491160332,0.923879330554364
"12052","LDLRAP1",60.2219397607224,-0.100583474086541,0.24680884838799
5,-0.407535932133273,0.68361440889343,0.923881082428307
"12053","PRPSAP2",80.5148505293232,-0.0946033509792627,0.2321418312710
25,-0.407523928200659,0.683623223433506,0.923881082428307
"12054","GIGYF2",338.451741476051,0.0813253025766724,0.199600427477153
,0.407440523071932,0.683684469373904,0.923887201064503
"12055","CCSER2",540.61870019076,0.0543684616324928,0.133473870503158,
0.407334120360332,0.683762605889245,0.923916141628362
"12056","UCHL5",176.235728968754,-0.0777010556757805,0.190798909088129
,-0.407240565719854,0.683831310259643,0.923932333511888
"12057","LRRC27",32.0738597870146,-0.119063800802628,0.292438883988038
,-0.407140798716419,0.68390457973025,0.923954690157257
"12058","MAP2K5",103.898282958904,0.0856288137790867,0.210365590693739
,0.407047623599953,0.683973010768276,0.923970506916938
"12059","PPP1R10",580.926976886865,-0.0799611517636212,0.1965778739408
35,-0.406765777656581,0.684180024004564,0.924096883168354
"12060","IPMK",134.619618457364,-0.102770609474965,0.252622231940017,-
0.40681538075939,0.684143589263365,0.924096883168354
"12061","MTIF2",122.837871927722,-0.0846588113840181,0.208356871723929
,-0.406316387280907,0.68451014616135,0.924226342036538
"12062","AC104083.1",2.62885542370909,0.540174869806837,1.329930038724
96,0.406167884082622,0.684619249861281,0.924226342036538
"12063","STEAP1B",10.6641369525294,-0.238763587383118,0.58728207098499
1,-0.40655691562772,0.684333446543205,0.924226342036538
"12064","RAD54B",5.84761005132085,0.24434445512979,0.601256635406994,0
.406389619242026,0.684456345851926,0.924226342036538
"12065","MAN1B1",323.009264341945,0.0748369785793781,0.184319616177819
,0.406017439333102,0.684729786708634,0.924226342036538
"12066","PTPRE",43.1788398397868,-0.194670964766263,0.479273947360382,
-0.406178900060018,0.684611156315356,0.924226342036538
"12067","LINC00237",8.34064251980957,-0.233625841689308,0.575246496363
248,-0.406131707305143,0.684645829546911,0.924226342036538
"12068","PRELID3B",123.884921251738,-0.0731505375455046,0.180153906619
358,-0.406044692109077,0.684709762671257,0.924226342036538
"12069","TGFBRAP1",92.9123293989266,0.09998177610855,0.246579073254355
,0.405475512536358,0.685128014613515,0.924444875490788
"12070","PHAX",439.691864626064,-0.0711286357875269,0.175363356149317,
-0.405607176718052,0.685031254838699,0.924444875490788
"12071","BTN2A2",29.8320799884695,0.114697794059011,0.283142549083479,
0.405088512589447,0.685412450093264,0.924444875490788
"12072","STEAP2",235.230472281826,-0.164976282417186,0.407051355750191
,-0.405295990509936,0.685259953357312,0.924444875490788
"12073","XRCC2",4.11880359967141,-0.27880198211922,0.68831097097588,-0
.405052358418662,0.685439024801223,0.924444875490788
"12074","ITGB1-
DT",4.56272443537113,-0.282668063495281,0.697194438960346,-0.405436486

149824,0.685156696028708,0.924444875490788
"12075","HIP1R",4.86108175318022,-0.308681338046356,0.761880747427196,
-0.405157026330887,0.685362090922236,0.924444875490788
"12076","SNX6",991.888064995107,0.0611755170206786,0.150992946417217,0
.405154800090073,0.685363727238311,0.924444875490788
"12077","CCDC144NL",5.51361562492114,-0.370015127224808,0.913561462274
876,-0.405024886123618,0.685459218256353,0.924444875490788
"12078","IL13RA1",1181.0139741208,-0.115786115223375,0.285587785708209
,-0.405430907824876,0.685160795708818,0.924444875490788
"12079","CPA3",9.50732371513092,0.384876741519975,0.950968754118382,0.
404720701761421,0.685682823373586,0.924669882434998
"12080","C1orf53",11.0261795574083,-0.291871962826881,0.72237766546270
2,-0.40404344815939,0.686180769553966,0.924728928209196
"12081","SEMA4F",29.9552494882793,-0.137441581730182,0.340117212205147
,-0.404100635892788,0.686138717385074,0.924728928209196
"12082","DCAF16",147.670552483223,0.0950874285314179,0.235152286546414
,0.404365315463984,0.685944101766843,0.924728928209196
"12083","AL645940.1",3.59728329706616,0.321771078654355,0.795679469793
432,0.404397864805901,0.685920170075865,0.924728928209196
"12084","LAPTM4B",346.244791770732,0.103735181950045,0.256659088498212
,0.404174979958943,0.686084051009004,0.924728928209196
"12085","PAK1",110.060827857782,0.0851149140420844,0.210563006948695,0
.404225392083345,0.686046983105453,0.924728928209196
"12086","MYL9",1606.0132078062,0.114746084348737,0.283989188122773,0.4
04050890483658,0.686175296893942,0.924728928209196
"12087","DUSP18",67.9335728131487,0.117085364046556,0.289561813744522,
0.404353607723494,0.68595270988357,0.924728928209196
"12088","NSUN2",65.1451327059359,0.0958711249740492,0.237390109328375,
0.403854757240258,0.686319527571535,0.924762907156318
"12089","KIF24",2.58010725293304,0.358833388335069,0.888506229622165,0
.403861420856511,0.686314627154614,0.924762907156318
"12090","MICU2",243.913818714606,-0.0757951197981255,0.187761380447286
,-0.403677900202726,0.686449592884383,0.924861655789389
"12091","PYHIN1",102.925903584527,0.123308324868569,0.30585445916285,0
.403160134418425,0.686830424823216,0.925068694389397
"12092","GPR176",32.3227637830953,-0.1315768415449,0.32627546833022,-0
.403269182995189,0.686750209780865,0.925068694389397
"12093","AC012181.1",4.04348374327645,0.414059941995136,1.026711076745
47,0.403287693464502,0.686736594014963,0.925068694389397
"12094","MRPL12",195.617893748548,-0.123157049554988,0.305453902871775
,-0.40319356995313,0.686805829603391,0.925068694389397
"12095","TRPM3",10.3228343309386,0.274898470920861,0.682048887939953,0
.40304804506193,0.686912880308251,0.925103258151394
"12096","LINC01266",11.9224906576507,0.186526655090769,0.4630991406388
77,0.402779100029084,0.687110737593893,0.925293221285294
"12097","AC097500.1",4.1302040974376,-0.286600389285521,0.712026662692
988,-0.402513563469038,0.687306108365191,0.925403306262242
"12098","MKI67",2.79720553952114,0.513357118761138,1.27524663641755,0.
402555163919719,0.687275499101712,0.925403306262242
"12099","ATRAID",1720.67036916281,-0.0995164411533563,0.24776325278595

,-0.401659406850505,0.687934702894996,0.925790513504345
"12100","PARP3",53.7404576341861,0.0958874396839835,0.23872408398472,0
.401666384402678,0.687929567071138,0.925790513504345
"12101","CCDC50",827.95648942862,0.0717234160230386,0.178511167450307,
0.401786717590118,0.68784099815227,0.925790513504345
"12102","ERH",1334.26967870246,-0.108520512897063,0.270007702926817,-0
.401916359128749,0.687745582793011,0.925790513504345
"12103","KEAP1",237.035398755666,0.082967874373944,0.206469379660911,0
.40184105996833,0.687801001897656,0.925790513504345
"12104","ZIM2",2.73631949985763,0.406870195456552,1.01212943443299,0.4
01994232767756,0.687688270673136,0.925790513504345
"12105","PLPP2",2.70270443551042,0.405959323301149,1.01097821654213,0.
401551009367602,0.688014490657741,0.925821399283266
"12106","C11orf49",128.688027260398,-0.0885599544128407,0.220625707999
766,-0.401403604392897,0.688122996103333,0.925890920496215
"12107","ID2-
AS1",4.40932663254231,0.288859625851185,0.72032348498151,0.40101375544
9886,0.688409996551725,0.926165210498343
"12108","FRMD6-
AS1",43.032209630079,0.203892934372083,0.508496390023815,0.40097223573
708,0.688440565333289,0.926165210498343
"12109","CNOT9",110.558122201168,-0.0837567863172898,0.209131130468681
,-0.400498893347842,0.688789098409917,0.926370505742038
"12110","ACOX3",67.9442436849809,0.0905630178996278,0.22617436671375,0
.400412386317172,0.688852802704387,0.926370505742038
"12111","CHD1",336.685492060226,-0.1257122450604,0.313961453738167,-0.
400406621779881,0.68885704782253,0.926370505742038
"12112","ZCCHC10",197.502985909919,-0.0898770681734702,0.2244004060474
89,-0.400520969442675,0.68877284179519,0.926370505742038
"12113","MIR222HG",179.132597094298,-0.309112894373238,0.7720510624816
79,-0.400378821291465,0.688877520784168,0.926370505742038
"12114","BRI3BP",55.3320791178896,0.100191452241052,0.250501902911633,
0.399962838910632,0.689183887621962,0.926629496118377
"12115","C19orf18",8.34584939279961,0.244682172211898,0.61165952424930
4,0.400030020806427,0.689134405378926,0.926629496118377
"12116","AC069148.1",7.98579302300165,-0.218281991892085,0.54588697932
7397,-0.39986663935644,0.68925474488482,0.926644288238452
"12117","MYH7B",6.33213263541391,0.246747528199186,0.61718754023893,0.
399793437345905,0.689308664779012,0.926644288238452
"12118","C8orf76",134.869442079578,0.0949657236179073,0.23772396756669
6,0.399478961208502,0.689540322813861,0.926802732759714
"12119","LAG3",17.1347232469497,0.197796984172442,0.495131714653635,0.
399483568348696,0.689536928765814,0.926802732759714
"12120","IMPDH2",866.493155656264,-0.0852062954333586,0.21417353683649
4,-0.397837644612497,0.690749866360085,0.927090378036346
"12121","WWTR1",346.394612536718,-0.0785193941856232,0.197473297103651
,-0.397620312909494,0.69091008497841,0.927090378036346
"12122","SLC25A48",21.9216102130111,-0.155496739465552,0.3900420084198
12,-0.398666646435138,0.690138847032555,0.927090378036346
"12123","SUPT3H",54.7764744559612,0.117200182260222,0.294659487102563,

0.397747866232548,0.690816049999752,0.927090378036346
"12124","MED23",106.244724043792,0.0716583998638918,0.179787245899497,
0.398573322069519,0.690207622075303,0.927090378036346
"12125","TTYH3",68.5090733274826,0.124736815554267,0.313228152192759,0
.39822989945522,0.690460728112908,0.927090378036346
"12126","TMED4",642.553202742558,0.0574811285756529,0.144534257972975,
0.397698991102861,0.690852081204305,0.927090378036346
"12127","CROT",87.8302824412114,-0.0804734732721429,0.201938124821879,
-0.398505598401119,0.690257532381698,0.927090378036346
"12128","AC026979.4",18.1517478951605,0.162206453599252,0.407001034606
835,0.398540641932137,0.690231706185309,0.927090378036346
"12129","NDUFAF6",61.9315746303714,-0.0974791735281641,0.2451024320087
27,-0.397707900037048,0.690845513402167,0.927090378036346
"12130","ZNF707",11.7253495140522,0.190139342356589,0.477158847444252,
0.398482273513338,0.690274722434215,0.927090378036346
"12131","ACBD5",185.604438840398,0.0842388467363941,0.21190295887894,0
.397535018774889,0.690972968260137,0.927090378036346
"12132","LHPP",55.1486274190212,0.145060606074656,0.364886170895997,0.
397550298270972,0.690961703262446,0.927090378036346
"12133","TMEM223",239.246058207857,-0.0960780822105794,0.2407975305398
05,-0.398999449849828,0.689893609634584,0.927090378036346
"12134","NUP107",154.317321876234,-0.0989265856107342,0.24844576048415
7,-0.398181822132733,0.690496164362411,0.927090378036346
"12135","FBXL3",493.316116171575,0.0647653124366465,0.16269060161962,0
.398088837289271,0.690564702427318,0.927090378036346
"12136","AC009093.2",11.8970619913888,-0.182919273572231,0.46018632740
1164,-0.397489587761638,0.691006463241407,0.927090378036346
"12137","ATPAF2",80.6449613593152,0.0977142252745676,0.245653040471507
,0.397773319178198,0.690797286126573,0.927090378036346
"12138","SMG8",44.2855421758333,0.116845388382482,0.293788313202324,0.
397719661169823,0.690836842951375,0.927090378036346
"12139","CEP89",166.962242259905,0.0852684231066156,0.213762086292961,
0.398894044240171,0.689971277783597,0.927090378036346
"12140","TSC22D3",5555.16489991331,0.147646764636502,0.370486841187715
,0.398520941157242,0.690246225113944,0.927090378036346
"12141","IL13RA2",2.46120443099253,0.463689375661647,1.1635910246766,0
.39849858397672,0.690262701878463,0.927090378036346
"12142","GMCL1",133.839594740373,-0.0799273584861223,0.201200647658885
,-0.397251994047409,0.691181644115168,0.927249036484267
"12143","ABCF1",350.23278682552,-0.0683021264586213,0.172173385266287,
-0.396705485885544,0.691584654293961,0.927663130187182
"12144","WDFY4",7.42714297740369,-0.22790063596268,0.574521607644861,-
0.396678963732824,0.691604214684335,0.927663130187182
"12145","COPB2",559.819547326056,-0.0755571344454369,0.190665416729806
,-0.396281275028023,0.691897539314883,0.927702664996086
"12146","PPM1A",319.947299873406,0.0738145273346652,0.186137036930232,
0.396560128774009,0.691691859349507,0.927702664996086
"12147","ADCK1",15.3445936451911,-0.162872564293694,0.411031830499282,
-0.396252923029956,0.69191845276183,0.927702664996086
"12148","IDH2",142.751688602636,0.102732338736535,0.259247988313752,0.

396270533880494,0.691905462341495,0.927702664996086
"12149","CWC25",247.780077555785,-0.0804540657732011,0.203016275708159
,-0.396293673955756,0.691888393496552,0.927702664996086
"12150","ODC1",125.855204472815,-0.0968153424607408,0.244835920921321,
-0.395429486394085,0.692525951498471,0.9277535343333
"12151","CAMKMT",38.1080529239864,-0.109798217176695,0.277296948458786
,-0.395958981110152,0.692135288668674,0.9277535343333
"12152","SPOPL",150.647373458531,0.0892165342717283,0.225395081946248,
0.395822896850095,0.692235684238601,0.9277535343333
"12153","KDM7A",230.716907429245,0.143016372995771,0.361640189744111,0
.395465927326734,0.692499062643993,0.9277535343333
"12154","DOLPP1",15.4491736343994,0.17819564002641,0.450096278650279,0
.395905606153359,0.692174665165802,0.9277535343333
"12155","DAGLA",19.509366063337,-0.142291412473152,0.359742103428022,-
0.395537278281418,0.692446415677925,0.9277535343333
"12156","IKZF4",93.0927005501929,0.114490890836871,0.289258923108052,0
.395807637001064,0.692246942462977,0.9277535343333
"12157","PSMA3-
AS1",323.655947932787,-0.0858503870698716,0.216949961807205,-0.3957151
51801517,0.692315176514758,0.9277535343333
"12158","CXXC1",137.152275791809,-0.0735143311204631,0.185894403733894
,-0.395462852263687,0.69250133164128,0.9277535343333
"12159","ZNF790",40.4358245834745,-0.11876928621088,0.299896364390231,
-0.396034431602296,0.692079627727827,0.9277535343333
"12160","STK40",125.294518554636,-0.0844058874655553,0.213898723431327
,-0.394606784517132,0.693133105735185,0.928032645020585
"12161","KTI12",59.6408638357403,-0.100957197824834,0.255680430972241,
-0.394856960467945,0.692948454931617,0.928032645020585
"12162","NEMP2",34.88678532584,0.110716573452465,0.280341439817604,0.3
94934739311106,0.692891051347847,0.928032645020585
"12163","CFAP20",285.950136369418,-0.0891563868814327,0.22591812099262
9,-0.394640263869499,0.693108394112527,0.928032645020585
"12164","RHBDL3",4.53944832524296,-0.286868700854314,0.726384924864319
,-0.394926561709548,0.692897086628981,0.928032645020585
"12165","AL031663.3",4.63257740648917,0.313469200144818,0.793999990763
014,0.394797485883573,0.692992350503514,0.928032645020585
"12166","NOL12",75.9479479217398,-0.10902042552544,0.276205825529492,-
0.394707190974143,0.693058995170458,0.928032645020585
"12167","KCNMB3",12.5996237119783,0.241053426018968,0.611233012678738,
0.39437239320983,0.693306122690915,0.92816321528469
"12168","ATP5ME",1803.05182538896,-0.114839359089692,0.291428244772314
,-0.394057066017789,0.693538908049678,0.92816321528469
"12169","C9orf153",2.46436402996119,0.377363138665247,0.95741527717320
4,0.394147814080659,0.693471911748768,0.92816321528469
"12170","AC156455.1",9.3791328612833,0.228382035139954,0.5793723356990
32,0.394188712625368,0.693441718485826,0.92816321528469
"12171","AC018557.1",4.48536930103031,0.344828296224357,0.874696011528
289,0.394226441734729,0.693413865483376,0.92816321528469
"12172","FGF13",4.07183839491924,-0.372241785886722,0.94474841320548,-
0.394011549195119,0.693572512520427,0.92816321528469

"12173","MYC",3184.98234749804,-0.152007355916318,0.38597113573118,-0.393830890044035,0.69370589673505,0.928189202556039
"12174","RAB27B",3.85863271231365,0.335796351647338,0.852620137479553,0.393840512188689,0.693698792276551,0.928189202556039
"12175","AGRN",15.1352850031979,0.227616327129676,0.579420758971455,0.392834263538856,0.694441896232184,0.928545737044314
"12176","LINC00339",48.9017055204539,-0.124476801331185,0.317126976045803,-0.392514073962624,0.694678414486079,0.928545737044314
"12177","LINC01355",16.886766075427,-0.226057935623122,0.581226392104441,-0.388932675277591,0.697325950059544,0.928545737044314
"12178","AL357079.1",3.13865228337685,-0.323534134224102,0.838152049148366,-0.386008880551972,0.699490091844998,0.928545737044314
"12179","POMGNT1",81.3412583309445,0.084488174832913,0.215761219549513,0.391581837594891,0.695367209843418,0.928545737044314
"12180","BEND5",135.110486980792,-0.0926478091437898,0.238435348767747,-0.388565745903873,0.697597410129229,0.928545737044314
"12181","PRKAA2",38.9673619383565,0.113470908552131,0.294046394725179,0.385894575099904,0.699574748485163,0.928545737044314
"12182","TNNI3K",14.4045263433562,0.152174652340056,0.395582580429374,0.384684917558509,0.700470870943614,0.928545737044314
"12183","CRYZ",98.6068513667177,0.0793803856030327,0.203224942331528,0.390603558265682,0.6960902950787,0.928545737044314
"12184","PTGFRN",261.451677378246,-0.130850147220021,0.334677372728406,-0.390973988331764,0.695816462913965,0.928545737044314
"12185","RNF115",751.760749456354,0.0483681363723402,0.124192939844137,0.389459629774788,0.696936168679991,0.928545737044314
"12186","FMO5",25.7639929345631,0.148762389794321,0.378406027351834,0.393129017620019,0.694224193015285,0.928545737044314
"12187","SHCBP1L",4.52113049178515,0.307781026914276,0.794311201579601,0.38748166499756,0.69839965607291,0.928545737044314
"12188","LINC02767",37.6408142649342,-0.158876109493297,0.404487941887666,-0.392783302147038,0.694479538501398,0.928545737044314
"12189","STUM",5.59825141187231,0.350098687273765,0.89512274190881,0.391118078987911,0.695709957821086,0.928545737044314
"12190","TRIM11",67.0796333351719,-0.103337692443191,0.264561046517302,-0.390600558183204,0.69609251298456,0.928545737044314
"12191","AL359921.2",4.46641548090382,0.270320952063039,0.689513875149833,0.392045703219965,0.695024445031111,0.928545737044314
"12192","RNF144A",97.514132141962,0.144547101783239,0.374707508910723,0.385759821583076,0.699674554119857,0.928545737044314
"12193","SPDYA",3.18298265677388,-0.377851778222485,0.969316623712415,-0.38981254316607,0.696675167956486,0.928545737044314
"12194","WDPCP",69.3839227183103,0.100824091396628,0.260938460571797,0.386390305115203,0.699207629097862,0.928545737044314
"12195","ANKRD36C",256.665700740461,-0.155017703509478,0.395920218647029,-0.391537729594152,0.695399805866965,0.928545737044314
"12196","POLR1B",33.4597738602481,0.110413447817407,0.286932181958523,0.384806775816342,0.700380578614148,0.928545737044314
"12197","NFE2L2",1533.68783620212,-0.066713237247997,0.173074379633141,-0.385459924163279,0.699896692748273,0.928545737044314

"12198","IRAK2",13.2545649418592,-0.196994846052346,0.509946109399172,
-0.386305224064654,0.699270631993481,0.928545737044314
"12199","SLC4A7",213.053223771341,-0.13285067569674,0.345080338754537,
-0.384984772462621,0.700248697485443,0.928545737044314
"12200","TMPPE",3.00507589457857,-0.31525177020113,0.817299757589325,-
0.38572355769563,0.699701413973216,0.928545737044314
"12201","SEC22C",270.714173032778,-0.0528266756177732,0.13709819504346
9,-0.385319993461794,0.700000350357319,0.928545737044314
"12202","KALRN",9.72315242945175,-0.235809196488548,0.612183172412238,
-0.385193855556938,0.700093795353708,0.928545737044314
"12203","OCIAD1",1189.38875637786,-0.0854489633021047,0.22067561136450
3,-0.38721525579446,0.698596856915356,0.928545737044314
"12204","LNX1",56.7847040012523,0.111564684763309,0.284638639420101,0.
391952002688749,0.695093678261496,0.928545737044314
"12205","ELMOD2",109.890839365322,0.0739907728345954,0.189603918138072
,0.390238627773053,0.696360100552522,0.928545737044314
"12206","PALLD",1201.20900915597,0.0906617157051967,0.232576621395621,
0.389814398202036,0.696673796140415,0.928545737044314
"12207","FYB1",38.7993089830871,-0.2066533408464,0.529915960449617,-0.
389973800130612,0.696555920661078,0.928545737044314
"12208","CKMT2-
AS1",51.6192057442676,0.0963246759566225,0.250149567494471,0.385068329
005812,0.700186791956803,0.928545737044314
"12209","LUCAT1",18.4756030265909,-0.267656402537439,0.693543003681005
,-0.385926180664851,0.69955134047102,0.928545737044314
"12210","LARS",324.61213971922,-0.0716173048850356,0.185782161944866,-
0.385490749678591,0.699873858630846,0.928545737044314
"12211","SLC36A1",39.1079612310309,-0.109229309140009,0.28033090417473
4,-0.389644193748702,0.696799668020556,0.928545737044314
"12212","TTC1",435.646804445725,-0.078612880295867,0.203563335194789,-
0.386183888275571,0.69936048526619,0.928545737044314
"12213","RUFY1",228.844377044694,-0.0604839628045118,0.156154923534658
,-0.387333049995619,0.698509661037634,0.928545737044314
"12214","MGAT4B",217.901787376967,-0.0765919125437464,0.19766955282809
4,-0.387474507064605,0.698404954236456,0.928545737044314
"12215","RPP40",31.9609970185874,0.160757069774029,0.409732626739811,0
.3923462748211,0.694802376552573,0.928545737044314
"12216","TOMM6",1133.83572033051,-0.0982303758352288,0.254645684510994
,-0.385753153539061,0.699679492963281,0.928545737044314
"12217","BAG2",117.104250352593,-0.124436550327772,0.323050160443994,-
0.385192659111354,0.700094681721652,0.928545737044314
"12218","AL078600.1",2.66650431185785,0.36469963236568,0.9383391326403
41,0.388665056885639,0.697523934495406,0.928545737044314
"12219","CALD1",7288.70238676924,0.07551973923551,0.193262608475942,0.
390762288841356,0.695972952187182,0.928545737044314
"12220","RHEB",876.580833843607,-0.0954575228205265,0.245732322609837,
-0.388461403069427,0.697674611654242,0.928545737044314
"12221","IMPA1",105.702159466711,0.0781035536848955,0.20099832291775,0
.388578136131294,0.697588243014252,0.928545737044314
"12222","AK3",795.43069142242,0.0685423962259602,0.178174654148315,0.3

84692180566292,0.700465489212743,0.928545737044314
"12223","CCDC107",712.459363617782,-0.119523542666479,0.30545958185949
3,-0.391290860607077,0.69558225362035,0.928545737044314
"12224","GLIDR",36.034037620495,0.135384583022158,0.348138683698773,0.
388881182590153,0.697364042816338,0.928545737044314
"12225","BX664615.2",17.8607994607668,0.230017998121892,0.589331489621
885,0.390303254064147,0.696312317345779,0.928545737044314
"12226","TLE4",185.348952359641,-0.120735386864279,0.310745394787713,-
0.388534758324448,0.697620336996328,0.928545737044314
"12227","AL353748.3",4.59984793987112,0.285284985468571,0.736686829898
995,0.387254086662151,0.698568112347898,0.928545737044314
"12228","TNFSF15",5.61125832101598,0.443167359829471,1.1466479100454,0
.386489484650893,0.699134188825834,0.928545737044314
"12229","CAMSAP1",50.6164189910642,0.108247727505905,0.278092583920582
,0.389250680402245,0.697090716245047,0.928545737044314
"12230","EGFL7",72.0754304091004,-0.102469467222379,0.265748679776458,
-0.385587869368058,0.699801918626597,0.928545737044314
"12231","AC069549.2",2.81800718460452,0.45450949729503,1.1677231514887
4,0.389227101231634,0.697108157160547,0.928545737044314
"12232","AL731563.3",3.23322444468596,0.354443136917493,0.918829806985
518,0.385754939840648,0.699678169895806,0.928545737044314
"12233","PRXL2A",310.997730465997,0.0985271579950106,0.254607147088527
,0.386977188667656,0.698773095660447,0.928545737044314
"12234","XPNPEP1",164.577724823967,0.0715095989955409,0.18236127708257
,0.392131488326673,0.694961062561188,0.928545737044314
"12235","CUZD1",2.29249706560178,-0.399760499320163,1.01782427221624,-
0.392759840998597,0.69449686816494,0.928545737044314
"12236","EEF1G",8004.314055057,0.0800679057494023,0.205035737288961,0.
390507073586693,0.696161625731524,0.928545737044314
"12237","EHBP1L1",128.144399241909,0.0730636414664168,0.18633646366757
8,0.392105978767321,0.694979910119165,0.928545737044314
"12238","MRPL11",585.408877991717,-0.0987243779498134,0.25337153525215
5,-0.389642734932963,0.696800746899624,0.928545737044314
"12239","RHOD",145.028010651293,0.101100785867136,0.262550649430385,0.
385071551285357,0.700184404668167,0.928545737044314
"12240","MRPL21",469.509419268259,-0.102637577465236,0.264826996809734
,-0.387564631633747,0.698338246845636,0.928545737044314
"12241","AP001527.2",8.36005571734571,0.221453746200255,0.570185261285
846,0.388389110060205,0.69772810188264,0.928545737044314
"12242","M6PR",324.019468626016,-0.0590010372946197,0.153043079435849,
-0.385519146060773,0.699852824143106,0.928545737044314
"12243","PRR4",20.0617197530494,0.20714589424664,0.533597090994087,0.3
88206565858014,0.69786317461454,0.928545737044314
"12244","KCNJ8",167.99648256278,-0.106372648167795,0.27461463692627,-
0.38735243452219,0.698495312233606,0.928545737044314
"12245","KLHL42",152.60688596436,0.0855063279223199,0.218069524317208,
0.3921058120801,0.694980033275463,0.928545737044314
"12246","LINC02422",4.41497646319013,-0.277170864492267,0.715470778736
872,-0.387396484565867,0.698462705934128,0.928545737044314
"12247","AC026356.2",14.8129698893973,-0.164902149316828,0.42341409517

3226,-0.389458336877907,0.696937124921132,0.928545737044314
"12248","YAF2",184.381048672864,0.0641901093004984,0.164328661448413,0
.390620289453581,0.696077926066529,0.928545737044314
"12249","SMARCC2",473.048311308214,-0.0700122950128,0.18031990315569,-
0.388267150700223,0.697818344081364,0.928545737044314
"12250","MDM1",70.9403281807615,-0.0953773467384411,0.243375276596902,
-0.3918941482968,0.695136426849644,0.928545737044314
"12251","METAP2",733.634559475382,-0.0806370150584576,0.20595132484665
7,-0.39153433520516,0.695402314359997,0.928545737044314
"12252","AC008149.2",10.4321987061735,-0.259514540726884,0.66042280265
0217,-0.392952120498377,0.694354844914918,0.928545737044314
"12253","UHRF1BP1L",162.811314013652,-0.0788907814772514,0.20211318484
881,-0.390329713206318,0.696292754406817,0.928545737044314
"12254","PARP4",277.997109592263,0.0685995298683665,0.175978858049232,
0.389816882714259,0.696671958822447,0.928545737044314
"12255","DNAJC15",971.077295744952,-0.0977711052939407,0.2501044612083
22,-0.390921076823589,0.695855574139415,0.928545737044314
"12256","LINC01551",6.79540256934495,0.304702881382467,0.7809618620347
44,0.390163586975405,0.696415585520085,0.928545737044314
"12257","PPP4R3A",254.572756281202,0.0875750230362028,0.22673230369922
2,0.386248547769259,0.699312602191004,0.928545737044314
"12258","AC022613.2",3.68557258976636,0.391791907571948,1.013173882278
89,0.386697598926166,0.698980093918847,0.928545737044314
"12259","GLDN",14.3414716700495,-0.167199320191231,0.43154420823911,-0
.387444245569828,0.698427353372039,0.928545737044314
"12260","HMG20A",142.317828976014,0.0850921074411464,0.219408803052768
,0.387824491347696,0.698145919848758,0.928545737044314
"12261","CHRNA5",2.57488911449083,-0.350435908919794,0.899099727578082
,-0.389763113224123,0.696711722216214,0.928545737044314
"12262","LINC01579",11.2828174861998,0.541951591107645,1.4069003539168
4,0.385209648713812,0.700082095279441,0.928545737044314
"12263","ZSCAN32",41.6998690551145,-0.111000748191323,0.28218747683907
3,-0.393358165410808,0.694054963553779,0.928545737044314
"12264","NPIPB4",21.1452820380158,0.20008501745887,0.519573702312935,0
.385094581515906,0.70016734235901,0.928545737044314
"12265","AC009133.1",20.012751506654,0.151595095042545,0.3875272877561
73,0.391185601200621,0.695660050615786,0.928545737044314
"12266","C16orf86",79.5038754945115,0.105175790688274,0.27159553240827
2,0.387251549227142,0.698569990672118,0.928545737044314
"12267","MON1B",146.674418677415,-0.0930871808698424,0.24000590887177,
-0.387853704550152,0.698124299827177,0.928545737044314
"12268","COX4I1",3605.2696840981,-0.0952280031607539,0.245264902054722
,-0.388265921307841,0.697819253775586,0.928545737044314
"12269","ALKBH5",306.586970365124,-0.0667989994960367,0.17222008347141
7,-0.387869975147951,0.698112258436874,0.928545737044314
"12270","AP001033.2",12.8201013651194,0.187251489801768,0.486524532326
409,0.384875740810827,0.70032948005502,0.928545737044314
"12271","ZCCHC2",108.202069137154,-0.123908926304665,0.321360664858828
,-0.385575896039104,0.699810787551139,0.928545737044314
"12272","CERS4",164.960201414306,-0.104155824542045,0.268843032531158,

-0.387422443354462,0.698443491229971,0.928545737044314
"12273","SCAF1",182.926945956649,0.0608507400954825,0.156134510560628,
0.38973280075614,0.696734139136078,0.928545737044314
"12274","LINC01431",7.86548877261626,0.206015295195833,0.5245465731100
15,0.39274929197302,0.694504660293505,0.928545737044314
"12275","CST3",23201.019927291,-0.112769799735689,0.289267157822476,-0
.389846536968072,0.696650029386335,0.928545737044314
"12276","LINC01270",3.47319572840914,0.301889413775211,0.7726338934117
13,0.390727634846771,0.695998569818494,0.928545737044314
"12277","BX322562.1",4.76586494364451,-0.285568732204241,0.72874841854
3516,-0.391861889422665,0.695160263340336,0.928545737044314
"12278","IL17RA",149.127113152222,0.120170985203647,0.308337065782326,
0.389739017911271,0.696729541353976,0.928545737044314
"12279","ESS2",65.5183990637619,0.0855533163375071,0.221153538318855,0
.386850316697887,0.698867024360718,0.928545737044314
"12280","ADORA2A",6.85696825595633,0.239249437755243,0.617251810130854
,0.387604270782978,0.698308907923332,0.928545737044314
"12281","DNAL4",109.40611336271,-0.10507886666336,0.269160788224177,-0
.390394408326083,0.69624492201094,0.928545737044314
"12282","PHETA2",127.390177584936,-0.0981120296602813,0.25363396262058
7,-0.386825284147959,0.698885557563724,0.928545737044314
"12283","EFCAB6",7.44631459888442,0.228315274045162,0.593260287985075,
0.384848402411364,0.700349735862223,0.928545737044314
"12284","ZNF674-
AS1",9.74953036941048,0.246766607422,0.635035916167825,0.3885868517660
13,0.697581794633992,0.928545737044314
"12285","SLC9A7",5.34966771058362,-0.296782254092459,0.770185177723096
,-0.385338828474781,0.699986397465293,0.928545737044314
"12286","SLC25A53",29.4400417566073,-0.133228984555243,0.3389070736654
33,-0.393113614048569,0.694235569356396,0.928545737044314
"12287","MTM1",34.6119441578152,0.102895533655018,0.263992299185569,0.
389767178711107,0.696708715694758,0.928545737044314
"12288","ABCD1",46.8228769853481,-0.115103918071264,0.297382174934484,
-0.387057220550031,0.698713847035401,0.928545737044314
"12289","HLA-
A",6496.01153360938,0.0999219254164194,0.259808546427529,0.38459830051
9308,0.700535053445491,0.928555251491057
"12290","GIMAP6",3.3763252376468,-0.338906410584441,0.881890070064082,
-0.384295528534315,0.700759421751869,0.928777072491147
"12291","UBR5-
AS1",17.6924413421754,-0.138700780120234,0.361102679332079,-0.38410343
6664564,0.700901784415459,0.928890177068051
"12292","STX12",438.041242721936,-0.0646256801826233,0.168380678110974
,-0.383806983720725,0.701121511520099,0.928910930419812
"12293","TMEM106B",514.260454689873,0.0520603175540725,0.1356007330066
49,0.383923570321113,0.701035096049361,0.928910930419812
"12294","GIMAP7",8.39194645273107,-0.299444369253921,0.780547344445033
,-0.383633832572738,0.701249860488683,0.928910930419812
"12295","C10orf143",24.0633551054217,-0.145126485981506,0.378383125975
936,-0.38354375768526,0.701316632224376,0.928910930419812

"12296","BTBD7",380.435735265716,0.102052319230866,0.265905500387067,0
.383791681940813,0.701132853681039,0.928910930419812
"12297","NF2",118.694751367017,0.0737602547096946,0.192279123476377,0.
383610312841668,0.701267295239517,0.928910930419812
"12298","FAM3A",259.275223018088,-0.103037732593624,0.268541554503199,
-0.383693811500585,0.701205399907476,0.928910930419812
"12299","SNRPE",998.826104804803,-0.0819947986476354,0.213867395847752
,-0.383390831139151,0.701430000634131,0.928985550071499
"12300","TMEM175",115.858049776575,-0.0976290876327837,0.2548217161105
08,-0.38312703141221,0.701625577826206,0.929007048691704
"12301","PPDPF",1986.39225510394,-0.113068739469497,0.295171451186411,
-0.383061231074446,0.70167436429824,0.929007048691704
"12302","WWC3",159.879110662438,-0.104059586309013,0.271568621407712,-
0.383179712625141,0.701586519178842,0.929007048691704
"12303","SEPTIN6",197.1682249105,-0.0731549302535934,0.190879457753684
,-0.383251980671458,0.701532939839892,0.929007048691704
"12304","FMO2",400.571806217674,-0.128860704486912,0.336623619657889,-
0.382803513959815,0.701865455836756,0.929102793253839
"12305","UBE2G2",350.247523952534,0.0833727738885164,0.217835381762831
,0.382732929856586,0.701917795676944,0.929102793253839
"12306","ADARB1",135.715012089437,-0.143203633635654,0.374104697024434
,-0.382790258381336,0.701875285064801,0.929102793253839
"12307","C2orf73",10.5069670999629,-0.221969366846691,0.58026542084052
4,-0.382530750367934,0.70206772454129,0.929225738608359
"12308","SNRPN",479.398615628751,-0.0921891867051656,0.241163185292404
,-0.38226890473929,0.702261916851407,0.929407244360787
"12309","MDH1",786.884540549231,-0.111977081045304,0.293057614779708,-
0.382099203016707,0.702387782951442,0.929422794191393
"12310","AC002377.1",3.50751454472063,-0.34067871789016,0.891543250163
998,-0.382122480123643,0.702370518066919,0.929422794191393
"12311","SENP7",199.716689904427,0.072349589797843,0.189498072117551,0
.381795914804678,0.702612749163755,0.929479171773854
"12312","BHMT2",400.287475532625,0.065080693755057,0.170431850107487,0
.381857579519394,0.702567006807679,0.929479171773854
"12313","STRN4",109.455630542952,-0.0894674918539765,0.234289220473034
,-0.381867726024015,0.702559480320835,0.929479171773854
"12314","ZSCAN22",15.0966631767645,0.205465527832856,0.538242591038207
,0.381734056824707,0.702658635964346,0.929479171773854
"12315","MC1R",10.549743500313,0.19852313346877,0.520193549754121,0.38
1633208567476,0.702733448423154,0.929502650537129
"12316","RPAP2",186.565800256935,0.0724514072565061,0.190123597413048,
0.381075301763325,0.703147373520465,0.929521795753519
"12317","RAB1A",790.972209389904,0.0640363777518609,0.167991978510824,
0.381187115715379,0.703064408880254,0.929521795753519
"12318","ENTPD4",119.615743040011,0.0942181342139387,0.247165552893322
,0.381194438751761,0.703058975396567,0.929521795753519
"12319","ZNF33A",183.280191387421,0.122319608420669,0.320633110118303,
0.381494002211864,0.702836720874967,0.929521795753519
"12320","AC009041.2",24.5301455980619,0.17790707843056,0.4667239473023
62,0.381182665811027,0.703067710589504,0.929521795753519

"12321","TIMM21",98.4289872730752,0.0667352466225407,0.175052479500565,0.381229942089024,0.703032633150106,0.929521795753519
"12322","ZCCHC3",125.128050535106,-0.0939301368474784,0.246476792236571,-0.381091201305977,0.703135576030785,0.929521795753519
"12323","MFSD10",594.962832728545,-0.0824275174793214,0.21637829681891,-0.380941705758531,0.703246504823352,0.929577401368788
"12324","SETD1A",63.7292881245542,-0.0839389034470422,0.220391746385704,-0.380862281930204,0.703305441518883,0.929579871543418
"12325","GPR158",6.78378166550826,-0.218962692887785,0.575182365558912,-0.380683946516713,0.703437782355006,0.929679353897014
"12326","RPS8",29410.1302769144,-0.083150079989965,0.21848792047591,-0.380570604584673,0.703521896881633,0.929715088293438
"12327","FOSB",16186.8948563187,0.123317083134367,0.324120220422476,0.38046710869698,0.703598707525788,0.929741165481266
"12328","BRI3",3223.43612698391,-0.0855046035799917,0.224883617302098,-0.380217130112812,0.703784244434694,0.929835473890561
"12329","L2HGDH",23.1426411108128,0.118448470005311,0.311474856821367,0.380282605196742,0.703735646388004,0.929835473890561
"12330","TMEM44-AS1",82.7082356221482,0.115519194237812,0.30427746208675,0.379650840537369,0.704204616119638,0.930062387027862
"12331","B4GALT7",168.759236611424,-0.110978384942958,0.292442597906013,-0.379487754990552,0.704325695590106,0.930062387027862
"12332","TRIM4",178.805970233278,0.07017256718392,0.184928620966108,0.379457581078164,0.704348098405032,0.930062387027862
"12333","IQCH",4.06136760192011,-0.276328117197579,0.728385935773436,-0.379370473297456,0.704412773574973,0.930062387027862
"12334","TMEM204",276.215420883591,0.0919824280348967,0.24243200415438,0.379415367850206,0.704379440317517,0.930062387027862
"12335","AC012184.3",5.22782658130543,-0.23898968206348,0.62945353364368,-0.37967803704279,0.704184425369367,0.930062387027862
"12336","AC140912.1",6.26632702997034,0.29275173750814,0.771063506892437,0.379672666247683,0.704188412643901,0.930062387027862
"12337","AL133227.1",4.53706142655638,-0.262717799694389,0.691646253271262,-0.379844173884872,0.70406108951021,0.930062387027862
"12338","CDNF",17.6995479983192,0.139381674967157,0.367766298517227,0.378995235640461,0.704691401809629,0.93022810550724
"12339","AC073592.1",3.88508404288028,0.369005266839625,0.973698788104716,0.378972708344318,0.70470813044523,0.93022810550724
"12340","RPL3",24623.0401677634,-0.0915527448203921,0.241582635975542,-0.378970717206932,0.704709609058833,0.93022810550724
"12341","TBC1D3L",3.69433721183191,0.316815171236108,0.836320274916243,0.378820388239227,0.704821246192926,0.930300079348235
"12342","LRRC28",88.2894020765952,-0.0852595630968528,0.225160288344991,-0.378661635777521,0.704939145674908,0.9303105921665
"12343","PTPN2",231.912710331629,-0.0744449387109134,0.196603161167839,-0.37865585816985,0.704943436620487,0.9303105921665
"12344","ISCA1",447.746862356164,-0.0763778919669636,0.201749976994124,-0.378576955025815,0.705002037782788,0.930312556176591
"12345","GSTM5",636.786122589065,0.111351788227424,0.294805446745982,0

.377712791457918,0.705643964348609,0.930575850655076
"12346","S100A11",8513.28842871496,0.116203528812356,0.307619899650045
,0.377750363174006,0.705616050592699,0.930575850655076
"12347","DPP4",234.653934415452,0.188048259948024,0.497451610069948,0.
378023221035674,0.705413343825693,0.930575850655076
"12348","AL359711.2",5.75771976815574,-0.238675375939745,0.63205137278
7478,-0.377620216038985,0.705712744575593,0.930575850655076
"12349","LIPT2",9.44595026478626,0.199700876283373,0.528846155298229,0
.377616201389904,0.70571572737075,0.930575850655076
"12350","WDFY2",257.77461739213,0.120578129609785,0.319098280800054,0.
377871448594042,0.705526093361024,0.930575850655076
"12351","PSMB5",1028.81566044795,-0.102537958233765,0.271526947789149,
-0.377634555496825,0.70570209071362,0.930575850655076
"12352","GNAL",63.2090143974546,0.130784463758584,0.34605658316926,0.3
77927975132946,0.705484099863136,0.930575850655076
"12353","AC011468.5",5.80541137894799,0.258143111140172,0.683039325489
387,0.377933014259782,0.705480356345409,0.930575850655076
"12354","AC016910.1",4.06290946355564,0.319020136229354,0.845658801367
741,0.37724450536479,0.705991908851903,0.930864675674975
"12355","AL513477.2",5.17803946501437,0.313325790902119,0.831220182729
389,0.376946803521161,0.706213138333003,0.930984164566784
"12356","RAB3IP",12.8565669494616,0.261676111241154,0.694090940111477,
0.377005513426132,0.706169507608759,0.930984164566784
"12357","TICAM1",57.8836200231496,0.0934114999764994,0.24789755917336,
0.376814924229144,0.706311148978839,0.930984164566784
"12358","GPATCH1",81.6461667041435,0.0953153138891386,0.25293163214970
9,0.376842204666287,0.706290874181163,0.930984164566784
"12359","COLCA2",59.6172555648979,0.18490009993663,0.49111070999075,0.
376493723665917,0.706549880633997,0.931223481321075
"12360","ACBD6",390.873319717939,-0.0673830986140682,0.179102309345292
,-0.376226855256009,0.706748251988363,0.931258880168132
"12361","ZCCHC24",910.10201642491,0.0703674745591236,0.186964407793205
,0.376368290573009,0.706643116361208,0.931258880168132
"12362","AC084824.4",4.5458218935267,0.276478179146094,0.7348644200419
93,0.37623018832548,0.706745774294238,0.931258880168132
"12363","TRG-
AS1",3.65156905764617,0.310440470527505,0.825406580303544,0.3761061250
72737,0.706838000926688,0.931301803534322
"12364","OSCP1",64.4788818638378,-0.0953074601107704,0.253476423251406
,-0.376001282045239,0.706915942907046,0.931329164834428
"12365","PPME1",112.860306872249,0.0669415627433962,0.178133473260756,
0.375794405835256,0.707069746996517,0.931456458457442
"12366","CCDC28B",134.761258103316,0.110738369906092,0.295220884894894
,0.375103441430023,0.707583537701414,0.931469623733604
"12367","EPM2AIP1",235.786806484359,0.0630052529211079,0.1679011028821
48,0.37525216832752,0.70747293536925,0.931469623733604
"12368","PROS1",803.840816857143,0.0659423596568966,0.175755165897538,
0.37519443209618,0.707515870796164,0.931469623733604
"12369","TRMT11",192.516493213108,-0.0718148844768053,0.19138962222297
1,-0.375228727883375,0.707490366694042,0.931469623733604

"12370","TBX3",202.05530902749,-0.0768423492653844,0.204797698703316,-0.375210999693427,0.707503550242184,0.931469623733604
"12371","SPRED1",260.29618528289,0.0793464479832227,0.211346455553355,0.375433066882881,0.707338416563037,0.931469623733604
"12372","FAM227B",10.3587658548667,-0.175086270162147,0.466031695529708,-0.375696056387619,0.707142870024409,0.931469623733604
"12373","EDC4",74.2073510772894,0.0828712292670526,0.220937603062931,0.375088840098658,0.707594396468759,0.931469623733604
"12374","ZNF337-AS1",33.3737056989831,-0.120050771357888,0.319728287024302,-0.375477479566152,0.707305392040122,0.931469623733604
"12375","SMIM1",32.4434705305027,0.167296789938831,0.447693935906013,0.373685628777317,0.708638218583738,0.931512896846348
"12376","RERE",493.314618347677,0.0862175711877844,0.231848011620448,0.371871083065093,0.709988835979363,0.931512896846348
"12377","AL137127.1",2.33177662840769,0.353828375251962,0.95026040765604,0.372348855536066,0.709633127918186,0.931512896846348
"12378","WASF2",2215.11166543953,0.0497892798188563,0.13401356310289,0.371524185060509,0.710247145841232,0.931512896846348
"12379","AL078459.1",13.5683599358496,0.175438621079951,0.468701995187348,0.374307391223767,0.708175633715203,0.931512896846348
"12380","EXTL2",226.435022637836,0.0828258570872335,0.225328773601727,0.367577809807946,0.713188063369891,0.931512896846348
"12381","BCAS2",404.621184983873,-0.0921284760449228,0.251117198312651,-0.366874418255572,0.713712694197835,0.931512896846348
"12382","POGK",93.0048874155387,0.0750466381689306,0.202343156701686,0.370887947940693,0.710720992130408,0.931512896846348
"12383","ANGEL2",159.560065408236,0.070677591595807,0.19151163419587,0.3690511643983,0.712089588623251,0.931512896846348
"12384","PPP1R21",93.7465063257384,0.0820351610872272,0.220785882227887,0.371559812880397,0.710220614845254,0.931512896846348
"12385","MRPL19",333.913756961748,-0.0694332589766243,0.187378229007776,-0.370551367382936,0.710971710338218,0.931512896846348
"12386","ZAP70",6.89490951878616,0.35959890445147,0.978190248534387,0.367616529596623,0.713159187808454,0.931512896846348
"12387","UBXN4",1460.28448408307,0.0500075678007747,0.135270712141782,0.36968510780338,0.711617129180912,0.931512896846348
"12388","LINC01124",7.75369236182561,0.241118106988351,0.648897623193601,0.371581122152474,0.710204746621112,0.931512896846348
"12389","TMBIM1",483.156946900727,0.0566603549953116,0.152645792951114,0.371188448105201,0.710497176580812,0.931512896846348
"12390","MKRN2",119.075704798293,-0.07362718034988,0.199872680605066,-0.368370405234931,0.712597061569397,0.931512896846348
"12391","LINC01985",29.3135133384388,-0.15432642814696,0.416786265205911,-0.370277144499268,0.711176001553934,0.931512896846348
"12392","GORASP1",70.0800336450572,-0.0897074002697948,0.244408999675844,-0.367038040288092,0.713590643160325,0.931512896846348
"12393","CCDC51",149.143849525472,0.107670959645974,0.292757980828528,0.367781466934758,0.713036189204836,0.931512896846348
"12394","RARRES1",577.301314640743,-0.174380142611036,0.4659015899728,

-0.374285356315733,0.708192025621332,0.931512896846348
"12395","NMD3",241.660202528664,0.0740939703316062,0.199097158858058,0
.372149812466334,0.709781310488202,0.931512896846348
"12396","POLR2H",333.083943893032,-0.078585292715358,0.210264814514755
,-0.373744379898823,0.708594503752273,0.931512896846348
"12397","CHRD",114.766046911387,-0.100158428056981,0.27194214293197,-0
.368307857609393,0.712643694178591,0.931512896846348
"12398","COMMD8",248.626079180255,-0.0718890797230223,0.19598332274012
1,-0.366812230336298,0.713759084127004,0.931512896846348
"12399","THAP9",18.389424037394,-0.142179887927892,0.385199402969339,-
0.369107238567576,0.712047793729018,0.931512896846348
"12400","NUDT6",48.0493974672405,-0.0953244831064054,0.259763232061436
,-0.36696680415441,0.713643779626797,0.931512896846348
"12401","AC110609.1",5.11874838628655,0.303749637984356,0.825230913110
254,0.368078356201586,0.712814808987302,0.931512896846348
"12402","LSM6",570.856236232211,-0.102081194508861,0.274189157794798,-
0.372302082729537,0.70966794811187,0.931512896846348
"12403","HMGB2",463.440360728463,0.0839564004116384,0.225677375647903,
0.372019570728372,0.709878278134967,0.931512896846348
"12404","SKP2",51.217236356576,0.103766694423308,0.279960649986291,0.3
70647426445071,0.710900152938321,0.931512896846348
"12405","AC104113.1",5.00069617992457,-0.261156570543108,0.69847318027
7758,-0.37389634694242,0.708481434385755,0.931512896846348
"12406","ERAP1",156.899595897311,0.103430866543211,0.281791576460945,0
.367047403766331,0.713583658856674,0.931512896846348
"12407","PCDHB9",19.8491692483994,0.145946586715405,0.397942674419323,
0.366752791538053,0.713803424300823,0.931512896846348
"12408","ABHD16A",203.737272585066,-0.0641643608473511,0.1742825137726
42,-0.3681629295929,0.712751749915363,0.931512896846348
"12409","DCBLD1",13.3334477486704,0.158674065416471,0.426282006304916,
0.372227922055364,0.709723158550618,0.931512896846348
"12410","ECHDC1",414.795265077684,0.0916559063218117,0.244719614386547
,0.374534368859525,0.70800679150024,0.931512896846348
"12411","FOXK1",167.297932520137,0.0882204996683451,0.239311752294439,
0.36864257113375,0.712394159807317,0.931512896846348
"12412","KDELR2",1638.99200487804,0.0859855631268911,0.234009975507928
,0.367444007206342,0.713287850787994,0.931512896846348
"12413","TCAF2",13.4899883480397,0.194165411768454,0.524140590500143,0
.370445287557635,0.711050735477547,0.931512896846348
"12414","ERI1",61.911572366932,-0.0841850663917934,0.226827238030617,-
0.371141786686263,0.710531928838919,0.931512896846348
"12415","PCMTD1",985.840959774385,0.0680038550766675,0.181977747240546
,0.373693246058142,0.708632550754418,0.931512896846348
"12416","CPQ",1672.58049245076,-0.0741109944801075,0.201572786798381,-
0.367663689415751,0.713124018588653,0.931512896846348
"12417","CNTLN",321.371252121651,-0.0667174948676809,0.178265318121087
,-0.374259533884,0.708211235262112,0.931512896846348
"12418","SMC5",342.956567014598,-0.0815063193061735,0.219369277839132,
-0.371548468906133,0.710229062333166,0.931512896846348
"12419","PSMD5",166.5621268246,0.0649700763405845,0.175202483357604,0.

370828512789827,0.710765262984993,0.931512896846348
"12420","PHPT1",1458.76235266898,-0.0998795844599224,0.268089692613579
,-0.372560330411089,0.709475702213444,0.931512896846348
"12421","IL15RA",385.809643328377,-0.0986715271387437,0.26818530403434
9,-0.367922946016856,0.712930690054396,0.931512896846348
"12422","AIFM2",147.986606148241,0.070455610990249,0.19091159642097,0.
369048356993939,0.712091681145437,0.931512896846348
"12423","MICU1",403.018328336057,0.0876221188101223,0.236867728024001,
0.369920037402664,0.711442071142718,0.931512896846348
"12424","CFAP70",15.9138257050883,0.16830198631276,0.455869324371899,0
.369189101601974,0.711986778651875,0.931512896846348
"12425","NT5C2",148.390533991385,0.0762212959193574,0.205426061689189,
0.37104004863161 4,0.710607702884755,0.931512896846348
"12426","SMNDC1",227.327992280841,-0.0595059433170697,0.16184811662618
3,-0.36766534302348,0.713122785429304,0.931512896846348
"12427","NRIP3",15.0546372707933,-0.222117171027171,0.599056353314099,
-0.37077842476484,0.710802572298909,0.931512896846348
"12428","DEPDC7",7.99614503409514,0.207367701650301,0.559646764425443,
0.370533191348284,0.710985250521529,0.931512896846348
"12429","LINC02696",4.60029724261113,0.294182664049095,0.7890556328633
52,0.372828799132394,0.709275867131038,0.931512896846348
"12430","TULP3",410.020585197207,0.0532487530340117,0.14405892021637,0
.369631765627803,0.71165687927755,0.931512896846348
"12431","AC125494.2",4.28677542854278,-0.261116001573294,0.70341223265
867,-0.371213336149074,0.71047864083417,0.931512896846348
"12432","SLC38A2",1514.72202217072,0.130252067272693,0.351728640267404
,0.370319764616462,0.711144248955863,0.931512896846348
"12433","HOXC6",376.689505077004,0.0527046159613625,0.143694621215817,
0.366782107189694,0.713781555280778,0.931512896846348
"12434","ARHGAP9",9.40211358935145,0.195326961243795,0.526188455451591
,0.371211035172103,0.710480354514345,0.931512896846348
"12435","CTDSP2",820.649133023071,0.0546047106028375,0.145976779501714
,0.374064360025125,0.70835643362247,0.931512896846348
"12436","GIHCG",149.282312692861,0.0989334148256255,0.266656801041671,
0.371014031665988,0.710627080663119,0.931512896846348
"12437","ELK3",297.839867806932,-0.0850699186106087,0.230075653944447,
-0.369747590204174,0.711570568869694,0.931512896846348
"12438","DNAH10",2.36432560718353,0.360282984707564,0.971737552260538,
0.370761615489124,0.710815093262207,0.931512896846348
"12439","SLC7A1",61.236500441368,0.132952724793831,0.358636816540084,0
.370716888679976,0.710848409934122,0.931512896846348
"12440","EBPL",541.313910793305,-0.11248565692899,0.305859633995821,-0
.367768886202638,0.713045570762428,0.931512896846348
"12441","LMO7-
AS1",34.8739977040837,0.387729181810948,1.04465469414594,0.37115535304
0304,0.710521824895585,0.931512896846348
"12442","C14orf119",359.046170632062,0.106602753997611,0.2887338658466
07,0.369207656625375,0.711972949269568,0.931512896846348
"12443","AP4S1",65.3015008375951,0.0885160822395419,0.240034281975381,
0.368764334457111,0.712303390907232,0.931512896846348

"12444","BCL11B",11.4384918289823,0.217738102968159,0.592911115157724,
0.367235657085357,0.713443244085778,0.931512896846348
"12445","IGHG3",104.883923916878,0.483123206076621,1.30927582537372,0.
369000325763075,0.712127481958604,0.931512896846348
"12446","AC116158.3",3.11337121191429,0.357452992410985,0.968248492531
954,0.369174850431475,0.711997400361934,0.931512896846348
"12447","PAK6",9.64385848746488,-0.237494713090564,0.639911044905963,-
0.371137074412373,0.710535438456147,0.931512896846348
"12448","UBE2Q2",303.818258372777,0.0635034057880466,0.170136885592248
,0.373248902299996,0.708963202869745,0.931512896846348
"12449","UBE2Q2L",10.6284786227658,0.18316397496634,0.489150550502166,
0.374453171479215,0.708067190276227,0.931512896846348
"12450","IQGAP1",1195.85855497084,0.0725241139464958,0.193532859445316
,0.374737985861198,0.707855338810136,0.931512896846348
"12451","NUDT16L1",139.867597277271,-0.103431710714045,0.2767540068896
47,-0.373731574391575,0.708604031839071,0.931512896846348
"12452","GSPT1",449.016005357552,-0.0481452084317258,0.129381703132977
,-0.3721175967381,0.709805295321581,0.931512896846348
"12453","ADAT1",45.5381947920298,0.117724639029387,0.320842964783345,0
.366922924767519,0.713676510836354,0.931512896846348
"12454","C16orf46",18.6494247448237,-0.155799499370916,0.4202267342068
52,-0.370751041494246,0.710822969703531,0.931512896846348
"12455","SNAI3-
AS1",15.4110852984943,-0.138245954892946,0.376677092039519,-0.36701450
0787434,0.713608201594159,0.931512896846348
"12456","AC015917.2",4.74020803518724,-0.244554544041711,0.66568682317
9252,-0.367371766311587,0.713341728703573,0.931512896846348
"12457","PSMC3IP",24.5481957013248,-0.149279273283019,0.39903476718994
5,-0.374100919411767,0.708329234690511,0.931512896846348
"12458","VPS25",316.861589845473,-0.095914961771482,0.256481113073293,
-0.373965009049509,0.70843034922558,0.931512896846348
"12459","HEXIM2",68.2506938967727,-0.127016582118537,0.344064483561527
,-0.369165049538813,0.712004705214707,0.931512896846348
"12460","PHB",845.187024360976,-0.0929075562442841,0.251529275004538,-
0.3693707471729,0.711851399118703,0.931512896846348
"12461","NACA2",5.54472916701947,0.281501337417212,0.758608610809089,0
.371075853089749,0.710581035559029,0.931512896846348
"12462","AC005332.1",2.75450730327966,-0.391401064947411,1.05776335154
175,-0.370027061702338,0.711362326916407,0.931512896846348
"12463","JMJD6",93.7202248178739,0.0774260267129153,0.210740725218017,
0.367399450831423,0.713321081168179,0.931512896846348
"12464","ME2",138.881767179033,-0.0827443218370218,0.225549340182997,-
0.366856856109125,0.713725794814008,0.931512896846348
"12465","LINC00909",95.0283133669017,-0.089577219357986,0.243281141738
854,-0.368204533724776,0.71272073002872,0.931512896846348
"12466","ZNF236-
DT",8.53454425253363,-0.214575302995154,0.577104853150534,-0.371813374
681816,0.710031804930117,0.931512896846348
"12467","ZNF763",17.4907462072353,0.153933254374287,0.417472834193488,
0.368726397902439,0.712331670409061,0.931512896846348

"12468","POU2F2",7.97833202923117,-0.204065026253255,0.55533926627706,
-0.367460107082444,0.71327584354901,0.931512896846348
"12469","LIPE-
AS1",38.4469822419182,0.14141893657817,0.382064682486533,0.37014396530
3977,0.711275225192077,0.931512896846348
"12470","ZNF221",14.6341973853347,0.152160457764097,0.412571790153264,
0.36880965057638,0.712269610886426,0.931512896846348
"12471","ZNF814",18.1184758107182,-0.150628224313336,0.409167777020375
,-0.368133154106697,0.712773950699313,0.931512896846348
"12472","SPAG4",2.71010271950458,-0.353109860108034,0.950511054492986,
-0.371494743210942,0.710269070590958,0.931512896846348
"12473","SERINC3",1160.80078345312,-0.062872217293986,0.16863918565898
4,-0.37282092562474 7,0.709281727503256,0.931512896846348
"12474","LRRC3",27.9278017483494,-0.127394017970019,0.346623084655546,
-0.36752894890603,0.713224502326134,0.931512896846348
"12475","LINC01547",8.51513777812333,0.23474965811975,0.63472109705644
7,0.369846944127766,0.711496535043577,0.931512896846348
"12476","DERL3",3.20494241563367,0.36261468478365,0.981383065396673,0.
369493521509954,0.711759901165003,0.931512896846348
"12477","SNRPD3",778.575278783868,-0.0812551100054706,0.21904919331328
,-0.370944575400746,0.710678813519663,0.931512896846348
"12478","CCDC117",143.860465531155,0.0730496279623607,0.19769298020192
8,0.369510479773972,0.711747263292615,0.931512896846348
"12479","HORMAD2-
AS1",2.83837011309193,0.443475454204611,1.19353537614828,0.37156456613
4415,0.710217075262489,0.931512896846348
"12480","POLA1",33.9748553086828,0.108743567109646,0.29403569868641,0.
369831172185733,0.711508287364232,0.931512896846348
"12481","PIN4",421.559348322968,-0.0843753394233967,0.228149150183416,
-0.369825350458526,0.71151262538918 5,0.931512896846348
"12482","TMEM187",106.6978077982,0.115599937453005,0.313241881830318,0
.369043682082159,0.712095165634618,0.931512896846348
"12483","AL691432.2",23.9487768356044,-0.134145192239967,0.36855247408
9619,-0.363978542190841,0.715874032829565,0.931518231368218
"12484","AL645504.1",2.46781919645479,0.417775213444288,1.145950586745
09,0.364566516459422,0.715435012999487,0.931518231368218
"12485","HS1BP3",137.981654718185,0.0740852513600256,0.202366912060728
,0.366093698844371,0.71429515923374,0.931518231368218
"12486","CGREF1",102.168042321602,0.151791511545731,0.415487948602615,
0.365333127124966,0.714862752958287,0.931518231368218
"12487","TMEM185B",140.29379708606,0.0703564324680753,0.19217214643364
1,0.366111498329806,0.714281877859247,0.931518231368218
"12488","XPC",314.208271975033,-0.0798364364240944,0.218689673015496,-
0.365067244937704,0.715061210734361,0.931518231368218
"12489","SCN11A",2.7677603700361,-0.366625759712655,1.00727512917016,-
0.363977774388906,0.715874606181887,0.931518231368218
"12490","AC073475.1",4.55955890580968,0.253906463109063,0.696925366326
936,0.364323750256426,0.71561626663838 7,0.931518231368218
"12491","AC097376.3",79.1189346929038,-0.0839872284244183,0.2300291411
13003,-0.365115602388651,0.715025114697109,0.931518231368218

"12492","CENPK",8.36680206588188,-0.207598600967943,0.570943682073371,
-0.363606091959986,0.716152177016649,0.931518231368218
"12493","NLN",41.7059173458979,0.101224973053779,0.27699319561803,0.36
5442092640309,0.714781425308593,0.931518231368218
"12494","SNX2",843.922477414547,-0.0694385013357553,0.189845102716375,
-0.365763985176353,0.714541196075967,0.931518231368218
"12495","KCNIP1",3.04252779037211,0.374093301423131,1.02302245209558,0
.36567457601427,0.714607919529082,0.931518231368218
"12496","DDX43",5.64010007354945,0.316337944947304,0.86474958177707,0.
365814510482012,0.714503491477084,0.931518231368218
"12497","SYNCRIP",541.777816693494,-0.0562329309720157,0.1545209597086
96,-0.363917821103534,0.715919376497833,0.931518231368218
"12498","DBNL",344.705371803546,-0.0752168532684199,0.205484516230635,
-0.36604633112111,0.714330503849635,0.931518231368218
"12499","SLC26A4",4.20130987651439,-0.281820424422891,0.77406692009656
6,-0.3640775973061,0.715800065252884,0.931518231368218
"12500","CASP2",90.001442183839,0.076017147632982,0.208163870495313,0.
365179353420475,0.714977529216938,0.931518231368218
"12501","ESYT2",438.809185511998,0.0558576768326527,0.153034337722334,
0.365000938116261,0.715110705973336,0.931518231368218
"12502","PPP1R3B",173.671344991013,0.0877879649834922,0.24141412122107
9,0.363640554825287,0.716126438719017,0.931518231368218
"12503","DERL1",380.563091300676,-0.0639282550018269,0.175709871445613
,-0.363828477454748,0.715986095978173,0.931518231368218
"12504","MRPL50",202.592475701063,-0.0745629356372779,0.20501047964779
1,-0.363703044670581,0.71607976953263,0.931518231368218
"12505","ZBTB26",22.5234407856644,0.118545347682311,0.323975334000954,
0.365908559205195,0.714433309306717,0.931518231368218
"12506","RPL12",25882.8070948723,0.0882680528817669,0.241865283388131,
0.364947179046443,0.715150835725527,0.931518231368218
"12507","KLLN",2.649864660119,-0.408880573319731,1.11514963700846,-0.3
66659827300495,0.713872775730724,0.931518231368218
"12508","RASSF7",2.60366840999139,0.432699253989992,1.18852422210287,0
.36406431265188,0.715809985168038,0.931518231368218
"12509","KCNQ1-
AS1",4.66877710584001,-0.261717922839391,0.715154597249069,-0.36595992
5093289,0.714394979455074,0.931518231368218
"12510","TPCN2",29.6698630779486,0.151718197091695,0.415365282165888,0
.365264511999109,0.714913966297897,0.931518231368218
"12511","RAB38",2.59578072678058,-0.36480084210827,0.995738925182616,-
0.366361937735202,0.71409501761604,0.931518231368218
"12512","PPHLN1",362.364048920292,-0.0538902860948417,0.14814297311536
1,-0.363772138236194,0.716028169735605,0.931518231368218
"12513","MTHFD1",85.6396429024105,0.0873338480245841,0.240187582689915
,0.363606840314194,0.716151618111253,0.931518231368218
"12514","AC015722.2",2.41661556402056,0.533280693047236,1.463683168529
85,0.364341617443667,0.715602926126519,0.931518231368218
"12515","MSRB1",103.581970871132,-0.0957868734683901,0.262494491479042
,-0.364910032696963,0.715178564972556,0.931518231368218
"12516","CMTM1",7.32907590826163,0.231650658738628,0.635044368830427,0

.364778699109267,0.715276606731391,0.931518231368218
"12517","FHOD3",5.44458868198735,0.281512158152454,0.769042048416198,0
.366055612605596,0.714323578188692,0.931518231368218
"12518","OAZ1",5367.1315471652,-0.0799369601479972,0.219438124866238,-
0.36428018238273,0.715648796898055,0.931518231368218
"12519","ZNF555",26.1307207109813,0.127695301345664,0.349031587764444,
0.365856002213312,0.714472528720197,0.931518231368218
"12520","SNRPB2",763.169976267582,-0.0763430397833274,0.20944927798538
4,-0.364494165449713,0.715489029894666,0.931518231368218
"12521","ENTPD6",89.2207728821826,0.0839499202529744,0.230319983755024
,0.364492558936033,0.71549022932576,0.931518231368218
"12522","AP000350.5",5.07394588985154,-0.260336176640891,0.71486657565
6465,-0.364174498439549,0.71572770870343,0.931518231368218
"12523","AL078622.1",3.29978649159012,0.324966440091225,0.893725454652
837,0.363608799994912,0.716150154532137,0.931518231368218
"12524","MAP3K20",1158.76154002807,0.0670197965811271,0.18450362036267
1,0.363243802205014,0.71642276954834,0.93169192435121
"12525","PSMC2",409.034039006603,-0.0774169303235712,0.213163698401973
,-0.363180648975147,0.716469942047611,0.93169192435121
"12526","ALG8",110.167822233671,0.0850772599501104,0.234154306261121,0
.363338438265727,0.716352082877568,0.93169192435121
"12527","MPPE1",183.694414444587,-0.0648455071277668,0.178578237756634
,-0.36312099359015,0.716514502814636,0.93169192435121
"12528","RALGAPB",110.044095601619,0.0813890138337998,0.22429321029005
9,0.362868825714994,0.716702875247641,0.931862478840105
"12529","AC026202.2",2.56414385492967,-0.344508001336558,0.94967692202
0988,-0.362763370729719,0.716781656501108,0.931890526198942
"12530","IFT140",56.924038935962,0.088562791537506,0.244386713467593,0
.362387914960238,0.717062169135721,0.932180819876437
"12531","TSGA10",40.9705781741647,0.096823876057776,0.267344811230405,
0.362168525404185,0.717226098369868,0.932260675143074
"12532","MTERF4",170.261669602113,-0.0793044188935855,0.21917552620957
6,-0.361830630750965,0.717478600823493,0.932260675143074
"12533","CDH23",17.4753313841699,0.177152414399568,0.48959473379057,0.
361834803712057,0.717475482258158,0.932260675143074
"12534","CMAS",252.604583686205,-0.0660667459340504,0.182455231562644,
-0.362098391853276,0.717278505341074,0.932260675143074
"12535","DCAF11",86.3215795185017,0.0700015522733789,0.193474716179986
,0.361812404512111,0.717492221834032,0.932260675143074
"12536","CDKL1",54.2277232260477,0.100156792342423,0.276911058728595,0
.361693002808488,0.717581456500943,0.932260675143074
"12537","DPH6-
DT",2.44378982440564,0.340859595599,0.942336292438063,0.36171757188414
1,0.717563094527696,0.932260675143074
"12538","ZNF486",46.1537615027259,-0.0964319016567979,0.26643339710147
7,-0.361936238871998,0.71739967850103,0.932260675143074
"12539","PROX2",2.81051400476159,0.352243233811884,0.974130370363613,0
.361597630592713,0.717652735538178,0.932278922496322
"12540","RHCE",3.18737974873386,0.418651637676445,1.1582315323532,0.36
145764122469,0.717757364878019,0.932340487759015

"12541","C4orf36",28.3529027679612,0.135543211236291,0.375198136658418,0.361257687587318,0.717906821245158,0.932460267224494
"12542","AL078645.1",2.75412108663549,0.319430863817719,0.88506451181965,0.360912520558512,0.718164843504621,0.932633516746173
"12543","ZFP36L2",10155.2426368145,-0.0688957457227236,0.190926549683091,-0.360849477650332,0.718211973360181,0.932633516746173
"12544","STRADA",107.591262002344,-0.0683044186708049,0.18925012185072,-0.360921398638165,0.718158206482416,0.932633516746173
"12545","BCDIN3D",62.5904128361021,-0.118585375659281,0.328772309631535,-0.360691494342037,0.718330083828464,0.932712533717166
"12546","MFSD14A",222.444432069472,-0.0542991403349419,0.150617466780889,-0.360510248216654,0.718465594184849,0.932814129099076
"12547","CHODL",3.60164948439415,-0.349498505359294,0.969727397225196,-0.360409024597386,0.718541278821837,0.932838040227059
"12548","MUS81",107.719431037391,-0.0709740177694303,0.196978335238421,-0.360313826815137,0.718612460475667,0.932856102063128
"12549","LINC00115",6.19057914710374,-0.215731171611102,0.607213198403735,-0.355280768234657,0.722379261629372,0.93307238876624
"12550","CPTP",70.8126300489848,0.0995708354500453,0.276859804982443,0.359643522310361,0.719113732242042,0.93307238876624
"12551","FBXO44",80.5771658207366,-0.0830843127867364,0.232231811454939,-0.357764564062998,0.720519511418975,0.93307238876624
"12552","AL450998.2",26.7258008323474,-0.127704461955756,0.357811027370582,-0.356904768682531,0.721163099579882,0.93307238876624
"12553","SFPQ",1434.3453049325,0.097603362759403l,0.272925071220997,0.357619629163237,0.720627986611123,0.93307238876624
"12554","WDR77",56.4906296050267,-0.0999268690920752,0.281620533103013,-0.354828065947604,0.722718401129251,0.93307238876624
"12555","VSIG8",3.20188466552104,-0.326745398793016,0.922408111780375,-0.354230838410942,0.723165894284626,0.93307238876624
"12556","BPNT1",67.5294455360319,0.0823565445735822,0.231230060040028,0.356167120137086,0.72171541382737,0.93307238876624
"12557","MARK1",51.5670223939351,0.0882965118771798,0.24925766514626,0.354237900067662,0.723160602543431,0.93307238876624
"12558","ERO1B",96.4129147213284,0.100566456005139,0.282875589853852,0.355514790290306,0.722203966637883,0.93307238876624
"12559","MGAT4A",12.9014477054627,0.18505814387698,0.51477269958647,0.359494868367421,0.719224916045352,0.93307238876624
"12560","PSMD1",385.7810261305,-0.0592917305541926,0.165632001028399,-0.357972675485738,0.720363762176521,0.93307238876624
"12561","D2HGDH",135.615401794042,0.0878087253308872,0.245823224735076,0.357202723320869,0.720940047293972,0.93307238876624
"12562","HACD2",119.995105728381,0.0724907217643062,0.20454276272044,0.354403748146218,0.723036326008493,0.93307238876624
"12563","OSBPL11",127.65459782449,0.0615501680839801,0.172903052467679,0.355980806616967,0.721854939017467,0.93307238876624
"12564","HAUS3",95.6921289000981,0.0849794794265129,0.237168934702192,0.358307800864477,0.720112980899017,0.93307238876624
"12565","REST",379.508709826332,0.0460786276949526,0.129028025436347,0.357121079231616,0.721001164642395,0.93307238876624

"12566","SMN1",35.5029986779552,0.107166928063295,0.301400484116231,0.355563224715881,0.722167688493115,0.93307238876624
"12567","COMMD10",203.976131605225,-0.0915471902265573,0.254424639156105,-0.359820458152984,0.718981403103925,0.93307238876624
"12568","AC011374.2",14.6083611075979,0.147150685196447,0.414869501798283,0.354691498311184,0.72282072074398 7,0.93307238876624
"12569","SAP30L",258.568413043351,-0.0659972806627269,0.183713179548356,-0.359240860263677,0.719414911886851,0.93307238876624
"12570","AL365272.1",7.52540243306407,-0.200998524346701,0.56562169928971,-0.355358580830808,0.722320974230106,0.93307238876624
"12571","GPSM3",25.1078387464699,-0.122827382343077,0.342588177735026,-0.35852779029076,0.719948374614121,0.93307238876624
"12572","GINM1",1217.36909395074,-0.0539805241554032,0.152401720268788,-0.354198916260255,0.723189815714518,0.93307238876624
"12573","AL357075.4",2.64865992287236,-0.331020005824514,0.933895538473215,-0.354450784041312,0.72300108147851,0.93307238876624
"12574","KLHL7",157.68484241257,0.0694830836120747,0.194264627671998,0.357672338215848,0.720588536355003,0.93307238876624
"12575","HOXA11",45.9388843503158,0.0890583790213612,0.249085438051339,0.357541491458065,0.720686470390467,0.93307238876624
"12576","TNFRSF10B",116.017693675515,0.112927570682037,0.316202083130944,0.35713733939973,0.72098899242059,0.93307238876624
"12577","LOXL2",64.8071584447942,0.153969224459239,0.428772624588643,0.359092944907466,0.719525559268988,0.93307238876624
"12578","AC011029.1",5.63205018458009,-0.244702983055866,0.688074580493433,-0.355634389051816,0.722114386422188,0.93307238876624
"12579","OTUD6B-AS1",499.679730683968,-0.0782307102674092,0.217681246695369,-0.359381947021316,0.719309378083997,0.93307238876624
"12580","ASTN2",22.0406684043114,-0.128739107294317,0.357606976268652,-0.360001666180029,0.718845887559625,0.93307238876624
"12581","AL158151.3",6.8183546930939,0.21074509350893,0.588176033352602,0.358302755567383,0.720116756176373,0.93307238876624
"12582","DIPK1B",3.75617749564687,-0.392194710632687,1.09331125057672,-0.358721919696524,0.719803128777848,0.93307238876624
"12583","AL137186.1",3.38404803688611,0.284647205530016,0.796340053006337,0.357444290859687,0.720759224466396,0.93307238876624
"12584","HPS1",234.026189839191,-0.0675358644409047,0.189798283960863,-0.355829689455099,0.721968113397584,0.93307238876624
"12585","PGGHG",38.7909279771216,0.140235653072271,0.393601042531572,0.356288825279273,0.721624277114841,0.93307238876624
"12586","CTSD",1742.90817874972,-0.0747400625440808,0.209647868190646,-0.356502850179784,0.721464017993297,0.93307238876624
"12587","RPL27A",16164.7543582985,0.0622125545725797,0.174509860619565,0.356498792398926,0.721467056294265,0.93307238876624
"12588","TMEM41B",169.091163341135,-0.064277581537929,0.180204858239768,-0.356691723884633,0.721322601923204,0.93307238876624
"12589","SYVN1",173.660888619842,0.0695154720408655,0.195881773144767,0.354884841630925,0.722675864940256,0.93307238876624
"12590","TTC12",28.5615060756613,0.113561334611299,0.319027914480833,0

.355960495795807,0.721870149805288,0.93307238876624
"12591","FAM216A",56.6534743865635,0.0863813726687649,0.241857629684247,0.357157939493324,0.720973571469452,0.93307238876624
"12592","N4BP2L2-IT2",10.0264522028448,0.228841575204433,0.638901142284045,0.35817994374894,0.720208655481393,0.93307238876624
"12593","RNASEH2B-AS1",2.6163942056713,0.346435667559655,0.970810544177195,0.356851982745279,0.721202618216551,0.93307238876624
"12594","AL096870.8",76.2533946002955,-0.0925306725797515,0.258576053411849,-0.357847029370397,0.720457793528895,0.93307238876624
"12595","MAX",364.370892995647,0.0579716912903437,0.162368278953526,0.357038281516409,0.721063147395147,0.93307238876624
"12596","AC116913.1",13.6171013461735,0.158427502650723,0.447086916042215,0.354355041416072,0.723072823133337,0.93307238876624
"12597","AP3B2",4.15189435790812,-0.279062102566505,0.778712480678345,-0.358363464681356,0.720071329424813,0.93307238876624
"12598","AF001548.2",10.3072871539343,-0.2605775377059,0.727525170382982,-0.358169790288805,0.720216253432176,0.93307238876624
"12599","CYLD",171.068146878378,0.0758016575261915,0.213650637139874,0.354792564819571,0.722744998914178,0.93307238876624
"12600","DLG4",67.5108964853735,-0.0915400759976728,0.258024572533465,-0.354772706718854,0.722759876939421,0.93307238876624
"12601","VAMP2",1101.92600476162,0.0889427129450454,0.249626885946958,0.356302617835582,0.721613949055745,0.93307238876624
"12602","NT5C3B",321.26889909737,-0.074466004813284,0.208694307848153,-0.356818571532224,0.721227632187423,0.93307238876624
"12603","MRPL10",251.898826523739,-0.0901893314230149,0.251662726636706,-0.358373814940064,0.720063584745079,0.93307238876624
"12604","NXPH3",210.707862094713,-0.0926677436995596,0.259470651143254,-0.357141523680062,0.720985860115806,0.93307238876624
"12605","FAM117A",123.860266531956,-0.0941970997984304,0.263172020155025,-0.357929766785019,0.720395873821243,0.93307238876624
"12606","TMEM200C",3.31903744962453,-0.305756226055127,0.862830254621234,-0.354364284768095,0.723065896818828,0.93307238876624
"12607","TRAPPC8",121.02379357835,0.0754692005068752,0.2122850086778,0.355508855650848,0.722208411818922,0.93307238876624
"12608","ZNF561-AS1",39.592792650079,0.102878632749809,0.287005462451443,0.358455312561217,0.720002604376923,0.93307238876624
"12609","YJEFN3",23.2074969559021,0.145283782952596,0.40767439316638,0.356372108201809,0.721561914469553,0.93307238876624
"12610","ZNF682",26.4282589882566,0.121340153001181,0.338983997055008,0.35795245219641,0.720378896646558,0.93307238876624
"12611","ZNF585A",143.3186086794,0.0664454774821143,0.186568606307827,0.356145006370917,0.721731973748642,0.93307238876624
"12612","AC011445.2",4.80637002578407,-0.252760554989833,0.708839420299097,-0.356583660207808,0.721403511652401,0.93307238876624
"12613","ZNF766",151.547318588007,-0.0826591423018668,0.231838689485727,-0.356537308269057,0.72143821735722,0.93307238876624

"12614","AC012313.5",3.14269042325457,-0.296316118578213,0.827797569852177,-0.357957222115458,0.720375326980149,0.93307238876624
"12615","MCM8",26.8258631033665,0.138649194614672,0.38645453852924,0.358772328414982,0.719765415092199,0.93307238876624
"12616","ERGIC3",884.255357435457,-0.0430182080667011,0.119979824673029,-0.358545348636197,0.719935237205615,0.93307238876624
"12617","NFS1",70.9652647819312,-0.0852379835060107,0.23720392974422,-0.359344735974332,0.719337211667209,0.93307238876624
"12618","SAMHD1",1488.92574313514,0.0892867797216169,0.250908494768134,0.355853953068139,0.721949941528042,0.93307238876624
"12619","PLCG1-AS1",16.1599302899677,0.216918932006068,0.610757290514077,0.355163884860852,0.722466818963748,0.93307238876624
"12620","ZFP64",65.1833037557793,0.0808923873145739,0.228075545484466,0.354673654918797,0.722834089790157,0.93307238876624
"12621","COL9A3",2.41012354102268,-0.392364071039521,1.10418858786159,-0.355341537987987,0.722333740444166,0.93307238876624
"12622","GRAP2",2.94388245809769,-0.321519743822422,0.897825389458418,-0.358109435974369,0.720261417828803,0.93307238876624
"12623","ARAF",114.214545977948,0.0740952780365714,0.208979538524612,0.354557573242249,0.722921065299873,0.93307238876624
"12624","HDX",41.3028509667848,0.0971583949063202,0.271640272026739,0.357673014319307,0.720588030328075,0.93307238876624
"12625","CAPN6",20.4522149540926,0.181780670629566,0.508945261051909,0.357171359163172,0.720963525747336,0.93307238876624
"12626","MAST4-AS1",3.77122545914623,0.28780199123501,0.812843398764515,0.354068190345713,0.723287780319122,0.933124873563929
"12627","LINC01001",7.75856672853694,0.204357869217801,0.577679110104777,0.353756723487431,0.723521208526981,0.933312050411286
"12628","RNF213-AS1",21.4311563175458,0.147883743500003,0.418079366541146,0.353721698163378,0.723547459794568,0.933312050411286
"12629","PIP4K2C",35.0200840994207,-0.1126786586111 7,0.318658630415885,-0.353603034269341,0.723636400079548,0.933352864114004
"12630","ADAMTS6",12.0345327673857,-0.185610784777002,0.525076990785238,-0.353492512592157,0.723719241013298,0.933385804977483
"12631","NRAS",110.442768975052,0.0759842563057224,0.215418483885521,0.352728581759505,0.724291929747998,0.933459232824206
"12632","AC239809.3",22.8109664486757,-0.134832148925835,0.38225328648589,-0.352729861828964,0.724290970001374,0.933459232824206
"12633","LINC01106",6.10720320890917,0.29410542177824,0.833359419963181,0.352915458483969,0.724151821387168,0.933459232824206
"12634","AC012510.1",10.4866239909037,0.178407927165306,0.50500838406369,0.353277158944763,0.723880667487838,0.933459232824206
"12635","GBA2",54.5822315804989,-0.104574515020652,0.296038797769629,-0.353245979272048,0.723904040409215,0.933459232824206
"12636","FAM13C",201.64158218949,0.136936925243782,0.387838305707965,0.353077360406203,0.724030445025232,0.933459232824206
"12637","DUT",1108.23734378543,-0.0813469876865391,0.230587084114961,-

0.352782064957216,0.724251830481943,0.933459232824206
"12638","AL021707.6",7.76590614431988,0.233050342073429,0.660182920259656,0.353008741852589,0.724081886868273,0.933459232824206
"12639","GSPT2",133.911771256308,0.0631989244750764,0.178966804517299,0.353132105395375,0.723989404786678,0.933459232824206
"12640","POLR3C",124.716576837604,0.062944212652871 3,0.178868813250617,0.351901550130367,0.724912096631942,0.933471333436822
"12641","ZNF687",44.8256846540317,0.0944941703732292,0.268506936855406,0.351924503254511,0.724894882316795,0.933471333436822
"12642","IFIH1",77.3227999575054,-0.0820698372561335,0.233472244343235,-0.351518603365461,0.725199318402608,0.933471333436822
"12643","HYAL3",4.01095869953813,-0.269676731708316,0.765971897675079,-0.352071312964423,0.724784781700564,0.933471333436822
"12644","SLC10A6",6.50678468059577,0.271354445728028,0.771182063059669,0.351868201720652,0.724937107419919,0.933471333436822
"12645","ZSCAN16-AS1",315.024208178702,0.0920337327534736,0.261151215283904,0.352415487147634,0.724526689003237,0.933471333436822
"12646","GLCCI1",39.6611693641103,0.109333102784218,0.310929981022767,0.351632552205419,0.725113849249472,0.933471333436822
"12647","AC007066.2",2.50344802086939,0.361475522096415,1.02879765207125,0.351357258027044,0.725320343914298,0.933471333436822
"12648","FUOM",271.87325240316,0.103524447100946,0.294355636420743,0.35169853840669,0.725064356807631,0.933471333436822
"12649","RNF26",87.0122550184093,0.0739320549571566,0.210170582095719,0.35177166195165,0.725009512397798,0.933471333436822
"12650","GALNT4",71.0976341668067,0.0855883030028344,0.243604934596922,0.35134059638181,0.725332842243837,0.933471333436822
"12651","GSTZ1",147.876013144022,-0.0925841739498482,0.263227784907821,-0.351726448567236,0.725043423352397,0.933471333436822
"12652","SPESP1",35.3129945218936,-0.122347932120076,0.348175662374452,-0.351397140413835,0.725290427405198,0.933471333436822
"12653","C16orf72",201.417942459168,0.0992714615959033,0.282343697783632,0.351597936752878,0.725139812762175,0.933471333436822
"12654","SCPEP1",1932.44090010242,0.0745658912996695,0.212218190518931,0.351364278044854,0.725315078039908,0.933471333436822
"12655","TPGS2",353.535457890978,0.0585124995744801,0.1660597434891,0.352358123317954,0.724569703378864,0.933471333436822
"12656","TPX2",2.56998273912504,-0.339426615733457,0.963269766642617,-0.352369219389596,0.724561382900836,0.933471333436822
"12657","AP000331.1",5.88995497421302,-0.271517427015863,0.77274970183869,-0.351365295088191,0.725314315133738,0.933471333436822
"12658","CAB39",196.9230898866,-0.0702468836618929,0.200050140296136,-0.351146385390712,0.725478530309876,0.93351957220767
"12659","AL355377.3",6.14176522373899,0.306982990595508,0.874252102984303,0.351137834896372,0.725484944722015,0.93351957220767
"12660","AGTRAP",508.210417158295,0.0962195144559946,0.274203517781717,0.350905470631457,0.725659267180314,0.933533139494877
"12661","PIGF",236.6617389857,0.0968616800554302,0.276043514781818,0.350892793594476,0.72566877805447,0.933533139494877

"12662","PKP4",58.7617731298216,-0.154982616333291,0.441837569643672,-0.35076830713667,0.725762175551511,0.933533139494877
"12663","C8orf58",63.5870659047801,0.0990418332803656,0.282649245616791,0.350405440015376,0.726034444340943,0.933533139494877
"12664","ERICD",3.88960768208885,0.258527414231625,0.737850440953142,0.350379155290148,0.72605416780995,0.933533139494877
"12665","LINC00595",2.49288172559944,-0.333984454140332,0.952076713951075,-0.350795738669326,0.725741594356155,0.933533139494877
"12666","PIDD1",30.9680262338206,-0.101836133981303,0.290784335804084,-0.350211897417732,0.726179678625695,0.933533139494877
"12667","USP2",4.47605790232006,-0.266133871804476,0.759579822175962,-0.350369854536268,0.726061146930825,0.933533139494877
"12668","CPM",62.3940515928009,-0.13129835290728,0.374997994693827,-0.350130813404697,0.726240526961697,0.933533139494877
"12669","TTC19",228.31116323124,-0.0599016011073262,0.170912470624963,-0.350481160843843,0.725977626142973,0.933533139494877
"12670","IFI35",343.014547784169,0.0883754674534681,0.252357139609636,0.35019998875472,0.726188615203012,0.933533139494877
"12671","DOK6",70.2598627632425,0.124373308379165,0.355011421668311,0.350336075934389,0.726086493986306,0.933533139494877
"12672","MED14OS",17.6444587367452,-0.139462915218827,0.398214854322172,-0.350220273566178,0.726173392963546,0.933533139494877
"12673","SCFD2",55.9216512467395,0.079886681701233,0.228255634878673,0.349987774644407,0.726347872561739,0.933592241240314
"12674","SLC9A3-AS1",10.7236930851572,-0.248821448560302,0.71108744674295,-0.349916806575617,0.726401133616535,0.933592241240314
"12675","DLEU2L",23.2456107381473,0.126002991504006,0.360817899368338,0.349214913463529,0.726927970833462,0.933630258200294
"12676","GADD45A",524.262168688183,0.15059362288081l,0.431499670692114,0.3490005511227,0.727088895815441,0.933630258200294
"12677","KCNC4",15.8892171892415,0.164006247069719,0.468994065705197,0.349697915309682,0.726565418358639,0.933630258200294
"12678","TRA2B",1242.82899084978,0.0571184291178379,0.163606605089576,0.349120557122771,0.726998804051185,0.933630258200294
"12679","AC105206.1",3.95579783708364,0.275505781285072,0.788980237591403,0.349192246089883,0.726944986996913,0.933630258200294
"12680","RBPMS",490.930249858735,0.0979569414642367,0.280175373923376,0.349627235586474,0.726618468388086,0.933630258200294
"12681","ERCC6L2",180.20651326373,0.0625017341786938,0.179132423556605,0.348913574314164,0.727154194026869,0.933630258200294
"12682","TRIM14",7.67139060247907,-0.235223823471716,0.673768521033861,-0.349116671569604,0.72700172098238,0.933630258200294
"12683","LPAR1",652.051011973471,-0.0691546106848967,0.198105740077335,-0.34907928794946,0.727029785514922,0.933630258200294
"12684","SPTLC2",119.246273166126,-0.0677137134190681,0.193962491794881,-0.349107256730217,0.727008788831224,0.933630258200294
"12685","SPG21",341.596139563502,-0.0610689338207862,0.174849517920151,-0.349265668828866,0.726889869793017,0.933630258200294
"12686","ZNF333",85.2983246408627,0.0742745297448042,0.212660977581929

,0.349262617849999,0.726892160082922,0.933630258200294
"12687","TBC1D22A",132.31923180819,-0.0581092433101721,0.166557128682653,-0.348884756658416,0.727175829442392,0.933630258200294
"12688","ARHGEF10L",158.789777669132,0.0657662189618185,0.18879806375998,0.348341596582406,0.727583658103882,0.933712298657066
"12689","LRPPRC",284.285528813673,0.0607039138094618,0.174196528866851,0.348479468588386,0.727480130378085,0.933712298657066
"12690","EAF1-AS1",4.92645232010888,0.306314254155639,0.879134853780952,0.348426925446367,0.727519584298411,0.933712298657066
"12691","LINC02043",2.38111280821709,-0.412648280064475,1.18406187429425,-0.348502294536281,0.727462990913851,0.933712298657066
"12692","PDSS2",93.861478318593,0.0702188993982126,0.201539023500879,0.348413414823886,0.727529729355153,0.933712298657066
"12693","AL162171.3",7.55981792510952,-0.240574570093716,0.690297833762233,-0.348508365994061,0.727458432022804,0.933712298657066
"12694","NECTIN3-AS1",2.99366634639586,-0.279995820540398,0.805349187535454,-0.347670085068624,0.728087965381646,0.933991563088804
"12695","CYFIP2",8.56024056008593,0.224804305634675,0.646419498739892,0.347768447692096,0.728014087373667,0.933991563088804
"12696","SLC13A4",3.72640004922357,-0.34764109200586,0.999743068347857,-0.347730434961015,0.728042637602248,0.933991563088804
"12697","ZNF34",62.7080306741234,-0.108441606218877,0.311870404231364,-0.347713680899418,0.728055221198045,0.933991563088804
"12698","GPR137C",3.66616930589848,0.300891342800631,0.865022270346812,0.347842307782428,0.727958614341864,0.933991563088804
"12699","LINC02716",9.91615882619042,0.228478479727631,0.657344173677221,0.347578162060081,0.728157009019987,0.934004932224748
"12700","ZCCHC18",8.91564886950131,-0.191307390945405,0.550519272526508,-0.347503530743682,0.728213066440807,0.934004932224748
"12701","ZNF211",16.4346315283523,-0.137352620956699,0.395566673102384,-0.347230012780041,0.728418524949185,0.934055931711995
"12702","UBE2L3",854.008982200543,-0.0766569861005324,0.220772526268281,-0.34722158321176,0.728424857298635,0.934055931711995
"12703","SASH3",4.13759879825604,-0.276735321009986,0.796761968505327,-0.34732496272271,0.728347199181964,0.934055931711995
"12704","SSX2IP",18.0852794617624,-0.124205487353031,0.357991741365907,-0.34695070584346,0.728628352098643,0.934092582506656
"12705","RO60",430.865958802736,-0.0423062352851073,0.122290534548495,-0.345948567820926,0.72938136770586,0.934092582506656
"12706","OBSCN",16.3651950993406,0.128620658355445,0.371980924379049,0.345772188641534,0.729513927663232,0.934092582506656
"12707","OST4",3106.76629946653,-0.0817906168771368,0.236039450743402,-0.346512486025275,0.72895760228051,0.934092582506656
"12708","UGP2",1210.58692473125,0.0513089889755688,0.147883072422445,0.346956471319442,0.728624020624359,0.934092582506656
"12709","NDUFB3",594.284903511829,-0.101167221762789,0.292309545225328,-0.34609619636199,0.729270421864871,0.934092582506656
"12710","NHEJ1",79.3648791480932,-0.0916632550062912,0.265284506236698

,-0.345528113596297,0.729697378621089,0.934092582506656
"12711","GTF2H2C",68.3217619291331,-0.102824685928185,0.296301785287705,-0.347026886214484,0.728571120173975,0.934092582506656
"12712","ATG10",118.058379666784,-0.0619127742677215,0.179002163196654,-0.345877240599061,0.729434973589229,0.934092582506656
"12713","IGIP",74.833176879596,0.100525864056804,0.290944883513594,0.345515146521238,0.729707125328299,0.934092582506656
"12714","PSORS1C1",38.0398712632295,-0.0972327105077928,0.281206074579908,-0.345770306182213,0.729515342492467,0.934092582506656
"12715","NCR3",3.43746729620528,-0.29485058294572,0.853641129699166,-0.345403440260229,0.729791091192838,0.934092582506656
"12716","WAKMAR2",10.9392788519369,-0.171420256171236,0.49582186267189 2,-0.345729523195052,0.729545994622601,0.934092582506656
"12717","ST7-OT4",9.36150637858121,-0.202416875318502,0.58425241257336,-0.346454496314272,0.729001175766275,0.934092582506656
"12718","MMP21",2.2988775309583,-0.355690962350174,1.02839291995588,-0.345870683712443,0.729439901475452,0.934092582506656
"12719","TMEM80",195.32174842107,0.0906111417596216,0.261889393174332,0.345990116901391,0.729350142156651,0.934092582506656
"12720","MALAT1",283842.878924629,-0.0443785015378306,0.128092820175019,-0.346455808195918,0.729000190008345,0.934092582506656
"12721","AC004466.1",2.52689900332056,-0.296973118655579,0.859594411785071,-0.345480513349164,0.729733157580156,0.934092582506656
"12722","AC012531.1",4.24282355792476,0.329918480173229,0.955717038593938,0.345205188199437,0.729940118620396,0.934092582506656
"12723","DDX55",113.99724794468,-0.067105384145915,0.19434344070797 2,-0.345292765742222,0.729874284725665,0.934092582506656
"12724","EAPP",661.340380946418,-0.0780472143813202,0.226135354754864,-0.345134950109528,0.729992919519235,0.934092582506656
"12725","AL133453.1",61.0019495096128,-0.136482883357339,0.39496680107794,-0.345555330181806,0.729676921405611,0.934092582506656
"12726","DDX24",1278.97665606045,-0.0546407910769132,0.158231729759381,-0.345321328155888,0.729852814184547,0.934092582506656
"12727","AHNAK2",764.743217430753,-0.104915106563176,0.304060408324161,-0.34504691729324,0.73005909926283,0.934092582506656
"12728","AC005291.1",3.0663516636173,-0.408825585531935,1.18420595642235,-0.345231826706102,0.729920093679802,0.934092582506656
"12729","CBR3",558.96700675397,-0.0969656825450805,0.281011831601427,-0.345059074532533,0.730049959790232,0.934092582506656
"12730","SYN3",3.09692444391557,0.286915436451752,0.82924242339907,0.345997055089975,0.729344927915339,0.934092582506656
"12731","AC093151.8",2.92137697000854,-1.07601132465102,3.11740618739634,-0.345162375375186,0.729972302651961,0.934092582506656
"12732","VBP1",351.191207829335,-0.0853673663119152,0.247572687007838,-0.344817384113186,0.730231663083955,0.93423999057293
"12733","LINC00839",3.7939444463716,0.350232831625849,1.0162252917406,0.34464093195906,0.730364329696438,0.934336336010781
"12734","CSNK1D",296.812906169087,0.0641203603818969,0.186227132098595,0.344312666255041,0.730611159702351,0.934578701145877

"12735","CCDC88A",386.471970917703,0.0709708127414507,0.206368020270794,0.343904121618861,0.730918392120846,0.934647255880097
"12736","PIGB",41.1446740157683,-0.0977190797718736,0.284160157483022,-0.343887336766106,0.730931015535169,0.934647255880097
"12737","CCDC137",120.043543314539,0.0966975982142091,0.281212195397555,0.343859902937373,0.730951647900826,0.934647255880097
"12738","TECR",476.572277150945,-0.0738601649800362,0.21479680844969,-0.343860625831114,0.730951104226203,0.934647255880097
"12739","GCAT",35.014955094616,-0.112499810947693,0.32689187579573,-0.344149914016195,0.730733546939523,0.934647255880097
"12740","PHTF1",58.9836538445145,-0.0809055762436931,0.235467615642701,-0.34359534334632,0.731150627206602,0.934754930269864
"12741","AC136475.5",2.77438152970899,0.316805862822608,0.921916661567787,0.343638287525747,0.731118327003801,0.934754930269864
"12742","CCNH",274.004081367795,-0.062795570007812,0.182867981556868,-0.343392919160558,0.73130288574126,0.934874610376491
"12743","GRTP1",39.1054802168305,-0.0959380251983402,0.27944338798246,-0.343318286723471,0.731359025110665,0.934874610376491
"12744","INE2",3.18368282617912,-0.338197319972168,0.985326325833603,-0.343233821227751,0.731422562754006,0.934882464273384
"12745","NSD3",919.400492594432,0.0436468069026057,0.127295955069859,0.342876620695865,0.731691280763629,0.935101379551159
"12746","JCAD",26.0055036553595,-0.161219671263708,0.470232453371824,-0.342851009341603,0.7317105491671,0.935101379551159
"12747","SLC4A11",2.74015552585068,-0.420576019154214,1.22696603036154,-0.342777231599711,0.731766055935823,0.935101379551159
"12748","LRP8",7.17182689420239,0.225254694724994,0.657659978869915,0.342509354320236,0.731967605513633,0.9351316177438
"12749","AC010883.1",4.13554433136542,-0.235208684569621,0.687029917555202,-0.342355810947232,0.732083139148329,0.9351316177438
"12750","COLQ",12.5983523303285,0.156778373065809,0.458030596569316,0.342287991763194,0.73213417159351,0.9351316177438
"12751","C15orf41",47.6535914276465,0.0960929251169286,0.280566244227164,0.342496387552331,0.731977362116847,0.9351316177438
"12752","AC025040.1",5.13637581202459,0.264434307381699,0.772088746240553,0.342492114629671,0.73198057720748,0.9351316177438
"12753","SLC1A5",227.377944285235,0.0960915870708498,0.280622849315481,0.34242253367908,0.732032932915612,0.9351316177438
"12754","RTCA",221.000414334465,0.0668610958913476,0.195404385127095,0.342167837471302,0.732224587965465,0.935173773982238
"12755","ZBTB48",53.4154399573019,-0.100575564173838,0.29435846318742,-0.341677161528735,0.732593861278572,0.935463753942129
"12756","ABL2",741.36090523389,0.245827198295468,0.719556258166566,0.341637218084708,0.732623924675532,0.935463753942129
"12757","AL355312.3",16.8026815492991,-0.230798586198178,0.675514424835047,-0.341663445979761,0.73260418422783,0.935463753942129
"12758","TMEM240",73.4001106001014,-0.0926084547787892,0.275730922378652,-0.335865321088699,0.736972429217331,0.935662034257295
"12759","YTHDF2",511.18858414436,-0.0598546638792296,0.176655979662921,-0.338820480311162,0.734744971851277,0.935662034257295

"12760","DMAP1",236.710993950941,-0.0764372338585091,0.226451045263401,-0.337544186513246,0.735706708470397,0.935662034257295
"12761","LAMTOR5",969.74824180343,-0.0843992624649017,0.249920603916974,-0.337704299453998,0.735586034430236,0.935662034257295
"12762","LYSMD1",36.9794005392696,0.130781891198188,0.384909410003274,0.339773172074634,0.734027352800013,0.935662034257295
"12763","YY1AP1",185.916511147176,0.0545142366785355,0.162026776879814,0.336452021871498,0.736530025762884,0.935662034257295
"12764","AL512343.2",5.20733619331224,-0.220094076807869,0.649372471449377,-0.338933488074459,0.734659836159305,0.935662034257295
"12765","RTKN",53.5277636300463,-0.0857201287001561,0.25450265299148З,-0.336814283437056,0.73625690495679,0.935662034257295
"12766","RNF149",333.151724005433,0.0583275554195669,0.172864162599277,0.337418436201715,0.735801488631413,0.935662034257295
"12767","HIBCH",252.199540440029,-0.0680197174517028,0.200323381505007,-0.339549567008495,0.73419576341273,0.935662034257295
"12768","ANKZF1",80.1724590125548,0.0759336582782124,0.224618557882406,0.338056031496586,0.735320963677254,0.935662034257295
"12769","CUL3",333.63194884778,0.0524214170161486,0.153986614829714,0.340428400703,0.733533933862631,0.935662034257295
"12770","C3orf35",8.67451129692221,0.216824447277269,0.643802386346891,0.336787268695274,0.736277271102023,0.935662034257295
"12771","BST1",430.494635089525,-0.0943446620398346,0.276690222639068,-0.34097577117101,0.733121821710495,0.935662034257295
"12772","MAPK10",132.297669482814,0.110170383436657,0.32723447580842,0.336671077106058,0.736364868898106,0.935662034257295
"12773","SPRY1",2760.87277512909,0.0742380460254837,0.217671769472952,0.341055003160199,0.733062174760165,0.935662034257295
"12774","TENT4A",92.4417479538048,0.0852870989036834,0.249975754979474,0.341181483423008,0.7329696198844,0.935662034257295
"12775","AC010457.1",5.12900937686975,-0.337301791445473,1.0042399468171,-0.335877687911676,0.736963103078039,0.935662034257295
"12776","AC010273.3",7.40927465464305,0.22486845638163,0.661395703229655,0.339990803211416,0.733863453804115,0.935662034257295
"12777","ARSK",113.286266589527,0.0601136127142139,0.178943450533617,0.335936367243129,0.736918852012915,0.935662034257295
"12778","SPATA9",8.31322860396849,0.177413949815772,0.520931524976333,0.340570576572097,0.733426883061173,0.935662034257295
"12779","AC010226.1",198.076743569051,-0.0978133194235878,0.290576173547981,-0.336618512898947,0.736404498612612,0.935662034257295
"12780","SMAD5-AS1",17.332151280135,-0.131904745178796,0.388615169060867,-0.339422533344642,0.734291445908907,0.935662034257295
"12781","RNF145",322.689752379201,0.075786841584314,0.225172160530143,0.336572875642719,0.736438906462132,0.935662034257295
"12782","SERAC1",20.9697070154683,-0.111149746957479,0.326806487534285,-0.340108753030243,0.733774630327542,0.935662034257295
"12783","AC004540.2",5.27492300002173,0.288185382364909,0.846236590819041,0.340549422574584,0.733442810558163,0.935662034257295
"12784","METTL27",74.3063403469645,0.160360309840664,0.473763757772427

,0.338481589631627,0.735000298565544,0.935662034257295
"12785","CLIP2",43.2530008179091,-0.114250398502511,0.338919515812672,
-0.337101858028321,0.73604011682009,0.935662034257295
"12786","CLDN15",32.2873267570395,0.104734984177923,0.307313195144989,
0.340808614249411,0.733247665066134,0.935662034257295
"12787","AC078846.1",22.1327529182174,0.12965042445878,0.3856095724996
92,0.336222007193256,0.73670345861016,0.935662034257295
"12788","DOCK5",14.4384070362259,0.190363713707318,0.562960980067172,0
.338147261440045,0.735252216515234,0.935662034257295
"12789","AC090739.1",3.30126839238016,0.302102185856194,0.888303020011
364,0.340089112668253,0.733789420482284,0.935662034257295
"12790","TRMT12",41.279148265338,-0.0974257585005821,0.286263035073203
,-0.340336496731645,0.733603135379583,0.935662034257295
"12791","ADCK5",34.0541629462012,-0.101569063012116,0.301589605722005,
-0.336779056986928,0.736283461864024,0.935662034257295
"12792","TSTD2",103.696438274623,0.0973251554531823,0.286746651341746,
0.339411654845063,0.734299639850008,0.935662034257295
"12793","ANKS6",59.9154976556439,0.0934277788887078,0.277848771539375,
0.336254065012009,0.736679285962128,0.935662034257295
"12794","SPOUT1",72.4117884804864,0.0759068695136391,0.225200596341425
,0.33706335927529,0.736069137891218,0.935662034257295
"12795","MIGA2",38.3200803243594,-0.110122373117312,0.32652940903869,-
0.337251010380703,0.735927686578407,0.935662034257295
"12796","PRTFDC1",188.469701747664,0.0704859666825341,0.20973092430249
,0.336078081555935,0.736811986477204,0.935662034257295
"12797","ATRNL1",2.86074840068737,0.351984430872659,1.03897822651186,0
.338779410281165,0.734775913236152,0.935662034257295
"12798","TIAL1",463.686153253853,-0.0483503331872888,0.143170628773414
,-0.337711258248431,0.735580789868808,0.935662034257295
"12799","NUP160",146.709942734975,0.0717310065047391,0.211930903334418
,0.338464119088619,0.735013462000632,0.935662034257295
"12800","SNHG1",118.327740615132,-0.0741841599763243,0.220537556710838
,-0.336378805872019,0.736585229737302,0.935662034257295
"12801","ATN1",525.535272490939,0.0675242232884716,0.198875253713393,0
.33953054504098,0.734210090606861,0.935662034257295
"12802","PPP1R12A",824.179225260974,0.0537930718039995,0.1580803672001
65,0.340289390496452,0.733638606099737,0.935662034257295
"12803","USPL1",70.167108774541,0.0717215382580366,0.21192971972269,0.
33842133303382,0.735045700105532,0.935662034257295
"12804","CMA1",5.60321721621211,-0.327773764463081,0.964949655798555,-
0.339679653226912,0.734097785991722,0.935662034257295
"12805","C14orf28",89.9876009111421,0.0732193640430082,0.2156604935609
12,0.339512178767818,0.734223924025041,0.935662034257295
"12806","NAA30",112.236841105825,-0.0635142833064242,0.188920718786065
,-0.336195435389742,0.736723494820038,0.935662034257295
"12807","PSMA3",741.196116492647,-0.0812515897734161,0.241448951769198
,-0.336516639140702,0.736481306256335,0.935662034257295
"12808","ARID3B",17.30333466049,0.160474388509455,0.4758500109404,0.33
7237332814844,0.735937996418559,0.935662034257295
"12809","MRPS11",289.731515239773,-0.0845490290311142,0.24924412532732

8,-0.339221752649445,0.734442683512108,0.935662034257295
"12810","TM2D3",410.369843513115,0.0775378596836542,0.227909319710818,
0.340213641908273,0.733695645536766,0.935662034257295
"12811","Z92544.2",2.70024596210642,-0.300765722830212,0.8878525158111
58,-0.338756400949573,0.73479324822014,0.935662034257295
"12812","NOMO3",7.92099291775856,0.193253093508372,0.568866555838666,0
.339716039772216,0.73407038140479,0.935662034257295
"12813","LAT",14.7851392496149,-0.149290612157564,0.441107852741834,-0
.338444693808114,0.735028098352546,0.935662034257295
"12814","CCDC189",14.7209252548952,-0.161215749932459,0.47636319393754
5,-0.338430323719753,0.735038925834449,0.935662034257295
"12815","NUDT7",79.6453506005725,0.125032994124051,0.37016271975922,0.
337778461875851,0.73553014185188,0.935662034257295
"12816","ANKRD40",188.237114061905,-0.0729901310821183,0.2152978167560
26,-0.339019374101829,0.734595135118317,0.935662034257295
"12817","TRIM25",271.579099610248,0.113690462596192,0.337238683386576,
0.337121653585241,0.736025194710266,0.935662034257295
"12818","AC090229.1",2.75806564554483,0.387838205353314,1.152086649992
63,0.3366397877762,0.736388458767279,0.935662034257295
"12819","KATNAL2",20.063115984892,0.177874503595399,0.526219594127915,
0.338023337747778,0.735345600868111,0.935662034257295
"12820","ZNF77",30.8078771894634,0.114040829594349,0.337228687023369,0
.338170606424256,0.735234625028669,0.935662034257295
"12821","CCDC159",38.8400662254764,-0.094366090308947,0.27704384327312
9,-0.340617893522053,0.733391257085978,0.935662034257295
"12822","SUGP2",243.347679144263,0.0879784547520304,0.259386187764712,
0.339179412405086,0.734474577520478,0.935662034257295
"12823","SIRPB1",2.45003977134261,-0.345543110326835,1.01244493369836,
-0.341295707870848,0.732880978772753,0.935662034257295
"12824","RALY-
AS1",21.2106427434533,0.142363058507167,0.423778658988165,0.3359373000
21384,0.736918148596278,0.935662034257295
"12825","CU638689.5",5.59120683399943,-0.231704666608873,0.68657501690
7762,-0.337479024000085,0.735755822069181,0.935662034257295
"12826","SPATC1L",193.907391579884,0.081966514175828,0.243192404130835
,0.337043891106611,0.736083813499727,0.935662034257295
"12827","PCNT",88.9580902679179,-0.0913897012014515,0.271473955442558,
-0.33664261108388,0.736386330189319,0.935662034257295
"12828","ZFX",346.442875989632,0.0616868105392414,0.181797774556993,0.
33931554272081,0.734372035071218,0.935662034257295
"12829","FUNDC1",146.498991737835,-0.0834385947821558,0.24618651281970
4,-0.338924313222887,0.734666748017143,0.935662034257295
"12830","STARD8",22.9064018279969,0.136494246194989,0.403514172932355,
0.338263821573056,0.735164384641994,0.935662034257295
"12831","TAF1A",39.7743880224479,0.098952337971863,0.294961559508315,0
.335475368847423,0.737266522094105,0.935761357427489
"12832","REC8",24.8659382623375,0.184691715614023,0.550616966402389,0.
335426851847226,0.737303115175133,0.935761357427489
"12833","PRR11",10.4568739481295,0.157190804690191,0.468619320229753,0
.335433896778144,0.737297801624831,0.935761357427489

"12834","HDAC8",156.944895749458,0.0708308432233914,0.211045588603816,
0.335618686426837,0.737158430847582,0.935761357427489
"12835","FAM122B",62.4369791605922,0.0805342665950435,0.24012788470021
6,0.335380735542585,0.737337898126455,0.935761357427489
"12836","LMBRD1",565.105472637926,0.044806835774959,0.133690305281324,
0.335153964086417,0.73750894698819,0.935896243948437
"12837","POLA2",23.8291740254557,-0.117558772006103,0.350929898055959,
-0.33499218122292,0.737630984290147,0.935896243948437
"12838","UBASH3B",5.80308904089484,0.217265142032725,0.648678233803723
,0.334935150758372,0.737674005528515,0.935896243948437
"12839","CHMP6",149.351729465882,-0.0887291439446477,0.26491423460773,
-0.334935357762231,0.737673849372552,0.935896243948437
"12840","DENND4B",63.5757595737634,0.0917924205844646,0.27416059761456
2,0.334812592995271,0.737766460418167,0.935940644373172
"12841","ITSN2",226.634021322243,0.0802386248864287,0.239835844665874,
0.334556433790006,0.737959713557048,0.936040007329914
"12842","AC100858.3",4.40444719161809,0.316478945636845,0.945776047021
005,0.33462355769496,0.737909071942827,0.936040007329914
"12843","SPRY4",71.7661617857601,0.154151705454964,0.461564054842014,0
.333976842082574,0.738397033667551,0.936254559590258
"12844","SPIN1",367.744163340106,0.0521793997934354,0.156126772049763,
0.334211737733257,0.738219787326218,0.936254559590258
"12845","TPM1",984.065206179278,-0.0912313844233871,0.273171547639912,
-0.333971034727402,0.738401415927665,0.936254559590258
"12846","FBXL22",5.82462254677405,0.212676453734333,0.636795041661543,
0.333979443651779,0.738395070512792,0.936254559590258
"12847","AC105429.1",13.9726981402034,0.208526721655285,0.624422409178
801,0.333951374246041,0.738416251891217,0.936254559590258
"12848","LRFN1",2.60246495398089,-0.365144951055995,1.09384238386916,-
0.333818616320569,0.7385164346807,0.936308702094795
"12849","AGT",21.5051489881501,0.25163875229886,0.754558242979418,0.33
3491489411407,0.738763312608063,0.936322209105439
"12850","UBE2K",509.339736671759,-0.0470645213658416,0.141155309278554
,-0.333423670752369,0.738814497736645,0.936322209105439
"12851","ROPN1L",3.23726218403013,0.326979447020285,0.980658576116618,
0.333428427572738,0.738810907559168,0.936322209105439
"12852","AC022075.1",8.01274814329878,0.223202047629649,0.668819307054
027,0.333725485008493,0.73858671678547,0.936322209105439
"12853","AC138150.1",38.1235640904202,0.107277095790588,0.321728348672
825,0.333439985108931,0.738802184611896,0.936322209105439
"12854","RAB2B",132.397516726456,0.0633685286728015,0.190218421824837,
0.333135603086616,0.739031925438315,0.936524897577775
"12855","AL513550.1",20.8504709409795,-0.10992888209638,0.330093363922
119,-0.333023605171041,0.739116464855042,0.936559167329738
"12856","ADRA2B",3.06445320079846,0.354754624323369,1.06605902453032,0
.332772028715449,0.73930637387373,0.93665408135873
"12857","AL136419.3",9.02547350970066,-0.236871169146321,0.71165865755
7631,-0.332843796152566,0.739252196739324,0.93665408135873
"12858","AC104506.1",20.2908534009479,0.131342100709574,0.398111199442
26,0.329913101901126,0.741465623381157,0.936758234652504

"12859","S100A2",10.2129016415096,0.153286077841707,0.46262570191471,0.331339303474251,0.74038820650325,0.936758234652504
"12860","COLEC11",13.0857682723708,0.227370347566113,0.688582272095857,0.330200699001529,0.741248318704375,0.936758234652504
"12861","SATB2-AS1",6.66922963830868,0.227634069246686,0.686861282284419,0.331411997033232,0.740333304133099,0.936758234652504
"12862","FARSB",89.6370998093381,-0.0768940068814877,0.232068948030755,-0.331341213609057,0.740386763842312,0.936758234652504
"12863","ITM2C",452.553840260253,0.101423029158143,0.306506920018291,0.330899638912201,0.740720294726161,0.936758234652504
"12864","PDHB",328.878998076969,-0.0678608379513764,0.204059002894762,-0.332554981592131,0.739470230305747,0.936758234652504
"12865","TERC",3.34330621301417,-0.269761993994626,0.816561296231308,-0.33036343412266,0.741125367276764,0.936758234652504
"12866","RPS3A",22202.3827786935,-0.0830869320483809,0.250483704101375,-0.331705938102681,0.740111316366962,0.936758234652504
"12867","HSD17B4",303.558045728697,-0.0519280620001517,0.157415364450099,-0.329879247693214,0.741491204542778,0.936758234652504
"12868","WDR55",130.087525463302,-0.0553425256447087,0.167224486726666,-0.33094749894594,0.740684142655362,0.936758234652504
"12869","LINC01600",3.30240630709778,-0.270952717605641,0.817946363980291,-0.331259761687956,0.740448282564947,0.936758234652504
"12870","PGBD1",27.300726571284,0.0977573306300926,0.296018937138151,0.33024012441633,0.741218530975047,0.936758234652504
"12871","AL035587.2",13.9017043504919,0.159360928915419,0.479445245898879,0.332386086374981,0.739597743370001,0.936758234652504
"12872","AL109615.4",6.1634904501585,-0.208275127973592,0.629710742734526,-0.330747300052648,0.740835370859832,0.936758234652504
"12873","SRSF12",6.9167766998653,0.200234343985426,0.605748070182511,0.330557130665057,0.74097903215195,0.936758234652504
"12874","AC025062.3",2.41118822393867,0.371259262524464,1.118027471475,0.332066315002678,0.739839185012577,0.936758234652504
"12875","BET1L",153.294100056847,-0.0758816583330948,0.229013958966026,-0.331340756151688,0.740387109344481,0.936758234652504
"12876","RASGRP2",10.5025603244565,-0.203889930297362,0.617708360608222,-0.330074746109317,0.741343484553956,0.936758234652504
"12877","MSANTD4",107.10167453332,-0.0717657446308196,0.217471726506613,-0.330000344337347,0.741399701936942,0.936758234652504
"12878","PIH1D2",14.2629857148213,0.153183020083551,0.46076966403576,0.332450315287386,0.739549250771629,0.936758234652504
"12879","PLBD1-AS1",15.961876867002,0.130639418396652,0.395406246248515,0.330392905109913,0.741103101739335,0.936758234652504
"12880","AC022364.1",8.74796265351622,-0.180260636421385,0.544367127518079,-0.331137989987095,0.740540256965766,0.936758234652504
"12881","AC139768.1",16.1765769930199,0.160406635028029,0.48356971405255,0.331713567592444,0.740105554774315,0.936758234652504
"12882","CARS2",222.60506497802,-0.0757753306479063,0.229164350085652,-0.330659330823423,0.740901825086579,0.936758234652504

"12883","SUPT16H",549.715370226405,-0.0646532803626226,0.195427157422928,-0.330830582684602,0.740772458791371,0.936758234652504
"12884","ZNF710-AS1",8.86933080402889,-0.175775246527128,0.531704374410059,-0.330588302423043,0.740955483184394,0.936758234652504
"12885","AC010538.1",5.00977380329709,-0.221078685477412,0.670248393770334,-0.329845901209525,0.741516402333439,0.936758234652504
"12886","CHD3",455.737682950443,0.0735880383415847,0.221888714225712,0.331643899052607,0.740158167188691,0.936758234652504
"12887","NEK8",18.7076945612508,0.124300104977165,0.376377563586411,0.330253758467267,0.741208229907501,0.936758234652504
"12888","TIMP2",7467.75830278469,0.0405918770171032,0.12291816572786,0.330234971997332,0.741222423844527,0.936758234652504
"12889","ZNF414",122.467340207105,-0.0865451531476497,0.261237966274549,-0.331288573333536,0.740426521616101,0.936758234652504
"12890","SLC25A42",45.5786615533283,0.0796821008106379,0.241126128691954,0.330458176568803,0.741053789466488,0.936758234652504
"12891","LINC00266-1",4.1013552094918,0.303368092393104,0.916662893120942,0.330948372263912,0.740683482981808,0.936758234652504
"12892","AP000692.2",66.1192574095264,-0.142706999678346,0.42950695124082,-0.332257718451527,0.739694663793551,0.936758234652504
"12893","AP000692.1",6.55765574491603,0.228692359835741,0.692107125297594,0.330429136584032,0.741075728903799,0.936758234652504
"12894","ZNF74",31.8775630365268,0.0873938023304813,0.264624175984408,0.330256304078697,0.741206306601995,0.936758234652504
"12895","C17orf100",7.61340112862912,-0.226561342794592,0.687771365581986,-0.329413747260149,0.741842978309113,0.937098121262284
"12896","AC104794.3",97.3380768561415,-0.0834123170933639,0.253307069517649,-0.329293285229658,0.741934018983788,0.93714044938174
"12897","EPS8",1003.38496124752,-0.0547919540653313,0.166431723286786,-0.3292158068382,0.741992576148207,0.937141744039555
"12898","SAP130",53.211755172552,0.0829126463096387,0.252195228085428,0.328763739659471,0.742334272437189,0.937209964635667
"12899","SOX2-OT",4.67539995528607,0.235818083324759,0.716742043564128,0.3290138836451,0.742145194099483,0.937209964635667
"12900","TMEM181",154.754701621489,0.0881126959777612,0.267855944029412,0.328955537264785,0.742189295454155,0.937209964635667
"12901","CCZ1B",80.0539366355858,-0.155676344652695,0.47343335080711,-0.328824203844739,0.742288567463748,0.937209964635667
"12902","AC109460.2",3.15366711511162,0.324631772438543,0.987068433214419,0.328884767777822,0.742242788001728,0.937209964635667
"12903","ANKRD16",101.423866441411,0.0706153580799726,0.214901871030469,0.328593500565477,0.74246296131776,0.937299788956444
"12904","NOL9",134.878666379296,0.0642829641068283,0.195941202212563,0.328072724781448,0.742856676166459,0.937327770029353
"12905","ARL13B",172.996887600398,-0.0583747711702538,0.177867412863548,-0.328192613983971,0.742766032041366,0.937327770029353
"12906","MIR4453HG",6.51588257104607,-0.183717767973894,0.560129690815292,-0.327991483019736,0.742918102310195,0.937327770029353

"12907","PCDHB14",81.8300014797504,0.0742774667679437,0.22656149647654
,0.327846822708619,0.743027482683766,0.937327770029353
"12908","CDX1",3.69688384089428,-0.2594875363836,0.791462482253557,-0.
32785829044575,0.743018811523357,0.937327770029353
"12909","RORB",8.1106038968129,-0.291553875571632,0.889500048458777,-0
.327772748384672,0.743083493677392,0.937327770029353
"12910","OLMALINC",23.390858538227,0.127032578146966,0.386853975674895
,0.328373459069015,0.742629308006377,0.937327770029353
"12911","UBE2L6",506.693985069161,0.10455203419861,0.319021753250906,0
.327726975145741,0.743118105602496,0.937327770029353
"12912","SLC38A6",96.7581716766782,-0.0669758005230136,0.2040535846972
96,-0.328226532370746,0.742740388156525,0.937327770029353
"12913","TDRD9",2.86184717689037,0.369805358850292,1.12778972804033,0.
327902754969114,0.742985190637655,0.937327770029353
"12914","AC100786.1",7.68630500978407,0.192007978070723,0.585731567575
982,0.327808826943267,0.743056212854663,0.937327770029353
"12915","GPAT3",14.6375777561696,-0.219607441881388,0.670870172508171,
-0.327347154309075,0.743405331221737,0.937472279962133
"12916","RNF227",15.6752598148148,-0.129262658928627,0.394840922333536
,-0.32737908260541,0.743381185239707,0.937472279962133
"12917","AC006305.1",3.86709882952224,0.268763095036678,0.820828229347
09,0.327429156829145,0.743343316790228,0.937472279962133
"12918","ABHD17B",58.9700744813211,0.105250025255009,0.321632644352359
,0.327236762508795,0.743488817684169,0.937504981518612
"12919","AKIRIN2",348.408519352196,0.0674123371276329,0.20608678931466
3,0.327106542596987,0.743587303511431,0.937518810111524
"12920","SEMA4D",22.374215847085,0.120040362942987,0.367017275817431,0
.327070061417762,0.743614895121916,0.937518810111524
"12921","BMP8A",2.79851976360317,0.33079494670699,1.01538447227726,0.3
25782947975458,0.744588581056613,0.937550670114467
"12922","AC092807.3",19.7918865743472,0.169547129458669,0.519443808367
564,0.326401290625637,0.744120761175735,0.937550670114467
"12923","LAMTOR2",614.114135557274,0.0854676223257147,0.26285615176998
7,0.325149789153511,0.745067708080638,0.937550670114467
"12924","IER5",538.330957384481,0.0669869713426856,0.205893800542196,0
.325347199217672,0.744918312384639,0.937550670114467
"12925","PUS10",24.1453603793664,-0.122199339090076,0.3743942118232,-0
.326392169619817,0.744127661175102,0.937550670114467
"12926","PCOLCE2",3230.03108484737,-0.0974718158410344,0.2989969748691
5,-0.32599599338318,0.744427386510429,0.937550670114467
"12927","DDX46",583.849150160766,-0.0611989920642369,0.188002859948662
,-0.325521601538129,0.744786336437346,0.937550670114467
"12928","GET4",5.10743497503225,-0.217697810271979,0.667535437514891,-
0.326121727832799,0.744332258487257,0.937550670114467
"12929","PSPH",31.8871808326123,0.117779901229217,0.361702753381972,0.
325626222438059,0.744707169971298,0.937550670114467
"12930","GTF2IRD1",20.9499740404255,0.126604912642815,0.38891055804371
1,0.325537350489172,0.744774419059118,0.937550670114467
"12931","AL499604.1",27.8870829237991,0.124201923476991,0.381523056227
564,0.325542379286533,0.744770613734095,0.937550670114467

"12932","SURF4",566.692256039191,0.0652612120732625,0.199791034088835,
0.32664735117315,0.743934625245824,0.937550670114467
"12933","LNX2",29.5670782726202,0.124997158912721,0.383292004633652,0.
326114704720209,0.744337571922744,0.937550670114467
"12934","CYSLTR2",3.05982715404484,0.371475757283692,1.14145237289338,
0.32544131153021,0.744847093585274,0.937550670114467
"12935","AC008870.2",5.01842245374251,0.242164343785229,0.743856884527
771,0.325552332474498,0.744763082107715,0.937550670114467
"12936","MT2A",21436.9731582496,-0.208075103853535,0.638380741302479,-
0.325942012957663,0.744468228154226,0.937550670114467
"12937","ZFHX3",546.690421168443,-0.0833210727717997,0.256266288050017
,-0.32513473935962,0.745079097834844,0.937550670114467
"12938","AC015922.3",106.198509253128,-0.0686943987384533,0.2106134377
90674,-0.326163417961621,0.744300717479263,0.937550670114467
"12939","LINC02693",57.4799822091127,-0.0851099969947391,0.26129004642
5303,-0.325729962389019,0.744628672780886,0.937550670114467
"12940","RAD51C",140.500259818473,0.0857457808384928,0.263293937301654
,0.325665610523551,0.744677365769375,0.937550670114467
"12941","AC110285.2",6.25616053045217,-0.198663661193941,0.61044209031
2224,-0.32544227265248,0.744846366274326,0.937550670114467
"12942","INSR",399.087796874422,0.0869979804053848,0.267005821836253,0
.32582802804479,0.744554471613625,0.937550670114467
"12943","PAF1",263.067558568103,0.0540770981748636,0.166174547073963,0
.325423472650082,0.7448605928589,0.937550670114467
"12944","DZANK1",18.5394785196575,0.131535345829787,0.402671173320946,
0.32665697110865,0.74392734841537,0.937550670114467
"12945","FGF16",44.646050987387,0.14456795814491,0.444564772133631,0.3
25189864799847,0.745037378916402,0.937550670114467
"12946","AC093772.2",5.32136267500456,-0.211941032626792,0.65248119110
5843,-0.324823206424677,0.74531487992699,0.93763006480775
"12947","CAMK4",3.19401603600435,0.271009980879839,0.834241984536584,0
.324857758184376,0.745288728429384,0.93763006480775
"12948","ATP5MC1",824.959008853718,-0.0894701621574614,0.2753248387198
7,-0.324962188567712,0.745209689060963,0.93763006480775
"12949","COLCA1",92.9984260053905,-0.136326519577543,0.420303736682201
,-0.324352385381292,0.745671263674215,0.937788697806462
"12950","RNF40",157.112841747977,0.0579268554907034,0.17848482263612,0
.324547794233462,0.745523344092849,0.937788697806462
"12951","B3GNT8",59.7753822746777,-0.0861627276820988,0.26556560572483
2,-0.324449875377977,0.745597465028925,0.937788697806462
"12952","RPS11",18585.7369655449,0.0525312102424014,0.161941829170107,
0.324383209153589,0.74564793023168,0.937788697806462
"12953","AC024560.4",3.20576958284641,-0.270411194651858,0.83413729249
3271,-0.324180679949685,0.745801248101848,0.937879759926736
"12954","RPS28",20456.2718925297,-0.0903273701075692,0.278712083719105
,-0.324088460400605,0.745871063121956,0.937895148000119
"12955","AC082651.1",8.65338708211908,0.192753374374569,0.596090232016
652,0.323362746144085,0.746420539533553,0.937900238969488
"12956","RAB43",46.1851679095438,0.0844789558501652,0.261072028611381,
0.323584860084404,0.746252351686874,0.937900238969488

"12957","MRPS27",157.274393912391,-0.0581049190400463,0.179645307360885,-0.323442453875629,0.746360182311163,0.937900238969488
"12958","AZIN1",452.228834268475,-0.0693854810558308,0.214601359427294,-0.323322653877868,0.746450899257195,0.937900238969488
"12959","CNTFR",386.681186876986,0.140777070711715,0.435367864664918,0.323352002151249,0.746428675344681,0.937900238969488
"12960","HEPN1",3.23040735168812,-0.278062206606155,0.859753250267901,-0.323420942601276,0.746376471177081,0.937900238969488
"12961","UBL3",1148.7370845928,-0.0696206395374557,0.215214515308091,-0.323494163197079,0.746321027205059,0.937900238969488
"12962","ACTR10",610.812749441472,-0.0793910188847789,0.245435195481131,-0.323470391967001,0.746339027070541,0.937900238969488
"12963","AL008727.1",4.93184131749832,0.234238375084002,0.723903310790173,0.323576880492949,0.746258393738162,0.937900238969488
"12964","PLAGL2",50.2784549382628,-0.0764704703751576,0.236377488988083,-0.323509953094615,0.746309070978477,0.937900238969488
"12965","CTH",12.1407802777306,-0.161495078282848,0.499641895208929,-0.323221651009305,0.746527385058729,0.937923993460983
"12966","EIF2B3",194.238369407006,-0.0688191542425593,0.213289152470743,-0.322656606983327,0.746955318402721,0.937955225212915
"12967","ABHD1",7.14837925592016,0.184912541140674,0.572754540630134,0.322847796085976,0.746810513525961,0.937955225212915
"12968","EFHD1",198.492836965015,0.105379922235331,0.326537088436167,0.322719611239294,0.746907598568727,0.937955225212915
"12969","CDKN1A",2372.98277030316,-0.127431848116266,0.394598148160255,-0.322940816398644,0.746740064024232,0.937955225212915
"12970","YAE1",105.230144508246,0.0765499405882123,0.237020311571606,0.322967850648048,0.746719589867264,0.937955225212915
"12971","TTF1",202.531478447531,-0.0805590672669244,0.249583280811172,-0.322774294035638,0.746866182248968,0.937955225212915
"12972","AHNAK",7137.94228176551,0.0531546534799764,0.164574307019409,0.322982696647221,0.746708346453133,0.937955225212915
"12973","SFMBT1",37.6342197728393,-0.0877394479928765,0.272391111752914,-0.322108336899278,0.747370622670377,0.938144060039844
"12974","AL080317.2",13.4280686941036,0.173240166342424,0.537850878609004,0.322097022115981,0.747379194178605,0.938144060039844
"12975","BLOC1S1",1445.74466267708,-0.0828740948635139,0.257371454308553,-0.322001890559935,0.747451262274977,0.938144060039844
"12976","SNAP23",279.424836550879,-0.0504013055148452,0.156357555243848,-0.322346466956725,0.747190234617403,0.938144060039844
"12977","MZF1-AS1",16.5260778377263,-0.138166126493844,0.429021669207746,-0.322049295899176,0.747415349493199,0.938144060039844
"12978","FBXO7",368.561387421021,-0.0497183920060656,0.154297498113953,-0.322224226664695,0.747282832237351,0.938144060039844
"12979","C1orf35",258.541853147474,-0.0596874477357894,0.185507770330479,-0.321751739182985,0.747640778102855,0.938197321189442
"12980","SLC29A3",47.1832806610829,-0.079575978392601,0.247347138492901,-0.321717804691259,0.747666488204319,0.938197321189442
"12981","AP002495.1",29.9610210621972,-0.112400581778295,0.34933639377

8081,-0.321754571754405,0.747638632048379,0.938197321189442
"12982","TUFT1",29.7830271023255,0.119153316277141,0.370656863360034,0
.321465290557436,0.747857811626508,0.938279278794849
"12983","KLHL6",3.43157556399633,-0.338804395877979,1.05451110936131,-
0.321290494590602,0.747990259102764,0.938279278794849
"12984","AL358976.2",3.63789076532877,0.267475905419374,0.832367484140
584,0.321343529769838,0.74795007217396,0.938279278794849
"12985","INTS13",69.6789968925126,0.0755884929429307,0.235293825331583
,0.321251494111284,0.74801981180121,0.938279278794849
"12986","GATA6-
AS1",13.2463069826945,-0.173245901703451,0.539162989901142,-0.32132380
1797331,0.747965020787232,0.938279278794849
"12987","CDHR3",25.0399803159964,-0.107406715047675,0.334652207790041,
-0.320950265820633,0.748248080667973,0.938348832550667
"12988","ANGPTL2",3479.99520654181,0.0829843786547695,0.25851159472573
8,0.321008342944192,0.748204068478277,0.938348832550667
"12989","PLEKHA5",820.528847548472,-0.0557626520073636,0.1737304260745
71,-0.320972286014129,0.748231393158638,0.938348832550667
"12990","MREG",22.1596649436818,-0.148038508664131,0.46174267463056,-0
.320608245236541,0.748507288427441,0.938463104647231
"12991","ALG9",88.4671646921579,0.0652887026032631,0.203630249921425,0
.320623790563809,0.748495506445291,0.938463104647231
"12992","SREBF2-
AS1",10.0108182816152,0.156304820862406,0.487535430236161,0.3206019730
43667,0.748512042211113,0.938463104647231
"12993","CLK3",172.85561189142,-0.060134544963914,0.187791961378073,-0
.320218951453667,0.748802357799779,0.938754837697268
"12994","AC026304.1",19.6171857234516,0.120956773822524,0.377983022120
235,0.320005838209441,0.748963904838302,0.938855366632732
"12995","IKBKG",117.107381080761,-0.0663178072801574,0.207268344365772
,-0.319961099139792,0.748997819963923,0.938855366632732
"12996","CELF6",21.0907332029489,0.179683294481033,0.561719688618502,0
.319880712963698,0.749058759135313,0.938859505044253
"12997","TIMM23B",47.8742768871401,-0.0804382925428326,0.2516031257636
05,-0.319703073237687,0.749193429857861,0.938956049777233
"12998","PARS2",15.1870922712639,0.142273940025001,0.44551845481242,0.
319344661232729,0.749465169407154,0.939079857267164
"12999","AC012470.1",8.20840996889533,0.191503986319235,0.599600026012
389,0.319386220832616,0.749433658301516,0.939079857267164
"13000","ZNF26",66.4786510464404,0.0862490032731967,0.270008046674485,
0.319431233014979,0.749399529868413,0.939079857267164
"13001","ASAH1",892.257508296916,0.0570223073455529,0.178787256506754,
0.318939439307292,0.749772436598184,0.939104043306661
"13002","PPP3CC",98.1884130609872,-0.063768188570813,0.199943530518282
,-0.318930992193229,0.749778842204945,0.939104043306661
"13003","AC055822.1",3.15364933934976,-0.28979339823969,0.908804446934
585,-0.318873217684145,0.749822654173296,0.939104043306661
"13004","MGMT",860.9418814671,0.0882721306722204,0.27683401735779,0.31
8863019489886,0.749830387822852,0.939104043306661
"13005","NXN",74.3746635312501,0.114862360024223,0.359994088313592,0.3

19067350695399,0.749675441076667,0.939104043306661
"13006","GPR174",2.84367326971803,-0.259393527902382,0.81327999147909,
-0.31894738665663,0.749766409988245,0.939104043306661
"13007","MRPL27",537.539512287475,-0.0816396748207614,0.25616103237141
,-0.31870450421355,0.74995059876759,0.939182386662971
"13008","CNRIP1",416.541836696783,0.0566153822381729,0.177789165229064
,0.318441127530069,0.750150345372799,0.93919457971222
"13009","EIF2AK3-
DT",11.277167736802,0.15235032587921,0.479409234211451,0.3177876332102
8,0.750646032082209,0.93919457971222
"13010","ZNF2",20.5955048280287,0.108795584503772,0.342537359540858,0.
317616696320668,0.750775707654734,0.93919457971222
"13011","UBE2E2",305.702006757694,-0.0694138175681271,0.21850350215299
1,-0.317678283799429,0.750728985555663,0.93919457971222
"13012","UBE2E1",452.59364948161,0.0510894950097968,0.160929575147664,
0.317464921925746,0.750890852140543,0.93919457971222
"13013","MRPS30",199.802202015573,-0.0634358833522921,0.19952904400598
3,-0.317928067406517,0.750539501631655,0.93919457971222
"13014","ZBED6CL",3.51057726642596,-0.243002174594582,0.76298710065853
9,-0.318487919893856,0.750114856512832,0.93919457971222
"13015","AL359644.1",11.0729354328342,0.167079235488769,0.525411698652
701,0.317996793594825,0.7504873691127,0.93919457971222
"13016","AL135925.1",102.105200722855,-0.095133338431781,0.29953793993
0394,-0.317600296155765,0.750788149463286,0.93919457971222
"13017","AC138123.1",3.13351114664494,0.314369122969712,0.988971965904
686,0.317874655508698,0.750580018222315,0.93919457971222
"13018","HPD",2.99539938733857,-0.265435803156052,0.834227620818739,-0
.318181508897469,0.750347258244191,0.93919457971222
"13019","PSPC1",161.082845251361,0.0559881009357059,0.175856198555678,
0.31837433878101,0.750201001061789,0.93919457971222
"13020","DGLUCY",143.448753854869,-0.0842373888195671,0.26519360638516
5,-0.317644870733502,0.750754333591047,0.93919457971222
"13021","LYSMD4",35.2465757394309,0.101765421966609,0.319709913375987,
0.318305494165051,0.750253217143098,0.93919457971222
"13022","KDM8",7.84586927415942,0.173626460694903,0.546959478759245,0.
317439348685879,0.75091025396693,0.93919457971222
"13023","ZNF700",32.7604697853821,0.0922936012118452,0.290736923607348
,0.31744712734352,0.750904352462383,0.93919457971222
"13024","PPP1R13L",29.3338685592706,-0.12050718017224,0.37967041296204
,-0.317399449780904,0.750940524659092,0.93919457971222
"13025","MBNL1",1418.03347555964,0.0726761263242797,0.22910851871392,0
.317212676037716,0.751082232153152,0.939299691327653
"13026","MRPL44",157.562701659973,-0.0682557012164094,0.21540616733745
6,-0.31686976310887,0.751342426187509,0.93944045902816
"13027","AP000919.2",4.33566613360122,0.299577400823903,0.945527369443
028,0.316836307975275,0.75136781264773,0.93944045902816
"13028","ADNP",303.664240895243,0.0475833565490139,0.150123029950275,0
.316962404534301,0.751272129326572,0.93944045902816
"13029","SMIM27",103.357635203471,0.0824289223735844,0.260411449872427
,0.316533402866753,0.751597675670504,0.939493789391116

"13030","AC018653.3",2.66919688843654,-0.302576391166775,0.95578809143
4498,-0.31657267325088,0.751567873644302,0.939493789391116
"13031","ATXN1L",120.205035407563,0.0656300863306881,0.207423645400952
,0.316406001851065,0.751694361981033,0.939493789391116
"13032","WFDC2",8.10459166583951,-0.234691717325211,0.741756167030438,
-0.316400089081538,0.751698849354436,0.939493789391116
"13033","ACOT8",90.1799526529888,-0.080109053867347,0.253045982057617,
-0.316579039176787,0.75156304262152,0.939493789391116
"13034","NRDC",376.157461173413,0.0459051757524453,0.145196212524732,0
.31615959503507,0.751881374420223,0.939505654183109
"13035","THRB-
IT1",19.0906608763538,0.154476541575738,0.488571703760534,0.3161798777
6765,0.75186598012642,0.939505654183109
"13036","GOLGA8A",155.342085908794,-0.138750927696733,0.43878796645083
7,-0.316214067625938,0.751840030753528,0.939505654183109
"13037","C2CD3",42.8770618917881,-0.0882818443141799,0.279701362162621
,-0.315628939493159,0.752284169182119,0.939936859078587
"13038","AF124730.1",2.70661321404803,-0.291091659002223,0.92310399059
6631,-0.315340050489957,0.752503479116169,0.940138761414579
"13039","NOC3L",195.611334356124,-0.0690762037689201,0.219213177537258
,-0.315109723534662,0.752678346018233,0.940213004470169
"13040","BNIP3",528.229372186788,-0.0619584749498594,0.196593012082349
,-0.315161125482456,0.75263931996144,0.940213004470169
"13041","GSDMB",6.71770024504166,0.220202826989792,0.699508098244697,0
.314796680041812,0.752916032623106,0.940437792761121
"13042","GGA1",140.256319600115,-0.0651423672653763,0.207101624591063,
-0.314543004643273,0.753108659604781,0.940606268693627
"13043","C1orf131",205.603959328658,-0.0604977596248824,0.192401850966
755,-0.314434395100158,0.753191136348012,0.940637155560283
"13044","AC004803.1",7.7716690386052,0.161665366828851,0.5145288931508
52,0.314200755255649,0.753368569100859,0.940721327586537
"13045","SLC16A2",95.6528234578158,0.0821475360645762,0.26145519298916
5,0.314193553110955,0.753374038821682,0.940721327586537
"13046","RTF1",881.968879112537,-0.0595556465215517,0.189716244406967,
-0.313919594538235,0.753582107826634,0.940909010761002
"13047","WDR75",120.528584523531,0.0528813624511595,0.168633280850639,
0.313587935812014,0.753834023509017,0.941110983738486
"13048","NSMAF",121.546815172649,0.0595632861834526,0.190053711165558,
0.313402384084814,0.753974973159018,0.941110983738486
"13049","GABARAPL1",834.941280421496,0.0713511861417143,0.227596491744
807,0.313498620276261,0.753901868760533,0.941110983738486
"13050","PAQR5",4.77876508827094,0.262792796082048,0.838495330175935,0
.313409969769192,0.753969210726095,0.941110983738486
"13051","HSD17B1",13.2753703786632,-0.140574438871402,0.44867487295020
4,-0.313310255034058,0.754044959694639,0.941126223926594
"13052","AL138762.1",16.6851668266232,0.153910434710272,0.491476295351
32,0.313159426336632,0.754159542224997,0.941197117936176
"13053","RNF138",93.7768467088148,-0.0892019787379349,0.28501200262024
1,-0.312976218256992,0.754298730215564,0.941298706541127
"13054","ATXN7L3",71.8525983152733,0.0666291749376197,0.21296153669878

3,0.312869525504323,0.754379791179969,0.941327747704192
"13055","GKAP1",54.3670114540776,-0.0990508320739555,0.316688072627946
,-0.312770958666079,0.754454680798309,0.941349084299016
"13056","CCNE2",4.14941856366215,-0.231453476537049,0.74059585166501,-
0.312523322965818,0.754642840907187,0.941439628975812
"13057","EMC10",304.458303621398,0.0589929003719091,0.188735633352555,
0.312568958622197,0.754608164642043,0.941439628975812
"13058","AC008033.3",3.06705002483536,0.305910216362533,0.979649635024
182,0.312264921483875,0.754839196665626,0.94156610735788
"13059","EVI2A",11.5187741157499,0.175036855793126,0.560724951395079,0
.312161703091035,0.754917635342496,0.94156610735788
"13060","ZGLP1",4.37401270967791,-0.249377609980023,0.798828890182926,
-0.312179007350269,0.754904485153225,0.94156610735788
"13061","FBXO32",108.410505852009,0.114374235406572,0.366671812713915,
0.311925355156245,0.755097252571744,0.941573845758335
"13062","NFIX",2007.71847029982,-0.0623743524409471,0.19993445366406,-
0.311974005969734,0.755060278353134,0.941573845758335
"13063","ZNF473",20.5224166779314,0.112433251283325,0.360395428195794,
0.311971913312516,0.755061868743881,0.941573845758335
"13064","C5orf22",43.9283255146152,0.097520264872181,0.312772145262686
,0.311793317753015,0.755197602741218,0.941626894599794
"13065","PROX1",14.6080322544313,0.205914174996005,0.66074163947104,0.
311640984456271,0.755313383190298,0.941627100779639
"13066","LCP2",38.1855534993891,-0.118548202625314,0.380390896111601,-
0.311648369708968,0.755307769925122,0.941627100779639
"13067","C1orf122",861.672468067194,0.0955692742884278,0.3071028317194
26,0.311196330406166,0.755651372549773,0.941690765669421
"13068","EEFSEC",80.2606367298375,-0.0722459570922763,0.23211908860940
3,-0.3112452212573,0.755614207464701,0.941690765669421
"13069","SLC26A1",3.06337925821114,0.31336511478859,1.00665890385675,0
.311292249626973,0.755578458705384,0.941690765669421
"13070","ZHX1",366.5424986954,0.0464800254558433,0.149262152639164,0.3
11398600609808,0.755497617609943,0.941690765669421
"13071","EIF4B",1556.84925030288,-0.0629436898951572,0.202265425539049
,-0.311193520728561,0.755653508383878,0.941690765669421
"13072","KIN",235.674876638546,0.0678850728144779,0.218310972540777,0.
310955844428746,0.755834189661016,0.941843873576215
"13073","PER3",320.239498079484,-0.26602320829988,0.857546463288321,-0
.310214337867823,0.756397967959795,0.94189790536634
"13074","PDGFA",36.1599234348213,0.133577169768345,0.430566382375164,0
.310235947896079,0.75638153570259,0.94189790536634
"13075","TSPAN4",2434.72428956454,0.064288078866436,0.207115273734212,
0.310397575742946,0.756258637443526,0.94189790536634
"13076","TM7SF2",8.28122235279224,0.16822837669401,0.541896425352153,0
.31044378376308,0.756223503014732,0.94189790536634
"13077","WSCD2",23.6985094542876,0.171697514668259,0.553356999615427,0
.310283442312261,0.756345421353859,0.94189790536634
"13078","SCIMP",3.98281230293488,-0.261413915721638,0.842676769099096,
-0.310218491013008,0.756394809901237,0.94189790536634
"13079","LSM14A",515.308139077651,0.0435565673260342,0.140380344048042

,0.310275399461395,0.756351537031588,0.94189790536634
"13080","ZNF180",48.9603570572984,-0.0749546419170614,0.24126416128311
,-0.310674579757026,0.756048024024863,0.94189790536634
"13081","AP000240.1",3.38974696700757,-0.286959565980734,0.92354953113
7721,-0.310713780155601,0.756018220397043,0.94189790536634
"13082","RPF1",178.15062133997,-0.0644949330938697,0.208349791429266,-
0.309551224656568,0.756902252510447,0.941961057805407
"13083","AL355816.2",3.98420528920062,-0.27465386558986,0.886138230750
043,-0.309944719750313,0.756602994731798,0.941961057805407
"13084","SHC1",303.207648327405,0.0577787055765,0.186706113198185,0.30
9463383853794,0.756969061482129,0.941961057805407
"13085","THNSL2",158.492466917661,-0.123002287465244,0.396992853029339
,-0.309835017246907,0.756686421145402,0.941961057805407
"13086","ACVR1",293.750875582682,0.0614231339620535,0.198362822783251,
0.309650432980427,0.756826799941648,0.941961057805407
"13087","LINC02569",4.30875664936583,-0.207688910392411,0.670173363282
122,-0.30990326051646,0.756634523261323,0.941961057805407
"13088","TBP",62.560695696053,0.0734190885481064,0.236963259099057,0.3
09833215610085,0.756687791275442,0.941961057805407
"13089","TBC1D2",13.3928593314814,-0.131894818144536,0.426001380812997
,-0.309611245608694,0.756856603493146,0.941961057805407
"13090","LRMDA",148.996966914533,0.071139460485941,0.229961978062644,0
.309353142137967,0.757052910458632,0.941961057805407
"13091","POLL",105.181846183841,-0.0580444787278467,0.187719315100224,
-0.309208877609939,0.757162641236246,0.941961057805407
"13092","FBXL19-
AS1",12.6550958368211,-0.201144744902152,0.650618715041223,-0.30915917
4570329,0.757200447596783,0.941961057805407
"13093","AC135048.3",2.78347891683677,-0.302596495865924,0.97864489421
4841,-0.30919948354576,0.757169786738148,0.941961057805407
"13094","ITPA",364.93398264853,-0.0783867651018845,0.253524951969421,-
0.309187575001845,0.757178844883996,0.941961057805407
"13095","C11orf68",248.165253101451,-0.0743975608237802,0.240778838020
582,-0.308987124596974,0.757331321006468,0.9420519196544
"13096","AC025449.2",10.6654808415144,0.170417858060006,0.551676349500
799,0.308909124370137,0.7573906558023,0.942053786832901
"13097","CMTM6",461.364166493835,-0.0520715951099656,0.168659103363602
,-0.308738716567867,0.757520290041534,0.942071156244202
"13098","CASC4",667.397276781015,0.0449552443224907,0.145606390339075,
0.308744995448367,0.757515513390977,0.942071156244202
"13099","TNFRSF1B",162.726508597879,-0.0572190072460928,0.186316494771
432,-0.307106503459544,0.758762307709834,0.942256164547166
"13100","AC231981.1",6.49430445481765,0.176542446616378,0.573992507652
309,0.307569252669264,0.758410119543122,0.942256164547166
"13101","DDX1",559.986628602308,0.0652245665475642,0.211660403774405,0
.308156676376195,0.757963116586517,0.942256164547166
"13102","ACTR2",834.141178511235,-0.0518328563636506,0.168704815407711
,-0.307239934072928,0.758660751488865,0.942256164547166
"13103","RGPD5",57.1718657574184,0.150061909671652,0.486709273734588,0
.308319396752411,0.757839308020888,0.942256164547166

"13104","CHN1",2.92724252110367,0.273097675749166,0.886185365533396,0.308172179738929,0.757951320321694,0.942256164547166
"13105","CTBP1-DT",67.9217317837222,0.0751534034119471,0.24455685433212,0.307304424638555,0.758611668157446,0.942256164547166
"13106","CC2D2A",186.762710765401,0.0752908975747268,0.244116687187348,0.308421756997483,0.757761428669967,0.942256164547166
"13107","ZNF131",263.810514796303,0.0544740304157271,0.176876295064233,0.307978128985259,0.758098974548838,0.942256164547166
"13108","SLU7",479.278747394095,-0.0552312033508958,0.179825023327126,-0.307138585770807,0.758737888958221,0.942256164547166
"13109","HDAC2",345.206183232257,-0.0553462893521414,0.179876567295742,-0.307690380043468,0.75831794045765,0.942256164547166
"13110","CCDC28A",162.220953943763,0.0667311299609984,0.217483722505351,0.306832755997896,0.75897067440318,0.942256164547166
"13111","RP9",150.744550888988,-0.0657385551924743,0.213861071570978,-0.307389066694434,0.758547249139867,0.942256164547166
"13112","TMEM225B",43.4129949190498,-0.110603035060493,0.360243688993126,-0.307022824937272,0.758825998924388,0.942256164547166
"13113","ZMIZ1",616.931792897835,-0.0880742587021446,0.285988047213815,-0.307964824265181,0.758109098502823,0.942256164547166
"13114","ARMH3",70.8088566812276,-0.0636424731475635,0.207301382856804,-0.30700457599709,0.758839889171501,0.942256164547166
"13115","MTRF1",31.5686669141748,-0.0881129803206311,0.286354361968156,-0.307706087363285,0.758305987289267,0.942256164547166
"13116","LINC02323",3.42604094347611,0.258472504933329,0.842026213359308,0.30696491490703,0.758870077622626,0.942256164547166
"13117","DPH6",100.621128957925,-0.0646548466375946,0.209872237523896,-0.308067648205414,0.758030857812126,0.942256164547166
"13118","AC100830.1",3.32221230585055,-0.27163892812712,0.884244252925104,-0.307198974976123,0.758691925684215,0.942256164547166
"13119","WFIKKN1",2.42821204283649,0.321723113002964,1.04792014828457,0.30701109576872,0.758834926614705,0.942256164547166
"13120","NUTF2",274.63583289861,-0.0703138145314508,0.22918836847764,-0.306794864846341,0.758999517160253,0.942256164547166
"13121","ATP2A3",12.1060308778052,0.143499831292729,0.467557683915473,0.306913641309488,0.758909105599275,0.942256164547166
"13122","TRIM3",58.4169015427412,-0.079286220675114,0.258554215735544,-0.30665220619032 5,0.759108111983564,0.942283667516277
"13123","MISP3",15.5092874341809,-0.156488297105439,0.510375950060945,-0.306613775760305,0.75913736686206,0.942283667516277
"13124","INAFM2",12.2743214605993,0.136407209021178,0.445472743996442,0.306207755377885,0.759446467905185,0.942523696434861
"13125","INTS14",81.6577563943676,0.0675353387144034,0.220538262701952,0.306229576160553,0.759429854887918,0.942523696434861
"13126","AC123912.4",3.17751763657989,-0.375500718537158,1.22663211865152,-0.306123337900168,0.759510739285645,0.942531649567566
"13127","SEMA4C",81.3792783776047,-0.0672514982499933,0.220070048173174,-0.305591327889713,0.759915824150672,0.942531703311528
"13128","KCNE4",366.842763355206,0.195627952737746,0.639881808321136,0

.305725135788774,0.759813933483125,0.942531703311528
"13129","CCDC80",24318.3273568086,-0.0754871649912446,0.246859550234726,-0.305789931641161,0.759764594899344,0.942531703311528
"13130","AL356417.2",6.36317014074338,-0.207644327879173,0.679202330218606,-0.305717926221397,0.759819423244374,0.942531703311528
"13131","HOXC-AS3",49.7691505387046,-0.0838341597175875,0.274294801833221,-0.30563524776004,0.759882380038539,0.942531703311528
"13132","SRP14-AS1",44.134621570619,-0.116992693914839,0.382590675351965,-0.30579076138542,0.759763963099709,0.942531703311528
"13133","NPIPB2",27.4573591506058,0.123047486573457,0.402167295062635,0.305960947307496,0.759634380291056,0.942531703311528
"13134","CTSK",5147.11633717293,-0.0653847019873829,0.214295902826646,-0.305114102159367,0.760279251025347,0.942604701503611
"13135","ZCWPW1",72.979194011705,0.0617111952219392,0.202080033777662,0.305379972817288,0.760076773132422,0.942604701503611
"13136","GNB2",1068.04018216639,0.0552572232267999,0.181249772393797,0.304867821333005,0.760466824635734,0.942604701503611
"13137","TM2D2",337.553962890435,0.0674013559580733,0.221092825726474,0.304855463928347,0.760476236714012,0.942604701503611
"13138","AC113139.1",5.79699525729041,-0.249302166086888,0.817530244512262,-0.304945496219068,0.760407663987193,0.942604701503611
"13139","RAPGEF1",168.869350462688,0.0631012684900098,0.207004655689308,0.304830189832629,0.760495486964237,0.942604701503611
"13140","AP001107.9",7.73006388765364,-0.167982261863425,0.550645144533672,-0.305064456721369,0.760317061093336,0.942604701503611
"13141","FSIP1",3.36059432537304,0.29181521156101,0.956017415856384,0.305240476502833,0.760183006649375,0.942604701503611
"13142","AC087477.2",63.7121335708074,0.0967922965716887,0.317434790134004,0.304920253167047,0.760426890064848,0.942604701503611
"13143","HIST1H2BN",7.68232222659534,0.188988828789865,0.620163867649809,0.304740147964541,0.760564069396371,0.942617981160883
"13144","SMIM11A",11.1095625262136,0.150512176023654,0.494030727641938,0.304661567797745,0.76062392330144,0.942620441772455
"13145","DHX16",108.415217029006,-0.0742302048048076,0.243711350332901,-0.304582468988054,0.760684173699947,0.942623393335751
"13146","MCEE",220.046023313874,-0.0717049711927925,0.235842719545363,-0.304037247073045,0.761099514457795,0.943062411195454
"13147","VPS50",86.5169877648155,-0.0668221506538679,0.219944693066888,-0.30381342564854,0.761270037743817,0.943062411195454
"13148","ERAL1",110.532557204695,-0.0741661800541469,0.244034289869227,-0.303917044173961,0.761191092244123,0.943062411195454
"13149","GNG7",8.54053690996596,-0.202918194988111,0.667820910060882,-0.303851215095394,0.761241246206875,0.943062411195454
"13150","CXCR5",2.85804195471417,0.289282653462363,0.952410791761978,0.303737269636755,0.761328061524544,0.94306256989911
"13151","DNM3OS",140.522071290122,-0.0882415440691664,0.290634893788658,-0.303616482242952,0.761420093009698,0.943104850964563
"13152","SLC33A1",144.963630096511,0.0599976536059912,0.19793452660433

7,0.303118685937619,0.761799414331381,0.943144383981444
"13153","GFM1",142.177014021422,-0.0553846549493141,0.182491800397383,-0.303491197022068,0.761515555088219,0.943144383981444
"13154","ZBTB42",8.35578751913258,-0.159982108859133,0.527669353432303,-0.303186280989234,0.761747903468638,0.943144383981444
"13155","CCBE1",390.913087079761,-0.247935911136552,0.81772050973777,-0.303203733040841,0.761734604290308,0.943144383981444
"13156","CC2D1A",84.5901157478824,-0.0959430987159671,0.316307459186246,-0.303322276884639,0.761644270887281,0.943144383981444
"13157","MMGT1",154.785789798271,-0.0506041886070437,0.166853051707912,-0.303285963840985,0.761671941998664,0.943144383981444
"13158","TMED5",421.741456320109,-0.0719446914974129,0.237843556700747,-0.302487452237074,0.762280497393871,0.943166503763674
"13159","SH3YL1",232.79399189422,0.0531434607018257,0.175672243276372,0.302514840766388,0.762259621826224,0.943166503763674
"13160","AC024060.2",7.14548453208961,0.193961244344123,0.6409307278921,0.302624349096235,0.762176156183312,0.943166503763674
"13161","HGSNAT",425.953839162882,0.0554941205754994,0.183371445057423,0.302632291293344,0.762170102864727,0.943166503763674
"13162","SAMD12",2.33382694085488,0.349297292057609,1.15393027340041,0.30270225169524,0.762116781648589,0.943166503763674
"13163","TAS2R14",20.6759100090694,0.0989944462358901,0.326860508430117,0.302864505447145,0.761993122220564,0.943166503763674
"13164","SALL2",36.291384398771,-0.0884748018416037,0.292168760707056,-0.30282088210763,0.762026368541613,0.943166503763674
"13165","ADSSL1",19.4679394105272,0.17665651124279,0.58353737886335,0.30273383958178,0.762092706906387,0.943166503763674
"13166","HMBS",35.9633479935892,-0.108090133665043,0.357637881900014,-0.302233457738857,0.762474100521295,0.943334393391416
"13167","ZMYND11",374.014909570657,-0.0543506045005496,0.179899780072131,-0.30211601414275,0.762563624995842,0.943373500991666
"13168","CARMIL1",237.832622768199,0.0632869480421938,0.209634354907109,0.301892063780466,0.762734345861135,0.943513043722055
"13169","NR4A3",56.6438719222487,-0.276224816381,0.91540357451439,-0.301751952987003,0.762841160381499,0.94353890865727
"13170","SNX21",567.532960358408,-0.0614420084314798,0.20364410735081,-0.301712675268506,0.762871104857035,0.94353890865727
"13171","PDE9A",44.5475634449898,-0.103015215047139,0.341548795185631,-0.301611999512839,0.762947859476781,0.943562195962136
"13172","UBP1",92.0318629154696,-0.0693755040591058,0.230108855600511,-0.30148993561355,0.763040923420714,0.943605648466445
"13173","NPRL2",93.2668263055127,0.0628929740970439,0.208762285456981,0.301265977996797,0.763211682066621,0.943745167325832
"13174","LHFPL5",5.40045220965932,-0.271502031953552,0.90185499417589,-0.301048432072663,0.76337756308725,0.943878634061653
"13175","C1orf52",272.798237813924,-0.0662604195446714,0.220731539188277,-0.300185554761857,0.764035622911708,0.943949457432059
"13176","RNPC3",186.666400798425,0.0735652732761291,0.246771937106959,0.298110369187739,0.76561892772316,0.943949457432059
"13177","AL592148.3",26.5600873769526,0.11535618494978,0.3881209695820

79,0.297217089491435,0.766300775553824,0.943949457432059
"13178","AC104335.1",4.45157305925407,0.218406893802657,0.731815708929092,0.298445211188845,0.765363386789937,0.943949457432059
"13179","AC016727.1",16.4008311807131,-0.13175198653277,0.442754732905505,-0.297573299032105,0.766028856113585,0.943949457432059
"13180","AC009237.14",6.23316391532215,0.246028665442274,0.827308839044239,0.297384306598854,0.766173123554478,0.943949457432059
"13181","CNOT11",87.5749938948363,0.0608151285406957,0.203050821059999,0.299506932418291,0.764553283360353,0.943949457432059
"13182","WDR12",80.5514725976206,-0.0613234145971701,0.205126666772248,-0.298953888161492,0.764975229318777,0.943949457432059
"13183","AC097639.1",2.87331253320765,-0.289846887170024,0.96534119134049,-0.300253309161642,0.763983944909971,0.943949457432059
"13184","CRIPAK",28.6065928831377,-0.104441620943127,0.350707814508391,-0.297802377427858,0.765853999916469,0.943949457432059
"13185","RPL9",22969.8108648625,-0.0637344353800782,0.213838232839401,-0.298049766563235,0.76566518047607,0.943949457432059
"13186","UGDH-AS1",11.5662711164584,-0.134170863214356,0.447932349622505,-0.299533765148751,0.764532813064433,0.943949457432059
"13187","USP46-AS1",32.3003758076779,0.102328573404622,0.343056353748632,0.298285026021121,0.765485631911084,0.943949457432059
"13188","TRIP13",5.17223027621561,-0.211343270479434,0.70826074815265,-0.298397547839096,0.765399760408273,0.943949457432059
"13189","SGTB",98.3912529828482,-0.0752499848912609,0.250589757836995,-0.300291542402982,0.763954783909629,0.943949457432059
"13190","HIST1H2BJ",5.6527042024092,-0.182534077137609,0.608658994863814,-0.299895472962575,0.764256887548985,0.943949457432059
"13191","TMEM14A",295.475372950425,-0.0644634771442916,0.215254893507092,-0.299475083209515,0.764577580867413,0.943949457432059
"13192","AL023581.2",3.61678299677676,0.278551247290045,0.930367157958941,0.299399269317649,0.764635419612523,0.943949457432059
"13193","SFT2D1",620.452868121137,0.0740330008829556,0.249121960278861,0.297175731918955,0.76633234852241,0.943949457432059
"13194","BRAT1",122.819580512934,-0.0484446909913888,0.161536731715818,-0.299898917582502,0.764254259995824,0.943949457432059
"13195","FAM221A",31.1481885211981,0.104391275564666,0.350909979247749,0.297487337887771,0.766094473577974,0.943949457432059
"13196","ADCYAP1R1",21.8630342346284,-0.179725222119396,0.602831594521848,-0.298135040951113,0.765600098134826,0.943949457432059
"13197","SUMF2",572.234698385759,-0.0567301511169737,0.190002966093597,-0.298575081659663,0.765264280583404,0.943949457432059
"13198","PMPCB",338.189733720898,-0.0607886979162214,0.204458880609288,-0.297315028503878,0.766226009044006,0.943949457432059
"13199","DMRT2",72.4382888603135,0.0864992249605773,0.288925174534382,0.299382790371159,0.764647991645586,0.943949457432059
"13200","RNF20",170.967391822133,-0.0651638142237294,0.218355865133757,-0.298429420175237,0.765375437423675,0.943949457432059
"13201","SLC16A12",22.5175331684155,0.166467600264245,0.55542409555864

6,0.299712600867291,0.764396386124718,0.943949457432059
"13202","ZFYVE27",58.5314694460115,0.0776798103580829,0.25958101393043
8,0.299250739420023,0.764748737517394,0.943949457432059
"13203","AC132217.1",104.073621108655,0.270156439419623,0.908620581152
834,0.297325908111014,0.766217703700954,0.943949457432059
"13204","C11orf74",255.263851062959,-0.0950817199501969,0.319640598874
463,-0.297464465668642,0.766111933109642,0.943949457432059
"13205","CDCA5",2.34607540570032,-0.264009752523381,0.883139201581787,
-0.298944664726144,0.764982266944641,0.943949457432059
"13206","MRGPRF-
AS1",5.12787069264216,0.1992845134534,0.670594774078237,0.297175762706
062,0.766332325018944,0.943949457432059
"13207","TSKU",278.604104462956,-0.114944062422638,0.383794961023068,-
0.299493412097532,0.76456359787679,0.943949457432059
"13208","HOXC5",66.8485062909037,0.0823917000631211,0.277204199532916,
0.297223852315187,0.766295612753344,0.943949457432059
"13209","MNAT1",283.219410985051,-0.0623500716353667,0.208007218500043
,-0.299749557178728,0.76436819447766,0.943949457432059
"13210","IFI27L1",233.937883953876,0.0857346069059168,0.28716920210952
4,0.298550841371974,0.765282778437967,0.943949457432059
"13211","TECPR2",90.9351229965204,-0.0685682194245261,0.22979592474480
4,-0.298387447473985,0.76540746842699,0.943949457432059
"13212","SENP8",12.1461438798237,0.139200815467866,0.467033428271841,0
.298053216410975,0.765662547482606,0.943949457432059
"13213","LINGO1",6.31067846889462,0.261016969952568,0.870458836934708,
0.299861359178948,0.764282909660764,0.943949457432059
"13214","AC048382.6",13.6294500219072,0.126482433587536,0.425311333764
148,0.297387874590888,0.766170399851384,0.943949457432059
"13215","THUMPD1",471.387792557546,0.0434476611621667,0.14444342102448
5,0.300793631541044,0.763571864945726,0.943949457432059
"13216","AC020978.5",3.50173517349782,-0.246396495469105,0.82853950076
8552,-0.297386540099233,0.766171418563816,0.943949457432059
"13217","DRG2",186.368413716346,-0.0632733691402489,0.212800162876497,
-0.297337033416516,0.766209210822918,0.943949457432059
"13218","MAPT",16.3422617563898,0.112844830554776,0.376035120163196,0.
300091200273534,0.764107591227654,0.943949457432059
"13219","AC005332.5",24.2234237323039,0.10116867316559,0.3401968051537
16,0.297382784414679,0.766174285547231,0.943949457432059
"13220","NACC1",32.9141033874462,0.0982748400240536,0.326941230744917,
0.300588701523329,0.763728148123027,0.943949457432059
"13221","REX1BD",1146.28397770372,-0.0622861989410147,0.20952845982989
7,-0.29726844263343,0.766261572363135,0.943949457432059
"13222","CCDC61",74.2222410854138,-0.0815461658030903,0.27330388200923
9,-0.298371780172276,0.765419424858045,0.943949457432059
"13223","SLA2",4.63636851444102,0.261095866704511,0.873915906396879,0.
298765435888447,0.765119025210953,0.943949457432059
"13224","IL2RB",8.90893890818634,0.172637854761067,0.577808508323715,0
.298780395709139,0.765107610031435,0.943949457432059
"13225","FASTKD2",151.554537752145,0.0509350902694676,0.17153473613435
2,0.29693746827799,0.766514249985543,0.944102126125861

"13226","RSBN1",279.871722220064,0.0615031717012743,0.207326024809951,
0.296649548736837,0.766734078227041,0.944230089985656
"13227","TRIP4",121.190432786266,-0.0737960552215109,0.248758318916736
,-0.296657637593264,0.766727902081766,0.944230089985656
"13228","RERE-
AS1",5.60514542656139,-0.194784204985839,0.658051955809262,-0.29600125
5320784,0.767229122726218,0.944508539184782
"13229","SLC16A1-
AS1",161.183013450017,0.0878783035215016,0.297297666410201,0.295590290
306047,0.767542989673051,0.944508539184782
"13230","MIA3",466.904854061463,-0.054170933543899,0.183508073919282,-
0.29519645859139,0.767843807157392,0.944508539184782
"13231","PAK2",501.011189164946,-0.0410089097766826,0.138944644894137,
-0.295145666160273,0.767882606100972,0.944508539184782
"13232","PPID",201.293043088063,-0.0699443132263802,0.2368469197255,-0
.295314430550517,0.767753693861692,0.944508539184782
"13233","ENPP6",3.03796445762087,0.252232535057001,0.85204092751036,0.
296033355808409,0.767204608178015,0.944508539184782
"13234","HCG27",6.80651249717443,-0.196709027831258,0.664892332188223,
-0.295850949557307,0.767343911553975,0.944508539184782
"13235","EPHB6",101.855762669108,-0.068834101725552,0.233013606598553,
-0.295408078224988,0.767682162991449,0.944508539184782
"13236","DNAJC14",73.5766199940975,0.0767182130217175,0.25955985864219
2,0.29557040685353,0.767558176260447,0.944508539184782
"13237","AL161891.1",7.80000727051009,0.174515445404508,0.591067944550
363,0.295254457653368,0.767799503977768,0.944508539184782
"13238","GTF2F2",233.396927157404,-0.0592468900490448,0.20067036360488
5,-0.295244843257973,0.767806847980056,0.944508539184782
"13239","RAB40C",48.3804019695766,0.0808790203216299,0.273814947517191
,0.295378397180278,0.767704834039217,0.944508539184782
"13240","KCTD5",56.348699399111,0.0831824272034543,0.281841826434977,0
.295138689156363,0.767887935688137,0.944508539184782
"13241","SPOP",925.274378410899,-0.0520525833117945,0.175901961265732,
-0.295918152004909,0.767292588272302,0.944508539184782
"13242","AC005828.5",5.49388966508177,0.225811117886404,0.763147881015
278,0.295894312889907,0.767310794361642,0.944508539184782
"13243","MAP7D2",18.5988901371698,0.122860811655852,0.414857737919342,
0.296151669418153,0.767114256276085,0.944508539184782
"13244","SHLD3",10.0235622559936,0.139756711076354,0.474040250709894,0
.294820346725964,0.768131122473976,0.944736322408532
"13245","UQCRHL",4.74372130674552,0.222982728771993,0.758694295867319,
0.293903262468956,0.768831825063297,0.94474212420459
"13246","UXS1",257.7636578323,-0.0619513296937565,0.210697680497512,-0
.294029481233363,0.768735375794346,0.94474212420459
"13247","THAP6",102.519455109604,0.0705099738586712,0.239653680835813,
0.294216110567389,0.768592770730917,0.94474212420459
"13248","UBE2B",1404.91233193924,-0.0540905920113459,0.183927016775894
,-0.294087257867356,0.768691227339763,0.94474212420459
"13249","PRIM2",62.1990463148831,-0.0710624588125124,0.241496349739655
,-0.294258935545491,0.768560048904814,0.94474212420459

"13250","WASL",530.691399912462,-0.0436185053252095,0.148326770490863,
-0.294070350084892,0.768704146887497,0.94474212420459
"13251","SORCS3",2.37504095728957,-0.294720474279764,1.00053681164679,
-0.294562349779697,0.768328227142143,0.94474212420459
"13252","PLBD2",409.287208655626,-0.049639546020027,0.168623420989194,
-0.294381087329548,0.768466717102062,0.94474212420459
"13253","IFI27",3398.24643097202,0.138880427191677,0.471680849549006,0
.294437281743506,0.768423782090241,0.94474212420459
"13254","ZFYVE21",545.999797358008,-0.0698542050110284,0.2376466840647
11,-0.293941425212595,0.768802662867165,0.94474212420459
"13255","BCKDK",193.163293027742,-0.0634680254819112,0.215402613561917
,-0.294648353761351,0.768262520101457,0.94474212420459
"13256","SNX5",594.950410960013,0.0477189315252882,0.162096163020974,0
.294386558176049,0.768462537102726,0.94474212420459
"13257","USP14",380.855580146601,-0.0466463185709525,0.158793165790653
,-0.293755202490574,0.768944968717629,0.944809881228141
"13258","EIF4EBP3",322.406634652999,0.0866137524763909,0.2950219893719
87,0.293584056770702,0.769075760037787,0.944899310247059
"13259","AC007563.4",2.79582266007348,-0.267025495377179,0.91113982206
4599,-0.293067528068427,0.769470536420419,0.945198380252251
"13260","AC022915.1",3.1049233042567,-0.250539813030442,0.855563217987
415,-0.292836119836708,0.769647418208838,0.945198380252251
"13261","POLR1E",173.213552462868,0.0628861329612035,0.214739034679459
,0.292849099629574,0.769637496511557,0.945198380252251
"13262","AL136418.1",5.06491701896467,-0.297952385945029,1.01756199674
471,-0.292810056682748,0.769667340884391,0.945198380252251
"13263","FPR1",4.46021258722635,0.243995838925522,0.832743920327847,0.
293002245911879,0.769520435006643,0.945198380252251
"13264","THOC2",558.079289680801,0.0572225698666361,0.195411880109205,
0.292830557869141,0.769651669764227,0.945198380252251
"13265","MRPL40",606.119427213459,-0.0755446586418492,0.25806615543621
2,-0.292733692700445,0.76972571438972,0.945198806007852
"13266","RPS18",42626.0817406161,-0.0748298100082903,0.255803845639698
,-0.29252808854832,0.769882887051121,0.945320544789364
"13267","AC022558.1",2.44906315931256,0.270260850711674,0.924222041082
596,0.292419828459297,0.769965649521503,0.945350905634715
"13268","ARF4",1294.83972962843,-0.0684249247687375,0.234058154407929,
-0.292341554780796,0.770025489668909,0.945353120381132
"13269","KLHL17",12.150933717951,-0.128595535911029,0.440599245710614,
-0.291865084116578,0.770389780530976,0.945526228368656
"13270","PDE8B",8.35552400439607,-0.159968426518628,0.549957104406609,
-0.290874370449728,0.771147403613959,0.945526228368656
"13271","SRPK1",151.007694036675,-0.0569446406589513,0.195653594006238
,-0.291048273087873,0.771014400173961,0.945526228368656
"13272","FRK",16.4764588128854,-0.150697836112675,0.516455551969932,-0
.291792460237601,0.770445310364111,0.945526228368656
"13273","EPHX2",111.636483275174,0.0717485783894236,0.246399013687769,
0.291188577890744,0.770907097786775,0.945526228368656
"13274","AC016405.3",7.57242233952761,-0.177026025308903,0.60672212678
3702,-0.291774467246377,0.770459068387293,0.945526228368656

"13275","CCDC3",26.6789730977861,-0.230606122388179,0.79275791285628,-0.2908909752251,0.771134703733133,0.945526228368656
"13276","DCLRE1C",132.888403925376,0.0709583102490051,0.243244004127244,0.291716585177926,0.770503327379265,0.945526228368656
"13277","GUCY1A2",18.5679633655268,0.216157729473577,0.741595076503176,0.291476759113369,0.770686716138166,0.945526228368656
"13278","SOCS4",83.5561067078826,-0.0758663543131699,0.260828623948033,-0.290866673928723,0.771153290188323,0.945526228368656
"13279","TYRO3",40.3080847839062,0.0898603668183435,0.308542761114209,0.291241209140152,0.770866847560375,0.945526228368656
"13280","AC138969.1",15.1408734983612,-0.140341850144037,0.480836969507411,-0.291869924826723,0.770386079258173,0.945526228368656
"13281","PWWP3A",378.548289590966,0.0662091051377147,0.227374121957389,0.291190152017927,0.770905893949754,0.945526228368656
"13282","ZNF225",22.6646913262801,0.0944748923709464,0.324348941371051,0.29127547625589,0.770840641803736,0.945526228368656
"13283","GTSE1",2.78581574108004,0.279235679277306,0.959740243424654,0.290949224220196,0.771090153462187,0.945526228368656
"13284","SHANK3",11.3079342642917,-0.166228052750056,0.570282133610901,-0.291483886576521,0.770681265767437,0.945526228368656
"13285","DCAF12L2",40.146941530511,-0.0812875507492017,0.279188467975512,-0.291156548616226,0.77093159276754,0.945526228368656
"13286","RNF123",42.0256674879611,-0.0723642846356592,0.249069532756404,-0.290538484714761,0.771404313287207,0.945742548152576
"13287","MIR4458HG",153.753861311389,0.0763910870936233,0.262978458820279,0.290484199490383,0.771445836902405,0.945742548152576
"13288","MSTO1",30.9008670549581,0.0996949145257492,0.343363880274613,0.290347704732414,0.771550246765466,0.945799365560106
"13289","RMC1",44.929588380694,-0.0841355180549085,0.290106876883109,-0.290015593421485,0.77180430820737,0.946039609932264
"13290","MIR22HG",554.013108833337,-0.10241781978169,0.353317521298229,-0.289874726295391,0.771912077377552,0.946100513800071
"13291","AC106881.1",3.08928822925979,-0.269126541841563,0.930280094313216,-0.289296249040185,0.772354682493438,0.946102746668699
"13292","LARP7",675.927006771328,-0.0585513604699162,0.20207286989407,-0.289753693806644,0.772004675745822,0.946102746668699
"13293","HSPA9",519.868181511824,-0.0529586540764972,0.182980587266743,-0.289422254390821,0.772258266834081,0.946102746668699
"13294","SDHAF4",75.3354776391206,0.0769897729500111,0.266121567837305,0.289303018825889,0.772349502359354,0.946102746668699
"13295","OSTM1",180.526574771062,0.0455739336089851,0.157468860805515,0.289415528732834,0.772263413024738,0.946102746668699
"13296","TBC1D31",17.8964748958422,0.117851338135636,0.407416447970636,0.28926504740459,0.772378557627869,0.946102746668699
"13297","AC009163.7",10.6279524545399,-0.143245152086233,0.494572643107359,-0.289634200521556,0.772096099700777,0.946102746668699
"13298","YWHAH",664.654825807421,0.0766237806001662,0.264677790758925,0.289498338264267,0.772200051351372,0.946102746668699
"13299","STIL",2.65649227686641,-0.287314074302338,0.9941600844914,-0.289001820515983,0.772579984405317,0.946116359218583

"13300","HOXD-
AS2",215.628471762313,-0.100068629448249,0.346194288402555,-0.28905338
0718659,0.772540528241485,0.946116359218583
"13301","EXOSC9",129.663907347928,0.0697564684735696,0.241416209994596
,0.288946912368192,0.772622003212327,0.946116359218583
"13302","MTCP1",2.51683615113906,-0.287642431443115,0.995058593534488,
-0.28907084800416,0.772527161632751,0.946116359218583
"13303","SSB",1083.48111224145,-0.0508348339390583,0.176054889569084,-
0.288744232344145,0.772777111152489,0.946138600794355
"13304","RNF8",195.467361747814,-0.0607767581634894,0.21063277195179,-
0.288543694318376,0.772930588788168,0.946138600794355
"13305","CTSH",617.382195413052,0.118267479145,0.409807996865938,0.288
592414129219,0.77289330127037,0.946138600794355
"13306","KLF2",5143.55203883767,-0.0786648214226515,0.272370932204066,
-0.288815039057524,0.772722922819537,0.946138600794355
"13307","SYNJ1",66.5804330430565,-0.0713530884749646,0.247282098331725
,-0.288549348927174,0.772926261028421,0.946138600794355
"13308","ZNF484",36.0254232686856,0.103211174014531,0.357992587136241,
0.288305338499234,0.77311302104159,0.946219700935191
"13309","GNB1L",30.7731020473453,-0.125536752968657,0.435372858287752,
-0.288343084735166,0.773084130074694,0.946219700935191
"13310","ARSD",114.263754243535,0.0975043927313807,0.33842847608528,0.
288109304096534,0.773263070818283,0.946332243467995
"13311","WDSUB1",77.7445415437416,-0.0693442196210356,0.24099074351522
6,-0.287746403075662,0.773540866961023,0.946458888449493
"13312","LEKR1",6.31251518288888,0.223780455794489,0.777555723480614,0
.287799895283092,0.773499917528913,0.946458888449493
"13313","TESK1",146.482509079701,-0.0673593114903243,0.234087347717602
,-0.287752892871361,0.773535898849621,0.946458888449493
"13314","YEATS4",146.608452427205,-0.0644426112667176,0.22403534809783
8,-0.287644837361,0.773618619428461,0.94648292713461
"13315","AL354696.1",2.88963181446973,0.274677414710056,0.955540355735
007,0.287457680946163,0.773761900823321,0.946587127488627
"13316","CHPF",189.304388920378,-0.0850108780719701,0.296148244069527,
-0.287055148137269,0.774070094104863,0.946758563054798
"13317","SLTM",588.729305402345,-0.0491555722207716,0.171242321519928,
-0.287052708608901,0.774071962002234,0.946758563054798
"13318","SMARCD2",403.016659112942,-0.0550783141775342,0.1918791415556
02,-0.287046907397038,0.774076403877696,0.946758563054798
"13319","ST3GAL6-
AS1",2.35639234051572,-0.306500779039661,1.06860713731013,-0.286822695
018841,0.774248084615253,0.946897443524128
"13320","PIGK",250.267499934494,0.0638841001342242,0.22304193955794,0.
286421918052004,0.774554989398767,0.946985613964108
"13321","CYP39A1",14.2055325239193,0.123067365863599,0.429788859761745
,0.286343778039808,0.774614831133633,0.946985613964108
"13322","AP5Z1",111.538504394451,-0.0519270371024043,0.181480551976279
,-0.286130037279101,0.774778526428914,0.946985613964108
"13323","SELPLG",7.07930165250862,0.203695181622356,0.711870937434176,
0.28614060626852 1,0.774770431837409,0.946985613964108

"13324","DOCK9",139.532464944284,0.0647037729535398,0.226109434682256,
0.286161314075487,0.77475457218597,0.946985613964108
"13325","LTBP4",3111.34743474134,0.112929999976522,0.394090158813973,0
.286558792324041,0.774450170606961,0.946985613964108
"13326","ITGB2-
AS1",5.45643702299476,-0.222154193391769,0.776433795133874,-0.28612123
1177817,0.774785270876031,0.946985613964108
"13327","GPRASP2",46.6092850700669,0.0654764326067576,0.22878223214654
2,0.286195444429522,0.774728432707647,0.946985613964108
"13328","ARHGAP18",360.514529419376,-0.0705362380260486,0.246817646802
835,-0.285782799324698,0.775044483098347,0.947160295985369
"13329","HSBP1",1279.33500362421,-0.0670823081012404,0.234705641139926
,-0.285814639032248,0.775020095313905,0.947160295985369
"13330","AC005899.5",12.4932375143473,0.206051511399201,0.721308537905
472,0.285663485971663,0.775135873736018,0.947171159930202
"13331","NFATC1",96.7306340214269,-0.124149632423311,0.434668117504787
,-0.285619366646885,0.775169668673923,0.947171159930202
"13332","NPHP4",38.8093044737603,0.0844502699267824,0.295948346295373,
0.285354762018154,0.775372361965497,0.947347765080706
"13333","HSPA1B",673.518692402067,0.0942260467132428,0.330617692838704
,0.285000012867467,0.775644132066855,0.947433978760586
"13334","COA1",203.094452100183,0.0544625162171254,0.191552863722581,0
.284321075439527,0.776164336454266,0.947433978760586
"13335","ZNF800",209.570579747669,-0.054475127691971,0.191624293527391
,-0.2842809055637,0.776195117911716,0.947433978760586
"13336","AC100810.1",453.386848468478,0.0604793581119574,0.21248345078
5212,0.284630910729574,0.775926927029291,0.947433978760586
"13337","ALG2",163.877049884163,0.052296382175056,0.183680379963944,0.
284714035246016,0.77586323693019,0.947433978760586
"13338","TGDS",79.0746018046338,0.0554942133428074,0.19521260786541,0.
284275764509371,0.77619905743508,0.947433978760586
"13339","TUBGCP4",139.231782763654,-0.059930142224062,0.21043888635519
6,-0.284786444473513,0.775807758126046,0.947433978760586
"13340","CD7",9.35536908967546,-0.146994474108028,0.51702937043513,-0.
284305848977821,0.776176004178191,0.947433978760586
"13341","ZNF57",11.7940783532355,0.145476214792457,0.510983794600416,0
.2846982944072,0.775875297463127,0.947433978760586
"13342","TIMM50",155.747210743304,-0.0570479400229512,0.20049417220060
6,-0.284536649603318,0.775999151834755,0.947433978760586
"13343","TSEN34",311.554115918084,-0.0614168305099633,0.21549517745913
4,-0.285003271229168,0.775641635740475,0.947433978760586
"13344","SRC",93.6714029760924,-0.0623484352255846,0.219149638458559,-
0.284501656786327,0.776025964540888,0.947433978760586
"13345","LINC01697",220.54651298748,0.114200929272241,0.40131011193279
9,0.284570275895178,0.77597338645895,0.947433978760586
"13346","AC137630.3",5.25240434062153,0.194133407201429,0.683451019578
01,0.284048749128057,0.776373022121248,0.94747012191277
"13347","ARRDC3",939.068039512405,0.0982669205841626,0.346091306852417
,0.283933513031191,0.776461333256404,0.94747012191277
"13348","TRHDE-

AS1",13.5687417660267,-0.237905089667851,0.837749555280051,-0.28398115
9009176,0.776424819430453,0.94747012191277
"13349","TEX26",4.23225919180908,-0.22102133002639,0.77809650538356,-0
.284053878274956,0.77636909146883,0.94747012191277
"13350","TRMT10C",239.293044579026,-0.069286957314913,0.24419142982756
,-0.283740331771025,0.776609384116891,0.947519858243022
"13351","LTBP3",1279.05580394789,0.0474071247746503,0.167086211819419,
0.283728527078501,0.776618431297353,0.947519858243022
"13352","AP000944.7",3.11509294463487,0.255429908769807,0.900546476466
335,0.283638785387392,0.776687210807541,0.94753280234027
"13353","CYP4Z1",5.86805262743988,-0.188688143920488,0.666147949461635
,-0.283252607882351,0.77698320357849,0.94761001820081
"13354","SLC9B1",12.9284979007028,-0.13625222436399,0.480902984571716,
-0.283325803197777,0.776927099209832,0.94761001820081
"13355","CNOT7",308.867710854325,0.0411852564380996,0.145361051641473,
0.283330754511059,0.776923304059431,0.94761001820081
"13356","TMC4",2.53504580718619,0.260145868076238,0.918117611046569,0.
283346997101706,0.776910854253528,0.94761001820081
"13357","PIGH",184.144275701548,-0.0574304173751629,0.203182687658576,
-0.282654088480549,0.777442013622465,0.94809859698258
"13358","SASS6",88.3133976829149,0.0537808835649737,0.19037010036285,0
.282506987507314,0.77755478944874,0.948132766080171
"13359","PIK3C3",149.009597041501,0.0491774235111545,0.174100515921144
,0.282465696617629,0.777586446194671,0.948132766080171
"13360","AC004865.2",2.5007288860716,-0.279906491249384,0.997497743070
152,-0.28060864617886,0.779010583697125,0.948210023675104
"13361","PPT1",353.313612420743,-0.0461014244706907,0.163862217280033,
-0.281342613544069,0.778447628784688,0.948210023675104
"13362","FRRS1",48.2975598677547,-0.0709000865451822,0.251981475352097
,-0.28137023345114,0.778426446510426,0.948210023675104
"13363","AMIGO1",12.3424472719957,0.186476126066098,0.662471331889108,
0.281485578454151,0.778337987836898,0.948210023675104
"13364","PPOX",67.6367279770906,-0.0880736383780574,0.314412881639682,
-0.280120960435044,0.779384704092203,0.948210023675104
"13365","PPP3R1",152.428813424528,0.0538974308539119,0.192314803829537
,0.28025627658745,0.779280893321859,0.948210023675104
"13366","AC019080.1",25.4297917230562,-0.0970948639481291,0.3464111110
07725,-0.280287960930802,0.779256586547897,0.948210023675104
"13367","CREB1",337.692937055877,0.0474855503471716,0.1688990554467,0.
281147518685543,0.778597255727233,0.948210023675104
"13368","CNTN4",173.565901070193,0.140087590032397,0.499026881730016,0
.280721530565136,0.778923993521335,0.948210023675104
"13369","HESX1",3.7961918780056,0.217136283026994,0.774430560762686,0.
280381862530258,0.779184550818661,0.948210023675104
"13370","B4GALT4",148.42481369573,0.0490762738576467,0.175573837486382
,0.279519286929372,0.779846338813331,0.948210023675104
"13371","EVC",43.4300296687059,0.0833334565168077,0.295797787624236,0.
281724407697969,0.778154837597805,0.948210023675104
"13372","HOPX",12.8487755257469,-0.154947046038623,0.552051359292872,-
0.28067505573593,0.778959642615592,0.948210023675104

"13373","ALPK1",123.513403484995,0.0650934120425903,0.232674806520855,
0.279761324467915,0.779660625877291,0.948210023675104
"13374","DLD",192.958982173776,0.0594238529490608,0.212150082055734,0.
280102898727372,0.779398560827256,0.948210023675104
"13375","PAXIP1-
AS1",95.9774037115914,-0.072412147560O887,0.259010653563431,-0.2795720
81548974,0.779805828968107,0.948210023675104
"13376","AC083843.3",20.4314150257243,-0.140601658634939,0.50059088881
4849,-0.280871389744656,0.778809045346817,0.948210023675104
"13377","DOCK8",39.0181726326582,-0.150432645596332,0.53341021501858,-
0.282020556338788,0.77792774812591,0.948210023675104
"13378","BAG3",474.223194558829,0.0934806896253056,0.334362604967025,0
.279578781348843,0.779800688186703,0.948210023675104
"13379","SPTY2D1OS",26.5046823557403,0.101555787456827,0.3620344534775
22,0.280514151295085,0.779083069954531,0.948210023675104
"13380","ARL14EP",273.245746914289,0.05813384331688,0.207278415785571,
0.280462599526133,0.779122615707635,0.948210023675104
"13381","INPPL1",110.196576060586,-0.0552499042966817,0.19594882878136
4,-0.281960880502779,0.777973506574627,0.948210023675104
"13382","ZNF202",17.2899214044525,-0.105977209675882,0.375420448710315
,-0.282289390575144,0.777721620009871,0.948210023675104
"13383","ETV6",118.306149344178,0.0690806014059867,0.246445032779938,0
.280308353659015,0.779240942296979,0.948210023675104
"13384","AC026356.1",4.01555226441255,0.198958947928437,0.711783497631
451,0.279521720566011,0.779844471446197,0.948210023675104
"13385","DNAL1",90.5315289712404,-0.0580288351752262,0.207730038525561
,-0.279347347100625,0.779978274125037,0.948210023675104
"13386","LPCAT4",68.2279016566716,0.0789721245861465,0.281317568415561
,0.280722334658777,0.778923376735426,0.948210023675104
"13387","ADAMTS17",6.52517839771828,-0.211269260780428,0.7513859438973
32,-0.281172761476751,0.77857789544319,0.948210023675104
"13388","AC087190.1",30.5482220099224,-0.123842688737025,0.44214962157
0404,-0.280092264462802,0.779406719345903,0.948210023675104
"13389","ZFPM1",213.67203113313,-0.0894451690087472,0.319096317701318,
-0.280307744235614,0.779241409813933,0.948210023675104
"13390","RNF135",152.840180752528,-0.0566304468834636,0.20160267526160
5,-0.280901266860563,0.778786128944428,0.948210023675104
"13391","HSPB9",3.40668376697824,0.254049709057043,0.904062170172184,0
.281009113575295,0.778703409751969,0.948210023675104
"13392","SRSF1",383.445003946175,0.0496763565382865,0.177462757984914,
0.279925529741342,0.779534640003631,0.948210023675104
"13393","AC116025.1",3.69321891153216,-0.256790064188939,0.91338026906
5935,-0.28114255681431,0.778601061314483,0.948210023675104
"13394","CD226",7.09635151567924,0.177797394725685,0.636363695050597,0
.279395880230956,0.779941032352,0.948210023675104
"13395","CEACAM1",2.65521036676186,0.263199502362473,0.941275059576537
,0.27962018082247,0.779768922429922,0.948210023675104
"13396","IQSEC2",45.6757112422716,0.0694868818045569,0.248098322694192
,0.280077999117338,0.779417663637541,0.948210023675104
"13397","NLGN3",5.94141284140392,-0.159093426621517,0.569351273457165,

-0.279429297936726,0.779915389654782,0.948210023675104
"13398","P2RY10",4.12575421713596,0.213884464607468,0.761372720887107,
0.280919526980507,0.778772123127116,0.948210023675104
"13399","MT-
ND6",395.252529517379,0.0877310267975366,0.312644288988631,0.280609721
294883,0.779009758995419,0.948210023675104
"13400","HYAL2",172.394703150376,-0.0880259144634817,0.315228500533561
,-0.279244783750478,0.780056977509471,0.948234933332222
"13401","AC012485.3",17.7520894119678,0.125228152280041,0.448845992131
296,0.279000268411463,0.780244618778395,0.948321488977859
"13402","SLA",20.9190222948214,0.12710861336492,0.455466277178292,0.27
907359937246,0.780188343195257,0.948321488977859
"13403","TIPARP",725.68964824338,-0.113264110738079,0.406367835752471,
-0.27872311923592,0.78045731875737,0.948466017402548
"13404","MAGED2",1063.52457124175,-0.0752092533833954,0.26986359771341
7,-0.278693584539195,0.780479986325972,0.948466017402548
"13405","GHRLOS",4.5035462246898,0.20201183164395,0.725135600916065,0.
278584903828674,0.780563399232754,0.948496621417556
"13406","MITD1",265.337273722023,0.0527451829104369,0.189878766639435,
0.277783471232441,0.781178580072564,0.948660214198866
"13407","AP1AR",89.143586209746,-0.0677790182145395,0.244133035435532,
-0.2776314892969,0.781295257091175,0.948660214198866
"13408","AC011379.2",28.46533989218,0.1051778481686,0.378101969771542,
0.278173235204648,0.780879379543149,0.948660214198866
"13409","G3BP1",538.257226995484,0.0441894463088777,0.159052975299938,
0.2778284796342,0.781144027923654,0.948660214198866
"13410","LINC00847",56.7527769265394,-0.0653854331700822,0.23534755523
1857,-0.277824994211929,0.781146703604861,0.948660214198866
"13411","HVCN1",62.2744371761909,-0.0653907478141073,0.235578702913434
,-0.277574954804534,0.781338660058444,0.948660214198866
"13412","HAPLN3",28.4005911113049,0.156217450954936,0.561513820317142,
0.278207668809833,0.780852948302566,0.948660214198866
"13413","PDXDC1",214.156532852832,-0.0627482515712046,0.22598383606110
7,-0.277666990103829,0.781268002567227,0.948660214198866
"13414","ROCK1",822.367660601711,-0.0637732641651179,0.229696666496674
,-0.277641226308529,0.781287781808534,0.948660214198866
"13415","C19orf73",2.30980593034147,-0.258621580134231,0.9316436222288
48,-0.277597113277617,0.781321648354074,0.948660214198866
"13416","GLA",218.332941403105,-0.0662571138059896,0.238398398129859,-
0.277926002547628,0.781069162801268,0.948660214198866
"13417","PUM3",266.990132326045,-0.0540180970519077,0.194836127903397,
-0.277248873877697,0.781589013966882,0.948824510542497
"13418","FAM122C",20.9325879585341,0.103463349529232,0.373181175637857
,0.277246968184792,0.781590477159999,0.948824510542497
"13419","LINC00645",20.9998351004371,-0.211737509733612,0.763950854567
947,-0.277161166150354,0.781656356857739,0.948833772774105
"13420","NTNG2",11.1793689059128,-0.12690777762971,0.458013564470792,-
0.277083011234273,0.781716366381663,0.948835908494107
"13421","OXSR1",121.995200425715,0.0585345092299517,0.211375842444016,
0.276921471030707,0.781840405573111,0.948915756380329

"13422","AC135507.1",17.4233903800866,0.118506341437264,0.428737632292026,0.27640760341874,0.782235017442281,0.949011534422333
"13423","AC024230.1",3.59437431791142,0.246673929283397,0.892133087023945,0.276499025617661,0.782164807933467,0.949011534422333
"13424","SEPTIN7-AS1",4.02504965544438,0.224327476818188,0.811593897947174,0.276403602079312,0.78223809039157,0.949011534422333
"13425","RNF125",38.5657595288462,0.113862022672649,0.412000937636726,0.276363503747764,0.78226888530227,0.949011534422333
"13426","FCHO1",2.94771875622699,0.239053258526294,0.864386199449245,0.276558393318415,0.782119216268967,0.949011534422333
"13427","FAM199X",84.2328598092437,0.0569507397747745,0.206016555311505,0.276437685741627,0.782211914924005,0.949011534422333
"13428","TNFSF8",2.59840293828705,-0.270675507169034,0.980585216435044,-0.276034660356278,0.782521444912367,0.949247230874109
"13429","LRRC34",52.1525221104952,-0.0828139645442917,0.300375015845209,-0.275701906535955,0.782777031162467,0.949425125576409
"13430","HSP90AA1",6689.8247464558,0.0524900567432608,0.190393856925808,0.275691966068605,0.782784666737747,0.949425125576409
"13431","MBIP",294.958968348506,0.0647950752566375,0.235158754761381,0.275537584481538,0.782903254615482,0.949498258836392
"13432","CRYM",5.59717550403163,0.199526007277037,0.724600333862908,0.275360081899696,0.783039609068936,0.949563158536453
"13433","TRAPPC2",117.271785193515,0.0735460103232232,0.267132930478533,0.275316151368816,0.783073356781888,0.949563158536453
"13434","CYTIP",25.5691556446229,-0.122526838262628,0.445467815198918,-0.27505205557424,0.783276245449835,0.949738481623669
"13435","GLOD4",315.060237567901,-0.0560704277937007,0.203910438514441,-0.274975759957134,0.783334861456827,0.949738858077428
"13436","PSMB10",450.115617373937,-0.0747563860035676,0.27211102163733,-0.274727519501959,0.783525586873909,0.949865152517423
"13437","TMEM86B",8.11036622838176,0.145227095473653,0.528697617628524,0.274688386388178,0.783555654391099,0.949865152517423
"13438","SELENBP1",884.624489790254,0.0620549613466471,0.226051961854183,0.274516358264019,0.783687834227292,0.949954690558741
"13439","CMTM7",62.6825413852008,0.0707604236672458,0.258087619013435,0.274172096816323,0.783952370308869,0.950064846250312
"13440","FAM13A",925.776368037308,-0.0697086577812643,0.254211308598974,-0.274215408297322,0.783919087690484,0.950064846250312
"13441","ECHS1",814.879283605236,-0.0674253275935922,0.245993010804639,-0.274094484932906,0.784012011989483,0.950064846250312
"13442","Z82215.1",5.25408595516309,-0.210041433065923,0.766144544860134,-0.274153793138693,0.78396643585106,0.950064846250312
"13443","SZRD1",337.752554335578,0.0423333188610356,0.15486310870883,0.273359609102448,0.784576798004652,0.950124036513985
"13444","GEMIN6",146.976561021475,0.065124690157168,0.238345102697105,0.273236955239353,0.784671074220357,0.950124036513985
"13445","TRABD2A",21.1004348755433,-0.17352248352576,0.634586053680597,-0.273442006043672,0.784513466496687,0.950124036513985
"13446","MYNN",98.9180064488289,0.0581419711229861,0.212891252713474,0

.273106435242965,0.784771400101922,0.950124036513985
"13447","FBXW11",257.689890871593,0.050119502724766,0.183677275515372,
0.272867193745867,0.784955305454037,0.950124036513985
"13448","STXBP5-
AS1",3.77635981812702,0.203917429755452,0.747559842649887,0.2727773993
7531,0.785024333641074,0.950124036513985
"13449","SORBS1",17.7264622833436,0.159302078779657,0.584134459054052,
0.27271474283101,0.78507250099065,0.950124036513985
"13450","DPP3",44.2285542885433,-0.0846016018439917,0.31028745651045,-
0.272655565247261,0.785117994654166,0.950124036513985
"13451","DLAT",86.3260478552856,0.0554366334792683,0.20241697732863,0.
273873437944216,0.78418188486009,0.950124036513985
"13452","KRT7",4.87828446236332,-0.248421903011536,0.908475141971785,-
0.273449312517624,0.784507850701588,0.950124036513985
"13453","SETDB2",87.9198798096809,0.0676358489151049,0.247612992540496
,0.273151453892482,0.784736795531214,0.950124036513985
"13454","AL161665.1",4.1704936147336,0.234655253567028,0.8589966644690
12,0.273173649297091,0.784719734704069,0.950124036513985
"13455","SAMD15",2.74408864426515,-0.242501305359772,0.888760297669434
,-0.27285344090603,0.78496587765079,0.950124036513985
"13456","AL357093.2",6.64671098846152,0.230904049673248,0.847049233356
593,0.272598144925116,0.785162138089732,0.950124036513985
"13457","ERCC4",110.540592958042,-0.0537109084226123,0.197039843024866
,-0.272589074362155,0.785169111395098,0.950124036513985
"13458","ZNF768",71.6568930452602,0.0569444211787681,0.208661661895891
,0.272903132570562,0.784927678450868,0.950124036513985
"13459","AC027601.6",5.99741179085615,-0.167640630097141,0.61281935119
013,-0.273556358446536,0.784425575912532,0.950124036513985
"13460","CRLF1",379.606091192519,-0.11847623966644,0.43343482935693,-0
.273342684163658,0.784589806939598,0.950124036513985
"13461","SIPA1L3",34.5942001924452,-0.0733079499745423,0.2678784802537
22,-0.273661213491686,0.784344987357925,0.950124036513985
"13462","ATP6V1FNB",3.91441243874327,-0.203574487976746,0.747379045175
429,-0.272384527357148,0.78532636847532,0.950204848822068
"13463","PRX",17.1352287803605,0.115457880277895,0.423931286892948,0.2
72350458311539,0.785352561832617,0.950204848822068
"13464","CDC123",329.995152505555,-0.0696180098946514,0.25575738518177
1,-0.272203322086566,0.785465687596235,0.950264945982155
"13465","MARK2",127.252237515729,0.0606256910741641,0.222778734505068,
0.272134102964773,0.785518908321549,0.950264945982155
"13466","MYL6",7619.61322433025,-0.0737912664314071,0.271680969957107,
-0.271609993305962,0.785921914690813,0.950681870518242
"13467","ACSF3",96.5648331701386,0.0636134662154591,0.234335714708505,
0.271462957725369,0.786034985835555,0.950748042197621
"13468","AL121987.1",3.8697177792069,0.217660549728063,0.8027066114131
97,0.27115828701705,0.786269293595669,0.950819987385256
"13469","CA3",17.2260218999058,-0.185218111830165,0.683016355767319,-0
.271176685984462,0.786255143274963,0.950819987385256
"13470","UTP20",97.0339527902767,-0.0696016650446943,0.256683143442749
,-0.271157911311065,0.786269582545239,0.950819987385256

"13471","IFT46",70.2603443462961,-0.0667633858848029,0.246345083528272
,-0.271015702560756,0.786378955174468,0.950881656954711
"13472","PREP",77.5316326470471,0.0597051305972329,0.220425337094104,0
.270863283614912,0.786496185136603,0.950952817672961
"13473","TMED3",561.956173649659,-0.0743002644906835,0.274481095303861
,-0.27069355872553,0.786626731311917,0.951040067270824
"13474","GLT8D1",751.329629391423,-0.0531499306357074,0.19648134084334
9,-0.270508794410574,0.786768852081666,0.951141296686823
"13475","GRIP2",8.27791793263419,-0.202948642009079,0.750608730460449,
-0.270378738979739,0.786868895020468,0.951161996364913
"13476","TNIP1",207.885293109265,-0.0468757001508817,0.173408469421014
,-0.270319554214354,0.786914423064297,0.951161996364913
"13477","ZNF250",29.1570289194064,-0.0794405864332666,0.29394263633653
9,-0.27025880771619,0.786961153232853,0.951161996364913
"13478","MORN1",22.1097839815235,0.0958259775581705,0.355432088374809,
0.269604182324474,0.787464782429209,0.951239336217002
"13479","PPIH",171.521333611302,-0.0661972350426268,0.246845550410489,
-0.268172689086535,0.788566396228016,0.951239336217002
"13480","DTWD2",24.2311004428233,-0.0902591503350897,0.336398962327572
,-0.268309835769347,0.788460835860869,0.951239336217002
"13481","MRPL14",406.73906664725,-0.0663986669297811,0.24683938182085,
-0.26899543516914,0.787933195455237,0.951239336217002
"13482","WIPI2",491.439279447099,-0.0381879397664837,0.142215148648611
,-0.268522306725843,0.788297306859066,0.951239336217002
"13483","AC027373.1",7.25493300495015,-0.179376141632333,0.66788275842
5715,-0.268574295966473,0.788257294581811,0.951239336217002
"13484","AC104248.1",23.8934174524271,0.14938000166142,0.5559084482689
13,0.268713314443387,0.788150305076194,0.951239336217002
"13485","PARD3-
AS1",3.55130855926347,-0.200925360529766,0.748119134889077,-0.26857401
6034969,0.788257510022911,0.951239336217002
"13486","TCTN3",181.199968777678,0.0536586822823198,0.199367661561408,
0.269144363043012,0.787818592744034,0.951239336217002
"13487","SLK",502.125036819871,-0.0566691685248024,0.211259494198209,-
0.268244363359281,0.788511228805593,0.951239336217002
"13488","FNBP4",451.08257900813,0.0697122684975742,0.259997593845232,0
.268126590967883,0.78860187833964,0.951239336217002
"13489","ATG2A",81.0606190345236,0.0603406270104849,0.2244682166839,0.
268815905885944,0.788071352611195,0.951239336217002
"13490","SMAD9",198.301318042629,-0.09306318927122,0.346134021220601,-
0.26886461187214,0.788033870152807,0.951239336217002
"13491","PPP1R13B",8.45883166377923,0.159943272114877,0.59524600058465
9,0.26870112853808,0.788159683266779,0.951239336217002
"13492","AC020659.1",3.70383219480886,0.202289397220452,0.750376445839
975,0.269583884651401,0.787480399653532,0.951239336217002
"13493","MAN2C1",165.111568119363,0.0546015503920253,0.202431221969591
,0.269728897848709,0.787368826984805,0.951239336217002
"13494","SNN",49.1458224789693,0.0916322206879595,0.341453528242355,0.
268359273250565,0.788422785301074,0.951239336217002
"13495","PIEZO1",103.789304308783,0.0582878667246987,0.216370096066258

,0.269389660514129,0.787629841887683,0.951239336217002
"13496","JSRP1",2.60371466817542,-0.253045662988632,0.942768125741092,
-0.268407104652289,0.788385971373404,0.951239336217002
"13497","ERVK9-11",3.87849736964507,0.196581583781467,0.73210734523442
2,0.268514699464628,0.788303161652805,0.951239336217002
"13498","AC008735.2",6.36940680409729,0.255400736963198,0.950277618319
542,0.268764340061849,0.788111036437684,0.951239336217002
"13499","NCOA5",64.3908069453785,0.0574254733665008,0.213772857947565,
0.268628458812981,0.788215610034923,0.951239336217002
"13500","LSS",143.329902122993,0.0658475675003432,0.245475881914327,0.
268244550083029,0.788511085086401,0.951239336217002
"13501","JADE3",14.7796156897611,0.120689325690219,0.447361393872003,0
.269780377438537,0.787329219810147,0.951239336217002
"13502","DLG3",37.550746118072,-0.0769305183673094,0.285550321045118,-
0.269411423127604,0.787613096651,0.951239336217002
"13503","TIMM8A",50.7351895224355,-0.0865385823792808,0.32155533784750
9,-0.269125006471887,0.787833487718366,0.951239336217002
"13504","PDZD4",4.3623934373195,0.193485770325268,0.717213038117336,0.
269774474308446,0.787333761510422,0.951239336217002
"13505","FAM27C",6.52774799857777,0.15887026928936,0.592711781651137,0
.268039668195542,0.788668784725412,0.95124959899239
"13506","AL050343.2",7.98312727820101,0.168329718601144,0.635079268691
17,0.265053083763943,0.790968568760433,0.951263122893875
"13507","DRAM2",403.919186608713,0.0705059336074387,0.264783823703241,
0.266277345123843,0.79002561942344,0.951263122893875
"13508","USF1",37.9708892256216,-0.0797206153649965,0.299086377758401,
-0.266547129168799,0.789817867911801,0.951263122893875
"13509","NUCKS1",3870.45290937586,-0.0414061550584323,0.15581723139401
8,-0.265735404794401,0.790442994316617,0.951263122893875
"13510","AL445524.1",27.8986047952423,-0.0900341561279351,0.3386544950
53501,-0.26585844110444,0.790348232726462,0.951263122893875
"13511","ZNF496",74.3429060316931,0.0706730229653228,0.264475795365968
,0.267219247294555,0.7893003576165,0.951263122893875
"13512","AC079779.2",6.90387948592348,0.239180320822026,0.899359063557
177,0.265945305400061,0.790281332401511,0.951263122893875
"13513","C2orf74",240.331703604542,-0.0871186507370422,0.3297481989474
51,-0.264197502867712,0.791627735152967,0.951263122893875
"13514","WDR92",110.635580133547,-0.062224344847377,0.232498610694624,
-0.267633189985405,0.788981680700877,0.951263122893875
"13515","LINC01412",4.40512314102525,0.213369639259261,0.7992430063927
14,0.266964662252447,0.789496369350059,0.951263122893875
"13516","AC010894.2",3.47743480394751,-0.210455440753459,0.78953287184
5814,-0.266556907581877,0.789810338169132,0.951263122893875
"13517","ATRIP",34.691666253831,-0.0801111617438409,0.300508494074853,
-0.266585348911589,0.789788437396353,0.951263122893875
"13518","MFN1",168.721336496018,0.0428334692314365,0.161404500531476,0
.26537964610896,0.790717014261125,0.951263122893875
"13519","CXCL10",18.2112014623394,-0.199169553042646,0.751772612680876
,-0.264933238698857,0.791060892170849,0.951263122893875
"13520","AC104118.1",3.28413096872748,0.228907873066836,0.865928604433

295,0.264349591750285,0.79151055019824,0.951263122893875
"13521","CD109",450.994278863015,-0.0683913973677223,0.258693361921703
,-0.264372447981181,0.791492939808747,0.951263122893875
"13522","COX19",154.759350039562,-0.0564545614684237,0.212951347361462
,-0.265105443886196,0.790928233726685,0.951263122893875
"13523","HOXA4",25.5331670238682,-0.107570893142297,0.407339017173496,
-0.264081977436695,0.791716751002042,0.951263122893875
"13524","ZNF777",52.4678454208056,0.0621627672782274,0.233367895237331
,0.266372404031879,0.789952416089789,0.951263122893875
"13525","RRM2B",102.914623169087,-0.0593886654120814,0.223890732086955
,-0.265257363976174,0.790811206956135,0.951263122893875
"13526","SLC25A32",109.637535473609,-0.0611810111130232,0.230121465623
464,-0.26586399033774,0.790343958824029,0.951263122893875
"13527","AKNA",105.864580450133,-0.0776691297823742,0.292268730442212,
-0.265745602223195,0.790435140220066,0.951263122893875
"13528","ZNF25",126.838953221986,0.0569428340591189,0.213785055461352,
0.26635554078481,0.789965402069459,0.951263122893875
"13529","AL162274.1",2.5768798702635,0.262918769518134,0.9843279287351
26,0.267104855854276,0.789388428952227,0.951263122893875
"13530","MTG1",197.967358017875,-0.0594362072279707,0.223805187247989,
-0.265571178035797,0.790569485291132,0.951263122893875
"13531","PITPNM1",21.5271748358331,-0.0958380005175262,0.3584640009942
5,-0.267357392239405,0.789194001762731,0.951263122893875
"13532","NECAP1",166.555940969484,-0.0484157886640115,0.18119802946145
8,-0.267198207441377,0.789316556248674,0.951263122893875
"13533","AC009318.4",2.50602351138005,0.297168698330887,1.117374130702
35,0.2659527280662,0.790275615754091,0.951263122893875
"13534","KIF21A",44.3088427792572,-0.0979671202912738,0.36953205322795
,-0.265111292607794,0.790923728264268,0.951263122893875
"13535","AC073655.2",3.05328465132045,0.243680841413351,0.922482666340
218,0.264157637107817,0.791658452623349,0.951263122893875
"13536","PWP1",347.346416346057,-0.0354371214693436,0.132656030682322,
-0.26713539736619,0.789364914414164,0.951263122893875
"13537","P2RX7",44.4603791779611,0.0964768152590711,0.362261068799176,
0.266318474626248,0.789993946028791,0.951263122893875
"13538","TTLL5",57.7843868831671,0.0773429073138845,0.289968736400611,
0.266728435189062,0.789678258708283,0.951263122893875
"13539","YY1",1134.95146692064,0.0334336309624892,0.124859007821103,0.
267771076720333,0.788875535389511,0.951263122893875
"13540","COMMD4",402.964205868557,-0.0704861761368734,0.26639181275378
,-0.264595880061907,0.791320794284997,0.951263122893875
"13541","ALDH1A3",267.165211524933,0.181482811931622,0.679062126361543
,0.267255093291712,0.789272759909206,0.951263122893875
"13542","AC040168.1",3.04532634084729,-0.232988443019033,0.87004172465
7811,-0.267789965028019,0.788860995461321,0.951263122893875
"13543","CTCF",312.578121990562,0.0504725222054267,0.1905326360229,0.2
64902240681541,0.791084772168445,0.951263122893875
"13544","AC009060.1",4.46376496118599,-0.170231227902976,0.64367182389
4814,-0.264468975623197,0.791418567855099,0.951263122893875
"13545","LINC00324",27.7941669984597,0.0990230411391666,0.373528007815

808,0.265102051431699,0.790930847049049,0.951263122893875
"13546","SUPT6H",251.387837063114,-0.0490994572375697,0.1857009213195,
-0.264400719655524,0.791471157047004,0.951263122893875
"13547","LRRC37A",5.10105964679251,0.194280434891108,0.735262559136993
,0.264232732208128,0.791600590418017,0.951263122893875
"13548","ABCA5",133.930956053605,0.0755075996412045,0.282085227092149,
0.267676547331344,0.788948303761347,0.951263122893875
"13549","MAP2K6",75.0463722689464,0.0745156015014537,0.280820232050706
,0.265349832372472,0.790739979200277,0.951263122893875
"13550","MRPS7",367.348803563648,-0.0586591196399896,0.222084663268863
,-0.264129538602919,0.79168010335175,0.951263122893875
"13551","ATP13A1",87.5573708066903,-0.0579976648739732,0.2190758730974
88,-0.264737800899529,0.791211455213782,0.951263122893875
"13552","LENG9",78.3341340413791,-0.0666275368596602,0.250764922773118
,-0.265697196094456,0.790472422984817,0.951263122893875
"13553","ZNF749",54.9304252531761,0.129409298878321,0.48658269872983,0
.265955405352738,0.790273553815383,0.951263122893875
"13554","CDK5RAP1",89.8071031992125,-0.0572361376967399,0.214426082232
284,-0.266927125193366,0.789525271248396,0.951263122893875
"13555","NELFCD",224.739364982335,-0.0485935869589752,0.18385725874881
,-0.264300617172613,0.791548284747746,0.951263122893875
"13556","MAPK8IP2",6.27514581074283,-0.1531079891843,0.579525683259923
,-0.264195347345166,0.791629396023607,0.951263122893875
"13557","SMC1A",282.453863794604,-0.0644542510396751,0.244000767488472
,-0.264155935668195,0.791659763628288,0.951263122893875
"13558","KIAA1143",264.7376723258,0.0447828562063971,0.170051665711562
,0.263348530101179,0.792281957730376,0.951280775563101
"13559","HSD17B8",61.3921391451617,0.0936201734871088,0.35586502477762
4,0.263077759736605,0.792490645490286,0.951280775563101
"13560","CFAP69",64.8968193688839,-0.113280326014855,0.429738439060727
,-0.263602963380353,0.792085874826736,0.951280775563101
"13561","RBM28",199.116897696898,0.0666675292232744,0.253129472277992,
0.263373239881205,0.792262914183697,0.951280775563101
"13562","STKLD1",6.41686152396509,0.200995371631458,0.763644908161256,
0.263205279683492,0.792392361640789,0.951280775563101
"13563","SPSB2",70.2723980894063,-0.078726029135455,0.298989895023052,
-0.263306654993759,0.792314230682769,0.951280775563101
"13564","NEK3",64.3989898045366,0.0869201328452131,0.330188519669852,0
.263243958124658,0.792362551536197,0.951280775563101
"13565","AC002310.1",5.55690775653429,0.1706001167324,0.64711553857633
2,0.263631618408861,0.792063792213839,0.951280775563101
"13566","AC027575.2",6.16809155673825,0.164272450536331,0.623723969441
266,0.263373637353534,0.792262607857302,0.951280775563101
"13567","CEBPA",259.508813895833,-0.0540723543294512,0.204875291586954
,-0.263928138481754,0.791835292799462,0.951280775563101
"13568","GPI",342.820348270179,0.0464372124201079,0.176041429307858,0.
263785704323608,0.791945050833671,0.951280775563101
"13569","SYCP2",4.91345691339617,-0.22403359459567,0.851032084446571,-
0.263249293052635,0.79235843984413,0.951280775563101
"13570","UQCR10",1489.81017290883,0.0729166484477363,0.277128903061197

,0.263114556591863,0.792462284595854,0.951280775563101
"13571","IPO13",42.5938639140615,0.0769300027348013,0.293247805478037,
0.262337863396434,0.79306097314273,0.951322852359558
"13572","SRBD1",122.59946248978,0.0591993839477187,0.225308376156325,0
.262748260662287,0.79274461661092,0.951322852359558
"13573","HK2",6.78314997921175,0.190525012719338,0.725328215283488,0.2
62674205559304,0.792801699790909,0.951322852359558
"13574","ZBTB20-
AS4",4.52044599968746,0.184804301437152,0.708164771889594,0.2609622912
25019,0.794121587117224,0.951322852359558
"13575","NDUFS4",1303.91669205883,-0.0676229973333485,0.25816108832807
2,-0.261941091786121,0.793366858662835,0.951322852359558
"13576","AC034236.2",18.1950252341539,-0.117134818963298,0.44907243510
5731,-0.260837249865312,0.794218017262382,0.951322852359558
"13577","DUSP1",10863.541791843,-0.0881769030351658,0.336798426175044,
-0.261809130275857,0.793468599589849,0.951322852359558
"13578","NEDD9",57.605779725951,0.0872518496690883,0.334049095834475,0
.261194688915795,0.793942373632949,0.951322852359558
"13579","PSMB8",760.164080893206,0.0719546690036974,0.275727871707467,
0.260962624337222,0.794121330229961,0.951322852359558
"13580","SNHG26",69.4574261663401,-0.109414956871489,0.418238553526843
,-0.261608969208686,0.793622928361977,0.951322852359558
"13581","AC016065.1",27.7333619058819,-0.0886989917550094,0.3398540649
44128,-0.260991410444337,0.794099131234808,0.951322852359558
"13582","MFSD3",32.1986284724837,-0.082384567216275,0.315940499819098,
-0.260759754648255,0.794277782067038,0.951322852359558
"13583","VSIG10L2",12.6235976367151,-0.192451536640855,0.7339445011355
95,-0.262215380513219,0.793155396212356,0.951322852359558
"13584","SOX5",140.808995260432,0.0697587608691649,0.267239117291957,0
.261034992092695,0.794065522672867,0.951322852359558
"13585","ATXN7L3B",510.544423565606,-0.0354889211551555,0.135624801370
441,-0.261669847966983,0.793575988588032,0.951322852359558
"13586","ARHGEF40",207.666547745064,-0.0699060293820133,0.266387907405
62,-0.262421932222208,0.792996165544087,0.951322852359558
"13587","IFI27L2",1570.72615942921,0.0690000045987751,0.26379510383893
2,0.261566661377101,0.79365554970498,0.951322852359558
"13588","AC023355.1",4.70018971548657,-0.205601471298104,0.78533628914
6304,-0.261800548554304,0.793475216128038,0.951322852359558
"13589","AC005632.6",2.27879980517065,-0.35261118769091,1.351072375191
76,-0.260986157489057,0.794103182146251,0.951322852359558
"13590","MVD",80.5312932002512,-0.0653908086210362,0.249737649732985,-
0.261838007568947,0.793446335202173,0.951322852359558
"13591","NCOR1",722.228211453713,-0.0327288731783834,0.12533205764958,
-0.261137284364159,0.793986640142892,0.951322852359558
"13592","KPNB1",738.922264673904,0.0545561373831823,0.208879132839365,
0.26118519663301,0.793949693394002,0.951322852359558
"13593","TWSG1",295.707337360905,-0.0377556046450731,0.144758959783893
,-0.260817048571208,0.794233596512581,0.951322852359558
"13594","PSTPIP2",54.3592951451691,-0.0928326560626841,0.3532075433826
18,-0.262827501286182,0.792683537556013,0.951322852359558

"13595","HSH2D",2.85486603930838,-0.234539458601971,0.893424232631785,
-0.262517458152083,0.792922527543544,0.951322852359558
"13596","SIRPA",276.252349957023,0.0477370045757312,0.181957399835368,
0.262352641986107,0.793049580410582,0.951322852359558
"13597","PRPF6",439.293755430224,-0.0492865076235044,0.188846447192858
,-0.260987211335624,0.794102369452971,0.951322852359558
"13598","SH3BGR",19.2111209800807,0.116360439004144,0.444996656262676,
0.261486097404421,0.793717669343801,0.951322852359558
"13599","AL022328.3",2.55503128940809,0.244783382781909,0.932825035308
849,0.262410820375189,0.79300473144497,0.951322852359558
"13600","ZDHHC9",274.690952175404,-0.0761276970901325,0.29165828354267
6,-0.261016749345963,0.794079590760678,0.951322852359558
"13601","LIN7C",207.693888621652,-0.0572211862102082,0.219558115702616
,-0.260619772706158,0.794385740092605,0.951382201335817
"13602","RB1-
DT",3.81552479745305,0.190199583931945,0.73003954226876,0.260533262815
953,0.794452460931497,0.951392158220347
"13603","AGAP1",178.765509796561,-0.0871637406950036,0.335580820767041
,-0.259739935362732,0.795064385587531,0.951822815143627
"13604","ULK4",27.0326229357512,-0.0881674461872347,0.339540004906732,
-0.259667329072029,0.795120395978454,0.951822815143627
"13605","TPD52",3.16110629092076,-0.234558237605391,0.903706020628845,
-0.259551482729055,0.795209765062694,0.951822815143627
"13606","RPS27L",2003.09666915276,-0.0657300653126775,0.25310156843409
5,-0.25969837215684,0.795096448401972,0.951822815143627
"13607","BCAR1",77.5771516430667,0.0612608529644476,0.235941118601933,
0.259644666124533,0.795137878984933,0.951822815143627
"13608","DSC2",3.7637260731543,-0.253937860671789,0.978427281435382,-0
.25953677446448,0.795221111872406,0.951822815143627
"13609","SAT1",4565.2381465937,0.0880646910788655,0.339194045288152,0.
259629236722162,0.795149781835419,0.951822815143627
"13610","NOL3",62.5493102060234,-0.0857504524011305,0.33066459487938,-
0.259327589736091,0.795382493811262,0.951880940754452
"13611","GRIPAP1",104.227376558675,0.0581480134257484,0.22423064154815
,0.259322334469002,0.795386548260105,0.951880940754452
"13612","SGCB",548.098612637007,-0.0428913621030199,0.165449999566588,
-0.259240629890468,0.795449584213013,0.951886444111502
"13613","RMI1",22.9029266687699,0.0927205128727749,0.357918706315721,0
.259054671456557,0.7955930581032,0.951900877792884
"13614","AL138995.1",9.74089280008252,-0.129060534384847,0.49823800340
8722,-0.25903390247607,0.795609082577975,0.951900877792884
"13615","CHCHD10",314.912088717536,-0.0849479575356663,0.3279872152058
43,-0.258997770636741,0.795636960596115,0.951900877792884
"13616","AKT3",370.381390003833,-0.06699290525164,0.258921198152679,-0
.258738588148105,0.795836944059592,0.952070210178224
"13617","NECAP2",258.106688773054,0.0503304168602553,0.19657444963541,
0.256037429857257,0.797921935494998,0.952109956383771
"13618","UROD",453.55874186014,0.0629800224001739,0.248522581115149,0.
253417706019209,0.799945446694248,0.952109956383771
"13619","GBP2",696.52302926206,0.0429900597822363,0.168709707302753,0.

254816752808952,0.798864635431693,0.952109956383771
"13620","HENMT1",44.0131649512987,-0.0700295518644248,0.274626827108792,-0.254998947486958,0.798723912150857,0.952109956383771
"13621","HSD11B1-AS1",18.9186576218126,0.137016675197941,0.534526202130878,0.256332944300441,0.797693760939193,0.952109956383771
"13622","AL353593.1",12.3405960563415,-0.118571578813023,0.460774733900494,-0.257330903995767,0.796923337580672,0.952109956383771
"13623","HNRNPU",1981.90686099036,0.0593338255940148,0.232783144757034,0.254888839378573,0.798808956522459,0.952109956383771
"13624","AC009309.1",3.90078951006937,-0.257040859044231,0.998490445490359,-0.257429462850792,0.796847261016975,0.952109956383771
"13625","RNF103",244.759087159125,0.0478025202199356,0.1863420720344,0.256531011478239,0.797540837680079,0.952109956383771
"13626","CCDC148",4.32185105322141,0.190741384986856,0.746338633808304,0.255569491309286,0.798283278591624,0.952109956383771
"13627","HOXD4",13.1955614039511,0.139614477075663,0.541166499217019,0.257988026379429,0.79641614807703,0.952109956383771
"13628","DUSP19",19.8638604674568,0.0947628898550217,0.371305742620416,0.255215255186336,0.798556849189227,0.952109956383771
"13629","CRELD1",151.192112566242,-0.101571698447165,0.400709357407554,-0.253479726813214,0.799897525386807,0.952109956383771
"13630","AC116036.2",6.08432050320888,0.172679877066422,0.681460873581985,0.253396612719318,0.799961744924077,0.952109956383771
"13631","FYTTD1",293.976508648412,0.0415476820426204,0.161111852929154,0.257880977018434,0.79649876657395,0.952109956383771
"13632","MCUB",581.581410688925,-0.0501992210023464,0.197504605334512,-0.254167344185845,0.799366277908169,0.952109956383771
"13633","PCDHB4",101.150646909712,0.0570290675550872,0.223623948830019,0.255022182791504,0.798705966167475,0.952109956383771
"13634","SH3PXD2B",285.397556440855,-0.0844888109633246,0.333079518748343,-0.253659580393353,0.799758563050072,0.952109956383771
"13635","ZNF165",5.65071558968946,-0.167110295155841,0.657612596573531,-0.254116627367791,0.799405458132786,0.952109956383771
"13636","HLA-F",195.718651678852,-0.108318859553947,0.419528221836798,-0.258192068890385,0.796258678580203,0.952109956383771
"13637","ADAT2",58.3603486670362,-0.0717119151344838,0.280956008300425,-0.255242504220813,0.798535804339845,0.952109956383771
"13638","LINC00574",4.24405719503774,-0.21291767588,0.828932491315005,-0.256857679136489,0.797288641830081,0.952109956383771
"13639","NUP42",170.973675017222,-0.0570612671329658,0.222542615867163,-0.256406023226698,0.797637337422755,0.952109956383771
"13640","YKT6",108.050659316475,-0.0616528931207522,0.242489311772417,-0.254249940626724,0.799302470824013,0.952109956383771
"13641","AGFG2",11.2101423941835,0.1248783682155,0.488920212317711,0.255416661183872,0.798401303690429,0.952109956383771
"13642","MSC-AS1",112.532517736753,0.0631361036565177,0.248995875214915,0.253562849593485,0.799833300491017,0.952109956383771

"13643","YWHAZ",1485.62404499748,-0.0398820508090785,0.155590670393803
,-0.256326749593252,0.797698543859967,0.952109956383771
"13644","C8orf33",282.741524110418,0.0630766946390428,0.24726864262599
3,0.255093787749099,0.798650662143567,0.952109956383771
"13645","FAM219A",59.8305170621899,-0.0550101909374968,0.2158496773826
01,-0.254854172610086,0.798835732642649,0.952109956383771
"13646","FGD3",5.78087137395821,-0.180170568281373,0.705140688862861,-
0.2555101005048,0.798329143383506,0.952109956383771
"13647","AL669970.3",7.93290291202878,-0.181713767935099,0.71475005527
0786,-0.254234003334572,0.799314782535202,0.952109956383771
"13648","KIAA1217",146.203792199504,-0.109322522829321,0.4246382429464
38,-0.257448603947596,0.796832486425575,0.952109956383771
"13649","PARD3",198.716346019371,0.0592707257097752,0.230868772553363,
0.256729071906316,0.797387927401768,0.952109956383771
"13650","ZNF488",3.6554331838979,0.205480098624805,0.807022600767696,0
.254615048487288,0.799020435165001,0.952109956383771
"13651","OAT",576.50272503426,0.045972496894852,0.178929064819664,0.25
693140989244,0.797231722712599,0.952109956383771
"13652","TSPAN32",9.19488836910325,0.142889354817694,0.55862801636218,
0.255786230966714,0.798115906411014,0.952109956383771
"13653","UBQLNL",3.49356179871156,-0.204949436321535,0.798826248495251
,-0.256563222237124,0.797515969204526,0.952109956383771
"13654","BTBD10",127.483823188001,0.0458493126845699,0.179976516261866
,0.254751640029853,0.79891492873752,0.952109956383771
"13655","CLP1",41.3455583429908,-0.0786624583056509,0.306094725128682,
-0.256987304412322,0.797188573639661,0.952109956383771
"13656","NFRKB",103.327056581982,0.0513555558929399,0.202458556472672,
0.253659597241433,0.799758550032844,0.952109956383771
"13657","WBP11",336.360033797769,0.0355690721041045,0.140247378840903,
0.253616662201254,0.799791722849887,0.952109956383771
"13658","GRASP",186.211841976991,-0.0600227569964575,0.236187704664664
,-0.254131590303048,0.799393898775352,0.952109956383771
"13659","TBX5-
AS1",9.96730672419987,-0.186344250988602,0.730975968439001,-0.25492527
666339,0.798780813135605,0.952109956383771
"13660","PAN3",146.29465524264,-0.0515090691145728,0.201570876826593,-
0.255538249996724,0.798307404736363,0.952109956383771
"13661","VWA8",53.6658918838549,-0.069501735624559,0.26985320867045,-0
.257553860363528,0.796751242615524,0.952109956383771
"13662","EDNRB",40.963683084412,-0.150662898287534,0.589229743878796,-
0.255694658751858,0.798186619801516,0.952109956383771
"13663","AL355075.4",77.6817445659406,-0.19432890997559,0.764614219779
378,-0.254152885139466,0.799377447892733,0.952109956383771
"13664","DHRS4-
AS1",203.852335014053,0.039010938221299,0.150927649852913,0.2584744296
9739,0.796040780741967,0.952109956383771
"13665","BAZ1A",212.510499569533,-0.0635889586654645,0.249489952520501
,-0.254875829760075,0.798819004939497,0.952109956383771
"13666","AL109628.2",14.8917162278709,0.146672008629452,0.570301282573
802,0.257183375018048,0.79703721728251,0.952109956383771

"13667","POLR2M",241.201251628557,0.0428724214132272,0.165889913594734
,0.258438988147066,0.796068130114375,0.952109956383771
"13668","OAZ2",376.700012299788,0.0460113464618485,0.18035215186353,0.
255119475905475,0.798630822165531,0.952109956383771
"13669","AC106028.3",2.53437485166598,0.256776114759204,1.009449052369
42,0.254372535351327,0.799207766890384,0.952109956383771
"13670","BRICD5",3.73247713369085,-0.187793483394303,0.737683072945216
,-0.254572038157976,0.799053658085511,0.952109956383771
"13671","AC007216.3",7.48294000364349,-0.162132327843459,0.63083226458
1457,-0.257013372565892,0.797168449934932,0.952109956383771
"13672","ITGAL",11.5708727682342,-0.17623703165857,0.69304735653982,-0
.254292913746139,0.799269273925334,0.952109956383771
"13673","ZNF720",188.596276270194,0.044644836523035,0.175461292006583,
0.254442652350696,0.799153603116763,0.952109956383771
"13674","ZFP1",59.376081127247,-0.0749355729891939,0.290483460093415,-
0.257968467344392,0.796431243167995,0.952109956383771
"13675","SMCR5",15.9571342752702,-0.133318629686032,0.522540079211886,
-0.255135701527638,0.798618290543505,0.952109956383771
"13676","SPAG9",780.45516159234,0.0419227029950979,0.164429160993411,0
.254959051921319,0.798754726075389,0.952109956383771
"13677","AC002546.1",10.657238988848,0.202389415690652,0.7904484699209
91,0.256043781969598,0.797917030678599,0.952109956383771
"13678","IMPACT",163.042640870168,0.0556327341206831,0.217877766203029
,0.255339198166929,0.798461127296019,0.952109956383771
"13679","LIN37",95.6039371645252,0.0673584456013416,0.265071410594766,
0.25411433639789,0.79940722798593,0.952109956383771
"13680","COX7A1",1095.97187367483,-0.0763009202730675,0.29607149530759
6,-0.257711132217563,0.796629853980642,0.952109956383771
"13681","AC005498.3",7.72947540779865,0.151982031980935,0.587693544375
347,0.25860762541211,0.795937999126309,0.952109956383771
"13682","RPN2",1120.74821938065,0.0479284246606805,0.189047118456478,0
.253526343337068,0.799861506918325,0.952109956383771
"13683","LZTR1",127.336758302372,0.0635551739072766,0.246097276926295,
0.258252243588664,0.79621224043224,0.952109956383771
"13684","AL031846.2",20.2237375447633,-0.124079264560371,0.48882996208
2017,-0.253829090246216,0.79962759850916,0.952109956383771
"13685","Z83745.1",6.18311247226606,0.17923238440472,0.706471146232214
,0.253700926585057,0.799726618163288,0.952109956383771
"13686","TCEAL8",846.679987976984,0.0508783755272993,0.200077216648349
,0.254293699100793,0.799268667239243,0.952109956383771
"13687","MIER1",675.214861893205,0.0376801360087365,0.149842452982855,
0.25146502382104,0.801454600486026,0.952286380284257
"13688","KLF11",245.808056802146,0.0532042134160547,0.210828400705723,
0.252357904523109,0.800764434655371,0.952286380284257
"13689","KIAA1841",46.1974750928024,0.0682119386494182,0.2698545581973
15,0.25277297187451,0.800443654892737,0.952286380284257
"13690","TTL",97.1326723511336,-0.0605690823071668,0.239823806571462,-
0.252556587992938,0.800610880352405,0.952286380284257
"13691","APOD",48807.5638876921,-0.0903219698917172,0.358509791362198,
-0.251937247093109,0.80108956892601,0.952286380284257

"13692","FAM218A",2.59947678903251,-0.288972427102359,1.1473580249312,-0.25185898457431,0.801150063345322,0.952286380284257
"13693","RBM27",411.263768890571,0.0411708246117258,0.163239180138728,0.25221166007289,0.800877465911006,0.952286380284257
"13694","EEF1A1",62799.611529891,-0.045982759843724,0.182032411593865,-0.252607540828041,0.800571502237584,0.952286380284257
"13695","POP7",242.702198820401,-0.0634229876199428,0.252021354656636,-0.251657196694117,0.801306044410883,0.952286380284257
"13696","CA13",9.00223456170301,-0.17521629688126,0.69654080617937,-0.251552091890132,0.801387293059117,0.952286380284257
"13697","PHF20L1",388.886605708601,0.0409212456338083,0.162551991754768,0.251742505225918,0.801240100357258,0.952286380284257
"13698","SAR1A",773.803482386168,0.0431185443348744,0.171006659050584,0.252145411028233,0.800928670680647,0.952286380284257
"13699","C10orf88",52.7706207174347,0.0626619554390005,0.247972739883381,0.252696951561973,0.80050240375232,0.952286380284257
"13700","TIMM8B",629.908726137975,-0.0690006004082817,0.274336888570238,-0.251517762587059,0.801413830936401,0.952286380284257
"13701","VCPKMT",40.232094305479,-0.0822510202161016,0.325818687765245,-0.252444145485492,0.800697781607517,0.952286380284257
"13702","HHIPL1",34.8705086092818,0.0799196811751278,0.316198983043472,0.252751227742374,0.800460458746354,0.952286380284257
"13703","EMC4",669.488915774095,-0.0567229583267642,0.225486699455116,-0.251557889950201,0.801382810957348,0.952286380284257
"13704","AC135050.6",26.1279124598512,0.0880749922107419,0.350156340276523,0.251530479617156,0.801404000154206,0.952286380284257
"13705","SMPD3",2.75867363319161,-0.235016728030424,0.929786182913199,-0.252764272420215,0.800450377815252,0.952286380284257
"13706","MAP1LC3B",1190.76610690722,0.0558095972117012,0.221537964093682,0.251918886408566,0.801103761039764,0.952286380284257
"13707","P4HB",2069.58275493027,0.0578066368360884,0.229048305239162,0.252377492056661,0.800749295912054,0.952286380284257
"13708","TMX3",331.183952605857,0.0425176469344631,0.168256899208013,0.252694820447744,0.80050405070381,0.952286380284257
"13709","PAXBP1",181.109136468354,-0.0735279627704108,0.291441692036979,-0.252290474490799,0.800816550311273,0.952286380284257
"13710","INPP5F",45.8829947429439,0.0627745518837357,0.249767772458513,0.251331672080163,0.801557690073086,0.952339402888439
"13711","AC233263.6",12.2377404606684,-0.142224661622118,0.566578390773614,-0.251023801715986,0.801795707124272,0.952388734038245
"13712","SP140",4.46475555156891,0.175224476521412,0.698173748974033,0.250975458156231,0.801833083590183,0.952388734038245
"13713","RCOR2",3.87051144427351,0.212954962126743,0.848272589890785,0.251045435941954,0.801778980929268,0.952388734038245
"13714","NPB",38.9927847450903,-0.121700165635051,0.484831076475622,-0.251015604279587,0.801802044879703,0.952388734038245
"13715","PPP1R15B",496.998369585674,-0.160340637219229,0.640175206110738,-0.25046367883153,0.802228789788243,0.952444444265475
"13716","AC026979.2",35.2051168042416,-0.0969533247290628,0.387148455695608,-0.250429320594504,0.802255357289447,0.952444444265475

"13717","SUPV3L1",78.6443100034438,0.0539230128016684,0.214973147524359,0.250836038931598,0.801940877072734,0.952444444265475
"13718","KLF5",83.1304119817392,-0.10266229559305,0.410140897045656,-0.250309823605865,0.802347760096436,0.952444444265475
"13719","MMP25-AS1",19.7082157188044,0.091459676689311,0.364832815018946,0.25068928266379,0.802054347342142,0.952444444265475
"13720","GSE1",210.297321911441,-0.0764729245925125,0.305419218973962,-0.250386746614764,0.802288277929493,0.952444444265475
"13721","UNC13D",8.83511485874056,0.137680383252781,0.549887196981019,0.25037932144751,0.802294019530116,0.952444444265475
"13722","GP1BB",70.405471172454,0.103091545537444,0.411808534345574,0.250338535847179,0.802325557680515,0.952444444265475
"13723","GOSR2",164.559447956611,-0.0533364359638778,0.213162818282532,-0.25021453738327,0.802421443552625,0.952462500475749
"13724","GTPBP2",79.3277620049322,0.0540235143529507,0.216051062497743,0.250049750870885,0.802548874722324,0.95254434715476
"13725","SMIM2-AS1",2.29683865900599,-0.240792567619644,0.963589686182953,-0.249891184051057,0.802671501094734,0.952551076885628
"13726","PMEPA1",110.207206225644,-0.0563627923598168,0.225537058177509,-0.249904795315085,0.802660974742685,0.952551076885628
"13727","UVRAG",225.980496613909,0.0477618327868734,0.191444456257445,0.249481409493758,0.802988419443434,0.952580173644607
"13728","KTN1-AS1",24.5353506253356,0.0937901449028033,0.37560119411279,0.249706727169347,0.802814155451611,0.952580173644607
"13729","NEURL4",25.7110186035182,0.0786731512668287,0.315115185094075,0.249664741619295,0.802846626948216,0.952580173644607
"13730","PDK2",179.395404237583,-0.0469200905645174,0.188000777509838,-0.24957391765075,0.802916871093508,0.952580173644607
"13731","HPR",2.27986345503846,-0.337639680756394,1.35321250422531,-0.249509725709849,0.802966518728636,0.952580173644607
"13732","EXO5",27.9611035681359,-0.0858995212646643,0.344913113973255,-0.249046840449578,0.80332454916951,0.952697456757996
"13733","FCHO2",275.564461630438,0.0481597903628254,0.193229031916146,0.249236824742386,0.803177595962141,0.952697456757996
"13734","FSCN1",196.198702851562,0.0875424374571666,0.352201609405316,0.248557743972209,0.803702898193295,0.952697456757996
"13735","NUDCD3",183.224981238125,0.0508788195655343,0.204291973077118,0.249049528472312,0.803322469930371,0.952697456757996
"13736","ARHGEF5",54.4037384031969,-0.0806588235790296,0.32446708617792,-0.248588615039988,0.803679015984927,0.952697456757996
"13737","NAP1L4",447.383041126546,0.0496935307744022,0.20002235908833,0.248439879425967,0.803794081229113,0.952697456757996
"13738","NUDT15",106.966459749081,0.0438619554978043,0.176597383574864,0.248372623704304,0.803846113176857,0.952697456757996
"13739","RNASEH2B",308.45564597059,-0.0494987714386676,0.19915069427772,-0.24854932903041,0.803709408119551,0.952697456757996
"13740","DHX38",193.621353158031,0.0555956227412377,0.223662253317991,

0.248569536953536,0.803693774987478,0.952697456757996
"13741","ARRDC2",74.7892338508229,0.0705832256254854,0.283486468779951
,0.248982697231569,0.803374165639471,0.952697456757996
"13742","MPST",443.765752967076,0.0534270557771728,0.215040089958505,0
.248451606337601,0.803785008869883,0.952697456757996
"13743","CHKB-
DT",20.0755749059009,-0.116717086479862,0.469931019987338,-0.248370678
920082,0.80384761775934,0.952697456757996
"13744","RENBP",60.0696874227521,0.0835887751321703,0.336304903269917,
0.248550569199053,0.803708448705476,0.952697456757996
"13745","ADRA2A",123.292991454488,-0.227814448604595,0.918090975517529
,-0.248139296300321,0.804026632128757,0.952840291796677
"13746","CRISPLD2",778.603479379706,0.0910887648740486,0.3673093009869
75,0.247989268524618,0.804142709986185,0.952908526332385
"13747","AC020741.1",3.23317619575427,-0.235196225972046,0.94890028969
6551,-0.247861897109611,0.804241261800682,0.952955984103536
"13748","FAM131A",55.4471876361324,-0.0573656755421589,0.2316076873243
03,-0.24768467836663,0.80437838744957,0.952979820580846
"13749","ATP6V0C",1459.98150797646,-0.0581469468122837,0.2347072261029
66,-0.247742465273628,0.804333673305761,0.952979820580846
"13750","STRIP1",75.8005050713245,0.0503186818982935,0.20324825403097,
0.247572517354202,0.804465176818423,0.953003778837894
"13751","AL136171.2",2.5172742407159,0.240906811088139,0.9742248148801
66,0.247280512063093,0.804691139673178,0.953003778837894
"13752","AC098829.1",4.8691459150617,0.23562223400832,0.95241484357711
4,0.247394541986937,0.804602897798095,0.953003778837894
"13753","LINC01503",79.0915549724602,0.0787617144304039,0.318471928722
123,0.247311324255288,0.804667295461906,0.953003778837894
"13754","AMER1",19.1559121431501,0.109930923063691,0.444308809868873,0
.247420084008989,0.804583132486186,0.953003778837894
"13755","AL353796.2",5.68354981052386,-0.181697534398881,0.73634575924
2225,-0.246755728702596,0.805097274500486,0.953138273445629
"13756","CHCHD1",400.534368147704,0.0611349603542417,0.247548868908692
,0.246961178306944,0.80493826882965,0.953138273445629
"13757","DYNLL2",258.0880075114,-0.0472518629488613,0.191396403738631,
-0.246879575717567,0.805001423373837,0.953138273445629
"13758","XRCC1",114.742148381657,-0.0494863389742248,0.200515080369323
,-0.246796095750392,0.805066032190189,0.953138273445629
"13759","FANCB",10.1187846733625,-0.11715120078521,0.474475040894353,-
0.246906982850748,0.804980212078977,0.953138273445629
"13760","INTS9",48.2040892356079,0.0664416753945283,0.269607368055716,
0.246438648445237,0.805342691419941,0.953359527655481
"13761","AL356488.3",22.7111141028388,-0.0999537280152207,0.4061325770
49684,-0.246111082103598,0.805596244602199,0.953521089109521
"13762","ZNF793",8.02341376010514,-0.173444832761357,0.704576966439476
,-0.246168752347734,0.805551603386159,0.953521089109521
"13763","LINC01473",45.3711103945893,-0.0708921138399533,0.28884958660
4361,-0.245429168424099,0.806124146376087,0.953528634351032
"13764","AC084198.2",14.8765824234864,-0.115547059754958,0.47061493687
9905,-0.245523570758325,0.806051059769993,0.953528634351032

"13765","RFC4",69.5751331867261,0.0601500503019752,0.244495583117284,0
.246016919958515,0.80566913472533,0.953528634351032
"13766","HCG11",278.167889258148,0.0405184524549675,0.164763831307869,
0.245918367722688,0.805745424989182,0.953528634351032
"13767","CNPY3",261.886720160583,0.0497332691533549,0.202534061945343,
0.245555086762523,0.806026660350203,0.953528634351032
"13768","NIPSNAP3A",96.5503558612063,-0.0596697250365961,0.24289550288
7141,-0.245660065037602,0.805945388433327,0.953528634351032
"13769","AL133415.1",3.4301587450104,0.298329153980013,1.2159493632475
,0.245346692055703,0.80618800124516,0.953528634351032
"13770","CD6",8.5678508153956,0.168933860058039,0.687587103071258,0.24
5690850371479,0.805921555489075,0.953528634351032
"13771","SMAD9-
IT1",7.00003087136523,0.166231357334138,0.676613723458711,0.2456813268
35047,0.805928928263428,0.953528634351032
"13772","TXN2",757.773355585095,-0.0572101871188305,0.233148360489643,
-0.245381039775195,0.806161408390442,0.953528634351032
"13773","LBH",118.859381347288,0.123917577855266,0.505334971626409,0.2
45218686243781,0.806287108487168,0.953576614401182
"13774","RAPGEF3",5.06857931025176,0.217578412683981,0.887783035021595
,0.245080615534278,0.806394011864679,0.953633807119482
"13775","APOL4",85.5211453400787,0.0761202701435982,0.310887569187924,
0.244848227101629,0.806573950362128,0.953777355894643
"13776","RIMS4",6.74135388778804,-0.261994129607041,1.07081453441589,-
0.244668073869537,0.806713450159415,0.953873068354145
"13777","TRAIP",18.5434541424807,0.0893149711895912,0.365221780647503,
0.244549958195933,0.806804915150733,0.953911973788945
"13778","TGOLN2",1125.13706623788,0.0346463397104591,0.141827477979631
,0.244285100489729,0.807010022070771,0.954085226412454
"13779","ROBO4",82.4480401223349,0.102501212562526,0.419923040984981,0
.244095233074368,0.807157064362149,0.954189812134048
"13780","ST7L",91.3721832149,0.0463539585436152,0.190948919774159,0.24
2755804004754,0.808194574807844,0.954224441341823
"13781","PCAT6",18.4270962722188,0.0923420922293746,0.380365184548871,
0.242772198877497,0.80818187343462,0.954224441341823
"13782","EEF1B2",5558.78763335892,0.0575732867002986,0.236665666561841
,0.243268436595363,0.80779745398157,0.954224441341823
"13783","EXOC3",217.330793545176,0.0400045774855533,0.164614170115843,
0.243020254315902,0.807989707043023,0.954224441341823
"13784","PPP2CA",261.139419393999,-0.0413214859115454,0.16949302510668
3,-0.243794609751857,0.807389895193069,0.954224441341823
"13785","RAD21",557.322725224755,0.044771205156158,0.184496755981561,0
.242666625317968,0.808263663864865,0.954224441341823
"13786","CUL2",186.307456477034,-0.0416688588643663,0.171546103224542,
-0.242901809374385,0.808081463865111,0.954224441341823
"13787","TRIM66",38.3579485249531,0.0906191367079829,0.372400244089077
,0.243338016412006,0.807743556435378,0.954224441341823
"13788","CRY2",206.652655428906,0.0538166090688254,0.221883085079493,0
.24254489272828,0.80835797569377,0.954224441341823
"13789","GRK2",140.762592798868,-0.0453981698986737,0.186329827233065,

-0.243644136705439,0.80750644201003,0.954224441341823
"13790","ZNF641",91.144313484113,-0.0792660749002768,0.32612360897165,
-0.243055310071609,0.807962550587795,0.954224441341823
"13791","SPATS2",217.053726246546,0.0397950314567026,0.163933741112826
,0.242750706392493,0.808198524020858,0.954224441341823
"13792","B2M",30662.8832027426,0.0682455567249955,0.279788591837913,0.
243918296584913,0.807294098468599,0.954224441341823
"13793","SAXO2",10.2958241719912,0.116482397775844,0.478610385176525,0
.24337624377474,0.807713945348081,0.954224441341823
"13794","AC009053.2",7.27304619184363,0.152211190365817,0.624817977215
621,0.243608852363878,0.80753377162732,0.954224441341823
"13795","ASPA",216.66738897188,0.0589989514763378,0.243209387341782,0.
242585009243195,0.808326895279257,0.954224441341823
"13796","SKA2",211.070304431699,-0.0627803387085443,0.258028069671259,
-0.24330817491496,0.807766671984796,0.954224441341823
"13797","ELP2",253.92206992902,0.0409591299171527,0.168348486511339,0.
243299662301353,0.807773265979004,0.954224441341823
"13798","ZNF407",86.0063291313255,0.0701677887035815,0.287753330080315
,0.243847008422099,0.807349311531236,0.954224441341823
"13799","AL021707.1",4.61777967654936,0.21871437403186,0.9003562308521
9,0.242919820552412,0.808067510811489,0.954224441341823
"13800","RHEX",2.89836588166702,-0.240966558214008,0.994877147929122,-
0.242207350641826,0.808619499614358,0.954325679555013
"13801","AC017104.1",2.50129293851366,-0.224993522080894,0.92868077163
8442,-0.242272187550459,0.808569263025988,0.954325679555013
"13802","WDR4",22.5113486375384,-0.0839796427540096,0.346521290508092,
-0.24235060024991,0.80850850876993,0.954325679555013
"13803","TADA2A",58.1113457387597,0.0657827616912995,0.271753133898855
,0.242068088590226,0.808727404562578,0.95438387980293
"13804","PRKACA",297.575459627331,0.0454233183906771,0.187737423274151
,0.241951325412334,0.808817879426065,0.954421503764936
"13805","LINC00910",38.0961295764083,0.1109853656623,0.459136099688583
,0.241726507102311,0.808992088853192,0.954523513980798
"13806","MOCS3",26.3002795482822,-0.0746641373375802,0.308927115861091
,-0.24168851973212,0.809021525816128,0.954523513980798
"13807","SNX8",78.0821896027922,0.0580388586025237,0.240260149202747,0
.241566730042887,0.809115904230968,0.954565724923462
"13808","LZIC",455.129790973486,-0.0373268530274228,0.154609105482817,
-0.241427262067506,0.809223985480502,0.954624094690896
"13809","RB1CC1",890.122310554455,0.0628991260074755,0.260991293580508
,0.241000859241586,0.809554450571506,0.954668243347517
"13810","CTSL",2082.14149039237,0.0780325461146101,0.323730685816504,0
.241041549452746,0.809522913914355,0.954668243347517
"13811","AL365203.2",6.22360130585908,0.168109370620846,0.697415866368
055,0.241046094199595,0.809519391560015,0.954668243347517
"13812","AP006621.3",66.5300805297711,-0.0562682719741388,0.2333048954
89286,-0.241179131094242,0.809416284538382,0.954668243347517
"13813","SLC39A11",51.785834983451,-0.0696132129369027,0.2886482601022
86,-0.241169695297088,0.809423597415353,0.954668243347517
"13814","ZNF223",24.6769607620539,0.087153247031632,0.361802266612571,

0.240886404188723,0.809643159799908,0.954703737511272
"13815","DNAJC8",1117.01697505556,-0.0482321625506706,0.20049696032615
9,-0.240563061266409,0.809893782315162,0.95476014888327
"13816","ZC3H8",121.227644481665,-0.0505881888406413,0.210388492344688
,-0.240451311176091,0.809980404142817,0.95476014888327
"13817","RAB14",876.48555839041,-0.0395731981186873,0.164582077035162,
-0.240446583440751,0.809984068845104,0.95476014888327
"13818","AP003071.4",8.97767809380068,-0.17311080360023,0.719058456746
125,-0.240746495609813,0.809751600292467,0.95476014888327
"13819","SETBP1",617.97958079329,-0.0648882010075872,0.269712021670355
,-0.24058327324725,0.809878115473819,0.95476014888327
"13820","VPS41",159.896401147777,-0.0523870679254701,0.218087337165667
,-0.24021141532704,0.810166364576674,0.954771292936685
"13821","MAP4K5",361.955859183505,0.0452103665063103,0.188130356371362
,0.240314042764405,0.810086809396179,0.954771292936685
"13822","MORF4L2-
AS1",6.41596091468628,-0.153486970850902,0.63897648272677,-0.240207542
844005,0.810169366503214,0.954771292936685
"13823","RARRES2",3376.75676757812,0.0619988020841221,0.25819604807741
2,0.240122970687505,0.810234927048084,0.954779478165828
"13824","GPHN",65.3021142925488,0.0572784029315459,0.238641660188487,0
.240018456485366,0.810315948555329,0.954805880064941
"13825","PASK",7.2484015139615,-0.132965343443523,0.555218946869593,-0
.239482719732822,0.810731294332346,0.954910890282089
"13826","AC124045.1",2.566423974998,-0.248454196126673,1.0368509407656
7,-0.239623832470269,0.810621887330763,0.954910890282089
"13827","ARL6",46.1640422305988,-0.0710148393663012,0.296402141169022,
-0.239589495157545,0.810648509267573,0.954910890282089
"13828","LEAP2",26.0936974635891,-0.148631396545911,0.619851018110623,
-0.239785677853618,0.810496410526209,0.954910890282089
"13829","SAYSD1",188.085364803682,0.0662261133821739,0.27692593362202,
0.239147386869758,0.810991298186654,0.954910890282089
"13830","PRRG4",3.15734962846499,0.208777312700207,0.872347576190344,0
.239328128372827,0.810851155773223,0.954910890282089
"13831","NTHL1",185.094432988343,-0.0688855298435451,0.287987626537501
,-0.239196144194671,0.810953492386982,0.954910890282089
"13832","PITPNC1",99.5153804575567,-0.07743708824587,0.323164173711615
,-0.239621512980499,0.810623685639026,0.954910890282089
"13833","ZNF581",232.731783560452,-0.0492945339154365,0.20609085777878
9,-0.239188358216004,0.810959529504646,0.954910890282089
"13834","POLDIP3",219.44738980564,0.0400994106880039,0.167605557992403
,0.239248692992756,0.810912747221683,0.954910890282089
"13835","GLIS2-
AS1",4.40214703960788,0.16174791865905,0.676690662767685,0.23902785653
5652,0.811083982327953,0.954950992998918
"13836","PRPF40A",697.434926216165,0.0403246368650091,0.16919766430829
1,0.23832856694484,0.811626266373375,0.954961852123943
"13837","MATR3.1",55.9863892154201,-0.0628120025031724,0.2636280139821
26,-0.23825996924375,0.811679467288108,0.954961852123943
"13838","CBWD5",61.0586900794673,-0.080989539879883,0.339099239619963,

-0.238837279525162,0.811231761728848,0.954961852123943
"13839","SLC18A2",2.85401230540108,0.293440243950807,1.23109828888974,
0.238356471289911,0.811604625420563,0.954961852123943
"13840","ETFRF1",342.958303675937,-0.058082787946176,0.243737360014811
,-0.238300718210152,0.811647864341568,0.954961852123943
"13841","LINC00346",12.0653948091755,0.16812886998771,0.70528642402391
6,0.238383817213542,0.811583417685901,0.954961852123943
"13842","GAS8",46.2583359691892,0.0577430545640776,0.242123999575845,0
.238485464742167,0.811504587630716,0.954961852123943
"13843","TUBG2",92.0695729476297,0.0534911714215256,0.224179837779984,
0.238608306399183,0.811409323579433,0.954961852123943
"13844","C1QTNF1-
AS1",4.41697879842777,-0.207481309624192,0.869383002045462,-0.23865351
5350583,0.811374264613021,0.954961852123943
"13845","SHFL",272.791422570081,-0.0420374848098801,0.175956458749209,
-0.238908449901212,0.811176573196216,0.954961852123943
"13846","AC006230.1",2.52703075278481,0.253508411405835,1.065705364783
21,0.237878516692469,0.811975318489019,0.955240933328588
"13847","BLOC1S6",690.50831291331,0.0345600361764333,0.145382708113492
,0.237717653116313,0.812100090896109,0.95531872467731
"13848","NAF1",86.8452002635874,-0.0496743711286052,0.209280498700783,
-0.237357859126792,0.812379179181203,0.955571177531483
"13849","LINC01569",8.680858484938,-0.128955153061427,0.54345019306298
6,-0.237289736405487,0.812432023920038,0.955571177531483
"13850","AL390195.2",3.79414423312886,-0.27566456625552,1.166057671932
67,-0.236407317485955,0.813116618571265,0.9556222269905
"13851","NFU1",310.565619773529,-0.0593044370565151,0.250862692489505,
-0.236401979377607,0.813120760395871,0.9556222269905
"13852","SH3BP5-
AS1",27.3640418151683,0.0901967682684924,0.380342567229601,0.237146130
987866,0.812543425538479,0.9556222269905
"13853","LETM2",8.25376017004284,-0.130707386243625,0.552335878723459,
-0.236644750555969,0.812932400148092,0.9556222269905
"13854","C8orf89",10.5934214326349,-0.119663355795617,0.50608400849894
7,-0.23644958897346,0.813083820416881,0.9556222269905
"13855","MVB12B",29.5891311420436,0.0887794266910026,0.374532024250949
,0.237040949618549,0.812625022184657,0.9556222269905
"13856","EIF3J-
DT",223.720745760182,-0.0462542012061142,0.195180688447619,-0.23698144
3061809,0.812671186541222,0.9556222269905
"13857","CHTF18",16.2660998535622,0.105603507695892,0.446550500567625,
0.2364872675356,0.8130545861578,0.9556222269905
"13858","RPRD1A",100.49417603254,-0.0539286083951772,0.227851845364548
,-0.236682780904824,0.81290289430622,0.9556222269905
"13859","PTPN1",198.353632191904,-0.0520867506078686,0.219947108879068
,-0.236814890967751,0.812800398792788,0.9556222269905
"13860","TRAPPC10",91.1872802448773,0.0587611813141228,0.2480245569039
33,0.2369167877876,0.812721345938908,0.9556222269905
"13861","EFTUD2",89.4550302311157,-0.047130483515565,0.199443874712859
,-0.236309506037321,0.813192511053367,0.95563760280992

"13862","AC010542.2",9.98036291232764,0.155069214563755,0.656753049331844,0.236114951763857,0.813343472080102,0.955746055166122
"13863","BRIX1",242.648925039595,-0.0483996780821692,0.205149565078404,-0.235923863955899,0.813491750113497,0.955782394518086
"13864","FIG4",71.8123333545341,0.0483161073207276,0.204732858534378,0.235995861468493,0.813435881559036,0.955782394518086
"13865","XRCC3",8.35460113288696,0.115523114595997,0.49036857925498,0.235584251281989,0.813755295191884,0.955954132654017
"13866","TMEM159",1128.99141625693,0.0404536998220989,0.171702837976677,0.235602977206433,0.813740763013792,0.955954132654017
"13867","MESP2",18.9752208300571,0.103003748069725,0.437437437823668,0.235470810596797,0.813843331748194,0.955988608267566
"13868","RAB42",75.3604649913078,0.0758484713518585,0.322489291419957,0.235196868143711,0.814055936686867,0.956017035695008
"13869","C3orf52",7.7308200092085,0.123321668581378,0.524635722246405,0.23506151669073,0.814160987117757,0.956017035695008
"13870","HIST1H4E",3.80834480644249,0.216991616070227,0.922445390755155,0.235235189253412,0.81402619510086,0.956017035695008
"13871","DDR1",15.6876883183273,0.0858354961688425,0.365061835654634,0.235125909600825,0.814111009380295,0.956017035695008
"13872","SMYD4",252.510533571243,0.0543916494958923,0.231293486336076,0.235162910800089,0.814082291712811,0.956017035695008
"13873","C1orf50",158.401520934339,0.0549120394762367,0.235140049103182,0.233529080586952,0.815350590673577,0.956038559576065
"13874","NFIA",4695.7785296644,0.047349813072013,0.201930570540316,0.234485610303168,0.814608003144123,0.956038559576065
"13875","PMF1",423.174570517315,0.0622366995643292,0.266002286677572,0.233970543417801,0.815007846958837,0.956038559576065
"13876","ATP6V1E2",67.7801805032691,-0.0630908147001276,0.269106435999119,-0.23444558085684,0.814639076071291,0.956038559576065
"13877","KRCC1",440.722816949635,-0.030309599775957,0.12981277937287,-0.233487025872057,0.815383243034001,0.956038559576065
"13878","OXNAD1",36.8207132932033,0.0679338028856659,0.289851532142882,0.23437448263057,0.814694266911717,0.956038559576065
"13879","MST1R",3.85268368667993,0.177316535484034,0.755537519150761,0.234689252339633,0.814449930177015,0.956038559576065
"13880","AC007370.2",3.03577226993741,0.235229494919092,1.00731979410379,0.233520175316693,0.815357504927293,0.956038559576065
"13881","SCD5",105.217330547854,-0.080219364182675,0.342431233220739,-0.23426415700510,0.814779910306191,0.956038559576065
"13882","FBXO8",203.888904731274,0.0429051960637464,0.183091574057316,0.234337359786502,0.81472308432469,0.956038559576065
"13883","FGF10",123.271216044074,0.0909456813785007,0.387816109164433,0.23450722966213,0.814591221200505,0.956038559576065
"13884","STK10",41.8715335538177,-0.0724401674687616,0.309738430463559,-0.233875297167182,0.815081791423234,0.956038559576065
"13885","AL355347.1",5.47689251127843,0.154161699141904,0.660692355805641,0.233333559541371,0.815502400952903,0.956038559576065
"13886","B3GAT2",18.2919522627158,0.0996872148620036,0.426907472413933,0.233510119413759,0.815365312575589,0.956038559576065

"13887","GNRH1",12.0836715421695,0.115773917741909,0.496407800492889,0
.233223405488302,0.815587931970593,0.956038559576065
"13888","JAK2",91.17739982038,0.0815171182260322,0.346954735389374,0.2
34950297290361,0.814247310421125,0.956038559576065
"13889","LZTS2",348.554367149954,-0.0334911126488334,0.142758463435033
,-0.234599839778149,0.814519333919796,0.956038559576065
"13890","POLD3",180.382578684364,0.0493084428575501,0.210848475636775,
0.23385724136082,0.8150958092414,0.956038559576065
"13891","AC009318.1",15.6661126052589,-0.115364109190999,0.49285243847
883,-0.234074339871515,0.814927266417248,0.956038559576065
"13892","BIN2",10.4489835580931,0.126529359295932,0.541614718946246,0.
23361506781445,0.815283828980582,0.956038559576065
"13893","MMP17",70.3430505103586,-0.0710882282357747,0.304726725321103
,-0.233285177599261,0.815539967677099,0.956038559576065
"13894","CERS1",5.64180534835113,-0.191256848243231,0.819379374386574,
-0.233416722731659,0.815437828913254,0.956038559576065
"13895","LIN7B",23.6993763008353,0.091902602086817,0.392695851874747,0
.234029979303499,0.814961704720366,0.956038559576065
"13896","PTGIS",1234.36474181322,-0.0833384506899942,0.355436673327282
,-0.234467788340054,0.814621837437896,0.956038559576065
"13897","AC124017.1",4.90698946661996,-0.180482201159636,0.77417599104
4065,-0.23312813009899,0.815661911964985,0.95605647866429
"13898","SIGMAR1",222.294378029013,-0.0519802075244257,0.2230459133265
39,-0.23304711908497,0.815724817166025,0.956061415082557
"13899","ZNF326",252.972209820746,-0.0457067043805075,0.19660414156493
7,-0.232480882735681,0.816164533869561,0.956370339702272
"13900","TRIM24",72.7020153940279,-0.0542663117929606,0.23341535859015
3,-0.232488179529973,0.816158867099891,0.956370339702272
"13901","LINC00921",33.4906221911074,-0.0678889219701235,0.29185043674
7625,-0.232615454431647,0.81606002557586,0.956370339702272
"13902","AC006460.2",6.95511436105839,0.136087574773704,0.586020155301
632,0.232223369013064,0.816364527976327,0.956535879456653
"13903","TMEM117",24.1277608228914,-0.072126798101761,0.31095285168620
6,-0.23195412973587,0.81657364134552,0.956643271280004
"13904","ACO2",192.839446608583,-0.0458908420509875,0.197823794638464,
-0.231978373152007,0.816554811379136,0.956643271280004
"13905","MEF2D",233.509256023632,-0.0420931348111316,0.181602706685493
,-0.23178693522465,0.816703504929671,0.956657801797742
"13906","RPTOR",41.9834943687383,-0.0647786000494696,0.279450401231363
,-0.23180714632733,0.81668780626267,0.956657801797742
"13907","COL4A3",2.31971209874011,-0.286389076975301,1.23626477024686,
-0.231656748511982,0.816804627243486,0.956702871694229
"13908","AC025171.4",5.08498927690948,0.159309871768769,0.687907526084
002,0.231586173617928,0.816859447450631,0.956702871694229
"13909","SELENOF",1523.16602143087,0.0416284402867253,0.18241342835432
4,0.228209297211744,0.819483534001828,0.956732816193771
"13910","FNBP1L",96.0192823988504,-0.0483446256117115,0.21025339986371
4,-0.22993504810409,0.818142241429412,0.956732816193771
"13911","SCAMP3",125.348301740552,0.0522206494850219,0.227624136509912
,0.229416134359494,0.818545497137491,0.956732816193771

"13912","TOMM40L",28.4356567181939,0.0720433996715513,0.31450439731989
2,0.229069610108738,0.818814813107319,0.956732816193771
"13913","BLZF1",166.398466015313,0.0404049618148796,0.177737154646814,
0.227329856242884,0.820167258851211,0.956732816193771
"13914","IL18RAP",4.23457341500043,-0.203795595820866,0.88182517765401
6,-0.231106574165912,0.81723200791552,0.956732816193771
"13915","BIN1",886.769597798907,0.0607488574301971,0.266142102038053,0
.228257224110717,0.819446276983125,0.956732816193771
"13916","FAM171B",154.143994959552,-0.0612739960570023,0.2659069497185
89,-0.230433977456584,0.817754561261522,0.956732816193771
"13917","MYOSLID",2.37631500311778,0.2261864827284,0.989873177411078,0
.228500466413253,0.819257193567,0.956732816193771
"13918","NAALADL2",158.217078428196,0.0442023361973205,0.1932846370924
84,0.22869037530474,0.819109575966243,0.956732816193771
"13919","STIM2",68.2220532040862,0.062566823173825,0.271793765645405,0
.230199625901106,0.81793665254017,0.956732816193771
"13920","CYP4V2",191.84421167978,0.046397690648297,0.203129806432585,0
.228413995282842,0.819324410345717,0.956732816193771
"13921","ICE1",213.583845185577,0.0418130001045852,0.182680337261111,0
.228886155628345,0.81895740116879,0.956732816193771
"13922","AC008608.2",28.5048045122586,-0.0825875831018971,0.3632889692
54741,-0.227333032630523,0.820164789109826,0.956732816193771
"13923","RARS",224.551730309621,-0.0362304041440235,0.15858922397267,-
0.228454388239311,0.819293011441893,0.956732816193771
"13924","SFXN1",152.958525074424,0.0528902310510104,0.231060825616777,
0.22890176606021,0.818945267892013,0.956732816193771
"13925","QRSL1",84.4055384851052,-0.0513770631780518,0.222782161319889
,-0.230615695950092,0.817613372666986,0.956732816193771
"13926","PTCD1",43.5060176293865,-0.063962515157875,0.277683362220821,
-0.230343347351904,0.817824979733743,0.956732816193771
"13927","POLR3D",132.359757257198,-0.0448955614821564,0.19465995214556
9,-0.230635839510445,0.817597722224713,0.956732816193771
"13928","TRAM1",1332.48377547743,0.0324118723752528,0.141934114951652,
0.228358575993471,0.819367490232156,0.956732816193771
"13929","PLAA",107.027125080046,0.0478851203916757,0.2074392512972,0.2
30839246151493,0.817439690488944,0.956732816193771
"13930","FAM122A",124.082108288223,0.0383622463761753,0.16634995087098
7,0.230611708481521,0.817616470720122,0.956732816193771
"13931","GOLM1",383.769541317115,0.040523272173594,0.175465366567482,0
.230947411254569,0.817355657321262,0.956732816193771
"13932","ZFP37",50.7987012325618,-0.055100929866239,0.240737570170274,
-0.228883800012046,0.818959232085676,0.956732816193771
"13933","GOT1",61.6522042020941,-0.0625745669751962,0.272476575398471,
-0.229651179679159,0.818362833915478,0.956732816193771
"13934","PHRF1",103.831603378152,-0.0507427028585818,0.223272657402652
,-0.227267877083005,0.820215449956063,0.956732816193771
"13935","SLC39A13",283.848047996509,-0.0468577317463175,0.205369164230
98,-0.228163424250081,0.819519194729307,0.956732816193771
"13936","POLD4",641.423578787252,-0.0472149313132866,0.205764462056509
,-0.229461058733844,0.818510583827957,0.956732816193771

"13937","CHORDC1",183.062758946219,0.0369316127393074,0.160629060985597,0.229918624392749,0.818155003806803,0.956732816193771
"13938","MPZL3",3.95378884063075,-0.20599679103524,0.894566289684126,-0.230275602166921,0.817877617866105,0.956732816193771
"13939","MRPL51",1165.26922582014,-0.0602383431634331,0.264751673610409,-0.227527714336854,0.820013421359558,0.956732816193771
"13940","CFAP54",2.51251752055632,0.24740017259817,1.08006045562165,0.229061411618642,0.818821185169471,0.956732816193771
"13941","ANKRD13A",122.855210139091,0.0419116438604944,0.184378008447133,0.227313681352143,0.82017983536563,0.956732816193771
"13942","AC079949.1",3.01000460748679,-0.202798797701553,0.880622154700323,-0.230290365304931,0.817866146810946,0.956732816193771
"13943","ZNF10",36.6069938477969,0.0737124690123424,0.321476098607541,0.229293777458494,0.818640589531828,0.956732816193771
"13944","AL136295.7",9.07037587713353,0.132778785387833,0.580518946880838,0.228724292464977,0.819083212578676,0.956732816193771
"13945","AC007686.3",12.8890919755613,0.0951756521490832,0.414194843212449,0.229784734669586,0.818259047324292,0.956732816193771
"13946","VIPAS39",92.3303439552784,0.0459575544100844,0.201632086605335,0.227927782645227,0.819702383704945,0.956732816193771
"13947","AL049840.5",31.5951660854092,0.0680682771119286,0.294328697454452,0.231266192187944,0.817108009514179,0.956732816193771
"13948","SERF2",10180.3559733165,0.0616583193138822,0.271249936888949,0.227311829160444,0.820181275509103,0.956732816193771
"13949","LINS1",76.2634014424752,-0.059537532847238,0.260944986424301,-0.228161244494764,0.819520889237,0.956732816193771
"13950","ITFG1",368.114790465267,-0.036625520016196,0.160313806541356,-0.228461420799385,0.819287544808584,0.956732816193771
"13951","PLCG2",8.10991786826803,0.189308007480349,0.830413612996873,0.227968333511726,0.819670858555017,0.956732816193771
"13952","LINC00311",6.24955623423799,-0.15076754598563,0.654715243147331,-0.230279572018003,0.817874533261918,0.956732816193771
"13953","CDT1",12.6375271273482,0.106176341851467,0.466337581017098,0.227681289635488,0.819894019146769,0.956732816193771
"13954","CASC3",237.47400117628,0.0471831843943073,0.20435314169235,0.2308904282242,0.817399927017457,0.956732816193771
"13955","ARL17A",46.8652554342031,0.0883378193309527,0.38554952175002,0.229121849068791,0.818774212022,0.956732816193771
"13956","MMD",101.002350637421,-0.0848525687300078,0.368464348805109,-0.230287052207834,0.817868721105748,0.956732816193771
"13957","VAPA",1131.79887373459,-0.0458010770541871,0.19993152612251,-0.229083816556885,0.81880377154513,0.956732816193771
"13958","ASXL3",15.2862882785887,0.139784607757773,0.610952014365926,0.22879801436263,0.819025910168325,0.956732816193771
"13959","MADCAM1",2.80196148020038,-0.233746790703692,1.01259831269376,-0.230838613666922,0.817440181870619,0.956732816193771
"13960","ADAT3",5.81258356398008,-0.209857794047838,0.911577262797322,-0.230213940839042,0.817925529544261,0.956732816193771
"13961","FBXW9",44.3956694059327,-0.0678407719491546,0.297799505653568,-0.227806865563015,0.819796389076778,0.956732816193771

"13962","ZNF580",244.736914898683,-0.05714258761908,0.248651650445233,
-0.229809806276214,0.818239564344556,0.956732816193771
"13963","PPIL2",106.68986491807,0.0454442296502754,0.199391499622018,0
.227914578788078,0.819712648741486,0.956732816193771
"13964","ADSL",231.934901367781,-0.0420769433150734,0.184477579115906,
-0.228087031045853,0.819578582114284,0.956732816193771
"13965","NFAM1",2.32227006112246,-0.252278512387449,1.11015879806721,-
0.227245429056336,0.820232904300203,0.956732816193771
"13966","AC084757.3",3.28118189284742,0.216549899173444,0.954590659254
404,0.226851056077359,0.820539561434673,0.957021975956565
"13967","YIPF1",84.5836359554696,0.0515489309730104,0.227790930331557,
0.226299312698619,0.8209686329137,0.957111226848787
"13968","CMTR1",85.4336301856985,0.0552878521574945,0.244139127215225,
0.226460431755187,0.82084333072912,0.957111226848787
"13969","CCPG1",991.683205275258,0.0283377265559504,0.125186676199393,
0.22636375863846,0.820918512800152,0.957111226848787
"13970","VKORC1",1697.8658759093,-0.0574808038241254,0.25396087438203,
-0.22633724176606,0.820939135091553,0.957111226848787
"13971","CYB5D1",43.2184517530844,0.115416063563975,0.509450635051498,
0.226550043562725,0.82077364166351,0.957111226848787
"13972","RPS6KB1",203.968900477338,0.0376454791498905,0.16611541349010
3,0.226622432915495,0.820717347142488,0.957111226848787
"13973","PPP3CA",547.432125243568,-0.0524187857719366,0.23183261088549
9,-0.226106178814619,0.821118839028969,0.957149332255823
"13974","IFFO1",145.486429538709,0.0523279470477384,0.231389823946804,
0.226146276250111,0.821087653490044,0.957149332255823
"13975","C16orf95",40.5492126632449,-0.0914839315908165,0.404972419037
16,-0.225901635001029,0.821277926146735,0.95726627112731
"13976","NFKBIA",4329.01275954979,0.0611248771513916,0.270772385071807
,0.225742655164713,0.821401580253262,0.957273402070929
"13977","F8A3",13.9301480882948,-0.11930250465111,0.528453956278947,-0
.225757614705293,0.821389944572,0.957273402070929
"13978","DAZAP1",242.440979260343,-0.0359327996168842,0.15923786701332
7,-0.225654866463873,0.821469863980369,0.957284490941209
"13979","SH3RF1",161.92881193149,0.0502668441212716,0.222932206117884,
0.225480404992229,0.821605567457666,0.957326163053788
"13980","BPGM",116.206541593646,-0.0458004749700158,0.203144353549114,
-0.225457779996541,0.821623166522927,0.957326163053788
"13981","GAS6",3853.14927141977,-0.0576618025201794,0.255995260879008,
-0.225245585883843,0.821788228073274,0.957381522463565
"13982","TMEM230",1363.65199010999,-0.0620530667636834,0.2754280357777
53,-0.225296842380109,0.821748355948454,0.957381522463565
"13983","FGD5-
AS1",694.082195522055,0.0311104248617646,0.138517076175895,0.224596314
913976,0.822293331928649,0.957433092269022
"13984","AC079298.3",13.5020811065151,-0.0964999409799716,0.4297443784
31835,-0.224551956519143,0.822327843471824,0.957433092269022
"13985","AP003168.2",3.39406098241175,0.180822160439866,0.804840544081
458,0.224668304510222,0.822237323599086,0.957433092269022
"13986","CRADD",48.2565250865906,0.0620666050416185,0.276548286534169,

0.224433157115041,0.822420273008051,0.957433092269022
"13987","TC2N",149.266448618602,0.0996640300680706,0.443224736626249,0
.224861163721809,0.822087282462246,0.957433092269022
"13988","FMN1",17.9139279973014,0.113295192374304,0.504763343577135,0.
224452099812575,0.82240553485019,0.957433092269022
"13989","MAPKBP1",48.486882017434,0.0766065677369039,0.340340661540223
,0.225087908656633,0.821910887140069,0.957433092269022
"13990","DAPK2",57.9512732230908,0.065736349634279,0.292773394883088,0
.224529792608134,0.822345087479138,0.957433092269022
"13991","ZNF497",3.99509320770895,-0.165626924860708,0.737470811755952
,-0.224587769740124,0.822299980184328,0.957433092269022
"13992","FLNA",1176.43619237121,0.0475672991959744,0.211782585184137,0
.224604393957211,0.822287046340347,0.957433092269022
"13993","RAB7B",120.677123892904,0.0506964222729256,0.22695248580394,0
.223379012983013,0.823240536896215,0.958065642560772
"13994","ITPRID2",139.675453204728,0.051137670796619,0.22869932832672,
0.223602190573833,0.823066858994051,0.958065642560772
"13995","MOB1B",64.6249901749959,0.0530984006311942,0.237507694407211,
0.223564970236948,0.823095823441879,0.958065642560772
"13996","AC139019.1",5.29357225608696,0.147217069427824,0.658880617543
887,0.223435119364424,0.823196873818331,0.958065642560772
"13997","COG1",82.7278200359975,0.0491030325908922,0.219841078546128,0
.223356949099889,0.823257707589363,0.958065642560772
"13998","ETV3",83.5715173598495,0.0562740300607064,0.252410920432299,0
.2229460990211,0.823577457357549,0.958202441679036
"13999","INSIG2",242.302791531436,-0.0368488333414485,0.16519228612582
,-0.223066307789835,0.823483900183736,0.958202441679036
"14000","FKBP10",795.447486464818,0.0487498867384881,0.218721487111614
,0.222885677041922,0.823624484072012,0.958202441679036
"14001","MRPL58",155.395829932978,-0.0636767757418521,0.28576650547863
3,-0.222827989008716,0.823669383534279,0.958202441679036
"14002","ZNF283",36.6853828158913,-0.0889545045032785,0.39891521954842
7,-0.222991001957697,0.823542509598226,0.958202441679036
"14003","RPS19",16262.2854377461,-0.0477594625950856,0.214506090441997
,-0.222648515464878,0.823809074178085,0.958296508554369
"14004","C1orf112",18.5467793554919,-0.083244159273388,0.3749695710849
28,-0.222002438844655,0.824311984780664,0.958365490284255
"14005","COPS7B",80.8578506776962,0.0489778830766364,0.220622102669018
,0.221998985977004,0.824314672711195,0.958365490284255
"14006","SPCS1",1405.00608453617,-0.0580508625633262,0.261287620988268
,-0.222172264969004,0.824179783929485,0.958365490284255
"14007","DNAAF5",88.1898870584929,0.0562802346494945,0.253076268541154
,0.222384481065408,0.824014591689556,0.958365490284255
"14008","NAA35",131.473763988476,-0.0562107744427207,0.25314537429597,
-0.222049384070517,0.8242754398526,0.958365490284255
"14009","PCGF5",205.952151099208,-0.038367068338528,0.172849809313964,
-0.221967663665964,0.824339056072976,0.958365490284255
"14010","LINC02823",12.2454302145956,0.0934395910885383,0.419992048443
938,0.2224794289195,0.823940685358394,0.958365490284255
"14011","MROH8",11.275707977679,0.105954846383982,0.476801010485617,0.

222220263912755,0.824142420139143,0.958365490284255
"14012","POP1",22.7545012169888,-0.0791915158161455,0.357060599522091,
-0.221787326639062,0.824479445657775,0.958460297624857
"14013","ARHGEF11",114.723196420856,-0.0500606410154467,0.225882852845
094,-0.221622139019898,0.824608046590635,0.958472989218985
"14014","AC004067.1",4.36116778908576,0.190336146831069,0.858655912718
837,0.221667543438198,0.824572698129854,0.958472989218985
"14015","TFAP2A",2.84947572835101,0.184549694485209,0.833459140890754,
0.22142620487427,0.824760590239558,0.958524073506949
"14016","FAM102A",189.793707284835,-0.0446735438736777,0.2019547293403
33,-0.221205732688706,0.824932245782376,0.958524073506949
"14017","MXI1",444.362176899036,-0.0592968177455668,0.268083601328609,
-0.221187784152759,0.824946220547235,0.958524073506949
"14018","RAB11FIP2",291.244508298738,0.0448277634923999,0.202537310899
397,0.221330891050817,0.824834798781819,0.958524073506949
"14019","AL049780.1",2.43125775340052,-0.235624377675927,1.06516751081
326,-0.221208753819412,0.82492989352961,0.958524073506949
"14020","MRPL13",309.813845314232,-0.0618354768530477,0.27975781988818
,-0.221032165884634,0.825067387557631,0.958596481877763
"14021","ZNF771",72.2077152513458,0.0528495473464437,0.239447870614011
,0.220714208946284,0.825314967151204,0.958815740669421
"14022","CTBP1-
AS",4.69981008037682,-0.162146188262425,0.735560954448754,-0.220438819
219192,0.82552941550379,0.958954833009968
"14023","TOR1B",84.4752602575326,-0.0463128499661088,0.210122071616792
,-0.220409258340891,0.825552435588359,0.958954833009968
"14024","RUVBL1",124.14612208706,-0.0615110293171504,0.279190033262385
,-0.220319574443196,0.825622276477957,0.958960480799828
"14025","ABHD13",149.905055250782,0.0407619493777613,0.185069750723417
,0.22025181975135,0.825675041022628,0.958960480799828
"14026","EN2",2.29009417943227,0.244671453789283,1.11246526538343,0.21
9936263542532,0.82592079348378,0.959129403537604
"14027","RUBCNL",5.04163013761993,-0.213474803518133,0.971388033355119
,-0.219762644986272,0.826056013397326,0.959129403537604
"14028","ALKBH1",30.3609549764633,0.0742261748333774,0.337664578030117
,0.219822213115754,0.826009619172706,0.959129403537604
"14029","AC007114.1",17.331188521619,0.0860417518064808,0.391328061037
651,0.219871152552494,0.825971503486302,0.959129403537604
"14030","PRDM16-
DT",3.68013162457143,-0.235430934045766,1.072172961247,-0.219582980130
319,0.826195947802423,0.959155141739981
"14031","CEBPZ",402.453982889214,-0.0442062234622645,0.201297804349241
,-0.219606088626626,0.826177949127803,0.959155141739981
"14032","CNPY4",339.421757323798,0.0656484621079827,0.299259950463402,
0.219369354323311,0.826362340338266,0.959279943113598
"14033","TMEM120A",183.489972472473,-0.0415242427965757,0.189376221468
682,-0.219268514676975,0.826440886762741,0.959302758104346
"14034","LYAR",202.989295850464,-0.044972338597523,0.205188355783897,-
0.219175880744848,0.826513043089707,0.959318152977643
"14035","AL021155.5",86.8083428472657,0.0884899139406148,0.40403342101

265,0.219016322260737,0.826637333099315,0.95932569954793
"14036","GZF1",53.0620935273086,-0.0862968087687659,0.393913397707628,
-0.219075586844643,0.826591167728217,0.95932569954793
"14037","AC008522.1",4.42541093765653,0.160277135341249,0.732541363436
491,0.218796020731661,0.82680894677588,0.959333442057567
"14038","TOPORS",440.89132613274,-0.0477998631942084,0.218791384445253
,-0.218472328402717,0.82706111631981,0.959333442057567
"14039","RFK",257.088494030413,-0.041370826723348,0.189471358653917,-0
.218348709890843,0.827157424896358,0.959333442057567
"14040","LRRC20",20.8910052081411,0.0791796625240122,0.362020395032668
,0.218716027081477,0.826871263435951,0.959333442057567
"14041","XPO4",63.5810430007494,-0.0486745589100478,0.222943006967827,
-0.218327363446174,0.827174055724632,0.959333442057567
"14042","CILP",4336.06632489193,0.0739329306121226,0.338405489774347,0
.218474383088235,0.82705951557961,0.959333442057567
"14043","AC007613.1",6.46981604777816,0.181930569156642,0.831952496611
485,0.218679035038225,0.826900081349284,0.959333442057567
"14044","VEZF1",381.698762138807,0.041408112284769,0.189415077044256,0
.218610434453927,0.826953523882939,0.959333442057567
"14045","C18orf21",132.598490435396,0.0537741878879042,0.2462942949698
14,0.218333063274952,0.827169615030316,0.959333442057567
"14046","DFFA",284.670651396429,0.0342204107896604,0.157434949826111,0
.217362223746744,0.827926068483546,0.959337598471975
"14047","GSTM4",125.122566285747,-0.0553201927783374,0.254403342879719
,-0.217450730608098,0.827857099565685,0.959337598471975
"14048","ZXDC",70.2423163555437,0.0628256544313162,0.288135400569896,0
.218042122929202,0.827396291550473,0.959337598471975
"14049","RHOH",12.946875033696,0.134098735993092,0.618073981048491,0.2
16962273295519,0.828237746166823,0.959337598471975
"14050","AC008393.1",18.6031841961767,-0.0827191589575116,0.3799875523
88007,-0.217689128071876,0.827671335091287,0.959337598471975
"14051","HSF2",98.8936614643029,-0.0509315666009347,0.234343394025687,
-0.217337325904531,0.82794547035109,0.959337598471975
"14052","MRPS24",1025.18333548896,-0.0536028532399794,0.24618417096757
8,-0.217734767549449,0.827635772919616,0.959337598471975
"14053","CAAP1",95.2239519828759,0.0456847638681894,0.210541981719141,
0.216986481722833,0.828218879994094,0.959337598471975
"14054","EXOSC2",75.5503138750505,0.0557860998792952,0.255883148931949
,0.218013965015458,0.827418230618509,0.959337598471975
"14055","TRIM68",26.5370213907154,-0.0666130384912578,0.30529559266601
,-0.218191942797326,0.827279562572547,0.959337598471975
"14056","AC007406.5",25.8719947013599,0.0907510973232447,0.41789612632
6563,0.21716185340356,0.828082211852852,0.959337598471975
"14057","PLBD1",957.419731039758,-0.0451130886045992,0.207451011498027
,-0.217463816053885,0.827846902852635,0.959337598471975
"14058","HECTD4",145.438026820251,0.0529272619276014,0.243793055735588
,0.217099136675185,0.828131086755848,0.959337598471975
"14059","ELL3",6.85160844411178,-0.127649426743105,0.587279491437063,-
0.217357201476164,0.827929982124626,0.959337598471975
"14060","AC040169.1",14.7063452266188,-0.0891202046928415,0.4104940669

7073,-0.217104732720038,0.828126725752772,0.959337598471975
"14061","DRC3",31.0271482839327,-0.0875501617558955,0.402636390490757,
-0.217442247704397,0.827863709805728,0.959337598471975
"14062","AC010336.8",7.85521952598569,0.140587616012427,0.646479943928
675,0.217466322556075,0.827844949687211,0.959337598471975
"14063","ZNF569",42.9905263973085,-0.055005938603811,0.253398892238003
,-0.217072529867838,0.828151821538597,0.959337598471975
"14064","SATB2",21.0218010118924,0.0974867852308138,0.450955826893138,
0.21617812525526,0.828848903927349,0.959363305341618
"14065","AC092902.4",5.59303161936894,0.147865276025068,0.683584893416
701,0.216308577689607,0.828747223307426,0.959363305341618
"14066","FNIP2",176.650378125778,-0.0608767718975231,0.281457014231489
,-0.21629154300434,0.828760500760838,0.959363305341618
"14067","RAPGEF2",92.5483672440482,0.0639236869408038,0.29510638821583
9,0.216612345558749,0.828510463797631,0.959363305341618
"14068","NEU1",739.443474343227,0.0642384410830736,0.297067104282658,0
.216242189582698,0.828798968889296,0.959363305341618
"14069","PDLIM2",2132.82491291255,0.0559222933590921,0.258061445681367
,0.216701465077199,0.828441006168595,0.959363305341618
"14070","TOX",3.16174246469068,0.205516403862832,0.950256623242384,0.2
16274634489351,0.828773679920924,0.959363305341618
"14071","TSC2",182.283134072239,0.0480540235844075,0.222252395205968,0
.216213748967135,0.828821136885337,0.959363305341618
"14072","AC067852.2",31.795384752259,0.0665860682612096,0.307798973534
637,0.21632972812275,0.828730737958472,0.959363305341618
"14073","HCCS",66.4043867693229,-0.0547076673582817,0.252852408842965,
-0.216362057251581,0.828705539709171,0.959363305341618
"14074","PHF24",3.11837943469092,-0.231162213163208,1.07072481747017,-
0.215893205603827,0.829070993361308,0.959484007876543
"14075","FGF14-
IT1",2.44825103802726,0.249376783832092,1.15508249816859,0.21589521460
8034,0.829069427333003,0.959484007876543
"14076","LBR",158.267132800133,-0.0400425313277547,0.185855114975219,-
0.215450251843184,0.829416294492062,0.959571153292455
"14077","LINC00852",6.14122130032742,-0.141492790411827,0.656434394141
853,-0.215547496710312,0.829340485192889,0.959571153292455
"14078","DECR1",756.346717465337,-0.064702885577953,0.300314475000357,
-0.215450439336552,0.829416148326101,0.959571153292455
"14079","SNUPN",263.741847242747,0.0456348312256262,0.211823731717406,
0.215437764482912,0.829426029393765,0.959571153292455
"14080","SCAPER",186.97984883126,0.0545085907189948,0.253168662596465,
0.215305441676555,0.829529187273008,0.959571153292455
"14081","AC002550.2",2.49031422038786,0.202977080000392,0.942905679537
688,0.215267639600933,0.82955865803084,0.959571153292455
"14082","ABI3",2.68593726769382,0.225794858296173,1.0488285648971,0.21
5282903091341,0.829546758479998,0.959571153292455
"14083","TMPO",210.11541969937,0.0419413046451507,0.195109652077685,0.
214962736074437,0.829796371679318,0.959709819531697
"14084","FAM81A",2.78759616868287,-0.198458309628337,0.923107018191511
,-0.21498949278616,0.829775510569949,0.959709819531697

"14085","AC006160.1",4.98719536663271,-0.150661922648339,0.701416248003348,-0.214796738851165,0.829925795578386,0.959791358478973
"14086","BDH2",1111.06886051107,-0.0495112306749858,0.230754748679272,-0.214562131259981,0.830108721141094,0.95986661167511
"14087","SGCE",1473.52692712287,0.0495438219371853,0.230867599546604,0.214598419329881,0.83008042641771,0.95986661167511
"14088","PTCH2",86.6498085618182,0.0529656047038538,0.247856495851358,0.213694640206718,0.830785191298336,0.959896546201846
"14089","FALEC",7.4132631954972,0.158309606716481,0.741637202221371,0.213459635307274,0.830968469900419,0.959896546201846
"14090","AL139011.1",4.42552817240188,-0.168740298144312,0.790498290946409,-0.213460674206254,0.830967659650465,0.959896546201846
"14091","CRBN",558.857467180366,-0.0419352524691584,0.196335882978167,-0.213589344102839,0.830867309822047,0.959896546201846
"14092","OGG1",124.115539358777,0.0412421854845964,0.1923249865675,0.214440079891136,0.830203889288223,0.959896546201846
"14093","RAB7A",1060.85477058265,-0.0355897562462497,0.16626486482079,-0.214054582636028,0.830504492678195,0.959896546201846
"14094","PRKAA1",408.57866745015,-0.035574362587733,0.166705014624472,-0.213397075474122,0.831017261406647,0.959896546201846
"14095","EPB41L4A-DT",12.3860830102186,0.0983844019476425,0.459633582471225,0.214049638015303,0.830508348560131,0.959896546201846
"14096","H2AFV",752.438630262285,0.0449003724039228,0.209951310575591,0.213860881748375,0.830655546293691,0.959896546201846
"14097","VPS37D",42.29297500184,-0.0644937219099008,0.301776248142342,-0.213713711091936,0.830770318458081,0.959896546201846
"14098","AC007384.1",3.80987058693871,-0.174610304450558,0.818247633895922,-0.213395428495388,0.83101854592292,0.959896546201846
"14099","AC023632.2",13.3206485580137,-0.0945826637544833,0.44203818226125,-0.213969443251813,0.830570886088156,0.959896546201846
"14100","UBAP1",181.542178033242,0.0433055388071769,0.202697989935777,0.213645625301454,0.830823416905108,0.959896546201846
"14101","CAVIN3",1738.57993721586,-0.0534133899955185,0.249219824307693,-0.214322396478271,0.830295653930796,0.959896546201846
"14102","AP001020.2",9.75244757880875,0.158617056474754,0.743069073656102,0.213462061735816,0.830966577499557,0.959896546201846
"14103","UCHL1",118.061424268728,0.0623935294770509,0.292710476234362,0.213157828444428,0.831203860349765,0.960042521537071
"14104","SLC35A3",250.03904323116,0.0384314566455079,0.180965725885128,0.212368703838998,0.831819399626028,0.960351186906294
"14105","AC011246.1",2.37042547979809,-0.206002970367819,0.975856864902548,-0.21109957594897,0.832809571118706,0.960351186906294
"14106","EMC3",545.474327087413,-0.0444353729618368,0.209647871715884,-0.211952416202231,0.832144157221747,0.960351186906294
"14107","ZNF330",257.095302432141,-0.036066777800557,0.170263058700051,-0.211829730276931,0.832239873403281,0.960351186906294
"14108","PJA2",891.102491650111,0.0274624976287544,0.12965589078116,0.211810643259604,0.832254764792399,0.960351186906294
"14109","INHBA",14.2916597295816,0.184732004505201,0.870474262909799,0

.212219949947382,0.831935443391913,0.960351186906294
"14110","FDFT1",247.502306034234,-0.0402830678859816,0.190307580881535
,-0.211673479844492,0.832361779290583,0.960351186906294
"14111","LY6E",2084.85742419966,0.0571074878201812,0.269336887472677,0
.212029953847204,0.832083665769138,0.960351186906294
"14112","CAMK1D",301.153991407276,0.0545513938609062,0.256921738108274
,0.212326890914605,0.831852017758401,0.960351186906294
"14113","NRP1",909.98473817406,-0.0634401082966035,0.299653070772839,-
0.211711857759323,0.832331836639733,0.960351186906294
"14114","FANK1",12.0386234358837,0.0965032478662623,0.457123493634902,
0.211109796827327,0.832801595744427,0.960351186906294
"14115","CSNK2A3",2.52109028947251,-0.215225448119122,1.01921373647987
,-0.211168119517759,0.832756086747719,0.960351186906294
"14116","FJX1",29.3143783255657,0.10318670915355,0.486698197953463,0.2
12013748124493,0.832096308677425,0.960351186906294
"14117","PACSIN3",45.534813344458,-0.08849043547267,0.416834534966091,
-0.212291516296433,0.831879613614235,0.960351186906294
"14118","NUAK1",413.339392729965,-0.0925596888663407,0.437620190701314
,-0.211506897609107,0.832491750444901,0.960351186906294
"14119","FBXW8",58.355049514887,0.0605646425158165,0.286931383926918,0
.21107709336962,0.832827114386292,0.960351186906294
"14120","USP12",136.164453723763,-0.0432519896534698,0.204088061407211
,-0.211928073377945,0.832163148625243,0.960351186906294
"14121","NLGN2",130.656900722856,-0.0430024322679651,0.203370932152003
,-0.211448272439566,0.832537492191876,0.960351186906294
"14122","ANKFN1",5.35838690672732,0.148699913119797,0.703631022268345,
0.211332230123145,0.83262803480915,0.960351186906294
"14123","WBP2",338.848819150471,-0.0355414860304819,0.168013265000792,
-0.211539761639144,0.832466108837132,0.960351186906294
"14124","KLHL26",49.9004583838099,0.0490098684647914,0.23126821495172,
0.211917874123008,0.832171105749388,0.960351186906294
"14125","WTIP",264.381388333336,-0.0421211759926642,0.19953003740884,-
0.211101930013461,0.832807734235384,0.960351186906294
"14126","KLF8",84.4039367522365,-0.0537152555353167,0.254083231854109,
-0.211408108844267,0.832568829793398,0.960351186906294
"14127","TMTC3",194.337474918069,0.0426590780930043,0.202367541885746,
0.210800001302032,0.833043337527124,0.960464533195025
"14128","GNAS-
AS1",3.93605828835232,-0.152426940372166,0.722977733441684,-0.21083213
6761043,0.833018260629663,0.960464533195025
"14129","ASXL1",394.215703836602,-0.0524119294704439,0.248776361511753
,-0.210678897110439,0.833137842679136,0.96050550777836
"14130","AL020997.5",13.4907200975255,0.109823193927334,0.523488431513
151,0.209791061876741,0.833830749422587,0.960759303903176
"14131","OVGP1",2.96252067657462,0.209719283125335,0.999292526756001,0
.20986775894957,0.833770886459581,0.960759303903176
"14132","CCDC121",63.5653127180995,0.0504038934196479,0.24013873566434
6,0.209894889636214,0.833749710871811,0.960759303903176
"14133","RBM45",42.2170936132236,-0.0625480628598615,0.297517228795014
,-0.210233414425073,0.833485501334579,0.960759303903176

"14134","AC083799.1",22.0202488354399,-0.0859336464682166,0.408913851023203,-0.210150979853555,0.833549837580552,0.960759303903176
"14135","TOR2A",72.6735734551828,0.0596846559722545,0.284122608379618,0.210066549482432,0.833615732608148,0.960759303903176
"14136","AC016394.1",21.5974669346663,-0.0890696009714146,0.424605535154846,-0.209770230477407,0.83384700873993,0.960759303903176
"14137","EDRF1-DT",47.8098063437834,-0.0636721853352645,0.303701982049053,-0.209653506064311,0.833938115756854,0.960759303903176
"14138","ALKBH8",48.6783454677168,-0.0530475723509255,0.252365300975841,-0.210201529868814,0.833510385575721,0.960759303903176
"14139","KIAA0556",32.8015092115067,0.0601938132404151,0.287127931606633,0.209641092399471,0.83394780513764,0.960759303903176
"14140","KANSL3",112.087876613673,0.0541930758526204,0.25898718706832,0.209250026868412,0.834253060922672,0.961022705860637
"14141","SMOC1",260.704672943632,0.14017879243211,0.670080247665216,0.209197022178373,0.834294436955938,0.961022705860637
"14142","VKORC1L1",258.672307772381,0.0324149156259841,0.155172474283672,0.20889604148946,0.834529394427047,0.961031977533946
"14143","NRAV",24.4041331888313,-0.0847341602155992,0.405282888336983,-0.209074112561951,0.834390383294646,0.961031977533946
"14144","CLIP1",684.517359659714,-0.0501600623859774,0.240133116752136,-0.208884401553629,0.834538481319766,0.961031977533946
"14145","LYSMD2",174.592953840225,0.0617394240508435,0.29538631815673,0.209012470300283,0.83443850372333,0.961031977533946
"14146","RAB29",169.49709392535,0.0732155267974924,0.350939133982065,0.208627421988321,0.834739101947151,0.961100462295651
"14147","TMEM144",21.5553217459291,0.092150359206877,0.441535055259888,0.208704514192282,0.834678915929314,0.961100462295651
"14148","REXO5",28.0089578051762,0.0856352278444032,0.410560052862837,0.208581490691236,0.834774961050946,0.961100462295651
"14149","AF235103.3",2.94466922872606,-0.17913861463179,0.859604575553453,-0.208396534553637,0.834919361958972,0.961130847134254
"14150","FBXO17",601.782560165058,-0.0390247885920092,0.187234500213461,-0.20842733869836,0.834895311835524,0.961130847134254
"14151","MAD1L1",105.293248323217,0.04498015966561,0.216157909719708,0.208089353398798,0.835159199968751,0.961339001363224
"14152","ATP8A1",24.7995712697763,0.103428893467554,0.497271267737424,0.207992900812778,0.835234510474431,0.961357754460006
"14153","LINC02298",7.20285413558085,-0.146839807372985,0.706341320950621,-0.207887890765562,0.835316504392626,0.961384196993676
"14154","OTUD3",50.6025550695986,-0.0605032138011097,0.291555482283146,-0.207518697049749,0.835604792338916,0.961436334856362
"14155","SEC22A",88.9819859197658,0.0426980365255339,0.205665137065809,0.20760950122466,0.83553388510323,0.961436334856362
"14156","LINC00689",4.20019930555023,0.170076381845226,0.819652608673503,0.207498127920891,0.835620854562109,0.961436334856362
"14157","NDRG3",153.592844697438,-0.0386614037500841,0.186354886020599,-0.207461175693621,0.835649710352161,0.961436334856362
"14158","APOBEC3G",117.203678348217,-0.0572680649366802,0.276054614565

912,-0.207451938547496,0.835656923623081,0.961436334856362
"14159","RNF220",144.394993434452,-0.0386985433887998,0.18684700007130
6,-0.207113538745772,0.83592118894817,0.961498125614319
"14160","ATPAF1",154.68575034141,0.0403863804424939,0.195669861220136,
0.206400618831418,0.836477987481154,0.961498125614319
"14161","XXYLT1",86.120435519895,-0.051738698708543,0.250160876469593,
-0.206821703851969,0.836149105384853,0.961498125614319
"14162","MRPL32",407.545364928624,-0.0534837619697728,0.25806944310190
3,-0.207245620895357,0.835818040310531,0.961498125614319
"14163","LRRC8A",150.303148434855,0.0354944131093326,0.17148466555341,
0.206983015039777,0.836023123302143,0.961498125614319
"14164","POSTN",648.780130081899,-0.194346335951092,0.939662666118108,
-0.206825643881295,0.836146028219609,0.961498125614319
"14165","NPTN",601.785138058446,-0.0442606714962943,0.213900002982947,
-0.206922257499094,0.836070573711464,0.961498125614319
"14166","ZNF843",12.6162554434228,-0.092001848804793,0.445730618794368
,-0.206406840646585,0.836473127818177,0.961498125614319
"14167","BEAN1",4.29006217550758,-0.169153106697046,0.817898701911781,
-0.206814250104154,0.836154926773182,0.961498125614319
"14168","B3GNTL1",17.415744486878,0.0889605296330451,0.430742280770485
,0.206528436154255,0.836378154667305,0.961498125614319
"14169","RALBP1",778.693561704858,0.0356159540685946,0.17224296376969,
0.20677410752276,0.836183698494552,0.961498125614319
"14170","FDX2",204.625462548231,-0.0586217204737833,0.2836917371663,-0
.206638801183762,0.836291955236619,0.961498125614319
"14171","HLCS",45.0480567734732,0.0772349318320868,0.374019778122926,0
.206499592667804,0.836400682889045,0.961498125614319
"14172","Z97832.2",3.69641389899724,0.155435844526358,0.75449743094620
1,0.206012423834802,0.836781206645465,0.961602436513135
"14173","DEF6",8.10006970285387,-0.134881629463895,0.654821997574368,-
0.205982129439041,0.836804870622733,0.961602436513135
"14174","SUGCT",13.547412041901,-0.0972605046003249,0.471940490029067,
-0.206086374564587,0.836723441849289,0.961602436513135
"14175","LINC00893",24.2155068788768,0.093395470115634,0.4529624755515
47,0.206188095386735,0.836643986565917,0.961602436513135
"14176","AC093673.1",65.7514321451075,-0.0696724185350895,0.3385762546
38308,-0.205780581421809,0.836962310354784,0.961653858826891
"14177","LIMA1",2574.6181393297,-0.0336178347372974,0.163372852256341,
-0.205773690506114,0.836967693326099,0.961653858826891
"14178","GOLIM4",846.011876461137,0.0414983263239466,0.202029325290828
,0.205407439064643,0.837253808660859,0.961914747445107
"14179","CARD9",8.05462093004063,-0.113260385712988,0.55173060636454,-
0.205282042370791,0.837351773406784,0.961959449680732
"14180","WSB2",164.80743819905,-0.0524375652599247,0.255838474084024,-
0.204963563231318,0.837600592957518,0.961973916568075
"14181","AC004812.2",30.896582643453,0.0857677184020891,0.418253144698
264,0.205061741888309,0.837523886768021,0.961973916568075
"14182","CD2BP2",349.387825527929,-0.0422032793150811,0.20571313931818
4,-0.205155973288628,0.837450265990584,0.961973916568075
"14183","KPNA2",121.473252611387,0.0856032266484511,0.417650023512945,

0.204964017309095,0.837600238186675,0.961973916568075
"14184","RASAL2-
AS1",7.41596882700198,-0.19277812752991,0.940971921807319,-0.204871285
808021,0.837672689968137,0.961988892194795
"14185","UGGT2",218.695758129755,0.0404520699506894,0.197553456883543,
0.204765184010603,0.837755589745663,0.962016270804872
"14186","ODR4",260.401792419037,0.0268231332727979,0.131779312030571,0
.203545859053925,0.838708405468762,0.9620176974112
"14187","TMEM182",14.4424321237426,-0.110566166291408,0.54447358098554
7,-0.203069846091106,0.839080440002844,0.9620176974112
"14188","AC104162.2",15.9788892063775,0.0854582445514301,0.41975878003
2951,0.203588938734579,0.83867473772422,0.9620176974112
"14189","ADCY5",5.43641002328684,0.145378259379212,0.71212782398189,0.
20414629857646,0.838239175050077,0.9620176974112
"14190","ATF6B",207.085785714622,0.0325084287362332,0.160117842572138,
0.203028146107997,0.839113032917523,0.9620176974112
"14191","FRS3",32.3789251791754,0.0593289992665165,0.291910714788853,0
.203243650406703,0.838944596656418,0.9620176974112
"14192","PTK7",157.580705280963,0.0560835571422437,0.275632013676482,0
.203472580685314,0.838765674848941,0.9620176974112
"14193","DNPH1",463.09065568602,-0.0612480537574573,0.300050849006123,
-0.204125580581868,0.838255364754861,0.9620176974112
"14194","APTR",94.1455484974576,-0.048518404544479,0.237971066964401,-
0.203883628221649,0.838444439148337,0.9620176974112
"14195","TCIM",78.4476399329175,0.190349734274514,0.93756621374848,0.2
03025377283464,0.839115197054351,0.9620176974112
"14196","AZIN1-
AS1",66.2539440968686,-0.0535962844809331,0.262497448334589,-0.2041783
06573927,0.838214163109928,0.9620176974112
"14197","RAB18",438.46865511367,0.031910652634418,0.157094554407883,0.
203130227872601,0.839033245790594,0.9620176974112
"14198","CD5",3.30374358472965,0.163015989465109,0.799240940639082,0.2
03963512348054,0.838382012427603,0.9620176974112
"14199","CRACR2A",27.4898376457902,-0.0684526502575144,0.3367890229450
27,-0.203250835371459,0.838938981078309,0.9620176974112
"14200","FAM222A",2.54445438311373,0.227935016490308,1.11869000074127,
0.203751724194614,0.838547519872841,0.9620176974112
"14201","CDIPT",786.130860415346,-0.0296359419721102,0.145697142398643
,-0.203407846469789,0.838816267411583,0.9620176974112
"14202","PLCD3",157.878120034182,0.0338999747233962,0.166425801263836,
0.203694225690729,0.838592454830105,0.9620176974112
"14203","EFCAB13",43.3556554161266,0.0715015309401272,0.35140032747282
4,0.20347599404459,0.838763007180932,0.9620176974112
"14204","STK11",144.245478581827,0.0386426870210686,0.189128597879209,
0.204319639940167,0.838103723238331,0.9620176974112
"14205","ZNF17",27.0674692361421,0.0814521576849795,0.40112607333671,0
.203058746611586,0.839089115384417,0.9620176974112
"14206","MAFB",1116.21672782385,0.0517756794335464,0.254464012013749,0
.20346955557216,0.838768039089625,0.9620176974112
"14207","FMR1",213.796961882559,-0.0479239558264959,0.235983456884153,

-0.203081845055022,0.839071061603962,0.9620176974112
"14208","ARMCX3-
AS1",3.00396969430535,-0.191347730347183,0.941109314966577,-0.20332147
1059904,0.838883774532158,0.9620176974112
"14209","AL162231.2",98.3841696930777,-0.0430905100138248,0.2124538442
72035,-0.202822924487306,0.839273439178946,0.962131399168545
"14210","CFAP53",7.43018832019229,0.122403738276947,0.604297844779116,
0.20255531164717,0.839482621979791,0.962303478496046
"14211","WDR31",23.5608579704315,0.0625595186341929,0.309302120957597,
0.202260231648296,0.839713287960482,0.962500158158349
"14212","ARFRP1",231.660153567536,-0.0476451690479617,0.23584612182413
1,-0.202018030567789,0.839902628430515,0.962649445152312
"14213","TFDP2",585.130468561884,0.0378727058682284,0.18754841028931,0
.201935627232491,0.839967049274196,0.962655545319593
"14214","MYCBP2-
AS1",7.06311945033565,0.129160660890163,0.640136342729036,0.2017705483
48377,0.840096106995587,0.96273571738083
"14215","ZFP91",504.725290872538,-0.0401279129544248,0.198979111918649
,-0.2016689720217,0.840175520919655,0.962758991224781
"14216","SHISAL2A",3.86775254801369,0.169862273317128,0.85084641855362
,0.199639170610698,0.841762790691415,0.962918992692712
"14217","AL445183.2",6.64716746084546,0.140244200444469,0.703955712661
643,0.199223044748381,0.842088273623804,0.962918992692712
"14218","FPGT",59.868596755471,0.0536005852964737,0.266225792761872,0.
201335057510439,0.840436591845732,0.962918992692712
"14219","SILC1",6.75267544501609,-0.145183621245083,0.721403813303661,
-0.201251530096877,0.84050190046589,0.962918992692712
"14220","CEBPZOS",315.087483006663,0.0354978207451381,0.17876636631554
9,0.198571025840953,0.842598320265401,0.962918992692712
"14221","C1D",482.707625387319,-0.0488322917968911,0.245711764250134,-
0.198738110671737,0.842467610611663,0.962918992692712
"14222","IGKC",1290.5233635668,-0.162086207351635,0.815838600051237,-0
.198674354635164,0.842517486138318,0.962918992692712
"14223","CMC1",360.926647603949,-0.0488781900129693,0.245789981778615,
-0.198861603956643,0.842371005202371,0.962918992692712
"14224","CBLB",535.654677664656,0.0533017604604454,0.268329151253886,0
.198643197026374,0.842541860564395,0.962918992692712
"14225","TPRA1",173.679814495808,-0.0418376775280575,0.210027161145546
,-0.19920127139682,0.842105304922005,0.962918992692712
"14226","IFT122",101.841385069319,0.0499573459917668,0.249626385342838
,0.200128467682433,0.841380109715554,0.962918992692712
"14227","C5orf51",94.146535286931,-0.0419072144603671,0.21041551902812
8,-0.199164085681175,0.842134392071457,0.962918992692712
"14228","PFDN1",481.90181003189,0.048728284544796,0.244852575326679,0.
199010708708223,0.842254367806827,0.962918992692712
"14229","PCDHGA3",10.4075542913439,-0.0975249740397857,0.4880682145915
89,-0.199818326873414,0.841622667600013,0.962918992692712
"14230","MALSU1",354.487417319692,-0.0498399727962066,0.24892326805361
7,-0.200222233887237,0.841306779109807,0.962918992692712
"14231","PINX1",73.1077979050352,-0.0543306744867032,0.273450564888261

,-0.198685544895123,0.842508732096292,0.962918992692712
"14232","GINS4",7.72094311652769,-0.106162111313509,0.534647495161896,
-0.198564684720653,0.842603280978534,0.962918992692712
"14233","SNX16",93.6861301760744,0.0382730668142618,0.190757938319343,
0.200636823565318,0.840982562465993,0.962918992692712
"14234","CPSF1",100.54645574623,0.0426761875964349,0.213985332246898,0
.1994351068287,0.841922400750943,0.962918992692712
"14235","WDR74",434.222021965752,-0.0635591036425466,0.316592270735361
,-0.200760124354633,0.840886144213997,0.962918992692712
"14236","XRRA1",251.00399078236,0.0680773092154914,0.338762264675586,0
.200958950610055,0.840730671891734,0.962918992692712
"14237","PHLDB1",413.181158015098,-0.0466586269275787,0.23353437193745
5,-0.199793403174393,0.841642160817311,0.962918992692712
"14238","NDUFA9",316.365937756869,-0.0536102551728707,0.26667228710462
2,-0.201034219771918,0.840671816741326,0.962918992692712
"14239","PHC1",133.296568341661,0.0422190745559678,0.210966613232021,0
.200122066279442,0.84138511603383,0.962918992692712
"14240","GPRC5A",548.05224667554,-0.145141984448825,0.72801874107436,-
0.199365725440851,0.841976669418145,0.962918992692712
"14241","PRIM1",59.6403926417861,-0.0600284319183071,0.301420210503085
,-0.199151980612437,0.842143860859829,0.962918992692712
"14242","NT5DC3",16.762970381333,0.113111049662531,0.569706937711557,0
.198542517521174,0.842620622619063,0.962918992692712
"14243","AC079949.2",4.38196699187616,0.14491985282786,0.7248693492700
41,0.199925480328003,0.841538862302632,0.962918992692712
"14244","AC051619.6",5.2250169331083,-0.164212075625739,0.817100525687
752,-0.20096924486436,0.840722622461841,0.962918992692712
"14245","ZNF710",23.6383079552039,-0.0723305394566859,0.36228547126520
3,-0.199650676589617,0.841753791394931,0.962918992692712
"14246","SRRM2-
AS1",5.37856112488963,-0.12404052042184,0.622644241611413,-0.199215719
237717,0.842094003691438,0.962918992692712
"14247","HGS",204.370467941637,-0.0330211590367276,0.164064141849078,-
0.201269812309771,0.84048760583031,0.962918992692712
"14248","FOXK2",101.088231579161,-0.0401031341286732,0.20039929861925,
-0.200116140151106,0.841389750661774,0.962918992692712
"14249","AP001094.2",7.80274587966768,0.136303787149479,0.683147685943
54,0.19952316307889,0.841853526079207,0.962918992692712
"14250","CTDP1",39.2909629814098,-0.0606123089649495,0.304957294210901
,-0.198756711564445,0.842453059491829,0.962918992692712
"14251","ZNF101",20.0533814428708,0.0729315191946502,0.363279251697771
,0.200758834570947,0.840887152781472,0.962918992692712
"14252","PDRG1",107.201169779328,-0.0547393898650735,0.274182178606205
,-0.199646053377135,0.841757407395928,0.962918992692712
"14253","CLIC6",80.1035596618321,0.0573642985475728,0.288863944901002,
0.198585872554058,0.84258670557805,0.962918992692712
"14254","C22orf23",6.46971244721797,0.1662024379909,0.829451687016502,
0.200376273377323,0.841186314291075,0.962918992692712
"14255","CT70",3.8100620558558,0.194741304440556,0.981573640469021,0.1
98397039622523,0.842734433450389,0.962981493263653

"14256","AC092794.1",3.11732147060784,0.170908933440457,0.862291004486026,0.198203312514351,0.842885995888959,0.963048030038654
"14257","DDX23",205.437544305382,-0.0403894137312242,0.20381043425444,-0.198171471833485,0.842910907008478,0.963048030038654
"14258","AMMECR1L",75.0774136693091,0.0495349660968534,0.251096906278708,0.197274298719839,0.843612890776082,0.963280842184025
"14259","ATG16L1",42.0871799756012,0.0586168197393286,0.29692344851158,0.197413912687473,0.843503643102582,0.963280842184025
"14260","AL583856.2",2.56050275824477,-0.192787532300928,0.975738364336795,-0.197581174777282,0.843372764788863,0.963280842184025
"14261","ZNF425",20.345829207163,0.0633866000280844,0.321477468678037,0.197172760780839,0.843692345917101,0.963280842184025
"14262","ZFPM2",230.757129156123,0.0374515791201434,0.189959833086796,0.19715525388481,0.843706045517803,0.963280842184025
"14263","AL391684.1",5.15494983265716,0.131415729445466,0.665416035794103,0.197494082463215,0.84344091176095,0.963280842184025
"14264","ZNF408",34.0462505247967,0.0621570337132463,0.314903151127874,0.197384603776181,0.843526577022257,0.963280842184025
"14265","MAP6",12.3836960702872,-0.101428280344143,0.513900376891449,-0.197369538737598,0.843538365310619,0.963280842184025
"14266","TRAF4",36.1588374445206,0.0640890628812697,0.324281698228725,0.197633919001083,0.843331494677551,0.963280842184025
"14267","CCR7",6.47562507726167,-0.156634874661675,0.794265662940926,-0.197207158725335,0.843665428770147,0.963280842184025
"14268","AC114760.2",14.6026386763633,-0.147410210959447,0.748572080474116,-0.196921866049403,0.843888682074215,0.963303037517939
"14269","PCDHB13",22.7760191428301,0.0723777953959044,0.367867519440779,0.196749621999602,0.844023476231834,0.963303037517939
"14270","GTPBP10",89.2999327092656,0.0505019611734433,0.256447594916379,0.196928971745321,0.843883121421248,0.963303037517939
"14271","AAAS",109.556834367903,-0.0419530428998325,0.213210205737813,-0.196768455593644,0.84400873727991,0.963303037517939
"14272","PHLDA1",280.642895177489,-0.152511546300094,0.775038283799959,-0.196779371403875,0.844000194718625,0.963303037517939
"14273","THSD1",85.2844939502052,0.0703156466908605,0.357793349292433,0.19652586284769,0.844198591722548,0.963303037517939
"14274","SPIRE2",13.8200771879998,-0.102447520776487,0.521125252491323,-0.196589054717116,0.844149136539107,0.963303037517939
"14275","AL035461.2",2.8144542987958,0.189853670577022,0.965657126439815,0.196605674393948,0.844136129759494,0.963303037517939
"14276","MAML2",438.838159881464,0.0535009129961217,0.272936141797625,0.196019891846318,0.84459459657863,0.963687404291769
"14277","DTYMK",206.063076508613,-0.056146809844259,0.286556457580823,-0.19593629233926,0.844660030615494,0.963694560390542
"14278","LINC01715",5.39338865071991,0.12983402878512,0.663415125277195,0.19570556027174,0.844840632104207,0.963798081513102
"14279","ARID4B",839.753033998925,-0.0482059702152274,0.246364645263012,-0.195669188506184,0.844869102211651,0.963798081513102
"14280","KHDC4",83.9873309277303,0.0552199086743167,0.282530565017747,0.19544755687176,0.845042589408992,0.963877302769784

"14281","LINC02580",32.1895457611784,-0.0920729680366062,0.47660046557
0374,-0.193186903261661,0.84681259521885,0.963877302769784
"14282","KCNIP3",21.056823284037,0.0773149517239482,0.398193673278398,
0.19416418921827 8,0.846047322092422,0.963877302769784
"14283","MGAT5",33.707671115728,-0.0832250054368405,0.428593105685671,
-0.194181857647233,0.846033487994094,0.963877302769784
"14284","DIS3L2",80.5810659065493,0.044675078286801,0.231736186196238,
0.192784213031664,0.847127967736032,0.963877302769784
"14285","AC016252.1",2.53983648994951,-0.245385206842606,1.27150417237
088,-0.192988125540362,0.846968267723858,0.963877302769784
"14286","AC005674.2",5.35956682249089,0.194612527597616,1.002222676511
17,0.19418092621 4003,0.846034217290285,0.963877302769784
"14287","GPRIN3",10.9837017586132,0.133244002706291,0.689765672760367,
0.193172852706721,0.846823598695778,0.963877302769784
"14288","ANKS1A",108.387338149025,0.0454796321256439,0.234898566075461
,0.193613919767538,0.846478198038775,0.963877302769784
"14289","PPARD",61.5144733851291,0.0447324719467531,0.22975931420386,0
.194692746632518,0.845633490604397,0.963877302769784
"14290","SRF",78.3906425991888,-0.0471568334647715,0.242722739041372,-
0.194282718014045,0.845954516843011,0.963877302769784
"14291","SND1-
IT1",30.231848766992,-0.0797669925858589,0.411945813301792,-0.19363467
2352943,0.846461947366152,0.963877302769784
"14292","HIST4H4",10.3640254448473,0.102250550204189,0.525248815474731
,0.194670691664051,0.8456507575854,0.963877302769784
"14293","CNPY2",842.215555132405,0.0435083143909433,0.223671770414435,
0.194518576529921,0.845769851550727,0.963877302769784
"14294","AC011611.4",7.76730537358271,0.10807470868868,0.5574582100022
55,0.193870512173895,0.846277273485645,0.963877302769784
"14295","EEA1",1505.9601503431,0.034356554615201,0.177707569676895,0.1
93331970481998,0.846698989793322,0.963877302769784
"14296","LINC00562",4.29899034612809,-0.148223460926707,0.767468430450
987,-0.193132974654875,0.846854828744042,0.963877302769784
"14297","GNPNAT1",130.145319658185,-0.0374310590632196,0.1926012622245
48,-0.194344827395679,0.845905887516425,0.963877302769784
"14298","PNMA1",240.675090617621,-0.0415228203112001,0.213094510661947
,-0.194856358252568,0.845505400298243,0.963877302769784
"14299","KLC1",377.472991372362,0.0339404853562868,0.175891970315902,0
.192962107908222,0.846988643839578,0.963877302769784
"14300","PDCD7",534.614334339395,0.0270688993391687,0.13893388966603,0
.194832948276602,0.845523727542,0.963877302769784
"14301","CSK",120.436035809432,0.0396018668229682,0.205311535128087,0.
192886711398178,0.847047692373893,0.963877302769784
"14302","TAOK2",65.8303632337685,0.0455820397695809,0.236321461848162,
0.192881507304096,0.847051768113166,0.963877302769784
"14303","FXR2",81.1389942937932,-0.0427208657063335,0.220300922441216,
-0.193920503068855,0.846238129303728,0.963877302769784
"14304","FAM106A",52.3905853035032,-0.102417025769821,0.52541564025764
2,-0.194925727219693,0.845451093088414,0.963877302769784
"14305","MTRNR2L1",3.32481729243163,-0.185997335657729,0.9525591323216

53,-0.195260671329035,0.845188884154203,0.963877302769784
"14306","AC104984.3",10.1776419741608,0.105376839128085,0.542589427573969,0.194211007020994,0.846010664602677,0.963877302769784
"14307","MED24",119.555698814447,0.0329798122494971,0.168812759261184,0.195363267526902,0.845108570781735,0.963877302769784
"14308","YPEL2",395.778794437449,-0.0680336425903338,0.352766799550593,-0.192857271934335,0.847070748809364,0.963877302769784
"14309","RAB11B-AS1",126.726144741757,0.0509498635311307,0.26426194848639,0.192800604941254,0.847115129703123,0.963877302769784
"14310","AC022137.4",20.6753773989686,-0.100785654598302,0.519967755849168,-0.193830585578729,0.846308537329362,0.963877302769784
"14311","DUXA",3.30115431042545,-0.17670119946251,0.910916414984321,-0.193981793011768,0.84619013818874,0.963877302769784
"14312","RIN2",344.990734711325,-0.0601329046941742,0.311243929686447,-0.193201855389608,0.846800885721632,0.963877302769784
"14313","CSTF1",88.6702412330512,0.0395901789792621,0.204196194751622,0.193883039923532,0.846267463893475,0.963877302769784
"14314","HAR1A",8.7557365876421,0.116100049258667,0.597113982419025,0.194435321692389,0.845835034911981,0.963877302769784
"14315","RPA4",16.7777900716405,-0.0892984068429381,0.458077495279756,-0.194941702578954,0.84543858648773,0.963877302769784
"14316","AL136169.1",28.107497808078,0.0818452642860749,0.420276299365039,0.194741565036449,0.845595270616071,0.963877302769784
"14317","AL359220.1",3.60089051974293,0.161583088768871,0.838551301526672,0.192693146471411,0.847199291189801,0.96389112622691
"14318","GORAB",59.2495718812182,0.0438167716119384,0.227997129323903,0.192181242552797,0.847600238309845,0.963908215332782
"14319","EGLN1",186.586811571473,0.0293420484682403,0.152634022056496,0.192237930134474,0.847555835997258,0.963908215332782
"14320","YPEL5",516.650004542126,0.0397893455994978,0.206915467462101,0.192297589385316,0.847509106554899,0.963908215332782
"14321","LINC02478",13.575833428607,-0.132511747370643,0.688150379041815,-0.192562194843448,0.847301854876547,0.963908215332782
"14322","MIOS",60.368437505259,-0.0476633073815288,0.248254125459194,-0.191994019408001,0.847746890101138,0.963908215332782
"14323","FLVCR2",26.8185033751466,0.0647305906541558,0.33685970307628,0.19215890194945,0.847617737415597,0.963908215332782
"14324","CORO7",48.5656690324736,-0.0480798417762314,0.250245817099982,-0.192130451303495,0.847640022547476,0.963908215332782
"14325","ZFP3",16.9980289568377,0.0817660785644267,0.425802366260054,0.192028239022253,0.84772008550136,0.963908215332782
"14326","LINC00665",32.8106044713796,0.0847690823042462,0.441373032729061,0.192057683678835,0.847697021319367,0.963908215332782
"14327","AC233266.2",6.24169811828533,-0.147512906877004,0.772203690897028,-0.191028492373102,0.848503270506844,0.964206282583184
"14328","AC021851.1",4.83205233532675,0.130380469859603,0.682362399722474,0.191072177940389,0.848469044824379,0.964206282583184
"14329","AC005618.4",3.31132159087169,-0.16724860225557,0.875847660327207,-0.190956269944351,0.848559854166649,0.964206282583184

"14330","TMEM254-
AS1",18.628191202295,-0.0896494744606947,0.469561777126232,-0.19092157
5877319,0.848587035990376,0.964206282583184
"14331","CASC2",17.523597444589,-0.0703791980179354,0.368356359747566,
-0.191062801430023,0.848476390875593,0.964206282583184
"14332","VPS18",62.9063415081435,0.0519891278293491,0.271574931139093,
0.19143566606564,0.848184279266046,0.964206282583184
"14333","PHF23",90.4546078775932,0.056260497570254,0.294706033933794,0
.190903785780283,0.848600974099854,0.964206282583184
"14334","NARF",192.120259364849,-0.0328575525156347,0.171810411838177,
-0.191243081045532,0.848335152652863,0.964206282583184
"14335","MAST3",16.9428298563275,0.0866750144665209,0.453649240164984,
0.191061742845637,0.848477220227086,0.964206282583184
"14336","MTG2",100.548521960309,0.0492045408630729,0.257606928559201,0
.19100627897811,0.848520673814989,0.964206282583184
"14337","SLC31A1",86.2483074157566,-0.0499072593600789,0.2617239521488
75,-0.190686633570665,0.848771111402538,0.964265074183006
"14338","IL12RB1",3.34313029066116,-0.147470714539999,0.77324948967658
,-0.190715566591167,0.848748442167665,0.964265074183006
"14339","CR1",2.51341018374598,-0.207446868119,1.08863065302659,-0.190
557621671096,0.848872194698934,0.964312656353368
"14340","CHN2",13.7387838865903,0.100692334106755,0.528793599633669,0.
190418972878097,0.848980831456483,0.964368811966154
"14341","NRP2",55.270891760112,0.162097045150793,0.852154692732221,0.1
90220210641649,0.849136574453072,0.964411208315518
"14342","SPATA5",49.6091257584493,0.0578246422400825,0.303929653218509
,0.190256665079369,0.849108009615244,0.964411208315518
"14343","ATXN10",562.094421192764,-0.0418234215581113,0.21995580263176
6,-0.190144661144171,0.849195773888481,0.964411208315518
"14344","CASZ1",4.09558655160775,-0.16478855865419,0.870987310668405,-
0.189197427604002,0.849938083729933,0.964636916572243
"14345","MCL1",4446.44645966588,-0.0609111673946447,0.322050638777652,
-0.189135372082583,0.849986718863123,0.964636916572243
"14346","RABGAP1L",353.320332713321,0.0360171217235034,0.1900478355736
39,0.189516084804594,0.849688349674995,0.964636916572243
"14347","C3orf38",175.145809071624,-0.041695816576345,0.22020850978055
4,-0.18934698126742,0.849820875517831,0.964636916572243
"14348","HERC6",26.713310960059,-0.0627949830861032,0.33109753235245,-
0.189657055550806,0.849577874646611,0.964636916572243
"14349","DCAF12",106.844332941626,0.0344656883577632,0.182206567313231
,0.189157223397515,0.8499695931404,0.964636916572243
"14350","GEMIN2",115.729320130743,0.0512804872270708,0.27108387116356,
0.18916834486302,0.849960876843868,0.964636916572243
"14351","TMEM8A",74.9074127927279,-0.0441070389759438,0.23258720366720
4,-0.189636567620694,0.849593930309712,0.964636916572243
"14352","CEP112",121.449884802879,0.0414782450009025,0.218657942723511
,0.189694664114493,0.849548402313475,0.964636916572243
"14353","AC004832.6",4.5712381383827,0.226551475180112,1.1964214707955
7,0.189357580677204,0.849812568673252,0.964636916572243
"14354","SEPSECS-

AS1",29.6071217952797,-0.0594296468402445,0.314860568122998,-0.1887490
93589353,0.850289471972267,0.964855514052279
"14355","ZNF823",31.0213379466627,-0.0562035629122832,0.29778541878801
8,-0.188738465237925,0.850297802456901,0.964855514052279
"14356","AL118558.3",22.5883732342266,-0.0783362114698928,0.4155684846
24566,-0.188503734927502,0.850481787952338,0.964929849129736
"14357","SPAG7",506.237124423336,0.0486170402777314,0.257873851485764,
0.188530322084305,0.850460948090555,0.964929849129736
"14358","PNPLA7",50.3977567721686,0.0836376111695836,0.443899917843194
,0.188415468910108,0.850550974421822,0.96494113541977
"14359","MAGI3",190.075125725962,-0.0497795549224171,0.266380465895205
,-0.186873893906322,0.851759508249366,0.964969764030631
"14360","AP001816.1",98.0265151418763,-0.0454439130727716,0.2417852427
68502,-0.187951557971145,0.850914625457008,0.964969764030631
"14361","PLA2G12A",307.527164979769,-0.0250698830766759,0.133755249101
454,-0.187431022297004,0.851322701301377,0.964969764030631
"14362","AC008549.2",9.48083408675859,0.118711603618156,0.631234249319
646,0.18806267838937,0.850827517366182,0.964969764030631
"14363","ZNF322",278.261559758413,-0.0311907640293714,0.16690128308731
2,-0.186881511348565,0.851753535617617,0.964969764030631
"14364","AL590062.1",21.523045482887,0.0696385759186196,0.372367721639
676,0.187015608152003,0.851648395310388,0.964969764030631
"14365","AP003469.4",13.3295346539409,-0.0840562201882007,0.4501704918
85368,-0.186720857327105,0.851879501919116,0.964969764030631
"14366","AL590399.3",3.67261465477686,0.159048451760009,0.849447655729
34,0.187237495668228,0.851474427368197,0.964969764030631
"14367","AL162390.1",49.4008378279147,-0.0711960290708252,0.3785555784
22131,-0.188072856745579,0.850819538570921,0.964969764030631
"14368","STX17-
AS1",7.10410757469148,-0.108328157645134,0.579498516296142,-0.18693431
406436,0.85171213466096,0.964969764030631
"14369","PPP1R26-
AS1",3.76144975068355,0.172051350774316,0.920407796277556,0.1869294800
30646,0.851715924858492,0.964969764030631
"14370","HECTD2",72.1229368495607,-0.0405922378551155,0.21601708084136
9,-0.187912167394412,0.850945504448735,0.964969764030631
"14371","ALKBH2",148.149162530547,-0.0515030626963564,0.27569160745329
6,-0.18681403896229,0.85180643919826,0.964969764030631
"14372","NBEA",37.7114895024844,-0.0796490680565937,0.426483447486779,
-0.18675770074069,0.851850613241655,0.964969764030631
"14373","CHMP4A",485.553339985521,-0.0433340748421296,0.23171075927179
6,-0.187017965753152,0.851646546826749,0.964969764030631
"14374","SNRPA1",229.280490834654,0.0436915744998412,0.233847079154017
,0.18683823059883,0.851787471003416,0.964969764030631
"14375","AC009061.2",27.9372628447546,0.070040899148146,0.373051677850
957,0.187751197237957,0.851071694266059,0.964969764030631
"14376","ALOX12-
AS1",73.680077346194,0.0429830756156948,0.228662835800132,0.1879757830
57572,0.850895635083334,0.964969764030631
"14377","GIT1",113.601508182977,0.0351623971330142,0.187538812781332,0

.187493973175639,0.851273348611465,0.964969764030631
"14378","FAM171A2",24.6374516722702,0.0805398417865305,0.427814036271107,0.188258997971474,0.850673625490847,0.964969764030631
"14379","TXNRD2",91.1798448161717,-0.0464052406363625,0.247819602677437,-0.187254116038446,0.85146139667987,0.964969764030631
"14380","TOM1",194.970541281951,-0.0425780977679593,0.227809995286632,-0.186901798204189,0.851737629280794,0.964969764030631
"14381","FBXW2",213.313310576378,0.0353900836359164,0.189649930424615,0.186607416921695,0.851968451040227,0.964978845731281
"14382","FCSK",25.28076136013,0.0613416101005662,0.328804488156556,0.186559527956805,0.852006001553642,0.964978845731281
"14383","GNPAT",158.350489552017,-0.0331146212940906,0.177959810091926,-0.186079212362528,0.852382643337446,0.965002841070591
"14384","KRTCAP3",3.63673103934514,0.15301646714035,0.821129862808939,0.186348681336357,0.852171333794585,0.965002841070591
"14385","AC061958.1",3.74054423650139,-0.183100843030591,0.983029195227431,-0.186261856636139,0.852239417980413,0.965002841070591
"14386","CYB5R4",80.2735489040357,-0.0429347577817538,0.230634026473952,-0.186159685273513,0.852319537767286,0.965002841070591
"14387","AK9",38.8656948409874,0.0595439203264175,0.319973239645717,0.186090313028509,0.852373938316471,0.965002841070591
"14388","ILF3",402.201426891302,-0.0365129661031381,0.196108001523947,-0.186188048521209,0.852297295987225,0.965002841070591
"14389","AC114730.2",4.52412868312594,0.133740132211231,0.719948681082992,0.185763424151358,0.852630288529886,0.965090421187707
"14390","ZSCAN26",92.6697756509962,0.0417843938461243,0.225128012435349,0.185602819454215,0.852756242381648,0.965090421187707
"14391","AC127024.6",5.02742875586089,0.126583312219502,0.681437444147522,0.185759255389697,0.852633557823815,0.965090421187707
"14392","AC006213.4",4.73218700872571,0.148878922956907,0.802126420775921,0.185605309962103,0.852754289178092,0.965090421187707
"14393","CEP250",56.6947273028196,0.0586851765129642,0.316006467590093,0.185708782989491,0.852673140309534,0.965090421187707
"14394","BVES-AS1",3.99467652578683,-0.144141764817079,0.77822308856667,-0.185219080408626,0.853057204012404,0.965251132333824
"14395","SNX10",51.6187569495929,-0.101160048514312,0.546235048303212,-0.185195089235942,0.85307602069358,0.965251132333824
"14396","MYO1C",609.855450026163,0.034674899159089,0.187204042148746,0.185225162667895,0.85305243360721,0.965251132333824
"14397","MB21D2",36.1175271274534,0.068456641688343,0.370641259504002,0.184697844433058,0.853466036982835,0.965432283343743
"14398","CLDN4",6.73839072596637,0.14546137433166,0.787602922607094,0.184688718333039,0.853473195417147,0.965432283343743
"14399","IL11RA",818.134888497503,0.0495589116357309,0.268196925894496,0.184785532013244,0.85339725621465,0.965432283343743
"14400","TMED1",383.043431934593,-0.0431326754562203,0.233404858069261,-0.184797676505178,0.85338773035273,0.965432283343743
"14401","GREB1",9.16746241977499,-0.113283271979791,0.6151584823207,-0.184152986970817,0.853893439610852,0.96565268547147

"14402","AC013643.2",6.28320836059957,0.144192060269216,0.782219210400389,0.184337150445858,0.853748971421879,0.96565268547147
"14403","FAT3",3.90803032951311,0.246858574452268,1.34031146795342,0.184180006181106,0.853872243915676,0.96565268547147
"14404","MYH11",11.7782713393921,0.116535252905721,0.632868989308242,0.184138036267348,0.853905167998714,0.96565268547147
"14405","GPR137B",145.537828457635,0.0562024349976321,0.305417148247382,0.184018596598607,0.853998866070767,0.965677264352232
"14406","P4HA3",22.4225722623796,-0.122134559284947,0.664467946118922,-0.183808052741025,0.854164038601938,0.965677264352232
"14407","RCAN1",129.986592044367,-0.048526587990286,0.263812697591737,-0.183943337198209,0.854057906648583,0.965677264352232
"14408","SLC16A8",24.4949543416902,0.0752900136128972,0.409568212024406,0.183827776185938,0.854148565205975,0.965677264352232
"14409","CCN2",4344.79490349376,0.083140367142696,0.453293924454846,0.183413813107433,0.854473338418078,0.965892866723947
"14410","PXMP4",125.182081577628,-0.0350306089106476,0.190979727751867,-0.183425797716926,0.854463935589851,0.965892866723947
"14411","IL22RA1",4.62630648564932,-0.149113360758873,0.815491841051059,-0.182850830937421,0.854915063573759,0.965932673252349
"14412","TOPBP1",153.269977745667,0.0395793471022677,0.216119248410603,0.183136612742013,0.854690828734642,0.965932673252349
"14413","EXOSC1",192.247409747809,0.041135798368496,0.22480867433934,0.182981366219006,0.854812639370533,0.965932673252349
"14414","TBC1D24",76.4203462351073,0.0472872404763942,0.258249477433141,0.183106819600967,0.85471420497335,0.965932673252349
"14415","CHD9",1308.43563840587,-0.0276887025946053,0.151404169501508,-0.182879392857998,0.854892652320618,0.965932673252349
"14416","FMNL1",168.58041727439,0.0414351053348673,0.226619618134426,0.18283988683755,0.854923650947211,0.965932673252349
"14417","HUWE1",347.887464312484,-0.0307613150431248,0.168154450531498,-0.182934884838881,0.854849110596295,0.965932673252349
"14418","PHC2",321.004733225396,0.0365001228321438,0.20009771876284,0.182411489035537,0.855259810120188,0.96596251854959
"14419","CRIPT",351.557629966644,0.0287131437564673,0.157521871139432,0.18228036239521,0.855362709046102,0.96596251854959
"14420","RBM5-AS1",10.4770426796347,-0.125028995958596,0.685069809960172,-0.182505482128697,0.855186052493687,0.96596251854959
"14421","PHYKPL",468.42612733221,0.0287506118349466,0.157772952172375,0.182227761090096,0.855403987526451,0.96596251854959
"14422","ZNHIT1",1111.40718680687,-0.0443321999969912,0.243133262283471,-0.182337042577514,0.855318230140431,0.96596251854959
"14423","C12orf66",28.813837921135,-0.0529174047308107,0.290433181331966,-0.182201649577794,0.85542447848719,0.96596251854959
"14424","IFNG",5.75175887438518,-0.143564845575623,0.7873948170376,-0.18232891869387,0.855324605205581,0.96596251854959
"14425","LINC00323",8.75598025880552,-0.120709750569686,0.661787378729749,-0.18239959607779,0.855269142771969,0.96596251854959
"14426","CDK16",154.174092514528,0.0304549399890666,0.16726498883509,0

.182075999294107,0.855523083676759,0.966006897962757
"14427","BRD3OS",93.9811613118744,0.0342928940029286,0.188503056733912
,0.181922217056331,0.855643768740082,0.966076200804547
"14428","NAA50",328.975166761634,-0.0246587708700449,0.135701977871711
,-0.18171268581919,0.855808209878386,0.966194894005339
"14429","NCAM2",92.5009080972416,0.0799392779299467,0.440415961779302,
0.181508584763795,0.855968395403431,0.966308766562234
"14430","AC091978.1",3.06033355162422,-0.150276820433435,0.82939948981
8913,-0.181187500448361,0.856220405421374,0.966332413375072
"14431","ELN",2336.58931497135,0.0486876063283777,0.268627221610229,0.
181245988535824,0.856174498679257,0.966332413375072
"14432","UGCG",415.818812718892,0.0662736836671672,0.365389278042909,0
.181378293370132,0.856070655672171,0.966332413375072
"14433","CCDC144A",107.337252188775,-0.148626344564783,0.8203262318802
09,-0.181179558557,0.856226638973689,0.966332413375072
"14434","EFHB",5.46333059836749,-0.201382781246411,1.11269220508818,-0
.180986961466537,0.8563778102486,0.966427703245517
"14435","TTC3",1506.85586736444,0.0319222448681917,0.176443186502904,0
.180920813667499,0.856429731496656,0.966427703245517
"14436","AC092445.1",3.35236969517006,0.152499908466779,0.844734478078
115,0.18052999187832,0.856736511067536,0.966573003794092
"14437","RTN4IP1",14.6544387093885,0.0788764087519831,0.43679654742949
9,0.180579286205815,0.856697815785868,0.966573003794092
"14438","TTI2",68.2556271104737,-0.0513053666071634,0.28417054830929,-
0.180544278470838,0.856725296278801,0.966573003794092
"14439","CAP2",31.3943776209362,-0.0972658795249724,0.539744761006138,
-0.180207176710079,0.85698992446947,0.96662309479013
"14440","APOM",37.0045841322432,-0.0679681283134563,0.377752705876288,
-0.179927574987949,0.857209426763558,0.96662309479013
"14441","POLR2J2",14.7618158876497,-0.0990010282248441,0.5497685143217
78,-0.180077661135209,0.857091599743232,0.96662309479013
"14442","DDB2",106.930815261487,-0.0438439242939472,0.243366623672186,
-0.180155863743275,0.857030207062527,0.96662309479013
"14443","C11orf24",140.284878921379,-0.0457167998260942,0.254055789526
019,-0.179947876454168,0.857193488655644,0.96662309479013
"14444","TIMM9",374.578084691807,-0.043308771457755,0.240515164099208,
-0.180066698164158,0.857100206253961,0.96662309479013
"14445","AC011939.2",60.9751226161985,0.0932010284467012,0.51733103486
7313,0.180157427575567,0.85702897939034,0.96662309479013
"14446","AC011815.2",4.8398403498342,-0.135104928230114,0.751130834367
192,-0.17986870202704,0.85725564659207,0.96662309479013
"14447","HCG18",217.561103817871,0.0457662278169626,0.254581732119301,
0.179770274308275,0.857332921058405,0.96664331356824
"14448","RCN1",992.36082868944,-0.0357945478140174,0.199291596946524,-
0.179608916594823,0.857459604095259,0.966719233880653
"14449","CDC34",166.342338517936,-0.0432543147073107,0.241065507685881
,-0.179429712373755,0.857600302849385,0.966810944225458
"14450","TPH1",7.32526794224071,-0.136620659100723,0.761933756990421,-
0.17930779132344,0.857696029407946,0.966851946230175
"14451","TMEM19",111.017820486423,0.0346514690005901,0.193548074537969

,0.17903287895428,0.857911885086406,0.966920940360872
"14452","FBXO33",143.585362183129,0.0490762415193768,0.273936183993668
,0.179152095951337,0.857818276985818,0.966920940360872
"14453","MYO19",65.4402925185433,-0.0598951329383994,0.334604000066265
,-0.179003039194205,0.857935315306998,0.966920940360872
"14454","C2orf92",5.72532617769681,-0.111434419862113,0.62325120824820
6,-0.178795353121458,0.858098394162253,0.966929942439011
"14455","ADH5",1860.65612667483,0.0351890454347651,0.196699122827533,0
.178897825922788,0.858017929910666,0.966929942439011
"14456","AC044802.2",4.03488537947856,0.172760774992043,0.966406944703
847,0.178766073587132,0.858121385468619,0.966929942439011
"14457","MXD3",12.138056052947,0.0762969026072912,0.427067711809197,0.
178652940734089,0.858210222441236,0.966963153721055
"14458","KCTD14",4.41660409416069,0.129011253989088,0.722693373434525,
0.178514510761287,0.858318926285493,0.967018743274616
"14459","AC005523.2",20.5509946041753,-0.0594190804435674,0.3331345607
73438,-0.178363602700405,0.85843743176274,0.967085367313319
"14460","CHRNE",3.18200975036521,0.154060648960351,0.865098348233917,0
.1780845487393,0.858656576388999,0.967265350816072
"14461","CIAO1",389.452410765288,0.0252385557686911,0.142267382240321,
0.177402264463246,0.859192428744693,0.967660247145306
"14462","STK36",30.7934227812541,0.0626463395357947,0.35281696807967,0
.177560449761726,0.859068187406311,0.967660247145306
"14463","ARHGAP42",111.638164936463,0.0389870463508688,0.2196757778491
58,0.177475399120423,0.859134987147053,0.967660247145306
"14464","PEX11G",73.8194899077674,0.0549988134080877,0.310139644456476
,0.177335643446919,0.859244755031598,0.967660247145306
"14465","SYNE1",1051.49366917809,0.0417464881408686,0.235634763732155,
0.177166083135007,0.859377935937394,0.96766354486479
"14466","TERF2",138.702117114133,0.0369095916819286,0.208405116254905,
0.177105017118599,0.859425901133213,0.96766354486479
"14467","Z95114.4",2.2890549243824,0.187675853495503,1.05881744081773,
0.17725043644026,0.859311680253447,0.96766354486479
"14468","STAMBP",205.868553345399,-0.0259870197890642,0.14709286641901
1,-0.176670836742261,0.859766949406733,0.967913735495631
"14469","AC022868.2",3.99446421992237,0.164897165651547,0.932969129082
254,0.176744503661931,0.859709082266686,0.967913735495631
"14470","GMPR",25.5644824289643,-0.0600895945305958,0.340321066854875,
-0.1765673664752,0.859848229065864,0.967938341620861
"14471","UBQLN4",48.3263152578426,-0.0440105651976696,0.24959662757004
9,-0.176326762208829,0.86003723821034,0.968017314345511
"14472","TBC1D14",89.5388558338106,-0.0344538514007695,0.1953665961023
63,-0.176354873802056,0.860015154446583,0.968017314345511
"14473","PLPPR4",6.68026093644908,0.137617211642512,0.781325568702351,
0.176132993920922,0.860189460702356,0.968080409283671
"14474","NR6A1",2.48509300564688,0.164060044606521,0.932409381143264,0
.175952803483552,0.860331021228462,0.968080409283671
"14475","AL079343.1",32.9797686453959,0.07038865254019,0.3999161363425
6,0.176008533148801,0.860287238621188,0.968080409283671
"14476","AC092376.2",38.7981969967711,-0.0527298164603976,0.2994601071

27813,-0.17608294128437,0.860228782413859,0.968080409283671
"14477","NBPF20",24.9334082647223,0.0753642598771767,0.428588372370794
,0.175842987667372,0.860417296513784,0.968087441977094
"14478","UBE2V2",450.554560646306,-0.0372617655853117,0.21205445930619
1,-0.175717906179509,0.86051556709352,0.968087441977094
"14479","PRPF39",76.968085448405,-0.0408554900308222,0.232426612182865
,-0.175778021488686,0.860468337082779,0.968087441977094
"14480","ANKH",215.306525987873,0.0299259450819789,0.170512926188426,0
.175505433816256,0.860682501480159,0.968208374765906
"14481","PGAP2",72.5675004081969,-0.0429077976684515,0.244657813645353
,-0.175378815943517,0.860781985034922,0.968253418564591
"14482","BX470102.2",10.7691001793005,-0.10377759446705,0.592528991632
909,-0.175143488221659,0.860966887692833,0.968394533464201
"14483","AC103706.1",14.900775598002,-0.115898616234229,0.662739746400
399,-0.174878022426933,0.861175479707498,0.968562272247147
"14484","KDM1A",208.245726133414,0.0308238159522668,0.176714181112758,
0.174427517690834,0.86152948980406,0.968566939588469
"14485","AL160408.2",2.85253661156615,-0.166740327452485,0.95597720797
8398,-0.174418726786479,0.861536398041459,0.968566939588469
"14486","TANK",570.957469289049,-0.0236751584270039,0.135539732486453,
-0.17467319724399,0.861336429462889,0.968566939588469
"14487","CBX3",800.705803651867,0.0242635575097,0.138982383854192,0.17
4580093079675,0.861409591767732,0.968566939588469
"14488","OXR1",482.172616441199,-0.02883877320069,0.165302652235512,-0
.174460438539138,0.861503619404509,0.968566939588469
"14489","AC005519.1",3.52223764644402,-0.143134153264339,0.81890952420
3991,-0.174786284728427,0.861247565639528,0.968566939588469
"14490","RASAL2",80.3944851862247,-0.0628594288434145,0.36075599634692
5,-0.174243614742207,0.861674010159613,0.968654793063488
"14491","TSHZ3",94.461554883753,-0.0353018358253267,0.20269464999781,-
0.174162642308064,0.861737643914045,0.968659477035117
"14492","NAGK",324.379409888829,-0.0369804067883446,0.212990828215709,
-0.173624409549186,0.862160647332468,0.96866704265406
"14493","AC011416.3",5.19742810375124,0.114330407010047,0.658479782813
012,0.173627816668312,0.862157969513255,0.96866704265406
"14494","STC2",26.1920040877124,-0.122386176092087,0.703842614295786,-
0.173882873253614,0.861957512744069,0.96866704265406
"14495","FAM162B",32.908187936284,0.103793063018824,0.597137049511759,
0.173817824741722,0.862008635514517,0.96866704265406
"14496","FAM20C",270.472896777615,-0.0296460670437773,0.17063429319767
3,-0.173740380601182,0.862069500981429,0.96866704265406
"14497","AC027807.2",6.17659793940902,0.11198523736549,0.6445626394148
73,0.173738331261566,0.862071111624574,0.96866704265406
"14498","APOBEC3A",3.40709348766284,-0.173434185894067,0.9988772459845
41,-0.17362912869551,0.862156938328235,0.96866704265406
"14499","AL592211.1",2.36064845301786,0.169564977802829,0.977344598927
864,0.173495589978028,0.862261894007072,0.968713979686958
"14500","SRSF4",632.151045230177,-0.0308916756165696,0.178258464050288
,-0.173297104186059,0.86241789978113,0.968794306491627
"14501","TIGD2",7.20228281018579,-0.104710098465939,0.605062703083941,

-0.173056607079304,0.862606932779185,0.968794306491627
"14502","AC067817.2",3.35308929837541,-0.16338412914368,0.944273488067302,-0.173026280212619,0.862630770541749,0.968794306491627
"14503","ZNF891",62.2514756632152,0.0595758184588028,0.344110336146184,0.173129988264851,0.862549253639512,0.968794306491627
"14504","RNF167",293.108076798857,0.0306514896664922,0.177007029115097,0.17316538117004,0.862521434341064,0.968794306491627
"14505","AL031432.4",4.1337704835172,0.16146194378566,0.934890191078816,0.17270685405239,0.862881856000659,0.968942682503429
"14506","NDOR1",8.11462303063682,0.0960704493220468,0.556125334017668,0.172749636539656,0.862848225952199,0.968942682503429
"14507","WARS2-IT1",23.0125267657036,-0.0759689645218282,0.441239780390122,-0.172171612574597,0.863302614484552,0.968967056258075
"14508","B4GALT3",83.9828734589577,0.0530192801638132,0.308526286103825,0.171846881616989,0.863557907526458,0.968967056258075
"14509","GFPT1",301.910263775121,0.0327938385127698,0.1899974083894,0.172601504361359,0.862964669337516,0.968967056258075
"14510","PRRT3-AS1",11.2756398793877,0.0782162913290638,0.45462509890641,0.172045695491101,0.863401604755807,0.968967056258075
"14511","IRF5",2.5229832614624,-0.185826619353432,1.08098146741609,-0.171905462725111,0.863511851888081,0.968967056258075
"14512","ACER2",5.84217215293192,-0.130069051645039,0.756671057569738,-0.171896427574212,0.863518955165638,0.968967056258075
"14513","MTRNR2L8",8.91517347632425,-0.135639232016903,0.787508223234577,-0.172238496075361,0.863250034597658,0.968967056258075
"14514","FOLR1",2.31311017061924,0.182554787721208,1.06092383399327,0.172071530369979,0.863381294376777,0.968967056258075
"14515","JPT2",49.2435857223403,0.0617889955027453,0.359274936809114,0.171982482417287,0.863451300801564,0.968967056258075
"14516","ENO3",5.99751155974354,-0.115094346842756,0.668208952451663,-0.172243048256797,0.863246455961522,0.968967056258075
"14517","APOL3",509.053052990428,-0.03257039460145,0.189400937909124,-0.171965329005274,0.863464786344799,0.968967056258075
"14518","MMP27",31.7645392319678,-0.103081334141091,0.600395495243911,-0.171689053228513,0.863681992279312,0.969039535214059
"14519","MACO1",236.0913005859,-0.0285932567140959,0.16700690959168,-0.171210022291918,0.864058627203415,0.969103521170351
"14520","IFI16",1814.8356042284,0.0329548530221176,0.192131176644206,0.171522673195014,0.86381280398006,0.969103521170351
"14521","LINC01139",15.5212562145407,-0.117114701118689,0.685326997620066,-0.17088878962217,0.864311211586163,0.969103521170351
"14522","CYTOR",807.059111737264,0.0449827454278623,0.263272761932562,0.170859853095569,0.864333964980224,0.969103521170351
"14523","GPR35",3.20738244178014,-0.143912054248886,0.841743435150122,-0.170969024811248,0.864248121573024,0.969103521170351
"14524","JMJD1C",955.570198007289,-0.0406973275940545,0.23736676355084,-0.171453353389713,0.863867305862103,0.969103521170351
"14525","AL158163.2",2.53016380254974,0.150157590875726,0.878028719249

946,0.171016719138752,0.864210619289065,0.969103521170351
"14526","KRAS",355.298713948219,0.03420544400942,0.199706202834283,0.171278826215547,0.864004528669089,0.969103521170351
"14527","ABCA3",15.0523229321928,0.0667013032176629,0.389294227527634,0.171339050263539,0.863957176765946,0.969103521170351
"14528","AC234772.2",11.4209452737139,-0.146565015020708,0.857595760541593,-0.170902214964483,0.864300654997854,0.969103521170351
"14529","SSBP3",385.869211964682,0.0331550277820416,0.19431354286881 9,0.170626438551556,0.864517507813815,0.969242596515881
"14530","C7orf31",21.8724525262203,0.0848959310776095,0.498056576893513,0.170454392163886,0.864652799186084,0.969327559940958
"14531","NEPRO",136.594924507054,0.0341580587826308,0.200636056410023,0.170248854537017,0.864814432119974,0.969442040107512
"14532","SMIM10L2B",4.76763834971465,-0.109498435567669,0.64448397466866,-0.169900943811619,0.865088038859289,0.969682016582643
"14533","PLA2G2A",20925.0467497078,-0.0844601220464045,0.49804973105887,-0.169581703953217,0.865339112255653,0.96972722733613
"14534","KIAA0232",178.966260002097,0.0353321492042123,0.208917511796804,0.169120093860666,0.865702179968807,0.96972722733613
"14535","ADAMTS12",78.8748566205401,0.0412458827471913,0.2432166929716 3,0.169584917232644,0.865336585031717,0.96972722733613
"14536","NCAPG2",36.3019239585181,-0.0438225162328249,0.259162620329083,-0.169092734813297,0.865723699424274,0.96972722733613
"14537","SGMS1-AS1",35.580277859252,0.0473771956465687,0.280027224522934,0.1691878199603,0.86564890994642,0.96972722733613
"14538","PTEN",879.795233622624,0.0373853668571068,0.22021725690657 2,0.169765836620913,0.865194295300063,0.96972722733613
"14539","OSBP",208.41701140624,0.0359788111231866,0.212525213210646,0.169291965784436,0.865566995149228,0.96972722733613
"14540","ALX1",5.90010179450268,0.177297600032962,1.04847696104045,0.169100139174276,0.86571787546107,0.96972722733613
"14541","FN3KRP",184.712811430161,0.0550563838445564,0.325224294571224,0.16928742644255,0.865570565490512,0.96972722733613
"14542","CLEC11A",633.401100512471,-0.0475220172235325,0.280646782212594,-0.169330347737726,0.865536806593711,0.96972722733613
"14543","CES4A",23.1123325748669,-0.0744952156187711,0.440773029720572,-0.169010376306366,0.86578847970105,0.969733104988682
"14544","ZNF354B",56.2653724027912,0.0423817926612852,0.252082888500084,0.168126416328616,0.866483827917656,0.96992034070995
"14545","IER3-AS1",10.4703745895882,-0.0880707526681664,0.523781026489016,-0.168144220989672,0.866469821240092,0.96992034070995
"14546","AC022400.4",3.59382077578784,0.156000217753942,0.927807789499279,0.168138508341401,0.866474315295934,0.96992034070995
"14547","WDHD1",13.8291754273401,-0.0702248721226235,0.417801745830402,-0.168081806319521,0.866518922171333,0.96992034070995
"14548","AL136040.1",7.37888333027734,0.105969058991139,0.632411433495963,0.167563477474377,0.866926705629585,0.96992034070995
"14549","TTC8",90.5490134178129,0.0368571458578255,0.218676609977282,0

.168546356474314,0.866153478061978,0.96992034070995
"14550","EEF2KMT",45.3822682125528,0.0594867629489844,0.35464938658308
8,0.167734007725536,0.866792540909034,0.96992034070995
"14551","AC009054.2",10.3552120373841,0.0933811160441692,0.55746220688
9098,0.16751111535485,0.86696790231057,0.96992034070995
"14552","UBB",4986.00636143499,0.0450301926858506,0.268529000606768,0.
167692102469753,0.866825509524692,0.96992034070995
"14553","APBA3",57.0025992395119,0.0435956458103155,0.260188448902089,
0.16755411700356,0.866934070093523,0.96992034070995
"14554","TRAPPC5",1150.43746342401,-0.0403539512298138,0.2406294074393
16,-0.167701660654218,0.866817989681205,0.96992034070995
"14555","C19orf54",27.4145868574676,0.0544799281850724,0.3246688983121
38,0.167801500138443,0.866739442294954,0.96992034070995
"14556","SMG9",88.2824183490958,0.0335481231473384,0.19970770371063,0.
167986124340744,0.86659419512667,0.96992034070995
"14557","ZNF614",34.3717367684713,0.0472633841774862,0.281977105228768
,0.16761426123281,0.866886751093082,0.96992034070995
"14558","U2AF2",169.747708606263,-0.0309968827287449,0.183750528310929
,-0.168690033240581,0.866040458751137,0.96992034070995
"14559","AC020915.2",84.5816501465536,0.0380793720544186,0.22710133253
9738,0.167675687450031,0.866838423969733,0.96992034070995
"14560","INTS6L",28.6107365615946,-0.0619701885079822,0.36861721447984
5,-0.168115286193099,0.866492583860728,0.96992034070995
"14561","RACGAP1",20.8239801703131,0.06077022958108,0.36309475194692,0
.167367413754203,0.867080963551235,0.969980208453132
"14562","TRIM26",92.8931011598821,-0.0364734687422461,0.21828924723787
,-0.167087793850427,0.86730097009777,0.970159696602292
"14563","IWS1",297.431355170365,-0.0395085543991997,0.236729996014925,
-0.166892895130657,0.867454323610372,0.97026460738099
"14564","Z99289.3",4.6743347848179,-0.136255364054407,0.81679636841766
3,-0.166816809333234,0.867514192080613,0.970264946086316
"14565","ZNF169",18.9779213549968,0.0586512552563471,0.352488958931222
,0.166391751486864,0.867848664832052,0.970572392821785
"14566","TMEM81",5.63042581369792,0.103233892956392,0.621138721526152,
0.166201026242164,0.867998751855828,0.97060261111583
"14567","TMED7",470.316689641324,0.0222221256555923,0.133813112717383,
0.166068371061109,0.868103144729734,0.97060261111583
"14568","AP006623.1",29.6063162191317,0.0680240586862938,0.40964889797
3399,0.166054538466526,0.86811403040988,0.97060261111583
"14569","CDK5RAP3",289.490843863378,-0.0299101559317662,0.180096534944
778,-0.166078464202198,0.868095201860691,0.97060261111583
"14570","ASPRV1",9.02040626525312,0.0969746917188912,0.584872494996323
,0.165804842163933,0.868310535375936,0.970755683647126
"14571","IFITM2",2998.5775744843,-0.0402983366904663,0.243283860545728
,-0.165643280240909,0.868437685047339,0.970764579449362
"14572","ZNF23",59.3157540734515,-0.0373586938790157,0.225511101481557
,-0.165662327191777,0.868422694868375,0.970764579449362
"14573","FKBPL",42.2832321385536,-0.0526035006593419,0.318205210696817
,-0.165313134075174,0.868697521552595,0.970788551630778
"14574","AC004908.2",6.46067585694832,-0.105248124301355,0.63628226752

102,-0.165411059955208,0.868620449001387,0.970788551630778
"14575","OSBPL5",103.509478070981,0.0295520399302164,0.17858911966648,
0.165475029976102,0.868570102078808,0.970788551630778
"14576","FUT8-
AS1",5.77196987497255,0.102642995706389,0.620768444999287,0.1653482816
87396,0.868669858485362,0.970788551630778
"14577","PABPC4",513.421017324255,0.0292620075987791,0.177483095294554
,0.164872082888882,0.869044666286018,0.970905730564612
"14578","RPL37A",22735.7942784963,-0.0447171059993886,0.27133956775833
8,-0.164801272327576,0.869100402563854,0.970905730564612
"14579","RNF32",3.66764370282954,-0.157870192384143,0.956812775296597,
-0.164995907726259,0.868947203073385,0.970905730564612
"14580","C9orf64",114.144622076759,0.0291224514950382,0.17670797274649
4,0.164805532214539,0.869097049511129,0.970905730564612
"14581","COG3",81.9434189055873,0.0387202772134867,0.234649255187517,0
.16501342474982,0.868933415488235,0.970905730564612
"14582","ABI3BP",2981.4887522954,-0.035082845785548,0.213418529771751,
-0.164385191028487,0.869427920835908,0.97106109734O517
"14583","HOTAIR",21.3722185544347,0.0796340401855309,0.484622923803016
,0.164321653545839,0.869477936319691,0.971061097340517
"14584","TSNAXIP1",4.54379704761265,-0.111472888189164,0.6775044147229
19,-0.164534556184041,0.869310345517293,0.971061097340517
"14585","DIAPH2",426.859606213599,-0.0371990417727595,0.22628560881166
,-0.164389781427597,0.869424307382286,0.971061097340517
"14586","AL109811.2",19.7479196680148,0.0621823965309228,0.37892124897
1888,0.164103746357956,0.869649472630408,0.971186086636276
"14587","ADAMTS7",49.7949805708746,0.0423402760046951,0.25817777459270
4,0.163996595258791,0.869733824114027,0.971213701308931
"14588","WWP1",238.360714700915,0.0341471650464804,0.208411383243861,0
.163845009399151,0.869853158059385,0.971213797493271
"14589","SYN1",3.87051160977211,-0.116942597249491,0.713538776848058,-
0.163891019022211,0.869816937284148,0.971213797493271
"14590","MYO9B",135.167533202326,0.0398516584949347,0.243354948413226,
0.16375939242158,0.869920560192509,0.971222481492514
"14591","AP003486.2",2.57805692113235,-0.168037064193705,1.02709241651
166,-0.163604619693731,0.87004240772333,0.971291945679208
"14592","TRIM6",23.980447203723,-0.0669836771769645,0.409675235343029,
-0.163504335625457,0.870121359753498,0.971313516243472
"14593","PCM1",773.885312292933,0.0332796724279508,0.203775657970199,0
.163315249522187,0.87027022771519,0.971413125419908
"14594","SCRG1",15.3954430181752,-0.0812952667951099,0.499186723325375
,-0.162855426629848,0.870632266600385,0.971540644329495
"14595","MTPN",1059.98143172985,0.0190056077732192,0.116669457635697,0
.162901312463152,0.870596137436883,0.971540644329495
"14596","ENG",499.860100332279,0.0268347190002855,0.164836214141559,0.
162796258941256,0.870678853906904,0.971540644329495
"14597","CPSF6",293.769960159244,-0.0291880638372544,0.179136091858381
,-0.162937929115533,0.870567306749157,0.971540644329495
"14598","AP1G2",27.3003393540093,0.0571936094336727,0.351330682662014,
0.162791387875149,0.870682689294736,0.971540644329495

"14599","NUDT1",433.455235100591,-0.0431320906524924,0.265348017158684,-0.162549134959989,0.870873438617998,0.971541197005868
"14600","POLR2J3",375.850980381552,-0.0432539596725769,0.266057129761772,-0.162573954365766,0.870853895536082,0.971541197005868
"14601","NUDT14",77.137530261955,-0.0667222763378377,0.410629535267817,-0.162487767214116,0.870921760616348,0.971541197005868
"14602","TMEM98",1012.99416367386,-0.0364160471696159,0.224085207112457,-0.162509822218388,0.870904394078448,0.971541197005868
"14603","ACBD4",73.7320162585257,-0.0493021682207289,0.30359851591,-0.162392652259684,0.87099665668812,0.971558210011147
"14604","FN1",3376.13147740924,0.0383194713991441,0.236290741410684,0.162170854305896,0.871171310851787,0.971686488802024
"14605","SLC25A40",48.9118567982019,-0.042508182853686,0.262273026104716,-0.162076075778811,0.871245945847906,0.971703198351013
"14606","DNAJC24",101.014716316936,0.0352194586369335,0.217493575644687,0.161933328524933,0.871358356814002,0.971721931344591
"14607","PRELID3A",11.4064365619277,0.0774499691273488,0.478371956984504,0.161903238675543,0.871382052375864,0.971721931344591
"14608","WDR53",27.3388921537782,-0.0596336929895518,0.368812082958138,-0.161691267030209,0.871548981945928,0.97172422632978
"14609","KIF18A",4.00309623589516,0.141337356368904,0.87443445743965,0.161632876159456,0.871594966285269,0.97172422632978
"14610","PAFAH1B1",610.397200984454,0.0213481844819946,0.132107047319815,0.161597620377611,0.871622731346578,0.97172422632978
"14611","RAVER1",59.672460225977,0.0408473216181606,0.252719998814454,0.161630744736393,0.871596644844941,0.97172422632978
"14612","TBCE",31.9722356733229,-0.056399150172018,0.349511701707838,-0.16136555627875,0.87180549322874,0.97180464493305
"14613","AC019131.2",7.73427081549229,0.10209164072857,0.632716341353007,0.161354518693568,0.871814186040067,0.97180464493305
"14614","AC007038.1",5.32780990770102,-0.1054094226565,0.654553393346151,-0.161040220290716,0.872061722828122,0.971814520844733
"14615","SLC35A5",132.756508798871,0.0303815220695429,0.189071252470057,0.160688215012245,0.872338971868555,0.971814520844733
"14616","ERCC8",35.7985552527008,-0.0434994281369527,0.270751990217879,-0.160661526816287,0.872359992865842,0.971814520844733
"14617","BHMT",2.72111278554535,0.171884557969161,1.0688397102662,0.160814157930522,0.872239773958162,0.971814520844733
"14618","RIOK1",153.885904520433,-0.0309227416809776,0.192087777954356,-0.160982348852646,0.872107302886091,0.971814520844733
"14619","GDAP1",22.2586295972378,0.0655024417364525,0.407608799931849,0.160699282614616,0.872330254481511,0.971814520844733
"14620","ZFP41",22.2898810874352,0.0612832031114705,0.38116091918686,0.160780394910915,0.872266366927608,0.971814520844733
"14621","BMERB1",404.587346753686,-0.0322008750617241,0.200034830698853,-0.160976340716391,0.872112034971834,0.971814520844733
"14622","BST2",833.543163682549,0.0698983839978233,0.433722571257049,0.161159203209642,0.871968012160393,0.971814520844733
"14623","KNOP1",267.379678939589,0.0454454744905291,0.283118585805351,0.160517453706744,0.872473473821379,0.971874472753637

"14624","NEXN",961.212979408188,0.0459102806521993,0.286923699900371,0
.160008673623478,0.872874241690139,0.972199903387595
"14625","CEP72",10.9756925789887,0.0745265762713026,0.46633453934695,0
.15981354581985,0.873027953212055,0.972199903387595
"14626","TLR4",116.658613607069,0.0581346577260182,0.36371669968895,0.
159834997336485,0.873011054589772,0.972199903387595
"14627","AL139317.3",2.9708109094521,0.134234967114566,0.8392987982043
29,0.159937041970941,0.872930668818397,0.972199903387595
"14628","NOL11",181.851947273304,0.0304629321708586,0.190670115228009,
0.159767733577074,0.873064042406148,0.972199903387595
"14629","ZNF195",93.745417598987,-0.0323032056220451,0.202432721743883
,-0.159575020005486,0.873215857937316,0.97228718276454
"14630","CCDC82",425.36307292712,-0.0290336988441264,0.182010402037791
,-0.159516700798772,0.873261801451607,0.97228718276454
"14631","TCF20",79.9902726945385,0.0434946067489163,0.272955549080103,
0.159346849314839,0.873395611846175,0.972369702779191
"14632","AC124242.1",8.0106626379916,0.094682011171528,0.5957530782109
26,0.158928278567796,0.87372538077148,0.972670361357753
"14633","SLC48A1",150.016095885742,0.0282941088274063,0.17845256549801
5,0.158552547274648,0.874021417528075,0.972933429243136
"14634","ATIC",175.424726900125,-0.0280407821316244,0.177112249993819,
-0.158322093094085,0.874202999922207,0.973069062849038
"14635","HMGCR",61.1749333900807,0.0366222695232497,0.231497952082208,
0.158196948153755,0.874301608494409,0.973107166791028
"14636","ATP5MF",1139.13222726685,-0.0444429421489142,0.28105850133737
4,-0.158127016039149,0.874356712698967,0.973107166791028
"14637","MRPL24",302.525764932432,-0.0357515314700695,0.22654493534980
7,-0.157812097696493,0.874604865534445,0.973250352144458
"14638","CFAP44",52.5209352223673,-0.0596419116318837,0.37791254375590
3,-0.157819348993102,0.874599151438805,0.973250352144458
"14639","AP002840.2",6.91422867177338,-0.0933331945877318,0.5918710578
97564,-0.15769176975686,0.874699686131686,0.973289376828952
"14640","MIEF2",57.208844903601,0.0411664253086409,0.261280640574354,0
.157556354799757,0.874806397723801,0.973341626538455
"14641","C2orf81",42.9877438345556,-0.0552654377218476,0.3520597952550
31,-0.15697741823037,0.875262645082853,0.97378274883919
"14642","JAK1",1366.11312395013,-0.0200778478645847,0.128141660139074,
-0.156684780287644,0.875493282487768,0.973972823278462
"14643","MAFK",116.460383411793,-0.0488546376979855,0.312291427920822,
-0.156439252986387,0.875686798635573,0.974121577748743
"14644","SIK2",248.342343824175,0.0357076443513103,0.228386527984229,0
.156347419729486,0.875759180350904,0.974135570113075
"14645","EAF1",67.8345455558113,-0.0378158641390028,0.242226786419568,
-0.156117598296915,0.875940327009928,0.974230833646368
"14646","TRAF3IP2-
AS1",67.8817098337151,0.0350325773046204,0.224442626658873,0.156087004
621746,0.875964441622242,0.974230833646368
"14647","RFXAP",86.9488503675351,-0.0359570998574677,0.230757731579852
,-0.155821863957893,0.876173436186619,0.974295521174514
"14648","FAM104A",174.952737726242,0.0248195318706304,0.15931855281332

,0.155785571939714,0.876202043691169,0.974295521174514
"14649","MYO15B",95.5914955721144,-0.0431669308159064,0.277085333978267,-0.155789302147952,0.876199103314051,0.974295521174514
"14650","NTNG1",43.0193354665002,-0.0667943567616979,0.430167433107165,-0.155275252427252,0.876604324674025,0.974366575348037
"14651","LEFTY2",15.3673159731468,-0.105127609131221,0.679194625063563,-0.154782745993289,0.876992593972305,0.974366575348037
"14652","MRPS5",309.737794855507,-0.0274833580553678,0.177362708383637,-0.154955674199117,0.876856261994692,0.974366575348037
"14653","AFAP1L1",30.0080821935335,-0.0738540219993494,0.478041028396944,-0.154493061499366,0.877220981662851,0.974366575348037
"14654","AC146507.3",5.42993642219552,-0.105648683011191,0.682224920811661,-0.154859020519945,0.876932460723157,0.974366575348037
"14655","BET1",224.481097447705,0.0345654702483907,0.222784299319867,0.155152182420013,0.876701344597659,0.974366575348037
"14656","OAS3",45.1815357268559,-0.0574218285739666,0.371212995312343,-0.154687010689513,0.87706807069776,0.974366575348037
"14657","IFT88",112.219902997749,0.0304795521141743,0.196332056088399,0.155244908658476,0.876628245440821,0.974366575348037
"14658","NUBPL",96.081469380789,0.0328388138389818,0.211323987130609,0.155395581376598,0.876509467404301,0.974366575348037
"14659","PACS2",214.44110031899,0.023550156848191,0.152025323670902,0.154909434030684,0.876892716150505,0.974366575348037
"14660","AC016355.1",7.96106092887952,-0.091693861138664,0.590029903221956,-0.155405447483178,0.876501689870522,0.974366575348037
"14661","TPSB2",36.2369001020502,0.144681430413903,0.936507142741594,0.154490471893629,0.877223023358031,0.974366575348037
"14662","SYDE1",194.377120551285,0.0305853596394873,0.197812798788524,0.154617698282431,0.877122716581771,0.974366575348037
"14663","EBF4",9.68982275160015,-0.0787638644202929,0.506211655556791,-0.155594727137721,0.8763524814562,0.974366575348037
"14664","MRPS26",490.00117163113,0.0373903691649262,0.240412682749722,0.155525776499282,0.876406834467342,0.974366575348037
"14665","HSPA12B",132.012433780471,-0.0348554165729572,0.225465460155635,-0.154593153864442,0.877142067535056,0.974366575348037
"14666","TMA16",350.879132492249,0.0341874387898983,0.221430080268017,0.154393832800486,0.877299216069496,0.974384762754399
"14667","AC011978.2",4.10631784980615,0.107606527455786,0.697897415063458,0.154186740247492,0.877462496916938,0.974433229634578
"14668","PPP4R1",130.516197329181,-0.0302469710469504,0.196107636548113,-0.154236579356917,0.877423201101536,0.974433229634578
"14669","UBE2I",1140.24595518064,-0.0298937901669994,0.194006426624498,-0.154086597475759,0.877541455738909,0.974454480368879
"14670","RWDD4",283.985521362285,-0.0324791655880987,0.211118514345169,-0.153843284132801,0.877733304264859,0.974468224725347
"14671","ZNRD1",270.077551434926,-0.0338823315957598,0.220190170659955,-0.153877584517999,0.877706258546272,0.974468224725347
"14672","CIAO3",74.8127791312733,-0.0354189485914991,0.230121282308274,-0.153914267451592,0.877677334355628,0.974468224725347
"14673","HR",2.36230869861171,-0.173923353845274,1.13125861967816,-0.1

5374322972651,0.87781219758524,0.974489394565935
"14674","PLCD4",9.29107975800879,0.105069532651569,0.684421843388197,0
.153515750069324,0.877991570767119,0.974566223939484
"14675","TOP1",705.823748567533,-0.0277473864988176,0.180760363233141,
-0.153503710672619,0.878001064295655,0.974566223939484
"14676","HMGCS1",146.297979651454,0.0445341463563222,0.291187658093571
,0.1529396769351,0.878445846288619,0.974993485295401
"14677","GBP5",10.5789400470261,0.104602460557086,0.686394489324376,0.
152394085593618,0.878876121608947,0.975193707934127
"14678","BTBD9",30.4272673194278,0.0491924679021281,0.32276948042062,0
.152407432815589,0.878865595024361,0.975193707934127
"14679","KLHL28",157.559132740706,0.0292535212928769,0.192003350304307
,0.152359431470924,0.878903452456428,0.975193707934127
"14680","AVP",6.75166458018677,-0.116376279011647,0.763942226933096,-0
.152336492091619,0.87892154425559,0.975193707934127
"14681","SERHL2",18.7507205849029,-0.0637848371584975,0.41872423787591
9,-0.152331370837432,0.878925583288165,0.975193707934127
"14682","FOXD1",18.2543163765538,0.107393515327046,0.706001272510047,0
.152115186627397,0.879096086400484,0.975316452212061
"14683","ATR",116.957368309819,0.0331674888193255,0.218589890697317,0.
151733864331598,0.879396846534165,0.975475431229332
"14684","OSTC",1533.941348537,0.0425607487166982,0.281666743062612,0.1
51103208898316,0.879894301232292,0.975475431229332
"14685","OCLN",33.0713975236925,-0.102826162690141,0.679863031154525,-
0.151245409704841,0.879782130511923,0.975475431229332
"14686","GUCA1B",10.0373968282086,-0.0797480042654652,0.52736330595358
4,-0.151220237292134,0.879801986817637,0.975475431229332
"14687","AFDN-
DT",15.9455063247201,0.0689469354580187,0.455145298575726,0.1514833519
62049,0.879594442497869,0.975475431229332
"14688","EBLN3P",360.844349493531,-0.0240620116993302,0.15933254144575
4,-0.151017560386572,0.879961863582327,0.975475431229332
"14689","RAB30-
DT",124.279437221785,-0.043587927099726,0.288313497932235,-0.151182401
838054,0.879831832026786,0.975475431229332
"14690","PPP2R5C",383.779288761011,0.0260085184614627,0.17199468071594
6,0.15121699318374,0.879804545815346,0.975475431229332
"14691","AC078909.1",11.419982732662,0.0758994805444963,0.499925003693
957,0.151821733227331,0.879327540201718,0.975475431229332
"14692","DET1",53.3198103341921,-0.0395517003793694,0.262024570290737,
-0.15094653274494,0.88001789317423,0.975475431229332
"14693","NKIRAS2",134.080092814899,0.0265952969499849,0.17615803725755
3,0.150974076255749,0.879996165622806,0.975475431229332
"14694","AC132938.6",4.58072548684293,-0.117248707965632,0.77497666570
7256,-0.151293210691226,0.879744424719791,0.975475431229332
"14695","CCNE1",4.16472539835024,-0.140046935193616,0.925677995371967,
-0.151291200497146,0.87974601037112,0.975475431229332
"14696","OS9",1110.76777277342,-0.0192938760038143,0.128158839328584,-
0.150546588162734,0.880333397493183,0.975692366589539
"14697","CCDC160",7.64870376123543,-0.114152993191914,0.75821846054356

3,-0.15055422563845,0.880327372338939,0.975692366589539
"14698","ZSCAN9",38.2890665621349,0.0442397607287847,0.294278930977702
,0.150332749211111,0.880502096443565,0.975746557518826
"14699","LINC02749",2.32712642264296,-0.133979236806971,0.891193733717
329,-0.150336825471292,0.880498880604465,0.975746557518826
"14700","INCENP",15.8769627611288,0.0571531285849156,0.380496592570694
,0.1502066764876,0.88060155858307,0.975790393725145
"14701","LRP12",96.83528950313,-0.0428453082176157,0.2854537976454,-0.
150095421994839,0.880689331789052,0.975821272397243
"14702","COPZ2",1754.08644904841,0.0383509413223411,0.255671552724309,
0.150000815161845,0.880763972130556,0.975835874479819
"14703","TNFRSF11A",14.5985255201813,-0.0872201396360571,0.58175128720
2701,-0.149926852857425,0.880822325647785,0.975835874479819
"14704","AC034213.1",2.79229160654164,0.159546665244182,1.067746787613
83,0.149423690237207,0.881219319467092,0.976159562612013
"14705","TPD52L1",21.3548674478875,-0.0846410024373482,0.5668100624761
61,-0.149328687051861,0.881294280052321,0.976159562612013
"14706","DGKA",51.0508186237702,0.0422248950826665,0.282708860058223,0
.149358230491858,0.881270969208894,0.976159562612013
"14707","PEX19",209.636584998917,-0.0311705975572411,0.208876504216225
,-0.149229793337473,0.881372311520425,0.976165563448128
"14708","NIBAN1",857.476566125291,0.0355748395881214,0.238485340815441
,0.149169921582946,0.881419553514339,0.976165563448128
"14709","PRPF4B",705.680268407646,0.0267561751923382,0.180087173114474
,0.148573464337354,0.881890212968804,0.976185106890608
"14710","ZSCAN23",3.16191170678898,0.130427972129736,0.876627518125544
,0.148783798629348,0.88172423515801,0.976185106890608
"14711","FKBP5",939.717261262539,0.0986680411231081,0.662617852460803,
0.148906403225749,0.881627488505829,0.976185106890608
"14712","DPY19L1",96.776785942548,0.0325874645586024,0.219491802569737
,0.148467797781417,0.881973597923625,0.976185106890608
"14713","AC090198.1",5.47262623923719,0.0986508579110456,0.66447644001
6014,0.148464041717819,0.881976561981035,0.976185106890608
"14714","NSMF",75.1357076554008,0.0360949771767186,0.243081291573466,0
.148489326114222,0.88195660910197,0.976185106890608
"14715","KLRG1",15.2338404915098,0.0675764718353327,0.454792244231396,
0.148587564305406,0.881879086319219,0.976185106890608
"14716","GNPTG",531.524939632012,-0.0288253715705744,0.193407749779796
,-0.149039382358739,0.881522557357345,0.976185106890608
"14717","PSENEN",444.311385912276,-0.0444546353674132,0.29915615116455
8,-0.148600104642207,0.881869190434592,0.976185106890608
"14718","RAB15",3.40755707436209,0.147501444709526,0.994598258178291,0
.148302536724416,0.882104013481934,0.976193510130255
"14719","DONSON",32.7323321622204,0.0437033664233967,0.294638184696812
,0.1483289291521,0.882083185715777,0.976193510130255
"14720","ZC3H6",375.957219809498,0.0257332629638227,0.173928643886708,
0.147952990311271,0.882379868126473,0.976432450537507
"14721","MCOLN2",3.70176412593914,-0.137619557764073,0.93126195218379,
-0.147777494228512,0.882518371308925,0.976519377097418
"14722","S100A16",830.337805586671,-0.0429576514011081,0.2909307158586

14,-0.147655950573416,0.882614296826445,0.976548536477887
"14723","UBE2Q1",363.162062646229,0.0544151689770877,0.368685988669567
,0.14759218047165,0.88266462659242,0.976548536477887
"14724","LRRCC1",142.820024028755,0.0383432684394593,0.259945074078538
,0.147505270393678,0.882733220061788,0.976558097092262
"14725","TENT4B",206.237285506922,-0.0267626770750598,0.18176737829492
4,-0.147235864466485,0.88294585331537,0.976726995222687
"14726","PCDH10",4.57705791614235,-0.132627259229459,0.901735363542092
,-0.147080024352697,0.883068856652497,0.976729821235872
"14727","WDR41",215.459617683333,-0.024664075216869,0.168361455123394,
-0.146494785274887,0.88353080625796,0.976729821235872
"14728","UQCRB",3405.74320765063,-0.034339721079497,0.233754257010256,
-0.14690522225651,0.883206829883144,0.976729821235872
"14729","ARHGAP19",51.2217049520278,-0.0379469001990778,0.259071024735
962,-0.146472961373246,0.883548033391281,0.976729821235872
"14730","ZBTB3",22.1682329496685,0.0529130978914054,0.360984368407437,
0.146580025403436,0.883463520785107,0.976729821235872
"14731","BORA",12.5308099470899,0.077896155189161,0.531342981153512,0.
146602397984167,0.883445860818748,0.976729821235872
"14732","PIGBOS1",258.723215455193,-0.0280027222811329,0.1909912539377
29,-0.146617825182,0.883433683276821,0.976729821235872
"14733","ITPKC",186.406342572647,0.0630708544752579,0.429989434163541,
0.146680009935476,0.883384597685133,0.976729821235872
"14734","CNFN",5.35345154218227,0.122312644483303,0.832939311105691,0.
14684460542622,0.883254676246262,0.976729821235872
"14735","OGFRP1",2.33480049356797,0.131209743678488,0.892284724762875,
0.147049187369376,0.883093196367415,0.976729821235872
"14736","ZNF503",1347.5917363022,-0.0232476419134534,0.158942221130167
,-0.146264735374589,0.883712403516751,0.976845232144704
"14737","GTF2H4",77.6755195451037,-0.0360083681528202,0.24649332571923
7,-0.146082527986315,0.883856239092924,0.976937930283276
"14738","CELF4",9.01109300044249,0.109513802818394,0.750150513935873,0
.145989105897961,0.883929988519974,0.976953153955886
"14739","CDV3",763.055045413331,0.026171516320681,0.18052091527902,0.1
44977751083465,0.884728438244367,0.977172601746846
"14740","PCDHGA9",9.54915250413155,-0.0795321760162199,0.5475408116261
94,-0.145253420982465,0.884510789280073,0.977172601746846
"14741","PEX3",118.15457736359,0.0375809888248082,0.258697271107268,0.
145270140129253,0.884497589334163,0.977172601746846
"14742","TOR1A",212.91211768885,-0.0291464051899021,0.200830629730041,
-0.145129282465932,0.884608798974194,0.977172601746846
"14743","APIP",199.071090160743,-0.0319635639129491,0.21966488217035,-
0.145510577736141,0.884307764809345,0.977172601746846
"14744","ALG1L9P",6.58688790884008,0.100685421179494,0.694442430686057
,0.144987426934763,0.884720798746351,0.977172601746846
"14745","MGST1",5610.69069707014,-0.0352630295325779,0.243192349262811
,-0.145000571109538,0.884710420875928,0.977172601746846
"14746","ZNF174",49.7745540829162,0.0358619344162334,0.246675700009938
,0.145380896516311,0.884410146787168,0.977172601746846
"14747","SLX1A",5.38610415648464,0.0995310000547172,0.683931253281216,

0.145527784521045,0.884294180376295,0.977172601746846
"14748","IFNGR2",742.234696808969,-0.0254990876350751,0.17542234194057
6,-0.145358267099825,0.88442801267459,0.977172601746846
"14749","GRWD1",105.889381019979,-0.0331931607728291,0.229140702753741
,-0.144859295506753,0.884821964851848,0.977209640346583
"14750","RPS20",13363.5023659345,-0.0231586659660763,0.160068986653082
,-0.144679281416756,0.884964098059251,0.977234098928014
"14751","TRDC",9.11484260915656,-0.0949250034238025,0.655806529122919,
-0.144745438187015,0.884911862417919,0.977234098928014
"14752","AC092802.1",19.956956617197,-0.0572933740196024,0.39661015599
9748,-0.144457657356709,0.885139090214782,0.977322103067604
"14753","CMTM3",643.049664336297,0.0365928400747924,0.253366741401638,
0.14442637527072,0.885163790690427,0.977322103067604
"14754","RRP1",120.476824064477,-0.0345446212694126,0.23955166450116,-
0.144205306781516,0.885338350542348,0.977448582891711
"14755","MSH5",5.6479175751662,-0.102262556434252,0.709935801172541,-0
.144044794283305,0.885465097728371,0.977522262073699
"14756","FBXO6",89.3775233333276,-0.0386510571740456,0.269299315539805
,-0.14352452807602,0.885875941177504,0.977578294549174
"14757","NUP54",289.326340886114,0.0227623776085431,0.158543004451263,
0.14357226096052 9,0.885838246227265,0.977578294549174
"14758","GTF2IRD2",165.128854380128,0.0418833771494313,0.2912743029700
05,0.143793588113897,0.885663466200586,0.977578294549174
"14759","FNTA",580.125713693159,0.0262993675223169,0.183123436211894,0
.143615520035817,0.885804084494617,0.977578294549174
"14760","SLC35G1",31.5092920466299,0.0452907383095425,0.31514030114372
1,0.143716110396453,0.885724649012374,0.977578294549174
"14761","MYPOP",58.2717061024939,0.0330712491326466,0.23011442535642,0
.143716540505547,0.88572430936079,0.977578294549174
"14762","CTSS",374.069028116478,-0.0399602934534216,0.279195027224178,
-0.143126809423205,0.88619003205728,0.977591788717229
"14763","ZNF852",10.3114108129646,0.0697323816569328,0.486503355420983
,0.143333814412424,0.886026551497971,0.977591788717229
"14764","TMEM132A",15.9870837453523,0.0843706110782237,0.5897116060585
88,0.143070969286369,0.886234132195211,0.977591788717229
"14765","ARMH4",92.5074029904431,0.0337383306679511,0.235749953138929,
0.14311065694282,0.886202788555213,0.977591788717229
"14766","AC100847.1",2.93144999488183,-0.125776015735047,0.87922621269
6084,-0.14305307771633,0.886248262262099,0.977591788717229
"14767","TMX4",591.928310068245,0.0190706568197083,0.133282022772034,0
.143084989431222,0.886223059654798,0.977591788717229
"14768","MAP10",28.5830230414758,0.0478441541143003,0.335836850454222,
0.142462490490816,0.88671470538045,0.977636193353525
"14769","TSN",272.113618274856,-0.0276944617287751,0.194311189650832,-
0.142526335094447,0.886664279306534,0.977636193353525
"14770","NXPE3",50.7460945420925,0.0417689240749724,0.293395034355814,
0.142364113853124,0.886792406621275,0.977636193353525
"14771","MUSK",4.11085431512332,0.12417460434012,0.870992925297733,0.1
42566719813107,0.886632382676793,0.977636193353525
"14772","MRPS16",396.951494433899,0.0277204664958636,0.19459350127684,

0.142453197634934,0.886722045150023,0.977636193353525
"14773","RAP1B",1630.17249755826,0.032944820071437,0.231487069489733,0
.142318187119727,0.886828681502345,0.977636193353525
"14774","MIDN",778.449835096129,-0.0603113651150497,0.422504198702179,
-0.142747374583046,0.886489700303928,0.977636193353525
"14775","SLC25A14",31.28230645802,-0.0404724331262577,0.28386990667381
8,-0.142573876887777,0.886626729900327,0.977636193353525
"14776","RTL8A",461.820793995955,-0.0312375567119331,0.218584136485788
,-0.142908617313883,0.886362352799807,0.977636193353525
"14777","USP1",310.191353063964,-0.0257504620397725,0.181166679040406,
-0.142136855276953,0.886971907394636,0.977727914972676
"14778","ABCA2",77.8611854897003,-0.0328951426690611,0.231704911626319
,-0.141969984313982,0.887103714548149,0.977747753666344
"14779","GOLT1B",198.776824834793,-0.0288820830680911,0.20344927459714
4,-0.141962084285046,0.887109954658659,0.977747753666344
"14780","C1orf56",181.06714256891,-0.0421429440625813,0.29722732081670
1,-0.141786912275708,0.887248322113294,0.97781576395114
"14781","DMWD",164.769582160795,-0.0299740700219199,0.21148417894901,-
0.141731973383914,0.887291718764921,0.97781576395114
"14782","ELOVL5",224.319011058376,-0.0335689472537298,0.23699405893174
4,-0.141644678373131,0.887360674457869,0.977825600476541
"14783","ATXN7L2",6.42794940077817,-0.0922240333265735,0.6518340710155
76,-0.141483910441941,0.887487669789317,0.977859808734813
"14784","EMC2",410.328680333285,-0.0351870475878344,0.248927217141531,
-0.141354762214806,0.887589689876249,0.977859808734813
"14785","AC015871.4",4.20421930171329,0.102968075911566,0.728830449806
832,0.141278504402301,0.887649930282118,0.977859808734813
"14786","SPN",7.53370998800858,0.101841864900442,0.720087600005014,0.1
41429827287309,0.887530392318093,0.977859808734813
"14787","NAGA",116.69434060243,0.02961247416987,0.209682346532794,0.14
1225404329585,0.887691877448688,0.977859808734813
"14788","LSM3",880.207219456937,-0.0373661553277932,0.265697850296774,
-0.140634014486969,0.888159075599599,0.9779115305152
"14789","AP3B1",245.934512148519,-0.0275951194675969,0.195754784200308
,-0.140967790801781,0.887895387425003,0.9779115305152
"14790","ACOT13",356.529960821623,-0.0443410663624377,0.31516581128396
1,-0.140691232281178,0.888113871824737,0.9779115305152
"14791","ZNF92",85.276921776445,0.0330508686025175,0.234810341275948,0
.140755592036197,0.888063026121329,0.9779115305152
"14792","KLRC1",2.93562052473361,0.157881793091426,1.12143042733984,0.
14078607931652,0.888038940621531,0.9779115305152
"14793","PINLYP",47.6011576419911,0.0416214040367456,0.29560955806298,
0.140798573325827,0.888029070159325,0.9779115305152
"14794","AL022322.2",4.6890680459516,-0.0991428839263115,0.70300799860
705,-0.141026679814105,0.887848865529988,0.9779115305152
"14795","ARHGAP30",10.6200246694883,-0.0982248625023952,0.699898349791
46,-0.140341611795001,0.888390088179003,0.978040768428114
"14796","TNRC6C",423.675744462337,0.0323300041804384,0.230379889087042
,0.140333447978281,0.888396538133856,0.978040768428114
"14797","ZNF502",37.4757591597481,-0.0423868598206217,0.30424437091259

9,-0.139318468550395,0.889198496407906,0.97811379834264
"14798","EXOSC7",301.041847410346,-0.0343528347326518,0.246898183294392,-0.13913765696562 8,0.889341371667671,0.97811379834264
"14799","PIK3CB",40.0164493256676,0.0413383700525411,0.295829653143013,0.139737073729241,0.888867733171191,0.97811379834264
"14800","MYL5",103.738029642436,0.0465518221149028,0.334641469854694,0.139109543521657,0.889363586914657,0.97811379834264
"14801","TAPT1",130.441630092193,0.0288031879412268,0.206707423948472,0.139342784071494,0.889179282833868,0.97811379834264
"14802","SPEF2",81.1083910611902,-0.0326309166833842,0.233966959447132,-0.139468054636824,0.889080297902318,0.97811379834264
"14803","XPA",395.218528092632,-0.0229183696308379,0.164106591230727,-0.139655387751096,0.888932276288744,0.97811379834264
"14804","CD3G",10.0492095112757,0.100055541193481,0.714757187333699,0.139985358617692,0.888671558607181,0.97811379834264
"14805","SORL1",11.3157119065991,0.084244925270186,0.605344151830735,0.139168644836834,0.8893168851486,0.97811379834264
"14806","LTBR",441.534141768436,-0.0229109343754432,0.163552980406933,-0.1400826467267,0.888594691299521,0.97811379834264
"14807","GSKIP",64.2881327779412,-0.032510872246663,0.232676278257744,-0.139725770457139,0.888876664261322,0.97811379834264
"14808","SYNM",37.7189943511066,-0.0709833038323316,0.509138606771606,-0.139418427297095,0.889119511686592,0.97811379834264
"14809","ADAMTS10",78.8213934524903,0.0435697013185625,0.312381734916955,0.139475828604849,0.889074155209871,0.97811379834264
"14810","CLASRP",103.081540366165,-0.0290359965256785,0.207321947675161,-0.14005269027847,0.888618359769284,0.97811379834264
"14811","ZNF628",29.7341433919826,0.0456277396919828,0.326842630922252,0.139601555535259,0.888974811519331,0.97811379834264
"14812","AC007743.1",2.818135944451,-0.127995135045026,0.921299185033158,-0.138928957199087,0.889506288320314,0.978204694197245
"14813","GBP3",215.723394843577,-0.0327655333668591,0.236514079471908,-0.138535234096923,0.889817425286099,0.978243441899711
"14814","C1QTNF7",437.67382440235,-0.0667581729338765,0.481873887215058,-0.13853868139587,0.889814701008168,0.978243441899711
"14815","MCM9",37.8637646032423,0.0445102512493071,0.320964131650449,0.138676714498995,0.889705619418235,0.978243441899711
"14816","CD3D",19.5392491113575,0.0686951415704101,0.495978079088045,0.138504390550324,0.889841799903494,0.978243441899711
"14817","C15orf40",315.120778293749,-0.0339184244940297,0.244609451416679,-0.138663589234136,0.889715991657438,0.978243441899711
"14818","COMP",399.028530580223,-0.155079214663279,1.12042041013506,-0.138411629474498,0.889915106488924,0.978258008476048
"14819","NBPF1",116.762151201086,0.038586610584514,0.282729536710598,0.1364788802523,0.891442719436951,0.978311344627399
"14820","LRRC42",123.922291772603,0.0359607088891617,0.260494453395785,0.138047887086964,0.890202571398585,0.978311344627399
"14821","PIAS3",75.8404048959437,-0.0321764486670338,0.237078955599296,-0.135720391486023,0.892042326806316,0.978311344627399
"14822","ZC3H11B",4.60250329139729,-0.0908470937327674,0.6657122087992

76,-0.136466017194766,0.891452887540613,0.978311344627399
"14823","EHD3",3.50219069190544,-0.13213152105497,0.956627908093129,-0
.138122168438878,0.89014386581481,0.978311344627399
"14824","GORASP2",316.531305540858,-0.0288435293414,0.210126244092775,
-0.137267619596651,0.890819263942195,0.978311344627399
"14825","CCDC174",273.284420299794,-0.0264502716420058,0.1920116435287
28,-0.137753477632457,0.890435253196395,0.978311344627399
"14826","GOLGA4",1205.02259939263,-0.0265572130116535,0.19343875462931
1,-0.13729003302645,0.89080154833111,0.978311344627399
"14827","ADAMTS9-
AS1",7.73234710364872,-0.0878457269752999,0.647495068413599,-0.1356701
09720723,0.89208207818559,0.978311344627399
"14828","TIMMDC1",418.810399761172,-0.0297290499736569,0.2169577556705
58,-0.137026905914345,0.891009527859376,0.978311344627399
"14829","PCCB",67.600863195762,0.0300325397554531,0.220408693324787,0.
136258417498978,0.891616995254519,0.978311344627399
"14830","CWC27",258.828308165756,0.0267084271241365,0.196072549170448,
0.136217064740249,0.89164968519572,0.978311344627399
"14831","AL513548.4",2.51402397692781,0.143565605475633,1.058627543360
14,0.135614840532061,0.892125772797311,0.978311344627399
"14832","AL358852.1",7.99786732774136,0.0751515946421889,0.55344911611
1235,0.135787721860025,0.891989097691239,0.978311344627399
"14833","BX322234.1",112.478483792103,-0.0761479180154947,0.5541084739
67051,-0.137424207701293,0.890695497591829,0.978311344627399
"14834","MKX",17.5555688209004,0.175954367695428,1.27385343653669,0.13
8127639058546,0.890139542331939,0.978311344627399
"14835","ASAH2B",7.1882297859321,-0.0739180264655518,0.544801583633809
,-0.135678802496353,0.892075205897118,0.978311344627399
"14836","AC067750.1",9.46236656203314,0.0798710291594768,0.58103033883
7929,0.137464472714489,0.890663672783972,0.978311344627399
"14837","DUSP5",112.004850899429,-0.119416677907537,0.868625858896424,
-0.1374776915567,0.8906532248657,0.978311344627399
"14838","CCS",463.53971962959,0.029150931411161,0.214029096131475,0.13
620078736049,0.891662552745365,0.978311344627399
"14839","AC006064.2",5.2614078396721,0.123716275748282,0.9006332019564
54,0.137365883779914,0.890741596177612,0.978311344627399
"14840","AMN1",37.6480179211203,-0.0452842931581791,0.331574124193679,
-0.136573664390433,0.891367794182337,0.978311344627399
"14841","ORMDL2",139.824270163227,-0.0397102774655809,0.29064325007105
3,-0.136628934117248,0.891324104847735,0.978311344627399
"14842","TMCO3",301.728773752068,-0.0238054767902424,0.172546445920285
,-0.137965616522987,0.89026759167016,0.978311344627399
"14843","LBHD2",44.9216836462457,-0.124036709162473,0.913204661471703,
-0.135825751220518,0.891959033180877,0.978311344627399
"14844","APBA2",9.56882858446682,0.0715820801320415,0.524807266624982,
0.136396892124576,0.891507530444053,0.978311344627399
"14845","ANTKMT",274.300668416652,0.0329783171108313,0.242285800662402
,0.136113288606553,0.891731722509321,0.978311344627399
"14846","NUPR1",5564.16265061841,0.026747810707006,0.196475027633631,0
.136138475353127,0.891711811726286,0.978311344627399

"14847","TRAPPC1",709.263227596025,0.0409239664883953,0.301157446049111,0.135888941234153,0.891909077995328,0.978311344627399
"14848","AC068234.2",5.23513731777859,0.0965317287845037,0.707460516265208,0.136448220876141,0.891466955361629,0.978311344627399
"14849","SEPTIN9",175.046865631485,0.0255181208838718,0.186823245536578,0.136589645526073,0.891355161461025,0.978311344627399
"14850","PPP5C",102.099110267733,-0.0260094107926888,0.190237022794802,-0.136721077793273,0.891251268313503,0.978311344627399
"14851","RCN3",805.984080015909,0.0419030749609894,0.305081742205766,0.13735032014052,0.890753897569393,0.978311344627399
"14852","TRAPPC2B",120.348705944569,-0.0439076940467454,0.32129034664892,-0.136660483281573,0.891299166171512,0.978311344627399
"14853","SRSF6",566.389809371483,0.0251428428199089,0.183896902657627,0.13672492095035,0.891250150361795,0.978311344627399
"14854","THOC5",110.63601995504,0.0263209514747555,0.192675568460768,0.136607623296645,0.891340950478537,0.978311344627399
"14855","KDM4C",56.0243462944215,-0.0382881775568564,0.282729272049091,-0.135423464572173,0.892277072975,0.978411392910789
"14856","GTF2H3",146.757371583432,0.0267761653749933,0.197859476571942,0.13532920352823,0.892351596382276,0.978427245117858
"14857","NDN",1416.63371613425,0.0288641538801713,0.214055980699211,0.134843949633581,0.892735256363289,0.978716152301899
"14858","DNM2",147.840769908133,-0.026779127564077,0.198554202384271,-0.134870615894848,0.892714172361425,0.978716152301899
"14859","KYAT3",159.575475868726,-0.0366258454193655,0.272348421113672,-0.134481577934607,0.89302177743718,0.978807601687003
"14860","AL513548.1",13.5193286875787,-0.0862892303245797,0.642164039547833,-0.134372566837188,0.893107973371958,0.978807601687003
"14861","TNFAIP3",280.350012322215,0.0486588630563606,0.361477130963864,0.134611179762918,0.892919301893303,0.978807601687003
"14862","FAM91A1",143.38560647906,0.0263852988027344,0.196240006775014,0.13445422896354,0.893043402365671,0.978807601687003
"14863","ZNF16",27.687934481721,0.0421287269677907,0.313706316937008,0.134293524526795,0.893170473540112,0.978807601687003
"14864","ZNF883",8.32364061022343,-0.0727042053803823,0.54142736501387,-0.13428247273486,0.893179212442483,0.978807601687003
"14865","VEGFA",253.467705149306,0.0520232237707959,0.387851338118815,0.13413186615037,0.89329830177446,0.978872252781983
"14866","AC134312.5",6.63957223067272,-0.0794324492047365,0.592839816766569,-0.133986360157069,0.893413360194582,0.978932478421199
"14867","AC017083.1",9.12595875728183,0.07201849648856,0.539503902739046,0.133490223375446,0.89380569569025,0.978961744664595
"14868","LRP2BP",8.71492624883446,-0.0796030608445526,0.595661533690256,-0.133638075219318,0.893688774552299,0.978961744664595
"14869","TBCA",1581.54470867322,-0.0279526373320307,0.208954146271097,-0.133774025693489,0.89358126704811,0.978961744664595
"14870","BPHL",135.410914165432,0.0311269967626349,0.233352191732884,0.133390633837567,0.893884452330174,0.978961744664595
"14871","PTRH1",118.52886695946,-0.0374610729189494,0.281026210460377,-0.133300993019764,0.893955342293756,0.978961744664595

"14872","TNS2",684.583156205176,0.0315853922080681,0.236433508850847,0.133591014072348,0.893725990224917,0.978961744664595
"14873","LINC01852",19.1904797829851,-0.0495887956644058,0.370803873420792,-0.133733219146101,0.893613536020946,0.978961744664595
"14874","NAA38",1053.77791631062,-0.0364823815812182,0.273750783886915,-0.13326859219621,0.893980965798137,0.978961744664595
"14875","ZNF563",32.2840617646991,0.0444494737058072,0.333525905145894,0.133271428155974,0.893978723035064,0.978961744664595
"14876","GCLM",173.549134755735,0.0381393017851907,0.286830348254394,0.13296815353501,0.89421856660984,0.979156105909362
"14877","UFM1",572.760406336071,0.0162680291216025,0.12280481884236,0.132470608848706,0.894612068863254,0.979521139323354
"14878","LANCL2",51.9152906570206,0.0316547486114778,0.239443724316608,0.132201203860419,0.894825148937058,0.979564131690665
"14879","CERS3-AS1",6.26828468410463,0.0879542907288179,0.664997532929166,0.132262581999965,0.894776602551276,0.979564131690665
"14880","ZNF808",38.8763685488316,0.0389283393968239,0.294481394633372,0.13219286551291l,0.89483174409461,0.979564131690665
"14881","AL604028.1",10.0733021768322,-0.0696402896512424,0.527944637878561,-0.131908318893204,0.89505680860414,0.979613005130205
"14882","AP003396.5",13.61221398797,0.0824566206460133,0.6248277184575,0.131966969790605,0.895010417498496,0.979613005130205
"14883","AC104590.1",3.05873040282732,-0.139375906968706,1.05622717318748,-0.131956373123879,0.895018799118367,0.979613005130205
"14884","GOLGA3",313.763024388171,-0.0307632064308552,0.233373826848232,-0.131819436850822,0.895127112325304,0.979624128773642
"14885","RHOA",2767.71091301992,0.0298811437159384,0.227089885125251,0.131582891503325,0.895314218452403,0.979763070498569
"14886","ING4",241.157174755242,0.0357494177022441,0.272068249880965,0.131398712337382,0.895459907238169,0.979856672645609
"14887","AC132153.1",2.45268438029017,-0.130904431399934,0.998097970027509,-0.13115389002978,0.895653571215872,0.980002755527328
"14888","THBS3",386.462833571488,0.0373915254042282,0.285714471753377,0.130870253700358,0.895877946370849,0.980182419964721
"14889","TFDP1",179.423228493075,0.022927451310961,0.175573624352589,0.130585965833444,0.896102845292779,0.980335072824368
"14890","TBC1D17",197.721593519868,-0.0261942285607535,0.20065788700019,-0.130541734254028,0.896137837458091,0.980335072824368
"14891","FBXL8",62.020133209374,-0.0450756027705759,0.345540879884092,-0.130449406697396,0.896210879613954,0.980349138273568
"14892","ACOXL-AS1",4.99690602161722,-0.0955268835030816,0.735721953387961,-0.129841012713003,0.896692214095921,0.980472111045185
"14893","AC073343.2",2.46615135399169,0.129797473353384,0.996737723754001,0.130222294451271,0.896390556345401,0.980472111045185
"14894","THAP1",111.780946476188,0.0261853996560669,0.201536424462936,0.129928868817868,0.896622703868266,0.980472111045185
"14895","AC009812.1",21.0916379577214,0.0527902990707729,0.406758294511556,0.129782968861556,0.896738137820324,0.980472111045185

"14896","OSBPL7",12.2621469985423,-0.0594882659184904,0.45839632192807
7,-0.129774745286513,0.896744644260001,0.980472111045185
"14897","NECTIN2",227.802558471327,-0.038912988872373,0.29937777186657
4,-0.129979552689422,0.896582604028298,0.980472111045185
"14898","VPS16",142.362340818882,0.0275478392108934,0.211835255594583,
0.13004369425463,0.896531857168859,0.980472111045185
"14899","ZNF444",86.2997135420785,0.0293192038709867,0.226453948680255
,0.129470932354482,0.8969850239609,0.980669109020679
"14900","AGBL5",31.44854837009,-0.0342306276989803,0.26496442748866,-0
.129189521866837,0.897207687069144,0.980725970836405
"14901","RNF122",35.9827233933675,0.071115092478013,0.550680512687469,
0.129140383288583,0.897246568286469,0.980725970836405
"14902","MORN4",51.3235033109843,-0.0537411510885705,0.416272699129979
,-0.129100830299203,0.897277865023939,0.980725970836405
"14903","GAS2",9.63635011194343,0.0728835594740833,0.564153550992826,0
.129190996574991,0.89720652020061,0.980725970836405
"14904","CD55",610.614196223409,-0.0421214300630024,0.326982681266736,
-0.1288185352809,0.897501238696872,0.980761392866836
"14905","ASAP2",316.283288384039,0.0328310761827684,0.256717922837181,
0.127887744727473,0.898237809694604,0.980761392866836
"14906","SF3B6",1227.74355677219,0.0312261483992545,0.243142548997328,
0.128427330091031,0.897810803953829,0.980761392866836
"14907","LINC01914",39.9129100555695,0.0538641838946845,0.420922556314
373,0.127966969426213,0.898175112666634,0.980761392866836
"14908","AC093297.2",32.5395176619156,0.0564037387855235,0.44173649454
0232,0.127686391055893,0.898397160293668,0.980761392866836
"14909","AC021097.1",6.31250801919379,0.0744963068245182,0.58227901054
5724,0.127939193196572,0.898197094212077,0.980761392866836
"14910","NAIF1",29.9668253815218,-0.0413109022725015,0.320896039169505
,-0.128736092783869,0.897566475116315,0.980761392866836
"14911","WASHC2A",228.189047988669,0.0292179178608151,0.22785202337885
2,0.128231987706487,0.897965386499864,0.980761392866836
"14912","H2AFJ",2069.03847762131,-0.0314488999409363,0.246016510714506
,-0.127832476973189,0.898281547995832,0.980761392866836
"14913","PUS7L",252.043104868163,-0.0227208996468714,0.17778517511322,
-0.127799742764839,0.89830745363896,0.980761392866836
"14914","MAP1LC3B2",9.57364278389764,0.0796844746585752,0.624116583198
694,0.127675624720913,0.898405680849368,0.980761392866836
"14915","DMXL2",90.7543989569531,-0.0439222276090208,0.344179683189313
,-0.127614236848669,0.89845426388108,0.980761392866836
"14916","ORC6",7.724699515431,-0.0786429123432174,0.610565362907376,-0
.128803428954334,0.897513192221069,0.980761392866836
"14917","TMEM231",26.1371620816403,-0.0407142527812849,0.3183269053846
15,-0.127900758913521,0.8982275104539,0.980761392866836
"14918","UBTF",472.538233675143,0.0215929791061761,0.168594025009171,0
.128076775585621,0.89808821506167,0.980761392866836
"14919","AC005253.1",11.7341546698924,-0.0708374246518604,0.5550551916
76032,-0.127622308041046,0.898447876229658,0.980761392866836
"14920","BAX",338.417987384607,-0.0331676879826637,0.258359070041686,-
0.128378260447029,0.897849634437645,0.980761392866836

"14921","COL18A1",607.251853239081,-0.0488619125391397,0.382212749688325,-0.127839567306386,0.89827593676577,0.980761392866836
"14922","PHKA2",29.7644752560888,-0.0453932898259715,0.354478564254058,-0.128056515692266,0.898104248097765,0.980761392866836
"14923","EMC1-AS1",45.413352065053,0.0339805841485289,0.266440616205134,0.127535300858061,0.8985167352444,0.980763861180461
"14924","S100A8",29.0643498898703,-0.0522084159073394,0.417473369211888,-0.125058074975894,0.900477573978426,0.980932201065644
"14925","RRNAD1",101.897089429869,-0.0262323252941747,0.207187735475513,-0.126611380900367,0.899247988782818,0.980932201065644
"14926","ZC3H11A",10.8701497388646,0.0777269039481595,0.612086964221535,0.126986700406231,0.89895092486651,0.980932201065644
"14927","RND3",2369.82456938226,-0.0497365415532653,0.392647584471096,-0.126669674080031,0.899201849032781,0.980932201065644
"14928","UBA3",182.474505179269,0.0206334689870213,0.163503703129143,0.126195728855903,0.899576992217216,0.980932201065644
"14929","DUBR",70.1267168214569,-0.0308156073258662,0.246133435261648,-0.125198786150724,0.900366178195833,0.980932201065644
"14930","CCDC58",84.7142221036919,-0.038857332177342,0.306981310400986,-0.126578820471467,0.899273760900595,0.980932201065644
"14931","IDUA",120.108759220433,-0.0307518394446257,0.245618856526025,-0.125201460016435,0.900364061415191,0.980932201065644
"14932","AC093323.1",94.2981340910698,0.0276796903770562,0.22130857868769,0.125072830620442,0.900465892393611,0.980932201065644
"14933","AC026748.3",10.0162710072978,0.071489108909423,0.566843130672124,0.126117976987136,0.899638537515287,0.980932201065644
"14934","AGPAT1",233.127631981505,0.0188629456958458,0.148269231226891,0.127220904430135,0.898765560471723,0.980932201065644
"14935","OGFRL1",505.996354998758,-0.0239124069266609,0.189564707643564,-0.126143770240308,0.899618120531329,0.980932201065644
"14936","UFL1",478.567300768677,0.0192701192850492,0.152276699058302,0.126546736330759,0.899299156132293,0.980932201065644
"14937","ZNF138",106.683154056886,0.025686974277755,0.202287810561622,0.126982313993309,0.898954396613442,0.980932201065644
"14938","EPS8L2",146.056965248136,-0.0227702020411171,0.181917526081158,-0.125167720404019,0.900390771618698,0.980932201065644
"14939","RELT",12.3917555893558,-0.0874082124648355,0.694728772170225,-0.125816312734228,0.899877328692961,0.980932201065644
"14940","CWC15",754.769581248331,0.0306675625330095,0.244147815953992,0.125610636380989,0.900040143033952,0.980932201065644
"14941","CCDC153",3.17206085707607,-0.172234446322203,1.37679653204164,-0.125097966412508,0.900445993218523,0.980932201065644
"14942","TIRAP",18.408752997479,-0.0497290863701889,0.392419264574061,-0.126724375838596,0.899158552245421,0.980932201065644
"14943","AC024941.2",13.3569396278718,0.0699271750277425,0.557079873379103,0.125524504419056,0.900108326738562,0.980932201065644
"14944","P2RX4",117.762569649193,0.0227341444363091,0.180611137770797,0.125873435696749,0.899832110642094,0.980932201065644
"14945","PCDH17",2.61288178780089,0.137812555722607,1.09945906631848,0

.125345781343247,0.900249809686974,0.980932201065644
"14946","IPO5",570.591798539458,0.0338669382508927,0.268536962121411,0
.126116486845415,0.899639717058315,0.980932201065644
"14947","GOLGA6L10",22.7029826896183,0.07575733061972,0.60337046453101
6,0.125556909184483,0.900082674421385,0.980932201065644
"14948","TRAPPC2L",806.913847946412,0.0405706619069696,0.3200949213646
13,0.12674572196900 5,0.89914165672980 2,0.980932201065644
"14949","VPS9D1",32.4700054592134,-0.0374419260146029,0.29863776660212
2,-0.125375723374221,0.900226106388852,0.980932201065644
"14950","ZNF599",53.186998819805,0.0319027262373049,0.251000959911515,
0.127102008886944,0.898859661497557,0.980932201065644
"14951","ZNF274",44.4686264211453,-0.031626344773299,0.251010746852504
,-0.125995978936643,0.899735107553622,0.980932201065644
"14952","SRPX",3163.60492387712,0.0406246987099413,0.324140447140012,0
.125330544424138,0.900261871870032,0.980932201065644
"14953","NSDHL",70.1190553487151,0.0298031732993443,0.236945111776214,
0.125780916415327,0.899905348280778,0.980932201065644
"14954","TRIM35",38.2746295181905,-0.0326629603816271,0.26145252553653
4,-0.124928838666211,0.900579887265823,0.980978051603116
"14955","MAPK8",123.348072760273,-0.0266211091636397,0.213690451240415
,-0.124577907010404,0.90085771977079,0.98108386690823
"14956","METTL3",102.900033346989,-0.0246781026606634,0.1980739820628,
-0.124590329348956,0.900847884800863,0.98108386690823
"14957","SHROOM4",8.46046241833896,0.0716764773119105,0.57525673023674
8,0.124599111221892,0.900840932056677,0.98108386690823
"14958","NIF3L1",96.6870335670296,0.0294572618365795,0.236667959460882
,0.124466623634571,0.90094582533009,0.981114223078074
"14959","USE1",346.767031558736,0.0315548573951659,0.253728157743702,0
.124364822870942,0.90102642436021,0.981136401257
"14960","NCKIPSD",62.7732207019058,0.028464011217192,0.229023533751583
,0.124284219839461,0.901090241168853,0.981140303368947
"14961","HLA-
DRB1",623.705578130011,0.0574370752514891,0.463564785290559,0.12390301
6523328,0.901392064514879,0.981236898484756
"14962","REEP4",35.2064066816305,-0.0409616598531974,0.33083628365225,
-0.123812477280313,0.901463752392142,0.981236898484756
"14963","COL13A1",2.47254995656222,-0.164837147833836,1.33003277085189
,-0.12393465142085,0.9013670165753,0.981236898484756
"14964","AC005520.3",3.65752424019514,-0.0975664565681357,0.7878466299
6943,-0.123839403326408,0.901442432590614,0.981236898484756
"14965","AL137077.2",3.37010421079893,0.111187219395355,0.898179442078
965,0.123791766084065,0.901480151379727,0.981236898484756
"14966","MAP3K6",101.645015810452,-0.0311690636644651,0.25343647362187
8,-0.122985706118089,0.902118416898127,0.981268339833674
"14967","AGO1",132.134052589827,0.034274510360702,0.279568854562617,0.
122597742206742,0.902425642415049,0.981268339833674
"14968","TM2D1",652.685471579462,-0.0232376661605126,0.189443997112703
,-0.122662457056837,0.902374394226397,0.981268339833674
"14969","CENPO",8.80523101704247,-0.0603756198592779,0.49233353330394,
-0.122631540967991,0.902398876864737,0.981268339833674

"14970","SLC4A1AP",174.209831736003,-0.020753564361195,0.168951692229671,-0.122837268377181,0.902235961828516,0.981268339833674
"14971","ALKBH4",119.758129251368,-0.0271858218770556,0.221627112049218,-0.122664693979401,0.902372622797448,0.981268339833674
"14972","LOXL4",83.3134019811423,0.0378029105798837,0.306559031185762,0.123313641857697,0.901858738640201,0.981268339833674
"14973","RPS26",6174.13183439293,-0.0849074586900897,0.689801193029447,-0.123089753320367,0.902036025242125,0.981268339833674
"14974","NAP1L1",1661.10780551023,0.0195323158106192,0.158107188274177,0.123538442646566,0.901680734594526,0.981268339833674
"14975","AL049775.3",7.29804622705331,-0.0740197154422782,0.602896097377084,-0.122773585306495,0.902286391858049,0.981268339833674
"14976","PARN",118.567358016667,-0.0272222767665521,0.220774112120001,-0.123303753801331,0.901866568414865,0.981268339833674
"14977","SRSF2",1216.94010504719,-0.0261954603906884,0.213356047417578,-0.122778148113228,0.902282778600851,0.981268339833674
"14978","AC011468.1",9.90731524820584,-0.0634775994789797,0.515542215917472,-0.123127840008239,0.902005865874343,0.981268339833674
"14979","GET1",247.331875415809,-0.0347595169718408,0.283507815495516,-0.122605145509262,0.902419779662114,0.981268339833674
"14980","EP300",388.324148391067,0.0245414840332252,0.200276400256417,0.122538072392974,0.902472895760837,0.981268339833674
"14981","RBMX",1292.67794409033,0.0195746949143101,0.159225674492531,0.122936800090166,0.902157144383785,0.981268339833674
"14982","COMMD1",548.953917857205,-0.0346586570791928,0.283332516349563,-0.12232502476501,0.90264161392407,0.981386280150125
"14983","PIWIL2",12.2262266547294,0.0728659460243225,0.596769858433886,0.122100580306696,0.902819362300669,0.981448517920155
"14984","WDR25",35.7092735922229,-0.0399925781644328,0.327484630181688,-0.122120473691376,0.902803607574331,0.981448517920155
"14985","MAP7D1",249.593800897578,-0.019442048026467,0.162132361110766,-0.119914666592591,0.904550746053011,0.981483119638225
"14986","SYDE2",202.016148818619,0.0359628513324105,0.299338402922856,0.120141121156709,0.904371358310484,0.981483119638225
"14987","SLC22A15",19.0521559415737,-0.0520620779517467,0.432873331364017,-0.120270929575853,0.904268531762659,0.981483119638225
"14988","GON4L",298.108610965857,0.0248562293618965,0.205716133581432,0.120827807373004,0.903827424363786,0.981483119638225
"14989","KLHL20",220.884764079692,-0.0172916121663514,0.14421804643298,-0.119899087486163,0.904563087342415,0.981483119638225
"14990","THUMPD2",120.02472417244,0.0267350366002512,0.223062810192137,0.119854298335176,0.904598568059959,0.981483119638225
"14991","ANKRD36",100.882941227692,-0.0389891807073269,0.323419907419394,-0.12055281636318,0.904045243261728,0.981483119638225
"14992","TMEM177",31.4797146724675,0.0444205798317685,0.369728460183353,0.120143793663436,0.904369241292635,0.981483119638225
"14993","ICA1L",103.959509353081,0.0392228984550027,0.325169307427615,0.12062300333722,0.903989647850611,0.981483119638225
"14994","ADPRH",14.4279540344772,-0.0561212418072204,0.462793636185337,-0.121266234924513,0.903480163235489,0.981483119638225

"14995","AL035587.1",2.38318118140059,0.128268319019276,1.06763402758036,0.120142591661281,0.904370193454799,0.981483119638225
"14996","PEX7",54.760757939232,-0.0327717140729978,0.272162332239706,-0.12041237963868,0.90415648522144,0.981483119638225
"14997","CUX1",265.539022063057,-0.0242076373101338,0.201305541075433,-0.120253209031453,0.904282568834686,0.981483119638225
"14998","VPS37A",196.963205538156,0.0177232216575122,0.146174892996137,0.121246688088771,0.903495645118484,0.981483119638225
"14999","PLIN2",1495.05701598137,-0.038327453884302,0.317176099689034,-0.120839665794109,0.90381803153495,0.981483119638225
"15000","RERGL",2.84355087307545,0.120488714829161,0.999498494778155,0.120549170867841,0.904048130887488,0.981483119638225
"15001","REP15",5.84649958931475,-0.0710154300372397,0.584035008728034,-0.121594474605047,0.903220189644622,0.981483119638225
"15002","AL138966.2",2.28384364888944,0.167289074132584,1.38102468053651,0.121134022070912,0.903584881867918,0.981483119638225
"15003","ZC3H13",743.59662865108,-0.027864788693617,0.230404064151086,-0.120938789844196,0.903739517794961,0.981483119638225
"15004","DNAJA4",66.4910934982339,0.0369853675193828,0.308013177173954,0.120077224808129,0.904421973805625,0.981483119638225
"15005","KIF7",59.1602398895235,-0.034984604810466,0.289348211041283,-0.120908315570939,0.90376365562262,0.981483119638225
"15006","LUC7L",217.780920002116,-0.0214670257997804,0.176567236616096,-0.121579893366375,0.903231738110522,0.981483119638225
"15007","AC092140.1",5.50283124457062,0.0886706721291588,0.735229135795353,0.120602772404058,0.90400567281356,0.981483119638225
"15008","AC009113.1",39.376524940378,-0.0323902372383745,0.269436994799794,-0.120214513461458,0.904313221072748,0.981483119638225
"15009","FANCA",5.54039458076656,0.0870118128859586,0.716922310177918,0.121368538334879,0.903399135403368,0.981483119638225
"15010","CDC27",329.078500113898,-0.0197545189430649,0.162035660810585,-0.121914638075611,0.902966622598952,0.981483119638225
"15011","LGALS3BP",3352.61695545102,-0.0319847639632051,0.262662757638632,-0.121771218161081,0.903080208863631,0.981483119638225
"15012","LINC00683",14.0540346967508,0.0535162612197823,0.439194423470286,0.121850957935496,0.903017056000304,0.981483119638225
"15013","CETN2",332.707428470469,0.0279134030658522,0.230303590440929,0.121202639578526,0.903530533451295,0.981483119638225
"15014","ABHD10",102.906654638167,-0.0258499196862535,0.21613398890614,-0.119601363103882,0.904798939876074,0.981572147565829
"15015","C4orf33",57.3098962010002,-0.0368397006746658,0.308153136240426,-0.119549978053519,0.904839647148409,0.981572147565829
"15016","AP003696.1",2.5596347644698,-0.120903554842033,1.01265255165935,-0.119392929632102,0.904964062565803,0.981572147565829
"15017","TSTA3",164.60572498768,-0.0341554414501766,0.286329104918579,-0.119287354528242,0.905047701589074,0.981572147565829
"15018","AC025159.1",50.4824518653504,0.0358516192293966,0.300722551462357,0.11921825967177,0.905102440692415,0.981572147565829
"15019","COQ5",161.566467696164,0.0339287814112734,0.284543137842226,0.119239499741815,0.90508561359752,0.981572147565829

"15020","ZNF521",379.124940043426,-0.0241245716315543,0.202037574397314,-0.119406361433109,0.904953421659975,0.981572147565829
"15021","THY1",3676.12153039136,-0.0477402552245485,0.400762257639795,-0.119123630817195,0.905177409358154,0.98158809806504
"15022","TNFRSF14",358.025391587762,-0.0167215322972344,0.141414854239427,-0.118244525210368,0.905873911379369,0.981624543803788
"15023","DNAJC27",88.6557488873018,0.0298804798671033,0.252444724097827,0.118364445816361,0.905778895827868,0.981624543803788
"15024","LINC01410",4.05317897995142,0.103777469943048,0.877196905775601,0.118305786602484,0.905825372555648,0.981624543803788
"15025","AL157394.3",19.4133166252782,-0.0488650994380257,0.412412263259363,-0.118486048527841,0.905682548886937,0.981624543803788
"15026","RAB3IL1",383.996286688483,-0.024978348533591,0.211103305640971,-0.118322867838329,0.9058118387585,0.981624543803788
"15027","IRAK4",82.0118933367555,-0.0262600716729819,0.22207264713405,-0.118249915115077,0.905869640818582,0.981624543803788
"15028","DGKH",64.5380558420073,-0.0452489271182257,0.382284556560086,-0.118364517587081,0.905778838962861,0.981624543803788
"15029","AC004584.3",2.39825987939958,0.128432074351342,1.08570665969737,0.118293530949825,0.905835082965735,0.981624543803788
"15030","PTOV1-AS1",5.27186782231929,0.0750653177852149,0.633790280359434,0.11843873298695,0.905720037257999,0.981624543803788
"15031","KMT5C",36.5539183727994,-0.0340496607336573,0.286911609972939,-0.118676482756724,0.905531668894716,0.981624543803788
"15032","UXT-AS1",4.16553129481816,-0.0984335207185547,0.83231355163248,-0.118264950180722,0.905857728161923,0.981624543803788
"15033","PDHX",102.296988326748,-0.0227444538541732,0.19272636887331,-0.118014229122557,0.906056383462034,0.981756963361476
"15034","AL645728.1",12.1884229818254,-0.0577996908251991,0.492652811500923,-0.117323375561596,0.906603801929195,0.98181983323138
"15035","EFNA3",6.60348989983122,0.0725278470905636,0.619284816441506,0.117115493816429,0.90676853193955,0.98181983323138
"15036","EIF2B4",136.172873759625,0.0244656297542063,0.209185458970936,0.116956646387193,0.906894408796399,0.98181983323138
"15037","P4HTM",303.746714407547,0.0220628679503473,0.188473371961365,0.117060928664607,0.906811771215884,0.98181983323138
"15038","MKLN1-AS",45.6704387693934,0.0310306142427093,0.264959449204352,0.117114578611524,0.906769257176669,0.98181983323138
"15039","ACTR3B",24.4479345818683,-0.0448283827668546,0.380736151055517,-0.117741335154481,0.906272613835455,0.98181983323138
"15040","ARHGAP39",8.62174388940378,-0.0710033097969596,0.604284780373028,-0.117499748633631,0.906464043225903,0.98181983323138
"15041","GBGT1",54.069037987648,0.0321703862071882,0.273457589343426,0.117643054941095,0.906350488887033,0.98181983323138
"15042","RNASEH2C",1070.78604476836,0.0257128382830928,0.218822159498718,0.117505641759483,0.906459373540203,0.98181983323138
"15043","HOXC4",106.785249196422,-0.027114075475343,0.23132481412576,-

0.117212135575747,0.906691950410993,0.98181983323138
"15044","NDUFB1",1146.56526264802,-0.0315241110785512,0.26858920252655
9,-0.117369241883184,0.906567457000448,0.98181983323138
"15045","ERVK13-1",37.446146918968,-0.0415954876412038,0.3558061672616
75,-0.116904909100726,0.906935407931594,0.98181983323138
"15046","PFAS",34.5961283291598,-0.0380126595720929,0.323672032083554,
-0.117441903544759,0.906509879578705,0.98181983323138
"15047","CLIC2",263.813682413885,0.0241098826311693,0.206285864737471,
0.116876077097443,0.906958255917034,0.98181983323138
"15048","KHDRBS1",1041.33805705101,0.0142605938277696,0.12220290051173
3,0.11669603395707,0.907100933246812,0.981830508823035
"15049","AC138696.2",8.73938623206705,0.0690528082012525,0.59203962701
3422,0.116635449808644,0.90714894455072,0.981830508823035
"15050","ITGB3",49.6810338295397,0.035434880920245,0.303675961997342,0
.116686486105723,0.907108499639008,0.981830508823035
"15051","STX2",273.508180779371,0.0262685489330864,0.225609064269169,0
.116433925286556,0.907308649740328,0.98187288038933
"15052","ATP6V0A1",135.99487326294,0.0331693437817851,0.28486281097286
3,0.116439712395259,0.907304063490371,0.98187288038933
"15053","TBC1D30",3.171875491854,-0.0914471608683448,0.786651967686224
,-0.11624856305555,0.907455549863186,0.981966614742672
"15054","AC091132.4",29.7521354094985,0.0739200145914438,0.63680430363
3594,0.116079640432167,0.907589424304718,0.982046242360804
"15055","PTPRC",76.3156981757935,0.0639535773458139,0.557883326087712,
0.114636115394062,0.908733552456043,0.982079647651473
"15056","AL512306.3",3.64623190504088,-0.0929442747974749,0.8110402892
51867,-0.114598838096206,0.90876310070748,0.982079647651473
"15057","TAF8",82.1336504749186,-0.0230247060030185,0.200316110389835,
-0.114941858436698,0.908491206710533,0.982079647651473
"15058","PNRC1",7166.27349477472,0.025890595736421,0.225593682066623,0
.11476649301187,0.90863020822714,0.982079647651473
"15059","AC092428.1",10.6006823667755,0.069531630662467,0.603531541336
494,0.115207948384093,0.908280298729929,0.982079647651473
"15060","C7orf26",104.058493658562,-0.0238036749839482,0.2077681615270
91,-0.114568444024299,0.908787192989191,0.982079647651473
"15061","MAP3K8",287.4873886906,-0.0390812546664019,0.338278243487747,
-0.115529908939643,0.908025115282552,0.982079647651473
"15062","LIPA",312.435033466572,0.0158720051526255,0.137529519304198,0
.115407988284454,0.908121747536138,0.982079647651473
"15063","ABCC8",3.46906024100652,0.113234831306281,0.980251700989587,0
.115516077342144,0.908036077904866,0.982079647651473
"15064","C2CD2L",18.397826906749,-0.0442910200909812,0.385485208706582
,-0.114896808205925,0.908526915023837,0.982079647651473
"15065","MAB21L1",32.3045609265366,0.0587894567556377,0.51113380003629
8,0.115017744378209,0.908431057404014,0.982079647651473
"15066","AL161669.3",28.4238213965185,-0.0453336183299779,0.3936095044
53181,-0.115174094672732,0.908307131470268,0.982079647651473
"15067","AC022613.3",12.5344905988702,-0.0915765388321636,0.7967325492
56683,-0.114940125036439,0.90849258065789,0.982079647651473
"15068","ARNT2",6.81022562154801,0.0734766285177426,0.635604882553391,

0.115601107755132,0.907968684932477,0.982079647651473
"15069","MGRN1",122.115945286634,0.0287843328950758,0.249326555974317,
0.115448323515289,0.908089778362362,0.982079647651473
"15070","MBD1",148.246369001752,-0.0193873947727883,0.169293630302248,
-0.114519339789542,0.908826116317657,0.982079647651473
"15071","TXNL1",767.579929334951,0.0276974100611838,0.240975747724493,
0.114938579183704,0.908493805949433,0.982079647651473
"15072","AC008946.1",7.27799337352017,-0.0747289758047881,0.6495985079
92462,-0.115038712197374,0.908414437820292,0.982079647651473
"15073","RAE1",104.248586068722,0.0308214804483857,0.268041527220879,0
.114987706449633,0.908454866238362,0.982079647651473
"15074","APEX2",45.6702263388116,-0.0309039188898124,0.269786316085724
,-0.114549615926379,0.908802117383647,0.982079647651473
"15075","KIAA1191",144.834688848224,0.0223487454630077,0.1955022886536
98,0.114314495328467,0.908988492213941,0.982189953543806
"15076","MYOZ2",6.11126738690123,0.0709052709268463,0.621106320139282,
0.114159635198924,0.90911124910601,0.98225743809285
"15077","MPV17L",42.6439483943176,0.0390945902123672,0.342824596876492
,0.114036713142994,0.909208690383357,0.98229756302013
"15078","DIMT1",200.170879711953,-0.0248270718938724,0.218138567150279
,-0.113813307835513,0.909385789018029,0.982366419324046
"15079","NFYA",51.3037418255032,-0.0271180793583332,0.238829180198192,
-0.113545921548737,0.909597758327344,0.982366419324046
"15080","HMGN3-
AS1",58.002826241681,-0.0269775253909212,0.238116185860579,-0.11329563
8821953,0.909796174695869,0.982366419324046
"15081","AC087623.2",9.54644015027718,0.0708734060367095,0.62293884354
5102,0.113772654845817,0.909418016082355,0.982366419324046
"15082","CSPP1",132.842026414291,-0.0193875969902755,0.171452737717761
,-0.113078375115775,0.909968419173249,0.982366419324046
"15083","ARRB1",160.182003117609,0.0182460217279301,0.1612620156706,0.
113145192015956,0.909915446956986,0.982366419324046
"15084","NAB2",83.3578827060477,-0.0311596898660566,0.275433982293207,
-0.113129431621427,0.909927941709276,0.982366419324046
"15085","ARHGEF25",152.366826586885,0.0234805930626543,0.2076402659719
66,0.113083042697626,0.909964718716873,0.982366419324046
"15086","AL135999.1",15.817660003624,0.0532938004934803,0.471445373098
464,0.113043426735147,0.909996126292623,0.982366419324046
"15087","AC091057.6",5.47449472589663,-0.0817242117022106,0.7213335149
31589,-0.113296013578353,0.909795877596431,0.982366419324046
"15088","SPC24",5.81545725277476,0.0736115966663744,0.647283442723271,
0.113723898693706,0.909456667007014,0.982366419324046
"15089","IGLC2",301.717784094054,0.100294437831527,0.884594258958204,0
.113379028651672,0.909730065204502,0.982366419324046
"15090","FAM229B",313.841842423972,0.0346174584220418,0.30655207154468
4,0.112925214459025,0.910089845942417,0.982402485126311
"15091","COA6",290.30808736646,0.0322556823495538,0.286402277732714,0.
112623693515652,0.910328899850316,0.982414271785015
"15092","AGFG1",255.72724993547,0.0169072523966016,0.149908858600765,0
.112783544310939,0.910202164831068,0.982414271785015

"15093","AC021086.1",2.72004902807064,0.100304252270171,0.890744916944075,0.112607156507039,0.910342011070232,0.982414271785015
"15094","MROH6",29.5835475055273,-0.0492207712457835,0.437026074836994,-0.112626623626845,0.910326576740313,0.982414271785015
"15095","AL161457.2",15.8278783938802,0.0476352186516015,0.424273994444392,0.112274660420755,0.910605632805129,0.982568571327685
"15096","RASGRP1",10.2558140172419,0.0691892183575196,0.616027157442225,0.112315208058029,0.910573483813583,0.982568571327685
"15097","AL031848.2",4.66115000022432,-0.0755802456710024,0.676340011628458,-0.111748890161066,0.911022513328378,0.982608718643288
"15098","DNAJB4",284.215028997182,-0.0230381009148613,0.205478618494823,-0.112119212615019,0.910728884010273,0.982608718643288
"15099","AC012511.1",20.4303565970528,-0.0498562676441368,0.44739914000716,-0.111435769955523,0.911270796366795,0.982608718643288
"15100","AC007319.1",2.81735151221009,-0.11333887734875,1.01619062793989,-0.111533086639975,0.911193629922211,0.982608718643288
"15101","TMF1",573.577740398625,0.0180726873983472,0.161806697532098,0.11169307373548,0.911066771324113,0.982608718643288
"15102","ERICH6-AS1",22.2295894388517,-0.0355493115868658,0.318529994674783,-0.111604282740033,0.91113717610531,0.982608718643288
"15103","SLC22A23",23.994161822546,0.0442841632363197,0.397241777769864,0.111479118548238,0.911236423362215,0.982608718643288
"15104","NIPSNAP2",413.695461664609,0.0211655189909016,0.18963362840617,0.111612687943553,0.911130511362258,0.982608718643288
"15105","MRPS31",268.519774939563,0.0212406193466102,0.189818672835323,0.111899525106455,0.910903073149691,0.982608718643288
"15106","AL442663.3",5.82372035141338,-0.086277767064446,0.770676730467416,-0.11195065797837,0.910862529765694,0.982608718643288
"15107","AKAP13",1419.05441255155,0.0274547205361156,0.246471904971259,0.111390872478213,0.9113063977251,0.982608718643288
"15108","SMAP2",128.190276982955,-0.0291718939639525,0.262766128867811,-0.111018471405148,0.91160169921349,0.982633925252803
"15109","SLC25A24",232.283516032864,0.0178339320010921,0.160766392385626,0.110930722127012,0.911671283209771,0.982633925252803
"15110","ATP8B2",185.350819969996,0.0275080178290319,0.248620953524004,0.110642395337672,0.911899927238679,0.982633925252803
"15111","COA5",349.151252466996,-0.0266126438204174,0.240433921514432,-0.110685895121583,0.911865431308844,0.982633925252803
"15112","MTLN",442.917381188918,-0.0373930281280844,0.337245051663604,-0.110877914868217,0.911713158981602,0.982633925252803
"15113","TMEM44",94.188604856195,0.0263511481806023,0.238116251618419,0.110665055415159,0.911881957463334,0.982633925252803
"15114","GEMIN5",113.69037993193,0.0199851560325341,0.18012812814063,0.110949668099206,0.911656259251519,0.982633925252803
"15115","ANKLE2",249.800179635212,0.0221605594287952,0.200398957853394,0.110582208940464,0.911947656157238,0.982633925252803
"15116","MCTP2",5.66935114003962,-0.0813979229308921,0.734958114512126,-0.110751784793784,0.9118131802045,0.982633925252803
"15117","COA3",508.853055032965,-0.0306176059301517,0.275961334668718,

-0.11094889784798,0.911656870052088,0.982633925252803
"15118","DENND1C",3.70753956050053,0.103069134045854,0.932544831028392
,0.110524588863134,0.911993350234629,0.982633925252803
"15119","AL160314.2",2.61438349931951,-0.103803176666266,0.94060865350
4145,-0.110357454483921,0.912125893376488,0.982711732072863
"15120","SLC35B4",153.830500584214,0.0190393802482933,0.172667693052,0
.110266025518505,0.912198400632773,0.982711958817052
"15121","PARP6",91.4092983459655,0.0273710824007007,0.248379795463288,
0.110198506080766,0.912251947034173,0.982711958817052
"15122","ITGB4",5.43440178451453,-0.0735532394889739,0.667882751764903
,-0.110128969934629,0.912307093205934,0.982711958817052
"15123","LRRC2",89.483066439948,0.0452147365799187,0.414408745165564,0
.109106617819695,0.913117925967755,0.982805464258541
"15124","LRRTM2",6.47304602039957,-0.0805355277901541,0.73767494958547
1,-0.109174817221881,0.91306383384347,0.982805464258541
"15125","TBC1D22B",26.0428504931912,0.0344490157929948,0.3143203985184
95,0.109598409633499,0.912727871989419,0.982805464258541
"15126","SNRNP35",215.863036136418,0.0250914528670301,0.22910341158400
5,0.109520206152975,0.912789895975386,0.982805464258541
"15127","AC092338.2",2.27861280446672,-0.113562090792778,1.03576151330
447,-0.109641157094621,0.912693968762355,0.982805464258541
"15128","PPP4C",413.253540675833,-0.0274998940016889,0.251912482076948
,-0.109164475594699,0.913072036244057,0.982805464258541
"15129","ADPRM",47.5529957924924,0.0302010784537096,0.27584853423205,0
.109484281066739,0.912818388709433,0.982805464258541
"15130","CCL4",291.626533495495,-0.0712749769449695,0.64900181115592,-
0.109822462310272,0.912550176448101,0.982805464258541
"15131","SSTR2",5.27716109380602,0.0996029282321105,0.908834506981596,
0.109594131238381,0.912731265214915,0.982805464258541
"15132","AC022966.1",9.29339345801158,0.0796874338616224,0.72895061069
7243,0.109318015092135,0.912950258249808,0.982805464258541
"15133","OXLD1",164.504654853417,0.026871331485547,0.245916081983869,0
.109270330223095,0.912988078707911,0.982805464258541
"15134","AC002470.1",2.81835324204517,-0.0894584108322782,0.8151413698
64241,-0.109745884750245,0.912610909401995,0.982805464258541
"15135","DRAM1",234.692092193711,-0.0195441609224295,0.179333335276295
,-0.108982308795619,0.913216522283404,0.982846642317435
"15136","CIR1",893.61357457388,0.0217070966861874,0.199474243059792,0.
108821551861614,0.913344029419014,0.982918928065957
"15137","RAB9A",462.889026083002,-0.0280326589384616,0.257927374774983
,-0.108684310701481,0.913452886378032,0.982971134717035
"15138","L1TD1",4.40425827638097,-0.100496979426806,0.926944170488872,
-0.108417510597002,0.913664511559927,0.983022527785427
"15139","ARL6IP6",138.025839086816,-0.0256267441409466,0.2362442883219
14,-0.108475613624262,0.913618423860734,0.983022527785427
"15140","ZFYVE28",43.7249418184899,0.031394857318883,0.289700472291537
,0.10837005915299,0.913702150569372,0.983022527785427
"15141","AL355304.1",10.1207582246182,0.069619986106209,0.643630624191
555,0.108167609634263,0.913862737936935,0.983022527785427
"15142","PRKCQ-

AS1",4.55913666681752,-0.0936395198821122,0.86564112835851,-0.10817360
3141614,0.91385798370582,0.983022527785427
"15143","DHX32",109.082950426778,0.0217350923091859,0.200771277753247,
0.108257976700726,0.913791056376728,0.983022527785427
"15144","RGP1",65.3912289569416,0.0263779765833179,0.245191915656508,0
.107580939251974,0.914328117499603,0.98302306214822
"15145","AP000880.1",4.64184170458089,-0.0888197599953901,0.8222731736
54621,-0.108017338812876,0.913981938299589,0.98302306214822
"15146","TRIAP1",181.006382974946,-0.0298771907096383,0.27745476258283
3,-0.107683106361235,0.914247070751965,0.98302306214822
"15147","AC023034.1",2.57018469218898,-0.0956645125530768,0.8884501028
05159,-0.107675729060112,0.914252922960334,0.98302306214822
"15148","PALB2",39.4474170839025,0.0293516459118121,0.272150925989833,
0.10785061930272,0.914114188633387,0.98302306214822
"15149","HIC1",1018.13545215327,0.019058132165037,0.176518540426433,0.
107966744564035,0.914022071960936,0.98302306214822
"15150","CCDC43",179.704616061239,0.0211055614600786,0.196119235670718
,0.107615968356692,0.914300329640206,0.98302306214822
"15151","AL162458.1",17.391051143071,-0.0393844295262035,0.36616798512
1822,-0.107558364265791,0.914346025821578,0.98302306214822
"15152","SH3GLB1",912.515335489091,0.0159097912598237,0.14889619862065
,0.106851561068781,0.914906741765473,0.983171653029277
"15153","CANX",1252.31254477645,-0.0154684432534663,0.144367525797619,
-0.107146279386617,0.914672932821129,0.983171653029277
"15154","ATF1",242.774672702267,0.0173221984854442,0.162028609662965,0
.106908270838563,0.914861751619317,0.983171653029277
"15155","COPZ1",594.143391975374,-0.0274347120176954,0.256267883151522
,-0.107054819668816,0.914745489788916,0.983171653029277
"15156","EFNB2",5.86432166603479,-0.0884762072768863,0.824682513326114
,-0.107285174412203,0.914562745760053,0.983171653029277
"15157","CHAMP1",102.434899925307,0.0244362404822837,0.228286110258878
,0.10704216938373,0.914755525589002,0.983171653029277
"15158","PLA2G6",32.9005028380371,-0.0307544480687383,0.28782017591504
3,-0.10685299587134,0.914905603475408,0.983171653029277
"15159","AL121658.1",7.29061731431137,-0.0568655164509168,0.5353197495
65149,-0.106227196917562,0.915402093004873,0.983224307962538
"15160","ENOPH1",145.533240151551,0.0258468569168912,0.243958819238951
,0.105947622625501,0.915623909276868,0.983224307962538
"15161","USP38",89.1522334033503,0.0251339962885681,0.237056447538795,
0.106025364631582,0.915562227544898,0.983224307962538
"15162","IBTK",256.625385930642,0.0173818838418288,0.163682684092145,0
.106192563607056,0.915429570967607,0.983224307962538
"15163","DUS4L",38.472182034734,-0.0268822268954839,0.253105943836167,
-0.106209386030399,0.915416224095703,0.983224307962538
"15164","LINC00513",55.7435938840609,-0.0563965692822848,0.53265138457
7008,-0.105878949938468,0.915678395690888,0.983224307962538
"15165","AC073140.1",5.23693983559351,-0.0682722021694597,0.6404426521
34578,-0.106601585546981,0.915105060670074,0.983224307962538
"15166","DNHD1",20.0110133555672,-0.0535843152236995,0.506464388263818
,-0.105800756115132,0.915740436865349,0.983224307962538

"15167","AP002381.2",5.69835511472797,-0.0730377381663235,0.686427831864598,-0.106402646827308,0.915262893136487,0.983224307962538
"15168","LATS2-AS1",8.97305891424246,-0.0972590362265376,0.919120122347322,-0.105817546435769,0.915727114910338,0.983224307962538
"15169","ABHD2",263.957717128719,0.029306421130546,0.27518426086452,0.106497446614413,0.915187681195784,0.983224307962538
"15170","EIF3C",39.4749432912553,0.0387571729964526,0.364368299346127,0.106368125509282,0.915290281733767,0.983224307962538
"15171","PELP1",115.782496876035,-0.0214101051016559,0.202134846660438,-0.105919911659875,0.915645895716251,0.983224307962538
"15172","ARIH2OS",49.2779920659311,-0.0341897316048016,0.324683568982509,-0.105301699472952,0.916136414490273,0.9834549924629
"15173","ZNF775",67.3281970121436,-0.0302639240246371,0.28724262092966,-0.105360144419683,0.916090040136998,0.9834549924629
"15174","CASP4",641.96592144976,-0.0187819245725771,0.178249772379858,-0.105368575352523,0.91608335046231,0.9834549924629
"15175","BACH1",193.017213914588,0.0345387022838641,0.328275774833469,0.10521246138672,0.916207222848275,0.983466191299872
"15176","NUDT22",158.656529739803,-0.0250787891966041,0.238590722523795,-0.10511217255777,0.916286800508494,0.983486801099292
"15177","AC093690.1",15.3245234280721,0.0510322783924342,0.487448196767995,0.104692721669301,0.91661963749026,0.983629515121855
"15178","USP19",103.736505527098,-0.0228678469589993,0.218434259894633,-0.104689836521204,0.916621926924539,0.983629515121855
"15179","LZTS3",22.324460881093,-0.0352957673781462,0.336850114285063,-0.10478181802924,0.916548937730196,0.983629515121855
"15180","BPI",3.33976307319963,0.156373927680438,1.49439617011224,0.10464020907434,0.916661307603275,0.983629515121855
"15181","CDK8",41.078012074406,0.0269812697154641,0.258879148653095,0.10422341797647,0.91699205031604,0.983919603952175
"15182","MAP3K2",493.505449381142,-0.0246471746009984,0.236927253673915,-0.104028448474402,0.917146772428876,0.983955988677729
"15183","RBM10",142.838295561819,0.0192592862275139,0.185132672304183,0.104029645269041,0.917145822677962,0.983955988677729
"15184","DEDD",123.039727444114,0.0188817830300539,0.181932522781927,0.103784539132052,0.917340336190123,0.984098836683411
"15185","ZNF76",75.7643922123672,-0.0246874167049086,0.238717719424396,-0.103416775111775,0.917632198916603,0.98434711150165
"15186","TMEM53",35.0598792807969,-0.038782954876974,0.376426969702846,-0.103029161028471,0.917939826948531,0.98454743143245
"15187","TRAPPC12",236.942687317627,0.0183014229855985,0.177564354933319,0.103069239276494,0.917908018471668,0.98454743143245
"15188","GABPB1-IT1",304.773676122434,-0.0201637113118961,0.196013693350285,-0.102868891286399,0.918067027842504,0.984619029268274
"15189","RBPMS2",13.3454157287377,-0.0462990923626484,0.450417805027055,-0.102791434632268,0.918128503427512,0.984620132486059
"15190","AC024560.5",33.6195884233775,-0.0356094819012974,0.347089939802394,-0.102594393607521,0.918284892613584,0.984656328768776

"15191","SDAD1",213.485959669324,-0.0215198857971362,0.209722548181726,-0.102611216503478,0.918271540351797,0.984656328768776
"15192","HACD4",173.413169810127,0.0315638552571563,0.307878706047425,0.102520423261407,0.918343602839661,0.984656328768776
"15193","C12orf49",124.988635618583,0.0215056599897228,0.209995132575216,0.102410278400238,0.91843102558491,0.984685248190127
"15194","TMEM69",98.3905154165239,0.020902021631040l,0.210130807714434,0.0994714761647218,0.920763934280605,0.984852828517747
"15195","OSBPL9",451.369216833919,-0.0171725138189857,0.169639883145962,-0.101229224522691,0.919368498057064,0.984852828517747
"15196","ATG4C",115.555943529827,-0.020995550252837,0.210196541484247,-0.0998853268687608,0.920435365550879,0.984852828517747
"15197","WDR78",22.7126057048983,0.0349524028626315,0.347961607462417,0.100449021136352,0.919987853293265,0.984852828517747
"15198","C1orf61",2.56105783868209,0.105780249997497,1.04481672968225,0.101242875417651,0.919357661889771,0.984852828517747
"15199","ARL8A",277.382824569659,-0.0195122126397564,0.193671883267356,-0.100748814492709,0.919749860118052,0.984852828517747
"15200","ST6GAL2",2.28951922994265,0.102639974369755,1.00799901276233,0.101825471126682,0.918895207692796,0.984852828517747
"15201","EIF4E2",465.319015915744,-0.0213874156779437,0.214753976514444,-0.0995903127153748,0.920669584922133,0.984852828517747
"15202","NCK1-DT",24.9893416454475,0.0344611055286792,0.339471328957069,0.101514038415413,0.919142414082495,0.984852828517747
"15203","RAI14",68.6444120126895,-0.0401862077575049,0.40289233849325,-0.0997442838148639,0.920547342421696,0.984852828517747
"15204","PPT2",32.7012819871645,-0.0304850036173085,0.300195915435218,-0.101550361113714,0.919113581759127,0.984852828517747
"15205","ZUP1",37.1979838392005,-0.0313177003518801,0.31053617322052,-0.100850409880077,0.919669209497395,0.984852828517747
"15206","AEBP1",5075.31518057321,0.0189036242091497,0.188460219192063,0.100305646943373,0.920101674492414,0.984852828517747
"15207","CALU",1457.63475798993,-0.0215595940402833,0.215070873253338,-0.100244136800838,0.920150506363744,0.984852828517747
"15208","EPHA1-AS1",16.5133045389091,0.0510366688609653,0.508505123641704,0.100366085783879,0.920053693403267,0.984852828517747
"15209","GAS1",4262.81510648617,-0.0253855532703996,0.25407731454545,-0.0999127108841455,0.920413625022119,0.984852828517747
"15210","ASB13",88.0285031065387,-0.0252648411684754,0.253235358908099,-0.0997682206679687,0.920528338369411,0.984852828517747
"15211","MYOZ1",2.58275389835311,-0.092316571287541,0.925821714265125,-0.0997131195619209,0.920572084551218,0.984852828517747
"15212","SLC1A2",51.0748934744086,-0.0654607546102844,0.641635978332483,-0.102021639716038,0.918739498692976,0.984852828517747
"15213","FDX1",371.820685579635,-0.0227102540907124,0.224108974397137,-0.101335763781009,0.919283926913568,0.984852828517747
"15214","MAGOHB",154.752336494104,-0.0357980804675972,0.352953129876047,-0.101424459616406,0.919213520622554,0.984852828517747

"15215","AC008147.2",40.0315542669242,-0.0541092749725925,0.538156295876677,-0.100545650747143,0.919911142377656,0.984852828517747
"15216","PHF11",298.252712072972,-0.0196341398455436,0.19530693945603,-0.100529658087054,0.919923838347525,0.984852828517747
"15217","ABCC4",12.3755096673234,-0.0482924147363241,0.485085362329121,-0.0995544670827618,0.920698044166813,0.984852828517747
"15218","RORA",471.175323204132,-0.0265891296330849,0.262172542808802,-0.101418437446655,0.919218300969422,0.984852828517747
"15219","CCL14",7.01341652745467,0.075342656556469,0.748445298248241,0.10066554861499,0.919815960631388,0.984852828517747
"15220","TMEM101",227.458587357007,-0.022976299049975,0.230849273443854,-0.0995294406051625,0.9207179137229,0.984852828517747
"15221","CEP95",219.434858675353,0.0189467581548919,0.190081894293819,0.099676816801946,0.920600906375124,0.984852828517747
"15222","SMIM7",760.616114460702,0.0224791518392914,0.221880982026482,0.10131755671824,0.919302984534131,0.984852828517747
"15223","BORCS8",129.187983895798,-0.0312046805237102,0.308225780071497,-0.101239683833299,0.919360195388399,0.984852828517747
"15224","CARD8-AS1",16.9037664656174,0.04754903552277,0.477087798319382,0.0996651679004768,0.920610154801126,0.984852828517747
"15225","ZNF579",71.9889914602247,0.0284298834684632,0.280610512280448,0.101314392099644,0.919300891737275,0.984852828517747
"15226","ZNF416",14.9773095275062,-0.0450022617494316,0.44899529522446,-0.100228804684767,0.920162678320635,0.984852828517747
"15227","AL031055.1",2.52255411806159,0.104959064244121,1.04517933855293,0.100422061910866,0.920009255425532,0.984852828517747
"15228","ZNF711",25.4675903298097,-0.0477694767100498,0.473059204763031,-0.100979911666615,0.919566406820911,0.984852828517747
"15229","MOSPD1",53.3569709721199,-0.028007400889678,0.278606228009511,-0.100526829890974,0.919926083547885,0.984852828517747
"15230","CCDC30",53.3903970799197,0.0249790730972139,0.252038139523782,0.0991083061651348,0.921052277194107,0.984902549938607
"15231","EGR1",12186.3889603399,0.0257270430532062,0.259465014341068,0.0991541889319534,0.921015847480805,0.984902549938607
"15232","ETS1",177.636045585868,0.0287064510970695,0.289176667160403,0.0992695966066528,0.920924217547232,0.984902549938607
"15233","CIDEB",37.2581631940494,-0.0253257715582627,0.255440258096573,-0.0991455761397166,0.921022685799286,0.984902549938607
"15234","IKZF5",88.5560587855084,-0.0225218000384818,0.228329451892478,-0.098637297342997,0.921426255409173,0.985173106292092
"15235","CCDC92",175.633602264828,-0.0164205506020188,0.166409983429471,-0.0986752733436707,0.921396102044493,0.985173106292092
"15236","GINS2",10.8285393507961,0.0452153226105228,0.458766842715143,0.0985583926312604,0.921488906984348,0.985175427006303
"15237","PM20D1",2.290468954379,-0.0959735576382002,0.97750582379006,-0.0981820827072766,0.921787709645534,0.985219015794833
"15238","AC010913.1",8.00647518706055,0.0542582534989445,0.553021362014066,0.0981124007603245,0.921843040653833,0.985219015794833
"15239","DUSP28",25.8796996341374,-0.0308406888343232,0.31358988497216

7,-0.0983472054178678,0.921656595330813,0.985219015794833
"15240","AC018647.2",47.2922647591888,-0.0257896993640316,0.262885210461072,-0.0981025114299863,0.921850893315524,0.985219015794833
"15241","TMED10",1183.32752723473,-0.0137168739102299,0.139896706990515,-0.098050012793796,0.921892580191838,0.985219015794833
"15242","ITGA2B",3.33361485615716,-0.0839448786281962,0.853614152804398,-0.0983405422138447,0.921661886151335,0.985219015794833
"15243","PGM5",100.955984131494,-0.03865886042243,0.395702582924464,-0.0976967603716895,0.922173088100793,0.985454138429037
"15244","DESI1",148.2546839137,0.0186032439479502,0.190631525207631,0.0975874474470476,0.922259892412464,0.985482247934048
"15245","CCDC86",44.7671856422417,-0.0345882367878736,0.355666778195854,-0.0972489951502498,0.922528659899365,0.985704778032191
"15246","AL591895.1",43.3725433804999,0.0301921165976819,0.311554098864186,0.0969081026625919,0.922799374102208,0.985929358831881
"15247","SLC35E2A",86.0309729077961,0.0476342129257751,0.493200719407359,0.0965817993595255,0.923058510920072,0.985931236448243
"15248","TMEM50A",2013.47799540325,-0.0215391804062408,0.223142465924138,-0.0965265859057211,0.923102360009067,0.985931236448243
"15249","FAM161A",57.1632043993462,-0.0279603726608513,0.290494570048668,-0.0962509304603075,0.92332128188768,0.985931236448243
"15250","HOXD8",139.858833818357,-0.0329785867885191,0.348335056928075,-0.0946749003082069,0.924573055059997,0.985931236448243
"15251","H1FX",1564.72282742829,-0.0215204844470626,0.228912368712933,-0.0940118900872953,0.925099711646341,0.985931236448243
"15252","DNAJB14",437.186305782843,0.0111302790381319,0.118856068081559,0.0936450213925499,0.925391144827772,0.985931236448243
"15253","GTF2H2",64.303542109989,0.0243503598759714,0.252934708923833,0.0962713262231799,0.923305083645615,0.985931236448243
"15254","RGMB",57.8519903412916,-0.0322630784877509,0.338815025007471,-0.0952232814558311,0.924137478081287,0.985931236448243
"15255","SNX24",91.9284394109578,-0.0252814082741257,0.270176217981553,-0.0935737736763042,0.925447743756244,0.985931236448243
"15256","NR3C1",1721.49283108609,0.013459550808797,0.14440986502075,0.0932038182215813,0.925741641093646,0.985931236448243
"15257","PPARGC1B",6.8743962421628,-0.0534569537823743,0.571355800649028,-0.0935615840806206,0.925457427164841,0.985931236448243
"15258","HNRNPH1",1085.9595236021,0.0205493389322827,0.220540947563792,0.0931769776056612,0.925762964016447,0.985931236448243
"15259","AGER",8.52174354203982,-0.0505076828812442,0.52867043058113,-0.0955371815021406,0.923888158742371,0.985931236448243
"15260","IRAK1BP1",79.5008778044238,-0.0202826875502455,0.215212125670692,-0.0942450964927561,0.924914462347311,0.985931236448243
"15261","MAGI2",154.104182632886,0.0164217844444771,0.173293778132491,0.0947626892404753,0.924503323134217,0.985931236448243
"15262","MEPCE",159.509334616553,0.0151584719450863,0.161093776529266,0.0940971915344748,0.925031951315095,0.985931236448243
"15263","KMT2E-AS1",175.684202562425,0.0314082535395448,0.332859649770531,0.0943588493264271,0.92482410338707,0.985931236448243

"15264","TEK",7.02842913568177,0.0596753568992376,0.627548018805141,0.
0950928934695071,0.924241042687406,0.985931236448243
"15265","HNRNPK",2212.07584220312,-0.0111765691783443,0.11660690816776
8,-0.0958482593695391,0.923641088333216,0.985931236448243
"15266","ENO4",13.7503096518359,0.0466201538439031,0.500069682859409,0
.0932273149960383,0.925722974654172,0.985931236448243
"15267","IPO7",258.103322503467,-0.0175709513122945,0.188586322487232,
-0.0931719282743006,0.925766975349983,0.985931236448243
"15268","KBTBD4",69.2031805408024,-0.0223471719189502,0.23655666451966
5,-0.0944685788680982,0.924736941226504,0.985931236448243
"15269","USP35",19.9978053030436,-0.0372556968155105,0.3953264819754,-
0.094240326702497,0.924918251226081,0.985931236448243
"15270","CBL",57.7181925119421,0.0310079186066116,0.326510339765749,0.
0949676467485161,0.924340524897037,0.985931236448243
"15271","FBXL14",40.6977472874403,0.0292504646005433,0.308724972503808
,0.0947460270651817,0.924516558078835,0.985931236448243
"15272","P3H3",263.436826260675,-0.0205449048302114,0.212931211257508,
-0.0964861126223784,0.923134502986498,0.985931236448243
"15273","AC025259.3",9.13449150049736,-0.0910895210065407,0.9545031302
50835,-0.095431348645869,0.923972217079417,0.985931236448243
"15274","AC079866.2",24.7687043614354,-0.0520049494807348,0.5458327106
51234,-0.0952763520139488,0.924095325551687,0.985931236448243
"15275","MRPL42",314.805302306418,0.021457646543219,0.223368563354699,
0.0960638606478624,0.923469853547464,0.985931236448243
"15276","OAS1",120.778981737766,-0.0333228615540533,0.353822735125282,
-0.094179537508392,0.924966539219011,0.985931236448243
"15277","LINC00571",3.86572786802691,-0.076162453628226,0.792327297659
857,-0.0961249900807055,0.92342130399173,0.985931236448243
"15278","TPT1",54830.5327091204,0.0227888222151804,0.244448325558401,0
.0932255198031044,0.925724400799978,0.985931236448243
"15279","SRP54",279.145654351326,0.0164797311893889,0.174835980517771,
0.0942582364372868,0.92490402465254,0.985931236448243
"15280","AC004816.2",32.4651475933475,0.0470015993088084,0.50178167424
4943,0.0936694218248089,0.925371761299303,0.985931236448243
"15281","ZNF410",132.41954742325,0.0187977904686095,0.195868963536398,
0.0959712561358211,0.923543401442553,0.985931236448243
"15282","IGHM",39.8479970828978,0.0899094316158994,0.939483292143193,0
.0957009372788246,0.92375809647763,0.985931236448243
"15283","EFL1",48.2327517317856,0.023354422908795,0.2499383644801,0.09
34407287067548,0.925553435209552,0.985931236448243
"15284","TMEM186",73.0904826904393,0.0275082781146009,0.28911493480797
8,0.0951465137311953,0.924198453015454,0.985931236448243
"15285","RRAD",44.3436178957113,-0.0877353576266432,0.923163797875606,
-0.0950376930167113,0.92428488770543,0.985931236448243
"15286","CHRNB1",84.184547857517,0.0287351679544515,0.298163726023802,
0.096373788782602,0.923223708728066,0.985931236448243
"15287","AC002553.1",4.80736631686183,0.072797245073675,0.764816482180
79,0.0951826310883124,0.924169765728661,0.985931236448243
"15288","RIOK3",341.032808927174,-0.0190020841401845,0.198396260713073
,-0.0957784389276669,0.923696541863542,0.985931236448243

"15289","PRR22",2.94577980756787,0.0765478257492984,0.800700523848587,
0.0956010686509476,0.923837416429021,0.985931236448243
"15290","ZFP14",103.77399484084,-0.0236612687748585,0.253234307056735,
-0.0934362687657379,0.925556978227218,0.985931236448243
"15291","AL357033.4",3.13493914157856,-0.0785521063319157,0.8229670418
59488,-0.0954498811452131,0.923957497476989,0.985931236448243
"15292","CBR3-
AS1",42.1851221616028,-0.0251561646395135,0.266474214465166,-0.0944037
481825544,0.924788438485677,0.985931236448243
"15293","STS",83.0497327855606,0.0208635565033916,0.217468848053227,0.
0959381386812937,0.923569703981326,0.985931236448243
"15294","ZNF674",19.412386340291,-0.0339452965585243,0.353799948004851
,-0.0959448884883918,0.923564343143966,0.985931236448243
"15295","SLC35A2",87.0287231655537,-0.0234516155137814,0.2507464358213
99,-0.0935272138044884,0.925484730954147,0.985931236448243
"15296","NFXL1",68.3019547709735,0.0253025642770234,0.272158489109785,
0.0929699615829976,0.925927425023786,0.986037645542132
"15297","ELF3-
AS1",3.03169352115067,0.0954402865720932,1.03515874539556,0.0921986960
904468,0.926540174002949,0.986101859645981
"15298","DBI",2295.24587942664,0.0267977578958376,0.290863280458305,0.
0921318010771699,0.926593322288902,0.986101859645981
"15299","DAPL1",6.01602531127514,-0.0573178490363517,0.621982878093145
,-0.0921534194189958,0.92657614641653,0.986101859645981
"15300","THRB",271.230578242638,0.0218733752376924,0.236437584642839,0
.0925122597184966,0.926291051146068,0.986101859645981
"15301","PODXL2",66.5062184069397,-0.0347044064785425,0.37594032318835
1,-0.0923136049472275,0.926448879341565,0.986101859645981
"15302","MFHAS1",83.8869260879849,-0.022973174475865,0.249194194784381
,-0.0921898461388432,0.92654720529687,0.986101859645981
"15303","ATP6V1G1",1501.63099568306,0.0193276510012396,0.2103995272970
55,0.0918616655157767,0.926807949121354,0.986101859645981
"15304","PCSK7",331.642919841487,-0.0268800671017462,0.290311161809882
,-0.0925905395237588,0.926228859827252,0.986101859645981
"15305","LTA4H",212.839050919884,-0.0197544229638753,0.213731956161824
,-0.0924261552583113,0.926359459474804,0.986101859645981
"15306","AC073575.2",2.60855798636612,-0.0819385767591238,0.8913206359
86422,-0.091929406154097,0.926754127647874,0.986101859645981
"15307","PARD6G",13.3319480397494,0.0418167893710283,0.45538516713092,
0.0918272978333664,0.926835255152555,0.986101859645981
"15308","ZNF835",26.5219141833166,-0.0294369288322329,0.31845311746167
2,-0.0924372449761802,0.926350648877829,0.986101859645981
"15309","AL121917.1",3.05074158475757,-0.0765713594819046,0.8306363341
303,-0.0921839755084601,0.926551869520765,0.986101859645981
"15310","SYP",15.5388035955338,0.0388565527808454,0.422835684921371,0.
0918951596719444,0.926781337212768,0.986101859645981
"15311","PLEKHB2",153.066366558593,0.0206231389077723,0.22478536575549
4,0.0917459143234651,0.926899916843803,0.986106246846627
"15312","LINC00954",12.167644944439,-0.0519365007647682,0.570369596343
75,-0.0910576249114568,0.927446803128901,0.986108418809835

"15313","AC007038.2",10.4579350194754,0.0490969169609326,0.538204268610079,0.0912235740673819,0.927314943651075,0.986108418809835
"15314","ACTR3C",2.89414766499285,-0.0810190765518246,0.889250276617571,-0.0911094195663292,0.927405648043589,0.986108418809835
"15315","APH1B",224.455013999847,0.0167789031019645,0.183681689238324,0.0913477177368184,0.927216303180296,0.986108418809835
"15316","TIMM22",162.028680622365,-0.0269561157327032,0.29451596864711,-0.0915268392967922,0.927073981060271,0.986108418809835
"15317","TEFM",92.0585525776833,0.0238841188476434,0.260973241413222,0.0915194167735592,0.927079878624739,0.986108418809835
"15318","AC142472.1",31.234792893633,0.0348072064866333,0.380783704250768,0.0914093909431345,0.9271673000977,0.986108418809835
"15319","JOSD2",513.727220826264,-0.0224318329302139,0.245940963747414,-0.0912082013033494,0.927327158420799,0.986108418809835
"15320","RRAGB",123.001724741592,0.0174876838608584,0.191867946504223,0.0911443739273746,0.927377874057453,0.986108418809835
"15321","DNALI1",70.4067876613591,0.0193418103807896,0.214152328936127,0.0903180015686799,0.928034515430628,0.986147527689112
"15322","AC093425.1",38.989901097753,0.0561463297483574,0.620073213560016,0.0905479038934862,0.927851828462833,0.986147527689112
"15323","MPV17",280.239761499924,0.0205887095876162,0.228154638755785,0.0902401533446542,0.928096376717897,0.986147527689112
"15324","PDE12",109.14624543825,-0.0217165496208645,0.241486262364481,-0.0899287164753378,0.928343861153012,0.986147527689112
"15325","ZBTB9",12.4277926435045,-0.0448121925110998,0.495357533651235,-0.0904643403337248,0.927918230019835,0.986147527689112
"15326","ADAP1",30.4078529155772,-0.0928930675765783,1.03126966133675,-0.0900764087796092,0.928226496063424,0.986147527689112
"15327","NOM1",99.1266792080284,0.0236554303237237,0.260602843091311,0.0907719579844922,0.927673792328038,0.986147527689112
"15328","GLI4",116.659529377268,0.0212968363261887,0.236871884277353,0.0899086710571872,0.928359790554758,0.986147527689112
"15329","CLN3",315.033085323013,-0.0188088914001492,0.20762850042709,-0.0905891597803746,0.927819045756419,0.986147527689112
"15330","MT1E",5983.57077952446,-0.0611461410565331,0.676532438896345,-0.0903816839238091,0.927983911228198,0.986147527689112
"15331","FAM157C",11.0757702596636,0.046294705569678,0.515138168131361,0.0898685215611374,0.928391696059459,0.986147527689112
"15332","SRP68",226.082220771978,0.0145108426989606,0.159717020542872,0.0908534522472229,0.927609036882683,0.986147527689112
"15333","NRSN2",85.0662631645072,0.0250532704351016,0.275702038241597,0.0908708205238132,0.927595236089864,0.986147527689112
"15334","LARGE1",78.8580714308766,-0.0210784169338044,0.233822636071787,-0.0901470331868679,0.928170374260404,0.986147527689112
"15335","AC240274.1",104.358540312942,-0.0313618433328463,0.348322469168238,-0.0900368081557743,0.928257964921442,0.986147527689112
"15336","C20orf96",52.9409445180399,-0.0257941112507284,0.287294138905947,-0.0897829358752522,0.928459708617451,0.986155463854307
"15337","FOXN3",1304.62103407148,-0.0197042459441525,0.220081842592054,-0.0895314475382527,0.928659562408297,0.986255299472116

"15338","TMEM164",27.4072923784883,0.029888568744537,0.333904689768074
,0.0895122759889871,0.928674797919044,0.986255299472116
"15339","LZTFL1",81.7878710457759,0.0210280554796692,0.235339407092782
,0.08935203729556,0.92880213963227,0.986294433825492
"15340","IMPG2",2.72258883388549,-0.0833270747838433,0.932972652449209
,-0.0893135233547263,0.928832746938612,0.986294433825492
"15341","DFFB",24.561905997137,-0.0294527708531427,0.338922128589154,-
0.086901291974493,0.930749972560032,0.986437114217266
"15342","AHDC1",240.686449174635,-0.0199033862044901,0.238553057901043
,-0.0834337919606402,0.933506620072728,0.986437114217266
"15343","ZFP69B",5.56082508337966,-0.0495366633505867,0.59605426675962
6,-0.083107639879648,0.933765951118771,0.986437114217266
"15344","LRRC8C-
DT",10.592910263322,0.0452054673892564,0.539278600862809,0.08382581344
21258,0.933194924087314,0.986437114217266
"15345","URB2",13.2840749496342,-0.0410265864719264,0.461721994216815,
-0.0888556035575408,0.929196667156661,0.986437114217266
"15346","TTC13",30.5384585665902,-0.0256954016016019,0.309139628331638
,-0.0831190803336171,0.933756854430693,0.986437114217266
"15347","REL",140.312968389737,-0.0199460908434605,0.235162722726868,-
0.0848182510058241,0.93240588362188,0.986437114217266
"15348","FAHD2A",218.059425308605,0.0162380893295768,0.191773425315543
,0.0846733028982441,0.932521120926739,0.986437114217266
"15349","CCNT2-
AS1",5.41061173559784,0.0605321763470789,0.707011073309524,0.085617013
1306817,0.931770873598306,0.986437114217266
"15350","TTC21B",110.919924189497,-0.0177286688000927,0.20957834910535
5,-0.0845920815569578,0.932585694505985,0.986437114217266
"15351","CNOT10",87.0834658555494,0.0177316866113488,0.205381333691299
,0.0863354341539171,0.931199771242313,0.986437114217266
"15352","GK5",150.197253734382,0.0245725958746818,0.294321133932398,0.
0834890636169057,0.933462673001883,0.986437114217266
"15353","LAP3",390.823677672764,0.0166747994112803,0.193577071126873,0
.0861403642188153,0.931354836848637,0.986437114217266
"15354","CCDC149",124.914988390571,-0.020625330208115,0.23836398479218
7,-0.0865287187831532,0.931046127392692,0.986437114217266
"15355","HERC5",43.9720889254537,0.0270873549988118,0.308821131739911,
0.0877121162214534,0.930105489389889,0.986437114217266
"15356","IRF2",326.342851924371,-0.015828739331786,0.179651863255447,-
0.088107849509354,0.929790957639154,0.986437114217266
"15357","BRD9",228.428110865218,-0.0199936944129047,0.231814859519505,
-0.0862485453018269,0.931268840879689,0.986437114217266
"15358","XRCC4",67.8210743138715,-0.0218057729943842,0.263286362599455
,-0.0828215057517351,0.933993468756393,0.986437114217266
"15359","NDUFA2",1016.68080444188,-0.0224883928704084,0.25802607530829
4,-0.0871555048982505,0.93054790666202,0.986437114217266
"15360","CREBRF",905.228798416373,-0.021394015563866,0.245707803099008
,-0.087070965162817,0.930615104155999,0.986437114217266
"15361","AL138724.1",21.0318311278631,-0.0390979338805559,0.4708180736
22738,-0.0830425509787985,0.933817705648135,0.986437114217266

"15362","KDM1B",62.2458636343055,0.0245125184356923,0.291430312900921,
0.0841110802500012,0.932968115098502,0.986437114217266
"15363","C6orf132",146.490109225028,0.0222278819640951,0.2513373104489
06,0.0884384492075392,0.929528202953994,0.986437114217266
"15364","ZNF318",83.3147069632639,0.0238025280653949,0.282018195858806
,0.0844006819946886,0.932737865069268,0.986437114217266
"15365","FBXL13",3.45379581281334,-0.077462649013636,0.888582115530739
,-0.0871755661741723,0.930531960771988,0.986437114217266
"15366","CNOT4",451.472317903389,0.0135273940902416,0.163093850300006,
0.0829423921586156,0.933897346079827,0.986437114217266
"15367","LINC01006",22.3835750642928,0.0378066911186847,0.440003781788
921,0.0859235594861805,0.931527183005626,0.986437114217266
"15368","GTF2E2",150.210578568712,-0.0169576906694174,0.19288077228901
8,-0.0879179944593312,0.929941854474703,0.986437114217266
"15369","PREX2",82.6523048285334,-0.030208110514073,0.3582632053473,-0
.0843182053395334,0.932803438177387,0.986437114217266
"15370","AC090578.1",7.76572505818193,-0.0606108751651199,0.7169951830
9156,-0.0845345639614707,0.932631423113127,0.986437114217266
"15371","TATDN1",383.321171321115,0.0234410942667019,0.268779697645145
,0.0872130390504786,0.930502175185641,0.986437114217266
"15372","AC105219.1",6.12327252119074,0.0568705149605366,0.64585982837
8457,0.0880539591745781,0.929833789432779,0.986437114217266
"15373","ZNF7",107.419595289931,0.0201723128018418,0.227103324332156,0
.0888243836199344,0.929221478955301,0.986437114217266
"15374","WASHC1",133.558111095515,-0.0215744021245948,0.25654424004825
2,-0.084096224965086,0.932979926055073,0.986437114217266
"15375","CEP78",84.6974079867902,0.0254845968027241,0.30162818619647,0
.0844901039391736,0.932666770620958,0.986437114217266
"15376","CDK20",21.0883227672524,0.0288445803990744,0.336739743701902,
0.0856583784318878,0.93173798966681,0.986437114217266
"15377","AL157938.3",28.5695729759379,0.0371314276833596,0.44209209118
2334,0.0839902554783442,0.933064179394497,0.986437114217266
"15378","SURF6",133.913797505279,0.0200300701317077,0.24042533670306,0
.0833109788110478,0.933604270815206,0.986437114217266
"15379","AC016396.2",5.2994630003764,-0.0553086354154048,0.64786470714
4573,-0.0853706565668232,0.931966720644893,0.986437114217266
"15380","KIF11",7.07967532306786,0.0493422099319503,0.595536207397222,
0.0828534173389714,0.933968094203814,0.986437114217266
"15381","AL391988.1",11.5139953712291,-0.0409559168595808,0.4778092300
02723,-0.0857160437427033,0.931692148006482,0.986437114217266
"15382","PDZD8",249.725129807876,-0.016830485810592,0.197033586653013,
-0.0854193749222634,0.931927990495129,0.986437114217266
"15383","LSP1",2208.72666231275,-0.0182648517476818,0.214440340425874,
-0.0851745138596976,0.932122651930097,0.986437114217266
"15384","AP5B1",70.8631863253682,-0.0216123758756118,0.260807991744052
,-0.0828670000910917,0.933957293875839,0.986437114217266
"15385","RBM4B",85.8736577908693,-0.0198929552511053,0.239408801167541
,-0.0830919964265807,0.933778389766184,0.986437114217266
"15386","AP001267.1",5.68855633958561,0.0616393982874175,0.73393114834
3951,0.0839852599613754,0.933068151207156,0.986437114217266

"15387","YBX3",4706.24276223371,-0.0158555698174036,0.184077881243792,
-0.0861351168878598,0.931359008109478,0.986437114217266
"15388","CD63",17421.6442540569,-0.0225196044518268,0.271882605598238,
-0.0828284119253443,0.933987977295077,0.986437114217266
"15389","KCNMB4",75.5216170872366,0.0233222663796737,0.271630430164084
,0.0858602858508359,0.931577482180614,0.986437114217266
"15390","UNG",94.4801509964682,0.0166211432755391,0.19950638457658,0.0
833113351776427,0.933603987460856,0.986437114217266
"15391","AL138963.4",4.50451064261685,-0.0913176332999825,1.0744365971
5322,-0.0849911791369858,0.932268403364766,0.986437114217266
"15392","RPS29",19846.6518381995,0.0251367139461434,0.2842942818474 9,0
.0884179371557955,0.929544505340003,0.986437114217266
"15393","WDR89",47.9009325520544,0.0264402114015651,0.303958046087897,
0.08698638427857,0.930682334852641,0.986437114217266
"15394","TMEM121",87.7925673554811,-0.0255224821561707,0.2898724569227
82,-0.0880472826811879,0.929839095893592,0.986437114217266
"15395","OIP5",5.4181398851239,-0.0567808519701075,0.65432958026632,-0
.0867771436330252,0.930848655814843,0.986437114217266
"15396","LCMT2",15.3265908033944,-0.0388253331913068,0.440589600326273
,-0.0881213109945292,0.929780258543541,0.986437114217266
"15397","MRTFB",100.138861333943,0.0195843591001795,0.225692963172407,
0.0867743452205862,0.930850880234165,0.986437114217266
"15398","POLR3E",96.3213330856926,-0.0181890295207727,0.21320018059281
8,-0.0853143251107801,0.932011503263109,0.986437114217266
"15399","EARS2",27.6238224108381,-0.0288779637274012,0.333894635204463
,-0.0864882531272704,0.9310782937274,0.986437114217266
"15400","NFATC2IP",249.315079128955,0.0167933047063636,0.2024705962435
62,0.082941943264503,0.933897703015643,0.986437114217266
"15401","AC004943.2",11.3246313244745,-0.0426343634791387,0.5073364053
15138,-0.0840356872333177,0.933028057800185,0.986437114217266
"15402","SERPINF1",11296.7713450828,-0.0240693505967263,0.283344405847
192,-0.0849473294690948,0.932303264266436,0.986437114217266
"15403","GOSR1",391.785581318902,0.0138824434890393,0.158059999063853,
0.0878302136610229,0.930011623524348,0.986437114217266
"15404","AC005562.1",87.582243474532,-0.0226622821569208,0.26395876834
4408,-0.0858553868055314,0.931581376672484,0.986437114217266
"15405","RPL23",13434.0787729886,-0.0121411085143262,0.138588252436761
,-0.0876056108714292,0.930190142498208,0.986437114217266
"15406","AC025048.2",6.80359650966581,0.0474567063157537,0.54855553649
9516,0.0865121271377335,0.931059316153807,0.986437114217266
"15407","GRB2",303.560401987372,0.013896647326614,0.158549507154246,0.
0876486314971299,0.930155948536876,0.986437114217266
"15408","TRIM65",88.0636180707391,-0.0170245262895631,0.19309362400960
7,-0.0881672109935442,0.929743777636188,0.986437114217266
"15409","TYK2",91.7260044021179,-0.018064432057447,0.202878927942205,-
0.089040455017552,0.929049759331014,0.986437114217266
"15410","TIMM29",124.800770060878,-0.0207086117512313,0.24040555096855
7,-0.0861403227496182,0.931354869813738,0.986437114217266
"15411","KANK2",807.906361925277,0.0194701916056038,0.234198544686726,
0.0831354081710798,0.933743871635309,0.986437114217266

"15412","ZNF20",16.0181664814765,0.0362457852542765,0.433729030637256,
0.083567810070316,0.933400061225105,0.986437114217266
"15413","AC011503.2",2.58917964127456,-0.0817080934854972,0.9291869297
88736,-0.087935043924988,0.929928303452628,0.986437114217266
"15414","CCDC97",126.93877375028,-0.0169090199859013,0.197314836464163
,-0.0856956338859614,0.93170837301617,0.986437114217266
"15415","ZNF230",35.0646780465388,-0.0226839417500584,0.2674283579772,
-0.0848224994598079,0.932402506017997,0.986437114217266
"15416","DMPK",49.5807634386303,-0.023451901292813,0.27415884861473,-0
.0855412889691898,0.931831071893649,0.986437114217266
"15417","FTL",53612.8370081549,-0.0230885112391903,0.275438039980487,-
0.0838247006144321,0.933195808881303,0.986437114217266
"15418","RAC2",22.4711044867449,-0.0438633380120993,0.517079177643821,
-0.0848290550239746,0.932397294218854,0.986437114217266
"15419","CPT1B",5.39664839477195,0.0656184442555949,0.770108897271304,
0.0852067084124052,0.932097057437514,0.986437114217266
"15420","PNPLA4",89.2848544688258,-0.025926559332435,0.30011299564332,
-0.0863893257166657,0.931156932051909,0.986437114217266
"15421","PDZD11",253.468622366527,-0.0257878825028174,0.30554377819567
7,-0.084399959492228,0.932738439493249,0.986437114217266
"15422","ABCB7",173.270150236679,-0.0189332407866748,0.217937786055546
,-0.0868745210701978,0.930771252174141,0.986437114217266
"15423","MECP2",381.160749270686,0.0194558125691564,0.224968336670961,
0.0864824484061172,0.931082907935995,0.986437114217266
"15424","AC092053.3",10.1216445523668,0.0514221088228639,0.62171043017
9179,0.0827107063461102,0.93408157161173,0.986466203318431
"15425","WBP1",289.248599046701,0.0148063571812521,0.179380003542925,0
.0825418490846947,0.934215841094555,0.986489703355703
"15426","C12orf81",65.6940816715387,0.0347997020448476,0.4216592084495
21,0.0825303974098164,0.934224947140099,0.986489703355703
"15427","COPG1",243.6924316882,-0.018606669935223,0.226028445856747,-0
.0823200365984713,0.934392221592827,0.986569384330698
"15428","AL627171.1",7.58258657629544,-0.0466759044739056,0.5672594547
67362,-0.08228316704399,0.934421539778624,0.986569384330698
"15429","VPS26C",113.471913914906,-0.0143995334149076,0.17520548978249
4,-0.0821865424010612,0.934498374848334,0.986586559589377
"15430","CYB5A",2090.8463360429,0.0236720421845069,0.290169601433739,0
.0815800210206114,0.934980689244765,0.987031785295397
"15431","CADPS2",100.298014753898,-0.019032191146544,0.233908773907512
,-0.0813658710984028,0.935150990007391,0.987034696156825
"15432","ACACB",222.040826975471,0.0200508344072433,0.246459174834934,
0.0813556014730324,0.935159156907456,0.987034696156825
"15433","LINC00519",20.9865119829944,-0.044864555889105,0.55151419567
9638,-0.081347962102432,0.935165232106838,0.987034696156825
"15434","SCP2",1635.67217902772,-0.0192588702476479,0.237638601583387,
-0.0810426846451964,0.935408006687733,0.987053175740495
"15435","AC018797.3",2.40343425820234,-0.10728096813289,1.326164558875
31,-0.0808956681996331,0.935524924952367,0.987053175740495
"15436","SCD",5.23672414587258,0.0581018071545224,0.717966450961772,0.
0809255182838843,0.935501185862579,0.987053175740495

"15437","GPR162",5.52143648492874,-0.0589176107266413,0.72586176339052
3,-0.0811691890910953,0.935307402185226,0.987053175740495
"15438","PRR14",97.3535249135565,-0.0163012206613591,0.201695244273292
,-0.080821046227899,0.935584270351746,0.987053175740495
"15439","PAXBP1-
AS1",15.1450072453764,-0.0341034556973097,0.42078191513054,-0.08104781
71019534,0.935403925006683,0.987053175740495
"15440","GDI1",282.626172039314,-0.0138620094366457,0.171575293759834,
-0.0807925729449683,0.935606914692937,0.987053175740495
"15441","ST7",93.3378019957601,-0.0164892721739669,0.204578354142643,-
0.0806012554117514,0.935759067781423,0.98714976070796
"15442","GOLPH3L",155.281681456023,-0.0156811681886615,0.1962841081449
83,-0.0798901568591525,0.93632461849527,0.987212853034608
"15443","PSMB4",862.839561375686,0.0178206367769556,0.223100471573582,
0.0798771811249985,0.936334938652101,0.987212853034608
"15444","PCGF3",150.823368783203,0.0178308025089585,0.221671472799991,
0.0804379665264678,0.935888931798027,0.987212853034608
"15445","HNRNPA1",3647.64160132488,0.0164653596165997,0.20553884700314
2,0.0801082610741123,0.936151152479183,0.987212853034608
"15446","AC005480.1",4.54887565660313,-0.0645117003997673,0.8068874561
64963,-0.0799512991643016,0.936275989546642,0.987212853034608
"15447","COPS2",581.43807142688,0.0161340674727541,0.202079430724632,0
.0798402262659753,0.936364330492031,0.987212853034608
"15448","PRAM1",3.14398758480762,-0.0746444446165522,0.929765561697956
,-0.080283081769812,0.936012113568644,0.987212853034608
"15449","PNMA8B",47.7067939507336,0.0236463144945939,0.295744636635912
,0.0799551760720671,0.936272906093079,0.987212853034608
"15450","MED25",131.053151412311,-0.0185743694331785,0.232570098737502
,-0.0798656815042377,0.936344084801702,0.987212853034608
"15451","C9orf85",171.557497294217,-0.0189650310784213,0.2381520777630
61,-0.0796341197463318,0.936528257746321,0.987306779799438
"15452","AL022344.1",6.74631124608569,-0.0505621684308386,0.6353962955
63797,-0.0795757998336677,0.936574643100307,0.987306779799438
"15453","SNX22",3.1082044523892,-0.074916924158922,0.944131737622889,-
0.0793500749668112,0.93675417776734,0.98743213626171
"15454","TNNT3",2480.26006953645,0.0186468625996686,0.2353143122898,0.
0792423648957831,0.936839848203012,0.987458540661244
"15455","SNHG3",146.444077354683,-0.0209624193873915,0.26502242780228,
-0.0790967751719131,0.936955648518727,0.987490430499289
"15456","SFSWAP",312.508724288319,-0.0161617441118448,0.20444475184280
3,-0.0790518903819627,0.936991349609983,0.987490430499289
"15457","NAV1",903.687803262276,0.0175549599948952,0.222302677366139,0
.0789687294947947,0.937057495612864,0.987496250633237
"15458","KCNIP2",2.60216883422828,0.0703894211634094,0.89274522150927,
0.0788460352040972,0.937155087198728,0.987535206066767
"15459","XKR8",55.1453290798464,-0.0226920208446161,0.288285115438067,
-0.0787138136151571,0.937260257896963,0.987569999891679
"15460","SPCS3",337.842915266643,-0.0123660469104361,0.157521116548908
,-0.0785040582580977,0.937427102164623,0.987569999891679
"15461","MTAP",231.996881145501,0.0126564683807955,0.161438282007884,0

.0783981855070626,0.937511316845957,0.987569999891679
"15462","CHKA",34.6859692619732,0.0244483983800238,0.312684363904716,0
.0781887462318837,0.93767791381783,0.987569999891679
"15463","SPATA5L1",41.4611444397162,0.0221102641552575,0.2830066599156
19,0.0781263033239213,0.937727584110159,0.987569999891679
"15464","C17orf80",70.1578979705886,0.0172413251316384,0.2205405770042
03,0.0781775642643294,0.937686808510641,0.987569999891679
"15465","NAT9",114.123984378915,-0.0187114882034666,0.239526850330969,
-0.0781185415230558,0.937733758261685,0.987569999891679
"15466","FUZ",122.290903821311,-0.0190774241570866,0.242638301540134,-
0.0786249493010528,0.937330942294072,0.987569999891679
"15467","HMGN1",718.862441392173,0.0158697103237552,0.202547879981668,
0.0783504143572945,0.937549315824455,0.987569999891679
"15468","GLRX",522.083625726172,0.0259591561952293,0.333208618376276,0
.0779066169468489,0.937902335849058,0.987683679121108
"15469","DCAF17",39.5404969844005,-0.0233843729823896,0.30122458301437
8,-0.0776310244946822,0.9381215628663,0.98774942317893
"15470","TUBB2B",24.6966655229795,0.0535480873400338,0.690057601973021
,0.0775994455925541,0.938146683406055,0.98774942317893
"15471","PIP5KL1",4.12305995289192,-0.0728247078730631,0.9367624194784
42,-0.0777408512113556,0.93803419785142,0.98774942317893
"15472","MNS1",192.355196735985,-0.0218987770046909,0.282992570117197,
-0.0773828690824704,0.938318968406694,0.987803120039853
"15473","TMC8",11.311153957959,-0.0509895077480194,0.658525765752826,-
0.0774297839200387,0.938281647757579,0.987803120039853
"15474","PLK1",5.41540290083603,0.0528246948652811,0.686559787248609,0
.0769411431406086,0.938670367044903,0.988045338209656
"15475","TTC28-
AS1",83.3488223777819,0.0169679124962477,0.220462866079072,0.076964945
6074925,0.938651431572917,0.988045338209656
"15476","ARRDC1-
AS1",58.479848944843,0.0204760028471607,0.266619421828399,0.0767986169
452405,0.93878375100855,0.988100834852564
"15477","C19orf57",3.48625813807705,0.0599527932973159,0.7819178923237
99,0.0766740266284748,0.938882867446898,0.9881413082537
"15478","SRSF10",832.076557938372,0.0117102424290139,0.154060138320413
,0.0760108523637636,0.939410464240477,0.988441124734392
"15479","COPS8",461.256194877825,0.0147527310451862,0.193764860919359,
0.0761372881294816,0.939309874586125,0.988441124734392
"15480","GOLGB1",890.060347753103,-0.0182487801243394,0.23998686905759
1,-0.076040744212385,0.939386682822744,0.988441124734392
"15481","POLE2",10.3155747578947,0.0376821669970289,0.495443671288091,
0.076057419199765,0.93937341652585,0.988441124734392
"15482","GPSM2",114.104648466679,-0.0218327665637715,0.290933646820321
,-0.075043800544855,0.940179862325504,0.988444554386378
"15483","AL353708.3",2.77626624010579,0.068184469366581,0.906971350777
616,0.0751781953290159,0.940072932858031,0.988444554386378
"15484","HLX-
AS1",7.29165466013435,0.0768492700626318,1.01816990245136,0.0754778449
820688,0.939834524389389,0.988444554386378

"15485","AC009228.1",3.09726516561929,-0.0633239445035423,0.834308791172,-0.0758998888344299,0.939498745303291,0.988444554386378
"15486","HAAO",77.3588704913396,-0.0201059026842677,0.267877058686728,-0.0750564560580036,0.940169793084531,0.988444554386378
"15487","HSPBAP1",40.8251656734035,0.0206809709339046,0.273213775377874,0.0756951984038925,0.93966159615385,0.988444554386378
"15488","GOLGA7",439.949603153152,0.0135506175925187,0.180255510836722,0.0751744982975477,0.940075874338741,0.988444554386378
"15489","CHMP5",911.624374300325,0.0167575072121945,0.221834304332634,0.075540648515151,0.939784557071215,0.988444554386378
"15490","KAZALD1",263.341995494485,-0.0263990615519552,0.350682845526081,-0.0752790217392937,0.939992712341182,0.988444554386378
"15491","LDB1",101.140963453818,0.0182130462018974,0.240838335499706,0.0756235346175594,0.939718612159268,0.988444554386378
"15492","SNX19",94.7105973747932,0.0177253687658936,0.235009207395938,0.0754241459826309,0.939877248208519,0.988444554386378
"15493","AL049840.6",4.30559002015197,0.0565889787216829,0.754366427134996,0.0750152401885141,0.940202586141723,0.988444554386378
"15494","NONO",585.46105041106,0.0101767335036359,0.134525199639349,0.0756492726338179,0.939698134851286,0.988444554386378
"15495","CAD",62.923454187838,0.0179139126721849,0.241615954949451,0.074142093289877,0.940897321808367,0.988536924338009
"15496","EIF4G1",408.585829481381,-0.0150185163651686,0.200729867928273,-0.0748195399128903,0.940358294789863,0.988536924338009
"15497","LINC02202",67.3913532269821,0.0180990683273518,0.243626959935512,0.0742900881418977,0.940779563755286,0.988536924338009
"15498","SH2B2",7.78151399187329,0.0457994088224459,0.615019651431904,0.0744682039278169,0.940637840487345,0.988536924338009
"15499","OPLAH",28.9883369700868,0.0236283565923998,0.318399789818485,0.0742097116517254,0.940843518380182,0.988536924338009
"15500","DPH7",107.851596635826,0.0173359256212922,0.233487988809655,0.07424761209205,0.940813361399435,0.988536924338009
"15501","RPS3",18011.1236218451,0.0179223863715905,0.239849665414573,0.0747234161891039,0.940434776330369,0.988536924338009
"15502","CCDC15",38.0901146728815,0.0213162291814239,0.285815647762467,0.0745803434776921,0.940548614191744,0.988536924338009
"15503","AL121672.2",9.37163496577144,0.0410084988218756,0.55163543153576,0.0743398565021601,0.940739963849635,0.988536924338009
"15504","SLC10A3",149.361697443576,-0.0174682184361205,0.234647887338339,-0.0744443882886237,0.940656790019658,0.988536924338009
"15505","GNE",91.7281111999342,0.0172245380342687,0.233376355852393,0.0738058402332881,0.941164879868469,0.98875425528394
"15506","L3MBTL4",32.2827703887174,0.0274104654012099,0.372874294053023,0.0735112766913133,0.941399270852793,0.988872942730454
"15507","AC119403.1",7.78802847008417,0.0431932131600809,0.587104286138161,0.0735699162480928,0.94135260960368,0.988872942730454
"15508","ZNF281",111.558593476164,-0.015924862346207,0.21795329972826,-0.0730654794676741,0.941754011602103,0.988911371762466
"15509","LINC01963",8.94928541951841,0.0360510918411209,0.493799568004542,0.0730075402593088,0.941800117266323,0.988911371762466

"15510","AL355353.1",5.59764790731201,-0.0494378314426738,0.675205192158557,-0.0732189740493945,0.941631867804213,0.988911371762466
"15511","PGM3",245.578344812487,0.0121403180714956,0.166034700874828,0.0731191612809184,0.94171129397386,0.988911371762466
"15512","ABLIM1",1195.33845287281,0.0221476214924635,0.301811448080238,0.0733823108213422,0.941501893560035,0.988911371762466
"15513","AC068870.2",7.80601379159712,-0.0441404928951723,0.603051731193442,-0.0731952013599531,0.941650784907105,0.988911371762466
"15514","CTBP1",265.902340065628,-0.0155279445478774,0.213309277892971,-0.0727954484739696,0.941968892940633,0.989024835446047
"15515","GAREM2",43.7541873707714,0.0215132685744062,0.297075960573357,0.0724167264590699,0.942270273952817,0.989200770038667
"15516","AGAP4",31.2548230163301,0.0247171437369134,0.341899693693194,0.0722935533223773,0.942368294977632,0.989200770038667
"15517","SPTBN5",2.59662139906112,0.0636705687779626,0.8808922023013,0.0722796371810597,0.942379369480019,0.989200770038667
"15518","INTS2",49.1962787016117,0.0210828081022891,0.291258237965698,0.0723852765488888,0.942295301668097,0.989200770038667
"15519","ALDH4A1",10.3997006625614,0.0344749835177517,0.480173970158105,0.0717968604303983,0.942763571387793,0.989268090783936
"15520","BHLHE40-AS1",8.87432308730515,0.0384786374631616,0.543874339547974,0.0707491320424163,0.943597417061208,0.989268090783936
"15521","LNP1",21.6084653292986,0.0263585942849352,0.36801279559588,0.0716241244879975,0.942901040813336,0.989268090783936
"15522","PCDHGA5",57.61183929311,0.0256926136236124,0.361693803407655,0.0710341548059505,0.943370572534022,0.989268090783936
"15523","MGAT1",642.00612200886,-0.0138886141988216,0.19513801183154,-0.0711732894501939,0.943259839422566,0.989268090783936
"15524","EIF3B",422.072877451474,-0.0118147256979231,0.166137909269222,-0.0711139664023197,0.943307052732146,0.989268090783936
"15525","RBM48",94.1992905742561,0.0198633531809667,0.276878563417804,0.0717403071432195,0.942808578323827,0.989268090783936
"15526","ZSCAN25",42.7400322356451,-0.0198368094117797,0.27721223792838,-0.0715582023363076,0.942953504462129,0.989268090783936
"15527","TRIP6",621.567664444453,0.0161709666743967,0.224566684853227,0.0720096424140818,0.942594234237892,0.989268090783936
"15528","FKBP2",134.889743355033,0.0282851612067514,0.399657658975449,0.0707734746764581,0.943578043014601,0.989268090783936
"15529","ISCA2",211.065811426121,0.020494183532397,0.284815728067578,0.0719559403248067,0.942636971440369,0.989268090783936
"15530","CDCA4",59.1384851024972,-0.0205033259455407,0.286211992323086,-0.0716368513391843,0.942890912273025,0.989268090783936
"15531","AFG3L2",208.778584670356,-0.0155380168558517,0.219393698135927,-0.0708225304002353,0.943539000181012,0.989268090783936
"15532","B4GALT6",27.6246946571478,0.0224965723655922,0.316191436033287,0.0711485821621806,0.943279503135798,0.989268090783936
"15533","AC104971.1",2.46211291395171,0.0732777783407233,1.0330330135744,0.0709345949043533,0.943449809960626,0.989268090783936
"15534","JAK3",15.5497804545935,0.0290910469039985,0.409758323168734,0

.0709956217094804,0.943401240069252,0.989268090783936
"15535","PRKD2",66.3893101319683,0.016717994073428,0.23269110830292,0.0718462952682504,0.942724229693772,0.989268090783936
"15536","STK35",74.7129700495478,-0.0193073407279373,0.271514000522004,-0.0711099268944425,0.943310267654046,0.989268090783936
"15537","XPNPEP3",105.632204950071,0.0163839136577137,0.229903818202892,0.0712642085972438,0.943187480178797,0.989268090783936
"15538","ADIPOR1",220.506053492978,0.0124949221713739,0.177189802644315,0.0705171628666225,0.943782040562686,0.989334298135375
"15539","KMT2E",925.16984305949,0.014248407108249,0.201850910182797,0.0705887681920562,0.943725049797784,0.989334298135375
"15540","DKK2",151.006390241575,0.0281872181817834,0.400830761199335,0.0703219934953191,0.943937377561026,0.989353901275966
"15541","PYROXD1",122.033546647744,0.0163492469304444,0.232680702212874,0.0702647309164764,0.943982953749835,0.989353901275966
"15542","ZBTB33",62.1935764205295,0.017718037184964,0.251753134631052,0.070378615983988,0.943892311010532,0.989353901275966
"15543","AC012368.1",3.32424006242478,-0.062435658046195,0.893306367801179,-0.0698927717260951,0.944279006388223,0.989375708874561
"15544","SRD5A3",135.411332008527,-0.0172463842857756,0.246881160258104,-0.0698570286519442,0.944307455698496,0.989375708874561
"15545","ZNF827",161.667972105577,-0.0177266281727221,0.253322043873912,-0.0699766506761068,0.944212244131392,0.989375708874561
"15546","COX16",757.004083306255,0.0161147605217271,0.230506683156152,0.0699101661655965,0.944265161499408,0.989375708874561
"15547","ATG4D",40.165241678404,0.0186103565313818,0.265578851484386,0.0700746931744147,0.944134209096552,0.989375708874561
"15548","OSTN",2.38047114593567,-0.0851996389120265,1.2344476572475,-0.0690184297501924,0.944974949660262,0.989462768115119
"15549","TTC33",134.668106359348,0.0131529648427828,0.188843744296201,0.0696499896875185,0.944472247501807,0.989462768115119
"15550","ARSB",39.4350113216915,-0.0229526278993233,0.33256636172048,-0.0690166852130848,0.944976338288233,0.989462768115119
"15551","SMIM29",100.343345431377,0.0195137031081838,0.282038384049771,0.0691881113059427,0.944839886233752,0.989462768115119
"15552","CAT",755.187412495204,-0.015375487083542,0.221658597638481,-0.0693656246468681,0.944698590546109,0.989462768115119
"15553","RARA-
AS1",96.1150568901757,-0.0208291822571136,0.30127623065722,-0.0691364938139187,0.944880972649219,0.989462768115119
"15554","APLP1",2.44387432646843,-0.0691414916324278,0.998905782534728,-0.0692172303347579,0.944816708176705,0.989462768115119
"15555","MICAL3",59.9022609404829,0.0277192489768059,0.4009498071511,0.0691339626118332,0.944882987435509,0.989462768115119
"15556","DDT",776.901152579938,0.0151581381247634,0.218939499772103,0.0692343690404962,0.944803066194828,0.989462768115119
"15557","BX537318.1",13.7437025291392,-0.0274979673006588,0.398582021549821,-0.0689894822494438,0.944997991501437,0.989462768115119
"15558","AL445228.2",3.68571062888662,0.0712981851122923,1.0409701370879,0.0684920561811197,0.94539394347681,0.989523088088106

"15559","TRAPPC4",299.197326726091,-0.0192409157141335,0.281056636517885,-0.0684592114689634,0.945420088398011,0.989523088088106
"15560","XPOT",158.305480186562,-0.0132999152897696,0.194129001873339,-0.0685107076295958,0.94537909664885,0.989523088088106
"15561","BATF",6.51444291785345,-0.054797534827524,0.797716815017346,-0.0686929669726625,0.945234016524051,0.989523088088106
"15562","GEMIN7",58.4722880954398,-0.0203951354905781,0.2970606477541,-0.068656470134209,0.945263068200129,0.989523088088106
"15563","AC245060.6",10.8302474931465,-0.0485981712142401,0.70762030664583,-0.0686783162634194,0.945245678557202,0.989523088088106
"15564","DDX10",120.921209628685,0.014670317642818,0.214665979394179,0.0683402078159749,0.94551481771944,0.989537620051927
"15565","AKAP8",126.853397832904,0.014475138905231,0.211968392997474,0.0682891383028202,0.945555470324037,0.989537620051927
"15566","ELP6",199.212205423603,-0.0153809814246047,0.225792051450768,-0.0681201190466103,0.945690014872695,0.989614843393378
"15567","MTOR",55.6379877370552,0.017289929555478,0.254150319269433,0.0680303278987756,0.945761492030185,0.989626064346353
"15568","TELO2",102.558385973846,-0.0199402890987317,0.294490936923353,-0.0677110450564443,0.946015656808728,0.989741795572463
"15569","AC007292.2",2.31529421410276,0.0700147162971931,1.03679988408374,0.0675296336081942,0.946160071655342,0.989741795572463
"15570","TCP10L",6.99581704947061,0.0410860001252663,0.607202562026586,0.0676644050844229,0.946052784971104,0.989741795572463
"15571","AP001062.1",8.52580683320099,0.0363998697485489,0.538923146115679,0.0675418564055063,0.946150341489416,0.989741795572463
"15572","AIFM1",99.3637749878337,-0.0143311010775668,0.212281945544127,-0.067509750020582,0.946175900340991,0.989741795572463
"15573","DZIP1L",41.7580825582654,-0.0225162266053569,0.335038161749592,-0.0672049610342166,0.946418535721809,0.989899658743956
"15574","UVSSA",59.5517227237294,-0.0224929043554914,0.336747570065229,-0.0667945557888792,0.946745257582939,0.989899658743956
"15575","PEX1",115.305094108125,0.014493355661732,0.216398366898758,0.0669753467618018,0.946601329555406,0.989899658743956
"15576","PCF11-AS1",15.0055624997029,0.0402745309917946,0.601982268417578,0.066903184868989,0.946658777562866,0.989899658743956
"15577","HSPB8",337.712002742887,-0.0181997296603457,0.272160540264623,-0.066871301925878,0.946684159628919,0.989899658743956
"15578","KMT5A",145.864013770249,0.0141407686104759,0.210619468206301,0.0671389436641494,0.946471091261107,0.989899658743956
"15579","TOP3A",77.7613606932807,-0.0181928222346201,0.272405462911004,-0.0667858200794003,0.946752212141451,0.989899658743956
"15580","ICMT",171.612788508099,0.0157728885231816,0.237097469156035,0.0665249130635022,0.94695992388257,0.990028116869339
"15581","AL365436.2",5.24430181890267,0.0565659155171087,0.850947498297792,0.0664740370354944,0.947000427423767,0.990028116869339
"15582","ANO10",105.767953693516,-0.0162585530408085,0.246264275584119,-0.0660207535268545,0.94736130257489,0.990028116869339
"15583","C1QTNF4",281.852669082284,-0.0281301689936932,0.4259627762698

39,-0.0660390310158774,0.947346751005224,0.990028116869339
"15584","LINC02334",3.39193016567896,-0.051076324126442,0.772220918260
796,-0.066142114152355,0.947264681995286,0.990028116869339
"15585","AC011330.2",4.92203860903813,-0.0443841759672344,0.6700503141
47239,-0.0662400644102695,0.947186700013729,0.990028116869339
"15586","ARPIN",196.826616651381,0.0140053979832286,0.211833684550726,
0.0661150657551577,0.947286216357511,0.990028116869339
"15587","CTPS2",63.9981239170634,0.0148926693271551,0.224847950935239,
0.0662344009149743,0.94719120892719,0.990028116869339
"15588","ZNF837",16.1435674911108,-0.0291760837319992,0.44339954831104
4,-0.0658008873557358,0.947536349761506,0.99014752381737
"15589","SEC22B",480.41948161593,-0.0125398966970732,0.19250250856383
,-0.0651414716131606,0.94806136096171,0.990196620987129
"15590","MRPL55",412.76326418227,-0.0170250298975928,0.262095998244346
,-0.0649572294565169,0.948208054244891,0.990196620987129
"15591","TATDN2",75.8488009174062,-0.0190578802325497,0.29296511238307
2,-0.0650517055683758,0.948132832310093,0.990196620987129
"15592","METTL6",86.8010192133339,0.0159131037402076,0.246669206228732
,0.0645119185467018,0.94856261725314,0.990196620987129
"15593","ABHD14B",507.15519109919,-0.0141189715908157,0.21659929312527
3,-0.0651847537777968,0.948026900033051,0.990196620987129
"15594","MAP6D1",3.3765738605929,-0.0584613913297754,0.89104085217042,
-0.06561022560065,0.947688147805761,0.990196620987129
"15595","ATP6V1G2",4.85452082069744,0.0462557872749935,0.7130103608481
73,0.0648739342580788,0.948274374312907,0.990196620987129
"15596","AC009403.1",20.8062183117476,0.0245001822473098,0.37904225598
5407,0.0646370737310436,0.948462965847137,0.990196620987129
"15597","PPP6C",216.278072608636,0.0118285428183537,0.183718418039417,
0.064384088131087,0.948664399569723,0.990196620987129
"15598","AGBL2",2.94045574626377,0.0559633158742713,0.869433227722809,
0.0643675834898197,0.948677541109039,0.990196620987129
"15599","CHST14",193.721697165072,0.0157146507825836,0.241250746281313
,0.0651382473414583,0.948063928106085,0.990196620987129
"15600","PRSS36",25.3343525293153,0.0333581593576052,0.514121063726422
,0.0648838604585086,0.948266471001942,0.990196620987129
"15601","WWOX",102.359464848524,0.0143759539700007,0.222822278184732,0
.0645175791537425,0.948558110131609,0.990196620987129
"15602","KIAA0753",40.2793994827067,0.0165662642316312,0.2572202406255
3,0.0644049791390597,0.948647765480702,0.990196620987129
"15603","AC005856.1",2.56107826612054,0.0647902761051211,0.98830808703
9379,0.0655567600374594,0.947730715508199,0.990196620987129
"15604","AL035071.1",38.3573204760485,0.0277071697718879,0.43044631681
0522,0.0643684675412946,0.948676837197765,0.990196620987129
"15605","DRG1",238.453790460076,0.012653486567734,0.195319711367348,0.
0647834592789045,0.948346411365523,0.990196620987129
"15606","CASK",142.754142171197,-0.0124750088790673,0.190537659473968,
-0.0654726677839331,0.947797667579565,0.990196620987129
"15607","AL592156.2",3.0260373127738,-0.0605787341606069,0.94268877882
9701,-0.0642616476625639,0.948761891038231,0.990221211195088
"15608","CAHM",16.2220261049062,-0.0292378418473334,0.455704088641216,

-0.0641597092852791,0.948843058595618,0.990242477028704
"15609","GPR155",39.4112146643635,-0.019901085402951,0.310949385506065
,-0.0640010443196801,0.948969395267611,0.990310877026979
"15610","EXOSC10",159.76419958084,0.010127715919794,0.161096395285086,
0.062867427305691,0.949872072761469,0.990387629461718
"15611","PLEKHM2",301.317187084669,-0.0129564990311447,0.2127699330996
73,-0.0608944075997575,0.951443303271293,0.990387629461718
"15612","ATP5IF1",1349.83242845961,0.0165683105749723,0.26498169550534
3,0.0625262456086828,0.950143761853101,0.990387629461718
"15613","TMEM59",4437.46586748348,0.0134269163344584,0.232985066219708
,0.0576299440660144,0.954043397388182,0.990387629461718
"15614","CCDC18",61.4862707984698,0.0148476747819128,0.23960184370973,
0.0619681157374578,0.950588223140238,0.990387629461718
"15615","PI4KB",193.582092963877,0.0110666281954357,0.182310777064138,
0.0607019967423119,0.951596541445229,0.990387629461718
"15616","CRTC2",105.39664420741,-0.0117436364760411,0.193480695407973,
-0.0606966832079991,0.951600773229222,0.990387629461718
"15617","SCYL3",71.4980172261888,0.0153879531841769,0.26751022400867,0
.0575228600746049,0.954128696549203,0.990387629461718
"15618","MXD1",101.488431706848,0.0207371705077407,0.337476296638122,0
.0614477837831003,0.951002598273382,0.990387629461718
"15619","PCBP1-
AS1",124.653068222734,-0.0131653890667936,0.214653184158705,-0.0613333
043178142,0.951093767709606,0.990387629461718
"15620","TTC30A",19.3292332198476,0.025815425625139,0.408827432587372,
0.06314504254707,0.949651007074698,0.990387629461718
"15621","OSGEPL1",57.4818648964337,-0.0164601034112736,0.2773660287496
43,-0.0593443381854484,0.952677846555666,0.990387629461718
"15622","ABI2",289.105998000334,-0.0103515293226157,0.179754258542838,
-0.0575871159133001,0.95407751265311,0.990387629461718
"15623","PSMD6-
AS1",6.36346825808114,-0.0398599936660353,0.642377215546826,-0.0620507
588085988,0.950522410162502,0.990387629461718
"15624","TMEM41A",149.972092625059,-0.0110724860236,0.17718414523879,-
0.062491404119018,0.950171507081591,0.990387629461718
"15625","SLAIN2",291.018896262683,0.00949081989055338,0.15814168145784
,0.0600146640851521,0.952143955491285,0.990387629461718
"15626","OCIAD2",6.35218543548424,-0.0344480429153603,0.59807488107017
7,-0.0575982105346408,0.954068675094908,0.990387629461718
"15627","AC068620.1",2.61422160388522,-0.0668531932721775,1.0673173050
2416,-0.0626366619912192,0.950055834675639,0.990387629461718
"15628","ADAMTS3",78.9494290765652,-0.0242241171420092,0.4191512210353
5,-0.057793263925542,0.953913303821148,0.990387629461718
"15629","PLAC8",7.05203575332838,-0.0365123693663325,0.622727901669556
,-0.0586329426840223,0.953244471342147,0.990387629461718
"15630","HERC3",113.547172015289,-0.0132454876477311,0.213745912675977
,-0.0619683786319239,0.950588013783161,0.990387629461718
"15631","AP002026.1",16.8986648525112,0.0264173824552305,0.41808966606
274,0.0631859254116703,0.949618452277897,0.990387629461718
"15632","AC010359.3",25.6338610314114,-0.0258704313514392,0.4418343045

6185,-0.0585523375716466,0.953308674614128,0.990387629461718
"15633","ACSL6",3.70360560924294,-0.0473148534768323,0.793267645125774,-0.0596455102733081,0.952437970910857,0.990387629461718
"15634","HSPA4",209.578083010281,0.0104352492510737,0.17008060321518 8,0.0613547286040072,0.951076705735505,0.990387629461718
"15635","SPDL1",50.3487275046843,-0.0161963603785724,0.265741366410866,-0.0609478328395853,0.951400755126814,0.990387629461718
"15636","FAF2",305.623741016825,0.00827018440582064,0.133995731468424,0.0617197601385497,0.950786003652954,0.990387629461718
"15637","SLC35B3",180.38111350314,0.0133108114944445,0.217831314073675,0.0611060514924063,0.951274749765338,0.990387629461718
"15638","MOCS1",59.7178026628719,-0.0194552065536962,0.332673808424166,-0.0584813293413543,0.953365234065815,0.990387629461718
"15639","KLHDC3",452.087551093259,-0.0123524199867937,0.209489236411149,-0.0589644613652153,0.952980414560855,0.990387629461718
"15640","EPM2A",19.9567864794089,0.0249073819546817,0.410945276490748,0.0606099726157637,0.951669831129099,0.990387629461718
"15641","TULP4",124.61233059077,0.0150616678948675,0.262309280229758,0.0574195006813136,0.954211029330972,0.990387629461718
"15642","AL135910.1",21.7032984468001,-0.0268308282963853,0.447139530053519,-0.0600054937955807,0.952151259160883,0.990387629461718
"15643","HNRNPA2B1",3251.86137540936,-0.00877979414204215,0.152833664143779,-0.0574467293657407,0.954189339785736,0.990387629461718
"15644","FAM133B",974.615712089485,0.0158203791503841,0.274477594417723,0.0576381441404916,0.954036865532749,0.990387629461718
"15645","SAMD9",158.762409174696,-0.0100326554617925,0.167965481755533,-0.0597304598357577,0.952370311597376,0.990387629461718
"15646","PPP2R2A",363.244118286852,-0.00919528204193475,0.150605491169575,-0.061055423481082,0.951315069789605,0.990387629461718
"15647","AL354733.3",10.4349848681551,0.04489919591734,0.732173175311681,0.0613231916045364,0.951101821327699,0.990387629461718
"15648","EHMT1",224.871549851675,0.0113606769312684,0.184658328986725,0.0615226889228761,0.950942945482207,0.990387629461718
"15649","MCMBP",227.391088169148,0.00850537581290987,0.147375547064371,0.0577122594781268,0.953977828322389,0.990387629461718
"15650","TUBGCP2",281.072461779167,0.0101096366447551,0.172773150298962,0.0585139336017295,0.953339264102137,0.990387629461718
"15651","SLC25A22",18.2101346351179,-0.0244765726186322,0.413112058498936,-0.0592492330230424,0.952753596207236,0.990387629461718
"15652","MADD",76.0765335088905,0.0195333365047965,0.332477736492874,0.0587508105380018,0.953150588241896,0.990387629461718
"15653","FAM111A-DT",42.7563630682398,-0.0145781434507733,0.246939722334534,-0.0590352305937397,0.952924047078564,0.990387629461718
"15654","NARS2",68.5371362725892,0.0148540849516368,0.236357425076572,0.0628458570608667,0.949889249361241,0.990387629461718
"15655","BCO2",27.7923549467436,0.0186671877706414,0.303429196771697,0.0615207368613468,0.950944500057206,0.990387629461718
"15656","DPAGT1",97.7718658794724,-0.0148730976456507,0.245194014768453,-0.0606584857289276,0.951631194354672,0.990387629461718

"15657","TLCD5",86.1974404867236,-0.0193650150968928,0.305499728630631
,-0.0633879944302877,0.949457547082774,0.990387629461718
"15658","A2M-
AS1",23.6613421994747,0.0209009457422426,0.331534718498353,0.063043007
4922799,0.949732257385591,0.990387629461718
"15659","AC092821.3",44.778068046808,-0.0159813801980246,0.26937104257
6778,-0.0593285011081675,0.952690460489922,0.990387629461718
"15660","EEF1AKMT3",82.9929437108158,-0.0142391200477752,0.22895084421
3045,-0.0621929134907461,0.95040920578213,0.990387629461718
"15661","ACSS3",105.300519879656,-0.0126665810552964,0.208537607763802
,-0.0607400324148874,0.951566249265094,0.990387629461718
"15662","DHX37",37.5409735601511,0.0198794645997079,0.312080045280371,
0.0636998901414806,0.949209192225398,0.990387629461718
"15663","ELF1",586.11330788291,0.0109365761823847,0.179954780208386,0.
0607740242838797,0.951539177690003,0.990387629461718
"15664","AL049838.1",13.1249667370679,-0.0338803404070321,0.5360954946
99745,-0.0631983307862113,0.949608573964047,0.990387629461718
"15665","AL359232.1",10.6909576824194,-0.0304622019797033,0.4904105521
49336,-0.062115714774479,0.950470682583856,0.990387629461718
"15666","FAM71D",5.27985151434157,0.0385235740568961,0.659943108182351
,0.0583740834312213,0.953450657987208,0.990387629461718
"15667","GREM1",72.2351717303429,-0.0343990907483908,0.593262951300514
,-0.0579828736532144,0.953762270417826,0.990387629461718
"15668","ULK3",60.3830976868677,-0.0171672100273539,0.276044898119863,
-0.0621899196264067,0.950411589925142,0.990387629461718
"15669","BOLA2",142.924616883491,0.0170226521548025,0.283664513834306,
0.060009804979504,0.952147825521487,0.990387629461718
"15670","ARMC5",35.1970014520671,0.0224943637962282,0.375846455294873,
0.059849876137797,0.952275201328899,0.990387629461718
"15671","LINC02182",3.13861753951296,0.0562373956563233,0.962877646672
812,0.058405547008646,0.953425596443034,0.990387629461718
"15672","MRM3",77.8624619515571,0.0177324732786364,0.28328368790096,0.
0625961678557219,0.950088081043121,0.990387629461718
"15673","CRK",443.25262990621,0.00873140410541558,0.149437037290964,0.
0584286483705839,0.953407195646698,0.990387629461718
"15674","ABHD15",48.7859678418482,0.0162224666982587,0.275966521189906
,0.0587841837782028,0.953124006190587,0.990387629461718
"15675","STAT5A",144.692892648391,-0.0129719434034267,0.21698720387217
6,-0.0597820662782876,0.952329209063887,0.990387629461718
"15676","TMEM106A",41.8397841077827,0.0268373140660746,0.4282440570262
18,0.0626682697068499,0.950030664815868,0.990387629461718
"15677","G6PC3",159.730637912163,0.0139035024000702,0.225361684802121,
0.0616941713595999,0.950806381710381,0.990387629461718
"15678","TOB1-
AS1",5.67412548442636,0.0362783768963655,0.624461626718181,0.058095446
2919111,0.953672601601426,0.990387629461718
"15679","SUPT4H1",524.414522707752,0.0124369339656281,0.20942207685678
2,0.0593869287913393,0.952643923997761,0.990387629461718
"15680","NUP85",119.807748790263,0.0121578652044317,0.202333281343599,
0.0600883113430331,0.952085299339053,0.990387629461718

"15681","RAC3",8.70497610851338,0.0352615472993697,0.585818950316922,0
.0601918857017062,0.952002808270524,0.990387629461718
"15682","RAB12",150.143026840294,0.0171995487687868,0.287209033670357,
0.0598851246041498,0.952247127476905,0.990387629461718
"15683","MAPRE2",163.065683505593,0.0108509962352367,0.17818232873269,
0.060898273764933,0.951440224232185,0.990387629461718
"15684","CNDP2",177.179141692061,-0.0107327209960834,0.182574911247578
,-0.0587853003610634,0.953123116824357,0.990387629461718
"15685","MYO1F",22.934280739706,0.030495758210527,0.492588643081917,0.
0619091784571566,0.950635158169157,0.990387629461718
"15686","AC008764.8",9.06306642785702,0.0306039399610835,0.52292591529
7612,0.0585244277741827,0.953330905288574,0.990387629461718
"15687","ZNF574",33.3959546438693,0.0180695259814853,0.311475215266029
,0.058012724916338,0.953738492580796,0.990387629461718
"15688","CALM3",1256.1061144532,0.0114353636883812,0.191888313196843,0
.0595938517456795,0.952479115263531,0.990387629461718
"15689","NDUFA3",685.196058616342,-0.0208228874208229,0.33217950421618
6,-0.0626856478395824,0.950016826280437,0.990387629461718
"15690","ZNF587B",16.3350889770566,0.023030253686371,0.395714245073776
,0.0581992030185247,0.953589955547142,0.990387629461718
"15691","LINC00654",15.9639598728298,0.0246830340214209,0.417415627675
205,0.0591329897227206,0.952846182607376,0.990387629461718
"15692","YTHDF1",138.304529511869,0.0106548781600342,0.175699096073702
,0.0606427602539556,0.951643718412306,0.990387629461718
"15693","CCDC134",9.17200052117999,-0.0286714835928144,0.4921215373984
57,-0.0582609811071934,0.953540747261105,0.990387629461718
"15694","MED12",81.5011821267723,-0.0146722098110335,0.231479423302579
,-0.0633845099564408,0.949460321711082,0.990387629461718
"15695","MRAS",103.474422574229,0.0132870677438677,0.232159242817497,0
.0572325597836,0.954359941702282,0.990441165101222
"15696","KIF1BP",233.081826401004,-0.0115007137036171,0.20184275436046
4,-0.0569785808762717,0.954562257401164,0.990441165101222
"15697","AP003778.1",3.08567272802266,0.048859033569557,0.856030222749
47,0.0570762950548959,0.954484419439194,0.990441165101222
"15698","TOX4",452.68734095253,0.00984511090830727,0.172802823341412,0
.0569730905892433,0.954566630911909,0.990441165101222
"15699","ST13",1930.53111789066,-0.00825016775756272,0.14451038244372,
-0.0570904845593068,0.954473116283452,0.990441165101222
"15700","MSH6",162.012708638084,0.0133972617851362,0.236456275982759,0
.0566585163766727,0.954817220036441,0.990574975936156
"15701","CLIP3",164.311138962107,0.0138652180894357,0.244560125385254,
0.0566945166044298,0.954788542107555,0.990574975936156
"15702","TESK2",25.3425440593194,0.0200693144723409,0.356220515650376,
0.0563395806546935,0.955071288089325,0.990586196223306
"15703","LEMD2",109.277996420697,0.0109642049971274,0.194405661080292,
0.0563985890956082,0.955024280906769,0.990586196223306
"15704","SEC23IP",141.387298096292,0.0107208184483061,0.19008855858921
1,0.0563990727683628,0.955023895605023,0.990586196223306
"15705","CCDC15-
DT",4.84765678882566,-0.0373752962321885,0.661109426727622,-0.05653420

55659224,0.954916246986951,0.990586196223306
"15706","CDK17",90.1785382680473,-0.0176918233207158,0.316483263595602
,-0.0559012919663333,0.955420441600921,0.99088523960508
"15707","AC239799.2",29.178466420666,0.0234611708052929,0.420400731814
245,0.0558066840275132,0.955495810160429,0.990887380453271
"15708","PMPCA",159.007960874178,-0.0100014085155071,0.17941039707488,
-0.0557459806040835,0.955544169203756,0.990887380453271
"15709","MBD4",299.148968193951,-0.00818795663514672,0.147946972077833
,-0.0553438608452165,0.9558645197885,0.990889375257082
"15710","RAP1GDS1",71.8308966586837,0.0114734400291701,0.2067761665879
81,0.0554872460327204,0.955750290488402,0.990889375257082
"15711","GIGYF1",208.981381881951,0.0129927852739471,0.235012903832608
,0.0552854122563476,0.955911083724587,0.990889375257082
"15712","AL512598.1",10.3917574334371,0.0300650917259106,0.54057282030
058,0.0556170983757437,0.955646843053664,0.990889375257082
"15713","ANKS3",106.125948543903,-0.0124883464142187,0.225645810677777
,-0.0553449070324292,0.955863686329296,0.990889375257082
"15714","ARHGAP45",15.6594211398462,-0.0367518564823988,0.661829459115
672,-0.0555307050422115,0.955715668596103,0.990889375257082
"15715","TXLNA",332.086887049769,-0.0105978714596483,0.194153086465937
,-0.0545851299742675,0.956468985657154,0.99096319217462
"15716","IFI44",152.854648972919,0.0162452787926075,0.297366841838959,
0.0546304311944951,0.95643289436538,0.99096319217462
"15717","CACNA2D2",4.27570973740361,-0.0420557688747935,0.763418013225
789,-0.0550887824837781,0.956067732855812,0.99096319217462
"15718","MITF",657.693720266666,-0.0155422744077641,0.284084016754257,
-0.0547101332392408,0.956369396298144,0.99096319217462
"15719","SMARCD3",143.669109343703,-0.0112632070536121,0.2063360489819
95,-0.0545867147751527,0.956467723051442,0.99096319217462
"15720","AL158212.2",34.30606658388,-0.0235433775179501,0.429552866941
495,-0.0548090336018098,0.956290603449019,0.99096319217462
"15721","FEZ1",622.174842048834,0.0162889432877862,0.296092590216429,0
.0550130054787249,0.956128102610532,0.99096319217462
"15722","DOK5",90.8930411511802,-0.0176246891600894,0.320876276399904,
-0.0549267442200181,0.956196825229427,0.99096319217462
"15723","SKI",520.501187540271,-0.0104836809584198,0.193803551208466,-
0.0540943697525078,0.956859977968195,0.99097432455615
"15724","LRRC47",183.249510261565,0.00846162126948328,0.16543713364261
7,0.0511470495358217,0.959208344921846,0.99097432455615
"15725","MFN2",81.2972325526756,0.0116751042660979,0.231472011964602,0
.0504385137840481,0.95977294586835,0.99097432455615
"15726","RCC2",118.940001383088,0.011065391940568,0.210803687189201,0.
0524914534850455,0.958137105079063,0.99097432455615
"15727","AL136115.2",2.78325106578522,-0.0539586189996621,1.0637234378
0478,-0.0507261728772446,0.95954372055246,0.99097432455615
"15728","TTC4",133.187398945765,0.0110594972051646,0.206588901596066,0
.0535338400065107,0.95730656886994,0.99097432455615
"15729","AC242426.2",23.9087551086756,-0.0165694012057637,0.3175594563
4676,-0.0521773194739024,0.958387404749356,0.99097432455615
"15730","KIFAP3",445.317157482353,-0.00924196907936042,0.1747691588350

24,-0.0528810068147352,0.957826717585576,0.99097432455615
"15731","ATP2B4",607.151390578244,0.00955526444605757,0.18392271882994
7,0.0519526054575795,0.958566457743785,0.99097432455615
"15732","JMJD4",86.98005077397,0.0120607504430874,0.222827091672351,0.
054126050618754,0.956834737272509,0.99097432455615
"15733","EIPR1",66.254839407003,0.0143404702943996,0.268346889103175,0
.0534400467332637,0.957381298103375,0.99097432455615
"15734","AC019171.1",13.2725997661364,-0.0240829992436303,0.4740256097
99495,-0.0508052703182367,0.959480691196181,0.99097432455615
"15735","FANCL",87.7067159852661,0.0115762023842009,0.217500529461874,
0.0532237894447523,0.957553601240035,0.99097432455615
"15736","DOK1",75.2821575365424,-0.0166813183654099,0.307982588446374,
-0.0541631864631025,0.95680515055625,0.99097432455615
"15737","BCL2L11",120.612258391668,0.0176244036946512,0.33198699536796
8,0.0530876327704241,0.957662085177645,0.99097432455615
"15738","SGO2",43.6151679619946,-0.0150261958058057,0.294627746916737,
-0.0510006133606015,0.959325031794832,0.99097432455615
"15739","GRM7",2.87675855910264,-0.0731967232874633,1.38768892065006,-
0.052747213152912,0.957933320703226,0.99097432455615
"15740","BTD",295.26726109124,0.0155794203646302,0.287042580547958,0.0
542756420837961,0.956715555742552,0.99097432455615
"15741","ZNF35",20.7049162672656,-0.0244717133502246,0.46203840149197,
-0.0529646740859698,0.957760054183362,0.99097432455615
"15742","CD38",25.8692445106493,-0.019902120795271,0.377204865849167,-
0.0527620998484926,0.957921459355535,0.99097432455615
"15743","NR3C2",56.2689272485809,-0.0179963959572929,0.332942084596081
,-0.0540526319438582,0.956893231270373,0.99097432455615
"15744","THBS4",142.551312707883,0.0665109769880226,1.26700924993881,0
.0524944683641693,0.958134702865501,0.99097432455615
"15745","SNCAIP",57.1979220044375,-0.0139080856591698,0.27544637501827
9,-0.0504928977854469,0.959729608933417,0.99097432455615
"15746","MSX2",20.7646431777225,-0.0202410071658419,0.391625034550924,
-0.0516846610407638,0.958779959619473,0.99097432455615
"15747","AL357054.4",17.0010122711538,-0.0256631092567056,0.5052554292
1111,-0.0507923473415716,0.959490988944399,0.99097432455615
"15748","HIVEP2",255.53543062095,-0.0114396971295584,0.222113038194424
,-0.0515039424184764,0.958923960427186,0.99097432455615
"15749","LINC01176",10.0577504302397,-0.0379359610977353,0.72923690550
9253,-0.0520214498349383,0.958511602056002,0.99097432455615
"15750","THAP5",181.105524107347,-0.0123375466596283,0.234631116862119
,-0.0525827384902171,0.958064370630991,0.99097432455615
"15751","AP001330.4",10.1243319567101,0.0241042077034367,0.47596195054
372,0.0506431400155011,0.959609886148722,0.99097432455615
"15752","SIT1",8.63527842098026,-0.0259255699713959,0.505698304702383,
-0.051266871433658,0.959112866141645,0.99097432455615
"15753","ZDHHC24",183.11784647702,0.0107382556339281,0.214052990399197
,0.050166342520615,0.95998983254512,0.99097432455615
"15754","NEU3",17.9511977212424,0.0203319778613727,0.404117417152996,0
.0503120553541377,0.959873717153842,0.99097432455615
"15755","CEP126",782.49032089262,-0.0115371571496702,0.228492711321645

,-0.0504924515225766,0.959729964546064,0.99097432455615
"15756","BICD1",65.5866549429133,-0.0157074914506325,0.292625455049078
,-0.0536778027325001,0.957191868153167,0.99097432455615
"15757","ANO4",3.52433953244672,0.0641326548731738,1.18003378373082,0.
054348151516824,0.956657786851124,0.99097432455615
"15758","CLIP1-
AS1",3.07866111389818,0.0540011223658952,1.06810940391414,0.0505576696
244835,0.959677994406041,0.99097432455615
"15759","RILPL1",163.373685911203,0.0110376085468816,0.220060522869947
,0.0501571495102042,0.959997158283879,0.99097432455615
"15760","WBP4",438.205425778345,0.00917955310544408,0.171422540512636,
0.0535492769970204,0.957294269575372,0.99097432455615
"15761","RBM23",244.408511904976,-0.0081160469445438,0.162104200027426
,-0.0500668517112491,0.960069115097342,0.99097432455615
"15762","MED6",247.057506653352,0.00947582801706627,0.185358575922901,
0.0511216056224327,0.959228619703738,0.99097432455615
"15763","INF2",180.32360789543,0.00935682024655113,0.185680074801128,0
.0503921611221489,0.959809882970157,0.99097432455615
"15764","GTF2A2",764.390330100049,-0.0147051246798918,0.28444778875715
5,-0.0516970961319238,0.958770051098592,0.99097432455615
"15765","AC015712.1",9.83259551608532,-0.0348913599720614,0.6512507772
38842,-0.0535759206614585,0.957273041479919,0.99097432455615
"15766","PKD1",340.664410044471,0.0106697297767604,0.206011213504303,0
.0517919854714004,0.958694441649508,0.99097432455615
"15767","SHCBP1",2.56795775353485,0.0492405974039592,0.965773901465456
,0.0509856368340893,0.959336965808019,0.99097432455615
"15768","TAF1C",69.8481106776183,-0.0112170267003743,0.212768018255973
,-0.0527195148609201,0.957955390036139,0.99097432455615
"15769","AC004771.3",2.42899904352471,0.0478346273966766,0.95481626286
709,0.0500982537237483,0.960044091319176,0.99097432455615
"15770","CCT6B",25.7149354317542,0.0183357586034523,0.357723089687885,
0.0512568495912587,0.959120851915648,0.99097432455615
"15771","HEXD",144.665270675727,0.00982493057325863,0.187015507683881,
0.0525353789904206,0.958102105887702,0.99097432455615
"15772","SLC25A23",134.818322777309,0.0114611315987096,0.2160801240151
54,0.0530411191262824,0.9576991454817,0.99097432455615
"15773","AC006504.7",2.97952990661881,0.0440976067215157,0.82801889088
2777,0.0532567640751551,0.957527328553259,0.99097432455615
"15774","CTU1",142.355898516979,0.0153107397648531,0.304598269978156,0
.0502653536605809,0.959910932626221,0.99097432455615
"15775","ZSCAN18",265.24701862909,0.00998648535682727,0.18979279029252
1,0.0526178330664482,0.958036407920693,0.99097432455615
"15776","FASTKD5",47.7152928008995,0.0149206019910674,0.29607124416600
1,0.0503953095245607,0.959807374096183,0.99097432455615
"15777","SHLD1",32.0220045458629,-0.0209391161315129,0.388884334986982
,-0.0538440719969186,0.957059396050674,0.99097432455615
"15778","MMP24OS",531.160457292563,0.0133166217367015,0.25965520444408
8,0.0512857878786286,0.95909779283113,0.99097432455615
"15779","AL021578.1",3.25959819435119,0.0563032026966007,1.09772568941
772,0.051290776228865,0.95909381793489,0.99097432455615

"15780","SNAP29",284.885572887889,-0.0107669585851259,0.210491772744333,-0.0511514461812414,0.959204841492261,0.99097432455615
"15781","PDK3",49.0276370029626,-0.0149246766976181,0.27985186796734,-0.0533306309728114,0.957468474934011,0.99097432455615
"15782","AP000550.1",2.61649339845026,0.0464143694898685,0.929519319794831,0.0499337329536231,0.960175195813369,0.991021021708527
"15783","HIST1H4A",2.58995847664373,0.0543519994427226,1.09204522160563,0.0497708321664639,0.96030501042509,0.991092207743413
"15784","GATA3",4.21173704544704,-0.0450330350557977,0.906505212822183,-0.0496776349642804,0.960379279162315,0.991106061725478
"15785","MEF2A",458.132503864185,-0.0121424302099104,0.245355198766175,-0.0494891906549013,0.960529451253454,0.991198240827844
"15786","HIST3H2A",102.413312515582,-0.0226870946361084,0.46129470010449,-0.0491813468287613,0.960774776349951,0.991259209475486
"15787","ENPP3",3.45815135369546,0.0368681866401744,0.752340833356743,0.0490046332799437,0.960915603554412,0.991259209475486
"15788","BAZ1B",387.930226166913,0.0112224311621531,0.228262771614201,0.0491645268424264,0.960788180542041,0.991259209475486
"15789","COMMD9",249.75825519085,-0.0137570128890434,0.280169953560416,-0.0491023848711059,0.960837702849623,0.991259209475486
"15790","LAIR2",2.34183639334622,0.0473978818535011,0.96128503222434,0.0493067927457749,0.960674806275765,0.991259209475486
"15791","PRDX4",1604.0701555125,0.013685493771921,0.279857060723014,0.048901727676851б,0.960997612024292,0.991259209475486
"15792","GNL3L",161.546010501192,-0.0140630530848027,0.287702622656232,-0.0488805175113269,0.961014515073785,0.991259209475486
"15793","RAB3A",15.9637182932684,0.02503786663103９,0.514136802829294,0.048698841423636,0.961159299191996,0.991345774997684
"15794","CDC42-IT1",9.01660077396876,0.0308038545451981,0.634062889463924,0.0485817023154305,0.961252652181702,0.991379286525753
"15795","SLC16A4",129.691565334145,0.012821224092331３,0.266261075126041,0.0481528292720298,0.96159444333324,0.991384009188823
"15796","SLC41A1",100.90397156979,-0.0162034419457046,0.336513858679248,-0.0481508904545566,0.961595988493424,0.991384009188823
"15797","HOXD9",10.6609845269567,0.0270714913654173,0.563011957979245,0.0480833328346737,0.961649829311543,0.991384009188823
"15798","U2SURP",747.565097374123,0.00687366128339638,0.142576132349144,0.0482104625096997,0.961548512007794,0.991384009188823
"15799","CCNJ",19.6580209443246,-0.0177900302658903,0.367194101656321,-0.0484485730725082,0.961358749014599,0.991384009188823
"15800","CCDC84",106.755966375768,-0.0112888150993186,0.234278677353721,-0.0481854141692735,0.961568474491586,0.991384009188823
"15801","AC005332.3",18.8883699152271,0.0220683339940332,0.459360979354811,0.0480413770125381,0.961683266572078,0.991384009188823
"15802","AC007786.1",3.33416827382083,-0.0399110892914208,0.83420015633456,-0.047843540891659,0.961840935654513,0.991483799574507
"15803","CENPT",144.72785301478,0.00948346236905081,0.198685581286652,0.047731004472683,0.961930624259707,0.991513506205554
"15804","ABHD6",44.3140244988204,-0.0132922378310088,0.279218974073223

,-0.0476050665078478,0.962030994120313,0.991554218123626
"15805","AC096677.1",2.6426464804011,-0.0407625071792795,0.859100168335247,-0.0474479096637456,0.962156245602846,0.991609670795864
"15806","B4GALT5",263.019135986062,0.00962362466538765,0.203095208073586,0.0473847943369535,0.962206547768398,0.991609670795864
"15807","AC097359.2",4.15340361410188,0.036290961490291,0.768847943034658,0.0472017410192318,0.962352439937852,0.991673204726341
"15808","AC009118.3",11.6215399884684,0.0261539489380738,0.554641788565322,0.0471546671694636,0.962389957659402,0.991673204726341
"15809","AL451085.1",2.40720006059038,-0.0529674370031867,1.12512838845901,-0.0470767936766147,0.962452022789836,0.991674425910787
"15810","GPANK1",194.45331086456,0.0103552985228913,0.220963871522491,0.046864215636434,0.962621448526693,0.991762836939284
"15811","AC064807.1",30.3574660959617,0.0134710923054408,0.289248653993914,0.0465727052466223,0.962853786474721,0.991762836939284
"15812","AL353751.1",4.13872342335554,-0.038016013650571,0.813231485379746,-0.0467468541663989,0.962714986915485,0.991762836939284
"15813","SEMA7A",2.77738033774513,-0.0422797126393576,0.909348198322074,-0.0464945251086129,0.962916097699593,0.991762836939284
"15814","LMAN1",1179.69415300721,-0.00570130526335753,0.122080006412898,-0.0467013840421553,0.962751227245079,0.991762836939284
"15815","FAM227A",8.65879961041041,0.0310307179727033,0.665900561786721,0.0465996272618283,0.962832329111227,0.991762836939284
"15816","CXorf38",107.430150115051,0.01068153525031,0.230035006073932,0.0464343902809168,0.962964026584303,0.991762836939284
"15817","PHYHD1",391.889893129028,0.0127589576511846,0.275420253244418,0.0463254154365396,0.96305088246221,0.991789582375099
"15818","CCDC127",258.976840572413,0.00971636504614337,0.211881523021611,0.0458575382486388,0.963423798117408,0.991860508665809
"15819","DOCK8-AS1",5.77784772643661,0.0369894414360773,0.806625483860926,0.0458570206076639,0.963424210701113,0.991860508665809
"15820","PPFIBP2",72.2147343998882,-0.0139036681075091,0.301239864827412,-0.0461548079483929,0.963186862092777,0.991860508665809
"15821","LENG8-AS1",19.9145186932535,-0.0168653449262644,0.367780191904527,-0.0458571323238705,0.963424121658148,0.991860508665809
"15822","KCNE5",7.25770402173332,-0.0352862445401789,0.76921608566115,-0.0458729935553155,0.963411479530474,0.991860508665809
"15823","PRNCR1",3.42187091930736,-0.0387408228601296,0.847770047746921,-0.0456973243665416,0.96355149642952,0.991928858329043
"15824","SON",2199.66456054258,-0.00838411808394397,0.184302823514533,-0.0454909909900693,0.963715955614709,0.99203546517998
"15825","FBXO36",32.3872830622039,0.0135339492072075,0.29806466213329,0.0454060844057901,0.963783631336834,0.992041964982043
"15826","AC016949.1",21.4455817369996,0.0202261795079703,0.446732012595858,0.0452758677186365,0.96388742247902,0.992041964982043
"15827","ATG12",501.934744191346,0.00608402271961477,0.134493742942783,0.0452364741027622,0.963918821865577,0.992041964982043
"15828","TBL3",96.2730817960533,-0.0100154097657607,0.221690535706881,

-0.0451774349943522,0.963965880148307,0.992041964982043
"15829","XYLB",29.1980689716227,0.0174761373457228,0.390202142480587,0
.0447873946427453,0.964276772629149,0.99217694066958
"15830","C3orf62",39.4294718967302,-0.0147187009225017,0.3286624795866
46,-0.0447836362124852,0.96427976841673,0.99217694066958
"15831","OGDH",201.803619753055,0.00834667777919088,0.186003347853759,
0.0448738040228892,0.964207897165987,0.99217694066958
"15832","LINC02453",3.97406197617563,-0.0414746254074986,0.93775070209
6897,-0.0442277732394735,0.964722843865188,0.992539458879295
"15833","BX890604.2",3.58646885912769,0.0434090622957757,0.98235580528
8958,0.0441887369749976,0.964753959876964,0.992539458879295
"15834","GAB3",26.0284813350455,-0.0173532640253392,0.394104438719518,
-0.0440321455950144,0.964878780228776,0.992605181959488
"15835","TIPRL",411.150594686199,-0.00747430132913164,0.17064436840056
6,-0.0438004570510447,0.965063462761414,0.992612361109988
"15836","ANKAR",91.6758650081286,-0.0120162438878375,0.273514832844238
,-0.0439326955795501,0.96495805314868,0.992612361109988
"15837","SACM1L",117.910658660555,-0.00921068280588612,0.2110549781873
11,-0.0436411540016444,0.965190446781331,0.992612361109988
"15838","AC132192.1",5.69707814430631,-0.0328775532446234,0.7514655836
87593,-0.0437512428490558,0.96510269240696,0.992612361109988
"15839","SYCP3",4.01359195813859,0.038797821077685,0.888064141884746,0
.0436880842811016,0.965153037515159,0.992612361109988
"15840","LRRC57",113.595946578768,-0.00964752622392992,0.2216096521443
3,-0.0435338719707329,0.965275964182781,0.992637637662458
"15841","GDPD3",19.7289433446773,-0.0192185040450291,0.443117761656327
,-0.0433710984032606,0.965405716147652,0.992708396586649
"15842","LY96",398.762825855863,-0.0137802183132453,0.319412568209742,
-0.0431423797456728,0.96558803657907,0.992833198323221
"15843","JMJD7",36.2400818757315,0.0141392349894552,0.328617873056944,
0.0430263724182924,0.965680511166034,0.992865609189139
"15844","ZNF845",34.3428100612887,0.0148367943728753,0.345964268672782
,0.0428853373494134,0.965792937097596,0.992918527668691
"15845","RLF",207.422914862804,-0.00878461299560275,0.206409918552115,
-0.0425590642989609,0.9660530278606,0.992948469817964
"15846","MRPS25",309.859157636818,0.00968920725936655,0.22730962569096
1,0.0426255915468336,0.965999994922179,0.992948469817964
"15847","UBL7-
AS1",59.5617412237879,-0.00948121807867533,0.222862397367477,-0.042542
9242019764,0.966065894141758,0.992948469817964
"15848","ZNF14",32.7393801592174,-0.0139427450368053,0.326112996305312
,-0.0427543372842213,0.965897364247507,0.992948469817964
"15849","AL049597.2",29.8569650984664,-0.0150887992815988,0.3573325588
53178,-0.0422262089131333,0.966318369498098,0.993065504963431
"15850","SIX2",29.0379135704428,-0.0138992509786213,0.330795138141167,
-0.0420177003106068,0.966484587769984,0.993065504963431
"15851","ATAT1",47.3119710208801,0.010606755411661,0.251990634197532,0
.0420918636338943,0.966425466303743,0.993065504963431
"15852","BCL9L",332.820338092784,0.0109519207079074,0.260013625286604,
0.0421205646274709,0.966402586515251,0.993065504963431

"15853","Z69706.1",6.51738674470303,0.0251498016336203,0.595025056482199,0.0422667942461221,0.96628601597215,0.993065504963431
"15854","SDCBP",3038.92726882898,0.011384603265223,0.272492514752537,0.0417795082391966,0.966674470803479,0.993135317244899
"15855","SLC27A5",61.2119609194625,-0.0102099912991304,0.244082875630421,-0.0418300188932136,0.966634204333477,0.993135317244899
"15856","EFCAB7",76.2714333660565,0.0101049769779924,0.245316983352517,0.0411915100206156,0.967143221952024,0.993333435718436
"15857","SLC16A1",218.0157923282,0.0136868455411665,0.333186420454434,0.0410786415679815,0.967233201788331,0.993333435718436
"15858","LINC02613",6.33084378151642,0.0307905369854151,0.744933448976705,0.041333272157011,0.967030208380745,0.993333435718436
"15859","AC007364.1",21.5174010552412,0.0191036711287114,0.463998618378974,0.0411718276133063,0.967158912929894,0.993333435718436
"15860","LINC00504",5.01989136041402,-0.0273070374270164,0.664290869249487,-0.0411070491724021,0.967210554928422,0.993333435718436
"15861","ITIH5",2216.77028376692,0.0111908198949669,0.272388294706464,0.0410840704701593,0.967228873804763,0.993333435718436
"15862","LIMS2",185.811239925164,-0.0107584785284496,0.262450688419978,-0.0409923806762271,0.967301970097729,0.993341431781737
"15863","ATG4B",150.913083218997,-0.00756087237645557,0.185595327597326,-0.0407384844992429,0.967504380854946,0.993410513091835
"15864","PRDM5",95.6852597887191,0.00979630407252526,0.242507402444354,0.0403958970892574,0.967777501229591,0.993410513091835
"15865","MTRES1",151.313792847915,0.0103646655930474,0.255832424111907,0.0405134948356416,0.967683748551809,0.993410513091835
"15866","AC104051.2",5.38112309745574,0.0324238914974297,0.796028798001036,0.0407320584115193,0.967509503878887,0.993410513091835
"15867","AL163051.1",4.90779528264733,-0.0281262187718187,0.696667586978441,-0.0403725094974013,0.967796146617615,0.993410513091835
"15868","LINC00524",3.73379265879454,0.0335860721687905,0.827751201162019,0.0405750811616359,0.967634650144684,0.993410513091835
"15869","CCT8",654.229724455446,0.0106768585671603,0.262834779828104,0.0406219396616498,0.967597293270203,0.993410513091835
"15870","GALNT3",4.33164052046912,-0.0309956405204914,0.771230210964694,-0.0401898681869847,0.967941755120929,0.99349736919753
"15871","PSAP",4795.38006238083,0.00561726414416381,0.140240962507335,0.0400543752961623,0.968049775826495,0.993545636597427
"15872","CAVIN4",2.67466285874401,-0.0372822260118178,0.933728132657424,-0.0399283524913309,0.968150247102263,0.993565107749884
"15873","PITRM1",90.7978064294843,0.0105547721142805,0.265188253291957,0.0398010544707659,0.968251735552929,0.993565107749884
"15874","AC021739.2",7.84325495484114,-0.0284781912091196,0.715175695182853,-0.039819853220597,0.968236748201902,0.993565107749884
"15875","PPFIA2",3.74693377636304,-0.0320112925935425,0.809163793553838,-0.039560955209018,0.968443156283307,0.993573759066498
"15876","LONP1",125.20237419187,0.00823933195433249,0.208189545747434,0.0395761080353577,0.968431075538043,0.993573759066498
"15877","LINC00630",27.3955563164512,0.0133221922751364,0.33633058762018,0.0396104094171216,0.968403728438537,0.993573759066498

"15878","MID2",9.08302037041467,0.0217275342947362,0.552778423540728,0
.0393060462735939,0.968646386114007,0.993719673977267
"15879","RAP1GAP2",4.11847786072539,-0.0385292291352004,0.982309181307
973,-0.0392231182079533,0.968712502151778,0.993724916402186
"15880","MYL6B",610.328094577692,-0.0106903365919269,0.274781988356206
,-0.0389047937817116,0.968966294579148,0.993922668287138
"15881","TARSL2",89.6883071562848,-0.0124995182825105,0.32196415752166
2,-0.0388227011935933,0.96903174554081,0.993927215106999
"15882","C9orf92",3.03007660570192,-0.0357988966266621,0.9336284921315
32,-0.0383438347569396,0.969413541384132,0.994131023394996
"15883","SLC11A2",127.817882221271,-0.00824374630106591,0.214911251096
729,-0.0383588400281355,0.969401577711385,0.994131023394996
"15884","EME2",17.021439244478,-0.0167913138339462,0.4379109627299,-0.
0383441276036355,0.96941330789798,0.994131023394996
"15885","LINC01309",5.0715856493267,0.0282700823894529,0.7409715763338
71,0.0381527217674471,0.96956591599006,0.994224690309228
"15886","IRX6",35.7475120754771,0.0174944291908357,0.460191988951282,0
.0380155013795508,0.969675322648444,0.994274287461948
"15887","FIBIN",824.702387678887,-0.0124350409022748,0.329898904758285
,-0.0376934894991116,0.969932066936707,0.994474944189087
"15888","LAMP1",2095.50991306175,-0.00616563757107473,0.16453961287942
2,-0.0374720558969167,0.970108620656784,0.994593361145415
"15889","MKRN1",357.310485401188,-0.00496927312453061,0.13361304702483
3,-0.0371915260910638,0.970332295139286,0.994760070207302
"15890","THAP3",146.544788310986,-0.00992013154465543,0.27249199596036
,-0.0364052217742885,0.970959250567315,0.995340165669666
"15891","LINC01772",4.40276363903535,0.0267049487055331,0.758102688262
254,0.0352260308781479,0.971899505469447,0.99536176608815
"15892","DR1",250.877245695169,0.00537625699441808,0.15069629354218,0.
0356761063463931,0.97154062279401,0.99536176608815
"15893","ASB3",138.465001871183,-0.00700572334463691,0.193851833318857
,-0.0361395774530207,0.971171064682919,0.99536176608815
"15894","CSTA",58.8110101918863,0.0165516316395546,0.471287767875151,0
.0351200111010293,0.97198404470304,0.99536176608815
"15895","DBR1",106.88727358102,-0.00640900039780963,0.181991668747242,
-0.0352158999471054,0.971907583770726,0.99536176608815
"15896","ZMAT2",474.038918365598,-0.0064419076314533,0.183655652186239
,-0.0350760107558289,0.972019130282043,0.99536176608815
"15897","RWDD2A",47.6080305829238,0.00962425601413657,0.26932032771277
2,0.0357353494103897,0.971493383789998,0.99536176608815
"15898","FSBP",18.2459624554598,0.0154273870904471,0.430751022094825,0
.0358150910830594,0.97142979984304,0.99536176608815
"15899","GRINA",988.424905013102,-0.00495637331524691,0.13821778900543
3,-0.0358591564147513,0.971394663365344,0.99536176608815
"15900","PPP3CB-
AS1",6.4696483281568,-0.0241357021064709,0.675411407228684,-0.03573481
56222345,0.971493809419476,0.99536176608815
"15901","TBK1",177.568485021708,0.00638975792671178,0.180410213919069,
0.0354179388622541,0.97174648054206,0.99536176608815
"15902","AC025164.1",13.8086253475377,-0.0222656960948219,0.6210144008

28243,-0.0358537516442877,0.971398972976959,0.99536176608815
"15903","HAUS2",92.9912445042884,0.00748293131968101,0.209710056212251
,0.0356822722516816,0.971535706243768,0.99536176608815
"15904","INO80E",163.087120080998,-0.00746579067691483,0.2069686146890
58,-0.0360720908729624,0.971224876096644,0.99536176608815
"15905","HLF",170.354471203943,-0.0148237908886272,0.421977809164535,-
0.0351293138328209,0.97197662677428 7,0.99536176608815
"15906","ZNF557",58.376720763952,-0.0107248914986759,0.303168360495731
,-0.0353760249952828,0.971779902025175,0.99536176608815
"15907","IL2RG",19.0197966924022,-0.0165704012236576,0.472295698124434
,-0.0350848023589067,0.972012119912613,0.99536176608815
"15908","TBCC",118.140815926627,0.0096288274380422,0.275186853312113,0
.0349901433231671,0.972087600550579,0.99536930634702
"15909","MARCO",7.02464571064936,-0.0250881477306656,0.71925272875581,
-0.0348808516501063,0.972174749490704,0.995395970485517
"15910","MUC1",21.2699108687638,-0.0192669606900107,0.554227478198457,
-0.0347636330710972,0.972268219697447,0.995429103120786
"15911","CIP2A",25.5602610121285,-0.0105600003358822,0.307183278517362
,-0.0343768722921725,0.972576625806246,0.995682273757648
"15912","USP34",430.911193127419,0.0068802315521482 8,0.202675012575,0.
0339471129900745,0.972919324085662,0.995699082408672
"15913","SNRNP200",387.419773824481,-0.0054369858253288,0.160330603504
607,-0.033911091871943,0.972948048241527,0.995699082408672
"15914","MARS2",4.65346090901993,-0.0244750714811282,0.71497189959148,
-0.0342322145739052,0.972691978072808,0.995699082408672
"15915","AC078802.1",2.48589464593763,0.0408883802656275,1.20875817966
39,0.0338267661419233,0.973015291860782,0.995699082408672
"15916","TCTN2",42.7436721840295,0.013337339624296,0.394366102550086,0
.0338196907342006,0.973020933991113,0.995699082408672
"15917","HSPH1",247.036522199772,0.00801022355566907,0.235999682168307
,0.0339416709466433,0.972923663707272,0.995699082408672
"15918","ZNF821",41.2827781133327,-0.0107570090301423,0.31489876462043
1,-0.0341602134994352,0.972749393038674,0.995699082408672
"15919","AC112220.2",10.8039860274307,0.018887196709103,0.563359502019
957,0.0335260107291736,0.973255123925055,0.995751065486793
"15920","DHRS4L2",406.495535515773,0.0102404915783422,0.30501880622674
2,0.0335733121017126,0.973217404124502,0.995751065486793
"15921","AC104938.1",2.61297550620745,0.034600148938755,1.031203949898
66,0.0335531578812857,0.973233475810963,0.995751065486793
"15922","POLG",89.1068401105357,0.00852769636663911,0.256251241665702,
0.0332786538367846,0.973452376100615,0.995888098640035
"15923","AC040977.1",13.6721655351771,-0.0142812640985957,0.4300974827
284,-0.0332047144475246,0.973511338611657,0.995888098640035
"15924","SPSB4",21.663177459411,-0.023332923121422,0.709250441361874,-
0.0328980029629859,0.973755925340011,0.995968909737759
"15925","ITGA5",231.884084190641,-0.0102330935902737,0.309892951112283
,-0.033021382233912,0.973657536381192,0.995968909737759
"15926","WRAP53",33.1065820317851,-0.0114694658453186,0.34887438035421
8,-0.0328756322939891,0.973773764901685,0.995968909737759
"15927","PDIA6",1510.79680204674,0.00742303873523234,0.227553480146976

,0.0326210732107363,0.973976764780209,0.996008801021672
"15928","AC034243.1",4.48380688492472,-0.0388364669082018,1.2084792943
5991,-0.0321366423814256,0.974363082097142,0.996008801021672
"15929","TNXB",7385.9081618534,0.00535470021613356,0.165051640284681,0
.0324425749837916,0.97411911041591,0.996008801021672
"15930","MRPS18A",209.819217011077,0.00918404602557884,0.2854874424682
58,0.032169702268428 1,0.974336717755323,0.996008801021672
"15931","RARS2",301.756482787995,-0.00680705855310215,0.21048408646103
1,-0.0323400151885706,0.974200898375984,0.996008801021672
"15932","REV3L",1131.87383348719,0.00923093959935816,0.282634298386395
,0.0326603658935207,0.973945430451768,0.996008801021672
"15933","EIF2AK1",292.12546064759,0.0049745738398551,0.15476369937881,
0.0321430274658852,0.974357990167414,0.996008801021672
"15934","CORO2B",125.148122198385,-0.00540351983560608,0.1681171362425
42,-0.0321413982915486,0.974359289389164,0.996008801021672
"15935","WDR24",39.8752667864435,0.00817008602417901,0.251564534564385
,0.0324770979276809,0.974091579599608,0.996008801021672
"15936","SCMH1",103.583114973419,0.00694126695124178,0.216739692797194
,0.0320258225969564,0.97445145800197,0.996036633998123
"15937","TFAP2E",4.11397616401711,0.0262977778676995,0.831549294743559
,0.0316250377866166,0.974771076107335,0.996050816053721
"15938","WNT2B",32.4869643547358,-0.0115941149904077,0.363268048419321
,-0.0319161430267728,0.974538924924303,0.996050816053721
"15939","TPR",1087.67879661014,-0.00559050344179768,0.175668237339804,
-0.0318242132240657,0.974612237053212,0.996050816053721
"15940","AC095055.1",2.67453184778293,-0.0304656728431732,0.9619936549
53055,-0.0316693074702867,0.974735771694106,0.996050816053721
"15941","POMP",1282.30579906391,-0.00757951014909441,0.239047237694422
,-0.0317071647520287,0.974705581115147,0.996050816053721
"15942","POLR2D",97.2986335526834,-0.00714248016908196,0.2296625352563
52,-0.0310998925493417,0.975189875362512,0.996316457933797
"15943","IGHG2",71.2053459999896,-0.0353805915300483,1.1387758885704,-
0.031068967902424,0.975214537743045,0.996316457933797
"15944","BLMH",134.737048155813,0.00547477025003123,0.175719982381658,
0.0311562189787854,0.975144955142228,0.996316457933797
"15945","ARHGAP44",36.4846076880277,-0.0102398561754358,0.330459284096
914,-0.0309867407823615,0.975280113919003,0.996320964291417
"15946","C12orf60",6.15047988191628,0.0189769227584588,0.6139516816446
28,0.0309094727253197,0.975341735391607,0.996321430314429
"15947","LINC00342",369.605437705515,-0.00945784440129965,0.3067871956
54247,-0.0308286803858619,0.975406167645816,0.996324767340734
"15948","AP3S1",1453.70508125861,0.00664083774185545,0.216771411052552
,0.0306352101949713,0.975560461644982,0.996357411733345
"15949","AC010632.2",7.10721559729824,-0.0244517254975064,0.7975385273
71828,-0.0306589897018311,0.975541497251791,0.996357411733345
"15950","VWA5A",109.669672409914,0.00580528695206087,0.190106720836529
,0.0305369895736237,0.975638793713508,0.996374941115946
"15951","TXNDC11",106.61476254676,0.00802372478348812,0.26453455792604
8,0.0303314804931127,0.975802690314074,0.996445462233858
"15952","QRICH2",3.58301982367794,0.0235478702337377,0.777234511429051

,0.0302969951635855,0.975830192986341,0.996445462233858
"15953","LMOD1",474.079466093795,-0.00737153220933317,0.245061597071539,-0.0300803238753939,0.976002992902629,0.996496975767264
"15954","ARIH1",311.689832730083,0.00843316077274976,0.279939206961013,0.0301249720048119,0.975967384986559,0.996496975767264
"15955","CDKN2A",19.2869128991178,0.0355065771952838,1.19006275137729,0.0298358865145481,0.976197938198277,0.996571083937812
"15956","AL731537.1",4.14810842875291,0.025162707678086,0.841706304862083,0.0298948784543191,0.976150890426928,0.996571083937812
"15957","FAM32A",451.58766850816,-0.00606330791962378,0.204307583706647,-0.0296773512251494,0.97632437506834,0.996637697906134
"15958","MOSMO",311.545564966498,0.00488990678865091,0.165390039308919,0.0295659086187014,0.976413254401656,0.996665966972589
"15959","COP1",152.497696833513,-0.00534106690774705,0.181413826957125,-0.0294413441209712,0.976512599243691,0.99667286902892
"15960","SFXN2",6.05630407270215,0.0191745140759187,0.652105837219892,0.02940399085778,0.976542389938091,0.99667286902892
"15961","TUT1",41.5406734211353,-0.00737458787329811,0.252927174246543,-0.0291569614663455,0.976739406401309,0.996686599691219
"15962","AP001107.2",4.44853016113015,0.0225423364044328,0.770145029679027,0.0292702485060869,0.976649054984154,0.996686599691219
"15963","EPN2",495.266783648121,0.00821201731996087,0.281055720309612,0.0292184671100609,0.976690352897141,0.996686599691219
"15964","TRAPPC9",56.7408895021468,0.00647479169248935,0.224866769383294,0.0287939018746377,0.97702896446413,0.996732310525222
"15965","VPS26A",443.201104087535,0.00527913296443908,0.182383003842598,0.0289453120806977,0.976908206931906,0.996732310525222
"15966","NLRC3",3.62049456543177,0.0303842813214795,1.05516537953016,0.0287957526951927,0.977027488335072,0.996732310525222
"15967","ACSS2",82.8297130376031,-0.0072110270834522,0.249382727074471,-0.0289155033632254,0.976931980896242,0.996732310525222
"15968","AL603839.3",3.03941805936683,-0.0279081734460911,0.982536349990286,-0.0284042147105978,0.977339762706007,0.996774672106724
"15969","TEC",7.92911976677899,-0.018330876800522,0.640495148267082,-0.028619852703206,0.977167778401647,0.996774672106724
"15970","TUBE1",147.595792542407,0.00546305569647581,0.192644587256123,0.0283582101853328,0.977376454226079,0.996774672106724
"15971","AGPAT4",114.823158747027,0.00637878049366544,0.224054077766957,0.0284698254869529,0.977287434042975,0.996774672106724
"15972","U2AF1",61.2390872115995,0.00935103291436401,0.327469647049441,0.0285554187956608,0.977219168218131,0.996774672106724
"15973","DARS",417.392552832328,-0.00418475574339214,0.148283466892102,-0.0282213238677321,0.977485630009365,0.996823604033215
"15974","RPL38",9894.83703133419,-0.00682385640767249,0.242565152843493,-0.0281320557700856,0.977556827377899,0.996833802501477
"15975","ABITRAM",77.372773338185,0.00897155285693924,0.320960877513813,0.0279521695180844,0.977700299419444,0.996917694976108
"15976","AL121821.2",28.7040382316153,0.0146456354626422,0.527405631420929,0.0277692056931286,0.977846226783649,0.996941677916934
"15977","ZNF224",154.046902535048,0.00515427937885825,0.18538742515221

8,0.0278027453837615,0.977819476310805,0.996941677916934
"15978","GPR153",130.024420784324,0.00649459509941224,0.23747383053084
4,0.027348677051675,0.978181632697784,0.997190303474773
"15979","GABRP",3.55195802027358,-0.0270685646214755,1.00243087488798,
-0.0270029238918847,0.978457401954527,0.997190303474773
"15980","MINAR1",3.05512287998829,-0.0230405600583459,0.84716004788783
6,-0.0271974110627517,0.978302280616357,0.997190303474773
"15981","SLCO3A1",424.887455774806,0.00570968810886103,0.2109897709523
34,0.0270614451264129,0.978410725822176,0.997190303474773
"15982","CDC42EP4",644.49078370979,0.00680057802411514,0.2508216942210
27,0.0271131970670862,0.978369448893419,0.997190303474773
"15983","AC092296.2",8.04601642733253,0.0174324578631599,0.64166151228
3664,0.0271676850324372,0.978325989796131,0.997190303474773
"15984","OTULINL",26.764358584773,0.0116012928371017,0.433418605570291
,0.0267669469838213,0.978645616252823,0.997194948276131
"15985","FAM110A",16.8619731355141,-0.0103472852538115,0.3863990083379
85,-0.0267787572704139,0.978636196384111,0.997194948276131
"15986","CXCR3",3.40385798721362,-0.0236870067611778,0.882362231486436
,-0.0268449916779353,0.978583367964936,0.997194948276131
"15987","PKD2",524.411751529566,-0.00777342188834812,0.292272039824415
,-0.0265965293601745,0.978781541451239,0.997240585726584
"15988","BFSP1",7.14042378681118,-0.0153783796542497,0.57906459716326,
-0.0265572782891335,0.978812848216381,0.997240585726584
"15989","FCMR",5.1829058519214,0.0218027841131192,0.829613381321366,0.
0262806562719527,0.979033483641375,0.997402990495613
"15990","RSPO3",139.5451006675,0.0117926590864881,0.450479515218247,0.
0261780140674651,0.97911535210489,0.99742401316051
"15991","PRCC",172.111810077456,0.0054388536243089,0.213193745308742,0
.0255113189011829,0.979647120248913,0.997425536925159
"15992","DNM3",10.0597512895276,0.0134779727843027,0.557427553569784,0
.0241788779510258,0.980709926158162,0.997425536925159
"15993","LINC02245",4.53741801395769,0.0169424822938632,0.688262862247
25,0.0246162959287738,0.98036102096689,0.997425536925159
"15994","SP3",470.762169336986,-0.00395007203117623,0.167589117537079,
-0.0235699792995347,0.981195618544554,0.997425536925159
"15995","POGLUT1",98.6180369398158,-0.00627655586671175,0.249770503395
843,-0.025129291815393,0.979951836065586,0.997425536925159
"15996","ETFDH",183.389274096404,0.00426716954624734,0.16947631364593,
0.0251785600857611,0.979912538107594,0.997425536925159
"15997","AC010245.1",3.37198376899117,0.020686496349717,0.818780974584
614,0.0252649939261373,0.979843595812745,0.997425536925159
"15998","PCDHGA1",5.1037936514443,-0.0154138809509881,0.62973581519849
5,-0.0244767418002572,0.980472335511023,0.997425536925159
"15999","HIST1H3D",3.80624309465091,-0.0237534109328551,0.972832578496
53,-0.0244167511017825,0.980520186861961,0.997425536925159
"16000","CYP21A2",7.41350578759337,0.016804543823411,0.646496345291701
,0.0259932541704142,0.979262719026792,0.997425536925159
"16001","CEP85L",129.324649990655,0.00537647360214612,0.22672934157761
7,0.0237131796208458,0.981081393145311,0.997425536925159
"16002","AC091729.3",36.8450200051207,-0.0090737245897224,0.3784320611

09993,-0.0239771560662908,0.980870830286378,0.997425536925159
"16003","CARD11",3.1378452838984,0.0205442217559672,0.87309856394779,0.0235302434390394,0.981227314383554,0.997425536925159
"16004","AC006043.1",2.58131961304614,0.0205514522999428,0.866712909965449,0.0237119489783094,0.981082374779973,0.997425536925159
"16005","IQUB",6.56900453877509,-0.0148694893912884,0.633366059055544,-0.0234769280397836,0.981269842169277,0.997425536925159
"16006","AC090114.2",71.9041261115642,0.0058611069396971,0.250254794801903,0.0234205730148317,0.981314794613491,0.997425536925159
"16007","GIMAP5",3.65438129433564,-0.0191748502658143,0.774243820159727,-0.0247659067680495,0.980241685170322,0.997425536925159
"16008","SOX7",2.87197132335455,0.020319611675797,0.83738831602016,0.0242654587926061,0.980640864903981,0.997425536925159
"16009","PRKDC",578.07001587809,0.00433300375033252,0.172315368883041,0.0251457764819199,0.979938687359636,0.997425536925159
"16010","CSGALNACT2",237.59992035179,-0.00481157858169539,0.196400877683234,-0.0244987631341229,0.980454770296004,0.997425536925159
"16011","AP000941.1",3.52656432260678,0.0230442947086072,0.889500421856727,0.0259070081838803,0.979331510201649,0.997425536925159
"16012","AL138820.1",2.8023649182957,0.0261162915503585,1.00464524871625,0.025995535820958,0.979260899148008,0.997425536925159
"16013","CLMN",26.9540588161592,0.00937805353397248,0.400557639221475,0.0234124945218863,0.98132123855133,0.997425536925159
"16014","TANGO6",35.1680798864566,0.00737230423895192,0.286107604411737,0.0257675927702448,0.979442710484672,0.997425536925159
"16015","RASL10B",2.70352459557185,-0.0265588038484665,1.12731927535373,-0.0235592563962262,0.981204171808445,0.997425536925159
"16016","SOX9",151.623893232425,0.0105768487870143,0.430534319083882,0.0245667959978669,0.980400504257068,0.997425536925159
"16017","GAMT",282.238436766158,0.00660652815888698,0.280830958875766,0.0235249282534038,0.981231554114502,0.997425536925159
"16018","MAP2K7",192.030966695471,-0.00611264579435444,0.255765504377755,-0.0238994144625787,0.980932841341697,0.997425536925159
"16019","SLC7A10",2.69896807120105,-0.0215469642405488,0.913766876932809,-0.0235803734896524,0.981187327485097,0.997425536925159
"16020","AC011462.5",8.90737272839914,0.0164991811743074,0.664071785439179,0.0248454783595357,0.980178215756093,0.997425536925159
"16021","GGT7",127.001857004224,0.0052825810952326,0.207932116898901,0.025405315802181,0.979731671080731,0.997425536925159
"16022","C21orf91",89.1940844998558,0.0057520077806984,0.240943206393838,0.0238728780395507,0.980954008304683,0.997425536925159
"16023","FAM207A",285.12476807724,-0.0055308004628354,0.226828932623428,-0.0243831348975988,0.980547000729168,0.997425536925159
"16024","DEPDC5",41.3198713211761,0.00663402818528188,0.257976296639692,0.0257156501263658,0.979484140989422,0.997425536925159
"16025","TEF",106.488865194412,-0.0192721556569742,0.752216017543679,-0.0256205068856502,0.979560029308587,0.997425536925159
"16026","SPIN2A",69.5512721886417,0.00686829600521792,0.292540166523742,0.0234781298131944,0.981268883557054,0.997425536925159
"16027","OASL",23.645687157926,0.0267445548947484,1.14804470573951,0.0

232957434157767,0.981414367055999,0.997457953763972
"16028","E2F6",70.062569905182,-0.00553876445469424,0.239074018504223,
-0.0231675716556226,0.981516605731151,0.99746949203862
"16029","SLC22A5",47.3787591078034,-0.00699647921273826,0.302511542278
305,-0.0231279744238705,0.981548191287804,0.99746949203862
"16030","AC099343.2",2.78993051123429,0.0230994004283183,1.00318966985
32,0.0230259552330703,0.981629569153388,0.997489959572023
"16031","GNAI3",773.995884533224,0.00434108905191111,0.196425769940598
,0.0221004049174603,0.982367863478445,0.997673848760106
"16032","LCLAT1",34.2383782069978,-0.00864728684730224,0.3926315500968
1,-0.0220239225430817,0.982428872734519,0.997673848760106
"16033","IKZF2",47.7440810418457,-0.00839885243722193,0.39356898025720
5,-0.0213402296891719,0.982974252486399,0.997673848760106
"16034","HES6",15.2179658636356,0.0110730932879451,0.497021550579571,0
.0222788997278547,0.982225480282918,0.997673848760106
"16035","ERAP2",353.034196415422,-0.022013642581984,0.973291364687733,
-0.0226177313193843,0.981955199897971,0.997673848760106
"16036","LINC01023",25.4579348601156,0.00784494065875469,0.35494022653
6155,0.0221021458607639,0.982366474745877,0.997673848760106
"16037","TRIM5",96.2368228260717,-0.00515663775839971,0.23797120677303
9,-0.0216691667379649,0.982711859374004,0.997673848760106
"16038","CCDC65",3.67241102404346,-0.016574723258224,0.769859799855342
,-0.0215295346780523,0.982823243653518,0.997673848760106
"16039","PRKCH",14.9924947448513,0.0100638086699222,0.466316861785404,
0.0215814813802583,0.982781805809804,0.997673848760106
"16040","RMDN3",126.100264319378,-0.00368852525077613,0.17238451625509
3,-0.0213970798010529,0.982928903014761,0.997673848760106
"16041","FURIN",120.15326189204,-0.00499701699699344,0.226753496198352
,-0.0220372213913841,0.98241826436346,0.997673848760106
"16042","AC008915.1",3.79382597549596,-0.018481907846745,0.84866009893
2658,-0.0217777504444822,0.982625242551087,0.997673848760106
"16043","AKAP8L",178.397050535049,0.00344436352053919,0.15452268414667
6,0.0222903422857303,0.982216352709331,0.997673848760106
"16044","LINC00662",136.862398056135,0.00454100945963885,0.21235632303
5884,0.0213839145202731,0.982939404986153,0.997673848760106
"16045","FXYD1",2870.87489836027,0.00641059124668386,0.28341067952659,
0.0226194413611799,0.981953835830998,0.997673848760106
"16046","EID2B",58.44539110116,0.0059206786649953,0.267763590752122,0.
0221115897361725,0.982358941464528,0.997673848760106
"16047","GSS",117.600653608944,0.00557781571068421,0.25441918182111,0.
0219237231672498,0.982508800970504,0.997673848760106
"16048","USP18",104.959328041137,0.00885134834341958,0.406995893323534
,0.0217480040674105,0.982648971106234,0.997673848760106
"16049","IGLC1",72.4062516462158,-0.0289576224650394,1.3404746321218,-
0.0216025143416575,0.982765027846156,0.997673848760106
"16050","ZNF652",340.530126418085,-0.00409046863793929,0.1942455840159
79,-0.0210582323333683,0.983199203269084,0.99783998891278
"16051","AL513314.2",2.59467895789037,-0.0176043888916171,0.8710827560
08504,-0.0202097777394703,0.983876027971288,0.997880509700493
"16052","AC017033.1",6.36082702281505,-0.0146143670776776,0.7042741404

06351,-0.020750963636469,0.983444314654433,0.997880509700493
"16053","ANKRD44",56.0271057631083,0.00744363453940962,0.370576478941601,0.020086635181675,0.983974261575868,0.997880509700493
"16054","UBE2J1",353.244963014583,0.0025219463052500З,0.124617311805684,0.0202375277456033,0.983853891197224,0.997880509700493
"16055","IGHG1",130.962818100432,0.0215684632403764,1.05466787063586,0.0204504790947815,0.983684015761364,0.997880509700493
"16056","ZSCAN2",13.0049560134741,-0.0095084142166305,0.455744050829014,-0.0208634960770949,0.983354546191835,0.997880509700493
"16057","CLCN7",112.16824264911,0.00454767232177363,0.219481989176842,0.0207200250864751,0.983468994739502,0.997880509700493
"16058","AC018553.1",6.1906266273713,-0.0137014249013342,0.675675871901129,-0.0202781029649362,0.983821523498364,0.997880509700493
"16059","NTN1",24.9178340282267,0.0122319512190278,0.600336194460976,0.0203751686669676,0.983744092271225,0.997880509700493
"16060","AC008569.2",4.58832100868948,-0.0182302555371803,0.904266354676157,-0.0201602718523229,0.983915519908286,0.997880509700493
"16061","ZFP28",26.9144785465964,-0.00753599774105898,0.366161743609343,-0.0205810625292934,0.983579847179565,0.997880509700493
"16062","FMR1-
IT1",2.71133029375034,-0.0181029718768708,0.880033818544197,-0.0205707684129887,0.983588058957545,0.997880509700493
"16063","NEDD1",124.188721687087,0.00392104497698208,0.197966004965398,0.0198066581061098,0.984197606525708,0.997933699935948
"16064","PLIN3",515.524496285973,0.00466269028353713,0.235602555379789,0.0197904911346182,0.984210503374732,0.997933699935948
"16065","SULT2B1",10.9004489726766,0.0126877826943369,0.637804582327661,0.0198928998722979,0.98412880910689,0.997933699935948
"16066","TMEM52",3.47004084429884,0.0149513299990261,0.783495617097092,0.0190828508453204,0.984775011983106,0.998009094145014
"16067","TTLL7",41.8735224453346,-0.00776467810391022,0.400617672694936,-0.0193817662902328,0.984536556069829,0.998009094145014
"16068","CCNJL",3.13097071555106,0.0171557377927839,0.888998781878419,0.0192978192349539,0.984603523604118,0.998009094145014
"16069","CDK10",114.983147528052,0.00335734140453332,0.175655200602424,0.0191132479597475,0.984750763017445,0.998009094145014
"16070","MED11",232.612792455789,0.00510652739216073,0.260450922763942,0.0196064860817905,0.984357289683744,0.998009094145014
"16071","TMEM256",583.306815331457,0.00495236396253711,0.258133752842912,0.0191852630971157,0.984693313785317,0.998009094145014
"16072","ZNF625",21.4982531157512,0.0077604157751478,0.400939751234711,0.0193555658954975,0.984557457039458,0.998009094145014
"16073","ZNF773",22.0622362367507,-0.00761380252742661,0.393844888122689,-0.0193319826079723,0.984576270260357,0.998009094145014
"16074","SVIL",466.783259900673,-0.00466068733809273,0.246728578168172,-0.018889937163728,0.984928907090181,0.998102959287791
[truncated: 550,961 more chars]
