# Supplementary material for: PPARγ agonist treatment reduces fibroadipose tissue in secondary lymphedema by exhausting fibroadipogenic PDGFRα+ mesenchymal cells
Source: JCI Insight. 2023 Dec 22;8(24):e165324. doi: 10.1172/jci.insight.165324 (PMC10807713; doi:10.1172/jci.insight.165324)
Supplement: Supplemental data set 4 [file jciinsight-8-165324-s186.pdf]

```
"","gene","baseMean","log2FoldChange","lfcSE","stat","pvalue","padj"
"1","PLP2",645.162184256591,-1.58178013620185,0.175392489878645,-9.018
51691196294,1.90652773383564e-19,2.98238133403909e-15
"2","ANOS1",93.2249297919357,3.91128739027876,0.444823367341979,8.7929
0000804244,1.45748177189242e-18,1.13996936788566e-14
"3","CAV1",2601.75707344832,-1.52233726557857,0.188442521383231,-8.078
52311890176,6.55558470699162e-16,3.41830038571567e-12
"4","VEGFD",706.729658242378,1.83159985272054,0.230510258847253,7.9458
4962022989,1.92864279583643e-15,7.54243981381733e-12
"5","STEAP4",305.629418999477,2.20684905836752,0.291174119540094,7.579
13877048281,3.47856288550646e-14,1.08830318435955e-10
"6","PDGFD",870.998882218195,2.15342342793014,0.303169978279807,7.1030
233275363,1.22056792534404e-12,3.18222400935946e-09
"7","CD9",1303.78555477962,-2.24232181142358,0.344668368077187,-6.5057
3716390889,7.73133024337973e-11,1.72773141424556e-07
"8","PERP",199.923479545768,-2.24569867971208,0.347355128282874,-6.465
13754040005,1.01206712899426e-10,1.97897076235715e-07
"9","C1QB",50.9259251261301,-2.32854174957271,0.369163401221066,-6.307
6180950514,2.83362355423087e-10,4.92515258431483e-07
"10","LMNA",3605.85069238526,-1.02849884538313,0.163921164053525,-6.27
435054723804,3.51096687716525e-10,5.4922054859496e-07
"11","TYROBP",74.5942074021764,-1.78943839302955,0.286543821204116,-6.
24490308501492,4.24062969454124e-10,6.03056093742806e-07
"12","EDN1",31.1695735679481,2.63676525835564,0.432663034450152,6.0942
6978596995,1.0993811816326e-09,1.43313498535657e-06
"13","LUZP2",29.4376566196634,3.63467073653645,0.604526642741053,6.012
42439879254,1.82769075970051e-09,2.19927435030731e-06
"14","CRIP2",285.362261692395,-1.75743233481736,0.293276106418574,-5.9
9241566685659,2.06746657439363e-09,2.31009854451711e-06
"15","FXYD5",395.83845433287,-1.79030466200533,0.301288960054978,-5.94
215155337467,2.81305156618730e-09,2.93363770999119e-06
"16","S100A3",24.012466483664,-2.47199677915064,0.416905386496987,-5.9
2939515586832,3.04052576825279e-09,2.97268403704865e-06
"17","ADAMTS16",71.4563178849952,3.8607178753803,0.652449354727507,5.9
1726828665915,3.27332527170934e-09,3.01203689560877e-06
"18","GPC1",91.230262165066,-1.26856264279937,0.215157240897495,-5.895
97931962577,3.72465623848786e-09,3.19670373341393e-06
"19","MS4A7",25.5453618096978,-2.34107244745924,0.397525417842446,-5.8
8911385884536,3.88271884771877e-09,3.19670373341393e-06
"20","LARP6",555.718394965409,-1.05857097430269,0.182033364395827,-5.8
1525797655894,6.0540446918015e-09,4.73517105569254e-06
"21","C1QA",69.8472027223623,-2.13688282346453,0.368286809692638,-5.80
222469886423,6.54407838635648e-09,4.87471515227497e-06
"22","ADAMTSL1",280.154796487338,2.13307736182868,0.368753464889859,5.
78456222090224,7.27014105084180e-09,5.16940074810537e-06
"23","VEGFC",273.987129508053,2.20365893130051,0.385909770446442,5.710
29577393492,1.12779998849009e-08,7.67051096519585e-06
"24","GSTM1",12.1924993453051,6.66056011684563,1.18055719775849,5.6418
7836852965,1.68204913910978e-08,1.09634561179559e-05
"25","MARCKS",6501.48137331662,1.70656711355763,0.302955593119956,5.63
```

306026465043,1.77039557328168e-08,1.10777191811381e-05
"26","ARHGAP20",307.354027000119,1.53177273468556,0.272850443785538,5.61396460798702,1.97742676618999e-08,1.17957417957294e-05
"27","MAF",1063.03960693786,0.905483739466732,0.161436447711252,5.60891764099206,2.03595875781305e-08,1.17957417957294e-05
"28","ADIRF",1997.56438092906,-2.02729055822447,0.362707911653313,-5.58931992683474,2.27960622706251e-08,1.27356715035496e-05
"29","AIF1",64.0175522877645,-1.94858124473553,0.353803624985542,-5.50752198996026,3.63919963829539e-08,1.90602697551555e-05
"30","TMCC3",50.2978624183049,1.64820526816068,0.2996211768959,5.50096386789551,3.77720617790591e-08,1.90602697551555e-05
"31","DYNLT3",131.648284812029,-1.4564523588624,0.264500995059127,-5.50641542402069,3.6621377590733e-08,1.90602697551555e-05
"32","SYNE2",562.538788274244,1.32443396608909,0.243534010222398,5.43839427141858,5.37628982709628e-08,2.6281656801646e-05
"33","TRIL",137.301442759969,1.33940587831682,0.247817693336907,5.40480326598757,6.48795643402656e-08,3.07548795446901e-05
"34","IGSF10",502.352108624719,2.01500664851065,0.376844189403886,5.34705510969429,8.93968033258273e-08,4.11304174831152e-05
"35","IL17RD",145.88009442056,1.22575901055108,0.230014845968026,5.32904302499446,9.87316078030064e-08,4.41273868817837e-05
"36","KLHL13",67.5358747111642,1.95836320481578,0.37065765009494,5.28348249203588,1.26750982431813e-07,5.50768227272457e-05
"37","PLXNC1",141.659981000741,2.16147491530442,0.409905831728121,5.27310115640917,1.34137578902395e-07,5.67111931559504e-05
"38","AQP7",50.2130524663863,-2.62798855379661,0.499986795941916,-5.25611591171282,1.47129579479503e-07,6.05670529420491e-05
"39","NT5DC1",81.3700152308069,-0.964850853909342,0.185051558386826,-5.21395692271041,1.84854412444226e-07,7.41455788170519e-05
"40","CYP1B1-AS1",32.8098704153159,2.15062741981353,0.414084798559995,5.19368841187231,2.06168087404768e-07,8.06271847818197e-05
"41","CIDEC",14.3586023562982,-2.61265924795463,0.50683678450896,-5.15483352394373,2.53856354163478e-07,9.68554865409583e-05
"42","VGLL3",685.233636665027,1.7685549638636,0.343878403949528,5.14296607042289,2.70434478218406e-07,0.00010072396530406
"43","MYO10",22.9382932644775,3.92952687906592,0.76687500332948,5.12407740766802,2.98998136806531e-07,0.00010393839675699
"44","SEMA3E",76.6917714722745,-2.9241800283408,0.570504849464241,-5.1256006519259,2.965906664597e-07,0.00010393839675699
"45","MRC1",18.1495185373648,-2.60729194003089,0.50855950145711,-5.1268178700045,2.94680332521039e-07,0.00010393839675699
"46","APBB2",206.947716649948,1.12794729709209,0.220326945192276,5.1194251166499,3.06468426318194e-07,0.000104219252019468
"47","ST8SIA4",14.8536147640394,-2.35864852752743,0.461225869045479,-5.11386868305703,3.15626815507362e-07,0.000105050005850674
"48","KANK4",8.59932997864118,6.16506276803684,1.20755018104632,5.10542987347731,3.30043368889906e-07,0.000105364661623363
"49","FBN1",7495.23071521749,1.71872784775402,0.336635140543914,5.1056103203516,3.2972855302297e-07,0.000105364661623363

"50","SERPINA5",216.268610322669,-1.41810433512135,0.27815894340876,-5
.09817990298238,3.42934826089033e-07,0.000107290589690215
"51","CYP26B1",555.614051022641,-1.9112086753271,0.379179530799092,-5.
04037934563443,4.64609969717321e-07,0.000142507720711531
"52","MUC12",20.6117174409446,-2.20575739335143,0.438176460462639,-5.0
3394771828347,4.80480764010283e-07,0.000144541549834863
"53","CAPG",249.511801755641,-1.93775177446773,0.386291666156483,-5.01
629194786399,5.26782625957441e-07,0.00015548038901608
"54","TNFAIP6",276.804818925983,-1.69789032135493,0.340657767224198,-4
.98415267378153,6.22339266014874e-07,0.000180282465523531
"55","MS4A6A",38.2463392782774,-2.22678242508421,0.448503866250727,-4.
96491244032972,6.87320722099051e-07,0.000195486510105372
"56","PRDM6",38.7863663844587,1.4719545529769,0.297319231731708,4.9507
5459600659,7.3926275661356e-07,0.000206505130387606
"57","GPX3",4839.20814940457,0.836669973461511,0.169359468373722,4.940
20193553777,7.80417056376324e-07,0.000214176561629734
"58","PLEKHG1",30.4861142542676,1.7149394524843,0.34882848261981,4.916
28275192603,8.82029781116154e-07,0.000236319264720476
"59","MS4A4A",18.321759428363,-2.70097041798288,0.549622159553057,-4.9
142312969682,8.91314748993676e-07,0.000236319264720476
"60","ABCG1",33.4260612670562,3.40606274701286,0.693674294113822,4.910
17582158519,9.09947690562818e-07,0.000237238528724569
"61","HPSE2",26.2703238309747,-2.72679598548262,0.556414666865963,-4.9
0065440014631,9.55179553255492e-07,0.00024494875002583
"62","LILRB5",14.3121017757935,-3.18201541156681,0.654922469070641,-4.
8586138998746,1.18210398167681e-06,0.000298252461054361
"63","PHACTR2",584.976880302115,1.09007575422439,0.224829463471733,4.8
4845596921259,1.244261404679e-06,0.000308952081799897
"64","MXRA5",603.491109932249,1.58126530345448,0.328158518909212,4.818
60202413928,1.44567595227519e-06,0.000353354826897512
"65","FREM1",115.240464021405,1.48933546320038,0.309524926905593,4.811
68181861694,1.49665491647991e-06,0.00036018727474608
"66","LINC00968",18.7325842609234,2.92228720169889,0.609740056536627,4
.7926770930841,1.64570391078715e-06,0.000390056761764293
"67","SUCNR1",37.5471288061912,-2.656844751828,0.555357317061793,-4.78
402763446867,1.71817046329688e-06,0.000401154336676913
"68","AL161431.1",37.8066030366754,-1.45310815611851,0.304882139167054
,-4.76613080742754,1.87797362071087e-06,0.000431691373127046
"69","NATD1",75.0382509848555,1.13111845490186,0.237463354226992,4.763
33899427959,1.90415551657394e-06,0.000431691373127046
"70","SYCE1L",68.4280899869542,-1.56411960735146,0.32861623685428,-4.7
5971492560498,1.93866585377138e-06,0.000433236427864938
"71","AC091938.1",4.61868309182165,-5.47860063335059,1.15560851450642,
-4.74087942809125,2.12792544777687e-06,0.000468832926472867
"72","ABCA1",580.230775367495,1.75699877877175,0.371668690396959,4.727
32523392056,2.27496721745053e-06,0.000494268224758038
"73","SERTAD4-
AS1",43.5273582043836,-2.18718912287745,0.465350278248458,-4.700092006
1977,2.6004430344411e-06,0.000549712572807596
"74","ACSM5",18.8853981634999,-2.04986789106422,0.436132825829146,-4.7

0009999171043,2.60034134769244e-06,0.000549712572807596
"75","IL7",38.5676110276022,-1.5578353297064,0.332254676998475,-4.6886
7840711705,2.74975124588335e-06,0.000573524783191377
"76","AL356218.2",18.9693830608011,-2.06293751113888,0.441394718400819
,-4.67367964576681,2.95850687072405e-06,0.000608946354983372
"77","EHBP1",345.416937815774,0.949985331952556,0.203432063942267,4.66
979154388444,3.0150552577607e-06,0.000612526096066891
"78","IL33",423.259932477974,0.861186003414326,0.185502243815654,4.642
45599244687,3.44291985345506e-06,0.000690481990610224
"79","DBNDD2",471.849415489377,-1.71002487710533,0.36889559919162,-4.6
3552528371875,3.56032201070901e-06,0.000704988825487608
"80","CRIP1",5079.28033159717,-1.05949812664972,0.230703731463062,-4.5
9246202881359,4.38047230568585e-06,0.000856546603473046
"81","PDZRN4",23.9839417277246,4.03831740483496,0.881417041868179,4.58
161938448079,4.61389082409634e-06,0.000881749623109372
"82","PTGDR",36.8018157951756,-1.94737410074841,0.425075040506234,-4.5
812478155133,4.62209736591245e-06,0.000881749623109372
"83","RBMS3",986.342688869495,0.822275946937994,0.180495699853402,4.55
565394414295,5.22228520707746e-06,0.0009842434637869
"84","PRELP",1477.01802584106,0.759234436998079,0.16697644093646,4.546
95544317532,5.44275150913508e-06,0.00100833327267131
"85","APP",2125.6872384521,0.55913270945325,0.123006428194256,4.545556
82708102,5.47902117094299e-06,0.00100833327267131
"86","NCKAP5",15.012238464248,2.36197318010433,0.524699066332469,4.501
57686884027,6.74511616853487e-06,0.00122690525842315
"87","CD36",519.145016774717,-2.25342757831541,0.501073153268043,-4.49
720278090805,6.88533133894005e-06,0.00123484049385437
"88","FOLR2",18.0614554382982,-2.66343685029406,0.592491365872875,-4.4
953175754219,6.94661915611997e-06,0.00123484049385437
"89","MAP4K3",137.714421532845,0.990817201406613,0.220782867530056,4.4
8774496178571,7.19810248980392e-06,0.00126516760952812
"90","F13A1",17.0862254639955,-2.38425862889149,0.531602693207147,-4.4
8503865642085,7.29007168451091e-06,0.00126709545956449
"91","IL6ST",2083.81812842359,0.982014682273986,0.219597366501052,4.47
188733599535,7.75322739017426e-06,0.00133278830840106
"92","PHLDB2",215.329638061944,0.858144578365659,0.192398663565535,4.4
6024188766445,8.18672015355518e-06,0.00139200938437026
"93","FHDC1",35.500069227292,1.41289170324726,0.31698544800121,4.45727
623194193,8.30076116234587e-06,0.00139622372970512
"94","ANTXR2",949.257250593883,0.758197745527779,0.170405702071273,4.4
4936839737124,8.61231820372601e-06,0.00141813151221985
"95","TRIM47",228.010481558307,-1.09248196789413,0.245490506467439,-4.
45020047257522,8.57901690037247e-06,0.00141813151221985
"96","RNASE1",48.2913676665334,-2.283825936563,0.513922086401886,-4.44
391474309406,8.83366213367013e-06,0.00143942684121877
"97","SPP1",32.5598520252493,-2.71641274623176,0.613507469109693,-4.42
767673256488,9.52535172515631e-06,0.00152045996976143
"98","C16orf89",202.571722768173,1.36457808506972,0.308156682952859,4.
42819565681289,9.50246830636814e-06,0.00152045996976143
"99","CDH13",54.886434189568,-2.41527398599109,0.546701139998562,-4.41

790552329459,9.96619639680886e-06,0.00157475969934627
"100","C1QC",21.8692877223911,-2.09447869188886,0.475445710621609,-4.40529516850722,1.05639932636659e-05,0.00163616382795569
"101","LRRTM4",7.43246841116757,5.93133934921497,1.34617076162856,4.40608243640605,1.05256918775962e-05,0.00163616382795569
"102","RASL12",76.9064004616439,-1.45663357332386,0.33090280260347,-4.40199829636797,1.07258399400905e-05,0.00164494425669446
"103","VIM",59486.1777826422,-0.992476334356752,0.22585894062191,-4.39423089306952,1.11165615205929e-05,0.00168831428996732
"104","UCP2",91.6629762929532,-1.73901951009118,0.395940501629283,-4.39212331887031,1.12248997045368e-05,0.00168837601998143
"105","EMILIN2",415.588963225969,1.02400434800585,0.233354931060035,4.3881838851839,1.14301117218166e-05,0.00170286893013692
"106","LAMC1",2439.52229623766,0.987951542386679,0.226030434754121,4.37087838839663,1.23747704676988e-05,0.00182621258892653
"107","MAP4K3-
DT",187.128005287334,1.15858884077908,0.265460139345199,4.36445503131631,1.27440060695807e-05,0.00186312604622852
"108","NTM",297.979225601839,1.49498796068472,0.342958771747192,4.35908944118429,1.30604729755565e-05,0.00187435760327184
"109","MGAT4C",14.6265194880132,3.88533941499867,0.891287548892721,4.3592434560828,1.30512854447101e-05,0.00187435760327184
"110","IGSF3",41.0275910636282,1.43446364425741,0.330909855805594,4.33490758613166,1.45821369320533e-05,0.0019517462627159
"111","NMUR1",14.7698860780091,2.48273534633936,0.572064971172131,4.33995345188217,1.42512900772956e-05,0.0019517462627159
"112","TRIO",458.109740097898,1.13440255301314,0.261704447068374,4.33467052516981,1.45978592813246e-05,0.0019517462627159
"113","HRH2",33.9918672128151,2.85725254881334,0.657323857851621,4.34679574563428,1.38140748342182e-05,0.0019517462627159
"114","NRN1",265.253209916755,-1.00162735455641,0.230842147475286,-4.33901419437992,1.43123279373385e-05,0.0019517462627159
"115","TWIST1",596.575328691601,-1.23160070483193,0.284014416538619,-4.33640207367593,1.44833907413461e-05,0.0019517462627159
"116","PILRA",40.9707178188825,2.21763040327415,0.511397097020337,4.33641570551576,1.44824929780915e-05,0.0019517462627159
"117","FLRT2",492.646559855938,1.11660214971116,0.257131580036256,4.34253213686828,1.40849874434822e-05,0.0019517462627159
"118","SCIN",192.246568932354,2.31253303421124,0.534099695021505,4.32977785939034,1.49259830909946e-05,0.0019787046906138
"119","ADD3",2252.06269021234,0.617381723247822,0.142756565831841,4.32471683281498,1.52727915611476e-05,0.00200766620496665
"120","EMCN",127.888376584247,-1.60440400298125,0.371390586805284,-4.31999102826594,1.56035544368006e-05,0.00203049228167451
"121","MIR497HG",114.10046311006,1.23434663828018,0.285824580252847,4.31854614179175,1.57060388725063e-05,0.00203049228167451
"122","SMOC2",1820.70830492826,1.53425306218143,0.355975557771169,4.30999552831009,1.63257847218981e-05,0.00209331352790698
"123","CEP152",72.8613833032314,1.30854264719774,0.304177972902553,4.30189811152744,1.693411819945e-05,0.00215366187799997

"124","STAC",62.7727133531044,1.36350452994766,0.318857314132861,4.2762215872505,1.90092022574337e-05,0.00239807218478255
"125","AFF3",132.291921953779,-1.79918392712225,0.421475511199142,-4.26877452975472,1.96549818341279e-05,0.0024597030466501
"126","AC024132.3",5.56597972700113,-4.48574113631345,1.05448440330412,-4.25396631970832,2.10016903943538e-05,0.00260737653046728
"127","MAZ",409.337460258411,-0.813396897655354,0.191653604636074,-4.24409913499874,2.19473317732114e-05,0.00269514207435229
"128","VSIG4",17.1713498737116,-2.46726660819069,0.581488358290468,-4.24301978365357,2.20531985883202e-05,0.00269514207435229
"129","AC007262.2",58.3158445043196,2.6449077442625,0.62548899641118,4.22854400227339,2.35208518780268e-05,0.00285222237153468
"130","PCSK6",25.5423856459438,1.92185790751171,0.454756727383406,4.22612309348289,2.37751969422127e-05,0.00286088773666949
"131","SEPTIN11",905.870716670935,1.10277540098146,0.261236133024709,4.22137392792119,2.42817748975323e-05,0.00289954049406181
"132","EYA2",28.345163389786,2.59027586678038,0.613932922855569,4.21915126286484,2.45223714393681e-05,0.00290608679110633
"133","WASF1",111.455739749122,0.942820012912571,0.223582151969529,4.21688406076824,2.47701245723045e-05,0.00290916970950441
"134","SCARA5",2614.02212762508,0.709806792552818,0.168379415520608,4.21551999309527,2.49203312071592e-05,0.00290916970950441
"135","ATP11A",162.992628576071,0.806541696066749,0.191708825221088,4.20711824370425,2.58647824025398e-05,0.00299218892206497
"136","MYADM",1726.34940302662,-0.89827147191333,0.213578343800071,-4.20581720005374,2.60140441987366e-05,0.00299218892206497
"137","LYVE1",13.019100083119,-2.81650793369409,0.670009971110324,-4.20368062437435,2.62609409080299e-05,0.00299853940601688
"138","FAM43A",174.466083688865,1.30914676254191,0.312167216985797,4.19373557282114,2.74397830266447e-05,0.00311043859337538
"139","PLCXD3",17.0309684396119,-2.74868506893417,0.6558553606829,-4.19099276107486,2.77736522355006e-05,0.00312563483395637
"140","KCNQ1OT1",2218.87905966874,1.50666651046322,0.359853104165378,4.18689318786812,2.82798827073345e-05,0.00315987289422024
"141","TNFRSF12A",30.9172021999591,-1.43481170554007,0.343347473135498,-4.17889111702836,2.9293388014254e-05,0.0032499040333828
"142","TAGLN2",1257.03215573579,-1.01914682139488,0.244158900935212,-4.17411291372626,2.99149371097852e-05,0.00329548845921388
"143","CRIM1",305.459907838371,1.14394366323394,0.274562410489074,4.16642489842748,3.09413761606293e-05,0.00338472690406101
"144","ANKRD33B",79.6792899609095,1.13573866470477,0.273092474232094,4.15880616226552,3.19915228783959e-05,0.00347530133601908
"145","MMP23B",437.345196263345,1.69219976892123,0.40710118125566,4.15670562217929,3.22869581203614e-05,0.00348320610949526
"146","DKK3",69.7101133405972,-2.30227884594701,0.554477517307288,-4.15215905800398,3.29353212966857e-05,0.00352881665098667
"147","CAV2",264.39400940119,-0.750564569708029,0.181063027250906,-4.14532210746672,3.3933635813287e-05,0.00361104670086564
"148","YDJC",38.61528051536,-1.10954320828592,0.267819309507395,-4.14287980327751,3.42971719824011e-05,0.00362507203595068

"149","STK3",224.820785858966,0.819517797101008,0.197946805067175,4.14
009105538681,3.47167990443157e-05,0.00364479790235054
"150","KCP",70.2878757964508,3.39018468138865,0.819643835813575,4.1361
6809308834,3.53153540429141e-05,0.0036829205552887
"151","DLGAP2",11.1271771302134,2.68625953864185,0.651716420714435,4.1
2182270272871,3.75886453460413e-05,0.00384313188985702
"152","TET1",205.029465491319,0.668282033391486,0.162089215188051,4.12
292719547174,3.74087961713441e-05,0.00384313188985702
"153","C11orf96",907.76113617931,-1.42095428361991,0.344735731288708,-
4.12186540196465,3.75816772239709e-05,0.00384313188985702
"154","DUXAP8",295.658236592234,1.1775940464216,0.28688352431315,4.104
78102303354,4.04698040301148e-05,0.00411083860027978
"155","PLAGL1",1512.86245435876,1.07663941394974,0.262793209201628,4.0
9690728775145,4.18706495018635e-05,0.00420003408681845
"156","CPNE2",67.0857741096814,-0.901170124500921,0.219967742141835,-4
.09682854279537,4.18848889307472e-05,0.00420003408681845
"157","SLC30A1",131.507264801591,0.666649755306046,0.162973749580206,4
.09053456169002,4.30380067345122e-05,0.00428817540985971
"158","LMO7",60.0286659253499,2.05721706445946,0.503537128930538,4.085
53202189635,4.39759347779927e-05,0.00435389587172241
"159","CDH12",10.9091257702616,-3.59431214289606,0.882381019256947,-4.
07342413816066,4.63269460353647e-05,0.0045546671047531
"160","PLEKHA2",200.727806257769,0.859080718010872,0.210966184531557,4
.07212520773617,4.65861239378953e-05,0.0045546671047531
"161","S100A6",29772.621071133,-0.998425770758548,0.246205920774074,-4
.055246793495,5.00813975140867e-05,0.00486598323796806
"162","STAB1",16.0746526334846,-2.22558811276194,0.549297603776356,-4.
05169820050422,5.08472264655987e-05,0.00489749050143114
"163","RGCC",85.5377170881903,-1.44674765906662,0.357272883738972,-4.0
4941915525734,5.13449109655889e-05,0.00489749050143114
"164","PARP16",70.8232407229715,-0.857901732939004,0.211790804979269,-
4.05070339584845,5.10639011330642e-05,0.00489749050143114
"165","ROR1",119.962491726971,1.12674472134921,0.278877681605563,4.040
28287549683,5.33867701467588e-05,0.00506138936609544
"166","STARD10",81.8829955848838,-1.28674839735164,0.318631426943282,-
4.0383599624675,5.38262114000801e-05,0.00507230978874369
"167","PCSK5",576.500284398795,1.79768105777797,0.446325381858233,4.02
773655912979,5.63163939005562e-05,0.00527519371129581
"168","APBB1IP",343.414086177989,1.37934293440327,0.343013965460337,4.
02124424453723,5.78915403077007e-05,0.00539046050615096
"169","H19",2210.64618664517,1.51789221283245,0.378028040102275,4.0152
9000976168,5.93727418946528e-05,0.00549566746424883
"170","TPP1",393.356106622525,0.691834183221084,0.172846819972844,4.00
258554557022,6.26540024762091e-05,0.00575167299667858
"171","TNFSF14",40.6136995491799,1.87924745301443,0.46960560020298,4.0
0175690452191,6.28738785675406e-05,0.00575167299667858
"172","MEG8",344.147318700473,1.69168065557394,0.425142736251169,3.979
08869498954,6.91799307441735e-05,0.00629175381762271
"173","AC022424.1",8.91361224447201,4.69732925283768,1.1817926943234,3
.97474893473343,7.04535377397173e-05,0.00633561867634218

"174","CYGB",1243.14932859942,-0.898375502578842,0.226024288664306,-3.
97468567598556,7.04722655298562e-05,0.00633561867634218
"175","PXDN",332.162225526519,0.965221786134703,0.243170273770404,3.96
932474997352,7.20765945911521e-05,0.00644113805278498
"176","PPP1R1A",48.413104581925,-2.03467077302165,0.51294070491686,-3.
96667831879601,7.28812526588852e-05,0.00644113805278498
"177","AP000525.1",26.1787660744344,1.41630468283304,0.357038305224388
,3.96681437848225,7.28396768159736e-05,0.00644113805278498
"178","CSF1",714.728911485255,0.646856689950437,0.163277856501297,3.96
169268638884,7.44202883937081e-05,0.00650366799632836
"179","LINC00882",95.5091326490833,1.41234022777862,0.356467894565501,
3.96204047912961,7.43119372460066e-05,0.00650366799632836
"180","C3AR1",10.7239188067444,-2.29071593545356,0.57882899825515,-3.9
575003020906,7.57381917327335e-05,0.00658206962930639
"181","SGIP1",18.710871697826,-2.37024540712142,0.599940316166058,-3.9
508020102209,7.78897409579186e-05,0.00673165313704265
"182","HMGA1",50.9665530029697,-1.47183387096014,0.373433041026203,-3.
94135951900643,8.10210815090703e-05,0.00688811292416515
"183","PNMA2",14.7626596566245,2.99384091210266,0.759193117045665,3.94
345107309841,8.03173833793222e-05,0.00688811292416515
"184","KCNS2",8.68890047597248,4.65147210159984,1.18011449748903,3.941
5430549298,8.09590988137107e-05,0.00688811292416515
"185","MAN1C1",448.787318722315,0.94398716648127,0.240592764561721,3.9
2358917443297,8.72394528038302e-05,0.00737668519032603
"186","SUSD1",44.396574535908,1.5038842416206,0.385027159596474,3.9059
1729476108,9.38686156898799e-05,0.00789455244750963
"187","LMO2",47.1487931054584,-1.56440029646522,0.402044596934474,-3.8
911135529125,9.97861215594094e-05,0.00830294840188214
"188","MID1IP1",333.947426409332,-1.42887755543567,0.367164415334847,-
3.89165587883172,9.95623910939523e-05,0.00830294840188214
"189","FCER1G",52.2828685341858,-1.70091375815698,0.437546974408669,-3
.8873854869085,0.000101329764863535,0.00838678048550414
"190","CADM1",7.84691557831226,-2.18494021334149,0.563720320438221,-3.
87592948865667,0.000106218442673687,0.00874513209865522
"191","MRPL49",82.2415760745964,-0.869076826021424,0.224331134842424,-
3.87408028150835,0.00010702812886247,0.00876565978950588
"192","AC009271.1",17.2425622282591,2.08641892115324,0.538867400877763
,3.87185960359574,0.000108008162121925,0.00879985250038163
"193","IL10",12.4531572622674,-2.60399529980959,0.674354969685714,-3.8
6146082829818,0.000112711085025229,0.00908834795386423
"194","TBC1D12",179.092004769514,0.949909143851386,0.24595965042101,3.
86205274818622,0.000112438281271698,0.00908834795386423
"195","NID1",1169.16869398293,1.04357525763432,0.270522179397624,3.857
63289338443,0.000114490432830748,0.00909544240169053
"196","SLC4A4",37.3493979728973,2.70908161928346,0.702285854873208,3.8
5751984108033,0.000114543383822351,0.00909544240169053
"197","TNMD",129.407065155692,-2.13981806950371,0.554465196859281,-3.8
5924685917983,0.000113737002953995,0.00909544240169053
"198","FCGR2A",31.5507136701944,-2.00035953358373,0.519376366960401,-3
.8514642960954,0.000117413650440652,0.0091614848741638

"199","HS6ST1",97.8883947131174,1.10609514745148,0.28741646211139,3.84840568743347,0.000118889051298041,0.0091614848741638
"200","LAMA4",1983.8562806878,0.765639075961451,0.198858914389914,3.85016220324032,0.000118039626232277,0.0091614848741638
"201","LINC02518",7.23513002165738,3.4316594912957,0.890496855178537,3.85364582855004,0.000116371898340041,0.0091614848741638
"202","ZDHHC12",95.3463534716572,-0.892338687857414,0.231848223749958,-3.84880536682384,0.000118695267233808,0.0091614848741638
"203","FAM107B",168.056668002368,0.843959419235717,0.219278954771771,3.84879351561176,0.000118701008992204,0.0091614848741638
"204","KCND2",145.627912085048,0.760166605895157,0.198010330277302,3.83902498839625,0.000123523859947707,0.00947197912334301
"205","FKBP1B",37.1822633332073,-1.40719732916173,0.367125855420147,-3.83301069207262,0.000126584449364473,0.00965931971418758
"206","DOCK7",185.779115956574,0.703784712352735,0.183871860368739,3.82758248565799,0.000129408023483005,0.00973235438146462
"207","SESN3",357.030574930542,0.908069268895177,0.237202254686526,3.82824889289199,0.000129058211484508,0.00973235438146462
"208","MEG3",2259.6125237019,1.49331097193323,0.389953148307918,3.82946253521222,0.000128423431775192,0.00973235438146462
"209","FSTL1",7347.70384863384,1.09369740871148,0.286563915507637,3.81659151597661,0.000135307902810932,0.010079150112721
"210","PIM3",127.949635312734,-0.844567812247285,0.221242784676184,-3.81738013957569,0.000134876275279954,0.010079150112721
"211","OSMR",238.016858572997,0.762578456743075,0.1999233085893,3.81435492501593,0.000136539117972707,0.0101226607698913
"212","SERTAD4",44.1613451659551,-2.3583645645158,0.619996149527178,-3.80383743723947,0.000142471695074627,0.0105126638021339
"213","ESR1",41.0675422872148,1.40685610506159,0.369997310321407,3.80234143821071,0.000143335001808581,0.0105217711345949
"214","PTGER2",37.9544195546541,-1.53159759824567,0.402914426884413,-3.80129748663741,0.000143940358166804,0.0105217711345949
"215","GPR34",6.21443842712015,-2.50719408510126,0.661142704545082,-3.79221319068537,0.000149310647072469,0.0108635648937425
"216","PTMA",4566.90161948363,-0.709341630859888,0.187113734295678,-3.79096507014809,0.000150063059681907,0.0108677613083522
"217","COL6A6",219.855800322934,1.69405891326008,0.44736188059507,3.78677528583053,0.000152615000919491,0.0110016426699705
"218","PNP",14.6382329680693,-1.58626456347465,0.420098942756049,-3.77593086302004,0.000159411206636314,0.0114388509422562
"219","SPATA18",10.4137517577392,2.38042087756275,0.631011337376908,3.77239002940594,0.000161691238667969,0.0114969820294684
"220","TUBA1C",142.660820179203,-1.15251892830482,0.305477706177471,-3.77284137270315,0.000161398910315328,0.0114969820294684
"221","MLIP",6.43943606822505,-3.3240343349668,0.883202570708068,-3.76361487750415,0.000167474739074369,0.0118009339790106
"222","ZNF98",7.69877040735203,3.17006964995763,0.842140369611769,3.76430077971331,0.000167015757538682,0.0118009339790106
"223","ELMO1",46.4085027165698,0.962646494188273,0.255974280381539,3.76071569672317,0.000169427907557496,0.0118850258202777

"224","ST3GAL5",578.666307693098,0.832441706439975,0.221967949871679,3
.7502788439557,0.000176638034827565,0.0121190735912614
"225","FGFRL1",39.935133516788,-1.02617957276746,0.273579977552444,-3.
7509308318104,0.00017617930025625,0.0121190735912614
"226","AIF1L",64.5188722975912,2.02701858214045,0.540209225727471,3.75
228427358081,0.000175230601423366,0.0121190735912614
"227","ADGRD1",260.727137796998,1.25398091390396,0.334154261777157,3.7
5270064560846,0.000174939711958545,0.0121190735912614
"228","FRMD6",723.313267433236,0.916357511605935,0.244143097542365,3.7
5336235523482,0.000174478356746045,0.0121190735912614
"229","GNS",509.037655758103,0.797936825077035,0.213355896574493,3.739
93331278021,0.00018406908285259,0.0125737670876116
"230","SLC6A6",70.4973752725409,-1.33200878254025,0.35639540224062,-3.
73744659489446,0.000185898559072973,0.0126435267807761
"231","SPAG17",37.872511959468,-2.43858744946065,0.654351182296424,-3.
72672582465964,0.000193983272955862,0.0131362785231539
"232","TGM2",5.12881113036994,-3.10010357081873,0.832853144542839,-3.7
2226915529077,0.000197440391706626,0.0133127588252877
"233","S100A4",17458.3746488688,-0.875243213712853,0.235441815585009,-
3.71745015446008,0.00020124367619315,0.0134532257550831
"234","RAB13",393.730939358642,-1.12625719039387,0.302935206769841,-3.
71781544444108,0.000200952985820701,0.0134532257550831
"235","FMO4",51.2502658710631,1.08725800359384,0.292681782570438,3.714
81270219534,0.000203354260100895,0.0135364710245034
"236","ZNF727",69.0274167609626,0.889942229646069,0.239787329954013,3.
71138137205476,0.000206131266126123,0.01354836721013
"237","AQP3",85.3239546281587,-1.58011214303194,0.425526079425202,-3.7
1331445810877,0.000204562451823573,0.01354836721013
"238","HRCT1",225.762467467099,-1.80175835653331,0.485384431425197,-3.
71202337751737,0.00020560899142404,0.01354836721013
"239","MME",420.702081582524,1.04871386426308,0.282656766244017,3.7102
0258314894,0.000207093462879,0.0135546570703606
"240","HELLPAR",574.827656254768,-0.738151478255993,0.199222497429063,
-3.70516125328078,0.000211256291213712,0.0137695090144004
"241","PLEKHG3",32.4023595971131,1.32185029684966,0.357002564108435,3.
70263530221637,0.000213371500440258,0.013849669632311
"242","AC097518.2",10.2112520887224,2.7143678207582,0.733580178591911,
3.70016516254346,0.000215459194240419,0.0139083776066485
"243","CCL3L1",30.4163527748265,-2.56840597781985,0.694264112193781,-3
.69946527943672,0.000216054194106987,0.0139083776066485
"244","P2RY14",5.97482824031737,-3.00287302630986,0.812375607466053,-3
.69640963947252,0.000218670039109097,0.0139889409661492
"245","CXXC5",219.582716727693,-0.729017274842878,0.197249341650286,-3
.69591740455788,0.000219094197833315,0.0139889409661492
"246","TBCK",91.7890532223254,0.894435070025448,0.242150750923662,3.69
371173375968,0.000221004326911916,0.0140535393735085
"247","TBC1D2B",515.536516176868,0.664687580882591,0.180232155730935,3
.68795223131488,0.000226066076036813,0.0143172130665744
"248","ATP23",44.9797218511505,-1.20290537500639,0.326528352564493,-3.
68392320470488,0.000229671461968521,0.0144868979015064

"249","ADAMTSL3",113.01814654256,1.40089266486202,0.380387228590836,3.68280678100497,0.000230680004444017,0.0144920775482641
"250","NID2",624.706105086697,1.18287043475972,0.321330896161776,3.68115997835515,0.000232175264528285,0.0145276706520638
"251","AC074387.1",7.27872938308404,-3.70894137186399,1.01139028432843,-3.66717124866069,0.000245248556182257,0.0149260641942659
"252","AC137936.1",12.2928798116807,-2.2732405153092,0.619111773805719,-3.6717772323008,0.000240869599070616,0.0149260641942659
"253","ENPP5",11.2837145690575,2.48608828724562,0.677517665771402,3.66940732743113,0.000243113448346425,0.0149260641942659
"254","CNTNAP2",11.0792534922858,6.50549840058065,1.77444973899905,3.66620606805709,0.000246175577710197,0.0149260641942659
"255","RERG",145.179316637645,-1.67639696411379,0.457039025305841,-3.66795146867814,0.000244501577209432,0.0149260641942659
"256","ZNF610",5.08631274127276,3.88988799592568,1.059547791798,3.67127186337174,0.000241346451238176,0.0149260641942659
"257","SNTA1",202.975070445461,-0.828403299700461,0.225805812699196,-3.66865356475126,0.000243831218634246,0.0149260641942659
"258","SELENOM",3317.56914172539,-0.767964279857498,0.209228594551372,-3.67045566359688,0.000242118465508498,0.0149260641942659
"259","SEPTIN1",164.644509859889,-1.00817424790474,0.275210888896298,-3.6632789202052,0.000249007138596296,0.0150394543207021
"260","SLC7A4",22.8367659203771,2.73231939883198,0.74615743617225,3.6618537407449,0.000250396809080422,0.0150652203247886
"261","TMC3-AS1",13.031136552701,2.06209683693168,0.563803430712116,3.65747479458777,0.00025471229808173,0.0152661474287069
"262","FABP4",1036.40264820522,-2.36847538851165,0.647760532121285,-3.65640583373515,0.00025577630862035,0.0152714076173593
"263","CYP27C1",43.0080309816982,1.41086488368357,0.386432722790407,3.65099744528827,0.000261223838684329,0.0155373555457755
"264","HAS2",223.758222149668,-1.10630304891669,0.303760886571412,-3.64201942324989,0.000270507657201399,0.0159612538695633
"265","ITGA7",30.6878430176664,-1.39002676696647,0.381748636081956,-3.6412095174272,0.000271360183671616,0.0159612538695633
"266","SREBF1",160.319192974139,1.29241336219441,0.355133549834244,3.63923195315574,0.000273452409195357,0.0159612538695633
"267","KCNJ12",65.7177239113115,1.00791802116424,0.276831596101425,3.64090672942897,0.000271679551890694,0.0159612538695633
"268","ABCA6",1996.76446685191,0.937619116401971,0.257637112296288,3.63930145018739,0.000273378627039823,0.0159612538695633
"269","TUSC1",286.215965569619,-0.861784292167275,0.236949843369849,-3.63699034323537,0.000275842268383179,0.0160408944398441
"270","EPB41L2",1352.14433634223,0.621052601025696,0.171058921264849,3.6306355519694,0.000282724171651608,0.0163802008042448
"271","ARSI",10.5364016488907,-2.40090449562495,0.664282299605951,-3.61428341090701,0.000301179469434072,0.0173211413248426
"272","PI16",1288.81019702644,1.42441708061133,0.394038588470552,3.61491773214434,0.000300443012329179,0.0173211413248426
"273","MYO5B",4.44175763459598,4.46815727245888,1.23684438562795,3.612

5460279228,0.000303205264856148,0.0173737727404568
"274","IER3",221.108416602134,-1.56843920179636,0.434319313897024,-3.6
1125824159925,0.000304715055446332,0.017396560629003‌5
"275","NBL1",767.444966082918,-0.827801454205207,0.229691750894105,-3.
60396684244376,0.000313397055041851,0.0177006926377632
"276","CYP11A1",27.1787313742756,1.55225340779601,0.430491212742189,3.
60577257293662,0.000311225620634496,0.0177006926377632
"277","ZNF528-
AS1",65.1024492716749,1.08706649059488,0.301633304215408,3.60393390054
357,0.000313436799888795,0.0177006926377632
"278","AFAP1",136.863647595051,0.686290898821742,0.19054539653094,3.60
171859995738,0.000316120444983753,0.0177880292118016
"279","VCAM1",395.198748916794,1.73026050883192,0.481724912474254,3.59
180200987507,0.000328399311442599,0.0184127255516006
"280","KCNB2",9.29060543299946,3.28133923695548,0.913937008860229,3.59
033413150392,0.000330254336350615,0.0184179994986253
"281","CCND1",310.791175556006,-1.0447599003851,0.291030344762912,-3.5
8986586514273,0.000330848165896165,0.0184179994986253
"282","LIFR",291.743342342681,0.875822935284008,0.244232068836712,3.58
602758210906,0.000335753462245339,0.0185689720777658
"283","PROSER2",20.8159565664525,1.54698532708997,0.431409373831091,3.
58588714323035,0.000335934226044092,0.0185689720777658
"284","PTPRD",286.54950307251,1.04811695547194,0.292618472711885,3.581
85505432503,0.00034116307152172,0.0187256629046114
"285","PLAUR",149.583244185354,-0.853756584997428,0.238319874212019,-3
.58239776611283,0.000340454870725739,0.0187256629046114
"286","ALPK2",38.8087493558953,2.24483827007793,0.627704531491738,3.57
626583440951,0.000348537232967059,0.0190635242493136
"287","ACP5",19.3402515747933,-2.09868500847642,0.587113171723117,-3.5
7458341858862,0.000350785962568824,0.0191196683361119
"288","VAMP8",46.0452752034624,-1.1842381390611,0.331532544944438,-3.5
7201172892262,0.000354249541870026,0.0191748290085565
"289","TMEM35A",40.4354729664252,1.22600607598893,0.343155614128755,3.
57274083684065,0.000353264336821728,0.0191748290085565
"290","ARHGAP12",365.463106901156,0.535693987284279,0.150040482038098,
3.57032968707909,0.000356532206909971,0.0192318390092851
"291","SAMD9L",188.062516754911,1.10493270543981,0.309996058065817,3.5
6434437371206,0.000364766795315283,0.0196084088629449
"292","SEMA3C",1106.54120928642,1.46531089161471,0.412207315290168,3.5
5479108997186,0.000378279522951739,0.020235469192528
"293","RDH10",139.161381784083,1.1122620243818,0.313173294837087,3.551
58643063867,0.000382916207204511,0.020235469192528
"294","PFKP",145.205070688191,-1.02025844004092,0.287274817403134,-3.5
5150670449888,0.000383032233857042,0.020235469192528
"295","GRK5",163.688572521025,0.794054319707648,0.223546039963916,3.55
208403528786,0.000382192777652877,0.020235469192528
"296","CEBPG",116.829587033091,-0.773631104645943,0.217880666576693,-3
.55071019747238,0.000384193207836145,0.020235469192528
"297","AL354822.1",24.2592958995303,2.19473208473646,0.617760617448938
,3.5527225639596,0.000381266340601104,0.020235469192528

"298","SCHLAP1",4.47424885163116,-3.20649695328735,0.903790755060813,-3.54783110507874,0.000388417205347434,0.0203892964538587
"299","FOXRED2",75.4055049952039,0.805807878600211,0.227492242554984,3.54213343518934,0.000396904643655542,0.0207651482966677
"300","IL17RE",32.3640495923384,-1.19837013095367,0.338941832675198,-3.53562179532453,0.000406816664242881,0.021212776929171З
"301","DUSP10",19.5492604543222,-1.24619753326664,0.353494797874377,-3.52536314752079,0.000422902621791362,0.0219339381212853
"302","NIPAL2",170.526354006018,0.767150678876395,0.217630183574279,3.52501967455521,0.000423451340064448,0.0219339381212853
"303","TREM2",6.87700749627002,-2.52441571488368,0.717431535850169,-3.51868518281985,0.000433691019421799,0.0223165415026816
"304","OGN",3398.2614447853,0.935654580744011,0.265899531527437,3.51882748859775,0.000433458466232779,0.0223165415026816
"305","SPI1",15.3599045428616,-1.66037768531033,0.472379428940454,-3.51492377437889,0.000439880228039913,0.022560807892552
"306","CMKLR1",73.5569800970164,-1.52272110048258,0.433453266847027,-3.51299947871883,0.000443078346100256,0.0225767901239293
"307","LINC01426",27.1437065495375,1.84521361184643,0.525153398125299,3.51366594681384,0.000441968248709372,0.0225767901239293
"308","GALNT16",271.180219997266,1.0766080441356,0.307158849576119,3.5050529900777,0.000456516410225426,0.0231859941725855
"309","TEAD1",457.009439206862,0.772622013201409,0.220583810668687,3.50262338319051,0.000460700357606111,0.0233227692363508
"310","PLXNB2",176.516894697969,0.751540227978779,0.214796940192126,3.49884047373561,0.000467286066818073,0.0235798578814036
"311","KAZN",147.607281313785,0.985393072541803,0.2817448312365,3.49746637131615,0.000469699926173033,0.0236254531997581
"312","KCNIP4",6.98288116575765,-3.96235109848827,1.13373478346398,-3.49495416060343,0.00047414317973972,0.0237725056431681
"313","C14orf180",14.88936139006,-1.93586209006091,0.554091325290641,-3.49375996645586,0.000476269027254103,0.023802799978709
"314","SSC5D",445.889007157364,-0.651802250694716,0.186725506278389,-3.49069746113285,0.000481761458024162,0.0240006193881273
"315","CXCL3",94.447452764302,-2.93342258947569,0.840927079686082,-3.48831980838426,0.000486066319948014,0.0241382077553866
"316","ECM2",1548.40444903048,0.564556956362714,0.162020431759085,3.48448001423782,0.000493094286182444,0.0244097275909873
"317","SLC25A33",40.5053559017456,-1.39201113239537,0.400338017709086,-3.47708953638999,0.00050688851484533,0.0248550415765271
"318","PXN",367.851593746974,0.737961606890823,0.212220949902863,3.47732684840305,0.000506440040565915,0.0248550415765271
"319","LHFPL6",4383.44256332393,0.551924278026886,0.158769235283864,3.47626715616599,0.000508445522245649,0.0248550415765271
"320","MBP",560.493694465708,0.579762513442602,0.166738550517595,3.47707540723417,0.000506915227919617,0.0248550415765271
"321","THBD",24.4943212631816,-1.5706944950431,0.452022988137436,-3.47481109647798,0.000511213211420723,0.0249124868107613
"322","TRIM7",31.0710486182325,-1.4019081448246,0.403785193076453,-3.47191568403836,0.000516758611324179,0.0251045184998265

"323","KRT222",635.528860750118,1.23484284746527,0.355758160526522,3.4
7101763073462,0.000518489955465052,0.0251106451186991
"324","AC087854.1",19.7575351542777,1.80589730575325,0.521054402849872
,3.46585173424506,0.000528554665759925,0.0255190760385262
"325","PODN",2572.32712574826,0.592297168511936,0.171593239416908,3.45
175119092469,0.000556961060855883,0.0268078211537495
"326","ABCB5",55.4779309742752,1.69826555899004,0.492389351445356,3.44
902982569579,0.000562604556329031,0.0269963897995553
"327","CD163",24.7785354479459,-1.9829760299087,0.575865916176539,-3.4
4346830434881,0.000574303906848177,0.0273897439476403
"328","APOC1",424.548681509506,-1.50280849345608,0.436323610592858,-3.
44425205735287,0.000572641583256641,0.0273897439476403
"329","ACADVL",750.431404906125,-0.567805556389305,0.164960351979848,-
3.44207289554443,0.000577274666000466,0.0274477434657911
"330","CCDC18-
AS1",309.542121002279,0.753163786955581,0.219295478061865,3.4344702116
6167,0.000593713322716753,0.0280990798294484
"331","CDC14B",186.135358190573,0.611602486179453,0.178139044590184,3.
4332871133696,0.000596310278457154,0.0280990798294484
"332","A1BG",54.174817358635,-1.47804786043568,0.430508134449654,-3.43
326349065428,0.000596362238916887,0.0280990798294484
"333","CTSO",439.461562834594,0.605277033207111,0.176520740725412,3.42
892869540274,0.000605968706116286,0.0284659713807119
"334","MUC20",38.3719910784874,1.98073060741052,0.57792527406143,3.427
31265841815,0.000609586759908954,0.0285501966624424
"335","KCTD16",25.6058034806267,2.44301979729946,0.713052651600575,3.4
261422236011,0.00061221972173018,0.0285879197224633
"336","C20orf194",109.984983698078,0.690096725689969,0.20163011740329,
3.42258753095736,0.000620281230806698,0.0288781526592535
"337","PLD5",11.5413737620978,2.42107655958995,0.707586838714555,3.421
59637110863,0.000622546578483548,0.0288976146208253
"338","MAOA",117.950933604608,-1.69205053171506,0.494789218822589,-3.4
1974009809975,0.000626809908700461,0.0290094301828441
"339","PPP1R3C",5.23237667947632,-2.47049379309671,0.723082844251114,-
3.41661237400172,0.000634054893702829,0.0291043752938855
"340","WDR81",87.0631667053282,0.703065199411064,0.205788485612955,3.4
1644576136967,0.000634443008068462,0.0291043752938855
"341","LYL1",9.25104863725571,-1.65487596058335,0.484310129806375,-3.4
1697573256409,0.000633209237154723,0.0291043752938855
"342","SCN9A",2.95860774705528,-4.27723006079324,1.25338990796555,-3.4
1252951983302,0.000643629555846458,0.0292683056456574
"343","IRX1",110.626738013067,-0.823340637237928,0.241244961793727,-3.
41288220535736,0.000642797199938605,0.0292683056456574
"344","CTHRC1",1974.75580510385,-1.0862070562309,0.318239886543791,-3.
41317069971186,0.000642117083036865,0.0292683056456574
"345","ASPH",1291.71378279264,0.571886872020877,0.168078525715988,3.40
249814534445,0.000667728084511488,0.0302761461623571
"346","AGTR1",950.546479628906,0.776394224488825,0.228439031228711,3.3
9869338577043,0.000677085763799148,0.0305234945334584
"347","MAN2A1",156.24047975519,0.915622035569324,0.269383245860169,3.3

9895687515995,0.000676433811810072,0.0305234945334584
"348","NRK",26.4374891562892,3.84375636572478,1.13139832735661,3.39735
022828372,0.000680418227212028,0.030585581403097
"349","SCUBE2",13.2123840752066,2.16922023865366,0.638713505778541,3.3
9623355233353,0.000683200375157344,0.0306226460417946
"350","SLC6A15",9.15740517739953,-2.34520893842118,0.691525582753176,-
3.39135528303116,0.000695478811567722,0.0310839287124396
"351","PRSS12",26.7759007021564,2.01748725780902,0.595364439651595,3.3
8865932098606,0.000702352133042958,0.0313016934962706
"352","TKT",449.509127041293,-0.561845033937978,0.166013866287717,-3.3
8432593916132,0.000713532443197864,0.0316703808572468
"353","SH2D3C",8.27220726099807,-1.80244804554646,0.532656088287723,-3
.3838870618012,0.0007146739399481,0.0316703808572468
"354","RECK",577.012063739769,0.68583696510813,0.202959649841364,3.379
17889415058,0.000727026825972705,0.0321267814652289
"355","TGFBR2",1871.01142067519,0.522416511476383,0.154806902773861,3.
3746331857018,0.000739141400959038,0.032570109676626
"356","CKB",617.270015104821,-0.783815132854589,0.232323803524131,-3.3
7380466816082,0.000741369547894944,0.0325765276340466
"357","CDA",39.4570209260392,-1.94901058071789,0.57837045874708,-3.369
8307913928,0.000752143544204939,0.0328381474058145
"358","ALPL",543.064414461839,2.51183334598678,0.745508220841866,3.369
28993640109,0.00075362110328501,0.0328381474058145
"359","PTPRT",10.7340554713967,-2.51719114382856,0.747077866788561,-3.
36938257138995,0.000753367843076102,0.0328381474058145
"360","NEGR1",1609.50861970457,0.64303652489296,0.191093030543475,3.36
504436118965,0.000765313534178968,0.0332549989310044
"361","EIF4G2",917.147452643503,0.42501450009661,0.126397193378134,3.3
6253115071252,0.000772314118484894,0.0334662320095823
"362","UBXN11",56.1771073580805,-1.07808746072186,0.321332556326323,-3
.35505207765822,0.000793500449205951,0.0342893025605765
"363","FLNB",197.652806440707,0.924049903440372,0.275867952167869,3.34
96094641616,0.000809255722658039,0.0348737941309634
"364","AL512308.1",17.1490415748788,1.61961546684458,0.483656593396344
,3.34868890232899,0.000811949092269984,0.03489373530324
"365","SPG11",169.043833937699,0.678698615696415,0.20283544405241,3.34
605531526852,0.000819700416338456,0.0351303386651575
"366","TGFBR3",1387.40458290549,0.556053770772378,0.166331997794315,3.
34303548412849,0.000828673027627821,0.0352253591608206
"367","RHOBTB3",1277.54748457855,0.713809980034219,0.213507128692089,3
.34326064149196,0.000828000902843753,0.0352253591608206
"368","ID1",271.695054815842,-1.31274877004502,0.392599851576612,-3.34
373221175007,0.00082659484115891,0.0352253591608206
"369","ANKRD36B",160.31924130007,-1.24957303452241,0.373957961257667,-
3.34147996293471,0.000833330310804371,0.0352318541943588
"370","NUDT11",23.0733963314892,-1.20617107783631,0.360939998282899,-3
.34174955276342,0.000832521414271999,0.0352318541943588
"371","AKAP12",796.273477729862,0.868558028709252,0.260042868832127,3.
34005709370157,0.000837611680682015,0.0353174111075708
"372","APCDD1L",10.4725264285251,2.50701769282139,0.751337294302898,3.

33674065141069,0.000847670076279995,0.0356454381807741
"373","PLXNA4",208.375829293666,1.06183025931206,0.318404393966381,3.3
348480091144,0.000853460337603094,0.0357927079386735
"374","DAB2",1727.00350601829,0.587718576996618,0.176361556055065,3.33
246422941016,0.000860805354419082,0.0360042196769457
"375","PLAT",219.027378319199,1.04310038785499,0.313273075663977,3.329
6841282774,0.000869445564443626,0.0361721727781692
"376","SERPINA3",157.527069057251,-1.44731500292557,0.434624251098869,
-3.33003738117764,0.00086834325595307,0.0361721727781692
"377","LINC01625",12.1773775834441,1.79328481632669,0.538773627377914,
3.3284569347876,0.000873285052748455,0.0362355386741222
"378","GPR88",9.00424986550483,3.23452084474357,0.972542828842694,3.32
583897471392,0.00088152839160006,0.0362888121836835
"379","DENND2A",158.3280821934,0.824830664410179,0.247949355718018,3.3
2660942804878,0.000879094955563232,0.0362888121836835
"380","FZD10-
AS1",29.0860773423505,-1.68951936220351,0.507886900116761,-3.326566134
73412,0.000879231529894891,0.0362888121836835
"381","IGSF6",11.9746549274029,-1.67291294179749,0.503283631100987,-3.
32399632814962,0.000887373642635835,0.0364335587710036
"382","PDE11A",9.97154335055716,-2.01576853451945,0.607305801433119,-3
.31919854834689,0.000902762166808042,0.0369683470559639
"383","KHDRBS3",289.275342447783,-1.002128130671,0.302284037259113,-3.
31518706630213,0.000915818160742243,0.0374050743824828
"384","AKR1C1",1820.21155847851,-1.11699447711131,0.337193446666626,-3
.31262214065404,0.000924257586711723,0.0376514620545091
"385","TRABD",105.004304053107,-0.641086194700841,0.193622170578966,-3
.31101646461186,0.000929577390009132,0.0377698158750983
"386","MIR4435-2HG",234.837795333462,0.936515801977474,0.2829447367489
31,3.3098894601757,0.00093332822401415,0.0378104293885271
"387","ARL3",325.348647053576,-0.677230250350675,0.204646700352496,-3.
30926542760852,0.000935411121483091,0.0378104293885271
"388","PDCD4",631.653641163077,0.52556245497813,0.158912430747557,3.30
72457107715,0.000942182099114851,0.037985965403231
"389","LRRN2",9.18231689121845,3.52194826921388,1.06521294746911,3.306
33257658184,0.000945258205444516,0.0380120157012045
"390","CFH",8983.82013419005,-0.968627092959385,0.293337478004151,-3.3
0209115981313,0.00095966875696043,0.0382989216850032
"391","CPB1",716.142871121214,1.28649385464741,0.389602117588742,3.302
07100158889,0.000959737729369127,0.0382989216850032
"392","LDLR",31.7232091346192,-1.38126191733975,0.418137448552151,-3.3
0336812003453,0.000955308916396325,0.0382989216850032
"393","SPRED2",131.995724441663,0.675334292536615,0.204611410474882,3.
30057004626103,0.00096488624293641,0.0384064007589167
"394","CDKN2C",326.866800831337,-0.713562192204415,0.216444624466242,-
3.29674249921464,0.000978131340586484,0.0388347933015086
"395","OLA1",200.342497343646,-0.637653845752376,0.193544997829861,-3.
2946025622058,0.000985609700362047,0.0390326393487683
"396","CYB5R2",25.9804080599801,-1.06410576640701,0.323069615437304,-3
.29373520616182,0.000988655867706053,0.0390544033801156

"397","PXYLP1",73.3008978290487,0.737341734911815,0.223963124347323,3.29224615463188,0.000993905774827864,0.0391628917774113
"398","ATP1B3",344.12216543374,-0.792556144535199,0.241101717467838,-3.28722728671944,0.00101179135448529,0.0397545861208425
"399","DOCK4",86.6588885659904,1.05857685621412,0.322087580295869,3.28661184402614,0.00101400497744782,0.0397545861208425
"400","CAB39L",640.08366356977,-0.976256624134338,0.297402678305867,-3.28260871655734,0.00102851317790638,0.0402225791049738
"401","EHD4",153.870974170486,0.673746059394595,0.205544895082329,3.27785352744827,0.00104599657128513,0.0407027969268987
"402","RIPOR3",178.342770379443,0.892304707462043,0.272204355156587,3.27806918059316,0.0010451977638317,0.0407027969268987
"403","SLC4A3",14.379076983531,-1.62735207299306,0.496705583892338,-3.27629107818888,0.00105180098205533,0.040716960970263
"404","KANK1",198.460641885081,0.775786388575015,0.236753158918306,3.27677312572927,0.00105000703154915,0.040716960970263
"405","CNTNAP4",17.6591940161161,-1.46260642698218,0.446507961036924,-3.27565587763671,0.00105416922540155,0.040716960970263
"406","AKT1",219.602603798852,-0.570116545964901,0.174129544528513,-3.27409428140753,0.00106001237544938,0.0408418068698391
"407","ATP6AP1L",27.5189743061647,1.38252651709927,0.422919063732977,3.26900968922075,0.00107924605737881,0.0414807028883947
"408","PDZRN3",131.541586905746,1.17961953425026,0.360947998523497,3.26811490595776,0.00108266400666899,0.0415100810203995
"409","SULF1",123.120635819945,0.807440260849406,0.247125156177777,3.2673333356178,0.00108565768721143,0.0415230885111207
"410","ENO1",1215.4416086697,-0.675874689020403,0.206947049716646,-3.26593053607586,0.00109105009786174,0.0416152378193458
"411","ENTPD1",58.6815137719771,2.77696423134436,0.850440526851983,3.26532443323656,0.0010933876330468,0.0416152378193458
"412","PLD1",101.461439188879,0.829755615137256,0.254282893879291,3.26312007260364,0.00110192822164058,0.0418385028425331
"413","ADM",2415.46923072491,1.21803124841217,0.373372739415916,3.26223936519198,0.00110535765465788,0.0418670939269085
"414","VPS51",331.105809414846,-0.756798089405114,0.232134350835817,-3.26017276926145,0.0011134436687189,0.0420714959173184
"415","SVEP1",3341.05856841213,1.30076214679914,0.399107604385938,3.25917655415379,0.00111736108686697,0.0421177818840001
"416","LRRC6",36.8983753221264,1.03432283438737,0.318027296751624,3.25230835513835,0.00114471761664556,0.043045234800929
"417","ZNF608",15.687093883154,1.43693275434796,0.441975377217513,3.25116019673827,0.00114935076125607,0.0431158128497092
"418","NR2F2-AS1",70.7334318329632,1.11180952386537,0.34229951893065,3.2480604335603,0.00116194588667921,0.0434840179553178
"419","ZMAT3",175.480594268858,0.764546636089716,0.235708208118305,3.24361481593369,0.00118023232378669,0.0438536205249292
"420","NR2F1-AS1",34.289203511313,1.45717401562554,0.449166395376852,3.24417416490601,0.00117791698366626,0.0438536205249292

"421","LAMB1",526.512000149416,1.39797706630008,0.430914692710739,3.24
420840121713,0.00117777540405312,0.0438536205249292
"422","ABCA8",3918.45045557589,0.921882412784482,0.284279263578377,3.2
4287604090517,0.00118329681592044,0.0438632987948896
"423","INKA1",20.8314521516544,-1.24266447173558,0.383551480040112,-3.
23988965341921,0.00119575964494793,0.0441161984101897
"424","CPZ",8.89663081399468,2.18007827316133,0.672818279187872,3.2402
1855617953,0.00119438114552217,0.0441161984101897
"425","MAST4",200.052696346265,0.914848135502552,0.283047529392283,3.2
3213609200821,0.00122868523835907,0.0452242898438847
"426","DDIT4L",25.6922887493773,-1.54118074378156,0.477588560538717,-3
.22700514862232,0.00125093209252801,0.0459350486465158
"427","SNHG18",121.40581325895,-0.803520823214753,0.249426629232312,-3
.22147168362832,0.00127534064133436,0.0467216713170805
"428","IL20RB",8.43078560260737,-1.76048672658395,0.546799412884157,-3
.21962073312781,0.00128360296294945,0.0469144886668652
"429","FBXO31",62.1383597266511,0.674768343577037,0.209730209156028,3.
21731593313315,0.00129396025372543,0.0471827977832795
"430","ZNF426",104.074694982834,0.64257050499056,0.199771870214412,3.2
1652144669266,0.00129754835038524,0.0472036019652937
"431","STK17A",139.338387087541,0.770928252633873,0.240048051865841,3.
2115580469894,0.00132017297021169,0.0479152338121148
"432","EXOC4",164.845109275807,0.520415327104069,0.162160369074492,3.2
0926333649997,0.00133075549727316,0.0481875190829723
"433","IRF4",55.3617822025503,1.29566277983585,0.404068781999506,3.206
54016730605,0.00134341548337544,0.0484943377453027
"434","CX3CL1",12.2808591214992,-2.17522638815686,0.678462957182179,-3
.20610928736789,0.00134542879124601,0.0484943377453027
"435","CLSTN1",367.832973808765,0.504597540822185,0.157644945840463,3.
20084819803128,0.00137023700218161,0.0492239208496211
"436","OSBPL10",91.1906484448109,0.954625148757426,0.298296755833686,3
.20025320452922,0.00137306904097094,0.0492239208496211
"437","MRC2",1691.26222634534,0.737644356057842,0.230526467439147,3.19
982501034317,0.00137511049103653,0.0492239208496211
"438","IQSEC3",10.3026812210486,1.89313864385678,0.592339768051653,3.1
9603502240575,0.00139330198445228,0.0497285487164608
"439","LASP1",398.653386452329,0.507651016865697,0.158861033906341,3.1
9556661808579,0.00139556561315133,0.0497285487164608
"440","FBLL1",5.49302495924132,3.41228225303829,1.06932413113783,3.191
06448052136,0.00141749640990577,0.0503952189548997
"441","BOP1",46.125344876907,-0.91962744534271,0.288281108644193,-3.19
00371469632,0.00142254507438908,0.0504600285684089
"442","OXT",34.2256554210315,-1.07738791471662,0.338438923418293,-3.18
34042722829,0.00145554233562879,0.0513974012556234
"443","NFATC2",29.8663296247092,-1.58761913152491,0.498714892686955,-3
.18342033655983,0.00145546157375788,0.0513974012556234
"444","C5orf38",179.139545743799,-0.963131213983917,0.302771528942215,
-3.18104947763346,0.00146742565423757,0.0517003142099961
"445","PRR16",52.4366622723332,-1.35761760367326,0.426918358563213,-3.
18004034364392,0.00147254549775602,0.0517641106098819

"446","PTPN3",3.7653491179032,-3.00428387794398,0.945677764443387,-3.1
7685790118187,0.00148879963258631,0.0522181449608692
"447","FCGR2B",17.2741785231842,-1.85550426565244,0.584433777844358,-3
.17487512870377,0.00149900993714407,0.0524586408204579
"448","AL731577.1",5.6846958903862,3.61630877141671,1.13950599218514,3
.17357591466631,0.00150573519473645,0.052459277619738
"449","GPR183",75.0870160138119,-1.59150533875122,0.50141238126204,-3.
17404475482925,0.00150330508937173,0.052459277619738
"450","CARF",73.3427949055341,1.10140959809143,0.347636104096981,3.168
28311303411,0.00153342114902781,0.0533051267427602
"451","ZBTB16",828.852878470513,-1.24464549428727,0.393106563920918,-3
.1661783559983,0.00154456057125664,0.0535733060225447
"452","GAB1",169.364103361392,0.635810024466111,0.200922613542452,3.16
44522896462,0.00155375133955223,0.0537728588597689
"453","EFHD2",109.978451782255,-0.638345816683366,0.201779853487686,-3
.163575578284,0.00155843881740944,0.0538160230038318
"454","SHMT1",63.3598815925104,-0.780842534082612,0.247030289724031,-3
.16091818114664,0.00157272668896244,0.0540706892207461
"455","SLITRK4",92.9231758475451,-0.916555320825677,0.289958430977735,
-3.16098868977552,0.00157234603690779,0.0540706892207461
"456","CXCL16",76.432333749391,-0.785930707974043,0.248785647975742,-3
.15906771298429,0.00158274712051069,0.0542958622941858
"457","NOTCH4",25.7328488398018,-1.38450569134298,0.438631144964513,-3
.15642358559604,0.00159716730387748,0.0546706523732065
"458","SLC9A9",198.408181104924,0.588664710488502,0.186542499684244,3.
15566003181538,0.00160135391865204,0.0546942780556199
"459","UPP1",116.42348334417,-1.12540313641054,0.35725694985616,-3.150
12244510192,0.00163202047109442,0.0556202532229412
"460","FPR3",8.61975579761617,-2.37345840056218,0.754378175686176,-3.1
4624478419369,0.0016538152749595,0.0562405051004161
"461","S100A10",20786.6534241185,-0.889812994353768,0.282945171252125,
-3.14482480975397,0.00166186315707327,0.0563915951542236
"462","GBA",138.231309981582,0.573270011745941,0.182401819822173,3.142
89633899944,0.00167285070891311,0.0566415663193242
"463","TMEM250",106.580742318766,-0.613306847191656,0.195415450436335,
-3.13847674696258,0.00169828409063554,0.0573785270622283
"464","LINC00963",260.884620230549,-0.509558831858921,0.16249002823188
7,-3.13593909363925,0.00171304775862056,0.0577525993278048
"465","STX16",386.877857693974,0.700519429895989,0.223451344220174,3.1
3499760916965,0.00171855512167869,0.0578136726202577
"466","AKIRIN1",165.430674009275,-0.58049454815719,0.185258271817496,-
3.13343389454185,0.00172773831314162,0.0578736840095811
"467","LINC00702",70.0371893355314,-1.69677368139986,0.541483696376728
,-3.13356374855535,0.00172697400781879,0.0578736840095811
"468","ABCC3",11.3483253526707,-1.56964679182283,0.502256873360357,-3.
1251872798098,0.00177691891359464,0.0593938943704293
"469","EBF1",2117.14687943726,0.466824271915633,0.149483192511815,3.12
292147412317,0.0017906551637109,0.0597055205078404
"470","SPON1",134.552747093467,2.44089623364069,0.781739055976027,3.12
239258737451,0.00179387551228569,0.0597055205078404

"471","COL5A1",1145.749097427,1.19108369517236,0.381778677925396,3.119
82770133933,0.00180956853346598,0.0600999587452407
"472","IRX5",83.9701837851178,-0.752352091008606,0.241744117707812,-3.
11218365163262,0.00185708930789495,0.0615475594139844
"473","RGL1",221.761321640757,0.505620481156496,0.162567418685638,3.11
022027196133,0.00186947873935132,0.0618140440558083
"474","SLC38A4",5.52663090659776,2.74905563669563,0.884037590869706,3.
10965921029573,0.0018730331063385,0.0618140440558083
"475","LSMEM1",21.0198521417489,-1.79990135681359,0.579216041617673,-3
.10747843203153,0.00188690753387404,0.062140830636614
"476","GTF2A1",173.842567653999,0.483912811956463,0.155801908547783,3.
1059491919385,0.00189669302073415,0.0623318674860176
"477","MAN1B1-
DT",16.5213309603648,-1.30730137836878,0.421097570276954,-3.1045094311
7285,0.0019059485126775,0.0625047223979332
"478","GALC",134.993050980584,0.736452650252281,0.237301825198117,3.10
344283967237,0.00191283181455826,0.0625992219145081
"479","SEMA3B",83.1846710454886,1.45471214289337,0.468898702524428,3.1
0240172357394,0.00191957272018844,0.0626886765384295
"480","FOLH1",35.0437368942448,1.67300619337798,0.539712605960858,3.09
980937058066,0.00193645229815713,0.0631081735418166
"481","BCL7A",77.8188338212543,1.09083959205486,0.352157448352027,3.09
759057251125,0.00195100767653238,0.0634402750663537
"482","ADGRG4",12.6237804167772,2.23878705301499,0.722883809170945,3.0
9702198972002,0.00195475372895113,0.0634402750663537
"483","TXNIP",13390.1571846271,0.730893119580545,0.236191540689659,3.0
9449321278146,0.00197149445461042,0.0635878098009708
"484","LRATD1",4.20100844785944,-2.94295981192314,0.951001998437087,-3
.09458846223217,0.00197086151711159,0.0635878098009708
"485","IGFBP6",9530.06741475039,-0.840299160774476,0.271451621058371,-
3.09557613801755,0.00196430934729008,0.0635878098009708
"486","HIVEP3",32.8857681617147,-1.00416461404916,0.324710480898382,-3
.09249215261213,0.00198483481414795,0.0636812361092813
"487","CTNND2",13.7468582700128,1.87362787549577,0.606097990894039,3.0
9129530809372,0.0019928532994804,0.0636812361092813
"488","POLR3G",32.6270739403108,0.97548626409212,0.315490129969131,3.0
9197078269157,0.00198832418284108,0.0636812361092813
"489","CDO1",441.6180550344,-1.08498253024236,0.351080665348937,-3.090
40809514247,0.00199881652685911,0.0636812361092813
"490","GAB2",55.6326962977295,1.00360868428125,0.324687635560077,3.090
99754460948,0.00199485283529059,0.0636812361092813
"491","APOL1",253.054985381886,0.897490930718676,0.290327858711057,3.0
9130145037816,0.00199281207229727,0.0636812361092813
"492","RASSF4",199.991814997946,0.808615371302219,0.262200710752698,3.
08395568029138,0.00204268023029509,0.0647600068821265
"493","PIEZO2",15.3018151605008,1.68012878854045,0.544858811331732,3.0
8360396050844,0.00204509642650198,0.0647600068821265
"494","AL592183.1",30.6649210596015,-1.50837166494869,0.48904194240203
9,-3.0843400824477,0.0020400425129283 2,0.0647600068821265
"495","PDLIM4",293.112316919234,-0.735159501539307,0.238546724966117,-

3.08182600974181,0.00205735052745875,0.0649268051915547
"496","PPIL1",35.770706228842,-1.17353455683343,0.380815450646358,-3.0
8163588121645,0.00205866492201056,0.0649268051915547
"497","SESN1",154.37042048048,0.637494875237506,0.207031079117625,3.07
922307102168,0.00207541219152553,0.0653232855372914
"498","IL6R",135.655968126016,0.865875878652143,0.281272551544371,3.07
842295274784,0.0020809933304468,0.0653674270445367
"499","LDHA",2237.88998697051,-0.67898108488722,0.220619016578802,-3.0
7761812837516,0.00208662118184585,0.0654128560072439
"500","RGS2",97.2153093765671,-0.93174976042243,0.302969909869363,-3.0
7538712614791,0.00210229485425071,0.0657723968100878
"501","RAP2B",233.370029289111,-0.539183880147041,0.175397382604153,-3
.07407027483359,0.00211159685736402,0.0659315561671564
"502","HDGF",369.486368903578,-0.458987433401886,0.149431093873781,-3.
07156577324914,0.00212939244560184,0.0663547530409355
"503","CRISPLD1",88.9676589827517,1.0269286064147,0.335226983828996,3.
06338288966187,0.00218849845531457,0.0680609966928146
"504","PHLDA2",12.3494084175953,-1.62982685474255,0.53227182535066,-3.
06201977470595,0.0021984892819904l,0.0682360472979682
"505","SLC39A8",110.531836267733,1.20882114863564,0.394977998684489,3.
06047717255576,0.00220984605544529,0.0684527165254072
"506","CD44",2264.50642189815,-0.526564909140399,0.172271000951979,-3.
05660793883227,0.00223856865743414,0.0690688944935745
"507","PSMG1",98.4606190075112,-0.795953832373204,0.260394002734438,-3
.05672874188641,0.00223766674735213,0.0690688944935745
"508","RNF24",341.719693959878,-0.853197841503765,0.279271309235973,-3
.05508590853079,0.00224996063845788,0.0692837288728279
"509","DPY19L4",180.058438900717,0.514779745692139,0.168560915783561,3
.05396861009664,0.00225835709376708,0.0694056581882091
"510","AC105446.1",5.74204253112349,-1.97155023169534,0.64641777212479
7,-3.04996291363523,0.00228869625848146,0.0702001481792656
"511","SMARCE1",912.527215695562,0.52081971149245,0.171284959055203,3.
04066226459848,0.00236058456033499,0.0722634525974957
"512","RASSF1",182.679069969673,-0.682707022849331,0.224744196666292,-
3.03770701524738,0.00238385603427096,0.0728170019845807
"513","SET",777.698419199914,-0.42499842340519,0.139931620360321,-3.03
718646515225,0.00238797685981525,0.0728170019845807
"514","LMO7DN",9.3906779927166,2.44465763856146,0.80556933428307,3.034
69550605117,0.00240778642743594,0.0731502127871842
"515","ZNF415",22.1442656799684,1.27892784855172,0.421443493015398,3.0
3463659956186,0.00240825670174518,0.0731502127871842
"516","KCNK15-
AS1",34.4654045906658,-1.22416874291588,0.403900004814867,-3.030870830
21204,0.00243849542728174,0.0737821740212151
"517","TYMP",838.024576654585,-0.748252191170611,0.246856824564516,-3.
03111810860654,0.00243649919860948,0.0737821740212151
"518","SNCG",231.135852751937,-1.40950422904664,0.465245500930483,-3.0
2959238988374,0.00244883991220188,0.0739521288543898
"519","GPSM3",21.1965947047985,-0.976457932172604,0.322627882694174,-3
.02657638892981,0.00247340315804631,0.0745499915246983

"520","TUBB6",400.923234794754,-0.801764613943061,0.265028871611398,-3
.02519725140988,0.00248471021869435,0.074746772982761
"521","SDC4",118.316070494301,-0.7668755208497,0.254255073783686,-3.01
616604710174,0.00255993031622903,0.0768617849074295
"522","ENPEP",59.2687386146275,1.47511923365603,0.489772647560525,3.01
18448651703,0.00259665263574218,0.0778150137565421
"523","PTP4A2",864.15892375779,-0.436334625070383,0.14491354793254,-3.
01099953244887,0.00260389253550137,0.0778827742501872
"524","DHRS11",20.0355048565029,-1.14677414767351,0.381687830844536,-3
.00448181734303,0.002660336595304,0.0794191705350008
"525","AL158151.1",21.3198343473643,1.49611363005165,0.498457911749177
,3.00148436766009,0.0026866683344301,0.0800524814390286
"526","FZD7",194.257606645938,0.558770899017299,0.186419201601036,2.99
738918640554,0.00272302836111281,0.0809816210130946
"527","SYK",7.49005289720952,-1.80113532309815,0.6011059837082,-2.9963
6897970474,0.00273215618581385,0.0810988979405808
"528","CMC4",150.412119801748,-0.796861862013937,0.266048062167777,-2.
99518010212536,0.00274282837799943,0.0812614854489491
"529","HFE",55.3915385200741,0.984277128493033,0.329115190231538,2.990
67669225652,0.00278360036750236,0.0823135360091482
"530","ROBO2",64.5625465067702,1.03199027042072,0.345344295983249,2.98
829395019392,0.00280539593258779,0.082591481057034
"531","DEPTOR",223.98288844643,1.08607731579238,0.363396283680623,2.98
868580820959,0.00280180083049862,0.082591481057034
"532","CLEC10A",21.8244758564981,-1.4394177019932,0.481745875705559,-2
.98791909714858,0.00280883896454274,0.082591481057034
"533","WNT10B",22.8708220257904,1.54237039286065,0.516357343580009,2.9
8702131777014,0.00281710077833089,0.082679000891989
"534","CDS1",19.8017834491182,1.77378587905941,0.594116136223893,2.985
58778479458,0.00283033888020732,0.0829118121423309
"535","IGF2R",360.135244016531,0.689657873610555,0.231039915982582,2.9
8501612016924,0.00283563379761855,0.0829118121423309
"536","GSK3A",120.548935672471,-0.578928915002615,0.194109819648078,-2
.98248133995599,0.00285922077449212,0.0834455048048138
"537","EGR3",34.5730865265203,-1.47921105013128,0.496199903441848,-2.9
8107887541061,0.00287234801299143,0.0836725139054467
"538","SH3BGRL3",2736.80867897241,-1.07461809315986,0.360908310758582,
-2.97753767681645,0.00290573927988077,0.0840193707119684
"539","DYRK3",46.03895771824,-1.00865640786259,0.338721725975648,-2.97
783203884332,0.00290295018950307,0.0840193707119684
"540","IL1RN",9.1846246998714,-2.72323366436666,0.914205280337195,-2.9
7879887913383,0.002893806529079,0.0840193707119684
"541","NRG2",10.0795860665629,-1.56251625482121,0.524736880753308,-2.9
7771380692372,0.00290407014727127,0.0840193707119684
"542","GEM",257.520175606517,-0.974426883684015,0.327369977017514,-2.9
7653099579099,0.00291529611239211,0.0841401791257376
"543","AC108463.3",27.873172479881,1.42914007188852,0.480384638930885,
2.97499119678166,0.00292996955956642,0.0842527827578999
"544","AHCY",115.998378868636,-0.629665187968762,0.211632802883207,-2.
97527216665108,0.00292728705244914,0.0842527827578999

"545","GTF2I",750.735368000815,0.529761612404307,0.178110233939711,2.9
7434684513202,0.00293612985975273,0.0842750080662605
"546","GASAL1",11.9254404088598,-1.789654496056,0.601863080245303,-2.9
7352430278094,0.00294401091998028,0.0843464520535741
"547","FO393415.1",2.68830333937257,3.66495673971833,1.23299253007614,
2.97240790217279,0.00295473841826064,0.084499036703567
"548","LINC01121",6.79292908633273,2.09990118469435,0.707560437115771,
2.96780469136202,0.002993484690309,0.0853069238200917
"549","AQP1",91.078867558331,-1.68995358032365,0.569305638852222,-2.96
844693780088,0.00299308774744884,0.0853069238200917
"550","NUDT2",193.955965898208,-0.734145633280085,0.247364349537336,-2
.96787162197467,0.00299869546146446,0.0853069238200917
"551","ART3",5.1013631285638,2.6356063349422,0.889119750619205,2.96428
724376744,0.00303384950033993,0.0861315929833348
"552","HPS4",168.873660693531,0.601600001958964,0.203038219797449,2.96
298895133694,0.00304667504645701,0.086339017666172
"553","FBXL7",167.712551425548,0.801965725283098,0.27077040512152,2.96
179238984106,0.00305853938537245,0.0864541558737839
"554","AL513534.2",30.7553746948908,1.0240587590287,0.345805169123678,
2.96137493150788,0.00306268853976543,0.0864541558737839
"555","A4GALT",346.862469927972,-0.826295456043668,0.27906809927832,-2
.96090974991587,0.00306731806622451,0.0864541558737839
"556","SBF2",284.115385326427,0.588920853816382,0.198962543799429,2.95
995840508585,0.00307680580747497,0.0865655993639045
"557","FZD8",117.772616559513,1.08725513559056,0.368030963528638,2.954
24908047433,0.0031343091977233,0.088025132459552
"558","LRRC17",320.378770454407,1.42296025549434,0.482097549059476,2.9
5160234328176,0.00316129763925545,0.08862397665031
"559","CCL2",425.197751794564,-1.96693224882648,0.666532935021874,-2.9
5099033442651,0.0031675682921528,0.0886409137641257
"560","ZNF43",126.193744531558,0.513571731406423,0.174498807448337,2.9
43124591602,0.00324917625748624,0.0907622574926024
"561","PKN1",275.066309847364,-0.785077850361943,0.26685234875972,-2.9
419934057573,0.00326106871178991,0.0909320817442594
"562","HLA-
DQA1",73.7517676714715,-1.14360809637127,0.389023527759422,-2.93968877
141665,0.00328542072419877,0.0914481074530986
"563","SIGIRR",298.96799307338,-0.692642262228505,0.235687551054599,-2
.93881564439544,0.00329468984159329,0.0915432205897759
"564","ANXA1",7089.43772343483,-0.654319272592779,0.222844274131612,-2
.9362175678175,0.00332241209769497,0.0921498092983022
"565","RIPOR2",49.7181398950514,0.96593899821116,0.329376806435051,2.9
3262603601578,0.00336108469070896,0.0928319955844678
"566","PPP1R9A",22.8846816631968,1.4259211090275,0.486374537393945,2.9
3173470113745,0.00337074560455013,0.0928319955844678
"567","CABLES1",71.3917678634202,0.660706359677054,0.225356859419014,2
.93182271611524,0.00336979051249104,0.0928319955844678
"568","DPY19L3",103.379365724264,0.580732529467895,0.198036635413464,2
.93244998964677,0.00336299080445143,0.0928319955844678
"569","AP003120.1",7.91356163359695,1.85922961772969,0.634442179031005

,2.93049497523845,0.00338422466793381,0.0930394138497163
"570","WLS",200.401026256764,0.861557370714674,0.294143123969844,2.929
04134248266,0.00340009195597053,0.0932214344452617
"571","SGMS1",150.181486926707,0.515175028188089,0.175941792512584,2.9
2809923572445,0.00341041176812728,0.0932214344452617
"572","EPB41L3",385.716292728637,0.448885165354771,0.153322955795728,2
.92771009419372,0.00341468272947229,0.0932214344452617
"573","HPRT1",43.6565653106378,-0.823478181511161,0.281262436048208,-2
.92779296475274,0.00341377278891119,0.0932214344452617
"574","LINC02762",251.837911720851,-0.783753124406705,0.26779696484967
4,-2.92666918329959,0.00342613102511759,0.0933710237385268
"575","ELP5",194.489467273812,-0.707511106274521,0.241810701539499,-2.
92588831581943,0.00343474219888485,0.093442908203749
"576","CXCL2",193.964886159908,-2.1004460054103,0.718317471047164,-2.9
2411933451691,0.00345432291222416,0.0938124536734765
"577","PGM1",165.314759604393,-0.576966126935925,0.197379026876738,-2.
92313796488741,0.00346522937890329,0.0939455514283953
"578","NES",59.4282419750335,-0.700366455667365,0.239656287464772,-2.9
2237880790136,0.00347368777124139,0.0940119339196006
"579","SYNPO",13.7326969830102,-1.58494423135347,0.542724623163081,-2.
92034701155842,0.00349641820900344,0.094463678831504
"580","ACTN1",262.94345941734,-1.26851684742366,0.434463471737888,-2.9
197318760757 9,0.00350332659565166,0.0944871343720325
"581","TTC28",240.611860851407,0.511660278825768,0.175571514677988,2.9
1425565111854,0.00356537799030432,0.0959951943241489
"582","LRRFIP2",231.282129630093,-0.439986361384704,0.151040234679775,
-2.91304076902112,0.00357927868022956,0.0962038769670635
"583","MLF1",121.222230464657,-0.706152302399488,0.242850882887025,-2.
90776090251232,0.00364026544722177,0.0976752528145629
"584","QPCT",26.5262372769511,-1.44344561137753,0.496708030883688,-2.9
060243072968,0.00366053011726433,0.097730526405762
"585","UBE2E3",981.839683401499,-0.839125908032097,0.288780858049805,-
2.90575321958277,0.00366370273285109,0.097730526405762
"586","SEC11C",47.6098355964765,-1.1101271571684,0.382085100832411,-2.
90544476806313,0.00366731566836171,0.097730526405762
"587","CXorf40A",38.8911329311994,-0.881524884717883,0.303334133296653
,-2.90611832943896,0.00365943033336445,0.097730526405762
"588","ENHO",11.4387862359286,-1.66840060599164,0.575014269778051,-2.9
0149426489124,0.00371387577517194,0.0984680656796858
"589","PTMS",1685.21919411859,-0.554238599324012,0.190977686486714,-2.
90211180960435,0.00370656221774457,0.0984680656796858
"590","MMP9",8.99719878188077,-2.24753878904352,0.774500062290122,-2.9
0192202489663,0.00370880843566348,0.0984680656796858
"591","SIN3B",105.324169121632,0.734846171176455,0.253407830744728,2.8
9985581351946,0.00373334350909164,0.0988167386002041
"592","LRRC61",14.1790614953827,-1.30647332991563,0.450770990022846,-2
.89830836241128,0.00375181512575944,0.0991379121828631
"593","MRPS6",1687.17847753191,-1.1672969324079,0.402830449259076,-2.8
9773758303252,0.00375864934976057,0.0991510148032119
"594","MBNL2",564.299588585094,0.602226583582882,0.207954031195788,2.8

9596013176531,0.00378000419621057,0.0995464741436396
"595","ANGPT1",585.762272346841,1.08163083893145,0.373639267830213,2.8
9485322357221,0.00379335858535866,0.0997302661357405
"596","TRIM61",14.7773561299235,1.72818842312602,0.597112218287963,2.8
9424394644122,0.00380072754274766,0.0997563438778551
"597","GSTM2",87.8584231093963,0.713228806955354,0.246500781195422,2.8
934139822864,0.00381078654856427,0.0998528207356632
"598","FAM43B",25.189763947868,-1.29395161896268,0.44803072768249,-2.8
8808677399394,0.00387592930269406,0.101220637866516
"599","MYO1B",240.944905708062,0.59376269563277,0.205561832194029,2.88
848707610429,0.00387099935906571,0.101220637866516
"600","CSF1R",9.16418669416938,-1.92955531254658,0.668320422592238,-2.
88717095470814,0.00388722958528729,0.101269755461992
"601","SLC25A3",2166.2916936725,-0.709613915166154,0.245806046416051,-
2.88688551609126,0.00389075772119526,0.101269755461992
"602","ARHGAP29",520.472777331494,0.7610270285859,0.263889569260704,2.
88388446242095,0.00392802839743372,0.101584499451842
"603","MAP1LC3C",14.1172761968433,1.81639109791817,0.629810111652375,2
.8840297485105,0.00392621661851496,0.101584499451842
"604","SNHG14",285.816169000661,0.910847303253865,0.315768447871187,2.
88454185145639,0.0039198365291114,0.101584499451842
"605","SULT1A1",255.243864158897,1.34760774293662,0.467299447214111,2.
88382053728209,0.00392882581144054,0.101584499451842
"606","SAA1",3.18015318422765,-2.66926324046251,0.926218429646609,-2.8
8189389783676,0.00395292816403708,0.102039035099063
"607","BTN3A1",97.1368661861367,0.858761167000481,0.29810076610737,2.8
8077477362528,0.00396699003511546,0.102233319801172
"608","LINC02066",7.86185822407308,1.93681261559059,0.672446771391902,
2.88024673176967,0.0039736406757529,0.10223628468882
"609","CCNI",3225.47385996355,-0.57121106517388,0.198468221937373,-2.8
7809836556165,0.0040008036741937,0.10276612787424
"610","FAM107A",23.7753581859994,-1.19362405573916,0.414810501268264,-
2.87751648545472,0.00400818966731868,0.102787067157158
"611","TM4SF19",2.81211808626756,-3.37853101696564,1.17606052657328,-2
.87275266929481,0.0040691254422531,0.104178935013364
"612","RTCA-
AS1",84.5037659504914,0.745233413055682,0.259558669118643,2.8711559339
7899,0.00408973726968253,0.104535555734712
"613","GREM2",9.60765542052861,1.6619230905452,0.579563970343664,2.867
54038481676,0.00413676005095963,0.105453716497926
"614","IFI30",88.8672003467495,-0.949806099011198,0.33124778820126,-2.
86735831254551,0.00413914095312451,0.105453716497926
"615","ABRACL",93.5961718063654,-1.01089054328358,0.352939912672936,-2
.8642001286501,0.00418063785616767,0.106170161603763
"616","Z83843.1",142.725514661115,1.0108069441724,0.352912575937821,2.
8641851072785,0.00418083612784747,0.106170161603763
"617","KCNAB1",33.9492561342373,3.33850767236792,1.16625453446816,2.86
258923219565,0.00420194923153226,0.106533374114845
"618","PHLDA3",528.435407715691,-0.574251735955335,0.200969553899906,-
2.85740663106285,0.0042711827389202,0.107237816615724

"619","NOSTRIN",40.382660745354,1.00552160672754,0.351912816485195,2.8
5730317176399,0.00427257530538457,0.107237816615724
"620","LRAT",5.6876178774911,2.9137209879817,1.01961177053867,2.857676
88464637,0.00426754705610972,0.107237816615724
"621","AC022893.2",3.15955356348014,3.91559778334797,1.37041651846915,
2.85723189306123,0.0042735349592242,0.107237816615724
"622","CCN3",92.7452644448602,-1.40390536996018,0.491492466355969,-2.8
5641279584413,0.00428457683211835,0.107237816615724
"623","BDKRB2",175.436392498752,1.14158856659544,0.399462936550524,2.8
5780847768602,0.00426577776995303,0.107237816615724
"624","CCL3",77.984181612685,-1.60757908510061,0.562440272929434,-2.85
822186367921,0.00426022406588291,0.107237816615724
"625","MAP7D3",944.517916318223,0.600418779455738,0.210187146408184,2.
85659132690123,0.00428216793450986,0.107237816615724
"626","JAG1",269.511349797121,0.602136923100771,0.210850301540199,2.85
575557019525,0.00429345528892472,0.107288372339695
"627","CKMT2",3.298074425936,-2.70770191431156,0.948610615055699,-2.85
438711241131,0.00431199532865321,0.107579813279302
"628","PTX3",360.218566319805,2.56624032340153,0.899499131518566,2.852
96587120558,0.00433132730143354,0.107890052510071
"629","CCDC71L",692.561362634052,-1.15954798696547,0.406704736926298,-
2.8510806039016,0.00435709228453059,0.108359291902881
"630","LIN7A",4.32453356396758,-2.1438640104722,0.752451388249219,-2.8
4917277574633,0.00438330696130657,0.108690545119555
"631","CCL4L2",66.2590882397684,-1.55842846941638,0.546989719287282,-2
.84910011004775,0.00438430825100296,0.108690545119555
"632","DDX39A",97.7849810408293,-0.651925737191862,0.229499544351646,-
2.8406406602401,0.0045023012697406,0.111439080320494
"633","MLH3",211.212075298231,0.490167541368364,0.172640135543376,2.83
924441918205,0.00452205043752444,0.111751082139328
"634","AC008969.1",47.1093211554193,1.04441968209729,0.367948064962505
,2.8384975531906,0.00453264668945052,0.111836265241442
"635","SCN2B",11.1059166468393,1.8209227578288,0.641667551867456,2.837
79778567474,0.00454259513043807,0.11190522145739
"636","BVES",14.9562410261487,-1.12174272276889,0.395479817938777,-2.8
3640952556155,0.00456239028447245,0.112216149716985
"637","IFRD2",92.4358986624662,-0.894171506871563,0.315683052688042,-2
.83249765629701,0.00461859047597123,0.11332962871103 8
"638","USP9X",250.989876997717,0.453821562795507,0.160233508563574,2.8
3225129914352,0.00462215068194349,0.113329628711038
"639","RGPD2",5.13122667833732,3.33093239138417,1.17716756224148,2.829
61618908495,0.00466038746551469,0.113772318889098
"640","VEPH1",10.5047551196593,2.86211959428402,1.01152704458779,2.829
50377807285,0.00466202495735548,0.113772318889098
"641","PRIMA1",25.4663196447854,1.47462481562819,0.521112349675502,2.8
2976370939288,0.00465823932649207,0.113772318889098
"642","C8orf82",183.317399928482,-0.643941865169356,0.227802822471468,
-2.82675103926779,0.00470228704050453,0.114576131113103
"643","CYP2U1",151.186845749541,0.4885675263746,0.172891358544642,2.82
586434907589,0.00471532280806441,0.114715077273019

"644","SORT1",49.4011614873655,-0.963996842290844,0.341449617054311,-2
.82324768909467,0.0047539828381815,0.115435955878256
"645","CCDC85B",575.395044142311,-0.870104521127178,0.308234933143226,
-2.8228614850831,0.0047597130691987,0.115435955878256
"646","AC013275.1",5.30516658198195,2.67467054409992,0.948488954694288
,2.81992798214715,0.00480344285865536,0.1161364090231
"647","TMOD2",206.803393886723,0.508267844194265,0.180235411713589,2.8
2002209977444,0.00480203421909443,0.1161364090231
"648","RNF126",115.442917998537,-0.593895910795408,0.210684339938768,-
2.81888967622374,0.00481900780926415,0.116332930802962
"649","CCL18",7.91051962169052,-2.30674548507916,0.819104274270948,-2.
81618050050136,0.00485983541600179,0.117137758724986
"650","KLF3-
AS1",75.3710558530459,0.67211372298208,0.238886415812472,2.81352843231
444,0.00490010526017427,0.117926687053702
"651","GTF3A",479.358232741609,-0.796605361093539,0.283497345850267,-2
.80992176030556,0.00495535447486988,0.119073133717956
"652","NFIC",1863.95148470304,-0.431478968200908,0.153592244785401,-2.
80924970400536,0.00496571149849831,0.119138995354308
"653","RAD23A",575.301758585183,-0.512601930870393,0.182537536782287,-
2.80820010999587,0.00498192590071035,0.119162487560875
"654","PRPS2",85.9468267761924,-0.612744802289433,0.218185584163722,-2
.80836520266912,0.00497937233707257,0.119162487560875
"655","RBL2",302.809926334236,0.492116462523513,0.175444433800547,2.80
497050754527,0.0050321183908487,0.120179279371063
"656","SMYD2",48.489728882796,-0.641456941576656,0.228735927296839,-2.
80435587516697,0.00504172225254075,0.12022509328734
"657","PRR5L",11.6200057033261,1.79439002667578,0.640015153646587,2.80
366803262697,0.00505248969731825,0.120298472351826
"658","FGF12",24.440481065451,1.9415087006311,0.692716480500369,2.8027
4651359339,0.00506694768169372,0.120459365630296
"659","APPBP2",223.59653392101,0.447078734333295,0.159550074462336,2.8
0212175293489,0.0050767709987703,0.12050975528644
"660","DIP2B",64.5036563440635,0.80759047617848,0.288315249742914,2.80
106750128061,0.00509338638585755,0.120720974596924
"661","AL020997.5",9.86885284977539,1.62153499447711,0.579358026362496
,2.79884789835041,0.00512852891626977,0.121370011856593
"662","SLCO2B1",11.5384225150863,-2.20887921969483,0.78990322034297,-2
.79639221971491,0.00516766440898321,0.122111441615898
"663","TXN",1644.02104218528,-0.786687788841742,0.281554410593813,-2.7
9408796041438,0.00520463198236369,0.122799484313899
"664","USP40",88.2275452280015,0.767440879258655,0.274793387776446,2.7
9279237928023,0.00522552196527697,0.123106686901849
"665","IMPDH1",66.0317967558472,-0.57398846522456,0.20562592850741,-2.
79142066076495,0.00524772212301168,0.123443785218454
"666","PPP1CC",650.897816963639,-0.489175086430801,0.175306541515708,-
2.79039836278425,0.00526432256442295,0.12364834515806
"667","PDE1B",26.46669202144,-1.52121293017487,0.545377578317928,-2.78
928395785291,0.00528247269937425,0.123888636336299
"668","LINC01278",93.759688540112,0.688928927450248,0.247048590962635,

2.7886373476805,0.00529302983085632,0.123950397670787
"669","ROR2",18.1680634154736,1.98730141182025,0.713047090107583,2.78705493562902,0.00531894612467671,0.124371112448906
"670","CDH11",736.186076549717,0.80710597244749,0.289678687228583,2.78621109536653,0.00533281310199037,0.124509246797665
"671","AL359555.4",8.96095634094113,-1.70407147362884,0.611886623826351,-2.78494643823501,0.00535365657568351,0.124809612240562
"672","AC093627.6",13.9859811872602,-3.74613453322721,1.34751486776392,-2.78003205964144,0.00543535321085037,0.126525640293649
"673","RCAN3",23.3915996857175,-1.1108790863077,0.400060464458826,-2.77677797482542,0.00549006672535377,0.127303967738645
"674","HLA-DQB1",77.5854203623178,-1.30943683668217,0.471659635555154,-2.77623255833825,0.00549928573559131,0.127303967738645
"675","AC005162.3",14.1137956597006,1.61992890053687,0.583826090579925,2.7746771284712,0.00552565348783093,0.127303967738645
"676","DENND4C",161.072363241094,0.491116763570375,0.176923109100688,2.77587685445251,0.0055053056161559,0.127303967738645
"677","OGA",471.239793951879,0.696108428460559,0.250721669856153,2.77641908200412,0.00549613141145559,0.127303967738645
"678","PSD",3.65683377332075,-2.33829352584904,0.8424816982509,-2.77548287482522,0.00551198021120609,0.127303967738645
"679","HRAS",151.371797643944,-0.606629500560722,0.218699936400286,-2.77379824862139,0.00554060271621367,0.127303967738645
"680","SERGEF",79.1326500935759,-0.839858620973875,0.302696294251365,-2.77459168454979,0.00552710523950042,0.127303967738645
"681","LRRC4C",61.3213265357615,-1.21033678995197,0.436359566180765,-2.77371434880054,0.0055420317093919,0.127303967738645
"682","C5AR1",30.7112299594581,-1.25661910824544,0.453258271176774,-2.77241296663586,0.00556423965814659,0.127626687642796
"683","FIGN",56.3968910137454,0.996990783073082,0.359773972777294,2.77115872328607,0.00558571914675261,0.127931778349416
"684","ACSL3",256.857615867435,0.701654418377438,0.253346895481926,2.76954022682112,0.00561354718710548,0.128381167613876
"685","C21orf62-AS1",44.9666687808856,0.833567051974869,0.301152260397192,2.76792560306693,0.0056414332111568,0.12883056893741
"686","ZNRF1",146.268211330263,-0.631085523507984,0.228128552968809,-2.7663592097315,0.00566860561235486,0.129262387163363
"687","PTGER3",61.5758350120254,-1.51715529083236,0.549240168001192,-2.76228029052142,0.00573991790670059,0.130094518296302
"688","TMEM64",327.954944121931,1.13809053944313,0.412037763710638,2.76210250534797,0.00574304446847876,0.130094518296302
"689","ZDHHC13",31.3064122545738,-0.910568295568212,0.329745623942736,-2.7614264737789,0.00575494730816094,0.130094518296302
"690","NEMP1",37.5250755954985,0.89653124674272,0.32466269055222,2.76142369552167,0.00575499627060288,0.130094518296302
"691","ZNF544",29.6635762095093,1.00068988971774,0.362370828530376,2.76150785585065,0.0057535132429758,0.130094518296302
"692","ADAMTS5",1263.21445586828,0.924805507182072,0.334812050646276,2

.76216314615006,0.00574197785597376,0.130094518296302
"693","ARHGAP28",74.378513481058,1.07595043429438,0.389722941378734,2.76080856438156,0.0057658462490254,0.130151706888174
"694","RFTN2",148.609724749547,0.534323754967105,0.193615464028916,2.75971631525933,0.00578515732608597,0.130399446760753
"695","CXCL12",10821.5078025017,0.556126288863002,0.201574470806382,2.75891230986873,0.00579940946595399,0.130519002150758
"696","NAV2-AS3",6.55327629997384,-2.64891837596653,0.960283115003719,-2.7584764686363,0.00580714859662007,0.130519002150758
"697","SLC24A1",14.8634500316255,1.30377522794197,0.472730310046271,2.75796833889148,0.00581618309246522,0.130534508056576
"698","INTS3",124.114462262123,0.681427771329024,0.247208023042063,2.75649537156437,0.00584244395534698,0.130936032655434
"699","CPPED1",79.8681940818641,-0.608468775532512,0.220936378021844,-2.75404521872063,0.00588636347161784,0.131731593399882
"700","ARHGEF10",358.187462216086,0.739650835535871,0.268700623892196,2.75269489449575,0.00591069531262691,0.132087152536318
"701","OLR1",8.1990991946546,-2.13035111092599,0.774229055581741,-2.75157732142368,0.00593090166692883,0.132336865973507
"702","HOXC10",228.04027246465,-0.390675569771533,0.142004872437779,-2.75114200706538,0.00593878922926559,0.132336865973507
"703","PEG10",198.223851141546,0.99020403546885,0.360272734875625,2.7484845219017,0.00598714616261378,0.133224647826127
"704","PTPRQ",10.3223897193459,1.72050634097256,0.626117416932417,2.74789727045441,0.00599787982938067,0.133273912174718
"705","GSN",68328.3460575551,-0.80910115465567,0.294520671421184,-2.74717951290625,0.00601102240511741,0.133376487210286
"706","SNHG16",478.801577593307,-0.500074487158727,0.182074112513085,-2.74654359291625,0.0060226881564874,0.133446049337015
"707","MSL1",294.836735067411,0.56278018560064,0.204961261158752,2.74578806950617,0.00603657450427769,0.133564547341465
"708","CAMK2N1",414.371211690948,-0.81101773042605,0.295566462693671,-2.74394369048087,0.0060705949997873,0.133938388690653
"709","ZNF135",28.0118289919889,1.06157852626796,0.38687342861768,2.74399441197354,0.00606965711174002,0.133938388690653
"710","ECHDC3",13.7043742618474,-2.06825121514968,0.754001785052842,-2.74303225290738,0.00608747060080458,0.134121552969558
"711","ACACB",215.34359669433,-0.537123656882601,0.195911281227629,-2.7416678279926,0.00611281241057742,0.134490470518513
"712","EPHB3",45.812844626285,1.21685165335906,0.444162591237588,2.73965362541789,0.00615039638660461,0.134560350595323
"713","ANXA5",3765.57929372289,-0.569974460375964,0.208046064453627,-2.7396550945236,0.00615036889818594,0.134560350595323
"714","SCRN1",203.288104080825,0.402332584527057,0.146812366709097,2.7404543196573,0.00613543099834312,0.134560350595323
"715","DENND5B",82.3262288400362,0.636805514898041,0.232435140072528,2.73971274179685,0.0061492903481176,0.134560350595323
"716","GPRC5B",317.663064480999,0.731686450190996,0.267189500758831,2.73845509689928,0.00617285893684386,0.134863173671855

"717","FRS2",169.480967508986,0.539167993212463,0.197087403310407,2.73567962313293,0.00622516009956181,0.135795540548
"718","ADAMTSL5",10.0622417182182,1.40880501498043,0.515051373270082,2.7352708644108,0.00623289638262891,0.135795540548
"719","POLR3F",59.8839241090135,-0.583895698482455,0.213593077434649,-2.73368268997905,0.00626303683448629,0.136262427262683
"720","RGS14",27.883913230219,-1.08435396994276,0.396732732401461,-2.73321024806566,0.00627202814590205,0.136268522619925
"721","PTGS1",65.0087592685488,1.19651015546907,0.437901056196347,2.73237558699237,0.00628794145823003,0.136424782567396
"722","ERI2",34.3991168594609,0.847480071116406,0.310265968096092,2.7314631904906,0.00630537841235729,0.136613621197375
"723","HSD11B1",119.893341043687,-0.707704833361062,0.259190237718546,-2.73044555840702,0.00632487787189412,0.136657547721049
"724","IGF1",2514.93588405159,-0.565034270834632,0.206924907450565,-2.73062473626874,0.006321440605461,0.136657547721049
"725","AKR1C2",1278.80956611464,-1.01824510394081,0.373367437781397,-2.72719311033487,0.00638756444336979,0.137809044285183
"726","MYH10",285.672546174979,0.670959135994925,0.246063852837356,2.72676839063565,0.00639579148187962,0.137809044285183
"727","VILL",45.1706233544495,-0.746485332362571,0.273879852305169,-2.72559418328736,0.00641858612172707,0.137853699227586
"728","AC106897.1",12.5195860084892,-1.21694270877713,0.446433855737309,-2.72591940135741,0.00641226542418697,0.137853699227586
"729","FZD6",36.5743358894393,-1.07961960901612,0.396147016780768,-2.72530036396459,0.00642430139595411,0.137853699227586
"730","EMX2",474.274379081947,-0.797009271178889,0.292531654792686,-2.7245231691036,0.00643944120819862,0.13798928605459
"731","SIRT7",76.7442364527775,-0.557086247122132,0.204568586949875,-2.72322478943765,0.00646480535734397,0.13834329713397
"732","GNG4",13.2645442109,1.64043585546413,0.602568186491036,2.72240701092594,0.00648082694665721,0.138423034900927
"733","PEX11A",53.3165809628293,-0.817854438932206,0.300545746150941,-2.72123112506626,0.00650392703779209,0.138423034900927
"734","ZNF781",18.2224918130264,1.23353057135371,0.453194600690402,2.72185628309458,0.00649163670319747,0.138423034900927
"735","CYBB",11.7812104407389,-1.63212061777584,0.599738502175985,-2.72138709096405,0.00650085885825036,0.138423034900927
"736","LAYN",31.2996860868232,-1.28114211142527,0.470890880848472,-2.72067725991391,0.00651483326707896,0.138466761952332
"737","NCOA1",337.443704270379,0.481453211549414,0.177059307370167,2.71916353170223,0.00654472424459626,0.138537376668768
"738","ANP32B",431.043891911446,-0.575988070979557,0.211821377975619,-2.71921595678531,0.00654368696929045,0.138537376668768
"739","ZNF528",51.5816741539981,0.750485437121189,0.275982977425523,2.71931785112984,0.00654167132510124,0.138537376668768
"740","FMNL2",176.637402254673,0.6341134999427,0.233366389516128,2.71724433521681,0.00658279914169334,0.138745311549526
"741","LSAMP",195.972661561162,2.25311457012007,0.829301830919123,2.71688121998106,0.00659002534560491,0.138745311549526

"742","MBNL1-
AS1",16.1792468655485,-1.07572162426407,0.395914926917411,-2.717052455
28281,0.00658661677542587,0.138745311549526
"743","HDAC9",10.1118735171546,-1.36192931762301,0.501278791013989,-2.
71690991527508,0.00658945403312985,0.138745311549526
"744","ADAM33",358.429972857194,0.632477500377012,0.232840942843367,2.
71635002269545,0.00660060933043507,0.138781359887091
"745","HLA-F-
AS1",108.663065339571,-0.883534621657388,0.325624678960078,-2.71335276
085051,0.00666061604255991,0.139667582779845
"746","IFNAR2",84.3700529322334,-0.635338805744626,0.23414963629293,-2
.71338796764046,0.00665990834734027,0.139667582779845
"747","TEX41",16.7395995100914,-1.04339802375187,0.385351060429044,-2.
70765577390696,0.00677602630116676,0.140342766472492
"748","DNAJC18",86.7206015312198,0.601226407822784,0.221843728808341,2
.71013479196522,0.00672558696955469,0.140342766472492
"749","CD24",15.4953294879029,1.79663946310013,0.663618630169671,2.707
33728894979,0.0067825309373652,0.140342766472492
"750","ITGB8",31.672512060195,1.01012797457474,0.373068901727504,2.707
61773467936,0.00677680290741489,0.140342766472492
"751","CKAP5",117.17002371004,0.490603629550276,0.181108934804388,2.70
888694740636,0.00675093389583187,0.140342766472492
"752","TNKS1BP1",308.569946179863,0.531242098015984,0.196202740945674,
2.7076181273282,0.00677679489071469,0.140342766472492
"753","IL17D",165.65455391958,-0.806776602440023,0.297813970348573,-2.
70899515390678,0.00674873254918592,0.140342766472492
"754","ABCA10",967.670394361607,1.0406820207288,0.384378193365421,2.70
744292650194,0.00678037280766255,0.140342766472492
"755","ZNF790-
AS1",28.7970498683555,0.970724229302039,0.35839347387276,2.70854326339
289,0.0067579300639479,0.140342766472492
"756","ZNF550",20.2646023174654,1.07390399135138,0.396574756415794,2.7
0794843589447,0.00677005400860652,0.140342766472492
"757","LINC01554",9.24375098027159,1.9766150841686,0.730516192737823,2
.70577860397681,0.00681444603275592,0.140790579537354
"758","SGSH",97.4019338258916,0.722769158279092,0.267157748141351,2.70
540219517303,0.0068221734507009,0.140790579537354
"759","AL110292.1",25.3692278848246,-1.12733068574201,0.41708407064966
,-2.70288597688723,0.00687403227279765,0.141517963088582
"760","ALYREF",119.406009064565,-0.853500335728541,0.315782076439749,-
2.70281437550617,0.00687551313349882,0.141517963088582
"761","SRSF8",193.044268948131,-0.567192147905828,0.209900821736294,-2
.7021911739746,0.00688841430443846,0.141597194434075
"762","ISOC1",59.6101129353348,-0.609363537876639,0.225771742753669,-2
.69902482234676,0.00695429890889773,0.142576799255422
"763","NDRG2",256.158253690245,-0.546185851360802,0.202355099419342,-2
.69914547707511,0.00695177802067078,0.142576799255422
"764","CMPK2",9.7605315345927,-1.29564521326844,0.480175579964103,-2.6
9827385508714,0.00697000764069841,0.142711818747965
"765","AC118549.1",136.0920118091,0.481291785358677,0.178527887959918,

2.69589133024827,0.00702005646107147,0.143174371865112
"766","CRAT",197.763752865537,-0.545628076177031,0.202369918892858,-2.69619160378231,0.00701373100135561,0.143174371865112
"767","RASA3",184.283409094474,0.535115686929186,0.198464784952339,2.6962752462997,0.00701196992835958,0.143174371865112
"768","FBLN2",6833.68684397234,0.513661621353424,0.190580855191637,2.69524250395935,0.00703374191177735,0.143266698861892
"769","SEC24B",88.3890410402571,0.561247741281527,0.208604694044989,2.69048471728294,0.00713483029202237,0.144948247088449
"770","KL",10.579137586335,1.72583736913134,0.641394307111316,2.69075878908887,0.00712897189456328,0.144948247088449
"771","SRM",385.210767627452,-0.731325994986303,0.271914006319742,-2.68954882054271,0.00715486806049912,0.145166797756664
"772","STARD4-AS1",30.7601571163322,1.1273116557408,0.419293046266277,2.68860088613271,0.00717521503869625,0.145204315299582
"773","MATN4",21.2001895346062,1.53109369918554,0.569506053899293,2.68845904043065,0.0071782641550618,0.145204315299582
"774","AL683813.1",41.4688166590473,1.22269475643563,0.454843450701098,2.6881661251821,0.00718456434455518,0.145204315299582
"775","ITGB1BP1",1045.99771767357,-0.681710011774123,0.253779895558297,-2.68622544064971,0.00722643122524251,0.145674051103697
"776","COCH",77.3968974273357,-0.934183293520607,0.347745529510849,-2.68639914604988,0.00722267493231208,0.145674051103697
"777","C1QTNF3",67.8126236687146,1.07024675605749,0.398523956374783,2.68552677684198,0.0072415571962809 2,0.145791092949064
"778","CAPN2",817.337258696547,-0.477911647335892,0.17805650762836,-2.68404482207071,0.00727373539315996,0.146179858579297
"779","CRAMP1",52.887099858502,0.868205116950734,0.323501172803778,2.68377733974198,0.00727955697968884,0.146179858579297
"780","ATP8B4",229.461179692057,0.957266056573524,0.356935339030356,2.68190328022441,0.00732046211904566,0.146812806318245
"781","RALGPS2",181.839745845605,0.75040156019255,0.28027619219108,2.67736461783011,0.00742038338371025,0.1478881490423
"782","KIAA1109",344.789965382503,0.572924267518922,0.213991629489941,2.67732092551711,0.00742135121128974,0.1478881490423
"783","CCND3",81.1606522863697,-0.64904262045825,0.242278142873842,-2.67891528620564,0.0073861077816297,0.1478881490423
"784","DCHS1",106.359162236675,-0.641199423063092,0.239453104452984,-2.67776617274549,0.0074114938485044,0.1478881490423
"785","NSMCE1-DT",12.6009246184994,1.42960229409879,0.533828332765312,2.67801876811827,0.00740590684267667,0.1478881490423
"786","SGMS2",164.355694409821,0.55378462175351,0.206977299524556,2.67558144311284,0.0074599745542093,0.148468679327603
"787","PTGDS",3616.18675222545,2.20498748336685,0.824353901264637,2.67480687600822,0.00747723092168175,0.148623028345448
"788","CYCS",257.456396341853,-0.607798827346734,0.227442918506759,-2.67231370111297,0.00753301899836007,0.149541898719983
"789","MIR34AHG",10.9940568645074,1.46416681072862,0.548240004315013,2

.6706675893854,0.00757005718543677,0.149648022496197
"790","MIIP",50.2165665145897,-0.777421390904185,0.291373372646496,-2.66812778341066,0.00762752420778589,0.149648022496197
"791","FCGR1A",4.83241369840646,-2.16733622076884,0.812128068195793,-2.66871236895401,0.00761426252337382,0.149648022496197
"792","TFPI",1457.54041977568,0.424759906671974,0.159237985478067,2.66745340564787,0.00764284860071569,0.149648022496197
"793","ZDBF2",53.9331611217493,0.652317048920757,0.244310084853887,2.67003733927268,0.00758428123768298,0.149648022496197
"794","AADAC",1275.32947531942,-1.2796523605251,0.479525309812452,-2.66858147909979,0.00761723004344535,0.149648022496197
"795","UTRN",526.771290411879,0.679703020102786,0.254621020255526,2.66946939188551,0.00759711970597722,0.149648022496197
"796","PRUNE2",17.6331314227899,-1.47022303469115,0.550594526562614,-2.6702463678123,0.00757956103993096,0.149648022496197
"797","FBLN5",3078.75610107164,0.630188719491986,0.235895638523872,2.67147253520846,0.00755192522504348,0.149648022496197
"798","PPL",930.216028334724,-1.04947689981175,0.393134339684228,-2.66951215875648,0.00759615228166914,0.149648022496197
"799","MT-
CYB",57504.5843024951,-0.428833612546777,0.160767154685431,-2.66742055232529,0.00764359585593951,0.149648022496197
"800","ASIC1",47.626826498347,0.688223853520075,0.258082672711659,2.66667981344485,0.00766046149943089,0.149790749044497
"801","IL4R",140.924779831828,0.59560094191866,0.22339039812127,2.6661886407281,0.007671663235767,0.1498225068628
"802","SNAI2",654.967730361652,0.664688074369886,0.249697681817122,2.66197134684134,0.00776844910469652,0.15152350292365
"803","KIDINS220",689.528307020893,0.448907096427458,0.168683793021285,2.6612343034687,0.00778547594135591,0.15166650081025
"804","TMEM131",202.124462615329,0.492975786193451,0.185381982324594,2.65924325552996,0.00783163950635819,0.152048882094318
"805","PCDHGB5",3.51850466989974,2.84258235840368,1.0689894729178,2.65913035667683,0.00783426446129389,0.152048882094318
"806","GRIA1",61.2571261643509,1.19764495298166,0.450338366706273,2.65943353159338,0.0078272172754735,0.152048882094318
"807","FNDC4",145.333589095119,-0.916385826063557,0.344883295341394,-2.65708962551069,0.0078818485661985,0.152782846494477
"808","GALNT2",238.744577882308,0.534966258315513,0.20156182837487,2.65410500901277,0.00795190764571308,0.153569989261592
"809","NOX4",161.431182454265,0.685635744950978,0.25828775546093,2.65454219356012,0.00794161070105149,0.153569989261592
"810","NMB",95.1286028482251,-0.735451228811954,0.277063018888967,-2.65445468601743,0.00794367079720398,0.153569989261592
"811","CACHD1",151.089311414009,0.575998467763245,0.217160252122566,2.65241204195209,0.00799189474534425,0.153962080666774
"812","TOMM34",35.4588537097103,-0.769521201724112,0.290118310867148,-2.65243927356413,0.00799125012502083,0.153962080666774
"813","LINC01615",7.43556176506909,-2.39763714939284,0.904972506198146,-2.64940330559376,0.0080634042682576,0.155148626037335

"814","AL360178.1",8.52858037998739,2.02843641968358,0.766113497049231
,2.64769701551574,0.00810421222775596,0.155404429950049
"815","C1GALT1",306.452481674113,0.578850298250203,0.218631958079727,2
.64760149126559,0.00810650225910889,0.155404429950049
"816","GAREM1",67.4009591629983,0.848155248276691,0.320286625843206,2.
64811322059979,0.00809424117607559,0.155404429950049
"817","NT5E",873.456382691663,0.700312332258463,0.264767894852001,2.64
500472253224,0.00816897769310533,0.155459024395677
"818","VEGFB",1113.56618811168,-0.662918469329175,0.250507224639357,-2
.64630479333898,0.00813764573728495,0.155459024395677
"819","AC092111.1",6.77787242487374,1.94627011542281,0.735761070866446
,2.64524747569322,0.00816311911019244,0.155459024395677
"820","FAM216A",40.9375373392044,0.70420989949351,0.266224773372484,2.
64517043463955,0.00816497800455992,0.155459024395677
"821","HELZ",251.55632022297,0.62856391731054,0.237457415969598,2.6470
5953589175,0.00811950568637775,0.155459024395677
"822","LILRB4",4.51678822368768,-2.19404477471756,0.829268110824802,-2
.64576045560865,0.0081507512672855,0.155459024395677
"823","BCLAF3",149.769895585449,0.605377964178971,0.228968566876878,2.
64393480920242,0.00819484375313856,0.155761775006496
"824","NR2F1",40.3095305202667,1.14749593533684,0.434579083766946,2.64
047667777821,0.0082789490630942,0.157169417711144
"825","DCBLD2",174.28961167725,0.49595567712836,0.187909157517535,2.63
933745263095,0.00830682483520906,0.157497661896553
"826","HPGDS",4.6409902814061,-2.07288539192452,0.785496897264804,-2.6
3894790564108,0.00831637593342408,0.157497661896553
"827","MTHFD2",35.1729967392408,-0.958783540521609,0.363503611926127,-
2.63761764413075,0.00834906602143131,0.157925561999093
"828","XPO1",326.259224427634,0.429717672103337,0.162962138947431,2.63
691723046147,0.00836632426845896,0.158060882284425
"829","HLA-
DPA1",342.223000600316,-0.70766423786195,0.26847650345406,-2.635851662
09169,0.00839264116194693,0.158176006863055
"830","RUNX1T1",971.542733629465,0.558094469710323,0.211728578839351,2
.63589579059035,0.00839154982958621,0.158176006863055
"831","NNAT",22.9676579067783,-1.56183149266703,0.593300091269751,-2.6
324477539258,0.00847720601090859,0.159577537459258
"832","C2orf27A",88.3714851431001,0.493306156786204,0.18754056937487,2
.63039703052275,0.00852852011622103,0.159965995417321
"833","ADAMTS2",371.454850087794,0.61139547104882,0.232371197224268,2.
63111555283998,0.00851050941294701,0.159965995417321
"834","DHRS4",118.367438721324,-1.06557789091016,0.405088981589685,-2.
63047858455328,0.00852647414444829,0.159965995417321
"835","SPAG1",21.37271594951,-0.85386935673558,0.324696678029964,-2.62
974466482463,0.00854490202678659,0.160081320245536
"836","DYSF",6.13671590904836,-1.98732056700976,0.756262693708994,-2.6
2781779868474,0.00859345300678874,0.160606195203341
"837","ZNF667",52.7674789594013,0.78969236902101,0.30048363750237,2.62
807111756553,0.00858705611816817,0.160606195203341
"838","MSANTD3",103.998314698189,-0.664765928905486,0.253025841893011,

-2.62726496207678,0.00860742820724406,0.160675416999903
"839","TRANK1",102.859494619816,0.64968659896254,0.24733254157061,2.62
677363373579,0.00861986558326348,0.16071580133372
"840","PAXIP1-
AS2",99.6069678782286,0.6568610600497,0.250124249797182,2.626139051220
86,0.00863595303564134,0.160824063495878
"841","RIN1",27.6871028397103,-0.789623827692324,0.301038680225382,-2.
6229979054557,0.00871598083970752,0.162101885698621
"842","ISLR",2777.90844669975,0.527791714031874,0.201244898887932,2.62
263399941277,0.00872529487682917,0.162101885698621
"843","IL16",108.556963160836,-0.914516049737487,0.348778754511636,-2.
62205205422561,0.00874020802897972,0.162186327636216
"844","BCL2A1",28.4254740305585,-1.70437856434242,0.650456071573156,-2
.62028235084394,0.00878569918310131,0.162837313176841
"845","TOB2",299.955698357208,0.447129709818859,0.170693902600291,2.61
948261190026,0.0088063262068703,0.163026462549198
"846","TMEM183A",191.008283924506,-0.474178128833795,0.181075725586386
,-2.61867308441394,0.00882724975327884,0.163149971406954
"847","TSPAN33",4.63403920184712,-1.98466775381493,0.75802697225692,-2
.61820202506233,0.00883944547468084,0.163149971406954
"848","DDX54",138.985723175184,-0.503774255577501,0.192426024203046,-2
.61801519656157,0.00884428663000044,0.163149971406954
"849","GNB4",302.187932132884,0.584954441208949,0.223553211517256,2.61
662284893543,0.00888044021911586,0.163532095128198
"850","MPEG1",10.4659449498458,-1.85462232739495,0.708841672931352,-2.
61641266056682,0.00888590940733672,0.163532095128198
"851","NECAB1",67.2845120187786,2.24088124515641,0.8567121801471,2.615
6757159349,0.00890510878653753,0.163692851642546
"852","HIST1H2AC",146.488148313035,1.2004363205608,0.459056958421054,2
.61500517210272,0.00892261043388548,0.163822059879426
"853","DOCK11",259.325259278885,-0.539795383537264,0.206550111673792,-
2.61338703311751,0.00896497154055368,0.164406857923659
"854","RUFY3",453.292401281461,0.609962089347784,0.233479940669344,2.6
1248177294862,0.00898874852638308,0.164506449262405
"855","AC025171.1",51.4690343500515,0.643339285561586,0.24626561819279
2,2.61237963416371,0.00899143477078288,0.164506449262405
"856","PCBP4",68.6530686007668,-0.736801303227344,0.2821826472889,-2.6
1107942074483,0.00902569301330892,0.164678104107735
"857","MBTPS1",479.166336243957,0.321167743931854,0.123013871372798,2.
61082543251193,0.00903239872942763,0.164678104107735
"858","ZDHHC7",230.311642628466,0.52301707263798,0.200288722519385,2.6
113156350446,0.00901946054881867,0.164678104107735
"859","PKM",723.171277351832,-0.613037845762193,0.234843439330013,-2.6
1041078052312,0.0090433557985206,0.164685931031732
"860","NEK6",128.591894562329,0.703781289068999,0.269748755951493,2.60
902515226264,0.00908005680259221,0.164970184161382
"861","TMEM25",46.9160558290463,-0.825184683482837,0.316236699102817,-
2.60938937771592,0.00907039673682229,0.164970184161382
"862","AL158071.1",51.2059186117869,1.04234428620109,0.399743455157672
,2.60753308841529,0.00911972559423485,0.165306914797933

"863","AXIN2",14.5171443238019,1.30207049668455,0.499337625726456,2.60759540158875,0.00911806581291643,0.165306914797933
"864","KDM5B",401.981509526875,0.366298204655588,0.140659636712572,2.60414581763845,0.00921035616516514,0.166013248996445
"865","ANKRD50",100.82579581638,0.607580456363134,0.233261568911907,2.60471735312983,0.00919500788589543,0.166013248996445
"866","ERLIN2",184.2840577706,0.564467650761225,0.216761648815885,2.60409373080881,0.0092117560652633,0.166013248996445
"867","SHTN1",61.9389358247207,-0.961676539135544,0.36924584374319,-2.60443429609566,0.00920260637530087,0.166013248996445
"868","AC005332.7",11.1341791989811,1.27387761975636,0.489078848376043,2.60464672309219,0.00919690338068127,0.166013248996445
"869","PTTG1IP",699.965595002695,0.476812846647478,0.183149180928465,2.60341238890777,0.00923008549587659,0.166152160428075
"870","IRAK1",107.863954534649,-0.643639832770627,0.247341302190826,-2.60223354154598,0.00926187568473125,0.166532783145116
"871","STXBP6",162.106788197382,-0.972236684527061,0.373723870739225,-2.60148403848001,0.00928213844251206,0.166705501327458
"872","MRPL23",102.321312101945,-0.97647991664175,0.375489520189173,-2.60055171752809,0.00930739886847026,0.166967477637019
"873","REEP5",1050.18975588532,-0.486879426281725,0.187284959943123,-2.5996717858689,0.00933129609761808,0.167204427096265
"874","PALMD",101.471367274686,-1.01779125814215,0.391649843926266,-2.59872759794528,0.00935699927351251,0.167385827437981
"875","RADIL",54.2741385313274,0.584110876257064,0.224820426221772,2.59812191478044,0.00937352073359786,0.167385827437981
"876","SIGLEC1",4.66877969406441,-2.79337769396548,1.07513614344395,-2.59816183373535,0.00937243104788277,0.167385827437981
"877","CSRNP1",84.6722344869828,-1.38011813686064,0.531393885793732,-2.59716600765774,0.00939964834198578,0.167661002296104
"878","NGF-AS1",8.28574589791778,1.90980665822376,0.736224481418403,2.59405481130476,0.00948513641531279,0.168002905560997
"879","PLEKHO1",132.539333766264,-0.795293065858627,0.306628741025101,-2.59366771425227,0.00949582125623866,0.168002905560997
"880","TTC14",457.705524485699,0.553336654947831,0.213368110233599,2.59334281182895,0.00950479765523415,0.168002905560997
"881","FAM198B-AS1",13.3081290382208,1.94444995380624,0.749096067258078,2.59572842362332,0.00943906384828011,0.168002905560997
"882","THEM6",27.5316912004594,-1.00938130572638,0.389084153232455,-2.59424933485619,0.00947977113077815,0.168002905560997
"883","MPDZ",259.884036985661,0.655266631764309,0.252714921977902,2.59290835157572,0.00951681274108673,0.168002905560997
"884","CCNB1IP1",203.04665782256,-0.647888054774146,0.249901979339405,-2.59256872029099,0.00952621474350214,0.168002905560997
"885","KIFC3",33.115908592863,-1.04023447295275,0.400784744906105,-2.59549418028985,0.00944550025297303,0.168002905560997
"886","WFIKKN2",2.90973034396096,3.10386793439635,1.19648535145654,2.59415456329478,0.00948238475056579,0.168002905560997

"887","ABCA9",1533.15939605202,0.736666972600885,0.284005425817529,2.5
9384823540022,0.00949083708881471,0.168002905560997
"888","LINC01137",9.44228439846226,-1.26163411731855,0.48755170708201,
-2.58769295439332,0.00966210639705478,0.168087665127079
"889","AC245297.3",472.405593314638,-0.442232537475795,0.1706950552960
28,-2.59077532567567,0.00957599871188203,0.168087665127079
"890","CEP170",280.156751027847,0.478335400425298,0.184841711945513,2.
58781091881631,0.00965879833780903,0.168087665127079
"891","RPL39L",42.555081804306,-0.78108972425803,0.301845016292871,-2.
58771780912945,0.00966140931531466,0.168087665127079
"892","IQGAP2",18.1848932020551,-1.39641945751406,0.539234938614176,-2
.58963089651207,0.0096078887643133,0.168087665127079
"893","STK38",214.600578061455,0.480638365295238,0.185524053862957,2.5
9070646251766,0.00957791494391778,0.168087665127079
"894","ZC2HC1B",11.8050996485437,2.06859213494542,0.798967779965053,2.
58908079501767,0.00962325126727646,0.168087665127079
"895","FKTN",75.3883081067962,0.658716538722177,0.254240314648402,2.59
092087591671,0.00957194965883609,0.168087665127079
"896","CNTN1",97.0620645050545,2.04498678887509,0.789547277070492,2.59
007515859309,0.00959549796751983,0.168087665127079
"897","AL161772.1",74.2164471671989,-0.801284888311976,0.3097347233835
35,-2.58700374164949,0.00968145408677992,0.168087665127079
"898","LINC02185",7.81387977096439,-1.94297413931064,0.750512672275773
,-2.58886253501753,0.00962935260795771,0.168087665127079
"899","ARHGAP23",85.5430503585697,0.581303820006426,0.224701384714139,
2.58700595346108,0.00968139194125632,0.168087665127079
"900","ZNF253",54.3430202219432,0.677610293880427,0.261654482483888,2.
58971406661131,0.00960556800419974,0.168087665127079
"901","SPIN4",19.6893995155072,1.12521627616105,0.434514538194309,2.58
959408087255,0.0096089162194138 8,0.168087665127079
"902","CD86",5.94412940154962,-1.78290275866133,0.689373102386429,-2.5
862667871569,0.00970218018985688,0.168260759101919
"903","NHSL1",184.180931915868,0.723264554461713,0.28002678156094,2.58
284064984804,0.00979905710144953,0.169752658070847
"904","NQO2",371.309522994047,-0.55092202380349,0.213358380452585,-2.5
8214382127784,0.00981886568022565,0.169907650260807
"905","LURAP1L",187.846765386747,0.805740754261042,0.312301082616845,2
.58001268362424,0.00987966864022437,0.17058240236096
"906","CSF2RA",3.14104392615082,-2.50060968328186,0.969125410924558,-2
.58027460129876,0.0098721778949837,0.17058240236096
"907","PTGS2",32.6447339175037,-1.51697243094803,0.58859487126637,-2.5
7727769133333,0.00995819142250741,0.170638753843056
"908","GALNT13",28.2454493706873,-1.28970604698653,0.500409101212429,-
2.57730333813221,0.00995745251741051,0.170638753843056
"909","FAXDC2",356.62071819568,0.501238096061894,0.194429756486133,2.5
7799065904628,0.00993766842817352,0.170638753843056
"910","LRRN4CL",806.28060109887,0.617013621655121,0.239408720480065,2.
57723954423163,0.00995929056183024,0.170638753843056
"911","MAP1A",571.196551396554,-0.500881412325601,0.194300444598541,-2
.57787064440594,0.00994112044679071,0.170638753843056

"912","PAQR4",8.00859434485152,-1.50791729654226,0.584986583105233,-2.57769552343904,0.00994615942242997,0.170638753843056
"913","DNASE1",88.6153199606606,0.942534201302896,0.365570566788525,2.57825516310817,0.00993006418503225,0.170638753843056
"914","MSX1",810.494983963109,-0.618221787417951,0.23993917810648,-2.57657708214536,0.00997839544281141,0.170779037102734
"915","STAT1",320.866770781634,0.817716716690041,0.31745482016859,2.57585226223932,0.00999933606610448,0.170950397903904
"916","TMEM192",125.79769208844,0.48872645768755,0.189833667479664,2.57449831832335,0.0100385574545372,0.171433574521098
"917","CD52",58.1143265980471,-0.98086425675063,0.381219905456947,-2.57296180684721,0.0100832334272377,0.171622914589607
"918","AC068491.3",26.100719986041,1.43271026557347,0.55684480346482,2.57290766953163,0.01008481076074,0.171622914589607
"919","ATXN7",164.272608341123,0.716355921101654,0.278338008837677,2.57369061485032,0.0100620204059427,0.171622914589607
"920","DUSP22",216.426138527113,0.403222803029199,0.156736943595537,2.57260856170401,0.0100935294650923,0.171622914589607
"921","RCC1",28.2734810608726,-0.755562027450006,0.293815459698783,-2.5715530020939,0.0101243516732785,0.171773571827652
"922","ADGRA2",228.763470145109,0.497640257796056,0.193490631526144,2.57190879925789,0.0101139530900523,0.171773571827652
"923","FRAS1",9.35761119290637,2.22782926888103,0.866727560815575,2.57039163123494,0.0101583603604635,0.171899465209084
"924","SLC43A2",67.4036479334918,1.19668057528769,0.465544885865703,2.57049451432037,0.010155343510872,0.171899465209084
"925","IL1RAPL1",3.96823616660076,3.60938600666281,1.40433516066537,2.57017420610098,0.0101647385615549,0.171899465209084
"926","NHLRC3",335.472092117695,0.49037495536656,0.190828013746003,2.56972205359357,0.0101780139460258,0.171938090883026
"927","DHCR7",44.3454653902094,-0.685699165240737,0.267178294980453,-2.56644786692311,0.0102746068941071,0.173195771168661
"928","RAB30",239.492192851909,0.422826402962354,0.164729958058708,2.56678510663897,0.0102646203102371,0.173195771168661
"929","VPS13B",151.770018705457,0.490963039267682,0.191346102567319,2.56583767675619,0.0102926982641057,0.173313970877724
"930","AGA",96.1828587651639,-0.714767359793403,0.278825433418022,-2.56349412258173,0.0103624455333466,0.174132449054695
"931","LGALS3",8625.24151376524,-0.728177109933178,0.284060647550639,-2.56345648794371,0.010363569012972,0.174132449054695
"932","PDGFB",4.15863465283503,-2.21776607603005,0.865354125047276,-2.56284220741294,0.0103819220150112,0.174253654593155
"933","GAL",149.559751868763,2.15323794382047,0.840385891164009,2.56220144395576,0.0104010970669569,0.17438838308511
"934","BBOX1-AS1",10.414497087567,-1.78816130688986,0.698302392862524,-2.56072630591987,0.0104453608767423,0.174943019480599
"935","LTBP2",736.174107120022,0.842518844820828,0.329103882728257,2.56003921265341,0.0104660353313585,0.175101808222931
"936","AP002387.2",19.2164856230525,-0.906470845149488,0.3543860256850

95,-2.557862837275,0.0105317623885311,0.175744092343203
"937","SLC39A9",110.054290528772,0.534225257745592,0.208873264032917,2
.5576526522868,0.0105381294264479,0.175744092343203
"938","FTX",928.44547259487,0.856419898793363,0.334809840849078,2.5579
2929091176,0.0105297500509461,0.175744092343203
"939","BBS2",154.603245333101,0.510412744333467,0.199659068836977,2.55
642154051225,0.0105754917409502,0.176179358150889
"940","AC114730.2",4.14374160534663,2.53129583173624,0.990533721366041
,2.5554867816568,0.0106039388516823,0.17646533559241
"941","TMEM72-
AS1",11.1605143629627,1.29174542516714,0.50573219892541,2.554208389166
97,0.0106429537909811,0.1769263827336
"942","HEBP2",543.938053395983,-0.556383612525139,0.217896541832577,-2
.5534302097949,0.0106667653070586,0.177133980571463
"943","ZMYM4",211.208163978505,0.370291650197406,0.145250564175825,2.5
4933020259507,0.0107930054559844,0.178871205553779
"944","DDX60L",105.876889065196,0.523100779512471,0.20519468785229,2.5
4929006685118,0.0107942477812931,0.178871205553779
"945","IQSEC1",177.496911937703,0.737901299655543,0.289576001399815,2.
54821289087672,0.0108276372292141,0.179234634049309
"946","CMPK1",555.069491729682,-0.531693255123836,0.208916080938907,-2
.54500875535435,0.0109274998307662,0.179581999566931
"947","PCYOX1",899.592844882509,0.431003926342066,0.169242788381641,2.
54666051335762,0.0108759180907634,0.179581999566931
"948","GRID1",26.2330496620221,0.83810194786557,0.329394247319886,2.54
437336014451,0.0109474000277977,0.179581999566931
"949","STAMBPL1",62.899276660194,0.938008033900038,0.368703166604108,2
.54407371257328,0.0109567959800763,0.179581999566931
"950","SERP2",48.4356345652485,-0.653608552645198,0.256935483872943,-2
.54386254009359,0.0109634219514428,0.179581999566931
"951","PCNX4",223.626341524561,0.536011012606594,0.210514925982867,2.5
4619006279021,0.0108905874177404,0.179581999566931
"952","TGFB1I1",411.463056336823,-0.573584169377458,0.225237358064798,
-2.54657652844803,0.0108785355728982,0.179581999566931
"953","SLC44A2",186.602878639738,0.707594911306055,0.278102936793227,2
.54436331908339,0.0109477147661293,0.179581999566931
"954","TMEM238",17.9333010719062,-1.1375722435131,0.446835155689694,-2
.54584320196841,0.0109014142978203,0.179581999566931
"955","CD93",8.35815543751633,-1.87973609160983,0.738490558953638,-2.5
4537592772102,0.0109160148742252,0.179581999566931
"956","PKN3",4.85232418444292,-1.87541841235294,0.737469909550186,-2.5
4304397788493,0.0109891396896615,0.179627076452848
"957","AL118506.1",10.1414664892307,1.37329576858434,0.540006385789639
,2.5431102385506,0.0109870559076813,0.179627076452848
"958","MID1",104.595203868677,0.924015434160436,0.363790052069424,2.53
996894336215,0.0110862312700357,0.181024964255917
"959","NT5DC2",71.0658604629067,-0.651611837931832,0.256590745923665,-
2.53949859176015,0.0111011492231845,0.181079538371507
"960","SLC35E2B",171.096216721632,0.628491247202769,0.247657960087296,
2.53773893228078,0.0111571179078565,0.181802911908958

"961","SCX",5.00654856551699,-1.80922895657363,0.713444882024848,-2.53590571907784,0.0112156925108821,0.182377419904085
"962","MT-CO2",40030.5984393338,-0.477245128561624,0.188177748424497,-2.53614007265641,0.0112081892809156,0.182377419904085
"963","HLA-DMB",27.8386259592736,-1.00358185925326,0.395988294045267,-2.53437254167554,0.0112648899498277,0.182797379133978
"964","CLIP2",31.2444700180739,-0.818703330222642,0.323027814909789,-2.53446697910915,0.0112618540604395,0.182797379133978
"965","ZDHHC17",197.486125320697,0.431027794356616,0.170170154284198,2.53292239270562,0.0113115993739768,0.183175309531179
"966","ENOSF1",189.142019304219,0.574365195670764,0.226759167413288,2.53293043109447,0.0113113399835169,0.183175309531179
"967","PDLIM3",109.731935057484,-1.38013758711376,0.545149441889339,-2.53166834828002,0.0113521308244639,0.183641553761208
"968","MMP14",894.791821277287,0.697473787150474,0.275627853585171,2.53049094305322,0.0113903025552217,0.184068701313361
"969","NCEH1",28.6068309790313,-0.907467869380676,0.358746048828506,-2.52955502184354,0.0114207265620136,0.18417982021606
"970","MEGF9",100.743598707477,0.643784718808637,0.254469932211629,2.52990486228933,0.0114093458605554,0.18417982021606
"971","CARD19",145.564446250049,-0.565798961935357,0.223781942065225,-2.52834950270673,0.0114600206774698,0.184623175548569
"972","SRGAP2",124.477141298087,0.561455751444356,0.222185663884369,2.52696659914364,0.0115052444126932,0.18516104768288
"973","PDE7A",100.892533046831,0.744855807122933,0.294840453215019,2.52630125547843,0.0115270588702857,0.185321461364727
"974","FGFR2",31.9320527739899,-1.70993818127918,0.677534020377433,-2.52376726459674,0.0116104766807203,0.186470930920439
"975","DPYS",8.40608192703866,2.3991956003463,0.951434118238005,2.52166235618023,0.0116801760917682,0.187397943183108
"976","C3orf14",120.033168828366,-0.509204375316508,0.202044732111218,-2.52025563842075,0.0117269631389726,0.187932914358626
"977","TRIP10",524.803690760179,-0.395615602220282,0.156994175159879,-2.51993809208143,0.0117375476141646,0.187932914358626
"978","ANGPTL1",620.407949656708,-0.546942493273969,0.217119707594883,-2.51908267256187,0.0117661026852589,0.188173572301966
"979","SASH1",1065.79895012988,0.620801717849998,0.246478795703163,2.5186820475934,0.0117794972667671,0.188173572301966
"980","FBXO10",19.1569646462013,0.970967215004053,0.385603008963912,2.51804885447594,0.0118006951625793,0.188173572301966
"981","AC093525.6",11.7759534557124,1.23101617553459,0.48886226817743,2.51812474733231,0.01179815265548,0.188173572301966
"982","GRK6",49.9955339065392,-0.659715888706452,0.262190880570412,-2.51616641765419,0.0118639148907916,0.188989023051581
"983","VIT",1117.07675563788,0.550682390196159,0.219020008111692,2.51430175235557,0.0119268336916147,0.189798025877852
"984","SLC25A37",298.883612685055,0.339884942709955,0.135234078096308,2.51330838716482,0.0119604731341412,0.189879466901433

"985","ENPP2",439.637570969671,-0.842738788576374,0.335341590122555,-2
.51307566194931,0.0119683663213459,0.189879466901433
"986","MT-
ATP6",76441.3059443206,-0.367384435945462,0.146170742249105,-2.5133924
2239985,0.0119576241009776,0.189879466901433
"987","KRIT1",257.050364004311,0.430748585796333,0.171472754333185,2.5
1205264341502,0.0120031181589649,0.190151707827079
"988","PRTG",27.8630757021066,0.895801826423573,0.356680271797384,2.51
149810419692,0.0120219931624996,0.190151707827079
"989","C17orf49",339.881743799605,-0.752783946159655,0.299733538548959
,-2.51151055635602,0.0120215690363407,0.190151707827079
"990","SLC7A7",39.1611088243614,-0.656619912988919,0.261518986902184,-
2.51079250790504,0.0120460477717924,0.190339722519342
"991","SLITRK2",67.3632316030499,-1.37919586204549,0.54974911214323,-2
.50877324143118,0.0121151228567903,0.191238008929133
"992","ADD1",680.77982463384,0.339629970401767,0.135427213145791,2.507
84138957469,0.0121471178649015,0.191535318891387
"993","CCT6A",283.320346597404,-0.485621115369722,0.193683093106721,-2
.50729739793107,0.0121658303408804,0.191535318891387
"994","FAM92A",251.127837409784,-0.52682652136276,0.210129114995339,-2
.50715623760395,0.0121706902114709,0.191535318891387
"995","GPR1",21.0949030616883,1.64203101554254,0.655154997221962,2.506
32449192206,0.012199360515801,0.191793564370527
"996","HERPUD1",908.628431647628,-0.517610688542759,0.206577475560336,
-2.50564921048993,0.0122226814924038,0.191967275688426
"997","ANKFY1",195.679318115801,0.456529627812625,0.18223074593046,2.5
0522833280197,0.0122372365601421,0.19200310081274
"998","ATP10D",135.632301216784,0.502580713591272,0.200883831778351,2.
50184750630306,0.0123547127394411,0.193652075534145
"999","BICC1",880.677722241362,0.651867077656075,0.26061092172997,2.50
130375706779,0.0123736997780446,0.193755541169121
"1000","TCEA3",209.27987924613,0.712228812096601,0.285026877571803,2.4
9881280728404,0.0124610114063706,0.194620282480029
"1001","SOS1",231.237531228817,0.525443581808603,0.210319580348088,2.4
983103377202,0.0124786897223978,0.194620282480029
"1002","WDR34",94.1071812140341,-0.516077242193884,0.206490286841482,-
2.49928095935121,0.0124445604402581,0.194620282480029
"1003","DDX17",1887.52325639976,0.576220162831297,0.230624685285815,2.
49851902070753,0.0124713449634127,0.194620282480029
"1004","HSP90AB1",2600.00026428186,-0.401774767227818,0.16090577092890
6,-2.49695685187908,0.012526419759498,0.194782091747343
"1005","AGAP2-
AS1",40.2254030423219,-0.919682066145848,0.368233448821312,-2.49755167
296638,0.0125054238010223,0.194782091747343
"1006","DUSP6",90.7895788324855,-0.699285661951731,0.28001656713195,-2
.49730103155723,0.0125142671242559,0.194782091747343
"1007","KYNU",3.58818473207198,-2.35372278745525,0.944100973097158,-2.
49308374265708,0.0126638974497696,0.195529901964787
"1008","ZBED3-
AS1",10.1511925790632,1.56776314551447,0.628308715752239,2.49521151976

617,0.0125882066564764,0.195529901964787
"1009","AVIL",20.0737895023535,1.10311404908892,0.44237071197967,2.49364168833043,0.0126440109420535,0.195529901964787
"1010","SLC12A6",97.4564431361465,0.538136944396404,0.215780508771769,2.49390896082088,0.0126344945129941,0.195529901964787
"1011","THOC6",62.6823395344924,-0.582286432379547,0.233550096602577,-2.49319713778754,0.0126598535380462,0.195529901964787
"1012","FBXL20",151.210608766107,0.496813617630367,0.199328508023125,2.49243634318845,0.0126870069995691,0.195529901964787
"1013","CD300A",5.79998351509214,-1.59497415991224,0.639507862668955,-2.49406497248634,0.0126289425367702,0.195529901964787
"1014","SMAD4",262.534597128454,0.366499345219543,0.147029080071762,2.492699709749,0.0126776013755783,0.195529901964787
"1015","TGFB1",176.163279149059,-0.604683854542008,0.242423795992475,-2.4943254933636,0.0126196762129532,0.195529901964787
"1016","VOPP1",100.569866335633,-0.445889122703441,0.17897810513625,-2.49130541617927,0.0127274661009716,0.195960386040846
"1017","ZKSCAN8",72.411425488248,0.70811513766565,0.284334905770605,2.49042633631813,0.0127589941676975,0.196252650703336
"1018","JAM2",85.8922039620541,-0.919555437921625,0.369302528697709,-2.48997872060152,0.0127750743799479,0.196306963188138
"1019","IL1B",104.838544410952,-1.93961758997429,0.779157829414904,-2.48937701290997,0.0127967184723908,0.196358339357978
"1020","SGCZ",3.42318199266579,3.31110341428608,1.33039808038388,2.48880651821948,0.0128172697615465,0.196358339357978
"1021","RIMKLB",132.099206091707,0.62717447394306,0.251964780637759,2.48913547502787,0.0128054159813524,0.196358339357978
"1022","ATF5",90.8889060366105,-0.670731894547032,0.269533520384969,-2.48849157458798,0.0128286276816373,0.196358339357978
"1023","C1orf162",32.5099208946856,-1.04117217116379,0.418518773354337,-2.48775500037675,0.0128552257876491,0.196381149410346
"1024","CLIP4",145.899863496729,0.569224417140112,0.228805421759639,2.48781000363744,0.0128532379046994,0.196381149410346
"1025","AP1S2",120.171395913474,-0.67931205460196,0.273133913508751,-2.48710255667389,0.0128788266353655,0.196549741519046
"1026","AMOTL2",523.898809010048,0.48323886210972,0.194446436246585,2.48520297639658,0.0129477585912278,0.1974091497491
"1027","SNX7",290.837005739592,-0.57478483430742,0.23159957307697,-2.48180437757714,0.0130719016240544,0.199107845282457
"1028","MAPKAPK3",38.8983962881235,-0.760084613975585,0.306649852360532,-2.47867268849014,0.0131872257839254,0.200374346671299
"1029","ARHGEF26",99.2968546454075,-0.916711773390761,0.370019937614967,-2.47746588818862,0.013231905651822,0.200374346671299
"1030","LOX",860.376235137895,0.892152007638907,0.360058912674904,2.47779453926316,0.0132197246250721,0.200374346671299
"1031","MDGA1",53.9968479464873,0.602478572060594,0.243114283838019,2.47817019448356,0.0132058135939957,0.200374346671299
"1032","SF3B3",176.11171274204,0.463437557736216,0.18701716545676,2.47804824014064,0.0132103283120588,0.200374346671299
"1033","SSBP4",436.994534952851,-0.551879663229553,0.222654560397777,-

2.47863624371138,0.0131885731381972,0.200374346671299
"1034","AL357568.2",19.2710192515975,1.217565676689,0.491745214197981,
2.47600920463416,0.0132860152863394,0.200417682858445
"1035","RPS6KA2",36.8440609244007,1.04807616845802,0.423230358876015,2
.47637284631807,0.0132724892468825,0.200417682858445
"1036","EGFR",1153.27349542611,0.395354342005684,0.159670347414612,2.4
7606614758016,0.0132838964285172,0.200417682858445
"1037","CHP1",227.990443927813,-0.529876777857938,0.213924568776554,-2
.4769327847116,0.0132516855460349,0.200417682858445
"1038","H2AFY",357.407059907699,-0.379986944087731,0.153498202877495,-
2.4755139601927,0.0133044560283242,0.200502510261152
"1039","HIST1H2BD",12.1190049723069,2.28858985354612,0.924719612354716
,2.4749013895341,0.0133272967848574,0.20065342021706
"1040","AL627171.2",173.437920755267,1.10353219695584,0.44608189502440
9,2.47383318907274,0.0133672094162289,0.20106082394045
"1041","AC091563.1",23.378657558797,1.08149403199481,0.4373401066901,2
.47289012704513,0.0134025340990119,0.201398502315892
"1042","ZEB1-
AS1",88.8495361467633,0.534968539693752,0.216521725104585,2.4707383955
8294,0.0134834414665956,0.20241984151819
"1043","BEX5",11.9585729762732,-1.32165603701605,0.535112286380839,-2.
46986673760549,0.013516339357284,0.202719172162986
"1044","FER",170.270431967881,0.486461180447292,0.197071805754819,2.46
844635428219,0.0135700990570645,0.202963272583826
"1045","SLC37A3",167.365481428765,0.521588326673407,0.211328587601215,
2.46813898958935,0.0135817572510287,0.202963272583826
"1046","FLCN",182.067271217208,0.488438109822324,0.197903151674182,2.4
680663531143,0.0135845136096188,0.202963272583826
"1047","EVA1C",32.4342991783458,-0.934771727282762,0.378632050099428,-
2.46881299942066,0.0135562039483063,0.202963272583826
"1048","CIZ1",277.408410750687,-0.365867609327,0.148494701337853,-2.46
384285789823,0.0137456360668831,0.205174604002148
"1049","KIF22",131.226815899427,-0.589493977884249,0.239291971988486,-
2.46349249824651,0.0137590774691168,0.205179455528497
"1050","NPLOC4",163.245061606648,0.522586269561874,0.212274174226334,2
.46184573072311,0.0138224106391195,0.205927590121663
"1051","MERTK",6.32020609779248,-2.01797981618152,0.820146027902843,-2
.4605128203103,0.0138738615115681,0.206105238010882
"1052","HDAC4",153.937308594683,0.519093757317913,0.210963354064097,2.
46058733575214,0.0138709807298643,0.206105238010882
"1053","INTU",117.467765152703,0.510237583111258,0.207316634590022,2.4
6115119570735,0.0138491989059391,0.206105238010882
"1054","LY86",4.86833845555851,-2.24575334627002,0.913129387365627,-2.
45940320982222,0.0139168218148425,0.206547289990116
"1055","USP53",1499.13127692508,0.797011212552357,0.324247367861533,2.
4580344870917,0.0139699758022507,0.206593988330622
"1056","NPY5R",12.9702732083537,1.57894458551157,0.642313369128991,2.4
5821535312694,0.013962941656792,0.206593988330622
"1057","ST6GALNAC4",83.089032916096,-0.732929352744701,0.2981857938404
43,-2.45796200853514,0.013972795477453,0.206593988330622

"1058","POLR2I",513.000089204858,-0.570271088870954,0.231950808310029,
-2.4585863400343,0.0139485232147159,0.206593988330622
"1059","MDGA2",5.55158373752767,2.510739629605,1.02198642685524,2.4567
2502454932,0.0140209960941623,0.20691551122734
"1060","MYZAP",7.70430738238823,-1.69846488979214,0.69130097707219,-2.
45691087691719,0.0140137447701797,0.20691551122734
"1061","LAMC2",46.6437861075774,0.665743417167849,0.271331186560146,2.
45361923046127,0.0141426646128518,0.207888437707379
"1062","VASH2",64.4472930637432,0.823089812712214,0.335317449686675,2.
45465845419414,0.0141018500297139,0.207888437707379
"1063","ABCF3",79.7822660657149,-0.487951357205437,0.198850425114271,-
2.45386127248676,0.0141331493281763,0.207888437707379
"1064","HSPB2",608.791543299162,-0.567036273622662,0.231127623067925,-
2.45334705603761,0.0141533712304775,0.207888437707379
"1065","A2M",231.943470184087,-1.06949266329009,0.435787932642854,-2.4
541585096313,0.0141214719126463,0.207888437707379
"1066","SLC25A45",85.22420753545,0.840086504485206,0.342576763521183,2
.45225769503558,0.0141962954281536,0.207933192305812
"1067","AC008014.1",15.3545600966461,1.16609642053951,0.47539654914427
8,2.4528920595627,0.0141712855538211,0.207933192305812
"1068","ZNF532",161.771212299061,0.436180016394653,0.177853842466465,2
.45246327178395,0.0141881862857136,0.207933192305812
"1069","NCSTN",309.269817093764,0.574251829481252,0.234229278812028,2.
4516654467527,0.0142196800095794,0.208080874078439
"1070","EBP",116.474038890108,-0.629999748317764,0.257088600639043,-2.
45051607403743,0.0142651593230108,0.208551296532578
"1071","ZNF260",74.8773637451347,0.482026370376764,0.196756050450166,2
.44986809439362,0.014290855632761,0.208731890441905
"1072","DTNA",12.9785047329822,-1.74395066563715,0.712072691438816,-2.
44911887031269,0.0143206177906338,0.208971477704183
"1073","SLC25A5",482.826966888469,-0.605822503678395,0.247428890154398
,-2.44847116802066,0.0143463911500234,0.209152466691347
"1074","C7",768.941923246503,-0.905115670358948,0.369723867989234,-2.4
4808558149485,0.0143617538239578,0.209181485165895
"1075","ADCY3",487.938112575821,0.576489581694089,0.235552382778845,2.
44739439649542,0.0143893285804647,0.209193556676774
"1076","LRRFIP1",230.012382876866,-0.584203411360432,0.238685514988549
,-2.44758636228285,0.0143816654449038,0.209193556676774
"1077","LEAP2",7.48496867077072,-1.66797921418518,0.681928630405491,-2
.44597328784005,0.0144461703107653,0.20982492309313
"1078","SAP130",37.6169228242078,0.646299888164308,0.264278268667455,2
.44552793320118,0.0144640243821347,0.209889363088806
"1079","DPYD",329.65324757252,0.410614684997545,0.168050694706986,2.44
339772420158,0.0145496929255359,0.210351983765396
"1080","LRBA",88.184552731815,0.613366494905074,0.250971807276642,2.44
396572491893,0.0145268065630078,0.210351983765396
"1081","AL445250.1",20.9859282759256,1.10092287328186,0.45050422748998
1,2.44375703068479,0.0145352117551378,0.210351983765396
"1082","SEC14L2",15.1777308407974,-1.03563690646216,0.423834074150026,
-2.44349609818198,0.0145457268836644,0.210351983765396

"1083","AC009236.1",10.2588233255723,1.87218589132252,0.766552926731162,2.4423439348228,0.0145922373074388,0.210383749493332
"1084","SOCS2-AS1",4.07992184488691,2.24365241949107,0.918578889013943,2.44252556457023,0.0145848966004273,0.210383749493332
"1085","RRBP1",1440.68659929113,0.529285291496687,0.216696299733989,2.4425211327855,0.0145850756756844,0.210383749493332
"1086","NR1H2",293.467171670225,-0.429345304407949,0.175948613949058,-2.44017440530822,0.0146801726729521,0.211456667700728
"1087","AC124016.2",13.6792961391925,-1.07905567559012,0.442522658495557,-2.43841903883201,0.0147516627005773,0.21196050592157
"1088","ASS1",159.597892468816,-0.687780134071473,0.281994887529049,-2.4389808627315,0.0147287482339367,0.21196050592157
"1089","APLP2",1936.69861591603,0.340153111465421,0.139503197883012,2.43831766315977,0.0147558007382592,0.21196050592157
"1090","AC015912.3",19.9610960032435,-1.99856171295446,0.819838830917419,-2.43774951561885,0.0147790108019056,0.212099143095604
"1091","TTC32",98.0932472796399,-1.09281158443022,0.448558567519153,-2.43627401985484,0.0148394383587981,0.212254602928862
"1092","TMEM178A",3.52619130358102,2.3672398894696,0.971709538806621,2.43615997881101,0.0148441178548984,0.212254602928862
"1093","SNX33",172.008869294252,0.515072131395557,0.211366176898517,2.43687111605779,0.0148149586631324,0.212254602928862
"1094","EMP2",1042.38889229701,-0.697323469788676,0.286169235050376,-2.43675204871663,0.0148198373325938,0.212254602928862
"1095","TJP2",66.0887392133167,-1.02599362723398,0.421387765642383,-2.43479690415289,0.0149001502279954,0.212473154071588
"1096","MDK",420.114703935819,0.755119667722436,0.310113893754234,2.43497528788849,0.0148928067648362,0.212473154071588
"1097","HMG20B",292.82605435711,-0.598688585265795,0.245880301038573,-2.43487820186081,0.0148968030770591,0.212473154071588
"1098","RABEP1",223.709857808034,0.437302559503106,0.179639667480347,2.43433182457293,0.0149193110064389,0.212552624839457
"1099","RFPL4B",4.94127842256727,2.46113320876905,1.01127289212209,2.43369838936799,0.0149454427727068,0.212731174971295
"1100","ITIH4",23.3034217907883,1.29077838690968,0.530770356839084,2.43189614920603,0.0150200131798036,0.213598241974243
"1101","ODF3B",162.410897208791,-0.80140449863529,0.329634971595381,-2.43118773095166,0.0150494146688507,0.213821974264152
"1102","AC016405.3",7.50197779382104,-1.26109681938848,0.519102898194649,-2.42937734267013,0.0151247816553611,0.214697785331047
"1103","BCAN",4.28389043107815,-2.21604286043428,0.912882099771157,-2.42752362105665,0.0152022968562347,0.215602474816028
"1104","AASS",253.630974643498,0.717594081170312,0.295649004794498,2.42718246817405,0.0152166005524772,0.215609857284784
"1105","AMPH",81.4745136326754,1.36994853193594,0.564767741682306,2.42568480957358,0.0152795339658436,0.216305655952663
"1106","AHCTF1",201.962051291418,-0.648484853401968,0.267382338042832,-2.42530923376879,0.0152953520065063,0.216333807809926
"1107","DIRC3",18.4441257430188,1.79425838722526,0.740057211788312,2.4

2448605140881,0.0153300722274139,0.216629015224422
"1108","AUP1",360.772223294449,-0.454046406453972,0.187333373824601,-2
.42373474188904,0.0153618215333506,0.21688174570957
"1109","LINC01341",13.9293119043858,1.13409417999211,0.469040552569021
,2.41790219157059,0.0156102721607505,0.217432206081947
"1110","HTR2B",26.0049886181915,1.58096494151625,0.6537208569204,2.418
40982244928,0.0155885088832953,0.217432206081947
"1111","DNAJC13",160.564107669458,0.424462646769584,0.17540429177756,2
.4199102682611,0.0155243373958775,0.217432206081947
"1112","LYPLA1",153.522703311772,-0.39859733286721,0.16472031004423,-2
.41984326498767,0.0155271980443587,0.217432206081947
"1113","ZNF706",815.947505660852,-0.517935687981974,0.214159212429926,
-2.41846092962938,0.0155863192834415,0.217432206081947
"1114","OLFML2A",169.55355217923,0.712282017159434,0.294553883552589,2
.41817221544888,0.0155986923040597,0.217432206081947
"1115","C14orf39",4.08101395670065,2.35736852264237,0.973823067321729,
2.42073596503085,0.0154891230205174,0.217432206081947
"1116","AC091057.2",6.22952326976892,1.61667373684442,0.66756254423835
1,2.42175620965815,0.0154457086606583,0.217432206081947
"1117","PHKG2",55.7747403104865,-0.57655139949831,0.238480808960228,-2
.41760082084619,0.0156232052442696,0.217432206081947
"1118","LINC00324",42.5662995056032,1.23592715550992,0.510325242923623
,2.42184209511049,0.0154420588782907,0.217432206081947
"1119","ZNF358",360.121776488956,-0.486191371443887,0.201002419041144,
-2.41883343376263,0.0155703681527833,0.217432206081947
"1120","CEBPA",176.099711056849,-0.427072656006406,0.176529756644732,-
2.4192672336024,0.0155518103626165,0.217432206081947
"1121","ZFP82",44.8710988384642,0.610517252534131,0.25234522451305,2.4
1937311757036,0.0155472836433822,0.217432206081947
"1122","LINC00205",54.3413079032725,0.827691578413827,0.34230621232355
1,2.41798585189417,0.0156066836157197,0.217432206081947
"1123","KREMEN1",250.748515536308,0.505165542859681,0.208846808301109,
2.41883295688843,0.0155703885639955,0.217432206081947
"1124","DCAF12L2",38.2510519634798,-0.665019243529565,0.27453474461445
3,-2.4223500178948,0.0154204897405892,0.217432206081947
"1125","AC092683.1",149.484413227177,-0.980229961195969,0.405539838491
804,-2.41709905700369,0.0156447589532085,0.217538634937815
"1126","APPL2",259.007028019317,0.442641149480031,0.18317388477654,2.4
1650795374037,0.0156701838747136,0.217698655730147
"1127","ELOVL6",32.660485748872,0.855905582780747,0.354294178166987,2.
4158048184956,0.0157004749208425,0.217925935391961
"1128","MXRA7",1194.53960208829,-0.531467172647769,0.220197923081066,-
2.41358848989738,0.0157962916208582,0.21906151580238
"1129","DNASE1L3",206.485121911409,1.78005659699081,0.738393817849122,
2.41071438297785,0.0159213111924704,0.220210671419563
"1130","PPIP5K2",206.297091819893,0.424652429824645,0.176175799844762,
2.41039024768912,0.015935465067247,0.220210671419563
"1131","ZNF572",6.92607149360006,1.54091420606716,0.639181736271381,2.
41076069390838,0.0159192898559795,0.220210671419563
"1132","UBAC1",223.522070540626,-0.429438902791477,0.178144298597399,-

2.41062389407138,0.0159252614195435,0.220210671419563
"1133","ZNF177",15.044438298004,1.35833914192581,0.563820492174306,2.4
091695154384,0.0159888695970542,0.22075365146224
"1134","TFRC",63.6784081531823,-0.848585211300331,0.352343922246475,-2
.40840030924876,0.0160226015467128,0.22087019213547
"1135","RANBP3L",18.7221451501474,-1.2353064927733,0.512930069092608,-
2.40833315730272,0.0160255493238994,0.22087019213547
"1136","Z95331.1",44.7617337836935,1.21137475663554,0.503131783587069,
2.40766891727465,0.0160547332420702,0.221077633895867
"1137","CREB3L1",201.14983362245,1.0016600924132,0.416194227123949,2.4
0671308522234,0.0160968104742788,0.22146209872396
"1138","PLCG1",51.0463379345064,0.624683683176484,0.259616246053225,2.
40618101784129,0.0161202748920286,0.221590035268896
"1139","DHFR",56.2205116829868,0.69758721118249,0.29008437307853,2.404
77349324034,0.0161824924017762,0.222178656683837
"1140","ZEB1",549.323602183538,0.549949380617001,0.228710067205153,2.4
0457006260112,0.0161915021811401,0.222178656683837
"1141","BECN1",165.388635957373,-0.426170001782288,0.177342948497367,-
2.40308399850821,0.0162574526010436,0.222888107833588
"1142","BPNT1",47.8176240942252,0.627161933406833,0.26108320627263,2.4
0215348340687,0.0162988682464397,0.223074785534289
"1143","SNED1",532.233567515507,0.799569249492148,0.332857431235685,2.
40213729500904,0.0162995895842033,0.223074785534289
"1144","ERMP1",24.1703667112726,0.851398452524498,0.354497207002948,2.
40170708176388,0.0163187697092551,0.22314205818346
"1145","PTPRS",134.955551509539,0.692820958222172,0.288548683374589,2.
40105395775715,0.0163479257332379,0.22334550414414
"1146","FAM117B",42.5209715570201,0.710151055143238,0.295868794203648,
2.40022289966287,0.0163850910526666,0.223462928802846
"1147","ADGRE5",158.214583409359,-0.453536055900568,0.188953222120587,
-2.40025573954557,0.0163836210297879,0.223462928802846
"1148","SWAP70",348.863124805069,0.500079724058269,0.20839573800944,2.
39966387429485,0.0164101326774633,0.223518772952661
"1149","NCF4",9.36231296188508,-1.46421326325193,0.610217595232553,-2.
39949368010918,0.0164177632246122,0.223518772952661
"1150","MCFD2",454.570488663554,0.359524313333375,0.149940090648802,2.
39778642108115,0.0164944797112079,0.223962633129163
"1151","AL442663.3",2.88066961665532,-2.48991069811851,1.0383833451822
9,-2.39787233652269,0.0164906115495959,0.223962633129163
"1152","ARID3A",56.8905608627959,0.570710352833316,0.238044499963711,2
.39749438831949,0.0165076338296954,0.223962633129163
"1153","NUDT10",3.22022571919126,-2.03375894600406,0.84809624499571,-2
.39802847613639,0.0164835837325121,0.223962633129163
"1154","KDM4A",106.213424032637,0.506439066340328,0.211336946134135,2.
39635840114247,0.0165588901094859,0.224463360470266
"1155","SERPINF2",16.1870092050711,1.21841985817723,0.508589325229836,
2.39568507975786,0.0165893366203698,0.224487017951942
"1156","LINC01894",29.0541677696698,1.06963686564975,0.446447315227878
,2.39588598512185,0.016580246860709,0.224487017951942
"1157","NEXN-

AS1",15.2109031340463,1.14375015878333,0.477944698976189,2.39305961805
492,0.0167085258721001,0.224932418431378
"1158","MPZL1",907.397609873639,0.374199094409575,0.156355706443865,2.
39325511630061,0.0166996249135892,0.224932418431378
"1159","KANSL1L",103.00993259448,0.466394002344461,0.194805824731047,2
.39414813693776,0.0166590189575466,0.224932418431378
"1160","ERLIN1",148.341655520829,0.437056420394696,0.182579728992808,2
.39378392555239,0.0166755692938529,0.224932418431378
"1161","ZMYM2",219.968121688685,0.528061919912333,0.220554059160285,2.
39425164933632,0.0166543178248278,0.224932418431378
"1162","C15orf56",4.90801532702329,-1.85380614306333,0.774534009645314
,-2.39344705329629,0.0166908901479099,0.224932418431378
"1163","ALDH2",658.108925544112,-0.616798949563151,0.257822429440794,-
2.39234015016056,0.0167413188460869,0.224986641502867
"1164","TUG1",346.483055744664,0.578840180085686,0.241938420681632,2.3
9251036877431,0.0167335552711478,0.224986641502867
"1165","NRM",42.222106411314,-0.863236955101683,0.361010493254029,-2.3
9116859823312,0.0167948385030685,0.2252100225088
"1166","TFPI2",2.64424807446761,-2.60874545821674,1.09104674861599,-2.
39104828599322,0.0168003431862742,0.2252100225088
"1167","NPAS3",13.1259945685857,1.22692629483384,0.513194025673673,2.3
9076496111435,0.0168133124942732,0.2252100225088
"1168","ADAP2",8.0312817525541,-1.32733838999664,0.55520524767954,-2.3
9071657831802,0.0168155281141902,0.2252100225088
"1169","LRRK2",195.253719794622,0.583166639419639,0.244055964509006,2.
3894791532461,0.0168722813678926,0.225776815601321
"1170","PARP15",13.5876181901325,1.17653353530073,0.492753156754278,2.
38767325825054,0.0169554086316434,0.226501671413149
"1171","FZD1",169.845941378024,0.670180765252578,0.280663088954286,2.3
8784789175372,0.0169473543979173,0.226501671413149
"1172","DAAM2",28.447987326819,1.01572527417442,0.425606438075075,2.38
653644143243,0.017007921670042,0.226998873802599
"1173","TNFSF12",504.971018924395,-0.590283272224056,0.247369642473995
,-2.38623974356962,0.0170216505127181,0.226998873802599
"1174","COL24A1",19.5360964683747,2.04408987946592,0.856846919003818,2
.38559517940778,0.0170515093852341,0.22701000962827
"1175","PRG4",306.761786281723,-1.96411902977646,0.823259086990626,-2.
38578481648612,0.0170427198450173,0.22701000962827
"1176","FSIP2",19.2258605076338,1.30637707376623,0.547995707224656,2.3
8391844414699,0.0171293980595904,0.227291165023022
"1177","CASD1",146.075892960679,0.459666870234735,0.192802122790045,2.
3841380145762,0.0171191807183265,0.227291165023022
"1178","TMEM245",208.88128760361,0.52997520198619,0.222294343844886,2.
38411465096025,0.0171202676507087,0.227291165023022
"1179","DDX21",340.280204025162,-0.468448500360942,0.196510398605905,-
2.38383568342559,0.0171332505788625,0.227291165023022
"1180","MCM5",64.5343744666733,-0.497243532910028,0.208612284924032,-2
.38357742493978,0.0171452774229474,0.227291165023022
"1181","SLC12A7",64.6689444198171,0.598283250713037,0.251095030036898,
2.38269650588114,0.0171863566943155,0.227450234999304

"1182","FAM184A",98.0741102764411,-0.733607434594867,0.30785909195928,
-2.38293249657184,0.0171753434503807,0.227450234999304
"1183","SOD3",5538.18387087799,-0.783407985093717,0.328938474707954,-2
.38162466640384,0.0172364554379759,0.227535757313297
"1184","RAB20",23.0604448976449,-0.922079632762391,0.387080058508252,-
2.38214191740062,0.0172122626895839,0.227535757313297
"1185","ZNF91",284.865466024442,0.509524334867309,0.21393299702349,2.3
817005415549,0.0172329047566454,0.227535757313297
"1186","BEND6",47.9063120823366,-0.911225420483655,0.382753232584901,-
2.38071253985172,0.0172791898968728,0.227907561177725
"1187","LINC00310",51.8727687620668,-1.20317537696847,0.50548601492165
4,-2.38023474725596,0.0173016122427668,0.228011053339175
"1188","EFEMP1",7487.92996560238,0.734318615493345,0.308594810615206,2
.37955594272447,0.0173335117645778,0.228123908073476
"1189","GARS-
DT",257.200577388842,0.493854198104143,0.207551297405274,2.37943199718
873,0.0173393419867905,0.228123908073476
"1190","AL022323.4",30.2513747347263,1.05294346195542,0.44269799331461
1,2.37846901918782,0.0173846977220154,0.22852842560125
"1191","AC245407.2",49.8642588078369,-0.750798711043985,0.315721002645
031,-2.37804487111717,0.0174047078441286,0.228599365915788
"1192","STON2",26.6560335107416,0.940449234134464,0.395833312239715,2.
3758718760005,0.0175075407852249,0.229757097737645
"1193","NLGN1",47.5228888693377,0.702781277257964,0.295971137189316,2.
37449260739379,0.0175730881047394,0.23023100269886
"1194","ZNF44",77.0504486123227,0.570377206183064,0.240209183862093,2.
37450207778286,0.0175726373086526,0.23023100269886
"1195","PINK1",316.399992379442,-0.587265261352734,0.247552483600892,-
2.37228587978755,0.0176784064173132,0.230305695746276
"1196","GABPB2",176.681045794745,0.741999870915665,0.312668295182138,2
.37312155517216,0.0176384580597779,0.230305695746276
"1197","FNDC3B",669.414606249905,0.507345448810686,0.213859135608179,2
.37233470231647,0.0176760703416517,0.230305695746276
"1198","HPF1",359.33312505862,-0.507461946214244,0.213933542257765,-2.
37205414755771,0.0176894981041511,0.230305695746276
"1199","PAM",1439.15760022118,0.558964056016351,0.235689740148927,2.37
160962400465,0.0177107919580033,0.230305695746276
"1200","PDE7B",71.4270135180508,0.782199289234344,0.329824457876755,2.
37156241920248,0.0177130545117395,0.230305695746276
"1201","LINC00242",5.53032191088493,-1.91589795026742,0.80790159406987
4,-2.37144964724716,0.0177184607628772,0.230305695746276
"1202","TMEM260",97.9228828326104,0.549360033057006,0.23168825284515,2
.37111733681281,0.0177344000275935,0.230305695746276
"1203","PIF1",7.5461797813817,1.63768138872293,0.690211335078674,2.372
7245634647,0.0176574258473137,0.230305695746276
"1204","ITGAM",10.953528063876,-1.7447484111304,0.735182420604376,-2.3
7321834993835,0.0176338360308906,0.230305695746276
"1205","NOL4L",21.4553881103146,0.868287240489215,0.366213679248817,2.
37098527359835,0.0177407379258622,0.230305695746276
"1206","NCOA3",209.734305884225,0.466044397191788,0.196616632104217,2.

37032031422836,0.0177726804235427,0.230529054614825
"1207","TP53INP1",109.228502761682,0.588095897635844,0.24820391513485,
2.36940620906938,0.0178166733336512,0.230717070329724
"1208","TM6SF1",4.48786787349873,-1.82696356304401,0.77105964534282,-2
.36941924542261,0.017816045266275,0.230717070329724
"1209","ISYNA1",52.3791062646084,-0.712964971863312,0.300992528487711,
-2.36871318848176,0.0178500897243201,0.230958605093085
"1210","COX15",103.792266203003,0.496585059960069,0.209705954837607,2.
36800648004782,0.0178842226599658,0.231060961771638
"1211","COL1A1",24662.49533272,0.93571820657304,0.395161647556968,2.36
79378106605,0.0178875423323822,0.231060961771638
"1212","CD1D",2.68207170435035,-2.26538722631992,0.957060706159592,-2.
36702563561539,0.0179316906958753,0.231249330218942
"1213","PREPL",201.942570724333,0.46451992787208,0.196234088330503,2.3
6717245114785,0.0179245785313713,0.231249330218942
"1214","KIRREL1",283.39697978093,0.645777514481882,0.272895818972347,2
.3663884515113,0.0179625863719541,0.231456951084413
"1215","C1QTNF6",100.385456346197,1.06195924900322,0.44904810326903,2.
36491200223817,0.01803435546406,0.232190471213408
"1216","TNIK",12.6385171496327,1.22617875476525,0.518601091173962,2.36
439678904173,0.0180594586377858,0.232299575042103
"1217","BDNF",11.408148568536,-1.86813279719258,0.790199257486702,-2.3
6412876814683,0.0180725297466112,0.232299575042103
"1218","CMTM8",141.170022942388,0.715624045048715,0.302760455355579,2.
36366418529872,0.0180952066132577,0.232400096101141
"1219","LINC00886",9.59153651648283,1.34214370127049,0.567898356364005
,2.36335197351798,0.0181104600553299,0.232405190029144
"1220","USP24",91.049946034379,0.618470463573476,0.261747525908603,2.3
6285123011796,0.0181349479254854,0.2325286806544
"1221","SGCD",258.845384341289,0.612628341426312,0.259506374542577,2.3
6074486611811,0.0182382733016342,0.233223280238374
"1222","ZDHHC21",122.964932838769,0.566596465091054,0.240012461850488,
2.36069602687549,0.0182406751588456,0.233223280238374
"1223","NEDD4",90.2061691767523,0.50314372015325,0.213069218793474,2.3
6140970057689,0.0182056050457563,0.233223280238374
"1224","TLCD2",94.0529398396311,0.605148873739686,0.256361251933108,2.
36053174641847,0.0182487563134802,0.233223280238374
"1225","PIK3R4",54.8178249580936,0.63200461060367,0.268251628539539,2.
35601406800226,0.0184722181152351,0.235348597635057
"1226","KCNIP4-
IT1",9.53374743107318,-1.88070052717173,0.798275763832185,-2.355953434
11564,0.0184752335163236,0.235348597635057
"1227","SLC7A8",75.1213100684749,0.79269685838127,0.336384354463161,2.
35652118733746,0.0184470152804275,0.235348597635057
"1228","SMURF2",276.369962552726,0.647054066077223,0.274579851764957,2
.35652420204199,0.0184468655455647,0.235348597635057
"1229","MIR99AHG",991.954627796124,0.38827068724031,0.164832679879024,
2.35554434669918,0.0184955892195031,0.235416193784123
"1230","LINC01750",11.7907871704483,1.40020759163282,0.594971845851813
,2.35340142797541,0.0186025393115817,0.236584977602497

"1231","GJA1",580.911595420289,0.764238804778084,0.324837065346163,2.35268350292376,0.0186384907646104,0.236657395317208
"1232","SURF1",279.248161057233,-0.508347649823873,0.216065452808141,-2.35274840663801,0.0186352380909638,0.236657395317208
"1233","WDFY3",156.136752233222,0.523930583652621,0.222844006177937,2.35110915765117,0.0187175419616224,0.23746837705244
"1234","IFIT5",104.268585489395,0.502970668846945,0.213967416710566,2.35068814018217,0.0187387317795301,0.237544555289456
"1235","AMPD2",59.1213956643617,-0.540481193133798,0.230138857208041,-2.34850037794883,0.018849180069452,0.238054173021607
"1236","CISD1",193.270937283687,-0.626269023267606,0.2666807334207,-2.34838495917754,0.0188550227177505,0.238054173021607
"1237","ACER3",100.627773413158,0.564826114541045,0.240439925057141,2.34913612789895,0.0188170259537978,0.238054173021607
"1238","ADAMTS15",52.5331713555984,1.43313818887701,0.61018785194311,2.34868358049618,0.0188399093696471,0.238054173021607
"1239","ZNF740",75.9575560351559,0.530989484080792,0.226055609364817,2.34893301507887,0.0188272935048049,0.238054173021607
"1240","HLA-DPB1",376.110467224226,-0.532990660976326,0.227267710298115,-2.34521067809053,0.0190163309459122,0.239674644097086
"1241","HERC4",219.729369399194,0.482080303262252,0.205558302025803,2.34522419435892,0.0190156415363391,0.239674644097086
"1242","AC108134.3",2.60086340097354,-2.52752959218703,1.0778581042087,-2.34495578065223,0.0190293363145548,0.239674644097086
"1243","ART5",7.26964376851757,-1.68380155834223,0.718929104963841,-2.34209680303167,0.0191757405895002,0.241324304136405
"1244","TNFAIP2",1027.26742024562,0.703397154221005,0.300449648212812,2.34114820371759,0.0192245341004484,0.241743880171474
"1245","STXBP5",103.78470294741,0.762867690845358,0.325962400530315,2.3403548679364,0.0192654245468764,0.241893215638514
"1246","HSF4",36.2004248045781,1.01890905766104,0.435372091187377,2.34031780696415,0.0192673366160959,0.241893215638514
"1247","POU2F1",145.118640888046,0.604317725188897,0.258284901840849,2.3397330656256,0.0192975268588026,0.242077957219125
"1248","TPT1-AS1",271.613642747174,0.562733931047556,0.240620239253167,2.33868078925597,0.0193519601205693,0.242566275774091
"1249","AC087071.1",8.53145136539415,1.48323300003406,0.634558837874745,2.33742391013209,0.01941715308711,0.24318857145049
"1250","TBXAS1",5.71187138376142,-1.5987867020998,0.684434114327929,-2.33592491757911,0.0194951550457264,0.243970168304238
"1251","KCNS3",34.1594707074727,-0.968227315386016,0.414817822863877,-2.33410249516628,0.0195903556843638,0.244876065587696
"1252","HS3ST3A1",6.09972858017058,-1.57238067393698,0.67374558826501,-2.33378993691978,0.0196067239925293,0.244876065587696
"1253","SDF2L1",158.307822927762,-0.741368100494579,0.317687238648197,-2.33364142560212,0.019614505541225,0.244876065587696
"1254","CAVIN1",2825.41191684283,-0.381420083924395,0.163621787836444,-2.33110815477497,0.0197476574060265,0.246341790113615

"1255","AC005229.4",16.0684892606794,-0.919861524204001,0.394741893374209,-2.33028604169962,0.0197910380017141,0.246489814857336
"1256","GPCPD1",106.790662923679,0.585963672246753,0.25144511704579,2.33038397854449,0.019785865787644,0.246489814857336
"1257","GPAT3",10.705205617316,-1.5493896762796,0.665008631207363,-2.32987904753445,0.0198125447163436,0.246561365948896
"1258","CSRP1",411.912858814476,-0.550265089412173,0.236213308756989,-2.32952619099999,0.0198312071588019,0.246597435282303
"1259","MT1H",9.85805407286234,-1.93995390162158,0.833042513482299,-2.32875738059532,0.0198719223967188,0.24690745198719
"1260","ZNF518B",52.0136166057459,0.626628367543107,0.269137750114749,2.32828121389861,0.0198971761711356,0.247025021305614
"1261","PLIN4",6.31367736615384,-2.50598570007033,1.07703638459717,-2.3267419150446,0.0199790055508398,0.247844237773027
"1262","WNT3",7.20838205344449,1.73638898656405,0.746414404845285,2.32630690845786,0.0200021837451394,0.247935150812373
"1263","SDS",3.77602927189931,-2.23215255271906,0.959689657356037,-2.32591081461551,0.0200233089868934,0.248000492859837
"1264","AL512625.1",19.9143395946714,0.864686974028378,0.371884031177711,2.32515220212608,0.0200638231430491,0.248305684673036
"1265","IVNS1ABP",124.784246104553,-0.624357614139811,0.268647829359126,-2.32407466544304,0.0201214926130928,0.248822536716688
"1266","NR4A2",102.079266140956,-1.30972869170268,0.564005421894108,-2.32219166848467,0.0202226174087385,0.249667947856159
"1267","DANCR",177.306720983762,-0.710388074956259,0.30594977054187,-2.32191079502392,0.0202377394286013,0.249667947856159
"1268","UBQLN2",167.665653170938,0.401453113340795,0.172853663653192,2.32250277405898,0.020205879223026,0.249667947856159
"1269","CAPN1",349.308983562384,-0.483174503851236,0.208223045112216,-2.32046603482742,0.0203156802495534,0.250431982776803
"1270","TMUB1",102.655060586607,-0.711397100239428,0.306685411686824,-2.31963136533498,0.0203608276010595,0.250596546504002
"1271","REPS2",39.9723428955882,0.831691307552408,0.358545293905953,2.31962689704292,0.0203610695267268,0.250596546504002
"1272","NET1",66.1358575587332,0.69828112293229,0.301446523144464,2.31643448943562,0.0205345574523433,0.252533083511797
"1273","CDC42EP3",17.0542787702349,1.2641134323381,0.546163290752487,2.31453386513115,0.0206384558533948,0.253213619540905
"1274","NBR2",28.2861233026395,0.708080831465291,0.30592070884165,2.31458940503373,0.0206354132528427,0.253213619540905
"1275","PAK3",92.6897107446134,0.615230282876869,0.265804637510874,2.3145957445972,0.0206350659821934,0.253213619540905
"1276","USB1",52.8449341466677,-0.622859326061688,0.269200538455704,-2.31373729649571,0.0206821367263399,0.25355067775089
"1277","MFAP5",10719.2301855792,0.910972181729398,0.39387522454599,2.31284458873861,0.0207311852735425,0.253952961028994
"1278","POLR2J",530.511328739378,-0.525445841406474,0.227384461149996,-2.31082563315467,0.0208424880511675,0.255116620175598
"1279","BMPR1A",177.289749041563,0.452526282237181,0.196021107674662,2.30855894860181,0.0209680681546189,0.25645308064324

"1280","AC005899.5",7.99290247169857,1.49004615541589,0.645797109268384,2.30729765437313,0.0210382322031126,0.257110208088509
"1281","AC004540.1",37.1481320491004,0.899042821196885,0.389864695126152,2.30603804970331,0.0211085063403515,0.257401479228531
"1282","OSGIN2",121.605078600115,-0.599090538368823,0.259864349588628,-2.30539717863262,0.021144339372797,0.257401479228531
"1283","KIF5B",501.212716015017,-0.406990786262797,0.176464480966501,-2.30636094036429,0.0210904726018314,0.257401479228531
"1284","GALNT8",32.6378753668486,0.997410708793017,0.432587575949928,2.30568505487654,0.0211282367941931,0.257401479228531
"1285","AC110048.2",13.3677805635167,1.58106255104978,0.685771853897727,2.30552266335149,0.021137318974768,0.257401479228531
"1286","KIAA1755",47.4722461888167,-0.753705786904221,0.327152270821913,-2.30383785816515,0.0212317470867103,0.258264556514315
"1287","C11orf52",15.5465439320783,-1.02207886969224,0.443812861245231,-2.30295009212787,0.0212816512844934,0.258670451471119
"1288","IGFLR1",19.8050175287368,-0.830016058925813,0.360543595416194,-2.30212398577676,0.0213281811270149,0.259034733982837
"1289","DUSP19",18.0867253921534,0.851408798262395,0.369947881701553,2.30142904007557,0.0213673920317784,0.259309630374793
"1290","EGR2",21.671835260882,-2.75302687415488,1.19640660171388,-2.30107964149571,0.0213871298446659,0.259332537585878
"1291","EMX2OS",567.235201338527,-0.415026770556584,0.180472586356505,-2.29966655288433,0.0214671184269895,0.259332537585878
"1292","AC073896.2",25.7377797665466,-0.73851483217582,0.3211712497513,-2.29944253337648,0.0214798230538209,0.259332537585878
"1293","UBAC2",213.450298279495,-0.57026704123679,0.247870892988938,-2.30066158377959,0.0214107671308995,0.259332537585878
"1294","PDPR",101.371787180556,0.473718550119249,0.206011204087883,2.2994795463511,0.0214777235172767,0.259332537585878
"1295","CMTR2",88.2024340534385,0.584202356762662,0.254073322860231,2.29934552036398,0.0214853269009332,0.259332537585878
"1296","FCHO1",3.55582692609505,-2.1554511637062,0.937348648586066,-2.2995191457923,0.0214754774629742,0.259332537585878
"1297","MSRA",55.5016584202791,-0.684917695629661,0.297996865792126,-2.29840570238561,0.0215387092678155,0.259776429511518
"1298","HSPA12A",126.9560394387,0.679420251579279,0.295656727338292,2.29800369399977,0.0215615788835901,0.259851909457627
"1299","ENPP4",9.23270910733816,1.40862353445292,0.613144164147078,2.29737738173273,0.0215972509129106,0.259854599302371
"1300","DDX11",31.9927156331304,1.1903294006575,0.518079679353941,2.29757978954487,0.0215857170274666,0.259854599302371
"1301","SYNGR1",109.599860174753,0.486482325256976,0.211778772683596,2.29712505692813,0.0216116367507757,0.259854599302371
"1302","ARAP1",248.682805389899,0.496327783413697,0.21613246869873,2.29640547022824,0.0216527084959962,0.260148478496827
"1303","TRNP1",3.45842918926752,-2.29461549546132,1.00033113695947,-2.29385591498818,0.0217987763931218,0.261514063961721
"1304","OMA1",94.9295985073497,0.47914160856375,0.20890854627981,2.29354718653775,0.0218165219887519,0.261514063961721

"1305","AC007098.1",13.2906923336614,1.08395285703646,0.472582412755444,2.29368006040756,0.0218088829081597,0.261514063961721
"1306","LRFN4",56.5875127577434,-0.646683765069447,0.282002761902225,-2.29318238129052,0.0218375070601031,0.261565178362322
"1307","NPR3",98.5907834972026,2.29789913893114,1.00220294800855,2.29284811374505,0.0218567508982102,0.26159537436932
"1308","COPS3",160.531412978508,-0.475806238486768,0.20760264381701,-2.29190837716963,0.0219109307656976,0.262043340953982
"1309","FGF11",7.69726310625216,1.4106610615556,0.615610291539638,2.29148388346066,0.0219354429494076,0.262136084077604
"1310","HSPA4L",9.0157218345852,1.43224184194821,0.625486944486916,2.2898029360518,0.0220327428900679,0.263097860327735
"1311","GDF10",656.019845768453,-0.882187560527323,0.385395477821704,-2.28904491955521,0.0220767425954058,0.263257776639413
"1312","PQBP1",221.923919558704,-0.557081711942668,0.243374206125002,-2.28899241547618,0.0220797930672448,0.263257776639413
"1313","ITGBL1",398.069367387997,-0.765191322620551,0.334358573385028,-2.28853507440768,0.0221063799609717,0.263374030258553
"1314","TSPAN9",94.7380488881457,0.47393993906518,0.207195384646249,2.28740586994421,0.0221721439941538,0.263616058520347
"1315","YPEL1",33.1184337902653,0.759397116346831,0.331974445386958,2.28751678600338,0.0221656767978444,0.263616058520347
"1316","PRR14L",142.986578908194,0.473734638756947,0.207113560622572,2.28731830659918,0.0221772507199883,0.263616058520347
"1317","DANT2",16.9465658619382,1.48461873980064,0.649163891605418,2.28697060788316,0.0221975387311973,0.263656870442004
"1318","WDR76",12.2699981364022,-1.29234891610778,0.565225985248202,-2.28642870256625,0.0222291907628139,0.263832497043018
"1319","CDK6",98.2514791903399,0.676707246926917,0.296107914251967,2.28533995329516,0.0222929019477197,0.264388070635466
"1320","ATPSCKMT",49.0520435888039,0.610483579620961,0.267221641125617,2.28455890417192,0.0223387049597789,0.264682041095427
"1321","SNHG30",105.615588347418,-0.605582982475511,0.265101893441819,-2.28434046476705,0.0223515295203644,0.264682041095427
"1322","RPL22L1",498.327608560813,-0.561779599443426,0.245976541807648,-2.28387469518429,0.0223788961950627,0.264805652934468
"1323","ANKFN1",4.66951263578316,1.75230209976358,0.767697598597896,2.28254211419176,0.0224573540488388,0.265533174139067
"1324","CXCL8",306.118064126885,-2.03702130526269,0.892891291628528,-2.28137660694103,0.0225261710829723,0.266145690521855
"1325","WDR26",177.958712568269,0.494666692637297,0.216913822804065,2.28047565730341,0.0225794929302242,0.266569373264468
"1326","PTK2",217.062827459194,0.366769728153634,0.160850143241835,2.28019522246991,0.022596112571034,0.266569373264468
"1327","PDE1C",32.5632639418288,1.44256182844651,0.632971987359127,2.27902949459917,0.0226653120674268,0.266983039661715
"1328","FKRP",38.0822855286119,0.668852661180516,0.293471577270599,2.27910541593537,0.022660799653858,0.266983039661715
"1329","FAM241A",67.8967925958919,-0.654301985077697,0.287280894046758,-2.27756874416856,0.0227522844961731,0.267604501032809

"1330","NRBP2",170.519118503617,0.512363336013518,0.224947729794289,2.27769951927083,0.022744486404062,0.267604501032809
"1331","COQ8B",73.1020578886479,-0.478452492888063,0.210151908730044,-2.27669829781404,0.0228042482469234,0.268014166285967
"1332","LINC02613",6.61944221253515,1.53939423112719,0.676828388702724,2.27442326123132,0.0229405501811199,0.269337103782887
"1333","CTSC",251.555256181268,0.812756443258059,0.357374146985655,2.27424521363232,0.0229512471611959,0.269337103782887
"1334","AJUBA",5.95684223686745,1.59121556743334,0.699803406218917,2.27380369014033,0.0229777922912519,0.269445761921595
"1335","RND2",11.0661417880361,-1.09328064547294,0.480876082841896,-2.27351844785425,0.0229949557096036,0.269445761921595
"1336","LINC02591",42.9077971809877,0.610517423913595,0.268879382446631,2.27059962113226,0.023171226431133,0.269914412702294
"1337","LINC01871",13.1009904952184,1.6918118063933,0.744575712389808,2.27218236942382,0.0230754976884586,0.269914412702294
"1338","BTN3A3",108.162573868674,0.627821269249646,0.276538570187837,2.27028464356058,0.0231903181822225,0.269914412702294
"1339","USP49",32.978390793446,1.00311350852041,0.442003498354954,2.26946961337136,0.0232397830575157,0.269914412702294
"1340","DMTF1",176.403150132345,0.709032695132485,0.312394749141028,2.26966905520041,0.0232276703055057,0.269914412702294
"1341","WDR86",161.088877208003,0.900651076544158,0.396779205727826,2.26990493337992,0.0232133517305761,0.269914412702294
"1342","GLUD1",455.601418627439,-0.3760282905642,0.165486475813944,-2.27225994580348,0.0230708145094775,0.269914412702294
"1343","AC068888.1",157.9702044226,-0.574810201215301,0.253164993256361,-2.27049638191183,0.0231774825709939,0.269914412702294
"1344","TBC1D4",159.222130727262,0.515423804145173,0.226979416508115,2.27079535261183,0.0231593694261196,0.269914412702294
"1345","PTPN21",92.4552208696896,0.518841143784237,0.228567867490108,2.26996536950536,0.0232096842942044,0.269914412702294
"1346","ECSIT",160.083876982329,-0.60513308884482,0.266645054985346,-2.26943300665402,0.0232420068982926,0.269914412702294
"1347","PSMF1",340.201428245605,-0.373668297062735,0.164601732748038,-2.27013586566991,0.023199340812334,0.269914412702294
"1348","LY6E-DT",15.2166114725478,0.933365773087782,0.411530126559769,2.26803753321867,0.0233269191285829,0.270699551875685
"1349","ALCAM",51.7422640568851,-0.820498610377085,0.361819736138967,-2.26769998544786,0.0233474987504341,0.270737526280979
"1350","EPB41L4A-AS1",729.701269280143,-0.639026948271043,0.281925845673862,-2.2666490429199,0.0234116736001642,0.271280600094347
"1351","PLEKHO2",58.4594131091388,0.635846614235182,0.280578373989668,2.26619965464122,0.0234391617856879,0.271398081283135
"1352","SEL1L3",21.872608309889,0.975770919256352,0.430690439246409,2.26559688894809,0.0234760757447355,0.271423690225349
"1353","NAV2",96.1043781235229,-1.27162437274159,0.561221672606905,-2.26581480154681,0.0234627247455682,0.271423690225349

"1354","LINC01232",11.0259299294456,1.26419270627638,0.558557404950745
,2.26331742283116,0.0236161290316303,0.272841289838842
"1355","USP25",130.061753662022,0.423445995713294,0.187137234144856,2.
26275651474853,0.0236507028806235,0.273039073919995
"1356","PTPMT1",261.828110225765,-0.581224682304992,0.257100109443587,
-2.26069402911913,0.0237782104747435,0.274308662578476
"1357","GALNT17",25.2173579478394,1.41150418846412,0.624481895864187,2
.26028039853872,0.0238038537332893,0.274402125239384
"1358","AC106707.1",38.8894469200377,-0.822148505568895,0.363845670911
993,-2.25960777136127,0.0238456048540824,0.274562033148628
"1359","PKIA",34.7623585749866,-0.852776124634572,0.377419489108867,-2
.25949149220693,0.0238528289362005,0.274562033148628
"1360","KCTD12",852.230864227202,0.849027758839147,0.375835486648117,2
.25904096074373,0.0238808370614696,0.274682304523948
"1361","MEGF6",227.784412898685,0.69508151469799,0.307738377307112,2.2
5867673957455,0.023903500393929,0.274740967422653
"1362","EIF2A",420.071266827691,-0.429370588252859,0.190152610006174,-
2.25803152656657,0.023943693983942,0.27500088472159
"1363","ACVR1B",70.4900437561513,0.678197533869725,0.300398357740457,2
.25766059099326,0.0239668279666 4,0.2750646294073
"1364","GNG10",492.195047269039,-0.438560037577534,0.194474879460315,-
2.25509864715987,0.0241271375485687,0.276688086360156
"1365","NIBAN2",63.9283599784018,-0.55933459575554,0.248060047934392,-
2.25483547396345,0.0241436577307175,0.276688086360156
"1366","GPD1",34.8652584370142,-2.44494396041446,1.08467984799761,-2.2
5406968233805,0.0241917846001296,0.277036666544529
"1367","NABP1",292.467614357507,0.94376207773968,0.418848086434591,2.2
5323239691358,0.0242444997181349,0.277437241470947
"1368","DLL1",21.6829962555552,0.921923524938312,0.409288121127244,2.2
5250496496011,0.0242903793170583,0.277759067000543
"1369","ACSL1",141.227583215674,0.574302725560722,0.255022705153918,2.
25196703648055,0.0243243551952774,0.277944403447571
"1370","VLDLR",72.7525856386288,1.34728256933245,0.598376894156896,2.2
5156182079984,0.0243499760491551,0.278034069589003
"1371","UAP1",455.772606087811,-0.53801325154687,0.239042617918336,-2.
25070013134926,0.0244045364509118,0.278258681436545
"1372","PENK",15.8628703143283,1.30194277650716,0.578529752265027,2.25
043357132433,0.0244214359255632,0.278258681436545
"1373","LINC00944",5.29447207934312,1.63759767054759,0.727688997584394
,2.25040872678258,0.0244230115458912,0.278258681436545
"1374","HLA-
DMA",54.2331459673284,-0.761147649222298,0.338423079756273,-2.24910088
806728,0.0245060780721638,0.278845582338388
"1375","ZNF134",40.4347643187044,0.646513892727068,0.287462608908542,2
.24903647532386,0.024510175523575,0.278845582338388
"1376","JUND",6013.58292886477,-0.576472024489491,0.256558748979215,-2
.246939645532,0.0246438845628114,0.280162998703531
"1377","LGALSL",61.1298386316136,-0.700113104812244,0.311754254300477,
-2.24572109331171,0.0247218782244031,0.280438245876967
"1378","AC004951.1",25.2643679970876,1.06905931144852,0.47600849258112

9,2.24588285316424,0.0247115124625683,0.280438245876967
"1379","DCT",4.21681094148513,2.18977785665153,0.974988804139193,2.2459517969387,0.0247070956087266,0.280438245876967
"1380","MDFI",18.8649910166101,1.73022966928012,0.770660414082106,2.24512591754295,0.024760050218933,0.280667728677369
"1381","TPMT",47.0765450044702,-0.624630606662294,0.278452346547691,-2.24322263542251,0.0248824613457897,0.281647136636895
"1382","BZW2",181.230742447377,-0.492921471894446,0.219722256147372,-2.243384355037,0.0248720398828181,0.281647136636895
"1383","MNDA",14.9333643113474,-0.978832191733762,0.43676610473961,-2.24109009630525,0.0250202395875074,0.282251973147087
"1384","CFAP36",344.110250932475,-0.489602828587847,0.218475215066385,-2.24099941240053,0.0250261130700639,0.282251973147087
"1385","ITPR3",96.2580654824934,0.658235542406743,0.293665877965959,2.24144373519297,0.0249973462420763,0.282251973147087
"1386","RSPH9",5.16968572978223,-1.6138671804825,0.7200514628968,-2.24132199383339,0.0250052253041302,0.282251973147087
"1387","KCNB1",9.89808402582587,1.1313995775145,0.504620249764658,2.24208120471217,0.0249561245059736,0.282251973147087
"1388","ZNF669",28.1275844194735,-0.691057699082337,0.308451899014362,-2.240406693201,0.0250645321566412,0.282278240839697
"1389","C11orf80",12.7699091763755,-0.943456835321366,0.42107429202222,-2.24059471973554,0.0250523390588182,0.282278240839697
"1390","AC009549.1",3.02562616552292,-2.72431932636519,1.21676850845874,-2.23897915456082,0.025157272520402,0.282915322815707
"1391","GSDMB",7.14917030664529,1.46514113077365,0.654340752757993,2.23911031767194,0.0251487391051999,0.282915322815707
"1392","RAB34",815.758077525747,-0.497968243314734,0.222500256792829,-2.23805693751803,0.0252173423593899,0.283387131126391
"1393","CDC23",74.4607380871895,0.574463892425387,0.25675179378728,2.2374289345816,0.0252583191912991,0.283440378127325
"1394","ZNF268",66.2798000252653,0.503267890020495,0.22491420332774,2.23759941601885,0.0252471896803074,0.283440378127325
"1395","SV2A",2.76932174361947,-2.25380548033305,1.0078034068715,-2.23635429783819,0.025328572314037,0.283852423067868
"1396","WDR11",213.050292240467,0.506313405882638,0.226405512851503,2.23631217944204,0.0253313291953426,0.283852423067868
"1397","CYP1B1",145.84849608235,1.08765847289765,0.48664240753885,2.23502608085141,0.0254156365630277,0.284389701541804
"1398","SLC43A1",164.271290999541,0.408853245766405,0.182918827247327,2.23516218597657,0.0254067030177459,0.284389701541804
"1399","HSD11B1L",51.8297598726147,-0.656520548994029,0.293850880642267,-2.23419629561456,0.0254701600791537,0.284796078712081
"1400","C1orf220",22.1081242053072,0.892498533361313,0.399625890590038,2.23333511260634,0.0255268536715324,0.28481923821953
"1401","AL157394.3",19.26866725138,-0.856198382720397,0.383355523456443,-2.2334317111194,0.0255204889450115,0.28481923821953
"1402","NYNRIN",96.5248680060286,0.469438738781932,0.210177796781193,2.2335315431565,0.0255139126093195,0.28481923821953
"1403","GCHFR",114.194750583766,-0.537610549121568,0.240776605149834,-

2.23281887701264,0.0255608909209993,0.284995735336559
"1404","ADAMTS14",4.20376780922893,3.26021944122743,1.4622941323438,2.22952371148606,0.0257790794402638,0.287019316501101
"1405","RBM26",319.81884479683,0.4491724614206,0.201445452479066,2.22974733801587,0.0257642212870615,0.287019316501101
"1406","BTBD8",86.1541138367995,0.632819178372679,0.283937471800699,2.2287272418093,0.0258320585465578,0.287095083005172
"1407","PABPC1",1407.8842404174,-0.474606813041442,0.21293919656167,-2.2288372488716,0.0258247355612976,0.287095083005172
"1408","MED16",101.142141065867,0.565920062533602,0.253935939444416,2.22859380902039,0.0258409433530194,0.287095083005172
"1409","SDK1",188.387769555182,0.661824172869514,0.297123037326659,2.227441462716,0.0259177839403437,0.287540350481416
"1410","CXorf40B",43.8141511701835,-0.805429190461882,0.361578540423224,-2.22753593042091,0.0259114772329589,0.287540350481416
"1411","FAM178B",3.54146967118848,-1.9238242494055,0.864222625901804,-2.22607484662648,0.0260091684487083,0.287869316667561
"1412","ADCY1",28.7750107266723,0.847300620892556,0.380564579077913,2.22643059147942,0.0259853533006242,0.287869316667561
"1413","TMEM123",499.268333341521,0.384350783555523,0.172719960268264,2.22528295489739,0.0260622489759718,0.287869316667561
"1414","GOLGA6L4",57.7610235783066,0.808527836812672,0.363124201881582,2.22658757698651,0.0259748499870078,0.287869316667561
"1415","ARRB2",41.8908406402797,-0.707059590995221,0.317730953668283,-2.2253405997497,0.0260583818818762,0.287869316667561
"1416","FDXR",36.5724515153882,0.660630985962281,0.296902892196477,2.22507426948574,0.0260762527467835,0.287869316667561
"1417","POLI",101.659541738003,0.463202205987948,0.208132721200582,2.22551362090515,0.0260467777687693,0.287869316667561
"1418","SLC30A7",164.247622225578,0.413661502033908,0.18599300299275,2.22407023585738,0.0261437190831927,0.288262105398707
"1419","MAP3K5",45.9057433132249,0.662553712264262,0.297911327300332,2.22399637592942,0.0261486880752263,0.288262105398707
"1420","FERMT2",749.904106023709,0.464827778155575,0.209072642097796,2.22328360847014,0.026196682101309,0.288384727734537
"1421","ZNF429",107.56869480874,0.594194892489476,0.267259751087213,2.22328611050594,0.0261965134942582,0.288384727734537
"1422","GATD3B",258.219109198936,-0.539785461250969,0.242891492552395,-2.22233169049562,0.0262608979948313,0.288888345522606
"1423","PLOD1",140.112585232652,0.554267025860911,0.249531854336898,2.22122753559385,0.0263355540809527,0.289032286267279
"1424","PDPN",191.647148522963,0.936517604739779,0.421588384528764,2.22140276892729,0.0263236936637608,0.289032286267279
"1425","AL162258.2",6.7160248190842,-1.55993260952037,0.70234162847618,-2.22104535210998,0.0263478898048418,0.289032286267279
"1426","LINC02482",44.319356726836,0.770684170812889,0.346896413316192,2.22165505675154,0.0263066260301616,0.289032286267279
"1427","PTGFR",783.902359583175,-0.750707212270894,0.338368148542229,-2.2186107513521,0.0265132174759279,0.28974079841896
"1428","PGAP1",61.4086976474795,0.620927875291643,0.279891151578276,2.

21846196919873,0.0265233498944498,0.28974079841896
"1429","AC117453.1",7.68763168480835,1.64952825423752,0.74353217835414
7,2.2185028466271,0.0265205657111309,0.28974079841896
"1430","FAM171A1",99.4563232955102,0.603877537894671,0.27220596238794,
2.21845815792251,0.0265236094953622,0.28974079841896
"1431","MCU",78.8080076643836,0.604602024291751,0.272369807058346,2.21
978357594619,0.0264334623393536,0.28974079841896
"1432","LINC02254",31.8727324252868,0.969874601552229,0.43716899576093
9,2.21853473360813,0.0265183940472374,0.28974079841896
"1433","RGS22",43.9819377348113,-0.78227515431587,0.352883402156792,-2
.21680914867255,0.0266361360812939,0.29056420970689
"1434","USF2",286.083551966495,-0.342200838520707,0.15434928295219,-2.
21705492876636,0.0266193382122856,0.29056420970689
"1435","SLFN12",54.6735063931191,0.574630105456795,0.259280289554006,2
.21625063148931,0.0266743420482406,0.290778210913329
"1436","PDZD2",8.46622313630227,1.39238501401587,0.628394068315057,2.2
1578319118979,0.0267063541767302,0.290924441773392
"1437","SPATA9",4.56466219201961,1.81731410341236,0.820291224313693,2.
21544989090044,0.0267292001343079,0.290970687335406
"1438","MLPH",10.867794794493,1.25033389845995,0.564499912672571,2.214
94081821973,0.0267641269278403,0.291148287574552
"1439","EDNRA",5.98407065858855,1.48856291158865,0.672550067702485,2.2
1331166715033,0.0268761658030827,0.291556076045508
"1440","JRK",53.4986292023165,0.906379005698997,0.409385737525867,2.21
399751534267,0.0268289498554828,0.291556076045508
"1441","AGPAT2",275.936130359159,-0.580863365352191,0.2623777255787,-2
.21384404514918,0.0268395090015438,0.291556076045508
"1442","KCNMA1",15.4066926274698,-1.00881359603133,0.455769468622198,-
2.21342951971092,0.0268680473606725,0.291556076045508
"1443","SLC25A38",82.180138148713,-0.486589815673818,0.219909741758436
,-2.21267967386512,0.0269197377081208,0.29182637350529
"1444","PICALM",270.486687687513,0.330737765267387,0.149557344293483,2
.21144449194262,0.0270050717201649,0.292346253922865
"1445","KCTD15",11.3914162991355,1.17854525796896,0.532928709892572,2.
21145011723338,0.0270046825615643,0.292346253922865
"1446","ACAP2",393.27878716067,0.298122562229591,0.134903361118304,2.2
0989721648337,0.0271122964721391,0.292620054883104
"1447","BBS9",73.9715881195928,0.477868532279149,0.216231683171946,2.2
0998387132357,0.0271062816732218,0.292620054883104
"1448","PLCE1",70.6061656792253,0.661736684023492,0.299464926560808,2.
20973017315643,0.0271238943668414,0.292620054883104
"1449","FGF7",2396.17602091166,0.392038990443365,0.17736201374165,2.21
0388696953,0.0270781975783204,0.292620054883104
"1450","SERPINB7",6.66551845843835,2.56711874684866,1.16134275537295,2
.21047467250465,0.0270722364039427,0.292620054883104
"1451","PCBP1",1122.07008123085,-0.286599653943971,0.129778593965634,-
2.20837385570584,0.0272182227462717,0.293233235826397
"1452","PELI3",48.4907726163814,-0.629315016913458,0.28493794340864,-2
.20860377310625,0.0272022126850656,0.293233235826397
"1453","ST3GAL6",11.3587444476573,-1.08516728431651,0.491595222937433,

-2.20744066191755,0.0272832882453055,0.293731918803382
"1454","GBE1",154.011299495917,-0.433172884571802,0.19627356248407,-2.20698538860504,0.0273150801579089,0.293751290463149
"1455","RNF213",538.610502346696,0.469497506556704,0.212742929731297,2.20687713170867,0.0273226444814858,0.293751290463149
"1456","ANKRA2",120.062471452171,0.483325184130585,0.219071954545432,2.20623943002413,0.0273672398310791,0.294028662553277
"1457","ACKR1",3.96323449966705,-2.01544204110171,0.913725948485748,-2.20574018330305,0.0274021966508339,0.294202170356208
"1458","TRAPPC11",124.991447428911,0.421841490921468,0.191309095309342,2.20502579994622,0.0274522841466398,0.294537778399099
"1459","PNISR",2297.18721716923,0.444325466050207,0.201571540120976,2.20430654934491,0.0275027926698364,0.294877440530672
"1460","LINC01719",17.6612463178325,1.07815857785616,0.489533175970772,2.20242188022929,0.0276355217202517,0.294951249659343
"1461","FH",157.655216391781,-0.567787407793443,0.257747565230149,-2.20288175093507,0.0276030841753059,0.294951249659343
"1462","SOD2",1640.07913516222,-0.731901600526597,0.332163956253499,-2.20343473982477,0.027564121915523,0.294951249659343
"1463","C1RL",327.895990855206,0.501458904325107,0.227618722628357,2.20306527747219,0.0275901480763214,0.294951249659343
"1464","CEP290",214.170802614308,0.512720695889681,0.232800950019277,2.20239949986125,0.0276371011855505,0.294951249659343
"1465","ELMO2",49.0585915363645,0.581432001919207,0.263948606727936,2.20282277344436,0.0276072423884993,0.294951249659343
"1466","DIP2A",149.360328590948,0.573856771599494,0.260567445779478,2.20233486912697,0.0276416628524322,0.294951249659343
"1467","PRXL2C",220.036033160777,-0.354280237355363,0.160975630472614,-2.20083149427786,0.0277479551173232,0.295883614110625
"1468","LHFPL2",123.766193191956,0.46664851343205,0.212099158583378,2.20014316204185,0.0277967393961154,0.296039278768584
"1469","SLC25A28",133.276387020455,-0.481691872088154,0.218941743474247,-2.20009151495961,0.0278004027687176,0.296039278768584
"1470","UBL4A",139.110979353117,-0.547986029050887,0.249145768381643,-2.19945950762237,0.0278452653274145,0.296315296269895
"1471","LINC02427",7.80886012228479,1.66424729748616,0.757229091570542,2.19781214960245,0.0279624952798106,0.297158501129128
"1472","KIF1C",40.8075155959634,-0.599923818350941,0.272953389068686,-2.19789840455132,0.0279563466321104,0.297158501129128
"1473","SMAD5",461.916236356631,0.354107747971143,0.161220425860703,2.19641987720028,0.0280619042229927,0.297810290203714
"1474","PCDHB3",16.9232481299812,0.968729135917992,0.440995747741974,2.19668588841994,0.0280428873837579,0.297810290203714
"1475","AC104506.1",9.29086563111334,-1.14625040021058,0.522040019143857,-2.19571365829467,0.0281124449434294,0.298144390678011
"1476","PCDHGA8",20.8696526882639,1.25910267372886,0.573621696929537,2.19500531529498,0.0281632164614413,0.29828796909833
"1477","NDE1",87.7135207135081,0.451941187320249,0.205896440352978,2.19499271840477,0.0281641200765987,0.29828796909833
"1478","MVK",35.4256960237714,-0.64884404919444,0.295861959087435,-2.1

9306345160342,0.0283028078169828,0.2995540072267
"1479","SHPRH",365.743421125383,0.584459723883931,0.266568471102916,2.19253132775138,0.0283411635789366,0.29968826736634
"1480","ADAM9",420.12492220955,0.47640296926843,0.217301826546098,2.19235602774543,0.0283538090968601,0.29968826736634
"1481","RASA2",136.111735374151,0.512320578946233,0.233787646001313,2.19139286317702,0.0284233751210988,0.30022070021563
"1482","SETX",346.974871225887,0.464091026998179,0.211840030699113,2.19076170573894,0.0284690411291264,0.30031937360675
"1483","C11orf54",175.817742777118,0.430956267095849,0.196717834190017,2.19073308157497,0.028471113664822,0.30031937360675
"1484","PLA2G4A",40.3231560006746,0.62794546176636,0.286790278686512,2.18956327474671,0.0285559249193769,0.30050293921698
"1485","NUP153",103.632680076119,0.495012263902678,0.226034894245785,2.18998162011399,0.0285255698261994,0.30050293921698
"1486","RGS17",15.3529362237853,-1.16521251080676,0.53217000675282,-2.18954938463485,0.0285569332613759,0.30050293921698
"1487","SYNGR2",9.40488880726139,-1.17729785495475,0.537718055690015,-2.1894333703264,0.0285653564287956,0.30050293921698
"1488","PRPH2",9.48082226599053,1.56149641966907,0.71391100698776,2.18724239349883,0.0287248336065523,0.301786654665783
"1489","MAP3K7CL",19.2176566361438,-0.987435735085819,0.451455568635282,-2.18722683623278,0.0287259687270568,0.301786654665783
"1490","ACVR2A",254.317775292394,0.73250050440243,0.334976683147148,2.18672087119764,0.0287629070264117,0.301971915848428
"1491","UGGT1",163.286681985108,0.454921735689417,0.208149722092055,2.18555053120957,0.0288485051861619,0.302667449112763
"1492","CENPA",31.7864602122078,2.65981538191584,1.21743820177774,2.18476418600294,0.0289061412392679,0.302711289933636
"1493","SRRM3",64.0966698734472,-0.744882000792214,0.340949905941562,-2.18472563802345,0.028908969203693,0.302711289933636
"1494","ZNF471",43.821877506528,0.706018669839306,0.323164816027494,2.18470153563759,0.0289107375286615,0.302711289933636
"1495","PSMC5",757.921395261347,-0.556359637791288,0.254770124431491,-2.18377111143931,0.0289790713774834,0.303223821777909
"1496","MAN2B1",215.292168889247,-0.362375133356642,0.166018728236967,-2.1827364731972,0.0290552223137722,0.303817408191403
"1497","ANPEP",557.634859222207,0.561107918659249,0.257102096074651,2.18243229917632,0.0290776427154875,0.303848740813876
"1498","N4BP2L2",1226.60011058973,0.404462683509375,0.185381660628843,2.18178368959138,0.0291255008945871,0.304145667886533
"1499","THEMIS2",13.0967736128673,-1.02820427869416,0.471556840385101,-2.18044611091734,0.0292244092899227,0.304163296421997
"1500","DISP1",42.6585766947388,0.887825238089934,0.406999513466834,2.1813913990398,0.0291544792542516,0.304163296421997
"1501","EMILIN1",866.983195536822,0.56963722334374,0.261203928901489,2.18081414678328,0.0291971657838265,0.304163296421997
"1502","NEDD4L",11.9694908989269,1.24840560051388,0.572413908027107,2.18094910519672,0.0291871810930321,0.304163296421997
"1503","LGALS2",14.2752679936875,-1.46978230531738,0.674003874459721,-

2.18067337742761,0.0292075835199287,0.304163296421997
"1504","IRF7",74.1555539482797,-0.647958768266907,0.297634346952876,-2
.17702954951465,0.029478362767166,0.305714006029413
"1505","NUMA1",644.716453566045,0.418173034577252,0.192098769946306,2.
17686471753117,0.02949066260297 8,0.305714006029413
"1506","SNRNP25",141.94743062332,-0.769055903014681,0.353229244714207,
-2.17721469703595,0.0294645522391277,0.305714006029413
"1507","GJC1",5.05460943519456,-2.33347681172124,1.07132855994681,-2.1
7811500501498,0.0293974757514251,0.305714006029413
"1508","SLC38A10",347.439408334594,0.366143361139044,0.168197263954358
,2.17686871076809,0.0294903645736967,0.305714006029413
"1509","TRO",16.784637123975,0.985386693421122,0.45247544259266,2.1777
683398129,0.0294232881045489,0.305714006029413
"1510","ZDHHC14",165.843696287144,0.636052959024573,0.292223772055838,
2.17659554029381,0.0295107582187237,0.305719729016884
"1511","ASAP3",187.522489843698,0.401426218746134,0.18454943648403,2.1
7516903001149,0.0296174520649755,0.30601440069512
"1512","FUT11",115.309373626937,0.496891030538857,0.228411917186267,2.
1754163997216,0.0295989266517223,0.30601440069512
"1513","LRTOMT",33.4532871246054,-0.702363425335517,0.322786777162972,
-2.17593617529414,0.0295600333453741,0.30601440069512
"1514","FAM20A",115.376808793195,0.622819484182987,0.286311776179558,2
.1753191311013,0.0296062098684583,0.30601440069512
"1515","GNAI1",220.744906905161,-0.46314913621156,0.213072127198723,-2
.17367302941318,0.0297296995528069,0.306601996272207
"1516","PTENP1-
AS",2.92025068179441,-2.2035359679641,1.01375970649006,-2.173627491660
13,0.0297331220574658,0.306601996272207
"1517","GANC",66.9972072740538,0.491078681027741,0.225889724984058,2.1
7397529286646,0.0297069907663383,0.306601996272207
"1518","MARK3",286.018579953554,0.445299703949318,0.204916093200759,2.
17308312389624,0.0297740616284651,0.306821901221396
"1519","ZNF146",124.805830827754,0.404412476768708,0.186202626769137,2
.17189458487134,0.0298636150267962,0.307542152642642
"1520","APLF",21.9611286726367,0.750269186134981,0.345490654187613,2.1
7160486699463,0.029885479603712,0.307564840421623
"1521","ODF2L",557.029022761061,0.382256895256114,0.176101569435183,2.
17066149087791,0.0299567701835845,0.307691238333429
"1522","GATM",5.2618084274669,-1.59087775424421,0.73285371713949,-2.17
079850594714,0.0299464069407641,0.307691238333429
"1523","ATP5MGL",3.0891398167971,2.28310787923941,1.05179178829601,2.1
7068425960829,0.0299550478391567,0.307691238333429
"1524","FAM131C",2.75510224733812,-2.42207037542438,1.11617295364052,-
2.16997766118998,0.0300085382810736,0.308008489046944
"1525","AC096642.1",29.1749798165327,0.998322070339504,0.4601525894845
,2.16954569669576,0.0300412789891857,0.308008489046944
"1526","CCNG2",435.531155125099,0.437991361639682,0.201888287682076,2.
16947385441899,0.030046727244495,0.308008489046944
"1527","DSTN",3984.18529753382,-0.302449292728525,0.139430879496651,-2
.1691700849939,0.0300697733938892,0.30804287177512

"1528","PKNOX2",20.1252318641611,0.888559768393549,0.40977553700123,2.
16840608616146,0.0301278030457157,0.308233631814343
"1529","ARFGAP3",266.333976220526,-0.394643569587903,0.18199157164889,
-2.16847168257481,0.0301228168857365,0.308233631814343
"1530","QSER1",177.765338415269,0.436094779731047,0.201275199731701,2.
1666592819799,0.0302608435153277,0.309392402032857
"1531","CCDC34",105.595657141969,-0.529405096737043,0.244435648595286,
-2.16582605597592,0.0303244814673568,0.309805001395386
"1532","SPATA20",142.227052576388,-0.494617850333322,0.228396282208971
,-2.16561252901994,0.0303408081658078,0.309805001395386
"1533","TLR5",105.119133770958,0.722620929205426,0.333817233894641,2.1
6472025957024,0.0304091146140589,0.310116119536465
"1534","TARS",190.815286032169,0.320813530886266,0.148225505303077,2.1
6436118892155,0.0304366400234467,0.310116119536465
"1535","LPCAT3",110.8370002744,0.680664609065664,0.314440728791809,2.1
646833464641,0.0304119432889549,0.310116119536465
"1536","METTL7A",1593.25462783684,0.468113560960013,0.216326532260137,
2.16392116153878,0.030470400545135,0.310116119536465
"1537","MARCH2",243.55416047293,-0.72972024270517,0.337187603516296,-2
.16413721944527,0.0304538197692806,0.310116119536465
"1538","PCNX1",180.037738163195,0.524232104870934,0.242362547659452,2.
16300789842968,0.0305405721267609,0.310628198815943
"1539","FXYD6",13.3443018384904,-1.36487594861562,0.631204937145841,-2
.1623340824729,0.0305924344627332,0.310953510266754
"1540","GPRC5A",126.534758554188,-1.0354129578363,0.479126435432651,-2
.16104326804954,0.0306919972598666,0.311010701616886
"1541","N4BP2L1",198.487591251821,0.46235116855568,0.213956638417148,2
.16095734152563,0.0306986347908058,0.311010701616886
"1542","G2E3",97.8014146844451,0.423561792449952,0.196020121756351,2.1
6080772042592,0.030710195452376,0.311010701616886
"1543","BCAS3",63.7390972363939,0.491917397379871,0.2276641615413,2.16
071512551453,0.0307173517866195,0.311010701616886
"1544","CHAF1B",7.45602717231873,-1.16494398645927,0.538918527604583,-
2.16163283833883,0.0306464881938723,0.311010701616886
"1545","XG",650.172692240368,-0.554167664009422,0.256442187361275,-2.1
6098478066993,0.030696515076534,0.311010701616886
"1546","STIM1",163.102713713908,0.377288067760855,0.17477316144196,2.1
587300055,0.030871119501851,0.312163492157373
"1547","LSM12",189.946730281286,-0.393539572841991,0.182288275957909,-
2.15888581300128,0.0308590267755993,0.312163492157373
"1548","N4BP2",90.7725966247718,0.600382509907514,0.278165438346769,2.
15836486903546,0.0308994748683679,0.312248375559353
"1549","TRIM59",4.62352028568488,-1.87448495636027,0.869166627875923,-
2.15664625888956,0.0310332372770043,0.313195439176889
"1550","TTPA",8.65726815658275,1.52092067044295,0.705208074983995,2.15
669775261361,0.0310292222201225,0.313195439176889
"1551","DUSP23",136.942266663885,-0.620740006035384,0.28788729492119,-
2.15619104068248,0.0310687508464835,0.313351688904927
"1552","MFNG",4.21160447272096,-1.64081026275755,0.761090896452176,-2.
15586636288278,0.0310941016920958,0.313405304619494

"1553","SPPL3",274.455220628969,0.286073266010716,0.132738626844881,2.
15516216199089,0.0311491467193779,0.31375795372262
"1554","QPRT",78.2317009440227,1.23711624143633,0.574178523059164,2.15
458466618553,0.0311943500224751,0.314011079408995
"1555","ASAP1",282.690605423328,0.563974923363438,0.262020908883283,2.
15240427096854,0.0313655276177396,0.315360390778349
"1556","ABCC9",358.440733053622,0.666140515866143,0.309529357434473,2.
15210770760949,0.031388872239461,0.315360390778349
"1557","CEMIP",6.51437719985403,1.96405443728514,0.912514975534069,2.1
5235310098406,0.0313695545093408,0.315360390778349
"1558","TSHR",11.5028698756378,3.37503951503514,1.56957060009526,2.150
2948098227,0.0315319026898829,0.316594065325955
"1559","MAP1B",1243.34846479687,-0.451030753563685,0.209876937433208,-
2.14902484798847,0.031632430291013,0.317399683798792
"1560","IL15",10.7379598392644,-0.94037733349696,0.437681456951136,-2.
14854277822866,0.0316706618596374,0.317579591968146
"1561","RUNX3",15.493220855239,1.23005731488758,0.57279776146111,2.147
45482201242,0.031757090195819,0.317631817092837
"1562","NOTCH2",565.678092395731,0.335869448537792,0.156392367364734,2
.14760767547234,0.0317449351654918,0.317631817092837
"1563","VPS8",97.3076889170448,0.401783624505446,0.187092707704244,2.1
4751087541362,0.0317526323208674,0.317631817092837
"1564","ENTPD1-
AS1",88.4599907243743,0.462692270362601,0.215438765861332,2.1476741593
4983,0.0317396495590128,0.317631817092837
"1565","LINC01359",43.7927479394238,1.08062429398139,0.503423708118135
,2.14655026482742,0.0318291030285351,0.317945503624122
"1566","TARBP1",81.3265152153636,0.57642737335791,0.268518010510767,2.
14669910692935,0.0318172439237943,0.317945503624122
"1567","WDR86-
AS1",72.3226368630138,1.17955015281919,0.549593747524249,2.14622192871
134,0.0318552768532111,0.31800389011792
"1568","AL359258.2",11.3133750869366,-0.943300649523724,0.439677210467
936,-2.1454390336033,0.0319177610227859,0.318424448775153
"1569","CACNA2D1",192.363938705962,0.396074966332194,0.184683219048368
,2.14461805665442,0.0319833973985557,0.318811377944627
"1570","PRRC2B",391.871001706654,0.394859253076766,0.18413127293693,2.
14444426945343,0.0319973063589506,0.318811377944627
"1571","SPEGNB",3.76673864326599,-1.62435391318786,0.757735212588582,-
2.14369595896003,0.0320572562098233,0.319205384398642
"1572","GCLC",46.8992278244411,-0.641457262111915,0.299394943069426,-2
.14251201284708,0.0321523030487753,0.319608522639611
"1573","CPB2-
AS1",12.9489832238049,1.16650864961763,0.544479681657607,2.14242824647
988,0.032159036926085,0.319608522639611
"1574","EGLN3",11.8274645484486,-1.50558562081805,0.702738847198264,-2
.14245395258942,0.0321569703143902,0.319608522639611
"1575","AC099518.5",49.367773677873,-0.943595074701533,0.4405946710403
2,-2.14163978078432,0.0322224799842651,0.319832648727068
"1576","LMNB2",42.8421308360225,-0.593327268494308,0.27701914184123,-2

.14182768941781,0.0322073503885118,0.319832648727068
"1577","RRP36",163.998199816271,-0.476603407510388,0.222582141872281,-2.14124728741206,0.032254101511069,0.319943506618676
"1578","IGF1R",192.838098134488,0.539736429583759,0.252158138552631,2.14046801218397,0.0323169632247357,0.320363913640393
"1579","LINC00174",33.3189018960549,0.722981625659068,0.337834443870497,2.14004711117085,0.0323509596418958,0.320431558177037
"1580","FTO",149.38109582619,0.45223140561416,0.211335290572748,2.13987642285654,0.0323647549651421,0.320431558177037
"1581","EYA4",4.17966091236468,2.35732620206071,1.1018188326865,2.13948621327608,0.0323963113070205,0.320477936488994
"1582","AC107959.1",35.9490516966115,0.571306220131531,0.267060910693609,2.13923564720774,0.0324165885392872,0.320477936488994
"1583","SPECC1L",251.221688119344,0.499601856771289,0.233561526551358,2.13905887732512,0.0324309003044223,0.320477936488994
"1584","GFPT2",334.292091130326,0.481160436481823,0.225051962290375,2.13799707225393,0.0325169808558088,0.320720763888662
"1585","CCN6",6.32216948695828,-1.29934314628476,0.607620675298692,-2.13841167541911,0.0324833457097122,0.320720763888662
"1586","RFX2",101.807421621024,-0.676978908506281,0.316606349723101,-2.13823541157136,0.0324976416681562,0.320720763888662
"1587","FMO3",97.6702334391384,1.01898484114104,0.477197171975761,2.13535389768152,0.0327321134562983,0.321751372784468
"1588","HECW2",85.6032824556269,0.638160762122101,0.298947193780566,2.13469393725277,0.0327860185526077,0.321751372784468
"1589","LVRN",138.347269447159,0.809904000521978,0.379229907189937,2.13565434889695,0.0327075979671823,0.321751372784468
"1590","CYP7B1",418.106918521384,0.491632152288885,0.230121784503949,2.13639987778058,0.0326468340167369,0.321751372784468
"1591","CBWD1",130.861372175638,-0.48524128128899,0.22718771573342,-2.13586055796418,0.0326907813208778,0.321751372784468
"1592","FAM225B",13.687911700222,1.06540117781664,0.498861987408952,2.13566317880872,0.0327068777233788,0.321751372784468
"1593","RXYLT1",166.075729150876,-0.427834057502149,0.200392447868505,-2.13498094390707,0.0327625667206919,0.321751372784468
"1594","SCO2",118.610479588872,-0.59390229242592,0.278213082435927,-2.13470296661084,0.0327852805285206,0.321751372784468
"1595","AL354743.1",4.05570609319789,-1.97230988143191,0.925161174681513,-2.13185543817365,0.0330187325198383,0.323831995490802
"1596","GABPB1-AS1",485.725786530938,0.80553523353481,0.378000635986918,2.13104200587294,0.0330856817531987,0.324285288010832
"1597","NBPF14",109.422585073188,1.01520809552522,0.476577405776965,2.13020609709796,0.0331546019186522,0.324351118082224
"1598","TNKS2",193.370596281665,0.372809045031421,0.174972366286327,2.130673848357,0.0331160209892377,0.324351118082224
"1599","ZPR1",102.043157547431,-0.523822755472301,0.245885385913832,-2.13035334949052,0.0331424521409746,0.324351118082224
"1600","B3GNT5",7.85502677529451,-1.15944628104198,0.544404546886652,-2.12975128086758,0.033192152825119,0.324515529152085

"1601","PLSCR4",937.116025998004,0.53552306485934,0.251519340147812,2.1291526311441,0.0332416345071908,0.324796307680191
"1602","SERTAD3",102.855774025416,-0.662380104917083,0.311144627393494,-2.12884956576606,0.0332667085804294,0.324838403447976
"1603","MAN2B2",231.282377856234,0.404348603279297,0.190189968176252,2.12602487479561,0.0335011883944084,0.326923948879433
"1604","GCK",6.83404578009563,-1.48372777555195,0.698277855125919,-2.12483865077518,0.0336000785024232,0.327684556118083
"1605","POU3F3",3.91328122868718,-1.77505540870498,0.835804496170227,-2.12376867657273,0.0336894914594151,0.327708292469054
"1606","PDGFC",14.6049692676788,-1.19471321197331,0.562507090127282,-2.12390782790484,0.0336778517060856,0.327708292469054
"1607","EXT1",212.973257536904,0.457463193491734,0.215333449857838,2.12444092542867,0.033633290900387,0.327708292469054
"1608","RRAS2",157.364957176624,0.638308491755336,0.300620095924401,2.12330612759786,0.0337282075609011,0.327708292469054
"1609","SYNRG",216.684087466222,0.406694039474793,0.191514534051644,2.12356749574486,0.0337063259489078,0.327708292469054
"1610","ZNF562",44.03202973072,0.726745643946351,0.342245864469461,2.12346070294503,0.0337152651220112,0.327708292469054
"1611","FLT3LG",115.177757100679,-0.535881940053185,0.252412337993775,-2.12304178279273,0.0337503507324459,0.327719886100342
"1612","AC079922.2",18.5028195077004,-0.920556166372719,0.433719920691787,-2.12246687886603,0.0337985511972355,0.327984327778135
"1613","KCNK1",50.5969491709046,0.744518072685992,0.350871200971901,2.12191274354721,0.0338450661027303,0.328031177829142
"1614","ID4",431.430125450334,-0.757320377690185,0.356905086470395,-2.12190973566583,0.033845318737853,0.328031177829142
"1615","SUN1",524.764728689721,0.304324219781844,0.143446980408181,2.12151011416123,0.0338788977095448,0.328153310755671
"1616","SNX18",283.53071117781,0.42950631296529,0.202600197665907,2.11996986139942,0.0340085870313762,0.329205647853848
"1617","PRIMPOL",71.8887507573579,0.485588946996161,0.229103722823296,2.11951574165684,0.0340469048420414,0.329252805812618
"1618","DOK2",13.7716158131995,-1.33524443178696,0.630006633195155,-2.1194132909603,0.0340555545486682,0.329252805812618
"1619","MYLK",127.757881330344,-1.60627204787161,0.758104371500046,-2.11880066684395,0.0341073163819652,0.329549567735072
"1620","C3",14514.9113606526,0.511576138877306,0.241621099355479,2.117266001363,0.0342372786413421,0.33039713126867
"1621","RINL",38.4839944062053,-0.538081126047854,0.254136663183164,-2.11729043463533,0.0342352062131804,0.33039713126867
"1622","WASHC5",145.498006665734,0.372484441920401,0.176013543757042,2.11622602425728,0.034325588870022,0.3305417684401
"1623","CDK12",266.641137307279,0.426068208105779,0.201346512546145,2.11609430289055,0.0343367879380657,0.3305417684401
"1624","ACE",261.956997900198,0.698518289044046,0.330065611444632,2.11630131956725,0.0343191885977509,0.3305417684401
"1625","AC002398.2",8.48213004829456,1.14949632331607,0.543057022120984,2.11671385598983,0.0342841401618907,0.3305417684401

"1626","GPATCH4",82.1885718369215,-0.626271694030722,0.296192526308682
,-2.11440748298302,0.0344804790892762,0.33055990060002
"1627","SH3RF3",47.7371416598115,0.749178059394492,0.35409199300787,2.
1157723817207,0.0343641711030862,0.33055990060002
"1628","AOX1",464.389585508956,0.349821123928233,0.165362718542787,2.1
154775817121,0.0343892636613287,0.33055990060002
"1629","POLN",11.3513431331719,1.32911709801267,0.628675616342957,2.11
415404615853,0.0345021123431026,0.33055990060002
"1630","LGI1",11.5707295770462,1.48990877031611,0.704439070536516,2.11
502858463169,0.0344275111253847,0.33055990060002
"1631","RILPL2",159.017399335537,-0.478163954922119,0.226082432188122,
-2.11499827869969,0.0344300940212771,0.33055990060002
"1632","GATA6",37.8659536070503,0.798558118467022,0.377609232048435,2.
11477382090222,0.0344492291310917,0.33055990060002
"1633","EMP3",2066.67886893288,-0.580291109437397,0.274487636219726,-2
.11408833355567,0.0345077234341132,0.33055990060002
"1634","HES1",292.195411145029,-0.641417962570985,0.303455966796442,-2
.11371016804308,0.0345400295193701,0.330666879909123
"1635","AL137026.1",77.8537952609418,0.582681540632513,0.2757429729425
35,2.11313287303225,0.0345893967498539,0.330936962298449
"1636","SLC35F5",229.526168659472,0.33430521271084,0.158252319418951,2
.11248223051829,0.0346451085062326,0.331241112645778
"1637","MT-
ND4",46283.6214579787,-0.320093505635104,0.151540252038368,-2.11226721
171126,0.0346635364956299,0.331241112645778
"1638","MEF2C",91.0472333441886,-0.587572243957381,0.278309820200498,-
2.11121635425615,0.0347537197591539,0.331638081555235
"1639","AC022364.1",9.05780599182119,1.1117102198807,0.526616752404973
,2.11104226138592,0.034768679521229,0.331638081555235
"1640","MON2",201.417452178548,0.463067891381367,0.219347243965683,2.1
1111789238534,0.0347621798906123,0.331638081555235
"1641","CRTAM",3.27852542548829,2.1420575142184,1.01502675683026,2.110
34586015019,0.0348285761360952,0.331804760351362
"1642","PTPRH",6.68032039965589,1.63126046939812,0.772898337236423,2.1
1057572620851,0.0348087959040117,0.331804760351362
"1643","LYZ",147.097494361622,-1.01480543256721,0.481096981121193,-2.1
0935730713215,0.0349137516682588,0.332413765883489
"1644","RBM43",336.757555622873,0.401807429739376,0.190556681552477,2.
10859795870617,0.0349792992379206,0.332835266410457
"1645","ENAH",424.828607168437,-0.349883185688089,0.165975334442966,-2
.10804326355082,0.035027247352743,0.333088893823075
"1646","FSTL3",317.917680605918,-0.506446135354741,0.240285784289207,-
2.10768247007565,0.035058464621799,0.333183209039369
"1647","AC108047.1",10.181190505421,-0.972414683820007,0.4615987525184
63,-2.10662329244729,0.0351502460747235,0.333852640769217
"1648","PRKACB",122.221554650115,0.367117668118168,0.174446146330662,2
.10447565532516,0.0353369760363051,0.333932079916967
"1649","ATF2",180.274008816872,0.377461788423228,0.179257710748885,2.1
0569345578668,0.0352309886620175,0.333932079916967
"1650","PLOD2",106.25469740065,0.534301276116952,0.253734598411388,2.1

0574860291884,0.0352261955357468,0.333932079916967
"1651","C6",83.1142825851223,-0.868506069944244,0.412381389589682,-2.1060748420495,0.0351978517668714,0.333932079916967
"1652","TMEM161B-AS1",228.003924852269,0.492683586506832,0.234126541255666,2.10434743478666,0.0353481511175712,0.333932079916967
"1653","TFCP2",101.046979898703,0.445316806304517,0.211620509136585,2.10431780984469,0.035350733512913,0.333932079916967
"1654","TPCN1",205.420784251467,0.552625531100457,0.262533293832695,2.10497313705525,0.0352936464744771,0.333932079916967
"1655","AC087392.1",7.62257656487273,-1.71117199390736,0.812840823766019,-2.10517476961754,0.0352760976402643,0.333932079916967
"1656","NBR1",295.765239949228,0.321485956263676,0.152730429851803,2.10492405852337,0.0352979190894885,0.333932079916967
"1657","PDCD6IP",371.294937195649,0.269249474381653,0.128034451588024,2.1029455044492,0.0354705334567214,0.334861529790883
"1658","SULF2",170.841757982897,0.889762624757004,0.423233979369091,2.10229487264553,0.0355274534568508,0.335196594949045
"1659","CLIC1",2293.39438208448,-0.546137538649732,0.259861588907021,-2.1016478077687,0.0355841386771729,0.335421018676986
"1660","SLC25A27",43.8043477619309,0.79908252945637,0.380237752049341,2.10153390911242,0.0355941245927122,0.335421018676986
"1661","AC004982.2",18.9055084119118,-0.73434668945349,0.349487203601523,-2.10121195250048,0.0356223646475892,0.335485039242768
"1662","RNF130",549.908022522464,-0.299609533745693,0.142612306663981,-2.10086731470955,0.0356526153340585,0.335567907142405
"1663","SNHG9",250.190969153211,-0.828677357859377,0.394500757736713,-2.1005722843565,0.0356785291190047,0.335609880341907
"1664","RTRAF",834.790980122583,-0.572821746911698,0.272756079493774,-2.10012458008209,0.0357178835990724,0.33577815693527
"1665","EDEM3",207.853770329892,0.485340426217011,0.231206481131362,2.09916445180125,0.0358024064677535,0.335914294017103
"1666","KHDC1",9.98608973840241,-1.00642185334166,0.479480346371108,-2.09898457978236,0.0358182600793024,0.335914294017103
"1667","PATZ1",64.7838827353209,0.459687314641793,0.218965918468665,2.09935554289274,0.0357855705828005,0.335914294017103
"1668","NAA10",221.51077682009,-0.576124280372683,0.274457973295866,-2.09913479085419,0.0358050203206742,0.335914294017103
"1669","KCND3",33.6010812365381,1.02705445060304,0.489712409044512,2.09726041577535,0.0359705289187708,0.336912667758312
"1670","AC008592.5",2.78269908536299,2.81719485359655,1.34387726558843,2.09631855953975,0.0360539414324244,0.336912667758312
"1671","TRERF1",91.8812467352903,0.52697138224772,0.251254477163615,2.09736116226323,0.0359616163748268,0.336912667758312
"1672","CLDN23",50.610677973731,-0.831013210608951,0.396386495129004,-2.09647205649248,0.0360403362235911,0.336912667758312
"1673","ZNF607",12.0469396373569,1.03976465237575,0.495897216832146,2.09673419628748,0.0360171115727127,0.336912667758312
"1674","LENG8",366.630534281792,0.486142073961842,0.231884366861438,2.09648490125398,0.0360391979261156,0.336912667758312

"1675","RBM5",407.841655592156,0.440206541858616,0.210258464948956,2.09364480029608,0.0362916335600399,0.33792756288633
"1676","LINC02615",8.4704395643562,1.67787198826045,0.801414235810246,2.09363886151097,0.036292162989774,0.33792756288633
"1677","LPCAT2",44.7429686832178,-0.553328203370819,0.264188079457799,-2.09444803303174,0.0362200877427714,0.33792756288633
"1678","HSDL1",80.2052265600264,0.451200958343973,0.215395205611766,2.09475859531075,0.0361924574814468,0.33792756288633
"1679","SAMD1",148.034563831066,-0.420269313200448,0.200694231806615,-2.09407768931501,0.0362530601721665,0.33792756288633
"1680","TTPAL",66.8793619204586,-0.544112694084075,0.25988744799872,-2.09364745498118,0.0362913969027846,0.33792756288633
"1681","SCAF8",121.817251317552,0.382935143952251,0.182945133639724,2.09316933625778,0.0363340409802699,0.338116242150126
"1682","PICK1",22.0284762095907,-0.699731050432885,0.334404504710276,-2.09246897268661,0.0363965844791894,0.338496891205683
"1683","HAUS4",163.119700034674,-0.512687799666328,0.245076162854022,-2.09195294106064,0.0364427255469864,0.338523489151727
"1684","CHMP4B",427.549691699798,-0.299030703714151,0.142929133922359,-2.09216060790369,0.0364241509859372,0.338523489151727
"1685","RPS6KA4",55.8295520157387,-0.583036939850282,0.278750495788346,-2.091608620108,0.0364735407900217,0.338608663844694
"1686","WTAP",430.767474252234,-0.352322303292228,0.168478030766998,-2.09120620468008,0.0365095833746461,0.338742237680658
"1687","WDR1",209.809071676466,-0.407755319882188,0.195100957361146,-2.0899708817287,0.0366204154639631,0.339367985250459
"1688","MGST2",146.233001794938,-0.681765223528975,0.326193703204484,-2.09006248996042,0.036612186625184,0.339367985250459
"1689","RABGAP1",162.051992757124,0.444221050412004,0.212695685917965,2.08852872823822,0.0367501667352663,0.339765282647618
"1690","NAALADL1",115.647890490216,-0.48518438525634,0.23230350625354,-2.0885796907723,0.0367455749447373,0.339765282647618
"1691","PPTC7",106.001253635335,-0.446566657253295,0.213808046872066,-2.08863353735466,0.0367407238286998,0.339765282647618
"1692","TPGS1",338.663587215484,-0.667960015532746,0.31978765043208,-2.08876113455361,0.0367292305915563,0.339765282647618
"1693","CLN8",96.837886992194,0.457442268280925,0.219177465624634,2.0870862201881,0.036880341448085,0.340767384094739
"1694","NCK1",285.448740310731,0.343364501187119,0.164566819900296,2.08647466965181,0.0369356473695573,0.340844763016666
"1695","CCDC170",40.205614214441,1.01969192175027,0.488762929762765,2.08627099081594,0.0369540828534338,0.340844763016666
"1696","CCDC134",6.07596617013046,1.49450588948663,0.716231709564159,2.08662346211405,0.0369221847407212,0.340844763016666
"1697","BORCS7",535.956785879164,-0.381808513107465,0.183092060861747,-2.08533625822132,0.0370387883829131,0.341424729919806
"1698","LTBP1",127.907722814305,0.735885525089447,0.353097185730865,2.08408776627959,0.0371521846455042,0.341732491560351
"1699","RNF4",123.971215569496,0.370605061596739,0.177832951414512,2.08400669644679,0.0371595581502369,0.341732491560351

"1700","NOX5",3.40230931351124,2.54236243558288,1.21984414654296,2.084
1698857907,0.0371447169398899,0.341732491560351
"1701","PAFAH1B3",26.4908609779246,-1.13025293084449,0.542325400232775
,-2.08408628907916,0.0371523189894474,0.341732491560351
"1702","TIFA",59.7610350613175,-0.805592326343652,0.386770267295995,-2
.08287036119851,0.0372630418845615,0.342146521695764
"1703","RANBP9",137.398694626028,0.428426517567319,0.205658269220948,2
.08319616415249,0.0372333466185842,0.342146521695764
"1704","LRIG3",240.803586260454,0.455699183887921,0.218792469311944,2.
08279190468025,0.0372701958044865,0.342146521695764
"1705","TNS1",312.009854462299,-0.417615652334326,0.200634604122609,-2
.08147370270743,0.0373905686928663,0.342247317766242
"1706","IFIT2",177.872435637535,0.637993363223919,0.306457604385068,2.
08183237777409,0.0373577832091735,0.342247317766242
"1707","FTH1",30069.5528258486,-0.605118073045745,0.290654617316774,-2
.08191453702677,0.0373502767072236,0.342247317766242
"1708","MYOM1",23.9184585809039,0.800769348907908,0.384642525823869,2.
08185339671617,0.0373558626857245,0.342247317766242
"1709","UXT",711.166078128188,-0.543731897629385,0.261197035049931,-2.
08169245690497,0.0373705700721152,0.342247317766242
"1710","CLCA2",2.71234873617621,2.51768691556786,1.21122079242613,2.07
863581215842,0.0376508367678418,0.343914103417298
"1711","NLRP3",31.2118536152804,-1.03193228404585,0.496320962602311,-2
.07916320647675,0.0376023521978993,0.343914103417298
"1712","MYO9A",346.553821902941,0.407481999500066,0.196043393453144,2.
07852961695166,0.0376606059677703,0.343914103417298
"1713","DSEL",270.829704408146,0.412937517246448,0.19866600548474,2.07
855146751902,0.0376585956955575,0.343914103417298
"1714","KLHL24",305.193473876814,0.528116482127842,0.254216144219492,2
.07743093480272,0.0377618035124907,0.344637043375667
"1715","ADGRL2",133.738033068344,0.520122008066995,0.25045990322214,2.
07666776747768,0.0378322334928837,0.344877405902786
"1716","MMS22L",44.615897886914,0.539580308123515,0.259823584889918,2.
07671797135785,0.0378275969253873,0.344877405902786
"1717","TLR2",8.58878977469029,-1.04575759696197,0.503735405176195,-2.
07600574868504,0.0378934193141815,0.345233988544986
"1718","FRZB",56.5466269713387,1.28348899053625,0.618322582095035,2.07
575952698907,0.0379161973476212,0.345240439527846
"1719","AXL",843.976149437358,-0.369263034022322,0.178037915031981,-2.
07406963823404,0.0380728438607884,0.346465093958297
"1720","MLF2",454.680319761348,-0.428156677508542,0.206502410612485,-2
.07337375015929,0.0381375099355198,0.346851783675196
"1721","ZFYVE1",76.8918769636991,0.467189993829224,0.225415369017594,2
.07257382611191,0.038211959012001,0.3471363168856
"1722","IGDCC4",106.044898660744,0.626435294661349,0.302251838262388,2
.07256074359268,0.0382131776306976,0.3471363168856
"1723","BHLHE40",109.670743643559,-0.600895019450587,0.290049510309362
,-2.0716981001267,0.0382936046396046,0.347665036202748
"1724","DMD",147.765318190596,0.643957753206952,0.310953887394021,2.07
091076623515,0.0383671358628421,0.348130572101183

"1725","IGBP1",563.39717483786,-0.565771102828778,0.273365474487376,-2
.06965090924423,0.0384850469669296,0.348998023016626
"1726","S1PR1",12.1126065913639,1.12508051456117,0.543741582394488,2.0
6914562172461,0.0385324237217944,0.349022990318489
"1727","OSR2",720.349959003859,0.777279651294326,0.375616752376659,2.0
6934234529265,0.0385139726426974,0.349022990318489
"1728","RFTN1",560.933368487665,0.439945251427709,0.212659862771372,2.
06877426560126,0.0385672743981761,0.349136500816359
"1729","CASP3",91.0657983526269,0.467098314687553,0.225839307903963,2.
06827730310874,0.0386139547701648,0.349190798759611
"1730","ANKRD10-
IT1",18.0658700118947,1.04466769459177,0.505101027576879,2.06823514021
215,0.0386179173978219,0.349190798759611
"1731","WASHC4",295.049896742174,0.396031217609919,0.191521448696611,2
.0678165307598,0.0386572786401057,0.349344777450707
"1732","SLC3A2",345.103119058281,-0.520017149024534,0.251517877096444,
-2.06751565744622,0.0386855903759375,0.349398781899994
"1733","NCOA2",218.393753962744,0.424467472007699,0.205381836325711,2.
06672352142448,0.0387602134793222,0.34966898469264
"1734","TLE2",143.509708071089,-0.531466713759039,0.25714933572579,-2.
06676292691561,0.0387564983999412,0.34966898469264
"1735","AC108734.4",89.3544242991078,0.644190349601071,0.3119276747368
51,2.06519139459019,0.0389048944324281,0.350771909859638
"1736","AKR1E2",6.48176045393742,1.37146770643648,0.664311580617357,2.
06449465349068,0.0389708403020575,0.35116408689233
"1737","PILRB",79.739083404132,0.456112585406704,0.221012822364381,2.0
6373811495297,0.0390425534406908,0.351537891261888
"1738","ALDH16A1",161.600201920622,-0.537254748119115,0.26035044022379
2,-2.06358302162771,0.0390572687472455,0.351537891261888
"1739","NPR1",221.338118453206,-0.556925192457722,0.270193301498792,-2
.06121021271955,0.0392829896365589,0.351944816607649
"1740","SRGAP3",14.7449167122965,0.877287899327588,0.42566545145793,2.
06097980543834,0.0393049667335909,0.351944816607649
"1741","MANBA",140.673775178304,0.483064090251635,0.234332883099415,2.
06144389068391,0.0392607112277516,0.351944816607649
"1742","GLIDR",30.2539225255677,0.657204014190022,0.318730220669827,2.
06194446453454,0.0392130235812161,0.351944816607649
"1743","SLC2A8",54.2417027329706,-0.704783492446986,0.341915831068539,
-2.06127774266676,0.0392765503584304,0.351944816607649
"1744","LATS2",268.938016550372,0.393525638810431,0.190931806396097,2.
06107953535014,0.0392954528453835,0.351944816607649
"1745","RECQL5",26.1743178090245,0.642479860440671,0.31155269997488,2.
06218678410578,0.0391899564495395,0.351944816607649
"1746","ZNF829",30.2452252170642,0.674712996142942,0.327347065689048,2
.06115486241707,0.0392882682003166,0.351944816607649
"1747","SYS1",230.244397141625,-0.397226795892488,0.192701964767499,-2
.06135311786652,0.0392693640593914,0.351944816607649
"1748","IQCA1",12.958458913693,1.302018134653,0.632147647375667,2.0596
7409680044,0.0394297073478047,0.352456521166691
"1749","HNRNPA0",892.371090881536,-0.348900627922897,0.169390271297888

,-2.05974419457257,0.0394230020443217,0.352456521166691
"1750","RPP25",69.9935582104293,-0.714070856497404,0.346661474373674,-2.05985063032326,0.0394128226299522,0.352456521166691
"1751","OLFML1",705.392368237667,0.286231043736965,0.139056723219438,2.05837615837733,0.0395540385627737,0.352583269190359
"1752","JHY",52.8548469349044,0.529700433950494,0.257330373279477,2.05844505333697,0.0395474306716647,0.352583269190359
"1753","KATNAL1",139.230023523992,0.358436235989117,0.17411845310182,2.05857696070566,0.0395347817142921,0.352583269190359
"1754","MAP2K3",131.982717251151,-0.537536616723143,0.261091697624655,-2.05880394364705,0.0395130237398367,0.352583269190359
"1755","ZNF8",28.4080587974766,0.731719713595138,0.355488544993235,2.05834962589037,0.0395565836111411,0.352583269190359
"1756","RHEBL1",7.58009275241595,1.36707515937279,0.664257436684788,2.05805021347697,0.0395853134750708,0.352638416110781
"1757","HNRNPAB",344.911530100557,-0.375880823269357,0.182684669528803,-2.05753895079901,0.039634412192677,0.352795614928171
"1758","LINGO2",6.77740304496948,5.79322234712075,2.81580205641143,2.05739687345206,0.0396480656551636,0.352795614928171
"1759","PM20D2",65.5436786786417,0.586634607888322,0.285251037611639,2.05655556172597,0.0397289965317854,0.353314777002114
"1760","CAMSAP2",149.694615827775,0.512289830136665,0.249240125248078,2.05540672725454,0.0398397362753658,0.353377760976642
"1761","NECTIN3",226.667211794912,-0.381724778200212,0.185686439355789,-2.05574935641261,0.0398066818052406,0.353377760976642
"1762","C9orf16",608.691006188052,-0.633479580473047,0.308216008758388,-2.05531043966517,0.0398490296210955,0.353377760976642
"1763","LINC01465",3.52153104443843,-1.73187592436617,0.842593444813978,-2.05541110606257,0.039839313691652,0.353377760976642
"1764","XYLT1",116.190968472603,-0.544178195977784,0.264667483379025,-2.05608255698898,0.0397745592611465,0.353377760976642
"1765","AC092155.1",7.73667665711662,1.23066781351773,0.598912785175039,2.05483643692471,0.0398948055490478,0.353583253939804
"1766","BRINP3",5.28544845997854,2.28011870638542,1.10989456580582,2.05435613132314,0.0399412356652234,0.353594085744816
"1767","ZNF622",153.598393822527,-0.539855957065801,0.262759675915969,-2.05456166431887,0.039921361621282,0.353594085744816
"1768","AC006427.2",3.61351216174171,2.03650864312755,0.991639605197955,2.05367820370689,0.040006847492157,0.353927531046766
"1769","KRT7",4.97261043129728,-1.57612632605056,0.767531851539811,-2.05349956863491,0.0400241515324253,0.353927531046766
"1770","BBC3",27.3957899121107,0.721591511641978,0.351448527246016,2.05319258924326,0.0400539028737408,0.35399050997397
"1771","VSIG10L",5.76309881122496,-1.31812732096171,0.642164167650908,-2.05263293619065,0.0401081907173054,0.354270145336425
"1772","WDR6",126.768603991767,0.450743320353285,0.219759912194239,2.05107162563337,0.0402599720569926,0.354944329868511
"1773","GTF2E1",34.1632980924059,0.533217037229792,0.259951273586849,2.05121917608781,0.0402456072665565,0.354944329868511
"1774","APC",243.251460651011,0.388930837278076,0.18963776738605,2.050

91444936873,0.0402752787519407,0.354944329868511
"1775","PDCD2",289.403971568043,-0.460621946193011,0.224585522855154,-2.05098681489852,0.040268230783024,0.354944329868511
"1776","PCDHA10",8.69624658564449,1.21914653287945,0.594572033859528,2.05046060603564,0.0403195042306287,0.355134011643989
"1777","AC007563.2",13.9100961562121,1.00767325986973,0.4917893634032,2.04899360347404,0.040462740407834,0.356195074957652
"1778","TRIM69",147.639705003372,0.606240657053013,0.29595461365187,2.04842441742142,0.0405184310288147,0.356484711239454
"1779","ZNF844",51.645842911865,0.764628331597211,0.373431101440879,2.04757538578577,0.0406016232121139,0.357015847052893
"1780","HOXB2",204.786129639767,-0.478427448071493,0.233703212096463,-2.04715820454371,0.0406425536518533,0.357174981334799
"1781","QRICH1",139.000380118855,0.359056300694692,0.175467644872951,2.04628209921361,0.0407286238639449,0.357730411624756
"1782","PARP9",139.389877625371,0.490986007407106,0.24016347880351,2.04438247585848,0.0409157771428029,0.358962407255973
"1783","TPRG1",47.8122010484173,-0.511202713397147,0.250088052990475,-2.04409090032229,0.040944567944243,0.358962407255973
"1784","PAWR",140.034839478772,0.915392776385644,0.447839951730501,2.04401767383296,0.040951801183135,0.358962407255973
"1785","AHSA1",302.543093295619,-0.47310355259926,0.231476174421287,-2.04385420565233,0.0409679523119924,0.358962407255973
"1786","ZNF559",94.3168209020141,0.469675709275937,0.229816859181778,2.0436956233243,0.0409836258619937,0.358962407255973
"1787","ASPHD2",11.0026203133809,-0.977816271403342,0.478784377268279,-2.04228942678186,0.0411228302862862,0.359980097464116
"1788","KHNYN",93.3938393995576,0.506585666812607,0.248091713957224,2.04192900573839,0.0411585740764443,0.360091484495424
"1789","TTLL3",294.586445250989,0.562953850301288,0.275763775045006,2.04143510223347,0.041207598373756,0.360117576179143
"1790","STOX2",13.629439149924,1.30652398569295,0.639962641822239,2.04156289806658,0.0411949087643406,0.360117576179143
"1791","SPHK1",18.9882033792781,-1.29347901149649,0.633746041660778,-2.04100527098651,0.041250303178165,0.360289498948094
"1792","ISM1",452.153121579314,0.660957232203772,0.323945434538259,2.0403350741642,0.0413169636908474,0.360670347665137
"1793","CD164",1244.74628924822,0.324018372596907,0.158899061814906,2.03914591373951,0.041435467014025,0.361503073340989
"1794","EVA1B",712.023831967675,-0.45998927854589,0.225663223354284,-2.0383883191446,0.0415111135351452,0.36175952592216
"1795","MAVS",223.967364878593,0.533440557877507,0.261687711034004,2.03846239385766,0.0415037119521109,0.36175952592216
"1796","COL1A2",31470.3877398105,0.66258621904533,0.325129768372819,2.03791311500446,0.0415586227640401,0.361878470784578
"1797","STRBP",25.63263010174,0.684983905437856,0.336140725682919,2.03778909576104,0.0415710293421906,0.361878470784578
"1798","BTK",3.56720624058254,-1.7487876558419,0.858340891372468,-2.03740457133019,0.0416095161514109,0.362012047361802
"1799","WDR5B",25.7099651510307,0.770485332308199,0.378336988244662,2.

03650543364252,0.0416996280388146,0.362594375437008
"1800","TUFM",634.628673890452,-0.501884451107058,0.246499454401454,-2.03604690454884,0.0417456455545207,0.362628476255839
"1801","GRWD1",101.599265672519,-0.415306943448885,0.203981357856988,-2.03600440654022,0.0417499127876216,0.362628476255839
"1802","TRIM52-AS1",116.52673075358,-0.506448079324428,0.248777599245475,-2.0357463085923,0.0417758363830996,0.36265227998936
"1803","EREG",23.4667673798281,-1.43693223882799,0.705947879145684,-2.03546505524873,0.0418041012407336,0.362696370332111
"1804","CDYL2",12.725088691366,0.98065446403281,0.481982193682191,2.03462799432677,0.0418883183765647,0.36322559000255
"1805","PBX1",549.990016761452,0.409536585350379,0.201363755591086,2.03381479526054,0.0419702722299929,0.363550962619699
"1806","C6orf120",231.995462183639,0.455492619356382,0.223961971623799,2.0337944699,0.0419723223480903,0.363550962619699
"1807","NBPF10",32.8930300626426,0.865443320271986,0.425649090886894,2.0332319246089,0.0420290971373049,0.363841265367383
"1808","ARHGAP42",98.8971204366386,0.456540386030265,0.224715293968005,2.03163913754471,0.0421902015233784,0.364897704632149
"1809","LINC02175",21.9413629637149,0.984454535026356,0.484579562353677,2.03156429100045,0.0421977848033981,0.364897704632149
"1810","STMN1",54.4123571872865,-0.686306968330659,0.337897450772487,-2.03111022815251,0.0422438141316114,0.365093914066739
"1811","ASPN",2014.94298731088,0.71042292572807,0.349909853052163,2.03030271806082,0.0423257781126171,0.365600302051722
"1812","C3orf70",13.6810922809434,-1.29697051319222,0.63906314174638,-2.02948727358609,0.0424086839288351,0.365606625159823
"1813","NDUFV1",418.287228001414,-0.492081147249375,0.242418082851888,-2.02988630823397,0.0423680971372462,0.365606625159823
"1814","CSAD",339.488464093738,0.58980486463428,0.290685814208054,2.02901151623497,0.0424571173493093,0.365606625159823
"1815","CEP83",97.1553188179667,0.514534875213687,0.253523573094175,2.02953464616308,0.0424038638282494,0.365606625159823
"1816","LINC01048",8.25508657998701,1.9541672812166,0.963484425435098,2.02822923716086,0.0425368572390768,0.365606625159823
"1817","APOE",4196.75467364907,-0.845419699508493,0.416705769379617,-2.02881688143444,0.0424769451935381,0.365606625159823
"1818","ZNF347",52.5887992795174,0.554670206175425,0.273430668147501,2.02855886625055,0.0425032417986259,0.365606625159823
"1819","TOX2",9.16940444194546,-1.57730341022113,0.777509256711172,-2.02866190544541,0.0424927385138044,0.365606625159823
"1820","CRKL",102.375089791834,0.413332574264418,0.203783229712165,2.02829533543183,0.0425301147626109,0.365606625159823
"1821","TPI1",1035.6393218796,-0.419597851634071,0.206945817659389,-2.02757347976312,0.0426037979719092,0.365980896032167
"1822","L1TD1",2.97583506509259,-2.29446111897532,1.13220202542352,-2.02654744246465,0.0427087161830994,0.366205710855563
"1823","SMIM3",139.642317826002,-0.652182664138156,0.321842691809297,-2.02640196821557,0.0427236094298666,0.366205710855563

"1824","NINJ1",92.101208504814,-0.541348485454177,0.267081509913047,-2.02690364312536,0.0426722678882576,0.366205710855563
"1825","ZNF215",13.23067029938,-1.26004125822277,0.62174755720303,-2.02661231817483,0.0427020758048275,0.366205710855563
"1826","POLR3GL",318.914754135438,-0.493434907916129,0.243683443165449,-2.02490124690626,0.0428775055554047,0.367122506515159
"1827","ACSF2",52.2976874776996,0.624751945739823,0.30852684566987,2.02495165172214,0.0428723290416535,0.367122506515159
"1828","Z82195.3",3.04056067284323,3.33005547691187,1.64516945025282,2.02414132866382,0.0429556122952945,0.367590067360663
"1829","GTF3C3",85.078275010982,0.46186616551822,0.228210033857006,2.02386440995676,0.0429841047366519,0.367632777690238
"1830","OBSL1",152.112207872642,0.494724765840627,0.244530851225541,2.02315889124486,0.0430567684626446,0.368053021344891
"1831","AC006994.2",3.64974608293535,1.88597005786705,0.932815418265857,2.02180412216293,0.043196591759594,0.368585075759578
"1832","JAGN1",333.339716617822,-0.487561265372827,0.241220033851203,-2.02123039943515,0.0432559202062681,0.368585075759578
"1833","AL359715.1",7.213146998975,1.25677207241017,0.621654531423966,2.02165673840003,0.043211826090615,0.368585075759578
"1834","RHOBTB2",37.8635299246655,0.552097734191848,0.273111048618709,2.021513728515,0.0432266126552836,0.368585075759578
"1835","SPRYD3",258.220917927442,-0.395174878073566,0.19554158404367,-2.02092501196734,0.0432875282763305,0.368585075759578
"1836","SDSL",17.0168298186635,1.37725186603516,0.68156085349366,2.02073205785721,0.0433075093809438,0.368585075759578
"1837","CSPG4",9.35852010123899,-1.37732717051549,0.681113301508267,-2.02217041932013,0.0431587490892138,0.368585075759578
"1838","HAS1",14.8159043311791,-1.50643314975157,0.745456235283058,-2.02082037610104,0.0432983627364786,0.368585075759578
"1839","NPAS2",45.6253693553893,0.9506852366609,0.470811519445066,2.01924803747676,0.0434614457287097,0.369694070437306
"1840","REEP6",8.02051851931534,1.30163226113806,0.64517392890646,2.01749048251882,0.0436443532545497,0.371048161935283
"1841","IGSF8",143.732504063238,-0.563528332312343,0.279544864859026,-2.01587796147331,0.0438127386637055,0.371339406397201
"1842","CISD2",175.374158941304,-0.385049360653062,0.191015790175056,-2.01579859078763,0.0438210409901703,0.371339406397201
"1843","RCAN2",87.818523519226,-0.517979320323103,0.256939343725583,-2.0159595366458,0.0438042071278063,0.371339406397201
"1844","GFRA1",480.65360689754,0.566804200442583,0.281119042931177,2.01624263704308,0.0437746099664427,0.371339406397201
"1845","BTG1",1865.8492037331,-0.563971450760419,0.27968141928888,-2.01647807778714,0.0437500083199043,0.371339406397201
"1846","RAI2",166.434374164621,-0.654189920883864,0.324480994034602,-2.01611167652582,0.0437882993330119,0.371339406397201
"1847","TCFL5",51.5808791813933,-0.528130954276902,0.26215205002795,-2.01459784205614,0.0439468037771608,0.372203492954048
"1848","KIAA0319L",119.705835851059,0.49203363157683,0.244347502436717,2.01366343699077,0.0440448812470125,0.37255596811018

"1849","UHRF1BP1",41.0650066828927,0.727183045928617,0.361150005552154
,2.01352079398931,0.0440598696544035,0.37255596811018
"1850","CENPV",62.158923326432,-0.514852542070866,0.255658575961355,-2
.01382856074693,0.0440275360193313,0.37255596811018
"1851","TSPAN17",44.8332230617503,-0.49876324795519,0.248011966443407,
-2.01104509233026,0.0443206940162925,0.374558950025318
"1852","AL356488.3",19.5331571972051,-0.855918221012565,0.425783287115
661,-2.01022033253283,0.0444078743671008,0.374864319158162
"1853","AL023581.2",4.52414997966911,-1.55031916195599,0.7712941098736
56,-2.01002333884015,0.0444287187700078,0.374864319158162
"1854","SNAI3-
AS1",10.3318601454719,1.00315871435679,0.49906206047546,2.010088110887
6,0.0444218641643633,0.374864319158162
"1855","ZNF718",30.9027299053645,0.923520706502729,0.459622790617379,2
.00930137790215,0.0445051818760822,0.374902832572727
"1856","AL157392.3",31.0669303600876,0.75269700841,0.374554051644908,2
.00958180829822,0.0444754682286104,0.374902832572727
"1857","MORC2",57.9342548892684,0.532079025281468,0.264799770831539,2.
00936361693442,0.0444985857505458,0.374902832572727
"1858","PAOX",35.576809267589,-0.626928084062241,0.31212823393506,-2.0
0855935446288,0.0445838854794816,0.375363681676819
"1859","ARNTL2",6.56345376480947,-1.26869268998165,0.631910515184418,-
2.00770941374728,0.0446741797028675,0.375628066495661
"1860","DGCR2",115.595768882591,0.34642982012028,0.172560406239685,2.0
0758579369066,0.0446873254330004,0.375628066495661
"1861","HAUS7",43.5086184888939,-0.611697658536013,0.304647684411718,-
2.00788546847883,0.0446554637119,0.375628066495661
"1862","HTRA2",194.705244732053,-0.508820915452197,0.253541816305606,-
2.00685205646271,0.0447654180697914,0.375648709244168
"1863","CD83",70.171578463243,-0.94078393428525,0.468896685393385,-2.0
0637787297637,0.0448159472611595,0.375648709244168
"1864","FLNC",146.044931255054,0.579091896259078,0.288669772752804,2.0
0607043382741,0.044848733804281,0.375648709244168
"1865","REPIN1",127.528751057609,-0.413771630893013,0.20626858552016,-
2.00598472059901,0.0448578782118588,0.375648709244168
"1866","AL162231.1",27.8595697387938,-0.698775375049719,0.348162754826
605,-2.00703655219447,0.0447457711168353,0.375648709244168
"1867","GLYR1",154.023284967203,0.43220010482926,0.215391429576847,2.0
0657986103881,0.0447944174502338,0.375648709244168
"1868","RETREG3",158.196927964748,0.357293565500713,0.177996403197447,
2.00730778309254,0.0447169009410694,0.375648709244168
"1869","MRPS30-
DT",42.9494369720482,0.627711600598191,0.313102616124055,2.00481110112
949,0.0449832453135204,0.376094551811545
"1870","CENPH",23.0762753218978,-0.791781774156654,0.394930612740863,-
2.00486300279839,0.0449776948924639,0.376094551811545
"1871","DACT2",5.12806303558878,2.13671981050563,1.06571095533007,2.00
497123522939,0.0449661222561239,0.376094551811545
"1872","ANKRD27",58.6633607529471,0.42448512211723,0.211782027926627,2
.0043491238279,0.0450326751253077,0.376306697107473

"1873","AL138828.1",2.94354913011506,2.52192405520486,1.25882302508069,2.00339841658298,0.0451345412953537,0.376942384867727
"1874","ZBTB44",360.634880572985,0.375581365105224,0.187491670438401,2.00318960424762,0.0451569410753769,0.376942384867727
"1875","PUS7",34.8026179878756,-0.76148189723409,0.380241525569236,-2.00262687273338,0.0452173532475961,0.377245363654478
"1876","CALCOCO1",469.970010974516,0.411012899599189,0.205301575868013,2.00199583398925,0.0452851795852992,0.37748990909249
"1877","NR2C1",113.830443162939,0.419863085172446,0.209751354451994,2.00171811175857,0.0453150573464988,0.37748990909249
"1878","PDE4A",174.678100748461,-0.367971348281638,0.183831171262881,-2.00168092143325,0.0453190595969888,0.37748990909249
"1879","AL162377.1",30.6529505327654,-0.74433006123689,0.371941965933562,-2.00119945962174,0.0453708991886369,0.37772058329316
"1880","BOLA3",174.931405180339,-0.516542438109485,0.258225455521288,-2.00035444633731,0.0454620036593061,0.378069047616231
"1881","PIP4P2",455.324558994773,-0.380774420903811,0.190416592942522,-1.99969138728761,0.0455335987795806,0.378069047616231
"1882","LUC7L3",1223.90744347191,0.537248541117705,0.268613356677231,2.00008126090048,0.0454914899002573,0.378069047616231
"1883","UTP18",92.4303943868959,-0.491471688742557,0.245689854627825,-2.00037437234454,0.0454598535782609,0.378069047616231
"1884","ARHGEF9",79.703227146507,0.50005005227096,0.250062609221299,1.99969941059212,0.0455327318797332,0.378069047616231
"1885","R3HDM4",83.4740973617062,-0.451679972533135,0.226028538158445,-1.99833160986296,0.0456807207096828,0.379089397380142
"1886","ZNF385B",45.7946498840556,0.942656468866544,0.471907091255262,1.99754673395371,0.0457658229945751,0.379393094384811
"1887","TSPAN15",144.335723997981,-0.724631272428081,0.362755384090829,-1.99757551288789,0.0457627002037603,0.379393094384811
"1888","POLH",46.4252957583892,0.624528748572519,0.312780119294107,1.99670218804819,0.0458575443075386,0.379952100425226
"1889","MBD2",415.712152568098,-0.40795018403549,0.20434927177239,-1.99633784107573,0.045897161739176,0.380079037102133
"1890","AL161421.1",10.5941003670946,-0.977842862978111,0.490108406730186,-1.99515627471461,0.0460258382932808,0.380942956836926
"1891","PLGLB1",9.93860988934436,1.21974437262465,0.611781845721441,1.99375705760977,0.0461786105572965,0.381432883270308
"1892","ANXA11",878.801792427337,-0.366604476477142,0.183831610880868,-1.99424067884995,0.0461257585880935,0.381432883270308
"1893","NR4A1",216.548465799594,-1.51207573910161,0.758361805845777,-1.99387116735822,0.04616613561542,0.381432883270308
"1894","LINC00665",31.7018943186493,0.947953295961198,0.475469412357165,1.99372088156348,0.0461825660623898,0.381432883270308
"1895","NPEPPS",489.643189372634,0.466923339145305,0.234230727672721,1.99343332868655,0.0462140173707529,0.381491226243107
"1896","PCDH18",1040.40429164309,0.398792589473398,0.200156802094708,1.99240088420628,0.0463270904166814,0.382021441954742
"1897","HCFC2",163.108657892079,0.371233799250862,0.186319339247455,1.99245983133193,0.0463206282791563,0.382021441954742

"1898","OTUD7B",80.5396592124818,0.536444432108636,0.269709185243626,1.98897353690076,0.0467041239657282,0.382509220521407
"1899","AL358472.4",8.11218767443403,-1.35450686898976,0.680686532854684,-1.98991283595576,0.0466005381329729,0.382509220521407
"1900","LPIN1",138.26525618033,0.405088669549451,0.203635188058962,1.98928620053701,0.0466696219478967,0.382509220521407
"1901","ERICH2",13.5860262310262,-0.869848950485978,0.437248515296473,-1.98936970636981,0.0466604108202654,0.382509220521407
"1902","ACADL",73.2050590127408,0.635732127574775,0.319585033368577,1.98924249009366,0.0466744440472392,0.382509220521407
"1903","EBF2",272.004481016741,0.457424796634413,0.229850263479149,1.99009907454776,0.0465800227415606,0.382509220521407
"1904","AC130895.1",6.281825672158,1.47362729654078,0.740304540501488,1.99056903736202,0.046528287074931,0.382509220521407
"1905","ZFYVE26",38.7395344125922,0.541898017170871,0.272441434675074,1.98904405938533,0.0466963400288367,0.382509220521407
"1906","GSC",278.176316427066,-0.652543982814938,0.328049546680234,-1.9891628853583,0.0466832270518709,0.382509220521407
"1907","STX4",296.777275338334,-0.430141066612802,0.216177048277001,-1.98976288205044,0.0466170620565834,0.382509220521407
"1908","SGSM2",76.9135652576017,0.585506936290541,0.294357689164428,1.98910019287275,0.0466901450614882,0.382509220521407
"1909","SHPK",56.2317236713254,0.623897841708563,0.313631865494536,1.9892680251887,0.0466716269861085,0.382509220521407
"1910","AC127024.5",7.70843007020458,1.26147567869794,0.63377152029205,1.99042657851939,0.0465439644880624,0.382509220521407
"1911","ZNF45",20.1964035439493,0.770025448500437,0.387244013439721,1.98847605586109,0.0467590645856715,0.382758789803066
"1912","NFKBID",13.5177731762573,-0.862512754085799,0.433852249602512,-1.98803337974165,0.0468079983999157,0.38295895343613
"1913","SCARB2",880.364669920086,0.33306565479953,0.167568487791735,1.98763895997848,0.0468516342070704,0.383115584893467
"1914","KCNMB2-AS1",2.63598079784803,2.76991874273006,1.39410337460608,1.98688188636853,0.0469354873701627,0.383600746568159
"1915","OXA1L",259.465153941716,-0.398848146960595,0.200810716461967,-1.98618955197113,0.0470122805395986,0.384027730799447
"1916","CRABP2",828.060991569604,-0.733717407437219,0.369695311121168,-1.9846543501244,0.0471829409916629,0.384089295326448
"1917","TBX19",33.3367101352122,0.9040273225155,0.455981813918098,1.98259512752822,0.0474126720753929,0.384089295326448
"1918","KLHDC8B",168.447205277867,-0.463077452222388,0.233544036702005,-1.9828271308561,0.0473867423850122,0.384089295326448
"1919","TCTA",98.2931423210989,0.514190867130914,0.259341946821904,1.98267528038579,0.0474037124985063,0.384089295326448
"1920","CCDC112",109.626618160877,-0.46941215105304,0.23661290206355,-1.9838823114006,0.0472689612667417,0.384089295326448
"1921","C8orf31",27.2609650835034,0.831463628791443,0.419142049673217,1.98372754401447,0.047286221253349,0.384089295326448
"1922","AK1",254.205853150057,-0.701312314822929,0.353388100526566,-1.

98453856759166,0.0471958330412535,0.384089295326448
"1923","ZSWIM8",118.935455404582,0.462572777758252,0.233270791810505,1
.98298627174044,0.0473689630108172,0.384089295326448
"1924","EPS8L2",117.996811451691,-0.35983392969093,0.181291195924144,-
1.98483951664973,0.0471623293918736,0.384089295326448
"1925","GLI1",29.2615384894282,-0.714735631597337,0.360115193665073,-1
.98474167202754,0.0471732199101686,0.384089295326448
"1926","APAF1",72.2102787939533,0.495379203172346,0.249773703537786,1.
98331207871691,0.0473325810363874,0.384089295326448
"1927","ZCCHC8",102.2173425,0.428272445861939,0.215778173852804,1.9847
8112134775,0.047168828780322,0.384089295326448
"1928","GZMH",13.5681833790466,1.36382877813855,0.686822740549089,1.98
570707930873,0.047065858488409,0.384089295326448
"1929","RTN1",6.7307067184924,-1.26020794235972,0.635557365622627,-1.9
8283901741136,0.0473854142136253,0.384089295326448
"1930","LAIR1",7.32718388920392,-1.31716899438974,0.663852488948927,-1
.984129029139,0.0472414577376062,0.384089295326448
"1931","PROCR",951.071736968543,0.812666160624449,0.409509155185861,1.
98448838159823,0.0472014220265789,0.384089295326448
"1932","POLR1D",646.110030609721,-0.389461374484753,0.196528150072502,
-1.98170783341254,0.0475119499775165,0.384694323756879
"1933","ACAD10",46.0468731381149,0.691074971776069,0.348790352712595,1
.98134772479077,0.0475522917797085,0.384821779777538
"1934","GANAB",406.138977746411,0.258440509340546,0.130456950823049,1.
98104054793595,0.0475867265635583,0.384901325560363
"1935","SMAGP",13.7922704536182,-0.97685220754561,0.493261385352148,-1
.98039464785637,0.0476592008923196,0.385288309849383
"1936","TRMT61B",68.9798909723312,-0.442916817246093,0.223846005076005
,-1.97866750892295,0.0478534533978602,0.38530931239561
"1937","PCNP",591.167663764348,-0.253694882753017,0.128184649074228,-1
.97913622719449,0.0478006706569832,0.38530931239561
"1938","TMEM70",181.943551479995,-0.49173487152266,0.248478267014962,-
1.97898543574859,0.0478176460613064,0.38530931239561
"1939","DLG2",37.9234271218413,0.603759874738971,0.304994388614412,1.9
7957699314354,0.0477510803008187,0.38530931239561
"1940","AL136040.1",8.69666012717269,1.10528459170996,0.55831760822657
7,1.97966995026497,0.0477406272651542,0.38530931239561
"1941","IDH3A",85.8838333306726,-0.393829332521769,0.199042469182743,-
1.97861960886443,0.047858850219566,0.38530931239561
"1942","AL162458.1",21.3491362920007,0.857430880493962,0.4331157134291
64,1.9796808425751,0.0477394025499169,0.38530931239561
"1943","BCAS4",4.60274167360045,1.55898228109501,0.787623054451729,1.9
7935074688771,0.0477765297495229,0.38530931239561
"1944","GAS2L1",89.8124798073447,-0.606403493726273,0.306609717891563,
-1.97776997381647,0.0479546622562218,0.385882089338517
"1945","HSPD1",549.973317572407,-0.33004270943706,0.166933008093582,-1
.97709675998912,0.0480306939551043,0.386295190508841
"1946","SCN7A",3.78715527654759,2.89433345428238,1.46465151139031,1.97
612430791469,0.0481406999599626,0.386296870203163
"1947","WEE1",118.258463768587,0.521345409874736,0.263833793319812,1.9

7603727450781,0.0481505556901768,0.386296870203163
"1948","AL590807.1",17.1494015327634,1.59002811062668,0.804722751085434,1.97587070637932,0.04816942272048,0.386296870203163
"1949","HEATR5A",105.385370835308,0.471286580249985,0.238531240898711,1.97578555527706,0.0481790701122784,0.386296870203163
"1950","OPRL1",4.95825214069624,1.46270530065191,0.740253944437498,1.97595070130073,0.0481603609887344,0.386296870203163
"1951","CCNQ",71.4149450038011,-0.670478038888502,0.339266815886837,-1.97625587735684,0.0481258041524592,0.386296870203163
"1952","RASGEF1A",3.10557298547533,-1.73447163854872,0.878199619609786,-1.97503118860312,0.0482646087479405,0.386784464469279
"1953","RAMP2",1557.64136408666,-0.758104337096526,0.384072884137962,-1.97385540194555,0.0483981871183087,0.387656344645009
"1954","COL4A5",4.32813257482326,-1.81195771514786,0.918138383204214,-1.97351265157252,0.0484371845874253,0.387770152764121
"1955","WDR19",92.9423936095728,0.60877881683291,0.30851448867802,1.97325843412256,0.0484661259964495,0.387803380543458
"1956","SPAG16",201.042438700209,-0.390313469327996,0.197847533766968,-1.97279926565939,0.0485184368783519,0.387890278454614
"1957","EFHD1",125.318449853147,0.641683582330316,0.325277299372553,1.97272783427586,0.0485265789769022,0.387890278454614
"1958","PRG2",34.9358079002141,-0.640619497024643,0.324790632526923,-1.97240755387716,0.0485631002115975,0.38798395128193
"1959","STX6",71.4182984378328,0.466537289595366,0.23663984787655,1.97150773118629,0.0486658295766643,0.38840794493253
"1960","KIF23",6.84343393017637,1.36821932970945,0.693980827298548,1.97155206006988,0.0486607644454308,0.38840794493253
"1961","SLC25A25",38.5042192977681,-0.952376652747111,0.483263174172999,-1.97072051760803,0.0487558523675699,0.388927995199335
"1962","TOP2B",239.154816656016,0.323162174079737,0.164021380986752,1.97024419704062,0.0488103904600899,0.389164596313551
"1963","TCEAL3",141.227672691784,-0.551568624225282,0.280090088808881,-1.9692543444536,0.0489238911255703,0.389870824695515
"1964","KCMF1",209.091334661378,-0.341848681878658,0.173627702505997,-1.9688602506668,0.0489691411976908,0.390032726963074
"1965","EIF1B-
AS1",33.8395861581308,0.566111792493692,0.287678959998223,1.96785956295584,0.0490841986005931,0.390154531864369
"1966","AC080013.1",16.6575838065409,1.14391644625033,0.581281527847582,1.96792155168963,0.0490770646534303,0.390154531864369
"1967","GUCY1A2",13.9927818028014,1.37421146758681,0.698251132411633,1.96807624620749,0.0490592654957154,0.390154531864369
"1968","DCAF15",50.2820085969784,-0.490422799914078,0.24913933973941,-1.96846792813629,0.0490142227805489,0.390154531864369
"1969","ASPSCR1",92.4890150431361,-0.43504299979363,0.221193870468739,-1.96679500599052,0.0492068485621616,0.390930793325492
"1970","PLAAT3",592.008591305912,-0.511684881755498,0.26028794072479,-1.96584167645522,0.0493169019374029,0.391407558095786
"1971","SUPT20H",153.914220350424,0.39010623520716,0.198427055066529,1.96599317102381,0.0492993994542554,0.391407558095786

"1972","UBLCP1",137.250863644652,-0.349964907746429,0.178050651250095,
-1.96553567925376,0.0493522703324389,0.39148963732776
"1973","MUSTN1",151.396731705734,-0.800157963295758,0.407344845378016,
-1.96432573622777,0.049492328949781,0.392004304689329
"1974","AL137781.1",2.59563338461193,2.21902115225126,1.12945374289891
,1.96468528808964,0.0494506737528542,0.392004304689329
"1975","XBP1",405.432586461813,-0.484946697964791,0.246869840193135,-1
.96438211158317,0.0494857957451329,0.392004304689329
"1976","PRRG1",129.22599453652,0.398786299238567,0.203055504930385,1.9
6392754471387,0.0495384950153439,0.392171395508616
"1977","SPSB4",18.3534522773517,1.34786098441503,0.686542422385988,1.9
6325957503212,0.0496160201073805,0.392586445391883
"1978","TRMT1",65.3201281046363,-0.534752848695253,0.272492296822948,-
1.96245125065942,0.049709970928752,0.39313097838143
"1979","ZNF280D",415.044831289465,0.351889444952131,0.179382649235547,
1.96166935013913,0.0498009924457684,0.393651806381584
"1980","TEX261",168.274551422027,0.348431014135567,0.177758905490974,1
.9601325355441,0.049980301303649,0.393874989064474
"1981","GLIS3",84.8098681139795,-0.561946325991594,0.286659933052041,-
1.96032392810745,0.0499579409846303,0.393874989064474
"1982","BATF2",7.53769710468523,1.34440522464768,0.685508028051729,1.9
6118086095738,0.0498579286705932,0.393874989064474
"1983","ETHE1",140.000337281114,-0.580853144946977,0.296273729906418,-
1.96052868113028,0.0499340290547985,0.393874989064474
"1984","NRSN2-
AS1",21.5719995677124,-0.622711383180389,0.31762635265629,-1.960515486
11345,0.0499355697357155,0.393874989064474
"1985","SMIM26",733.878153788281,-0.495085955235479,0.25257520173532,-
1.96015266674633,0.0499779489882949,0.393874989064474
"1986","DOCK9-
DT",5.49146501704793,-1.23674205512818,0.631030302073006,-1.9598774433
8955,0.0500101166651094,0.393911508052521
"1987","WDR7",45.2421088887604,0.542838395432971,0.2770676269946,1.959
22707145989,0.0500862001004355,0.394312243669407
"1988","LSM10",237.113230969277,-0.501173801228745,0.255972436490606,-
1.95792097031954,0.0502392868116709,0.394323216761718
"1989","P4HA1",352.238550112281,0.52298016866242,0.267041946916022,1.9
5841954682455,0.0501808029794365,0.394323216761718
"1990","ZDHHC5",114.160999006668,0.326136580978738,0.166560804155675,1
.95806319879386,0.0502225973599434,0.394323216761718
"1991","AC005520.2",32.7612917207786,0.632375483658975,0.3230365611725
37,1.95759724956092,0.0502772903618657,0.394323216761718
"1992","NF1",338.165850216493,0.382492852689699,0.195399109340766,1.95
749537436556,0.0502892550942675,0.394323216761718
"1993","AP005329.3",25.3425603166302,-0.647938123437342,0.330997090738
051,-1.95753419461356,0.0502846955688274,0.394323216761718
"1994","CEP192",79.0343545109383,0.460479287779927,0.235130121812115,1
.95840194455341,0.0501828667821503,0.394323216761718
"1995","IZUMO4",12.0026272404262,0.94190327908178,0.480912498460111,1.
95857517136229,0.0501625596592779,0.394323216761718

"1996","EIF3I",831.094509012431,-0.452358918816317,0.231123692695532,-1.95721569494058,0.0503221143133896,0.394383183469115
"1997","LINC00377",22.993467525019,1.44765183211522,0.739880585138487,1.95660199928649,0.0503942797851952,0.394750985818632
"1998","PTCD2",46.1605774440118,0.575220986457007,0.294094390480873,1.95590601206798,0.0504762269848331,0.395195004366238
"1999","ATP9B",99.3280883558701,0.452064644299977,0.231155513351553,1.95567320781336,0.0505036628236867,0.395212004777855
"2000","NAMPT",687.276761086482,-0.759993472781284,0.388732132820455,-1.95505698812993,0.0505763442514427,0.395582876562659
"2001","STUB1",774.821938720843,-0.471567782663017,0.241287852019542,-1.95437846835665,0.0506564751576076,0.396010491246021
"2002","CNN2",295.479865389202,0.546917121025494,0.279872466663102,1.95416550811999,0.0506816469650665,0.396010491246021
"2003","SHKBP1",58.315898931293,-0.616948231833967,0.3158099854995,-1.95354251024765,0.0507553452149121,0.396388350073325
"2004","SDR39U1",117.673706226096,-0.517831585837904,0.265140197368472,-1.9530482023375,0.0508138837890832,0.39664749706219
"2005","ZBTB37",114.517030238225,0.590275388677189,0.302365103714692,1.95219415675086,0.0509151576731255,0.397239806224789
"2006","POLD2",223.086674898449,-0.492970015184561,0.252589350728106,-1.95166587096226,0.0509778871017822,0.397391533918714
"2007","TMEM205",358.820471651648,-0.431091110215562,0.220890833774178,-1.95160253076086,0.0509854125535294,0.397391533918714
"2008","RHOBTB1",28.5579789040328,0.84078962158357,0.430959455558822,1.95097151423056,0.0510604344655426,0.397656687248031
"2009","ACSS1",46.5753452627371,0.599639259182836,0.307367213363563,1.9508888167378,0.0510702732648018,0.397656687248031
"2010","CEP128",33.95538915763,1.03584088217901,0.53118978617936,1.95003915574018,0.0511714522717896,0.398246282531147
"2011","CD300E",4.15079502631708,-1.76312485984289,0.904284873937641,-1.94974494283587,0.051206526717578,0.398321082766321
"2012","SULT1C4",3.81973653026056,1.80352853718707,0.925951863022576,1.9477562594883,0.0514441348275905,0.399573287541211
"2013","GSAP",18.6855086282349,-0.849645851851546,0.436194018710528,-1.94786222507878,0.0514314508111878,0.399573287541211
"2014","ZNF285",3.78816315190674,1.81334573590236,0.930898324892928,1.94795251791965,0.0514206448790004,0.399573287541211
"2015","USP46",109.342780414397,0.327896322255584,0.168371850410243,1.94745333888447,0.0514804086802289,0.399638835252394
"2016","TNRC18",260.949081197213,0.376012069154127,0.193098110072349,1.94725918867484,0.0515036688530862,0.399638835252394
"2017","KLF13",326.597078378301,-0.557195440555674,0.286205964725198,-1.94683378136674,0.0515546655438164,0.399836208776361
"2018","FAM87B",11.1772859516532,-1.28677291931083,0.661108544104846,-1.94638676324044,0.0516082984091434,0.400053821612602
"2019","AC006942.1",12.3208104742922,-0.900771625770981,0.462966758718218,-1.94565075960287,0.0516967052420006,0.400540643932944
"2020","LINC00517",4.31934634260193,-1.82756012700022,0.939660526765516,-1.94491529115416,0.0517851743409894,0.401027466443612

"2021","TRAFD1",91.7089154365114,0.45295868309384,0.232945789152331,1.94448109468781,0.0518374630394732,0.401233762655359
"2022","PON2",151.7122912423,0.456816670784832,0.23498960742826,1.94398669704699,0.0518970553226668,0.401496358265319
"2023","GMPS",163.813493350398,-0.307592716319838,0.158279126972922,-1.94335615947933,0.0519731403975367,0.401496747507451
"2024","EPB41L4A",58.7896097407057,0.626880914614414,0.322577766701074,1.94334817624096,0.0519741043088019,0.401496747507451
"2025","CLEC7A",14.3842141632355,-1.23101128775793,0.633352199667323,-1.94364413418717,0.0519383797856696,0.401496747507451
"2026","TRAK1",86.8057636415786,0.530596082804844,0.273327034653545,1.94124991506017,0.0522279713413821,0.403258714557374
"2027","MBTD1",105.239156729819,0.405618385047314,0.208985444149109,1.94089299711184,0.0522712576065048,0.40339382473535
"2028","LURAP1",33.4313113254802,-0.699207409974094,0.36043318148227,-1.93990854864867,0.0523908049804859,0.403666266556053
"2029","MTUS1",319.793321794351,0.474223301085087,0.24450294031035,1.93954027907865,0.0524355848393466,0.403666266556053
"2030","HSD17B12",394.805660013969,-0.306126687682735,0.157776961082561,-1.94024961301255,0.0523493616712581,0.403666266556053
"2031","EMSY",80.6207781767435,0.435529093822384,0.224532336435051,1.93971657150758,0.0524141445124941,0.403666266556053
"2032","CISD3",228.665764430906,-0.495241850458959,0.255322327161038,-1.93967310248821,0.0524194304452948,0.403666266556053
"2033","POMGNT2",86.0769287603409,0.416515062413568,0.214814409955826,1.93895308279933,0.0525070513518382,0.4040176115577
"2034","WDR13",345.057429330989,-0.448725788515506,0.231453611201178,-1.93872882858361,0.0525343663743875,0.404029052701349
"2035","LATS1",149.36556744939,0.37534416490321,0.193631222091907,1.93844856654911,0.0525685200629309,0.404093051274904
"2036","TBC1D9",103.889725867489,0.373796826218884,0.192919996759773,1.93757429243762,0.052675181378561,0.404220174835221
"2037","FILIP1",346.91783674815,0.410825889539034,0.212042901682513,1.93746589147396,0.0526884188767509,0.404220174835221
"2038","PAPSS2",227.593793617195,0.40914297012672,0.211173536442009,1.93747273934145,0.0526875825599069,0.404220174835221
"2039","ANO8",31.7214615602401,0.612183277098909,0.315925016287178,1.93774866040501,0.0526538940823318,0.404220174835221
"2040","TLR1",14.8230895951612,-0.778232520486487,0.401795213523587,-1.93688848023273,0.0527589769212028,0.404420251952933
"2041","PLAU",353.452601335544,0.751618127798133,0.388066258670667,1.93682937128527,0.052766204323719,0.404420251952933
"2042","EBNA1BP2",230.074401215749,-0.394699843712159,0.203877927736705,-1.93596162220115,0.0528724015056533,0.40483748250266
"2043","PCBP2",1654.07418333274,-0.326468120638933,0.168619715604547,-1.93612069305453,0.0528529206798442,0.40483748250266
"2044","CCDC77",41.4367886157889,0.510763835366912,0.263965992847313,1.93496074951729,0.0529951124254019,0.405578543870138
"2045","JTB",992.081269641462,-0.427263473195656,0.220999977947512,-1.93331907615454,0.0531969032618266,0.406365949020737

"2046","SIPA1L2",116.965077755537,0.716561615088342,0.370534008779224,
1.93386193469569,0.0531301053825876,0.406365949020737
"2047","AC107223.1",13.9777444040351,0.873943306343597,0.4520882256492
96,1.93312556434848,0.0532207315369446,0.406365949020737
"2048","FAM53C",156.076281950264,0.531615012777446,0.274988063080176,1
.9332294166618,0.0532079424673356,0.406365949020737
"2049","ZNF626",141.246307894572,0.325141975796068,0.168200013989908,1
.93306747177546,0.05322788656546,0.406365949020737
"2050","AC073343.2",3.31596042709846,2.21004079042734,1.14381578586803
,1.93216496723742,0.0533391476700629,0.406417408116663
"2051","UNKL",132.715788744307,0.518696094359662,0.268438443240952,1.9
3227202518858,0.0533259393785466,0.406417408116663
"2052","PGP",105.89140388456,-0.554222709018004,0.286863942598115,-1.9
3200547966549,0.0533588295417897,0.406417408116663
"2053","SPATA33",79.9229643160041,-0.487247929391512,0.252204035582614
,-1.93195928949323,0.0533645308618312,0.406417408116663
"2054","SMCHD1",321.236007073897,0.346033408365813,0.179037948385806,1
.9327377881931,0.0532685075878068,0.406417408116663
"2055","COL8A2",96.3278820489201,-0.5626849025532,0.291527403054292,-1
.93012696802437,0.0535911077276187,0.406793124645362
"2056","ANKRD35",414.797422336783,-0.53270971766038,0.276206201664467,
-1.92866675132628,0.0537722464020888,0.406793124645362
"2057","ACTR1B",117.919908605833,-0.45779601444021,0.237140110642947,-
1.93048747931765,0.0535464651099374,0.406793124645362
"2058","EFHB",5.25187096737586,-1.65135461893968,0.855821306689603,-1.
92955539436997,0.0536619501881637,0.406793124645362
"2059","CHCHD6",80.1827269464941,-0.61169832064009,0.317069054991025,-
1.92922743803366,0.0537026333185457,0.406793124645362
"2060","AC025171.2",72.8912558631433,0.487607630682471,0.2527423184001
,1.92926785577147,0.0536976180892153,0.406793124645362
"2061","BCL7B",128.297078923522,-0.562349575346037,0.291340636713413,-
1.930213312121,0.0535804127879655,0.406793124645362
"2062","BDNF-
AS",51.2550878560985,0.436131047746555,0.226027791627103,1.92954611734
683,0.053663100653269,0.406793124645362
"2063","HEBP1",716.328486939987,-0.528572875654342,0.274067781447956,-
1.92862098879986,0.0537779314560256,0.406793124645362
"2064","VDR",40.7224538210859,1.01430805516355,0.525745100182033,1.929
27723874622,0.0536964538600385,0.406793124645362
"2065","PELI2",88.2366742450121,-0.691630395248445,0.35843041805548,-1
.92960853880819,0.0536553600202969,0.406793124645362
"2066","GABPB1",53.6876230479739,0.533325054710821,0.276208386590189,1
.93087929477723,0.0534979812742848,0.406793124645362
"2067","NEO1",95.91273287717,0.48863187534784,0.253001509963159,1.9313
3975927256,0.0534410495675962,0.406793124645362
"2068","HOMER3",326.072415070185,-0.512145431421492,0.265518277286182,
-1.92885189168914,0.0537492516355878,0.406793124645362
"2069","TPPP",26.8887460587308,-1.20586257761669,0.625412396622155,-1.
92810789189587,0.0538417075676171,0.407078700570437
"2070","ANKMY2",123.268161401628,0.364742863275894,0.189243176769501,1

.9273765612176,0.0539327184770846,0.407569814075862
"2071","GIPC2",5.90051022253147,-1.2084170458862,0.627152009407501,-1.
92683277380845,0.054000473653364,0.407873792896737
"2072","C8orf88",40.442179320244,-0.570882158597567,0.296310436280584,
-1.92663534151387,0.0540250910235913,0.407873792896737
"2073","CUL7",61.6174157511473,0.511667102472208,0.265753529611129,1.9
2534452212514,0.0541862711774541,0.408439465056335
"2074","TPST1",478.14372919048,0.58116009101804,0.301964730640025,1.92
459592809481,0.0542799292241,0.408439465056335
"2075","AC004889.1",136.237518724215,0.47218091178975,0.24532204699732
6,1.92473900152519,0.0542620185971287,0.408439465056335
"2076","ANGPT2",38.6142130286239,-0.78503308220747,0.407768037116595,-
1.92519523540537,0.0542049379551013,0.408439465056335
"2077","RSU1",278.29250039383,0.371274963827285,0.192868411141428,1.92
501696690514,0.0542272356374528,0.408439465056335
"2078","RBM3",1314.41044989749,-0.569649783855652,0.295987606038504,-1
.92457309777203,0.0542827876911155,0.408439465056335
"2079","TSR2",193.630626410945,-0.40021091832562,0.207815211310996,-1.
92580184963796,0.0541291204330087,0.408439465056335
"2080","KLHL8",96.6776550708784,0.451798895768562,0.234807546106975,1.
9241242594594,0.0543390099345362,0.40846954945024
"2081","TNKS",129.005492476198,0.414875384558674,0.215601506876217,1.9
2426941058842,0.0543208227465652,0.40846954945024
"2082","ZNF431",151.689623361151,0.474183012026513,0.246490697349571,1
.92373593456158,0.0543876913580503,0.408639123877993
"2083","BNC2",265.694029378319,0.54683892088944,0.284319363086196,1.92
332634314342,0.05443907823188,0.408764679755459
"2084","METTL21C",3.5644920586524,1.81799617243905,0.945508851239088,1
.92277012537383,0.0545089255238693,0.408764679755459
"2085","AC024337.2",9.67067877983949,0.955240471761896,0.4967240058421
18,1.92308094742157,0.0544698846984752,0.408764679755459
"2086","AC046158.1",2.74050514677802,2.23242816500423,1.16098459895573
,1.92287491755896,0.0544957604866005,0.408764679755459
"2087","ZKSCAN1",436.228964266874,0.480941005167727,0.250344289765949,
1.92111833514304,0.0547167905816653,0.408998629831019
"2088","GPAM",58.4379709567719,0.568483593164708,0.295799352076016,1.9
218554373933,0.0546239504126136,0.408998629831019
"2089","CDKN1C",2578.21817866773,0.553388029952126,0.288093806272537,1
.92086055966306,0.0547492891942821,0.408998629831019
"2090","PDE3A",39.3461956118738,0.73176415770835,0.380917996517298,1.9
2105430669806,0.0547248613586568,0.408998629831019
"2091","EXOSC6",232.979639382813,-0.306799123609109,0.159706189270297,
-1.92102212826493,0.0547289178207183,0.408998629831019
"2092","AC009283.1",15.0564557273721,0.876360470446233,0.4561585037952
51,1.92117534399752,0.054709605458542,0.408998629831019
"2093","MAPK11",10.1883854247298,-1.09425022553817,0.569607610654839,-
1.92105969981719,0.0547241815184173,0.408998629831019
"2094","MED14",56.016534028596,0.484478581906045,0.252170004308301,1.9
2123794911676,0.0547017159155537,0.408998629831019
"2095","TMEM173",313.743079869212,-0.40741381567782,0.212184676583939,

-1.92009065987689,0.0548464488721288,0.409333492226484
"2096","SRGAP1",387.543448089577,0.404460879431523,0.210646485617962,1
.92009317527885,0.0548461311999225,0.409333492226484
"2097","TMEM59L",4.23539109584047,1.61910279057027,0.843690428561499,1
.91907213328337,0.0549752055931341,0.410098779729803
"2098","YWHAQ",1234.0326070087,-0.318746632520924,0.166116662580396,-1
.91881192151124,0.0550081405696922,0.410148876516537
"2099","STRAP",371.510476091119,-0.45819669471119,0.238863375252581,-1
.91823754573794,0.0550808974842417,0.410495702403999
"2100","GCC2",770.981363626583,0.290246395152575,0.151351286992351,1.9
1770021200576,0.0551490348495812,0.410738010133657
"2101","AC079140.6",4.86137780951266,1.40711590508151,0.73380269307131
5,1.91756710402909,0.0551659246494159,0.410738010133657
"2102","LINC02328",26.7947790658581,0.72760825034726,0.37951662742373,
1.91719729195129,0.0552128719822225,0.410891986878167
"2103","COL5A2",1792.48890599296,0.6556516579987,0.342097070146353,1.9
1656613053776,0.0552930743132826,0.411153475601589
"2104","RSRC1",439.508271851734,-0.260892452485018,0.136143812030266,-
1.91630048104588,0.0553268596906825,0.411153475601589
"2105","DDB1",355.385167285251,0.287523887722194,0.150033831512348,1.9
1639368816979,0.0553150036235659,0.411153475601589
"2106","MLLT3",93.3702869022317,0.414764097854738,0.21653861372804,1.9
1542788010852,0.0554379584231646,0.411588032089969
"2107","SLC7A6",91.8523183077006,0.664719738283726,0.347026972542846,1
.91546995155155,0.0554325976641586,0.411588032089969
"2108","ZNF589",38.9270807057742,0.487368794929426,0.25459003007984,1.
91432788933874,0.0555782730439769,0.412238466205278
"2109","TKFC",42.362394141988,0.753436070806983,0.393547201675741,1.91
44744711659,0.0555595580217322,0.412238466205278
"2110","ZBTB2",34.0640262889288,-0.638131829561065,0.333434823844166,-
1.91381278717097,0.055644081037424,0.412419053762333
"2111","CARS",89.1321560615819,-0.419541375864819,0.219227660517289,-1
.91372464074501,0.0556553488775993,0.412419053762333
"2112","ELL2",82.0139885518095,0.885260963317154,0.462645239309215,1.9
1347686758639,0.0556870321347451,0.412458448713929
"2113","BBIP1",187.491578867765,-0.329798656595014,0.172380798405461,-
1.91319833557846,0.055722666527127,0.412527057493539
"2114","EXOC6B",85.4164313290836,0.399189656299628,0.2089083524698,1.9
1083626662239,0.0560256254945127,0.41309351846069
"2115","WDFY3-
AS2",173.257254287828,0.370181588598358,0.193569699625782,1.9123942916
3763,0.0558256396252406,0.41309351846069
"2116","C4orf46",41.9706050388719,0.714085559199543,0.373560313953772,
1.91156697466506,0.0559317586833926,0.41309351846069
"2117","FIGNL1",48.1673319335165,0.526993547539868,0.275859604458108,1
.9103686767589,0.0560857609326556,0.41309351846069
"2118","PCED1B",14.4760117383485,0.757307616153304,0.396053867680505,1
.91213286361395,0.0558591545770304,0.41309351846069
"2119","SSH1",69.0610186577751,0.59999466560156,0.313829066246533,1.91
185180129308,0.0558952053297576,0.41309351846069

"2120","KLHL33",17.3173301570041,0.894973748519322,0.468489167617644,1
.91034032455955,0.0560894089582708,0.41309351846069
"2121","AC004846.1",37.9854758524728,-0.545685377205916,0.285466263498
48,-1.91155820137332,0.055932884923742,0.41309351846069
"2122","STXBP2",8.6851497103763,-1.02866801249363,0.538474255992872,-1
.91033833288261,0.0560896652311261,0.41309351846069
"2123","ZNF787",100.469700636903,-0.537512064180192,0.281322416299537,
-1.91066204837328,0.0560480249421111,0.41309351846069
"2124","BGN",608.056698483385,-0.789975157395746,0.413377745226763,-1.
91102488345713,0.0560013832088983,0.41309351846069
"2125","LINC00115",10.8445836961425,1.02246632017662,0.535285711073012
,1.91013191464989,0.0561162307450211,0.413094681197348
"2126","TSBP1-
AS1",14.2784305568978,-0.801965940424388,0.419983949873617,-1.90951568
664878,0.0561956000924518,0.413484370764922
"2127","PRKCE",71.6331791524522,0.580501598767143,0.304216116392214,1.
90818818427991,0.0563668982283691,0.414549783256407
"2128","BEGAIN",5.1347798278304,2.15006858331812,1.12692347631121,1.90
791001209415,0.056402848019368,0.414619244157412
"2129","SNHG7",898.788969164527,-0.591535662310351,0.31015295735105,-1
.90723850374516,0.0564897095606543,0.415062718016588
"2130","ZNF529-
AS1",19.6682266230734,0.68309760603837,0.358304687360111,1.90647130817
976,0.0565890847659776,0.415402652742462
"2131","F8A1",113.827432676406,-0.526826606111637,0.276317202600086,-1
.9066008238152,0.0565722983434863,0.415402652742462
"2132","FAM72A",7.81522013134413,1.15094190232726,0.603995772752711,1.
90554628732225,0.0567090965884566,0.415698405779394
"2133","KERA",3.2073376924858,2.30344628085108,1.20862424321743,1.9058
4153327852,0.0566707685088694,0.415698405779394
"2134","SPIN3",58.3075049840614,0.462118180682754,0.242488039654583,1.
90573597502387,0.0566844693364465,0.415698405779394
"2135","TMEM68",74.3828499504771,0.467500365569423,0.245467149888827,1
.90453331853633,0.0568407616288422,0.416468400074931
"2136","EFCAB11",54.7874153772944,0.450221670310019,0.236429316140238,
1.9042548430964,0.0568770022317239,0.41653883235527
"2137","HPS3",86.9507902244709,0.380168153857534,0.19970128029676,1.90
368410904826,0.0569513372489766,0.416693062949365
"2138","MTA1",152.982065169553,-0.30372530243298,0.159537129925434,-1.
90379068856848,0.0569374497097155,0.416693062949365
"2139","FRY",5.98185915250269,-1.10531646289224,0.580687473548873,-1.9
0346186759824,0.0569803048498835,0.416710102275235
"2140","MAP3K13",118.253783079668,-0.418918186061825,0.220146529318465
,-1.90290615690705,0.0570527914564731,0.417045241473649
"2141","ACTR3",435.808227404041,-0.279493280877041,0.146961131599474,-
1.90181769720424,0.0571949918358141,0.417096931629649
"2142","TASOR",269.614883837831,0.326033937044056,0.171464834730936,1.
90146240513788,0.0572414722671426,0.417096931629649
"2143","SLC30A9",119.982594652451,0.351243676528478,0.184659395379726,
1.90211646586515,0.0571559302770317,0.417096931629649

"2144","ZBED3",107.045983153939,-0.385099109088093,0.202531956401125,-1.90142393294905,0.0572465072050665,0.417096931629649
"2145","CDK9",121.935639536713,-0.318732326201358,0.167550560130082,-1.90230534564553,0.0571312472407791,0.417096931629649
"2146","CTNND1",241.009755328123,0.364812319750812,0.191859363600309,1.90145694692706,0.0572421865724872,0.417096931629649
"2147","TOMM40",145.210586932686,-0.450103629055881,0.236613747365521,-1.9022716730003,0.0571356469728864,0.417096931629649
"2148","PHIP",596.733587813489,0.367439925854251,0.193312822583017,1.90075299167729,0.0573343739702618,0.417542649914714
"2149","WEE2-AS1",9.74816710632679,0.96278572181605,0.506585673863458,1.90053878640783,0.0573624499953642,0.417552724652155
"2150","ATP13A3",152.829994399325,0.413583873440381,0.217811888429286,1.89881221095355,0.0575891711096484,0.418460392063375
"2151","CLK4",206.233460111319,0.393289356102534,0.20712800757414,1.89877438936769,0.0575941458871346,0.418460392063375
"2152","LAMA2",1424.085044696,0.480631608525062,0.253074122218684,1.89917327110096,0.057541697854448,0.418460392063375
"2153","AC084782.1",3.34176598017344,1.68820721796846,0.888836696867369,1.89934464218051,0.0575191768676846,0.418460392063375
"2154","CREBL2",304.466743623053,0.29728563566203,0.156636444605204,1.89793401153433,0.0577047752987029,0.419069545031388
"2155","AC018638.7",9.76933493003034,1.17838766932943,0.621059457078212,1.89738302170484,0.0577774047938166,0.419377748870289
"2156","PLPP7",29.407859856923,0.638184061552869,0.336453156327533,1.89679915183082,0.057854451324048,0.419377748870289
"2157","UBE3B",86.9415046151886,0.413037501310336,0.217750075167185,1.89684206075801,0.0578487862252262,0.419377748870289
"2158","ZNF28",24.6173921741838,0.662129116857891,0.349006050218247,1.89718521052525,0.0578034980808701,0.419377748870289
"2159","FCN1",17.1199223182469,-1.11374932417077,0.587525568998901,-1.89566102811239,0.0580048817715979,0.420273443980132
"2160","MAPKAPK2",100.146616343863,-0.407147215749365,0.214873150196845,-1.894825925791,0.0581154672809659,0.420490404568062
"2161","KPNA4",263.712338764039,-0.282426584641307,0.149044492275965,-1.89491460119423,0.0581037164431866,0.420490404568062
"2162","LPIN2",287.284732821325,0.271939968699378,0.143504922961698,1.89498703659079,0.0580941191180544,0.420490404568062
"2163","COX5A",401.10663075541,-0.538051314306587,0.284021671877914,-1.89440232060132,0.0581716286532029,0.420702166908022
"2164","PYCARD",329.660865342459,-0.575892075609176,0.304148261094338,-1.89345838617355,0.0582969372354671,0.421413580949359
"2165","TMOD3",573.60915810813,-0.354389807363375,0.187213496246051,-1.89297147091152,0.0583616635217221,0.421686606221847
"2166","AGPS",290.301844474914,-0.434834099595072,0.229989639887581,-1.89066820491401,0.0586686493122075,0.422417523119585
"2167","SLC16A14",5.91908058287898,1.39068994987133,0.735584449272049,1.89059182973156,0.0586788517380846,0.422417523119585
"2168","IFT57",467.181567575128,-0.402286755826841,0.212745600710588,-

1.89092867012606,0.05863386666572,0.422417523119585
"2169","OR2A1-
AS1",22.7114332169633,-1.09999475084526,0.581400439251609,-1.891974406
24777,0.0584943909307515,0.422417523119585
"2170","TRMT112",1044.45042622379,-0.451089339551993,0.238523804009813
,-1.89117116182432,0.0586014996057796,0.422417523119585
"2171","FAM89B",421.351922248726,-0.519758438518949,0.27476987344160 1,
-1.89161363292405,0.0585424781391488,0.422417523119585
"2172","VPS4A",198.640313662877,-0.358642763941285,0.18962534578498,-1
.89132292656677,0.0585812500565396,0.422417523119585
"2173","EIF3G",1356.80877494819,-0.539050976960958,0.285028331307716,-
1.89121893422938,0.0585951248392622,0.422417523119585
"2174","RTN4",3117.5072865726,-0.292667773848644,0.154838842322363,-1.
89014442021809,0.0587386476417234,0.422459156349186
"2175","MALL",42.7641251721564,0.625169081000087,0.330740951991935,1.8
9020766020935,0.0587301926010718,0.422459156349186
"2176","MTX3",98.4405694586901,0.516678002208017,0.273473504858563,1.8
8931648963667,0.0588494333120375,0.422676468019771
"2177","AL356599.1",78.2107070453139,0.579684988868418,0.3068231893802
4,1.88931283205594,0.0588499231187791,0.422676468019771
"2178","KCNE3",36.2695853778971,0.5609333050863,0.296848872251345,1.88
96258585458,0.0588080162698072,0.422676468019771
"2179","DST",1309.85899847425,0.502328455445801,0.265996902224585,1.88
847483276958,0.0589622334682136,0.423094595478562
"2180","ASL",78.0066848866402,-0.444864191041875,0.23556424256785,-1.8
8850475009483,0.0589582208275132,0.423094595478562
"2181","SWT1",45.843278544928,0.493766362145223,0.261584356418566,1.88
759897153459,0.0590798082683841,0.423355676015727
"2182","PCDHGA5",53.3894285970165,0.603209799616537,0.319535695377929,
1.88776968689865,0.0590568763393813,0.423355676015727
"2183","AC100793.4",52.9271321793531,-0.797566590085488,0.422492665906
529,-1.88776434349263,0.0590575939987619,0.423355676015727
"2184","ANAPC16",718.432679391053,-0.357365077765177,0.189440668654057
,-1.88642217272666,0.0592380869839868,0.424295510389425
"2185","ADGRL4",9.05712203202389,-1.11527571229039,0.591438539644041,-
1.88570009820737,0.059335379663044,0.424797869139129
"2186","AC005089.1",4.84767557820545,1.62119680141179,0.85997026749557
4,1.88517773542692,0.0594058457142081,0.42510779712139
"2187","ACKR4",671.672691328143,0.70668344034908,0.374919265334424,1.8
8489497790605,0.0594440182896172,0.425186455466155
"2188","ZNF566",87.5940950656501,0.440387612940763,0.233684477569721,1
.884539433345,0.0594920460784369,0.425335501281987
"2189","ZNF32",260.223827309898,-0.447119963859841,0.237336042816394,-
1.88391092458611,0.0595770252674115,0.425604907580887
"2190","RSL24D1",898.894224287837,-0.464947118592235,0.246832121135003
,-1.88365726654326,0.0596113502850939,0.425604907580887
"2191","GTPBP6",238.572470859674,-0.511790549498323,0.271691904071474,
-1.88371659894469,0.0596033199522858,0.425604907580887
"2192","CDKN2AIPNL",108.959413331066,-0.463715459633945,0.246405735187
7,-1.88191828928295,0.059847110794152,0.425796594399702

"2193","AC011346.1",21.383811477251,0.765186753178915,0.406342214471028,1.88310917726091,0.059685573776559,0.425796594399702
"2194","CLU",4552.54388247321,-0.85066211023951,0.451951516690545,-1.88219771109202,0.0598091763317119,0.425796594399702
"2195","FBXL15",180.020860723004,-0.513843096236821,0.273007250845964,-1.88215915381215,0.0598144097033658,0.425796594399702
"2196","CIT",4.68722986586804,1.68952225465925,0.897812899171307,1.88181998300393,0.0598604616544811,0.425796594399702
"2197","TSC22D1-AS1",14.6402394878841,1.11203712258453,0.591049903237401,1.88146062877853,0.0599092861499339,0.425796594399702
"2198","SEPHS2",210.113862797841,-0.517578907173683,0.275025288570188,-1.88193205746458,0.0598452411507494,0.425796594399702
"2199","MED1",167.664527604154,0.420531043066777,0.223463296502231,1.88187970753657,0.0598523502413721,0.425796594399702
"2200","ZNRF3",50.9510643955161,0.518936183255833,0.275782644592734,1.88168542665975,0.0598787395979009,0.425796594399702
"2201","AL022322.2",4.92951630490086,1.47464554471206,0.783780385755891,1.88145247254419,0.0599103946988266,0.425796594399702
"2202","AC093525.4",13.158557059559,1.00479320525318,0.534380311660666,1.88029608001581,0.0600677372551947,0.426720987231158
"2203","PSMD7",496.206974253161,-0.35190815637214,0.187272727318629,-1.8791212228858,0.0602279429069833,0.42766487103674
"2204","LINC00622",7.91889081876351,1.25023137380723,0.665773233534873,1.87786367915276,0.0603998162020636,0.427667941791323
"2205","ST6GAL2",3.16438632609209,2.62969995086827,1.39979458348874,1.87863275218154,0.0602946558381193,0.427667941791323
"2206","TET2",95.8073497156993,0.469147080027921,0.249801898714435,1.87807651760179,0.0603706982110922,0.427667941791323
"2207","TLE1",36.8522738007565,0.84050951666772,0.447549117065726,1.87802742675121,0.0603774131969065,0.427667941791323
"2208","UBXN1",804.66747236822,-0.529199998165315,0.281789424983086,-1.877998076745,0.0603814281893181,0.427667941791323
"2209","C15orf61",647.625717375621,-0.441987003317162,0.235385184797744,-1.87771802077069,0.0604197501348094,0.427667941791323
"2210","DUS3L",51.7752606443596,-0.53999756965507,0.287516645087549,-1.87814367926643,0.0603615123772734,0.427667941791323
"2211","PPWD1",160.585992951086,0.349702466651086,0.18629895192776,1.87710377880541,0.0605038716005003,0.427725411133932
"2212","AC008883.3",5.73782686555833,1.28181534518452,0.68301826711219,1.87669262581226,0.060560233907842,0.427725411133932
"2213","PRELID1",688.057515591333,-0.51425841927963,0.273996473692302,-1.87687969976264,0.0605345837558853,0.427725411133932
"2214","ZNF189",50.8084909700941,0.577053195612863,0.307374863178768,1.8773597477854,0.0604688044302283,0.427725411133932
"2215","PTER",108.664472667654,0.398899228188296,0.212557967591067,1.87666090671192,0.0605645838817144,0.427725411133932
"2216","ZMYND15",5.75710058274445,-1.15940519563824,0.617932199711642,-1.87626603077049,0.0606187590542515,0.427914823053094
"2217","AC003681.1",64.8843160064507,0.932273552065363,0.4969435311478

31,1.87601506736996,0.06065321095217,0.427964898026521
"2218","ESCO2",4.49817300688067,-2.0553675909749,1.09631283133021,-1.87480026889864,0.0608202061933484,0.428177536220769
"2219","RET",8.62431766682606,-1.4215197530792,0.758083086748584,-1.87515033368716,0.0607720446562316,0.428177536220769
"2220","RELA",201.434655324527,-0.294218813740424,0.156885658077193,-1.87537100169895,0.0607417016294945,0.428177536220769
"2221","C1RL-AS1",74.090727100922,0.756129973462244,0.40314566988568,1.87557508350929,0.0607136504682701,0.428177536220769
"2222","MT-ND5",10276.2095158351,-0.392712093195104,0.209458011838638,-1.87489649953157,0.0608069637357437,0.428177536220769
"2223","OTUB1",206.628432425238,-0.322141214132215,0.171909244495043,-1.87390279724895,0.0609438238550336,0.42866262122149
"2224","E2F4",117.619762158397,-0.361766617828097,0.193055235655255,-1.87390213272493,0.0609439154635683,0.428662621221493
"2225","ATM",312.322126743672,0.402767564127595,0.214965141031248,1.87364128991057,0.0609798829876864,0.428722835764665
"2226","COQ9",139.328426355414,-0.456431175820899,0.243677790302364,-1.87309305150274,0.0610555367100256,0.429061887131595
"2227","EPAS1",123.50882682853,0.524808135699072,0.280285875145961,1.8724030792696,0.0611508593543925,0.429153383975218
"2228","SOCS2",51.9809126265803,0.5726617471601,0.305841691059491,1.87241230970276,0.0611495833171934,0.429153383975218
"2229","ZNF605",94.9137646520915,0.468939105238819,0.250396153499624,1.87278877364833,0.0610975588282799,0.429153383975218
"2230","ATL2",89.7208901049807,0.458249604657294,0.244773694479978,1.87213583400311,0.0611878135442478,0.429220164696264
"2231","ZFHX4",1153.23989719319,0.459697406347725,0.245771527578546,1.87042580105546,0.0614247118406865,0.430505911054734
"2232","DHRS13",7.5775528549818,-0.970053366996565,0.518629888905457,-1.87041546919676,0.0614261454627736,0.430505911054734
"2233","CD58",117.427611702467,-0.475004023942368,0.254056817217744,-1.86967635485749,0.061528774969184,0.430703358164531
"2234","FAHD2B",84.2547066047349,-0.583185795715766,0.312059113593539,-1.86883116150671,0.0616463079913763,0.430703358164531
"2235","ZCWPW2",46.7675521206951,0.462746852626956,0.247521808856233,1.86951951735183,0.0615505708435404,0.430703358164531
"2236","PAQR8",33.8158055832727,0.563472011753828,0.301387684251867,1.86959202779812,0.0615404931956344,0.430703358164531
"2237","ALDH1A1",875.381432424994,0.732537734401445,0.391977532379682,1.86882582262874,0.0616470510087825,0.430703358164531
"2238","LINC00937",6.19081886039032,-1.08047911449209,0.578093981249266,-1.8690371281105,0.0616176490561133,0.430703358164531
"2239","ZFP36",6293.21210881103,-0.792641025027312,0.424069558288314,-1.86912974424921,0.0616047657082632,0.430703358164531
"2240","SEMA6C",61.4012717003682,0.465328196094413,0.249134536158366,1.8677787643164,0.0617929146083141,0.431148540495141
"2241","PCDHB13",16.2184018718692,0.807738080147047,0.432434961348788,

1.86788338673559,0.0617783270346648,0.431148540495141
"2242","TMEM135",182.200406834034,0.354124563776827,0.189597026299407,
1.86777488386132,0.0617934557175801,0.431148540495141
"2243","PLP1",160.014778636334,0.600684819883394,0.32165261217 5249,1.8
6749554378286,0.0618324185418728,0.431228053165634
"2244","FKBP15",131.955651485936,0.388462077274755,0.2080510205573,1.8
6714814584516,0.0618809025561563,0.431373867507109
"2245","HIST1H1D",8.18827251249763,-1.97225047051256,1.05726347273795,
-1.86542949923836,0.0621212256811445,0.432086408772852
"2246","RIC1",93.182188506519,0.446113093921526,0.239144489599085,1.86
545420582099,0.0621177654281497,0.432086408772852
"2247","PRICKLE1",354.07816782318,0.455246403159956,0.244010304342476,
1.86568515779155,0.0620854274186438,0.432086408772852
"2248","SMARCA1",469.683732543573,0.468904500076205,0.251343688346776,
1.8655909092464,0.0620986224639345,0.432086408772852
"2249","AC009509.1",7.95729594571261,6.02522122327397,3.22990899486002
,1.86544612645815,0.0621188969567916,0.432086408772852
"2250","SYT11",42.9027355083732,0.52523694645223,0.281640623243373,1.8
6491898932619,0.0621927602810566,0.432391710700697
"2251","AL359711.2",3.02759059350373,-1.56272288184538,0.8381322466329
18,-1.86453019571005,0.062247285232037,0.432578535266439
"2252","HIST2H2BF",6.76642810281057,1.4668102234607,0.787322896336584,
1.86303514134515,0.0624573222538712,0.4326342866573
"2253","AL592295.5",48.4743739860315,-0.691897479545151,0.371268584747
053,-1.86360362274267,0.0623773885402482,0.4326342866573
"2254","EAF1",47.7486008123696,-0.451960783267219,0.242553627261185,-1
.86334374121951,0.062413919766 9015,0.4326342866573
"2255","TENT2",135.083873609703,0.369608772327549,0.198405222653312,1.
86289840249515,0.0624765616287694,0.4326342866573
"2256","GNG11",1321.38591885034,-0.503943052423337,0.270394099247894,-
1.8637353915084,0.0623588727250037,0.4326342866573
"2257","PHF21A",156.786973522792,0.481521651218924,0.258309911569009,1
.86412378949804,0.0623043224657907,0.4326342866573
"2258","ATG14",171.924786386848,0.503156190025595,0.270075396554622,1.
86302120239166,0.0624592832625894,0.4326342866573
"2259","ABHD12",146.254345157749,-0.457565197508466,0.245517236184029,
-1.86367851243444,0.0623668646715353,0.4326342866573
"2260","R3HCC1",178.446000752115,-0.526131117410392,0.28247171709469,-
1.86259751178566,0.0625189146936261,0.432736009978935
"2261","CAMKK1",26.5115912271067,-1.16438257385363,0.625251743792235,-
1.86226201752192,0.0625661665151228,0.432871535955801
"2262","KIAA0355",157.484256256838,0.475616481850117,0.255425179775288
,1.86205793128361,0.0625949249585299,0.432879050011619
"2263","TRIM17",3.77016543479199,1.59729752175529,0.858293347983646,1.
86101584674721,0.0627419388838447,0.432880939776301
"2264","CCDC110",11.2913832185715,-0.87464564983948,0.46979297109869,-
1.86176827591519,0.0626357599882594,0.432880939776301
"2265","COG5",103.749171717286,0.33889320250488,0.182152832148517,1.86
048824225014,0.0628164804224225,0.432880939776301
"2266","FAM66D",19.7367699177502,1.00163077679818,0.538370050711536,1.

86048755029069,0.062816578232577,0.432880939776301
"2267","PIWIL4",17.5904143839666,0.755840155576085,0.40617536251147,1.86087149871071,0.0627623255338807,0.432880939776301
"2268","ENGASE",52.5746370170037,0.598583432899438,0.321532037547088,1.86166031063631,0.0626509863504039,0.432880939776301
"2269","MFSD12",80.8305277190056,-0.593438683281703,0.318927607587875,-1.86073161796817,0.0627820864815517,0.432880939776301
"2270","ZNF524",174.350433348712,-0.620527254635153,0.333415729870726,-1.86112171395077,0.0627269904631068,0.432880939776301
"2271","HECTD3",94.1158606905136,0.367759879042483,0.197739996853025,1.85981533779344,0.0629116566029651,0.432898001496759
"2272","AC016773.1",7.14833188789752,-1.16826818323602,0.628252034900372,-1.8595533612896,0.0629487430106999,0.432898001496759
"2273","DCP1B",60.7204062753457,0.396845605089464,0.213343191614822,1.86012781605867,0.0628674445943924,0.432898001496759
"2274","POLR3K",85.2338942098918,-0.608513589462141,0.327247208447732,-1.8594920712955,0.062957422067706,0.432898001496759
"2275","RANGRF",176.548578949817,-0.50896921662873,0.273705513879022,-1.85955046873368,0.0629491525929659,0.432898001496759
"2276","RREB1",169.009383285476,0.526079255683977,0.28298111192254,1.85906137731193,0.0630184390070984,0.432936952739588
"2277","TMEM140",299.026045004743,0.478792452514778,0.257531681961743,1.85915942018312,0.0630045448589473,0.432936952739588
"2278","OSBPL6",6.9080405355948,-1.28654536736001,0.692303840146844,-1.85835364872036,0.0631188099311329,0.43329982140293
"2279","ZFAND2B",111.540609776118,-0.466444463777422,0.25100622926635,-1.85829835833463,0.0631266568418639,0.43329982140293
"2280","ERV3-1",82.8444741358654,0.487890404078368,0.262606662411213,1.85787519478232,0.0631867396762878,0.433331945969386
"2281","ZNF681",24.6685353259879,0.609629609352957,0.328128428630105,1.85789939597152,0.0631833021991269,0.433331945969386
"2282","MTFR1L",190.488142606893,-0.329776778189579,0.177722088928319,-1.85557563597279,0.0635140691125555,0.43386488346188
"2283","MCM6",89.2266889163193,0.365474885019472,0.196805368066796,1.8570371764220 8,0.0633058651082585,0.43386488346188
"2284","PARD3B",179.744208909062,0.475890803280279,0.256446309868395,1.85571320376769,0.0634944477795667,0.43386488346188
"2285","CAND1",248.258832939289,0.293257451169853,0.157961024834452,1.85651778011187,0.0633797911064373,0.43386488346188
"2286","FUT8",214.179987232053,0.368134778285601,0.198321602918349,1.85625152715797,0.0634177147013282,0.43386488346188
"2287","SERPINB2",3.57896794454939,2.31021967570956,1.24438410276984,1.856516545468,0.0633799669190107,0.43386488346188
"2288","C19orf44",31.8424957293262,0.665151937155798,0.358397098285843,1.85590770778311,0.0634667141640208,0.43386488346188
"2289","NAPB",47.3306870376687,0.542413438889221,0.292235505243984,1.85608329294678,0.0634416867148322,0.43386488346188
"2290","CLDN5",5.9973631036384,-1.5411619315747,0.830492420047287,-1.85572064762127,0.0634933862034966,0.43386488346188
"2291","CYSTM1",845.776641094879,-0.460552698072978,0.248296448025331,

-1.85485012667597,0.0636176315995914,0.434289099173421
"2292","SH2B3",37.616225570995,-0.830401432430187,0.447715693297977,-1
.8547516758085,0.0636316956661434,0.434289099173421
"2293","RLIM",128.181843600615,0.385790918250902,0.208085810767779,1.8
5399915942101,0.0637392802520648,0.434833650668578
"2294","AL021918.5",3.75742909186915,1.82407548413012,0.98409526787023
,1.85355579249737,0.0638027371497884,0.435076816579835
"2295","RNF5",204.334235278714,-0.409895610450665,0.221206131151963,-1
.85300293584122,0.0638819378574696,0.435427082311284
"2296","ZDHHC9",188.254487382861,-0.344472269465928,0.185986053473666,
-1.85214032467602,0.0640056753007582,0.436080478540836
"2297","SPSB1",186.648011399188,-0.536933163713239,0.289944903389915,-
1.85184549697422,0.0640480122796361,0.436178953456834
"2298","TSPAN12",16.5261797376363,0.86917263318697,0.469480144355842,1
.85135120970778,0.0641190433434751,0.436472669722359
"2299","DLG1",186.485704409054,0.48177873673029,0.260414312159879,1.85
004707588616,0.0643067648510361,0.437179801201546
"2300","SYNJ2",51.0705626278701,-0.876664253906166,0.473826405484062,-
1.8501802427211,0.0642875755840211,0.437179801201546
"2301","ZFAND1",216.420999636686,-0.537966363025987,0.290729275331498,
-1.8504031367759,0.0642554672522951,0.437179801201546
"2302","HBEGF",17.2519519318868,-1.01870434309026,0.551025563672666,-1
.84874243637709,0.0644950128769918,0.438269107921278
"2303","ARHGAP21",774.542627644668,0.313956341891071,0.169870686697045
,1.84820788092176,0.0645722757812253,0.438407101637012
"2304","TSPAN18",25.0959382522736,0.650949621421385,0.3522887098646,1.
8477731564874,0.0646351657413116,0.438407101637012
"2305","TP53BP1",205.950985877007,0.346619445422439,0.187585514798273,
1.84779430221565,0.0646321054986821,0.438407101637012
"2306","CRNDE",533.672601113859,-0.355390032073027,0.192318861370138,-
1.84792084115474,0.0646137950854695,0.438407101637012
"2307","SNF8",483.440546833789,-0.50836805997255,0.275165074658774,-1.
84750212432651,0.0646744005260191,0.438407101637012
"2308","ZNF83",271.225648405803,0.367399254839941,0.198869442002888,1.
84743946148653,0.0646834744344578,0.438407101637012
"2309","VPS13D",170.910781007293,0.435349861273658,0.235843549317248,1
.84592651583631,0.0649028759900882,0.438753538942502
"2310","MMAA",33.8854489036908,0.532211553848706,0.288298321163355,1.8
4604458222684,0.0648857323987143,0.438753538942502
"2311","SLC38A9",60.8247584595522,0.421269438693704,0.228185712894633,
1.84616921607283,0.064867639246898,0.438753538942502
"2312","ABCF2",70.5716188132037,-0.425228609177119,0.2303479647887,-1.
84602720309331,0.0648882556661248,0.438753538942502
"2313","CIB2",45.578070355735,-0.675194331917622,0.365730658729563,-1.
84615184918607,0.0648701601560841,0.438753538942502
"2314","CRYZL1",134.250021865573,-0.310896541459793,0.168395954752134,
-1.84622333664374,0.0648597838354213,0.438753538942502
"2315","RNF139",214.365417070917,0.323946639951172,0.175610757788144,1
.84468562194794,0.0650832836079473,0.43958548367675
"2316","ANAPC15",54.6703374358599,-0.703139592307977,0.381208692644666

,-1.8445004163727,0.0651102452009863,0.43958548367675
"2317","TMEM87A",471.05772531682,-0.273723415922914,0.148379142659688,
-1.84475668895532,0.06507294036165,0.43958548367675
"2318","MICU3",168.727356702216,0.506334976576555,0.274619188280517,1.
84377129561444,0.065216477642768,0.440112752271709
"2319","PITHD1",335.745369859833,-0.368076429549093,0.199733962866082,
-1.84283345840327,0.0653533300866959,0.440348277605594
"2320","TPRKB",245.346729384607,-0.477663710453434,0.259207856682609,-
1.84278253200603,0.0653607682160993,0.440348277605594
"2321","KPNA5",111.549723905,0.466964345012448,0.253347012405992,1.843
18078424438,0.0653026195153083,0.440348277605594
"2322","LRP11",259.68831175612,0.400095161511852,0.217117280521386,1.8
4276056033431,0.0653639775362902,0.440348277605594
"2323","PPP1R12B",225.817862944175,0.559178904078877,0.303486431410636
,1.84251698331209,0.0653995646300427,0.440377165249336
"2324","LDLRAD3",27.3870553478348,-0.834725069711408,0.453167957539499
,-1.8419772532983,0.0654784771971448,0.440377165249336
"2325","AATF",151.237445551953,-0.377620815642508,0.205031643223365,-1
.84176846903149,0.0655090240705239,0.440377165249336
"2326","LPAR4",38.9699580195596,0.798672583286139,0.433589156555121,1.
84200312948695,0.0654746921138117,0.440377165249336
"2327","G6PD",141.848146160186,-0.435523050131416,0.236460395792611,-1
.84184353016728,0.0654980406500824,0.440377165249336
"2328","ZNF117",97.764736827839,0.692694001895352,0.376231718361041,1.
84113664024102,0.0656015374602589,0.440620373761628
"2329","ZNF66",36.7139280610825,0.730058541935464,0.396517909054771,1.
84117419481959,0.0655960356492345,0.440620373761628
"2330","ACVR2B",47.8032818506866,0.595428599345564,0.323539469689642,1
.84035845739851,0.0657156282406833,0.441048559916156
"2331","SPOCK1",603.214833599706,-0.509626797642338,0.276923341745951,
-1.84031723158198,0.0657216769906386,0.441048559916156
"2332","AC051619.8",3.35356309828607,2.21474463195467,1.20403153434773
,1.83944071959418,0.0658503895611054,0.441722831862938
"2333","TAPT1-
AS1",6.35453984465504,1.27608555463841,0.694275096223585,1.83801141878
702,0.0660607227167128,0.442754021190034
"2334","GUCY1B1",25.8857258694058,1.07593223007756,0.585336472549374,1
.83814315446882,0.0660413136153644,0.442754021190034
"2335","SLC3A1",4.97117673044286,2.36581910379347,1.28818036635543,1.8
3655889003101,0.0662750409052414,0.443732931559558
"2336","IGFBP5",6687.96517054079,0.799492626255487,0.435262425038953,1
.83680598246894,0.0662385424485656,0.443732931559558
"2337","SLC45A4",12.3013675759238,0.918419502461217,0.500107291293077,
1.83644493581878,0.0662918788630497,0.443732931559558
"2338","KIF9",43.0934203884536,-0.539352007239576,0.293733137682757,-1
.83619734393774,0.066328475367853,0.443787998365836
"2339","AC245140.2",7.73091906001134,1.51379052157253,0.82460833262346
6,1.83576913024448,0.0663918088286663,0.444021832196164
"2340","EXD3",47.3787157046441,0.456414589059644,0.248690000677478,1.8
3527519327792,0.0664649247232489,0.444201280198235

"2341","TRPT1",96.9264792713991,-0.563351506121564,0.306969378845513,-1.8352042416748,0.066475432905713,0.444201280198235
"2342","GNL1",217.758817630821,-0.355834445406705,0.193945100827035,-1.83471737047922,0.0665475771633948,0.444219370203099
"2343","PA2G4",684.103844461269,-0.357418695656816,0.194788706991239,-1.83490460600928,0.0665198250967013,0.444219370203099
"2344","SDR42E2",20.4847762694884,0.854664944717382,0.465856192837308,1.83461110501081,0.0665633320818299,0.444219370203099
"2345","FAM204A",504.322120367704,-0.296689164588195,0.161794088497464,-1.83374539418259,0.0666917968554786,0.444886899023561
"2346","UBAP2L",181.558453725125,0.379626284467788,0.207071948177467,1.83330619047654,0.066757049304547,0.445071792187018
"2347","PLSCR1",320.766098081343,0.292864664597309,0.159758081764387,1.83317589547194,0.0667764173280656,0.445071792187018
"2348","LINC01354",52.5113160591515,0.681561621597372,0.371908021512028,1.83260801642949,0.0668608851503596,0.445255353940858
"2349","VDAC2",836.958465697182,-0.459764464520897,0.250874531691284,-1.83264702646925,0.0668550798818202,0.445255353940858
"2350","FAAP20",331.442530995225,-0.435317233627299,0.237618713391523,-1.83199895081508,0.0669515769244016,0.445323479319859
"2351","TMEM214",171.059601629077,-0.349312608496685,0.190676382685319,-1.83196578190372,0.066956518785419,0.445323479319859
"2352","NBDY",322.172954565658,-0.484817273092686,0.264625978222346,-1.83208495382615,0.0669387646698648,0.445323479319859
"2353","WAC-AS1",196.733469370491,0.372603727436409,0.203453637143104,1.83139378911339,0.0670417876731114,0.445701098414995
"2354","ZNF423",118.005676413294,0.517400669248092,0.282559557128722,1.83112075381823,0.0670825214696898,0.445782448322157
"2355","BCORL1",52.1603489780544,0.424862182861016,0.23210090644038,1.83050634905707,0.0671742582584982,0.446202514623222
"2356","SLC25A6",2387.88145498803,-0.523205371810852,0.285972198183006,-1.82956726260512,0.0673146727807933,0.446945427126464
"2357","HTRA1",2986.0869737401,-0.371624243372352,0.203166971731512,-1.82915677782242,0.0673761252993956,0.447163652124924
"2358","ZNF439",13.7858998785706,1.19035016871951,0.651014488045832,1.82845419046298,0.0674814147989378,0.447672507082181
"2359","TRIM4",136.38662871847,0.290503933627248,0.15893147957645,1.82785647249643,0.0675710951540987,0.448077423270694
"2360","TTN",12.0529274257406,1.22508933731341,0.670349166127043,1.82753913813511,0.0676187471734938,0.44820341611651
"2361","AC090136.3",9.18291079623051,1.15085050674878,0.62981991210732,1.82726916794063,0.0676593085885762,0.448282322850952
"2362","CTDSP1",456.888512654829,-0.291956709595813,0.159827829576145,-1.82669507788516,0.0677456287435086,0.448369568515498
"2363","UBR2",226.960572105904,0.4146216800937,0.226997095818474,1.82655059351626,0.0677673676696613,0.448369568515498
"2364","MDN1",97.4784812389975,0.542316934106367,0.296929042828834,1.82641929849546,0.0677871271200635,0.448369568515498
"2365","ERCC6",83.6947705047237,0.486816273848396,0.266519747547016,1.

82656736819293,0.0677648434797085,0.448369568515498
"2366","C11orf65",11.2682926227834,0.882024017290386,0.482993273859505
,1.82616211244994,0.0678258464717589,0.448370282314901
"2367","JUNB",5771.81188546633,-0.820557390332664,0.449411767120472,-1
.82584758648009,0.0678732230724084,0.448370282314901
"2368","XIST",3316.71036171415,0.543387715633743,0.29758578488667,1.82
598680189204,0.0678522498959646,0.448370282314901
"2369","GLB1",175.324751348046,0.376498715635311,0.206302406055493,1.8
2498460795478,0.0680033521110047,0.448745377648559
"2370","LSM2",435.555851835447,-0.466440870680625,0.255582147644659,-1
.8250135034045,0.0679989916316099,0.448745377648559
"2371","AC004160.1",44.7236383301473,0.80410015728606,0.44062687842773
4,1.82490037864982,0.0680160640800827,0.448745377648559
"2372","CREM",129.030328187084,-0.446056698666362,0.244471838722012,-1
.82457292831004,0.0680655017726055,0.448746201504208
"2373","SMS",288.889047761133,-0.41255056228948,0.226114628692092,-1.8
2451955751728,0.0680735623709957,0.448746201504208
"2374","PNO1",66.5615226060693,-0.441681534172727,0.242140398017801,-1
.82407205814643,0.0681411791526343,0.448769136342035
"2375","ODF3L1",7.83815613861851,-1.05678327884037,0.579399979769629,-
1.82392702060596,0.068163106050545,0.448769136342035
"2376","CFAP410",157.623859974677,-0.477811905013765,0.261943036258724
,-1.82410615620193,0.0681360250219323,0.448769136342035
"2377","AL050403.2",30.7332021884537,0.686992085493731,0.3767452388374
92,1.8234924152288,0.0682288447995392,0.449012965586534
"2378","AC087741.1",16.9475989897025,1.04210617370676,0.57165667609591
8,1.82295810979369,0.068309735684509,0.449356263798475
"2379","MCC",115.533932218704,0.522963232243272,0.286936824711053,1.82
257273101805,0.0683681288267921,0.449551340579029
"2380","COL21A1",49.388852797653,1.65381963872605,0.907533572786215,1.
82232336997591,0.0684059342294436,0.449610936618146
"2381","MFSD4B",71.127659422047,0.639004471159275,0.350759910317229,1.
82177168018248,0.0684896364916631,0.44970126608965
"2382","BX088651.4",4.35562378528916,1.39239693513968,0.76443369197666
5,1.82147509948081,0.0685346684368552,0.44970126608965
"2383","SLX4",21.9030853501018,0.705344297965327,0.387205607373464,1.8
216272815621,0.0685115585162928,0.44970126608965
"2384","DYNLL2",153.591152108394,-0.359583176652142,0.197386677852353,
-1.82171958393825,0.0684975448714682,0.44970126608965
"2385","CEP350",288.281338330178,0.414018717532794,0.227432137518819,1
.82040551546298,0.0686972731267011,0.450390378676021
"2386","TCF19",32.1331229715995,-0.540704803966719,0.297016328149615,-
1.82045481248543,0.0686897717291807,0.450390378676021
"2387","VDAC1",525.35414715302,-0.392977811274161,0.216019708017593,-1
.81917573577201,0.0688846234314318,0.451429478147418
"2388","ARHGEF6",117.004528090605,0.350198722719671,0.192548653558175,
1.81875446152557,0.0689488987486016,0.451661483720425
"2389","STUM",4.06862346537582,1.82329838837589,1.00274410217561,1.818
30876334247,0.0690169541470915,0.451831792130483
"2390","MED20",32.4165236241936,0.59869371698587,0.329277179160757,1.8

1820592156367,0.0690326652938601,0.451831792130483
"2391","EMC8",110.914130762144,-0.463692968371468,0.255079764091567,-1
.81783517803872,0.0690893282018696,0.452013534530258
"2392","LRRC23",52.014408706887,-0.513686133160578,0.282649924306535,-
1.81739349274859,0.0691568834223865,0.452266357598826
"2393","PARVA",663.362727877478,0.231376108750536,0.12734218032412З,1.
81696361850893,0.0692226842498411,0.452507500927817
"2394","PPP1R15B",176.148817573749,-0.76769632946188,0.422688334463519
,-1.81622312912006,0.0693361515011921,0.453059907240245
"2395","AL356275.1",3.11286263612015,1.78358789247718,0.98294106337718
7,1.81454205031289,0.0695943149630979,0.454556939026196
"2396","FOXN2",107.285678535384,-0.448367443850687,0.247127632658346,-
1.81431529541075,0.0696291980695335,0.45459496886549
"2397","PRR5",99.5691709617671,-0.503378590233716,0.277560153160915,-1
.81358377454808,0.069741830307636,0.455140363580455
"2398","ZNF518A",178.972467127684,0.449460616142957,0.247885451994427,
1.81317867800109,0.0698042673266838,0.455168050767534
"2399","RALGAPA2",52.6834716885821,0.570639023780788,0.314713327919921
,1.81320259790837,0.0698005793073227,0.455168050767534
"2400","LYST",199.528161125873,0.409733381890367,0.226054063617226,1.8
1254596946402,0.0699018776883328,0.45523525090699
"2401","NAV3",193.191074722849,0.403167965517038,0.222424005545002,1.8
1260994976313,0.0698920021144149,0.45523525090699
"2402","RCVRN",12.6001242692783,-0.732551536759077,0.404103613259897,-
1.81278145683874,0.0698655350709346,0.45523525090699
"2403","WSB1",1808.75251258774,0.558389673540508,0.308285188782658,1.8
1127635662761,0.0700980836729031,0.456323064042956
"2404","ANTXR1",418.34313161674,0.406172634849427,0.224318480758176,1.
81069626308363,0.0701878816407543,0.456402751099279
"2405","CPED1",406.317164272876,0.279734288898156,0.154495395439428,1.
81063188389864,0.0701978532982718,0.456402751099279
"2406","TMEM100",153.66058725701,-0.761219475970796,0.420399212543449,
-1.81070623649687,0.0701863369680971,0.456402751099279
"2407","ADRA1B",28.3478740402079,-0.949927484215831,0.524848220535883,
-1.80990893566512,0.0703099102050756,0.456941389837141
"2408","MX1",97.779599003668,-0.61830772264068,0.341731736735119,-1.80
933655313361,0.0703987335020698,0.457328649573454
"2409","LINC01578",798.241913713836,-0.369521892297726,0.2042580210239
89,-1.80909366714332,0.0704364527739025,0.457383740449214
"2410","TADA3",285.219944139543,-0.465618972213758,0.25751356996632,-1
.80813373165017,0.0705856893887223,0.45806828333178
"2411","HSPA8",1099.23601257849,-0.440042512353456,0.243381100394816,-
1.80803896292527,0.0706004366881622,0.45806828333178
"2412","TTC21B",99.8215290841877,0.405386901483255,0.224294530576241,1
.80738647724385,0.0707020409257874,0.458347296395397
"2413","ADAM10",146.537190174253,0.391020901084526,0.21634068726732,1.
80743116805098,0.0706950779067854,0.458347296395397
"2414","RNF207",33.3226295626767,0.751557556203545,0.41602943402683,1.
80650092213206,0.0708401302355168,0.459052260676964
"2415","KLRD1",23.5187640669341,0.788262698828396,0.436437935392475,1.

80612782461162,0.0708983754938496,0.459239456666786
"2416","LINC01816",2.92660515225977,1.71613991003562,0.95069380373275,
1.8051447304037,0.0710520370517115,0.459555362164837
"2417","BOK",58.5502052041226,-0.564655817351683,0.312800978372049,-1.
80516001033755,0.0710496466496892,0.459555362164837
"2418","NSD2",42.9640999833995,0.583516661725098,0.323300077214508,1.8
0487634507411,0.0710940341647322,0.459555362164837
"2419","EP400",127.098804154837,0.562910027284831,0.31186186835517,1.8
0499793146801,0.0710750057250914,0.459555362164837
"2420","IRF8",24.8751304447415,-0.763133162616873,0.422784609553369,-1
.80501642059073,0.0710721125174628,0.459555362164837
"2421","BRAF",159.07578865788,0.43171154584902,0.239284754082676,1.804
17489406725,0.071203893787519,0.460075386418075
"2422","CREBBP",360.866257572278,0.359686503636813,0.199387520168612,1
.8039569544405,0.0712380553054967,0.460106069010687
"2423","AC090337.1",3.61826904159521,1.89189423484396,1.04924725865779
,1.80309666690395,0.0713730345357475,0.460407579069154
"2424","LIME1",18.0489557194347,0.669069864102869,0.371004257893776,1.
80340211700328,0.071325085394318,0.460407579069154
"2425","LGALS1",9976.56501785383,-0.590287972755447,0.327366961252681,
-1.80313850394887,0.0713664654522584,0.460407579069154
"2426","MTERF1",39.6058680552457,0.491790636138729,0.272805013313779,1
.80271846974115,0.0714324400149842,0.460600848785819
"2427","AC007563.4",2.73628634124699,1.97198307315145,1.09424314874021
,1.80214340425322,0.0715228463157197,0.46094434887042
"2428","HIST1H2BK",6.19892323860339,1.22092225265748,0.677676322817223
,1.8016303824544,0.0716035778146853,0.46094434887042
"2429","RPL41",27221.926316032,-0.489872225591712,0.271862049261836,-1
.80191470976483,0.0715588255264564,0.46094434887042
"2430","ZFC3H1",249.061042090969,0.355776204082183,0.19746656402862,1.
80170352298538,0.0715920635184285,0.46094434887042
"2431","FAM131A",40.5465334856644,-0.460137894427495,0.255491849433884
,-1.80098854600279,0.0717046853155587,0.461405344463712
"2432","PLEKHM1",105.904254151175,0.351912040550888,0.195433096064503,
1.80067781577149,0.071753676172907,0.461530738640125
"2433","TSC1",138.711034425481,0.453577575368139,0.251986124738875,1.8
0001012293104,0.0718590398263987,0.462018479245522
"2434","RTL8B",81.2228239548988,-0.386658165148736,0.214847863555604,-
1.7996835469982,0.0719106204881723,0.46216016281696
"2435","AREG",77.2905724077686,-0.839350875359055,0.466565743058133,-1
.79899807872194,0.0720189846290362,0.462666520144564
"2436","ARSA",186.273103395291,0.563885462186253,0.31349215860409,1.79
872270074348,0.0720625561576945,0.462756389973241
"2437","AC104794.5",4.90052339567849,-1.31077657345521,0.7288162154907
83,-1.79850083682967,0.0720976761551944,0.462791936026141
"2438","ZNF512",86.2750743198856,0.422098037220007,0.234782386931411,1
.79782667148417,0.0722044793293708,0.462907651700552
"2439","FP565260.1",23.7789920172978,-0.758411467464026,0.421763041911
541,-1.79819327939856,0.0721463842105003,0.462907651700552
"2440","ADAMTS1",530.074204850436,0.784171042938761,0.436134809127176,

1.7980015044158,0.072176769368191,0.462907651700552
"2441","TMEM37",582.8209062063,0.739916543710499,0.41160885063819,1.79762058702886,0.072237153653991,0.462927404592127
"2442","ILF2",326.466781752912,-0.499595781588854,0.278040013334209,-1.79684850247914,0.0723596739579322,0.463469421174884
"2443","GOLGA8B",54.2132236120483,-1.01289240812425,0.563747063037891,-1.79671429712828,0.0723809880413119,0.463469421174884
"2444","MCPH1-AS1",6.40956117427755,1.12782352223823,0.627866651853857,1.79627874630415,0.0724501963055644,0.463722758104723
"2445","AC009948.2",5.60204905782888,1.30365394607816,0.726246564138482,1.79505695510537,0.0726446259801425,0.464558520748708
"2446","FAN1",79.1507408181308,0.429164287032433,0.239151897800266,1.79452595183193,0.0727292602003187,0.464558520748708
"2447","LONP2",276.446707506957,0.345013715755967,0.192214696530709,1.79493931516753,0.0726633691250635,0.464558520748708
"2448","NECAB2",3.46484550975631,2.28548541574993,1.27355529027314,1.79457101957448,0.0727220739231288,0.464558520748708
"2449","APMAP",206.882246513064,0.405337165688558,0.225836331777974,1.79482708781799,0.0726812535910749,0.464558520748708
"2450","PAPOLB",7.17055836727654,1.2196195197145,0.679717293365649,1.79430409027185,0.0727646456108074,0.464594837261168
"2451","AC097376.3",81.8694246435928,0.395298177740528,0.220359690913715,1.79387698404112,0.0728328058234776,0.464840302528217
"2452","STX5",223.664650654182,-0.411406566087926,0.229365305299517,-1.79367391921237,0.0728652304673561,0.464857585726285
"2453","ZNF197",61.0494396912061,0.397266230755035,0.22163474676341,1.79243659469653,0.0730630572653801,0.465739773758085
"2454","KCTD2",60.7038449081923,0.438105853875374,0.244412359912166,1.79248649304321,0.0730550708875108,0.465739773758085
"2455","AL133243.2",17.8262259693547,0.719645681626397,0.401561880404747,1.792116524858,0.0731143023775323,0.465876591483396
"2456","SFMBT2",39.3045489740408,0.78965160241315,0.440893865093269,1.79102424626866,0.0732894042225535,0.466356295503351
"2457","TSC22D1",969.149390146248,-0.58955813708047,0.329195989139841,-1.79090315960693,0.0733088365813936,0.466356295503351
"2458","RAB15",4.97127872782326,-1.40571222978658,0.784799814698377,-1.79117298890652,0.0732655393151548,0.466356295503351
"2459","PLCB1",105.598475379177,0.521442387702791,0.291128610394277,1.7911066418261,0.0732761835400935,0.466356295503351
"2460","CEP83-DT",4.00433743440976,1.43998193361974,0.804164389690677,1.7906561793586,0.0733484857947356,0.466418846864654
"2461","CCNT2",140.369179215436,0.335863354231185,0.187642829573364,1.78990774651408,0.0734687433125415,0.466689908393904
"2462","SNX15",151.075939323308,-0.489255799717998,0.273348866563901,-1.78985852719321,0.0734766574765982,0.466689908393904
"2463","KDM2A",221.115628979195,0.409097885498378,0.228567511368757,1.78983392280264,0.0734806139726514,0.466689908393904
"2464","ANKRD28",548.705372071138,-0.465649204462652,0.260318155820156

,-1.78876960385487,0.073651928077,0.466830271842995
"2465","HCLS1",24.3111658754572,-0.825642477990101,0.461475750663559,-1.78913513180899,0.0735930554567845,0.466830271842995
"2466","DCUN1D4",117.542029138106,0.329068650623301,0.183889879390849,1.78948755479839,0.0735363299907855,0.466830271842995
"2467","AC067852.5",4.56355955023358,-1.71939122867357,0.961095083809022,-1.78899180490994,0.073616135368836,0.466830271842995
"2468","SLC25A39",126.969185480045,-0.522554095443919,0.292103271030172,-1.78893613070818,0.0736251021743634,0.466830271842995
"2469","AL121933.2",20.3263568673637,-0.959487498336301,0.536555603173108,-1.7882349800506,0.0737381049441693,0.467187191430393
"2470","KLHL2",35.3783288305285,0.501613557024816,0.280641835125361,1.7873798352293,0.0738761184967034,0.467682768775367
"2471","CCDC124",315.870078406504,-0.471940326226587,0.264037198177116,-1.78740090216383,0.0738727159260858,0.467682768775367
"2472","RAB3GAP1",215.300251955713,0.290639099898044,0.162728726325799,1.786034380409,0.0740936916344231,0.468682296713232
"2473","SETD9",37.2284798854767,-0.62856842036009,0.352122685314733,-1.78508357051255,0.0742477624666718,0.468682296713232
"2474","FOXP4",28.9443721959137,-0.565878459220349,0.31700231457998,-1.78509251571278,0.0742463117523261,0.468682296713232
"2475","PHKG1",68.7759444717793,-0.728581342604102,0.408143703988832,-1.78510984117505,0.0742435020100397,0.468682296713232
"2476","ZBTB34",36.6678136920781,0.566651534480605,0.31738321717136,1.78538594299604,0.0741987371547909,0.468682296713232
"2477","TMEM9B-AS1",28.6053529340839,0.859213781315061,0.481163371424314,1.78570072524777,0.074147727889284,0.468682296713232
"2478","CDKN1B",318.591133427944,0.344446620890659,0.192891868938458,1.78569798087526,0.0741481724805217,0.468682296713232
"2479","LINC02381",254.32275342351,0.441338147049887,0.24725883188525,1.78492369184494,0.0742736951704981,0.468682296713232
"2480","SLCO1C1",4.21784063612367,-1.21236286254165,0.679357179279109,-1.78457356383299,0.0743305126298955,0.468851697205425
"2481","TRMT10A",59.1003808003312,0.404388689214416,0.226706928650501,1.78375090528369,0.0744641503421302,0.469505321967732
"2482","MGA",139.855539010946,0.406413990813133,0.227879796207022,1.78345775965114,0.0745118181352282,0.46955020498205
"2483","PFKL",253.972437083936,-0.306063579515246,0.171623989742742,-1.78333798191048,0.0745313021140721,0.46955020498205
"2484","PHYHIP",27.967206547528,-0.589347879986612,0.330647668170261,-1.78240446469182,0.0746832979666965,0.470309550001762
"2485","LIG4",43.9228810794143,0.577004801307388,0.32376515264858,1.78217080061633,0.0747213828926152,0.470309550001762
"2486","SAE1",123.864623619015,-0.373546066038851,0.209616613746384,-1.7820441775231,0.0747420278274231,0.470309550001762
"2487","UBE2D2",676.050658602838,-0.400683647391937,0.224919741825055,-1.7814516597818,0.0748386952710729,0.470580395198988
"2488","MCRIP2",56.1294265494532,-0.715921315225711,0.401884279949871,-1.78141159269781,0.0748452357767105,0.470580395198988

"2489","WBP1",218.096860468411,0.3916807860363,0.220027043456311,1.780
14838486922,0.07505167989617,0.471688802175888
"2490","NUDC",648.607316903088,-0.379921800978809,0.213488728091042,-1
.77958716779085,0.0751435478080388,0.472076513398053
"2491","NOVA1",5780.62730010046,-0.359893182014335,0.202311658532295,-
1.77890480768752,0.0752553697806265,0.472253298441693
"2492","CAMTA2",75.8329720342616,-0.382137907658252,0.214793175129966,
-1.77909706594276,0.0752238496618991,0.472253298441693
"2493","PIAS2",133.524633894459,0.422804328009256,0.237682369312178,1.
7788628127227,0.0752622561538797,0.472253298441693
"2494","NOC4L",80.4831177617382,-0.503063776617494,0.282887754537553,-
1.77831584629696,0.075351995211067,0.47262680877575
"2495","PABPC1L",16.3505123047295,0.790387277794079,0.444713215564212,
1.77729658155382,0.0755194558077716,0.473487313507403
"2496","TMEM52",3.96163745040404,-1.28109035981567,0.721157570333084,-
1.77643612508146,0.0756610612657225,0.473907133397984
"2497","AC008149.2",6.47928150284445,-1.17121208649888,0.6593409569014
51,-1.7763375295279,0.0756773009798736,0.473907133397984
"2498","TSPAN6",162.817296530055,0.372638161960835,0.209766896731856,1
.77643931319238,0.0756605361981345,0.473907133397984
"2499","RAD23B",443.853521826529,0.356765101560375,0.20089713981659,1.
77585953630841,0.0757560717677388,0.474210576495694
"2500","PHC3",218.959762873951,0.392031475237222,0.220961585269859,1.7
7420647466136,0.0760290036362444,0.474783269406249
"2501","AC079298.3",9.23758877265569,-1.08924534225285,0.6139094148348
03,-1.77427697952141,0.076017346442957,0.474783269406249
"2502","SCNN1A",5.55699515152859,-1.15297082205721,0.64953351976025,-1
.77507516853447,0.0758854764482834,0.474783269406249
"2503","SLFN13",15.4894812882016,0.896948725725038,0.505368066815283,1
.77484250514167,0.0759238958259449,0.474783269406249
"2504","HSD17B10",280.783773846265,-0.552878856774729,0.31154312000422
,-1.77464633713381,0.0759563011018333,0.474783269406249
"2505","TCEAL5",2.64328392325787,-1.82711109169862,1.02982107938895,-1
.77420245930753,0.0760296675741644,0.474783269406249
"2506","APLNR",6.35953205959042,1.45207800746186,0.818552006728357,1.7
7395937646726,0.0760698700826066,0.474844763648131
"2507","STAT2",308.508829326577,0.575799472369094,0.324636382766124,1.
77367511140584,0.0761169055510518,0.474948844649024
"2508","ZC3H3",40.4016569584746,0.449232213752089,0.253341746180422,1.
77322616791375,0.0761912375927757,0.475150784872996
"2509","MEX3B",77.9277510425273,0.517585777921904,0.291907982305905,1.
77311279339905,0.0762100184904652,0.475150784872996
"2510","STRADB",62.6311950100144,0.380875655252652,0.214960847929854,1
.7718373318705,0.0764215635966922,0.475957425022852
"2511","LTC4S",63.5752880594969,-0.611405851227742,0.34508133802069,-1
.77177315566999,0.0764322203538955,0.475957425022852
"2512","PAK1IP1",134.104463564502,-0.378517786489532,0.213658807295038
,-1.77159926745656,0.0764611013924712,0.475957425022852
"2513","PHLPP1",47.2362787358257,0.473702482982043,0.267353900438631,1
.77181811151761,0.0764247550987597,0.475957425022852

"2514","FAM172A",270.661605386208,0.274658014377278,0.155089687103487,
1.77096246376464,0.0765669438949139,0.476389845784667
"2515","AC092490.1",4.84405842670002,1.42749861629821,0.80620856256097
,1.77063192155101,0.0766219300641963,0.476389845784667
"2516","DLX3",4.57527954158965,-1.53582008518681,0.867321240895294,-1.
77076268027456,0.0766001743160939,0.476389845784667
"2517","IGF2-
AS",7.23781114301921,1.34427877818194,0.759640059049171,1.769625972417
19,0.076789469251744,0.476673677581362
"2518","AQP5",6.45531365195548,-1.06797446894508,0.603480141277208,-1.
76969281322964,0.0767783277690369,0.476673677581362
"2519","ALDH6A1",183.127014590193,0.37597807328879,0.212451371626114,1
.76971356038342,0.0767748697598887,0.476673677581362
"2520","DICER1-
AS1",16.5198430581222,0.68630393785057,0.387820177711542,1.76964474076
704,0.0767863406820523,0.476673677581362
"2521","SLC5A6",21.1504935025116,-0.619520575722147,0.35018732802626,-
1.7691119185092,0.0768751993164572,0.476675551347178
"2522","LXN",215.703053099564,0.551975369085373,0.312013367331124,1.76
907602968045,0.0768811874991326,0.476675551347178
"2523","VENTX",3.74920109515927,-1.99636960639596,1.12830293732334,-1.
76935603051067,0.0768344784208299,0.476675551347178
"2524","OSER1",195.335757435908,-0.38454856628312,0.21741133460787,-1.
76876043273781,0.0769338623802784,0.476813157375077
"2525","FCGR3B",2.95503986311176,2.15176347222536,1.21708394374094,1.7
6796636196803,0.0770665273252159,0.477446212652813
"2526","NR4A3",27.356250921786,-1.24410878139878,0.703824059839179,-1.
76764173376377,0.07712081643343,0.477593401214626
"2527","ZNF529",45.9886811350568,0.488971917655689,0.276661433332836,1
.76740180864831,0.0771609602693502,0.477652909178253
"2528","HAX1",368.208060761151,-0.394360864229301,0.22322663751728,-1.
76663891287963,0.0772887197751706,0.478089557929002
"2529","CHRDL1",1844.09445242667,0.350507117474723,0.198405987397565,1
.76661562522495,0.0772926223871665,0.478089557929002
"2530","PER2",187.714107696709,-0.74869192777213,0.423892314614609,-1.
76623142708501,0.0773570305922064,0.478238207104249
"2531","ARHGAP10",418.578577155405,0.294312156766281,0.166644521889098
,1.7661076009576,0.0773777985156847,0.478238207104249
"2532","SORBS2",24.9430194745864,0.922897428427257,0.522726420889528,1
.76554578369456,0.077472082691038,0.478492773767763
"2533","STK32A",21.4096360045916,-0.963956020809838,0.545996771000383,
-1.76549765861008,0.077480163392811,0.478492773767763
"2534","HIST2H4B",3.76285626840499,1.57893577006678,0.894657759376728,
1.76484890844378,0.0775891623172695,0.478976821676814
"2535","LINC01257",4.19933252994758,1.64089442722539,0.930050500272513
,1.76430680564614,0.0776803387830802,0.479350508711528
"2536","C12orf10",294.547711192091,-0.4924453282049,0.27931074881226,-
1.76307331636528,0.0778881249924254,0.480443193713135
"2537","ABAT",19.8093682908135,0.779844546218441,0.442556422913769,1.7
6213586752167,0.078046344513126,0.481229391887596

"2538","STBD1",44.9077090287877,-0.571566051390498,0.324665358781551,-1.76047747605581,0.0783268832198452,0.482290812770749
"2539","SERPINE1",32.8336324887891,-1.04177338846388,0.591695522470137,-1.76065788720998,0.0782963245693136,0.482290812770749
"2540","AUXG01000058.1",209.895165159698,1.36346396323286,0.774604139852331,1.76020743123422,0.0783726424639292,0.482290812770749
"2541","PAAF1",101.285076882047,0.346756447297546,0.196927818273355,1.76083018812616,0.0782671487188624,0.482290812770749
"2542","ZNF512B",55.4195316199834,0.516612688926384,0.293484287960794,1.7602737526971,0.0783614022402395,0.482290812770749
"2543","WDR45",366.858925045466,-0.492661865960976,0.279942432861557,-1.75986848769232,0.0784301074255222,0.482454648233364
"2544","CASTOR2",57.861481451184,0.426855386276391,0.242614170913756,1.75940005758414,0.0785095821617797,0.482682923292302
"2545","ZNF500",57.8204426433832,0.486471857938123,0.276545195624398,1.75910435485867,0.0785597853801829,0.482682923292302
"2546","COLEC12",1059.14698907726,0.379438382092505,0.21569705514839,1.75912639062906,0.0785560433351586,0.482682923292302
"2547","PSMC4",282.43248551147,-0.486626651495414,0.276677489909104,-1.75882270601517,0.0786076268753556,0.482787242721315
"2548","CARMN",8.03164938883353,1.13897659864422,0.647867375565417,1.75803974949378,0.0787407461696292,0.482874467711761
"2549","ZMYM5",129.615070431278,0.385214121652724,0.219116308370474,1.75803491998148,0.0787415678584674,0.482874467711761
"2550","AL132800.1",4.64176163688491,1.40426220951564,0.798942916686273,1.75765024032007,0.0788070393190645,0.482874467711761
"2551","PCGF2",146.073111531658,-0.366283542279418,0.208371111966074,-1.75784224033442,0.0787743558905994,0.482874467711761
"2552","LZTS3",18.0483128364416,0.78406581731338,0.446065656168616,1.75773634771155,0.0787923802228395,0.482874467711761
"2553","COMMD7",241.366370620312,-0.355005455593879,0.20192783987663,-1.75808078673437,0.0787337644122686,0.482874467711761
"2554","DCTN5",114.388912998827,0.350120017431735,0.199254154867167,1.75715290687485,0.0788917497919212,0.483204245103768
"2555","LBX2",14.1256239029728,0.801718964033189,0.456495176386806,1.75624848958724,0.0790459884147544,0.483391867385459
"2556","KLHL9",159.744517905068,0.360360933583789,0.20517805828599,1.75633270240571,0.0790316164780206,0.483391867385459
"2557","PPP1R15A",1126.34535232311,-0.518146869357906,0.295019616883677,-1.75631327445662,0.0790349319040524,0.483391867385459
"2558","LINC01260",5.19687448979955,-1.11643162724864,0.635541894521686,-1.75666094851053,0.0789756175944444,0.483391867385459
"2559","SFRP2",2970.1727764971,0.876296906787027,0.49913413761631,1.75563408860775,0.0791509077218438,0.483612728923935
"2560","EIF4A1",2134.23940751666,-0.431141265405976,0.245562518898999,-1.75572912079187,0.0791346719765034,0.483612728923935
"2561","NLE1",65.6831178426901,-0.481081883250381,0.274043597781277,-1.75549396937325,0.0791748512928592,0.483612728923935
"2562","CDC37",522.399452676114,-0.302055590492997,0.172082112982417,-1.75529917234256,0.0792081479910417,0.483627267378558

"2563","FUCA1",136.021758497914,-0.343005524309446,0.195501745892022,-1.75448829239045,0.0793468742618804,0.483719077972952
"2564","ENSA",580.411389067608,-0.405636957020388,0.231196155916038,-1.75451427993336,0.0793424252208683,0.483719077972952
"2565","FZD2",34.5742837656773,0.875183016014207,0.498751435812555,1.75474786270716,0.0793024451916537,0.483719077972952
"2566","ZNF714",46.7810870057378,0.788519274384709,0.44938435491457,1.75466561254588,0.0793165212607868,0.483719077972952
"2567","FAHD1",133.14259858661,-0.353628116078571,0.201647236425366,-1.75369681403721,0.079482471755115,0.484356955849343
"2568","ITGB3BP",106.839580825211,-0.49313344155555,0.281297581794232,-1.7530667644213,0.0795905476168444,0.484400485919925
"2569","AC253572.1",6.40257040858361,-0.998098483244893,0.569269980527672,-1.75329547909715,0.0795513011313459,0.484400485919925
"2570","TNIP2",170.608798359021,-0.408558918317551,0.233060612579395,-1.7530157232311,0.0795993082204205,0.484400485919925
"2571","NDST1",121.571003594764,0.441511659127163,0.25187021742675,1.752933171845,0.0796134788275988,0.484400485919925
"2572","FBLIM1",40.9642515305552,-0.57407612175972,0.327584766670939,-1.7524506026142,0.0796963567437872,0.484716216385328
"2573","ANXA2",8381.33646652631,-0.433813863530033,0.24761087642043,-1.75199841703819,0.079774080111937,0.485000363463285
"2574","AC091607.2",26.3548984779026,-0.810274378436836,0.462688744086334,-1.75122993328242,0.0799063113262833,0.485615550923485
"2575","RBM8A",553.65995066031,-0.387287097353059,0.221271871382949,-1.75027713614259,0.0800705041960489,0.485822040642559
"2576","FAM228B",128.655442482316,-0.35343021892493,0.201886687212478,-1.75063657641258,0.0800085306838278,0.485822040642559
"2577","STN1",85.1118905129679,-0.492282994322701,0.281239653313515,-1.75040392961203,0.0800486384305162,0.485822040642559
"2578","EXT2",304.576690342231,0.297837238828626,0.170211918182333,1.74980249332234,0.0801524003110571,0.485822040642559
"2579","ETNK1",218.039927649993,0.323676630297131,0.184885833342579,1.7506837838537,0.0800003942283071,0.485822040642559
"2580","ZNF668",40.6376679511533,-0.589605907674138,0.336982344922384,-1.74966408940486,0.0801762936974777,0.485822040642559
"2581","MNT",99.5968784137571,0.496195520888337,0.28360642032228,1.74959198851873,0.0801887431400043,0.485822040642559
"2582","PRDM15",13.7870182212627,0.881513183913689,0.503755201692893,1.74988403286224,0.0801383264256705,0.485822040642559
"2583","CCT3",478.757181774797,-0.370803897272529,0.212052355317431,-1.74864314389461,0.0803527236622068,0.485930410484365
"2584","GMPPA",100.70159465473,-0.48110829166649,0.275088431095234,-1.74892230018911,0.0803044512621523,0.485930410484365
"2585","CYREN",75.6168218856341,-0.499801703589339,0.285827615341149,-1.74861236900711,0.0803580467744938,0.485930410484365
"2586","GSDMD",292.050302956556,-0.263979518048145,0.150919657692123,-1.74913939035474,0.0802669277780537,0.485930410484365
"2587","WHAMM",86.2355275380663,0.319274845245083,0.182589903989017,1.74858980847204,0.0803619492375537,0.485930410484365

"2588","FNDC5",62.5167339645234,0.899472738228673,0.514527144203737,1.74815410296897,0.0804373466517002,0.486069940602456
"2589","ACKR2",26.8701170384869,0.652381966677545,0.373233755149703,1.7479179138443,0.0804782424189965,0.486069940602456
"2590","TRPM3",9.9086847062604,1.03434149668004,0.591725876300628,1.74800788356016,0.0804626623164294,0.486069940602456
"2591","ZNF667-AS1",172.629342681249,-0.387706239136898,0.221846009728109,-1.74763674862606,0.0805269477415192,0.486112030911032
"2592","PNMA6A",23.0878617685837,0.703030491464165,0.402302072020063,1.74751894250523,0.0805473620227191,0.486112030911032
"2593","SLAMF8",2.99796623275932,-1.41695215114414,0.811060506643129,-1.74703630560033,0.0806310406621335,0.486284964676634
"2594","PRDX6",1695.56929564412,-0.452774906387319,0.259173542337879,-1.74699509179469,0.0806381895014504,0.486284964676634
"2595","MAMDC2",89.2163721654286,-1.31133564678264,0.750798033925175,-1.74658907925874,0.08070864289468,0.486343574417576
"2596","DCTPP1",183.480563836197,-0.480982094314426,0.275384973982355,-1.74658074970076,0.0807100888057295,0.486343574417576
"2597","GLS",453.14938842085,0.452809999714944,0.259458750233017,1.74520997772586,0.0809483250591271,0.486683641924914
"2598","PTPRG",381.755565081894,0.343434916948631,0.196756920031745,1.74547821186274,0.0809016618569063,0.486683641924914
"2599","CHMP2B",405.237128059448,-0.324844411014562,0.186063953158457,-1.74587503651455,0.0808326685345361,0.486683641924914
"2600","PRDM8",127.843869884986,0.325835379813153,0.186705674223727,1.74518198853833,0.0809531954413237,0.486683641924914
"2601","PSMG4",189.99739078363,-0.405824699152486,0.232443310301815,-1.74590827598155,0.0808268915736581,0.486683641924914
"2602","CREB5",313.41974662349,0.635085363078038,0.36389947566398,1.74522197900738,0.0809462367960042,0.486683641924914
"2603","ZNF165",8.00216756815938,-0.860358516656002,0.493195801120373,-1.74445628835761,0.081079557179878,0.487256055691445
"2604","ZNF687-AS1",3.7304795577405,-1.40993940317359,0.808920355442785,-1.74298915052251,0.0813355099920596,0.488601762339738
"2605","CFP",5.32275276013416,-1.09745332571539,0.629701595842031,-1.74281490306196,0.0813659522403002,0.488601762339738
"2606","BOLA3-AS1",42.0213919144528,-0.479642470676384,0.275283748592297,-1.74235665246897,0.0814460559566586,0.48870757703491
"2607","ZBTB25",100.254773910674,0.373516068644301,0.21437154444501,1.74237709399026,0.0814424813480982,0.48870757703491
"2608","ARFIP1",219.096151783213,0.249767094818098,0.143381085240278,1.74198078079504,0.0815118073283552,0.488914571333382
"2609","NBPF12",88.2446611443919,0.741142793368885,0.425837303088768,1.74043651881383,0.0817823977886746,0.4901617044476
"2610","IL18",22.2106500490902,-0.731267593583527,0.420146343415068,-1.74050686158441,0.0817700562856717,0.4901617044476
"2611","DUSP2",68.2520357796062,-0.776991747792368,0.446533576366395,-

1.74005223552287,0.0818498462271245,0.490230562396723
"2612","UFD1",310.321271878787,-0.461657746187393,0.265318412683457,-1
.74001397610569,0.0818565638931305,0.490230562396723
"2613","GULP1",320.652154609309,0.557260891433516,0.320403903023748,1.
73924501597664,0.0819916743206777,0.490563289602678
"2614","CGRRF1",117.481026831699,-0.343157014381083,0.197311656589482,
-1.73916240080555,0.0820062010043472,0.490563289602678
"2615","ALDOA",64.579478056724,-0.527683773029064,0.303374966887467,-1
.7393780984731,0.0819682780763092,0.490563289602678
"2616","GPN2",63.5770321087546,0.41705754126556,0.239939984034308,1.73
817441450661,0.0821800859144052,0.490792420379006
"2617","BRK1",1222.84305187807,-0.417851861532415,0.240397075208439,-1
.73817364945106,0.0821802206794168,0.490792420379006
"2618","RTN4RL2",32.0480334661332,-0.555988337309416,0.319789588500236
,-1.73860675051028,0.0821039583184528,0.490792420379006
"2619","MEG9",10.9742344530843,1.4205461785911,0.817275665967976,1.738
14813990407,0.0821847143046324,0.490792420379006
"2620","AC103691.1",10.8641049735288,1.02082990278977,0.58734087796982
8,1.73805355812848,0.0822013770627753,0.490792420379006
"2621","HRNR",4.12990104033549,1.87046491045241,1.07662471172215,1.737
34161039314,0.0823268909924877,0.490979624779064
"2622","FRMD4A",68.9600753301778,0.524877102686102,0.302085790863357,1
.73751006687872,0.0822971786703081,0.490979624779064
"2623","WBP2",251.143345501169,-0.334297997711383,0.192411913442923,-1
.73740800000177,0.0823151801679471,0.490979624779064
"2624","SF3B1",883.523598333209,0.271873871337082,0.156539008904517,1.
73678032868417,0.082425952525477,0.491383069876538
"2625","CENPX",326.757126392428,-0.52942898266207,0.304899847999611,-1
.73640290782547,0.0824926184760153,0.491593154598212
"2626","SYBU",17.2744632019079,-0.944774975210248,0.544185741868409,-1
.73612592635458,0.0825415710622905,0.491697561358496
"2627","TOP1MT",35.5728119981011,-0.555786595467165,0.320177776674527,
-1.73586874529441,0.0825870452848856,0.491781176015023
"2628","AP4E1",39.5265803977089,0.707272608617341,0.407498814180378,1.
73564335405469,0.0826269151929293,0.491831367718034
"2629","SLC48A1",108.913944236027,0.406883646376668,0.234516739161644,
1.73498765090801,0.0827429925573,0.492334968647335
"2630","HMCES",94.0457896292514,-0.356995806218759,0.205820782785537,-
1.73449834067896,0.0828296999146654,0.492663496488635
"2631","RRP9",48.9431429167775,-0.52547590825433,0.303086632672954,-1.
73374821456196,0.0829627676480054,0.493080005439874
"2632","PCMT1",525.449332541101,-0.451487604323201,0.260393953902921,-
1.73386362300686,0.0829422836356399,0.493080005439874
"2633","FAM180A",133.418197437624,0.382590376104843,0.220803462495528,
1.73271909679674,0.0831456085631773,0.493769901968062
"2634","MUSK",3.42225222020622,-1.52661980512974,0.880981143450002,-1.
73286320198791,0.0831199860959542,0.493769901968062
"2635","ANGPTL4",884.472196873049,-0.586608617736365,0.338578707511385
,-1.73256204457759,0.0831735403494114,0.493769901968062
"2636","RN7SL832P",21.1841157285088,-0.950108851977382,0.5486231577345

17,-1.73180595565954,0.0833081174099263,0.493819204487866
"2637","ARHGEF3",91.1136418426416,0.500106216847146,0.288741781630325,
1.73201887867905,0.0832702011883534,0.493819204487866
"2638","AC138331.1",6.03617690386026,-0.990403041648077,0.571778888560
192,-1.73214342373156,0.0832480293351774,0.493819204487866
"2639","RTN4RL1",8.44956201395021,1.82260193458919,1.05239428536464,1.
73186224966784,0.0832980915066354,0.493819204487866
"2640","UBE4B",112.575299144318,0.364313871031773,0.210548825320555,1.
7303058826242,0.0835756390908101,0.493947255911491
"2641","WNT2B",26.509320991051,0.727101470297647,0.420094923715113,1.7
308028001565,0.0834869423109481,0.493947255911491
"2642","ZNF438",93.7681676084175,0.361587611404763,0.208861325104525,1
.73123296629381,0.0834102218294476,0.493947255911491
"2643","AC090559.1",3.58835864666922,-1.55316208665012,0.8974880184639
07,-1.73056581781273,0.0835292327148764,0.493947255911491
"2644","APEX1",494.271525935817,-0.409266368072798,0.236498157454934,-
1.73052666658,0.0835362210713882,0.493947255911491
"2645","LINC01303",3.06826227673731,-1.58735410132183,0.91740338512844
6,-1.7302684152398,0.0835823298854259,0.493947255911491
"2646","ZNF689",42.6386937684175,-0.464014124794426,0.26801996235185,-
1.73126703221188,0.0834041485861286,0.493947255911491
"2647","N6AMT1",64.972006239417,0.419515340469201,0.242370764848742,1.
73088260348153,0.0834727050065618,0.493947255911491
"2648","ZNF678",52.8440230698202,0.417406329656839,0.241623471746664,1
.72750737599896,0.0840765816467733,0.493981538904187
"2649","CHMP3",568.961472512706,-0.293259016990726,0.169762568216139,-
1.72746571916463,0.0840840566847992,0.493981538904187
"2650","TSC22D2",203.113070171244,-0.66741420591886,0.386196953906278,
-1.72817055952447,0.0839576502657093,0.493981538904187
"2651","SERP1",822.378991705145,-0.301974214817643,0.174567600088283,-
1.72984113125762,0.0836586634511375,0.493981538904187
"2652","AL512329.2",5.155270336757,1.34570783392811,0.77853884005437,1
.7285044299577,0.0838978274930619,0.493981538904187
"2653","RBAK",48.6375155181561,0.506030990537247,0.292674912183364,1.7
2898656315377,0.0838114999599314,0.493981538904187
"2654","GGCT",188.338523793763,-0.40953483289295,0.23697665556233,-1.7
2816529932516,0.0839585930623878,0.493981538904187
"2655","HAS2-
AS1",58.4211131012487,-0.450237447459875,0.260620708326462,-1.72755822
18735,0.0840674584264166,0.493981538904187
"2656","ENDOG",173.002058197984,-0.482460234303144,0.279200214827638,-
1.72800810558468,0.0839867711850335,0.493981538904187
"2657","RAD9A",64.2524553441678,-0.430477035181644,0.249043044743992,-
1.72852462362143,0.0838942103073669,0.493981538904187
"2658","PTPN11",127.375134029389,-0.305162432793771,0.176486381212551,
-1.72909904264086,0.083791370487941,0.493981538904187
"2659","NCBP3",231.663696797353,0.387407685905621,0.22393855865309,1.7
2997311510683,0.083635078734974,0.493981538904187
"2660","THOC1",128.461855585209,0.362470024508816,0.209672966313911,1.
72873990806304,0.0838556553751984,0.493981538904187

"2661","LRRC25",3.05989137079996,-1.54811783829134,0.896205586985282,-1.72741373271171,0.0840933860577798,0.493981538904187
"2662","SLC35E4",22.4041805183754,-0.576494928861054,0.333576649439675,-1.7282232729102,0.0839482028070635,0.493981538904187
"2663","PGK1",615.411828512806,-0.403985676497948,0.233733163142275,-1.72840546487637,0.0839155564177618,0.493981538904187
"2664","ARFGEF1",197.051402538522,0.330982166640679,0.19170812593979,1.72649002236156,0.0842592928060016,0.494214405186848
"2665","ZBTB5",23.502855573582,0.663790424490078,0.384454550516001,1.72657710410545,0.0842436408352142,0.494214405186848
"2666","AL138756.1",6.72005727294622,1.36365489809886,0.789956841079695,1.7262397477754,0.0843042899694849,0.494214405186848
"2667","RASSF8",300.191461455866,-0.307423395111488,0.178084751661995,-1.72627578859181,0.0842978089502207,0.494214405186848
"2668","SLFN12L",11.2813987192488,0.91370345683715,0.529333955266347,1.72613800370581,0.0843225882147732,0.494214405186848
"2669","GSTT2B",12.2731633515418,-1.40175877660656,0.811976928772271,-1.72635296267106,0.0842839325214932,0.494214405186848
"2670","EEF1D",3937.00927557215,-0.492241544887301,0.285223391784968,-1.72581057187064,0.0843814972767042,0.494374442658983
"2671","STK40",89.503543837392,-0.355964965973165,0.206407251285004,-1.72457587491272,0.0846039342476055,0.494465577019091
"2672","P3H1",223.496402848556,0.419814041046549,0.243418304389714,1.72466093747175,0.0845885945927057,0.494465577019091
"2673","RAB6B",25.6615922825529,0.662731776206303,0.384110034594333,1.72536959860012,0.0844608864779703,0.494465577019091
"2674","HPGD",165.493148294278,0.59596928678458,0.345765942740472,1.72362055690345,0.0847763649917025,0.494465577019091
"2675","LYSMD3",115.894814774125,0.365061861733917,0.211795479017984,1.72365275890954,0.0847705480436487,0.494465577019091
"2676","NAPEPLD",68.9201267103833,0.436476157058208,0.253119920896839,1.72438485091063,0.0846383905345245,0.494465577019091
"2677","ITGB1",2548.40306438124,0.2814979945089,0.163201052588784,1.72485403766474,0.0845537804520891,0.494465577019091
"2678","HIF1AN",75.3361312322127,0.500308185030279,0.290112474060747,1.72453179288499,0.084611884612684,0.494465577019091
"2679","TNFRSF19",31.6460939152981,0.618539232510957,0.358773896553523,1.72403633166406,0.084701284550984,0.494465577019091
"2680","ATG2B",84.0246260597851,0.468634951075729,0.271729664733861,1.72463669557287,0.0845929659972888,0.494465577019091
"2681","MEIS2",293.876890338828,0.310545216542531,0.180145537126114,1.72385739606265,0.0847335900746754,0.494465577019091
"2682","ZMYM3",50.7768600778989,0.468965382405759,0.272041917636819,1.72387177123136,0.0847309943741842,0.494465577019091
"2683","PYURF",659.799726722251,-0.363011560298958,0.210720242411883,-1.72271802720025,0.084939528711304,0.494519171812094
"2684","RICTOR",245.863407314585,0.411282691813254,0.238705308998602,1.72297253688506,0.0848934915184881,0.494519171812094
"2685","TAPBP",507.438069548604,0.221746774144842,0.128700491845816,1.72296757350777,0.0848943891300682,0.494519171812094

"2686","ALDOC",15.8335835246917,-0.752895829055488,0.436897630960821,-1.72327743549382,0.084838366256466,0.494519171812094
"2687","CLCN5",35.3613272167961,0.643166533138594,0.373348952671711,1.72269542618521,0.0849436178903725,0.494519171812094
"2688","PUM2",209.520971441613,0.326670724880435,0.189755414274933,1.72153572602217,0.0851536550570683,0.495557524575044
"2689","CPXM2",163.830348413173,0.626378526093646,0.363965560252737,1.72098295690034,0.0852539166024232,0.495780852257636
"2690","PLD3",1057.39606405482,0.379216469210319,0.220349823866913,1.72097468722897,0.0852554172839635,0.495780852257636
"2691","SLC45A1",11.4668236423755,-0.830227171043378,0.482476373978439,-1.7207623332878,0.085293960066252,0.495820667899064
"2692","AC239799.2",9.57684847046307,1.01949965827489,0.592694467268793,1.72010996318705,0.0854124550291053,0.496325049784656
"2693","CHRAC1",161.77977883431,-0.328131156918286,0.190783350795057,-1.71991505312626,0.0854478838401984,0.496346545455709
"2694","DYNC2LI1",235.016051967339,-0.323637636616062,0.1882500918058,-1.7191897943398,0.0855798185212059,0.496822419923929
"2695","AL589863.1",4.12762561697772,1.5127261339103,0.879944401014529,1.71911558521903,0.0855933274752278,0.496822419923929
"2696","ZDHHC11",13.1483676260448,-1.16630428135456,0.678561377910416,-1.71878966195529,0.085652678629017З,0.496982511792922
"2697","MAD2L1BP",61.809306731272,-0.438937140394608,0.255401765850428,-1.71861435230508,0.085684616554661,0.496983484154454
"2698","MTF1",73.3996852928975,0.400057424159356,0.232848496806905,1.71810181145861,0.0857780464925696,0.496984754098538
"2699","PPM1G",354.830027410588,-0.259012529034143,0.150938703405487,-1.71601135553896,0.086159964066845,0.496984754098538
"2700","STON1",38.6219960180144,0.731538522483548,0.426264252368858,1.71616202488997,0.0861323915477304,0.496984754098538
"2701","CP",754.200958736226,-0.562465561006494,0.327416453986862,-1.71789033250315,0.0858166205086572,0.496984754098538
"2702","NSD1",269.066187909328,0.316879029915167,0.184637773424831,1.71621994804965,0.086121793495888,0.496984754098538
"2703","CSPP1",113.742986915397,0.345612504546333,0.20121660914336,1.71761419704721,0.0858670090482265,0.496984754098538
"2704","PTP4A3",18.5601385750798,0.767979848182238,0.447326938675445,1.71682002978909,0.0860120600595626,0.496984754098538
"2705","NACC2",50.5366672270561,0.529940632279729,0.308439133321778,1.7181368219151,0.0857716618952034,0.496984754098538
"2706","RPS6KB2",84.0372685885675,-0.392820941649846,0.228759475293144,-1.71717888907756,0.0859464915565592,0.496984754098538
"2707","KCTD21",28.6206533556306,0.502018187772602,0.292570869942371,1.7158857540106,0.0861829546188651,0.496984754098538
"2708","HCFC1R1",665.631093475404,-0.502614471605396,0.292927896525013,-1.71582999628196,0.0861931623006669,0.496984754098538
"2709","RPGRIP1L",54.5363747833623,0.540764083991651,0.315038681940004,1.71650059180553,0.0860704597444815,0.496984754098538
"2710","LGI4",146.543866045218,0.641020484963335,0.37348566682694,1.71631883603276,0.0861037026499527,0.496984754098538

"2711","SLC2A4RG",596.173598925452,-0.472495841731586,0.275284947531095,-1.71638822234628,0.0860910107553977,0.496984754098538
"2712","TUBA8",5.12592651129339,-1.12230314956979,0.653921232785815,-1.71626656744054,0.0861132644307754,0.496984754098538
"2713","CYSLTR1",15.1463329497945,0.990886988959835,0.576779154959239,1.71796601947215,0.0858028135040385,0.496984754098538
"2714","LST1",29.7199787166903,-0.582191008246017,0.339342144530989,-1.7156460452346,0.0862268455225855,0.49699577911194
"2715","AC010198.2",8.72104062382274,1.16077792368463,0.6766544642318,1.71546629046843,0.0862597706355708,0.497002428011872
"2716","CMYA5",201.992846794811,0.708800131016953,0.413371262698598,1.71468167958681,0.0864036042552656,0.497281670848094
"2717","H1F0",324.712334794053,-0.276470218978271,0.161208336936678,-1.71498710446265,0.0863475912443445,0.497281670848094
"2718","TOMM22",328.685557148668,-0.366633211302246,0.213812096572796,-1.71474494277464,0.0863919997734483,0.497281670848094
"2719","SART1",119.175366639101,-0.367285236667691,0.214261789368756,-1.71418915967128,0.0864939911385397,0.497438505107405
"2720","PEAK1",227.769829243661,0.522473476361028,0.304793809222157,1.71418664209223,0.0864944533588277,0.497438505107405
"2721","SLF2",169.717301745807,0.341877545879557,0.199487565714007,1.71377872428242,0.0865693722478949,0.497668589751904
"2722","CARM1",152.99361721854,0.360360877649767,0.210291875401736,1.71362244481078,0.0865980886853343,0.497668589751904
"2723","AC046134.2",23.3162236322824,0.607762385564615,0.354788695155528,1.71302635586569,0.0867076909807957,0.497932602794636
"2724","MFSD13A",5.46337166208493,-1.28989368651016,0.752938357147248,-1.71314646712559,0.0866855972343767,0.497932602794636
"2725","RFX7",49.9743026351933,0.434233844107361,0.253585029867913,1.71237964770059,0.086826727226091,0.498433208806511
"2726","CLTB",699.749358572268,-0.485397084453516,0.283572827054838,-1.71171931208927,0.0869484078991611,0.498765656313376
"2727","AL442125.1",12.9425928267966,0.888824319117978,0.51921677065233,1.71185595180465,0.0869232178800905,0.498765656313376
"2728","OASL",8.54602183415488,-1.36380312539292,0.796872180479773,-1.71144527165174,0.0869989459917282,0.498792227935516
"2729","CLN6",43.9002832690571,-0.573036085528099,0.33485055029885,-1.71131893024118,0.0870222536538075,0.498792227935516
"2730","LINC00683",9.85115588398421,1.00033203667764,0.584646616972026,1.71100286504439,0.0870805839347883,0.498792227935516
"2731","LDOC1",78.6609765852679,-0.457167937340007,0.267169324334426,-1.71115429691978,0.0870526330305666,0.498792227935516
"2732","TMEM225B",31.315854957727,-0.615901582551127,0.3600870532846,-1.71042412364751,0.0871874732738215,0.499039021010753
"2733","AL118558.4",3.38313137749793,1.65858101626854,0.969641488255574,1.71050954023471,0.0871716907921841,0.499039021010753
"2734","PNRC2",755.553654065872,0.240805855264133,0.140908933510506,1.70894668822526,0.0874608251173433,0.499236646901517
"2735","PPCS",432.784267761248,-0.359474567121937,0.2103595704778,-1.70885767785818,0.0874773156783903,0.499236646901517

"2736","DESI2",331.950718071225,-0.257277718018286,0.150512073051532,-1.70934937511756,0.0873862524178989,0.499236646901517
"2737","PGM5-AS1",13.7679362230964,-1.49546068906243,0.875110023818363,-1.70888305282723,0.0874726143133423,0.499236646901517
"2738","PFN1",1262.99887653251,-0.44643559298246,0.261129387019719,-1.70963367270781,0.0873336348759544,0.499236646901517
"2739","SHISA6",3.43354156927445,1.61960591062053,0.947664606402633,1.70904969931146,0.087441743835422,0.499236646901517
"2740","PROCA1",11.968594690754,0.831605773157391,0.486419881228466,1.70964593605251,0.0873313657624597,0.499236646901517
"2741","SAMD4B",429.4403081915,0.249016231839544,0.145696772837759,1.70914033982645,0.0874249567972123,0.499236646901517
"2742","FBLN7",133.382046475953,0.424472449879271,0.24846385945899,1.70838709019302,0.0875645410898722,0.499370075198276
"2743","UBN1",201.309998562791,0.330272658084573,0.193323117827848,1.70839712185212,0.0875626809478228,0.499370075198276
"2744","SKIL",227.150686799565,-0.524157383365442,0.306989122265303,-1.70741353797045,0.0877452158390925,0.500218079945672
"2745","MAF1",547.21553139117,-0.356158945445458,0.208728317412109,-1.70632787089576,0.0879470517071879,0.500909217123722
"2746","AC021092.1",5.48729294677051,1.3049944041391,0.764813959339235,1.70628999144649,0.0879541006122352,0.500909217123722
"2747","AC010624.5",2.95580446991869,1.98143067528568,1.1612816053675,1.70624477829272,0.0879625148270067,0.500909217123722
"2748","ZNF688",165.741914936994,-0.46159292638158,0.270734163737023,-1.70496741161174,0.0882005024478141,0.502081681146709
"2749","AL158071.3",2.67148250859821,1.98246903088435,1.16327205064013,1.70421788247507,0.088340389571409,0.5025122596602
"2750","NFATC1",43.6702853120123,-0.550319369159682,0.322891364103953,-1.7043483670951,0.0883160239414847,0.5025122596602
"2751","DIPK1A",82.7096677157981,0.455446665828792,0.267278221537963,1.70401712196406,0.088377888528814,0.502542824520624
"2752","EHHADH",6.11903538378109,1.05841878424186,0.621240760120972,1.70371754750246,0.0884338682617656,0.502678416140552
"2753","AC019080.6",3.05513293904258,1.69959023699639,0.997899525843522,1.70316769672752,0.0885366900430745,0.502714861104833
"2754","PRPF39",75.1009211347953,0.397574438796591,0.233398379362629,1.7034155930401,0.0884903216518822,0.502714861104833
"2755","MT-CO3",63239.6469379464,-0.257924939195601,0.151425351705078,-1.70331411676656,0.0885093001706179,0.502714861104833
"2756","CD33",4.42647578198601,-1.4402368822056,0.845962152762232,-1.70248382566873,0.0886647079673069,0.503259080817338
"2757","PEAR1",92.0435928145238,0.843271252811745,0.495818356073759,1.70076650547867,0.0889868411606723,0.504229703925853
"2758","AC092535.2",4.57362082446881,1.30497887422285,0.767222598751389,1.70091297668581,0.0889593295091266,0.504229703925853
"2759","ABCG2",3.63552014191456,1.44894200550583,0.85196147317114,1.70071306172171,0.0889968811953768,0.504229703925853

"2760","PTTG1",20.5210223749341,-0.78027552112569,0.458749321592087,-1
.70087558585972,0.0889663519674984,0.504229703925853
"2761","NUBP2",229.954179770845,-0.443741729764539,0.260827942614648,-
1.70128140917835,0.088890157220591,0.504229703925853
"2762","TAF1",133.613784473573,0.420929318749445,0.247546142277091,1.7
0040750737403,0.0890543006785767,0.504372348122728
"2763","MSC-
AS1",91.7184927867679,0.418458661950717,0.246141611005727,1.7000728167
8586,0.0891172296617546,0.504546081649955
"2764","CDC42BPA",273.700397298913,0.400365482480446,0.235548273376487
,1.69971733072534,0.0891841078623856,0.504628010335128
"2765","ZNF654",69.3359455763029,0.392045304647629,0.230710889391754,1
.69929258944481,0.089264068168562,0.504628010335128
"2766","UNC119",54.3523366579645,-0.434401431314826,0.255628376003796,
-1.69934745940871,0.0892537352945119,0.504628010335128
"2767","KRT24",12.9226427472984,0.917423472945774,0.539970922412056,1.
69902384529825,0.0893146908197557,0.504628010335128
"2768","CRLS1",151.493756481242,-0.277893464792908,0.163546252724783,-
1.69917353753466,0.0892864908220483,0.504628010335128
"2769","DIDO1",198.257376177909,0.374538598904339,0.220450678062964,1.
69896777907557,0.0893252547860365,0.504628010335128
"2770","CCDC39",23.560938117035,0.803408005937353,0.472953516999077,1.
69870394671136,0.0893749794429831,0.504726643836312
"2771","GRHL1",9.13340164902552,-1.10374840664246,0.650094197698596,-1
.69782842324982,0.0895401496730198,0.505112355331788
"2772","GTDC1",71.2522950169682,0.359873583189744,0.211958257099002,1.
69785120955044,0.0895358478532651,0.505112355331788
"2773","AC246817.1",12.6898482222613,1.25431868541544,0.73873498996722
6,1.69792781234187,0.0895213872523958,0.505112355331788
"2774","FAM76A",184.551703909392,-0.421920698149594,0.248559031036238,
-1.6974667803886,0.0896084464189211,0.505133307146372
"2775","HAND2",94.0155419011917,-0.605700319335483,0.356794667308352,-
1.69761595346945,0.0895802697990606,0.505133307146372
"2776","FUBP1",180.234288233825,0.387352781355148,0.22826151014126,1.6
9696932748511,0.0897024595994862,0.505299090930775
"2777","BAG1",467.658481939371,-0.451494613883134,0.266050135023135,-1
.69702832078575,0.0896913063595589,0.505299090930775
"2778","INPP4A",55.1680197117563,0.472661914030538,0.278704095802252,1
.69592740526463,0.0898996289047994,0.505527578022947
"2779","GNAI2",1218.57709380303,-0.28900824887565,0.170517424858833,-1
.6948898279159,0.0900963225357074,0.505527578022947
"2780","PROS1",664.474724992613,0.283195288341343,0.167103779319122,1.
694727010336,0.0901272192947509,0.505527578022947
"2781","SRP19",305.620575869056,-0.332268154233087,0.195902516415547,-
1.69608926068251,0.0898689771482358,0.505527578022947
"2782","RUFY2",196.656948444125,0.359031728271117,0.211688039938595,1.
69604162982123,0.0898779964827054,0.505527578022947
"2783","GLIPR1L2",24.3759870072268,0.539267260149209,0.318302486244034
,1.69419744882472,0.0902277694713333,0.505527578022947
"2784","RPUSD1",41.6315306974702,-0.52194721421141,0.308001898406696,-

1.69462336729566,0.0901468913544815,0.505527578022947
"2785","RNF112",12.3949470045255,-0.856530566823186,0.505547544974643,
-1.69426313180128,0.0902152930534574,0.505527578022947
"2786","PIP4K2B",146.645060507097,0.356696669615168,0.210539684136054,
1.6942015994698,0.0902269810192185,0.505527578022947
"2787","CEP131",82.816986424416,0.643135141923957,0.379594050721553,1.
69427086831696,0.0902138236011827,0.505527578022947
"2788","NR2C2AP",84.5365704596652,-0.47076225293246,0.277841389000663,
-1.69435610232763,0.0901976357651956,0.505527578022947
"2789","ZNF728",7.02811047778779,1.20949521800678,0.713268006135563,1.
6957093373075,0.0899409393517604,0.505527578022947
"2790","ZNF530",8.66655015294402,-0.916082447358504,0.540250569186512,
-1.69566216050065,0.0899498784611171,0.505527578022947
"2791","SS18L1",40.3699674356447,0.595355453506929,0.351310293956947,1
.69467124575601,0.0901378033111994,0.505527578022947
"2792","DYRK1A",122.930173590005,0.342156816213889,0.201799884467015,1
.69552533252226,0.0899758088105861,0.505527578022947
"2793","SOAT1",131.69443861662,0.304640037017446,0.18005038206122,1.69
197106681986,0.0906514903817583,0.505941509972343
"2794","FGFBP2",163.919692026879,-1.60364625034097,0.947273597751673,-
1.69290715390694,0.0904731414079147,0.505941509972343
"2795","NDUFB2",1058.79412995233,-0.406373121305712,0.240135170915196,
-1.69226823274973,0.0905948419180085,0.505941509972343
"2796","AC022034.1",34.0477651884082,0.544760314248357,0.3217784453730
89,1.69296707744588,0.0904617340336973,0.505941509972343
"2797","PBLD",32.5995684838835,0.599899514318318,0.354583193572624,1.6
9184418549,0.0906756863297308,0.505941509972343
"2798","ANKRD13D",132.450482106247,-0.323609900167537,0.19121315295772
9,-1.69240397515476,0.0905689749467607,0.505941509972343
"2799","DEXI",309.47225360784,-0.460675420267488,0.272147526039594,-1.
69274153240131,0.0905046760477679,0.505941509972343
"2800","TTC39C",166.665193370237,-0.383875170110028,0.226868296768466,
-1.69206176260845,0.0906341981080043,0.505941509972343
"2801","PIGN",78.2501527310177,0.541447067965828,0.320047612447025,1.6
9177037074585,0.0906897650043118,0.505941509972343
"2802","YWHAB",1687.0906324326,-0.331156771361128,0.195602603548025,-1
.6930079935251,0.0904539456888137,0.505941509972343
"2803","RNF215",61.1203641113489,-0.396407167699983,0.234161718414095,
-1.69287777005023,0.0904787355032405,0.505941509972343
"2804","DNASE1L1",14.1545450925367,-0.788838361122395,0.46593313823366
,-1.69302909879486,0.0904499285275104,0.505941509972343
"2805","TYW5",71.8007273094728,0.355512566691204,0.210166363515458,1.6
9157690481263,0.0907266730749339,0.50596696859579
"2806","ADO",56.2951810639534,-0.419971024211795,0.248348111343707,-1.
69105785399175,0.0908257536321429,0.506285777044018
"2807","UBE2M",449.629530618473,-0.442589118481185,0.261741717180637,-
1.69093839242959,0.090848569722084,0.506285777044018
"2808","AL353746.1",2.83026111294445,4.53561724721283,2.68293141343922
,1.69054535814566,0.090923668287736,0.506523840108638
"2809","CASTOR3",40.4702043545267,0.70021466257965,0.414321661565215,1

.69002668104389,0.0910228503118266,0.506754496765947
"2810","CKAP4",389.177570512565,0.371637434288378,0.219905104745368,1.68999002873855,0.0910298622970217,0.506754496765947
"2811","NABP2",133.797465671807,-0.388217321045406,0.229822939974696,-1.68920178763769,0.0911807665481945,0.506976374488259
"2812","MIR22HG",146.6584823153,-0.698700728951691,0.413635561347187,-1.68916987377986,0.091186880507652,0.506976374488259
"2813","CXXC1",91.1644134378954,-0.307678359847775,0.182145299734927,-1.68919187206881,0.0911826661073179,0.506976374488259
"2814","LILRB2",9.74805772996007,-1.37500637286996,0.814044459502098,-1.6891047618099,0.0911993554823219,0.506976374488259
"2815","ANKRD37",33.59923212686,-0.497031646712861,0.294520995672072,-1.68759325826221,0.0914893342759887,0.508227150596339
"2816","AL118522.1",9.22193001712917,-0.882256672336613,0.522755208115856,-1.68770517947873,0.0914678370499525,0.508227150596339
"2817","RHBDF2",3.59749104760938,-1.43371258050454,0.850077556759662,-1.68656679511642,0.0916866812666401,0.509142618052556
"2818","TST",72.3562229371545,0.605959486630853,0.359328057344072,1.68636841528526,0.0917248610326985,0.509173882588539
"2819","MYO18A",39.8374029903051,0.575152288934982,0.34117321099949,1.68580729785329,0.0918329216943338,0.509592903180015
"2820","AC092803.1",22.5009535626591,-0.860545273955095,0.510632574538665,-1.68525338347747,0.0919396954886418,0.509605591468122
"2821","C3orf67",10.6399351405747,-0.895022856070968,0.531131704107783,-1.68512413992395,0.0919646231155664,0.509605591468122
"2822","KBTBD11",16.5458815126858,0.883783962803527,0.524463671637322,1.68511950512119,0.0919655171459764,0.509605591468122
"2823","AL121894.1",5.58121766220823,-1.28081842223519,0.759851422971577,-1.68561692919682,0.0918696063466553,0.509605591468122
"2824","CCDC136",34.9535454147511,0.736868749726045,0.437428189051953,1.68454792847959,0.0920758249775303,0.509855621282657
"2825","AC127502.2",68.5584729321228,-0.514180564342932,0.30521224816583,-1.68466556448142,0.0920531138766628,0.509855621282657
"2826","ACER2",7.00582790100602,1.0859515399671,0.644767121633227,1.6842539011858,0.0921326103083449,0.509989533989186
"2827","AL355574.1",4.48470547914707,1.25784847160867,0.747132319527039,1.68356854433086,0.092265082114487,0.51036162642041
"2828","TRAPPC8",96.3729523101082,0.378460709659843,0.224779446685901,1.68369802150412,0.0922400439126914,0.51036162642041
"2829","DTNB",14.4682545940527,0.665896317533162,0.395586344930252,1.68331472020494,0.09231418227958,0.510452723011477
"2830","UBE2D3",991.163106794444,-0.247883572033717,0.147349016738648,-1.68228860646818,0.0925128893798989,0.510468828419668
"2831","PLPP1",1105.00407796851,0.602657205781222,0.358164766704202,1.68262560085632,0.092447592517834,0.510468828419668
"2832","HDAC3",177.520883110543,-0.44152612965641,0.262437957299668,-1.68240194444224,0.0924909246032021,0.510468828419668
"2833","PANK3",121.059221046166,0.374506532353959,0.222595370942188,1.68245427013496,0.0924807853536523,0.510468828419668
"2834","ZNF345",48.790043892814,0.416490445052139,0.247488237647575,1.

68286965478022,0.0924003271397586,0.510468828419668
"2835","RPS5",8212.63166316176,-0.460680771053563,0.273711552483191,-1
.68308851736119,0.0923579570197245,0.510468828419668
"2836","RPS13",11555.846817382,-0.488144243978983,0.29031491433971,-1.
68143012937938,0.0926793973587454,0.511207268294378
"2837","AMMECR1L",44.4189391964279,0.443498506302724,0.263824136002329
,1.68103841074953,0.0927554539834435,0.511437262262082
"2838","BAZ2B",342.971034010003,0.485738036306404,0.288978641592341,1.
68087867542691,0.0927864827910113,0.511437262262082
"2839","NDC1",30.3362317014465,0.512562567486202,0.305201221465441,1.6
7942502007398,0.0930692404743829,0.511939860674895
"2840","AC096537.1",2.93389639721401,-5.3907050274259,3.20967986068999
,-1.67951486173049,0.0930517449035175,0.511939860674895
"2841","AC092645.2",7.06024534999158,1.20128304449349,0.71530470287284
8,1.67940045643322,0.0930740244044877,0.511939860674895
"2842","RCE1",64.1952239906701,-0.485473822620348,0.289070000438366,-1
.67943343094801,0.0930676024468704,0.511939860674895
"2843","STARD9",165.391247230246,0.610722378223448,0.363604463207248,1
.67963388797939,0.0930285700531267,0.511939860674895
"2844","EIF5A",677.512019862501,-0.355236743601447,0.211521170394712,-
1.67943824695444,0.0930666645344281,0.511939860674895
"2845","NUBP1",123.224717674735,-0.437372241188311,0.260498047788811,-
1.67898471754727,0.0931550222190473,0.512205276826909
"2846","HMBOX1",121.795877728437,0.503815695907642,0.300146245254517,1
.67856737798075,0.0932363887767782,0.512472533251982
"2847","PLEK",22.7743624196334,-1.20902617767195,0.720519698335361,-1.
67799184458828,0.0933486911164306,0.512611238697219
"2848","FARP2",66.4782764445993,0.4095533079351,0.244130681021014,1.67
759867879878,0.093425470915155,0.512611238697219
"2849","NRARP",4.24088950792539,-1.53970897899468,0.91773396232496,-1.
67772910473317,0.0933999949339478,0.512611238697219
"2850","GPR135",21.8383506497546,0.723314363382925,0.43108022894877,1.
67791124437972,0.0933644270867065,0.512611238697219
"2851","GLRX5",499.544119136306,-0.487498073276028,0.290501818258313,-
1.67812400004515,0.0933228943697456,0.512611238697219
"2852","PLEKHA8",50.6994914658178,0.48278011835543,0.287887342589408,1
.67697584066412,0.093547206221786,0.512739645034127
"2853","BTBD1",104.918614542404,-0.334022730506714,0.199154784503078,-
1.67720163660719,0.0935030591316094,0.512739645034127
"2854","ZNF808",37.7138882576836,0.583167708584618,0.347742236761498,1
.6770114381722,0.093540245170902,0.512739645034127
"2855","SDHB",298.531209122607,-0.396817740975579,0.236772685098117,-1
.6759439156216,0.0937491786464177,0.51312750229738
"2856","NOCT",7.62910314329311,-1.35545713229137,0.808690135631418,-1.
67611433918759,0.0937157985857916,0.51312750229738
"2857","CPLANE1",156.009407601358,0.459807528404031,0.274352181474073,
1.67597547769994,0.0937429960115011,0.51312750229738
"2858","SPG7",302.317286427681,0.371541820509729,0.22162574584728,1.67
643799274907,0.0936524323315663,0.51312750229738
"2859","TRABD2B",19.4139549378332,0.914263043817047,0.545786470454196,

1.67512954847747,0.0939088165788109,0.513462292115462
"2860","DHX57",67.7083519718384,0.419156271422236,0.250174848368275,1.67545328459721,0.0938453294792455,0.513462292115462
"2861","ZNF207",461.383071727786,0.352586549366006,0.210471026074605,1.67522606765373,0.0938898848363209,0.513462292115462
"2862","COL3A1",21716.1067802254,0.71120231260942,0.424615519736903,1.67493244959603,0.0939474860242808,0.513494243143894
"2863","LINC02664",7.38269837867448,1.15236501858735,0.688132685863438,1.67462619093789,0.0940075972015162,0.513643326239371
"2864","PTCH2",67.9944269821622,0.564502887212401,0.337253081969367,1.67382573323281,0.0941648533672926,0.513745137765843
"2865","FAM160B1",194.805254184895,0.35464306753667,0.211892215520259,1.67369559408265,0.0941904401401545,0.513745137765843
"2866","GLRX3",249.610531695431,-0.414442592989318,0.247590826072569,-1.67390124894145,0.0941500087125513,0.513745137765843
"2867","SPTSSA",190.814725778696,-0.361779244095985,0.216148278423168,-1.67375491831448,0.0941787756594787,0.513745137765843
"2868","FPR1",3.40024799954717,1.79334120979697,1.07115505881984,1.67421251949537,0.0940888398724533,0.513745137765843
"2869","TENT5A",797.835651971424,-0.301596184457866,0.180216370503375,-1.67352268617694,0.0942244443381847,0.513751475351071
"2870","RCOR3",172.659024596743,0.271318131049452,0.162157312535656,1.67317851293196,0.0942921589976021,0.513884571029533
"2871","FAM111A",181.989084182502,0.31698812880024,0.189465551082534,1.67306471804025,0.09431455625045,0.513884571029533
"2872","AC147067.1",41.8302923452584,-0.531182340876986,0.317537882345898,-1.67281565573447,0.0943635918868258,0.513972725586914
"2873","FAM86C1",9.12692549254794,-0.844933288673371,0.505158858583633,-1.67260907003076,0.0944042801909185,0.514015368961551
"2874","PSMD3",330.703869387455,-0.357680235929441,0.21392136251544,-1.67201737930042,0.0945208950842978,0.514323219932126
"2875","PTPRM",108.625608747982,0.329755006169018,0.197223242999377,1.6719885605474,0.0945265778498281,0.514323219932126
"2876","RUSC2",74.2442668644755,0.375786155834419,0.224822678596425,1.67147797624538,0.0946273053010603,0.514513360036318
"2877","NAE1",241.132307270703,-0.28501099772959,0.170505212573876,-1.67156765137665,0.094609608075547,0.514513360036318
"2878","MRTO4",168.960045635466,-0.361593055361033,0.216713972011313,-1.6685267313644,0.0952112099631084,0.514887266472388
"2879","RSRP1",1039.66169854811,0.282918272869481,0.169353830993605,1.67057498026226,0.0948056578257944,0.514887266472388
"2880","AL049597.2",21.3563622420976,-0.595115523841645,0.35668975341601,-1.66844020088112,0.0952283734945742,0.514887266472388
"2881","BMPR2",362.70168006621,0.325738065740977,0.194975088137712,1.67066505189066,0.0947878555152667,0.514887266472388
"2882","SCAP",101.482525583824,0.377143055567559,0.225831646166336,1.6700186265736,0.0949156783780895,0.514887266472388
"2883","TWF2",350.247341081099,-0.442460765160993,0.264932794405852,-1.67008680881984,0.0949021896440736,0.514887266472388
"2884","EXOC1",285.348272957475,0.314254447746258,0.188277478705836,1.

669102698349,0.0950970286402244,0.514887266472388
"2885","SYNPO2",183.164302653588,0.676272303696173,0.405365434597287,1
.66830283486805,0.0952556254664126,0.514887266472388
"2886","CCNA2",4.1523565604879,1.48877910159043,0.892053361838158,1.66
893502707356,0.0951302569440028,0.514887266472388
"2887","AC079781.5",14.7319562739062,0.791046480561361,0.4739759036291
87,1.66895927515385,0.0951254509984806,0.514887266472388
"2888","KLF6",2419.86154722068,-0.496335718965533,0.29730155981552,-1.
66946893677085,0.0950244815672098,0.514887266472388
"2889","GVQW3",56.0170747031337,0.510184812086423,0.305772823246402,1.
66850934190217,0.0952146590062139,0.514887266472388
"2890","IKBIP",396.500308327091,-0.311890178480365,0.186817867690541,-
1.66948794746444,0.0950207170065856,0.514887266472388
"2891","CFL2",340.610706734604,0.270589298052214,0.162085745758297,1.6
6942069326514,0.0950340354426215,0.514887266472388
"2892","EPS15L1",103.710524584276,0.318054075890852,0.190575980476381,
1.66890956087863,0.0951353045280351,0.514887266472388
"2893","ZNF584",37.728436694254,-0.626563984275199,0.375158891264365,-
1.67012964070649,0.0948937168464962,0.514887266472388
"2894","MRTFA",88.0674065421755,0.341779875004072,0.204558371747942,1.
67081831989363,0.0947575688416541,0.514887266472388
"2895","PXDC1",477.779521797901,-0.311761097828398,0.186922397968394,-
1.66786378313589,0.0953427706013016,0.514896379789394
"2896","SNAPC3",150.150323751363,0.33213236502192,0.199156563241365,1.
66769480059463,0.0953763280940641,0.514896379789394
"2897","CCL19",25.3652098407854,-2.45575028248817,1.47259800340766,-1.
66763113681089,0.0953889732551085,0.514896379789394
"2898","PRTN3",4.38475559377786,-1.29380941430213,0.775609642685027,-1
.66811930009428,0.0952920465825239,0.514896379789394
"2899","PCDH9",201.106659151869,-0.758688524777634,0.455055749817894,-
1.66724302479696,0.0954660906942007,0.514957260941166
"2900","ACTG1",9166.81534788905,-0.465814866450845,0.27937026352617,-1
.66737454649396,0.0954399518840327,0.514957260941166
"2901","GRB10",53.9060649865018,0.486769352336291,0.292146685391847,1.
66618132833991,0.0956773037421644,0.515385696432052
"2902","TCAF1",355.764734491255,0.406884961961868,0.24419952617405,1.6
66198818387,0.0956738212577602,0.515385696432052
"2903","ITGAE",283.39769642023,-0.40376111862129,0.242261992054539,-1.
66663006110506,0.0955879876008581,0.515385696432052
"2904","LPAR2",13.6560820335862,-0.860520752564542,0.51638757944574,-1
.66642418759989,0.0956289565421422,0.515385696432052
"2905","BAHD1",31.0711880660599,0.528712860587754,0.317420352944243,1.
66565519722872,0.0957821103981508,0.515772651620748
"2906","ZNF595",74.628533192506,0.365730836900389,0.219708499835359,1.
66461851578093,0.0959888889299886,0.516491324763787
"2907","SHMT2",152.214542880195,-0.442906274146223,0.266049357318046,-
1.66475227984391,0.0959621880256324,0.516491324763787
"2908","GPS2",360.146904107469,-0.289133333006247,0.173706901088604,-1
.66448961552061,0.0960146245869139,0.516491324763787
"2909","LRIG2",102.234032491786,0.402917811698406,0.242116641952767,1.

66414753008597,0.0960829506311353,0.516681195160828
"2910","SLC39A14",89.925305314712,-0.348369037710774,0.209365498281541
,-1.6639276316784,0.0961268923318474,0.516700680155256
"2911","AC062004.1",158.676522589862,0.701083942748869,0.4214143570562
83,1.66364512981041,0.096183367512198,0.516700680155256
"2912","LINC00173",9.73685633344961,1.54178071337732,0.926754955582548
,1.6636336326987,0.0961856664713997,0.516700680155256
"2913","SERBP1",1252.33517735404,-0.203416032298786,0.122300534744932,
-1.66324728442952,0.0962629461316238,0.516938299463437
"2914","TNNI3K",9.03209143386357,0.880233817578585,0.529327460030331,1
.66292868601252,0.0963267114383267,0.517103207628601
"2915","TMTC2",18.6181233021316,0.784016022483242,0.471519117795782,1.
66274492993687,0.0963635043322717,0.51712325841157
"2916","CLPTM1L",230.276458412406,-0.324400799722941,0.195145025093416
,-1.66235751881274,0.096441111270779,0.517174559171449
"2917","PLK2",96.2142742971683,-0.623719978004303,0.375252224349542,-1
.66213532534138,0.0964856440514415,0.517174559171449
"2918","MOSPD3",145.601797312216,-0.521957250181127,0.313972331222007,
-1.66243072486555,0.0964264426635765,0.517174559171449
"2919","BMP4",211.688805506911,0.394259704721735,0.237214724050008,1.6
6203723778386,0.0965053083309761,0.517174559171449
"2920","TPM3",683.099656364124,-0.244275643704205,0.147094242043154,-1
.66067441057645,0.0967788554383958,0.518311337211438
"2921","HSD17B14",34.336327188476,-0.877572135023241,0.528450892516008
,-1.66065030346535,0.0967836998014837,0.518311337211438
"2922","PTDSS1",70.2193800502096,-0.386465533452426,0.232749881706211,
-1.66043278140198,0.0968274199836471,0.518368011911086
"2923","LAPTM5",47.2713845026277,-0.744930740840549,0.44872535690591,-
1.66010395752328,0.0968935409184127,0.518544529793613
"2924","HOXB5",12.3012617988594,-0.9344503441004,0.562968246771622,-1.
65986332170432,0.0969419515996295,0.518626179505131
"2925","TIA1",248.941163120052,0.392907307996616,0.23674504421469,1.65
962210233348,0.0969904990893547,0.518669408602476
"2926","ADAMTS9",10.0263693048625,1.04997792476473,0.632772575481847,1
.65932906299738,0.09704950194844,0.518669408602476
"2927","QDPR",201.597699547039,-0.47125254666809,0.283997319640794,-1.
65935561386333,0.0970441548048375,0.518669408602476
"2928","TIMM10",244.787728547887,-0.521959104828841,0.314605860732961,
-1.65908894263061,0.0970978710745497,0.518750682110376
"2929","FAM3B",7.49586173125867,-1.00946651637182,0.608539752377048,-1
.65883413931249,0.0971492189674238,0.518847808913421
"2930","PARP8",47.1314797532619,0.457402664796885,0.275769368745067,1.
65864202713437,0.0971879477127693,0.518877496952509
"2931","WDR82",281.019646752714,0.2640006052713,0.159201721213777,1.65
827733053714,0.0972615024733966,0.519093034183331
"2932","NAALAD2",90.6903524345061,0.510105965712585,0.307724428282467,
1.65767134107516,0.0973838214043042,0.519568594211299
"2933","CHCHD7",106.963529690373,-0.354262329358891,0.213895235273556,
-1.65624226694819,0.0976727668556456,0.520932523669576
"2934","DZIP3",114.107273461997,0.316147362905758,0.19094687446302,1.6

5568231370546,0.0977861708258996,0.521004451713061
"2935","ING1",107.398029919992,-0.421661048422388,0.254668185192217,-1.65572722837024,0.0977770706489871,0.521004451713061
"2936","ITPKC",63.2577134633703,-0.521973077912421,0.3152281385136,-1.65585813618571,0.0977505512282639,0.521004451713061
"2937","KLF7-IT1",4.13256283192941,1.24696751590636,0.753649084775959,1.65457311777543,0.0980111202293292,0.521770789039517
"2938","PTRH2",82.0769770177631,-0.390240125795036,0.23586877866736,-1.65447978320769,0.0980300676971899,0.521770789039517
"2939","ZNF772",6.62183594528783,1.04954928788169,0.63433560632286,1.65456467746743,0.0980128335413121,0.521770789039517
"2940","DCTD",265.804160935504,-0.325393255491634,0.1967389919123,-1.65393373387155,0.0981409775794268,0.521921302967755
"2941","SYNM",47.5423846771315,-0.730300281889825,0.441548667055269,-1.65395195678036,0.098137274648278,0.521921302967755
"2942","PARD6G-AS1",13.7666759088868,-0.693999352305238,0.419627096116683,-1.6538478061299,0.0981584397705769,0.521921302967755
"2943","PROX1",7.6258379792177,-1.22207888601244,0.739297128224741,-1.65302804428172,0.0983251561561456,0.522148196436568
"2944","ING2",150.611006776849,0.418908759280249,0.253426162631384,1.65298150329319,0.0983346280574141,0.522148196436568
"2945","TMEM86A",20.0845641663159,0.731090138232667,0.442195200387829,1.65331993108804,0.0982657687245763,0.522148196436568
"2946","PSMC3",516.688864380783,-0.40600463706013,0.245574551217713,-1.65328465448436,0.0982729445971734,0.522148196436568
"2947","ATRX",922.680275362711,0.284069071316624,0.171874008129258,1.65277504381575,0.0983766549416686,0.522194100187486
"2948","ARPC5L",141.959890430857,-0.459509232213885,0.278114645407322,-1.65222953843691,0.0984877670218609,0.522429345379101
"2949","ITGA3",2.71909824637442,-1.95885830924778,1.18547657868213,-1.65238043878134,0.0984570206373781,0.522429345379101
"2950","Z99572.1",12.940209596369,0.868844413223609,0.525962140845491,1.65191435989467,0.0985520102982675,0.522497326621159
"2951","ARC",15.6444152235532,-0.83217557234579,0.503837308284524,-1.6516751710571,0.0986007868174686,0.522497326621159
"2952","MX2",324.827137456229,0.436828294014177,0.264463980940454,1.65174967290737,0.0985855919812717,0.522497326621159
"2953","CAMTA1",315.761232474712,-0.415708689157834,0.251779749008112,-1.65108071953968,0.098722093666098,0.522962990592201
"2954","MICOS10",983.192697145995,-0.422850498796767,0.256321422023333,-1.64968848666217,0.0990066662194043,0.523004031133067
"2955","HP1BP3",1251.92777355857,0.290898412113903,0.176243247197805,1.65055068343932,0.0988303560266418,0.523004031133067
"2956","S100PBP",106.948454685747,0.419445001694521,0.254273333685598,1.64958313014904,0.0990282277224895,0.523004031133067
"2957","UBR3",144.229952242535,0.399572418322396,0.242228105940425,1.64957083229916,0.0990307447558745,0.523004031133067
"2958","ATP2C1",161.198025134172,0.280893724634326,0.170239285393008,1

.64999356045148,0.0989442531541798,0.523004031133067
"2959","SNHG8",761.79713051081,-0.454138515148446,0.275282873312094,-1
.64971583478635,0.099001069962419,0.523004031133067
"2960","AC006333.2",50.7296137438325,-0.433487779648714,0.262706528314
109,-1.65008377382388,0.0989258030039098,0.523004031133067
"2961","ZFAT",15.1242548570733,0.704464271607014,0.426782218534866,1.6
5064110221233,0.0988118808660519,0.523004031133067
"2962","CTXN1",17.0596359337787,-0.783080750613951,0.474558016052438,-
1.65012648427673,0.0989170689566423,0.523004031133067
"2963","PKIB",3.85914892239157,-1.43796808631447,0.871870852571509,-1.
64929023842615,0.0990881885191728,0.523078028299296
"2964","SURF2",136.558441939423,-0.524350883263315,0.317947241870586,-
1.64917575689096,0.0991116330549839,0.523078028299296
"2965","TSR3",295.297565436597,-0.399696950399856,0.242412969924468,-1
.6488265892885,0.0991831660108609,0.523279010424249
"2966","FABP5",332.614032590233,-1.07805131656629,0.654238000420583,-1
.64779685049364,0.0993943655589466,0.524216473512678
"2967","CTNNAL1",100.801109460235,-0.415794572275096,0.252376169719474
,-1.6475191486473,0.0994513836300244,0.524340409209461
"2968","CEMIP2",38.4567565179818,-0.928624599930947,0.56385608396808,-
1.64691776205702,0.0995749504826171,0.524815010242446
"2969","EMC1",131.020004962024,0.341926549440869,0.207661174598686,1.6
4655983527809,0.0996485517877475,0.524849257783075
"2970","BRIP1",3.09840539683359,2.04313221132255,1.24076743498043,1.64
666814563422,0.0996262751029837,0.524849257783075
"2971","FKBP9",1003.99304424531,0.385381549435934,0.23408133319542,1.6
4635746120859,0.0996901856329962,0.524891812136304
"2972","CRYBG3",289.546188378309,0.395985235926409,0.2405771960532,1.6
4597992836712,0.0997678914821968,0.524947570284563
"2973","HOXA9",68.8502000524877,-0.519194386086364,0.315405613465313,-
1.64611650497293,0.0997397749720143,0.524947570284563
"2974","DTX2",65.4164443699732,0.387727498903998,0.23561160121578,1.64
562142485041,0.099841725324365,0.525159418039355
"2975","AC046143.1",4.38220034193748,1.39423327841035,0.84773476424042
8,1.64465742968508,0.100040476391993,0.526003307226669
"2976","PHF19",127.798382189703,-0.38246249957612,0.232591297337057,-1
.64435429852682,0.100103039417873,0.526003307226669
"2977","SCAI",79.7662066206121,0.475768364830714,0.289308921123184,1.6
4449946093483,0.100073075559386,0.526003307226669
"2978","COA6-
AS1",33.4847191783324,-0.674257252977337,0.410165748701586,-1.64386532
788697,0.10020402346669,0.526015537757516
"2979","FOXP1-
IT1",8.36730563986546,1.02238742884658,0.621890229053974,1.64399982678
913,0.100176238154965,0.526015537757516
"2980","ANGPTL5",257.489333722873,-0.584005626621068,0.355429632699155
,-1.64309774113681,0.100362712165595,0.526015537757516
"2981","KCNA1",21.5655980829097,-1.14430237396713,0.696502737818264,-1
.64292588073891,0.100398269528582,0.526015537757516
"2982","NFATC3",91.8201609784106,0.377076771105756,0.229433434694917,1

.64351273216636,0.100276893243998,0.526015537757516
"2983","GLG1",1001.97345372847,0.296098704814026,0.180148783708757,1.64363421566433,0.100251781887331,0.526015537757516
"2984","COTL1",433.43972940187,-0.419058873817822,0.255075945309375,-1.6428788426504,0.100408003307802,0.526015537757516
"2985","FAM174C",226.054808651222,-0.463751133036679,0.282198676922633,-1.64334977787235,0.100310584733247,0.526015537757516
"2986","PRKCSH",438.151035594182,0.236132479507942,0.143706959511555,1.6431527068037,0.100351342065073,0.526015537757516
"2987","ATG101",140.914433899705,-0.456356326338701,0.27784977924809,-1.6424570412605,0.100495321972489,0.526296726352742
"2988","ISY1",184.520168453042,-0.276754763408316,0.168553510252335,-1.64194007584889,0.10060242342971,0.526681295083988
"2989","PIM1",134.760549838679,-1.28401762107754,0.782150291863005,-1.64165076000821,0.100662401651239,0.526818985958628
"2990","AC010680.3",7.39259280504377,1.00474662965391,0.612392450529259,1.64069075114424,0.100861625787869,0.527031091927344
"2991","COQ10B",186.245620957586,-0.341094890706038,0.207848327702052,-1.64107594454642,0.100781651504213,0.527031091927344
"2992","AC008780.1",3.66404067590733,2.81236967080822,1.71418667575971,1.64064375868621,0.100871385874223,0.527031091927344
"2993","AC027307.3",5.06933882402959,-1.18918339251398,0.724775897870213,-1.64076012462397,0.100847218656405,0.527031091927344
"2994","NKAP",280.075833511462,-0.318501956548997,0.194100254695357,-1.64091467602085,0.100815128104012,0.527031091927344
"2995","CLCN6",71.4062756164199,0.398152861994627,0.242825690267022,1.63966531530004,0.101074774424333,0.52712649971609
"2996","STK16",138.668656260534,-0.454063252719304,0.276897155387403,-1.63982635388229,0.101041276970674,0.52712649971609
"2997","CCRL2",2.85915467067439,-1.56050697792932,0.951305444105324,-1.64038478660966,0.100925186513847,0.52712649971609
"2998","MIR155HG",11.5366086117914,-0.859892735621625,0.524458084272297,-1.63958333641621,0.101091830157148,0.52712649971609
"2999","AL021707.2",8.3294662848366,0.996547620666492,0.607780073852935,1.63965168247294,0.101077610579421,0.52712649971609
"3000","MAOB",64.027387532154,-0.481871160924846,0.293876629728455,-1.6397056185451,0.101066390161968,0.52712649971609
"3001","GFER",150.648168822523,-0.408428070041392,0.249151378448147,-1.63927678259421,0.1011556290946,0.527283407506441
"3002","BTG3",186.684478710491,-0.360109377703085,0.219756125335978,-1.63867731628652,0.101280480660702,0.527637015768162
"3003","PARVG",10.3842700773124,-0.882471136118358,0.538542959484714,-1.63862718948683,0.101290926187547,0.527637015768162
"3004","NUTM2A-AS1",273.146449063035,-0.37287701270859,0.227617391054482,-1.63817453043093,0.101385291087257,0.527952765804912
"3005","GNA13",168.654766019071,0.281930799277026,0.172197450344288,1.63725304128104,0.10157760836325,0.528778212188459
"3006","MDM2",179.531420674833,0.34862908209657,0.212959633390285,1.63706650197715,0.101616574950919,0.528805083818106

"3007","PTPN12",397.761885892524,0.258372494605115,0.157883521177974,1
.63647537550081,0.10174013522884,0.529272010437225
"3008","SBDS",796.142245412901,-0.282145383652222,0.172442634661413,-1
.63616952504934,0.101804112592952,0.529428767716604
"3009","NBEAL1",405.671100149158,0.333072440459079,0.20374171381082,1.
63477784803728,0.102095626139106,0.530044587964829
"3010","FBXL2",29.2278704390539,0.571760236355364,0.349658590090956,1.
63519573823893,0.102008021279163,0.530044587964829
"3011","CPE",4472.64857613992,0.528897256381172,0.323472171739124,1.63
506261925901,0.102035921322641,0.530044587964829
"3012","FBXO38",61.2185212476091,0.405072717168902,0.247809606069743,1
.63461265119359,0.102130273866106,0.530044587964829
"3013","KIF13A",169.130327055074,-0.330244232919769,0.202055125785857,
-1.63442640534529,0.102169347549118,0.530044587964829
"3014","DYNC1I1",4.38427189835539,-1.3143325771636,0.804212116882255,-
1.63431083612488,0.102193599520675,0.530044587964829
"3015","PRKAR2B",56.881737355248,-0.500075047676551,0.305880824781529,
-1.63486890044095,0.102076533170704,0.530044587964829
"3016","LINC01089",68.6956321074484,0.506145892258815,0.30954286624077
2,1.63513990293389,0.102019722908866,0.530044587964829
"3017","MST1R",3.35652032158978,1.77997779388205,1.08955036639392,1.63
36810566851,0.102325838000917,0.530203075140226
"3018","NDUFS6",694.684740428351,-0.433953132793541,0.265605363114211,
-1.63382669576194,0.102295245213696,0.530203075140226
"3019","ULBP3",3.3974062403087,-1.28680382481695,0.787530101971818,-1.
63397414472799,0.102264279657996,0.530203075140226
"3020","BTN3A2",184.086861609934,0.537734502366503,0.329215588267928,1
.63338104734237,0.102388880584078,0.530266066225955
"3021","SH3GLB2",197.605650014289,-0.428676907129184,0.262483380078701
,-1.63315828606235,0.102435710604235,0.530266066225955
"3022","AL158151.4",5.88994790990941,1.30590941788107,0.79970082061412
2,1.63299747132711,0.102469528494487,0.530266066225955
"3023","NPIPA9",5.18336190220617,-1.27340995954064,0.779885276829754,-
1.63281702754677,0.102507484770651,0.530266066225955
"3024","ARHGDIA",534.10636156521,-0.30599791157593,0.187395557616752,-
1.63289842869026,0.102490360688453,0.530266066225955
"3025","ZNF441",49.8659764035045,0.535394236606014,0.327981496341145,1
.63239159092418,0.102597019468308,0.530553777038925
"3026","CCDC152",105.415336071394,0.623751297087655,0.382156027088497,
1.63219013406587,0.102639438502284,0.530597731821293
"3027","AC087645.2",4.33394291449916,-1.45038488042588,0.8889436314380
39,-1.63158250887022,0.102767465406011,0.531084063873879
"3028","PDIA4",385.134915748981,0.435046573002187,0.266718566792995,1.
63110719374791,0.102867703034353,0.531251065885239
"3029","AL139383.1",3.1957200827394,1.46733333143427,0.89954670088696,
1.6311919436617,0.102849824712323,0.531251065885239
"3030","MOV10",50.9982379965987,0.423766556923969,0.260075696702057,1.
62939698825237,0.103229005955917,0.531349033037044
"3031","ECT2",15.9702812325009,0.675944451597054,0.414979427756641,1.6
2886255651558,0.103342118214864,0.531349033037044

"3032","CAMLG",935.276662934617,-0.354093193224277,0.217238415950805,-1.6299750284704,0.103106774811925,0.531349033037044
"3033","TCP1",419.896563142928,-0.279479234337834,0.171392563656586,-1.63063804155364,0.102966717220959,0.531349033037044
"3034","ZNF277",138.424526531312,-0.352523509748629,0.21636463421861,-1.62930282493602,0.103248928436501,0.531349033037044
"3035","SLC52A2",143.093612564923,-0.398511414984303,0.244520466970945,-1.62976711079018,0.103150727379012,0.531349033037044
"3036","CCDC6",229.589792755341,0.344825335677413,0.211639143225822,1.62930793624259,0.103247846940037,0.531349033037044
"3037","AGAP5",17.7676933082788,0.901143658683408,0.55327091488268,1.62875660809767,0.103364553852771,0.531349033037044
"3038","NCKAP1L",10.8640806747426,-0.997413817556284,0.612302673563883,-1.62895551598832,0.103322436304761,0.531349033037044
"3039","N4BP2L2-IT2",11.8812022226813,1.01724967629899,0.624135196959361,1.62985468734145,0.103132212398773,0.531349033037044
"3040","DCLK1",1524.65703758521,0.334214870821553,0.205038555332552,1.63000987926144,0.103099409018426,0.531349033037044
"3041","AL133467.1",94.1237210448273,-0.512604908868607,0.314750470044086,-1.62860728626333,0.10339618081984,0.531349033037044
"3042","AP001269.4",5.47429948388209,-1.15199423375635,0.707326374083574,-1.6286600867229,0.103384996590407,0.531349033037044
"3043","ZSCAN30",86.339815898191,0.429383602604188,0.263446416191858,1.62987072973307,0.103128821085922,0.531349033037044
"3044","CR381653.1",5.92552439187421,1.47862287019291,0.907097984933267,1.63005859868787,0.103089112758783,0.531349033037044
"3045","CPD",158.951434318185,0.314775219805404,0.193321501400208,1.62824733682244,0.103472451186336,0.53139184304263
"3046","THOP1",42.0647210476629,-0.443026127641485,0.272075803548037,-1.62831873273606,0.10345731941701,0.53139184304263
"3047","ALMS1",60.0069448606871,0.557526811889803,0.342508386745933,1.62777565007004,0.103572465474392,0.531556455845118
"3048","AP000787.1",19.6262124851311,0.633388793742867,0.389083332937452,1.62790009266392,0.103546071780948,0.531556455845118
"3049","TRMT1L",85.8634007041937,0.312291551750859,0.191996764018961,1.62654591261766,0.10383357465053,0.531949767342805
"3050","ADRA2B",3.60641645025849,1.58144265425469,0.972636311794069,1.6259342110492,0.103963651423049,0.531949767342805
"3051","MMADHC",258.233121562603,-0.320770501717258,0.197311590853834,-1.62570531375868,0.104012359121222,0.531949767342805
"3052","RBSN",116.254747210459,0.359806031290297,0.221241778054825,1.62630238490099,0.103885344693338,0.531949767342805
"3053","FRG1",468.528873208348,-0.351221409385512,0.21603068007385,-1.62579411991596,0.103993459657194,0.531949767342805
"3054","TMEM161B",76.0251832381973,0.404261462943488,0.248595633976038,1.62618086439304,0.103911185651939,0.531949767342805
"3055","SNTB1",20.4198434828233,0.66203186739094,0.407320227879161,1.62533511001409,0.104091174188859,0.531949767342805
"3056","GNA14",13.3436895248145,0.734562697211294,0.45179270935677,1.6

2588435359462,0.103974259186944,0.531949767342805
"3057","DLEU2",135.491041352136,0.566123354410017,0.348272952519031,1.6255162805934,0.104052597687473,0.531949767342805
"3058","AC108134.2",55.6578908947589,0.975605355898926,0.600240640571084,1.62535704841763,0.104086502258234,0.531949767342805
"3059","ZNF319",24.3741924568671,0.534164367896076,0.328307858391257,1.62702279047946,0.10373225753023,0.531949767342805
"3060","LINC01915",43.9967312972002,-0.650692475718285,0.40000648017356,-1.62670483597154,0.103799801178949,0.531949767342805
"3061","MGME1",56.4703158000005,0.382960084231295,0.235610971234528,1.62539156060817,0.104079152992243,0.531949767342805
"3062","ALG14",111.78450462835,-0.369349567618119,0.227397938762298,-1.62424325228473,0.104323903180492,0.531972107089685
"3063","FEZ2",468.197751297035,0.232647863831072,0.143204420297432,1.62458577289632,0.104250850616501,0.531972107089685
"3064","ANKIB1",274.712184025575,0.362835571836186,0.223275426483335,1.62505824107458,0.10415014961123,0.531972107089685
"3065","TYSND1",81.4587544596277,-0.446093961780052,0.274602462300782,-1.62450823653369,0.104267383960398,0.531972107089685
"3066","RHOT2",123.705441074516,-0.341394730501971,0.210139994153539,-1.62460616731783,0.104246502190144,0.531972107089685
"3067","AC092376.3",3.61984535116297,1.40775648011416,0.866739542551586,1.62419782530042,0.104333594869984,0.531972107089685
"3068","HOXB7",31.9347971580003,-0.819110703971403,0.504296117196229,-1.62426533943087,0.104319191222858,0.531972107089685
"3069","TTC27",67.7488510546263,0.513803452724308,0.316489113698127,1.62344747571439,0.104493782833586,0.532095131792247
"3070","LIMD1",44.6157923761734,0.450794463796312,0.277647251013234,1.623623004194,0.104456292744069,0.532095131792247
"3071","IKZF3",6.27551533142989,1.23222061503106,0.758899990587314,1.62369301662192,0.104441342184203,0.532095131792247
"3072","TXNL4A",328.646844629992,-0.403625699615588,0.248602430742387,-1.62357905516154,0.104465678555295,0.532095131792247
"3073","EIF5A2",59.8765748787402,-0.358952591208281,0.221155857489574,-1.62307521619772,0.104573326902549,0.532326896432338
"3074","AGO4",67.7028439625604,0.456013034165073,0.280988899894736,1.62288629314505,0.104613714194838,0.532359248910169
"3075","SLC35F2",29.8695566698187,-0.533331441767428,0.328746819621173,-1.62231665809575,0.104735563708219,0.532805991248021
"3076","MTREX",239.637602029457,0.283854676253627,0.175062169875544,1.62145069066279,0.10492101692939,0.533402491981293
"3077","SMCR8",48.4942261308228,0.464225988964537,0.286288037687605,1.62153470579549,0.104903013067282,0.533402491981293
"3078","SLC35B1",157.471216992513,-0.458275099694305,0.282678215961134,-1.62119000976472,0.104976894656523,0.533513178398958
"3079","AKR1B1",312.36085997005,-0.356323062351713,0.219824409645412,-1.62094402039555,0.105029644870987,0.533607903448148
"3080","ZNF638",525.762631446265,0.403657334527552,0.249135614341608,1.62023135710364,0.105182587904309,0.534058586245232
"3081","EXOC6",21.126219265698,0.643179245129875,0.397105998323664,1.6

1966640606029,0.105303956305489,0.534058586245232
"3082","AC009090.6",3.71115879102148,1.58003775537764,0.975580971706187,1.61958648354357,0.105321135024687,0.534058586245232
"3083","CHMP1B",243.985868516901,-0.280446660099842,0.173119759179262,-1.61995754516643,0.10524139703621,0.534058586245232
"3084","C20orf27",114.897864051723,-0.45596040798779,0.281530569997835,-1.61957690062325,0.105323194947679,0.534058586245232
"3085","SHOX",6.00529562460117,1.62570688991291,1.00344776486054,1.62012109333747,0.105206267139634,0.534058586245232
"3086","RNASE6",8.26144125596794,-1.01672950580155,0.627894105139596,-1.61926907336629,0.105389381812056,0.534221030358392
"3087","TTYH2",15.2321939260879,0.76834359581215,0.474577736660409,1.61900472032035,0.105446247492966,0.534336135255093
"3088","ZRANB3",20.686425832673,0.572021355330142,0.353548680904518,1.61794226997732,0.105675039576739,0.534422150541865
"3089","COPS7B",91.4756120920337,0.407028800367468,0.251629704328576,1.61757055453188,0.105755179118075,0.534422150541865
"3090","DUSP28",21.1167551710967,-0.516719963265857,0.31952788829258,-1.617135724919,0.105848986854514,0.534422150541865
"3091","CHL1",1353.13952845799,0.336585177040426,0.208041221109824,1.61787733817782,0.105689034995729,0.534422150541865
"3092","H2AFZ",708.656428915592,-0.461326272472765,0.285034406526361,-1.61849328330157,0.105556333170243,0.534422150541865
"3093","AMACR",60.2605241888182,0.380115291704848,0.235005854171304,1.6174715861664,0.105776524207292,0.534422150541865
"3094","AL080317.1",5.5592679905041,1.04839209729042,0.648346810901041,1.61702360474853,0.105873185739899,0.534422150541865
"3095","LMTK2",37.6442311929629,0.537760138605896,0.332551734013167,1.61707212323422,0.10586271346139,0.534422150541865
"3096","STK32C",63.265270268871,-0.366506638531657,0.22641216397847,-1.61875860418219,0.105499212066392,0.534422150541865
"3097","UQCRC2",435.792675369007,-0.315296391369947,0.194979615570797,-1.61707361278217,0.10586239196887,0.534422150541865
"3098","DDX19A",125.229768948154,0.31386865288305,0.194018953516309,1.61772160500117,0.105722607762335,0.534422150541865
"3099","AC245060.5",125.270285604359,0.701620566749161,0.433767897050596,1.61750228986476,0.105769901794556,0.534422150541865
"3100","SATB1",111.100545824775,0.448709340655208,0.277527754038087,1.61680889253919,0.105919539302086,0.534483662355656
"3101","CHST2",5.88365672292713,-1.11576087142532,0.690358480119292,-1.61620506382787,0.106049984321887,0.534805718926519
"3102","GIMAP2",25.4626265886603,0.525079899618756,0.324886085102084,1.61619694932077,0.106051738164678,0.534805718926519
"3103","FGR",10.048717840778,-0.901265938604921,0.558047098591656,-1.61503561416133,0.106302982615954,0.535258763603107
"3104","MZT2A",712.678653495662,-0.446514655098977,0.276497569199021,-1.61489540899934,0.106333346644598,0.535258763603107
"3105","GMPPB",54.6018009021684,-0.609417707954494,0.377395210150046,-1.61479979492109,0.106354057590137,0.535258763603107
"3106","SFXN4",68.7292668436974,-0.459068298372086,0.284202163159617,-

1.61528784041752,0.106248375648935,0.535258763603107
"3107","LCTL",5.30966849029233,1.41886956368149,0.878819788391833,1.61451708578149,0.106415313865989,0.535258763603107
"3108","AC137932.3",12.5313103627327,0.753423603827506,0.46634672888808,1.61558676657582,0.10618368695803 8,0.535258763603107
"3109","TP53",87.5309785520655,-0.408239417121505,0.252833060525722,-1.61465995100737,0.106384354910325,0.535258763603107
"3110","PSMD12",330.220069033831,-0.241476766984161,0.149500908557846,-1.6152193944074,0.106263192006101,0.535258763603107
"3111","KIAA0930",368.89053870888,-0.3608173246131,0.223558615452043,-1.61397190568351,0.106533520262124,0.535681085651046
"3112","THUMPD2",105.467930934478,0.322201262001055,0.199728204376813,1.61319861161512,0.106701365003715,0.536008173652253
"3113","NPM1",2114.15078408016,-0.446956951511772,0.277058053523179,-1.61322490296922,0.106695654982699,0.536008173652253
"3114","FAH",60.4367930733155,-0.442250049296549,0.274109925589808,-1.61340399602441,0.106656765555273,0.536008173652253
"3115","HEXD-IT1",8.18110358885472,1.00412659048538,0.622540584804771,1.61294960520569,0.10675545683863,0.536107740393803
"3116","PGR-AS1",9.21173091859205,-0.895721561358965,0.555570103123846,-1.61225659250295,0.106906114878386,0.536519844415332
"3117","FGF9",5.88226190200762,1.48717544907145,0.92235128104346,1.61237424356261,0.106880526167667,0.536519844415332
"3118","HTATSF1",243.220692919479,-0.289017708534282,0.179287339034003,-1.612036355113,0.106954028820727,0.536588156780832
"3119","AC116667.1",12.2833723912536,0.869873302365409,0.539826366852967,1.61139461830388,0.107093739475836,0.537116821616062
"3120","ADAM17",163.723295764866,-0.399596902985847,0.248103514819551,-1.61060557032607,0.107265718955286,0.537345839296547
"3121","ADAM32",26.8422024199502,0.616475449403013,0.382725515997564,1.61075084789209,0.107234038096869,0.537345839296547
"3122","PTPRO",6.39692484301489,1.43170514773472,0.888889097455186,1.61066791327914,0.107252122842407,0.537345839296547
"3123","F7",4.60883801944844,1.43530494843912,0.891186688808044,1.61055474286631,0.10727680471285,0.537345839296547
"3124","TRIP12",275.267148792631,0.294277931078695,0.18274264037714,1.61034080754974,0.107323475169399,0.537407529473401
"3125","C12orf29",135.181587865304,-0.361897778045916,0.224855963936889,-1.60946488458492,0.107514727423487,0.538192921947395
"3126","PUSL1",49.7440930988723,-0.489204231489757,0.304213676968874,-1.60809414081606,0.107814562260551,0.538392705965124
"3127","RNASEH1",190.594280792195,-0.357901060302155,0.222505257232045,-1.60850608544907,0.107724384486793,0.538392705965124
"3128","COX17",326.030218335436,-0.39506716135032,0.245673003109341,-1.60810164873708,0.107812918185698,0.538392705965124
"3129","PLXNA1",189.215423376273,0.288102165645899,0.179136419009372,1.60828360441226,0.107773079836266,0.538392705965124
"3130","XAF1",286.42933228867,0.518477787028146,0.32229646662912,1.608

69832812899,0.1076823215643,0.538392705965124
"3131","SNAPC2",152.947685102794,-0.4515630910575,0.280802771446495,-1
.60811479434968,0.107810039624223,0.538392705965124
"3132","ZNF234",66.6275534230576,0.384864264770254,0.239302722670124,1
.60827365637952,0.107775257610559,0.538392705965124
"3133","STK26",18.8199177812564,-0.719365018509452,0.447359698610894,-
1.60802374631235,0.107829978123681,0.538392705965124
"3134","UQCRHL",2.86582952123903,1.6077628991802,1.00029236559771,1.60
729298200682,0.107990113082928,0.538657628014856
"3135","SMARCA2",493.027879528316,0.338400914614381,0.210548757567485,
1.60723301587717,0.108003262025868,0.538657628014856
"3136","CCNY",316.317236646288,-0.24127828025462,0.150186405986783,-1.
60652542864534,0.108158512407765,0.538657628014856
"3137","AC020656.1",4.37247039800202,-1.3986313062573,0.87003010411883
1,-1.60756656538206,0.107930139760658,0.538657628014856
"3138","DNAJC3",536.542495045296,-0.27033162281789,0.168214543955285,-
1.60706450501539,0.108040218664238,0.538657628014856
"3139","VASH1",25.4483636589685,0.656371307584476,0.408517289462932,1.
60671610361312,0.108116659396597,0.538657628014856
"3140","MIR762HG",26.7116832516708,-0.50079073499067,0.311697436339296
,-1.60665657334935,0.108129724867881,0.538657628014856
"3141","KSR1",44.546574708825,0.54523210815245,0.339340372861282,1.606
74105340049,0.108111183885707,0.538657628014856
"3142","EN1",103.818549781634,0.601290427581582,0.374420573639263,1.60
592251044649,0.108290936882599,0.538973950255963
"3143","ST6GALNAC6",358.620858931371,-0.3306295060988,0.20587877320297
5,-1.60594266691512,0.108286507659623,0.538973950255963
"3144","CETN3",188.594794186742,0.380231864519477,0.236866927648614,1.
60525518819386,0.108437656583029,0.539532526058626
"3145","CMTR1",74.3097708934902,0.400740370398929,0.249694405028877,1.
60492330756304,0.108510683512231,0.539552645321626
"3146","CHEK1",19.5805152462983,-0.550968152045391,0.343268048240424,-
1.60506681256711,0.108479101933993,0.539552645321626
"3147","SMPDL3A",163.496967987182,0.578526771108567,0.360511338784569,
1.60473945995435,0.108551154038602,0.539582364990738
"3148","BCL9",43.302315344368,0.532329623825841,0.331838961573371,1.60
418059802824,0.108674250109728,0.539851157340894
"3149","NPR2",119.704869984445,0.339318626248851,0.211501575431138,1.6
043314361,0.108641015331865,0.539851157340894
"3150","ITGAX",9.99859567674272,-0.901056002926444,0.56177721354083,-1
.60393832502954,0.108727647958356,0.539944951432559
"3151","AC025031.4",4.11930409222052,1.45889712016917,0.90967436475412
7,1.60375753862592,0.108767507459489,0.539971475464545
"3152","CPT1B",4.40419087548573,-1.65380691074906,1.03131807415402,-1.
60358569503949,0.108805405977315,0.539988250540333
"3153","AC005332.6",98.5362409882954,0.404631991691172,0.2523748357234
24,1.60329769222557,0.108868945763957,0.540132229173988
"3154","SIX1",244.190689344716,-0.367426481272265,0.229196300229477,-1
.60310825656605,0.108910755456865,0.54016834103099
"3155","UBXN7",242.487224593293,0.368618466705392,0.230243298648216,1.

60099542036442,0.109377933478394,0.540600004234603
"3156","CRIP3",3.66990246616562,1.26755225084592,0.791567518713094,1.60131918109357,0.109306242776675,0.540600004234603
"3157","IPCEF1",3.69884762367526,1.40358881117279,0.876659544684955,1.60106488280715,0.109362549200243,0.540600004234603
"3158","HSPA14.1",70.3011595645939,-0.394168936309148,0.246035064378018,-1.60208439112374,0.109136949136144,0.540600004234603
"3159","AC012531.1",8.15790729514843,1.14973247501772,0.717869547099974,1.60158970339719,0.109246369214898,0.540600004234603
"3160","RPUSD2",38.450541438961,-0.478986238433072,0.298962515608514,-1.60216152000907,0.109119896794684,0.540600004234603
"3161","MTFMT",46.334966716194,-0.395854533888149,0.247227432548797,-1.60117560501712,0.109338030402405,0.540600004234603
"3162","STAP2",19.4408689133231,-0.780306791541462,0.487306551344852,-1.60126472625496,0.109318298173103,0.540600004234603
"3163","KXD1",247.100424894024,-0.323184850718785,0.2018465885004,-1.60114101070449,0.109345690647481,0.540600004234603
"3164","MCM3AP",104.697975811653,0.388980578588829,0.242941620050517,1.60112778744105,0.109348618796901,0.540600004234603
"3165","PHF8",44.3332642889213,0.466727178280955,0.291361652241848,1.60188265919615,0.109181559792734,0.540600004234603
"3166","MECR",106.900078393444,-0.300303044562269,0.187605832271959,-1.60071273331706,0.10944055958383,0.540738684008166
"3167","SLC2A12",17.8003605327447,0.5890511072869,0.36813188908204,1.60010888694195,0.109574429842257,0.541059254117459
"3168","TAF9B",51.3558383081875,0.373112035854887,0.233179275820053,1.60010804795029,0.109574615933268,0.541059254117459
"3169","AL020997.2",8.51844371783752,-1.10065603864884,0.68814253127088,-1.59945939777348,0.109718563375336,0.541166878986128
"3170","FAM200A",59.3011861331829,0.464632265423039,0.290440189861961,1.59975196836177,0.109653618070779,0.541166878986128
"3171","HOXC12",3.39305671655444,1.78579538324827,1.11665912773875,1.59923054304363,0.109769386116665,0.541166878986128
"3172","LRRC4B",7.54649027492409,1.08550159989657,0.678753879009521,1.59925656923036,0.109763605433419,0.541166878986128
"3173","PRPF31",271.065615873465,-0.336866697360508,0.210591304580281,-1.59962301402662,0.109682239822095,0.541166878986128
"3174","SH3GL1",299.339931437458,-0.369929880201421,0.231364346774964,-1.59890616405661,0.109841454209057,0.541351565277969
"3175","PKNOX1",76.9061147673067,-0.318960403507851,0.199518447354421,-1.59865119109139,0.109898128435223,0.541460290743997
"3176","ZNF593",162.481619333569,-0.433610631700666,0.271686345857999,-1.59599714270256,0.110489431273435,0.541528888503183
"3177","TAF1A",34.7048302774231,0.451296335671292,0.282853442537643,1.59551296820873,0.110597572381464,0.541528888503183
"3178","AC108488.1",46.5487478869755,-0.422651009054696,0.264829184699188,-1.59593818760864,0.110502594515954,0.541528888503183
"3179","AC097534.2",48.2185608292742,-0.506982811148843,0.317298023868409,-1.59781269661831,0.110084668121132,0.541528888503183
"3180","RAD17",118.830025659297,0.271876773024464,0.170358802031953,1.

59590681421597,0.110509599938505,0.541528888503183
"3181","DPYSL3",469.321025328549,-0.402627912658407,0.252240551277332,
-1.59620612395398,0.110442780804472,0.541528888503183
"3182","GM2A",98.4576622094647,0.373903653699856,0.234336115086687,1.5
9558697796769,0.110581036779491,0.541528888503183
"3183","AL133351.1",3.88947167751816,-1.30108661494403,0.8148363816653
66,-1.59674585502044,0.110322369687282,0.541528888503183
"3184","YTHDF3",175.715348534924,0.26791576057303,0.167695322785716,1.
59763406708354,0.110124440102154,0.541528888503183
"3185","AC027031.2",10.0807734784191,-0.822537077189473,0.514703254336
747,-1.59808019525621,0.110025130600998,0.541528888503183
"3186","RC3H2",136.50052656794,0.33801249687431,0.211817252032803,1.59
57741573475,0.11053922501195,0.541528888503183
"3187","DDX31",21.8221780872988,0.509872806251628,0.319305316067499,1.
59681903367949,0.110306051905896,0.541528888503183
"3188","CACNA1C",56.2702948247356,0.654079135983932,0.409956982932023,
1.59548236331027,0.110604410839843,0.541528888503183
"3189","EMP1",2372.6357959838,-0.394937434161215,0.24731296386241,-1.5
9691359479616,0.110284968969791,0.541528888503183
"3190","FAM155A",7.64915761757832,-1.0156781353783,0.635940233600487,-
1.59712828614076,0.110237114137633,0.541528888503183
"3191","ERVK13-1",44.941316915029,0.670315542415607,0.419390013667688,
1.59831069069457,0.109973849340865,0.541528888503183
"3192","AC090617.10",4.47719686248964,-1.11029467405207,0.695402509958
784,-1.59662160856721,0.110350079214524,0.541528888503183
"3193","AP001033.3",3.13133564256911,-1.7775294724678,1.11388274792381
,-1.59579585533664,0.110534378964839,0.541528888503183
"3194","C2CD2",91.8204483143402,0.387908138373456,0.24305543055217,1.5
9596573296968,0.110496444150793,0.541528888503183
"3195","ASMTL-
AS1",2.77480378806839,2.1480356546276,1.34530762566202,1.5966873402435
1,0.110335419007221,0.541528888503183
"3196","ZFAS1",2196.04160869905,-0.432086464143678,0.270864037608782,-
1.59521532632455,0.110664092624292,0.541651564743991
"3197","ZMYND12",3.72293281581008,1.54345632230705,0.967753310997514,1
.59488611898019,0.110737704254229,0.541842323318392
"3198","ANKRD53",12.0244836008197,-0.676948335017238,0.424567747372146
,-1.59444126221833,0.11083723681792,0.542159754703791
"3199","HDAC1",79.7500621103277,-0.409664273970676,0.257242141222129,-
1.59252396214869,0.111267023383822,0.542728973414559
"3200","AKR1A1",598.871097344017,-0.390199652832024,0.245054588067361,
-1.59229686703423,0.111318016575256,0.542728973414559
"3201","ACOT11",6.37644518508625,-0.970376036179838,0.609177502821639,
-1.59292822155311,0.111176294367794,0.542728973414559
"3202","DPT",4037.00681328422,-0.415182433734895,0.260738515518085,-1.
5923325823572,0.111309995637296,0.542728973414559
"3203","BATF3",7.62553274472706,-0.938912095671733,0.589564847689305,-
1.59255101343242,0.11126095036823,0.542728973414559
"3204","AAK1",285.73038327317,0.348625225544851,0.218955416290156,1.59
222014897708,0.111335247438939,0.542728973414559

"3205","AC009404.1",20.2283097192292,0.529719140838259,0.332494868538131,1.59316486046012,0.111123211969546,0.542728973414559
"3206","FIS1",1127.39870989989,-0.459918724145663,0.288651588301307,-1.59333515832097,0.111085023452119,0.542728973414559
"3207","MAPK8IP1",39.7936168445341,-0.520880115458373,0.327059465730903,-1.59261593085014,0.111246377469456,0.542728973414559
"3208","AC068338.3",6.99670875167926,-1.18661444895165,0.744668205495019,-1.59348074779539,0.111052383892486,0.542728973414559
"3209","HMOX1",63.9586370917352,0.582566744745303,0.365625716474093,1.5933418206008,0.111083529676901,0.542728973414559
"3210","ERN1",63.750097659502,-0.411224609483713,0.258307497837918,-1.59199641096654,0.111385510982514,0.542804843707001
"3211","MGST3",2434.23600159581,-0.422277167588921,0.265453687142583,-1.59077529543638,0.111660154612033,0.543296982455998
"3212","PGF",14.7360793169556,-0.709496073683333,0.445965045497213,-1.59092305741654,0.111626892770679,0.543296982455998
"3213","AC027796.1",14.5487182377165,0.910976114074138,0.572448339449092,1.5913682533359,0.111526724561445,0.543296982455998
"3214","EXOSC5",73.2553993714707,-0.499416535924867,0.313905343206772,-1.5909781299769,0.111614497706457,0.543296982455998
"3215","ATRN",117.282095021503,0.318651651320538,0.200259215359792,1.59119594445648,0.111565485304867,0.543296982455998
"3216","DGKD",34.2190147164321,0.55692109490547,0.350203562594396,1.59027821070596,0.11177210787422,0.543334705865887
"3217","GALNT11",370.570933985599,0.272166367161271,0.171135060650818,1.59036007073148,0.11175366529746,0.543334705865887
"3218","CTTN",736.406950161805,-0.267772944724183,0.168359364167929,-1.59048441438098,0.111725656006003,0.543334705865887
"3219","CCNL2",342.918045933541,0.408678969410585,0.257047007385538,1.58989973688983,0.111857406988998,0.543411473086597
"3220","COL16A1",507.603391502648,0.356275001928826,0.224260696443585,1.58866447656133,0.112136163019364,0.543411473086597
"3221","SLC39A1",568.60969806921,-0.32533812736714,0.204666940595247,-1.58959784331039,0.111925483525882,0.543411473086597
"3222","GSK3B",146.650067902298,0.300898156675749,0.189344140681353,1.58916011656325,0.112024248273867,0.543411473086597
"3223","SLC1A3",128.532319264104,0.765906598510515,0.482212665005037,1.58831705198476,0.112214663503846,0.543411473086597
"3224","PELO",68.924234263917,-0.376338827667989,0.236692660820602,-1.58998942494981,0.111837188764262,0.543411473086597
"3225","CCNJ",13.0262678041159,0.662285841053338,0.416950303379767,1.58840474676454,0.112194844805589,0.543411473086597
"3226","AP000662.1",9.31020670218208,0.932900625662738,0.587173892019566,1.58879786438402,0.1121060355871,0.543411473086597
"3227","C1R",10589.1178331427,0.37130940124991,0.23370491420882,1.58879586467805,0.112106487200427,0.543411473086597
"3228","GPALPP1",156.188958038062,0.298060425363344,0.187573548202464,1.58903229277095,0.112053102254705,0.543411473086597
"3229","ZFP30",31.1363821642,0.557768011457817,0.351080728445171,1.5887172557947,0.112124241356911,0.543411473086597

"3230","CCDC8",136.240953328698,0.376891620580075,0.237258634695007,1.58852646633731,0.112167341203757,0.543411473086597
"3231","ZNF674-AS1",17.8768689802325,-0.628625247481057,0.3958080882291,-1.58820717962994,0.112239498148871,0.543411473086597
"3232","NKD1",4.36682320487086,1.67026871430102,1.05179841229039,1.58801220346383,0.112283579630634,0.543456694357056
"3233","LRP1",5267.22078543656,0.304510584170408,0.19180196117704,1.587630190545,0.112369987176356,0.543538561966523
"3234","GMFG",38.0360844949202,-0.689553459819087,0.43432316897707,-1.58765064604576,0.112365359016126,0.543538561966523
"3235","MN1",90.1472579100352,0.522490349940536,0.329176203708895,1.58726646717937,0.112452306522735,0.543768603071142
"3236","PHOSPHO2",37.3797020034237,-0.507577037545237,0.319996544569171,-1.58619537041757,0.112694997668318,0.544437260199351
"3237","RAB33B",79.5310035039536,-0.300024294130844,0.189123166039118,-1.58639631735431,0.112649435292937,0.544437260199351
"3238","AC004241.1",11.6656293319236,0.712137409926103,0.448924236934994,1.58631980930275,0.1126667808896,0.544437260199351
"3239","OSR1",235.013905388791,-0.5772611944733,0.364042983797688,-1.58569515185082,0.112808479484462,0.5444810381288
"3240","ICAM2",78.5464234278132,-0.404055473654467,0.25479675323059,-1.58579522121618,0.112785770097789,0.5444810381288
"3241","ZGPAT",96.7669739529365,-0.343935238076824,0.216863330746475,-1.58595386731795,0.112749774899081,0.5444810381288
"3242","SALL2",22.0717511191558,0.529727353948984,0.334180327479899,1.58515421282794,0.112931300669169,0.544832355204936
"3243","AC011043.1",27.8036130385498,0.592857878747455,0.374026821034736,1.58506782242869,0.112950925521294,0.544832355204936
"3244","AC116407.2",17.2472086072441,-0.647634396714307,0.408665995529376,-1.58475234983859,0.113022612596096,0.545010089038449
"3245","FBXW5",303.891826051669,-0.355080050124401,0.224093355803009,-1.58451842024507,0.113075793223673,0.545098500276708
"3246","RNF19B",45.4356038797918,-0.522723534847435,0.329942334527818,-1.58428755617404,0.113128296277519,0.545183591703398
"3247","ITGA2",7.58049752609927,-1.14169056953648,0.720735807480207,-1.58406250624343,0.113179495568156,0.545262349606608
"3248","AC009275.1",12.6453808739749,0.888721475101437,0.561248449816552,1.58347248066685,0.113313814198893,0.545472026909164
"3249","LINC02550",12.687819324375,1.07367519512144,0.677964380181094,1.5836749341236,0.11326771176136,0.545472026909164
"3250","KDM6B",108.75307094992,-0.490071320323081,0.309503384255062,-1.58341183086778,0.113327628169455,0.545472026909164
"3251","AC132938.6",3.43542031602323,-1.53986157131727,0.972625277169912,-1.58320126719087,0.113375597744013,0.545535058600307
"3252","LINC00857",7.16157254889145,0.941655363819052,0.594843425719522,1.58303063143049,0.113414482870427,0.545554352872724
"3253","FAM136A",176.132951402153,-0.28222533035102,0.178401661260657,-1.58196582003051,0.113657373528293,0.546386691488349
"3254","ZCCHC4",33.290305665666,0.446657910932217,0.282319133499226,1.

5821028684669,0.113626088904351,0.546386691488349
"3255","ILKAP",121.719766054594,-0.319558611138367,0.202038090044015,-1.58167507457999,0.113723765678702,0.546537900618106
"3256","DNAJB4",148.10884665619,0.263025928219816,0.166338227483182,1.5812716787932,0.11381593227955,0.546792918688694
"3257","NR2F2",610.878048529796,0.409166876731583,0.258792028533876,1.58106445182883,0.113863301708584,0.546792918688694
"3258","LINC00629",4.88946753144785,1.42650848446981,0.902291530736086,1.58098401223612,0.113881693350877,0.546792918688694
"3259","LRRC8C",29.5198147023468,0.775728047864325,0.490836435127861,1.58042067040572,0.114010560925518,0.547075829618983
"3260","MRPL41",526.689657981013,-0.396746141165806,0.25101680350476,-1.58055610471624,0.113979569095979,0.547075829618983
"3261","SFRP1",2168.14573750538,1.07909897520889,0.683183876071431,1.57951470022116,0.114218047399484,0.547137091037608
"3262","DEAF1",132.11802867434,-0.380158554116717,0.24060604755885,-1.58000415190617,0.114105915652254,0.547137091037608
"3263","GALNS",89.9141675192428,0.45746424968891,0.289590648122917,1.57969275822311,0.114177244871921,0.547137091037608
"3264","NDUFS7",479.091168495997,-0.465716190538112,0.29485997630705,-1.57944864667947,0.114233186685983,0.547137091037608
"3265","SELENOW",1435.96776881116,-0.442745988647364,0.280235359944186,-1.57990764882613,0.114128017323987,0.547137091037608
"3266","ZNF880",50.5922967221177,0.455583623643871,0.288371406081413,1.57985020024923,0.114141176116567,0.547137091037608
"3267","SRP9",845.447448928405,-0.362599882473725,0.2297093549812,-1.57851595771274,0.114447125043049,0.547180513713056
"3268","SNRNP27",273.599522972005,0.299701478506843,0.189875231289991,1.57841271065586,0.114470827022066,0.547180513713056
"3269","ZNF502",30.6107933234631,0.489537337656525,0.310368789730074,1.57727630436769,0.114731962218169,0.547180513713056
"3270","AC007036.1",3.00086524100905,1.53417750303708,0.972430040313376,1.57767390910988,0.1146405432302,0.547180513713056
"3271","GTF3C5",102.436805981457,-0.278738213600012,0.176698973823875,-1.57747499924841,0.114686270271454,0.547180513713056
"3272","FAM149B1",65.9556636449247,0.486863944428684,0.308450288868867,1.57841947956699,0.114469272994177,0.547180513713056
"3273","CPT1A",159.889324715292,0.375520238996816,0.238049847442966,1.57748573683412,0.114683801460201,0.547180513713056
"3274","POC1B-AS1",34.6668365031582,0.510816056507052,0.323640838757441,1.57834239482333,0.114486971334,0.547180513713056
"3275","MZT1",119.49912088178,-0.385623627594724,0.244225105480116,-1.57896800509774,0.114343395958243,0.547180513713056
"3276","AC011330.2",3.8643347227714,1.27760061955903,0.809940067543324,1.57740142852075,0.114703186953071,0.547180513713056
"3277","SGF29",109.378521375921,-0.447193456036752,0.283448597223273,-1.57768801968879,0.114637299918854,0.547180513713056
"3278","HSD3B7",231.445084611912,-0.595781209010875,0.377517315117989,-1.5781559815996,0.114529779943632,0.547180513713056

"3279","ATP6V0D1",304.836640804046,-0.448901997525776,0.284300898725134,-1.57896791581999,0.11434341643706,0.547180513713056
"3280","LINC01002",9.4514883855197,0.923412912929207,0.585301817083435,1.57766965004582,0.114641522188685,0.547180513713056
"3281","ZNF644",327.429173093416,0.206198566602635,0.130761507050496,1.57690570607302,0.114817223406485,0.547301950490933
"3282","MTR",244.804867138961,0.402568709068562,0.255424817841591,1.57607515381777,0.11500848431317,0.547301950490933
"3283","ZNF445",98.9510980905911,0.451873950970628,0.286580352104914,1.57677924411651,0.114846329107805,0.547301950490933
"3284","NIT2",237.669608058603,-0.427562118922723,0.271244115388499,-1.57630007312908,0.114956664805436,0.547301950490933
"3285","ARL9",4.23770132378387,-1.07126380349218,0.679452397479538,-1.57665762526717,0.114874325624695,0.547301950490933
"3286","CEP120",73.1368395240688,0.544735264114446,0.345721681934162,1.57564680660724,0.115107222705464,0.547301950490933
"3287","CSF2",3.5695806354045,-1.87854198769258,1.19223569272035,-1.57564649268826,0.115107295091426,0.547301950490933
"3288","PRMT7",78.9180297227122,0.340901212161783,0.216233836511262,1.57653962794129,0.114901493602568,0.547301950490933
"3289","ULK2",100.627816550363,0.365646891279685,0.231997463873856,1.57608141560762,0.115007041401117,0.547301950490933
"3290","PRR22",3.25037218331677,1.49216040572992,0.946849588683198,1.5759212694015,0.115043948566624,0.547301950490933
"3291","YIPF4",474.718717574249,0.231311965757888,0.146842898695592,1.57523426609415,0.115202380511589,0.547309279419507
"3292","IL7R",30.3776694154422,0.754380992220354,0.478921609710443,1.57516590799997,0.115218154163844,0.547309279419507
"3293","MACORIS",6.10333273411051,-0.985635138829606,0.625654132407291,-1.57536742391078,0.115171659178926,0.547309279419507
"3294","TRIM34",70.0336304970992,0.396480717524835,0.251752712505057,1.57488161132274,0.115283773936411,0.547309279419507
"3295","SLC2A10",191.373567611629,0.323026782716339,0.205095321695007,1.57500805014317,0.115254586405437,0.547309279419507
"3296","LINC00294",49.4881124534875,0.596805125192385,0.379140517028831,1.5741000984788,0.115464309817213,0.547841597328493
"3297","AC091057.6",4.51289745422254,1.25707322783595,0.798613380890881,1.57406982892478,0.115471306801921,0.547841597328493
"3298","AC009271.2",5.16033815674329,1.53995987846035,0.978409830531493,1.57394154310961,0.115500964520192,0.547841597328493
"3299","HOXA-
AS2",3.23352580581026,1.48742788491743,0.945271385453992,1.5735458703249,0.115592475741152,0.547943363036011
"3300","FHOD3",5.48818496884005,1.15506688539329,0.734035446043887,1.57358461586367,0.115583512154158,0.547943363036011
"3301","ITPKB",42.2229032359867,0.441063824591577,0.280448343402789,1.57270968064913,0.115786057251051,0.548030648586443
"3302","LINC02511",322.675960990804,-0.327073848757857,0.20790668581791,-1.57317619426784,0.115678025936818,0.548030648586443
"3303","PCDHB5",26.8304762076256,0.633789130406962,0.402905685349462,1

.57304588506177,0.115708193871998,0.548030648586443
"3304","ARL10",55.9275646864932,0.490283798723345,0.311689622267581,1.
57298724017973,0.115721772789454,0.548030648586443
"3305","ZNF676",29.1404804954019,0.664911704450665,0.422744814777995,1
.5728441395546,0.115754912252854,0.548030648586443
"3306","PTPN23",122.847689642589,0.285016783980705,0.181283734111419,1
.57221377515056,0.115900981832654,0.548110279747729
"3307","ZNF317",36.5779381678054,0.471929532912615,0.300174584290865,1
.57218351456205,0.115907997532794,0.548110279747729
"3308","ZNF546",36.6861188567278,0.494734761162072,0.314658864976292,1
.5722892828694,0.115883477365756,0.548110279747729
"3309","RPL14",11451.3591432003,-0.429495439469896,0.273216312945669,-
1.57199778753805,0.115951064320996,0.548148231844468
"3310","PCGF6",65.9444232762223,-0.390990246391548,0.248853202224066,-
1.57116823451402,0.116143576446177,0.548726658516324
"3311","TBXA2R",30.1845582098679,-0.535600597493331,0.340885609705638,
-1.57120330763107,0.116135432040882,0.548726658516324
"3312","NIFK-
AS1",81.330676706362,0.326028088983772,0.207550735988587,1.57083561969
012,0.116220835975274,0.548739896700668
"3313","RPS15",12890.8318907679,-0.403172080677348,0.256638204036741,-
1.57097452497614,0.116188566247158,0.548739896700668
"3314","ADRM1",271.567767674045,-0.440973776982032,0.280749277591856,-
1.57070315822185,0.116251615269834,0.548739896700668
"3315","SRPX2",557.39463603818,0.325211449781324,0.207083501518626,1.5
7043630900781,0.116313640904695,0.548867054199741
"3316","LYRM4",213.582992684998,-0.366585784362075,0.233499182682906,-
1.5699660279321,0.116423014921508,0.549217497713256
"3317","BX284613.2",3.67133790983364,1.39397089101715,0.88835386574294
9,1.56916173247171,0.116610258436613,0.549769220230239
"3318","C5AR2",7.25580081649473,-1.07136790516345,0.682712885758363,-1
.56928033366965,0.116582632697627,0.549769220230239
"3319","FAT3",2.89978320683028,1.74382598226003,1.11169792083562,1.568
61495337625,0.116737686019096,0.550204164626913
"3320","DNAH1",7.08180959389573,1.13482803137159,0.723619367444731,1.5
6826652578266,0.116818944558877,0.550421310160998
"3321","FZD5",10.7091034808225,-0.807304257261207,0.514883555295187,-1
.56793560205739,0.116896162057099,0.55055467338986
"3322","ABHD16A",170.627502643527,-0.300267188137892,0.191550318785849
,-1.5675629779222,0.116983157868161,0.55055467338986
"3323","ITPRIP",33.9466957960654,-0.477567984327877,0.3046702073292,-1
.56749157889225,0.11699983305917,0.55055467338986
"3324","USP8",451.467319184244,0.242520487094402,0.15472873018273,1.56
739143924979,0.11702322374361,0.55055467338986
"3325","MIDN",241.077145088059,-0.498597009342855,0.318043476600911,-1
.567700789438,0.116950977367127,0.55055467338986
"3326","P2RY13",3.15827126024956,-1.58119411463085,1.00914536026784,-1
.56686457361423,0.117146349854877,0.550968235351728
"3327","CR1",4.10725215974708,-1.17456477299971,0.749793160371792,-1.5
6651838810758,0.117227307193665,0.551017658182244

"3328","RAB37",3.09123396671572,1.44042859426947,0.919431893729249,1.5666506721091,0.117196366686359,0.551017658182244
"3329","KIAA2026",264.252659429466,0.37817040453631,0.241471701894904,1.56610651090247,0.117323684096133,0.551305013612439
"3330","SPATS2L",441.83606040933,-0.248127158031852,0.158452998030518,-1.56593539482329,0.117363742560196,0.551327635095839
"3331","RNPEPL1",150.546896353515,-0.296810371357222,0.189565056549709,-1.56574411317937,0.117408534513043,0.551372472346904
"3332","COLGALT1",197.331164150939,0.273970747541513,0.175087510813927,1.56476464979089,0.11763810334935,0.552284769115809
"3333","FOS",14631.5105086137,-0.604492743507578,0.386416595997836,-1.56435502452116,0.117734216647145,0.552570162319619
"3334","CAPZA2",448.332460689796,-0.286944501269249,0.183449800713887,-1.56415815199916,0.117780432170063,0.552621265877713
"3335","AC007405.3",59.0954745021299,-0.541165308321738,0.346158417495829,-1.56334580056329,0.117971280993586,0.552687855220926
"3336","LINC01473",36.9500149855741,0.470305654512057,0.300808797982841,1.5634704093292,0.117941990437292,0.552687855220926
"3337","STAG1",358.147056105493,0.36298930792741,0.232168071660564,1.56347643037717,0.11794057527332,0.552687855220926
"3338","NAT14",55.268347150925,-0.429076907071205,0.274415366463519,-1.56360379012612,0.117910644248525,0.552687855220926
"3339","PCMTD2",307.627716442806,0.341128020737062,0.218149662998321,1.5637338882329,0.117880075831197,0.552687855220926
"3340","PTPN13",239.305822387753,0.350561580280387,0.224378874168645,1.56236446759646,0.118202152761212,0.552776166111699
"3341","SPACA9",45.5967741914721,0.417553920693993,0.267174202657542,1.56285268764966,0.118087248173589,0.552776166111699
"3342","MACROD1",126.394007469504,-0.379902113354858,0.243140008637615,-1.5624829310633,0.118174263844583,0.552776166111699
"3343","FIBP",325.753923666572,-0.491914215855052,0.314838030667392,-1.56243581759261,0.118185354777957,0.552776166111699
"3344","BRMS1",136.605981518621,-0.429600943982155,0.274911342034329,-1.56268905023391,0.118125751142589,0.552776166111699
"3345","REM1",46.4862772007768,-0.660389629001172,0.42267190328729,-1.56241667322823,0.118189861766245,0.552776166111699
"3346","PDXK",196.791922726173,-0.232918492318179,0.149139617514433,-1.56174795269029,0.118347377340855,0.553289905482067
"3347","MED27",67.1039579196606,-0.451942377763942,0.289451422478557,-1.5613755631048,0.118435164186964,0.553369555966751
"3348","AC005498.3",5.9734582572853,-0.994810386675616,0.63708207311695,-1.56151056300873,0.11840333351043,0.553369555966751
"3349","NGFR",55.8133203324933,0.848494318740027,0.543530954119837,1.56107819123941,0.118505303075574,0.553531936700865
"3350","GPC4",252.892738350886,0.397097494008813,0.254473065204962,1.56046964612532,0.118648937608315,0.554037412240858
"3351","THNSL1",23.5506512769271,0.507405926479471,0.325300175076834,1.55980834120247,0.118805179731006,0.55460144032591
"3352","CDK2AP2",74.5020491053315,-0.411351621494498,0.263796766337034,-1.55935050761367,0.118913443488001,0.554941228067663

"3353","NOG",3.39642593041463,-1.38832478695198,0.890792338739643,-1.5
585279829823,0.119108139870347,0.555647912204155
"3354","CD320",144.837944796431,-0.495317597796084,0.317835078338952,-
1.55841073422317,0.119135913669548,0.555647912204155
"3355","GCNA",10.4015991161536,0.793667529686517,0.509362315357286,1.5
5815910552748,0.119195536423355,0.55576029098973
"3356","SNRPG",492.039245043373,-0.347200117474112,0.222916153344023,-
1.55753682389398,0.119343084830659,0.555953243227181
"3357","CHUK",35.1949549596761,0.457594154052956,0.293737480454124,1.5
5783372740008,0.119272668575452,0.555953243227181
"3358","SIX5",111.592001541527,0.330902610601386,0.212452773412624,1.5
5753490663409,0.119343539650762,0.555953243227181
"3359","MRPL9",218.396487885142,-0.346669167538337,0.222636257095845,-
1.55711011342189,0.119444344297075,0.556257183042318
"3360","ZNF616",48.235696696151,0.535635125051477,0.344072026844457,1.
55675289840874,0.119529164069083,0.556320950173361
"3361","CDC42EP1",201.458127615086,-0.299989355895564,0.19269428006951
1,-1.55681505329243,0.119514402164201,0.556320950173361
"3362","ANXA3",12.8734676126574,1.28394137409817,0.824956765847329,1.5
5637413650327,0.119619151630458,0.55657417874933
"3363","HTR2A",11.8115414272399,-0.919312638984707,0.590860906851893,-
1.55588672109449,0.119735031547921,0.556947695065158
"3364","ECM1",1442.09879676047,-0.540504040390344,0.347559460982985,-1
.55514120910898,0.119912442364996,0.557554874684668
"3365","USP5",99.323244669061,-0.392493369964376,0.252401028773388,-1.
55503870912018,0.119936850560245,0.557554874684668
"3366","MAML3",44.6508584883011,0.431318227223195,0.277444995331779,1.
55460806459099,0.120039441925275,0.557700323741337
"3367","PLCXD1",15.5011886252377,0.941828214914626,0.605826932044588,1
.55461595564277,0.120037561441958,0.557700323741337
"3368","SEC61G",968.474093704082,-0.41059960503782,0.264235767679442,-
1.55391379692374,0.12020498024865,0.557751207444701
"3369","GDI2",828.14768776553,-0.26444762913805,0.170199085220108,-1.5
537547031822,0.12024293906563,0.557751207444701
"3370","SCD",5.28471855506389,1.12204789218186,0.722043518787007,1.553
98928594615,0.120186972300969,0.557751207444701
"3371","UBE4A",185.00962293004,0.300644287091717,0.19344640192364,1.55
414773343984,0.120149181434305,0.557751207444701
"3372","SLC39A6",305.271812479031,0.30785912665149,0.19815025030434,1.
55366509090272,0.120264324152079,0.557751207444701
"3373","APBA3",44.3621824232975,-0.431033160750524,0.27735010786287,-1
.55411210787644,0.120157677576452,0.557751207444701
"3374","ZNF783",53.0667437962986,0.48785619349624,0.314068811435444,1.
55334173828501,0.120341513822667,0.557943776149371
"3375","STARD13",36.0523772024713,0.561523708668579,0.361552242326511,
1.5530914842494,0.120401280261188,0.558055474703932
"3376","WASHC2C",157.750297148468,0.315817330722103,0.20338834732138,1
.55277986611037,0.12047573434214,0.558235163600147
"3377","GDPD5",91.0771569039152,0.508090374908677,0.32726499572632,1.5
5253504512769,0.120534254034793,0.5583409345177

"3378","TEAD3",40.271706833641,0.450551174985856,0.290282708263462,1.5
521116558446,0.120635509513225,0.558644545682471
"3379","IL1RL1",3.51990668881657,-1.37011752065892,0.883113704957736,-
1.55146218767434,0.120790962161915,0.558662831216834
"3380","SYPL1",707.834136311834,-0.368101635372111,0.237278388199527,-
1.55134919014443,0.12081802454814,0.558662831216834
"3381","GOLGA8Q",3.41126369201689,1.44882563521989,0.933741596338363,1
.55163445743598,0.120749713479108,0.558662831216834
"3382","BTBD3",32.6034594858753,0.473952559997834,0.305491741873743,1.
55144147953339,0.120795921310932,0.558662831216834
"3383","MIR503HG",21.000494413849,0.803765591075204,0.517981017824421,
1.55172788850663,0.120727346731623,0.558662831216834
"3384","CATSPERE",5.57717201807602,1.01869424887899,0.657166630894749,
1.55013082069004,0.121110119866881,0.559262925714958
"3385","NR1H3",156.636268841637,0.461911378061076,0.297995361035385,1.
5500623112258,0.121126560910457,0.559262925714958
"3386","NIN",179.839736648123,0.353094527730089,0.227776442025554,1.55
018018803927,0.121098273670179,0.559262925714958
"3387","POLDIP2",154.568885307724,-0.324022750086787,0.209033123396278
,-1.5501024183259,0.121116935713366,0.559262925714958
"3388","ZNF576",101.717338352932,-0.362647204805817,0.23392281770292,-
1.55028572401336,0.121072952282019,0.559262925714958
"3389","ZNF462",147.847454057301,0.426431079643234,0.275152179756179,1
.54980084119671,0.121189325080588,0.559373149313905
"3390","EIF3F",1488.61634061829,-0.422092648362442,0.272376711931494,-
1.54966496720396,0.121221950787837,0.559373149313905
"3391","COL8A1",233.209845934313,1.42811802956069,0.921895840362916,1.
54910996127121,0.121355288714991,0.55982329146818
"3392","HMCN1",502.804089757097,0.615951075856982,0.397714579297247,1.
54872641819004,0.121447500397236,0.55997262182045
"3393","HINT3",57.5489401106175,0.499512450355831,0.322541287546102,1.
54867754809354,0.121459253713277,0.55997262182045
"3394","PIGB",32.7772431403069,0.440586384035908,0.284533439594023,1.5
4845203665532,0.121513501005255,0.560057659465292
"3395","HMCN2",149.944933808417,-0.720255712587888,0.4653374399387,-1.
54781380299588,0.121667132271455,0.560600574410126
"3396","AEBP2",145.242769216969,0.328672173990187,0.212399975292679,1.
547420961501,0.121761769892741,0.56087142710016
"3397","GUCY1A1",11.4201969720672,1.57789436842006,1.02008476328365,1.
54682671990984,0.121905035246931,0.561284164283957
"3398","TEX10",45.8851576163472,0.396897479653443,0.256674667776656,1.
54630561360579,0.122030776879738,0.561284164283957
"3399","C17orf58",819.279508593573,0.681491144323873,0.440646972688414
,1.54656944575394,0.121967102184586,0.561284164283957
"3400","SLC25A19",35.5767509792498,-0.41068934423976,0.26558731289225,
-1.54634398671889,0.122021514087984,0.561284164283957
"3401","EIF3K",1654.99634777078,-0.475852938137946,0.307703214532096,-
1.54646723096976,0.121991768177482,0.561284164283957
"3402","TOR1AIP1",315.760223823961,0.227519534687525,0.147164068544082
,1.54602639719336,0.122098192796696,0.561391832698237

"3403","TMSB15A",5.31435902835439,-1.34489822434351,0.86997107503375,-1.54591142503368,0.122125960920034,0.561391832698237
"3404","F10",1403.68720696846,0.444689537412003,0.287742444746643,1.54544296655139,0.122239154238813,0.561747088647988
"3405","RALB",175.225304733661,-0.250251414544146,0.161975388009951,-1.5449965431092,0.122347099530149,0.562078025829699
"3406","ZBTB40",62.8120370136242,0.427311792992076,0.276608306469689,1.54482632299006,0.122388278405845,0.562102125397131
"3407","SENP1",41.1766133265111,0.374273947118319,0.24248708659585,1.54347991215762,0.122714377731536,0.563434402951108
"3408","TPP2",178.150465633319,0.325502300438823,0.210935472591274,1.54313684863021,0.122797575782094,0.563650961842518
"3409","COL14A1",2503.22046738665,0.532475369183572,0.345138403173271,1.54278794908908,0.122882234340356,0.563874095566499
"3410","RTEL1",11.8844426728194,0.651208709976915,0.422148765347671,1.54260479582499,0.122926693718054,0.56391268910015
"3411","COX10",34.9021880564488,0.429190311251105,0.278265299637009,1.54237812551897,0.122981733997045,0.563974518280832
"3412","CLTC",590.473250730315,0.278631720161874,0.18066545068428,1.54225237369149,0.12301227746431,0.563974518280832
"3413","INPP5B",55.8568666520416,0.477674141088135,0.309850646710584,1.54162705858189,0.123164246277548,0.564051965810246
"3414","FOXJ3",148.56015774242,0.364981447273837,0.236779361139809,1.54144113539664,0.12320945900234,0.564051965810246
"3415","CCDC96",9.66823553380286,-0.703310747588913,0.456187337731227,-1.54171475053804,0.123142925880255,0.564051965810246
"3416","EFR3A",216.631437657358,-0.271964188617401,0.176406385418811,-1.54169129406356,0.123148628530928,0.564051965810246
"3417","GPATCH2L",232.304854498374,0.397331593793987,0.257759173943959,1.54148381108784,0.123199079999219,0.564051965810246
"3418","CACNB4",172.930381263871,0.583649025908937,0.378699512691428,1.54119296790462,0.123269828466284,0.56416323191869
"3419","TXNL4B",37.4527994139832,0.565763190047185,0.367146847147481,1.54097248673886,0.123323482300789,0.564243706823998
"3420","RELL1",144.519518080562,0.322191440684466,0.209122686546992,1.54068143444621,0.123394337457132,0.564312139307799
"3421","DDAH2",1911.28191915655,-0.40716762893144,0.264289071674367,-1.54061470022913,0.123410588031195,0.564312139307799
"3422","AP000941.1",2.83947126931078,1.49206887488242,0.968658985049746,1.5403448457207,0.123476317847143,0.564447703121815
"3423","MSS51",11.1466609322942,0.775387593411464,0.503611985713882,1.53965277913776,0.123645012901461,0.565053735558738
"3424","ARL2BP",413.772709408357,-0.285041640972112,0.185185279518979,-1.53922407716484,0.123749601578589,0.56536653548302
"3425","PPP1CB",545.166171086047,-0.33719042526033,0.219259744307268,-1.53785833476024,0.124083256629739,0.565474055264266
"3426","PRKG2",35.9940518939878,0.883548133669154,0.57455900847764,1.53778484129979,0.124101231190335,0.565474055264266
"3427","SAP30L",211.615411383312,-0.247517030554956,0.160927666475677,-1.53806387661978,0.124032997300926,0.565474055264266

"3428","MTURN",754.981893701172,0.338130489455027,0.219884428397773,1.53776459715167,0.124106182731497,0.565474055264266
"3429","AC093799.1",3.02407808356833,1.54143646868832,1.00209686885816,1.53821104185737,0.123997022049289,0.565474055264266
"3430","FAM131B",5.11804876516726,1.1563324210848,0.75201357566175,1.53764833309992,0.124134622884197,0.565474055264266
"3431","FRMPD1",17.3430136543757,0.83924255802562,0.545506473783162,1.53846489154447,0.123934986406104,0.565474055264266
"3432","DNAJC4",223.624462380124,-0.482473400950195,0.313669593987894,-1.53815801785625,0.124009983083472,0.565474055264266
"3433","VRK3",105.145368662535,0.294382195792492,0.191395385224974,1.53808408414061,0.124028056992735,0.565474055264266
"3434","Z82215.1",4.9086658690032,-1.17529550550845,0.763876147630888,-1.53859432468674,0.123903364932235,0.565474055264266
"3435","EEPD1",40.4847506235488,-0.556986654339928,0.362350339750003,-1.53714952971815,0.124256696334831,0.56586535684593
"3436","AC108075.1",12.1088116716655,-1.06299886364904,0.691758024300773,-1.53666285942041,0.124375890669131,0.566078573679724
"3437","ZCCHC7",226.667669005045,-0.245550660234621,0.159781013111809,-1.53679498866861,0.124343521016007,0.566078573679724
"3438","ADCY9",87.6353037605113,0.330111720410972,0.214847497327994,1.53649320804985,0.124417462308633,0.566103072394982
"3439","INSYN1",22.8425342131245,-0.716094627683042,0.466143463039401,-1.53621081161126,0.124486685213091,0.566253334338002
"3440","UBR5",256.398998264847,0.342079618828857,0.22276163886932,1.53563073321405,0.12462897213828,0.566735759057882
"3441","CCDC14",240.208560793365,0.340485619506214,0.221754180024331,1.53541917211597,0.124680897329187,0.566739733259744
"3442","GPX4",6275.09406406133,-0.509410346408136,0.331791662524057,-1.53533196866031,0.124702305304612,0.566739733259744
"3443","PRAC2",9.37452768122089,-1.39603596978655,0.909462193233552,-1.53501264832462,0.124780721182486,0.566766789041123
"3444","EIF6",392.320239484258,-0.487456304870179,0.317533801404708,-1.53513201654049,0.124751403287439,0.566766789041123
"3445","ARHGAP19",46.634245519908,0.403539489598787,0.262927061745678,1.53479633066116,0.124833864413774,0.566829758395574
"3446","TRIR",1180.28353236671,-0.390903158775741,0.254716247713092,-1.53466126438878,0.124867055387787,0.566829758395574
"3447","PRRG3",232.744247244208,0.434989718975605,0.283504278987562,1.53433211142005,0.124947969739107,0.567032518314145
"3448","SMPD4",84.0776245458831,0.340360077157092,0.221888051974052,1.53392701467718,0.125047609153025,0.567237237744884
"3449","WDR46",190.502899260896,-0.382182784225251,0.249165052973058,-1.53385388386138,0.125065603335812,0.567237237744884
"3450","GSPT2",107.664358441202,0.291536623261026,0.190111696873596,1.53350176793629,0.12515227138302,0.567465791665098
"3451","NDUFAF2",324.875581352724,-0.378353129818375,0.246749500086711,-1.5333491240526,0.125189856926714,0.567471727587538
"3452","FLAD1",43.7605805730796,-0.416614762575262,0.271826704945783,-1.53264839324142,0.125362510977227,0.567570989590894

"3453","MPC2",528.949639160036,-0.401628739126834,0.262120583070017,-1
.53222892465317,0.125465953195535,0.567570989590894
"3454","PCNX2",142.939671926066,0.438753974234594,0.28623301569818,1.5
3285592566736,0.125311357439401,0.567570989590894
"3455","PPM1B",146.080211879475,0.317025663568058,0.206942404658991,1.
53195119236421,0.125534479408176,0.567570989590894
"3456","CLHC1",28.0544375536492,0.744103547275514,0.485729127223056,1.
53193108169074,0.125539442542773,0.567570989590894
"3457","CBWD2",63.6199806212533,-0.728805287568102,0.475770050035561,-
1.53184356079923,0.125561043697875,0.567570989590894
"3458","LRCH3",207.983243320861,0.340835884536634,0.222339857216769,1.
53295000187185,0.125288174467495,0.567570989590894
"3459","WFS1",92.3835447377718,0.364265081328467,0.237755267218065,1.5
3210099439929,0.12549751441518,0.567570989590894
"3460","PMPCB",248.863110932713,-0.314614490960353,0.205390266006245,-
1.53178871169478,0.125574582559233,0.567570989590894
"3461","DUS1L",150.870618668629,-0.329320686207113,0.214977835562202,-
1.53188204423905,0.125551545188876,0.567570989590894
"3462","ALDH1L2",218.157852503576,0.366455962979236,0.239280007257303,
1.53149428228317,0.125647278467461,0.567735521971834
"3463","C19orf53",907.608144759692,-0.409630621631416,0.26757448070364
,-1.53090317340507,0.125793324829893,0.568231296654352
"3464","SLC16A9",5.72765935230431,1.48529468626644,0.970368504223445,1
.53065013940768,0.12585588281709,0.568349761809393
"3465","ARHGEF18",47.6981304825597,0.387028423382022,0.252953735303711
,1.53003640336575,0.126007718414705,0.568871208993139
"3466","MAP3K2-
DT",18.9560203121361,-0.649903927201318,0.424896154481176,-1.529559447
28398,0.126125813750505,0.569240076312508
"3467","AL358852.1",8.03218777638109,-0.799337044432616,0.522676055191
646,-1.5293163643005,0.126186034765498,0.569347603644849
"3468","TFEC",4.62190375446623,-1.24690999111246,0.81543516235616,-1.5
2913444094019,0.126231118836191,0.569386791221032
"3469","RASGEF1B",29.6621103366067,-0.693209512779774,0.45349235790367
9,-1.52860241346561,0.12636303735645,0.569817524752652
"3470","UBALD2",151.408530265256,-0.386194307873305,0.252701289315793,
-1.52826409757922,0.126446980069578,0.57003173176611
"3471","ZNF704",340.586562001912,0.349670984090657,0.228837452303774,1
.52803214932878,0.126504555999385,0.570126986314715
"3472","FBXO2",6.23284511569504,-0.936907114003789,0.6132247911116,-1.
5278363294893,0.126553179753519,0.570154514510827
"3473","ZBTB41",143.865205208994,0.269862939427249,0.17666189954315,1.
52756729167476,0.126620007889191,0.570154514510827
"3474","CLEC12A",11.459891233075,1.2473231706375,0.816516965045231,1.5
2761451878517,0.126608274837837,0.570154514510827
"3475","TRIB1",21.3070289153553,-1.20038591323021,0.785905947314447,-1
.52739130850467,0.126663736408673,0.570187288817518
"3476","SMDT1",736.141618363315,-0.446495667815172,0.292373378448453,-
1.5271420065144З,0.126725703403578,0.570302122653096
"3477","CCNK",155.236686942091,0.244044579366061,0.159851967481322,1.5

2669112061181,0.126837836427774,0.570617208777265
"3478","MBTPS2",68.7741688307114,0.319683587580936,0.20941337629423,1.52656718132358,0.126868673024824,0.570617208777265
"3479","MASP1",210.003448107188,-0.712265203085483,0.466713103171201,-1.5261307176632,0.12697731341113,0.570786157208125
"3480","PDE10A",39.2448781953571,0.562443970113262,0.368579690777943,1.52597656405359,0.127015701159719,0.570786157208125
"3481","LINC01572",3.23087367629515,-1.40753837114233,0.922313684347942,-1.52609507484152,0.12698618848033,0.570786157208125
"3482","CEP70",45.4760591096892,-0.411115387841316,0.269593680836216,-1.52494445183631,0.127272953250836,0.571137307994198
"3483","TRIM27",111.680919125382,0.303060589712406,0.198704106174782,1.52518533988337,0.12721287610665,0.571137307994198
"3484","COMMD6",1912.60260320836,-0.438205315799008,0.287339855437354,-1.52504188857485,0.127248650005478,0.571137307994198
"3485","LINC02308",3.10863804722322,1.55738853241719,1.02126757066741,1.52495641411528,0.12726996935428,0.571137307994198
"3486","ERCC1",335.680864328661,-0.373512140449032,0.244960641078812,-1.52478430332349,0.127312906282412,0.571137307994198
"3487","BCL2L13",113.322871502355,0.262147484967478,0.17191690870609,1.52484992279408,0.12729653469278,0.571137307994198
"3488","C2orf68",151.612383079959,0.314294327886582,0.206153743747797,1.52456279557591,0.127368182983459,0.571221469727709
"3489","ATAD3C",3.20086558899152,1.60004558044701,1.04975776026329,1.52420457463034,0.127457615631592,0.571294980322347
"3490","CLCF1",14.0729565790482,-0.808369969457835,0.530331987268945,-1.52427156736425,0.127440886661317,0.571294980322347
"3491","DEGS1",440.43922685929,-0.373362793195515,0.24511486713595,-1.52321561542995,0.127704770602322,0.572238821693532
"3492","CCDC71",44.4964373230128,-0.386826407020735,0.253985005233834,-1.5230285215641,0.127751569922599,0.572284595732882
"3493","NNT",143.456013602477,0.292446077063554,0.192066804279579,1.52262687016888,0.127852083327118,0.572462017907559
"3494","GPR150",9.03014571838226,-0.940243658604403,0.617593351747946,-1.52243163878509,0.127900962257043,0.572462017907559
"3495","URGCP",91.2664586011297,0.319446018235512,0.209817325426441,1.52249590250117,0.12788487132539,0.572462017907559
"3496","NOTCH2NLA",47.320911827673,0.464116300725663,0.304890322339292,1.52224018514165,0.127948909487632,0.57251281210384
"3497","SLC38A1",25.8429588844451,-0.623314777954622,0.409533927923529,-1.52201010821016,0.128006547939201,0.572606928628228
"3498","RFLNB",261.613650010714,0.742146826898238,0.487695722207214,1.52174151444148,0.128073861098086,0.572744256477235
"3499","BHLHE41",133.8135487472,-0.69738186069456,0.458529502516426,-1.52090946573187,0.128282558224745,0.573513591971904
"3500","EMC1-AS1",35.1633617169134,-0.584804611726791,0.384897009500338,-1.51937946331661,0.12866700823636,0.573514159635453
"3501","TMEM61",4.65526203586802,1.35635567368213,0.892517722478617,1.51969606823648,0.128587380233236,0.573514159635453

"3502","MIGA1",251.009558177913,0.328950450293201,0.216549022351529,1.
5190576559575,0.128747983946907,0.573514159635453
"3503","SLC16A1-
AS1",148.768074228973,0.511449377450559,0.336650414855094,1.5192299040
2583,0.12870463660292,0.573514159635453
"3504","ADAMTS9-
AS1",8.9709296551809,-0.982812997622009,0.646316645802817,-1.520636988
08378,0.128350959374446,0.573514159635453
"3505","Z99289.3",10.4008685554945,-1.09733218651425,0.721837330354973
,-1.52019317977725,0.128462431032591,0.573514159635453
"3506","RPL30",17082.2991298767,-0.440144626746394,0.289736382284264,-
1.51912101364807,0.128732038254225,0.573514159635453
"3507","SHARPIN",296.339456169278,-0.358408600409095,0.235798420923207
,-1.51997879801671,0.128516304402793,0.573514159635453
"3508","C9orf24",3.18067737887467,-1.43646290876364,0.945655629604983,
-1.51901269742737,0.128759299919434,0.573514159635453
"3509","HSDL2",214.530723360924,-0.240735395715907,0.158320411499176,-
1.52055817336705,0.128370749832131,0.573514159635453
"3510","TRIAP1",152.763717177303,-0.420657273242077,0.276805184380805,
-1.51968711923897,0.128589630432768,0.573514159635453
"3511","AC022467.1",9.67563642683935,1.01417680829696,0.66707843486418
7,1.52032617948958,0.128429017503959,0.573514159635453
"3512","PGPEP1",80.0828983275038,0.463696751018522,0.305091179946595,1
.51986285247476,0.128545448418702,0.573514159635453
"3513","DAB1",4.70796097049611,-1.58067585945795,1.04071325773104,-1.5
1883897674576,0.128803032331891,0.573545640412118
"3514","ZNF436",147.807428419275,0.359837906835844,0.237102600611764,1
.51764639403956,0.129103564523328,0.574371426370832
"3515","CNBP",1578.60931555381,-0.217990920075175,0.14367697209998,-1.
51722935755831,0.129208786639325,0.574371426370832
"3516","PAPSS1",189.751028323359,0.32754608382945,0.215850801286702,1.
51746522077715,0.129149268013167,0.574371426370832
"3517","FAM177A1",312.296027018674,-0.246257322237274,0.16224613255978
1,-1.51780087668061,0.129064604040705,0.574371426370832
"3518","EIF3D",686.021949204428,-0.404532548198326,0.266619015236823,-
1.51726818073723,0.12919898838542,0.574371426370832
"3519","ATF4",879.442394040407,-0.318558936434175,0.209871129113439,-1
.51787879438142,0.129044956681917,0.574371426370832
"3520","TLR3",162.148428275571,0.262958049089978,0.173375761296974,1.5
1669441635246,0.129343854702746,0.574808499748594
"3521","AC011815.2",5.42314672031565,1.1341667753063,0.747923567009887
,1.51642069501911,0.129413009387178,0.574952486749115
"3522","DAP",210.038729034729,-0.325393168144849,0.214648422426383,-1.
51593552128923,0.129535657289182,0.575154030359206
"3523","CCDC125",44.8891433572565,0.509742057867821,0.336374405828388,
1.51540084214339,0.129670924273067,0.575154030359206
"3524","MIOS",47.0889329421896,-0.375551718948814,0.247810450874221,-1
.51547974520021,0.129650955913965,0.575154030359206
"3525","ZNF487",43.2258771747021,-0.398052543870237,0.26270215174852,-
1.51522376661492,0.129715746283148,0.575154030359206

"3526","NIPA2",134.953287393776,-0.309105843113688,0.203993825765829,-1.51527058210341,0.129703895004714,0.575154030359206
"3527","PKIG",397.055825797331,-0.41406100331574,0.273123224227306,-1.51602268348714,0.129513616758656,0.575154030359206
"3528","ZC3H12B",10.2262811194702,0.713689971090793,0.470940768594708,1.5154559101359,0.129656987711505,0.575154030359206
"3529","CANT1",93.4859496461712,0.31958316632896,0.210956140491397,1.51492706296451,0.129790875966106,0.575324078418192
"3530","SMIM17",3.80401963941341,1.175513152622,0.776191405445164,1.51446298474255,0.12990845506288,0.575682142365052
"3531","AC005670.3",114.503715352534,0.373503991358183,0.246651116606262,1.5143008330849,0.129949557376872,0.575701196841238
"3532","CHST15",5.50630530850968,1.41852170765106,0.936941724949543,1.51399139335741,0.130028022384132,0.57588571748442
"3533","DMXL1",155.577546900677,0.305936787428769,0.202108201913416,1.51372771877825,0.13009491168061,0.576018880107494
"3534","TAFA2",22.4180907529597,1.12556393986571,0.743704840145223,1.51345517617704,0.130164078693249,0.576162049518531
"3535","MTG1",165.981504028446,-0.303327619323651,0.200544299085973,-1.51252177551861,0.130401177312377,0.576885072595451
"3536","ARMC6",53.852359175351,-0.442588288151377,0.292613171715387,-1.51253713411734,0.130397273274126,0.576885072595451
"3537","FAM214B",40.4906595387259,-0.392366769039464,0.25950198740524,-1.51199909088458,0.130534093816309,0.577146644875216
"3538","PTPN20",12.9469815119221,-0.664106400311643,0.43920690091722,-1.51205820975206,0.130519054867123,0.577146644875216
"3539","ITPRID2",96.2965835045043,0.364374648799792,0.241146161487999,1.51101160620351,0.130785493861024,0.577511435858041
"3540","BMP1",226.566161357824,0.461250197769835,0.305184801917445,1.51137997328781,0.130691668776131,0.577511435858041
"3541","TMEM126A",196.088106803112,-0.39350800003391,0.260393838062592,-1.51120319498237,0.130736688646624,0.577511435858041
"3542","RNF111",75.9328595896344,0.333967127052581,0.220952445399083,1.51148871174234,0.130663982488446,0.577511435858041
"3543","MPND",52.1247090337226,-0.378875376144177,0.250753086395333,-1.51095000101753,0.13080119013265,0.577511435858041
"3544","TMEM108",21.1565542098459,1.13597981806039,0.751961913432865,1.51068797204688,0.130867968335232,0.577643236080145
"3545","PLEKHA4",111.100435386036,-0.40786391895522,0.270043148057023,-1.51036573928954,0.130950125749676,0.577768047668394
"3546","USP27X",26.6117173334312,-0.513723778454004,0.340149689541835,-1.51028736538306,0.130970114238453,0.577768047668394
"3547","ZNF354C",57.6772430595664,0.418773045636262,0.277350817380008,1.50990377310656,0.131067979783729,0.578036765648962
"3548","HLX",38.2908801498875,0.514384035622124,0.340914846369845,1.50883436464979,0.131341116413587,0.5785235329816
"3549","CLASP2",116.313564672705,0.319902734558201,0.211958889170669,1.50926783872988,0.13123035006159,0.5785235329816
"3550","CLCN3",233.155079571612,0.249214237251966,0.165187471211326,1.50867517629799,0.131381812269772,0.5785235329816

"3551","LPCAT1",89.5033538997762,0.334811167561774,0.221998395322584,1
.50816931390546,0.131511198829034,0.5785235329816
"3552","H2AFY2",22.1496814460155,0.67166179031694,0.445132366715698,1.
5089035094721,0.131323442864991,0.5785235329816
"3553","NUDT21",242.602772054108,0.216695288094272,0.143618624426772,1
.50882442273189,0.131343657738406,0.5785235329816
"3554","CAPS",58.8605733553189,-0.527490904490352,0.349662700102364,-1
.50857070066647,0.131408526353738,0.5785235329816
"3555","MOCS3",23.4307692210516,0.506610603101381,0.335899838790134,1.
5082192504948,0.131498421944552,0.5785235329816
"3556","GABRE",158.933508074607,0.520680945522778,0.345224860725061,1.
50823710792211,0.131493853137899,0.5785235329816
"3557","GRIK2",2.66584470888147,1.4598561228668,0.968173892179001,1.50
784495911288,0.131594212378956,0.57854380759763
"3558","AUTS2",222.845322164309,0.365348120874527,0.242319378540854,1.
50771318032631,0.131627950704382,0.57854380759763
"3559","ST3GAL4",219.605456769796,-0.557207074418352,0.369605270152621
,-1.50757340172196,0.131663744489392,0.57854380759763
"3560","TASP1",163.789077000558,0.428989326547018,0.284501190526745,1.
50786478521499,0.131589137032208,0.57854380759763
"3561","USP33",546.122124762574,0.343450051593678,0.227909564762859,1.
50695760378043,0.131821524687348,0.578911878350418
"3562","ACIN1",297.572898110034,0.377326096040643,0.250365889372234,1.
50709865863416,0.131785370578522,0.578911878350418
"3563","WDR77",59.1788187131097,-0.351438570543682,0.233240707765081,-
1.50676343727121,0.131871304544188,0.578967953125099
"3564","RSL1D1",702.160182383332,-0.278740477807558,0.185034757283609,
-1.50642226303637,0.131958809107562,0.579189576562736
"3565","DVL1",110.918832351031,-0.370997372352539,0.24656233918349,-1.
50467980463328,0.132406417676407,0.579246817690029
"3566","AL162595.1",11.4766499355179,0.676723777729195,0.4494402348636
42,1.50570359579513,0.132143280490204,0.579246817690029
"3567","CEP63",188.884634947138,-0.222782648825465,0.14806358076797,-1
.50464177395917,0.132416200221156,0.579246817690029
"3568","AC018647.1",7.29131750320013,0.925059934955615,0.6143018981234
13,1.50587184864887,0.132100074538947,0.579246817690029
"3569","UBE2H",470.788297665282,0.262457408971467,0.17437714372028,1.5
0511359099033,0.132294875389948,0.579246817690029
"3570","RBM33",266.235652748153,0.353872510319059,0.235152663532063,1.
5048628622946,0.132359337997675,0.579246817690029
"3571","ZFP37",40.919271969701,0.422009986207203,0.280437000502889,1.5
0482990992787,0.132367811893943,0.579246817690029
"3572","EBF3",398.875279009353,0.373442359368675,0.248188861432768,1.5
0467010168318,0.132408913491243,0.579246817690029
"3573","ITPRIPL2",330.17113709367,-0.399508650754725,0.265393209876099
,-1.50534616519103,0.132235102065965,0.579246817690029
"3574","RUVBL2",141.357723829681,-0.388023208123839,0.257838985198376,
-1.50490511675456,0.132348472627909,0.579246817690029
"3575","SRXN1",30.065559029227,-0.446214020517924,0.296387905116168,-1
.50550684699172,0.13219381785267,0.579246817690029

"3576","ROMO1",639.10422856736,-0.418784797903693,0.27817039411231,-1.50549737415481,0.132196251445693,0.579246817690029
"3577","EPHA4",35.6843679452966,0.588222838525609,0.391105962555884,1.50399864702027,0.132581715108197,0.579651899633149
"3578","BBS12",17.1264092002945,-0.686979004811796,0.456813484152968,-1.50385010216063,0.132619967320018,0.579651899633149
"3579","CYB5R3",1342.49189761656,-0.332998466047805,0.221419445426941,-1.50392602332518,0.132600415576043,0.579651899633149
"3580","GPR157",22.3521409372292,-0.577055430529237,0.387220870762727,-1.49024878073484,0.136158834431313,0.579653701205753
"3581","AZIN2",30.8346216917027,-0.493216633369562,0.328994435843442,-1.49916405760816,0.133831076826929,0.579653701205753
"3582","ELOVL1",273.734445432457,-0.290779490838276,0.194418787021735,-1.4956347341359,0.134748866543376,0.579653701205753
"3583","SLC35D1",50.3074394564976,0.424861030859099,0.283536050619673,1.49843742949285,0.13401963784423,0.579653701205753
"3584","TRMT13",140.184421002591,0.380162598010679,0.255001468867416,1.49082513014205,0.136007412143287,0.579653701205753
"3585","S100A13",5430.21564614772,-0.389442469015152,0.260025217731132,-1.49771038522055,0.134208512449698,0.579653701205753
"3586","YY1AP1",126.483468872839,0.25855264892859,0.173351681016777,1.49149202022199,0.13583236471491,0.579653701205753
"3587","ACP1",368.261950787121,-0.320141709330156,0.213344388072694,-1.5005865034569,0.133462544320071,0.579653701205753
"3588","PUS10",20.8111241893825,0.533356146533002,0.357616318345667,1.49142004760943,0.135851247936149,0.579653701205753
"3589","CCT4",514.531668745751,-0.252440074903204,0.169252144348307,-1.49150296367119,0.135829493695121,0.579653701205753
"3590","GPAT2",37.1919547621062,-0.957749044824892,0.641574276067558,-1.49281085690543,0.135486704551741,0.579653701205753
"3591","PSD4",6.22817575863843,-1.32982420300583,0.891513113364933,-1.49164850529964,0.135791315226254,0.579653701205753
"3592","ERCC3",78.7523088078226,0.320747124188387,0.214560987687254,1.49489955115191,0.134940659451671,0.579653701205753
"3593","IGFBP2",13.6326078499939,1.43543165541606,0.957707087559928,1.49882116783044,0.133920031617961,0.579653701205753
"3594","SLC19A3",34.7829696980925,0.643876416474593,0.429186280046403,1.50022600071227,0.133555870322272,0.579653701205753
"3595","RPSA",5445.26365182195,-0.402124470526854,0.26790771392217,-1.50098130673339,0.13336039659023,0.579653701205753
"3596","MAP4",429.042261335468,-0.284365511275455,0.189625150545338,-1.49961917212804,0.133713078537253,0.579653701205753
"3597","PTPRG-AS1",11.8543298525695,0.815371018255505,0.546275995960692,1.49259902372532,0.135542179065786,0.579653701205753
"3598","EEFSEC",63.4128427892597,-0.31463486531377,0.210970799951133,-1.49136688767663,0.135865196637063,0.579653701205753
"3599","SIAH2",74.5291642545952,-0.291453798698679,0.194850939045303,-1.49577826068837,0.134711448249049,0.579653701205753
"3600","BCHE",55.7706638776285,0.802086798204348,0.538503877205698,1.4

8947265220519,0.136362949590051,0.579653701205753
"3601","AC107294.2",11.488007611423,0.865505204309446,0.580331099964689,1.49139896924723,0.135856778581522,0.579653701205753
"3602","LPP",1051.26736294085,0.287743654405969,0.19246044826786,1.4950794149949,0.134893717488684,0.579653701205753
"3603","LINC00887",7.10249068603441,-0.954198776730451,0.635896657691021,-1.50055636429228,0.133470344734073,0.579653701205753
"3604","FAM184B",12.9594301675122,1.38374281186056,0.926416854155982,1.49365029970361,0.1352670451353,0.579653701205753
"3605","TBC1D19",47.573074779186,0.438214491637752,0.293733944266674,1.49187555674501,0.135731771657369,0.579653701205753
"3606","FGF2",175.675555912205,-0.259739855784881,0.173779748718921,-1.49464973738106,0.135005878315895,0.579653701205753
"3607","CEP44",125.472780526709,0.279145598928773,0.186349708967266,1.49796637985523,0.134141985626238,0.579653701205753
"3608","FGF10",89.542246913832,-0.533081712684746,0.354604863952947,-1.50331190255608,0.132758632222742,0.579653701205753
"3609","PRRC1",234.782687099925,0.257762951337683,0.172668606263713,1.49281885639366,0.13548461000303,0.579653701205753
"3610","PCDHB2",47.6837996834319,0.42105236944634,0.28080674412471,1.49943823734997,0.13375998015203,0.579653701205753
"3611","RANBP17",10.6600867074605,-0.854213847453433,0.570199135999448,-1.49809740759456,0.134107944549923,0.579653701205753
"3612","NHP2",478.102263461726,-0.378693529903031,0.252542665518621,-1.49952297812865,0.133738012202854,0.579653701205753
"3613","C6orf47",92.1133256178438,-0.318180734863671,0.213170035806984,-1.49261472729577,0.135538066039382,0.579653701205753
"3614","C6orf226",119.310383206625,-0.443138926716382,0.296517301997226,-1.49447915427386,0.135050426429856,0.579653701205753
"3615","HMGCLL1",54.1209666290612,-0.658726656520121,0.44159224666561,-1.49170793077564,0.135775729122728,0.579653701205753
"3616","CCNC",201.714672002092,-0.29121515762248,0.194391008086685,-1.49808965182494,0.134109959316951,0.579653701205753
"3617","REPS1",60.5893921510389,0.345585670212806,0.231969724311364,1.48978782140095,0.136280034327329,0.579653701205753
"3618","UST",225.299845131411,0.304279068963413,0.202624613779086,1.50168858209476,0.13317755403392,0.579653701205753
"3619","CCZ1",211.804284599442,-0.489210965895811,0.326143141439875,-1.4999885134362,0.1336173779876,0.579653701205753
"3620","KLHL7",119.375712274341,0.268785649534762,0.17886804108634,1.50270360150598,0.132915493592962,0.579653701205753
"3621","DENND11",60.850030512283,0.459390035460244,0.305907134113085,1.50173037576306,0.133166755743579,0.579653701205753
"3622","KMT2C",409.325755488044,0.359670023884786,0.239341826760172,1.50274621345306,0.132904500664099,0.579653701205753
"3623","RNF170",201.351704708405,0.303920595345639,0.202804885747378,1.49858616189462,0.133981024860826,0.579653701205753
"3624","RRS1",37.1187902155682,-0.448913808892404,0.301277486460571,-1.49003436720837,0.136215199758967,0.579653701205753
"3625","MSC",47.7129822060634,-0.362983552697108,0.242306296729424,-1.

49803598831953,0.13412390047827,0.579653701205753
"3626","NDRG1",867.883038900577,0.288423707953154,0.192673160987931,1.49695840600872,0.134404081244717,0.579653701205753
"3627","RMRP",10.9558831423383,0.956869487265474,0.640936202400051,1.49292469934196,0.135456898933622,0.579653701205753
"3628","TMEM8B",76.7318388677811,0.308002058504287,0.205854013380183,1.49621595152216,0.134597389028875,0.579653701205753
"3629","AGTPBP1",20.6893118083261,-0.764593104760756,0.510851983333288,-1.49670184261949,0.134470856608427,0.579653701205753
"3630","WNK2",6.33666010975603,-1.08989684122325,0.729054689313385,-1.49494524512242,0.134928732764458,0.579653701205753
"3631","ERCC6L2",131.78327667348,0.350512562801441,0.233834294824315,1.49897842429311,0.133879229404065,0.579653701205753
"3632","CDC26",425.9042036132,-0.382853534476295,0.256751535891098,-1.49114408662655,0.135923669712951,0.579653701205753
"3633","GPR107",134.813052078123,0.25072688051823,0.167974355384943,1.49264975563469,0.135528891881914,0.579653701205753
"3634","NUP214",140.210929843401,0.294583883727506,0.196495771908528,1.49918688257903,0.133825157041208,0.579653701205753
"3635","VSIR",752.918422146886,0.48952603861505,0.328470079833679,1.49032155033092,0.136139708751171,0.579653701205753
"3636","DDIT4",1326.38599718207,1.11252845634471,0.740904391632484,1.50158167357248,0.133205179227194,0.579653701205753
"3637","HPS6",39.5566934750679,-0.582166315060799,0.389066610598153,-1.49631528176056,0.134571514648866,0.579653701205753
"3638","TAF5",23.7340267643763,0.480957273898542,0.321270251751288,1.49704889038676,0.134380537129516,0.579653701205753
"3639","IGF2",271.606976406952,1.11678834476473,0.749778256451305,1.48949150652952,0.136357988266699,0.579653701205753
"3640","ABTB2",3.50475741884793,-1.29582927978261,0.863540805181448,-1.50059993923545,0.133459067076755,0.579653701205753
"3641","SYT7",5.42885809657371,1.23987056279335,0.831129425243231,1.49178999700377,0.135754207078053,0.579653701205753
"3642","FADS2",23.8593483850306,1.07027332348764,0.717074580400817,1.49255510199427,0.1355536834099,0.579653701205753
"3643","MUS81",93.1727739852079,-0.314030818133221,0.209898826508961,-1.49610563982745,0.134626128458425,0.579653701205753
"3644","PPFIA1",238.760375840627,0.276059931830442,0.184819906967193,1.49366989931147,0.135261919738432,0.579653701205753
"3645","FZD4-
DT",8.457409938144,-0.741035195565813,0.497289532905685,-1.4901483874714,0.136185223710747,0.579653701205753
"3646","KDM5A",410.045919322882,0.293054212235679,0.195240446658183,1.50099130201614,0.133357811289181,0.579653701205753
"3647","C12orf57",1995.80987393986,-0.419627346853457,0.280009629520214,-1.49861755673357,0.133972875427486,0.579653701205753
"3648","HDAC7",452.638357351375,0.298949055337978,0.199211372989904,1.50066259195518,0.133442853180927,0.579653701205753
"3649","LETMD1",329.158390590167,-0.341055312129745,0.227251873177581,-1.50078108206938,0.133412193294636,0.579653701205753

"3650","ANKRD10",286.705109236296,-0.335116459598695,0.224590948597567
,-1.49211916905508,0.135667907476562,0.579653701205753
"3651","MDP1",198.381886674072,-0.432063874794889,0.287570167253063,-1
.50246417742863,0.132977272737451,0.579653701205753
"3652","YLPM1",155.835482451162,0.394390256881639,0.262775668849193,1.
50086291706094,0.13339102128027 6,0.579653701205753
"3653","AVEN",102.855052281737,-0.39226452308029,0.262682254780408,-1.
49330423331491,0.135357567967847,0.579653701205753
"3654","AC078909.1",12.2863256438656,0.711345423340737,0.4756130500173
4,1.4956389933262,0.13474775602945,0.579653701205753
"3655","ICE2",177.640818488706,0.286687025039095,0.191897496002267,1.4
9395917618283,0.135186289818064,0.579653701205753
"3656","SNX1",250.111014179088,0.196316949278624,0.130769553207806,1.5
0124355756311,0.13329257769851,0.579653701205753
"3657","NDUFAB1",558.678152274569,-0.420712295381157,0.281926505370764
,-1.49227648825666,0.135626677798386,0.579653701205753
"3658","ZNF48",12.1568114857035,-0.733267503735009,0.488564271163912,-
1.50086190704887,0.13339128257062,0.579653701205753
"3659","COG4",128.267410976068,0.270394949794767,0.18030030118277,1.49
969216923641,0.133694159949621,0.579653701205753
"3660","BORCS6",57.0114526590727,-0.543696647952836,0.363175817297335,
-1.49706181429946,0.134377174576283,0.579653701205753
"3661","NSRP1",403.565988357531,-0.288670458367354,0.193780852885511,-
1.48967482632511,0.136309756793023,0.579653701205753
"3662","RAMP2-
AS1",159.128148145811,-0.516250411229929,0.346292577818955,-1.49079259
648421,0.136015956134838,0.579653701205753
"3663","AC008105.3",48.8181474271707,0.524591220825346,0.3499247317465
05,1.49915445589417,0.133833567146469,0.579653701205753
"3664","CCDC40",12.8444843541292,-0.844805879005671,0.565883325585977,
-1.49289763597623,0.13546398406039,0.579653701205753
"3665","GAA",336.989625230224,0.403741513211361,0.269728029293748,1.49
684670988222,0.13443314907406,0.579653701205753
"3666","FN3K",36.2232119956103,0.507048456457946,0.340182490126664,1.4
9051897488654,0.136087831058494,0.579653701205753
"3667","AC017100.1",2.71851832734152,-1.37970317015724,0.9229993739678
22,-1.49480401511663,0.134965598162053,0.579653701205753
"3668","INO80C",46.4286679124254,-0.515909279726051,0.344723340780548,
-1.49658934772996,0.134500143570119,0.579653701205753
"3669","RELCH",56.9474241871559,0.399468172820595,0.266442285643663,1.
4992671747113,0.133804334430639,0.579653701205753
"3670","SAFB2",218.883389752583,0.343399277824159,0.230267653840159,1.
49130488845181,0.135881466087843,0.579653701205753
"3671","GPR108",217.829785681086,-0.365719374135481,0.243260662000083,
-1.5034053230331,0.132734554774381,0.579653701205753
"3672","OSCAR",8.15300104636229,-0.904774880573014,0.602746162178171,-
1.50108775027847,0.133332866734698,0.579653701205753
"3673","TRMT6",72.9153664295904,-0.29899357224241,0.19888039499764,-1.
50338384155944,0.132740090938623,0.579653701205753
"3674","RTCB",258.853526965498,-0.389247489459771,0.259429933940496,-1

.50039543836545,0.133512000461015,0.579653701205753
"3675","TAB1",61.133433651681,0.339369026159963,0.22681331536649,1.496
24824985078,0.134588975265341,0.579653701205753
"3676","RRP7A",163.412991880647,-0.518602303518395,0.345651231337145,-
1.50036295693839,0.133520409508687,0.579653701205753
"3677","ZBED1",191.427216552885,0.314580138124112,0.210076169971228,1.
49745750870837,0.13427425399584,0.579653701205753
"3678","MOSPD2",116.805352258942,0.315906448429616,0.211845315848578,1
.49121280857311,0.135905631892863,0.579653701205753
"3679","MED12",68.7751288125667,0.326975950081009,0.219429696863043,1.
49011712979347,0.13619344088075,0.579653701205753
"3680","PLXNA3",60.1814581843979,0.399264524162094,0.267192613485626,1
.49429476718514,0.135098592254908,0.579653701205753
"3681","MORF4L2",777.461070744662,-0.300153233160099,0.201543267606014
,-1.48927442094892,0.136415120559207,0.579717938306895
"3682","NBPF9",55.5143971300026,0.645335532958883,0.433487248736016,1.
48870707233162,0.136564521848431,0.580047503844216
"3683","TBC1D5",313.759509411752,0.253187083546196,0.170179159151426,1
.48776786069855,0.136812124227375,0.580047503844216
"3684","SEMA3D",355.879669573438,0.517388735152903,0.347774380714475,1
.48771377031847,0.136826394501384,0.580047503844216
"3685","SINHCAF",25.8660172138417,-0.524161268238657,0.352201498940122
,-1.48824258220369,0.136686931143523,0.580047503844216
"3686","HMGA2",3.28320216984622,-1.79628693471355,1.20716038023434,-1.
4880267478335,0.136743839805889,0.580047503844216
"3687","MESD",620.06691974094,0.267566092519369,0.179787008506705,1.48
8239304618,0.136687795202005,0.580047503844216
"3688","LSM4",253.258115975386,-0.42407055592487,0.285001900677498,-1.
4879569396442,0.136762249918503,0.580047503844216
"3689","ATP7A",89.1381168301998,0.332511158203522,0.223428897166727,1.
48821912662173,0.136693114749623,0.580047503844216
"3690","ARMCX2",154.504396974586,0.352519104106208,0.236868731210521,1
.48824668543059,0.136685849430068,0.580047503844216
"3691","FAM149A",79.1113237053761,0.382588973948011,0.257246429536518,
1.48724697418473,0.136949593675486,0.580071472454218
"3692","CD2AP",123.039953699878,0.292728565760845,0.196795362950079,1.
48747694748835,0.136888887278482,0.580071472454218
"3693","AL021368.2",90.0590098253839,0.527154325171299,0.3544805849849
28,1.48711762364563,0.136983747655677,0.580071472454218
"3694","AP003486.1",14.6792406927564,0.666341205112347,0.4481163819061
28,1.48698247155788,0.137019440507744,0.580071472454218
"3695","AC009148.1",5.16386043879821,1.08000689137362,0.72615124864539
1,1.48730294602996,0.13693481679183,0.580071472454218
"3696","ISOC2",245.267986573388,-0.447588471344768,0.301031437895156,-
1.48684959442892,0.137054539550648,0.580071472454218
"3697","UBIAD1",53.2476969024608,-0.371262934819854,0.249910383395285,
-1.48558427135309,0.137389117908788,0.580545103038134
"3698","EIF4G3",244.306224494572,0.298851738538188,0.201092965971601,1
.48613720571605,0.137242832890693,0.580545103038134
"3699","LGALS9",37.8739204471956,-0.419259602399306,0.282174201404731,

-1.48581833602126,0.137327178792629,0.580545103038134
"3700","LRRC59",179.038199249477,-0.342826692923375,0.230738595804147,
-1.48577957549143,0.137337434268922,0.580545103038134
"3701","MKKS",95.2452436645957,-0.393945182856157,0.265177888595244,-1
.48558835332405,0.137388037536975,0.580545103038134
"3702","PHKA2",30.5716361808278,-0.423470670857919,0.285035826441525,-
1.48567524351117,0.137365041938389,0.580545103038134
"3703","MAGED1",170.20289781404,0.487668153216972,0.328313304525786,1.
48537432536083,0.13744469297185,0.58062309807147
"3704","DNAJB11",222.218420697959,-0.343242171231358,0.231114423488221
,-1.48516118574854,0.137501131153243,0.580641806633851
"3705","ALDH3B1",12.3722930474784,-0.65554679925481,0.441422689244036,
-1.48507726319523,0.13752335828028,0.580641806633851
"3706","DUSP14",135.24178330862,-0.447347875451326,0.301273919958378,-
1.48485429974532,0.137582424242317,0.580734447496643
"3707","MRPL20",819.074797447167,-0.344157680376687,0.232038647726411,
-1.48319120003867,0.138023618582078,0.581676542869482
"3708","DGUOK",388.436827370045,-0.357975127833711,0.24132052590243,-1
.48340107620371,0.137967881671253,0.581676542869482
"3709","NPHP3",270.889085765224,0.449862719994961,0.30327279345045,1.4
8335996406635,0.137978798474173,0.581676542869482
"3710","C8orf48",26.3378014157771,-0.564258027125786,0.380406047784685
,-1.48330456471913,0.137993510116199,0.581676542869482
"3711","ZNF511",259.627145496491,-0.391183510663306,0.26374791059969,-
1.48317197953858,0.138028723846546,0.581676542869482
"3712","KMT2A",604.744736365666,0.351318444406741,0.23679969943196,1.4
8361017876919,0.137912367459718,0.581676542869482
"3713","DCAF6",242.198614994656,0.249834974404608,0.168529745818756,1.
48243844545574,0.138223670721959,0.582181841781584
"3714","FLVCR1-
DT",22.3783912625172,-0.603519549188171,0.407292306922752,-1.481784799
09918,0.13839756503254,0.582181841781584
"3715","SPAST",87.8700810489744,0.341277293773751,0.230343023992832,1.
48160464275389,0.138445522958537,0.582181841781584
"3716","BAD",572.932888905523,-0.413495095522311,0.279086582100646,-1.
48160148872078,0.13844636268155,0.582181841781584
"3717","GPR137",76.3850512091868,-0.38984586100073,0.263061153079995,-
1.48195906706978,0.138351186781985,0.582181841781584
"3718","SLC24A4",3.7728689558486,1.19206285476809,0.804467456791018,1.
48180370095165,0.138392534069228,0.582181841781584
"3719","PATL2",13.4021661120246,0.789205643597803,0.532426832326535,1.
48227999732701,0.138265808365803,0.582181841781584
"3720","PIAS1",254.375991621433,0.282144378768262,0.190427383943168,1.
4816376349132,0.138436739431986,0.582181841781584
"3721","ACTR5",21.4587886206205,-0.489519628407983,0.33049547173454,-1
.48116894261466,0.138561560003669,0.582509670286856
"3722","GRAMD2B",103.738422004036,0.369248541385546,0.249400523140906,
1.48054437390626,0.138728027785897,0.583052804582157
"3723","MATN2",177.495040227623,-0.650328139951231,0.439293840390885,-
1.48039439699989,0.138768024414775,0.583064250851551

"3724","AC092164.1",27.3215160807115,0.491690922709252,0.332169009614801,1.48024321498095,0.138808351417975,0.58307108336367
"3725","ZFP36L2",5786.25094632933,-0.226716185291849,0.153193110130452,-1.47993721844793,0.138890001996032,0.58307108336367
"3726","DALRD3",223.274058482859,-0.363989062132829,0.246068803122523,-1.47921661549104,0.139082430186604,0.58307108336367
"3727","IGF2BP2",89.3537750704001,-0.478725160127625,0.32359749355811,-1.47938463572079,0.139037544100265,0.58307108336367
"3728","POLK",282.227320631515,0.236426060005887,0.159864881559373,1.47891180163969,0.139163888748852,0.58307108336367
"3729","RXRA",325.302837605851,0.241765147208994,0.163441409608622,1.47921599420812,0.139082596181124,0.58307108336367
"3730","FAM160A2",67.5141509464986,0.359793438039409,0.243093351728943,1.48006284614723,0.138856475657126,0.58307108336367
"3731","KLF12",98.7100725402077,0.362602991137549,0.2450332451689,1.47981140635675,0.138923583791272,0.58307108336367
"3732","MRPL28",228.003379359894,-0.394944093397383,0.267061125378004,-1.47885280135164,0.139179660249309,0.58307108336367
"3733","CHMP1A",186.148650412263,-0.28533260612533,0.192897115629234,-1.4791958147978,0.13908798780377,0.58307108336367
"3734","TMSB4X",19035.8774485094,-0.437505644845563,0.29579574546566,-1.47908024896306,0.139118868286412,0.58307108336367
"3735","FLG-AS1",11.7572344325384,0.873084202136521,0.590729402522434,1.47797654629755,0.139414055997306,0.583584179279065
"3736","PHF1",155.798579551112,-0.279435430199787,0.189032917681833,-1.47823687866953,0.139344386116909,0.583584179279065
"3737","NEK9",113.335116482064,0.291204331218008,0.197012328705419,1.47810207174105,0.13938045966096,0.583584179279065
"3738","RACK1",9400.04561528588,-0.434279519261628,0.293896693674433,-1.47766044534923,0.139498686649348,0.583782224519998
"3739","ARPC4",406.022937919321,-0.317303587853608,0.214802444915438,-1.47718797138702,0.139625257236228,0.583899719794369
"3740","AC091978.1",3.76284637202078,-1.24351862496996,0.841843428740889,-1.47713765115425,0.139638742680479,0.583899719794369
"3741","INTS10",222.911315913872,-0.229558446732668,0.155402127555276,-1.47718985797678,0.139624751663844,0.583899719794369
"3742","NANOS1",16.7989984684234,1.00811113732376,0.682628003961956,1.47680893762446,0.139726860100003,0.584112045041246
"3743","SLC25A12",110.506057876064,-0.314213801332481,0.212790934560953,-1.47663152089064,0.139774437541215,0.58415482940348
"3744","NDUFS5",1368.37949082587,-0.356332731889725,0.241381061441919,-1.47622489420308,0.139883528723189,0.584454604651934
"3745","ATP11C",135.78183486718,0.359123407838459,0.243385102648181,1.47553570013519,0.140068577646837,0.585071498031904
"3746","RASA1",124.066951987338,0.309400659267861,0.209780827518697,1.47487576880821,0.140245945965829,0.585090663093358
"3747","TRMT9B",17.3421684895067,0.774541669438964,0.525034511901356,1.47522048909517,0.140153274696985,0.585090663093358
"3748","CCDC89",36.3048590370381,-0.421149466443587,0.285559360388287,

-1.47482283848421,0.140260179415719,0.585090663093358
"3749","USP32",69.7410713566067,0.372677248122063,0.252664171356784,1.47499048290392,0.140215102106115,0.585090663093358
"3750","CD99L2",141.776321017363,0.260824954177899,0.1768447475231,1.47488097798228,0.140244545231172,0.585090663093358
"3751","SLC39A4",72.2849490301062,-0.355367799326423,0.241015710112643,-1.47445906808455,0.140358030676806,0.585251362473914
"3752","OSGEP",138.049151405133,-0.290908476370748,0.197306149145731,-1.47440147015327,0.140373528862886,0.585251362473914
"3753","LINC01985",17.2388898566062,-0.700344646589279,0.475258660761519,-1.47360733093659,0.140587346389842,0.585686390045055
"3754","TSSC4",183.945078750327,-0.409915953419436,0.278173760710485,-1.47359676330531,0.140590193352885,0.585686390045055
"3755","AL121603.2",34.1124671106267,-0.428049946108708,0.290450669837516,-1.47374404868189,0.140550518067567,0.585686390045055
"3756","SH3D19",788.475300066015,0.233017572779633,0.158182731278883,1.47309109468348,0.140726474369218,0.585786119892942
"3757","RPAP3",128.340084055649,0.322210527408221,0.218709325637437,1.47323634449114,0.140687318197708,0.585786119892942
"3758","AC012645.1",11.0205193393482,-0.693863354711797,0.471008002655867,-1.47314557459601,0.140711786794342,0.585786119892942
"3759","AL357078.2",4.67733839163548,1.08622329190594,0.737818309943071,1.4722097259822,0.140964251809475,0.586463774216919
"3760","STK25",272.625804061727,-0.278712605003026,0.189307369596694,-1.47227551466593,0.140946492544953,0.586463774216919
"3761","IRF1",657.024766921096,-1.12790714780996,0.766433056412605,-1.47163165572383,0.141120372414717,0.586672755965018
"3762","TCERG1",180.285391824578,0.382552100752038,0.259962624158941,1.47156577600227,0.141138173117455,0.586672755965018
"3763","AL121929.3",5.25967806603895,-1.01867353683813,0.692093008482993,-1.47187375735954,0.141054971368479,0.586672755965018
"3764","RBM4B",58.299688119225,0.432502046744311,0.293925512662443,1.47146820575938,0.141164539770753,0.586672755965018
"3765","NCLN",90.314208236203,0.470893189719785,0.320046025254262,1.4713295981279,0.14120200257037,0.586672755965018
"3766","ADA",270.487863729197,-0.366763391470641,0.249317790354274,-1.47106787265153,0.141272762434243,0.586810892925881
"3767","DAG1",173.471068630349,0.313043786610151,0.212947503551502,1.47005145112885,0.141547819686063,0.587089533131054
"3768","ABHD10",77.6051540649735,0.317749960199839,0.216228220394956,1.46951197960861,0.141693974950272,0.587089533131054
"3769","TSTD3",108.879216980478,0.264159557798653,0.179762988458185,1.46948801899841,0.141700469119703,0.587089533131054
"3770","FPGS",69.1137565647027,-0.377013487759124,0.256573418004859,-1.46941756745816,0.141719565293554,0.587089533131054
"3771","ZMIZ1",377.032967198709,-0.380738676900663,0.259121157572173,-1.46934615632309,0.14173892358701,0.587089533131054
"3772","UBQLNL",4.35187629176279,1.12791404474007,0.767164349873834,1.47023782443171,0.141497353794775,0.587089533131054
"3773","EIF3M",575.558160677559,-0.383443198486826,0.260970798837887,-

1.46929541617037,0.14175267957783,0.587089533131054
"3774","DRAP1",686.278848242738,-0.411325466166778,0.279938329169691,-1.46934314921,0.14173973880666,0.587089533131054
"3775","AKAP11",187.952501859132,0.288075000991609,0.195971976698698,1.46998058520641,0.141567012285833,0.587089533131054
"3776","BRD7",379.580432744312,-0.255852666918353,0.17412421313986,-1.46936868976884,0.14173281494976,0.587089533131054
"3777","LRRC3",20.2809311200569,0.64897052360388,0.441516819312541,1.46986591499357,0.14159807262575,0.587089533131054
"3778","ZNF316",44.9913705056265,0.468085179517933,0.318621829219355,1.46909325285331,0.141807497573275,0.587161112900672
"3779","XRN1",293.37406788698,0.258614261367323,0.176065492685335,1.46885262650256,0.141872766314049,0.587211984123603
"3780","DUSP5",22.7784452958007,-1.10540897136741,0.752683923992593,-1.46862306491654,0.141935055296432,0.587211984123603
"3781","RESF1",188.838864793338,0.280166872148276,0.19078509561828,1.46849454481932,0.141969936965765,0.587211984123603
"3782","LINC02384",50.6221227580844,0.759537852011792,0.517146476916172,1.46870932301626,0.141911647657614,0.587211984123603
"3783","RNF19A",507.069969122044,0.34514363885178,0.235113223081471,1.46798905790247,0.142107195165622,0.587469041748368
"3784","TGFBR1",140.584377557474,0.359550680155682,0.244912953707932,1.46807539050981,0.142083745488972,0.587469041748368
"3785","AC067838.1",8.47081654021493,0.946156387580432,0.64497918474109,1.46695646923899,0.142387897482273,0.587852700004011
"3786","CES2",140.520604174689,0.327700434784621,0.223333674569399,1.46731313769161,0.142290891462721,0.587852700004011
"3787","NOB1",221.92873187851,-0.412914672297427,0.281465739125881,-1.46701574969576,0.142371770976959,0.587852700004011
"3788","TXNDC17",306.102833868567,-0.381107704544874,0.259711267160063,-1.46742845896629,0.142259537456651,0.587852700004011
"3789","AC022150.4",8.36543527618437,0.966356462923908,0.658661675757473,1.4671514959673,0.142334848187428,0.587852700004011
"3790","POGLUT3",442.60552492943,0.237815456470215,0.162139637415165,1.46673238118375,0.142448870500627,0.587949256264195
"3791","ADH1C",347.543989374089,-0.599599636707258,0.408853999093396,-1.46653729213075,0.142501969384754,0.588013270136034
"3792","AURKAIP1",654.593156995548,-0.372913586234487,0.254360794659748,-1.46608122817561,0.142626159091088,0.588231330945215
"3793","TMEM219",708.033903275827,-0.367895341461122,0.250940325271647,-1.46606704627034,0.142630022263965,0.588231330945215
"3794","RAB28",135.122796945229,-0.305068899351681,0.208313330366815,-1.46447132698849,0.143065211842285,0.588728603557585
"3795","LCA5",201.600813904508,0.293187072737955,0.200191133289886,1.46453575600378,0.143047620842145,0.588728603557585
"3796","GATAD1",454.885407144448,0.308948919852666,0.21092331627845,1.46474522259459,0.142990441823535,0.588728603557585
"3797","ZNF782",23.357739191653,0.576427690246522,0.393461125957647,1.46501814847287,0.142915966359837,0.588728603557585
"3798","FRMD6-

AS1",51.4467732040051,0.509457447193164,0.347657015443928,1.4654024643
8758,0.142811145484699,0.588728603557585
"3799","USP22",355.688064033803,0.234026357284095,0.159755463426726,1.
46490362372761,0.142947214015811,0.588728603557585
"3800","PIGS",90.4850238303152,0.316454389354665,0.215991250992003,1.4
6512596182139,0.142886554674315,0.588728603557585
"3801","AC008982.2",5.30047667843808,-1.09755224259025,0.7490063315764
93,-1.4653444120828,0.142826975271433,0.588728603557585
"3802","SLC5A3",1454.22888437104,-0.983518576642598,0.671626574609574,
-1.46438305722839,0.143089314755861,0.588728603557585
"3803","HGH1",44.8346268918536,-0.425292865448163,0.290478080164586,-1
.46411345464412,0.143162951646958,0.588824231780447
"3804","HMOX2",208.03611600252,-0.405124154122105,0.276719923117172,-1
.46402235718518,0.143187839780913,0.588824231780447
"3805","RPL5",10886.996042105,-0.415282513136349,0.28375271752923,-1.4
6353669051141,0.143320581588287,0.588905662670234
"3806","AC079610.1",5.33842156645553,-0.95640791391134,0.6533826572512
83,-1.46377915498225,0.143254299706716,0.588905662670234
"3807","AASDH",136.714915282363,0.257686483657428,0.176057276566726,1.
46365142459627,0.14328921409815,0.588905662670234
"3808","PTPDC1",43.9436902922173,0.441559702888216,0.301837434487013,1
.46290569835603,0.143493184554641,0.589305299550604
"3809","SNRPB",559.471154917061,-0.339934179699565,0.232365942586475,-
1.46292600333656,0.143487627804355,0.589305299550604
"3810","GZMA",26.6759449302133,0.681635568324876,0.466084362222438,1.4
624725126469,0.143611771389056,0.589328158404774
"3811","APTR",65.8973427503338,-0.385661275874728,0.26366765656552,-1.
46267949925399,0.14355509835805,0.589328158404774
"3812","HPS5",58.8234859147906,0.343521739442656,0.234881993553741,1.4
6252905233478,0.143596289092553,0.589328158404774
"3813","VPS37B",51.8336841117565,0.433319031265519,0.296332805817428,1
.4622715499562,0.143666811485326,0.589399405209799
"3814","ACADSB",75.7486607427164,0.399479983383096,0.273320492201941,1
.46158079902747,0.143856119218433,0.589557577399514
"3815","UEVLD",71.4332174999248,0.384715467435849,0.263205578123279,1.
4616539291415,0.14383606807409,0.589557577399514
"3816","DNAJA2",378.322251578084,-0.273978236117951,0.187447552932197,
-1.46162610197986,0.143843697598111,0.589557577399514
"3817","NUP88",51.5561202171175,0.367663186482627,0.251539757031602,1.
4616504000059,0.143837035659181,0.589557577399514
"3818","HERC1",209.920107063975,0.267422745243468,0.1829872069121,1.46
142864168601,0.143897845273327,0.589574120903786
"3819","SDC2",1385.85992072887,-0.294968557850856,0.201869075245871,-1
.46118744285916,0.143964008158181,0.589690751405715
"3820","MRPS18C",391.68853026768,-0.364391586461486,0.249445941066085,
-1.46080383150011,0.144069284053929,0.589967489648066
"3821","AC108519.1",20.6431774952642,-0.761813955601469,0.521690431947
124,-1.46027971561243,0.144213214525079,0.590275584416208
"3822","UBFD1",109.994526769687,0.32858677987888,0.225020127939833,1.4
6025505756862,0.144219988725868,0.590275584416208

"3823","SLC6A9",6.77927721546313,-0.782785618927325,0.536159299288786,
-1.45998702244965,0.144293640625692,0.59032031084096
"3824","AC007285.1",4.87717099890915,-1.0217183869285,0.69983557123976
4,-1.45994063308117,0.144306390631965,0.59032031084096
"3825","NOC2L",153.791310568002,-0.27491553268487,0.188477884079359,-1
.45860897169833,0.144672762869154,0.590420746509994
"3826","NOL9",105.074393701192,0.310139063244003,0.212833563208474,1.4
5719057919552,0.145063780375853,0.590420746509994
"3827","SF3B4",154.479987561005,-0.382726385059755,0.262670501894571,-
1.45705887147302,0.145100130137969,0.590420746509994
"3828","DDX59",100.802184751053,-0.356898231259211,0.244618032129876,-
1.45900213549965,0.144564519928076,0.590420746509994
"3829","NKTR",801.806288269129,0.416800497177992,0.285794305383128,1.4
583932896047,0.144732169236217,0.590420746509994
"3830","HYAL1",12.8010186773933,-0.765127222521239,0.524709851675344,-
1.45819107470208,0.144787883244542,0.590420746509994
"3831","HADH",174.741152009295,-0.310087448066332,0.212477084545269,-1
.45939242685847,0.144457129201253,0.590420746509994
"3832","MIR3945HG",2.82544720651986,1.9173713628166,1.31518489601593,1
.45787209739472,0.144875800892936,0.590420746509994
"3833","C5orf49",6.7389963340384,-0.902876013447515,0.619495512626153,
-1.45743753593962,0.144995642058687,0.590420746509994
"3834","ATXN1",195.439862230244,0.36841349352271,0.252848431596916,1.4
570527141336,0.145101829660976,0.590420746509994
"3835","RMI1",18.7577769054217,0.538425674858182,0.36916190671534,1.45
850821838278,0.144700511518565,0.590420746509994
"3836","POMT1",69.4459616295826,0.302691759202271,0.207506947774434,1.
45870662379606,0.144645872222943,0.590420746509994
"3837","ARL2",855.757947961721,-0.429590455182131,0.294429523922296,-1
.45906038721683,0.144548487777644,0.590420746509994
"3838","MPG",558.284282330134,-0.393971417700239,0.27033487160651,-1.4
5734590346779,0.145020921691396,0.590420746509994
"3839","LUC7L",186.253963518068,0.274274560394786,0.1879690351239,1.45
914756765122,0.144524496355188,0.590420746509994
"3840","ACD",69.1608861921705,-0.336563258012712,0.230604060137783,-1.
45948539592763,0.144431557290115,0.590420746509994
"3841","RNF213-
AS1",15.4287708070339,0.753894675100868,0.517233526697427,1.4575518333
3637,0.144964114341647,0.590420746509994
"3842","OLFM2",65.2189289715337,-0.793177085056354,0.544400298396283,-
1.45697400863469,0.145123554965859,0.590420746509994
"3843","PRMT2",522.696917373812,0.239754144470693,0.164464973760261,1.
45778240186375,0.144900530456879,0.590420746509994
"3844","WBP2NL",6.82204383216063,0.933855682281571,0.640147245997847,1
.45881387152716,0.144616343621911,0.590420746509994
"3845","MPP1",56.8006642929546,-0.431514721549076,0.296126907566986,-1
.4571952447498,0.145062492866396,0.590420746509994
"3846","IPO9",135.186760906652,0.315684088654758,0.216826412967072,1.4
5593004253914,0.145411959676575,0.590492248229543
"3847","SEPTIN2",1110.50469472227,0.279772637368668,0.192068595332255,

1.45662874706141,0.145218887948052,0.590492248229543
"3848","SH3BP5",1543.29683909206,0.216445487029989,0.148745969976197,1
.45513513451575,0.145631854098931,0.590492248229543
"3849","DUBR",80.8349659712813,0.398516967858467,0.273724103655804,1.4
5590747229037,0.145418199747486,0.590492248229543
"3850","AC105345.1",6.71446619747847,0.888836890464039,0.6104462059879
96,1.45604458139841,0.145380295902006,0.590492248229543
"3851","GTF2H2",72.4640431899052,0.400417936533821,0.275133901390436,1
.45535659004667,0.145570567561823,0.590492248229543
"3852","ZDHHC16",64.9922979430363,-0.321591535719819,0.220995327823959
,-1.45519608439864,0.145614984601481,0.590492248229543
"3853","KLRC3",3.14881483017944,1.80584214733151,1.24046715226071,1.45
577586963139,0.145454588451107,0.590492248229543
"3854","MYEF2",3.46119132504732,1.37793875809932,0.946393518979169,1.4
559892163945,0.145395600659667,0.590492248229543
"3855","PARD6A",16.6073879622551,-0.646424078042069,0.44413812418855,-
1.45545730671759,0.145542701339346,0.590492248229543
"3856","CRISPLD2",354.613239370817,-0.309415310378902,0.21248851213970
4,-1.45615076910827,0.145350945483547,0.590492248229543
"3857","EIF1AX",1360.19156667339,-0.20448941358292,0.140506191153375,-
1.45537653468738,0.145565048967353,0.590492248229543
"3858","DLG3",30.4761254940152,-0.554445978706917,0.380784417613157,-1
.45606267762297,0.14537529376166,0.590492248229543
"3859","ZNF853",51.7919631128877,-0.375804808973531,0.258331216852661,
-1.4547402112377,0.145741195854774,0.590781970445495
"3860","PDHA1",210.178181843711,-0.265004901033374,0.182183499382288,-
1.45460429694182,0.145778840754306,0.590781970445495
"3861","NELFA",103.262875898552,0.295772765450501,0.203410789052932,1.
45406626082914,0.145927936515332,0.590926924905344
"3862","RPS10",7055.84567884236,-0.541016411131887,0.372038881712253,-
1.45419319787744,0.145892750338773,0.590926924905344
"3863","BRD3OS",70.3450899551779,-0.288550246096933,0.198443192620557,
-1.45406976317233,0.145926965600007,0.590926924905344
"3864","MATR3.1",53.4038421069499,0.456981393023739,0.314308028174323,
1.45392847799066,0.145966136430108,0.590928641867541
"3865","KLF15",29.0066896399252,0.685547365710348,0.472030493270226,1.
45233703221349,0.146407915333591,0.591130473661644
"3866","PCDHB12",33.9261721933274,0.489819458012393,0.337248506130805,
1.45239919260728,0.146390640683369,0.591130473661644
"3867","ADHFE1",14.7505210977046,0.640890140331166,0.441021243445157,1
.45319562233483,0.146169447285563,0.591130473661644
"3868","VIM-
AS1",23.8371857244584,-0.554459316073876,0.38174921802142,-1.452417686
53149,0.146385501440502,0.591130473661644
"3869","SIDT2",189.076008271422,0.28201779669276,0.194161850577644,1.4
5248819916857,0.146365908078898,0.591130473661644
"3870","ARHGDIB",186.096123879168,-0.407435109871759,0.28050948752866,
-1.45248245776404,0.146367503369165,0.591130473661644
"3871","FGD4",148.367348652631,0.443934338092766,0.30549462990221,1.45
31657667268,0.146177734503147,0.591130473661644

"3872","LINC00648",31.5417200279688,0.492214972368727,0.338932305193241,1.45225157008298,0.146431668186337,0.591130473661644
"3873","CDK5R1",5.31494313968055,1.05596208399446,0.726789470884681,1.45291329373428,0.146247829470201,0.591130473661644
"3874","BCL2L1",115.179891111197,0.334853546855397,0.230368228009058,1.45355785278789,0.146068929236631,0.591130473661644
"3875","KIAA1671",54.4696156516541,0.564525259720886,0.388615210033198,1.45265868433884,0.146318543618735,0.591130473661644
"3876","KCNA2",8.55631397053612,0.983184758734426,0.677201299301331,1.45183531063626,0.146547403096056,0.591306529375063
"3877","SRGAP2B",123.008049866408,0.324558150113384,0.223824698329683,1.45005512142063,0.147043148611766,0.591306529375063
"3878","RAB4A",400.058111298409,-0.296663545773388,0.204604781017345,-1.44993457287902,0.14707676516584,0.591306529375063
"3879","PFN2",160.495424482289,-0.256683064984839,0.177011368676611,-1.45009366858116,0.147032400466434,0.591306529375063
"3880","ZDHHC11B",8.04889587422725,-0.931947168811157,0.642577389341594,-1.4503267377118,0.146967426352671,0.591306529375063
"3881","PRRT1",5.16695468060217,-0.920855956262223,0.635106938160648,-1.44992268377534,0.147080080917878,0.591306529375063
"3882","HLA-DOA",6.2775834066007,-0.941266940086856,0.649142719433592,-1.45001540016987,0.147054224758429,0.591306529375063
"3883","ELP1",61.6417567605689,0.34704986020942,0.239132284337382,1.45128819043012,0.146699628362057,0.591306529375063
"3884","PKP3",4.63681212145098,-1.03016149323056,0.710024585623699,-1.45088144000767,0.146812876901691,0.591306529375063
"3885","DCTN2",584.276061687583,-0.318972179509247,0.219959236610902,-1.45014223737053,0.147018858835149,0.591306529375063
"3886","RDH11",284.25901755334,-0.228060669673343,0.157212312114471,-1.45065400162352,0.146876230044936,0.591306529375063
"3887","AC124068.2",4.33959322465412,1.29607410335003,0.89303032452791,1.45132149239745,0.146690359299479,0.591306529375063
"3888","LRRC75A",142.132376164385,-0.643093235761251,0.443435664627795,-1.45025149544758,0.146988399700571,0.591306529375063
"3889","MRPL4",241.561500365962,-0.375540930209473,0.258792685529462,-1.45112652407913,0.146744631935719,0.591306529375063
"3890","FTSJ1",68.2579530158627,-0.340714239106472,0.234802658949329,-1.45106635772808,0.146761383329246,0.591306529375063
"3891","PLS3",864.419573221805,0.273705552948726,0.188617892380775,1.45111128904027,0.146748873506084,0.591306529375063
"3892","AL031280.1",13.6103941323468,0.756844860900876,0.52230197160402,1.44905610556392,0.147321914858675,0.591366875579743
"3893","TRAPPC3",245.046976919848,-0.328996855441616,0.2269572749941,-1.44959819177495,0.147170600560478,0.591366875579743
"3894","KIAA0040",44.906055957937,0.518099272801735,0.357511445352746,1.44918233957668,0.147286668126664,0.591366875579743
"3895","NDUFC1",779.408661537238,-0.353614287671279,0.244002618057896,-1.44922333410118,0.147275223129277,0.591366875579743
"3896","TAS2R4",8.82366610767262,0.908613199560733,0.626845978034463,1

.44949992725451,0.147198020636329,0.591366875579743
"3897","AATK",9.21294720824954,0.755536301392421,0.521287746524122,1.4
4936516622583,0.147235631176933,0.591366875579743
"3898","DAAM1",382.108875470833,-0.330608618829695,0.22825785122026,-1
.44839976834212,0.14750527955832,0.591951023122319
"3899","B3GALT4",131.794795567986,-0.346635456680025,0.239352977130372
,-1.44821869707189,0.147555897168553,0.592002282484656
"3900","ZNF143",79.8519723463008,-0.323378129880067,0.223366826214756,
-1.44774466002913,0.147688474837555,0.59224645295115
"3901","ST8SIA1",130.356916097657,0.619497330553812,0.427989326441277,
1.44745976659026,0.147768197012615,0.59224645295115
"3902","SLC25A10",11.6507146760474,-0.630766745843086,0.43575155264077
1,-1.44753757507109,0.147746420480532,0.59224645295115
"3903","VPS4B",494.702284125122,0.289020682637821,0.199642664090176,1.
44768997125421,0.147703775932809,0.59224645295115
"3904","BDP1",381.225748041206,0.333233753053044,0.230242465991451,1.4
4731664342675,0.147808259799001,0.592255278697688
"3905","SLC27A3",411.04759709993,-0.396371810345005,0.273953704862851,
-1.44685690797078,0.147937004298606,0.592467628838476
"3906","DHX33",60.0568150767038,0.346533161913882,0.239494644181247,1.
446934912046,0.147915153972806,0.592467628838476
"3907","AC073316.1",7.56857431199014,1.2159321259097,0.840482978901449
,1.44670642527345,0.147979164140861,0.592484787472611
"3908","PRRX2",584.779481958754,-0.362103110576224,0.25044497817709,-1
.4458389751388,0.1482223714852,0.593306693230035
"3909","C6orf163",5.27727304746014,1.25324203639992,0.867252551779183,
1.44507160437738,0.148437773959788,0.593561374757914
"3910","KANSL1",367.228844668932,0.281475405805964,0.194752648363535,1
.44529693522087,0.148374498371702,0.593561374757914
"3911","CDC25B",65.4648470170048,0.359400090426265,0.248704023636854,1
.4450915798252,0.148432163783437,0.593561374757914
"3912","ITGB2",39.7257181850887,-0.677767323353651,0.468923088972199,-
1.44536991095748,0.148354010331598,0.593561374757914
"3913","RARB",3.03849809511444,1.6619936961375,1.15030368175337,1.4448
3037175382,0.148505537797255,0.593680584656905
"3914","FAM120AOS",281.556820907717,-0.247711363855742,0.1714653482754
89,-1.44467302779889,0.14854974948206,0.593705603256991
"3915","ALKBH7",496.609332967243,-0.431979152921129,0.299059916904349,
-1.44445687470479,0.148610502188149,0.593796701335687
"3916","AC114980.1",12.9899686022058,0.690532624706114,0.4781564363766
12,1.44415628897282,0.14869501734686,0.593982675269899
"3917","MKNK1",141.653136939986,0.306697024196993,0.212513495380813,1.
44318846032535,0.148967389347557,0.594210099243758
"3918","RNF2",70.249904420807,-0.3948541499753,0.273503762246651,-1.44
368818451284,0.148826706523107,0.594210099243758
"3919","ARF4-
AS1",14.1720199987096,-0.630794897901013,0.437015512508425,-1.44341534
761618,0.148903503245999,0.594210099243758
"3920","TMEM186",59.1682409405414,-0.35328202567342,0.244773043418954,
-1.44330446171207,0.148934723483503,0.594210099243758

"3921","WDR59",85.6539147785409,0.343493075573148,0.238017159661572,1.
44314416683884,0.148979863787894,0.594210099243758
"3922","AC060766.4",3.84269565242142,1.20951850063527,0.83801745879018
,1.44330942983146,0.148933324588623,0.594210099243758
"3923","CERK",102.867200026703,0.365067895494378,0.25299240672833,1.44
29994173161,0.149020635371932,0.594221208035465
"3924","EXOSC10",130.250128674421,0.297519383499097,0.206310956258385,
1.44209201922598,0.149276416183324,0.594238498680515
"3925","AC024560.1",2.72425484200156,-1.37949422545034,0.9566263960452
06,-1.44204072891289,0.149290884089652,0.594238498680515
"3926","DDIT3",294.201677903192,-0.410579306054004,0.284663415983399,-
1.44233253379475,0.149208586412967,0.594238498680515
"3927","TINF2",190.176010679206,-0.289437604841238,0.20062337360572,-1
.44269134567572,0.149107438235257,0.594238498680515
"3928","DACT3",37.940759815235,-0.574386870404438,0.398284324029491,-1
.44215284345941,0.149259260347798,0.594238498680515
"3929","FAM110A",9.53053394782099,-0.704989360165566,0.488807525189282
,-1.44226372106807,0.149227990532916,0.594238498680515
"3930","PARVB",107.56944342011,-0.427063094286609,0.296098092349131,-1
.44230275480146,0.149216983390096,0.594238498680515
"3931","LINC00891",60.2765496877282,0.394336781393609,0.27350859288142
,1.44177108747942,0.149366961804286,0.594390074664066
"3932","MPHOSPH6",87.9209026863262,-0.370561744463442,0.25711981885856
1,-1.44120257282572,0.149527461750915,0.594877437479545
"3933","EIF2AK3-
DT",9.54199237478961,0.750036130290384,0.520537411772183,1.44088803864
619,0.149616315843423,0.594928324539569
"3934","UBE2R2",512.726345378618,-0.265531777902812,0.184273514322993,
-1.44096550651001,0.149594427880321,0.594928324539569
"3935","COX20",310.813208403697,-0.284439414449706,0.197443645910769,-
1.44061062657976,0.149694716581089,0.595088805966447
"3936","BRWD1",235.152182389176,0.322896778096131,0.224177443450657,1.
44036247860593,0.149764873408865,0.595216441751745
"3937","Z97200.1",200.034993450025,0.482774670735845,0.335272672906107
,1.43994637723142,0.149882570604344,0.595381679015682
"3938","NDUFA8",285.693739111793,-0.385150171642061,0.267461612271231,
-1.44002037664935,0.149861634193016,0.595381679015682
"3939","STK38L",56.8217812120246,-0.339525634066834,0.23591646813633,-
1.43917733572813,0.150100285076376,0.596095140759012
"3940","AIDA",509.655052605098,0.254485497918199,0.176892640946148,1.4
3864378165778,0.150251475069413,0.59654411789615
"3941","AC006504.1",3.76274682872847,1.21328289442701,0.84343389641082
9,1.4385038348471,0.150291150168904,0.596550231436734
"3942","ZNF891",82.104791130699,0.499878575281433,0.347572260458526,1.
43820043239924,0.150377192582523,0.596740340834198
"3943","DENND5A",165.753937079152,0.276898520294985,0.192574464072868,
1.43787766269057,0.15046876861594,0.596952307242999
"3944","LYPLAL1-
AS1",49.609436008691,0.924345127777041,0.643751094215633,1.43587348601
449,0.151038344338617,0.597114434491166

"3945","PTPN18",159.784771583599,-0.274228546526514,0.190876013235641,
-1.43668416936167,0.150807755138816,0.597114434491166
"3946","GRPEL1",122.618669459938,-0.360570943317428,0.250831924114861,
-1.43750020891405,0.150575913505926,0.597114434491166
"3947","RBM17",368.595579313904,-0.226179648929734,0.15746912466423,-1
.43634283490185,0.15090481092697,0.597114434491166
"3948","ZNRD2",195.453943070674,-0.417499799612768,0.290783971515483,-
1.43577308417956,0.15106692112942,0.597114434491166
"3949","NFATC4",178.454395561321,0.434543181683423,0.302548872721463,1
.43627433734806,0.150924293405092,0.597114434491166
"3950","GNB5",74.348367390235,-0.376557993443365,0.26225566274933,-1.4
3584313679163,0.151046982027126,0.597114434491166
"3951","EPOP",4.26723706159182,-1.05174345645652,0.732449682472132,-1.
43592588218036,0.151023432758287,0.597114434491166
"3952","AC090360.1",5.1102983824309,1.01052579975981,0.703266586597241
,1.43690290285117,0.150745584996179,0.597114434491166
"3953","FKBP8",1044.37425835261,-0.35768154188236,0.248859364223229,-1
.43728383699284,0.15063735955384,0.597114434491166
"3954","UQCRFS1",426.910226919716,-0.378450957971266,0.263395132429983
,-1.4368183439072,0.150769616683752,0.597114434491166
"3955","ZNF180",39.4100824592488,-0.371586291979394,0.258597567108986,
-1.4369288007369,0.150738225390182,0.597114434491166
"3956","AC012313.1",13.1429614709076,0.747378382337023,0.5205602059371
35,1.43571939194153,0.151082204929747,0.597114434491166
"3957","SLA2",3.05537316693622,1.32522514201345,0.921937062561471,1.43
74355862552,0.150594263271053,0.597114434491166
"3958","ETS2",34.0348969687064,-0.49387323802839,0.343957260176389,-1.
43585641359953,0.15104320327018,0.597114434491166
"3959","AC092069.1",3.40317517022331,-1.48747218390052,1.0362267054901
3,-1.43546984074007,0.151153256560702,0.597244352710044
"3960","NCS1",51.6170915415025,-0.394514316208253,0.274886251129711,-1
.43519115483914,0.15123263344343,0.597407092160498
"3961","LRRC10B",10.0968491003749,1.03440785535741,0.720886493506673,1
.4349108558348,0.151312501810103,0.597571690435607
"3962","MUTYH",48.6536554147075,-0.352625944472166,0.245866896385927,-
1.43421481157294,0.15151097155299,0.597656461585161
"3963","TRAF5",83.0092244646008,0.334734241656289,0.233391584221968,1.
43421727382397,0.151510269118544,0.597656461585161
"3964","GADD45G",59.2494314587305,-0.534848744309019,0.372882460997318
,-1.43436283615621,0.151468747298947,0.597656461585161
"3965","TAS2R31",3.09190361373411,1.27474696659105,0.88881218148992,1.
4342141041026,0.151511173381584,0.597656461585161
"3966","ZNF664",105.800056955324,0.340294747737823,0.237277156721386,1
.4341656501616,0.151524996908953,0.597656461585161
"3967","SLC2A1",6.21241337177591,-0.816314531510173,0.570399670537034,
-1.43112728438572,0.15239373890029,0.598449921521845
"3968","CEPT1",152.347411117045,0.268521867180656,0.187389628368824,1.
4329601350836,0.151869230211616,0.598449921521845
"3969","NOTCH2NLC",45.6332890771273,0.489208675144064,0.34171926607911
4,1.4316098730904,0.15225550248119,0.598449921521845

"3970","MR1",166.826837779718,0.292125681922274,0.204054848700113,1.43160372705278,0.152257262399188,0.598449921521845
"3971","AC108471.3",6.8903354104434,1.03119021810197,0.720736079493345,1.43074593799559,0.152503042243655,0.598449921521845
"3972","SEPTIN8",175.762385994124,0.272639187193217,0.190507714791233,1.4311188787918,0.152396147509373,0.598449921521845
"3973","PTK7",121.89256440232,0.30570831330289,0.213464487008952,1.43212727131549,0.152107401004171,0.598449921521845
"3974","GPER1",23.9553266312989,-0.65414252821075,0.45683731622798,-1.43189381640686,0.152174212188629,0.598449921521845
"3975","ABCB4",28.3969254224008,-0.492110998287134,0.343945258879703,-1.43078290972824,0.152492442616066,0.598449921521845
"3976","PDAP1",698.003493196585,-0.270637659581619,0.189088829819433,-1.43127259204078,0.152352105810844,0.598449921521845
"3977","FAM71F2",8.97221979295467,0.851845792563287,0.594766927081723,1.43223463473826,0.152076682750479,0.598449921521845
"3978","PLEC",403.980159046526,-0.35414500083811,0.247329257602133,-1.43187669858212,0.15217911192428,0.598449921521845
"3979","NOL8",200.634138792975,0.299558004370238,0.209045075357717,1.43298283328433,0.151862743273279,0.598449921521845
"3980","SLC11A2",105.847315234397,0.318336013965799,0.222389867697184,1.43143218376864,0.152306390070214,0.598449921521845
"3981","ALKBH2",104.165869149155,-0.384463624780947,0.268546711941366,-1.43164525084518,0.152245372361475,0.598449921521845
"3982","RPL6",10878.6148345605,-0.408857050661803,0.285667720242803,-1.43123293844434,0.152363466374811,0.598449921521845
"3983","AC123768.3",10.3436293030157,0.642229441806137,0.448615425925831,1.43158127137678,0.152263692714126,0.598449921521845
"3984","AC022087.1",2.64218748066933,1.79331319068683,1.25221547866539,1.43211230114975,0.152111684563898,0.598449921521845
"3985","SOX9",108.163485878778,-0.541040083299564,0.378185112490654,-1.43062237362116,0.152538471656339,0.598449921521845
"3986","LRRC8E",10.9346554643606,-0.872687694226795,0.609922008052732,-1.4308185025377,0.152482238849198,0.598449921521845
"3987","DONSON",20.0683481925156,0.52827480618464,0.369288638786662,1.43052006127333,0.152567812250151,0.598449921521845
"3988","EWSR1",332.95976418344,0.284357142597288,0.198522923088211,1.43236427397826,0.152039597352728,0.598449921521845
"3989","MMACHC",16.5259984830112,0.523445789910529,0.366289604554088,1.42904899129681,0.152990152866366,0.59879814595796
"3990","UQCRH",1269.32719869699,-0.364715554878403,0.256265684177651,-1.42319310542402,0.154680183727389,0.59879814595796
"3991","FOXD2",4.29527974230652,-1.30872952976728,0.91932041052673,-1.42358367635657,0.154567023925415,0.59879814595796
"3992","MEX3A",6.8995256397752,0.857435055446557,0.601735390830551,1.42493705457988,0.154175397235329,0.59879814595796
"3993","CD1C",7.93721059347867,-1.14284920998483,0.801525619269466,-1.42584239668653,0.153913839867761,0.59879814595796
"3994","LHX9",5.39985665385785,1.4660085174984,1.02980587926759,1.42357753729377,0.154568802104524,0.59879814595796

"3995","PLEKHA3",274.464146060398,-0.219372406860971,0.15390848459664,
-1.42534316698588,0.154058027806686,0.59879814595796
"3996","ARPC2",1359.05746372476,-0.306699967733484,0.214721777787749,-
1.42835985661713,0.153188307405233,0.59879814595796
"3997","HACL1",90.0947286765308,-0.409426851074331,0.287197655801744,-
1.42559259382313,0.153985975312035,0.59879814595796
"3998","NOA1",162.246376033876,-0.317424324201839,0.223143025074065,-1
.42251510705513,0.154876769068971,0.59879814595796
"3999","RAP1GDS1",62.9973633931767,-0.344537853658787,0.24207798640069
,-1.42325148511647,0.154663265427089,0.59879814595796
"4000","SAP30",148.641993595711,-0.310840785508857,0.218060602330356,-
1.42547889067068,0.154018817821551,0.59879814595796
"4001","CARD6",216.907440646535,0.305747147182397,0.21441007325251,1.4
2599245709095,0.153870519353065,0.59879814595796
"4002","MOCS2",252.810719060274,-0.304167413949648,0.213284728832386,-
1.42610966858619,0.153836688344821,0.59879814595796
"4003","LINC01023",25.4265175542007,-0.440563080599918,0.3089344257015
73,-1.42607312085542,0.153847236588952,0.59879814595796
"4004","AFF4",390.654767561885,0.297391836617161,0.208383822113946,1.4
2713495510484,0.15354099868278,0.59879814595796
"4005","BRD8",146.829121343846,0.36960023341015,0.259215791869745,1.42
5839956525,0.153914544387701,0.59879814595796
"4006","RPS14",17272.3806391299,-0.415813647084221,0.291074514083372,-
1.42854707975264,0.153134453757664,0.59879814595796
"4007","SPARC",5658.84960039028,0.495694008589956,0.347587053295442,1.
42610032189152,0.153839385894103,0.59879814595796
"4008","CLINT1",504.321825422768,0.237370382566907,0.166558659141717,1
.42514585425991,0.15411504413013,0.59879814595796
"4009","HIST1H4H",21.9764577547825,0.58358041816519,0.409448214884972,
1.42528504692379,0.154074820760837,0.59879814595796
"4010","TRAF3IP2",101.024513168493,-0.368272392581919,0.25807539684302
5,-1.42699535518266,0.153581233463056,0.59879814595796
"4011","HECW1",4.63669337851717,1.1588563836715,0.81385761389964,1.423
90556269269,0.15447381133799,0.59879814595796
"4012","MDH2",480.899374797713,-0.404406841625961,0.284272242760436,-1
.42260404216378,0.154850971564441,0.59879814595796
"4013","ARPC1A",417.224846278548,-0.415229099632582,0.291849786195852,
-1.42274937064348,0.154808822996848,0.59879814595796
"4014","TPM2",595.673726046582,-0.373898781281286,0.26247646043314,-1.
42450405138912,0.154300613132558,0.59879814595796
"4015","COL15A1",571.542897084848,-0.360132577787602,0.253119270286402
,-1.4227781921942,0.154800465126473,0.59879814595796
"4016","FNBP1",247.678547541527,0.349821795383244,0.245499507718275,1.
42493888739152,0.154174867386881,0.59879814595796
"4017","RIC3",3.51824731320056,-1.05859101292302,0.741696337563822,-1.
42725662688327,0.153505937598213,0.59879814595796
"4018","SPTY2D1",164.978032185508,0.283634303205517,0.198974800470947,
1.42547851554163,0.154018926184257,0.59879814595796
"4019","FAU",9303.1051100858,-0.40950892329256,0.287412624305704,-1.42
481188598379,0.154211585562654,0.59879814595796

"4020","NCKAP5L",102.219801902967,0.304454994270877,0.21330258662733,1
.42733850106939,0.153482348061999,0.59879814595796
"4021","GPR180",62.8294388606766,0.406522040643474,0.284557161114328,1
.42861293334363,0.15311551477847,0.59879814595796
"4022","FGF14-
AS2",58.4884373764415,-0.422255208886431,0.296783205596051,-1.42277325
982239,0.154801895425074,0.59879814595796
"4023","AL589743.4",3.02829072576468,4.6250990874805,3.23642006560157,
1.42907873320851,0.152981605235904,0.59879814595796
"4024","DHRS7",672.025949753528,-0.321484449586,0.22510710670368,-1.42
813993877673,0.153251583891058,0.59879814595796
"4025","AC015712.2",36.6372886848976,0.455237875922473,0.3198821983512
19,1.42314226383626,0.154694918647501,0.59879814595796
"4026","JMJD8",191.592626666725,-0.357946402115956,0.251590698252826,-
1.42273305254017,0.154813555183991,0.59879814595796
"4027","PAM16",161.696803126345,-0.338524792308986,0.237704027432307,-
1.42414411722743,0.154404757723896,0.59879814595796
"4028","CDIP1",82.9889374465841,0.402075336743634,0.282504924674932,1.
42325071750975,0.154663487868629,0.59879814595796
"4029","LITAF",346.598577883329,-0.256969577290926,0.180601349536345,-
1.42285524416424,0.15477812275212,0.59879814595796
"4030","AC009152.6",3.50178303095642,-1.11645250740929,0.7815025470476
16,-1.42859740077247,0.153119981664238,0.59879814595796
"4031","AC008870.2",7.6064092419662,-0.985695486018395,0.6909150786636
94,-1.4266521551749,0.153680182904627,0.59879814595796
"4032","TUBB3",35.2941134233777,-0.737558333327947,0.516598664642287,-
1.42772017004469,0.153372418223788,0.59879814595796
"4033","ARL4D",177.003047041507,0.563543020986647,0.395812072348331,1.
42376410512993,0.154514769683756,0.59879814595796
"4034","NFE2L1",571.282777031443,0.341256658540259,0.239280149721134,1
.42618039539833,0.153816277047033,0.59879814595796
"4035","MEX3D",122.093496683857,-0.379939678052169,0.265679896938499,-
1.43006558806412,0.152698195584918,0.59879814595796
"4036","CDKN2D",62.809620559507,-0.437759814087608,0.306507032916359,-
1.42822110775861,0.153228227000912,0.59879814595796
"4037","RBM42",263.990069639682,-0.311133530002523,0.217748199105623,-
1.42886844199157,0.15304204935033,0.59879814595796
"4038","PAK4",37.1213351518725,0.371114712997538,0.26033795461925,1.42
55113647962,0.154009437311837,0.59879814595796
"4039","MED29",224.752322426825,0.266431377256333,0.18693824828297,1.4
2523736957797,0.154088597481321,0.59879814595796
"4040","ZNF581",170.942137416877,-0.370425130845234,0.25966472641133 9,
-1.42655159969028,0.153709183659229,0.59879814595796
"4041","EPN1",289.367240837869,-0.282715205834677,0.198160033737657,-1
.42670144176985,0.153665969898922,0.59879814595796
"4042","BIRC7",2.78544401445422,-1.38540690892914,0.973384753296776,-1
.42328807209778,0.154652663320767,0.59879814595796
"4043","AP001347.1",34.6581786882925,0.549889198822442,0.3852923077426
03,1.42720004467309,0.153522241637958,0.59879814595796
"4044","THAP7-

AS1",41.8697111577684,-0.531629720869416,0.372941710812646,-1.42550351
826023,0.154011703830602,0.59879814595796
"4045","FBLN1",11064.5481474603,0.365527287567299,0.256902075972674,1.
42282730173881,0.154786224794388,0.59879814595796
"4046","BX537318.1",12.7770355853874,-0.642626149316831,0.451341588784
01,-1.42381328307939,0.154500529513443,0.59879814595796
"4047","RAB8B",337.173902692332,0.237810261099988,0.167218291335845,1.
42215459325777,0.15498137713573,0.599054529907148
"4048","TCF23",5.3182288701875,1.50834800366621,1.06091179274169,1.421
74685396627,0.155099752982291,0.599154316218523
"4049","POLE4",473.077524729321,-0.415336496416225,0.29223460398643,-1
.42124338032025,0.15524601736774,0.599154316218523
"4050","BMPER",242.956956517058,0.461760907708781,0.32490853085002,1.4
2120278128964,0.15525781637402,0.599154316218523
"4051","ANK3",4.48180560967827,1.07488141557733,0.756381946468442,1.42
108285449694,0.155292673817108,0.599154316218523
"4052","AC010201.1",3.07999055684297,1.3980965620317,0.983479006106464
,1.42158251813293,0.155147482761588,0.599154316218523
"4053","ADORA2B",4.52187151873198,-1.11286393818486,0.783149504209042,
-1.42101084429444,0.155313606869917,0.599154316218523
"4054","AC008764.7",3.76994341381312,1.24036384309597,0.87257948696440
3,1.42149094910659,0.155174082952136,0.599154316218523
"4055","IDH3B",256.63631219254,-0.345492760650417,0.243026654763465,-1
.42162496943671,0.155135152116049,0.599154316218523
"4056","PIK3CD-
AS2",8.12007311157594,-1.09367275012207,0.769847608963895,-1.420635379
50582,0.155422787557115,0.599186093218164
"4057","TF",6.65766539898774,0.991988429373631,0.698720849348281,1.419
72066569774,0.155689019291963,0.599186093218164
"4058","COQ2",52.2706958188297,-0.409051815044448,0.28810827480675,-1.
4197850281073,0.155670274998927,0.599186093218164
"4059","SMARCAD1",122.605608628202,0.286900973462356,0.202050746354235
,1.41994513081067,0.155623655647024,0.599186093218164
"4060","AC106791.1",37.6488882180808,0.587826712070491,0.4139150654080
9,1.42016264010815,0.155560337388792,0.599186093218164
"4061","LARP1",321.898454280551,0.2611768555322,0.183876640188738,1.42
03917107911,0.155493674684725,0.599186093218164
"4062","PCOLCE-
AS1",4.37069360289545,1.10472752580903,0.777913086781966,1.42011690583
458,0.155573649285778,0.599186093218164
"4063","FBF1",17.9987438728194,0.490731699464229,0.345604215956633,1.4
1992393844468,0.155629825917212,0.599186093218164
"4064","RABAC1",1897.3960906517,-0.392640748321064,0.27657258279818,-1
.41966620244343,0.155704881987588,0.599186093218164
"4065","FAM50A",347.526872825491,-0.273461793511358,0.19256787277964,-
1.42008004535776,0.155584378911724,0.599186093218164
"4066","AC002546.1",9.68349567271698,-1.06703729281406,0.7517950184459
48,-1.41931945095854,0.155805903859948,0.59942738664072
"4067","MAGEF1",213.293989431379,-0.276666434319323,0.194954374190809,
-1.41913427419966,0.15585987324959,0.599487582307189

"4068","EDA2R",27.8516705930511,0.677476522879208,0.477444346705342,1.
41896438308299,0.15590940014639,0.599530665312187
"4069","PDE3B",4.10005089681941,-1.20065215313839,0.846244942491152,-1
.41879979761408,0.155957391720612,0.599567824695389
"4070","LSM7",595.036526755717,-0.391261295863139,0.27579771209l005,-1
.41865316030625,0.156000159221176,0.599584887149103
"4071","APCDD1",32.718792419189,0.805355462286922,0.56782209987185,1.4
1832356026418,0.156096321191775,0.599704402332045
"4072","EIF2S3",639.234891389667,-0.217669904212,0.153474154030058,-1.
41828378587687,0.156107928549261,0.599704402332045
"4073","CCM2",180.624495859119,-0.347663158903903,0.245155383003206,-1
.41813389795873,0.156151676216495,0.599708489986794
"4074","ZBTB6",42.389064616746,0.377796073697912,0.266425544149999,1.4
1801746113808,0.156185666956862,0.599708489986794
"4075","ABHD18",46.3161508514093,0.440705947870327,0.31090057054801,1.
41751411743477,0.156332669808416,0.599879842022916
"4076","LIMK1",53.5815612485671,-0.370310671442133,0.261247485373324,-
1.41747075924178,0.156345337590451,0.599879842022916
"4077","PTGES3",1297.19153073689,-0.281777472862165,0.198763372606931,
-1.41765290640042,0.156292125650938,0.599879842022916
"4078","FOPNL",148.149809292487,0.26778931104109,0.188946436036725,1.4
1727632792736,0.156402153349167,0.599950682893825
"4079","CFL1",1892.77837820749,-0.416136869506646,0.293657477605543,-1
.41708248977615,0.156458811363542,0.600020884079405
"4080","SNHG26",38.5001148709082,-0.584545796585637,0.412729392149122,
-1.41629311530698,0.156689702731008,0.60075907348558
"4081","ASMTL",171.49762987435,-0.382221583478603,0.269909711099294,-1
.41610904595423,0.156743579991311,0.600818383191393
"4082","RPS7",8698.1166639853,-0.431857142218888,0.305074497777518,-1.
4155792941232,0.156898717202022,0.601077612885581
"4083","COBLL1",210.499515200639,-0.340285859289767,0.240558939125928,
-1.4145633520259,0.157196560320982,0.601077612885581
"4084","ABHD5",257.805720520668,-0.650512643799186,0.459994670135793,-
1.41417430686132,0.157310729857033,0.601077612885581
"4085","TBC1D1",44.7301249343963,-0.387831910149279,0.274077018843999,
-1.41504717099257,0.157054666009651,0.601077612885581
"4086","NKD2",5.65607769638391,1.05027957748781,0.742582856167299,1.41
436011990451,0.157256193160965,0.601077612885581
"4087","ADCK2",40.668325964377,-0.457589898707988,0.32355982894687,-1.
41423581597679,0.157292675156782,0.601077612885581
"4088","PLEKHA7",2.84573717848996,1.64190288835011,1.16031908169076,1.
4150442876089,0.157055511360044,0.601077612885581
"4089","IPO8",95.894103283288,0.331759531312496,0.234590315065751,1.41
420813224753,0.157300800935784,0.601077612885581
"4090","AC073611.1",10.9787168506814,0.832845222078286,0.5884679870650
07,1.41527702506319,0.156987288491769,0.601077612885581
"4091","IRAK3",165.207492661748,0.350970335134732,0.247967058287205,1.
4153909699095,0.156953895768412,0.601077612885581
"4092","PDS5B",290.395365525323,0.236247886699354,0.166991219613103,1.
41473238680877,0.15714697480072,0.601077612885581

"4093","ZNF155",15.2990470882219,0.696740779083407,0.492528875937457,1
.41461914848599,0.157180191393483,0.601077612885581
"4094","TMEM191C",5.68107323334619,-0.860187956308693,0.60817645383184
4,-1.41437234356746,0.15725260598093,0.601077612885581
"4095","MACO1",175.352359086341,0.206635182980289,0.146294188437314,1.
41246337388742,0.157813567729108,0.60175774264758
"4096","LMCD1-
AS1",17.8853575914071,0.544425074906977,0.385674275690067,1.4116188432
1391,0.15806222106171,0.60175774264758
"4097","PRRT3",31.0268841664011,-0.396776152408941,0.281021004986508,-
1.41190923585229,0.157976687936364,0.60175774264758
"4098","LDB2",294.073850925306,0.293759694356668,0.207950111480575,1.4
1264504387683,0.15776011777663,0.60175774264758
"4099","ARAP2",7.47222510610475,-0.867337386699229,0.614196901029625,-
1.41214875106866,0.157906166786752,0.60175774264758
"4100","TMEM176B",2536.05036909315,0.449519296671503,0.318258253301918
,1.41243563052256,0.157821731437752,0.60175774264758
"4101","GALNT12",109.48108586665,0.406525347801168,0.287791068292233,1
.4125711065792,0.157781869549493,0.60175774264758
"4102","YBX3",2487.86430307354,-0.252854096192056,0.17901644762097,-1.
4124629303751,0.157813698233663,0.60175774264758
"4103","DYNLL1",2793.87303597794,-0.399150692532164,0.282720795946088,
-1.41181935766861,0.158003157181291,0.60175774264758
"4104","RB1",105.512397821315,0.343666907412501,0.243254310370601,1.41
278856226194,0.157717902278341,0.60175774264758
"4105","TDP1",23.8903410106229,0.513625685349194,0.363682377337415,1.4
1229192656939,0.157864022604035,0.60175774264758
"4106","C16orf74",46.6641830087659,0.512224763134665,0.36289492023056,
1.41149609592008,0.158098385964896,0.60175774264758
"4107","MINK1",98.0035030631983,0.292531786937599,0.207252355591067,1.
41147629470035,0.15810422056393,0.60175774264758
"4108","CBX8",13.8300436914001,0.778347327548795,0.551364790128563,1.4
1167397970281,0.1580459782967,0.60175774264758
"4109","B9D2",29.9704113031805,-0.45746329697819,0.324041071960446,-1.
41174479583881,0.158025118288678,0.60175774264758
"4110","LINC00893",25.0327171072407,0.606657911785064,0.42926887676258
3,1.41323525795836,0.157586562864121,0.60175774264758
"4111","CCL22",4.76171923971804,-1.29332343422207,0.916516127648507,-1
.41113003383839,0.158206275654271,0.601853300111809
"4112","KATNB1",38.6080687976947,-0.413520264114247,0.293019893854654,
-1.41123614057196,0.158174997018446,0.601853300111809
"4113","ANKRD36C",415.380156971912,-0.565668670849946,0.40101231512921
5,-1.41060174340949,0.158362077280023,0.602006798272514
"4114","TRIM22",348.449969190193,0.258809096634162,0.183465692868345,1
.41066753455581,0.158342668047035,0.602006798272514
"4115","CBX6",312.757139952439,-0.194907768062393,0.138162688028182,-1
.4107120442144,0.158329538147732,0.602006798272514
"4116","AC012358.3",19.37416329246,0.497166368160325,0.352832058375102
,1.40907368352503,0.158813381525787,0.602843745842211
"4117","PTGER4",312.306338824564,0.419874547010891,0.298078241625549,1

.4086051525302,0.158951954442361,0.602843745842211
"4118","LRCH4",36.8581028251403,-0.393729991930862,0.279517703067312,-1.40860484903185,0.158952044234802,0.602843745842211
"4119","AC104389.5",7.37080092261124,0.919488729293071,0.65244130039156,1.40930491178477,0.158745027068668,0.602843745842211
"4120","CSNK1G1",112.65507002743,0.31873439663101,0.226221344082793,1.40894926569952,0.158850170472616,0.602843745842211
"4121","ANP32A",309.824896243667,-0.248569452555117,0.176393705497029,-1.40917416443357,0.158783675162473,0.602843745842211
"4122","GPT2",5.32072696700915,0.975556891476709,0.692595477729446,1.40855221098888,0.15896761820617,0.602843745842211
"4123","CCL5",56.6909452072326,0.71632587035789,0.508519681235901,1.40864925545642,0.158938906643082,0.602843745842211
"4124","HCST",37.6117244152371,-0.51669848557264,0.366573709622272,-1.40953503213599,0.15867702223683,0.602843745842211
"4125","TIMP3",8173.29340510353,-0.301429439687951,0.213863846570771,-1.40944551648754,0.158703473150052,0.602843745842211
"4126","PHACTR1",66.1862002943184,0.342849898651951,0.24349775107428,1.40802080158582,0.159124910519897,0.603001689743881
"4127","AC022075.1",6.27841448438505,0.83646073506739,0.594021850046318,1.40813125813835,0.159092206701266,0.603001689743881
"4128","NUP50",113.453887559951,0.32231391894737,0.22888731300427,1.40817730225771,0.15907857552608,0.603001689743881
"4129","PNPLA6",49.034185829821,-0.328935206107891,0.233651590497895,-1.40780212711993,0.159189670347738,0.603100996185438
"4130","ALOX5",8.25966214089809,-1.04547870348903,0.743016055655737,-1.40707417495354,0.15940539502127,0.60333379973814
"4131","RAB1B",319.330406677118,-0.305779769406964,0.217270906186831,-1.40736638316418,0.159318774150175,0.60333379973814
"4132","SIAE",45.8650752646893,0.384087076226726,0.272949875633945,1.4071707317494,0.159376768224142,0.60333379973814
"4133","PPAN",58.9017844838485,-0.402107088357506,0.285740015938459,-1.40724807842143,0.159353839578183,0.60333379973814
"4134","PPP1R32",8.84145824522605,0.711411408878082,0.505723686932755,1.40671957288146,0.159510559488852,0.60337838208839
"4135","ASB16-
AS1",34.1216719813534,0.487841176130394,0.346766120258667,1.40683056281996,0.159477637520857,0.60337838208839
"4136","CUEDC1",164.64197420961,-0.275780454587068,0.196055572970542,-1.40664430196282,0.159532889363778,0.60337838208839
"4137","SQLE",61.7969965777715,-0.551355681391964,0.392006735711072,-1.40649542766617,0.159577061383875,0.603399557947293
"4138","YBX1",3463.21672925382,-0.305520455129407,0.217453934920524,-1.4049893152822,0.160024455528702,0.604360829994082
"4139","SNN",36.0133669204237,0.420419642110806,0.299171761644126,1.40527849219576,0.159938481379062,0.604360829994082
"4140","ZNF737",90.1682642889222,0.338409094450771,0.240791041399806,1.40540566826522,0.159900682177988,0.604360829994082
"4141","CLEC11A",471.483812700397,0.38087179580916,0.271065032232678,1.40509379860633,0.159993387936006,0.604360829994082

"4142","PIGA",13.1618332926579,-0.552734122053086,0.393391748981015,-1
.40504757276889,0.160007132394512,0.604360829994082
"4143","AC242426.2",28.9365340982003,0.509840674890193,0.3629708192420
45,1.40463268081672,0.160130533354825,0.60443449528782
"4144","GPD2",62.5926887082816,0.37193028710606,0.264828596244484,1.40
441890483271,0.160194144644226,0.60443449528782
"4145","ZC2HC1A",340.436598274237,-0.292786351791094,0.208456712976619
,-1.40454268711382,0.160157309598565,0.60443449528782
"4146","PNKP",125.388861853257,-0.291151903019387,0.207336527197305,-1
.4042479969886,0.160245013807772,0.60443449528782
"4147","AL022323.3",2.70178483248314,1.62482691901575,1.15706246418426
,1.40426897363858,0.16023876964161,0.60443449528782
"4148","HMGN5",40.6457065535776,-0.379587700033855,0.270333768747472,-
1.40414459426429,0.160275796615347,0.60443449528782
"4149","SLC46A3",44.4938016655,0.495325250036521,0.352965770271816,1.4
0332375475128,0.160520317682921,0.605210732589523
"4150","FAM66B",22.1177472035161,0.573975534342671,0.409167439520061,1
.40278888030759,0.160679803847892,0.605666065443995
"4151","SLC29A1",342.597971868745,-0.384896652250223,0.274437414127241
,-1.40249336437694,0.160767970635826,0.605852412588828
"4152","VLDLR-
AS1",8.67777636750698,1.0480907902175,0.747793429908716,1.401577960300
41,0.16104131222716,0.606298254433084
"4153","LPXN",22.9733269505254,-0.569241996989723,0.406118678317029,-1
.40166416218206,0.161015557199569,0.606298254433084
"4154","FUS",1287.72265330032,0.32458583036594,0.231563520084715,1.401
71401025167,0.161000665227624,0.606298254433084
"4155","GMIP",12.9795978397825,-0.583405633944583,0.416188597963289,-1
.40178187677319,0.160980391966004,0.606298254433084
"4156","PLPP3",4658.68475340077,0.3310403563903,0.236347417810427,1.40
065146239857,0.161318323789256,0.60646540231563
"4157","TRIM52",97.751238865582,0.405619116093349,0.289558710144534,1.
40081821711004,0.161268439628038,0.60646540231563
"4158","CBLN3",28.5543456731815,0.51516383338369,0.367789203751167,1.4
0070406670292,0.161302586114627,0.60646540231563
"4159","TNFSF13",80.754634219774,-0.565894867979592,0.403984724199608,
-1.40078283677871,0.161279022571976,0.60646540231563
"4160","DSG3",5.55997990910963,-1.85695507191742,1.32539280665908,-1.4
0106017068121,0.161196080675097,0.60646540231563
"4161","LINC01415",5.80193986662029,0.886664135626816,0.63303527754451
2,1.40065517211949,0.161317213910879,0.60646540231563
"4162","KDM7A-
DT",75.0080721211846,-0.404713593763702,0.288989468848017,-1.400444090
14969,0.161380374818218,0.606483943910535
"4163","RPL36AL",2284.50159150243,-0.391745926476408,0.279743416784394
,-1.40037585505841,0.161400796426488,0.606483943910535
"4164","PIK3CA",199.403461673511,0.258291732585928,0.184492165482574,1
.400014639702,0.161508934581511,0.606566542097371
"4165","CSNK2B",624.483239598611,-0.371810692553614,0.265610658514071,
-1.39983348045469,0.161563189379218,0.606566542097371

"4166","SOBP",273.843055935544,-0.337485616743529,0.241098277952851,-1
.39978443483336,0.161577880260803,0.606566542097371
"4167","FEN1",23.3652229832054,-0.496390816270808,0.354531502244571,-1
.40013176016267,0.161473865876106,0.606566542097371
"4168","H6PD",504.576362670965,0.303584107425243,0.217086410041929,1.3
9844823711723,0.161978506448411,0.6067123474132
"4169","RPS6KC1",88.0342984998489,0.312470287442985,0.223421841858385,
1.39856642861732,0.161943039421018,0.6067123474132
"4170","KIAA1841",53.9956296084436,0.396858041998003,0.283787590406724
,1.39843338966734,0.161982962300991,0.6067123474132
"4171","PSMD6-
AS2",6.71596200042582,0.953048434574517,0.681499426885937,1.3984581600
1549,0.161975528549421,0.6067123474132
"4172","AP2M1",946.795678849481,-0.280423074118762,0.200501960041544,-
1.39860515109508,0.161931420814844,0.6067123474132
"4173","AL355347.1",4.4899121693883,1.02073739079971,0.729704157729833
,1.39883729589167,0.161861779400838,0.6067123474132
"4174","TM9SF3",806.892093313456,0.206325870873409,0.147499285636243,1
.3988262382656,0.161865096079314,0.6067123474132
"4175","GOT1",51.9712259758439,-0.389609270919261,0.27858249320855,-1.
39854183381005,0.161950419373152,0.6067123474132
"4176","ESAM",3.3188592686169,-1.29504981853911,0.925476002839268,-1.3
993337639939,0.161712919182553,0.6067123474132
"4177","TAF15",174.734239978503,-0.309896110449722,0.221613748085854,-
1.39836139736992,0.16200456914562,0.6067123474132
"4178","NCF2",12.2319119134279,-0.838800453344162,0.599994199474194,-1
.3980142709367,0.162108781753282,0.60685008182834
"4179","SUPT7L",96.5732597193403,0.305825161531479,0.218763004872195,1
.39797477050632,0.162120643585572,0.60685008182834
"4180","AP4M1",86.3108774363203,-0.366304829230678,0.262096922353743,-
1.39759301994508,0.1622353156176,0.60685008182834
"4181","AL512506.1",5.76819770270196,-0.783515464275023,0.560590444889
442,-1.39766111145463,0.162214857485332,0.60685008182834
"4182","SULT1A2",8.52173680327127,1.01598929169151,0.726850645675964,1
.39779650432401,0.162174184405938,0.60685008182834
"4183","CREB3",187.615822082203,-0.344730064547579,0.246689521673973,-
1.39742483672727,0.162285854703414,0.606894005528451
"4184","TRIM62",8.54294358522022,0.750703854621938,0.537266288963664,1
.39726588107729,0.162333631822979,0.606927581885005
"4185","NANS",209.722832850082,-0.396982199761947,0.284206268499339,-1
.39681014728523,0.162470670047602,0.607294788901945
"4186","USP48",265.42919465549,0.242349551549581,0.173581819874972,1.3
9616897509279,0.162663616975897,0.607435416651697
"4187","ERICH1",338.937084250429,0.265788608967111,0.190358729746019,1
.39625122168935,0.162638856978624,0.607435416651697
"4188","ATP5MG",1753.35168780473,-0.37812364959549,0.270819164244137,-
1.39622190567954,0.162647682115856,0.607435416651697
"4189","ZNF726",9.19325173866852,0.688462837536208,0.493103794460836,1
.39618239662702,0.162659576284467,0.607435416651697
"4190","C11orf1",62.755326065734,-0.401048242157188,0.287297054730951,

-1.39593579381718,0.162733830691958,0.607552580791002
"4191","DBT",122.836205564609,0.279086399421249,0.200032603585482,1.39520455375158,0.162954164218139,0.607783083778365
"4192","ANKRD17",308.489191149591,0.294259501157784,0.210945154892244,1.39495738268129,0.163028691397687,0.607783083778365
"4193","RIPK2",91.5501124906286,-0.375937011411711,0.269393581668878,-1.39549357146077,0.162867052003491,0.607783083778365
"4194","HPS1",178.293581635222,-0.275855265384658,0.197750760538475,-1.39496437148208,0.163026583776938,0.607783083778365
"4195","CSNK2A1",345.238944421754,0.229925358328117,0.164789087119905,1.39527053852066,0.162934272794095,0.607783083778365
"4196","LAGE3",132.242101714637,-0.32180373564015,0.230657672505724,-1.39515730018547,0.162968410156836,0.607783083778365
"4197","CARD16",19.5920038333456,-0.54291823265486,0.389381080493434,-1.39431076611853,0.16322378121287,0.6083654061265
"4198","TASOR2",189.62128098567,0.332314661316509,0.238497138992482,1.39336959227416,0.163508055998774,0.608669025339021
"4199","DCN",88968.3968285451,0.293890202810516,0.210875833081767,1.39366469128096,0.163418883333024,0.608669025339021
"4200","NLRC3",3.79022673423079,1.1242727721044,0.806786623579486,1.39351935102285,0.163462797496064,0.608669025339021
"4201","B3GNT9",71.6355407913565,0.45404769699781,0.325745652132543,1.39387185684695,0.16335630419542,0.608669025339021
"4202","SPATA2L",32.6282273246657,-0.592680377215373,0.425373088137219,-1.39331893282394,0.163523367899062,0.608669025339021
"4203","PHF5A",223.652810908115,-0.391966436807003,0.281328774824842,-1.39326820390501,0.163538701879429,0.608669025339021
"4204","PEF1",193.482718113735,-0.316362012089486,0.227145259938788,-1.39277399922297,0.163688143307369,0.609079797169196
"4205","DARS2",24.1206283738994,0.447343663844838,0.32148491590178,1.39149192300366,0.164076307221705,0.609079797169196
"4206","PPARG",211.158376961794,-0.395219474342334,0.283937704310123,-1.39192318717442,0.163945659504044,0.609079797169196
"4207","IMPDH2",586.041879847039,-0.372885505310086,0.267897611859333,-1.39189559295467,0.163954016584128,0.609079797169196
"4208","NUDT16",132.412121863336,-0.349776889148734,0.251415304382883,-1.3912314924793,0.164155240354493,0.609079797169196
"4209","AC144652.1",10.6470153295782,-0.718537599193992,0.516428565675711,-1.39135912873804,0.16411655187697,0.609079797169196
"4210","PLPP5",146.119391920379,-0.314865036002169,0.226216445380851,-1.39187509321912,0.163960225263609,0.609079797169196
"4211","ERCC5",208.421799595029,0.242593916245869,0.174297319436985,1.39183962799598,0.16397096690361,0.609079797169196
"4212","GON7",79.8456984188548,-0.413121249247965,0.296930363689588,-1.3913068509216,0.164132397241904,0.609079797169196
"4213","PYGO1",64.1181005773969,0.387248410263273,0.278265935090446,1.39164864048991,0.16402882195646,0.609079797169196
"4214","PEMT",139.049532410666,-0.361616269997169,0.259759270330489,-1.39212074909622,0.16388583602965,0.609079797169196
"4215","TOM1L2",80.5589241599674,0.267755486489372,0.19233910591428,1.

39210112897531,0.163891776437727,0.609079797169196
"4216","DDX42",334.058819440099,0.23405826622821,0.16821821565835,1.39139667670462,0.16410517182907,0.609079797169196
"4217","PRRX1",4855.76085335338,0.24449505185961,0.175915046664852,1.38984729558362,0.164575252744321,0.609597226654467
"4218","STK17B",221.70403717465,0.513860268854062,0.369566027596294,1.39044238507602,0.164394583293301,0.609597226654467
"4219","SUCLG2-AS1",72.8881622531217,0.425941988483653,0.30648841582089,1.38974906226985,0.164605090798376,0.609597226654467
"4220","MBOAT1",154.641548626954,-0.374066487249672,0.269131129156456,-1.38990420179976,0.164557969527044,0.609597226654467
"4221","KIF13B",102.971585908629,-0.483775218422933,0.348022673276064,-1.3900681063937,0.164508197037709,0.609597226654467
"4222","CCDC81",2.93926127237465,-1.37127597564775,0.986294800396391,-1.39033073589827,0.164428468646696,0.609597226654467
"4223","CPEB1",52.1027184527129,0.392794981181989,0.282503472583649,1.39040762079724,0.164405133634399,0.609597226654467
"4224","MFGE8",950.929360469238,0.205908776015793,0.148164693850075,1.38972902832134,0.16461117654611,0.609597226654467
"4225","MAFG",198.815026351555,-0.286608351247633,0.206249989997518,-1.38961631586543,0.164645418565181,0.609597226654467
"4226","DDI2",175.814100579754,0.401228111928188,0.288786475315809,1.38935908092446,0.164723586572666,0.609605295494365
"4227","AGO3",256.106639071828,0.349118908867281,0.25135127727112,1.38896811131262,0.164842447097178,0.609605295494365
"4228","THRB-AS1",4.08628049698993,-1.08837299538138,0.783407756304931,-1.38928034171485,0.164747519261665,0.609605295494365
"4229","PPA2",102.378081309242,-0.299360102475748,0.215524078359318,-1.38898681184318,0.164836760390164,0.609605295494365
"4230","ECI1",359.833828924609,-0.375128448001992,0.270065421959865,-1.38902805579361,0.164824218901171,0.609605295494365
"4231","HDLBP",999.220860894609,0.228808929730521,0.164803578967627,1.388373548462,0.165023326722188,0.609606798225149
"4232","PIGX",100.777916348172,-0.264698837345053,0.190639517184662,-1.38847832419054,0.164991440707435,0.609606798225149
"4233","RPL18A",14309.7439041557,-0.338948529689779,0.244054388551979,-1.38882374416959,0.164886353157477,0.609606798225149
"4234","ZNF486",43.7009238985017,0.369035103794434,0.265747884209263,1.38866619725874,0.164934277597865,0.609606798225149
"4235","PHLDB3",16.9035943105593,-0.61788385015238,0.445056644286539,-1.38832631325591,0.165037703156908,0.609606798225149
"4236","AC007878.1",15.1490306391212,0.679766373272866,0.490150596765257,1.38685207721663,0.165486873428063,0.609733995953395
"4237","TTN-AS1",94.6425695894413,0.403688930391229,0.29094854938235,1.38749250081574,0.165291636270063,0.609733995953395
"4238","PBRM1",289.241693429872,0.240237320261037,0.173194318139492,1.38709700665554,0.165412184667165,0.609733995953395

"4239","EMB",18.6368240498776,-0.800332303074269,0.57669214520469,-1.3
8779816879628,0.165198512604664,0.609733995953395
"4240","JAZF1",218.719222081963,0.210851434465341,0.152063763210591,1.
3865988189003,0.165564128679765,0.609733995953395
"4241","LMBR1",72.7019894047694,0.325112363982483,0.234475665211581,1.
38655055606352,0.165578854107909,0.609733995953395
"4242","KLF9",1176.34861482665,-0.409934241785816,0.295471157264491,-1
.38739173590085,0.165322343589993,0.609733995953395
"4243","WDR31",20.9323627492279,0.487035653757408,0.350891020208844,1.
38799691558801,0.165137984338439,0.609733995953395
"4244","AL138762.1",18.3281276779641,0.578906147226485,0.4173228377950
13,1.38719019137611,0.165383775589466,0.609733995953395
"4245","PIDD1",26.644811632937,-0.49220000076168,0.354818561091938,-1.
3871878608801,0.165384486039311,0.609733995953395
"4246","METTL9",555.190913013495,-0.321477616997006,0.231752570733181,
-1.38715879603824,0.165393346626052,0.609733995953395
"4247","AC015813.1",26.5407455598397,0.572704534939501,0.4129677730043
32,1.38680200339384,0.165502146059015,0.609733995953395
"4248","ZNF236",65.9869005633876,0.386811466743152,0.278913162363905,1
.38685268011292,0.16548668954977,0.609733995953395
"4249","ZKSCAN7",15.7933111862629,0.549034970149166,0.396149538099994,
1.38592858843769,0.165768710248249,0.610101674511632
"4250","AL137026.3",39.4777808127485,0.653468766529359,0.4715351440333
42,1.38583258278444,0.165798030639663,0.610101674511632
"4251","HAGHL",27.0713878724805,-0.660329506820052,0.476490934991009,-
1.3858175640477,0.165802617756184,0.610101674511632
"4252","CLCN4",62.5990089960428,0.522927914205126,0.377371143501646,1.
38571250931603,0.165834706899153,0.610101674511632
"4253","DPEP2",3.65950635085103,-1.4142280223177,1.02083012608536,-1.3
8537057849275,0.165939182589114,0.610342495471786
"4254","RNF175",10.3161271691635,-0.940991489188951,0.679328534429106,
-1.38517880745248,0.165997799182678,0.610414568080543
"4255","DNMT3A",91.9995188689531,0.382921527249604,0.276577784318157,1
.3844984990158,0.166205867379645,0.610748974258817
"4256","DHRS9",9.92250814259705,-1.09689703254301,0.792221321849886,-1
.38458408312173,0.166179681221872,0.610748974258817
"4257","CDC37L1",151.571311143957,-0.252377867067042,0.182279327612765
,-1.38456659003699,0.166185033326443,0.610748974258817
"4258","AC020659.1",2.6772296935873,1.23770840446342,0.894524765357525
,1.38364912006514,0.166465919989436,0.611417324816798
"4259","AC008764.6",2.86327843834777,1.4141955716108,1.0219934929708,1
.38376181584085,0.166431398563445,0.611417324816798
"4260","VSTM5",3.61189592160925,-1.2628666058794,0.912835782501189,-1.
38345431904425,0.166525604921864,0.611492966618007
"4261","TMEM120B",38.7853130708855,0.400990872922048,0.289943886644027
,1.38299474965084,0.166666475776901,0.611866622994149
"4262","ZNF516",77.5905193927336,0.32700940768372,0.236480831742463,1.
38281570338795,0.166721382699771,0.611924586948033
"4263","TMEM53",32.7290462603829,-0.578372295616085,0.418548472984671,
-1.38185259999089,0.16701696449604,0.612023635265268

"4264","IDH1-
AS1",3.53727926047894,1.29555258700749,0.937559253323589,1.38183542257
711,0.167022239912257,0.612023635265268
"4265","SCARA3",378.040549800589,-0.393854810339434,0.284973284393721,
-1.38207625734938,0.166948287723697,0.612023635265268
"4266","ASAP1-
IT2",11.7472213095624,0.803958934817173,0.581712649680768,1.3820551010
1658,0.166954783129715,0.612023635265268
"4267","TEK",6.85512663104792,0.921091984069991,0.66633553229252,1.382
32457888143,0.166872062375688,0.612023635265268
"4268","NOSIP",280.999143481472,-0.294134748248749,0.212783491154446,-
1.38231940200311,0.166873651215127,0.612023635265268
"4269","HCK",5.62017760961629,-1.05875275890595,0.766148023719602,-1.3
8191671338624,0.166997275510628,0.612023635265268
"4270","CAMK2D",620.382098533989,0.28637258956967,0.207322619707438,1.
38128965365083,0.167189918136262,0.612207839280325
"4271","FZD10",4.08050423353514,-1.10265543612852,0.79821302535223,-1.
38140496472348,0.167154480234818,0.612207839280325
"4272","PLA2G6",24.5987985122098,0.455887962674976,0.330034352468434,1
.3813348800367,0.167176018293431,0.612207839280325
"4273","CGNL1",5.98375498007374,-0.955409231708594,0.691983265813905,-
1.38068256691851,0.167376583779165,0.612747928869055
"4274","HEG1",272.125424668867,0.341024585361834,0.247163030451832,1.3
7975564039014,0.167661895002943,0.612912980430071
"4275","THSD7A",15.9482005389104,0.622944682715336,0.451539422114321,1
.3796019842485,0.167709226174429,0.612912980430071
"4276","SND1",219.523109141724,-0.257504072940522,0.186543070753483,-1
.38040009688066,0.167463490376673,0.612912980430071
"4277","C9orf72",40.6716454130256,-0.378874474464834,0.274507870926348
,-1.38019530436885,0.167526519387318,0.612912980430071
"4278","RNF41",123.467796912349,-0.249289637717401,0.180631101499456,-
1.38010362361739,0.167554741754488,0.612912980430071
"4279","SFSWAP",257.528946691011,0.259899790002484,0.188398992632295,1
.37951793887634,0.167735119172865,0.612912980430071
"4280","TTC23",69.0719419918867,0.276971204079379,0.200761182781073,1.
37960536116891,0.167708185863464,0.612912980430071
"4281","MALT1",124.960826802113,0.360649917873354,0.26137901697507,1.3
7979674897833,0.167649233900016,0.612912980430071
"4282","GGCX",173.562528615501,0.366497230386705,0.26573976539129,1.37
915840275939,0.167845920238733,0.612936335628375
"4283","MRPL2",222.249197646744,-0.375826989419589,0.272513007780976,-
1.37911578049019,0.167859059121138,0.612936335628375
"4284","EIF1",12433.9615472043,-0.332685810664655,0.241196329903554,-1
.37931539338797,0.167797532446719,0.612936335628375
"4285","EI24",399.653866829896,-0.257442128791956,0.186714323292754,-1
.37880224854686,0.167955733258719,0.61314621595476
"4286","ANKRD13C",115.134909124352,0.229896106795765,0.166949251042917
,1.37704185768804,0.168499306966042,0.61349235785118
"4287","FAM126B",120.438569095346,0.353152687216464,0.256330722258968,
1.37772282660553,0.168288880919379,0.61349235785118

"4288","SRFBP1",168.978551338297,-0.280504275890468,0.203620399165701,
-1.37758435323663,0.168331654556855,0.61349235785118
"4289","HLA-
DRA",753.194902323734,-0.545560828995637,0.395938336522388,-1.37789342
094887,0.168236196513271,0.61349235785118
"4290","FANCE",11.4176671502114,-0.707554675277517,0.513653391896412,-
1.37749440856454,0.168359442327012,0.61349235785118
"4291","AL158206.1",36.5794955132463,0.485186533173785,0.3521990039764
85,1.37759200819937,0.168329289769249,0.61349235785118
"4292","TUBB4B",678.202272124567,-0.376404459746997,0.273331119891039,
-1.37710063858461,0.168481135311265,0.61349235785118
"4293","LINC02656",2.78087943130127,1.7282924003536,1.25514061043009,1
.37697114250919,0.168521169960143,0.61349235785118
"4294","KMT2D",66.0004349024887,0.401263166216052,0.291173783102576,1.
37808823974614,0.168176046048678,0.61349235785118
"4295","C14orf93",35.6679056186344,-0.381082072656501,0.27675129800437
5,-1.37698386748118,0.168517235626668,0.61349235785118
"4296","AL359397.2",3.586290995884,1.17158127958855,0.850734486275064,
1.37714092762162,0.168468681119935,0.61349235785118
"4297","ASIP",21.1520758731414,-0.57900724671419,0.420427625914623,-1.
37718649067026,0.168454597452928,0.61349235785118
"4298","FAM151B",4.79617075322478,1.15866887892768,0.841908744432727,1
.37624046143907,0.168747199109909,0.614172274470988
"4299","RABGGTB",134.225264639262,-0.333920207906945,0.242706087566074
,-1.37582131233539,0.168876961481338,0.614358676384317
"4300","CAHM",15.5374536967811,0.599985350125463,0.436085860136711,1.3
7584224798614,0.168870478337279,0.614358676384317
"4301","FKBP14",231.415824998281,0.397285113270399,0.288865276545813,1
.37533011243527,0.16902912497509,0.614630137952579
"4302","HDGFL2",198.159037212792,-0.257606533310258,0.187305694050976,
-1.37532675990165,0.169030163873425,0.614630137952579
"4303","C6orf132",84.3722181849037,-0.329835670055762,0.23986362809348
8,-1.37509664419487,0.169101484622215,0.614746577723754
"4304","RAET1G",16.2951354627012,0.816365945458801,0.5939507033638,1.3
7446751192543,0.169296589510156,0.61477159780693
"4305","FGFR1",2019.24152008253,0.202006545686708,0.146944284430086,1.
37471522945025,0.169219747844423,0.61477159780693
"4306","RECQL",320.750831453438,-0.222891724966446,0.16212584720603,-1
.37480684793705,0.169191334532552,0.61477159780693
"4307","RNASEH2B",239.746413089972,-0.259437060234047,0.18875021874209
9,-1.37449938846711,0.16928669997999,0.61477159780693
"4308","STAT5B",146.953423609146,0.295310504821386,0.214878453765903,1
.37431417457568,0.169344167675642,0.61477159780693
"4309","TCN2",104.814290923336,0.472755285660906,0.343969262534377,1.3
7441142902604,0.169313989987873,0.61477159780693
"4310","SSBP2",413.269724339118,-0.255240358565156,0.185763521004912,-
1.37400689427289,0.169439542098004,0.614822142502809
"4311","COG1",66.9098445640162,0.314945756827201,0.229212995329766,1.3
7403098098383,0.169432064550217,0.614822142502809
"4312","SCAMP4",147.237656403223,0.313460886843954,0.228155826789383,1

.37388946517381,0.169476000669444,0.614822142502809
"4313","C1orf174",142.72011577737,-0.26817774319139,0.195252256509655,-1.37349369469709,0.169598920064246,0.615090585441809
"4314","ZFYVE19",91.5130033396702,-0.330725169106623,0.240807951478694,-1.37339804219831,0.169628638087065,0.615090585441809
"4315","TSSK3",7.13294085707787,0.751058424465827,0.547049269910653,1.37292647257987,0.169775205885194,0.615194242682904
"4316","JMY",108.541040847621,0.374845493722385,0.273009178667252,1.37301425377808,0.169747915562129,0.615194242682904
"4317","TTC38",51.9742611976405,-0.299423802886337,0.218056128065126,-1.37315014048543,0.169705676189265,0.615194242682904
"4318","NCDN",41.2620189077888,0.354179607338505,0.258072002813637,1.37240616369483,0.169937032404307,0.615495484579898
"4319","PLA2R1",294.459805678687,0.429004651324188,0.312567834341171,1.37251695213118,0.169902565289082,0.615495484579898
"4320","PCDHGA10",63.2269173158067,0.420481269494087,0.306455393120968,1.37207984891984,0.170038581836853,0.615578230889583
"4321","RPL28",14904.8857274822,-0.335265762352443,0.244336966615469,-1.3721450626015,0.170018283648461,0.615578230889583
"4322","SPATA6",314.784707937447,0.277469466485756,0.202289964232844,1.37164227369371,0.170174826996186,0.615861505058247
"4323","SLC25A29",75.4386687975653,0.376274168921042,0.274337154265061,1.37157568003891,0.170195569031951,0.615861505058247
"4324","PRKCA",43.7941252935435,0.421863072353137,0.30763807256679,1.37129669560502,0.170282485370432,0.616033514951356
"4325","TTF2",76.7027733829233,0.295095584980762,0.215732508528101,1.36787722441156,0.171350509967078,0.616286968227836
"4326","SFXN5",45.2598375695673,0.370290894569732,0.270460998041402,1.36911013880474,0.170964849526233,0.616286968227836
"4327","EIF5B",546.142267832718,-0.296062876673155,0.216038164303222,-1.37041933136227,0.170556041017309,0.616286968227836
"4328","C2orf69",102.466158403658,-0.271658460200395,0.198553810182154,-1.36818558128487,0.171253993707904,0.616286968227836
"4329","ADGRA3",75.3515516942454,0.32596998496401,0.237973428234181,1.36977471553351,0.170757237158836,0.616286968227836
"4330","LIMCH1",168.839344242804,0.363036669813718,0.265136702511069,1.36924336153936,0.170923215882445,0.616286968227836
"4331","LINC01094",3.02803870149669,1.42599299279148,1.04111953848562,1.36967268414315,0.170789099272846,0.616286968227836
"4332","AP002026.1",16.2770135963191,0.610755512449549,0.445846790894344,1.36987755642338,0.170725126762161,0.616286968227836
"4333","LINC02227",2.82296750344273,1.31880722501834,0.964160486045229,1.36782957205371,0.171365428873552,0.616286968227836
"4334","HIST1H2BC",11.2064223959684,0.758760291675493,0.554437361920788,1.3685230177253,0.171148422141767,0.616286968227836
"4335","RWDD1",1124.32349734297,-0.349678016160767,0.255181083408551,-1.37031323595772,0.170589143076922,0.616286968227836
"4336","ZNF736",44.9013692158211,0.437021155823945,0.319241278873165,1.36893686607982,0.171019010617164,0.616286968227836
"4337","TMEM243",443.996085688041,-0.334517151210785,0.244435288245321

,-1.36853051624467,0.171146076681929,0.616286968227836
"4338","RPL8",13091.6678500966,-0.382143379779597,0.279386842609455,-1
.36779304354637,0.171376865805222,0.616286968227836
"4339","WAPL",179.062586126584,0.237422094891232,0.17354112866153,1.36
810274729914,0.171279916895789,0.616286968227836
"4340","NPM3",80.3876187272505,-0.330094016492171,0.240837045153519,-1
.37061147001599,0.170496105475408,0.616286968227836
"4341","CD82",33.618724837378,-0.512264389067695,0.374073231422343,-1.
36942273875066,0.170867170427236,0.616286968227836
"4342","AP003119.3",2.73342242149876,1.28785776647299,0.94118219566148
5,1.36834055341203,0.171205502523647,0.616286968227836
"4343","MANSC1",100.957735843269,0.351528930423149,0.256627179309155,1
.36980397543811,0.170748100768683,0.616286968227836
"4344","ZBTB4",260.155094376364,0.226365346976492,0.165461860783938,1.
3680817192796,0.171286498156206,0.616286968227836
"4345","NAGLU",362.627685307305,0.405539979020897,0.296245974804693,1.
3689299214555,0.171021181615686,0.616286968227836
"4346","MED13",263.143120442932,0.252710462026837,0.184635309200483,1.
36870061918891,0.171092876693948,0.616286968227836
"4347","ZNF99",11.8752485853278,0.787201019291566,0.57539260476083,1.3
6811111713676,0.171277297392167,0.616286968227836
"4348","HSPA12B",86.3816927297117,0.400683680061708,0.292872362500031,
1.36811707544329,0.171275432642423,0.616286968227836
"4349","NIPSNAP1",64.7713227010096,-0.310289061386621,0.22640103690363
8,-1.37052844646947,0.170522001822061,0.616286968227836
"4350","SH3KBP1",349.612542999289,0.311857156952239,0.227734596263669,
1.36938858684069,0.170877839948812,0.616286968227836
"4351","MBLAC1",20.440890879775,-0.597805213422526,0.437165369419439,-
1.36745784373639,0.171481842227595,0.61652274372932
"4352","AL513477.2",4.98272435366818,0.989828283234799,0.7247434589098
42,1.36576366584073,0.172013154088974,0.616526792457919
"4353","PIGM",109.197845837622,0.364958660076884,0.267228498123362,1.3
6571758865481,0.172027621561597,0.616526792457919
"4354","ZIC1",43.7481459274092,-0.624654479450867,0.457463001438129,-1
.36547541000505,0.172103676612035,0.616526792457919
"4355","RPL35A",12942.3537725459,-0.374270394409208,0.273806819289646,
-1.36691407241135,0.171652240544356,0.616526792457919
"4356","AC009570.1",2.84288293202488,-1.57550906915593,1.1522860472013
8,-1.3672898955797,0.171534457600589,0.616526792457919
"4357","SNCAIP",43.4112137642794,0.436296411159673,0.319119058125245,1
.36719008172943,0.171565733349697,0.616526792457919
"4358","MZB1",5.84518885255147,1.22354573442005,0.895931239851331,1.36
566923888387,0.172042803567152,0.616526792457919
"4359","AC146507.3",4.50999125092915,0.956164015997708,0.7001035185498
74,1.3657466226969,0.17201850524754,0.616526792457919
"4360","CFAP70",18.4925185934661,0.736206283675064,0.538718124935521,1
.36658903719488,0.171754155355527,0.616526792457919
"4361","EXOC3L4",24.1805939610944,0.645464617506903,0.472530362177935,
1.36597490694968,0.171946839536149,0.616526792457919
"4362","ZWILCH",36.2723719526329,0.418025060137292,0.305962332846357,1

.36626314830463,0.171856383294261,0.616526792457919
"4363","AC025280.2",10.3417792424199,2.15414669333161,1.57595912264753
,1.36687980187757,0.171662983937136,0.616526792457919
"4364","AP000915.1",5.96456223275874,-1.04799513500006,0.7668119430669
24,-1.36669120046373,0.171722117177688,0.616526792457919
"4365","MICOS13",681.335795859868,-0.40911720068634,0.299622117321667,
-1.36544392764945,0.172113565343203,0.616526792457919
"4366","HMGXB4",234.796668604122,0.210567439390381,0.154117117224609,1
.36628197556733,0.171850476139698,0.616526792457919
"4367","OGT",438.162127661593,0.329385163939927,0.241193768520934,1.36
564541430654,0.172050284940586,0.616526792457919
"4368","PPARGC1A",9.75249115657441,1.01011443374274,0.740002774440568,
1.36501438728573,0.172248528124075,0.61673826437062
"4369","BX005266.2",2.87044607896836,-1.30642753973788,0.9570862096412
5,-1.36500508165046,0.172251452856565,0.61673826437062
"4370","MARK1",38.0696658374593,0.390276295734505,0.285947356818315,1.
3648536572502,0.172299050299912,0.616767515753209
"4371","CRYGS",7.15537859312384,0.833014591144337,0.610889605453359,1.
36360904442977,0.172690644024922,0.616843275992165
"4372","C4orf3",1049.13560791353,-0.351508844689635,0.257840008189609,
-1.36328278593268,0.172793405062818,0.616843275992165
"4373","PCDHGA1",5.15866673995459,0.970672403701543,0.711320635654351,
1.3646059948881,0.17237691956064,0.616843275992165
"4374","LINC02202",51.2289807878192,0.391480509971624,0.28715015422331
7,1.36333031417134,0.17277843233643,0.616843275992165
"4375","SVIP",9.48407113881521,-0.746095491906348,0.546965312011336,-1
.3640636353387,0.172547538572644,0.616843275992165
"4376","SARNP",305.358650895717,-0.317418942345107,0.232667533935092,-
1.36425970988139,0.17248584159031,0.616843275992165
"4377","KIAA0513",19.9679366567231,0.552269643827096,0.405096611675558
,1.3633035377482,0.172786867540375,0.616843275992165
"4378","AKAP10",85.8491833696154,0.329641592105382,0.241579552168475,1
.36452605010003,0.172402061181642,0.616843275992165
"4379","MAP2K2",924.34169917258,-0.352012776802518,0.258092268947431,-
1.36390283303766,0.172598149078648,0.616843275992165
"4380","ZNF492",5.88591865400987,0.882987096302601,0.647412152821828,1
.36387167348341,0.1726079574412,0.616843275992165
"4381","CD177",3.53264554558815,2.20058392561211,1.61400570226601,1.36
343008114691,0.172747006100336,0.616843275992165
"4382","NSFL1C",252.707436509618,-0.254030075507283,0.186290379733557,
-1.36362422939183,0.172685862358638,0.616843275992165
"4383","LRP5",153.569039798889,-0.307187116960947,0.225357014561866,-1
.36311318091506,0.172846843302323,0.616893262554927
"4384","FAM53B",46.7584115281867,0.404874987421116,0.297058376726195,1
.36294755220553,0.172899040632729,0.61693879849858
"4385","ZNF587",67.4836728921991,0.498578539921132,0.365882559737312,1
.36267369584134,0.172985371395888,0.616965381839005
"4386","DOP1B",35.25037261165,0.449958628623857,0.33019811359614,1.362
6929110025,0.172979312937563,0.616965381839005
"4387","SRP72",566.827032552336,-0.222218278903243,0.163092380482877,-

1.36253010867405,0.173030648839022,0.616977447193654
"4388","AC015908.2",14.089722361578,-0.696141774820067,0.510962432801742,-1.3624128314149,0.173067636533002,0.616977447193654
"4389","XCL2",5.43343192223428,1.39089416937173,1.02154933429607,1.36155359577437,0.173338808379697,0.617252568413784
"4390","VHL",107.702666603646,0.384525236865629,0.282628478163571,1.36053252440855,0.173661467784431,0.617252568413784
"4391","GPD1L",62.9678996805886,0.38344977093017,0.281731210571905,1.36104824932878,0.173498442233726,0.617252568413784
"4392","CTDSPL",345.705475167625,-0.257667966536259,0.189438067436433,-1.36016994906644,0.173776149798268,0.617252568413784
"4393","CISH",13.9541064328824,-0.695127943861213,0.511018516549782,-1.36027936630256,0.173741535338158,0.617252568413784
"4394","PSMD2",388.12792237405,-0.286689536459332,0.210658198353404,-1.36092275876383,0.173538100462147,0.617252568413784
"4395","DMGDH",34.214204780285,0.414751185712729,0.304767854820884,1.3608757588837,0.173552955369947,0.617252568413784
"4396","CRYGN",3.22588043987431,1.16320751484342,0.854410425865277,1.36141540368661,0.17338245095991,0.617252568413784
"4397","PTGES2",87.1363066402555,-0.45530515783722,0.334698764035092,-1.36034311076656,0.173721371966873,0.617252568413784
"4398","CCDC186",438.008881340875,-0.261913772558427,0.192314453313373,-1.36190373654154,0.17322826683315,0.617252568413784
"4399","ARID2",101.372871015253,0.31811210862385,0.233699890595657,1.36119921927667,0.17345074084389,0.617252568413784
"4400","RIOX1",17.045562775624,-0.594815953362224,0.436945773531613,-1.36130382622682,0.173417694339672,0.617252568413784
"4401","TEDC1",25.268806143972,-0.51505487276067,0.378509698072269,-1.36074419066095,0.173594544227143,0.617252568413784
"4402","AC116913.1",11.198277521558,-0.633268710154993,0.465551182684843,-1.36025582945128,0.173748980854449,0.617252568413784
"4403","YIPF2",217.069297810228,-0.312250439742431,0.229303249527796,-1.36173578170152,0.173281284609179,0.617252568413784
"4404","SPTLC3",45.4433743671156,0.37529797401724,0.275615809674099,1.3616707055412,0.173301830255627,0.617252568413784
"4405","PRDM4",63.6055401646031,0.347809217236148,0.255748513700929,1.35996574213868,0.173840765032567,0.617341904064573
"4406","PLSCR3",154.265706998946,-0.256336160785407,0.188537035642979,-1.35960640258933,0.173954510968828,0.617605632111978
"4407","SLC44A3",15.2015256207272,-0.749702871965437,0.551984547518271,-1.35819539756341,0.174401690613389,0.61790484308277
"4408","AC008443.8",3.78476686251584,1.1178923389953,0.823047606025104,1.35823533269739,0.174389022467489,0.61790484308277
"4409","THBS2",1186.91897130763,0.311707962341051,0.229602823734386,1.3575963800064,0.174591792266563,0.61790484308277
"4410","IFT22",210.066493694699,-0.338557715898596,0.249256003199388,-1.35827306685879,0.174377053141212,0.61790484308277
"4411","C9orf153",3.05679023986959,1.4292990079563,1.05246793822582,1.35804517747659,0.174449349292416,0.61790484308277
"4412","SPOCK2",10.4108305125624,0.833960910701981,0.613999393174296,1

.3582438679467,0.174386315021349,0.61790484308277
"4413","BUB3",307.492872622152,-0.197060683552179,0.145027242225409,-1
.35878391210043,0.174215072712324,0.61790484308277
"4414","AC127035.1",3.26139069995884,-1.22954427778236,0.9055287134146
55,-1.35781920503202,0.174521059406317,0.61790484308277
"4415","SOX5",110.045429147641,-0.338032060858274,0.248926311424698,-1
.35796034948492,0.174476266034453,0.61790484308277
"4416","SIVA1",700.310750248853,-0.365620399902322,0.269005916019952,-
1.35915375138145,0.17409787304425,0.61790484308277
"4417","OIP5-
AS1",376.806414508625,0.221724932680153,0.163313159196204,1.3576672802
8189,0.174569283589694,0.61790484308277
"4418","TTBK2",115.822713407597,0.379875192621443,0.279529373109662,1.
35898130631308,0.174152512437789,0.61790484308277
"4419","ZNF594",25.9019163744425,0.504404150069008,0.371402770790162,1
.35810551169525,0.174430206548362,0.61790484308277
"4420","CFAP298",252.489016798091,-0.26412872493825,0.194408414752412,
-1.35862804742598,0.174264482860798,0.61790484308277
"4421","LINC01914",27.1624925265069,-0.497845789540803,0.3669544444615
45,-1.35669644299124,0.174877683164521,0.618051557887966
"4422","PSMD14",234.400396209557,-0.343573025827771,0.253246412793622,
-1.35667479763182,0.174884563736584,0.618051557887966
"4423","SLC11A1",13.1401889874412,-0.55311694881203,0.407857061824842,
-1.35615390925748,0.17505020335812,0.618051557887966
"4424","TMED2",874.243444461248,-0.221292329495843,0.163183088976525,-
1.35609842223098,0.175067854823345,0.618051557887966
"4425","TRAP1",128.794718785225,-0.25524009654784,0.188181324866234,-1
.3563518947976,0.174987231263864,0.618051557887966
"4426","CHMP6",121.574708343934,-0.429802612385387,0.316742538535543,-
1.35694628947718,0.174798277221656,0.618051557887966
"4427","RAC3",11.7944579295335,-0.781693122892736,0.576272496792974,-1
.35646439356893,0.174951456975936,0.618051557887966
"4428","GATAD2A",98.1457643770957,0.298014246892693,0.219602800237092,
1.35706032241367,0.17476204434198,0.618051557887966
"4429","PSMD8",896.980776667052,-0.380462605188416,0.280304283118347,-
1.35731998439632,0.174679560137685,0.618051557887966
"4430","PTPRA",1025.36488777365,0.251553777099904,0.185476256987907,1.
35625864563519,0.175016888369019,0.618051557887966
"4431","SLC23A2",77.5381317273799,0.299717760807615,0.220998179174293,
1.35620013670447,0.175035498553269,0.618051557887966
"4432","AC159540.2",26.0859562483178,-0.691569859800473,0.510138279480
419,-1.35565176662462,0.175209992744303,0.618068388258526
"4433","THOC3",80.5208926067675,-0.366690707594587,0.270520615904528,-
1.35550004707959,0.175258293603371,0.618068388258526
"4434","WBP1L",259.782999376167,0.323414548515479,0.238597705700429,1.
35548054649587,0.175264502455734,0.618068388258526
"4435","CASP1",50.7119856024353,0.491835595473073,0.36276962044942,1.3
5577944719781,0.175169352538493,0.618068388258526
"4436","ATP1B2",5.68390808695764,1.13706608830979,0.838876692309114,1.
35546272620816,0.17527017645687,0.618068388258526

"4437","FIZ1",22.9079833521601,0.473486018434017,0.349396946764164,1.3
551521351834,0.17536909102691,0.618277820809995
"4438","FJX1",17.7441365216202,-0.555404124651166,0.409908223443435,-1
.3549475050427,0.175434282768569,0.618368293228645
"4439","SMIM11B",5.03138065974762,-0.931353058700052,0.687768744014828
,-1.35416601409263,0.175683419092108,0.619106944099538
"4440","EDEM2",79.4879456688605,0.48154329025647,0.355637361343323,1.3
5402897051528,0.175727135242008,0.619121526259174
"4441","SPECC1",68.9785148311837,-0.544525677443665,0.402235405101203,
-1.35374875144733,0.175816548843174,0.61929706677635
"4442","MXD4",612.955020215526,0.249492192390356,0.184369837147254,1.3
5321588525941,0.175986671859742,0.619477837061645
"4443","MPP7",8.62470279440933,0.864521731504008,0.638761927156983,1.3
5343340726621,0.17591721089919 5,0.619477837061645
"4444","LIMK2",29.968538074337,-0.399119710056868,0.294925494858781,-1
.35328995632602,0.175963016566501,0.619477837061645
"4445","RGS16",102.34456688426,-1.29848609655914,0.959691838916142,-1.
35302400614933,0.176047961289511,0.619489452194573
"4446","BCL7C",676.823214428331,-0.399190059502984,0.295049939740677,-
1.35295760390203,0.176069174995658,0.619489452194573
"4447","PRKD3",196.054635082936,0.265596399873544,0.19638266313913,1.3
5244321279714,0.176233573492566,0.619789071525227
"4448","ATP5MC2",1714.71994150346,-0.380234680828447,0.281126598347869
,-1.35253897376847,0.176202959787903,0.619789071525227
"4449","KCNN4",2.91690216668418,-1.23218068129559,0.911190977430778,-1
.35227489276713,0.176287393121589,0.619838995414928
"4450","FAF1",115.665359603271,-0.240989698680548,0.178272549673617,-1
.3518048579086,0.176437749690875,0.62022825132907
"4451","AC058791.1",89.1062551216957,-0.711861669593264,0.526754816677
394,-1.35140989138641,0.176564167000112,0.620533198019041
"4452","VEZT",178.191917795459,0.260500616317033,0.192785624573422,1.3
5124502614469,0.176616955540087,0.620579298183645
"4453","ZSCAN23",4.49575848315366,0.959188911865765,0.71005292347102,1
.35086960444705,0.176737206469601,0.620862367124179
"4454","ATG4A",33.3024770688719,-0.42741906085611,0.316449729439,-1.35
066969914569,0.176801262814418,0.620947946611121
"4455","COPS6",631.956851417863,-0.312718557041429,0.231558992490876,-
1.35049195748142,0.176858231716037,0.621008601287084
"4456","CADM3",126.592303862504,-0.994030662792289,0.736131259135867,-
1.35034431761418,0.176905562932909,0.621035395188395
"4457","FHL3",119.577092211051,-0.399198974508745,0.295752983282583,-1
.34977159005467,0.177089260465343,0.621201702984915
"4458","CCDC18",48.8295786250775,-0.364943006658246,0.27037679580345,-
1.34975712532499,0.177093901744402,0.621201702984915
"4459","RUSC1-
AS1",9.86599690785352,-0.691811982896865,0.512498480338281,-1.34988104
245738,0.177054143551789,0.621201702984915
"4460","IFT46",54.5639420984444,-0.314086259346429,0.23270795900361,-1
.34970140553532,0.177111781327924,0.621201702984915
"4461","ATP6V1C1",168.814843467725,0.244106030038204,0.180931848875786

,1.34916009290209,0.177285549831839,0.621393200990243
"4462","SNRPF",533.504821314071,-0.314960984528695,0.233429493020362,-1.34927673642859,0.177248094990196,0.621393200990243
"4463","COPE",807.983888621025,-0.393365303035184,0.291561342220105,-1.34916824034314,0.177282933454752,0.621393200990243
"4464","PTRHD1",211.557291578228,-0.391356386256225,0.290134629311468,-1.34887857814481,0.17737596996334,0.621570855317321
"4465","SAMD11",30.6952806711229,-0.605014745202319,0.448653637080884,-1.34851184788957,0.177493812092663,0.621844502254318
"4466","URM1",234.574524209698,-0.409203881755297,0.303554501433669,-1.34804089487276,0.177645229817213,0.622235631444392
"4467","RNF44",56.7166865758947,0.382477663605169,0.2837561023461,1.34790991433431,0.177687358910456,0.622243867346375
"4468","RCC1L",163.903658225374,-0.272203501792853,0.201976852219981,-1.34769652463137,0.177756010341326,0.622344957423761
"4469","HARS",159.96733348086,-0.242377991965654,0.179905359215982,-1.34725276124027,0.177898840498863,0.622408813383876
"4470","AP001107.9",5.35801872632078,-1.09972237117521,0.816335308030687,-1.34714541972729,0.177933402381429,0.622408813383876
"4471","CAMKK2",241.681291327064,-0.333797381935411,0.247760379425081,-1.34725892295603,0.177896856697735,0.622408813383876
"4472","FUNDC2",501.73392253321,-0.30816111754109,0.228728610762226,-1.34727840349381,0.177890584930774,0.622408813383876
"4473","RNF187",386.579708461506,-0.36562281820983,0.271686502849318,-1.34575260226531,0.178382315859123,0.622725857394391
"4474","PPP1R3G",45.7466216978581,-0.404629960102885,0.300484159025747,-1.34659331598314,0.17811248038954,0.622725857394391
"4475","STEAP1",309.556788596658,-0.413798786560138,0.307427109422452,-1.34600617147109,0.178300526285439,0.622725857394391
"4476","INTS9",36.167551331161,0.377002278722607,0.279944553242512,1.3467033894959,0.178075780205167,0.622725857394391
"4477","ITPK1",53.7036402508432,-0.378520186863306,0.281255052207441,-1.34582537768647,0.17835883905391,0.622725857394391
"4478","PSMG2",814.176924423238,-0.350250818075946,0.260147019933147,-1.34635721818361,0.17818734109027,0.622725857394391
"4479","SAFB",241.172707847848,0.273910090604098,0.203460157724069,1.34625910875176,0.178218968347498,0.622725857394391
"4480","STX10",280.187520530392,-0.375504453090451,0.278949140700647,-1.34613948674401,0.17825753620449,0.622725857394391
"4481","LAMP2",978.236890823504,0.261930804722947,0.194622184167058,1.34584248884039,0.178353319457813,0.622725857394391
"4482","AL353622.1",8.6271151155947,0.804192311217988,0.598019698785429,1.34475889816221,0.178703107443776,0.622726136805829
"4483","INKA2",5.6322881540734,-0.912859062953697,0.678601460412526,-1.34520645210337,0.178558573190659,0.622726136805829
"4484","SMG5",52.6289906027262,0.325430587879488,0.241929596265201,1.34514583128041,0.178578145145547,0.622726136805829
"4485","QARS",245.180341889653,-0.271411857113404,0.201858882954445,-1.3445623652572,0.17876660379316,0.622726136805829
"4486","LAMTOR4",1190.24305426913,-0.376730974607129,0.28016630343119,

-1.34466911257105,0.17873211351838,0.622726136805829
"4487","ADRB1",12.3036259213541,-1.02824907460092,0.764769569804015,-1
.34452142867612,0.178779831799273,0.622726136805829
"4488","VWCE",27.1411642317227,0.642209328251195,0.477524553243969,1.3
4487184771646,0.178666623008069,0.622726136805829
"4489","PPP1CA",299.903319105585,-0.246899716095221,0.183594050944444,
-1.34481327050153,0.178685543653759,0.622726136805829
"4490","NME3",676.420332281981,-0.330493340456562,0.245830307936921,-1
.34439623507027,0.178820290643213,0.622726136805829
"4491","AC027097.2",23.6891170909951,0.541801846575189,0.4027424687054
71,1.34528113788619,0.178534462436686,0.622726136805829
"4492","NUMBL",242.04520003258,-0.317510547970755,0.236159978734332,-1
.34447229235203,0.178795710383587,0.622726136805829
"4493","AL132780.2",3.42162672463526,1.08042600611503,0.80380665164644
7,1.34413668249943,0.178904191997981,0.622879651774852
"4494","AC005070.3",7.06976511194456,1.02936750542472,0.76610894045288
3,1.34363071760553,0.179067831237138,0.623310655105152
"4495","TMEM200A",10.5942429006802,-0.668985218339384,0.4979834100303,
-1.34338856448788,0.179146187806775,0.623436927359383
"4496","ZBTB42",5.19476481133861,-0.963277707624704,0.717112756599791,
-1.34327230795909,0.179183815470676,0.623436927359383
"4497","ANXA4",984.405034149336,-0.343568519535927,0.25594869120747,-1
.34233356660314,0.179487864450921,0.623437933041479
"4498","PLCL1",33.5468538473306,0.480949894151053,0.358335993755025,1.
34217578622552,0.179539005571979,0.623437933041479
"4499","SEMA3G",4.75160356226145,-1.02638477910052,0.764793807147972,-
1.34204117437622,0.179582645674417,0.623437933041479
"4500","UBA6",124.313733234984,0.267286024695965,0.199051065482881,1.3
428012758814,0.179336330163797,0.623437933041479
"4501","SIL1",360.569190078535,0.306819604907904,0.228610920514848,1.3
4210388645006,0.179562313932553,0.623437933041479
"4502","GALNT10",90.868045760232,0.380677465733095,0.283516552278908,1
.34269926278803,0.179369373525047,0.623437933041479
"4503","AC021546.1",17.1058944963613,-0.748860990241831,0.557548996390
676,-1.34313037076494,0.179229762922598,0.623437933041479
"4504","PALM2-
AKAP2",168.415258825445,-0.42589911495539,0.317344322530449,-1.3420725
8399755,0.179572462217104,0.623437933041479
"4505","CEP295NL",5.64571129358615,1.16385093926621,0.867077051584496,
1.34226933712453,0.179508681749994,0.623437933041479
"4506","ZNF628",19.1653717092731,-0.482039318333262,0.359126694439894,
-1.34225421222186,0.179513584115672,0.623437933041479
"4507","TRAM2",364.653701950252,0.345302052719877,0.257345206458577,1.
34178544637263,0.179665572398619,0.623587430448545
"4508","PDCL3",73.5397315138883,-0.388478890028934,0.289578093093012,-
1.34153411219597,0.179747102035963,0.623593683111237
"4509","NSMCE4A",191.611043062529,-0.316605743807945,0.236000166229123
,-1.34154881696382,0.179742331237508,0.623593683111237
"4510","LEPR",815.038675119408,0.860328151316541,0.641450770764336,1.3
4122241414005,0.179848251147287,0.62366796557238

"4511","OPA3",27.7138812344057,-0.436740607038629,0.325621542874651,-1
.34125218860827,0.179838587209985,0.62366796557238
"4512","ADSS",204.460024429336,-0.300025684847452,0.223751875779947,-1
.34088567437315,0.17995757407079,0.623770514334006
"4513","COLEC11",8.18685135009615,1.02792335327986,0.766553358179626,1
.34096777779556,0.179930914556214,0.623770514334006
"4514","PTN",39.4899965934619,0.876931729261535,0.654105194512888,1.34
065856167766,0.180031334327754,0.623887940383044
"4515","EPHA2",27.5379267016066,0.490233901277766,0.365863384907888,1.
3399370407104,0.180265814493936,0.624009324215235
"4516","RBM14",97.0756594507134,0.346389214941903,0.258429803405048,1.
34036094280888,0.180128027148434,0.624009324215235
"4517","AC090630.1",5.96250414120495,-1.4138611305966,1.05513113578939
,-1.33998617104482,0.180249840903708,0.624009324215235
"4518","CIDEB",29.0989175406007,-0.393783479319937,0.293821889339095,-
1.34021151455288,0.180176589153937,0.624009324215235
"4519","TAX1BP3",709.308659949033,-0.394561634601511,0.29444466137279,
-1.34001965857335,0.180238953812133,0.624009324215235
"4520","SIKE1",185.994044302971,0.20262703733371,0.151302358828431,1.3
3921928846779,0.180499294927754,0.624126982881268
"4521","SP3",368.193620601098,0.235192061195105,0.175585448222842,1.33
947353596532,0.18041656408865,0.624126982881268
"4522","AL023806.1",6.28237972736933,-0.798984249101752,0.596554328118
459,-1.33933191235367,0.18046264421395,0.624126982881268
"4523","LRWD1",32.1682145982293,-0.448788296900078,0.334989867195648,-
1.3397070802681,0.18034059478609,0.624126982881268
"4524","TSG101",278.337829009405,-0.271782646108203,0.202910776827147,
-1.33941947469712,0.180434152991383,0.624126982881268
"4525","KCTD10",131.394385738059,0.2725851695195,0.203577701021962,1.3
3897361130969,0.180579263787594,0.624265507940185
"4526","ATP6V0B",493.307399763782,-0.356907789119416,0.26682708302103,
-1.33759956102839,0.181027007627592,0.624746968647243
"4527","B4GALT2",146.688152748197,-0.382833552978202,0.286072346248444
,-1.33824033674938,0.180818103950098,0.624746968647243
"4528","CLOCK",123.378971794639,0.346999571582488,0.259521177473003,1.
33707612982215,0.181197787940455,0.624746968647243
"4529","PAIP1",386.105468134335,-0.255898528165872,0.191306658283097,-
1.33763524208965,0.181015370291687,0.624746968647243
"4530","ZBTB22",48.2731741922648,-0.414271754044749,0.309797920938578,
-1.33723219571536,0.181146855689797,0.624746968647243
"4531","SPA17",30.4123349882541,-0.46487953692829,0.347524227739421,-1
.33768957621241,0.180997650348689,0.624746968647243
"4532","ROBO3",389.101315861347,-0.338146146647406,0.252856061686,-1.3
373068630141,0.181122491705733,0.624746968647243
"4533","CCND2-
AS1",18.6225987757164,-0.559046043166909,0.41802103336571,-1.337363430
41337,0.181104035349272,0.624746968647243
"4534","CHFR",38.8698641867846,0.510356975579494,0.381668972978266,1.3
3717176850149,0.181166574906773,0.624746968647243
"4535","NLRC5",26.0913419626312,0.434741339033424,0.325110440537888,1.

33721125139554,0.18115369027074,0.624746968647243
"4536","TTI1",45.3818178309371,0.337158522370691,0.252023652537442,1.3
3780507891258,0.180959985826347,0.624746968647243
"4537","SPATA2",26.2911341911746,-0.38903432331794,0.290723165309558,-
1.33816073068585,0.180844047125334,0.624746968647243
"4538","HOXD3",6.65920763453188,0.816845006522218,0.611174034354353,1.
33651785024725,0.181380070029776,0.625237645543365
"4539","DIXDC1",337.046793797009,-0.327313328364713,0.244927842786608,
-1.33636635443641,0.181429557913928,0.625270450418061
"4540","TSNAX",316.989576466748,-0.247695350435138,0.185374430672749,-
1.33618940614527,0.181487372836084,0.625331932439396
"4541","GRIP2",9.5485153287289,-0.766579147600148,0.573813297480751,-1
.33593827637963,0.181569448795724,0.625365194370218
"4542","MYOZ2",2.64744307932152,1.30498282926062,0.977113814635425,1.3
3554843838487,0.181696912894754,0.625365194370218
"4543","RPL36",8363.14111337177,-0.32641432705916,0.244372498317322,-1
.33572447516293,0.181639346486041,0.625365194370218
"4544","AL110114.1",4.49585544306919,0.999451242661966,0.7481872218989
91,1.33583040903216,0.181604711200194,0.625365194370218
"4545","PMM1",286.365440787901,-0.33243029299921,0.248887937622881,-1.
33566253219918,0.181659601130062,0.625365194370218
"4546","PLPBP",235.601934205246,-0.218748448831585,0.163806819357161,-
1.33540501970574,0.181743822749678,0.625389049554159
"4547","GXYLT2",235.347334044814,0.324918539816335,0.243452895311717,1
.33462590124594,0.181998816395905,0.626128762894468
"4548","MFSD10",504.739579250491,-0.322513465491527,0.24181216147409,-
1.33373550579707,0.182290554147258,0.626994533536843
"4549","FAM200B",426.160856327217,-0.353918182514861,0.265447831992152
,-1.33328714670129,0.182437590064169,0.627020678091288
"4550","AC093849.2",39.399923583486,-0.536262956250509,0.4023109992172
59,-1.33295623856636,0.18254616523221,0.627020678091288
"4551","FZD3",19.2429886393646,0.545628122883489,0.409286834109211,1.3
3311916585594,0.182492700730429,0.627020678091288
"4552","COX11",294.38665115597,-0.252575035617519,0.189497534272427,-1
.33286713511749,0.182575409392533,0.627020678091288
"4553","RELB",44.0193412648162,-0.3728911896317,0.279768340337348,-1.3
3285699583471,0.18257873737172,0.627020678091288
"4554","TSPO",2022.7072473598,-0.397480707512246,0.29815838321917,-1.3
3311934154159,0.182492643085545,0.627020678091288
"4555","SMIM10",182.334376207453,-0.351309700517057,0.263520402158041,
-1.33314042343623,0.182485725923311,0.627020678091288
"4556","AC254633.1",38.5733209609062,0.692841315219306,0.5201330798047
83,1.33204624377927,0.182844993115438,0.627057583395674
"4557","TIMM17A",135.432651466724,-0.272760132589401,0.204673144697066
,-1.3326620499876,0.182642732469835,0.627057583395674
"4558","CFLAR",782.523085575353,0.351421274093364,0.263835748912533,1.
33196989241161,0.182870082174203,0.627057583395674
"4559","RBMS3-
AS3",16.1565646249204,0.731549561063992,0.54905911528892,1.33236939464
896,0.182738833993707,0.627057583395674

"4560","MAFK",70.3360292231407,-0.439901966144447,0.330188190615928,-1
.33227649760538,0.182769347159409,0.627057583395674
"4561","SMO",77.075730691451,0.306940491940596,0.23039590677185,1.3322
3066434312,0.182784403044088,0.627057583395674
"4562","HCAR1",3.7420542528134,-1.1372845797982,0.85371174556844,-1.33
216461610346,0.182806101015005,0.627057583395674
"4563","AC007364.1",14.111919055411,0.639736275597929,0.48059974616764
5,1.33112071052733,0.183149295367184,0.627740518075766
"4564","RPL19",16641.9306149205,-0.38982213011561,0.292852754227241,-1
.33111990407686,0.183149560680243,0.627740518075766
"4565","ZNF799",21.1856743448294,0.476543146496652,0.358090865729868,1
.33078833364083,0.183258667727085,0.627740518075766
"4566","RTL5",40.2246475470566,0.378795185295308,0.284639582008319,1.3
3078886155839,0.183258493971496,0.627740518075766
"4567","MT-
ND3",57917.4687045749,-0.516355111443316,0.388016886180972,-1.33075422
702734,0.183269893629868,0.627740518075766
"4568","DENND10",236.524092697397,-0.220816352454801,0.165991742925795
,-1.33028516095234,0.18342433444815,0.628003935916468
"4569","FSD2",2.77739265411704,-1.27982887790958,0.962077130038301,-1.
33027679169406,0.183427090916215,0.628003935916468
"4570","PNMA1",187.811697590095,-0.256778316569859,0.193062930196665,-
1.3300239269563,0.183510387986057,0.62815164097722
"4571","AC018926.2",24.7994284121612,-0.703420428011585,0.529057430788
464,-1.32957291038,0.183659028514476,0.628522901564636
"4572","RBP7",8.34108782795563,-1.18272611291749,0.890125222304918,-1.
32871879515435,0.183940761688337,0.629074187820433
"4573","MICA",130.299829064456,-0.407193080957365,0.306402051053006,-1
.32895024546335,0.183864385325572,0.629074187820433
"4574","RPL18",11334.8253642758,-0.389649499186663,0.293247327955102,-
1.32874015222509,0.183933713077822,0.629074187820433
"4575","HOXA11-
AS",26.6227085175389,0.426507696784675,0.321024288798387,1.32858388497
992,0.18398529157787,0.629088943421339
"4576","ACSF3",78.1099576775376,0.366416205634929,0.275820290691681,1.
3284599356924,0.184026210636066,0.629091348990381
"4577","AC010680.4",3.19644187648043,1.18512440615006,0.89219169231601
9,1.32832934486716,0.184069329537582,0.62910127200271
"4578","KCNMB1",15.2851502144062,-0.646217282334602,0.486645998849504,
-1.32790012424297,0.184211103693665,0.62944829512451
"4579","HIST2H3PS2",8.42461533108711,0.873733734682686,0.6582696962224
11,1.32731878696035,0.184403251775455,0.629461346129408
"4580","HABP4",105.884501302111,-0.321956397049982,0.242561422563619,-
1.32731905035534,0.184403164682529,0.629461346129408
"4581","MRGPRF",341.177168985862,-0.274627333400738,0.206902983222169,
-1.32732418413633,0.184401467177178,0.629461346129408
"4582","ATF7IP",280.865526042838,0.312201397962067,0.235218964855551,1
.32727987368617,0.184416118986836,0.629461346129408
"4583","TLCD1",7.41998303963344,-0.769967291676078,0.579998899018354,-
1.32753233321519,0.184332651588009,0.629461346129408

"4584","CLASP1",100.183123284388,0.258001538984528,0.194404565580989,1
.32713724193398,0.184463287827507,0.629484993779602
"4585","AC093525.7",6.42181116723761,0.913486439114877,0.6883847424396
65,1.32699983424595,0.184508737496033,0.629502765681668
"4586","ELOA-
AS1",30.1327060458121,-0.476969208743619,0.360142081453552,-1.32439177
009959,0.185372966427626,0.629642666426072
"4587","SHISAL2A",3.37061245840412,1.13609074675112,0.85657446070688,1
.3263187251854,0.184734146980881,0.629642666426072
"4588","ZNHIT6",191.38956419634,0.302201106061486,0.227971850638552,1.
32560710989106,0.184969869956275,0.629642666426072
"4589","ATP5PB",571.172720845014,-0.322633844790563,0.243680161670165,
-1.324005378933,0.185501258305122,0.629642666426072
"4590","VPS45",95.5402294596684,0.273215207033128,0.206380604204526,1.
32384149220906,0.185555692769959,0.629642666426072
"4591","UFC1",590.962569871627,-0.319938359238692,0.241544724541919,-1
.32455121860121,0.185320044525887,0.629642666426072
"4592","ANGEL2",131.672493836967,0.27945177167507,0.210990654515033,1.
32447464233616,0.185345459246785,0.629642666426072
"4593","RPS27A",16501.7489150954,-0.380440333478832,0.287186624389234,
-1.32471466694496,0.185265806652698,0.629642666426072
"4594","SEMA4C",57.6885557411236,0.305683945114881,0.230567910251863,1
.32578703073192,0.184910250064773,0.629642666426072
"4595","AC097448.1",56.7454172570757,-0.53613460404131,0.4044456865377
67,-1.3256034663909,0.184971077440159,0.629642666426072
"4596","ZNF148",405.028027732675,0.248947259261418,0.187738545776256,1
.32603168002649,0.184829204090749,0.629642666426072
"4597","SMAD1",58.5581371829269,0.335293903171974,0.253253185189774,1.
32394742802829,0.185520505167817,0.629642666426072
"4598","COL12A1",2123.59110233334,0.541742587053942,0.409097568270186,
1.32423810130338,0.18542398058736,0.629642666426072
"4599","AC007036.3",12.1230971378219,0.777242485820846,0.5865220222943
92,1.32517187126305,0.185114152639511,0.629642666426072
"4600","NOD1",39.4401224332359,0.3806083199396,0.287503147679405,1.323
84053187486,0.185556011776781,0.629642666426072
"4601","PUF60",314.996866216522,-0.354346859958932,0.267442578318319,-
1.32494557219373,0.185189204232305,0.629642666426072
"4602","AL136084.3",4.21201886200354,-0.912804054593716,0.689461076357
11,-1.32393848745846,0.185523474673482,0.629642666426072
"4603","LINC00863",43.9150354386941,-0.501148616756572,0.3784480000309
56,-1.32422054473951,0.185429809584025,0.629642666426072
"4604","DYNC2H1",247.776349973705,0.326077470055918,0.24629169240152,1
.32394831054361,0.185520212052586,0.629642666426072
"4605","TMEM106C",230.671257477749,0.524227618106052,0.395651220865144
,1.32497409450616,0.185179743630086,0.629642666426072
"4606","CACNB3",27.9999525804103,-0.458110556462261,0.345484602367963,
-1.32599413496971,0.184841640087834,0.629642666426072
"4607","NUTF2",195.65381309868,-0.315954089392437,0.238394886014743,-1
.32533920787587,0.185058670289351,0.629642666426072
"4608","LINC01229",2.81911527303663,1.43090143592147,1.07897486968843,

1.32616752819708,0.184784212462331,0.629642666426072
"4609","NAT9",99.0023097806888,-0.26154331439415,0.197256266817921,-1.32590623666,0.184870756944542,0.629642666426072
"4610","ESF1",305.833177112598,-0.22543833051175,0.170247815227882,-1.3241775244516,0.185444093425366,0.629642666426072
"4611","AC006001.2",6.88670687448193,0.955070016730254,0.721620097137606,1.32350806264772,0.185666476821558,0.62988087116019
"4612","DCAF11",65.7751109881914,-0.312202300203014,0.235942943540919,-1.32321100821085,0.185765216199343,0.630079201432421
"4613","HEATR5B",72.0094787481663,0.34845013146716,0.263588379512533,1.32194800131768,0.18618546671699,0.630204705463798
"4614","AC092944.1",46.482001080992,0.489187012017182,0.369909610089126,1.32245013017996,0.186018305243066,0.630204705463798
"4615","LINC02062",6.22690512173036,0.952942196283917,0.720492566088072,1.3226259938502,0.185959785494501,0.630204705463798
"4616","POM121",116.548392336895,0.320192527191483,0.242114235963038,1.32248533803838,0.186006588515796,0.630204705463798
"4617","ATP6V1H",141.894357145726,0.22465511127262,0.169949030819589,1.32189698399107,0.186202456878158,0.630204705463798
"4618","RBM7",267.173823686812,-0.283893813599427,0.214642236303214,-1.32263723342122,0.185956045918887,0.630204705463798
"4619","IFNG",5.22861593023857,1.30389844779834,0.986390201544906,1.32188909191935,0.186205085255621,0.630204705463798
"4620","ST20-AS1",12.0907768479722,-0.604916272396358,0.457486114233317,-1.32226149292009,0.186081090614866,0.630204705463798
"4621","OTOA",2.9831374919885,-1.35736220299904,1.02673718818135,-1.32201523293739,0.186163078503931,0.630204705463798
"4622","ELANE",160.863946613305,-0.503819396910395,0.380999649900827,-1.32236183692436,0.186047690514556,0.630204705463798
"4623","DCTN4",145.839152075905,0.275214630768656,0.208224859512561,1.32171841255128,0.186261935062571,0.630260750634611
"4624","LERFS",30.1462605817659,1.96139519012406,1.48450146307012,1.32124840488043,0.186418551300711,0.630505463753011
"4625","SSH3",73.0053500890043,-0.307044605127939,0.232378927875726,-1.32131001693985,0.186398015353109,0.630505463753011
"4626","GOLGA8N",27.2976229613438,0.557267592814085,0.421847196972484,1.32101765002467,0.186495479178238,0.630505463753011
"4627","PSMD11",311.428197083568,-0.216160686835369,0.163630921764009,-1.32102590699281,0.18649272610771,0.630505463753011
"4628","RYBP",169.89088434362,-0.271828379676063,0.205812967801445,-1.32075438481751,0.186583273833989,0.630665979383124
"4629","MMP11",45.2850520524334,0.45846569481855,0.347205364086852,1.32044531058531,0.186686384016864,0.630878182150746
"4630","MARCKSL1",202.881810653635,-0.395112508420225,0.299332825993648,-1.31997720967833,0.186842627199156,0.63090515824737
"4631","NIFK",327.425654576046,-0.310913693607517,0.235562059680551,-1.31988017947012,0.186875026120265,0.63090515824737
"4632","FAM162A",396.031156040131,-0.409401741737201,0.310318424199318,-1.31929563252178,0.18707029737405,0.63090515824737

"4633","RNF144B",9.29078040761575,-0.879431437641517,0.666590666995334
,-1.31929755573321,0.187069654667279,0.63090515824737
"4634","PGAM1",623.215657118236,-0.343323904653506,0.260082773908153,-
1.3200563016709,0.18681622101667,0.63090515824737
"4635","PYROXD2",70.3047894157137,0.344066443299364,0.260778414434561,
1.31938237313619,0.187041311657751,0.63090515824737
"4636","SH3PXD2A",289.752110436116,0.413178295634118,0.313229079372113
,1.31909303076956,0.187138012802391,0.63090515824737
"4637","LRP6",447.849452223484,0.355036894884109,0.268961735189899,1.3
2002752969093,0.186825826703619,0.63090515824737
"4638","POLR2C",300.791156250956,-0.301610740468839,0.228640470461315,
-1.31914852983068,0.187119461593897,0.63090515824737
"4639","CENPB",94.1039975641673,-0.454787432170621,0.344631848320952,-
1.31963262938799,0.186957703254998,0.63090515824737
"4640","FAM118A",86.268552042962,0.409903709001338,0.310631301520387,1
.31958275613263,0.186974363266222,0.63090515824737
"4641","ZNF490",28.0452893010594,0.445813646557577,0.338054631848466,1
.31876213060561,0.187248648232192,0.631142125467826
"4642","HECA",110.788482052204,-0.381257613267755,0.289142582038341,-1
.31857995657381,0.187309578157393,0.631211488822943
"4643","LRRC8D",30.9378248556264,0.504152971226227,0.382608588021593,1
.31767290910306,0.187613167157432,0.631977764966936
"4644","MED15",128.640941499025,0.314251683200444,0.238492388512114,1.
31765917210595,0.187617767723995,0.631977764966936
"4645","BTF3",4401.23708642609,-0.358986620484165,0.272471404811378,-1
.31752034945714,0.187664264569409,0.631998297235578
"4646","ZNF862",23.4180545770733,0.461216438094004,0.350100737784698,1
.31738207983337,0.18771058464063,0.632018225469948
"4647","GRINA",776.587572860819,-0.211316949996316,0.160421955707097,-
1.31725703669979,0.1877524811278,0.63202325420318
"4648","MED8",209.95586874436,-0.267243917598596,0.202927756321946,-1.
31694117375748,0.187858343726707,0.632243560868519
"4649","USP31",38.7634490992222,0.429764259521009,0.326377559697487,1.
31677024584456,0.187915649196256,0.632300387261138
"4650","TMEM30A",423.692094658119,0.185714699867742,0.141072864520485,
1.31644523203663,0.188024649247116,0.632461443242407
"4651","EDNRB",51.7474203678009,-0.594359035771203,0.451508025203324,-
1.31638642636208,0.188044375920248,0.632461443242407
"4652","STXBP1",185.729870153675,0.239316403314407,0.181827963045285,1
.31616941259362,0.188117187548325,0.632499063921809
"4653","AC104564.5",7.95052744541305,0.957389471869627,0.7274376465974
49,1.31611207688764,0.188136428078257,0.632499063921809
"4654","STARD7-
AS1",10.5524564689415,0.695719311575658,0.528943293983904,1.3153003724
3809,0.188408973021038,0.633279236134098
"4655","TUB",75.6692432653593,0.350542500443919,0.266581137956575,1.31
495612604452,0.188524648092934,0.633395848392992
"4656","PGM2L1",38.7304175553638,-0.509039071901945,0.387102578616186,
-1.31499788433768,0.188510613510531,0.633395848392992
"4657","SLC25A46",145.077628289822,0.262903365388513,0.199990328560422

,1.31458039636693,0.18865096208449,0.633412105577951
"4658","FAM50B",89.4077102612129,-0.318322448400957,0.242127454270889,
-1.31468960989786,0.188614239901367,0.633412105577951
"4659","IFT140",44.717583516796,0.345323830468668,0.262680752726512,1.
31461413477903,0.188639617248346,0.633412105577951
"4660","UPF1",191.789601243209,0.269720543128647,0.205213316806546,1.3
1434230159104,0.188731037840965,0.633544983894037
"4661","C1orf167",38.701413106005,0.390254515853979,0.297367752875601,
1.31236326763796,0.189397594469042,0.633691964653382
"4662","SLC66A1",36.3265093737739,0.447614546717668,0.341096256315148,
1.31228220313302,0.189424934674922,0.633691964653382
"4663","KIAA1614",46.1821538254298,0.377543119599035,0.287769587482024
,1.31196323733348,0.189532538857608,0.633691964653382
"4664","LINC00467",101.107156693263,0.383614521246984,0.29237536228893
9,1.31206172176669,0.189499309997734,0.633691964653382
"4665","ORC4",176.668525561668,0.29183383921852,0.222420817492907,1.31
207969878012,0.18949324497808,0.633691964653382
"4666","SETD5",363.339043993396,0.292311928179854,0.222619591683873,1.
31305571970928,0.189164173261555,0.633691964653382
"4667","ARL13B",148.236213742345,-0.20588108108828,0.15698763838777,-1
.31144772418157,0.189706544171309,0.633691964653382
"4668","GASK1B",121.001693641035,0.995384648578925,0.758726714291762,1
.31191459300082,0.189548953144352,0.633691964653382
"4669","REEP2",8.03674230656204,-0.860653294298941,0.65623886877988,-1
.31149393192622,0.189690942497589,0.633691964653382
"4670","TPBG",273.138915217124,-0.269802278630494,0.205701843283863,-1
.31161818641642,0.189648993656674,0.633691964653382
"4671","FDFT1",164.19803970169,-0.244940374763968,0.186400861472195,-1
.31405173146426,0.188828796003368,0.633691964653382
"4672","TMEM65",140.38110610649,0.266395334722993,0.203033991713906,1.
3120725868325,0.189495644364096,0.633691964653382
"4673","PPP1R16A",36.9526378707194,-0.434097316883082,0.33090133080125
9,-1.31186331536334,0.189566257133715,0.633691964653382
"4674","PPP1R14B",864.207295017971,-0.44039731148329,0.335208467262241
,-1.31380127441339,0.188913088653479,0.633691964653382
"4675","ATXN2",295.332605674278,0.281715384041419,0.214707881062892,1.
3120868346649,0.189490837538789,0.633691964653382
"4676","MED13L",538.162840543958,0.299345398751909,0.228220326758393,1
.31165090771609,0.189637947944801,0.633691964653382
"4677","CHAMP1",80.346539243629,0.294982659879675,0.224605660204397,1.
31333582426744,0.189069812055355,0.633691964653382
"4678","ARG2",3.59082149121601,-1.03773770874686,0.790248007309929,-1.
31317978552,0.189122373873978,0.633691964653382
"4679","ELOF1",243.546784198196,-0.337829767656101,0.257109392484174,-
1.31395342811872,0.188861877209097,0.633691964653382
"4680","CALR",1637.25201207433,-0.306195045006615,0.233475667341212,-1
.31146448147476,0.189700886096637,0.633691964653382
"4681","TP53INP2",20.2516315589492,-0.665489530162394,0.50719320362624
2,-1.31210261770937,0.18948551288022,0.633691964653382
"4682","HUNK",31.011471259805,0.525255857335634,0.40003631777356,1.313

02042839259,0.189176064609866,0.633691964653382
"4683","AGPAT3",86.5197151665446,0.333616099279878,0.254092037087686,1
.31297345286247,0.18919189378864,0.633691964653382
"4684","LENG8-
AS1",25.676793862521,0.477012455704056,0.36378798644012,1.311237516037
58,0.189777531196511,0.633793749040781
"4685","TYW3",218.271301582999,-0.272250443704045,0.207660564756086,-1
.31103584363177,0.189845654106911,0.633797255118026
"4686","LINC00623",185.179918015042,-0.254029443464451,0.1941924858227
14,-1.30813219877295,0.190828473938313,0.633797255118026
"4687","ROCK2",603.107724856407,0.249658839299027,0.190724832024731,1.
30900017920401,0.190534290031903,0.633797255118026
"4688","LINC00342",603.696017278734,-0.467925211199858,0.3572393264419
84,-1.30983678605678,0.190251055712995,0.633797255118026
"4689","ANAPC1",75.2071039415273,0.282906186077407,0.216489939843833,1
.30678675545609,0.191285144564252,0.633797255118026
"4690","AC017071.1",4.82954552416495,1.05089751568669,0.80403523118152
9,1.30702918843792,0.191202798576454,0.633797255118026
"4691","NEMP2",32.1161798544457,-0.375493688673644,0.287365890263089,-
1.3066745267849,0.191323273545681,0.633797255118026
"4692","WNT5A",63.402594791248,0.646591170225685,0.49342683705445,1.31
040940960074,0.190057372112792,0.633797255118026
"4693","FAM193A",114.125291519116,0.355550167835681,0.271672900189852,
1.30874359417967,0.190621219400178,0.633797255118026
"4694","ELOVL2",22.1825884534903,0.612785866735245,0.468284983689145,1
.30857466730563,0.190678466684809,0.633797255118026
"4695","NOL7",728.566844265761,-0.303434726461283,0.232270845301961,-1
.30638318410908,0.191422281478789,0.633797255118026
"4696","EYS",3.82240069303122,1.27718276157991,0.977770903445125,1.306
21882598451,0.191478152465473,0.633797255118026
"4697","AL356417.2",7.73727322281075,-0.734525225870945,0.560707447769
905,-1.30999727004227,0.190196759119143,0.633797255118026
"4698","MAP3K4",138.046883864215,0.323307126464006,0.247601437374059,1
.3057562584969,0.191635459477874,0.633797255118026
"4699","BX322234.1",59.3589175818046,-0.553426066702459,0.423479552076
217,-1.30685428372904,0.191262204954934,0.633797255118026
"4700","AC016831.6",10.3258237117564,0.899889764081238,0.6881323818723
15,1.30772768116617,0.190965690929634,0.633797255118026
"4701","LINC-
PINT",362.545553680038,-0.41668281201293,0.317920729145376,-1.31065002
629128,0.18997602952212,0.633797255118026
"4702","MTFR1",63.5013817112474,-0.297031488533913,0.227311986695647,-
1.30671282606674,0.191310260978323,0.633797255118026
"4703","TRPA1",3.72228153971488,1.16947711042539,0.893573282356201,1.3
087646346606,0.190614089920703,0.633797255118026
"4704","DNLZ",19.5221361206803,-0.582027574654614,0.445568443359543,-1
.30625851836854,0.191464658558553,0.633797255118026
"4705","IPMK",81.9454913864454,0.339885413827096,0.260215142269132,1.3
0617077416488,0.191494489199996,0.633797255118026
"4706","TRIM8",351.34738476402,-0.217011883303045,0.165626449558847,-1

.31024896012119,0.190111627727589,0.633797255118026
"4707","CALHM2",570.449235076542,-0.264769229449558,0.202380269662562,-1.30827590007178,0.190779746291663,0.633797255118026
"4708","PTDSS2",81.3535902602548,-0.306120167515654,0.233780788267399,-1.30943252345232,0.19038788059376,0.633797255118026
"4709","APIP",148.332919973436,-0.317203833630801,0.242182318235766,-1.30977288491392,0.190272678582767,0.633797255118026
"4710","EED",86.2186012613403,0.274396083742599,0.209653797647778,1.30880569215154,0.190600178325942,0.633797255118026
"4711","SIK3",131.654997794654,0.245350002731688,0.18787420463949,1.30592703347694,0.191577372341773,0.633797255118026
"4712","NLRX1",36.8092134014186,0.353099360569617,0.270047500918668,1.30754537393761,0.191027555357106,0.633797255118026
"4713","ANO6",512.366030626069,0.22470889138717,0.1720936562345,1.30573605270479,0.191642333101596,0.633797255118026
"4714","CCNT1",112.696157452085,0.241438990773546,0.184477797179029,1.30876991413356,0.190612301024056,0.633797255118026
"4715","NAA25",71.0720857106936,0.275185838748171,0.210020508885166,1.31028079214223,0.190100862897375,0.633797255118026
"4716","HIF1A",217.880289034576,0.256433802716304,0.195940614226084,1.30873225915491,0.190625060307556,0.633797255118026
"4717","SRP14-AS1",35.6797616280671,-0.476872001784317,0.364152899732797,-1.30953784010571,0.190352228620885,0.633797255118026
"4718","LACTB",104.212950780224,-0.434796421268936,0.331767978212088,-1.31054366250798,0.190011983572321,0.633797255118026
"4719","AC105339.6",6.25319119494257,0.886021999897504,0.67850139861748,1.30585139795271,0.191603097293255,0.633797255118026
"4720","MRPL34",498.957848044454,-0.354796920472574,0.27127283460904,-1.30789697753521,0.19090825483421,0.633797255118026
"4721","TIMM50",124.71112967748,-0.280698666193653,0.214591927405534,-1.30805790127972,0.190853671053976,0.633797255118026
"4722","ZNF611",43.8049764684596,0.454578827324526,0.347546924176429,1.30796389121189,0.190885556965793,0.633797255118026
"4723","LTN1",254.664777056401,0.217275666891973,0.166297741144828,1.30654611058577,0.191366908997286,0.633797255118026
"4724","SETD4",70.1369571747687,0.282783661436304,0.216294290848833,1.30740233746595,0.191076103908195,0.633797255118026
"4725","LINC01637",18.595504994276,-0.70494086800125,0.53812629467282,-1.30999149266596,0.190198713582509,0.633797255118026
"4726","PPARA",100.287413002181,0.337000599604642,0.257943953596038,1.3064876881449,0.191386763196276,0.633797255118026
"4727","SPIN2B",95.4689586649781,-0.362369994901123,0.277476024641193,-1.30595065058218,0.191569340275889,0.633797255118026
"4728","WDR44",57.3944905083849,0.278866179860867,0.213369516381285,1.30696354657589,0.191225092240804,0.633797255118026
"4729","GPC3",8653.67279864042,0.309345977688755,0.236656575511175,1.30715141559271,0.191161292187877,0.633797255118026
"4730","MT-ND1",48146.9244979954,-0.202478501218553,0.154919405714411,-1.30699249

90018,0.191215259016681,0.633797255118026
"4731","AC009506.1",16.9257106388533,-0.517999626767636,0.396774521161
56,-1.30552643665523,0.191713651169132,0.633899100663439
"4732","INTS11",220.983073695478,-0.313909594715211,0.240497473614506,
-1.30525111136251,0.191807355274771,0.633972193802721
"4733","RTN4IP1",11.5664079134586,-0.551522184163664,0.422561273756262
,-1.30518866355413,0.191828613423942,0.633972193802721
"4734","SMAD6",82.8280195216914,0.346848300206123,0.265762900393805,1.
3051042854069,0.191857339734199,0.633972193802721
"4735","NAXE",258.776856960623,-0.368423882679438,0.282348204461142,-1
.30485647458807,0.191941724541814,0.634117084901288
"4736","SMIM10L1",204.77837035911,-0.330502225594641,0.253418443485622
,-1.30417589599548,0.19217361630618,0.634369106412553
"4737","BTBD6",308.14816043746,-0.344336150915065,0.26399689752919,-1.
30431893002452,0.192124863607278,0.634369106412553
"4738","ZNF609",106.349588024246,0.363813009961966,0.278959038550624,1
.30418075661651,0.192171959429508,0.634369106412553
"4739","CNTROB",34.0727073444905,0.377379164741602,0.28936646686289,1.
30415652108167,0.192180220884043,0.634369106412553
"4740","SORBS3",437.49869895374,-0.213732228539999,0.163909940549934,-
1.30396135721181,0.192246758228046,0.634454860540365
"4741","NNT-
AS1",231.076251426715,0.211577446742701,0.162289116957611,1.3037069318
5769,0.19233352505754,0.634529550579052
"4742","CALN1",3.41298204441743,-1.70154098580008,1.30529849144452,-1.
30356466122707,0.192382056243274,0.634529550579052
"4743","CYTH4",6.54049383946097,-0.955319526095786,0.732898312709098,-
1.30348168296981,0.192410365842158,0.634529550579052
"4744","MAGT1",315.199484944661,0.254366867335983,0.195153518626163,1.
30341932406173,0.192431642776131,0.634529550579052
"4745","DHCR24",25.0161770151253,-0.62787555675704,0.481964168791747,-
1.3027432274293,0.192662438791087,0.634653187917149
"4746","CNIH3",32.6004259970105,-0.418768307950685,0.321548942829629,-
1.30234702146904,0.192797784312831,0.634653187917149
"4747","RIF1",403.190990946685,0.335177048837495,0.257391303537404,1.3
0220813302958,0.192845245687758,0.634653187917149
"4748","BLOC1S4",213.115119333351,-0.267633650969259,0.205385446677173
,-1.30307991777981,0.19254747892989,0.634653187917149
"4749","LINC01091",72.167411073511,-0.339731900481833,0.26083249223140
8,-1.30249071952441,0.192748688416608,0.634653187917149
"4750","SETD7",397.979609672583,0.239953274182019,0.184215949475874,1.
30256514088344,0.192723265217006,0.634653187917149
"4751","LY6G5C",5.88120416765688,-0.9829201072615,0.754860505699975,-1
.30212151760417,0.192874848517428,0.634653187917149
"4752","BMI1",279.592181439861,0.182414578837448,0.140001857200578,1.3
0294399292222,0.192593883167612,0.634653187917149
"4753","ART4",3.2926103453271,1.51889816641564,1.16644263212637,1.3021
6276787377,0.192860749865061,0.634653187917149
"4754","AL118516.1",62.4000690998643,-0.488631499883816,0.375011379004
106,-1.30297779545102,0.192582342342975,0.634653187917149

"4755","LYPLA2",75.7185968374319,-0.355865262983598,0.273520977938468,
-1.3010529052132,0.193240346332372,0.634718519754807
"4756","PLXND1",220.552552055758,0.306209812708224,0.235330222260938,1
.30119204310568,0.193192728168843,0.634718519754807
"4757","DNAH5",4.37141171072526,-0.927243537228315,0.712416824636039,-
1.30154637729398,0.193071500754028,0.634718519754807
"4758","MARCH3",168.206550887587,0.353769731206317,0.271743614257826,1
.3018511296853,0.192967281338356,0.634718519754807
"4759","CRTAC1",6.15216063269514,-1.1981775432822,0.921211484675235,-1
.30065415294362,0.193376861953108,0.634718519754807
"4760","CNNM2",32.3999235692299,0.388655590462209,0.298756419455061,1.
30091126132495,0.193288830996089,0.634718519754807
"4761","LRRC32",216.05147752386,-0.302332606411389,0.232272025878541,-
1.301631590235,0.193042355414576,0.634718519754807
"4762","GLIPR1",191.090401531692,0.329840364683558,0.253371012104763,1
.3018078190696,0.19298209020988,0.634718519754807
"4763","NUMB",260.298221143585,0.25060921802381,0.192653035118293,1.30
083192237242,0.193315992601519,0.634718519754807
"4764","MT1A",365.966100479978,-1.13689656707153,0.874052364536271,-1.
30071905666054,0.193354636887818,0.634718519754807
"4765","FASN",49.2648769370131,0.352637022950074,0.271048217124123,1.3
010121471804,0.193254296880146,0.634718519754807
"4766","AC010642.2",186.774688662456,-0.363091020603425,0.279163286600
431,-1.30064029917774,0.193381606159394,0.634718519754807
"4767","TMEM232",6.18288156048065,0.836648789837749,0.643745393844438,
1.29965790487648,0.193718243963765,0.634757957755587
"4768","UBTD2",209.285658902594,0.264265249063817,0.203248856683662,1.
30020534125375,0.193530600481279,0.634757957755587
"4769","ZSCAN31",18.5598895650611,0.559983716426937,0.430791856572567,
1.29989392297764,0.193637328217993,0.634757957755587
"4770","GALT",130.832095442894,0.313295019536431,0.241032045199079,1.2
9980650198469,0.193667296477584,0.634757957755587
"4771","ACAD8",67.8851119745295,-0.29993294893485,0.23067412732885,-1.
30024529585524,0.193516910564579,0.634757957755587
"4772","ARGLU1",1483.98977359488,0.28229051367586,0.217095713859305,1.
30030440793874,0.193496657894022,0.634757957755587
"4773","AC091057.3",4.94143919689324,1.11293513199516,0.85608087695667
8,1.30003503401639,0.193588961984528,0.634757957755587
"4774","ZNF426-
DT",4.34947559836908,1.03890952282517,0.799341669724242,1.299706448662
4,0.193701599328468,0.634757957755587
"4775","AC095057.3",5.01548594755867,1.01815961764007,0.78364158409777
2,1.29926695864706,0.193852329426176,0.63479840678536
"4776","HIVEP1",159.873993301394,0.301331577952984,0.231891343091472,1
.29945160494465,0.193788991552731,0.63479840678536
"4777","SLC14A2-
AS1",5.24671462390224,-1.32056323469351,1.016309160122,-1.299371575608
92,0.193816441566543,0.63479840678536
"4778","POLR2H",246.811992262605,-0.254089375288237,0.195586281047517,
-1.29911655320297,0.19390393314019,0.634834496884052

"4779","NEIL2",45.1545800580978,-0.345020483078641,0.265631608515232,-
1.2988683274824,0.19398912078973,0.63494543907318
"4780","HACD3",323.601552233465,0.270638149760319,0.208393313059592,1.
29868922273397,0.194050604134193,0.63494543907318
"4781","AC027682.6",8.87350579655866,0.665614767010688,0.5125384640573
24,1.29866305397178,0.19405958858332,0.63494543907318
"4782","ABHD4",63.7104209830149,0.385970656187603,0.297294838399501,1.
29827567227703,0.194192622991975,0.634982274553401
"4783","CALM1",3291.73526814763,-0.255412198992818,0.196730346590572,-
1.29828571656193,0.194189172744111,0.634982274553401
"4784","IER2",819.38825140615,-0.58834036998156,0.453108805734859,-1.2
9845273924302,0.194131806448852,0.634982274553401
"4785","CYTH2",174.751010809658,-0.314880468155464,0.242592556436513,-
1.29798074920682,0.194293950180105,0.635180828143653
"4786","GATAD2B",157.305846869067,0.295664931172773,0.227826900470647,
1.2977612852652,0.194369376931415,0.635294643405375
"4787","PPM1F",86.3132751616843,0.298060374160579,0.22972101518515,1.2
9748849455654,0.19446316131335,0.635468400339406
"4788","RPS19BP1",726.953902397912,-0.324599154300393,0.25020722392769
6,-1.29732127316274,0.194520667789928,0.635523560200051
"4789","DDHD2",127.939127757664,0.315419130668653,0.243220075997711,1.
29684660846673,0.194683970228121,0.635791512793007
"4790","POFUT1",99.4547572984619,0.292127492050313,0.225253616374939,1
.29688258395843,0.194671589789736,0.635791512793007
"4791","IMP4",180.313604580361,-0.33058926642375,0.254972520609609,-1.
29656821697237,0.19477979411247,0.635796537745481
"4792","CCL27",3.17329409692423,-1.12258302131112,0.865864625085247,-1
.29648791368582,0.194807441374039,0.635796537745481
"4793","PCNA",164.351011428477,-0.322301935385957,0.248587568086619,-1
.29653279875063,0.194791987739782,0.635796537745481
"4794","AP000866.6",9.78161512357587,0.80272687416058,0.61926476717817
8,1.29625794443044,0.194886632392646,0.63592231758827
"4795","MFAP4",5004.31735850214,-0.29459106444764,0.227283588515383,-1
.2961387417891,0.194927689685942,0.635923639156868
"4796","STAT4",6.76173824206659,0.960733865460694,0.741364172998746,1.
29590004541846,0.195009923603819,0.636059264998863
"4797","TREM1",5.26500280697745,-1.07474133555241,0.82954247094595,-1.
29558325606506,0.195119100830006,0.636240566667862
"4798","RAD51B",67.0019430771002,0.360299627556557,0.278115679955576,1
.29550274768438,0.195146854111897,0.636240566667862
"4799","GNA12",186.984623385176,0.212708484378263,0.164242143685572,1.
29509077028047,0.195288918488069,0.636438448314346
"4800","RAB11FIP1",24.4886336686457,-0.630408981122288,0.4867581539902
44,-1.2951174540261,0.195279714692887,0.636438448314346
"4801","P2RY12",7.58732457743289,0.73385304263132,0.566940153202405,1.
29441006865733,0.19552381461445,0.636673403833021
"4802","PLA2G7",25.4016826762164,0.761865587281837,0.588548865379694,1
.29448144767101,0.195499173468699,0.636673403833021
"4803","PGAP2",60.0337222759125,-0.273836187230503,0.211552732456942,-
1.29441101540127,0.195523487768817,0.636673403833021

"4804","TEX26-
AS1",19.4506593489409,-0.696939346502468,0.538334594693855,-1.29462113
96628,0.195450956208026,0.636673403833021
"4805","CEP135",58.9491229995301,0.439415933011385,0.339578735402424,1
.29400309030142,0.195664353374261,0.636998434928941
"4806","SENP2",122.506682766593,0.311600143905061,0.240864459346764,1.
29367422968974,0.195777970403419,0.637135696492728
"4807","NREP",135.465267209702,0.363721913258905,0.281166057074317,1.2
9361956789388,0.19579686002282,0.637135696492728
"4808","PNN",733.306484322983,0.252721473248494,0.195373880889205,1.29
352742597057,0.195828704771274,0.637135696492728
"4809","NBPF15",44.7136078719535,0.466386166078333,0.360748230714479,1
.29283008583198,0.196069832384562,0.637558556944512
"4810","NHEJ1",58.5629966284379,-0.424464897039462,0.328302837334959,-
1.29290657517648,0.196043373134101,0.637558556944512
"4811","MB21D2",24.7928717166413,0.535272084654171,0.414041559033961,1
.29279796429872,0.196080944669184,0.637558556944512
"4812","CD200R1",3.64703893908529,-1.08023848063223,0.835891169318264,
-1.29231952709017,0.196246512269887,0.637809666517352
"4813","AL109615.4",6.30581090056592,0.81888452724747,0.63387624289762
4,1.29186814685485,0.196402810433682,0.637809666517352
"4814","EMG1",158.257434975703,-0.290951713998189,0.225162797909764,-1
.29218377413657,0.196293509478082,0.637809666517352
"4815","SERPINA1",11.1615532099963,-0.719169314640416,0.55662319043469
1,-1.29202183272096,0.196349583826392,0.637809666517352
"4816","BABAM1",234.955371483634,-0.306118529861436,0.236951537901402,
-1.29190353678487,0.19639055276875,0.637809666517352
"4817","ZNF217",206.803345903118,0.22363236487349,0.173046236178711,1.
29232724046386,0.196243842173832,0.637809666517352
"4818","ZNF235",12.2091792955571,0.577437206072394,0.447062787692004,1
.29162440258886,0.196487248980683,0.637951439560986
"4819","CTNNBL1",215.414519329007,-0.270824990675304,0.209707927443192
,-1.29143897408775,0.196551503429669,0.638027633980142
"4820","DDIAS",2.60245775767335,-1.17731644312099,0.911855979810211,-1
.2911210423449,0.196661708547284,0.638252926723064
"4821","CCDC74B",26.610135444183,-0.537532246576544,0.416443649792693,
-1.29076826323112,0.196784045815436,0.638517491949981
"4822","ASCC2",155.306213313579,-0.265335253713044,0.205618262153192,-
1.29042649682236,0.196902617218939,0.638769730642028
"4823","AL139317.5",25.791471380256,-0.741068336594739,0.5744114882991
24,-1.2901349497537,0.197003807016843,0.63896548894142
"4824","ZNF665",37.5161603277863,0.350289423421219,0.271543912412212,1
.28999181130406,0.197053501266545,0.63899417916927
"4825","COL5A3",202.660921274773,0.525302413761237,0.407299032144927,1
.28972173342735,0.197147290845606,0.639165817761205
"4826","CAP2",23.6447673178909,-0.586326280213032,0.454668732916263,-1
.2895680695972,0.197200668059951,0.639206392553216
"4827","SGCG",54.3351020502802,0.537711226294685,0.417009093890823,1.2
8944724269122,0.19724264635258,0.639210009714813
"4828","KIT",14.7707812340912,0.895357289479551,0.694584816754864,1.28

905393248114,0.197379337468492,0.639274546561001
"4829","AC010491.1",3.85289409387864,1.13872283485208,0.88339021548566
,1.28903718299172,0.197385160128469,0.639274546561001
"4830","PAGR1",219.841400926946,-0.295015170556682,0.228850837707451,-
1.2891155370548,0.197357922823439,0.639274546561001
"4831","CNNM4",16.8102893722128,0.483874275110766,0.37549732086998,1.2
8862244340303,0.197529377016673,0.639486341017543
"4832","SPART",615.762564724118,0.242927915519094,0.188518762036203,1.
28861399732957,0.197532314760389,0.639486341017543
"4833","AC068587.4",41.8442327792046,0.530965350644938,0.4121376993838
71,1.28832026635445,0.197634501219261,0.639684771895902
"4834","MT-
ND2",31896.788792801,-0.242640318380794,0.188389048616282,-1.287974646
94996,0.197754788741816,0.639941696377376
"4835","RGPD8",7.28776039979329,0.864823037181324,0.671635946261781,1.
28763661622757,0.197872486946885,0.640057757094733
"4836","RNF139-
AS1",15.0186354117708,-0.53950423369961,0.418959303897464,-1.287724675
59677,0.197841820805436,0.640057757094733
"4837","SPTBN1",2600.30037051767,0.241491194810689,0.187600667268277,1
.28726191823906,0.198003012129828,0.64021519610312
"4838","TMEM167A",442.246550639388,-0.254803889275976,0.19792796763385
7,-1.28735666981299,0.197969999688721,0.64021519610312
"4839","KIF1B",290.363056385617,0.315320669454489,0.245099259774477,1.
28650192474928,0.198267947521267,0.640347864300096
"4840","ZBTB20",1686.68137447906,0.289864381475244,0.225310042577736,1
.28651336690967,0.198263956837847,0.640347864300096
"4841","SEPSECS-
AS1",31.0991778462897,0.436402814230997,0.339090245827293,1.2869813260
6642,0.198100797002273,0.640347864300096
"4842","PNPLA8",226.461385950439,-0.231342839171161,0.179813089371503,
-1.28657396399655,0.198242823365164,0.640347864300096
"4843","AC233723.1",18.0912815397398,-0.873677777246987,0.679094672116
941,-1.28653310520531,0.198257072848585,0.640347864300096
"4844","MSN",431.123804457855,-0.262853824893324,0.20432658190693,-1.2
8643969100923,0.198289653817661,0.640347864300096
"4845","AL591846.2",10.1138269596594,-1.3002576228151,1.01239349567925
,-1.2843401586087,0.19902296127914,0.641016202662872
"4846","METTL5",357.430351604443,-0.37824310730172,0.29448880088533,-1
.28440574366359,0.199000024319747,0.641016202662872
"4847","CEP19",29.051561397044,-0.398106185488739,0.309635361599987,-1
.28572584032907,0.198538759698468,0.641016202662872
"4848","ZNF721",204.812379448405,0.278272527090881,0.216741930516939,1
.28388875390742,0.199180882761701,0.641016202662872
"4849","PRDM5",83.2128380160343,0.290324011005118,0.226147811833253,1.
28377988118313,0.199218984995227,0.641016202662872
"4850","ARMC10",222.695709094145,-0.290949775247608,0.226561384272782,
-1.28419843558734,0.199072532450054,0.641016202662872
"4851","UBN2",174.113513597541,0.339304809163828,0.264075515717939,1.2
8487795713042,0.198834934868611,0.641016202662872

"4852","FSBP",14.8388456806735,0.70484680013779,0.5483992759234,1.2852
8032600146,0.198694342639379,0.641016202662872
"4853","MTDH",1526.49917438581,-0.165847891509866,0.129167727956025,-1
.2839731265253,0.199151358503593,0.641016202662872
"4854","MRPL48",84.8533051240641,-0.317937797057813,0.247348311703935,
-1.28538494913348,0.19865779804321,0.641016202662872
"4855","AMIGO2",41.1291571926796,0.506156861846287,0.393949672159236,1
.28482620399719,0.198853023274762,0.641016202662872
"4856","PGAM5",31.7498770948738,-0.360014453127217,0.280450756708931,-
1.28369934655182,0.199247173161212,0.641016202662872
"4857","CBFB",113.84848369924,0.274025745322642,0.213298941140165,1.28
470279251209,0.198896162078855,0.641016202662872
"4858","SLC16A5",26.9650838032929,0.408322466756498,0.317939055164774,
1.28427904695408,0.199044335502157,0.641016202662872
"4859","SNRPD1",398.454367490464,-0.339101697302341,0.264176219986954,
-1.28361931031902,0.199275189768558,0.641016202662872
"4860","SERPINB8",34.317075707178,-0.364439037701836,0.283697833593447
,-1.28460282225523,0.198931111955536,0.641016202662872
"4861","NINL",40.5278928418901,0.469811332831292,0.365821955983743,1.2
8426226241098,0.199050206305614,0.641016202662872
"4862","LINC01422",6.12237120827669,0.775071580954578,0.60307063459035
4,1.28520862482561,0.198719390517387,0.641016202662872
"4863","RBM10",103.533932610909,0.288185249250591,0.224219997890793,1.
28527897583405,0.198694814281569,0.641016202662872
"4864","DIPK2A",125.496623032833,0.368922327640348,0.287490955109072,1
.28324846776617,0.199405040421171,0.641046984303929
"4865","AC145124.1",89.0551256493669,-0.237522301753351,0.185087982763
468,-1.28329402161615,0.199389086394616,0.641046984303929
"4866","GLIS2",126.47629707045,-0.33776931493097,0.263216509506279,-1.
2832375733746,0.199408856030778,0.641046984303929
"4867","POLR2E",610.034460821679,-0.357889858605513,0.278920736904701,
-1.28312388163449,0.199448678169611,0.641046984303929
"4868","RNF144A",71.0609764736271,0.474326148631272,0.369702115807253,
1.28299549380606,0.199493654805807,0.641059827881519
"4869","PSME2",478.298263030907,-0.40015388738854,0.311924670839029,-1
.28285424269965,0.199543146246637,0.641087171233547
"4870","C1orf21",993.768089333745,0.257385280894126,0.200695062910024,
1.28246941983579,0.199678025653832,0.641135111949856
"4871","DSTYK",151.454353383479,0.27218364952888,0.212243810917289,1.2
8241030140073,0.199698752410434,0.641135111949856
"4872","FNDC3A",408.130146901443,0.173313795294531,0.135153905151345,1
.28234397001296,0.199722009878645,0.641135111949856
"4873","SUSD2",27.0905400171783,0.666141979135987,0.519347373561826,1.
28265206111933,0.199614002061113,0.641135111949856
"4874","DAP3",179.557762773657,-0.256804383321913,0.20035425893339,-1.
28175155691245,0.199929812692413,0.641670508811534
"4875","RRM1",107.06381491396,-0.26815657044761,0.209239893965325,-1.2
8157477699759,0.199991852978613,0.641737960234757
"4876","EIF3H",1014.90601559959,-0.341645322406495,0.266660663364231,-
1.28119880186394,0.200123846865014,0.642029806503162

"4877","MOB1A",429.122135504788,0.166360652444248,0.129975616254533,1.
27993740086188,0.200567152586077,0.642135073250921
"4878","KIF5C",4.72503824155167,-0.901274018521949,0.703838159267317,-
1.28051315015395,0.200364722901968,0.642135073250921
"4879","HIST1H2BN",6.21019391357685,1.12554134538803,0.878763127175606
,1.28082450273668,0.200255315526518,0.642135073250921
"4880","SYTL3",8.52185738640173,-0.828993445937022,0.647429392532283,-
1.28043838524938,0.20039100132371,0.642135073250921
"4881","STAG3",6.92589775864322,0.755036539315583,0.589709881562701,1.
28035253083224,0.20042118059842,0.642135073250921
"4882","AL117336.2",18.3532574230489,0.561313605506915,0.4385418851861
37,1.27995437714842,0.200561181699816,0.642135073250921
"4883","CUEDC2",394.716565052233,-0.303708376189106,0.237172986469413,
-1.28053527811134,0.200356945834266,0.642135073250921
"4884","MGP",63383.4904455985,-0.416575240743735,0.325435809582505,-1.
28005348052555,0.200526327726682,0.642135073250921
"4885","ITGAL",11.361423269438,1.06117706349448,0.828485933275114,1.28
08631032509,0.200241754583535,0.642135073250921
"4886","PIK3IP1",346.582300631963,0.292805042765539,0.228761169226086,
1.27995954801297,0.200559363033148,0.642135073250921
"4887","HSCB",135.57580066206,-0.36072536102398,0.28188252994877,-1.27
970101974585,0.200650305848829,0.642269845384334
"4888","PHB2",723.820759213439,-0.344236698422913,0.269047215407832,-1
.27946575436993,0.200733091600294,0.642403386232284
"4889","SH3BP5L",38.1994991322268,0.362652054189377,0.283504781149452,
1.2791743854161,0.200835653762537,0.642600149684468
"4890","ELOA",154.569486495209,0.225229930167513,0.176117860805474,1.2
7885910683576,0.200946675205398,0.642678495068093
"4891","HSPA5",1105.09015957777,-0.30659967350988,0.239764228005335,-1
.2787548670649,0.20098339179653,0.642678495068093
"4892","BPTF",798.615535147949,0.279417653488542,0.218472133652667,1.2
7896244164836,0.200910282200334,0.642678495068093
"4893","AC010359.3",15.859160424561,0.560102686815372,0.43810974154532
8,1.27845293930179,0.201089768060912,0.642887235188402
"4894","SSRP1",208.309431073377,-0.231254183680534,0.180932214924806,-
1.27812608593026,0.201204972499437,0.64312410805245
"4895","TUBB2A",251.146226885755,-0.410783225578855,0.321465220438257,
-1.27784655838921,0.201303534416882,0.64317630491897
"4896","RPL39",16109.5137522923,-0.321340648175056,0.251453216296836,-
1.27793413386178,0.201272651359014,0.64317630491897
"4897","DYNC1H1",865.613773357777,0.214077924341183,0.16757089354764,1
.27753644925406,0.201412920661223,0.64321939468353
"4898","SLC38A7",50.5498923008454,0.321771938849103,0.251853701452755,
1.27761449203662,0.201385388184586,0.64321939468353
"4899","SNAI1",6.91327870051595,-0.89838287339858,0.703257899637863,-1
.2774586305553,0.201440376817402,0.64321939468353
"4900","AC087190.1",16.2110073056524,-0.723332304498525,0.566371184013
308,-1.27713472174377,0.201554688329151,0.643453059088349
"4901","CDC5L",335.656489371297,-0.19881250891478,0.155688164311555,-1
.27699179827778,0.201605142864817,0.643482809596884

"4902","AC121761.2",11.43019642087,0.567448272083328,0.444411747975696
,1.2768525464686,0.201654310097625,0.643508439995339
"4903","BRCA1",52.6752394656632,0.340011660284384,0.266319987030922,1.
27670350271121,0.20170694437257,0.643545121521541
"4904","C1QBP",506.291485680755,-0.315274101314758,0.246969679551656,-
1.27657007081639,0.201754073871147,0.643564228704395
"4905","PELI1",59.9581263779256,-0.505100679446719,0.395891696522416,-
1.27585570468796,0.202006531798153,0.644019437578824
"4906","AL162426.1",4.75158264111369,0.933940884067067,0.7323009494652
67,1.27535118553245,0.202184968225379,0.644019437578824
"4907","ARHGEF17",54.0717983029602,0.333599820361769,0.261532741895657
,1.27555662034417,0.202112296957983,0.644019437578824
"4908","TRMT61A",70.2445166483309,-0.313875709039868,0.246074418508555
,-1.27553164990597,0.202121129076252,0.644019437578824
"4909","KLF16",16.1084595467654,-0.544356689682922,0.426701035462618,-
1.27573322875288,0.202049838069073,0.644019437578824
"4910","MAP1S",93.604679961724,0.269368854082804,0.211203378906557,1.2
7540030598649,0.202167590442779,0.644019437578824
"4911","CST7",18.6894188165089,0.641970149785911,0.503152443104258,1.2
7589592097616,0.201992313194904,0.644019437578824
"4912","ILK",493.042304071039,-0.308436767286833,0.241886740051493,-1.
27512887734637,0.202263629795252,0.644138835685491
"4913","AC002074.1",20.6882007276714,-0.589680534363656,0.462491342779
485,-1.27500880518063,0.202306125428178,0.644143032785057
"4914","FAM161B",37.7668786898904,0.383864542972638,0.301123866338804,
1.27477289541952,0.202389636955361,0.644277796274463
"4915","LINC01128",55.9201168028252,-0.298975290174731,0.2347569001738
25,-1.27355272604705,0.202821975180246,0.644437897394734
"4916","AL109811.2",27.1436051718463,0.47684429551746,0.37434368993671
9,1.27381416686379,0.202729283212228,0.644437897394734
"4917","MAD2L2",114.581542739476,-0.371415331242613,0.291617437940079,
-1.2736389629722,0.202791397084971,0.644437897394734
"4918","GTF3C6",371.486699526754,-0.35540326886891,0.278993072736827,-
1.27387847082555,0.20270648943276,0.644437897394734
"4919","UBTD1",54.1501585862716,-0.582354291717092,0.457142508549947,-
1.2739009845405,0.202698509453558,0.644437897394734
"4920","GLT8D2",644.058157229809,0.339314872176342,0.266290660344259,1
.27422746159282,0.202582815486453,0.644437897394734
"4921","SACS",132.820444983763,0.385867922736759,0.302826274574261,1.2
7422207098524,0.202584725370197,0.644437897394734
"4922","CORO1A",35.0878932268585,-0.566776362638998,0.444920376087195,
-1.27388268351172,0.202704996230064,0.644437897394734
"4923","MLKL",25.8352564131271,-0.443828083374242,0.348519130881161,-1
.27346835237455,0.202851895849369,0.644437897394734
"4924","WDR83OS",1237.41519929274,-0.333299180663658,0.261599270096236
,-1.27408299167289,0.202634005486247,0.644437897394734
"4925","CYP20A1",193.875855453498,-0.212843577063764,0.167188056869658
,-1.27307883738191,0.202990067697141,0.644615028214855
"4926","ALPK1",100.172683104898,0.290126006715017,0.227877089076877,1.
27316882925927,0.202958138977252,0.644615028214855

"4927","SCRN3",46.5854326535143,0.325075794840592,0.255456939969274,1.27252677057704,0.203186018618882,0.64502408097573
"4928","RBPJ",1936.15248296763,0.288207499437717,0.226496657506169,1.27245806896672,0.203210413243538,0.64502408097573
"4929","ZNF623",60.7593139126929,0.293208955013057,0.230464637820906,1.27225138652685,0.203283815074496,0.64502408097573
"4930","MORC3",233.234431933415,0.202197606523589,0.158916987807341,1.27234733877991,0.203249735898702,0.64502408097573
"4931","TRIB2",19.42384476623,-0.490170417682689,0.385877584574304,-1.27027440120275,0.203986903364922,0.645118211605904
"4932","EHD3",3.11969038543719,-1.0736693291891,0.845241616740059,-1.27025137892529,0.203995101339122,0.645118211605904
"4933","ARL4C",148.331324540244,0.647995410477251,0.509547658242111,1.27170717006682,0.203477182102982,0.645118211605904
"4934","LINC02447",13.138901355218,0.53438077599176,0.420277822903983,1.27149410906187,0.203552921850444,0.645118211605904
"4935","TIGD6",26.4743559318244,-0.39333923920819,0.309387732357383,-1.27134723866114,0.203605143855202,0.645118211605904
"4936","RPP21",327.40483137091,-0.315945979003338,0.248535821485435,-1.27122914159822,0.20364714213541,0.645118211605904
"4937","RAET1E",2.81482410123681,1.24244655309794,0.978152850812478,1.27019673056817,0.204014561964739,0.645118211605904
"4938","ZFAND2A",104.764840853388,-0.378637052320977,0.2979981916479,-1.27060184569293,0.203870330015645,0.645118211605904
"4939","AIMP2",76.5823948294179,-0.358567974268624,0.281894242142614,-1.27199467269438,0.203375012085277,0.645118211605904
"4940","ZYX",402.050451527803,0.336591231814185,0.264674225257956,1.27171896502629,0.203472989784737,0.645118211605904
"4941","AC040970.1",4.81377958945843,-1.0030587107162,0.789423396438222,-1.27062196945501,0.203863167346855,0.645118211605904
"4942","GAPDH",3065.85821755555,-0.440967183407702,0.347130673749227,-1.27032042039092,0.203970517167905,0.645118211605904
"4943","LMO7-AS1",16.7626164009749,0.933249954652641,0.734283939940658,1.27096604445422,0.20374072875652,0.645118211605904
"4944","LINC00638",2.85980658910485,1.25115231896559,0.984750542448239,1.27052716909913,0.203896911323879,0.645118211605904
"4945","LCAT",51.0092018706318,0.358686133130527,0.282131948411569,1.27134177873142,0.203607085404253,0.645118211605904
"4946","RALGAPB",80.771200745728,0.281515118599705,0.221574992958117,1.27051845897122,0.203900011878524,0.645118211605904
"4947","AR",498.225411745853,0.256368478279763,0.201717818804217,1.2709262860342,0.203754873997104,0.645118211605904
"4948","MIF4GD",97.4461755776889,-0.369100006503343,0.290673798619922,-1.26980831521719,0.204152918052297,0.645334485279639
"4949","ZNF708",127.797925166232,0.337042906544737,0.265435538242367,1.26977310113232,0.204165464913951,0.645334485279639
"4950","KLF4",1650.58841905868,0.511866633768882,0.403197564127207,1.26951817002394,0.204256314203475,0.64549121678484
"4951","FRAT2",30.7935932488993,-0.363107104813339,0.286062274480161,-

1.26932887418722,0.204323792207196,0.645574041910151
"4952","ADPGK",186.724508021699,0.207722475429705,0.163674692505586,1.26911785963866,0.204399031360608,0.6456813504794
"4953","C3orf35",5.93252065025635,0.85429821953492,0.673392944625687,1.26864741656863,0.204566844657128,0.645704627963705
"4954","PPP4R2",255.036325440829,0.176209748138571,0.138909857903387,1.26851866957583,0.204612787880591,0.645704627963705
"4955","LAMTOR1",845.074619762669,-0.346381351927295,0.273020008349213,-1.26870317681716,0.204546949002115,0.645704627963705
"4956","AC005921.2",65.9557760830014,-0.580761328254235,0.457786272875179,-1.26862984467991,0.204573114724206,0.645704627963705
"4957","IFT52",208.366616236924,-0.29742069593879,0.234459556334732,-1.26853731444484,0.204606134014834,0.645704627963705
"4958","RPL13",32245.7648020667,-0.351909189084147,0.27746494542765,-1.26830143729241,0.204690323995626,0.645819027483577
"4959","ADNP2",78.0221887664385,0.282398242038519,0.222683629426149,1.26815896959401,0.204741186195852,0.645849238891252
"4960","CYFIP2",5.87072270839155,1.12646984404865,0.888413292690886,1.26795698951861,0.204813310579887,0.645946495443783
"4961","IRX2",64.7307591381911,-0.463522928637703,0.365657926946164,-1.26764085906429,0.204926233622986,0.64609305632112
"4962","SPIRE1",47.2863145657295,-0.391094355736656,0.308558852250431,-1.26748707056778,0.204981183844094,0.64609305632112
"4963","ZNF160",120.001583649954,0.286312762901924,0.225891334754424,1.26748006165525,0.204983688456288,0.64609305632112
"4964","SMARCB1",253.564530520919,-0.309665097194974,0.244351223194416,-1.26729505646301,0.205049807507717,0.64617126084674
"4965","C2orf49",223.217928587983,0.284876439661166,0.224854963546304,1.26693418356541,0.205178824579195,0.646187306803371
"4966","MBD5",170.958287293619,0.327480384058828,0.258471158424059,1.26699004274028,0.205158850289359,0.646187306803371
"4967","EVI2B",16.870816027149,-0.756454291995684,0.596993765405482,-1.26710584905672,0.205117444425543,0.646187306803371
"4968","DPH2",24.0150075728315,-0.414410165772534,0.327129113412101,-1.26680918567612,0.205223526817156,0.646197993156355
"4969","NIPBL",438.950174163096,0.260782351464021,0.205889084517769,1.26661572212447,0.205292727972554,0.646204516711855
"4970","CNOT3",100.291625907362,0.28953992252646,0.228601157641393,1.26657242471476,0.205308217609021,0.646204516711855
"4971","PARP14",299.65155237524,0.277578085815344,0.219210372383102,1.26626346553637,0.205418772329164,0.646422421151703
"4972","CNNM3",101.289816814623,0.307489041774206,0.242882510303416,1.26599910956982,0.205513401045329,0.646590131245392
"4973","NDUFA11",1323.14766808326,-0.316259675573319,0.249840735332092,-1.26584511990385,0.205568537700532,0.646633548210221
"4974","RBMXL1",53.5839068916026,-0.329020399110914,0.259973507450078,-1.26559203027291,0.20565918087073,0.646658606303683
"4975","ZNF577",34.7329694093725,0.39136175876562,0.309206574325272,1.26569675829054,0.205621669374112,0.646658606303683
"4976","COX7A2L",719.404196344539,-0.319451680367589,0.252480528006613

,-1.26525274202224,0.205780741334793,0.646855270136373
"4977","SLC22A4",22.1715067732846,0.537834041706443,0.425102528491274,1.26518664477312,0.20580442878404,0.646855270136373
"4978","NCOA5",56.4360693423246,0.292384781424813,0.231161075435867,1.26485300725265,0.205924025598429,0.647101151554083
"4979","RPS25",9908.51424894403,-0.304310486556781,0.240709451007738,-1.26422325871616,0.206149904925045,0.647680852127432
"4980","AC027644.3",148.051625730014,-0.419571572099381,0.331959818515606,-1.26392276624183,0.206257749499912,0.647689423724757
"4981","DNAJC7",361.298506865471,-0.193172205916736,0.152837631781865,-1.26390473121461,0.206264223443807,0.647689423724757
"4982","GABPA",199.435595925253,0.206677699207711,0.163527712462762,1.26386956739687,0.206276846448682,0.647689423724757
"4983","LUCAT1",8.85588722560518,-0.816501786875371,0.646402545877428,-1.2631475418573,0.206536161111371,0.648141740990382
"4984","CIB1",607.718749090472,-0.320356553190091,0.253622734721443,-1.26312238349587,0.206545200974049,0.648141740990382
"4985","MYO1D",187.75071346359,-0.427073085078941,0.338071183769861,-1.26326379053255,0.206494394551391,0.648141740990382
"4986","UBXN10",7.14660218378445,-0.890121986631166,0.705537560391404,-1.26162239489753,0.207084693787826,0.648154486552982
"4987","RXRG",10.4822253701435,-0.596045735269479,0.472252418039446,-1.26213379222908,0.206900647491355,0.648154486552982
"4988","NAB1",100.160709077667,-0.31042593108281,0.245880125674394,-1.26250924197871,0.206765602838686,0.648154486552982
"4989","RNF38",113.404193767852,0.273464236313694,0.216639264570658,1.26230227403907,0.206840038742218,0.648154486552982
"4990","ECPAS",174.960189173521,0.212475797243332,0.16833761792767,1.26220033204122,0.206876709273665,0.648154486552982
"4991","MVB12B",21.9081361582603,-0.431057613892642,0.341671695800681,-1.2616134704471,0.20708790665421,0.648154486552982
"4992","SNHG10",44.1407046970696,-0.359104909218446,0.284549441233933,-1.26201235068748,0.206944342218598,0.648154486552982
"4993","LRRC37B",23.0916099188518,0.419950731901731,0.332614238976916,1.26257592938129,0.206741622894422,0.648154486552982
"4994","SNHG25",147.99931534506,-0.522295386223094,0.413691517225272,-1.26252379968111,0.206760367901252,0.648154486552982
"4995","DNMT1",192.133492839316,-0.286672136583619,0.227057891738395,-1.26255086043831,0.20675063713157,0.648154486552982
"4996","TUBGCP6",57.9917892597706,0.3599764822737,0.285295623949291,1.2617665749324,0.207032792950574,0.648154486552982
"4997","AL353804.1",2.72023134955785,1.14995127057998,0.911042361793517,1.26223688250471,0.206863560814099,0.648154486552982
"4998","FAM199X",72.9900002010897,0.296455637567939,0.234936123897911,1.26185634056328,0.20700048456724,0.648154486552982
"4999","TMED2-DT",10.3608731828411,0.856239915795939,0.678845507453632,1.26131779085895,0.207194373888269,0.648357989744788
"5000","KIF21A",37.1593199053523,0.427774457836018,0.339209695912644,1.26109148114145,0.20727588950141,0.648409719967786

"5001","ZCCHC2",65.3445768092166,-0.30575285087136,0.242460530184845,-
1.26104174827244,0.207293806147088,0.648409719967786
"5002","ORC3",103.647174450486,0.315063706817062,0.249902200498265,1.2
6074802938459,0.20739964353451,0.648611080329935
"5003","PAFAH1B1",493.67742352174,0.176249946252657,0.139812839868058,
1.26061344880045,0.207448150812246,0.648633104768331
"5004","LYRM7",157.449581165076,0.269289053915217,0.213653278692152,1.
26040215981534,0.207524322929482,0.64874160343443
"5005","MRPS15",440.932031991074,-0.358369290197944,0.284652633570123,
-1.25897057653486,0.208040959735257,0.649189055084506
"5006","NUDT17",12.7342639352301,0.540452647082762,0.428969217325166,1
.25988678267581,0.20771020743673,0.649189055084506
"5007","COL6A3",10337.8986885795,0.294501365784926,0.233795859214524,1
.25965176104637,0.207795014335219,0.649189055084506
"5008","AL159169.2",2.97084802436618,1.41124500655699,1.12078047003065
,1.2591627390852,0.207971557029539,0.649189055084506
"5009","AAAS",83.3693057261842,-0.246392734470945,0.195689509482003,-1
.25910037345975,0.207994079573441,0.649189055084506
"5010","HVCN1",48.164764567845,-0.322431953573142,0.256041799596944,-1
.25929420149643,0.207924086862937,0.649189055084506
"5011","METRN",156.96644961836,-0.332477596851539,0.263993501609767,-1
.25941583722392,0.207880172042728,0.649189055084506
"5012","SLC26A11",71.2032479260945,0.324330150204946,0.257605623219489
,1.25901813070519,0.208023783191029,0.649189055084506
"5013","RBM41",96.4881053749488,0.412159835766119,0.327338763573564,1.
25912321310975,0.207985831122201,0.649189055084506
"5014","TUSC2",134.421751550956,-0.304242295205746,0.241742607707291,-
1.25853815382902,0.208197197811172,0.649288031371644
"5015","RBM12B",125.961553743369,0.302859106521985,0.240626011383008,1
.25862995767286,0.20816402117483,0.649288031371644
"5016","ZNF14",27.7096603687508,0.458590001910622,0.364370038935302,1.
2585831789316,0.208180925884363,0.649288031371644
"5017","GBP1",154.799042707808,0.395120490789324,0.314151141162109,1.2
5774010983278,0.20848576136561,0.650058354602797
"5018","INPP5K",58.8952751284469,-0.285802330534593,0.227327503201448,
-1.25722724487642,0.208671360400771,0.650507391540308
"5019","LRIF1",64.2356641354449,-0.318638279765272,0.253579342974013,-
1.256562447194,0.208912120040059,0.650787651795961
"5020","LRR1",31.7631946150114,-0.400248692741464,0.318472173350684,-1
.25677759702017,0.208834180489952,0.650787651795961
"5021","AC005225.2",5.8311837448557,-0.785060286468753,0.6247895662572
71,-1.25651952091896,0.208927672909245,0.650787651795961
"5022","RPS16",11535.8952211449,-0.303436205514053,0.241468916755719,-
1.2566263583359,0.208888965575586,0.650787651795961
"5023","GDE1",161.617204368628,-0.268959230760105,0.214093200050998,-1
.25627171108675,0.209017474736515,0.650937757774898
"5024","ATIC",120.248914500144,-0.240211864474108,0.191242563976563,-1
.2560585859095,0.209094729833036,0.65104873781413
"5025","HEXA",704.156211799819,0.386431876710698,0.307683751158904,1.2
5593852536959,0.209138259320302,0.651054684686067

"5026","TLCD4",76.0717521565569,-0.487149240315406,0.388267855012988,-
1.25467311812128,0.209597448195164,0.651070390072391
"5027","GAS5",1854.96088968245,-0.324104868719456,0.258251288361526,-1
.25499807097087,0.209479460238294,0.651070390072391
"5028","SH3TC1",3.03304088911614,-1.25622796008701,1.00131882400311,-1
.25457339857531,0.209633665266554,0.651070390072391
"5029","WWC2",138.243637771598,0.325548069094225,0.259510209056272,1.2
5447114500083,0.209670807375387,0.651070390072391
"5030","SKP1",2086.99038152102,-0.277663735908448,0.22117679046042,-1.
25539273506248,0.209336225343533,0.651070390072391
"5031","CCDC167",101.129008539842,-0.446081852247283,0.355599047272739
,-1.25445176433542,0.209677847654506,0.651070390072391
"5032","AVL9",94.0054889478206,0.42210333948988,0.336364020566361,1.25
490038672731,0.209514923601328,0.651070390072391
"5033","ATP2B1",461.999108069499,0.297485941885372,0.237067442217385,1
.25485785438468,0.209530365934316,0.651070390072391
"5034","GNPTAB",167.556529855024,0.288289533952766,0.229784181944085,1
.25461000628371,0.209620369212441,0.651070390072391
"5035","KLHL36",195.580724152728,0.182121320221886,0.145151929406479,1
.25469444992273,0.20958970130159,0.651070390072391
"5036","ARL17B",38.6647977970694,0.57655988832488,0.45961762655937,1.2
5443380542413,0.20968437161572,0.651070390072391
"5037","SOCS6",77.698677551401,0.292620258948725,0.233101519084708,1.2
5533398537136,0.209357542792973,0.651070390072391
"5038","MBD3",135.347773395829,-0.247970433912677,0.197587384657449,-1
.25499122498421,0.209481945468904,0.651070390072391
"5039","APOL6",499.101120856578,0.353737357603031,0.28201624594508,1.2
5431553213398,0.209727340606363,0.651074576127275
"5040","POU5F2",32.1622935730346,0.53003801348837,0.422728442809008,1.
25384989466594,0.209896569854285,0.651470643299719
"5041","PURB",159.257713799913,0.319152933760112,0.254587605448782,1.2
5360750849404,0.209984700738144,0.651614892609955
"5042","MED28",298.465252775835,0.161731181572512,0.12904279935449,1.2
5331426768125,0.210091358018707,0.651687311815712
"5043","GRN",1240.40226965972,0.363215866156959,0.289792796293119,1.25
336402699801,0.210073256843659,0.651687311815712
"5044","PSMD4",526.152552584324,-0.296463697569098,0.23663581075535,-1
.25282685077451,0.210268727596882,0.652108189095565
"5045","CRYAB",1294.0270614665,-0.334799102269205,0.267268447036026,-1
.2526697632365,0.210325914374122,0.652156249465686
"5046","GAL3ST4",50.0291834519749,0.337461428454304,0.269498835286081,
1.25218139846905,0.21050377252655,0.652449081361764
"5047","YIF1A",430.273692843609,-0.356601576717558,0.284765429162624,-
1.25226428561281,0.210473578092837,0.652449081361764
"5048","HOMER2",7.59031375501279,0.816479210617892,0.65221190230115,1.
25186186841603,0.210620201439386,0.652680628192613
"5049","PREB",136.501072044712,0.32082986307891,0.256544281596367,1.25
058278860289,0.211086733260594,0.653425260426248
"5050","SH2D4A",85.3777796643361,0.396819667238603,0.317297385842648,1
.25062381520972,0.211071757616697,0.653425260426248

"5051","MOB3B",68.2499847357396,-0.685217421533127,0.547989287849726,-1.25042119750529,0.211145725157041,0.653425260426248
"5052","PRPF40B",40.3540439447608,0.385828774377019,0.308418824246259,1.25098970635123,0.210938233016008,0.653425260426248
"5053","RDH5",62.7610302308033,-0.410276140714981,0.328023847500235,-1.25075095558315,0.21102535337094,0.653425260426248
"5054","NUDT15",79.2514561171855,-0.319678628698331,0.255671718677935,-1.25034802578624,0.211172441802686,0.653425260426248
"5055","NPTN-IT1",12.2899353528267,0.576477208963773,0.461075841181342,1.2502871707326,0.21119466321774,0.653425260426248
"5056","RGN",48.0087546502232,-0.404088632899598,0.323177661270534,-1.25036065707937,0.211167829657996,0.653425260426248
"5057","PLA2G12A",222.730472489711,-0.185754634633047,0.148588456994721,-1.25012829657183,0.21125268458864,0.65347552798499
"5058","NEAT1",10973.1818150269,0.466537834377688,0.373256019177741,1.24991376001234,0.211331052341501,0.653487984187269
"5059","COPRS",721.675510117301,-0.357783625740567,0.28625242219156,-1.24988855291201,0.211340261586869,0.653487984187269
"5060","ODC1-DT",4.60687585553114,-0.944010994775626,0.755346161470613,-1.24977267765245,0.211382599569942,0.65348972432265
"5061","TMEM44",84.4860898097243,0.298618227332203,0.23896376403008,1.2496381137293,0.211431773628087,0.653512593334155
"5062","SLIT2",299.014400619691,0.360412031442187,0.288507869054796,1.24922773379791,0.211581790944223,0.653588853819211
"5063","GSTCD",29.0448151334189,0.502091704534549,0.401886145510345,1.24933817735109,0.211541409949138,0.653588853819211
"5064","LTB4R",23.4380703886534,-0.414338185970413,0.331645656847985,-1.24934000314779,0.211540742437861,0.653588853819211
"5065","ORC5",43.2442135263864,0.328254045324494,0.262875750826509,1.24870416648332,0.211773296398319,0.654051268619724
"5066","BTNL9",4.72115925985021,-1.0621846020724,0.851284735848953,-1.24774303748455,0.21212517499574,0.654491935396127
"5067","AHI1",509.580742086499,-0.258769676756378,0.207348048191188,-1.24799668486764,0.212032271237417,0.654491935396127
"5068","MEDAG",1071.13157712923,0.336095019368232,0.269339267737909,1.24785005243009,0.21208597490579,0.654491935396127
"5069","SENP3",28.9854978445964,-0.410019313492466,0.328571945556936,-1.2478829036894,0.21207394238135,0.654491935396127
"5070","THRA",358.049989395668,0.204756278292957,0.164066104958985,1.2480108450441,0.212027085637701,0.654491935396127
"5071","TRNAU1AP",174.823921907563,-0.195731574117324,0.157009439617093,-1.24662297117081,0.212535774839669,0.654815607951702
"5072","CSF3R",23.4743039361281,0.771167220642516,0.618376290762663,1.24708406865246,0.212366673821028,0.654815607951702
"5073","CMTM6",330.343928917932,-0.22245358087517,0.178393959969628,-1.2469793310998,0.21240507631252,0.654815607951702
"5074","SLC25A36",543.75492636741,0.181593705675866,0.145640976705136,1.24685860932889,0.21244934571159,0.654815607951702

"5075","PCYOX1L",27.8206345550154,0.472387193583176,0.378763584779831,
1.24718218056197,0.212330705200971,0.654815607951702
"5076","MICAL1",48.7034678419174,-0.391388846935721,0.313852373337526,
-1.24704759366216,0.212380046969913,0.654815607951702
"5077","AP3S2",121.171199362805,0.255266470140967,0.20477944546607,1.2
4654341923815,0.212564959226411,0.654815607951702
"5078","ZBTB14",65.2449639129613,-0.2682139289911,0.215157829447346,-1
.2465915355255,0.212547306960706,0.654815607951702
"5079","LNX2",20.4513087791722,0.519279708818503,0.416623585152808,1.2
4640017349964,0.212617517592377,0.654848558318084
"5080","PIP5K1A",89.5045969356729,0.274154770093426,0.220115347589568,
1.2455050185988,0.212946171914861,0.655428305220396
"5081","ETNK2",40.4764463412378,-0.354082452922626,0.284382961387084,-
1.24509025152415,0.213098577097291,0.655428305220396
"5082","INHBB",280.963660758101,0.548275085244606,0.440279079532586,1.
24528988710223,0.213025211649313,0.655428305220396
"5083","SRA1",153.748881948349,-0.405342548386851,0.325611679009878,-1
.24486489434107,0.213181417068898,0.655428305220396
"5084","PMS2",60.5393031204754,0.350897018714196,0.281876462071992,1.2
4486101512292,0.213182843250104,0.655428305220396
"5085","DUS4L",31.1478244013442,0.400081549281644,0.321195190221587,1.
24560255402839,0.212910344183137,0.655428305220396
"5086","COQ4",209.325699029902,-0.261685373253472,0.210173340600116,-1
.24509308605112,0.213097535289894,0.655428305220396
"5087","ZNF248",88.7528452139823,0.31693489625408,0.254578246891343,1.
24494099603628,0.213153439935373,0.655428305220396
"5088","ARHGEF12",276.561734831837,0.280544586178537,0.225216678770321
,1.24566523096915,0.212887323332393,0.655428305220396
"5089","GNL2",114.555016571555,-0.242807174114254,0.195126813352465,-1
.24435575994193,0.213368657449146,0.655473472419821
"5090","SPICE1",66.4121542388411,0.2706267178743,0.217443308098483,1.2
4458517597483,0.213284272220641,0.655473472419821
"5091","DXO",43.8891891138135,-0.393822729831714,0.316529946968627,-1.
24418789944936,0.21343041620051,0.655473472419821
"5092","LARP4B",106.38157055218,0.277319950458986,0.222942164603469,1.
24390983173701,0.21353275046036,0.655473472419821
"5093","PARG",59.0639547288681,0.337395551303293,0.271124642070086,1.2
4442967901116,0.21334146544777,0.655473472419821
"5094","SLC36A4",76.2330150814163,-0.264951376404684,0.212994835363112
,-1.24393333741166,0.213524098550758,0.655473472419821
"5095","WDR73",147.389783145326,0.277676889179723,0.223220642541658,1.
24395703738691,0.213515375379602,0.655473472419821
"5096","ABHD17A",412.246885659655,-0.276399586684063,0.222168194608581
,-1.24410061112045,0.213462536170284,0.655473472419821
"5097","ARID1A",224.703435988561,0.239385250833263,0.192480703017222,1
.24368441657159,0.213615733553262,0.655599552672882
"5098","ZC3H7A",191.417706324011,0.242249784415678,0.194808702052276,1
.24352650504633,0.213673880088053,0.655649373522444
"5099","SLX4IP",32.4472825190028,0.400008784240869,0.321838997554639,1
.24288475691315,0.213910303531001,0.656246102791812

"5100","ETAA1",95.4735927531391,0.263179052125016,0.211792800279736,1.24262511179516,0.214006011781161,0.65628230587977
"5101","ELOC",431.717096827049,-0.326416454652318,0.262664235805185,-1.24271373927899,0.213973339174576,0.65628230587977
"5102","AL365295.1",47.7003390755779,0.421899478760701,0.339585489366703,1.24239548499998,0.214090680658567,0.656413272744406
"5103","LIMD1-AS1",9.62644173845995,0.853084729663265,0.68733967897922,1.24113994252477,0.214554056355181,0.657576235024312
"5104","ARL6IP4",1403.79514750866,-0.314772994629801,0.253601997762977,-1.24120865531981,0.21452867823851,0.657576235024312
"5105","FAM193B",62.9051854131389,0.351882340100692,0.283585382880523,1.2408338417391,0.214667136727903,0.657793931407363
"5106","PIK3C2A",193.761492460601,0.267187413866772,0.215377558578298,1.24055363813422,0.214770687771933,0.65785350867757
"5107","CYP46A1",45.8438458400208,0.402445710411976,0.324385902479674,1.24063871868535,0.214739241901248,0.65785350867757
"5108","NUDT8",85.7206841171281,-0.393935517672751,0.317622754830419,-1.24026226610583,0.214878404361162,0.658054596597819
"5109","SWI5",284.692888387267,-0.275604339346242,0.222239359887762,-1.2401238893301,0.214929574157181,0.658082467907767
"5110","MAPRE1",180.648965584181,-0.256934927513088,0.207230215595261,-1.23985262851294,0.215029908093999,0.658260832155465
"5111","AL355297.4",4.11916805619803,-0.975636498836923,0.78710159568078,-1.23953058180892,0.21514907053233,0.658496754125853
"5112","ARHGAP5",269.882700728232,0.300858672918008,0.242836531974169,1.23893497601922,0.215369579582223,0.659042709977447
"5113","CLDN11",105.907682069919,1.07536891158677,0.868203540521936,1.23861382889581,0.215488544303116,0.659097964794847
"5114","POLR2B",224.865895697496,0.220848672204295,0.17832936524571,1.23843132565408,0.215556171315632,0.659097964794847
"5115","C16orf70",33.7564706774175,0.36943303694591,0.298289430037246,1.23850528964362,0.21552876194031,0.659097964794847
"5116","CNTNAP1",41.1363397777441,0.467267812133641,0.377293124298407,1.23847423141502,0.215540271108924,0.659097964794847
"5117","THAP8",31.3146036471598,0.49358392205018,0.398647467070904,1.23814638953266,0.215661785641269,0.659292029076876
"5118","MAU2",120.739699874455,0.287799226291186,0.232515655506445,1.23776278919511,0.215804029669443,0.659597975013502
"5119","NPHP4",33.6208282540938,-0.382833918280344,0.30944867608997,-1.23714834756326,0.216032013442032,0.659930813511124
"5120","EFCAB14",402.880633114625,0.224784224754754,0.181758200954672,1.2367212239893,0.216190596453993,0.659930813511124
"5121","RNF212",117.719128110457,-0.272008433209997,0.21989862994825,-1.23697193235815,0.216097502959403,0.659930813511124
"5122","C1QTNF2",119.625103221098,-0.357792949003295,0.289296873152764,-1.2367674254618,0.21617343866816,0.659930813511124
"5123","GIGYF1",153.842151521127,0.313639773111457,0.253582753239661,1.23683400824597,0.216148713623373,0.659930813511124
"5124","AC092376.1",2.67272432417845,-1.08454112616196,0.8769824239842

31,-1.236673730854,0.216208234944992,0.659930813511124
"5125","PRELID3B",93.2873202928857,-0.304776861060227,0.24638368444226
3,-1.23700098791098,0.216086715864444,0.659930813511124
"5126","ZBED5",158.805863138307,0.261614282645267,0.211577613806106,1.
23649320898862,0.216275288472424,0.660006698707399
"5127","PIFO",33.3239551561827,0.441617807609952,0.357198689006841,1.2
363365857748,0.216333477147974,0.660055506734106
"5128","TBKBP1",24.2808919500347,0.417790767495001,0.337983593533404,1
.23612736087945,0.216411225987878,0.660163964143599
"5129","LRRC8B",11.4563021446479,-0.59564191189817,0.482419460638512,-
1.23469710593723,0.216943253385668,0.660718290876449
"5130","PFDN2",396.86026592026,-0.318661345212634,0.258140669050572,-1
.23444843613621,0.217035849724304,0.660718290876449
"5131","LBR",136.00094798365,-0.271193266514163,0.219577852219922,-1.2
350665778557,0.216805726860697,0.660718290876449
"5132","PXK",416.525217454032,0.27295079932313,0.221133159437813,1.234
32776892011,0.217080792414654,0.660718290876449
"5133","GRK4",60.7721409425687,0.296684900385487,0.240212900927036,1.2
3509145112736,0.216796470672138,0.660718290876449
"5134","LINC00865",8.3122990567672,0.790759305652691,0.640071992630276
,1.23542244428348,0.216673323952745,0.660718290876449
"5135","FBXO21",200.391024645247,-0.26600182626441,0.215493005718844,-
1.23438728499367,0.217058624724832,0.660718290876449
"5136","EIF5",1235.52314203824,-0.224402180511507,0.181759048114061,-1
.23461353280573,0.216974370060496,0.660718290876449
"5137","AL049840.6",4.53919982267645,-0.868318967116589,0.703433471278
121,-1.23440098114591,0.217053523609826,0.660718290876449
"5138","BRF1",58.3471922537918,0.28599056207005,0.231601073125097,1.23
484126481389,0.216889586445168,0.660718290876449
"5139","RGMA",182.935440530545,-0.300310462462761,0.243249946532077,-1
.23457565662058,0.216988473508335,0.660718290876449
"5140","CTSZ",990.673076455734,-0.223545541437798,0.181114598715329,-1
.23427676743585,0.217099790008627,0.660718290876449
"5141","SRSF11",1146.89944678009,0.283632149456851,0.229848921297648,1
.23399382453292,0.217205205416013,0.660910528753684
"5142","PSMA5",364.565004637393,-0.325532578663314,0.26403482282485,-1
.23291532223103,0.217607358241991,0.661106450362606
"5143","TMEM63A",79.3316156036178,0.396758475572168,0.321725822238915,
1.23321924491822,0.217493977161683,0.661106450362606
"5144","TBL2",124.739106972943,-0.261412836245298,0.212028369437901,-1
.23291442998085,0.217607691166468,0.661106450362606
"5145","TRRAP",106.297586671282,0.26371931850455,0.213875742514352,1.2
3304922477056,0.217557399464123,0.661106450362606
"5146","RBMS2",127.807587916842,0.233354367662678,0.18921190615276,1.2
3329642625279,0.217465190738733,0.661106450362606
"5147","CYP19A1",39.618990509699,-0.6126716927332,0.496869954378605,-1
.23306246903059,0.217552458504638,0.661106450362606
"5148","FLII",241.973463062613,-0.220878659137463,0.17911609924431,-1.
23315916363381,0.217516387653329,0.661106450362606
"5149","PORCN",40.1739482972899,-0.315889563412532,0.256068767392927,-

1.23361223092003,0.217347433201659,0.661106450362606
"5150","NR2C2",105.780084567499,0.29985660939535,0.243306522243592,1.23242322741822,0.21779102872317,0.661287368286709
"5151","CYC1",403.287336441066,-0.353315436692762,0.28663413080977,-1.23263561005317,0.217711744925253,0.661287368286709
"5152","RPAIN",239.428339874969,-0.249723683003345,0.202646966810713,-1.23230900976971,0.217833675485057,0.661287368286709
"5153","LINC00892",4.25433200711779,1.226324496422,0.995149412908978,1.23230188403293,0.21783633630259,0.661287368286709
"5154","RAB3GAP2",175.00418409127,0.218161687865059,0.177076725766537,1.23201785508892,0.217942414424893,0.66148102228339
"5155","LINC01135",4.14677094592088,0.931603139354729,0.756311731106186,1.23177137288636,0.218034499812151,0.661632139779144
"5156","ST7-AS1",26.3079770236672,-0.40913549639255,0.332227476876854,-1.23149204947971,0.218138888423956,0.661820525914652
"5157","TAB3",37.6323295206804,0.445149532862986,0.361569532991259,1.23115885672189,0.218263456036215,0.661941691115647
"5158","TMLHE",58.4456364373822,-0.359447949759255,0.291946865532274,-1.23121016937076,0.218244268934198,0.661941691115647
"5159","TTC9C",126.876825980584,0.27059033908963,0.219817325519618,1.23097821543407,0.21833101204458,0.662018224736066
"5160","CDK13",269.696650956893,0.300299347122608,0.244050111003483,1.23048232138818,0.218516543129665,0.662195715648461
"5161","ANXA7",613.682540837235,-0.229779274665978,0.186721776901656,-1.23059708663227,0.218473595418674,0.662195715648461
"5162","VPS35",446.875209906104,0.19186149326482,0.15591184586256,1.230576754469,0.218481203725033,0.662195715648461
"5163","OR2C3",4.10610008663265,1.35312055498833,1.09996244219443,1.23015159707531,0.218640341460363,0.662314264419919
"5164","PSMB6",788.040465183229,-0.374771555897786,0.304638271685614,-1.23021823168873,0.21861539441609,0.662314264419919
"5165","SFT2D2",619.409937829728,0.379448548603844,0.308646824593654,1.2293939816274,0.218924125644751,0.662601360281969
"5166","TRIM23",155.816953310362,0.240542718016045,0.195633752308805,1.22955632746006,0.218863292614664,0.662601360281969
"5167","SLC39A7",405.981127962154,-0.243127732885162,0.197788286562359,-1.22923221142577,0.218984755056819,0.662601360281969
"5168","OSTF1",191.945634834322,-0.348874018682351,0.283718860804454,-1.22964690360435,0.218829357868453,0.662601360281969
"5169","TLK2",142.75967640429,0.269267244565872,0.2190347100858,1.229335955294,0.218945871789006,0.662601360281969
"5170","CARD8",74.8375569853216,0.295402255539959,0.240316795045444,1.22922018614678,0.218989262459744,0.662601360281969
"5171","SLC9B1",10.6109807169922,0.572164168258789,0.465977664537539,1.22787895601527,0.219492410349172,0.663264912372393
"5172","PABPC4L",14.8894785323732,0.584213238627682,0.475639076915897,1.22827006228293,0.219345605454524,0.663264912372393
"5173","NPY1R",168.514049749491,0.912587171242663,0.743243309803686,1.2278444477135,0.219505366704077,0.663264912372393

"5174","TRIM73",73.3055993768491,0.392020100467705,0.319269699976612,1
.22786503227968,0.219497638035582,0.663264912372393
"5175","TP53TG1",210.150840271769,-0.371570849260599,0.302600459896036
,-1.22792559333274,0.219474900952773,0.663264912372393
"5176","RABGGTA",48.6541123386938,-0.345352142950881,0.281136028738627
,-1.22841652313428,0.21929064833743,0.663264912372393
"5177","ZSCAN5A",18.7623542393475,-0.470606583364154,0.383231161617261
,-1.22799665188541,0.219448224837369,0.663264912372393
"5178","NCKAP1",387.618145576103,0.184209279311029,0.15006870307575,1.
22749964206758,0.219634856261226,0.663525319829477
"5179","AC008273.1",2.99194645019184,-1.1326977855253,0.92291517764948
8,-1.22730432108624,0.219708232128615,0.663525319829477
"5180","ADH1B",5868.10729708051,0.316210417356339,0.257699606877822,1.
22705044523509,0.219803631487333,0.663525319829477
"5181","AC018553.1",5.08472968152263,0.872831469565599,0.7112643776632
74,1.22715476407398,0.219764427823238,0.663525319829477
"5182","RBFADN",6.39560382997132,0.730284386824805,0.595153339249531,1
.2270524899443,0.219802863024759,0.663525319829477
"5183","RABIF",61.6309124899035,-0.266551438907587,0.217294185028654,-
1.22668463894898,0.219941143405044,0.663808835042892
"5184","AC098818.2",22.9143814200505,-0.954168305426605,0.777984382159
003,-1.22646203099691,0.220024855187457,0.663808835042892
"5185","RAB5IF",335.682032779325,-0.291449011106722,0.237625622987431,
-1.22650498478499,0.220008700623757,0.663808835042892
"5186","RPL29",13587.0787250822,-0.379101971788531,0.309205086339786,-
1.22605347886139,0.220178550805907,0.663811821703102
"5187","PGRMC2",572.322246923042,0.203447001308998,0.165970346349228,1
.22580331838865,0.220272698148766,0.663811821703102
"5188","STOML2",433.392873368419,-0.321299400316252,0.262061790577056,
-1.22604443634746,0.220181953433142,0.663811821703102
"5189","TBC1D13",73.1598769683192,0.269528062223465,0.219840782209446,
1.22601484362752,0.220193089205836,0.663811821703102
"5190","SCAF11",894.466926873522,0.202881157610067,0.165490204683413,1
.22594058058109,0.220221036250119,0.663811821703102
"5191","MAP3K10",26.1673426471979,-0.416728945966863,0.339969672241002
,-1.22578270943958,0.220280455568676,0.663811821703102
"5192","INCA1",6.38697254697774,-0.755903996545148,0.616824794660451,-
1.22547602348128,0.220395918468447,0.664031847573559
"5193","AP2A2",265.453431145858,0.259766591295071,0.212045128224744,1.
22505333402306,0.220555126170615,0.66418836424028
"5194","BCAT1",65.9542263641294,-0.423270606689813,0.345496894899583,-
1.22510683290753,0.220534971040657,0.66418836424028
"5195","CTSA",654.653232339574,0.296949456020333,0.242407731886571,1.2
2499993589018,0.220575244660759,0.66418836424028
"5196","NDUFV2",850.673499910064,-0.346520818932519,0.282919286605386,
-1.22480451258823,0.220648884332868,0.664215448459605
"5197","ZSWIM9",27.7919804282172,-0.391656502701169,0.319784670609176,
-1.22475071101776,0.220669161007771,0.664215448459605
"5198","AC105942.1",21.9453557836663,-0.516615067604311,0.421858616080
028,-1.22461660829586,0.22071970730546,0.664239780950232

"5199","AC100793.2",16.0181859764401,-0.454848250015924,0.371498689005
291,-1.22436030994835,0.220816334946685,0.664402755832083
"5200","PRDM2",350.164533545264,0.241753406705111,0.19766849589725,1.2
2302446633062,0.221320455508085,0.664768086142395
"5201","IFI44L",136.654264023821,-0.450404385480224,0.368316679485047,
-1.22287262719121,0.221377808713887,0.664768086142395
"5202","TSPAN2",2.69178559692183,-1.07376441699327,0.878119348066354,-
1.2228000890286,0.221405211839281,0.664768086142395
"5203","AOAH",5.71546727900064,-0.866119396008503,0.708212212669438,-1
.22296591404979,0.221342570816881,0.664768086142395
"5204","SNHG15",92.8354500867073,-0.396873003378385,0.324407278798792,
-1.22337884910572,0.221186638250908,0.664768086142395
"5205","CHCHD2",2960.42949052257,-0.357439544969943,0.292291106024397,
-1.22288888578125,0.221371666953792,0.664768086142395
"5206","ZNF618",119.019959800566,0.284821386322603,0.23285233398343,1.
22318458849062,0.221259985147983,0.664768086142395
"5207","CCDC7",49.0129999845278,0.391613828002803,0.320183261297113,1.
22309275761735,0.221294663758733,0.664768086142395
"5208","SGPL1",67.1318247695538,0.332117550149423,0.271587377352803,1.
22287550101413,0.22137672310466,0.664768086142395
"5209","TDG",180.86212890412,0.253618949931669,0.207250637545158,1.223
73061398379,0.221053866890824,0.664768086142395
"5210","RRAD",12.241266255278,-0.977689257325899,0.79911351092035,-1.2
2346730966904,0.221153244009494,0.664768086142395
"5211","AL133330.1",29.3593074976834,-0.725732938309196,0.593763489759
477,-1.22225928475861,0.221609590950213,0.665254045525653
"5212","AC096921.2",12.8590292926559,0.544389964367669,0.4456457743753
03,1.22157551057403,0.221868194273472,0.665380090219091
"5213","NDUFB4",1112.51958957677,-0.290811537582898,0.238104468832575,
-1.22136110678117,0.221949326264989,0.665380090219091
"5214","CSRNP2",43.0817860028063,0.356395504116212,0.291685463644184,1
.22184869846982,0.221764848722489,0.665380090219091
"5215","SUCLA2",285.896899999778,-0.240351024946297,0.196742141707351,
-1.22165501941019,0.221838112938409,0.665380090219091
"5216","ARHGAP44",30.4324291210252,0.458545751439635,0.375322870142296
,1.22173677097211,0.221807186138066,0.665380090219091
"5217","GCDH",89.2857229035719,-0.387367556943705,0.317016538084195,-1
.22191592679882,0.221739421885878,0.665380090219091
"5218","ARFGEF2",91.4645219391936,0.247469916618395,0.202605292925011,
1.22143855693834,0.221920016097504,0.665380090219091
"5219","HLA-
C",3490.87469686522,-0.355804254200429,0.291369494147521,-1.2211444964
1144,0.222031314819147,0.665498344072796
"5220","PAXX",196.049250061035,-0.26466570319645,0.216791732005492,-1.
22082932198607,0.222150649304332,0.665709438193315
"5221","KIF3B",96.8578037642745,-0.282078885192202,0.231073230812536,-
1.22073372238018,0.222186855258409,0.665709438193315
"5222","TSC2",168.00693746161,0.303592815917524,0.248722781544608,1.22
060719179869,0.22223478204155,0.665725525751812
"5223","TGFB2",52.1757842227468,0.514403097129614,0.422025737592785,1.

21889034555984,0.22288581448845,0.6665328011209
"5224","AFF1",302.993919328982,0.257189436390353,0.210974281052561,1.2
1905587310085,0.222822986633884,0.6665328011209
"5225","BHMT2",294.408225181648,0.255092037114319,0.209283062639262,1.
21888524516681,0.222887750601766,0.6665328011209
"5226","MYOZ3",3.03960424769456,-1.05322898795184,0.86358929363441,-1.
21959477232439,0.222618529136717,0.6665328011209
"5227","USP2",3.45842074374198,-0.948406348639356,0.777839434052607,-1
.21928293568003,0.222736823079196,0.6665328011209
"5228","APRT",1167.95684536189,-0.37034747818815,0.303787382165898,-1.
21910092363844,0.222805889386241,0.6665328011209
"5229","TBC1D16",211.486427507587,0.266774990637889,0.218801460826408,
1.21925598499336,0.222747048822718,0.6665328011209
"5230","UBXN6",353.157215834833,-0.224083889578658,0.183803885139788,-
1.21914664321832,0.222788539188603,0.6665328011209
"5231","ICAM3",76.159987116108,-0.313228426215085,0.256945025329944,-1
.21904841634068,0.222825816659058,0.6665328011209
"5232","YY1",806.486651110991,-0.152392452984695,0.125063300769094,-1.
21852255655766,0.223025458352484,0.666606633206562
"5233","TLCD3A",51.3136000355502,0.368161410239147,0.302147220087018,1
.2184835264515,0.223040281161104,0.666606633206562
"5234","AC005838.2",28.9346769562965,0.434911163415604,0.3568981217948
87,1.21858630476501,0.223001249647927,0.666606633206562
"5235","POP7",197.16581952077,-0.287106583752577,0.235719512699928,-1.
21800092179075,0.223223622454011,0.66670843249993
"5236","PRH1",127.666504329289,0.237593274275072,0.195076262365818,1.2
1795072036762,0.223242700141776,0.66670843249993
"5237","CORO1C",105.356306577329,0.252143440131038,0.207042707321382,1
.21783299394191,0.223287443448644,0.66670843249993
"5238","ANKRD29",26.9681477687668,-0.462117013906954,0.379370912357329
,-1.21811398516417,0.223180660060826,0.66670843249993
"5239","ZNF85",37.9482854642785,0.420337500603038,0.345141360825481,1.
21787055482921,0.223273167296645,0.66670843249993
"5240","TUT4",364.528427449274,0.239299233637003,0.19657512592199,1.21
734239016572,0.223473972236783,0.666734983314601
"5241","NBAS",151.654334307709,0.265524091926708,0.218171396421724,1.2
1704355512054,0.223587644680931,0.666734983314601
"5242","MRPL36",326.219935406204,-0.367886690380363,0.302132123005146,
-1.21763514161021,0.223362653995452,0.666734983314601
"5243","C7orf31",15.5642576581733,-0.568295218595039,0.466961012442067
,-1.21700785173269,0.223601228488292,0.666734983314601
"5244","CELF1",288.348264545165,0.290664859825463,0.238767647299393,1.
21735445782985,0.223469382743856,0.666734983314601
"5245","DTX3",75.2121975488244,0.269455221988706,0.221465974999961,1.2
166890285912,0.223722554971447,0.666734983314601
"5246","MMAB",120.000710550051,-0.3346466521497,0.275025253388217,-1.2
1678517891345,0.22368596051077,0.666734983314601
"5247","CCL8",20.2440120117864,-1.53596314685702,1.2622480364799,-1.21
684732514256,0.223662310161245,0.666734983314601
"5248","PRMT1",443.016917602425,-0.327443955781575,0.269036053961739,-

1.21710064862958,0.223565923956884,0.666734983314601
"5249","APEX2",41.3744867341718,-0.325639010396472,0.267470868367309,-1.21747468195035,0.223423663595939,0.666734983314601
"5250","HSPB7",7.8136908379039,-0.862924496129789,0.709347685209797,-1.21650428149994,0.223792881054365,0.66681753111113
"5251","AC091488.1",3.00808841449005,1.24579577552693,1.02418037365157,1.21638317583184,0.223838989882426,0.666827903014815
"5252","PEX19",162.810618329555,-0.23235636894929,0.191080095750244,-1.21601555639263,0.223978996049405,0.667086044594431
"5253","EIF4E",154.554530131211,-0.230788005673349,0.189803424467593,-1.2159317268417,0.22401093091188,0.667086044594431
"5254","HYMAI",394.490947757909,0.340118131505369,0.279840814427463,1.21539859080681,0.224214105242161,0.667183041335956
"5255","WASHC2A",174.819404808729,0.294248220922415,0.242092480137083,1.21543726081785,0.224199363948161,0.667183041335956
"5256","ADCK1",12.709682604327,-0.482998406941924,0.397313026883442,-1.2156621461184,0.224113649739734,0.667183041335956
"5257","PLIN1",9.15204682709919,-0.986931820708915,0.811997387822177,-1.21543718675736,0.224199392179903,0.667183041335956
"5258","AC027097.1",26.8686258395166,0.379466729747817,0.312260252719937,1.2152258458849,0.224279965343822,0.667252091645761
"5259","NCBP2AS2",238.578460480501,-0.311546261564012,0.256428207447001,-1.21494536293712,0.224386930706286,0.667398945743763
"5260","DTD1",253.751544000223,-0.314387603773075,0.258782347440889,-1.21487267923051,0.224414655412146,0.667398945743763
"5261","EXOC7",326.084855235452,0.196751854074052,0.161974076967507,1.21471199439847,0.224475956238206,0.667454359139757
"5262","DNPEP",266.260116136878,-0.244829461412627,0.201600749512242,-1.21442733722456,0.224584581551752,0.667650438847216
"5263","PGBD1",25.8749973209102,0.461705276379077,0.380243965938897,1.21423432779278,0.224658255409142,0.667732079256471
"5264","GKAP1",38.0539852741225,-0.35306941379666,0.290824097681476,-1.21403080628951,0.224735960546224,0.667732079256471
"5265","AL441992.1",6.57320989633878,-0.799364909871153,0.658505246854012,-1.21390818629023,0.224782786509274,0.667732079256471
"5266","PHKB",233.345708195701,0.184694204674971,0.152148367383866,1.21390855420087,0.224782646001621,0.667732079256471
"5267","ATAD3A",42.6841492838983,-0.372864923890385,0.307351529129596,-1.21315460816584,0.225070714992512,0.667796979329612
"5268","MYSM1",198.921969250124,0.304782405775629,0.251249038814836,1.21306894232637,0.225103463018925,0.667796979329612
"5269","METAP1D",47.5002713666141,0.483871196563903,0.398768374187306,1.21341417194891,0.224971510667951,0.667796979329612
"5270","AC069224.1",10.5480688409314,-0.604359147957365,0.498086364293642,-1.21336216223151,0.224991386088095,0.667796979329612
"5271","NDUFS8",397.332625821964,-0.345444404558984,0.284668821073522,-1.21349575010101,0.224940338250164,0.667796979329612
"5272","COA8",331.672049908392,-0.251283592527963,0.207113530989581,-1.21326497272939,0.225028530243841,0.667796979329612
"5273","CRELD2",238.680275980354,-0.308713561058357,0.254398184927457,

-1.21350536029331,0.224936666233243,0.667796979329612
"5274","CDH5",14.5081043058965,0.827429715545624,0.682204151916094,1.2
1287698590171,0.225176855781894,0.667888046074357
"5275","AL392172.1",29.1490812092325,-0.366829946596735,0.302542669141
672,-1.21248995269807,0.225324886312266,0.668073767358374
"5276","ATP5F1D",1285.18201347121,-0.359267663713114,0.296288440412622
,-1.21256051438519,0.225297893051106,0.668073767358374
"5277","LINC01285",4.93504669992338,0.945209487925288,0.77968230055857
4,1.21230081438059,0.22539725226705,0.668161686036283
"5278","MIF",2271.72033638404,-0.349446413315078,0.288285968246548,-1.
21215200115541,0.225454201157868,0.668203878119084
"5279","HMGCL",208.154111948325,0.30239227135669,0.24949736681704,1.21
200586288527,0.225510136376351,0.668232303967608
"5280","TOMM20",1188.73176271798,-0.264232274081555,0.218054875073536,
-1.21176962446928,0.225600578870875,0.668232303967608
"5281","AC087672.2",12.9883686356173,-0.648225447693146,0.534895960104
596,-1.21187202005861,0.225561374139667,0.668232303967608
"5282","ZNF284",23.1465287091117,0.530908795023064,0.438159023863258,1
.21168061390595,0.225634662760142,0.668232303967608
"5283","IDH3G",420.616630532142,-0.349495676912432,0.288466850916586,-
1.21156270053883,0.225679819760084,0.668239526879992
"5284","FARS2",80.8227753538648,-0.327547914356183,0.270500640359035,-
1.21089515322932,0.225935590414772,0.668743697418784
"5285","ZFAND4",15.0701490993492,0.522410242173015,0.43141286458695,1.
21092875307089,0.225922711697565,0.668743697418784
"5286","PPP6R3",192.719073286564,0.255068892942555,0.210684039928145,1
.21067021986833,0.22602182018465,0.668872367224457
"5287","SNX14",197.06103860882,0.180219264943821,0.14891020622636,1.21
02546192829,0.226181205348006,0.669090884126107
"5288","AC022966.1",12.536022157704,0.650154986838163,0.53719854772029
7,1.21026944245702,0.226175519198365,0.669090884126107
"5289","UPK3BL1",185.667920113154,-0.442158794183145,0.365423307860502
,-1.20999067293194,0.226282471893591,0.669153143835939
"5290","CTCF",239.733035687605,0.206079073153109,0.170316552656583,1.2
0997677523826,0.226287804825936,0.669153143835939
"5291","CDC14A",49.0705763160655,0.371276795243863,0.306933230135745,1
.20963375350288,0.226419460248508,0.669164333517709
"5292","SIPA1L1",33.4246253148331,-0.422967252224585,0.349611060030628
,-1.20982228705102,0.226347092287654,0.669164333517709
"5293","SPPL2B",140.918235922774,0.240559293754986,0.198869725250799,1
.20963255443538,0.226419920559307,0.669164333517709
"5294","RHOB",1992.09397009367,0.461165631601805,0.381301390403338,1.2
0945174397079,0.226489339646985,0.66924305630861
"5295","EGFLAM",34.0658930250295,-0.435044230014385,0.36000303406474,-
1.20844600975266,0.226875751235335,0.670194148180156
"5296","CES1",191.260091824499,0.569993301434016,0.471696083135112,1.2
0839099965718,0.226896900131823,0.670194148180156
"5297","RPL32",19171.249264175,-0.350347273271457,0.289980428146086,-1
.20817558450862,0.22697973105675,0.670312239554606
"5298","ZNF346",73.8765454361566,0.331376859801249,0.274381539459923,1

.20772286814015,0.227153878757809,0.670699910420614
"5299","PPP1R11",119.688984748864,-0.296805751445673,0.245785684336253
,-1.20757949042964,0.227209052129648,0.670721610144514
"5300","DHTKD1",61.5373883782064,0.393042255225812,0.325555261998855,1
.20729811833664,0.227317355078637,0.670721610144514
"5301","MYLPF",5.21742839629918,-0.781612506067139,0.647330096958757,-
1.20744039206466,0.227262587890791,0.670721610144514
"5302","CBFA2T2",94.7597341160452,0.313279338708366,0.259496557507972,
1.20725816834291,0.227332735216149,0.670721610144514
"5303","SERPINE2",161.902818054766,0.583083099527945,0.483098728282198
,1.20696467490459,0.227445748451545,0.670802006603983
"5304","AC008760.2",32.7254907791863,0.49541345143154,0.41044999171164
6,1.20700075876621,0.227431851760927,0.670802006603983
"5305","ALG1L",11.3419819920109,0.575382188143826,0.476955405218438,1.
20636474992942,0.227676881473203,0.671088220332472
"5306","CRPPA",18.3915104457047,0.436679313119405,0.361955417664785,1.
20644502556893,0.227645943990619,0.671088220332472
"5307","BANF1",552.507734887901,-0.3312217734129,0.274584032692749,-1.
20626742263461,0.227714394523094,0.671088220332472
"5308","ZNF507",102.117004115988,0.284095811809468,0.235511002753243,1
.20629528339757,0.227703655644718,0.671088220332472
"5309","ZNF395",166.174938044641,0.287013741994933,0.237986652211166,1
.20600772912367,0.227814510256843,0.671256806168355
"5310","TMEM160",353.177032749896,-0.324097193443322,0.268796602485703
,-1.20573396555695,0.227920084162713,0.671441408014561
"5311","IDNK",57.250426747234,-0.419383324249585,0.347904720583493,-1.
20545453808793,0.228027878233515,0.671560327926704
"5312","LZTR1",93.7791303519921,0.246149234549337,0.20420429206438,1.2
0540676232081,0.228046312212917,0.671560327926704
"5313","WDR48",148.395787441222,0.212905175010904,0.176648132939829,1.
20525007237651,0.228106777493556,0.671611955643083
"5314","LIN9",17.3086307970931,0.4558796173806,0.378364984646375,1.204
86735263484,0.228254513712062,0.671733084833808
"5315","ZNF514",75.5788034917848,0.348208060578296,0.289132569625886,1
.20431973827386,0.228466020515705,0.671733084833808
"5316","NEPRO",104.368040895386,0.209187229714747,0.173706911403857,1.
20425392417692,0.22849144949183,0.671733084833808
"5317","ALAD",73.6644114550149,0.265737663934393,0.220654049678094,1.2
0431809124768,0.22846665686222,0.671733084833808
"5318","EMC6",200.534702776604,-0.337331551690142,0.280068080896324,-1
.20446268139716,0.228410797690576,0.671733084833808
"5319","TCF4",2909.64366242407,0.276318087832736,0.229344419341794,1.2
048171419464,0.228273900933266,0.671733084833808
"5320","IL27RA",4.15494127314669,-1.07737749948151,0.894446573009525,-
1.20451856152401,0.228389212258995,0.671733084833808
"5321","LRP3",82.0174596796934,-0.398711439138737,0.330992197518048,-1
.20459467663734,0.228359812773716,0.671733084833808
"5322","IGFBP7",7367.70088475863,-0.451024113815922,0.374624668504327,
-1.20393597041171,0.228614327530508,0.67196804313411
"5323","PCOLCE",4024.26450352322,0.449062575977277,0.373065501772403,1

.203709734199,0.228701788581333,0.671972591806498
"5324","SAMM50",179.239049226964,-0.269254345766243,0.223682155707848,-1.2037363683043,0.228691490819191,0.671972591806498
"5325","RPS21",7251.5031911311,-0.360779363166611,0.299795449658058,-1.20341840937916,0.228814447436947,0.672177352348575
"5326","CHTOP",197.378584475081,0.208617014921185,0.173382267223871,1.20322001933346,0.22889118989406,0.672276545909274
"5327","GLMP",216.947531152311,0.306518852519674,0.254834739697208,1.20281423515442,0.229048214887372,0.672416543469015
"5328","AAMP",309.733111571546,-0.236893687153924,0.196957810488409,-1.20276360996542,0.229067810531636,0.672416543469015
"5329","CTDSPL2",163.017456088988,0.227178324780071,0.188873260448197,1.20280829716698,0.229050513260272,0.672416543469015
"5330","ZNF672",41.6690567318611,-0.346635626670986,0.288360639740767,-1.202090642407 4,0.229328411474396,0.672573131412489
"5331","BIRC6",258.963533816989,0.258825789441833,0.215408248281257,1.20155932517443,0.229534308522042,0.672573131412489
"5332","GCA",51.6683956778468,-0.304339304046423,0.25324792324711,-1.20174452032706,0.229462526423256,0.672573131412489
"5333","AC114296.1",8.07953664819336,-0.744813803914311,0.619774956895031,-1.20174878902126,0.229460872055179,0.672573131412489
"5334","SMC5-AS1",6.7674526820146,0.713395670523392,0.593424625833329,1.20216728370784,0.229298722144241,0.672573131412489
"5335","SLC35D2",116.904522989673,-0.238253431676715,0.198309944789355,-1.20141948468489,0.229588521626847,0.672573131412489
"5336","NAGPA",68.3633001438565,0.316760509712027,0.263482845526619,1.2022054380008,0.229283942942507,0.672573131412489
"5337","VPS35L",131.852689997064,0.217561795088911,0.181044500781967,1.20170341628284,0.229478457073081,0.672573131412489
"5338","MPV17L2",14.64698716301,-0.599127148942277,0.498401427181085,-1.20209757891523,0.229325724295313,0.672573131412489
"5339","NRIP1",278.679492137996,0.314882379508925,0.262095092659848,1.20140509428609,0.229594100987195,0.672573131412489
"5340","AL035681.1",21.6858675474411,0.565526431838941,0.470697719767503,1.20146414161147,0.229571208121407,0.672573131412489
"5341","RGS3",22.8608645405105,-0.616972539511178,0.513657226137568,-1.20113668827457,0.229698183458066,0.672626110788941
"5342","AL133482.1",52.9075521899437,0.60491657281982,0.503597211343572,1.20119126793005,0.22967701584349,0.672626110788941
"5343","FOSL2",188.907851018385,0.298766701939888,0.248764993728805,1.20099977678368,0.229751287915201,0.672655698457326
"5344","LINC02802",859.026186064747,0.302566680317771,0.251989586548213,1.20071104708083,0.229863307391858,0.672738285537688
"5345","LCLAT1",23.0401896521854,0.417365638782362,0.347600374583197,1.20070537692262,0.229865507651917,0.672738285537688
"5346","C1orf54",138.165538641894,0.44985069641507,0.374756210786393,1.20038223108057,0.229990926595409,0.672979435976803
"5347","LRPPRC",226.025690498575,0.205711830423421,0.171452012850867,1.19982161190697,0.230208628819002,0.673124689266344

"5348","PUS3",65.4532544123707,-0.354785497678293,0.2957011146721,-1.1
9981116091399,0.230212688587543,0.673124689266344
"5349","CTDP1",33.356633190751,0.337194880474753,0.281021218668574,1.1
9989117573513,0.230181607512447,0.673124689266344
"5350","TMEM221",92.1539584669094,0.635191055355626,0.529363652209325,
1.19991437399343,0.230172596904758,0.673124689266344
"5351","IL4I1",2.61259691365852,-1.09914349894693,0.916342595448281,-1
.19948969349092,0.230337589945087,0.673364029062044
"5352","CPSF3",58.9256378536229,-0.268005424498427,0.223503324552948,-
1.1991157936685,0.230484562182423,0.673541940261468
"5353","TENM4",6.67631395111059,1.55866354405784,1.29982905513896,1.19
912963777472,0.230477541398252,0.673541940261468
"5354","NDFIP1",1946.31947372824,-0.287643049612057,0.239909010566949,
-1.19896726234794,0.230540675506005,0.673580087213381
"5355","SLC38A5",7.39963630039898,-0.752735473438222,0.627905095448256
,-1.19880453096315,0.230603960348572,0.673639169324503
"5356","RPL23A",11371.4011295205,-0.311847779892221,0.260197210404754,
-1.19850546978241,0.230720294877915,0.673853168927414
"5357","HEATR1",107.010655375456,0.280838970238882,0.234461383717498,1
.1978047974726,0.230993019100476,0.673861589209555
"5358","COPS9",878.257576357075,-0.276636486233668,0.230933953511567,-
1.19790304555546,0.230954763981661,0.673861589209555
"5359","EEF1AKMT4",8.64370615441328,-0.580716446007103,0.4848502175427
48,-1.19772338960723,0.231024720509547,0.673861589209555
"5360","UNC5C",5.17899005779113,0.955574628143189,0.797438974221404,1.
1983043957391,0.23079853594557,0.673861589209555
"5361","UBE2QL1",8.36003741026873,0.760464325046587,0.634676164111186,
1.19819266587955,0.230842019937396,0.673861589209555
"5362","SEMA4D",15.238174885911,0.514732782387262,0.429633459030654,1.
19807424577362,0.230888114052195,0.673861589209555
"5363","CHSY1",77.1376177705224,0.26923941942798,0.224783303982626,1.1
9777320938743,0.23100531959414,0.673861589209555
"5364","CSTB",1639.00931910134,-0.303543271957201,0.253487755619832,-1
.19746719605833,0.231124506182459,0.674026966855372
"5365","PEX2",280.201887215946,0.241380845298704,0.201702452420211,1.1
9671745386531,0.231416701666759,0.674125039883261
"5366","DCAF10",188.672131267956,0.25279820526748,0.21122607029426,1.1
9681346585346,0.231379268469994,0.674125039883261
"5367","LINC01220",10.6112239805751,0.967520723824023,0.80845490650141
7,1.19675286282937,0.231402895903426,0.674125039883261
"5368","FAM214A",180.386430434412,0.348955884149058,0.291516438750334,
1.19703672851162,0.231292239354653,0.674125039883261
"5369","AC009269.4",3.98721546660034,-0.878526658204923,0.733895203568
562,-1.19707371561102,0.231277823808302,0.674125039883261
"5370","HOXB6",9.70832336789327,-0.702218960172945,0.586609503557932,-
1.19708077675833,0.231275071832225,0.674125039883261
"5371","RAC1",2270.80487509006,-0.264430459451568,0.220984246219716,-1
.19660321482218,0.231461246839511,0.674129265371528
"5372","C1GALT1C1L",51.7569161248428,-0.394022819678105,0.329325838433
387,-1.19645279444967,0.231519909468076,0.674156237604394

"5373","RWDD4",189.854647064527,-0.218259236347057,0.182436319675161,-1.19635847037301,0.231556700418616,0.674156237604394
"5374","SPR",96.8168424784596,-0.359799636272573,0.300932112686273,-1.19561728743675,0.231845942105765,0.674542655775522
"5375","AC012306.2",38.6567693601683,-0.509461669134006,0.426122223602875,-1.1955763884514,0.231861910126253,0.674542655775522
"5376","BTBD2",196.523972914244,-0.259278976506272,0.216836456646972,-1.19573516610447,0.231799923596828,0.674542655775522
"5377","HDHD5",74.3043160279107,-0.323349879951257,0.270380420543266,-1.19590715667045,0.231732792045351,0.674542655775522
"5378","ARF1",1034.0437153143,-0.201204261079057,0.16833509941861,-1.1952602979056,0.231985346379086,0.674776268763117
"5379","SDCCAG8",273.912287072397,0.204957960854342,0.17157796799209,1.1945470811485,0.232264035423516,0.67485614488661
"5380","TMSB10",18201.2989024262,-0.311195479370632,0.260565753380185,-1.19430690846228,0.232357936224463,0.67485614488661
"5381","MYLK4",3.1056832538572,-1.01662892407609,0.850947430202109,-1.19470238465216,0.232203330438992,0.67485614488661
"5382","MRE11",148.01090849782,0.243498991347703,0.203801212277533,1.19478676611653,0.232170352160795,0.67485614488661
"5383","COL4A2",1220.29482683308,-0.323962808122408,0.271254218754772,-1.19431435798345,0.232355023265292,0.67485614488661
"5384","ARHGAP17",108.621604260167,0.282949980987519,0.236836316143014,1.19470689966592,0.232201565780308,0.67485614488661
"5385","KCNK6",29.6639793128742,-0.611958594423959,0.512314387375141,-1.19449816266013,0.232283158999345,0.67485614488661
"5386","PSMA7",1661.58047780512,-0.294352211892323,0.246381360925153,-1.19470162347932,0.232203627938529,0.67485614488661
"5387","DTX3L",130.043421222154,0.295412521557038,0.247376756889768,1.19418059025115,0.232407333920769,0.67487431307269
"5388","ECE1",295.332955424026,-0.297596872044744,0.249502197490909,-1.19276252889752,0.232962389069023,0.675220203275238
"5389","ASPRV1",6.76974979131211,0.695878210034449,0.5830377078735,1.19353894377177,0.232658369726146,0.675220203275238
"5390","FBXL4",122.661876676892,0.299668641645734,0.251265569679308,1.19263710514816,0.233011527440199,0.675220203275238
"5391","ZBTB10",143.412275373987,0.259575349563188,0.217555340859659,1.19314629802923,0.23281208209657,0.675220203275238
"5392","RABEPK",109.527662180207,-0.251938395725073,0.211189076763508,-1.19295183058732,0.232888238591606,0.675220203275238
"5393","ZFPL1",142.463601393406,-0.332744328110446,0.278976350135708,-1.19273310425268,0.232973916362317,0.675220203275238
"5394","TAGLN",2285.47907740216,0.489673329943511,0.410452584014555,1.19300827675176,0.232866131569833,0.675220203275238
"5395","AVPR1A",4.91981809638907,-0.806503479330709,0.676120509370765,-1.19283983868687,0.232932104391144,0.675220203275238
"5396","SECTM1",54.415126387447,0.448030955524311,0.375438726977214,1.19335306491038,0.232731128328463,0.675220203275238
"5397","OCEL1",156.447087043699,-0.365055594253493,0.306112639398299,-1.19255315615537,0.233044420985937,0.675220203275238

"5398","C19orf48",102.834894362414,-0.424172496830627,0.35538377700793
,-1.19356178945997,0.232649428347324,0.675220203275238
"5399","AAR2",65.5314572462956,0.283209149635165,0.237424610399676,1.1
9283821992344,0.232932738483108,0.675220203275238
"5400","RANBP1",532.092266356083,-0.304684838393566,0.255616431430787,
-1.19196108281507,0.233276505281098,0.675767476317077
"5401","DHX15",190.574175625106,0.179656418480635,0.1508004636298,1.19
135189744293,0.23351546826665,0.676334469560305
"5402","CCL20",15.2072992452493,-0.668318484395473,0.56113378111984,-1
.19101452609345,0.233647882372601,0.67655910791922
"5403","WDR83",67.2987294413808,-0.286592464401553,0.240645144328013,-
1.19093391724917,0.233679528229083,0.67655910791922
"5404","AC010168.2",8.62450897745051,0.767018922398284,0.6441827664365
25,1.19068525636173,0.233777168008152,0.676591348594175
"5405","DICER1",329.369373384573,0.291969932380087,0.245202401192154,1
.19073031487682,0.233759473078956,0.676591348594175
"5406","AC017048.3",2.79708468997897,1.13022273888823,0.94969498208060
4,1.1900902502529,0.234010921901562,0.676873825398561
"5407","AC008914.1",4.41813034955956,1.01146289426241,0.84976154036460
2,1.1902902711135,0.233932323301856,0.676873825398561
"5408","AC084824.4",4.4832001583011,1.03628599779278,0.870831284567019
,1.1899962899335,0.234047850257676,0.676873825398561
"5409","SNRPN",342.731452687565,-0.283058019323115,0.237855096266011,-
1.19004395435173,0.234029116635769,0.676873825398561
"5410","ORC2",63.6774332639615,0.316875194429046,0.26640646429176,1.18
944258830752,0.234265550360627,0.676927182389704
"5411","LAMB2",1801.57156347179,0.217776573319821,0.183091743705303,1.
18943961596841,0.234266719388825,0.676927182389704
"5412","GLCCI1",29.9628700583212,0.42162159335806,0.354449889068806,1.
18950973426942,0.234239142791882,0.676927182389704
"5413","LPCAT4",57.2028362443793,0.346705549703604,0.291496402668642,1
.18939906815152,0.234282667356508,0.676927182389704
"5414","INTS6L",21.3956475573957,0.525923512976101,0.442099444459331,1
.18960455519071,0.234201854640429,0.676927182389704
"5415","AC023157.3",25.3830605903309,-0.634423793731096,0.533533898723
064,-1.18909744113635,0.234401325207297,0.677144954795522
"5416","TICAM2",69.3486691256048,0.33572310246094,0.282535864432306,1.
18824950997107,0.234735123135152,0.677358703413242
"5417","DPM2",195.478283590175,-0.347100884725549,0.292065941650617,-1
.18843327901878,0.23466275176316,0.677358703413242
"5418","PEX16",120.517223763956,-0.323182500134198,0.271932184462192,-
1.18846726720989,0.234649368368077,0.677358703413242
"5419","TIMELESS",6.40051425848985,0.944469971707537,0.794753784999533
,1.18838058972955,0.23468350008402,0.677358703413242
"5420","NDUFB11",889.443339333735,-0.337115644968325,0.283700482926169
,-1.18828012378131,0.234723065799627,0.677358703413242
"5421","BCAP31",551.989216343349,-0.240926372608206,0.202689478146991,
-1.18864765359692,0.234578347384661,0.677358703413242
"5422","LINC00894",14.9586838916908,0.735497762472455,0.61906811413296
6,1.18807243610431,0.234804872782922,0.677435010133393

"5423","TSPAN5",29.1525388247677,-0.729189443143515,0.613994447966329,
-1.1876156951561,0.234984851438917,0.677538947079822
"5424","ZNF879",13.0220867505818,0.565281522981291,0.476088691621158,1
.18734499039752,0.235091568670578,0.677538947079822
"5425","TRBC2",27.1301859696348,0.621612408358334,0.523541713730905,1.
18732164420777,0.235100773812522,0.677538947079822
"5426","IKBKB",83.2891617045631,0.382585389871627,0.322198807055288,1.
18742025573663,0.235061894125045,0.677538947079822
"5427","AP002884.3",4.4084663150225,1.07295017529019,0.903590567341772
,1.18742958821122,0.235058214834731,0.677538947079822
"5428","ZC3H4",87.5146184522495,0.305763736919048,0.257420953956887,1.
18779661181062,0.234913549597932,0.677538947079822
"5429","CD46",608.692415504261,0.214528408117991,0.180765603655806,1.1
867767085074,0.235315708367342,0.67790858673855
"5430","EDF1",2116.77035649052,-0.350790711580715,0.295580001319437,-1
.18678770557826,0.235311369507537,0.67790858673855
"5431","AL450384.2",7.32960845622361,0.655014855178356,0.5523608349895
64,1.18584594288031,0.235683144268095,0.678842096443713
"5432","WDR91",33.7964405063478,0.363165726868767,0.306304045531987,1.
18563803569105,0.235765274681407,0.678920088243323
"5433","NR1D1",68.0448154860828,-1.19368787192139,1.00685769457123,-1.
18555767945908,0.23579702355213,0.678920088243323
"5434","PLA2G5",127.736956127244,-0.616256848994612,0.519870623899144,
-1.1854042537979,0.235857650667686,0.678969677842219
"5435","GLRX2",128.516965491108,-0.242761241553616,0.204875085185756,-
1.1849232000734,0.236047813568713,0.678970357746943
"5436","TIMP4",81.3216316478682,-0.42330561403415,0.357349083047333,-1
.18457170905369,0.23618682824972,0.678970357746943
"5437","SELENOK",732.509116050224,-0.253284159456145,0.213862202620322
,-1.18433344626965,0.236281094091348,0.678970357746943
"5438","AC007370.2",3.5535618844718,1.06756358351868,0.900703397676128
,1.18525541956771,0.235916473987654,0.678970357746943
"5439","ANKHD1",368.498177898443,0.262902419848702,0.221987151978801,1
.18431367538698,0.236288917401688,0.678970357746943
"5440","AL669970.3",7.70645941464354,-0.77333792910712,0.6530411645078
45,-1.18421007914552,0.236329913282775,0.678970357746943
"5441","INTS4",49.5297876252001,0.302660490014049,0.255409327321521,1.
18500171151951,0.236016770204855,0.678970357746943
"5442","SNHG19",178.516999022412,-0.367108113332766,0.309938946784597,
-1.18445299353714,0.236233793309026,0.678970357746943
"5443","MT1G",167.182024484476,-0.876410852089077,0.74008911158017,-1.
18419638713215,0.236335331965231,0.678970357746943
"5444","NME1",198.150204201426,-0.432878564101568,0.365457001748695,-1
.1844856221943,0.236220884424039,0.678970357746943
"5445","SLPI",353.203874236701,0.559127591161038,0.472098035910331,1.1
843463616257,0.236275983602126,0.678970357746943
"5446","RGS12",109.501120267807,0.312931937214022,0.264331925648401,1.
18385978707039,0.236468570745507,0.679012143977685
"5447","AP006623.1",30.8335141213697,0.41748879796146,0.35269187651826
7,1.18372104876035,0.236523503965633,0.679012143977685

"5448","HECTD4",120.138048881253,0.296528372747778,0.250497598274315,1
.18375734853575,0.236509130254015,0.679012143977685
"5449","HNRNPM",583.58443418971,-0.228601474060239,0.193111003688038,-
1.18378274512795,0.236499074269968,0.679012143977685
"5450","AC079779.2",2.72715073225841,1.66453974712739,1.40686075640261
,1.18315884464904,0.236746200178871,0.67916031853478
"5451","BICRAL",135.77819108496,0.232528722578296,0.196551409290987,1.
18304276431845,0.236792199532615,0.67916031853478
"5452","PSMB7",652.501394963388,-0.324695367042915,0.274415564018157,-
1.18322504120586,0.236719971183029,0.67916031853478
"5453","COX8A",932.671791012494,-0.303525150082962,0.256552251191041,-
1.18309291255036,0.236772326430795,0.67916031853478
"5454","RPLP0",10237.0575490643,-0.246190285493141,0.208097790293714,-
1.18305093555132,0.23678896129731,0.67916031853478
"5455","AC009315.1",4.29190310798084,1.10732141495117,0.93651607567496
4,1.18238377718514,0.237053457244992,0.679162496645313
"5456","TMCC1",88.7712137543996,0.221695567401739,0.187494801329834,1.
18240914323667,0.237043397018497,0.679162496645313
"5457","RPS2",17383.432962943,-0.308487940141277,0.260815518774975,-1.
18278215034985,0.236895496495272,0.679162496645313
"5458","NUP85",99.6151190891846,0.277205016511706,0.234396528929832,1.
18263277096006,0.236954718873967,0.679162496645313
"5459","NAGA",93.0465446006856,0.335091778617683,0.283364173528228,1.1
8254814800821,0.236988272806757,0.679162496645313
"5460","USP27X-
AS1",3.97883953073057,-0.843931558641477,0.713753984442135,-1.18238437
478018,0.237053220234141,0.679162496645313
"5461","AL031293.1",9.4447504220261,0.936831134782276,0.79255132223718
8,1.18204475659421,0.23718794245209,0.67930286550733
"5462","FHIT",62.7083344256704,0.357723423494448,0.30263190839036,1.18
204133000089,0.237189302013747,0.67930286550733
"5463","SLC39A10",212.933348853461,0.243341029838644,0.205968957394048
,1.181445169784,0.237425922977099,0.67975361164363
"5464","TCF21",16.196573344607,-0.965511970170305,0.817291397917841,-1
.18135584521026,0.237461391006121,0.67975361164363
"5465","AC016396.2",4.9595752216269,-0.8361010897338,0.707770653804636
,-1.18131641265334,0.237477049647283,0.67975361164363
"5466","GNAS",5372.04205403163,-0.265024233261795,0.224401063702036,-1
.18102930926254,0.237591080195869,0.679955592298569
"5467","CHSY3",15.6548051880091,0.598527406527075,0.506855670007868,1.
18086359084783,0.237656917159631,0.680019600352682
"5468","RMND5A",116.80222738922,0.232232805970527,0.196720355203339,1.
18052250226206,0.237792466111035,0.680034286540206
"5469","ANKS6",44.1187599113581,0.32411757698163,0.274539354968476,1.1
8058693996293,0.237766854320794,0.680034286540206
"5470","TMEM88",3.15056275003273,-1.23866052488345,1.04906899581033,-1
.18072360333809,0.237712541734114,0.680034286540206
"5471","MFF",334.841231166148,-0.226871616342859,0.192203074926698,-1.
18037454098683,0.23785128304919,0.680078161348652
"5472","FNDC10",22.2737828278674,0.70505941251839,0.59774485771861,1.1

7953237642121,0.238186252393409,0.680349438419027
"5473","PRDM1",19.9525930227801,0.558341505718536,0.473366377405736,1.17951238695596,0.23819420720932,0.680349438419027
"5474","DPY19L1",76.329297728068,0.275674111249719,0.233695557144566,1.17962923479621,0.238147710219456,0.680349438419027
"5475","DEPP1",2034.70175225702,0.551203327400819,0.467335713698413,1.17945902965278,0.238215441688556,0.680349438419027
"5476","COMTD1",106.72156648503,-0.38963033641619,0.33040200562931,-1.17926141420985,0.238294097877507,0.680349438419027
"5477","AC079414.3",12.6926789291894,0.641715782522575,0.544135067722441,1.17933178835279,0.238266084999914,0.680349438419027
"5478","ZNF653",9.0741610018746,-0.571928708638753,0.484731177914758,-1.1798884303236,0.238044592019174,0.680349438419027
"5479","DNMT3B",3.85174504039668,0.909083671637818,0.770788548669315,1.17942031340146,0.238230850339798,0.680349438419027
"5480","CNN3",988.585684331211,0.304046506618675,0.257907412146722,1.17889790017243,0.238438833986179,0.680540765222393
"5481","DUSP12",115.162560899441,-0.293265522722662,0.2487673639383,-1.17887458418943,0.238448119554046,0.680540765222393
"5482","PTOV1",288.738805760232,-0.256749198784558,0.217854075404612,-1.17853750639141,0.238582388981905,0.680799764838369
"5483","TTC17",294.895171194704,0.19977998421475,0.169557510888165,1.17824320001087,0.238699664740404,0.680886005750208
"5484","SLC30A4",60.8781736232979,0.270310086554707,0.229399768525766,1.17833635269927,0.238662540683017,0.680886005750208
"5485","ARHGEF35",38.5333429984079,-0.357284784389139,0.303402926631924,-1.17759175349875,0.238959399032971,0.681502621526484
"5486","SLIT3",3882.61945308286,0.347029771847663,0.29473237317702,1.17744029305947,0.239019815464864,0.681550669580179
"5487","PCDHGB7",41.4420311704211,0.302662495674935,0.257133875988899,1.17706192741403,0.239170789762112,0.681608428538661
"5488","PITPNA-AS1",158.826204010687,-0.330982893289581,0.281167169291996,-1.17717475380581,0.239125763085173,0.681608428538661
"5489","TNFAIP1",56.6554071250577,0.296505748592197,0.251892855107703,1.17711059515927,0.23915136673922,0.681608428538661
"5490","LINC01138",27.3481293665827,0.363432529585886,0.30881454036412,1.17686339884568,0.239250032922414,0.68163404993002
"5491","AL359532.1",3.45689981745165,0.959718280270579,0.815517584116193,1.17682107530601,0.23926692886056,0.68163404993002
"5492","SHE",49.4693181850387,0.287958754422137,0.244832885191535,1.17614410415849,0.239537296231309,0.681795545895873
"5493","RNF7",785.767127697243,-0.310701614238676,0.264172407463894,-1.17613197086506,0.239542143965912,0.681795545895873
"5494","STMP1",550.971131895532,-0.185971200478062,0.158135494707304,-1.17602440124072,0.239585125346136,0.681795545895873
"5495","AC048382.6",12.7944134267439,-0.539235696966575,0.458320740628427,-1.17654657353538,0.239376532869147,0.681795545895873
"5496","MCRIP1",625.310363846425,-0.350193700714831,0.297694994816675,-1.17635065020319,0.239454783461562,0.681795545895873

"5497","DHPS",235.007070654896,-0.319288321513801,0.271478723371576,-1
.1761080851879,0.239551687448635,0.681795545895873
"5498","IFNGR1",738.048852228396,-0.249739802148012,0.212408656662793,
-1.17575152572281,0.239694182065719,0.68184689955337
"5499","IQCH-
AS1",105.906984128449,0.439060955284906,0.373461641337459,1.1756520795
8847,0.239733935149494,0.68184689955337
"5500","MANBAL",276.580155263868,-0.29297819268741,0.249164633865763,-
1.17584180444024,0.23965809763487,0.68184689955337
"5501","MRPL37",163.633732849762,-0.308820997627835,0.262888856706236,
-1.17472076031327,0.240106450876364,0.681883835186324
"5502","MZT2B",1867.99054759764,-0.331632342157393,0.282387778557116,-
1.17438631321758,0.240240324915503,0.681883835186324
"5503","YEATS2",63.5848356553267,0.287953931237994,0.245059301590536,1
.17503775359292,0.239979611873462,0.681883835186324
"5504","KLHL3",12.1154534287691,0.821894939946215,0.699883468201883,1.
17433112409099,0.240262421330968,0.681883835186324
"5505","HCG18",199.759254337461,0.315716691574259,0.268853326279786,1.
17430829643411,0.240271561401279,0.681883835186324
"5506","AARS2",41.6054594814073,0.363303265979686,0.309318176011064,1.
17452931691505,0.240183076294526,0.681883835186324
"5507","SEC23IP",112.572961721393,0.249633567055843,0.212593119361427,
1.17423163931963,0.240302256288093,0.681883835186324
"5508","SCART1",3.20393897201152,1.31457682636448,1.11854174775961,1.1
7525950998031,0.239890908233968,0.681883835186324
"5509","MED21",241.507366425108,-0.29419317408142,0.250391566333079,-1
.1749324403765,0.240021745770912,0.681883835186324
"5510","AC037198.1",10.4763014612896,1.04264808011594,0.88749069658777
,1.17482705353951,0.240063914341876,0.681883835186324
"5511","SLC25A11",249.624374109924,-0.289081623035717,0.24596610971375
7,-1.17529046327616,0.239878528602688,0.681883835186324
"5512","ZNF433-
AS1",29.7779517637605,-0.438955761801717,0.373832839576313,-1.17420332
119354,0.240313596073784,0.681883835186324
"5513","ZNF333",71.3919593342827,0.273602050730905,0.232785419291431,1
.1753401547387,0.23985865566784,0.681883835186324
"5514","ARID5A",38.9985271250409,-0.465367482729717,0.396562285791024,
-1.17350413643458,0.240593699073878,0.682307330422893
"5515","BLVRA",173.249514947124,-0.397752419572504,0.338928600090986,-
1.1735581460689,0.240572053880355,0.682307330422893
"5516","CDC42EP4",288.750356480912,-0.213088304132748,0.18157947859542
2,-1.17352635760967,0.240584793429309,0.682307330422893
"5517","GPAA1",422.585929765951,-0.318395526790289,0.271373364270029,-
1.17327478931746,0.240685628748967,0.682320639818791
"5518","BBOF1",68.7905525902713,0.297058388295242,0.25318736487248,1.1
7327493196534,0.240685571563439,0.682320639818791
"5519","ADAMTSL2",41.2843733150769,-0.727711606253492,0.62051274671989
8,-1.17275851318165,0.240892659303484,0.682435545326168
"5520","AC074032.1",45.3503491227744,-0.330475795555993,0.281798353989
683,-1.17273855889198,0.240900663638119,0.682435545326168

"5521","ZNF519",85.8874028368357,0.293655607807786,0.250336546686393,1
.17304329589423,0.240778443821148,0.682435545326168
"5522","NXT1",127.850420587381,-0.319161022445181,0.27212207990654l,-1
.17285970530137,0.240852070634868,0.682435545326168
"5523","SMIM4",138.963955462948,-0.344817004635113,0.294099175431838,-
1.17245144985124,0.241015853438158,0.682493800280511
"5524","SH3BGRL2",27.9402061344836,0.430784408218636,0.367415967423082
,1.17247056854931,0.2410081817012,0.682493800280511
"5525","LINC00562",3.91861923598811,0.941027064992497,0.80267681672070
5,1.17236108654167,0.241052115741854,0.682493800280511
"5526","CAPN10-
DT",15.1075504903879,0.681319973483341,0.581420388886914,1.17181988541
488,0.241269377215656,0.682563384842206
"5527","NDUFAF3",608.142864074714,-0.313362899140592,0.267364929727809
,-1.1720418959196,0.241180235952208,0.682563384842206
"5528","HIGD2A",853.594724836695,-0.369459811203602,0.315212905689337,
-1.17209608025291,0.241158483475899,0.682563384842206
"5529","NME2",3929.41477622911,-0.334949097553853,0.285852153268261,-1
.17175642626528,0.241294861482925,0.682563384842206
"5530","L3MBTL1",23.0757822837556,0.609745123325934,0.520348168610048,
1.1718021895891,0.241276483410194,0.682563384842206
"5531","AC007405.1",7.70810410235937,0.765878135316407,0.6539464809709
53,1.1711633254441,0.24153313373492,0.682743745525161
"5532","LINC01305",4.73761701634978,1.08445567453141,0.925941091116631
,1.17119294622039,0.241521229960222,0.682743745525161
"5533","IRAK2",8.60641301965375,-0.563321158267968,0.480941356641628,-
1.17128866230509,0.241482767123497,0.682743745525161
"5534","C16orf91",86.0283155905416,-0.354567903431372,0.30284199365383
5,-1.17080164198319,0.241678517670216,0.682743745525161
"5535","MTHFSD",37.599722819529,0.332684881894071,0.284169029525281,1.
17072885264745,0.241707783846982,0.682743745525161
"5536","VMO1",18.4938559614182,-0.578798141404962,0.494332967218914,-1
.17086696576448,0.241652255268625,0.682743745525161
"5537","TSPAN10",33.2258712887502,0.389618086164095,0.332720259885691,
1.17100800022803,0.241595561424479,0.682743745525161
"5538","BICRA",27.9345157781944,0.393134832174435,0.335752746199632,1.
17090578297365,0.241636650369987,0.682743745525161
"5539","AKNAD1",19.4299003904235,-0.403439874494206,0.344653303150188,
-1.17056726515225,0.2417727617313,0.68280399201349
"5540","AC244033.2",16.7317807566159,0.476240674723317,0.4069388240270
21,1.17030041520859,0.241880094871017,0.682857690322517
"5541","ST5",298.992808665146,0.229785664822165,0.196365372071559,1.17
019443091232,0.241922733476148,0.682857690322517
"5542","MIEN1",221.843756657409,-0.336381135122426,0.287450400489881,-
1.17022322650849,0.241911148179538,0.682857690322517
"5543","CKS1B",634.459648878604,-0.336084395096474,0.287308708827146,-
1.16976751755435,0.242094538799367,0.683031651043296
"5544","LPGAT1",144.143199129922,0.217629379077789,0.186040011323401,1
.16979878430277,0.242081953020446,0.683031651043296
"5545","WDR36",111.750726209399,0.239565593817019,0.204806665599102,1.

16971580547069,0.242115355432786,0.683031651043296
"5546","CHCHD5",266.746513546727,-0.311632292055198,0.266467033014983,
-1.16949661100356,0.242203605867386,0.683034253936096
"5547","AP4S1",45.4883853469312,0.303899934450804,0.259850273601051,1.
16951939376205,0.242194432190208,0.683034253936096
"5548","SLC41A1",46.3869535115659,-0.353718772457609,0.302569824629473
,-1.1690484102001,0.242384127551185,0.683203415457443
"5549","RAB24",66.330406559275,-0.377411349134624,0.322866605370194,-1
.16893894523991,0.242428231090601,0.683203415457443
"5550","AMBRA1",37.20202429178,0.333993322557974,0.285659610797636,1.1
6920036971757,0.242322912268156,0.683203415457443
"5551","ZNF841",29.3640428394,0.474883663494114,0.40626057219728,1.168
91398278125,0.242438289279823,0.683203415457443
"5552","ZNF547",4.72224072985731,0.951216551021612,0.813900588473604,1
.16871343317927,0.242519107910254,0.683308070072065
"5553","CLEC3B",1868.15021477339,0.506421932519901,0.433446759597413,1
.16836017643843,0.242661511416456,0.683586173795717
"5554","AGO1",90.429762351579,0.283262671648043,0.242602614464528,1.16
759941879959,0.242968384754332,0.68383491232674
"5555","RPLP2",10564.1122635105,-0.224329858053171,0.192089420747974,-
1.16784077529963,0.242870997179947,0.68383491232674
"5556","TAF10",415.686373116879,-0.315646736417587,0.2703297346836,-1.
16763602341795,0.242953612990738,0.68383491232674
"5557","TRDC",11.3948714293982,-0.700740447934731,0.600091325628999,-1
.16772300816086,0.242918512902089,0.68383491232674
"5558","CD37",23.1026792008494,-0.429638008633673,0.367876474794675,-1
.16788660887726,0.24285250638931 7,0.68383491232674
"5559","PTCD3",190.646187470216,0.219518153960913,0.18807000455198,1.1
6721512547335,0.243123504118424,0.683953529062122
"5560","C2orf88",28.864486147436,0.592482173826261,0.507622832337931,1
.16717006423351,0.243141697571723,0.683953529062122
"5561","FADS3",85.3248709696402,-0.271897645155235,0.232916046829521,-
1.1673633004524,0.243063685290442,0.683953529062122
"5562","C4orf48",84.8197723397638,0.54309873817306,0.465488143378478,1
.16672947721351,0.243319634782691,0.684022223330808
"5563","JDP2",723.956917963626,-0.257105729378295,0.220374477194491,-1
.16667652557327,0.243341026199319,0.684022223330808
"5564","PRPF8",374.405710939032,0.241232376021514,0.206718103055055,1.
16696299190239,0.243225315225339,0.684022223330808
"5565","SUMO3",722.509692375806,-0.225087844228851,0.192898128574209,-
1.16687417287337,0.243261187334012,0.684022223330808
"5566","AL390728.5",4.55727012773269,1.01058180417683,0.86661195747402
7,1.16612954097984,0.243562074508294,0.684421411974708
"5567","ITGA4",50.8317816251126,-0.41117955893359,0.352673747984932,-1
.16589216317614,0.243658047899197,0.684421411974708
"5568","AL049775.1",19.8529154351839,0.504616675704288,0.4327507444810
78,1.16606772406454,0.243587064931366,0.684421411974708
"5569","PCBP3",19.1971417463528,0.789703357880801,0.677327123024466,1.
16591131675717,0.243650302996497,0.684421411974708
"5570","ACBD5",135.654874528432,0.211791651921773,0.181689224138542,1.

16568086481715,0.243743499548287,0.684538521262809
"5571","USP10",101.468042039111,0.225747099993526,0.193714329960059,1.16536086948277,0.243872949666402,0.684779133303091
"5572","AC007325.4",45.6530820073847,-0.411676316176855,0.353350698867937,-1.1650643892761,0.243992930123553,0.68499307356833
"5573","PRSS27",3.0724916179401,-1.0285837614733,0.882971649242888,-1.16491142422893,0.244054848665503,0.685043961542162
"5574","MGLL",485.994673323271,-0.293253872725329,0.251816666220351,-1.16455307397612,0.244199948049009,0.685082458273071
"5575","TMTC4",30.3847194436145,0.437458411367366,0.375602834663047,1.16468346614,0.244147144027918,0.685082458273071
"5576","SEPTIN4",35.938949077142,-0.404935456638585,0.347715777276505,-1.16455876638746,0.244197642664803,0.685082458273071
"5577","ZNF37A",260.459652483443,0.312669453540769,0.268655894402876,1.16382874917269,0.24449341890352,0.685407100153208
"5578","SFXN3",205.108687950082,-0.24118035392824,0.2071718612794 5,-1.1641559449182,0.244360820086157,0.685407100153208
"5579","GLB1L2",2.88978576388225,-0.966713542774231,0.830756567900176,-1.16365440867678,0.244564092473993,0.685407100153208
"5580","UBR1",108.386389294569,0.244206390909343,0.209801021570655,1.16399047574276,0.244427871544167,0.685407100153208
"5581","LRRC36",9.28487327595432,-0.787828300112499,0.676904380024316,-1.1638694080901,0.244476938788711,0.685407100153208
"5582","AP2A1",304.656634875568,-0.213386827609253,0.183382085357467,-1.16361872095247,0.244578561213016,0.685407100153208
"5583","PMVK",338.819559283646,-0.343377128281684,0.295214314095478,-1.16314525375836,0.244770574092961,0.685680593705874
"5584","GSTP1",3222.12530866077,-0.342697028801144,0.294680857019928,-1.16294296231794,0.244852644892296,0.685680593705874
"5585","TRIM9",10.7321116801439,-0.755102190891255,0.649146696459159,-1.16322272763621,0.244739147609213,0.685680593705874
"5586","GAPLINC",8.61426620825888,0.865311224496915,0.744038406027596,1.16299268624693,0.244832469818882,0.685680593705874
"5587","ALG13",196.919804853247,-0.192376884854379,0.165437420012703,-1.16283779594487,0.244895319122593,0.685680593705874
"5588","SLC16A4",98.5725753211943,0.265365307383698,0.228229654201108,1.16271177955634,0.244946460710025,0.68570105312937
"5589","CCDC12",274.241623651967,-0.259591053945664,0.223313431618253,-1.16245159131058,0.245052077351851,0.685762670267124
"5590","MCUR1",197.45503346945,-0.253715428266836,0.218309065664239,-1.16218457302663,0.245160499688082,0.685762670267124
"5591","SENP6",400.548964766999,0.215242313679035,0.185211554765307,1.16214300966147,0.245177379450977,0.685762670267124
"5592","ROM1",32.2196067863754,-0.354458310388167,0.304929685463953,-1.16242637986806,0.245062312978154,0.685762670267124
"5593","CHST14",143.347961994969,0.235892661214404,0.202985173939959,1.16211768887209,0.245187663159498,0.685762670267124
"5594","AL451085.2",25.6222057266635,-0.39763744136852,0.342340308950494,-1.16152679358019,0.24542773351372,0.685943547499576
"5595","CLK1",412.061517600496,0.255528393731097,0.219990000754139,1.1

6154549231843,0.245420134020306,0.685943547499576
"5596","ANKS1A",80.8295257010461,0.26761646457757,0.23038648010428,1.1
6159795686118,0.245398812396136,0.685943547499576
"5597","DSE",761.014271063141,0.283383211498004,0.243970143176393,1.16
154873628579,0.245418815632179,0.685943547499576
"5598","AC004943.2",7.41226424554032,0.681014346221037,0.5865701674764
56,1.16101087982517,0.245637474968766,0.686407113422008
"5599","SEC61B",1453.24215698511,-0.316774817437274,0.272943328875108,
-1.16058823911473,0.245809390502821,0.686764832226404
"5600","UQCRC1",288.252745674343,-0.256525565982743,0.2210914208817 9,-
1.16026919977098,0.245939220472938,0.686821124833826
"5601","TOMM6",834.94067600204,-0.307427548261962,0.264930671982328,-1
.16040753590988,0.245882920019095,0.686821124833826
"5602","MTCL1",7.32109140883443,-0.787992287420136,0.679188532834748,-
1.16019668961617,0.245968734481668,0.686821124833826
"5603","AVP",5.89119787551451,-0.917197799393789,0.790614693956838,-1.
16010720064338,0.246005162848809,0.686821124833826
"5604","PPP2R1B",78.4185044298567,-0.250021472798505,0.215548803749817
,-1.15992976276825,0.246077403849205,0.686900219202912
"5605","ATAD2",57.8625429571002,0.374574169196269,0.322980138198349,1.
15974366499972,0.246153186563216,0.68698916992121
"5606","SLC35A3",197.919041725951,0.258480086506635,0.222915339580199,
1.15954373975974,0.246234618324631,0.687069055842414
"5607","KCNK15",46.2028994598935,-0.485729788848774,0.418928412177359,
-1.15945773724016,0.246269653909635,0.687069055842414
"5608","C2CD5",87.9530210416357,0.286946197195381,0.247513813621126,1.
15931387019318,0.24632827007482,0.687110044361699
"5609","STOML1",83.5045471081655,0.363915566136775,0.314029372231739,1
.15885836904524,0.246513920704216,0.687382756074161
"5610","NDEL1",122.988455136659,-0.19451362892081 6,0.167847718902795,-
1.15886965990562,0.246509317653294,0.687382756074161
"5611","TRIP4",89.8111312582651,-0.233223235117382,0.201346801533258,-
1.15831606631635,0.246735077264211,0.687876815833907
"5612","CTBP1-
DT",57.6396050010252,0.312823332411925,0.270168567017862,1.15788204329
204,0.246912176374987,0.688157504161406
"5613","GTF2H4",57.9770271534219,-0.343966287550547,0.297127629893914,
-1.15763817613783,0.247011723190328,0.688157504161406
"5614","NPC2",1488.01261688187,-0.227548675775951,0.196532962433067,-1
.15781430737578,0.246939823424366,0.688157504161406
"5615","SECISBP2L",248.518207637143,0.211721669153506,0.18288605061110
4,1.15766986298873,0.246998786997487,0.688157504161406
"5616","TFB2M",49.3936774926124,-0.312204006823009,0.26975161176593,-1
.15737587174795,0.247118827478533,0.688182860170326
"5617","ZNF141",150.293937271212,0.298108260809646,0.257585332522968,1
.15731846176864,0.247142273493875,0.688182860170326
"5618","TMEM208",413.369975238457,-0.304343258602979,0.262994058652712
,-1.15722484440179,0.24718050980884,0.688182860170326
"5619","AF165147.1",9.60675588354665,0.650860341794385,0.5624514301875
7,1.15718497075798,0.247196796733175,0.688182860170326

"5620","WDR75",105.004343152024,0.23194023038424,0.200544954157073,1.1
5654981876322,0.247456334414787,0.688217318984605
"5621","CRIPAK",25.8431531809316,-0.405250983057546,0.350405140604132,
-1.15652122671161,0.247468022269021,0.688217318984605
"5622","ZBED8",24.7261545713991,0.392839617331712,0.339738397579948,1.
15630031850983,0.247558338117051,0.688217318984605
"5623","NFIB",2899.68641609359,0.244219670472425,0.211115921508905,1.1
5680365898942,0.247352586594715,0.688217318984605
"5624","ISCU",1170.84182717483,-0.268873583936107,0.232406848596406,-1
.1569090392987,0.247309525231887,0.688217318984605
"5625","LPAR6",130.36694995059,0.217060770092045,0.187721175215147,1.1
5629347538056,0.24756113622236,0.688217318984605
"5626","STARD5",35.0177324625155,0.435941366142726,0.376955585443655,1
.15647939167594,0.247485124284611,0.688217318984605
"5627","AP1G1",136.211380894522,0.228000395881346,0.197115525889805,1.
15668410619672,0.247401445589699,0.688217318984605
"5628","IDE",67.7238792569734,0.283378785063119,0.245159888635864,1.15
589375831387,0.247724616014575,0.688427103982235
"5629","AC051619.6",4.16590638901454,-0.985351520849048,0.852393002241
507,-1.15598264915116,0.247688254128085,0.688427103982235
"5630","LRTM1",7.34856489769442,-0.717369563262813,0.620750188079745,-
1.15564936916404,0.247824605649076,0.688582647632059
"5631","BCL10",165.810846004013,-0.274079052446556,0.237310066939298,-
1.15494068996518,0.248114715110789,0.688862983525416
"5632","DMRT3",15.7001396286076,0.582133339445464,0.504022192890959,1.
15497560951925,0.248100414654511,0.688862983525416
"5633","ACSL5",125.199645728394,-0.282218164552881,0.244288834551416,-
1.15526427997053,0.247982218764177,0.688862983525416
"5634","PARP6",65.7342869152996,-0.24917751455091,0.215747487258572,-1
.15494978744421,0.248110989403386,0.688862983525416
"5635","FTSJ3",126.31321858825,-0.251903093686033,0.218125211984011,-1
.15485546762241,0.248149618275336,0.688862983525416
"5636","AC020910.5",18.2551588606013,-0.599461084134479,0.519122889899
816,-1.15475756472647,0.248189719053202,0.688862983525416
"5637","AC007877.1",3.04341047910225,1.03720704075704,0.89834742404586
3,1.15457228795269,0.248265620358611,0.688951410195096
"5638","MAPRE3",53.951689339433,-0.28208184486504,0.244459200998754,-1
.15390152513211,0.24854054384793,0.689217855041952
"5639","ITGAV",490.568223833139,0.200582644647516,0.173845156284639,1.
15380059435823,0.248581930457501,0.689217855041952
"5640","AC005072.1",4.90630641770994,0.834222270467999,0.7228056909954
26,1.15414457974056,0.248440899214641,0.689217855041952
"5641","CD248",2405.6298701352,0.385009210341527,0.333622370597564,1.1
5402696063792,0.24848911580329,0.689217855041952
"5642","DDHD1",181.513064292046,0.269145786048807,0.233260342336209,1.
15384288367748,0.24856458915821,0.689217855041952
"5643","UBXN2A",230.400221760908,0.162436192725345,0.140904443629475,1
.15281100113841,0.248987967948982,0.68986753556955
"5644","NEK10",3.23207921065234,1.15894950287334,1.00519408276269,1.15
296092838914,0.248926421890981,0.68986753556955

"5645","TM4SF19-
AS1",3.84873864118979,0.897927974423505,0.778747308111472,1.1530415130
4363,0.248893345790542,0.68986753556955
"5646","DOCK2",10.543371350399,-0.641830774177465,0.556788578015936,-1
.15273696250122,0.249018365188589,0.68986753556955
"5647","TRPV1",41.656628350556,0.402952725140261,0.349575311804815,1.1
5269217113721,0.249036755952263,0.68986753556955
"5648","IBA57",40.6819275030082,0.334674827615014,0.29053500260743,1.1
5192601446107,0.249351477168633,0.689883795266482
"5649","CCT7",534.73119100181,-0.275495797196105,0.239205510134197,-1.
15171175213125,0.249439541394056,0.689883795266482
"5650","RASA4",13.2461745787387,-0.498322358097167,0.432466759015409,-
1.15227898493676,0.249206449726689,0.689883795266482
"5651","PRKAG2",221.170598112245,0.300961048872907,0.261294622476399,1
.15180728183601,0.249400274927015,0.689883795266482
"5652","APBA1",20.9871873733195,0.425088677784166,0.368963894694059,1.
15211456702734,0.249273997922644,0.689883795266482
"5653","LINC01001",6.89385300409488,0.752988057546641,0.65371263595965
5,1.15186400893299,0.24937695990282,0.689883795266482
"5654","CSNK1G2",161.834463703459,-0.198154457754295,0.172009667458954
,-1.15199605162646,0.249322695819674,0.689883795266482
"5655","TP53TG5",2.69915898049934,-1.25610247160872,1.09022113075974,-
1.15215384858059,0.24925785862643,0.689883795266482
"5656","CCDC117",108.387047783636,0.245796041393247,0.213398861402332,
1.15181514923754,0.249397041308016,0.689883795266482
"5657","CFLAR-
AS1",3.63808940503162,1.32392935459836,1.15052116076566,1.150721429336
68,0.249846857593395,0.690086277264993
"5658","KCTD6",57.9132943719498,-0.270352687665571,0.234855003614457,-
1.15114723341976,0.249671668999359,0.690086277264993
"5659","GAR1",109.111570452221,-0.221526449157553,0.192518574768442,-1
.15067571751973,0.249865669911713,0.690086277264993
"5660","NCF1",6.94610668063606,-0.706309538355894,0.61373306427022,-1.
15084159462022,0.24979740928934,0.690086277264993
"5661","LINC00476",56.3199527996995,0.282614342197946,0.24552091971478
,1.15108049662838,0.249699120847082,0.690086277264993
"5662","CAPRIN1",395.17840772873,0.192765161789274,0.167472620922307,1
.15102493009111,0.249721979475225,0.690086277264993
"5663","RPLP1",29241.586866045,-0.303573949424698,0.263785995081443,-1
.15083421821151,0.249800444502328,0.690086277264993
"5664","TNRC6B",911.955291974605,0.245747566377997,0.213506938364652,1
.15100505988373,0.249730153916604,0.690086277264993
"5665","DIP2C",145.920369735169,0.323420864239295,0.281138593523186,1.
15039653640659,0.249980586055846,0.690159955466219
"5666","MOK",88.0867137219316,-0.216437201684593,0.188134168799988,-1.
15044068318443,0.249962411963168,0.690159955466219
"5667","ISG15",267.927677130081,-0.445895088684475,0.387724513773939,-
1.15003068633533,0.250131232638511,0.690265121349671
"5668","CD247",13.4208883269303,0.713876944363343,0.621119032221053,1.
14933999335135,0.250415813057558,0.690265121349671

"5669","EDIL3",70.8737810805802,-0.626974021594044,0.545378786397778,-1.14961204438332,0.250303695184532,0.690265121349671
"5670","TNC",342.239983253949,1.29530037171936,1.1265231143674,1.14982138865986,0.250217443935553,0.690265121349671
"5671","AC022387.2",5.8021561553725,-0.795014498803228,0.691404142155655,-1.14985498398164,0.250203604370203,0.690265121349671
"5672","MPP5",62.2900233517158,0.324758703244782,0.282405336587754,1.14997367673279,0.250154713269259,0.690265121349671
"5673","AC022467.2",3.30157634576924,1.38446096128428,1.2041836439276,1.14970915629504,0.250263681842791,0.690265121349671
"5674","CTSH",522.343645816507,0.509402025007358,0.4432045271514,1.14936105973788,0.250407129910341,0.690265121349671
"5675","SMG6",222.630201954157,0.209817722051195,0.182542061382506,1.14942123728697,0.25038232707474,0.690265121349671
"5676","AL136441.1",8.85930024918749,0.79479759189814,0.691707965595049,1.14903634399296,0.250540994663997,0.690488509430745
"5677","GRSF1",164.798876959983,0.21708407167247,0.188974108221518,1.14875034318458,0.25065894047949,0.690570237041327
"5678","TMEM14C",1179.9125537993,-0.298477417015851,0.259804041223259,-1.14885594392798,0.250615386558773,0.690570237041327
"5679","HEYL",4.14784890595203,-0.947320861693692,0.828320132425484,-1.14366514178492,0.252762534933553,0.690729559969186
"5680","ERI3",223.723348316387,-0.337214061772888,0.294209985138591,-1.14616797119934,0.251725657607282,0.690729559969186
"5681","KYAT3",128.4282889436,-0.308700947954551,0.269109158564813,-1.14712167211583,0.251331338064326,0.690729559969186
"5682","RHOU",142.396975228164,0.316334438939377,0.275682344511108,1.14745991260471,0.251191591892897,0.690729559969186
"5683","TBCE",26.0512782382451,0.405983003511756,0.354247942325203,1.1460419525572З,0.251777793842729,0.690729559969186
"5684","EIF2AK3",59.7117685875038,0.267417487126041,0.233461551780377,1.14544551377611,0.252024653727386,0.690729559969186
"5685","DENND6A-AS1",3.092241446448,-0.993988681943478,0.869692461927529,-1.14291973939899,0.253071916092247,0.690729559969186
"5686","AC108673.3",18.0099685915191,-0.457390659744671,0.400331311757946,-1.14253031504372,0.253233652523383,0.690729559969186
"5687","OPA1",145.920264814335,0.195984748680133,0.171344086492934,1.14380806884873,0.25270324281475,0.690729559969186
"5688","NAP1L5",57.7680688084752,0.329903219889142,0.288651948550184,1.14291007403952,0.253075929455919,0.690729559969186
"5689","NFKB1",251.062805706657,0.197235674817195,0.172628045141942,1.14254711425956,0.25322667395705,0.690729559969186
"5690","MDC1",56.1368906905928,0.371404435249115,0.323945057230898,1.1465044054813,0.251586505427657,0.690729559969186
"5691","XPO5",36.2385499714631,0.294794350092397,0.257977529413334,1.14271328500117,0.253157652144068,0.690729559969186
"5692","SRI",1348.45606591586,-0.291540810579295,0.254595152652225,-1.14511532345448,0.252161388659632,0.690729559969186
"5693","HILPDA",285.755070387157,0.45221940677857,0.39565684328769,1.1

4295863814935,0.253055764546153,0.690729559969186
"5694","KLF10",273.495816523662,-0.261179153056208,0.228571722064419,-1.14265732741253,0.253180893606353,0.690729559969186
"5695","B4GALT1",443.022637096191,-0.260429538223304,0.226819549633191,-1.14817941682922,0.25089450458529,0.690729559969186
"5696","PITRM1",71.1866486200962,0.30021038769537,0.261670841299374,1.14728254093815,0.251264867342607,0.690729559969186
"5697","BBS1",85.493652196149,0.215708774740198,0.187937642137424,1.14776780365514,0.251064431928894,0.690729559969186
"5698","NARS2",52.6408136539793,0.289121506063369,0.252541042375314,1.14484957907828,0.252271473472679,0.690729559969186
"5699","SORL1",8.30364204361923,0.762391309631153,0.663956195073314,1.14825543505467,0.25086313056601,0.690729559969186
"5700","RPUSD4",74.5848295671591,-0.279263188380924,0.244399487878267,-1.14265046463609,0.253183744098814,0.690729559969186
"5701","CLEC4E",5.79758413626623,-0.781690753565001,0.682862654169858,-1.14472617413129,0.252322605456408,0.690729559969186
"5702","AC026356.1",3.49659908725597,0.88989193810286,0.777660577557471,1.14431921044255,0.252491279230317,0.690729559969186
"5703","ADCY6",76.9286474957113,0.258468087945518,0.225272878824945,1.14735555071576,0.251234703874616,0.690729559969186
"5704","MSRB3",144.135677540656,-0.261614292580522,0.22871388548897,-1.14384962688739,0.252686004622484,0.690729559969186
"5705","MRPL57",436.403784115568,-0.268103590734806,0.234296998429621,-1.14428948100818,0.252503604234271,0.690729559969186
"5706","SPRY2",662.662953118103,0.276969159781041,0.241359378284544,1.14753842071352,0.25115916353139,0.690729559969186
"5707","MIR17HG",7.79503868751258,-0.733638421003009,0.641768891429893,-1.14315048734822,0.252976115391584,0.690729559969186
"5708","FARP1",223.667786637631,0.273437712226592,0.238968005049369,1.14424402618293,0.25252244936242,0.690729559969186
"5709","SEL1L",381.383575384647,0.193195804936722,0.168910570298379,1.14377569500502,0.252716671988915,0.690729559969186
"5710","TRIP11",528.770326849119,0.206442319926667,0.180261405190017,1.14523860339961,0.252110331220589,0.690729559969186
"5711","ATXN3",350.573489613436,0.254209705523818,0.221734694770935,1.14645886060561,0.251605340048278,0.690729559969186
"5712","ADAL",48.9810870972486,0.369137053691072,0.322881129589335,1.1432599178545,0.252930691451447,0.690729559969186
"5713","SPPL2A",668.243378591112,0.202300097411467,0.176867508754402,1.14379457728655,0.252708839264259,0.690729559969186
"5714","AC009269.5",3.48942281993936,0.955798869665175,0.836200702523793,1.14302567168434,0.253027932554702,0.690729559969186
"5715","CHRNA5",2.63501330379405,-1.03106634359062,0.9008408340528,-1.14455995400647,0.2523914890299,0.690729559969186
"5716","NFAT5",413.182929166251,0.291692186624562,0.254044916510875,1.14819139320221,0.250889561549983,0.690729559969186
"5717","DDX5",3481.24571551348,-0.242071029546304,0.211059737385793,-1.14693135007472,0.251409994647627,0.690729559969186
"5718","LAMA1",6.85729971169198,0.801600703958281,0.701516634536002,1.

14266813428947,0.253176404955068,0.690729559969186
"5719","MADCAM1",7.29201215324534,0.866878706152961,0.755165789697493,
1.14793164359341,0.250996783839021,0.690729559969186
"5720","ZNF558",35.9214013512454,0.35401051072242,0.309704570314518,1.
14305872323068,0.25301421050141,0.690729559969186
"5721","DDX49",145.667375924406,-0.321708905242592,0.281429217357843,-
1.14312546601561,0.252986502393368,0.690729559969186
"5722","EID2",96.7831040939024,-0.229789438589636,0.200538904365825,-1
.14585964911054,0.25185322946573,0.690729559969186
"5723","SYMPK",117.17133935544,0.243410544706547,0.212444089911749,1.1
4576284427429,0.251893292893611,0.690729559969186
"5724","ZNF845",31.3322352103533,0.361796373522749,0.316020429715681,1
.14485121689206,0.252270794904473,0.690729559969186
"5725","TMEM86B",5.99430957087851,0.691345144737697,0.603926638805671,
1.14475020692067,0.252312647070442,0.690729559969186
"5726","AP000331.1",7.9533796362223,-0.766451299501142,0.6683763062287
66,-1.1467361909128,0.251490668155313,0.690729559969186
"5727","AP000350.5",4.71480827594441,0.82451105902065,0.72098713477795
6,1.14358636825687,0.252795217627986,0.690729559969186
"5728","ANKRD54",63.4722304037368,-0.243151575986095,0.211820720369482
,-1.14791213797197,0.251004836873844,0.690729559969186
"5729","EIF3L",731.821161766353,-0.259017773073184,0.226296649168961,-
1.14459393908123,0.252377404149573,0.690729559969186
"5730","TAFA5",20.7554536076464,0.654888133029343,0.570943506776077,1.
14702790251048,0.251370089163852,0.690729559969186
"5731","MAP7D2",12.9236773973697,0.550907562586389,0.48086715874453,1.
14565437162464,0.25193819041564,0.690729559969186
"5732","LANCL3",9.11093209742515,-0.984998601044102,0.860180115334324,
-1.14510738330805,0.252164677385972,0.690729559969186
"5733","ARMCX6",230.747906647053,0.300805934815312,0.263234293950129,1
.14273079810908,0.253150378543452,0.690729559969186
"5734","RADX",3.99524838888027,-0.771840799008958,0.674148343667962,-1
.14491240134102,0.252245446278553,0.690729559969186
"5735","MTM1",22.5958415440938,0.374733659145889,0.327462516742166,1.1
4435588803876,0.252476074287727,0.690729559969186
"5736","CCT5",401.198951264151,-0.23330836757713,0.204251276938603,-1.
14226148827094,0.253345344140493,0.690825979510647
"5737","SMAD9-
IT1",5.05762011282325,-0.776211896181338,0.67955673699108,-1.142232655
38978,0.253357325605867,0.690825979510647
"5738","RPA2",272.982920532986,-0.265188572370687,0.232231245833479,-1
.14191598731223,0.25348894257064,0.690988346887201
"5739","ARSB",28.1054344088315,-0.396759738715545,0.347495146825372,-1
.14177058971972,0.253549390214955,0.690988346887201
"5740","DUSP1",4152.47413561085,-0.441598267876167,0.386736323154843,-
1.14185878449115,0.25351272289338,0.690988346887201
"5741","ACAD11",81.9895324816193,0.343378759453501,0.300932378441351,1
.14104956479591,0.253849297649869,0.691685170377442
"5742","LNPEP",282.151019139664,0.23205604608559,0.203408474091363,1.1
4083765252258,0.253937488645974,0.691776048730584

"5743","CD276",523.395357727657,0.19699987420747,0.172692246719077,1.1
407569126594,0.253971095560937,0.691776048730584
"5744","MANEAL",7.39484736013788,-0.642897583126039,0.563685677122514,
-1.14052495782381,0.25406766095669,0.691859452832675
"5745","SF3B5",985.521843065793,-0.318010726012017,0.278841597368579,-
1.14047089463364,0.254090171739674,0.691859452832675
"5746","MAK16",55.1092229513469,-0.275637224571082,0.24180140261704,-1
.13993228156592,0.254314514769291,0.692219995963349
"5747","LINC00327",4.05682454107584,0.835192952672675,0.73273189438074
8,1.13983430921691,0.254355337006797,0.692219995963349
"5748","FAM122C",19.7050376535739,0.396391940071045,0.347707119697438,
1.14001674862445,0.254279323454321,0.692219995963349
"5749","PYM1",96.8128004759982,-0.289827447905777,0.254335747998627,-1
.1395466433108,0.254475225395528,0.692425804637718
"5750","S1PR2",187.640515466887,-0.234008987889368,0.205420957221188,-
1.13916803355851,0.254633075688982,0.692734817913522
"5751","TRPC1",74.3062522734331,0.282254636583577,0.247826158894611,1.
13892188719112,0.254735735763768,0.692805334882126
"5752","TMEM41A",93.6880038953128,0.222244508919939,0.1951407283337,1.
13889350940563,0.254747573115258,0.692805334882126
"5753","MAGI3",145.547201977254,-0.284855457880763,0.250354018239813,-
1.13781060868734,0.255199574175991,0.692878517467455
"5754","C1orf56",140.94044205384,-0.349939834714933,0.307378926873386,
-1.13846397433445,0.254926793707315,0.692878517467455
"5755","SUCO",156.107516998542,0.208936662417163,0.18363227737791,1.13
779922244921,0.255204329734181,0.692878517467455
"5756","G0S2",291.78338306587,-0.478568267342575,0.420718369509026,-1.
13750266692909,0.255328210342424,0.692878517467455
"5757","DNAJC27",41.3240956565359,-0.286111567755974,0.251513284381634
,-1.13756046110806,0.255304064609388,0.692878517467455
"5758","CCDC191",60.3701709692134,0.368182833213961,0.323833897121457,
1.13694964142642,0.25555933821207,0.692878517467455
"5759","AC093788.1",6.23208068252037,0.979892507130658,0.8619549239769
62,1.13682569688163,0.255611158726771,0.692878517467455
"5760","GPX8",658.717429063589,0.227366896487637,0.199968800410429,1.1
3701185395409,0.255533330257764,0.692878517467455
"5761","KDM3B",128.1642509038,0.224853216109798,0.197747106425506,1.13
707462108683,0.255507092315064,0.692878517467455
"5762","NKAPL",15.7821282288643,0.554169616692208,0.487270583205161,1.
137293396714,0.2554156542751,0.692878517467455
"5763","TSPYL1",299.630699255352,-0.228711998599315,0.2010860739505,-1
.13738357960888,0.255377968639521,0.692878517467455
"5764","HGF",77.9274972949991,-0.403185746404844,0.354589817718792,-1.
13704829145599,0.255518098410198,0.692878517467455
"5765","ECD",76.754178818505,0.262848001952642,0.231257702081342,1.136
60215243421,0.255704639860616,0.692878517467455
"5766","AP006621.3",57.6397744247136,0.281398020513029,0.2472329805910
87,1.13818965350116,0.255041298081694,0.692878517467455
"5767","NOMO2",6.03771724844975,0.883943436037802,0.777677315369982,1.
13664551937877,0.255686502947138,0.692878517467455

"5768","MAPK3",422.77132758612,-0.216037836332826,0.18985135974953,-1.13793146711113,0.255149100435673,0.692878517467455
"5769","KAT8",155.58367955678,-0.244546950698991,0.215053937790807,-1.13714239883797,0.255478761934341,0.692878517467455
"5770","ALDH3A1",4.40056987031272,0.804335080338276,0.707542700205656,1.13680076143035,0.255621584980532,0.692878517467455
"5771","SUMO2",3235.21475442594,-0.288368628756231,0.253477465591988,-1.13764996064939,0.255266675878721,0.692878517467455
"5772","DEDD2",56.3656964604101,-0.31192751844513,0.273964178932507,-1.13857044983233,0.254882359357348,0.692878517467455
"5773","TCEA2",268.850485643361,-0.285780209126508,0.251033144683117,-1.13841624175665,0.254946715213182,0.692878517467455
"5774","ENTR1",102.519152707593,-0.252319147148864,0.222036086867006,-1.13638801110738,0.255794211103206,0.692982423362643
"5775","EHD1",95.7462590995856,0.275364959469635,0.2423576005985,1.13619279440638,0.255875885529798,0.692982423362643
"5776","AC026471.1",21.2090386481347,0.420604031229292,0.370167374061395,1.136253653623,0.255850421412089,0.692982423362643
"5777","HIST2H2AB",3.29909180506626,1.05895907773586,0.932374444124967,1.13576587647647,0.256054561852769,0.693152869330883
"5778","AL122125.1",4.10524268641008,0.967841781753871,0.852259254625891,1.13561897568213,0.256116063717478,0.693152869330883
"5779","RAB31",156.295660976934,-0.301658650174289,0.265610078315152,-1.13571989469603,0.256073811600137,0.693152869330883
"5780","EMD",211.564118055594,-0.181716454400575,0.16001484891703,-1.1356224477317,0.256114609981877,0.693152869330883
"5781","KNSTRN",17.8478844428702,-0.538825653765116,0.474595135117767,-1.13533749904834,0.256233936116199,0.693351922273949
"5782","NFIA-AS2",5.50861208884016,-0.767083489471961,0.676162499044821,-1.13446618313731,0.256599051043135,0.693523501836434
"5783","GPR89A",47.4486580616083,0.290540497867964,0.255974736541118,1.13503583124616,0.256360305683191,0.693523501836434
"5784","RNASEH1-AS1",48.2753344949215,-0.464499835438546,0.409475990324025,-1.13437624284388,0.25663676004895,0.693523501836434
"5785","RPL15",12955.5252012456,-0.300609687897664,0.264963854503092,-1.1345309286107,0.256571907778118,0.693523501836434
"5786","MEST",921.554551063686,0.54813895333501,0.483154423951724,1.13450053680928,0.256584648694223,0.693523501836434
"5787","SLC8B1",120.595849240622,0.27477568969993,0.242101439953703,1.13496098888332,0.25639166407852,0.693523501836434
"5788","GLCE",145.324301549943,0.269598358052055,0.23766982926165,1.13433984822388,0.25665202020911,0.693523501836434
"5789","UNC45A",160.447670741189,-0.210202257152666,0.185232195681281,-1.13480411102155,0.256457403383676,0.693523501836434
"5790","SEM1",1243.11419099981,-0.285857433143697,0.25203367966003,-1.13420330778526,0.256709276840371,0.693558414095669
"5791","GNLY",44.4258325875529,0.695186821968305,0.61321491956286,1.13367564909197,0.256930627677427,0.694036575506475

"5792","SREK1",414.341217636754,0.199661442520304,0.17619544044441,1.1
3318166472814,0.257137972300481,0.694237021176461
"5793","SNAPC5",131.066900967037,-0.255677436042531,0.225617276654286,
-1.13323518408702,0.257115502517737,0.694237021176461
"5794","ZNF19",15.2118026509572,0.458254707417984,0.404349750592932,1.
13331269957755,0.257082960518867,0.694237021176461
"5795","AREL1",32.3939487171058,0.323999418862631,0.285998175933682,1.
13287232621288,0.257267872928479,0.69446787510271
"5796","TMEM176A",1674.99982954515,0.346261592803183,0.305732055796037
,1.13256554632984,0.257396744076323,0.694576033739163
"5797","RGS10",289.153907894338,-0.378417061304032,0.33409830037127,-1
.13265185989726,0.257360481211564,0.694576033739163
"5798","ERO1A",134.776299457735,0.204414510582589,0.180554702132365,1.
13214725603066,0.25757253033662,0.694800158196906
"5799","AC026401.3",9.06557473128312,0.574243732088889,0.5072596537125
16,1.13205086958155,0.25761304849083,0.694800158196906
"5800","PITPNC1",75.1531510178834,-0.317080568563578,0.280092226213444
,-1.13205772559338,0.25761016627009,0.694800158196906
"5801","VPS41",120.748368591645,-0.189944020228975,0.167818276896901,-
1.13184346628506,0.257700250000402,0.694827270938958
"5802","UBALD1",51.0372188630633,-0.293834605091224,0.259613479361586,
-1.13181567387714,0.257711936712129,0.694827270938958
"5803","SLC25A16",62.3365344721716,0.286386852501798,0.253148654263585
,1.13129913068234,0.257929210116405,0.695293233474224
"5804","POLR1A",69.9874063311802,0.307790744811095,0.272137735977678,1
.13101089676274,0.258050505066928,0.695302783508685
"5805","COX6A1",1178.08135074847,-0.295136965656042,0.260995493873443,
-1.1308124951734,0.258134019646426,0.695302783508685
"5806","TMEM251",111.441598882493,-0.289005395744936,0.255584484801955
,-1.13076267508522,0.258154993710825,0.695302783508685
"5807","DCXR",131.764270329611,-0.240310742001539,0.212490885492517,-1
.13092258731258,0.258087675472177,0.695302783508685
"5808","ARHGAP35",157.072444715424,0.235326090008386,0.208111202593927
,1.13077089111614,0.258151534712228,0.695302783508685
"5809","KLF7",216.152499244456,0.227069671370084,0.200863914029908,1.1
3046523297497,0.258280240275335,0.695520364714592
"5810","NCBP1",56.4234605826901,0.287625026587133,0.254477327262358,1.
13025796710998,0.258367540437073,0.695635703107941
"5811","FRYL",118.375569197018,0.285997972572453,0.253085874869654,1.1
3004320260761,0.25845802059395,0.695759562235616
"5812","RMDN2",58.0175006880467,0.293394555166939,0.259688458446255,1.
12979435791006,0.258562886182425,0.695922097135525
"5813","AL603840.1",16.2652427024633,-0.70681766281663,0.6258257452439
97,-1.12941608457008,0.258722350746222,0.695932982039107
"5814","SRPRB",207.654355475806,-0.251190275461884,0.22241610357721,-1
.12937090175526,0.258741402527981,0.695932982039107
"5815","HSD17B11",538.001383226765,-0.275606600520396,0.24405993583057
7,-1.12925785865861,0.258789072529212,0.695932982039107
"5816","AP003071.4",8.04042080033669,-0.753363307124344,0.667131759260
141,-1.12925714698373,0.258789372660071,0.695932982039107

"5817","AC002553.1",5.52264644272649,0.737459508460478,0.652828089472332,1.12963813958519,0.258628733177451,0.695932982039107
"5818","PCDHB4",85.3106198828489,0.301848822105725,0.267342175353434,1.12907296316667,0.258867055629654,0.696022232934801
"5819","LINC01560",21.9685025250749,-0.405011206077354,0.358778367102962,-1.12886183564441,0.258956122485338,0.696142056029926
"5820","SLC2A5",6.80063084290774,0.888995742867961,0.787914022518724,1.12829029241809,0.259197341863417,0.696394657921415
"5821","UBR4",102.394906454781,0.307048794644373,0.272384294011542,1.12726321375696,0.259631210694983,0.696394657921415
"5822","ZDHHC18",59.291491191136,0.251077257407873,0.222700322554066,1.12742206445129,0.259564074543688,0.696394657921415
"5823","PRDX1",2192.11798474429,-0.314758648622419,0.279157037396106,-1.12753255858564,0.259517382744629,0.696394657921415
"5824","CXCL1",13.4409581158334,-1.60044664948874,1.41983290225801,-1.12720774884389,0.259654655040963,0.696394657921415
"5825","RIOK1",123.388502386078,-0.240806894959774,0.213514924847261,-1.12782230624879,0.259394970932514,0.696394657921415
"5826","ZFAND3",219.980920516181,0.194904324539079,0.172931901099048,1.12705824258213,0.259717856825004,0.696394657921415
"5827","AKAP9",1136.84089353604,0.229346540215399,0.203383212174409,1.12765718351781,0.259464726637068,0.696394657921415
"5828","USP6NL",29.4953841975396,-0.315024371609417,0.279313937091401,-1.12785052865561,0.259383049743742,0.696394657921415
"5829","ALG10B",50.4155431744105,0.330889782454825,0.293444512315907,1.12760596490081,0.259486366459384,0.696394657921415
"5830","IRF9",262.348023307618,-0.316128176243175,0.280432783156067,-1.12728680536342,0.259621239253418,0.696394657921415
"5831","TLE5",1286.51794220832,-0.23104825428553,0.204933030167174,-1.1274329672335,0.25955946706746,0.696394657921415
"5832","FKBP1A",1458.753075072,-0.320586481048796,0.284434847755807,-1.12709987393678,0.259700256653501,0.696394657921415
"5833","RP2",62.1363802592381,-0.267771300434619,0.237577466708454,-1.12709047766394,0.259704228972217,0.696394657921415
"5834","MECP2",266.598212316548,0.221430344260651,0.196294047843444,1.12805429758754,0.259296988688423,0.696394657921415
"5835","SORCS2",34.8313897940579,0.676653162424939,0.600474673074799,1.12686378421268,0.259800077450271,0.696495734628036
"5836","ZC3H7B",134.334931275762,0.216017924044862,0.191740636216998,1.12661524602635,0.259905190263137,0.696658137643292
"5837","ACOXL-AS1",4.31261892916824,0.825974163932403,0.733289776277052,1.12639530872217,0.259998231621012,0.696788133844012
"5838","SSU72",559.210709010847,-0.269566247878773,0.239372821840069,-1.12613556462511,0.260108142366904,0.69695183928674
"5839","AL391684.1",8.72680107040963,0.628006949137156,0.557712631525198,1.12604039004769,0.260148423550168,0.69695183928674
"5840","MFAP3",19.9127188407855,0.436339880329355,0.387613779234434,1.12570786619392,0.260289193074048,0.697072331920306
"5841","AOPEP",41.6141938072622,-0.406035968221962,0.360722580633777,-

1.12561838382441,0.26032708323713,0.697072331920306
"5842","PCIF1",76.5171158929297,-0.259497936530131,0.230533427373216,-1.1256412550967,0.26031739832678,0.697072331920306
"5843","ICOSLG",21.358885815816,-0.586147168984582,0.520829860503216,-1.12541006849772,0.260415306478565,0.697189224583979
"5844","FAM160B2",84.1296256239,0.279805139765893,0.248701348891052,1.12506482579821,0.26056156512992 7,0.697266246367608
"5845","TRIM66",37.2403803690955,0.445836911480527,0.39624398052869,1.12515756298851,0.260522272356116,0.697266246367608
"5846","SLC25A30",19.8858652128272,0.411360007485474,0.365644720794505,1.12502651916219,0.260577796858981,0.697266246367608
"5847","DHRS3",1438.35588143456,0.34738805969415,0.308833612048992,1.12483889751949,0.260657308166518,0.697359718085999
"5848","ERO1B",67.0243912523974,0.371361981084557,0.330394991843912,1.12399397766901,0.261015580807668,0.697489807991654
"5849","PKDCC",595.562816438703,0.308025051477723,0.274072153201442,1.12388306465898,0.261062636693162,0.697489807991654
"5850","AC007319.1",2.73905529997944,1.05092586358873,0.934529661648992,1.12455056989241,0.260779529908704,0.697489807991654
"5851","KLF3",388.3284383599,0.175496507271177,0.156137961179063,1.12398359723625,0.261019984554885,0.697489807991654
"5852","ERBIN",203.825867002658,0.224790007563945,0.200005591611086,1.12391861524078,0.261047553386394,0.697489807991654
"5853","WDR61",318.817136612197,-0.23548335505454,0.209505689807281,-1.12399503455565,0.261015132441804,0.697489807991654
"5854","MRPL38",231.620347924486,-0.276521934271683,0.245947529422593,-1.12431271385758,0.26088038663763,0.697489807991654
"5855","DKC1",132.448737885584,-0.210721469325151,0.187392751517785,-1.1244910361709,0.260804771120037,0.697489807991654
"5856","RBM34",20.7750368071318,-0.430937067496692,0.383528686614486,-1.12361104276369,0.261178069371472,0.697679053821369
"5857","AL662884.3",12.841894377287,-0.463424551718074,0.412530096122347,-1.12337149719286,0.261279749918296,0.697831505544119
"5858","MATN1-AS1",5.79675211936116,-0.734178159917272,0.653648721972257,-1.12319987056967,0.261352617555449,0.697871812958417
"5859","CD55",585.584263752116,0.451114147581708,0.401697069936689,1.12302075704164,0.261428678893839,0.697871812958417
"5860","SENP7",163.635935301521,0.199367757690154,0.177524092613585,1.12304620040569,0.261417873326295,0.697871812958417
"5861","FOXO6",7.72775450675849,0.622267151527838,0.554417482532875,1.12238010368106,0.261700860375327,0.698002823333546
"5862","ASH1L-AS1",13.8107770166424,-0.571425011655706,0.509015074737044,-1.12260921142837,0.261603501497268,0.698002823333546
"5863","AL353593.1",9.65143306686794,0.578748148235683,0.515460163223707,1.12277958516943,0.261531117749744,0.698002823333546
"5864","AC007620.2",12.0572436900855,0.596795515333291,0.531672971149119,1.12248609148481,0.261655817958284,0.698002823333546
"5865","GALNT7",53.5216340726863,-0.292201749821121,0.260320704039147,

-1.12246834495799,0.261663359456911,0.698002823333546
"5866","AIP",480.098222870778,-0.294529424640973,0.262516549463554,-1.
12194612203625,0.261885348641519,0.69830313383578
"5867","ZNF84",115.486294508128,0.218764686170454,0.194993908223344,1.
1219052336747,0.261902735166817,0.69830313383578
"5868","DNM1",485.902126126841,-0.230323147683522,0.20534643871129,-1.
12163205327047,0.262018917245198,0.698493851817763
"5869","UBE2J2",200.794558590368,-0.241974505756152,0.216213316950709,
-1.11914709588085,0.263077390798271,0.698562915499653
"5870","ZC3H12A",23.6842980530421,-0.588341573626475,0.526187029908169
,-1.11812253093573,0.26351466483861,0.698562915499653
"5871","GBP4",104.349975502941,0.226365967367248,0.201892755043705,1.1
2121887344717,0.262194707934146,0.698562915499653
"5872","VPS72",143.618445859369,-0.233440358005534,0.208706404313848,-
1.11851075568573,0.263348915437462,0.698562915499653
"5873","OAZ3",4.40365220677926,-0.800136631862225,0.713737612176228,-1
.12105151558787,0.262265934864006,0.698562915499653
"5874","ARIH2OS",36.0121008539799,0.413519694720704,0.369182864530111,
1.12009449638738,0.262673495687112,0.698562915499653
"5875","HEMK1",65.3698579988793,0.360735199298176,0.32177009122419,1.1
2109611532179,0.262246952064298,0.698562915499653
"5876","AC139887.4",9.46570366262949,0.641759330608834,0.5735784427650
05,1.11886933461996,0.263195887002261,0.698562915499653
"5877","AC124852.1",90.1900994963429,0.327511813596787,0.2928679080590
72,1.11829191449248,0.263442339056756,0.698562915499653
"5878","FNIP1",344.477009417936,0.217793401872563,0.194656039817095,1.
11886280064676,0.263198674915072,0.698562915499653
"5879","TMEM242",262.014395792493,-0.228607846395449,0.20434912618808,
-1.11871213085121,0.263262968291068,0.698562915499653
"5880","SLC29A3",32.5399725852182,0.325564343323099,0.29037757737353,1
.12117590575634,0.262212993560596,0.698562915499653
"5881","SHOC2",205.917245071613,0.214385961361163,0.191410952550855,1.
1200297501482,0.262701084619283,0.698562915499653
"5882","SPTY2D1OS",20.4865539564255,0.503134485463178,0.44941949021910
1,1.11952084058012,0.262918005054639,0.698562915499653
"5883","AC011603.2",13.3814928295479,0.557759885730088,0.4987756929077
81,1.11825795374758,0.263456838997007,0.698562915499653
"5884","PPFIA2",5.65492792468275,-0.761103034024164,0.679777194605225,
-1.11963602201478,0.262868898658524,0.698562915499653
"5885","EXOSC8",231.876727018675,-0.255411897898303,0.228363144240533,
-1.11844623066356,0.263376458884955,0.698562915499653
"5886","TOGARAM1",147.790972388847,0.218844244797172,0.195726311466497
,1.11811356969567,0.263518491616918,0.698562915499653
"5887","TEX9",15.3959782639651,-0.433008676486336,0.387128077035181,-1
.118515298096,0.263346976518233,0.698562915499653
"5888","FLYWCH1",94.7059411083517,0.233612956935593,0.208455802388099,
1.12068339791596,0.262422651509456,0.698562915499653
"5889","MT1M",1656.8499891195,-0.541854502743812,0.484134897022831,-1.
1192221549736,0.26304537603204,0.698562915499653
"5890","GABARAPL2",1331.45638255412,-0.28691931805822,0.25600345903901

2,-1.12076344255371,0.262388569058879,0.698562915499653
"5891","MVD",65.3814839535024,-0.294009659942977,0.262789880924268,-1.
11880129824217,0.263224917732848,0.698562915499653
"5892","ZNHIT3",234.408845223868,-0.281021540930002,0.250979345916722,
-1.11969986973848,0.262841680579463,0.698562915499653
"5893","PSMB3",655.364193325134,-0.338402198780991,0.302087361204556,-
1.12021303185817,0.262622991882754,0.698562915499653
"5894","AC004687.1",7.63982609822809,-1.07371461828228,0.9582516431151
92,-1.1204933756145,0.262503573929853,0.698562915499653
"5895","CIRBP",2857.82043729292,-0.239816892201804,0.214406259873411,-
1.11851627999759,0.263346557396692,0.698562915499653
"5896","ZNF561-
AS1",33.7661725202619,0.314033671103356,0.280665925529825,1.1188877684
7614,0.26318802176247,0.698562915499653
"5897","AC093227.1",15.9442562154491,0.507033793514624,0.4532013988513
74,1.11878249890597,0.263232939691009,0.698562915499653
"5898","DMAC2",94.5636711162463,-0.318754107399466,0.284981744440334,-
1.11850711007983,0.263350471564568,0.698562915499653
"5899","VASP",86.0874057017596,-0.248442274921193,0.222088501946254,-1
.1186633830387,0.263283772136027,0.698562915499653
"5900","MTMR3",146.273668444999,0.216668955021545,0.193461563290286,1.
11995866949776,0.262731375002804,0.698562915499653
"5901","ADSL",156.042668565532,-0.26403404638305,0.235652092500239,-1.
12044006731144,0.262526278765121,0.698562915499653
"5902","SATB2-
AS1",7.72234649002335,-0.683907889680761,0.611901164036962,-1.11767705
288995,0.263704946735942,0.698624746934399
"5903","CUTA",780.336521250478,-0.365040038818791,0.326662923970242,-1
.1174823098444,0.263788159225391,0.698624746934399
"5904","RAB23",82.3787007842294,0.29905296342874,0.26758700869251,1.11
759148880202,0.263741505506844,0.698624746934399
"5905","UBE3C",147.086326693562,0.224579170867879,0.200910173240449,1.
11780885579698,0.263648638451655,0.698624746934399
"5906","TRMT5",83.7820903406999,0.212851012105902,0.190482343163342,1.
11743171871515,0.263809779463114,0.698624746934399
"5907","TBC1D24",64.351190715267,0.312661643155371,0.27971803061641,1.
117774361797,0.263663374032021,0.698624746934399
"5908","TRIP13",4.55263837834027,0.770540750361345,0.689739994974712,1
.11714668712171,0.263931611225355,0.698829078266457
"5909","TMEM43",641.491929219392,-0.201982130531725,0.180819689963238,
-1.11703615116689,0.26397886832403,0.698835917615976
"5910","SHB",6.46429983913918,-0.700412230595725,0.627265388101903,-1.
11661227270193,0.264160141882656,0.699197478759795
"5911","ERMAP",39.6483556852157,0.346884591831181,0.31094614259119,1.1
1557772976538,0.264602929233172,0.699217972190431
"5912","FALEC",7.65706658248301,0.720403662065251,0.645856492804002,1.
11542373591012,0.264668882763347,0.699217972190431
"5913","LAMB3",18.1939370943451,-0.670862537360447,0.601698812947619,-
1.11494741708731,0.264872955532238,0.699217972190431
"5914","PYCR2",141.129322445349,-0.218568072673804,0.195783675542459,-

1.11637536719145,0.26426149298958,0.699217972190431
"5915","SH3RF3-
AS1",7.88364050292898,0.881545738616503,0.789719511434311,1.1162770146
2689,0.264303577307927,0.699217972190431
"5916","SMARCAL1",92.5035427117312,0.222989642088061,0.199922806343781
,1.11537871124425,0.264688168372396,0.699217972190431
"5917","AL021368.3",7.90162663821791,0.780946509590787,0.6999350303680
94,1.11574142700086,0.264532832296286,0.699217972190431
"5918","SSNA1",355.752901459,-0.27991606909569,0.251029905015773,-1.11
507060912963,0.264820165068927,0.699217972190431
"5919","LINC01197",7.44075292625724,0.798102271879045,0.71572981776217
2,1.11508875566261,0.264812389498514,0.699217972190431
"5920","PHLPP2",40.7887451669024,0.323666145167784,0.290272945786993,1
.11504068796441,0.264832986270769,0.699217972190431
"5921","GGA3",83.8133681089907,0.289022133260996,0.259035352142551,1.1
1576327659687,0.264523477029999,0.699217972190431
"5922","GIPC3",3.39519441344782,-1.14401729528831,1.02617248831017,-1.
11483917988505,0.264919343506485,0.699217972190431
"5923","GPI",252.995013792616,-0.175397913661311,0.157333008813023,-1.
11481954730655,0.264927758177631,0.699217972190431
"5924","MRPS12",239.972980317575,-0.316990934762315,0.284151207735059,
-1.11557130898376,0.264605678942621,0.699217972190431
"5925","NORAD",1041.66021855978,0.133377276045988,0.119512132023559,1.
1160145316435,0.264415914412097,0.699217972190431
"5926","RAB22A",290.44769880293,-0.155395675392302,0.139268555820339,-
1.1157987133346,0.264508304688925,0.699217972190431
"5927","SLC25A43",122.570305710229,-0.187399280854291,0.16803218099000
7,-1.11525827820705,0.264739758734068,0.699217972190431
"5928","LINC01358",25.5430211053358,-0.403015762738265,0.3615853065372
64,-1.11458003257312,0.26503043082739,0.699370956382062
"5929","GNPAT",123.816677161557,0.224819038693246,0.201737423748042,1.
11441414545886,0.26510155763377,0.699440658806724
"5930","F2R",55.786838322336,-0.499943222329183,0.448703527395724,-1.1
1419499024412,0.265195544169466,0.699526165608545
"5931","SOX12",30.8976585595339,0.356381727217981,0.319874443885605,1.
11413004080261,0.265223402686459,0.699526165608545
"5932","ANKRD65",185.912938005344,-0.291416888535029,0.261799396021093
,-1.113130484501,0.265652392836563,0.699618934251155
"5933","ZC3H11B",4.44226750427912,0.880848947190446,0.791340270593731,
1.1131022568023,0.265661092466398,0.699618934251155
"5934","TMEM131L",28.1534853876602,0.353928930893544,0.317775679634067
,1.11376972366516,0.265377988761251,0.699618934251155
"5935","ITK",7.49470069704428,0.715593586423263,0.642858648624192,1.11
314297156106,0.265647030687321,0.699618934251155
"5936","EIF3E",2146.92342557677,-0.291132936174855,0.261519193474823,-
1.1132373586296,0.265606501736512,0.699618934251155
"5937","AL590399.3",3.55315206289926,0.942170751893002,0.8460847500341
02,1.11356545766252,0.265465652116986,0.699618934251155
"5938","CSTF3",90.51773049444,0.282612286503631,0.253872519730894,1.11
320550488569,0.26562017896906,0.699618934251155

"5939","ATMIN",192.945149851691,0.169539988764719,0.152287763519225,1.11328700905977,0.265585184001481,0.699618934251155
"5940","TCF4-AS2",4.63233777753291,0.830955930390521,0.746475476450525,1.11317244384464,0.265634375098924,0.699618934251155
"5941","MAP4K4",354.198315119931,0.302276739861767,0.271723219755752,1.11244353770531,0.265947493942855,0.699683957370723
"5942","GIN1",43.5373106524961,0.290191228652626,0.260790349361213,1.11273760460626,0.265821139998076,0.699683957370723
"5943","IGFBP3",2122.36924567302,-0.358945055304083,0.322527242243123,-1.11291391327964,0.265745403942118,0.699683957370723
"5944","SCARB1",32.0029040822122,0.30713268157813,0.276092178212749,1.11242804329452,0.265954152689786,0.699683957370723
"5945","ALKBH5",226.494976400908,-0.156041189153328,0.140238980447134,-1.11268057323157,0.265845641868012,0.699683957370723
"5946","HIC2",47.4767539391842,0.35459531498081,0.318734349182982,1.11251051507235,0.265918711636456,0.699683957370723
"5947","AL355388.2",2.72913102390767,1.12045135679432,1.00732699661774,1.1123015272661 3,0.266008527442767,0.69970933155998
"5948","PPP2R5A",138.524315686629,-0.210905142577719,0.189841465545429,-1.11095403721085,0.266588133942582,0.700193088314238
"5949","SNX17",409.220678800427,-0.29353262878281,0.264124297646213,-1.11134277080403,0.266420835797672,0.700193088314238
"5950","FAIM",158.977896831596,-0.27359408208654,0.246105635180168,-1.11169369155748,0.266269873113168,0.700193088314238
"5951","WDR37",42.2251373159879,0.354380511790156,0.318866291483929,1.11137652757509,0.266406311404353,0.700193088314238
"5952","RRP12",23.6114786279709,-0.49286647793587,0.443649179815545,-1.11093742614556,0.266595284408336,0.700193088314238
"5953","UACA",584.944133696037,0.304766481866865,0.274256800346265,1.11124494080759,0.26646293180391,0.700193088314238
"5954","CMIP",62.3495558963005,0.262799192230486,0.236501222341351,1.11119591530558,0.266484029077947,0.700193088314238
"5955","METTL4",59.3683809129377,0.24967468783856,0.224680805777103,1.11124173235453,0.26646431247086,0.700193088314238
"5956","URB1",55.9912364098735,0.29712842553945,0.267421023050446,1.11108850811404,0.266530253916299,0.700193088314238
"5957","B3GNT10",3.36019586138346,-0.830856643156541,0.748161356299483,-1.11053135284356,0.266770125392173,0.700534677104208
"5958","MTERF4",145.841662576148,-0.209370732595664,0.188607172362011,-1.1100889217182,0.266960710556637,0.70068617557406
"5959","MSL2",72.4426247347466,0.274893202303184,0.247601683641856,1.11022347772402,0.266902738230208,0.70068617557406
"5960","FBRS",94.1457762239433,-0.22517854653875,0.202847934685543,-1.11008547801004,0.266962194363063,0.70068617557406
"5961","DPY30",338.281091662876,-0.281178300272797,0.25336009825383,-1.10979709200735,0.267086472698398,0.700894764707437
"5962","ADCY4",3.21575178671519,-1.07811478869186,0.971774067739553,-1.10942946975285,0.267244955132407,0.701193028033586
"5963","CCDC93",150.659573270221,0.208939536318161,0.188369609025572,1

.10919981943476,0.267343990491942,0.701323864129736
"5964","TCIRG1",158.965800624404,-0.175232910215889,0.157994746363524,
-1.10910593072951,0.267384486714169,0.701323864129736
"5965","SUCLG1",435.824256921853,-0.23366925445162,0.210752099079646,-
1.10873986770264,0.267542417863381,0.701620459788242
"5966","THOC7",549.95839193391,-0.249374883448307,0.225037563547209,-1
.10814781104752,0.267797985503193,0.702172961318546
"5967","KHK",22.2724934547497,-0.555492346469053,0.501368068150666,-1.
10795318201661,0.267882035854315,0.702275630445625
"5968","TNFAIP8",48.7471911685584,-0.365027008246676,0.329567037804858
,-1.10759562205615,0.268036495003576,0.70232729569592
"5969","PIP4K2A",155.496054444286,0.201867905671546,0.182274844714961,
1.10749185378391,0.268081332391507,0.70232729569592
"5970","CCT6B",18.4989961019733,0.443446679113105,0.400349873402804,1.
10764785647113,0.268013926906907,0.70232729569592
"5971","RGL3",5.19347271253282,-0.938969956456197,0.847825636367473,-1
.10750361416203,0.268076250573225,0.70232729569592
"5972","AC092903.2",21.6352823546206,0.47655390124375,0.43065924021076
9,1.10656838806133,0.268480581076395,0.70260821041259
"5973","ZNF451",232.119958656935,0.255019200563601,0.230480819493095,1
.1064660440052,0.268524853354699,0.70260821041259
"5974","ZNF292",525.841145053743,0.223497692903423,0.201968727489971,1
.10659553922535,0.268468836791324,0.70260821041259
"5975","DPH7",84.9304180378589,-0.226032965369632,0.204238313092303,-1
.106711869812,0.268418521780455,0.70260821041259
"5976","ATP5MD",1104.03066078804,-0.315945219055046,0.285584965474896,
-1.10630900520153,0.268592795388819,0.70260821041259
"5977","TMEM132A",14.2829793447508,-0.632090461598696,0.57104186859516
3,-1.10690738518652,0.268333972557046,0.70260821041259
"5978","SCPEP1",1389.42214850535,0.242215309074933,0.218922153795123,1
.10639926054084,0.268553745435901,0.70260821041259
"5979","PRDX2",1189.18798761755,-0.304158141264367,0.27473437875262,-1
.10709894642724,0.268251151015139,0.70260821041259
"5980","LSM14B",71.5169368056673,0.213877746137202,0.1932541216704,1.1
0671764352833,0.268416024716005,0.70260821041259
"5981","AC026904.2",6.76911824526029,0.64330808242621,0.58165833453201
2,1.10598962351979,0.268731010656088,0.70273473749468
"5982","ZNF343",29.5807197481363,0.367918946400484,0.332635698892186,1
.10607174042295,0.268695469171545,0.70273473749468
"5983","YAF2",138.735774574619,-0.230618976186768,0.208555754934365,-1
.10579051754936,0.268817200247526,0.70284263136755
"5984","CAP1",617.940616963268,-0.208669898696594,0.188734526085874,-1
.10562652750483,0.268888203004089,0.702910788702032
"5985","AC084198.2",15.279555916355,0.536381626003278,0.48531992507134
3,1.10521245531889,0.269067541102774,0.703003503824225
"5986","ADRB2",3.78872368511726,-0.952511274066203,0.861746479086736,-
1.10532656318556,0.269018111845403,0.703003503824225
"5987","NTMT1",170.973354487193,-0.272060804385155,0.246203104838732,-
1.10502588731917,0.269148372077177,0.703003503824225
"5988","FLYWCH2",267.73050464739,-0.326285688633327,0.295257309888664,

-1.10508928214635,0.269120904266933,0.703003503824225
"5989","CIAO2B",653.998330823896,-0.293233736765727,0.26531806362545,-1.10521587847741,0.269066058167912,0.703003503824225
"5990","TAF5L",47.6942086752591,-0.30113568859315,0.27262205990813,-1.10458972884732,0.269337403667861,0.703145027632901
"5991","GAS6-DT",22.943398042907,0.447318575082847,0.404951630250523,1.10462223551517,0.269323312098619,0.703145027632901
"5992","AC009120.2",60.3815146284521,0.407500462554686,0.36885505195439,1.10477126555684,0.269258714379669,0.703145027632901
"5993","TCF12",255.433776117311,0.207825032770528,0.188183226822437,1.10437596527465,0.269430082312162,0.703269610814142
"5994","LINC00853",6.7877434232379,0.710988974416983,0.644006497892653,1.10400900727479,0.269589230461448,0.703567622974378
"5995","CHD6",294.335876778431,0.21353330149453,0.193461911201702,1.10374853720897,0.269702234371483,0.703745129653563
"5996","MED12L",6.44885065869967,0.748415130355978,0.678196580130546,1.10353716353438,0.269793961888792,0.703749699153972
"5997","HNRNPL",256.725026574277,-0.259801912578104,0.235417389073663,-1.1035799589843,0.269775388688728,0.703749699153972
"5998","ECRG4",21.1643011481527,-0.737921208270938,0.668814239051692,-1.10332759858288,0.269884925619217,0.703752273955894
"5999","KAT5",149.850100097993,-0.259049490478596,0.234770190419606,-1.10341730360058,0.269845985698235,0.703752273955894
"6000","NMRK1",87.8477818899171,-0.435615241330032,0.394915052057641,-1.10306061787296,0.270000841694009,0.703937194436565
"6001","MRGBP",67.5066108841101,-0.271103029202905,0.24583735940391,-1.1027739228092,0.270125355244689,0.704144464604678
"6002","CREG1",769.165965815875,0.203126884397217,0.184250415987131,1.10245007214206,0.2702660530753,0.704376661120593
"6003","SQOR",227.573614816139,0.236243449311712,0.21430667006904,1.1023616261482,0.270304487419735,0.704376661120593
"6004","CAPZA1",366.265588943528,0.214594003001881,0.194763318935491,1.10181939892367,0.270540195018394,0.704492858682143
"6005","LINC00877",2.62951731342834,-0.977954300003401,0.887727847965937,-1.10163751452002,0.270619292195934,0.704492858682143
"6006","AC104596.1",6.48457218308066,-0.728738118291163,0.661499671338875,-1.1016454729542,0.270615830931636,0.704492858682143
"6007","ZNF391",22.0007154821096,0.368257506443428,0.334184819801094,1.10195761334286,0.270480099471182,0.704492858682143
"6008","NT5C",501.207358340285,-0.303285378457723,0.275218559541604,-1.10198010978208,0.270470318898869,0.704492858682143
"6009","KMT5C",34.8315687024813,0.301799347244775,0.273924862079507,1.101759602811,0.270566197168958,0.704492858682143
"6010","VANGL2",25.9593165360642,0.342654396078639,0.311107657265556,1.10140135762122,0.270722014834172,0.704643007995168
"6011","SLC66A2",176.570097609354,-0.235772568720534,0.214109833284161,-1.10117580824802,0.270820148399397,0.704714834331461
"6012","MAMLD1",5.5815948061908,0.722876225747596,0.656485305713008,1.10113085465406,0.270839710030093,0.704714834331461

"6013","AL359715.2",3.11744630861417,1.08445724134613,0.985264066750244,1.10067674032106,0.271037372932102,0.704742501229608
"6014","ZHX2",133.326731028874,0.247341211540127,0.224688457308567,1.10081850444347,0.270975656479504,0.704742501229608
"6015","CH25H",36.1702995858387,-1.14200240738623,1.03761762837285,-1.10060042944439,0.271070598559229,0.704742501229608
"6016","AMN",5.51194440580235,-0.760780294096599,0.691140056397616,-1.10076139713557,0.271000516762812,0.704742501229608
"6017","DENND4A",88.3390975021638,0.293159922676223,0.266379060326908,1.10053666499329,0.271098363633955,0.704742501229608
"6018","LINC02076",4.23283012175133,0.915000631028224,0.83147729560572,1.10045173315485,0.271135348690001,0.704742501229608
"6019","AP3D1",191.681818458099,0.216900783590412,0.1971140727083,1.10038203061936,0.271165704462124,0.704742501229608
"6020","AC009950.1",27.0535808431002,0.46791197192671,0.425321851458432,1.10013621525025,0.271272776761476,0.704743862146157
"6021","SLC4A7",173.080380862084,0.379997144453674,0.345494740555422,1.09986376013361,0.271391486643767,0.704743862146157
"6022","KCNMB2",9.13980152724301,0.896093586875761,0.814488887334979,1.10019129887431,0.271248780909787,0.704743862146157
"6023","AL662884.5",23.608977042152,-0.521783032309674,0.474323962833932,-1.10005623412359,0.271307621198066,0.704743862146157
"6024","SPSB3",325.077486799094,-0.237078190415067,0.215541581238506,-1.09991858207967,0.271367597623687,0.704743862146157
"6025","PARL",217.816550632363,-0.297782082601572,0.270860425199915,-1.09939310027217,0.271596639015056,0.705159539271789
"6026","ZNF136",89.2938712533599,0.248894281090822,0.226500867114612,1.09886679137912,0.271826173578262,0.705638372599528
"6027","TMEM54",268.051689767951,-0.289654226213928,0.263654997958182,-1.0986107923502,0.271937868230941,0.705811195078248
"6028","USP38",55.270442523901,0.245813780733967,0.223838606480459,1.09817419165995,0.272128433478125,0.706188633858378
"6029","HCG25",21.4867072311409,0.430031573181704,0.391851394819534,1.09743535142896,0.272451126893851,0.706557191313081
"6030","PHF2",100.013587365462,0.2288669742161,0.208503212221346,1.09766641855443,0.272350178651855,0.706557191313081
"6031","TRUB1",71.5035760642891,0.236554614367342,0.215552090356414,1.09743595608932,0.272450862697378,0.706557191313081
"6032","GZMB",22.1148708814186,0.495441364005392,0.451382802536974,1.09760797536102,0.272375708803445,0.706557191313081
"6033","LRRC40",91.0245758110768,0.212004394823308,0.193239128839708,1.09710903840374,0.272593729621267,0.706642753009239
"6034","TMEM150C",155.306752143577,-0.369136911923006,0.33648146539864,-1.0970497631591,0.272619639098047,0.706642753009239
"6035","ATP5F1B",821.44336034804,-0.252773325424984,0.230384144198659,-1.09718195366352,0.272561860343393,0.706642753009239
"6036","SEMA3B-AS1",3.42457682611846,-1.03077858311187,0.939859503255321,-1.09673688412113,0.272756427855872,0.706763094409377
"6037","MYO1G",5.60739576054892,0.99262135411036,0.904999169165422,1.0

9682018274751,0.272720005632129,0.706763094409377
"6038","TCAIM",32.5940145518867,-0.320841178622093,0.292649909637197,-1.09633103601448,0.272933932052921,0.70687575955638
"6039","HYAL3",3.74332779983965,-0.889122026325751,0.811395820374186,-1.09579320474651,0.273169282918715,0.70687575955638
"6040","WRNIP1",93.3333400520822,-0.201031317800278,0.18348890895809,-1.09560473677565,0.273251787894741,0.70687575955638
"6041","DTNBP1",146.956552194729,-0.230058130299973,0.209937904817267,-1.09583893628079,0.273149265750716,0.70687575955638
"6042","GARS",181.136430376995,-0.190105715420224,0.173495044070081,-1.09574147457165,0.273191926963189,0.70687575955638
"6043","CYHR1",318.390011339369,-0.235791208837752,0.215095740200739,-1.0962151487412,0.272984631714596,0.70687575955638
"6044","GAS1",2540.71523076715,-0.264386331311425,0.241250784717527,-1.09589832680124,0.273123271399752,0.70687575955638
"6045","NPIPB5",72.8189033150824,0.469457506343767,0.428375524247447,1.09590179590323,0.2731217530775,0.70687575955638
"6046","PRR12",109.882397171119,0.263315942908259,0.240137326692149,1.0965223380113,0.272850253205866,0.70687575955638
"6047","SMTN",71.0032019000581,0.300834135403166,0.274580507388619,1.09561358985104,0.273247911932977,0.70687575955638
"6048","TIGD7",13.8987096881267,0.503947484521468,0.460036760419972,1.09545046804827,0.273319334266321,0.706933588943131
"6049","NVL",58.0862557792272,0.273429679679829,0.249677986817604,1.0951293030073,0.273459992604853,0.706946730179759
"6050","BTRC",58.2679302707202,0.309549042329842,0.282659061874743,1.09513220724908,0.273458720433315,0.706946730179759
"6051","TSPOAP1",3.44026081140205,0.968161236603164,0.883965473640579,1.09524779583961,0.273408091392983,0.706946730179759
"6052","USP44",38.9163740834979,0.50413203934873,0.460493616794635,1.09476444615639,0.273619846366368,0.70724310256264
"6053","PLIN2",1149.5701686118,-0.301590833450128,0.27554746000301,-1.09451501910718,0.273729163928471,0.707408774381806
"6054","TTC9",2.82600338094178,1.11205727988131,1.0166880821646,1.09380379232307,0.27404104052726 5,0.708097786086556
"6055","TSLP",12.5058308889742,0.826321486735897,0.755582991940722,1.093621078756,0.274121200550017,0.708108699909409
"6056","ALDH5A1",23.698957583077,0.420493164495603,0.384539030109115,1.09349931104857,0.274174631324962,0.708108699909409
"6057","ACACA",47.1145214238302,0.288015896938319,0.263392720500194,1.09348465056804,0.27418106471593,0.708108699909409
"6058","DYNLRB1",1097.14459651008,-0.308105682130301,0.281890983787383,-1.09299587376193,0.274395611424434,0.70854581537016
"6059","ZDHHC4",186.355462583741,-0.243361702103806,0.222771375552359,-1.09242806217986,0.27464499410077,0.708721815031071
"6060","KCTD7",69.6057696172179,0.353895843311203,0.323925627810567,1.0925219029541,0.274603768587922,0.708721815031071
"6061","GCN1",87.992159955683,0.255671886399018,0.233991583932524,1.0926541976516,0.274545656928946,0.708721815031071
"6062","SYT17",4.80578228751679,0.845705902772568,0.774113409580146,1.

09248321022013,0.274620766313729,0.708721815031071
"6063","KDELR3",334.051902334194,-0.297321793529635,0.272222233393729,
-1.09220246202154,0.274744120575096,0.708860675928787
"6064","SEC16B",18.437882647412,0.500312354614989,0.458309894487223,1.
09164641792157,0.274988545148025,0.709023539105085
"6065","IRS1",137.737586490733,0.274055056980774,0.250983688856262,1.0
9192377492597,0.274866606650313,0.709023539105085
"6066","CASC1",4.24886328850173,0.927444143542017,0.849558910763282,1.
0916772595661,0.274974983962129,0.709023539105085
"6067","POLE",39.9070498834482,0.281083049817857,0.257465584293016,1.0
9173057280527,0.274951543009218,0.709023539105085
"6068","DIAPH1",167.430832952443,-0.367669420935791,0.336883031916194,
-1.09138598891278,0.275103074958402,0.709085088412305
"6069","RAD51-
AS1",95.20501269296,0.364363905622225,0.333852280554967,1.091392591407
6,0.275100170953056,0.709085088412305
"6070","TNPO1",329.082128093064,0.168089285213706,0.154049851520515,1.
09113565222308,0.27521319712216,0.709135240089267
"6071","AC004825.2",9.41938064698578,-0.597864737749146,0.547926017659
945,-1.09114135572988,0.275210687836179,0.709135240089267
"6072","TRIM33",168.826893730304,0.236509804192597,0.216860394431057,1
.09060856784426,0.275445157904716,0.709334420975888
"6073","PARP1",233.517500080205,0.183893704205892,0.168630736821837,1.
09051118243159,0.275488030150238,0.709334420975888
"6074","LIPC",22.3312983749311,-0.470312202746256,0.431303052990262,-1
.0904448727769,0.275517224435818,0.709334420975888
"6075","MRPS23",184.829207505328,-0.299105280950312,0.274231751874516,
-1.09070258606369,0.275403772327617,0.709334420975888
"6076","MIS18A",83.0208227350596,-0.291948742193739,0.267657556187257,
-1.09075471790338,0.275380826409986,0.709334420975888
"6077","PAPLN",97.435113190894,0.472675140492012,0.433628347324969,1.0
9004668031488,0.275692581836917,0.709441235620585
"6078","ZSCAN29",44.4412528381761,0.308356202745041,0.282884772685217,
1.09004171492881,0.275694768991724,0.709441235620585
"6079","RDH13",58.2765313865639,0.265365948298399,0.243400603271628,1.
09024359320202,0.27560585513025,0.709441235620585
"6080","PCGF1",69.4000007706008,-0.307935495181061,0.282638097250914,-
1.08950455786464,0.275931446015166,0.709828810242919
"6081","PPP2R5D",108.012345137811,0.212176698892395,0.194747943228139,
1.08949391390408,0.275936137255462,0.709828810242919
"6082","GDAP2",60.1929906463676,0.34524423440488,0.31726640255067,1.08
818403596877,0.276513870782601,0.709885222625104
"6083","GPN1",92.7301390206775,-0.21991079329505,0.201996118254442,-1.
08868821438461,0.276291400783919,0.709885222625104
"6084","AC009309.1",3.54628332604842,-1.24628283005389,1.1444532270284
1,-1.08897664021612,0.276164187053278,0.709885222625104
"6085","ARMC9",31.0636823710505,0.344615473728209,0.316800888944173,1.
08779831671791,0.276684152806064,0.709885222625104
"6086","MIER3",45.3988793934895,0.292618695488963,0.26890471261996,1.0
8818730857468,0.276512426343324,0.709885222625104

"6087","CXCL14",35335.3359075682,-0.461612108047236,0.424263208064881,
-1.08803238007064,0.276580813215378,0.709885222625104
"6088","C5orf58",21.1495887909011,0.413450080584473,0.379907018247421,
1.08829282094285,0.276465858809093,0.709885222625104
"6089","ZSCAN26",70.0683809134562,0.249737717331498,0.229490431418116,
1.08822714650134,0.276494843378035,0.709885222625104
"6090","AIG1",309.020272810668,-0.226422523916795,0.208127190363546,-1
.08790458143067,0.276637233392216,0.709885222625104
"6091","AC083862.3",25.998573767455,-0.339103634151619,0.3116491923483
44,-1.08809405728409,0.276553586942705,0.709885222625104
"6092","GTF3C4",85.3281224426319,0.244425967041603,0.224430849576053,1
.08909255346723,0.276113073354099,0.709885222625104
"6093","TUBA1A",2283.27680385167,-0.350712335714856,0.322212270510253,
-1.08845121000349,0.276395964359516,0.709885222625104
"6094","TTC5",115.111893712292,-0.220613769634859,0.202653570688152,-1
.08862512950411,0.276319230464607,0.709885222625104
"6095","RBM39",1478.28274837338,0.160823264463289,0.147841511705015,1.
08780857695893,0.276679622330983,0.709885222625104
"6096","MICAL3",48.3078166890604,-0.442227238924024,0.405959107692218,
-1.08933937075087,0.276004257040723,0.709885222625104
"6097","SCML1",124.129509700045,0.279819514593406,0.257229272484955,1.
08782142829321,0.276673947813423,0.709885222625104
"6098","NBPF1",103.267526241924,0.30902102257312,0.284313212193579,1.0
869034899536,0.277079463807317,0.710162428100707
"6099","NENF",1548.199381875,-0.257921090510533,0.237460575683167,-1.0
8616383906466,0.277406512560665,0.710162428100707
"6100","ARL5A",498.546456127099,-0.162225879425369,0.149363338280311,-
1.08611578512606,0.277427769489447,0.710162428100707
"6101","HOMER1",13.5885091293793,-0.496612856688186,0.457227170124428,
-1.08614030210199,0.277416924129253,0.710162428100707
"6102","AC011389.1",6.00857819220218,1.16237254651812,1.06959697667702
,1.08673881084568,0.277152256627094,0.710162428100707
"6103","NUS1",109.69809163084,0.21931051219894,0.201695994095335,1.087
33201758721,0.276890103541571,0.710162428100707
"6104","ZNF34",53.3230266522355,-0.304520462988017,0.280335539586162,-
1.08627134268298,0.277358961750556,0.710162428100707
"6105","SELENOH",790.464096016132,-0.286535190210651,0.26374587870819,
-1.08640632268486,0.27729926549777,0.710162428100707
"6106","OIP5",3.35491655098507,-0.812338907543098,0.747304537411469,-1
.0870252579449,0.277025647292018,0.710162428100707
"6107","AARS",123.599964471252,0.211866214793981,0.195053279823084,1.0
8619662784521,0.2773920088968,0.710162428100707
"6108","SGCA",38.1076557342752,1.54708038770119,1.42392094276403,1.086
49317615773,0.277260858311011,0.710162428100707
"6109","TANC2",71.9452115220191,0.333479948364706,0.306805868691006,1.
08694123025581,0.277062783362128,0.710162428100707
"6110","ZNF90",23.1667569685041,0.364587247480016,0.335360179049747,1.
08715127870305,0.276969958726636,0.710162428100707
"6111","ZNF549",14.0662471825212,0.555132323637684,0.511107227968807,1
.08613671116301,0.277418512603468,0.710162428100707

"6112","ALG6",66.1072903469618,0.298683071244882,0.275065329189653,1.0
8586230087524,0.277539918029294,0.710217068171477
"6113","SUFU",43.9423398768217,0.344436001401583,0.317171722366795,1.0
8596062357431,0.277496413645394,0.710217068171477
"6114","LMX1A",7.31815863778873,0.722740395100302,0.665699799803463,1.
0856851621612,0.277618307488573,0.710221601663409
"6115","PTPA",100.51936505234,-0.213446824257759,0.196606837287642,-1.
08565310953799,0.277632493394601,0.710221601663409
"6116","CEP41",45.4401987650391,0.28746195085306,0.264828465761521,1.0
8546469891919,0.277715890475203,0.710293159577276
"6117","CHD3",271.691313743856,0.243628631079177,0.224462918461443,1.0
8538476087321,0.2777512789832,0.710293159577276
"6118","ZKSCAN3",20.6415566895048,0.363369898884127,0.335160750609673,
1.08416602547625,0.278291192445509,0.71077476403963
"6119","PSMG3",170.548276180509,-0.304758545173731,0.281098389823927,-
1.08417036954436,0.278289266706938,0.71077476403963
"6120","BUD23",222.454502622853,-0.287541721977544,0.265301365751774,-
1.08383053801004,0.278439942454958,0.71077476403963
"6121","LINC00484",2.8872774273755,1.0795910836345,0.995350012314971,1
.08463462126614,0.278083514786804,0.71077476403963
"6122","PDCD4-
AS1",5.50996400880613,0.836180806610191,0.771019507959155,1.0845131646
844,0.278137333176775,0.71077476403963
"6123","AC007406.5",15.8142062040053,0.463961853157534,0.4279534198910
33,1.08414101066343,0.278302281764417,0.71077476403963
"6124","AL355073.2",3.29795366370075,-0.987485584434383,0.910894922729
295,-1.08408287256185,0.278328056133154,0.71077476403963
"6125","LTBP4",2503.5135800581,0.385875407801237,0.355743096525554,1.0
8470244839598,0.278053463124053,0.71077476403963
"6126","CIC",90.8403292071837,-0.27959007980798,0.257936324920633,-1.0
8395000159055,0.278386967929395,0.71077476403963
"6127","SHLD1",22.3183712344607,-0.361231796521032,0.333354421947234,-
1.08362683299942,0.278530288535634,0.71077476403963
"6128","KIF16B",87.3913539784102,0.250097578673264,0.230757440938377,1
.08381154538827,0.278448365110792,0.71077476403963
"6129","ZNF341",9.07193428527433,0.551389221394457,0.508713618567828,1
.08388924783805,0.278413907510474,0.71077476403963
"6130","GPR82",10.7648784322084,-0.508081562265806,0.468851406081113,-
1.08367289865375,0.278509856015035,0.71077476403963
"6131","CTPS1",18.6497486954499,-0.453223994165504,0.418453418941088,-
1.08309306042332,0.278767118900106,0.710846634079802
"6132","AC012485.3",6.69302300626413,0.887081195481342,0.8195333876025
23,1.08242227699401,0.279064934020547,0.710846634079802
"6133","AC083855.2",4.51072056290239,-0.902160593211067,0.834002859028
054,-1.08172362174207,0.279375353684209,0.710846634079802
"6134","ACTRT3",11.0700770920359,-0.469911900406318,0.434466038766181,
-1.081584884611,0.279437023936813,0.710846634079802
"6135","SH3BP2",67.1572136977781,0.271114994541264,0.250582593806382,1
.0819386551276,0.279279787155631,0.710846634079802
"6136","MEA1",216.054925286095,-0.253265339588024,0.23380379491546,-1.

08323878865867,0.278702446927159,0.710846634079802
"6137","COQ3",37.920392335038,-0.39270747351951,0.362704434559095,-1.08272035327301,0.27893256710293,0.710846634079802
"6138","ARHGAP18",247.874212644517,-0.291020921912096,0.269037842497773,-1.08170998997847,0.27938141275014,0.710846634079802
"6139","AC027702.1",4.10692455363439,-0.906292660862071,0.837982965658651,-1.08151680643022,0.279467288857047,0.710846634079802
"6140","TOMM5",722.241128778103,-0.28093947117554,0.259586547492478,-1.08225743548472,0.279138153633963,0.710846634079802
"6141","LZTS2",230.742703655873,-0.176842006532153,0.163492926283262,-1.08164928325867,0.279408396845358,0.710846634079802
"6142","MED19",158.835072428133,-0.208664774687397,0.192882772695221,-1.08182172918632,0.279331749381356,0.710846634079802
"6143","NCAPD2",41.4277745643851,0.302143145970625,0.278937400687985,1.08319338039791,0.278722597327335,0.710846634079802
"6144","CLYBL",30.4218995562464,-0.309554443381784,0.286119119331272,-1.08190757788324,0.279293597331346,0.710846634079802
"6145","EXOC3L1",5.76957676671484,-0.800227629538725,0.739666622103832,-1.08187608528642,0.279307592556017,0.710846634079802
"6146","GTF2F1",191.372406930228,-0.22713419889441,0.209689570447539,-1.08319263761969,0.278722926951334,0.710846634079802
"6147","NFKBIB",49.9506546784894,-0.317536764259027,0.293574996152235,-1.08162060264276,0.279421145975727,0.710846634079802
"6148","ARVCF",20.9603619389322,0.351664815063732,0.325095498029386,1.08172772983754,0.279373527729532,0.710846634079802
"6149","RPS4X",20445.7087685644,-0.320593248027794,0.296360074257252,-1.08176936057016,0.279355024276791,0.710846634079802
"6150","RPL10",39213.4831539467,-0.327796155672247,0.302795816474071,-1.08256500862295,0.279001545768176,0.710846634079802
"6151","GNL3",277.73172994772,-0.255485274671119,0.23627028207073,-1.08132632014481,0.279551983516589,0.710946460437327
"6152","ARMC1",177.307780286721,-0.168150492742558,0.155584881497876,-1.08076370354052,0.279802237891417,0.711467231361417
"6153","RPL22",7534.26986782306,-0.294873257256648,0.272963993930716,-1.08026429790404,0.280024503268563,0.711916675545284
"6154","TMEM237",194.995524759848,-0.245971882545533,0.227740072751205,-1.08005534368229,0.280117535985071,0.712029430766454
"6155","NUDCD2",330.009270883019,-0.260838014973632,0.241548433652308,-1.07985802693753,0.280205406622661,0.712029430766454
"6156","ACOX1",138.380107572885,0.189747376066456,0.175698993554878,1.07995710292552,0.280161282987473,0.712029430766454
"6157","AC067930.8",6.48680387485745,0.723104327197789,0.669718687673858,1.07971352824177,0.280269767790436,0.712033803522858
"6158","ABHD2",201.903842499456,-0.294751893163296,0.273006948434675,-1.07964978493514,0.280298162890351,0.712033803522858
"6159","LETM2",8.69411104157787,0.531964891369081,0.492769648347715,1.0795407005135,0.280346760181726,0.712041625186352
"6160","CHERP",90.4371698443233,0.236320874598843,0.218933989485014,1.07941610690385,0.280402273843774,0.712067008074376
"6161","TTC30A",13.4325140087249,-0.446833958216162,0.414046492288779,

-1.07918788478593,0.280503979366406,0.712094084587582
"6162","TOLLIP-
AS1",18.5732420100268,-0.452832086206852,0.419601647265188,-1.07919520
611572,0.280500716280225,0.712094084587582
"6163","AL139220.2",3.41567297371433,-0.9038510501197,0.83828059388882
9,-1.07822017676287,0.280935509729071,0.712327213361325
"6164","AC119674.1",17.6662610483231,0.514102881436458,0.4766143955635
82,1.07865579852775,0.280741197021287,0.712327213361325
"6165","CWC22",226.529890461128,0.174427745880337,0.161755671602167,1.
07834083437481,0.280881680258129,0.712327213361325
"6166","DCP2",169.368963461493,0.207735609766117,0.192693385301653,1.0
7806300377626,0.28100564045597,0.712327213361325
"6167","GLIS2-
AS1",3.39708791121879,0.912222300442335,0.846121694052704,1.0781218669
3031,0.280979374272173,0.712327213361325
"6168","AFMID",90.4066973564271,-0.274522749711965,0.25458075181755,-1
.07833270092904,0.280885308645222,0.712327213361325
"6169","METRNL",1010.72319899662,0.324291109024868,0.300632239998384,1
.07869704535552,0.280722803261909,0.712327213361325
"6170","ACTN4",388.955996387118,-0.200978880317767,0.186418727818619,-
1.07810455885803,0.280987097385751,0.712327213361325
"6171","TM9SF4",159.907781950607,0.21115051004934,0.19574307547033,1.0
7871253959809,0.280715893914854,0.712327213361325
"6172","SEC61A1",325.544187935945,0.194155093481605,0.180146444352485,
1.07776256245008,0.281139730714579,0.712338838956333
"6173","RANBP10",46.9280585879997,0.348758312339096,0.323579681090799,
1.07781276983591,0.281117319585603,0.712338838956333
"6174","BCL3",91.6581693967943,-0.277492282433898,0.257474504811679,-1
.07774663995124,0.281146838312114,0.712338838956333
"6175","AL133453.1",46.3910347228117,-0.488784719485968,0.453570347564
145,-1.0776381703763,0.281195260973071,0.712346148567086
"6176","AC026150.3",2.79038873268183,1.14655660405392,1.06424268389072
,1.07734506556557,0.281326136253956,0.712562297509818
"6177","UBA2",348.808446551258,-0.181999663247238,0.168996373223195,-1
.07694419576016,0.28150519699264,0.71290040416964
"6178","HINT1",1620.26210366813,-0.283384689108164,0.263260100907848,-
1.07644374567554,0.281728846820119,0.713351303141326
"6179","TP53BP2",101.785967470156,0.270994808878278,0.2522473272661,1.
07432182459718,0.282678466659851,0.713364446614215
"6180","CCNYL1",34.0989786602919,-0.339837143032259,0.316084177512481,
-1.07514759424755,0.282308653773584,0.713364446614215
"6181","HLTF",222.134516405259,0.164593464737218,0.153200302473691,1.0
7436775306291,0.282657889424636,0.713364446614215
"6182","AC078881.1",3.35606917394254,0.888112863072065,0.8258116102797
25,1.07544245202757,0.282176684107603,0.713364446614215
"6183","FAM174A",134.628721015761,-0.275911970539471,0.256384874412072
,-1.07616321427767,0.281854268275792,0.713364446614215
"6184","DDO",4.56874413409472,0.792576050885225,0.737080999341177,1.07
529030268539,0.28224477645117,0.713364446614215
"6185","TRAPPC3L",4.66692762204878,0.827139128364235,0.769361007589891

,1.0750988420317,0.282330477861935,0.713364446614215
"6186","AC002429.2",2.86541904382676,1.02138179438042,0.95047728100695
,1.07459885132483,0.282554366357299,0.713364446614215
"6187","RBIS",967.259544217003,-0.266058618547782,0.247668188212263,-1
.07425430964011,0.282708717089309,0.713364446614215
"6188","ZNF484",29.7690481780163,0.372998284250826,0.346635675116638,1
.07605278690751,0.281903649113788,0.713364446614215
"6189","MRRF",64.0831089466907,-0.234954916353426,0.218556479624758,-1
.0750306591542,0.282361002067949,0.713364446614215
"6190","ASB13",58.8162108430421,-0.238416460923728,0.22186667232825,-1
.07459339621316,0.282556809739731,0.713364446614215
"6191","ATP5F1C",952.498684148808,-0.302163810120278,0.281137631687222
,-1.07478962637221,0.282468925896741,0.713364446614215
"6192","PATL1",69.9561898339865,0.269860229716913,0.25110259211261,1.0
7470109108189,0.282508575109279,0.713364446614215
"6193","AC138123.1",2.63189466974862,1.3231336322782,1.2311905448862,1
.07467819483661,0.282518829466077,0.713364446614215
"6194","AC007216.3",4.07479239013037,-0.763774669531996,0.709728700694
541,-1.07615018074451,0.281860096297065,0.713364446614215
"6195","POLR2A",277.629429889693,0.263163108453512,0.244651032368014,1
.0756672714859,0.282076089647253,0.713364446614215
"6196","IFT27",138.692285724506,-0.287066211211599,0.26709420911673,-1
.07477512208488,0.282475421167618,0.713364446614215
"6197","PPP2R3B",46.8426152000684,-0.252301765699051,0.234876179131879
,-1.07419052298781,0.282737299048017,0.713364446614215
"6198","MSL3",95.780126496938,-0.24234741855691,0.22552405363112,-1.07
459676542223,0.282555300645882,0.713364446614215
"6199","UBE2A",308.918729531623,-0.253063267095976,0.235204004392008,-
1.0759309466271,0.281958140378532,0.713364446614215
"6200","RAP2C",119.031046192223,0.235645912854777,0.219367643741972,1.
07420542444241,0.282730621727168,0.713364446614215
"6201","AC016747.1",71.0029812253872,0.293529250687205,0.2734070391676
59,1.07359800091763,0.283002893977931,0.713492604477391
"6202","AL137186.1",4.07072975754377,-0.809867346633959,0.754360863329
07,-1.07358080993218,0.283010602270345,0.713492604477391
"6203","FAM234A",189.690635372498,0.244257327415498,0.227469408934433,
1.07380297227529,0.282910997515877,0.713492604477391
"6204","SRRM2-
AS1",4.9384109592092,0.778988411547451,0.72551271945984,1.073707449439
93,0.282953821522753,0.713492604477391
"6205","TMEM265",75.3833320659476,-0.475339249524118,0.442765669495117
,-1.0735684409908,0.283016148487004,0.713492604477391
"6206","RNF13",597.879408137659,-0.165509772043387,0.154205872599001,-
1.07330394915491,0.283134763895732,0.713653846986182
"6207","SERPINB9",44.5671547440651,-0.327237474238678,0.30497547478991
2,-1.07299603177633,0.2832728965785,0.713653846986182
"6208","Z94721.3",7.2250158808381,0.688039157809697,0.641257939331175,
1.07295226399429,0.283292534645239,0.713653846986182
"6209","RALGAPA1",217.379685661376,0.246124049175357,0.229365425878895
,1.07306516765657,0.283241878036324,0.713653846986182

"6210","NME4",353.3170559224,-0.321309730078286,0.299472963895014,-1.0
729173208134,0.283308213883794,0.713653846986182
"6211","POGZ",252.853024633946,0.230466655580317,0.214916443378737,1.0
72354687976,0.283560751929128,0.713871805330435
"6212","AC093495.1",14.8337566797093,-0.54476425942847,0.5081228188486
31,-1.07211138571353,0.283670005481989,0.713871805330435
"6213","C6orf89",375.639301658898,0.137979489532046,0.128650589830414,
1.07251346234735,0.283489470532891,0.713871805330435
"6214","TAF6L",29.295769255955,-0.439862590374125,0.410198364354801,-1
.07231678279845,0.283577771167894,0.713871805330435
"6215","NUSAP1",7.02271493642147,0.906191487967774,0.845317617214146,1
.07201301559791,0.283714186136887,0.713871805330435
"6216","DLGAP1-
AS1",94.5759894895323,0.325198180041405,0.303262437591366,1.0723325401
73,0.283570696100007,0.713871805330435
"6217","ZNF468",27.7137478040159,0.324355744884731,0.302548920355827,1
.07207701981966,0.283685439596275,0.713871805330435
"6218","MIR29B2CHG",31.0463150191151,0.47302064162533,0.44142977524281
7,1.07156487431129,0.283915517360797,0.713919054504895
"6219","FAT4",185.36032992351,0.256325769253124,0.239206679839327,1.07
156610101898,0.283914966120108,0.713919054504895
"6220","SUGT1",421.699690563828,-0.188588919514812,0.175955997683967,-
1.07179591487149,0.283811708379296,0.713919054504895
"6221","STX8",354.479375984861,-0.311071571864835,0.290258342961059,-1
.07170587653554,0.283852160504888,0.713919054504895
"6222","SLC9B2",63.449556479152,-0.303160721511017,0.282996201097319,-
1.07125367879678,0.284055381442727,0.713926467208962
"6223","SLC10A7",59.0139651528659,0.266141724204115,0.248398147779151,
1.07143199972948,0.283975230965959,0.713926467208962
"6224","AC132217.1",49.2571964143956,1.11644228740046,1.04209172960833
,1.07134742142141,0.284013244738124,0.713926467208962
"6225","MED4",429.971103070264,-0.191513196448619,0.178797640395055,-1
.07111702383471,0.284116814546733,0.713966157422416
"6226","RTL3",3.14630554856811,1.45937347979937,1.36322387890411,1.070
53104217376,0.284380343986705,0.714513607610669
"6227","AC103740.1",7.31146302380244,0.718188719508995,0.6710787285328
81,1.07020039374382,0.284529117176287,0.714531817188313
"6228","TSPAN3",659.732927819208,0.210358237632888,0.196576480182119,1
.07010888300575,0.284570301162385,0.714531817188313
"6229","TRADD",223.228688880609,-0.278715583052184,0.260407196663321,-
1.07030676042542,0.284481252409659,0.714531817188313
"6230","C17orf82",4.91120292623634,0.771729582225714,0.721054066497812
,1.07027977246427,0.28449339641539,0.714531817188313
"6231","MRPL39",93.6801504173931,-0.296526346247232,0.277160581807984,
-1.06987200096392,0.284676927566893,0.714684830352899
"6232","KPNA3",136.289672237941,-0.208764503913092,0.195167751608833,-
1.06966700283308,0.284769224057136,0.714780744042363
"6233","FAM104B",122.777293004233,-0.336106150447048,0.314240016480723
,-1.06958418030654,0.284806519057473,0.714780744042363
"6234","AC026356.2",12.4668603595202,0.539478288542883,0.5044513036270

53,1.0694358100851,0.284873338458907,0.714833755776817
"6235","AL357079.1",3.95237971711389,-0.774833479685619,0.724633375412736,-1.06927655553305,0.284945071481161,0.714899078296679
"6236","ZMYM6",71.609223264315,0.301178955979292,0.281897715685423,1.0683980011934,0.285341018277233,0.714956702400105
"6237","NRDC",338.068485632025,0.185063707831707,0.173331727813598,1.06768512704567,0.285662569595704,0.714956702400105
"6238","CZIB",602.360279914331,-0.290791931671093,0.27232847678053,-1.06779847303829,0.285611427016952,0.714956702400105
"6239","AL445426.1",78.7439148296064,0.289407553556191,0.271148989690867,1.06733775363183,0.285819345689356,0.714956702400105
"6240","CPOX",62.7508192404879,-0.244638832550383,0.228849753520356,-1.06899320968079,0.285072729146753,0.714956702400105
"6241","PARM1",2.61739098356537,-1.06954419406737,1.00081452030209,-1.06867373761177,0.285216709376947,0.714956702400105
"6242","RNF150",25.1370436185841,0.770273540043649,0.721213815001167,1.06802382874821,0.285509763183345,0.714956702400105
"6243","SELENOP",7867.55752872058,0.246839735502436,0.231246980659569,1.06742900944433,0.285778154610724,0.714956702400105
"6244","COX6C",1764.70779100464,-0.291573822866267,0.273004181682044,-1.06801962178678,0.285511660829173,0.714956702400105
"6245","ZNF510",48.3642559197668,0.273476934441326,0.256096618156985,1.06786624676819,0.285580849948585,0.714956702400105
"6246","PBX3",176.154745976418,0.196322427409992,0.183943074090636,1.06729991537086,0.285836426312744,0.714956702400105
"6247","HSP90B1",2655.77875494954,0.243943858339598,0.228444159225753,1.06784896215503,0.285588647933911,0.714956702400105
"6248","PSMD9",454.513143070027,-0.267567150057834,0.25034114427323,-1.06881012641615,0.285155235418115,0.714956702400105
"6249","BRCA2",17.6264603317415,0.476483992809718,0.446348959366505,1.06751451484503,0.28573956279578,0.714956702400105
"6250","ICAM4",3.41823550748692,-1.00675453275074,0.943069009415176,-1.06753007754444,0.285732539140985,0.714956702400105
"6251","ATG4D",29.6991419031479,-0.28728309225163,0.268732781326542,-1.06902883538628,0.285056676338457,0.714956702400105
"6252","AC006946.2",9.13402693611603,0.574405753923233,0.537645261964091,1.06837313477822,0.285352230481947,0.714956702400105
"6253","CASTOR1",42.6097804572038,-0.321037661039927,0.300375419582157,-1.06878805691395,0.285165182105429,0.714956702400105
"6254","DNAL4",84.1759715157995,-0.358883038144673,0.335966730188806,-1.0682100514625,0.285425771726053,0.714956702400105
"6255","AL359504.2",30.6518518151966,0.390399029859923,0.365865577973654,1.06705591715446,0.285946586462149,0.715117898005978
"6256","YRDC",43.5836140674634,-0.311727483945739,0.292238794809247,-1.06668754964314,0.28611295111986,0.715128765644165
"6257","MARC2",106.276154017808,0.307194861329979,0.288002002242722,1.06664140852424,0.286133794295648,0.715128765644165
"6258","KIF3C",25.9668595375554,0.382743648111347,0.358790022696584,1.06676223947013,0.286079213904375,0.715128765644165
"6259","TBRG1",242.847470656343,-0.148794550458137,0.139488718027306,-

1.06671387164809,0.286101061226711,0.715128765644165
"6260","PRADC1",108.839085456236,-0.380082085550597,0.356495841783813,
-1.06616134328177,0.286350713448774,0.71532804383251
"6261","NAB2",68.0896332482438,0.212438367093743,0.199246338953105,1.0
6620963883177,0.286328885912151,0.71532804383251
"6262","RPS15A",12475.7947152908,-0.279047506536712,0.261687312932977,
-1.06633945455422,0.28627022029198,0.71532804383251
"6263","EYA3",70.7020691194938,0.238873882364842,0.224369404431301,1.0
6464552495607,0.287036370201927,0.715426345443224
"6264","NBPF11",58.8255110147588,0.345346519051924,0.324181884549323,1
.06528629609278,0.286746392274863,0.715426345443224
"6265","AL391001.1",4.9090457548995,0.796963182928298,0.74858843325946
8,1.06462128924194,0.287047341842756,0.715426345443224
"6266","IFIH1",58.9576903088783,-0.224546056407247,0.210985447976448,-
1.06427271909441,0.28720517277796,0.715426345443224
"6267","MAIP1",97.3768565306814,-0.274056643111831,0.257415313113912,-
1.06464778569935,0.28703534676547,0.715426345443224
"6268","GOLGB1",752.839614435133,0.261985417618132,0.246132350944418,1
.06440870780654,0.287143590757425,0.715426345443224
"6269","SOX4",1220.91431304025,0.355391751499908,0.333679883171359,1.0
6506795711565,0.286845178313772,0.715426345443224
"6270","SNX3",1676.7293651651,-0.256205955584451,0.240460778903076,-1.
06547918855292,0.28665913845978,0.715426345443224
"6271","ANK1",10.7090851099072,-0.578471140431361,0.542883234787895,-1
.06555351752089,0.286625520956834,0.715426345443224
"6272","TMEFF1",9.62185286512528,-0.570863148556541,0.536005763214621,
-1.06503173610087,0.286861568496289,0.715426345443224
"6273","HECTD2",70.4502288467878,0.2692225041081,0.252898662874843,1.0
6454696536429,0.287080990435457,0.715426345443224
"6274","AP001267.3",10.0741950640312,-0.47073288565402,0.4419847344231
58,-1.06504331256685,0.286856330021288,0.715426345443224
"6275","RHNO1",31.3641295341435,0.427827521970097,0.401639516276588,1.
06520276176081,0.286784184122291,0.715426345443224
"6276","GAS2L3",3.66794334122971,-0.879012982429558,0.825609126219205,
-1.06468418833366,0.287018867664689,0.715426345443224
"6277","AC092376.2",24.8236610771237,-0.369357797691294,0.346541039155
728,-1.06584143278139,0.28649532778353,0.715426345443224
"6278","AC087393.2",3.42316627983434,-0.935062863177647,0.877426386414
43,-1.06568810518538,0.286564656427861,0.715426345443224
"6279","ZNF600",33.2081577950222,-0.434004804604354,0.407801593860611,
-1.06425480218378,0.287213287053855,0.715426345443224
"6280","CCDC22",65.6873434187968,-0.331720978191527,0.311559968583477,
-1.06470988458406,0.287007235619101,0.715426345443224
"6281","YOD1",22.2936830039923,0.46434479437044,0.436376587404735,1.06
409190541601,0.287287067434963,0.715496194218296
"6282","TXNRD3",35.5848216889346,-0.286198586248334,0.268992156776274,
-1.06396628689204,0.287343972219007,0.715523998316128
"6283","TMEM158",7.84181849547887,-0.649596819638562,0.610780112845299
,-1.06355266973647,0.287531393202538,0.715860977049936
"6284","DYNLT1",578.213585673179,-0.278470424013087,0.261851821080217,

-1.06346567636732,0.287570822718264,0.715860977049936
"6285","NIPA1",25.4924539920269,0.386465033187023,0.363474451043666,1.06325226457415,0.287667566503845,0.71598786679708
"6286","ZNF548",36.1927275881071,0.302516889846185,0.284592023834517,1.06298442862225,0.287789012903652,0.716176189763256
"6287","TOR3A",141.081207609207,-0.323176756304488,0.304136372211531,-1.06260475836712,0.287961228231599,0.716338314700029
"6288","KCTD18",87.9756439230222,-0.205514956350699,0.193440924808319,-1.0624171516670‌8,0.288046350751839,0.716338314700029
"6289","FAM114A1",820.170652744824,-0.195149497794059,0.183744964265046,-1.06206718956696,0.288205183904471,0.716338314700029
"6290","CBR4",155.836036412751,0.244047073905049,0.229755192871415,1.06220482268548,0.288142710946966,0.716338314700029
"6291","CWF19L1",37.4152928348238,0.273033918983403,0.257081442963756,1.06205222685753,0.288211976168375,0.716338314700029
"6292","TMEM258",1387.06613955487,-0.276809154065004,0.260586750880438,-1.06225336909784,0.28812067745589,0.716338314700029
"6293","ZNF569",33.8143913423205,-0.291461923635465,0.274437614357889,-1.06203343997655,0.288220504552962,0.716338314700029
"6294","HIF3A",74.3442855990883,-0.504890045126985,0.475257391675298,-1.06235074713353,0.288076484451934,0.716338314700029
"6295","CD302",1106.95422631415,0.219084231199095,0.206321461630266,1.06185866205087,0.288299853891569,0.716389048569687
"6296","PPIC",1373.7641897123,0.381941457637974,0.359724452494328,1.06176117578217,0.288344119145327,0.716389048569687
"6297","ZNF300",17.7149903627269,-0.37799281501764,0.356065346011055,-1.06158271017451,0.288425166280305,0.716389048569687
"6298","EYA1",8.02565099510924,0.853455065201534,0.804020488461855,1.06148422515233,0.288469898161507,0.716389048569687
"6299","WDR74",263.5616494946,-0.2511910174129,0.236613199532555,-1.06161033242923,0.288412621096408,0.716389048569687
"6300","MFSD14C",137.204393837095,0.244184198736165,0.230111500360343,1.06115599765238,0.288619012804161,0.716398646633835
"6301","RPL7A",16104.1293041394,-0.302039963795238,0.28462051680579,-1.06120235879318,0.288597947661898,0.716398646633835
"6302","THAP11",130.747211976678,-0.24816681703013,0.233870982063907,-1.06112701473292,0.288632182318931,0.716398646633835
"6303","NACC1",29.8517529648235,0.327845788679129,0.308975857721829,1.06107250934242,0.288656950056451,0.716398646633835
"6304","ZSCAN12",13.7083658263859,0.42757590409308,0.403109519760458,1.06069413678734,0.288828925421692,0.716484440274585
"6305","BAALC-AS1",10.1589280986698,-0.699407986718493,0.659372780064959,-1.06071710550379,0.288818483866087,0.716484440274585
"6306","AC018529.1",2.85848584036119,-1.23512074166778,1.16439601577895,-1.06073940904162,0.288808344943467,0.716484440274585
"6307","YIPF1",65.2218634515206,-0.262458435680277,0.247516810221116,-1.06036610380448,0.288978076649169,0.71674077263722
"6308","FGD2",3.13527904630709,-1.00695103989452,0.949794869362774,-1.06017738395459,0.289063907988646,0.716839998837411

"6309","WARS2-
AS1",54.5807904700838,0.340335241521709,0.321113974515332,1.0598580832
0982,0.289209167704251,0.716965587419935
"6310","GTF2IRD2B",129.460455413756,0.231283576241366,0.21821892529473
2,1.05986946791617,0.289203987606422,0.716965587419935
"6311","KDM4B",119.477140558106,0.259037643242916,0.244429591710102,1.
05976384213798,0.289252050259363,0.716965587419935
"6312","FAM110B",124.751076410734,0.263911946210999,0.249089837210199,
1.05950507321698,0.28936982001822,0.717089099807144
"6313","TRIM28",289.458846779845,-0.179649788997862,0.169568450279341,
-1.0594529153384,0.289393561790098,0.717089099807144
"6314","EID2B",56.0920536657621,-0.258913589235147,0.244444696778463,-
1.05919086258515,0.289512865574133,0.717271104874274
"6315","RPS12",22685.261460633,-0.288554675209959,0.272494966956879,-1
.05893579772291,0.289629019813035,0.717318412677073
"6316","P4HA3",19.9402343620305,-0.694695450246297,0.656086800018669,-
1.05884686329085,0.289669527129136,0.717318412677073
"6317","RNF40",114.152895058999,0.239628770847815,0.226306721663912,1.
05886722712411,0.289660251597049,0.717318412677073
"6318","OLFML3",2086.43282127275,0.262818750103306,0.248585326375176,1
.05725769873741,0.290393993297602,0.717401630833864
"6319","PBXIP1",211.134656629516,0.168981333641314,0.159631777534486,1
.05856951699237,0.289795875638007,0.717401630833864
"6320","LANCL1",90.9640547118578,0.207097463514917,0.19602597227699,1.
056479715975,0.290749103185751,0.717401630833864
"6321","EPM2AIP1",229.680601810597,0.213564039851324,0.201981726416482
,1.05734337279086,0.29035490526082,0.717401630833864
"6322","CCDC51",102.955014149579,0.342833591685845,0.324107234524252,1
.05777827572742,0.290156539208765,0.717401630833864
"6323","AC116351.2",4.41877952537486,0.759358063968729,0.7180864559810
86,1.05747442754822,0.290295119525821,0.717401630833864
"6324","LMBRD2",65.5697790920954,0.275124647110392,0.260498182843371,1
.05614804720467,0.29090058202357,0.717401630833864
"6325","FST",991.030826999957,-0.386693139998243,0.365674976184501,-1.
05747771978561,0.29029361775011,0.717401630833864
"6326","NME5",13.3093528309267,0.651626439258915,0.61594797964014,1.05
792446894561,0.290089878677439,0.717401630833864
"6327","CCNG1",339.49127369249,-0.201362753728815,0.190502419326261,-1
.05700890540374,0.29050752314841,0.717401630833864
"6328","LHFPL5",4.21740264103324,1.01218859241843,0.957792470863307,1.
05679322317714,0.290605967838551,0.717401630833864
"6329","AL080250.1",11.1660945716346,0.483395693281949,0.4577356086505
49,1.05605874689769,0.290941376079399,0.717401630833864
"6330","SEC63",636.538912423559,0.139971369083461,0.132269602644163,1.
05822778843615,0.289951605292606,0.717401630833864
"6331","AC102953.2",4.04811432509365,0.784301015545732,0.7415250293673
11,1.05768650346829,0.29019839038101,0.717401630833864
"6332","SEPTIN7-
AS1",5.25105856000953,0.794969776232965,0.752627258724165,1.0562596119
367,0.290849622566288,0.717401630833864

"6333","TOPORS",268.324042452173,-0.213545164206304,0.201874945214235,
-1.05780915001455,0.29014246043039,0.717401630833864
"6334","GSN-
AS1",7.688855723706,0.886107829392072,0.838575178038717,1.056682635735
21,0.290656452404223,0.717401630833864
"6335","HK1",98.802371895274,-0.265060758439882,0.250968708759926,-1.0
5615062431323,0.29089940480914,0.717401630833864
"6336","SPINDOC",20.8575245162872,-0.361921198239311,0.342558560878718
,-1.05652358332813,0.290729072184049,0.717401630833864
"6337","KBTBD6",66.8724341519787,0.272959638510111,0.258225891549515,1
.05705758966378,0.290485305103787,0.717401630833864
"6338","SPRYD7",94.223714783635,-0.198604972392388,0.188005117215028,-
1.05638067375175,0.290794331916717,0.717401630833864
"6339","CLEC16A",30.5916705658303,0.344968630848687,0.326164139187056,
1.05765346156233,0.290213459572861,0.717401630833864
"6340","CTU2",44.2845201289177,-0.309100156012044,0.292493211748268,-1
.05677719549289,0.290613284313518,0.717401630833864
"6341","ZNF573",39.2932132612601,0.316710801187864,0.299385728327893,1
.05786873327841,0.290115291562333,0.717401630833864
"6342","PHF20",298.458306252583,-0.155964748290067,0.147339418442256,-
1.05854054494718,0.289809076348805,0.717401630833864
"6343","PLTP",2927.99464162337,-0.300611330435398,0.284294154471207,-1
.05739539736419,0.290331171232557,0.717401630833864
"6344","FMR1",162.608938912766,0.236331532684766,0.223733769520914,1.0
5630693654708,0.29082800790314,0.717401630833864
"6345","TIPARP",190.908928026475,-0.459821797565372,0.435533256453958,
-1.0557673627708,0.291074512574174,0.717616800661592
"6346","MAP3K7",112.367333854135,0.209940272624151,0.198877595188899,1
.05562555915232,0.291139318968121,0.717658457459255
"6347","CDCA7L",35.7958257089356,0.366268833919042,0.347000048221519,1
.05552963406282,0.291183163683046,0.717658457459255
"6348","AC079848.1",7.79896328500675,-0.605086735905947,0.573436184835
024,-1.05519454807343,0.291336357095983,0.717696792764168
"6349","MED10",505.257261992036,-0.206277590403207,0.195450996156826,-
1.05539288342994,0.291245676304674,0.717696792764168
"6350","FOXJ2",83.682149527581,0.238110058493881,0.225651896801711,1.0
5520964755336,0.291329452804205,0.717696792764168
"6351","PTGR1",329.642317593771,-0.293619114038106,0.278319617783939,-
1.05497095884216,0.291438606955266,0.71783563668733
"6352","LINC02018",11.042908800077,-0.597736357407682,0.56690165833097
3,-1.05439161911696,0.291703657724197,0.718081598617666
"6353","SEC24B-
AS1",14.2806978299802,0.480918095409525,0.456518370213212,1.0534474115
1362,0.292135984418756,0.718081598617666
"6354","CENPK",5.26099301446708,-0.657933518529364,0.624372483217388,-
1.05375162457358,0.291996646648592,0.718081598617666
"6355","PCDHGA12",31.1775269048065,0.465153192740084,0.441406512328134
,1.05379775727979,0.291975520527136,0.718081598617666
"6356","HIST1H3D",3.39705328491545,-0.910509652977068,0.86457492570392
2,-1.05312983977155,0.292281488470819,0.718081598617666

"6357","CUX1",193.66477040231,0.189742530651825,0.180191273438605,1.05300621406882,0.292338144079421,0.718081598617666
"6358","ZNF79",5.52710979625258,0.709780463928874,0.674062435923248,1.05298919818415,0.292345942754543,0.718081598617666
"6359","JMJD1C-AS1",9.80151819947908,-0.567202444460395,0.538680305103179,-1.05294817554496,0.292364744716225,0.718081598617666
"6360","ARCN1",272.290479474123,0.200127907203507,0.189913975831607,1.05378188375645,0.291982789570363,0.718081598617666
"6361","C12orf4",56.4276804184556,0.252770491731925,0.2398033026174,1.05407427242658,0.291848914048068,0.718081598617666
"6362","EPSTI1",338.842620616119,0.363351306907494,0.344589179582019,1.05444781333016,0.291677941503147,0.718081598617666
"6363","MYCBP2-AS1",5.66176645809091,0.72264711607711,0.685264811455633,1.05455161858095,0.291630440996508,0.718081598617666
"6364","SHF",47.4655173143281,-0.266196180100964,0.252643874885061,-1.05364193065068,0.292046884269484,0.718081598617666
"6365","RPS17",3940.09321422655,0.252005318376081,0.239111205314912,1.05392517278388,0.291917176902876,0.718081598617666
"6366","CDR2L",34.6751054443064,0.280646954290142,0.266382956605741,1.05354696060947,0.292090383337182,0.718081598617666
"6367","ITGB4",4.46051161716191,-0.908623743057387,0.862118380148473,-1.05394312890175,0.291908955417175,0.718081598617666
"6368","AC012313.5",2.84972848442909,1.16993191120039,1.11085297603177,1.05318339730219,0.292256946232871,0.718081598617666
"6369","HSF2BP",2.98854373163265,0.922712053549873,0.875866048883848,1.05348535284102,0.292118603831838,0.718081598617666
"6370","MATN3",4.65592403825154,0.906729075478609,0.861460192403691,1.05254901326155,0.292547735704295,0.71841824640852
"6371","ALG3",125.218853379348,-0.311192231870126,0.295784603585382,-1.05209070417452,0.29275793664312,0.718458504055301
"6372","PPM1K",91.8848604083294,0.293014570737549,0.278536439043748,1.05197930921895,0.292809042640917,0.718458504055301
"6373","ZNF696",32.6424025785107,0.306281695015454,0.291047263015753,1.05234349858455,0.292641981341821,0.718458504055301
"6374","TSTD2",88.5863898352718,0.283154190465424,0.269180366611028,1.05191249284012,0.292839699664809,0.718458504055301
"6375","TSPAN11",28.3226783017917,0.488247015583216,0.464029933593267,1.05218862025219,0.292713019457511,0.718458504055301
"6376","RAB5C",590.879461705283,-0.281017411396258,0.267095968735642,-1.05212150047235,0.29274380891352,0.718458504055301
"6377","AC008440.2",6.2737380767007,-0.78715667415812,0.748408751737433,-1.05177374306585,0.292903368410954,0.718502021648511
"6378","IPP",86.4460905449349,0.276556327506079,0.262984818593562,1.05160567436971,0.292980503310193,0.71857855335236
"6379","NRIP2",3.15715973673729,0.890801912695087,0.847460952487069,1.05114213236707,0.293193315940242,0.718871775726939
"6380","NKIRAS2",101.871504773822,0.198492945906442,0.188832578494028,1.05115837261482,0.293185858271901,0.718871775726939

"6381","IRF2BP1",101.104976999883,-0.277220502537208,0.263757024375475
,-1.05104500323209,0.293237921173279,0.718871775726939
"6382","ZBED4",25.8985472733217,0.432593332119738,0.411669920555186,1.
05082569923091,0.293338650299135,0.719006033630425
"6383","AC093627.4",4.30580975334381,-0.917849818727262,0.873656725233
38,-1.05058404773577,0.293449670798978,0.719029762361294
"6384","ADAM28",2.77301739729333,-1.06667530818096,1.01540721512737,-1
.05049017998869,0.293492803497646,0.719029762361294
"6385","ZNF205",83.2584438494104,-0.260782391106708,0.248237219700527,
-1.05053702833651,0.293471275916136,0.719029762361294
"6386","SERPIND1",3.8625060397013,-1.4797069946593,1.40870210998203,-1
.0504044710192,0.293532190912179,0.719029762361294
"6387","VWA1",118.311994192396,0.302053762478936,0.287718760322115,1.0
4982296649955,0.293799513996886,0.719174264000748
"6388","AC007389.5",4.9984277490587,0.784517185790762,0.74726142011734
3,1.04985640188352,0.293784139013924,0.719174264000748
"6389","PCDH7",138.760377026521,0.426603204447703,0.406316149177891,1.
04992923690299,0.293750648304912,0.719174264000748
"6390","COX7A2",1067.18224567943,-0.24173915438144,0.230245499862429,-
1.04991912773921,0.293755296507341,0.719174264000748
"6391","AL353150.1",5.5620838574372,0.743932268697791,0.70865801524779
7,1.04977612994,0.293821052306385,0.719174264000748
"6392","LINC02580",24.1246504558914,0.517778585786951,0.49368711939787
,1.04879905803186,0.294270611113958,0.719267217832798
"6393","DPP4",261.880595635921,0.46771520442941,0.445783022535149,1.04
919923098357,0.294086432525128,0.719267217832798
"6394","AC069148.1",7.459378401311,-0.593703493103326,0.56566385670426
7,-1.04956943256447,0.293916117028332,0.719267217832798
"6395","FAM13A-
AS1",19.0104950607113,0.510520533059403,0.48680011899515,1.04872721500
812,0.294303684870105,0.719267217832798
"6396","LINC00271",28.1479855203399,0.386036679512719,0.36798098779536
4,1.04906691464015,0.294147322231885,0.719267217832798
"6397","TCEA1",821.96860339151,-0.159825084643857,0.15233076429314,-1.
04919768101665,0.294087145744334,0.719267217832798
"6398","PRR26",2.82405948976207,1.07791665569741,1.02741334537397,1.04
915578579043,0.294106424324162,0.719267217832798
"6399","ANKDD1A",35.7968499547974,0.306112226594535,0.291865181944843,
1.04881378640219,0.294263831048342,0.719267217832798
"6400","WDR93",3.02795938956984,-0.83853614265167,0.799356880695997,-1
.04901347933799,0.294171914635441,0.719267217832798
"6401","FBXO27",21.5813973934486,0.451007648789559,0.430065883895647,1
.04869431795942,0.294318830233826,0.719267217832798
"6402","FLOT1",449.491113129947,-0.185818115435217,0.177244800618054,-
1.04836990866455,0.294468211941375,0.719474514019163
"6403","RELN",26.9712335644069,-0.816161650672388,0.778623808427398,-1
.0482104988811,0.294541634454946,0.719474514019163
"6404","ZNF641",63.7651680505117,-0.308982140066965,0.294744637604865,
-1.04830453431756,0.294498321211929,0.719474514019163
"6405","TRAF2",35.8990885016099,-0.281934936154682,0.269025768840723,-

1.04798487286027,0.29464557646225,0.719530622933768
"6406","PPP6R2",151.996312825298,0.252020020443862,0.240486079484589,1
.04796095052151,0.294656598511393,0.719530622933768
"6407","CD38",13.8963550455111,0.437892060083424,0.417935450376934,1.0
4775045928382,0.294753592779442,0.719655135297145
"6408","AL445183.2",4.17850471985014,0.787395623219989,0.7515843969216
7,1.047647644689,0.29480097747564,0.719658503534869
"6409","LRRC45",38.5172375352753,-0.308299420543557,0.294309994339405,
-1.04753296345084,0.2948538372353,0.719675234181899
"6410","CLIC4",413.097863035437,0.182977279783153,0.174786079776016,1.
04686414397322,0.295162241178714,0.719918887958015
"6411","RAB1A",582.305269788611,0.203640892928744,0.194601642957534,1.
04645001878624,0.295353309570697,0.719918887958015
"6412","FHL2",151.249885803691,0.527619532323722,0.503962402826653,1.0
4694225077978,0.295126213672796,0.719918887958015
"6413","LARS2",25.1402157395581,0.35282091588279,0.337179456019146,1.0
4638912479518,0.295381411723568,0.719918887958015
"6414","UFSP2",251.158757459417,-0.193623223273308,0.185051848802711,-
1.04631877242002,0.295413881084351,0.719918887958015
"6415","PBX2",92.363407287591,0.246977610176795,0.235858308785528,1.04
714398847563,0.295033173876524,0.719918887958015
"6416","ARL5B",101.655741881156,-0.224910641898145,0.214893009504589,-
1.04661683698623,0.295276333305026,0.719918887958015
"6417","AP004609.3",9.99868515175258,0.506554411314115,0.4839412739670
98,1.04672702776857,0.295225494454892,0.719918887958015
"6418","CDC16",171.590429443647,0.192064882015477,0.183476904985821,1.
04680685577468,0.295188667776865,0.719918887958015
"6419","FAM3A",200.057904895928,-0.288989422355987,0.276138702981259,-
1.04653719031772,0.295313083632391,0.719918887958015
"6420","HES4",31.8664596808311,-0.454117678914544,0.434334168118505,-1
.04554905473299,0.295769280965493,0.720672719960002
"6421","SLU7",351.94647831445,-0.172661531413728,0.165164377147816,-1.
04539207785225,0.29584179664685,0.72073714763225
"6422","CENPC",310.531647077105,0.221975323731872,0.212411983328694,1.
04502260302508,0.296012523011306,0.720936546085919
"6423","AP003307.1",28.3463379526017,-0.399534997665407,0.382324479836
525,-1.04501547438511,0.296015817650697,0.720936546085919
"6424","AC109460.2",3.39067074101608,0.88080027177532,0.84339100974595
6,1.04435577519451,0.296320816676038,0.721567019810595
"6425","RASAL2-
AS1",4.60764131688682,-0.750309420741876,0.718713085383814,-1.04396237
664323,0.296502796856651,0.721596582365557
"6426","EAF2",18.1811828466113,-0.427311136301035,0.409272756117366,-1
.04407422657397,0.296451049176195,0.721596582365557
"6427","PFDN6",191.289067773673,-0.253652199378248,0.242926994660583,-
1.04414990904016,0.296416037901152,0.721596582365557
"6428","MRPS2",172.598602284786,-0.253806794128314,0.243126104591492,-
1.04393065711626,0.296517473083539,0.721596582365557
"6429","SDCBP2",17.1202906866573,-0.512066172001466,0.490598782136651,
-1.04375752783429,0.296597586393959,0.721679272664598

"6430","MTX2",200.895359895482,0.284349900541651,0.272516344849254,1.04342328787267,0.296752292520742,0.721943407760804
"6431","ZIC4",23.4743004406984,-0.547125720870567,0.52450627973073,-1.04312520557704,0.296890308252782,0.722054585198736
"6432","SMC2",155.865300384804,-0.176843064614253,0.169530638652859,-1.04313335937091,0.296886532375979,0.722054585198736
"6433","AL356488.2",3.27475054514447,0.890617640280486,0.854772471890752,1.04193533316585,0.297441661295069,0.72216046991134
"6434","SLC33A1",138.293062835698,0.204807042397385,0.196550768923177,1.04200580602885,0.297408987119526,0.72216046991134
"6435","HMGN4",257.094838016862,-0.230097890194701,0.220662067824573,-1.04276141551266,0.297058805637447,0.72216046991134
"6436","AC021097.1",9.58787344121989,0.581207493529945,0.557448379453804,1.04262119139968,0.297123770595649,0.72216046991134
"6437","ANKRD26",192.904473415676,0.265914091882563,0.255206029433549,1.04195850103065,0.297430919436353,0.72216046991134
"6438","BUD13",55.335935422133,-0.242737008497041,0.23281316571141,-1.04262578001251,0.297121644569575,0.72216046991134
"6439","NACA",9352.89229885469,-0.313280604305677,0.300510647960769,-1.04249418924609,0.297182618099792,0.72216046991134
"6440","AC008033.3",3.35276124246882,0.826983387995157,0.793546641863628,1.0421358296634,0.297348709005227,0.72216046991134
"6441","LUM",16706.2539431902,-0.265424934485936,0.25462871688334,-1.04239984293501,0.297226339291887,0.72216046991134
"6442","AKAP8L",143.008718724493,-0.167865029252003,0.161072106260696,-1.04217318037869,0.297331394965739,0.72216046991134
"6443","STRN4",80.3059728730695,-0.256913977261061,0.246389432108752,-1.04271508344426,0.297080269945438,0.72216046991134
"6444","PHRF1",82.1192061632322,0.270812551278666,0.259979328257609,1.04166955539758,0.29756490860457,0.722238843527493
"6445","PLEKHH3",190.993650433755,-0.167535222204818,0.160833824455187,-1.04166659452594,0.297566281821562,0.722238843527493
"6446","ECI2",486.905407265724,-0.218279811919206,0.209604549105681,-1.0413887143697,0.297695178178679,0.722298754469782
"6447","AC105020.1",4.24566229185682,0.795295129715227,0.763741337353521,1.04131476302048,0.297729487235259,0.722298754469782
"6448","SS18",224.999586380721,0.17124414529333,0.164428303028199,1.04145175824118,0.2976659316336,0.722298754469782
"6449","LIMA1",2021.57536597991,-0.149173722270865,0.1432932188739,-1.04103825319285,0.297857794866614,0.722497981872918
"6450","AL390728.6",69.7051623173508,0.44199300164356,0.424657656244329,1.04082193066421,0.29795819980584,0.722517457690709
"6451","AL080276.2",17.2033834259229,-0.3758422826874,0.361076348025111,-1.04089421736719,0.297924645805886,0.722517457690709
"6452","ATAD3B",26.6983207368319,-0.349608589378166,0.33594256272048,-1.04067965234002,0.298024249850764,0.722565613827573
"6453","KLHDC2",372.397179299354,-0.212144868638266,0.203882394006091,-1.04052568968721,0.29809573514452,0.722571572795252
"6454","CD40LG",3.39553689653767,1.11002057108181,1.06683981017604,1.04047539330075,0.298119090380398,0.722571572795252

"6455","ADI1",567.679151880274,0.283234958224208,0.272346224762613,1.03998121681726,0.29834862730138,0.722731905288601
"6456","PAXIP1-AS1",98.3188465761924,-0.212985962417731,0.204841005920641,-1.03976233401356,0.298450332501786,0.722731905288601
"6457","GAS6-AS1",4.72832808598475,0.797703841569108,0.76685750085301,1.04022434504688,0.298235683456392,0.722731905288601
"6458","STXBP4",67.5269884056503,0.273915796083792,0.263450532608173,1.03972382736149,0.298468227241836,0.722731905288601
"6459","TIMP2",5666.62587167908,0.148989303189905,0.143253611931307,1.04003872001041,0.298321911939277,0.722731905288601
"6460","ZNF571",35.8653504925276,0.319684989423815,0.307496786710598,1.0396368457817,0.298508651797587,0.722731905288601
"6461","MAGIX",3.25536829340234,-0.809705797188503,0.778764067153367,-1.03973184092615,0.298464503133871,0.722731905288601
"6462","HNRNPUL1",443.794284562356,-0.171550449652869,0.165026812654477,-1.03953077014249,0.298557955235719,0.722739414074954
"6463","AC253572.2",16.4601654421064,-0.501863278725108,0.483088968530039,-1.03886304887524,0.2988684336446,0.722812240458113
"6464","AC007383.2",5.84879864346476,-0.704156052245494,0.677746533790605,-1.03896665956694,0.29882024241064,0.722812240458113
"6465","AC104232.3",7.96231849489889,0.578708735747185,0.556960932434922,1.03904726892995,0.298782753104575,0.722812240458113
"6466","SORBS1",11.3479104104165,-0.555012361183625,0.534095397234752,-1.03916334807821,0.298728773245478,0.722812240458113
"6467","BET1L",107.548813780877,-0.213968098041074,0.206001768428816,-1.03867117099536,0.298957693266253,0.722812240458113
"6468","WDR90",48.2119963886498,0.292737736404879,0.281817853879734,1.03874801533974,0.298921943929657,0.722812240458113
"6469","AL132655.2",3.79817977721957,-0.877148772722241,0.843939002787983,-1.03935091259504,0.298641564568551,0.722812240458113
"6470","GMEB2",28.4051250018326,0.337036308631594,0.324463549403117,1.03874937339373,0.298921312164786,0.722812240458113
"6471","PNKD",441.444034958978,-0.277679166321435,0.26752155761552,-1.03796930907719,0.299284343793517,0.722931592026248
"6472","ANKRD20A1",3.6956744163799,1.12279657907143,1.08159679806416,1.03809162627054,0.299227399548148,0.722931592026248
"6473","ENO3",4.13851527733594,0.933526103760658,0.898948579250152,1.0384644075408,0.299053897419451,0.722931592026248
"6474","AC024075.2",49.6590307610483,-0.301638806002151,0.29056054598052,-1.03812719990681,0.299210839751323,0.722931592026248
"6475","CLIC6",53.7247418049767,0.283686278392717,0.273224271053771,1.03829091500033,0.299134637019984,0.722931592026248
"6476","TRIOBP",673.783708732547,0.232873140250854,0.224337400128635,1.03804867185465,0.299247395968758,0.722931592026248
"6477","CDK14",236.033796417543,0.156803442680659,0.151083370446952,1.03786036952171,0.299335066224177,0.722942479688868
"6478","NPIPA5",7.32377584626872,1.03594030989305,0.998449718038597,1.03754880308655,0.299480163710202,0.723069640518396

"6479","PEX11G",60.2744236824656,0.33456523335487,0.322431316951491,1.
03763256161995,0.299441152482591,0.723069640518396
"6480","DEFB1",36.8525792691206,2.77560468822297,2.6759834359529,1.037
22790318192,0.29962965686448,0.723318938631337
"6481","MRO",5.47575759389699,0.680751813043717,0.656412083048458,1.03
70799542297,0.299698596536826,0.723373730230762
"6482","BOC",736.695969837719,0.293428692486732,0.283071051770858,1.03
659025057871,0.299926858869071,0.723812998039011
"6483","HOXA3",54.1992310909105,0.465894228494629,0.449518807272906,1.
03642877885592,0.300002150026494,0.72388302219103
"6484","ADAMTS6",9.23409231220594,0.74250721129613,0.716627625799117,1
.03611301681003,0.300149420208325,0.723993049712871
"6485","SRPK2",339.02398634061,0.196153352167561,0.189311119323118,1.0
3614279430024,0.300135530045558,0.723993049712871
"6486","MAP3K12",150.487736349087,0.205701057060986,0.198546757302849,
1.03603332461998,0.300186595949478,0.723993049712871
"6487","SDHAF3",150.101115710609,-0.288128084048463,0.278140189072938,
-1.0359095713885,0.300244331882802,0.724000869158625
"6488","RHBDL1",8.19477309349232,0.564673733496264,0.545142385273282,1
.03582797586577,0.300282403573558,0.724000869158625
"6489","MS4A4E",3.26363133061322,-0.980974154755894,0.947145040803615,
-1.03571693087637,0.300334221273517,0.724014212264081
"6490","FDPS",396.312214537917,-0.276769652966319,0.267279166045912,-1
.03550776912697,0.300431840068945,0.724056919433006
"6491","IMPA2",187.776238882439,-0.375423728096886,0.36255987664641,-1
.03548062617812,0.300444509623451,0.724056919433006
"6492","FGL2",1643.18785973012,-0.230809538870395,0.223143761621374,-1
.03435353600442,0.300970917578328,0.724120825941209
"6493","SDHD",758.638044922222,-0.260716189333933,0.251969623458174,-1
.03471277908708,0.300803066274612,0.724120825941209
"6494","C1S",11953.4944804899,0.235973364443873,0.228140474600192,1.03
43336264966,0.300980221841702,0.724120825941209
"6495","C12orf60",7.40514536723579,0.557397645539621,0.538773603724015
,1.03456747265804,0.300870951157278,0.724120825941209
"6496","DCAF5",243.74131367334,0.222720411526424,0.215238833224401,1.0
3475942602896,0.300781275719658,0.724120825941209
"6497","TOB1-
AS1",4.51427602876709,0.717170760590262,0.692747905184063,1.0352550404
3164,0.300549820395177,0.724120825941209
"6498","ANKRD12",1207.4276646455,-0.238670736305767,0.230665363171981,
-1.03470557097823,0.300806433549336,0.724120825941209
"6499","RFX1",95.1009800616731,-0.266897396151536,0.257856523207368,-1
.03506164137991,0.30064012495287,0.724120825941209
"6500","ZNF888",9.89088831573962,-0.507575197315308,0.490522723013916,
-1.03476388248972,0.300779193992933,0.724120825941209
"6501","TGIF2",28.8301105381596,0.413085552684526,0.399135017223737,1.
03495192067543,0.300691365298167,0.724120825941209
"6502","GEMIN8",82.0915909775629,-0.320855743567187,0.310191210585193,
-1.03438051310956,0.30095831073705,0.724120825941209
"6503","EPG5",92.3533934170269,0.218861798662959,0.211657379964399,1.0

3403811716734,0.30111834404802,0.724341727809192
"6504","ABHD17B",42.7680090599903,0.266074351952156,0.257419211543871,
1.03362274461326,0.30131256215807,0.724363364044673
"6505","GBF1",119.510195052168,0.213646396217762,0.206673959636058,1.0
3373640585385,0.301259408636169,0.724363364044673
"6506","AC136469.1",2.69304540292699,-1.09668096551622,1.0610002406943
5,-1.03362932773561,0.301309483399631,0.724363364044673
"6507","DDX27",303.128676820311,-0.212567219720259,0.205617203392321,-
1.0338007531144,0.301229319497306,0.724363364044673
"6508","ESD",1184.16998482469,-0.261075449325448,0.252617155041327,-1.
03348265988799,0.301378081177487,0.724401966151451
"6509","TACO1",80.889581936887,-0.266485943048058,0.257875380374687,-1
.03339040222009,0.301421236187419,0.724401966151451
"6510","CD14",122.112458555126,-0.562244577550142,0.544295247686309,-1
.0329771937935,0.301614571566861,0.724592859398285
"6511","AP000880.1",4.57346006115969,-0.727165615182432,0.703945770990
14,-1.03298527407819,0.301610790105123,0.724592859398285
"6512","MAP1LC3A",496.023864310561,-0.228388885834393,0.22111722141770
3,-1.03288601570727,0.301657243840627,0.724592859398285
"6513","ZSWIM1",13.5490114994512,0.586945073573695,0.568291096209015,1
.03282468701185,0.301685948555969,0.724592859398285
"6514","TDRD10",17.7088717781642,0.438508743867161,0.424989496513079,1
.03181077994869,0.302160768290141,0.724980242477587
"6515","PAPPA2",11.6230291300798,1.01932580812768,0.987919368490671,1.
03179048881792,0.302170275840483,0.724980242477587
"6516","COQ8A",157.102536660701,0.232789547349384,0.225617713471143,1.
03178754791856,0.302171653835829,0.724980242477587
"6517","TBX5-
AS1",11.3141388480012,-0.689124314694908,0.667704479521725,-1.03207981
349552,0.302034729567652,0.724980242477587
"6518","SLIRP",532.031972696965,-0.307906487084668,0.298325519382218,-
1.03211581671689,0.302017865180755,0.724980242477587
"6519","ERBB2",108.201483304703,0.244572016639656,0.236928758300485,1.
03225973239381,0.301950459425924,0.724980242477587
"6520","PTGIR",89.5122033754494,-0.604236205270001,0.585559986062165,-
1.03189463018713,0.302121481789024,0.724980242477587
"6521","TBCD",120.101311626458,0.218547598811113,0.211835096519169,1.0
316873945925,0.302218584431146,0.724981646412577
"6522","ECHDC2",402.767286351117,0.22426542543457,0.217424055323381,1.
03146556208334,0.302322549635128,0.725041656002549
"6523","ZNF442",7.19313051762768,0.558801692676944,0.541770472033582,1
.03143622903521,0.30233629879848,0.725041656002549
"6524","ARPC5",825.891467915571,-0.184471415248662,0.178869669518647,-
1.03131747123528,0.30239196792258,0.725064002791679
"6525","RFX8",18.0769704509658,0.484604652025877,0.47003695869258,1.03
099265507508,0.302544264171015,0.725206853268034
"6526","ESCO1",154.418125558188,0.198311994834614,0.192347647946115,1.
03100816127562,0.302536992633874,0.725206853268034
"6527","TIMM13",612.629098956491,-0.287658610676967,0.27905318292676,-
1.03083794873777,0.302616819119827,0.725269634057216

"6528","MINDY1",70.6505950737211,0.264384958906713,0.256553944887692,1
.03052384956485,0.302764162511896,0.725511610627083
"6529","ZNF174",38.5899216249678,0.265668508038893,0.257831954120783,1
.0303940368634,0.302825071361879,0.725546422317947
"6530","CFAP44",45.9846057718092,0.411131712184644,0.399048594926208,1
.03027981406793,0.302878672078873,0.725563716283278
"6531","DCAF8",259.146497233535,0.198444555423793,0.192738315741573,1.
02960615101499,0.303194926632678,0.726210111363495
"6532","UBA52",7790.9060509461,-0.263889500825492,0.256351248442671,-1
.0294059515162,0.303288953602678,0.726324112248421
"6533","PIGL",64.7985518323135,0.249873314582068,0.242787640308369,1.0
2918465810162,0.303392910225505,0.726461854378934
"6534","SARAF",922.981074139506,-0.202613088007184,0.196889349038342,-
1.02907084104244,0.303446387088894,0.726469470407878
"6535","CCDC102A",112.540564780999,-0.225771993768169,0.21941334771152
5,-1.02898021530123,0.303488972007638,0.726469470407878
"6536","CORT",2.83033563263912,-0.906885939296661,0.882039317976156,-1
.02816951672576,0.303870094885149,0.726522565863572
"6537","MAGOH",376.341744252289,-0.283490761894868,0.275555007001322,-
1.02879916783188,0.303574057871289,0.726522565863572
"6538","KHDC4",115.22310165596,0.329415148706972,0.320334402354908,1.0
2834770878591,0.303786296585367,0.726522565863572
"6539","ZBED6",6.42138667235571,-0.629617225356327,0.612968173362087,-
1.02716136451738,0.304344487347339,0.726522565863572
"6540","INTS7",25.830285427313,0.36872776501601,0.35899018730863,1.027
12491330301,0.304361648905765,0.726522565863572
"6541","NAA80",43.7040479972915,-0.343120003260304,0.333916127670902,-
1.02756343532611,0.304155231439004,0.726522565863572
"6542","ZBTB11-
AS1",16.6048551301823,-0.425522374726921,0.41431494767521,-1.027050501
35072,0.304396684714535,0.726522565863572
"6543","RNF217-
AS1",7.83830539212019,-0.573372364587311,0.558227227357791,-1.02713077
486601,0.304358889186017,0.726522565863572
"6544","FOXP2",100.577537965869,0.31171978625976,0.303194528380422,1.0
2811811256911,0.303894271548256,0.726522565863572
"6545","CHCHD3",230.856947002764,-0.253568222339062,0.246642155452375,
-1.02808143998735,0.303911520363456,0.726522565863572
"6546","ZNF239",9.87177123108732,0.56609532185381,0.550866258787109,1.
02764566321457,0.304116536159622,0.726522565863572
"6547","FERMT3",29.6626045957054,-0.338693981631811,0.329613540209046,
-1.02754875123457,0.30416214190853,0.726522565863572
"6548","GXYLT1",84.0977776927358,0.218182678443755,0.212264869980471,1
.02787935876449,0.304006580195718,0.726522565863572
"6549","HNRNPC",849.066156216029,0.146433349348568,0.142584156718853,1
.02699593502036,0.304422378196364,0.726522565863572
"6550","CHMP4A",376.246624798359,-0.240973274913991,0.234468730680624,
-1.02774162769801,0.304071380767854,0.726522565863572
"6551","MTHFD1",66.2632962619679,-0.295368407211322,0.287279075617911,
-1.02815844340912,0.303875302835491,0.726522565863572

"6552","INTS14",56.961040232847,0.279372707469169,0.271618415688523,1.0285484758498,0.303691900343833,0.726522565863572
"6553","TAOK1",346.939735413358,0.194664597268336,0.189505356349829,1.02722477621679,0.304314634082068,0.726522565863572
"6554","LYPD3",3.53749839067313,-0.805849530985111,0.784695364284822,-1.02695844484766,0.304440031914321,0.726522565863572
"6555","SLC25A1",187.472230145463,-0.216436990874196,0.210483081120511,-1.02828688045609,0.303814900570531,0.726522565863572
"6556","PLK1",3.00106334704226,1.07024983689761,1.04226472170425,1.02685029494964,0.30449096234333,0.726533270887235
"6557","VPS53",124.032303754123,0.385517765036542,0.375487186979162,1.0267135028976,0.304555389164087,0.726566369253106
"6558","EGFL6",8.97660430494699,1.74103734999368,1.69588670648268,1.02662362016189,0.304597727390006,0.726566369253106
"6559","MYLK-AS1",10.4580380915351,-0.484599444410539,0.472123962571319,-1.02642416574511,0.304691692046702,0.726568923580268
"6560","NHLRC2",172.748565843849,0.267542095304505,0.260633795050353,1.0265057731781,0.304653243771796,0.726568923580268
"6561","TNFRSF6B",11.8934594864328,-0.443602586933112,0.432289838705096,-1.02616935957112,0.304811761371075,0.726744457114422
"6562","SSR2",2002.62872932316,-0.277161728497176,0.270204467307011,-1.02574813532694,0.305010318787616,0.727107043095807
"6563","TADA1",24.3827249276024,0.468789877826545,0.457139760711258,1.02548480380959,0.305134492046178,0.72729222292829
"6564","AGL",293.838505251986,-0.192655867913872,0.187973209150839,-1.02491130935194,0.305405037844244,0.727715309519804
"6565","PEPD",412.399190497533,-0.27663663860907,0.269891205454231,-1.0249931565702,0.305366416718534,0.727715309519804
"6566","IMMT",177.0980395982,-0.164939625004885,0.160991415853148,-1.02452434579082,0.305587677636699,0.728039604214268
"6567","AC009951.6",62.5225746389902,-0.315633753608067,0.308331544256199,-1.0236829785596,0.305985037655539,0.728134488412272
"6568","CDKL3",36.7198128646523,0.346407430077089,0.338566525288922,1.02315912591027,0.30623261518023,0.728134488412272
"6569","FGF1",6.76594683782847,-0.926285788821009,0.905160753832721,-1.0233384345254,0.306147857357764,0.728134488412272
"6570","MTCH1",1167.57378906345,-0.199960033637028,0.195406117578295,-1.02330487967915,0.306163717296317,0.728134488412272
"6571","AC073140.1",4.64543228964156,0.875536242356345,0.855477028695392,1.02344798631419,0.30609608071114,0.728134488412272
"6572","ATAD1",214.149333595037,-0.197971684660617,0.193340751406551,-1.02395218400868,0.305857860343956,0.728134488412272
"6573","NTAN1",751.445244580255,-0.265179916128954,0.25899787756949,-1.02386907034713,0.305897120935199,0.728134488412272
"6574","PIEZO1",89.0470663521423,0.244573635252979,0.239032798686457,1.02318023550312,0.306222636028155,0.728134488412272
"6575","MRPL12",142.820317518702,-0.334277820437121,0.32662951842655,-1.02341583224754,0.306111276846508,0.728134488412272
"6576","CYBC1",203.292115587897,-0.211230100175597,0.206426338060156,-

1.02327107170811,0.306179697426886,0.728134488412272
"6577","PNMA8B",35.4353725651508,0.338705250064491,0.331005719646108,1.02326101925555,0.306184449061028,0.728134488412272
"6578","ZNF81",11.1911751150394,0.442232312280099,0.431774336491786,1.02422092955613,0.305730935260127,0.728134488412272
"6579","TSIX",67.7580210793101,0.873068115008977,0.852988025205618,1.02354088124334,0.306052181020208,0.728134488412272
"6580","AC073349.1",16.8480547539743,-0.48919477883446,0.478412390086178,-1.02253785431088,0.306526405841043,0.72850084572644
"6581","MCRS1",124.228503928602,-0.247748174687628,0.242275137637436,-1.02259017208106,0.306501658299457,0.72850084572644
"6582","MNS1",143.017843538151,0.255980325063791,0.25032466374557,1.0225933043656,0.306500176696857,0.72850084572644
"6583","GORAB-AS1",6.92751219072574,-0.726855211018987,0.711145400786091,-1.02209085542215,0.306737900857675,0.728711736160152
"6584","SDHAF1",210.706869198124,-0.287686115562675,0.281478128880257,-1.02205495221641,0.30675489245123,0.728711736160152
"6585","TBC1D20",172.301469336242,0.20258262437796,0.198181807921759,1.02220595574513,0.306683432546423,0.728711736160152
"6586","CDS2",124.927489144516,0.200245049140557,0.195983160054814,1.02174620046208,0.306901038407607,0.728948214972699
"6587","MTX1",224.465470516818,-0.2584245551687,0.253303668990263,-1.020216391649,0.307625845323041,0.729101383530557
"6588","FN1",3326.99031681621,0.264619341996804,0.25923642934651,1.02076449156418,0.307366031353161,0.729101383530557
"6589","ARID1B",591.474152162065,0.228500781087994,0.223949177411157,1.02032427057537,0.307574696349772,0.729101383530557
"6590","ZNF655",217.884905956756,0.198775273074798,0.19460737552445,1.0214169557506,0.307056935397094,0.729101383530557
"6591","GGH",63.471554856176,0.430753400999168,0.42188327516023,1.02102507105921,0.307242560694254,0.729101383530557
"6592","MARCH8",81.2564081108443,0.238609878593404,0.233743984861684,1.02081719336906,0.307341056951207,0.729101383530557
"6593","PSMA1",661.402060993795,-0.244508636666091,0.239705829231433,-1.02003625631491,0.307711266001965,0.729101383530557
"6594","SRPRA",288.162821469821,-0.173989011109442,0.170371372339531,-1.02123384181412,0.307143662325214,0.729101383530557
"6595","WNK1",343.764523670991,0.221648235500138,0.217249271276802,1.02024846480489,0.307610637789109,0.729101383530557
"6596","POP5",170.349369984688,-0.303681308952293,0.297502805590766,-1.02076788267344,0.307364424329316,0.729101383530557
"6597","MIA2",93.7523387377854,-0.202096330450013,0.197959525982199,-1.02089722354753,0.307303134712177,0.729101383530557
"6598","MRPL46",118.507774536026,-0.289039199298311,0.283344169239508,-1.0200993374033,0.30768135098756,0.729101383530557
"6599","TP53I13",376.670152444526,-0.2580546128382,0.252950312396527,-1.02017906360073,0.307643545098511,0.729101383530557
"6600","ZNF383",55.8754425489776,0.225791420641052,0.221267185211586,1.02044693353486,0.307516544616445,0.729101383530557

"6601","CPT1C",13.0950754301422,-0.473164617599401,0.463722497161567,-1.0203615750705,0.307557010362626,0.729101383530557
"6602","POLR2F",518.62531690832,-0.266366438675036,0.260864331793608,-1.02109183284505,0.307210932172054,0.729101383530557
"6603","SRSF2",780.416964910831,-0.202922419420306,0.1989631141224,-1.01989969505338,0.307776034194573,0.72914440449882
"6604","SNX13",188.118642711188,0.185610018736029,0.182007460177866,1.01979346645815,0.307826422466799,0.729153350491844
"6605","ZNF606",38.0635713344209,0.301323424010946,0.295542583765138,1.01956009239738,0.30793713985003,0.72930517466677
"6606","JPX",691.220468433531,0.172441847306102,0.169178935615495,1.01928674913774,0.308066852933196,0.7295019346706
"6607","KIF2A",161.458701708197,-0.173498674924886,0.170512840577333,-1.01751090614315,0.308910446718635,0.729824831754648
"6608","PITX1",4.25797622215173,0.935188657501849,0.918640039771164,1.01801425696055,0.30867118077011,0.729824831754648
"6609","CDK19",82.8921396596853,0.243996316202986,0.239693986353366,1.01794926070142,0.308702069609473,0.729824831754648
"6610","TRMT11",180.184260379582,0.167446059746392,0.164461731286014,1.01814603578013,0.308608560441527,0.729824831754648
"6611","AC007566.1",26.1202818238743,0.469821123200508,0.461654389172239,1.01769014704466,0.308825231166749,0.729824831754648
"6612","FAM86B1",16.3390692014724,0.390136264662472,0.383036803458387,1.0185346711856,0.308423932669901,0.729824831754648
"6613","PTBP3",179.058539913285,0.240747781714259,0.236550661154118,1.01774300921276,0.308800102154257,0.729824831754648
"6614","NCOA4",307.467967895724,0.151923393309359,0.149320972338267,1.01742836877057,0.308949692250801,0.729824831754648
"6615","SAC3D1",81.4337821955427,-0.35228707818379,0.346145411215237,-1.01774302581967,0.308800094260064,0.729824831754648
"6616","AC021054.1",28.7055697858233,0.365571563331609,0.358868675589631,1.01867782896059,0.308355941581428,0.729824831754648
"6617","VAMP1",30.1431957176476,0.415343923666347,0.407702939421862,1.01874154808724,0.308325682126009,0.729824831754648
"6618","PEBP1",2682.26124637237,-0.230493204206863,0.2264201764657,-1.01798880207913,0.308683277711213,0.729824831754648
"6619","CARHSP1",1040.56999700058,0.226455701806492,0.222423666203069,1.01812772746827,0.308617259914529,0.729824831754648
"6620","NUFIP2",417.888970085954,0.21864476201747,0.214590217176556,1.01889436011698,0.308253121514177,0.729824831754648
"6621","CCL13",16.4639891361601,-0.670858865510574,0.659160193862906,-1.01774784302297,0.308797804380341,0.729824831754648
"6622","GOSR2",132.421903545968,-0.173228699658903,0.170236957498194,-1.01757398748589,0.308880454517675,0.729824831754648
"6623","FAM102B",173.215540177631,0.235327193468841,0.231379908700408,1.01705975592524,0.309125003471769,0.729982654977593
"6624","CCDC36",57.5502519644249,-0.354760960481458,0.348823257951515,-1.01702209469865,0.309142918745286,0.729982654977593
"6625","CU633967.1",3.83616807695473,0.939663715686829,0.923962315186805,1.01699355075629,0.309156497425465,0.729982654977593

"6626","ADAM19",28.2557568635526,0.447337017177779,0.439918245741077,1
.01686397758794,0.309218141821132,0.73001801879082
"6627","AP000944.7",3.4133411465426,0.876806424019389,0.86258435704987
4,1.01648774042014,0.309397182575214,0.730330485441991
"6628","AC139887.2",63.5318824773806,0.391768183565267,0.3854654062850
41,1.01635108411146,0.309462230448741,0.730373818785403
"6629","PPP2R3C",155.284671368607,-0.226893133092389,0.223295359959719
,-1.01611217149035,0.309575973636682,0.730381297568818
"6630","AC105114.2",4.85969118369237,-0.751569088418657,0.739696790093
731,-1.01605022285337,0.309605471084756,0.730381297568818
"6631","AC012615.2",2.97320359625588,1.18970463809841,1.170894564969,1
.01606470274282,0.309598576177806,0.730381297568818
"6632","FGD6",28.9872482248706,0.389166145499836,0.383128368530041,1.0
1575914880164,0.309744093692738,0.730487993010025
"6633","MRPL54",416.051587782969,-0.288745497033794,0.284263770646989,
-1.01576608365042,0.309740790527904,0.730487993010025
"6634","HDHD2",115.111623114353,0.183311822554059,0.180527553436472,1.
01542295934657,0.309904253307204,0.730755537305487
"6635","PARTICL",17.1667469877148,-0.458602089863942,0.451865303719333
,-1.01490883696791,0.31014928537114,0.731002752909558
"6636","NGLY1",115.392329743458,-0.184215232457724,0.181506657836841,-
1.01492272874815,0.310142662831421,0.731002752909558
"6637","C22orf31",3.92203448760831,0.862627159907319,0.849955186981808
,1.01490898946156,0.31014921267331,0.731002752909558
"6638","AL109659.2",3.63645403832051,0.850109855044631,0.8405836945026
16,1.01133279244448,0.311857176030743,0.731605912966152
"6639","AL513314.2",3.49386370445672,0.982628634163653,0.9714165556214
34,1.01154198832348,0.311757095328475,0.731605912966152
"6640","CAD",51.8688704023039,-0.265826995294344,0.262384747107579,-1.
01311908647401,0.311003282638592,0.731605912966152
"6641","DDX18",550.072232548618,-0.228043909732079,0.22524958994711,-1
.01240543783287,0.311344239406886,0.731605912966152
"6642","MARCH7",318.376538545296,0.191259028663173,0.188759125430383,1
.01324388014137,0.310943685831483,0.731605912966152
"6643","AC021851.1",5.1739310313726,0.679806807018695,0.67177098621499
3,1.0119621433027,0.311556154361646,0.731605912966152
"6644","TEX264",371.847316564956,-0.296567423602431,0.293068069601383,
-1.01194041372643,0.311566544532474,0.731605912966152
"6645","ADAMTS9-
AS2",36.6395346870865,0.448692238204695,0.443327928777677,1.0121000935
8492,0.311490197659315,0.731605912966152
"6646","SLC25A26",135.83802020174,-0.26055437852067,0.257252335163123,
-1.01283581490311,0.311138590554746,0.731605912966152
"6647","PCCB",52.3345912483969,0.259709608602175,0.256834500931369,1.0
1119439818397,0.311923396405398,0.731605912966152
"6648","COMMD2",284.118333683261,-0.177981412772163,0.176111496729985,
-1.01061779654876,0.312199394703652,0.731605912966152
"6649","NMD3",177.053517181949,-0.164597302715735,0.162693262031001,-1
.01170325470744,0.311679958870131,0.731605912966152
"6650","PSMG3-

AS1",37.3662491270374,0.328700083580947,0.324592929392145,1.0126532460
103,0.311225817256958,0.731605912966152
"6651","UCK1",119.488674601852,-0.232384406455396,0.229177604770442,-1
.01399264857562,0.310586260161555,0.731605912966152
"6652","CSGALNACT2",179.354643665309,0.17625991344072,0.17422218706298
7,1.01169613590602,0.311683363648617,0.731605912966152
"6653","SUPV3L1",61.579875403283,0.269001987408241,0.266077261510354,1
.01099201743616,0.312020250408626,0.731605912966152
"6654","MGST1",4532.29866721384,-0.256987018135169,0.253396087937821,-
1.01417121403322,0.310501061748377,0.731605912966152
"6655","IQCD",12.2111425805718,-0.47089012976479,0.464983122805706,-1.
01270370185361,0.311201709145342,0.731605912966152
"6656","CCDC92",134.061206235519,-0.187990917506263,0.185402310330347,
-1.01396210851582,0.310600833195676,0.731605912966152
"6657","LINC00539",16.0504755248918,-0.459039659724518,0.4533784339493
66,-1.01248675576788,0.311305376055104,0.731605912966152
"6658","GTF2F2",180.227124831065,-0.213789821265513,0.211326676846574,
-1.01165562462674,0.311702739839537,0.731605912966152
"6659","PSME1",1134.27066363357,-0.242896654405006,0.239712022825225,-
1.01328523927272,0.310923935913447,0.731605912966152
"6660","TYRO3",29.3339225027845,0.37304049734914,0.368395219030418,1.0
1260949675446,0.311246721917018,0.731605912966152
"6661","BNIP2",282.164154411705,0.151985887500315,0.149944000254672,1.
01361766554297,0.310765224932828,0.731605912966152
"6662","AC027348.1",3.00091532746975,0.977586828269007,0.9669556217176
08,1.0109945134116,0.312019055780951,0.731605912966152
"6663","TPPP3",1106.57513300735,-0.402483049095419,0.398179939248499,-
1.01080694787146,0.312108837091945,0.731605912966152
"6664","ASPA",188.525437091555,0.257248521244458,0.25447886458005,1.01
088364123668,0.312072124502081,0.731605912966152
"6665","CCR7",4.70273852221403,-0.861775497795402,0.852716293535824,-1
.01062393709168,0.31219645460087,0.731605912966152
"6666","SPAG9",708.109377765882,0.173545424838261,0.171714428252143,1.
01066303283163,0.312177735919502,0.731605912966152
"6667","METTL23",194.928763639505,-0.262349384424202,0.259236794704775
,-1.01200674357578,0.311534829099798,0.731605912966152
"6668","AP005205.2",3.83426831616931,-0.801327860758245,0.791226175528
328,-1.01276712720376,0.311171405871215,0.731605912966152
"6669","OAZ1",3841.64481038809,-0.242676822430072,0.239459180953958,-1
.01343711885798,0.310851417086337,0.731605912966152
"6670","ZNF738",51.1724319532923,0.270804791000477,0.267615327483797,1
.01191808984436,0.311577219113312,0.731605912966152
"6671","BCAM",19.1240491671866,-0.46150656997043,0.45625670189751,-1.0
115063911414,0.311774123762452,0.731605912966152
"6672","CPNE1",479.825106274462,0.24070142302668,0.238121502175258,1.0
1083447243468,0.312095660944183,0.731605912966152
"6673","RPRD1B",78.655966993384,0.215395301854177,0.212908156210976,1.
01168177719193,0.311690231205259,0.731605912966152
"6674","STAG2",522.139435353099,0.175961289706852,0.174123315539924,1.
01055558907334,0.31222918078131,0.731605912966152

"6675","MBNL3",69.6642357119041,0.294308528790605,0.291164056314898,1.01079965884356,0.312112326447898,0.731605912966152
"6676","MT-ND4L",2775.64246427488,-0.345208738491637,0.340820219463695,-1.01287634587774,0.311119228089419,0.731605912966152
"6677","FAM71A",9.25880442778101,0.886966198161386,0.877969738956266,1.01024688984818,0.312377019321944,0.73162355341416
"6678","DCTN3",735.130171301092,-0.312249889497869,0.309077496543204,-1.01026406965937,0.31236879056053,0.73162355341416
"6679","ARPC3",1330.87040138861,-0.279305025080934,0.276429132734355,-1.0104037237976,0.312301904520193,0.73162355341416
"6680","NDUFB10",1162.69622582797,-0.286017189387577,0.283179196316392,-1.0100218981765,0.312484798694094,0.731766423049658
"6681","FAM49A",98.2284285801352,0.23885143044178,0.236573197573844,1.0096301393873,0.312672524171686,0.731848394611349
"6682","COX7C",2595.80268706136,-0.241135532708459,0.238852500763662,-1.00955833385666,0.312706940457857,0.731848394611349
"6683","UBL7-AS1",68.9999427155973,0.213392225333527,0.211361784322645,1.00960647175358,0.312683867758374,0.731848394611349
"6684","N4BP1",215.813764868616,0.151038200751942,0.149584730271248,1.00971670355696,0.312631037408248,0.731848394611349
"6685","SEPTIN10",548.977645488495,0.185553912417704,0.183827888734566,1.00938934617059,0.312787945854632,0.731928472251909
"6686","FANCD2",7.91057768532726,-0.564657726290034,0.559729176609039,-1.00880523990344,0.313068047597568,0.731983396056126
"6687","CLSTN2",74.8908275332552,0.37571735066501,0.372462839234111,1.00873781512698,0.313100391037088,0.731983396056126
"6688","SEMA6A-AS1",3.05618859491825,0.934071925176015,0.926220070632848,1.00847730986633,0.313225375207839,0.731983396056126
"6689","DGAT1",93.2063144857546,-0.198491020225121,0.196815768230694,-1.00851177733109,0.31320883666058,0.731983396056126
"6690","TMEM268",25.8236018398585,0.322758343536093,0.320019133682484,1.00855951899528,0.313185929694718,0.731983396056126
"6691","ARFIP2",114.774224174893,-0.24605871082654,0.244014056719926,-1.00837924722083,0.313272431818783,0.731983396056126
"6692","NBEA",33.0784611436241,0.379599673265412,0.376286128982711,1.00880591663493,0.313067722982888,0.731983396056126
"6693","PPP1R13B",8.75298514080407,-0.526668350847643,0.522191779200601,-1.00857265821744,0.313179625547633,0.731983396056126
"6694","GGA2",122.97062595236,0.221407388085971,0.219570715116006,1.00836483576143,0.31327934773354,0.731983396056126
"6695","TMEM47",264.382695318359,-0.2010693317532,0.199270664535277,-1.0090262519178,0.312962044298182,0.731983396056126
"6696","GOLM1",273.53512550938,0.178802821388356,0.177359120372285,1.00813998746183,0.313387263192746,0.732126188489265
"6697","EIF4B",816.735357941635,-0.204766753395006,0.203142901975848,-1.00799364094617,0.313457515032812,0.732180962768147
"6698","GUSB",482.127184251525,-0.261290975504518,0.25927821775484,-1.

00776292650847,0.313568287701937,0.732221036650455
"6699","SLFN11",154.424901916481,0.227147603868067,0.225384263488713,1
.00782370673116,0.313539102861419,0.732221036650455
"6700","APH1A",464.415475609132,-0.181040729459285,0.179692377706456,-
1.00750366693367,0.313692796452141,0.732290869540614
"6701","PRRT3-
AS1",9.04388575653779,-0.570869806160936,0.56683624200146,-1.007115924
95433,0.313879069249493,0.732290869540614
"6702","AC008875.2",4.8571880312748,0.749584857541886,0.74424103356694
5,1.00718023292713,0.31384817040963,0.732290869540614
"6703","KCNMB4",56.3933474623981,0.287266563360586,0.285181675330545,1
.0073107363144,0.313785471999112,0.732290869540614
"6704","PAN3-
AS1",8.5579139534888,-0.537282078695243,0.533414483869331,-1.007250637
06155,0.313814344767082,0.732290869540614
"6705","COIL",155.626678276586,0.165312464715415,0.164103507143285,1.0
0736704286932,0.313758422897708,0.732290869540614
"6706","MRPL22",305.558343980696,-0.288392495582945,0.286463146019293,
-1.00673507077773,0.31406210394569,0.732333847388174
"6707","MRAP2",11.1608817854884,-0.959798905384346,0.953603783655618,-
1.00649653643883,0.314176776904201,0.732333847388174
"6708","AC004812.2",26.9380688230242,-0.361257506402068,0.358961648689
729,-1.0063958300858,0.314225198725911,0.732333847388174
"6709","SLC7A1",48.7734164985584,-0.368859835366907,0.366413126379341,
-1.00667745998006,0.314089797229023,0.732333847388174
"6710","GREB1L",8.29466574823012,0.634461414688648,0.630391539617441,1
.00645610674546,0.314196215797218,0.732333847388174
"6711","DNM2",110.625990799113,0.175324831422408,0.174208414726879,1.0
0640851188089,0.314219100770784,0.732333847388174
"6712","COX7B",756.311461835232,-0.248601610755607,0.246939325192175,-
1.0067315546527,0.314063794087113,0.732333847388174
"6713","CTNS",52.5953627864665,0.251705129852183,0.250131483551919,1.0
0629127640359,0.314275475620462,0.732341913471011
"6714","ARSJ",25.5707182912775,0.506807584917828,0.503762320331497,1.0
060450424008,0.314393903454489,0.732399677101798
"6715","TMEM168",124.198394377512,0.223274942559737,0.221926288512014,
1.00607703601392,0.314378514261134,0.732399677101798
"6716","SNRNP40",174.550393357762,-0.311329159323807,0.309744331361673
,-1.00511656809074,0.31484072307606,0.732676797244689
"6717","PIGC",221.190962433424,-0.260032551229708,0.25869953298716,-1.
00515276632762,0.314823295196274,0.732676797244689
"6718","CLDN12",60.9256192386667,0.263097770126716,0.261697484232404,1
.00535078087747,0.314727971007534,0.732676797244689
"6719","ARPC1B",1077.51609427248,-0.27859453471236,0.277160775082664,-
1.00517302504032,0.314813541782237,0.732676797244689
"6720","HMBS",30.1796008262988,-0.39985661503904,0.397624218889731,-1.
00561433645954,0.314601124805765,0.732676797244689
"6721","PRKAB1",41.4589367815409,0.299425596760161,0.297779739055004,1
.00552709768092,0.314643108082031,0.732676797244689
"6722","GYS1",26.7828298995611,0.390240838310612,0.388241441589822,1.0

0514987970527,0.314824684956369,0.732676797244689
"6723","DNAJC19",518.517968717556,-0.226018402069324,0.224901468229389
,-1.0049663252478,0.314913065261052,0.73272162287623
"6724","EEF2K",248.2785409126,0.257069264763978,0.255820346053127,1.00
488201478154,0.314953665679203,0.73272162287623
"6725","EVA1A",3.72543821923933,0.795787866492525,0.792021428388812,1.
00475547500195,0.315014608436231,0.732754426731296
"6726","TMEM115",240.395109951673,-0.241637281266836,0.24051697206588,
-1.00465792160667,0.315061596365495,0.732754765379935
"6727","CMBL",674.140826940757,-0.201016189727538,0.200128881104628,-1
.00443368602279,0.315169619965773,0.732897036587571
"6728","KIAA1586",103.470976551257,0.190901703820152,0.190097623338554
,1.00422982921867,0.315267847377128,0.733016488781275
"6729","C12orf76",208.041392706924,-0.233024852553056,0.23207401627390
9,-1.00409712510867,0.315331801015212,0.7330562287533
"6730","RSAD2",17.885514512464,-0.547889034229127,0.545793167320776,-1
.00384003874332,0.315455722015861,0.733235343164059
"6731","PLCG2",8.16027877499422,0.912017695559896,0.908936579996606,1.
0033898025793,0.315672822313306,0.733525257817347
"6732","LRRC37A2",14.9605397640644,0.511186029824773,0.509460549857305
,1.00338687650683,0.315674233563024,0.733525257817347
"6733","USF3",126.198709389015,0.197561052330491,0.196984222172482,1.0
0292830639758,0.315895453912405,0.733836754031264
"6734","RUNX1",227.177374246302,0.218090590857806,0.217456810187655,1.
00291451286168,0.315902109675032,0.733836754031264
"6735","MFSD6",17.1529099156657,-0.498321826391794,0.497099251415121,-
1.00245941826143,0.316121757041774,0.734010797141605
"6736","INPP5A",52.2434214035634,-0.240879941457641,0.240265444451314,
-1.00255757546713,0.316074373860636,0.734010797141605
"6737","CUL5",279.996942500883,0.16792247631905,0.167541614620711,1.00
227323640876,0.316211644949005,0.734010797141605
"6738","ATP5PD",1057.11248129741,-0.269524964032737,0.268898144594138,
-1.0023310664324,0.316183723032255,0.734010797141605
"6739","BCL2L12",56.5192064722061,-0.327711144313302,0.326851409487522
,-1.00263035373514,0.316039244800476,0.734010797141605
"6740","TMEM184B",90.9714461789653,0.216426472238684,0.215965349973996
,1.00213516781624,0.316278314785548,0.734056628811621
"6741","ING5",118.488904296506,0.217643733253851,0.217204486339555,1.0
0202227367261,0.316332835372017,0.734074253630688
"6742","PFKFB2",12.1761068600757,-0.453272876550096,0.452474084016067,
-1.00176538847693,0.316456917329991,0.734144380512093
"6743","KLHL26",30.5779829040579,-0.346797144695429,0.346168161418629,
-1.00181698765774,0.316431991075336,0.734144380512093
"6744","BCL6",435.316051228774,0.332214458600693,0.331748566213841,1.0
0140435388213,0.316631360027119,0.734351646941392
"6745","ZNF169",24.4089487120634,0.389920637583138,0.389393710685233,1
.0013531982758,0.316656082211976,0.734351646941392
"6746","RNPS1",368.118222497834,-0.143530636172397,0.143345858778286,-
1.00128903196567,0.316687093924863,0.734351646941392
"6747","SLC19A2",104.629330374118,0.422231512590066,0.421928237714389,

1.00071878307392,0.316962783954526,0.734352196401828
"6748","PJVK",28.3599842870821,0.328752485342451,0.328650442216623,1.0
0031049136931,0.317160271546898,0.734352196401828
"6749","AP1S3",3.5931374398309,0.865021884797535,0.864257825804611,1.0
008840637251,0.316882861899461,0.734352196401828
"6750","TMEM39A",106.592578245162,0.224393392895799,0.22438642336266,1
.00003106040479,0.317295476679051,0.734352196401828
"6751","HAUS3",86.0418009128394,0.212628944082508,0.21253646569037,1.
00043511776612,0.317099982152378,0.734352196401828
"6752","SEC24D",189.093881480892,0.208269178375148,0.208262205844974,1
.00003347957516,0.317294305980018,0.734352196401828
"6753","VCAN",2893.33157983589,0.300095418329009,0.29980521241448,1.00
096798155106,0.31684228819215,0.734352196401828
"6754","SFR1",68.6671789511487,-0.298420297986485,0.298470244234923,-0
.999832659203378,0.317391497786407,0.734352196401828
"6755","TMEM41B",130.348681187583,0.190489771776279,0.190519569442727,
0.999843597870107,0.317386203255284,0.734352196401828
"6756","CPM",52.0034005804005,-0.291945020693899,0.291855837766938,-1.
00030557184548,0.317162651575037,0.734352196401828
"6757","SETD1B",61.9628549407403,0.269484543503296,0.269440236515852,1
.000164440872,0.317230934652121,0.734352196401828
"6758","GNG2",155.143487202898,-0.29289522230541,0.292632008158383,-1.
00089947148531,0.316875412095081,0.734352196401828
"6759","HOXB13",3.53874382236418,-0.906487346636996,0.905592637619794,
-1.00098798177021,0.316832618722933,0.734352196401828
"6760","AC023043.1",8.28468461556547,0.594166931034989,0.5940611029993
93,1.00017814335102,0.317224304590441,0.734352196401828
"6761","IDS",414.660367868659,-0.252148886903388,0.252142608147537,-1.
00002490160587,0.31729845709373,0.734352196401828
"6762","MRPS22",225.525850411893,-0.248504234077378,0.248589011468256,
-0.999658965654287,0.317475576660616,0.734438101996748
"6763","UQCR11",1093.15277952397,-0.233986592999946,0.234105494597745,
-0.999492102489932,0.317556362938636,0.734516366323981
"6764","POLG2",79.760450937338,0.377263967666222,0.377670315042423,0.9
98924068532748,0.3178314758084,0.735004079272333
"6765","GIPC1",196.305452972392,-0.282026704410198,0.282375223113054,-
0.998765760327644,0.317908176203836,0.735004079272333
"6766","ETV2",10.235287439468,-0.505017048157478,0.50559319092501,-0.9
98860461774658,0.3178622918533,0.735004079272333
"6767","RNASEH2A",21.5141284207725,-0.527191847934593,0.52789639640847
9,-0.998665366009922,0.317956823579722,0.735007919500162
"6768","SLC10A6",4.12615621075846,0.867227807507506,0.868813033308858,
0.998175412038521,0.318194307154319,0.735148586639522
"6769","NRF1",36.2868637560354,0.266925186321309,0.267382507444832,0.9
98289637089973,0.318138931214578,0.735148586639522
"6770","POLR2G",372.481866892315,-0.254173061876079,0.254592939683196,
-0.998350787701972,0.31810928818836,0.735148586639522
"6771","AL357093.2",4.85802417259941,0.727128467084771,0.7284746855951
68,0.998152003718157,0.31820565621276,0.735148586639522
"6772","AC016877.3",4.05580692268833,0.778135266089352,0.7797735562020

12,0.99789901811926,0.318328328166888,0.735232967527395
"6773","SLC6A8",32.6000763019244,-0.296943364343414,0.297573379510157,
-0.997882824170026,0.31833618161881,0.735232967527395
"6774","CCND2",198.458083989631,-0.264589007469644,0.265182490327527,-
0.997761983239733,0.318394788899208,0.735259770113715
"6775","LMO3",584.996780248195,0.220493842374226,0.221057065885691,0.9
97452135224866,0.318545096013209,0.735498293274485
"6776","NAAA",201.382786024544,-0.29234826292165,0.293130639465443,-0.
997330962927589,0.318603889270327,0.735525478136913
"6777","MAP3K11",37.6307146667473,0.285873185621848,0.286736195680122,
0.996990229795623,0.31876925235782,0.735798644626439
"6778","CCDC57",41.2595631137741,0.34978992796672,0.350903283781628,0.
996827171855135,0.31884840678899,0.735872768869898
"6779","AC002310.1",5.45130937211364,0.630292885874103,0.6324432819906
11,0.996599859342739,0.318958774267147,0.736018897456996
"6780","WIPF2",206.502658643781,0.216429395349918,0.217247652662805,0.
996233527484152,0.319136692694844,0.736320838322338
"6781","SZT2",81.1040383932971,0.311114950450574,0.312372929002322,0.9
95972831078016,0.319263346118798,0.736395830630545
"6782","TNF",7.67305392556139,-0.742010135883136,0.744955275917432,-0.
996046554565751,0.319227525902485,0.736395830630545
"6783","AKR7A2",570.807874974845,-0.282990648588964,0.284221668958615,
-0.995668801839911,0.319411093358344,0.736589598785996
"6784","SYF2",698.636828716879,-0.20149539997991,0.202464136581183,-0.
995215268157456,0.319631577498105,0.736589598785996
"6785","S100A14",3.36833128910052,0.883851405195603,0.888962175258413,
0.994250857679827,0.320100753854581,0.736589598785996
"6786","AL137796.1",6.35999051418198,-0.686701308054914,0.690626865318
168,-0.994315950536553,0.320069072645901,0.736589598785996
"6787","SLC12A8",3.33905618250206,-0.895416557987435,0.900336485981859
,-0.994535456386555,0.319962252526666,0.736589598785996
"6788","NCAPG",3.7179865263183,0.745156994882238,0.748916586561058,0.9
94979959388958,0.319746011422522,0.736589598785996
"6789","AC008467.1",7.97092716248305,-0.543842264807455,0.546979364151
021,-0.994264684283957,0.320094024165379,0.736589598785996
"6790","CCAR2",64.115794999581,0.215310438641269,0.216274430280795,0.9
9554273874043,0.31947236859512,0.736589598785996
"6791","TRIM68",22.5651557048727,0.338486328701533,0.340157280735675,0
.995087707573015,0.319693608657475,0.736589598785996
"6792","ARHGAP32",94.2763517688071,-0.28394416935112,0.285469512126639
,-0.99465672265961,0.319903249621177,0.736589598785996
"6793","UBE3A",340.009823459995,0.187815866093463,0.188856851027512,0.
994487968382479,0.31998536006751,0.736589598785996
"6794","RAB40C",35.5204283897656,0.263922190293135,0.265251359397115,0
.994989020576555,0.319741604345017,0.736589598785996
"6795","HAGH",153.179581796321,-0.246258512976831,0.247625107926074,-0
.994481193927835,0.319988656588689,0.736589598785996
"6796","TMEM102",3.1193542877497,-0.761552125188133,0.765593683889529,
-0.99472101352657,0.319871971362088,0.736589598785996
"6797","NOP53",2252.41231713427,-0.289897764582427,0.291479607523804,-

0.99457305794112,0.31994395648093,0.736589598785996
"6798","OPHN1",163.618561588119,0.235586893785047,0.236817190388883,0.994804867831529,0.319831178267311,0.736589598785996
"6799","PABPC4",324.562349141019,-0.197405258601714,0.198920165771194,-0.992384345932916,0.321010072254948,0.736678263905992
"6800","CCN1",1512.21439101605,0.497262639183544,0.500622655213434,0.993288326057763,0.320569464944529,0.736678263905992
"6801","KLHL29",69.7448101796047,0.326410996831622,0.32882651506705,0.992654125732728,0.320878537975145,0.736678263905992
"6802","SEC22C",204.067164129564,-0.167738679394562,0.169019648703941,-0.992421181092244,0.320992110768382,0.736678263905992
"6803","TMA7",2308.84102002041,-0.246783391383437,0.248393214853811,-0.993519052155587,0.320457070502753,0.736678263905992
"6804","ARL6IP5",2763.6280013404,-0.169091051075364,0.170417535908307,-0.99221626562153,0.321092039573048,0.736678263905992
"6805","ZBTB24",75.5098983096697,-0.248791142750531,0.250441243091006,-0.993411227639232,0.320509592244496,0.736678263905992
"6806","ACO1",213.643744843047,0.21336509035636,0.214973813303707,0.99251656226059,0.320945558431464,0.736678263905992
"6807","NDUFB6",470.197951066245,-0.255970635526926,0.257841722457035,-0.992743273228714,0.320835080818431,0.736678263905992
"6808","INIP",105.486417146518,-0.168536461993422,0.169841524865031,-0.992315996499409,0.32104340240823,0.736678263905992
"6809","C10orf143",19.2230741503477,-0.337787530928225,0.339956436869356,-0.99362004743577,0.320407880411441,0.736678263905992
"6810","AHNAK",5378.4688668281,0.170203514234479,0.171418488011506,0.992912236065544,0.320752726247398,0.736678263905992
"6811","PFDN5",4082.08449244222,-0.287866484225777,0.290068418373136,-0.992408914559851,0.320998092077404,0.736678263905992
"6812","ZSWIM4",9.10020837595845,-0.498847547227037,0.502471875967153,-0.992787001793603,0.320813765651419,0.736678263905992
"6813","POP4",135.507566623081,-0.264741236336231,0.266493694424563,-0.993424016684086,0.32050336235687,0.736678263905992
"6814","TBCB",474.099529332629,-0.285537582323362,0.287517260540554,-0.993114576100684,0.320654121396372,0.736678263905992
"6815","USP16",427.144653413137,-0.141613233607672,0.142487630354224,-0.993863349791291,0.320289399448565,0.736678263905992
"6816","C21orf58",31.6972839015275,0.393803185495417,0.396628563702365,0.992876513530507,0.320770136700011,0.736678263905992
"6817","CLTCL1",7.55858014144708,0.582333876884043,0.585922933209717,0.993874524920859,0.320283958183134,0.736678263905992
"6818","SREBF2",62.2050416988761,0.265087471748706,0.26703959070917,0.992689776990446,0.32086115842438,0.736678263905992
"6819","HDAC6",68.5149747129608,0.261521371164927,0.263592671908926,0.992142039727438,0.321128241486605,0.736678263905992
"6820","TXNDC12",348.239666350216,0.247702602346201,0.249863701188203,0.99135088917788,0.321514271993538,0.736777370320144
"6821","GPX7",180.036083276717,0.243441509189368,0.245449673832523,0.991818426108294,0.321286107479464,0.736777370320144
"6822","LINC01140",348.490495969321,-0.294047837700016,0.2966549920850

97,-0.991211493301509,0.321582319586979,0.736777370320144
"6823","C1orf43",437.291493745224,-0.224921790347028,0.226944236623952
,-0.991088355857765,0.321642438273749,0.736777370320144
"6824","AL080317.2",11.0622451818975,0.607049538279412,0.6119859940892
85,0.99193371113465,0.321229863042847,0.736777370320144
"6825","TMEM120A",148.852650661105,-0.195062537506717,0.19680721716978
9,-0.99113508290925,0.321619624129964,0.736777370320144
"6826","FMC1",266.909104019628,-0.284622122317593,0.287179385340295,-0
.991095241673871,0.32163907625763,0.736777370320144
"6827","DDX58",73.7156313386558,0.260518658208298,0.26272602157906,0.9
91598230896599,0.321393552622686,0.736777370320144
"6828","GINS3",14.6435448427025,0.448852674706081,0.452675699027388,0.
991554606687477,0.321414842016412,0.736777370320144
"6829","FAM217B",145.584501090174,0.186675441279864,0.188260999161566,
0.991577873862546,0.321403487104534,0.736777370320144
"6830","NUDT12",80.6934533058273,0.22004907985849,0.222106566056359,0.
990736490890829,0.321814267724108,0.736847276055067
"6831","AC104041.1",12.2534269032836,0.720995995915638,0.7276983693719
51,0.99078962694104,0.321788315468232,0.736847276055067
"6832","IRX3",187.996476302365,0.281383647900595,0.284012429467366,0.9
90744131967391,0.321810535650637,0.736847276055067
"6833","SSH2",137.576839817551,0.216835739264668,0.218890123547822,0.9
90614540985879,0.321873834621307,0.736875807841519
"6834","TFR2",3.92357612786936,-0.848850067260586,0.85710246854034,-0.
99037174482322,0.321992450761754,0.736972816271317
"6835","REXO2",933.702834448062,-0.250518100931201,0.252963002897818,-
0.990334942506971,0.322010432731219,0.736972816271317
"6836","INO80D",176.263811951102,0.230850997818465,0.233143913148987,0
.990165236142981,0.322093361389598,0.736991997101546
"6837","MPC1",435.701955699357,-0.223186652320805,0.225412608634838,-0
.990124969816397,0.322113039965689,0.736991997101546
"6838","BLVRB",1141.4703964674,-0.324360187397555,0.327730147925568,-0
.989717270292819,0.322312331186577,0.737340128217553
"6839","WDFY1",179.256569763892,0.181323085793269,0.183372095093739,0.
988825948138825,0.322748306410076,0.737462885736388
"6840","AC007920.2",52.1268211641765,-0.40217793900632,0.4066979106153
78,-0.988886169584154,0.322718837994883,0.737462885736388
"6841","PDGFRL",2152.46864604844,-0.170220972966301,0.172132190086665,
-0.988896805882722,0.322713633472146,0.737462885736388
"6842","LINC01230",13.2467206197905,0.462910991402318,0.46807130611219
4,0.988975366269004,0.322675194227365,0.737462885736388
"6843","AL139384.1",19.257100789385,0.393167498981008,0.39734756778420
7,0.989480069485491,0.322428316428043,0.737462885736388
"6844","DYNLRB2",13.2880083618764,-0.384766225241676,0.388893738353671
,-0.989386527205433,0.322474063740626,0.737462885736388
"6845","SOCS3",2078.88047808689,-0.776222409601099,0.784916754599552,-
0.988923226638361,0.32270070558158,0.737462885736388
"6846","STAU1",484.880991024146,-0.147125360769203,0.148800832299797,-
0.988740173662345,0.322790281828105,0.737462885736388
"6847","U2AF1",33.9735482110564,-0.352610221753887,0.356514966926009,-

0.989047457934824,0.322639922726167,0.737462885736388
"6848","AL355999.1",3.62001236247247,-0.735627365032952,0.744218625114415,-0.988455999632981,0.322929373383576,0.737565219424628
"6849","KANSL3",77.5724531601916,0.268466975591936,0.271580863930813,0.988534213000847,0.322891087244094,0.737565219424628
"6850","ZNF219",50.309774441565,-0.308359207092424,0.312053759071242,-0.988160527244363,0.323074036460094,0.737731867320241
"6851","RAC2",19.0975343460307,-0.439040656375776,0.44432171741502,-0.988114330602681,0.323096658122545,0.737731867320241
"6852","SEPSECS",38.9280273693961,0.261654838388773,0.26485704503944,0.987909679162243,0.323196884662949,0.737774310470659
"6853","ZP1",8.67204005194161,-0.713225617933253,0.721973208461911,-0.987883774042954,0.323209572949909,0.737774310470659
"6854","SGTA",115.17272377512,-0.222074138637651,0.224951750515953,-0.987207870702489,0.32354074411261,0.738422506587914
"6855","CALM2",2796.42964677908,-0.202972949302504,0.205731229175584,-0.986592799332737,0.323842301645686,0.738902377936019
"6856","PC",13.5984633065463,-0.449902144526584,0.456019030159809,-0.986586336909928,0.323845471017666,0.738902377936019
"6857","AL031118.1",4.48751008848703,-0.663568845641555,0.672703150755672,-0.986421492000065,0.323926322890686,0.738979067956687
"6858","SLC4A5",11.6408697377046,0.465464563205773,0.471969538112888,0.986217384000832,0.324026450442584,0.7390997031603
"6859","HLA-B",5200.0949770756,0.412036363228326,0.418157232188159,0.98536227885429,0.324446151332379,0.739883072288139
"6860","AL356124.1",6.31234146351192,0.789332328976768,0.801088340285891,0.985324950173501,0.324464480975301,0.739883072288139
"6861","SRSF9",823.615809979915,-0.234503615887064,0.238041272559954,-0.985138473530891,0.324556057400784,0.739984026515152
"6862","FAM98A",117.513780526611,0.190998088746353,0.194009503732902,0.984478002733848,0.324880541950828,0.740002636753216
"6863","ALKBH3",93.2159160368411,-0.278137971112825,0.282565113001267,-0.984332312501642,0.324952146926248,0.740002636753216
"6864","PRDX5",1808.19415136655,-0.287973258736305,0.292572645928923,-0.984279503717735,0.324978104336449,0.740002636753216
"6865","PIP4P1",97.7979899278071,-0.250622178541287,0.254514081086508,-0.984708498136505,0.324767277242586,0.740002636753216
"6866","YWHAE",1171.81973510183,-0.164103714497048,0.166712073360862,-0.984354109386139,0.324941433369605,0.740002636753216
"6867","AC006213.4",3.12141549442555,0.846729251231029,0.859876701991867,0.984710074443949,0.324766502738614,0.740002636753216
"6868","LINC01684",29.3204184890659,0.334356968018026,0.339678961368974,0.984332284432633,0.324952160722813,0.740002636753216
"6869","TIMM17B",113.107280702436,-0.245742059674997,0.249673071590036,-0.984255362863107,0.324989970881199,0.740002636753216
"6870","AMOT",60.4761027316942,0.24834015776754,0.252196532204008,0.984708852248021,0.324767103253088,0.740002636753216
"6871","E2F5",11.1493433017704,-0.46718282727643,0.474772287557748,-0.984014525531054,0.325108370983674,0.740164495313288

"6872","TMCC2",3.73877556962444,0.771884220019722,0.78459523765964,0.983799267405913,0.325214219623437,0.740297735385538
"6873","ENOX1",7.7259836018277,0.617509433687196,0.627865793770763,0.9835054558055,0.325358731458825,0.740312996704464
"6874","GZF1",25.9234098473022,-0.308176326292091,0.313296192509683,-0.983658064349333,0.325283665422262,0.740312996704464
"6875","TFE3",113.595195090661,0.187677474419023,0.190826690948603,0.983496980878692,0.325362900488601,0.740312996704464
"6876","RPS23",20113.4853825094,-0.270309997235015,0.274942308442744,-0.983151697408936,0.32553278366452,0.740417066644974
"6877","SEMA6A-AS2",3.22812986197154,0.822359397074875,0.836458896299288,0.983143822981867,0.32553665863993,0.740417066644974
"6878","CATSPERZ",9.43657870054898,-0.641121659492746,0.652132644209431,-0.983115421663896,0.325550635068985,0.740417066644974
"6879","BCAR3",11.2265298248765,0.52694067563856,0.536256122063248,0.98262873645368,0.325790195908936,0.740461441568936
"6880","PRRC2C",1191.78559573654,0.151376068050704,0.15400404501943,0.982935662706819,0.325639104392513,0.740461441568936
"6881","ZKSCAN5",20.3742910116227,0.367979972363617,0.374498123502274,0.982594969828689,0.325806821090519,0.740461441568936
"6882","TSTA3",128.877765547547,-0.284009263137557,0.289030969146835,-0.982625716461802,0.325791682795912,0.740461441568936
"6883","AC010542.2",8.64360351588241,-0.582980259400016,0.593299493310398,-0.982607040749683,0.325800877843524,0.740461441568936
"6884","MYO15A",4.154002607419,0.817716990948449,0.8323008802491,0.982477623601352,0.325864601433623,0.740485177255398
"6885","SMIM8",152.307063442495,0.20173004418442,0.205390534849099,0.982177899934054,0.326012213206735,0.740660896795093
"6886","PPP1R3E",36.9112355305498,0.396172323204848,0.403429467321169,0.982011368270866,0.326094247647859,0.740660896795093
"6887","FANCM",45.8740473682151,0.273793106927772,0.278805961892696,0.982020273415627,0.326089860583538,0.740660896795093
"6888","ZNF710",16.4836454742572,0.408977408107511,0.416501033989356,0.981936117157307,0.326131321173982,0.740660896795093
"6889","PRDM16",7.09796704920406,-0.793783135916794,0.808624610056742,-0.981646027148609,0.326274263758418,0.740662938321424
"6890","AASDHPPT",177.405879622585,0.203969544714714,0.207757338508559,0.981768182914565,0.326214066234064,0.740662938321424
"6891","USP14",297.486701288683,-0.153422776416485,0.156285175856531,-0.981684766809401,0.326255172337387,0.740662938321424
"6892","IL1R1",750.362151871532,0.195900202638328,0.199676702295597,0.981086928951389,0.326549875650597,0.740722781597995
"6893","PAX8",7.4993856442958,-0.807851424218009,0.824123779888376,-0.98025496161199,0.326960280487376,0.740722781597995
"6894","TBL1XR1",607.978741257648,0.185636143883964,0.189172083890408,0.981308341412084,0.326440710536128,0.740722781597995
"6895","FBXO45",56.8199200120177,-0.227633042455078,0.23195833060536,-0.98135316744609,0.326418612412319,0.740722781597995
"6896","AC097460.1",3.96712053298518,0.716134619612236,0.7306464518220

12,0.980138366273335,0.327017823067245,0.740722781597995
"6897","CCL28",25.0484781240987,-0.503314697139716,0.513170644078099,-0.980794016469729,0.32669432954817,0.740722781597995
"6898","AL590617.2",4.71048782941594,-0.777453654618746,0.793190981798459,-0.980159472887564,0.327007405962346,0.740722781597995
"6899","AGFG2",16.3005364239272,-0.374103123303964,0.381790628287097,-0.979864605326687,0.327152956470021,0.740722781597995
"6900","DNAL1",61.0372399644948,-0.229360957679176,0.234034462741643,-0.980030697155801,0.327070966184536,0.740722781597995
"6901","ANKRD11",796.955506707497,0.217991390507983,0.222388288527954,0.980228733945142,0.326973223893094,0.740722781597995
"6902","DAPK3",129.476907788704,-0.231511341935291,0.236263240043149,-0.979887272742936,0.327141766042747,0.740722781597995
"6903","AC011447.3",11.567414844922,0.487113483348247,0.496728146052567,0.980644014677391,0.326768321105497,0.740722781597995
"6904","ZNF730",3.81004575043576,0.803489403442226,0.819994930682283,0.979871183805583,0.327149708787571,0.740722781597995
"6905","ZNF780B",42.3847367687389,0.307820609882527,0.31380039634796,0.980943980520654,0.326620367488387,0.740722781597995
"6906","AC010487.2",3.39256499121854,0.800186921612765,0.81566326539143,0.981026062548939,0.326579889354136,0.740722781597995
"6907","UQCC1",76.6675655242613,0.225105042525412,0.229656148399718,0.980182956537335,0.326995815932101,0.740722781597995
"6908","LINC00685",40.4173824301338,0.416391998890734,0.424896336342426,0.979984912261427,0.327093566304587,0.740722781597995
"6909","TXLNG",191.341019859444,0.214213337450327,0.218478667951937,0.98047713059772,0.326850652999551,0.740722781597995
"6910","HMGB1",4211.25886440113,-0.193961319364516,0.198052589466471,-0.979342506386934,0.327410774543143,0.740783946705494
"6911","SLC9A3R2",130.820022684336,-0.266796301252661,0.272377894028769,-0.979507908319762,0.327329083019459,0.740783946705494
"6912","NXN",58.786634088489,0.294166578027948,0.300433779596856,0.979139490980949,0.327511061339846,0.740783946705494
"6913","ACAP1",29.3963838524383,0.330871793879272,0.337921276693988,0.979138683175905,0.327511460424164,0.740783946705494
"6914","ZNF112",15.7936788864517,0.47314053405134,0.483198987568062,0.979183620463806,0.327489260291109,0.740783946705494
"6915","ZNF813",24.1397938009927,0.366019669377161,0.373611301950269,0.979680399031081,0.32724390446602,0.740783946705494
"6916","ZRSR2",80.3425920374851,-0.234700802293622,0.239657402260824,-0.979317976743286,0.327422890788181,0.740783946705494
"6917","RC3H1",161.394976531597,0.271727977151823,0.277574727535324,0.978936301458651,0.327611454110281,0.740794412374181
"6918","HSPA1A",1443.56826014796,-0.409401957274305,0.418251407336055,-0.978841792504382,0.327658156313812,0.740794412374181
"6919","AC139530.1",43.0453273233966,-0.276175719123168,0.282127372673482,-0.978904373957352,0.327627230805762,0.740794412374181
"6920","AC107204.1",11.3768563376948,0.487997915220417,0.498608080765258,0.978720430024807,0.327718134694884,0.740822945235849
"6921","CAVIN2",49.5730715226497,0.380662211983617,0.389019745107837,0

.978516429488938,0.327818969567309,0.740891330000194
"6922","UTP23",211.570908197029,0.161885408284598,0.165447899542919,0.978467594522732,0.327843111056788,0.740891330000194
"6923","RAB6A",306.700876069421,0.1402849477105,0.143425353821395,0.978104247071928,0.32802276752491,0.741190257459507
"6924","GLYCTK",11.3363724964464,-0.466349147912162,0.476841750267638,-0.977995629892754,0.328076485484512,0.741204572853008
"6925","SLC25A5-AS1",6.47737652329233,-0.558623366761979,0.571327032326452,-0.977764634183782,0.328190746221733,0.741355645219722
"6926","FCER1A",12.9097716548027,-0.79345566290613‌5,0.811692714367005,-0.977532074468487,0.328305806658443,0.74150848015565
"6927","MRPS35",149.984972225294,-0.20368827061191,0.208469200371452,-0.977066493510678,0.328536234485598,0.741921801076687
"6928","YTHDF2",332.241550848102,-0.232130286328988,0.237783635539239,-0.976224818005537,0.328953067139507,0.742056038376852
"6929","AC093110.1",5.86032619443525,0.792436377861067,0.812214470105893,0.97564917522062,0.329238346650293,0.742056038376852
"6930","AC112220.2",6.20689812055113,-0.870409186700297,0.892084470076744,-0.975702655854347,0.329211835736135,0.742056038376852
"6931","USP13",58.5099521515693,-0.234124580999825,0.239787421651938,-0.976383912829537,0.328874250515265,0.742056038376852
"6932","SHROOM1",28.6594060905682,0.336649179796998,0.345017727487536,0.975744586368131,0.329191051305704,0.742056038376852
"6933","SERPINB1",520.187156007457,-0.184513657188147,0.189044964741941,-0.97603053030278,0.32904933515153,0.742056038376852
"6934","CELF2-AS2",3.18837507067548,1.06187590967195,1.08837752596742,0.975650345892697,0.32923776632099,0.742056038376852
"6935","MYOF",727.399983024427,0.214301268963296,0.219452688398909,0.976526059110066,0.328803840666582,0.742056038376852
"6936","DEPDC7",7.48926868074744,0.536889024503341,0.549808738214686,0.976501439840157,0.328816034721874,0.742056038376852
"6937","BRMS1L",83.9279717930265,-0.211817531876323,0.217113825919725,-0.975605910766088,0.329259794308875,0.742056038376852
"6938","MYCBPAP",6.58916113975379,0.645776947172138,0.661336573793699,0.976472454060261,0.328830391908541,0.742056038376852
"6939","SNRNP70",520.687086570016,0.183520774391392,0.187875660070645,0.976820383877211,0.32865808274646,0.742056038376852
"6940","BID",82.9464254821405,-0.322779281705413,0.330679014658865,-0.976110570664419,0.329009673577297,0.742056038376852
"6941","AMMECR1",64.0581906742567,0.232553405617851,0.238309357885208,0.975846721595675,0.329140427711125,0.742056038376852
"6942","DOK1",60.6218614615699,-0.297855235798905,0.305422341369598,-0.975224125593563,0.329449097420013,0.74226879316452
"6943","TBCEL",44.3869268207489,0.240587163947798,0.246678600867736,0.975306180193536,0.329408405788103,0.74226879316452
"6944","ASH1L",595.328828896448,0.189147680845011,0.194076300682477,0.974604731128252,0.329756365904341,0.742406639999011
"6945","AC072022.2",23.510559828188,0.416678034441088,0.42751749099006

,0.974645583450003,0.329736094216916,0.742406639999011
"6946","SNX25",13.953831952124,0.471221387505842,0.483399832518167,0.974806683426255,0.329656161257786,0.742406639999011
"6947","RPP38",100.223011839093,-0.222624816644826,0.228444015137087,-0.974526806978204,0.329795035565629,0.742406639999011
"6948","B4GAT1",224.737816372802,0.241288382975305,0.247584142880057,0.974571231293266,0.32977298975239,0.742406639999011
"6949","RNF138",53.7348379350813,-0.249686983635587,0.25619464669227,-0.974598754733319,0.329759331571151,0.742406639999011
"6950","MAP3K8",156.084851180823,-0.300179600179707,0.308113582751239,-0.974249812355926,0.32993251721513,0.742502426528021
"6951","ERVK3-1",193.680490374396,-0.202570031923156,0.207917627580955,-0.974280219912006,0.329917423119955,0.742502426528021
"6952","KRT27",51.8459755975854,0.934675775000904,0.959602706971939,0.974023695650367,0.330044773969992,0.742648216227357
"6953","Z95114.1",7.74747233289993,-0.786719946980857,0.807944561186683,-0.973730110671638,0.33019056257608,0.742869404627877
"6954","SLC50A1",86.3001044708671,-0.266832003593112,0.274400707253373,-0.972417331806391,0.330842973001495,0.743229204383461
"6955","C1orf198",70.7562085736011,0.374105487724601,0.384832276736312,0.972126067224187,0.33098783543963,0.743229204383461
"6956","MYBL1",9.99116924752886,0.655201682453015,0.673860921106134,0.972309955854852,0.330896372393568,0.743229204383461
"6957","PHYHIPL",13.2034427171989,0.778381812608698,0.800134890895105,0.972813236200621,0.33064613301349,0.743229204383461
"6958","SMPD1",143.946375031546,0.27518986860501,0.282804283567881,0.973075319557375,0.33051586929857,0.743229204383461
"6959","ARNTL",76.5354003976562,0.907760434087019,0.933842520301094,0.972070144968698,0.331015653451357,0.743229204383461
"6960","LINC02739",5.01356414214516,0.619100484682821,0.636881181579444,0.972081610493612,0.331009949908186,0.743229204383461
"6961","FCHSD2",45.0571952918636,0.283615051801772,0.291702677114954,0.972274422047917,0.330914045024156,0.743229204383461
"6962","CD27-
AS1",176.770338932505,0.231437313991825,0.237991773715275,0.972459301340004,0.330822102547538,0.743229204383461
"6963","HOXC9",364.524521289321,-0.204513849921897,0.210284151947678,-0.972559501168606,0.330772278992863,0.743229204383461
"6964","NGRN",407.798876054926,-0.152380565181298,0.156652605603725,-0.972729209284693,0.330687904079398,0.743229204383461
"6965","ZNF720",150.240867115847,0.196836565084795,0.20226894927583,0.97314276753558,0.330482350894959,0.743229204383461
"6966","KRT10",1387.9491045122,-0.265799983275046,0.27320474435577,-0.972896659982294,0.33060466515251,0.743229204383461
"6967","AC145207.5",11.999084283017,-0.44369827600783,0.456251935739447,-0.972485246092665,0.330809201263228,0.743229204383461
"6968","ATG12",437.523738372532,0.148052355737534,0.152339081286009,0.971860631478883,0.331119887456092,0.743356544126816
"6969","EVI5",242.607128880757,0.188453674570086,0.193942480475647,0.971698794961791,0.331200416459775,0.743430637778771

"6970","DHRS1",129.431197949535,-0.210136429257401,0.216327676036334,-0.971380237182906,0.331358966142424,0.743679814543177
"6971","ELP3",71.283033705714,0.217698571518381,0.224199282506108,0.971004764533312,0.331545905980097,0.743885916128321
"6972","ZNF480",100.828533909262,-0.222614446600765,0.22924599030533,-0.971072367740292,0.331512242750851,0.743885916128321
"6973","IKBKE",23.6102352652403,-0.321091456985247,0.330813719003952,-0.970611067618426,0.331741992502712,0.743997578638226
"6974","KCTD3",202.773239252582,0.137497591402864,0.141673722345262,0.970522896742837,0.331785917572094,0.743997578638226
"6975","EPDR1",202.355677921341,0.265308812832697,0.27333370247574,0.970640687297773,0.331727237379873,0.743997578638226
"6976","AL352979.4",4.83084756001493,-0.62424050650872,0.643089206254071,-0.970690380802465,0.331702483379873,0.743997578638226
"6977","OS9",914.051773906824,0.157147805561402,0.161964609677878,0.970260144323778,0.331916838169405,0.744184477495199
"6978","ID3",1470.41152562884,-0.326577308460824,0.3367734100395,-0.969724149013191,0.332184009910442,0.744215553966668
"6979","SNHG3",94.3194742736815,-0.215450356800336,0.222198786364331,-0.9696288639807,0.33223152014372,0.744215553966668
"6980","CDC42SE1",115.081881790089,-0.197098001747134,0.203224611802884,-0.969853011397595,0.332119764598865,0.744215553966668
"6981","SMARCC1",117.199471818715,0.22250669304176,0.229373002020503,0.970064877216329,0.332014154751952,0.744215553966668
"6982","TJAP1",65.6933765468818,0.205699949843191,0.212255628336702,0.969114230115436,0.332488198496426,0.744215553966668
"6983","PLAG1",8.8937766868578,-0.629787447822012,0.649567651597545,-0.969548662519008,0.332271512934314,0.744215553966668
"6984","AL161908.1",3.41457760642936,0.852155918070425,0.879134607643281,0.969312219837212,0.332389434154429,0.744215553966668
"6985","HELB",30.4685964817444,0.360482631906288,0.371876830710209,0.969360288506922,0.332365458645021,0.744215553966668
"6986","MMP2",6441.0422865941,-0.239307676905669,0.246883758525732,-0.969313163144858,0.33238896364426,0.744215553966668
"6987","AC103702.1",20.6433034291609,0.471215632881366,0.486294650454241,0.968992014288478,0.332549173574571,0.744215553966668
"6988","CU638689.5",8.37935275001895,-0.492921156823483,0.508441477366502,-0.969474715903574,0.332308389475527,0.744215553966668
"6989","AL732509.1",13.9998977450117,0.381017206314821,0.393205961920695,0.969001600213954,0.332544390769699,0.744215553966668
"6990","CHST7",219.889145484956,-0.365552591724886,0.377143881073042,-0.96926560411062,0.332412686037351,0.744215553966668
"6991","MED14OS",14.3206886373382,-0.449702111533393,0.464377020402473,-0.968398718661053,0.33284527915852,0.744771663835892
"6992","DYNC1LI2",466.830511620059,-0.242447979334687,0.250468455438274,-0.96797809892047,0.333055307702656,0.744921958592029
"6993","BCAT2",69.3483600840433,-0.261207087259387,0.269822972855081,-0.968068376445023,0.333010222112583,0.744921958592029
"6994","AIFM1",70.4364758237572,-0.237696392337597,0.245529771671225,-0.968096010189277,0.332996422303953,0.744921958592029

"6995","ARHGEF19",18.2116262375292,-0.40560132439565,0.419339388019598,-0.967238795075205,0.333424672433497,0.744997383939025
"6996","IPO9-AS1",10.0126402555343,-0.529505742159328,0.547251356723454,-0.967573192197506,0.33325757104509,0.744997383939025
"6997","AL133245.1",4.64712987621045,0.85810323465066,0.886965006576034,0.967460078231508,0.333314089108121,0.744997383939025
"6998","DNER",19.7268267912252,0.45649578816611,0.47214231070959,0.966860579574791,0.33361373542316,0.744997383939025
"6999","CENPE",16.0452462295764,-0.391232554467263,0.404681333481936,-0.966766989475503,0.333660530069476,0.744997383939025
"7000","AC025181.2",52.2498889925443,-0.278879437215008,0.288460780076003,-0.966784590756255,0.333651729181677,0.744997383939025
"7001","AL590004.3",5.24180903600313,-0.851327220147603,0.880426598192096,-0.966948547324392,0.33356975578774,0.744997383939025
"7002","NINJ2",179.278112337456,0.290913141929557,0.300809050244237,0.967102358434211,0.333492866633914,0.744997383939025
"7003","NEK3",54.5035621439114,0.267894674720811,0.276940293499769,0.96733729619245,0.333375444882006,0.744997383939025
"7004","FNTB",26.5902676653528,0.309330062193798,0.319860747816955,0.967077280675953,0.333505402058661,0.744997383939025
"7005","SEC14L5",8.07762545198516,0.557358803107541,0.57615051690508,0.967384019893824,0.333352095584787,0.744997383939025
"7006","NLRP1",59.3985740538768,0.274156908682136,0.283462133166468,0.967172954001347,0.333457580207174,0.744997383939025
"7007","SPEN",239.449777546403,0.222793072819313,0.230703101285079,0.965713384771578,0.334187620015591,0.745070885608497
"7008","COL11A1",6.79102258811373,1.21170950006083,1.25508905499054,0.96543707017664,0.334325941550833,0.745070885608497
"7009","SDE2",73.8348891056957,0.188624617121025,0.195264355260795,0.965996158741303,0.334046103162652,0.745070885608497
"7010","PLGLB2",11.8928102579475,0.480323738597972,0.497308568051415,0.965846497437207,0.334120997734028,0.745070885608497
"7011","LMCD1",253.585857936359,-0.422791656786311,0.437579478083805,-0.966205404873531,0.333941408879715,0.745070885608497
"7012","DCLK2",12.6926516964554,0.489214718071362,0.506993391195731,0.964933126480329,0.334578307965469,0.745070885608497
"7013","S1PR3",104.552288237288,-0.429866921268417,0.44519810379712,-0.965563234888149,0.334262779626121,0.745070885608497
"7014","GATD1",91.5142426759412,0.215528035282695,0.223389929436601,0.964806407461008,0.334641786004421,0.745070885608497
"7015","COA4",332.203846663486,-0.234619336367529,0.242736734444908,-0.966558839575964,0.333764619302705,0.745070885608497
"7016","CACNA2D4",19.2751719317464,0.463980717726871,0.480576321864934,0.965467287123798,0.334310813319485,0.745070885608497
"7017","UBE2N",280.185970892312,-0.244657303528405,0.25355053583523,-0.964925207996387,0.334582274386818,0.745070885608497
"7018","SMAD9",119.658493432769,-0.339867674846508,0.352268253253221,-0.964797910989161,0.334646042465339,0.745070885608497
"7019","VTI1B",410.847886853175,-0.217964435553866,0.225875263266899,-

0.964977007226837,0.334556328356587,0.745070885608497
"7020","AAGAB",47.8368494112403,-0.314454103051236,0.325825561521558,-0.965099550762013,0.334494951946341,0.745070885608497
"7021","AC068338.2",6.14562091458942,-0.553873466591753,0.57391486481668,-0.965079492702583,0.334504997607285,0.745070885608497
"7022","AC012645.4",2.91435071550161,1.06095986271998,1.09833072747315,0.965974852730243,0.334056764606884,0.745070885608497
"7023","RNFT1-DT",15.263138131074,-0.474247248646373,0.491468738833183,-0.964959134068841,0.334565280804393,0.745070885608497
"7024","EIF4ENIF1",62.1165996131504,0.247081462406686,0.255745803761253,0.966121276567825,0.333983499121584,0.745070885608497
"7025","RBX1",890.086302795651,-0.269545349817296,0.278909235382203,-0.966426764061523,0.333830676983872,0.745070885608497
"7026","KDM6A",83.4607004925754,0.24016841928794,0.248835295238739,0.965170230603806,0.334459554970869,0.745070885608497
"7027","ACOT7",22.6760679915694,-0.328650211173548,0.341590517853052,-0.962117488621067,0.335990590117459,0.745814076156737
"7028","KDM1A",162.482431125658,0.164627294600148,0.171058044521222,0.962406036272232,0.335845682699979,0.745814076156737
"7029","PHTF1",44.6345177893877,-0.254954118122332,0.264668767813861,-0.963295065859977,0.335399468738999,0.745814076156737
"7030","HSPE1",730.658410823228,-0.271861332642836,0.28205671735595,-0.963853423493375,0.335119418038197,0.745814076156737
"7031","PLCL2",89.8522885246479,0.235311923981538,0.24448793709642,0.962468442313114,0.335814347940518,0.745814076156737
"7032","UBA7",109.41634083581,0.207603209381526,0.215476908467221,0.96345919782447,0.335317130826485,0.745814076156737
"7033","ARHGEF26-AS1",5.52962158334471,-0.604943229887383,0.628717317173599,-0.962186364782996,0.335955997142701,0.745814076156737
"7034","SENP5",132.330889501173,0.265687443871235,0.276138906622466,0.96215142994855,0.33597354283746,0.745814076156737
"7035","C4orf36",17.5624183276611,0.348956071681699,0.362061839753674,0.963802404360284,0.335145001017119,0.745814076156737
"7036","MYLIP",221.63455172433,0.274366493393977,0.285213668897,0.961968248068292,0.336065553882147,0.745814076156737
"7037","PRKAR1B",11.9092041003034,-0.510493166533088,0.530273498825652,-0.96269786754124,0.335699167188035,0.745814076156737
"7038","WRN",121.57739029355,0.253336644447354,0.263000220757799,0.963256394680578,0.335418870293374,0.745814076156737
"7039","EXOSC4",78.9899996336293,-0.320878695248469,0.333218620111871,-0.962967481051152,0.33556384280189,0.745814076156737
"7040","MAPKAP1",160.687264741314,-0.171990197547978,0.178735872589793,-0.962258974966391,0.33591953124212,0.745814076156737
"7041","KLLN",2.79094414732511,1.04886758341954,1.08885218404085,0.963278210571316,0.335407925045956,0.745814076156737
"7042","CPSF7",149.62389777991,0.184829662489499,0.19187242585288,0.963294551929101,0.335399726576353,0.745814076156737
"7043","LMBR1L",99.567201395714,0.20988765570055,0.21790944635367,0.96

3187503858366,0.335453435007898,0.745814076156737
"7044","MRPL52",263.681046941573,-0.264864524175471,0.275088393022987,
-0.962834241259093,0.335630714114542,0.745814076156737
"7045","ORAI3",269.558963651095,0.341514632346925,0.355024515452667,0.
961946619127087,0.336076419026327,0.745814076156737
"7046","DRG2",145.17640206288,-0.201427686875965,0.209173486242956,-0.
962969497204852,0.335562830986555,0.745814076156737
"7047","XYLT2",60.8253244876568,-0.293640637885654,0.304887662593553,-
0.963110922192701,0.335491861154944,0.745814076156737
"7048","AL117379.1",5.0979852354745,0.688279784316064,0.71545682231709
3,0.962014426093509,0.336042357437445,0.745814076156737
"7049","FBXO7",280.868521034288,-0.154298012798911,0.16006653441492,-0
.963961726059138,0.335065115086422,0.745814076156737
"7050","NUP58",108.931035553016,0.217288915493311,0.225917405509855,0.
961806882488444,0.336146620177116,0.745841494020852
"7051","KDELR1",1383.87197879871,-0.219139569820145,0.227859236365255,
-0.961732222558965,0.336184131837949,0.745841494020852
"7052","ACYP2",94.8119412644633,0.243618796490168,0.253406024175164,0.
961377288812082,0.336362499335766,0.74604897663056
"7053","AGAP9",30.3908933851578,0.365412241354387,0.38010073169144,0.9
6135632185792,0.336373037919539,0.74604897663056
"7054","SNAPC4",47.273355383712,0.242954724492887,0.252758320699151,0.
961213556969571,0.336444801235066,0.746102356920915
"7055","CUL9",35.9311004931184,0.268378927214178,0.279280767031719,0.9
60964587954231,0.336569973511122,0.746168380900578
"7056","SRRM2",2016.13578459056,0.196762233725732,0.204746191314971,0.
961005586780577,0.33654935878009,0.746168380900578
"7057","WDR70",178.512222804074,0.236268648537255,0.245922698634352,0.
960743558237174,0.336681124054724,0.746203290392186
"7058","ZNF428",488.28465853426,-0.272650895300609,0.283780763037034,-
0.960780048593453,0.336662772302847,0.746203290392186
"7059","AC093849.4",5.39862723294831,0.64721461833574,0.67377218258343
8,0.960583762680927,0.33676149610945,0.746275688290145
"7060","ATP6V1A",127.001418912109,0.163187882875822,0.169936965770324,
0.960284786397659,0.336911904755859,0.746503247322366
"7061","C2CD3",31.5029436142059,0.299988770580528,0.312457551657457,0.
960094479999645,0.33700766637561,0.746609676407544
"7062","TRIM13",153.492709259546,0.239092993714752,0.249085520476014,0
.959883148798989,0.337114028119198,0.746633830081922
"7063","SETD6",41.0416523530937,-0.236471962014626,0.246346109844275,-
0.959917581666334,0.337096696789202,0.746633830081922
"7064","CHMP7",74.8520742942175,0.208652719476424,0.217522286959852,0.
959224557596412,0.337445631579846,0.747156689993423
"7065","KIF7",50.401772475922,-0.252273588255553,0.262991464520689,-0.
959246296131058,0.337434682791234,0.747156689993423
"7066","AC087501.4",3.88850901839442,-0.678303181813286,0.707224772799
917,-0.959105517652995,0.337505591057305,0.747183691042941
"7067","ENO4",11.2900312968224,0.463988346111146,0.483966148039443,0.9
58720662572728,0.337699486360318,0.74750715510605
"7068","EEF1E1",108.187066943386,-0.240127099385507,0.250674458170057,

-0.957924078657449,0.338101043661519,0.747772106435199
"7069","KMT2E-
AS1",113.712717395277,0.362815229882491,0.378874194935339,0.9576139909
56579,0.338257441506404,0.747772106435199
"7070","FOCAD",67.1096884828168,0.230163362144302,0.240418241062497,0.
957345670308233,0.338392810934908,0.747772106435199
"7071","GOLGA1",80.8772856573298,-0.195658638794668,0.204245529180376,
-0.95795800074466,0.338083937316098,0.747772106435199
"7072","C9orf78",322.867081538426,-0.135919881593453,0.141848247407054
,-0.958206280853166,0.337958750702668,0.747772106435199
"7073","PTPRJ",11.100062878852,0.4771390075705,0.498218374727185,0.957
690506360331,0.338218845384236,0.747772106435199
"7074","AP001372.2",32.2296898815001,-0.309807910585479,0.323291750886
469,-0.958292037257316,0.337915517936272,0.747772106435199
"7075","MT2A",8054.27451642015,-0.42529210041731,0.443929994341868,-0.
958016141819414,0.338054619030196,0.747772106435199
"7076","RPL27",6161.06770484995,-0.238335897695744,0.24893096835195,-0
.957437715659284,0.338346369566515,0.747772106435199
"7077","RTTN",29.8596411053801,0.316266454505255,0.330317009788137,0.9
57463421905237,0.338333400242899,0.747772106435199
"7078","SNRPD2",1070.14825659881,-0.228163850823863,0.238243264452597,
-0.957692765619661,0.33821770580507,0.747772106435199
"7079","NAA20",325.653157459999,-0.240154951191501,0.250728901554353,-
0.957827157949081,0.338149922228725,0.747772106435199
"7080","BIVM",106.118454271717,0.229368769219973,0.239651964975628,0.9
57091126890195,0.338521261810303,0.747950296398102
"7081","CHD4",350.463440192439,0.250460370050831,0.261782604081292,0.9
56749478941907,0.338693717654801,0.748225649664462
"7082","IQCC",7.75804840895767,0.517903859924278,0.541407532119003,0.9
56587836702724,0.338775330501111,0.748300267583858
"7083","FGGY",34.1659031594668,0.316178840791506,0.330722329850993,0.9
56025076788616,0.339059565319972,0.748432362912558
"7084","HACE1",41.0617346384901,0.325803773304471,0.340901717493631,0.
955711739148273,0.339217889985938,0.748432362912558
"7085","CELF2",1289.02651560522,0.21652081835353,0.226487449449808,0.9
55994775337487,0.339074874103668,0.748432362912558
"7086","DACT1",49.3007658633903,-0.342327785589373,0.358156790770548,-
0.955804257830432,0.339171136792376,0.748432362912558
"7087","AL121839.2",23.522012080201,-0.402172578814645,0.4207208358801
61,-0.955913148378512,0.339116115571284,0.748432362912558
"7088","SAXO2",9.59804973563322,-0.439038984468398,0.459262736109625,-
0.955964745120537,0.339090046292999,0.748432362912558
"7089","AC005523.2",18.3380257487154,-0.396952777852778,0.415182576537
742,-0.95609209125059,0.339025710103746,0.748432362912558
"7090","MAGEE1",21.9157393154978,0.304130144380754,0.318190714559557,0
.955810872110879,0.339167794504343,0.748432362912558
"7091","LINC01127",3.41704891170519,1.19179206547124,1.24794000262527,
0.955007502735778,0.339573902811079,0.748900967386677
"7092","AL022344.1",8.3951365406099,0.542145500292293,0.56759182261337
9,0.955167919432097,0.339492786248768,0.748900967386677

"7093","RAB36",12.9938141977611,0.439497758890906,0.460190088691549,0.
955035255410483,0.3395598684603,0.748900967386677
"7094","LINC01376",8.24411591928885,0.510000084629326,0.53433099786883
3,0.954464716932856,0.339848460911847,0.748976998409897
"7095","MAP6D1",2.85803436843918,-0.76592718482746,0.802694434556409,-
0.95419521034892,0.339984838664389,0.748976998409897
"7096","AL138724.1",14.4130053821972,-0.485643523794268,0.509064017504
904,-0.953993028567551,0.340087171239883,0.748976998409897
"7097","NUP43",64.9272942875109,0.316473122441081,0.331640635523971,0.
954265215241413,0.339949410888922,0.748976998409897
"7098","ATXN7L1",43.0423960277984,0.285302304857375,0.298958544148431,
0.954320625523665,0.339921370769107,0.748976998409897
"7099","FBXL6",38.5475065020505,-0.292592315543324,0.306647593501851,-
0.954164721144496,0.340000269249893,0.748976998409897
"7100","SP1",224.983680534355,0.235982232489744,0.247357406539469,0.95
401320619882,0.34007695761807,0.748976998409897
"7101","RAN",814.453710550656,-0.261743052409094,0.274318524352949,-0.
954157408897129,0.340003970044822,0.748976998409897
"7102","UBAP1L",18.8670598548226,0.56208164505915,0.589111542380882,0.
954117522103724,0.340024157568473,0.748976998409897
"7103","ARMCX3",495.23357658171,0.106348649381908,0.111427323393353,0.
95442164581557,0.339870253728279,0.748976998409897
"7104","ZNF785",61.5169392577227,0.26218877316527,0.274866262152522,0.
953877609831147,0.340145598294825,0.749000224398361
"7105","DLAT",57.4546002084241,0.232144014068397,0.243433360767256,0.9
53624488183223,0.34027375558133,0.749101420165597
"7106","SNX12",71.4127875850107,0.200110251271646,0.209847669441016,0.
953597682569896,0.340287329265277,0.749101420165597
"7107","CEP162",130.461025051251,0.172448241035634,0.180904581698454,0
.953255243270089,0.340460762385694,0.749111817033675
"7108","SCML4",3.06962461825009,0.962819262589245,1.00980124978296,0.9
5347402550372,0.340349950565148,0.749111817033675
"7109","AC007066.2",6.01549279621905,0.596237683017933,0.6256795987154
46,0.952944101489071,0.340618393541979,0.749111817033675
"7110","LINC02716",4.87095039492368,0.764522350833703,0.80219481370014
6,0.953038261750064,0.340570684984402,0.749111817033675
"7111","XAB2",138.102713897577,-0.192457887611408,0.201892304079187,-0
.953270053998304,0.340453260115958,0.749111817033675
"7112","CHMP2A",866.645908905807,-0.248229368333007,0.260491596816121,
-0.952926587141426,0.340627268079047,0.749111817033675
"7113","POFUT2",128.250107263217,0.220998908003545,0.231897043811211,0
.953004421149334,0.340587830644659,0.749111817033675
"7114","MSH3",116.710404331522,0.204152644473558,0.214265517298774,0.9
52802144961503,0.340690327318099,0.749145177148864
"7115","TCF25",984.461518326693,-0.138757334462145,0.145683415374149,-
0.952458000149049,0.340864756557503,0.749423385358963
"7116","LINC02021",2.64324379960103,-0.83420913113685,0.87611443532869
8,-0.952169143091306,0.341011207430388,0.749433206345908
"7117","RB1CC1",556.962437101201,0.234680544342541,0.246470275382745,0
.952165708331785,0.341012949099928,0.749433206345908

"7118","CD81",10530.7321323631,-0.231560766606572,0.243172628398348,-0
.95224848344052,0.340970977765668,0.749433206345908
"7119","E2F3",43.0662824610765,0.343722686143451,0.361204310567666,0.9
51601838868587,0.341298948600158,0.749956377714885
"7120","CDK17",58.5659357607514,0.253204674551304,0.266215108364506,0.
951128116307405,0.341539343460433,0.750379206425781
"7121","CITED4",116.494590055035,0.53000523221658,0.557536352965802,0.
950620043692631,0.34179728997825,0.750439530634336
"7122","PSMD6",175.328565539324,-0.207328483720917,0.218066307764436,-
0.950758903777478,0.341726778870658,0.750439530634336
"7123","THG1L",75.2016332436511,-0.224714847110699,0.236439238494649,-
0.950412666448273,0.341902610421972,0.750439530634336
"7124","PHF14",482.295776216367,0.14105970916157,0.148349147433056,0.9
50862958111875,0.34167394771199,0.750439530634336
"7125","BNIP3",389.945488610757,-0.198662643025317,0.208994123820934,-
0.950565687653164,0.341824893710177,0.750439530634336
"7126","PRR13",361.35134393654,-0.182337532773176,0.191822935717478,-0
.950551257549971,0.341832222016781,0.750439530634336
"7127","NLK",17.2167662703496,0.373636116896315,0.393116595650438,0.95
0446053487283,0.341885652782573,0.750439530634336
"7128","ATP2A2",229.723753482514,0.181305110889536,0.19080285859877,0.
950222193844558,0.341999363815827,0.750478932839518
"7129","AC015726.1",5.74803148295753,0.671126061920254,0.7063083866532
94,0.950188436952102,0.342016512958699,0.750478932839518
"7130","HP",158.116588898689,1.07529367533577,1.13224463074525,0.94970
083861472,0.342264283536071,0.750917277328857
"7131","SUMF1",198.148204676539,0.146417967844,0.154212752636114,0.949
454343698107,0.342389582327608,0.750930946322177
"7132","SUGCT",8.87944351077848,0.522453973654286,0.550346400473051,0.
949318416919253,0.342458689446445,0.750930946322177
"7133","C12orf65",167.40964933781,0.168847099788032,0.177836193965458,
0.949452954559001,0.342390288540698,0.750930946322177
"7134","HDAC5",146.530749957122,-0.189299594343595,0.199427215716776,-
0.949216453046386,0.342510535192018,0.750930946322177
"7135","CA5B",89.2077428471759,-0.296599958315985,0.312444536002003,-0
.949288350858161,0.342473976666474,0.750930946322177
"7136","AL358781.1",5.69764954486381,0.814436163154641,0.8583152836014
24,0.948877619582084,0.34268285847058,0.751203469038016
"7137","ZNF619",22.9678941711357,0.31333548677562,0.330434245182701,0.
948253673290953,0.343000328857897,0.751296185473097
"7138","QKI",887.958257275583,0.130942368220994,0.138033792929495,0.94
8625444842173,0.34281114498697,0.751296185473097
"7139","PCM1",644.305429080971,0.193406677620483,0.203933299075667,0.9
48382037151871,0.342935000633818,0.751296185473097
"7140","OTUD1",133.238070805073,-0.220605142499574,0.232649855995346,-
0.948228149790848,0.343013319481484,0.751296185473097
"7141","DPH6",88.2190935522427,0.20281477359281,0.213827480600107,0.94
8497232552197,0.342876381005393,0.751296185473097
"7142","TMSB15B",14.3111265889411,0.391022584618142,0.412224174614163,
0.948567815034463,0.342840466773081,0.751296185473097

"7143","LINC01772",4.89363168839758,0.751617869576038,0.794003050414757,0.946618365236029,0.343833282183603,0.751303818019012
"7144","RPL11",19678.4433091248,-0.277625997612299,0.292888631837709,-0.947889291128694,0.343185817210765,0.751303818019012
"7145","PIGK",205.092300472957,0.216468376734769,0.228508118393112,0.94731153648716,0.343480053943509,0.751303818019012
"7146","NCL",1298.78657493774,-0.180225573548268,0.190331336918553,-0.946904364074273,0.343687513959385,0.751303818019012
"7147","RPUSD3",102.735717802866,-0.212330274979486,0.224118628250978,-0.947401278673316,0.34343433980955,0.751303818019012
"7148","MAT2B",179.927107265149,0.231946202405582,0.244847914194964,0.947307242408817,0.343482241418818,0.751303818019012
"7149","PPP1R18",162.103494568998,-0.189384796740296,0.200006343843749,-0.946893948965185,0.34369282165192,0.751303818019012
"7150","PPP1R3B",130.259147114787,-0.269100431165789,0.284258000464358,-0.946676718777281,0.343803537277031,0.751303818019012
"7151","POLR2L",1767.84243819739,-0.305216537750272,0.322149015857786,-0.947438988561153,0.343415131777463,0.751303818019012
"7152","USP47",265.288283470601,0.168590308641609,0.17786155970285,0.947873778478436,0.343193715330967,0.751303818019012
"7153","SNHG1",98.6184078646515,-0.217066874861281,0.229211796972926,-0.947014410811155,0.343631435724748,0.751303818019012
"7154","CDK2AP1",924.05043571303,-0.241536793673359,0.255079801730152,-0.946906779898161,0.343686282827535,0.751303818019012
"7155","IGHA2",16.4517583037754,-0.749746384562391,0.790946901410999,-0.947909882730295,0.343175333369577,0.751303818019012
"7156","IGHG2",52.754523591917,0.977311308361944,1.03207018050944,0.946942685505682,0.343667985245822,0.751303818019012
"7157","LAMA3",6.56331161743039,-0.545317629224523,0.576043790018201,-0.946660025980477,0.343812046030394,0.751303818019012
"7158","ZNF382",4.7622395716655,0.674311352050596,0.71166176032091,0.947516628891973,0.343375586805003,0.751303818019012
"7159","ADA2",17.3035786996688,-0.436780508294956,0.460931988203225,-0.947602942459222,0.343331627660451,0.751303818019012
"7160","STAMBP",154.904375853182,0.154943185174554,0.163733497414588,0.946313293377124,0.343988815072863,0.751538691925252
"7161","HSBP1L1",57.6961749613744,-0.376611293374662,0.398062746331109,-0.946110372914417,0.344092293620656,0.75165978901022
"7162","DENR",339.618077495568,0.14730072590273,0.155727052317835,0.945890413452977,0.344204483584287,0.751694923455117
"7163","AC010618.3",22.0649788230689,0.383833058921155,0.405782116624977,0.945909253255467,0.344194873459716,0.751694923455117
"7164","AC123768.4",8.60936125125333,0.580171156981048,0.61345740715964,0.945739916430857,0.344281257794678,0.751757637588239
"7165","NBPF19",141.024547674987,0.440110342828425,0.465550995106121,0.945353672218233,0.34447834555653,0.752072001082719
"7166","PML",191.664411495428,0.171072095364194,0.180977088635188,0.945269352349012,0.344521380793886,0.752072001082719
"7167","ZC3HAV1L",9.79687147735681,0.58031548556132,0.614012464684998,0.945120040615193,0.344597595042417,0.752133414154951

"7168","WNT9A",16.9237802424315,0.413616862861938,0.437813083397293,0.944733902542153,0.344794744116453,0.752265310511564
"7169","VAMP5",928.071422067399,-0.264951347421806,0.280568664718685,-0.944336915483635,0.344997507309049,0.752265310511564
"7170","MRPL47",222.049151122908,-0.23774092893447,0.251683752261465,-0.944601813976012,0.344862200594434,0.752265310511564
"7171","SGCE",1258.13185432409,0.244625873484193,0.259054635946442,0.944302241843564,0.345015220660246,0.752265310511564
"7172","MED30",216.226232607106,-0.252848009710439,0.267714324456076,-0.944469483372468,0.344929789119097,0.752265310511564
"7173","SLC25A15",8.44871684481059,-0.467230379721272,0.494697401591953,-0.944477125244057,0.344925885764715,0.752265310511564
"7174","PHF12",86.2103818131895,0.278436074125565,0.294875884531426,0.944248372728125,0.345042741348876,0.752265310511564
"7175","AC020916.1",332.44842242796,-0.62194344381662,0.658616444252683,-0.944318122093543,0.345007108015008,0.752265310511564
"7176","MSMO1",85.6129183587197,-0.278851220997432,0.295364150127426,-0.944092981078206,0.34512213577909,0.752333552117099
"7177","CPT2",56.8453501378792,0.220829874263245,0.234471559355343,0.941819446547784,0.346285087192003,0.752352447075626
"7178","HIST2H2BE",12.7124633116883,0.422441071732635,0.447681732393071,0.943619185608686,0.345364284533018,0.752352447075626
"7179","DDR2",1026.71037289639,0.243649535051646,0.258678048883952,0.941902631873308,0.346242492548965,0.752352447075626
"7180","LINC01088",36.2617623029358,-0.470350339164747,0.498752768636032,-0.943053089110745,0.345653748744225,0.752352447075626
"7181","DAPP1",6.17555267294711,-0.760094601714867,0.80679869016604,-0.942111843982342,0.346135381253108,0.752352447075626
"7182","TRAPPC13",47.7499758626032,-0.237170039031717,0.251524943559147,-0.942928505124369,0.345717473486339,0.752352447075626
"7183","GFOD1",7.03591161513083,0.541401204535563,0.574326093443876,0.942672134725966,0.345848630566879,0.752352447075626
"7184","SMIM19",686.712877433924,-0.302265120536917,0.320398726212723,-0.94340300321991,0.345474807627435,0.752352447075626
"7185","AP003117.1",13.2956156229635,-0.478265854180689,0.507773842566434,-0.94188753749779,0.346250221303003,0.752352447075626
"7186","PTPRD-AS1",52.7210858298847,0.38744133434907,0.410781945446985,0.943180046356426,0.345588817781163,0.752352447075626
"7187","MEN1",44.302278488322,0.262214215008272,0.278313035060013,0.942155709493559,0.346112925900472,0.752352447075626
"7188","AP002381.2",3.91651007454352,0.757007286231403,0.803132257820138,0.942568647766774,0.345901582662728,0.752352447075626
"7189","RACGAP1",14.1399716083648,0.392104309002635,0.415854527690748,0.94288815653878,0.345738113403512,0.752352447075626
"7190","CERS5",149.711410860929,-0.1714519471348,0.18187405072169,-0.942696038574309,0.345836400205861,0.752352447075626
"7191","USP30",41.9864235282111,0.250389342881523,0.265854462068731,0.94182862658438,0.346280386434905,0.752352447075626
"7192","TCTN2",38.5549255641194,0.343636612484831,0.364163332001771,0.

943633206000982,0.34535711739574,0.752352447075626
"7193","KIAA0556",31.6247344631844,0.275942707281219,0.292490562979812
,0.94342430904434,0.345463914038264,0.752352447075626
"7194","FAAP100",57.1703575430926,0.224218702131667,0.238047181110545,
0.941908663171877,0.3462394043815,0.752352447075626
"7195","MISP3",15.712538875649,-0.419209058880871,0.445096140966791,-0
.941839347270704,0.346274896819528,0.752352447075626
"7196","CDH22",4.61257544965458,-0.846107660541061,0.897842847266709,-
0.94237834952615,0.345998967743914,0.752352447075626
"7197","KANTR",68.4915711257692,0.354854075796289,0.376528292996469,0.
942436683767657,0.345969113352728,0.752352447075626
"7198","CHIC1",48.8088674290915,0.275568796781869,0.29257090749019,0.9
41887213413758,0.346250387244546,0.752352447075626
"7199","TBC1D8B",108.614505648816,0.214695043338131,0.227733609114277,
0.942746413992834,0.345810626615957,0.752352447075626
"7200","APLN",7.45436965307315,0.607797736763929,0.644736714207804,0.9
42706880762541,0.345830852910602,0.752352447075626
"7201","TNK1",2.99835721001666,-1.19814750544916,1.27241010858403,-0.9
41636267557232,0.346378894872482,0.752451750102796
"7202","FBXO46",24.5486812052362,-0.332643743330295,0.353312707160601,
-0.941499517533881,0.34644893639263,0.752499404608431
"7203","BCS1L",78.4013673078395,0.233046725663443,0.247728061920827,0.
940736079136337,0.346840124965204,0.752931008768137
"7204","CSNK1A1",786.557194987812,0.115122485722464,0.12237491480727,0
.940735982564499,0.346840174466739,0.752931008768137
"7205","BCKDK",139.004538222752,-0.245883032502464,0.261322526591168,-
0.940917860047865,0.346746954280261,0.752931008768137
"7206","TRIB3",7.90337971160812,0.687352480741578,0.730606572376407,0.
9407970126875,0.346808892073974,0.752931008768137
"7207","LCMT1",151.968114588598,-0.226256781532743,0.24055887311217,-0
.940546397668074,0.346937362019899,0.753037484955914
"7208","SLC35A5",106.087212793487,0.21126560961483,0.224706298005672,0
.94018552879856,0.347122403353376,0.753247264733126
"7209","IFNGR2",494.504029657522,-0.185896625875716,0.197726587760549,
-0.940170100446178,0.347130315889606,0.753247264733126
"7210","DPM3",359.290230890312,-0.220945586427892,0.235082428572059,-0
.939864318103067,0.347287162138934,0.753274137179609
"7211","SMARCA5",391.376074701792,0.157406510753929,0.167476948276096,
0.939869709677503,0.347284396225072,0.753274137179609
"7212","MRPS34",422.65991496368,-0.2325745018294,0.247449791334013,-0.
939885625183117,0.347276231545657,0.753274137179609
"7213","JAKMIP3",3.79165914567844,0.769733580158966,0.819090665206567,
0.939741609635908,0.347350116195416,0.753306234249951
"7214","PTPRU",42.6833149691136,-0.335984691536313,0.357610118122899,-
0.939527923035014,0.347459762783713,0.753439571558862
"7215","LUZP1",214.773698817293,0.150485557845625,0.160410173051306,0.
938129764360354,0.348177727378073,0.753570982543327
"7216","MACF1",734.208979858906,0.241396561587504,0.257434244026137,0.
937701829454341,0.348397663405722,0.753570982543327
"7217","PRKAB2",123.734719704583,0.200755998023502,0.214006729597962,0

.93808264067512,0.34820194214788,0.753570982543327
"7218","LHCGR",10.159701617517,0.543105541360296,0.578721522401883,0.938457479698061,0.348009358622946,0.753570982543327
"7219","SP140L",70.3075452692408,0.224263596043801,0.239130594310672,0.937828957813922,0.348332316900425,0.753570982543327
"7220","LINC00884",11.5861445930573,0.483579854722054,0.515481704345868,0.938112547244917,0.348186574365245,0.753570982543327
"7221","PLK4",4.09456038276633,0.74822785804747,0.797744079087067,0.937929691566921,0.348280543280032,0.753570982543327
"7222","CWC27",186.379794839159,-0.181230476069925,0.193102094837023,-0.938521543346705,0.347976450995606,0.753570982543327
"7223","NSA2",1028.16448566476,-0.23324347885129,0.248710609211129,-0.937810733491029,0.348341684086514,0.753570982543327
"7224","LINC02604",12.6082635045816,0.426956625084562,0.455067511572356,0.938226997592806,0.348127766919664,0.753570982543327
"7225","EPHB4",41.5059209019794,-0.270739124188097,0.288748996844976,-0.937627929954167,0.348435652799072,0.753570982543327
"7226","BACE1-AS",26.6085147138366,0.334980657866301,0.357184734039884,0.937835875787727,0.348328761147798,0.753570982543327
"7227","SC5D",119.821616067647,0.220277219267071,0.23484378947996,0.937973364144971,0.348258098624754,0.753570982543327
"7228","IST1",256.415097958907,0.170030922786987,0.181305083219925,0.937816633528903,0.348338651487037,0.753570982543327
"7229","YJU2",86.815800896202,-0.235261627821591,0.250645025057941,-0.93862476531184,0.347923433045622,0.753570982543327
"7230","IRF3",245.698830370303,0.180195752492719,0.192040479144749,0.938321718916861,0.348079101514507,0.753570982543327
"7231","LENG1",104.945949646827,-0.26205063686289,0.279436458955836,-0.93778255651424,0.348356167188539,0.753570982543327
"7232","SLC2A11",43.5643935938228,0.266572513936324,0.283819193092037,0.939233569908289,0.347610836923971,0.753570982543327
"7233","PRAF2",131.700064522663,-0.223951715171077,0.238778590294032,-0.937905341074769,0.348293058131726,0.753570982543327
"7234","EML4",103.603582898938,0.218225624059711,0.232822038392848,0.937306560693759,0.348600889175766,0.753718869705482
"7235","ZNF354A",62.2067050928157,-0.223201679889273,0.238202108960391,-0.937026464053632,0.348744945274454,0.753718869705482
"7236","TMEM132C",86.8835691152686,-0.317820344304857,0.339163693413093,-0.937070654899842,0.348722215031778,0.753718869705482
"7237","DIO2",5.83018401010708,-0.874633337674541,0.933356744075984,-0.937083642697008,0.348715534737147,0.753718869705482
"7238","RCOR1",114.046309445509,0.209724974350093,0.223783712143067,0.93717711776992,0.348667458079399,0.753718869705482
"7239","GUK1",1764.61987040548,-0.289056324024268,0.308598726510325,-0.93667374228324,0.348926406890429,0.753871663045627
"7240","AC108488.3",6.04005062296423,0.565574845448665,0.603854362462165,0.93660803102023,0.348960219402505,0.753871663045627
"7241","GEMIN5",79.3626727556336,0.202766409739479,0.216464639981776,0.936718393159038,0.348903432438621,0.753871663045627

"7242","RBM6",360.960968608176,0.271392863507914,0.289849338648509,0.9
3632390114584,0.349106445762494,0.753979308444386
"7243","MPHOSPH9",39.1337397555893,0.290383714017105,0.310118252853447
,0.936364471762751,0.349085563867553,0.753979308444386
"7244","AL359541.1",13.1043988881852,0.448157968904458,0.4787089963992
37,0.936180377380457,0.349180324513319,0.75403476205989
"7245","MPHOSPH8",926.238641690366,0.146068691029207,0.156042327251029
,0.936083776770536,0.349230055194465,0.754038061201796
"7246","FILIP1L",994.440624304504,-0.288392049258073,0.308237722343881
,-0.935615689945738,0.349471093312304,0.754189307153401
"7247","LINC02848",3.77357610509595,0.74697341259632,0.798388682981591
,0.935601203422298,0.349478554733194,0.754189307153401
"7248","INSIG1",163.935543827467,0.223747480185423,0.23915549213643,0.
935573246454163,0.349492954519913,0.754189307153401
"7249","PGR",54.2449912421525,-0.26092699759728,0.278819212339427,-0.9
35828615998076,0.349361435402769,0.754189307153401
"7250","ITSN2",166.639046245639,0.235607477978798,0.251915509603125,0.
935263884109322,0.349652322828173,0.754221081908592
"7251","SMIM20",359.597973104162,-0.256311880786668,0.274032011089926,
-0.935335546264181,0.34961540189626,0.754221081908592
"7252","ZFAND5",927.257419265596,0.303129294524353,0.324098689566222,0
.935299352583207,0.349634048869248,0.754221081908592
"7253","AC008124.1",123.826858213122,-0.172764300261179,0.184774462426
003,-0.93500096275678,0.349787803267408,0.754317578521428
"7254","ELOB",1801.10148602741,-0.24999044793494,0.267372348511781,-0.
934989909489182,0.349793499622479,0.754317578521428
"7255","TTC39B",68.5085571615092,0.245716253772861,0.262838031387096,0
.934858066300844,0.349861450188121,0.754360119268473
"7256","MTCH2",116.482849406451,-0.268225472660087,0.286997457327666,-
0.934591808434918,0.349998702174458,0.754552053213209
"7257","CMAS",179.729903539255,-0.160216810515657,0.171471707759848,-0
.934362948901437,0.350116703172911,0.75462056220484
"7258","CCNDBP1",441.669079651474,-0.194262265648118,0.207913211359819
,-0.934343057747897,0.350126960332591,0.75462056220484
"7259","FYCO1",93.0014783595113,0.210828550086677,0.225744617710468,0.
933925035400302,0.35034256367294,0.754642172309803
"7260","AC096751.2",3.96090227869733,-0.706582679564002,0.756748788746
14,-0.933708372014365,0.350454345249885,0.754642172309803
"7261","NELFE",312.085849855289,-0.222316465192087,0.238068959627729,-
0.933832220461356,0.350390446245488,0.754642172309803
"7262","PSMC2",305.06581657015,-0.203566789828799,0.217923403433846,-0
.9341208269565,0.350241569801751,0.754642172309803
"7263","ZNF488",3.325458114704,0.790400725733281,0.846298287613027,0.9
33950519931449,0.350329417109259,0.754642172309803
"7264","WARS",96.5515919128787,0.248829853365778,0.266507576188074,0.9
33668967032215,0.350474677608561,0.754642172309803
"7265","FCGRT",3304.23790689118,0.280117351571806,0.299976139982805,0.
933798773421989,0.350407702354556,0.754642172309803
"7266","CDC42",1596.84585168617,-0.216294896741248,0.231720999030253,-
0.933428121087156,0.350598966633783,0.754702027666475

"7267","COPS4",234.547258720464,-0.233760842907184,0.250410544037587,-0.933510383141439,0.350556511995962,0.754702027666475
"7268","CSMD2",8.36127977054588,0.502768592819343,0.539558750269558,0.931814362325075,0.35143247089513,0.754972045820706
"7269","GADD45A",327.656588914032,0.405705500422916,0.435224915298759,0.932174345176036,0.351246431527911,0.754972045820706
"7270","TSTD1",7.42193234881833,-0.560066350067982,0.600381906878219,-0.932850146967513,0.350897345313109,0.754972045820706
"7271","ALMS1-IT1",6.7104040038605,0.646406460494339,0.693163011046571,0.93254609693954,0.35105437549168,0.754972045820706
"7272","MRPL3",298.209932087434,-0.220497759290676,0.236742790099691,-0.93138109590508,0.351656466024859,0.754972045820706
"7273","SDAD1",175.065065351287,-0.155926697584225,0.167209146578209,-0.932524929258544,0.351065309445128,0.754972045820706
"7274","LARP1B",109.171255570256,-0.223487360423663,0.239836635297123,-0.931831620080871,0.351423550651512,0.754972045820706
"7275","TMEM14B",623.243294441738,-0.220474679215543,0.23663102776589,-0.931723456966384,0.351479460713497,0.754972045820706
"7276","VPS50",78.1326081808084,0.189793077955987,0.203700817529862,0.931724674733639,0.351478831212069,0.754972045820706
"7277","CERCAM",531.623884649153,0.292785726990665,0.314268649812248,0.931641533972868,0.351521810874114,0.754972045820706
"7278","RSF1",590.52108578746,0.163003084676331,0.175024293755878,0.931316911375093,0.35168965658093,0.754972045820706
"7279","BCDIN3D-AS1",4.47614202666857,0.65136263247589,0.699392153078517,0.93132676654833,0.351684560228966,0.754972045820706
"7280","PSPC1",113.624930069424,0.233511147883081,0.250424507383184,0.932461244800521,0.351098206315059,0.754972045820706
"7281","USP3",134.44251439759,0.183250262769892,0.196675456768348,0.931739352641908,0.351471243805069,0.754972045820706
"7282","IREB2",249.033505867402,0.19024164400031,0.204253803517607,0.93139829331947,0.351647573381802,0.754972045820706
"7283","TYK2",76.7025844869213,0.215191805655494,0.230787277924493,0.93242490483335,0.351116978978945,0.754972045820706
"7284","PDCD5",741.506417828282,-0.237355648656165,0.254555706068468,-0.932431067140659,0.351113795581697,0.754972045820706
"7285","ZNF221",14.370983470054,0.420020144132867,0.450289481477144,0.93277804925627,0.350934576988058,0.754972045820706
"7286","HM13",591.759642350424,-0.196299662007008,0.21072839499378,-0.931529241765456,0.351579865677383,0.754972045820706
"7287","RBMX",930.045129122982,0.165988319753232,0.178098256067367,0.932004183636953,0.351334363341149,0.754972045820706
"7288","HS1BP3",104.840236007098,0.188738724852891,0.202731479530805,0.930978875553525,0.351864491519003,0.755243721299638
"7289","CAMK1",194.128935900377,-0.196792555770496,0.211539620026497,-0.930286987117807,0.352222513556156,0.75553426123845
"7290","PSORS1C1",39.6070718168689,-0.257370324706377,0.276588206547876,-0.930518072041613,0.35210291156474,0.75553426123845

"7291","OSTM1",149.539869519566,0.17081278223524,0.183625279520766,0.930224763611172,0.35225472280038,0.75553426123845
"7292","HOOK3",522.935979983993,0.181098730888788,0.194752287128425,0.929892704003862,0.352426640954506,0.75553426123845
"7293","AF117829.1",139.556957789153,0.318009246961531,0.342059107948588,0.929690920580101,0.352531136788304,0.75553426123845
"7294","AL353796.2",4.14053372882505,-0.733831057421097,0.78918134738722,-0.929863661692748,0.352441679636789,0.75553426123845
"7295","BEAN1",3.22770207454629,-0.715915939836015,0.769431509479694,-0.930447909938251,0.35213922245401,0.75553426123845
"7296","RUNDC1",50.6937913270675,-0.279669337407266,0.300757819361038,-0.929882182286815,0.352432089260482,0.75553426123845
"7297","SLC35C2",204.526024447958,-0.200636376532168,0.215745712509594,-0.929966923552404,0.352388210451593,0.75553426123845
"7298","PRPS1",88.7517871021722,-0.198966536501889,0.213886802662196,-0.930242231055876,0.352245680801063,0.75553426123845
"7299","LONRF3",24.867902759008,-0.440131057390355,0.473370029956596,-0.929782262368217,0.352483831979017,0.75553426123845
"7300","THAP5",157.519304279589,-0.204181001296427,0.219689297863217,-0.929408047102754,0.352677659031352,0.755548801501485
"7301","NELFB",174.44650583482,-0.190654989309885,0.20513150081111,-0.929428140271079,0.352667249940717,0.755548801501485
"7302","TCEANC",15.3477020178923,0.396790641823294,0.426932923658405,0.929398085355373,0.352682819699792,0.755548801501485
"7303","TNFSF4",8.90555550950416,0.544444237274188,0.585871723809031,0.929289151786499,0.352739255691349,0.755566229875363
"7304","UNC50",374.41055201629,-0.266654684267805,0.287190309767341,-0.928494713083556,0.353151008983254,0.75600407038058
"7305","LINC01124",7.2388577815646,-0.605080933519333,0.65165179238719,-0.92853413523616,0.353130569529584,0.75600407038058
"7306","VPS52",63.0190913386553,0.209096438329427,0.225166286554681,0.928631197542302,0.353080248208444,0.75600407038058
"7307","ARL14EP",230.214190781126,0.160552125489167,0.172928894419682,0.928428566133793,0.353185306292993,0.75600407038058
"7308","AL031663.3",6.68779376997924,-0.560194172440847,0.603265994906121,-0.928602270260606,0.353095244895013,0.75600407038058
"7309","DIABLO",4.49719649924845,-0.587971194717682,0.633575314901568,-0.928020995908006,0.353396678644096,0.756353022852591
"7310","SPN",6.52536315762679,-0.609942251723457,0.657476018344844,-0.927702660941078,0.353561827800531,0.756602964744694
"7311","OTUB2",5.127256406627,0.710502807593625,0.766007047992365,0.927540822836797,0.3536458065747,0.756679161844895
"7312","NNMT",3361.90903902108,-0.275711275670125,0.297391462074482,-0.92709882707081,0.353875225091574,0.757066486065028
"7313","FCRLB",5.62866223188048,-0.550118284355621,0.594491386874273,-0.925359553564,0.35477891036702,0.757239254314545
"7314","IAH1",566.916338661724,-0.223638821193166,0.2415893101841,-0.925698330868798,0.354602775549799,0.757239254314545
"7315","KLF11",152.434045828329,0.19906134682052,0.215107053467798,0.925405948393597,0.354754785815642,0.757239254314545

"7316","ZNF620",10.9892351846387,-0.469444186108013,0.507248074737061,
-0.925472583314103,0.354720138560632,0.757239254314545
"7317","FRMD4B",50.6688716252755,-0.259815437398228,0.280542430989249,
-0.926118150762671,0.354384582241259,0.757239254314545
"7318","JCHAIN",66.9249695520417,-0.459734183282477,0.496738094167973,
-0.925506194672914,0.354702662929348,0.757239254314545
"7319","SLC12A2",48.1878137754641,-0.311974699033674,0.336746600804732
,-0.926437559542219,0.354218632490325,0.757239254314545
"7320","WASL",381.01341631054,0.143618695725687,0.154994205505887,0.92
6606870604798,0.354130686351374,0.757239254314545
"7321","MSR1",45.6947680966261,-0.352736078679786,0.381091682322189,-0
.925593748282257,0.354657143531728,0.757239254314545
"7322","C9orf40",46.1498092282042,0.288378499393876,0.311410103132893,
0.926040923183573,0.354424713422867,0.757239254314545
"7323","STOM",810.686450301867,0.154943684338755,0.167286694341172,0.9
26216427128125,0.354333517243396,0.757239254314545
"7324","SPTAN1",687.518942730972,0.138999611696393,0.150117829083131,0
.925936729470153,0.354478862059183,0.757239254314545
"7325","CORO1B",312.641478797828,-0.239491135063939,0.25861680801471,-
0.926046287951698,0.354421925537368,0.757239254314545
"7326","RARG",258.65732387988,-0.197630256845756,0.213487358507028,-0.
925723463102615,0.354589711168359,0.757239254314545
"7327","SMG1",268.565807266071,0.224817299032169,0.242710592578662,0.9
26277245025084,0.354301918222045,0.757239254314545
"7328","CDT1",8.82517308070081,-0.601298152927367,0.64883925210463,-0.
92672900256411,0.354067255268413,0.757239254314545
"7329","MMP28",111.28495550435,-0.37672915165525,0.40655045507265,-0.9
26647964489252,0.354109342818677,0.757239254314545
"7330","AP002495.1",30.3914532050341,0.343074437533297,0.3708360597644
3,0.925137748878123,0.354894259482339,0.757382114745188
"7331","CARMIL1",202.381796009202,0.167093594502003,0.180666839092903,
0.924871411604647,0.355032798965394,0.7575076073484
"7332","ZNF592",65.2313760421764,-0.204169526181794,0.22076235206359,-
0.924838516501147,0.355049912234128,0.7575076073484
"7333","SRBD1",98.7989367702646,0.19337029320039,0.209116865898032,0.9
24699652368932,0.355122160310823,0.757558428166127
"7334","ACTR2",632.596002560712,-0.143388315082149,0.155082840888413,-
0.92459174890484,0.355178306611033,0.757574890962148
"7335","AGRN",14.7976359407787,0.483615890237887,0.523229773622532,0.9
24289699513118,0.355335504251993,0.75774479981096
"7336","LMO4",682.872573756338,-0.154738755313095,0.167487870250211,-0
.923880368661505,0.355548605166046,0.75774479981096
"7337","HOXA4",18.2520367304992,0.440792288675488,0.477096481596062,0.
923905972227832,0.355535273381447,0.75774479981096
"7338","CAPRIN2",91.0466065592528,0.255472156922879,0.276492961434589,
0.923973455227782,0.355500136474304,0.75774479981096
"7339","DGCR11",6.47191101835491,0.544406830894283,0.58925269559756,0.
923893662195641,0.355541683179507,0.75774479981096
"7340","MCTS1",346.969900575255,-0.228240251691161,0.247029823387125,-
0.923938043438104,0.355518574337643,0.75774479981096

"7341","SETDB1",62.5603480891996,0.248162475307816,0.268638210441879,0
.923779513344798,0.355601123627226,0.757753490927761
"7342","OAF",1332.22344341429,0.308065531400798,0.333527825334625,0.92
3657662120749,0.355664581829557,0.757785488090406
"7343","SCG2",7.05498292544776,2.48806723074267,2.69478803551452,0.923
288658682061,0.355856796586073,0.757895305216424
"7344","MKLN1-
AS",29.2855363681768,0.29242738565081,0.316678258269685,0.923421100168
415,0.355787799969651,0.757895305216424
"7345","AC009630.1",7.12622988009362,-0.503770706790246,0.545631747194
754,-0.923279683376695,0.355861472659633,0.757895305216424
"7346","TAF7",590.693365716586,0.13698365071491,0.148398029551627,0.92
3082679256563,0.355964120237099,0.758010717787768
"7347","NAT10",67.8450727693958,0.241613482314455,0.261785228161123,0.
922945438944887,0.35603563934516,0.758059821189102
"7348","ARHGAP26",118.363205714027,0.287318762350465,0.311402737515581
,0.922659719187887,0.356184563598031,0.758170516854537
"7349","WASF3",182.911441702275,-0.211867396886377,0.229617980317212,-
0.922695150413254,0.356166093828301,0.758170516854537
"7350","PSME4",70.4685452134679,0.23647774469362,0.25643794519896,0.92
2163623289626,0.35644323440857,0.758263343510832
"7351","FKBP5",440.577865350249,-0.692007331317472,0.750564321427311,-
0.921982715620583,0.356537591456377,0.758263343510832
"7352","CDNF",13.9605956521652,-0.378820302288457,0.410901249000352,-0
.921925409596727,0.35656748416964,0.758263343510832
"7353","RNASE4",2438.22397857384,-0.194053956073365,0.210405492919851,
-0.922285599013735,0.356379623716357,0.758263343510832
"7354","IGHG4",160.538524202212,0.861149042655604,0.933679743961079,0.
922317366554652,0.356363058026548,0.758263343510832
"7355","SNX11",45.6515122507424,-0.269780974158239,0.29245167672798,-0
.922480517727282,0.356277987898397,0.758263343510832
"7356","RGS19",36.3087285869475,-0.36965144042952,0.400928106860519,-0
.921989339495521,0.356534136326434,0.758263343510832
"7357","CTSF",1307.35389107106,-0.214346012887298,0.232563597334929,-0
.92166622525453,0.356702703000699,0.758437784450067
"7358","UBE2V1",672.965990667067,-0.148610221531831,0.161255503357584,
-0.921582323936495,0.356746482003682,0.758437784450067
"7359","PDE4DIP",642.604342060701,0.247130929445919,0.268326972473458,
0.921006662758678,0.357046948462006,0.75894832069725
"7360","SLC30A6",75.2163266598554,0.204636680206985,0.222227035841442,
0.9208451142416,0.357131297374323,0.75894832069725
"7361","CLTA",1329.97156121164,-0.253650250390398,0.275482058574214,-0
.92075052619829,0.357180690211159,0.75894832069725
"7362","RPS9",14001.3906312657,-0.285044000816249,0.309558650787363,-0
.920807737374609,0.357150814649453,0.75894832069725
"7363","PIN1",389.261046668731,-0.251849316249243,0.27361165528864,-0.
92046267540599,0.357331029197567,0.759164646168347
"7364","AC010273.3",5.86802478703353,0.615916818484394,0.6692938198811
06,0.920248775931931,0.357442770619961,0.759195826314738
"7365","AC006141.1",36.7326274240587,0.389677525508537,0.4234278350093

93,0.920292652701713,0.357419847532897,0.759195826314738
"7366","NPAT",94.3647512310972,0.228659732245537,0.248544300399519,0.919995879519188,0.357574912483206,0.759373385280314
"7367","LSM8",503.078439673021,-0.176889709521099,0.192298896306713,-0.919868563566599,0.35764144846053,0.759411589285744
"7368","B4GALT7",125.527937242447,-0.29927287179562,0.325387086355647,-0.919744158096416,0.357706470932218,0.759446569597269
"7369","ZNF324",51.0553374206952,-0.260941724760434,0.283767746619899,-0.919560901013745,0.357802266675434,0.759546866278167
"7370","CEP72",7.65865436288191,-0.502545760424509,0.546697248815246,-0.919239600187455,0.357970262323402,0.759800381753728
"7371","KIF15",2.72297156864794,0.807274655278003,0.878329662593563,0.919102120375,0.358042160305566,0.759849886536422
"7372","NOL6",42.0128607215866,-0.229250833588782,0.249464084818374,-0.918973301329894,0.358109537200818,0.759889784377699
"7373","HNRNPR",662.657740664674,-0.164838102094141,0.179415065681382,-0.918752845353978,0.358224861942843,0.760031400430205
"7374","BCAS2",266.196179777351,-0.221376790654793,0.241055783452465,-0.918363324389799,0.358428684919554,0.760057580611372
"7375","CEBPD",4683.44486602171,-0.341204261954331,0.371507503264112,-0.918431684303726,0.358392909242218,0.760057580611372
"7376","UGGT2",182.240787289313,0.187878009774252,0.204580393864879,0.918357845661114,0.35843155227067,0.760057580611372
"7377","LINC00526",29.4843471984527,-0.26421597879722,0.287660931225076,-0.918497961026514,0.358358225932668,0.760057580611372
"7378","WARS2-IT1",16.7228564059902,0.434430300326856,0.473392352719583,0.917696067186354,0.358778007210984,0.760327884108552
"7379","SMYD3",70.1859872619766,-0.219648322776073,0.239264385955151,-0.918015114950059,0.358610952724643,0.760327884108552
"7380","PID1",532.67631776964,0.274832044823307,0.299543987122433,0.917501457677312,0.358879929427155,0.760327884108552
"7381","SYCP2L",3.15648049600163,0.963251245735063,1.04991440786945,0.917456926502945,0.35890325415561,0.760327884108552
"7382","HOXA10-AS",29.9181567121571,0.294344648482075,0.320809041438243,0.917507334464379,0.358876851329738,0.760327884108552
"7383","AC079174.2",6.39547338437965,0.618783007943367,0.674517171482964,0.917371764729041,0.358947863206652,0.760327884108552
"7384","WDR89",32.3845836368906,0.246937131621843,0.269127122877249,0.917548290866517,0.358855399967686,0.760327884108552
"7385","BTG3-AS1",5.78364459722445,-0.631796876756073,0.688658332611125,-0.917431543099965,0.358916550009112,0.760327884108552
"7386","AL356512.1",8.95297425761824,-0.482210542419754,0.526028633490196,-0.916700178886252,0.359299772341531,0.760564058832064
"7387","LDAH",35.5392105454488,0.26056834783262,0.284159594992344,0.916978882376433,0.359153706170999,0.760564058832064
"7388","MRFAP1L1",195.642305621252,0.129830370256332,0.141639877843554,0.916623003584725,0.359340225870738,0.760564058832064

"7389","KAT6A",221.098059949073,0.216446979501023,0.236203255176684,0.916359003346999,0.359478630400314,0.760564058832064
"7390","HIPK3",314.630057076152,-0.162740267724159,0.177525560858614,-0.916714567395562,0.35929223053032,0.760564058832064
"7391","MPI",85.0432027910097,0.208613087585045,0.227578506591183,0.916664278669307,0.359318590058755,0.760564058832064
"7392","GAN",13.3181631090219,0.398608497780925,0.435052232162401,0.916231358702989,0.359545561277448,0.760564058832064
"7393","MAP3K3",138.244145468903,0.225264368551008,0.245817385399826,0.916389083646836,0.359462858838697,0.760564058832064
"7394","ZNF585B",31.2663467056252,0.25300394359347,0.276083092103284,0.916405063656776,0.359454480451718,0.760564058832064
"7395","AL022328.4",6.09304094011681,0.564866611202811,0.616486440253762,0.916267697583579,0.359526506072171,0.760564058832064
"7396","EGFL7",36.965883918233,-0.31390941808821,0.342710259620645,-0.915961542661969,0.359687065943281,0.760760515488202
"7397","BIN3",109.430341852925,-0.203074500317425,0.221778443656508,-0.915663835354299,0.359843238733033,0.760782238613438
"7398","MIR100HG",364.648077346756,0.194497118409422,0.212383738407917,0.915781593578784,0.359781459443972,0.760782238613438
"7399","ACLY",180.581751158198,0.180070736975505,0.196646017159557,0.915710064086361,0.359818985040789,0.760782238613438
"7400","STAM",178.723918696482,0.163071364162367,0.17812732033596,0.915476434804067,0.35994156808879,0.760887290488236
"7401","MED18",31.6145727004264,0.306645102938556,0.335036119701504,0.9152598329152,0.360055240450668,0.760916757403793
"7402","TMEM18",290.918727603564,-0.21533067514484,0.235266797985404,-0.915261639078369,0.360054292485634,0.760916757403793
"7403","PKP4",32.7723657632634,-0.418403809103911,0.457394036660322,-0.914755715135467,0.360319888006578,0.760916757403793
"7404","AC007038.1",6.34074877445836,0.771061966351519,0.843129918966404,0.914523312488741,0.360441933923295,0.760916757403793
"7405","CDK7",52.650187793291,-0.224941037321503,0.245906948518634,-0.914740468606391,0.360327893904315,0.760916757403793
"7406","ADAP1",20.8423930712481,-0.635370517202894,0.694302935052287,-0.915120022004582,0.360128624985536,0.760916757403793
"7407","TBRG4",80.1721879650237,-0.222729797226478,0.243503111549856,-0.914689737674567,0.360354533338525,0.760916757403793
"7408","ZNF252P-AS1",4.72252975301799,0.63373160098097,0.692957598111312,0.914531571207581,0.360437596424368,0.760916757403793
"7409","RBM4",517.009907073035,-0.201725621164889,0.220515882033361,-0.914789534906928,0.360302129764412,0.760916757403793
"7410","ZADH2",167.983865868404,0.151296745992276,0.165375671023808,0.914867011910687,0.360261449851554,0.760916757403793
"7411","ALKAL2",30.3983144830424,1.16358355071858,1.27523261357773,0.912448080710616,0.361532888865487,0.760917080595628
"7412","PPIL3",176.61743708974,-0.222079501297592,0.243618593111388,-0.911586831125211,0.361986257275977,0.760917080595628
"7413","AC079834.2",3.89978825497115,-0.703804505227348,0.770973928016

622,-0.912877179955915,0.361307140637941,0.760917080595628
"7414","TFG",507.649614766436,-0.169961531552254,0.187010773971888,-0.908832833223835,0.363438372490553,0.760917080595628
"7415","MELTF",93.0001253037681,0.50896406873002,0.5566383133863,0.914353281996967,0.360531241625206,0.760917080595628
"7416","PCGF3",120.611059735267,0.224244937685492,0.246588197454096,0.909390392568305,0.363144091333194,0.760917080595628
"7417","SLC2A9",2.84328144453549,-0.933321889568349,1.02556097440001,-0.910059872465774,0.362790935337219,0.760917080595628
"7418","TRAM1L1",25.3915240723835,0.319485226701678,0.351338092557621,0.909338422076395,0.363171515179251,0.760917080595628
"7419","NUP155",46.7396839154406,0.228203199527802,0.250648544171557,0.910450927541023,0.362584750175755,0.760917080595628
"7420","AK6",278.625242148989,-0.24943297314234,0.274331802587344,-0.909238268366364,0.363224368054039,0.760917080595628
"7421","CHD1",173.74221552902,-0.202776787244148,0.222780042236549,-0.910210740640936,0.362711380866571,0.760917080595628
"7422","CTNNA1",558.286840763988,0.150259450918202,0.16485769325637,0.911449432235679,0.36205861804533,0.760917080595628
"7423","AC011379.1",17.0229494538107,-0.432764404799975,0.474722450514754,-0.911615627891028,0.361971092682572,0.760917080595628
"7424","ANXA6",769.588943411715,0.170362256151663,0.187360300529785,0.909276168270132,0.363204367041968,0.760917080595628
"7425","TRIM41",38.2668756822201,0.260194965264987,0.285717002084965,0.910673720381583,0.362467314687381,0.760917080595628
"7426","GMDS-DT",88.1011658053649,0.242044177601038,0.266112280314377,0.909556587599394,0.363056402056671,0.760917080595628
"7427","SSR1",512.778789214697,0.14793246745642,0.162627949849404,0.909637412224699,0.363013761451006,0.760917080595628
"7428","NFKBIL1",185.883008603715,-0.302399814408724,0.330888088389109,-0.913903597681388,0.360767503081951,0.760917080595628
"7429","CDKN1A",516.062276924518,-0.654902681063007,0.718007252230803,-0.912111512840945,0.361710018392228,0.760917080595628
"7430","AL451064.1",2.98318623567958,-0.868306712718176,0.955396620596783,-0.908844237041359,0.363432352034121,0.760917080595628
"7431","TRGC2",3.31706521294348,-0.814700307053312,0.895148731106688,-0.910128427536376,0.362754784130134,0.760917080595628
"7432","AL590399.1",13.0444220490177,0.431025932936898,0.473193223729668,0.910887796616337,0.362354496220319,0.760917080595628
"7433","ISCA1",324.909902300117,-0.201065632906093,0.221175062529819,-0.909079127666053,0.363308359301508,0.760917080595628
"7434","TRUB2",118.548515035543,-0.199297645034422,0.218699576028002,-0.911285008659112,0.362145223207577,0.760917080595628
"7435","SLC27A4",28.4560585333387,-0.327688161584644,0.359807501363947,-0.910731878414022,0.362436663148925,0.760917080595628
"7436","TMEM203",264.721287682955,-0.208520332456997,0.22834238508665,-0.913191531996431,0.361141816786691,0.760917080595628
"7437","DTX4",49.0309401742411,0.313811426254494,0.343994509786139,0.912257077735311,0.361633403570782,0.760917080595628

"7438","CLNS1A",295.902608508478,-0.213215346146409,0.234447106881476,
-0.909439015829698,0.363118434930938,0.760917080595628
"7439","CD3E",11.0223354375142,0.481309915608743,0.52778513342811,0.91
1942919806215,0.361798766273743,0.760917080595628
"7440","STAT6",369.861986516556,0.186509637243694,0.204693609921474,0.
911164922614069,0.362208483111942,0.760917080595628
"7441","GLTP",218.487335664236,-0.16226026369197,0.177712607582394,-0.
913048690801189,0.361216933867117,0.760917080595628
"7442","DTD2",57.928404491024,-0.197755435790649,0.217029077897417,-0.
911193272839327,0.362193547925293,0.760917080595628
"7443","TXNDC16",42.2251657460028,0.243778228867496,0.26779498115936,0
.910316645263891,0.362655542703728,0.760917080595628
"7444","ABCD4",194.153209792958,0.199419186293128,0.218262960598442,0.
913664809394835,0.360893000500706,0.760917080595628
"7445","C15orf48",8.33177038406568,-0.614340431891635,0.67524304197902
7,-0.909806386291822,0.362924626017816,0.760917080595628
"7446","TRPM7",212.519607501107,0.190061950721745,0.208226680549366,0.
912764638135241,0.361366340104627,0.760917080595628
"7447","AQP9",4.25510235155784,-0.821566619021258,0.90129794001554,-0.
91153722043023,0.362012383574649,0.760917080595628
"7448","TIPIN",45.2204135185908,-0.220996542148039,0.243008633317057,-
0.909418480864018,0.363129270210098,0.760917080595628
"7449","AC027807.2",6.2408976301319,0.626650446536332,0.68922234742580
4,0.909213766612221,0.36323729879355,0.760917080595628
"7450","KDM8",7.24237467172554,0.517303860593994,0.566541441925501,0.9
13090945008075,0.361194712277507,0.760917080595628
"7451","OSGIN1",20.6690670400582,-0.467681464764545,0.513047718260413,
-0.911574982440831,0.361992496997858,0.760917080595628
"7452","MRM3",61.8663352713258,-0.220779877221159,0.242936730983881,-0
.908795785334776,0.36345793174011,0.760917080595628
"7453","ABR",123.193941630557,0.21992522070717,0.241213062910032,0.911
746727370225,0.361902059742667,0.760917080595628
"7454","CYB5D1",28.8567832787363,-0.331517078083927,0.363425545781501,
-0.912200812331562,0.361663016399535,0.760917080595628
"7455","ALDH3A2",496.88128771639,0.164983306405086,0.180979481856635,0
.911613320540828,0.361972307735866,0.760917080595628
"7456","SUPT6H",187.572576792979,0.167225028946432,0.183950664970915,0
.909075425048735,0.363310313612804,0.760917080595628
"7457","BLMH",112.105247848166,-0.174095869512665,0.191426119584483,-0
.909467683357759,0.363103308841874,0.760917080595628
"7458","AC005899.7",7.08062786567835,0.548754698086252,0.6004366980765
96,0.913925980613943,0.360755740925994,0.760917080595628
"7459","OXLD1",150.146307794398,0.222876874012001,0.244712515454777,0.
910770230111866,0.362416451205162,0.760917080595628
"7460","NPC1",54.2725540213604,0.22895059231361,0.251772995089917,0.90
9353253838221,0.363163688606777,0.760917080595628
"7461","AC036176.1",80.3724892316079,0.280976369051681,0.3077886693527
11,0.91288730557425,0.361301814638758,0.760917080595628
"7462","DOHH",62.613730838496,-0.280870622973384,0.307688299271167,-0.
912841416585206,0.361325952296302,0.760917080595628

"7463","SMARCA4",121.437374830166,0.24014532421439,0.26410778983072,0.90927012932224,0.36320755394505,0.760917080595628
"7464","MYH14",4.40848001214321,-0.902911927159903,0.9883809927762,-0.913526194614256,0.360965863357461,0.760917080595628
"7465","ZNF761",42.6551016757055,0.250056973268773,0.274401401040079,0.911281692881187,0.362146969827135,0.760917080595628
"7466","ZNF551",16.8067203820571,0.427732010954821,0.468433822369215,0.913110861191587,0.361184238605705,0.760917080595628
"7467","RHBDD3",64.0689429893049,0.284042057917078,0.312502427684923,0.90892752424778,0.363388383826078,0.760917080595628
"7468","MCAT",71.4883464776228,-0.249271302542704,0.274105430984048,-0.909399356473205,0.363139361381096,0.760917080595628
"7469","NR0B1",28.7869652270164,-0.642683158763007,0.704942395062596,-0.911681810122853,0.361936242130468,0.760917080595628
"7470","ZCCHC12",3.02123282215556,-0.838422624162145,0.920981685285755,-0.910357542997183,0.362633980833756,0.760917080595628
"7471","UPF3B",159.689349165518,-0.19064469696046,0.208599552664701,-0.913926681649687,0.360755372537948,0.760917080595628
"7472","CCL17",2.84885220324835,-1.08020703608728,1.18523937810187,-0.911383013461133,0.362093600570201,0.760917080595628
"7473","RAB8A",269.529607770531,-0.158510958112279,0.174476902282714,-0.908492505531964,0.36361807157093,0.761048634410497
"7474","HCFC1",107.237804080844,0.184857578807872,0.20345888817507,0.908574604265054,0.363574716892392,0.761048634410497
"7475","NAPRT",174.671772745507,-0.260586596907365,0.286954422728833,-0.908111449997115,0.363819341619217,0.761312468662133
"7476","TLE3",191.35730874191,-0.230636400767218,0.254010652322035,-0.907979246771181,0.363889186430477,0.761312468662133
"7477","ZNF264",162.313407948905,0.184163939759592,0.202828757648963,0.907977457902322,0.363890131572382,0.761312468662133
"7478","PPP1R35",144.382124116253,-0.241634995475568,0.266205393815082,-0.907701350497124,0.364036030250336,0.761515862691362
"7479","MAPK1",263.401605183809,0.150324632382857,0.165630108655592,0.907592427506274,0.364093596608435,0.761534447352018
"7480","PLAAT5",29.4429948349338,-0.322767000313533,0.355808012214794,-0.907138089174576,0.364333778119673,0.761635974064711
"7481","HOXC6",282.208614171618,0.14754002737972,0.162609600452609,0.907326670559771,0.364234074396805,0.761635974064711
"7482","SETDB2",73.0895671382879,0.217487728166983,0.239753067893364,0.907132200968189,0.364336891512257,0.761635974064711
"7483","RASAL3",7.63313716198054,-0.597708971977558,0.65877587755382,-0.907302456484871,0.364246875519279,0.761635974064711
"7484","TMEM87B",109.420363570251,0.221130199032343,0.243836368276787,0.906879480674232,0.364470533189639,0.761813542315008
"7485","JAML",5.98485522905265,-0.57059830936319,0.629332484289716,-0.906672265626308,0.364580133974741,0.761940819741734
"7486","IFI16",1272.44531746616,0.169855269999033,0.187682240319654,0.905015145331497,0.365457363636575,0.762104492523646
"7487","AC073195.1",6.02649596808772,-0.576299388367244,0.636047045559106,-0.906064091313651,0.364901930213533,0.762104492523646

"7488","TTC30B",19.80138416997,-0.348754357119984,0.385383584156846,-0
.904953847172808,0.365489838293002,0.762104492523646
"7489","PIKFYVE",92.9409510146616,0.23888024852807,0.263656166307274,0
.906029439302666,0.36492027057247,0.762104492523646
"7490","CYP4V2",171.603615984748,0.196229756116173,0.216735086469009,0
.905389890087002,0.365258869811938,0.762104492523646
"7491","PYCR3",25.0274673685942,-0.291265891615108,0.321726491567699,-
0.905321443055051,0.365295119631214,0.762104492523646
"7492","MOB2",187.715729539111,-0.225812817218687,0.249509861489832,-0
.905025620511953,0.365451814255555,0.762104492523646
"7493","CS",97.3940558495566,-0.204679207335447,0.226077663696647,-0.9
05349091054337,0.365280476870835,0.762104492523646
"7494","AC025159.1",73.9006713274093,0.228086861775517,0.2518915780060
21,0.90549618046406,0.365202582459886,0.762104492523646
"7495","CNOT2",340.466866938886,0.138463891382974,0.152997122693641,0.
905009773682029,0.365460209367346,0.762104492523646
"7496","VPS36",409.173341725762,0.227572176154576,0.251436220573451,0.
905089074420352,0.365418199738241,0.762104492523646
"7497","ZNF839",27.6694979121705,0.296424137849852,0.327382419270921,0
.905436945911718,0.365233950163769,0.762104492523646
"7498","BFAR",307.664010284167,-0.17684998704304,0.195168808745133,-0.
906138579110686,0.364862507814968,0.762104492523646
"7499","NIP7",92.8070468979227,-0.225792472752131,0.249204419274645,-0
.906053244999994,0.364907670807935,0.762104492523646
"7500","ZCCHC14",166.269188538088,0.194254514916488,0.214392372536389,
0.906070083643097,0.36489875869632,0.762104492523646
"7501","USP36",119.268013010849,0.239503646603584,0.264380475673142,0.
905905195887787,0.36498603393755,0.762104492523646
"7502","LIPE",9.93855362944336,0.504328059976785,0.557152074564465,0.9
05189234682517,0.365365144068883,0.762104492523646
"7503","BEX3",975.859066351182,0.267687318155868,0.2958302789298,0.904
867882774738,0.365535383711879,0.762104492523646
"7504","ABCD2",8.57763673531375,0.853164719200088,0.94300815495256,0.9
047267669101,0.365610157009355,0.762158806782695
"7505","DIMT1",136.450117713127,-0.189980977527979,0.210013140799975,-
0.904614715080731,0.365669536898291,0.762181021412387
"7506","TMEM79",23.5553008367775,0.323803791815634,0.358011953055165,0
.904449667259405,0.365757012040402,0.762203550625956
"7507","TFEB",54.9573183375148,-0.241644091738504,0.26723841704589,-0.
90422662433676,0.365875245263076,0.762203550625956
"7508","AC083837.1",5.3897097973297,-0.670117054755456,0.7410592494246
08,-0.904269200169575,0.365852674321874,0.762203550625956
"7509","MRPL11",418.502277422748,-0.244560331408735,0.270426220239646,
-0.904351401990574,0.36580909871397,0.762203550625956
"7510","MTERF2",138.765300525954,0.179765237879673,0.198829536481256,0
.904117371397784,0.365933168051025,0.762212876667276
"7511","TIAF1",3.54915695387741,0.686637409979914,0.759525773800281,0.
904034377325116,0.365977172962214,0.762212876667276
"7512","ITGB2-
AS1",5.11153030269705,0.757629270276084,0.838175948163434,0.9039024227

98173,0.366047144361956,0.762257119176528
"7513","CCDC88B",6.39717470907457,-0.628754198931367,0.695857999528717
,-0.903566818743483,0.36622514240757,0.762526274814538
"7514","AP001885.2",4.65242261764444,-0.715592352213482,0.792119777230
983,-0.903389074206658,0.366319436616846,0.762621100212579
"7515","AC246817.2",7.47021752308105,0.707512441794386,0.7835255338203
59,0.902985813805781,0.366533424068442,0.762965050259831
"7516","AL360012.1",34.7127174098444,0.421096988862344,0.4673868221907
63,0.900960337068455,0.367609408507472,0.763038998305983
"7517","SERINC2",4.18173332579613,-0.658542877477134,0.730512164369225
,-0.901481056165254,0.367332601647115,0.763038998305983
"7518","ZNF691",24.7107069893081,0.273245138314124,0.30367622881098,0.
899791002357984,0.36823148353332,0.763038998305983
"7519","CERS2",430.503601273914,-0.243173243030602,0.269584284894264,-
0.902030484180408,0.367040674383182,0.763038998305983
"7520","UCN",6.28430038241383,0.634528890836581,0.705195369742699,0.89
9791629471558,0.368231149739297,0.763038998305983
"7521","IFT172",49.6640531371174,0.236216772653421,0.262279083724333,0
.900631378221893,0.36778434529848,0.763038998305983
"7522","AC007038.2",13.2169089641131,0.430802289361318,0.4785391553673
98,0.900244597603659,0.367990097392187,0.763038998305983
"7523","RETREG2",277.976051939434,-0.155294103039858,0.172646787707885
,-0.899490254649934,0.368391584354707,0.763038998305983
"7524","GIGYF2",241.863993865097,0.199292863998016,0.221572303270351,0
.899448446653775,0.368413843962089,0.763038998305983
"7525","AC099778.1",22.5378419327587,0.329517272533745,0.3659564374231
49,0.900427588742564,0.367892744364037,0.763038998305983
"7526","UTP3",112.309308883406,-0.183784489003257,0.204349841627854,-0
.899362032968692,0.368459855390531,0.763038998305983
"7527","HARS2",40.0804418030713,-0.278193733008055,0.308966020876757,-
0.900402355633222,0.367906167665088,0.763038998305983
"7528","AL357054.4",11.8528512402687,-0.49533163601745,0.5502041528738
94,-0.900268806460608,0.367977217129205,0.763038998305983
"7529","HLA-
E",3274.66574242103,-0.165004448997795,0.183315417106111,-0.9001122306
16605,0.368060527886591,0.763038998305983
"7530","RPL10A",10591.5209477974,-0.260568185833468,0.288951466682071,
-0.901771459496234,0.367178283795713,0.763038998305983
"7531","KLHDC10",147.408794173248,0.216162196128194,0.240380820180387,
0.89924893328004,0.36852008132722,0.763038998305983
"7532","RGP1",40.4022448499767,0.242817824808426,0.269800989454017,0.8
99988637179592,0.368126297682255,0.763038998305983
"7533","KIF27",44.6297344594729,0.306072348281611,0.339359059276344,0.
901913003101453,0.367103083378738,0.763038998305983
"7534","NMT2",188.094170564614,0.217569938447369,0.241234135171953,0.9
01903614479288,0.367108071143653,0.763038998305983
"7535","KAT6B",381.935835934666,0.193656819031613,0.214948097789171,0.
90094688449655,0.367616561424508,0.763038998305983
"7536","TMEM254",66.058699030157,0.250079989048888,0.277343142477812,0
.901698837096342,0.367216870930343,0.763038998305983

"7537","KCNQ1-
AS1",3.35333663242769,0.798640159961892,0.886251216433191,0.901144218651796,0.367511644838512,0.763038998305983
"7538","BTBD10",84.5289592101167,-0.182181308209071,0.202495613993894,-0.899680267714657,0.368290427264462,0.763038998305983
"7539","ATG16L2",28.3506666415825,0.345524611579002,0.383135984460253,0.901832836364257,0.367145673824331,0.763038998305983
"7540","LDHB",682.69652864181,-0.243600449008563,0.27063261062144 1,-0.900114913901894,0.368059100067279,0.763038998305983
"7541","AC022509.1",4.04805566127538,0.965876974385459,1.07289571290601,0.900252431589384,0.367985929308568,0.763038998305983
"7542","EID3",60.9786345579546,-0.340061011081643,0.377996194333726,-0.899641361948235,0.368311138085792,0.763038998305983
"7543","PIBF1",166.008084337535,0.168790104570225,0.187606250849216,0.899704054668657,0.368277765042219,0.763038998305983
"7544","TGFB3",78.7858426498262,-0.235216277194732,0.261106492508721,-0.900844230010387,0.367671147077949,0.763038998305983
"7545","CALML4",23.8726403608635,0.286187214234395,0.318249835861027,0.899253297209451,0.36851775740759,0.763038998305983
"7546","BOLA2B",24.0896210541198,-0.457195303152146,0.507498130676176,-0.900880762935998,0.36765172042563,0.763038998305983
"7547","PYCARD-
AS1",5.54756144352606,0.712454225979519,0.792194235196833,0.899342855988468,0.368470066760995,0.763038998305983
"7548","YES1",207.170001092932,0.131527190809201,0.146025407775327,0.900714422325513,0.367740178442361,0.763038998305983
"7549","PTPN2",162.115023919399,-0.159750150562427,0.177231856955019,-0.90136250506573,0.367395610303582,0.763038998305983
"7550","LDLRAD4",62.0539353271214,-0.329147172918421,0.365135839736789,-0.901437594172322,0.36735570045525,0.763038998305983
"7551","ATP5F1A",687.62517838827,-0.179205048595021,0.198845504121625,-0.901227560495454,0.367467340110814,0.763038998305983
"7552","KDSR",804.087829939061,-0.146447460292787,0.162751889222145,-0.899820339983254,0.368215868184089,0.763038998305983
"7553","ABCA7",3.23390176421283,0.786383194876281,0.872522618382723,0.901275426342406,0.367441896014021,0.763038998305983
"7554","AP2S1",789.134381458528,-0.24934008769553,0.277239811599913,-0.899366098456866,0.368457690619463,0.763038998305983
"7555","MCM8",21.3168438136566,0.355062565538553,0.394263709887037,0.900571258867025,0.36781632172992,0.763038998305983
"7556","SFRP4",1094.45092660532,0.741930524030722,0.825626470766403,0.898627345780243,0.368851187949117,0.76311852044545
"7557","ZC3H12C",48.3795291888746,0.277564066921428,0.308813909499069,0.898806881372891,0.36875553410371,0.76311852044545
"7558","Z69720.1",2.98460828942643,1.10948399229429,1.23414629361599,0.898989040467439,0.368658498270374,0.76311852044545
"7559","PIK3C3",114.909905333864,0.175968328834099,0.195808205774105,0.898676989242758,0.368824737120152,0.76311852044545
"7560","SPC24",4.10720652563285,0.634360686085981,0.705700620126352,0.898909067094772,0.368701097990535,0.76311852044545

"7561","RTF2",522.598773767698,-0.179306343474213,0.199519938632501,-0.898688846353748,0.368818419636799,0.76311852044545
"7562","RAB21",317.220470994748,0.156171284839476,0.173826489653785,0.898432023511071,0.368955270225642,0.763232913533419
"7563","PRKCI",120.611990223237,0.199372077346296,0.222053317999824,0.897856781164847,0.369261908374642,0.763362499366265
"7564","MAGI2-AS3",647.03718778932,0.165504712032135,0.184280538219469,0.898112810127717,0.369125410250155,0.763362499366265
"7565","SLC25A13",93.8314886740021,0.214066057758217,0.238406104354344,0.897905103302427,0.369236143725997,0.763362499366265
"7566","MARVELD1",323.601291815669,0.160533939834791,0.178789542700014,0.897893341022443,0.369242415096681,0.763362499366265
"7567","AP000897.1",2.62274209921525,0.875617608329438,0.975014766117411,0.89805574105941,0.369155833077413,0.763362499366265
"7568","YPEL2",238.36339075509,-0.265790425083926,0.296076007851999,-0.897710108334033,0.369340119003404,0.763423293019325
"7569","ST3GAL5-AS1",5.58686513217525,0.59092635415158,0.65841761741559,0.89749474880559,0.369454974230896,0.763479568719562
"7570","AC107214.1",6.5549271672262,0.536217805099534,0.597494902474689,0.89744331353898,0.369482408896298,0.763479568719562
"7571","SEMA6A",112.952280814431,0.367888722625541,0.410055534958767,0.897168044963797,0.369629253830968,0.763479568719562
"7572","LINC01003",164.10857012544,-0.267443377969156,0.29816687139576,-0.896958728906457,0.369740939972829,0.763479568719562
"7573","JCAD",16.8486309712592,-0.464442983858546,0.517818992070658,-0.896921493746934,0.369760809978243,0.763479568719562
"7574","TXNRD1",316.758477781798,0.17069894375963,0.190301416261898,0.896992503328032,0.36972291731423,0.763479568719562
"7575","VPS13C",523.052673832942,0.17857984449498,0.199028973753107,0.897255515754738,0.369582587680256,0.763479568719562
"7576","AC009053.2",6.91045565517546,0.543917512988153,0.606125537299202,0.897367755550709,0.369522712493219,0.763479568719562
"7577","ZNF185",14.8508170696319,0.365708825356973,0.407776844147995,0.89683568502052,0.369806603093276,0.763479568719562
"7578","ZCCHC9",212.124242913697,0.148798119136291,0.165964575443362,0.896565539596555,0.369950793266256,0.763575703795229
"7579","NUDT13",8.62783820143729,0.517320780670938,0.576949017709724,0.896649036208627,0.369906223203723,0.763575703795229
"7580","PGBD2",20.2850061313472,-0.297463698517773,0.331838742798158,-0.896410395029451,0.370033617498839,0.763635286425547
"7581","CA3",15.6179803318779,-0.536215091129293,0.598234951944435,-0.896328590274507,0.370077293766674,0.763635286425547
"7582","PPM1D",44.2787462329713,0.233912810704712,0.261076238765467,0.895955954516577,0.370276287748516,0.763945129154581
"7583","RPS27",23656.3948016906,-0.270197864283565,0.301647839113309,-0.895739432703412,0.370391944688792,0.76398154558159
"7584","PDE5A",93.294215886816,-0.294581248315948,0.329018586331865,-0.895333153060291,0.370609022902129,0.76398154558159

"7585","PIK3R1",2636.46930407589,0.220604590067734,0.246359632519444,0
.895457538281245,0.370542554571351,0.76398154558159
"7586","DHX37",27.8973749696222,0.283922886948331,0.317031581022509,0.
895566574259276,0.370484294578592,0.76398154558159
"7587","CYFIP1",307.085428345214,0.123221027726352,0.137554064819368,0
.895800701259993,0.370359215300167,0.76398154558159
"7588","ARPIN",129.701009406499,-0.154340488677005,0.172377185402215,-
0.895364942390003,0.370592034780391,0.76398154558159
"7589","ANKRD40",126.39743795199,-0.155006042444276,0.17315403044139,-
0.895191651324242,0.37068464686852,0.76398154558159
"7590","CCDC130",133.7875286962,-0.158853983108719,0.177443589858577,-
0.895236526917237,0.370660662589479,0.76398154558159
"7591","AUH",116.28392147517,0.147511710212131,0.164832935201184,0.894
916480326508,0.370831736254625,0.764184013994348
"7592","SLAMF7",2.87931589003026,0.802330988933086,0.896945949783239,0
.894514311734148,0.371046775857534,0.764526437663252
"7593","AC009063.3",4.76805318359592,-0.68968943100831,0.7712348521055
16,-0.894266421084859,0.371179361567203,0.764698900697453
"7594","MRPS10",280.140501482605,-0.175218913670797,0.195970662182737,
-0.894107881859431,0.371264172576243,0.764772906453802
"7595","TXNDC9",170.834904168404,-0.255472685513952,0.285787746168289,
-0.893924560934517,0.371362255614594,0.764773534041481
"7596","EDRF1",92.3059095495042,0.188746038110863,0.211130858016396,0.
893976559770366,0.371334432804925,0.764773534041481
"7597","ZNF814",17.0265916097157,0.438425592258803,0.490569731637554,0
.893706977793574,0.371478690985962,0.764912618545926
"7598","MRPL27",421.563343087113,-0.220117554346712,0.246387523433999,
-0.893379466942349,0.371653994719444,0.765172866464367
"7599","PAIP2B",38.5048577837651,0.319178624204118,0.357321998772333,0
.893252095590907,0.371722185471247,0.765173372490657
"7600","MKLN1",332.819173857278,0.186979587259183,0.20933762431368,0.8
93196279800163,0.371752069994822,0.765173372490657
"7601","PUM1",462.891458593661,0.147277041035344,0.164929223043286,0.8
92971168588423,0.371872612680983,0.765320784129539
"7602","AL592435.1",8.07418413433505,0.497008008777699,0.5567238589484
39,0.892737037921216,0.371998010820088,0.765356642900869
"7603","TMEM42",186.522793221141,0.230488458804371,0.258156650463917,0
.892824021345858,0.371951420272021,0.765356642900869
"7604","CCT8",465.511271001856,-0.240447403697843,0.269359180492723,-0
.892664594754139,0.372036815995539,0.765356642900869
"7605","HOTAIRM1",47.8138539546497,-0.374844069601007,0.42004630563441
7,-0.892387492933335,0.37218527254215,0.765561369937784
"7606","PDS5A",220.717526446769,0.158881808768926,0.178104890053544,0.
8920687619591,0.372356077197445,0.765812005732267
"7607","TMED4",504.098315893337,0.138013552358406,0.154778362547872,0.
891685052655342,0.372561767506115,0.766134314328664
"7608","PBX4",26.4720677128901,-0.293849841260448,0.329599235685542,-0
.891536779960252,0.372641269074287,0.766197078355555
"7609","ATP8B2",141.271269049807,0.230178246532432,0.258511311580497,0
.890399128475881,0.373251610187918,0.766211745189579

"7610","NDUFS2",326.0332033443,-0.204785781731771,0.230127075358913,-0
.889881303242484,0.373529624234909,0.766211745189579
"7611","AC069243.1",3.92153946832769,-0.663950688942025,0.746285940765
163,-0.889673317791944,0.373641325155061,0.766211745189579
"7612","ME1",45.7950924736384,-0.293340105755555,0.329111857335704,-0.
891308226115779,0.372763836762378,0.766211745189579
"7613","TBPL1",100.331311073883,-0.239024023714519,0.268458893001356,-
0.890356139974519,0.373274685311853,0.766211745189579
"7614","RPA3",240.774086228141,-0.226032575890348,0.254031785927908,-0
.889780682620931,0.373583661087628,0.766211745189579
"7615","DNAJB9",142.727938183276,-0.223086921543732,0.250502896352641,
-0.890556256202665,0.373167275572051,0.766211745189579
"7616","MTMR9",100.178580793022,0.206396491373623,0.231889749139152,0.
890063024087232,0.373432045937066,0.766211745189579
"7617","AC103706.1",13.878764803294,0.486242673784009,0.54660041009129
1,0.88957612326489,0.373693531653559,0.766211745189579
"7618","AL117339.4",7.24630692083299,0.540350601188081,0.6064341409522
67,0.891029321567521,0.372913440097262,0.766211745189579
"7619","CTSD",1452.35334292145,-0.235407949281706,0.264427563267138,-0
.890254958193921,0.373329000580009,0.766211745189579
"7620","FICD",27.9547504717101,-0.291742173929316,0.327783496137779,-0
.89004534202261,0.373441539948749,0.766211745189579
"7621","EIF2S1",222.078883994909,-0.189270393391338,0.212578588715563,
-0.890354924900678,0.373275337545132,0.766211745189579
"7622","BAG5",222.68242066518,-0.158957535496679,0.178440028031788,-0.
890817700770381,0.373026977494747,0.766211745189579
"7623","AXIN1",49.5134965801739,-0.217158299735919,0.243929178613954,-
0.890251428590248,0.373330895390566,0.766211745189579
"7624","PIGQ",59.2907757682671,-0.24611091106286,0.276573306156881,-0.
889857790264323,0.373542251107309,0.766211745189579
"7625","CRYM",7.84999510475089,0.571322234423217,0.642266602947175,0.8
89540623475649,0.373712600927721,0.766211745189579
"7626","GRB2",260.454388837794,-0.116305544806813,0.130613710957521,-0
.890454332506016,0.373221979377086,0.766211745189579
"7627","ZNF334",10.0996537854192,0.516144030197151,0.58025285867699,0.
889515704194874,0.373725987073866,0.766211745189579
"7628","RBM38",31.2602080285788,0.336025419372125,0.377250829663281,0.
890721485416079,0.373078605402094,0.766211745189579
"7629","APCDD1L-
DT",8.13692370787977,0.629332070701342,0.70630333904219,0.891022363783
204,0.372917172701584,0.766211745189579
"7630","KCND1",8.85717946553745,-0.427308799397677,0.479602890713672,-
0.890963769550724,0.372948607345521,0.766211745189579
"7631","ASAH1",747.798848587278,0.195252825556861,0.219642346897618,0.
888958018864526,0.374025642257556,0.766418822520695
"7632","AL583785.1",52.8896772162079,0.738642859376962,0.8309597025231
74,0.888903345293526,0.374055027429707,0.766418822520695
"7633","CARNMT1",44.3873690632281,0.244727802263947,0.275339771438889,
0.888821113582799,0.374099226847583,0.766418822520695
"7634","AL157786.1",4.76250171456637,0.647188410627796,0.7280125045612

35,0.888979799897598,0.374013936095964,0.766418822520695
"7635","CAPN3",18.6432012067459,0.387198518175226,0.43559388226157,0.888897971121451,0.374057915940251,0.766418822520695
"7636","LINC00899",62.7208223784232,0.248069545970115,0.279112214533927,0.888780687668406,0.374120956898807,0.766418822520695
"7637","HMGCS1",80.8348179132083,-0.208124464421313,0.234415440416464,-0.887844521041606,0.374624390827169,0.766591604202247
"7638","KIAA0825",30.6008940658089,0.267482501918107,0.301082222843061,0.888403504505587,0.374323740945211,0.766591604202247
"7639","AL662844.4",47.4857350417632,-0.343175955260251,0.38643490340182,-0.888056312303166,0.374510461097712,0.766591604202247
"7640","HOXA10",372.300894328498,-0.199541308021827,0.224758362703391,-0.887803708933211,0.374646347511742,0.766591604202247
"7641","IER5L",404.404984077657,-0.231908924455481,0.261146386193882,-0.888041867381255,0.374518230832122,0.766591604202247
"7642","SPCS2",1438.73224995126,-0.202704052839684,0.228251908780399,-0.888071665743244,0.374502202792542,0.766591604202247
"7643","COQ10A",58.6522079232495,-0.269347632968508,0.303327070947173,-0.887977562066715,0.374552821028516,0.766591604202247
"7644","INF2",146.314667662426,-0.192536180540203,0.216769032761815,-0.888208883377548,0.374428401200003,0.766591604202247
"7645","AC004494.1",3.08280868601789,0.742416825081626,0.835596579582327,0.88848715184153,0.374278763970939,0.766591604202247
"7646","ZBTB39",21.5244231769009,0.420241155949104,0.473615508980843,0.88730446528959,0.374915002170056,0.767040985998716
"7647","IFRD1",351.109761465868,-0.370230200607252,0.417420416007161,-0.886947993940242,0.375106900565724,0.767132598450729
"7648","UPF3A",484.04375196227,-0.191508908750084,0.215909643937287,-0.886986358079074,0.375086245173699,0.767132598450729
"7649","LRRC75B",56.4643211098948,0.2286685586361,0.257807951791565,0.886972481054333,0.375093716533207,0.767132598450729
"7650","GNG12",737.485587157124,0.153608476243623,0.173461867279195,0.885546077953739,0.375862179301207,0.767198283469696
"7651","CSDE1",888.581629962714,-0.138603874082902,0.156506431908981,-0.885611360455203,0.375826987686268,0.767198283469696
"7652","CACYBP",492.466257983766,-0.207264348465246,0.233765985645152,-0.886631765067248,0.375277186115812,0.767198283469696
"7653","CDK15",11.6022863432401,-0.444287079637032,0.501316462040094,-0.886240754650302,0.375487806676921,0.767198283469696
"7654","HRH1",44.0339821335189,-0.2552428264186,0.288598101969163,-0.884423094528433,0.376467860620716,0.767198283469696
"7655","NMNAT3",75.0387035550323,0.24436904407531,0.276233269588375,0.884647401232489,0.376346832650898,0.767198283469696
"7656","GYG1",151.201463922047,-0.217130879710268,0.24533597667809,-0.885034810834816,0.376137856676193,0.767198283469696
"7657","ERICH6-AS1",17.7857457996186,-0.339071081641475,0.382840969451975,-0.885670836448996,0.375794927936719,0.767198283469696
"7658","ETFDH",132.907556094044,0.166204376674454,0.187466082491522,0.886583719388122,0.375303062330886,0.767198283469696

"7659","AC099343.3",36.0055803355557,-0.425632628023404,0.480310763230175,-0.886160920402761,0.375530818940074,0.767198283469696
"7660","PCDHGA6",33.7341350608487,0.36230747858769,0.409216278082654,0.885369175159035,0.375957551912332,0.767198283469696
"7661","UBE3D",54.4776374970211,0.25369519790686,0.286405314106464,0.88579081955356,0.375730257767316,0.767198283469696
"7662","MRPL18",527.626660960067,-0.224932601816841,0.254217581472834,-0.884803484140129,0.376262629998832,0.767198283469696
"7663","C7orf61",2.87971529167899,0.788845367842293,0.889565715537698,0.886775821126915,0.37519960769037,0.767198283469696
"7664","SMIM30",223.771371842989,0.232909451465427,0.26336105897284,0.884373158179952,0.3764948077774,0.767198283469696
"7665","GFRA2",4.15049520575184,-0.676643203348343,0.763914425024897,-0.885757856092703,0.375748024189112,0.767198283469696
"7666","SNX16",71.5475562179871,0.180445972989733,0.203932068840494,0.884833729269275,0.376246314886496,0.767198283469696
"7667","GRHPR",549.853157350255,-0.230269939775536,0.260319870528796,-0.884565359178236,0.37639109685851,0.767198283469696
"7668","VSIG10L2",9.09595028389388,0.745728686500026,0.842282255479361,0.885366730272165,0.375958870115132,0.767198283469696
"7669","MLXIP",205.983748883858,0.231213450645713,0.260926687223119,0.88612419490844,0.375550606540752,0.767198283469696
"7670","LINC02298",5.87946139995418,0.523974455165605,0.591100947819181,0.886438191477727,0.375381446791586,0.767198283469696
"7671","RCN2",421.894244779759,-0.187639439730666,0.21217761894706,-0.884350765466378,0.376506891946132,0.767198283469696
"7672","CDRT4",17.6516749746433,0.375249131239251,0.424326368771652,0.88434082549602,0.37651225610157,0.767198283469696
"7673","AC018521.5",66.5057359650393,-0.279652583643218,0.316087869589334,-0.884730514987958,0.376301993503296,0.767198283469696
"7674","SARS2",31.3407059800928,0.256010366399207,0.288876522519781,0.886227666291836,0.37549485807762,0.767198283469696
"7675","CRNKL1",162.794490699424,0.214297411812902,0.242066629450653,0.885282751692084,0.37600415034299,0.767198283469696
"7676","SNX21",458.306398729,-0.227542572753824,0.256894817836429,-0.885742167437202,0.375756480136083,0.767198283469696
"7677","ATP5PO",1067.17756843392,-0.279376491969267,0.315650541791532,-0.885081617106104,0.376112613349735,0.767198283469696
"7678","STAM2",174.821616310487,0.149663761999835,0.16930554542841,0.883986177895863,0.376703674331213,0.767312980440611
"7679","HHIP",14.3668042710816,-0.788626447027313,0.892097545011085,-0.884013694956995,0.376688820067828,0.767312980440611
"7680","BOD1",392.536801235867,-0.203099958527277,0.22978361788956,-0.883874840132826,0.376763780385066,0.767312980440611
"7681","AC018645.3",16.6670472715761,0.388948033335187,0.440049661966263,0.883873041959084,0.376764751183554,0.767312980440611
"7682","RAB9B",46.8447266729995,0.268734674758721,0.304150605680266,0.883557914203941,0.376934906262758,0.767559585871951
"7683","SLC44A1",572.118785097898,0.150359151422618,0.170200113449486,0.883425682716971,0.377006319542588,0.767605083509658

"7684","AC093726.2",3.33554542243177,0.682025757702359,0.772227095014214,0.883193249894715,0.377131868036168,0.767760777159003
"7685","MRPS36",396.923759867944,-0.241939206157109,0.274043368938834,-0.882850065279664,0.377317286189187,0.767976909548549
"7686","FITM2",42.7090681098615,0.234843773622227,0.266016965704133,0.882815022720852,0.37733622238638,0.767976909548549
"7687","CHST11",36.9991119364728,-0.296069339817549,0.335436098299043,-0.882640065630628,0.377430773959562,0.76806941551313
"7688","SOX13",64.486404270037,0.221962246152644,0.251550293488766,0.882377210037149,0.377572855725159,0.768091728267155
"7689","ASB6",37.0391579921154,0.239747739277874,0.271669147727257,0.882498956114696,0.377507044020415,0.768091728267155
"7690","TM7SF3",233.079574149487,-0.145194492737435,0.164554816447901,-0.882347268050971,0.37758904240711,0.768091728267155
"7691","PSPH",28.5236961377275,0.255301558871427,0.289583366442727,0.881616793145196,0.377984070692037,0.768495557296013
"7692","SYTL2",66.3494729498334,0.231321352220831,0.262358490995959,0.881699507199843,0.377939327574476,0.768495557296013
"7693","FAM210A",77.296068283748,0.204398475914615,0.23182137099501,0.881706785864082,0.377935390429679,0.768495557296013
"7694","KDM5C",141.007551491974,0.178053897183485,0.201919321205421,0.881807130296084,0.377881115112014,0.768495557296013
"7695","AC092040.1",111.499751156034,-0.313951097754556,0.356391905536749,-0.88091534312971,0.378363642328571,0.768992560836001
"7696","NDUFAF6",56.4166458221091,-0.217820828681836,0.247298373313787,-0.880801704285582,0.378425157151156,0.768992560836001
"7697","GLIPR2",366.890053967444,-0.239934297243766,0.27239161433475,-0.88084318538861,0.378402701943987,0.768992560836001
"7698","GTF3C1",134.47319353592,0.160629427107011,0.182350967760735,0.880880584729197,0.378382457044392,0.768992560836001
"7699","AP1G2",21.651509960824,-0.337851600331885,0.383640506715791,-0.880646319712462,0.378509279680226,0.769063600732275
"7700","SCLY",23.1160118634505,0.286317690319114,0.325233012004807,0.880346335552437,0.37867171849857,0.769293726295211
"7701","GPR89B",30.8033470409475,0.253341703356785,0.287947490679548,0.879819104375264,0.378957313568016,0.769713606825456
"7702","CCT2",348.351346480701,-0.167124538534977,0.189961063820654,-0.879783125939756,0.378976807503015,0.769713606825456
"7703","AL034417.2",10.5081515643932,-0.528001045322303,0.601424336604014,-0.877917658443452,0.379988405543095,0.769787072126577
"7704","LINC02606",8.79656688803115,-0.448239027603322,0.510395549753258,-0.878218918288014,0.379824927310044,0.769787072126577
"7705","SLC25A34",4.89173967993308,0.689837768707014,0.786908163621602,0.876643299177582,0.380680413121646,0.769787072126577
"7706","HYI",418.728277560255,-0.243326523619529,0.277036975874052,-0.87831785938261,0.379771246497207,0.769787072126577
"7707","CYB5RL",31.6744409097508,0.277293999531359,0.316752765122182,0.875427241888158,0.381341482576562,0.769787072126577
"7708","PKN2",420.267738636871,0.133839627334508,0.152440009624931,0.877982280792374,0.379953334672714,0.769787072126577

"7709","CD2",13.3409846498418,0.502261808203645,0.572496434516016,0.87
731866597257,0.380313576431026,0.769787072126577
"7710","CENPL",37.7789934446598,-0.30223466470198,0.344982056683923,-0
.876088071383061,0.380982157040645,0.769787072126577
"7711","CNIH4",465.365248448652,-0.221190512658652,0.252128267126553,-
0.877293590201164,0.38032719286881,0.769787072126577
"7712","SMC6",144.657718513686,0.166370293313235,0.189576516666422,0.8
77589145737807,0.380166721790344,0.769787072126577
"7713","TTC31",66.888843582581,0.230682495051786,0.262734119154234,0.8
78007377931632,0.379939714866766,0.769787072126577
"7714","AC022007.1",6.02587650351187,0.522100640663262,0.5941938977065
85,0.878670485641839,0.379579965912313,0.769787072126577
"7715","EVC",43.3628557309234,0.291989632895701,0.333351010267452,0.87
5922447816895,0.381072195200536,0.769787072126577
"7716","WDR17",10.3490468776268,-0.574392943613155,0.653587651800718,-
0.878830776607588,0.379493036363148,0.769787072126577
"7717","CAMK4",3.10528539676463,0.829276030979935,0.943952038523918,0.
878515006203806,0.379664297755663,0.769787072126577
"7718","REV3L",871.906699773039,-0.228476536179792,0.261114931696197,-
0.875003718460731,0.381571882456657,0.769787072126577
"7719","PHF10",198.174260085818,0.213777271898676,0.243403022494216,0.
878285198384321,0.379788966312345,0.769787072126577
"7720","TOMM7",3208.97295630665,-0.223961318164662,0.255321414593648,-
0.877174045589177,0.380392111112275,0.769787072126577
"7721","DNAJC2",184.894405959191,-0.187939381566261,0.214441839992743,
-0.876411905310178,0.38080614851429,0.769787072126577
"7722","LZTS1",3.07010668397202,-0.717666651288373,0.820156974791926,-
0.875035722851037,0.381554468848439,0.769787072126577
"7723","PSIP1",630.174883124695,-0.136732680897529,0.15593003698474,-0
.876884810274945,0.380549207402474,0.769787072126577
"7724","RPS6",13554.433201052,-0.247350846835776,0.282662005624559,-0.
875076387748821,0.381532343756299,0.769787072126577
"7725","CBWD3",22.9540268371547,-0.322098720029648,0.367707810831411,-
0.875963769443359,0.381049730246675,0.769787072126577
"7726","PAPPA",35.5437418850306,0.43997393364034,0.501887899193974,0.8
76637859464102,0.380683368674384,0.769787072126577
"7727","ZER1",99.4695317649939,0.158104782783463,0.180595200018776,0.8
75465033218078,0.381320927963686,0.769787072126577
"7728","SFTA1P",13.9268098156517,-0.432252785991356,0.493472498254804,
-0.875940984593965,0.381062117378513,0.769787072126577
"7729","PLXDC2",742.190020189718,0.271013448156163,0.309255419762864,0
.876341790109853,0.38084425292591,0.769787072126577
"7730","CUTC",543.949672179965,-0.234739523604363,0.268112542841743,-0
.875526079892951,0.381287726261476,0.769787072126577
"7731","ADD3-
AS1",19.5272136196744,0.36966854658628,0.420794249523673,0.87850189731
617,0.379671408525713,0.769787072126577
"7732","EIF3A",558.410047902362,-0.174557880133112,0.198716411305173,-
0.878427096114573,0.379711985170936,0.769787072126577
"7733","RHOG",198.78007845308,-0.223275319964778,0.25410642207179,-0.8

78668544243635,0.379581018853129,0.769787072126577
"7734","UNC93B1",94.8146325103283,-0.224841490293517,0.256958877170191
,-0.875009623211414,0.381568669641535,0.769787072126577
"7735","USP28",72.8609684275758,0.208316050569072,0.237981851348009,0.
875344272637177,0.381386611736722,0.769787072126577
"7736","VPS11",114.404609153278,0.155889842546135,0.177266846344187,0.
879407772863822,0.379180219138628,0.769787072126577
"7737","DIS3",157.194501964173,0.199879737786917,0.227853036378977,0.8
7723095975981,0.380361203240958,0.769787072126577
"7738","NEDD8",1316.13679852944,-0.236279576102545,0.269434657878035,-
0.876945742479431,0.380516109156456,0.769787072126577
"7739","LINC01588",7.54200995228588,0.540781035822642,0.61679626606498
4,0.876757959759872,0.380618118010108,0.769787072126577
"7740","FBXO22",148.823880136855,0.182931911232853,0.208182874999806,0
.878707776674827,0.379559741017785,0.769787072126577
"7741","MRPL45",147.744770705699,-0.191402700890406,0.21865446607005,-
0.875366071091758,0.381374754665877,0.769787072126577
"7742","TTC25",6.44836192232914,-0.496522631603143,0.566260694764005,-
0.876844598599721,0.380571051269008,0.769787072126577
"7743","CCDC47",331.920429605392,-0.14303015469963,0.1627411953262 5,-0
.878881062738267,0.379465767538631,0.769787072126577
"7744","RPL17",12942.5674230112,-0.242968004767318,0.277013379509933,-
0.877098446281383,0.380433168540592,0.769787072126577
"7745","BCL2",109.795532660274,0.238426274530617,0.272379226748493,0.8
7534676332264,0.381385256939599,0.769787072126577
"7746","ZNF440",17.2592493837508,0.329506543471271,0.375497529008709,0
.877519871678378,0.380204330219417,0.769787072126577
"7747","MEF2B",29.6956175962573,-0.278921989932408,0.318157505490289,-
0.876678956551982,0.380661039793897,0.769787072126577
"7748","ZNF565",40.6869013494132,0.223241407424222,0.254806672590723,0
.876120727744042,0.38096440556144,0.769787072126577
"7749","ZNF527",22.8194566418755,0.284822870708065,0.325077709336764,0
.876168566861049,0.380938401894139,0.769787072126577
"7750","ZNF784",13.9111412862583,0.419992524109405,0.478483859635629,0
.87775693088004,0.380075641820363,0.769787072126577
"7751","ZNF417",20.7917828761762,0.366231590010459,0.416679840605484,0
.878928026559389,0.379440301402112,0.769787072126577
"7752","C19orf18",6.07593520915193,0.552495440700554,0.629210468933873
,0.878077317493932,0.379901761396662,0.769787072126577
"7753","LINC00649",19.1041505858649,-0.512988040298656,0.5860351755467
12,-0.875353667670357,0.381381501366244,0.769787072126577
"7754","TRMT2A",73.2982919371729,-0.215409287534759,0.245458626267144,
-0.87757880344494,0.380172336407609,0.769787072126577
"7755","ALS2",59.0889885878996,0.2418305994195,0.276572644837913,0.874
383652661044,0.381909356879322,0.769872173925675
"7756","LAMTOR3",200.046904586656,0.156741640379202,0.179252324844114,
0.874419009714446,0.381890108676312,0.769872173925675
"7757","CAST",2190.67153468091,-0.172105251958066,0.19674302023589,-0.
874771830541772,0.38169806738104,0.769872173925675
"7758","FGFR1OP2",397.778280460256,-0.15237886515046,0.174269086648548

,-0.874388384543297,0.381906780831803,0.769872173925675
"7759","HOXC4",77.9025417985048,0.247739741479372,0.283322671467388,0.874408462253569,0.381895850598971,0.769872173925675
"7760","GJD3",6.5007153106211,-0.620904464285372,0.709929132186358,-0.87460062721075,0.381791246399078,0.769872173925675
"7761","AC037459.2",8.05106844981256,0.541640323862947,0.619637781226814,0.874124109718681,0.382050668975501,0.77005780373454
"7762","SIRT3",47.9590175175049,-0.234770808844681,0.26860649389002,-0.874032512932498,0.382100547891373,0.770059117581132
"7763","MT1F",111.007396568134,-0.436177797368861,0.49919764124515,-0.873757729064787,0.382250205063389,0.77026149140881
"7764","EIF1B",773.992483933168,-0.194713165112055,0.222995438748603,-0.873171066658307,0.382569842759413,0.770409378255084
"7765","PLGRKT",178.939801800165,-0.250896031586126,0.287312425427173,-0.873251587407321,0.38252596206154,0.770409378255084
"7766","COQ6",81.0393538916723,-0.172453418573509,0.197501470629301,-0.873175364335359,0.382567500613487,0.770409378255084
"7767","TSEN34",220.903619515545,-0.21754097852461,0.249070593448756,-0.873410929457504,0.382439135893251,0.770409378255084
"7768","NDUFA1",1002.99430213877,-0.233381510571986,0.267205415026632,-0.873416096558991,0.382436320519693,0.770409378255084
"7769","NXF1",91.4506167906791,0.219028601255645,0.250917270001624,0.872911622441245,0.382711250821553,0.770594940996468
"7770","ARID5B",1917.98806305279,-0.21450575786946,0.245788668336918,-0.872724358372058,0.382813337555038,0.770701292068655
"7771","LRIG2-DT",19.6063085988047,0.338652154247618,0.388608972303938,0.87144708018412,0.38351008887262,0.770778601991157
"7772","RGS1",93.1492980366322,-0.446691706868294,0.512719619416314,-0.871220234124868,0.383633913851778,0.770778601991157
"7773","CD34",1973.12616679622,-0.184979252864819,0.212677114438056,-0.869765669679966,0.384428476170492,0.770778601991157
"7774","PFN4",10.8899460743206,0.488312112022402,0.560094329523059,0.871839056892823,0.383296184245547,0.770778601991157
"7775","AC008280.3",10.9943124285737,0.438808629184794,0.50412886119818,0.870429493248735,0.384065734783896,0.770778601991157
"7776","DCAF17",32.5351427342076,0.262094086929969,0.301219965235591,0.870108615559329,0.38424104981853,0.770778601991157
"7777","PDGFRA",2650.70447087984,0.17598114578951,0.202276656949613,0.870002245653818,0.384299176983792,0.770778601991157
"7778","AC106881.1",3.09753878105695,0.770164328061395,0.884144738669248,0.871083991542597,0.383708294264352,0.770778601991157
"7779","ATG10",90.0565910013464,-0.19318356818799,0.221939373740815,-0.870433961004112,0.384063294122464,0.770778601991157
"7780","AL359715.3",3.09500266626195,-0.809074110910968,0.927556774108432,-0.872263707726841,0.383064531583587,0.770778601991157
"7781","TBX18",123.703719166269,0.187302064975693,0.214991445999959,0.871207057120446,0.383641107333218,0.770778601991157
"7782","GLI3",307.398362130271,0.173154143124625,0.198753833734582,0.871199009704926,0.38364550055246,0.770778601991157

"7783","AC083862.2",21.389739513868,0.336400785442186,0.386264216135235,0.870908490587202,0.383804120442676,0.770778601991157
"7784","AC124067.2",4.98193673465999,-0.738882012234987,0.849252596592995,-0.870037978334373,0.384279649813551,0.770778601991157
"7785","C9orf116",19.8059219349647,-0.352367747641455,0.403944531579391,-0.872317162615682,0.38303537731187,0.770778601991157
"7786","PIK3AP1",4.65615200464023,-0.657228296398778,0.754790219965726,-0.870742994561617,0.383894497196833,0.770778601991157
"7787","TMEM223",186.684717568851,-0.230243607490112,0.264702232957112,-0.869821175733778,0.384398137278889,0.770778601991157
"7788","AP002490.1",4.28414751599704,0.705910239133233,0.80941874904263,0.87211995023364,0.383142943597853,0.770778601991157
"7789","PTS",99.648063661525,-0.247662156439253,0.284335073197172,-0.871022183983304,0.383742040456379,0.770778601991157
"7790","GSEC",29.8275580157793,0.363839992535689,0.417951431323328,0.870531753854007,0.384009873871285,0.770778601991157
"7791","VCPKMT",36.0809958844522,0.26600975123598,0.305261576436424,0.87141576853968,0.383527179023726,0.770778601991157
"7792","GALK2",127.085551041142,0.190932358873965,0.219338075796172,0.870493452543078,0.384030795775835,0.770778601991157
"7793","SPG21",249.320785420833,-0.156339587924195,0.179386054292628,-0.871525874966637,0.383467084100462,0.770778601991157
"7794","ZNF768",42.1290281377805,-0.234939874460958,0.269579811744104,-0.871503963672075,0.383479042593703,0.770778601991157
"7795","AC132938.3",6.48162706450635,-0.49266653673558,0.565435829191291,-0.871304065467892,0.383588151271175,0.770778601991157
"7796","ZNF407",75.5728535176326,0.239290084600468,0.27475210859498,0.870930839527103,0.383791916769284,0.770778601991157
"7797","ERF",44.7912906385718,-0.225122632425441,0.258593378649489,-0.870566112717772,0.383991106102865,0.770778601991157
"7798","NECAB3",107.021197399523,-0.262199101858276,0.301426328466138,-0.869861313019744,0.384376199669688,0.770778601991157
"7799","EPB41L1",102.005230813229,-0.183219207493541,0.210563849887312,-0.870136101669847,0.384226030564306,0.770778601991157
"7800","PARD6B",5.38930739155772,-0.527496906689836,0.606448853879546,-0.869812686288972,0.384402777404814,0.770778601991157
"7801","ZFX",249.102003643761,0.147461972567089,0.169467624697979,0.870148341489369,0.384219342466924,0.770778601991157
"7802","NAP1L2",4.34531491433311,0.745542925355485,0.856103773752672,0.87085578666176,0.383832900443698,0.770778601991157
"7803","EFNA1",6.53148655611998,-0.579084954168978,0.666436947079033,-0.868926845528425,0.384887144921411,0.770988486747682
"7804","AC112715.1",6.88057345658681,-0.568666402981271,0.654142199686796,-0.869331474492166,0.384665852189111,0.770988486747682
"7805","MRPS18B",323.595341648297,-0.176514827579915,0.20313609529207,-0.868948609680222,0.384875240064468,0.770988486747682
"7806","FAM78A",5.35872508207198,0.563023069045177,0.647857979913125,0.869053228487941,0.384818017363476,0.770988486747682
"7807","MORN3",3.58320453552707,-0.664194025739147,0.764449105633077,-0.868853166083694,0.384927448794949,0.770988486747682

"7808","PPP4R3A",191.348367743696,0.185343959854013,0.213226895574965,
0.86923349586943,0.384719429836097,0.770988486747682
"7809","VPS18",42.4926081203185,0.234467145535934,0.269797284472284,0.
869049316024604,0.384820157245664,0.770988486747682
"7810","AC022898.2",3.71822642913161,-0.778218856626447,0.895683327773
39,-0.868854909425464,0.384926495128396,0.770988486747682
"7811","UTP25",75.3399700091895,0.196042826843174,0.225699735049068,0.
86860016384403,0.385065864841342,0.771166985496494
"7812","SLC1A2",22.6554396839398,0.529614136715312,0.609806214398004,0
.868495801142569,0.385122969927897,0.771182618866115
"7813","ADNP",234.153589379685,0.141693558745494,0.163185461305159,0.8
68297687871381,0.385231387603832,0.771300985061659
"7814","CNOT10",70.8737417610241,0.1892667654464,0.218036779684343,0.8
68049719503314,0.385367114811683,0.771375275367774
"7815","KRT25",9.70909238031326,0.661045009438125,0.761498018808714,0.
868085002338237,0.385347800724053,0.771375275367774
"7816","EDEM1",87.1418662915661,-0.22458102863513,0.258821932999956,-0
.867704780781343,0.38555596800707,0.771632641968576
"7817","AC006449.6",10.3715065511744,-0.42181097040374,0.4861619442250
66,-0.867634695422531,0.385594346498009,0.771632641968576
"7818","SYTL1",7.56059735976295,-0.447844571681066,0.516456047668199,-
0.867149438375417,0.38586013553249,0.772065758523247
"7819","AGPAT5",35.0654999061819,-0.242916767749349,0.280249908578425,
-0.866786251533678,0.386059136451187,0.77206891734926
"7820","POLL",92.4144703885859,-0.186047513519232,0.214615615941025,-0
.866887121440207,0.386003860531823,0.77206891734926
"7821","CAPS2",36.9059014715622,0.278983486095714,0.321782051465882,0.
866995175227461,0.385944653263908,0.77206891734926
"7822","SNHG22",7.6009822233016,0.498199590578917,0.574730985838069,0.
866839622110239,0.386029889190809,0.77206891734926
"7823","PLRG1",219.504633117929,-0.180509017097513,0.208285866348256,-
0.866640738818547,0.386138884795284,0.772129691276062
"7824","DNM1L",176.360523862019,-0.204826055849249,0.236410342366889,-
0.866400571982488,0.386270530394269,0.772294211011957
"7825","WDYHV1",51.3950587477866,-0.217390066244704,0.250942705510524,
-0.866293625879424,0.386329160891819,0.772312723812233
"7826","CYP4B1",227.774502924505,0.268352161053279,0.309989320946494,0
.8656819539264,0.386664598955931,0.772374484267315
"7827","COPA",517.717578390378,0.141329279680149,0.163220312421697,0.8
65880462935331,0.386555718060683,0.772374484267315
"7828","PMS1",91.0552120849613,0.194469408008021,0.224582750042975,0.8
65914269777214,0.386537177093476,0.772374484267315
"7829","TAF9",221.059828591274,-0.219981950234491,0.254162337684924,-0
.865517496566289,0.386754816917024,0.772374484267315
"7830","POR",247.712197885524,-0.196876883219111,0.227360480868982,-0.
865923939229184,0.386531874095406,0.772374484267315
"7831","CIAO3",62.4302902729862,0.197962998004272,0.228722236478903,0.
865517061444665,0.386755055632927,0.772374484267315
"7832","ABCC1",66.5253839346178,0.226655760665762,0.261868279943593,0.
865533468637685,0.386746054400606,0.772374484267315

"7833","PLEKHG4",58.4628172780577,-0.210613504037338,0.243262833295319
,-0.865785788911101,0.386607643810679,0.772374484267315
"7834","RGS13",2.7521199857235,-0.862038972584322,0.997614687944048,-0
.864100121020543,0.387532892540372,0.772500397076626
"7835","AC137630.4",2.81786569601933,0.742299350070098,0.8590219936822
58,0.864121472476134,0.387521164473726,0.772500397076626
"7836","AC107027.3",4.31644302144091,-0.60072911960567,0.6950991340089
81,-0.864235171954491,0.387458714527608,0.772500397076626
"7837","CNPY3",207.012500854435,-0.196044499693613,0.226751883770506,-
0.864577159994083,0.387270913056384,0.772500397076626
"7838","TMEM74",9.1339386807255,-0.694721570553084,0.803752743631553,-
0.864347370578371,0.387397094947769,0.772500397076626
"7839","C9orf106",3.54961216768543,0.878223296354625,1.015671854293,0.
864672278396404,0.38721868900462,0.772500397076626
"7840","ABI1",234.346362491081,0.145996268779877,0.168983213075187,0.8
6396906605698,0.387604884004784,0.772500397076626
"7841","PPP3CB-
AS1",4.88720601906824,-0.671511009525878,0.777206754196751,-0.86400562
7717285,0.387584799035504,0.772500397076626
"7842","FUT8-
AS1",4.34616720560512,0.588916719672941,0.681260151173196,0.8644520285
80813,0.387339622070231,0.772500397076626
"7843","SPIRE2",12.4794063976508,-0.399854114182415,0.462622018071484,
-0.864321408326548,0.387411352907224,0.772500397076626
"7844","NOTCH3",107.311600734103,0.365685159168129,0.422761093329665,0
.864992462499314,0.38704292586628,0.772500397076626
"7845","PGLS",851.266426382647,-0.242839467925835,0.28088273726101,-0.
864558179309455,0.38728133477423,0.772500397076626
"7846","LINC00323",5.83625659206765,-0.596218683658587,0.6896264694264
61,-0.864553073426028,0.387284138289042,0.772500397076626
"7847","SFI1",36.6604577809919,0.279628595006383,0.32358787563396,0.86
4150408783227,0.387505270495766,0.772500397076626
"7848","ZFP92",2.63921100449266,-1.21758779947109,1.40930554497976,-0.
863962966589038,0.387608234779418,0.772500397076626
"7849","F8",28.1293717270803,0.29104797101174,0.336585340586165,0.8647
07804876108,0.387199184556684,0.772500397076626
"7850","TMX2",233.278033642737,-0.229714609136942,0.265967002194184,-0
.863695899272596,0.387754966919771,0.772595968351291
"7851","SELENOS",780.63146132036,-0.186322202956561,0.215707205225118,
-0.863773663759215,0.387712238057741,0.772595968351291
"7852","AC097468.3",6.70394236065065,0.541715186886572,0.6275543972795
02,0.863216303216024,0.388018551584943,0.772960958222315
"7853","BMP5",42.4164652823399,0.926521861809911,1.07338857244497,0.86
3174702614425,0.388041420304941,0.772960958222315
"7854","SUMO4",14.2373748158405,0.355860809990836,0.412308811060876,0.
863092906201062,0.388086387897338,0.772960958222315
"7855","AP000547.3",13.4024821002811,-0.474395502107945,0.549736279176
026,-0.862951055038598,0.38816437812511,0.773017869765894
"7856","PTGES",686.493761928464,-0.264079620975197,0.306105309168638,-
0.862708398271238,0.388297813735589,0.773086763429531

"7857","AC023509.2",4.9854675293462,0.863198166129595,1.00052074696817
,0.86274889225966,0.388275544373895,0.773086763429531
"7858","P2RX4",99.4218992623301,0.180317616250275,0.209083703959079,0.
862418317811925,0.388457364030888,0.773262987579849
"7859","AL117332.1",14.0043126212872,-0.340329845253605,0.394664767784
238,-0.862326392001787,0.388507933471238,0.773262987579849
"7860","GNAS-
AS1",3.82017089618834,0.674623338245065,0.782373470186348,0.8622778812
83962,0.388534621388328,0.773262987579849
"7861","FBXL18",41.7038961961261,0.26422971785586,0.306468420554272,0.
862176002923825,0.38859067286817,0.773276160243835
"7862","RPL26L1",196.173123867423,-0.263019350624778,0.305146491773157
,-0.861944533907027,0.388718040892019,0.773416822764274
"7863","AP5B1",50.9962174584105,-0.192403731472684,0.223240409254845,-
0.861867849619652,0.388760242753659,0.773416822764274
"7864","AL049840.4",11.6722784084359,0.574448831245399,0.6667471345290
01,0.861569253913927,0.38892459627386,0.77348269014911
"7865","TRIM37",80.3132265883444,0.186689620591413,0.216712280724838,0
.8614630419974,0.388983067801076,0.77348269014911
"7866","AC008735.2",5.38045010110759,0.677440139501726,0.7862290060404
06,0.861632087212655,0.388890007957173,0.77348269014911
"7867","ARSD",91.2374711259654,0.288586068004905,0.335000995606405,0.8
61448389078723,0.388991134910379,0.77348269014911
"7868","AC025164.1",20.7470976919172,-0.402001062402341,0.466755970656
84,-0.861266031233895,0.389091539855707,0.773485698050937
"7869","CCDC160",11.7299363563824,-0.573534679653091,0.665862363906269
,-0.861341188122512,0.389050157091519,0.773485698050937
"7870","AC096586.1",4.18752866756844,0.766525650939768,0.8903815678075
62,0.860895686359757,0.389295497671464,0.773792817036177
"7871","FAM8A1",244.460954531654,0.165376541019484,0.192137185413676,0
.860721159537255,0.389391636330753,0.773799487438355
"7872","POLR3A",55.6106972483017,0.22948408177627,0.266621838553634,0.
860709996679834,0.389397785917965,0.773799487438355
"7873","AL022345.4",12.1945022855888,-0.455663203271645,0.529476228980
044,-0.860592370972747,0.389462589185457,0.773829960958733
"7874","AP003555.1",9.02388264963191,-0.466681286660367,0.542369093123
952,-0.860449632135864,0.389541236783962,0.773859251504996
"7875","EEF2",3652.15964783299,-0.171692882918889,0.19955330768417,-0.
860386053788818,0.389576270894448,0.773859251504996
"7876","PRPF4",91.0070412800397,-0.1735059619684,0.201761821399674,-0.
859954379697527,0.389814190572532,0.773886556484518
"7877","DAZAP2",531.883895683101,0.111630753994835,0.129786409245703,0
.860111275468783,0.389727706339316,0.773886556484518
"7878","ITM2B",12606.7693375657,-0.179511040883223,0.208750746349939,-
0.859930055446602,0.389827599653636,0.773886556484518
"7879","DAD1",1941.38609087671,-0.254812602975877,0.296238644104196,-0
.860159901644203,0.389700904942483,0.773886556484518
"7880","TIAM1",35.0268103745245,0.336343613880385,0.39113710221791,0.8
59912322234779,0.389837375509685,0.773886556484518
"7881","CD160",2.75188094452956,-0.765721171944516,0.890730133290343,-

0.859655627811708,0.389978901130561,0.774069274252679
"7882","OMG",24.3161422951519,-0.551613895466439,0.641751448834591,-0.859544448973446,0.390040208042622,0.774092739712096
"7883","SEC11A",1809.13272742111,-0.230683690335223,0.268521940850125,-0.859086931983625,0.390292556509496,0.774495301468735
"7884","ZNF771",54.4184879651375,-0.216724935130916,0.252302445051902,-0.858988643912398,0.390346781308814,0.77450465499921
"7885","TMEM222",193.111250896652,-0.240989823578812,0.280794424187244,-0.858242909474981,0.390758346598454,0.774635935712809
"7886","AGPAT1",161.429219739103,0.142064511271125,0.165433500302983,0.858740889910092,0.390483485667799,0.774635935712809
"7887","LUC7L2",348.979371372463,-0.122759935629307,0.142974406674271,-0.858614758297147,0.390553093058737,0.774635935712809
"7888","MED22",60.7671057390998,0.229335583932042,0.26712996002009,0.858516895352337,0.390607105206792,0.774635935712809
"7889","DERA",262.71541320073,-0.209347959110654,0.24400053311358,-0.857981564381274,0.39090264336723,0.774635935712809
"7890","AC106739.1",8.6686685348213,-0.714225471707713,0.832458169106165,-0.857971605317536,0.390908142716673,0.774635935712809
"7891","IFI35",257.056374852002,-0.222726039620227,0.259487748322394,-0.858329694022805,0.390710437303021,0.774635935712809
"7892","AC116025.1",2.98019892113785,-0.942323690250346,1.09818828240012,-0.858071157152462,0.390853172761606,0.774635935712809
"7893","UBE2S",127.910631496818,-0.293664189687451,0.342226910845262,-0.858097888801713,0.390838413034184,0.774635935712809
"7894","MICALL1",12.4414862225808,0.383910924946498,0.447352886391597,0.85818363226215,0.390791072556958,0.774635935712809
"7895","CNST",114.295897728856,-0.213633167197303,0.249085718883334,-0.857669272068398,0.391075112119127,0.774689335179944
"7896","NRL",4.19659010476234,-0.599297705071563,0.698918934094839,-0.857463828545016,0.391188597000418,0.774689335179944
"7897","AC015871.7",18.4659925999598,0.340409270393448,0.397032055951008,0.857384851654026,0.391232228339932,0.774689335179944
"7898","CERS3-AS1",8.01431954262279,0.542292036484512,0.632345879582796,0.857587681036683,0.391120179769201,0.774689335179944
"7899","DBF4B",15.7480816765512,0.380816533626392,0.443966026594442,0.857760528542117,0.391024709398366,0.774689335179944
"7900","AC145343.1",2.75519155171222,0.927275731210986,1.08148715818317,0.857407990649424,0.391219444732989,0.774689335179944
"7901","KDM2B",41.5569615017142,0.275694553746824,0.32160502250132,0.857245796730964,0.391309057391913,0.774743397643551
"7902","NT5M",9.27290477856299,-0.444396498624746,0.518580607051963,-0.856947777417015,0.391473746498076,0.774971376419819
"7903","STK36",31.1878205899649,0.342815937717808,0.400096426120881,0.856833291518163,0.391537023985341,0.774984535104123
"7904","FAM227B",7.32892111011487,-0.543297155281311,0.634132525146677,-0.856756488173579,0.39157947743163,0.774984535104123
"7905","ARV1",81.8231587656908,-0.237986977401661,0.277823558921008,-0.856611938620103,0.391659385522155,0.775044625897921

"7906","ACADM",206.78197412066,-0.141981403305533,0.165861435822485,-0.856024202379929,0.391984392483203,0.775113839799851
"7907","RPAP2",171.363183050531,0.177215276115506,0.207029503791986,0.855990440345951,0.392003067213637,0.775113839799851
"7908","ZNF687",35.329839154994,0.246134345784583,0.287557588347408,0.855948010967526,0.392026536861344,0.775113839799851
"7909","AL358472.2",5.67892587120265,0.586129650746295,0.684997449184309,0.855666910065511,0.392182048274981,0.775113839799851
"7910","AC098487.1",70.1476309029992,0.268500683239635,0.313705508372373,0.855900441891256,0.392052850526674,0.775113839799851
"7911","TXNDC5",490.195477023365,0.171922047273083,0.20088318126195,0.85583096699817,0.392091283702178,0.775113839799851
"7912","ATP6V1F",1002.89862681043,-0.246591437525509,0.288191006976952,-0.855652784284243,0.392189863965724,0.775113839799851
"7913","AGAP3",116.670013613105,0.19927085445629,0.232732575888293,0.856222441983952,0.391874751471823,0.775113839799851
"7914","GTPBP3",64.1602966294027,-0.175911633191853,0.205535844878839,-0.855868392666742,0.392070579719026,0.775113839799851
"7915","ZNF506",190.098951073164,0.152859829725853,0.178534112779049,0.856193963979478,0.391890500748208,0.775113839799851
"7916","CAVIN3",1350.87788431487,-0.235570366835547,0.27534341007716,-0.855551134379909,0.392246108894941,0.775127069409243
"7917","GMPR",19.231219463334,-0.34454071150814,0.402982037650383,-0.854977838508662,0.392563416577616,0.775168303131666
"7918","L3MBTL3",30.2519062798665,0.331172746004127,0.387328122795886,0.855018591507358,0.392540855479561,0.775168303131666
"7919","FNTA",441.010921714328,-0.158563861201513,0.185459960465653,-0.854976248260759,0.392564296964077,0.775168303131666
"7920","RPL35",8795.5123727842,-0.21666289141478,0.25335142555717,-0.855187180961366,0.392447531726144,0.775168303131666
"7921","RNF114",242.69635057482,-0.173269255169869,0.202659213909514,-0.854978423271849,0.392563092843741,0.775168303131666
"7922","THOC2",463.552858480321,0.16174625005028,0.189154496333574,0.855101270048796,0.392495086571454,0.775168303131666
"7923","SLC35E3",181.064599246895,0.154990584859231,0.181306331967278,0.854854781835217,0.392631546241236,0.775185020931713
"7924","MIB1",192.517721260005,0.17726942269766,0.207385547595311,0.854781949625443,0.392671872777785,0.775185020931713
"7925","COX6B1",1492.03077455537,-0.238759711428296,0.27935361658556,-0.854686308867488,0.3927248320145,0.775191740971966
"7926","LYRM2",226.686589993333,-0.143989405054799,0.168531422488701,-0.854377201168247,0.392896024084668,0.775330758172684
"7927","LARP4",112.435527482832,0.199106050903524,0.233065930255285,0.854290675112562,0.392943952617339,0.775330758172684
"7928","LINC00667",253.753907182386,-0.159973323460278,0.187250560414009,-0.85432760845457,0.392923494058645,0.775330758172684
"7929","ALDH7A1",220.655186880323,0.138056373653725,0.161671688456397,0.853930425121769,0.393143540457165,0.775457513428427
"7930","SMURF1",44.3582218646323,0.227388223545933,0.266334994714622,0.853767728831805,0.393233698551674,0.775457513428427

"7931","AKR1B10",7.21506810179285,-0.60446258291416,0.708101819104658,-0.853637946698763,0.393305626257185,0.775457513428427
"7932","ADAMTS17",3.39182536219853,0.720173977286544,0.843357103457648,0.853937168886027,0.393139803672797,0.775457513428427
"7933","COG7",71.5710170817511,0.206981226404873,0.24239396242219,0.853904215833409,0.393158063479212,0.775457513428427
"7934","BAIAP2-DT",29.1097710659774,-0.245780905597217,0.287893031011441,-0.853723012098373,0.393258480508718,0.775457513428427
"7935","BTF3L4",372.09927826363,-0.196577304116004,0.230367567613646,-0.8533202227741,0.393481748482574,0.775706993259344
"7936","TMEM44-AS1",58.8036582351403,0.224521287871938,0.263156586910876,0.853185134020521,0.393556645987424,0.77575688170127
"7937","AC239868.1",7.22648441753829,0.497964783009017,0.583775684642131,0.853007064373161,0.393655386657673,0.775787250627483
"7938","TMEM127",120.490317080342,0.176263527499942,0.206645938632205,0.852973586931514,0.393673951771667,0.775787250627483
"7939","TMEM45A",303.032306638389,0.568226195382812,0.666236942388843,0.852889053773263,0.393720832495786,0.775787250627483
"7940","IMP3",425.326306109638,-0.21560492828527,0.25289440077736,-0.852549236450204,0.393909323798038,0.77606090077742
"7941","ETV5",53.524827442398,0.308948705461378,0.362899604464087,0.851333817014265,0.394583944735472,0.776353549064743
"7942","CCDC146",383.331403367895,-0.36112238131782,0.423865279946553,-0.851974432450226,0.394228282820864,0.776353549064743
"7943","SMIM27",66.0790851352572,0.23021150280125,0.270387598303453,0.851411646556963,0.394540724363031,0.776353549064743
"7944","AL596244.1",10.0175305456104,-0.379307060546089,0.445337300707318,-0.851729823537453,0.39436406381947,0.776353549064743
"7945","DRD4",5.00654541435398,0.58311849468868,0.684291491298407,0.852149269870714,0.394131248914824,0.776353549064743
"7946","DNHD1",18.7205996501778,0.466096496847645,0.547384516094085,0.851497408391311,0.394493102339784,0.776353549064743
"7947","SLC37A4",35.5866670757019,-0.259556202326848,0.304774554737414,-0.851633439512282,0.39441757380706,0.776353549064743
"7948","LLPH",254.191650024561,-0.184143885888646,0.216238454989988,-0.85157788376342,0.3944484189577,0.776353549064743
"7949","AC015922.3",73.5848665246558,-0.201863329584077,0.236989164613397,-0.851782949289596,0.394334571615342,0.776353549064743
"7950","TEN1",121.212902911024,-0.269544437933969,0.316627563141323,-0.851298084284788,0.394603788826553,0.776353549064743
"7951","YBEY",179.44148406236,0.296451940815253,0.348149207340908,0.851508303234386,0.39448705287589,0.776353549064743
"7952","THNSL2",114.858501685713,-0.316452419478247,0.371898058963223,-0.850911726617914,0.394818390237329,0.776519334683745
"7953","IFT80",107.929254096343,0.160860890000246,0.18911233463348,0.850610248728679,0.394985894398681,0.776519334683745
"7954","RFC1",557.476281258923,0.135803548405014,0.159616235610757,0.850812875553507,0.39487330815349,0.776519334683745

"7955","SCRG1",13.7384451896225,0.462192107461635,0.543299453327649,0.850713367427041,0.394928595773655,0.776519334683745
"7956","RGMB",40.1142976438324,-0.228263545311397,0.268328253900828,-0.850687700579462,0.394942857266966,0.776519334683745
"7957","HDGFL3",203.198891059973,-0.158230320490408,0.185923297784062,-0.851051602334323,0.394740688467186,0.776519334683745
"7958","SLC26A2",148.895959687017,0.213672616557429,0.251257482723144,0.850412947871769,0.395095540006061,0.776539707540496
"7959","SCG5",15.2991460057693,-0.4919877526714,0.57851694576918 2,-0.850429285208344,0.395086460192264,0.776539707540496
"7960","CMSS1",168.083030872394,-0.219521750138873,0.258185429182095,-0.850248408030987,0.395186993469828,0.776569513694909
"7961","POU2AF1",2.64887691454094,0.808058225601941,0.95042524121886,0.850207034238335,0.395209991595293,0.776569513694909
"7962","PRSS53",12.7153418156515,-0.381850966263204,0.449235151669268,-0.850002420434647,0.39532374055827,0.776695462641674
"7963","PPOX",59.2305240090508,-0.212178788615721,0.249884774404436,-0.849106509675982,0.395822028472909,0.776713751033279
"7964","AC012065.4",2.70960782055622,0.951465374130962,1.12048844660536,0.849152329069994,0.395796535426798,0.776713751033279
"7965","PPP1R21",80.7133395062297,0.17063074854296,0.20079632029903,0.849770296033578,0.395452807174407,0.776713751033279
"7966","GAK",111.344408454075,0.178044802050192,0.209692165313936,0.849077035299036,0.395838427984218,0.776713751033279
"7967","PHYKPL",393.79579831846,0.138698812779517,0.163229037536866,0.849718989173055,0.395481338429181,0.776713751033279
"7968","SMIM13",67.6663513117712,-0.221173697867896,0.26040995268456,-0.849328896948143,0.395698305667353,0.776713751033279
"7969","ERMARD",47.9376616454507,-0.247686994281539,0.291530216785296,-0.849610023320338,0.395541937424616,0.776713751033279
"7970","WBP4",302.802592765302,0.146653121357092,0.172716672077769,0.849096497708446,0.395827599074739,0.776713751033279
"7971","CLN5",316.97145359936,-0.145535873263401,0.171419595770389,-0.849003712844719,0.395879226298557,0.776713751033279
"7972","LRRC49",43.9519603650272,-0.230187270670139,0.270987950413515,-0.849437291654054,0.39563800988987,0.776713751033279
"7973","LSS",109.392823944849,0.187144920553384,0.220310578750742,0.849459529426949,0.395625640564207,0.776713751033279
"7974","AL033543.1",4.14359616033605,0.756830954311904,0.891816848650684,0.848639443689572,0.396081951756862,0.777014042053246
"7975","ZNF432",39.5694158350238,0.242499511638329,0.285835151113711,0.848389397502259,0.396221145379949,0.777189639771604
"7976","MXI1",314.605238954086,-0.232590946788199,0.274195049281203,-0.848268221464726,0.396288611267913,0.777224516808421
"7977","WDR18",115.232491902914,-0.244122906975284,0.287833543007643,-0.848139186365788,0.396360460385968,0.777267980671643
"7978","RPL24",10769.9535305856,-0.237969997146302,0.280743155639956,-0.84764309428612,0.396636766816541,0.777712326812628
"7979","IFI6",998.290503386458,0.323716128483516,0.382049704184495,0.847314171266028,0.396820029833382,0.777731535435776

"7980","NOP58",179.974206794289,-0.143795050233087,0.169716018038617,-0.847268583690007,0.396845433474932,0.777731535435776
"7981","AC027288.3",11.1148640273709,0.55177475009029,0.651206367801311,0.847311662435466,0.396821427851762,0.777731535435776
"7982","MEGF8",65.6566430439578,0.207396517005074,0.244766028156493,0.847325580952245,0.39681367194807,0.777731535435776
"7983","AC087239.1",19.665104259997,0.355117895918629,0.419229964222512,0.847071836998144,0.39695508167259,0.777751545917375
"7984","DTX1",114.236843538622,-0.298896915339586,0.352827160646671,-0.847148260331657,0.396912488286205,0.777751545917375
"7985","PHTF2",131.332685385269,0.187064524230083,0.22089771741944,0.846837741989363,0.397085568135954,0.777812364431596
"7986","ZNF835",18.7262476267955,0.294210832116062,0.347389427513107,0.846919361427491,0.397040069859161,0.777812364431596
"7987","AC003986.2",3.37524624506095,-0.659097511065674,0.778873083533087,-0.846219396972748,0.397430363093486,0.778292835487157
"7988","TCP11L1",40.618936495392,0.21625462998626,0.255541705346449,0.84625963379666,0.397407921174739,0.778292835487157
"7989","CHD2",365.43553108688,0.192856430057996,0.227941408240525,0.846078961899246,0.397508696150803,0.778348796330832
"7990","CYP4X1",70.8848863527969,0.27046487674884,0.320190908920614,0.844698800664256,0.39827902927698,0.778367948477574
"7991","TCEANC2",32.871886794038,0.26178370677846,0.309801008688495,0.845005985896203,0.398107496942163,0.778367948477574
"7992","RHOC",650.412533685511,-0.247987651664664,0.293634272472165,-0.844546004718071,0.398364367161763,0.778367948477574
"7993","RBAKDN",4.07003363395178,-0.657651611475008,0.778061608342356,-0.845243621358109,0.397974831762679,0.778367948477574
"7994","VCPIP1",118.302705582769,0.164341487927235,0.19431591747245,0.845743828220019,0.397695667444715,0.778367948477574
"7995","FANCF",40.2029800647231,-0.271985637743243,0.322033883613845,-0.844587018890796,0.398341459302886,0.778367948477574
"7996","THSD1",50.3542203535659,0.364429822604509,0.431089697426095,0.845368898353193,0.397904903873462,0.778367948477574
"7997","CHD8",187.502698477564,0.184515104487362,0.218442953533667,0.844683252549613,0.398287712532937,0.778367948477574
"7998","FAM98B",80.7977912227742,0.218757868632563,0.258878788500148,0.845020443350996,0.398099424991547,0.778367948477574
"7999","ARID3B",12.6860430167276,0.524196744363765,0.619654831281669,0.845949580155031,0.397580872027019,0.778367948477574
"8000","OGFOD1",71.1235874439124,0.249487541731103,0.295348475202972,0.844722633355897,0.398265719501202,0.778367948477574
"8001","DLGAP1",3.14551191067963,0.697469759386735,0.825611859113206,0.844791352846955,0.398227343421916,0.778367948477574
"8002","CACNA1A",34.5990018053923,-0.359729925254302,0.425815138914786,-0.844803043337289,0.398220815143843,0.778367948477574
"8003","ABHD8",70.9777363084022,-0.308096792278629,0.364350677252427,-0.845605103857609,0.397773077366127,0.778367948477574
"8004","NCOA6",114.885878304167,0.160191616348447,0.189513764162498,0.845276948913808,0.397956228058213,0.778367948477574

"8005","NPEPL1",212.169211022377,-0.153124564300225,0.181160228745049,
-0.845243822890735,0.397974719264003,0.778367948477574
"8006","ATP5PF",1505.95267696102,-0.228622717836955,0.270337940937902,
-0.845692310312712,0.397724414018369,0.778367948477574
"8007","CTBS",235.151613393058,0.151469789120157,0.17939494424429,0.84
4337000455787,0.398481115722256,0.778479157783013
"8008","CLBA1",18.9234613436683,-0.311860197369914,0.369386211664545,-
0.844265940422072,0.398520814135803,0.778479157783013
"8009","BABAM2",122.265249059024,0.222097468019727,0.263190249309396,0
.843866627287691,0.398743938790893,0.778623322245155
"8010","CTBP1",209.519114100997,-0.136847941314018,0.162159968281149,-
0.843907055265046,0.398721345382471,0.778623322245155
"8011","PRDX3",545.519135934427,-0.196211913134916,0.232475423290419,-
0.844011424337958,0.398663021687468,0.778623322245155
"8012","NDUFA4",1959.45622995406,-0.251109127563737,0.297607075387827,
-0.843760610316486,0.398803190647969,0.778641826174011
"8013","LINC02035",17.2388996964064,0.328595370477529,0.38958543425269
8,0.843448808880239,0.398977484164749,0.778690553311188
"8014","SAT2",944.715083845632,-0.231917349491609,0.274954784474306,-0
.843474500489302,0.398963121109403,0.778690553311188
"8015","BSG",1064.32895220239,-0.234165374962049,0.277587186503274,-0.
843574150204109,0.398907414255582,0.778690553311188
"8016","AC090578.1",7.11926715387694,0.603559277815216,0.7159783242463
26,0.842985405250295,0.399236606307767,0.778710502802045
"8017","IQCH",3.93390142523228,-0.63744067151023,0.756010576378263,-0.
843163695624401,0.399136899412521,0.778710502802045
"8018","AC091271.1",56.3729877368122,-0.345796882988963,0.410131391155
506,-0.843136834795099,0.39915192007164,0.778710502802045
"8019","QTRT1",155.344722579409,-0.205156079000901,0.243368476633321,-
0.84298542621034,0.399236594585216,0.778710502802045
"8020","PEG3",69.9920257773447,0.225561027286297,0.267466225939852,0.8
43325270297945,0.399046553531333,0.778710502802045
"8021","COX5B",1461.55806061086,-0.221389093724185,0.262717543060523,-
0.842688657731484,0.399402592264654,0.778937133873081
"8022","NDUFB9",654.819267992334,-0.231940673417401,0.275307161706201,
-0.842479621597787,0.399519541710677,0.779068086634271
"8023","SLTM",480.499441522161,-0.129968719102456,0.154294736405809,-0
.842340588732899,0.399597337833729,0.779122666799579
"8024","RNF103",152.982657978653,0.172188848868139,0.204640294440405,0
.841422014852913,0.400111556662035,0.779445215549715
"8025","RAB2A",1160.84847238154,-0.137552217524831,0.16347097237027,-0
.841447356251531,0.400097365181109,0.779445215549715
"8026","AC007773.1",3.52002689099727,-0.684195423776456,0.813066613991
379,-0.841499837778003,0.400067975872042,0.779445215549715
"8027","RAB4B",50.1348503207885,-0.258521292644416,0.307174319806714,-
0.841611020110951,0.400005718785429,0.779445215549715
"8028","AL121832.2",9.77392851340432,-0.437206264749205,0.519499282509
812,-0.841591662334871,0.400016557847224,0.779445215549715
"8029","DNAJC5",50.8356251829714,0.2230371856772,0.265062272089622,0.8
41452025287805,0.400094750498959,0.779445215549715

"8030","LCA5L",6.20863561447569,-0.689481857276114,0.818911975005911,-0.841948681079108,0.399816679599442,0.779445215549715
"8031","AJM1",9.78621660615968,-0.457510551993826,0.543827833926341,-0.841278293335376,0.400192048124896,0.779504944442504
"8032","ARHGEF11",89.4754143346883,-0.172294043739864,0.204871330107276,-0.840986601930327,0.4003554402783,0.779726114575878
"8033","AC022306.2",9.09843879990016,0.462582301367508,0.550124937448523,0.840867719091163,0.400422044493433,0.779758750405922
"8034","MTHFR",94.8605068353323,0.194413957837249,0.23136175064119,0.840302933818815,0.400738556889814,0.780016240850323
"8035","KLHL12",51.1026988245856,0.206622902596909,0.2459153554921,0.840219603950459,0.400785268661155,0.780016240850323
"8036","PAX3",6.49992476903372,-0.72145126208603,0.858770380792984,-0.840097979881241,0.400853452675046,0.780016240850323
"8037","CFI",79.5431348148295,0.751380302420082,0.893965009957962,0.84050303317287,0.400626401639721,0.780016240850323
"8038","CCNH",173.703356474664,-0.150894128711426,0.179596684629672,-0.840183264087359,0.40080564052485,0.780016240850323
"8039","EZH1",307.536986545627,0.178496765599831,0.212390124242632,0.840419328517923,0.400673315621014,0.780016240850323
"8040","CFAP97",429.231303447483,-0.123110884447753,0.14656342861354,-0.839983654942827,0.400917551055045,0.780043936710705
"8041","PLAC9",8152.24539698925,-0.217479956937084,0.258994886679007,-0.839707531394722,0.401072390190134,0.78009270583872
"8042","ARHGEF7",157.72842286791,0.199685150729993,0.237813229924136,0.83967216960004,0.401092222275831,0.78009270583872
"8043","TLNRD1",156.941579043314,0.267144820704646,0.318137283301672,0.839715540197556,0.401067898665498,0.78009270583872
"8044","IP6K2",434.398224157317,-0.174684587396863,0.208115371511497,-0.839364176361252,0.401264980159095,0.780208375849637
"8045","NAALADL2",141.994170411256,0.150034754347702,0.178761899840647,0.839299394789645,0.401301322769685,0.780208375849637
"8046","CLDN4",4.56808591510651,1.09771252017408,1.30766076429003,0.839447469979005,0.401218255151873,0.780208375849637
"8047","LYPLAL1",215.928848627298,0.190921473851792,0.227517997391577,0.839148885102047,0.401385766679103,0.780275574519846
"8048","HDX",34.1386904815775,0.265515525231716,0.316459535355336,0.839018880987684,0.401458714460704,0.780320411320674
"8049","MARS2",4.71412894837177,-0.540682003230744,0.644503821560599,-0.838912020601427,0.401518681814727,0.780330121041233
"8050","IMMP2L",125.595517116512,-0.250187113660371,0.298270297515822,-0.83879325478964,0.401585336501227,0.780330121041233
"8051","MYCBP2",1062.22331253248,0.176450258845308,0.21037456125616,0.838743324248483,0.401613360896437,0.780330121041233
"8052","TRAK2",95.2066044888534,0.219072562669063,0.261319615599597,0.838331872509462,0.40184434011093,0.780585000913359
"8053","C1GALT1C1",277.288576526444,-0.21964306117906,0.261997303034777,-0.838340924257164,0.401839257817897,0.780585000913359
"8054","LCOR",149.853316863862,0.199447726244642,0.237959007086333,0.838160020445374,0.401940837362597,0.780675505197803

"8055","BRF2",81.628095060843,-0.20321315070545,0.24249535000111,-0.838008443067132,0.402025961642007,0.780743900430281
"8056","USP11",167.309488960775,0.14990896214174,0.178986428422553,0.83754373704711,0.402287003102399,0.781153871590223
"8057","ZNF579",59.0842374148464,-0.23514510219417,0.280855125204373,-0.837246968603686,0.402453761382733,0.781380686274059
"8058","SOS2",146.817793409083,0.160246029011554,0.191422449930376,0.837132891517367,0.402517873896879,0.78140817837787
"8059","POP1",23.122224440885,0.318866182137024,0.381094409309581,0.836711781510271,0.402754595136688,0.781679348221023
"8060","GSTO1",791.383704457653,-0.220543918425028,0.263604177525053,-0.836648039859185,0.402790433905062,0.781679348221023
"8061","FCSK",22.0662649127919,0.324297684553963,0.38762946012163,0.836617744307167,0.402807468261182,0.781679348221023
"8062","HLCS",35.6678379127581,0.225253569620982,0.269322304481477,0.836371759311433,0.402945794846953,0.781850789976543
"8063","ZC3H12D",4.98257775617483,-0.543122645997847,0.649559162816904,-0.836140381181785,0.403075933420914,0.782006303671507
"8064","IGHA1",142.838360824407,0.587096262863718,0.702270495813882,0.835997334877803,0.403156402376193,0.782065426881298
"8065","ABCC4",9.95188503770922,-0.452981934894232,0.542182147063798,-0.835479252401371,0.403447923901628,0.782242919384379
"8066","RMI2",9.82987051354028,0.431998579942163,0.517060053360502,0.835490146907494,0.403441792337098,0.782242919384379
"8067","LINC01480",3.46627932825866,0.715323191127787,0.855910872083657,0.835744952492987,0.403298400479372,0.782242919384379
"8068","C22orf39",164.504255830865,-0.203321010699539,0.243332229192814,-0.835569588845668,0.403397083106355,0.782242919384379
"8069","KLHDC9",15.5374967324834,-0.407577498831391,0.487998458664609,-0.835202430652575,0.403603741374812,0.782448051347898
"8070","AC004160.2",3.06916821881205,0.719948065414014,0.862267735349344,0.834947239586008,0.403747415282492,0.782532625110151
"8071","SMIM25",4.20535761869689,-0.637506799290972,0.763462777654446,-0.83502014498933,0.403706366034353,0.782532625110151
"8072","URI1",419.818309897805,-0.116159822523007,0.139156185729615,-0.83474422580617,0.403861734970654,0.782657224993303
"8073","RAB7B",118.926526866902,0.180564482635392,0.216336351359103,0.834646981429707,0.403916501401319,0.782666398045439
"8074","CAPZB",790.021774042353,-0.213128479969428,0.255473632603652,-0.834248441991198,0.404140998686481,0.782670427251355
"8075","ARNT",227.987259418549,0.150963522251244,0.180954401568695,0.834262780803007,0.404132920338385,0.782670427251355
"8076","FASTKD2",120.951875626567,0.169397216824737,0.20308728762359,0.834110390694196,0.404218780397858,0.782670427251355
"8077","CDKL1",42.7689072751989,0.214271942800804,0.25685558245677,0.83421174167732,0.404161675729084,0.782670427251355
"8078","SLC7A5",5.98194716366797,0.680832931429544,0.816219075867941,0.83413013927121,0.404207652984029,0.782670427251355
"8079","UBE2O",50.7752055299796,0.225814652205683,0.270619180375044,0.834436982229909,0.404034784655521,0.782670427251355

"8080","AC009318.3",4.33710340781665,-0.586157597263931,0.702850343726
523,-0.833972128627148,0.404296689840197,0.782724395936906
"8081","PRMT5",90.4311243819492,-0.177263840365222,0.212610408052041,-
0.833749589163259,0.404422107711497,0.782773450993682
"8082","INAFM1",224.540870974861,-0.162730341257882,0.195178435529178,
-0.833751642781021,0.404420950235694,0.782773450993682
"8083","PIGT",1044.52509033186,0.167308836567328,0.200726505911659,0.8
3351640984057,0.4045535469185,0.782930982858605
"8084","RGS5",10.550097506259,-0.445975607622091,0.535406356510865,-0.
832966591073784,0.404863571519828,0.783173417864456
"8085","DISC1",17.0891566037598,0.38416691159166,0.461162533108154,0.8
33040162656848,0.404822078708723,0.783173417864456
"8086","MPHOSPH10",305.491146164549,-0.155466097411177,0.1866273872501
35,-0.833029383853548,0.404828157565989,0.783173417864456
"8087","AC027307.2",16.9006601128539,0.303466259174152,0.3643668843592
74,0.832859055530762,0.404924223858045,0.783173417864456
"8088","RWDD2B",92.5346263294051,0.196200578371042,0.235577234941577,0
.83285033216261,0.404929144261825,0.783173417864456
"8089","EGR1",2099.91068573941,-0.303085189402497,0.364003071499207,-0
.832644593228814,0.405045201387418,0.783189666191143
"8090","AC007036.5",3.43190498193452,0.727743171064288,0.8741865046754
41,0.83248044573106,0.405137811086027,0.783189666191143
"8091","HEXIM1",181.873241172928,0.237255508508991,0.284998243460203,0
.832480599278224,0.405137724450974,0.783189666191143
"8092","C18orf25",56.8561184601724,-0.200275628928465,0.24052495798106
8,-0.832660488165346,0.405036234360966,0.783189666191143
"8093","HECTD1",416.935659184149,0.144185293410842,0.173251241414634,0
.832232382484177,0.405277788878933,0.783220971620814
"8094","IL34",103.420233502829,0.293486052932739,0.352594769863466,0.8
32360766571737,0.40520534035405,0.783220971620814
"8095","ZNF778",17.0163108372957,0.309150038132245,0.371491709550366,0
.832185564804188,0.405304210526785,0.783220971620814
"8096","WDPCP",58.5984713480684,-0.225537905116807,0.271098332223352,-
0.831941322792761,0.405442065669473,0.7833905920538
"8097","ERLEC1",601.892394624559,0.130238620286338,0.156718549923467,0
.83103512857884,0.405953784824543,0.783406075053706
"8098","ARHGAP31",18.5528165924167,0.349982227577871,0.421400628070073
,0.830521371505108,0.406244069745906,0.783406075053706
"8099","USO1",344.684069766974,0.149493353370813,0.179996890141161,0.8
30532978950773,0.406237509896063,0.783406075053706
"8100","AC095055.1",3.32604616003781,-0.679383127155099,0.818057024943
398,-0.830483825014651,0.406265289260863,0.783406075053706
"8101","AL137003.2",4.15966986376228,0.654950714255637,0.7875228647747
9,0.831659299749899,0.405601280041724,0.783406075053706
"8102","AC002467.1",16.2010190568922,-0.376409708845384,0.453265889776
719,-0.830439080758547,0.406290577479386,0.783406075053706
"8103","ESRRA",132.523588470079,-0.21330612437175,0.256514148157775,-0
.831556956618825,0.405659066462045,0.783406075053706
"8104","NDFIP2",142.928644363132,-0.191000861347194,0.229980246880754,
-0.830509854379925,0.406250578614393,0.783406075053706

"8105","MIPOL1",71.6662841980956,-0.246304348776417,0.296602191567726,-0.830419854534946,0.406301443898914,0.783406075053706
"8106","DMXL2",81.503313399687,0.238881572361536,0.287549125866142,0.830750473130421,0.406114606592272,0.783406075053706
"8107","DET1",47.5917748267163,0.207242874413961,0.24933868542494,0.831170157413653,0.405877510895302,0.783406075053706
"8108","ASB7",45.4969877348312,0.261779847693122,0.315180187691874,0.83057202805223,0.40621544209054,0.783406075053706
"8109","NKG7",49.7366959776377,0.291712850550345,0.351040630586415,0.830994549158136,0.405976708652117,0.783406075053706
"8110","ZCCHC3",81.5668610404506,0.19503051759705,0.234726328816808,0.830884709781583,0.406038762182063,0.783406075053706
"8111","PAXBP1-AS1",13.0514582346337,-0.360523224121148,0.433898781629646,-0.830892455533264,0.406034386049964,0.783406075053706
"8112","GCAT",26.8153888054264,-0.304630547577439,0.366376309245952,-0.831469011204371,0.405708727369723,0.783406075053706
"8113","ADGRG2",5.11985253092324,0.724571662721913,0.871563818336497,0.831346652394154,0.40577782683217,0.783406075053706
"8114","SRGAP2C",149.270829176167,0.163535515646087,0.197054171768607,0.829901311798261,0.406594583161469,0.783443538511508
"8115","USP46-AS1",21.9875644848921,-0.293825738175875,0.354220422896889,-0.829499710301588,0.406821700653901,0.783443538511508
"8116","SRD5A1",27.2697467551769,-0.249444800804853,0.300511566866286,-0.830067219728166,0.406500779423873,0.783443538511508
"8117","ANXA2R",47.2826659912334,-0.259309318663713,0.312327900727775,-0.830247051446509,0.406399117816053,0.783443538511508
"8118","DOCK5",10.6495926711173,-0.4805050020126,0.579019110081597,-0.829860351146073,0.406617744152751,0.783443538511508
"8119","TMEM233",25.2399025444646,-0.486772796753914,0.586475575168306,-0.829996708071295,0.40654064487884,0.783443538511508
"8120","AL109628.2",13.0428127952936,0.492979899351595,0.594291860774639,0.829524905000402,0.406807450083687,0.783443538511508
"8121","TTC7B",46.2482134392149,0.197492717988339,0.238056236682176,0.829605309824365,0.406761973675619,0.783443538511508
"8122","C16orf58",130.824642465682,-0.170936949166566,0.205988776003637,-0.829836229346535,0.406631384069265,0.783443538511508
"8123","ZNF582-AS1",23.9415665443109,0.366448419067454,0.441700396223627,0.82963117579348,0.406747344708624,0.783443538511508
"8124","VPS28",1200.1929542258,-0.236440530569612,0.285130162784138,-0.829237174562316,0.406970213262671,0.783597143822125
"8125","SECISBP2",225.470463117797,0.148630400706714,0.179249513895884,0.829181611019848,0.407001648887986,0.783597143822125
"8126","ZNF354B",51.2144483351039,0.196427654935399,0.236919691676463,0.829089610683945,0.407053702175034,0.783600918425309
"8127","HIBADH",345.477507450147,-0.177627914205081,0.214286455597961,-0.82892739865156,0.407145490528218,0.783681174890231
"8128","RHBDD2",103.635243831593,-0.26581823462876,0.320715044859839,-

0.828829950103929,0.407200638128981,0.783690893485686
"8129","NADK",119.763246741661,0.154231746436088,0.186259812237809,0.828046289658937,0.407644285306252,0.783737697398181
"8130","ZNF436-AS1",22.1958618979576,0.273148720133352,0.329703215312716,0.828468475426532,0.407405240998245,0.783737697398181
"8131","POMGNT1",71.416158398993,-0.184833772220651,0.223226483731539,-0.828010051186128,0.407664807658816,0.783737697398181
"8132","LINC02609",144.762277633382,-0.176807502060332,0.213463297481689,-0.828280571630813,0.407511623018305,0.783737697398181
"8133","CASP10",18.903638990124,0.309278454663115,0.373538074550707,0.827970361616058,0.407687285122448,0.783737697398181
"8134","DBN1",686.237057697015,-0.208798043810881,0.252262822957334,-0.827700417220003,0.407840182800638,0.783737697398181
"8135","CCDC91",151.306678519171,0.164558770862084,0.198864588907136,0.82749156984871,0.407958498300826,0.783737697398181
"8136","BICD1",59.6763915599739,0.246712890594156,0.29807245747101,0.827694355551621,0.407843616548166,0.783737697398181
"8137","ESYT1",131.612607388082,0.178178335880438,0.215249440936568,0.827776068105771,0.407797330364461,0.783737697398181
"8138","B3GLCT",51.2733653566101,0.228720401048347,0.276170734580425,0.828184787196345,0.407565857893426,0.783737697398181
"8139","AC018362.1",12.6461960467718,0.430542302042646,0.519911778810664,0.828106458806421,0.407610212038935,0.783737697398181
"8140","ZFP1",41.331989669834,0.213887959108524,0.258440092530404,0.827611370257347,0.407890626881288,0.783737697398181
"8141","RNASEK",1759.79798857381,-0.229588229424662,0.277380157063285,-0.827702427799409,0.407839043873438,0.783737697398181
"8142","NEK8",18.9460002994478,-0.331385901085933,0.400485785399754,-0.827459832950509,0.407976479569992,0.783737697398181
"8143","RABL2B",82.3010019904672,0.189865753147795,0.229302825247646,0.828013143504625,0.407663056411691,0.783737697398181
"8144","SLC27A1",221.644619635377,0.15800387860405,0.190983799443191,0.827315610353896,0.40805819814579,0.783798427504247
"8145","PHF13",53.7426411307405,0.24361919341342,0.294776520563531,0.826453860530299,0.408546681040738,0.784002243563784
"8146","PLXNB1",34.409777881255,0.262856055855074,0.317875888290063,0.826914105593366,0.408285747815116,0.784002243563784
"8147","TRA2A",355.902436039189,0.175596468452078,0.212484989566247,0.826394696446695,0.408580230970341,0.784002243563784
"8148","RFC2",92.3639150126717,-0.243663933887512,0.29484740822236,-0.826406904359669,0.408573308145836,0.784002243563784
"8149","NUCB2",808.251834278631,-0.167916230543945,0.203197087651672,-0.82637124618534 5,0.40859352926598,0.784002243563784
"8150","PCED1B-AS1",14.8745455200885,-0.450708208444874,0.545185564646239,-0.826706056931881,0.408403687481672,0.784002243563784
"8151","MTIF3",446.421063126363,-0.156578887029341,0.18938625695272,-0.826770060028331,0.40836740292543,0.784002243563784
"8152","GMPR2",198.643771703304,-0.156350283061905,0.189108908300164,-

0.82677375945578,0.408365305709294,0.784002243563784
"8153","LINGO1",7.4239576201754,-0.675357706272855,0.81729512169555,-0
.826332726508592,0.408615373763059,0.784002243563784
"8154","MTIF2",90.4112310643135,-0.175543705722823,0.212484697448772,-
0.826147519470878,0.408720414326977,0.784011458162711
"8155","POLR2K",475.124309151066,-0.220891831095293,0.267369859498061,
-0.82616578962931,0.4087100516533,0.784011458162711
"8156","BMP2K",77.4056062048567,0.223059771305988,0.270101813572144,0.
8258358889042,0.408897192538294,0.784044119667226
"8157","LINC00847",51.7178172548848,-0.222161595943199,0.2691949017690
72,-0.825281587738906,0.409211742582569,0.784044119667226
"8158","IQCE",48.0574491498978,-0.235478953242828,0.28511229400282б,-0
.825916518494618,0.408851449546083,0.784044119667226
"8159","NUDT22",128.478021317278,-0.213720774935,0.258892501338297,-0.
825519371284258,0.409076789597726,0.784044119667226
"8160","ME3",59.5248549061889,-0.218038966440346,0.264084997201985,-0.
825639353808424,0.409008704108814,0.784044119667226
"8161","AC007529.1",7.41331800737095,0.563682017026793,0.6826295101997
41,0.825751023951275,0.408945341637732,0.784044119667226
"8162","GSTZ1",115.6890934028,-0.257052702935402,0.311459723319163,-0.
82531603186455,0.409192192333055,0.784044119667226
"8163","SOX8",3.16310960415172,0.720853938814796,0.873320752099665,0.8
25417164405742,0.409134793407641,0.784044119667226
"8164","AC016590.3",4.56529708154642,0.590244919620637,0.7151094296430
64,0.825391045277153,0.409149617157596,0.784044119667226
"8165","PRICKLE3",20.1997221062451,-0.269976767033441,0.32715170291549
1,-0.825234179212513,0.409238652245918,0.784044119667226
"8166","CDC42-
IT1",4.28477828661986,0.585784183211655,0.710352054893147,0.8246392463
80121,0.409576432944797,0.784135972453218
"8167","GNB3",7.74712609684058,0.47091370443243,0.571128650499413,0.82
4531747830631,0.4096374842989,0.784135972453218
"8168","BBS10",68.4914565333605,0.183895615968976,0.222968205020718,0.
824761610974481,0.409506945345095,0.784135972453218
"8169","MMD",73.3167851331751,0.265001905314949,0.321310744597221,0.82
4752703639408,0.409512003347207,0.784135972453218
"8170","RAVER1",47.1998989212268,-0.193480957639181,0.234609883098254,
-0.824692272482621,0.40954631997009,0.784135972453218
"8171","DYRK1B",53.7334210191577,0.219594354023148,0.266298807015247,0
.824616364167847,0.409589427923002,0.784135972453218
"8172","MIATNB",27.140145176294,0.251418707601755,0.30478745877487,0.8
24898467320021,0.409429236572582,0.784135972453218
"8173","MFSD1",171.54950561303,-0.169548038031068,0.205669321352086,-0
.82437204011005,0.409728196655394,0.784213652303967
"8174","EVL",275.436065154544,-0.141574158075304,0.17177235704872,-0.8
24196398697314,0.409827972977206,0.784308659320093
"8175","KAT2B",84.5128038991927,0.197495142020713,0.239653240259856,0.
824087092695133,0.409890073545932,0.784331549905689
"8176","FRS3",30.577698398229,-0.231262006264456,0.280730124912804,-0.
823787637099108,0.410060233415051,0.784561182890368

"8177","FHAD1",3.57004720636673,-0.646355434743676,0.784736296908938,-0.82365940926864,0.410133109244986,0.784561214757508
"8178","HPN",7.91880031563682,0.572888120435362,0.695580851190338,0.823611115019894,0.410160558351141,0.784561214757508
"8179","STARD4",6.59039441210756,-0.716633918321952,0.870368867733973,-0.823368051051419,0.410298725717959,0.784729547182544
"8180","PEBP4",14.7235353292999,0.322553439286446,0.391794270762721,0.823272475777961,0.410353062134676,0.784737524568794
"8181","AL391840.3",42.2413466482805,0.218440804515337,0.26541799097659,0.823006774000498,0.410504141268991,0.784834549238674
"8182","SLC31A2",162.663034173444,-0.19643475285822,0.238661034591978,-0.823070063339209,0.410468151690213,0.784834549238674
"8183","ZNF697",26.4908278157025,0.287033027830327,0.349006660552629,0.822428510034246,0.410833058761626,0.785069569254503
"8184","CACNA2D3-AS1",3.85369666489602,-0.704856562773344,0.856858126383084,-0.822605914643817,0.410732134089482,0.785069569254503
"8185","NDUFB5",575.926029698743,-0.242465924630885,0.294747037900108,-0.822623787361212,0.410721967198759,0.785069569254503
"8186","HSPA9",347.950109241149,-0.0973749944811418,0.118386410161052,-0.822518347745098,0.410781948657685,0.785069569254503
"8187","FBL",349.13299141534,-0.194756754609353,0.236829656840665,-0.82234951993526,0.410878000606445,0.785069569254503
"8188","LINC01686",5.96976368630959,-0.546143153028607,0.664343757762034,-0.82207915201671,0.411031849995688,0.785267614738953
"8189","TTC7A",108.835955538083,0.20097782466715,0.244514181989185,0.821947516631322,0.411106767798865,0.785297252019461
"8190","DINOL",5.27460364978163,-0.576091793939403,0.700947774779168,-0.821875487258519,0.411147765392788,0.785297252019461
"8191","LINC01252",16.6554298603846,0.482369763050415,0.587150222980826,0.82154403450881,0.411336452560225,0.785561729630032
"8192","SEC31A",1003.43272057612,0.140437430613246,0.170992431425637,0.82130787568993,0.41147092279702,0.785662956052394
"8193","PPP2CA",188.772818264798,-0.133085864458544,0.162066935456964,-0.821178385852088,0.411544666153756,0.785662956052394
"8194","DNAJB6",551.27102541903,-0.133961916606224,0.163148283649661,-0.821105276803828,0.41158630459596,0.785662956052394
"8195","WASHC3",464.888827800606,-0.220049946716501,0.267994688163115,-0.821098165134408,0.411590355101283,0.785662956052394
"8196","LAP3",279.145837051021,-0.15842412820814,0.193003929306908,-0.820833693785681,0.411741003560925,0.785854626488964
"8197","PCYT1A",117.611818354267,0.154987873795034,0.18887771766038,0.820572567875455,0.411889778477622,0.786042674725563
"8198","C1orf50",127.793704158451,-0.201136245853566,0.245193351376174,-0.820316883490791,0.412035484010069,0.786224820245122
"8199","LOXL3",32.8990707472905,-0.270347568159688,0.329608501590882,-0.820208116158514,0.41209747595136,0.786247202867072
"8200","PLK3",128.074409781962,-0.399104897168998,0.486690511377429,-0.820038377241941,0.412194229697284,0.786335894531051
"8201","HIST1H4C",990.852597220076,-0.300723749796246,0.36681530512334

4,-0.819823343235708,0.412316821667197,0.786473849693934
"8202","LINC02019",3.93339551739509,-0.722565955595272,0.881981523472049,-0.819252939393544,0.412642116472808,0.786605333420139
"8203","PPM1L",47.5363466960248,-0.387203577847407,0.472556922187348,-0.819379760760117,0.41256977854794,0.786605333420139
"8204","PIGG",85.5174314591638,0.188957081152611,0.23055113684283,0.819588589933634,0.41245068038448,0.786605333420139
"8205","TMEM179B",574.028390838526,-0.187007173898854,0.228202979453708,-0.819477354531161,0.412514116934852,0.786605333420139
"8206","AMDHD2",108.093653684322,0.16192067296935,0.197622339430415,0.819343974148047,0.412590190192941,0.786605333420139
"8207","IGLC2",369.435459514013,0.771278019379845,0.941532006812388,0.819173446892211,0.412687462211793,0.786605333420139
"8208","ITCH",150.465904447613,0.192921568063979,0.235615576265759,0.818798023125498,0.412901659020848,0.786917720767925
"8209","HK2",5.21457153691298,-0.562222643932099,0.687061420298744,-0.818300412920342,0.413185670393741,0.786956386495334
"8210","TNFRSF10D",9.05884900279158,-0.523662976967918,0.639994301984277,-0.818230686342553,0.413225476127031,0.786956386495334
"8211","LOXL4",62.6620167879635,0.280371527108692,0.342509107925992,0.818581230748683,0.413025379200604,0.786956386495334
"8212","RIC8B",53.0834808543171,0.237663977627423,0.290379969763653,0.818458579704597,0.413095384049733,0.786956386495334
"8213","LAT",14.6078044831972,0.364115943703212,0.444930758533619,0.818365412414389,0.413148565318645,0.786956386495334
"8214","AC005726.1",9.10873008750605,0.460777929637343,0.563198009582717,0.818145522173883,0.413274098002887,0.786956386495334
"8215","DDX52",166.905923736874,0.162655939755189,0.198797009415631,0.818201140114335,0.413242344258031,0.786956386495334
"8216","AKR1C4",10.5199265718427,-0.63550479639149,0.777132912036232,-0.817755607244004,0.413496751261873,0.787284527749451
"8217","CENPS",48.89326845756,-0.224378447517775,0.274578223672332,-0.817174954797354,0.41382845303091,0.787325603734944
"8218","ADAMTSL4",558.974316867829,0.191747667144563,0.234701746461626,0.816984406956315,0.413937339126527,0.787325603734944
"8219","STK11IP",24.9753580392603,0.246059115687296,0.300944583299556,0.81762267653899 6,0.413572674959563,0.787325603734944
"8220","AC023481.1",5.39554238993084,0.558988421045308,0.68425919795921,0.816924964563838,0.413971310180827,0.787325603734944
"8221","RBMS3-AS2",3.60601426672517,0.751632432703874,0.920472085134725,0.816572761784363,0.414172626295138,0.787325603734944
"8222","SETD2",251.277772173183,0.185301757075279,0.226909481527577,0.816632940271203,0.41413822468168,0.787325603734944
"8223","EBLN2",14.3670697426883,0.394339046445403,0.482516178587221,0.817255594620692,0.413782377553816,0.787325603734944
"8224","IL1RAP",21.4241226425112,0.348284350814866,0.426493246764159,0.816623366154868,0.414143697704705,0.787325603734944
"8225","AC093673.1",53.5115354399046,-0.278443265898011,0.340633888016571,-0.817426790738054,0.413684570654587,0.787325603734944

"8226","APTX",123.359431103888,-0.162735972348858,0.199276820840868,-0
.816632720565182,0.414138350275676,0.787325603734944
"8227","ENO2",45.2196414624442,-0.20722421720996,0.253758943495602,-0.
816618379456451,0.414146548357085,0.787325603734944
"8228","MFSD5",94.3646503545842,0.247205288411201,0.302694324208213,0.
816682932717156,0.414109647302481,0.787325603734944
"8229","ZNF469",31.7820983517726,0.239831723477484,0.293420425386608,0
.817365468547337,0.41371960336507,0.787325603734944
"8230","SLC35A2",69.5492510685436,-0.20569453674951,0.251950845132964,
-0.816407409314134,0.41426716033651,0.78740962201021
"8231","ANKRD13A",104.865887226853,0.184199209420289,0.22566926109374,
0.81623526628l015,0.414365590185634,0.787501023845689
"8232","SCAMP1",244.648545893842,0.123419415678138,0.151231316919988,0
.816096944678696,0.414444691264715,0.78751854809573
"8233","EBAG9",223.175482677689,-0.130531021527904,0.159956042605147,-
0.816043079098435,0.414475497441165,0.78751854809573
"8234","AC012368.1",3.11261567073475,-0.695084452048813,0.851908912597
83,-0.815914051103429,0.414549295138828,0.787563107099427
"8235","IL6R-
AS1",2.74935650628651,0.716997901379917,0.879400270649031,0.8153259958
07058,0.414885732376341,0.787657861974461
"8236","UBXN4",1217.46456286472,0.0959176101723103,0.117638854525576,0
.815356546603035,0.414868249731014,0.787657861974461
"8237","MTERF3",52.8840565175453,-0.223068675845103,0.273533019333717,
-0.815509134467431,0.414780938083057,0.787657861974461
"8238","CARD9",5.74909507413528,-0.632131825040712,0.775320361358835,-
0.815316940642229,0.414890914263951,0.787657861974461
"8239","LSM14A",359.195062723501,0.124069864569824,0.152165578366669,0
.815360910802418,0.414865752360275,0.787657861974461
"8240","MAP4K1",2.96813319629452,-0.794016320403849,0.973905502750028,
-0.815290927263247,0.414905800832651,0.787657861974461
"8241","MYH9",395.817646872751,0.16416497947647,0.201377327294584,0.81
52108367012,0.414951635909451,0.787657861974461
"8242","SLC8A1",8.22993769916007,0.436029909830015,0.53510084355254,0.
814855582987326,0.415154980351532,0.787727312231037
"8243","LINC01266",9.18364576245641,0.413554349779353,0.50799488818515
9,0.814091557607596,0.415592501939701,0.787727312231037
"8244","POC1A",4.73860282719185,0.542759202691874,0.666633155170162,0.
814179730609606,0.415541995490239,0.787727312231037
"8245","MUC4",2.68594109751016,-0.708832315070611,0.870613827794828,-0
.814175346681557,0.415544506566003,0.787727312231037
"8246","TMEM38B",68.99470108943,-0.259078105710686,0.318127509126285,-
0.814384478796649,0.41542472749865,0.787727312231037
"8247","NRBF2",227.610910254675,-0.19155356699831,0.235241122522539,-0
.814286060805362,0.415481093226244,0.787727312231037
"8248","TMED8",63.8713696072209,0.23080385801923,0.283462937900812,0.8
14229400599777,0.415513545578985,0.787727312231037
"8249","RPAP1",45.0569784389867,0.207442608275969,0.254731245411121,0.
814358709475034,0.415439485609213,0.787727312231037
"8250","KRBA2",44.0777636351599,0.235203589390922,0.288819747632055,0.

814361176198248,0.415438072901503,0.787727312231037
"8251","TYMS",14.4203976211924,-0.35751190215877,0.438786388218369,-0.814774368025402,0.415201475427575,0.787727312231037
"8252","ZNF543",18.5949687036682,-0.338834632950864,0.416023538760331,-0.814460244149947,0.415381338415745,0.787727312231037
"8253","SH3BGRL",1064.19687235038,-0.187270548100009,0.229908854245962,-0.81454256607126,0.415334197571408,0.787727312231037
"8254","TNFRSF4",3.27590460434482,-0.690024437168192,0.848123164526736,-0.813589895935969,0.415879928162546,0.788131326270081
"8255","TBC1D31",13.4645995002416,0.327170913940542,0.402198779145408,0.813455760944165,0.415956800465754,0.788131326270081
"8256","YIF1B",105.895542407051,-0.23630183842568,0.290483947654041,-0.813476408366321,0.415944966952782,0.788131326270081
"8257","GGPS1",185.798497217054,-0.187072800854378,0.230037908948602,-0.813225966578303,0.416088514321991,0.788285409899347
"8258","TMEM147",472.125517552948,-0.212770003546484,0.261679498812584,-0.813093897351398,0.416164225085824,0.788333370430799
"8259","PHLDA1",143.706207201575,-0.449824960593073,0.553493170060917,-0.812701917430284,0.416388981622715,0.788472683636863
"8260","MOSMO",223.846635766498,-0.148059160113988,0.182166469213738,-0.812768457076855,0.41635082354908,0.788472683636863
"8261","ZFPM1",168.859117328062,-0.259732652795607,0.319564759651534,-0.812770009680759,0.416349933211697,0.788472683636863
"8262","LINC01355",36.5022629261723,0.405212317652283,0.498807257281713,0.812362514251533,0.416583649139926,0.788650371958835
"8263","PAX8-AS1",148.799820618427,-0.691914683750571,0.851671840419903,-0.812419350872789,0.416551046280661,0.788650371958835
"8264","REP15",2.80144277694812,0.721696905707203,0.889574501894169,0.811283264269036,0.417203018653637,0.789636519339817
"8265","SAP18",1414.94393773391,-0.223726722315322,0.275770412157805,-0.811278920623647,0.417205512519567,0.789636519339817
"8266","INTS5",45.987754296866,0.21452169010523,0.264510272221337,0.811014590487141,0.417357291889959,0.789637169452664
"8267","LIPT2",6.87114901353424,-0.460772898793275,0.56812738658384,-0.811037999002145,0.41734384931821,0.789637169452664
"8268","ARPP19",281.600721740949,-0.124388842133567,0.153347130217227,-0.811158591343454,0.417274602014943,0.789637169452664
"8269","ADCY2",7.16731064159788,-0.586984700256406,0.72397191766429,-0.810783796904942,0.417489841053594,0.789792427573029
"8270","ZNF433",15.8915913282576,0.306064996054499,0.377551645464742,0.810657296110452,0.417562503401042,0.789834370097038
"8271","SLC40A1",487.343366208399,0.238277519132307,0.294413043226661,0.809330716196779,0.418324942696581,0.789986134906273
"8272","ITGB5",770.063838397936,-0.121486896327745,0.150087656129665,-0.809439626552559,0.418262316630082,0.789986134906273
"8273","GATA2",5.37531927629569,-0.916230292780278,1.13183217626252,-0.809510731357549,0.418221432640655,0.789986134906273
"8274","AP001816.1",71.565013112035,-0.251757851568358,0.311131238870093,-0.809169315439503,0.418417762155151,0.789986134906273

"8275","CYB5R4",55.9585425819351,-0.19021098639704,0.234886877422946,-0.809798267506186,0.418056128504162,0.789986134906273
"8276","ZNF282",49.6113523038754,0.182321580430922,0.225021268517359,0.810241545753516,0.41780136385089,0.789986134906273
"8277","THAP1",96.7413474972413,-0.177424863607436,0.219307251497989,-0.809024153991842,0.418501252954566,0.789986134906273
"8278","EDRF1-DT",39.4914461752916,0.299021397532713,0.369415112162611,0.809445492855441,0.418258943521924,0.789986134906273
"8279","SERPINH1",1009.90636277429,0.238980204292757,0.29499552745961,0.81011466970623,0.417874273789967,0.789986134906273
"8280","MAX",250.983248062345,0.111837747724482,0.138091942274666,0.80987888128936,0.418009790658367,0.789986134906273
"8281","DLST",178.833174609118,0.154977654907909,0.191421995763061,0.809612575034153,0.41816287844965,0.789986134906273
"8282","GDPD3",20.4149473891144,0.362599714496772,0.447948845036706,0.809466791832135,0.418246696803062,0.789986134906273
"8283","SRR",34.8697290373891,0.293119629498157,0.362280809652265,0.809095104373613,0.418460444034364,0.789986134906273
"8284","TRIM25",191.339738111334,0.185423886195415,0.22903391700954,0.809591385487643,0.418175060808813,0.789986134906273
"8285","CCDC106",60.9261969204617,-0.310221226396619,0.383126384410813,-0.809709899968621,0.418106926783833,0.789986134906273
"8286","SOGA1",104.390319136667,0.182866757541538,0.225937737277597,0.809367924743175,0.41830354626921,0.789986134906273
"8287","PI4KA",101.809177525645,0.195715530287482,0.241877635948777,0.809150997031118,0.418428297597607,0.789986134906273
"8288","TVP23B",56.8133358806459,-0.258232860339748,0.319287765253468,-0.80877781250011,0.418642960727097,0.790158281208251
"8289","THAP9-AS1",61.4542222459448,-0.199753138151689,0.247112624904806,-0.808348574778966,0.418889946891289,0.790400223240883
"8290","MYO6",122.458926339306,-0.205541821697929,0.254291666768619,-0.808291613759222,0.418922729073078,0.790400223240883
"8291","BAZ2A",197.189773363688,0.198357393374765,0.245397535143803,0.808310455353705,0.418911885199595,0.790400223240883
"8292","PLEKHA1",210.999890155972,-0.178107088582265,0.220384277117086,-0.808166040300778,0.418995004387792,0.790441251041755
"8293","KTI12",43.1908768267598,-0.27810989716004,0.344254137964007,-0.807862176486365,0.41916992721136,0.790619587186661
"8294","LIAS",110.498141100516,0.211200052661056,0.261442427910318,0.807826236732716,0.419190619198757,0.790619587186661
"8295","RSPH3",25.5096130791246,-0.361589470650092,0.447662997110302,-0.807726957519783,0.419247781427574,0.790632072920017
"8296","CDK11B",114.979242094253,-0.177840361746734,0.220272531121875,-0.807365134640127,0.419456147843844,0.790779573659938
"8297","SQSTM1",2149.85198631415,-0.148811207659775,0.184486034066859,-0.806625869608344,0.419882064745857,0.790779573659938
"8298","CDC40",340.694865632168,0.122995359388664,0.152447748836632,0.806803382321311,0.419779770171831,0.790779573659938

"8299","FAM24B",23.8052719812953,-0.257000792560671,0.318472361005009,
-0.806979895365643,0.41967806619888,0.790779573659938
"8300","RNF121",87.5191111675684,-0.167123648093456,0.207100340547448,
-0.806969450903277,0.419684083726657,0.790779573659938
"8301","CBL",42.1853819666022,-0.271553130618989,0.336639719842216,-0.
806658022250811,0.419863535178866,0.790779573659938
"8302","RBM25",940.103289411207,0.215394373578162,0.266822770766754,0.
807256340825764,0.419518811887271,0.790779573659938
"8303","MT1X",1780.89075342496,-0.322931392958578,0.399942179306577,-0
.807445199999856,0.419410034590823,0.790779573659938
"8304","CYB5D2",244.696282480265,-0.250362010314882,0.310294510437567,
-0.806852850737931,0.419751265806064,0.790779573659938
"8305","AC011815.1",3.40356367913728,-0.665176236733331,0.824579675462
102,-0.806685219788567,0.41984786161378,0.790779573659938
"8306","ZBTB45",34.2170905059228,0.228538575080705,0.283200416723247,0
.806985306465988,0.419674948638859,0.790779573659938
"8307","MRPL20-
AS1",43.6701975330936,-0.235513336479346,0.292229225475581,-0.80591985
998685,0.420289059191961,0.790784430230917
"8308","LINC01943",18.6055418918822,-0.427949370108006,0.5309588280422
66,-0.805993511184147,0.420246590550436,0.790784430230917
"8309","SP100",661.545643672112,0.123642952481545,0.15331957886586,0.8
06439421476107,0.419989524272653,0.790784430230917
"8310","CHIC2",215.297948881429,-0.165355288022592,0.205143390228632,-
0.806047359548385,0.420215542183788,0.790784430230917
"8311","FAM13C",168.732517136071,0.29949260537365,0.371468551198414,0.
806239463360872,0.420104788246121,0.790784430230917
"8312","PCID2",262.723381602297,-0.137563303444452,0.170585553293623,-
0.806418250481441,0.420001727213581,0.790784430230917
"8313","MRPL58",107.856832505069,-0.219415536007979,0.272183910882532,
-0.806129705817453,0.420168064848953,0.790784430230917
"8314","ZNF100",58.1777816231873,0.265167299556095,0.328881829544552,0
.806269230268236,0.420087628215186,0.790784430230917
"8315","AC090198.1",5.40614165286549,0.541798174130519,0.6724748027915
32,0.805678029691883,0.420428520736439,0.79095169571619
"8316","CTNNBIP1",101.599799143561,-0.201914075442401,0.25070883516811
5,-0.805372795525959,0.420604585568286,0.791018339047889
"8317","LINC01118",8.27342449822487,0.439966528909139,0.54665713385787
2,0.804830855867927,0.420917293250454,0.791018339047889
"8318","EIF4E3",88.9637007409703,-0.203861631763805,0.253346280494338,
-0.804675842747809,0.421006763344942,0.791018339047889
"8319","PGM2",60.8080723707923,-0.228593858776227,0.283859508255542,-0
.805306329814527,0.420642929985354,0.791018339047889
"8320","MARCH6",420.187851980335,0.165348079124199,0.205494146015394,0
.804636445029495,0.421029504602678,0.791018339047889
"8321","DBNL",251.434641663297,-0.1718346772423,0.213418100463041,-0.8
05155124469201,0.420730168786643,0.791018339047889
"8322","LINC00535",12.6488168926257,0.494559133067866,0.61444143812763
4,0.804892219793832,0.420881878453131,0.791018339047889
"8323","PPA1",896.930481025149,-0.20605280134398,0.256013694773938,-0.

804850699592168,0.420905840703959,0.791018339047889
"8324","GAS2",7.18339100688344,0.466448598558178,0.579217325262682,0.805308436426066,0.420641714638627,0.791018339047889
"8325","TGDS",66.0751200521696,-0.213682741982412,0.265542391532101,-0.804702935563418,0.420991125174844,0.791018339047889
"8326","ATPAF2",67.2104356186054,-0.207048394152916,0.25734203567816,-0.804564996959364,0.421070747890543,0.791018339047889
"8327","GAGE10",2.8144588146738,-0.755560951512328,0.93879141781404,-0.804823027964655,0.420921811072985,0.791018339047889
"8328","ZFAND6",309.830659912595,-0.142187823769387,0.176775380991376,-0.80434177526295,0.421199617543761,0.791165419937206
"8329","MIEF1",74.2686245957807,0.210088442638929,0.261328683282071,0.803924161712342,0.421440775035825,0.791523357412104
"8330","ZNF703",265.767650747879,0.236863025085601,0.29472750803027,0.803667858044911,0.421588821707919,0.791611323727882
"8331","SEPHS1",131.586990736684,-0.181779406013987,0.226183872785703,-0.803679783952647,0.421581932363049,0.791611323727882
"8332","C1orf109",74.1479756104867,0.169610444842617,0.211180800071601,0.803152771393566,0.421886439390387,0.792075080578952
"8333","GATA3",3.81868327696061,0.715088080739843,0.890820975433726,0.802729280584887,0.422131225133423,0.792142454347973
"8334","NTF3",9.13636873562102,0.555017942089576,0.691630531161367,0.802477503642885,0.42227679644619,0.792142454347973
"8335","GLDN",18.3188486401647,-0.386563786455883,0.481591935077238,-0.802679111297587,0.422160229450016,0.792142454347973
"8336","DDX28",19.1184414349304,-0.348190842379679,0.433893832255832,-0.802479354383583,0.422275726285548,0.792142454347973
"8337","IFT20",357.546044030751,-0.173512818031226,0.21609112683923,-0.802961327330752,0.421997087391381,0.792142454347973
"8338","AC080038.1",45.534571879703,-0.21574077716672,0.26882035949631,-0.802546271312761,0.422237033729669,0.792142454347973
"8339","RTL8A",345.705852703184,-0.211707785469576,0.263760111321771,-0.802652775693233,0.422175455292388,0.792142454347973
"8340","RPL37",17604.1796755879,-0.22312587767922,0.27812935183965,-0.802237794045766,0.422415418047311,0.792286392229367
"8341","TRMT12",30.1763025905903,-0.254809577277815,0.317650478958001,-0.802169661804618,0.422454823089251,0.792286392229367
"8342","MTA3",104.731891711054,0.168516566053956,0.210274889773025,0.801410792490992,0.42289386919624,0.7924447527356
"8343","ARMC8",118.571011349982,-0.136715507924665,0.170585206299385,-0.801449966796788,0.422871198245812,0.7924447527356
"8344","UBXN8",58.0867431139422,0.225168055248526,0.28078265120572,0.801930084645981,0.422593402313372,0.7924447527356
"8345","TUBA1B",1800.26796953735,-0.267810451618205,0.334115941544248,-0.801549457294416,0.422813624314068,0.7924447527356
"8346","SNHG29",3262.06966638173,-0.250958731801741,0.31312144712406,-0.801474105676033,0.422857228950391,0.7924447527356
"8347","ZDHHC8",118.941202126751,-0.168879690036212,0.210689127010368,-0.801558639653487,0.422808310826819,0.7924447527356
"8348","EFCAB6",6.36150093516368,0.476054820955533,0.593794152715859,0

.801716922907009,0.422716724384365,0.7924447527356
"8349","WDR27",104.742766862129,0.202671226446499,0.252949961244333,0.801230509977156,0.422998211449802,0.792545337370854
"8350","LINC00663",14.6002306739727,0.361732033466533,0.451523619657273,0.801136458245761,0.423052651827983,0.792552411083249
"8351","N4BP3",4.47228180544615,0.715606506761541,0.893495654114162,0.800906533195189,0.423185757630151,0.792706838295827
"8352","NT5DC3",13.3953533790513,0.490653126329044,0.612748086595014,0.800741996691593,0.423281024427372,0.79279035741348
"8353","CTC1",21.1336768751802,0.308032407366738,0.384733439353657,0.800638509312382,0.423340950151961,0.792807671881614
"8354","CPSF2",94.5947921214399,0.202061351243809,0.252410230232464,0.800527581856388,0.423405189664326,0.792833059841879
"8355","GSTM3",860.767886165887,0.259685846890849,0.32458372715412,0.800058121113212,0.423677123558608,0.79300197227082
"8356","GALNT15",815.19386610626,-0.553779480385967,0.692217616831993,-0.800007782119728,0.423706288352133,0.79300197227082
"8357","SLBP",240.154318621826,-0.178804188971346,0.223523593469554,-0.79993429863904,0.423748864425736,0.79300197227082
"8358","AKR1C3",903.188221977151,-0.287722206748708,0.359579511204255,-0.800162961969406,0.423616385909606,0.79300197227082
"8359","THAP7",190.419195867216,-0.166959432702749,0.208627224542141,-0.800276344897763,0.42355070530065,0.79300197227082
"8360","NPFF",14.880513000703,0.324680371314672,0.405952399034044,0.799799119520521,0.423827193274482,0.793053682343627
"8361","LAMA5",8.79576563944572,-0.710980630121475,0.889259260891269,-0.799520074054542,0.423988911527121,0.793261397323137
"8362","TMX1",238.981812601879,-0.137831440895872,0.172431558996014,-0.79933999146327,0.424093295906796,0.793361806729253
"8363","STX17-AS1",3.74582919609667,-0.569992738727659,0.713186029528287,-0.799220280723476,0.424162694223365,0.793396750655997
"8364","ANKRD9",98.1160168548082,-0.271061972500964,0.339204390526285,-0.79911103768558,0.424226030032691,0.79342034765679
"8365","PDK4",3956.52817156613,0.273897639630524,0.342813983977392,0.798968689820387,0.424308567316139,0.793479846805303
"8366","ITM2C",334.787076395007,0.261135293962287,0.32705565987804,0.798442974690197,0.424613473040507,0.793699587085238
"8367","AL390208.1",5.45937292922742,0.557081986951851,0.697733852462691,0.798416165398308,0.424629025398987,0.793699587085238
"8368","BCLAF1",713.606541346161,0.179740414839009,0.225087248000496,0.798536640505784,0.424559139107166,0.793699587085238
"8369","KIAA0895L",18.1956832672821,0.326437868845501,0.408810204832065,0.798507143381115,0.424576249444976,0.793699587085238
"8370","CLCC1",159.842324443113,0.144071387694931,0.180806735911417,0.796825333794622,0.425552479741291,0.793702518315569
"8371","AC010680.2",5.72174845909677,0.534629788535673,0.671036555788124,0.796722300631978,0.42561232937388,0.793702518315569
"8372","C2CD6",17.1440308828922,0.406300168390647,0.509639156057222,0.797231067435933,0.425316846023292,0.793702518315569

"8373","GLB1L",52.4098665140594,0.248821005460028,0.312330906887725,0.
796658287645778,0.425649515543475,0.793702518315569
"8374","AZI2",409.468761986382,0.132204547811282,0.166222708713635,0.7
95345887661123,0.426412327652987,0.793702518315569
"8375","ACY1",59.1352743414806,0.265202315184185,0.333062495725711,0.7
96253912066367,0.425884467624653,0.793702518315569
"8376","GK5",141.802532026958,0.290924190720009,0.365623921775471,0.79
569244076613,0.426210821615572,0.793702518315569
"8377","U2SURP",660.524588665778,0.0930799049429538,0.116704698352542,
0.797567760826371,0.425121365953709,0.793702518315569
"8378","EIF4G1",293.024604994604,0.169023290032461,0.212313993852716,0
.796100562969554,0.425973586980753,0.793702518315569
"8379","FAM47E",2.81797432047436,-0.708352385398703,0.889292899072794,
-0.796534399563129,0.425721489502872,0.793702518315569
"8380","DROSHA",70.7451627680342,0.192687225732328,0.241429453167533,0
.798109854470068,0.424806743291735,0.793702518315569
"8381","LACTB2",103.982222543057,-0.194256864292044,0.244139472125296,
-0.79567987347965,0.426218127992683,0.793702518315569
"8382","MTBP",13.3233224702904,0.383387115146406,0.480253063403242,0.7
98302279280824,0.424695095670986,0.793702518315569
"8383","UBQLN1",203.00188965032,0.140349055932917,0.17634009796629,0.7
9589984099786,0.4260902536877,0.793702518315569
"8384","YPEL4",50.6318105202392,-0.269648137841046,0.338164677263588,-
0.797387060124156,0.425226272049102,0.793702518315569
"8385","PANX1",43.2785055179016,-0.224020466089608,0.280831648694495,-
0.797703774239884,0.42504241311089,0.793702518315569
"8386","AMOTL1",93.1499699974685,0.185487559717093,0.232805680522167,0
.796748426847047,0.425597152781794,0.793702518315569
"8387","CPNE8",113.262053241148,0.21426753400257,0.268487655806929,0.7
9805357664805,0.424839399728066,0.793702518315569
"8388","DYRK2",159.058041189474,-0.201775036350112,0.253719303146337,-
0.795268762951532,0.426457179980973,0.793702518315569
"8389","KMT5A",101.387891231032,0.149531255329558,0.187870119422526,0.
795928888474585,0.426073369113614,0.793702518315569
"8390","DDX51",65.4841925742595,-0.21195340826255,0.265787133215276,-0
.797455488903886,0.425186543827959,0.793702518315569
"8391","POLE2",5.00618560492028,0.602360303943069,0.757387312312252,0.
795313433630283,0.426431201150031,0.793702518315569
"8392","ARMH4",70.6976598431574,0.195895310983635,0.24557150875603,0.7
97711884314124,0.425037705658917,0.793702518315569
"8393","BDKRB1",12.724195922134,0.385678716587927,0.484551089171774,0.
795950571996762,0.42606076527719,0.793702518315569
"8394","USP7",249.383942139688,-0.130909165125932,0.164356501769704,-0
.79649520229727,0.425744263006285,0.793702518315569
"8395","SNTB2",745.810548971602,-0.166289001876942,0.20836041543503,-0
.798083462877302,0.424822057407662,0.793702518315569
"8396","RNF135",108.087090101438,-0.175164963121491,0.220091062841109,
-0.795874947670858,0.426104723868723,0.793702518315569
"8397","RAB11B-
AS1",96.5138722541183,-0.21914261937538,0.27520875229576,-0.7962777983

88739,0.425870586985526,0.793702518315569
"8398","ZNF257",10.3710425051258,0.489485322033531,0.615059043062672,0
.795834688644118,0.426128126546415,0.793702518315569
"8399","A1BG-
AS1",6.87104900994038,0.545290918462893,0.684105882660943,0.7970855569
05159,0.425401343901167,0.793702518315569
"8400","SNHG11",63.5327374540659,-0.240648551594074,0.302331189119306,
-0.79597659869326,0.426045637200022,0.793702518315569
"8401","PPDPF",1360.44871213861,-0.235495979799169,0.295835743036869,-
0.79603626452203,0.426010957482666,0.793702518315569
"8402","ZBTB21",46.7624207694325,-0.252160344262271,0.316959900281373,
-0.795559135519735,0.426288326428282,0.793702518315569
"8403","THOC5",91.1816352516982,-0.156325423381908,0.196507310695999,-
0.795519631448963,0.426311296012515,0.793702518315569
"8404","INE2",3.59226699654564,0.799413811861496,1.00285290926528,0.79
7139644783172,0.425369933958365,0.793702518315569
"8405","NHSL2",93.9041413281341,0.328945504949782,0.413419550172432,0.
795669930975889,0.426223908423691,0.793702518315569
"8406","LINC02478",9.15625851944043,-0.589489375819531,0.7416131318329
04,-0.794874511408128,0.426686502256625,0.794034850678133
"8407","EIF2S2",780.829685285031,-0.138151098180122,0.173848171050019,
-0.794665237751474,0.426808258595756,0.794166954824957
"8408","WDR33",349.340751114776,-0.127320594094238,0.160309369333618,-
0.794218046165924,0.427068504505011,0.794376545487189
"8409","RPL34",18195.1224511226,-0.21940121384658,0.276311481512076,-0
.794035820176337,0.427174578558889,0.794376545487189
"8410","GPBP1",544.650841232374,0.102125245847239,0.128612432363317,0.
794054229211262,0.427163861931796,0.794376545487189
"8411","TMEM109",703.475489896975,-0.148693300218119,0.187269952276254
,-0.794005116201301,0.427192452905267,0.794376545487189
"8412","VPS39",91.9687071311868,0.192884696746962,0.24294367933382,0.7
93948199335232,0.42722558826208,0.794376545487189
"8413","AC138150.2",7.10584379069965,0.45192631149823,0.56901918240188
1,0.794219818021967,0.427067473179994,0.794376545487189
"8414","NDUFA13",2008.64320957872,-0.218155046125369,0.274826063846523
,-0.793793147098295,0.42731586279532,0.79444996930202
"8415","NT5C3A",200.686270487642,-0.169705346492623,0.213880347332695,
-0.793459280429554,0.427510284411453,0.794663001263472
"8416","MIAT",8.05880862229055,0.47171581024621,0.594533377606148,0.79
3421913746112,0.427532047473847,0.794663001263472
"8417","MRPL30",102.950196420736,0.186324393886352,0.234925667914002,0
.793120630626702,0.427707544028135,0.794893046661431
"8418","FAP",255.579587262357,-0.321151349491892,0.40505401316093,-0.7
92860554536209,0.427859071334735,0.794893046661431
"8419","AC008269.1",6.95019393888819,0.503874500685197,0.6354969801134
87,0.79288260440705,0.4278462232761,0.794893046661431
"8420","AC096992.2",4.39010260084438,0.636822369136322,0.8031477794167
57,0.792908086726928,0.427831375471642,0.794893046661431
"8421","PDZD4",3.31454266067036,0.69909579611674,0.88184581794088,0.79
2764201965754,0.427915216860055,0.794902949452777

"8422","HERC2",261.261994232389,0.215636076371033,0.27214691110034,0.792351732008189,0.428155615357443,0.795255080863985
"8423","SDF4",929.691694492573,0.156987853873961,0.198455076224982,0.79104982780077,0.428914915242774,0.795258864165591
"8424","ADAR",376.052437761882,0.131562284468234,0.166198139420291,0.791599021066849,0.428594517912463,0.795258864165591
"8425","PPP3R1",127.272590386083,-0.159340386553638,0.201287887698535,-0.791604444636425,0.428591354516293,0.795258864165591
"8426","HES6",9.287158420832,-0.475229837120773,0.601011001168761,-0.79071736822889,0.429108938585896,0.795258864165591
"8427","FGD5",106.89747067259,0.204259594453607,0.25831069021722,0.79075161109995,0.42908895209621,0.795258864165591
"8428","SFMBT1",30.9304289782842,0.258553029525938,0.326734835463124,0.79132373246782,0.428755102862956,0.795258864165591
"8429","ARHGEF37",3.82761947153052,-0.582966295645091,0.736625741865757,-0.791400927923764,0.42871006866873,0.795258864165591
"8430","HMGXB3",99.9803056850187,0.181042271251179,0.22873041374005,0.791509394360348,0.428646796295247,0.795258864165591
"8431","HLA-A",5073.61160529497,0.197903503617012,0.250270932672867,0.790757046786952,0.429085779505989,0.795258864165591
"8432","BLOC1S2",188.788431238475,-0.211120508331751,0.266768835430134,-0.791398695396123,0.428711371039446,0.795258864165591
"8433","LGR4",134.686035545136,0.293303847232507,0.37074590780924,0.791118232337768,0.428875000696378,0.795258864165591
"8434","DDX47",16.1424940266541,-0.351019696026394,0.443793243744087,-0.7909532219666,0.428971289130942,0.795258864165591
"8435","FAIM2",18.2015694273148,0.413315699738281,0.522726354044397,0.790692293473262,0.429123574277425,0.795258864165591
"8436","DLGAP1-AS2",11.8084257996069,0.371921273830049,0.470294054639349,0.790827079698597,0.429044905353223,0.795258864165591
"8437","TIMM21",79.5485554428978,0.181966703658648,0.229927194586404,0.791410098252934,0.428704719075714,0.795258864165591
"8438","ZNF443",10.5984655530402,0.370000985453617,0.467512843563981,0.791424215499613,0.428696483730296,0.795258864165591
"8439","MEIS3",108.961104731478,-0.198407942020194,0.250803394833034,-0.791089539088093,0.428891743153913,0.795258864165591
"8440","CPXM1",1030.35200034573,0.324843959932619,0.410179828175544,0.791954985640095,0.42838692387151,0.795258864165591
"8441","PABPC5",69.9685431919426,0.179690422256144,0.227088022208326,0.791280933748674,0.4287800719148,0.795258864165591
"8442","GIMAP7",5.73763409324731,-0.493769774972825,0.62459006633048,-0.790550156959372,0.429206542331859,0.795318401053928
"8443","C3orf52",6.15890495282984,-0.497519007286772,0.629541470603783,-0.790287900826628,0.429359651189731,0.795344132396196
"8444","ABTB1",215.026797576266,-0.162173752086207,0.205214364510313,-0.790265108747088,0.429372959028695,0.795344132396196
"8445","RPL3",15007.6247173689,-0.233912881858175,0.295986691090847,-0.790281755561705,0.429363239263295,0.795344132396196

"8446","RER1",836.629468512166,-0.172033561648355,0.218046405142393,-0
.78897683057884,0.430125548460592,0.795388179606685
"8447","DFFB",22.5465493532768,-0.301117461643431,0.38115893921918,-0.
790004983905881,0.429524857694296,0.795388179606685
"8448","AL604028.1",6.83162620921388,0.475473504970719,0.6019785516775
61,0.789851239127533,0.429614650906134,0.795388179606685
"8449","MTMR11",55.6684722098197,-0.215907682258824,0.27367539732726,-
0.788918859230308,0.430159432300233,0.795388179606685
"8450","ANKRD39",92.5682705934888,-0.172206527748839,0.218172617315507
,-0.789313204689686,0.42992897077851,0.795388179606685
"8451","QRSL1",62.0301478164419,-0.174636573738287,0.221135147915277,-
0.789727799423341,0.429686752612915,0.795388179606685
"8452","PIP5KL1",4.94022615155939,-0.572388960479094,0.725524162520689
,-0.788931630464854,0.430151967471656,0.795388179606685
"8453","LRRC20",14.2659786087844,0.34429977249429,0.435849452490792,0.
789951141447318,0.429556302642038,0.795388179606685
"8454","VWA5A",87.6389338905153,0.20611776748406,0.261186680675692,0.7
89158799946582,0.430019198730673,0.795388179606685
"8455","KRT8",3.73784506011903,-0.583629205179014,0.739661832955136,-0
.789048696547269,0.430083545466178,0.795388179606685
"8456","TARBP2",39.2574954238457,-0.251250995730204,0.31831805143718,-
0.789308035142295,0.429931991477202,0.795388179606685
"8457","RPL21",14693.6348025096,-0.198937078815505,0.251919824234003,-
0.789684096598592,0.429712281321474,0.795388179606685
"8458","WDFY2",218.83438615262,-0.249595629473497,0.316327141395067,-0
.789042724480516,0.430087035827069,0.795388179606685
"8459","SCAMP2",256.102595506379,-0.17615004162733,0.223229056379109,-
0.789099969710823,0.430053579658626,0.795388179606685
"8460","ZNF493",143.046793541344,0.19350476457381,0.244973333457694,0.
789901340862586,0.429585388254641,0.795388179606685
"8461","GOPC",393.078804641962,0.11670655790372,0.147987321517939,0.78
8625381597798,0.430330991662759,0.7956113583005
"8462","FAM3C",785.344346545917,-0.177631952743717,0.225299847707629,-
0.788424646315025,0.430448359139179,0.795734304185084
"8463","DCLRE1B",16.6958817528684,0.350010949018615,0.444154809361783,
0.788038183176614,0.430674371740117,0.795775950523346
"8464","EIPR1",47.5434244406153,-0.23670755789446,0.300320769406281,-0
.788182443599952,0.430589996847575,0.795775950523346
"8465","PPP2CB",305.354244608296,0.1329892035717,0.168758889937818,0.7
88042654349663,0.430671756500832,0.795775950523346
"8466","KIAA1958",5.81764055420985,0.542873707930598,0.688669111164739
,0.788293970398122,0.43052477374005,0.795775950523346
"8467","WRAP73",91.2045385104369,-0.16600166734055,0.210840671716772,-
0.787332282661024,0.431087376426965,0.795972543165481
"8468","ARHGEF2",107.120534467611,-0.177140615383776,0.22517816740342,
-0.786668696288026,0.43147583354223,0.795972543165481
"8469","LIMS1",834.634663986731,-0.115950813201072,0.147466072879246,-
0.786288065703218,0.431698742590786,0.795972543165481
"8470","PRKRA",220.908606016022,-0.165189561728107,0.210169281545409,-
0.785983377368188,0.431877225591276,0.795972543165481

"8471","ARIH2",198.774564454668,0.145899620847371,0.185636117795005,0.785944150202958,0.431900207529796,0.795972543165481
"8472","SLC66A1L",295.811615799066,0.341155643635833,0.43394131683573,0.786179214561812,0.43176250145701,0.795972543165481
"8473","NEK1",161.253030753236,0.137215055536928,0.174546719498993,0.786122225217298,0.431795884780618,0.795972543165481
"8474","TNIP1",134.527556931228,-0.205461328855712,0.261347248042181,-0.786162205245606,0.431772465049126,0.795972543165481
"8475","NOP16",39.9673755520461,-0.274048404491581,0.348082188596959,-0.787309473076483,0.431100725572624,0.795972543165481
"8476","SNX8",55.3716767581437,0.270788382144721,0.344062934791048,0.787031542090326,0.431263401943717,0.795972543165481
"8477","LINC00265",7.40032870356411,0.489460113697478,0.621771842129603,0.787202122278569,0.431163555075791,0.795972543165481
"8478","DLX5",3.13160695254834,0.84879342908196,1.07831161387592,0.787150410103653,0.431193822700677,0.795972543165481
"8479","SSBP1",783.042722490308,-0.20444161650489,0.25961728642873,-0.78747305049355,0.431004998368324,0.795972543165481
"8480","SPAG8",18.1777330659571,0.295761742492871,0.376194579805186,0.78619352422896,0.431754119348652,0.795972543165481
"8481","MSRB2",475.455841997152,-0.200852751080606,0.255075096392442,-0.787425953851594,0.431032558548688,0.795972543165481
"8482","RAB3IP",8.63454121078916,0.601026697047673,0.763392580466825,0.78731011071674,0.431100352394983,0.795972543165481
"8483","CSRP2",60.5314802016348,-0.329403181183663,0.418608441438574,-0.786900474466423,0.431340129759614,0.795972543165481
"8484","DZIP1",126.682976330006,0.165538377440082,0.210585368591992,0.786086794856157,0.431816639997039,0.795972543165481
"8485","SMARCD2",295.191169589813,-0.133943078897513,0.170049949492087,-0.787669030761726,0.43089032491461,0.795972543165481
"8486","POLRMT",38.3465103041606,-0.258777943741858,0.328934147401992,-0.786716568607285,0.43144780275501,0.795972543165481
"8487","BRD1",187.139756307298,0.14047796390482,0.178610922042055,0.786502652238387,0.431573065870143,0.795972543165481
"8488","Z93943.1",3.12830275811726,0.706924414520919,0.899022537469499,0.786325575897926,0.431676772496146,0.795972543165481
"8489","PROSER1",41.3755796370659,0.216975454571579,0.276216931936141,0.785525539838167,0.432145502045017,0.796330791434821
"8490","AC012020.1",6.67646244056878,0.464379288514722,0.591245028459969,0.785426119732968,0.432203771432325,0.796344357657934
"8491","CAMSAP1",41.0468080331205,0.20822397618631,0.265220738296944,0.785096887684475,0.432396764380008,0.7965073009819
"8492","ARHGEF25",92.3340590115499,0.218607884140817,0.278476172348257,0.785014682934633,0.432444959869544,0.7965073009819
"8493","ZBTB33",50.557132739217,0.18600976058492,0.236942053986281,0.785043251949228,0.432428209905155,0.7965073009819
"8494","AL008727.1",3.91051873083308,0.610939097753515,0.778499720760297,0.78476469735514,0.43259154201156,0.796683481479495
"8495","MILR1",41.79301193531,0.39822598272076,0.507568965959653,0.7845751206791,0.432702721819195,0.796794429360525

"8496","LBH",65.7090532264527,0.352043389384776,0.449002967394636,0.78
4055819113016,0.43300735794416,0.796885407044821
"8497","AC012360.3",18.2856688683306,0.329855491573194,0.4206253143406
11,0.784202662862288,0.432921202909226,0.796885407044821
"8498","MST1",5.61922266794658,-0.485432003552529,0.61887288304142,-0.
784380794270542,0.432816704336245,0.796885407044821
"8499","CNTRL",104.055078809391,0.209120419516464,0.266736856123136,0.
783995217443539,0.433042916581236,0.796885407044821
"8500","TMEM216",116.240310571666,-0.205426530219684,0.261944661864076
,-0.784236367932861,0.432901429129352,0.796885407044821
"8501","SIAH1",151.036543147861,-0.170664156399034,0.217692234187619,-
0.783969887745038,0.433057779536407,0.796885407044821
"8502","UBL5",1422.91220466825,-0.20246300373939,0.258308867308047,-0.
783801988098003,0.433156307118174,0.796972960744483
"8503","JARID2",45.0465522501657,0.211887494689656,0.270411853305059,0
.783573249840569,0.433290557137006,0.797032477104209
"8504","NCAM2",62.019489165797,0.369774341664645,0.471900569017724,0.7
83585284574548,0.433283493165848,0.797032477104209
"8505","KDM4A-
AS1",16.9133867495835,0.432727686405493,0.552433582469734,0.7833116959
88723,0.433444096625623,0.797221164434406
"8506","OGFOD2",40.9144279987544,0.2220525520478,0.283548851611708,0.7
83119207803669,0.433557112715879,0.797241555685258
"8507","C19orf25",148.770215955474,-0.19864202381106,0.253651651742769
,-0.783129234311096,0.433551225405784,0.797241555685258
"8508","CCDC85C",7.60716439892877,0.56535527131498,0.722013316741135,0
.783026099666356,0.433611785653282,0.797248373645309
"8509","HSPBP1",60.2936859249718,-0.221910840687668,0.283519352795583,
-0.782700857982226,0.433802798289077,0.797505837776005
"8510","GNG5",908.176861582087,-0.206966097841944,0.26451733396731,-0.
7824292447599,0.433962352491872,0.797610231844839
"8511","PCDHB11",54.1778709754744,-0.376259469056346,0.480938771000895
,-0.782343807036604,0.434012548325978,0.797610231844839
"8512","FAM49B",56.6647066917232,-0.212576453910523,0.271672804414346,
-0.782472336046965,0.433937037042714,0.797610231844839
"8513","SLC13A4",4.07862064103689,0.746784226486932,0.954930856295378,
0.782029632369464,0.434197159104621,0.797762057772326
"8514","DEF8",130.517861119569,-0.191188175579304,0.244452548111201,-0
.782107517620692,0.434151389069379,0.797762057772326
"8515","C3orf18",30.2670482880805,0.250091810210752,0.320015839028868,
0.781498225118137,0.434509520208122,0.797918683132043
"8516","MALINC1",22.0210508318587,-0.287616937715556,0.368031065064329
,-0.781501794326201,0.43450742179531,0.797918683132043
"8517","RIC8A",220.832477257252,-0.155363679760688,0.198772015710492,-
0.781617468663058,0.43443941757815,0.797918683132043
"8518","NDUFA4L2",9.83848519489452,-0.454062627028514,0.58105087248195
4,-0.781450727522341,0.434537445605183,0.797918683132043
"8519","MAN2A2",75.577574302827,0.196806032787848,0.251839041189898,0.
781475468846973,0.434522899238236,0.797918683132043
"8520","CYP2R1",23.8422396469098,-0.254201969383861,0.325374495125382,

-0.78125966599166,0.434649787426705,0.798031293980746
"8521","STT3B",280.936031852669,0.159638235293207,0.20442409046027,0.780916940531687,0.434851347787763,0.798072790205671
"8522","FBXO30",59.0226676488047,0.211675153611827,0.271164497805727,0.78061529191583,0.435028795076164,0.798072790205671
"8523","TCP11L2",150.859688232907,-0.263877037644772,0.337965429420193,-0.780781152964296,0.434931220773142,0.798072790205671
"8524","GABARAP",3152.18314619173,-0.20758966848396,0.265816527630571,-0.780950945128839,0.434831346923983,0.798072790205671
"8525","TMEM161A",108.094132821329,-0.212319500109834,0.272002047997098,-0.780580520158803,0.435049252535578,0.798072790205671
"8526","RAE1",77.3216873140889,0.210980211531861,0.270105000336888,0.781104427051394,0.434741078356,0.798072790205671
"8527","PHKA1",6.95261308857089,-0.474164300692449,0.60731181878183,-0.780759217964086,0.434944124177113,0.798072790205671
"8528","RPL36A",5804.24491618008,-0.212208483175109,0.27187834141139,-0.780527356734194,0.4350805315396,0.798072790205671
"8529","MAPK8IP2",6.84638730958632,-0.480703273891504,0.615965195304862,-0.780406551466902,0.435151612833166,0.798109588410038
"8530","KIF9-AS1",10.8466808077218,0.377615338245482,0.484058042724609,0.780103427514613,0.435329999151609,0.798156021651269
"8531","AC135050.6",20.2941182245988,0.312173151257147,0.400094891792839,0.780247780366022,0.435245043249778,0.798156021651269
"8532","RTN2",166.241767912626,0.236345205529588,0.302956790985531,0.780128429406542,0.435315284117265,0.798156021651269
"8533","SP110",224.941241858082,0.137102242765293,0.175783503650375,0.779949425959689,0.43542064414749,0.798187555924126
"8534","EXOSC9",97.1262527247674,-0.164302940994977,0.210671580744895,-0.779900831493422,0.435449249009557,0.798187555924126
"8535","AL096803.2",23.7231898918907,0.283937948540396,0.364174589093678,0.779675345407905,0.43558199432449,0.798260758823962
"8536","AC109322.1",4.35474188983028,0.563872316241248,0.723304546588212,0.779578006112367,0.435639305895327,0.798260758823962
"8537","ZNF597",10.814074824765,0.355200088416995,0.455634179938936,0.779572964575658,0.435642274377048,0.798260758823962
"8538","USP1",243.313002889793,0.123699726605296,0.158771809325137,0.779103841740451,0.435918547268214,0.798526606834907
"8539","C5orf34",11.1758975179733,-0.356474286756728,0.45765096253149,-0.778921745919413,0.436025813213214,0.798526606834907
"8540","C8orf33",224.816403112948,-0.202213278338591,0.259583723925397,-0.778990590321855,0.435985257721078,0.798526606834907
"8541","MRPL43",534.949441599621,-0.196845663706777,0.252724825013804,-0.778893263437911,0.43604259257072,0.798526606834907
"8542","ZSCAN22",13.6716600316619,-0.336286735149388,0.431735726167354,-0.778918015737787,0.436028010684937,0.798526606834907
"8543","HNRNPF",744.424535305004,-0.170957580067272,0.219521491798957,-0.778773771380156,0.436112990773213,0.79856204081299
"8544","FMN2",3.13789928351504,0.807540158743505,1.03798673970078,0.777986970215339,0.436576694673842,0.798791318644328

"8545","C2orf74",168.897681041444,-0.241979898324709,0.310956184594765
,-0.778180046941515,0.436462877966008,0.798791318644328
"8546","AC016727.1",15.0619626092557,0.352457946315774,0.4531458915686
49,0.777802365361132,0.436685533290857,0.798791318644328
"8547","IL1R2",5.54093592589417,0.539909563360397,0.694598332792847,0.
777297522712909,0.436983256277293,0.798791318644328
"8548","AC022382.1",3.01586790248633,0.641758050630264,0.8260347115377
49,0.776914143759849,0.437209426031131,0.798791318644328
"8549","RNF123",29.1309267380294,0.217813715701351,0.280368213625763,0
.776884486599076,0.437226924720426,0.798791318644328
"8550","WDR53",21.5527372373183,0.303395144303534,0.390592602679528,0.
776755991337762,0.437302745760826,0.798791318644328
"8551","GHR",350.301983068131,0.18216804527768,0.234401236831749,0.777
163327889941,0.437062415217041,0.798791318644328
"8552","AC008906.1",5.38278987587161,0.682319090054479,0.8774804856740
59,0.777588905045949,0.43681140388318,0.798791318644328
"8553","AC011379.2",20.2297659179365,0.358070386896713,0.4612137893189
99,0.776365310814793,0.437533320618446,0.798791318644328
"8554","MAPK14",206.068035856386,0.145276724595905,0.186977204050202,0
.776975596217063,0.43717316836624,0.798791318644328
"8555","NDUFA5",578.901633305319,-0.183389085905448,0.236049687558621,
-0.77690882712948,0.437212562986283,0.798791318644328
"8556","LINC00513",30.5627800208542,-0.397901749600925,0.5122963892930
06,-0.776702233154617,0.437334469031124,0.798791318644328
"8557","SLC20A2",68.1207459151483,0.185178435528049,0.23833620940403 7,
0.776963080813823,0.437180552459416,0.798791318644328
"8558","PNPLA7",47.988764924868,0.328763340934004,0.422653431183682,0.
777855606219193,0.436654142147577,0.798791318644328
"8559","AP003119.2",7.65109124680053,0.461716864345241,0.5947492286511
01,0.776321922085416,0.437558932430605,0.798791318644328
"8560","ALG11",39.9071357332,0.267467302573074,0.343671580537084,0.778
264243307756,0.436413250444067,0.798791318644328
"8561","FCF1",131.875833655457,0.210973950071095,0.271765018126519,0.7
76310179748289,0.4375658639304,0.798791318644328
"8562","GCOM1",2.98422344649875,-0.643831761500734,0.829323733464372,-
0.776333457636894,0.437552123057874,0.798791318644328
"8563","CHRNE",3.08057769308704,-0.644163858820616,0.829018482676895,-
0.77701990037739,0.437147029470007,0.798791318644328
"8564","TEX2",42.7582769844144,0.214742561256978,0.276360341110124,0.7
77038269653197,0.437136192092669,0.798791318644328
"8565","MYO1F",22.3987110785028,-0.361711242776149,0.464944088665056,-
0.777967182709413,0.436588360167292,0.798791318644328
"8566","PPP1R12C",165.72822078006,-0.147548599151154,0.190057425706783
,-0.776336933968521,0.43755007101035,0.798791318644328
"8567","RBM12",100.270099821284,0.162928559778764,0.209565903010372,0.
77745738900426,0.436888965010044,0.798791318644328
"8568","STMN3",211.311215585573,-0.213041216654115,0.273715778978885,-
0.778330052614721,0.436374462995894,0.798791318644328
"8569","AKAP17A",132.052222554274,0.177217777807439,0.22789513389694,0
.77762861706201,0.436787985419248,0.798791318644328

"8570","ZNF717",41.8384713836027,0.246103969080691,0.317107826294104,0
.776089231088357,0.437696301669999,0.798936201519697
"8571","WDR43",168.120437146503,-0.151558134353666,0.195424506355298,-
0.775532901068816,0.438024831918904,0.79900551383932
"8572","TCF7",26.08182586033,-0.504308663360775,0.650477552154453,-0.7
7528988001269,0.438168387936334,0.79900551383932
"8573","GPC2",6.13416612655329,-0.499861605831642,0.644346654722797,-0
.775765036052972,0.437887731722932,0.79900551383932
"8574","LHPP",38.0979831332383,0.312029327115048,0.402424845241932,0.7
75372919451482,0.438119332308375,0.79900551383932
"8575","COX14",453.076115120177,-0.206559766561526,0.266317035710913,-
0.775616047280381,0.437975722472476,0.79900551383932
"8576","STK4",197.193015883921,0.138437678531894,0.178569881129979,0.7
75257717907796,0.438187388579102,0.79900551383932
"8577","RCAN1",85.6926630796732,-0.161874400694717,0.208625879106798,-
0.775907578617567,0.437803557535376,0.79900551383932
"8578","RRP1B",168.434182482401,-0.118878601741217,0.153292968907891,-
0.775499376051926,0.438044634017855,0.79900551383932
"8579","MT-
ND6",449.131858492384,0.24659468294831,0.318085485383966,0.77524657451
9555,0.438193971950874,0.79900551383932
"8580","HTATIP2",169.290968393963,0.277635350237865,0.358200573986398,
0.775083487857302,0.43829032797959,0.799088065336216
"8581","CEP104",74.0825846778137,0.248110161704126,0.320613102345919,0
.773861579232756,0.439012652225008,0.799270379238959
"8582","AL109936.2",2.93495797801537,0.800965072844227,1.0339685034975
6,0.774651326548956,0.438545719518015,0.799270379238959
"8583","FBXO48",39.0602671944791,0.198251363149186,0.256059039330805,0
.774240830033978,0.438788387145663,0.799270379238959
"8584","LONRF2",11.8196682455231,0.401957079019027,0.519557968760839,0
.773652033434704,0.439136592670367,0.799270379238959
"8585","MYD88",87.160101404758,-0.151545040289901,0.195881195842949,-0
.773657928918325,0.439133105382906,0.799270379238959
"8586","ZGRF1",9.40362991294571,0.364749259843271,0.470931393950127,0.
774527382393833,0.438618981764052,0.799270379238959
"8587","FGF18",137.903821562264,-0.27802251305578,0.358899284845231,-0
.774653293543541,0.43854455690237,0.799270379238959
"8588","AL589843.1",11.7404253875747,0.367485820649747,0.4746034544057
8,0.774300770966474,0.438752947872026,0.799270379238959
"8589","SART3",115.38008286672,0.144969061662653,0.187261322341571,0.7
74153786002985,0.438839853691714,0.799270379238959
"8590","AL358334.2",3.02744312602238,0.811022758136893,1.0481997091693
3,0.773729234078502,0.43909092832314,0.799270379238959
"8591","CHURC1",929.822902216513,-0.153397732929218,0.198251750431872,
-0.773752224608641,0.439077329902082,0.799270379238959
"8592","HSP90AA1",3624.24081103307,-0.118324183678175,0.15284106197071
8,-0.774164888365171,0.438833289002264,0.799270379238959
"8593","PRPSAP2",63.0357990599099,-0.210182876526171,0.271550284631725
,-0.774010886459649,0.438924353461052,0.799270379238959
"8594","IGFBP4",7624.89301909517,-0.234929951463987,0.303676957263341,

-0.773617970823717,0.439156741645391,0.799270379238959
"8595","DOK6",36.4749247660497,0.303957785399388,0.392461059817018,0.774491577689532,0.438640146903654,0.799270379238959
"8596","THBS3",307.233042310567,0.19783081497643,0.255817821397398,0.773326947652765,0.439328911533794,0.799304741000597
"8597","LHX4",3.40359181200646,0.828678473600247,1.07150179790495,0.773380385567731,0.439297294651978,0.799304741000597
"8598","TRMO",41.3253143443544,-0.21351913163679,0.27610142910581,-0.773335843745172,0.439323648013561,0.799304741000597
"8599","POLG",72.5793219382002,0.195627595347936,0.253034358351089,0.773126608665925,0.439447454998894,0.799427437905302
"8600","ZNF792",7.49362442838187,-0.450025264608832,0.582164866579909,-0.773020308237824,0.439510361950358,0.799448906045285
"8601","MUC1",16.7668429471951,0.458642548625999,0.593623694007286,0.772614963412107,0.439750286193959,0.799565410708495
"8602","LRPAP1",1013.2896699548,-0.165851407063197,0.214674685126792,-0.772570864446551,0.439776392974395,0.799565410708495
"8603","PSMC1",560.312114763868,-0.177804580685379,0.230110508097842,-0.772692138899529,0.439704600108383,0.799565410708495
"8604","C16orf95",33.541890660582,-0.307298996272914,0.397763715521591,-0.772566687914081,0.439778865545988,0.799565410708495
"8605","SLC25A32",85.7328223098722,-0.171992588177781,0.222726968855986,-0.772212673935281,0.439988476274364,0.799769486681706
"8606","FGFBP3",23.5719156676095,-0.260564518935373,0.337467187328683,-0.772118086495891,0.440044490946075,0.799769486681706
"8607","PBDC1",307.94966512441,-0.182497574629433,0.236334290710246,-0.772200995805474,0.439995391840239,0.799769486681706
"8608","DNAJC25",75.4430687044106,0.179723618610124,0.232804749763047,0.771992920217693,0.440118620691602,0.799811289902269
"8609","MRPL24",221.429589852382,-0.189707821986226,0.245902312993656,-0.771476362612011,0.440424627783453,0.799842658577612
"8610","CPEB2",45.1476492734229,0.268304698401863,0.347850305121213,0.771322302874992,0.440515915899157,0.799842658577612
"8611","NFX1",124.770727745147,0.183600586309723,0.238048661215985,0.771273341223034,0.440544930403676,0.799842658577612
"8612","CDK5RAP2",130.762118353369,0.184860159724774,0.239584251217035,0.771587275815191,0.440358912868535,0.799842658577612
"8613","SETD3",111.018157305546,0.139506114358964,0.180846132907866,0.771407782493404,0.440465263601197,0.799842658577612
"8614","SOX9-AS1",3.8886418080491,-0.567963364752804,0.736255818610829,-0.771421223976796,0.4404572989418,0.799842658577612
"8615","AC023421.1",2.74764997114362,-0.683672403909243,0.886216289793066,-0.771450955916058,0.440439681777152,0.799842658577612
"8616","P2RY11",5.77897133247831,-0.485450618033225,0.629010762097667,-0.771768381854567,0.440251621519352,0.799842658577612
"8617","PINK1-AS",4.52859624786157,-0.546298233727996,0.708812005761137,-0.77072373109901,0.440870702625899,0.799878116565755
"8618","UHMK1",147.475999546265,0.174451498233765,0.226463541981095,0.

770329284385777,0.441104589405903,0.799878116565755
"8619","IGKC",1075.81322053556,0.631682605376447,0.819976759971919,0.770366474042605,0.44108253480142,0.799878116565755
"8620","OTUD4",107.979089642751,0.18807276037553,0.244170706947126,0.770251119501637,0.441150945633555,0.799878116565755
"8621","DDX60",80.0175354219219,0.213749863241063,0.277324714030701,0.770756634467855,0.440851195818897,0.799878116565755
"8622","TMTC1",52.2083294874921,0.278687032794278,0.361739602326258,0.770407859692749,0.44105799258731,0.799878116565755
"8623","VPS29",641.649065977947,-0.198489085989085,0.257617611087287,-0.770479491488771,0.44101551587532,0.799878116565755
"8624","HNRNPA1L2",21.4208059112709,-0.321608870044455,0.417523137470101,-0.7702779587095,0.441135028140198,0.799878116565755
"8625","AC024075.1",17.754766909957,0.453193745712797,0.587826142085173,0.770965619366977,0.440727310386718,0.799878116565755
"8626","NAPA",232.893136695509,-0.200618876163043,0.260236034161771,-0.770911210698561,0.440759561711292,0.799878116565755
"8627","MBOAT7",107.019895712389,0.153173327824502,0.198873349060617,0.770205402322734,0.441178059817767,0.799878116565755
"8628","TSPYL2",121.562514719024,0.265932651218313,0.344989295730406,0.770843195744042,0.440799880211121,0.799878116565755
"8629","SLC35F6",314.66743593851,-0.165994206949229,0.215546121835683,-0.77010992141984,0.441234691211607,0.799888083743559
"8630","ATAD2B",114.037343565926,0.245513528832557,0.318923065630749,0.769820546993028,0.441406349680881,0.800106550180535
"8631","NR3C2",40.5197562279962,0.254354245389471,0.330451985768259,0.769716195828359,0.441468260736546,0.800126057548579
"8632","ZNF660",38.9098525262539,0.224493954877608,0.291856889240711,0.769191899021631,0.441779398824438,0.80025345454551
"8633","DVL2",108.027363343411,0.140602693848454,0.182760991980483,0.769325512653534,0.441700095388195,0.80025345454551
"8634","COX10-AS1",43.5341360538597,0.264290631303861,0.343482953387712,0.769443224757471,0.441630236708502,0.80025345454551
"8635","TVP23C",25.5408332104206,0.282107659190142,0.366770490962761,0.769166729988606,0.441794338263442,0.80025345454551
"8636","RRAGB",122.74546734104,0.164252151545387,0.21347829085056,0.769409155802014,0.441650454983305,0.80025345454551
"8637","PPP1R8",110.312387355778,0.127937850544514,0.166449963240028,0.768626487228614,0.442115076792217,0.800683433355257
"8638","ZRANB1",137.789139862891,0.177314994735294,0.230704404364239,0.768580882640397,0.442142158030269,0.800683433355257
"8639","MAGEH1",334.234066768532,-0.197606400196459,0.257129824005991,-0.76850828549494,0.44218527013719,0.800683433355257
"8640","TMEM150A",81.4419117952939,-0.194416741270428,0.253083801758507,-0.768191167982929,0.442373619848213,0.80070768417736
"8641","XRCC5",862.922986252994,-0.123442805431886,0.160721339372759,-0.768054857641437,0.442454594516979,0.80070768417736
"8642","PSMA2",790.143262961174,-0.209921571718652,0.273310055187497,-0.768071162162843,0.442444908428197,0.80070768417736

"8643","AC007032.1",24.7581966741462,-0.462544068757819,0.602089305661
465,-0.768231663323865,0.442349565369925,0.80070768417736
"8644","FBXL12",141.513789970218,-0.171657218561348,0.223433730798193,
-0.768269043121288,0.442327362207566,0.80070768417736
"8645","KLHDC1",69.9162724813181,0.177919350066579,0.23173411853442,0.
767773650215226,0.442621671563744,0.800824752286797
"8646","NOMO1",51.2721986829636,-0.271588880420725,0.353727326615826,-
0.767791629272935,0.442610988376967,0.800824752286797
"8647","NCAM1",3.40628002085337,-0.698368014155568,0.909847811830688,-
0.767565745693662,0.442745219497385,0.800955645726563
"8648","AC097662.1",10.4814067574562,0.37404699111126,0.48738790781218
9,0.767452341586193,0.442812618565578,0.800968718658457
"8649","C11orf68",186.111700039799,-0.175043862251882,0.22810546136471
5,-0.76738128585184,0.442854851861983,0.800968718658457
"8650","TSKS",33.0090711511688,0.257490849582679,0.335589680435998,0.7
67278806809993,0.442915766240973,0.800986281076017
"8651","LGALS8",134.694719119628,0.166124535026413,0.216579326496747,0
.767037822646976,0.443059028069406,0.800991738882951
"8652","TM9SF2",496.608467179311,0.107530550784597,0.14017817726298,0.
767099079786615,0.443022609017161,0.800991738882951
"8653","AC016876.1",94.1289689257552,-0.24709377071012,0.3221544100223
71,-0.767004153979953,0.443079045745581,0.800991738882951
"8654","ZNF227",44.4743946027447,0.201961170516789,0.263337432458315,0
.76692921561297,0.443123602141089,0.800991738882951
"8655","CTTNBP2NL",136.733622799574,0.145253135155931,0.18942658944882
,0.766804362463464,0.443197842249871,0.80103337334659
"8656","ZNF329",28.8159835082609,0.286256852979525,0.373393777640936,0
.766635305998045,0.44329837783732,0.801122519005222
"8657","NEMF",333.750882530054,-0.139555873265416,0.182089356083061,-0
.76641422797803,0.443429869599978,0.801267581165814
"8658","AC138696.2",5.87922060134151,-0.469626333602461,0.612901003001
708,-0.766235217926625,0.443536356695997,0.80136743217781
"8659","YTHDC1",380.337283707041,0.17744405473873,0.231736041256512,0.
765716259657317,0.443845150174931,0.801462505389177
"8660","MRNIP",167.044007191221,0.121120619893817,0.158149228924453,0.
765862854454226,0.443757910071998,0.801462505389177
"8661","TUSC3",244.466123560998,0.189318403663516,0.247222031437136,0.
765782897919663,0.443805491831186,0.801462505389177
"8662","SMU1",235.134886467496,-0.121118765074721,0.158138155247572,-0
.765904755149722,0.443732976327872,0.801462505389177
"8663","PPP1R9B",81.0772013414677,-0.154988047752963,0.202402801255548
,-0.765740626076015,0.443830648784672,0.801462505389177
"8664","LBP",10.4258159029903,-0.551195446434025,0.720072457687824,-0.
765472197345156,0.443990415944074,0.801632280310844
"8665","AKT2",256.817977207508,0.127982988000513,0.167225642023064,0.7
65331120587722,0.444074397140227,0.801691378472541
"8666","STXBP3",226.552549886445,-0.135289572588053,0.176931828329382,
-0.764642370259091,0.444484531624537,0.801826131235518
"8667","SNAP47",94.1597512881361,-0.178083799112941,0.233104197668755,
-0.763966504653002,0.444887203591046,0.801826131235518

"8668","MRPL33",541.474317139008,-0.189342639342697,0.247674990107581,-0.764480253983066,0.444581099436666,0.801826131235518
"8669","LIN54",36.5767845946856,0.249752543824603,0.326707102619944,0.764453976732603,0.444596753135332,0.801826131235518
"8670","AC026748.3",9.19081742910415,-0.407449207311638,0.532657531535143,-0.764936536497196,0.444309336169757,0.801826131235518
"8671","ANKH",152.49956997788,-0.11907314868401,0.155800262323118,-0.764267960198047,0.44470757458747,0.801826131235518
"8672","BAG2",93.0853988385096,0.208134200210393,0.272450104187374,0.763935109627455,0.444905913395481,0.801826131235518
"8673","CROT",76.9194892681058,-0.159633865996533,0.208991633338107,-0.763828979403579,0.444969164818483,0.801826131235518
"8674","CREB3L2",258.925930374827,-0.154714852112475,0.20241514551322,-0.764344247660907,0.444662123563588,0.801826131235518
"8675","DNAJA1",917.03736980211,-0.192080173093341,0.25139314547852,-0.764062889335037,0.444829766130827,0.801826131235518
"8676","AP000442.1",3.10593054946524,-0.606451795919449,0.793155470056485,-0.764606459659492,0.444505921461581,0.801826131235518
"8677","EIF2B1",247.366979688089,-0.140627824561881,0.184027668652112,-0.764166745098129,0.44476788124597,0.801826131235518
"8678","COG6",100.902277337764,0.186821544967295,0.244493467791381,0.764116713035067,0.444797693407563,0.801826131235518
"8679","AL160314.2",4.32152833073961,0.579948171345167,0.758954593707433,0.764140801246311,0.444783340036536,0.801826131235518
"8680","MTHFS",147.09035175631,-0.181223749538464,0.237059308795616,-0.764465864931331,0.444589671142323,0.801826131235518
"8681","LSR",15.0498005001933,-0.387534956073204,0.507315177425109,-0.76389387370618,0.444930488544359,0.801826131235518
"8682","SNHG6",1322.93045450361,-0.198474556014534,0.259934335980588,-0.7635565161709,0.44513157076095,0.801934027572676
"8683","AP003392.5",3.55246514434982,0.617956502636764,0.809242152447485,0.763623719757808,0.445091509879395,0.801934027572676
"8684","ALG5",317.272282295311,-0.222138288836049,0.291309753156395,-0.762550125524941,0.445731738412745,0.802922798709186
"8685","AC104248.1",13.1308522424836,-0.477519104989362,0.62645241320505,-0.76225918349693,0.445905329463186,0.802958106226848
"8686","UBC",7727.4914317139,-0.18676877145054,0.244998894616812,-0.762324955558081,0.44586608308387,0.802958106226848
"8687","ARMCX4",57.2360095251087,0.215756689694855,0.282996758887901,0.762399861195299,0.445821389069884,0.802958106226848
"8688","DBP",169.180756069406,-0.407362824698557,0.534496568552315,-0.762143012071901,0.44597465409584,0.802990505757508
"8689","AJ009632.2",3.38157666509891,0.865124391033863,1.13533554195339,0.761998862068019,0.446060683318891,0.803052971476281
"8690","AC078802.1",3.52813390064194,-0.67890286214132,0.891143790287476,-0.761833128997411,0.446159605069576,0.803138630851943
"8691","C5orf56",206.627372352273,0.141066993336765,0.185228613642036,0.761583162358406,0.446308827268289,0.80316657157179
"8692","INVS",48.6657691680628,0.212468012729403,0.278982830095869,0.761580964163246,0.446310139647485,0.80316657157179

"8693","AC068205.2",10.8579594451373,0.44862632911701,0.589097042880806,0.761549110691736,0.44632915723797,0.80316657157179
"8694","GALM",120.225947706672,0.149211019586075,0.196040736183755,0.761122522240553,0.446583889302482,0.803532525921179
"8695","B3GNT2",31.1354587649016,0.236085865246284,0.310363960920134,0.760674224373096,0.446851674021585,0.803628948550032
"8696","MRM2",96.4641918336203,-0.192320720244359,0.25278263365673,-0.760814607642405,0.446767808135074,0.803628948550032
"8697","AC007938.3",15.5824023800236,0.616778683527813,0.810907716462516,0.760602804741375,0.446894344015644,0.803628948550032
"8698","MYOM2",14.8487414022628,0.355338055236137,0.467081471589719,0.760762472608341,0.446798952901139,0.803628948550032
"8699","BAHCC1",44.7944134876217,-0.222519300292062,0.292556390885698,-0.760603108407229,0.446894162584148,0.803628948550032
"8700","ORMDL1",461.339096107038,0.13013420199384,0.171125343300196,0.760461305638129,0.44697889016429,0.803688595268964
"8701","ADPRHL2",139.704968342644,-0.186259832953014,0.245123473800673,-0.759861265284102,0.447337517203592,0.803809123898064
"8702","CMC1",261.566771795018,-0.182200114888902,0.239683858635297,-0.760168481625364,0.447153882305692,0.803809123898064
"8703","EXOSC7",240.746100838183,-0.182446954247518,0.240004492607425,-0.76018141271191,0.447146153843563,0.803809123898064
"8704","AL590062.1",19.4090274647666,-0.277726021529179,0.365481450706653,-0.759890880897513,0.447319812957084,0.803809123898064
"8705","KCNQ1",3.01108388909748,-0.734712809339274,0.96693946181663,-0.759833307411963,0.447354230816119,0.803809123898064
"8706","PLBD1-AS1",16.0311131411002,0.351316182026927,0.462349017350526,0.759850608183686,0.447343888127507,0.803809123898064
"8707","KLC4",30.8982773326064,0.269550736580133,0.354836714563403,0.759647256095788,0.447465463974913,0.803916647864886
"8708","RBBP4",373.404081057097,0.111450137892704,0.14681084121843,0.759141061843553,0.44776817825536,0.803990093391306
"8709","PPIE",230.555783269371,-0.170387310745065,0.224470504388855,-0.759063250688382,0.44781472119916,0.803990093391306
"8710","PRICKLE2",193.588043683005,0.192275824500946,0.253302826843024,0.75907492584006,0.447807737501675,0.803990093391306
"8711","HERC3",86.5918153983793,0.18322033260747,0.241278579905007,0.759372558805695,0.447629724005471,0.803990093391306
"8712","CCDC180",30.6847387896156,0.30760357736897,0.405178883888251,0.759179684827327,0.447745076838653,0.803990093391306
"8713","FOSL1",3.6673461110691,-0.67859585133043,0.893489015234356,-0.75948986474382,0.447559574683424,0.803990093391306
"8714","AC245014.3",3.02467640153457,0.640163845853695,0.843590776385754,0.758855909492499,0.447938756294203,0.80412049170418
"8715","CYBRD1",3201.02133957386,0.147927078466599,0.194985828508585,0.758655537164259,0.448058641036438,0.804143647284842
"8716","CDK5",27.3434137363835,-0.285256506088662,0.375981060304658,-0.758699137285049,0.448032553102216,0.804143647284842
"8717","TMEM101",161.90384253892,-0.206929069782208,0.272816885034839,

-0.758490698828201,0.448157279104344,0.804143647284842
"8718","PACSIN2",84.2225728518829,0.147423082643538,0.194349236319196,
0.758547270036156,0.44812342591263,0.804143647284842
"8719","PPIA",2685.94444167611,-0.195467985326515,0.25779890715906,-0.
758218828313002,0.44831999139907,0.804251103836658
"8720","AC060780.1",98.0864804878047,0.22112297657585,0.29162731539601
9,0.75823822015977,0.448308384425261,0.804251103836658
"8721","THRAP3",739.990031259299,-0.108573721280656,0.143219872894457,
-0.758091171891117,0.448396404304102,0.804295946855758
"8722","S100A2",8.32297418374413,-0.451141089910245,0.59526889630406,-
0.757877814062378,0.448524133083393,0.80443281515977
"8723","ATP5MC3",1412.08144164662,-0.237123880726467,0.312918877905171
,-0.757780682053724,0.448582288965614,0.804444886654717
"8724","AC024941.2",13.5714714520903,-0.339108259301451,0.447721927067
555,-0.757408200939608,0.448805344397081,0.804660401421609
"8725","CHODL",3.80317413612079,-0.701969096795423,0.926794096146451,-
0.757416452817474,0.448800402186459,0.804660401421609
"8726","EXOC8",39.4921062747101,0.221633589169852,0.292735380040058,0.
757112410326089,0.448982519562405,0.804701369559429
"8727","RNF122",14.4940985269842,-0.429570931236679,0.567310577459804,
-0.75720592617915,0.44892650035521,0.804701369559429
"8728","NOXA1",7.12724035867647,-0.551143198460828,0.727895213103955,-
0.757173819169101,0.448945733109604,0.804701369559429
"8729","ZAP70",6.06515261640215,0.533279047081029,0.704556027880611,0.
756900836808105,0.449109274002645,0.804770191780915
"8730","SUCLG2",361.75243529243,-0.125080766049223,0.165357318072781,-
0.756427157304097,0.449393130659756,0.804770191780915
"8731","FAM53A",2.68766300552356,0.699818002884641,0.925205917707796,0
.756391619952502,0.449414430836418,0.804770191780915
"8732","SMCO4",76.5139318195501,-0.200452645129181,0.26494366151281,-0
.756585924662666,0.44929797657231,0.804770191780915
"8733","GADD45GIP1",815.378377695444,-0.200956777215276,0.265510277857
333,-0.756870049766043,0.449127720320004,0.804770191780915
"8734","SUPT5H",191.45105707152,0.163655172972404,0.216371634378358,0.
75636149554719,0.44943248708036,0.804770191780915
"8735","PPIL2",94.5255065160972,0.165671309946118,0.219024762287417,0.
756404473247253,0.449406726835265,0.804770191780915
"8736","HCCS",46.4518635192306,-0.220217617968545,0.291117234193816,-0
.756456822552566,0.449375350509502,0.804770191780915
"8737","TBX15",257.708175750502,-0.155207737189756,0.20525393171351,-0
.756174246671156,0.449544731265238,0.80484696186537
"8738","MCCC2",70.4094932892583,0.187811225316435,0.248388673599968,0.
756118314874962,0.449578262020048,0.80484696186537
"8739","TMEM99",125.049992701676,-0.245772402584889,0.325137062178808,
-0.755903989960171,0.449706761566174,0.804984880556089
"8740","ASCC3",128.102366704591,0.140401187773778,0.185865867966301,0.
755389837359669,0.450015109136897,0.805260392613645
"8741","HEATR3",22.4315258228206,-0.252358130299929,0.334020515690235,
-0.755516857335677,0.449938921581638,0.805260392613645
"8742","AL121672.3",3.9683779826531,-0.658441596175037,0.8716035987300

88,-0.755436986646654,0.449986827779949,0.805260392613645
"8743","PDP1",165.701564432067,-0.176849613403978,0.234146312025147,-0
.755295318873032,0.450071806793674,0.805269732777473
"8744","ZNF496",55.0462788817873,0.198952767541909,0.263489979176442,0
.755067680993983,0.450208373776928,0.805421956883861
"8745","FAM41C",3.45194662142259,0.640688090312455,0.849911752139564,0
.753828957770721,0.450951933400642,0.805443312136862
"8746","PAQR7",41.9526113106179,-0.305696052838742,0.405548448602123,-
0.753784298503519,0.450978753679332,0.805443312136862
"8747","TMEM35B",994.051020117741,0.189267009109358,0.251153919003329,
0.753589710486855,0.451095624728699,0.805443312136862
"8748","MDM4",398.616176697996,0.211331928258075,0.280211911983138,0.7
54186097094945,0.450737484617007,0.805443312136862
"8749","MRPL35",125.323578854015,-0.158513052709645,0.21002720900463,-
0.754726273138022,0.450413238883,0.805443312136862
"8750","MFSD9",36.9023570537469,0.251674163780798,0.333806523624347,0.
753952202755698,0.45087792287912,0.805443312136862
"8751","DZIP1L",42.6139581027791,0.262740857024711,0.348122108772434,0
.754737634881052,0.450406420309243,0.805443312136862
"8752","PDLIM5",258.543136989313,0.113845678593269,0.1509471787202,0.7
54208720947983,0.450723901785918,0.805443312136862
"8753","GTPBP2",59.145929324758,0.199403067740185,0.264318855149249,0.
754403493566857,0.450606974457629,0.805443312136862
"8754","AFDN-
DT",22.60518975397,0.35640701881501,0.472286126826152,0.75464215137998
9,0.450463725069632,0.805443312136862
"8755","AHR",377.217291462201,0.177548063350691,0.235392673979118,0.75
4263335172621,0.450691113640573,0.805443312136862
"8756","SRGN",219.270285584741,-0.259057206634116,0.34330535102087,-0.
754597054382551,0.450490791627625,0.805443312136862
"8757","TSPAN8",221.816333954296,0.521680422973596,0.692209936009801,0
.753644806055211,0.451062532169976,0.805443312136862
"8758","RNF6",102.786496726692,-0.141259546693979,0.187346448325197,-0
.754001732921995,0.450848181184595,0.805443312136862
"8759","AC020765.2",7.62921035075323,-0.407103898375576,0.539447612492
779,-0.754668088147346,0.4504481586186,0.805443312136862
"8760","MLYCD",92.9205072156832,0.194693909830419,0.258093994807692,0.
754352730971081,0.450637446976563,0.805443312136862
"8761","OSBPL7",8.88237290241529,0.459764426215876,0.610046721170528,0
.753654450160313,0.451056739683603,0.805443312136862
"8762","PLOD3",192.665710516206,0.122284257111934,0.16232921343543,0.7
53310230019538,0.451263512818095,0.805467267345215
"8763","TMX4",477.258906072263,-0.0909817514166915,0.120763559265237,-
0.753387461998078,0.451217114880661,0.805467267345215
"8764","PIGU",65.4467787252136,0.195789986877588,0.259870239635927,0.7
53414423875109,0.45120091788028,0.805467267345215
"8765","HS3ST1",2.76978122633498,0.707203879797359,0.939318046238843,0
.752890762217439,0.451515559731748,0.80553384403089
"8766","NHLRC1",11.8937718052923,-0.337000089342711,0.447656549145646,
-0.752809469638894,0.451564415464525,0.80553384403089

"8767","BCAP29",414.584239952251,-0.115148225724254,0.152936266468858,-0.752916416641447,0.451500142392943,0.80553384403089
"8768","EXTL3",32.4277026605923,0.219600872961763,0.291737687455273,0.752733988115373,0.451609781509359,0.80553384403089
"8769","ANAPC2",50.9890700329585,-0.196314418870543,0.26068467243568,-0.753072349963269,0.451406438768679,0.80553384403089
"8770","DOCK9",116.925047500837,0.210332247418739,0.279413590551821,0.752763124382563,0.451592269670713,0.80553384403089
"8771","AC108863.2",35.1285645699891,0.278624285262756,0.370295357942491,0.752437964145442,0.451787723259543,0.805759360956452
"8772","EID1",4136.01568404365,-0.152518484556969,0.202837821763821,-0.751923301239931,0.45209718452256,0.806211825284385
"8773","ELP2",202.508145494837,0.117563711159952,0.156367027836115,0.751844636218119,0.45214449550725,0.806211825284385
"8774","POT1",51.2312089842128,0.172052856948632,0.22889307472979,0.751673492750892,0.45224743485888,0.80630346746039
"8775","YARS",83.2889039228109,-0.160881352301706,0.214136625420405,-0.751302361218474,0.45247070850398,0.806332410009567
"8776","PPIL4",367.764858480523,-0.128024828475006,0.170365529267456,-0.751471433367372,0.452368986559894,0.806332410009567
"8777","LAT2",4.24609045682592,-0.627832258492882,0.835752284427262,-0.751218118324539,0.452521397907945,0.806332410009567
"8778","RNF166",63.6141049752001,-0.193380749105249,0.257323108571169,-0.751509455093361,0.452346112638993,0.806332410009567
"8779","SLC39A3",170.981894227023,-0.205209263152458,0.273165303911173,-0.751227407779383,0.452515808235457,0.806332410009567
"8780","CCDC17",12.461914990403,0.335945042050818,0.447349837435979,0.750967171411782,0.452672413030237,0.806388247400989
"8781","AL359644.1",8.24484627348653,0.433649757657946,0.577402358756129,0.751035653183229,0.452631199166995,0.806388247400989
"8782","TMBIM4",1766.93694852097,-0.188953740788714,0.251662022710682,-0.750823420846224,0.452758932233132,0.806388247400989
"8783","CSTF2",16.3920334109532,-0.301190144430164,0.40113455026468,-0.750845680661091,0.452745534099514,0.806388247400989
"8784","MARCH5",162.211135226145,-0.129050156158013,0.171931621278668,-0.75059000315508,0.452899439312878,0.806546667710765
"8785","THPO",5.30321404995264,0.544562760228417,0.725614074083966,0.750485388415168,0.45296242072829,0.806561611935934
"8786","EIF3J",294.582760512208,-0.152112851782849,0.202747489443707,-0.750257634263251,0.453099553104637,0.806561611935934
"8787","CYTH1",90.6381851221593,0.301413823453822,0.401668855408468,0.750403770158645,0.453011560960019,0.806561611935934
"8788","GRAMD1A",130.595545295915,-0.142124970860438,0.189440976497769,-0.750233521215577,0.453114073112126,0.806561611935934
"8789","AC012511.1",13.7254749747169,0.332257838099396,0.443009194500698,0.750002126872048,0.453253423793184,0.806717864193512
"8790","CCDC28A",128.50030649611,-0.174769190097366,0.233147409663177,-0.749608114239192,0.45349076237977,0.80704846369815
"8791","PARK7",2071.44779696298,-0.21047686219993,0.280835764308662,-0.74946601875322,0.45357637262883,0.807108997501171

"8792","PCSK4",2.67805794124779,0.826337880211843,1.10272607791523,0.7
49359153430095,0.453640763277421,0.807131762960498
"8793","HIST1H1A",4.9905791135909,-0.611388059390357,0.816272946240413
,-0.748999537723608,0.45385748405341,0.807241912796758
"8794","DMAC1",440.825545182238,0.180863952282407,0.24145996082153,0.7
49043243720595,0.453831141715561,0.807241912796758
"8795","PGBD4",9.86210518391886,0.338932214360101,0.452480523493284,0.
7490537089717,0.453824834261063,0.807241912796758
"8796","AC008115.3",2.69212217847309,-0.772757731549939,1.031847464716
79,-0.748906944072434,0.453913294649539,0.807249393838419
"8797","SEMA5A",43.5625693253825,0.705819854175776,0.942717781514248,0
.748707479604391,0.454033534516836,0.807371442588026
"8798","SMAP1",312.386861413401,-0.143597403982927,0.191840637950905,-
0.748524429009018,0.4541438956829,0.807384130033823
"8799","ZNF682",27.4935145923572,0.284990546685736,0.38070716210231,0.
748582046925474,0.454109156222048,0.807384130033823
"8800","HSD17B1",14.9716398153481,-0.370304729966123,0.494901542364256
,-0.748239191571508,0.454315895416224,0.80750636882127
"8801","TSPAN7",7.78651205763579,-0.501881295972069,0.670747139471801,
-0.748242171211182,0.454314098487457,0.80750636882127
"8802","INO80B",66.2222758983488,-0.202904201669225,0.271273555780923,
-0.747968968391033,0.454478875335841,0.807678233233733
"8803","HAVCR2",5.43572972026188,-0.539545004967855,0.721571051321718,
-0.747736489677015,0.454619116769355,0.807678233233733
"8804","MAP2K4",83.4943217830721,0.152403572434274,0.203796127553223,0
.747823691568782,0.454566509877211,0.807678233233733
"8805","SF3A2",138.74586695067,-0.250842005830119,0.33544336830643,-0.
74779241305785,0.454585379087641,0.807678233233733
"8806","TALDO1",691.053029369274,-0.203317587618446,0.272023827089401,
-0.747425656766548,0.454806663007318,0.807685704368678
"8807","TRAF6",46.7291050390436,0.190345167635566,0.254722253751286,0.
747265560163508,0.454903276974608,0.807685704368678
"8808","PLD6",18.765121328194,0.328557148817802,0.439629679694931,0.74
7349790045558,0.454852445079268,0.807685704368678
"8809","FAM210B",408.820541133038,0.1261432422839,0.168817505358517,0.
74721659946349,0.454932825740142,0.807685704368678
"8810","PRR34-
AS1",27.0168059853864,-0.299522164761423,0.400850781530954,-0.74721611
7722085,0.454933116486123,0.807685704368678
"8811","ATP6AP2",1339.63826263661,-0.134227676656835,0.179618859469121
,-0.747291665549803,0.454887522291689,0.807685704368678
"8812","PPID",167.230480001674,-0.167888109372647,0.224750648296239,-0
.746997219564668,0.455065239187451,0.807697989123056
"8813","FAM133B",589.720664888883,-0.126043610141877,0.168706785913354
,-0.747116421307509,0.454993288637539,0.807697989123056
"8814","RPS6KA5",177.881738053378,-0.280801218547176,0.375931402765699
,-0.746948024244166,0.455094935506656,0.807697989123056
"8815","EXTL2",177.899641469935,0.193731096615238,0.259439574715098,0.
746729163536376,0.455227062059079,0.807840831740236
"8816","PDE1A",91.503250382271,-0.266544033179213,0.357097681725447,-0

.746417708150074,0.455415125395855,0.808052786457883
"8817","TMEM128",174.180666255486,0.17360847615497,0.232660126261013,0
.746189211468943,0.455553124322193,0.808052786457883
"8818","C5",22.101581833903,0.244719525081673,0.327942834162187,0.7462
26169895956,0.455530801950842,0.808052786457883
"8819","PNPO",50.4520869886418,0.182921436433807,0.245102340778277,0.7
46306362693126,0.455482368743667,0.808052786457883
"8820","FAS",145.38489039715,-0.136244185103723,0.182703279982542,-0.7
45712858120235,0.455840890157645,0.80819665020243
"8821","GOLGA8R",13.8885418740063,0.354038895437837,0.47467988975269,0
.745847681944756,0.455759432490476,0.80819665020243
"8822","AC005332.1",3.11647227199919,-0.590271942653387,0.791550079723
993,-0.745716484368507,0.455838699148922,0.80819665020243
"8823","LINC00654",16.7836087827537,0.299367929470389,0.40133810535809
4,0.745924509718003,0.455713018458867,0.80819665020243
"8824","CASZ1",4.36111048758815,0.715649133738271,0.959968708131683,0.
745492147479565,0.455974256477365,0.80824354305218
"8825","EXO5",22.5266384735487,0.26551587019665,0.356199879584127,0.74
5412577080729,0.456022342963533,0.80824354305218
"8826","RNF113A",68.5840436573837,-0.191816881100747,0.257294129164694
,-0.745515965418567,0.45595986322466,0.80824354305218
"8827","FAAH",12.787256829072,0.377322375411455,0.506689629514551,0.74
4681464613672,0.456464306953205,0.808332495503003
"8828","CENPF",26.3103701545988,-0.294003264208503,0.394922052734868,-
0.744458968985161,0.456598855314351,0.808332495503003
"8829","MORN2",173.935240215376,-0.219955918227936,0.295388589433481,-
0.744632413356875,0.456493967491262,0.808332495503003
"8830","SS18L2",240.022553284833,-0.201764847333231,0.271109686044795,
-0.744218512723643,0.45674428995404,0.808332495503003
"8831","YTHDC2",111.869983994396,0.171142822098969,0.229914235566501,0
.744376796318329,0.456648552702614,0.808332495503003
"8832","NSMAF",95.9974819574401,0.147304119985524,0.197687903951272,0.
745134715080151,0.456190284652857,0.808332495503003
"8833","SURF6",103.662817126887,-0.16895671917228,0.226900101432196,-0
.744630425926756,0.456495169282423,0.808332495503003
"8834","ZBED5-
AS1",47.2873630403784,-0.243641877519929,0.327159040170092,-0.74471999
121057,0.456441011274232,0.808332495503003
"8835","TDRD3",147.543137823856,0.168471402952181,0.226196544995744,0.
744800955979903,0.456392056902298,0.808332495503003
"8836","AL049840.3",2.60952474139049,0.700262604615854,0.9407330829087
03,0.744379694238747,0.456646800011224,0.808332495503003
"8837","UNC13D",6.83203983174165,0.467056971502517,0.62749309607635,0.
744322088040452,0.456681641528258,0.808332495503003
"8838","CEBPA-
DT",18.559786428284,-0.322512877029674,0.433312848940938,-0.7442956695
5591,0.456697620518205,0.808332495503003
"8839","GPRASP1",63.7771081317729,0.227524753510957,0.305533153353724,
0.744681063293795,0.456464549620746,0.808332495503003
"8840","TBX21",3.7520300184961,0.736247699496114,0.989493223377451,0.7

44065428748535,0.456836892963283,0.808404922695095
"8841","ZCCHC17",283.471330716809,0.133114401638741,0.178925929994473,
0.743963726458498,0.456898420179787,0.808411641889429
"8842","LINC00571",4.30173474210098,0.498913772945143,0.67068371109257
,0.743888310828948,0.456944047662618,0.808411641889429
"8843","FAM102A",132.563301977377,0.162203525831346,0.218123260521083,
0.743632409692815,0.457098890413621,0.808522763373952
"8844","RPS6KA3",222.192600767749,0.192920717108675,0.259436753706018,
0.743613672129447,0.457110229449545,0.808522763373952
"8845","ZFP41",19.211366745935,0.395264962121863,0.531729573156243,0.7
43357116241714,0.457265500128112,0.808705960260492
"8846","ADK",305.850788696665,0.151607930894841,0.203991443669403,0.74
3207304030569,0.457356181958364,0.808707308200455
"8847","RAB11B",377.431584909839,-0.163355357837178,0.219804420790832,
-0.743185042636736,0.457369657715874,0.808707308200455
"8848","DCTN6",322.215715110067,-0.185249091879295,0.249303077720389,-
0.743067809564166,0.457440627501437,0.808741380651558
"8849","CXCR5",3.77599596202949,0.657754664564604,0.885536848652532,0.
742775035918007,0.457617891882651,0.808963349838435
"8850","AC124017.1",4.49044182935135,-0.632850506200943,0.852526844560
249,-0.742323259659209,0.457891502524248,0.808971532797808
"8851","WWP1",172.006998525902,-0.168400655197341,0.22689176103595,-0.
742207008436321,0.45796192295611,0.808971532797808
"8852","MRPL13",239.91340485891,-0.226775867359524,0.305561621680886,-
0.74216083195277,0.457989896545139,0.808971532797808
"8853","LOXL1",442.554814012921,0.267587671621442,0.360366581684738,0.
742542969357624,0.457758427600773,0.808971532797808
"8854","AC145285.6",3.34771812696079,0.640794574628925,0.8630516205302
97,0.742475373877628,0.45779936687405,0.808971532797808
"8855","SPAG5",6.09933325442897,0.483284483080675,0.651148365077628,0.
742203327229516,0.457964152986084,0.808971532797808
"8856","OSBPL1A",837.577313505243,0.123670136417614,0.166619289497039,
0.742231807559184,0.457946900110146,0.808971532797808
"8857","GET3",419.819672461692,-0.242887100332246,0.327303902614177,-0
.742084339332058,0.458036237677567,0.808971532797808
"8858","DTYMK",171.327160290116,-0.203616138934963,0.274533372475853,-
0.741680827721124,0.458280738585731,0.809074744274804
"8859","SAMD4A",141.610127755399,0.223092063750697,0.300806479565223,0
.741646470093158,0.458301560379661,0.809074744274804
"8860","CDC42BPB",254.454585077619,0.191148368032875,0.257691655620741
,0.741771663395257,0.458225691890408,0.809074744274804
"8861","RGS9",198.601247557656,-0.216926643890723,0.292429344201988,-0
.741808741809736,0.458203223324193,0.809074744274804
"8862","DCAF13",158.117363456222,-0.150226647298872,0.202649737122136,
-0.74131182913073,0.458504390986929,0.809158865998254
"8863","CDK8",31.7089674067068,0.210083772318072,0.283360368218876,0.7
4140139511605,0.45845009884754,0.809158865998254
"8864","DUT",880.127551240271,-0.173790302282854,0.234421163786529,-0.
741359267549377,0.458475634830813,0.809158865998254
"8865","PRPSAP1",159.425933033129,-0.208194450733209,0.280914288281077

,-0.741131581476886,0.458613662485568,0.809260408602565
"8866","CHID1",495.127295999916,0.225981157569694,0.304953748293397,0.741034202184251,0.458672702788288,0.809273301344145
"8867","YIPF3",625.544662481512,-0.175361871372032,0.236673859751974,-0.740943133964205,0.458727920592458,0.809279447595333
"8868","PTEN",646.623865388235,-0.156472578676532,0.211268821309552,-0.740632610655162,0.45891622960131,0.809520363064197
"8869","EEF1AKMT2",167.113774636966,-0.149766017224428,0.202248208723248,-0.74050602559039,0.458993006340607,0.809564505376719
"8870","FDXACB1",12.61614101559,0.318819023741284,0.430764159495127,0.740124304015805,0.459224572786007,0.80982872582878
"8871","AC130371.2",6.79720807375398,0.456857975571043,0.617301888379068,0.740088414067024,0.459246348323666,0.80982872582878
"8872","NDUFB8",1013.1187863809,-0.225395471981177,0.304638860333678,-0.739877610276954,0.459374261153287,0.809871696970682
"8873","ZNF570",60.7026365911736,0.207778361146032,0.280825961974535,0.739883021089312,0.459370977697547,0.809871696970682
"8874","MAP10",20.8113028828258,-0.276128122728849,0.37339798347555,-0.739500840788365,0.459602929373697,0.810090758856704
"8875","SLC22A18",52.4072218852001,-0.25231493753874,0.34123177611135,-0.739423920052526,0.459649621793198,0.810090758856704
"8876","TAF1D",482.570253113958,0.131401394365637,0.177709479080608,0.73941691262307,0.459653875574513,0.810090758856704
"8877","BACH1",128.984189412962,0.175149126233368,0.23702768459525,0.738939531609795,0.459943716245912,0.81051025720793
"8878","RALGDS",191.19159461662,-0.163550855511228,0.221389348372119,-0.738747625908025,0.460060260124751,0.810624312810484
"8879","SLC35E2A",93.719506457092,0.347488830378899,0.47101782160095,0.737740302900736,0.46067227595945,0.810938943560835
"8880","PRDM16-
DT",3.1740324813886,-0.692777986902466,0.938904123919957,-0.737858072249266,0.460600699741689,0.810938943560835
"8881","PHACTR4",187.525158612135,-0.143916671879566,0.19509021286537,-0.737692935825957,0.460701065815497,0.810938943560835
"8882","ACTL6A",152.531585804758,-0.191301211746001,0.259141084867997,-0.738212591196983,0.460385272686075,0.810938943560835
"8883","ETF1",170.027244579331,-0.132675304463549,0.179853401180645,-0.73768582408008,0.460705388443722,0.810938943560835
"8884","OSBPL3",6.01587663195827,-0.476729684698876,0.645955562746686,-0.73802241546115,0.460500827903935,0.810938943560835
"8885","IL10RA",12.9024219365475,-0.464732700025165,0.62995141334262,-0.737727847230664,0.460679846457147,0.810938943560835
"8886","AC048382.5",14.3897759460316,-0.312456401066634,0.423310942438761,-0.738125027589703,0.460438476363647,0.810938943560835
"8887","CLASRP",73.8586693347351,-0.159644969919663,0.216245218637168,-0.738258958629401,0.460357101211027,0.810938943560835
"8888","THYN1",305.890190092363,-0.216687523606337,0.293858460324569,-0.73738739176338,0.460886800624482,0.811089520983757
"8889","PPCDC",51.5705548651766,-0.213442466914365,0.289462769153641,-0.737374507742217,0.46089463351177,0.811089520983757

"8890","TNFRSF14-
AS1",5.63875425945989,0.457578019288552,0.622040351385014,0.7356082580
00826,0.461969135532749,0.811195034893538
"8891","METTL18",49.8858999814566,-0.248074032075216,0.337134274868328
,-0.735831538256099,0.461833225316285,0.811195034893538
"8892","MEMO1",28.7081756357366,0.269717928152857,0.365967553346649,0.
736999566454398,0.461122613015443,0.811195034893538
"8893","STEAP3",5.5420683058374,0.53746332972645,0.729113755956017,0.7
37146056203159,0.461033533808996,0.811195034893538
"8894","AC135507.1",11.3181495159542,0.355232473960185,0.4828718267442
61,0.735666183623348,0.461933874184829,0.811195034893538
"8895","NDNF",100.284340557687,-0.243310029537464,0.330171964476305,-0
.736919107966616,0.461171543249392,0.811195034893538
"8896","MARCH1",8.89409485712524,-0.470747995551266,0.639154521810256,
-0.736516725592399,0.461416292645982,0.811195034893538
"8897","MIR4458HG",115.676753379635,0.189022985179408,0.25674052366292
8,0.736241332231503,0.461583842691805,0.811195034893538
"8898","OTULINL",21.562565951735,0.279923000902128,0.380174011685026,0
.736302304466945,0.461546744094667,0.811195034893538
"8899","TBC1D22B",19.5137138009292,0.265958983475559,0.361009022434277
,0.736710073566036,0.461298679533007,0.811195034893538
"8900","SIM1",65.6661811441584,0.43586585869453,0.591399037580108,0.73
700806223495,0.461117446538723,0.811195034893538
"8901","LSM5",411.210087127901,-0.194961322780651,0.264983746132558,-0
.735748232207122,0.461883930920077,0.811195034893538
"8902","TMEM67",65.8068958353007,-0.17573631892982,0.238911380798589,-
0.735571149195158,0.461991725747397,0.811195034893538
"8903","UROS",232.217373810795,-0.172085165245174,0.233800600314489,-0
.736033889620899,0.461710073757402,0.811195034893538
"8904","THUMPD1",333.409748870629,-0.112708015218124,0.153212746503062
,-0.735630799594543,0.461955413499408,0.811195034893538
"8905","NMT1",514.252107078133,0.152525620062034,0.207060502433266,0.7
36623442277177,0.461351375068931,0.811195034893538
"8906","H3F3B",7101.02750479216,-0.17930651096285,0.243653384281653,-0
.735908148747813,0.461786597817542,0.811195034893538
"8907","TANGO2",47.5907308506619,-0.198750250325066,0.270197189016089,
-0.735574826106839,0.461989487377178,0.811195034893538
"8908","LMF2",354.292882922009,-0.155666887491618,0.211422750539153,-0
.736282576471308,0.461558747424456,0.811195034893538
"8909","ZNF449",38.2129569525027,0.201281712272215,0.273196417087722,0
.736765563830893,0.461264928030403,0.811195034893538
"8910","RUNX2",22.4787698970761,0.331699705232394,0.451038543286606,0.
735413215055593,0.462087876026981,0.811272799628514
"8911","MIR3936HG",26.8314002588367,-0.255102132747703,0.3471681197223
66,-0.734808636667763,0.4624560464508,0.8114381092316
"8912","ADIPOR2",111.579148387336,0.148617917826776,0.202180366013884,
0.735075916405109,0.4622932607875,0.8114381092316
"8913","LGMN",734.920495737025,-0.107509841067557,0.14629754945292,-0.
734871099820815,0.462418000652098,0.8114381092316
"8914","ZNF598",57.9209613712217,-0.173363541845837,0.235828545483046,

-0.735125349184246,0.462263157453596,0.8114381092316
"8915","AC139099.2",5.79081036082874,-0.514379872326366,0.699881296255064,-0.734953020003132,0.462368106382115,0.8114381092316
"8916","PLA2G4C",54.8090278471846,0.365081259840661,0.496879873256684,0.734747530520448,0.46249326739813,0.8114381092316
"8917","CLN3",257.878336243261,-0.150166566130177,0.204403335207279,-0.734658101238408,0.462547743532349,0.811442677142147
"8918","SVBP",493.972570623115,0.207538582734757,0.282581254502269,0.734438606341067,0.462681464726254,0.811586247220541
"8919","MROH6",20.6769716993574,-0.362075533239241,0.493114110214442,-0.734263177100701,0.462788355638585,0.811594417923795
"8920","TPCN2",27.2716005541851,-0.21395135559176,0.291383382294542,-0.734260663415214,0.462789887354104,0.811594417923795
"8921","FBP1",17.1544309265514,-0.369621032128068,0.503702920741304,-0.733807601480837,0.463066007042749,0.811746032924759
"8922","PARPBP",15.0773303780465,-0.299317849915669,0.407913231707596,-0.733778231862381,0.46308390959666,0.811746032924759
"8923","ERG28",123.25934705857,-0.185585392702463,0.252834140712873,-0.734020303504898,0.462936363845583,0.811746032924759
"8924","GHDC",104.152758700745,0.158398428357867,0.215850579911956,0.733833693763883,0.463050102546093,0.811746032924759
"8925","SMIM15",298.366990188299,-0.150639089607474,0.205321869400126,-0.733672891482947,0.463148124085189,0.811767630819564
"8926","NCKIPSD",45.939272680331,-0.201187570617621,0.274448214863278,-0.733062048582996,0.463520585900627,0.811783757864253
"8927","AC110772.2",7.88065206714467,-0.415203842790139,0.56629240341933,-0.733196914320405,0.463438337086258,0.811783757864253
"8928","AP003469.4",11.8243948239236,0.382460130644666,0.521646296853688,0.733179039037517,0.463449237985196,0.811783757864253
"8929","CCDC122",70.9825306495146,-0.248807990598867,0.33926944981578,-0.733363969947683,0.463336468318141,0.811783757864253
"8930","IL32",304.976767087513,0.231727931628368,0.315993695748089,0.733330869401589,0.463356651692398,0.811783757864253
"8931","ATP5MC1",625.151890701941,-0.205284981384645,0.279920170831364,-0.733369734574498,0.463332953331952,0.811783757864253
"8932","ZNF175",29.7133691920302,0.239397944985034,0.326563297319452,0.733082826361986,0.463507913896687,0.811783757864253
"8933","NME6",121.296413690086,-0.16756398015624,0.228765580721553,-0.732470241492287,0.463881599724339,0.811991532388215
"8934","TFB1M",43.9284513394676,-0.200163074048599,0.273185595829518,-0.732699956016388,0.46374145081138,0.811991532388215
"8935","AEBP1",3897.01825335222,0.112398592874337,0.153454020630723,0.732457790368468,0.463889196833227,0.811991532388215
"8936","ELF1",412.139039406986,0.111481050922681,0.152204588818068,0.732442114842776,0.463898761423862,0.811991532388215
"8937","AC005944.1",3.25742855458403,-0.647126396895608,0.883500526867444,-0.732457284649362,0.463889505401466,0.811991532388215
"8938","BAMBI",15.8423064654606,0.407701094171092,0.556770721708846,0.732260297236485,0.464009707415775,0.812094859376255
"8939","DOP1A",64.0033620172381,0.174938723378258,0.239015148465342,0.

731914794947087,0.464220575306482,0.812352728016347
"8940","GDAP1",19.9840464868315,0.315368965794201,0.430971073293249,0.731763650363634,0.464312839045845,0.812352728016347
"8941","ASCL2",3.32634506779741,0.718198393356262,0.981383334721428,0.731822487652215,0.464276921572032,0.812352728016347
"8942","STX18-AS1",34.7775270932759,0.291429500192149,0.398741475939456,0.730873304577925,0.464856543156577,0.81307925359065
"8943","TULP4",96.336480599631,0.178623449255112,0.244382335928445,0.730918004267759,0.464829238125295,0.81307925359065
"8944","ZSWIM3",10.931443633772,0.338832860917681,0.463628536371327,0.730828312617721,0.464884027623523,0.81307925359065
"8945","NMNAT1",58.0570917592541,0.164087634530582,0.224549375527915,0.730741887590702,0.464936825053475,0.813080688016938
"8946","ADAM22",75.3111671460949,-0.25367769064177,0.347253557682663,-0.730525821922874,0.465068835110701,0.813129740431061
"8947","COL6A2",16087.4199206525,-0.155227622001282,0.212465653243826,-0.730601015417502,0.465022891632212,0.813129740431061
"8948","CLCN2",12.7864578783404,-0.316568694639875,0.433432007246091,-0.730376828077987,0.46515987830654,0.81313472768545
"8949","EFNB1",95.2298729603627,0.247001275425262,0.338195290280269,0.730351020620565,0.465175649047951,0.81313472768545
"8950","ATP5F1E",3116.51722194193,-0.21178224380829,0.290083476312016,-0.730073448170126,0.465345290257385,0.813340377150422
"8951","WDR54",59.9913166815782,-0.219688169918901,0.301121217450058,-0.729567221397598,0.465654764392205,0.813568595765588
"8952","OXNAD1",25.3153115952131,0.233421875262182,0.319922647013596,0.72961972977256,0.465622658871259,0.813568595765588
"8953","TRIM2",166.114758144411,0.165720882259262,0.227164407416202,0.729519576346457,0.46568389736528,0.813568595765588
"8954","TLN2",362.174073307796,0.177069736930648,0.242655477318592,0.729716629054972,0.465563414372096,0.813568595765588
"8955","AK4",69.0903325360692,-0.215420267703597,0.295336275103602,-0.729406733487206,0.465752900133624,0.813589218832411
"8956","SPRY3",4.18901064228577,0.623953419440939,0.855515711727383,0.729330169963923,0.465799721528036,0.813589218832411
"8957","CTBP2",244.570701084621,0.150336952207951,0.206176578992108,0.729166003931543,0.465900123873576,0.813673734258607
"8958","CNOT9",82.3977353575405,-0.164400022461236,0.225537577587463,-0.728925193840401,0.466047422724555,0.813840124322417
"8959","TRIM38",313.33085777001,0.164394071360737,0.225600535875866,0.728695393929352,0.466188010974126,0.813895476363193
"8960","BUD31",563.109444011615,-0.202903643343476,0.278434763918899,-0.728729561236027,0.466167106421766,0.813895476363193
"8961","AC100814.1",5.77381841295578,0.461109170662137,0.632854265001413,0.728618255675511,0.466235208316216,0.813895476363193
"8962","AC009126.1",7.01084414742503,-0.415151474242299,0.56990835946458,-0.728453035207849,0.466336308030825,0.813976431106428
"8963","BIRC3",33.6624914805954,-0.379651391963637,0.521232532056902,-0.728372403129647,0.466385651857503,0.813976431106428

"8964","ABCC5",100.752836250467,0.161012851355449,0.221260717901025,0.727706449128822,0.466793301683391,0.814413455161948
"8965","NIPSNAP2",309.521870578707,-0.124491931529006,0.171113961753564,-0.727538128702188,0.466896366802554,0.814413455161948
"8966","PRKAG2-AS1",109.568009689183,-0.220610142469275,0.303131302559715,-0.727770905236076,0.466753837585651,0.814413455161948
"8967","WNT11",1200.20129978723,-0.269466002317345,0.370377131340887,-0.7275449252004,0.466892204960318,0.814413455161948
"8968","SP2-AS1",13.6132508965537,0.366019035098824,0.502959776975609,0.727730231828408,0.466778740197886,0.814413455161948
"8969","DCUN1D1",162.422705304298,0.151678263138866,0.208621690255621,0.727049344452231,0.467195728262234,0.814693236755976
"8970","MTHFD2L",38.6853908753805,-0.199405252401976,0.274393041019108,-0.72671395623364,0.467401202138008,0.814693236755976
"8971","ZFR",334.601468940172,0.106523185147742,0.146605328673854,0.726598317478067,0.467472059242226,0.814693236755976
"8972","CSTF2T",120.725018899702,-0.161210492818732,0.221948720205901,-0.726341168668051,0.467629647307544,0.814693236755976
"8973","LTBP3",1061.51974221675,0.146161168121633,0.201195446358105,0.726463599287841,0.467554614688782,0.814693236755976
"8974","POLD4",474.288588566393,-0.164030454451183,0.22562854510105,-0.726993361490321,0.467230022454436,0.814693236755976
"8975","ATP7B",11.0227063455591,0.381902273283969,0.525294063879664,0.72702567865198,0.467210225350997,0.814693236755976
"8976","L2HGDH",18.9741713909025,0.304285378849468,0.418860062917608,0.726460710362169,0.467556385113921,0.814693236755976
"8977","NDUFB7",1177.17334297387,-0.200659212510095,0.276128343275917,-0.726688213638358,0.467416975277132,0.814693236755976
"8978","YJEFN3",21.9988433575978,0.318859711805296,0.438953903548315,0.726408192814259,0.467588570178137,0.814693236755976
"8979","NUCB1",1225.70593549986,0.169177722390721,0.232836526813288,0.726594425308469,0.467474444254435,0.814693236755976
"8980","PACRG",5.48679286286381,-0.506861082678592,0.69812803879513,-0.726028829257971,0.467821097324869,0.814845276188947
"8981","GCH1",13.3754148634777,0.347196814042941,0.478204345130605,0.72604278396533,0.467812542789905,0.814845276188947
"8982","GTPBP10",70.59520030494,0.161768824806654,0.222905038699615,0.725729780494787,0.468004441460718,0.815016115658954
"8983","ZSWIM8-AS1",4.3036278522288,0.569353390989002,0.784558717800981,0.725698890434648,0.468023382149484,0.815016115658954
"8984","ORAI2",89.03375296735,-0.201814771407094,0.278168473226423,-0.725512740772823,0.468137531505397,0.815124154645917
"8985","AC135803.1",4.76668245721776,0.52676931239017,0.726500769869885,0.725077431761722,0.468404528742861,0.815330382034561
"8986","FAM78B",15.9372152742093,-0.305688668859789,0.421700738360698,-0.724894791619552,0.46851657636698,0.815330382034561
"8987","MRPS28",162.542958935527,-0.201237588848892,0.277591812974738,

-0.724940648257536,0.468488442452733,0.815330382034561
"8988","FBXL19",17.6875096046581,0.277451495638529,0.382727800189564,0
.724931649859528,0.468493963066257,0.815330382034561
"8989","TFIP11",58.3336952922972,0.169368089774493,0.233630101951826,0
.724941214165187,0.468488095263534,0.815330382034561
"8990","ALX3",2.67574074922606,-0.70293695658071,0.969922255854769,-0.
724735361352472,0.468614397127419,0.815409901475441
"8991","AC007684.2",4.19971217921748,0.761761271599107,1.0512393224754
5,0.724631637451803,0.46867804450526,0.815429946635056
"8992","AL513166.1",10.5473483401432,0.335091199153704,0.4625942495134
94,0.724373896792093,0.46883622083167,0.815599137663953
"8993","CYB561D2",118.86671371522,-0.194734121243486,0.268888692567806
,-0.72421833504352,0.468931703902678,0.815599137663953
"8994","CORO6",20.3467081728168,0.267442202019946,0.369263767931334,0.
724257902469538,0.468907416582144,0.815599137663953
"8995","ACAT2",95.6138564410851,-0.240641204514729,0.332426153752743,-
0.723893718343582,0.469130986754579,0.815612873023694
"8996","ZNF710-
AS1",5.5871226799728,0.470790040938558,0.650383085322663,0.72386575168
2324,0.469148157736189,0.815612873023694
"8997","AC134312.5",6.08457287075044,-0.441705743821444,0.610025808834
304,-0.724077141367998,0.46901837723118,0.815612873023694
"8998","ZXDB",33.7459705462708,0.22144545168582,0.305910708424917,0.72
3889179381804,0.469133773564474,0.815612873023694
"8999","AL139420.1",3.84494298913476,-0.525204581372635,0.725826174896
061,-0.723595537799177,0.469314081665812,0.815706943841057
"9000","ATF3",657.391708691244,-0.588515860335018,0.813339850497302,-0
.7235792762093,0.469324068075699,0.815706943841057
"9001","NFKB2",71.1892630760118,-0.226871846163602,0.313567007869074,-
0.723519504508359,0.469360775502791,0.815706943841057
"9002","SCARF2",34.8670315829403,-0.404021454037623,0.55847421503298,-
0.723437972895067,0.469410848843393,0.815706943841057
"9003","DDX56",124.629776448694,-0.144042802963409,0.199145548565705,-
0.723304156185465,0.469493039926922,0.815759149569793
"9004","CCDC74A",60.9855054835827,-0.208793824011222,0.288815206578736
,-0.7229322392147,0.469721515502227,0.815793679027572
"9005","Z97832.2",6.83053767983356,-0.473593840100416,0.65499138992663
6,-0.723053535334964,0.469646994267077,0.815793679027572
"9006","SLC35A1",148.560950199885,-0.156799096182697,0.216867958505961
,-0.723016425584082,0.46966979285539,0.815793679027572
"9007","AC034111.1",2.94532627173963,-0.643854566883319,0.890478658570
984,-0.723043231509399,0.469653324420069,0.815793679027572
"9008","COL25A1",7.14944443102893,0.415473255263278,0.57508520440976,0
.722455128522564,0.470014703515111,0.815868924822708
"9009","ANO1",3.60542106086787,-0.802075591956533,1.11033966289502,-0.
722369576409852,0.470067286602704,0.815868924822708
"9010","HSPA2",15.7100463462503,-0.393507672612958,0.544456605627205,-
0.722753050556974,0.469831616389962,0.815868924822708
"9011","SLC25A35",23.7130640758866,-0.287383895661404,0.39783801785502
8,-0.722364084787209,0.470070662042088,0.815868924822708

"9012","ZNF473",13.46306321789,0.296924891820276,0.41095642060568,0.72
2521603100054,0.469973848339587,0.815868924822708
"9013","MYL9",1267.12001170055,0.251770700307834,0.348542706503036,0.7
22352514083208,0.470077774047629,0.815868924822708
"9014","TRIM44",330.557406815567,0.148765736776992,0.206025587112096,0
.722074082458749,0.470248931710588,0.815894414235661
"9015","USE1",276.627319702027,-0.192498331563587,0.266580931303031,-0
.722100904301997,0.470232442272247,0.815894414235661
"9016","SELENOO",72.1267704075738,-0.159243058201102,0.220529974242189
,-0.722092580604119,0.470237559451741,0.815894414235661
"9017","AL139274.2",9.2584814903953,-0.402453411743484,0.5574676216538
37,-0.721931455946315,0.470336620499789,0.815956055725651
"9018","FCGR3A",17.3355832691984,-0.392822054692156,0.544335649194079,
-0.721654103077305,0.470507166802218,0.815957924291667
"9019","POLR3B",36.6734961878791,0.198432925264879,0.27505120855214,0.
721439932256335,0.47063888536187,0.815957924291667
"9020","AC090826.1",4.05047149954414,0.588094737338656,0.8152854634495
78,0.72133597826012,0.470702826108036,0.815957924291667
"9021","G6PC3",131.861328627352,-0.186230636629257,0.258134832192058,-
0.721447140813204,0.470634451650415,0.815957924291667
"9022","CA11",35.694931652334,-0.265539252304449,0.368085746837293,-0.
721405962023916,0.47065977948098,0.815957924291667
"9023","ZNF701",60.5658193958219,0.172628299602994,0.239232114475217,0
.721593336169242,0.47054453737671,0.815957924291667
"9024","LINC01431",8.11745421504418,0.409497040188289,0.56746714348442
7,0.721622467291849,0.470526622046664,0.815957924291667
"9025","MYC",752.787585622321,-0.512844141625868,0.711138773522123,-0.
721159020884007,0.470811681308129,0.816056191767652
"9026","MRPL21",365.846654894838,-0.205297366130154,0.284834373618692,
-0.720760502048764,0.471056880747542,0.816329163417041
"9027","NACA2",4.18314599413786,0.559999567747085,0.776985695452545,0.
720733433092254,0.471073538206586,0.816329163417041
"9028","C5orf15",467.495537488466,0.163160004753935,0.226509770186527,
0.720322150428988,0.471326669732242,0.816406056319507
"9029","HOXA11",42.1557276975707,-0.208035041484208,0.288719110887252,
-0.720544756614492,0.471189653323312,0.816406056319507
"9030","BLOC1S6",439.578618678834,-0.0922354753288654,0.12801818361922
1,-0.720487298923186,0.471225017022514,0.816406056319507
"9031","MED31",145.340165571571,-0.220531730139528,0.306136551081043,-
0.720370466580277,0.471296928774475,0.816406056319507
"9032","FAM120C",41.1440978634484,0.184920242772988,0.256755278038136,
0.720219830283378,0.471389656210828,0.816424755547606
"9033","LIN37",85.4062940403087,-0.194035781211458,0.269536512408841,-
0.719886814136479,0.471594687219539,0.816689437858436
"9034","GIT2",136.554092695698,0.164455904491224,0.228613187812083,0.7
19363156890164,0.47191719134948,0.816976607379362
"9035","AC068790.9",5.26091736915422,0.442396822782688,0.6148448007872
29,0.719526004312399,0.47181688569219,0.816976607379362
"9036","EFS",182.706223614964,0.119417923839568,0.165994980525771,0.71
9406836648461,0.471890285706026,0.816976607379362

"9037","MAP7",3.65413655013931,-0.595818542710868,0.828614338222687,-0.719054106629209,0.472107582765596,0.817162860073742
"9038","AL138995.1",9.12466723360771,-0.338913783060118,0.471355854116562,-0.719018932511885,0.472129254576902,0.817162860073742
"9039","EPHX1",2571.79735106772,-0.195658252732942,0.272175661248704,-0.718867557206584,0.472222527622983,0.817198079886992
"9040","SBNO1",206.678833454306,0.125317373257128,0.174338513373521,0.718816346613182,0.472254084394196,0.817198079886992
"9041","DAGLA",12.7071067986031,-0.353921876063015,0.492544727661559,-0.71855784091593,0.472413397392504,0.817344967691076
"9042","RAB2B",101.288165717113,-0.15190985390828,0.211423716803299,-0.718509050002234,0.47244346978602,0.817344967691076
"9043","CCDC115",298.104158153625,-0.178079641664707,0.248048520008563,-0.717922613118433,0.4728050040057,0.81751836826143
"9044","DOCK1",286.211178345015,0.136511968922012,0.190118401032397,0.718036592884821,0.472734724358966,0.81751836826143
"9045","ITFG2",121.199245889359,0.155862204892022,0.217038588047389,0.718131306945245,0.472676328275123,0.81751836826143
"9046","RAB11FIP4",5.2730156137809,-0.491028901732179,0.683664677371922,-0.718230615072501,0.472615103978525,0.81751836826143
"9047","SLC17A9",3.35049902610366,-0.65304706411964,0.9095307042991,-0.718004418138791,0.472754562646498,0.81751836826143
"9048","STK19",172.696397241824,-0.176140540940976,0.245434482506234,-0.717668271965421,0.472961850876679,0.81756102224617
"9049","MCM7",82.749168499953,-0.177845577592526,0.247824063863678,-0.717628364331701,0.472986463678824,0.81756102224617
"9050","GHITM",615.706958048453,-0.126713738675226,0.176555118569372,-0.717700736755685,0.472941828925133,0.81756102224617
"9051","SPARCL1",746.429215996665,-0.646883574186626,0.901914502939841,-0.71723380883451,0.473229841430486,0.817602406940621
"9052","AC109454.2",3.76027174909516,0.588021145175643,0.819627243692181,0.717425085245799,0.473111845818143,0.817602406940621
"9053","IL6",56.1844961814053,0.794140411904054,1.10758581760128,0.717001246570616,0.473373327486932,0.817602406940621
"9054","LINC01004",19.4200486784748,-0.319212522836047,0.44500051703538,-0.717330678540914,0.47317008190615,0.817602406940621
"9055","RELT",4.47348189841169,-0.566119406528093,0.789570694912314,-0.716996476915806,0.473376270514684,0.817602406940621
"9056","DHX40",225.944357528754,0.171330576200819,0.238834841705178,0.717360059267703,0.473151957573764,0.817602406940621
"9057","MATK",7.17280751831804,-0.481982986640734,0.672112085839739,-0.717116976164091,0.473301921755261,0.817602406940621
"9058","C1orf229",6.82341814691654,-0.404463253374449,0.564214268464643,-0.716861086259102,0.473459815026128,0.817656423763934
"9059","MANF",328.334424246992,-0.190724649351877,0.266128230911334,-0.716664476740242,0.473581149845691,0.817775684627016
"9060","SMG7",89.849335427292,0.168011348617677,0.234475356876206,0.716541605292793,0.47365698692560,0.817816362745835
"9061","MTRNR2L12",56.3445370862641,-0.24980471019569,0.348727448517633,-0.716332228098354,0.473786231314818,0.817948443543701

"9062","CTSB",923.743207161426,0.13718966637614,0.191539263438558,0.716248271572516,0.473838061458353,0.817948443543701
"9063","CA2",2.70031313229832,-0.780285199979931,1.08959304704179,-0.716125347989676,0.473913953343101,0.817964822469309
"9064","RBM19",75.8779867606993,-0.15263740178444,0.213161818516771,-0.716063518535008,0.473952128802775,0.817964822469309
"9065","KCTD20",240.32608700665,0.119484826759687,0.166909573331758,0.715865629362032,0.474074323194631,0.817978433146818
"9066","BICD2",157.329975869053,0.134809571132383,0.188397332833205,0.715559870753242,0.474263159816306,0.817978433146818
"9067","ZCRB1",573.353913245439,-0.154396286805175,0.215728257023632,-0.715698021832445,0.474177832544843,0.817978433146818
"9068","SPNS1",217.404740751296,0.142536232193029,0.199099271739677,0.715905341830662,0.474049799792695,0.817978433146818
"9069","TTLL1",35.9446829507823,0.199026427699713,0.278122865962111,0.715606129726948,0.474234587605269,0.817978433146818
"9070","TMEM255A",23.5532094980375,0.334950227064198,0.46810654465413,0.715542713276276,0.47427375750442,0.817978433146818
"9071","HHIPL1",22.4633383647608,0.290025848288266,0.405399328804488,0.715407815655602,0.474357084481006,0.818031955962559
"9072","PCDHGB6",41.9774959263759,0.242302621698921,0.338900825449795,0.714966159723372,0.474629953958046,0.818322095201776
"9073","TMEM185A",140.011392879027,0.140149480674961,0.196012072570057,0.715004330281083,0.474606367536813,0.818322095201776
"9074","AL160408.2",2.91555908345041,0.748763420752627,1.04829774194818,0.714265986456394,0.475062720492014,0.818510267874687
"9075","KRBOX1",27.1846058544641,0.307073838608018,0.429756106206747,0.714530484088783,0.474899213046659,0.818510267874687
"9076","BCKDHB",68.8916927306533,0.167310279012978,0.234341083828736,0.713960506964511,0.475251600593024,0.818510267874687
"9077","PTRH1",95.1385933407253,-0.21952228957041,0.307199429197751,-0.714592114131496,0.474861118955584,0.818510267874687
"9078","ZNF33B",54.338121336524,-0.240885954292337,0.337404719515475,-0.713937714440562,0.475265695021366,0.818510267874687
"9079","KDM4D",6.91024528365151,0.405829133657626,0.56822279431982,0.714207767999549,0.475098714191946,0.818510267874687
"9080","SLC2A3",366.100245066109,-0.319629072448853,0.447748481161621,-0.713858529725484,0.475314663004133,0.818510267874687
"9081","SCYL2",79.0113041466226,0.191587823437404,0.268259177718874,0.714189259307211,0.475110157548715,0.818510267874687
"9082","CLUH",41.1668164662314,0.179023269884983,0.250560973513208,0.714489840037063,0.474924336423416,0.818510267874687
"9083","C19orf54",19.0567956452158,-0.263128935842009,0.36842141123278,-0.714206416401125,0.475099549838675,0.818510267874687
"9084","FP565260.6",9.97390423945802,-0.411898413994977,0.576949650495892,-0.713924366954635,0.475273948938807,0.818510267874687
"9085","ARHGEF1",124.130422569107,-0.146294130409943,0.205035348540005,-0.713506873091199,0.475532161595584,0.818794672959792
"9086","AL121929.2",3.33551286528123,0.593462666046785,0.831898609724301,0.713383408878955,0.475608536792603,0.818836048981586

"9087","NDUFS3",407.692446692137,-0.195070687755458,0.273492114213756,-0.713258911746883,0.475685557767671,0.818878527584425
"9088","CD8A",7.03220336734544,-0.493756112877845,0.692408802727092,-0.713099127182032,0.475784419568946,0.818958591034003
"9089","PRKAG1",217.49323165824,-0.205348862180827,0.28803900099909,-0.712920338803273,0.475895052752022,0.81905889649025
"9090","SPINT2",5.0025949222438,-0.501850758605919,0.704160518622191,-0.712693690336224,0.47603532174256,0.819210180200095
"9091","CAVIN4",2.94862626669958,0.723938729275424,1.01608000983027,0.712482011526191,0.476166346723943,0.819255407149433
"9092","NADSYN1",74.7984470732954,-0.175981701344621,0.246989818592347,-0.712505893350509,0.476151563359684,0.819255407149433
"9093","RAD54B",7.12473510299359,0.379343267688775,0.532540516861749,0.712327523780232,0.476261984046711,0.819265440398709
"9094","PDXP",12.3741375150932,-0.340851199050859,0.478519690480201,-0.712303392800431,0.476276923543174,0.819265440398709
"9095","TMEM9B",284.874516737466,-0.154353163149937,0.216734409040431,-0.712176547477252,0.476355457741387,0.81931043710264
"9096","HAUS1",139.99860240556,-0.149290059643084,0.209695666144247,-0.711936791008303,0.476503918399042,0.819475681125354
"9097","TAP1",103.164764838419,0.220557116864479,0.309907790006036,0.711686262743454,0.47665907618438,0.819539800793172
"9098","IPO7",155.564918579541,-0.133778639017153,0.187998730297016,-0.711593311326083,0.476716650123341,0.819539800793172
"9099","ZNF26",53.7481287130023,0.228148958878581,0.320669510801441,0.71147692934191,0.476788742281972,0.819539800793172
"9100","RFNG",336.917818520595,-0.191037518695741,0.268461223610594,-0.711601907070361,0.476711325775153,0.819539800793172
"9101","PRKX",30.8415589355725,0.26019689734913,0.365725710269722,0.7114536660746,0.476803153296596,0.819539800793172
"9102","P3H3",201.133233827094,0.167884337761335,0.236119468559068,0.711014380922751,0.477075324277789,0.819917523366013
"9103","C11orf71",77.0575484812653,-0.211004272700313,0.296865953583059,-0.710772893130963,0.477224980780295,0.82008462862201
"9104","ADGRB1",3.02128031534313,0.583379968966431,0.821041062958416,0.710536896734942,0.477371258936724,0.82024589230527
"9105","PSMB1",1708.63781917917,-0.202209536490505,0.284730614648058,-0.710178414570723,0.477593504642563,0.820464209088919
"9106","IGHG1",119.004917569462,0.752744680275984,1.05996079873942,0.710162754293557,0.477603214726312,0.820464209088919
"9107","SF3A3",223.372177493608,-0.113372166532478,0.15971268543965,-0.709850731145072,0.477796705772198,0.820589835089001
"9108","SIX2",22.7175865951285,0.283031715221031,0.399145656545082,0.709093812196008,0.478266262615099,0.820589835089001
"9109","FBXO36",29.4242531815523,-0.217864532152404,0.306984321128434,-0.709692701410816,0.477894719143286,0.820589835089001
"9110","ACAA1",304.371314441431,-0.163477214105041,0.230476571988932,-0.709300787903471,0.478137839593338,0.820589835089001
"9111","COX18",49.7201337470082,0.211709908665493,0.298500890885476,0.709243808410334,0.478173192000474,0.820589835089001

"9112","RNF145",226.471758563192,0.173095355682276,0.243876239918495,0.709767198887949,0.477848512868489,0.820589835089001
"9113","LINC00472",54.6214110971989,-0.254932436177048,0.359593834238382,-0.708945515478578,0.478358288447011,0.820589835089001
"9114","AL499604.1",18.7087365290456,-0.381883361245897,0.538217121286253,-0.70953402658997,0.477993143671138,0.820589835089001
"9115","MYO5A",79.15911921945,-0.183369950502475,0.258302159002699,-0.709904830878935,0.477763154503367,0.820589835089001
"9116","AC015961.2",5.11658562898004,0.569514245383494,0.802664691452963,0.709529460368531,0.477995976220226,0.820589835089001
"9117","MKNK2",246.836418473353,-0.112234333876288,0.158209376281607,-0.709403807246638,0.478073925807787,0.820589835089001
"9118","SLC16A8",17.3824817739559,0.307132249296075,0.433206135113975,0.70897483761459,0.478340091767965,0.820589835089001
"9119","MID2",7.79551965255511,-0.388888708465396,0.548440328689081,-0.709081167307561,0.478274109049181,0.820589835089001
"9120","AC011416.3",4.7296645335562,0.453118229898909,0.639249738188063,0.708828182211323,0.478431106702691,0.820624759007697
"9121","TP53I3",406.851841589236,0.209142614423564,0.295174927139026,0.708537870918348,0.478611302964605,0.820629928624775
"9122","AC084036.1",7.13364800846386,-0.439101954103034,0.619775705325316,-0.708485263830327,0.478643960159333,0.820629928624775
"9123","CDKN2B",15.3081186055324,0.454051122976999,0.640723320012571,0.708653967781431,0.47853923717633,0.820629928624775
"9124","ZBTB26",19.2843356363082,0.244348843896453,0.344781418138114,0.708706534174562,0.478506609144208,0.820629928624775
"9125","LINC01133",390.938116572178,0.225473950955011,0.318409695595306,0.708125267773206,0.478867469576654,0.820653355235276
"9126","DNAJB2",155.246220578671,-0.16747620538581,0.236431447303424,-0.708349956386639,0.478727961305337,0.820653355235276
"9127","IL20RA",16.4269038235294,0.361917822077977,0.511033495705701,0.708207632413984,0.47881632711958,0.820653355235276
"9128","ARHGEF7-AS2",3.88986047161476,0.560748545916096,0.791862476834525,0.708138802280027,0.478859065426558,0.820653355235276
"9129","RAB11FIP3",51.5581394508941,-0.220805838031716,0.311924555450983,-0.707882191937321,0.479018419399396,0.820822120129779
"9130","ABCB11",32.8041291830899,-0.485551011169585,0.686047693136056,-0.707751102478078,0.479099836589316,0.820830125360015
"9131","SEC24A",86.9833269516353,0.187761457988653,0.265323649828478,0.707669512725434,0.479150514259395,0.820830125360015
"9132","GSTA4",223.41861079462,0.189683752836992,0.268058314850484,0.707621223922088,0.479180509159858,0.820830125360015
"9133","PCBP1-AS1",132.131007526304,0.176283852102455,0.249466110539973,0.706644488587592,0.479787434574735,0.821188696753353
"9134","LINC01886",43.7793399815976,-0.254211011915291,0.359752722237824,-0.706627069654912,0.479798262183994,0.821188696753353
"9135","PASK",6.40178609673976,-0.422667343268699,0.598279267037399,-0.706471653884469,0.479894874532293,0.821188696753353

"9136","FAM13A",672.989836655739,-0.193035261253045,0.273214770422579,
-0.706533036096395,0.479856715749421,0.821188696753353
"9137","DEK",1403.28459008169,-0.131990864175183,0.186753802776154,-0.
706763997375676,0.479713151501249,0.821188696753353
"9138","NEU1",411.404158169716,0.212907641247963,0.301205424982094,0.7
0685194750599,0.479658488349212,0.821188696753353
"9139","AC007036.2",3.60564131264502,0.574738539646124,0.8132669798756
49,0.706703399828189,0.479750816332788,0.821188696753353
"9140","ERH",1015.81468746774,-0.191199959031806,0.270652939147398,-0.
70643961833231,0.479914790367522,0.821188696753353
"9141","DDX19B",142.724219270513,-0.154856720570079,0.219043674011656,
-0.706967326350812,0.479586782734959,0.821188696753353
"9142","DNASE2",371.948954172865,-0.138780892637304,0.196356715757234,
-0.706779455452323,0.47970354368329,0.821188696753353
"9143","R3HCC1L",65.2613879458959,0.157245466586657,0.222635777279965,
0.706290195168946,0.48000768958843,0.821193217505289
"9144","SMAD7",146.962151797711,-0.233070863379871,0.330004133563281,-
0.706266496917008,0.480022424142962,0.821193217505289
"9145","BTAF1",102.82197658756,0.231088411170184,0.327281680277767,0.7
06084162651746,0.480135800000635,0.821297355867681
"9146","SRSF3",1272.56095437836,-0.144795733580662,0.205181612328521,-
0.705695466262476,0.480377541018184,0.821341261713423
"9147","DUSP4",10.5141651959033,-0.476062824553397,0.674551985584949,-
0.705746680354919,0.480345685766137,0.821341261713423
"9148","DPAGT1",86.4617080196173,0.157214385083433,0.222802939246871,0
.705620785860616,0.480423994417811,0.821341261713423
"9149","AC008147.2",34.741678103543,-0.338993735657949,0.4803513902763
6,-0.705720317501146,0.480362083361943,0.821341261713423
"9150","COA3",394.096651745044,-0.203700428783868,0.288649363209802,-0
.705701985685,0.480373485862404,0.821341261713423
"9151","FAM207A",191.976367276818,-0.174594074526669,0.247500308559366
,-0.705429724685737,0.480542851213131,0.821454684900776
"9152","CENPN",9.51035073248668,-0.402378365216193,0.570471218373514,-
0.705343849534468,0.480596278302,0.8214562479762
"9153","RBPMS2",11.1759458199277,0.390388785283812,0.553715291621843,0
.705035225125092,0.480788315201319,0.821694702796267
"9154","GIMAP6",2.64337672275468,-0.712007609405742,1.01004749148673,-
0.704924882648548,0.48085698429066,0.821722285914223
"9155","PLAC8",7.12289265592024,0.525623826478283,0.745736857446499,0.
704838202952833,0.48091093114153,0.821724707356303
"9156","ZFYVE28",30.3745692008996,0.206956468746655,0.29370700617531,0
.704635791436058,0.481036918851937,0.8218502098734
"9157","REEP3",288.502893393555,-0.117800578792869,0.167220347870704,-
0.704463184611682,0.481144369319308,0.821854266134738
"9158","TUBGCP5",79.996433777915,-0.162390452823885,0.230503588869906,
-0.704502926050043,0.481119628489923,0.821854266134738
"9159","CLEC2B",1180.04964100427,-0.178932252082732,0.254136204968467,
-0.704080129413023,0.481382873886897,0.822082128407503
"9160","RPL26",16486.173128283,-0.198745249737285,0.282273048685301,-0
.704088649847902,0.481377568044167,0.822082128407503

"9161","RBMS1",977.662001244994,0.143689258280813,0.204149400351016,0.703843646044285,0.481530149442431,0.822243873783206
"9162","ACP6",8.56288944581596,-0.377414476024521,0.5363239309345,-0.703706201151434,0.481615757733173,0.822300294501204
"9163","MLLT6",241.426816642411,0.166850150149905,0.237247741721276,0.703273923449708,0.48188505819624,0.822670300705421
"9164","PCF11-AS1",11.0962022079783,0.335943547209402,0.477750214624713,0.703178223526902,0.481944688427643,0.822682317882324
"9165","SARS",479.845536283305,-0.140082169020982,0.199434391772534,-0.702397253432365,0.482431457676758,0.823016607921578
"9166","AP1AR",70.58557092064,-0.175674236479871,0.250107473941014,-0.702394989288895,0.482432869278463,0.823016607921578
"9167","KIF3A",108.053763092642,-0.150966068196532,0.214992616018299,-0.702191875202277,0.482559511814156,0.823016607921578
"9168","TOR1B",59.5176909182441,0.16147995424352,0.22996657254239,0.702188811435865,0.482561422224427,0.823016607921578
"9169","KAZALD1",191.421697740732,0.266968955188981,0.379911969570817,0.702712671808086,0.482234829042977,0.823016607921578
"9170","INTS6-AS1",33.6811892533497,0.211879319191165,0.301662459497688,0.70237217963407,0.482447090299164,0.823016607921578
"9171","MYDGF",985.079936664325,-0.192082591267941,0.273543051323571,-0.702202415080646,0.482552939708773,0.823016607921578
"9172","KCTD17",69.7690240397237,-0.193057853517529,0.274859628341537,-0.702387086391742,0.482437796432183,0.823016607921578
"9173","ZXDC",65.9910420626586,0.21215096946762,0.302205501716614,0.702008958349673,0.482673576743426,0.823118146843717
"9174","AL162171.3",7.00525739680497,0.410348253812618,0.584770389385772,0.701725431487113,0.482850409912056,0.823254034966127
"9175","AC032011.1",2.73616535547095,0.667527315688791,0.951283292197336,0.70171243536391,0.482858516321308,0.823254034966127
"9176","OXSM",20.8280429621275,0.296686605373485,0.42308612945593,0.701243989622185,0.483150761494335,0.823341033747499
"9177","SNHG32",833.180402444851,0.193296748388409,0.275544723465725,0.701507711551056,0.482986223773107,0.823341033747499
"9178","AC004540.2",4.11523839798996,0.684685222258657,0.976294731503986,0.701309963236099,0.483109597299491,0.823341033747499
"9179","TLN1",1019.48824826713,0.137992731854054,0.196752881669591,0.701350499586513,0.483084305613792,0.823341033747499
"9180","ZKSCAN2-DT",20.2584125934167,0.321544352210651,0.458557201698094,0.701208815432256,0.483172709186348,0.823341033747499
"9181","SAMD8",152.835634400443,0.163939482798373,0.233843214624551,0.70106581053287,0.483261945741987,0.823403400200621
"9182","EBPL",421.23405040265,-0.24429779412785,0.348522578012754,-0.700952562444634,0.483332620083796,0.823434129380398
"9183","ATP10A",37.7656186184696,-0.247926047791306,0.35378434332232,-0.70078298395876,0.483438458831963,0.823524753512839
"9184","SUMO1",826.024174337369,-0.181403155263737,0.259136302128697,-

0.700029883013632,0.483908642464241,0.823877110804106
"9185","ADAM23",14.8827433368541,0.477707384772708,0.682334716044136,0
.700107108051363,0.483860417133098,0.823877110804106
"9186","TTC1",341.860727618893,-0.128044837085315,0.182882887058209,-0
.700146630146755,0.483835737465673,0.823877110804106
"9187","ALG1L9P",8.98652331941618,0.383502596845427,0.547613151115414,
0.700316630570841,0.483729588078987,0.823877110804106
"9188","RALY-
AS1",15.5364147024395,0.281939762877143,0.402727023500066,0.7000765938
84434,0.48387947224511,0.823877110804106
"9189","MTOR",53.4727296811447,0.198429736919459,0.283550773019042,0.6
99803195056476,0.484050219147178,0.82396663814583
"9190","NSUN5",104.176425527813,-0.141286554548108,0.201902251954241,-
0.699777011799397,0.484066573199525,0.82396663814583
"9191","GCLM",103.629628102764,0.15025661568999,0.214765789223268,0.69
9630123742776,0.484158324979096,0.824033149564575
"9192","LFNG",14.1856431515821,0.459756933511053,0.657495734981205,0.6
99254624859575,0.484392918501336,0.82434273543477
"9193","HMGCR",49.8904594757625,0.161155914206571,0.230547358446992,0.
699014359965546,0.484543056695846,0.824508543010238
"9194","SP2",59.9505668289145,0.164203585026511,0.234952084917775,0.69
8881157338855,0.484626304032826,0.824560504022787
"9195","C1orf216",83.5497857616205,-0.157640219791775,0.22571718855378
7,-0.698397055190196,0.484928917491563,0.82464264870341
"9196","SAPCD1",3.27558203334806,0.56308576494514,0.806257979716516,0.
698394036537937,0.484930804779677,0.82464264870341
"9197","SRP54",231.525781027868,0.120554792940459,0.172559653022667,0.
698626769518502,0.484785309732666,0.82464264870341
"9198","AC007952.4",87.3163149792065,-0.316299603717458,0.452903260032
001,-0.698382263124158,0.48493816566021,0.82464264870341
"9199","SH2D3A",8.41172205349424,-0.391862059293058,0.560931104556589,
-0.698592137447648,0.484806958774992,0.82464264870341
"9200","AL355353.1",4.97719742302568,-0.518041469687427,0.742298764529
409,-0.69788809363821,0.485247180971201,0.824719866572414
"9201","CYP3A5",46.9946634796086,-0.186473706063569,0.267116511238743,
-0.698098762966035,0.485115431658475,0.824719866572414
"9202","OSBPL5",74.803896916375,0.154220372629138,0.220876851877204,0.
698218809795769,0.485040364908605,0.824719866572414
"9203","IGHMBP2",25.4256720877837,0.247294015766643,0.354285480173489,
0.698007763811113,0.485172338734241,0.824719866572414
"9204","CLPP",232.604402899899,-0.18638713183105,0.267066770650189,-0.
697904615303806,0.485236847879079,0.824719866572414
"9205","LIPA",252.389698016158,-0.108860788062878,0.156038886476496,-0
.697651659282226,0.485395066400966,0.824785036600486
"9206","TRAPPC6B",161.518076936703,0.114307988451773,0.163826578830718
,0.697737749683996,0.485341215581321,0.824785036600486
"9207","NPIPB11",9.5452363414861,0.365770872728957,0.524347122428704,0
.697573910646742,0.485443702101942,0.824785036600486
"9208","SLC1A7",24.1103806218487,-0.442771041121687,0.635008376384519,
-0.697268032341001,0.485635070043173,0.824806940962677

"9209","SAXO1",6.43956244666152,0.546002637505721,0.782840018534814,0.697463880969749,0.485512535733917,0.824806940962677
"9210","B2M",22898.4402614636,0.197859315403958,0.283777395229266,0.697234236166365,0.485656216591549,0.824806940962677
"9211","ZNF77",17.8677570340828,0.257972050526899,0.370002949445826,0.697216200339154,0.485667501963001,0.824806940962677
"9212","GMEB1",71.8144583293643,0.140104468739634,0.201154926391314,0.696500310745976,0.486115562736259,0.824835512093119
"9213","TMEM39B",91.8776021555716,-0.148832422613108,0.213521496434816,-0.697037184068931,0.485779523654986,0.824835512093119
"9214","CGREF1",74.1868203004527,0.28810725143697,0.413801783008807,0.696244586821508,0.486275669328473,0.824835512093119
"9215","TMEM184C",78.956366384582,0.146355486717887,0.210004101675829,0.696917277091129,0.48585456477831,0.824835512093119
"9216","MTRF1L",83.1351430927474,-0.191381563622342,0.274981344720806,-0.695980172097329,0.486441247135742,0.824835512093119
"9217","BRD3",251.855796903533,0.129253212412461,0.185722349751351,0.695948616768569,0.486461009274076,0.824835512093119
"9218","UBE2D1",180.444261428871,0.176532981346665,0.25366532966679,0.695928693048259,0.486473487114923,0.824835512093119
"9219","MTRNR2L8",7.99634674135174,-0.460290237891029,0.661271083947476,-0.696068902851934,0.486385680101054,0.824835512093119
"9220","AC124947.1",3.4887457861437,0.702090794639991,1.00832309823138,0.696295459135539,0.486243816329981,0.824835512093119
"9221","TMEM116",54.0820193594949,-0.229837716985592,0.329743371877846,-0.69701997549396,0.485790292874621,0.824835512093119
"9222","AL139089.1",7.88128338270782,0.409517718650792,0.588154586438173,0.696275652853114,0.4862562176267,0.824835512093119
"9223","ALDH1A2",3.38924872047414,-0.648279158435163,0.931534791445757,-0.695925868135342,0.486475256317274,0.824835512093119
"9224","LINC00923",2.92508132249414,0.739602411787316,1.06140498767629,0.696814524497867,0.485918875199977,0.824835512093119
"9225","GATA6-AS1",11.008849608032,0.489190972238334,0.702378055989368,0.696478154559172,0.486129433409787,0.824835512093119
"9226","FAM182A",46.8524135535971,-0.23863809512182,0.342771941138951,-0.696200786823103,0.486303094997852,0.824835512093119
"9227","RND3",1245.43377731983,-0.292267700191808,0.420153838547333,-0.695620683134332,0.486666409779621,0.825041597109343
"9228","CTSW",9.62339471173341,0.427426165291402,0.614557159334295,0.695502702717518,0.486740318015219,0.825041597109343
"9229","CCDC59",382.255294890905,-0.140342371925108,0.201792328772525,-0.695479222519468,0.486755027790201,0.825041597109343
"9230","HOXA5",37.0349870836676,0.25880590670385,0.37233762596054,0.695083947092895,0.487002694320956,0.825234541894148
"9231","AP001830.1",4.28651645427608,0.52003279679091,0.748198735324161,0.695046345628481,0.487026257702213,0.825234541894148
"9232","CBR1",518.023161048057,-0.157739105699227,0.22694805920473,-0.695044964261756,0.48702712336296,0.825234541894148
"9233","KIAA2013",189.173033914055,-0.116158155898978,0.16714467188311

9,-0.694955780464275,0.487083013910439,0.825239855583342
"9234","AC100830.1",2.76889031059371,0.713604524045918,1.0275036268851
5,0.694503168041546,0.487366714750257,0.825631093658032
"9235","DHX9",260.485652085469,-0.141276413156714,0.203540308884669,-0
.694095503396158,0.487622318335409,0.8259746535702
"9236","CCDC90B",381.166247670332,0.159746651844846,0.230203579128886,
0.693936438561661,0.48772207076837,0.826054174212821
"9237","WDR3",61.3017498496965,0.151905428998463,0.21894501636446,0.69
3806287627931,0.487803698962485,0.826076833496453
"9238","SMG8",33.0160244051191,0.21539871831668,0.310497182991397,0.69
3721972745395,0.487856583590187,0.826076833496453
"9239","PUDP",29.9643994597724,0.188028228287396,0.271065859678606,0.6
93662523603433,0.487893873596735,0.826076833496453
"9240","LCP2",28.028164724469,-0.224456395234452,0.32367361943076,-0.6
9346521236176,0.488017649861072,0.826196980170643
"9241","FBXO44",64.883817517833,-0.170797517714661,0.246868004533396,-
0.691857650964065,0.489026727851143,0.826266057869874
"9242","TACSTD2",3.18646518407188,0.792709781149482,1.14459405074312,0
.69256849678261,0.488580386187242,0.826266057869874
"9243","GASK1A",51.298528695301,-0.208628528247408,0.301041646888992,-
0.693022146282435,0.48829565348959,0.826266057869874
"9244","RTP4",51.7525865056165,0.198776806526852,0.287285568088643,0.6
91913651804181,0.488991556830628,0.826266057869874
"9245","G3BP2",253.764420419617,0.124889541743079,0.180358017987706,0.
692453505180966,0.488652574777254,0.826266057869874
"9246","TCOF1",72.2954873538642,-0.187244634627356,0.270291494307362,-
0.692750747141273,0.488465986160861,0.826266057869874
"9247","ADTRP",4.40548935175008,-0.650145407500336,0.938004125610238,-
0.693115722787861,0.488236931414951,0.826266057869874
"9248","DBF4",33.3178131674794,-0.255940767573965,0.36935226195535,-0.
692944903651099,0.488344128435645,0.826266057869874
"9249","AC073389.1",7.74404431390204,0.386894409063047,0.5582842655951
74,0.693006113383096,0.488305714998808,0.826266057869874
"9250","AC010201.2",5.52789031568167,0.573079252618366,0.8279755101165
58,0.692145172914221,0.488846165731052,0.826266057869874
"9251","MGAT2",128.400382881221,0.138440883123663,0.200045863019468,0.
692045719086875,0.488908618094647,0.826266057869874
"9252","METTL26",513.119111106617,-0.181268417466753,0.261819768241711
,-0.692340454978199,0.488723550213612,0.826266057869874
"9253","AF001548.2",6.7581963434312,0.467908795004672,0.67588796643252
,0.692287506573603,0.488756794312175,0.826266057869874
"9254","CBX1",386.047456252193,0.137255839647118,0.198403520030381,0.6
91801433896436,0.489062036042777,0.826266057869874
"9255","SRSF1",316.978648059856,0.136715721033165,0.197524978597575,0.
692143960747876,0.488846926889087,0.826266057869874
"9256","WIPI1",179.828960597491,0.135840274927626,0.196334657341048,0.
691881284574535,0.489011884725499,0.826266057869874
"9257","ARMC7",28.9302574044411,0.222838780077944,0.321683408845231,0.
692726991665138,0.488480896847125,0.826266057869874
"9258","ZNF416",9.37843425859077,0.359699434651123,0.519648408070952,0

.692197703417212,0.488813180760906,0.826266057869874
"9259","MEI1",3.60851783173042,-0.523940303321746,0.757180904668804,-0.691961854942605,0.488961284205243,0.826266057869874
"9260","CEBPB",1256.70215637147,-0.265623233240986,0.384087469203743,-0.691569641133186,0.489207632370739,0.826422785440116
"9261","LANCL2",40.389365482487,0.214919910060479,0.310837407185206,0.69142228410245,0.489300204094522,0.826489913902451
"9262","NCAPD3",37.0274579216743,0.243553378302416,0.352490747044202,0.690949706750387,0.489597147344777,0.826902199947565
"9263","MIR210HG",3.02265158008847,-0.571290709813302,0.827398764827982,-0.690465993059676,0.489901188526633,0.827237078165168
"9264","ARHGAP27",18.935514881116,0.306906060940763,0.444461955463511,0.690511431109336,0.489872623841513,0.827237078165168
"9265","PAPOLG",67.0346138957155,0.174500233062803,0.252793899033351,0.690286568347051,0.490013992846367,0.827248962885356
"9266","YME1L1",438.651570586349,0.103449910746776,0.149847707055341,0.690366991792344,0.489963428897096,0.827248962885356
"9267","TSGA10",29.362988231159,-0.217842064726144,0.315991509602755,-0.689392145377582,0.490576524971753,0.827573447658054
"9268","TIGD2",7.16116217310816,-0.369129654543173,0.535077340154484,-0.689862243907769,0.49028082121832,0.827573447658054
"9269","OAS2",30.3443179509058,0.267636708818659,0.388204744546727,0.689421529690874,0.490558038693812,0.827573447658054
"9270","AC079949.2",4.19557785582093,0.488934129205731,0.708852927703555,0.689753981534242,0.490348912466889,0.827573447658054
"9271","HOXB3",82.0381922035133,0.244695448480311,0.354917032894275,0.689444083550655,0.490543849849178,0.827573447658054
"9272","TMEM94",52.58906644185,0.174566122553902,0.253144179174568,0.689591690881904,0.490450994140461,0.827573447658054
"9273","AL078622.1",3.8061109770603,0.77729737902137,1.12735535349385,0.689487459843432,0.490516562036932,0.827573447658054
"9274","PRKCD",33.4371435706264,-0.185202797340646,0.268714323708262,-0.689218180798268,0.490685977350141,0.827642153133302
"9275","AC073111.4",40.3624540483844,0.221137095661392,0.320879537764499,0.689159231535948,0.490723069124297,0.827642153133302
"9276","AC008915.2",32.7992000635593,-0.240491090719983,0.34901068577612,-0.689065121846301,0.49078228749823,0.827652794667401
"9277","GRAMD1B",3.62515808791158,0.630003209460426,0.914469555373814,0.688927483433711,0.490868903167896,0.827709631589458
"9278","CKS2",29.8315748354011,-0.244036724974026,0.3544821292860 8,-0.688431672043698,0.491180984693568,0.828146598788693
"9279","LINC01873",4.49891470279599,0.569584751719657,0.827478218841267,0.688338059843143,0.49123991953836,0.828156704530507
"9280","ANKRD36",142.438877621437,-0.22871575127735,0.332617279940402,-0.687624381145596,0.491689350572377,0.828646467464307
"9281","STX1A",5.62470356098552,-0.402114412891341,0.584768716037049,-0.687646930937845,0.491675146721695,0.828646467464307
"9282","PDCD11",94.2091470386028,0.204964960817095,0.298044340097946,0.687699557554883,0.491641998687289,0.828646467464307
"9283","UTP4",52.4247084318835,-0.163008721789657,0.237123599007101,-0

.687442002703303,0.491804236716071,0.828708367172753
"9284","IL2RB",7.46287512178774,0.511838181312387,0.744602500373717,0.687397881494482,0.49183203227206,0.828708367172753
"9285","PHF3",634.96650602905,0.151823061221552,0.220948415058319,0.687142567560299,0.491992891941668,0.82880684714554
"9286","PEX7",42.4490510394932,-0.196784691386005,0.286393374916446,-0.68711328061767,0.492011345884069,0.82880684714554
"9287","GEMIN2",84.0387209253703,-0.1764072844822,0.256778388244756,-0.68700207088321,0.492081423430866,0.82880684714554
"9288","ZNF236-DT",9.73244453263095,-0.435020152045043,0.633245894412821,-0.686968768188252,0.492102409786344,0.82880684714554
"9289","TOR1AIP2",317.629392189863,0.139855654474355,0.203917675212838,0.68584370790017,0.492811670912131,0.828841303954249
"9290","CCDC142",37.1330277163728,0.236634581559783,0.344989791114992,0.685917634823311,0.492765049049807,0.828841303954249
"9291","CHL1-AS2",48.8650689185191,0.290981599022922,0.423855534339954,0.686511264919664,0.492390763167747,0.828841303954249
"9292","TIAM2",39.7059408737816,0.235913148577205,0.343546752314802,0.686698817519402,0.492272542287256,0.828841303954249
"9293","PLPP6",22.6626821986374,-0.267156636860544,0.389521662345847,-0.685858227374636,0.492802514048815,0.828841303954249
"9294","NOP10",646.033464196522,-0.185291733204742,0.269963618222587,-0.686358163461745,0.492487279589134,0.828841303954249
"9295","DCUN1D3",130.398165784611,0.236832934133075,0.34482941829879,0.686811859908838,0.492201295118929,0.828841303954249
"9296","PSMB10",363.081217786602,-0.190945892279296,0.278401643044213,-0.685864818150413,0.492798357535011,0.828841303954249
"9297","RARA",183.118003755842,0.126292394147207,0.183955201113453,0.686538860455036,0.492373367793652,0.828841303954249
"9298","AC103810.2",3.79916145154576,0.500065850475856,0.728763214418822,0.68618426476788,0.492596919062435,0.828841303954249
"9299","AC011825.4",2.63855793791168,-0.672798829044024,0.980699436115061,-0.686039783717269,0.492688021280659,0.828841303954249
"9300","RIMS4",2.73781777825149,-0.936905016404911,1.36502615066204,-0.686364152035119,0.492483504152489,0.828841303954249
"9301","ARHGAP4",12.5570285128265,-0.480268656693939,0.700044097548978,-0.686054861937232,0.492678513319173,0.828841303954249
"9302","OXSR1",91.2498200162331,0.177012021740169,0.258346030310446,0.685174150063229,0.493234033830785,0.828931804347936
"9303","ENC1",10.5454161136185,-0.425685300770136,0.621432885050175,-0.685006073883209,0.49334008818572,0.828931804347936
"9304","SMARCD3",104.989217978315,0.147278588148578,0.214973619597711,0.685100750613898,0.49328034663249,0.828931804347936
"9305","LRSAM1",38.3668945809223,-0.173407612169507,0.253022519587,-0.685344579022269,0.493126507366599,0.828931804347936
"9306","PER1",250.165562317544,-0.219346474819075,0.32002010556714,6-0.685414669276309,0.493082289904214,0.828931804347936
"9307","PWWP3A",333.534607906771,0.166296060627464,0.242589991146959,0

.685502562744737,0.493026844025585,0.828931804347936
"9308","SLC7A10",5.22352731131799,-0.508938700324485,0.742973583441798
,-0.685002416865004,0.49334239586264,0.828931804347936
"9309","ZNF586",14.7642323032214,0.32363015457427,0.472258696193876,0.
685281514522732,0.49316629434433,0.828931804347936
"9310","ZNF70",22.6814101449968,0.284304997737499,0.414957010806137,0.
685143256611521,0.493253526367613,0.828931804347936
"9311","C7orf25",52.3665924367986,-0.221530527896148,0.323445779681416
,-0.684907770675964,0.493402122166263,0.828943120722463
"9312","RERE",382.008794156607,0.136142207216333,0.198920071836478,0.6
84406585818291,0.493718458478335,0.829064172949661
"9313","MLLT11",13.3204903433295,-0.313093496828386,0.457407027482638,
-0.684496472543308,0.493661716064758,0.829064172949661
"9314","CCDC150",11.1466237012108,0.3924960881315,0.573511289977892,0.
684373777797174,0.49373916992898,0.829064172949661
"9315","AC009065.4",7.22849568892484,-0.356058037398138,0.520080794720
784,-0.684620622434817,0.493583350227539,0.829064172949661
"9316","CHCHD10",231.180419659869,-0.244708790900108,0.357512924445471
,-0.684475369050418,0.493675037664066,0.829064172949661
"9317","DPP8",146.767960487969,0.136409415805841,0.199378379337505,0.6
84173561140896,0.493865575237766,0.829187420139998
"9318","HSPB11",237.720430278912,-0.167030822483427,0.24428744218507,-
0.683747068573774,0.49413489590101,0.829307117199255
"9319","AC016394.1",19.2087699735097,-0.22638243734154,0.3310208274309
92,-0.683891823660957,0.494043477444441,0.829307117199255
"9320","TSEN54",24.6880403439107,-0.223888653203553,0.32745431371574,-
0.683724855119509,0.494148925360497,0.829307117199255
"9321","AC012615.1",23.2365820358214,-0.223215094750312,0.326376870883
378,-0.683918238894054,0.494026796175721,0.829307117199255
"9322","ACKR3",1757.24893375698,0.283713848073859,0.415010180510352,0.
683631056291118,0.494208168692819,0.829317569498152
"9323","AC022506.2",4.93364299112221,-0.511149059087589,0.748171407644
87,-0.683197799146868,0.494481863165639,0.829687845704182
"9324","RPIA",143.97651191885,0.148025571331727,0.216740919896624,0.68
2960888983625,0.494631556829086,0.829826744923087
"9325","CD28",3.2935303360229,-0.575634633583746,0.843248165114169,-0.
682639651526321,0.494834572085825,0.829826744923087
"9326","TNPO3",136.405987085157,0.103699944960742,0.151959072960242,0.
682420226318926,0.494973269754832,0.829826744923087
"9327","AP002851.1",2.82155276658941,-0.620854535257141,0.910209440633
752,-0.682100742467424,0.495175251170254,0.829826744923087
"9328","RAD21",434.787073134734,0.128054536045509,0.187624282748623,0.
682505132968719,0.49491959818648,0.829826744923087
"9329","CCDC171",63.0787228295785,0.188129355275317,0.275571194636316,
0.682688753168123,0.494803538007147,0.829826744923087
"9330","GBA2",55.4305501702303,0.227827182723772,0.333886618029625,0.6
82348948479142,0.495018328620521,0.829826744923087
"9331","PTHLH",19.9487410232835,-0.304682799276176,0.446179005285467,-
0.682871214617637,0.494688224645258,0.829826744923087
"9332","ACOT2",104.07908495884,-0.287129175403839,0.420973686886723,-0

.682059673437739,0.495201218682926,0.829826744923087
"9333","FLVCR2",17.3923387649422,0.280158295944639,0.410708445926296,0
.682134245651512,0.495154068000409,0.829826744923087
"9334","ATF7IP2",21.4780144450145,-0.352084644235714,0.516140361719242
,-0.682149024468721,0.495144723902278,0.829826744923087
"9335","LINC00630",26.5568286666948,0.277377377970825,0.40667109778556
4,0.682068087654178,0.495195898403704,0.829826744923087
"9336","HNRNPLL",114.832759428936,-0.134683103449014,0.197643812634745
,-0.681443560785355,0.49559086748629,0.830035111358463
"9337","LNPK",307.362555504297,0.118549277483718,0.173953933618426,0.6
81498112849574,0.495556360460514,0.830035111358463
"9338","TCF7L2",998.741482893994,0.1263781573503,0.185455056104872,0.6
81448972083215,0.495587444500855,0.830035111358463
"9339","PDIA3",1495.67747004223,-0.162632075836876,0.238592140435285,-
0.681632159132199,0.495471574644681,0.830035111358463
"9340","THSD4",3.16951068207183,0.625828724427057,0.918173403051224,0.
681601887342126,0.495490721223009,0.830035111358463
"9341","NSL1",228.392955795205,0.116819927660363,0.171593995915616,0.6
80792629351737,0.496002714527055,0.830279710851467
"9342","EPRS",403.726752201458,0.118790038379878,0.174458088130159,0.6
80908748072782,0.495929232346021,0.830279710851467
"9343","PSMB9",430.50049641658,0.207255638626264,0.304507574019825,0.6
80625561756209,0.496108448336551,0.830279710851467
"9344","RHEB",658.015712253795,-0.165908107943752,0.243651065453299,-0
.680925025446081,0.495918932172454,0.830279710851467
"9345","PEX5",44.4643412734307,-0.189821682373178,0.278875745301154,-0
.680667593261623,0.496081846287852,0.830279710851467
"9346","HYKK",50.6772450028359,-0.176186170533093,0.258851235056231,-0
.68064643575998,0.496095236931018,0.830279710851467
"9347","LINC01273",3.74230396820573,-0.53654522955118,0.78816590735610
4,-0.680751634324068,0.496028658363959,0.830279710851467
"9348","CD74",703.477252893108,-0.262213712883915,0.385353007827674,-0
.680450671352161,0.496219145929745,0.830287314127608
"9349","ATP2A3",8.48327164396735,0.400896333059063,0.589126329222477,0
.680492982868653,0.496192363474983,0.830287314127608
"9350","MYOC",4393.04372654969,-0.426557353093598,0.627184732087853,-0
.680114376626516,0.496432042066038,0.83035815126747
"9351","ARF4",844.372357562149,-0.183347048093142,0.269533750483335,-0
.680237809789532,0.49635389529742,0.83035815126747
"9352","ZFP28",18.8339183016992,0.31618386321622,0.464943169335577,0.6
80048410363916,0.496473808655926,0.83035815126747
"9353","TBX1",25.279031315045,-0.52250215619802,0.76825264746362,-0.6
8011761225043,0.496429993476476,0.83035815126747
"9354","ROCK1",630.134791835856,-0.143935308846926,0.211689478663491,-
0.679936054241649,0.496544951334576,0.830388355112975
"9355","AC008494.3",4.17736816203833,-0.48162230436276,0.7087339741963
45,-0.679553008459749,0.496787532618861,0.830705224239107
"9356","CRTAP",902.797465609303,-0.0914068065536551,0.134662725531278,
-0.678783280176698,0.497275189245058,0.830994524275098
"9357","TLL1",6.42500274412193,-0.555591235664985,0.818412260986538,-0

.678864750871718,0.497223561923989,0.830994524275098
"9358","AC092171.4",6.58519944068936,0.585839702923196,0.862743663192876,0.679042603170329,0.497110868280024,0.830994524275098
"9359","KIFC2",12.0388226473358,-0.334381632847821,0.492623818033764,-0.678776828498582,0.497279277743348,0.830994524275098
"9360","ISG20",48.2736255721441,-0.182087076596021,0.268171268754976,-0.678995469728679,0.497140732406727,0.830994524275098
"9361","C22orf23",3.57213581228842,-0.506638727688002,0.746069446929878,-0.679077168717808,0.49708896787937,0.830994524275098
"9362","CD96",3.56995047413423,0.563812086900212,0.830947946469643,0.678516734165623,0.497444117248292,0.831181192706156
"9363","SAAL1",30.9326911748329,0.215661162630565,0.318007919983052,0.678162866641994,0.497668433931598,0.831467191283989
"9364","FGD5-AS1",491.014435531084,0.0991288238470604,0.146287274709475,0.677631216002413,0.498005548562238,0.831497576706061
"9365","UBR5-AS1",12.0912669295353,-0.316674022685168,0.467151415158388,-0.677883042648602,0.497845852528119,0.831497576706061
"9366","DHRS4L2",302.098564387645,-0.192457443114854,0.284010087050675,-0.677642984844106,0.497998084736382,0.831497576706061
"9367","AC021739.2",3.32487993687329,-0.588814195585739,0.86861991770497,-0.677873237285967,0.497852070095318,0.831497576706061
"9368","MLX",171.416095789213,-0.129342415433265,0.190845611771313,-0.677733243289102,0.497940844606521,0.831497576706061
"9369","ZNF766",117.292379924272,0.14402930153715,0.212450664572777,0.677942344058008,0.497808250465323,0.831497576706061
"9370","PURA",546.595283004545,-0.118207616557089,0.174499586863814,-0.677409148534789,0.498146395406831,0.831629573437473
"9371","SDHAF2",181.133142505142,-0.151397321385306,0.223517817834383,-0.67733893813103,0.498190930939242,0.831629573437473
"9372","CPA1",2.80917000401236,-0.718131071687191,1.06051236668089,-0.67715483029655,0.498307723411493,0.831735778630599
"9373","PEX10",82.499149337328,-0.183940281159464,0.271676267627819,-0.677056861703695,0.498369877675879,0.831750773123202
"9374","ZNF775",50.9777931269897,-0.222091543249644,0.328110357074087,-0.676880624038015,0.498481698607224,0.831848646395648
"9375","OTUD6B",63.6799343909587,0.218081880828185,0.322270148203903,0.676705186762142,0.498593024949103,0.831856942115916
"9376","FBXL22",6.24555144593555,-0.41850064984547,0.618374032821206,-0.676775911718261,0.49854814378233,0.831856942115916
"9377","ZNF485",16.6897614495416,0.272936856217595,0.403392014409312,0.676604509926301,0.498656916906884,0.831874816164486
"9378","C6orf62",318.68894380053,0.118128547406694,0.17468113539828,0.676252459301664,0.49888037097528,0.831944868633048
"9379","CLIC3",29.628642681035,-0.360205018710192,0.532639504224416,-0.676264182159549,0.498872929369989,0.831944868633048
"9380","IL15RA",316.180786347474,-0.193167450075872,0.285727749212886,-0.676054218072987,0.499006222343524,0.831944868633048
"9381","SLC35C1",85.7801476866943,0.133293428112408,0.197157020679905,

0.67607751249608,0.498991433248301,0.831944868633048
"9382","ANKLE2",213.696190856103,0.148416635643212,0.219539656959338,0
.676035654326911,0.499018008207114,0.831944868633048
"9383","SLC25A53",25.3218470972172,-0.206268846929612,0.30494411331937
2,-0.676415244368347,0.498777041194512,0.831944868633048
"9384","TMEM234",64.0613836808334,-0.17007620517794,0.2516897494418,-0
.675737512374408,0.499207314638524,0.83208902616277
"9385","CCDC50",646.379663801325,0.123060015535952,0.182134555159023,0
.6756544107103,0.499260087172104,0.83208902616277
"9386","NRSN2",62.7677889681543,0.191111180528161,0.282856064314549,0.
675648163992118,0.499264054181663,0.83208902616277
"9387","NSMF",53.3057316196944,0.198467228188763,0.293828595894162,0.6
7545239286462,0.499388388097654,0.832207580165293
"9388","LDLRAD2",20.7732946424268,0.332053933826988,0.492473323823412,
0.674257706486563,0.50014748764955,0.832488896574804
"9389","RFX5",28.7654574798786,0.208661361610216,0.309475146030387,0.6
7424271152853,0.500157019281903,0.832488896574804
"9390","RHOQ",463.327066449584,-0.168880156935502,0.250308586012984,-0
.674687830831107,0.499874117639536,0.832488896574804
"9391","PRPF4B",603.31030135254,0.125184100720869,0.185629251707599,0.
674377015310378,0.500071651735161,0.832488896574804
"9392","STX7",284.152928321981,0.11125875439146,0.16487237109894,0.674
817458194332,0.499791747171179,0.832488896574804
"9393","DNAAF5",66.4348581876034,0.162552642326812,0.241051732975133,0
.674347536607757,0.500090388628111,0.832488896574804
"9394","SEC24C",81.6272622555352,0.176417639141904,0.261636404864189,0
.674285519377468,0.500129808478706,0.832488896574804
"9395","NUP107",131.098513116156,0.167021880983875,0.247572725927091,0
.674637645800542,0.499906009171803,0.832488896574804
"9396","ACADS",217.536717424819,-0.194849029528224,0.288660937579216,-
0.675010034825902,0.499669389495359,0.832488896574804
"9397","SLC38A6",99.1949270055146,0.12756386505991,0.189212891481126,0
.674181680018532,0.500195815310783,0.832488896574804
"9398","AC012146.1",39.7856518275502,-0.211764753466451,0.313687986181
24,-0.6750808535718,0.499624397292291,0.832488896574804
"9399","FDX2",151.854702201666,-0.190385587577867,0.282292394378612,-0
.674426911135694,0.50003993840883,0.832488896574804
"9400","PPIL6",24.7497012156371,-0.279754154078073,0.415213751892009,-
0.673759365635926,0.500464312522608,0.832847153275656
"9401","DNAJC10",320.122773052625,0.135838600304413,0.201653246834845,
0.673624662317815,0.500549969689568,0.832901093059665
"9402","MFSD8",95.4892016422208,0.182699368592633,0.27147772257255,0.6
72981071379839,0.500959333223579,0.832969373150165
"9403","SCLT1",73.5857915233561,0.142668143253361,0.211989703799974,0.
672995625240259,0.500950074110463,0.832969373150165
"9404","NAA15",139.785394029422,-0.125106999212964,0.185845613967872,-
0.673177034108495,0.500834670058333,0.832969373150165
"9405","PDLIM1",931.674709505724,0.176802836423451,0.262718619839482,0
.672974136859714,0.500963744971985,0.832969373150165
"9406","MRVI1",9.77453318131674,-0.456085075633865,0.677301096635993,-

0.673385999076541,0.500701753537669,0.832969373150165
"9407","VPS37C",44.8182882539521,0.171727042603836,0.255122597387771,0
.673115766153875,0.500873644362146,0.832969373150165
"9408","RFC5",48.1888280302413,-0.170941984940276,0.253999046058374,-0
.67300246828876,0.500945720619216,0.832969373150165
"9409","SCNM1",131.321293612923,-0.186006550725141,0.276587634134457,-
0.672504941543115,0.501262295303553,0.83302306230038
"9410","CRTC2",69.8900628916035,-0.157936928716261,0.23480788812427,-0
.672621903709955,0.501187863122416,0.83302306230038
"9411","NUDT18",53.1714226884079,-0.241011718598967,0.358361549046971,
-0.672537885942047,0.501241329611689,0.83302306230038
"9412","DNAH9",4.1041087520221,0.492857488098409,0.732653715202375,0.6
72701820616948,0.501137009106805,0.83302306230038
"9413","SLC14A2",5.91473784648705,-0.705712775625164,1.04906443130823,
-0.672706799090604,0.501133841214584,0.83302306230038
"9414","AC010883.3",11.9469915814057,0.565354542959811,0.8420221397564
12,0.671424795461272,0.501949953426686,0.833474688899367
"9415","METAP1",70.5625138687203,0.157500103336759,0.234610852408741,0
.671324884248583,0.502013585553272,0.833474688899367
"9416","EXOC2",55.0431194045106,0.183976751343411,0.274035503474484,0.
671361006186343,0.50199057947745,0.833474688899367
"9417","GSTK1",676.551732913545,-0.172066114066641,0.256274647322762,-
0.671412938674087,0.501957504634015,0.833474688899367
"9418","KDM4C",50.3639124067052,0.200436452790416,0.298547610773139,0.
671371819963161,0.501983692287415,0.833474688899367
"9419","LINC02666",3.60456791739088,0.506420151181062,0.75435013843978
2,0.67133301284797,0.502008408396601,0.833474688899367
"9420","CCDC82",337.522300375886,0.0977182602860983,0.145506196121632,
0.671574564456438,0.501854575534686,0.833474688899367
"9421","RIPOR1",104.392809573678,0.138637039852654,0.20638455718143,0.
67174134414902,0.501748375952102,0.833474688899367
"9422","RALY",437.205924234539,-0.175497515802804,0.261170727942279,-0
.671964722790798,0.50160615474049,0.833474688899367
"9423","TNFSF10",1092.70172572433,-0.181129988451908,0.269901325977011
,-0.671097067775561,0.502158694805295,0.833627131788096
"9424","AL357075.4",4.37570278108714,-0.543826900379594,0.810575177592
238,-0.670914821244585,0.502274793946784,0.833731388127073
"9425","AC114760.2",15.5595854918501,0.39415772087236,0.58779658663244
5,0.670568237101437,0.502495622621124,0.833744062861926
"9426","CGAS",37.9230519610943,-0.193230682496458,0.288133612095671,-0
.670628744390637,0.502457066251374,0.833744062861926
"9427","PDSS2",67.0370924183065,0.130141891222983,0.194053804002861,0.
670648493038889,0.502444482384145,0.833744062861926
"9428","KNTC1",24.4179577242528,0.242099805971512,0.360994978594868,0.
67064591012833,0.502446128209015,0.833744062861926
"9429","NMI",180.113690880159,-0.13614463792925,0.203103984799507,-0.6
7031987611491,0.502653899248459,0.833788679893393
"9430","LINC01151",9.69525452190385,-0.513444184722304,0.7660200412826
59,-0.670275132570382,0.502682416421057,0.833788679893393
"9431","OGFOD3",194.380472066667,0.171186001021983,0.255366135116273,0

.670355139079183,0.502631425099743,0.833788679893393
"9432","MCMBP",162.330152440177,0.115159038300215,0.171833979855687,0.670176168863285,0.502745493699941,0.833804893760409
"9433","CD109",415.586541015559,-0.15649159764894,0.2336802716221,-0.669682539149101,0.503060184837391,0.833863008415252
"9434","AL512662.2",2.78581272168437,0.612366794757296,0.914159216723226,0.669868862617067,0.502941390571344,0.833863008415252
"9435","NT5C2",100.743649847997,0.177590434862753,0.265187536134602,0.669678663829107,0.503062655782795,0.833863008415252
"9436","SRP14",4507.7517140658,-0.1568053404034,0.234200041541772,-0.669535920536684,0.503153674898137,0.833863008415252
"9437","RILP",136.1600078677,-0.216624979240242,0.323413577481024,-0.669807931155741,0.502980237015921,0.833863008415252
"9438","SLC13A3",9.65872740898833,-0.317762544737075,0.474589187986237,-0.669552852827086,0.503142877704218,0.833863008415252
"9439","AL391097.1",6.0565074672614,0.657373556948829,0.981698448714223,0.669628802826187,0.503094448262629,0.833863008415252
"9440","WNT5A-AS1",2.61827801278274,0.678139221792055,1.01344665312631,0.669141508041012,0.503405214085388,0.834103142033442
"9441","HAUS2",64.6350588467862,-0.156522708156597,0.233914187220281,-0.669145852231686,0.503402443187291,0.834103142033442
"9442","WDTC1",114.172587983181,0.139475499771354,0.208660221002757,0.668433585956528,0.50385686261219,0.834497925023026
"9443","MBD4",256.72106642452,-0.102646795693452,0.153524489667712,-0.668602096744435,0.503749334710388,0.834497925023026
"9444","CTSL",1565.73556724292,0.202374525089009,0.302750555784904,0.668453025839498,0.503844457270023,0.834497925023026
"9445","ZC2HC1C",2.68748648647309,-0.563086960199165,0.842302196192215,-0.668509428973004,0.503808465158605,0.834497925023026
"9446","GAS7",606.208468593637,-0.184406734205012,0.275921574928929,-0.668330246565573,0.503922810185551,0.834518793111642
"9447","PHPT1",1084.83077881808,-0.193305707180249,0.289297125162426,-0.668190902594407,0.504011741830304,0.834577715407161
"9448","SUDS3",198.364165473885,-0.122279467976888,0.183025069203143,-0.66810229062756,0.504068299771715,0.83458302427275
"9449","ZNF629",44.3805075290772,0.171225980995475,0.256320978250433,0.668013918190428,0.504124708164497,0.834588084434038
"9450","OSBPL9",305.096433798947,0.114267875183322,0.171127182961594,0.667736552462078,0.50430177317124,0.834623054934219
"9451","FANK1",9.11998144014574,0.352097942220689,0.52729671627118,0.667741579561839,0.504298563674498,0.834623054934219
"9452","MC1R",7.35083633184508,0.351730484673754,0.526755475851756,0.667730096407657,0.504305894984225,0.834623054934219
"9453","GLOD4",263.06870825827,-0.125673539577547,0.188238203976724,-0.66763035835747,0.504369574150886,0.834640140531292
"9454","C1orf159",18.4441047138869,0.262943010242557,0.393991234294567,0.667382894224413,0.504527589433291,0.834725021840821
"9455","AL035587.2",12.3047228910731,-0.286931347738609,0.429887233420073,-0.667457243277164,0.504480111985945,0.834725021840821

"9456","SLC9A5",2.97901453793513,0.64592668695348,0.967991311586858,0.667285624593668,0.504589706946221,0.83473950780031
"9457","SNRPE",694.854932652533,-0.153087139074614,0.22948395632741,-0.667092992140162,0.504712736161182,0.834759975922583
"9458","TFAM",222.7795012749,-0.105436036209603,0.158071319879932,-0.667015599602068,0.504762169165231,0.834759975922583
"9459","CNKSR2",69.9889206471002,-0.346542841921752,0.519495742417359,-0.667075422618848,0.504723958133098,0.834759975922583
"9460","USP37",58.7071892493526,0.159953482553325,0.23987084748604,0.666831689760191,0.504879648297533,0.834793642418246
"9461","AL031651.2",5.34604088355572,0.472620465932353,0.708771244923625,0.666816648273141,0.504889257234484,0.834793642418246
"9462","GTF2IRD1",12.8290234059839,0.28096303368395,0.421481562022301,0.666608124767944,0.505022478009272,0.834880739497763
"9463","BPGM",87.0814680587694,-0.192413677491911,0.288762696911176,-0.666338414033783,0.505194817336488,0.834880739497763
"9464","VCP",583.159091743473,0.106336131252298,0.159612359625785,0.666214894019519,0.505273754296462,0.834880739497763
"9465","AC090559.2",6.96438828299703,0.39557630648282,0.593602084967418,0.666399792892461,0.505155594850248,0.834880739497763
"9466","REC8",10.0731888169069,0.378429104063746,0.568089463394425,0.666143501065081,0.505319381790431,0.834880739497763
"9467","AC092140.1",7.43136061636774,-0.562805243923182,0.8447576308042,-0.666232802641152,0.505262309172417,0.834880739497763
"9468","SLC16A11",4.08715727275167,0.571268540859506,0.857675548369753,0.666066022221757,0.505368901253233,0.834880739497763
"9469","C1QTNF1",113.310557540851,-0.257931128288945,0.387140359120101,-0.666247065728757,0.505253193950666,0.834880739497763
"9470","AC015982.2",9.12486781314694,-0.333680255059516,0.501301322687973,-0.665628116180357,0.505648830492167,0.834995295207267
"9471","LBX2-AS1",33.0664401173381,0.18320994768262,0.275245595922652,0.665623539110523,0.505651756792076,0.834995295207267
"9472","MON1A",22.4865188872648,-0.211711468896282,0.318061593867328,-0.665630409261524,0.505647364438969,0.834995295207267
"9473","FRG1-DT",4.1182114514459,0.49927364030248,0.749950102979848,0.665742478491127,0.505575717084682,0.834995295207267
"9474","MROH1",51.9143849703827,0.207029719081568,0.311138623959069,0.665393824936383,0.505798633506599,0.835149675315995
"9475","AC068620.1",2.77332968903684,-0.63785137210255,0.958790617221225,-0.665266597989013,0.505879990677677,0.835195851627536
"9476","PSMC6",428.629182870162,-0.11490040353042,0.172801695825065,-0.664926365345042,0.506097591351218,0.835449151348655
"9477","DLX4",2.7959589621904,-0.787013933785443,1.18372932713646,-0.664859707150531,0.50614022932501,0.835449151348655
"9478","AC092296.2",6.05650968154315,0.449783180607581,0.676618815979865,0.664751215876572,0.506209629906183,0.835475547649549
"9479","TNFRSF25",3.76008930444119,-0.56447237997475,0.849347095340034,-0.664595644197459,0.506309155998769,0.835505581375991

"9480","SLC25A51",33.7215223309417,0.200411007923564,0.301571378092977
,0.664555798334999,0.506334648817004,0.835505581375991
"9481","ZDHHC2",88.5497808775125,-0.169507476259796,0.255162433574739,
-0.664312037963638,0.506490617944707,0.835586662783068
"9482","ABHD15",39.3472729796224,-0.18764418476533,0.282462557371672,-
0.664315251236723,0.506488561780095,0.835586662783068
"9483","TRAF3IP2-
AS1",61.8168846158668,0.140759029151173,0.211917137514862,0.6642173011
67071,0.506551241732602,0.835593885038771
"9484","ECHS1",640.0860291727,-0.165103628140966,0.248618732376207,-0.
664083621386717,0.506636792328905,0.835593885038771
"9485","TMCO3",244.554431855793,0.104398802476767,0.157214139881892,0.
664054788934358,0.506655245131544,0.835593885038771
"9486","TUFT1",26.2955417727746,0.279629444902175,0.421329636866078,0.
663683302656102,0.50689302837492,0.835776269659183
"9487","BATF",5.15448677170908,0.625880854418371,0.943172315883638,0.6
63591205846618,0.506951987342979,0.835776269659183
"9488","LINC01270",3.43899551935766,0.628601254500075,0.94698683222055
1,0.663790913571727,0.506824142078895,0.835776269659183
"9489","ZNF630",8.84490999152383,0.352047689030853,0.530553333654347,0
.663548161324363,0.506979545023076,0.835776269659183
"9490","LRRC28",74.7801843959274,-0.174874705890899,0.263654010265081,
-0.66327345339856,0.507155435259042,0.835978132113508
"9491","S100A9",53.6879210743117,-0.305113125027928,0.46121640464152,-
0.661540053557022,0.508266036947203,0.836264681602972
"9492","SOWAHC",107.268398839788,0.136459670306844,0.206275603267333,0
.661540522220618,0.50826573649878,0.836264681602972
"9493","ITGA9-
AS1",74.9958813218171,0.168055310795913,0.253690378078515,0.6624425887
52423,0.507687617055729,0.836264681602972
"9494","NSUN3",75.0506121568963,0.133501091231226,0.201910991241127,0.
661187835345705,0.508491861506832,0.836264681602972
"9495","THAP9",13.9480777796431,0.335421692624315,0.506238638361207,0.
662576238175222,0.50760199273107,0.836264681602972
"9496","PPP3CA",414.346033221334,-0.14064108591763,0.212471898546912,-
0.661927939080273,0.508017405179877,0.836264681602972
"9497","INPP4B",3.99630984888664,0.608800517624392,0.920678468340236,0
.661252042444214,0.508450691231777,0.836264681602972
"9498","ITGA1",23.9693869078099,0.359024062437484,0.541833200049446,0.
662609936793686,0.507580404448613,0.836264681602972
"9499","ERCC8",28.5241203113104,-0.192426585779104,0.290668303770154,-
0.662014341719438,0.507962030415514,0.836264681602972
"9500","MACC1",2.71900951120118,0.605796966219615,0.914058743341412,0.
662754960370575,0.507487503768986,0.836264681602972
"9501","MALSU1",245.508638456976,-0.183179044104381,0.276764829900218,
-0.661858098698533,0.50806216763982,0.836264681602972
"9502","ARF5",469.295234077968,-0.296339574324627,0.447549835194659,-0
.662137601270115,0.507883039831854,0.836264681602972
"9503","RARRES2",2174.18840493431,0.203835494250107,0.308345995205917,
0.661060942640048,0.50857323160085,0.836264681602972

"9504","MFSD3",30.3320161945209,-0.211743901536386,0.319955747654017,-0.66179121046875,0.508105039931031,0.836264681602972
"9505","RECQL4",4.87681008977451,0.460907134249521,0.695845057545753,0.662370349909707,0.507733901011385,0.836264681602972
"9506","TOR1A",162.456588720038,-0.156644451051933,0.236636565240806,-0.661962156577655,0.507995475079123,0.836264681602972
"9507","AP000821.2",12.2636989943174,-0.280597263426464,0.423989932735357,-0.661801712168494,0.508098308696972,0.836264681602972
"9508","OSBPL8",806.300430935248,0.115350736068051,0.174335998317385,0.661657587539958,0.508190691789752,0.836264681602972
"9509","LINC00345",4.86480063441391,-0.540771354036182,0.818085483399761,-0.661020596269317,0.508599105187914,0.836264681602972
"9510","SGPP1",85.1913495104776,0.194936453843511,0.294316450393596,0.662336249240632,0.507755750329329,0.836264681602972
"9511","PECAM1",9.54846816589483,-0.431243706738895,0.65241103042214,-0.661000023957076,0.508612298201794,0.836264681602972
"9512","MRI1",136.45325352921,0.190744938855721,0.288421515254769,0.661340880506893,0.50839373020267,0.836264681602972
"9513","ERGIC3",584.927863742331,-0.12806701405387,0.193514903011429,-0.6617940637177,0.508103211090406,0.836264681602972
"9514","HMGN1",540.047616558547,0.139768808444045,0.211325466640623,0.661391221161878,0.508361454357544,0.836264681602972
"9515","OFD1",183.655569130151,0.15629818379453,0.236620157443387,0.660544669918603,0.508904362453504,0.836656956580155
"9516","ALDH18A1",75.2363611340067,0.150359016249159,0.227701356006423,0.660334303169093,0.509039321439164,0.836702963672674
"9517","SRP68",170.811847741618,0.110233470093629,0.166929504680035,0.660359415220943,0.509023210031939,0.836702963672674
"9518","KCNAB2",23.4547176843289,0.205258324136205,0.310897328572682,0.660212569463168,0.509117427219214,0.836707528409529
"9519","LCORL",71.2811188122628,0.156372729787903,0.236869789608501,0.660163248535643,0.509149073894413,0.836707528409529
"9520","CYP4F12",49.5362577974143,0.232366415520163,0.35209292869163,0.659957631828812,0.509281018547941,0.836748553003407
"9521","BFSP1",4.25978059513604,0.484006202810673,0.733349876606588,0.659993569577325,0.509257955931188,0.836748553003407
"9522","EEF1AKMT1",156.691085708117,-0.209617653095333,0.317688569764347,-0.659821199267011,0.509368577479162,0.836804521897347
"9523","WDR4",18.6629067927487,-0.247079993433247,0.374612241648994,-0.659561984268409,0.509534956754841,0.836989953640237
"9524","POMC",10.1304880253673,-0.553189145718246,0.839972955999948,-0.658579709938043,0.510165695700735,0.837130885580455
"9525","ATG16L1",35.1173157740015,0.183978586136676,0.279534342193134,0.658160942563411,0.510434719130124,0.837130885580455
"9526","ROBO1",223.823697269605,0.122513002603886,0.185974776637552,0.658761391296911,0.51004900351501,0.837130885580455
"9527","AL359643.3",14.1764535476616,-0.287667226065045,0.436988594234206,-0.658294586770998,0.510348855713641,0.837130885580455
"9528","RSPO3",166.802390569747,0.308425686986066,0.468664270945235,0.658095157038559,0.510476987633572,0.837130885580455

"9529","CDHR3",27.7026907724151,-0.210634708953214,0.320020611280041,-0.658191071227263,0.510415361477507,0.837130885580455
"9530","AC010997.5",10.1107401128913,0.44122965712049,0.669748358668266,0.658799161520657,0.510024745817705,0.837130885580455
"9531","AL450306.1",10.5771271781934,-0.501081282937085,0.760549343417549,-0.658841253725185,0.509997713065588,0.837130885580455
"9532","AP003068.2",7.32677189726015,-0.345658571197897,0.524394486511623,-0.659157523751416,0.509794619881241,0.837130885580455
"9533","LTBR",375.877954831431,0.11163462360204,0.169332554012178,0.659262622318989,0.509727140078429,0.837130885580455
"9534","CDK2",21.6211177697084,-0.230244284495934,0.349424797507114,-0.658923711592755,0.509944758565762,0.837130885580455
"9535","SOCS1",282.04570982664,-0.327716446876142,0.497898534906814,-0.658199259287792,0.510410100718734,0.837130885580455
"9536","CYB5A",1687.84684072022,-0.184883453580098,0.280805963979245,-0.658402873500804,0.510279289449686,0.837130885580455
"9537","VAV1",3.02158607420248,-0.568442265852478,0.863720560396877,-0.658132145877459,0.510453221346331,0.837130885580455
"9538","AC123912.4",4.14373109127507,-0.674332584263557,1.02359411280685,-0.658789041307043,0.510031245404372,0.837130885580455
"9539","APOBEC3D",32.6490079836357,0.208200571005477,0.316010438614239,0.658840802596498,0.509998002788657,0.837130885580455
"9540","KIF21B",5.11252830572116,0.73317671008636,1.11527174274868,0.657397369612708,0.510925442512155,0.837602881703798
"9541","MRPL53",25.4125693854689,-0.251542959602276,0.382602271443833,-0.657452865224828,0.510889768990919,0.837602881703798
"9542","DDX55",101.055937525892,-0.136864534581908,0.208178223423397,-0.657439247637109,0.510898522486896,0.837602881703798
"9543","HIBCH",243.502356531679,-0.112898431654113,0.171791275658169,-0.657183731953648,0.511062784573234,0.837652466374591
"9544","PCDHB14",72.9479738860569,0.18840971530389,0.286692317242625,0.657184388880711,0.511062362222455,0.837652466374591
"9545","EEA1",1290.32962105821,-0.101570389493461,0.154583113819947,-0.657060056454595,0.511142301128902,0.837695025307429
"9546","RPN1",409.187585326637,0.125616001683044,0.19122441480269,0.656903574852919,0.511242919467735,0.837699116954793
"9547","AP001033.2",14.840010587635,-0.270913705492467,0.412418923432528,-0.656889609326543,0.511251899863671,0.837699116954793
"9548","ZRANB2-AS2",9.12457143328233,0.421728893303555,0.642534757031597,0.656351876203354,0.511597746567416,0.838035963823546
"9549","ETFA",329.404698106729,-0.123031301153804,0.187452985868048,-0.656331509386616,0.511610848023618,0.838035963823546
"9550","AL022311.1",32.3123300430456,-0.22326631103258,0.340179001121066,-0.656320085298628,0.511618196926092,0.838035963823546
"9551","BAG6",225.390059464288,-0.128446717694722,0.195749649528577,-0.65617853214072,0.511709260000345,0.838097367206094
"9552","ZNF331",158.337860724025,-0.144967331741211,0.220966392172762,-0.656060545297172,0.511785169007247,0.83813394040833
"9553","PLEKHA6",62.0880690541648,-0.361335207680434,0.550986019978067

,-0.655797415140983,0.511954479799322,0.838310955732057
"9554","TJP1",219.981186778959,-0.157802865101422,0.240653666323192,-0
.655725996251795,0.512000439242094,0.838310955732057
"9555","MRPL10",191.61347220315,-0.173799918622935,0.265083849656079,-
0.65564129556902,0.512054948567105,0.838312460537438
"9556","CYP4Z1",5.52981802182823,0.495458602099846,0.755983742321891,0
.655382615210903,0.512221441663408,0.838497280445866
"9557","C5orf63",27.6439504460008,-0.241854318153465,0.369165422389727
,-0.655138058672623,0.512378870277859,0.838667224835884
"9558","TRABD2A",28.5234280782127,-0.402153995874233,0.614331565728937
,-0.654620433506548,0.512712164813414,0.838918022218024
"9559","TAX1BP1",667.644315897895,-0.103586489908167,0.15820284688463,
-0.654770074926068,0.512615800339624,0.838918022218024
"9560","CCDC183",16.3441242681184,0.302950698312264,0.462779550569075,
0.65463285475715,0.512704165550596,0.838918022218024
"9561","RPS27L",1615.55625143029,-0.155602341260587,0.237717992725392,
-0.654566949167941,0.5127466093733,0.838918022218024
"9562","CNIH1",410.79841059477,-0.131884617126401,0.201516224971106,-0
.654461531051957,0.512814503443649,0.83894135927306
"9563","LIN52",35.7551535619176,-0.211981267567916,0.324069293020484,-
0.654123275896173,0.513032386804899,0.839210041492109
"9564","OST4",2171.98088612332,-0.168604163796625,0.258206437408141,-0
.652982030537511,0.513767863200121,0.839369464794632
"9565","RGPD5",30.989553020581,0.326358226386663,0.499570169654912,0.6
53278050232867,0.513577040407338,0.839369464794632
"9566","XXYLT1",68.2021395740947,0.142508759929875,0.21835736886217,0.
652640030755402,0.513988372004588,0.839369464794632
"9567","TMEM154",12.7896181185226,0.306530739457189,0.468858267075792,
0.653781240478026,0.513252754206365,0.839369464794632
"9568","SIT1",7.20123318780274,-0.341292719086758,0.522547368917738,-0
.653132595028885,0.513670800396749,0.839369464794632
"9569","AK8",5.40568524922503,0.515688486450018,0.789964282005889,0.65
2799750819839,0.513885384290083,0.839369464794632
"9570","AC004803.1",6.61602309218142,-0.472406880788938,0.723820794238
349,-0.652657238572477,0.513977275864713,0.839369464794632
"9571","TWF1",165.927733376478,-0.128490633799909,0.196799804000503,-0
.652900212235886,0.513820612133298,0.839369464794632
"9572","PSEN1",138.873539811942,0.115691468770576,0.177015175258888,0.
653568083083133,0.513390112693208,0.839369464794632
"9573","CYLD",138.000480343472,0.14903915347435,0.228162953222789,0.65
3213641255866,0.51361855714228,0.839369464794632
"9574","NRN1L",3.51292105452045,0.490083716837894,0.750581926384394,0.
652938339720837,0.51379603067899,0.839369464794632
"9575","AC009060.1",5.38298037155476,0.525627923768008,0.8052133996789
35,0.652780895074018,0.513897541941648,0.839369464794632
"9576","PLCG1-
AS1",9.42866006255796,-0.412481446323255,0.631237659961359,-0.65344872
8563667,0.513467033026076,0.839369464794632
"9577","AURKA",4.70055922268197,-0.422626169666998,0.647323398224225,-
0.652882578980415,0.513831980807485,0.839369464794632

"9578","SON",1821.01368084773,0.120339052592993,0.184272073946423,0.653050948067051,0.513723433687589,0.839369464794632
"9579","NOL12",76.5219668792682,-0.166691754240647,0.255028606450559,-0.653619829401225,0.513356765640325,0.839369464794632
"9580","RDH14",345.377377692193,-0.135981159969972,0.208548531285748,-0.65203604710912,0.514377918527949,0.839479789205291
"9581","FBXO4",79.3999157526007,-0.162813959248884,0.249670655281191,-0.6521149194146,0.514327040172944,0.839479789205291
"9582","LRP12",73.5492297991374,0.158635045197602,0.243195490581183,0.652294353067568,0.514211302207065,0.839479789205291
"9583","AC092868.2",4.36426556616098,0.608123836997933,0.932496120193936,0.652146238282963,0.514306837958175,0.839479789205291
"9584","ZNF823",26.527918323299,0.224700677098815,0.344446010788149,0.652353837934319,0.514172936376885,0.839479789205291
"9585","C19orf57",2.79721008207126,-0.559839377172105,0.858375817127422,-0.652207769605652,0.514267148421933,0.839479789205291
"9586","MAP3K1",50.6629361708869,0.224432098266396,0.344284429269016,0.651879896929725,0.514478654431985,0.839485538527501
"9587","IPO11",35.2309756085412,0.169494431766612,0.260048118012575,0.65178103599433,0.514542437090179,0.839485538527501
"9588","TIGAR",8.99305734662488,-0.383773393618957,0.588744999243486,-0.651849942015798,0.51449798017598,0.839485538527501
"9589","OSBPL11",107.166572113528,0.153525968167288,0.235603813278001,0.651627688156876,0.514641381497228,0.839559404605396
"9590","PHYH",405.340420629568,-0.175051867949301,0.268780984619945,-0.651280700518393,0.514865304334524,0.839749552257842
"9591","ECH1",731.675305525915,-0.176280684692352,0.270639230985253,-0.65134934078333,0.514821004438314,0.839749552257842
"9592","FARSA",71.813840106397,-0.170913080726677,0.262468123891037,-0.65117652457344,0.514932542467547,0.839771659906156
"9593","MPV17",209.365806268982,0.146547944413516,0.225277749497988,0.650521166604715,0.515355633824929,0.839811328480978
"9594","PODXL",7.1078940881833,-0.451388358300817,0.693538917473525,-0.650847914844013,0.515144666466318,0.839811328480978
"9595","DENND3",13.2153610626024,0.364059750649915,0.559494081981118,0.650694551336115,0.515243681136219,0.839811328480978
"9596","INTS6",298.36800476802,-0.169380785055347,0.26028532515764,-0.650750421495189,0.515207609055429,0.839811328480978
"9597","LRFN5",41.0106686563296,0.351416024052239,0.539869456845129,0.650927774476875,0.515093111339522,0.839811328480978
"9598","PSMA3",562.68407988189,-0.184096931274711,0.283019839451141,-0.650473590938814,0.515386355137594,0.839811328480978
"9599","BBS4",118.293431297093,0.115515573514392,0.177522564477628,0.650709242818254,0.515234195581861,0.839811328480978
"9600","MAPK8IP3",42.4760002077547,0.210302249546111,0.323241185913068,0.650604745654749,0.515301666139026,0.839811328480978
"9601","WNT5B",65.6727961687991,0.184049031262314,0.283037887062459,0.650262878841018,0.515522430921091,0.839829879444662
"9602","ANG",551.414683656963,-0.157489536173761,0.2422146204757,-0.650206564180385,0.515558801528293,0.839829879444662

"9603","SMAD3",336.002641849473,0.127250798189701,0.195674634406854,0.650318313231731,0.515486630133612,0.839829879444662
"9604","LINC01409",17.6495191545954,0.282293773721934,0.434274208205168,0.650035780132186,0.515669109893049,0.839922103920966
"9605","EPB41L5",35.6407834124574,0.17668143280715,0.272040431137876,0.649467551819914,0.516036213208542,0.839995263602625
"9606","THUMPD3-AS1",239.676395937869,-0.19092671180989,0.293937493878568,-0.649548682240472,0.515983790690099,0.839995263602625
"9607","ENG",375.164193458965,0.117473287836782,0.180875448307668,0.649470610499665,0.516034236788848,0.839995263602625
"9608","CLPX",171.110849978391,0.134477362506949,0.20703399477379,0.649542422508353,0.515987835325161,0.839995263602625
"9609","UBE2Q2L",8.82936659824269,-0.328717043470007,0.50612969452765,-0.649471957532121,0.516033366381391,0.839995263602625
"9610","VEZF1",254.608373998146,-0.124550939934773,0.191737392564317,-0.649591288736196,0.515956261558146,0.839995263602625
"9611","AL590560.3",9.57783410219958,0.487343544453899,0.750526709673541,0.649335377638833,0.5161216236298,0.840046879454891
"9612","DEDD",83.0130663421902,-0.144096826296596,0.22205635554983,-0.648920072293362,0.516390039964676,0.840049261573351
"9613","BET1",189.855935976221,0.144737402153167,0.223081433619181,0.648809718518514,0.516461374969692,0.840049261573351
"9614","SLC38A2",763.07387736151,-0.208993024743722,0.322103395570191,-0.648838315950566,0.516442888496792,0.840049261573351
"9615","AC127070.1",2.84234498253768,0.6380939852189,0.983353423877953,0.648895879878582,0.516405678014125,0.840049261573351
"9616","TEP1",31.6038845050211,0.260776018978381,0.402005476421646,0.648687727589223,0.516540238420493,0.840049261573351
"9617","PTBP1",324.123577921437,-0.10525323849447,0.162260454511564,-0.648668455979015,0.516552697505214,0.840049261573351
"9618","SPACA6",30.7431241256938,-0.216581044480615,0.333713956995501,-0.649002056822977,0.516337046744626,0.840049261573351
"9619","AC005261.1",171.318679066117,0.19134350412429,0.294811372014968,0.649037053138423,0.516314426659389,0.840049261573351
"9620","KIAA1328",116.555036959756,0.136641708208502,0.21082532966181,0.648127568104326,0.516902444720321,0.840354357533718
"9621","PARD6G",9.13170068400287,0.323417670828765,0.499065901867192,0.648046018809818,0.516955186508149,0.840354357533718
"9622","MLLT1",136.858322119888,0.123245696631688,0.190176962404231,0.648057972288585,0.516947455453423,0.840354357533718
"9623","CABIN1",194.767352573986,-0.148061908634554,0.228461667186482,-0.648082063209749,0.51693187454528,0.840354357533718
"9624","PRKCQ-AS1",2.99674888952851,0.840216640634943,1.29672274238855,0.647953963610811,0.51701472631069,0.84036381584353
"9625","SNHG12",92.2338224820215,-0.164223396668307,0.253543298471258,-0.647713418806546,0.517170323534343,0.840472592105061
"9626","TBC1D10B",92.0058737764645,0.143765033737075,0.221967731260984,0.647684386015732,0.517189105133498,0.840472592105061

"9627","DLGAP4",357.323378112753,0.115445559412518,0.178298787414674,0
.647483704664931,0.517318937538859,0.840596254276553
"9628","TOB1",181.318538318881,0.209624276567617,0.323998799140762,0.6
46990905903159,0.517637829216431,0.841027062986355
"9629","APEH",95.5194191635211,-0.136417126731745,0.210898782748551,-0
.646836956353566,0.517737471317655,0.841101595578158
"9630","KDM7A",154.760405698477,0.235468183911576,0.36410204749266,0.6
46709310022001,0.517820096483808,0.841148470331901
"9631","AL136454.1",14.6905852171562,0.358165973549499,0.5541788341025
68,0.646300348387555,0.518084862305756,0.841257673639319
"9632","GCC1",26.7862527819063,0.202054211828895,0.312645194195303,0.6
46273205474817,0.518102437373982,0.841257673639319
"9633","TTLL11",16.0731702421757,0.243024885216584,0.375922662926102,0
.646475749360067,0.517971297401607,0.841257673639319
"9634","ALKBH8",35.068402730841,-0.180949454469183,0.279955525937507,-
0.646350715397472,0.518052250423892,0.841257673639319
"9635","MRPS25",252.821692350259,0.123820841426655,0.191720010908916,0
.645842032032229,0.518381664032358,0.841398793231556
"9636","RBM5-
AS1",9.48753138636976,0.50335890768511,0.779433186988596,0.64580122592
6597,0.518408093975495,0.841398793231556
"9637","P2RY1",26.7642750635714,0.283492428301714,0.438854031894442,0.
645983419767014,0.518290093178606,0.841398793231556
"9638","CPEB3",19.7960767338139,0.245191164192721,0.379795483907762,0.
645587361044737,0.518546624751222,0.841398793231556
"9639","AC007906.2",17.0370062833021,-0.287787260564127,0.445547963812
916,-0.645917575520483,0.518332736670825,0.841398793231556
"9640","ATXN1L",98.9489024737306,0.132620182282068,0.20540293322796,0.
645658658315668,0.518500439885495,0.841398793231556
"9641","TMEM220-
AS1",20.4118229358676,-0.231597464409204,0.358755656641838,-0.64555766
612042,0.518565861122894,0.841398793231556
"9642","TIPARP-
AS1",12.2846394269228,-0.339607668336116,0.526240686934596,-0.64534665
7466499,0.518702563146403,0.841533312103213
"9643","TBX5",32.5167326293114,-0.347679639780491,0.539172856512929,-0
.64483891498005,0.519031580564622,0.841716263332004
"9644","CDC27",236.123662692021,-0.105521494803276,0.163577833941127,-
0.645084314059655,0.518872548366526,0.841716263332004
"9645","AC011468.1",11.2512595130485,0.334782252415008,0.5190603805604
94,0.644977472666094,0.518941784414535,0.841716263332004
"9646","SBF1",71.9941459919071,0.144783099363192,0.224533827378492,0.6
44816422779514,0.519046158015762,0.841716263332004
"9647","BX842570.1",5.03248822617682,0.442853033599604,0.6868521217474
72,0.644757466094607,0.519084369517602,0.841716263332004
"9648","FMO5",25.4625843940328,0.220919836954165,0.343204067539506,0.6
43698189645538,0.519771164093755,0.841775397539402
"9649","TUBA4A",23.1768441080999,0.266240554588268,0.413230142354122,0
.644291224912897,0.51938660482684,0.841775397539402
"9650","ZBTB47",93.8439635165283,-0.141124551595522,0.219193389251125,

-0.643835802154775,0.519681914889393,0.841775397539402
"9651","PRIM2",50.2597484320187,-0.166169715083807,0.25810323937065,-0.64381104045416,0.519697973616999,0.841775397539402
"9652","ZNF658",24.7054800863867,0.211602286680848,0.328509072710337,0.644129201470879,0.519491655861442,0.841775397539402
"9653","STAM-AS1",6.48658404237926,0.376202309541621,0.584235416018249,0.643922465545755,0.519625713019811,0.841775397539402
"9654","FZD4",395.264625928527,0.167838254677702,0.260718565424132,0.64375260121835,0.519735874281356,0.841775397539402
"9655","PARP4",245.462957575458,0.105638021413823,0.164130456764641,0.643622295923451,0.519820388686993,0.841775397539402
"9656","ORMDL3",76.0719982723859,0.132593035357642,0.205783574147708,0.644332454165994,0.519359874792762,0.841775397539402
"9657","SPOP",637.2124268379,-0.113994212404678,0.177107118038996,-0.64364557261656,0.519805291191165,0.841775397539402
"9658","PPP6R1",84.3928468215613,0.142659047439102,0.221512259020271,0.644023261150739,0.51956035024682,0.841775397539402
"9659","DHRSX",149.82574488444,-0.176362865334659,0.273692860030208,-0.644382412150589,0.519327486638701,0.841775397539402
"9660","UTP14A",76.8089279760209,-0.153741704725249,0.238685803876963,-0.644117506060392,0.519499239231493,0.841775397539402
"9661","LINC02289",10.6546465440452,0.342564998059666,0.532338959254402,0.643509162920304,0.519893771104877,0.841776318522409
"9662","ARMCX1",456.467335817229,-0.133077792897958,0.206817399024181,-0.643455500000744,0.519928580806975,0.841776318522409
"9663","LINC01139",19.5154644101139,-0.395787550821085,0.615339270483606,-0.643202164734307,0.520092928855767,0.841853279837679
"9664","CSTA",43.8091113934826,-0.302399305168239,0.470088970598189,-0.643281004409516,0.520041779743243,0.841853279837679
"9665","BCKDHA",129.999020539353,-0.160653077995041,0.249797455216957,-0.643133365211864,0.520137566300017,0.841853279837679
"9666","DEF6",6.2992535883891,0.369309413937361,0.574369160265962,0.642982666002389,0.520235347536342,0.841924430117008
"9667","NCK2",129.280442634372,-0.136794828574305,0.2129239328004,-0.642458678905482,0.520575410493655,0.842126281939218
"9668","FCHO2",219.369626796015,0.156025599628909,0.242810377443972,0.642582089247447,0.520495307971477,0.842126281939218
"9669","CTTNBP2",13.9689249156379,-0.490953317155082,0.76409431375343,-0.642529735293267,0.520529288819249,0.842126281939218
"9670","AL157893.2",10.9468993592248,0.320954155723743,0.499417621208184,0.642656850888231,0.520446785176588,0.842126281939218
"9671","RNF115",531.732740953924,-0.0942462896431177,0.146969467452912,-0.641264415503945,0.521350904585011,0.842201482062827
"9672","AC064836.3",11.8540812965439,-0.273532107417837,0.426292927991062,-0.64165293266036,0.521098556012026,0.842201482062827
"9673","PECR",30.5593733685633,-0.230390690017704,0.359286804925372,-0.641244506782149,0.521363837335896,0.842201482062827
"9674","CNTN4",104.070927901196,0.322869978312757,0.502830825311715,0.642104584802658,0.520805279245518,0.842201482062827

"9675","MAGI1",26.2842896263575,0.281378672820312,0.438704553302211,0.641385348527436,0.521272349766007,0.842201482062827
"9676","SCRIB",60.1311117252755,-0.144319821339102,0.224954350106252,-0.641551591560404,0.521164372736004,0.842201482062827
"9677","UAP1L1",51.1403394756352,0.205758744330845,0.320614757824657,0.641763173120602,0.521026964392839,0.842201482062827
"9678","GRASP",136.147465181409,0.15604308480173,0.243090759569826,0.641912860356617,0.520929763621116,0.842201482062827
"9679","C15orf41",42.0426781216761,0.190596253497906,0.296898975146034,0.641956589456594,0.5209013694939,0.842201482062827
"9680","SLC7A6OS",147.911367267013,0.133257082636558,0.207603512671101,0.641882600742275,0.520949412224528,0.842201482062827
"9681","FAM157C",10.5105558331712,0.36204678258254,0.564037029929371,0.641884775947911,0.520947999776317,0.842201482062827
"9682","RBL1",45.0241212457348,0.176170072372897,0.27460896482936,0.641530667006331,0.521177962874817,0.842201482062827
"9683","KRBOX4",105.042839493849,-0.143831885586994,0.22421786985449,-0.64148270465836,0.521209114285131,0.842201482062827
"9684","GAB3",17.6918052208329,0.293119267976659,0.45712293981442,0.641226336389194,0.521375641008529,0.842201482062827
"9685","XPO6",95.6254743396452,0.142538321418329,0.22233244913349,0.641104445049982,0.521454826421605,0.842242421240389
"9686","RBM15B",136.090431929584,-0.123054538510257,0.191986369419347,-0.640954557776309,0.521552207579259,0.842312738298817
"9687","EPHX2",84.5902660393146,0.151276287490696,0.236105323701569,0.640715275365436,0.521707687786766,0.842427256570908
"9688","PAMR1",224.252276549652,0.240965354127806,0.376109189623516,0.640679251599811,0.521731097265922,0.842427256570908
"9689","ALG8",97.4265503200569,0.149718753211097,0.233717608517039,0.640596804669862,0.521784676143676,0.842427256570908
"9690","GFPT1",228.055783981394,0.143015540847775,0.22339732191999,0.640184670159101,0.522052547880871,0.842429793089001
"9691","AMT",35.8368215977172,0.208546057356764,0.325720984837785,0.640259814579105,0.522003701599635,0.842429793089001
"9692","HELQ",89.1338379772421,-0.1609501654269,0.251409717722233,-0.640190708955506,0.522048622382841,0.842429793089001
"9693","RABGEF1",121.985034526068,0.15306864661284,0.2390152327364,0.640413771375203,0.521903632088007,0.842429793089001
"9694","C12orf75",136.75046082889,0.275559062796077,0.430440671662148,0.640178962949773,0.522056257845855,0.842429793089001
"9695","ZNF224",153.204137554674,0.112331482358611,0.175491271060584,0.640097263412216,0.5221093680239,0.842429793089001
"9696","AC008532.1",2.63083685160859,-0.5877491571694,0.918695030831751,-0.639765251192526,0.522325226807045,0.84268705414646
"9697","IL10RB-
DT",4.07144290908228,-0.51901156763768,0.811353236207047,-0.6396863221548,0.522376549514685,0.84268705414646
"9698","MAP7D1",185.891390420975,-0.111760275306918,0.175087134095066,-0.63831232308729,0.523270391784624,0.842785679303903
"9699","CCDC148",2.7089104943786,-0.783744700213712,1.22694348602426,-

0.638778158196454,0.522967259177107,0.842785679303903
"9700","RAPGEF4",10.745794575226,0.413096058633205,0.647007510470371,0.638471813616022,0.523166596455622,0.842785679303903
"9701","DPH3",110.515149730159,-0.183741245917113,0.287435120340463,-0.639244243012036,0.522664054339139,0.842785679303903
"9702","ADAMTS3",33.7809115574013,0.37091569840136,0.581204633989338,0.638184344566262,0.523353686959639,0.842785679303903
"9703","UBE2D3-AS1",36.3979325738666,-0.181062603574684,0.283825566218405,-0.637936201403913,0.523515211007864,0.842785679303903
"9704","HCG11",215.382803204969,0.11921341223844,0.186590648602882,0.638903466658507,0.522885732666643,0.842785679303903
"9705","SNRPC",328.544073478947,-0.168744948446815,0.264261796539987,-0.638552188232328,0.523114293218298,0.842785679303903
"9706","NFKBIE",22.9719532457309,-0.220807975995298,0.345739309072587,-0.63865453016492,0.523047698783721,0.842785679303903
"9707","TMEM181",112.206607479044,0.156852176344289,0.245666470853445,0.638476124964812,0.523163790806462,0.842785679303903
"9708","HOXA7",81.4814677324887,-0.248122096714615,0.388701249536209,-0.638336246695037,0.523254821793056,0.842785679303903
"9709","ENTPD4",90.720664513205,0.164247675097796,0.257032505047046,0.639015190190566,0.522813050104796,0.842785679303903
"9710","TRAPPC9",42.5537890758725,0.167752599786569,0.262491209435038,0.639078924386169,0.522771589681457,0.842785679303903
"9711","ABRAXAS2",90.9069787378111,-0.127193399133964,0.199362744895928,-0.637999838938623,0.523473784932821,0.842785679303903
"9712","MRGPRF-AS1",4.2218997951167,0.464640804247139,0.728037066271705,0.638210368362941,0.523336748748383,0.842785679303903
"9713","ADPRHL1",8.02525422770223,-0.348555612536555,0.545205927095015,-0.639310020699409,0.522621270873771,0.842785679303903
"9714","ZNF106",959.601368832827,0.132253228408821,0.207284187940074,0.638028542954058,0.523455100053263,0.842785679303903
"9715","NARS",393.459354186454,-0.137133562341228,0.214963883406655,-0.637937685940512,0.523514244601103,0.842785679303903
"9716","PIK3R2",104.620164602363,0.127378619399308,0.199225919410455,0.639367707656933,0.522583751303747,0.842785679303903
"9717","ZNF304",18.7674757307602,0.235857868988272,0.369229294069891,0.63878428059835,0.52296327575048,0.842785679303903
"9718","RPS20",8725.2561798377,-0.119090064071098,0.186717223566409,-0.637809741364017,0.523597537590413,0.842806464185203
"9719","COMMD4",324.370666271419,-0.178032370205486,0.279229077560461,-0.637585353792308,0.523743632189756,0.842806464185203
"9720","ZNF555",26.20995616131,0.216224269067282,0.339116109908466,0.637611315857703,0.523726727699566,0.842806464185203
"9721","FBXW9",39.1600961239749,-0.201708377208078,0.31630605184403,-0.637700025124843,0.52366896919341,0.842806464185203
"9722","ITPR1",25.4251833893066,0.297618602748204,0.466929307914269,0.637395420899235,0.523867310319737,0.842832082210393
"9723","ERAP1",130.397907298464,0.16190074003217,0.253978486145025,0.6

37458481186957,0.523826245848327,0.842832082210393
"9724","CNOT1",183.057691156115,0.124250778252277,0.19506266929607,0.636978773068433,0.524138670299848,0.843095241079745
"9725","AC005899.8",2.8377859724967,-0.641488535674011,1.00703888560618,-0.637004732233229,0.52412176116297,0.843095241079745
"9726","CFD",179711.09556059,0.186667214201344,0.293251289377097,0.636543541199252,0.524422210858999,0.843464594331412
"9727","RNF217",120.037754642182,0.173440687152417,0.272576676564229,0.636300542433048,0.524580551474067,0.843545802498851
"9728","SPATA22",9.74209150246083,-0.457602417546825,0.719125066480395,-0.636332174855846,0.524559938060134,0.843545802498851
"9729","AL133243.3",33.4196220237344,0.232559149245676,0.365975364240853,0.635450284278222,0.525134781535389,0.843916518138288
"9730","ACVR1",221.83347008838,0.121321720030644,0.190843678923524,0.63571254083433,0.52496380094799,0.843916518138288
"9731","ZNF501",23.2680808042632,0.204756737434483,0.322144292271114,0.635605665991938,0.525033475549338,0.843916518138288
"9732","SRD5A3-
AS1",3.80290801745975,0.484163059394863,0.761637592328523,0.63568692547679,0.524980499861324,0.843916518138288
"9733","PCDHB9",16.2307415281724,0.276657218417186,0.435252084461654,0.63562525785344,0.525020702730431,0.843916518138288
"9734","CSNK1D",173.626106725458,0.102928966735814,0.161975449144966,0.63546029524323,0.525128254270988,0.843916518138288
"9735","PRPF38A",180.494171655298,-0.119204851376683,0.187808575041127,-0.634714636169188,0.525614546180002,0.843973865619532
"9736","HS2ST1",99.001809851875,0.125175939136191,0.197324251689703,0.634366724131981,0.52584152061458,0.843973865619532
"9737","SHISA4",124.654961272801,-0.198011782730511,0.311915774298251,-0.634824523306005,0.52554286729255,0.843973865619532
"9738","RNPEP",144.117633976789,-0.130056982127926,0.204969019215838,-0.634520195420228,0.525741391259866,0.843973865619532
"9739","CCDC66",286.685251787445,-0.115545919517092,0.18210257130072,-0.634510093359868,0.525747981852753,0.843973865619532
"9740","SLC41A3",161.134385300429,0.133408419555094,0.210104656082593,0.634961747361994,0.525453363675775,0.843973865619532
"9741","LEKR1",4.03527962608039,0.547145757124673,0.861707400888793,0.63495538805902,0.525457511322968,0.843973865619532
"9742","NDUFAF4",86.9447896049644,-0.158822471553859,0.250261848421787,-0.634625183804215,0.525672899248695,0.843973865619532
"9743","OXR1",364.119358471802,-0.0881500713991326,0.138864123592207,-0.634793704225556,0.525562969936277,0.843973865619532
"9744","MARK2",92.0498246997981,0.145651156435969,0.229647635178747,0.634237562788753,0.525925796973675,0.843973865619532
"9745","MAP4K5",274.031826077381,0.124757737302946,0.19653527511921,0.634785471601844,0.52556833997167,0.843973865619532
"9746","AC120049.1",20.9366465729269,-0.241044554798614,0.380030320691184,-0.634277165990892,0.52589995558491,0.843973865619532
"9747","SLC25A23",75.6155966984277,0.152224577035259,0.239893560124777,0.634550493794341,0.525721624827835,0.843973865619532

"9748","APOO",18.5841901523305,-0.346994997004621,0.547081877255291,-0
.634265201299474,0.525907762568076,0.843973865619532
"9749","NUDT19",39.6616804116192,0.189077706758599,0.298285143057882,0
.633882414726598,0.526157562775882,0.844259180890668
"9750","KBTBD2",94.5251418629884,0.143536993265348,0.226480644077318,0
.633771569531328,0.526229909842633,0.844288664581365
"9751","POLA2",19.6495447482287,-0.214891849086959,0.339278540239861,-
0.633378842455041,0.52648627806533,0.844613357376265
"9752","DMAP1",174.76886891814,-0.154205712461036,0.243643583718703,-0
.632915138200697,0.526789061572529,0.844752566907131
"9753","ZC3H6",329.182822806187,-0.086434792156425,0.136552064656029,-
0.632980485312702,0.52674638669378,0.844752566907131
"9754","TCTEX1D2",88.1477830128837,0.230028895947117,0.363420830287379
,0.632954626638268,0.526763273469209,0.844752566907131
"9755","GGACT",28.950834881799,0.228238387822256,0.360501034339005,0.6
33114377163277,0.526658954242432,0.844752566907131
"9756","FOXN3-
AS1",25.1776496873408,0.236987971431187,0.374619309506583,0.6326101335
86888,0.526988267932474,0.844985391068848
"9757","EIF4H",604.589060624376,-0.0912089592642076,0.144247632212776,
-0.632308190193844,0.527185512794015,0.845052770450018
"9758","WRAP53",28.9849870610798,-0.253896473548022,0.401494203555369,
-0.632378926768261,0.527139300668676,0.845052770450018
"9759","ARSG",68.7814556034807,-0.187258435406623,0.296155481315657,-0
.632297719342339,0.527192353565283,0.845052770450018
"9760","DNAH14",28.6615100154935,-0.243223538627288,0.385029232358084,
-0.631701487021343,0.527581956179088,0.845224656299229
"9761","ATG9A",101.513256285765,0.131337759441066,0.207936603940077,0.
631624047678085,0.527632568987941,0.845224656299229
"9762","AC026202.2",2.71885540676834,0.570002951158947,0.9026269307912
59,0.631493401885618,0.5277179620812,0.845224656299229
"9763","HNRNPUL2",268.357227896037,0.133312512120893,0.211086786806657
,0.631553088365496,0.527678948751702,0.845224656299229
"9764","TBC1D15",229.828359535166,0.124371950381659,0.196955000956518,
0.63147393961891,0.527730683669093,0.845224656299229
"9765","CRADD",38.8135955977284,0.213321672038516,0.337816428742111,0.
631472166208251,0.527731842873782,0.845224656299229
"9766","AC007216.1",5.45950700597092,0.474204035653837,0.7508881048970
01,0.631524234518115,0.527697808528623,0.845224656299229
"9767","VAT1",1082.90477426647,0.119235578598317,0.188717031368054,0.6
31822033941244,0.527503174045511,0.845224656299229
"9768","PSMB2",531.818925712014,-0.163839859038088,0.259725322524136,-
0.630819734655879,0.528158398278662,0.845314062612871
"9769","MGAT5",29.2327008288342,-0.216204205622015,0.342671972015711,-
0.630936356861141,0.52808213856588,0.845314062612871
"9770","FYN",1315.46274930114,-0.0943260401063867,0.149433857720051,-0
.631222679689478,0.527894934756937,0.845314062612871
"9771","LINC02649",5.65128096911643,0.450649498287776,0.71404432756847
2,0.631122580053773,0.527960378130944,0.845314062612871
"9772","TNNT3",1174.82266427842,0.145580307053353,0.230708037385201,0.

631015324404518,0.52803050456867,0.845314062612871
"9773","C18orf54",22.9843417315752,0.235232251134957,0.372740167952302,0.6310890839247,0.527982278231838,0.845314062612871
"9774","ATP6V1E1",321.160757491241,0.112332470665112,0.178077056890481,0.6308082165475,0.528165930318878,0.845314062612871
"9775","MSX2",14.4311628347358,-0.265906344614332,0.421631443701018,-0.630660612691134,0.5282624577989,0.845382059063753
"9776","HAR1A",7.32044027871582,-0.322727652682164,0.511807410447193,-0.630564634459239,0.528325228845219,0.84539602647563
"9777","SOX7",2.88327342819234,0.639965464342218,1.01507496074564,0.630461285215939,0.528392824879389,0.845417710912169
"9778","RNF10",252.903703893145,0.0899323554497117,0.142683949256827,0.630290624265214,0.528504456078012,0.845509839070193
"9779","HMGN2",1369.52026570168,0.117814801325717,0.187380415822174,0.628746610518381,0.529514957936987,0.845672667320094
"9780","SESN2",60.4830607664641,0.194993685051871,0.309856637584836,0.629302914314636,0.529150763786875,0.845672667320094
"9781","AL078459.1",10.2382909152059,0.373098295485355,0.592671979434965,0.629519039926698,0.529009307699393,0.845672667320094
"9782","PYGO2",45.2009030142892,0.173542269280968,0.27593948626453,0.628914229095146,0.529405210047673,0.845672667320094
"9783","SCAMP3",107.951699081995,0.131390237080565,0.208973329295263,0.628741655806807,0.529518202199358,0.845672667320094
"9784","ENPP1",130.210850952004,0.277906430684974,0.441748456340984,0.629105606812712,0.529279920058721,0.845672667320094
"9785","RNF216",212.207356679168,0.116509750615855,0.185210011554855,0.629068318919397,0.529304330284696,0.845672667320094
"9786","CFAP69",47.9153269413103,-0.240717175701467,0.382676151760016,-0.629036261063965,0.529325317165276,0.845672667320094
"9787","LPL",75.8490562341538,0.282230318573488,0.448233981734159,0.629649535899924,0.5289239062708,0.845672667320094
"9788","YWHAZ",1024.08270166769,-0.0948867597086193,0.150877859006527,-0.628897840501,0.52941593993291,0.845672667320094
"9789","LINC01503",51.219925617248,-0.225101749336037,0.357703198192001,-0.629297558629071,0.529154269373843,0.845672667320094
"9790","CDAN1",21.0773136719745,0.212416577088044,0.337849768918593,0.628730863922026,0.529525268580216,0.845672667320094
"9791","TAOK2",59.0423131778852,0.160617360751211,0.255227042413871,0.629311687476901,0.529145021301396,0.845672667320094
"9792","ZNF286A",66.1249680517153,-0.142327812849962,0.226061301875069,-0.629598306607196,0.528957431792756,0.845672667320094
"9793","PRKAR1A",674.920590944682,0.101586206967127,0.161344542694143,0.629622826225374,0.52894138550623,0.845672667320094
"9794","NDUFA6",443.83209079216,-0.180538373717602,0.286712630455517,-0.629684061810497,0.528901312401902,0.845672667320094
"9795","FUNDC1",116.80332053516,0.143942825314053,0.228809023780658,0.629095928716695,0.529286255693108,0.845672667320094
"9796","ZZEF1",100.311138963257,0.168057186370723,0.267358263059108,0.628584224208432,0.529621291042225,0.845739674946257
"9797","AL355488.1",15.4083844172053,0.359827417632969,0.5733600320536

8,0.627576736285798,0.530281252465153,0.845820023429285
"9798","SELL",6.08753309299437,-0.476211298886072,0.759012650544365,-0
.627408908856173,0.530391229467274,0.845820023429285
"9799","GMCL1",92.0293234652011,0.144265140295547,0.229728503442243,0.
62798102165767,0.530016372575297,0.845820023429285
"9800","FGFR1OP",48.400519234248,0.19066542116576,0.303496812191118,0.
628228744115094,0.529854102874606,0.845820023429285
"9801","ZNRF2",72.3270346207878,-0.16203568871338,0.258140992427171,-0
.627702276921769,0.530198993492755,0.845820023429285
"9802","PLEKHF2",62.2225368622936,-0.173285198933554,0.275753036844939
,-0.628407218706335,0.529737209388059,0.845820023429285
"9803","AZIN1",335.173413533223,-0.128426757202723,0.204747808130005,-
0.627243624123087,0.530499551564036,0.845820023429285
"9804","ZNF250",32.2230770877841,-0.187376928865466,0.298875032431948,
-0.626940722819094,0.53069809211596,0.845820023429285
"9805","ZBTB43",117.09493122657,0.156381464836677,0.249315356312184,0.
627243612867798,0.530499558940759,0.845820023429285
"9806","MIR1915HG",5.47770721949916,0.389677718480388,0.62113405386842
,0.627364923970079,0.530420054596605,0.845820023429285
"9807","AL512598.1",8.11033293879063,0.373574931851063,0.5946947988219
36,0.628179248567666,0.529886522735688,0.845820023429285
"9808","TMEM134",152.195252882498,-0.152600875090622,0.243406090307841
,-0.626939428251873,0.53069894073761,0.845820023429285
"9809","CLPB",38.7933633462774,-0.175426379194188,0.279758270784437,-0
.627064138987906,0.53061719284978,0.845820023429285
"9810","HIST4H4",11.4365359414699,0.382908399059465,0.610579805007118,
0.627122606937518,0.530578869309646,0.845820023429285
"9811","CEP112",95.1558212565252,0.153812539990111,0.245058378612666,0
.62765672759642,0.530228838384099,0.845820023429285
"9812","SMAD2",213.279149750795,0.0964411286713418,0.153782374254653,0
.627127322872789,0.530575778252831,0.845820023429285
"9813","BMP7",6.53293250113678,0.770783312399184,1.22850510282567,0.62
7415637612181,0.53038681990313,0.845820023429285
"9814","AP000692.1",5.29790038574888,-0.436347292702658,0.695775221813
574,-0.627138304185792,0.53056858059354,0.845820023429285
"9815","MIR222HG",59.9977283620809,-0.304703539665807,0.48539124310668
9,-0.627748324661955,0.530168822896713,0.845820023429285
"9816","RBBP5",88.1790013000891,0.13932142454577,0.222340793312923,0.6
26612069111802,0.530913555062805,0.84598968542808
"9817","PDK2",141.436584372553,-0.17385717480572,0.277437136438186,-0.
626654300998583,0.530885865664157,0.84598968542808
"9818","TOE1",34.6456415895353,-0.162686758500816,0.260393573722974,-0
.624772555538924,0.532120345495949,0.846067406136827
"9819","DLEU2L",28.9308240638732,0.244269948586916,0.391693011766338,0
.6236260062067,0.532873227393333,0.846067406136827
"9820","PDE4B",34.5573785058557,0.310302111844248,0.496895340068765,0.
624481831126261,0.532311198756386,0.846067406136827
"9821","NGF",3.73080493604252,0.491841951726352,0.791203081895488,0.62
1638063577867,0.534179886357273,0.846067406136827
"9822","ALDH9A1",488.786872083159,0.138525448890687,0.221424288755543,

0.625610901447319,0.531570186634974,0.846067406136827
"9823","GLUL",1534.53678458966,0.170835434390909,0.273348713773507,0.6
24972519652902,0.531989094206925,0.846067406136827
"9824","NPL",21.384611392755,-0.214189088193542,0.344492660276691,-0.6
21752254522662,0.534104785782423,0.846067406136827
"9825","AC010969.2",19.3629376477475,0.21523474134106,0.3452852698460
76,0.623353383817993,0.533052324220183,0.846067406136827
"9826","SDC1",3.75204345849333,0.520791810003649,0.834991376378526,0.6
23709207947028,0.532818574821689,0.846067406136827
"9827","GTF3C2",69.4220589942139,0.135418352282604,0.217515680178609,0
.622568231271456,0.533568293162041,0.846067406136827
"9828","ARHGEF4",37.5918379991419,0.168598839482165,0.270258425541839,
0.623843046314442,0.532730666613874,0.846067406136827
"9829","MRPL44",121.974331319476,-0.149928050778688,0.241256055094806,
-0.621447825298192,0.53430501323202,0.846067406136827
"9830","PSMD1",300.895168790468,-0.114336970147352,0.183637164083043,-
0.622624351221455,0.533531405138222,0.846067406136827
"9831","NDUFA10",507.590896779659,-0.135526286956001,0.217566547920813
,-0.622918772445333,0.533337901190822,0.846067406136827
"9832","PLCD1",56.2320437067574,0.146724766348935,0.235001392855614,0.
624357007275626,0.532393153106797,0.846067406136827
"9833","HIGD1A",202.410830406575,-0.182676331431931,0.293555904259175,
-0.62228805069664,0.533752477032639,0.846067406136827
"9834","HSPBAP1",37.5286113195751,-0.179941935889144,0.287241509107204
,-0.626448233225186,0.53102098123775,0.846067406136827
"9835","AC098829.1",2.63268026480834,-0.740198492756773,1.189374440402
58,-0.622342693446675,0.53371655370138,0.846067406136827
"9836","ZNF454",14.3444961050862,0.253085281096584,0.405968723254083,0
.623410786594478,0.533014611452285,0.846067406136827
"9837","PPP1R10",355.504993950255,-0.144603817757032,0.231761418234908
,-0.623933952675697,0.532670961349512,0.846067406136827
"9838","HCP5",8.42161330573201,0.39768771643558,0.640074213562243,0.62
1315010055327,0.534392379584447,0.846067406136827
"9839","RPS18",27945.9931253064,-0.182447180910718,0.292821267010495,-
0.623066701313668,0.533240690546927,0.846067406136827
"9840","Z99289.2",3.90600822994437,-0.467466733521712,0.75012017038226
,-0.623189126194929,0.533160246483694,0.846067406136827
"9841","AC078846.1",22.3981798978325,0.208986523835784,0.3340888113878
86,0.625541822150831,0.531615508556042,0.846067406136827
"9842","MRPS33",285.312130459233,-0.177960374230078,0.285560571632638,
-0.623196589125116,0.533155342871569,0.846067406136827
"9843","GAS1RR",96.0233720020688,0.16641792362643,0.267686432821904,0.
621689795302964,0.534145863013919,0.846067406136827
"9844","CDC123",254.811417489447,-0.158215870206896,0.254075420289444,
-0.622712224687676,0.53347364790197,0.846067406136827
"9845","AC069544.1",10.8996700615287,0.339215035319874,0.5461732880094
73,0.621075843082957,0.534549722673873,0.846067406136827
"9846","ASCC1",72.9564074548799,0.169158671641274,0.272368523684792,0.
621065420309133,0.534556580136442,0.846067406136827
"9847","IKZF5",69.9748188193813,0.187647081785168,0.301856182693026,0.

621643989899642,0.534175988630085,0.846067406136827
"9848","NUP98",108.216096868283,0.143080922479121,0.228625146113068,0.
625831956421626,0.531425168846228,0.846067406136827
"9849","APBB1",81.9741623896478,-0.134171576755855,0.215073754379283,-
0.623839841095828,0.532732771791554,0.846067406136827
"9850","KCNC1",7.00189124070843,0.485798675763884,0.777778333183608,0.
624597851389623,0.532235030217618,0.846067406136827
"9851","AP000766.1",23.3657229076491,0.256971326576843,0.4136019519189
64,0.621301048954409,0.534401563666465,0.846067406136827
"9852","ZW10",44.5771386764634,0.198518100164979,0.317532954345125,0.6
25188968415575,0.53184704130524,0.846067406136827
"9853","JAM3",232.194209427877,0.130490661918826,0.210070704439537,0.6
21174962339331,0.534484511293508,0.846067406136827
"9854","KLRG1",11.4669564830926,0.283122192396854,0.454997608349036,0.
622249847475389,0.533777593386012,0.846067406136827
"9855","FKBP11",316.523046877675,0.175005523300577,0.281340759069859,0
.622041128626947,0.533914824197782,0.846067406136827
"9856","ZC3H10",33.7425787179505,-0.185229220780686,0.297596430424122,
-0.622417481677132,0.533667388273738,0.846067406136827
"9857","UBAC2-
AS1",8.60251050402715,-0.310488078029102,0.49920713093414,-0.621962425
592964,0.533966575378504,0.846067406136827
"9858","ABHD13",109.438970366356,0.114266348650081,0.183762690394205,0
.621814735107317,0.534063696095934,0.846067406136827
"9859","PSMB5",735.546220684753,-0.181860555967489,0.291968206273489,-
0.622877943761926,0.533364733113521,0.846067406136827
"9860","C14orf28",69.7043415788279,0.140379999121452,0.224681030443357
,0.624796845752595,0.532104401150869,0.846067406136827
"9861","FAM219B",147.609350925533,-0.11610712169738,0.185729519764259,
-0.625140913758625,0.531878577379181,0.846067406136827
"9862","CLCN7",98.8969539204055,0.117010819713068,0.187393251173643,0.
624413200476696,0.532356258106798,0.846067406136827
"9863","LYRM1",235.08450982402,0.15060081311692,0.241725297460452,0.62
3024626297376,0.533268339001253,0.846067406136827
"9864","C16orf54",3.58694556390243,-0.552598563569065,0.88958526844943
9,-0.621186729555732,0.53447676981091,0.846067406136827
"9865","CIAPIN1",105.703184495348,-0.15683266513193,0.250637972435129,
-0.625733856718465,0.531489522316761,0.846067406136827
"9866","WWP2",56.0562160763862,0.159771064686877,0.255551150311243,0.6
25201899863442,0.531838555149105,0.846067406136827
"9867","AC027279.1",33.4231033897117,0.233564067052051,0.3731626129456
28,0.625904254470644,0.531377743806725,0.846067406136827
"9868","PIGW",13.5982429107201,-0.246959661600636,0.394673312273244,-0
.625731849407792,0.531490839155142,0.846067406136827
"9869","NDUFAF8",438.768895186435,-0.167474020328311,0.267941609245786
,-0.625039241944259,0.531945303064374,0.846067406136827
"9870","TYMSOS",3.63840345782113,0.533105615510171,0.852945936527724,0
.625016888737875,0.531959973706745,0.846067406136827
"9871","TMEM241",29.4791717793652,0.205118428528141,0.32766489266539,0
.626000627835364,0.531314529523141,0.846067406136827

"9872","ZNF699",34.968730395886,0.196507515706642,0.315040232831702,0.623753715328221,0.532789340505708,0.846067406136827
"9873","REX1BD",890.644385725341,-0.1440950269324,0.232029396534067,-0.621020565000884,0.53458609232605,0.846067406136827
"9874","ZNF420",58.9556404912927,0.167602768057187,0.268570440739398,0.624055155123406,0.532591363560647,0.846067406136827
"9875","AC011445.2",4.95300143341978,-0.471319707164099,0.755204532004034,-0.624095443274672,0.532564906288227,0.846067406136827
"9876","POLD1",23.0264524792511,-0.23085138720194,0.369893601310275,-0.624102137436806,0.532560510289176,0.846067406136827
"9877","ZNF582",21.5692108682458,0.232624372729919,0.374198101752252,0.621661017628395,0.534164789614861,0.846067406136827
"9878","DGCR6L",336.715352415777,-0.177935546930686,0.285060445325359,-0.624202865913564,0.532494364976949,0.846067406136827
"9879","RNF185",92.2498578015572,0.114672854614451,0.183143822819726,0.626135530256603,0.531226049239937,0.846067406136827
"9880","RBFOX2",490.237253787337,-0.0898019425127651,0.143633494241327,-0.625215887054055,0.531829376247728,0.846067406136827
"9881","JADE3",12.4215205579342,-0.306633543618506,0.491493714055952,-0.623880905999946,0.53270580080795,0.846067406136827
"9882","WAS",14.3384724964346,-0.329895342057376,0.528816858357227,-0.623836658842908,0.532734861889602,0.846067406136827
"9883","APOOL",151.883456158923,0.114454406254137,0.182999760479939,0.62543473255903,0.531685772350068,0.846067406136827
"9884","AL359851.1",6.78829189115877,-0.365097611520668,0.587453285285767,-0.621492160594636,0.534275850917109,0.846067406136827
"9885","HADHA",831.45478246458,-0.104871714683698,0.169080666013492,-0.620246638224913,0.535095420658597,0.846274154823823
"9886","TADA2B",67.2995382120013,0.14196925326758,0.228779040302578,0.620551834992468,0.53489453864339,0.846274154823823
"9887","VEGFA",191.716276000708,-0.197717166607183,0.318648561452099,-0.620486612919812,0.534937464937002,0.846274154823823
"9888","LONRF1",109.396990758998,0.132437355399724,0.213410738674649,0.620574935554809,0.534879335288031,0.846274154823823
"9889","DDN-AS1",3.90362159013041,-0.479093874465309,0.772328738638216,-0.620323769525987,0.535044648864883,0.846274154823823
"9890","AC073508.3",21.1802626339898,0.267149152478867,0.430600717087691,0.620410375267587,0.534987643401703,0.846274154823823
"9891","ZNF780A",60.116278106978,0.129617924634851,0.208892846959653,0.620499583979944,0.534928927818517,0.846274154823823
"9892","CD200",55.46067501873,0.195200351630159,0.31488045826358,0.619918913693783,0.535311172902736,0.846396859643757
"9893","FNBP4",390.624459541786,0.160342773016856,0.258631917832221,0.619965139495557,0.535280738223844,0.846396859643757
"9894","PVR",21.7030229743804,-0.242863423067009,0.391789621312512,-0.619882227235643,0.535335327578811,0.846396859643757
"9895","HEPH",54.9307502171653,-0.207133476966956,0.334212392724636,-0.619765997539226,0.535411857826445,0.846432308436491
"9896","HHAT",16.6964792968025,0.250845138751996,0.405085559797393,0.6

19239893116552,0.535758334815178,0.846672567596453
"9897","HLA-
DRB5",70.2796814818531,1.0655856471379,1.72094883646379,0.619184966200
077,0.535794514585835,0.846672567596453
"9898","EPHA1-
AS1",17.4228214713216,-0.298091946242808,0.481405785375882,-0.61921139
150843,0.535777108365103,0.846672567596453
"9899","NFIL3",284.669611416286,-0.281310407935899,0.454554242807125,-
0.618870932099655,0.536001389105442,0.846672567596453
"9900","LRRC8A",103.523347795308,0.127649372486724,0.206250958691851,0
.618903171632955,0.535980148992844,0.846672567596453
"9901","RPS24",16115.432876424,-0.17120152715882,0.27657510797993,-0.6
19005551183743,0.535912701906149,0.846672567596453
"9902","AL354950.2",3.81034948650325,0.527174106723357,0.8512060963970
72,0.61932604683489,0.535701588763955,0.846672567596453
"9903","ZFHX2",7.95689286454155,0.352570307570276,0.569844165731703,0.
618713551480451,0.536105080997434,0.846672567596453
"9904","HAPLN3",16.535411346244,0.312836297238978,0.505563616942852,0.
618787204527696,0.536056552648135,0.846672567596453
"9905","SSR4",1755.48535933167,0.165499784069789,0.267396925850128,0.6
189292698247,0.535962955241338,0.846672567596453
"9906","PTGFRN",230.636805743931,0.187782466725322,0.303707143238635,0
.618301119699953,0.536376863953569,0.846697012563032
"9907","POPDC2",7.58017929426316,-0.316219934784932,0.511508044230124,
-0.618211068920485,0.536436214544375,0.846697012563032
"9908","ADAMTS13",6.52260509879537,0.352528315824325,0.570058811344569
,0.618406923652026,0.536307134997372,0.846697012563032
"9909","CENPJ",54.8258901742496,0.211201912650782,0.341616164794575,0.
618243322232086,0.536414956679515,0.846697012563032
"9910","ADAT1",36.4919295060486,0.189685608151089,0.306790666065924,0.
618290023563914,0.536384177006934,0.846697012563032
"9911","TCEAL7",107.247285565244,-0.208226820799769,0.336829091139817,
-0.618197258719957,0.536445316851768,0.846697012563032
"9912","TWISTNB",203.921947293717,-0.135605052068818,0.219410984042312
,-0.618041310286763,0.536548107904952,0.846773814765654
"9913","FMNL3",35.721378925297,-0.214497192536095,0.347109086320407,-0
.617953263079084,0.536606147267531,0.846779982014122
"9914","RCHY1",102.28825513765,0.119056222399713,0.19269627600481,0.61
7843919291627,0.536678229403393,0.846808305684616
"9915","UIMC1",88.9045259632665,-0.118789280565001,0.192329437973299,-
0.617634418406051,0.536816351185122,0.846814942082145
"9916","IBTK",193.846325128928,0.115837125155084,0.187562779824587,0.6
17591215396879,0.536844836708344,0.846814942082145
"9917","WFDC2",6.52427277460065,0.409281180843453,0.662536957509058,0.
617748453433041,0.536741166846406,0.846814942082145
"9918","AC067750.1",9.07823649583451,0.301201270248867,0.4878058630419
42,0.617461357209988,0.5369304621346,0.846864611733368
"9919","HSPG2",2507.64776866616,0.0824755669710035,0.133610060306797,0
.617285605452329,0.537046359633509,0.846893495328421
"9920","GPANK1",147.213286183867,-0.137805373086564,0.223249970041073,

-0.617269391172644,0.537057052589525,0.846893495328421
"9921","RNH1",1877.003756927,-0.162893775394209,0.264030242546602,-0.616951201586151,0.537266913184088,0.847085412217244
"9922","AC074212.1",20.761919485133,0.223034670164035,0.361528936423892,0.616920660266395,0.537287058749568,0.847085412217244
"9923","TMEM185B",94.4058659858209,0.140897874701141,0.228598988687252,0.616353884635532,0.537660982308567,0.847589513882185
"9924","FEM1B",178.556045545951,0.127128778318811,0.206301197291658,0.616228989398847,0.537743398042021,0.847634016079336
"9925","GNB1",651.55251334845,0.0957151259119872,0.155378530735456,0.616012556296788,0.537886232703093,0.847773736843777
"9926","MEAF6",433.933811433133,-0.137264372332916,0.223119405216394,-0.615205890315946,0.538418758293161,0.847787614572718
"9927","HENMT1",27.1672611653825,-0.213339850841947,0.347033349717393,-0.614753167139932,0.538717742149538,0.847787614572718
"9928","CYS1",220.791087651298,-0.192960956840313,0.313552590427278,-0.615402209171244,0.538289132827399,0.847787614572718
"9929","HIST1H1E",74.1566824623013,0.211304035135774,0.343624185193388,0.614927715337774,0.538602458550476,0.847787614572718
"9930","MINDY4",81.0868675885115,-0.149725692765779,0.243182678651703,-0.615692258987831,0.538097647318149,0.847787614572718
"9931","LSM1",223.020700001565,-0.13639892438372,0.221700266772607,-0.615240235699045,0.538396079584481,0.847787614572718
"9932","TIGD5",56.7901397660984,-0.168410595084976,0.273971703412668,-0.614700689842075,0.5387524041744,0.847787614572718
"9933","DAB2IP",54.1122792011777,-0.202673597497821,0.329432030888183,-0.615221285408682,0.538408592651464,0.847787614572718
"9934","RNF26",73.1496683264209,-0.132336434602916,0.21530694700799,-0.614640802082456,0.538791962284064,0.847787614572718
"9935","SUOX",43.7143045121718,0.154900723250143,0.251788121943049,0.615202663472661,0.538420889044895,0.847787614572718
"9936","AC073578.1",7.89352798387877,0.30139078756515,0.489774670196858,0.615366220233501,0.538312894440599,0.847787614572718
"9937","ZNF140",50.6294502158797,0.161620995532301,0.262967755140707,0.614603853030658,0.538816369243877,0.847787614572718
"9938","KIAA0586",130.04809887204,0.108597607340835,0.176684730391733,0.614640592314118,0.538792100846472,0.847787614572718
"9939","AL121612.2",4.02659191471904,0.546059635021314,0.887761857823637,0.615096976975321,0.538490678375121,0.847787614572718
"9940","NUDT7",66.5553486119002,0.202913335284829,0.330014940000779,0.614861058364055,0.538646481926532,0.847787614572718
"9941","GOSR1",274.562563323157,-0.0903752935621796,0.14684017893576,-0.615467062333921,0.538246315004855,0.847787614572718
"9942","SLC19A1",13.4958961339644,-0.260749664518009,0.424094338275236,-0.614838824725792,0.538661166460093,0.847787614572718
"9943","FXR1",514.556978595365,0.100691152028258,0.163876217473426,0.614434196619078,0.538928444112092,0.847793408210524
"9944","TXN2",557.615062302246,-0.163165974044261,0.265525554203135,-0.614501962095274,0.538883676899863,0.847793408210524
"9945","CTBP1-

AS",6.82114592453655,0.393433213016165,0.640463887122245,0.61429414042
992,0.539020973904642,0.847853704855737
"9946","ACTR8",58.1857616075221,-0.159465280922922,0.259643973921237,-
0.61416900425078,0.539103653348812,0.847898496816356
"9947","YTHDF3-
AS1",17.4351444513029,-0.254553282232041,0.41467850864005,-0.613856944
423902,0.539309863871294,0.84805229197212
"9948","MT-
CO1",44467.0363336732,-0.12905915834209,0.210232462275372,-0.613887869
386425,0.539289426754716,0.84805229197212
"9949","CDK4",432.309967182195,-0.154861417475898,0.252336247174161,-0
.613710551734621,0.539406614426046,0.848119174737827
"9950","EFTUD2",67.0101897805334,0.139629220940284,0.227577662119535,0
.613545370138058,0.539515792959442,0.848205582840658
"9951","DIS3L2",59.3498679752235,0.158633996876988,0.258686251564297,0
.613229330579862,0.539724713504789,0.848206446325154
"9952","UQCRQ",886.500716230503,-0.158237791570198,0.257989540990143,-
0.613349637984912,0.539645178532562,0.848206446325154
"9953","STAT5A",109.926070788121,-0.124861991598434,0.203542807579879,
-0.613443398384064,0.539583197799118,0.848206446325154
"9954","CDPF1",34.7107035817964,-0.178803012475349,0.291602940565214,-
0.613172871743938,0.539762040345462,0.848206446325154
"9955","ABCB7",128.253963404057,-0.11130009777749,0.181526419417107,-0
.613134430431018,0.539787455933447,0.848206446325154
"9956","SLC47A1",7.38262685736519,0.50288885740673,0.820958833223379,0
.61256281929778,0.540165448996425,0.848715158562785
"9957","NCOA7",663.699324742462,0.0931045692144056,0.152109144487166,0
.612090545432401,0.540477852520691,0.848787777120207
"9958","BX664615.2",16.6620418147229,0.325793558153653,0.5323417172181
71,0.612000802522362,0.540537226597935,0.848787777120207
"9959","AL354920.1",8.50267811733311,-0.360616388977408,0.589049576305
443,-0.612200404657307,0.5404051738918,0.848787777120207
"9960","RAPGEFL1",7.21761451796997,0.371121867691313,0.606401056648959
,0.612007290591106,0.540532933970149,0.848787777120207
"9961","SH2D1A",4.82793151579619,-0.453535173507152,0.740645554606509,
-0.612351172144828,0.540305439890138,0.848787777120207
"9962","ZNF275",53.0004139172451,-0.152549669052643,0.24924791681212,-
0.612039895874568,0.540511361962056,0.848787777120207
"9963","PPP2R2A",248.868296328477,0.095154569151486,0.155591471687622,
0.611566740255056,0.540824449032195,0.849153553770012
"9964","PPP1R12A",567.36504768279,0.0962773794569442,0.157513233784672
,0.611233590623631,0.541044948503238,0.849329265372419
"9965","AC015712.1",8.54411317289735,-0.486394568405469,0.795658601458
092,-0.611310639405044,0.540993948760212,0.849329265372419
"9966","ETFB",926.905390923858,-0.178628614256765,0.292353485915287,-0
.611002169847651,0.541198143823271,0.849484503695307
"9967","FUCA2",428.627296699521,-0.122805981662113,0.20105569454778,-0
.610805786617144,0.541328162072453,0.849603334935224
"9968","EVC2",91.5122238952271,0.19748235436017,0.323733523943227,0.61
0015150592815,0.541851771414881,0.849680997559979

"9969","CANX",975.545369156386,0.0913166702687216,0.149721322513379,0.609910924748622,0.541920815230833,0.849680997559979
"9970","PRPF18",182.837266656918,-0.114770741784595,0.188132459393008,-0.610052843379033,0.541826803124432,0.849680997559979
"9971","SLK",360.628626536613,-0.13541424546262,0.221872247282244,-0.610325298099855,0.541646341935919,0.849680997559979
"9972","SMNDC1",167.594233813727,-0.098401268326873,0.161299205893289,-0.610054263949524,0.541825862127572,0.849680997559979
"9973","H2AFX",94.6651021217162,-0.222429090762366,0.364677743447406,-0.609933276047172,0.541906008370888,0.849680997559979
"9974","RUBCNL",3.96747484829764,-0.598397005747912,0.980213624433974,-0.610476115442139,0.541546460525526,0.849680997559979
"9975","KTN1",844.145266865929,-0.121098275594426,0.198342905979987,-0.610550072341104,0.541497484641931,0.849680997559979
"9976","PLCB2",9.06946269037891,-0.379413911804992,0.622004419852086,-0.609985877423857,0.541871162815389,0.849680997559979
"9977","HSD11B2",41.3446701915343,-0.377294231358635,0.617870025108961,-0.610636891297808,0.541439994042852,0.849680997559979
"9978","KLHDC3",292.69440621519,-0.154351355900426,0.253124953979068,-0.609783245286793,0.542005401743745,0.849728452543336
"9979","SH3BP5-AS1",18.4290995756359,-0.255403359996074,0.418975256273372,-0.60959055737036,0.542133068251323,0.849784397456406
"9980","ATAD5",25.4536861863837,0.275848357392505,0.452532828938701,0.609565405540712,0.54214973384996,0.849784397456406
"9981","GTF2H5",603.663646792798,0.162123192437451,0.266047793388262,0.609376196557486,0.542275111884951,0.849873341621524
"9982","ZNF107",68.719550017944,0.160691401271001,0.263724331052999,0.609315798164668,0.542315137509816,0.849873341621524
"9983","AC010623.1",4.43135574934951,0.567258839515217,0.931443373585335,0.60901054814713,0.542517447257962,0.850105221622388
"9984","ZRANB2",870.578886578022,0.14107642240028,0.232349378625883,0.607173659058648,0.54373567116424,0.850215491475507
"9985","GSTM5",443.062303969809,0.175239323382928,0.288575246197094,0.607256948377484,0.543680404287764,0.850215491475507
"9986","AC239809.3",22.4085946316133,0.26359605075227,0.43430494415843,0.606937715763403,0.543892247215713,0.850215491475507
"9987","FMO2",296.379897940703,0.249995145438219,0.411104771558189,0.608105677028936,0.54311738726407,0.850215491475507
"9988","XPR1",53.4108163817283,0.210757082336567,0.346473200809114,0.608292594764585,0.542993431574975,0.850215491475507
"9989","ATG4B",115.777630484566,0.106494124651894,0.175446968115152,0.606987546128462,0.543859177055622,0.850215491475507
"9990","PEX6",52.0829277845346,-0.16792088737677,0.276607734401604,-0.607072277787316,0.543802946781444,0.850215491475507
"9991","RAB32",485.364492472258,0.206687925410809,0.340089968937089,0.607744844862044,0.543356715270065,0.850215491475507
"9992","ATP6V0E2",38.1214397737495,-0.195378673207929,0.321398619326972,-0.607901407968287,0.543252865707962,0.850215491475507
"9993","PPP3CC",64.9511661928278,0.146757287304432,0.241701813964084,0

.607183226710244,0.543729322378327,0.850215491475507
"9994","BEND7",66.4803817980169,0.231217661581123,0.380026292588794,0.
608425432898432,0.542905347685461,0.850215491475507
"9995","NDST2",61.2455346565106,0.185420860414189,0.305329774453001,0.
607280638602543,0.543664685078698,0.850215491475507
"9996","MALAT1",269573.358963631,0.0987252481115381,0.162281159825338,
0.60835927114272,0.54294921811946,0.850215491475507
"9997","VPS33A",69.0781028435552,0.136295974686716,0.224097299535667,0
.608199987099903,0.543054843177413,0.850215491475507
"9998","LINC00346",9.14176136455366,-0.310244766302436,0.5104187946103
65,-0.607823946881237,0.543304245032108,0.850215491475507
"9999","AL121821.2",18.4926406244069,0.406996240454006,0.6702522031187
16,0.607228500794526,0.543699280452167,0.850215491475507
"10000","FAM174B",22.4990294447156,0.190896880763325,0.314498659783138
,0.606987899073901,0.54385894282526,0.850215491475507
"10001","MGRN1",103.630182517528,0.130611210219872,0.214571557127135,0
.608707006504518,0.542718662048795,0.850215491475507
"10002","ZNF460",40.9154922684202,0.161672745054436,0.266139121420799,
0.607474557634881,0.543536022206988,0.850215491475507
"10003","ZNF776",17.8433581280289,0.234498421455397,0.38618397092182,0
.607219457854892,0.543705280890182,0.850215491475507
"10004","SYNJ1",45.9951056322273,0.167413701569693,0.275800156134187,0
.607010902083173,0.543843677108853,0.850215491475507
"10005","JOSD1",186.599417915079,0.116464489987278,0.191495044343,0.60
8185399193256,0.543064517278706,0.850215491475507
"10006","NUP50-
DT",12.1611866261856,-0.322694938457727,0.530137773874684,-0.608700142
416199,0.542723212615863,0.850215491475507
"10007","CXCR3",2.6728195406627,0.682674750593177,1.12362855467986,0.6
07562657383408,0.543477574123618,0.850215491475507
"10008","ADGRB2",5.65959568565451,0.419513924983651,0.691759077777914,
0.606445131636327,0.544219206830665,0.850310080059335
"10009","ARHGAP15",27.4143990221297,0.299086703622921,0.49293537057170
5,0.60674628253201,0.544019302096731,0.850310080059335
"10010","ATG7",76.0678709487998,0.125140435199174,0.206351196356831,0.
606443952875255,0.544219989367021,0.850310080059335
"10011","NLGN2",89.993175594861,0.128843290553703,0.212459448505745,0.
606437094042534,0.544224542706262,0.850310080059335
"10012","UBOX5",30.52766711222,0.185608403163232,0.305986525649794,0.6
06590119512858,0.544122958949358,0.850310080059335
"10013","ATAT1",44.600763900519,0.151331733878842,0.249587947125745,0.
60632628947663,0.544298104607903,0.850340082930334
"10014","SP5",81.8014971764209,-0.281750086756628,0.46503691486684,-0.
605866067293399,0.544603693812973,0.850392851099654
"10015","AIMP1",367.270124473027,0.0839740656120326,0.138567296671734,
0.606016481731379,0.544503808701845,0.850392851099654
"10016","NFRKB",77.802774790509,0.157008326461007,0.259079724697595,0.
606023210207868,0.544499340762246,0.850392851099654
"10017","CTF1",44.3745480359554,0.191305923064933,0.315731703970939,0.
605912933857733,0.544572570345646,0.850392851099654

"10018","TUBD1",52.1496916641539,-0.144160642543716,0.23784129447876,-0.606121165206618,0.544434297340383,0.850392851099654
"10019","PCDHB15",18.6745286889524,-0.288427928807018,0.476126234469544,-0.605780374879695,0.54466060330005,0.850396827769507
"10020","AC009961.4",8.34765280113063,-0.32988860055593,0.545017465070725,-0.605280787677367,0.544992444705523,0.850830021210429
"10021","SDK2",3.58767495164429,0.475971718395141,0.786589350285989,0.605108266749463,0.545107061803365,0.850924036302767
"10022","MYCBP",31.8825842619834,-0.234703924926542,0.387928222892948,-0.605018947000693,0.545166407539587,0.85093176143901
"10023","CNBD2",26.3716917729858,-0.195826209126255,0.323896360557052,-0.604595274826381,0.545447947052774,0.851286265164776
"10024","KCNJ14",3.31298715373367,0.522455561669537,0.864295236368324,0.604487378485203,0.545519658077779,0.851313249332672
"10025","TRPV2",125.142980169388,-0.121829998724393,0.201660537426254,-0.604134057556726,0.545754518105938,0.851594805658972
"10026","SLC12A9",57.6648562145434,-0.133854919151891,0.221599763521955,-0.604039088420007,0.545817654685884,0.851608375448961
"10027","H3F3A",5627.35865467487,-0.14597307509235,0.241732496392209,-0.603862026293351,0.545935377305694,0.851626075162869
"10028","RAD50",442.553443915692,0.103635908121012,0.171622903922058,0.603858259897979,0.545937881591335,0.851626075162869
"10029","ATP6V1E2",48.8489042308866,-0.180997552964223,0.299851212236623,-0.603624549702977,0.546093287221726,0.851698633301043
"10030","SUN2",240.291950383995,-0.13236673888533,0.21926495691152,-0.603683966420331,0.546053775987615,0.851698633301043
"10031","LINC00339",41.2077725974713,0.159031291194072,0.263605324012117,0.603293168641624,0.546313676692538,0.851787585418257
"10032","ZNF213-AS1",34.4353153424231,-0.215487920642423,0.35717732111676,-0.60330795910746,0.546303839152978,0.851787585418257
"10033","FOXK2",72.4324618334312,-0.137669939968177,0.228187631669514,-0.603319027244939,0.546296477492285,0.851787585418257
"10034","TAF1A-AS1",5.16028130097213,0.540761514580374,0.896647512861409,0.603092638772487,0.546447063204102,0.851855895592277
"10035","SRRD",88.9225537465114,-0.150568165009759,0.24967212881744,-0.603063568700752,0.546466401091127,0.851855895592277
"10036","LAPTM4A",7733.18624314984,-0.153611514910637,0.254948309401141,-0.602520233499336,0.5468278989233,0.852033390760906
"10037","RPL9",14682.7280378225,-0.150377817521714,0.249630647345472,-0.602401264110818,0.546907068761124,0.852033390760906
"10038","LSM6",412.546369875032,-0.158551147448646,0.263108405764296,-0.602607685558638,0.546769706350352,0.852033390760906
"10039","UCHL3",140.942815713538,-0.191668748161011,0.318167210895871,-0.602415150264305,0.546897827734232,0.852033390760906
"10040","AC023355.1",6.54814740913235,-0.365195313647767,0.606011170458546,-0.602621422591002,0.546760565697825,0.852033390760906
"10041","LINC01547",7.16181397931556,0.360068506499036,0.597419983187021,0.602705829453847,0.546704402840557,0.852033390760906

"10042","MIS18BP1",234.3056832586,0.154812720633198,0.257143343693114,
0.602048329969444,0.547141967086405,0.852314458388034
"10043","USF1",30.5361034453906,-0.192094616728912,0.319128438705103,-
0.601935125269174,0.547217322013598,0.8523469648769
"10044","AP001160.3",16.45642719981,-0.253204533475414,0.4207605272948
,-0.601778249265311,0.547321755316688,0.852384782738289
"10045","ZNF765",62.2599078596393,0.144687647073107,0.240450794717052,
0.601734950568021,0.547350581257183,0.852384782738289
"10046","XCL1",6.06161013995748,-0.53107703597341,0.882736863776672,-0
.60162553277912,0.54742342907454,0.852413368605717
"10047","SKI",401.885674839604,-0.117870067583077,0.196205927176777,-0
.600746721972771,0.548008694746402,0.852617684391123
"10048","PPT1",294.296130588299,-0.118664051324079,0.19744881415906,-0
.600986396547747,0.547849046904652,0.852617684391123
"10049","COL9A2",3.67871147645784,-0.555453069251781,0.923849025669034
,-0.601237922884134,0.547681529302588,0.852617684391123
"10050","PCDHGA7",6.29183180202927,0.380618836674086,0.633541983704798
,0.600779185064139,0.547987069652871,0.852617684391123
"10051","LINC02823",9.74953176062388,0.308552508293016,0.5135217496340
17,0.600855773904258,0.547936052127871,0.852617684391123
"10052","EML1",198.34002648783,-0.136949318268428,0.227933258877615,-0
.600830782408813,0.547952699259211,0.852617684391123
"10053","LYRM9",121.78185877385,-0.145390838398859,0.241918621526048,-
0.60099068637924,0.547846189646472,0.852617684391123
"10054","CATSPERG",4.61506030406844,0.453983933274413,0.75556167324293
6,0.600856223061017,0.547935752941517,0.852617684391123
"10055","TIMM8A",41.427704310199,-0.174356993955045,0.290260249096438,
-0.60069194627169,0.548045184207169,0.852617684391123
"10056","BSDC1",266.937336380863,0.110876830130303,0.184630417876828,0
.600533928294916,0.548150456420159,0.85262917856053
"10057","HNMT",663.126390291303,-0.142802142855031,0.237798578292613,-
0.600517227143858,0.548161583378078,0.85262917856053
"10058","OPLAH",22.0526096690201,-0.196953021524075,0.328132882406207,
-0.600223360974414,0.548357386731708,0.852848936234253
"10059","ZBTB8OS",185.9341029944,-0.144351792839653,0.241068911522479,
-0.598798874263771,0.549307013116313,0.853009460940775
"10060","AL356055.1",4.02415742721046,-0.439797418334881,0.73334752659
453,-0.599712145177856,0.548698092682724,0.853009460940775
"10061","SLC25A24",187.604850471462,-0.117252910409288,0.1957840021104
28,-0.598889128556856,0.549246821520206,0.853009460940775
"10062","SLC16A1",123.407434629957,0.155731594772384,0.260119973562792
,0.598691413963224,0.549378683833401,0.853009460940775
"10063","AL596325.2",5.07465803946515,-0.595735985143796,0.99500117026
1315,-0.598728929120092,0.549353662551294,0.853009460940775
"10064","ANKZF1",75.685855765392,0.143951049243126,0.240481027788209,0
.598596282488877,0.549442135679809,0.853009460940775
"10065","STIM2",63.5758561304007,-0.144134569382183,0.240417556974444,
-0.599517652521126,0.548827742103014,0.853009460940775
"10066","OCIAD2",3.77528198351023,-0.559026220603718,0.932366134451512
,-0.599577998328499,0.548787513779079,0.853009460940775

"10067","HSF2",75.5177923101167,-0.120306103834289,0.200979221806682,-0.598599709725262,0.549439849680907,0.853009460940775
"10068","NFE2L3",2.59750372069331,0.603338143948618,1.0056116690871,0.599971303531442,0.548525360403469,0.853009460940775
"10069","LINC01176",5.23116461823389,-0.429103601463047,0.715847561245872,-0.599434327493172,0.548883291456386,0.853009460940775
"10070","ATP6V1B2",213.589904756699,-0.101828049643507,0.169855351236646,-0.599498625755027,0.548840426217375,0.853009460940775
"10071","CHST3",71.4232853259334,0.138443660362439,0.231083354505826,0.599107022046231,0.549101519327475,0.853009460940775
"10072","KIAA1549L",14.584427598628,-0.441208045187643,0.7368230388238,-0.598797841462651,0.549307701921742,0.853009460940775
"10073","POLD3",141.430176456875,-0.127892467482129,0.21344699666227,-0.599176701860505,0.549055057371391,0.853009460940775
"10074","AP000866.1",18.1293298917361,0.272317577502813,0.454629327229043,0.598988145271189,0.549180789910043,0.853009460940775
"10075","PRRT2",76.5132999076052,-0.239918904632425,0.400443759367268,-0.599132584839169,0.549084474031474,0.853009460940775
"10076","MEAK7",61.6462143746314,0.140554918678522,0.234685352333198,0.59890793047436,0.549234282720887,0.853009460940775
"10077","AL162274.2",6.92738558924035,0.423195802666103,0.707186121070234,0.598422098592165,0.549558324152796,0.853105176612304
"10078","MIR181A2HG",2.6325631634467,0.576803798857802,0.96427508396373,0.59817349680633,0.549724173674413,0.853277956815721
"10079","AL021368.1",2.91919203165443,0.576906215704174,0.964860750107223,0.597916554943357,0.549895613025193,0.853290057985626
"10080","MICU1",326.07577484811,0.144175188515744,0.241085884402579,0.598024180772823,0.54982379862352,0.853290057985626
"10081","BX890604.2",4.37835681176988,0.486778875509429,0.81403136379756,0.597985405916725,0.549849670998042,0.853290057985626
"10082","CASC15",8.14248828665857,-0.377519832961149,0.631650464180287,-0.59767205815493,0.550058773104879,0.853458578424878
"10083","AC010654.1",2.76945837204452,0.603364375951578,1.00972234048536,0.597554745259525,0.55013706800301,0.853495403627005
"10084","DARS",276.030754435262,0.09293270232363,0.155567483898029,0.597378706623199,0.550254566894304,0.853508397613049
"10085","PLA2G4B",17.6682385451221,0.296801327733763,0.496820435983968,0.597401608784346,0.550239279899924,0.853508397613049
"10086","LEMD3",108.529276798026,0.1260189968716,0.211017242350906,0.597197629291542,0.550375441809893,0.853611246899876
"10087","CEBPZ",300.839653601184,-0.124670891784627,0.2089089549659,-0.596771410804181,0.550660007885932,0.853947124047932
"10088","ARL1",692.199930424757,-0.116834802032016,0.195798379380551,-0.596709750109515,0.550701181831844,0.853947124047932
"10089","SIRT4",11.586089881965,-0.267233564177398,0.44793554411955,-0.596589325597426,0.550781599701621,0.85398717059495
"10090","EPS15",470.77819521164,0.103251194315648,0.173389310842567,0.595487656153139,0.55151754784461,0.854292748555177
"10091","RABL2A",58.477784064355,0.136243935030701,0.228641967645237,0.595883321132447,0.551253176193164,0.854292748555177

"10092","MOB4",277.783570473003,-0.146228499281412,0.245392503252187,-0.595896359275224,0.55124446555185,0.854292748555177
"10093","POGLUT1",95.1497658105545,-0.140322437828054,0.235751703972656,-0.595212825457792,0.551701218262709,0.854292748555177
"10094","RPS3A",14146.2189058945,-0.18052052207875,0.303382163074651,-0.595026814527428,0.551825547139116,0.854292748555177
"10095","HSD17B4",275.74388557685,-0.0814212821810384,0.136691760556365,-0.595656108675725,0.551404985228095,0.854292748555177
"10096","AC009403.1",26.0707832667461,0.225285297923221,0.378612001003355,0.595029469024213,0.551823772788502,0.854292748555177
"10097","TCTN3",162.60268627695,-0.125899702490622,0.211443147086716,-0.595430517495036,0.551555731356533,0.854292748555177
"10098","GRK2",103.858552855555,-0.117300875279778,0.197148604267047,-0.594987094714039,0.551852097468407,0.854292748555177
"10099","ETFBKMT",57.0223396513192,0.147111031063751,0.246845434174697,0.595964156904913,0.551199171784136,0.854292748555177
"10100","POLR2M",160.116833844393,0.110848063581438,0.18630348294101 5,0.594986533969058,0.551852472297517,0.854292748555177
"10101","ANKS3",103.708593474932,0.113212530054014,0.190202129028182,0.595222201940964,0.551694951427554,0.854292748555177
"10102","ZNF747",40.1484712277507,0.151049985995136,0.253360137505172,0.596186864605143,0.551050399656717,0.854292748555177
"10103","ZFP3",13.8593662013284,0.257704809929127,0.433074315284678,0.595059094556848,0.551803970324446,0.854292748555177
"10104","PPP5D1",31.1539200701284,0.190738777410824,0.320305485091402,0.595490200101929,0.551515847854164,0.854292748555177
"10105","SNU13",831.209992390465,-0.147859247685322,0.248327417103759,-0.595420551664425,0.55156239126117 7,0.854292748555177
"10106","KLHL21",93.4210410836148,0.144705464459576,0.2433261648732,0.594697510376592,0.552045686310293,0.854422743737204
"10107","AC008393.1",20.6950343323784,0.195211243754487,0.328247681784154,0.594707151299403,0.552039240759795,0.854422743737204
"10108","DAPL1",3.57288145631106,0.475862823274533,0.800618685101618,0.594368870137142,0.552265424643212,0.8545937320896
"10109","CNP",206.720209596959,0.123110831221104,0.207106143458311,0.594433507212136,0.55222203040927,0.8545937320896
"10110","ZNF318",68.5140928886669,0.201417996824283,0.338971544851724,0.594203259487132,0.552376172962684,0.85468056119241
"10111","INTS2",35.1593353769643,0.193334566215781,0.325507018648998,0.59394899384415,0.552546228484474,0.854774589812365
"10112","XIAP",310.236271354183,0.129782953619434,0.218500663855959,0.593970523151319,0.552531828463242,0.854774589812365
"10113","DLG5-AS1",6.15667725292285,-0.380655354068338,0.641024904148885,-0.593823034962658,0.552630480615602,0.854820390415294
"10114","NDUFS1",311.976045796424,0.0930591080205158,0.15677985530513,0.593565466937072,0.552802783877699,0.854833328212618
"10115","AL928654.1",13.8966986206304,0.237641142773336,0.400308989882638,0.593644281740979,0.552750056958818,0.854833328212618
"10116","CARD8-

AS1",13.6892570712436,-0.275648400509073,0.46426672339008,-0.593728532
806068,0.552693695921999,0.854833328212618
"10117","GPBP1L1",175.905807388046,0.0909589344759819,0.15337124072799
7,0.593063823727533,0.553138439813729,0.85499906659774
"10118","SLC4A1AP",140.224319781487,-0.100519808946769,0.1695124492900
13,-0.592993667236755,0.553185390387672,0.85499906659774
"10119","ICA1L",81.2076036488335,0.167539460939698,0.282569009924617,0
.592915199668901,0.55323790526768,0.85499906659774
"10120","TMEM198",11.653656002627,-0.298722733763574,0.503551341898948
,-0.593231928718643,0.553025947468564,0.85499906659774
"10121","AC004837.4",3.15663508096178,-0.575356613164977,0.97007168097
6868,-0.593107318198991,0.553109333147232,0.85499906659774
"10122","POLR2J2",15.9705655599499,-0.317910317159459,0.53601974162701
8,-0.593094418863163,0.553117965353739,0.85499906659774
"10123","STK39",45.2782985093298,0.167936186094429,0.283379895464415,0
.592618561804491,0.553436453973742,0.855196685336014
"10124","XYLB",22.752809836982,0.239672631711447,0.404469262135333,0.5
92560805353001,0.553475116176041,0.855196685336014
"10125","TAF12",272.175521979915,-0.150741052895372,0.254715000447839,
-0.591802809533554,0.553982641625138,0.855220612152574
"10126","PEA15",338.567607895136,0.107471298863668,0.181554701615989,0
.591949962777517,0.553884095537038,0.855220612152574
"10127","PCDHB16",51.9622035471769,0.199000889110189,0.336250380676576
,0.591823535514741,0.553968761259815,0.855220612152574
"10128","AL133346.1",3.3593899166065,0.495425795386068,0.8368917865599
64,0.591983101450322,0.553861904301334,0.855220612152574
"10129","HINT2",290.571476448901,-0.186297770686808,0.314677663173182,
-0.592027310766827,0.553832300312421,0.855220612152574
"10130","AL359546.1",3.8755557636051,0.474073423574406,0.8010119174653
82,0.591843158931395,0.55395561944822,0.855220612152574
"10131","AL442128.2",2.60801649290675,-0.709428505287069,1.19837226855
811,-0.591993426333752,0.553854990355939,0.855220612152574
"10132","SLC25A21",7.15115273820523,-0.317458285815907,0.5359635321627
14,-0.592313220518759,0.553640864600988,0.855220612152574
"10133","LINC00640",12.4154180075159,0.391659075219218,0.6612284754598
61,0.592320339723471,0.553636098230627,0.855220612152574
"10134","PRR7",5.3116781534998,-0.404373547163742,0.684162197859767,-0
.59104924011985,0.554487429048127,0.855236020495298
"10135","GOLGA2",474.589513885735,-0.108937309365443,0.184299553908573
,-0.591088296499536,0.55446126114108,0.855236020495298
"10136","BACE1",314.187046981585,0.0929440136691429,0.157234111632474,
0.591118636434278,0.554440933698663,0.855236020495298
"10137","PTPN6",9.29885498729258,-0.396371931247774,0.670712137449493,
-0.590971758398545,0.554539343852446,0.855236020495298
"10138","YARS2",81.8713511290749,-0.141634096547012,0.239590239596833,
-0.591151362364946,0.55441900807235,0.855236020495298
"10139","SMARCD1",111.145030218163,-0.154336867879079,0.26105622112513
4,-0.591201646962857,0.554385319366503,0.855236020495298
"10140","UNC119B",52.6606131446959,0.133716418885704,0.226018393607269
,0.591617419943487,0.554106806177311,0.855236020495298

"10141","LINC02588",6.20500042388132,-0.333004347515266,0.563110106554
266,-0.591366312980878,0.554275006610977,0.855236020495298
"10142","UBE2Q2",248.135156134885,-0.0912291859728753,0.15419570797213
1,-0.591645430165696,0.554088045476525,0.855236020495298
"10143","TSNAXIP1",4.12361273549853,-0.415944230698094,0.7034784973104
23,-0.591267867160623,0.554340955962435,0.855236020495298
"10144","JAK3",9.39950792488021,-0.291893259485326,0.49403644983445,-0
.59083344879338,0.554632020858163,0.855294627591113
"10145","ACTA2",281.161793164684,0.160785159840662,0.272477167945947,0
.590086725624505,0.55513250784638,0.855982042409159
"10146","EPHB2",60.3974511420531,-0.198594459837197,0.336893020931821,
-0.589488198027669,0.555533826856098,0.856263243029849
"10147","GNE",61.9258594549275,0.151048757640321,0.256134595000736,0.5
8972415514541,0.555375598226775,0.856263243029849
"10148","PIR",95.0129106218996,0.175339117176679,0.297413253497073,0.5
89547086805952,0.555494335031713,0.856263243029849
"10149","MT-
ATP8",593.623193870514,-0.225666299171312,0.38281621708651,-0.58948991
4739727,0.555532675580158,0.856263243029849
"10150","AL442003.1",5.76859931302835,-0.403823927773228,0.68531740292
6234,-0.589250945691648,0.555692946227391,0.856424114072422
"10151","AL160006.1",17.0243164080277,0.222812651717409,0.378430397438
972,0.588781063110399,0.556008150701059,0.856468321474734
"10152","HEXB",716.415475381887,-0.10530407755051,0.178880499113485,-0
.588683943036761,0.556073311220308,0.856468321474734
"10153","ASF1A",128.005498896126,-0.151101870786051,0.256800430071575,
-0.58840193820523,0.556262537103877,0.856468321474734
"10154","CCN2",1823.41099642529,-0.267848304585876,0.45533419598487,-0
.588245528114861,0.556367502168996,0.856468321474734
"10155","HTRA4",4.22688806030611,0.639206326446806,1.08622580815435,0.
588465420033529,0.556219937913566,0.856468321474734
"10156","SPOUT1",56.0387283909689,-0.135385049578475,0.229996589548298
,-0.588639378715852,0.556103211891591,0.856468321474734
"10157","EMC7",665.844823914747,-0.15069700975324,0.256093155464988,-0
.588446065571799,0.5562329254679,0.856468321474734
"10158","SPATA5L1",40.0577108132241,0.172126443145328,0.29256584201154
4,0.588334037773748,0.556308103138845,0.856468321474734
"10159","AC027237.3",3.82635758935191,0.452042794219567,0.767509780326
847,0.588973334029779,0.555879161861607,0.856468321474734
"10160","TRMU",75.8905397710094,-0.136644573561784,0.232083026956473,-
0.588774523297698,0.556012538323646,0.856468321474734
"10161","CLTRN",2.79467176939147,0.490433413369196,0.833745822096388,0
.588228930654236,0.556378641106325,0.856468321474734
"10162","ZMAT1",65.8395096341736,0.252061759513593,0.42797223211413,0.
588967555835199,0.555883038067577,0.856468321474734
"10163","ERRFI1",790.508144354343,0.188994339962859,0.323459763854472,
0.584290106784006,0.559025147108053,0.856638075158076
"10164","GON4L",272.77241108276,0.106421456119306,0.181518200968657,0.
586285317678318,0.55768379593846,0.856638075158076
"10165","SPATA17",7.87348112394942,0.313441742009431,0.533626052454803

,0.587380883237479,0.556947929912681,0.856638075158076
"10166","PLEKHB2",105.080069678398,0.110310904081844,0.187834956606198
,0.587275691782518,0.557018564042917,0.856638075158076
"10167","TLK1",298.488106331637,0.0793476458168619,0.135232960038774,0
.58674782977546,0.557373079566601,0.856638075158076
"10168","COLQ",10.2235838631029,0.328254422157412,0.558908461042164,0.
587313388574106,0.556993250837927,0.856638075158076
"10169","NR1D2",204.060514421751,-0.477521855951,0.816906552131551,-0.
584548936111486,0.558851051532323,0.856638075158076
"10170","LNP1",18.6378720984054,-0.216587994350924,0.370108151666905,-
0.58520190213441,0.558411966054964,0.856638075158076
"10171","KCNMB3",13.2924664426743,0.296989851553165,0.508570804784782,
0.58396952549969,0.559240815215176,0.856638075158076
"10172","DCK",51.9826554454888,-0.145040852812099,0.248114780151901,-0
.584571595143592,0.558835811710844,0.856638075158076
"10173","LINC00603",9.70979437209977,-0.474229363845594,0.809491780126
215,-0.585835922597822,0.557985780993479,0.856638075158076
"10174","ZSWIM6",53.0928921114983,0.189249893107114,0.322528666046083,
0.586769217840852,0.557358713069018,0.856638075158076
"10175","DTWD2",16.8428664429315,-0.221811562335362,0.379950229423888,
-0.583791099880873,0.559360866899143,0.856638075158076
"10176","CENPQ",75.6331364971137,0.135790809107716,0.231388028058167,0
.586853219016068,0.557302290697751,0.856638075158076
"10177","EFHC1",275.878685368527,-0.099882266089361,0.170837516341546,
-0.584662363562299,0.558774765474229,0.856638075158076
"10178","HIVEP2",164.565384386118,0.152103401196708,0.258994678313656,
0.587283886244575,0.5570130614554,0.856638075158076
"10179","RBM48",77.0986984673248,-0.137806458139394,0.234629327648603,
-0.587336883758124,0.556977474224127,0.856638075158076
"10180","TSPYL5",90.5283917234058,0.123381290975689,0.210005000784489,
0.587515966356941,0.556857230479324,0.856638075158076
"10181","NUDCD1",68.8608748875411,-0.122920029098985,0.210200502668609
,-0.584775143438996,0.558698919960173,0.856638075158076
"10182","HSF1",153.105596675063,-0.0912553630123838,0.156212996289282,
-0.584172669240612,0.559104147446476,0.856638075158076
"10183","CD72",10.6473347051334,-0.33108847785422,0.56707970141457,-0.
583848226322202,0.559322428650288,0.856638075158076
"10184","NXNL2",3.00824780021353,-0.555314343195983,0.944708581764908,
-0.587815495608755,0.556656141956969,0.856638075158076
"10185","PTPRE",44.4334957292505,-0.271713856618765,0.463456145177841,
-0.586277384485864,0.557689126205136,0.856638075158076
"10186","CAT",662.236340587166,-0.131616103883095,0.225236572486156,-0
.584346060812059,0.558987508690895,0.856638075158076
"10187","OSBP",152.15085422104,-0.0950680484750164,0.16189981500904,-0
.587202946894708,0.557067413451472,0.856638075158076
"10188","SF3B2",628.23156260883,-0.0871460161049007,0.148747366908449,
-0.58586594113318,0.557965606622684,0.856638075158076
"10189","KITLG",252.900987509077,-0.154239591514277,0.262963566146777,
-0.58654358006457,0.557510284458917,0.856638075158076
"10190","GATC",121.954449851171,-0.132673718679196,0.22705514595163,-0

.584323769114044,0.559002503428658,0.856638075158076
"10191","IFT88",88.9089117561262,-0.138836640716643,0.237385240851146,
-0.584857930589293,0.558643248016494,0.856638075158076
"10192","UBL3",814.96136690535,-0.103180441163143,0.176618574736611,-0
.584199262829604,0.559086257441113,0.856638075158076
"10193","RFC3",42.5868976730067,0.194172234895288,0.332157130234694,0.
584579457192717,0.558830523967056,0.856638075158076
"10194","BAZ1A",149.570858559981,-0.143531690612681,0.245763534362794,
-0.584023545172496,0.559204471148535,0.856638075158076
"10195","WDHD1",10.236179522751,0.263987992035886,0.451737902002733,0.
584383092199088,0.558962599582905,0.856638075158076
"10196","NDUFB1",850.016166635843,-0.166429008818247,0.283958184818536
,-0.586103932607556,0.557805673727243,0.856638075158076
"10197","CEP170B",33.8787721334782,-0.162081366737653,0.27706444962785
6,-0.584995176953796,0.558550959778403,0.856638075158076
"10198","ARNT2",4.25676460221189,0.394557114371295,0.671994074466614,0
.587143740343945,0.557107173180639,0.856638075158076
"10199","E4F1",58.127526686136,0.140697415825938,0.239723166579413,0.5
86916224383048,0.557259972731841,0.856638075158076
"10200","ZDHHC1",119.993930393178,-0.150070434814413,0.256981936145038
,-0.583972698881505,0.559238680153882,0.856638075158076
"10201","ZC3H18",147.090584635907,0.16632343441841,0.282923767653699,0
.587873672819142,0.556617088877859,0.856638075158076
"10202","PLXDC1",228.23243792328,0.179496753423269,0.306370626537463,0
.585881079566614,0.557955432764238,0.856638075158076
"10203","AC067852.2",25.0273969725549,0.193021624252028,0.329264684145
109,0.58622024634431 4,0.557727517728222,0.856638075158076
"10204","SEC14L1",162.992509187439,0.0861597529159012,0.14758489189865
2,0.583797920013846,0.559356277818989,0.856638075158076
"10205","ZBTB7A",625.68851070111,-0.100913712973528,0.172872674673033,
-0.583745887916607,0.559391289250128,0.856638075158076
"10206","PRX",9.79450111282046,0.275309309699147,0.46974107742756,0.58
6087363717096,0.557816807481981,0.856638075158076
"10207","AP5S1",50.5068297762861,0.158802064812622,0.270295051355244,0
.587513770660607,0.556858704687506,0.856638075158076
"10208","PRNP",868.600684140925,0.108398581734027,0.185053853016885,0.
585767764176929,0.558031589117784,0.856638075158076
"10209","TTC3",1150.38264545726,0.111078034325055,0.189930796602133,0.
584834246537391,0.558659174575282,0.856638075158076
"10210","AP001505.1",15.6064403886612,-0.277527378837505,0.47372044261
0766,-0.58584632174199,0.557978792065019,0.856638075158076
"10211","COMT",1156.00227019213,0.17251676531626,0.294706763596304,0.5
85384479171902,0.558289222557172,0.856638075158076
"10212","KLHL22",70.3107538918995,0.136089239846371,0.232538187216024,
0.585233941468488,0.558390425596039,0.856638075158076
"10213","ZNRF3-
AS1",4.47426323949376,0.457283592133578,0.781787791090712,0.5849203547
87836,0.5586012713464,0.856638075158076
"10214","PHETA2",106.946906909786,-0.131400312833631,0.224396890254853
,-0.58557100628443,0.558163837036451,0.856638075158076

"10215","SRPX",2913.63756294275,-0.182098705512513,0.3112214743525,-0.585109706492367,0.558473952631404,0.856638075158076
"10216","LINC01703",12.9232749913652,-0.246285989227965,0.422059150042986,-0.58353429656218,0.559533676094338,0.856664241367377
"10217","BACH2",36.7864557931538,0.245576217154496,0.42077421763118,0.583629430854888,0.559469654896027,0.856664241367377
"10218","COPB1",459.676107313482,0.106672040377416,0.182821529026311,0.583476360500542,0.559572666259148,0.856664241367377
"10219","TPD52L2",212.413633385998,0.117741618658176,0.20193144079662,0.583077197853315,0.55984133299555,0.8569167942552
"10220","KMT5B",250.835301770057,0.0847717763650604,0.145485602018145,0.582681551913898,0.560107694434406,0.85731552485689
"10221","POC1B",42.1767199323841,0.161015663401207,0.276428638868338,0.582485461927477,0.560239731217463,0.857349844984815
"10222","SLC12A4",133.70425383994,0.115048288944875,0.197501673888452,0.58251804493492,0.560217790472489,0.857349844984815
"10223","INPP1",133.010653139345,-0.11719197637203,0.201304855705669,-0.582161696801682,0.560457770796484,0.857515738318604
"10224","AC026124.2",12.7240642178392,-0.277023274026761,0.475803836574623,-0.582221606326443,0.560417421635898,0.857515738318604
"10225","RBM27",289.627653605905,0.0952183852758769,0.1636135237854,0.581971361980857,0.560585970939517,0.85762800424517
"10226","RHOF",22.2804305363483,-0.229943641315913,0.395186396281568,-0.58186122670093,0.56066015911578,0.857657624589101
"10227","FAM168B",152.345935413967,0.10443301576503,0.179537130158318,0.581679208489855,0.560782778742278,0.857761318848681
"10228","CHROMR",65.8721003125866,0.168510191236345,0.289839520648041,0.581391353600018,0.560976723526657,0.857806342729961
"10229","XPA",322.464298813505,-0.105876944582821,0.182104050143436,-0.581409059817319,0.560964792868712,0.857806342729961
"10230","GLUD2",3.84723029587653,-0.43999801773585,0.756686728494989,-0.581479760601832,0.560917155071018,0.857806342729961
"10231","PHF24",2.64421301500975,-0.633937209266407,1.09108553300879,-0.581015135924544,0.561230252456351,0.858110139690617
"10232","ZFYVE16",289.130764215162,0.11480416378126,0.197654235009077,0.580833311140476,0.561352802034557,0.858213631961158
"10233","CLP1",34.9616627286815,-0.201559429208706,0.347121056410545,-0.580660335886738,0.561469399058775,0.858274782279846
"10234","AC084757.3",3.27476958922108,-0.484198416747972,0.834010802886555,-0.580566121052792,0.561532911158122,0.858274782279846
"10235","RASD1",1930.68464852667,0.217654860225505,0.374924528553049,0.580529796397966,0.561557399260642,0.858274782279846
"10236","AC092807.3",10.1736207426516,0.331624367565063,0.571807738146319,0.579957817010522,0.561943064661156,0.858291010501197
"10237","ARHGAP30",10.5659954243709,-0.368111301158467,0.634327356857052,-0.5803175555637,0.56170049078627,0.858291010501197
"10238","OARD1",169.674588133843,-0.130228319766692,0.22456267959027,-0.579919691038166,0.561968776200867,0.858291010501197
"10239","AL360020.1",6.46904114738389,0.353295921271472,0.609195803727182,0.579938205598162,0.561956290209764,0.858291010501197

"10240","AL691447.2",15.7629771914877,0.381750976369903,0.657990326531
594,0.580177186467456,0.56179513649101,0.858291010501197
"10241","IFITM10",4.95641779905378,-0.4407446725662,0.760404078645803,
-0.579619027492749,0.562171558754412,0.858291010501197
"10242","AP002360.1",39.7312525143634,-0.184340006118349,0.31772298595
5217,-0.580190965926312,0.561785845168874,0.858291010501197
"10243","DDX10",82.5020866757832,0.137917620620777,0.237938697332028,0
.579635099995197,0.562160717759534,0.858291010501197
"10244","AC103858.3",4.58004882998056,-0.419970786090901,0.72437865079
8496,-0.579766929392465,0.562071801888325,0.858291010501197
"10245","P3H4",140.770378453756,0.13800950517954,0.2380102623418,0.579
846868036927,0.56201788843614,0.858291010501197
"10246","ZHX3",207.185342084346,0.144417737638295,0.248939190470278,0.
580132591278502,0.561825206995738,0.858291010501197
"10247","MAML1",106.513911150997,0.109715345767837,0.189334290066002,0
.579479531835411,0.562265653615471,0.858350894847937
"10248","CUL2",144.834544774193,0.0939634206190918,0.162189525661941,0
.579343334506964,0.562357530973332,0.858407382612786
"10249","GBP5",8.30280047472867,0.345647955079549,0.597335348142022,0.
578649758723751,0.562825522712196,0.858870709373
"10250","CDCA4",47.4073570251229,-0.147654414920083,0.255109541122054,
-0.578788289417366,0.56273203381263,0.858870709373
"10251","AP000692.2",18.2508750313813,-0.393716244854947,0.68040554077
9314,-0.578649380785462,0.562825777778088,0.858870709373
"10252","MBOAT2",74.2878307835705,0.188580537041362,0.3262138053007,0.
578088768706556,0.563204189354671,0.858965900714842
"10253","DYNC1I2",943.664825094008,0.11548973212335,0.199756849139743,
0.57815155085149,0.563161805483016,0.858965900714842
"10254","TMED9",855.023227135722,-0.130955661456827,0.226527375268417,
-0.578100820272408,0.563196053291287,0.858965900714842
"10255","SIX4",7.96262740996327,0.353469472265854,0.611466018217622,0.
578068873387585,0.563217620893187,0.858965900714842
"10256","PDCD7",402.551268497944,-0.0767838319450227,0.132778074616584
,-0.578286981241047,0.563070382430247,0.858965900714842
"10257","AC027020.2",10.5328703604611,-0.366073214168087,0.63314328675
2643,-0.578183835835418,0.563140010671561,0.858965900714842
"10258","SND1-
IT1",23.1405830976205,-0.239075557447595,0.413841320201031,-0.57769861
4849429,0.563467614500326,0.858988909663332
"10259","HIKESHI",357.136966961128,-0.174903077413307,0.30279869004540
6,-0.577621644885845,0.563519590304025,0.858988909663332
"10260","CCDC113",26.8217653134174,-0.188725097487566,0.32671231857177
1,-0.577649163375846,0.563501007521902,0.858988909663332
"10261","AC018557.1",4.91864811631692,0.615907724784085,1.066116972397
46,0.577711208741985,0.563459110394005,0.858988909663332
"10262","MYO1C",453.531702921,-0.101290205078132,0.175376505603921,-0.
57755857735523,0.563562179880763,0.858988909663332
"10263","ZNF335",54.4803183585989,0.178890417716546,0.309677603340452,
0.57766630672925,0.563489212297726,0.858988909663332
"10264","VAMP4",137.714538617016,0.094183144192009,0.163185078388146,0

.577155369365257,0.563834503364595,0.85931751022543
"10265","KRBA1",14.0596089568064,-0.244530085306803,0.423739303193628,
-0.577076715480095,0.563887632964523,0.85931751022543
"10266","TRIM16L",15.2781920581134,-0.259634519944647,0.45004234554331
1,-0.576911311826013,0.563999368688472,0.859404064328246
"10267","D2HGDH",109.882165810203,0.158736770383881,0.275209605099675,
0.576784993846382,0.564084707907547,0.859450383344478
"10268","GSR",57.6532104774244,0.151180721691023,0.262408478675173,0.5
76127427186394,0.564529054066933,0.860043629993088
"10269","MOGS",90.5994272630924,0.112979829469669,0.196167498093134,0.
57593551718761,0.564658767677369,0.86015747422116
"10270","ACBD4",60.7400356494139,0.15018630710227,0.260821740789016,0.
575819740516796,0.564737029054156,0.860192925559315
"10271","NIPAL3",38.5447606384994,-0.173383299581189,0.301203955928779
,-0.575634204559339,0.564862456387908,0.86030020497284
"10272","ASB1",86.5095631106241,0.123621125401635,0.214889010204755,0.
575278955791381,0.565102651558791,0.860539797610366
"10273","AC079848.2",2.87181438473181,-0.506282224204878,0.88012527546
5741,-0.575238819197603,0.56512979229376,0.860539797610366
"10274","EGLN2",229.700387052131,-0.129554803448413,0.225274848597572,
-0.575096617553823,0.565225955399856,0.860602454771263
"10275","SMG9",74.191077004278,-0.135679513823716,0.236000676168465,-0
.574911546977364,0.565351120171921,0.860709252832055
"10276","SLC49A3",88.0313340192623,-0.162885966764773,0.28340792270524
6,-0.574740343212566,0.565466918562536,0.86071801178104
"10277","TRAPPC4",249.726850855775,-0.151395426122794,0.26341317580193
2,-0.574745077431633,0.565463716289629,0.86071801178104
"10278","TM9SF1",340.058363429187,-0.127663984327602,0.222236427942346
,-0.57445120725537,0.56566250951675,0.860848198888074
"10279","SEMA6D",8.59305042081835,0.418264492744893,0.728036491601478,
0.574510340580357,0.565622505126372,0.860848198888074
"10280","AC008443.5",19.3518263944356,0.194946033069306,0.339410185529
59,0.574367067874308,0.565719433140811,0.860851079048804
"10281","RASSF5",60.6491789946167,0.19040873720415,0.331637576871221,0
.57414705233505,0.565868295369318,0.86085567095595
"10282","GALNT5",34.9650411745557,-0.672356897800252,1.17111149709552,
-0.57411860396535,0.565887544872469,0.86085567095595
"10283","NUFIP1",67.2180493613495,-0.118005643789249,0.205500639322051
,-0.57423492295961 2,0.56580883996292,0.86085567095595
"10284","VSIG10",132.783412427215,-0.17529135393948,0.305420127061824,
-0.573935174560377,0.566011669372082,0.860960768571323
"10285","CYBA",1766.46767002382,0.182790902076908,0.318593374980521,0.
57374357545283,0.566141336166452,0.861074275318601
"10286","CDK3",17.6584858784323,0.273577671656894,0.476900530084274,0.
573657721891291,0.566199443129471,0.861078931448018
"10287","TMCO4",71.1522105335533,-0.113514226046423,0.197986508160831,
-0.573343239905073,0.566412313638236,0.861318928963053
"10288","C8orf89",5.84283917094536,-0.323586306278565,0.56449891767306
9,-0.573227505222553,0.566490663244195,0.861354339534306
"10289","XPNPEP1",128.498608689348,0.111695413455151,0.194918715317711

,0.573035858937871,0.566620414615331,0.861384173549817
"10290","SIRT2",236.90566593328,-0.126651221708829,0.221017490629377,-0.573037099227635,0.5666195748491,0.861384173549817
"10291","AP001527.2",8.59971144297234,-0.303267816323649,0.529480812257152,-0.572764506858771,0.566804154024279,0.861412356106266
"10292","RNFT1",138.974654640261,-0.134373212288563,0.234596930799287,-0.572783334508019,0.566791404420953,0.861412356106266
"10293","SYDE1",129.142185405795,-0.116164892657711,0.202805825462174,-0.572788737172531,0.566787745899886,0.861412356106266
"10294","ICAM1",77.5967280893917,-0.240094320847265,0.419466551818044,-0.572380133306584,0.567064472030435,0.861724260343122
"10295","KLHL17",9.47127221579378,0.27652602385872,0.483358735213764,0.572092741297884,0.567259146454442,0.861788463968875
"10296","INPP5D",6.56315210079687,-0.471189271115875,0.823651234057117,-0.572073775443648,0.567271994725277,0.861788463968875
"10297","SPHK2",49.2826339659318,0.173215327405563,0.302722237012361,0.572192281330461,0.567191716117398,0.861788463968875
"10298","GPR161",67.0496636581404,-0.166022363560712,0.290429268211586,-0.571644740156699,0.567562678591517,0.861829180397095
"10299","AL078645.1",3.03849214136151,-0.498294920224166,0.871805340500438,-0.571566721463454,0.567615546184726,0.861829180397095
"10300","HOXD-AS2",154.484780045427,-0.201269581428579,0.352163162232337,-0.571523665771131,0.567644722905021,0.861829180397095
"10301","TBC1D32",38.1220094425193,0.215331667911796,0.376651625964659,0.571699823040192,0.567525354348141,0.861829180397095
"10302","NUTM2B-AS1",175.424040649081,0.165462419553489,0.289457980800223,0.571628459149958,0.567573710850812,0.861829180397095
"10303","RCBTB2",76.4671476965131,-0.112419566967631,0.196598548748786,-0.57182297470202,0.567441910851395,0.861829180397095
"10304","AL008721.2",6.61795177335369,0.419323527242827,0.733769342843281,0.571465040523485,0.567684451499819,0.861829180397095
"10305","AC120053.1",16.6201198752396,0.265710425130804,0.465090022210001,0.571309665746449,0.567789750824711,0.861905392736629
"10306","TBC1D9B",262.494477029537,0.10619236819127,0.185920920650336,0.571169547890672,0.56788471836937,0.861939332433393
"10307","AGO2",270.690840819343,0.143306361712851,0.250924228657224,0.571114086829037,0.567922310259604,0.861939332433393
"10308","EAPP",475.845337711535,-0.121373976764937,0.212583722181105,-0.570946709934526,0.568035766692436,0.861968023916549
"10309","AFG3L2",167.404118938781,0.143265686215094,0.250936695276019,0.570923619032712,0.568051419712057,0.861968023916549
"10310","AC010226.1",134.89659657892,-0.177116151051486,0.310325980292173,-0.570742259106797,0.568174368403053,0.861987357669379
"10311","TMEM259",347.938349383122,-0.0972763224135831,0.170430113614797,-0.570769568536728,0.568155853804127,0.861987357669379
"10312","CDC42EP2",169.325609847694,0.243201466414563,0.426176428450357,0.570659121854488,0.568230733599989,0.861989271305724
"10313","GALE",9.95594210398957,-0.276265868133783,0.484513835058613,-

0.570191908143061,0.568547543777786,0.862150737630797
"10314","URB2",10.6959119734136,0.265581624992659,0.465993385553451,0.569925739776829,0.568728066052953,0.862150737630797
"10315","BBS7",107.841332989814,-0.109757987172249,0.192732080821557,-0.569484782732513,0.569027194797671,0.862150737630797
"10316","ZSCAN25",35.7408611582262,-0.17863260683641,0.313190207371363,-0.570364598356033,0.568430435458038,0.862150737630797
"10317","TRIP6",417.082860140864,-0.152711658122465,0.26797807161624,-0.569866247642669,0.568768418903197,0.862150737630797
"10318","TONSL",6.79031249675886,-0.333193773824707,0.584634144960026,-0.569918429665939,0.568733024345809,0.862150737630797
"10319","SEC16A",107.919062589865,0.117229322921567,0.205767102300124,0.569718490522265,0.568868646826167,0.862150737630797
"10320","CTR9",196.163131390713,0.0940584836469508,0.165123373999079,0.569625494979745,0.56893173271543,0.862150737630797
"10321","ELAC2",90.9415059139886,0.106886738735723,0.187556251713819,0.569891633891339,0.568751199526873,0.862150737630797
"10322","NDUFV2-AS1",17.1311450840471,-0.218927605772253,0.384457334033109,-0.569445778223595,0.56905365761286,0.862150737630797
"10323","SULT2B1",7.55907161319254,-0.395813675569846,0.695078467919908,-0.569451786867111,0.569049580978615,0.862150737630797
"10324","SEPTIN5",21.006234051254,-0.259669358212541,0.455683016644059,-0.569846469427173,0.568781834548921,0.862150737630797
"10325","CETN2",282.158352977818,0.157406426269824,0.276104465355061,0.570097358140892,0.568611666899612,0.862150737630797
"10326","CDIPT",565.529612457917,-0.102080642694633,0.179510021992173,-0.568662638229104,0.569585107419736,0.862872345086862
"10327","ZNF724",17.4405518237401,0.211929511365147,0.372852023145865,0.568401130231327,0.569762623156179,0.863057685100427
"10328","AAMDC",530.062272001761,-0.140393675072999,0.247042888903054,-0.568296766996167,0.56983347392682,0.863073941995506
"10329","AC016957.2",3.97206765700705,-0.419332496474389,0.737971984718282,-0.56822278508915,0.569883701775336,0.863073941995506
"10330","TSPAN32",9.12132468192724,-0.270523860242248,0.476415907976037,-0.567831291342722,0.570149530132557,0.863392942871596
"10331","RPS8",18539.7162397891,-0.143827139854873,0.253666853222862,-0.566992249982746,0.570719447038257,0.863420146036697
"10332","ZNF142",28.8152713563544,0.168972426279698,0.29798836761081,0.567043699170116,0.570684492489063,0.863420146036697
"10333","SLC22A23",19.6288864216324,0.258826957226724,0.456244520475898,0.567298774255409,0.570511209680114,0.863420146036697
"10334","VARS",77.0977349189891,-0.159858165604819,0.281748565569669,-0.567378809122244,0.570456843929567,0.863420146036697
"10335","TYW1",71.3777877428737,0.140973106710643,0.248321437034492,0.567704135390702,0.570235883073335,0.863420146036697
"10336","PARP11",47.4663502145208,0.147807867560183,0.26066784705049,0.56703528736919,0.570690207392187,0.863420146036697
"10337","ITGA5",174.668098138939,-0.1402403748454,0.247336338405991,-0.567002712780531,0.57071233853668,0.863420146036697

"10338","KRR1",423.311361219505,-0.0778824363155183,0.137332550079215,
-0.567108353195182,0.570640568026436,0.863420146036697
"10339","TMEM11",210.583417587109,-0.158013787424068,0.278519993115231
,-0.567333733053388,0.570487462709982,0.863420146036697
"10340","VMA21",130.450982005528,-0.103669263662199,0.182683630090489,
-0.567479766035129,0.570388269859933,0.863420146036697
"10341","MDH1",640.717819175226,-0.157241858056705,0.277639359123697,-
0.566352906709632,0.571153901548193,0.863546433173789
"10342","FBXL17",93.1108085349532,-0.116173374379462,0.204976063352796
,-0.566765565106544,0.570873468759647,0.863546433173789
"10343","UBE2V2",339.276730710002,-0.116991924963069,0.206589738107943
,-0.566300756439028,0.57118934629222,0.863546433173789
"10344","WASHC1",116.260767021394,0.162747319185428,0.28733674932342,0
.566399249551765,0.571122404791901,0.863546433173789
"10345","IFT81",89.2169894424194,-0.142769991626085,0.251972056592078,
-0.566610415285919,0.570978897179972,0.863546433173789
"10346","SLC27A5",46.8931511337279,-0.169722114352631,0.29966483331919
8,-0.566373145866753,0.57114014597166,0.863546433173789
"10347","PDE9A",26.234621319992,0.202937888167656,0.358184165621692,0.
566574147172087,0.571003543662389,0.863546433173789
"10348","SAP25",9.38499611280932,-0.283412889668779,0.500613487664516,
-0.56613115038304,0.571304628928945,0.863553803298434
"10349","AC016065.1",24.1253443858569,0.232395635992069,0.410480916471
262,0.566154543772411,0.57128872759085,0.863553803298434
"10350","SILC1",4.53846127519774,0.409387277724421,0.723807438247316,0
.565602473933874,0.571664045698218,0.863763224848569
"10351","CHPF",152.277692554178,-0.16925772681666,0.299219586611728,-0
.565663928398817,0.571622260812801,0.863763224848569
"10352","PRKCH",12.7735921741067,-0.260350576199837,0.460193102877671,
-0.565742021277194,0.571569165025704,0.863763224848569
"10353","NEIL1",52.60737453478,-0.199112286139277,0.352008044523419,-0
.565646976644678,0.571633786715595,0.863763224848569
"10354","THAP6",96.9933955546188,0.137423286610093,0.243287494002085,0
.564859641362889,0.5721692365261,0.863974125988166
"10355","HERPUD2",153.388857675609,-0.113479994690126,0.20109541373563
1,-0.564309213134575,0.572543712416665,0.863974125988166
"10356","CHPF2",97.530775195131,0.154622514249253,0.27375062458954,0.5
64829813561498,0.572189526457147,0.863974125988166
"10357","GDF6",15.2839208724731,-0.305071565722377,0.540544622087424,-
0.564378135045135,0.572496816017827,0.863974125988166
"10358","ZNF517",12.0541509965959,0.252651935066702,0.447482188673249,
0.564607802191627,0.572340557224923,0.863974125988166
"10359","PPP1R26",10.1108479019376,-0.269836800163198,0.47781294673187
8,-0.564733128327339,0.572255297540926,0.863974125988166
"10360","FRA10AC1",266.798627571588,0.0863535552390975,0.1529592197362
21,0.564552796412107,0.572377979699632,0.863974125988166
"10361","KBTBD4",55.9444307696736,-0.141393092309782,0.250551478806103
,-0.56432751059196,0.572531262136198,0.863974125988166
"10362","C1QTNF4",171.601188496302,0.294178597447929,0.520778639555564
,0.564882226542515,0.572153873511312,0.863974125988166

"10363","FXYD2",6.59060268323346,-0.338054090499102,0.599172449900817,
-0.564201659397158,0.572616898636817,0.863974125988166
"10364","TRIM29",8.64083075349181,-0.335921067927561,0.595119942052073
,-0.564459437822315,0.57244149767559,0.863974125988166
"10365","PPP4C",286.226657670054,-0.16903000839618,0.299093079769432,-
0.565141823162488,0.571977303349286,0.863974125988166
"10366","CMC2",120.774537029603,-0.151506092068484,0.268542432310079,-
0.564179339425746,0.572632087083379,0.863974125988166
"10367","TNNC2",63.7679925376052,-0.234285107225907,0.415162573966132,
-0.564321357264298,0.572535449078191,0.863974125988166
"10368","MPST",327.788489600232,-0.146868917742286,0.260083965673012,-
0.5646980864902,0.57227913598294,0.863974125988166
"10369","SLC9A3-
AS1",9.2234783614812,0.33429778490272,0.592716297970214,0.564009773389
966,0.572747480782798,0.863981566237735
"10370","PPP4R1",96.1366367992412,-0.105071762040354,0.186293604552412
,-0.564011643302509,0.572746208202883,0.863981566237735
"10371","MAN1A2",365.146195819049,0.0809142340374,0.143621302667532,0.
563386019584487,0.573172054957623,0.864071257353679
"10372","CD47",889.441910825659,-0.110901518223215,0.196761415567177,-
0.563634480385974,0.573002915792678,0.864071257353679
"10373","AL008729.1",3.05507766021404,0.626643091241011,1.111584642892
18,0.563738528818265,0.57293209207818,0.864071257353679
"10374","AP002813.1",5.6261870243267,0.377849719051732,0.6707140907796
35,0.563354377440499,0.573193596980063,0.864071257353679
"10375","NSMCE3",206.330286466188,-0.124277851773195,0.220567863817424
,-0.563444962572005,0.573131927501036,0.864071257353679
"10376","TVP23A",7.10864459458846,0.514427153309896,0.913088739607884,
0.56339228707476,0.573167788085746,0.864071257353679
"10377","RTL8C",559.131826594338,-0.100446850974387,0.178284003767353,
-0.563409217045982,0.573156262332551,0.864071257353679
"10378","IL18R1",14.6633074486789,0.326840667495319,0.580668811439451,
0.562869334561118,0.573523863002266,0.864221652268002
"10379","MARCO",5.52709387695804,0.362124938685933,0.643403857271944,0
.562826807133789,0.57355282425687,0.864221652268002
"10380","SERPINI1",66.4352203789338,-0.248791295528172,0.4420651786126
55,-0.562793243089086,0.573575681921216,0.864221652268002
"10381","MCM4",58.5528806357745,-0.127697114670714,0.226906259214971,-
0.562774756027041,0.573588272097763,0.864221652268002
"10382","PCGF5",136.560820410201,-0.112602127785295,0.200201058280374,
-0.562445217585213,0.573812718438069,0.864221652268002
"10383","COPS7A",211.645337908861,0.0988750638807673,0.175631331469117
,0.562969391928531,0.573455726489192,0.864221652268002
"10384","INTS13",51.3740364872807,0.132323689059304,0.235285360803401,
0.56239660898355,0.573845828939956,0.864221652268002
"10385","TRPV3",13.3157606494356,0.279022144657798,0.496125988249732,0
.562401791613763,0.573842298668006,0.864221652268002
"10386","DOT1L",29.1200621343595,0.183408752168633,0.326102332152086,0
.562426987130826,0.573825136288351,0.864221652268002
"10387","ELAVL1",234.183672049886,-0.0807905208486965,0.14359063727873

8,-0.562644768348411,0.573676800831546,0.864221652268002
"10388","DBX2",38.1054354190393,0.196261866874485,0.349055275469466,0.
562265866374662,0.573934890784346,0.864272573790868
"10389","AGAP4",23.6094225151155,0.20124640952712,0.358341992344918,0.
561604316061884,0.574385639111485,0.864739348046146
"10390","ANAPC11",842.332349768835,-0.140488910673686,0.25014964643022
7,-0.561619465302229,0.574375315278772,0.864739348046146
"10391","CHM",194.212842499137,0.0869323536826587,0.154803023588432,0.
561567543498256,0.574410699069712,0.864739348046146
"10392","AC074117.1",16.9205275059768,0.220552031518771,0.393092243908
899,0.561069405301939,0.57475022386077,0.864756178599237
"10393","INTS12",55.5607011323346,0.140808360900507,0.250843616957745,
0.561339222453592,0.574566308065621,0.864756178599237
"10394","GET4",5.09880395248558,-0.347589205451403,0.619517366229056,-
0.561064506661284,0.574753563184572,0.864756178599237
"10395","PSMD13",521.901949696576,-0.126866401425007,0.225982464951398
,-0.561399316766868,0.574525349697676,0.864756178599237
"10396","ZNF414",87.3925609949715,-0.169117849370933,0.301347332110883
,-0.561205729568911,0.574657297503165,0.864756178599237
"10397","CD99",5080.47846789698,-0.151540576012493,0.269982757832196,-
0.561297237013486,0.574594924822864,0.864756178599237
"10398","AC027117.1",4.36391888767343,0.572671459448542,1.021163500494
65,0.56080290685198,0.574931904858057,0.864774979586017
"10399","PCYT2",38.1390212777905,-0.149337916676818,0.266218622643197,
-0.560959692429066,0.574825015543963,0.864774979586017
"10400","ACOT9",255.475898315128,-0.102649330843494,0.183036950800283,
-0.560812067698276,0.574925659148898,0.864774979586017
"10401","MAMDC2-
AS1",4.54612937928862,-0.497469838664292,0.887444842832554,-0.56056423
4140415,0.575094639159507,0.864936586902429
"10402","GMNN",86.5164203108679,0.145071674016979,0.258946854053263,0.
560237252340355,0.57531762062638З,0.864939311817252
"10403","RNF20",138.476083101295,-0.121769035195072,0.21726242032617,-
0.560469845692889,0.575159002077444,0.864939311817252
"10404","AC104564.3",7.54319542800461,0.352529829883012,0.629200574917
556,0.560282116603602,0.575287023541189,0.864939311817252
"10405","ZNF419",17.6734531121344,0.202848132674969,0.362043817898736,
0.560286138435613,0.575284280720008,0.864939311817252
"10406","AC023509.3",9.07758750881461,-0.294346301125265,0.52559178264
7467,-0.560028354405787,0.575460097550237,0.865001720826674
"10407","ZNF121",51.6408158595309,0.153374394732052,0.273875883662098,
0.560014239593588,0.57546972502993,0.865001720826674
"10408","TIPRL",311.2901971662,-0.0961925923505757,0.171888141797447,-
0.559623202302863,0.575736475344901,0.865143227657154
"10409","PPP1R7",337.123466700883,-0.145130626879803,0.259312342680287
,-0.559674967183257,0.575701160018941,0.865143227657154
"10410","FOXD1",9.58907566943783,0.354369530074277,0.633181581570347,0
.559664937181856,0.57570800266263,0.865143227657154
"10411","EIF4EBP2",573.738782605658,0.105801530587355,0.18910442867248
,0.559487323116045,0.575829180470144,0.865143227657154

"10412","APOLD1",25.3203196277545,-0.584688176634689,1.045206032472,-0
.559399925440398,0.57588881225594,0.865143227657154
"10413","DGCR6",26.0403263021165,0.303962243315046,0.543381781814332,0
.559389831400176,0.575895699648018,0.865143227657154
"10414","FAM218A",4.18329381503235,-0.389947846977324,0.69767380075064
3,-0.558925742313629,0.57621240012402,0.865291869457464
"10415","PHC1",102.311331079826,0.124323380591734,0.222499301885639,0.
558758519861041,0.576326535055764,0.865291869457464
"10416","AC127024.6",4.45788111327912,-0.406550376099727,0.72750529371
1606,-0.558828065738983,0.576279066366313,0.865291869457464
"10417","ABHD3",41.9364804113339,0.144566121035467,0.25858507355717,0.
559065993434093,0.576116682255966,0.865291869457464
"10418","ZNF563",38.0081132925835,0.151112254300966,0.270309586712299,
0.559034017767934,0.576138504184798,0.865291869457464
"10419","TCEAL1",155.965469650585,-0.125879614266585,0.225277259014517
,-0.558776393219849,0.576314335379916,0.865291869457464
"10420","TMSB15B.1",24.9923844091728,0.176171690754998,0.3154203291773
14,0.558529918520132,0.576482580391875,0.865443090697706
"10421","KRI1",57.5499576261015,0.150027773908746,0.268651821436638,0.
558446889012178,0.576539262015548,0.865445127694963
"10422","TNXB",6802.30718753851,0.0772906368502154,0.138491137632303,0
.558090850949783,0.576782347755423,0.865573080244303
"10423","TIMM23B",47.3836932614058,-0.150938326724025,0.27046054496435
5,-0.558078912190011,0.576790499806087,0.865573080244303
"10424","TUBGCP4",104.413890289165,0.109742321989353,0.196589363165023
,0.558231229922809,0.576686497945266,0.865573080244303
"10425","CREBZF",346.964308628756,0.125853739348506,0.225569472354976,
0.55793781860008,0.576886845760681,0.865634621413366
"10426","ZFYVE9",113.397995432052,0.10614497366903,0.190291387036359,0
.557802301628864,0.57697939085447,0.865663784083738
"10427","MANEA",76.9158459349237,0.131088946483348,0.235102024766681,0
.557583230571677,0.577129010170421,0.865663784083738
"10428","AC016885.2",12.3856773243661,0.325181493815545,0.583207100332
799,0.557574648233851,0.577134872035706,0.865663784083738
"10429","AL954642.1",9.97010296572939,1.07420775112517,1.9269849975220
3,0.557455170905082,0.577216479754104,0.865663784083738
"10430","P2RX7",30.8917872641802,0.168200367621494,0.30174625926583,0.
55742320726937,0.57723831309707,0.865663784083738
"10431","PPIP5K1",6.9731371446487,-0.353687158356751,0.634497947065065
,-0.557428373082635,0.57723478446797,0.865663784083738
"10432","C2orf15",2.93029032111329,-0.496752034126677,0.89151906629940
9,-0.557197319613855,0.577392620858222,0.865726331680489
"10433","COMMD5",169.177746295566,-0.165051314369456,0.296329609896241
,-0.556985562216508,0.577537293634454,0.865726331680489
"10434","TTLL5",43.9975323531504,0.148868533782053,0.267327922212862,0
.556876111368253,0.57761207720701,0.865726331680489
"10435","AC087286.2",3.32018156904968,0.486539397274588,0.873455009509
099,0.557028572711528,0.577507907458244,0.865726331680489
"10436","NQO1",130.70218754978,0.225158971555656,0.40415491077684,0.55
7110567140877,0.577451888134141,0.865726331680489

"10437","AC011484.1",12.4903324254203,0.307447281662948,0.552073382572
726,0.556895679755847,0.577598706542609,0.865726331680489
"10438","ARL16",270.920262469773,-0.136767236748291,0.245651301317451,
-0.556753560900329,0.577695816657147,0.865768888672901
"10439","MXD3",10.1602288805251,0.277575885663312,0.498899217702416,0.
556376670505988,0.577953383866501,0.865988963967785
"10440","PITPNM2",28.1527273217788,0.209584835108915,0.376670405943936
,0.556414392534225,0.577927602167974,0.865988963967785
"10441","MIB2",107.457268975733,-0.099593966038955,0.17923920997741,-0
.555648320763673,0.578451291722871,0.8660655953607
"10442","HIPK1",152.688896483012,0.109114521818272,0.196455838471315,0
.555415011675533,0.578610826998678,0.8660655953607
"10443","MCL1",1522.32553455325,-0.217100002944283,0.391569319455116,-
0.554435682668874,0.579280711424886,0.8660655953607
"10444","CNPPD1",162.770408114755,-0.118326615463181,0.213380443866569
,-0.554533551993047,0.5792137500999,0.8660655953607
"10445","CNOT6L",141.711753621611,0.146981921254872,0.265005274523518,
0.554637720019523,0.579142483250941,0.8660655953607
"10446","SUPT3H",43.1027451771426,0.162468534125718,0.292453960196415,
0.55553542176896,0.578528488757918,0.8660655953607
"10447","TRIM56",443.014470175312,0.173734892991655,0.313220192679812,
0.554673348181146,0.579118109088656,0.8660655953607
"10448","GNRH1",10.1219820199612,0.285847617609065,0.514786198722643,0
.555274438822075,0.578706959806398,0.8660655953607
"10449","PTAR1",142.978460117997,0.140566042918049,0.252925675017526,0
.555760275853011,0.578374744884769,0.8660655953607
"10450","RAB14",662.80159693344,-0.0885669829273552,0.159649951938628,
-0.554757341620761,0.579060648874178,0.8660655953607
"10451","AP003168.2",3.77735579073222,0.46753770499424,0.8432353744008
59,0.554456939530601,0.579266167359409,0.8660655953607
"10452","BLID",4.32610114530185,-0.496962227245097,0.894061166991402,-
0.555848129404189,0.578314680280047,0.8660655953607
"10453","TIRAP",17.3942182771171,0.222644164199951,0.401393143861302,0
.554678542982,0.579114555229018,0.8660655953607
"10454","AC079866.2",18.2162070013167,0.31084987520951,0.5605239148109
33,0.554570227952469,0.579188657669279,0.8660655953607
"10455","METTL17",119.790541966258,-0.120657051191311,0.21765152350403
7,-0.554358863419918,0.57933327302016,0.8660655953607
"10456","AC116158.3",2.80960486178451,0.627296003875788,1.130625030773
76,0.55482232110719,0.579016198010352,0.8660655953607
"10457","APH1B",180.312869076375,-0.102733625274531,0.185178414837276,
-0.554781859239954,0.579043876787416,0.8660655953607
"10458","DNAJA3",67.7379325327641,0.11769157566829,0.211839322389063,0
.555570015712843,0.578504833914962,0.8660655953607
"10459","IQCK",113.431446006599,-0.138076215909312,0.248830348070799,-
0.554901027868296,0.578962358802628,0.8660655953607
"10460","LINC02693",48.893701277108,-0.138480077086775,0.2493866107501
71,-0.555282726166484,0.578701292176197,0.8660655953607
"10461","EVI2A",11.4966918491606,-0.257420135496621,0.463716384370666,
-0.555124089147679,0.578809786941583,0.8660655953607

"10462","MYO19",50.5364940550289,0.172914219235846,0.31138313468484,0.
555310162866905,0.578682528683103,0.8660655953607
"10463","EDA",39.841859147134,-0.171373175480864,0.308131897065478,-0.
55616824195402,0.578095847309925,0.8660655953607
"10464","HMGB3",11.9524215063104,-0.277207305204591,0.498506759657779,
-0.556075318607298,0.578159366867663,0.8660655953607
"10465","TENT4A",69.0669512913198,0.127971046189085,0.231052488054252,
0.553861363998977,0.579673728382931,0.86631357716318
"10466","TRAM2-
AS1",67.5172133693171,-0.127628232909898,0.230462077749474,-0.55379277
2139449,0.579720675429532,0.86631357716318
"10467","RPARP-
AS1",22.6792280802968,-0.221457473379535,0.399781039039979,-0.55394691
5319785,0.579615176093677,0.86631357716318
"10468","BRWD3",58.444510881781,0.169844774840492,0.306647389557208,0.
553876473840995,0.57966338683482,0.86631357716318
"10469","GUCA1B",9.10607261415042,-0.284988429730499,0.515307395756682
,-0.553045487173767,0.58023226304258,0.866995251769517
"10470","SURF4",469.178333943811,0.0986771324645628,0.178642758059757,
0.552371299773343,0.580693989884298,0.867602300263617
"10471","TLR6",4.6480534455924,-0.442081761832855,0.80045362509203,-0.
552289037084475,0.580750340433651,0.867603626721765
"10472","PPM1M",117.299810787583,-0.121990212065206,0.220929147999256,
-0.5521689336602,0.580832616763761,0.86764368067566
"10473","TNFRSF10B",54.3261806475121,0.126270506879661,0.2287545901832
85,0.55199113940616,0.580954423963982,0.867742772278103
"10474","AKTIP",109.522063084647,-0.114732600768516,0.207986911380718,
-0.551633754291871,0.581199305387381,0.86794359780914
"10475","CENPBD1",28.6585874927174,0.16450820205605,0.298220397018831,
0.551632965754728,0.581199845748945,0.86794359780914
"10476","STYXL1",53.2472832407656,0.14564755746627,0.264296975984259,0
.551075383756735,0.5815819993021,0.868280200464839
"10477","BCCIP",132.02208473847,-0.112626488541268,0.20436643126996,-0
.551100725502678,0.581564628118845,0.868280200464839
"10478","AP000759.1",3.71156322832214,-0.517155603292493,0.93851873341
4618,-0.551033863129107,0.581610461260519,0.868280200464839
"10479","SETBP1",384.160224549279,-0.153693713772635,0.278945998798041
,-0.550980169763647,0.58164726846967,0.868280200464839
"10480","WAKMAR2",10.2157349639574,-0.27163677915474,0.493079227682536
,-0.550898849321696,0.581703016322706,0.868280561482452
"10481","DNM3",9.39918385783341,-0.394766121658154,0.716803224261067,-
0.550731509425209,0.581817741214593,0.868368946266567
"10482","PDCD2L",32.7569405642581,-0.180539231718288,0.327915674064054
,-0.550566032665527,0.581931199174625,0.868455423458181
"10483","BRD2",507.307876143073,0.158855489544453,0.288596321634229,0.
550441837390391,0.582016359569941,0.868499657803357
"10484","B4GALT1-
AS1",22.5089682973508,-0.221339776777268,0.402232621514665,-0.55027803
5490461,0.582128687033507,0.868501578566061
"10485","AC048341.1",41.2794896465153,0.206575419591726,0.375398753051

348,0.550282647218783,0.582125524393531,0.868501578566061
"10486","KCTD13",42.5452419257506,-0.145039924073701,0.263620337452723
,-0.550184881315206,0.58219257220249,0.868514057501769
"10487","PAFAH2",23.3140157149383,0.176162226377016,0.320465098192441,
0.549707994320272,0.582519672764741,0.868557304388895
"10488","ABCB8",67.8130840278505,-0.171159991773709,0.311277587861248,
-0.549862882675651,0.58241342421035,0.868557304388895
"10489","BORA",8.84977999068576,-0.285406272732679,0.519272915470399,-
0.549626726581984,0.582575423503323,0.868557304388895
"10490","LINC02323",3.73470966347588,0.449320224302879,0.8175761479763
21,0.549575994132221,0.582610227894437,0.868557304388895
"10491","CFDP1",762.475652729881,0.0908893549957803,0.165252500224141,
0.550002903874387,0.58231738184653,0.868557304388895
"10492","ELAC1",55.2047981576961,-0.178299928963198,0.324218179186225,
-0.549938098507383,0.582361831772442,0.868557304388895
"10493","HIRA",38.3065781984238,0.148966804226484,0.271038762977864,0.
549614389432003,0.582583887168386,0.868557304388895
"10494","TDRD6",15.2060306809906,0.246405221762389,0.448666634112481,0
.549194441993241,0.582872018270971,0.868781989691549
"10495","NDUFA12",609.192744027465,-0.149979098243353,0.27306545471305
3,-0.549242299436801,0.582839179334332,0.868781989691549
"10496","METTL24",3.15386562344844,-0.533708538108133,0.97235294655326
8,-0.548883550978055,0.583085367234595,0.868936427747679
"10497","ETS1",144.367124657411,0.157510822823751,0.287030279040695,0.
548760302746387,0.583169956414262,0.868936427747679
"10498","BTBD7",287.867667641041,0.10699795393543,0.194995648326755,0.
548719701457814,0.583197823622252,0.868936427747679
"10499","TOM1",143.992875545028,-0.139366391270827,0.253931215764331,-
0.548835206618197,0.58311854682371,0.868936427747679
"10500","HIST1H1C",166.737973150507,0.157413789796551,0.28702906585466
4,0.548424562257597,0.583400414803522,0.86907272533773
"10501","CDK20",20.1940140066783,0.186003044184562,0.33912262585297,0.
54848314445762,0.583360199857055,0.86907272533773
"10502","UTP6",134.839427027958,-0.102041329472743,0.186093600686582,-
0.548333360718839,0.583463024532042,0.869083231075484
"10503","PRIM1",45.5206533384543,0.15092570241987,0.275523513300064,0.
547777939574622,0.583844387954395,0.86956848145964
"10504","CACNA2D2",2.6025008189391,0.476132925889611,0.869913193086483
,0.547333837069736,0.584149401235063,0.869739786279745
"10505","BTBD9",26.6423043775553,-0.223029565889247,0.407439641735377,
-0.547392897115543,0.584108834030477,0.869739786279745
"10506","CTDNEP1",509.141809806808,-0.110857535208581,0.20255844311119
9,-0.547286667027369,0.584181802367914,0.869739786279745
"10507","TMEM38A",11.7507293199156,-0.2906230497926,0.530879405593854,
-0.547437038864791,0.584078514772019,0.869739786279745
"10508","OSGEPL1",46.9305556477252,0.133848931430859,0.244610909699827
,0.547191176367034,0.584247397525343,0.869753141666852
"10509","ZNF212",30.9741882476429,0.172739050444912,0.31581838557912,0
.546956916799377,0.584408331374154,0.869753141666852
"10510","AC013643.2",6.43163710361007,0.325118968087474,0.594437672062

669,0.546935336314281,0.584423157976947,0.869753141666852
"10511","RFX3-
AS1",11.3006305794658,-0.262471950081986,0.480025055336702,-0.54678802
1091693,0.584524373735448,0.869753141666852
"10512","RPS19",10889.3106346408,-0.135014085434678,0.246841661820721,
-0.546966360697806,0.584401843116722,0.869753141666852
"10513","MOSPD1",44.4507644097806,-0.177820322656463,0.325162362712052
,-0.546866252211153,0.584470622524075,0.869753141666852
"10514","CDKN2AIP",117.265083556011,0.12011725109647З,0.21971260712179
2,0.546701678478963,0.584583700878903,0.869758686784161
"10515","HCG17",3.14957389152366,0.592560323937529,1.08454049623672,0.
546369938230681,0.5848116700094,0.869932384362594
"10516","EEF1A1",36543.8458664696,-0.131032003759528,0.23980742970425,
-0.546405104800662,0.584787501877532,0.869932384362594
"10517","SH3YL1",191.861909338889,-0.0951541961688014,0.17443790542230
7,-0.545490361962537,0.585416307469172,0.870245509363184
"10518","MAATS1",13.9189255882601,-0.318215928548764,0.583295139863614
,-0.54554873991093,0.585376168362832,0.870245509363184
"10519","GFM2",44.7569834918058,0.142863875227325,0.261711268798123,0.
545883545188599,0.585145989955545,0.870245509363184
"10520","EZR",27.4026266451247,-0.238938136048129,0.438083953366557,-0
.545416316237913,0.58546722115567,0.870245509363184
"10521","DNAJC15",682.438419466527,-0.143350141402812,0.26264352354740
8,-0.54579735858949,0.585205239165776,0.870245509363184
"10522","UBR7",77.1491574603725,-0.117925644277955,0.216111450504507,-
0.545670504744938,0.585292450253728,0.870245509363184
"10523","MED24",86.2319298949967,0.12905543588314,0.236507983670838,0.
545670526128005,0.585292435552518,0.870245509363184
"10524","PPP1R37",68.2691321796653,-0.124221899097573,0.22770856348252
8,-0.5455302040369,0.585388912992505,0.870245509363184
"10525","DCLRE1A",10.6873680876459,0.282103632905158,0.517788529092702
,0.544824029608141,0.585874550201507,0.870520097720571
"10526","BLOC1S1",1073.28087220571,-0.151466885140602,0.27794626739313
2,-0.544950240063358,0.585787741362781,0.870520097720571
"10527","FBXW8",49.9941104047114,0.159024700030726,0.291862032882178,0
.544862579282187,0.585848034709168,0.870520097720571
"10528","PLAGL2",30.1242582445688,-0.152197239005949,0.279350709521704
,-0.544824959516073,0.585873910579514,0.870520097720571
"10529","SMN1",27.6262149553013,-0.208044637650148,0.381995856250541,-
0.544625378118491,0.586011196814464,0.870640436106815
"10530","AC015871.4",7.07060502143891,0.338092165330015,0.621095068306
347,0.544348494429287,0.586201681758337,0.870840731979645
"10531","AC002451.1",62.4109164305521,0.130592885411792,0.240021373974
507,0.544088566985963,0.586380527609605,0.871023700825852
"10532","GTPBP4",132.634179762844,-0.106236620196313,0.195323396872258
,-0.543901150079793,0.586509497506968,0.871096233766281
"10533","XPNPEP2",93.3668582738165,0.166527714402309,0.306198305555176
,0.543855767262897,0.586540729416367,0.871096233766281
"10534","VTA1",141.360721501109,0.090553479324382,0.166586515896263,0.
543582287180865,0.586728951440957,0.871213416475184

"10535","CD4",8.99054202657726,-0.317541320364711,0.584167444900061,-0
.543579282168036,0.586731019789431,0.871213416475184
"10536","ENDOD1",82.4587059147132,-0.159140746280705,0.292985998066039
,-0.543168435799568,0.58701383689322,0.871550631218739
"10537","CDK11A",135.945025002954,-0.137299295943866,0.253063742160601
,-0.542548271718562,0.587440862990767,0.871759709954686
"10538","CREB3L4",32.2372770938079,0.188355533989524,0.347128236672392
,0.542610810907,0.587397793893328,0.871759709954686
"10539","ZNF692",35.8569419470351,-0.145676935359253,0.268539637024228
,-0.542478335688335,0.587489027829847,0.871759709954686
"10540","UBA6-
AS1",40.1843145650867,0.181820894095285,0.33498461259193,0.54277386859
1912,0.587285507219136,0.871759709954686
"10541","ZFHX2-
AS1",33.2450583074195,0.207903941642532,0.38309610318601,0.54269396089
7811,0.587340532940706,0.871759709954686
"10542","LEO1",151.135197721754,-0.106877782922396,0.196912117872488,-
0.542768947270203,0.587288896051346,0.871759709954686
"10543","POU3F1",5.32087654972874,-0.658511253597394,1.2141879832772,-
0.542347035769546,0.5875794588226,0.871777709859876
"10544","UBE2W",114.945171764363,0.0989040153490675,0.182379157361143,
0.542298894128675,0.587612617321648,0.871777709859876
"10545","ANAPC4",90.0099725395362,-0.116604332167024,0.215373271986172
,-0.541405770046111,0.588227930991735,0.872013414134135
"10546","LINC01366",5.05446532280838,0.347531588015113,0.6422655291566
14,0.541102662743664,0.588436823009612,0.872013414134135
"10547","ARHGEF5",42.807475936825,-0.175808395554684,0.324833007117832
,-0.54122700496046,0.588351126119628,0.872013414134135
"10548","CUBN",21.7199089148791,0.253781194377201,0.468483326833294,0.
541708060546427,0.588019636026963,0.872013414134135
"10549","ZFYVE27",59.9086888306389,0.131801573394521,0.243606476557958
,0.541042977415108,0.588477960298661,0.872013414134135
"10550","CD6",6.63289879972112,0.35605240887275,0.657645100297964,0.54
1405096322364,0.588228395262943,0.872013414134135
"10551","AL356019.2",8.71724282186426,-0.301773485953768,0.55758936664
8725,-0.541210977116574,0.588362172216063,0.872013414134135
"10552","ATP5MPL",1029.04001845102,-0.135532172524855,0.25028792735193
5,-0.54150503365782,0.588159529083199,0.872013414134135
"10553","RMDN3",91.0046664752966,0.102658956192626,0.189770325181491,0
.540964221326202,0.588532243879038,0.872013414134135
"10554","SEH1L",46.0670227370022,0.142821022173478,0.263557074805078,0
.541897887882942,0.587888851868546,0.872013414134135
"10555","PROSER3",31.758292043697,-0.189151120349706,0.349673957606462
,-0.54093568089673,0.588551916283846,0.872013414134135
"10556","SLC24A3",6.57441131820818,0.334309055354194,0.617142738912315
,0.541704591620731,0.58802202611625,0.872013414134135
"10557","KRTAP13-4",25.5433051739884,-0.286806106205458,0.529918561507
282,-0.541226760183068,0.588351294815002,0.872013414134135
"10558","PAXBP1",164.673646059827,0.176971017959061,0.32695028166581,0
.541278071569153,0.58831593258728,0.872013414134135

"10559","RNF181",427.810184964271,-0.158897889420785,0.293867827213067
,-0.5407120981147,0.58870603838317,0.87201794728582
"10560","SNX10",43.9382671976312,0.300811480143343,0.556348808837002,0
.540688638791486,0.5887222106556,0.87201794728582
"10561","EXPH5",11.513273386698,-0.25265324346404,0.467216949359674,-0
.540762153021855,0.588671532464195,0.87201794728582
"10562","OPN3",37.9657972747772,0.166599093985846,0.308180794664792,0.
540588826007332,0.588791021393055,0.87203008612046
"10563","YWHAG",312.520403286395,-0.0761387626016105,0.140863358301684
,-0.540515031869011,0.58884189731448,0.87203008612046
"10564","ZNF621",122.96532759926,0.17379928818208,0.32169385991919,0.5
4026299484155,0.589015674600454,0.872204865370589
"10565","S1PR5",2.61308040661394,0.640008874135632,1.18489285304935,0.
54014071608969,0.589099993236972,0.872247155154373
"10566","PPIH",133.55855128755,-0.146493229976139,0.27139004871842,-0.
539788509814275,0.589342892005406,0.872299892656212
"10567","TACC3",5.81056185306903,0.33226798734953,0.615304830103222,0.
54000549173942,0.58919324513579,0.872299892656212
"10568","UGDH",407.023009583164,0.100597604006342,0.186420489293917,0.
539627400332246,0.589454016423208,0.872299892656212
"10569","IK",450.617766385694,-0.0996807134481761,0.184728511594157,-0
.539606542530758,0.589468403688488,0.872299892656212
"10570","AC083880.1",3.80157796223561,-0.474312054433407,0.87864326870
4399,-0.539823238084784,0.589318939629448,0.872299892656212
"10571","DNAJC14",43.5776740955179,0.138343699930204,0.256380070597422
,0.539603954425289,0.589470188919569,0.872299892656212
"10572","NFU1",239.915277067594,-0.141571184796022,0.262455307486113,-
0.539410637765489,0.589603542507144,0.872332187216425
"10573","PPT2",28.6015743261772,-0.175543588967507,0.325408101423485,-
0.539456725876212,0.58957174877037,0.872332187216425
"10574","PRCD",52.0789854155789,-0.152520821972561,0.282865905917536,-
0.539198322533171,0.58975001770263,0.872466382345587
"10575","AC009093.2",11.0461512259948,-0.236968668635789,0.43959433058
9879,-0.539062158326309,0.589843965520543,0.872522851313272
"10576","GREM1",31.8744490436101,0.384339551320115,0.71313315716579,0.
538945002708327,0.589924803697118,0.872559174938657
"10577","MYL12B",3116.53255135359,-0.157766629029971,0.292819775253089
,-0.53878406570598,0.590035859649755,0.872559174938657
"10578","APOL3",343.37384679218,-0.0927910622209799,0.172200289822522,
-0.538855435821944,0.589986608903341,0.872559174938657
"10579","SELENOT",282.369542912708,-0.0991565311930566,0.1841145176319
07,-0.538559003757087,0.590191181719027,0.872573542947326
"10580","FBXO32",64.7498444851892,-0.191158382467908,0.355028569891885
,-0.538430984656025,0.590279539980009,0.872573542947326
"10581","ELK3",225.718480700872,-0.10628676881504,0.197424786541754,-0
.538365879365209,0.590324477722403,0.872573542947326
"10582","AC010776.2",3.72227106247485,-0.646553907037011,1.20029072527
801,-0.538664419728192,0.590118428619367,0.872573542947326
"10583","APOBEC3C",282.223230279538,0.0840090985853883,0.1560163419463
02,0.538463455413554,0.590257128219162,0.872573542947326

"10584","CROCC",175.139032045782,-0.12021927549176,0.223384634897805,-0.538171640796863,0.590458556719189,0.872689267078446
"10585","PSKH1",72.0654573828207,0.115362568713269,0.214496853359589,0.537828722922435,0.590695300279513,0.872956691759322
"10586","LNCTAM34A",3.46147607565485,0.452327106208008,0.842125830279827,0.537125320164691,0.591181051936272,0.873179604894638
"10587","HADHB",549.840711026706,-0.12594308241827,0.234463277354497,-0.537154832259085,0.591160667960815,0.873179604894638
"10588","AC093772.1",30.1498764273742,-0.309811530810024,0.576683487269189,-0.53722975887015,0.591108917673644,0.873179604894638
"10589","CCDC126",99.5076364575572,0.128054636149299,0.23838328669365,0.537179589749777,0.59114356823484,0.873179604894638
"10590","DENND1A",63.2134527047664,0.131814532916219,0.245250970337079,0.537467936355357,0.590944427162493,0.873179604894638
"10591","ZNF875",60.2584485223601,0.147070977307106,0.273719733118233,0.537304985766512,0.591056962081115,0.873179604894638
"10592","MARF1",180.579850379039,0.14398228582295,0.268172001526263,0.536902752723979,0.591334789461739,0.873241773959217
"10593","LINC02246",12.1322154132748,-0.288000936648302,0.536401988358353,-0.536912507594766,0.591328050940677,0.873241773959217
"10594","NT5C3B",229.170360781126,-0.133706162585045,0.249136146589768,-0.536679098618348,0.591489296103481,0.873387489045379
"10595","TIE1",6.4076824259987,0.388037363712566,0.723560596243744,0.536288689194802,0.591759046520761,0.873620872473034
"10596","TMEM175",102.227269285777,-0.147907477989473,0.275785940715301,-0.536312611171724,0.591742516188261,0.873620872473034
"10597","SMIM14",923.672836819126,0.0890560134206334,0.166188049402432,0.535874954552118,0.592044974966852,0.87365691093813
"10598","NIPBL-DT",96.1078040016719,-0.122520649476425,0.22855391018576,-0.536068927356545,0.59191091413696,0.87365691093813
"10599","AC016831.5",71.9652075142853,-0.255538034414717,0.476876792849888,-0.535857559533528,0.59205699790337,0.87365691093813
"10600","KIN",209.917050369725,0.104428781862957,0.194877923109477,0.535867686789187,0.592049998221917,0.87365691093813
"10601","TEKT3",36.1361980218319,0.229762239349303,0.428781448502929,0.535849300737023,0.592062706185202,0.87365691093813
"10602","RNF168",177.456781120631,-0.130798049577707,0.244507060324736,-0.534945900555962,0.592687267152983,0.873691380147998
"10603","TEC",6.92905060475912,-0.391264696517236,0.731365689096446,-0.534978195382146,0.592664935089095,0.873691380147998
"10604","DNAJC21",297.546331457423,-0.0942691365735834,0.176238974262416,-0.53489381090711,0.592723288267173,0.873691380147998
"10605","TFAP2A",2.93344219055507,-0.525505277191457,0.982625228532407,-0.534797257318868,0.592790059807528,0.873691380147998
"10606","UBE2D4",122.81397671006,-0.156774415130327,0.292808787151442,-0.535415677430619,0.592362451678732,0.873691380147998
"10607","IDO1",13.5548426258443,0.286663740375896,0.535668433086055,0.535151453156181,0.592545132898348,0.873691380147998
"10608","DPCD",140.207587054737,-0.153318030214346,0.286375903426533,-

0.535373362003826,0.592391706275641,0.873691380147998
"10609","INCENP",17.2323891837762,0.213785871336055,0.399775115405904,
0.534765329550318,0.592812140183523,0.873691380147998
"10610","SSSCA1-
AS1",10.4200436751164,-0.235012574078392,0.439234263649197,-0.53505064
0462079,0.59261484029 0104,0.873691380147998
"10611","C16orf87",81.1925995840582,0.129559803969371,0.24209074289225
8,0.53517041759433,0.592532020272346,0.873691380147998
"10612","TNFRSF11A",10.3590289857526,0.290545668249614,0.5427317766655
03,0.535339334716499,0.592415231382048,0.873691380147998
"10613","CTU1",104.635448539351,-0.166088897750313,0.310291194680253,-
0.535267840653562,0.592464660915668,0.873691380147998
"10614","KAT14",37.1253187744478,-0.15691568429229,0.293215235511717,-
0.535155289657586,0.592542480206434,0.873691380147998
"10615","AC093157.1",44.5134132965805,0.17277073981123,0.3232369573085
18,0.534501813313156,0.592994395238067,0.873732442009223
"10616","AC019171.1",9.81505895573892,0.291632793224707,0.545962061159
738,0.534163111270438,0.593228688560415,0.873732442009223
"10617","LINC00607",3.42633856715248,0.431449190444058,0.8075146952459
99,0.534292679729652,0.59313905603072,0.873732442009223
"10618","TMEM165",502.392835553435,-0.0857583970091379,0.1605314226850
9,-0.534215641864508,0.593192348335712,0.873732442009223
"10619","AC034236.2",22.1368034826036,-0.196433349227079,0.36751647097
9157,-0.534488559665723,0.59300356250216,0.873732442009223
"10620","TTC8",71.8254459492203,-0.162561989568706,0.304193130722579,-
0.534403880792959,0.593062134597853,0.873732442009223
"10621","ZNF254",135.438536981303,0.100230474693722,0.187641368159501,
0.534159794702217,0.593230982968737,0.873732442009223
"10622","TMEM9",402.974349769837,-0.107150405469393,0.200754084787238,
-0.533739602772975,0.59352170543357,0.873749297769371
"10623","KLHL23",31.0509175484517,0.180204691197671,0.337595997116007,
0.533787997301841,0.593488218895228,0.873749297769371
"10624","DBR1",72.5165062548592,0.102799219853264,0.192505068484498,0.
534007861001036,0.593336095355549,0.873749297769371
"10625","TSPYL4",101.224688502707,-0.11384268434248,0.213241894948309,
-0.533866407302732,0.593433965021889,0.873749297769371
"10626","ZNF12",148.021339308144,0.105769907733428,0.198122881807,0.53
3860131493865,0.59343830733001,0.873749297769371
"10627","DISP2",4.7262938667444,1.07184817011569,2.00890488551942,0.53
3548490942394,0.593653953495895,0.873851456743434
"10628","GZMM",9.85222988922758,0.326847541519884,0.612673099289304,0.
533477872456004,0.593702824411508,0.873851456743434
"10629","VGLL4",433.108751814246,-0.0977727196013849,0.183409700077601
,-0.5330836894669,0.593975649172481,0.874010409086028
"10630","DLD",139.517142842544,0.1065342449845,0.199820612278892,0.533
1494272263,0.593930146297983,0.874010409086028
"10631","HOXC11",27.862277019723,-0.165506546159694,0.31047244356587,-
0.533079664844973,0.593978435018447,0.874010409086028
"10632","ANAPC10",142.633833272431,-0.145752350900574,0.27361356943614
8,-0.532694161334667,0.594245308504385,0.874320857875667

"10633","SAV1",673.621750539403,0.106196967706706,0.199411687795504,0.532551370888605,0.594344172318152,0.874384076702046
"10634","HOXC-AS1",51.8254152817455,-0.178180013130142,0.334731736190615,-0.532306900916849,0.594513453433879,0.874550870045719
"10635","DNAH7",5.37338669974997,-0.377458002851983,0.709551191344868,-0.531967259665307,0.59474867164137,0.874565104780405
"10636","PPP2R3A",70.9468261655114,0.122070323611716,0.22949942091163,0.531898177027293,0.594796519955226,0.874565104780405
"10637","C9orf170",5.5362852010276,-0.357904057028834,0.672683762231669,-0.53205395629213,0.594688625938886,0.874565104780405
"10638","CCDC97",91.617538040362,-0.105862234646095,0.198923318629662,-0.532176093659385,0.594604038834934,0.874565104780405
"10639","SCML2",12.0955808669666,0.243872597149114,0.458502544424708,0.531889299447849,0.594802668909974,0.874565104780405
"10640","ZFP2",8.46934504616014,-0.258180507289511,0.485632213244662,-0.531637935557293,0.59497678530725,0.874656691341162
"10641","TAPBPL",137.645942839114,0.17433335108616,0.327881935258855,0.5316955048119,0.594936905801659,0.874656691341162
"10642","DIRAS3",5.58729190108197,0.381800554546947,0.718837101567192,0.531136405890228,0.595324257707997,0.874718079770744
"10643","ZNF326",215.724641195398,0.113480077918418,0.213932593576104,0.530447820135685,0.595801478071724,0.874718079770744
"10644","SEMA4A",10.3435888967747,-0.369424207429938,0.696122901790545,-0.530688196695895,0.595634866675106,0.874718079770744
"10645","ODR4",179.161893081013,0.0798790527811944,0.150424113892264,0.531025583028561,0.595401051071325,0.874718079770744
"10646","C2orf92",5.70500009275291,-0.357232179071581,0.673321194612891,-0.530552404899362,0.595728984974936,0.874718079770744
"10647","MDH1B",4.37173352606658,-0.378093110376556,0.712700297036217,-0.530507861367346,0.595759859903918,0.874718079770744
"10648","GLT8D1",573.796629496215,0.0982186941707592,0.185166960723708,0.530433149558002,0.595811647327849,0.874718079770744
"10649","AC021086.1",2.68609229546583,0.542907684018605,1.02250315262167,0.530959423085008,0.595446897959448,0.874718079770744
"10650","AC069287.2",2.80331792846339,-0.48568638620805,0.91404319968583,-0.531360428451289,0.595169037784698,0.874718079770744
"10651","OAS1",90.2447830299219,-0.205844917031519,0.387728927217925,-0.530899044619961,0.595488739859204,0.874718079770744
"10652","BCL2L2",65.5733530620877,-0.126270711532243,0.238042561403333,-0.530454347272348,0.595796953658797,0.874718079770744
"10653","LIMD2",37.3569591541743,-0.195769605743664,0.368880282539097,-0.53071312024630 7,0.595617592714348,0.874718079770744
"10654","C17orf80",65.3331157985918,0.12104460447999,0.228186888943556,0.530462574078619,0.595791251108827,0.874718079770744
"10655","TBL1X",95.0052163293764,0.126720929730708,0.238881698761407,0.530475672216632,0.595782171964185,0.874718079770744
"10656","PPP1R3F",25.6787657299686,-0.192585226209535,0.363116718263894,-0.530367280058898,0.595857307296366,0.874718079770744
"10657","CHN1",4.19862451258395,0.454675095729766,0.858372438843719,0.

529694425350192,0.596323813678787,0.874970134897184
"10658","TTYH3",60.1377335402283,0.168165170132717,0.317608759679037,0
.529472708191861,0.596477571961537,0.874970134897184
"10659","TPD52",3.68372291399472,0.477656625014553,0.902271183255605,0
.529393638940187,0.596532409939172,0.874970134897184
"10660","AKR1C8P",13.0355315618993,0.247268024510177,0.46683064256579,
0.529673937321563,0.596338021132377,0.874970134897184
"10661","IGSF9B",6.05418378525653,0.457618377602221,0.864080786785494,
0.529601380566079,0.596388336961011,0.874970134897184
"10662","CRTC3",147.362675727989,0.0991752979959454,0.18721067971644,0
.529752352516222,0.596283644834549,0.874970134897184
"10663","ATP6V0C",966.995621078877,-0.133895860661106,0.25287681771400
1,-0.529490452590794,0.596465265761684,0.874970134897184
"10664","CCDC43",126.824700017176,0.100457880303385,0.189660156630006,
0.529673085208721,0.596338612034621,0.874970134897184
"10665","ZNF497",2.82868498699116,0.456549034012474,0.86169055159194,0
.529829453472615,0.596230182085507,0.874970134897184
"10666","RPS28",13375.5664732254,-0.154739768053562,0.292377350497807,
-0.529246768910448,0.596634276807861,0.875037501603728
"10667","LINC00824",11.8574709316143,-0.264747243544555,0.500421693849
64,-0.529048294265402,0.596771948450715,0.875157362858773
"10668","STPG1",17.0864290549842,-0.193602648649983,0.366483613682352,
-0.528270955158687,0.597311287720416,0.875211795504975
"10669","SNX4",159.291853478534,-0.0948909026422953,0.179477790826983,
-0.528705541811412,0.597009732219156,0.875211795504975
"10670","FYB1",33.7032852100127,-0.250222836698585,0.473728555429841,-
0.528198762414783,0.597361388278884,0.875211795504975
"10671","MAD1L1",71.6745003895267,-0.123985696805795,0.234679766503922
,-0.528318647375692,0.597278191166428,0.875211795504975
"10672","USP42",73.4512736264195,-0.135541905774868,0.256393583008509,
-0.528647808515432,0.597049788808754,0.875211795504975
"10673","LAPTM4B",256.815090225595,0.162439741588064,0.30755492921795,
0.528164975281375,0.597384836641445,0.875211795504975
"10674","TSPAN4",1747.05416477134,0.12078460648614,0.228643978475292,0
.52826497899306,0.59731543500701,0.875211795504975
"10675","INO80E",134.246035839236,-0.101564666928841,0.192252829451521
,-0.528286981359888,0.597300166063609,0.875211795504975
"10676","ZNF430",145.248559302299,-0.0980678623172708,0.18542020348528
1,-0.528895236192832,0.596878126828717,0.875211795504975
"10677","SCAND1",599.010222518016,-0.164606889391822,0.311691825833388
,-0.528107815954761,0.597424506322452,0.875211795504975
"10678","AL672277.1",3.04116534961982,-0.507213284062814,0.96038515637
857,-0.528135280615351,0.597405445165989,0.875211795504975
"10679","ACVRL1",445.667466551319,-0.12649660159882,0.239569036663184,
-0.528017323777382,0.597487312096798,0.875221839416632
"10680","AC008875.3",86.1732762808238,-0.161190700262958,0.30552913308
2713,-0.527578822472948,0.597791694867991,0.875339837388372
"10681","SLC35B2",195.352279119841,0.1159209438622,0.219692330623171,0
.527651300040301,0.597741380186205,0.875339837388372
"10682","KIAA1217",125.314002107837,0.237358932156341,0.44986692070753

6,0.527620327769444,0.597762881223645,0.875339837388372
"10683","TIMM8B",490.801138391139,-0.149857168227951,0.283992049113391
,-0.527680858305006,0.597720861083491,0.875339837388372
"10684","ZNF503",995.114635615165,-0.0908687666908979,0.17236608471058
9,-0.527184723395389,0.598065316218734,0.875576248131041
"10685","SLC16A7",112.527917012584,-0.166219478329904,0.31538666173619
5,-0.527033950690465,0.598170012118716,0.875576248131041
"10686","TMEM121",67.6730925881823,-0.151385743931115,0.28724648357561
8,-0.527023836973317,0.598177035336984,0.875576248131041
"10687","FANCB",9.61511064738462,-0.274414123128579,0.520654683605981,
-0.527055900521292,0.598154769735716,0.875576248131041
"10688","AC079949.1",2.85699452586731,0.45077294479709,0.8556248477887
27,0.526834799108588,0.598308314849131,0.875686467925239
"10689","AC104162.2",15.6881316063535,0.220748937668905,0.419123798001
932,0.526691490011471,0.598407846207832,0.875750204717852
"10690","AL162231.2",78.4157729642151,-0.133270577367894,0.25314383529
0926,-0.526461871823708,0.598567336813464,0.875777833015244
"10691","MTMR10",54.1415747928518,-0.149244639802694,0.283507330390329
,-0.52642250765515,0.598594680726139,0.875777833015244
"10692","KANSL1-
AS1",38.8025985542213,0.172807367395583,0.328229196831634,0.5264838383
16873,0.59855207826245,0.875777833015244
"10693","KRTCAP2",1868.97403974319,-0.1527699985915,0.290469594597954,
-0.525941445964259,0.598928891021407,0.87580093226004
"10694","LMOD1",381.742236788221,-0.122468118430275,0.232940181817295,
-0.525749217995942,0.59906246213025,0.87580093226004
"10695","HACD2",104.967859912104,0.100649962913328,0.19155666539109,0.
525431796945497,0.59928305418386,0.87580093226004
"10696","PCDHGB2",5.79487507073447,0.317698636669362,0.604641624247009
,0.525432957191805,0.599282247802605,0.87580093226004
"10697","FAM135A",57.6820607697452,0.163775023290502,0.311608575469712
,0.525579320285493,0.599180528124784,0.87580093226004
"10698","RBM18",96.9996759391975,0.103384780179163,0.196566343121409,0
.525953622260283,0.598920430681458,0.87580093226004
"10699","DNA2",5.98012067713401,0.313833529510178,0.596911527582746,0.
525762219371233,0.599053427597862,0.87580093226004
"10700","C11orf58",1331.88717676556,-0.0799999698544279,0.152008195602
382,-0.526287214563676,0.59868866499352,0.87580093226004
"10701","DPF2",191.486005782405,0.109645340635314,0.208668872281347,0.
525451349962154,0.599269464733227,0.87580093226004
"10702","YPEL3",975.439756773175,-0.117732260321795,0.224101535829026,
-0.525352313567441,0.599338296991864,0.87580093226004
"10703","ZNF709",23.0261981843893,0.17905445867364,0.340778060969411,0
.525428362859639,0.59928544090622,0.87580093226004
"10704","GPR173",16.4033154531741,0.194695656253235,0.37051808521585,0
.525468699158944,0.59925740706603,0.87580093226004
"10705","CUL4B",138.106758869618,0.0944297173842798,0.179578883719017,
0.525839761494631,0.598999545589648,0.87580093226004
"10706","EML3",74.9074163159153,0.121938717514552,0.232170387973339,0.
52521218825096,0.599435692984374,0.875861437077766

"10707","RUSC1",137.762677843609,-0.109827087921838,0.209510224797033,-0.524208725508436,0.600133372691537,0.875899080893237
"10708","C1orf112",16.3182101088689,0.194865213060859,0.371177443129852,0.524992066914711,0.599588705764237,0.875899080893237
"10709","UBE2T",9.35326132487701,0.303015612299124,0.577963674748566,0.524281413413994,0.600082822475939,0.875899080893237
"10710","GAREM2",34.0269825617485,-0.146198149610831,0.278553652672766,-0.52484736138994,0.599689304441324,0.875899080893237
"10711","TATDN2",57.2858731997045,0.143654527774345,0.273695081045976,0.524870696343326,0.599673081562336,0.875899080893237
"10712","STIMATE",72.5673235424395,0.119657003950939,0.227903555240978,0.525033511760785,0.599559894887916,0.875899080893237
"10713","SIRT5",40.9245611595885,0.135583175318107,0.258516707542894,0.524465813474011,0.599954591589455,0.875899080893237
"10714","NDUFC2",1338.02418547714,-0.144546673430233,0.275664348592991,-0.524357517277838,0.600029898724102,0.875899080893237
"10715","RAPGEF3",4.71263837591084,0.489595057794652,0.933432295778653,0.524510518876187,0.599923505542193,0.875899080893237
"10716","AL355075.4",41.9910264318645,-0.362904837945802,0.691840334955206,-0.524549985899995,0.599896062624217,0.875899080893237
"10717","ARL6IP1",589.528920744216,0.0848844736651867,0.161889124077607,0.524337098917742,0.600044097746235,0.875899080893237
"10718","YIPF6",283.530711085019,-0.0996961278470623,0.190038062030961,-0.52461136880474,0.599853381892279,0.875899080893237
"10719","ACAP3",116.225032689574,-0.116489632720934,0.222587626129146,-0.523342805468202,0.600735717871987,0.875960927914941
"10720","LRRC2",74.044532707029,-0.238343421372011,0.455370468845332,-0.523405529516156,0.600692077036678,0.875960927914941
"10721","PINX1",61.3613655290805,-0.125225872922136,0.239145269028402,-0.523639348714289,0.60052940779733,0.875960927914941
"10722","LYN",26.6300823487972,-0.24437064973972,0.466558559992549,-0.523772728001437,0.600436624049604,0.875960927914941
"10723","AL353801.3",8.27595401641604,0.319156319364443,0.609139443472822,0.523946237243925,0.600315934066279,0.875960927914941
"10724","CKAP2",42.7643451534201,0.203883652140715,0.389498738820529,0.523451379478431,0.600660177408632,0.875960927914941
"10725","COX16",593.71560738354,-0.122255663445424,0.233509048639058,-0.523558569391451,0.600585604143471,0.875960927914941
"10726","MSRB1",78.4924909864536,-0.16610515156101,0.316980467074274,-0.52402330368858,0.600262331518792,0.875960927914941
"10727","RFWD3",29.6314488901705,0.175686602806802,0.335610174361724,0.523484137931545,0.600637386529012,0.875960927914941
"10728","ITGB3",44.3542040457447,0.201630039164984,0.385075373672503,0.523611876921701,0.600548519034782,0.875960927914941
"10729","TIMM9",278.715834003626,-0.128131395597987,0.244945534672862,-0.523101577536872,0.600903567779839,0.87612401069811
"10730","SERPINB9P1",9.7594130630595,-0.432591646777157,0.827222928912891,-0.522944458691027,0.601012904752616,0.876201758531703
"10731","NTNG1",34.9147299982756,0.232904247080194,0.445446940392875,0.522855195446571,0.60107502589198,0.876210663500908

"10732","ARHGAP11A",5.76621336902516,0.327908999199885,0.627316524117315,0.52271698033346,0.601171219918571,0.876269231567854
"10733","HLA-DQA2",10.9894322150618,-1.2992071233215,2.48712832870455,-0.522372371512575,0.601411088715841,0.876394421812633
"10734","OLMALINC",24.4446291153773,-0.162557609671566,0.311203116427485,-0.522352126603605,0.601425181752772,0.876394421812633
"10735","DRC3",26.3296702150154,0.190886457802928,0.365362288745874,0.522458019567801,0.601351468402294,0.876394421812633
"10736","ZXDA",11.7251533089386,-0.21912815814728,0.419660793975713,-0.522155420027065,0.601562122359394,0.876512321168778
"10737","ARMH3",52.4232695829705,0.139724368583285,0.267793447984627,0.521761714615609,0.601836249279893,0.876748411946858
"10738","LINC02352",8.68988579109074,-0.327907739738758,0.628434756576532,-0.521784857230164,0.601820134116243,0.876748411946858
"10739","CNTN3",187.769689513859,0.232090142761901,0.444977373055293,0.521577403292058,0.601964599854682,0.876789306041484
"10740","LETM1",65.2442366708623,-0.117043140779585,0.224469645011709,-0.521420795107861,0.602073668235815,0.876789306041484
"10741","CCDC127",199.769686127985,0.0913813749658013,0.17525472093643,0.521420333087335,0.602073990019091,0.876789306041484
"10742","FNDC1",268.772533551056,0.223948299641015,0.429513861124566,0.521399470216554,0.602088520456282,0.876789306041484
"10743","CYTIP",24.3969817215214,-0.227499117980603,0.436426227135815,-0.52127737481232,0.60217355984217,0.876831517882441
"10744","AL049775.3",6.4469079253848,-0.301341213601936,0.578521071789396,-0.52088200118601,0.602448974505659,0.877002600483994
"10745","FAM98C",114.019447732023,-0.117012769114265,0.224649882692315,-0.520867261143989,0.602459243418845,0.877002600483994
"10746","YTHDF1",100.910091571159,0.0984838121346813,0.189049764035997,0.520941206337232,0.602407728975391,0.877002600483994
"10747","RBM22",223.347463392157,-0.113001627311115,0.217099702632575,-0.520505675230527,0.602711173379743,0.877124466013519
"10748","WDR60",273.634419834681,-0.127038115210938,0.244040673945656,-0.520561237424,0.602672458109422,0.877124466013519
"10749","AC005052.2",3.24119481989878,0.426935332349834,0.820122037209931,0.520575369249039,0.602662611350898,0.877124466013519
"10750","USPL1",47.3373041971596,0.143343004882037,0.275496258504088,0.5203083543144,0.602848673987186,0.877161362401781
"10751","ATP8B1",33.3378276600508,-0.181495529913443,0.348797128968377,-0.520346972035708,0.602821762600844,0.877161362401781
"10752","DDA1",124.940008632314,-0.130666319476557,0.251180793987945,-0.520208242843713,0.60291844080941,0.877181284373288
"10753","KCTD14",3.23643842954807,0.447048049963709,0.859809489763031,0.519938492522242,0.603106445570438,0.877321212947391
"10754","EML2",27.1210729129262,0.183635329225019,0.353206464970674,0.519909309248532,0.603126786680064,0.877321212947391
"10755","EEF1AKNMT",83.5197133959362,0.0997701269546672,0.19195969439665,0.519745185406007,0.603241188815275,0.877398739061416
"10756","SHC3",54.4850029173025,0.203620979142408,0.391826017538069,0.

519671920771887,0.60329226090549,0.877398739061416
"10757","PDLIM2",1323.79343572003,-0.151330342794295,0.291378737361849
,-0.519359594198409,0.603510002645905,0.877470673054177
"10758","MAP1LC3B2",4.7970626187467,0.34845308347818,0.670745740523464
,0.519501000790911,0.603411415182729,0.877470673054177
"10759","EVI5L",16.632936916504,-0.221463989432905,0.426417125054134,-
0.519359979749382,0.60350973383323,0.877470673054177
"10760","BANK1",14.5957698704627,-0.354438405075435,0.683101423732827,
-0.518866441733639,0.603853880962044,0.877731411170689
"10761","MBD6",96.3416743491239,0.110310484164509,0.212576212929769,0.
518922049857729,0.603815100663996,0.877731411170689
"10762","RPA4",11.1540018439886,-0.231938249105116,0.447014213401496,-
0.518861016387405,0.603857664579618,0.877731411170689
"10763","MFSD11",135.037107219161,-0.103619757102743,0.199775607989425
,-0.518680724566876,0.603983405525019,0.877832612898622
"10764","HEATR6",61.8641674353099,-0.113167861646611,0.218251667000078
,-0.518520033327261,0.604095486346048,0.877913943971687
"10765","AC004522.5",5.42111879703734,-0.33311541320155,0.642657126540
163,-0.518340806387575,0.60422050668286l,0.878014062799814
"10766","LINC00957",4.16346752970771,0.43097613285527,0.83176781169426
5,0.518144759626362,0.604357273042176,0.878131230001743
"10767","XKR8",39.7541567156485,-0.1583383767247,0.305755930767523,-0.
517858725838059,0.604556841173193,0.878339617950428
"10768","MRPS11",221.136063981965,-0.11852857389624,0.228919347822236,
-0.517774382217277,0.604615694056902,0.878343545888941
"10769","MRPS21",791.10665895744,-0.127090562173654,0.245835706354442,
-0.516973567665623,0.605174610570276,0.879073863232504
"10770","ZNF202",18.0239212417569,0.173021951208066,0.3347391241619,0.
516885953027652,0.605235773944053,0.879081078162193
"10771","SERINC1",987.24114753783,0.0813976684552972,0.157520443941566
,0.516743518609512,0.605335212664681,0.879143880021689
"10772","CYB561A3",171.290279731337,-0.106342845919319,0.2058980395518
26,-0.51648304253306,0.605517079550776,0.879326371649905
"10773","AC025171.4",5.72774462148333,0.329680507446663,0.638637381973
664,0.5162248824643,0.605697353521284,0.879390869966884
"10774","TMEM63B",59.4295197410316,-0.120228366831068,0.23298819590897
9,-0.516027717035231,0.60583505092974,0.879390869966884
"10775","ZNF181",71.0259345271565,0.141404952945767,0.274031608242721,
0.516016943638556,0.605842575313758,0.879390869966884
"10776","DMWD",129.247889301013,0.129383020941539,0.250727635013175,0.
516030157324859,0.605833346582056,0.879390869966884
"10777","ATP6AP1",336.77345387207,0.092346041720661,0.178874403871884,
0.516261911831736,0.60567149432387,0.879390869966884
"10778","COPS5",363.555226422381,-0.112639467853577,0.218437514361913,
-0.515659904767788,0.606091963000641,0.879508031281914
"10779","LCP1",42.5308800002464,-0.266420909156966,0.516546680773405,-
0.515773150953297,0.606012856823845,0.879508031281914
"10780","PXMP4",101.340088539474,-0.103996312733719,0.201656932165295,
-0.5157090887829,0.606057605784558,0.879508031281914
"10781","MAP3K21",5.87011122983294,0.353740757080657,0.686468011466945

,0.515305522138944,0.606339540740079,0.879732601179426
"10782","ZNF397",203.650756803562,0.110171569113943,0.213810214122088,0.515277390120539,0.606359196184656,0.879732601179426
"10783","GALNT3",4.8669365387286,0.389533081933448,0.756378009434379,0.51499789400903,0.606554491652212,0.879934333016373
"10784","NDC80",7.73881163988954,-0.29037330411394,0.563923075341132,-0.514916513991356,0.606611360513948,0.879935229276678
"10785","ZNF251",61.4698402072257,0.141430782532571,0.274963056758432,0.514362853686287,0.60699832501753,0.880379051655651
"10786","AC132192.2",13.642411413005,0.258966806621849,0.503515243806835,0.514317708961355,0.607029882449521,0.880379051655651
"10787","LINC01597",9.01271153756179,0.277763096226328,0.540317891277408,0.514073475467648,0.607200621232073,0.880545037353603
"10788","AC006449.2",8.96283448359561,-0.288340905482563,0.561256056840382,-0.513742171631594,0.60743226342435,0.880799304481564
"10789","AC083798.2",21.6544071672747,-0.260117741914397,0.506763620252163,-0.513292058701774,0.607747038152354,0.8808474861315
"10790","EFNA5",168.715333579048,0.127786502000475,0.248931219176541,0.513340602368756,0.607713086906071,0.8808474861315
"10791","REEP4",28.6579899579324,0.179926986752129,0.350482360714076,0.513369592653805,0.607692811620297,0.8808474861315
"10792","TMPO",186.19529894767,0.128001196169247,0.249355723608432,0.513327684309543,0.60772212166234,0.8808474861315
"10793","VIPAS39",73.0511067045523,-0.107829054756733,0.209955133738638,-0.513581415403556,0.60754467574362,0.8808474861315
"10794","TPRG1-AS1",4.6251499872661,-0.408019064220971,0.795238922290444,-0.513077331584571,0.607897227584747,0.880901929699694
"10795","PDCD6",702.551778109485,-0.113743995159799,0.2216777286788,-0.513105199325678,0.607877734746842,0.880901929699694
"10796","LINC00900",13.1599126369113,0.213642108890746,0.416682967898451,0.512721002176437,0.608146496295074,0.881181515519066
"10797","RFX3",45.3440824740516,0.171480318499845,0.334519447679778,0.512616888761564,0.608219336987124,0.881205435629302
"10798","CHI3L2",608.979005861836,-0.410101362094578,0.800872921974069,-0.512067958401841,0.608603448474722,0.881230669828208
"10799","R3HDM1",79.2557341223124,-0.103508687789508,0.202148140150541,-0.512043730466303,0.60862040434973,0.881230669828208
"10800","NKIRAS1",82.7376220956059,0.11972298543329,0.23367800467253,0.512341696862172,0.608411887746208,0.881230669828208
"10801","DNAJB14",356.914044168476,0.0713457400899728,0.139317528575109,0.512108855358488,0.608574827293962,0.881230669828208
"10802","ARHGEF28",11.778577395848,0.355075745669099,0.692881106151563,0.512462733529104,0.608327195490621,0.881230669828208
"10803","SPATA7",104.484548950021,0.107239708024585,0.209440912505959,0.51202846063580 9,0.608631091019879,0.881230669828208
"10804","TANGO6",31.1497397817166,0.150307257827827,0.293460070315585,0.512189810580321,0.608518173647002,0.881230669828208
"10805","OCRL",66.1039949112185,0.150918371588549,0.294812629372155,0.51191284413409,0.608712008540097,0.88126626095259

"10806","Z69706.1",5.20508533643143,0.377235121682911,0.73742486339553
1,0.511557367276583,0.608960829196851,0.881544905712229
"10807","PGD",223.802856491362,0.148524573979393,0.290387227088396,0.5
11470753960472,0.609021462172608,0.881551099543454
"10808","RAB27B",3.37288904242471,-0.429827500502998,0.840527547083881
,-0.511378243335673,0.609086226479937,0.881563271727023
"10809","AC110609.1",3.1383921610456,0.461650511281212,0.9030352329768
84,0.51122092961906,0.609196364798747,0.881607382161902
"10810","VCL",403.260136673386,0.101171073985125,0.197919161445515,0.5
11173719847113,0.60922941898422,0.881607382161902
"10811","AC087284.1",3.28951840255505,0.447694015729294,0.876016379639
785,0.511056672151934,0.60931137402207,0.881644419926671
"10812","AL590822.3",3.62841429020673,0.394964434363934,0.773947519712
779,0.51032456891975,0.609824092904491,0.88189314254059
"10813","AL031848.2",3.60930642288313,-0.403347215943364,0.79145166243
3962,-0.509629627541555,0.610310963225435,0.88189314254059
"10814","CAPN7",168.441524927952,0.111618160739263,0.219085610069274,0
.509472807018085,0.610420854274719,0.88189314254059
"10815","ST6GAL1",5.4878131940506,-0.310248459824147,0.608860331734022
,-0.50955604044784,0.61036252785027,0.88189314254059
"10816","SAR1B",250.341459107968,-0.0875651368054723,0.171718607490629
,-0.509933885937499,0.61009778101421,0.88189314254059
"10817","PSMB8-
AS1",47.4942770679601,0.138712417507503,0.272232187746132,0.5095371662
54777,0.610375753859928,0.88189314254059
"10818","TMEM209",54.4875220335938,0.122722637190245,0.240416951558309
,0.510457504742468,0.609730978805003,0.88189314254059
"10819","ZNF33A",164.907914743167,0.170879948128964,0.334685117919437,
0.510569305236026,0.609652673711343,0.88189314254059
"10820","RPS3",11436.6849043316,-0.149576259119708,0.293156631862865,-
0.510226421177052,0.609892843955828,0.88189314254059
"10821","C12orf49",89.4247735041836,0.0929061464394654,0.1822681182221
96,0.509722420715437,0.610245943096226,0.88189314254059
"10822","LACC1",30.8400517120571,-0.163737420583581,0.321389530117654,
-0.509467189312732,0.610424791011248,0.88189314254059
"10823","AL136038.5",27.0585305507681,0.206669799164865,0.405584744388
822,0.509560090767952,0.61035968962244,0.88189314254059
"10824","METTL16",102.530534194349,0.108759798294117,0.21300344625191,
0.510601120347563,0.609630391216178,0.88189314254059
"10825","PMP22",4193.69987226648,0.088240477509458,0.172936826670734,0
.510246887306801,0.609878507448585,0.88189314254059
"10826","ERAL1",86.9028511189426,-0.141741562059842,0.278140359870108,
-0.509604439017897,0.610328613341643,0.88189314254059
"10827","AC025682.1",2.83014518508272,0.473987025922289,0.930402266150
768,0.509443112045775,0.610441663838746,0.88189314254059
"10828","AL022322.1",12.3221999759702,-0.256483436718555,0.50283200363
5733,-0.510077789130462,0.609996965074824,0.88189314254059
"10829","AL135925.1",72.9521134979261,-0.156096253134341,0.30646180685
7201,-0.509349777497968,0.610507072625583,0.881906190514544
"10830","RAB42",76.9628362160913,0.164088127238508,0.322417437215942,0

.508930685186882,0.610800810551943,0.881985690529867
"10831","GZMK",19.0590969666987,0.261897025695298,0.514635465840069,0.508898129023792,0.610823631488929,0.881985690529867
"10832","EEF2KMT",38.044025837832,-0.180351124057885,0.354320393266114,-0.50900576846682,0.610748180766856,0.881985690529867
"10833","FAM192A",146.948919365771,-0.111015459830682,0.218185024925535,-0.508813379234303,0.610883040434877,0.881985690529867
"10834","ZNF805",55.3861113071161,0.124963746180219,0.245610342546469,0.50878861567193,0.610900399980254,0.881985690529867
"10835","ZFP64",43.1457567805174,-0.131738174278805,0.258772004700459,-0.509089746517591,0.610689318556093,0.881985690529867
"10836","NBPF3",12.3564233140885,0.272538126869728,0.536366531924231,0.508119188369171,0.61136975926043,0.882187125074165
"10837","PTH1R",149.18229522247,0.134209281717839,0.264071347449413,0.508231139099816,0.611291255500723,0.882187125074165
"10838","ZCCHC10",143.05288307707,-0.10247208614321,0.201706142239128,-0.508026602490501,0.611434687083941,0.882187125074165
"10839","AC010864.1",6.83373222580946,-0.29954685464615,0.589612317755824,-0.508040360802302,0.611425038580937,0.882187125074165
"10840","AC091132.4",21.7640056167963,0.372274307415293,0.732705919703766,0.508081479082091,0.611396203334184,0.882187125074165
"10841","RBMX2",202.093338068659,-0.118562169858449,0.233162649000485,-0.508495551781976,0.611105857833188,0.882187125074165
"10842","RENBP",49.253164570755,0.203554433261998,0.400366893530819,0.508419743368039,0.611159009706279,0.882187125074165
"10843","B3GALT6",71.7367220443654,0.139177731067669,0.274410483907915,0.507188096772472,0.612022846140402,0.882276725026641
"10844","CD53",39.0648479625187,-0.219217380907723,0.431964892610797,-0.507488883142268,0.611811834615846,0.882276725026641
"10845","MSH2",72.1871193996833,0.132246929721734,0.260726983821906,0.5072237931923,0.611997802244761,0.882276725026641
"10846","DIRC1",3.13187775489232,-0.636555627422243,1.25364587919096,-0.507763506416216,0.611619205489749,0.882276725026641
"10847","PDIA5",84.1774789889638,0.183425271659709,0.361871741832935,0.506879234975996,0.612239556343517,0.882276725026641
"10848","RAI14",49.7119138926631,0.183963570253038,0.363121864007592,0.506616616864446,0.612423846756467,0.882276725026641
"10849","SREK1IP1",356.999350147835,-0.112357680424866,0.22145010354523,-0.5073724447454,0.611893516158357,0.882276725026641
"10850","PRRC2A",211.661955834839,0.0750280810389709,0.14794601722046,0.507131468954442,0.612062576017392,0.882276725026641
"10851","HDDC2",361.180938505501,-0.144066088677801,0.284083107711531,-0.507126558274952,0.612066021386529,0.882276725026641
"10852","SLC25A40",33.9203448522848,0.152435542355062,0.300714876501792,0.506910546389791,0.612217585415596,0.882276725026641
"10853","CT70",3.83692328808357,0.451399985106164,0.890330322116213,0.507002821192518,0.612152839072707,0.882276725026641
"10854","AC022400.4",4.04344234568547,-0.352661380919636,0.694940668318806,-0.507469769718314,0.611825242352827,0.882276725026641
"10855","AP3M1",79.2849988842823,0.0947108469270577,0.186873423072332,

0.506818173338637,0.612282403729757,0.882276725026641
"10856","SCAPER",137.21219427437,0.152648369116396,0.301263320476849,0
.506694173305862,0.612369419517213,0.882276725026641
"10857","UBE2I",853.696806953996,-0.100787734823143,0.198876308579453,
-0.50678602968376,0.61230495969429,0.882276725026641
"10858","DCAF7",224.047424090778,0.103766273718688,0.20484038324611,0.
506571370714613,0.612455600400453,0.882276725026641
"10859","SREBF2-
AS1",6.00046529960646,0.317050073913611,0.625744018960943,0.5066769546
43654,0.612381502974185,0.882276725026641
"10860","PRKN",22.7308826600179,0.174011055350665,0.343720262923743,0.
506257774477704,0.612675701519634,0.882350027515341
"10861","NPIPA1",92.5650625812918,0.185707916076773,0.366819850743909,
0.506264630172435,0.612670889397883,0.882350027515341
"10862","TRIM65",77.4781548450313,-0.105586758609365,0.208539408000003
,-0.506315614981337,0.61263510286927,0.882350027515341
"10863","AGAP1",149.653409898921,0.167346947155339,0.33072674526384,0.
505997623572405,0.612858317939924,0.882531774605011
"10864","SLC7A2",41.4565527558687,0.2346954878343,0.46418970475646,0.5
05602527219846,0.613135707105497,0.88283710653664
"10865","AC010186.3",6.18093851543543,0.313098310649523,0.619340698257
42,0.505534855904767,0.613183223328044,0.88283710653664
"10866","KALRN",9.352443127306,-0.337430675106408,0.66764087579309,-0.
505407453828488,0.613272684629247,0.882843304839516
"10867","CEP57L1",135.42721734269,0.0853321591025689,0.168876742785962
,0.505292544697649,0.613353378367993,0.882843304839516
"10868","PSMD5",128.929598568199,-0.0874899778089891,0.173256134600506
,-0.504974776279776,0.61357655231717,0.882843304839516
"10869","TRAF1",10.5219299805954,0.259736344983698,0.514082191599744,0
.505242837094665,0.613388286465637,0.882843304839516
"10870","AL391988.1",6.36050005971499,0.288969688887531,0.572437693108
776,0.504805487769688,0.613695461025692,0.882843304839516
"10871","LBHD2",33.7975008272353,0.363394102908678,0.719767910676914,0
.504876776969565,0.613645386059605,0.882843304839516
"10872","ZNF568",52.8798967112921,-0.126076729872106,0.24955835559268,
-0.505199393435193,0.613418796309273,0.882843304839516
"10873","AL031846.2",25.7105022396044,0.226315296494135,0.448292710039
489,0.504838226064838,0.61367246476574,0.882843304839516
"10874","YY2",15.1813275975304,0.230563717457095,0.456671026987483,0.5
04879232164238,0.613643661513648,0.882843304839516
"10875","NICN1",42.5357566636481,0.133434505932224,0.264430014039385,0
.504611802169894,0.61383151878044,0.882931514624886
"10876","TMEM144",16.7360415979109,0.286375114829158,0.567576763083379
,0.504557503858009,0.613869663942995,0.882931514624886
"10877","AC009163.7",11.7627744428028,0.263796027834182,0.523155481049
45,0.50424020657302,0.614092589676771,0.883170946061758
"10878","AC114971.1",2.81113760504933,-0.494777355642262,0.98187104803
8971,-0.503912765969065,0.61432267926996,0.883207853013873
"10879","AL512625.3",5.010253441189,-0.48901304201401,0.97025394090924
,-0.504005210796411,0.614257715266632,0.883207853013873

"10880","ZNF830",137.880641657021,-0.123661749021827,0.24535290518617,-0.504015833552059,0.614250250502727,0.883207853013873
"10881","PNPLA4",67.6313891139601,-0.161105018299619,0.319727483991212,-0.503882294660811,0.614344093117941,0.883207853013873
"10882","SPRYD4",45.204745483527,0.13468047413834,0.267379160997561,0.503705949393597,0.61446802699053,0.883288040159774
"10883","MACROD2",15.9044641102455,0.263532066146397,0.523252504796535,0.503642244863922,0.61451280068137 9,0.883288040159774
"10884","CTSK",3553.47257095464,0.0923400547019024,0.18358766058223,0.502975278453112,0.614981653296121,0.883637182190798
"10885","SLC66A3",589.065556399369,-0.116649859061885,0.231914332291623,-0.502986848243611,0.614973518821906,0.883637182190798
"10886","VPS26A",330.627428540183,0.0804864024323879,0.159982078443908,0.503096366888416,0.614896520931476,0.883637182190798
"10887","POU6F1",27.6952202690534,-0.17566184658121,0.349242160313549,-0.50298007097282,0.614978283771458,0.883637182190798
"10888","AC016831.1",27.1307965458668,-0.270594240296767,0.538590628532605,-0.502411713018482,0.615377941490707,0.883881841772026
"10889","ALDH1B1",32.0971673068203,-0.192189423345248,0.382408813488233,-0.502575820866016,0.615262532475521,0.883881841772026
"10890","ATP2B1-AS1",88.051364252837,-0.179229924143394,0.356686668234671,-0.502485627036318,0.615325960202549,0.883881841772026
"10891","TBX3",164.91157192581,-0.104481401846204,0.207938073527162,-0.502464027265096,0.615341150408798,0.883881841772026
"10892","RAB10",377.664940384848,-0.0755819986251374,0.150532986490776,-0.502095921878019,0.615600048711726,0.88394580750388
"10893","RPL37A",14839.866602734,-0.13610192428827,0.27110477206906,-0.502027032757652,0.615648505577879,0.88394580750388
"10894","HOXA2",3.13018806072675,-0.431953892481684,0.860240162852526,-0.502131743127803,0.615574852572345,0.88394580750388
"10895","PDCD1LG2",71.4866463372592,0.150008648300807,0.298665560536066,0.502262959383604,0.615482560861873,0.88394580750388
"10896","AL020996.1",4.45650213465571,-0.347413799362099,0.69254020351125,-0.5016514530141,0.615912719251374,0.88409078408503
"10897","RFT1",98.0663209104077,-0.102456683694753,0.204296370865031,-0.501510052581607,0.616012204843712,0.88409078408503
"10898","ULBP2",3.43317701148857,0.526302442481158,1.04983083564087,0.501321188722632,0.616145095448123,0.88409078408503
"10899","SLC1A1",9.176710867928,0.304257612945235,0.606893411069479,0.501336161170488,0.616134559899421,0.88409078408503
"10900","NCOR1",559.19120634319,0.0750769985728189,0.149673478847164,0.501605221920993,0.615945245445425,0.88409078408503
"10901","PIH1D1",663.428460575773,-0.133252895115463,0.265769920364834,-0.501384411496005,0.616100608497286,0.88409078408503
"10902","ADARB1",90.6921430126221,0.178232507105602,0.355401922321703,0.50149561921691,0.616022360172987,0.88409078408503
"10903","EPB41",16.4954533995111,-0.232528924468828,0.464968271553297,-0.500096326340785,0.617007252756129,0.884093012528386
"10904","NDUFAF7",78.8266598670134,0.105958104509906,0.211726554758756

,0.500447875471437,0.616759749922298,0.884093012528386
"10905","NUP35",63.1234718496847,-0.126984640025499,0.253785684422831,
-0.500361713917364,0.616820406630693,0.884093012528386
"10906","DENND6A",129.612288292602,0.119264066108333,0.238498852522325
,0.500061383302335,0.61703185625048,0.884093012528386
"10907","NUDT6",46.4978796024813,-0.121583461354659,0.242681300173148,
-0.50100053554976,0.616370746010822,0.884093012528386
"10908","PJA2",702.16536910911,0.0721546163638444,0.144320163779997,0.
499962129157763,0.617101743725329,0.884093012528386
"10909","SAMD3",4.39446930662819,0.353430714911649,0.706310949888886,0
.500389686677305,0.616800713853984,0.884093012528386
"10910","MRPL32",307.544471062674,-0.124357895986292,0.248270496000404
,-0.500896795993389,0.616443757539568,0.884093012528386
"10911","CNNM1",5.97274309309378,-0.426652932810878,0.852425829080204,
-0.500516195375322,0.616711655382356,0.884093012528386
"10912","VWF",3.28805706640618,-0.451539370634237,0.903161747607199,-0
.499954046803385,0.617107434878057,0.884093012528386
"10913","MAPK6",139.876420197513,-0.114729936512553,0.229434416631181,
-0.500055476406502,0.617036015355387,0.884093012528386
"10914","RNF227",12.5302611477451,-0.200728991267101,0.401326210945348
,-0.50016417017536,0.616959484943902,0.884093012528386
"10915","AC243829.1",6.18685924900664,-0.433870768700347,0.86640578710
175,-0.500770857211961,0.616532397902118,0.884093012528386
"10916","AP1M1",177.743532687946,0.11647021590203 8,0.232892320925381,0
.500103289963586,0.617002349700568,0.884093012528386
"10917","MVB12A",397.378289708263,-0.139221288472974,0.278446468527057
,-0.499993011976182,0.617079997942348,0.884093012528386
"10918","MAMSTR",12.7756390680155,0.268856307033629,0.537151725751864,
0.500522094864919,0.616707502449578,0.884093012528386
"10919","SCAF4",74.9871086160735,0.127937254947497,0.255895227294985,0
.49995951976086,0.617103581117665,0.884093012528386
"10920","NAV1",667.815292314368,-0.107258053039846,0.214603194470752,-
0.499797094373935,0.617217956769711,0.884170375251702
"10921","LAMTOR5",732.605350193298,-0.130582704846698,0.26161028082438
9,-0.499149744555926,0.617673895579407,0.884359533807858
"10922","NEK7",182.824756030456,0.110423569522988,0.221232166712324,0.
499129810840645,0.617687937552492,0.884359533807858
"10923","TM4SF1",4.51218689052497,-0.446504821377109,0.894526833277763
,-0.499151959188309,0.617672335527205,0.884359533807858
"10924","WDR20",105.492390035765,-0.0938428840952917,0.187999598834149
,-0.499165342252026,0.61766290814011,0.884359533807858
"10925","VMP1",1104.19348350335,0.166993882424789,0.33467887190557,0.4
98967507192706,0.61780227484832,0.884359533807858
"10926","IER3IP1",505.534069589869,-0.114588718495761,0.22953011213796
2,-0.499231745361959,0.617616132949562,0.884359533807858
"10927","SCN1B",224.837373427932,-0.147389386824599,0.295355175181132,
-0.499024223070442,0.617762319425119,0.884359533807858
"10928","MRPL40",480.055971317224,-0.142415970879559,0.285340568259032
,-0.499108737844364,0.617702782224983,0.884359533807858
"10929","ZNF587B",19.9614152971828,0.215094745656379,0.431471741752878

,0.498514096850339,0.618121735782825,0.8847358690503
"10930","SLC37A1",20.2478728521282,0.205412078316337,0.412125761992755
,0.498420863872005,0.618187434226476,0.884748950924498
"10931","ATPAF1",126.459084505722,-0.102340060305023,0.205514464179882
,-0.4979701098578,0.618505109922023,0.884798850892566
"10932","LINC00954",17.7042438701553,-0.212921633762292,0.427518698764
947,-0.498040517005218,0.618455484721244,0.884798850892566
"10933","PRMT3",55.6730399433208,0.135543731793191,0.272129384596111,0
.498085614658491,0.618423699372579,0.884798850892566
"10934","CLEC4A",7.44666224908339,-0.285189832872662,0.572375261809329
,-0.498256741514565,0.618303093676427,0.884798850892566
"10935","AC104938.1",3.92180110716989,0.474982462232847,0.953657548035
317,0.498063967732846,0.61843895628754,0.884798850892566
"10936","AC126118.1",3.05428803265607,0.596746053625389,1.199172665051
62,0.497631467941859,0.618743819840608,0.884820239436444
"10937","C10orf25",8.41232218954003,-0.257644017302277,0.5175729196917
91,-0.497792692584654,0.61863016698475
8,0.884820239436444
"10938","MPPE1",149.872759452959,-0.0898742377503791,0.180586800109815
,-0.497678887359024,0.618710391298172,0.884820239436444
"10939","CBY1",270.761489026099,-0.107237565404904,0.215497481418305,-
0.497627929102077,0.618746314594084,0.884820239436444
"10940","RNF167",220.921254969548,0.104159622264357,0.209365028013745,
0.497502487652897,0.618834749096108,0.884865811710275
"10941","AKT1S1",135.274969990082,-0.123873490694812,0.249040740783367
,-0.497402514565139,0.618905232704588,0.88488571019083
"10942","DCAF16",122.09371933087,0.121980898052573,0.245281035182776,0
.49731076013144,0.618969925034385,0.884897325654622
"10943","RPL23",7370.48831297937,-0.0974789578749222,0.196098412915251
,-0.497092028567567,0.619124155694505,0.884971224186269
"10944","REXO1",64.8438224406975,0.11215899618827
2,0.225637072173681,0
.497076987871654,0.619134761714155,0.884971224186269
"10945","AC009237.14",5.48241417412873,0.337922702403141,0.67995073186
7272,0.496981158436498,0.619202338166982,0.884986950748844
"10946","ZNF513",25.7471881493876,-0.185315671093143,0.373060353637925
,-0.496744479240486,0.619369252037852,0.885144638190035
"10947","TSR1",72.471569284468,0.11309708481217,0.227753294163707,0.49
6577163581557,0.619487260326199,0.885232411919497
"10948","ATG13",98.1008127276656,-0.108368325653351,0.218462217063892,
-0.496050654020678,0.619858673226044,0.885520477194065
"10949","SLC39A11",49.6170946871603,0.13744629962158,0.277009738884145
,0.496178582656491,0.619768420268676,0.885520477194065
"10950","RBBP9",60.5879129821985,0.112831438989145,0.227432960189354,0
.496108562695598,0.619817818260985,0.885520477194065
"10951","TSN",217.743217061774,-0.0988311540351275,0.199318337037034,-
0.495845768654814,0.620003230669872,0.885565242637766
"10952","SNX5",425.683111791912,-0.079435864407963,0.160185881875352,-
0.495898037192665,0.619966351060951,0.885565242637766
"10953","CBR3",414.325125231225,-0.159997372381084,0.322879065847603,-
0.495533434355889,0.620223627267275,0.885799160169997
"10954","DHRS4-

AS1",180.865091315383,0.0826580454508072,0.166884231600282,0.495301711
001602,0.620387163337947,0.885863402173406
"10955","GOLGA8O",8.4497236522371,0.265287658129823,0.535686865352917,
0.495228976643002,0.620438498639125,0.885863402173406
"10956","NDRG3",113.392603208214,-0.0960879472997376,0.194022598193968
,-0.495241009006986,0.620430006169739,0.885863402173406
"10957","JUN",14335.9760136082,-0.199450257632655,0.403103864335225,-0
.494786270435727,0.620750996845185,0.885991677652969
"10958","ESRG",35.240715704757,0.151207038304585,0.305456173732212,0.4
95020403277706,0.620585718227105,0.885991677652969
"10959","ZGLP1",2.95599067856672,0.480007619171036,0.970142062647534,0
.494780751863379,0.620754892736466,0.885991677652969
"10960","Z83745.1",5.10168908672284,0.370703938627296,0.74909320511097
8,0.494870245916029,0.620691714828524,0.885991677652969
"10961","AC103591.3",148.408865095157,-0.254206722467699,0.51508211599
1918,-0.493526594256066,0.621640553567838,0.886007922271035
"10962","PSRC1",9.20330798250626,0.344683498351612,0.69804228321917,0.
493785987808691,0.621457330053933,0.886007922271035
"10963","TRIM45",11.8867187325835,0.20022720320708,0.405610746085588,0
.493643733898585,0.621557808679605,0.886007922271035
"10964","AC097634.1",5.48954650495934,0.322624113635018,0.653440382994
372,0.493731520168071,0.621495801510133,0.886007922271035
"10965","AC026304.1",18.5968096890524,0.174347504830159,0.353259126587
228,0.493539987245337,0.621631092810146,0.886007922271035
"10966","ATP6V1G2",5.09032624084212,0.35942578600664,0.727382143760411
,0.494136114131816,0.621210054356985,0.886007922271035
"10967","MRPS24",746.806069091532,-0.148156870675028,0.299647823590465
,-0.494436665348575,0.62099782505466,0.886007922271035
"10968","SIGMAR1",182.873947078325,-0.108007022522791,0.21893861998715
3,-0.493321016315573,0.621785780904649,0.886007922271035
"10969","CAMK2G",132.658167247929,-0.0964379358689284,0.19504410507092
3,-0.494441684530077,0.620994281109808,0.886007922271035
"10970","C2CD2L",13.893763345555,-0.216144146669216,0.437378340880861,
-0.494181184724216,0.621178226489485,0.886007922271035
"10971","NDUFA9",225.288327410627,-0.133632448259745,0.270490196529252
,-0.494038046385513,0.62127931010561,0.886007922271035
"10972","AC023794.1",2.95804225371275,0.404730208678998,0.819899795365
308,0.493633747644332,0.621564862565225,0.886007922271035
"10973","PRKD1",26.1932481521722,0.182161584549231,0.36917208542003,0.
493432715374389,0.621706871015233,0.886007922271035
"10974","ZFP36L1",3782.57515223783,0.128952994954913,0.261100571593722
,0.493882468995766,0.621389186227832,0.886007922271035
"10975","AC009041.2",22.772366998066,0.225810801099893,0.4576654718005
62,0.493397066227218,0.621732054911278,0.886007922271035
"10976","RRN3",89.9272662155254,0.0980529696817907,0.198573961633575,0
.493785634708372,0.62145757945159,0.886007922271035
"10977","ZNF101",16.891665564747,0.184653823639842,0.373582360001411,0
.494278754594152,0.621109327207855,0.886007922271035
"10978","ZNF615",32.7473690274517,0.141840960157626,0.286815526169822,
0.494537245078019,0.620926809373548,0.886007922271035

"10979","DSN1",56.71401906979,-0.115852411920202,0.234882581541873,-0.493235433465076,0.621846243920022,0.886013370401758
"10980","BROX",187.506989794153,-0.0960730053975108,0.194960800014631,-0.492781140569288,0.622167237842624,0.886067197885302
"10981","AC079305.3",6.28447102278705,-0.328016630122708,0.665406958659674,-0.492956416902267,0.622043382709174,0.886067197885302
"10982","FAM20C",226.731410697381,-0.0916925712718868,0.186060836216155,-0.492809626875821,0.622147107897742,0.886067197885302
"10983","CFAP300",36.4527092544615,-0.142128923202325,0.288417765832336,-0.492788378663704,0.622162122991939,0.886067197885302
"10984","ZIK1",6.15859164734011,0.30605507084003,0.621073898912619,0.492783662903681,0.622165455415089,0.886067197885302
"10985","AC093690.1",11.4210548017173,0.257151746344203,0.522403954243301,0.49224693698325,0.622544786937437,0.886132327066594
"10986","TTC33",101.665047134943,-0.0956992096821487,0.194397287421605,-0.492286754365035,0.622516642524256,0.886132327066594
"10987","BAG4",123.994611416173,-0.0986038768291704,0.200261870404435,-0.49237469234676,0.622454486627714,0.886132327066594
"10988","LRRC24",10.9157783992368,0.256073159098658,0.520025044385397,0.492424666587555,0.622419165277124,0.886132327066594
"10989","MAFG-DT",10.8770814686636,-0.242745319466561,0.493148719660459,-0.492235526097877,0.622552852679273,0.886132327066594
"10990","AC002470.1",2.69419766552172,-0.427437163183134,0.867752195578975,-0.492579754175029,0.622309556279004,0.886132327066594
"10991","PINLYP",39.5370494963374,-0.155947494455194,0.316870708538081,-0.492148659542171,0.622614255465821,0.886139095464639
"10992","RPRD2",198.854345599907,0.0835915620877058,0.169910888556464,0.491972955929349,0.62273846193868,0.886187804968538
"10993","SMIM2-AS1",2.85315085262095,-0.55606309271803,1.13034745357511,-0.491939970280193,0.622761780989525,0.886187804968538
"10994","POM121C",129.162140389313,0.107244577554848,0.218048636539023,0.491837872765851,0.622833960749296,0.886209900673207
"10995","ALS2CL",3.6808978611407,0.37424287956535,0.76141037388887,0.491512714298757,0.623063861799865,0.886243929748102
"10996","FEM1C",108.940614185748,-0.145383342798044,0.295739320297492,-0.491592875278806,0.623007181118526,0.886243929748102
"10997","ZNF567",69.5993934277704,0.0970896710054127,0.197499160061271,0.491595361596941,0.62300542311428,0.886243929748102
"10998","ZNF226",100.773800395119,0.127686307702419,0.259797731512426,0.491483535899588,0.623084493982588,0.886243929748102
"10999","SERTAD2",170.133658918579,-0.0983733274614499,0.200293189013888,-0.491146643307121,0.623322733739354,0.886356208429002
"11000","AC090018.2",2.88316225072074,-0.524758606911173,1.0684026027204,-0.491161857501109,0.623311973893748,0.886356208429002
"11001","ZNF821",37.8233939959204,0.125626918324375,0.255790778814825,0.49113153690079,0.623333417434472,0.886356208429002
"11002","NFIA",3213.71949775696,0.100386437583395,0.204548590549981,0.490770615008785,0.62358869558397,0.886589106835852

"11003","RPF2",211.864723966415,-0.114942090596168,0.234247643747677,-0.490686218897379,0.6236483950326,0.886589106835852
"11004","MAPKAPK5-AS1",153.648946849031,-0.129000992821641,0.262913424968129,-0.490659588179183,0.623667233370946,0.886589106835852
"11005","AC092802.1",22.3214141310473,-0.195090138611385,0.398149395214191,-0.48999230177515,0.624139346299421,0.886669818701055
"11006","PSMB4",633.588581075551,-0.121970765632395,0.249111163800368,-0.489623844116994,0.624400100569706,0.886669818701055
"11007","SRD5A3",108.747469070393,-0.111809496111718,0.22801930887322,-0.490350999940468,0.623885543955805,0.886669818701055
"11008","PDGFRB",1720.71269197178,-0.0830870455292742,0.169659326416769,-0.489728724521577,0.624325872831372,0.886669818701055
"11009","FAF2",223.360396169703,0.0707850486470854,0.144335941110504,0.490418728020709,0.623837626931327,0.886669818701055
"11010","AFG1L",14.0605167518299,0.189714084932507,0.38701927446529,0.490192859760325,0.623997432945881,0.886669818701055
"11011","AKAP7",56.4492785377315,-0.124934867823253,0.255168009480001,-0.489618068024492,0.62440418863458 6,0.886669818701055
"11012","NPDC1",435.250706066757,0.145959326787543,0.297696731447205,0.4902953622567,0.623924908321126,0.886669818701055
"11013","GIHCG",117.652390911783,0.137225465689518,0.280245370107899,0.489661847532694,0.624373203707543,0.886669818701055
"11014","TEX26",3.47950050618203,-0.408803523241425,0.83421791876966,-0.490044044899379,0.6241027319106,0.886669818701055
"11015","COPS2",426.163302354026,-0.0945264966330078,0.193005280141255,-0.489761194946721,0.624302893084344,0.886669818701055
"11016","TRAPPC2B",89.7875725243421,0.148384285667201,0.303020210137165,0.489684452400167,0.624357205382876,0.886669818701055
"11017","AL713852.1",3.18381239009778,0.397643221098273,0.812920660016245,0.489153789116796,0.624732822681673,0.886676513999738
"11018","AL391832.2",9.09729468764453,0.266089741239546,0.545159661176187,0.488095066801999,0.625482505188503,0.886676513999738
"11019","HMGB2",326.967609702898,0.104895289874346,0.214639659508331,0.48870413843661,0.625051173472371,0.886676513999738
"11020","AC113370.1",6.52564894434077,0.360125828973098,0.737647742936901,0.488208406277065,0.625402230841355,0.886676513999738
"11021","AL354740.1",34.4142615160249,-0.195663946539469,0.400995553163956,-0.487945427313673,0.625588496355028,0.886676513999738
"11022","EIF3B",304.533860339858,-0.0982375321051446,0.201308572003074,-0.487994778998504,0.625553539200956,0.886676513999738
"11023","ABHD11",70.3480722657579,-0.13269170856803,0.271328971050158,-0.489043643420963,0.62481079889843,0.886676513999738
"11024","ZFHX4-AS1",40.0270196641693,0.196220519451063,0.401639302465648,0.488549099270097,0.625160956773431,0.886676513999738
"11025","MINCR",57.8619609850242,0.130054484919842,0.266368519122274,0.488250208201748,0.625372625135664,0.886676513999738
"11026","TUT7",125.323332004224,0.125044950273982,0.256200706921066,0.488074181280492,0.625497298150327,0.886676513999738

"11027","TRIM21",43.0613073482328,0.125261607902346,0.256628149859154,
0.488105486366532,0.625475125193575,0.886676513999738
"11028","TC2N",103.381220607054,0.228871777506592,0.467646883885557,0.
489411531206957,0.624550373930357,0.886676513999738
"11029","GOLGA6L9",17.2819438616784,-0.276606409465735,0.5653181456272
5,-0.489293350311311,0.624634028178077,0.886676513999738
"11030","AC005736.1",20.3596230497472,0.276882815070878,0.566470134048
857,0.488786254434725,0.624993030467775,0.886676513999738
"11031","TMC5",7.54220464595154,0.424491002789672,0.869982905106313,0.
487930280351656,0.625599225533153,0.886676513999738
"11032","GOT2",60.9131446265504,-0.137710139798721,0.281691001976615,-
0.488869501803088,0.624934088767938,0.886676513999738
"11033","NPIPB15",51.7146769141382,0.151896438937756,0.311064890771967
,0.488311099850567,0.625329500445765,0.886676513999738
"11034","AP2B1",526.763866623204,0.103837527216352,0.212327370766094,0
.489044473360633,0.624810211337699,0.886676513999738
"11035","SHFL",231.799871187239,-0.0912321254601729,0.186506502202632,
-0.48916324301151,0.624726130115286,0.886676513999738
"11036","ZNF578",7.92102294409664,0.247654886214063,0.507172141673463,
0.488305381673572,0.625333550119123,0.886676513999738
"11037","SOD1",1973.13643167732,-0.1193957228758,0.244355720131851,-0.
488614397123077,0.625114718321661,0.886676513999738
"11038","SLC1A5",143.27998184147,-0.117733746255327,0.24133993429196,-
0.487833671624767,0.625667659090815,0.886693168251279
"11039","MORN4",42.3183405345078,-0.22874363413261,0.469115183236661,-
0.487606545911375,0.625828558094389,0.886840849195627
"11040","AFAP1L1",18.0001167978432,-0.266636261547109,0.54781277576731
2,-0.486728811999019,0.626450524409665,0.886918775757117
"11041","SRRT",110.678802499386,-0.142156884965836,0.292024052264723,-
0.486798549172139,0.626401098601874,0.886918775757117
"11042","RMDN1",151.776221844226,-0.100203489403665,0.205698760659918,
-0.487137059465959,0.626161205281883,0.886918775757117
"11043","OMD",710.415428945457,0.0702695236559588,0.144226466185492,0.
487216566518271,0.626104866465732,0.886918775757117
"11044","KBTBD3",67.0080873325095,-0.127637107805538,0.262097929196738
,-0.486982511447963,0.626270724481563,0.886918775757117
"11045","NUDT4",223.339214614871,-0.162968608171302,0.334748377862922,
-0.486839127381932,0.626372339808569,0.886918775757117
"11046","CRY1",48.967915673988,-0.132612494733141,0.272377940280237,-0
.486869438092904,0.626350858219444,0.886918775757117
"11047","SNURF",4.0576030356795,0.396491447104437,0.81427549551798,0.4
86925431609875,0.626311175730459,0.886918775757117
"11048","NUP93",48.8098334883349,-0.126758435437531,0.260171978530618,
-0.487210175951419,0.626109394750305,0.886918775757117
"11049","HSPB6",762.827679103467,0.155884534721837,0.320019231043687,0
.48710989715664,0.626180453034973,0.886918775757117
"11050","ARMH1",5.74212075960105,-0.336980465386956,0.693303978203606,
-0.486050096322962,0.626931647711856,0.886922857677449
"11051","TOMM40L",17.6193629287748,-0.187073777875351,0.38539277168675
2,-0.485410707254793,0.627385038654883,0.886922857677449

"11052","DENND1B",82.4482254319343,0.124055537551665,0.255148374025468
,0.486209398846814,0.62681870817076,0.886922857677449
"11053","CYTOR",479.51164654636,0.167887209798543,0.345521283866905,0.
485895421317122,0.627041314883267,0.886922857677449
"11054","AC021016.2",2.64171764190341,0.416243516439267,0.855672244619
418,0.486452048733218,0.62664669527201,0.886922857677449
"11055","AC010210.1",8.79417919920515,0.291318857295629,0.599806543589
355,0.485688027930342,0.627188373205077,0.886922857677449
"11056","CALD1",5259.95782551101,0.105241813483461,0.216631293315583,0
.485810761098799,0.627101343887416,0.886922857677449
"11057","AL136366.1",14.960345446712,0.261031824892852,0.5373840093885
6,0.485745426608165,0.627147671517871,0.886922857677449
"11058","SNX32",4.20000981393438,0.426473074645866,0.877983113530113,0
.485741773473458,0.627150261940003,0.886922857677449
"11059","TMPO-
AS1",4.70546706727351,0.32492969647667,0.668346930447537,0.48616920595
2799,0.626847202606831,0.886922857677449
"11060","TCHP",82.5420037834205,0.1292099070455,0.266154090403407,0.48
5470303498465,0.627342772994227,0.886922857677449
"11061","CCDC9B",16.4719803886661,0.228951768117721,0.471452942859952,
0.485630160093692,0.627229408723776,0.886922857677449
"11062","RCCD1",42.2686431882613,0.1505497130088,0.309924296345483,0.4
85762861395602,0.627135308648295,0.886922857677449
"11063","C17orf97",49.0979579209132,-0.285695840759148,0.5879909024282
78,-0.485884797841743,0.627048847407874,0.886922857677449
"11064","CD70",45.6716104323521,0.483143809598017,0.994039733723386,0.
48604074184067,0.62693827996237,0.886922857677449
"11065","AC008569.2",5.87305413134837,0.321672259650584,0.662851823706
705,0.485285320408074,0.627473967008651,0.886922857677449
"11066","AC010632.2",10.3051604184592,-0.282947794495942,0.58299518172
5699,-0.485334704925691,0.62743894128652,0.886922857677449
"11067","RPS6KA6",10.4291928462216,0.245965187066236,0.506672199809573
,0.485452304584068,0.627355537696838,0.886922857677449
"11068","HMGN3-
AS1",60.4806986579847,-0.128698420666248,0.265476119551608,-0.48478341
8122959,0.627829986300234,0.887097040856551
"11069","AC106845.1",12.1394598174643,0.361499049134137,0.745759697260
844,0.484739320805232,0.627861270426246,0.887097040856551
"11070","PRDM11",30.3145291958083,0.141234738466239,0.291400309152165,
0.484676007644482,0.627906188089377,0.887097040856551
"11071","CNPY2",722.52793714056,0.112565650031102,0.232096277293452,0.
484995499901012,0.627679537670084,0.887097040856551
"11072","C19orf12",184.974398276349,0.095189130751369,0.19644770394617
2,0.484552014807217,0.627994159077252,0.887097040856551
"11073","ZNF837",9.96844033425536,-0.24273907883408,0.500869516125893,
-0.484635361144773,0.627935025565784,0.887097040856551
"11074","AL035461.2",3.02767519748808,0.429496662229004,0.886308925184
01,0.484590248416865,0.627967032362703,0.887097040856551
"11075","INMT",341.313106923585,0.200467058513748,0.414043000190443,0.
48416965972505,0.628265467331408,0.887354180699718

"11076","TAF4",30.2432360542963,-0.151121674784291,0.312147418474077,-
0.484135590558604,0.628289644277318,0.887354180699718
"11077","LIMS2",149.017484068532,0.147105354355958,0.303964750523383,0
.483955307655458,0.62841758741766,0.887391250110795
"11078","ZBTB20-
AS4",3.83550338712417,0.358325900641219,0.740582796818987,0.4838431329
7626,0.628497201159271,0.887391250110795
"11079","NGDN",127.642224857522,-0.103078661907962,0.213136298954236,-
0.483627905775424,0.628649966471654,0.887391250110795
"11080","ZNF276",76.9694834382449,0.128956711939448,0.266649367418123,
0.483619043195538,0.628656257350114,0.887391250110795
"11081","RHOT1",103.019134078247,0.0849044066380477,0.175488363918476,
0.483817871123868,0.628515130851409,0.887391250110795
"11082","AC245060.6",21.0669062469208,0.264185225059968,0.546203992919
035,0.483675016083467,0.62861652686214,0.887391250110795
"11083","MTF2",144.25392468197,0.104458702913543,0.216052434631044,0.4
83487738020304,0.628749464170757,0.88740070449276
"11084","HOXA13",13.7498416344387,0.286396448284085,0.592449883192559,
0.483410422398547,0.62880434933987,0.88740070449276
"11085","NECTIN1",31.2449969883118,0.202799542571969,0.419622917176697
,0.483289959319771,0.628889868312136,0.88740070449276
"11086","AC009831.1",4.09915195881541,-0.361085293414395,0.74705940256
8475,-0.483342144109214,0.628852820751611,0.88740070449276
"11087","EBF4",7.65960168428383,-0.280626031265335,0.580862907664675,-
0.483119213780711,0.629011092264726,0.887491703463255
"11088","QSOX1",190.875770575973,-0.0975988234568918,0.202243297413719
,-0.482581250923925,0.629393094317755,0.887570843925325
"11089","SLC26A6",15.0829793336526,0.239179540576521,0.496156038359078
,0.48206516112865,0.629759657746863,0.887570843925325
"11090","PDE12",69.1491493835306,-0.109719027492591,0.227439014307763,
-0.482410758886434,0.629514179713151,0.887570843925325
"11091","NDUFS4",945.081804259055,-0.12655390821476,0.262243647427466,
-0.482581406475301,0.629392983847936,0.887570843925325
"11092","AL021368.5",3.46524425902239,0.48599375651545,1.0074087261293
,0.482419641512092,0.629507870924847,0.887570843925325
"11093","AC064807.1",28.9601582150547,-0.139752203531226,0.28990055775
8577,-0.482069453787007,0.629756608421396,0.887570843925325
"11094","ZMIZ1-
AS1",10.7242451502716,0.218796198785807,0.453412454251504,0.4825544528
70063,0.629412125942376,0.887570843925325
"11095","AP001160.2",7.97015423077738,0.283396050917152,0.587544465749
816,0.48233975032934,0.629564613740662,0.887570843925325
"11096","POMT2",32.5151477684309,0.135827421637473,0.281471797738581,0
.482561388845156,0.629407200080684,0.887570843925325
"11097","CSNK2A2",275.544948498894,-0.101958168212972,0.21147960905125
,-0.482118198867404,0.629721982391928,0.887570843925325
"11098","AP001094.2",3.08635052507586,0.407448391284385,0.844639292972
005,0.482393365635062,0.629526533159136,0.887570843925325
"11099","AC008622.2",6.41559939039874,-0.263446193624393,0.54656702456
4346,-0.482001624291878,0.629804792403702,0.887570843925325

"11100","AP001469.3",4.78830099183212,0.388385639652277,0.805646619540464,0.482079400859163,0.629749542460444,0.887570843925325
"11101","SAYSD1",152.617710122068,-0.135138271438974,0.280733919330861,-0.481374932395347,0.630250049501327,0.887898930590447
"11102","ABCA2",70.07275623866,0.103973145818175,0.215902824340211,0.481573810513649,0.63010873441852,0.887898930590447
"11103","PRR33",3.16260724110004,-0.453056329335196,0.941021056947192,-0.481451850615305,0.630195392790728,0.887898930590447
"11104","TSFM",114.146292721038,-0.128897428953883,0.267781657043406,-0.481352719887716,0.630265833688355,0.887898930590447
"11105","AC068234.2",2.9832502498143,-0.482727787127099,1.00301960411063,-0.481274528582249,0.630321397699093,0.887898930590447
"11106","PLEKHM3",66.9802419432883,0.125855255181271,0.261713564463449,0.480889309040181,0.630595171479713,0.888204598186309
"11107","BGLAP",34.8487860766478,-0.168394330230132,0.350459297154999,-0.480496113520582,0.630874666058014,0.888205738652377
"11108","AL354953.1",4.79492409141108,-0.335192030264901,0.700555488175315,-0.478466068602147,0.632318519263272,0.888205738652377
"11109","CCDC121",57.2120761513026,0.129699201588493,0.271032609989928,0.478537256433136,0.632267863722532,0.888205738652377
"11110","ARHGAP25",8.10644198329402,0.269000144140061,0.560678597343138,0.479776016803137,0.631386668217599,0.888205738652377
"11111","CXCR4",136.91718648136,0.180927857556736,0.378727082881479,0.477726219577901,0.632845080067416,0.888205738652377
"11112","CTLA4",3.85641399189953,-0.352520581026659,0.736822741042344,-0.478433361771607,0.632341793232219,0.888205738652377
"11113","CHCHD4",45.4842242907152,-0.125930526156579,0.263635284189002,-0.4776694688041,0.632885478069226,0.888205738652377
"11114","CGGBP1",359.813446881697,0.0630029735421105,0.13162830121265,0.478643065067953,0.632192576042725,0.888205738652377
"11115","UBA5",142.88285304634,-0.0835368089979243,0.174258040590298,-0.479385678359197,0.631664280067742,0.888205738652377
"11116","AC114956.3",3.96285341168429,0.501533505040398,1.04419611022418,0.48030585455133,0.631009926515398,0.888205738652377
"11117","AP3B1",190.583916651672,0.0921080492101061,0.191788019793084,0.480259660167926,0.631042769268114,0.888205738652377
"11118","CSNK1G3",297.908272939008,0.12575872164142,0.262417072393676,0.479232240853439,0.631773420286129,0.888205738652377
"11119","SLC35B3",132.898408853224,-0.102073226582686,0.213230317308615,-0.478699407622006,0.632152487295975,0.888205738652377
"11120","HSPA1B",473.508667758363,0.202107353612356,0.421802765228335,0.479151324441765,0.631830979425616,0.888205738652377
"11121","RAET1E-
AS1",3.9916976120281,-0.376060567634844,0.782285837892847,-0.480720152940254,0.630715405980438,0.888205738652377
"11122","TYW1B",36.5550699755353,-0.220523340196645,0.460943086236803,-0.478417719630043,0.632352924207667,0.888205738652377
"11123","AC011029.1",4.28439519751109,-0.343775470769781,0.719677454388558,-0.477679922684051,0.632878036399223,0.888205738652377
"11124","MTAP",182.261950057421,-0.0713874208257154,0.14880723026836,-

0.479730861847067,0.631418780128224,0.888205738652377
"11125","IL11RA",616.446424128826,-0.145441594943413,0.303930354857489,-0.478535929758019,0.632268807736716,0.888205738652377
"11126","WDR5",57.3050641472043,-0.109432672291786,0.229095324285593,-0.477673093647981,0.632882897693701,0.888205738652377
"11127","ARHGAP22",11.3663530916669,-0.304974795333763,0.638014180783064,-0.478006295972063,0.632645723908782,0.888205738652377
"11128","DLG5",103.349001542434,0.113581234506015,0.237808975421798,0.477615423490885,0.632923951208722,0.888205738652377
"11129","CEP295",79.7080783012563,0.136725977046991,0.285899934134648,0.478230180293075,0.63248638401532,0.888205738652377
"11130","NKAPD1",220.388572699932,-0.0801528014726332,0.167670102513302,-0.478038721699202,0.632622645256582,0.888205738652377
"11131","TNFRSF1A",989.345164679563,0.1282133664632,0.268410925280369,0.47767566215598,0.632881069282439,0.888205738652377
"11132","ATF1",176.697987005929,-0.0820532417376455,0.171438886160953,-0.478615112213286,0.632212465405885,0.888205738652377
"11133","MARS",55.1794222375194,-0.152239703884892,0.318169080056716,-0.478486796572923,0.632303769561962,0.888205738652377
"11134","CIPC",26.0646507309977,-0.148562341807895,0.310419019597802,-0.478586466771209,0.632232847843528,0.888205738652377
"11135","GPR176",27.3811629587571,-0.181818929927689,0.379096182623273,-0.479611608509316,0.631503590387617,0.888205738652377
"11136","IVD",113.184015348983,0.103989433498311,0.216393468944582,0.480557172106438,0.630831260411045,0.888205738652377
"11137","RPL4",3547.9494738273,-0.101965328826282,0.213160208138687,-0.478350672091392,0.6324006362978,0.888205738652377
"11138","IQGAP1",885.599261184134,0.0777072524130356,0.162451182560202,0.478342177559949,0.632406681250099,0.888205738652377
"11139","ZNF213",36.5100142575114,0.138470707757662,0.288999661644935,0.479137958049886,0.631840487699374,0.888205738652377
"11140","FBXL8",49.8513636038016,-0.129991231562425,0.271128820376187,-0.479444536298516,0.631622416499444,0.888205738652377
"11141","GFOD2",64.447588473571,-0.106920756460083,0.22253917009375,-0.480458143234019,0.630901659216195,0.888205738652377
"11142","KLF2",1638.09004931541,0.180788210049565,0.376673080254475,0.479960526851102,0.631255461275215,0.888205738652377
"11143","EHD2",582.420737547647,0.0995705480067411,0.207588049362316,0.479654528825765,0.631473065876691,0.888205738652377
"11144","ZNFX1",148.00738690787,-0.100081270654356,0.208574400609691,-0.479834871210487,0.631344814997519,0.888205738652377
"11145","PRR34",6.09584500690557,-0.291969234716152,0.610772612037274,-0.478032624518425,0.63262698483017,0.888205738652377
"11146","MORF4L2-AS1",10.3169860224823,-0.241014954526547,0.504341382049826,-0.477880584668612,0.632735200918388,0.888205738652377
"11147","RAP2C-AS1",41.3400959155851,-0.122356321550809,0.25576059597985,-0.478401768974797,0.632364274807772,0.888205738652377
"11148","RABGAP1L-

IT1",3.29914758746749,-0.415266769409508,0.870206193573545,-0.477205026206713,0.633216132379244,0.888413531993659
"11149","AC034206.1",2.93202636770092,-0.470645192610089,0.986329890965302,-0.477168133016305,0.633242401184511,0.888413531993659
"11150","AL731569.1",5.52939190908795,0.341993351018823,0.716614107245792,0.477235024486508,0.633194773250783,0.888413531993659
"11151","AL109627.1",3.33885082295484,-0.359708591298529,0.754486711942579,-0.476759345929879,0.633533497992981,0.888425931389542
"11152","SSX2IP",11.0602697485318,0.233000751854422,0.489193001353515,0.476296167790111,0.633863395169673,0.888425931389542
"11153","SDHC",376.034453098591,-0.105948210193268,0.22221228015234,-0.476788277050367,0.633512894309637,0.888425931389542
"11154","GPATCH2",193.439373596569,-0.0884229672995102,0.185584447187702,-0.476456775551232,0.633748994549356,0.888425931389542
"11155","ZNF860",5.73663386679049,0.29395799318064,0.61634996526617,0.476933576290046,0.633409421816274,0.888425931389542
"11156","PAK2",443.531367806837,-0.0772305352430203,0.162105353361893,-0.476421867886168,0.633773858472128,0.888425931389542
"11157","SLC36A1",26.7273883015911,-0.152061497474565,0.319134429536476,-0.476481016778496,0.633731728326924,0.888425931389542
"11158","GMDS",40.2788012392828,-0.168946059408797,0.354529753645837,-0.476535629721978,0.633692830068813,0.888425931389542
"11159","MAP4K2",11.4572253628923,-0.265414753601734,0.556800020640881,-0.476678778309382,0.633590876796189,0.888425931389542
"11160","MAGEL2",3.49460466570149,0.412554892086365,0.866205132219119,0.476278512723015,0.63387597137625,0.888425931389542
"11161","CENPT",127.062806294744,0.0808772708327865,0.169638844759862,0.476761504402339,0.633531960798218,0.888425931389542
"11162","UCK2",20.1745430499423,-0.170703855629182,0.358599614143625,-0.476029111288483,0.634053638410451,0.888436139883079
"11163","MOXD1",42.0069886009585,-0.494750093131499,1.03908924487012,-0.476138210046951,0.633975916723171,0.888436139883079
"11164","CHTF8",7.11873054539615,-0.251714315147401,0.528750359041439,-0.476055119099549,0.634035110143336,0.888436139883079
"11165","CAMK1D",250.157062515466,0.14562788975958,0.30598279282973,0.475934899517756,0.634120757892192,0.888450605974703
"11166","NUP188",45.9420654745293,-0.115889660069608,0.243586479360022,-0.475763927349688,0.634242571624994,0.888462124825806
"11167","PMPCA",124.440464222138,0.103109744442602,0.216720537921538,0.475772833675469,0.634236225828288,0.888462124825806
"11168","SNHG4",7.72069345745315,-0.31049594057052,0.652905406238578,-0.475560376133662,0.634387610100238,0.888585725716156
"11169","KLHL18",31.726640478759,-0.145765884909874,0.306650648928392,-0.475348366029098,0.63453869080354,0.888717767055222
"11170","AC017083.1",8.05169027013914,0.27729797957094,0.583495476557615,0.475235868505587,0.634618863944396,0.888750482424547
"11171","TARS2",56.2653057261372,-0.123985208632472,0.261350126770689,-0.474402710893874,0.635212760277078,0.889084487059155
"11172","AC016949.1",14.9343624456586,0.227304701884753,0.479025444949591,0.474514880746414,0.635132789015846,0.889084487059155

"11173","SLC4A2",106.282184194708,-0.0865427331361461,0.18238310395107
8,-0.474510693487047,0.635135774237159,0.889084487059155
"11174","GABARAPL1",426.35440803793,-0.0957382125375383,0.201833228070
668,-0.47434316664656,0.635255213952578,0.889084487059155
"11175","EIF2AK4",299.416092620817,0.0824243646395892,0.17372989344975
3,0.474439735171014,0.635186363425747,0.889084487059155
"11176","NPIPB4",17.3263183203167,0.232839133397211,0.490767450568279,
0.474438826632854,0.635187011172121,0.889084487059155
"11177","CHRNB1",91.088742998834,-0.143954244240929,0.303285676713494,
-0.474649003543015,0.635037172036766,0.889084487059155
"11178","AC099791.2",19.362959624645,0.250213683397987,0.5279075139187
98,0.473972574363612,0.635519464656962,0.889190526741035
"11179","NRAS",82.1052680643325,0.10114994856936,0.213456563201747,0.4
73866659577758,0.635594995690277,0.889190526741035
"11180","B3GALNT2",74.5488399699422,-0.103179560627979,0.2178259439720
17,-0.473678932575791,0.635728878800213,0.889190526741035
"11181","AP000487.1",10.4320602436866,0.217819670574469,0.459532800654
696,0.474002443925965,0.635498164455131,0.889190526741035
"11182","METTL22",36.6343251849773,0.129307983678203,0.272915694001032
,0.473801934152287,0.635641155212695,0.889190526741035
"11183","TPM4",721.22288095918,-0.108890336189575,0.229832351247409,-0
.473781587311688,0.635655666038495,0.889190526741035
"11184","CBR3-
AS1",41.2163181734938,-0.149391866250979,0.315376448481905,-0.47369379
3465208,0.635718279879751,0.889190526741035
"11185","TBK1",129.39608167468,0.0871224631249496,0.184048771352741,0.
473366176174977,0.635951956793236,0.889423018338542
"11186","ARF6",349.914941461129,-0.1053871106525,0.222683034244655,-0.
473260619112609,0.636027254339115,0.889448805616554
"11187","ICK",244.008319803206,0.11141353829695,0.235528013078792,0.47
3037312379815,0.63618655924032,0.889592057405589
"11188","THAP3",124.869713850065,-0.134068258475237,0.283689045715511,
-0.472588774575677,0.636506592690427,0.88996001335863
"11189","DPH5",110.188863283797,0.123850669103097,0.262240980027147,0.
472278089756512,0.636728307280716,0.890182509804726
"11190","AC132153.1",2.7078646434765,0.405221225642838,0.8582144315078
76,0.472167806512957,0.636807016743613,0.890182509804726
"11191","SLFN5",575.497169715648,0.130432442788871,0.276312494792727,0
.472046850022885,0.636893348445598,0.890182509804726
"11192","AC138150.1",48.3528704451304,0.133469822383616,0.282709432126
376,0.47210954859105,0.636848597241786,0.890182509804726
"11193","SEMA4F",26.2463699006123,-0.157203013676641,0.33316343926274,
-0.471849534344215,0.637034191470841,0.890220283828691
"11194","AC084018.2",3.07014496695439,0.389602702385318,0.825603543057
982,0.471900472885877,0.636997830517868,0.890220283828691
"11195","OSER1-
DT",102.583288466027,0.131568676574571,0.27889410272029,0.471751375490
808,0.637104261694331,0.890238674915981
"11196","NUP62",140.532925599606,0.0869523313629977,0.184431832436692,
0.471460540266796,0.637311892062174,0.890449261122597

"11197","TPRN",29.617921447761,-0.149015388840703,0.316403700062047,-0
.470966012127801,0.637665006332659,0.890863061004
"11198","NTPCR",304.884533397441,0.125907654064286,0.267827290972811,0
.470107633941859,0.638278120370541,0.891088612927658
"11199","LINC00852",4.90073706695184,0.425624116090598,0.9056769522673
57,0.469951360719791,0.638389768327688,0.891088612927658
"11200","PHAX",328.11266314291,-0.0636505129754395,0.135493933017619,-
0.469766516904949,0.638521838865796,0.891088612927658
"11201","RNGTT",55.7870107592347,0.107051908319921,0.227544198728503,0
.470466436490657,0.638021808248316,0.891088612927658
"11202","GSDME",40.4530233654298,0.207382711218268,0.441527013812628,0
.469694276296933,0.638573457753699,0.891088612927658
"11203","AC036214.2",3.99041354778247,0.359683368029807,0.764851177076
322,0.470265822698624,0.638165112230728,0.891088612927658
"11204","AS3MT",33.6009231303507,-0.180046253961868,0.383333177471967,
-0.46968607087247,0.638579320977993,0.891088612927658
"11205","AP003059.1",2.61732802501271,0.427888416835725,0.911214471707
535,0.469580356898744,0.638654861411429,0.891088612927658
"11206","SCN4B",4.28421712108296,-0.474169812194329,1.00892970770908,-
0.469973089870651,0.63837424364611,0.891088612927658
"11207","PRR4",19.0789794918455,0.236684569987778,0.504073357065214,0.
469543900050161,0.63868091338905,0.891088612927658
"11208","AC020978.5",2.81319238652153,0.520413673661471,1.107166034039
59,0.470041220251943,0.638325568014579,0.891088612927658
"11209","TIMM22",135.510644665602,-0.125248748982357,0.266628358343011
,-0.469750291232064,0.638533432624402,0.891088612927658
"11210","AC005332.4",31.4569701412366,0.173850259076237,0.369584901354
767,0.470393293770833,0.6380740545505,0.891088612927658
"11211","TMX3",268.763698156568,0.0697745954937162,0.148354168363886,0
.470324469229415,0.638123217992401,0.891088612927658
"11212","ZNF211",12.5303515826412,0.235401998415464,0.500274441271331,
0.470545722498325,0.637965175787882,0.891088612927658
"11213","ZBTB7C",23.238596469769,-0.150801336293559,0.321237872528604,
-0.469438223788919,0.638756431914765,0.891114497854514
"11214","EMC2",292.734546660438,0.116976978568742,0.249336709562456,0.
469152652146635,0.638960526282632,0.891319735387838
"11215","IL10RB",354.869902027189,0.08127152685193,0.17326204257798,0.
469067117313663,0.639021662246599,0.891325533885291
"11216","SRPK1",128.63951631914,-0.087504199179822,0.186609068315372,-
0.468917185910487,0.639128831533502,0.891395534208147
"11217","EFCC1",4.35227812590783,-0.465729845592905,0.993627626958161,
-0.468716683148863,0.639272160436086,0.891515949514281
"11218","SLAIN2",192.053921080407,-0.0881042376023779,0.18806933210360
3,-0.468466796882349,0.639450809870098,0.891685596255833
"11219","AMD1",267.387213266376,-0.146894646602431,0.313722305513475,-
0.468231439144903,0.639619091650897,0.891761270115418
"11220","FGF14",67.702958636272,-0.128283201245948,0.273956298620129,-
0.468261550809704,0.63959756064806,0.891761270115418
"11221","AC099063.4",7.61938446875358,-0.247327361400505,0.52839059523
178,-0.468076766756257,0.639729693164343,0.891835985221444

"11222","ATRIP",28.2370640999622,0.162515973717636,0.347287673873596,0.467957793908883,0.639814772472047,0.891836798708812
"11223","SEC22A",60.4294741148205,-0.100280667280084,0.214313165638291,-0.467916504249362,0.63984430044806,0.891836798708812
"11224","HIPK2",57.2130490872888,0.116173824743218,0.248369645153729,0.467745664617396,0.639966481133383,0.891839625781857
"11225","API5",159.235134979075,-0.0996768076524339,0.213132859162125,-0.467674520223145,0.640017364893379,0.891839625781857
"11226","NPB",28.5097057078167,0.183042101430106,0.391380647971764,0.46768306603476,0.640011252684577,0.891839625781857
"11227","FGD3",3.37808389978229,-0.377496155222899,0.808298348379949,-0.467025765893874,0.640481443831607,0.891955239277971
"11228","KIF1BP",160.270555634931,0.0929418052886062,0.198982347015823,0.467085682134483,0.640438577542315,0.891955239277971
"11229","EFEMP2",1096.40450454533,0.137143982632687,0.293695129980945,0.466960356617368,0.640528241410971,0.891955239277971
"11230","PRCP",409.157713053292,0.0725978839558158,0.155482197809501,0.466920875692557,0.640556489039745,0.891955239277971
"11231","FAM118B",30.8853788969331,-0.161219322628186,0.344888914483893,-0.467452898187354,0.640175883840139,0.891955239277971
"11232","TMEM220",169.648660518825,-0.0969277285476967,0.207487268054551,-0.467150247128481,0.640392386706191,0.891955239277971
"11233","DAZAP1",174.980089372767,-0.0853675698246462,0.182693796676845,-0.467271310670975,0.640305779654267,0.891955239277971
"11234","PPFIA3",6.52031438745208,-0.346344823344292,0.741426754643121,-0.46713289097721,0.640404803439313,0.891955239277971
"11235","ABCF1",278.280441997056,0.0811689885595737,0.173896719967659,0.466765494913702,0.640667665215142,0.892030644144233
"11236","ILVBL",177.578774809767,-0.133848995095092,0.28693006756172,-0.466486472583779,0.640867328137136,0.892229228733465
"11237","AC009812.4",3.80786387655999,-0.368876918798258,0.791040192182149,-0.46631880711482,0.640987318769155,0.89231686637945
"11238","PRPF38B",345.712373016962,-0.0713253639364459,0.152996767449902,-0.466188698789344,0.641080437908555,0.892367084018822
"11239","SLC35G1",28.0132767702946,-0.134285780298745,0.28812586981677,-0.46606637711547,0.641167989256243,0.892409543192046
"11240","KLHL25",6.28644773128072,-0.269898293839319,0.579204777949773,-0.465980779362155,0.641229258543623,0.892415417384154
"11241","AC012306.3",7.06282875596812,-0.26714178153001,0.573538412040945,-0.46577836099832,0.641374155583845,0.892537667093504
"11242","ADCYAP1R1",22.4266321436596,-0.296228552924974,0.636344196822305,-0.465516232259588,0.641561815373686,0.892687461059007
"11243","AC006441.4",3.496018164525,0.410189133946084,0.881239138237808,0.465468584119322,0.64159592946918,0.892687461059007
"11244","GLI2",69.7795575535706,0.141379109216786,0.303847297839357,0.465296582270521,0.641719081965834,0.892719110170132
"11245","RNASEH2C",782.690562030627,-0.101058213431393,0.217200630739915,-0.465275874600953,0.641733909222481,0.892719110170132
"11246","CCDC15",31.7232095610578,-0.18846096049652,0.405397933028401,-0.464878937809772,0.642018154408617,0.892719110170132

"11247","AC011939.2",15.478006604766,-0.286000002487703,0.615115303373569,-0.464953482573349,0.641964769136533,0.892719110170132
"11248","AMFR",196.557084616309,-0.0663013762938111,0.142593752872695,-0.464966907442319,0.64195515510609,0.892719110170132
"11249","MYH3",4.92573507856819,0.353479839684647,0.760163515494608,0.465005005475239,0.641927872064312,0.892719110170132
"11250","COL6A1",11055.6652712027,0.132045855150599,0.283855344878635,0.465187136803983,0.641797449525013,0.892719110170132
"11251","AC092375.2",4.17698999241003,0.362018133858208,0.778940987777548,0.464756816676328,0.642105615521167,0.892761367309361
"11252","AC099661.1",5.1412783832916,0.332306549991753,0.716422418606163,0.463841640576062,0.642761207255628,0.893275903082781
"11253","BTN2A1",98.3718453861556,0.128307735703263,0.276503284166437,0.464036932111194,0.642621285649514,0.893275903082781
"11254","SCYL1",163.63550585313,-0.0863853561354608,0.186181079456667,-0.463985687415497,0.642657999993152,0.893275903082781
"11255","LATS2-AS1",4.12958639597,0.526216484738007,1.13435900696037,0.463888840754265,0.642727388321241,0.893275903082781
"11256","PITPNA",125.971264056351,-0.101062866524403,0.217838372141037,-0.463935097986186,0.642694245726436,0.893275903082781
"11257","MRPL51",901.698275543948,-0.13169690533867,0.284233837784304,-0.463339996269587,0.643120681212309,0.893457976572305
"11258","GALK1",197.013954711213,-0.124192808151631,0.267908926632595,-0.463563531505416,0.642960487476214,0.893457976572305
"11259","B3GNTL1",14.1961997013628,0.18291547764186,0.394765454532908,0.463352290686856,0.643111870140404,0.893457976572305
"11260","MIER2",26.2948598496648,0.145122389916425,0.313107874131438,0.463490068140239,0.643013132248883,0.893457976572305
"11261","CIAO1",307.384423832654,-0.0731127893597246,0.158109331963333,-0.462419190896841,0.643780739768626,0.89373998155845
"11262","UCHL1",75.8120378685759,-0.138358430103406,0.299009183222778,-0.462723012758846,0.643562920825493,0.89373998155845
"11263","CPSF4",80.1709713893642,0.0862350882203289,0.186471946327296,0.462456095508162,0.643754280120198,0.89373998155845
"11264","TTC26",35.8678859956658,0.136211878944929,0.294277474993976,0.462868858541475,0.643458370515832,0.89373998155845
"11265","GBGT1",32.6751006690761,-0.152542597715737,0.329717504577822,-0.462646342999155,0.643617884772143,0.89373998155845
"11266","PWP1",259.1425021352,-0.0745266085293414,0.161070737656183,-0.46269489799211,0.643583075853851,0.89373998155845
"11267","NUPR1",4058.11963043352,0.0982986095725588,0.212450638290032,0.462689170358548,0.643587181935249,0.89373998155845
"11268","ZNF57",12.2291394968507,0.215346742697501,0.465590587163224,0.462523832385825,0.643705715712185,0.89373998155845
"11269","AC016831.7",9.75692052098091,-0.272969781812474,0.590789363776902,-0.46204247833337,0.644050858723615,0.893906268669531
"11270","ARID4A",298.06739122385,0.0985464859548778,0.213290817252284,0.462028732527737,0.644060715941133,0.893906268669531
"11271","ZNF624",54.2733059592573,0.120484294921092,0.260781143450928,

0.46201306324038,0.644071952577784,0.893906268669531
"11272","NRAV",18.2996554258624,-0.17739692748997,0.384190552949904,-0
.461742034331339,0.644266323577122,0.894096708633509
"11273","IPPK",22.6563125666424,0.159237022091611,0.345083980372047,0.
461444260379551,0.644479903087057,0.89431376953702
"11274","MAPK13",52.0459875158606,0.12952244389277,0.280948790247849,0
.461017980460096,0.644785705072648,0.894579404385937
"11275","C18orf21",103.612890372921,-0.119338168945809,0.2588456470698
24,-0.461039891134878,0.64476998546609,0.894579404385937
"11276","NAF1",63.8033203683734,-0.109554357294127,0.237716413241023,-
0.460861561052788,0.644897931310925,0.894655759089819
"11277","LRRC41",202.207976779855,0.085827646908511,0.186370025615727,
0.460522804699707,0.645141006575563,0.894683269469565
"11278","MAP2K1",127.996329425034,0.091417106213035,0.198481715787679,
0.460582002983219,0.645098525999585,0.894683269469565
"11279","NAA38",752.4335470285,-0.131091882017176,0.284659251353814,-0
.460522120372744,0.645141497654066,0.894683269469565
"11280","ZNF652",276.129481911052,0.0887641359577587,0.19274967611929,
0.460515097845476,0.645146537084747,0.894683269469565
"11281","ZNHIT1",834.797755381721,-0.119410038003784,0.259463933611147
,-0.460218252077922,0.645359571262017,0.894698826831212
"11282","BEST1",30.7719812570635,0.166888708502052,0.362658200312764,0
.460181814055559,0.645385723359893,0.894698826831212
"11283","CLIP1",451.937419424349,0.11736567989805,0.255042604065793,0.
460180683646775,0.645386534677708,0.894698826831212
"11284","TCEAL8",597.365490008998,0.108221827272846,0.235160403359502,
0.460204293438815,0.64536958952822,0.894698826831212
"11285","AC124242.3",3.45576859703786,0.374363232491019,0.813850458129
325,0.459990196911005,0.645523256983486,0.894809065927573
"11286","NUDT16L1",116.29460752359,-0.120832192966837,0.26275693299135
1,-0.459863005673058,0.645614555466494,0.894856325639055
"11287","MINPP1",79.7108987571208,0.125461961867177,0.272940762600826,
0.459667367642937,0.645754995810304,0.894971684190714
"11288","BBS5",64.2004687021503,-0.100863173551866,0.219547701353059,-
0.459413480215247,0.645937269785856,0.894986422609401
"11289","GOLIM4",669.164704604597,0.0917525373006458,0.19967762711944,
0.459503343585722,0.645872751541504,0.894986422609401
"11290","HDHD3",58.4537180926924,0.139177934211289,0.30289697352295,0.
459489352410923,0.645882796460758,0.894986422609401
"11291","AL161669.3",49.6619948292862,0.161696116081358,0.352032834009
16,0.459321121384805,0.646003582444578,0.894999029331373
"11292","DDX20",42.8065997230914,-0.130643688380257,0.284836480064217,
-0.458662065866012,0.646476858937597,0.895536265864996
"11293","NUDCD3",158.030024094272,0.104378111700744,0.22762675901612,0
.458549390905977,0.646557786644903,0.895536265864996
"11294","RNMT",366.918282605836,0.0621323780990919,0.135499865135028,0
.458541992179666,0.646563100855287,0.895536265864996
"11295","PCDHGA9",12.7413248118861,-0.262020230342731,0.57207355479620
1,-0.458018428130409,0.646939201821235,0.89559200066853
"11296","CCAR1",271.349945753895,0.0979656962460263,0.213779285021263,

0.458256262931567,0.646768342582591,0.89559200066853
"11297","ANKRD49",116.358850010691,0.113949578341181,0.248773622318098
,0.458045259297942,0.646919925523552,0.89559200066853
"11298","HSPH1",157.406330230004,-0.107966894583004,0.235683997317381,
-0.458100235111049,0.646880430027034,0.89559200066853
"11299","AC020915.2",56.3244628941268,0.127519553352376,0.278422243102
996,0.458007779591097,0.64694685210985,0.89559200066853
"11300","IL17RA",106.682400599347,0.120967478944955,0.26411456528208,0
.458011389170301,0.646944258855847,0.89559200066853
"11301","CHST12",297.626056442651,-0.0852131147502524,0.18614627276762
2,-0.457775025431904,0.647114080279146,0.895664975916359
"11302","TLE4",98.9077367118385,-0.179143437601678,0.391334652239705,-
0.457775554953786,0.647113699810402,0.895664975916359
"11303","AC005726.5",5.75998114650545,0.291415659849432,0.636838092524
62,0.457597721100778,0.647241481060883,0.895702039917215
"11304","ZNF791",227.187040969307,0.0962335563127433,0.210310538795523
,0.457578383203648,0.64725537679628,0.895702039917215
"11305","AC121247.1",18.0163169839395,-0.234676874779878,0.51382785012
1095,-0.456722761766555,0.647870328252798,0.89577955642514
"11306","NCK1-
DT",22.9763214156585,-0.1495260742751,0.326964406749238,-0.45731606006
2091,0.647443887874076,0.89577955642514
"11307","DDX46",518.985139988242,-0.0897629019551682,0.196658321575715
,-0.456440903369597,0.648072957910418,0.89577955642514
"11308","MCM9",30.6423384244969,0.141163768269668,0.309160577639329,0.
456603391504694,0.647956141052058,0.89577955642514
"11309","TAB2",156.297602932369,0.0832522205394074,0.182338437705552,0
.456580749440481,0.647972418489007,0.89577955642514
"11310","TAF3",148.373173549004,-0.108501599108664,0.237810978962671,-
0.456251429525866,0.648209186423853,0.89577955642514
"11311","NEU3",13.6133208708814,0.1915727815951,0.419248246847354,0.45
694354844816,0.64771162162754,0.89577955642514
"11312","KLHL35",16.5073826271031,0.194170970205902,0.42551564921903,0
.456319222482825,0.648160443072398,0.89577955642514
"11313","PAN2",46.2833347216056,0.166649522463192,0.364535875855943,0.
457155340532377,0.647559395574499,0.89577955642514
"11314","LINC00519",21.9872204983729,0.228646163261893,0.5001539230282
02,0.45715159420833,0.647562088125593,0.89577955642514
"11315","AL138976.2",2.70648100868629,0.482239083432673,1.057196195712
33,0.456149090763369,0.648282771175058,0.89577955642514
"11316","ZNF770",140.510600888502,0.106915582990753,0.233927432872379,
0.457045938041315,0.647638027029458,0.89577955642514
"11317","GPATCH8",314.975277821085,0.0865276666265601,0.18929226469585
,0.457111476613112,0.647590921658548,0.89577955642514
"11318","SUGP2",251.848289721281,0.142770351565114,0.312711568872829,0
.456556027267333,0.647990191513679,0.89577955642514
"11319","COX7A1",788.446635892164,-0.136403896667693,0.299035503788794
,-0.456146159701604,0.648284878750177,0.89577955642514
"11320","EMC10",237.945670954708,0.0996236460821621,0.218205147758565,
0.456559559229059,0.647987652337766,0.89577955642514

"11321","STARD8",15.7017059633023,0.197054388741818,0.431663543626678,
0.456499956160856,0.648030502352551,0.89577955642514
"11322","CEP57",305.767552938688,0.0706923150053439,0.155177919647298,
0.455556532566101,0.648708906446405,0.896173391503049
"11323","TMEM263",314.646802840946,0.0957694089051136,0.21021572647862
9,0.455576804406447,0.648694326152264,0.896173391503049
"11324","MAPKBP1",31.6641750798292,-0.1574023223646,0.345607587619566,
-0.455436535548125,0.648795215715073,0.896173391503049
"11325","C15orf40",257.243678881192,-0.10546333100126,0.23160859214580
4,-0.455351548162205,0.648856346747013,0.896173391503049
"11326","LINC00632",384.570626850314,0.0936300354734829,0.205598705285
139,0.455401873001243,0.648820148038344,0.896173391503049
"11327","CNOT8",129.437268119638,0.0882587609292401,0.193889767000164,
0.45520071685457,0.648964844830871,0.896244112976897
"11328","C5orf22",30.4033739069235,-0.15833859071844,0.348054600328122
,-0.454924573814481,0.649163503204074,0.896254218882294
"11329","CFB",864.577237094355,-0.196784669174649,0.432539172080065,-0
.45495224912999,0.649143592349336,0.896254218882294
"11330","AL157938.3",18.9388056398561,-0.162581686328343,0.35748553387
5483,-0.454792350800332,0.64925863378982,0.896254218882294
"11331","AL158063.1",2.76633836756677,0.383705201774218,0.843425975184
155,0.454936429590562,0.649154973599968,0.896254218882294
"11332","AC005899.6",15.8221120583553,-0.207656998696552,0.45657443423
3201,-0.45481521330756,0.649242184476431,0.896254218882294
"11333","GMFB",128.064711878558,0.0860116933631885,0.18919867168187,0.
454610450478288,0.649389515091756,0.896355791456837
"11334","PIGZ",46.4588739871039,-0.120355113806011,0.26486124072412,-0
.454408177946174,0.649535067358632,0.896424394561289
"11335","AC244517.1",4.74600684802085,0.347302902649712,0.764478495195
442,0.454300421571601,0.649612612684873,0.896424394561289
"11336","ACTR6",133.0500022153,0.0953293095970138,0.209873433797721,0.
454222851706393,0.649668437073536,0.896424394561289
"11337","B9D1",52.0073515595132,0.109254405592482,0.240511726319206,0.
45425812397804,0.649643052577727,0.896424394561289
"11338","AL589935.1",2.85709580466234,-0.367613141223934,0.81049564564
52,-0.453565843566368,0.650141342300109,0.896997796577933
"11339","RAB43",35.1953647472325,-0.13735168192316,0.302984912363871,-
0.453328454052547,0.650312246608083,0.897042566058492
"11340","RBP1",29.4370395734681,-0.185774657584159,0.409974104968053,-
0.453137540476209,0.650449704745691,0.897042566058492
"11341","ATP8A1",21.8065808546381,-0.204519045215249,0.451681600505908
,-0.452794723066374,0.650696563778857,0.897042566058492
"11342","UTP15",34.3485958121599,-0.127615410817439,0.281444156923484,
-0.453430663519277,0.650238660486848,0.897042566058492
"11343","DOLK",57.1272321272111,-0.155564268115163,0.343475279205754,-
0.452912560330068,0.650611706142337,0.897042566058492
"11344","ABCC2",168.784378909906,0.330088700201311,0.728564422858531,0
.453067278396883,0.650500296570166,0.897042566058492
"11345","IFITM1",459.401072346034,-0.320563262122962,0.70802210259628,
-0.452758834713596,0.650722408801875,0.897042566058492

"11346","IRF2BPL",238.880100319894,-0.0946592360462566,0.2088820781312
94,-0.45317069273295,0.650425834204522,0.897042566058492
"11347","MLST8",85.3798669603814,-0.129058838890524,0.285071559870892,
-0.452724357873421,0.650747237718581,0.897042566058492
"11348","SMOX",30.5250112575273,-0.16757089618578,0.369927461639435,-0
.452983121185824,0.650560895627728,0.897042566058492
"11349","ARHGEF10L",115.677137089263,-0.0880063625048804,0.19483569602
1544,-0.451695270948447,0.651488525531654,0.897104569111891
"11350","TRAPPC12",184.491376794666,-0.0816877948739888,0.180867676729
448,-0.451643966191824,0.651525491192433,0.897104569111891
"11351","POLR1B",25.20388117051,0.149013351677942,0.3297583482186,0.45
1886517757422,0.65135073757913,0.897104569111891
"11352","HHIP-
AS1",32.8901448672228,-0.460145877806595,1.0179626873063,-0.4520262712
42042,0.651250056649691,0.897104569111891
"11353","RCL1",56.6622143098164,-0.112244031455429,0.248386445966176,-
0.451892739230681,0.651346255382406,0.897104569111891
"11354","FAM225A",11.4738529980207,0.195091067677707,0.431607479110396
,0.452010396297621,0.65126149292564,0.897104569111891
"11355","MAP6",6.98590556338574,0.330039913808355,0.730097527841951,0.
452049077311492,0.651233627349754,0.897104569111891
"11356","RSRC2",732.529720393846,0.0711464800817986,0.157533805982597,
0.45162674537082,0.651537899181879,0.897104569111891
"11357","ATP6V0A2",50.3036570903812,0.133021107776905,0.29447523033539
,0.451722569757062,0.651468856778666,0.897104569111891
"11358","DUOX1",6.68209103249513,-0.297000996445228,0.657442695161774,
-0.451751914852664,0.651447713949295,0.897104569111891
"11359","MORF4L1",2041.40825894492,-0.0671455559725068,0.1487010258205
46,-0.451547362245763,0.651595097759337,0.897104569111891
"11360","PELP1",88.181922642681,-0.111362241074693,0.246314583765843,-
0.452113875565559,0.651186948158771,0.897104569111891
"11361","TTYH1",3.32959503992204,0.440472961434859,0.974322343013215,0
.452081351303759,0.651210377727582,0.897104569111891
"11362","HSPA13",93.3515170825527,-0.0846063802804322,0.18700059184044
6,-0.452439104324444,0.650952681404783,0.897104569111891
"11363","GINS4",6.85207463162264,0.257902279412647,0.571319730978476,0
.451414970337097,0.651690495753612,0.897156950195701
"11364","RHCE",2.72773965707512,-0.398609982468408,0.883525108909256,-
0.451158635390121,0.651875219922202,0.897332283108325
"11365","AL049840.2",45.9899352680258,-0.153412175354196,0.34010642410
4609,-0.451071089756923,0.65193831334422,0.897340170316201
"11366","TM2D1",524.836099925132,0.0827955763487207,0.183610820968267,
0.450929721418924,0.652040201609476,0.897401449390906
"11367","SMAP2",102.20266214808З,0.11757489450925,0.26148059153959,0.4
49650560360798,0.652962425205582,0.897616577421397
"11368","Z99127.4",22.5669246555153,0.236146359354303,0.52528068737353
8,0.449562234117271,0.653026124466695,0.897616577421397
"11369","LINC02767",34.7453937221635,0.189579662867861,0.4220458357714
06,0.449192118010954,0.653293072892212,0.897616577421397
"11370","C1orf35",191.436987008056,-0.0757572631286543,0.1686449764776

81,-0.449211501646361,0.653279091229165,0.897616577421397
"11371","CCDC138",19.9735694170126,-0.146030951139595,0.32500094006676
,-0.449324703828851,0.653197439485186,0.897616577421397
"11372","MTLN",343.158422845366,-0.145957912551571,0.324789073190473,-
0.449392927901776,0.653148232063839,0.897616577421397
"11373","FASTKD3",32.4342470855849,-0.141934399900765,0.31540300652024
5,-0.450009660550443,0.652703474813167,0.897616577421397
"11374","BRAT1",96.3230142996391,-0.0862700627226155,0.191491467899796
,-0.45051648341721,0.652338071471491,0.897616577421397
"11375","AC008267.5",30.5323325574238,0.205135101256317,0.456397459276
446,0.449465914165101,0.653095591526502,0.897616577421397
"11376","KCTD9",164.790962829953,0.0975714947050164,0.21669010649875,0
.450281262405395,0.652507648050972,0.897616577421397
"11377","EFCAB1",2.6595859534453,-0.439413287113739,0.975610118798078,
-0.450398451847837,0.652423161070522,0.897616577421397
"11378","RIDA",93.5935271310686,-0.11848450835726,0.263617555759546,-0
.449456061512584,0.653102697542632,0.897616577421397
"11379","AZIN1-
AS1",58.4847992569397,-0.11187700918172,0.248717195811996,-0.449816140
844107,0.652843018425529,0.897616577421397
"11380","CD3G",9.49081175090199,0.24789954647887,0.550884132539001,0.4
50003062052834,0.652708232672147,0.897616577421397
"11381","AC007541.1",50.656970220103,-0.120322374937477,0.267258234015
753,-0.450210169877816,0.652558903927668,0.897616577421397
"11382","AC135178.6",3.18831028418433,-0.360883557273473,0.80147150633
429,-0.450276216211422,0.652511286172432,0.897616577421397
"11383","POU2F2",6.1359183819752,-0.29628304131288,0.658894816174635,-
0.449666675225978,0.652950803734296,0.897616577421397
"11384","HDAC10",56.5041166480558,-0.109189423835481,0.24288577110741,
-0.449550516432659,0.653034575235212,0.897616577421397
"11385","KCNE5",7.15297108731468,-0.32511836236944,0.723505639957042,-
0.449365346189621,0.653168125519034,0.897616577421397
"11386","TENM1",82.7651425397828,0.261535201413898,0.582326393748473,0
.449121324778667,0.653344137986321,0.897616577421397
"11387","RNASEL",92.6039227043867,0.0898412372166216,0.200199459506771
,0.448758640197942,0.653605777753163,0.897897179361792
"11388","SELENON",250.068319494521,0.0985859193722896,0.22039121677406
5,0.447322360733437,0.654642324236659,0.89812987834009
"11389","CEP85",8.78963072037178,-0.230109127654822,0.5134494458995,-0
.448163162882958,0.654035445977452,0.89812987834009
"11390","ZMYM1",102.163112003387,0.0962842136079356,0.215424745815801,
0.446950573126189,0.65491074771479,0.89812987834009
"11391","AP4B1",34.7623875158426,0.122769849829017,0.274270338596569,0
.447623503355215,0.654424937794121,0.89812987834009
"11392","ACBD6",324.234907356754,-0.0816962020309984,0.182701987567999
,-0.447155518768466,0.654762775396548,0.89812987834009
"11393","NPRL2",82.1507593690704,-0.106712880664089,0.238325121644889,
-0.447761779906248,0.654325129631589,0.89812987834009
"11394","GTPBP8",107.563757780507,-0.103248068189609,0.231005811298799
,-0.446950090169205,0.65491109642943,0.89812987834009

"11395","AC099522.2",5.95790237440381,0.28000922283865,0.627206818948339,0.446438422509742,0.655280583619302,0.89812987834009
"11396","CASP8AP2",171.042067642535,0.0810900535462943,0.181520047360207,0.446727811751723,0.655071598501177,0.89812987834009
"11397","UFSP1",4.64000737592827,0.298411670457623,0.666051366896222,0.448031015758156,0.654130812636335,0.89812987834009
"11398","SELPLG",5.97558758428528,0.316781707078645,0.708319017818083,0.447230272108836,0.654708806288872,0.89812987834009
"11399","AC068790.7",5.35609062981983,0.30287268791826,0.677017088111897,0.447363431790072,0.654612674463666,0.89812987834009
"11400","RFXAP",84.9820802393768,-0.0901527431867536,0.201797454768168,-0.446748663358139,0.655056541363959,0.89812987834009
"11401","PARP2",79.1545667048754,0.135196348311511,0.30220243547781,0.447370148085514,0.654607825927422,0.89812987834009
"11402","AL096870.8",60.0891818780281,-0.155591438927138,0.347700779803185,-0.447486597571653,0.65452376262157,0.89812987834009
"11403","SRSF5",1633.03416401673,0.0794505734293796,0.177990069404156,0.446376439401088,0.655325348806098,0.89812987834009
"11404","ZNF75A",68.0337733940137,0.165157443878663,0.36859471616709,0.448073281125915,0.654100310360297,0.89812987834009
"11405","CCR10",16.4278510535025,0.202106194236127,0.451945627075299,0.4471913923452,0.654736875794647,0.89812987834009
"11406","PLCD3",115.987145422435,-0.0979213809409275,0.218829858827565,-0.447477238552204,0.654530518609005,0.89812987834009
"11407","FAM104A",131.335609707262,-0.0901538681177365,0.201178942382228,-0.448127756564351,0.654060997113176,0.89812987834009
"11408","AC027601.6",20.1429950583423,-0.208578513517129,0.465958684335026,-0.447633063894481,0.654418036787823,0.89812987834009
"11409","BRD4",490.210327762739,0.0928192259929538,0.207309860145589,0.447731844147544,0.654346736770227,0.89812987834009
"11410","ZNF526",35.008139540043,0.147814935297582,0.331048375454912,0.446505544981035,0.65523210810011,0.89812987834009
"11411","NAPA-AS1",23.1499758134416,0.206142548691853,0.461760101370588,0.446427805434,0.655288251352334,0.89812987834009
"11412","AL117335.1",13.068376120947,0.218245896308257,0.488687962898006,0.446595604716801,0.655167069683504,0.89812987834009
"11413","MMP24OS",373.654979039976,-0.120077803088935,0.26859071086334,-0.447066105536431,0.654827330765472,0.89812987834009
"11414","CDKL5",70.0307870168232,0.105865135662548,0.237053195653268,0.446588097539903,0.655172491039616,0.89812987834009
"11415","KIAA0232",155.922039098197,-0.0869195220035467,0.195287017708037,-0.445086022735497,0.656257589343165,0.898661600360634
"11416","TUBB",2234.98069364911,0.123300237603451,0.27705277668055,0.445042417840913,0.656289100352023,0.898661600360634
"11417","SEC61A2",28.5206523654507,-0.160175545028201,0.359804196917044,-0.445174198635406,0.656193871035778,0.898661600360634
"11418","MNAT1",212.230709890034,-0.0740293934705977,0.166150605126587,-0.445555966613521,0.655918023956657,0.898661600360634
"11419","ENTPD5",65.3265674822073,0.131243921598784,0.294953547244927,

0.444964716731481,0.65634525245287,0.898661600360634
"11420","TRAF7",136.760186642962,-0.0978655715533581,0.219756378480983
,-0.445336659758557,0.656076478752055,0.898661600360634
"11421","AC022916.1",2.68662158311313,0.407248767274303,0.915203677479
209,0.44498156781451,0.656333074550501,0.898661600360634
"11422","PLPPR2",90.9098350340246,-0.109550872865419,0.246163382762691
,-0.445033179329639,0.65629577662625,0.898661600360634
"11423","BAX",264.764529729034,-0.12570780069824,0.282237991155243,-0.
445396454898571,0.656033273703676,0.898661600360634
"11424","SLC25A18",15.0869683947802,0.215068375969268,0.48310110369647
8,0.445182953058187,0.656187544987867,0.898661600360634
"11425","DIAPH2",374.266036840908,-0.0937015226995433,0.21036244466582
5,-0.445428949299361,0.656009795318907,0.898661600360634
"11426","CD151",1089.05154658133,-0.138822308404085,0.312063213916325,
-0.444853165042735,0.656425870676516,0.898693321809271
"11427","RRM2B",75.2163335442234,-0.0951882566897505,0.214033588213045
,-0.444735134725685,0.656511175350241,0.898731453225153
"11428","SELENBP1",739.272444423458,0.110228623424368,0.24849202973525
9,0.443590176883359,0.657338909036325,0.899603089165126
"11429","PARP10",118.161322318066,0.0966299379209817,0.217789026245544
,0.443685981735542,0.65726963192227,0.899603089165126
"11430","PPP3CB",275.408086925023,0.0689564620782182,0.155515669067627
,0.443405236859008,0.657472648702163,0.899603089165126
"11431","OAT",438.284603631078,0.078107046805011,0.1761522613175,0.443
406438389283,0.657471779778278,0.899603089165126
"11432","AC026471.2",6.55760363380487,-0.328671764238791,0.74129157716
816,-0.443377173519716,0.657492943708041,0.899603089165126
"11433","AC132872.1",29.6358705536795,-0.143300023516662,0.32312108494
6902,-0.443487070923305,0.657413468918446,0.899603089165126
"11434","LSG1",107.148284656423,-0.0875044799345453,0.197494405413607,
-0.443073208839953,0.657712782797871,0.899745599737899
"11435","WDR41",171.036346662508,-0.0662313815405046,0.1495351744965,-
0.442915065057518,0.657827170248824,0.899745599737899
"11436","SEPTIN7",1916.44763091993,-0.0601244627488527,0.1357454710601
29,-0.442920579812349,0.657823181220213,0.899745599737899
"11437","ORMDL2",111.618605331801,-0.133217151076269,0.300761837120228
,-0.442932362535795,0.657814658367913,0.899745599737899
"11438","RNF180",13.2611312943366,0.231580159176668,0.523001637059414,
0.442790505358132,0.657917271537412,0.89978121735642
"11439","TRIM39",30.6513796736798,0.134618711321043,0.304071876943328,
0.442720032757692,0.657968250676986,0.89978121735642
"11440","RUFY1",171.577479995936,0.0740802454740713,0.167432603725125,
0.442448148245304,0.658164943983443,0.899971522616521
"11441","LRRCC1",88.2631312717883,0.103927834216554,0.235039494433871,
0.442171791029759,0.658364897291241,0.900166251929628
"11442","LINC00578",5.25118804479687,0.342738875109986,0.7758928376439
19,0.441734809862079,0.658681117040414,0.900243504121618
"11443","Z99289.1",3.93929661786753,0.455829464873028,1.03179687427266
,0.441782172672654,0.658646840173293,0.900243504121618
"11444","CWF19L2",352.824424025928,0.119984722682059,0.27164543066272,

0.441696082976028,0.658709144548746,0.900243504121618
"11445","LINC00645",13.7984414730863,-0.308303550354372,0.697680752501871,-0.441897743701257,0.658563203451479,0.900243504121618
"11446","CCDC157",3.83275263227367,0.385506917096053,0.872411750051637,0.441886433869368,0.658571387989478,0.900243504121618
"11447","DGLUCY",120.667772900825,0.100519512117501,0.22774950446095,0.441359959730391,0.6589524245484,0.900382465513899
"11448","GSPT1",356.451741971784,-0.0706546080410351,0.160058189951353,-0.441430757542049,0.658901179355973,0.900382465513899
"11449","ZNF846",53.27209776062,-0.155790877798347,0.353013517266085,-0.441317032290632,0.658983497261946,0.900382465513899
"11450","NMNAT2",4.41986468964949,-0.360813163727583,0.818802546904341,-0.440659552283654,0.659459482885821,0.900722295366548
"11451","KCNT2",63.5418693322574,0.209605421723739,0.475648243836109,0.440673174851374,0.6594496193665,0.900722295366548
"11452","DNAJC27-AS1",16.2915225951399,0.211322897915416,0.479998129681102,0.440257752787107,0.659750435358301,0.900722295366548
"11453","HNRNPD",731.754039048083,-0.0672316803773724,0.152621624813653,-0.440512151927752,0.65956621297264,0.900722295366548
"11454","CPVL",299.013382751409,-0.150405856874515,0.341171003796067,-0.440851816833823,0.659320277818768,0.900722295366548
"11455","EXOSC1",156.348664894567,0.109494397939542,0.248535519890572,0.440558347506029,0.659532762795595,0.900722295366548
"11456","MS4A2",4.15606140233315,0.456486240819604,1.03647637928561,0.440421267616573,0.659632024208261,0.900722295366548
"11457","M6PR",271.237540513709,-0.0655893588028274,0.148966083029092,-0.440297264109564,0.659721821997192,0.900722295366548
"11458","TCTN1",217.192780301443,-0.103636426532511,0.235362927699203,-0.440326042616871,0.659700981453493,0.900722295366548
"11459","MAP11",10.848601502707,-0.209990082657869,0.477174361635686,-0.440069918966418,0.659886467906812,0.90082939326872
"11460","AC073475.1",5.94987201564606,-0.281265222800139,0.639733298442558,-0.439660126313395,0.660183285958379,0.900998703738171
"11461","VIRMA",193.763322364387,0.0737206496026859,0.167652173358802,0.439723793170948,0.66013716773029,0.900998703738171
"11462","DDB2",110.658741254063,0.117002254026197,0.266050158599562,0.439775171125909,0.660099952130134,0.900998703738171
"11463","HBP1",471.517066708367,0.0820893487919538,0.18689979615805,0.439215828371133,0.660505157057229,0.901218423852758
"11464","HOXC-AS3",35.5833380093489,-0.12064458285056,0.274612984636618,-0.439325849832639,0.660425446290827,0.901218423852758
"11465","PPP1R36",3.44160204598074,-0.333192022538516,0.758635099072693,-0.439199323819566,0.660517114969755,0.901218423852758
"11466","ZNF446",34.9642036396473,0.137594757524203,0.313345891641633,0.439114605279609,0.660578496789426,0.901223567528082
"11467","MCPH1",98.2814548237511,0.100418923650118,0.228817534434119,0.438860264351947,0.660762790228179,0.901396383320782
"11468","SFT2D1",477.355305196332,0.10595017446276,0.241611123235672,0

.438515301132948,0.661012780738328,0.901580166456506
"11469","AC093249.6",7.3257134983768,0.254167206904625,0.579515357702575,0.438585800231841,0.660961687851704,0.901580166456506
"11470","PDCD10",309.211612279577,-0.0819352420329738,0.18692907288355,-0.438322625630399,0.661152426716455,0.901692014919399
"11471","FAM189B",43.355700086147,0.132592771291669,0.302723477559742,0.437999630423451,0.661386551337164,0.901713453338227
"11472","AL139243.1",6.23287606594609,-0.251767409241957,0.574668408363071,-0.438109013090019,0.661307261077742,0.901713453338227
"11473","NRIP3",5.66892886902805,-0.262529526935067,0.599405958148253,-0.437982845125699,0.661398719146124,0.901713453338227
"11474","AP002748.4",9.97560776639068,-0.234424099740033,0.535158058558895,-0.438046472422192,0.661352595636278,0.901713453338227
"11475","GPR146",67.274037686438,-0.152944083674717,0.349282307791943,-0.437880992717848,0.661472554764407,0.901735527161623
"11476","UFL1",396.036187744671,-0.0567322311402194,0.129675077258285,-0.437495256141018,0.661752215689705,0.901959563477743
"11477","DNAJC28",6.05890874804895,0.253564041515713,0.579501390293459,0.437555536126165,0.661708509289967,0.901959563477743
"11478","SCOC",308.425622566003,-0.107025713107581,0.245227726458886,-0.436433981805578,0.662521888296579,0.902055879239727
"11479","POLR1C",60.0861902294808,-0.117755768165444,0.269652630590586,-0.436694305216081,0.662333059783693,0.902055879239727
"11480","C7orf26",73.9913389139208,0.0893942259371742,0.204724472818772,0.43665627614686,0.662360643258081,0.902055879239727
"11481","AGBL3",13.0121398858098,0.183045548131271,0.418907096290484,0.436959769247598,0.662140524568754,0.902055879239727
"11482","C8orf58",36.5850019152336,-0.135451806529092,0.310343264489734,-0.436458019321934,0.662504451514581,0.902055879239727
"11483","MLLT10",162.583951145754,0.112739245059601,0.258070690817903,0.436854122032596,0.662217145492126,0.902055879239727
"11484","MRPL17",209.828523133181,-0.118519437842181,0.271467678374194,-0.436587657698287,0.662410415164883,0.902055879239727
"11485","SBF2-
AS1",32.5394852441327,0.122939623938306,0.281736291055087,0.436364174022109,0.662572527805694,0.902055879239727
"11486","ACSM3",5.29685240365931,0.29936560492907,0.685152931346499,0.436932531749869,0.662160278297979,0.902055879239727
"11487","RPS6KB1",142.137543188333,0.0936930216389252,0.214329353247632,0.437145077047258,0.66200613813813,0.902055879239727
"11488","EMID1",14.7136048163243,0.273110307422389,0.625343389740286,0.436736538521365,0.662302427404229,0.902055879239727
"11489","DENND6B",24.9921701739336,-0.146674057693983,0.3357532914984,-0.436850691885716,0.66221963327454,0.902055879239727
"11490","MAFIP",6.23537862758592,0.296634066131106,0.679182069075749,0.436751910330575,0.662291278166285,0.902055879239727
"11491","HOMEZ",26.4134012429651,0.153036327414059,0.350838444799841,0.436201703896415,0.662690391802232,0.902137829515475
"11492","NOP14",80.7497203907767,0.109610187013928,0.251418078195734,0.435967802317678,0.662860090434543,0.902290323239432

"11493","NSMCE2",151.098736635591,-0.0928145221390736,0.21305322173506
8,-0.435640078019981,0.6630978877763,0.902535478855361
"11494","BBX",624.735405872272,-0.0566636029420867,0.130147334958193,-
0.43538043218702,0.663286311367957,0.902713395574122
"11495","TMEM33",139.079691654543,-0.078086321272887,0.179480345196441
,-0.435068927393814,0.663512396836854,0.902863989537135
"11496","AP000254.2",8.05517886534603,0.231753884183898,0.532627506647
151,0.435114374101276,0.663479410394149,0.902863989537135
"11497","PUM3",221.413220262904,0.0790464179331345,0.18172981807484,0.
434966692700817,0.663586603910771,0.902886426457006
"11498","TNFAIP3",200.959967795253,-0.159189753507612,0.36611628192059
,-0.434806539257219,0.663702857955853,0.902955927946958
"11499","PHB",612.280711460472,-0.118095515932231,0.271648003278972,-0
.434737286881328,0.663753130183601,0.902955927946958
"11500","LYSMD1",27.815709690293,0.194681570845975,0.447997359050111,0
.434559639500462,0.66388209628399,0.903006598024845
"11501","PPP2R1A",432.136619120134,0.0914438189520265,0.21044452825747
1,0.434526949734461,0.66390582905349,0.903006598024845
"11502","TPRG1L",198.507903211118,-0.0729734452792925,0.16806622233095
6,-0.434194594649681,0.664147137972254,0.9030223078435
"11503","AC233266.2",7.33247102383003,-0.267299888899356,0.61554628104
2367,-0.434248239542136,0.66410818633559,0.9030223078435
"11504","RBM47",46.4989200583922,0.134576192292781,0.310002311660595,0
.434113512160261,0.664206013811118,0.9030223078435
"11505","TAF11",213.515063363888,-0.0892812121946296,0.205592313272791
,-0.434263376744861,0.664097195354184,0.9030223078435
"11506","PARP12",35.1381570542558,-0.15276277988961,0.351886494109531,
-0.434125158102998,0.66419755729988,0.9030223078435
"11507","UQCC3",264.241864895043,-0.12304740354393,0.283501302038368,-
0.43402764875936,0.664268363437793,0.903028592096758
"11508","SYDE2",133.234447963514,0.117727664687984,0.271498220843888,0
.433622232668984,0.664562787325996,0.903221888844419
"11509","TMEM167B",184.880021202414,0.0905126950586734,0.2087368003071
64,0.433621167544394,0.66456356091584,0.903221888844419
"11510","PPM1A",223.461535932711,0.0867104674690358,0.200017759119118,
0.433513843225274,0.664641511378131,0.903221888844419
"11511","LINC00910",29.4209003023792,0.143307150133732,0.3305453801374
53,0.433547581497402,0.664617006625775,0.903221888844419
"11512","PFKM",91.327811277922,-0.110981726372192,0.256211849361566,-0
.43316390966592,0.664895695948553,0.903410351057345
"11513","NXT2",41.1413092280918,0.108421658612672,0.250299562253318,0.
433167591811219,0.664893021112988,0.903410351057345
"11514","LAMTOR5-
AS1",6.50884662842338,0.306045750616357,0.707147202351896,0.4327893111
9077,0.665167839309407,0.903533878413265
"11515","CDKAL1",69.990436698119,0.0944506375948209,0.218311672731596,
0.432641261976603,0.665275408269768,0.903533878413265
"11516","STAU2",103.228097652712,0.0788107367409101,0.182134677076301,
0.432705830685356,0.665228493368128,0.903533878413265
"11517","LRFN3",46.612593275767,-0.135970495139779,0.314247351289383,-

0.43268620907028,0.665242750075563,0.903533878413265
"11518","OSBPL2",91.5082836978472,0.118139646461322,0.273015661688041,
0.432721133032702,0.665217375045812,0.903533878413265
"11519","CD2BP2",251.753393205886,-0.103221752941682,0.238687727859334
,-0.43245521614129,0.665410594420966,0.903639024961123
"11520","SP140",2.96365745758602,0.380506665994388,0.880294906906618,0
.4322490826756,0.665560389512847,0.90376399072478
"11521","BTG2",556.317098225787,0.169225621022601,0.391690837512105,0.
432038753057049,0.66571324766409,0.903893093760034
"11522","NFYC",139.217198755371,-0.0749402233525373,0.173656307289932,
-0.431543342836487,0.666073344517408,0.903989703998422
"11523","RPP40",30.1263297458057,-0.173091015449287,0.401023778220825,
-0.431622823507423,0.666015567531569,0.903989703998422
"11524","RPL7L1",549.210667899549,-0.0666721963338996,0.1544193727989,
-0.431760569450873,0.665915440390886,0.903989703998422
"11525","ITGB1-
DT",4.50006350610472,-0.361794882845234,0.837876343857969,-0.431799853
877439,0.665886885742414,0.903989703998422
"11526","ZNHIT2",35.8612460951788,-0.14877703303574,0.344699379424136,
-0.431613869697969,0.666022076236674,0.903989703998422
"11527","SPIN2A",53.0405790264658,0.126633725091005,0.293542515439525,
0.431398241925518,0.666178828019498,0.904054429314566
"11528","ITGA9",54.7520774291746,0.148806488464935,0.345191231902251,0
.431084206991309,0.666407143505338,0.904207385363345
"11529","SNRPA",198.084371475393,-0.0936402228758511,0.217214071170195
,-0.431096486389598,0.666398215329272,0.904207385363345
"11530","NFYC-
AS1",5.83943599529833,0.267329834204861,0.620381004590204,0.4309123461
66123,0.666532106029109,0.904298502568374
"11531","FBXO28",79.9400232810376,0.0858098376527195,0.199325283241504
,0.430501521218218,0.666830860364983,0.904625370626089
"11532","CUL3",243.562616618828,0.0772511502759318,0.179640766387646,0
.430031288717794,0.667172881079629,0.904932400826206
"11533","PRPF6",355.651054649635,0.0794379014252656,0.184706272910175,
0.43007690087438,0.667139702328066,0.904932400826206
"11534","SLC35G2",66.0297796885167,0.11626604095285,0.270904988202185,
0.429176449368577,0.667794820340108,0.905237733452485
"11535","CERT1",362.536684637387,0.0874779388614883,0.203911732626369,
0.428999046473583,0.667923918608822,0.905237733452485
"11536","AL133338.1",12.1154827772438,0.182225163013151,0.424614820493
437,0.429154033769689,0.6678111319047,0.905237733452485
"11537","AL450326.1",34.7439487413599,0.115475301057695,0.268868103559
696,0.429486798652768,0.66756899898811,0.905237733452485
"11538","ZFP91",341.783466396654,-0.0919821326193932,0.214279162757166
,-0.429263076427236,0.66773178433536,0.905237733452485
"11539","TSKU",209.602992363649,0.159446852224636,0.371678875778279,0.
428990891372994,0.667929853412069,0.905237733452485
"11540","TK1",3.25484750118428,-0.40413607071869,0.942203436274334,-0.
428926551485237,0.667976676931665,0.905237733452485
"11541","KHSRP",300.761162062746,0.0750803562058868,0.174986848667084,

0.429062851167341,0.667877486016787,0.905237733452485
"11542","ILF3",322.906524528707,0.0743256564955452,0.173237375378206,0
.42903938213841,0.667894564986257,0.905237733452485
"11543","Z68871.1",9.55572595445853,0.210351286495717,0.48985925055688
3,0.429411685614969,0.667623651188161,0.905237733452485
"11544","VPS25",257.31290630656,-0.109705466550913,0.255832405573182,-
0.428817710974193,0.668055888836711,0.905266655325075
"11545","HAT1",201.254994233592,-0.0959646484772613,0.223911199535884,
-0.428583512911251,0.668226345947988,0.905307946423198
"11546","ZNF852",10.8114149632515,0.204234904840028,0.476690027323836,
0.428443837993873,0.668328014127217,0.905307946423198
"11547","ASH2L",121.027287700354,0.0749396536062663,0.17488726891052,0
.428502623850847,0.668285223662501,0.905307946423198
"11548","GNAO1",8.17465744237795,-0.265650016221074,0.619829409616625,
-0.428585691642775,0.668224760116125,0.905307946423198
"11549","SNPH",7.26719320268013,0.251254160177438,0.586524023157455,0.
428378293569039,0.668375725451737,0.905307946423198
"11550","MITF",316.382050666386,0.142210941811493,0.332040721988851,0.
42829367723236,0.668437321649367,0.905312988966324
"11551","GID4",79.2339225434401,0.0993663247383604,0.232105182393724,0
.428109031059046,0.668571742003818,0.905338275637615
"11552","GNG7",5.75933136774755,0.304456515486553,0.711158955437967,0.
428113170984472,0.668568728067822,0.905338275637615
"11553","ABHD6",30.336951760075,-0.115279581921909,0.2695506959409,-0.
427673100674109,0.668889136612411,0.905376201784887
"11554","HTT",168.256542958184,-0.122795415914487,0.286972979208033,-0
.427898878331225,0.668724743774584,0.905376201784887
"11555","PLIN3",422.263097557002,-0.0974959024366052,0.227905322642758
,-0.427791248164178,0.668803109285533,0.905376201784887
"11556","XRN2",396.800784977151,-0.0598844135776519,0.140001857795667,
-0.427740135170586,0.668840325913375,0.905376201784887
"11557","FGF16",34.2446909842388,0.185833067917085,0.434382036280362,0
.427810204833477,0.668789306675211,0.905376201784887
"11558","TRIM35",31.0174494371882,0.137089871703246,0.320722069090187,
0.427441342256669,0.669057900661763,0.905435003039561
"11559","PHF23",67.6693243688348,-0.103314885593342,0.241706363546043,
-0.42743965892177,0.669059126509753,0.905435003039561
"11560","RASSF2",101.128369504089,-0.189416333182416,0.443208747683242
,-0.427374987909288,0.669106222280721,0.905435003039561
"11561","WASF2",1803.77575592349,0.048662728663414,0.11390121688781,0.
427236249032755,0.669207261341372,0.905493399287525
"11562","HSPA6",4.90239784746827,-0.561644732773081,1.31851798008561,-
0.425966684759667,0.67013212211868,0.905615299956608
"11563","AL445524.1",20.080266482031,-0.177762257833834,0.416823051582
707,-0.426469354703053,0.669765873819253,0.905615299956608
"11564","NDUFB3",475.313549661265,-0.120505398230365,0.282516186432746
,-0.42654334164691,0.669711973119626,0.905615299956608
"11565","FGF10-
AS1",17.0855305867276,0.239249938885831,0.56067714252301,0.42671605589
1313,0.669586154586095,0.905615299956608

"11566","MEF2C-
AS1",4.05206328773225,-0.32017506575422,0.749993692355815,-0.426903678014296,0.669449486498111,0.905615299956608
"11567","MANEA-
DT",28.056441694277,0.1695307835954,0.397887248878868,0.426077448003395,0.670051412623354,0.905615299956608
"11568","EPM2A",16.5836370221962,-0.209951731516775,0.492920676550302,-0.425934113752579,0.670155856253876,0.905615299956608
"11569","AC073332.1",41.2837706880845,0.165487673562845,0.388613670970488,0.425841101136693,0.67022363533834,0.905615299956608
"11570","UGCG",178.177652957771,-0.124943085883643,0.293137117344483,-0.426227449514061,0.669942117578711,0.905615299956608
"11571","TTF1",156.055608671101,0.0770803633985829,0.180699886635732,0.426565643364055,0.669695726287278,0.905615299956608
"11572","CACFD1",16.8561608470919,-0.158516100254408,0.371570859055478,-0.426610689162631,0.669662910830952,0.905615299956608
"11573","TMEM119",418.663920419186,-0.0990705736554012,0.232646011540074,-0.425842562266906,0.670222570579567,0.905615299956608
"11574","METTL3",91.9575383829828,0.0930873254963201,0.218492070187531,0.426044411664109,0.670075484707513,0.905615299956608
"11575","GOLGA6L10",17.9788832872678,0.259096637109967,0.607364263439289,0.426591837397206,0.669676644094613,0.905615299956608
"11576","AC092279.1",38.0221336848469,0.142863500817226,0.335281031399398,0.426100755598792,0.670034429638088,0.905615299956608
"11577","C21orf91",64.7772224059757,0.109282952087354,0.256142758208651,0.426648611312028,0.669635285377445,0.905615299956608
"11578","PDPK1",152.808781382144,0.0745039365496782,0.175106622420142,0.425477549163831,0.670488584503512,0.905895053324273
"11579","MRPL55",330.16181788485,-0.111963359887904,0.263483887985451,-0.424934369778568,0.670884518771789,0.906128739699133
"11580","C2orf73",6.53787777875458,0.294630016730887,0.69316189458585,0.42505224108853,0.670798592259056,0.906128739699133
"11581","FBXO25",134.648320280085,0.0934545194084289,0.220010360339524,0.424773266423491,0.6710019677932,0.906128739699133
"11582","CEP78",65.017578505721,0.139539501621651,0.328511056436385,0.424763486304979,0.67100909804224,0.906128739699133
"11583","CD79B",4.24234971437912,-0.300788167024838,0.70749211488315,-0.425147023828691,0.670729500108758,0.906128739699133
"11584","TEPSIN",32.727671841089,0.114723661476649,0.27002130386993,0.424868926386313,0.670932227934533,0.906128739699133
"11585","KHDRBS1",677.677656438846,-0.0589485803215746,0.138997744506819,-0.424097387556404,0.671494790667249,0.906279503821973
"11586","NBPF26",92.3152553287268,0.199869723166904,0.471148088425941,0.424218474141854,0.671406488951032,0.906279503821973
"11587","EPHA3",8.50864223369,0.328380992366742,0.774130712827816,0.424193210429802,0.671424911997549,0.906279503821973
"11588","TUBE1",118.01575023219,0.0867126555533741,0.204535163288408,0.423949868371062,0.671602374336722,0.906279503821973
"11589","PURG",9.4015248768966,0.248704184985384,0.58662210104767,0.423959793777313,0.67159513566623,0.906279503821973

"11590","AC090739.1",3.52099696070965,-0.336648766826591,0.79393113122
3361,-0.424027668883386,0.671545634677382,0.906279503821973
"11591","UNC13B",30.2891086046304,-0.133534279102466,0.31498802164674,
-0.423934467108801,0.671613606648868,0.906279503821973
"11592","ATL3",513.44475605188,0.0577449104107124,0.136266819608401,0.
423763544028237,0.67173826767847,0.906279503821973
"11593","TOX4",304.051143132225,0.0617248618325957,0.145583523040291,0
.423982470979997,0.671578597132767,0.906279503821973
"11594","AP000894.4",4.86480777949324,-0.267346789992116,0.63092705316
532,-0.42373645043568,0.671758028946863,0.906279503821973
"11595","ZFP14",110.835001366376,0.106323424828995,0.250866660230597,0
.423824452126331,0.671693843934068,0.906279503821973
"11596","VAMP3",322.3183785397,-0.0802068208624862,0.192398110122752,-
0.416879463167872,0.676766575763993,0.906286957409876
"11597","TNFRSF1B",125.448606083134,0.0694672338629899,0.1647693295197
57,0.42160294070178,0.67331485947507,0.906286957409876
"11598","TRIT1",42.7369306935972,-0.102637353509677,0.242526493124631,
-0.423200583933454,0.672148920792481,0.906286957409876
"11599","RWDD3",159.650186473456,-0.0772138191327173,0.184955787864746
,-0.417471764599127,0.676333372708423,0.906286957409876
"11600","S100A1",3.32413829570787,-0.425749986815091,1.02189120094512,
-0.416629467424053,0.676949452142269,0.906286957409876
"11601","TMCO1",815.35407598927,0.07865421851347,0.188956347189447,0.4
16256027825366,0.677222665395056,0.906286957409876
"11602","TAF1B",122.704324443247,0.082059866405831,0.196204929812149,0
.418235497366946,0.675774944678819,0.906286957409876
"11603","PLEKHH2",606.578068133944,0.189667482947815,0.455891471152419
,0.416036480060411,0.677383309214107,0.906286957409876
"11604","AFTPH",113.672255557762,0.092843284524891,0.220497597688979,0
.42106256711173,0.673709394818363,0.906286957409876
"11605","TET3",11.7199698431811,-0.230640537763694,0.552227188386013,-
0.417655165508573,0.676199256917103,0.906286957409876
"11606","C2orf76",103.943258966428,-0.131889031876667,0.31234064709657
5,-0.422260224862398,0.672835087019975,0.906286957409876
"11607","ANKAR",110.727920695672,0.122255642964462,0.292389378121068,0
.418126143124941,0.675854891652588,0.906286957409876
"11608","P4HTM",241.906219521042,-0.0874108590441249,0.208067495482882
,-0.420108190571824,0.67440641953749,0.906286957409876
"11609","ZBTB11",121.823809338702,0.0832929783816104,0.199269084243652
,0.417992478350359,0.675952616596367,0.906286957409876
"11610","UMPS",98.1004446332331,0.109285325231019,0.260276827222124,0.
41988111810566,0.67457230210137,0.906286957409876
"11611","AC078795.1",3.17165860059227,0.34317010309638,0.8226732958152
31,0.417140199933576,0.676575862439229,0.906286957409876
"11612","SLC7A14-
AS1",3.81043331363552,-0.3621155399015,0.866708728156967,-0.4178053458
29999,0.676089442032793,0.906286957409876
"11613","PDE4D",27.1155694774152,0.175905011240517,0.415530509684218,0
.423326343411453,0.672057176631455,0.906286957409876
"11614","MRPS27",110.562349769138,-0.0743049146282938,0.17671464225133

3,-0.420479670963616,0.674135077208561,0.906286957409876
"11615","POC5",45.5700428282419,0.113265444586185,0.272245166780913,0.416042076799603,0.677379213879407,0.906286957409876
"11616","XRCC4",65.8093478583428,-0.10241004219578,0.245957680004751,-0.416372614157858,0.677137364760809,0.906286957409876
"11617","MBLAC2",39.1223602148398,0.113616290301355,0.271823853962436,0.417977630164336,0.675963472733984,0.906286957409876
"11618","C5orf24",356.273586722957,0.086184752473432,0.206619746738229,0.417117694866896,0.676592322729625,0.906286957409876
"11619","PRELID2",69.5387466781634,-0.123849046272671,0.294044909820452,-0.42119092062602,0.673615673744943,0.906286957409876
"11620","C6orf136",37.1792334758431,-0.149318792452153,0.357988890747389,-0.417104542379552,0.676601942579866,0.906286957409876
"11621","MICB",5.89872378195673,0.270766698631183,0.643648185879829,0.420674996948932,0.67399242124512,0.906286957409876
"11622","ZBTB9",7.44742557228722,0.226094369028872,0.542099488386505,0.417071725527385,0.676625945357553,0.906286957409876
"11623","ELOVL4",35.0915476711589,-0.114988979420217,0.27286659512232,-0.421410980588041,0.673455002316258,0.906286957409876
"11624","SLC22A3",6.70193041151851,0.324980377475789,0.775237275181407,0.41920117605251,0.675069112679547,0.906286957409876
"11625","AL139393.3",43.2631947863975,-0.130706861583907,0.312047579515212,-0.418868371890496,0.675312333021033,0.906286957409876
"11626","FAM120B",75.2159431629272,0.0976190332303816,0.234361869990833,0.416531209766333,0.67702133459951,0.906286957409876
"11627","LINC00997",10.5452364689269,0.206513256842392,0.488904880221916,0.422399663404168,0.67273332353976,0.906286957409876
"11628","VKORC1L1",220.455401787985,-0.0586943670857612,0.140217284128663,-0.418595806148287,0.675511555105426,0.906286957409876
"11629","ELN",1332.6472992517,0.119096291000023,0.285217734775642,0.417562712549087,0.676266863814013,0.906286957409876
"11630","CBLL1",75.9867017379013,0.0968455572703358,0.230848107743294,0.419520689240516,0.67483563758781,0.906286957409876
"11631","TPK1",45.2892383027999,-0.113649591251522,0.270525802793188,-0.420106289596357,0.674407808185695,0.906286957409876
"11632","LOXL2",79.2940588879769,0.199928760250092,0.477675868494527,0.418544819691646,0.675548824338483,0.906286957409876
"11633","LINC02605",3.44816708544067,-0.383776462594587,0.921548932958321,-0.416447188932879,0.677082804141868,0.906286957409876
"11634","AC023632.2",14.514508566347,-0.21267636682091,0.506043906959144,-0.420272557175716,0.674286355170096,0.906286957409876
"11635","BNC2-AS1",17.0566203928352,-0.207872498366287,0.495579631437282,-0.419453272854282,0.674884897572955,0.906286957409876
"11636","CHMP5",663.130400070851,-0.0963110212579149,0.227442069889678,-0.423452975540677,0.671964800788204,0.906286957409876
"11637","PIGO",41.5993478695147,-0.129016721810353,0.307765097984467,-0.419205175165329,0.675066190249327,0.906286957409876
"11638","AL161457.2",16.7423271770941,0.228313972300989,0.548669188703592,0.416123188620185,0.677319862523178,0.906286957409876

"11639","TRIM14",6.39478602068767,-0.297130553835653,0.704788569434435
,-0.421588213432701,0.673325610897305,0.906286957409876
"11640","AL158151.3",5.7247946207099,0.367711498353097,0.8765578317400
02,0.419494852522366,0.67485451586539,0.906286957409876
"11641","TMEM141",221.081233325357,-0.129762043212094,0.31099451851043
2,-0.417248650663087,0.676496543334509,0.906286957409876
"11642","GPR158",6.59677268281493,0.282367600923443,0.666647952689796,
0.423563291215617,0.671884331539686,0.906286957409876
"11643","PCBD1",625.484995990525,-0.154613075152688,0.367575246544757,
-0.420629725766537,0.674025483924086,0.906286957409876
"11644","SHLD2",161.166713161892,0.0734270780868405,0.173348650593218,
0.423580326905142,0.671871905271478,0.906286957409876
"11645","IFIT1",452.698707515166,0.114910663677485,0.275030181236267,0
.417811104079412,0.676085231621664,0.906286957409876
"11646","ELOVL3",2.84267094221372,0.438653256708748,1.04677625744865,0
.419051591576883,0.675178427990949,0.906286957409876
"11647","CASP7",156.848688163436,0.102374047709856,0.24237940642534,0.
422371063695915,0.672754195371087,0.906286957409876
"11648","PPP2R2D",152.574722586341,-0.0781204778667724,0.1869053683787
47,-0.417968079485381,0.675970455675443,0.906286957409876
"11649","IFITM3",6619.09153119494,0.100407979125957,0.241198175115738,
0.416288303498881,0.677199050416532,0.906286957409876
"11650","LSP1",1420.41691921434,0.0933255690051688,0.223796904487642,0
.417010097699193,0.676671021837932,0.906286957409876
"11651","GTF2H1",136.705720914925,0.0699861699335402,0.16625217948124,
0.420963924514669,0.673781425038684,0.906286957409876
"11652","ANKRD42",47.5422915222385,-0.108860660399882,0.25919653799857
2,-0.419992725367657,0.674490768007921,0.906286957409876
"11653","ETFRF1",251.312631930226,-0.101438733470833,0.242282666048032
,-0.418679285338237,0.675450536591299,0.906286957409876
"11654","KANSL2",61.0067541318913,-0.0880891457482727,0.21159963642334
8,-0.416301026018932,0.677189741881926,0.906286957409876
"11655","RASSF9",61.2972795229969,-0.104514519610983,0.248596405265196
,-0.420418467030888,0.674179779796029,0.906286957409876
"11656","LINC02397",2.60645332074529,-0.585582609147553,1.399365360645
62,-0.418462987305464,0.675608642479285,0.906286957409876
"11657","ERP29",942.103426272091,-0.10722257259202,0.25621075532689,-0
.41849364385667,0.675586232799943,0.906286957409876
"11658","KCNRG",4.71160586023774,-0.285595487020023,0.680033602748345,
-0.419972609979556,0.674505462915729,0.906286957409876
"11659","OBI1",95.5177227858499,0.074546273881173,0.176353500055131,0.
422709352850205,0.672507330885317,0.906286957409876
"11660","PIGH",137.54485269476,-0.0701950591792031,0.16633159827826,-0
.422018786002238,0.67301130540297,0.906286957409876
"11661","IGHG3",83.5240707398907,0.536625508046137,1.27640762446747,0.
420418601205099,0.674179681795605,0.906286957409876
"11662","OTUD7A",15.4168842396188,0.223315889471494,0.533221741043091,
0.418804921634745,0.675358707663152,0.906286957409876
"11663","INO80",139.793498945902,-0.0872427967086488,0.209020969072757
,-0.41738777260324,0.676394797059169,0.906286957409876

"11664","SNRPA1",191.154730431804,-0.0973708912576289,0.232629932986875,-0.418565616244762,0.675533622723524,0.906286957409876
"11665","NTHL1",131.279090213694,-0.129094405977032,0.305690153818252,-0.422304756514289,0.672802586775517,0.906286957409876
"11666","MYH11",10.2069143681575,0.219337485564118,0.523259537241289,0.419175323053836,0.675088005384196,0.906286957409876
"11667","RSPRY1",144.74855337933,-0.0742374614516334,0.176603062189837,-0.420363387424353,0.674220010235575,0.906286957409876
"11668","CD68",411.530059652266,-0.103184270117465,0.247734889206321,-0.416510853388642,0.6770362271039,0.906286957409876
"11669","NTN1",24.1736720704001,-0.239775895884052,0.571356198546827,-0.419660968926025,0.674733142118255,0.906286957409876
"11670","LINC00672",9.25656914079718,-0.231819181568732,0.551096039327552,-0.420651147940743,0.674009838694962,0.906286957409876
"11671","FKBP10",568.32560369554,0.10421411525238 7,0.248185639836205,0.419903888561665,0.674555666955196,0.906286957409876
"11672","PSMC3IP",23.1195244872761,-0.154673199352484,0.369595009597175,-0.418493744060732,0.675586159552093,0.906286957409876
"11673","CDK5RAP3",206.310804484219,-0.0872340863361444,0.207785448899935,-0.419827696299151,0.674611330471068,0.906286957409876
"11674","MKS1",32.4729091228026,-0.112341079010464,0.268901682551121,-0.417777523534488,0.676109785737274,0.906286957409876
"11675","TSPOAP1-AS1",3.88905241944398,0.345415177842192,0.818175995440637,0.422177110752517,0.672895747282305,0.906286957409876
"11676","LRRC37A3",14.8024462682407,0.206414079234329,0.492102455670326,0.419453463106901,0.674884758556703,0.906286957409876
"11677","HOOK2",92.2307278675221,0.107973232248768,0.255370829320091,0.422809576709448,0.672434199900856,0.906286957409876
"11678","TSHZ3",69.4710084974285,0.100285029464779,0.240443628027919,0.417083331703574,0.676617456374584,0.906286957409876
"11679","ZNF302",273.044779268426,0.0851027818028336,0.201587203814048,0.422163610550081,0.6729056005133,0.906286957409876
"11680","AC011462.5",8.17097252849395,0.305287845022553,0.72295678630303,0.422276754027993,0.672823023575753,0.906286957409876
"11681","GEMIN7",47.440156426378,-0.157298670995888,0.376022827413214,-0.418322132403498,0.675711609914581,0.906286957409876
"11682","PPM1N",7.46863303809618,-0.312433792948995,0.745356976286612,-0.419173366439191,0.675089435236049,0.906286957409876
"11683","ZNF613",10.8008047717186,0.20065442963798,0.474300431861553,0.423053440728367,0.672256271027025,0.906286957409876
"11684","PCED1A",108.758021078426,0.0995087817949973,0.238703246209319,0.416872344114408,0.676771783215927,0.906286957409876
"11685","AL353194.1",23.8386641768004,0.181610538641527,0.429849968502573,0.422497503662002,0.672661922432948,0.906286957409876
"11686","AP000253.1",2.97152061223883,0.372048646067214,0.890678865569387,0.417713567088373,0.676156551683772,0.906286957409876
"11687","TMEM50B",304.455037818118,-0.0653096326073395,0.15649030891504,-0.417339789665804,0.676429888521485,0.906286957409876
"11688","XRCC6",737.982303653494,-0.0772346572153028,0.183060493326058

,-0.421907839381468,0.673092287760282,0.906286957409876
"11689","RIBC1",6.07779940638951,-0.249033479213513,0.590711995450871,
-0.421581889535583,0.673330227584176,0.906286957409876
"11690","NAP1L3",27.2131775905252,0.123465456547226,0.295486184956724,
0.417838338416086,0.676065318110519,0.906286957409876
"11691","AC002377.1",3.78542727726311,-0.323194712785303,0.76878337371
984,-0.420397635840498,0.674194994899501,0.906286957409876
"11692","SMIM10L2A",4.77237851093365,0.336329420383209,0.8068500162724
55,0.416842552643189,0.676793575277012,0.906286957409876
"11693","LMBRD1",466.52512230115,0.0662952533248481,0.159505341419882,
0.415630302626245,0.677680549255067,0.906374558139279
"11694","DHX32",81.4620762176764,-0.0828695481612836,0.199307950447584
,-0.415786464991408,0.677566263928662,0.906374558139279
"11695","AC022167.2",3.62922861686558,0.32609594514599,0.7844360736267
67,0.415707482240479,0.67762406546526,0.906374558139279
"11696","AOC3",34.2893543633856,-0.174962986272142,0.420935727465201,-
0.415652497177509,0.677664306018987,0.906374558139279
"11697","LRRC42",76.5685409948343,-0.129153162125479,0.311297230995194
,-0.414886960968414,0.678224655503421,0.906500710159305
"11698","MCMDC2",5.35978196807783,0.2615564577593,0.629819805011435,0.
415287762750729,0.677931258433365,0.906500710159305
"11699","AC009812.1",17.6899512684806,0.154714377636791,0.372924258495
607,0.414868097508367,0.678238465237135,0.906500710159305
"11700","NTNG2",11.0107663400232,-0.239333619359646,0.576809228242649,
-0.414926820933185,0.67819547481269,0.906500710159305
"11701","LRMP",5.02710982629842,0.376204758315141,0.906066438276437,0.
415206592389379,0.677990673254567,0.906500710159305
"11702","NTN4",111.764772710345,0.121710981157804,0.293342406302259,0.
414910965966485,0.678207081861981,0.906500710159305
"11703","TERF2",108.105905583506,0.102360438491463,0.246601702199395,0
.415084071109522,0.678080359781074,0.906500710159305
"11704","IFNAR1",395.163515026015,0.0588380595142619,0.14175397444108,
0.415071674330499,0.678089434573528,0.906500710159305
"11705","BLZF1",120.945138878773,0.0731137340766778,0.176330355197643,
0.414640655573607,0.678404981507263,0.90664580313696
"11706","CREB1",246.235760175468,0.0809579715628387,0.195391369028199,
0.41433750101395,0.678626953412194,0.90664692617608
"11707","DOCK10",21.5743549245482,0.146265089565695,0.353021990838318,
0.414322884583934,0.678637656369988,0.90664692617608
"11708","LY6E",1598.56407411844,-0.118567275922713,0.28603715516823,-0
.414517043609172,0.678495487690871,0.90664692617608
"11709","CAPN5",81.2611593403503,-0.128403147498865,0.309904793641792,
-0.414330949805448,0.678631750561079,0.90664692617608
"11710","NEB",6.25569612543001,0.370962714370729,0.8960210188461,0.414
011174479429,0.678865923161589,0.906725504714169
"11711","MINDY2",304.44551617627,0.095194541637724,0.229864746698934,0
.414132845531139,0.67877681936555,0.906725504714169
"11712","AC104984.3",12.8983780277442,0.23310693765663,0.5631609460786
57,0.413925964290983,0.678928328115902,0.906725504714169
"11713","RANGAP1",57.1656907491165,-0.10207125198316,0.246566212764732

,-0.413970960735623,0.678895374023253,0.906725504714169
"11714","LINC01960",8.71726303867267,0.255594842995233,0.6181411775234
2,0.413489429743659,0.679248065226846,0.907075079762981
"11715","WDR92",97.1231378286584,0.107279286000804,0.259582566237798,0
.413276159318527,0.679404294861262,0.907128831044275
"11716","MRFAP1",1412.06767393739,0.0880488079759762,0.213031093685166
,0.41331434981084,0.679376317693069,0.907128831044275
"11717","RBP5",33.2830516798492,-0.235051930689465,0.568983996917575,-
0.413108157633325,0.679527372919854,0.907215728820114
"11718","AL356776.2",6.86885412692618,0.227928359080864,0.552182990424
674,0.412776856645962,0.679770109100213,0.907350719503225
"11719","CCDC86",36.9542336376097,-0.138870213220678,0.336465289767713
,-0.412732657554514,0.679802495210497,0.907350719503225
"11720","NOP9",27.1152188797876,0.133987878428855,0.324528025212801,0.
412869977380214,0.679701878446678,0.907350719503225
"11721","TSC22D4",210.13665454314,0.0818500524039109,0.198414781944733
,0.412519932243302,0.679958374220726,0.907403928334313
"11722","AL359091.5",8.22152415211618,0.263059387330431,0.637630486587
765,0.41255773188979,0.679930674723739,0.907403928334313
"11723","BCO2",28.2577732647831,0.134524415634258,0.326173752503446,0.
412431762524597,0.680022986473451,0.907412742250635
"11724","EIF2D",183.693364753048,-0.0794352478722977,0.192939447859834
,-0.411710766011964,0.680551432971385,0.907577803137692
"11725","AC093702.1",7.49700967676811,0.253573896046619,0.615827834851
141,0.411761017765481,0.680514596410028,0.907577803137692
"11726","CDYL",179.84166784576,-0.0938660074873544,0.227861009902482,-
0.411944138786739,0.680380367768536,0.907577803137692
"11727","TXLNB",9.22708087716197,-0.328239101980563,0.797191865587197,
-0.411744168687406,0.680526947378638,0.907577803137692
"11728","CLDN15",27.7952046094039,0.127480546468225,0.309532695735293,
0.411848403172387,0.680450541198795,0.907577803137692
"11729","PLBD1",652.894112620319,0.0970610806977648,0.235540683408354,
0.412077774816892,0.680282418268119,0.907577803137692
"11730","IRGQ",59.6635605496753,0.109551276393374,0.266089171916777,0.
411708885424459,0.680552811532642,0.907577803137692
"11731","PIK3CD",12.3185317557336,-0.204599956101865,0.497363658610968
,-0.411368930076776,0.68080203271524,0.907746993122393
"11732","HNRNPA3",828.346702933905,-0.0800722666352803,0.1946733989732
15,-0.411315911971606,0.680840903417663,0.907746993122393
"11733","TBC1D22A",119.138748647033,0.0915526763004161,0.2225943100082
31,0.411298367406745,0.680853766560445,0.907746993122393
"11734","BHLHE22",71.8100284193744,0.168945860926887,0.411000812865787
,0.411059675889392,0.68102877716111,0.907748224363603
"11735","PCCA-
DT",36.392860970187,-0.12024951727028,0.292489453428486,-0.41112428451
9481,0.680981403882199,0.907748224363603
"11736","STARD3",103.031756540123,-0.108324742954066,0.263492441443638
,-0.411111386575531,0.680990860999627,0.907748224363603
"11737","TMEM230",997.671854670613,-0.124758587033004,0.303576133412,-
0.410963093938902,0.681099596728891,0.907765271502943

"11738","ZNF575",16.8531274198746,-0.179569803242462,0.437106190838119
,-0.41081505365584,0.681208154029598,0.907832608066537
"11739","GORASP1",44.6875553655378,-0.105779534849865,0.25755689702679
9,-0.410703561313905,0.681289915241493,0.907864225583326
"11740","DFFA",215.954096025089,0.0682733703401181,0.167195110683853,0
.408345495636026,0.683020042455224,0.907990241873488
"11741","SZRD1",250.127785486076,-0.061616704783533,0.15062453114465,-
0.409074832069421,0.682484745443324,0.907990241873488
"11742","LCK",10.2677308157942,0.260136032575872,0.637644249231851,0.4
07964210277517,0.683299950606323,0.907990241873488
"11743","DENND2C",41.8572107308276,0.117941275567802,0.288149686202938
,0.4093055839205,0.682315418194231,0.907990241873488
"11744","ISG20L2",101.556761999596,-0.0925249091608747,0.2259887174817
05,-0.409422692388903,0.682229489335164,0.907990241873488
"11745","CEBPZOS",270.964169376021,-0.0768964091476479,0.1877354990976
81,-0.409599726835028,0.682099597345628,0.907990241873488
"11746","SOCS5",203.45967922025,-0.102723453434687,0.251231589389247,-
0.408879526991059,0.68262807410403,0.907990241873488
"11747","VPS54",78.4670054819527,0.0971140495799403,0.237489604485478,
0.408919160021081,0.682598987661511,0.907990241873488
"11748","MXD1",55.7386490382159,-0.128133706706848,0.313605620358023,-
0.408582303341905,0.68284621977316,0.907990241873488
"11749","SNRNP200",336.042805518827,0.0807475896963253,0.1979102752894
94,0.408000997311592,0.68327294271234,0.907990241873488
"11750","RIOX2",93.7406801435327,-0.0849297535570214,0.207229477569186
,-0.409834327400004,0.681927483015031,0.907990241873488
"11751","ATP5ME",1312.32818016461,-0.115589408884481,0.282116067085624
,-0.409722884905377,0.6820092405608,0.907990241873488
"11752","PCDHGC3",84.7676265941636,-0.107019546943285,0.26191140088479
5,-0.408609730549144,0.682826088614831,0.907990241873488
"11753","MRPL14",329.295125440052,-0.11893511710012,0.28976382397785,-
0.410455368332009,0.681471937229521,0.907990241873488
"11754","PEX3",91.4994585025227,-0.0990780864893513,0.242734266338367,
-0.408175112578619,0.683145118209693,0.907990241873488
"11755","ZNF3",80.3828526652318,0.0875275254817532,0.213689546771307,0
.409601343651246,0.682098411114376,0.907990241873488
"11756","PVT1",14.1198177635167,0.202339668347346,0.494579100996425,0.
409114877558905,0.682455358649251,0.907990241873488
"11757","ZNF707",10.2751657936321,-0.211804861027491,0.517251898613893
,-0.409481070238845,0.682186655867073,0.907990241873488
"11758","REXO4",173.015948247246,0.0818831873672863,0.199539154728759,
0.410361502626355,0.681540782133255,0.907990241873488
"11759","UNC5B",11.5834551660203,-0.288197640331034,0.702371254159067,
-0.410320950102217,0.681570525814445,0.907990241873488
"11760","PAK1",89.743610422761,-0.10045869286277,0.246172094381,-0.408
083187151548,0.683212602957816,0.907990241873488
"11761","HMGA2-
AS1",23.1481285164155,-0.228677134909748,0.557808287061322,-0.40995650
3361537,0.681837855367883,0.907990241873488
"11762","LRCH1",40.9098978629899,0.115469177388679,0.282786792366965,0

.408325920818953,0.683034411604816,0.907990241873488
"11763","GPC6",175.408091534528,0.151752264486174,0.371177425125058,0.408840231689859,0.682656913154743,0.907990241873488
"11764","FES",101.191468610124,-0.0850935150931775,0.207794562353673,-0.40950790111796,0.682166969640934,0.907990241873488
"11765","ARRDC4",39.2316163817127,0.139765441869448,0.342335752303197,0.408270070914656,0.683075409586262,0.907990241873488
"11766","AL031668.2",4.37115417583243,0.300852395328892,0.737082675201856,0.408166418029702,0.683151500986434,0.907990241873488
"11767","MYH7B",3.82136248577241,0.316755901599773,0.773987639102452,0.409251886718884,0.682354820131895,0.907990241873488
"11768","LPIN3",117.691591222147,-0.101725855163092,0.248876438116873,-0.408740401191862,0.682730181423042,0.907990241873488
"11769","ADNP-AS1",12.571587033887,0.187781824061505,0.458367656949646,0.409675118247127,0.682044284752368,0.907990241873488
"11770","DUSP18",57.9632480521165,-0.115563271803725,0.283216956031953,-0.408037969981879,0.683245798938472,0.907990241873488
"11771","SGSM3",147.593021710639,-0.083723313401143,0.204958168624308,-0.408489761413752,0.682914145823698,0.907990241873488
"11772","BCOR",29.9067974337522,0.172755810609906,0.421233907814213,0.41011848145449,0.681719036006142,0.907990241873488
"11773","GEN1",11.5441304481235,-0.214753778900236,0.526545357476487,-0.407854282353684,0.683380658677387,0.90802035536315
"11774","SHC1",214.775330859229,-0.0849714919584858,0.208399619701605,-0.407733430992587,0.68346939081146,0.908057069424667
"11775","LINC00653",15.5286224756343,-0.187014127712395,0.458751906511976,-0.407658529714498,0.683524387424116,0.908057069424667
"11776","UQCC2",202.706683545452,-0.124748710835785,0.306327461404919,-0.407239723998777,0.683831928412072,0.908311357404265
"11777","DHRS7B",218.236456098412,-0.0907192544511909,0.222744186897749,-0.407280009030429,0.683802343682813,0.908311357404265
"11778","STIP1",140.408388506365,-0.0998856344350702,0.245326475308568,-0.407153913206618,0.683894948202062,0.90831793808158
"11779","JMJD4",59.627216782199,0.114390011599256,0.281331492030415,0.406602228473197,0.684300160248895,0.908430823640003
"11780","CCDC149",81.6936741314124,0.102934594642736,0.25318188912115,0.406563814655323,0.684328378557424,0.908430823640003
"11781","AL451165.2",38.1879733797662,0.158071656017581,0.388538993280882,0.406836015821219,0.68412843250192,0.908430823640003
"11782","AC005076.1",11.1313366443229,-0.21309387276769,0.524091504633186,-0.406596693294686,0.684304226294387,0.908430823640003
"11783","ZNF786",12.7973938340813,-0.172199025574799,0.423344043923776,-0.40675906050022,0.684184957983353,0.908430823640003
"11784","AC009118.3",14.0924520501755,-0.193358933958834,0.475450181425369,-0.406686003103745,0.684238621985386,0.908430823640003
"11785","AC007950.2",5.59330334415367,-0.289606950879416,0.712555442143215,-0.406434269884095,0.684423543769558,0.908480058989155
"11786","MAP3K20",713.608658470809,-0.0759197924520785,0.186941143650426,-0.406116015819643,0.684657358466116,0.908559132888145

"11787","NDST1-
AS1",4.58134067130506,-0.514582316586941,1.26676336730882,-0.406218185
548061,0.684582293179386,0.908559132888145
"11788","NOM1",97.1813467426131,0.10650292011741,0.262201454599464,0.4
06187373293188,0.684604930973029,0.908559132888145
"11789","CIART",4.45246377325667,-0.441677140286923,1.08840465149122,-
0.405802326994638,0.684887848750387,0.908710824258041
"11790","LIG3",41.0596278563309,-0.107757434303654,0.265497909286302,-
0.405869238644183,0.684838681370579,0.908710824258041
"11791","TEX35",3.07319730968239,0.375310170913853,0.929132961737939,0
.403935912694172,0.686259846827325,0.908784026076973
"11792","ATOH8",155.45841662033,0.137159981877447,0.338490355437197,0.
405210900914121,0.685322492849941,0.908784026076973
"11793","RAPGEF6",78.6631809388345,0.107480793509441,0.265994928940732
,0.404070836754146,0.686160629627481,0.908784026076973
"11794","RMND5B",113.623578072102,-0.10581159979555,0.261317874377927,
-0.404915278173896,0.685539787595462,0.908784026076973
"11795","AC006480.2",15.4166544613941,0.187876571663991,0.464041371638
389,0.404870304991679,0.685572846994766,0.908784026076973
"11796","HACD1",8.23320705544288,-0.231945746045714,0.574026360778844,
-0.404068108877454,0.686162635534057,0.908784026076973
"11797","FAR1",164.002499927881,0.0787304975959208,0.194921094817566,0
.403909580282254,0.686279211151779,0.908784026076973
"11798","UCP3",2.85299942953608,0.376872736064022,0.931707029609831,0.
404497040471879,0.685847253757664,0.908784026076973
"11799","LINC00621",3.84386280964848,-0.330461690496107,0.816690860452
053,-0.404634980625583,0.685745841725904,0.908784026076973
"11800","TNFSF13B",385.481868168545,0.117742783262466,0.29111743539279
1,0.404451155952239,0.685880988788787,0.908784026076973
"11801","ARHGEF40",176.14467723044,-0.10958122729601,0.27029094703608,
-0.405419524766335,0.68516916149238,0.908784026076973
"11802","IPO4",27.5011871907642,-0.126125862769079,0.312234016615516,-
0.403946578711155,0.686252003312238,0.908784026076973
"11803","PRORP",63.9122985458911,0.0985074058837121,0.243250417225837,
0.40496294726704,0.685504747112463,0.908784026076973
"11804","DMAC2L",105.607249228757,-0.0808116209791614,0.19964748440269
5,-0.404771546312911,0.68564544576206,0.908784026076973
"11805","DNAAF4",43.2531502903553,-0.114350735893658,0.282023558496134
,-0.405465190579904,0.685135600419483,0.908784026076973
"11806","TPM1",812.414832996734,0.0953389599528615,0.235825415268649,0
.404277714699463,0.686008511221168,0.908784026076973
"11807","PKD1",258.40107175391,0.0812803245408332,0.201035644876741,0.
404308025030425,0.685986224948906,0.908784026076973
"11808","APOBR",8.82734223967948,0.357779058564026,0.885255705007415,0
.404153349749979,0.686099955876783,0.908784026076973
"11809","CKLF",379.659898862137,-0.114743708986729,0.282982909443426,-
0.405479289234845,0.68512523905249,0.908784026076973
"11810","GSE1",142.028560571403,-0.11411496321506,0.281695697892719,-0
.405100127792933,0.685403912553961,0.908784026076973
"11811","PEX12",22.3708086234435,0.142524207787526,0.352492639756802,0

.404332436234297,0.68596827632544,0.908784026076973
"11812","ZNF75D",42.7170957881545,0.10901337302174,0.26953687945334,0.404446965635406,0.685884069607796,0.908784026076973
"11813","NSDHL",50.0577278986211,-0.109435776218074,0.269971336391045,-0.405360723404945,0.685212377158813,0.908784026076973
"11814","GOLPH3L",111.486120553573,-0.0809895006884012,0.200610588817423,-0.40371498416821,0.68642231959213,0.908819665288167
"11815","GRPEL2",33.7028527452134,-0.111823646030681,0.276978646945709,-0.403726595041818,0.686413780493559,0.908819665288167
"11816","SH3PXD2B",214.187434948188,-0.133046595062004,0.32963218010297,-0.403621378897058,0.686491162096089,0.908833890374841
"11817","KLF5",33.5897956102333,-0.184796443438863,0.458141923412202,-0.403360692386574,0.686682899109871,0.909010797222282
"11818","FOXO3",465.446005554373,0.114597267153315,0.284206181860522,0.403218770271348,0.686787292437913,0.909072060890698
"11819","TNFRSF11B",32.4216141520436,0.417020608419008,1.0344818327285,0.403120282276096,0.686859740540466,0.909091033190161
"11820","TRIM11",46.7693626472161,0.112373334290758,0.278836960987966,0.403007312562152,0.686942844986207,0.909124105255435
"11821","CCDC32",203.339367647947,-0.102263645933385,0.253806387356527,-0.402919906778128,0.687007146313354,0.909132289127807
"11822","CILP",3396.97080880501,0.137615525679342,0.341694218547398,0.40274467114009,0.687136067761663,0.90914907451541
"11823","C16orf86",62.1685657083011,0.120495237781497,0.299179982356247,0.402751670858841,0.687130917870543,0.90914907451541
"11824","MIR181A1HG",2.93972928556092,0.334422796560475,0.831068407537823,0.402401046083869,0.687388900283393,0.909329773119079
"11825","PCDHGA3",10.9984040842535,0.190118786411529,0.472411958669448,0.40244278943954,0.687358184481544,0.909329773119079
"11826","AL121890.5",3.13411446039973,-0.311514252981263,0.774589093952687,-0.40216710435674,0.687561049985927,0.909480594024172
"11827","RAPGEF2",72.5317690360833,0.115769170496074,0.288055373878874,0.401899013155553,0.687758349032419,0.909510850783171
"11828","AC012291.3",8.63366251533717,0.239077440629371,0.594795200165089,0.401949176057598,0.687721430527682,0.909510850783171
"11829","TRPM4",19.0595181541259,0.165659468908405,0.412123779593259,0.401965324766993,0.687709545683804,0.909510850783171
"11830","STRIP1",64.234577601214,0.0960312235903114,0.239046508981327,0.401726107607842,0.687885608424129,0.909525363247286
"11831","TMEM81",3.01079824571461,-0.391044798116426,0.973332195698519,-0.40175882380608,0.687861528452141,0.909525363247286
"11832","ANKS1B",16.7462023614015,-0.172378733883008,0.429280688741611,-0.401552500272765,0.688013393228744,0.909617436636007
"11833","HSD17B13",5.06098977244656,-0.279502070691505,0.696575794001876,-0.401251483468506,0.688234979711793,0.90962604663004
"11834","IER3-AS1",4.76630556906766,0.261035142268714,0.650215162655911,0.401459635611192,0.688081750522346,0.90962604663004
"11835","AC002558.3",29.6765581165169,-0.16228259564398,0.404323262697018,-0.401368436140633,0.688148884556095,0.90962604663004

"11836","AL031595.3",10.4427139604164,-0.245862755234579,0.61277615286154,-0.401227681734105,0.688252501944202,0.90962604663004
"11837","HDAC11",41.8946864910693,-0.117978339174462,0.29434812145284,-0.400812271510842,0.688558343339722,0.909953380490265
"11838","RAB3D",35.4714088369418,0.116716885258742,0.291396312397611,0.400543453341584,0.688756285025379,0.910138077939856
"11839","AC008875.1",4.71930401333982,0.302968605135655,0.757722359549478,0.39984118367022,0.689273495118536,0.910206422770493
"11840","TRDMT1",63.397882348195,0.103205439737421,0.257827007344202,0.400289484024612,0.688943312481696,0.910206422770493
"11841","EIF5AL1",19.2189704870508,-0.180132451823818,0.450301538014727,-0.400026285981565,0.689137156196898,0.910206422770493
"11842","TBC1D10C",10.2127855973093,0.292282959314898,0.730969563261666,0.399856538500316,0.689262184984057,0.910206422770493
"11843","SNHG21",40.0728910306544,-0.106606070166289,0.266532293007479,-0.399974310667478,0.689175438104749,0.910206422770493
"11844","FAM106A",47.7135669549743,-0.235038952936149,0.587605935374467,-0.399994177707488,0.689160805137306,0.910206422770493
"11845","GPRASP2",37.362196336746,0.116928813486266,0.292407581437484,0.399882974687047,0.6892427126534,0.910206422770493
"11846","PHF6",111.148987923423,0.0790956649534706,0.197616879137663,0.400247515792268,0.688974220454514,0.910206422770493
"11847","METTL14",113.439417795119,0.0794871980264579,0.198913888096255,0.399606074705019,0.689446681479194,0.910358271155484
"11848","SEC23A",338.911579694001,0.0699669493562807,0.175242599682776,0.399257654719428,0.689703365116427,0.910620335965249
"11849","MMS19",74.3095728052007,0.0871366908254714,0.218369412107483,0.399033408500372,0.689868587890355,0.910761610293596
"11850","FAM89A",64.5471098250484,0.133530266695288,0.335086381135185,0.398495057432421,0.690265300879693,0.911049737459649
"11851","SOS1-IT1",2.68144065014291,0.358228485095686,0.899298589079311,0.398342096213489,0.690378034126874,0.911049737459649
"11852","CACNB1",17.1146606837732,-0.147617602532862,0.370499852620059,-0.398428235501192,0.690314548159996,0.911049737459649
"11853","NAPSA",2.65817830347021,0.482721298216167,1.21179301550344,0.398352929947876,0.690370049379872,0.911049737459649
"11854","ZNF133",39.5675837427698,0.11437383003945,0.286955301103485,0.398577163759046,0.690204790906061,0.911049737459649
"11855","DNM3OS",181.616193640162,-0.134919139619791,0.339078055825917,-0.397899944575176,0.690703940898353,0.91109551795876
"11856","CATSPER2",9.5652447602238,0.260890660410202,0.655658934795327,0.397906055366488,0.690699436287417,0.91109551795876
"11857","U2AF1L4",63.5387863538596,-0.105790014323362,0.265813769606444,-0.397985456058172,0.690640906529077,0.91109551795876
"11858","DMPK",34.5799569684096,0.116448439393172,0.292543878339283,0.398054609975871,0.690589931627511,0.91109551795876
"11859","SDCBP2-AS1",17.1632109094147,0.161572542751062,0.406051914714886,0.397911047567679,0.690695756260846,0.91109551795876

"11860","TRMT10B",98.2347349422766,0.0949186231985594,0.238763857868833,0.397541839228882,0.690967939787519,0.911366904055325
"11861","LINC02245",4.37297887608613,-0.306722447637857,0.772462858924922,-0.397070802944156,0.691315249738542,0.911622572486988
"11862","LINC01116",218.813327899987,0.183758399436558,0.462684573293122,0.397156961877229,0.691251717175455,0.911622572486988
"11863","GYG2",3.99934536767366,0.293498036707575,0.739211864268802,0.397041837251748,0.691336609180664,0.911622572486988
"11864","DHFR2",60.8894326673433,0.100667244188305,0.25362926358626,0.396907055459189,0.691436001159095,0.911676784063699
"11865","AC007365.1",5.12584508591936,0.267168282487866,0.673450513797618,0.396715537391592,0.691577241247293,0.911677699622145
"11866","DCTN1",222.122387682404,-0.082667564525911,0.208445904539154,-0.396590015566282,0.691669816474821,0.911677699622145
"11867","TFAP4",56.1912181027612,-0.115736823777192,0.291812353280806,-0.396613859817717,0.691652230437429,0.911677699622145
"11868","CDH26",3.46390007216311,-0.37347974860978,0.941296699026454,-0.396771548222845,0.691535933460883,0.911677699622145
"11869","AC015802.6",17.8989859769584,0.240691522693495,0.607082936242327,0.396472225332683,0.69175669366752,0.911715389589773
"11870","TDRKH",4.27886181583385,0.31971117902716,0.80737195631152,0.395989948038029,0.692112443708051,0.911749860580345
"11871","NIT1",129.900211457224,0.0853494210827524,0.215518758089043,0.396018526830456,0.692091360774251,0.911749860580345
"11872","TAPT1",101.124098540766,0.102361513924158,0.258443091595301,0.396069839949317,0.692053507041393,0.911749860580345
"11873","AC108471.2",9.89261526697153,0.225555104100583,0.569092858150023,0.396341477265776,0.691853132772152,0.911749860580345
"11874","ARMC2",23.8072592461525,0.141568397097301,0.357641970009293,0.395838321474469,0.692224304517398,0.911749860580345
"11875","ITPR2",117.002578848943,0.126189143222587,0.318604968484966,0.396067719290892,0.692055071437737,0.911749860580345
"11876","PLEKHH1",3.4249937340856,0.368121597385513,0.930058737910137,0.395804675963467,0.69224912702888,0.911749860580345
"11877","ATXN10",410.298919388312,-0.0964523963262046,0.243670633686362,-0.395831023488667,0.692229688694554,0.911749860580345
"11878","KLRF1",5.05201261906989,-0.24498935962114,0.619612125776518,-0.395391486753284,0.692553990897461,0.912074598384323
"11879","ZNF131",172.798622144364,-0.0653628886494898,0.16546026410978,-0.395036772128701,0.692815750132402,0.912188938584392
"11880","MYMX",9.0611786864858,0.208199269535246,0.526852428977952,0.395175685037904,0.692713235928358,0.912188938584392
"11881","DMC1",9.05191522427631,-0.278362604594519,0.704535541510373,-0.395100868861447,0.692768447670057,0.912188938584392
"11882","SPART-AS1",5.83244352587462,-0.280631380450206,0.71070561421045,-0.394863041516803,0.692943966835306,0.91220760874607
"11883","GNPTG",354.707439619045,0.0783011426100981,0.198301260737026,0.394859529985217,0.692946558507291,0.91220760874607
"11884","MYL12A",1778.48471677092,-0.114510784096199,0.290064729261625

,-0.394776656878248,0.693007723718886,0.91221136167406
"11885","MFSD14B",152.414049393176,-0.0718902568146349,0.1822468842645
3,-0.394466314772694,0.693236792162826,0.912214362648609
"11886","FAM234B",24.537634636902,0.150929754856254,0.382586261765555,
0.394498626688122,0.69321294091589,0.912214362648609
"11887","ELL3",4.90529426634131,0.260027291688426,0.658808108558,0.394
693520481365,0.693069085264556,0.912214362648609
"11888","ZNF222",18.3222543737891,0.140035918555401,0.355008842976582,
0.394457550356396,0.693243261725159,0.912214362648609
"11889","ZNF677",172.5496687202,-0.0679842014446294,0.172435830579929,
-0.394257975363866,0.693390586527857,0.912331478261861
"11890","ZBTB48",42.0683960341233,-0.105964250190916,0.268958503488918
,-0.393979921870298,0.693595862912707,0.912524817791714
"11891","HSPB8",149.824533525387,-0.120325894351985,0.305491743260841,
-0.393876093237801,0.693672521435107,0.912548923791892
"11892","ABL2",210.950831740969,0.123990402288902,0.314893716492363,0.
393753180184238,0.693763274376675,0.91259156584883
"11893","AC090825.1",15.8066942055576,0.165695565734088,0.421120376035
674,0.393463663035986,0.6939770569271,0.912719278754887
"11894","TMUB2",167.981958002258,0.066277287466826,0.168445470375543,0
.39346434973326,0.693976549833688,0.912719278754887
"11895","GPM6B",17.8376953681614,-0.164678912839848,0.418765132570112,
-0.393248864415131,0.694135682258088,0.912851154061645
"11896","SIMC1",33.9791342226468,0.114142091107169,0.290356768724854,0
.393109799397623,0.69423838668577,0.912909472337382
"11897","MSTO1",29.2196111480029,0.136546443806831,0.347420767030284,0
.39302902061386,0.694298047249039,0.912911183753611
"11898","KLHL6",3.21963048766369,-0.391454740539659,0.99717327538943,-
0.392564412024362,0.694641228699608,0.913285656458898
"11899","TMBIM6",2466.57380609507,0.0676637148893237,0.172434513581174
,0.392402387921435,0.694760921940568,0.913289504362714
"11900","RPH3AL",13.1946378251924,0.208927788987134,0.53239983063143,0
.392426475303992,0.694743127212057,0.913289504362714
"11901","GLMN",48.7225193470517,0.108945591531732,0.277795287485998,0.
392179408504988,0.694925657585221,0.913429296832671
"11902","PEX11B",54.1737676504301,0.100091528917128,0.255434470292136,
0.391848166782873,0.695170403264044,0.913484143508902
"11903","S100A16",665.289273332029,-0.115225543685334,0.29408765343374
1,-0.391806804331875,0.695200967108194,0.913484143508902
"11904","NUP133",95.380232561408,0.0843796657545246,0.215299711407405,
0.391917226469736,0.695119374280254,0.913484143508902
"11905","MMUT",139.829421749374,0.0735751912135743,0.18775216013185,0.
391874006466322,0.695151309867967,0.913484143508902
"11906","HAAO",51.1360439165868,0.126340454318405,0.322683381992076,0.
391530712051071,0.69540499191678,0.913522026247413
"11907","BAK1",34.9470836422222,-0.151562960436645,0.387064658806337,-
0.391570134313084,0.695375858569789,0.913522026247413
"11908","SPATA13",77.2116513727542,0.0998914221506804,0.25508792970462
9,0.391596036183862,0.695356717138027,0.913522026247413
"11909","WDR25",25.424095140239,-0.166841500436096,0.426487092145896,-

0.391199413789108,0.695649841579445,0.913766938603347
"11910","CHML",42.1452370130234,0.108972744606623,0.278671097081794,0.391044301858969,0.69576448968425,0.913840798667567
"11911","BX255925.3",14.5706538859947,0.153273052423822,0.392142268381564,0.390860829811602,0.695900108620995,0.913924599488946
"11912","DHX8",98.4019085818159,0.0848090702526436,0.217014046622332,0.390799911676852,0.695945140261608,0.913924599488946
"11913","TTLL7",40.402047695956,0.136591262094453,0.350024978153034,0.390232899421063,0.696364336023574,0.913954464628959
"11914","PRPF3",87.0638805118699,0.106901221567098,0.273941427275187,0.390233863605125,0.696363623117404,0.913954464628959
"11915","AC019205.1",45.0554622007202,0.118416713463992,0.303526021983139,0.390136940122288,0.696435288523761,0.913954464628959
"11916","PPP2R5B",30.1357900083095,0.121472138984199,0.311330985261647,0.390170412630505,0.696410538581567,0.913954464628959
"11917","MSANTD2",62.1294312047308,-0.0842110585606829,0.215783745578541,-0.390256728257745,0.696346717345166,0.913954464628959
"11918","NANOG",3.58530838536778,0.2906270031452,0.744932914133625,0.390138491173002,0.696434141652594,0.913954464628959
"11919","AP000915.2",13.4503784833381,0.189358790172654,0.485025738204606,0.390409776754516,0.696233559526913,0.913954464628959
"11920","URB1-AS1",33.685316966212,0.153415921753296,0.392877033224898,0.390493484676349,0.69617167216263,0.913954464628959
"11921","BLCAP",252.949141640352,-0.0938059055978451,0.240594385881731,-0.389892329590588,0.69661616606177,0.914115148536554
"11922","ZNF195",78.0221951493295,-0.0755807515770484,0.193899868207104,-0.389792691846087,0.696689848251361,0.914135153178665
"11923","KIF11",5.84662288844385,0.24597387861986,0.631758433517378,0.389347993742443,0.697018737724867,0.914489986935343
"11924","FABP3",29.6368651259373,-0.183567831574684,0.473298014633882,-0.387848302547143,0.698128297707332,0.914640845172865
"11925","EFCAB7",68.9115181722296,0.0800263612098256,0.205854193869257,0.388752639456315,0.697459138527612,0.914640845172865
"11926","FNBP1L",75.0357208275286,-0.107430126022065,0.27703272727081,-0.38778857314229,0.698172502425072,0.914640845172865
"11927","PDE6D",61.2907879030296,-0.112527551451085,0.290250542456044,-0.38769109783189,0.698244644445078,0.914640845172865
"11928","TRAF3IP1",84.2656541905196,-0.0845986693086941,0.218086990358915,-0.387912498445993,0.698080788555032,0.914640845172865
"11929","ABHD14B",365.492756657607,-0.0929950947118805,0.23900020806197,-0.389100475961795,0.697201821404324,0.914640845172865
"11930","NAA50",243.280287958955,-0.0551212397578664,0.14184144542464,-0.388611661371938,0.697563439030962,0.914640845172865
"11931","GAP43",4.43263846432362,-0.359726160449825,0.92549445505913,-0.388685376215292,0.69750890150618,0.914640845172865
"11932","RNF146",349.862592835153,-0.0582424214819077,0.150208580843163,-0.387743637247463,0.6982057593923,0.914640845172865
"11933","AC007384.1",3.86866485247396,0.449622460280323,1.15780701356476,0.388339727616596,0.697764641246992,0.914640845172865

"11934","AL365203.2",4.23574321675365,-0.296293712595252,0.763964491739402,-0.387837020959767,0.698136646940038,0.914640845172865
"11935","IMMP1L",77.9542305350564,-0.115306896142539,0.296995066317719,-0.38824515697236,0.69783461850124,0.914640845172865
"11936","AP002761.3",2.89723883030965,0.361941130726142,0.933268410647411,0.387821045475077,0.698148470076256,0.914640845172865
"11937","PPME1",91.6339776043251,0.0756510048138308,0.194977347341228,0.387998943700032,0.698016815297906,0.914640845172865
"11938","LINC02356",8.40286821851422,-0.25694035999108,0.6626194177146 7,-0.387764609852892,0.698190237536227,0.914640845172865
"11939","LINC00598",4.66625568256161,0.257617733805671,0.664303786080178,0.387801092216834,0.698163237185882,0.914640845172865
"11940","LAMP1",1652.40324014835,0.0726273989738801,0.186833103332236,0.388728751375126,0.697476811360984,0.914640845172865
"11941","AC009159.3",3.89993473685571,0.311534426136837,0.801801817595257,0.388542928315108,0.697614292215682,0.914640845172865
"11942","PLEKHJ1",363.959151800357,-0.116341901534074,0.299111095458664,-0.388958829346241,0.697306602350525,0.914640845172865
"11943","CD8B",4.40551628468981,-0.284968101345534,0.735955371960906,-0.387208399044978,0.698601932672175,0.914684826706402
"11944","AC019080.1",23.5571152801247,-0.137266065963897,0.354302552833844,-0.387426127376141,0.698440764331528,0.914684826706402
"11945","SHQ1",34.8688596236075,0.111052489471503,0.286830034924688,0.387171760100582,0.698629055135722,0.914684826706402
"11946","TRMT10C",206.589424950138,0.0719593775877641,0.185706859977269,0.38748906527509,0.698394178546047,0.914684826706402
"11947","H1FX-AS1",29.4081063730843,-0.126915973339428,0.327755115332351,-0.38722804741196,0.698587387866949,0.914684826706402
"11948","SSPN",2227.91421753258,-0.0995967118387887,0.257216100813303,-0.387210254427578,0.698600559210883,0.914684826706402
"11949","ATRAID",1288.38775132625,-0.104847802734352,0.270877589081239,-0.387067099533681,0.698706533624836,0.914709708385079
"11950","CORO2B",94.0262738273716,-0.091035979783933,0.235371628924404,-0.3867755013633,0.698922415487009,0.914915761126634
"11951","POMZP3",18.4112199385731,0.161022036481212,0.416576027982746,0.386536972040746,0.699099026451201,0.915008278605288
"11952","AC004076.2",2.81641651107872,-0.34182524145125,0.884361562546252,-0.386522047008768,0.699110077727444,0.915008278605288
"11953","MEIS1-AS2",16.5933475163552,-0.176462727318163,0.456821606108004,-0.386283671697531,0.699286591945743,0.915162733858216
"11954","MRPS9",124.910368717161,-0.0898956636392778,0.232886013491615,-0.386007138391393,0.699491382091918,0.915306541500194
"11955","ACYP1",111.320953331939,-0.107188484519706,0.277744852355568,-0.385924288463441,0.699552741883193,0.915306541500194
"11956","AC090409.1",4.42208127794275,-0.262967406033991,0.681442307749721,-0.38589826760591,0.699572013691512,0.915306541500194
"11957","RAVER2",28.2918825579021,-0.13437405281788,0.348565249675475,-0.385506165468263,0.699862439427931,0.915456822474381

"11958","ZNF92",61.7815103918835,-0.0835843119413196,0.21679462428544,
-0.385546054090664,0.69983289235787,0.915456822474381
"11959","NEDD1",90.5938448030929,-0.0791334461258464,0.205226556028243
,-0.385590674312909,0.699799840944322,0.915456822474381
"11960","TMBIM1",367.142599947021,0.0567382952098554,0.147217947138019
,0.385403385340393,0.699938574801026,0.91547986000104
"11961","VARS2",27.6367808859682,0.114049807477125,0.296009956179881,0
.385290444108637,0.700022240587905,0.915507245608053
"11962","INHBA",13.5882475507665,0.291975538348173,0.758144863365564,0
.385118402111217,0.700149694654867,0.915507245608053
"11963","LRRC14",37.4065247311603,0.104292512940018,0.27086377908861,0
.3850367638336,0.700210177786798,0.915507245608053
"11964","AC005906.2",6.23121383465234,-0.37738518060769,0.980473019345
869,-0.384901137676859,0.700310662976792,0.915507245608053
"11965","NFKBIA",2061.25445235678,-0.118546662187695,0.307954189745074
,-0.384949015585172,0.700275189716422,0.915507245608053
"11966","VAPA",809.801151094647,-0.0731875522205504,0.190095522642332,
-0.385004082175328,0.700234391092287,0.915507245608053
"11967","GLE1",59.4659996602558,0.0907054516356838,0.236042218691422,0
.384276389785436,0.700773605344035,0.915959350634754
"11968","ZNF836",53.3800394783992,-0.0957052131435063,0.24900969987730
5,-0.384343313496074,0.700724009108241,0.915959350634754
"11969","NME7",100.263111721404,-0.092122308277018,0.240044493963878,-
0.383771803117803,0.701147588589873,0.916261216706848
"11970","YAP1",287.708633619122,0.0647939675320088,0.168824605915117,0
.383794572958082,0.701130710762932,0.916261216706848
"11971","AL157700.1",6.59429434120613,0.23148638234749,0.6032568864606
89,0.383727707951453,0.701180273937076,0.916261216706848
"11972","SEC13",288.979331384612,-0.0863186103148801,0.225080053692907
,-0.383501820346333,0.701347720798381,0.916326935308534
"11973","FAM215B",34.880974854873,-0.121347796402333,0.316374521419851
,-0.383557423833432,0.701306501478376,0.916326935308534
"11974","LARP7",484.510120512843,-0.0680881012278954,0.177611439052397
,-0.383354256860725,0.70145711507913,0.916393323132022
"11975","AC034243.1",3.39392576344281,-0.455759263170109,1.18961581617
256,-0.383114663552858,0.701634747658296,0.916451954105185
"11976","TAP2",80.0174317053609,-0.0870569200952034,0.227256811849744,
-0.383077274501074,0.701662469075954,0.916451954105185
"11977","SOX15",23.3474777998721,-0.176418070329603,0.46055345599435,-
0.383056663745386,0.701677750707524,0.916451954105185
"11978","LINC00710",23.2470368360043,-0.185214764351404,0.483864729636
103,-0.382782114519272,0.701881323896328,0.916564784181506
"11979","LRCH2",48.5398695576558,0.11813089922993,0.308579845182025,0.
38282117602414,0.701852359204956,0.916564784181506
"11980","RBM15",34.1129817056597,0.123192551065064,0.322226261018372,0
.38231691816714,0.702226307245481,0.916632687269781
"11981","AC010883.1",4.59394073762045,-0.261210622444553,0.68312758912
3707,-0.382374576292,0.702183545423998,0.916632687269781
"11982","KDM3A",101.811225992998,0.102367013634775,0.267613551430713,0
.38251804920753,0.702077143641349,0.916632687269781

"11983","DNPH1",330.683975860476,-0.133669999877852,0.349496172130268,
-0.38246484664796,0.702116598819639,0.916632687269781
"11984","UHRF1BP1L",122.739725851558,0.0826761055703852,0.216208702967
821,0.382390275856241,0.702171902094461,0.916632687269781
"11985","CEP164",161.758165588879,-0.0900867826176636,0.23570200094170
8,-0.382206270026291,0.702308371452067,0.91666331703168
"11986","MAFTRR",8.50532163919063,0.239914246569082,0.628125172659495,
0.381952924372192,0.702496282934495,0.916832083592884
"11987","PYGL",157.226562804524,-0.0812740086916343,0.212835878740489,
-0.381862349396137,0.702563468598931,0.916843275155842
"11988","SGTB",71.0645514114038,-0.0878896222902695,0.230266585365552,
-0.38168639253821,0.702693994495819,0.9169371167749
"11989","MRPS16",303.977456544564,-0.0872872118837526,0.22914947276574
5,-0.380918231363266,0.703263923884776,0.917604267355873
"11990","GHRLOS",3.59300209475508,0.329182344413997,0.865035248265447,
0.38054211672191,0.703543039169604,0.917635708451382
"11991","C4orf47",3.12870652207596,-0.318450070834157,0.83690479374162
2,-0.380509316251417,0.703567382346399,0.917635708451382
"11992","C11orf53",13.2499134885686,-0.542407241437795,1.4249263371786
1,-0.380656338005355,0.703458271144374,0.917635708451382
"11993","KIAA0100",144.542567913065,0.0719807834401834,0.1890590073287
23,0.380731838473202,0.70340224135482,0.917635708451382
"11994","GDI1",209.757530056927,-0.0647042572784099,0.170054843684518,
-0.380490528093677,0.703581326290729,0.917635708451382
"11995","CTH",7.86570742755846,0.209986849391025,0.552136479985417,0.3
80316927069502,0.703710171898699,0.917727237933417
"11996","ARMCX5",47.9244430761171,0.0899744906645104,0.236638266804793
,0.380219530337973,0.703782462876791,0.917745003899769
"11997","PUS7L",203.661519049737,0.0606960840719022,0.159853306454816,
0.37969864632771,0.70416912512611,0.918172678531944
"11998","AL139260.1",14.1795873629007,-0.165476982486838,0.43750788141
7719,-0.378226289205624,0.705262497559141,0.918217203080357
"11999","SNAPIN",183.593767105523,-0.0936041145898874,0.24701462812178
3,-0.378941584559675,0.704731243016823,0.918217203080357
"12000","GORASP2",241.560240104413,-0.0735725295474356,0.1939610695565
2,-0.37931596126818,0.70445324836885,0.918217203080357
"12001","BARD1",33.172800378575,0.125532511177917,0.331028164504117,0.
379220032126167,0.704524477106473,0.918217203080357
"12002","NISCH",173.72644102541,0.083702740270774,0.221410503381737,0.
378043222847749,0.705398485253332,0.918217203080357
"12003","AC080013.3",4.07190814154337,0.41373965204973,1.0904974552071
2,0.379404509450365,0.704387502398067,0.918217203080357
"12004","USP45",43.3680857993284,0.107056926933749,0.282660713255634,0
.378747105321737,0.70487566977839,0.918217203080357
"12005","HDAC2",267.859203587914,0.0603919682708009,0.159384032659482,
0.378908522159342,0.704755795506077,0.918217203080357
"12006","ZNF800",151.778537477599,0.064436828644349,0.16988522468652,0
.379296249943166,0.704467884093458,0.918217203080357
"12007","C9orf64",87.677643432027,0.0749369787790788,0.198174671402472
,0.378136006224982,0.705329561541464,0.918217203080357

"12008","ANKRD2",8.70065499099838,0.269409411600988,0.71273687013423,0
.377992808973457,0.705435935985408,0.918217203080357
"12009","GSKIP",51.0523997054912,-0.102089611567585,0.269507554784879,
-0.378800555884501,0.704835974549313,0.918217203080357
"12010","TMEM92-
AS1",4.69854323088627,0.287982493070302,0.760968475705637,0.3784420804
07154,0.705102212868828,0.918217203080357
"12011","ZNF627",16.5727577686625,0.165647864032703,0.436623296562704,
0.379383934244365,0.704402779046529,0.918217203080357
"12012","ZNF470",18.192106908055,0.155223872215036,0.410289292707239,0
.378327865177305,0.705187047657514,0.918217203080357
"12013","C20orf96",45.4352328405753,-0.106153024217205,0.2805731036028
94,-0.378343550590105,0.705175396904051,0.918217203080357
"12014","DUSP15",5.30217988403373,-0.260875497864139,0.688127974469088
,-0.379108984873654,0.704606934489764,0.918217203080357
"12015","GGT7",91.5206461355535,-0.0772438729821974,0.203845898300102,
-0.378932682120878,0.70473785403206,0.918217203080357
"12016","SAT1",2075.81739397568,-0.150890763177726,0.398731391588969,-
0.378427097441256,0.70511334144497,0.918217203080357
"12017","TRMT2B",22.805422490705,-0.119676601060077,0.316234695587078,
-0.378442349084758,0.705102013309505,0.918217203080357
"12018","MTMR1",25.4220630075784,0.118051116826701,0.312079999258467,0
.378271972273781,0.705228564145766,0.918217203080357
"12019","MRPS17",151.612011616686,-0.0975597623178004,0.25836056096492
4,-0.377610893680655,0.705719670887852,0.918474130032825
"12020","SUMF2",474.286771453494,-0.0739251988861336,0.195792523873307
,-0.377569058428242,0.705750753883178,0.918474130032825
"12021","VANGL1",94.3616986253759,0.0918750315104011,0.243699394026424
,0.377001477075643,0.706172507223225,0.918506933812023
"12022","ANKRD23",7.39037682318625,-0.200102696707217,0.53065599230394
5,-0.377085531133706,0.706110043379726,0.918506933812023
"12023","ASNSD1",168.313834243208,-0.0805978507042226,0.21379760724421
6,-0.376982005285763,0.706186977750892,0.918506933812023
"12024","THAP4",103.84560378353,-0.082335414852291,0.218379703858723,-
0.377028695421057,0.706152279994177,0.918506933812023
"12025","AC026329.1",13.3057322998941,0.188513368246253,0.499790087894
753,0.377185087924254,0.706036061413284,0.918506933812023
"12026","SLC18B1",62.5218654919268,0.116446391887957,0.308775285072826
,0.377123421197693,0.706081886445146,0.918506933812023
"12027","ABCA3",15.1382568511739,-0.156366231110966,0.414372690989791,
-0.377356506620797,0.705908684425062,0.918506933812023
"12028","SH3D21",14.8027072627698,0.208401295006766,0.553714548039446,
0.376369549517271,0.706642180552927,0.918613043332182
"12029","STARD7",139.124697813337,0.061457281934077,0.163265968763955,
0.376424324060638,0.706601465540947,0.918613043332182
"12030","OXCT1-
AS1",4.70984984437879,0.262579772341849,0.696973095608475,0.3767430536
36254,0.706364564140124,0.918613043332182
"12031","RING1",157.396881471483,-0.0700361635187423,0.186067024131957
,-0.376402878723279,0.706617406188499,0.918613043332182

"12032","RGL2",114.685970516431,-0.0841545065490172,0.223583538367981,
-0.376389546221926,0.706627316505719,0.918613043332182
"12033","CLK3",125.446255295551,0.0663209447890132,0.176072748077263,0
.37666785753756,0.706420452408918,0.918613043332182
"12034","DDT",565.359175033623,-0.0879754446564981,0.233778797569078,-
0.376319176808594,0.706679624334174,0.918613043332182
"12035","AC004148.1",51.4617913563666,-0.149349450440623,0.39699970147
4753,-0.376195372152239,0.706771655644608,0.918656336456054
"12036","ATP6V1D",252.906451231773,-0.0797244874860577,0.2120017920340
73,-0.376055724440501,0.706875469177736,0.91871493555561
"12037","RNLS",39.4974056739418,0.0949578210668276,0.252726117157428,0
.375734103522338,0.707114581576645,0.918720382026865
"12038","AL606834.1",20.5835455709477,0.190079430322836,0.505868347083
352,0.37574881175856,0.707103645955565,0.918720382026865
"12039","AC079915.1",6.51060766129632,0.232098366038571,0.617591513393
039,0.375812103964036,0.707056588686237,0.918720382026865
"12040","RMC1",35.1837151010589,0.119148403674488,0.316908519117866,0.
375970971074382,0.706938477139269,0.918720382026865
"12041","ATXN7L3B",378.632033075452,-0.0496695263334656,0.132264943104
404,-0.37553054624806,0.707265933271778,0.91876440742156
"12042","ATP13A1",81.1113978431679,0.0872080718581549,0.23219570153218
1,0.375580044258779,0.707229128767423,0.91876440742156
"12043","OSBPL10-
AS1",5.52120122215834,-0.246329429553851,0.656455448242537,-0.37524165
6099168,0.707480752696477,0.918890851414064
"12044","LZTFL1",66.6975517030531,-0.0850330031488393,0.22659434529739
,-0.375265336110833,0.707463143305169,0.918890851414064
"12045","ZNF362",84.9962339139749,0.0734528657556674,0.195959923386502
,0.374836162855568,0.707782317750613,0.918928944516735
"12046","AC079807.1",7.16142966953289,0.219389295017872,0.585220487216
685,0.374883142012491,0.707747377034452,0.918928944516735
"12047","SPDYE16",3.58247672348155,0.295162170269045,0.787483517172929
,0.374816950237485,0.707796607299581,0.918928944516735
"12048","GNL3L",129.643931419883,-0.102592440348643,0.273571984816726,
-0.37501076880139,0.70765245780342,0.918928944516735
"12049","ZDHHC15",25.7401983316361,-0.129234722746452,0.34480312968595
6,-0.374807278762691,0.707803800580588,0.918928944516735
"12050","RETSAT",83.2997596044777,-0.0753137286651446,0.20131622521203
9,-0.374106600627045,0.708325008094248,0.918946680512384
"12051","DKK2",103.5816806255,0.142261296351545,0.380298320196684,0.37
4078161265528,0.708346165928424,0.918946680512384
"12052","GYPE",7.9327549302673,0.208415305727862,0.55655484627067,0.37
4473975250416,0.708051715189213,0.918946680512384
"12053","SKAP1",4.76061121385862,0.303250449782359,0.810387922183511,0
.374204058921906,0.708252504431367,0.918946680512384
"12054","AC127496.5",2.75192186767187,-0.329919595967715,0.88167816428
7948,-0.374195040016854,0.708259213894457,0.918946680512384
"12055","EPOR",35.2656335188617,-0.108906000465195,0.290810158158383,-
0.374491734246374,0.70803850508318,0.918946680512384
"12056","CLDND2",25.27038037571,-0.131944149102271,0.352692441073583,-

0.374105406684185,0.708325896339095,0.918946680512384
"12057","PYGB",97.6802356533546,-0.108289619276614,0.28901129311727,-0
.374689923388822,0.707891087193442,0.918946680512384
"12058","CDR1",15.9477448528644,0.161385757655595,0.431335057570139,0.
37415404758597,0.708289709810968,0.918946680512384
"12059","SEC31B",30.0669398499567,0.166307805762421,0.444803499716925,
0.373890506410717,0.708485779848866,0.918975377626518
"12060","FAM222B",60.0743913339963,0.0990427171503616,0.26489214879576
2,0.373898273696008,0.708480000847696,0.918975377626518
"12061","FAM161A",56.757268910243,0.101511558632792,0.271714984053172,
0.373595732993978,0.70870510902021,0.919029466627515
"12062","AC012459.1",7.65264537995898,0.283912971309974,0.760103906160
967,0.373518632135341,0.708762480687486,0.919029466627515
"12063","PODNL1",7.25259969917974,0.231069944500003,0.618577645238927,
0.373550428597773,0.708738820360847,0.919029466627515
"12064","NHS",12.7344695458165,0.206635999970775,0.553200659069876,0.3
73528116033344,0.708755423516893,0.919029466627515
"12065","ADAMTS12",69.4820701199819,-0.0917988587636608,0.246177418743
336,-0.372897153736793,0.70922499047606,0.919198147497729
"12066","AL035530.2",13.8217809603473,0.173103739000081,0.464246781960
276,0.372870089199439,0.709245134584005,0.919198147497729
"12067","ACAT1",367.369371251132,0.0812151897227825,0.217706503788551,
0.373048982503818,0.709111988246983,0.919198147497729
"12068","NXPH3",162.291532095663,0.107765377819407,0.288777908264132,0
.373177361340393,0.70901644418905,0.919198147497729
"12069","CBX4",62.4150852109352,0.11528776189186,0.3090682272696,0.373
017190768349,0.709135649484718,0.919198147497729
"12070","ARMCX5-
GPRASP2",31.9388831772946,0.112229956189099,0.300851798062304,0.373040
669565343,0.709118175186742,0.919198147497729
"12071","JAK1",1039.40154091375,0.0553200573296234,0.148759798134855,0
.371875049732686,0.709985882481443,0.919724005920731
"12072","EFNA3",5.10826998596702,0.260042085115321,0.698623986775636,0
.372220378970231,0.709728774240841,0.919724005920731
"12073","STK32B",14.7191593201965,-0.238413267578445,0.641076140651003
,-0.371895399720133,0.709970730373928,0.919724005920731
"12074","C7orf50",206.298086143657,0.110369106747363,0.296682981884212
,0.372010238155274,0.709885226607577,0.919724005920731
"12075","ACTB",8616.72794637834,-0.120904794599642,0.325112658809141,-
0.371885841180426,0.709977847416683,0.919724005920731
"12076","ZSCAN21",52.309686886096,-0.0826779602067328,0.22234152531389
3,-0.37185118744693,0.710003649907226,0.919724005920731
"12077","ZC3H15",517.323263282788,-0.06095472904639,0.163964249501718,
-0.371756216563241,0.710074365069396,0.91973944628472
"12078","AL390195.2",2.74662962668018,-0.302670631937223,0.81444396711
8147,-0.371628551695461,0.710169428051598,0.91978641853048
"12079","TAS2R14",20.5858439854929,0.138140380914016,0.372163571628569
,0.371181898081859,0.710502054838106,0.91998871317213
"12080","CLEC14A",78.3144678664246,-0.147829487588835,0.39825951898333
1,-0.371188836782136,0.710496887106477,0.91998871317213

"12081","SMIM7",627.157659697269,0.0685672028315782,0.18468470732393,0
.371266272259966,0.710439216435404,0.91998871317213
"12082","HDAC8",136.594792271442,0.0778129175046351,0.209692553308076,
0.371080976778006,0.710577219441337,0.920009886088465
"12083","RARA-
AS1",70.2493391466038,-0.12559601715711,0.338801779597836,-0.370706485
975943,0.710856158911205,0.920294868314821
"12084","LINC01715",4.38811916473874,0.247012557276126,0.6669575369698
58,0.37035724702709,0.711116324436981,0.920441658845827
"12085","KPNA6",193.11486348733,0.072649893739143,0.196182809555646,0.
370317327515572,0.711146064617443,0.920441658845827
"12086","FAM66C",37.4140492452223,0.118674789324457,0.320362867685967,
0.370438653460883,0.711055677711989,0.920441658845827
"12087","CREBRF",596.574371164189,0.0882363193154163,0.238354246080363
,0.370189836205673,0.711241049048442,0.920488436358466
"12088","RAP1B",1245.59240922688,0.0921537434574541,0.249164699338917,
0.369850720033601,0.711493721471499,0.92051081672141
"12089","PRC1",16.5046782452767,-0.180300128660245,0.48725147528178,-0
.370035059526452,0.71135636783303,0.92051081672141
"12090","KIAA0753",35.771057826424,0.107217376395613,0.289806765050832
,0.369961606578806,0.711411097411168,0.92051081672141
"12091","RBCK1",396.827905083633,-0.083409948019338,0.22548768761995,-
0.369909101910353,0.711450219398807,0.92051081672141
"12092","AL139339.1",16.7832506863408,-0.172937293057805,0.46782915614
5192,-0.369659074869916,0.711636528584601,0.920619435713606
"12093","YAE1",84.7491782024781,-0.0816198905524803,0.221060818396681,
-0.369219163958844,0.711964372700986,0.920674167710876
"12094","CLEC2D",57.9587961300272,0.109715841366983,0.297136232752504,
0.369244236391626,0.711945686009782,0.920674167710876
"12095","PIP4K2C",23.2322135816618,0.152016862488493,0.411738354327131
,0.369207436933877,0.711973113008916,0.920674167710876
"12096","CRLF1",177.755698792865,0.256113710619861,0.693682470628561,0
.369208854863798,0.711972056204846,0.920674167710876
"12097","POLDIP3",159.031746228574,0.0723946937802372,0.19595205951087
6,0.369451048184666,0.711791554072273,0.920674167710876
"12098","FBXO42",86.8321816042972,0.0766138134733878,0.207714577284366
,0.368841775454699,0.712245664361902,0.920722165739463
"12099","GCFC2",87.1413023033638,-0.0758643160673146,0.205630932929869
,-0.36893435723111,0.712176653647925,0.920722165739463
"12100","AC079322.1",3.24255867460805,0.328191836609058,0.889645010600
943,0.36890201450955,0.712200761734786,0.920722165739463
"12101","SETD1A",45.0452978710468,0.0949595735365635,0.2573266684232,0
.369023444474059,0.712110249994271,0.920722165739463
"12102","AL583856.2",2.72555646561413,0.325748751756927,0.883700976422
425,0.368618752777312,0.712411915761866,0.920725243574842
"12103","FIBIN",518.471523899042,-0.138718269291271,0.37624001543139,-
0.368696214123367,0.712354170948793,0.920725243574842
"12104","SPTLC2",81.9194812563603,0.0816509987589543,0.221515518351338
,0.368601709562626,0.712424621123179,0.920725243574842
"12105","PEX1",100.905202417748,0.0783023663270042,0.212504383186359,0

.368474123464719,0.712519736434909,0.920772097236785
"12106","RSBN1L",453.04863252535,-0.0850208559681059,0.23087663273422,-0.368252321429082,0.712685100279749,0.920833651910144
"12107","PPP1R13L",20.5128045380105,0.160521888594579,0.435883427856409,0.36826793205696,0.712673461380828,0.920833651910144
"12108","AL645728.1",7.01502851196544,-0.19555730098339,0.531301678241121,-0.368072055843649,0.712819506698523,0.920922413921686
"12109","ZNF7",85.5063312317797,-0.0818111182443053,0.222359154734669,-0.367923319109244,0.71293041185141,0.920922413921686
"12110","NSMCE1",281.560889828723,0.110734216286769,0.300928602284889,0.367975046060718,0.712891841098311,0.920922413921686
"12111","ITGB7",5.17737145774686,0.247161738130097,0.67209875633371,0.367746162005062,0.713062516497404,0.921017004836008
"12112","AL034417.4",5.3889774787917,-0.30171934455493,0.821835864264125,-0.367128471358561,0.713523190805316,0.92102920004434
"12113","FKBP7",322.196151659832,0.0963844331481004,0.262568677511382,0.367082753592809,0.713557291315705,0.92102920004434
"12114","SNRK",111.60917998908,0.0796783885111316,0.216949265853508,0.367267380222128,0.71341958332318,0.92102920004434
"12115","REST",266.974422871091,0.0575308719824431,0.156650504205584,0.36725621966043,0.713427907418166,0.92102920004434
"12116","HMGN3",880.611372276853,-0.0797412908037847,0.21696797052964,-0.367525633433951,0.713226974926836,0.92102920004434
"12117","PTPRN2",91.6022473584915,0.0946176292904733,0.257797546266693,0.367023001811627,0.713601860547044,0.92102920004434
"12118","CRY2",129.330508596371,-0.0862731818553205,0.234718603490146,-0.367560050939646,0.713201307311381,0.92102920004434
"12119","TMEM143",24.0319121253358,0.121313410462336,0.330281240560179,0.367303363208219,0.713392745677448,0.92102920004434
"12120","PJA1",100.914820409403,-0.0831011724878184,0.226174048442367,-0.36742134236941,0.713304754275328,0.92102920004434
"12121","HAUS6",72.3797529191116,0.081317232904412,0.221715756574778,0.366763436936815,0.713795482971773,0.921127102798833
"12122","DGKA",39.3732569369456,0.119962264506249,0.32706977318037,0.366778817069389,0.713784009647626,0.921127102798833
"12123","SGCB",403.534281511041,0.0638549613739596,0.174196511476745,0.366568542806233,0.713940876370452,0.921238730434957
"12124","VAV2",31.5416526849095,-0.127056230834986,0.346738809360083,-0.366432102219743,0.714042668894069,0.921294083595341
"12125","SLMAP",143.979172460017,0.0821663067939601,0.224427493099554,0.36611515665557,0.714279148150918,0.9215231929505
"12126","NASP",444.864193555714,-0.0703250286929232,0.192339226234501,-0.365630194473084,0.714641040990644,0.921702704143818
"12127","HSD11B1-AS1",14.4944048702078,-0.194028135874476,0.531168500175326,-0.365285471202512,0.714898322514817,0.921702704143818
"12128","ZNRD1",207.207896665704,-0.0825000045952027,0.225542408602202,-0.365784887669224,0.714525597469111,0.921702704143818
"12129","MTHFD1L",32.8244304250236,-0.124040130746698,0.339634518902409,-0.365216501395547,0.714949801482735,0.921702704143818

"12130","TIMM10B",115.323136999843,-0.0723096696541434,0.197824628748425,-0.365524101380218,0.714720219290114,0.921702704143818
"12131","FADS1",25.4590179711519,0.182022965810743,0.498403905640813,0.365211756470308,0.714953343136005,0.921702704143818
"12132","BORCS5",75.4877225338876,0.0869578970770857,0.237930263283932,0.365476404207207,0.714755817151406,0.921702704143818
"12133","RNF34",91.8262549807026,-0.0736982440536132,0.201775586093973,-0.365248568869426,0.714925866203562,0.921702704143818
"12134","ABCB9",7.82141853922927,-0.202262334429484,0.553932126598328,-0.365139201568913,0.715007499506823,0.921702704143818
"12135","Z95115.1",9.51746938501019,-0.184217672147366,0.504133964194417,-0.365414126464853,0.71480229787232,0.921702704143818
"12136","MYL3",29.3469232507721,-0.126201421388706,0.345762495662779,-0.364994535184609,0.715115485554545,0.921765947637587
"12137","WASF3-AS1",4.33483019238274,0.291852332934296,0.800000872748157,0.364815018178327,0.715249493734714,0.921862719823032
"12138","AC012184.3",4.11808054052099,0.264032126643169,0.724283700411255,0.364542411341369,0.715453009599539,0.922049054965035
"12139","ODC1",65.6520959499634,-0.119986484987827,0.329435403081324,-0.364218550482284,0.715694815672237,0.92213277337623
"12140","EZH2",12.8569151824807,-0.188769462389392,0.51824905091888,-0.364244685165742,0.715675301526755,0.92213277337623
"12141","WNK3",7.80070762402787,0.211884703095196,0.581551847792131,0.364343615964112,0.715601433939255,0.92213277337623
"12142","THY1",3835.34174129918,-0.146014298277241,0.401056824097326,-0.364073840673028,0.715802870400544,0.922196038681907
"12143","CCDC189",7.43155252586946,-0.188188889259031,0.517088321138044,-0.363939546816396,0.715903152636385,0.922249280794777
"12144","PIK3R3",5.04817869323502,-0.32204655997281,0.885223257389214,-0.363802642197428,0.716005389471165,0.92230503190855
"12145","ZSCAN16",10.1909328312966,-0.209445884229449,0.57601355702875,-0.363612768612311,0.716147190590496,0.922372946414041
"12146","RRAS",1084.10298472336,0.103415215497279,0.284502293613624,0.363495190790007,0.716235004800317,0.922372946414041
"12147","ZSCAN18",220.220688616942,0.0667849224227778,0.183703221184805,0.363547911637283,0.716195629227314,0.922372946414041
"12148","LRP10",864.319068307985,-0.0539601254437269,0.14854536014781,-0.363256889276339,0.716412994255518,0.922526215767127
"12149","TARDBP",217.699598491808,0.0775975612700105,0.21366627375845,0.363171781418974,0.716476565782597,0.922532135857862
"12150","HFM1",7.73252786600465,0.213316606562764,0.587953956591092,0.362811754511451,0.716745510514972,0.922650594221997
"12151","LARS",264.702752681799,-0.0582935748159494,0.160642888902923,-0.362876783491964,0.716696930429159,0.922650594221997
"12152","POLR3D",88.4925847885217,0.0863094005691735,0.237835099451194,0.362895976112579,0.716682592746142,0.922650594221997
"12153","AL022068.1",3.37431334780027,0.332407038495679,0.917004478082872,0.362492273964271,0.716984196015669,0.92273005168845
"12154","AGK",53.1975030212683,0.0823115648750759,0.226982970143123,0.

362633217915753,0.716878892681897,0.92273005168845
"12155","SNAP23",245.61002276764,-0.0629993591086736,0.173777867023311
,-0.362528095135515,0.71695743246467,0.92273005168845
"12156","FOXO3B",9.88202053615649,0.174565204100639,0.482396911629862,
0.361870484433326,0.717448817293951,0.923176100101116
"12157","QPCTL",4.90739565174788,0.260486919893709,0.719807005882727,0
.361884390905953,0.717438424783521,0.923176100101116
"12158","COMMD1",403.384866341985,-0.105754532820769,0.292316226645848
,-0.361781260090273,0.717515497194918,0.923185961722331
"12159","BLOC1S5",159.656699917625,0.0605403129137591,0.16751531518066
6,0.361401659594325,0.717799207544949,0.923399095692898
"12160","ANAPC7",108.628740923309,0.0723514143248644,0.200161205859344
,0.361465719664513,0.717751326833909,0.923399095692898
"12161","TAF6",98.5850116441803,-0.0669014178592524,0.18517109977092,-
0.361295136995015,0.717878828672346,0.923425583169272
"12162","CCNT2-
AS1",3.65631886555545,0.277011107715451,0.768012097572566,0.3606858649
63575,0.718334292552506,0.923613040687778
"12163","GABBR1",110.399334522795,-0.0979168346322953,0.27156669572492
7,-0.360562750048984,0.718426339788999,0.923613040687778
"12164","ABCC10",23.9484757145774,0.125962658741069,0.349209406061601,
0.360708092492929,0.718317674508826,0.923613040687778
"12165","VSTM4",172.565990761165,0.101944094819348,0.282485415340333,0
.360882683789277,0.718187148855682,0.923613040687778
"12166","BCL9L",253.150084713456,0.093147590084627,0.258086760163663,0
.360915802211468,0.718162390223462,0.923613040687778
"12167","AC009113.1",39.5827151814026,0.104268822058165,0.289195927411
005,0.360547338932536,0.718437862244383,0.923613040687778
"12168","SOCS7",42.7758622307006,0.124826891574881,0.345959179149114,0
.360813931521901,0.718238547541923,0.923613040687778
"12169","PIK3C2B",34.2663825677956,-0.119946031750605,0.33314915912902
4,-0.360037023848982,0.718819446528967,0.923648151967523
"12170","KRCC1",313.322520618402,0.0573240112183383,0.159089272089071,
0.360326063885964,0.718603310388983,0.923648151967523
"12171","AC018809.2",10.9243944662157,-0.217351031712721,0.60358936892
3624,-0.360097514806004,0.718774211203831,0.923648151967523
"12172","CCP110",115.635308175894,0.0940538009071367,0.26116202744683,
0.360135819998889,0.718745566971397,0.923648151967523
"12173","ENDOV",120.304098975928,0.074469179320247,0.206632070345953,0
.360395069340239,0.718551713347919,0.923648151967523
"12174","APOBEC3G",90.7916189790612,-0.103854939438266,0.2883124435419
67,-0.360216639151577,0.718685132529978,0.923648151967523
"12175","PLD2",59.3566058540288,-0.0853687132628545,0.237472675137668,
-0.359488573636374,0.719229624239565,0.924099302831993
"12176","LMF1",97.6610496365744,-0.088355159623744,0.24589199351164,-0
.359325077494081,0.719351916212801,0.924180521133118
"12177","ARID4B",607.755265386965,0.0701369250284811,0.195659390381483
,0.358464395149821,0.719995808465605,0.924255150628893
"12178","AC108865.2",4.18345373611933,-0.296572334668925,0.82706283951
6556,-0.358585007690928,0.719905564030518,0.924255150628893

"12179","ADIRF-
AS1",27.74705380569,-0.184478674223247,0.514594800653297,-0.3584930784
16347,0.719974346784951,0.924255150628893
"12180","FBXW4",80.1822015371222,-0.0706288492790989,0.196968495057335
,-0.358579422859172,0.719909742614181,0.924255150628893
"12181","KRAS",249.605545192497,0.076483479669615,0.213344623795917,0.
358497337822669,0.71997115978815 6,0.924255150628893
"12182","NELL2",5.03975302424451,-0.287601868361018,0.80199667962923,-
0.358607305573858,0.719888880787833,0.924255150628893
"12183","PLBD2",298.0929224192,0.0691779806776601,0.193072823206667,0.
358299938482856,0.720118864137552,0.924255150628893
"12184","EDC3",45.9284608008251,-0.0902823681309074,0.251635827891334,
-0.358781851087973,0.719758290704874,0.924255150628893
"12185","ENKD1",75.8303801938343,-0.08292857479636,0.231106526769375,-
0.358832681861538,0.719720262075903,0.924255150628893
"12186","GCSH",444.447141643033,-0.105583870086793,0.294668756075707,-
0.358313760484556,0.720108521464029,0.924255150628893
"12187","TOP2A",4.02728634418497,0.300173656037999,0.836607963130693,0
.358798468657542,0.719745858329325,0.924255150628893
"12188","TMEM104",23.2010447312258,0.117647318489643,0.328348885200893
,0.358299734800875,0.720119016548293,0.924255150628893
"12189","CCNB1",18.6968997323808,-0.143281217200693,0.40017524414174,-
0.3580461792632 6,0.720308755197313,0.924271171975356
"12190","PRSS36",17.6050896601876,-0.213406800032841,0.595953328394103
,-0.358093142306801,0.720273610888593,0.924271171975356
"12191","ZNF700",27.5759702709186,0.105708776000861,0.295123808055299,
0.358184508045699,0.720205239974732,0.924271171975356
"12192","DERL1",292.2639447341,-0.062097024694744,0.17347479871732 6,-0
.357959917831811,0.720373309588417,0.924271471188792
"12193","SPESP1",25.0683137159153,-0.145841010580012,0.407504654047464
,-0.357887963073485,0.72042715899795,0.924271471188792
"12194","AC100786.1",3.80536370712844,-0.325359512383847,0.90963979511
2699,-0.357679505813108,0.720583171801745,0.924395814047457
"12195","RAB7A",748.845236326753,-0.0762688111805395,0.213417420281171
,-0.357369192636935,0.720815436775214,0.924516200133139
"12196","FN3KRP",140.045160558797,-0.117188490611125,0.327996970026913
,-0.357285284072927,0.720878245560182,0.924516200133139
"12197","NDUFA7",215.480449161691,-0.0929968821585225,0.26026721943804
,-0.357313081375819,0.720857438002058,0.924516200133139
"12198","AC008555.4",7.29235876585001,-0.296893554300804,0.83107986347
6946,-0.35723829603897,0.720913418731959,0.924516200133139
"12199","ATP6V1G1",1132.05823221308,-0.0774634035698057,0.216980323373
16,-0.357006581820717,0.721086878419113,0.924662844422508
"12200","LRRC27",28.5554151917036,-0.104974754374436,0.294192160457393
,-0.356823765158211,0.721223743856191,0.924762543044459
"12201","JADE2",104.50132442427,-0.0830526234282804,0.232876528400634,
-0.356638017573841,0.721362812668115,0.92483527543827
"12202","CCDC107",549.946527918584,-0.11114672113704,0.311762291400982
,-0.356511111839646,0.721457832012607,0.92483527543827
"12203","RAP2A",119.713339808189,0.0703885813472432,0.19741431800761,0

.356552564462572,0.721426794320809,0.92483527543827
"12204","RAB5B",213.388068065955,0.0672416251802066,0.188721032881686,
0.356301701794744,0.721614634998364,0.924960483061243
"12205","NSUN2",52.8040881347915,0.0940710865242691,0.264507681984284,
0.355645952580911,0.722105725469009,0.925514122368841
"12206","PPP1R12A-
AS1",7.49865432761129,-0.187972839591487,0.52893156247299,-0.355382157
027329,0.722303314230395,0.925615691366107
"12207","NUBPL",70.988124622877,-0.0703815244174081,0.198006552615561,
-0.355450481247745,0.722252136085211,0.925615691366107
"12208","CHN2",12.3374583944322,0.17140629194119,0.482496830306119,0.3
55248534653465,0.722403407441744,0.925645441622632
"12209","TMEM246",3.37285472065641,0.343703977996746,0.967653661529393
,0.35519317671316,0.722444876096063,0.925645441622632
"12210","ULK1",106.613531551841,0.109358055383179,0.307962330626123,0.
355102051477664,0.722513139820725,0.925657088142146
"12211","OSMR-
AS1",29.1841728472999,0.106472929068955,0.29996954207681,0.35494579993
622,0.722630196096228,0.925660251968031
"12212","CPEB1-
AS1",3.80105135765285,0.353370450479168,0.995797394406296,0.3548617946
42323,0.722693131578697,0.925660251968031
"12213","DLG4",46.3234580169531,0.104163758979586,0.293514473446484,0.
35488457436692,0.722676065171721,0.925660251968031
"12214","RPS10-
NUDT3",7.37595492666835,0.361082878835517,1.01927420140964,0.354254898
570124,0.723147864556304,0.9261119075883
"12215","SLC25A17",63.8929410017902,-0.0749000108206332,0.211442690136
328,-0.354233153070183,0.723164159764181,0.9261119075883
"12216","CPS1",10.6891147729388,-0.183763498842662,0.519004027814868,-
0.354069504270228,0.723286795655557,0.926193135595929
"12217","TRNT1",76.5282785848513,0.0820945403373773,0.231930546433887,
0.353961742425242,0.723367554603738,0.92622072985727
"12218","CHD1L",11.2744417324001,-0.183731573585945,0.519608565196092,
-0.353596121951162,0.72364158107588,0.926495764672614
"12219","FHL1",3195.96534891016,0.0899719106906438,0.254563655899093,0
.35343580517365,0.723761747012832,0.926573779239031
"12220","FMO1",169.593920673649,-0.18988351562556,0.537778245520208,-0
.353088874842604,0.724021813023828,0.926639345253805
"12221","ZNF483",57.8984000747054,-0.115365347221772,0.326742822124961
,-0.353076913737529,0.72403077987903,0.926639345253805
"12222","ME2",112.917183881487,-0.0746143922090911,0.21130971467894,-0
.353104410379138,0.72401016658903,0.926639345253805
"12223","CCDC159",32.7629448913105,-0.0967845495857459,0.2741372790402
22,-0.353051397914931,0.724049908395912,0.926639345253805
"12224","HNRNPDL",907.345467848977,0.0805502453778689,0.22861773988189
1,0.352335936045397,0.724586340780124,0.926643568412646
"12225","AC093827.4",35.6960647849656,0.117898152248958,0.334099455424
735,0.352883401438281,0.724175855078152,0.926643568412646
"12226","HERC6",22.7596334497378,0.118485701855766,0.336178499838969,0

.352448779182847,0.724501725306758,0.926643568412646
"12227","FBXW11",205.320110508005,0.0823407024195756,0.233517830152954,0.352609915763788,0.724380902808723,0.926643568412646
"12228","POLM",61.6498730702289,-0.0793307422621316,0.224857231087675,-0.35280494151064,0.724234678914981,0.926643568412646
"12229","TIAL1",369.826143589201,0.0495197179332139,0.140527002196723,0.35238578464722,0.724548961370513,0.926643568412646
"12230","FBXO33",90.4130056374605,-0.0826535057079153,0.234347071288393,-0.352696985942701,0.724315619208047,0.926643568412646
"12231","KANK2",473.580459877979,0.0768024431019271,0.217918040100278,0.35243728819599,0.724510341674135,0.926643568412646
"12232","CRTC1",66.6723208966265,0.0735051127062462,0.20846674918389,0.352598738139322,0.724389283728827,0.926643568412646
"12233","IMPA1",76.2101992294351,0.0837769050486578,0.238055012380136,0.35192245780097,0.724896416354258,0.926686127760391
"12234","ENY2",851.643587432453,-0.0798008941848651,0.226861293203566,-0.351760730347502,0.725017711273013,0.926686127760391
"12235","CDKN3",7.57320159797759,0.201440632233501,0.57203465104254,0.352147604810956,0.724727568526735,0.926686127760391
"12236","CAPN15",48.0847830944815,-0.0901149178258142,0.256075486345301,-0.35190763126893,0.724907535904356,0.926686127760391
"12237","ZFHX3",356.219035826858,-0.0824532687643922,0.234416311027124,-0.351738615811813,0.725034297619346,0.926686127760391
"12238","TBX2-AS1",13.0925962663117,-0.191897510858018,0.544964631136989,-0.352128376584096,0.724741988112622,0.926686127760391
"12239","CASK",119.967001560592,0.0799094171329023,0.227145380813178,0.351798556707723,0.724989341041523,0.926686127760391
"12240","AL357873.1",5.72713726002231,0.254731901729932,0.724902760944429,0.351401478176271,0.725287173595393,0.926756988241031
"12241","USP39",70.6028670133696,-0.0872371619153644,0.248166741632174,-0.351526402537311,0.725193468399647,0.926756988241031
"12242","FASTK",196.018193659188,-0.0868968765269831,0.247323700431749,-0.35134876429266,0.72532671527424,0.926756988241031
"12243","SMCR5",9.95532024236236,0.204760796799433,0.582633376716227,0.351440210915281,0.725258119903755,0.926756988241031
"12244","FAHD2A",178.626312354496,-0.074362014620294,0.211963098751419,-0.350825285430945,0.725719426378766,0.927043324406782
"12245","SMUG1",102.14964467509,0.0801923476224216,0.228641456549859,0.350734065608676,0.725787866394545,0.927043324406782
"12246","MPV17L",32.4006058388693,-0.121603918015564,0.346685411723719,-0.350761566259594,0.72576723309391,0.927043324406782
"12247","SF3A1",178.841531793118,-0.0590025170076282,0.168190792515853,-0.350807057420023,0.725733102237289,0.927043324406782
"12248","MRPS26",374.61984095155,-0.0906813417956676,0.258795525475091,-0.350397641648544,0.72604029604177,0.927290035187901
"12249","RBKS",19.9568955091336,0.132754161745775,0.378960146104416,0.35031167026518,0.726104807892805,0.927296718904984
"12250","TM7SF2",5.68861496745145,-0.210266579996391,0.600695430494474,-0.350038587480691,0.726309738685042,0.92748271365307

"12251","AMIGO1",9.03320530200427,-0.171658117245019,0.491305735966644
,-0.349391640843112,0.726795308033078,0.927814123800878
"12252","LY75",9.77229884170026,0.306521762794677,0.878530868252954,0.
348902666794425,0.727162383033866,0.927814123800878
"12253","PCSK1",3.60802633608744,-0.542430642292927,1.55378473748333,-
0.349102825640768,0.727012115318787,0.927814123800878
"12254","TMED7",360.601733673982,0.0580518696754355,0.166050669229593,
0.349603346645771,0.726636398989712,0.927814123800878
"12255","TNS3",36.4735779383349,-0.191589549838372,0.548397690242066,-
0.3493624303082,0.726817234751658,0.927814123800878
"12256","UPF2",267.357711538924,0.0790529057529212,0.226287130011645,0
.349347776644889,0.726828234523301,0.927814123800878
"12257","LINC00924",2.94029782057933,0.343063655274674,0.9828988564921
93,0.34903251032259,0.727064902807219,0.927814123800878
"12258","AC130650.2",9.88482710379442,0.218189753058941,0.625113728665
598,0.349040091512149,0.727059211354087,0.927814123800878
"12259","ADPRM",49.9446386900622,-0.0912812793435845,0.261527017762705
,-0.349031928419755,0.727065339661806,0.927814123800878
"12260","INE1",8.52014111254721,0.185293191833462,0.531065079270303,0.
348908634866483,0.727157902397834,0.927814123800878
"12261","RCSD1",6.07478844885849,-0.239465657554914,0.687017907576589,
-0.348558101490562,0.727421087366398,0.92784485194739
"12262","AL133215.2",3.16975389967622,0.278804884348553,0.799619038030
249,0.348672143969146,0.727335459275575,0.92784485194739
"12263","HYPK",17.1472209123079,-0.155213122439849,0.445305054034568,-
0.348554594279993,0.727423720787751,0.92784485194739
"12264","ZNF267",61.290683458597,-0.0772187757119619,0.221475476202715
,-0.348656099699654,0.727347505810461,0.92784485194739
"12265","COLCA2",44.9539381116702,0.123636243976103,0.354879408540392,
0.34838945568754,0.727547720264503,0.927927353289655
"12266","SH3RF1",111.593747532313,0.0944372280970099,0.271177821999317
,0.348248346419892,0.727653682223925,0.927986837683749
"12267","SSB",850.341407673393,-0.0634958959283724,0.182649904721755,-
0.347637169726974,0.728112687936665,0.928074475572977
"12268","CCR1",12.7684652268713,0.260301780218481,0.748712010819455,0.
347666093847732,0.728090963153415,0.928074475572977
"12269","TXNDC15",428.412267262217,-0.0684052681823995,0.1965783674455
22,-0.347979633116836,0.727855479173783,0.928074475572977
"12270","LINC01277",6.20038543547746,-0.210915178352711,0.606497817797
215,-0.347759171036674,0.728021054758065,0.928074475572977
"12271","SERAC1",15.3416896346464,0.133453150235061,0.383923085832735,
0.34760386952402,0.728137699847033,0.928074475572977
"12272","ADGRL1",31.3503364818259,0.101615752673753,0.292094601060382,
0.34788644605159,0.727925464729522,0.928074475572977
"12273","ZCCHC18",13.0782243162157,-0.150491324118825,0.43290454928891
6,-0.347631653134669,0.728116831450328,0.928074475572977
"12274","ICMT",145.335159531945,0.08966755158536,0.25878286257816,0.34
6497255235662,0.728969046615491,0.92815096827333
"12275","FAM229A",30.8938024460772,0.101357558126146,0.292067712981119
,0.347034449962289,0.72856543784992,0.92815096827333

"12276","NUP210",3.08389227159175,0.310253297611466,0.894754073390426,
0.346747007740178,0.728781391527473,0.92815096827333
"12277","RUBCN",38.5460369656585,0.100531773391504,0.28943270191256,0.
347340755647838,0.728335335869486,0.92815096827333
"12278","SCFD2",38.9892804741049,-0.105583506224064,0.304091323983387,
-0.347209860646444,0.72843366340083,0.92815096827333
"12279","TES",41.4428739091522,-0.171924361177279,0.495971838659515,-0
.346641377143401,0.728860756592703,0.92815096827333
"12280","PPP1R26-
AS1",6.91952718353637,0.22861886763107,0.659688704630543,0.34655568001
4421,0.728925146873429,0.92815096827333
"12281","SNRNP35",157.270845845947,0.0721851537041137,0.20829779847268
,0.346547847521208,0.728931032071882,0.92815096827333
"12282","NOL3",56.4514664853891,0.114116496987346,0.329229468496007,0.
346616897656993,0.728879149557532,0.92815096827333
"12283","FMNL1",148.565008109388,0.0795388706703763,0.229025023617206,
0.347293363031448,0.728370936399352,0.92815096827333
"12284","MCM3AP-
AS1",14.1760396246832,0.179887514696555,0.518930933384555,0.3466502054
97094,0.728854123338277,0.92815096827333
"12285","AC008079.1",8.31556042359554,0.18194199781681,0.5246958633371
92,0.346757065435232,0.7287738348759,0.92815096827333
"12286","ZNF74",23.1556733383247,0.114614980752357,0.330700930275939,0
.34658197258992,0.728905391208726,0.92815096827333
"12287","IRX6",25.1533301897348,0.197363395537687,0.569881548265119,0.
346323540634922,0.729099579122461,0.928166073910535
"12288","METTL2A",80.5522948934818,-0.0887174756827836,0.2561500150837
16,-0.346349679713229,0.729079937212018,0.928166073910535
"12289","FKBP4",112.427253874631,-0.0922452092041955,0.266489377660869
,-0.346149666504102,0.729230239367495,0.928256866663335
"12290","TIGD1",15.2678738381906,0.151653193491823,0.438216295820522,0
.346069269760645,0.729290657328977,0.928258238616532
"12291","HTRA3",953.070887761168,-0.120826233300984,0.34961741729222,-
0.345595577693985,0.729646669909458,0.928560271509409
"12292","AC022613.3",7.97681798931758,-0.220924736264613,0.63916586500
261,-0.34564539247369,0.729609227889253,0.928560271509409
"12293","FAM171A2",20.4610921230392,0.222263615941751,0.64343753159834
,0.345431537681108,0.729769970995236,0.928641638028023
"12294","AC087623.2",4.6761683772412,-0.239964338517122,0.695606355614
108,-0.34497145775109,0.730115828494323,0.92900617416111
"12295","AC010245.2",5.92931835270233,0.256661069732782,0.744850213781
074,0.34458078279913,0.73040955504786,0.929228746715491
"12296","AC092747.4",32.4294677053297,0.104361722656444,0.302825619149
267,0.344626465058107,0.730375207080765,0.929228746715491
"12297","ZNF789",25.5730724630073,0.117502674407876,0.341083735280618,
0.344498028647434,0.730471778323154,0.929232335391485
"12298","NAP1L1",959.845151950312,-0.0630632611478119,0.18311170602984
2,-0.344397758696729,0.730547174195279,0.929252678967047
"12299","WSB2",107.316848360043,0.0896302276333849,0.260348396559086,0
.344270327061698,0.730642997479444,0.929299000696881

"12300","ATP1A1-
AS1",31.3478988012394,0.108576647401287,0.316054866834875,0.3435373373
25717,0.731194256838089,0.929429016465483
"12301","AC017002.3",4.93685829639884,-0.296954554963897,0.86370427923
459,-0.343815078960887,0.730985359424337,0.929429016465483
"12302","AL031963.3",13.9325477830776,0.206716516822749,0.601790614139
282,0.343502394297737,0.731220539899041,0.929429016465483
"12303","TMEM106B",407.101382461338,0.057256040763174,0.16667980026478
6,0.343509175510276,0.731215439256799,0.929429016465483
"12304","NAA35",98.4977984388233,0.0791989838919889,0.230244240399538,
0.343978132762655,0.730862731296223,0.929429016465483
"12305","DCPS",117.73389095318,0.100677961718831,0.292845604897561,0.3
4379195055377,0.731002754198512,0.929429016465483
"12306","ZC3H14",204.77351435075,0.0701520513126955,0.204195359065874,
0.343553602949733,0.731182022448231,0.929429016465483
"12307","NPIPB13",5.62194926375419,0.218125743986391,0.634894384781322
,0.343562251005765,0.731175517733988,0.929429016465483
"12308","AC116036.2",3.14592559082245,-0.296565228106206,0.86423470853
0212,-0.343153572957709,0.7314829297719З,0.92947196982691
"12309","NCBP2",248.865529202967,-0.0656177671567951,0.19131805707899,
-0.342977386236487,0.731615472627747,0.92947196982691
"12310","STC2",21.4725478640899,-0.26126679381463,0.761977901297498,-0
.342879751984598,0.731688924986703,0.92947196982691
"12311","AK3",565.565878595046,0.0682181384938672,0.198924077847259,0.
342935552257517,0.731646944933627,0.92947196982691
"12312","MYO7A",2.69944822169821,-0.313282854533874,0.913825764318317,
-0.342825587509642,0.731729675153001,0.92947196982691
"12313","FLI1",27.967225394181,-0.133131246025222,0.388117713130081,-0
.343017701901695,0.731585142998492,0.92947196982691
"12314","LINC01550",34.1231400381742,0.157637719225373,0.4595239101185
98,0.34304573876186,0.731564051008532,0.92947196982691
"12315","TNFAIP8L1",7.84798924381062,0.232207589974313,0.6762757454784
14,0.34336229197462,0.731325923685386,0.92947196982691
"12316","TBC1D8-
AS1",5.74474374764287,-0.217747744999713,0.637292037884261,-0.34167655
0239999,0.732594321361395,0.929809677921616
"12317","BRIX1",184.07692212973,-0.0668845765130769,0.195931866796729,
-0.341366504625135,0.732827687725858,0.929809677921616
"12318","AGGF1",205.474070682057,0.0523560964101323,0.153054555472427,
0.342074734388187,0.732294650798891,0.929809677921616
"12319","SMIM29",82.5930320203759,-0.0936477461486979,0.27431142324015
6,-0.341392075628986,0.732808439906294,0.929809677921616
"12320","AP1S1",228.612140612346,-0.111711845532097,0.326657842055303,
-0.341984275746193,0.732362725749131,0.929809677921616
"12321","COL27A1",28.9589445951164,0.139122977198194,0.406880989318638
,0.341925479072317,0.732406974509849,0.929809677921616
"12322","DGKH",50.8082803816112,0.143160804926035,0.418409398754019,0.
342154849657664,0.732234361546769,0.929809677921616
"12323","TEX30",75.9465537906096,0.0955037419198944,0.279619462446922,
0.34154897904513,0.732690339162855,0.929809677921616

"12324","PPIB",3410.74732451075,0.0868285265377735,0.254020160362447,0.34181746210176,0.732488267431264,0.929809677921616
"12325","AC013564.1",2.9722305449991,0.336321275872302,0.983363157535311,0.342011263382344,0.732342415893832,0.929809677921616
"12326","ITGA11",445.324121931648,-0.124589889716599,0.364309952376477,-0.341988707428579,0.732359390622829,0.929809677921616
"12327","BRICD5",4.15884524631968,-0.295584823982512,0.865464223158633,-0.341533267433903,0.732702164961674,0.929809677921616
"12328","BMERB1",298.770823002391,0.0800805557087503,0.234254004432587,0.341853518802048,0.732461131042026,0.929809677921616
"12329","CYB561",155.047452221287,-0.0873477116612062,0.25580021758603,-0.341468480697557,0.732750929243745,0.929809677921616
"12330","WNT2",27.4884186383642,0.183138589389752,0.536683799205558,0.341241136141706,0.732922057588091,0.929853994067356
"12331","ADCK5",29.3463379460335,0.0980674332046401,0.287466004040699,0.341144454739614,0.732994836296339,0.929870912674043
"12332","DSCR9",3.38363253566176,0.319061141487512,0.935756711195772,0.340965913116236,0.733129243104823,0.929966003072393
"12333","ZEB2",740.534148830312,0.0656380229064567,0.192660017767044,0.34069353707744,0.733334304577513,0.930075281863632
"12334","AC093772.2",6.45923137392631,-0.216941279820316,0.636743357273344,-0.340704425640653,0.733326106630643,0.930075281863632
"12335","SNW1",439.052760914144,-0.0633263310533443,0.185977943145355,-0.340504524258827,0.733476616264695,0.930180357375648
"12336","ABCB10",44.9424412337518,0.0952467241258231,0.279891629102844,0.340298580672541,0.733631685906136,0.93030159392264
"12337","PRKRIP1",135.773535795023,0.0581025480327174,0.170948668130776,0.339883010894645,0.733944631171333,0.930472150531904
"12338","IRAK4",70.9799747501813,0.0666275990774496,0.195996498515493,0.339942802968916,0.733899601962248,0.930472150531904
"12339","FMN1",9.59706722340429,0.206094720541593,0.606187981670592,0.339984834363785,0.733867948808624,0.930472150531904
"12340","KIAA1549",12.8546213753336,0.174088290175229,0.512374745314307,0.339767507605078,0.734031618899583,0.93050701899888
"12341","KLHL11",14.8495620383782,0.13864805324086,0.408263603505936,0.339604246007308,0.734154580142509,0.930547797827399
"12342","TBC1D17",156.579230393032,0.0681158145358745,0.2005961958498,0.339566831002505,0.734182760390319,0.930547797827399
"12343","LZIC",325.542779764994,-0.0552548882880032,0.16294742876582,-0.339096411072633,0.734537101993398,0.930770667191797
"12344","ADAMTS4",11.7969482335554,-0.262129624955747,0.772855481192023,-0.33917029940895,0.734481442207153,0.930770667191797
"12345","AC005562.1",74.7519665267145,0.0849707520811875,0.250510097864702,0.33919092605632,0.734465904485774,0.930770667191797
"12346","NUF2",3.24366879458464,0.291506923329174,0.861116174917195,0.338522178331171,0.734969716719203,0.930923177425931
"12347","AGT",15.4123029978472,0.271941583911657,0.803006880274631,0.338654114418861,0.734870311432197,0.930923177425931
"12348","BRPF1",35.2526482410618,0.0911689671316804,0.269363418461985,0.33846083351718,0.735015937570982,0.930923177425931

"12349","RAF1",179.552209106759,0.0769653304092004,0.227369339875587,0
.338503557477515,0.734983746682575,0.930923177425931
"12350","FRAT1",19.3843338232188,-0.139911977013806,0.413185709730449,
-0.338617657191194,0.734897779145258,0.930923177425931
"12351","LINC02696",3.46268963826385,0.284924515714942,0.8420159993546
89,0.338383731346322,0.735074032318935,0.930923177425931
"12352","COMP",209.706282751937,-0.376851071053044,1.11367724560008,-0
.338384457922534,0.73507348485306,0.930923177425931
"12353","HSPB1",1735.12812834834,-0.080738943522674,0.238710929220507,
-0.338228935668431,0.735190671949272,0.930995521841048
"12354","AP3M2",74.5724114200162,0.0807178484573232,0.238723374997449,
0.338122936047573,0.735270546930879,0.931021302059232
"12355","STX12",330.616080853929,-0.0577462182034681,0.171095857868325
,-0.337507984839175,0.735733993879136,0.93114857328576
"12356","LRP8",5.12109436644981,-0.229759497418922,0.680546650137662,-
0.337610209928219,0.73565694710358,0.93114857328576
"12357","AC009948.3",8.26805186242246,-0.192540730425849,0.56991810671
2031,-0.337839293326991,0.735484297217238,0.93114857328576
"12358","TCAF2",14.2312817354496,-0.157312587961266,0.465719280492153,
-0.337784142831761,0.735525860451893,0.93114857328576
"12359","AC087623.3",6.10080251908002,0.231325044934125,0.685696624687
553,0.337357712734158,0.735847258387686,0.93114857328576
"12360","MRTFB",92.503003819074,0.0854182322869757,0.253087287130124,0
.337505029413264,0.735736221415263,0.93114857328576
"12361","IGLC3",49.9228211668603,0.342095687106508,1.01357708213454,0.
33751324209706,0.735730031433115,0.93114857328576
"12362","AC244090.1",53.0969517236722,0.117239552793258,0.347520102706
839,0.337360491896952,0.735845163598764,0.93114857328576
"12363","RRP8",92.0689109485873,0.0647039619006192,0.191897820089663,0
.337179243987174,0.735981782994405,0.931243470952154
"12364","SPDL1",42.2296482168516,0.0939233066857846,0.278752940692278,
0.336941043393221,0.736161344361138,0.931347629790192
"12365","MGAT1",438.421743717192,0.0628365853284199,0.18650736712098,0
.336912081803505,0.736183177290527,0.931347629790192
"12366","KLRB1",25.0564846541975,0.15291640144867,0.454025145575444,0.
336801613168053,0.736266456923542,0.931377663404089
"12367","YKT6",90.5778033009604,-0.0824526296319178,0.244915470605982,
-0.336657498311191,0.736375106287049,0.931439782295488
"12368","HIST3H2A",72.253071932661,0.158710154888697,0.472753226447296
,0.335714588520932,0.737086103636697,0.931890045629674
"12369","AC025171.3",4.18225411115842,-0.243591082137093,0.72555419833
3405,-0.335731062816012,0.737073679326766,0.931890045629674
"12370","POLB",29.105794515771,-0.0972013515289686,0.28945062056726,-0
.335813242820053,0.737011703204232,0.931890045629674
"12371","L3HYPDH",105.40538205181,-0.0664327203412436,0.19776373587108
2,-0.335919626763876,0.736931476199342,0.931890045629674
"12372","CNDP2",143.478397368829,-0.0632729868682358,0.188518703666386
,-0.335632410141157,0.737148080586945,0.931890045629674
"12373","CC2D1A",67.3756488307659,0.087379985587664,0.260266965514173,
0.33573214109228,0.737072866132695,0.931890045629674

"12374","GP1BB",56.2075126900576,0.142391517797958,0.424190851187941,0
.335677955805018,0.737113730935537,0.931890045629674
"12375","TBC1D2",13.6479942454387,0.134276163878118,0.400271873652074,
0.335462401224909,0.737276302633505,0.931901519238519
"12376","AC012470.1",5.4695966419281,0.27229200729783,0.81160370022157
3,0.335498725823321,0.737248905724499,0.931901519238519
"12377","CHAC2",4.00987130770656,-0.280687962048785,0.837211892999583,
-0.335265139441736,0.737425088169284,0.931938976751665
"12378","GNPNAT1",103.91647250578,-0.0704724962003661,0.21017645042787
4,-0.335301581394583,0.737397600930764,0.931938976751665
"12379","AC233263.6",6.34260460002374,0.22964163749288,0.6855382382927
6,0.334980056057517,0.737640130905241,0.932060142790847
"12380","NEURL4",17.447508576907,0.139355730073315,0.416011581814161,0
.334980409597269,0.737639864212104,0.932060142790847
"12381","SLX1A",2.99484611989923,0.306421479118921,0.915291308900272,0
.334780278299691,0.737790838548169,0.93217527561659
"12382","DIO3OS",649.893197905462,0.0972252249269476,0.290530075271234
,0.334647711897571,0.737890849088667,0.932226340841061
"12383","MEOX2",186.659058610213,-0.124401991992995,0.372009205247574,
-0.334405682005111,0.738073452350412,0.932381734241904
"12384","SLC2A6",6.80915542670549,0.200163601164686,0.599142675364453,
0.334083365106531,0.738316652353164,0.932538344187367
"12385","PHLDB1",370.409061331195,-0.0720201925756636,0.21555916449918
6,-0.334108701631823,0.738297534060465,0.932538344187367
"12386","GPAT4",118.187301808213,-0.0664004951714044,0.198905670057287
,-0.333829071601028,0.738508544676826,0.932626044820235
"12387","SCARF1",11.8887365204488,0.156671710072585,0.469347704082212,
0.33380734306339,0.738524941995806,0.932626044820235
"12388","AC110597.1",6.48730009156701,-0.227437207317835,0.68145094821
2786,-0.333754333916954,0.738564945549644,0.932626044820235
"12389","AL513320.1",5.32297751385865,-0.294874824149047,0.88861259525
9332,-0.331837322273145,0.740012100488175,0.932635240637719
"12390","AL590560.2",3.6092568873679,-0.291338030613234,0.873661346155
837,-0.333467918542052,0.738781102241405,0.932635240637719
"12391","AC068193.1",4.39313663687366,0.239826047901638,0.721150464883
407,0.332560345697638,0.739466180613126,0.932635240637719
"12392","PHF7",37.5647379116546,0.0911669062357661,0.27406615625831,0.
332645619146935,0.739401803420505,0.932635240637719
"12393","PPAT",52.7769753860345,-0.0893745637506028,0.268428428376856,
-0.33295491200778,0.739168318094097,0.932635240637719
"12394","CA13",4.38970085571079,-0.244519978208854,0.737830320981818,-
0.331404079305761,0.740339283993555,0.932635240637719
"12395","RRAGA",631.838464047827,-0.0800074079605018,0.240473648500596
,-0.332707589623083,0.739355019953745,0.932635240637719
"12396","AL731563.3",8.44135564477493,0.202774711339133,0.608692213277
636,0.333131764980611,0.739034822504715,0.932635240637719
"12397","PPIF",78.0563708573407,0.0815643372463093,0.245404000478275,0
.33236759420118,0.739611705086807,0.932635240637719
"12398","PNPLA2",166.452715840926,0.0619942310769958,0.186236015301614
,0.332879926455657,0.739224922515552,0.932635240637719

"12399","ARRB1",114.806569436834,-0.0810643558759968,0.2438216733063,
-0.332473280684406,0.739531912230589,0.932635240637719
"12400","PCSK7",236.727185025083,-0.104709686090441,0.315935898056438,
-0.331426997484584,0.740321975091333,0.932635240637719
"12401","TRHDE-
AS1",29.498731935244,0.235393685729999,0.710934718313585,0.33110450181
4707,0.740565551297311,0.932635240637719
"12402","AL355001.2",41.2201908952463,-0.138973952090642,0.41905792907
8669,-0.331634226313738,0.740165471961298,0.932635240637719
"12403","PAN3",103.775380954254,-0.0766373982574562,0.231224410867114,
-0.331441641347722,0.740310915415978,0.932635240637719
"12404","AL161891.1",6.96756912206717,0.202650138139947,0.611704953646
076,0.33128739097509,0.74042741462736,0.932635240637719
"12405","DLEU1",19.8733147736348,-0.126190695003947,0.378928141789875,
-0.333020119350024,0.73911909610886,0.932635240637719
"12406","IPO5",390.836168833146,0.0739234381073692,0.221843799473977,0
.333222917578279,0.738966020005078,0.932635240637719
"12407","FKBP3",412.929946366426,-0.0631662122354412,0.190799127171266
,-0.331061327019288,0.740598162555918,0.932635240637719
"12408","AC007686.3",13.6642182066776,0.14709603564094,0.4427379352571
76,0.332241770869477,0.739706704834853,0.932635240637719
"12409","PIGBOS1",200.805545168648,-0.0628898505480801,0.1893575148906
23,-0.332122285109237,0.739796923235094,0.932635240637719
"12410","ZSCAN2",9.64783455520334,0.183673795313435,0.551545271227042,
0.333016716660102,0.73912166461455,0.932635240637719
"12411","LYSMD4",28.7773433071508,-0.125642876541566,0.377664925232286
,-0.332683466605454,0.739373231065854,0.932635240637719
"12412","TELO2",76.9733530343969,-0.0839985109467947,0.253530529548956
,-0.331315171771354,0.740406432450049,0.932635240637719
"12413","HNRNPA1P48",70.9606337777718,-0.114566263258738,0.34526845439
0991,-0.331817928344535,0.740026745706965,0.932635240637719
"12414","ALOX12-
AS1",84.8789924081236,-0.0705590172773526,0.211814505008129,-0.3331170
22720633,0.739045950242761,0.932635240637719
"12415","MIEF2",53.4505928283583,-0.0839087446738066,0.252633252715249
,-0.332136580485637,0.739786129241577,0.932635240637719
"12416","JUP",131.971201999305,0.104421759779151,0.315162909097655,0.3
31326297495226,0.740398029508526,0.932635240637719
"12417","STK11",123.599707686617,-0.0565925249758438,0.170315218960241
,-0.332281080465597,0.739677024684474,0.932635240637719
"12418","CLPTM1",271.392504634671,0.0645011609013132,0.194223927732117
,0.332096882472053,0.739816104101759,0.932635240637719
"12419","DZANK1",14.0983975259978,0.151980992479632,0.458835826451495,
0.331231747213398,0.740469441623147,0.932635240637719
"12420","B4GALT5",157.16295019834,-0.0590728561000772,0.17843559978652
7,-0.331059811891515,0.740599306987262,0.932635240637719
"12421","NKILA",2.70591922363075,-0.352854455973736,1.0631989514948,-0
.331879988667823,0.73997988152608,0.932635240637719
"12422","GRIA3",76.5425369094444,-0.109366783492638,0.328207903272441,
-0.333224100949982,0.73896512680312,0.932635240637719

"12423","HSPA4",176.680995164816,0.0604293141671599,0.18257735212645,0
.330979245034223,0.740660162903914,0.932636796933585
"12424","MMRN2",8.75418568630389,-0.197414746608655,0.596925801485578,
-0.33071907114309,0.740856695496209,0.932809182843464
"12425","PYHIN1",81.4521855121749,0.122693894910863,0.371182804925395,
0.330548434040536,0.740985602133608,0.93281217525136
"12426","FSCN3",4.59790908181879,-0.336702973799866,1.01860242454394,-
0.330553870368626,0.740981495184412,0.93281217525136
"12427","ALOX5AP",16.9726129573213,0.154978795755733,0.468951851192919
,0.330479121388471,0.741037965981503,0.93281217525136
"12428","AADAT",29.44387119269,-0.118107903006526,0.357653949938308,-0
.330229550175243,0.741226520266535,0.932899385029319
"12429","SLC39A13",214.447256859217,-0.0730032236873579,0.221023178231
219,-0.330296687757277,0.741175795425065,0.932899385029319
"12430","COMMD8",189.227576807092,-0.0766914387138061,0.23251766984769
8,-0.329830583473677,0.741527977053063,0.932952459714269
"12431","TENM3",19.456687155553,0.135286316324529,0.410099613176539,0.
329886476304212,0.741485742383426,0.932952459714269
"12432","FAM111A-
DT",39.669418319669,0.0846182947462377,0.25657064360152,0.329805053136
393,0.741547268969933,0.932952459714269
"12433","DDX23",162.624108898957,0.0593198528830587,0.179711295008899,
0.330084165717694,0.741336367271765,0.932952459714269
"12434","TMEM170A",137.999309607944,-0.0783455966500756,0.237569937278
746,-0.329779085466319,0.741566891522548,0.932952459714269
"12435","GJC2",20.5340326204995,-0.173193685955315,0.525829451410345,-
0.329372357312408,0.741874258868509,0.933139394615236
"12436","ZNF461",6.86471285028398,0.193138101052213,0.586429773347669,
0.32934566052073 5,0.741894435263677,0.933139394615236
"12437","ZBTB46",7.27496092578237,-0.16730531501678,0.507832601895032,
-0.329449732830193,0.741815782468646,0.933139394615236
"12438","MCOLN3",99.2984455298192,0.157577913391803,0.479723149515661,
0.32847677572136 6,0.742551201318532,0.933140138353614
"12439","ELF3-
AS1",6.71400656939264,-0.201025113303183,0.611596501276494,-0.32868911
5918115,0.742390681894108,0.933140138353614
"12440","ECHDC1",328.363118099501,0.068256429598873,0.207380269895179,
0.329136564598809,0.742052467951074,0.933140138353614
"12441","AC008264.2",6.31197388660998,-0.2179275897668,0.6633681491738
23,-0.328516812328438,0.742520934627926,0.933140138353614
"12442","MKX",7.96534661562383,0.317142299213687,0.965475609918271,0.3
28482973526938,0.742546515903858,0.933140138353614
"12443","HNRNPH3",575.109357709032,-0.0503159057955011,0.1530955147913
49,-0.328656955522672,0.742414992954463,0.933140138353614
"12444","CD5",3.73848754843011,0.335075660103728,1.01780515785605,0.32
9213953689866,0.741993976751999,0.933140138353614
"12445","RDX",644.389429769495,-0.0383133370665363,0.116576112049424,-
0.32865512833618,0.742416374190253,0.933140138353614
"12446","KATNAL2",22.3833893532149,0.142021210254577,0.431807285862791
,0.328899522783192,0.742231635029855,0.933140138353614

"12447","INSR",274.822046257089,-0.0952843775499813,0.289846366097559,-0.328740976928135,0.742351479057281,0.933140138353614
"12448","DHX35",32.761041990174,0.0922296197547777,0.280691803463214,0.328579668578976,0.742473417651017,0.933140138353614
"12449","OSCP1",44.3022258344241,0.103108439346683,0.314169581267142,0.32819357918362,0.742765302301397,0.933195548020921
"12450","PLPPR4",7.59874086039991,-0.210068279546442,0.642551403807871,-0.326928364488104,0.743722067107131,0.933195548020921
"12451","ID2-AS1",3.91872068130865,0.282189446397136,0.865962786472382,0.325867867309488,0.744524327996978,0.933195548020921
"12452","AC006460.2",7.69650182660657,-0.230475406277823,0.702592942001142,-0.328035470469399,0.742884843603606,0.933195548020921
"12453","PARP3",43.533349076051,0.09726824917325,0.297987491357914,0.326417222179372,0.744108709077161,0.933195548020921
"12454","ADH5",1458.07734229373,0.0668636123777962,0.204681624465916,0.326671300133884,0.743916509519484,0.933195548020921
"12455","SPATA5",33.670665580005,0.109696797202051,0.336157261539952,0.326325829463046,0.744177847819606,0.933195548020921
"12456","FBXW7",144.437456212702,0.0811291942133684,0.24847435380885,0.326509327702209,0.74403903318212,0.933195548020921
"12457","TMA16",266.753244430043,0.0724566746623523,0.222578438192919,0.325533215394164,0.744777548118186,0.933195548020921
"12458","RETREG1",72.774240752104,-0.148886915688183,0.455682940768857,-0.326733573648755,0.743869404591718,0.933195548020921
"12459","SPEF2",78.8835273149569,0.0807220310706654,0.245890397362226,0.328284601337042,0.742696485968196,0.933195548020921
"12460","SCAMP1-AS1",56.6772281261128,0.0889556269026036,0.273373316756256,0.325399815746896,0.744878494944859,0.933195548020921
"12461","LINC01184",120.339335434043,-0.0552747565568559,0.169146027093363,-0.326787199833822,0.743828841444588,0.933195548020921
"12462","PCDHGA4",4.05626368536752,0.256621681694054,0.784518297207761,0.327107325103081,0.743586711686489,0.933195548020921
"12463","MGAT4B",209.667174516454,-0.0742479932877661,0.226912013858598,-0.327210499017626,0.743508680527188,0.933195548020921
"12464","GPLD1",4.87397299682118,0.204414938158176,0.628671655846089,0.325153736862953,0.745064720444948,0.933195548020921
"12465","IRAK1BP1",66.8416968953678,0.0807247380671231,0.247589352171173,0.326042850224485,0.744391935160242,0.933195548020921
"12466","ATG5",162.581183057758,-0.057006066443938,0.174742781020917,-0.326228449100362,0.74425151849558,0.933195548020921
"12467","AGPAT4",98.0825075071365,0.0744823093568818,0.228138988427457,0.326477775106666,0.744062901822538,0.933195548020921
"12468","HOXA-AS3",3.63869350282048,-0.247307982052687,0.760361896244732,-0.325250362063234,0.744991595464352,0.933195548020921
"12469","ESYT2",357.336123043427,0.059670867191245,0.182920087906579,0.326212762491783,0.744263386025554,0.933195548020921
"12470","GAPVD1",140.705009324085,-0.0670547283752161,0.20507647939538

3,-0.326974251620226,0.743687359988971,0.933195548020921
"12471","NAIF1",21.2736085116753,-0.11475803549885,0.351961143863306,-0.326053138250454,0.744384151429978,0.933195548020921
"12472","DPYSL4",4.92878286478249,-0.282575588732268,0.866918341538485,-0.325954101087298,0.744459082200839,0.933195548020921
"12473","AKIP1",120.89095398385,-0.0745959769892345,0.227507184799501,-0.327884049266289,0.742999334489725,0.933195548020921
"12474","C11orf91",2.84040191407607,0.305436167535741,0.931379804535944,0.327939435714867,0.742957455655684,0.933195548020921
"12475","UBE2L6",370.077017352172,0.106896348133952,0.326765757568119,0.327134485967881,0.743566169477447,0.933195548020921
"12476","RASSF3",15.8030588316084,-0.126417366318986,0.387436549753352,-0.326291792551491,0.744203597327009,0.933195548020921
"12477","ACSS3",80.5422200025613,0.0678060268206462,0.208006059822963,0.325981016506716,0.744438717958067,0.933195548020921
"12478","RBM26-AS1",24.6624744123804,-0.123696674534877,0.380389393037465,-0.325184342147767,0.745041558424314,0.933195548020921
"12479","CMA1",6.54190550711903,-0.293898715302393,0.896475111702769,-0.327838120061315,0.743034063084015,0.933195548020921
"12480","FBXO34",81.7169059847362,-0.0646895814053746,0.198890724676799,-0.325251876428609,0.744990449426144,0.933195548020921
"12481","DIO3",60.0952325095859,0.158685713381457,0.486839026169261,0.325951094410181,0.74446135706791,0.933195548020921
"12482","DAPK2",44.6248877124083,0.125164170575928,0.383861559836061,0.326065914569259,0.744374485139697,0.933195548020921
"12483","SNUPN",197.400143542572,0.0676191922973042,0.207327851950141,0.326146205930718,0.744313739330061,0.933195548020921
"12484","TMEM199",66.5241465136103,-0.100816388685794,0.308155150114986,-0.32716113505867,0.743546014508907,0.933195548020921
"12485","EIF4A3",347.281642983865,-0.0598812948594563,0.18423472435356,-0.325027190555781,0.745160492893264,0.933195548020921
"12486","SCGB1B2P",3.26386123566137,0.314967615598722,0.962343756577107,0.32729221075742,0.743446883231281,0.933195548020921
"12487","ARHGAP33",25.2431907480134,-0.108746647003191,0.333460728683491,-0.326115304289428,0.744337118309669,0.933195548020921
"12488","SEC23B",46.7573259475994,-0.0908222472143019,0.279193315408311,-0.325302370085321,0.744952237267177,0.933195548020921
"12489","CCN5",14377.1997688068,-0.0894274301506211,0.275123954990136,-0.325044142934872,0.745147662809102,0.933195548020921
"12490","SMIM11A",14.1082508068086,0.143566260195527,0.441352586255186,0.325287003331433,0.744963866321714,0.933195548020921
"12491","PWP2",4.18642296706047,1.01351922964877,3.11233755931641,0.32564566353509,0.744692459221685,0.933195548020921
"12492","ZNF41",18.7292708969219,0.11040487145858,0.339810107409981,0.324901670230123,0.745255492752431,0.933239807326792
"12493","CDADC1",157.048653010089,-0.0610672019686302,0.188061691375548,-0.324718987274674,0.745393762979519,0.933338240157577
"12494","GPS1",177.41788126206,-0.0609100999605855,0.187712007262508,-0.324486967290297,0.745569387552108,0.933483426402883

"12495","ZNF124",19.5161926513888,-0.178062892994094,0.549302523629668
,-0.324161796704472,0.745815543535822,0.933502738431923
"12496","LINC01119",8.70850913491724,0.166542182630796,0.5151077997378
39,0.323315202595566,0.746456541757147,0.933502738431923
"12497","RTKN",39.2074505990573,0.0972707965687995,0.300919912743109,0
.323244798531622,0.746509856060767,0.933502738431923
"12498","CNOT11",65.922328922685,0.0725582991530046,0.224648557389769,
0.32298582281619,0.746705978898755,0.933502738431923
"12499","HOXD4",8.48744574886585,0.191005274617945,0.589259695092874,0
.324144475192454,0.745828656747118,0.933502738431923
"12500","METTL6",79.7473679262647,0.0673129973421507,0.20823550345515,
0.323254182045131,0.74650275021496,0.933502738431923
"12501","UGDH-
AS1",11.1302683015931,0.150502778902063,0.464594821047556,0.3239441596
93201,0.745980310488637,0.933502738431923
"12502","APOM",32.6416195785643,-0.126036582241439,0.388803638986803,-
0.324165130166688,0.745813019954277,0.933502738431923
"12503","AL117344.2",2.63136790829274,0.286037664051682,0.885650254956
281,0.322969098073371,0.746718645144707,0.933502738431923
"12504","GPNMB",3805.30438688645,-0.0490603817093082,0.151862980523252
,-0.323056886808543,0.746652160358554,0.933502738431923
"12505","TSNARE1",8.87126118959789,0.189225396080627,0.584906281626131
,0.323514043231936,0.746305973906365,0.933502738431923
"12506","CDC37L1-
DT",6.98224416300629,0.216252959112668,0.668432386062606,0.32352256357
0721,0.746299522277059,0.933502738431923
"12507","LPAR1",409.983691743125,-0.0570040489489415,0.176019809446553
,-0.323850191226631,0.746051455002921,0.933502738431923
"12508","PDCL",204.797961172242,0.0548175589124064,0.169560960783514,0
.323291155340848,0.746474751686527,0.933502738431923
"12509","PUS1",21.1629199087854,-0.108720946130462,0.335500052336109,-
0.324056420776782,0.74589531928073,0.933502738431923
"12510","TK2",337.010975411396,0.0616906950932569,0.190580076010081,0.
323699603782264,0.746165470855397,0.933502738431923
"12511","AC026254.2",2.70631903779655,-0.317983540035126,0.98382857696
4473,-0.323210310698882,0.746535972819593,0.933502738431923
"12512","FTL",35651.1153928783,-0.101418963868685,0.313951532681195,-0
.32304019350551,0.74666480250377,0.933502738431923
"12513","AL136169.1",17.467720075819,0.156479066049209,0.4831257770600
45,0.323888878381583,0.746022164288816,0.933502738431923
"12514","CDC42-
AS1",2.75945588537981,0.303693459164073,0.940723799582545,0.3228295694
21801,0.746824317849269,0.933538280811891
"12515","AL353708.3",3.57885618127418,0.304063773868865,0.943596449439
956,0.322239209409206,0.747271482538844,0.933538280811891
"12516","AC092620.1",3.1045780389666,0.343809455623943,1.0684326945642
7,0.321788594988808,0.747612855017293,0.933538280811891
"12517","PIK3CB",34.9248044005873,0.117679674347317,0.365476783663453,
0.321989465836172,0.747460674942381,0.933538280811891
"12518","MYNN",69.6751281653536,0.0694033653914689,0.215629961319295,0

.321863274318821,0.747556276688508,0.933538280811891
"12519","CC2D2A",134.864508589054,0.0857849130216192,0.266603771858786
,0.321769314903232,0.747627462158417,0.933538280811891
"12520","AC124016.1",13.4864649654246,0.149437695084356,0.464294474813
955,0.321859731680495,0.747558960620749,0.933538280811891
"12521","MAD2L1",10.511190067174,0.150100035453911,0.465663186972702,0
.322336056731729,0.747198120271713,0.933538280811891
"12522","RIOK2",103.458791916201,-0.0830706525567505,0.257937137328163
,-0.322057744058248,0.747408949493337,0.933538280811891
"12523","FCHSD1",35.0682860766904,-0.0976532017330081,0.30350755846171
2,-0.321748829676435,0.747642982448094,0.933538280811891
"12524","CPQ",1451.78967549274,0.067818769229239,0.210280543296425,0.3
2251566486414,0.747062072394268,0.933538280811891
"12525","METTL1",31.5508051309829,0.128640816806624,0.399330836430366,
0.322140954494147,0.747345913403109,0.933538280811891
"12526","HIP1R",3.38065577097637,-0.281499038081835,0.872832848373904,
-0.322511966187192,0.747064873953808,0.933538280811891
"12527","PXMP2",90.0859351562586,-0.0780634466222651,0.241920578430306
,-0.32268212621174,0.746935989855945,0.933538280811891
"12528","LRRK1",110.875290028685,0.066671141670028,0.207264979704141,0
.321671040448788,0.74770191908791,0.933538280811891
"12529","CHKB-
DT",27.3048912850171,-0.104171820577708,0.323838013390596,-0.321678790
846774,0.747696046969439,0.933538280811891
"12530","AC093297.2",23.7118044276565,0.120454281556839,0.374577009183
326,0.321574145245753,0.747775333342008,0.933555430125221
"12531","MAPK7",60.7962112587902,0.0811131527200943,0.252424472396308,
0.321336326664659,0.747955530221763,0.933705878162879
"12532","EIF2B4",108.482840129171,-0.0752133880905702,0.23419875492136
5,-0.321151955380053,0.748095239170722,0.933805763353623
"12533","CASP8",70.3230040218377,0.0767324865580594,0.239096187352975,
0.320927269512583,0.748265508029706,0.933897805729633
"12534","THRB-
IT1",15.5304194379132,0.181728062475502,0.566312588047811,0.3208970916
60904,0.748288377997521,0.933897805729633
"12535","AL139011.1",3.28792053618396,-0.279563753173626,0.87308035276
2899,-0.320203921997482,0.748813750274057,0.93393934915746
"12536","RAD18",45.7797032546571,0.0853973948949428,0.266668755218281,
0.320237722732238,0.748788129064666,0.93393934915746
"12537","TGFBI",112.936113752005,0.11903764843828,0.371714386368622,0.
320239551665436,0.74878674272755,0.93393934915746
"12538","EIF4EBP3",252.97831136329,0.100489637482865,0.313965878282178
,0.320065473460621,0.748918698192877,0.93393934915746
"12539","NUDT3",149.964510761712,0.0766487028271352,0.239298550561532,
0.320305754662004,0.74873656119466,0.93393934915746
"12540","HMG20A",100.212027603939,0.0592395279363589,0.185028489286428
,0.320164360444271,0.748843738579087,0.93393934915746
"12541","TENT4B",135.529747569856,-0.0728967829857217,0.22747971536397
,-0.320453992432187,0.748624201582016,0.93393934915746
"12542","LINC00311",3.86490440041363,0.271394171999188,0.8477381753822

2,0.320139141872223,0.748862854867814,0.93393934915746
"12543","COX4I1",2396.64168822508,-0.0922033095378886,0.28745372335341
9,-0.320758793666856,0.748393188512063,0.93393934915746
"12544","KAT2A",40.5588930943173,-0.12416688778732,0.387516834526387,-
0.320416757994717,0.748652423624019,0.93393934915746
"12545","ZNF764",21.1671100308655,0.12819528951715,0.400861524319324,0
.31979943631365,0.74912037495055,0.93411638304914
"12546","VPS9D1",27.7202533460008,0.101733895955617,0.318270763925369,
0.319645746599185,0.749236891475439,0.934187206547927
"12547","PRKAA2",35.5882443826875,-0.117234973982313,0.367083292900051
,-0.319368863279303,0.74944681900527,0.934225562969116
"12548","AC010336.8",3.57921729764816,0.343857490777232,1.076271305738
22,0.319489601686797,0.749355275217446,0.934225562969116
"12549","AC004000.1",6.35076919653474,0.253611513009224,0.793924552938
215,0.319440319701211,0.749392640362784,0.934225562969116
"12550","KIAA0408",183.322770843066,0.108692199730586,0.34047024200758
2,0.319241408851718,0.749543458705455,0.934271579643779
"12551","ZNF398",53.9688730715915,0.0799555376773759,0.250749579714661
,0.318866088502963,0.749828060479599,0.934402959458485
"12552","UFM1",420.920569395436,-0.0474851236979774,0.148904014670173,
-0.318897538143336,0.749804211214818,0.934402959458485
"12553","SLC16A3",8.96195150449889,-0.182710874145443,0.57275726210876
3,-0.319002282874149,0.749724781671648,0.934402959458485
"12554","SLC35A4",100.094339427982,0.0613522651309826,0.19255902846689
8,0.318615364958229,0.75001820068749,0.934565454305752
"12555","ZNF662",65.5882912088874,-0.097343995207762,0.305760670445189
,-0.318366633177605,0.750206845418573,0.934726059966765
"12556","SGO2",35.2322576602994,-0.0981400922515586,0.308749394570662,
-0.317863270268203,0.750588654797657,0.934779071216063
"12557","CCDC58",66.0313670185932,0.0983831799294181,0.309461858328875
,0.31791698162965,0.750547910897753,0.934779071216063
"12558","AL391834.2",12.54138715697,0.137589862606581,0.43300056701895
8,0.317759081827154,0.750667691147234,0.934779071216063
"12559","CCSER2",368.657805971657,0.0525963633108861,0.165318980286433
,0.318150784742062,0.750370562662395,0.934779071216063
"12560","C14orf119",305.454269336661,0.0915052457659709,0.287947537642
375,0.317784435717657,0.75064845768875,0.934779071216063
"12561","SYNE3",545.159150347379,-0.0868304275955965,0.272956760014994
,-0.318110559309199,0.75040107418916,0.934779071216063
"12562","AC099850.1",15.9835570924126,0.117821599208545,0.370615931922
372,0.317907540016988,0.750555072984209,0.934779071216063
"12563","LINC00641",15.1144402720451,-0.157649247026377,0.496288937276
199,-0.317656178055488,0.750745755527461,0.934801866888169
"12564","EAF1-
AS1",4.13074368192636,-0.233994127605725,0.736815604618648,-0.31757488
0525003,0.750807430885993,0.934804253529893
"12565","CABLES2",7.5328612535083,-0.165148695320469,0.520245304209922
,-0.317443894224618,0.750906805360173,0.934853573915574
"12566","IFT43",185.079339526773,-0.0834115908667748,0.262830239477812
,-0.31735918603771,0.750971072537082,0.934859182532037

"12567","SIPA1L3",25.6834244120731,0.0974430407124042,0.30730665138163
1,0.317087314167482,0.751177350426884,0.935041560653119
"12568","AC136475.1",18.3205743059879,-0.128308081114496,0.40503051549
5837,-0.316786207966138,0.751405830093407,0.935251543614829
"12569","PCDHB10",24.4398673981843,-0.13317232606778,0.420534361652161
,-0.316674065692477,0.751490929326422,0.93525514118525
"12570","CASC4",460.086428142888,0.0427767431659556,0.135102302694047,
0.316624826616227,0.751528295384427,0.93525514118525
"12571","TBC1D8",23.0795015373345,0.136383580056691,0.431189605329722,
0.316296075719176,0.751777789504204,0.935362556994563
"12572","RBPMS",356.821719334583,0.0846660666826706,0.267697857698718,
0.316274726329556,0.751793992782244,0.935362556994563
"12573","CCBE1",197.585965360472,-0.283016854546252,0.894778554023184,
-0.316298209510862,0.751776170053148,0.935362556994563
"12574","RXRB",105.772402972887,-0.0625377913274843,0.197838285906914,
-0.316105606358262,0.751922351507865,0.93537346677038
"12575","CITED2",930.559520659589,0.0965506138665635,0.305422744440973
,0.316121230733106,0.751910492627491,0.93537346677038
"12576","MRPL1",188.21640572761,0.0716269539897328,0.226914083091535,0
.315656714708356,0.752263084695609,0.935398475582021
"12577","ZNF76",58.08272737062,0.0858373787850739,0.271737620389852,0.
315883309281674,0.752091081046006,0.935398475582021
"12578","ARFGAP2",239.956583189333,-0.0635124607925631,0.2012286466816
19,-0.315623356017751,0.752288407685074,0.935398475582021
"12579","AP3B2",3.35715179440314,-0.244271756470213,0.773975785784423,
-0.315606458182208,0.752301235140153,0.935398475582021
"12580","ERCC2",30.6140698236823,0.0886177450691165,0.280614014661476,
0.315799427109947,0.752154752979357,0.935398475582021
"12581","MROH8",9.08524937113238,-0.16128995300063,0.510700950341253,-
0.315820741850697,0.752138573568002,0.935398475582021
"12582","PET117",74.770396455901,-0.0813198571504717,0.257778797774058
,-0.315463714830993,0.752409596950321,0.935458855912722
"12583","AL355816.2",3.35435904926249,0.325080006461061,1.030771525146
98,0.315375423680536,0.752476624503477,0.93546784050766
"12584","LINC02593",5.65784260610468,0.224729379627579,0.7134258398150
24,0.315000336525303,0.752761398353082,0.935652880063957
"12585","KIFAP3",321.311969229781,0.0515146039544625,0.163690831522513
,0.314706715552223,0.752984344890696,0.935652880063957
"12586","AC107068.1",5.84641254764951,-0.221879880372436,0.70494299168
1376,-0.314748686050804,0.752952475409765,0.935652880063957
"12587","HACD4",127.605900335435,0.101521297666705,0.322534181720731,0
.314761359943573,0.75294285181881,0.935652880063957
"12588","FECH",79.8802079126594,-0.0677228979286758,0.2150383598592,-0
.314934033039587,0.752811740818013,0.935652880063957
"12589","RPGR",53.7133487071946,-0.116359500138162,0.369379447335519,-
0.315013466443543,0.752751429281737,0.935652880063957
"12590","RAI1",49.2606243720033,0.0751717757571134,0.239133713448331,0
.3143503886304,0.753254931753654,0.935914765482319
"12591","SENP8",12.285226858596,-0.132380369487344,0.421407500961577,-
0.314138616862004,0.75341576096012,0.936040246898511

"12592","WDR78",18.2484671176431,-0.12754734648007,0.406845694807137,-0.313503001526251,0.753898540661308,0.936061645199781
"12593","AC007390.2",17.7947575640497,0.133466266565918,0.426658703985651,0.312817400229123,0.754419394901458,0.936061645199781
"12594","MRPS5",269.247504207631,0.0626204268508094,0.199901035183183,0.313257141432139,0.754085308765263,0.936061645199781
"12595","NLN",37.7654233352229,-0.0928077116048746,0.295845100865066,-0.313703729869111,0.753746067654443,0.936061645199781
"12596","HLA-DRB1",466.294858297844,0.133949824046922,0.42799968185396,0.312967111252732,0.754305649254184,0.936061645199781
"12597","AL590428.1",11.6758410203325,0.14915185355132,0.475990266241147,0.31335063787997,0.754014282309228,0.936061645199781
"12598","WIPI2",339.451082588069,-0.0488959627821399,0.156021073310452,-0.31339332402134,0.753981855615712,0.936061645199781
"12599","ZNF777",41.1112726163221,0.0821138229165433,0.261939258437616,0.313484215410573,0.753912811063211,0.936061645199781
"12600","AC105219.1",4.8003238527901,-0.210414659349388,0.672548908638359,-0.312861498467663,0.754385889916718,0.936061645199781
"12601","TMEM126B",383.385532574501,0.0752163897487897,0.240479445177716,0.312776793431156,0.75445024756625,0.936061645199781
"12602","POSTN",647.726705928758,0.273268756275397,0.873503027806158,0.312842368688433,0.754400424294488,0.936061645199781
"12603","AL606834.2",2.75566444594942,0.306315263189341,0.976040275417392,0.313834655089769,0.753646622187879,0.936061645199781
"12604","EFL1",36.3063170526645,0.0850137317610136,0.270875125974564,0.31384842537737,0.753636163075571,0.936061645199781
"12605","CLDN7",5.38754716722363,-0.211109986255511,0.674003260486719,-0.313218048979558,0.754115006749465,0.936061645199781
"12606","GNAL",52.5101677922764,0.0912617020000022,0.291595778865561,0.312973330255504,0.754300924370064,0.936061645199781
"12607","ZNF283",31.5084907048753,0.104250037612836,0.332462198205216,0.313569597312495,0.753847953518586,0.936061645199781
"12608","CEBPB-AS1",9.57851085756564,-0.174206727349413,0.555566005977913,-0.313566210810131,0.753850525930303,0.936061645199781
"12609","VPS37D",33.9788894880469,-0.12972912423044,0.41513244177416,-0.312500568917268,0.754660130761559,0.93624779328282
"12610","AKT3-IT1",3.70842114435817,-0.251426783662793,0.80591425369743,-0.311977090005891,0.755057934529903,0.936252373536485
"12611","RALA",363.952020704509,0.0555914373162677,0.17802949249919,0.312259707848803,0.754843158598849,0.936252373536485
"12612","VIPR2",32.1307363347932,0.188877608136642,0.604583173118656,0.312409634496349,0.754729229270291,0.936252373536485
"12613","RTKN2",9.01788132306891,0.168332237146516,0.539465085505339,0.312035461922124,0.75501357313634,0.936252373536485
"12614","FLOT2",93.9346775926206,0.0690062809466317,0.221110319799475,0.31208982470476,0.754972259324235,0.936252373536485
"12615","ZNF521",303.526208517354,-0.0567297553715886,0.18183009264231

2,-0.311993216013946,0.75504567903187,0.936252373536485
"12616","LINC01697",169.267327349444,-0.123417199450827,0.395638455941573,-0.311944396702055,0.755082781086511,0.936252373536485
"12617","TCTE3",18.080442658602,-0.133626914840522,0.428715342881649,-0.311691468614901,0.755275012253245,0.936343913047542
"12618","SNHG20",48.9466596743242,-0.0852639413613999,0.27355388616093,-0.311689746243414,0.755276321347177,0.936343913047542
"12619","HIST2H2AA4",22.558687023724,0.216542594785821,0.696794929643803,0.310769475456023,0.755975876502759,0.936392460940569
"12620","LEFTY2",6.38276860606399,-0.25698187344584,0.826209888900281,-0.311037034170449,0.755772467801212,0.936392460940569
"12621","CAMKMT",31.6309423836119,0.0833547814891228,0.268057072858105,0.310959082707161,0.755831727835362,0.936392460940569
"12622","SMC4",105.678957720128,0.0916042012259779,0.294557254500217,0.31098945901504,0.755808635076314,0.936392460940569
"12623","NOP14-AS1",47.8335958733593,0.0776334850118559,0.249554248819398,0.311088612512621,0.755733257854147,0.936392460940569
"12624","MTMR12",67.1683384773147,0.0722158677482078,0.232654080135726,0.310400177405349,0.756256659247083,0.936392460940569
"12625","SERF1A",5.09330410421896,-0.189239865127766,0.60922647135378,-0.310623182061099,0.756087101672381,0.936392460940569
"12626","LRRN3",71.3730734932499,0.293042269538388,0.942019307902792,0.311078835730857,0.755740690132637,0.936392460940569
"12627","AC105206.1",2.95765253130229,-0.312115071509303,1.00610581974425,-0.310220918500045,0.75639296403791,0.936392460940569
"12628","ITIH5",2098.77361426388,-0.0827930753344615,0.266178582313254,-0.311043340207687,0.755767673906781,0.936392460940569
"12629","LRMDA",114.285134721985,0.0949014630927275,0.305685605913578,0.31045447105402,0.756215376967663,0.936392460940569
"12630","RTN3",859.180680581643,0.061544013173677,0.198049046159453,0.310751373799229,0.755989638708164,0.936392460940569
"12631","AP001107.4",2.68582049777632,0.318468505935772,1.02462503367679,0.310814683878033,0.755941506087045,0.936392460940569
"12632","NUAK1",224.706750571873,-0.133312649579694,0.429514051941031,-0.310380181922423,0.756271863025999,0.936392460940569
"12633","CHAF1A",35.5149113182854,-0.101447347406116,0.326862719767881,-0.310366833752585,0.756282012501961,0.936392460940569
"12634","ZNF230",30.3385043111668,0.0877724897807291,0.282005469372451,0.311243927204852,0.755615191150033,0.936392460940569
"12635","LMTK3",4.17213677993639,0.228433765799838,0.736205951103056,0.310285138903831,0.756344131289954,0.936392460940569
"12636","SPATC1L",141.054580153641,-0.0775028389370525,0.249622340873562,-0.310480378742658,0.756195678252379,0.936392460940569
"12637","GTF2B",189.985670491614,-0.0791036657994036,0.256738930868833,-0.308109352686436,0.75799912478617,0.936841736803569
"12638","ATP1A1",486.284344292625,-0.047952400406207,0.155693686736082,-0.307991938603726,0.75808846644665,0.936841736803569
"12639","TATDN3",74.1788559466371,-0.0727593427108443,0.235808523463335,-0.308552641109927,0.757661850918124,0.936841736803569

"12640","ZNF2",19.5406030031304,-0.114136720514612,0.369912271041285,-0.3085507820363,0.757663265289085,0.936841736803569
"12641","AC009961.1",5.71831614749222,0.226119627251596,0.733766130404149,0.308163075239044,0.757958247785605,0.936841736803569
"12642","C3orf62",35.3995725401883,-0.117063364870531,0.378837473543969,-0.309006824946383,0.75731633517098,0.936841736803569
"12643","TIMMDC1",344.433238935154,-0.0730245713977476,0.237312392762759,-0.307714951366869,0.758299241865599,0.936841736803569
"12644","LINC02104",10.5696767669805,0.175000589696793,0.566954907641642,0.308667563042608,0.757574420644669,0.936841736803569
"12645","AC016596.1",4.62501332855875,0.209919339013663,0.67909645230398,0.309115646682392,0.757233557337237,0.936841736803569
"12646","GINM1",935.512981896359,-0.0468592713469426,0.151638479188458,-0.309019660430025,0.757306571411192,0.936841736803569
"12647","AC093627.5",7.67614954310811,-0.216092885911018,0.701901738240207,-0.3078677172859,0.75818299132409,0.936841736803569
"12648","NUP42",140.15897810737,-0.0637930591664355,0.207227850488755,-0.307840181790127,0.758203944653342,0.936841736803569
"12649","BMT2",41.9596978154593,0.0803695476315507,0.260911794188389,0.308033402175452,0.758056915992082,0.936841736803569
"12650","UHRF2",116.578085739053,0.0697379438984155,0.225210679995448,0.309656468777703,0.756822209510406,0.936841736803569
"12651","UBAP1",117.330170568861,0.0644402348497484,0.209290809263905,0.307898063352092,0.75815989947725,0.936841736803569
"12652","HOXC8",248.823496151483,0.0590148855501401,0.191650272251966,0.307930089828164,0.758135529156091,0.936841736803569
"12653","RPS26",3900.48212902894,-0.218719051695272,0.708875171645289,-0.308543817647934,0.757668563757059,0.936841736803569
"12654","TRAF3",60.3714977076453,-0.089459338865908,0.289793462705889,-0.308700334474764,0.75754948937259,0.936841736803569
"12655","FANCI",12.234516366904,-0.133618414886675,0.432562977194778,-0.308899332423699,0.757398104639889,0.936841736803569
"12656","TMEM256",449.96835658861,-0.0923060705188262,0.298209042285599,-0.309534780740898,0.75691475910104,0.936841736803569
"12657","EML2-AS1",4.94673720591459,-0.205786792331159,0.666590443836286,-0.308715485248841,0.757537963319198,0.936841736803569
"12658","LIG1",21.3330623774446,-0.116567829133303,0.378420047579091,-0.308038196916457,0.758053267604492,0.936841736803569
"12659","CEP250",41.6065356916466,0.0898894985833503,0.292211258390586,0.307618190614679,0.758372876887016,0.936841736803569
"12660","PPP1R3D",36.3633835350166,-0.0929951871169383,0.302285167385894,-0.307640589583483,0.758355831056168,0.936841736803569
"12661","COL18A1",575.292713112827,-0.116087166346293,0.375838134095735,-0.308875432839187,0.757416285402109,0.936841736803569
"12662","DEPDC5",35.8305531266046,-0.0830759418604182,0.269681579169513,-0.308051970461799,0.758042787145145,0.936841736803569
"12663","APOL4",49.9848656160191,-0.104579645934256,0.339306493741669,-0.308215869319253,0.757918077910507,0.936841736803569
"12664","SHROOM4",8.39562528733466,-0.17028020740401,0.553990257748758

,-0.30737040051205,0.758561455374899,0.937000698549396
"12665","MCM2",14.4908250439977,-0.129745660386312,0.422353570850951,-0.307196788048701,0.758693590177815,0.937028538663905
"12666","UVSSA",60.2259945309072,0.112617579547968,0.366691545293936,0.307118015109117,0.758753545841114,0.937028538663905
"12667","ZNF23",46.8124274766322,-0.0729343845448555,0.237577182260595,-0.306992379701072,0.758849172469906,0.937028538663905
"12668","ZSWIM7",242.147213917078,-0.0876733075049317,0.285629852820515,-0.306947283833193,0.758883497815829,0.937028538663905
"12669","TXNL1",552.060398531209,-0.0790081590560349,0.257315549928927,-0.307047743822157,0.758807031950324,0.937028538663905
"12670","BIN2",8.15099809434777,-0.22266781551386,0.726679579748764,-0.306418154189559,0.759286287667708,0.937451886186737
"12671","CAND2",8.27453100931785,-0.169421219958034,0.554950274491874,-0.305290812069893,0.760144673040938,0.937632804294925
"12672","CXCL10",6.76704231149072,0.269344837179329,0.882201863890422,0.305309757555425,0.760130245049234,0.937632804294925
"12673","AC008771.1",28.5298975577553,-0.118612809720676,0.387928212928978,-0.305759688951501,0.759787622971982,0.937632804294925
"12674","CDC42SE2",98.0430718921451,-0.0620016000976713,0.202885126930303,-0.305599533271705,0.759909575880561,0.937632804294925
"12675","METTL2B",117.660491235423,0.0735918973504901,0.240715985000441,0.305720857509131,0.759817191199163,0.937632804294925
"12676","LMX1B",21.0914739999859,-0.113914754119092,0.372168292670974,-0.306083985020726,0.759540701230418,0.937632804294925
"12677","PLCB3",52.9697885495695,-0.0760561731764967,0.249033914114005,-0.305404882090392,0.760057803934002,0.937632804294925
"12678","PIH1D2",13.4968199142931,0.152281743893181,0.498418853561099,0.305529662060653,0.759962782216766,0.937632804294925
"12679","MPRIP-AS1",3.87650647949021,-0.293143842198394,0.958669272691161,-0.305782036150471,0.759770606841865,0.937632804294925
"12680","CASKIN2",30.6787950660179,-0.0856005767460444,0.280399232852654,-0.30528106612555,0.760152095126782,0.937632804294925
"12681","CCDC61",55.864448440779,-0.0849390252588271,0.278099196337971,-0.30542707917646,0.760040900273288,0.937632804294925
"12682","AP001062.1",5.14415274288133,-0.194304094284895,0.635474554614534,-0.305762194369461,0.759785715231894,0.937632804294925
"12683","GPR155",38.2139613493609,0.0998145742133135,0.327178039516043,0.305077242839886,0.760307323104346,0.937643583292125
"12684","STEAP1B",5.15718856472783,0.228972981664415,0.750600406962521,0.305053100878279,0.760325709807335,0.937643583292125
"12685","DDX24",964.737926221944,-0.0416311017209592,0.136480434487535,-0.305033478807993,0.76034065422621,0.937643583292125
"12686","MELTF-AS1",25.2382930779861,-0.115777618095891,0.38030695445968,-0.304432029807032,0.760798768818029,0.93811074402692
"12687","AL365272.1",3.82945080018733,-0.260199787339974,0.85485552872676,-0.304378668203177,0.760839417596978,0.93811074402692
"12688","AL391244.2",2.65473967377475,0.284089897366167,0.933910744370

873,0.304193841947437,0.760980216099642,0.938210397260932
"12689","LMLN",44.1447770520183,0.0891252743431082,0.293203169573116,0
.303971046673434,0.761149949483385,0.938345705711135
"12690","FAM229B",219.22426343109,0.0973019228830023,0.320187954058872
,0.303890017252528,0.761211683436475,0.938347861623072
"12691","IFFO2",22.2706803902851,0.146524931531446,0.483392675165195,0
.303117815100058,0.761800077960721,0.938471242511691
"12692","TENT5C",24.1392531053716,0.114120238758478,0.376777080491612,
0.302885299205502,0.76197727498763,0.938471242511691
"12693","AC097382.3",45.7731120186677,-0.0967269916478785,0.3193783280
53057,-0.302860222976086,0.761996385967933,0.938471242511691
"12694","AC004067.1",3.66595306631228,0.236646346427979,0.780616684503
133,0.303153072597476,0.761773209818036,0.938471242511691
"12695","MED7",91.3919705983892,0.0813382974683239,0.268608202732282,0
.30281390010041,0.76203168972598,0.938471242511691
"12696","RBM28",182.247025615605,0.0696328223298992,0.22953088480242,0
.303370164715912,0.761607780071365,0.938471242511691
"12697","ZNF425",15.7451803868534,0.115673004252943,0.381406459343349,
0.303280139649686,0.761676380152555,0.938471242511691
"12698","SPIDR",93.6537923424171,0.0587417162332369,0.193702337031,0.3
0325765364326,0.761693515026772,0.938471242511691
"12699","AC104051.2",8.59219253851805,0.237855727858458,0.783960335010
397,0.303402758068498,0.761582944043537,0.938471242511691
"12700","AC016405.2",2.85591232568489,-0.271245948100676,0.89559401478
3477,-0.302867084441441,0.76199115672518,0.938471242511691
"12701","AP003396.5",15.9240666886709,0.175786090378877,0.579000508937
899,0.30360265261482,0.761430630427465,0.938471242511691
"12702","DHX38",146.157550667246,0.071302092054433,0.235348395756131,0
.302964002900264,0.761917294633542,0.938471242511691
"12703","TRIM5",86.8379634784353,-0.0698357401832345,0.230742220209887
,-0.302656965507702,0.762151296967423,0.938544653897615
"12704","CHD9",983.132017012781,-0.0445355091303945,0.147190324426899,
-0.30257090134014,0.762216892933623,0.938551547241866
"12705","LRRC47",129.420161558068,-0.0513902017606171,0.17013609773330
3,-0.302053488032703,0.762611288465968,0.938772291174407
"12706","PMF1",305.22338931582,-0.0866433924618579,0.286878899399743,-
0.302020792198897,0.762636212762537,0.938772291174407
"12707","BRI3",2322.40747994055,-0.0761338133389397,0.251971412470463,
-0.302152583868475,0.762535748418564,0.938772291174407
"12708","RBPMS-
AS1",19.6057325432383,-0.107345570126718,0.355422687350938,-0.30202227
9238262,0.762635079175149,0.938772291174407
"12709","COQ7",101.518448196704,-0.0690887777736426,0.228982912700922,
-0.301720233002191,0.762865342978212,0.938980451664817
"12710","SIN3A",121.206553600519,0.0606804055928566,0.201171552100312,
0.301635121662723,0.762930231075885,0.938986436248628
"12711","SNX24",63.1014727595447,0.0831571507176039,0.275777583031046,
0.301537020535285,0.763005024534322,0.939004610084997
"12712","MUL1",91.0872489173543,0.0727787884783463,0.241540736419551,0
.301310617650561,0.763177645212241,0.939069291595617

"12713","ARRDC1",62.1797943330925,-0.0855542626066636,0.283904571788447,-0.301348661163564,0.76314863814625,0.939069291595617
"12714","DR1",193.786485888182,-0.0505773544228972,0.167987605364258,-0.301077893891201,0.763355097509242,0.93913989699859
"12715","ATN1",368.121446317869,0.0518343242994812,0.172153254836594,0.301094070795709,0.763342762187414,0.93913989699859
"12716","SH3GLB1",716.154508013903,0.0414784584862162,0.137839068089251,0.300919463989403,0.763475907716559,0.939195139897165
"12717","ATXN7L3",57.4109839348306,-0.0787898904903554,0.261880898719165,-0.300861540019564,0.763520078889743,0.939195139897165
"12718","CCDC88A",363.533091406495,0.0674244715612864,0.2243326344225,0.300555787323843,0.763753249958509,0.939257531419042
"12719","UBE2F",140.462364466373,0.0714379227675014,0.237777779821488,0.30043985952402,0.76384166364285,0.939257531419042
"12720","ANO7",4.69760493848707,-0.277988690980588,0.92889954454098,-0.299266688862419,0.76473656896894,0.939257531419042
"12721","DVL3",190.651505719029,0.0551572221221952,0.184246266512856,0.299366837473185,0.764660162407508,0.939257531419042
"12722","LINC01948",3.24464044735414,0.279472618653583,0.929929202688299,0.300531070371449,0.763772100371273,0.939257531419042
"12723","IQUB",6.73603403577859,0.161658633679649,0.538876568043727,0.299991952269358,0.764183294242323,0.939257531419042
"12724","VDAC3",524.252280358782,-0.0757851125495338,0.253274977205662,-0.299220686487301,0.764771666412091,0.939257531419042
"12725","AL353748.3",4.01158577415662,0.221457169045598,0.739372793568829,0.299520310960674,0.764543077046323,0.939257531419042
"12726","ASTN2",16.5697242127859,0.12370468836138,0.412496304043816,0.299892840611342,0.764258895506103,0.939257531419042
"12727","USP20",33.2688529514851,0.0828385774269752,0.276230018476013,0.299889845006721,0.76426118055478,0.939257531419042
"12728","STK24-AS1",3.54263771688611,-0.24676260181506,0.821854491567437,-0.300250962119141,0.763985735041326,0.939257531419042
"12729","LINC02256",69.3448247878739,0.116900230984295,0.389910480731208,0.29981300006368,0.764319798616769,0.939257531419042
"12730","EARS2",21.0950831445284,0.118042725222338,0.394463861918281,0.299248515816621,0.764750434009262,0.939257531419042
"12731","SH2B1",91.658256264488,0.0616672893325926,0.20527781505153,0.300408932729104,0.763865250842943,0.939257531419042
"12732","ORC6",6.47506789088256,0.20158052448345,0.67113179030685,0.300359076108264,0.763903275874767,0.939257531419042
"12733","B4GALT6",25.5968206074366,0.104781661433125,0.349402157996144,0.299888420936088,0.764262266837278,0.939257531419042
"12734","ARHGAP45",14.9633699256149,0.148046923082331,0.49405156398115,0.299658849147939,0.764437390490142,0.939257531419042
"12735","FUZ",87.6392543588819,-0.0755081685202116,0.252040898109452,-0.299586968173003,0.76449222574526,0.939257531419042
"12736","JOSD2",406.409597208719,-0.0850313692000935,0.283913814771002,-0.299497117703405,0.764560770903263,0.939257531419042
"12737","E2F1",8.75673661152686,0.181635272426628,0.606819297444549,0.

299323494146501,0.764693230129299,0.939257531419042
"12738","LINC01436",62.0833496310379,-0.317326035548161,1.06079752756739,-0.299139116845275,0.764833901035982,0.939260222476517
"12739","PATJ",25.5950199409338,-0.137746064636802,0.461613066581923,-0.298401571811564,0.765396689550003,0.939264122159956
"12740","GNAI3",482.116895134456,-0.0517808381918702,0.173441577624643,-0.298549165090811,0.765284057619367,0.939264122159956
"12741","WDR35",56.4360654538624,0.064241209284298,0.214873474571011,0.298972264550354,0.764961207903642,0.939264122159956
"12742","CCDC85A",35.3943885619513,0.181195392064275,0.607328841622044,0.2983480770983,0.765437513858922,0.939264122159956
"12743","AL354892.3",5.21874060446446,0.226798932425644,0.760165633702629,0.29835462479532,0.765432516971161,0.939264122159956
"12744","ACTR1A",160.764504870693,-0.0617110231308643,0.206831834608804,-0.298363272982531,0.765425917105172,0.939264122159956
"12745","EIF2B2",88.8393271427611,-0.0826004944433094,0.276401896308583,-0.2988419961891,0.765060605959077,0.939264122159956
"12746","AC003102.1",9.39218088131176,-0.173163358973316,0.579751823632434,-0.29868532001911,0.765180158966337,0.939264122159956
"12747","DOK5",54.1800802247888,0.0994016583414019,0.332532537898984,0.298923103794426,0.764998718349477,0.939264122159956
"12748","VAMP7",120.770974022071,0.0589231431214505,0.197356391681544,0.298562122155787,0.765274170012824,0.939264122159956
"12749","DNAJA4",32.7528692302252,-0.0799898284021444,0.268200223676979,-0.298246687886749,0.765514890479349,0.939285389581023
"12750","PPP2R5C",271.870543324689,-0.0495191582483422,0.166182728212975,-0.297980173877515,0.765718295597347,0.939461278276808
"12751","NFYB",317.860357558598,-0.0591522697022374,0.198831491517394,-0.297499501969297,0.766085188162394,0.939690315880525
"12752","ITPA",296.765058247082,-0.0891997636672521,0.299721812617493,-0.297608515337152,0.766001974631259,0.939690315880525
"12753","ESS2",45.356168166101,-0.0912956915895603,0.306830412456335,-0.297544467182023,0.766050864392229,0.939690315880525
"12754","TMEM97",37.5711794891753,-0.103580550114846,0.348389130417485,-0.297312806489464,0.766227705302674,0.939791437513701
"12755","IDH1",190.157509265713,0.0492229946805221,0.16568337758642,0.297090724474443,0.766397245652652,0.939915903029782
"12756","RAD51AP1",7.01421475083162,-0.179507988004077,0.604358278081356,-0.297022469145218,0.766449354922195,0.939915903029782
"12757","TMEM240",59.1412792057978,-0.0690109192409428,0.23354489998554,-0.295493154614875,0.767617180837916,0.940054077160953
"12758","RERE-AS1",4.56601399459806,0.204482633888889,0.691326867192816,0.295782854092168,0.767395917884249,0.940054077160953
"12759","LRRC8C-DT",15.937618086101,-0.155465626243839,0.525491616294205,-0.295847966786208,0.7673461895542,0.940054077160953
"12760","ZNF281",92.845877712396,-0.0754821814434787,0.255492297276775,-0.29543818834471,0.767659164409673,0.940054077160953
"12761","NUCKS1",2950.77621218277,-0.0433164951305074,0.14661235420853

1,-0.295449147954455,0.767650793338612,0.940054077160953
"12762","FOXP1",883.683681042822,0.0847417616413964,0.285655457766146,
0.296657246824847,0.766728200447769,0.940054077160953
"12763","ACOX3",49.7185628547354,0.0730280662735987,0.246852204794234,
0.29583720483465 8,0.767354408682348,0.940054077160953
"12764","ARRDC3",615.511193169003,0.104844180300975,0.355039975674583,
0.2953024658752,0.767762832977961,0.940054077160953
"12765","SPATA24",7.27575311932461,-0.185211997209866,0.62456206136964
6,-0.296546986545583,0.766812389592038,0.940054077160953
"12766","CCDC69",131.99174035303,0.100682372587904,0.341023872107966,0
.295235556283957,0.76781394189974,0.940054077160953
"12767","TMEM80",180.992980672393,0.0707396736092896,0.239225088283258
,0.295703406849741,0.767456595194796,0.940054077160953
"12768","ZNF214",26.2879273780881,0.105318048425767,0.355527760455465,
0.296230168611431,0.767054310976975,0.940054077160953
"12769","LTO1",47.2612978536316,0.0700822426948087,0.237012020546783,0
.295690668064556,0.767466324491083,0.940054077160953
"12770","AQP11",5.17795742063749,0.189720783198524,0.640430292613191,0
.296239552355329,0.767047145247115,0.940054077160953
"12771","MYL6",5564.03108906949,-0.0856890598288493,0.290106294845761,
-0.295371253058839,0.76771029090911,0.940054077160953
"12772","RIN3",46.1332671014183,0.10154188725252,0.34310755692567,0.29
594768521673,0.767270033741783,0.940054077160953
"12773","NOL11",148.439801349116,-0.0495527107237919,0.167464120914193
,-0.295900461861812,0.767306098338957,0.940054077160953
"12774","ZNF17",21.2975626898286,-0.0986683247146706,0.332657905549929
,-0.296605981906723,0.766767343390776,0.940054077160953
"12775","ZNF324B",15.4722643385195,0.113009781020734,0.382505737000144
,0.295445976593787,0.767653215656157,0.940054077160953
"12776","SIRPA",221.999879184938,0.0532487363223603,0.180368224191835,
0.295222379445984,0.767824007152432,0.940054077160953
"12777","LAS1L",89.3614792880319,-0.0608537692140164,0.20522726980673,
-0.296518923977913,0.766833817181684,0.940054077160953
"12778","NBN",293.946989890077,-0.051996866570784,0.176230848272335,-0
.295049743450315,0.767955880411847,0.940068380724823
"12779","SLC26A7",11.7853631558377,-0.273569853559228,0.92697369707252
3,-0.295121484485687,0.767901078022641,0.940068380724823
"12780","IQSEC2",37.4046067617021,-0.0942793266598158,0.31972366495956
5,-0.294877536424272,0.768087432682039,0.940155845809478
"12781","CEP97",71.4847550368642,0.0740809403624397,0.251310343137297,
0.294778716377653,0.768162926239434,0.94017468548341
"12782","NADK2",115.296427426223,-0.0632651856263385,0.214938372609144
,-0.294341046963184,0.768497310142081,0.94030005162873
"12783","IFT74",157.563885381153,-0.052272089667746,0.17761586209400 1,
-0.294298544350063,0.768529784868413,0.94030005162873
"12784","RAB18",340.053835179204,-0.0532556992160673,0.180858745527772
,-0.294460182506847,0.768406285100294,0.94030005162873
"12785","ADAMTS7",55.9990152062083,-0.0982735596565972,0.3338101848047
41,-0.294399524430572,0.768452630265055,0.94030005162873
"12786","ZNF843",9.1684281952637,-0.135263479222785,0.459932910019089,

-0.294093934737506,0.7686861254381,0.94030005162873
"12787","ALKBH6",42.5043280146415,0.0821870448205434,0.279381972232517
,0.294174474336314,0.768624584651232,0.94030005162873
"12788","SUV39H1",6.01524952368397,0.177315517232565,0.602824373856682
,0.29414125394127,0.768649968376182,0.94030005162873
"12789","RPE",129.576908787829,-0.0520839485985987,0.177303091195637,-
0.293756573827183,0.768943920754632,0.940366077629753
"12790","ACOX2",94.6754289415267,0.0950312270942016,0.323611938547708,
0.293657976651538,0.769019268897788,0.940366077629753
"12791","AC092910.3",7.24174406640865,0.158136902823603,0.537990876261
784,0.293939748425499,0.768803944183111,0.940366077629753
"12792","FAM13B",109.205684234532,0.0662624944824891,0.225787629770537
,0.29347265193328,0.769160900292315,0.940366077629753
"12793","RND1",4.81736767172917,0.309378548182444,1.05412096039125,0.2
93494351983678,0.76914431598431,0.940366077629753
"12794","ATXN2L",150.955325835859,-0.0525990754559969,0.17922367788750
6,-0.293482848226181,0.769153107741407,0.940366077629753
"12795","RNF165",6.14419554768316,-0.191265602568676,0.65141001610474,
-0.293617841052543,0.769049941221263,0.940366077629753
"12796","CTDSP2",576.892545092105,-0.044896915317781,0.153050242544258
,-0.293347560718815,0.769256503568427,0.940409462747804
"12797","SKAP2",326.413928368566,-0.106086535125956,0.361832490329283,
-0.293192396927687,0.769375095242475,0.940440485419282
"12798","ACAA2",591.874136607378,-0.0619859743134416,0.211442897515099
,-0.293157041650053,0.769402118033367,0.940440485419282
"12799","TRAF3IP3",6.50951264680751,0.207897505338762,0.70989790441190
3,0.292855499427047,0.769632604548052,0.940501744625043
"12800","TMEM273",13.5687978949772,0.137708207518919,0.470160179813816
,0.292896364752651,0.769601367575522,0.940501744625043
"12801","TULP3",307.328517535495,0.0529701291043423,0.180823226978197,
0.29293874459352,0.769568973320325,0.940501744625043
"12802","IL17RC",145.985770973202,-0.0678751781841713,0.23185959402787
8,-0.29274259048349,0.769718912754973,0.940533740995629
"12803","CBFA2T3",3.91901447551255,-0.21714467944689,0.742530760483601
,-0.292438631505941,0.769951274815532,0.940744184327061
"12804","TMCO6",25.1812269409738,0.104546984473351,0.357736475291306,0
.292245805765873,0.770098691555171,0.9408508147452
"12805","ATP1B1",91.1926772175401,0.136924628292277,0.469732120435154,
0.291495135920088,0.770672663417992,0.940885600527737
"12806","MAPK9",121.500315149899,-0.0494550950238846,0.169950172621731
,-0.290997615718579,0.771053143022492,0.940885600527737
"12807","ATF6B",208.307044527065,0.0458809891613946,0.157324123836972,
0.291633527283705,0.770566838140719,0.940885600527737
"12808","CALHM6",23.7549568409291,-0.109134250643578,0.374744971071332
,-0.291222722299867,0.770880985469237,0.940885600527737
"12809","AL078581.2",2.69365216464491,-0.244742150885302,0.84125029520
1302,-0.290926674595386,0.771107399882948,0.940885600527737
"12810","AC072061.1",4.06680122985778,0.225306895712621,0.773531103635
828,0.291270634953929,0.770844344170702,0.940885600527737
"12811","TRPS1",87.0275844104231,0.0833700866474093,0.28562706235673,0

.291884410249913,0.770375003537859,0.940885600527737
"12812","LYNX1",13.5267874033628,-0.133035366315879,0.45684351940821,-0.291205545584212,0.770894121521577,0.940885600527737
"12813","STK24",338.752370156943,-0.065705854679607,0.225212093147417,-0.291751005735726,0.770477007925834,0.940885600527737
"12814","COL4A1",1460.39831662092,-0.102333144909884,0.350978454235055,-0.291565318825385,0.770618995334443,0.940885600527737
"12815","INAFM2",7.89617451153709,0.154998417014484,0.532849773681017,0.290885770568558,0.771138684419818,0.940885600527737
"12816","AC009560.3",4.04412838779508,0.244727339080393,0.84130462011897,0.290890283053224,0.771135233127828,0.940885600527737
"12817","PAQR5",4.53318510259395,0.219508878229568,0.753065515062047,0.291487093538577,0.7706788134074,0.940885600527737
"12818","BANP",62.5370814409232,-0.066485785130577,0.227953160387333,-0.291664239344634,0.770543353783404,0.940885600527737
"12819","MED11",186.759200880222,0.0742288359299866,0.255188095647302,0.290878913225556,0.771143929142817,0.940885600527737
"12820","LLGL1",34.1195315751568,0.139930591391463,0.481073724614705,0.290871407503153,0.771149669779845,0.940885600527737
"12821","DPM1",221.373206493813,0.0664102137145452,0.228181173822007,0.291041599103826,0.771019504416478,0.940885600527737
"12822","DRAM2",294.555634685952,0.0764368194846455,0.262890290175427,0.290755582618282,0.771238258261007,0.940892906006196
"12823","XPO7",102.713641482434,0.0508301470874092,0.174878870204422,0.290659168989325,0.771312002348961,0.940892906006196
"12824","FRMD8",46.7147035913133,-0.0758689651098441,0.261052111661123,-0.290627662910197,0.771336100915647,0.940892906006196
"12825","AC018647.2",37.0045805850986,-0.0964147821604301,0.332032256923215,-0.290377757431944,0.771527257994426,0.941052701505404
"12826","DNAH10OS",3.34900210954282,0.257593985068025,0.887465396491299,0.290258060862377,0.771618820911675,0.941091003861012
"12827","GLO1",467.580473528053,0.0567422443292385,0.195632364668183,0.290045281748143,0.77178159597281,0.94121614608269
"12828","TTC4",107.330794415628,-0.0564663795144767,0.194926268231629,-0.289680708643014,0.77206051610001,0.941294149592521
"12829","AC008966.1",14.6462699853497,-0.110697860244609,0.382493433872836,-0.289411138705746,0.772266772094254,0.941294149592521
"12830","SLC25A48",12.977136322687,-0.147900465515076,0.510602537698545,-0.289658696530793,0.772077357619511,0.941294149592521
"12831","RAD52",22.7450897106845,0.108241711119436,0.373988531460976,0.289425214983445,0.772256001516283,0.941294149592521
"12832","GOLGA3",242.379689309205,0.0647336259505117,0.2234948002887,0.289642648808347,0.772089635837866,0.941294149592521
"12833","EIF3CL",2.71073873476265,-0.312392077992301,1.07851160807979,-0.289651104032614,0.772083166682863,0.941294149592521
"12834","PES1",71.2661518301279,0.0599441088640014,0.20708951018273,0.289459899784921,0.772229462346585,0.941294149592521
"12835","MAPK12",6.04191245289827,0.196995884488244,0.681018326068862,0.289266642243523,0.772377337270146,0.941355565790175
"12836","ALDH4A1",8.23389115718865,-0.149993160837099,0.52017261470422

,-0.288352667166817,0.773076795732082,0.941474528270686
"12837","SASS6",71.9684117610544,-0.0641842941625091,0.22231304619168,-0.288711325142695,0.772802295390047,0.941474528270686
"12838","MPZ",4.41947855839844,0.209608716823292,0.726492193702499,0.28852163676397,0.772947470624058,0.941474528270686
"12839","RNF149",257.548779984199,-0.0468814046793291,0.162221570410292,-0.288996121543863,0.772584345548728,0.941474528270686
"12840","AC024560.5",25.1962779799236,-0.0938894567885387,0.325364042520535,-0.288567403027066,0.772912443354674,0.941474528270686
"12841","FAM114A2",96.7627267095474,0.0645977674646711,0.223983901681121,0.288403617312806,0.7730377990996,0.941474528270686
"12842","PARD3",159.497892449292,0.0651072469777949,0.225513978819241,0.288706036400435,0.772806342944078,0.941474528270686
"12843","LINC00662",147.226229408856,0.0717715153512947,0.248839932018729,0.288424429186441,0.773021870105218,0.941474528270686
"12844","BMP2",4.86851080402743,0.25245706837171,0.874002134457834,0.288851775548941,0.772694808842494,0.941474528270686
"12845","RTL10",41.2651257597811,0.0698740358327584,0.242274746932207,0.288408250209876,0.773034253164002,0.941474528270686
"12846","GBP2",489.740006189102,0.0503473836563068,0.174830526432962,0.287978219156207,0.773363411333617,0.941526679186875
"12847","AL353807.5",77.0579483637822,0.100631047511933,0.349840210972635,0.287648601720643,0.773615737625591,0.941526679186875
"12848","TAMM41",65.3224633399165,-0.0701526969024028,0.244052920220204,-0.287448709235298,0.773768769492464,0.941526679186875
"12849","LSM3",659.524203382636,-0.0738983511516054,0.256458462177243,-0.288149396686833,0.773232382228332,0.941526679186875
"12850","CHDH",11.0030498326745,0.170219127951849,0.591860881514627,0.287599895969205,0.773653024520256,0.941526679186875
"12851","AC008608.2",25.7561108124671,-0.0993084906252662,0.345691855952379,-0.287274602844408,0.773902067441337,0.941526679186875
"12852","WDR55",102.844296430333,0.0553959014604296,0.192605086325733,0.287613907385313,0.773642297967687,0.941526679186875
"12853","FAM221A",21.9460655661404,0.102773856054279,0.357619747439494,0.287383056417116,0.773819033297161,0.941526679186875
"12854","LINC02334",2.92000143098587,0.268397053094166,0.933273604034403,0.287586675476436,0.77366314561472,0.941526679186875
"12855","BOLA2",118.148219525684,-0.068600587275154,0.23875844016214,-0.287322145464544,0.773865667577128,0.941526679186875
"12856","TTLL12",34.0026708259056,0.0765618291318348,0.265800867941721,0.288042058420296,0.773314544341886,0.941526679186875
"12857","GK",29.429202583644,0.102869519102641,0.358073018454959,0.287286429864252,0.773893012321453,0.941526679186875
"12858","PDZD11",209.377470410409,-0.0873532293122916,0.303523342428626,-0.287797401719879,0.773501826391027,0.941526679186875
"12859","ACTR10",470.538175456515,-0.0664067213959887,0.231242452935458,-0.287173572815038,0.773979420305257,0.941547559828536
"12860","STX3",33.0044077037572,-0.109026291060033,0.37977393924541,-0.287082076449644,0.774049475729399,0.941559560562597
"12861","TMEM71",4.33821629277822,-0.201422267619544,0.702820596322675

,-0.286591298936647,0.77442527753445,0.94178763739257
"12862","CBWD5",63.7752706740582,0.102167195166975,0.356384179933098,0
.286677133609449,0.774359547754342,0.94178763739257
"12863","XPO4",49.2365326584661,0.0865752751993327,0.30215850212345,0.
28652271768266,0.774477796293423,0.94178763739257
"12864","AC007611.1",6.84224112759818,-0.15692899587811,0.547372144817
973,-0.286695253610862,0.774345672173252,0.94178763739257
"12865","ABL1",253.275231726551,0.0548098613393332,0.191558289859351,0
.286126282394652,0.774781402230643,0.941937162904635
"12866","TNRC6A",698.659146762458,0.0673609366435325,0.235422769432269
,0.2861275347579,0.774780443066724,0.941937162904635
"12867","RAD51C",103.338281392026,0.069876363264774,0.244205979335119,
0.286136987534135,0.7747732033557,0.941937162904635
"12868","CFAP57",9.81581110674249,-0.203395538463812,0.713126410660981
,-0.285216667652638,0.775478151535951,0.941978934964815
"12869","AL135910.1",18.2571541364786,-0.138459963940735,0.48524401367
8185,-0.285340900738163,0.775382980449918,0.941978934964815
"12870","MPP6",55.0593653672896,0.0880763378255304,0.308328338416059,0
.285657615118984,0.775140370722745,0.941978934964815
"12871","DECR1",571.967546864669,-0.0849218413563506,0.297283058182959
,-0.285659875390836,0.775138639386887,0.941978934964815
"12872","CASC2",14.2894802807978,0.118734288732459,0.416101937741968,0
.285349040614388,0.775376744862765,0.941978934964815
"12873","STYX",54.4841944695128,-0.0657404046975391,0.230009433438032,
-0.285816123777595,0.775018958071141,0.941978934964815
"12874","ANGEL1",17.2780666002726,0.124721084207017,0.437167487757779,
0.285293595017114,0.775419219487423,0.941978934964815
"12875","AC015917.2",3.61220988194925,-0.222268057039006,0.77846983362
5531,-0.285519165211384,0.775246423499546,0.941978934964815
"12876","NECTIN2",145.75703550143,0.094809719225632,0.331913554281891,
0.28564581952899 4,0.77514940599383,0.941978934964815
"12877","DDRGK1",284.512241176273,-0.0593611076145939,0.20780471761330
4,-0.28565813277183,0.775139974207982,0.941978934964815
"12878","TOP3B",42.7801022707657,0.0810189176453209,0.283908278544706,
0.285370042961122,0.775360655991712,0.941978934964815
"12879","MT1E",2599.40108536577,-0.130935264481624,0.459367929003269,-
0.285033534591162,0.775618450202804,0.942076202851344
"12880","DNAJC3-
DT",20.0220342076496,0.114602742316207,0.402353083995395,0.28483127599
817,0.77577340949478,0.942118115420142
"12881","GRIPAP1",84.068138828427,-0.0720167299425051,0.25280846590281
2,-0.28486676538036,0.775746218856763,0.942118115420142
"12882","DNAJC30",107.456740601152,-0.0716546758858181,0.2517041773714
,-0.284678135397366,0.775890743250189,0.942139299648807
"12883","UNK",98.9500830709556,0.0651004649023843,0.228702507368801,0.
284651295044197,0.775911308404755,0.942139299648807
"12884","EMC4",476.343913524304,-0.0626248773439277,0.220219052607637,
-0.284375382612812,0.776122722397531,0.942225175870308
"12885","SLC46A1",29.0166656643698,0.0853886689002535,0.30039420892842
9,0.284255376309861,0.776214680705059,0.942225175870308

"12886","CHEK2",116.696115878254,-0.0907437768345351,0.319223885721243
,-0.284263743702987,0.77620826884583,0.942225175870308
"12887","ZNF280C",27.4582346286722,-0.0891590729959739,0.3136702819839
16,-0.284244565446419,0.776222964996526,0.942225175870308
"12888","RPL31",4375.79954349054,0.0614882687409133,0.216395063940203,
0.284148203851381,0.77629680735906,0.942241694407028
"12889","CCDC24",6.67949681502642,-0.188160503046125,0.662574695708525
,-0.283983834977149,0.776422768698475,0.94225327974124
"12890","KMT2E",709.632652197717,0.0528019450659411,0.185936387870321,
0.283978545946408,0.776426821956439,0.94225327974124
"12891","FBRSL1",92.834730411807,0.0589032216166086,0.207498057107219,
0.283873605554638,0.776507244467894,0.942277777147721
"12892","ENOPH1",121.31179754322,-0.0686445434072263,0.242312232869386
,-0.283289632530553,0.776954823959622,0.942601544222147
"12893","WWOX",88.645539839428,0.0594001395337695,0.209605368929063,0.
283390353201651,0.776877622456765,0.942601544222147
"12894","U2AF2",127.878892038843,-0.0540869140170496,0.190921142435947
,-0.283294523209736,0.776951075246429,0.942601544222147
"12895","SMIM1",23.4869162009983,0.111505201948174,0.393742860038834,0
.283192949675777,0.777028932584156,0.942611635382142
"12896","TGFBRAP1",65.7447773553675,0.0749681793491852,0.2647914914213
55,0.283121557066539,0.777083657219721,0.942611635382142
"12897","BX470102.2",7.88502337023225,0.159368705347734,0.563236321970
398,0.282951754230277,0.777213820771368,0.942696425395558
"12898","PRTFDC1",141.159538376895,-0.0605572556055347,0.2141682608918
39,-0.282755508931913,0.777364261767709,0.942805795226567
"12899","OGFRL1",361.230210075339,-0.0591349037397233,0.20931760156118
8,-0.282512809714365,0.777550325730369,0.942958349127852
"12900","EIF4E2",338.788144331335,-0.0638016896797954,0.22593829205518
2,-0.282385465072971,0.777647958872997,0.94300364501165
"12901","NFYA",34.718526831634,-0.0919295894169101,0.325801412178957,-
0.282164490331965,0.777817385078263,0.94313598595297
"12902","C2orf42",25.7425055567143,0.0936782225140123,0.33216172685828
3,0.282025937786566,0.777923621758191,0.943191692385939
"12903","HSBP1",951.345035529373,-0.070259958839503,0.249260060267197,
-0.281874114786729,0.778040038470947,0.943259731984889
"12904","ZMYND19",42.9251016178808,-0.0742471738027723,0.2636025956310
38,-0.281663287969651,0.778201707120817,0.943382618140959
"12905","TNFAIP8L3",13.8915815116206,-0.149458389436557,0.530986556196
24,-0.281473019782672,0.778347619007064,0.943413280964474
"12906","GRK3",26.9239696110696,-0.108090646676636,0.383933471137288,-
0.281534835596517,0.778300213157209,0.943413280964474
"12907","ATF6",177.213292592485,0.0485520879431456,0.172844371645526,0
.280900601396021,0.778786639368215,0.943420515638281
"12908","KIAA1143",203.399930551374,-0.0514544852302545,0.183078125736
706,-0.281052064648367,0.778670466673291,0.943420515638281
"12909","NUDT9",190.771729789458,0.0545615095384826,0.19402194651415,0
.281213081915471,0.778546971448111,0.943420515638281
"12910","ZNF503-
AS2",23.1781580753112,-0.102847129811278,0.366076298802284,-0.28094451

934684,0.778752953680718,0.943420515638281
"12911","IFIT3",389.893879261378,0.0779311420507405,0.277379117502772,0.280955332010391,0.77874466027908,0.943420515638281
"12912","C11orf24",99.1192639515245,-0.0737944282325478,0.262555983706668,-0.281061688980557,0.778663084953405,0.943420515638281
"12913","RBBP6",472.277765941016,0.0535044033079674,0.19034893449477,0.281085909148798,0.778644508532346,0.943420515638281
"12914","SERHL2",15.6915168610351,-0.117851538254975,0.419645162288781,-0.280836165517082,0.778836063348,0.943420515638281
"12915","RPS6KA1",4.4569933918562,0.23855795285576,0.849973025882881,0.280665321829437,0.778967109188216,0.943506193498355
"12916","AC091729.3",26.3683006801201,0.0938870990684526,0.334937825874678,0.280312021561822,0.779238128480321,0.943546307454841
"12917","ARHGAP39",6.26416721245509,-0.186686251837875,0.665727960100265,-0.280424231858548,0.779152048207681,0.943546307454841
"12918","AC004816.2",26.4836436546051,-0.148695006026578,0.530714790490055,-0.280178748908194,0.779340369909148,0.943546307454841
"12919","HDC",4.73731120784135,-0.270915680566574,0.966595094340412,-0.280278352489924,0.779263957679924,0.943546307454841
"12920","AC138969.1",10.1319635949062,0.144067403590839,0.513892081113954,0.280345638482202,0.779212339531915,0.943546307454841
"12921","CDC34",121.400832071116,-0.0660895215918325,0.235907305080797,-0.280150381817117,0.779362132495302,0.943546307454841
"12922","AC083799.1",16.6262369668991,-0.107453533790213,0.383752055525078,-0.280007708735755,0.779471590655708,0.943605795745801
"12923","XPNPEP3",82.0085063215254,0.0715765758430121,0.255799025519306,0.279815670515953,0.779618928436987,0.943711127256813
"12924","AC016252.1",2.89251731900261,-0.281009711365697,1.00461584277525,-0.279718574404928,0.779693426650756,0.943728278636473
"12925","NFKBIZ",439.13122365897,0.185861156803949,0.66482297749277,0.279564881323571,0.779811353738624,0.943797988900061
"12926","CSE1L",124.141570429023,-0.0549176483505904,0.196523540864856,-0.279445648642957,0.779902843213182,0.943835693670416
"12927","EXOG",105.854443091751,0.0580366091805879,0.207979982089906,0.279049015185989,0.780207209442221,0.943865039383481
"12928","AC092171.3",3.35267419099534,-0.251648599014463,0.901379412241 08,-0.279181658241778,0.780105418849501,0.943865039383481
"12929","FEZ1",405.431750418989,0.0830552666599325,0.297568981295742,0.279112649101646,0.780158376155512,0.943865039383481
"12930","GINS2",10.1984084995563,0.159699251150778,0.571948941635848,0.279219418946764,0.780076441884958,0.943865039383481
"12931","ZNF337",54.6220453565772,0.0686270995857002,0.245956837566991,0.279020905718909,0.7802287811972,0.943865039383481
"12932","CCDC88C",4.611091683628,0.204237350388037,0.732564488431044,0.278797776323364,0.780400021061265,0.943926198829457
"12933","FAM32A",358.188342182031,0.0671710271227912,0.240871065256434,0.278867148494072,0.780346780493124,0.943926198829457
"12934","PLAA",71.5138295807609,0.0705482751869094,0.25321838950524,0.278606444519108,0.780546866460681,0.944030820476607
"12935","NEK11",89.8118609787169,0.059872749961592,0.215117704074041,0

.278325534475696,0.780762476516394,0.944165973864412
"12936","PSMA6",627.340537605194,-0.0679230323253045,0.244121965239089
,-0.278234005935442,0.780832732091834,0.944165973864412
"12937","CCDC144A",119.264915962295,-0.216263004145013,0.7772955177164
35,-0.278224946903538,0.780839685730608,0.944165973864412
"12938","MAN1A1",823.681327825318,-0.0494308986009929,0.17778038955811
3,-0.278044719802096,0.780978030221266,0.944260266405261
"12939","FOXD2-
AS1",4.68938300739165,0.207085195054136,0.745670560280827,0.2777167372
36006,0.781229811368893,0.944319161624372
"12940","ARL8B",247.183696772927,-0.0413879759340271,0.149116231249035
,-0.277555136602843,0.781353875157029,0.944319161624372
"12941","FKBPL",25.9648641529621,-0.113325931633178,0.407949874770207,
-0.277793764974222,0.781170677712117,0.944319161624372
"12942","TNKS2-
AS1",3.45225232546375,0.28823922485971,1.0392097276363,0.277363863322
675,0.781500726374848,0.944319161624372
"12943","DCUN1D2",26.6582170043061,0.0909676692591473,0.32803429659075
4,0.277311458602257,0.781540961775838,0.944319161624372
"12944","USP3-
AS1",5.82357672813851,0.186826065646539,0.673796848449566,0.2772735819
0593,0.781570043185498,0.944319161624372
"12945","AC087482.1",2.74165139548487,-0.270298289750002,0.97339045867
8113,-0.27768742475355,0.781252314689304,0.944319161624372
"12946","REXO5",19.7206965246412,0.117969237889273,0.425022719114114,0
.277559839942579,0.781350264237684,0.944319161624372
"12947","MVP",470.563070733275,-0.0458608892516188,0.165331490134537,-
0.277387503217324,0.781482576280689,0.944319161624372
"12948","CLEC2L",10.4374187532987,0.178332351587597,0.644151628652228,
0.276848405957966,0.781896510658892,0.944640648458221
"12949","CPAMD8",4.5054278966172,0.223992003488356,0.809451408542276,0
.27672075324662,0.781994535132571,0.94468611576792
"12950","AC245297.2",11.339147051769,0.121958341140834,0.4413803881283
16,0.27631119193583,0.782309060493739,0.944736408310174
"12951","GTF2H2C",68.5438910016074,0.0756428433606025,0.27379829852560
4,0.276272145473281,0.782339048346409,0.944736408310174
"12952","KDM1B",50.8765109460694,0.0778265167469668,0.281507950776501,
0.27646294370121,0.782192517551945,0.944736408310174
"12953","ZNF467",4.98096175332542,-0.231098719627003,0.837200652748984
,-0.276037433640527,0.782519314867667,0.944736408310174
"12954","ZNF385A",157.705053069959,-0.119116765459525,0.43139395279274
1,-0.276120619420813,0.782455424075348,0.944736408310174
"12955","TAOK3",276.260413334552,-0.0592789125847612,0.214736482579724
,-0.27605422177274,0.782506420633873,0.944736408310174
"12956","IFI27L1",184.080777915606,0.094178097119185,0.340617478011635
,0.276492262431607,0.782170001796879,0.944736408310174
"12957","AC027682.4",3.573679471916,0.257428511628794,0.93216555757007
7,0.276161792868475,0.782423801374663,0.944736408310174
"12958","MTMR2",75.3154718785915,-0.0638438956942164,0.231625978501144
,-0.275633571447173,0.782829521844861,0.944864071545558

"12959","KLHL42",119.920924446226,-0.0668045936371929,0.242409930743318,-0.275585218115221,0.782866664405931,0.944864071545558
"12960","TEX22",3.45558100156472,0.220655874165546,0.800532783730466,0.275636274553672,0.782827445471058,0.944864071545558
"12961","GET1",192.96820484238,-0.0650254854881745,0.235939850106685,-0.275601961511681,0.78285380292753,0.944864071545558
"12962","RSAD1",102.902618645647,0.0548594235113436,0.199280463675332,0.275287514388369,0.783095356111989,0.945067169854949
"12963","KLHL15",31.9220537038739,0.0876544666173966,0.318548150660151,0.275168656404829,0.783186666335834,0.945104452788047
"12964","AL691432.2",21.7074520615312,-0.0914867538043853,0.33278475005815,-0.274912698939477,0.783383310581639,0.945128666015255
"12965","LINC01142",5.49270341862228,-0.234095403550051,0.854146465713865,-0.274069393185866,0.784031294281865,0.945128666015255
"12966","RABGAP1L",239.185558085286,0.0576816534971541,0.21047403453237,0.274055912052576,0.78404165420329,0.945128666015255
"12967","METTL8",101.473727028421,-0.0551570086843696,0.200830387682861,-0.274644735394676,0.783589193555082,0.945128666015255
"12968","MCUB",382.333848298569,-0.0586144122217667,0.21367844818883,-0.274311296804104,0.783845403771295,0.945128666015255
"12969","LEMD2",80.0558195247191,0.0681779128349074,0.248973821301602,0.273835668659791,0.784210911286776,0.945128666015255
"12970","MRPL15",197.645459349861,-0.0793906736938805,0.288829536974549,-0.2748703422977,0.783415853221094,0.945128666015255
"12971","RALGPS1",20.561355373158,-0.109496775997217,0.398948019879193,-0.274463766057479,0.783728245314949,0.945128666015255
"12972","HELLS",20.6875527707705,-0.123816336217815,0.451611879312288,-0.274165366080189,0.783957542564674,0.945128666015255
"12973","AC079630.1",18.2607793995341,-0.109685456835797,0.399915436809537,-0.274271625298714,0.783875888441045,0.945128666015255
"12974","ANKRD52",37.2749105341194,-0.0824209490087254,0.301038928102982,-0.273788342019773,0.784247283150131,0.945128666015255
"12975","LINC01852",11.5713465651076,-0.122703164851699,0.447859044224771,-0.273977195356397,0.784102146824484,0.945128666015255
"12976","AC092338.3",43.4588967282109,-0.0821092611120308,0.29984119758371,-0.273842493205449,0.784205666469021,0.945128666015255
"12977","AC009054.2",9.44552403584843,-0.142106376360072,0.517810986407471,-0.274436773437339,0.783748986278754,0.945128666015255
"12978","FXR2",62.1970291732258,0.0600799909905586,0.21877304578092,0.27462245532168,0.783606312564927,0.945128666015255
"12979","PLEKHF1",306.37948132492,0.0794961923790897,0.289758406949974,0.274353359462024,0.783813082036882,0.945128666015255
"12980","PRDX4",1201.48342147929,0.0872513799960581,0.31875304054311,0.273727208522918,0.784294266671612,0.945128666015255
"12981","ABCD1",38.6531050748001,-0.0844678535735491,0.30715140653136,-0.27500396149065,0.783313194783426,0.945128666015255
"12982","LDLRAP1",44.3444176210926,-0.066162653915745,0.242380056791417,-0.272970700607939,0.78487573807627,0.94542016062906
"12983","AL513548.1",10.3112914662719,0.214585842987955,0.786199432463196,0.272940724868814,0.784898780674398,0.94542016062906

"12984","POLE3",195.21904506149,-0.0578171361070932,0.211791280050708,
-0.272991107534032,0.784860051211585,0.94542016062906
"12985","AL353796.1",4.39744353886616,-0.193459027359016,0.70832554674
0678,-0.273121629241254,0.784759720854871,0.94542016062906
"12986","TMEM218",161.23570914677,-0.080619037114313,0.295175690559044
,-0.273122210577794,0.784759273996953,0.94542016062906
"12987","JPT2",37.5032398875477,0.0829700892652765,0.303945836130353,0
.272976561619002,0.784871232690717,0.94542016062906
"12988","SLC22A17",133.348885356169,0.0675981175666827,0.2478107159904
11,0.272781252806269,0.785021371334311,0.945495019385789
"12989","CRCP",191.211814609725,-0.0664173764675979,0.243558432035462,
-0.272695861574308,0.785087016168602,0.945501285235618
"12990","DIS3L",113.004225630826,0.0535052887884674,0.196463164519259,
0.272342598773635,0.785358604527865,0.945755554320969
"12991","RFFL",41.7456787751085,0.0765106482071053,0.281028567333604,0
.272252208852067,0.785428100478254,0.945766436439175
"12992","NDUFA3",484.40129757785,-0.0936236498905145,0.344043028195177
,-0.272127734666379,0.785523804785661,0.945808873018942
"12993","RSBN1",229.034815355701,0.052065325693267,0.191547686898888,0
.271813909821581,0.785765109278227,0.945953794400438
"12994","C12orf66",18.2388451831937,-0.0956156123784211,0.351743620479
482,-0.271833252435629,0.785750235865156,0.945953794400438
"12995","SPRTN",57.4603556624969,0.0589903506755945,0.217199582620352,
0.271595138277522,0.785933338081796,0.946010711573833
"12996","RBBP8",43.9964464147979,-0.069555355612359,0.256052268430166,
-0.271645145105711,0.785894883438274,0.946010711573833
"12997","AL445423.1",6.00662684769419,-0.163581122777966,0.60256021971
1947,-0.271476804187582,0.78602433763982,0.946047450465469
"12998","AL031432.4",9.30240206434306,0.138904160383268,0.512342323491
352,0.271115919990187,0.786301877595795,0.946133016581253
"12999","COMMD10",149.715602122701,0.0659532051339361,0.2433174956944,
0.271058211189101,0.786346261382673,0.946133016581253
"13000","PITPNM1",16.2031294806726,-0.10740449419323,0.395932198901667
,-0.271269915634987,0.786183443083542,0.946133016581253
"13001","AL118558.3",25.2244934908616,0.106251738214612,0.392119398763
167,0.270967818857608,0.786415783438696,0.946133016581253
"13002","ELK1",53.4064966720841,0.0649001706756926,0.239561369791035,0
.270912504517334,0.786458327341689,0.946133016581253
"13003","SASH3",3.4637966870676,0.277286784861737,1.02266944115254,0.2
71140188318562,0.78628321305373,0.946133016581253
"13004","SLF1",47.5195293932863,0.0759357275050815,0.280738770791978,0
.270485360076426,0.786786878286641,0.946455485776525
"13005","RARRES1",408.163693417725,0.134885675902595,0.49902324887252,
0.270299382257946,0.786929940563784,0.946554791252539
"13006","FMOD",334.539193364589,-0.0813146115067713,0.300977148987036,
-0.27016872138115,0.787030454930051,0.946602906848439
"13007","EIF2B3",154.384273312087,0.0531150659320597,0.196995756350175
,0.269625432121713,0.787448432722991,0.946817002361794
"13008","PARS2",11.034131738071,0.121328580978887,0.44978506042519,0.2
69747912178749,0.787354197746478,0.946817002361794

"13009","RAPGEF1",128.701854346672,0.0572748638196351,0.212465738052989,0.269572234772981,0.787489363220313,0.946817002361794
"13010","RCBTB1",29.3063683892485,0.0743005824690759,0.275652895987588,0.269543993734865,0.78751109235628,0.946817002361794
"13011","MYO1E",138.419033540651,-0.0976213750637885,0.362118761353174,-0.269583864417835,0.787480415221474,0.946817002361794
"13012","KCNC4",16.2062246901802,-0.106145178453038,0.394102084139304,-0.269334222590709,0.787672499025474,0.946865511584991
"13013","SUZ12",183.353293576257,0.0434554754590249,0.161320200324234,0.269374048455709,0.787641854642022,0.946865511584991
"13014","PHF20L1",313.855696733375,0.0435113789494016,0.161615500620152,0.26922775836748,0.787754420529236,0.94689122486083
"13015","AL445228.2",4.18056508832005,0.185808773394212,0.690936242481002,0.268923182733927,0.787988796674156,0.947024165022989
"13016","CLDND1",291.967844923179,-0.0516760823913242,0.19243123144284,-0.268543115396911,0.788281291895357,0.947024165022989
"13017","ATG3",235.175984487707,0.0468223479543688,0.174333637225606,0.268578965594435,0.78825370074153,0.947024165022989
"13018","PAIP2",721.985367514245,0.0426987227490899,0.158939432682536,0.268647761152991,0.788200754808324,0.947024165022989
"13019","TMEM248",436.721124263982,0.0463417483372245,0.172573521964446,0.268533363691632,0.788288797082679,0.947024165022989
"13020","SPRED1",201.87396291472,-0.0477165569071913,0.177396954741562,-0.268981826529697,0.787943667780096,0.947024165022989
"13021","CNEP1R1",66.093987020859,0.0660289199977474,0.24573760964879,0.268696843320469,0.788162981148666,0.947024165022989
"13022","ENTPD7",12.8530196219054,-0.112070208754029,0.417695242806464,-0.268306165042813,0.788463661130103,0.947093580516258
"13023","STRADA",100.476337635615,-0.0547812483243738,0.20417835243135,-0.268300961742713,0.788467665988828,0.947093580516258
"13024","PWWP2A",144.87765459001,0.0580476217490343,0.216964128750511,0.267544787626086,0.78904973496386,0.94752128049269
"13025","UBXN2B",30.6211995791614,-0.0826222860601566,0.308841070037789,-0.267523636186234,0.78906601808977,0.94752128049269
"13026","TGIF1",190.731293572462,0.0574987356596297,0.214818722594357,0.267661658933727,0.788959764962278,0.94752128049269
"13027","PRELID3A",13.9516484703385,0.120629636877252,0.450617526573145,0.26769850208583,0.788931402908867,0.94752128049269
"13028","SIRT6",61.8779148731405,-0.0890643236157779,0.333029798659949,-0.267436499599004,0.78913309988569,0.947529097444876
"13029","ANKRD6",46.8739535617438,0.0717035160485187,0.268233098578759,0.267317927684696,0.789224384568691,0.947565971894085
"13030","GTF2IRD2",166.024251434686,0.0941059400671719,0.352168489570586,0.267218512882624,0.789300923040429,0.947585137307861
"13031","ZNF384",73.8028393358307,0.0559358623852561,0.209499556068421,0.266997522261994,0.78947106881362,0.947643947932125
"13032","LCMT2",12.6325180789273,0.108963344870047,0.408048155397819,0.267035504090971,0.789441825013231,0.947643947932125
"13033","DENND4B",47.387775826385,0.0729657575822805,0.273406579714283,0.266876377512684,0.789564345209583,0.94768319282694

"13034","KYAT1",10.2243961368392,0.127685905224137,0.478850491305163,0
.266650880687444,0.78973797637468,0.947818870985816
"13035","ELMOD2",94.8864077199585,-0.054393432453283,0.204127987766408
,-0.26646729362525,0.789879344991247,0.947915810793869
"13036","GALNT1",392.688975466711,0.037421360567124,0.14054614083639,0
.266256763397624,0.790041469323273,0.948037642269404
"13037","C1D",361.208119078522,-0.0640450260419036,0.24077218242611,-0
.26599844465653,0.7902404068714,0.948081565863437
"13038","MTMR6",116.655495355967,-0.0503389067765173,0.189211313827534
,-0.266045966058886,0.790203808456678,0.948081565863437
"13039","CCL4",238.49655237806,-0.1337180154477,0.502750071750699,-0.2
65973140455378,0.790259894987749,0.948081565863437
"13040","TRBC1",18.5911682959534,-0.144165497847826,0.542590282195636,
-0.26569863592921,0.790471314006331,0.948262481978607
"13041","RAD1",111.802471626814,0.0504845134418334,0.190063442880275,0
.265619272579602,0.790532441269781,0.948263091694133
"13042","UBE2G2",295.426779862179,0.0544959028348763,0.205238720276465
,0.265524471997623,0.790605460292003,0.948277964679328
"13043","TMEM98",765.392285027912,0.0598049969605659,0.225390294850374
,0.265339716602561,0.790747771221626,0.948375939984658
"13044","NIPSNAP3A",84.8483613441087,-0.0579479510378244,0.21847717354
9334,-0.265235722782451,0.790827877267891,0.948399301142412
"13045","NOMO3",5.58443900115708,0.163018299019836,0.614954058560628,0
.265090207553715,0.790939970807014,0.948461016736996
"13046","IRF2",274.015755953913,0.0511051140270044,0.192909754020085,0
.264917211089718,0.791073239364053,0.948475410697622
"13047","RBP4",3.55906084426709,-0.230966191603807,0.871835024287631,-
0.264919606541992,0.791071393975562,0.948475410697622
"13048","SEC22B",386.530256782821,0.0459003738405116,0.173849538018999
,0.264023559472994,0.791761764849113,0.948523895475545
"13049","ZNF322",227.755822695077,-0.0419646331015499,0.15905234678609
,-0.263841646788074,0.791901941757375,0.948523895475545
"13050","PRR3",44.8412044114355,0.0635760435153543,0.240542435676461,0
.26430281765695,0.791546589280375,0.948523895475545
"13051","PSMB8",556.647913416479,-0.0791829933845605,0.299444800087837
,-0.26443268796564,0.791446526297211,0.948523895475545
"13052","RWDD2A",35.7487758461603,0.0758787611211031,0.2867523762312,0
.264614236570177,0.791306651778401,0.948523895475545
"13053","GIMAP1",5.84187843949484,0.169338156292062,0.64039106748805,0
.26442929155195,0.791449143135557,0.948523895475545
"13054","NCALD",236.595061241651,-0.0874952204279205,0.331618539644566
,-0.263842970063432,0.791900922053506,0.948523895475545
"13055","LINC01569",6.65791031734673,-0.163628202449017,0.620037127881
237,-0.263900652220876,0.791856473057404,0.948523895475545
"13056","DOK4",53.3024950470836,0.0622653283918174,0.235746074176263,0
.264120319328257,0.791687207110339,0.948523895475545
"13057","ARL17A",49.3850887164865,0.0929864301095131,0.352412191013397
,0.263857018799836,0.791890096247883,0.948523895475545
"13058","RNF152",18.4844186013611,0.132738146094216,0.502482448348314,
0.264164741535816,0.791652978477075,0.948523895475545

"13059","TMED1",317.840473885341,-0.0676690427453789,0.256330373032554
,-0.263991511988261,0.791786459270384,0.948523895475545
"13060","LARGE1",70.6509332416129,0.0581244889066557,0.21971349502484,
0.264546740290506,0.791358653649995,0.948523895475545
"13061","TM6SF2",7.96459322326516,0.142799709067137,0.541415795028633,
0.263752388420041,0.791970724259949,0.948533652828909
"13062","PHGDH",316.180967996161,-0.0679966418446884,0.258709147307646
,-0.262830450922671,0.792681263986542,0.948540061452778
"13063","LIX1L-
AS1",3.66679681567089,-0.2328543812509,0.885925484044292,-0.2628374343
49341,0.792675881192759,0.948540061452778
"13064","CD84",8.85617314304302,-0.209475815925802,0.796359359355158,-
0.263041820837546,0.792518345386567,0.948540061452778
"13065","AL513548.4",4.93728154995984,0.235470928823861,0.894170012222
297,0.26334022121659,0.792288361321154,0.948540061452778
"13066","AC092428.1",6.9751475360867,0.193214780236973,0.7352123569589
84,0.262801323193418,0.79270371561543,0.948540061452778
"13067","KDELR2",1296.48308700656,-0.0615747332821588,0.23366643686861
,-0.263515522842428,0.792153260734035,0.948540061452778
"13068","SGK3",110.073233373044,0.0614363921905728,0.23335166178664,0.
263278143040378,0.792336204917531,0.948540061452778
"13069","EXOSC3",114.707584369728,-0.0649920569226483,0.24661571618920
7,-0.263535746735562,0.792137675085874,0.948540061452778
"13070","AP001528.1",264.160111695106,-0.0829899491839708,0.3156298692
99769,-0.262934396443802,0.792601144268691,0.948540061452778
"13071","BAIAP2",39.5894151092052,0.0740747968390909,0.281811580268485
,0.262852210574594,0.792664491777542,0.948540061452778
"13072","ZNF350",50.0128328076341,0.0751394011810802,0.285529186709057
,0.26315839038075,0.792428500397084,0.948540061452778
"13073","PEX26",102.980168446861,-0.0611265749740214,0.232166129539683
,-0.263288082095426,0.792328544843876,0.948540061452778
"13074","PRXL2B",13.2407827464189,-0.136298278059119,0.518827904176033
,-0.262704216488855,0.792778566622022,0.948547709771087
"13075","AC097500.1",3.82594377309134,0.217423172158371,0.830979915074
985,0.261646723601919,0.793593818240574,0.948547709771087
"13076","FAM171B",126.155809240562,0.0707064790505819,0.27030262903551
9,0.261582653867902,0.793643218694292,0.948547709771087
"13077","ABI2",237.141342975269,0.0517596023317297,0.19709351471637,0.
262614436635396,0.792847771703193,0.948547709771087
"13078","UBE2E1",280.033537548561,-0.0456901753694229,0.17427222582048
7,-0.26217703454644,0.793184958059297,0.948547709771087
"13079","AC078785.1",7.79024998241039,-0.148397540710066,0.56653023387
5192,-0.261941078934117,0.793366868571423,0.948547709771087
"13080","COPG1",192.777229335726,0.056254742205734,0.214604264165703,0
.262132453073243,0.793219327384797,0.948547709771087
"13081","ABRAXAS1",240.488382709444,-0.0563246901983687,0.215245614014
288,-0.261676366583849,0.793570962537686,0.948547709771087
"13082","FAM162B",22.3930559859545,-0.148442108245517,0.56656022785403
6,-0.262005875011334,0.793316912769118,0.948547709771087
"13083","INSL6",2.77095804196704,-0.233327231663154,0.892146845751155,

-0.261534558771769,0.793680302575833,0.948547709771087
"13084","HSPA14",34.6100327413524,-0.0716018941111976,0.27343170954609
6,-0.261863900972052,0.793426371536278,0.948547709771087
"13085","PDSS1",13.2495626769237,-0.115090055446175,0.43974771783684,-
0.261718368914599,0.793538577678584,0.948547709771087
"13086","RASSF8-
AS1",218.97911426672,-0.0501126461729074,0.191458036787764,-0.26174219
172872,0.793520209844713,0.948547709771087
"13087","POMP",929.517679139284,-0.069567172893635,0.265255178145217,-
0.262265088961051,0.793117075192304,0.948547709771087
"13088","TMC8",9.03840344894346,0.18294101662087,0.698620083468436,0.2
61860517539981,0.793428980135391,0.948547709771087
"13089","SLC25A42",36.9701294176746,0.0740155474298349,0.2823654590781
72,0.262126775957196,0.793223704089303,0.948547709771087
"13090","SAMD15",2.8411600720464,-0.243196914089817,0.931151612941055,
-0.261178642349849,0.793954747593105,0.948803217463632
"13091","AL513217.1",4.00334120845549,0.219776463807983,0.842389913108
069,0.260896362109916,0.794172430335263,0.948845896871192
"13092","STXBP5-
AS1",3.40117358701098,0.221287423475977,0.847799554556839,0.2610138473
02205,0.794081828708706,0.948845896871192
"13093","NOP2",23.920738434421,-0.102510440524013,0.392866738837004,-0
.260929293295414,0.794147034352774,0.948845896871192
"13094","LRP5L",13.8958540292099,-0.136115895732746,0.5220329363145,-0
.260741969067527,0.794291498590511,0.948915679887838
"13095","UBQLN4",36.0896918968501,-0.0817404380394072,0.31506820105892
4,-0.259437282990422,0.795297866512398,0.949074296761659
"13096","ELMOD3",104.177308556533,0.0520171303949196,0.200233636477887
,0.259782179007992,0.79503179822117,0.949074296761659
"13097","PRPF40A",498.455366583551,0.0413634125089402,0.15887601074689
7,0.260350271349874,0.794593598235998,0.949074296761659
"13098","CDHR4",6.07555668439961,-0.216567796203632,0.834911841739562,
-0.259390016258971,0.795334332049388,0.949074296761659
"13099","RNF14",86.0037431321551,-0.0500931044984915,0.192428328500359
,-0.260320841992856,0.794616297081414,0.949074296761659
"13100","CCDC25",351.037505423467,-0.0545980259106226,0.21028666291531
8,-0.259636180220375,0.795144425340713,0.949074296761659
"13101","COPZ1",438.178320075193,-0.0686975324712984,0.264815021907049
,-0.259417052614982,0.795313473872888,0.949074296761659
"13102","ZDHHC20",105.165632685068,0.0731166704132841,0.28083802534624
7,0.260351746609592,0.79459246037364,0.949074296761659
"13103","OLFM4",37.4363333485365,0.0900237402534681,0.346179815810893,
0.260049073174865,0.794825920423142,0.949074296761659
"13104","PSMA4",842.046176832882,-0.0569366913029131,0.218847459777814
,-0.260166105472362,0.794735648127349,0.949074296761659
"13105","FHOD1",46.6059230152458,-0.0804340522623289,0.310045256775281
,-0.259426811101409,0.795305945370867,0.949074296761659
"13106","SAP30BP",218.968826741187,-0.0496845135234362,0.1912289556612
44,-0.259816895153948,0.795005017945673,0.949074296761659
"13107","CELF4",6.06888060474247,-0.17511819045686,0.673627386774073,-

0.259962991254679,0.794892321048988,0.949074296761659
"13108","ZNF266",40.3629522049382,0.093904033848259,0.361771381586455,
0.259567336245524,0.795197534787649,0.949074296761659
"13109","AURKC",6.6194495864023,-0.146991389720423,0.565436844668694,-
0.259960756194707,0.794894045116998,0.949074296761659
"13110","CADPS2",74.3644010178637,0.059986603486469,0.231399448393203,
0.259233995167255,0.795454703031284,0.949145531618488
"13111","SELENOF",1114.84476813705,-0.0574357280306166,0.2222999910416
97,-0.258370356928369,0.796121091849501,0.949534687114144
"13112","AC239799.1",3.12603139793737,0.197899963484307,0.768042339891
881,0.257668038863802,0.796663114645597,0.949534687114144
"13113","CCSAP",18.8461381332781,0.10472671210534,0.405441314413479,0.
258303010527774,0.796173063021709,0.949534687114144
"13114","KCNIP3",17.0952578422619,0.095290353549039,0.369180680393659,
0.258113055773749,0.796319655848659,0.949534687114144
"13115","SNX2",607.009495717202,-0.0489474661672655,0.18949575345878,-
0.258303762875155,0.796172482430745,0.949534687114144
"13116","MAN1B1",267.031498684131,0.0506372782592518,0.196549282323891
,0.257631458433958,0.79669134874213,0.949534687114144
"13117","ABCC8",5.21701847780237,-0.200498931918125,0.776210240276342,
-0.258304930178124,0.796171581616301,0.949534687114144
"13118","RCN1",851.543563833789,-0.0500589814335984,0.193751010120024,
-0.258367589426182,0.796123227511268,0.949534687114144
"13119","CDON",493.467084644449,0.0636352962576787,0.246602323815018,0
.258048242503233,0.796369675512791,0.949534687114144
"13120","KBTBD7",87.3156521606886,0.0602832743051092,0.233452349217916
,0.258225177459395,0.796233127874618,0.949534687114144
"13121","EIF3J-
DT",233.546381572633,-0.0454158090295008,0.176016708121964,-0.25801987
5011136,0.796391568397933,0.949534687114144
"13122","TM2D3",354.213055703062,-0.0509518547643325,0.197617567719191
,-0.25783059346593,0.796537652353787,0.949534687114144
"13123","CCNF",5.75523515372246,0.168994598171452,0.655716167845145,0.
257725226948136,0.79661897535051,0.949534687114144
"13124","LRRC29",20.9057574856057,-0.0999168812474203,0.38762109587220
4,-0.257769461753862,0.796584834188971,0.949534687114144
"13125","ACSL4",200.641732133389,-0.0479839225198251,0.186092001241101
,-0.257850537367574,0.796522259687461,0.949534687114144
"13126","MEF2A",330.118460795704,0.056644148652011,0.220001215207299,0
.257471980773549,0.796814442466815,0.949609044911503
"13127","RRNAD1",71.7164612908093,-0.0657588420317639,0.25557265399731
2,-0.257299992793656,0.796947198016282,0.949694905048274
"13128","C3orf38",119.969182427555,0.0561296732428644,0.21839222499242
9,0.25701314799467,0.797168623295502,0.949797198310354
"13129","ZBTB49",16.8108513529013,0.10152932757627,0.395166628579417,0
.256927888726985,0.797234440978811,0.949797198310354
"13130","DNAAF2",60.5359947383701,-0.0671360264913769,0.26135763312938
1,-0.256874175387647,0.797275906860146,0.949797198310354
"13131","MBD1",103.272831459564,0.0479421772919099,0.18647407010573,0.
257098358311839,0.797102844841562,0.949797198310354

"13132","PACC1",21.7033011821329,-0.110536576817783,0.430561056821145,
-0.256726833666472,0.797389655365703,0.949860370003479
"13133","UBE2L5",9.33788163806753,-0.130791203866663,0.510095192403706
,-0.256405482377397,0.79763775500327,0.950083560611905
"13134","RASGRP1",10.5695652956794,0.159605937468526,0.622910781340788
,0.256225999371822,0.797776333966331,0.950176274724784
"13135","CXCL9",48.8048494768361,-0.112111806535809,0.43827559576995,-
0.255802074351993,0.798103672087507,0.950213401427217
"13136","FAM120A",417.810336629781,0.0405800068719379,0.15876984881744
4,0.255590133606522,0.798267337656104,0.950213401427217
"13137","PPP6C",150.844491534326,-0.0489027660424245,0.19141583613914,
-0.255479207095891,0.798353001220189,0.950213401427217
"13138","NEK5",14.757161806518,0.109248493498746,0.426591640953073,0.2
56096188979859,0.797876564638976,0.950213401427217
"13139","AC009093.1",29.6484451931445,0.122949143243954,0.481011762874
932,0.255605273578147,0.798255645924071,0.950213401427217
"13140","WDR45B",258.332711316619,0.0552371213153336,0.216211163661209
,0.255477656102379,0.798354199000058,0.950213401427217
"13141","CEP76",18.9035726409014,0.101792242655737,0.397946765947659,0
.255793617051597,0.798110202841477,0.950213401427217
"13142","FNDC11",3.01262559937561,-0.248311356283174,0.969926225947306
,-0.256010560020329,0.797942683266804,0.950213401427217
"13143","USP51",68.6051396799457,0.057248075654866,0.223818460578149,0
.255779060882591,0.798121443194056,0.950213401427217
"13144","STRN",216.17695597851,0.0507374834063577,0.198662270712464,0.
255395668359158,0.798417516038718,0.950216464043949
"13145","PLEKHA5",580.487742030333,-0.0479189334026795,0.1878518201617
07,-0.255089002392577,0.798654358077831,0.950426026885623
"13146","TNFRSF18",4.30556008961553,-0.186810517603146,0.7334790141207
66,-0.254691019111268,0.79896175326673,0.950530521787141
"13147","UTS2B",3.81753337966504,0.203571908240661,0.799385062937234,0
.254660635629922,0.798985222206681,0.950530521787141
"13148","SYNCRIP",393.158557588149,0.0405609619794551,0.15919117094177
4,0.254794042530731,0.798882176823936,0.950530521787141
"13149","CHPT1",578.270349501644,-0.0724758666200398,0.284413583675012
,-0.254825615863887,0.798857789646003,0.950530521787141
"13150","HAUS5",35.6647151499007,0.0754767526379914,0.296553629158857,
0.254512996020562,0.799099265216388,0.950593901580225
"13151","AL162390.1",24.2439455056892,-0.118664290255526,0.46661427843
8225,-0.254309170848135,0.7992567153466,0.950708904126444
"13152","NBEAL2",16.0655099810046,0.131623718743353,0.518232957013321,
0.253985619714205,0.799506667711714,0.950754334901799
"13153","SLC35B4",118.805218813492,-0.0472198394915466,0.1859161406792
02,-0.253984615424136,0.799507443586073,0.950754334901799
"13154","AVPI1",56.4081702979691,0.073386404683682,0.289043100117442,0
.253894331516179,0.799577194132659,0.950754334901799
"13155","ZFYVE21",433.99455837146,-0.0571235783236514,0.22486851875518
8,-0.254031016168347,0.799471596432697,0.950754334901799
"13156","C15orf65",14.8925190673943,-0.111096696901732,0.4378901944985
07,-0.253709030934035,0.799720356621995,0.950754334901799

"13157","AC006305.1",5.47468564287658,-0.23019148309092,0.907158825327
563,-0.253749924119187,0.799688762131614,0.950754334901799
"13158","ZNF30",11.9190372470263,-0.11191557669793,0.440866340768388,-
0.253853756453424,0.799608541688243,0.950754334901799
"13159","BOD1L1",840.88864331678,0.0507102267454575,0.200009614713082,
0.253538945206221,0.799851770100084,0.950806402512202
"13160","SAR1A",554.318869277438,-0.0451473145006641,0.178148810789572
,-0.253424731271385,0.799940018488741,0.950806402512202
"13161","NDN",1105.40660630453,-0.0657388367314739,0.25941040434392 3,-
0.253416345800526,0.799946497696291,0.950806402512202
"13162","TOLLIP",146.536798915625,-0.0633679066418199,0.25026815285500
9,-0.253200041311415,0.800113634592085,0.950932805494908
"13163","PTPN22",4.80482316593841,0.219372157608718,0.869875591335281,
0.252187967789712,0.800895777894203,0.950953578216172
"13164","WARS2",121.415140024813,-0.0578343416270703,0.229617532583386
,-0.25187249848209,0.801139617442443,0.950953578216172
"13165","DHX36",583.489725064303,0.0419814730075453,0.166497647460008,
0.252144541667647,0.800929342626718,0.950953578216172
"13166","HIST1H4E",7.61470022483329,-0.238037922594845,0.9421348733178
42,-0.2526580103723,0.800532498126306,0.950953578216172
"13167","RNF8",142.255808113286,-0.0496815060382008,0.19690593284446,-
0.252310863977091,0.800800791491035,0.950953578216172
"13168","SOGA3",5.86288849130324,-0.151946908730327,0.601474827569081,
-0.252623886762536,0.800558869640928,0.950953578216172
"13169","AL356056.2",5.0196138437259,0.172410190050192,0.6827015162853
45,0.252541097298707,0.800622852197606,0.950953578216172
"13170","AP001160.1",6.4966289563164,-0.225234301521684,0.893867796912
329,-0.251977196515756,0.801058689762181,0.950953578216172
"13171","FAM168A",116.40834657923,0.0510870460444185,0.202918351360478
,0.251761586381431,0.801225350692907,0.950953578216172
"13172","ALG9",69.0477668199215,0.0555394303802134,0.219579924088772,0
.252934919304189,0.800318504912264,0.950953578216172
"13173","SPSB2",52.5578212271105,0.087462441413214,0.346371078386384,0
.252510809564902,0.800646259938483,0.950953578216172
"13174","HOXC5",51.7089527101301,-0.0653352255056562,0.258242381419269
,-0.25299962440937,0.800268503435979,0.950953578216172
"13175","EEF1AKMT3",60.4710837881598,0.0657375551606164,0.260046523652
583,0.252791516830429,0.800429323433755,0.950953578216172
"13176","EXD2",58.8654866010323,0.0644944680002614,0.256002467157563,0
.2519290877012,0.80109587581882,0.950953578216172
"13177","TRIM16",32.4577717442296,-0.0823232528236261,0.32607278702255
2,-0.252468945891926,0.800678614387387,0.950953578216172
"13178","MTMR4",62.7449872350286,-0.0675538239087074,0.2682056109116,-
0.251873268717608,0.801139022071284,0.950953578216172
"13179","RBBP7",465.848228890137,-0.0567012820182765,0.224035464726554
,-0.253090652801257,0.800198161779065,0.950953578216172
"13180","KLF8",85.1481269911514,-0.0734290849612817,0.291602744090885,
-0.251812050638302,0.801186342345972,0.950953578216172
"13181","BTBD19",21.5051326225732,0.0949686266152274,0.377590473151862
,0.251512242410396,0.801418098267571,0.950954758953939

"13182","H1FX",1178.19473898308,-0.0599530440103187,0.240009276274329,
-0.2497946951925,0.802746122354201,0.950954758953939
"13183","TMCC1-
AS1",12.843839470327,0.110532758554219,0.442273163010719,0.24991966006
2079,0.802649479043028,0.950954758953939
"13184","ZNF330",172.965649819371,-0.0434555507125873,0.17393327737838
9,-0.249840348940534,0.802710815046573,0.950954758953939
"13185","AC113410.4",2.72131447482249,0.245376616419535,0.981935603499
08,0.249890741862447,0.802671843064528,0.950954758953939
"13186","FOXF2",50.0286151824333,0.0869719033439859,0.346969563031234,
0.250661477577953,0.802075846387281,0.950954758953939
"13187","AL122017.1",4.93010360910706,-0.182196507508313,0.72726717160
334,-0.250522111573717,0.802183607204889,0.950954758953939
"13188","ZNF596",17.7906327939566,-0.0978501935898761,0.38937996318508
6,-0.251297454520957,0.801584143033629,0.950954758953939
"13189","LINC01301",3.74840217957996,0.183275033130046,0.7332465586222
38,0.249950076103212,0.802625956833379,0.950954758953939
"13190","AC083843.3",12.8369291298938,-0.116880205260431,0.46584554081
322,-0.2508990534854,0.801892156221424,0.950954758953939
"13191","WSCD2",23.8300914513357,0.118114747016823,0.471938865681245,0
.250275524238344,0.802374283098275,0.950954758953939
"13192","AC000403.1",4.5622040767498,0.170968971829284,0.6840576146532
65,0.249933584784294,0.802638710353282,0.950954758953939
"13193","CA12",172.441311402122,-0.106261818000477,0.424586851947403,-
0.250271098864928,0.802377705165926,0.950954758953939
"13194","NPRL3",143.131971005952,-0.0606388061153052,0.242171455292206
,-0.250396175065876,0.802280987280715,0.950954758953939
"13195","ZNF263",59.6650665919088,0.0617358571203848,0.245967261082614
,0.25099217208281,0.801820161290089,0.950954758953939
"13196","MMP15",2.59906799928828,-0.258588352891685,1.02813633413716,-
0.251511734685167,0.80141849076105,0.950954758953939
"13197","CRLF3",37.2744704392928,0.0724056988840861,0.289422378466247,
0.25017311815275,0.802453472991664,0.950954758953939
"13198","C17orf113",6.52129792509703,-0.141207177248087,0.563729190304
418,-0.250487609435008,0.802210285588059,0.950954758953939
"13199","STAT3",692.821311349748,0.0417509763677661,0.166764664373869,
0.250358650764078,0.802310003442971,0.950954758953939
"13200","TUBG1",40.5643573356641,0.0871219807321499,0.34860435375715,0
.249916502169798,0.802651921205557,0.950954758953939
"13201","RALBP1",607.990424944671,0.0350544269490923,0.140209862689978
,0.25001398815005,0.802576531109793,0.950954758953939
"13202","RYR1",5.10264585230406,-0.305291225184321,1.21964353981816,-0
.250311845401843,0.80234619668992,0.950954758953939
"13203","ZNF256",8.66074662181602,-0.124367329715342,0.497749231246262
,-0.249859410940629,0.802696073158128,0.950954758953939
"13204","FOXO4",15.7629714156533,0.113377676923093,0.451393940103528,0
.251172350468617,0.801680860592648,0.950954758953939
"13205","F8A3",13.3630313721024,-0.120748507859677,0.483019359270772,-
0.249986890881504,0.802597486407088,0.950954758953939
"13206","LY6K",19.4876190642474,0.156793904593019,0.627959331241121,0.

249687992187529,0.802828644987461,0.950980500798035
"13207","CUL1",154.381180975893,-0.0505460847254339,0.20250374265728,-0.249605681663764,0.802892304314191,0.950983896144991
"13208","ABHD1",8.1865291371684,-0.147271073298104,0.590272104476211,-0.249496922150485,0.80297642141332,0.951011520303495
"13209","CLK2",48.6926348417749,0.0814948673285574,0.326794115360549,0.249376789538101,0.803069337380881,0.95104956050035
"13210","LRRC57",91.3293053173886,-0.0616505772879922,0.247349849330916,-0.24924445054144,0.803171697537469,0.951098778544938
"13211","TRAPPC6A",180.665358338046,0.0799926846163817,0.321154145223186,0.249078786016574,0.803299838727002,0.951178516176406
"13212","PAG1",10.3574169161493,-0.125748933903599,0.505119562148798,-0.248948849592477,0.803400347999952,0.951225525564884
"13213","CSTF1",63.4909682595324,-0.0594937863448206,0.239179265177763,-0.248741404488401,0.803560819032392,0.951343517151571
"13214","TXLNA",204.661120634371,0.0475233684087726,0.192319289009248,0.247106614492982,0.804825714821373,0.951347517750348
"13215","HCN3",11.8072840594021,0.115415568519952,0.466745594927809,0.247277252906486,0.804693661802911,0.951347517750348
"13216","CEP68",162.65371396186,0.0459534115238294,0.185485382580709,0.247746808317006,0.804330312787231,0.951347517750348
"13217","SNRK-AS1",2.99866018186206,0.251409199108277,1.01494973353687,0.247706059522941,0.804361843141203,0.951347517750348
"13218","ANK2",805.557152770089,-0.0573153390789438,0.231785678744713,-0.247277309751611,0.804693617812746,0.951347517750348
"13219","LINC00240",5.97304490850021,0.155060753212348,0.624289158313003,0.248379698970528,0.803840639402657,0.951347517750348
"13220","SKIV2L",47.0601280788511,-0.070507786912795,0.283600119487049,-0.248616915395957,0.803657122669406,0.951347517750348
"13221","AL353135.2",13.0620063048151,-0.139684670301802,0.566056816442137,-0.246767932554488,0.805087829225853,0.951347517750348
"13222","ATP5MF",851.861887548477,-0.0699240600611548,0.28293840126378,-0.247135276614381,0.804803533496663,0.951347517750348
"13223","PAXIP1",43.7942240767681,0.0770986243410638,0.312662802844941,0.246587133613394,0.805227763009192,0.951347517750348
"13224","AC069185.1",5.20723361134785,0.156400658931765,0.630040957800372,0.248238875576911,0.803949589120683,0.951347517750348
"13225","PSD3",109.388237980699,0.079925970063591,0.323555025562853,0.247024350570827,0.804889378907875,0.951347517750348
"13226","AC067930.3",2.90949232665298,0.292557772833902,1.18705016560278,0.246457800446321,0.805327867419939,0.951347517750348
"13227","DCAF12",81.2168956738546,0.0495951968332119,0.200108706091522,0.247841274884506,0.804257218245729,0.951347517750348
"13228","NOLC1",128.353604624713,-0.0493411415804336,0.200144766153623,-0.24652726388339,0.805274102033157,0.951347517750348
"13229","TRIM6",19.0965714254978,0.106577738844273,0.431808510057454,0.246817133895977,0.805049749719014,0.951347517750348
"13230","ING4",232.14913217383,0.0590016847626956,0.238101312048013,0.247800754456985,0.804288571160874,0.951347517750348

"13231","ASB8",229.255589885192,-0.0518508718479921,0.20943959162191З,
-0.247569580548051,0.804467449329867,0.951347517750348
"13232","NAXD",81.8644572841945,0.0551299134584076,0.223619899782051,0
.246534022741891,0.805268870659028,0.951347517750348
"13233","CIAO2A",129.989190124342,0.0542065349584415,0.219294018806508
,0.247186563744222,0.804763843299774,0.951347517750348
"13234","C16orf72",114.020674393957,-0.0555638013428145,0.224333107597
212,-0.247684356259081,0.804378636690578,0.951347517750348
"13235","DNAAF1",2.65934118575016,-0.207617885437108,0.836513736782605
,-0.248194233170213,0.803984128052575,0.951347517750348
"13236","CRK",319.625694912041,-0.0427535658369494,0.173430025818168,-
0.246517669793661,0.805281527898628,0.951347517750348
"13237","SHBG",2.60752048169962,0.281848793185927,1.14251555243086,0.2
46691428038992,0.805147041013915,0.951347517750348
"13238","ANKRD13B",67.291599259936,-0.0661308409841189,0.2675179820502
89,-0.247201479606285,0.804752300274066,0.951347517750348
"13239","CNTD1",6.23315169706602,0.153093618764917,0.620849391229057,0
.246587370347335,0.80522757977906,0.951347517750348
"13240","ZNF564",31.6813128450615,0.0668509380933483,0.271137323624053
,0.246557490498951,0.805250706621464,0.951347517750348
"13241","MED26",32.1652991640001,0.0737411035312797,0.298010807323874,
0.247444393689853,0.804564320919095,0.951347517750348
"13242","BX322562.1",3.4704257010983,0.258870812727295,1.0414509949547
8,0.248567444825894,0.80369539348305,0.951347517750348
"13243","AL122035.2",3.61763043897049,-0.18697128042378,0.758889940926
054,-0.246374698544065,0.805392190322829,0.951351659987919
"13244","RAB29",135.261044416511,-0.0739722900953904,0.300983830972699
,-0.245768318704469,0.80586158297377,0.951652854309068
"13245","ZNF208",87.4850018327372,0.136371935954392,0.554545173942647,
0.245916730254505,0.805746692585172,0.951652854309068
"13246","AC092329.4",20.6982251162218,-0.0920429150020714,0.3744406698
7201,-0.245814417097195,0.805825896203713,0.951652854309068
"13247","PIN4",336.710590438431,-0.0647512495915404,0.263504663814683,
-0.245730943256012,0.805890517230213,0.951652854309068
"13248","GPATCH11",140.690769113531,0.0479910296034589,0.1954841751643
83,0.245498284263179,0.806070636493886,0.951670966596044
"13249","COASY",97.6646262972855,-0.0594328587144514,0.241990988201879
,-0.245599471104561,0.805992298693989,0.951670966596044
"13250","IQCN",23.3656707733184,0.0916432978125258,0.37332988873689,0.
245475384043287,0.806088365875956,0.951670966596044
"13251","DDAH1",18.6879498910683,0.108397215167108,0.442058040266841,0
.245210368986108,0.806293548149842,0.951697726832263
"13252","RGS11",39.630337964665,-0.120527298743885,0.491438917903042,-
0.245253874597828,0.806259863939715,0.951697726832263
"13253","BCAR1",55.6320236484936,0.0627963768377603,0.255963860427187,
0.245332980730004,0.806198616955531,0.951697726832263
"13254","TINAGL1",2.79845940825622,-0.222932588913168,0.91362631406407
8,-0.244008502690228,0.807224234734575,0.951986826725907
"13255","TWIST2",646.049293614593,-0.0509988100693682,0.20931381287870
4,-0.243647609147141,0.807503752430162,0.951986826725907

"13256","SLC49A4",45.3360909379396,0.066450292920007,0.272957542100679
,0.243445527859777,0.807660278286005,0.951986826725907
"13257","RUVBL1",101.999270199578,-0.0542213780180835,0.22196887649223
5,-0.244274687852377,0.80701808593208,0.951986826725907
"13258","EIF2B5",95.9740471903439,-0.0463784005918106,0.19004222731371
1,-0.244042606989928,0.807197821693221,0.951986826725907
"13259","TNFRSF21",18.8038029581149,-0.15709309447789,0.64544665808725
8,-0.243386641652814,0.807705891152563,0.951986826725907
"13260","SH2B2",4.81271661452309,-0.155014018501439,0.635178406211659,
-0.244047998145868,0.807193646379849,0.951986826725907
"13261","NUB1",384.660610536736,-0.0386771336049563,0.158293272072205,
-0.244338455441832,0.806968702703915,0.951986826725907
"13262","AL135791.1",8.77095790935101,0.144531135227434,0.592780860897
005,0.243818828780551,0.80737113707874,0.951986826725907
"13263","PSAP",4251.19944184182,0.0336993065331816,0.138467722885198,0
.243373010193295,0.807716450084163,0.951986826725907
"13264","DUSP8",5.22920199586463,0.202415031013507,0.831764443842023,0
.24335619599045,0.807729474417708,0.951986826725907
"13265","PPP1R14B-
AS1",6.12677885513399,0.195191555074131,0.802452070860327,0.2432438798
05633,0.807816476248782,0.951986826725907
"13266","RASGRP2",9.7983893019546,-0.165770737247224,0.67844595523425,
-0.244338898284075,0.806968359758413,0.951986826725907
"13267","IFFO1",113.538373026933,0.0629933174266937,0.258408798856778,
0.243773887365219,0.807405945177241,0.951986826725907
"13268","H2AFJ",1526.87156879067,-0.0659640307562153,0.271091963472315
,-0.243327134863413,0.807751985382279,0.951986826725907
"13269","FBXL3",357.229276038546,0.038024024958646,0.155892375430387,0
.24391202490609,0.807298955878814,0.951986826725907
"13270","SUSD6",57.4571957316285,0.0764704213686316,0.312820978997734,
0.244454261391419,0.806879021625955,0.951986826725907
"13271","PTPN9",110.246312038242,-0.0434918924089017,0.178255850727382
,-0.243985777922188,0.807241834697189,0.951986826725907
"13272","LDHD",16.4042029468634,-0.114571722315387,0.47088377929415,-0
.243312102377212,0.807763629720065,0.951986826725907
"13273","ASXL3",10.837190700494,-0.137965059188196,0.566981000119851,-
0.243332773336377,0.80774761776685,0.951986826725907
"13274","AC006115.2",50.5285180802334,-0.0709834655104615,0.2910935214
68253,-0.243851065981911,0.80734616890374,0.951986826725907
"13275","ZNF599",53.7121910392475,0.0715504062347225,0.294289050347484
,0.243129692220077,0.807904930132104,0.952019346294275
"13276","APPL1",561.838475527069,0.0363286415533414,0.149487439258482,
0.243021365096265,0.807988846557176,0.95204628542208
"13277","AC067852.3",10.3869767088228,0.11559732207579,0.4758206527915
33,0.242943053012951,0.80804951298018,0.95204628542208
"13278","SEMA4G",3.71188368498544,0.198864725530463,0.818870631254422,
0.242852433510558,0.808119715095145,0.952057290498069
"13279","MAT2A",277.027360862304,0.055947384553521,0.230840698651474,0
.242363607805533,0.808498430611457,0.952093086276343
"13280","SOX2-

OT",3.80968875652369,0.191474292346834,0.78981913843462,0.242428023112
134,0.808448522573728,0.952093086276343
"13281","HSD17B8",44.9256728886013,-0.100355879677975,0.41419275776147
6,-0.242292695363272,0.808553373435906,0.952093086276343
"13282","ABLIM1",833.84114104733,0.079633095021289,0.328704734277447,0
.242263304166693,0.808576145955457,0.952093086276343
"13283","AP001107.2",2.91471786878969,0.228189800980938,0.941509355116
452,0.242365941178263,0.80849662273478,0.952093086276343
"13284","COQ5",125.364024286396,-0.0650278968553005,0.268146125457022,
-0.242509179442621,0.808385644944995,0.952093086276343
"13285","PNMA8A",5.87997012716508,0.185328177870212,0.764169729878224,
0.242522270412027,0.808375502548174,0.952093086276343
"13286","SKA2",132.567925393982,-0.0595431558358539,0.24623705423979,-
0.241812330072263,0.808925584386953,0.95238914531772
"13287","CD40",224.632642630774,-0.0606491939791354,0.250842812312259,
-0.241781669644322,0.808949343082308,0.95238914531772
"13288","BRD9",212.212898215991,-0.0653440660974178,0.270355935935352,
-0.241696435742558,0.809015391562781,0.952395226536468
"13289","LCNL1",2.6433549874472,0.245443766046664,1.01713832229472,0.2
41308149213108,0.809316295331341,0.952606080351254
"13290","SERPING1",7495.54062914076,0.0381044868069065,0.1578864920413
19,0.241341018564999,0.809290822039103,0.952606080351254
"13291","ERGIC2",427.068255120664,-0.0432838607428726,0.17954051433166
4,-0.241081300808321,0.809492105209199,0.952741328853172
"13292","CCDC13",6.80550254366194,-0.155733156187453,0.646254810782692
,-0.240977944905108,0.809572210300234,0.952763924595739
"13293","S100A8",28.6192407600095,-0.109262801531925,0.454116001073758
,-0.24060548686585,0.809860897197942,0.952805238154156
"13294","MYL6B",412.009710734979,-0.076078711661751,0.316256007292829,
-0.240560526622054,0.809895746990467,0.952805238154156
"13295","SLC17A7",76.484284872261,0.0552074128070787,0.229311905564705
,0.240752492423473,0.809746952200715,0.952805238154156
"13296","STK35",43.6370021235594,0.0760984072310879,0.316365221697562,
0.240539737025318,0.809911861646475,0.952805238154156
"13297","TTC28-
AS1",65.2922097568523,0.0604604571285444,0.251040240681252,0.240839703
485273,0.809679356225364,0.952805238154156
"13298","LYAR",153.341859218482,-0.0456507211156927,0.189988043843045,
-0.240282073504615,0.810111591254805,0.952901950524462
"13299","ZNF774",4.49737943683019,0.159285192990257,0.66292452175105,0
.240276513787,0.810115901043586,0.952901950524462
"13300","C6orf52",4.08975332294026,0.19375622952472,0.807643708053749,
0.239903100330753,0.810405377321388,0.952955823305907
"13301","ANGPTL2",2666.85471479298,0.0604320687842686,0.25183416468291
3,0.239967713913477,0.810355285948523,0.952955823305907
"13302","AKAP13",898.929781950161,-0.049885386497005,0.207862732822664
,-0.239991968832452,0.810336482638369,0.952955823305907
"13303","L3MBTL2",60.593819088015,-0.0693087631882154,0.28880793300608
1,-0.239982200166074,0.810344055656634,0.952955823305907
"13304","MRS2",75.9275383945659,-0.0574684209442631,0.239976977426125,

-0.239474726120151,0.810737492020999,0.953211782346305
"13305","AL136418.1",4.39608026131054,-0.175079653850226,0.73112791484
9251,-0.239465147335163,0.810744918757118,0.953211782346305
"13306","PON3",42.3139701955172,-0.0741371008741176,0.309838736382009,
-0.239276411141542,0.810891255394058,0.953240543182479
"13307","THAP10",14.0482606480789,-0.102219620957834,0.427152400538364
,-0.239304802756583,0.81086924152852,0.953240543182479
"13308","RAMAC",103.110174420134,0.0673789646726241,0.281967006281229,
0.238960456974251,0.811136245296097,0.953346925347186
"13309","VAMP2",772.017037519262,0.064327449336599,0.26919568870984,0.
238961662591618,0.81113531042888,0.953346925347186
"13310","ACO2",142.086110689088,-0.0477505950984032,0.199856911274024,
-0.238923912082941,0.811164583287799,0.953346925347186
"13311","NAIP",73.3840417263941,0.0843257846453573,0.353377208591706,0
.238628249346968,0.811393858030627,0.953377540133774
"13312","C2",534.664190761879,0.07177933243271,0.301064095418596,0.238
418773693054,0.811556307896269,0.953377540133774
"13313","DDX43",3.81911026335128,-0.205127339509209,0.860061685940006,
-0.238503054911712,0.811490946233412,0.953377540133774
"13314","RP9",128.758874352999,0.0542033250467392,0.227314079692784,0.
238451243847258,0.811531126528271,0.953377540133774
"13315","MAFB",844.195317635895,-0.0547430100696377,0.229293719832292,
-0.238746225189584,0.811302370496121,0.953377540133774
"13316","ZNF711",16.8243361471994,0.0974631797576348,0.4084571648595,0
.238612976200723,0.811405702190257,0.953377540133774
"13317","ANAPC13",261.960321524319,0.049673646377463,0.208471342220247
,0.238275658651362,0.81166729929873,0.953436326719984
"13318","C1orf74",6.34423088935001,-0.137523456923591,0.57743960323514
8,-0.238160763745863,0.811756407603201,0.953469401121555
"13319","SPRY1",2118.70046666132,0.0577702513690795,0.242786339532249,
0.237946877408257,0.81192229655112,0.953592648468292
"13320","PDHB",251.039455453762,-0.0577563609250437,0.243182946967449,
-0.237501690168985,0.812267608144313,0.953843054840655
"13321","AC090114.2",50.2262828909219,-0.0759962797788781,0.3201596397
94021,-0.237369956524724,0.812369794973756,0.953843054840655
"13322","JMJD1C",623.778015513129,-0.0484262408320564,0.20405958326258
,-0.237314219983202,0.812413031211804,0.953843054840655
"13323","PDHX",72.9037992988645,-0.0541785462270068,0.22816751509564,-
0.237450744047842,0.812307127074269,0.953843054840655
"13324","SIPA1",63.5003838785082,-0.0661774845527601,0.278901606926251
,-0.237278964729161,0.812440379894962,0.953843054840655
"13325","SERPINB6",534.087829099606,-0.0488264569624255,0.205929773397
402,-0.237102465354539,0.812577299819377,0.953932210211971
"13326","RANBP6",61.3976280904555,0.0591210966782056,0.249597767080981
,0.236865487097984,0.812761145430441,0.954076436887917
"13327","TMED5",293.059201380192,-0.0600043743563932,0.254148795485729
,-0.236099385172032,0.813355551007754,0.95434834812542
"13328","GPSM2",133.789661432392,0.0789719422622559,0.335161646546029,
0.235623446405913,0.813724878047488,0.95434834812542
"13329","RPP14",81.507658572002,0.0523977294257838,0.22197272287875,0.

236054812259097,0.813390137693059,0.95434834812542
"13330","AC011374.2",7.96693611717504,0.134132186636215,0.569030820093
559,0.235720424799066,0.813649619745793,0.95434834812542
"13331","MTPN",794.600821886316,-0.0297533827840436,0.125871521234705,
-0.236378987813806,0.813138599551117,0.95434834812542
"13332","AC021097.2",11.6527663082819,0.105074564507818,0.444631113300
903,0.236318515201858,0.813185520715907,0.95434834812542
"13333","USP12",101.070234642678,0.0523702956754997,0.222171285982348,
0.235720360729517,0.813649669465223,0.95434834812542
"13334","LINC01290",7.91043376081561,0.13575154770394,0.57595000198509
,0.235700229596412,0.81366529172027,0.95434834812542
"13335","CEP95",159.918553822973,0.0555900281307338,0.235483579568039,
0.23606753486891,0.813380265451664,0.95434834812542
"13336","FAAP24",30.0419572217324,-0.0705551301737676,0.29924859065030
7,-0.235774310650694,0.81360780336336,0.95434834812542
"13337","AC010332.3",8.9452244087407,-0.145415222143494,0.616745715988
076,-0.23577824437828,0.813604750741363,0.95434834812542
"13338","CASS4",3.17382969795243,-0.210054791719223,0.888417514846892,
-0.236437022243335,0.813093570821509,0.95434834812542
"13339","AC100810.1",367.315288830306,-0.0632633457141624,0.2687821822
05701,-0.235370310617341,0.813921327530316,0.954435631675917
"13340","MED25",104.872207254394,0.0523642852733909,0.222425705263845,
0.235423712431418,0.813879883349238,0.954435631675917
"13341","SNX6",756.91171152197,0.0317462051059381,0.134924044656241,0.
235289456277574,0.81398407811097,0.954437668382423
"13342","LINC02268",2.84774770436326,0.269660186865269,1.1473775386391
,0.23502306589086,0.814190830577347,0.954536997880645
"13343","MRPL42",247.421535936881,-0.0515481290383712,0.21929953535980
1,-0.235058085981793,0.814163649840056,0.954536997880645
"13344","TACC2",74.3432725050613,-0.072586503031392,0.308973414328169,
-0.234927989481632,0.814264624971865,0.954551973054173
"13345","ZMIZ2",69.9096764347106,-0.0549603662912957,0.234178179333652
,-0.234694651942738,0.814445738948289,0.954621211926277
"13346","RTL6",36.9599499927126,-0.0678046280552672,0.288816755839652,
-0.23476694715355,0.814389623156893,0.954621211926277
"13347","MARC1",11.7041677929027,0.103059304770729,0.440546515360885,0
.233935126433371,0.815035342756317,0.954659510323129
"13348","SLC1A4",10.5052297684801,0.103588050193202,0.442603715794025,
0.234042432308474,0.814952037081275,0.954659510323129
"13349","TOPBP1",119.287337701806,0.0477756281089199,0.204055491324883
,0.234130568105392,0.814883615439548,0.954659510323129
"13350","NR3C1",1159.062218531,-0.0354698063990426,0.151566008212286,-
0.2340221717086,0.814967766003465,0.954659510323129
"13351","PDGFA",29.4122755358797,-0.111789413195421,0.477638696273014,
-0.234045972547256,0.814949288693489,0.954659510323129
"13352","PIK3CG",4.57136078380188,-0.179766397112789,0.766872850588509
,-0.234414866786369,0.814662918119505,0.954659510323129
"13353","IFITM2",1880.40457016421,-0.0580737763647422,0.24823953047410
3,-0.233942500027411,0.815029618284944,0.954659510323129
"13354","KLRK1",8.86924513214402,0.15937297950198,0.67988819530272,0.2

3441057015414,0.814666253429074,0.954659510323129
"13355","HNRNPA1",2031.5911001936,-0.0601477613932292,0.25667394404512
7,-0.234335283298777,0.814724696250734,0.954659510323129
"13356","AMZ2",100.896874232841,-0.0474807402262496,0.20302505457635,-
0.233866407893976,0.815088692698057,0.954659510323129
"13357","UBP1",74.6561167204847,0.0668693151135919,0.286288889696286,0
.233572861260984,0.815316598574853,0.954711995771123
"13358","ZDHHC3",236.291966604436,-0.041367105903198,0.176998914412272
,-0.233713896158957,0.815207098920192,0.954711995771123
"13359","NAP1L4",378.935073889661,-0.0342676682210015,0.14669451061186
1,-0.233598844824333,0.815296424635687,0.954711995771123
"13360","DNAJC1",383.117506408277,0.0453419712320283,0.194203897495116
,0.233476113594314,0.815391715659013,0.954728488626792
"13361","ZNF385D",74.1944777757669,0.101105788773241,0.433502087508844
,0.233230223536533,0.815582637915441,0.954780482358902
"13362","TMF1",421.118790228414,-0.0393176206037853,0.168572563111983,
-0.233238552454509,0.815576170716656,0.954780482358902
"13363","CSGALNACT1",71.9074518596844,-0.141883059257172,0.60846201565
2158,-0.233183100353601,0.815619228137953,0.954780482358902
"13364","MAST3",13.9548811377693,0.0893445814454955,0.383384286335315,
0.23304184503627,0.815728912515697,0.954837427303431
"13365","SRSF10",661.200255049762,-0.0336141603387515,0.14490459241042
4,-0.231974430758852,0.816557873445775,0.954868833139153
"13366","AL645568.1",27.6955383630986,0.0775420191327866,0.33415456605
3628,0.232054345534042,0.816495803986699,0.954868833139153
"13367","PCAT6",14.998492293383,0.0933244026212583,0.400986672134723,0
.23273691897147,0.815965699100242,0.954868833139153
"13368","EIF2AK2",519.3261722207,-0.0444023636215797,0.191651827028784
,-0.231682443679031,0.816784668323349,0.954868833139153
"13369","UBA3",137.047929808692,0.0428128720005639,0.185114138628332,0
.231278239024857,0.817098651179487,0.954868833139153
"13370","ZFP62",65.6072194823291,-0.0587999460913553,0.253864151197129
,-0.231619729741582,0.816833382069155,0.954868833139153
"13371","MTA2",37.334741974237,-0.0706915847367741,0.305300734965208,-
0.231547378177226,0.816889582827518,0.954868833139153
"13372","C12orf73",58.8247812254092,-0.0659210275821329,0.284203632492
717,-0.23194998249652,0.816576862534904,0.954868833139153
"13373","IRS2",247.115097891109,-0.0753667711275249,0.323972671340956,
-0.232633113205426,0.816046312026117,0.954868833139153
"13374","TMEM255B",4.01003605066916,0.16023835017297,0.692305981770131
,0.231455966570249,0.816960590262973,0.954868833139153
"13375","ADSSL1",12.8256984744977,0.170155806128342,0.73149421126206З,
0.232614015953411,0.816061142679851,0.954868833139153
"13376","PFAS",27.4962265195142,0.0756778318124756,0.325390504776142,0
.232575415390622,0.816091119527924,0.954868833139153
"13377","KAT7",93.0865684047084,-0.0528331840857668,0.228406163943341,
-0.231312426834824,0.81707209323239,0.954868833139153
"13378","GDPD1",9.2447931750213,0.13940411721891,0.602730870845804,0.2
31287501539926,0.817091455805039,0.954868833139153
"13379","RIOK3",273.415202354635,0.0499661332127889,0.215165428000709,

0.232221940471888,0.816365637464423,0.954868833139153
"13380","AC027575.2",6.09419240250438,0.159979573044053,0.690888371036071,0.23155632624724,0.816882632155242,0.954868833139153
"13381","GADD45B",1766.75572946337,0.145152945581972,0.625962457288333,0.231887621840412,0.81662529885983,0.954868833139153
"13382","PIAS4",52.930882592291,0.0613545221251199,0.264403423212429,0.232048894752116,0.816500037548793,0.954868833139153
"13383","DNAJB1",672.742228580142,-0.0921311376339762,0.397450011353085,-0.231805598194156,0.816689008748996,0.954868833139153
"13384","WTIP",177.272542995193,-0.0444689326004687,0.191944454224618,-0.2316760480531,0.816789636164249,0.954868833139153
"13385","ZNF816",17.1335132838732,0.0913423253662335,0.394740457572684,0.231398438173555,0.817005278399024,0.954868833139153
"13386","TBC1D25",39.4305648382215,-0.0569010403578639,0.245872879520725,-0.231424630763588,0.816984931886026,0.954868833139153
"13387","AL391121.1",28.8764695937515,-0.135621921728936,0.587070690042181,-0.231014635936248,0.817303431710744,0.954965460281683
"13388","MMP27",28.3492834191887,-0.137549631915635,0.595340912351434,-0.231043472843738,0.817281029099798,0.954965460281683
"13389","SNRPD3",609.017615540292,-0.0514149426671361,0.22264216987272,-0.230930837120969,0.817368533583389,0.954970197239895
"13390","LY6G5B",22.6595071780245,0.0960227688833566,0.416033646758213,0.230805295753308,0.817466066891541,0.955012821836026
"13391","ADIPOR1",155.231294411616,0.0476691199483652,0.207239769934385,0.230019170371874,0.818076873273901,0.955023489222776
"13392","SATB2",18.5768790885892,0.102099358228418,0.444695041707561,0.229594100794044,0.818407191319018,0.955023489222776
"13393","ZBTB38",552.428656984402,0.0460010571540795,0.199974064175132,0.230035116522876,0.818064482280284,0.955023489222776
"13394","CPA3",6.77829995378756,-0.156300409388601,0.681223188944325,-0.229440823397125,0.818526309824894,0.955023489222776
"13395","SDHA",358.462210288914,-0.0453846054949405,0.197339092090133,-0.229982843309178,0.818105101471919,0.955023489222776
"13396","TRIM26",59.9478443813796,0.056989560732411,0.248026825325268,0.22977176221835,0.818269128174432,0.955023489222776
"13397","LTB",16.5006212058655,0.10958461500999,0.477907790314697,0.22930075054401,0.818635170171208,0.955023489222776
"13398","INTS1",147.431917282365,0.0464213228018871,0.202118287392912,0.229674035935429,0.81834507192038,0.955023489222776
"13399","SEMA3A",102.451183970494,0.099747556380052,0.434981868422774,0.229314285539608,0.818624651033087,0.955023489222776
"13400","ERP44",514.741164449615,0.0480152805141164,0.208364857224855,0.2304384777434,0.817751064632557,0.955023489222776
"13401","LAMC3",3.17915698205276,-0.221505600105855,0.964633319290985,-0.229626735544096,0.818381829982033,0.955023489222776
"13402","PI4K2A",69.4654664830744,-0.0523059786276832,0.227555107624373,-0.229860710109945,0.818200007657285,0.955023489222776
"13403","EEF1G",5030.79239952454,-0.0592092926751085,0.257513350927853,-0.229927079360236,0.81814843369713,0.955023489222776
"13404","BIRC2",334.912879749229,0.0331628886733642,0.143966950655505,

0.230350705647151,0.817819262374357,0.955023489222776
"13405","ELMSAN1",245.083797747236,0.0456687609834648,0.19894978979005
5,0.229549179376653,0.818442101264567,0.955023489222776
"13406","SULT1A3",12.4493425239125,0.151686043100225,0.657929796624516
,0.230550499275826,0.817664027407717,0.955023489222776
"13407","AC022137.4",22.1603567940177,-0.115289817718606,0.50258678044
9369,-0.229392857519101,0.818563587047553,0.955023489222776
"13408","SYCP2",3.83916202633419,0.202075833622829,0.879025505844039,0
.229886200433736,0.81818019964205,0.955023489222776
"13409","TSC22D3",3568.89650462832,-0.0835713786043075,0.3636226404332
12,-0.229829964670908,0.81822389947074,0.955023489222776
"13410","RNPC3",216.80582243551,0.0638815672528587,0.279240439408996,0
.228769040000303,0.819048431187922,0.955063269799212
"13411","AC010896.1",9.52416390197076,-0.118278710737766,0.51825545008
4404,-0.228224731102206,0.819471536086459,0.955063269799212
"13412","LYG2",5.21886038764426,-0.191024256781027,0.836087160454241,-
0.228474094348303,0.819277693276858,0.955063269799212
"13413","AP3S1",991.917738667188,0.0527333631501883,0.230489912811248,
0.228788160431873,0.819033569356119,0.955063269799212
"13414","HOXA1",2.71039092146471,0.207429192969377,0.908093989056135,0
.228422603242839,0.81931771904325,0.955063269799212
"13415","STX2",231.72663532741,0.0515416005498199,0.225260723745471,0.
228808643126169,0.8190176487438,0.955063269799212
"13416","AC010809.2",5.36786836741622,-0.149710003936152,0.65571774159
6182,-0.228314706830595,0.81940159207651,0.955063269799212
"13417","KCTD5",46.6919127581248,-0.0664279490119354,0.290812103703523
,-0.228422229219377,0.819318009785976,0.955063269799212
"13418","MPRIP",240.439281035175,0.0458510170414601,0.200757427034365,
0.228390140871906,0.819342953376884,0.955063269799212
"13419","FAM117A",96.1848072243373,0.0692097251168346,0.30334225937720
4,0.228157215084143,0.819524021640019,0.955063269799212
"13420","RBFA",189.965356447141,-0.0423336627796985,0.184761838530446,
-0.229125576560674,0.818771314964984,0.955063269799212
"13421","TICAM1",39.0562365384383,-0.0653710182561678,0.28633078898137
1,-0.228305934156528,0.819408411585794,0.955063269799212
"13422","TSHZ2",1601.32472522273,-0.0480704452929713,0.210221616878737
,-0.228665567350764,0.819128859010361,0.955063269799212
"13423","TMSB15B-
AS1",22.5523214645147,-0.11233750221714,0.490641571019735,-0.228960424
172092,0.818899676004302,0.955063269799212
"13424","MORN1",17.1198740177354,0.111687403877805,0.490146350698566,0
.227865419621355,0.819750866682126,0.955098139965843
"13425","EFNA4",60.5518527750475,0.0812236951062982,0.357103964799746,
0.227451115396734,0.820072977307381,0.955098139965843
"13426","ZNF746",40.9570445660594,-0.0579115865630836,0.25464388347969
2,-0.227421863709136,0.820095720872116,0.955098139965843
"13427","GOLGA7",328.794714607562,-0.0400606171366226,0.17615882168659
,-0.227411927220402,0.820103446654811,0.955098139965843
"13428","THBS1",123.375541875224,0.177955437855779,0.780952876005764,0
.227869623537267,0.819747598403951,0.955098139965843

"13429","MTSS2",15.978939545955,-0.0943747358166916,0.414653004263104,
-0.227599305555277,0.81995775987151,0.955098139965843
"13430","KPNB1",529.357685944028,0.0410083687995236,0.180272879621951,
0.227479412796434,0.820050975857801,0.955098139965843
"13431","AC006504.7",4.9826579242706,-0.172716248777913,0.758484021577
411,-0.227712442008623,0.819869799209638,0.955098139965843
"13432","PTGIS",873.695780075195,-0.0807924707126584,0.354949503920099
,-0.22761680132069,0.819944157219023,0.955098139965843
"13433","PYROXD1",85.8771585697087,0.0536827068477558,0.23631010752218
,0.227170591265197,0.820291094671169,0.955174452429738
"13434","EMC9",30.3688171281447,-0.0828836528155925,0.364793267986421,
-0.227207188534734,0.820262638220092,0.955174452429738
"13435","FRK",14.4693700619051,-0.0994753693001543,0.438222939599974,-
0.226997174978925,0.820425938877418,0.955260361880123
"13436","GIT1",85.7646686971131,-0.0449630750978486,0.198164554871127,
-0.226897666573569,0.820503316544317,0.955279352538163
"13437","U91328.1",10.1063870739197,-0.106432582496246,0.4695774932611
8,-0.226656055759998,0.820691200214537,0.955426988535834
"13438","MFAP2",412.017439187048,-0.078680836890774,0.347313256286108,
-0.226541416046494,0.820780351025217,0.955459668930456
"13439","GNG12-
AS1",7.60619031434076,-0.128140827614436,0.56661795741189,-0.226150311
578084,0.821084515053681,0.95574261991106
"13440","ASGR1",2.72404274064486,-0.235542215487636,1.04264475118676,-
0.225908407652306,0.821272658494644,0.955819373322797
"13441","GGT5",1200.66464453882,-0.0658731028490163,0.291581580532297,
-0.225916543592231,0.821266330510664,0.955819373322797
"13442","ZFP69B",5.22899294777097,-0.164855495137797,0.73076670303381,
-0.225592510514494,0.821518366449639,0.955998695021879
"13443","ZNF93",19.3776310082511,0.0839185689728211,0.372056699034475,
0.225553172918532,0.821548964851954,0.955998695021879
"13444","ZNF138",93.6397032194196,0.0495570786243976,0.220036091304579
,0.225222500229744,0.821806186417211,0.956084647785545
"13445","DNAJB12",236.675405413677,-0.0451898150535459,0.2006281387140
33,-0.2252416602337,0.821791281832246,0.956084647785545
"13446","IMPACT",120.20952324244,0.0551063361780272,0.24459726641436,0
.225294162056064,0.821750440945243,0.956084647785545
"13447","DRG1",149.497354817945,-0.0475536513059462,0.211239803742376,
-0.225116907246997,0.821888328450895,0.956109104034904
"13448","NEDD9",43.6109362644943,-0.0855577592351946,0.380444801513777
,-0.224888758881087,0.822065814436747,0.956176052516358
"13449","NAA60",114.549911835521,0.0501397867358793,0.22295667468987,0
.22488578467374,0.82206812825497,0.956176052516358
"13450","MALRD1",7.8339606458961,0.129049672243847,0.574329847376901,0
.224696092033606,0.82221570498465,0.956220194565455
"13451","CDC6",21.2516442254041,-0.0749989525844295,0.333803630870636,
-0.224679858600744,0.822228334533014,0.956220194565455
"13452","TNPO2",120.438917952953,-0.0370482996039594,0.165208958012498
,-0.22425115471739,0.822561881167876,0.956536983876679
"13453","IER5",162.266544682217,0.0988089783527018,0.442229225575692,0

.223433849773435,0.823197861832692,0.956701849400924
"13454","MRPL19",253.373272156004,-0.0378010907737015,0.169296753289796,-0.22328302249834,0.823315239828373,0.956701849400924
"13455","CALU",1036.54990157365,0.0485116668584317,0.217196721265079,0.223353587364818,0.823260323787288,0.956701849400924
"13456","ZBTB3",15.2269602451906,0.0846982809506956,0.378646511926194,0.223686943581842,0.823000906055249,0.956701849400924
"13457","NUP37",113.59887730815,0.0591258414291438,0.264130675823171,0.223850718001142,0.822873463923826,0.956701849400924
"13458","C17orf75",58.2676782525667,0.0580561502758637,0.259804329818801,0.22346105746719,0.823176688466991,0.956701849400924
"13459","HAUS8",28.428045290787,-0.0777561775459694,0.347176180726844,-0.223967489310989,0.822782600421244,0.956701849400924
"13460","BLOC1S3",25.7321647856273,-0.0659391881506982,0.295190648530248,-0.223378309844871,0.823241084095473,0.956701849400924
"13461","SYAP1",369.627704586883,-0.0387200135791556,0.173293115838536,-0.223436536366699,0.82319577108667,0.956701849400924
"13462","ENOX2",75.208485823901,-0.0687734587910017,0.30753232549074,-0.223630015743085,0.823045205917542,0.956701849400924
"13463","STMN2",6.79916951594367,-0.204187121224667,0.914892695888036,-0.22318149673987,0.823394252233763,0.956722594346933
"13464","JAK2",78.0670886878771,0.0612903115161912,0.274831219234309,0.223010732503201,0.823527153511543,0.956734887662909
"13465","UBE2G1",130.450752251392,-0.0569195821802077,0.255174927929727,-0.223061029710109,0.823488008002981,0.956734887662909
"13466","HSD17B7",45.4323921398738,-0.0604270429144309,0.271350678251687,-0.22268985396964,0.823776898454294,0.956953959789137
"13467","AC016355.1",7.38952189681471,-0.133567630894398,0.600106861394789,-0.222573077374845,0.82386779199373,0.956988480742401
"13468","PGM5P4-AS1",4.36147564989661,0.204376616074943,0.91897991550211,0.222395084623014,0.824006337923336,0.957007286668257
"13469","NIF3L1",80.049479059478,0.0462579480185306,0.207926328592509,0.222472778371354,0.823945862018692,0.957007286668257
"13470","TADA2A",45.4527010728148,-0.0511880288275194,0.230349862645939,-0.222218621012153,0.824143699014733,0.957095759739233
"13471","NSUN4",49.2290876746058,0.0583742811601168,0.26303710388536,0.221924132747288,0.824372943705183,0.957148813061692
"13472","AC073263.2",3.03285804249709,-0.241383482293657,1.08717031098984,-0.222029133663413,0.824291203915168,0.957148813061692
"13473","ANKRD44",40.9968948582291,-0.0845674943693543,0.381012675289621,-0.221954543389066,0.824349269816012,0.957148813061692
"13474","HBS1L",155.622801520039,0.045043190991605,0.203040747043558,0.221843111037913,0.824436017766474,0.957151003853419
"13475","GYPC",1490.95066233568,-0.059788108800399,0.26994663870851,-0.221481212310847,0.824717763778469,0.957294511508815
"13476","SARDH",14.6387132509192,-0.115647612809018,0.522228651159354,-0.221450149378589,0.824741947998192,0.957294511508815
"13477","PPFIBP2",54.7257629628159,0.0792159054639672,0.357717022961852,0.22144852042005,0.824743216237569,0.957294511508815

"13478","AC093425.1",33.9928191751569,0.124722259650238,0.564504545727835,0.220941107727361,0.825138288908995,0.957567530681955
"13479","ABCA5",119.325493043732,0.0712594829120774,0.32257164299467,0.220910561915869,0.825162073361424,0.957567530681955
"13480","SH3BGR",17.4278183619238,-0.0809397397427723,0.366358524123532,-0.220930412186834,0.825146616958067,0.957567530681955
"13481","SMC1A",245.867382828021,0.0581729022208699,0.263605365855348,0.220681783286582,0.825340216615009,0.957703212559052
"13482","DOCK8-AS1",5.50374587721584,0.181201802799854,0.821793501125903,0.2204955412 1759,0.825485244529958,0.957800450985175
"13483","NACAD",25.1390179869091,-0.0800403309057643,0.363393491657609,-0.220258019868938,0.825670212595394,0.957944013619354
"13484","ARL6",33.3890538375071,0.0604706552102851,0.274907389939322,0.219967368733275,0.825896568270623,0.95813556937536
"13485","ROGDI",43.9364564563607,0.0928224961057692,0.422354462590252,0.219773920551234,0.82604723145513,0.958239291186697
"13486","C3orf33",38.5859145881352,-0.0751510071275819,0.342143771021727,-0.21964745084548,0.826145733277902,0.958282493375814
"13487","CTNNB1",465.324855960579,-0.0435998061390248,0.198744516980753,-0.219376145824678,0.826357050337043,0.958456538772326
"13488","SSR3",794.728681418586,-0.0599379201926421,0.273552750429323,-0.219109184969164,0.826564996041726,0.958538652155095
"13489","MOCS1",37.3470576069837,0.0692928469331418,0.316171012441965,0.219162555093063,0.826523423100916,0.958538652155095
"13490","AC092828.1",20.6342017686804,0.0914496466469062,0.41748438633084,0.219049261819425,0.826611674076087,0.958538652155095
"13491","AL591848.4",3.21788204455632,0.193591541155398,0.885203318561474,0.218697260952432,0.826885882785278,0.958747367270002
"13492","BSCL2",316.043497708121,-0.0568776976516568,0.260211953533011,-0.218582186096386,0.826975530689391,0.958747367270002
"13493","AC002553.2",5.18394350515521,0.154449186067437,0.706417773144805,0.218637174684699,0.826932692103774,0.958747367270002
"13494","LRRTM2",5.84233123760277,0.176356443869685,0.808561439307095,0.218111370758437,0.827342338096511,0.958864943371933
"13495","SAP30L-AS1",10.0177115630053,0.100733606053781,0.461956521980395,0.218058629461358,0.827383430648492,0.958864943371933
"13496","LRATD2",3.20072867074952,0.187890455266307,0.860462687325887,0.218359794136136,0.827148789288406,0.958864943371933
"13497","MMP17",58.94376254688,-0.0827357878275255,0.379220764210613,-0.218173147769871,0.827294206110951,0.958864943371933
"13498","TMEM164",19.6616265009867,0.0750710999976208,0.34406232778591,0.21819040893176,0.827280757632096,0.958864943371933
"13499","AC104825.1",20.8675075327601,0.0735022172080305,0.337221297305209,0.217964339131006,0.827456896653799,0.958879045437097
"13500","ZNF132",6.75382012635816,-0.132194959416084,0.606950834009261,-0.217801759234533,0.827583573799049,0.958954803328779
"13501","ZMYND8",169.428304192645,-0.0537251021677789,0.247137196652149,-0.217389785493918,0.827904590866546,0.959255722903887

"13502","TOX",2.87641402860081,0.178816247273464,0.823010065319623,0.2
17271033257679,0.827997130004459,0.959291890435473
"13503","DNAJC16",75.0744112944004,0.0470616976411605,0.21782775305962
1,0.216050053219249,0.828948731945404,0.959478979410185
"13504","ATP13A2",25.6313315830341,-0.0722040536785631,0.3341446744680
47,-0.216086202162314,0.828920554731321,0.959478979410185
"13505","SNX27",308.917580610109,0.033330179654924,0.153846367012355,0
.216645867576759,0.828484337378246,0.959478979410185
"13506","TRA2B",651.921349914616,-0.0467753127555829,0.215905553502559
,-0.216647103313295,0.828483374272462,0.959478979410185
"13507","ICE1",187.965329702336,0.0442166351120413,0.204171271108619,0
.216566389933078,0.828546281041214,0.959478979410185
"13508","Z97989.1",24.8952561725189,-0.0743919950613892,0.343968638302
013,-0.216275516944283,0.82877299209973,0.959478979410185
"13509","ADAT2",50.8807390740854,0.0762413188900474,0.352748347842752,
0.216135155150419,0.828882397429171,0.959478979410185
"13510","AL353743.2",3.56402392523849,-0.175785369035062,0.81239757841
676,-0.216378499524384,0.828692724198262,0.959478979410185
"13511","COMMD3",412.95779198432,0.0522106947891516,0.241698248742604,
0.216016024364136,0.828975256804035,0.959478979410185
"13512","AL158163.1",3.1538268509661,-0.180727590840002,0.836848777102
131,-0.215962066008905,0.829017316736435,0.959478979410185
"13513","SF1",823.472122693015,-0.0277325912308214,0.128136551408708,-
0.216429979782778,0.828652599516438,0.959478979410185
"13514","ANAPC5",525.140687961392,-0.0340123147301539,0.15733089905968
7,-0.216183311310326,0.828844861623254,0.959478979410185
"13515","GGNBP2",405.387115061938,0.0410075517713722,0.189478904196959
,0.216422783027844,0.828658208775738,0.959478979410185
"13516","ZNF649",30.7136413491372,-0.0682649090629593,0.31576555227451
9,-0.21618858856273,0.828840748239247,0.959478979410185
"13517","AC092053.4",3.18346887087931,0.193199782584355,0.895658240425
677,0.215707034071984,0.829216117900253,0.959496096775919
"13518","FAM153CP",26.7033172056848,0.101783071223766,0.47164507655326
8,0.215804375543546,0.829140237493393,0.959496096775919
"13519","TRAPPC5",880.153444332629,-0.0621103042556171,0.2878827233310
15,-0.215748633808084,0.82918368954437,0.959496096775919
"13520","TRAIP",11.2000207259808,-0.105495017494649,0.489725987062593,
-0.215416417101765,0.829442671455014,0.959556479679054
"13521","AC104113.1",3.65407954059096,0.173757126188166,0.806771237935
411,0.215373476417954,0.829476147525605,0.959556479679054
"13522","ACOT13",265.974193553243,-0.0742820118657346,0.34497569135515
2,-0.215325351110787,0.829513665837745,0.959556479679054
"13523","POLA1",24.7133055683183,0.0702665682250131,0.326019200204396,
0.215528926458809,0.82935496190368,0.959556479679054
"13524","AGMO",9.87318435433548,-0.117171214998611,0.544479364271441,-
0.21519863320329,0.829612456510917,0.959599797190201
"13525","FASTKD5",32.5598001450512,0.0658298950848969,0.30602374903548
2,0.215113680857704,0.829678687801602,0.959605450150127
"13526","FBXW2",159.177818625857,-0.0401485300497789,0.186858211147087
,-0.214860935483192,0.82987574251646,0.959691079766606

"13527","SIK2",198.566897988292,0.0539275594632119,0.251154153317124,0.214718963437246,0.829986436718625,0.959691079766606
"13528","MAPK1IP1L",221.832917578356,-0.0304028769431873,0.141603697211755,-0.21470397695706,0.829998121726166,0.959691079766606
"13529","RAP1GAP2",5.91402740075906,0.171096554551406,0.796038972652585,0.21493489694515,0.82981807683258,0.959691079766606
"13530","IFI44",115.557479451896,-0.048584608453017,0.226800093904297,-0.214217761627199,0.830377245790128,0.959846028975878
"13531","IKZF2",39.8911849094525,0.0851432493175732,0.397461125209068,0.214217803748196,0.830377212944772,0.959846028975878
"13532","IGHM",29.5041514114209,0.221548364285454,1.03422233117097,0.2142173472841 3,0.830377568888996,0.959846028975878
"13533","TAF4B",28.2798669959174,-0.0801071247294348,0.373784804802776,-0.214313486530579,0.830302601632592,0.959846028975878
"13534","PPRC1",38.8099584226013,0.0639630593340779,0.29883713754578,0.21403986083985,0.830515972945438,0.959935079413735
"13535","TSPAN14",246.590567872962,0.051253818557216,0.239684901944866,0.213838327493008,0.830673135168285,0.959999143672174
"13536","HOTAIR",30.5308475502634,-0.143999753126576,0.67348961044793,-0.213811394998066,0.830694138512213,0.959999143672174
"13537","EPN2",382.66906288743,0.0608899344793526,0.284934704359759,0.21369785269285,0.830782685967646,0.96003055009174
"13538","ZNF460-AS1",4.51247210999009,-0.15541145028482,0.72782976654066,-0.213527197470205,0.830915777721647,0.960113422285399
"13539","C1orf52",215.544928221948,-0.0408099106410082,0.191303889541861,-0.21332504393267,0.83107344087032,0.960179516894416
"13540","ELOVL5",153.842442982983,-0.0515598657235725,0.241728659610403,-0.213296453166423,0.831095739867698,0.960179516894416
"13541","AC026979.3",2.64694782509867,0.188081235720232,0.88307892235568,0.21298349553912,0.831339835998029,0.960319676156932
"13542","DOLPP1",13.9166387372658,-0.0942472199949498,0.442391757739322,-0.21304018066829 5,0.831295622349577,0.960319676156932
"13543","AC011611.4",7.8028999742964,-0.113152989788469,0.531518840466213,-0.212886131541864,0.831415779869477,0.960336487078065
"13544","PAF1",157.690111599397,0.0356966737055216,0.167817585464644,0.212711162579813,0.831552259532206,0.96042321292545
"13545","AL161729.1",10.5541029537052,-0.114942331197435,0.540836281469754,-0.212527034771175,0.83169588878327,0.960518182963211
"13546","SNRNP48",109.329964767785,-0.0482702748713293,0.227468349279955,-0.212206555435636,0.83194589270734,0.960577261227052
"13547","AC127070.2",2.9460837918955,0.20015567537326,0.94282363242591,0.212293867579724,0.831877779363986,0.960577261227052
"13548","MED9",46.0542645830291,-0.0571642077295517,0.26945616266881,-0.21214659617866 2,0.831992668437341,0.960577261227052
"13549","SLC9A3R1",30.2674679873018,-0.0894780730346458,0.421687244504734,-0.212190608562838,0.831958333207291,0.960577261227052
"13550","KIAA1522",29.6326298910024,0.0709056990537598,0.336748873478121,0.210559573136528,0.833230960974632,0.960591321444795
"13551","AC118553.1",8.97446898237285,-0.116754590568478,0.55237104615

1485,-0.211369859774401,0.832598673835899,0.960591321444795
"13552","RIT1",79.7228681644011,0.054437851775045,0.257828319473078,0.211139923986237,0.832778087554963,0.960591321444795
"13553","AL592148.3",19.7749706564327,-0.0799003557525342,0.376876157960702,-0.212006926054647,0.832101630933982,0.960591321444795
"13554","LRP1B",144.331147883026,-0.0760116069060969,0.361344944458875,-0.210357466104657,0.833388687024935,0.960591321444795
"13555","NUP54",215.345243816341,-0.0335279968901542,0.159592627153492,-0.2100848735193,0.83360143122247,0.960591321444795
"13556","SCD5",82.6729912755247,0.05170106638014,0.245816813180506,0.210323556437026,0.833415151075685,0.960591321444795
"13557","IGIP",45.3592117330927,0.0673260288320272,0.319629483249067,0.210637730123176,0.833169968395424,0.960591321444795
"13558","SRF",47.6542431965421,-0.0469937681860334,0.222056033343658,-0.211630224490703,0.832395527636517,0.960591321444795
"13559","SNX9",1203.67000557988,-0.0408791237771474,0.194540179938708,-0.210132034369593,0.833564623783469,0.960591321444795
"13560","PRKDC",543.75714569214,0.037666711631548,0.178770528384649,0.210698664773776,0.833122416574139,0.960591321444795
"13561","SLC16A12",19.2883292636973,0.120760714909915,0.57069687993786,0.211602199267435,0.832417393416839,0.960591321444795
"13562","EIF1AD",48.4338788854198,-0.0588204349443618,0.27968976769061,-0.210305995210481,0.833428856421051,0.960591321444795
"13563","STT3A",227.551547542196,0.0396596208173879,0.188759514450042,0.21010660539649,0.833584470186443,0.960591321444795
"13564","GPR162",4.61028736080412,0.17019669367998,0.806908249218358,0.210924468605752,0.832946210443024,0.960591321444795
"13565","ORAI1",60.4388219875982,0.0769183072790837,0.363095821728002,0.211840243473537,0.832231671199235,0.960591321444795
"13566","AP5M1",96.2905816984911,0.0425990668794742,0.201168878046092,0.211757739533219,0.832296039757894,0.960591321444795
"13567","ALDH1A3",203.827926913489,-0.102332242807475,0.486861726738834,-0.210187486892698,0.833521345442562,0.960591321444795
"13568","WDR24",27.7727209357688,0.0591772316451016,0.281388221277004,0.210304579831174,0.833429961030737,0.960591321444795
"13569","KARS",178.457030895843,0.0355503879117786,0.168724928895672,0.210700269038256,0.833121164655986,0.960591321444795
"13570","AC007114.1",12.5631989066182,0.128167639531808,0.60846167607171,0.21064209065601 5,0.833166565528388,0.960591321444795
"13571","SUPT4H1",362.458453155726,-0.0465893200457572,0.221669487709456,-0.210174708874783,0.833531318098532,0.960591321444795
"13572","CDC42EP5",325.058168250666,0.0460632219742106,0.218197706079391,0.211107727949489,0.832803210093049,0.960591321444795
"13573","MZF1",63.1436841383772,0.0489258184967907,0.232211654983074,0.210694930451949,0.833125330724503,0.960591321444795
"13574","LINC01638",14.5651652463043,0.102549286262564,0.486981768660054,0.210581366412817,0.833213953716854,0.960591321444795
"13575","FAM122B",57.8219998637888,-0.0539783504668027,0.255715262581094,-0.211087714991923,0.832818826271631,0.960591321444795
"13576","COL4A4",35.7118870633403,-0.200764518283029,0.956463490587138

,-0.209902960498562,0.833743411561184,0.9606134040695
"13577","PPDPFL",65.1594124441959,-0.113949184642409,0.542674579977364
,-0.209977008038892,0.833685617930579,0.9606134040695
"13578","TMEM170B",18.4927649244184,0.0739224986196702,0.3524437815022
84,0.20974266772491,0.833868522863538,0.960616047069322
"13579","VRK1",46.7408505725812,-0.0586489107056239,0.279542995569964,
-0.2098028268194,0.833821566635779,0.960616047069322
"13580","ASTE1",51.4014404073516,-0.0534472291664813,0.255070947001431
,-0.20953867853159,0.834027744375461,0.960657978445279
"13581","PPHLN1",282.045970785576,0.0360861922042716,0.17216413999624,
0.209603418023403,0.833977211706671,0.960657978445279
"13582","ZC3H11A",12.678162511627,0.105438863319997,0.5035104103925,0.
209407514013075,0.834130127465172,0.960705167422889
"13583","B3GALNT1",59.0096733603485,0.0492221627936162,0.2355422983554
77,0.208973773021993,0.834468712691935,0.960894561647307
"13584","RAB30-
DT",130.574689202114,0.0619936565467806,0.296612719223006,0.2090053882
69177,0.834444032281358,0.960894561647307
"13585","HEXIM2",51.5772308825848,-0.0832689749058249,0.39849090812164
6,-0.208960789841673,0.834478848045686,0.960894561647307
"13586","SLC41A2",119.487902645082,0.0545446695342602,0.26120379852324
1,0.208820353465905,0.834588481827562,0.960950067807195
"13587","XPOT",114.050409608739,0.0427843214936035,0.20506940290048,0.
208633374303853,0.834734454929565,0.961011009758617
"13588","GAS6",2549.75041430421,0.0661478330450358,0.317111041702424,0
.208595174390391,0.834764277990161,0.961011009758617
"13589","MRPS31",236.435892954868,-0.042623693466271,0.204458448494359
,-0.20847117729863,0.834861085417088,0.961051730015418
"13590","SHLD3",9.55770491267369,0.104679168488344,0.502711236602951,0
.20822921961265,0.835049994640821,0.961106517456583
"13591","USP35",11.4324901965101,0.0917315902002646,0.440593234928976,
0.208200178595688,0.835072669146051,0.961106517456583
"13592","MPP2",12.6770034800911,0.0982733550826812,0.472072827215548,0
.208174140550161,0.835092999122284,0.961106517456583
"13593","TTC13",27.8282525021567,-0.0603066782211865,0.29021633300917,
-0.207799049749833,0.835385874609601,0.961121747601389
"13594","MLH1",85.2433391587642,0.0544474446222533,0.262458526447127,0
.207451612867383,0.835657177946352,0.961121747601389
"13595","TFDP2",468.43848014476,-0.0334510066816107,0.16098820927095,-
0.207785444866408,0.835396497898339,0.961121747601389
"13596","TAGAP",15.4970162774395,-0.109322886830295,0.526571351549507,
-0.20761267491784,0.835531406851371,0.961121747601389
"13597","MKRN1",245.940236957813,-0.0343981770257701,0.165548377976165
,-0.207783231985048,0.835398225815847,0.961121747601389
"13598","DNAJC9",111.803283387302,-0.0472305863520025,0.22767321821559
7,-0.207449021550163,0.835659201503963,0.961121747601389
"13599","AMPD3",13.5103724563414,0.0995198394660364,0.479322718043884,
0.20762595996321,0.835521032933581,0.961121747601389
"13600","SH3BP1",16.8230085031173,-0.0849921764412468,0.40945488636509
7,-0.207573970348133,0.835561630322941,0.961121747601389

"13601","HNRNPH2",276.697301580327,-0.0399594151078987,0.192608872358593,-0.207464041601903,0.835647472372309,0.961121747601389
"13602","H2AFV",516.147383182408,0.0490404181532184,0.236666318296306,0.207213339465652,0.835843250008279,0.961134371266874
"13603","PGM5",73.2223037238393,0.095859246699085,0.462643540710396,0.207198930199728,0.835854502762549,0.961134371266874
"13604","AC090517.5",29.81708394055,0.0673321011911332,0.324740885761189,0.207341003684545,0.835743553539193,0.961134371266874
"13605","C8orf37",28.1955466169186,0.0676385785934066,0.32666532531136,0.207057723463417,0.835964778353646,0.961190520234184
"13606","AC109347.1",5.76394833952923,0.145250613436309,0.702099202251351,0.206880470694951,0.836103208705827,0.961255166070706
"13607","TBP",52.6498540210535,0.0625342042125616,0.302383718279963,0.206804138027908,0.836162824334386,0.961255166070706
"13608","ZNF287",39.6155489351067,-0.0544722608705668,0.263469616450019,-0.206749687514349,0.836205350629046,0.961255166070706
"13609","APOBEC3F",34.824890507756,-0.0610381256711461,0.295886514837052,-0.206288974354781,0.83656519051011,0.961598153806279
"13610","SCNN1D",8.7434285762029,0.110970855881391,0.538727254621768,0.205987083314175,0.836801000973196,0.961727377969375
"13611","ESRRG",2.68643407991671,0.243977544280636,1.18460123334916,0.205957530190012,0.83682408603688,0.961727377969375
"13612","TMEM177",26.690761846693,-0.0772218461915176,0.375029130255528,-0.205908928031543,0.836862051327695,0.961727377969375
"13613","AC004854.2",4.0553207679444,-0.153616368471898,0.746575154588962,-0.205761426063628,0.836977273950087,0.96178913512093
"13614","SCP2",1276.9365383116,0.0468303051217611,0.228058890456155,0.205343036739734,0.837304121932224,0.96182586177647
"13615","GATB",42.9742183965757,0.0569950568602834,0.277368199238392,0.205485189061985,0.837193068623784,0.96182586177647
"13616","PCBD2",65.5933675507804,0.0516728936946684,0.251455222340922,0.205495408739654,0.837185084855141,0.96182586177647
"13617","THRIL",2.67176015854324,-0.179398686578592,0.873721928763502,-0.205326981815002,0.837316664685288,0.96182586177647
"13618","CCDC120",15.7824388398221,0.0760409948624922,0.370222643162516,0.205392609735955,0.837265393774304,0.96182586177647
"13619","ACTR3B",22.2647257637899,-0.0728763291647519,0.355370104006514,-0.2050716375495,0.837516155453742,0.961843126037948
"13620","AC092139.1",2.9358084640602,0.175382145214613,0.85501552636512,0.205121591136719,0.837477127792577,0.961843126037948
"13621","ARFRP1",185.607573615082,-0.0542142233510546,0.264181876264007,-0.205215528475073,0.83740373765767,0.961843126037948
"13622","MREG",18.5421671630193,-0.094745973670155,0.462774953043692,-0.204734446077962,0.837779606336749,0.961933821339237
"13623","AC092119.2",2.92786939572135,-0.182328978105083,0.890353761068295,-0.204782622455948,0.837741964563052,0.961933821339237
"13624","ARMC5",24.2439339475428,0.071566228834194,0.349351589067602,0.204854453432429,0.837685841370548,0.961933821339237
"13625","ZNF639",112.423479662767,-0.037974713897493,0.185698728250857,-0.204496359534534,0.837965636559603,0.962076803868028

"13626","MTO1",63.687997480022,0.0488001424881043,0.238825393907067,0.
204333976759162,0.838092520425323,0.962108077152184
"13627","AC005332.3",16.5078664015135,0.0805064447683505,0.39405207014
7815,0.20430407772798,0.838115883612658,0.962108077152184
"13628","MIA3",409.656148465399,0.0420111607279905,0.205983683890317,0
.203953827480631,0.838389580767065,0.962210433744623
"13629","PRSS23",1076.40530468235,-0.0710752468868301,0.34839939032046
4,-0.204005083997001,0.838349526003909,0.962210433744623
"13630","TBC1D10A",78.6380717582556,-0.0486763194764145,0.238489996012
269,-0.204102143864811,0.838273679039021,0.962210433744623
"13631","CAAP1",81.9727454035401,-0.0446587903037527,0.219162279446626
,-0.203770422613389,0.838532907212163,0.962304325986345
"13632","ABI3BP",2533.99351275634,0.0400271674954099,0.197109210087207
,0.203071015695821,0.839079525837947,0.962379651549227
"13633","ITGA8",9.19010079440125,0.159596682727484,0.784180302497948,0
.203520392209675,0.838728308522245,0.962379651549227
"13634","CCDC84-
DT",8.48707439375974,0.111561760645177,0.549394578230365,0.20306308992
8053,0.839085720634833,0.962379651549227
"13635","ALG10",21.4205457540014,-0.0657182988405422,0.323645066466421
,-0.203056698988367,0.839090715814097,0.962379651549227
"13636","MARCH9",84.4302903082332,-0.0464635387602262,0.22834796461748
5,-0.203476912255641,0.838762289564951,0.962379651549227
"13637","MAPT",13.386928475212,-0.0844777272825567,0.415825243218409,-
0.203156803633938,0.83901247444179,0.962379651549227
"13638","UCKL1",111.697548223813,-0.0371162204331512,0.182629034851504
,-0.20323285650242,0.838953032901465,0.962379651549227
"13639","BRWD1-
AS2",3.05151920234858,-0.171863553855073,0.845319146876393,-0.20331203
2491089,0.838891151363262,0.962379651549227
"13640","UTS2",3.15530440529539,0.210126385571519,1.03579866141497,0.2
02864121570184,0.839241237990833,0.962440476604827
"13641","RORB",6.42704148608773,0.149076191907741,0.735394224971045,0.
202716022026976,0.839356999414049,0.962440476604827
"13642","CACNB2",4.47634920809744,0.148718142798518,0.734350467565573,
0.202516576712383,0.839512900548032,0.962440476604827
"13643","MTPAP",71.5551404475264,-0.0408676531974691,0.201706626044559
,-0.202609373816213,0.839440362719538,0.962440476604827
"13644","COLCA1",66.5496817539064,0.0755456103478192,0.372480387135495
,0.202817686398985,0.8392775334849,0.962440476604827
"13645","ZNF10",25.6366974231696,0.0653973839834617,0.322850952792524,
0.202562152652182,0.839477274484508,0.962440476604827
"13646","AKIRIN2",226.68974590072,0.0387803163400607,0.191758752141574
,0.202234922301901,0.839733073084763,0.962551871037552
"13647","FAM227A",8.50528823526021,0.12108586235604,0.598738863249444,
0.202234846922896,0.839733132011089,0.962551871037552
"13648","PTPRK",77.2278109470895,0.0629636668702203,0.311555455256957,
0.202094573559274,0.839842790016132,0.962607031376198
"13649","CCDC28B",110.993026371914,0.0753846572519936,0.37337230264503
2,0.201902114104223,0.839993249297044,0.96263841749111

"13650","FGD1",35.9747586078438,0.0554735443498409,0.274741989556463,0.201911416741926,0.839985976627236,0.96263841749111
"13651","PODXL2",53.2605262719886,-0.0766770135257682,0.379996229116419,-0.201783616916569,0.84008588990747,0.962653607179464
"13652","NKX3-2",3.71585861573733,0.195167498947295,0.968095728932111,0.201599380220983,0.84022992979179,0.962653607179464
"13653","AC011365.1",18.2113240663107,-0.119682356724748,0.593749932186176,-0.201570308032003,0.840252659491684,0.962653607179464
"13654","VPS13A",105.178711456632,0.0537371676928959,0.266414661655016,0.201704993858337,0.840147358311234,0.962653607179464
"13655","SLC9A1",64.4570554525138,-0.0481966940088294,0.240226825825043,-0.200629941486763,0.840987944156213,0.963021914596692
"13656","AK2",199.60889339264,0.0354617281274776,0.176545540144283,0.200864480057079,0.840804542429066,0.963021914596692
"13657","RNF25",38.7616321721852,0.0538599713820675,0.27026909161546,0.199282763190323,0.84204156173688,0.963021914596692
"13658","SHISA5",387.141836632482,0.0291907363658952,0.145766978128117,0.200256167348403,0.841280241516019,0.963021914596692
"13659","MRAS",74.9084695113673,0.0413008972291908,0.20707225717382,0.19945162038062,0.841909484307412,0.963021914596692
"13660","PAICS",101.116544143713,-0.0443303189798097,0.221858972143223,-0.199813054895035,0.841626790889188,0.963021914596692
"13661","PDE8B",6.76192609893238,0.12616219980378,0.62915185186999,0.200527423433303,0.8410681127354,0.963021914596692
"13662","NDUFA2",766.270180654034,-0.0495247418547911,0.247718098206025,-0.199923793269242,0.841540181746522,0.963021914596692
"13663","ATP6V0E1",2093.76150781631,-0.0506162312781038,0.252876743173233,-0.200161670238806,0.841354143235044,0.963021914596692
"13664","LINC01600",3.06105274900995,0.19387141247107,0.965640273492799,0.200769808170719,0.840878571803769,0.963021914596692
"13665","ZNF311",3.88567084386779,-0.154145860296056,0.773366110583575,-0.199318095513312,0.842013924983609,0.963021914596692
"13666","B3GAT2",18.3176333520882,0.100723166057303,0.504060704510997,0.199823483869899,0.841618634244013,0.963021914596692
"13667","IKZF1",16.5563566912925,0.103864067506516,0.521241108949524,0.199263000794079,0.84205701986194,0.963021914596692
"13668","SPAAR",10.2360076146499,0.135487569314923,0.676428737453703,0.200298363763998,0.841247242147198,0.963021914596692
"13669","ASAH2B",6.75692768930534,-0.113069927407361,0.567394439221779,-0.199279230798321,0.842044324765632,0.963021914596692
"13670","PACS1",177.308371197192,0.0316369243101775,0.158568069662423,0.199516361506636,0.841858845999809,0.963021914596692
"13671","MSANTD4",87.0124952976371,-0.0421151848083077,0.210691673130902,-0.199890124666396,0.841566513958715,0.963021914596692
"13672","AL132639.2",4.75469915719115,0.137911895728935,0.691219028879056,0.199519819285915,0.841856141460818,0.963021914596692
"13673","FURIN",97.7470991471432,0.0428407089350179,0.213252944883337,0.200891523249324,0.840783396065354,0.963021914596692
"13674","TPSB2",29.7520416061647,0.20387519312602,1.01667314204879,0.200531699613086,0.841064768752332,0.963021914596692

"13675","RAB40B",153.707968132679,-0.0487761700033855,0.244712536112816,-0.199320275038542,0.842012220177054,0.963021914596692
"13676","S1PR4",3.22030410268116,-0.165471167165637,0.825411017073324,-0.20047123644212,0.84111205136652,0.963021914596692
"13677","MYO9B",108.388424179487,0.0476649885216415,0.237467092110673,0.200722500528313,0.840915564888408,0.963021914596692
"13678","ENTPD6",75.1517574928005,0.0470416607881508,0.236163372746401,0.199191179568161,0.842113198860075,0.963021914596692
"13679","BACE2",227.691725615551,0.0375204561591574,0.187848927929368,0.199737398412331,0.841685963377326,0.963021914596692
"13680","NECAP2",207.295992179913,-0.0500740801358211,0.252124910810943,-0.198608221515126,0.842569221894296,0.963111339648236
"13681","ABCB6",127.002592428076,-0.0534911804779077,0.268873807565314,-0.198945300631092,0.842305532938519,0.963111339648236
"13682","AC112206.2",8.73222067621654,0.138371544392705,0.696540320818939,0.198655469406305,0.84253225993l029,0.963111339648236
"13683","TERF1",314.945952537069,-0.0287115506446228,0.144429365635244,-0.198793026046612,0.842424651527426,0.963111339648236
"13684","ZNF22",550.164368541394,0.0314392572534567,0.158307931289536,0.198595591499178,0.842579102397952,0.963111339648236
"13685","ADAM8",7.00254206999451,-0.171214581267734,0.862366982451139,-0.198540278966948,0.842622373868552,0.963111339648236
"13686","NTN5",3.89686671240715,0.167814624426759,0.844911224673515,0.198618055395826,0.842561528833744,0.963111339648236
"13687","PCMTD1",772.809907731549,0.037720477052245,0.190203164652869,0.198316768919628,0.842797232543037,0.963240820389474
"13688","COLGALT2",9.44962017687815,0.145075506404308,0.735945790113116,0.197127979198046,0.843727388762588,0.963350653020961
"13689","CCDC174",205.76667080073,0.0378583497924231,0.191683215231348,0.197504772375248,0.843432547180887,0.963350653020961
"13690","SPCS1",1099.5623623614,-0.0528316984058625,0.267287933343085,-0.197658374416958,0.843312359500966,0.963350653020961
"13691","CBLB",400.384316771737,0.0516353602695205,0.261664835767785,0.197333967776031,0.84356619948079,0.963350653020961
"13692","AC024230.1",2.80455112952183,-0.198407667084518,1.00495499888201,-0.197429404605422,0.843491520890767,0.963350653020961
"13693","SDHAF4",60.2842899521837,-0.0471057013306105,0.23814466600548,-0.197802882259503,0.843199291012744,0.963350653020961
"13694","SMPD2",32.7315745762877,-0.0686050174846317,0.346876527915007,-0.1977793594078,0.843217695975535,0.963350653020961
"13695","DNAJB5",4.83468790999145,-0.139472504697104,0.706172309815902,-0.197504918783157,0.843432432620602,0.963350653020961
"13696","AL132656.3",4.1487478165027,-0.195075606924287,0.98898509380862,-0.197248278205128,0.843633252113817,0.963350653020961
"13697","TMEM254-AS1",15.9203642087576,-0.126892202249419,0.640988503805572,-0.197963304327699,0.843073774408411,0.963350653020961
"13698","FBXO3",166.811657658664,0.0315327741506464,0.159946300815546,0.197146004564436,0.84371328337024,0.963350653020961
"13699","SYCP3",4.30285926258407,-0.161855122500542,0.821215865320059,

-0.197092055007317,0.843755500673796,0.963350653020961
"13700","GNA11",345.63477196143,0.0577915190633575,0.291767616272337,0
.198073795172027,0.842987327051798,0.963350653020961
"13701","SNAP29",201.583933033113,0.0496307366665865,0.250859842343213
,0.197842493254398,0.843168298412692,0.963350653020961
"13702","PER3",267.182620603956,-0.170239859448994,0.869977128544175,-
0.195683143686633,0.844858178728776,0.963387548221264
"13703","AHDC1",157.339758705663,0.0444034812747352,0.226711373643125,
0.195859080915068,0.84472046559832,0.963387548221264
"13704","ST7L",85.9424574070574,-0.0380064702348166,0.19386698568948,-
0.19604405618444,0.844575683147813,0.963387548221264
"13705","HNRNPU",1254.89988439842,0.0372001738297241,0.18915259170462,
0.196667534367257,0.84408771769709,0.963387548221264
"13706","MEIS1",91.4148291032527,0.0466566701937769,0.238205055822281,
0.19586767389432,0.844713739650066,0.963387548221264
"13707","TMEM182",11.3137604237514,0.110476345388775,0.561841710440048
,0.196632509363264,0.844115128446152,0.963387548221264
"13708","NRP2",37.8660405268575,0.0963457676825054,0.492530772989248,0
.195613701653132,0.84491253510088,0.963387548221264
"13709","AC008080.4",2.675223324778,-0.201278683299531,1.0221092797155
3,-0.196924817428084,0.843886372434241,0.963387548221264
"13710","TRIQK",200.658854860053,-0.0458056346943017,0.233531741001819
,-0.196143078871428,0.844498179012895,0.963387548221264
"13711","CDKN2A",19.1623964642325,-0.134501642789912,0.68565099324965,
-0.196166335517784,0.844479976470639,0.963387548221264
"13712","AEN",11.4218735810841,0.0945622197502849,0.481856943330129,0.
196245423168051,0.844418076666964,0.963387548221264
"13713","AC009107.2",90.2354689181893,-0.066998741038822,0.34175533493
6139,-0.19604299974232,0.844576510023335,0.963387548221264
"13714","FANCA",5.90273096200396,0.11406819441312,0.582688843796447,0.
195761761405832,0.844796640898199,0.963387548221264
"13715","LIPE-
AS1",30.9435844433538,0.0641658773461291,0.326413874997899,0.196578277
64381,0.844157570842388,0.963387548221264
"13716","AC245884.8",6.72929673944677,-0.147313514897674,0.74966847033
8663,-0.196504882793223,0.84421501125617,0.963387548221264
"13717","PDRG1",102.196625500229,0.0456875799282996,0.233478336340295,
0.195682308879013,0.844858832177512,0.963387548221264
"13718","SRSF6",386.259346772638,0.0334899980066057,0.171214559474959,
0.195602512480861,0.844921293594516,0.963387548221264
"13719","MTG2",84.9717887900812,0.0492167593429617,0.251676440505515,0
.195555687469615,0.844957946787429,0.963387548221264
"13720","TMEM187",83.3807057575699,-0.0706547525128496,0.3597420188374
31,-0.196403947309749,0.844294006924018,0.963387548221264
"13721","PPARGC1B",7.53283950684523,0.106068598804946,0.54287218321795
8,0.195384110816304,0.84509225463437,0.963470456908785
"13722","UMAD1",62.1399435430182,-0.0501721225284705,0.256955272051417
,-0.19525624879349,0.845192346197277,0.963498416369931
"13723","ARFGAP1",70.9679585495471,0.0446762623471404,0.22887966547734
1,0.195195419627889,0.845239964702715,0.963498416369931

"13724","ELF2",399.744781521415,-0.030869733367216,0.158530762427136,-0.19472393177573,0.845609075675605,0.963699503599892
"13725","ZKSCAN4",29.981032895427,-0.0564055422452398,0.289769398171307,-0.194656656642168,0.845661745735663,0.963699503599892
"13726","AL662797.1",5.34851966513498,-0.120415399024548,0.618432839056915,-0.194710551283428,0.845619551279269,0.963699503599892
"13727","NFATC2IP",201.025796394033,0.0494721750244669,0.254298204413416,0.194543941584579,0.845749992499829,0.963699503599892
"13728","MAP2K7",130.171253280007,0.0588567147287929,0.302630035187036,0.194484049451395,0.845796883978337,0.963699503599892
"13729","Z93930.2",23.2731191440912,-0.0704616540435645,0.36230152813839,-0.194483458034574,0.845797347020322,0.963699503599892
"13730","KLHL4",7.13772187188569,0.129965264224434,0.668479373345777,0.194419258703451,0.845847611355016,0.963699503599892
"13731","RABL3",95.8983063864524,0.034511461582845,0.17759530579777,0.194326429000008,0.845920292712041,0.963712121396435
"13732","PRF1",10.3467150505497,0.136763610470264,0.70411042986355,0.194236024165652,0.845991076769122,0.963722576019471
"13733","SEC62",1927.02555613307,-0.0336100068765714,0.173208790915306,-0.194043308650574,0.84614197092358,0.963824281013439
"13734","SACM1L",99.2029820377706,0.0399797391173081,0.20624798601771,0.19384305218803,0.846298775536233,0.963932703197414
"13735","RNF220",108.810078686713,-0.0480065438026199,0.248402623477114,-0.193261017660076,0.846754554157074,0.964014286104828
"13736","NRBP1",392.21339852812,-0.0310720926744633,0.161136921819608,-0.192830372602304,0.847091815975731,0.964014286104828
"13737","HPSE",12.0936326047527,0.0884614064508187,0.45785196622767,0.193209624454972,0.846794801527468,0.964014286104828
"13738","DHX29",351.502282765496,0.0301314321559265,0.155886073241864,0.193291366761008,0.846730787165774,0.964014286104828
"13739","CRYBG1",48.7880088985987,-0.0696418677684002,0.36095156805365,-0.192939646013808,0.847006235305941,0.964014286104828
"13740","CALHM5",50.180217658307,-0.0601430704166332,0.310758260953756,-0.193536513661927,0.846538812810802,0.964014286104828
"13741","GRIN3A",4.78011830499929,0.15642958244053,0.81058090947326,0.192984538140902,0.846971077245798,0.964014286104828
"13742","ADRA2A",63.5286755812911,-0.109929232259298,0.569101736733402,-0.193162707410248,0.846831543847911,0.964014286104828
"13743","IKZF4",41.540742179368,-0.0552365731913101,0.286485953483987,-0.19280726513664,0.84710991349466,0.964014286104828
"13744","CLUAP1",196.449290273064,-0.0333057919089789,0.172612153491437,-0.192951604132736,0.846996870064694,0.964014286104828
"13745","FXYD1",1998.72214501118,0.0570713580611454,0.294719844870898,0.193646132265526,0.846452973510449,0.964014286104828
"13746","GRAMD4",42.1183596870442,0.0601551500685534,0.311459846671866,0.193139342715753,0.846849841652573,0.964014286104828
"13747","DDOST",739.679875481766,-0.0480046482131207,0.249769368530275,-0.192195898542707,0.847588758503252,0.964093483443104
"13748","PEX13",85.6209873895537,0.0382671845286018,0.199481785500689,0.191832975790512,0.847873039700021,0.964093483443104

"13749","NPHP1",73.6840233997642,0.0477657441721173,0.249077457841848,
0.191770642698812,0.847921867856911,0.964093483443104
"13750","BASP1",1726.33327169031,0.0388648876393009,0.202270883449498,
0.192142769025897,0.847630374172741,0.964093483443104
"13751","BRPF3",64.3017810243177,0.0509756775723509,0.265679894076485,
0.191868781601049,0.847844991752611,0.964093483443104
"13752","EXOSC2",65.4737093440264,0.0491342141554912,0.255445221829489
,0.192347360438351,0.847470122674767,0.964093483443104
"13753","ITFG2-
AS1",6.2974384440401,0.123234736801085,0.642845187362127,0.19170204463
4993,0.847975604307036,0.964093483443104
"13754","FAR2",20.3147305657435,0.104628761812662,0.544373790743264,0.
192200219025619,0.84758537434305,0.964093483443104
"13755","KLHL28",115.992670560812,-0.0353488363538268,0.18423999084476
8,-0.191862994520066,0.847849524963093,0.964093483443104
"13756","ZNF410",79.3289965197879,0.0409307323877756,0.213425690175117
,0.191779782247356,0.847914708424023,0.964093483443104
"13757","AC009133.1",13.3719976535388,0.0850573494621883,0.44376264070
4132,0.19167307398213,0.847998298746444,0.964093483443104
"13758","TOP3A",72.5801847135314,0.053274212812796,0.278024672803925,0
.19161685283366,0.848042340483099,0.964093483443104
"13759","TNFSF9",49.1788040324135,-0.107205945472763,0.557707599068446
,-0.192226079852295,0.847565118059582,0.964093483443104
"13760","AL021707.6",4.53148591375226,0.159097403133646,0.830076257413
308,0.191666008650129,0.848003833461299,0.964093483443104
"13761","TCHH",4.99742635942005,-0.175372384595124,0.917734178774007,-
0.191092789885414,0.848452896389187,0.964139812452133
"13762","IL18RAP",3.54789554892501,0.19324856701754,1.00947200660689,0
.191435290679433,0.848184573342659,0.964139812452133
"13763","TDP2",118.400215054074,-0.036636659962045,0.191658514560277,-
0.191155921489325,0.848403436308865,0.964139812452133
"13764","DCBLD1",10.7261480263646,0.0854379502349572,0.446750357066082
,0.191243160489113,0.848335090414674,0.964139812452133
"13765","DDX6",425.832948606313,-0.0344266032539003,0.180124720931229,
-0.191126476565336,0.848426504685512,0.964139812452133
"13766","AC008686.1",2.61103620289602,0.211730414324622,1.106934751915
06,0.191276327677234,0.848309106450634,0.964139812452133
"13767","SPATA1",6.54562405680599,0.113942139725941,0.597028520916553,
0.190848737931343,0.848644103053992,0.964217352926288
"13768","TMEM229B",4.15514340459444,0.138334996703836,0.72484252573127
6,0.190848345389604,0.848644410604688,0.964217352926288
"13769","AC025034.1",3.95722403993071,0.14819557434758,0.7768563307130
48,0.190763167510725,0.848711146768447,0.964223143939197
"13770","AC068888.2",3.94593006482788,0.143547348409055,0.753662805519
955,0.190466276639486,0.848943766784996,0.964347349053641
"13771","LONP1",96.6621505016613,-0.0392199481130893,0.20583971699416,
-0.190536348795126,0.848888862643756,0.964347349053641
"13772","THEM4",98.8701211635013,-0.0404666058943899,0.212981451455178
,-0.19000061093539,0.849308651794169,0.964490106588154
"13773","TATDN1",280.870775247593,-0.0470157360796525,0.24748600810253

8,-0.189973309764539,0.84933004537069,0.964490106588154
"13774","BRAP",70.1570660195737,-0.0459740507344416,0.242080213119875,
-0.189912468028421,0.849377722198965,0.964490106588154
"13775","AL109809.5",5.54897340923598,-0.137689209681698,0.72498714547
1222,-0.189919518631194,0.849372197174265,0.964490106588154
"13776","LINC00237",4.61699847155147,0.157980302969109,0.8308612372125
73,0.190140418030707,0.849199098752784,0.964490106588154
"13777","AC025048.2",8.30582035908787,-0.105597156294894,0.55717617677
5197,-0.189522023188545,0.849683695861706,0.964685491541311
"13778","L3MBTL4",21.4301901967613,0.0782433197336459,0.41298761105228
7,0.189456820591502,0.849734794345569,0.964685491541311
"13779","AC011468.5",5.46237993560248,0.113003544103495,0.596092958253
215,0.189573694067179,0.849643202469768,0.964685491541311
"13780","VASH1-
AS1",9.52379845248206,-0.0910096471465979,0.481432326563283,-0.1890393
35593172,0.85006198722237,0.964916890364961
"13781","ZNF625",20.8978965564505,0.0705800168128088,0.373312562805383
,0.189064135110834,0.850042550531304,0.964916890364961
"13782","ZC4H2",27.5260822288545,0.0608609897791957,0.322102968114977,
0.188948863574181,0.850132895691818,0.964927360855254
"13783","PGGHG",31.0844466066651,-0.0804616464832173,0.426140965573294
,-0.188814624698123,0.850238109164566,0.964976764250258
"13784","C2orf81",30.0530973788146,0.0660325504499385,0.35055119077340
8,0.188367782474946,0.850588353454523,0.965094191128534
"13785","MPLKIP",318.030555084892,-0.041072319080012,0.21803381056788,
-0.188375917354456,0.850581976901966,0.965094191128534
"13786","C12orf45",196.523479265796,0.0416014780193593,0.2206408733016
47,0.188548374545655,0.850446798054916,0.965094191128534
"13787","TRAPPC10",80.385153931484,0.0494997673263817,0.26269227652972
4,0.188432518764139,0.850537609967911,0.965094191128534
"13788","FZR1",65.3324367035837,0.0492957650609687,0.261928720240486,0
.188202977572328,0.850717538433141,0.965170761075546
"13789","DCAF1",48.8388084726964,0.0569889393909845,0.303062203645387,
0.188043704247816,0.850842391202644,0.965201543042521
"13790","PSMA3-
AS1",262.622390481067,0.0397999408260787,0.211689492328408,0.188010941
820081,0.850868073806582,0.965201543042521
"13791","MGAT4A",12.2844485413119,0.0976377308498536,0.520017102691879
,0.18775869167462,0.851065819045186,0.965337554000442
"13792","INPP5F",39.2088645057993,-0.0548621579927557,0.29228553828816
1,-0.187700555812884,0.851111394539034,0.965337554000442
"13793","OGDH",156.956707311218,0.0314298499713924,0.167672469699912,0
.187447885914982,0.851309480381796,0.965422227172135
"13794","AQR",121.01640042012,0.0409922311353264,0.218674512206322,0.1
87457745860431,0.85130175029567,0.965422227172135
"13795","OCLN",26.3052921655827,-0.112856680739272,0.604399594844033,-
0.186725275301343,0.851876037804183,0.965784668746981
"13796","AC005618.4",3.05298183223498,-0.143802491820572,0.76961109476
2028,-0.186850855970362,0.85177757172756,0.965784668746981
"13797","CD27",4.60331020608871,0.145979892031353,0.781577975744231,0.

186775851625488,0.851836381325748,0.965784668746981
"13798","SPAG7",367.651406422562,-0.0522638073692975,0.279844954762609,-0.186759869991698,0.851848912352451,0.965784668746981
"13799","CRIPT",296.536655403902,0.0313916291115087,0.168644680805816,0.18614064174164,0.852334471315132,0.965884391102768
"13800","XPC",288.658037108533,0.0424931416479032,0.228251121550627,0.186168380506635,0.8523127191610 8,0.965884391102768
"13801","LINC01504",60.0064128693839,0.0450222738241456,0.24153948909332,0.186397155981194,0.8521333225476,0.965884391102768
"13802","SLC31A1",68.4811139880784,-0.045679314320308,0.245289811975762,-0.186225893168452,0.852267619303465,0.965884391102768
"13803","CRYL1",249.14733203957,-0.0534800903450287,0.287143658236233,-0.186248551242704,0.852249851593493,0.965884391102768
"13804","GPR132",2.59604275576619,0.181177851568849,0.972153827925532,0.186367472270784,0.8521565988991,0.965884391102768
"13805","PACRGL",74.9805067426212,0.0377261427489861,0.202781980222743,0.186042875740469,0.852411138245202,0.965901299208236
"13806","AC005726.3",3.40992154548878,0.144872747302242,0.779555871049825,0.185840108044011,0.852570150694694,0.966011507121331
"13807","AC007114.2",6.1521669663572,0.121774548597854,0.65588349518953,0.185664907702343,0.852707549375372,0.966097211188451
"13808","DHX30",141.810427967381,0.0337407305425834,0.181886305169929,0.185504513443499,0.852833340489388,0.966169752699558
"13809","ST3GAL3",58.127767935343,0.0389111146448191,0.209931326422149,0.185351635260823,0.852953240514759,0.966235610208732
"13810","NPIPB2",21.8996961861424,-0.0781661489529093,0.421909155719566,-0.18526772385301,0.853019052395592,0.966240190921379
"13811","MRPS30",148.509907633709,-0.0384487068220359,0.207685694653909,-0.185129297836847,0.85312762242596,0.966264416759584
"13812","TMEM147-AS1",12.7007678251462,0.0842766158630748,0.455345122322065,0.185082944192529,0.85316397905027,0.966264416759584
"13813","TMEM189",115.175865568803,-0.045617541578163,0.246898396605862,-0.184762405124019,0.853415396477123,0.966479189683025
"13814","PTPN7",5.00128364036241,0.164908122423205,0.894819586957837,0.184292034759601,0.853784362250546,0.966546259743205
"13815","CCZ1B",61.6800522018955,-0.0813477493258759,0.441087259780402,-0.184425524705419,0.85367964742275,0.966546259743205
"13816","ATF7",287.975350905083,0.0323105588261921,0.175241962432711,0.184376837474635,0.853717839326278,0.966546259743205
"13817","MFAP1",177.689587735816,-0.0368228706465476,0.199589065709347,-0.184493426609708,0.853626383454872,0.966546259743205
"13818","AC129507.1",3.4552438810758,0.17224426541068,0.934994166001632,0.184219615131138,0.853841172168176,0.966546259743205
"13819","LRRC37A",2.7178118992857,-0.206791265693147,1.12255820169323,-0.184214293193199,0.853845347017283,0.966546259743205
"13820","AL354733.3",15.3667426782407,0.126345937055898,0.686309460682283,0.184094704057107,0.853939161027652,0.966582510561184
"13821","ATR",129.775555574764,0.050664843772839,0.275345407248371,0.184004680808559,0.854009782868461,0.966592506577769

"13822","GCNT1",5.39993297276679,-0.138412854651928,0.752909850732964,
-0.183837221039387,0.854141155569064,0.966601323632126
"13823","TMEM159",698.209117761371,-0.033910615891098,0.18440895760284
,-0.183888116563898,0.854101227446597,0.966601323632126
"13824","RFK",170.076906507109,0.039026647880951,0.21281862124835,0.18
3379854883134,0.85449998131332,0.966858612910978
"13825","CD7",10.7029412749777,-0.10934848443056,0.59642298896547,-0.1
83340492324468,0.854530864541138,0.966858612910978
"13826","TIMP1",5376.07203582529,-0.0524459023506958,0.286103270899288
,-0.183311089683967,0.854553933523441,0.966858612910978
"13827","IL13RA1",815.689276864768,-0.0505726448732431,0.2762199583375
14,-0.183088308236758,0.854728729389613,0.966986440575809
"13828","MOB1B",53.3851461235749,-0.0485315100818485,0.265219221547081
,-0.182986398190726,0.854808691094658,0.96700696809327
"13829","PTPRC",63.1156661321075,-0.0994568619466897,0.543858608968797
,-0.182872644298614,0.854897947600767,0.967038006675739
"13830","TARSL2",81.6983491275429,0.0576687857611823,0.315610458200416
,0.182721403118277,0.855016621222892,0.967102314229191
"13831","ACBD3",414.634498221147,-0.0351966854844668,0.192735546190275
,-0.182616472052952,0.85509895885763,0.96712359753135
"13832","MED6",204.664842088306,0.0303262752374945,0.166135098385764,0
.182539845777063,0.855159087198979,0.96712359753135
"13833","SSBP3",285.984308648639,0.0315240942024522,0.172924093372287,
0.182300184940592,0.855347153554702,0.967128026600246
"13834","ADAM15",131.200372072772,0.0396452051960421,0.217536926161181
,0.182245864624692,0.855389780868562,0.967128026600246
"13835","PREX2",70.2412327216362,-0.0760913390003926,0.417464618043293
,-0.182270151077813,0.85537072227002,0.967128026600246
"13836","HARBI1",7.10563361553311,-0.104712570803737,0.574650070483496
,-0.182219712799538,0.855410303397111,0.967128026600246
"13837","MYL5",73.139715798361,-0.0702086270854542,0.385551573611814,-
0.182099184365261,0.855504888813994,0.967165062926741
"13838","ABLIM3",27.6760403582683,0.0732834604959167,0.403500663523887
,0.181619182124538,0.8558815940555,0.967178576966053
"13839","FSCN1",160.304441179304,0.0656989927598273,0.361756164663679,
0.181611259675165,0.855887811860965,0.967178576966053
"13840","NTRK2",3686.0998040802,0.0511309786321256,0.281383536021747,0
.181712758873617,0.855808152543823,0.967178576966053
"13841","MRPS7",282.16866689383,-0.0443222085712944,0.243926436634113,
-0.181703177330373,0.855815672335415,0.967178576966053
"13842","PIP5K1C",76.1400103337256,0.0441768567186785,0.24286435211267
7,0.181899304423988,0.855661750380743,0.967178576966053
"13843","ZNF557",40.3622459859338,0.0629807768061573,0.346067568713066
,0.181989826554295,0.855590709813097,0.967178576966053
"13844","AC012313.6",6.65566076185835,0.11517711748307,0.6350884050871
06,0.181356038876624,0.856088122535581,0.967333644404848
"13845","DDX3X",1019.27941371471,0.0526464642624897,0.29041703712394,0
.181278842260284,0.856148712317658,0.967333644404848
"13846","NBPF20",25.4048390077868,-0.0736955546475836,0.40781387683958
1,-0.180708795930877,0.856596154304142,0.967350104084587

"13847","POLR3C",86.948308832561,0.0349752887216521,0.193475220977086,
0.180773995476115,0.856544975400179,0.967350104084587
"13848","SMYD5",34.1371746277196,0.0585964501744213,0.323568659403188,
0.181094331825896,0.856293533589355,0.967350104084587
"13849","KCNE4",265.164771129156,0.0863571835016376,0.477143717101961,
0.180987782939168,0.856377165455781,0.967350104084587
"13850","AC097359.2",4.10423869067691,-0.134085229841873,0.74157246418
0864,-0.180812039710755,0.856515112552899,0.967350104084587
"13851","ENPP3",3.30021695256497,0.151386474691803,0.837192798909218,0
.180826298182504,0.856503920408938,0.967350104084587
"13852","EFCAB13",42.8620088582633,0.0697513134834251,0.38557153261535
2,0.180903691230258,0.856443171475334,0.967350104084587
"13853","C17orf67",22.6796161301392,0.0668359281631705,0.3711344616440
76,0.180085481329587,0.857085460488399,0.967832805776368
"13854","ABHD17C",11.6291803973313,0.0754567175872397,0.41951743237478
5,0.179865511571469,0.857258151360477,0.967957937182903
"13855","AC093151.2",2.84725596532724,0.146540280610475,0.817830537933
358,0.179181717744093,0.857795018533902,0.96799742817197
"13856","AC012467.2",10.4879358609269,0.0909748657460086,0.50776556433
395,0.179167064756238,0.857806523757751,0.96799742817197
"13857","PAQR3",49.8425183536147,-0.0618156024430968,0.344301078552489
,-0.179539380773891,0.857514198272799,0.96799742817197
"13858","LTV1",158.219840003531,0.0368258083035969,0.205592315884889,0
.179120548086124,0.857843047887858,0.96799742817197
"13859","HOXA6",25.8383517871416,-0.0814700948820768,0.454384145072725
,-0.179297838107968,0.857703844287563,0.96799742817197
"13860","TUBGCP2",249.22952600485,0.0283572518198835,0.158321667643636
,0.179111629140444,0.857850050933199,0.96799742817197
"13861","AL049840.5",25.6211368742364,-0.0572952683436071,0.3197299132
2249,-0.179198961292487,0.857781479294039,0.96799742817197
"13862","CMTM4",20.5969983110478,0.0762202748855754,0.424618802196005,
0.179502825808434,0.857542898693029,0.96799742817197
"13863","SLC6A16",6.14825449492601,0.105599441172977,0.58950117571505,
0.179133554814183,0.857832835186635,0.96799742817197
"13864","TSEN15",57.5669401053012,-0.0463061252397759,0.25926863665969
8,-0.17860288014919,0.858249532823037,0.96801284998997
"13865","WDSUB1",54.0563177541737,-0.0453447118139657,0.25390589532306
3,-0.178588652919107,0.858260704906996,0.96801284998997
"13866","AC024560.4",4.30197944230918,-0.145863697569132,0.81727649238
2008,-0.178475337206877,0.858349688290201,0.96801284998997
"13867","AC139795.3",7.65768391738125,-0.110930823525788,0.62140341888
3103,-0.178516596714534,0.858317288245027,0.96801284998997
"13868","DDX50",227.762869529203,-0.0342977884354653,0.192183478201017
,-0.178463771998085,0.858358770198228,0.96801284998997
"13869","B4GALNT1",3.87755621160956,-0.136167578656252,0.7619043320726
35,-0.17872004781208,0.858157526747564,0.96801284998997
"13870","PMAIP1",10.2166630659664,-0.121031736038016,0.677510932667878
,-0.178641746135995,0.858219013037639,0.96801284998997
"13871","AC004832.6",2.70217306684811,-0.281576074761643,1.57684892734
207,-0.178568834261293,0.858276267766449,0.96801284998997

"13872","IARS",145.900254398214,0.0411591140706422,0.230778131635821,0
.178349281965733,0.858448677752521,0.968044454014035
"13873","GREB1",7.39614239049353,0.118806268306992,0.667297613033461,0
.178040901070981,0.858690854443656,0.968072174232801
"13874","AC008443.1",56.422044362369,-0.0400545468356975,0.22512170206
3498,-0.177923969428766,0.858782686302409,0.968072174232801
"13875","ZNF713",15.7692271330134,0.0813520899188021,0.457139262427009
,0.177959096068217,0.858755099517398,0.968072174232801
"13876","ZNF614",24.4471486999549,0.0580917287503496,0.326433078698823
,0.177959075048111,0.858755116025534,0.968072174232801
"13877","STS",59.102282593191,0.0401163839634155,0.225292928910481,0.1
78063218217361,0.858673327967543,0.968072174232801
"13878","EEF1B2",3283.73535051694,-0.0543558569477603,0.30578707964066
8,-0.1777572061306,0.858913656584033,0.968119256758304
"13879","NDUFAF1",72.2340754134412,0.0498146526143842,0.28030926739751
9,0.177713184715152,0.858948230169949,0.968119256758304
"13880","ZNF670",25.5363402145496,-0.0611450709657679,0.34490430907156
4,-0.177281261374676,0.859287468916844,0.968184330248611
"13881","MCEE",182.135603015482,-0.040381953694971,0.227476981989748,-
0.177521054401851,0.859099128844513,0.968184330248611
"13882","HSPA1L",5.46781695751252,0.118024878052393,0.665139361370452,
0.177443833438477,0.859159779449151,0.968184330248611
"13883","CD63",13878.8119590202,0.0539062487736314,0.303891851385682,0
.177386292287307,0.859204973748546,0.968184330248611
"13884","CDH24",3.44988941693176,-0.1326833666925,0.748584552260055,-0
.177245664891047,0.859315428061862,0.968184330248611
"13885","GPATCH3",31.2949840761223,-0.0516520732407234,0.2925347738243
1,-0.176567293404047,0.859848286466438,0.968428048709562
"13886","ALKBH4",102.689133730404,-0.0340163557309689,0.19261598134791
5,-0.176601938701682,0.859821071158957,0.968428048709562
"13887","PCAT1",6.24526859760209,-0.118369032601452,0.670114320393578,
-0.176640058269357,0.85979112686174,0.968428048709562
"13888","AL445222.1",8.95625488623133,0.0936154917076274,0.53018991604
6379,0.176569732607736,0.859846370366081,0.968428048709562
"13889","RHOD",105.074142764563,0.0570526857335931,0.323129238230149,0
.17656305584132,0.859851615257829,0.968428048709562
"13890","ULK3",48.8853430513622,-0.0477504258230498,0.270544638824445,
-0.17649740179858,0.859903189706311,0.968428048709562
"13891","SDC3",127.292786942046,0.0549710557821033,0.313079794812541,0
.17558161431343,0.860622647593492,0.968765877646164
"13892","GDF11",42.3579444749969,-0.0486612505025737,0.278090688885016
,-0.174983386526451,0.861092687806919,0.968765877646164
"13893","SBNO1-
AS1",16.891989826831,-0.0909431465565548,0.518038689075746,-0.17555280
8070784,0.860645280136954,0.968765877646164
"13894","AL136295.2",7.40128187510727,-0.127880352069012,0.72748564816
7956,-0.175784020469759,0.860463623968961,0.968765877646164
"13895","CTSG",27.0315990117485,-0.133518482832808,0.761236734279014,-
0.175396794217067,0.860767859386102,0.968765877646164
"13896","RHOJ",98.984210111717,0.0541066558667506,0.309298916218218,0.

174933221649497,0.861132105649166,0.968765877646164
"13897","TMEM202-
AS1",11.2633147296369,-0.0891050337755583,0.509228842895102,-0.1749803
35499012,0.861095085189907,0.968765877646164
"13898","ODF3L2",2.73879902092825,0.213217454701268,1.218586379396,0.1
74971145506279,0.861102306349424,0.968765877646164
"13899","MCOLN1",169.912664570116,-0.0388769392119205,0.22144210538212
7,-0.175562543287932,0.86063763134024,0.968765877646164
"13900","PSENEN",345.270666109413,-0.0583267107378568,0.33195774161810
9,-0.175705228182197,0.860525527713988,0.968765877646164
"13901","ZNF540",20.2799970889201,-0.0747046250279698,0.42699408012417
,-0.174954708988578,0.861115221591665,0.968765877646164
"13902","XRCC1",90.2749040839872,-0.041596203332279,0.237605719859379,
-0.175063981443278,0.86102935980606,0.968765877646164
"13903","GEMIN7-
AS1",7.39722900979748,0.108455746022382,0.616750519873269,0.1758502709
40457,0.860411574458881,0.968765877646164
"13904","RPS11",11667.4217553443,-0.0366848760463599,0.209396688537288
,-0.175193200535391,0.860927826895572,0.968765877646164
"13905","PITPNB",286.532168784046,0.0274362076786957,0.156447897645158
,0.175369615646252,0.860789213786758,0.968765877646164
"13906","CASP9",26.7886377446614,-0.0634249936359999,0.364260563790785
,-0.174119847001687,0.861771275796284,0.968818553553137
"13907","UTP11",159.070426856109,-0.0354867929447619,0.203841948284675
,-0.17408974572399,0.861794931873023,0.968818553553137
"13908","TUBB2B",12.6114643212614,-0.10960002040035,0.62852140785441,-
0.17437754550715,0.861568760046807,0.968818553553137
"13909","NUDT1",316.132078268606,-0.0523526966741121,0.300072340946536
,-0.174466918573611,0.861498527175316,0.968818553553137
"13910","MLXIPL",140.038385614363,0.0830240385163775,0.476422981418339
,0.17426539389265,0.861656894745944,0.968818553553137
"13911","C11orf49",93.721830752558,-0.0428542725219895,0.2452256641893
12,-0.174754435526399,0.861272592474187,0.968818553553137
"13912","AP000873.2",49.4855612024617,0.0607022476599843,0.34755464530
4726,0.174655262071846,0.861350523031409,0.968818553553137
"13913","PGS1",70.9183324897778,0.0350691233987813,0.201447685617629,0
.174085511537455,0.861798259457387,0.968818553553137
"13914","GART",88.3823408516623,-0.0464179422079349,0.266033725356199,
-0.174481420149964,0.861487131369384,0.968818553553137
"13915","DGCR8",88.4895936917594,-0.0453426233096799,0.260375991390803
,-0.174142873417328,0.861753179815725,0.968818553553137
"13916","OTUD3",44.3025727678742,0.0555348783853598,0.319297492770344,
0.173928325911733,0.86192179104319,0.968855559304151
"13917","FAM219A",44.0936690493545,0.0451359305153742,0.25957195022113
5,0.173886009165944,0.861955048189981,0.968855559304151
"13918","AL359921.2",2.77778327084912,0.147816225901426,0.851186392541
8,0.173659056578688,0.862133416635148,0.968964988492848
"13919","PABPN1",483.788094624081,-0.0310619244299193,0.17892348876874
1,-0.173604509076318,0.862176288105348,0.968964988492848
"13920","DPH6-

DT",2.72906492677572,0.168176638254413,0.969725430010261,0.17342706816
7774,0.862315750141997,0.968982492599042
"13921","CFAP53",7.85754153400836,-0.093075799067795,0.53667842828835,
-0.173429365075554,0.86231394482942,0.968982492599042
"13922","SCO1",136.457231693782,-0.0380167181924425,0.219465309679005,
-0.173224270605895,0.862475146832067,0.969091992665854
"13923","ADAMTSL4-
AS1",23.4817933153826,0.0991503065879075,0.572915944125162,0.173062571
577248,0.862602244532265,0.969149325469652
"13924","CIR1",699.920336815714,0.0304060397708906,0.17616839036955,0.
172596455624687,0.862968638088737,0.969149325469652
"13925","LMNB1",6.90336467583008,-0.0926598509935766,0.536699823751042
,-0.172647440697798,0.862928559494408,0.969149325469652
"13926","SMAD5-
AS1",14.1911670445667,-0.0734294180229591,0.425058653939237,-0.1727512
59955423,0.862846949837952,0.969149325469652
"13927","HLA-
F",167.196872636272,-0.0727963682659745,0.421793113693866,-0.172587853
861478,0.862975399839384,0.969149325469652
"13928","RABL6",180.775291953437,-0.0295981301074289,0.171110759810603
,-0.172976440173546,0.862669946473426,0.969149325469652
"13929","CD69",55.291427480422,0.0878458612758793,0.509398995709313,0.
172450008766818,0.86308375970835,0.969149325469652
"13930","MBIP",211.025600154965,0.0353125106229884,0.204347239981301,0
.172806398687938,0.862803607289144,0.969149325469652
"13931","SAMSN1",30.5447060132714,-0.0717797683420979,0.41610740108793
9,-0.172502983975832,0.863042115653155,0.969149325469652
"13932","ULK4",25.1888676493905,0.0583834324834181,0.339200805145661,0
.172120559850519,0.863342749550466,0.969231421789719
"13933","RAB38",3.39257957615565,-0.14596247766977,0.847533242157725,-
0.172220357160465,0.863264294289207,0.969231421789719
"13934","AL139317.3",2.89749137949326,0.14589331794644,0.8473071356583
22,0.172184691721128,0.863292332378753,0.969231421789719
"13935","ASB3",98.1696422675231,0.0394864503560479,0.229838322831286,0
.171800985447641,0.863593990769371,0.969281487389578
"13936","FANCL",66.8868126235845,-0.0387414685589865,0.225623961692438
,-0.171708130060217,0.863666993886595,0.969281487389578
"13937","MAMDC4",4.8852890939328,-0.111912562681744,0.650757252082791,
-0.171972824464976,0.863458893614551,0.969281487389578
"13938","GALNT4",61.882525389262,-0.05040117915922,0.29349426428315З,-
0.171727986856311,0.863651382330387,0.969281487389578
"13939","NCOR2",358.844738247844,0.0348273286165221,0.202873983436765,
0.171669762808092,0.863697158647531,0.969281487389578
"13940","ZNF24",250.991825424149,0.0276240744209409,0.161061895118463,
0.171512165559849,0.863821065443025,0.969350999047721
"13941","CRBN",459.054917074817,-0.0295887265177616,0.172657079533421,
-0.171372796283364,0.863930643753766,0.969404422942412
"13942","LINC01011",21.9673360672304,0.0601341346877416,0.351314683984
291,0.17116886207475,0.864090990595881,0.969514801742316
"13943","FBXO6",68.2087025438189,0.047657625414585,0.27943645183446,0.

170549064381971,0.864578351713044,0.969644332940002
"13944","CNRIP1",304.960554872971,0.0382830200565347,0.224025422780593
,0.170886944800138,0.864312662204825,0.969644332940002
"13945","STARD3NL",114.657268554865,0.0328879473333863,0.1927484090140
86,0.170626297262889,0.864517618916743,0.969644332940002
"13946","U47924.2",5.96070374211727,0.0954935378613024,0.5591875021848
48,0.170771945882538,0.864403088944205,0.969644332940002
"13947","GOLGA5",134.96371533783,-0.0275446951900679,0.161488686426869
,-0.170567336941846,0.864563982842892,0.969644332940002
"13948","AC015871.1",25.745786526821,0.0712717027258044,0.417431188767
528,0.170738805924481,0.864429148113498,0.969644332940002
"13949","WIPF1",348.350990078647,0.0348203812072013,0.204478096338507,
0.17028905213181,0.864782820649265,0.969797411180071
"13950","PANK2",133.243623357033,0.0294293931809635,0.172892546770753,
0.170217824484854,0.86483883436438,0.969797411180071
"13951","Z95118.2",4.02960243298385,0.118575019399725,0.69724644410592
6,0.170061848865752,0.864961496605569,0.969865435553072
"13952","BCL11B",11.3219000527834,0.10236488075901,0.602446250848691,0
.169915375213646,0.865076689278769,0.969925075285822
"13953","KLHL5",43.9257498672115,-0.0532479237780564,0.313629734715257
,-0.169779577266167,0.865183488716812,0.969962518879187
"13954","LINC01239",64.4854892389154,0.092127686509136,0.5428368896646
44,0.169715228023746,0.865234097579759,0.969962518879187
"13955","PPFIBP1",382.408258866878,0.0360166404340687,0.21244880611022
8,0.169530914734261,0.865379057837858,0.969985998979479
"13956","LINC01315",58.2310951314029,-0.0755105338287681,0.44523998586
5039,-0.169595131223584,0.865328551801622,0.969985998979479
"13957","AC025449.2",10.3325617574448,0.0847071110040811,0.50016180561
2373,0.169359415400322,0.865513944137278,0.969998182271058
"13958","SMC5",286.343603895686,0.0388239462763304,0.229210182875627,0
.169381420097714,0.865496636957586,0.969998182271058
"13959","AC093388.1",3.02412955211577,0.134154300547456,0.794608212397
565,0.168830750116051,0.865929770410748,0.970047221321637
"13960","SGK1",172.946589873957,-0.0800216300609079,0.473157079657283,
-0.169122757539355,0.865700084838496,0.970047221321637
"13961","DGKZ",81.8659470042553,0.0380738128477473,0.22534203344242,0.
16896010152263,0.865828024503793,0.970047221321637
"13962","MCTP2",6.40677100480591,0.131507325574128,0.778747052162289,0
.168870399199562,0.865898582790713,0.970047221321637
"13963","TRPC4AP",165.205448912366,0.0260744534973828,0.15410549455334
8,0.169198726969182,0.865640331086186,0.970047221321637
"13964","APOL2",143.979508293009,-0.0451408731794262,0.267346558486667
,-0.168847781078422,0.8659163739801,0.970047221321637
"13965","HIST2H2AC",156.948404620758,0.0551963745029277,0.327582350029
708,0.168496179656572,0.866192948897596,0.970137727268255
"13966","SH2D2A",8.34782396484149,0.104928518738741,0.627084540285378,
0.167327548357339,0.867112329245574,0.970137727268255
"13967","OTULIN",114.865560765092,0.0509526099961475,0.304392518924825
,0.167391137522441,0.867062298027696,0.970137727268255
"13968","AL357497.1",3.23573304135696,0.140839736953005,0.838518311623

816,0.167962625264873,0.866612682020655,0.970137727268255
"13969","PTK2B",30.5758095450489,-0.059826825312668,0.357116527824966,
-0.16752746135007,0.866955041815461,0.970137727268255
"13970","PRNCR1",3.7556730730427,0.161171888524899,0.963323909711185,0
.167308095335472,0.867127634763995,0.970137727268255
"13971","AL137186.2",46.2361941850291,0.0580038750193594,0.34534585326
5012,0.167958799768325,0.866615691573533,0.970137727268255
"13972","ELP4",68.9809616102514,0.0371596139442627,0.221775312140051,0
.167555232301043,0.866933192619095,0.970137727268255
"13973","ZNF408",29.6285172047322,0.0599751939767649,0.358238801369287
,0.167416800602066,0.867042106764992,0.970137727268255
"13974","SLC2A13",100.109799224146,0.0374488833808633,0.22298801504471
3,0.167941238336752,0.86662950733606,0.970137727268255
"13975","CBX5",409.695151075067,-0.0367881797195339,0.219005503766004,
-0.167978334274376,0.866600323620483,0.970137727268255
"13976","TRHDE",45.7370297546488,-0.106551968920387,0.635880756112346,
-0.167565959334617,0.866924752997878,0.970137727268255
"13977","MTRF1",26.399529522373,0.0502491963753649,0.299785110582503,0
.167617385258819,0.866884293251221,0.970137727268255
"13978","ATL1",162.255829018211,0.0323692174201977,0.193513282248294,0
.167271295510689,0.867156588776917,0.970137727268255
"13979","ACOT1",3.37704501613845,-0.197250359021377,1.17951370665561,-
0.167230238960649,0.867188892181296,0.970137727268255
"13980","ISCA2",173.183372054525,0.04458181589215,0.265049571410301,0.
16820180336431,0.86642452227932,0.970137727268255
"13981","NHLRC4",26.4880896339093,0.0658028379378711,0.391113905535086
,0.168244690374395,0.866390784167876,0.970137727268255
"13982","VKORC1",1248.677322,-0.0477766821088241,0.284269273592839,-0.
168068400446455,0.866529468490729,0.970137727268255
"13983","CAPNS1",762.391850376177,-0.0412043253448148,0.24491340673168
6,-0.168240382977303,0.866394172675754,0.970137727268255
"13984","ALG2",119.748383541509,0.0348946792315151,0.208775142311936,0
.167140009318629,0.867259885886409,0.970147768515524
"13985","PIWIL2",11.6309198195893,-0.091938470573683,0.550647074644128
,-0.166964422054001,0.867398043005052,0.970232934338793
"13986","ZFP69",9.89709308207016,0.0857886957492505,0.515236428646301,
0.166503552504325,0.867760687701745,0.970372801945515
"13987","VRK2",69.4153343849197,0.0349989843269264,0.21052575123057,0.
166245621366268,0.867963658280361,0.970372801945515
"13988","ELP6",149.418656565496,-0.0392890126193801,0.236446922482562,
-0.166164195358803,0.868027735655221,0.970372801945515
"13989","ZSCAN16-
AS1",238.13673878964,0.0490859971072762,0.295505471305949,0.1661085897
67719,0.868071494412188,0.970372801945515
"13990","CACUL1",184.380539682624,0.0329451750211705,0.198350164293228
,0.166096030918666,0.868081377640193,0.970372801945515
"13991","DNAJC24",87.828281326519,-0.0340329382680242,0.20463322416287
6,-0.166311890003433,0.867911509485302,0.970372801945515
"13992","AP001160.4",4.59167782746965,0.119117611651984,0.715586154581
583,0.166461593603127,0.867793705310573,0.970372801945515

"13993","DUS2",21.2440619935495,0.0610945304535619,0.367295848414215,0
.166336022357277,0.86789251914886,0.970372801945515
"13994","CLIP3",104.525280434815,-0.0447420875414816,0.26878357512311,
-0.166461390064436,0.867793865476457,0.970372801945515
"13995","ETV3",43.9070639477539,-0.0437096526384841,0.263781456156156,
-0.165704038772947,0.868389867518523,0.970417564596146
"13996","ANKDD1B",3.20471756593352,-0.150451138866287,0.90726011552599
8,-0.165830213729897,0.868290568194097,0.970417564596146
"13997","AL359220.1",3.6068933388863,-0.12255842837857,0.7384776041011
16,-0.165960927857453,0.868187698746756,0.970417564596146
"13998","GRIN2D",6.51736148656584,0.112384937568558,0.677933463905269,
0.165775763481506,0.868333420126584,0.970417564596146
"13999","SLC10A3",114.556578554635,-0.0421684828954986,0.2545621768724
25,-0.165651014669911,0.86843159795317,0.970417564596146
"14000","AL163051.1",4.9198078068526,-0.123177282109523,0.744138653891
481,-0.16553001442052,0.868526827584249,0.970448468160222
"14001","SLC9A7",7.54746915931998,0.122955381585687,0.743120395266837,
0.165458225031674,0.868583328179458,0.970448468160222
"14002","SYNC",163.103250178936,0.0386060912366859,0.235650804600145,0
.163827538387544,0.86986691207069,0.97046946572592
"14003","ZSCAN20",11.0882134146143,0.0804059832114655,0.51521730347640
9,0.156062272499252,0.875983936113098,0.97046946572592
"14004","SNIP1",91.3796927219826,-0.0382572099938045,0.2352804212983,-
0.162602607487259,0.87083133384095,0.97046946572592
"14005","RLF",124.59520347345,0.0389465365811154,0.244971735940896,0.1
58983796361356,0.873681640084971,0.97046946572592
"14006","ZSWIM5",7.86978852281628,0.0927528572739562,0.573745105962603
,0.161662132382532,0.871571926189435,0.97046946572592
"14007","DNTTIP2",183.389372972111,-0.0319374700673265,0.1977453245271
22,-0.16150809200521,0.871693238528049,0.97046946572592
"14008","GSTM4",100.965032486933,-0.0349135300591878,0.222494896233431
,-0.15691834127538,0.875309204684357,0.97046946572592
"14009","DENND2D",22.5590279661731,0.0827237756672151,0.50925663000788
4,0.162440252699183,0.87095917467793,0.97046946572592
"14010","UBE2Q1",261.107894943734,0.0559616181896423,0.35625843216519,
0.15708152604145,0.875180596911808,0.97046946572592
"14011","SCYL3",56.3386916896668,-0.0420319230782996,0.266333804416868
,-0.157816703629972,0.874601236011158,0.97046946572592
"14012","TPR",756.103217927987,-0.0326086946914316,0.205804209447992,-
0.158445227038333,0.874105977831159,0.97046946572592
"14013","ATP2B4",544.268069853603,-0.032359764582848,0.205157346746642
,-0.157731444162273,0.874668421782284,0.97046946572592
"14014","COG2",87.9376029032184,-0.0281082100576896,0.179582378606284,
-0.156519867237721,0.875623260443812,0.97046946572592
"14015","E2F6",43.3487947573082,0.0435676998539109,0.271688450039389,0
.160359043042111,0.872598250957489,0.97046946572592
"14016","FAM228A",5.28043778069849,0.139529553151144,0.891454870112748
,0.156518919610027,0.875624007336251,0.97046946572592
"14017","TMEM17",24.6621598934852,0.0505589567956354,0.31100654152967,
0.162565573530909,0.870860494691789,0.97046946572592

"14018","AC005041.4",13.6852133805373,-0.0671540386239437,0.411680243416254,-0.163121839577916,0.870422504603765,0.97046946572592
"14019","MITD1",242.158996264444,-0.027506346247866,0.174654994240566,-0.157489606108711,0.874858998800536,0.97046946572592
"14020","SLC23A3",6.35750633992655,0.0970154097268121,0.609618078553573,0.159141293770353,0.87355755503628,0.97046946572592
"14021","SETMAR",87.1964374913964,-0.0435783064565459,0.278773260047795,-0.156321687557388,0.875779462282947,0.97046946572592
"14022","DYNC1LI1",238.750198316293,-0.0291204645796345,0.185945694028864,-0.156607361798409,0.87555430026687,0.97046946572592
"14023","GOLGA4",951.698376582322,0.030984416207911,0.193927184617011,0.15977345450099,0.873059535658991,0.97046946572592
"14024","COL7A1",7.54608380599327,-0.0882893938625113,0.560243913384211,-0.157590991625755,0.87477910251002З,0.97046946572592
"14025","USP4",110.133513107691,-0.034375479461615,0.219942263089931,-0.156293197035803,0.875801918423432,0.97046946572592
"14026","ABHD14A",501.436141299628,-0.043473861054063,0.277208421592077,-0.156827346024994,0.875380920507888,0.97046946572592
"14027","DUSP7",40.4730932818875,0.0518769955990169,0.32137094705069,0.161424036849337,0.871759436260079,0.97046946572592
"14028","ADCY5",2.60313989927084,0.146634909629964,0.926731974326951,0.158227959854799,0.874277172489802,0.97046946572592
"14029","CDV3",543.010729873281,0.0218866862695027,0.135856908331158,0.161101018257775,0.872013838267517,0.97046946572592
"14030","LRRC34",57.5338457258636,-0.0379918397678308,0.235057289128011,-0.161628001023787,0.871598805601867,0.97046946572592
"14031","LPP-AS2",29.8645192404074,0.059042149202852,0.369112314997318,0.159957137174578,0.872914838954547,0.97046946572592
"14032","DGKQ",24.3059176628269,0.0524883868549137,0.329090258846259,0.159495413321957,0.873278571702106,0.97046946572592
"14033","NSUN7",9.94591484073302,0.088219157263732,0.559677924657734,0.157624864903653,0.874752409146996,0.97046946572592
"14034","JADE1",206.487590183692,-0.0504861046557439,0.305951589163991,-0.165013376115145,0.868933453768355,0.97046946572592
"14035","C4orf33",48.3536598106846,0.0410175579585494,0.25903458951576,0.158347802257713,0.874182742501943,0.97046946572592
"14036","FBXO8",139.253879944401,-0.0342074073779935,0.212729517872244,-0.160802354652714,0.872249070628174,0.97046946572592
"14037","C5orf17",2.84266645821145,-0.165916993063562,1.00892669308268,-0.164449007248107,0.869377686461576,0.97046946572592
"14038","SUB1",1304.62412533593,0.0445471752237054,0.276115986650736,0.161335009117215,0.871829551129575,0.97046946572592
"14039","TBCA",1123.23275629723,-0.0348031038849553,0.222344683621246,-0.156527708772388,0.875617079979729,0.97046946572592
"14040","PCDHB6",8.00383477229673,-0.114286247149732,0.703342270064542,-0.16249022988373,0.870919821459468,0.97046946572592
"14041","DELE1",104.396436386239,0.0356103612727964,0.221889955127396,0.160486585579555,0.872497787822619,0.97046946572592
"14042","AC091982.3",2.7723781946667,-0.141729130550557,0.874577383481

602,-0.16205441991462,0.871262999294954,0.97046946572592
"14043","BNIP1",54.3137812143994,-0.0517827548468194,0.325309061038527
,-0.159180179861902,0.873526918931132,0.97046946572592
"14044","LMAN2",718.062233377146,-0.0402711428649854,0.253706150878845
,-0.158731440784881,0.873880466047914,0.97046946572592
"14045","RIPK1",89.2617343630098,0.0328497352844425,0.198799260458738,
0.165240731824858,0.86875450653665,0.97046946572592
"14046","HULC",17.6969546128832,0.0538232304717309,0.344401786157053,0
.15628034648806,0.875812047217912,0.97046946572592
"14047","DDR1",11.6873393520298,0.0715511673414705,0.443782764780021,0
.161230162638105,0.87191212553635,0.97046946572592
"14048","CCHCR1",64.2297827649766,-0.0385603007561234,0.23981604440994
4,-0.160791163289342,0.872257885349501,0.97046946572592
"14049","UBE2J1",251.263395419648,0.0211892069550622,0.129278652502508
,0.163903371089446,0.869807213246398,0.97046946572592
"14050","THEMIS",3.36412850613606,0.139998082178946,0.886001096227493,
0.15801118393086,0.874447985928199,0.97046946572592
"14051","CNKSR3",13.3725100073999,-0.0715159800414698,0.45748180521838
8,-0.156325299117263,0.875776615669831,0.97046946572592
"14052","COX19",124.070811762516,-0.0404920921020912,0.252400983270333
,-0.160427632164659,0.872544224191268,0.97046946572592
"14053","HUS1",61.9567942949245,0.0378484583121623,0.229856890960018,0
.164660968631764,0.869210840069864,0.97046946572592
"14054","STEAP2",183.870900708102,-0.0688535625624595,0.41935539928882
2,-0.164189045089743,0.86958232482677,0.97046946572592
"14055","CYP51A1",179.12834093194,0.0301498446853711,0.192245353549697
,0.156830030628423,0.875378804684497,0.97046946572592
"14056","ZC3HAV1",280.221584307041,0.039432489513341,0.251312804069663
,0.156906010655989,0.875318922719792,0.97046946572592
"14057","EIF4EBP1",215.366033310044,-0.0470990858910109,0.296385406673
869,-0.15891162260508,0.873738503544842,0.97046946572592
"14058","FAM91A1",100.558523237125,0.0418613930469935,0.25927800568956
6,0.161453698842138,0.871736075821889,0.97046946572592
"14059","AC139019.1",5.09054305116907,0.105864656006576,0.660928066584
104,0.16017576096249,0.872742622799958,0.97046946572592
"14060","AL162253.2",37.3353601767694,-0.0497658039752796,0.3080003898
12593,-0.161577081137983,0.871638906730832,0.97046946572592
"14061","LINC01410",3.84848128340722,0.117866494193502,0.7401673899436
98,0.159243025016905,0.873477407264872,0.97046946572592
"14062","HNRNPK",1690.80669811862,0.0207089259918053,0.126071963331824
,0.1642627388716,0.869524313284235,0.97046946572592
"14063","DAPK1",16.0802098746362,-0.100370102387368,0.614938154973286,
-0.163219832068687,0.870345351860396,0.97046946572592
"14064","SPIN1",270.456170278622,0.0269380662980287,0.168198705136796,
0.160156204984575,0.872758027352597,0.97046946572592
"14065","AL158152.1",71.2822706879937,-0.0484827936300484,0.3042343759
71773,-0.159360011422728,0.873385242499939,0.97046946572592
"14066","WHRN",10.502374436551,0.0790002254233145,0.498276470163951,0.
158546971718975,0.874025810612659,0.97046946572592
"14067","AL356481.1",11.3041928846259,0.0723932635182696,0.45490222173

5014,0.159140272479125,0.873558359655194,0.97046946572592
"14068","TIMM23",68.5983824673779,0.0423785605347517,0.261339562338474
,0.162158994051827,0.871180650351918,0.97046946572592
"14069","MAPK8",92.8183624421468,-0.0351799992014032,0.218822981297726
,-0.160769216253104,0.872275171672655,0.97046946572592
"14070","COMMD9",207.71510940626,0.0473309683072666,0.291817047409363,
0.162193979849541,0.871153100415151,0.97046946572592
"14071","SLC43A3",538.497209502922,0.0418524284580025,0.26405762759852
4,0.158497328172755,0.874064925862316,0.97046946572592
"14072","NRXN2",48.3696026932229,-0.0861107942095822,0.541661584815673
,-0.158975265412048,0.873688361320845,0.97046946572592
"14073","RNF169",176.870124760176,-0.0392978269628834,0.24154872763879
9,-0.162691094865329,0.870761658841247,0.97046946572592
"14074","FDX1",262.186609952869,-0.0343539895870035,0.219015635984193,
-0.156856333259617,0.875358074771385,0.97046946572592
"14075","TTC12",27.7815202172909,0.0498834266408325,0.307943818373665,
0.161988725424918,0.871314732415377,0.97046946572592
"14076","SNX19",78.8389567559616,-0.0392407119773538,0.244536245134801
,-0.160469921159222,0.872510913992524,0.97046946572592
"14077","EPS8",707.837915935157,-0.0305609356054059,0.192338958488289,
-0.158891031986464,0.87375472637301,0.97046946572592
"14078","AC009318.1",13.7881548092627,-0.0761135130511831,0.4870832732
58403,-0.156263861294215,0.875825040868183,0.97046946572592
"14079","ARF3",306.027030305075,-0.0254876548924834,0.155151172089909,
-0.164276263911905,0.869513666485937,0.97046946572592
"14080","PCBP2-
OT1",6.31172657698974,0.112914678928198,0.706667702361915,0.1597846888
30125,0.873050685652758,0.97046946572592
"14081","AC023794.3",5.92130245634325,0.0884714204144355,0.56110019736
7284,0.15767490517656,0.874712975870401,0.97046946572592
"14082","SLC26A10",3.87280274832602,-0.14455501137538,0.89412915771799
8,-0.161671286667705,0.871564716955614,0.97046946572592
"14083","YEATS4",114.225302476414,-0.0453451148377935,0.27635587255545
3,-0.164082327683102,0.869666333720115,0.97046946572592
"14084","PXN-
AS1",41.2337468323889,-0.0480525177677754,0.299996326315954,-0.1601770
20691803,0.872741630492938,0.97046946572592
"14085","AC156455.1",9.36842050812849,-0.0890438980304139,0.5439669818
48558,-0.163693571488139,0.869972378493708,0.97046946572592
"14086","AL354696.2",4.40723419626689,-0.117060655953565,0.72723378204
0532,-0.16096702167095,0.872119374781485,0.97046946572592
"14087","SYNJ2BP",167.84339673905,-0.0267354333720907,0.17062408152733
7,-0.156692028069949,0.875487570148879,0.97046946572592
"14088","IFI27L2",1165.40501736476,0.0421859468001525,0.27021251538894
4,0.15612136521297,0.875937357851233,0.97046946572592
"14089","LINC00926",15.1208785822157,0.0651664524885009,0.413831881754
348,0.157470836254187,0.874873790419548,0.97046946572592
"14090","ARIH1",192.164872974565,-0.0381498256462219,0.231026039896362
,-0.165132145550934,0.868839971830658,0.97046946572592
"14091","MESP1",21.3911708391812,0.0695142734442641,0.429821828302075,

0.161728113527566,0.87151996458806,0.97046946572592
"14092","CIITA",13.4477013527854,-0.0815893886846791,0.505289186161539
,-0.161470680392901,0.87172270197422,0.97046946572592
"14093","AC007336.1",5.47605742715014,0.134487409294971,0.850995978394
941,0.158035305347303,0.874428978629083,0.97046946572592
"14094","CDK10",96.1215535262307,-0.029937950284433,0.191902905757031,
-0.15600571636126,0.876028515388822,0.97046946572592
"14095","CALCOCO2",555.555126618603,-0.0260913681857727,0.163414700594
077,-0.159663531438238,0.873146129988985,0.97046946572592
"14096","UBE2Z",234.891511776897,0.0281135438717061,0.178137463201571,
0.157819379295271,0.8745991275604,0.97046946572592
"14097","PYCR1",56.4102809486587,-0.0659236331378257,0.411713508589667
,-0.160120160651635,0.872786420169292,0.97046946572592
"14098","AC124283.1",10.1577553890409,-0.0847921343071211,0.5199355857
271,-0.163081998298971,0.870453873318919,0.97046946572592
"14099","AP005482.3",3.84321295845766,0.125661031538681,0.787289952156
645,0.159612136792111,0.873186617782512,0.97046946572592
"14100","ZNF396",20.6779946628079,0.0525247045010946,0.334546961253969
,0.157002485702511,0.875242889091601,0.97046946572592
"14101","GAMT",184.448395630795,0.0514591374648987,0.312205730403908,0
.164824448924511,0.869082159772192,0.97046946572592
"14102","PSPN",7.12454488138309,-0.0927278203363874,0.567436605121454,
-0.163415295205603,0.870191460925557,0.97046946572592
"14103","CERS4",139.428018302631,0.0424330203786246,0.262435283210839,
0.161689464387051,0.871550401568112,0.97046946572592
"14104","ILF3-
DT",248.048713275522,-0.0346328540990712,0.212965698972944,-0.16262174
7380413,0.870816262995827,0.97046946572592
"14105","AC005253.1",11.2134277164625,0.103054609412983,0.645277510541
074,0.159705874960016,0.873112772760808,0.97046946572592
"14106","ZNF793",15.2640374211886,-0.108276344918075,0.664132547597604
,-0.163034239640487,0.87049147598868,0.97046946572592
"14107","C19orf47",14.4611884180011,-0.0636265388373032,0.404070904878
451,-0.157463796747362,0.874879337927359,0.97046946572592
"14108","TEAD2",77.3592873332887,-0.0391302016159032,0.24144635420037,
-0.16206582097914,0.871254021236635,0.97046946572592
"14109","DKKL1",2.85750980013904,0.139554312776921,0.851932524760495,0
.163809114831194,0.869881416013169,0.97046946572592
"14110","PTOV1-
AS1",6.59601409816458,-0.0891197369085335,0.571344158795019,-0.1559825
81665821,0.876046750975882,0.97046946572592
"14111","ZNF274",31.9508013285848,-0.0528459836508122,0.32241895382195
1,-0.163904705428686,0.869806162802748,0.97046946572592
"14112","NANP",30.8522997934375,0.0510012348700799,0.30871296369038,0.
165206003208958,0.868781840316092,0.97046946572592
"14113","NEURL2",23.6060826704764,0.0549970720863575,0.344065794779293
,0.159844637045761,0.873003460842252,0.97046946572592
"14114","PIGP",340.369365660918,0.045555253653372,0.291212031512725,0.
156433281333644,0.875691505378872,0.97046946572592
"14115","ADORA2A",5.1171525276413,-0.107267880926263,0.665860725086405

,-0.161096573029358,0.872017339319505,0.97046946572592
"14116","GTPBP1",56.5598454014258,0.0377184139357807,0.23289798849763,
0.161952510535163,0.871343251165221,0.97046946572592
"14117","CBX7",183.201979258365,0.0390080075327381,0.24342456293679,0.
160246801153207,0.872686663725898,0.97046946572592
"14118","Z98885.3",5.19680224966636,0.138691253195352,0.84273183753690
1,0.16457341115854,0.869279760637792,0.97046946572592
"14119","OTUD5",143.097124699001,0.0268803666358584,0.169109603517718,
0.158952336689986,0.873706426102276,0.97046946572592
"14120","AMER1",13.8080367329984,0.0658712211282088,0.418544195965323,
0.15738175744209,0.874943989740268,0.97046946572592
"14121","CLIC2",180.787097317915,-0.0313858625520395,0.200513877640241
,-0.156527133789471,0.875617533163883,0.97046946572592
"14122","ZNF16",23.0930873631955,0.0553116091016637,0.354893937382685,
0.155853913734291,0.876148172801217,0.970513090718697
"14123","RARS2",231.332552082968,0.0331322441736699,0.213446368762649,
0.155225147964512,0.876643823360426,0.970832204364576
"14124","AP002807.1",9.47449882095108,-0.0855622657007578,0.5507962021
99887,-0.155342875203244,0.87655101632058,0.970832204364576
"14125","TOP1",526.08075381057,0.0297158666506949,0.191598062351617,0.
155094818214607,0.876746567222295,0.970832204364576
"14126","CSNK1E",370.847286476848,0.0272494542376003,0.175626444992233
,0.155155758227672,0.876698525650861,0.970832204364576
"14127","FLNA",966.020197264143,0.0354497458378107,0.228498319963128,0
.155142260317411,0.876709166581198,0.970832204364576
"14128","CD48",28.5365509688464,-0.0612183509194479,0.395067353942408,
-0.154956744232459,0.87685541842036,0.970883556056283
"14129","COG8",68.0172374044602,0.0393217872283831,0.253887900344116,0
.154878539603845,0.876917072397828,0.970883556056283
"14130","SNHG5",1403.98221152689,0.0629832206885021,0.407202468136472,
0.15467298363082,0.877079129579966,0.970909879870651
"14131","AC068473.5",13.2294705923487,-0.0797316843074908,0.5153333205
86658,-0.154718666778084,0.877043113240514,0.970909879870651
"14132","DOCK6",33.4043996856365,0.0489473017253553,0.31658110167376,0
.154612203528801,0.877127048669184,0.970909879870651
"14133","RPL38",6459.98255226524,-0.0407145494983254,0.264045632413267
,-0.15419512576751,0.877455885304016,0.971136437937649
"14134","ZNF675",58.630431127424,-0.0385070042283283,0.249664531882141
,-0.154234980587897,0.87742446165172,0.971136437937649
"14135","TMEM69",69.2058017741835,0.038999428262065,0.25565037301143,
0.15255187139689,0.878751681682678,0.971196308927662
"14136","PIAS3",71.482159180048,0.0379226518455616,0.247966480668453,0
.152934589156293,0.878449858550401,0.971196308927662
"14137","TCF7L1",472.46195105624,0.0288839465717321,0.188607644237402,
0.153143032396798,0.878285481153757,0.971196308927662
"14138","SESTD1",991.032571694881,-0.0386120949333765,0.25177849837304
1,-0.153357396214858,0.878116440289285,0.971196308927662
"14139","SCOC-
AS1",12.7238168015088,0.0736104692379361,0.482300237215006,0.152623746
699758,0.878694997237345,0.971196308927662

"14140","HIST1H4J",8.26869050889056,-0.101374891993977,0.664332273743245,-0.152596668866877,0.878716352092635,0.971196308927662
"14141","KATNA1",176.586648741921,-0.0268614280643702,0.175879048941772,-0.152726707507175,0.878613798297601,0.971196308927662
"14142","AC092849.3",3.03767188737457,0.124295191372895,0.809326028429686,0.15357864075379,0.877941979322634,0.971196308927662
"14143","LINC00689",2.60114632574262,0.157346158194289,1.02962107317408,0.152819481160411,0.878540634441624,0.971196308927662
"14144","PLAAT4",624.086197752146,0.041101835841491,0.269120668049514,0.1527264187451,0.878614026024939,0.971196308927662
"14145","CRACR2A",21.2861349419818,0.0591130808902533,0.385741318930155,0.153245395266968,0.878204760177994,0.971196308927662
"14146","TSPAN31",301.189348890258,0.034910492666042,0.227712253742698,0.153309679616491,0.878154067684147,0.971196308927662
"14147","AC025165.5",2.61656054427146,-0.145965579448175,0.948698601136871,-0.153858748472125,0.877721110676561,0.971196308927662
"14148","MICU2",175.872550246777,-0.0274753537902005,0.178739486115348,-0.153717314440915,0.877832632094889,0.971196308927662
"14149","RASL11A",87.952220990761,0.0652515509729386,0.425179328781474,0.153468305150074,0.878028983017065,0.971196308927662
"14150","AC040169.1",10.6828311832043,0.0768680618459453,0.503475281560466,0.152674946836916,0.878654618637145,0.971196308927662
"14151","P4HB",1453.54344982819,0.0346994904331958,0.227449365215789,0.152559187845062,0.878745911538657,0.971196308927662
"14152","KCTD1",58.0341715750383,0.0588127639148438,0.383231433686071,0.153465396481597,0.878031276629411,0.971196308927662
"14153","RNF125",31.3823270164082,-0.061239242374813,0.397656294188466,-0.154000435224569,0.877609392419603,0.971196308927662
"14154","SCAF1",125.443801256422,-0.0278621220319091,0.181152718467002,-0.153804603473198,0.877763803983776,0.971196308927662
"14155","GEMIN4",60.6113372358327,-0.0368757186435266,0.24192315307483,-0.152427405871816,0.878849842867662,0.971236177462299
"14156","AC025774.1",3.4496619162434,0.126289177740213,0.831247694410725,0.151927251755856,0.879244313995277,0.97146622431333
"14157","SFXN1",131.072689826994,-0.0332236053288591,0.218596506539709,-0.151985984839258,0.879197989706935,0.97146622431333
"14158","AC048341.2",15.3896481837072,-0.0633269524106445,0.416630855462178,-0.151997749519523,0.879188710651761,0.97146622431333
"14159","POLR3H",95.9616248804227,-0.0347639102036275,0.229422463149807,-0.151527926805177,0.879559282770726,0.971745593642381
"14160","YPEL5",363.829990135479,0.0309580814600577,0.204445918531263,0.151424306645299,0.879641016604994,0.97176726149378
"14161","CARS2",169.710715368177,-0.0316406586202387,0.20914070996848,-0.151288855359663,0.879747860228121,0.971783399883791
"14162","FXYD7",6.68466058071381,-0.101867426874092,0.673511329211132,-0.151248275204824,0.87977987017543,0.971783399883791
"14163","AKT3",271.588332172479,-0.0431844116041363,0.286347525892783,-0.150811191643772,0.880124657591369,0.971963941919708
"14164","NOTCH1",16.440138118945,0.0606037643781188,0.401782856988372,0.150837108462975,0.880104212826906,0.971963941919708

"14165","LINC01551",3.39831937064445,0.187928856949014,1.2461731439822
8,0.150804772078836,0.880129721747278,0.971963941919708
"14166","AGBL5",25.0669689117069,-0.0524586130251785,0.349831305703211
,-0.149954026897991,0.880800886261604,0.972046691961599
"14167","CERS6",19.1468617005846,-0.060407546576498,0.402407959001689,
-0.15011518839329,0.880673737164199,0.972046691961599
"14168","SHOX2",316.00851681656,-0.0303563778608197,0.202480959341758,
-0.149922135688732,0.880826047341026,0.972046691961599
"14169","FYTTD1",217.825487154623,-0.0275126891870073,0.18315359262583
4,-0.150216486570445,0.880593819079076,0.972046691961599
"14170","BYSL",29.4785155239889,-0.0477641733533466,0.318378466378957,
-0.150023253446086,0.880746269283805,0.972046691961599
"14171","RPL7",8840.98121772582,-0.0315996881802404,0.20989521098287,-
0.15054982928038,0.880330840595992,0.972046691961599
"14172","SLA",17.1985681507608,0.0844712274442013,0.561852608915124,0.
150344104670629,0.880493137910716,0.972046691961599
"14173","CMTM3",526.555389159884,0.0471508748744968,0.313087354607466,
0.150599742150596,0.880291464806251,0.972046691961599
"14174","CES4A",19.0179803038832,0.0565816447170274,0.37668292693268,0
.150210271481562,0.880598722370231,0.972046691961599
"14175","NARF",142.104173120917,-0.0268915055254395,0.178826663537826,
-0.150377494012527,0.88046679652756,0.972046691961599
"14176","AICDA",5.46894333949774,-0.0890540332406113,0.59470996932467,
-0.14974363611516,0.880966879653792,0.97211439538416
"14177","SPATS2",161.43754554966,0.0277202954092148,0.185188583322596,
0.14968684846477,0.881011684674375,0.97211439538416
"14178","MBNL1",996.009500577454,-0.0318702023753519,0.213536685805617
,-0.149249306999002,0.881356914296368,0.972426732285095
"14179","DNAJC11",56.5181231955209,0.0346947095060674,0.23265416692764
6,0.149125674232421,0.881454467294873,0.972431122348056
"14180","KRTCAP3",3.25956152517211,-0.118142949567779,0.79536007374094
5,-0.148540206465354,0.881916457708373,0.972431122348056
"14181","PTPN4",74.4932321763709,0.0297191223143746,0.199596913327599,
0.148895700934997,0.881635933557706,0.972431122348056
"14182","AC026726.1",2.63512540163787,0.172287073478446,1.161779079441
49,0.148295899390158,0.882109251392825,0.972431122348056
"14183","HIST1H2AG",2.6039124801526,-0.161815517269228,1.0917857353921
3,-0.148211789203409,0.882175628135364,0.972431122348056
"14184","PNRC1",2705.66788460402,-0.0371399711834455,0.250839627011819
,-0.148062615248967,0.882293352904555,0.972431122348056
"14185","PREP",56.7733822551787,0.0327389874426026,0.220608142286319,0
.148403350408127,0.882024456107963,0.972431122348056
"14186","OTUD6B-
AS1",391.839388433745,-0.0296385004952409,0.200100333165688,-0.1481181
96638381,0.882249489003362,0.972431122348056
"14187","AC023794.2",17.5324747263643,0.0787293667859795,0.52870311754
2229,0.148910350958336,0.881624373399829,0.972431122348056
"14188","MIR193BHG",117.640571782797,-0.035716189927499,0.240136399877
554,-0.148732928226253,0.881764377247808,0.972431122348056
"14189","ADCY7",89.2073170137605,0.0350524710333711,0.236598997625522,

0.14815139280028,0.88222329132087,0.972431122348056
"14190","PLLP",4.63508796371089,-0.11359179487253,0.765580956983703,-0
.148373328563537,0.882048147798285,0.972431122348056
"14191","TTC19",167.961720121552,-0.027169659649019,0.183159504131439,
-0.148338792343102,0.882075402131567,0.972431122348056
"14192","USP18",70.3120517066389,-0.0632795589268676,0.425434956020141
,-0.148740854580534,0.881758122501979,0.972431122348056
"14193","SEPTIN6",141.85878881567,-0.0292981670961618,0.19726289531821
5,-0.148523456724588,0.881929675457753,0.972431122348056
"14194","PACS2",158.186015545928,0.0291345617497167,0.196969340291702,
0.14791419673016,0.882410484081246,0.972491700893542
"14195","ADAMTS10",64.0052261317741,-0.0451734437014302,0.305640653685
349,-0.147799198688848,0.882501241747068,0.972523207090481
"14196","AL021155.5",43.5622095127554,0.0663495989297345,0.45025868057
129,0.147358844576966,0.882848788104436,0.972545252885374
"14197","C1orf53",10.5564101941157,0.104800035519769,0.714524184951859
,0.146671082276705,0.88339164471318,0.972545252885374
"14198","ASXL2",120.590773370032,0.0300964776323357,0.205002100213448,
0.146810581945254,0.883281532007766,0.972545252885374
"14199","TTL",77.0725131329605,-0.0387733318341989,0.263125262816467,-
0.147356933420885,0.882850296520009,0.972545252885374
"14200","CCDC80",17136.5588574278,-0.0367388840159508,0.24964696762073
4,-0.147163349773849,0.883003088222878,0.972545252885374
"14201","CBWD6",6.55904949244047,0.0851131564640437,0.579525542116949,
0.14686696319395,0.883237028657734,0.972545252885374
"14202","TP53I11",169.635443431549,-0.0292315431501701,0.1982374151901
23,-0.147457245253804,0.882771124109559,0.972545252885374
"14203","NAA40",25.1450084063621,0.052398963647331,0.355882317327354,0
.1472367720904,0.882945136943443,0.972545252885374
"14204","AC006064.2",3.3363197213664,0.116323216467541,0.7925843925619
61,0.146764455065203,0.883317941562064,0.972545252885374
"14205","AC079601.1",3.6774277735429,0.117144395296342,0.7982767319019
49,0.146746598785659,0.883332036212118,0.972545252885374
"14206","TMEM117",22.2951321201713,-0.0517549635407551,0.3526362777137
06,-0.146765851421484,0.883316839365759,0.972545252885374
"14207","AACS",60.8729974641378,-0.0396381575714285,0.269004924592529,
-0.147351048057837,0.882854941655203,0.972545252885374
"14208","ALPK3",8.86352257209288,-0.105823902970431,0.720624906244521,
-0.146850188015182,0.883250269752937,0.972545252885374
"14209","ZKSCAN2",12.3180184246355,-0.0616495884891025,0.4200245460813
54,-0.146776156451489,0.88330870522665,0.972545252885374
"14210","SAMD13",5.75186340919128,-0.111258297716101,0.759262390058608
,-0.146534714708459,0.883499287303405,0.972587128470559
"14211","KLHDC4",57.2862820047955,0.0371704427494512,0.25378315653052,
0.14646536538362,0.88355402945056,0.972587128470559
"14212","NIBAN1",669.661457652761,0.034983611222227,0.240463356161599,
0.145484167653041,0.884328615139386,0.972674559512791
"14213","RRP15",156.503842452317,-0.0384802506262201,0.264485309406873
,-0.145491069853803,0.884323165956679,0.972674559512791
"14214","SPOPL",122.934108898178,0.029880539691443,0.205455264120681,0

.145435746410915,0.884366843128707,0.972674559512791
"14215","SPCS3",286.802727857328,-0.031969394460615,0.219228437148993,
-0.145826859308803,0.884058071893789,0.972674559512791
"14216","TMEM60",170.966832832405,0.0326722390871582,0.22351150919432
,0.146177221819843,0.883781486749717,0.972674559512791
"14217","JMJD7",31.2939568921605,0.0471070904895595,0.323939131993327,
0.145419573731925,0.884379611324581,0.972674559512791
"14218","AC005332.5",18.4879164765462,-0.0609248171624597,0.4186576727
76938,-0.145524186284102,0.884297021113872,0.972674559512791
"14219","AC093462.1",3.58470131084612,-0.140014617748855,0.95866114579
1549,-0.146052250436464,0.883880140738643,0.972674559512791
"14220","ZNF763",15.4041085498068,0.0562717784279075,0.386126184965963
,0.145734168307875,0.884131246910313,0.972674559512791
"14221","PLEKHG2",30.6323990520713,-0.0560298443239008,0.3845104495939
3,-0.145717351460987,0.884144523095247,0.972674559512791
"14222","SRC",65.2029302473215,-0.0358601185341298,0.246079177246835,-
0.145725936405255,0.884137745643037,0.972674559512791
"14223","UPRT",39.7292042121957,-0.0415197921965804,0.284186568844215,
-0.14610047323996,0.883842072834876,0.972674559512791
"14224","BST1",276.611733134309,0.0465602922421691,0.320355937146168,0
.145339251886333,0.884443025200079,0.972675916985717
"14225","METTL21A",68.3395846330037,0.0302418976318884,0.2081945187295
58,0.145257895435625,0.884507256648117,0.972678173338945
"14226","TWNK",11.6787101249385,-0.064679578398264,0.445721797648599,-
0.145111993040234,0.884622449441158,0.972736466793761
"14227","TESK1",119.105877753624,-0.0384956711840365,0.265459456777204
,-0.145015256383748,0.884698826267063,0.972752072769781
"14228","FCMR",4.46915613769681,-0.125783469559538,0.868600503667646,-
0.144811647044205,0.884859586150911,0.972860451655798
"14229","PLCD4",7.62932967310604,-0.089822749632186,0.624055162525075,
-0.143933990176032,0.885552594860503,0.972938561694258
"14230","AC012510.1",6.63615723258922,-0.0774647584377633,0.5379759141
30932,-0.14399298630851,0.88550600803462,0.972938561694258
"14231","AL024507.2",3.45240961005965,-0.13843222819667,0.960537650841
011,-0.144119523139425,0.885406088424518,0.972938561694258
"14232","PTCD1",27.1729035239915,0.0451627118853486,0.313039967425729,
0.14427139210607,0.885286167739158,0.972938561694258
"14233","DSCC1",43.4912334396655,0.0398883680569358,0.277015108983459,
0.143993474591733,0.885505622459039,0.972938561694258
"14234","AKNA",80.269582629459,-0.0415504496310455,0.288574597447199,-
0.143985125505193,0.885512215365712,0.972938561694258
"14235","JRKL",90.3555872285423,0.0346819627954003,0.240948285047848,0
.143939446543531,0.885548286174296,0.972938561694258
"14236","HYLS1",22.8248657050818,-0.0547710569091774,0.379655040920505
,-0.144265322479007,0.885290960464102,0.972938561694258
"14237","TMEM91",185.152267042287,-0.0258544193215992,0.17943616200372
3,-0.14408700583477,0.885431765489247,0.972938561694258
"14238","AC010331.1",7.52864915393388,0.0824986720063348,0.57116289875
2883,0.144439830014289,0.885153166750776,0.972938561694258
"14239","MOB3C",50.7634688262252,-0.0332167770217983,0.231324084201896

,-0.143594114449437,0.885820988479893,0.973008330145622
"14240","AC110769.2",11.0452245864806,-0.0621529672893772,0.432543267229671,-0.143691907834911,0.885743761495772,0.973008330145622
"14241","CMTM7",56.5625891772393,-0.037332628034707,0.260373639542249,-0.143380981655208,0.885989302217876,0.973008330145622
"14242","TNK2",23.0567672290613,-0.0574209897766893,0.400476721870213,-0.14338159159048,0.885988820536721,0.973008330145622
"14243","AP002840.2",6.88798813757673,0.0850932757338896,0.593364139680052,0.143408187390247,0.88596781720875,0.973008330145622
"14244","AL078587.2",8.07156201327406,0.0769266283169762,0.536104589656727,0.143491829395143,0.885901763674422,0.973008330145622
"14245","DHRS12",187.352787598631,-0.0346429268648197,0.241847561649867,-0.143242820512591,0.886098412616397,0.973037467178907
"14246","SARM1",21.5168653876391,-0.0504859222339071,0.352580292436701,-0.143189858641832,0.886140238920329,0.973037467178907
"14247","DYM",160.383549326257,-0.0282735305748458,0.197574710474932,-0.143102983711233,0.886208848536555,0.973044501835988
"14248","CYB5R1",226.835217411396,-0.0365176958441465,0.255980768736165,-0.142657966160672,0.886560315227819,0.973293775781372
"14249","LNX1",39.6755738485123,-0.043800277655193,0.307014141896909,-0.142665342334298,0.886554489479262,0.973293775781372
"14250","PRMT6",44.3739085995136,0.0402517671599446,0.282723482073124,0.142371503296404,0.88678657014785,0.973461969433627
"14251","LINC01963",8.80969478658456,-0.0754386963763015,0.530328858676018,-0.142248899229502,0.886883408464113,0.973461969433627
"14252","AL606970.4",9.91307365000221,-0.0868230386405695,0.610451290745617,-0.142227627260026,0.886900210213389,0.973461969433627
"14253","TNFRSF14",289.748072026746,0.0222958088635044,0.1571900147642,0.141839854757634,0.887206502760958,0.973549379020953
"14254","RHBDD1",56.7349219763775,0.0308723693746616,0.217740266019992,0.141785301997505,0.887249594079578,0.973549379020953
"14255","GUF1",61.1369417243934,-0.0339856813761173,0.239755675683325,-0.1417513111181,0.887276443701957,0.973549379020953
"14256","ANKRD46",78.3231066829954,0.040630844007067,0.286672026691242,0.141732852263357,0.887291024528653,0.973549379020953
"14257","CDR2",83.8908107890512,0.033883606094199,0.238675039684317,0.141965436096775,0.887107307113629,0.973549379020953
"14258","HERC5",40.0540057373671,0.044692035762159,0.315936393825618,0.141458966537508,0.887507373974213,0.973581896990085
"14259","C8orf76",106.710629875625,0.0396001252933372,0.279883133070963,0.141488073464208,0.887484381258775,0.973581896990085
"14260","PREX1",8.46274269039446,0.0880875810272764,0.622200475223908,0.141574274747342,0.887416288003039,0.973581896990085
"14261","TANK",395.025305681152,0.0214388474481561,0.151761831517946,0.141266399026167,0.887659493070508,0.973612217788666
"14262","CENPU",7.07001641727036,0.0885384381695685,0.626477253271616,0.141327458750017,0.887611258369457,0.973612217788666
"14263","BX284668.5",129.749149006888,0.0410393523179952,0.291618036017625,0.14072981520085,0.888083390365511,0.973629816114726
"14264","NEXN",553.502592277578,0.0455309688360325,0.323408198794137,0

.140784831695052,0.888039926262902,0.973629816114726
"14265","KLHL20",132.962413276457,-0.0219694235264977,0.156165533423462,-0.14068036041554,0.888122460896921,0.973629816114726
"14266","SFT2D3",72.4664245487525,0.0363587085793533,0.257963936232796,0.14094492862189,0.887913448486309,0.973629816114726
"14267","PIGY-DT",2.69733937133275,-0.152199955330516,1.08026899339355,-0.140890793183276,0.887956215570871,0.973629816114726
"14268","ZNF827",113.493164722546,-0.0411079622507161,0.29137296713668,-0.141083651838685,0.887803858409162,0.973629816114726
"14269","PGM3",190.45321464766,0.0251522438919751,0.178872098333239,0.140615803841673,0.888173462632304,0.973629816114726
"14270","TGS1",211.46545058225,-0.0236933134319385,0.168412395870288,-0.140686279709405,0.88811778449071,0.973629816114726
"14271","AHCYL1",619.094715854141,0.0202096759544656,0.144148277810049,0.140200606358244,0.88850149283286,0.973651334929063
"14272","IFT122",85.6490698312579,0.0317535346049184,0.226701751708061,0.140067442645126,0.888606703971066,0.973651334929063
"14273","GNB2",804.114439759077,-0.0280910986976629,0.200319319632421,-0.140231599973528,0.888477005410952,0.973651334929063
"14274","OLFM1",126.863874638297,-0.0486933405317795,0.347907196992166,-0.139960716400115,0.888691028582571,0.973651334929063
"14275","INPP5E",47.0495449855632,-0.0397202471152641,0.283784680372454,-0.139966142862727,0.88868674109362,0.973651334929063
"14276","BAG3",160.26880478753,0.0328004839446201,0.234289365654043,0.139999883703873,0.888660082274163,0.973651334929063
"14277","PGRMC1",1075.98130115313,-0.0316723417494991,0.225518590372914,-0.140442265522883,0.888310565687933,0.973651334929063
"14278","AC240274.1",90.6007317872842,0.0482434412372054,0.344331760957153,0.140107439125282,0.888575102999815,0.973651334929063
"14279","EHBP1L1",107.2679992985,-0.027911533281837,0.199812004746355,-0.139688970726601,0.888905741046495,0.973818370137287
"14280","ZNF580",174.903862182337,-0.0344300996361036,0.246704767412483,-0.139559928238182,0.889007703319355,0.973861869959711
"14281","ZNF684",47.5447965284535,-0.0363170591177293,0.260639965878191,-0.139338028975579,0.88918304019784,0.97386566087527
"14282","PRUNE1",45.7651566724017,0.0360693065233555,0.260326212680185,0.138554262945727,0.889802387478908,0.97386566087527
"14283","USP21",29.6280200573801,0.0661412749070845,0.477361095966261,0.138556064719106,0.889800963604987,0.97386566087527
"14284","COA5",270.13631314978,0.0315862422790785,0.228210095738126,0.138408610613459,0.889917492229493,0.97386566087527
"14285","PRKAR2A",236.485082871711,-0.0257744782371269,0.185488137679533,-0.138954860184415,0.889485819260964,0.97386566087527
"14286","TBC1D14",73.0509117971288,0.029066704088844,0.209745475992402,0.138580839235345,0.889781385270074,0.97386566087527
"14287","IDI1",152.043305916301,-0.0258868711761174,0.187079276713387,-0.138373804041253,0.889944999182509,0.97386566087527
"14288","CHCHD1",313.139149895067,0.0374591498671332,0.270370561177621,0.13854744282801,0.889807777165356,0.97386566087527

"14289","LINC02422",7.40418859180581,0.0927943317724462,0.667006518174769,0.13912057715174,0.889354868128409,0.97386566087527
"14290","TFDP1",118.634508519135,0.0289491615817481,0.208098860758158,0.13911254235741,0.889361217230125,0.97386566087527
"14291","TMEM30B",32.7002479093349,0.0595183815771198,0.427877560268084,0.139101432521558,0.889369996244112,0.97386566087527
"14292","ACOT4",15.2172861891481,-0.0714490679737652,0.515334593218138,-0.138645976641279,0.889729910036791,0.97386566087527
"14293","LINC02193",7.18504595419116,-0.0762535515729362,0.550710353427342,-0.138463987644998,0.889873729118078,0.97386566087527
"14294","LINC00909",92.9052196023147,0.0342816174511954,0.246857494593747,0.138872095042577,0.88955122196962,0.97386566087527
"14295","DDTL",8.78567807889081,0.0921068055631006,0.665320493982024,0.138439754067743,0.889892880279232,0.97386566087527
"14296","ABT1",118.045174220813,-0.0333106124909697,0.240974539798306,-0.138232912567653,0.890056344310675,0.973919375633177
"14297","AC011921.1",4.81011216442385,0.108073660971447,0.782377888723608,0.138134861080701,0.89013383470399,0.973936040866932
"14298","RINT1",54.0229311664424,0.0378542440762085,0.274304497888762,0.138000814305128,0.890239773983291,0.973939724154135
"14299","PRKG1",106.408037734227,0.0377907903620772,0.2740562860526,0.137894265832764,0.890323982318233,0.973939724154135
"14300","CSKMT",116.479800492376,-0.0457487976288964,0.331617141908291,-0.137956673064712,0.890274659930268,0.973939724154135
"14301","SFPQ",771.668892607204,-0.0344542642783613,0.250332948119376,-0.137633757510542,0.890529874777672,0.974036663451863
"14302","RARS",171.746816215125,-0.0277616282060167,0.20190599541346,-0.13749779024227,0.890637339288134,0.974036663451863
"14303","AC007529.2",3.12345892650361,-0.132842345088098,0.966357985524538,-0.137467012305995,0.890661665538288,0.974036663451863
"14304","RSKR",8.79608978574599,0.0792072436236765,0.575749965742774,0.137572294114671,0.890578453380327,0.974036663451863
"14305","FOSB",4397.23147321089,-0.0857997853505544,0.625350900682792,-0.13720262536901,0.890870635792766,0.974197088829517
"14306","BAP1",87.3410578635511,-0.0264885209173768,0.193671875262875,-0.136770095716909,0.891212521636815,0.97429851673525
"14307","FIG4",54.4806118662772,-0.032924501406522,0.240635155432776,-0.136823322208711,0.891170448552195,0.97429851673525
"14308","SP4",38.541096954598,-0.0438732811684108,0.320736646536197,-0.13678911232072,0.891197489854214,0.97429851673525
"14309","PLA2G15",61.8324663648719,0.0437095544013678,0.319122523199239,0.136967939345599,0.891056136929134,0.97429851673525
"14310","PTAFR",4.10799596119287,0.11058998104738,0.810456295681386,0.136453972455606,0.891462408788898,0.974435501410435
"14311","SERF2",7426.84031870848,0.0423851403240936,0.310497179349678,0.136507328062907,0.891420231823028,0.974435501410435
"14312","UQCRB",2156.93214443068,-0.0352437147805856,0.258656891230918,-0.136256624027548,0.891618413015254,0.974537928647123
"14313","FANCG",44.2043192442086,-0.0415541543596914,0.305378834587577,-0.136074114028929,0.891762691174913,0.974627525888993

"14314","AL603839.3",2.85598407542046,-0.123717122656492,0.912803375560244,-0.135535347446058,0.892188618866692,0.974889572748799
"14315","ZBTB7B",40.3551103252074,-0.0416266811675348,0.307072846893388,-0.135559628891535,0.892169422239728,0.974889572748799
"14316","EFCAB2",86.8058887075996,-0.0259761801143234,0.191732885596652,-0.135481088877833,0.892231515284789,0.974889572748799
"14317","RAB11FIP5",57.9467518886086,-0.0306289087688992,0.226117861049024,-0.135455503721834,0.892251742827115,0.974889572748799
"14318","TMEM50A",1514.11995532312,-0.0318441907686118,0.235631761859793,-0.135143880932147,0.892498116428571,0.974918835546168
"14319","IL1RL2",2.84021958701723,0.112377481414488,0.832206471620243,0.135035577403884,0.892583745235963,0.974918835546168
"14320","AL136295.7",6.95161470567195,0.0887288442638826,0.657538876223344,0.134940833876634,0.892658654027963,0.974918835546168
"14321","AARSD1",148.018847266096,0.0338287116847326,0.250971560914183,0.134791016007985,0.892777109198929,0.974918835546168
"14322","CCDC102B",149.028132292405,0.0568836760103364,0.421774829993601,0.134867403090885,0.892716712599704,0.974918835546168
"14323","AC119403.1",6.64189444570021,-0.0764321188212202,0.566137687332023,-0.135006237054123,0.892606943026026,0.974918835546168
"14324","CACTIN",45.4213343871323,-0.0344478121011814,0.255493710325693,-0.134828415373783,0.892747538748554,0.974918835546168
"14325","B3GNT8",42.3404881338819,0.0376652081401557,0.279307192619991,0.134852267092888,0.892728680055395,0.974918835546168
"14326","PANK4",40.9983182315551,-0.0455817029121233,0.340393026263997,-0.13390903865572,0.893474502848669,0.974957643227667
"14327","MFSD14A",164.839436282047,0.0234243400780232,0.17453776219257,0.13420786300777,0.893238208383973,0.974957643227667
"14328","CTSS",294.067919786872,0.0340938552392974,0.253545150862931,0.134468575412546,0.893032058567606,0.974957643227667
"14329","SCCPDH",261.682815170584,0.0371651214831096,0.277602248054307,0.133879036440076,0.893498227533838,0.974957643227667
"14330","ANKMY1",20.7769377141329,-0.0523330293004873,0.390747982598313,-0.133930388974741,0.893457619833712,0.974957643227667
"14331","IQCB1",69.3915186590382,-0.0291163429205145,0.217471096349695,-0.13388603547432,0.89349269293794,0.974957643227667
"14332","SLCO4C1",4.42123641781491,-0.134468041946766,0.998773363249856,-0.13463318796291,0.892901900292677,0.974957643227667
"14333","LIN7C",166.513981993141,-0.0249725636967075,0.186511852856692,-0.133892636388613,0.893487473166379,0.974957643227667
"14334","RAD51",2.75871224278707,0.123185947275477,0.916492275554001,0.134410240611154,0.893078184278827,0.974957643227667
"14335","TRAPPC1",535.428895278822,-0.0401084179643822,0.298692965029413,-0.134279754330446,0.893181361948453,0.974957643227667
"14336","NELFCD",192.183397724406,0.0259256390940347,0.193384533144917,0.134062629892985,0.893353049853787,0.974957643227667
"14337","LAMTOR2",470.636827626131,-0.0369399527023441,0.276487583672682,-0.133604381837541,0.893715419052625,0.975091170824509
"14338","JKAMP",228.241108806153,0.0303355235319021,0.227119016932814,0.133566638063056,0.893745266718776,0.975091170824509

"14339","AL627309.5",6.90917023258906,0.0863164108190438,0.64800837703
5208,0.133202615703769,0.894033142250426,0.975198508606676
"14340","NAGK",278.584163624022,0.022716289667103,0.170737445398037,0.
133048082183407,0.894155354404242,0.975198508606676
"14341","TGOLN2",845.965285893242,0.0184664583222277,0.138546942644882
,0.133286653387655,0.893966682474146,0.975198508606676
"14342","ANKRD16",75.0223226951889,0.0272890187761117,0.20506603939824
9,0.133074295754623,0.894134623339877,0.975198508606676
"14343","RAB27A",53.0836363429296,0.0417798719595618,0.313781841045557
,0.133149425793241,0.894075206973077,0.975198508606676
"14344","CFAP20",210.719327254382,-0.0299697927211261,0.22559487108134
,-0.132847846129978,0.894313713941929,0.975303222754713
"14345","SNX29",308.867431780893,-0.0341191768544862,0.257033491298449
,-0.132742144543605,0.894397311247748,0.975326395249113
"14346","RAD54L2",71.9349872587623,0.0346817876307472,0.26247144436958
3,0.132135469875771,0.894877140968972,0.975344300343036
"14347","MTRES1",117.153938203927,0.0274035150254012,0.207460929530271
,0.132090004067019,0.894913102232539,0.975344300343036
"14348","TPD52L1",15.5043935574876,-0.073441875051123,0.55520305640516
,-0.132279306109455,0.894763374863304,0.975344300343036
"14349","DPP7",843.194028654761,-0.0328700158537072,0.24796018929308,-
0.132561666239317,0.894540050699795,0.975344300343036
"14350","ZNF25",101.610342455065,0.0276434121204996,0.208911380399961,
0.132321236246567,0.894730210967859,0.975344300343036
"14351","LINC00839",3.41258297350908,0.140122593704895,1.0583193381901
,0.132401051977872,0.894667082644717,0.975344300343036
"14352","SIRT1",107.816714837256,0.0311961904697121,0.236313479559431,
0.132011895926853,0.894974882511279,0.975344300343036
"14353","AC023908.3",2.86188287261456,-0.125072894014102,0.94707513911
165,-0.132062271354118,0.894935037577956,0.975344300343036
"14354","C17orf53",3.05248882212631,-0.103736756378508,0.7822536416108
25,-0.132612685784233,0.894499699232406,0.975344300343036
"14355","HAND2-
AS1",122.106839703131,0.0410915782842779,0.311608472642754,0.131869258
675093,0.895087704267522,0.975399300442832
"14356","BDH2",852.072767036803,0.0296701033536072,0.22547273892191,0.
131590645926748,0.895308084651831,0.975535512602754
"14357","CCDC15-
DT",2.83342364187782,-0.1108885746676,0.843421304943499,-0.13147471378
498,0.895399788400584,0.975535512602754
"14358","AC005828.5",6.19344919334889,0.101283023500117,0.770310302524
888,0.131483407619159,0.895392911422421,0.975535512602754
"14359","TMEM14A",222.487033944885,0.0281203160598957,0.21407151009424
3,0.131359451089573,0.89549096399473,0.975566902275198
"14360","UBXN10-
AS1",3.91033549271111,-0.124097195042129,0.951519760896553,-0.13041998
7205731,0.896234154140442,0.975576101069115
"14361","DDX1",438.73319132408,0.0296878119703105,0.227197449668734,0.
13066965326238,0.896036639655866,0.975576101069115
"14362","UGP2",837.541387486119,0.0169557173320925,0.130024752024167,0

.130403765960969,0.896246987228072,0.975576101069115
"14363","AC016575.1",5.12538095198484,-0.0878632199090236,0.6722115127
1391,-0.130707698763288,0.896006541866743,0.975576101069115
"14364","ERI1",47.5565343390883,-0.0307388998050496,0.235872031148932,
-0.130320240408837,0.896313067095285,0.975576101069115
"14365","ZHX1",271.930324735224,0.0188205558349288,0.143474358919781,0
.13117713838646,0.895635180593143,0.975576101069115
"14366","IL18BP",15.4192415311225,-0.0627260287063444,0.47952399010057
3,-0.130808948042805,0.895926444308684,0.975576101069115
"14367","POGLUT2",59.5346882858546,0.0308482073345766,0.23648978936763
9,0.130442026343138,0.896216718396137,0.975576101069115
"14368","APBA2",7.66038369440386,0.0727291572739458,0.558110612887665,
0.130313159424876,0.896318669132398,0.975576101069115
"14369","ERCC4",88.316992897198,0.028750494891714,0.220230153520791,0.
130547495118555,0.896133279953413,0.975576101069115
"14370","ST3GAL2",85.8923612186182,0.0304838449774599,0.23404985099221
6,0.130245094573779,0.896372518101795,0.975576101069115
"14371","PCTP",53.2872316404107,0.0307615498380359,0.235292847452336,0
.130737292574384,0.895983130311442,0.975576101069115
"14372","CDK5RAP1",65.3591139230388,-0.0281706436589566,0.215621062556
898,-0.130648849073003,0.896053097909733,0.975576101069115
"14373","GSS",86.7720099306628,-0.0347418008442107,0.26588028796634,-0
.130667079947683,0.896038675410077,0.975576101069115
"14374","DTWD1",549.069698075396,0.0206804961429751,0.159388862212234,
0.129748690441356,0.896765258731746,0.97593564368587
"14375","LINC01807",3.0770423961658,-0.120346084199695,0.9287262263667
72,-0.129581873304574,0.89689724532177,0.975943694466515
"14376","BHLHE40-
AS1",7.41420124513409,0.0771873910532046,0.59566612772735,0.1295816355
17363,0.896897433462291,0.975943694466515
"14377","RFESD",16.3421380379686,-0.0642556050471324,0.496477895238713
,-0.129422892062974,0.897023034765882,0.975944591239581
"14378","AL135999.1",11.8930844054694,0.0686012990049194,0.52976400813
7427,0.12949407274026,0.896966714733154,0.975944591239581
"14379","PHC2",226.84493056879,-0.0254023839127483,0.196642134054539,-
0.129180778244112,0.897214605499032,0.976012268308262
"14380","AL365436.2",4.13542986912031,-0.101119048255444,0.78326899610
6909,-0.129098749928872,0.897279511144247,0.976012268308262
"14381","SAMD9",134.338102170953,0.025541790712113,0.197963337774953,0
.129022833213437,0.897339581543363,0.976012268308262
"14382","AC011472.4",3.32603924839022,0.120615379839455,0.935365406060
829,0.128950011469219,0.89739720354649,0.976012268308262
"14383","NOP56",291.411268701066,-0.0204899234542278,0.158783705481743
,-0.129042985815593,0.897323635394958,0.976012268308262
"14384","NUP160",123.531168953271,0.026817626127448,0.208211467998743,
0.128799947405442,0.897515947148189,0.97607355125411
"14385","ZBTB8A",148.824178996664,0.022776753664655,0.178162734556747,
0.127842411721629,0.898273685720898,0.976171544725392
"14386","NOL10",95.9164537320238,-0.0264313129470067,0.206042257746801
,-0.128281029513312,0.897926577316002,0.976171544725392

"14387","SERINC5",71.69748144479,-0.0454324049683444,0.355733722199522
,-0.127714641972747,0.898374802368577,0.976171544725392
"14388","YIPF5",400.36523987831,0.0234156664833665,0.183728717313548,0
.127446959983973,0.898586650547487,0.976171544725392
"14389","STK10",37.2683008787365,-0.037871948558297,0.295635164025152,
-0.128103666839426,0.898066934241803,0.976171544725392
"14390","SYNE1",765.213949603873,0.0364484273310729,0.283708807846013,
0.12847125758202,0.89777604283875,0.976171544725392
"14391","SLC29A4",2.80241204009133,0.105295720325681,0.820428437588867
,0.128342358091744,0.897878045351527,0.976171544725392
"14392","TAF2",100.333363920215,-0.031752552891955,0.249107810701321,-
0.127465103573272,0.898572291167031,0.976171544725392
"14393","GLI4",88.1803636588625,0.0354505373306366,0.277681786885136,0
.127666051592,0.898413257102958,0.976171544725392
"14394","CLSTN3",110.050702780446,0.0501664041423447,0.393695423658138
,0.127424402539935,0.898604503231199,0.976171544725392
"14395","MDM1",68.8355985613384,0.0305487135360768,0.238253124378105,0
.128219571583021,0.897975212025239,0.976171544725392
"14396","AL049838.1",7.21051046450024,0.075297930402702,0.589801167874
643,0.127666634967915,0.898412795414157,0.976171544725392
"14397","PARN",93.9361709960382,0.02419541497157,0.189645372004593,0.1
27582417202272,0.898479446446106,0.976171544725392
"14398","MPDU1",121.695558197164,-0.0388789747011614,0.302901797682232
,-0.128355047737117,0.897868003532373,0.976171544725392
"14399","PSMD10",118.407172484044,-0.0264488978262703,0.20719944715129
2,-0.127649461376014,0.898426386759361,0.976171544725392
"14400","RAB33A",7.61298370412083,0.0853727883516842,0.668643485932079
,0.127680580380853,0.898401758902644,0.976171544725392
"14401","FAXC",4.73963296982,0.159286351607917,1.25105131192763,0.1273
21997178906,0.89868555075903,0.976191797133776
"14402","BCR",48.4272488730012,0.0398474163735462,0.313448372109912,0.
127125931793238,0.898840727368026,0.976292563409112
"14403","CRELD1",116.885693110058,-0.049579501248354,0.391379863762415
,-0.126678722741983,0.89919468693899,0.976321781001512
"14404","AL355312.3",11.1177781555132,0.0947119906775078,0.74666105310
0949,0.126847369745831,0.899061202887382,0.976321781001512
"14405","CDH23",19.2092969294121,-0.0568741277602364,0.448908045171229
,-0.126694382896485,0.899182291811357,0.976321781001512
"14406","CCPG1",762.444286442853,0.0162609805482914,0.128424676275941,
0.126618816724537,0.899242103219957,0.976321781001512
"14407","AC138028.4",2.68483869473719,0.161851924268879,1.275569604491
5,0.126885999555783,0.899030627806516,0.976321781001512
"14408","HEXD",122.414103535554,-0.024062875991158,0.189991682643718,-
0.126652260016466,0.899215632437029,0.976321781001512
"14409","AC026401.1",2.60997497057269,-0.131909828615404,1.04463378347
462,-0.126273753254132,0.899515231802678,0.976550542791956
"14410","TESK2",21.1943920158592,-0.0467402526298755,0.378043220804796
,-0.123637325198883,0.901602437971761,0.976552485249161
"14411","CDC73",196.929046872396,-0.0182214187329166,0.148246315835147
,-0.122913130287698,0.902175888004238,0.976552485249161

"14412","CRIM1-
DT",46.4221784866364,0.0484780751712214,0.399814530320102,0.1212514090
78126,0.903491905900973,0.976552485249161
"14413","THADA",108.256491745302,0.0313223933011229,0.25278694902812б,
0.123908269084089,0.90138790562549,0.976552485249161
"14414","THORLNC",10.4449438670765,0.0646757558558303,0.51540755598239
8,0.125484687030935,0.900139847191976,0.976552485249161
"14415","AC008063.1",8.42619499386605,-0.093206729243463,0.74143452112
6705,-0.125711342792379,0.899960422852559,0.976552485249161
"14416","CAB39",144.084853052063,-0.0242112734673656,0.196807055353481
,-0.12302035322809,0.902090980841287,0.976552485249161
"14417","WWTR1",182.098276175709,-0.0264551264040547,0.213101891010431
,-0.124143086101286,0.901201984222218,0.976552485249161
"14418","STX18",136.804002565379,0.02570150247064,0.204837347121213,0.
125472736450892,0.900149307604698,0.976552485249161
"14419","LRP2BP",3.94041417810457,0.085073712803845,0.690088619846565,
0.12327940261174,0.901885850741208,0.976552485249161
"14420","AC008522.1",4.06783127895098,0.1156550907214,0.92922612890705
6,0.124463881420803,0.900947996418007,0.976552485249161
"14421","EGFL8",50.533056989347,0.0357004577533176,0.294191781235809,0
.12135096909693,0.903413050774714,0.976552485249161
"14422","ILRUN",105.048132292342,0.0239952144169055,0.196697653222464,
0.121990344184569,0.902906665379496,0.976552485249161
"14423","COL28A1",2.7154332991422,0.112480832166281,0.90097414004395,0
.124843574490155,0.900647389773985,0.976552485249161
"14424","AC004908.2",14.2904405379403,0.0633751514138314,0.51594840866
3155,0.122832342051484,0.9022398629258,0.976552485249161
"14425","TCIM",28.0876497553625,-0.0865623537420217,0.714060234886918,
-0.12122556265261,0.903512377357995,0.976552485249161
"14426","INTS8",78.6168401453503,-0.0294341298602495,0.238826224182336
,-0.123244965920399,0.901913119303357,0.976552485249161
"14427","RPL12",17380.8307661919,-0.0345595925256415,0.282848683523097
,-0.12218403174155,0.902753272520732,0.976552485249161
"14428","ODF2",54.3691709414075,-0.0337980316751966,0.27136835274721,-
0.12454669578468,0.900882430279148,0.976552485249161
"14429","NUDT5",255.745442196069,-0.0275004338927412,0.226000666895528
,-0.121682976738531,0.903150095636367,0.976552485249161
"14430","SUV39H2",14.9634763957805,-0.049927669091036,0.41163053839049
1,-0.121292432010164,0.903459414143651,0.976552485249161
"14431","MINDY3",96.437651904286,0.0234306198990061,0.190398778630287,
0.123060767866076,0.902058977791528,0.976552485249161
"14432","SKIDA1",9.10491463048308,-0.0631329328074701,0.51337213116025
6,-0.122976938122421,0.902125360042008,0.976552485249161
"14433","MASTL",19.8214150043608,0.0478319561838293,0.380052246221489,
0.125856264919833,0.899845702848762,0.976552485249161
"14434","MGMT",655.556183905686,0.038406130774972,0.307723450288011,0.
124807292843708,0.900676113686334,0.976552485249161
"14435","PWWP2B",9.17382969319143,0.0754202582809609,0.606870813542898
,0.124277287023673,0.901095730200438,0.976552485249161
"14436","CD59",2516.77686734069,-0.0280051064373486,0.230669523070236,

-0.12140791754627,0.903367945976849,0.976552485249161
"14437","ARHGAP1",328.148926994201,-0.0222454097555989,0.179691475551855,-0.123797802245658,0.90147537198274,0.976552485249161
"14438","MRPL16",275.400188338738,-0.0280615904604896,0.22529105815336,-0.124557053841824,0.900874229601856,0.976552485249161
"14439","PCNX3",43.1408568753362,0.0307046037298862,0.253150658688535,0.121289843324736,0.903461464478391,0.976552485249161
"14440","FADD",84.0595125109103,-0.0316536582426947,0.255847502254678,-0.123720802289427,0.901536340359629,0.976552485249161
"14441","DCUN1D5",162.421624070728,-0.0291290972978945,0.238843606085043,-0.121958874157689,0.902931588734353,0.976552485249161
"14442","DYRK4",25.4351009826972,0.0784289148630546,0.643755164426526,0.121830346686106,0.903033379782725,0.976552485249161
"14443","LAG3",10.0628136994951,-0.0722970954362016,0.583108643263268,-0.123985635046675,0.901326648869351,0.976552485249161
"14444","AC092821.3",46.9715196003016,-0.0378524597550963,0.305038030769052,-0.124090952395883,0.901243261732496,0.976552485249161
"14445","AC084824.5",4.83562873722969,0.0807521442607245,0.643973729854153,0.125396643554098,0.900209545198338,0.976552485249161
"14446","SMARCC2",355.639061149807,-0.0215904159320246,0.175828287262376,-0.122792619254754,0.902271319010682,0.976552485249161
"14447","AL356966.1",6.80523326998152,-0.0893185187569367,0.722413562291726,-0.123639039214034,0.901601080798426,0.976552485249161
"14448","RNF31",47.1348597138094,-0.0320438044500727,0.261560311326865,-0.122510193872756,0.90249497326267,0.976552485249161
"14449","STRN3",196.22317257462,-0.0257124326803169,0.211737023359413,-0.121435695431835,0.903345945211516,0.976552485249161
"14450","AL049828.1",6.89863408591132,-0.0755497294218831,0.615059413163704,-0.122833221970013,0.902239166128867,0.976552485249161
"14451","DEGS2",7.59305164093336,0.0814648584848788,0.665693338222104,0.122375955725275,0.902601279922633,0.976552485249161
"14452","BMF",32.1517185749963,0.0445347118882096,0.35582354830171,0.125159540735195,0.900397247128535,0.976552485249161
"14453","LINC00921",32.0841869761259,-0.0414979984370297,0.331429921470674,-0.125208968016189,0.900358117669274,0.976552485249161
"14454","CMTM1",6.20939610576671,0.0721016508936247,0.591581412105935,0.121879507060498,0.902994445614092,0.976552485249161
"14455","TRAPPC2L",567.256892608778,-0.039490512117414,0.320535527074932,-0.123201669648875,0.901947403454362,0.976552485249161
"14456","KCTD11",37.0057269963061,-0.0365465690997922,0.299791338889816,-0.121906687615229,0.902972919183677,0.976552485249161
"14457","NAPG",126.540102438485,0.0219666656604102,0.180921973699896,0.121415133889968,0.903362230448859,0.976552485249161
"14458","AC005306.1",5.62635142506998,0.0766518883875569,0.610895071618912,0.125474720534943,0.900147736947402,0.976552485249161
"14459","NFIX",1283.46748711591,-0.0237203164656221,0.190655775065038,-0.124414361209517,0.900987203092299,0.976552485249161
"14460","SLC35E1",168.36980532288,0.0224736883952541,0.183220674774289,0.122659129069028,0.902377029675676,0.976552485249161
"14461","BISPR",10.9020296542042,0.0795915553688284,0.649387439061944,

0.122564051260062,0.902452322696389,0.976552485249161
"14462","CERS1",3.58546587199607,0.106054752142086,0.85299888618931,0.124331641997653,0.9010526949751,0.976552485249161
"14463","ZNF850",13.4264160886273,0.0514367461556871,0.41178953505302,0.124910280075632,0.900594579792645,0.976552485249161
"14464","CD3EAP",33.4821260272671,-0.0411614131782121,0.337415544691119,-0.121990269345452,0.902906724649808,0.976552485249161
"14465","SERINC3",794.652437454923,-0.0224728249505891,0.180769106605152,-0.1243178404354,0.90106362225187,0.976552485249161
"14466","ATP9A",101.713885450163,-0.0290130334659483,0.23330680646287,-0.124355709573204,0.901033639709092,0.976552485249161
"14467","LINC00266-1",4.64630616340367,-0.0896454182992038,0.722880880082493,-0.124011328517879,0.901306305444502,0.976552485249161
"14468","YWHAH",484.595765525484,0.0350618175342044,0.287492085772787,0.121957505160385,0.902932672942958,0.976552485249161
"14469","CTPS2",48.7244623117509,-0.0313030481847923,0.252354660520248,-0.124043867944656,0.901280541662419,0.976552485249161
"14470","AL592156.2",2.95167425182761,0.11166349909993,0.889584726764855,0.125523174735717,0.900109379347046,0.976552485249161
"14471","AC234772.2",3.72373122964034,0.105997382714393,0.863426467849145,0.122763647700586,0.902294261391024,0.976552485249161
"14472","ARAF",85.6201845491961,0.0289501436246515,0.234506408011907,0.123451396787339,0.90174965954558,0.976552485249161
"14473","TCEAL9",774.18661143932,0.0305927336766952,0.244641508924179,0.125051279364765,0.900482953859629,0.976552485249161
"14474","UBA1",309.006832587967,-0.0252923657659099,0.208827614951698,-0.121116001692401,0.90359915496254,0.976578802064323
"14475","CASP4",427.363111522567,-0.0223008245588728,0.18427447355063,-0.121019608029137,0.90367550438032,0.97659384559733
"14476","PGGT1B",136.9843257668,-0.0256860730685572,0.212431176945491,-0.120914799032292,0.903758520245038,0.976616090922432
"14477","MMGT1",119.601808515786,-0.0218636277998531,0.18130398303633,-0.120590995485587,0.904015001352432,0.9768257695763
"14478","OXCT1",82.386973227736,-0.0297936343735046,0.247529064251168,-0.120364186176024,0.904194660393583,0.976952415564084
"14479","AL050341.2",49.0809805120771,0.0346890430556808,0.288395620252157,0.120282835867447,0.9042591003834,0.976954562283136
"14480","FLVCR1",29.330355110708,0.03821208937213,0.318016337848469,0.120157629732651,0.90435828110453,0.976970306342143
"14481","ST7-
OT4",7.4605899219325,0.0662720325807693,0.551776063305988,0.120106755236351,0.904398581227423,0.976970306342143
"14482","CC2D1B",22.5928375560479,-0.0463877531441179,0.387791790243486,-0.119620255794977,0.904783973136053,0.977078651146922
"14483","LEPROT",2027.63954588683,-0.0195734880358763,0.164180571555517,-0.11921927089441,0.905101639567004,0.977078651146922
"14484","CDC7",5.7349003178835,0.0757154284842364,0.634784328510945,0.119277406645887,0.905055582584382,0.977078651146922
"14485","FAM20B",108.717868969049,0.0244146202647341,0.205393887786935,0.118867316490258,0.905380475797757,0.977078651146922

"14486","MFHAS1",61.8153819460374,-0.0348679483085847,0.29257324329008
,-0.119176818483069,0.905135271912779,0.977078651146922
"14487","LEPROTL1",159.994718474318,0.0225551352765591,0.1897622794240
29,0.118859951224338,0.905386311061827,0.977078651146922
"14488","TNFSF15",6.92712341990103,-0.217877032239823,1.81842268097765
,-0.119816495097105,0.904628514880151,0.977078651146922
"14489","FUT4",63.951287105712,-0.0305171847824994,0.255767732522511,-
0.119316007854171,0.905025001661669,0.977078651146922
"14490","HOXC-
AS2",30.8856370364427,-0.037613958802788,0.316722233583759,-0.11876008
3171871,0.905465433822286,0.977078651146922
"14491","AC020663.3",2.7324007824036,0.118448320386696,0.9915886687428
18,0.119453079810674,0.90491641068353,0.977078651146922
"14492","LGALS3BP",2823.71953835249,0.0331095784600367,0.2766114519802
57,0.119697063238003,0.904723126830744,0.977078651146922
"14493","AC144831.1",17.3424127913069,-0.0469344511667977,0.3937625168
59192,-0.119194817071888,0.905121012754683,0.977078651146922
"14494","CCDC9",37.9701924503868,-0.0418761219487628,0.351911410160358
,-0.118996203986909,0.905278363193289,0.977078651146922
"14495","TFPT",54.4476967904292,0.0369685302198615,0.310003008409491,0
.119252166001656,0.905075578982521,0.977078651146922
"14496","ZNF865",42.8346940216252,0.0310203316008328,0.261367318965265
,0.118684813861351,0.90552506828415,0.977078651146922
"14497","ZNF749",44.3426818073172,0.0472493004499826,0.397226042522293
,0.11894814385774,0.905316439201428,0.977078651146922
"14498","ITSN1",245.708902622834,0.0301071745048476,0.253769556289864,
0.11863982009906,0.905560716251875,0.977078651146922
"14499","C12orf81",46.2582858301221,0.0620489173349747,0.5235210909950
85,0.118522287644677,0.905653836571519,0.977111729463292
"14500","AC008549.2",4.14373571356508,0.0999430246258113,0.84590314876
8281,0.118149488828997,0.905949211607229,0.977326000367043
"14501","PYGM",22.5209396597779,-0.047231281832141,0.399879035397306,-
0.118113923589952,0.905977391249919,0.977326000367043
"14502","GDF15",30.3503837523492,-0.0637539701346772,0.541010155601514
,-0.117842464646146,0.906192482019957,0.977490621723775
"14503","REL",100.903293803428,-0.0250537897436118,0.212931134167232,-
0.117661467598886,0.906335899038637,0.977557683168176
"14504","CCNL1",1276.97208037908,0.0478071834873818,0.406764139090913,
0.117530477475785,0.906439693868874,0.977557683168176
"14505","AC087294.1",6.11793236366934,0.0793253943077595,0.67495230701
205,0.117527406727337,0.906442127108253,0.977557683168176
"14506","PI4KB",160.210191288252,0.0205449715385805,0.17860526769312,0
.11503004252865,0.908421309597569,0.977602816870856
"14507","UCHL5",131.126486377166,0.0235477273290674,0.203905747554394,
0.115483391770435,0.908061983845263,0.977602816870856
"14508","ZBTB18",11.8254475491158,0.0515314787798583,0.447487272446064
,0.115157417770065,0.908320349764013,0.977602816870856
"14509","AC104695.4",20.8563468938341,0.0604594471185019,0.52397102541
7295,0.115387004596965,0.908138378989875,0.977602816870856
"14510","REV1",202.613456783433,0.021258536432044,0.184172753375386,0.

115427152184201,0.908106558462538,0.977602816870856
"14511","AC017033.1",4.18953208526424,-0.0920417365411581,0.790409198068131,-0.116448210327158,0.907297328934413,0.977602816870856
"14512","HOXD9",10.3634528323201,0.0733585413489379,0.624884466382824,0.117395367136549,0.906546755177676,0.977602816870856
"14513","AC064836.4",12.7168559169448,-0.0831581837083202,0.709225036597778,-0.117252182899857,0.906660216088766,0.977602816870856
"14514","EIF4A2",1711.31588268433,-0.0252853833926776,0.216409024168293,-0.116840707035461,0.906986285106862,0.977602816870856
"14515","HOPX",10.6309907996558,-0.0675912398600409,0.577284914790869,-0.117084715238968,0.906792921893913,0.977602816870856
"14516","EPB41L4A-DT",8.45990109181384,0.070845247662291,0.606206175152084,0.116866588573628,0.906965775135778,0.977602816870856
"14517","HEIH",127.690131788139,-0.033435886341708,0.286918705743193,-0.116534355106268,0.90722906013152,0.977602816870856
"14518","DAGLB",32.993792180268,0.034264571986579,0.297386374306103,0.115219038083131,0.908271508962651,0.977602816870856
"14519","AC005082.1",4.91809847613443,-0.0913153634930655,0.787665872902847,-0.115931598199797,0.907706752791648,0.977602816870856
"14520","VPS37A",154.444084104863,-0.0186417525755158,0.16081654581079,-0.115919369375393,0.907716444639549,0.977602816870856
"14521","IMPAD1",273.845000445432,-0.0191154701340468,0.164511015014249,-0.116195685330803,0.907497456198825,0.977602816870856
"14522","TLR4",89.9806722178761,0.0429342382527911,0.369540301057093,0.11618283074938,0.907507643673109,0.977602816870856
"14523","EHMT1",188.344755025781,0.0219434137806093,0.190106784458278,0.115426778918693,0.908106854307914,0.977602816870856
"14524","MCAM",3.48887115598668,0.112924900266777,0.966843098034623,0.116797545016692,0.907020489230347,0.977602816870856
"14525","NECAP1",148.013591957833,-0.0203644410094037,0.176947592075735,-0.115087415265236,0.90837583478468,0.977602816870856
"14526","FOXO1",282.200329432276,0.028052697053165,0.241063880120452,0.116370387132025,0.907359003561095,0.977602816870856
"14527","AL079343.1",59.8754384767066,-0.0337422793526873,0.291651428401029,-0.11569385940497,0.907895173056865,0.977602816870856
"14528","AL359232.1",7.70378140298519,-0.0603607730535097,0.522460103007648,-0.115531832394532,0.908023590795572,0.977602816870856
"14529","DCAF4",32.1791373391622,0.0374461213327037,0.32359220427417,0.115720097202888,0.907874377993202,0.977602816870856
"14530","KNOP1",245.993918241463,0.0314876046738621,0.269613857027797,0.116787783168785,0.907028225116964,0.977602816870856
"14531","LINC02126",5.42512107593854,-0.091988758633555,0.793430662132925,-0.115937993102344,0.907701684572997,0.977602816870856
"14532","AC022706.1",9.60938499496295,0.069216708025131,0.59984032311502,0.115391889070883,0.908134507602756,0.977602816870856
"14533","AC142472.1",39.4785724429644,-0.0434650109241518,0.372894533844328,-0.116561137209636,0.907207835744781,0.977602816870856
"14534","ZNF20",10.9811791987927,-0.0534159768678181,0.461953528862098,-0.115630628473376,0.907945287706601,0.977602816870856

"14535","OSM",3.52132561482411,0.119456978514124,1.03243175395326,0.11
5704479309856,0.907886756123461,0.977602816870856
"14536","ITM2A",5374.8638736571,-0.0390190819251033,0.339072382054838,
-0.115075966047842,0.908384909646263,0.977602816870856
"14537","ZNF773",21.1915292333366,-0.0478558029476697,0.41686317309804
5,-0.11479978572349,0.908603818902319,0.977731962515579
"14538","QTRT2",49.764964495089,-0.02863910992969,0.249690344148243,-0
.114698507975489,0.908684096587272,0.977751088383181
"14539","FXN",55.60189574523,0.0297802437773691,0.259983799689256,0.11
4546536410976,0.908804558414094,0.977813447092075
"14540","ATP5IF1",1068.62110118729,0.0322774391889574,0.28228473720809
2,0.114343550799785,0.90896546032131,0.977852052527767
"14541","AC092718.4",3.63327542172291,-0.0880959434573512,0.7699275342
19704,-0.114421084507172,0.908904000738688,0.977852052527767
"14542","TAF13",63.7000713923812,0.0285816200313957,0.250606827156569,
0.114049646434968,0.909198437997101,0.977968174763711
"14543","MRM1",8.40533014770006,-0.0572372305590213,0.50182757909643,-
0.114057562683343,0.909192162693047,0.977968174763711
"14544","TANC1",34.6108448184422,0.0487701853022643,0.429296945723338,
0.113604780532714,0.909551097572313,0.977983950851537
"14545","ERGIC1",520.425445726491,0.0159346807744456,0.140358878616657
,0.11352812826302,0.909611864105314,0.977983950851537
"14546","ZNF883",5.42034301103838,-0.067829056928519,0.596490797361915
,-0.113713501077477,0.909464909635196,0.977983950851537
"14547","TPH1",8.34380638053351,0.061222433461127,0.539259289439011,0.
113530605146953,0.909609900532888,0.977983950851537
"14548","PACSIN3",29.2488011816093,-0.0410598759315232,0.3618276710791
58,-0.113479092986618,0.909650737383485,0.977983950851537
"14549","DECR2",40.4345524745827,-0.0429950615358639,0.37857369242236,
-0.11357118150697,0.909577733330437,0.977983950851537
"14550","RRP1",84.2431989020761,0.0274774040824487,0.241763892831586,0
.113653878420917,0.909512175213634,0.977983950851537
"14551","MAML2",376.716082075617,0.032137233225928,0.283685050616973,0
.113284902239418,0.909804686450819,0.978056927552311
"14552","AC007448.4",5.17539252427136,0.0687335375432486,0.60699509014
1779,0.113235738903908,0.909843662324441,0.978056927552311
"14553","ATOX1",554.234238221071,-0.0331749563617067,0.294248904748515
,-0.112744536432719,0.910233091361769,0.978139468858429
"14554","HNRNPA2B1",2239.30606857359,0.0144105139854245,0.127802095166
265,0.112756476853349,0.910223624649771,0.978139468858429
"14555","PANK1",3.72497048094187,0.0851458835911093,0.7548694682259,0.
11279550594518,0.910192681342032,0.978139468858429
"14556","KCTD21-
AS1",19.7848732101216,0.0547346135558016,0.484422851926291,0.112989330
165064,0.910039014336115,0.978139468858429
"14557","NCAPH2",78.1761023869332,0.0292452023295863,0.25938537902797,
0.112748075620842,0.910230285389386,0.978139468858429
"14558","CNTLN",254.283596190077,0.0217957490293842,0.193692187648489,
0.112527765285707,0.910404956032518,0.978256953373861
"14559","IQCG",39.7141582989884,0.0295021169958253,0.26259288481004,0.

112349262689151,0.91054648304276,0.978274631472383
"14560","PCNT",72.2267301213252,0.0360480137320535,0.32075081538675,0.
112386351032617,0.910517077061048,0.978274631472383
"14561","GTF2H3",113.512469287642,0.0200301980465512,0.178562168554143
,0.112174926014508,0.910684709812935,0.978352502567471
"14562","CEP89",144.048118505745,-0.023889855018133,0.213111831841919,
-0.112100087600269,0.91074404796954,0.978352502567471
"14563","DGKE",25.7002120581203,-0.0384990516399795,0.343690780981432,
-0.11201653861661,0.910810293182484,0.978356479863599
"14564","AL627171.1",9.79138309041998,-0.0577327232945051,0.5159191517
67286,-0.111902655865248,0.910900590756426,0.978386290936746
"14565","SMN2",15.9650350886717,0.060453237734745,0.541077996377941,0.
111727400004118,0.911039553284641,0.978468364712094
"14566","METTL27",53.889631521396,0.0644011726499878,0.577991823771842
,0.111422290076217,0.911281485189462,0.978498241914356
"14567","AL451123.1",4.48904106144305,0.0743974754897503,0.66761637347
027,0.111437463858222,0.911269453193771,0.978498241914356
"14568","CPSF6",213.292814898114,0.0182489815541052,0.163965210851134,
0.111297887273623,0.911380130709721,0.978498241914356
"14569","HDDC3",119.29412637955,0.0297062974287993,0.266616581444403,0
.111419542129993,0.911283664165681,0.978498241914356
"14570","CD226",4.35191808790687,-0.0870042999250043,0.781435946734914
,-0.111339003904972,0.911347527020623,0.978498241914356
"14571","EIF3C",37.153328485242,0.0485602431480041,0.437263114937682,0
.111054972370411,0.911572754639014,0.978637883523306
"14572","PLXNA2",14.7511915204656,0.0570317044408714,0.514420932373642
,0.110865831562716,0.911722740989383,0.978659792094329
"14573","WIZ",91.7151302275648,0.0211928321789259,0.191046601047655,0.
110930171291765,0.911671720016846,0.978659792094329
"14574","ARRDC2",56.4827731246819,0.0350504061469968,0.316360659495494
,0.1107925562011 8,0.911780848301653,0.978659792094329
"14575","PDE8A",119.186312655217,-0.0212986111145914,0.192827645053863
,-0.110454136950342,0.912049220654944,0.97868756097525
"14576","NMRAL1",120.094682971098,-0.0275061413914412,0.24886548964255
4,-0.110526137757984,0.911992121920754,0.97868756097525
"14577","ZMAT5",133.241495371996,-0.0394769379213631,0.357205985686821
,-0.110515891399352,0.912000247553382,0.97868756097525
"14578","ARHGAP8",4.53081766884002,0.0997979922019212,0.90360425395570
7,0.110444358539743,0.912056975253927,0.97868756097525
"14579","DOCK8",32.5116932529223,0.0632648403935074,0.573950788757086,
0.110226942157375,0.912229395708166,0.97880543501357
"14580","COA6",223.56014042362,-0.0326543639503797,0.296470571082562,-
0.11014369430039,0.91229541589996,0.978809135179909
"14581","ZC3H8",107.400431637466,0.0271954784253126,0.248155486220888,
0.109590478290314,0.912734162395181,0.978877039788003
"14582","CHRD",85.146107451892,0.0290527359899232,0.264772856209981,0.
109727018115794,0.912625872433489,0.978877039788003
"14583","DDX41",92.0252138052314,0.0269714666253347,0.24553071748166,0
.109849663219224,0.912528603781956,0.978877039788003
"14584","MED23",99.8395441757787,0.021031282953995,0.191778798180111,0

.109664275475557,0.91267563352401,0.978877039788003
"14585","AL355312.2",16.0882178316103,0.051816863052035,0.472512872549866,0.109662331043831,0.912677175655262,0.978877039788003
"14586","SLC25A14",23.6057101717294,-0.0407843439554969,0.370907793569148,-0.109958174680127,0.912442545478904,0.978877039788003
"14587","PTCH1",59.8739672604824,0.0400209002113041,0.365582004334719,0.10947174570759,0.912828330725835,0.978909803208036
"14588","C10orf88",37.4825196738575,0.027605645250302,0.25235027261214 5,0.109394156640088,0.912889868260489,0.978909803208036
"14589","DNMBP",131.547245696133,0.0235797417315193,0.215929375809864,0.10920117581538 5,0.913042927722425,0.978913013148633
"14590","FAM180B",205.365061507473,0.03753600401583,0.343882159175481,0.109153682487714,0.913080596743061,0.978913013148633
"14591","AP001267.1",5.51955443262395,0.0763782716610785,0.699009635111923,0.109266407535068,0.91299118993123,0.978913013148633
"14592","RIPK3",15.2985183042734,-0.0523530455812563,0.481020237097552,-0.108837511488397,0.913331370665907,0.979114763660005
"14593","ARHGEF33",7.31707762464697,-0.0611533512387661,0.56585806875895,-0.108071890488173,0.913938665673166,0.979121442005505
"14594","USP19",78.0361413006906,-0.0234045673549505,0.215864113003358,-0.108422687909159,0.91366040487046,0.979121442005505
"14595","AL391422.4",51.9203217389908,0.0321801591005392,0.297137165615381,0.108300686768325,0.913757177859344,0.979121442005505
"14596","GPSM1",51.824511980763,0.0285867717823524,0.263898151829035,0.108325016996982,0.913737878684027,0.979121442005505
"14597","TPT1",34609.6872433349,-0.0305566313889888,0.282825551163293,-0.108040561622901,0.913963516982956,0.979121442005505
"14598","NAA30",80.3553455423347,0.0249057606233862,0.229293288502147,0.10861966691691,0.913504161105087,0.979121442005505
"14599","AC090517.2",13.4947366115367,0.0564402618233925,0.522044269666575,0.108113938037171,0.913905312006705,0.979121442005505
"14600","PRR14",72.2655564615186,-0.0215486634303435,0.199189194286322,-0.108181889622831,0.913851410623634,0.979121442005505
"14601","P2RY8",3.46748398704532,-0.11652653062517,1.07811277387797,-0.108083804819439,0.913929214770847,0.979121442005505
"14602","CXorf38",93.0545806872738,-0.0228152960517196,0.211132498074093,-0.108061507630687,0.91394690176195,0.979121442005505
"14603","CYB5B",194.486659581867,-0.0203062753694537,0.188478954247455,-0.107737627527334,0.914203820767217,0.979256470541695
"14604","AC110285.2",9.37722286767147,0.0677111190165412,0.628562079518284,0.107723837028848,0.914214760326722,0.979256470541695
"14605","SRRM1",896.387617923057,0.0158216113063357,0.150962203393204,0.10480511645107 5,0.916530450071022,0.979334625055622
"14606","PIGV",82.0350746452845,-0.0266258451587143,0.256716648593829,-0.103716861779546,0.91739404505879,0.979334625055622
"14607","MAST2",79.0794785213118,0.023041368526341,0.215635071378044,0.106853529804276,0.914905179883682,0.979334625055622
"14608","CYB561D1",39.5146498820222,-0.0299800696152875,0.280577202284952,-0.106851409776479,0.914906861792118,0.979334625055622
"14609","COP1",109.318669575824,-0.0172837947391529,0.166417581749555,

-0.103857985180698,0.917282049700679,0.979334625055622
"14610","AL591895.1",31.3615127219201,0.0351296381888359,0.33259064532
9509,0.105624252161488,0.915880481943234,0.979334625055622
"14611","BCL2L11",92.6020824287092,-0.0380914897349936,0.3645035603449
72,-0.104502380440244,0.916770679306287,0.979334625055622
"14612","CALCRL",26.7211237861426,0.0500767771427032,0.48161765644067,
0.103976207003682,0.917188230270353,0.979334625055622
"14613","HESX1",4.16618937353653,-0.0793368065281889,0.757525775948196
,-0.104731494355928,0.916588870499695,0.979334625055622
"14614","TPRA1",148.379843677786,0.0225855392197284,0.217268567755601,
0.103952170592546,0.917207305186522,0.979334625055622
"14615","TMEM129",123.76880607151,0.0235430529438946,0.219677765932109
,0.107170850195056,0.914653440389711,0.979334625055622
"14616","GNPDA2",176.938624690375,0.0225543803230975,0.213147340550839
,0.105815912433202,0.915728411377016,0.979334625055622
"14617","CPEB4",65.8114451756541,0.0255612832435573,0.240283298380011,
0.106379775106682,0.915281039139971,0.979334625055622
"14618","KIAA1191",107.296760737345,0.0193851237466373,0.1857813932929
88,0.104343731108023,0.916896574890656,0.979334625055622
"14619","CNOT6",55.5778408287766,-0.0289404660414268,0.275705943546204
,-0.104968596865148,0.916400727096505,0.979334625055622
"14620","HIST1H2BJ",5.11765281468206,0.076846654150998,0.7378890984876
07,0.104143907680036,0.917055146989995,0.979334625055622
"14621","AL121944.1",28.9091943531977,0.0348170457681354,0.32840845250
2674,0.106017508084241,0.915568461030202,0.979334625055622
"14622","CYP39A1",10.0690683136548,0.0711423936871915,0.67951082598029
8,0.104696483068622,0.916616652730636,0.979334625055622
"14623","RMND1",94.9957271143439,0.0282883611337985,0.265477866798185,
0.106556382552611,0.91514092317932,0.979334625055622
"14624","LINC01006",16.2111227765518,-0.0519967296233692,0.48489493091
0985,-0.107232982464225,0.91460415005516,0.979334625055622
"14625","UBAP2",121.555990094874,0.0222755066881388,0.210082014229181,
0.106032431047801,0.915556620964183,0.979334625055622
"14626","MYORG",14.9247000285806,0.0530423358790981,0.498236575010066,
0.106460140703292,0.915217278745254,0.979334625055622
"14627","SNX30",55.6954234149098,-0.0265615354398866,0.255613643227024
,-0.103912823684047,0.9172385303744,0.979334625055622
"14628","MADD",59.8111897425996,-0.0283545111256123,0.270974965300789,
-0.104638858774788,0.91666237910419,0.979334625055622
"14629","SLC15A3",45.7154146136379,-0.0261168887924706,0.2490499971193
77,-0.104866047358161,0.916482100664409,0.979334625055622
"14630","SYVN1",135.981325540221,0.0186388855604583,0.176404042351252,
0.10566019526550 9,0.91585196307955,0.979334625055622
"14631","CHORDC1",146.55795811609,-0.0193781754926358,0.18242729049160
4,-0.10622410408232,0.915404546846794,0.979334625055622
"14632","CEP126",638.54504480186,0.0238378356447491,0.227345811638021,
0.104852759208529,0.916492644944412,0.979334625055622
"14633","KCNJ8",108.798157651095,-0.0352001627976677,0.337164112031011
,-0.104400680682261,0.916851382529014,0.979334625055622
"14634","ARHGAP9",7.78067549259014,0.0816622635884901,0.76459397156029

6,0.106804744251178,0.914943883639348,0.979334625055622
"14635","OAZ2",288.495058940338,0.0212893259206152,0.204271821724523,0
.104220571104152,0.916994309488311,0.979334625055622
"14636","RAB11A",403.019345176032,-0.0206651255971122,0.1976179168233
6,-0.104571113435965,0.916716137142369,0.979334625055622
"14637","CSK",96.2639337267349,0.0237394739758293,0.224574530641211,0.
105708665662342,0.915813504673888,0.979334625055622
"14638","MESP2",16.2599898548176,0.0578883496042361,0.55647184468768,0
.104027454680526,0.917147561082767,0.979334625055622
"14639","GDPGP1",9.75208932386621,-0.0504140102422868,0.47437671599709
,-0.106274208961378,0.915364793954707,0.979334625055622
"14640","TMEM204",203.700973489796,0.0302589866555453,0.28299945308212
,0.106922420965827,0.914850525795528,0.979334625055622
"14641","C17orf100",5.75949000324495,-0.0634497635378437,0.61162685541
3596,-0.103739335472667,0.917376209853689,0.979334625055622
"14642","AC005697.2",3.28221980454702,0.0937215630113872,0.87325948566
3798,0.107323841939313,0.914532070647077,0.979334625055622
"14643","CASC3",171.397441535981,-0.0244364773920759,0.232340545286225
,-0.105175260572674,0.916236741030417,0.979334625055622
"14644","DHX58",43.2018209609211,0.0295373378591095,0.284375318279773,
0.103867445451262,0.917274542100242,0.979334625055622
"14645","LMAN1",962.604739273639,-0.0135288999288085,0.129215349874047
,-0.104700408596934,0.916613537732016,0.979334625055622
"14646","AC016588.2",9.15750139534459,0.0550775949177395,0.51786074774
4405,0.106355994652299,0.91529990616296,0.979334625055622
"14647","PET100",605.887573766786,-0.0315079880282639,0.29434605471608
,-0.107044030396996,0.914754049197936,0.979334625055622
"14648","ZNF561",64.3130122242323,0.0210614269576551,0.203125653934272
,0.103686691216611,0.917417988593306,0.979334625055622
"14649","AC020911.2",4.8996990324324,0.0779302716653188,0.726493795224
714,0.107269012037761,0.914575567477844,0.979334625055622
"14650","NDUFAF5",46.4748526841643,-0.0311270612401992,0.2979737931271
4,-0.104462412326704,0.916802395656783,0.979334625055622
"14651","ZNF337-
AS1",29.2372915819678,0.0388337581991862,0.363702558580665,0.106773398
435066,0.914968751780073,0.979334625055622
"14652","ACTL10",6.81341519837195,0.0651457618316181,0.624569628263286
,0.104305042838484,0.916927276138491,0.979334625055622
"14653","TCEAL4",775.178325426458,-0.0199501876849445,0.19057034574838
6,-0.104686737102766,0.916624386388249,0.979334625055622
"14654","ELF4",34.7364431543195,0.0318515437188768,0.301376821786623,0
.10568677289134,0.915830875274106,0.979334625055622
"14655","AC010913.1",7.34817889960671,-0.0672783516805796,0.6515538117
34657,-0.103258319526152,0.917757954933904,0.979537913866601
"14656","AGFG1",197.00923556341,-0.018368162869895,0.178570921132402,-
0.102862004370106,0.918072493811522,0.979537913866601
"14657","AC024060.2",11.9573518414827,0.0492017227250859,0.47829984273
7399,0.102867946691129,0.918067777543341,0.979537913866601
"14658","FNIP2",120.864203115179,-0.022351648395298,0.217689108906751,
-0.102676925398562,0.918219387698921,0.979537913866601

"14659","AGER",8.35960306646735,0.0511065186443188,0.497564107182334,0
.102713435126442,0.918190410356187,0.979537913866601
"14660","ZNF394",114.720910863115,-0.021192269786732,0.206436143127061
,-0.102657749102047,0.918234607744029,0.979537913866601
"14661","PRXL2A",206.507741708634,-0.028947239481733,0.28155089235756,
-0.102813524188625,0.918110971399046,0.979537913866601
"14662","FBXL14",29.3855150988162,0.0343487823808312,0.334214478454268
,0.102774668948196,0.918141810034158,0.979537913866601
"14663","SDF2",457.182082134909,-0.0251481079566696,0.243790642037522,
-0.103154525319306,0.917840330842482,0.979537913866601
"14664","AL035071.1",14.3971700034333,-0.0450584338574577,0.4380876274
72494,-0.102852559697744,0.918079989812298,0.979537913866601
"14665","SLC17A5",147.330172233047,-0.0205801080718576,0.2010531779575
16,-0.102361515898079,0.918469729038279,0.979654278206929
"14666","CNTFR",245.350873681871,0.045972240329622,0.449292405496776,0
.102321427576305,0.918501547822788,0.979654278206929
"14667","ZC3H13",498.658078727444,-0.0209454948933171,0.20490810606188
5,-0.102218966813305,0.918582873270339,0.979654278206929
"14668","AC011477.2",8.26669666391924,0.0538918422177621,0.52729315824
3712,0.102204706006926,0.91859419246559,0.979654278206929
"14669","PIGF",191.381782207335,0.0263603967071752,0.258848319604031,0
.101837233278159,0.918885871386433,0.979791829106232
"14670","CASP2",75.1323315900293,0.0277104893176136,0.271901681115783,
0.101913637326184,0.918825225304967,0.979791829106232
"14671","RCN3",705.077330763971,-0.0290962129297547,0.285802022970887,
-0.101805482785958,0.918911073631498,0.979791829106232
"14672","SLC25A44",33.2786383048942,-0.0299626225027306,0.297307454508
247,-0.100779923437471,0.919725164463253,0.979796194999797
"14673","ANO10",93.9372031738771,-0.0216817426157809,0.215174714365595
,-0.100763431612797,0.919738256392675,0.979796194999797
"14674","TOMM70",189.654311173311,-0.0160128914498835,0.15949556592261
,-0.100397094786028,0.920029076139654,0.979796194999797
"14675","AC034213.1",3.38298705624758,0.102140975654184,1.015182029216
26,0.100613459177403,0.919857312048297,0.979796194999797
"14676","LRRC1",6.91550415893295,0.0615885417791109,0.614519510913965,
0.100222272336823,0.920167864267853,0.979796194999797
"14677","COPG2",36.0335020339106,-0.0285769139866507,0.282340543549519
,-0.101214347848837,0.919380307272496,0.979796194999797
"14678","AC004918.1",11.8591548326989,0.0494597409442109,0.48883047703
3479,0.101179740764861,0.919407778704451,0.979796194999797
"14679","CHD7",7.42110596864722,0.0530212224169515,0.52627104597757,0.
100748887521377,0.919749802144578,0.979796194999797
"14680","LY96",283.727916229627,0.0335311825307157,0.330839747833315,0
.101351735244368,0.919271248802616,0.979796194999797
"14681","TRIM3",42.4733859909697,0.0254537725655389,0.251379914553173,
0.101256190697586,0.919347092150249,0.979796194999797
"14682","ERC1",203.689983887482,0.0252459011308035,0.25110072583297,0.
100540932516447,0.91991488800099,0.979796194999797
"14683","TNS2",536.778650993804,0.025413580180442,0.250525554433899,0.
101441069506334,0.919200335849051,0.979796194999797

"14684","SMOC1",221.579365478203,-0.0474099998286577,0.470644169793508
,-0.100734276278103,0.919761401221668,0.979796194999797
"14685","ANTKMT",222.109728267491,0.0304810312771823,0.303883316481626
,0.100305050076763,0.920102148333366,0.979796194999797
"14686","SCIMP",3.08309664082175,-0.0851547505521657,0.846397685044635
,-0.100608439811216,0.919861296704493,0.979796194999797
"14687","MAP2K6",56.8391607816462,0.0325170366385848,0.323764166654132
,0.100434328402136,0.919999517413495,0.979796194999797
"14688","KCNJ15",28.7642575776912,-0.0501471927107617,0.50013658922201
2,-0.100266994639941,0.920132359873182,0.979796194999797
"14689","GGA1",119.946886126274,0.0224007803297259,0.223482019385043,0
.100235269000013,0.920157546387045,0.979796194999797
"14690","PDK3",38.9049212773763,0.0300896551721318,0.29912790597683,0.
100591267384001,0.919874929161726,0.979796194999797
"14691","GLA",153.366543097119,0.0306772494990467,0.30377176048205,0.1
00987825367195,0.919560124714848,0.979796194999797
"14692","RRAGC",258.096636366061,0.0186862566386935,0.186625930650626,
0.10012679681515,0.920243661446835,0.979810209366516
"14693","AC253576.2",3.68007279550902,0.0857435523043871,0.85790705425
8385,0.0999450370279422,0.920387960949387,0.979816926081934
"14694","BNIP3L",785.109521142066,-0.0156974968649316,0.15729006423682
8,-0.0997996722876041,0.920503368229438,0.979816926081934
"14695","TUT1",32.1500569950048,0.0283371686316159,0.283540835881748,0
.0999403438432198,0.920391686913997,0.979816926081934
"14696","NAA16",78.0510275555222,0.022419984747314,0.22481951165549,0.
0997243725965836,0.920563150458747,0.979816926081934
"14697","VPS33B",24.6434848226308,-0.0318322277404051,0.31890853510192
4,-0.0998161674482354,0.92049027238607,0.979816926081934
"14698","SLCO3A1",324.310561784181,0.0228483602985931,0.22938209312971
2,0.0996082997885658,0.920655304322027,0.979848341645766
"14699","PRCC",118.841884724243,0.0197784017573342,0.198872466522872,0
.0994526899733481,0.920778849533788,0.9799131603005
"14700","RTCA",152.739007836284,-0.017972639796402,0.181425545580053,-
0.0990634463241654,0.921087894891277,0.97999714989847
"14701","DPYSL2",1914.27752710005,0.0235587446364111,0.237729563269809
,0.0990989270008179,0.921059724024944,0.97999714989847
"14702","AC139768.1",13.7606638055205,-0.0479765974808046,0.4848140206
67002,-0.0989587665282431,0.92117100889261,0.97999714989847
"14703","PGAP3",32.3910826489587,-0.0307988796279577,0.310409437096286
,-0.0992201780849988,0.92096345411486,0.97999714989847
"14704","C19orf38",4.07345711377372,0.0707117025052853,0.7143043903136
58,0.0989937951721606,0.921143196641261,0.97999714989847
"14705","BEND3",4.35532512104078,0.0756153424305775,0.765184068135174,
0.0988198076508039,0.921281341239336,0.980013286621194
"14706","CCL26",58.4406352620456,-0.0511065440551719,0.518975961328525
,-0.0984757442798398,0.921554531590424,0.980013286621194
"14707","CENPP",22.3618041174568,0.031660937623052,0.3211445344364,0.0
985878139841804,0.921465545912998,0.980013286621194
"14708","NRP1",756.013281839519,-0.0323240816466493,0.327795392561974,
-0.098610542979309,0.921447498737763,0.980013286621194

"14709","AC009318.2",4.57026961418773,0.0670234405500113,0.679650959750415,0.0986145014414811,0.921444355661431,0.980013286621194
"14710","CINP",213.650362668089,0.0215810633430991,0.219700308330473,0.0982295541917803,0.921750015090305,0.980013286621194
"14711","PAK6",10.0882085306448,-0.0638184320645531,0.649176116418205,-0.0983068083537453,0.921688672086927,0.980013286621194
"14712","CHTF18",14.0767424516387,0.0549164070974127,0.558357935958801,0.0983534101706843,0.92165166853994,0.980013286621194
"14713","LENG9",48.744422587698,0.0277312435640945,0.281799466242396,0.0984077221077516,0.921608543089616,0.980013286621194
"14714","AL513550.1",16.6593476403507,0.0384960071976548,0.392936533251246,0.0979700382632535,0.921956084889387,0.980099152968038
"14715","AC073655.2",3.13603406794749,-0.0866491426073221,0.884278909712609,-0.0979884758706765,0.921941444250603,0.980099152968038
"14716","MAGI2",129.770035798564,-0.0190800884716595,0.195249752460288,-0.0977214476906455,0.922153484273405,0.980242386143577
"14717","RASAL2",54.0460143629016,0.0331415206464535,0.339799135376678,0.097532680916669,0.922303382320501,0.980286648871458
"14718","SKP2",39.7185506869367,-0.0268658029159587,0.275602288841809,-0.0974803330874339,0.922344951763658,0.980286648871458
"14719","ACOT8",72.0135739713393,-0.0238482609551713,0.244767591490097,-0.0974322654808495,0.922383122466214,0.980286648871458
"14720","TERF2IP",717.836031294084,-0.0151080494952974,0.155274222731213,-0.0972991474666736,0.922488833012721,0.980332392311005
"14721","PPM1J",2.88342135324515,-0.0838783456175598,0.866757508131023,-0.0967725630648709,0.922907013147169,0.980341313421838
"14722","CHST10",20.1123625920404,0.0356028262542445,0.368832198222346,0.0965285200853906,0.923100823929927,0.980341313421838
"14723","CYB561D2.1",4.57933590157995,-0.0778180705349559,0.802785232759819,-0.0969351046324465,0.922777930602198,0.980341313421838
"14724","LINC02614",37.3998159501507,0.0294403427392629,0.305833331151264,0.0962627017416284,0.923311933174195,0.980341313421838
"14725","FBXL5",247.844083158616,0.014070523094459,0.145721922619235,0.0965573528097392,0.923077925705257,0.980341313421838
"14726","FAM122A",87.0615134799743,-0.0234838292774968,0.243030277294218,-0.0966292329456003,0.923020840586411,0.980341313421838
"14727","DCLRE1C",101.717475137252,0.0228905460639795,0.235931815826891,0.097021870423677,0.922709026296555,0.980341313421838
"14728","AL445223.1",4.78654515792339,0.0684081237318088,0.709768981293309,0.0963808302909471,0.923218116449856,0.980341313421838
"14729","RPRD1A",86.0077674945349,0.0234356054560465,0.243162787488127,0.0963782563036739,0.923220160678043,0.980341313421838
"14730","AC022098.1",12.3006293976763,-0.0395416123174441,0.408462086302258,-0.0968060773410037,0.922880397585701,0.980341313421838
"14731","RAB3A",11.6948683740222,0.0491380243924827,0.506751296242278,0.0969667463247885,0.922752802557646,0.980341313421838
"14732","ZNF225",19.2856171916089,-0.0315096510661213,0.325760582341501,-0.0967264082094781,0.922943667441035,0.980341313421838
"14733","MHENCR",26.722927285374,-0.0290097202111423,0.301292275478741,-0.0962843145083856,0.923294768412445,0.980341313421838

"14734","TECPR2",70.4792649447415,0.0229047164971207,0.238207451756508
,0.0961544919280424,0.923397873456281,0.980366019714714
"14735","TMEM201",19.4687730965146,-0.0351651252467237,0.3787064831414
98,-0.0928558839421422,0.926018053776169,0.980419655852495
"14736","MFN2",57.83677594069,-0.0216388454466938,0.229339606598397,-0
.0943528497656583,0.924828869082136,0.980419655852495
"14737","UROD",353.658964119447,0.0285707845894956,0.299180045158285,0
.0954969592787511,0.923920105367818,0.980419655852495
"14738","COA7",36.2218352445698,0.0291877144347225,0.313157894656016,0
.0932044662861953,0.925741126253969,0.980419655852495
"14739","HHLA3",68.1570777830373,0.0274141389629435,0.28833980420411,0
.0950758048775588,0.924254615914194,0.980419655852495
"14740","CRYZ",93.3855545324297,-0.0242763110155511,0.255879343412852,
-0.0948740554503538,0.924414864295551,0.980419655852495
"14741","OVGP1",2.62140001828515,0.0806599474173638,0.863439522057026,
0.0934170203666407,0.925572269347281,0.980419655852495
"14742","IWS1",224.388589671453,-0.0184298661798719,0.197941906590033,
-0.0931074500461533,0.925818198868113,0.980419655852495
"14743","TMPPE",3.2058016562281,-0.0747051503872195,0.786111538346002,
-0.0950312350641756,0.924290017190076,0.980419655852495
"14744","LINC00960",8.73550620478238,-0.0626534355693222,0.66187963038
3952,-0.0946598636567458,0.924584998931982,0.980419655852495
"14745","CIP2A",18.3990746656103,-0.0346319818233142,0.364231776620758
,-0.095082263674576,0.92424948578052,0.980419655852495
"14746","IDUA",102.008392888618,0.027627835468402,0.297264421399521,0.
092940269603507,0.925951013664703,0.980419655852495
"14747","AC110792.4",2.66062018001443,-0.0910042845359069,0.9771270772
08806,-0.0931345437646283,0.925796674734533,0.980419655852495
"14748","CBX3",609.237298415914,0.0136555413231625,0.14415603484547,0.
0947275036927916,0.924531271416803,0.980419655852495
"14749","JAZF1-
AS1",2.6049804700191,0.0887437873413973,0.934530746422135,0.0949608000
391151,0.924345963203513,0.980419655852495
"14750","XRCC2",5.16678577491552,-0.0635500227388418,0.680957899506865
,-0.0933244519005704,0.925645807043191,0.980419655852495
"14751","DLC1",875.043014848825,0.0218256567327687,0.228447365457936,0
.0955391045504853,0.923886631358345,0.980419655852495
"14752","MMP16",32.5984831237561,-0.0408466183586654,0.433865436352424
,-0.0941458224975684,0.924993320910626,0.980419655852495
"14753","C8orf37-
AS1",3.35272850880779,0.0781909068100516,0.836678748846394,0.093453917
5494305,0.925542957881741,0.980419655852495
"14754","CPSF1",73.2731236166308,0.0282309843627629,0.296463061099982,
0.095225976072756,0.92413533781376,0.980419655852495
"14755","TRIM32",42.6375307893099,-0.0314192172818537,0.33056459341109
9,-0.0950471342306763,0.924277388652871,0.980419655852495
"14756","EPC1",490.209499098184,0.0120288788145638,0.129459711811127,0
.0929160017914542,0.925970293151946,0.980419655852495
"14757","BMS1",128.11613769443,-0.0182078103523889,0.19530820575335,-0
.093226038722526,0.92572398855749,0.980419655852495

"14758","AL139407.1",4.61988753588168,-0.0740374575370053,0.777218482531307,-0.0952595173700376,0.924108696837505,0.980419655852495
"14759","RAB3IL1",261.937069534439,0.0204631983163606,0.218796613403033,0.09352611998005,0.925485599891057,0.980419655852495
"14760","AP001462.1",12.503509667273,0.047388448788996,0.494160920869742,0.0958967955329017,0.92360253964648,0.980419655852495
"14761","PCF11",321.63073243089,0.0138941564883988,0.147687247341052,0.0940782412736908,0.925047004658073,0.980419655852495
"14762","PCCA",86.6250029032829,-0.0174976409803868,0.187393411197976,-0.0933738324550855,0.925606578354657,0.980419655852495
"14763","MCF2L",42.4879142345305,0.0437122051314241,0.462871438569989,0.0944370325947741,0.924761999484742,0.980419655852495
"14764","AL136419.3",7.78811161445651,-0.0588810474134234,0.630968228607199,-0.0933185614486447,0.925650486522841,0.980419655852495
"14765","CLMN",19.1675139007979,-0.0407045330679488,0.427437415254844,-0.095229223309055,0.924132758615072,0.980419655852495
"14766","JMJD6",57.8397745795496,0.0223225627893252,0.237678781097696,0.0939190393279142,0.925173469491092,0.980419655852495
"14767","TNRC6C",253.663582587545,-0.0253931552617566,0.272798620206374,-0.0930838845245864,0.925836920131154,0.980419655852495
"14768","MARK4",60.5992525922741,-0.023108771905406 4,0.245934847662266,-0.0939629829813338,0.925138561897058,0.980419655852495
"14769","VPS16",109.892729148674,0.0232398555423207,0.248838591611611,0.093393293185784,0.925591118494043,0.980419655852495
"14770","SNHG17",80.9980295189658,-0.0318458735197842,0.333627297509192,-0.0954534408831065,0.923954670126488,0.980419655852495
"14771","OGFR",186.713744454928,0.0192914374344139,0.20517035995691,0.0940264346100746,0.925088157974123,0.980419655852495
"14772","LINC01311",3.63486759768807,0.0759084199615646,0.791968160583686,0.0958478178032052,0.923641439037538,0.980419655852495
"14773","UBE2L3",633.094784485572,-0.0217128091462384,0.229130845601176,-0.0947616157452302,0.924504175822379,0.980419655852495
"14774","MAGED2",824.466757690044,-0.0260725520350887,0.276471807155278,-0.094304559670510 2,0.924867227966459,0.980419655852495
"14775","PCDH19",4.402537850895,-0.0718241155949758,0.773145445243067,-0.092898582067434,0.925984132223912,0.980419655852495
"14776","RBM45",44.2026635735592,0.0253030401242616,0.273365244641447,0.0925612916062161,0.92625209650280 5,0.980506373904832
"14777","THUMPD3",145.421959760888,-0.0161535185473065,0.174410934459721,-0.092617579266723,0.926207377542364,0.980506373904832
"14778","AC019131.2",7.20081423735668,0.0521257162010859,0.563423193833344,0.0925160993931398,0.926288000611494,0.980506373904832
"14779","ZEB2-AS1",5.34596956357781,-0.0589590922886703,0.639668444991261,-0.0921713314926388,0.926561915217258,0.980597255919326
"14780","PPIG",865.862355050293,-0.0128318072077474,0.139173873438832,-0.092199828104857,0.926539274616971,0.980597255919326
"14781","CYP21A2",4.74923400784417,-0.0664651735135857,0.720805926574109,-0.0922095269519848,0.926531568882241,0.980597255919326
"14782","ZNF583",33.8928361094645,-0.0253008545326466,0.27486864197614

5,-0.0920470751073976,0.926660637792681,0.980635391489035
"14783","HPCAL1",67.4149635315574,-0.0299972845482373,0.326313115477949,-0.091927915628829,0.926755311900399,0.980649909943989
"14784","EP300",268.544902574499,0.0178611458533835,0.194413371970056,0.0918720027968784,0.926799735895412,0.980649909943989
"14785","CCDC30",48.7582833217904,0.0259694120781398,0.286190707197849,0.0907416328517846,0.927697888839571,0.980665337537129
"14786","CCDC163",4.05075926070642,-0.0746283280219348,0.815218103366059,-0.0915440024133324,0.927060344124529,0.980665337537129
"14787","OLFML2B",597.375347756231,0.0179152199635873,0.199743125689252,0.0896912967681234,0.928532532239865,0.980665337537129
"14788","RANBP2",192.26812335797,0.0169299079055912,0.188386108727141,0.0898681331653413,0.928392004705586,0.980665337537129
"14789","BZW1",894.788040194478,-0.0162323696217899,0.178482267980935,-0.090946679496048,0.927534958979562,0.980665337537129
"14790","TTLL4",19.5261438925241,0.031933878793295,0.35589841034082,0.0897275117433485,0.928503752908394,0.980665337537129
"14791","AC080013.5",4.1828239986725,-0.0644133075176547,0.717685442235011,-0.0897514478168307,0.928484731430961,0.980665337537129
"14792","ZSCAN9",22.9641769991676,-0.0312984576583947,0.347378405601506,-0.0900990307794163,0.928208519414669,0.980665337537129
"14793","KIFC1",4.38255813208034,-0.0733493240509098,0.811879223514809,-0.0903451177545399,0.928012967935686,0.980665337537129
"14794","RNASET2",308.816146225483,-0.0215288860764764,0.237714151913816,-0.0905662784615438,0.927837227669111,0.980665337537129
"14795","LINC00574",3.85625728258917,0.0651361836655083,0.722477207610032,0.0901567315610966,0.928162667459043,0.980665337537129
"14796","POMK",6.21396114781594,-0.0548491677326315,0.605011332978643,-0.0906580831512583,0.927764278218355,0.980665337537129
"14797","WDFY4",6.42113516755007,0.0644183349483729,0.713960566626137,0.0902267407467411,0.928107034944212,0.980665337537129
"14798","RPL27A",10417.5314825001,0.0194000214462884,0.213159176051663,0.0910118992090047,0.927483136096958,0.980665337537129
"14799","ATG2A",63.742376281253,0.0247267425882495,0.273226863979714,0.0904989437278957,0.927890733293418,0.980665337537129
"14800","TAS2R30",6.16021373042398,-0.0539715261766672,0.592495117799456,-0.0910919340181553,0.927419541718605,0.980665337537129
"14801","DEPDC4",3.81837659824467,-0.0712531298063128,0.794615667324115,-0.0896699281632077,0.9285495134955,0.980665337537129
"14802","GRTP1",30.9150957329387,-0.0257006186851514,0.28590222122321,-0.0898930360708405,0.928372215158548,0.980665337537129
"14803","KATNBL1",254.393977529569,-0.0125770010607976,0.138819555921255,-0.0905996347368514,0.927810722177792,0.980665337537129
"14804","SHC4",4.42154064540486,0.0620556207277506,0.692240527038284,0.08964459361149,0.928569646461674,0.980665337537129
"14805","CELF6",20.1599236999728,0.040843929057699,0.452092899482282,0.0903441064977391,0.928013771515703,0.980665337537129
"14806","SERPINF1",9024.88861909603,-0.0259245513644775,0.285257873592998,-0.0908811071117577,0.927587062400901,0.980665337537129
"14807","TWSG1",228.778470623959,-0.0151826897813628,0.167848766070639

,-0.0904545808515096,0.927925985165173,0.980665337537129
"14808","MEX3C",72.3458792586295,-0.0214624491572636,0.238366518529294
,-0.0900396972262948,0.928255669101541,0.980665337537129
"14809","SHC2",158.45375195658,0.0252087246882645,0.280311745831539,0.
0899310323707036,0.92834202079277,0.980665337537129
"14810","NFS1",46.0326279405907,-0.0219313540782405,0.244415878263855,
-0.0897296617307527,0.928502044358456,0.980665337537129
"14811","FGF13",2.78558327372406,0.0896599865635616,0.998694226815807,
0.0897772152437784,0.928464254662401,0.980665337537129
"14812","IKBKG",82.0648545380342,-0.0233704626397455,0.257537737166045
,-0.0907457792279877,0.927694594103102,0.980665337537129
"14813","ING3",113.173021716348,-0.0166645982880311,0.186277717052059,
-0.0894610399555937,0.928715515003831,0.980753176345435
"14814","VTN",3.70906423116065,0.072021419962292,0.808772055112909,0.0
890503319284905,0.929041909877517,0.981031631984204
"14815","PFDN4",278.43415960442,0.0226724305454185,0.255228604858743,0
.0888318554966307,0.929215540734093,0.981148748140628
"14816","BTD",276.373118640653,0.0214398359163773,0.241745186179637,0.
08868774702486,0.929330070618431,0.981203448615289
"14817","NXPE3",36.6420969847072,-0.029752912213743,0.336053235076416,
-0.0885363064782797,0.929450429230101,0.98126429536657
"14818","AL139424.2",3.52507233508107,-0.0792044712315005,0.8993274900
73915,-0.0880707774483696,0.929820422323237,0.981340723976762
"14819","ID2",992.977574569111,0.0167663038142893,0.192952044379186,0.
0868936313592019,0.930756061812755,0.981340723976762
"14820","EMC3",430.433615048183,-0.0184909374066311,0.211854996012496,
-0.087281101482924,0.930448075518688,0.981340723976762
"14821","AC006059.1",11.4777775757597,-0.0413584900648778,0.4712212830
72008,-0.0877687225739286,0.930060497571031,0.981340723976762
"14822","OSTN-
AS1",4.39195958062988,0.07320193606385,0.831691722646437,0.08801570830
94707,0.929864191170192,0.981340723976762
"14823","ETV7",20.7239255214248,0.0395093207338848,0.447524751788064,0
.0882841017754374,0.929650874558501,0.981340723976762
"14824","MCM3",124.984628220641,-0.0181045461996979,0.206370895704608,
-0.0877281950920641,0.930092709571221,0.981340723976762
"14825","AL009176.1",14.5510245765129,-0.0572493641201174,0.6530772511
21082,-0.087660937541221,0.930146167382632,0.981340723976762
"14826","TOR2A",60.3498749129955,-0.0237633566342883,0.271704788255666
,-0.0874602055666671,0.930305715535755,0.981340723976762
"14827","PFKFB3",60.4522401454163,-0.0340004027114471,0.39039642717572
8,-0.0870919925098153,0.930598390253068,0.981340723976762
"14828","C11orf98",7.79158213220439,0.0504893228608978,0.5802634976181
5,0.087011027004361,0.930662747070394,0.981340723976762
"14829","DPP3",29.2983508723022,-0.0353096243976713,0.40648069553473,-
0.0868666698949161,0.93077749291333,0.981340723976762
"14830","AMN1",24.4025775159849,0.0315150385804926,0.360047178858128,0
.0875303027798773,0.930249999724224,0.981340723976762
"14831","UNG",83.2465560935446,0.0200934607183023,0.230034558271843,0.
0873497480954875,0.930393511839619,0.981340723976762

"14832","WDR66",24.7515232339911,0.0474001855851633,0.544652266884745,
0.0870283453629576,0.930648981238616,0.981340723976762
"14833","AKAP5",4.34139426170242,0.0599168939654766,0.687394595477126,
0.0871652095604385,0.930540192818369,0.981340723976762
"14834","FEM1A",40.4611944831661,-0.024176045934015,0.277484935730974,
-0.0871256159197558,0.930571664243718,0.981340723976762
"14835","KEAP1",167.500189966673,-0.0205225131171054,0.235926796274614
,-0.0869867833631647,0.930682017631631,0.981340723976762
"14836","FBXO17",374.820081070423,-0.016280172867082,0.184545374005152
,-0.0882177239870969,0.929703630532808,0.981340723976762
"14837","AC007193.1",5.93245989792511,0.0815947429829714,0.93032517841
8506,0.0877056161391626,0.930110655794749,0.981340723976762
"14838","FUT10",70.3665483995626,0.0187089960086764,0.21620009604081,0
.0865355582688776,0.931040690665372,0.981552063895297
"14839","TMEM8A",55.9165660845682,0.0231204024010596,0.2679218737607,0
.0862953146621767,0.931231662941741,0.981687236565648
"14840","RAPH1",85.1895797088446,-0.0267483366709975,0.31054198328487,
-0.0861343654344488,0.931359605462498,0.981755950690692
"14841","WBP11",243.007128600193,-0.0138583237099398,0.161527677925007
,-0.0857953502951604,0.931629102777998,0.981927455638941
"14842","COPZ2",1142.76350357989,0.0239972453041547,0.27978022984667,0
.0857717692108058,0.931647848660305,0.981927455638941
"14843","RCC2",75.9582362626304,-0.018047352228564,0.219075102021735,-
0.0823797504235489,0.934344738131674,0.982312634661508
"14844","SRSF4",457.290059052704,0.0143771066797154,0.175583815800868,
0.0818817304666664,0.934740762623787,0.982312634661508
"14845","ATG4C",88.6612526790383,0.0179742479420615,0.218993276944446,
0.0820767111796823,0.934585712414473,0.982312634661508
"14846","DCP1A",188.422281936487,0.0129788985037411,0.159943439804556,
0.0811468011417082,0.935325206452759,0.982312634661508
"14847","CACNA2D3",14.1382162187053,0.0479135077253082,0.5673220437399
06,0.0844555720229949,0.932694224976306,0.982312634661508
"14848","ZBTB20-
AS2",20.1958796062629,0.027316585878081,0.329510180900039,0.0829005823
233362,0.933930591009565,0.982312634661508
"14849","ADPRH",9.6130705738316,0.0552955774483635,0.651628662756173,0
.0848574972354371,0.932374682149242,0.982312634661508
"14850","C5orf51",77.4501307343246,0.0166404246544992,0.20491453974131
,0.0812066565676916,0.935277605827151,0.982312634661508
"14851","C5orf30",30.3904751949867,-0.0273958022989932,0.3390129046357
52,-0.080810499908339,0.935592657662968,0.982312634661508
"14852","G3BP1",421.110715808432,-0.0121032813563151,0.147981757860857
,-0.0817890092081181,0.93481449617277,0.982312634661508
"14853","MICALL2",41.7941431338494,-0.0330444878647834,0.4060989139904
13,-0.0813705398521787,0.935147277192077,0.982312634661508
"14854","TECPR1",29.6619821773473,0.0283618325719198,0.343703818504591
,0.0825182353088722,0.93423461810563,0.982312634661508
"14855","ZC3HC1",34.9591215548233,-0.024010459422641,0.293552618258513
,-0.0817926938110172,0.934811566102124,0.982312634661508
"14856","NUP205",49.3876099044553,-0.0209171106018377,0.25588676178840

4,-0.0817436214974433,0.934850589469449,0.982312634661508
"14857","AC019270.1",14.2689539001806,0.055504191236751,0.686545104493725,0.0808456587534495,0.935564696450419,0.982312634661508
"14858","ST3GAL1",95.9013232424181,0.015643157350621,0.192128715749993,0.0814201942148861,0.935107789765756,0.982312634661508
"14859","PHYHD1",303.073690039276,-0.0223634205258483,0.276518024304624,-0.0808750915318699,0.935541289139343,0.982312634661508
"14860","SVIL",333.122340723509,0.0170404592386125,0.205457859092525,0.082938950663058,0.933900082567432,0.982312634661508
"14861","AFAP1L2",13.1983390527569,0.0528630658443767,0.646328012997589,0.0817898416613638,0.934813834189058,0.982312634661508
"14862","RNF141",106.084443400926,0.0163773141511498,0.194749605723532,0.0840942095379599,0.932981528457133,0.982312634661508
"14863","RELA-DT",9.12446590533917,0.0470856328161492,0.558352803774607,0.0843295359096225,0.93279442977464,0.982312634661508
"14864","CD3D",19.9968515086881,0.0405600364537865,0.480580135062619,0.0843980711947273,0.932739940780159,0.982312634661508
"14865","MMP19",135.817682493791,0.0311442970413369,0.368069082134804,0.0846153576950819,0.932567189182001,0.982312634661508
"14866","R3HDM2",286.205679447928,-0.0137636657756844,0.164776954559261,-0.0835290700237721,0.933430863616689,0.982312634661508
"14867","UTP20",85.7501029413022,-0.0179785731359854,0.219582238724541,-0.0818762630366424,0.934745110403209,0.982312634661508
"14868","BRI3BP",44.3081764144751,0.0208849423667924,0.258259208144199,0.0808681421927514,0.935546815807343,0.982312634661508
"14869","LINC00545",7.9975198664766,0.0762173951913591,0.906141967822067,0.0841119801288416,0.932967399634415,0.982312634661508
"14870","SCFD1",368.811619066868,0.013825818458273,0.163532623050722,0.0845447116321539,0.932623355324975,0.982312634661508
"14871","C14orf132",180.957264528711,0.020356014156539,0.240858699073701,0.0845143407102364,0.932647501395645,0.982312634661508
"14872","GABRB3",4.44818369993777,-0.0565339743689379,0.689269589042971,-0.0820201199467302,0.934630713956406,0.982312634661508
"14873","C15orf39",29.749798058274,-0.0257582068199495,0.309547010112535,-0.0832125847721328,0.93368250624465,0.982312634661508
"14874","SPATA41",21.6012915796251,-0.0345402680778526,0.42737861624021,-0.0808188963259666,0.935585980132147,0.982312634661508
"14875","VASN",507.996271735911,-0.0232265214987688,0.286105831710462,-0.0811815731259682,0.93529755365667,0.982312634661508
"14876","AC027277.1",7.952755085672,-0.0576868600372359,0.710960240164862,-0.0811393616383655,0.935331122808287,0.982312634661508
"14877","AC005632.3",11.9955420867961,-0.0369764852801151,0.442691006201639,-0.0835266241285978,0.933432808362689,0.982312634661508
"14878","SRCAP",84.1570074742878,-0.020716558549981,0.252876730149897,-0.0819235464556227,0.934707510009024,0.982312634661508
"14879","TMEM231",25.7486336274681,-0.0283462695828384,0.338424463554113,-0.0837595169248336,0.933247635680087,0.982312634661508
"14880","AC010538.1",5.8605127042584,-0.0615203527962106,0.752326778703676,-0.0817734454464262,0.934826872800163,0.982312634661508

"14881","DCAKD",69.5563337991741,-0.0220315185219163,0.261106741114251
,-0.0843774405359995,0.932756343157068,0.982312634661508
"14882","HOXB4",65.9473132540389,-0.0260938807000448,0.318664767190979
,-0.0818850509582895,0.934738122121778,0.982312634661508
"14883","TBX2",10.4348580122307,-0.0418410823972395,0.515847984503167,
-0.0811112646636358,0.935353467301428,0.982312634661508
"14884","MYO15B",73.8855555539106,0.0210170387964666,0.25622412414044,
0.0820259952764903,0.934626041864372,0.982312634661508
"14885","RPTOR",31.9318276559861,0.0243664737784908,0.288855440212842,
0.0843552531347045,0.932773983251885,0.982312634661508
"14886","TSHZ1",169.319641665613,0.0189294973294255,0.229830092761131,
0.0823630060885869,0.934358052930565,0.982312634661508
"14887","AC008946.1",4.20708850561892,-0.0563102522694942,0.6690536747
00467,-0.0841640280874388,0.932926018105507,0.982312634661508
"14888","NR2F6",158.774919337297,0.0214894265661519,0.257896643564404,
0.0833257318480201,0.933592540381557,0.982312634661508
"14889","PPP5C",85.6427570875691,0.0163919692076346,0.194781826781263,
0.0841555368820034,0.932932769156437,0.982312634661508
"14890","KIZ",179.259134534893,-0.0140075608428785,0.168962075680349,-
0.0829035793178743,0.933928207957049,0.982312634661508
"14891","GID8",214.222056762091,-0.0159972797540445,0.193546477839914,
-0.0826534273967834,0.934127117650927,0.982312634661508
"14892","U2AF1L5",7.59866237858085,0.0798595696780991,0.97893533567528
9,0.0815779824956573,0.934982310349035,0.982312634661508
"14893","AC245452.1",51.345568297544,-0.0274222977262695,0.33571892551
58,-0.0816823111301746,0.934899345022265,0.982312634661508
"14894","AP1B1",76.1050785863847,-0.0213446179977027,0.263628261586201
,-0.0809648323335144,0.935469920384924,0.982312634661508
"14895","UQCR10",1139.67494851108,0.0239797632779615,0.290399664162484
,0.0825750379123868,0.934189450333127,0.982312634661508
"14896","TEF",76.9096985874064,0.0467952296292627,0.576869302555163,0.
0811192923977575,0.935347083133817,0.982312634661508
"14897","TCF20",55.3270991292466,-0.0248541011773489,0.304208370759657
,-0.0817009115011667,0.934884553511578,0.982312634661508
"14898","RAB9A",346.102148306616,-0.0216853261139741,0.256017666544991
,-0.084702459820925,0.932497940343519,0.982312634661508
"14899","TRAPPC2",96.4303603802954,0.0181063203533137,0.21948584844976
3,0.0824942495436465,0.934253690949684,0.982312634661508
"14900","FRRS1",43.2191047180401,-0.0203660592211074,0.253340520211053
,-0.0803900584246879,0.93592703354259,0.982343835028575
"14901","FARSB",66.5077834760056,-0.017617503780308,0.219182260833343,
-0.0803783285806308,0.935936362415514,0.982343835028575
"14902","APBB3",34.2223967623024,0.0259018529510304,0.322020948320621,
0.0804353042437513,0.935891049131582,0.982343835028575
"14903","MSH5",5.2438591112344,0.0534009338816556,0.662709079022816,0.
0805797529745584,0.935776168620296,0.982343835028575
"14904","ALKBH1",22.7912547736468,-0.0272797722275052,0.33854454771901
2,-0.0805795645249827,0.935776318493941,0.982343835028575
"14905","ETV6",80.8218170267607,0.0174810555790669,0.218662290190894,0
.0799454517914625,0.936280640188523,0.982507368831038

"14906","GPN3",157.228780971548,-0.0176301220766451,0.220744787222056,
-0.0798665386327349,0.93634340308976,0.982507368831038
"14907","GABPB1-
IT1",259.828013953074,-0.0135708621173509,0.16966613440352,-0.07998568
58002966,0.936248640568448,0.982507368831038
"14908","MIS12",38.3246891153512,0.0226214619319085,0.282858465313451,
0.0799744915070525,0.936257543800446,0.982507368831038
"14909","PCK2",44.6252066302739,-0.0295255120835897,0.371745077906582,
-0.0794240834333505,0.936695313337987,0.982802715565571
"14910","BST2",578.859622142053,0.03479407574995,0.438462888123103,0.0
793546653375626,0.936750526694539,0.982802715565571
"14911","PPP4R3B",286.793102112346,-0.0134325141409084,0.1696304544404
96,-0.0791869253974107,0.936883943956011,0.982815350975563
"14912","ZWINT",12.4038080575032,-0.0366324183706586,0.464055742525891
,-0.078939694122231,0.937080590392307,0.982815350975563
"14913","AC004263.2",3.73264685170617,0.0814038205976748,1.03218432016
566,0.0788655853487581,0.937139536863229,0.982815350975563
"14914","PCOTH",4.98768244147562,-0.0564237265245674,0.71311970596888,
-0.079122377424569,0.936935284698118,0.982815350975563
"14915","ZNF646",32.3925620303408,-0.0270572288734129,0.34225770305164
6,-0.0790551348652335,0.936988768963306,0.982815350975563
"14916","HDHD5-
AS1",4.77677527134399,-0.0541071759134546,0.685619067997107,-0.0789172
565919403,0.937098437274242,0.982815350975563
"14917","PTPN14",82.7886227975372,0.0280433388718341,0.357853960136054
,0.0783653165698435,0.937537462025953,0.982848674836089
"14918","AL121658.1",7.73887628076415,0.0508483799148237,0.64845830891
5434,0.0784142622829047,0.937498528802676,0.982848674836089
"14919","FAM126A",250.111876775845,-0.0178557791050599,0.2273963728563
11,-0.0785227085233356,0.937412267200438,0.982848674836089
"14920","AL354811.1",4.10714669884943,0.0742050687108936,0.94361220902
6459,0.0786393690130951,0.937319472542162,0.982848674836089
"14921","AC105429.1",10.8195687136006,0.0430776328613364,0.54979830420
3563,0.078351701945932,0.937548291625911,0.982848674836089
"14922","PTPN1",150.194286439483,-0.0171873316303102,0.218745060527884
,-0.0785724330818401,0.937372714944499,0.982848674836089
"14923","AKR7A3",3.40022727165602,-0.0596522275063965,0.77602389020355
6,-0.0768690606815588,0.938727710698588,0.982868174551741
"14924","DHDDS",92.0428921231822,-0.0155393103163619,0.203520439099354
,-0.0763525785671873,0.93913859623969,0.982868174551741
"14925","B4GALT3",61.9054093869825,0.0243038126452932,0.31462904081386
,0.0772459293090866,0.938427904466983,0.982868174551741
"14926","PSEN2",28.4223650305202,0.0264083919919468,0.34145927192359,0
.0773398005658969,0.938353229435283,0.982868174551741
"14927","IRF2BP2",900.216962157988,0.013019613797659,0.169477030705328
,0.0768222911593039,0.938764917358503,0.982868174551741
"14928","LMAN2L",68.9353008423297,0.0217854983292556,0.284446027167478
,0.0765892164014252,0.938950337819822,0.982868174551741
"14929","HOXD8",106.891069538167,-0.0265257402432815,0.343803356391094
,-0.077153814092225,0.938501183086931,0.982868174551741

"14930","TBC1D23",195.070516240252,0.0108664007610047,0.140185251633214,0.0775145789903486,0.938214193811441,0.982868174551741
"14931","NFXL1",57.5266514520813,0.0198412342736154,0.260501821064116,0.0761654340555722,0.939287482406925,0.982868174551741
"14932","AC105285.1",8.64756916752139,0.0387729172416323,0.505500973208095,0.0767019635898328,0.93886064242615,0.982868174551741
"14933","SLC25A4",205.406075645599,-0.0185483939744888,0.242840417268893,-0.0763810002597323,0.939115985039217,0.982868174551741
"14934","CNPY4",271.398681897425,0.024597648666715,0.317173169473489,0.0775527410075301,0.938183836310972,0.982868174551741
"14935","HGSNAT",368.296167883946,0.0167802458622269,0.220166412793866,0.0762161932389644,0.939247099819719,0.982868174551741
"14936","RAB11FIP2",221.100438700717,0.0139960592811442,0.183720785401509,0.0761811422183763,0.939274985415073,0.982868174551741
"14937","PSTK",26.3407268395232,0.023977094229799,0.314563921756668,0.0762232810930766,0.939241460933574,0.982868174551741
"14938","PAFAH1B2",273.530613502179,-0.0129669020898886,0.166058104261586,-0.0780865357192215,0.937759217429338,0.982868174551741
"14939","FOXRED1",75.3657134275774,0.0202217257317282,0.259828283325008,0.0778272691215598,0.937965454610667,0.982868174551741
"14940","AC026124.1",12.5850659663957,-0.035496054135131,0.462365105760314,-0.0767706163222702,0.938806026509613,0.982868174551741
"14941","ANO4",2.84344832000673,0.0659056777651442,0.865849171399191,0.0761168110360864,0.939326165668256,0.982868174551741
"14942","OAS3",28.9737348307789,0.0293697212020345,0.379424591353801,0.0774059506718904,0.938300607036624,0.982868174551741
"14943","TUBGCP3",87.6349532976095,0.0184458382017462,0.2386863998959,0.0772806419209102,0.938400290355885,0.982868174551741
"14944","TMED10",932.703227299535,-0.0129272606825318,0.167354407590897,-0.0772448175618587,0.938428788870441,0.982868174551741
"14945","PDP2",16.688549054454,0.0355643768326353,0.456330714585616,0.0779355316131427,0.937879335215742,0.982868174551741
"14946","SBNO2",47.9396671958454,0.0181289772167717,0.234513387975692,0.0773046578417552,0.938381185576193,0.982868174551741
"14947","TCF3",130.31314344632,-0.0134489805286051,0.175173095782016,-0.0767753773407124,0.938802238945097,0.982868174551741
"14948","AKAP8",75.5221765665555,0.018795320324626,0.246325812761995,0.0763026826700719,0.939178291606304,0.982868174551741
"14949","CHADL",34.0199840736895,-0.0277347398393491,0.35462062321486,-0.0782096077433855,0.937661319555415,0.982868174551741
"14950","TAZ",102.70626926386,0.0145759573191572,0.186794040709923,0.0780322394855869,0.937802407770049,0.982868174551741
"14951","MIR4453HG",11.0994583703858,-0.0343129155372826,0.451815125525137,-0.0759445923759221,0.939463179688998,0.982945790908634
"14952","WAC",515.949775462346,-0.0144096408788963,0.190456177696514,-0.0756585638395917,0.939690742726417,0.983052383365835
"14953","TLCD5",78.2436393175346,-0.0166667403203447,0.220174157011452,-0.0756979862967197,0.93965937810101,0.983052383365835
"14954","SORD",49.83299115318,-0.0183696249864522,0.24358706268122 5,-0.0754129746639784,0.939886136317616,0.983136673115001

"14955","HSH2D",3.55007507439181,0.0668090369217811,0.886069650927237,
0.0753993061966044,0.939897011214911,0.983136673115001
"14956","ZBTB1",180.648231626073,0.0149995694829986,0.199329901010357,
0.0752499720662542,0.940015825070147,0.983195209385686
"14957","ASAP2",269.531813105754,-0.0177173189153694,0.236528815130296
,-0.0749055412365277,0.940289867676907,0.983309411737138
"14958","SF3B6",897.893139173527,-0.0187243531533087,0.249968145199295
,-0.0749069571980066,0.940288741068213,0.983309411737138
"14959","ACCS",97.6747295463927,0.024310388916633,0.325648001167114,0.
0746523510953705,0.940491320142711,0.983309411737138
"14960","DPP9",79.9473527549345,-0.0178282934650397,0.23886119928301,-
0.0746387170396653,0.940502168280448,0.983309411737138
"14961","EFHC2",5.87322926859708,-0.0471895630661379,0.632062125163883
,-0.0746596911718171,0.940485479907077,0.983309411737138
"14962","P2RY10",3.96240994187147,-0.0624729560097309,0.83534464101681
7,-0.0747870435054042,0.940384150731508,0.983309411737138
"14963","AGTRAP",434.744129993611,0.0217042702935789,0.293304617276706
,0.0739990747336343,0.94101121499387,0.983394001445402
"14964","ZDHHC24",139.712558279319,0.017890777720956,0.240508833578079
,0.0743871959079121,0.940702296761045,0.983394001445402
"14965","GOLT1B",158.115304495177,-0.0116796033871484,0.15775754972326
1,-0.0740351470191875,0.940982418712038,0.983394001445402
"14966","RHBDF1",146.489133040048,0.0193798334359795,0.261887116118122
,0.0740007134495244,0.941009817568324,0.983394001445402
"14967","PRR11",8.3855938207712,-0.0394068747002557,0.532640612645404,
-0.0739839842563604,0.94102312904406,0.983394001445402
"14968","VPS26C",86.9017339165004,-0.0151951673797072,0.20468997053337
2,-0.0742350362360811,0.940823367866025,0.983394001445402
"14969","CHKB",167.106805256589,-0.0156469316279605,0.211306566756211,
-0.0740484873147018,0.940971803832522,0.983394001445402
"14970","ZMAT2",359.84464162517,-0.0129443367458619,0.175498696217957,
-0.0737574524758058,0.94120338277061,0.983516667780938
"14971","DBI",1829.06317149637,-0.0215002589701804,0.294810877509063,-
0.0729289880748021,0.94186262620954З,0.983679867926017
"14972","SH3BP4",67.9917483634192,-0.0226574510091755,0.30840302376984
1,-0.0734670196556974,0.941434487632865,0.983679867926017
"14973","B4GALT4",111.961644075235,0.0163828543547426,0.22373826816037
9,0.0732233001061721,0.941628425350754,0.983679867926017
"14974","PRKAA1",342.662984023455,-0.0158352621681792,0.21698906255584
8,-0.0729772366480617,0.941824231724656,0.983679867926017
"14975","AC146944.4",14.5452948157787,0.0282395108370363,0.38666944163
1924,0.0730326935530578,0.94178010127479,0.983679867926017
"14976","ARRDC1-
AS1",48.5021885732984,-0.0218954254530668,0.300176754668643,-0.0729417
75512353,0.941852450412008,0.983679867926017
"14977","LINC02709",7.67889195468392,-0.0487130306275008,0.66454021182
5693,-0.0733033603695274,0.941564717708221,0.983679867926017
"14978","VBP1",254.520391589058,-0.0178450969481951,0.244464257887723,
-0.0729967525820932,0.941808701682395,0.983679867926017
"14979","IP6K1",105.389990165419,-0.0143722240881854,0.198120043163704

,-0.0725430090700608,0.942169779320051,0.983838017448883
"14980","COPB2",422.821549675176,0.0131412525605935,0.181001023226597,
0.0726031948678091,0.942121884295554,0.983838017448883
"14981","NF2",85.4624440412774,0.0173352224126612,0.239101239187751,0.
0725016000400102,0.942202732174245,0.983838017448883
"14982","ZNF680",133.840773129175,0.0136510759918699,0.188761477372421
,0.0723191838816601,0.942347898139335,0.983923920077
"14983","TMEM59",3421.76842384382,0.0176739115658559,0.245494407850638
,0.0719931330436209,0.942607372709284,0.983961899625093
"14984","AL121749.1",3.54473931595368,0.0588456092875593,0.81661355028
5253,0.0720605349580641,0.942553733080575,0.983961899625093
"14985","AC087477.2",46.7314305980614,-0.0268177021886302,0.3733689061
74048,-0.0718262869381228,0.942740152892959,0.983961899625093
"14986","AC026471.4",6.78036049158266,0.0500123011394992,0.69443430226
1882,0.0720187654564315,0.942586973953765,0.983961899625093
"14987","TMC6",9.77329208820819,0.0476460399102526,0.663309584248751,0
.0718307726010253,0.942736583072627,0.983961899625093
"14988","ZNF69",20.7200407873863,-0.0275341995010351,0.38348874396309,
-0.0717992377468196,0.942761679446455,0.983961899625093
"14989","U73166.1",5.05438404330284,-0.0563625713763332,0.791936195344
959,-0.0711705964541528,0.943261982687354,0.984040847367337
"14990","AC093418.1",3.67764456138989,0.0683138987888786,0.96012708389
441,0.0711508923504042,0.943277664531945,0.984040847367337
"14991","APOD",34371.5631534228,-0.0268977445608406,0.377570850547793,
-0.071238933095117,0.943207595985022,0.984040847367337
"14992","C11orf74",165.751546353798,0.0227061427036282,0.3182271956090
05,0.0713519869355429,0.943117621035342,0.984040847367337
"14993","NUDT14",58.0609666092325,-0.0286087932013847,0.40100876657012
1,-0.0713420642797398,0.94312551804415,0.984040847367337
"14994","RPN2",772.247629310951,-0.0163579611476255,0.228491978806149,
-0.0715909645191681,0.94292743089427,0.984040847367337
"14995","NDUFA6-
DT",21.8973293022973,-0.0260314756980404,0.365301445735361,-0.07126025
91693509,0.943190623375139,0.984040847367337
"14996","MAP9",107.848211227468,0.0125476021485754,0.176844120308366,0
.0709528941459625,0.943435245974805,0.984129987899574
"14997","PDLIM7",186.124446204302,-0.0221082026660852,0.31223457960305
4,-0.0708063876018841,0.943551848016225,0.984129987899574
"14998","SNRPB2",598.518521314312,0.0154491402615164,0.218001770415016
,0.0708670403552479,0.943503575384982,0.984129987899574
"14999","TMEM200B",87.8095397053553,-0.014133926071446,0.2008290965212
39,-0.0703778800794995,0.943892896725013,0.984232472995102
"15000","EGLN1",153.392284896383,0.0119407827387437,0.169201429105456,
0.0705714059383122,0.943738868410042,0.984232472995102
"15001","EOGT",23.3015345278065,-0.0277262233654218,0.394109655819725,
-0.0703515454544064,0.943913856762914,0.984232472995102
"15002","GFM1",114.980101077797,-0.0143186087464043,0.204173646383287,
-0.0701295637318664,0.94409053616899,0.984232472995102
"15003","EHMT2",125.928241461247,0.0122576936581442,0.17465547020198,0
.0701821342553363,0.944048694056925,0.984232472995102

"15004","CYTH3",169.436713436876,0.018167460467275,0.2580096442222,0.0
704138813184403,0.943864242978293,0.984232472995102
"15005","MED17",109.540381177157,0.0130541779575479,0.185864792061518,
0.0702348078555258,0.944006770058515,0.984232472995102
"15006","LINC01309",3.56588906786863,0.0683460901352936,0.978517052418
017,0.06984660100343,0.944315755485056,0.9844016635381
"15007","CENPO",7.32881439402511,0.0409282212108936,0.589040772820872,
0.0694828322577593,0.944605297497487,0.98446189861446
"15008","POLR2D",71.1710497921719,-0.0169139936835234,0.24407054617525
6,-0.0692996100864144,0.944751136099231,0.98446189861446
"15009","EXOC3",162.336715821586,-0.0123137433370298,0.177108559585191
,-0.0695265286210335,0.944570516956083,0.98446189861446
"15010","ZNF585A",109.417685259438,0.0137378447946538,0.19771649160145
3,0.0694825438352703,0.944605527070498,0.98446189861446
"15011","Z82217.1",17.4529483005379,0.0349727363636697,0.5043615686550
42,0.0693406051078195,0.944718505298866,0.98446189861446
"15012","Z93241.1",18.7812095728863,0.0392071439439519,0.5651536017207
58,0.0693743149200067,0.944691673374762,0.98446189861446
"15013","MAEA",177.922804369853,-0.0156086600397113,0.225684994247711,
-0.0691612665332074,0.94486125407821,0.984462738469158
"15014","PFDN1",394.322126647281,0.0180788956955272,0.262428355363452,
0.0688907860985093,0.945076552723833,0.984462738469158
"15015","DAXX",141.857752056957,-0.0141230036472069,0.20478407650755,-
0.0689653408998633,0.945017207742807,0.984462738469158
"15016","SDCBP",1933.64910812518,0.0182898526041047,0.265747337035094,
0.0688242178008707,0.945129540774137,0.984462738469158
"15017","XRRA1",225.220631982618,0.0256295067362908,0.372376950169178,
0.0688267808322906,0.945127500608745,0.984462738469158
"15018","RABEP2",54.9521383099083,-0.0179479942170547,0.26052206604885
5,-0.0688924147165672,0.9450752563546,0.984462738469158
"15019","RAB12",121.710358067073,-0.0213165836992236,0.310518538667179
,-0.0686483447678183,0.945269536044579,0.984543002353375
"15020","ARL4A",247.188765596708,-0.0244292935984078,0.356580162910286
,-0.0685099625257452,0.945379689762136,0.984592176228302
"15021","PPP1R2",318.892948125197,0.0123365114413248,0.180733097115462
,0.0682581753880065,0.945580117644549,0.984735355856047
"15022","GPR137B",116.216204377047,-0.0235506568140765,0.3471993679603
8,-0.0678303562371805,0.945920678621506,0.984798740659029
"15023","CEP85L",90.4154523431791,0.017880763157076,0.263008981974614,
0.0679853707764316,0.945797279767879,0.984798740659029
"15024","ROBO4",57.446899334307,0.0332297081724393,0.49046564554706,0.
0677513470599459,0.945983574136698,0.984798740659029
"15025","A2M-
AS1",22.6317630596795,-0.0239405274468677,0.353589040704346,-0.0677072
100401597,0.946018709702949,0.984798740659029
"15026","JPT1",166.927894711977,-0.0216283940506709,0.319176847296082,
-0.0677630418180285,0.945974264464855,0.984798740659029
"15027","MOB3A",116.104972412939,-0.0123121556257559,0.180960999158169
,-0.0680376196143479,0.945755687531878,0.984798740659029
"15028","SLC9A6",39.7410890734258,-0.0174898336395025,0.25920899198831

8,-0.0674738692718293,0.946204463906726,0.984926565670276
"15029","MSH6",122.965537687798,0.0138039647390964,0.205517163578492,0
.0671669679492454,0.94644878147666,0.984958986703788
"15030","AL022069.1",2.89671352681148,-0.0691328904907827,1.0312296457
718,-0.0670392775985819,0.946550434514993,0.984958986703788
"15031","AC124242.1",8.12421080990875,-0.0487324400036817,0.7240531194
93272,-0.0673050618686472,0.946338847240407,0.984958986703788
"15032","FBH1",197.068114691409,-0.0111884702297282,0.166875603475222,
-0.0670467701492955,0.946544469744659,0.984958986703788
"15033","PRKD2",50.2641889767377,-0.0163899043894367,0.244023718715019
,-0.0671652103153853,0.946450180705858,0.984958986703788
"15034","AC145212.1",3.54909112843477,0.0607021822471467,0.90704246007
2569,0.0669231981072751,0.946642845016974,0.984989625156347
"15035","PNPT1",138.355230758775,-0.0159900812458687,0.240953973309046
,-0.0663615587088076,0.947089974415636,0.985134855540153
"15036","TBCCD1",36.8474939395003,-0.019197211607627,0.288920750832659
,-0.0664445580744942,0.94702389634513,0.985134855540153
"15037","TRPV4",5.80489976222336,0.040204004728878,0.604523719491736,0
.0665052560099382,0.946975573285315,0.985134855540153
"15038","AC026461.3",3.24780979881584,-0.0648899459456159,0.9779599536
95321,-0.066352354920488,0.947097301826271,0.985134855540153
"15039","NONO",411.990300318401,0.0104824423964607,0.15767158208468,0.
0664827628280595,0.946993480615855,0.985134855540153
"15040","OBSCN",13.6570887169046,-0.0319432368851916,0.485373259918253
,-0.0658116948811139,0.947527745254821,0.98525501987644
"15041","PCDHB7",23.2795389278558,0.0307585640985912,0.466433504489986
,0.0659441566750734,0.947422285126335,0.98525501987644
"15042","LGR5",7.37000686762141,-0.0640842314999706,0.973286116501067,
-0.0658431579506665,0.947502695689244,0.98525501987644
"15043","AC008915.1",3.7451442955275,-0.0491781536554864,0.74415452690
7904,-0.0660859430094856,0.947309402238516,0.98525501987644
"15044","MTFP1",8.01272806239223,0.038974669097713,0.591167780639076,0
.0659282700684057,0.947434933280156,0.98525501987644
"15045","ANP32E",209.277111448078,0.0103291589894628,0.157519219184253
,0.0655739600726471,0.947717021331709,0.985291252513911
"15046","STKLD1",8.45825698023632,-0.0369089688522718,0.56205785776324
4,-0.0656675613417346,0.947642498946944,0.985291252513911
"15047","TMTC3",156.055121110646,-0.011906465658973,0.181693237503696,
-0.0655305933371946,0.947751548716795,0.985291252513911
"15048","IPO13",33.4447083982749,0.0198243076365562,0.303351956480198,
0.0653508481256501,0.947894657885821,0.985309065938461
"15049","AC104211.1",5.66286984904314,-0.0485689139649394,0.7430533279
77169,-0.0653639680171538,0.947884212060938,0.985309065938461
"15050","AC107029.2",6.20354651851016,0.0466361155621821,0.71826008827
1622,0.0649292872090448,0.948230301966944,0.985380140176675
"15051","ACSL6",2.98777282172839,0.0534840010147346,0.823543933398171,
0.064943713195779,0.948218815937254,0.985380140176675
"15052","CDKN2B-
AS1",3.43042247199466,0.056094993358448,0.864063395668115,0.0649199973
516688,0.94823769859511,0.985380140176675

"15053","C9orf85",128.279082840012,0.0149856483765617,0.231294714466237,0.0647902759522382,0.948340983849635,0.985380140176675
"15054","SLC15A4",159.883256619237,-0.00986311314234383,0.152083587780277,-0.0648532381850013,0.948290852688987,0.985380140176675
"15055","TECR",380.918462581407,0.012981135499976,0.200273266228736,0.0648171158558428,0.948319613624413,0.985380140176675
"15056","PDIK1L",32.6836095747716,0.0177579213544685,0.274428247726076,0.0647087954742684,0.948405859726222,0.985382097748226
"15057","CNFN",7.58074529895021,-0.0558796783844825,0.865203761141893,-0.064585570352506,0.948503973911698,0.98541858696292
"15058","CPLANE2",6.2963198888602,0.0394750143268657,0.614217017534758,0.0642688385374015,0.948756165385969,0.9854342969732
"15059","ABCE1",244.209711594812,-0.0110294118437665,0.171773464804195,-0.0642090549686415,0.948803767452717,0.9854342969732
"15060","AC012640.2",5.28610323484209,-0.0409456961581092,0.639012800215137,-0.064076488208568,0.948909323056822,0.9854342969732
"15061","ERAP2",286.221692135481,0.0591433086008935,0.923294823636946,0.0640567964714915,0.948925002578199,0.9854342969732
"15062","HNRNPH1",681.227206554382,-0.0151020398217681,0.234259816109062,-0.0644670523208181,0.948598340959233,0.9854342969732
"15063","AC026979.4",16.1839309957072,0.0308335427025924,0.478852782740507,0.0643904427705941,0.948659339800043,0.9854342969732
"15064","SMIM10L2B",4.48201261459665,0.0429882470661561,0.671557407337636,0.0640127658431784,0.948960061983269,0.9854342969732
"15065","S100A11",6528.43158755966,-0.0202335351119199,0.317759576146552,-0.0636756108416639,0.94922852505585,0.985445959548688
"15066","AL646090.2",5.18592133706898,0.0429723471434448,0.674832090931197,0.0636785768207132,0.949226163339926,0.985445959548688
"15067","AC044849.1",20.5870169113958,-0.0323372169325266,0.508771504746029,-0.0635594105229396,0.949321052069401,0.985445959548688
"15068","AC107375.1",22.8804511714334,0.0280425346291774,0.441337482519498,0.0635398889509423,0.949336596609823,0.985445959548688
"15069","MYPOP",40.5820672304146,-0.0184570530936507,0.290552553856582,-0.0635239747462732,0.949349268707967,0.985445959548688
"15070","SAMHD1",1008.50830760917,-0.0169342749506699,0.265552937072891,-0.0637698650119682,0.949153473639804,0.985445959548688
"15071","AC082651.1",7.90731114701938,0.0387615025215721,0.61211700958132,0.0633236814446382,0.949508758537444,0.985488843512591
"15072","SLC30A5",142.065826926359,-0.0134911934240566,0.213084361542666,-0.0633138599491038,0.949516579263681,0.985488843512591
"15073","RORA",307.477780077689,-0.0171809387757771,0.271737575413322,-0.0632262165055542,0.949586368787093,0.985495891125622
"15074","LSM11",30.8749836953737,-0.0204628598626856,0.324669437505557,-0.0630267512085592,0.949745202280228,0.985595342926204
"15075","FASTKD1",21.3280200924046,-0.0205061190826719,0.327387608836329,-0.0626355992994335,0.950056680919351,0.985853178084339
"15076","TTC37",361.340332619144,-0.00966142074601452,0.154745793482219,-0.0624341413656885,0.950217107105071,0.985954245585343
"15077","ARMT1",118.540135501499,0.0131564217642453,0.211042841370333,0.0623400522795212,0.950292033380119,0.985966590048763

"15078","CCS",386.381953867085,-0.0139747962591919,0.225309078869868,-0.0620250028506987,0.950542920935764,0.986030697095369
"15079","DUSP16",66.1001451889776,-0.0138279171061704,0.222730090538082,-0.0620837403368547,0.95049614534961,0.986030697095369
"15080","CST3",17729.3748539983,0.0204347443805067,0.328725338934286,0.0621635814469165,0.950432564170033,0.986030697095369
"15081","PISD",54.1087523796306,0.0175737354534713,0.283746163513926,0.0619347068373979,0.950614828481421,0.98603990199157
"15082","SFXN2",5.19966095886684,0.0578876003606142,0.943438379634569,0.0613581147536487,0.951074009059733,0.986185393640548
"15083","RBM23",175.492884164486,-0.0113742085913007,0.185839606809906,-0.0612044374530736,0.951196395783966,0.986185393640548
"15084","GOLGA8A",170.24058723131,-0.0247809645938934,0.403604196916182,-0.0613991746945084,0.951041309619826,0.986185393640548
"15085","SEMA4B",19.1237016123269,0.0255249078721013,0.416719541909001,0.0612520059778604,0.951158512668969,0.986185393640548
"15086","AC005332.2",6.78442976370092,0.03809765158207,0.619884505676516,0.0614592738376189,0.950993447831091,0.986185393640548
"15087","WFDC3",13.5074245517634,0.0398789876697777,0.647451760286511,0.0615937589730706,0.950886347125418,0.986185393640548
"15088","GUCD1",124.903041969969,-0.0105792101602553,0.171771838142891,-0.0615887346530859,0.950890348356263,0.986185393640548
"15089","TACC1",1417.16022788435,-0.0128690217541415,0.210868218359676,-0.0610287403869982,0.951336320190565,0.986189355506587
"15090","C11orf95",99.6512686761156,-0.0128144691355915,0.209844246869386,-0.0610665735504661,0.95130618989094,0.986189355506587
"15091","GAS8",33.657195471887,-0.0166984530248371,0.273915063591055,-0.0609621566843334,0.9513893475644,0.986189355506587
"15092","EPC2",177.544148994532,0.0113247678487017,0.186899951817963,0.0605926739870528,0.951683608116496,0.986217899347853
"15093","CAPN10",38.5649168692706,-0.0163967186261203,0.270197027352641,-0.0606843042899971,0.951610632003456,0.986217899347853
"15094","AC233280.1",2.97106857149722,-0.0543796089454984,0.893841375557588,-0.0608380977123327,0.951488148914256,0.986217899347853
"15095","SPTLC1",361.900456000056,-0.00983237610440201,0.16243330665102,-0.0605317733605361,0.951732110755942,0.986217899347853
"15096","NOC3L",164.247658628132,0.0137590484514415,0.226989268113838,0.060615413961074,0.951665497531601,0.986217899347853
"15097","MAP3K6",69.3145292785279,0.0183382848799639,0.305101124358039,0.0601055958694003,0.952071533164043,0.986392494733966
"15098","WWC2-AS2",14.7937036068833,0.023049317800171,0.384640240464784,0.059924353656599,0.952215883332968,0.986392494733966
"15099","EXOC3-AS1",28.4415015621579,-0.0224537620598574,0.373192264936835,-0.0601667402288142,0.952022835158081,0.986392494733966
"15100","GPHN",48.683394055373,0.0147466111374697,0.245782568735179,0.059998604511936,0.952156746127828,0.986392494733966
"15101","KPTN",30.4494929294668,-0.0232413746806794,0.387216409374509,-0.0600216677754501,0.952138377410607,0.986392494733966

"15102","FBXO11",215.773319887583,0.0106550289099371,0.179986411841594
,0.0591990739796214,0.952793547208014,0.986403008535735
"15103","ESPNL",7.12477851473945,0.0373139179616554,0.629428200688037,
0.0592822468406516,0.952727301212085,0.986403008535735
"15104","AHCYL2",36.4743365637698,0.0201181934063634,0.337880149071106
,0.0595423953187898,0.952520098775401,0.986403008535735
"15105","NAT1",10.6325533890038,0.0281717899157626,0.474939991394082,0
.0593165250899814,0.952699999171082,0.986403008535735
"15106","C10orf55",6.10786205464746,-0.0368242009942773,0.619364905771
198,-0.0594547748042422,0.952589886195177,0.986403008535735
"15107","KMT2B",124.504759529736,0.016474387644051,0.278251159461722,0
.0592068966609906,0.952787316540124,0.986403008535735
"15108","MZF1-
AS1",13.7228603198069,-0.0257356259988506,0.434116494318045,-0.0592827
647318003,0.952726888720205,0.986403008535735
"15109","HELZ2",70.7255557972981,0.0160882679258611,0.269418400023659,
0.0597148076168823,0.952382778008768,0.986403008535735
"15110","ZNF182",31.0583714496994,-0.0175660237877615,0.29377435320454
8,-0.0597942726999408,0.952319487140161,0.986403008535735
"15111","FOXC1",39.2477137732425,-0.0251675054468307,0.430836253416764
,-0.0584154774516741,0.953417686600728,0.986436005082951
"15112","AC016405.1",2.76247109795086,-0.0606593541089405,1.0273792986
9101,-0.0590428035548572,0.95291801525126,0.986436005082951
"15113","CEL",2.87632263333999,0.054513655729977,0.937853049738237,0.0
58126009981193,0.953648256445401,0.986436005082951
"15114","SLC25A22",14.7455393087405,-0.0234803487722747,0.406698586681
346,-0.0577340309044935,0.953960486153224,0.986436005082951
"15115","HINFP",32.8321238274273,0.0216313770267779,0.37393221435097,0
.0578483912233218,0.953869392066707,0.986436005082951
"15116","VPS26B",152.121439734006,-0.0112280007428167,0.19313761036976
1,-0.0581347191845272,0.953641319237327,0.986436005082951
"15117","AC005480.1",2.89551305826526,-0.0617071290853859,1.0490374704
7049,-0.0588226167533466,0.953093394084755,0.986436005082951
"15118","GPR65",9.80071996127819,0.0387014835353664,0.660603800810056,
0.0585850149331707,0.953282646532119,0.986436005082951
"15119","KLC1",293.558136284545,0.0101686827026363,0.176027397797016,0
.0577676136209329,0.953933735668024,0.986436005082951
"15120","EME2",11.4240327625994,0.0351021415021054,0.600103233827604,0
.0584935049894922,0.953355535905967,0.986436005082951
"15121","ALG1",62.120864893492,-0.0162718120117274,0.279236374147036,-
0.0582725372417248,0.953531542438307,0.986436005082951
"15122","AC106886.3",3.14443862743446,-0.0465708224622778,0.7905639390
63077,-0.0589083566314324,0.9530251019834,0.986436005082951
"15123","C18orf32",665.808407177069,-0.0155735909836276,0.264363451202
186,-0.0589097733170266,0.953023973591453,0.986436005082951
"15124","ADAT3",3.81289975791148,0.0473010474446434,0.81107176398864,0
.0583191889359199,0.953494382966968,0.986436005082951
"15125","TP53RK",67.6401290756332,0.0159491917903253,0.275112437859003
,0.0579733577821711,0.953769850234071,0.986436005082951
"15126","GNB1L",20.7492642477532,0.0281013532606009,0.48487523583051,0

.0579558434500536,0.953783801192685,0.986436005082951
"15127","SHANK3",8.85453022198753,0.0307494101169935,0.528432453602907
,0.0581898592854031,0.953597398154284,0.986436005082951
"15128","MORC4",203.985550603459,0.0133666402099132,0.229962007810772,
0.0581254283573317,0.95364871973081,0.986436005082951
"15129","COA1",172.967048771731,-0.0109103317273676,0.189407214002502,
-0.0576025141641305,0.954065246986994,0.986479123446199
"15130","KTN1-
AS1",13.9793299891345,-0.0270467591237889,0.470572899973852,-0.0574762
361480905,0.954165835615003,0.986517922440548
"15131","C16orf46",12.4336375834097,0.0285188030388107,0.4981515379696
67,0.0572492522155924,0.954346644870044,0.986639651424367
"15132","ZFP90",137.838489992472,0.0106732252726508,0.186963411154605,
0.0570872407961408,0.954475700217869,0.986707862708705
"15133","HLA-
DOB",2.8041004349985,-0.0652094580560733,1.1440387936361,-0.0569993416
47164,0.954545719579702,0.986715039409587
"15134","PALLD",534.346406182798,0.0126060430468468,0.222829454626361,
0.0565726064715478,0.95488565645503,0.986954832742372
"15135","THAP2",61.6573375659081,-0.0149880144186451,0.265041307353917
,-0.0565497301846281,0.954903879917906,0.986954832742372
"15136","AK9",38.066820048141,0.0192246036155151,0.34057120087981,0.05
64481188246438,0.954984824756887,0.986973283144291
"15137","IARS2",320.993880446001,-0.0100437973835932,0.179237017075837
,-0.0560364011154213,0.955312808809505,0.986986214134276
"15138","AC068870.2",6.8071861542675,0.0327241498158621,0.583374486885
071,0.05609458512763,0.95526645759057,0.986986214134276
"15139","TRAF4",19.4478731683355,-0.0190121635804202,0.338944590137555
,-0.056092246737747,0.955268320422775,0.986986214134276
"15140","CILP2",6.01945467502548,-0.0391709856093464,0.695163990071244
,-0.0563478347106731,0.955064712750861,0.986986214134276
"15141","AL390198.1",14.6208885257657,-0.0334646099242575,0.5967559010
72311,-0.0560775517495929,0.95528002690100l,0.986986214134276
"15142","MLEC",565.524761772996,0.0121426327362478,0.217575954358559,0
.0558087072261538,0.955494198393349,0.987108423290659
"15143","DHODH",33.9723922381745,0.0155366083101618,0.278901012307809,
0.0557065325134598,0.955575595392624,0.987127322110996
"15144","MORC2-
AS1",14.9041243233089,0.0275127020556743,0.494631213206405,0.055622656
4784005,0.955642415183631,0.987131160903166
"15145","LINC00504",4.132688883187,-0.044879140951051,0.80914733834792
7,-0.0554647328417131,0.955768225795955,0.987195929754118
"15146","TESPA1",3.47404092816214,0.0505869714239566,0.919209389669434
,0.055033131724371,0.956112068480215,0.987447490449987
"15147","LTA4H",149.660248777807,-0.0124170371356507,0.225762054943046
,-0.0550005497548435,0.956138025816401,0.987447490449987
"15148","C1orf131",179.548070101275,-0.0100478082904681,0.183395785412
139,-0.0547875637811851,0.956307708186618,0.987525391152424
"15149","RAB5A",208.160107254687,-0.00871673245718164,0.15956789155777
9,-0.0546271080734645,0.956435541878904,0.987525391152424

"15150","MSI2",69.0102606674088,-0.0167776647052471,0.306976563377343,
-0.0546545460039682,0.95641368223444,0.987525391152424
"15151","HUWE1",243.640536718357,-0.0120051077672784,0.219918426748495
,-0.054588912556235,0.956465972086581,0.987525391152424
"15152","SYNGAP1",70.9845345589548,-0.0150706220383657,0.2767227602813
84,-0.0544610859729833,0.956567811448212,0.987565356024577
"15153","LINC02731",6.99745843378943,0.0322038780089155,0.596397049813
069,0.053997379797585,0.95693725181652,0.987751199615033
"15154","PALB2",33.7992294615046,-0.0168677695811225,0.312179616227539
,-0.0540322580473289,0.956909463566633,0.987751199615033
"15155","SYP",15.5907531678334,0.0298932926247601,0.552400801999363,0.
0541152230709372,0.956843363762806,0.987751199615033
"15156","AL606760.1",3.09805021184297,0.0544369187602151,1.01565888264
36,0.0535976396115637,0.957255737127894,0.988014746363924
"15157","FUBP3",151.684731791766,0.00835070654961957,0.156735838767651
,0.0532788583343652,0.957509724877538,0.988211692700358
"15158","TPST2",48.61206649513,0.01507200352563,0.284329889219336,0.05
30088608236442,0.957724847725071,0.988368504615602
"15159","AL512353.1",5.9397623558246,-0.0337282410389186,0.64959242952
2071,-0.0519221584274523,0.958590718215758,0.988531911818575
"15160","SLC22A5",39.1330731029118,-0.0160025684887307,0.3085985061993
93,-0.0518556252452858,0.958643732597045,0.988531911818575
"15161","AL355336.1",3.69488688897636,0.0521198145362408,1.00072985188
373,0.0520818025345531,0.958463512758823,0.988531911818575
"15162","TBC1D7",39.6959406929727,-0.0184046257260626,0.35635468177864
9,-0.0516469311815993,0.958810023539161,0.988531911818575
"15163","FAM185A",30.5935607162649,-0.0153469021602803,0.2955798667678
55,-0.0519213379723646,0.958591371962404,0.988531911818575
"15164","NDOR1",6.87480034638544,0.0288922774998788,0.552684069175296,
0.0522762987234123,0.958308538367277,0.988531911818575
"15165","HYOU1",59.8408424797625,-0.0200198414168164,0.382373818818826
,-0.052356726406266,0.958244454120772,0.988531911818575
"15166","RAB35",88.0210863751202,-0.0114739257022655,0.22020343610504,
-0.0521060247978704,0.9584442123951,0.988531911818575
"15167","POLR3E",67.2856403342053,-0.0116577177391096,0.22264861220286
9,-0.0523592652285997,0.958242431208296,0.988531911818575
"15168","ADGRG1",2.7196753944278,-0.0503081587301478,0.969576580895365
,-0.051886730477432,0.958618947578518,0.988531911818575
"15169","VAC14",52.794022080425,-0.0132146643139292,0.25147770233997,-
0.0525480557161464,0.958092005275628,0.988531911818575
"15170","TAF1C",53.5732326244013,-0.0125271956050023,0.237593683634276
,-0.0527252888771453,0.957950789436217,0.988531911818575
"15171","CWC25",199.590660252581,-0.00955995703805669,0.18519589895823
2,-0.0516207815174827,0.958830860158121,0.988531911818575
"15172","HOXB-
AS1",149.171038654459,-0.0133338724075934,0.254878727959847,-0.0523145
753053742,0.958278039805944,0.988531911818575
"15173","GPKOW",61.7567634905802,-0.012737286920247,0.24674863246557,-
0.0516204965068015,0.958831087260962,0.988531911818575
"15174","MON1B",94.3028189933644,0.0133549587661656,0.260374733982289,

0.0512913006646568,0.959093400045716,0.988672030109729
"15175","MPP3",20.1046002871792,-0.0184112118084593,0.358858530060432,
-0.0513049301220701,0.959082539611077,0.988672030109729
"15176","SPON2",2017.30384972984,-0.0128499268668579,0.251983068514935
,-0.0509951995687213,0.959329345762285,0.988850089335756
"15177","ZNF674",16.7805483305707,0.019807974964255,0.389540890195102,
0.050849539709,0.959445414929017,0.988904567815419
"15178","LINC01060",7.45203953780543,0.110001403051755,2.1753363515851
9,0.0505675377380584,0.959670130848897,0.988930281298771
"15179","ASNS",7.38413092349564,0.0286760836581301,0.568726627014262,0
.0504215598426886,0.959786455966115,0.988930281298771
"15180","NPTN",497.600738915489,0.0102367412259855,0.202714886825286,0
.0504982213507008,0.959725366754699,0.988930281298771
"15181","FBXL19-
AS1",5.9540960836618,-0.0420365572726602,0.830944718568908,-0.05058887
35234487,0.959653129115589,0.988930281298771
"15182","AL022238.2",4.47957823303373,-0.0360167542568187,0.7127317511
49709,-0.0505333938030964,0.959697338982083,0.988930281298771
"15183","AC133552.5",19.3565345028723,0.0198186639348101,0.39435508423
5301,0.0502558854369551,0.959918477639807,0.989001168788744
"15184","SCMH1",68.8739316862384,0.0114948110893275,0.231617078572361,
0.0496285125439762,0.960418424897815,0.98945109461779
"15185","CCDC84",97.8481972027375,0.0130176662019242,0.2631508002726,0
.0494684651858902,0.960545967555586,0.989517324364309
"15186","ZNF35",19.3338646591137,0.0172239368972946,0.349425419959788,
0.0492921691251792,0.960686460066341,0.989596884947831
"15187","ALAS1",59.3850583923482,-0.0107444104506312,0.219621510224034
,-0.0489223958057248,0.960981140957758,0.98977008085345
"15188","MAB21L1",27.0984117677951,0.0179372818969628,0.36625273003503
9,0.0489751486500777,0.960939100671785,0.98977008085345
"15189","GTF2E2",118.875301282627,0.00996112736914004,0.20438017152332
4,0.0487382278569194,0.961127910637386,0.989790908894051
"15190","AC006504.5",28.9273016871909,-0.0139160177292513,0.2851456922
43854,-0.0488031841538412,0.96107614461244,0.989790908894051
"15191","TSEN2",34.9747678393548,-0.014521356265672,0.300365114467334,
-0.0483456818593067,0.961440748236114,0.989818998400217
"15192","RHOH",8.4853821596711,-0.0306230618733253,0.633595669545705,-
0.0483321830391965,0.96145150616016,0.989818998400217
"15193","ARSK",98.2843904876077,0.00940533719736967,0.195280051669753,
0.0481633280867595,0.961586076199219,0.989818998400217
"15194","TM2D2",294.615150313899,0.0104980991129237,0.216960060468876,
0.0483872427498229,0.961407626207331,0.989818998400217
"15195","LINC01235",30.9211611838236,0.0347660296597048,0.722662064272
973,0.04810828100501,0.961629946561371,0.989818998400217
"15196","AL158211.1",2.87335716238572,0.0404001476123155,0.84110898968
8507,0.0480320007366431,0.961690739131324,0.989818998400217
"15197","IGSF22",3.68524828298668,-0.0356590496295771,0.74306049290273
3,-0.0479894301610311,0.961724666412126,0.989818998400217
"15198","CD79A",3.13197700307163,0.0444964702983806,0.921269074473676,
0.0482991034121074,0.961477869095089,0.989818998400217

"15199","TXNRD2",71.7910419216098,0.0132253564103416,0.274487788993383
,0.0481819481254244,0.961571236786034,0.989818998400217
"15200","C1orf122",592.593428771587,0.0168653675686814,0.3531795587192
4,0.0477529549836958,0.96191313023427,0.989892860234778
"15201","RRAGD",38.5409513563163,0.0240128185353932,0.502985473127054,
0.0477405806297065,0.961922992292326,0.989892860234778
"15202","CYP27A1",303.638245267438,-0.0086595996945912,0.1889390539858
73,-0.0458327672966897,0.963443541718285,0.990345860029744
"15203","C1QTNF7",285.085352975543,0.0229753952976998,0.49041563468315
3,0.0468488230652428,0.962633716546779,0.990345860029744
"15204","OSTC",1214.44767445346,0.0142446994240173,0.303087904007288,0
.0469985744586983,0.962514363692006,0.990345860029744
"15205","MTRR",69.0159761632767,0.0105732931595041,0.228154049941608,0
.0463427809508975,0.963037041651453,0.990345860029744
"15206","ARL15",34.84437901407,0.0122038966814055,0.265954942505929,0.
0458870836030183,0.963400249125051,0.990345860029744
"15207","UBE2B",1046.8504763529,-0.00861659213706572,0.188119101708401
,-0.0458039192129573,0.963466535011085,0.990345860029744
"15208","PCDHGA11",5.61438561083171,-0.0288628379645627,0.617861976364
235,-0.0467140543821842,0.962741128797842,0.990345860029744
"15209","KIAA1324L",37.5447250719724,0.0206588912865315,0.444201812206
106,0.0465078951027465,0.962905441515193,0.990345860029744
"15210","AL158212.2",32.3625950679165,0.022652815213178,0.484484116971
535,0.0467565693479874,0.96270724378888,0.990345860029744
"15211","METTL15",90.1051267570211,0.00973554294640824,0.2127593503794
96,0.0457584727958751,0.963502758025486,0.990345860029744
"15212","PRPF19",139.474530641484,0.00899403803663736,0.19624005799941
6,0.0458318150143642,0.963444300732039,0.990345860029744
"15213","SNAPC1",137.558259608244,-0.0083027321898660 7,0.1795598098967
84,-0.0462393683455039,0.963119464614743,0.990345860029744
"15214","FAM71D",2.85777169220756,-0.0472226687316033,1.02838683405532
,-0.0459191689039682,0.96337467571582,0.990345860029744
"15215","FOXN3",859.359146763854,0.0107083503571993,0.23024280599019,0
.0465089465494768,0.96290460348891,0.990345860029744
"15216","LYSMD2",128.609189280858,-0.0138022914057144,0.29793050044225
8,-0.0463272185466941,0.963049445331417,0.990345860029744
"15217","SLC9A8",32.978945634165,0.0127914398679184,0.279282321838623,
0.0458011082968209,0.963468775446368,0.990345860029744
"15218","IGLC1",63.2634607741987,0.058480898079532,1.2634606458841,0.0
46286283842747,0.963082071500541,0.990345860029744
"15219","PIM2",8.20523243852053,0.0324049187999593,0.695269702750148,0
.0466076957931308,0.962825898341963,0.990345860029744
"15220","ZDHHC6",120.843103207757,0.00965160879672998,0.21233990032224
4,0.0454535807075488,0.963745773857585,0.99048242040521
"15221","NRDE2",131.067145292388,-0.0117505248687987,0.259087687238088
,-0.0453534669827847,0.963825570760603,0.99048242040521
"15222","MOCOS",5.39078829526745,0.0287268334619284,0.632877520577578,
0.0453908260728105,0.963795793185777,0.99048242040521
"15223","ZNF790",43.1721231385987,-0.0109683469485098,0.24316815074603
4,-0.0451060178516767,0.964022804754472,0.990620031187953

"15224","RHOA",2011.27074576037,0.0103513700590019,0.230044357440941,0
.0449972786733507,0.96410947806304,0.990644020319241
"15225","USP34",350.272558750644,-0.00837033279514215,0.18686055226107
9,-0.0447945416721621,0.964271075842124,0.990653502842739
"15226","WDR12",62.8210835903788,0.0109679763570605,0.244801056458236,
0.0448036316335573,0.964263830376591,0.990653502842739
"15227","RPP25L",111.625177987148,-0.0140035976392588,0.31294810210903
9,-0.0447473480263497,0.964308693203758,0.990653502842739
"15228","CKMT2-
AS1",44.6176945308038,-0.012898074141994,0.292605788001776,-0.04408003
76167396,0.964840604989951,0.991134855782625
"15229","HIP1",78.9751289298507,-0.0101816650431267,0.234662681083489,
-0.0433885140837721,0.965391833513427,0.991463830468859
"15230","AC011978.2",3.10879276558606,-0.0346765739592445,0.7971418205
30105,-0.0435011350128191,0.9653020597748,0.991463830468859
"15231","NIPSNAP3B",22.0991421141847,-0.0198487404902263,0.45701064329
8165,-0.0434316810369698,0.965357423704766,0.991463830468859
"15232","MAP3K14",22.3907609940498,0.0142714656981849,0.32913717666485
1,0.0433602361264619,0.965414374845085,0.991463830468859
"15233","ANKRD31",4.59028977079204,-0.0288675122983857,0.6701292324961
55,-0.0430775302710756,0.965639730915152,0.991604430337194
"15234","MEPCE",90.0223796631136,-0.00829969587477267,0.19314546630658
5,-0.0429712176707184,0.965724477624067,0.991604430337194
"15235","LINS1",66.1702361016503,0.0117757995012512,0.274175077271343,
0.042949926807523,0.965741449606032,0.991604430337194
"15236","GBP3",154.8672934534,-0.0115146804440138,0.277865538085518,-0
.0414397572413962,0.966945318308847,0.991648430600638
"15237","SELENOI",32.8950884691882,-0.0126684925881678,0.3120439862232
87,-0.0405984192853589,0.967616044346919,0.991648430600638
"15238","ARL6IP6",118.381956352346,-0.0116583380114783,0.2833445617813
18,-0.0411454447482076,0.967179945588066,0.991648430600638
"15239","TIGIT",3.20630680750271,-0.031685274047131,0.772897463264691,
-0.0409954431902176,0.967299528617416,0.991648430600638
"15240","AC092902.4",7.74201299316898,0.026820874293434,0.632404278034
459,0.0424109627733615,0.966171089184155,0.991648430600638
"15241","GNPDA1",182.508551929385,-0.00889553117284015,0.2121255956308
22,-0.0419352089331158,0.966550348405069,0.991648430600638
"15242","ZNF184",24.8341925422146,-0.0144556346033132,0.34610095952332
5,-0.041767103515756,0.966684359708914,0.991648430600638
"15243","HCG27",9.99810946088529,0.0216019084642414,0.529547270955731,
0.0407931636117283,0.967460789471577,0.991648430600638
"15244","EIF2AK1",234.573245712916,-0.006204280209431,0.15255630858573
6,-0.0406687882457789,0.967559944372021,0.991648430600638
"15245","ETV1",63.1947676917922,-0.0122760819836072,0.300486804114464,
-0.0408539803263072,0.967412305171915,0.991648430600638
"15246","POLR2J3",335.920338017804,-0.0108728478490433,0.2542503069727
95,-0.0427643450208566,0.965889386525371,0.991648430600638
"15247","GIMAP4",6.26425441122229,-0.0281334166130681,0.66744566133099
9,-0.0421508719630679,0.966378426216945,0.991648430600638
"15248","OPTN",709.925697572761,-0.00600450013606876,0.144700861241781

,-0.0414959529925382,0.966900519121159,0.991648430600638
"15249","AC010997.4",2.9593856380435,-0.0406440154134243,0.98159861070
9291,-0.0414059422761973,0.966972275610202,0.991648430600638
"15250","KIF20B",64.3483062125861,0.0101287840722568,0.248840914754953
,0.0407038532318172,0.967531989708365,0.991648430600638
"15251","AP002433.1",9.79613333974779,-0.022071995008737,0.54377791462
994,-0.0405900909450465,0.967622683928156,0.991648430600638
"15252","DDX11-
AS1",3.70446914282682,-0.0318240959954727,0.752164606367761,-0.0423100
152892767,0.966251561390618,0.991648430600638
"15253","C12orf43",101.508763605031,0.00840433720773727,0.200585046956
192,0.0418991212718503,0.966579116907891,0.991648430600638
"15254","SOCS4",56.199250580493,0.0103034556428174,0.242567232329349,0
.0424767003517926,0.966118685408681,0.991648430600638
"15255","TMED3",392.981034918541,0.0126356783716141,0.299861140392634,
0.0421384323259397,0.966388342800662,0.991648430600638
"15256","ZNF200",26.0872656868735,0.0126900790213695,0.304277571450335
,0.0417056011091529,0.966733388808524,0.991648430600638
"15257","TPGS2",255.678841857141,-0.00652627284832905,0.15631089316099
5,-0.04175187484603,0.96669649983944,0.991648430600638
"15258","PSTPIP2",43.1648865504963,-0.0145575569999056,0.3541100660673
31,-0.0411102603254934,0.967207994962932,0.991648430600638
"15259","AC008736.1",9.32555949680625,0.0213042671028518,0.50315224309
1132,0.0423415922226012,0.966226389200801,0.991648430600638
"15260","CALM3",837.909418145836,-0.00953608099256832,0.22867304956344
8,-0.0417018140562401,0.966736407813108,0.991648430600638
"15261","LIN7B",19.1270331672756,-0.0172764771546938,0.407826612519265
,-0.0423623094333445,0.966209874076338,0.991648430600638
"15262","ZNF71",33.3132426786915,-0.0113648899295814,0.279601203399729
,-0.0406467847469657,0.967577486119272,0.991648430600638
"15263","ALG12",93.2134315027573,0.00792413049666053,0.19493066927229,
0.040651019802285,0.967574109824553,0.991648430600638
"15264","NLGN3",5.65370040836491,0.0272561047956867,0.644023552914759,
0.0423215962713311,0.966242329373091,0.991648430600638
"15265","DNAJC8",882.833854961506,-0.00740394950689453,0.1829400709160
34,-0.0404719942974812,0.967716834054821,0.991679949893192
"15266","NCAPG2",28.4365699924813,0.0127071452898078,0.315548407825401
,0.0402700345642019,0.967877843346174,0.991779975335006
"15267","LRRC4",3.24406878395804,-0.0335786611013474,0.838104634875581
,-0.0400649986935488,0.968041306380257,0.991882501847537
"15268","ZCCHC24",542.080601878677,0.00797158716779226,0.1998270168325
06,0.0398924394416298,0.968178878758248,0.991958488368828
"15269","Z84485.1",9.903939075358,-0.019122937528584,0.48058926997192,
-0.0397906044171591,0.968260066889546,0.991976699610529
"15270","MRPS14",209.595980809171,-0.00968954107655106,0.2443479990839
69,-0.0396546773981206,0.968368435428895,0.99202275281036
"15271","DESI1",108.151420114146,0.00864557776283599,0.218564130782201
,0.0395562516680804,0.968446906230739,0.992038173935397
"15272","ZSCAN32",38.5773110089358,-0.011967079490554,0.30435873807674
6,-0.0393189943097228,0.96863606305598,0.992166967940328

"15273","RPS29",12783.2409363094,0.0118344720354975,0.301645725149995,
0.0392330175725605,0.968704609676566,0.99217221300141
"15274","OXER1",10.6114662356077,-0.0187106929081133,0.483794128029615
,-0.038674906998805,0.969149579751431,0.992497995158863
"15275","RNF214",118.432998050772,-0.00662572393076437,0.1710568970801
44,-0.0387340355394152,0.969102437325537,0.992497995158863
"15276","AC008026.3",3.2700801868755,-0.0394066907533267,1.02264389192
697,-0.0385341281206624,0.969261821376187,0.992547962279896
"15277","MTSS1",195.157647622192,0.0142340218760338,0.370397976495336,
0.0384289947010902,0.969345643622196,0.99256822621065
"15278","FSD1L",25.3111837700192,0.0139495676231724,0.365623557323597,
0.0381528141274176,0.969565842351081,0.99266434137692
"15279","FAM124A",7.74583109832595,0.0257990157468546,0.67609518129371
2,0.0381588516834094,0.96956102858375,0.99266434137692
"15280","CU638689.4",22.8320276014034,-0.0181816155961504,0.4788163730
19221,-0.0379720005844924,0.969710006218862,0.992746965136234
"15281","PI4K2B",45.950159628153,0.0093153629900754‌1,0.250950854446785
,0.0371202680724514,0.970389111579347,0.992783691435333
"15282","AF213884.3",2.79235021505977,0.0363948394909331,0.97247474023
1713,0.0374249715547997,0.97014616219322,0.992783691435333
"15283","AL645933.2",8.88001160487996,-0.0217583473929954,0.5758484700
76407,-0.0377848488337711,0.969859224625242,0.992783691435333
"15284","FBXO5",25.9993302507505,0.0121926252005283,0.332639252543864,
0.0366541985267375,0.970760728361221,0.992783691435333
"15285","ERICD",2.61514385394223,-0.0305091178667496,0.828883050418493
,-0.0368075060183049,0.970638489168423,0.992783691435333
"15286","STX17",235.039961850899,0.00800302842857882,0.217193178669773
,0.0368475127883591,0.970606590023462,0.992783691435333
"15287","MRPL50",147.757818680124,0.00961747625581283,0.25598966923816
7,0.0375697827355093,0.970030700788985,0.992783691435333
"15288","CCDC3",10.7599270341136,0.0283800936898946,0.753887574960872,
0.0376449946019704,0.969970732823775,0.992783691435333
"15289","MAP2K5",74.0873287967454,-0.00942914686736539,0.2527780575659
75,-0.037302078187322,0.970244148493764,0.992783691435333
"15290","PDXDC1",180.208375688006,0.00833078925790657,0.22521265335575
6,0.0369907690965609,0.970492365898593,0.992783691435333
"15291","MYBBP1A",37.2077416759088,0.0105690602112304,0.28275039720658
,0.0373794707828776,0.970182441171955,0.992783691435333
"15292","AC040977.1",10.0655581754853,-0.020341663269422,0.54410487467
4442,-0.0373855560136144,0.97017758925165,0.992783691435333
"15293","CCL14",6.19164371835342,0.0308555406477527,0.841818060823299,
0.0366534552817458,0.970761320986694,0.992783691435333
"15294","PALM",543.204351535217,-0.00654600874462324,0.177161202491743
,-0.0369494485957123,0.970525312365134,0.992783691435333
"15295","PMEPA1",70.5817176230785,-0.0087630372433127,0.23646113481294
1,-0.0370591017007719,0.970437881726206,0.992783691435333
"15296","BEX4",278.227948318943,0.010567491019945,0.285363189885109,0.
03703172446383,0.970459710617161,0.992783691435333
"15297","PLA2G2A",15482.4792064679,0.014513473882708,0.410961457842733
,0.0353159003252854,0.9718278446135,0.992975481750823

"15298","BEND5",104.525341085375,-0.00939745250128335,0.268498510187606,-0.0350000172988563,0.972079727080444,0.992975481750823
"15299","MEF2D",158.920053708569,-0.00649985935366797,0.18400228296562,-0.0353248842835415,0.971820680921767,0.992975481750823
"15300","ELK4",195.01661675267 8,-0.00740981567442182,0.209854270160144,-0.0353093395181677,0.971833076117395,0.992975481750823
"15301","SRSF7",1021.32918064163,-0.00767578920908884,0.214558085911405,-0.0357748773553952,0.97146186520652 3,0.992975481750823
"15302","MTMR14",91.2328826592041,-0.00891108196698874,0.25011923725832,-0.0356273354447563,0.971579511620855,0.992975481750823
"15303","MFN1",145.19775628252,-0.00681533562908902,0.189523692409856,-0.0359603358420776,0.971313985896413,0.992975481750823
"15304","SPRY4",36.3414515703916,-0.0209022497412469,0.58245655785428,-0.035886366904768,0.971372966500321,0.992975481750823
"15305","TDRP",11.7616021386317,0.0181193758453895,0.507302967197581,0.0357170705022359,0.971507958944047,0.992975481750823
"15306","ABITRAM",57.2383945268528,0.00891773173647834,0.251020233460253,0.0355259479028825,0.971660355996687,0.992975481750823
"15307","MAGOHB",132.668776697538,-0.00987684689621963,0.282314054016565,-0.0349853177895285,0.972091448413482,0.992975481750823
"15308","MIPEP",31.519093844594,0.0104359621039081,0.298123804326563,0.0350054640134559,0.972075383892393,0.992975481750823
"15309","MOAP1",99.0819019598316,-0.00801901296665853,0.22796170865834,-0.0351770172887988,0.971938588433377,0.992975481750823
"15310","AHNAK2",703.362287479628,-0.0106566405310583,0.300080847760872,-0.0355125647323897,0.97167102748800 4,0.992975481750823
"15311","AC004477.1",4.31675187800318,0.0303886391041602,0.865374157329535,0.0351161851168941,0.971987095514877,0.992975481750823
"15312","DHX34",15.8120022528408,0.0136913296391625,0.389635961965748,0.0351387730487929,0.971969084068704,0.992975481750823
"15313","RPL13A",18674.4184897037,0.00797849229338826,0.224894092291257,0.0354766646473551,0.971699653573362,0.992975481750823
"15314","VAPB",156.979092148647,0.00594718756067642,0.166248378150732,0.0357729057379693,0.97146343732333,0.992975481750823
"15315","BAZ1B",308.795890340433,0.00745221435982792,0.213659337650538,0.0348789547031957,0.972176262114942,0.992997275107022
"15316","RITA1",42.1003695362298,-0.0115508205328865,0.33219076235032,-0.0347716488296123,0.972261827911812,0.993019833770206
"15317","AL592295.4",5.7023721528889,0.022493459952437,0.649087584069457,0.0346539673604819,0.972355667584615,0.993035315179625
"15318","PMM2",100.850351283617,0.00864546233096813,0.249916376583609,0.0345934206039347,0.972403947958927,0.993035315179625
"15319","F3",1207.40339350205,-0.0131464895427171,0.382549909709524,-0.0343654232011176,0.972585755464862,0.993156144182834
"15320","IL2RG",17.9338780753065,-0.0171910549655246,0.501802256147001,-0.0342586242985893,0.972670918513635,0.993178275346527
"15321","ITFG1",295.383687768073,0.00598136933632809,0.175190337846549,0.0341421188511391,0.972763822062428,0.993208306802595
"15322","LRRC58",344.91341273464,0.0062994567259012,0.187972933313594,0.0335125734054054,0.973265839336924,0.993234630395005

"15323","GLRX",378.872167217323,-0.0107351236364665,0.320453871466853,
-0.0334997470535379,0.973276067542027,0.993234630395005
"15324","DDX39B",702.342126338738,0.0057883920235726,0.172057926607171
,0.0336421119195991,0.973162540804251,0.993234630395005
"15325","FOXK1",130.243230917405,0.00709132155616933,0.212703832801168
,0.0333389458139111,0.973404296741783,0.993234630395005
"15326","TMOD1",6.89222472441734,-0.0227520538672632,0.680551734997309
,-0.0334317770380133,0.973330269407615,0.993234630395005
"15327","GARNL3",32.2662993806633,-0.0090967607402256,0.26894971585431
4,-0.0338232770067442,0.973018074195737,0.993234630395005
"15328","RNF208",19.0982483236803,-0.014884792056989,0.437585887074601
,-0.0340157041089659,0.972864627879862,0.993234630395005
"15329","PPP2R5E",300.469988072658,-0.00548073843306439,0.163897541075
077,-0.0334400284294308,0.973323689428902,0.993234630395005
"15330","CTIF",75.1211978499744,-0.00697124422665228,0.209082329394253
,-0.0333421013954128,0.973401780351009,0.993234630395005
"15331","ASB9",3.29485454649578,-0.0327517650205888,0.983136223608303,
-0.0333135574034526,0.973424542516514,0.993234630395005
"15332","THRB",191.318286144327,-0.00719292745818345,0.217982028500447
,-0.0329978003584304,0.973676341747287,0.993330763718091
"15333","GPRIN3",9.18814913247457,-0.0243362133036208,0.73843388740059
2,-0.0329565228774755,0.973709258508803,0.993330763718091
"15334","ZNF444",67.6636752976075,0.00756000570011151,0.22909161534371
,0.0329999231476416,0.973674648928476,0.993330763718091
"15335","UNC5CL",4.97742003878081,-0.0203901817481188,0.62554388361106
5,-0.0325959253736329,0.973996819186268,0.993398070121701
"15336","AC022819.1",7.00390589585014,0.020258949160879,0.622294296257
376,0.0325552544555223,0.974029252670629,0.993398070121701
"15337","SCRN2",221.270419833788,-0.00851285073604913,0.26102855131482
1,-0.0326127187741313,0.973983427111347,0.993398070121701
"15338","AP001020.2",6.48466127315281,-0.0227923861872947,0.6950814644
23126,-0.0327909566775903,0.97384128986205,0.993398070121701
"15339","PRKCB",6.31258873143923,0.0230589709860524,0.711339091882461,
0.0324162853541902,0.974140075478437,0.993416155248973
"15340","AC008764.8",17.1157131081954,-0.0142384331483394,0.4398141467
85868,-0.0323737498040773,0.974173996133686,0.993416155248973
"15341","RAD51D",70.987195403954,-0.00867564476043733,0.26934064910757
,-0.0322106774049261,0.974304041260751,0.993484004787297
"15342","DUSP11",159.489184536642,-0.00664296629546853,0.2097367822001
95,-0.031672872186662,0.974732928888054,0.993791775180592
"15343","TMEM184A",4.53345967943213,-0.0271643914261022,0.855965021259
048,-0.0317353989373839,0.974683064825809,0.993791775180592
"15344","LINC01579",5.26656578936248,-0.0406771703945799,1.28906673245
856,-0.0315555194857901,0.974826516016334,0.993822418537768
"15345","THBS4",101.551313140313,0.0481725117521427,1.53094124081436,0
.0314659442621836,0.974897951230502,0.993830475796595
"15346","AL121672.2",5.66746760167434,-0.0207704078333552,0.6664358305
26603,-0.031166403248371,0.975136833215026,0.994009219469742
"15347","LIPT1",77.1931478644373,0.00746476315069311,0.246853475664992
,0.030239651803905,0.975875925413518,0.9941006948804

"15348","HDAC4-
AS1",19.9736140164314,0.0131495693619858,0.434479511678139,0.030265108
039725,0.975855623568298,0.9941006948804
"15349","MAPK10",119.731336943915,-0.0108258056298451,0.35092497233977
1,-0.0308493452536726,0.975389687305472,0.9941006948804
"15350","MDFIC",295.859826886869,-0.00772126648252844,0.25389358715241
7,-0.030411427752577 4,0.975738931043448,0.9941006948804
"15351","AC021321.1",9.64195413164832,-0.0149326212001565,0.4948287992
23825,-0.0301773486579185,0.975925613455111,0.9941006948804
"15352","PRDM10",20.6317438366923,0.0121994951781046,0.401372753354247
,0.0303944278134332,0.975752488765018,0.9941006948804
"15353","OVCH1-
AS1",3.29772025161949,-0.0393306726415879,1.28473615341967,-0.03061381
3222971,0.97557752595474,0.9941006948804
"15354","TMEM62",27.5377336692476,0.01004849008648,0.327403515166228,0
.0306914544927175,0.975515606280544,0.9941006948804
"15355","DPH1",5.76360364176444,-0.0184042015711461,0.606822905045769,
-0.0303287852487342,0.975804839819041,0.9941006948804
"15356","ST13",1298.64948224945,0.00509793033358766,0.166127338356621,
0.0306868838327143,0.975519251422645,0.9941006948804
"15357","CDK16",107.935649812556,-0.0061424871105037,0.203124470664597
,-0.0302400153482556,0.975875635479689,0.9941006948804
"15358","AL022069.3",12.5582190246116,-0.0168045201336203,0.5600458997
54205,-0.0300056122917702,0.976062577225827,0.994110742596758
"15359","VTI1A",92.702853941899,0.00603543456540613,0.200884639050219,
0.0300442811055222,0.97603173788028,0.994110742596758
"15360","AC092835.1",9.75341158624135,0.0158639892215966,0.53039351336
4052,0.0299098477298079,0.976138952011697,0.994123803796809
"15361","PRMT9",43.7535840237369,-0.00877131287451458,0.29582624860628
5,-0.0296502183827112,0.976346014421649,0.994269950107275
"15362","DHX16",99.6317274991648,-0.00820970116396583,0.27792711108313
4,-0.029539044003196,0.976434679905586,0.994295514761299
"15363","INSIG2",199.642341432223,-0.005728227242257,0.196883452883996
,-0.0290945082400199,0.976789215735039,0.994379620249163
"15364","PCDHGB3",3.58991701325574,0.0232051191084669,0.79466270529615
,0.0292012182701074,0.976704109610263,0.994379620249163
"15365","MRPS18A",166.835772599955,0.00922173487012509,0.3145326040505
29,0.0293188520088799,0.976610291636032,0.994379620249163
"15366","LOH12CR2",21.9052396490145,0.00933184341930127,0.320346126358
93,0.029130501827406,0.976760509174905,0.994379620249163
"15367","MAPRE2",132.483915696906,0.0050967475904631 8,0.17552618690119
3,0.0290369641159711,0.976835109912989,0.994379620249163
"15368","LINC01106",2.94489184952899,0.0245921447575417,0.862398962235
304,0.0285159721131851,0.977250629305283,0.994673146868537
"15369","TRIM24",57.2613392795462,0.00777304306594481,0.27190172275032
1,0.0285876933302205,0.977193427374005,0.994673146868537
"15370","TXNDC11",77.1500697942099,-0.00764778954178021,0.269539508950
868,-0.0283735381560492,0.977364229194155,0.994724049270278
"15371","TRMT44",41.8044876279173,-0.00684804817075693,0.2420348539798
17,-0.0282936447298949,0.977427949343216,0.994724182654085

"15372","AC007389.1",3.01453589700741,-0.0239990686038657,0.867583217716815,-0.0276619788324434,0.977931748586984,0.994812817383891
"15373","MCCC1",58.9625089649661,-0.00678364599091865,0.24507384083408,-0.0276800084734923,0.977917368521101,0.994812817383891
"15374","TTI2",50.7545267592336,-0.00706199273635063,0.255759419395104,-0.0276118578664784,0.977971724062361,0.994812817383891
"15375","ALX1",7.48819756735693,-0.0304548412016747,1.10557675545212,-0.0275465643172106,0.978023800968283,0.994812817383891
"15376","MAN2C1",135.060996339037,-0.00583411231982475,0.209993909860783,-0.0277822929421739,0.977835788696921,0.994812817383891
"15377","DERL2",311.441209920006,0.00629702029629056,0.226254121275596,0.0278316269369533,0.977796441079429,0.994812817383891
"15378","UBTF",373.302872738072,-0.0049281805649532,0.176978366224251,-0.0278462315484858,0.977784792799989,0.994812817383891
"15379","LILRB3",5.26767202959269,0.0221444096718021,0.794765301831695,0.0278628289644326,0.977771555114446,0.994812817383891
"15380","AL450998.2",30.6803126203558,-0.00962847794247652,0.350648659249402,-0.0274590467936972,0.978093603444632,0.994819131253861
"15381","CENPW",51.2582732156271,-0.0105369695655487,0.387058131139229,-0.0272232223478555,0.978281693813138,0.994911851536862
"15382","ZMYND11",251.338153249552,-0.00418422989426428,0.153915353463087,-0.0271852664475592,0.978311967035736,0.994911851536862
"15383","NEK4",41.8610538083063,0.00733948442720436,0.272953597471612,0.0268891287573804,0.978548164378643,0.99502268170665
"15384","AJ011932.1",7.32427286534494,-0.0165940488013518,0.615395589376848,-0.0269648484451359,0.978487770707417,0.99502268170665
"15385","RPP30",190.51943384273,0.00625018514479253,0.235178081546261,0.0265763931047422,0.978797602181375,0.995099435423987
"15386","MMP25-AS1",22.1696311217568,0.00962182953751918,0.360354865474944,0.0267009841114194,0.978698228205919,0.995099435423987
"15387","SYTL4",62.6783858206297,-0.00817591034098771,0.307883310193179,-0.0265552242369286,0.978814486535121,0.995099435423987
"15388","ATP11B",139.51116354187,-0.00505164113515643,0.190892389582939,-0.0264632924664689,0.978887811706571,0.995109308456323
"15389","PTPRF",68.1250809518259,-0.0116902585681508,0.451190565450112,-0.0259098027825305,0.979329281182767,0.995332575205391
"15390","CNOT7",208.662204511971,-0.00388316964823378,0.150574234199142,-0.0257890712105372,0.979425578861892,0.995332575205391
"15391","MAPKAPK5",92.4977067664318,-0.00528712927024247,0.204893822921116,-0.0258042394585903,0.9794134803772,0.995332575205391
"15392","XRCC3",7.03442819923398,-0.0151030903440608,0.582606273708843,-0.0259233225346429,0.979318497603393,0.995332575205391
"15393","ZNF232",21.4306069396652,-0.00927151101332273,0.359224533916755,-0.0258097934242745,0.979409050429152,0.995332575205391
"15394","AL359091.4",6.34756686709446,-0.0139802480191013,0.545734424313021,-0.0256173101718842,0.979562579080265,0.995407134244029
"15395","PDZD8",184.543900193708,0.00491327360294838,0.192642150420133,0.02550466547551,0.979652427187752,0.995433771906334
"15396","ZFPM2",158.381086604492,-0.004711716463857,0.186056159294556,

-0.0253241627781729,0.979796400999638,0.995450743705744
"15397","HYDIN",8.1177840590727,0.0202325407685891,0.798533018294057,0.025337137356966,0.979786052104211,0.995450743705744
"15398","SLC20A1",112.22957278511,0.0111192813831543,0.441291484676826,0.0251971356104851,0.979897721683929,0.995489028464846
"15399","MIER1",486.93982830071,-0.00341135264749974,0.138655953104169,-0.0246030016824224,0.98037162502922,0.995517905247133
"15400","RPF1",138.178684982273,-0.00571457065157711,0.231927475857566,-0.0246394724490797,0.980342534386373,0.995517905247133
"15401","SHCBP1L",6.28381441405306,0.0149862025144998,0.601466358461917,0.0249161109406398,0.980121876551355,0.995517905247133
"15402","MAP3K2",374.846088623909,0.00552499156054325,0.222546864812183,0.0248261936433301,0.980193597988657,0.995517905247133
"15403","AC002456.1",13.4976551888804,0.0116633619607546,0.471650452798577,0.0247288259590318,0.980271262316953,0.995517905247133
"15404","CPNE3",711.62809385515,0.00328525363361359,0.132561777060377,0.0247828122590516,0.980228200678875,0.995517905247133
"15405","HIRIP3",89.8539714006825,-0.0059921627072293,0.243548713843529,-0.0246035489683556,0.980371188490367,0.995517905247133
"15406","RO60",337.865224421205,-0.00363051952128599,0.149057354242742,-0.0243565273228561,0.98056822419913,0.995652909979683
"15407","PIK3R5",7.8212050100767,0.0150579564245086,0.623894323676876,0.0241354278329792,0.980744584222369,0.99576734802301
"15408","AL157392.4",3.05790503487658,-0.0191581491003247,0.803650322350093,-0.0238389117350206,0.980981101784057,0.995942846262202
"15409","ZNF273",74.5926900781006,0.00605710125775843,0.258036762270967,0.0234737918909315,0.981272343764626,0.996044596295507
"15410","METAP2",488.222522576879,0.00427552634158022,0.181767140637562,0.0235219981267433,0.981233891370572,0.996044596295507
"15411","TMEM106A",39.9695120431424,0.0108125526517995,0.459097631997743,0.0235517499943294,0.981210159389256,0.996044596295507
"15412","TIMM44",142.226799259565,0.00519194937094577,0.22204711955957,0.0233821964511136,0.98134540629817,0.996054126052574
"15413","PEX14",47.6683838779833,0.007429656822803,0.32795966566545,0.0226947593301697,0.981893756209146,0.996080323216004
"15414","ZBTB17",51.6918427318629,0.00541436265995121,0.236185349195586,0.0229242104914287,0.98171072828892,0.996080323216004
"15415","ATXN7L2",4.11102388547405,-0.0156774732201982,0.752239533058388,-0.0208410652873536,0.983372439482074,0.996080323216004
"15416","BOLA1",136.730343402621,-0.00617429586065834,0.299718704175793,-0.020600302132085,0.983564499451423,0.996080323216004
"15417","AC104794.3",67.4238067125999,0.00550548454432088,0.249142662641688,0.0220977189773345,0.982370006025362,0.996080323216004
"15418","NFE2L2",1005.95726428423,-0.00394506091079826,0.180470280696994,-0.0218598923632304,0.982559718380948,0.996080323216004
"15419","AC012063.1",25.9657400618778,-0.00662674503241796,0.317439318351428,-0.0208756277162922,0.983344868651923,0.996080323216004
"15420","MKRN2",106.687716464555,0.00384926584223146,0.189160111764547,0.020349247028479,0.98376477045913,0.996080323216004
"15421","LRIG1",41.3389931200619,0.00816683381732437,0.381974279324632

,0.0213805857079281,0.982942060387034,0.996080323216004
"15422","KPNA1",97.9221126997057,0.00481470042059446,0.223253480531221
,0.0215660710378987,0.982794098623008,0.996080323216004
"15423","RYK",275.875778556013,-0.00435964110255744,0.189986002738451,
-0.0229471700005144,0.981692414068791,0.996080323216004
"15424","AC020741.1",3.52307850255416,0.0172562506369008,0.80492543379
8858,0.0214383220014054,0.982896004046387,0.996080323216004
"15425","GOLPH3",307.590975821689,0.00334196125736975,0.15560494482034
5,0.0214772175860365,0.982864977003733,0.996080323216004
"15426","P4HA2",413.588580868034,-0.00405922706187058,0.19896603919498
1,-0.0204016076225585,0.983723001420801,0.996080323216004
"15427","AC139795.2",3.75252000965046,0.0185293831136096,0.83415864188
0787,0.0222132603839375,0.982277839944312,0.996080323216004
"15428","CNOT4",330.358781264966,0.00296949196070489,0.14357720929321,
0.0206821958395964,0.983499171644894,0.996080323216004
"15429","SMC3",571.954550218197,0.00359815905028791,0.169658985699457,
0.0212081843791161,0.983079585561397,0.996080323216004
"15430","AL158163.2",2.99507527469317,0.0197428504088451,0.94518359527
859,0.0208878470886135,0.983335121128825,0.996080323216004
"15431","AL158212.3",15.3677491019762,-0.00863836877438966,0.416446670
290304,-0.0207430372017812,0.983450637673312,0.996080323216004
"15432","ATE1",138.193031565503,0.00363973055557727,0.157371856709393,
0.0231282176602802,0.981547997265127,0.996080323216004
"15433","LRP4",31.4845320406531,-0.00930423819342358,0.425879852485675
,-0.0218470964031729,0.98256992564226,0.996080323216004
"15434","INPPL1",77.5541975706538,0.00512399896575264,0.23702899099451
9,0.0216176044299624,0.982752990508691,0.996080323216004
"15435","UVRAG",173.3493637065,-0.00438317727481767,0.197538821616682,
-0.0221889410848217,0.982297239156189,0.996080323216004
"15436","FAM76B",112.390709281225,0.00587209394514023,0.25241583432510
6,0.0232635720371532,0.981440029147671,0.996080323216004
"15437","AC008035.1",4.16326411679123,0.0165019203859524,0.74435038505
4328,0.0221695598165748,0.982312699367837,0.996080323216004
"15438","TMEM19",93.6487677676714,-0.00583257484053669,0.2551814141269
73,-0.02285658170087,0.981764674121905,0.996080323216004
"15439","VWA8",41.6827594861932,0.00616349059543315,0.272653546975056,
0.0226055764313862,0.98196489561648,0.996080323216004
"15440","COG3",65.0371581718634,-0.0058274611860022,0.252731963078106,
-0.0230578717271359,0.981604110234885,0.996080323216004
"15441","UBL7",87.3947042349166,-0.0053037174597588,0.235478346431175,
-0.0225231641895743,0.982030634334233,0.996080323216004
"15442","AC009061.2",37.3655702211103,-0.00593899058214457,0.292323565
053383,-0.0203164961437851,0.983790896483236,0.996080323216004
"15443","SMPD3",2.97936455242436,0.0197409759505743,0.953804893189925,
0.0206970797607802,0.983487298535457,0.996080323216004
"15444","HGS",166.054083773478,0.00361058735747651,0.168519930989059,0
.021425283859812,0.982906404589392,0.996080323216004
"15445","BORCS8",95.4623887450271,-0.0060658640707061,0.29294055529412
,-0.0207068088084145,0.983479537541803,0.996080323216004
"15446","AL133227.1",3.40403817686684,-0.0161180868640413,0.7563175195

61531,-0.0213112699985921,0.982997353722722,0.996080323216004
"15447","ZNF831",3.74277420490917,0.0166955213557316,0.800979113748961
,0.0208438910193159,0.983370185363826,0.996080323216004
"15448","AP000550.1",2.83253689594354,-0.0188838393613151,0.9247077653
35259,-0.0204214131958421,0.983707202151226,0.996080323216004
"15449","NKRF",38.4326734555904,0.00594110790144485,0.2792162064739,0.
0212778046678326,0.983024049140153,0.996080323216004
"15450","BRCC3",84.283135168344,-0.00396481466846398,0.194943828309335
,-0.0203382415480866,0.983773549745099,0.996080323216004
"15451","LIX1L",193.300181516061,0.00336303921263141,0.17273930416361,
0.019468870902977,0.984467069755492,0.996189448992184
"15452","WDCP",31.6076640781188,0.00580882627324441,0.304079191562625,
0.0191030048567071,0.984758934339321,0.996189448992184
"15453","HYAL2",131.313811415556,-0.0058095840571028,0.30803558376731
4,-0.0190918150876773,0.984767860855321,0.996189448992184
"15454","OCIAD1",939.956664475929,-0.00425659425124584,0.2163829223705
11,-0.0196715813087934,0.984305361222298,0.996189448992184
"15455","LIFR-
AS1",33.0705104783108,-0.00690069985633764,0.352681879097345,-0.019566
3578576799,0.984389301233361,0.996189448992184
"15456","NSD3",754.56249938782,-0.0028302489560811,0.143500824105521,-
0.0197228759745653,0.98426444193915,0.996189448992184
"15457","TDRD7",29.8808020480154,-0.00577618970763209,0.29734969540657
,-0.0194255780209703,0.984501605946318,0.996189448992184
"15458","USP54",24.3988573687086,0.00726144876983878,0.361339101336272
,0.0200959396394831,0.983966839190847,0.996189448992184
"15459","FKBP2",99.7575668864847,0.0075024661182742,0.392696315596785,
0.0191050076618942,0.984757336623564,0.996189448992184
"15460","AC009779.2",30.8691672258926,0.00717995964085065,0.3766285539
95753,0.0190637687044079,0.984790234559556,0.996189448992184
"15461","EXOC5",177.287534897308,0.00293662550829273,0.153340932932887
,0.0191509563175673,0.984720681606893,0.996189448992184
"15462","CORO7",36.203976842174,-0.00554172832123848,0.288335078659538
,-0.019219750669956,0.984665801756219,0.996189448992184
"15463","ELL",52.73988469203,0.00436870659251032,0.228841499196598,0.0
190905347493689,0.984768882231341,0.996189448992184
"15464","NDUFV3",296.404406039301,-0.00419470395803586,0.2184181783538
23,-0.0192049214477044,0.984677631580224,0.996189448992184
"15465","ZNF525",23.0011709176195,-0.00826335961157131,0.4373825933614
77,-0.0188927491331188,0.984926663863539,0.996263032836556
"15466","AC092794.1",3.41710083538091,-0.0147089618825704,0.7856380224
9837,-0.0187223141718563,0.985062627238757,0.996336135904298
"15467","MXRA8",3415.08271779922,-0.00409424162007395,0.22581941173139
7,-0.0181306008579275,0.985534666005989,0.996450352907915
"15468","ZMPSTE24",217.565808715695,0.0027348424016696,0.1573184033800
14,0.0173841225369125,0.986130175622869,0.996450352907915
"15469","WDR47",54.9815415354341,-0.00424728690837518,0.24382223123098
,-0.0174196047953954,0.986101869162919,0.996450352907915
"15470","ENTPD3-
AS1",68.6942045809571,0.00422935371865078,0.234978772014442,0.01799887

57384043,0.985639750297323,0.996450352907915
"15471","ACAD9",100.494859451133,0.00430593241968629,0.245405029634819,0.0175462272557895,0.986000854495653,0.996450352907915
"15472","PCOLCE2",2790.28757848038,0.00596072959208569,0.334061417650118,0.0178432146819442,0.985763929908262,0.996450352907915
"15473","AC096733.2",15.221893350506,-0.0081740374147806,0.453940988981584,-0.0180068282291913,0.985633406155864,0.996450352907915
"15474","TMEM267",68.8002108375774,0.00543676107832804,0.309464982399523,0.0175682593751726,0.985983278116983,0.996450352907915
"15475","USP15",237.777358080956,-0.00274001647460404,0.149614973295356,-0.0183137851396394,0.985388530360096,0.996450352907915
"15476","RILPL1",114.956085634897,0.00373297786149429,0.212899000183035,0.0175340319037898,0.98601058348202,0.996450352907915
"15477","IFI27",2760.58602176828,0.0083438629000646,0.474580326455219,0.017581560875874,0.985972666693879,0.996450352907915
"15478","PAPOLA",528.669007275546,-0.00221131106817221,0.127364119703111,-0.0173621195147177,0.986147728845366,0.996450352907915
"15479","SEPTIN9",146.82884935086,0.00401162832693542,0.220304082062218,0.0182095051956526,0.985471719844876,0.996450352907915
"15480","TIMM29",97.378608747676,0.00350981961363172,0.202842376076094,0.0173031872408902,0.986194742934242,0.996450352907915
"15481","TMC4",2.83974196527421,0.0179065788205838,1.01974383315239,0.0175598794897619,0.985989963266921,0.996450352907915
"15482","ARHGAP6",260.292559978543,0.00440027331669463,0.251965926215519,0.017463763385732,0.986066641064285,0.996450352907915
"15483","SLC22A15",15.372672825993,0.0081590801549228,0.487773820809314,0.0167271792926182,0.986654264252408,0.99650066322616
"15484","THAP12",270.82501041575,-0.00323632482732546,0.188997050753919,-0.0171236789908393,0.986337948577651,0.99650066322616
"15485","EDC4",57.9166966324523,0.00401762752039684,0.240403566297085,0.016712012980069,0.986666363527821,0.99650066322616
"15486","CCDC144NL-
AS1",11.7744845137147,-0.00965942213611758,0.580014056744854,-0.0166537724798051,0.986712826257561,0.99650066322616
"15487","AC104532.2",17.4120143533489,0.00768130152156174,0.449935333253792,0.0170720122512116,0.986379166646292,0.99650066322616
"15488","DNTTIP1",121.042382578671,0.00451264615003202,0.266435037409712,0.016937134822447,0.986486767706378,0.99650066322616
"15489","AP001627.1",6.79960837846509,-0.010636746690127,0.62963499334746,-0.0168935125946171,0.986521568229407,0.99650066322616
"15490","WWC3",115.057066838699,-0.00457608578043177,0.275635185527848,-0.0166019652812773,0.986754156707359,0.99650066322616
"15491","TCP10L",5.37753266944018,0.00995250737308313,0.608823814573453,0.0163471059029712,0.986957477476386,0.996641651291918
"15492","PLEKHM2",225.53711020498,0.00313642670203188,0.196458874193526,0.0159648003426013,0.987262473371834,0.99673807711514
"15493","MICAL2",4.90839780883235,0.0109383830086932,0.686184904734997,0.0159408680272813,0.987281566167087,0.99673807711514
"15494","IDH2",100.228093145795,0.00410211131627959,0.257865694102445,0.0159079373879408,0.987307837684529,0.99673807711514

"15495","AL121601.1",6.2121511050862,0.0101666122410812,0.630238541492171,0.0161313718088558,0.987129585683914,0.99673807711514
"15496","ABCD3",99.2390957697552,0.0032354811879395,0.204462243927818,0.0158243454917854,0.987374525973463,0.996741075748766
"15497","ARL8A",199.411861972046,-0.00332294117461835,0.222847781658955,-0.0149112598289345,0.988102976877269,0.996931928147542
"15498","PDIA6",1160.58629925043,0.00367841959867245,0.23988915871332,0.0153338300838695,0.987765853148357,0.996931928147542
"15499","UBE2K",373.086008161784,0.00215682374905924,0.142741305366408,0.0151100183897212,0.98794440835583,0.996931928147542
"15500","PKD2",340.442581619724,0.00449156825052378,0.303719642803783,0.0147885339553937,0.988200887160761,0.996931928147542
"15501","DUSP3",101.415650652919,-0.00320792256934271,0.211778868145901,-0.0151475102186903,0.987914497627514,0.996931928147542
"15502","RANBP3",119.394858861325,-0.00301967673415179,0.20311951924669,-0.0148665019755407,0.988138684519431,0.996931928147542
"15503","PRKACA",230.161399904851,0.00304519505231685,0.204000617052532,0.0149273815751875,0.988090115016356,0.996931928147542
"15504","SUGP1",47.8054916497498,0.00418543417721854,0.279961726312708,0.0149500227489794,0.988072051988978,0.996931928147542
"15505","ZNF552",24.4086121738381,-0.00595862657321892,0.401456819945052,-0.0148425092741842,0.988157825813475,0.996931928147542
"15506","RIN2",234.619714191019,-0.00465550829291373,0.304557362863118,-0.0152861459304339,0.987803895139473,0.996931928147542
"15507","POGK",72.740008550816,-0.00392602884673982,0.271891360969381,-0.014439696916968,0.988479189126155,0.997019792088493
"15508","BIN1",650.460012920944,0.0049398160848963,0.3403761539099,0.0145128148025431,0.988420855606713,0.997019792088493
"15509","UBE2E2",243.338010032782,0.00311408034174325,0.21490695210299,0.0144903657665336,0.988438765462664,0.997019792088493
"15510","KLF14",10.7671473457855,0.00670357182660652,0.483881901040569,0.0138537354097989,0.988946671978349,0.997086850541389
"15511","TRAM1",1062.19340017174,0.00218655443332965,0.160336141147951,0.0136373148167012,0.989119334315749,0.997086850541389
"15512","AP001330.4",5.00294493202303,0.00889934202169207,0.635762542749347,0.0139979023979723,0.988831654517526,0.997086850541389
"15513","DMRT2",45.6525096465332,0.00456494134896636,0.323036587757211,0.0141313446277401,0.98872519355295,0.997086850541389
"15514","AIFM2",109.805198665369,0.00295158942383081,0.211317212260365,0.0139675769534293,0.988855848356256,0.997086850541389
"15515","DNAJC17",103.367042087244,0.00317354684619712,0.231288124794395,0.0137211836924972,0.989052422895227,0.997086850541389
"15516","MAP1LC3B",771.147040682613,0.00303430583522845,0.213937733980315,0.0141831259907971,0.988683882143009,0.997086850541389
"15517","UBB",3555.1729640045,0.00388574555043891,0.283967402143518,0.0136837732821003,0.989082269282015,0.997086850541389
"15518","PSME3",79.846331950307,-0.00317656873670619,0.232016531473842,-0.0136911310436701,0.989076399187553,0.997086850541389
"15519","SLC25A20",53.5616426069703,-0.00435480078480213,0.325637085414701,-0.0133731720981849,0.989330070492959,0.997170766283592

"15520","RFC4",52.1664851371928,0.00436611029459353,0.324806302261281,
0.013442196977697,0.989275001557279,0.997170766283592
"15521","PPP1R1C",10.9578335785414,-0.008650584475208,0.65468364841614
3,-0.0132133809911644,0.989457554085681,0.997218850801775
"15522","NSUN6",85.8309721826777,0.0034437931159525,0.261814376262576,
0.0131535676730704,0.98950527406157,0.997218850801775
"15523","AC026979.2",28.6036233928762,0.00445413210135543,0.3464756112
29647,0.0128555429501882,0.989743043279384,0.997394216711937
"15524","AC231981.1",7.28351557431362,-0.00698305658269685,0.551880951
345281,-0.0126531937108442,0.989904481481645,0.997492643894446
"15525","UXS1",219.838237710699,-0.00279480664877247,0.227286322418289
,-0.0122964136998486,0.990189128593155,0.997659126989978
"15526","ZUP1",31.7833497992363,-0.00366703167899072,0.298466737145547
,-0.0122862323422074,0.990197251527609,0.997659126989978
"15527","TBCE.1",30.8737858697026,0.00344804895712983,0.28662928686447
3,0.0120296463590623,0.99040196239124,0.997672605942828
"15528","AKAP1",51.2907638397034,-0.00335574324371088,0.27862560311113
9,-0.0120439155850739,0.990390578020848,0.997672605942828
"15529","TMEM191B",9.95566981667727,0.00823961780166115,0.678366022318
858,0.012146271379418,0.990308915886316,0.997672605942828
"15530","FAT1",196.030323168328,0.00435157678979279,0.367934917739659,
0.0118270285857235,0.990563616483151,0.997706950785263
"15531","BPHL",109.293783290832,-0.00235585873873767,0.198357527559115
,-0.0118768305278234,0.990523883073194,0.997706950785263
"15532","METTL25",49.3789031384419,-0.00285966320204034,0.245990066733
15,-0.0116251165749002,0.990724707883476,0.997754467119724
"15533","RPA1",159.248698038136,-0.00200028344737557,0.17231927964061 5
,-0.0116080072499567,0.990738358228644,0.997754467119724
"15534","FBXO9",272.535677313583,0.00211032516995295,0.187745982935649
,0.0112403212945242,0.991031710030534,0.997985646968434
"15535","MIGA2",27.3806066313951,-0.00329082195049383,0.30060311946623
8,-0.010947397872441,0.991265414723781,0.997998020599225
"15536","HOGA1",2.67512799416801,0.0093467217960318,0.857099284320665,
0.0109050631204762,0.991299190952551,0.997998020599225
"15537","DRAM1",177.316155499985,-0.00176435076725393,0.15927866669185
4,-0.0110771316956545,0.991161908385593,0.997998020599225
"15538","RHBDL3",4.14961547873199,0.00979689588086358,0.89023789537841
6,0.0110048066159879,0.991219611932781,0.997998020599225
"15539","TTC21A",16.9880272478675,0.00481885819312051,0.45365050185317
2,0.0106224024297016,0.991524708488538,0.99803236695748
"15540","AC004130.2",28.2366395478725,0.00398119476650111,0.3707706621
27356,0.0107376207806151,0.991432782788067,0.99803236695748
"15541","HLF",128.900819975527,0.00487707195911038,0.457353524112911,0
.0106636807239433,0.991491775040178,0.99803236695748
"15542","PCLAF",3.50500544949223,-0.00772750883700424,0.75780011092284
,-0.0101972917734121,0.991863879337662,0.998309526732663
"15543","PCDHGA2",13.5996889775768,0.00388958069138824,0.3953280743498
5,0.00983886787647191,0.992149845879286,0.998340411622905
"15544","ACP2",114.509902757604,0.00319697517876293,0.320342491327059,
0.00997986612865205,0.992037351074245,0.998340411622905

"15545","PHETA1",33.7152132378519,-0.0027177685185004,0.27542767870605
4,-0.00986744880277954,0.9921270427064,0.998340411622905
"15546","PHF11",238.612139663841,0.0015050653228901,0.15230061049293,0
.00988220150936274,0.992115272323479,0.998340411622905
"15547","GPR153",90.8640137576754,0.0022452165907852,0.24038732293541
6,0.00925390587121842,0.992616556758298,0.998444978449384
"15548","FPGT",61.5802917923022,-0.00207019935739048,0.242646644411127
,-0.00853174525621236,0.993192734767706,0.998444978449384
"15549","RAP1A",436.586713520519,-0.00186625124218651,0.19765170736179
3,-0.00944212052148085,0.992466389756299,0.998444978449384
"15550","TAF8",65.7555470046396,0.0021510212284875,0.233571500132661,0
.00920926237689871,0.992652175595363,0.998444978449384
"15551","STX11",25.8678489996547,-0.00323972432391433,0.37900089481961
2,-0.00854806510537737,0.993179713886838,0.998444978449384
"15552","VPS13B-
DT",34.4897477529395,0.00248739142309024,0.281258881714905,0.008843779
11169953,0.992943777168465,0.998444978449384
"15553","EBLN3P",312.72621797922,0.00113300392469001,0.135477325744505
,0.00836305203445429,0.993327327682437,0.998444978449384
"15554","FANCC",14.922932805009,-0.00358714505158625,0.408275870911618
,-0.00878608143943629,0.992989811461816,0.998444978449384
"15555","AP003778.1",2.61289714672711,0.00904402333112682,1.0223120508
7742,0.00884663672248075,0.992941497214133,0.998444978449384
"15556","TMEM138",105.894759311859,-0.00195232549698136,0.233849385477
79,-0.00834864497502298,0.993338822451433,0.998444978449384
"15557","SLC25A21-
AS1",46.6555368557666,0.00328045220036285,0.365083809692068,0.00898547
706930574,0.992830723048217,0.998444978449384
"15558","AC025040.1",3.96829838887695,0.00742367255941499,0.7978567280
38051,0.00930451834086802,0.992576175588707,0.998444978449384
"15559","HIC1",679.759433892238,-0.00152893209591136,0.18278200212498,
-0.00836478470602336,0.993325945258897,0.998444978449384
"15560","ATP6V0A1",118.25634016531,-0.00213537994040179,0.243209103741
471,-0.00878001648602627,0.992994650407857,0.998444978449384
"15561","GPATCH1",69.4922111174493,-0.00207670835135727,0.242077570699
718,-0.00857868965453764,0.993155279927787,0.998444978449384
"15562","NEXMIF",5.44875299888948,-0.00547927894739098,0.6032779337636
31,-0.00908251179221451,0.992753303700256,0.998444978449384
"15563","CXorf56",65.3455910873968,-0.00222694230204662,0.259814647486
919,-0.00857127311176227,0.993161197255207,0.998444978449384
"15564","LINC01117",22.3413619124676,-0.00372991895687523,0.4520387527
50629,-0.00825132565334033,0.993416469361056,0.998458868556605
"15565","SMIM12",117.820059720767,-0.00176817064621769,0.2210396666734
47,-0.0079993363762618,0.993617521077265,0.99846076295285
"15566","AC103703.1",6.18567029692147,0.00529305767216836,0.6675595633
25948,0.00792896688618619,0.993673666026336,0.99846076295285
"15567","ZNF223",21.3103583699955,0.00286826086645221,0.36154814362053
1,0.00793327504804626,0.993670228718612,0.99846076295285
"15568","LIF",5.63897996084816,0.00509737530654119,0.642582282637423,0
.00793264216003507,0.993670733674295,0.99846076295285

"15569","CUL4A",132.166734085867,-0.00152677703973808,0.19678114541171
,-0.0077587567474705,0.993809469890135,0.998533080961615
"15570","RNF11",271.245494083056,-0.00107826022377663,0.15047173054461
1,-0.00716586577341817,0.994282515266436,0.998577973413966
"15571","GNAQ",298.654393000022,0.00137966658661759,0.188623859378396,
0.0073143800109764,0.994164021155127,0.998577973413966
"15572","KLC2",27.4065868716581,-0.00201558938720769,0.282188709146208
,-0.00714270033448919,0.994300998139483,0.998577973413966
"15573","ARHGAP5-
AS1",12.7342682162083,-0.00317070892143774,0.433025498556431,-0.007322
22220633167,0.994157764156032,0.998577973413966
"15574","ZNF554",18.6712724393282,0.00255425843492923,0.35457566031759
2,0.0072037049374494,0.994252324760907,0.998577973413966
"15575","LINC01534",26.2906894269992,-0.00227415823422003,0.3041103789
72622,-0.00747806846284837,0.994033420238941,0.998577973413966
"15576","ACSS2",65.0677780076844,-0.00166716901265252,0.23012238288517
8,-0.0072447060201196 3,0.994219611483721,0.998577973413966
"15577","ZYG11B",240.658819335828,-0.00131862144684389,0.1897374301984
5,-0.00694971701400572,0.994454972728757,0.998668494472359
"15578","CPTP",55.9267326987447,0.0015780532349803,0.283746120998627,0
.00556149712082912,0.995562590187385,0.998731307768157
"15579","AMY2B",18.6010625716977,-0.00267510351366031,0.42840743936914
6,-0.00624429752573752,0.995017803788186,0.998731307768157
"15580","HIPK1-
AS1",3.64893041456942,0.00489663712890341,0.734340127035572,0.00666807
784108223,0.994679683066839,0.998731307768157
"15581","GORAB",63.0692418074105,0.00145718011712117,0.222283687645168
,0.00655549731317787,0.994769507368464,0.998731307768157
"15582","HLX-
AS1",4.84972698852911,-0.00730525027131525,1.23282484922715,-0.0059256
1893597041,0.99527206780642,0.998731307768157
"15583","OGG1",119.227198918923,0.00108525662086292,0.183773381357992,
0.00590540704449916,0.995288194280407,0.998731307768157
"15584","TBCC",94.8166887209712,-0.001483208398520 1,0.275315475195829,
-0.00538730486350287,0.995701573417327,0.998731307768157
"15585","ZCWPW1",62.3564031123856,-0.0014081335052309,0.23729731410869
3,-0.00593404738068765,0.995265342998741,0.998731307768157
"15586","ST7",64.8796363022591,0.00146367185907776,0.219484740055696,0
.00666867254054354,0.99467920857587,0.998731307768157
"15587","LRRC69",7.88539085080969,-0.00278287213882331,0.5229059307217
47,-0.00532193646184549,0.995753729108056,0.998731307768157
"15588","FAM27C",8.07926327697363,-0.00268382125891052,0.5232425903963
76,-0.0051292102519357 7,0.995907500275668,0.998731307768157
"15589","SUPT16H",435.559647377703,0.00134570253766125,0.2097892374048
23,0.0064145451611728 4,0.994881968549438,0.998731307768157
"15590","PTGR2",61.3096779497492,-0.00162671953584945,0.27948347352992
3,-0.00582044982947904,0.995355979165554,0.998731307768157
"15591","GTF2A2",572.000213252106,-0.00149691787076022,0.2605074024760
49,-0.00574616251412604,0.995415250876296,0.998731307768157
"15592","LOXL1-

AS1",16.9861612037241,-0.00297365901349685,0.489178300069821,-0.006078
88578269398,0.995149780758695,0.998731307768157
"15593","AC087500.1",18.0200607012678,0.00235645197324047,0.4079247651
72262,0.00577668279650873,0.995390899618354,0.998731307768157
"15594","TMEM107",103.355753342367,-0.00120965493365312,0.210744067008
225,-0.00573992402645388,0.995420228387206,0.998731307768157
"15595","ZNF18",22.1985955559855,-0.00179801594298263,0.33531605947820
8,-0.00536215278737486,0.995721641580678,0.998731307768157
"15596","CCDC137",91.7821433101721,-0.00136055792645173,0.250866591571
038,-0.00542343210361855,0.995672748471323,0.998731307768157
"15597","ATP8B3",7.17842451512818,0.0031536975147359,0.61064252916344
,0.00516455596215407,0.995879298852732,0.998731307768157
"15598","SWSAP1",16.2514801187589,-0.0020064018986665,0.39257378614540
5,-0.00511089117377632,0.995922116593715,0.998731307768157
"15599","CAPN6",21.4028955071506,-0.00353256939360574,0.58597521188341
,-0.00602853042580342,0.99518995778431,0.998731307768157
"15600","FIGNL2",5.36727778388629,-0.00336050433667001,0.6844467569796
16,-0.0049098111758167,0.996082553205521,0.998828165371408
"15601","SAMD5",2.92508661324107,-0.00440998420123085,0.96294847789161
3,-0.00457966786643306,0.996345966488665,0.999028264456264
"15602","ARHGAP24",329.762447564825,-0.000856180536958877,0.1913997528
44245,-0.00447325832053508,0.996430868152645,0.999049357166506
"15603","GEMIN6",108.445114210783,-0.00104668475477055,0.2444754799399
87,-0.00428134860407061,0.996583988485285,0.999110319261216
"15604","AP5Z1",84.7498764547803,0.000863352884960873,0.20769125167568
8,0.00415690539680991,0.996683278915251,0.999110319261216
"15605","POLR1E",119.828300682102,0.00126760179271346,0.30076318947364
3,0.00421461747008288,0.996637231746409,0.999110319261216
"15606","TEFM",90.7914713512098,-0.000798843146160994,0.19938788707647
5,-0.004006477815047,0.996803301760338,0.999166605756566
"15607","DNALI1",51.3397671373157,-0.000957964461311034,0.307952909679
628,-0.00311074982960099,0.997517984741421,0.999186880177008
"15608","PTBP2",101.128214911282,-0.000914365247007734,0.2767949222308
98,-0.00330340325479304,0.997364270338617,0.999186880177008
"15609","FIP1L1",312.010540068639,0.000461904479160578,0.1449299292924
95,0.00318708828062953,0.997457075771943,0.999186880177008
"15610","CASP6",104.095637799231,-0.000771322817580737,0.2332387152247
11,-0.00330701023128864,0.99736139240348,0.999186880177008
"15611","BTN2A2",30.7201411524042,0.00108858221910969,0.32561981423734
8,0.00334310804045916,0.997332590678082,0.999186880177008
"15612","AFDN",143.935344334203,0.000801449335267097,0.263387396214721
,0.00304285378414133,0.997572157691402,0.999186880177008
"15613","EPHB6",80.9752944739354,0.000738853121447578,0.25193394864692
1,0.00293272552355801,0.997660026937985,0.999186880177008
"15614","QSOX2",26.8078460585173,-0.000880335503988042,0.3166332025307
73,-0.0027803006663601,0.99778164388193,0.999186880177008
"15615","B3GAT3",197.365002634324,-0.000779833088386346,0.271695990502
859,-0.00287024143029501,0.997709881821433,0.999186880177008
"15616","CHKA",27.1370752878618,-0.00116654937864,0.33466323427626,-0.
00348574106493286,0.997218786653473,0.999186880177008

"15617","CWC15",601.207803178412,0.000799933737506537,0.21237454091238
4,0.00376661785386296,0.996994680874242,0.999186880177008
"15618","CCDC144NL",5.12802784443342,-0.00292072237010046,1.0368238318
8676,-0.00281699000377478,0.997752370140705,0.999186880177008
"15619","VMAC",52.1577144476387,-0.000895417183486238,0.27010255873452
5,-0.0033151007072329,0.997354937173019,0.999186880177008
"15620","ZNF574",26.4809632736167,0.0011826801155O435,0.30518105385493
6,0.00387533924719494,0.996907934386363,0.999186880177008
"15621","MIRLET7BHG",66.2060220278512,0.00140376942316258,0.4121048557
59633,0.00340634040959069,0.99728213883431,0.999186880177008
"15622","ST3GAL6-
AS1",2.77831750758869,0.00261113892590177,1.27941657012516,0.002040882
52948477,0.998371612469739,0.999254957559917
"15623","PPARD",48.5055584329245,-0.000634410830231075,0.2693542871648
14,-0.00235530251591239,0.998120742224048,0.999254957559917
"15624","SGMS1-
AS1",25.431349178635,-0.000870637463343381,0.376324651660952,-0.002313
52758715307,0.998154073703912,0.999254957559917
"15625","FUOM",169.389123158039,-0.000766772517823119,0.37281447394477
1,-0.00205671338269101,0.998358981302892,0.999254957559917
"15626","BCDIN3D",52.7520618974998,-0.000475258786869458,0.26881423132
0247,-0.00176798224013396,0.998589355001713,0.999254957559917
"15627","RTF1",646.468973582223,-0.00052602799711912,0.205447573927444
,-0.00256040014035353,0.997957098490626,0.999254957559917
"15628","SMYD4",163.510509117399,0.000368732978999525,0.21260289263496
8,0.00173437423371575,0.998616170270036,0.999254957559917
"15629","TUBG2",66.6602324292223,-0.000557870391798117,0.2835992039397
,-0.00196710845463703,0.998430475546837,0.999254957559917
"15630","DSC2",3.84611258129652,-0.00194559181736415,0.947696106297514
,-0.00205297014985663,0.998361967964272,0.999254957559917
"15631","RFXANK",168.643136345668,0.000555593014674418,0.2521794570636
18,0.00220316524249735,0.998242129890212,0.999254957559917
"15632","ASXL1",237.165603575477,0.000430048696782018,0.23447055140187
5,0.00183412669186301,0.998536579450502,0.999254957559917
"15633","SLC16A2",73.3768338029421,-0.000683742958697822,0.30376811829
5937,-0.00225087136376737,0.99820406600699,0.999254957559917
"15634","LDB1",70.6718187604376,0.000414144281487459,0.268348685980386
,0.00154330653781449,0.998768620029707,0.999343579578145
"15635","TBL3",68.5725705992479,0.000278679518066095,0.223513775461202
,0.00124681137657427,0.999005188710143,0.999516352222115
"15636","PSTPIP1",8.06339453091814,0.000581585987272202,0.547562364880
689,0.00106213652466587,0.999152537824846,0.999599843258766
"15637","PDK1",18.0372350773604,0.0002960999568718,0.412659446052484,0
.000717540722027094,0.999427485385275,0.999619191424671
"15638","COPS8",360.433239025121,0.000166539010254457,0.22247303492470
5,0.000748580655227818,0.999402719108461,0.999619191424671
"15639","FGF14-
IT1",3.762692876376,-0.000633094632046479,0.741198114861907,-0.0008541
50353801738,0.999318486702966,0.999619191424671
"15640","NSF",96.7508742768646,0.000209600402352203,0.236699949755299,

0.000885510971036911,0.999293464560124,0.999619191424671
"15641","AC093323.1",65.638396174126,-4.30252516668558e-05,0.242862555046065,-0.000177158852910425,0.999858647687193,0.999858647687193
"15642","CLMP",655.705190968423,5.07016278075114e-05,0.18426338208484,0.000275158456519413,0.999780455318539,0.999858647687193
"15643","AL050309.1",71.9650351969403,5.45493871123549e-05,0.245468015447271,0.000222226048525954,0.999822689268332,0.999858647687193
"15644","AL627309.1",1.42214890320063,0.910555714696953,1.26736060044066,0.718466168492496,0.472469900775075,NA
"15645","AL627309.4",0,NA,NA,NA,NA,NA
"15646","PLEKHN1",0.56610483358578,-0.632615996136567,2.06114735235441,-0.306924197056529,0.758901070820303,NA
"15647","AL645608.7",1.77800150704377,-0.571900947888741,1.27046327033142,-0.450151500829735,0.652601204032011,NA
"15648","AL390719.2",0,NA,NA,NA,NA,NA
"15649","TTLL10",0.234567758772022,-1.27115276807931,3.34637793772629,-0.37985929615081,0.704049863502534,NA
"15650","C1QTNF12",2.11663879737539,1.38914666530519,1.06226484725424,1.30772158082411,0.190967760791012,NA
"15651","AL162741.1",0.208911410899576,0.729207474685882,3.34439623453418,0.218038600556985,0.827399035978092,NA
"15652","LINC01786",1.77154412403659,0.0427789024483617,1.07527995974082,0.0397839670132724,0.968265358581525,NA
"15653","TAS1R3",0.37494329422847,0.576284367808218,2.22422971227367,0.259093907714741,0.795562785286341,NA
"15654","AL691432.4",1.16318984344628,0.985719918202645,1.62352802729941,0.607146844173856,0.543753464855978,NA
"15655","FO704657.1",1.10199278312225,0.670383322700255,1.35925465306474,0.493199211191757,0.621871835187995,NA
"15656","AL109917.1",1.04549057154426,-0.747046049110553,1.41079314193944,-0.529522030482495,0.596443365962581,NA
"15657","CALML6",0.485045069170394,1.00465661106878,2.10030791679838,0.478337772777733,0.632409815829176,NA
"15658","CFAP74",0.0707302101443525,-1.18973004036761,3.34835011343899,-0.355318291116717,0.722351154009636,NA
"15659","PRKCZ",0.966555920575204,0.20009493074284,1.50807151626545,0.132682653696922,0.894444361904298,NA
"15660","AL590822.2",0.209068386788914,0.729207474685882,3.34439623453418,0.218038600556985,0.827399035978092,NA
"15661","PRKCZ-AS1",1.15850041812687,-0.522866487426888,1.45579226860551,-0.359162841225787,0.719473272926317,NA
"15662","AL590822.1",0.378065537872613,-1.68970642452469,2.88941888732371,-0.58479109136362,0.558688195236461,NA
"15663","PLCH2",0.737362699484682,1.72592559073496,1.69371706355752,1.0190164743985,0.308195145410721,NA
"15664","AL139246.1",0,NA,NA,NA,NA,NA
"15665","HES5",0,NA,NA,NA,NA,NA
"15666","AL139246.5",2.07645137371329,-1.52886157247582,0.980325109433753,-1.55954545870901,0.11886733401682,NA

"15667","AL139246.3",0.058206321172094,0.078062690925409,3.35247519753
738,0.0232850912611527,0.98142286394086,NA
"15668","MMEL1",0.980597710682104,0.593565887314824,1.77557188140024,0
.334295611195831,0.738156501894704,NA
"15669","TTC34",1.75162219249088,0.917189883016846,1.09621285719504,0.
836689587242871,0.402767073655364,NA
"15670","TP73",0.173731163804746,0.729207474685882,3.34439623453418,0.
218038600556985,0.827399035978092,NA
"15671","AL365330.1",0.225469641685237,0.752409996525134,3.34370795211
989,0.225022641719685,0.821961660372312,NA
"15672","LINC01134",0.452067310975647,0.581094713248316,2.421205558509
12,0.240002221705674,0.810328534264801,NA
"15673","AJAP1",0.66062474237233,-1.0019969909514,1.77976930694051,-0.
562992623282102,0.573439907079412,NA
"15674","AL365255.1",0.416887063880817,0.501819747339869,2.90334542477
376,0.17284190267473,0.862775699117178,NA
"15675","CHD5",0.20218318213956,-1.83828517007581,3.33484116516166,-0.
551236199576747,0.581471767876117,NA
"15676","AL031847.1",1.90206807479968,-0.682929785765246,1.03656263047
795,-0.658840831885239,0.509997983978891,NA
"15677","LINC00337",0,NA,NA,NA,NA,NA
"15678","HES2",0.896030029718359,-1.2711512714153,2.08807319717435,-0.
608767582063439,0.542678504132292,NA
"15679","ESPN",0.863999252464756,0.753930134963155,1.58362012676617,0.
476080167345887,0.634017265698154,NA
"15680","PLEKHG5",1.28186337729166,-0.597606268736048,1.22457038140556
,-0.488013002609222,0.625540631129704,NA
"15681","CAMTA1-
DT",0.440889886100425,-1.03251168638286,2.43890343402923,-0.4233507862
49491,0.672039345638377,NA
"15682","Z98884.2",0.389743395985852,0.560726757975734,2.2108845739310
4,0.253621000656194,0.799788370821431,NA
"15683","Z98884.1",0.058206321172094,0.078062690925409,3.3524751975373
8,0.0232850912611527,0.98142286394086,NA
"15684","TNFRSF9",1.01704531075262,-0.0161361811747472,1.4401734106809
6,-0.0112043320999222,0.991060423445112,NA
"15685","AL034417.3",16.2160137280323,-0.993817862782531,0.89466141452
5593,-1.11083125598919,NA,NA
"15686","ENO1-
AS1",2.16561871404117,0.0952856869956142,1.03589831196384,0.0919836299
520294,0.926711045852148,NA
"15687","CA6",0,NA,NA,NA,NA,NA
"15688","BX323043.1",0.881132133301246,1.07045156300154,1.533652554959
53,0.697975274477852,0.485192657083762,NA
"15689","PIK3CD-AS1",0,NA,NA,NA,NA,NA
"15690","AL139424.3",1.62509285287408,-0.154491569711375,1.15807348200
891,-0.133403943801026,0.893873926585647,NA
"15691","AL354956.1",0.435622913306408,-1.26528470650514,2.37503571560
68,-0.53274344389464,0.594211188486345,NA
"15692","AL139423.2",0.0951446972952032,0.078062690925409,3.3524751975

3738,0.0232850912611527,0.98142286394086,NA
"15693","AL139423.1",0.549645509749051,0.200843649353287,2.02659911059037,0.0991037883633431,0.921055864219183,NA
"15694","C1orf127",0,NA,NA,NA,NA,NA
"15695","MASP2",0.190289394590406,0.752410175041879,3.34370794685893,0.22502269546259,0.821961618563685,NA
"15696","AL109811.1",1.68265355157772,0.183002618798576,1.34058740621783,0.136509277910403,0.89141869049785,NA
"15697","ANGPTL7",0.128936531316447,-0.612660855905215,3.34835003754319,-0.182973957034297,0.854818452904503,NA
"15698","DISP3",0,NA,NA,NA,NA,NA
"15699","DRAXIN",1.06274539347128,0.455311039268689,1.4359322493532,0.317083928906658,0.751179919044748,NA
"15700","AL096840.2",0.33405882840697,-0.0228335752588019,3.03005219941498,-0.007535703597189,0.993987435350817,NA
"15701","TNFRSF8",1.19019682439582,-0.617877076943704,1.1992425990329,-0.515222755964453,0.606397369113099,NA
"15702","AADACL4",0.130481920279372,0.078062690925409,3.35247519753738,0.0232850912611527,0.98142286394086,NA
"15703","KAZN-AS1",0.296199851829589,0.0447176491070698,2.6798488591241,0.0166866310220516,0.9866866125766,NA
"15704","TMEM51-AS1",0.713031151534391,1.41569842803526,1.94988988111418,0.726040194242313,0.467814130339119,NA
"15705","TMEM51",1.11329562316519,-1.40663649379684,1.45233376021033,-0.968535285989036,0.332777105229568,NA
"15706","AL031283.1",0.61301989676623,-0.161948455441793,1.99307956314505,-0.0812553891156458,0.935238850952305,NA
"15707","CTRC",0,NA,NA,NA,NA,NA
"15708","CELA2A",0,NA,NA,NA,NA,NA
"15709","AL121992.3",0,NA,NA,NA,NA,NA
"15710","AGMAT",1.48968900475162,0.200193311906871,1.32638536635044,0.150931484156602,0.880029764210944,NA
"15711","AL121992.1",1.15393900095738,-0.375533853611561,1.4813716398955,-0.253504146763639,0.799878657148323,NA
"15712","AL121992.2",0.36612533414267,-1.58641169504468,2.96011018159318,-0.535929947780137,0.592006965966638,NA
"15713","AL450998.3",0,NA,NA,NA,NA,NA
"15714","CLCNKB",0.801938528801877,-0.0739780549084803,1.65816401940176,-0.0446144374397717,0.964414634706018,NA
"15715","AL451042.2",0.979334318465333,1.3188110683156,1.66250417466535,0.793267823571671,0.427621799319007,NA
"15716","SPATA21",1.41787534343772,-0.71779098216407,1.34649727620919,-0.533080159051548,0.593978092928006,NA
"15717","AL137802.2",0.950740843975963,-1.17090589327298,1.48605167663599,-0.787930804616154,0.430737181432867,NA
"15718","AL137798.1",0.51737476623301,-1.30587583199328,2.01482537973157,-0.64813350334472,0.516898606228312,NA
"15719","BX284668.2",0.423114679278783,1.83916952926597,2.786235705542

94,0.660091149362247,0.509195338039984,NA
"15720","BX284668.6",1.80690125376319,0.860573872515683,1.466169987874
19,0.586953681791995,0.557234814960364,NA
"15721","PADI2",0.34482458835095,-1.2643395936432,2.51846217071244,-0.
502028423673139,0.615647527186121,NA
"15722","PADI4",0.24689456262356,1.0044965898707,3.28047835550152,0.30
6204303462667,0.759449095993732,NA
"15723","PADI6",0,NA,NA,NA,NA,NA
"15724","IGSF21",0.271942340568077,-1.05950017282437,3.21845068887203,
-0.32919571410171,0.742007762222031,NA
"15725","PAX7",0,NA,NA,NA,NA,NA
"15726","AL137127.1",2.32823342638648,0.156689567749937,1.016558736870
08,0.154137249592064,0.877501518291412,NA
"15727","HTR6",0.365049679051394,-0.690220716572571,2.9169182990715,-0
.236626688101713,0.812946413994204,NA
"15728","PLA2G2D",0.201055154534386,-0.612660855905215,3.3483500375431
9,-0.182973957034297,0.854818452904503,NA
"15729","Z98257.1",0.338768542670294,0.182422513001829,2.5587663493460
4,0.0712931499386225,0.943164446915701,NA
"15730","PLA2G2C",0.60117656047864,1.15427224638881,1.79331023778358,0
.64365452338878,0.519799485691041,NA
"15731","VWA5B1",0,NA,NA,NA,NA,NA
"15732","KIF17",0.625282206473709,-1.38796043678129,1.80092099548921,-
0.770694794639929,0.44088785805387,NA
"15733","AL031005.1",0,NA,NA,NA,NA,NA
"15734","AL031728.1",0.469135517544044,-2.45275093572063,3.30514534594
697,-0.742100778934998,0.458026277935231,NA
"15735","LINC02596",0,NA,NA,NA,NA,NA
"15736","RAP1GAP",0.0707302101443525,-1.18973004036761,3.3483501134389
9,-0.355318291116717,0.722351154009636,NA
"15737","LINC01635",1.10854957484466,-0.132371575362966,1.459693207193
96,-0.0906845183019177,0.92774327254431,NA
"15738","WNT4",1.76094985493527,1.45031915102708,1.32565682079017,1.09
403816152253,0.273938241617931,NA
"15739","AL445253.1",0.0951446972952032,0.078062690925409,3.3524751975
3738,0.0232850912611527,0.98142286394086,NA
"15740","TEX46",0.116412642344188,0.528219805776366,3.3480090272049,0.
15777132065183,0.874636998383046,NA
"15741","AL109936.6",0.0707302101443525,-1.18973004036761,3.3483501134
3899,-0.355318291116717,0.722351154009636,NA
"15742","E2F2",0,NA,NA,NA,NA,NA
"15743","MDS2",0,NA,NA,NA,NA,NA
"15744","CNR2",0.462074759269145,0.186454920869812,2.4456366364102,0.0
762398297825214,0.93922829529979,NA
"15745","MYOM3",0.130324944390033,0.078062690925409,3.35247519753738,0
.0232850912611527,0.98142286394086,NA
"15746","IL22RA1",2.52336687401756,0.126485559304242,0.942322196103957
,0.134227507138426,0.893222675178001,NA
"15747","IFNLR1",0.214499643960916,-1.83828489322608,3.33484116993403,
-0.551236115770529,0.58147182531866,NA

"15748","RCAN3AS",0.131452971995207,-1.27115276807931,3.34637793772629
,-0.37985929615081,0.704049863502535,NA
"15749","NCMAP-
DT",1.10878188668118,-0.452092560685724,1.32301338325366,-0.3417142762
18357,0.732565927308791,NA
"15750","AL031432.1",0,NA,NA,NA,NA,NA
"15751","AL031432.5",0.130481920279372,0.078062690925409,3.35247519753
738,0.0232850912611527,0.98142286394086,NA
"15752","AL031432.3",0.982590887369274,-0.836231713794012,1.4395355972
7869,-0.580903810489182,0.561305284063558,NA
"15753","RHD",2.58836841716672,0.181334656718683,0.928478368060164,0.1
95303049544966,0.845155709850223,NA
"15754","AL606491.1",1.26552677580647,1.0607895002666,1.66434340256063
,0.637362156532451,0.523888972535736,NA
"15755","AUNIP",0.130481920279372,0.078062690925409,3.35247519753738,0
.0232850912611527,0.98142286394086,NA
"15756","SLC30A2",0,NA,NA,NA,NA,NA
"15757","TRIM63",0.415711774384641,-1.65437532246923,2.37145002858753,
-0.697621835807605,0.485413722179778,NA
"15758","FAM110D",0.898457178022401,-1.66045505297951,1.47054429968763
,-1.12914317054727,0.258837442448353,NA
"15759","CNKSR1",1.46640959378799,-0.707818812010223,1.33693140015082,
-0.529435401046286,0.596503445809515,NA
"15760","CRYBG2",0,NA,NA,NA,NA,NA
"15761","ZNF683",0.544648305701087,-1.42519595409201,1.98250408951928,
-0.718886766300486,0.472210690977506,NA
"15762","AL513365.2",0.461862019203791,0.876228869343173,2.45413702059
142,0.35704154331693,0.72106070556151,NA
"15763","AL512408.1",0.279798596933267,0.0289122040223894,2.7089597762
9476,0.0106728066896345,0.991484493987396,NA
"15764","SFN",2.28512195649015,-1.38054480303806,1.01436226550595,-1.3
6099779140489,0.173514387414141,NA
"15765","TENT5B",1.81637823503848,1.36076486375541,1.28598201346848,1.
05815232989552,0.289986000214061,NA
"15766","FCN3",0.143769433816564,-1.27115276807931,3.34637793772629,-0
.37985929615081,0.704049863502535,NA
"15767","GPR3",0.279641621043928,0.0289208376955327,2.70936934569688,0
.0106743799037462,0.991483238815646,NA
"15768","BX293535.1",0,NA,NA,NA,NA,NA
"15769","LINC02574",1.05971171474589,1.18863626671148,1.48957043337367
,0.797972516156472,0.424886439427863,NA
"15770","AL445490.1",0,NA,NA,NA,NA,NA
"15771","AL020997.3",1.70857003408623,-0.101883442108178,1.03004693324
062,-0.0989114561873834,0.921208572791887,NA
"15772","SMPDL3B",0.274251354095936,-0.692629117022953,3.1641665760259
8,-0.21889780464493,0.826729656640071,NA
"15773","MATN1",1.08992755853549,-0.156948875574671,1.37655503977663,-
0.114015692100578,0.909225354065274,NA
"15774","AL137857.1",0,NA,NA,NA,NA,NA
"15775","LINC01778",0.575373660254202,-0.374155156285336,1.77605843044

61,-0.210666017441418,0.83314789362985,NA
"15776","NKAIN1",2.26921834230234,0.436194899019304,0.960746548993138,
0.454016618093956,0.64981686594246,NA
"15777","LINC01226",0,NA,NA,NA,NA,NA
"15778","AC114488.2",0.236605117583908,-1.05745329221536,3.33856898350
371,-0.316738488088871,0.751442041982034,NA
"15779","AC114488.3",0.389743395985852,0.560726757975734,2.21088457393
104,0.253621000656194,0.799788370821431,NA
"15780","SPOCD1",1.08895399976371,-1.29668379879158,1.39896639357452,-
0.926887025126031,0.353985194510772,NA
"15781","AL354919.1",0.724300150574891,-1.28074172488482,1.69682659744
663,-0.754786450667418,0.450377124937916,NA
"15782","AL136115.2",1.0831078882899,0.625978672526889,1.4038901544701
6,0.445888640598908,0.655677688076584,NA
"15783","AL136115.1",0.0707302101443525,-1.18973004036761,3.3483501134
3899,-0.355318291116717,0.722351154009636,NA
"15784","AL445248.1",0.797807186866417,-0.570861672478101,1.9484474187
7974,-0.292982847253644,0.769535262605498,NA
"15785","AL049795.2",2.09738558821087,-0.391675315720646,1.10159849497
057,-0.355551789067312,0.722176253916999,NA
"15786","AL049795.1",2.27256086218351,1.05445467496262,0.9837695231087
11,1.07185133325796,0.283786812130749,NA
"15787","DCDC2B",0,NA,NA,NA,NA,NA
"15788","FAM167B",1.19745586411796,0.930356374088858,1.46086219379578,
0.636854302917853,0.524219750860921,NA
"15789","HPCA",2.0710475545076,-1.36703406097388,1.21941432309367,-1.1
2105790057123,0.262263217191815,NA
"15790","AL031602.2",0,NA,NA,NA,NA,NA
"15791","AL020995.1",0,NA,NA,NA,NA,NA
"15792","AL662907.3",0.260649888780067,0.752410175041879,3.34370794685
893,0.22502269546259,0.821961618563685,NA
"15793","AL513327.1",0.933519290341048,-1.12965835535608,1.90195491559
265,-0.593945916433083,0.552548286846511,NA
"15794","GJA4",0.212190630433057,-1.94617432154672,3.33246873969809,-0
.584003774247868,0.559217772758466,NA
"15795","DLGAP3",0.559891812015786,0.696846473867359,2.01207930416843,
0.346331515076817,0.729093586800757,NA
"15796","AL590434.1",0,NA,NA,NA,NA,NA
"15797","AC004865.2",1.92804009058262,-0.281162680035962,1.08340408860
683,-0.259517831797659,0.795235725409806,NA
"15798","TFAP2E",2.21007270904048,0.690040506503572,1.06743706042927,0
.646446082943823,0.51799050427932,NA
"15799","CLSPN",1.66313536595171,1.01739616795306,1.14357009343777,0.8
89666644651916,0.373644909382052,NA
"15800","AL139286.3",1.23953269471398,-0.993636968481126,1.38554592430
732,-0.717144737716203,0.473284793621509,NA
"15801","AL139286.2",1.00001807355128,0.163642660167067,1.353457492998
52,0.120907129343623,0.903764595202217,NA
"15802","TEKT2",0.476434536235496,-1.84295096042902,2.04032469214549,-
0.903263567570257,0.366386027525604,NA

"15803","AL591845.1",2.44136172268416,0.359353006979563,1.08140585557229,0.332301702573443,0.739661454410848,NA
"15804","CDCA8",2.51231137671531,0.134733872224404,0.871546235768679,0.154591766557941,0.877143161297276,NA
"15805","AL929472.2",0.689140373858575,0.0100408506925111,1.69079123143573,0.00593855143428022,0.995261749347239,NA
"15806","GJA9",1.60934238346123,2.16749743639893,1.2948018628326,1.67399931882795,0.0941307332248054,NA
"15807","RHBDL2",2.00761330758342,1.59524560302342,1.22461727183809,1.30264829649923,0.192694861209139,NA
"15808","AL442071.2",0.0785864665095423,0.078062690925409,3.35247519753738,0.0232850912611527,0.98142286394086,NA
"15809","BMP8A",1.2724438816833,1.42139172676481,1.37699552087118,1.03224135824751,0.30195906476331,NA
"15810","PABPC4-AS1",0,NA,NA,NA,NA,NA
"15811","HPCAL4",0.0707302101443525,-1.18973004036761,3.34835011343899,-0.355318291116717,0.722351154009636,NA
"15812","BMP8B",1.66686615182166,1.96498847220382,1.58957576219708,1.23617163707117,0.216394771093638,NA
"15813","OXCT2",0.201975754988658,-0.694084893685918,3.34637783400456,-0.207413785327198,0.835686717551155,NA
"15814","AL033527.4",0.36612533414267,-1.58641169504468,2.96011018159318,-0.535929947780137,0.592006965966638,NA
"15815","MYCL",0.35826907777748,-2.45050898641548,2.89622920268952,-0.846103265632384,0.397495139143923,NA
"15816","MFSD2A",1.14119291088222,-0.752613637408467,1.43562120239847,-0.524242492484152,0.600109889482005,NA
"15817","AL031985.3",1.16076590536976,-0.994620200409235,1.4296235380591,-0.695721757463199,0.48660309710125,NA
"15818","AL603839.2",0,NA,NA,NA,NA,NA
"15819","RIMS3",0.728719365807048,0.114982374909158,1.57513160503338,0.0729985828115751,0.941807245256122,NA
"15820","KCNQ4",1.16730632992257,3.29457124775341,1.5337889206855,2.14799520541651,0.0317141363485806,NA
"15821","SLFNL1-AS1",0.780447501657358,-1.64602238603202,1.88308566533627,-0.874109137110382,0.382058822017672,NA
"15822","SLFNL1",0.4268387479983,0.768065587606909,2.48413528347625,0.309188308992614,0.757178286578382,NA
"15823","EDN2",0,NA,NA,NA,NA,NA
"15824","RIMKLA",0,NA,NA,NA,NA,NA
"15825","AC098484.4",0.555197752697095,2.20946657600392,2.60587747735838,0.847878150527509,0.396505833989524,NA
"15826","AC098484.2",0.985425398944798,-0.370537123929209,1.47580648462129,-0.251074329724396,0.80175664225281,NA
"15827","TMEM269",0.712874175645053,1.41542770097864,1.94991583646903,0.725891689531451,0.467905171530191,NA
"15828","SLC2A1-AS1",2.16711219161868,0.829411758049562,1.15905397457096,0.715593730961994,0.47424224570506,NA

"15829","AL139289.2",2.51277791777835,-0.562117612355923,0.969272886663464,-0.579937415035827,0.56195682335252,NA
"15830","CDC20",0.790072229081008,0.864406803320574,1.50841483898586,0.573056417226533,0.566606495238166,NA
"15831","AL139289.1",0.190289394590406,0.752410175041879,3.34370794685893,0.22502269546259,0.821961618563685,NA
"15832","SZT2-AS1",0.598237445527676,-0.280856238268485,1.76714665392095,-0.158932048817408,0.873722410300682,NA
"15833","HYI-AS1",0.33245767526803,-0.153235300279338,2.55540708347022,-0.0599651230798211,0.952183412432077,NA
"15834","AL451062.1",0.520001850657632,0.502383285683405,2.75249876728963,0.182518986621782,0.855175455442837,NA
"15835","ARTN",0.220046886798247,-1.05745329221536,3.33856898350371,-0.316738488088871,0.751442041982034,NA
"15836","ERI3-IT1",0,NA,NA,NA,NA,NA
"15837","KIF2C",0.861639787675334,1.08287087633374,1.6885229168647,0.641312513746893,0.521319660538476,NA
"15838","AL592166.1",0.36612533414267,-1.58641169504468,2.96011018159318,-0.535929947780137,0.592006965966638,NA
"15839","BEST4",1.4834031723294,0.373138116768153,1.33820940709052,0.278833876664651,0.780372315259701,NA
"15840","TCTEX1D4",0.920346722209478,1.88654051628726,1.51522787119356,1.2450539962687,0.213111902746622,NA
"15841","LINC01144",1.5320706876474,-0.642157368357058,1.13626975505087,-0.565145173936545,0.57197502441836,NA
"15842","HPDL",0.732156406940081,-0.610590102094511,1.72584703224424,-0.35379155318332,0.723495104204058,NA
"15843","TSPAN1",1.75576012147725,-1.44801652970187,1.11460392907956,-1.29913101140568,0.193898972130624,NA
"15844","RAD54L",0.46201899509313,0.876665499896169,2.45400871536725,0.357238136281424,0.720913538320426,NA
"15845","TMEM275",0,NA,NA,NA,NA,NA
"15846","MKNK1-AS1",0.225469641685237,0.752409996525134,3.34370795211989,0.225022641719685,0.821961660372312,NA
"15847","TEX38",0.058206321172094,0.078062690925409,3.35247519753738,0.0232850912611527,0.98142286394086,NA
"15848","CYP4A22-AS1",0.604447277727109,2.27308633517066,1.96493722480466,1.15682389568279,0.247344316904499,NA
"15849","CYP4A22",0.744126605554256,1.44878817819202,1.97090986113798,0.735085965501997,0.462287141048668,NA
"15850","PDZK1IP1",0.638840447509495,1.24743431434294,2.23062982656239,0.559229639758451,0.576005007235948,NA
"15851","TAL1",0.0707302101443525,-1.18973004036761,3.34835011343899,-0.355318291116717,0.722351154009636,NA
"15852","STIL",2.48698621045696,0.92970459792921,1.01452300097631,0.916395781105529,0.359459347317767,NA

"15853","SLC5A9",0.188531265562127,0.606231064780358,3.34687063621681,
0.18113370090265,0.856262632601339,NA
"15854","AGBL4",0.578464438180054,1.00630221990004,2.03004167204419,0.
495705203374828,0.620102415391598,NA
"15855","DMRTA2",0,NA,NA,NA,NA,NA
"15856","LINC01562",0.94841298613291,-0.423214765072988,1.870962366482
68,-0.226201645022188,0.821044591225934,NA
"15857","TTC39A",1.11504739901931,-1.40798303635674,1.34581795398738,-
1.04619130112299,0.295472718328859,NA
"15858","TTC39A-AS1",0,NA,NA,NA,NA,NA
"15859","AC104170.2",0.696055559390979,-0.336297729577302,1.6191245528
3918,-0.207703433925201,0.835460536268582,NA
"15860","AC104170.1",0,NA,NA,NA,NA,NA
"15861","AL050343.2",1.71458388850636,0.237978986753418,1.502047782646
19,0.158436362346719,0.874112962608088,NA
"15862","AL050343.3",1.14739948771278,-1.67250634590541,1.383563898371
39,-1.20883925048502,0.226724608883285,NA
"15863","RAB3B",1.79593535298943,-0.642900698360403,1.18363027020254,-
0.543160068262186,0.587019597567708,NA
"15864","AL513218.1",0.741935076711535,-1.76298778969122,1.72476054763
364,-1.02216379665573,0.306703382523458,NA
"15865","AL513218.2",1.74790829342034,-0.532449166649956,1.20495280147
029,-0.441883836445923,0.658573267661644,NA
"15866","ORC1",0.408735128249713,-0.0503540500456734,2.18169844110953,
-0.0230802062727171,0.981586294586995,NA
"15867","AL445183.1",1.31047841188623,-0.800877278515385,1.29827772113
816,-0.616876701706998,0.537316055217127,NA
"15868","AL606760.2",1.82156550183329,1.65269695415227,1.2819504732634
3,1.28920499552922,0.197326828785453,NA
"15869","AL606760.3",0.866127480959784,0.0432746937155772,1.8629729184
2545,0.0232288367090984,0.98146773644231,NA
"15870","LINC01771",0,NA,NA,NA,NA,NA
"15871","LINC02812",0.058206321172094,0.078062690925409,3.352475197537
38,0.0232850912611527,0.98142286394086,NA
"15872","GLIS1",0.530767610601611,-0.631998870912514,2.07043557517381,
-0.305249232813949,0.760176338141371,NA
"15873","DIO1",0.143769433816564,-1.27115276807931,3.34637793772629,-0
.37985929615081,0.704049863502535,NA
"15874","SSBP3-
AS1",0.773972349877108,0.270623405469812,1.97238272268778,0.1372063354
42358,0.890867703314883,NA
"15875","FAM151A",2.3255377239897,0.378765493982585,1.62660204764431,0
.23285688993883,0.815872535115027,NA
"15876","MROH7",1.02803388621315,-1.54382390652551,1.55547447297451,-0
.992509959725201,0.320948823407569,NA
"15877","TTC22",0.201055154534386,-0.612660855905215,3.34835003754319,
-0.182973957034297,0.854818452904503,NA
"15878","LEXM",0,NA,NA,NA,NA,NA
"15879","MIR4422HG",0,NA,NA,NA,NA,NA
"15880","LINC01767",0.0707302101443525,-1.18973004036761,3.34835011343

899,-0.355318291116717,0.722351154009636,NA
"15881","AL136985.3",0.165874907439556,-0.612660855905215,3.34835003754319,-0.182973957034297,0.854818452904503,NA
"15882","AL136985.2",0,NA,NA,NA,NA,NA
"15883","LINC02777",2.48540224028748,-0.0330611038053464,0.933935273432388,-0.0353997806334486,0.971760959632779,NA
"15884","AC093424.1",1.45440792945553,-0.27443103020969,1.19248808968813,-0.23013314143998,0.817988312789714,NA
"15885","HOOK1",0.481010727356992,0.23379027428217,2.43233788915663,0.0961175153026262,0.923427240513671,NA
"15886","AC099792.1",0.173731163804746,0.729207474685882,3.34439623453418,0.218038600556985,0.827399035978092,NA
"15887","NFIA-AS1",0.789906832413823,-0.407116141044442,2.24050275390735,-0.181707494148109,0.855812284406726,NA
"15888","AC099791.4",2.02225204775439,0.162923080572471,1.23558145503798,0.13185944148657,0.895095469445091,NA
"15889","ANGPTL3",1.06351681583909,0.0270356445069629,1.63060984303764,0.01658008175432,0.986771614832709,NA
"15890","AC103923.1",0.449550870296886,0.917344182770861,2.11941104700857,0.432829763752359,0.665138448660239,NA
"15891","ROR1-AS1",1.2229638333894,3.29037927204701,1.94212405655082,1.69421683488678,0.0902240869631747,NA
"15892","DNAJC6",0.720229818648192,1.53522638596408,1.73480674122825,0.884955280308132,0.376180751084003,NA
"15893","AC119800.1",1.77923405651722,1.17164768235389,1.10410656239236,1.06117264606705,0.288611448133697,NA
"15894","AL139147.1",0.758375822812058,1.06276591585607,1.99381201762576,0.533032154717183,0.594011322012128,NA
"15895","TCTEX1D1",0.131452971995207,-1.27115276807931,3.34637793772629,-0.37985929615081,0.704049863502535,NA
"15896","IL23R",0,NA,NA,NA,NA,NA
"15897","IL12RB2",0.392416812553951,-0.0241020878329992,2.92421906854024,-0.00824223058125084,0.993423725931894,NA
"15898","DEPDC1",0.261934892274579,-0.693029938548298,3.21145314678967,-0.2157994860492,0.829144048953336,NA
"15899","LRRC7",0,NA,NA,NA,NA,NA
"15900","AL353771.1",0.0707302101443525,-1.18973004036761,3.34835011343899,-0.355318291116717,0.722351154009636,NA
"15901","AL354872.2",0.518712728045034,-0.0921539082213315,1.89368447188961,-0.0486638136338395,0.961187214227541,NA
"15902","AL031429.1",1.37160746560868,-1.78663690444059,1.54521307400704,-1.15623983157707,0.247583071546768,NA
"15903","AL031429.2",0.0707302101443525,-1.18973004036761,3.34835011343899,-0.355318291116717,0.722351154009636,NA
"15904","ZRANB2-AS1",0.618857505955023,-0.126067299199052,2.06154670428241,-0.0611518036128774,0.951238312995902,NA
"15905","NEGR1-IT1",0,NA,NA,NA,NA,NA

"15906","LRRIQ3",0.606250677782205,0.427692575063264,1.77090647689737,
0.241510537480547,0.809159450437648,NA
"15907","AC105271.1",0.435008466827936,-0.18214117261146,2.45580913540
409,-0.0741674790543079,0.940877122512563,NA
"15908","ERICH3",0.43469500436349,1.78300076673086,2.41372115536281,0.
738693764509371,0.460092972994471,NA
"15909","LHX8",0.0951446972952032,0.078062690925409,3.35247519753738,0
.0232850912611527,0.98142286394086,NA
"15910","SLC44A5",1.74032188356112,0.691975047323251,1.33599057288654,
0.517949049466844,0.604493818438799,NA
"15911","ST6GALNAC3",0.503125548975208,-1.21796792159655,2.05212417814
715,-0.593515701713645,0.552836077965909,NA
"15912","AC093433.2",0.676065600386737,0.44543723551551,1.876368381885
87,0.237393275124268,0.812351706318915,NA
"15913","AK5",1.97949636299079,1.68304466245893,1.37326963137254,1.225
57480629408,0.220358723406603,NA
"15914","AC093575.1",0,NA,NA,NA,NA,NA
"15915","AC096531.2",1.77102995710628,-1.04219678123513,1.187162015741
5,-0.877889258092688,0.380003819184183,NA
"15916","LINC01781",0,NA,NA,NA,NA,NA
"15917","LINC01725",0.778452439908176,-1.6951748841614,2.1184942929075
4,-0.800179112984461,0.423607029564799,NA
"15918","TTLL7-
IT1",1.01780774152119,0.727285456430066,1.71077082220674,0.42512149902
8101,0.670748106211455,NA
"15919","DNASE2B",0.42878085142997,-1.80978081927633,2.37254035184633,
-0.762802966814851,0.445580911505679,NA
"15920","AL359762.1",0.972572784241856,-1.39797217145935,1.56515242891
515,-0.893185957886749,0.371757596649054,NA
"15921","LPAR3",0,NA,NA,NA,NA,NA
"15922","MCOLN2",2.42837171144334,0.877290518786821,1.07506627399781,0
.816033894844896,0.414480750127807,NA
"15923","WDR63",1.53611319123996,0.718809384467853,1.30023476291001,0.
552830461830684,0.580379507523407,NA
"15924","AL360219.1",0.668802259004843,-2.45377611268987,2.12899711997
485,-1.1525502264272,0.249095042915274,NA
"15925","AC092807.2",0.835951746401564,-0.336036250887048,1.5955928090
8625,-0.210602760913348,0.833197257735664,NA
"15926","LINC02795",1.21468733909964,2.52712947892334,1.85267375349546
,1.36404451898527,0.172553554623583,NA
"15927","CLCA4-
AS1",0.961837836706573,1.99168830010125,1.48607797009576,1.34023136078
984,0.180170138856061,NA
"15928","AL121989.1",0,NA,NA,NA,NA,NA
"15929","LINC02801",0.260963840558744,0.75241017504188,3.3437079468589
3,0.22502269546259,0.821961618563684,NA
"15930","PKN2-
AS1",0.777705315998088,1.64455160946783,1.66575807337825,0.98726918137
1935,0.323510694716205,NA
"15931","GBP7",0.554864026855699,-1.22264759251233,1.84014750856734,-0

.664429121480717,0.506415699357895,NA
"15932","GBP6",0.215379254191179,1.0128453861209,3.3356918571358,0.303638774053483,0.761403107966425,NA
"15933","AC099568.1",0.565028451038536,-1.33504934240776,1.98482925053889,-0.672626797516855,0.501184748937678,NA
"15934","AC099568.2",0.564362672305873,0.114070418817057,2.25705006645767,0.050539604996926,0.959692389490806,NA
"15935","LINC01763",0.222355900326106,-0.694084893685918,3.34637783400456,-0.207413785327198,0.835686717551155,NA
"15936","BRDT",0.0785864665095423,0.078062690925409,3.35247519753738,0.0232850912611527,0.98142286394086,NA
"15937","EPHX4",0.2805622214982,-0.156179234616825,2.68884551896339,-0.0580841232846414,0.953681620823927,NA
"15938","AC104836.1",0.642824650865073,1.32286814046904,1.72871058602912,0.765234013813548,0.444132208834666,NA
"15939","GFI1",2.36380279377374,0.36488576993732,1.05027059558798,0.347420723259461,0.72827526688423,NA
"15940","AC093577.1",0.864289931619956,-0.645442592285578,1.68298062580209,-0.383511599830787,0.701340471122823,NA
"15941","AL049796.1",1.68727132780109,0.017841693495561,1.14123811518193,0.01563362917713,0.987526676753894,NA
"15942","ABCA4",0,NA,NA,NA,NA,NA
"15943","ARHGAP29-AS1",0.803609251425357,0.659243996674156,1.8530778919858,0.355756225642354,0.722023133976327,NA
"15944","AC118469.2",0.752509064449245,-0.0179010653351611,1.57223517762511,-0.0113857427883029,0.990915687890708,NA
"15945","AC092802.4",0.651938673728844,2.35596059061864,2.58625448504398,0.9109546660017,0.362319260471911,NA
"15946","DPYD-AS1",1.56397060291955,-1.92466693121239,2.03841120425567,-0.944199544819117,0.345067687792215,NA
"15947","DPYD-IT1",0.676646465769365,-1.27821782388462,1.9190824959112,-0.666056736283089,0.505374836395815,NA
"15948","PLPPR5",0.61633818870944,2.34849424330242,2.55843074113006,0.917943255428948,0.35864857434417,NA
"15949","LRRC39",1.30092870076085,0.160993952179511,1.20825948978702,0.13324451704318,0.894000005194147,NA
"15950","AL445928.2",1.2500591782767,1.28578261879283,1.39508777500187,0.921649979185798,0.356711179798612,NA
"15951","AC093157.2",1.05163237628517,-0.195567447926978,1.65786963111554,-0.117963104128631,0.906096892353001,NA
"15952","AL109741.1",0.2805622214982,-0.156179234616825,2.68884551896339,-0.0580841232846414,0.953681620823927,NA
"15953","VAV3",2.12848750500917,-1.35376272328523,1.15453373445077,-1.17256229323541,0.240971377838469,NA
"15954","VAV3-AS1",0,NA,NA,NA,NA,NA
"15955","LINC02785",0.594874195361388,-2.34786490627832,2.12562098380443,-1.10455482146968,0.269352536502951,NA

"15956","AL160171.1",0.297327879434763,-1.26373379883152,2.62728352491535,-0.481003967347693,0.630513679037775,NA
"15957","FNDC7",0,NA,NA,NA,NA,NA
"15958","SPATA42",0.153351018467297,0.606231064780358,3.34687063621681,0.18113370090265,0.856262632601339,NA
"15959","KIAA1324",1.24324326920411,-1.00528216889392,1.42915656403572,-0.703409405373447,0.481800646824751,NA
"15960","CELSR2",1.5758287340161,-1.24890640802709,1.25116717953756,-0.998193070001003,0.318185746217962,NA
"15961","SYPL2",1.25736455014231,1.59549509244202,1.48211877184565,1.07649610999474,0.281705439698406,NA
"15962","GNAT2",0.165874907439556,-0.612660855905215,3.34835003754319,-0.182973957034297,0.854818452904503,NA
"15963","AC000032.1",2.2313477093518,2.86410566356618,1.46549782182314,1.95435682053973,0.0506590334418517,NA
"15964","UBL4B",0.455805973523136,1.88181858068652,2.04897386053561,0.918420003754759,0.358399022015783,NA
"15965","RBM15-AS1",0.0707302101443525,-1.18973004036761,3.34835011343899,-0.355318291116717,0.722351154009636,NA
"15966","AL365361.1",2.16282548224248,1.38822394050949,1.22317184856403,1.13493777848078,0.256401389606306,NA
"15967","KCNA3",0.742945619631571,0.120281862330847,1.9549161704633,0.0615278875627393,0.950938805410975,NA
"15968","AL360270.2",0.602418421200236,-1.42108165329253,1.82047400929156,-0.78061078929962,0.435031444093873,NA
"15969","AL360270.1",0.540818201668026,0.50250593246059,2.07100297516163,0.242638923501002,0.808285125490117,NA
"15970","AL360270.3",1.42719808464605,-1.16069762970807,1.22063970573131,-0.950892900057414,0.341658746356703,NA
"15971","AL355816.1",0,NA,NA,NA,NA,NA
"15972","AL390195.1",0.657571794304944,-0.888640774814439,1.88659858929695,-0.47102800768318,0.637620734340294,NA
"15973","TMIGD3",1.19341609634777,-2.03862415383412,1.61022390172579,-1.26605011368245,0.205495141175287,NA
"15974","ADORA3",0.396054263388116,0.747655985455963,2.15170369894396,0.347471627168233,0.7282370304461,NA
"15975","LINC01160",0.820705050716305,0.944139109464009,1.97700037896202,0.477561420579854,0.6329623951554,NA
"15976","INKA2-AS1",0.625074779322807,-0.592287246215255,2.01270055366503,-0.294274896052832,0.768547853862295,NA
"15977","AL049557.1",0.538903096750408,0.475143390391347,2.06847682818628,0.229706895391219,0.818319536427979,NA
"15978","AL354760.1",1.18477906961955,-0.544817107384652,1.32900633140365,-0.409943199299311,0.681847614940655,NA
"15979","AL603832.1",0.331380098924419,0.0562416534272768,3.08130406907474,0.0182525489748778,0.98543738158498,NA
"15980","TAFA3",0.47181735488999,-0.847815027623758,2.40877406140485,-0.351969510635338,0.724861128220079,NA

"15981","AL390242.1",0.998818243313399,0.439564127458629,1.74975365888
728,0.251214864004435,0.801647993167566,NA
"15982","AL137856.1",0.0707302101443525,-1.18973004036761,3.3483501134
3899,-0.355318291116717,0.722351154009636,NA
"15983","AP4B1-
AS1",2.11339927687226,1.87487959097194,1.31655434204863,1.424080671105
86,0.15442312097892,NA
"15984","BCL2L15",1.35908457067138,0.66569914776821,1.32150128096939,0
.503744610281335,0.614440855529624,NA
"15985","AMPD1",0,NA,NA,NA,NA,NA
"15986","LINC01765",0,NA,NA,NA,NA,NA
"15987","CASQ2",0,NA,NA,NA,NA,NA
"15988","MAB21L3",2.32697725926224,1.49044435506252,1.11191060947375,1
.34043541123142,0.180103829627478,NA
"15989","LINC01779",1.1635100634539,-0.549107964318242,1.4021530014830
9,-0.391617721987142,0.695340691521207,NA
"15990","LINC01762",0.21003943850475,-0.694084893685918,3.346377834004
56,-0.207413785327199,0.835686717551155,NA
"15991","AL390066.1",0.153351018467297,0.606231064780358,3.34687063621
681,0.18113370090265,0.856262632601339,NA
"15992","AL157904.1",0.260389503311654,-1.26323271127984,2.73006768494
753,-0.462711132857542,0.643571437305483,NA
"15993","CD101",0.40226728495811,-0.422685492943438,2.52488789309826,-
0.167407627918389,0.867049323662297,NA
"15994","AL445231.1",0.495374623441428,-0.032926234943703,2.3839330287
4632,-0.0138117281595861,0.988980185708246,NA
"15995","AL358072.1",2.05564443882627,1.56528105955345,1.2965367847480
6,1.2072785577446,0.227324885515182,NA
"15996","AL157902.1",0,NA,NA,NA,NA,NA
"15997","AL139420.2",1.15986227058955,-2.57569870174676,1.581025254945
42,-1.62913191531255,0.103285096225962,NA
"15998","AL359915.1",0.935511227007101,1.15184349690932,1.562944734362
65,0.736970074235557,0.461140548153258,NA
"15999","HAO2",0.0951446972952032,0.078062690925409,3.35247519753738,0
.0232850912611527,0.98142286394086,NA
"16000","HSD3B2",0,NA,NA,NA,NA,NA
"16001","ADAM30",0,NA,NA,NA,NA,NA
"16002","AC245008.1",0.492587373756906,1.97664496117054,2.033427860466
39,0.972075282138199,0.331013097950552,NA
"16003","FCGR1B",0.708693281479659,-2.4781436072417,1.82820961009602,-
1.35550299788193,0.175257354102402,NA
"16004","AC244453.2",0.760060047831263,0.31384160178693,1.910478507883
66,0.164273819617364,0.86951559061235,NA
"16005","FAM72B",1.42718362602793,1.22734818163452,1.36279389125225,0.
900611742914938,0.367794788783811,NA
"16006","LINC02798",1.43026777909031,1.92115489459221,1.44854804241072
,1.32626246306264,0.184752775744383,NA
"16007","AL592494.1",0.131452971995207,-1.27115276807931,3.34637793772
629,-0.37985929615081,0.704049863502535,NA
"16008","AL592494.2",0.187142852488541,-0.1625046647162,3.343878261475

95,-0.048597661759514,0.961239933410772,NA
"16009","AC239800.2",2.57738666088061,-0.257985534593034,0.90081429941
1986,-0.286391473538372,0.774578304471533,NA
"16010","FAM72C",0.0707302101443525,-1.18973004036761,3.34835011343899
,-0.355318291116717,0.722351154009636,NA
"16011","AC246785.3",1.01188946946958,-2.4974893703969,1.5250597756286
5,-1.63763375725218,0.101498116725616,NA
"16012","FP700111.1",0.641347739954441,-2.44316322471159,2.18081292272
994,-1.12029931556588,0.26258623363998,NA
"16013","FAM72D",1.21253827542523,1.31710366439511,1.46140861017435,0.
901256264145029,0.367452081950956,NA
"16014","PDZK1",1.6672816196292,0.656133145027974,1.18201573211929,0.5
55096795413683,0.57882845459006,NA
"16015","ITGA10",1.18954384357675,0.837547377210574,1.36351260520454,0
.614257157589631,0.539045408388072,NA
"16016","ANKRD34A",0.704934980079303,0.354879130312088,1.6369784861051
1,0.216789122963038,0.82837268900974,NA
"16017","LINC00624",2.22993871193118,1.5957982512361,1.26064303305801,
1.2658605246602 5,0.20556302147811,NA
"16018","AC239803.1",1.56101964823977,0.851714139904709,1.276162945145
9,0.667402343207304,0.504515169571248,NA
"16019","LINC02804",0.53982369720468,0.109487109172759,1.8362475520554
7,0.0596254622914003,0.952453938467398,NA
"16020","LINC02805",0.165874907439556,-0.612660855905215,3.34835003754
319,-0.182973957034297,0.854818452904503,NA
"16021","LINC01731",0.131452971995207,-1.27115276807931,3.346377937726
29,-0.37985929615081,0.704049863502535,NA
"16022","AC245100.6",0.658462281196462,1.53089993933465,1.758924684267
26,0.870361279836379,0.384102999830184,NA
"16023","PPIAL4G",0.405547350201804,-1.52667881960836,2.44470022532275
,-0.624485081563244,0.532309064734737,NA
"16024","NUDT4B",1.42052996686316,1.76402526186946,1.29262859647832,1.
36468067214004,0.172353436990405,NA
"16025","AC239802.2",0.721158216905342,1.14979509713207,2.247696945019
24,0.511543649013694,0.608970432380646,NA
"16026","AC239804.1",0.486489246419996,1.04317563352526,2.409857236250
83,0.432878602862052,0.665102965483294,NA
"16027","AC243772.2",1.01491708225759,0.576286790446113,1.376208751480
19,0.418749546408774,0.675399181419411,NA
"16028","HIST2H3D",0.707088938970158,1.16773259019429,1.92302967138839
,0.607235867219464,0.543694392488982,NA
"16029","HIST2H4A",0.893945327508305,0.479070431906257,1.6194343661805
6,0.295825778377265,0.767363135336188,NA
"16030","AC242988.2",0.131452971995207,-1.27115276807931,3.34637793772
629,-0.37985929615081,0.704049863502535,NA
"16031","AL356356.1",1.3444171110632,-0.131044447948532,1.196617400260
87,-0.109512403814255,0.912796084119746,NA
"16032","HORMAD1",0.81921606934805,-1.9056088612948,2.11786597003998,-
0.899777836866055,0.368238491179085,NA
"16033","ANXA9",0.964058214607363,2.08491748281555,1.7172285741131,1.2

1411762781338,0.224702809472464,NA
"16034","BNIPL",0.5326696048019,-0.277819042222564,2.33430072504813,-0
.119015960215167,0.905262711232174,NA
"16035","AL592424.1",0.624326809702507,1.51901679899216,1.941162364447
21,0.782529492026666,0.433903460126161,NA
"16036","TNFAIP8L2",0.99197323435038,-0.768849268410805,1.737303201592
33,-0.442553301983276,0.658088868302127,NA
"16037","TMOD4",0.522747069858436,0.858680586942629,2.04670013447714,0
.419543914850034,0.674818667360067,NA
"16038","AL391069.2",2.23220578009603,-0.393225535884905,1.04246081553
701,-0.377208936800507,0.706018339410669,NA
"16039","CGN",0,NA,NA,NA,NA,NA
"16040","AL391335.1",0.42309902433415,-0.149835739621135,2.42316133055
455,-0.0618348179016394,0.950694375852862,NA
"16041","AL589765.5",0.261019604734759,0.0605108493027948,3.2785886274
0842,0.0184563713778956,0.985274782227795,NA
"16042","TDRKH-
AS1",0.521959992545992,-0.546572332978196,2.06379441268307,-0.26483855
6408152,0.791133833350357,NA
"16043","LINGO4",0,NA,NA,NA,NA,NA
"16044","RORC",0.19499910885373,0.927978805117746,3.33878404161865,0.2
77939151963797,0.781059068583634,NA
"16045","C2CD4D",0.280769648649102,-1.26351312461569,2.67096069862218,
-0.473055678156355,0.636173456634656,NA
"16046","C2CD4D-
AS1",0.116412642344188,0.528219805776366,3.3480090272049,0.15777132065
183,0.874636998383046,NA
"16047","AL450992.2",0.769312025758686,-0.116425391008312,2.2710013904
7077,-0.0512661029169067,0.959113478524019,NA
"16048","AL450992.1",1.10416827350828,2.1739867517821,1.62426228243367
,1.33844562869782,0.180751213193924,NA
"16049","AL589986.2",0.544325851637397,2.10432302061548,2.303375818275
38,0.913582144919393,0.360936451970079,NA
"16050","FLG",0.130481920279372,0.078062690925409,3.35247519753738,0.0
232850912611527,0.98142286394086,NA
"16051","FLG2",0.522590093969098,0.858340153093931,2.04676934051862,0.
419363401679859,0.674950567048898,NA
"16052","SPRR2D",0,NA,NA,NA,NA,NA
"16053","SPRR2A",0.0707302101443525,-1.18973004036761,3.34835011343899
,-0.355318291116717,0.722351154009636,NA
"16054","SPRR2B",0.20218318213956,-1.83828517007581,3.33484116516166,-
0.551236199576747,0.581471767876117,NA
"16055","SPRR2E",0,NA,NA,NA,NA,NA
"16056","SPRR2F",0.0785864665095423,0.078062690925409,3.35247519753738
,0.0232850912611527,0.98142286394086,NA
"16057","LOR",0.591719463778248,-0.906425545182174,2.25176574552795,-0
.402539894295112,0.687286734304173,NA
"16058","S100A12",1.51563789541656,0.927754239601254,1.41212501527229,
0.656991576218455,0.511186332940595,NA
"16059","S100A7",0.709973174351275,-0.3262023704554,1.76965238209653,-

0.184331326172061,0.853753540234786,NA
"16060","BX470102.1",0.954984587089741,-0.667764238896807,1.4681008047
0445,-0.454849038129392,0.649217848216345,NA
"16061","S100A5",1.82030806767422,-0.232272243570462,1.31799327779669,
-0.176231736142657,0.860111889118902,NA
"16062","AL358472.5",0.959777240553625,-0.582674669189658,1.3616701546
3393,-0.427911757635831,0.668715366596294,NA
"16063","AL358472.3",1.10159887451201,0.554509443409757,1.551194766217
16,0.357472482170642,0.720738123175751,NA
"16064","NUP210L",0,NA,NA,NA,NA,NA
"16065","C1orf189",0.21003943850475,-0.694084893685918,3.3463778340045
6,-0.207413785327199,0.835686717551155,NA
"16066","AL162591.2",2.23142678266059,-0.296367213233885,0.91248209910
1936,-0.324792358694564,0.745338228170783,NA
"16067","UBE2Q1-AS1",0,NA,NA,NA,NA,NA
"16068","CHRNB2",0.0951446972952032,0.078062690925409,3.35247519753738
,0.0232850912611527,0.98142286394086,NA
"16069","KCNN3",1.13748698199576,0.709233394655242,1.38392079001721,0.
512481205406577,0.608314270737407,NA
"16070","AL451085.1",1.44597696633237,0.981069627298543,1.424523848878
99,0.688700036907477,0.491012052964311,NA
"16071","DCST2",1.37442092270764,1.58743341685258,1.47802126302813,1.0
7402610270998,0.282810982639356,NA
"16072","DCST1-
AS1",2.1551506365176,0.435165108240909,0.968802298828739,0.44917844308
0713,0.653302936866767,NA
"16073","TRIM46",0.429878898152095,-0.30443563352693,2.14190392669269,
-0.142133188016985,0.886974804036957,NA
"16074","AC234582.1",1.83051426432312,-0.40547754896131,1.042659256869
98,-0.388887880954069,0.697359087523018,NA
"16075","AC234582.2",0,NA,NA,NA,NA,NA
"16076","ASH1L-
IT1",0.426524796219622,0.767151628407815,2.48443680512942,0.3087829108
08573,0.757486669524485,NA
"16077","PAQR6",1.16068884099514,0.637956580850391,1.42424130297164,0.
447927313664693,0.654205655314331,NA
"16078","TSACC",0.492587373756906,1.97664496117054,2.03342786046639,0.
972075282138199,0.331013097950552,NA
"16079","C1orf61",1.12077708823521,0.730867904972059,1.33927570944631,
0.545718779051265,0.585259261310498,NA
"16080","IQGAP3",0.274094378206597,-0.692634286879323,3.16476374960416
,-0.218858133396515,0.826760560514704,NA
"16081","TTC24",0,NA,NA,NA,NA,NA
"16082","HAPLN2",0.50635312345076,-1.72326132257384,2.26254683816244,-
0.761646695444065,0.446270897327239,NA
"16083","NTRK1",2.41457958126262,0.823817170471857,1.0745140642329,0.7
66688122467697,0.443266967199407,NA
"16084","INSRR",1.23067161615027,1.12052692273161,1.48925110644665,0.7
52409662736586,0.451804737479053,NA
"16085","LRRC71",0.283675962857331,1.19208447107321,2.67004879895283,0

.446465424729589,0.655261082574057,NA
"16086","FCRL5",0.636637958609412,1.4788032452688,1.9561878184026,0.75
5961790252007,0.44967210506941,NA
"16087","FCRL3",0.246737586734221,1.00448814052217,3.28114163691741,0.
306139829265606,0.759498183427876,NA
"16088","AL356276.1",0,NA,NA,NA,NA,NA
"16089","FCRL2",0.136792787681636,0.606231064780358,3.34687063621681,0
.18113370090265,0.856262632601339,NA
"16090","FCRL1",0.190289394590406,0.752410175041879,3.34370794685893,0
.22502269546259,0.821961618563685,NA
"16091","KIRREL1-
IT1",0.359148688007742,0.264128727365081,2.52571220720674,0.1045759396
5434,0.916712307375039,NA
"16092","AL138899.1",0.130324944390033,0.078062690925409,3.35247519753
738,0.0232850912611527,0.98142286394086,NA
"16093","CD1A",0,NA,NA,NA,NA,NA
"16094","CD1B",1.21110593015137,0.370449959323318,1.83631760287965,0.2
0173523291526,0.840123716956219,NA
"16095","CD1E",1.78346902090806,-1.55099282067809,1.27342692902604,-1.
2179676629458,0.223236261444565,NA
"16096","SPTA1",0.696321257773843,1.42842447572537,1.75523609473265,0.
813807601160879,0.41575517990232,NA
"16097","AIM2",1.0604187294246,0.42242542864818,1.39501859408843,0.302
809891164363,0.762034745054147,NA
"16098","CADM3-
AS1",1.14933475020517,0.0371548443094702,1.24863635283939,0.0297563371
633224,0.97626138122855,NA
"16099","FCRL6",1.53801941416261,-0.0434092137178329,1.2920348635306,-
0.0335975560281813,0.973198071176558,NA
"16100","VSIG8",1.13230751300383,0.143079413162539,1.56750857620293,0.
0912782330729756,0.927271513267815,NA
"16101","CFAP45",1.2039036205431,-0.186259074653366,1.30957493238637,-
0.142228649958927,0.886899402429452,NA
"16102","IGSF9",0.814332929713081,-1.06814098520726,1.83243721336407,-
0.582907276395201,0.559955722003673,NA
"16103","SLAMF9",1.46654792512355,-3.03883786012149,1.61474644639501,-
1.88192881111819,0.0598456819823842,NA
"16104","KCNJ10",0.890509480171638,0.0447288601279514,1.58131872889878
,0.0282857967280892,0.977434208637507,NA
"16105","AL121987.1",2.53191567292578,1.42833899583679,1.1008054731056
,1.29753987487649,0.194445494422111,NA
"16106","KCNJ9",0.130481920279372,0.078062690925409,3.35247519753738,0
.0232850912611527,0.98142286394086,NA
"16107","ATP1A2",0.201212130423725,-0.612660855905215,3.34835003754319
,-0.182973957034297,0.854818452904503,NA
"16108","ATP1A4",0,NA,NA,NA,NA,NA
"16109","AL121987.2",0.475306508630322,-0.628198346349813,2.1300803145
4614,-0.294917680831046,0.768056764997786,NA
"16110","NHLH1",0.572572676877383,-0.222597939857041,1.84748445905385,
-0.120487043214989,0.904097342888929,NA

"16111","SLAMF6",0.821012628463869,1.46947342579921,1.88451203384074,0
.77976335486929,0.435530179730897,NA
"16112","SLAMF1",1.94781104038466,0.716341915033241,1.14681739233389,0
.624634680134569,0.532210852908332,NA
"16113","AL121985.1",0.130324944390033,0.078062690925409,3.35247519753
738,0.0232850912611527,0.98142286394086,NA
"16114","LY9",0.84364690775933,-0.749778032316606,1.67978391500051,-0.
446353858744016,0.655341657223544,NA
"16115","CD244",1.26564954671709,-0.248383279561972,1.34056255159,-0.1
8528287193117,0.85300717165265,NA
"16116","ITLN1",0.143769433816564,-1.27115276807931,3.34637793772629,-
0.37985929615081,0.704049863502535,NA
"16117","F11R",2.27125831868235,0.0992689750713124,1.04642294300484,0.
0948650598067526,0.924422009553742,NA
"16118","NECTIN4",0.143769433816564,-1.27115276807931,3.34637793772629
,-0.37985929615081,0.704049863502535,NA
"16119","AL590714.1",0.810950300600525,-0.78871009153987,1.68175856711
941,-0.468979380845852,0.639084374376012,NA
"16120","NR1I3",0.271785364678738,-1.05948928335938,3.21905293552203,-
0.329130742669059,0.742056868265265,NA
"16121","CFAP126",0,NA,NA,NA,NA,NA
"16122","FCRLA",0.201975754988658,-0.694084893685918,3.34637783400456,
-0.207413785327198,0.835686717551155,NA
"16123","NOS1AP",2.40284593467835,-1.57863690198165,1.00019477326222,-
1.57832948559887,0.114489935449302,NA
"16124","C1orf226",0.565208390361154,-1.06154435331346,2.2614221411047
6,-0.469414504270693,0.638773382906723,NA
"16125","SH2D1B",0.579277668296338,-1.3464345852625,2.27591379973905,-
0.591601749335532,0.554117302180648,NA
"16126","AL596325.1",1.2619743842787,-1.45625962975598,1.8143871095875
6,-0.802617932006258,0.422195600549145,NA
"16127","RGS4",1.2495830192014,-2.512824476853,1.72495480538728,-1.456
74800812467,0.145185952384516,NA
"16128","AL499616.1",0,NA,NA,NA,NA,NA
"16129","AL357568.1",0.759503850417232,0.163878350707508,1.95406795651
589,0.0838652259564728,0.933163587792621,NA
"16130","AL356441.1",1.09719470370159,-2.07089640353892,1.790938284126
4,-1.15631924443956,0.247550599558649,NA
"16131","AL451074.5",0.296356827718928,0.0447067733798088,2.6795359287
6928,0.0166845209649205,0.986688295924262,NA
"16132","TMCO1-
AS1",1.20161841615873,0.39569332792259,1.41882079870371,0.278888869041
185,0.780330110990503,NA
"16133","AL358115.1",1.68857568938492,-1.32817359756776,1.235348954497
87,-1.07514042306178,0.282311863906517,NA
"16134","AL596087.2",0.390714447701687,-0.730825083633851,2.1740393936
2358,-0.336160000493712,0.736750214437084,NA
"16135","LINC01675",0.130324944390033,0.078062690925409,3.352475197537
38,0.0232850912611527,0.98142286394086,NA
"16136","ILDR2",0.0951446972952032,0.078062690925409,3.35247519753738,

0.0232850912611527,0.98142286394086,NA
"16137","GPA33",0,NA,NA,NA,NA,NA
"16138","DUSP27",0,NA,NA,NA,NA,NA
"16139","AL451050.2",0.468172886606056,0.956755906455499,2.10791526495004,0.453887270690729,0.64990996595284,NA
"16140","AL136984.1",1.88106270305043,0.927047425517785,1.15874728515438,0.800042802598041,0.423685998489205,NA
"16141","AL359962.3",0.663541711414478,0.982384261788634,1.87917107971213,0.522775319604816,0.601130616496385,NA
"16142","AL359962.2",0,NA,NA,NA,NA,NA
"16143","AL031733.2",0.149316676653895,-0.612660855905215,3.34835003754319,-0.182973957034297,0.854818452904503,NA
"16144","ADCY10",0.404158937128217,-1.84257376585777,2.12046546940135,-0.86894778172359,0.384875692947524,NA
"16145","Z99943.1",0.5611244429964,-0.32950570374316,2.27735396533269,-0.14468796188871,0.884957244158986,NA
"16146","AL023755.1",0.53624201054653,-1.20659434913627,2.03132419925207,-0.593993981650265,0.552516138298808,NA
"16147","CCDC181",0.461270031437873,-0.925423814673601,2.4185280245648,-0.38263927697928,0.701987243760388,NA
"16148","F5",1.45097816871403,-1.87725838128383,1.45695886675458,-1.28847726872721,0.197579876644194,NA
"16149","SELP",0.143769433816564,-1.27115276807931,3.34637793772629,-0.37985929615081,0.704049863502535,NA
"16150","SELE",0.495111471010467,-2.91581396096329,3.17821762015852,-0.917436849657218,0.358913770403175,NA
"16151","LINC01681",0.274094378206597,-0.692634286879323,3.16476374960416,-0.218858133396515,0.826760560514704,NA
"16152","MROH9",0.260963840558744,0.75241017504188,3.34370794685893,0.22502269546259,0.821961618563684,NA
"16153","AL021026.1",2.19299480295265,0.492505878684239,1.19352421572755,0.412648417346117,0.679864222384101,NA
"16154","MYOCOS",0.143769433816564,-1.27115276807931,3.34637793772629,-0.37985929615081,0.704049863502535,NA
"16155","FASLG",1.20588521517897,0.382263986655029,1.51361327100833,0.252550630981436,0.800615484170196,NA
"16156","ANKRD45",2.24810099911158,0.494433775841227,0.967139038679878,0.511233396716275,0.609187636024161,NA
"16157","GAS5-AS1",1.68706505051417,-0.949366697627624,1.19309826771473,-0.795715427067081,0.426197458014144,NA
"16158","SERPINC1",0.0785864665095423,0.078062690925409,3.35247519753738,0.0232850912611527,0.98142286394086,NA
"16159","RC3H1-IT1",1.96579464029506,0.493503027962471,1.17224975315981,0.42098795639089,0.673763876346967,NA
"16160","RABGAP1L-DT",0.794109760264971,-1.20674122221914,1.66635113731881,-0.724181833704517,0.468954109811508,NA
"16161","GPR52",1.24157282048841,-0.0460069436127254,1.26445596969627,

-0.0363847731477566,0.970975555408475,NA
"16162","Z99127.1",0.0951446972952032,0.078062690925409,3.35247519753738,0.0232850912611527,0.98142286394086,NA
"16163","TNN",0.397599652351042,1.65564772342944,2.14892136502339,0.770455238789725,0.441029897144077,NA
"16164","TNR",0,NA,NA,NA,NA,NA
"16165","LINC02803",0.70018416254964,-0.931487772240077,2.22760375208659,-0.418156851894129,0.675832440639345,NA
"16166","AL590723.1",1.26308830191968,-1.99546940449241,1.49282539924584,-1.33670649327142,0.181318461577848,NA
"16167","AL591043.2",0.837988925089416,0.108700984919746,1.88981305737491,0.0575194379653296,0.954131422484,NA
"16168","ASTN1",0.859962034084531,1.85583834503842,1.77966795648352,1.04280033715132,0.297040775168806,NA
"16169","CLEC20A",0.190289394590406,0.752410175041879,3.34370794685893,0.22502269546259,0.821961618563685,NA
"16170","AL449106.1",0.35098040111621,-0.237522332986343,3.00335770580659,-0.0790855956075846,0.936964540664927,NA
"16171","AXDND1",0.790877884129658,-1.74181257223229,2.15874267600282,-0.806864380639136,0.419744622285687,NA
"16172","AL160286.2",0.987190656337689,-1.61971203685332,1.78295808269137,-0.908440895261187,0.363645327565284,NA
"16173","AL353708.1",1.97747128163809,0.105791348654393,1.03527862965727,0.102186354111661,0.918608758895173,NA
"16174","AL162431.1",0.989938028087401,1.38132601967274,1.6003207204418,0.863155742488541,0.388051843354765,NA
"16175","KIAA1614-AS1",1.09025628426617,0.948573009610491,1.45637461490504,0.651324871981748,0.514836796186478,NA
"16176","LINC01732",0,NA,NA,NA,NA,NA
"16177","TEDDM1",0.0951446972952032,0.078062690925409,3.35247519753738,0.0232850912611527,0.98142286394086,NA
"16178","RGSL1",0,NA,NA,NA,NA,NA
"16179","SMG7-AS1",2.23956891331023,-1.35306531508894,1.21841435981201,-1.11051327013061,0.266777913010991,NA
"16180","AL713852.2",1.74343567609279,3.80263624669162,1.40897318759247,2.69887055351936,0.00695752330621852,NA
"16181","AL078644.2",0.697449285379017,0.27786103179921,1.78424850793117,0.155730006534454,0.876245843927052,NA
"16182","OCLM",0,NA,NA,NA,NA,NA
"16183","AL096803.4",0.350371831188281,-0.433874515519438,2.5830263161033,-0.167971387985691,0.866605788315891,NA
"16184","PACERR",0.722251522515445,0.0119991949159927,1.75288543256196,0.00684539599285453,0.994538206880853,NA
"16185","ERVMER61-1",1.16221165992778,-1.95572314686309,1.54670078459052,-1.26444828007303,0.206069173210045,NA
"16186","RGS18",1.14839367937239,-0.686116426753905,1.40645888443226,-0.487832551913428,0.625668452266128,NA
"16187","AL390957.1",1.82153188717963,0.0565732121055767,1.07230279685

468,0.0527586165694238,0.957924234744572,NA
"16188","AL136987.1",0.143769433816564,-1.27115276807931,3.34637793772
629,-0.37985929615081,0.704049863502535,NA
"16189","B3GALT2",1.17547775501213,-1.07507461089984,1.30278329370411,
-0.825213691406156,0.409250281702885,NA
"16190","LINC01031",0.222355900326106,-0.694084893685918,3.34637783400
456,-0.207413785327198,0.835686717551155,NA
"16191","AL138778.1",0.0951446972952032,0.078062690925409,3.3524751975
3738,0.0232850912611527,0.98142286394086,NA
"16192","AL136456.1",0,NA,NA,NA,NA,NA
"16193","CFHR3",1.13691264474867,-0.291868774675818,1.29415192547379,-
0.225528988467846,0.821567776648536,NA
"16194","CFHR1",0.558347484169522,-0.435416686402464,2.31333092078347,
-0.188220665919686,0.850703672945019,NA
[truncated: 481,833 more chars]
